# A high-density SNP genotyping array for *Brassica napus* and its ancestral diploid species based on optimised selection of single-locus markers in the allotetraploid genome

**DOI:** 10.1007/s00122-016-2746-7

**Published:** 2016-06-30

**Authors:** Wayne E. Clarke, Erin E. Higgins, Joerg Plieske, Ralf Wieseke, Christine Sidebottom, Yogendra Khedikar, Jacqueline Batley, Dave Edwards, Jinling Meng, Ruiyuan Li, Cynthia Taylor Lawley, Jérôme Pauquet, Benjamin Laga, Wing Cheung, Federico Iniguez-Luy, Emmanuelle Dyrszka, Stephen Rae, Benjamin Stich, Rod J. Snowdon, Andrew G. Sharpe, Martin W. Ganal, Isobel A. P. Parkin

**Affiliations:** 1 Agriculture and Agri-Food Canada, 107 Science Place, Saskatoon, SK S7N 0X2 Canada; 2 TraitGenetics GmbH, Am Schwabeplan 1b, Stadt Seeland OT, 06466 Gatersleben, Germany; 3 National Research Council Canada, 110 Gymnasium Place, Saskatoon, S7N 0W9 Canada; 4 School of Plant Biology and The UWA Institute of Agriculture, The University of Western Australia, 35 Stirling Highway, Crawley, Perth, 6009 Australia; 5 National Key Laboratory of Crop Genetic Improvement, Key Laboratory of Rapeseed Genetic Improvement, Ministry of Agriculture P. R. China, Huazhong Agricultural University, Wuhan, 430070 China; 6 Illumina, Inc., 9885 Towne Centre Drive, San Diego, CA 92121 USA; 7 BIOGEMMA 6, chemin des Panedautes, 31700 Mondonville, France; 8 Bayer BioScience NV, 9052 Ghent, Belgium; 9 DNA Landmarks Inc, 84 Rue Richelieu, St-Jean-sur-Richelieu, QC J3B 6X3 Canada; 10 Genomics and Bioinformatics Unit, Agri Aquaculture Nutritional Genomic Center (CGNA), Conicyt-Regional, Gore La Araucania, R10C1001, Temuco, Chile; 11 Syngenta France SAS, 12 Chemin de l’hobit, B.P. 27, 31790 Saint-Sauveur, France; 12 Max Planck Institute for Plant Breeding Research, Carl-von-Linné-Weg 10, 50829 Cologne, Germany; 13 SYNGENTA France SAS, 346, route des Pasquiers, 84260 Sarrians, France; 14 Department of Plant Breeding, IFZ Research Centre for Biosystems, Land Use and Nutrition, Justus Liebig University, Giessen, Germany

## Abstract

****Key message**:**

**The*Brassica napus*Illumina array provides genome-wide markers linked to the available genome sequence, a significant tool for genetic analyses of the allotetraploid*B. napus*and its progenitor diploid genomes.**

**Abstract:**

A high-density single nucleotide polymorphism (SNP) Illumina Infinium array, containing 52,157 markers, was developed for the allotetraploid *Brassica napus*. A stringent selection process employing the short probe sequence for each SNP assay was used to limit the majority of the selected markers to those represented a minimum number of times across the highly replicated genome. As a result approximately 60 % of the SNP assays display genome-specificity, resolving as three clearly separated clusters (AA, AB, and BB) when tested with a diverse range of *B. napus* material. This genome specificity was supported by the analysis of the diploid ancestors of *B. napus,* whereby 26,504 and 29,720 markers were scorable in *B. oleracea* and *B. rapa*, respectively. Forty-four percent of the assayed loci on the array were genetically mapped in a single doubled-haploid *B. napus* population allowing alignment of their physical and genetic coordinates. Although strong conservation of the two positions was shown, at least 3 % of the loci were genetically mapped to a homoeologous position compared to their presumed physical position in the respective genome, underlying the importance of genetic corroboration of locus identity. In addition, the alignments identified multiple rearrangements between the diploid and tetraploid Brassica genomes. Although mostly attributed to genome assembly errors, some are likely evidence of rearrangements that occurred since the hybridisation of the progenitor genomes in the *B. napus* nucleus. Based on estimates for linkage disequilibrium decay, the array is a valuable tool for genetic fine mapping and genome-wide association studies in *B. napus* and its progenitor genomes.

**Electronic supplementary material:**

The online version of this article (doi:10.1007/s00122-016-2746-7) contains supplementary material, which is available to authorized users.

## Introduction

*Brassica napus* is an economically important oilseed crop that is primarily grown to extract the healthy edible oil from the seed, but it is now also grown as a renewable feedstock for biodiesel. In addition, there are vegetable types of the species that have been bred for both human and animal consumption. It is a temperate crop widely grown in both the Northern and Southern hemispheres due to available genotypic variation for flowering time and response to photoperiod. It is believed that *B. napus* emerged from a small number of hybridisation events between the diploid progenitors *Brassica oleracea* (C genome) and *Brassica rapa* (A genome) (UN 1935) that probably occurred in the southern Mediterranean and possibly regions of Asia around 7000–10,000 years ago (Chalhoub et al. 2014). The progenitors, *B. oleracea* and *B. rapa,* are also important predominantly vegetable crop species that each display a wide range of genetic and morphological diversity (Dixon 2006).

There are extensive worldwide breeding efforts in *B. napus* and its diploid relatives in both the public and private domains that contribute to developing higher value crops with improved yields (Iniguez-Luy and Federico 2011; Snowdon and Iniguez Luy 2012). Such breeding efforts are benefiting from access to a burgeoning collection of genetic and genomic resources for the *Brassica* species culminating in the recent release of the diploid and amphidiploid genomes that complete one axis of U’s triangle and define the *B. napus* genome (Chalhoub et al. 2014; Parkin et al. 2014; Wang et al. 2011). The now available genome sequences can be exploited to identify candidate genes for traits of interest, but their primary utility in breeding is in the development of genetic markers for marker assisted selection and, more recently, genomic selection. Genomic selection or predictive breeding is showing potential for application in crop species, where traits can be controlled by multiple small-effect QTLs, as more sophisticated algorithms have been developed to overcome the statistical challenges of working with disproportionately larger numbers of marker loci than samples tested (Jannink et al. 2010).

The availability of genome sequences and access to relatively economical next-generation sequencing technologies has provided the impetus to identify extensive nucleotide variation among different plant species. The abundance of single nucleotide polymorphisms (SNPs) across plant genomes has made them highly desirable for marker development (Ganal et al. 2009, 2012). High-throughput (tens of thousands or higher) SNP screening can be achieved effectively by either genotyping-by-sequencing (GBS) or high-density SNP arrays. GBS requires no former knowledge of available SNPs within a species, but is heavily reliant on bioinformatics capacity, and although common SNP will be found across experiments, the SNP profile identified is dependent on the genotypes queried (Deschamps et al. 2012). In comparison, high-density SNP arrays provide a common platform that can be continuously used and replicated across multiple labs with minimal computational requirement (Ganal et al. 2012). However, such SNP genotyping arrays involve significant development costs to identify sufficient numbers of robust, informative loci that fulfill assay design criteria. Identifying high-quality SNP loci for array design requires sequence data from sufficient numbers of genotypes to be able to assess polymorphism levels and associated allele ratios across the diversity of a species to minimise ascertainment bias. In addition, genome duplication in polyploid genomes, such as *B. napus,* confounds the design of SNP assays, since nucleotide variation among closely related orthologous or paralogous sequences is often misinterpreted as allelic variation (Parkin et al. 2010). Furthermore, since the SNPs are evaluated through hybridisation, multiple homologous and homoeologous loci may hybridise to a single SNP oligonucleotide probe leading to highly compressed and often irresolvable SNP patterns.

The current manuscript describes the development of a high-density (>50,000) Illumina Infinium^®^ SNP array designed for genotyping in *B. napus*, that can also be applied to the diploids, *B. oleracea* and *B. rapa*. Next-generation sequence data from both genomic and transcriptome sources were utilised to identify millions of preliminary SNP loci across the *B. napus* genome. Extensive filtering of these data led to the development of a highly effective tool for Brassica breeding with the majority of the SNP assays targeting single loci within the amphidiploid genome. The efficacy of the array was tested through the generation of cluster files, which define common allele clusters across a range of genotypes in all three species, and a high-density genetic map for *B. napus*.

## Materials and methods

### Reference mapping and variant calling

Pseudo-genome sequences of the diploid A and C genomes (283.8 and 488.6 Mb, respectively) were combined into a single reference sequence set for mapping. Sequence reads from each genotype were aligned independently using the CLC Genomics Server v3.6. Default parameters for the mapping algorithm were used except for the mapping identity parameter which was increased to 98 % to facilitate resolution of homoeologous sequence reads. Mapped reads were interrogated for sequence variation using the CLC Genomics Server v3.6 variant discovery algorithm. A minimum depth of coverage of 3× for 454 and 8× for Illumina data was required for SNP calling. Mapping data and variant calls were exported from CLC in the form of SAM alignment files and tab-delimited text files, respectively. Data from these files were combined using a custom Perl script to determine a missing, reference, or variant call in each genotype at each covered position of the genome.

### SNP filtering

Combined SNP results were filtered using custom Perl scripts and eliminated based on the following criteria: (1) SNP positions without suitable flanking sequence (60 bp on at least one side of the SNP with no variation); (2) SNP positions with more than two variations within the surveyed genotypes; (3) SNP positions with high levels of heterozygous calls, biased allele ratio, or missing data; (4) Illumina Assay Design Tool (ADT) score less than 0.6; (5) SNP positions where the variation was the result of a transversion.

### Probe matching and SNP selection

Probe sequences for all filtered SNPs were obtained from Illumina and then aligned to the reference sequences using the open source alignment tool BLAT with default parameters (Kent 2002). These alignments were parsed using a custom Perl script to determine the number of times the probe sequence from a particular SNP matched to the reference sequence set. A probe alignment was considered to be matched if 35 consecutive base pairs of the probe were fully aligned. SNPs were ranked based on the number of times their probe sequence matched the reference sequence set and SNPs with fewer probe matches preferentially selected.

### Experimental SNP data collection

The cluster file for *B. napus* was generated at AAFC through analysis of 437 genotypes and at TraitGenetics through the analysis of 432 genotypes. The cluster files for *B. oleracea* and *B. rapa* were generated with 129 and 121 samples, respectively. In both laboratories, DNA was extracted from young leaf tissue of greenhouse grown plants using a cetyltrimethylammonium bromide (CTAB)-based method (Murray and Thompson 1980). DNA was quantified and 200 ng were hybridised to the Brassica 60 K Infinium array as described in the manufacturer’s protocol (Illumina Inc., San Diego, CA). The arrays were scanned using an Illumina HiScan or BeadArray Reader, and SNP data were analysed using the Genotyping module of the GenomeStudio software package with the setting for the No Call threshold set to 0.05.

### Generation of the genetic map

DNA from 124 lines of a doubled-haploid (DH) population (derived from a cross between DH12075 and PSA12 and named SG DH, Parkin et al, unpublished) was hybridised to the Brassica 60 K Infinium array, and allele calls were made using the newly generated cluster file. The genetic linkage map was generated using the MSTmap software package (Wu et al. 2008). The map order was checked manually to ensure the optimal placement of the SNP loci, and a bin map was generated. Final-map distances were calculated using the Kosambi mapping function and the Mapmaker v3 software (Lander et al. 1987).

## Results

### Array design

A set of 54,866 SNP assays, previously identified and tested on the Illumina platform, were provided from a number of different sources (Bus et al. 2012; Dalton-Morgan et al. 2014) (Cheung, Dryszka, Laga, Pauquet, Rae, unpublished data). The remainder of the SNP assays that were used in the array design was processed using a single pipeline (Supplementary Figure 1). Next-generation sequencing data were collated from two previously published data sets described in Harper et al. (2012), which contributed RNASeq data from 42 different *B. napus* genotypes, and Clarke et al. (2013), which contributed Illumina and Roche 454 sequence capture data from nine *B. napus* genotypes. In addition, Roche 454 (1.16 Gb) data from genomic material and Illumina HiSeq (417.85 Gb) data from both genomic and transcriptome sources were generated for an additional 13 *B. napus*, four *B. oleracea,* and three *B. rapa* genotypes (Supplementary Table 1).

The array was designed prior to the release of the *B. napus* genome sequence (Chalhoub et al. 2014). Thus, high-quality sequence reads were reference mapped using CLC Genomics Server v3.6 to a pseudo *B. napus* genome derived from concatenating the genome sequences of *B. rapa* (Wang et al. 2011) and *B. oleracea* (Parkin et al. 2014). Considering only uniquely matching reads, over 570 Gb of sequence data were aligned to the pseudo-genome providing an estimated 738× depth coverage, although it should be noted that the inclusion of transcriptome data can bias the overall distribution with over-representation of some genic regions. SNP calling was completed using the SNP Discovery algorithm of the CLC Genomics Server, and all relevant data were exported for further filtering. Custom Perl scripts were used to generate an output file that included the SNP id, reference id, and position, flanking sequence where available, the reference allele, and for each individual surveyed, the SNP call, depth, and frequency data. These data were then filtered in three steps. First, SNPs were excluded if there was insufficient SNP-free flanking sequence (60 bp on at least one side). This step removed the largest number of the identified SNP loci (76 %) (Table 1). In the second step, SNPs were excluded if they were multi-allelic (more than two alleles), since these cannot be efficiently assayed using the Illumina platform. The final step identified high-confidence SNP loci, SNPs were excluded when the frequency of individuals with missing data was greater than 70 %, the frequency of individuals that showed heterozygous calls was greater than 40 %, and finally, if the allele frequency was higher than 0.8 or lower than 0.2. Table 1 shows the attrition at each filtering step. A final set of 180,398 SNP loci consisting of filtered and previously tested SNPs were submitted to the Illumina Assay Design Tool, which returned 161,917 SNPs with a minimum recommended score at or above 0.6.

**Table 1:** SNPs and filtering steps used for array design

Filter step	SNPs excluded	SNP count
None	0	24,528,374
Flanking sequence	18,619,172	5,909,202
Multi-allele SNP	7671	5,901,531
Confidence^a^	5,742,443	159,088
Illumina ADT score (<0.6)	33,556	125,532
Transversions	1318	124,214

^a^SNP positions were filtered for high levels of heterozygous calls, biased allele ratio, or missing data as described in the Results section

The specificity of each Illumina SNP assay is reliant on a single 50 bp probe sequence flanking one side of the SNP, the length of which can lead to ambiguous matching across genomes with any level of redundancy. To filter potential designs to reduce the impact of high copy probe sequences, the 50 bp probes for each possible assay design were matched using BLAT to the pseudo-genome. More than half (74 %) of the filtered SNP assays had probe sequences that mapped to multiple regions of the Brassica genome. The final SNP list submitted for Illumina bead design contained 15,141 previously tested SNP loci, 32,294 newly designed SNP loci that matched the pseudo-genome uniquely, and 11,029 SNP loci that matched twice. Once synthesized, 52,157 SNP markers on the Brassica 60 K array passed bead representation and decoding quality metrics, including 1213 A/T or C/G SNPs, which are represented by Infinium I bead types that require two beads per assay.

### Cluster file generation for reliable scoring of the SNP markers in *Brassica napus* and its diploid ancestors

**Fig. 1:**
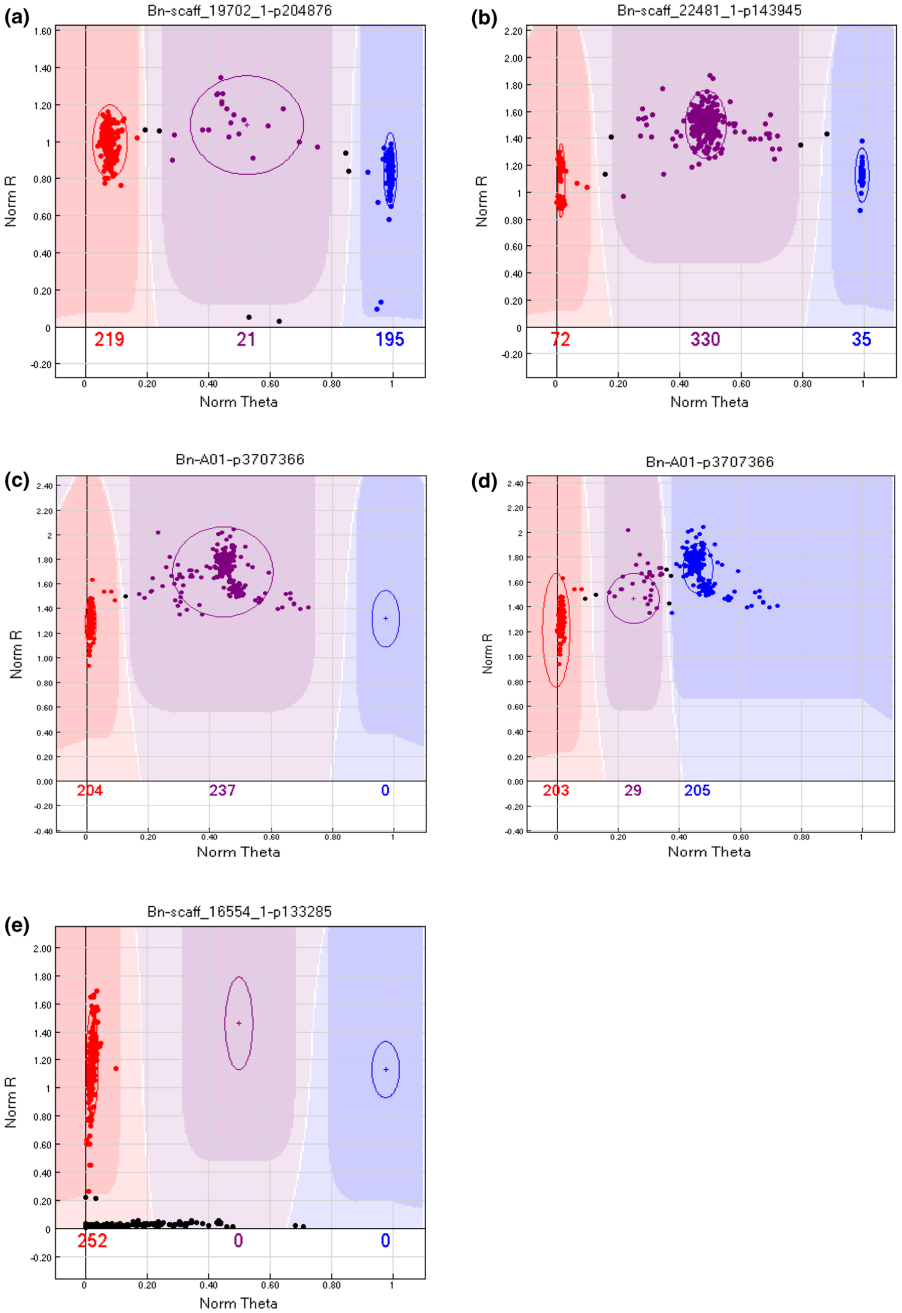
GenomeStudio images showing representative SNP cluster patterns across *Brassica napus* genotypes. One cluster representing one parental allele is coloured in *red* (AA), the second in *blue* (BB), and heterozygote genotypes in *purple*. **a** Shows a genome-specific SNP marker in *B. napus*, almost 60 % of the SNP loci show this clear separation of the expected three genotypes. **b** SNP locus likely resulting from hybridisation of two segregating homoeologous loci reveals five clusters and an excess of heterozygotes. **c** and **d** Show a SNP locus called automatically by the software and after manual adjustment of the cluster profile, respectively. **e** SNP locus where one parental allele shows no hybridisation (presence/absence marker)

The most efficient high-throughput application of an Illumina array can be achieved with the development of a robust cluster file that defines the expected intensity level of the three genotype classes (AA, AB, and BB) for each SNP locus. The cluster file is applied to intensity data to automatically call the genotypes for experimental samples, thus allowing easy comparison of data across labs (Fig. 1a). At AAFC Saskatoon, the first data set included 327 *B. napus* genotypes of both annual and biennial types, from diverse origins, ten F_1_ lines, and a subset of lines from two DH mapping populations. Independently at TraitGenetics, a second data set was generated that consisted of 432 mostly winter-type *B. napus* genotypes, including 67 hybrids, 88 F_2_, and 20 resynthesized *B. napus* lines. The two data sets were analysed independently and the resultant cluster files compared. After filtering out 173 SNP from the cluster file that displayed low intensity across the majority of the samples, 51,984 SNP remained. Based on the genotypes tested at AAFC, 1678 loci were monomorphic and for the genotypes tested at TraitGenetics, 2444 markers were monomorphic. Due to the strong sequence similarity between the A and C genomes of *B. napus,* it was anticipated that some of the SNP loci would display cluster patterns reflecting co-hybridisation of homoeologous loci. In such instances, when both homoeologous loci are polymorphic, the resultant SNP patterns are not automatically resolvable, generally these result in four-to-five clusters, and the Illumina software will identify exceptionally high numbers of heterozygotes (Fig. 1b). However, when one of the homoeologous loci is monomorphic, the genotype cluster intensities are shifted to one side of the theta space (actual genotype would be, for example, AAAA, AAAB, and AABB), leading to false cluster assignment with the routine analysis tools (Fig. 1c); yet, the cluster definition of such an SNP locus can be optimised manually to reflect the correct genotype positions, rendering it perfectly scorable (Fig. 1d). The two labs independently assayed for such loci, and manually adjusted the cluster assignments where necessary. By assessing the number of polymorphic markers that showed a cluster pattern that was indicative of a single copy locus, with three possible allelic states (homozygous allele AA, heterozygous AB, and homozygous allele BB) distributed over the entire theta space (difference between mean AA θ and mean BB θ >0.6), between 34,248 (TraitGenetics) and 37,536 (AAFC Saskatoon) loci were determined to be effectively genome specific. For a small number of markers, fluorescence was observed for only one allele, which could be informative in certain populations, but would be unable to detect heterozygous individuals (Fig. 1e). In total, 47,304 markers were defined as scorable and are retained in the current *B. napus* cluster file.

**Fig. 2:**
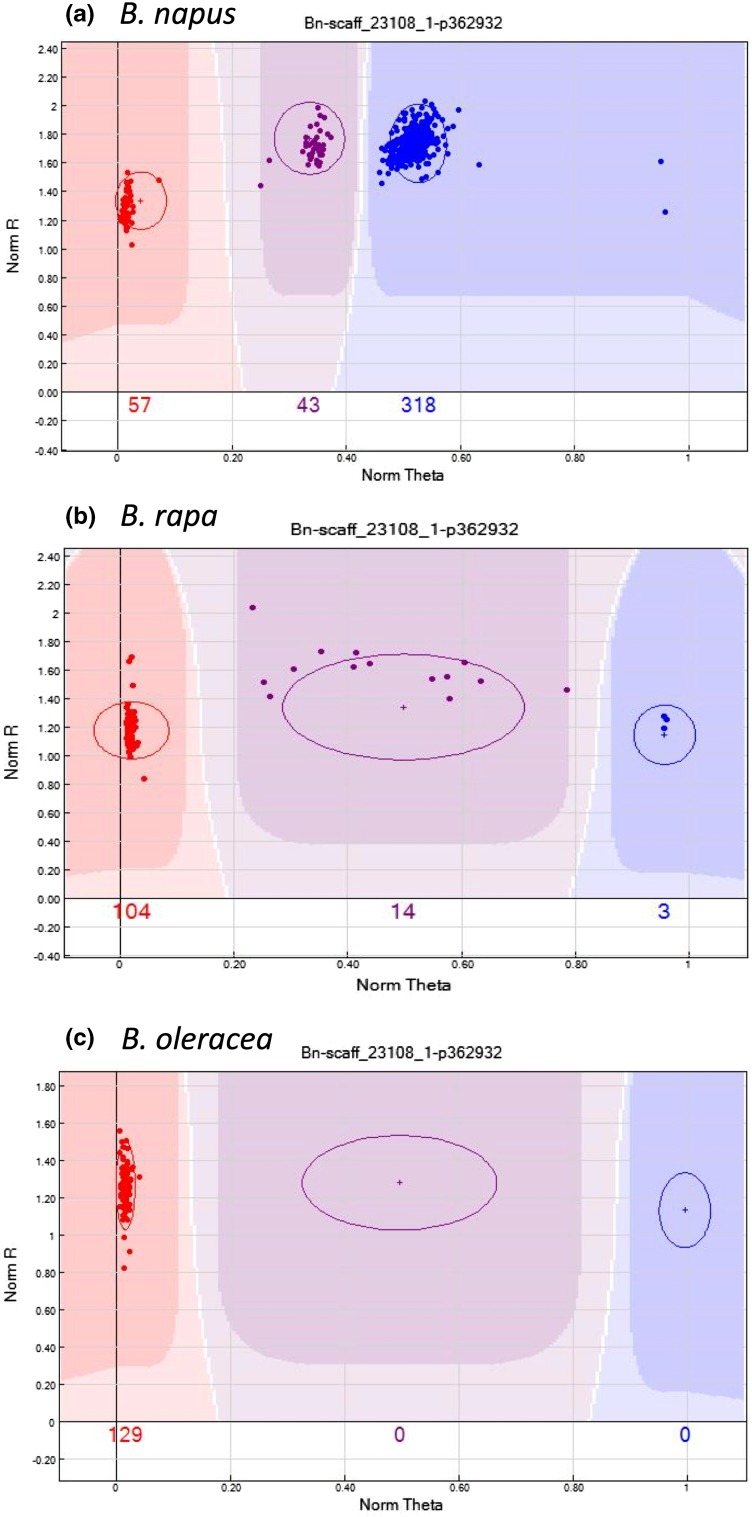
GenomeStudio images showing representative SNP cluster patterns in the different *Brassica* species for the marker Bn-scaff_23108_p362932. One parental allele is coloured in *red* (AA), the second in *blue* (BB), and heterozygotes in *purple*. The SNP marker is polymorphic, but not genome-specific in *B. napus*, **a** resulting in condensed clusters due to the detection of the homoeologous locus on the other genome. In the diploid *B. rapa*, **b** this marker is polymorphic and shows widely distributed clusters (no second homoeologous locus detected, typical diploid pattern). In *B. oleracea* material, this marker is monomorphic

Diploid samples included in the initial analysis indicated that a subset of the loci could not be scored accurately in the diploids using the *B. napus* cluster file (Fig. 2a–c). To facilitate the use of the Brassica array for scoring in the diploid ancestors, 129 *B. oleracea* lines, hybrids and representative segregating material, and 121 *B. rapa* lines, F_1_s and representative segregating material were analysed with the array. Based on these results, cluster files for the two ancestral species were generated, mainly based on modified cluster positions for those markers that were not genome specific. The final cluster file for *B. oleracea* contained 26,504 scorable markers of which 21,113 were polymorphic in the investigated material, and the *B. rapa* cluster file contained 29,720 scorable markers of which 22,695 were polymorphic in the investigated material (Supplementary Table 2).

### Physical and genetic positions of SNPs on *Brassica* genomes

**Fig. 3:**
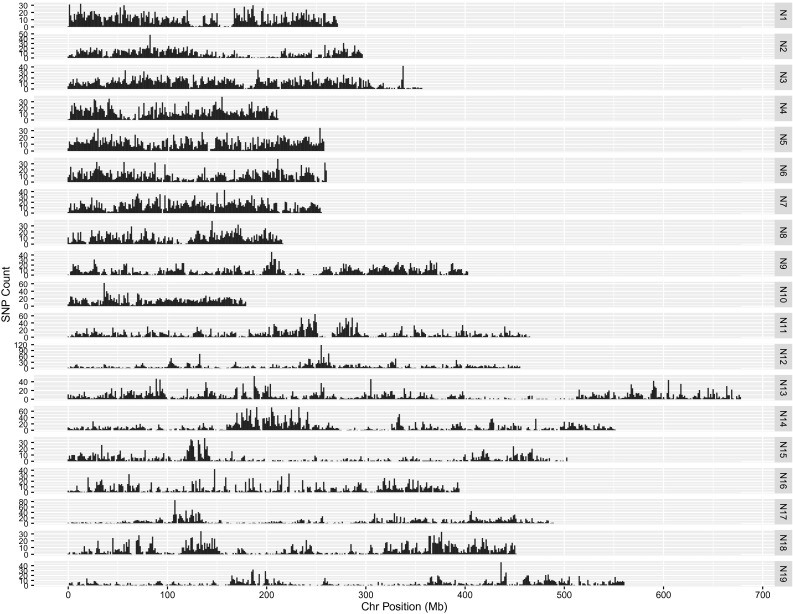
Physical distribution of SNP loci across the *B. napus* genome. The SNP loci were aligned to the genome of spring-type DH12075 based on BLAT scores, with the numbers of SNP loci per 125 Kb window indicated on the *y* axis for each chromosome

The physical positions of the assayed loci in the diploid A and C genomes were determined during the SNP calling process, based on unique read mapping to the reference sequence set, consisting of the A genome of *B. rapa* (Wang et al. 2011) and the C genome of *B. oleracea* (Parkin et al. 2014). The physical position was also determined in two recently completed *B. napus* genomes, one a winter type (Chalhoub et al. 2014) and the second a spring type (Parkin et al. unpublished), by aligning the DNA flanking each of the SNP loci to each genome using BLAT (Kent 2002). The best hit and associated percent identity of the match for each genome were then extracted from the BLAT results. Based on a percent identity of at least 85 %, 50,255 SNPs were positioned in the spring-type genome sequence and 49,794 were positioned in the winter-type genome sequence. Taking both genome sequences together, a total of 51,172 SNPs could be matched to one or both *B. napus* genomes (Fig. 3). It was recognized that the length of the query sequence could lead to ambiguities or erroneous matches due to the highly redundant nature of each genome. The latter would be particularly true for matches to the *B. napus* genome, where in addition to the strong homology between the two constituent genomes, there are also regions of effective identity resulting from homoeologous exchanges between the A and C genomes (Chalhoub et al. 2014). Based on the BLAT scores, 22,258 and 23,191 SNPs could be unambiguously positioned on the A and C genomes, respectively, while 2138 were placed on either the A or C with equal probability (Supplementary Table 3). In addition, 4570 SNPs could not be positioned on the pseudo-chromosomes as a result of either missing data in *B. napus* or the alignment of the SNP sequence to an unanchored scaffold in one or both *B. napus* genomes. The SNP loci were largely found in non-coding regions, although 17,955 lay within annotated gene sequences, only 8681 of which were positioned within an exon (Supplementary Table 4).

To genetically position 21,766 (46 %) of the SNP loci, the highly polymorphic SG DH population derived from a cross between a resynthesized *B. napus* and an established *B. napus* line was used (Supplementary Tables 5 and 6). Based on informative recombination events, these loci were placed in 1310 bins across the 19 linkage groups and covered a length of 1815 cM (Table 2). The loci were distributed with on average one marker every 0.15 cM or less (Table 2). There were a number of genetically defined bins with a higher than average density of markers, which tended to cluster together and were associated with regions of low recombination, predominantly found in the vicinity of the presumed centromeric regions (Fig. 3).

**Table 2:** Distribution of genetically and physically positioned SNP loci in the *B. napus* genome

Linkage group	Number of genetically defined bins^a^	Number of physically positioned SNP loci^b^	Physical length of pseudo-molecule (Kb)	Number of mapped SNP loci	Map distance (cM)	Markers/cM	CM/marker	Kb/marker
A1	60	2672	27,105	913	75.9	12.03	0.08	10.19
A2	74	2425	29,627	946	98.2	9.63	0.10	12.22
A3	94	3185	35,753	1330	114.3	11.64	0.09	11.23
A4	50	2112	21,080	1085	57.6	18.84	0.05	9.98
A5	78	2332	25,706	1121	99.6	11.26	0.09	11.06
A6	89	2302	26,146	1019	100.1	10.18	0.10	11.32
A7	46	2529	25,458	1333	60	22.52	0.04	10.07
A8	55	1863	21,685	953	85	11.21	0.09	11.64
A9	89	2452	40,546	1279	127.5	10.03	0.10	16.43
A10	63	2053	17,911	841	75.6	11.12	0.09	8.72
C1	46	3418	45,604	1882	71.3	26.40	0.04	13.65
C2	47	3743	47,311	1241	69.8	17.78	0.06	12.20
C3	118	3870	67,777	1804	165.3	10.91	0.09	17.51
C4	88	4399	55,069	1443	136.9	10.54	0.09	12.55
C5	65	1600	48,717	651	124.4	5.23	0.19	31.46
C6	40	1982	40,797	980	42.6	23.00	0.04	19.88
C7	82	2784	48,823	1360	101.7	13.37	0.07	17.54
C8	68	2151	44,716	742	104	7.13	0.14	20.97
C9	58	1782	55,995	843	106	7.95	0.13	31.42
Total	1310	49,744	725,833	21,766	1814.9	11.99	0.08	14.59

^a^ The genetic map position is based on mapping data from the SG DH population

^b^The physical position is based on the original reference mapped position in the diploid genome sequences

**Fig. 4:**
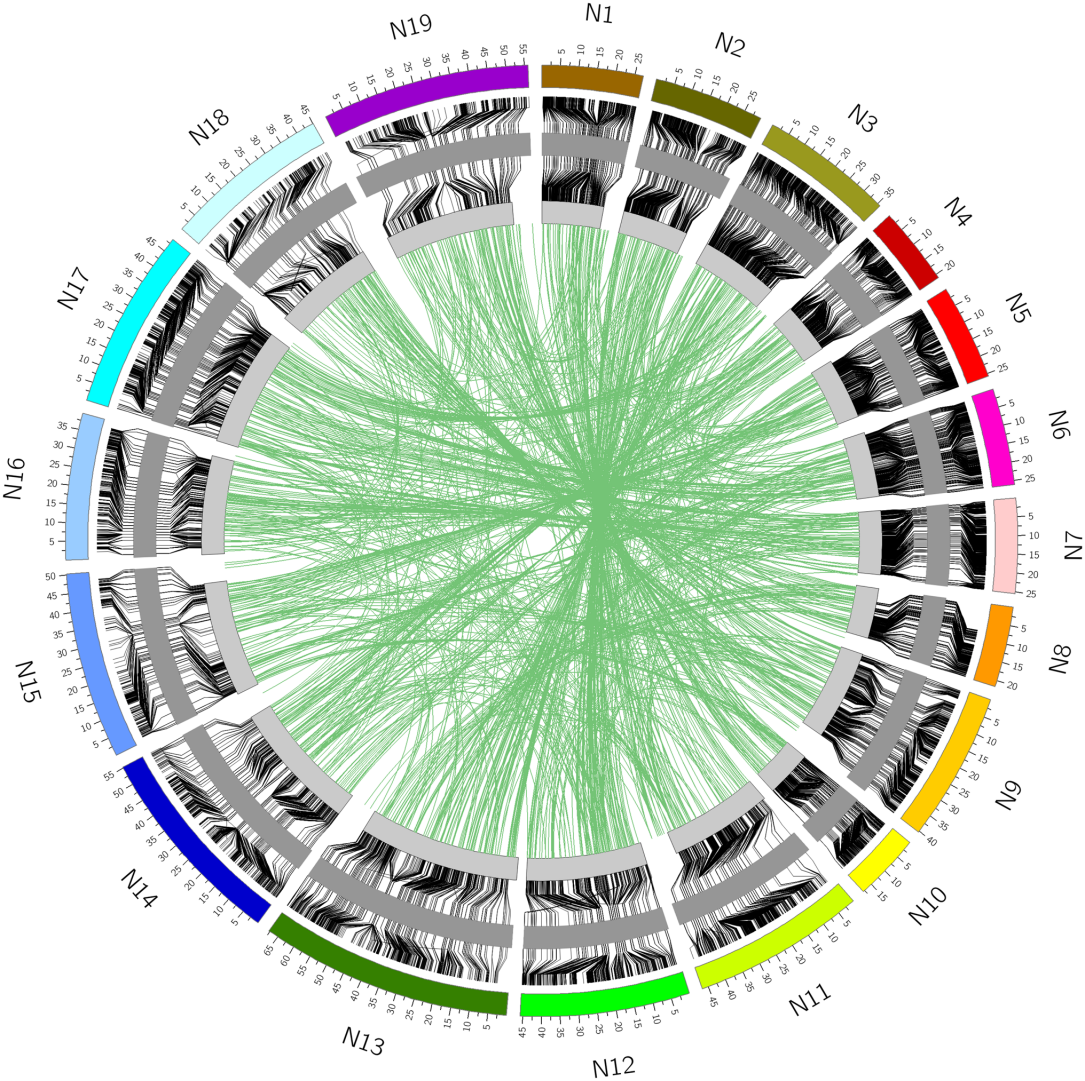
Relationship between the physical and genetic positions of the SNP loci in *B. napus*. The *inner circle* represents the genetic map which is flanked to the outside by the physical position in the spring-type DH12075 and to the inside by the physical position in the winter-type Darmor bzh. The *green lines* connecting across the centre of the *circle* represent those loci that are genetically positioned to an alternate (mostly homoeologous) position compared to their physical coordinates in the genome sequence

**Fig. 5:**
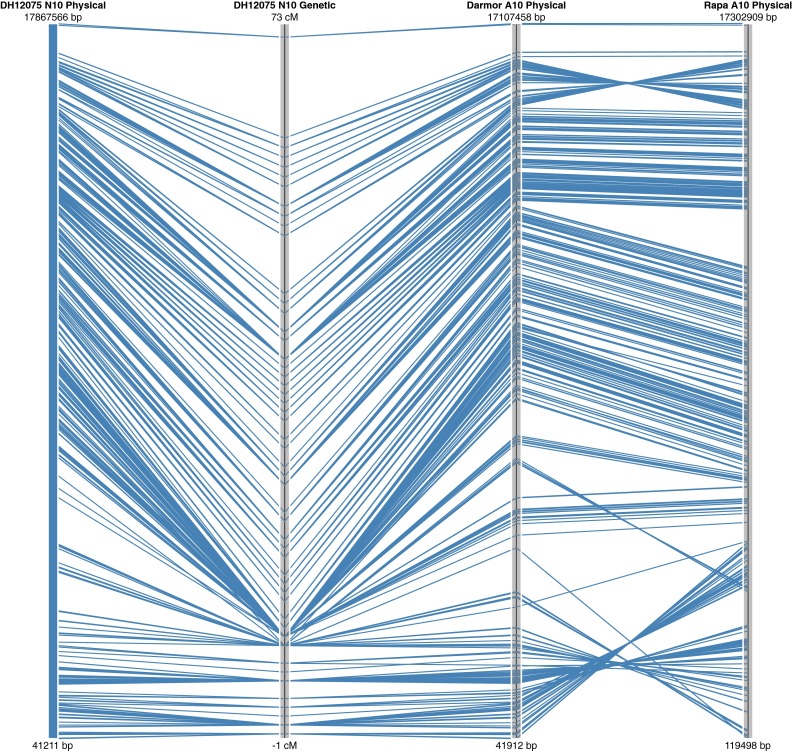
Alignment of the genetic map for linkage group N10 of *B. napus* with the genome sequence of the equivalent chromosome in the two independent *B. napus* assemblies and in *B. rapa*

Only markers that were positioned both genetically and physically could be definitively positioned on the *B. napus* genome. In general, there was good correspondence between the two, with 20,138 of the 21,766 (93 %) SNP loci genetically mapping to the position expected based on sequence alignment. In addition, 3 % of loci that were physically mapped to one position were genetically mapped to the homoeologous region of the genome (Fig. 4). The remaining 4 % did not match either the expected physical position or the homoeologous position. Some of these loci were genetically mapped to unanchored *B. napus* scaffolds, but others mapped to a different position in *B. napus* than expected based on the diploid genome from which the SNP probe was designed, suggesting inconsistencies with the original assemblies. This was particularly true for the *B. rapa* genome where genomic regions of varying sizes (0.1–1.1 Mb) were found to be anchored to the wrong chromosome relative to the two *B. napus* genome sequences (Supplementary Table 3). This is likely due to the relatively low marker density that was used to anchor the *B. rapa* genome assembly (Wang et al. 2011). It is possible that some differences between the genomes could reflect true chromosomal rearrangements; indeed, the relatively large inversion found to differentiate both ends of A10 from N10, could be evidence of such an event, since it appears common to both *B. napus* genotypes (Fig. 5). However, a similar but smaller inversion at the top of A7/N7 is specific to only the Darmor bzh *B. napus* genotype (Supplementary Table 3). The physical and genetic positions of the SNP loci have been imported into a web tool that visualizes these alignments (http://aafc-aac.usask.ca/Bn60). Particular regions of the genome can be selected to identify potentially useful SNP loci in any region of interest.

## Discussion

The recent release of three Brassica crop genome sequences has provided opportunities for the development and application of new breeding tools. The complexity of the *B. napus* genome becomes evident when carrying out genetic mapping even for relatively simple quality-based traits, where multiple loci control their expression. To dissect and follow genetically, complex traits within Brassica breeding programs robust sequence-based markers are required. The high-throughput genotyping array described here offers an excellent platform for facilitating such analyses and allows ready access to a set of well-characterised markers.

The SNP pipeline used to develop the majority (approximately 74 %) of the assays on the Brassica array included a number of steps to limit the impact of genome duplication and allopolyploidy on the resultant design. Reference mapping of short-read sequences to the constituent diploid genomes was optimised to prevent matching to homoeologous regions, sequence variation was avoided in the immediate proximity of the target SNP, and regions where multiple alleles were identified were excluded, all limiting the calling of paralogous SNPs. The final step of remapping the oligonucleotide probe for each SNP assay back to the reference genomes and selecting those with the lowest copy number further facilitated the design of the array, since 81 % of the SNPs that matched only once in the *B. napus* genome produced genome-specific three cluster patterns. Approximately 58 % of all assays produced clear genome-specific genotype calls for a wide range of *B. napus* genotypes, including F_1_ individuals. This is a marked improvement over similar arrays designed for hexaploid and tetraploid wheat where not more than 25.5 % of the SNP assays produced clear genome-specific cluster patterns (Wang et al. 2014). However, the stringency of the design pipeline limited the variation available to be employed for the array design, effectively reducing the number of initial SNPs by 99 %. This high level of attrition could cause some marker selection bias. Thus, to achieve an optimal density of markers across the genome, pre-validated SNP assays, which matched two or more regions of the reference genome, were also included.

Upon testing *B. napus* cultivars originating from multiple continents and covering the range of annual and biennial types, the SNP assays proved to be highly polymorphic, with only 3.5 % monomorphic loci identified indicating the value of the selected SNPs. This was also confirmed independently in a recently published work that used the array to assess diversity within a collection of predominantly Asian *B. napus* lines (Qian et al. 2014). The array was tested through the generation of a dense SNP map for *B. napus* with 21,766 (46 %) of the loci mapped in one DH population. Due to the design process, each of the SNP loci was physically anchored to a specific base-pair position in one or other of the diploid progenitor genomes (Supplementary Table 3). On extending this analyses to *B. napus,* however, the alignment of only the isolated short SNP regions to the genome sequence introduced a level of ambiguity, with some SNPs being equally likely to align, or in the case of the assays, hybridise to the A or C genome of *B. napus*. In addition, due to the prevalent homoeologous recombination events which have occurred during the evolution of *B. napus* (Chalhoub et al. 2014), it was expected that some SNPs may map to alternate orthologous positions in different *B. napus* genotypes. Comparing the genetic and physical position for each of the SNP loci, it was found that 3 % of the loci were genetically mapped to a homoeologous region relative to their physically defined coordinates (Fig. 4). Further studies with the array are likely to uncover additional ambiguities that should be considered when utilising the array for analyses, especially when the loci cannot be genetically anchored in the population or specific genotypes being queried.

Although not specifically designed for this purpose, assessment of the array using DNA from the diploid genomes of *B. oleracea* and *B. rapa* demonstrated its value for genetic analysis of these two important vegetable crop species. Although, the genome specificity of many of the markers could be seen as a disadvantage, since those specific for the other genome result in failed assays, there are still 26,504 and 29,720 clearly scorable markers for *B. oleracea* and *B. rapa*, respectively. In addition, despite the analysed sample number being lower, 21,113 and 22,695 markers were shown to be polymorphic in *B. oleracea* and *B. rapa,* respectively, demonstrating the utility of the array for these two diploid species. Indeed, this was confirmed through the recent generation of a genetic map for *B. oleracea* using the array (Brown et al. 2014).

Although the array offers relatively good coverage of the *B. napus* genome with the SNP loci physically distributed across each of the chromosomes at an approximate density of 1 marker every 15 Kb based on the diploid genome length, there was a significant sub-genome bias observed with a higher density in the A compared to the C genome (one marker every 11 Kb cf. 19 Kb) (Table 2). The genetic map was based on a highly polymorphic cross allowing almost 50 % of the SNP loci to be positioned on the *B. napus* genome. The genetic map showed only small gaps with five ≥9 cM. However, when considering the physical distribution of the mapped loci, each chromosome apart from N3 and N5 had at least one interval greater than 500 Kb, these larger intervals were also biased to the C genome with 89 % (140/158) of such intervals being localized to the C genome. This could reflect differing levels of genetic variation between the sub-genomes of *B. napus*, as observed by others (Delourme et al. 2013; Qian et al. 2014) or may suggest further optimisation of the array should focus on selection of C genome loci. Nevertheless, any bias in distribution of loci should be considered in downstream applications using the array. Once aligned to *B. napus*, this distribution did not change markedly and no large physical gaps were observed based on the overall marker selection (Fig. 3). The saturated coverage is partly a reflection of the genome organisation, with extensive blocks of repetitive elements largely limited to the pericentromeric regions. In addition, although 34.4 % (17,955) of the SNP loci fall within annotated genes, the array design did not focus on functional SNPs, which can bias the marker distribution. In maize, a similar high-density array was developed that targeted genic regions and even bearing in mind the greater genome size, there were significant gaps in the physical SNP coverage on many chromosomes of at least 1 Mb per chromosome (Ganal et al. 2011). Alignment of the physical and genetic maps for *B. napus* showed good collinearity; however, a number of rearrangements were noted on comparison with the diploid genomes. Although, some of these can be attributed to artifacts of the genome assembly process in each species, the larger rearrangements that are common to the two *B. napus* genomes may indicate chromosomal changes that have occurred since the fusion of the two progenitor genomes in the *B. napus* nucleus (Fig. 5).

The Brassica 60 K Infinium array provides a robust and efficient tool for genetic studies in *B. napus.* Due to the natural and breeding bottlenecks created in modern *Brassica* germplasm much emphasis is now placed on capturing the wider allelic diversity within the species gene pool (Bus et al. 2011). Genome-wide association studies (GWAS) in a number of other crop species have suggested the value of such analyses for exploiting untapped variation to identify causative loci for key economic traits (Cook et al. 2012; Zhao et al. 2011). An essential prerequisite for GWAS is the ability to query genome-wide polymorphisms that are spaced, such that the analyses are not limited by the observable linkage disequilibrium (LD) in the species of interest. More recent estimates for *B. napus* suggest LD breakdown across the genome, ranging from 0.3 to 1.7 cM (Delourme et al. 2013) and 0.25–2.5 Mb (Qian et al. 2014) with LD decaying more rapidly in the A genome. The distribution of SNP loci across the genome, which lie well within the current LD estimates, should facilitate the use of the array for GWAS or QTL mapping to identify genes underlying traits of interest. The demonstration of the utility of the array within *B. napus* as well as its diploid ancestors *B. oleracea* and *B. rapa* indicates that the developed array can be used in the entire crossing range of these three species, providing a valuable tool for Brassica breeding applications.

### **Author contribution statement**

WEC completed bioinformatics analyses to generate the array design. EEH, JP, and RW carried out experimental work to complete the cluster files. EEH created the genetic map. YK assisted with SNP data analyses. CS, RL, and FI-L generated data that were used in the SNP design. JB, DE, JM, JP, BL, WC, ED, SR, RJS and BS contributed SNP assays to the array design and were members of the consortium that agreed the array design. JB and JM also provided material for cluster file optimisation. CTL, AS, and MWG participated in study design, and helped edit the manuscript. All authors read and approved the manuscript. IAPP coordinated the design process, analysed data, and wrote the manuscript.

## Electronic supplementary material

Below is the link to the electronic supplementary material.

Supplementary Figure 1 Workflow for the design of the SNP genotyping array. The process started with independently mapping sequencing reads from multiple individuals to the reference sequence set and calling variants in the alignment (a). SNP reports and SAM files were then combined to generate an SNP summary table (b). This table is filtered to remove SNPs according to user-defined criteria (c) resulting in a final set available for array design (d). (PDF 120 kb)

Table 1 List of genotypes and available sequence data used for SNP discovery (PDF 47 kb)

Table 2 Scoring metrics for SNP loci at TraitGenetics in *B. napus*, *B. oleracea* and *B. rapa* (PDF 9064 kb)

Table 3 Physical position of each SNP locus in the *Brassica napus* genome based on BLAT analyses. SNP ID in *B. napus* genome is based on chromosome (or scaffold) name followed by base-pair position in the associated pseudo-molecule or scaffold. (PDF 6437 kb)

Table 4 List of SNP loci which fall within annotated *B. napus* genes. (PDF 1071 kb)

Table 5 Complete scoring matrix for genetic map for *Brassica napus* based on SG DH population (DH12075 x PSA12). (PDF 26859 kb)

Table 6 Genetic map for *Brassica napus* based on SG DH population compressed into genetic bins according to recombination events. (PDF 1685 kb)
