# Supplementary figures and images for: A high-density SNP genotyping array for Brassica napus and its ancestral diploid species based on optimised selection of single-locus markers in the allotetraploid genome
Source: Theor Appl Genet. 2016 Jun 30;129(10):1887–99. doi: 10.1007/s00122-016-2746-7 (PMC5025514; doi:10.1007/s00122-016-2746-7)

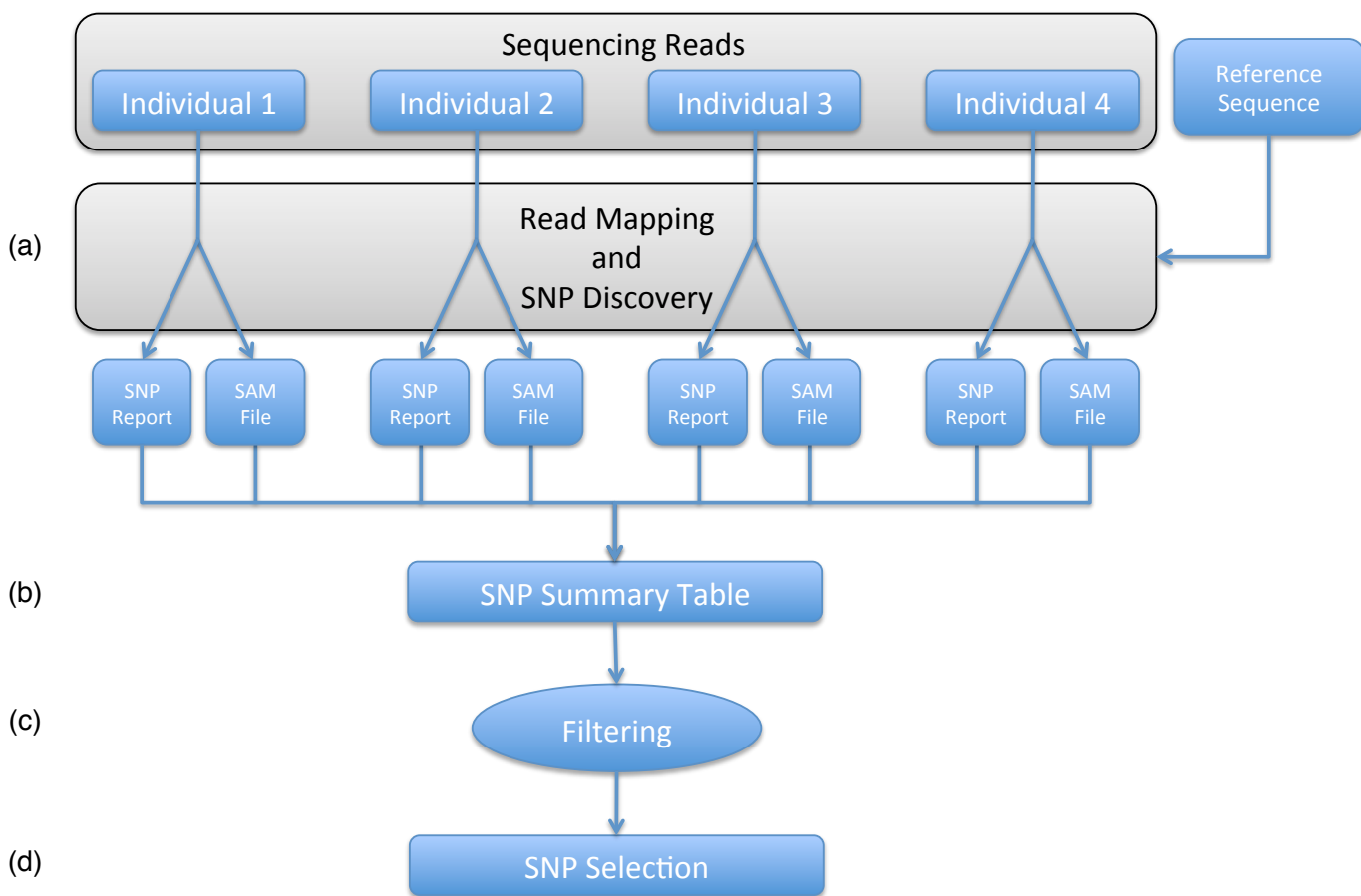

Supplement: Supplementary file 1 — Workflow for the design of the SNP genotyping array. The process started with independently mapping sequencing reads from multiple individuals to the reference sequence set and calling variants in the alignment (a). SNP reports and SAM files were then combined to generate an SNP summary table (b). This table is filtered to remove SNPs according to user-defined criteria (c) resulting in a final set available for array design (d). (PDF 120 kb) [file 122_2016_2746_MOESM1_ESM.pdf]
