# Supplementary material for: A high-density SNP genotyping array for Brassica napus and its ancestral diploid species based on optimised selection of single-locus markers in the allotetraploid genome
Source: Theor Appl Genet. 2016 Jun 30;129(10):1887–99. doi: 10.1007/s00122-016-2746-7 (PMC5025514; doi:10.1007/s00122-016-2746-7)
Supplement: Supplementary file 2 — List of genotypes and available sequence data used for SNP discovery (PDF 47 kb) [file 122_2016_2746_MOESM2_ESM.pdf]

Supplementary Table 1: List of genotypes and available sequence data used for SNP discovery

Line	Species	Platform
Badger	Brassica oleracea	Illumina
Reward	Brassica rapa	Illumina
Candle	Brassica rapa	Illumina
Maleksberger	Brassica rapa	Illumina
Gower	Brassica oleracea	Illumina
Gower_rnaseq	Brassica oleracea	Illumina RNASeq
Reward_rnaseq	Brassica rapa	Illumina RNASeq
Maleksberger_rnaseq	Brassica rapa	Illumina RNASeq
Candle_rnaseq	Brassica rapa	Illumina RNASeq
Badger_rnaseq	Brassica oleracea	Illumina RNASeq
D102_MAT	Brassica napus	Illumina RNASeq
D108_MON	Brassica napus	Illumina RNASeq
D10_BAL	Brassica napus	Illumina RNASeq
D113_NEWH	Brassica napus	Illumina RNASeq
D117_NOR	Brassica napus	Illumina RNASeq
D127_PRI	Brassica napus	Illumina RNASeq
D128_Q10	Brassica napus	Illumina RNASeq
D131_Raf	Brassica napus	Illumina RNASeq
D132_RAGJ	Brassica napus	Illumina RNASeq
D134_RAPCR	Brassica napus	Illumina RNASeq
D137_ROCxLIZ	Brassica napus	Illumina RNASeq
D145_SHEJ	Brassica napus	Illumina RNASeq
D146_SIBB	Brassica napus	Illumina RNASeq
D14_Bol	Brassica oleracea	Illumina RNASeq
D170_TIN	Brassica napus	Illumina RNASeq
D173_TOP	Brassica napus	Illumina RNASeq
D181_WESD	Brassica napus	Illumina RNASeq
D182_WILR	Brassica napus	Illumina RNASeq
D19_BRU	Brassica napus	Illumina RNASeq
D26_CAPxMOH	Brassica napus	Illumina RNASeq
D31_CES	Brassica napus	Illumina RNASeq
D32_CHE_DZA_trimmed_20	Brassica napus	Illumina RNASeq
D39_COR	Brassica napus	Illumina RNASeq
D3_ALT	Brassica napus	Illumina RNASeq
D41_CUBR	Brassica napus	Illumina RNASeq
D46_DIP	Brassica napus	Illumina RNASeq
D4_AMBxCOM	Brassica napus	Illumina RNASeq
D55_EUR	Brassica napus	Illumina RNASeq
D56_EVV	Brassica napus	Illumina RNASeq
D59_FID	Brassica napus	Illumina RNASeq
D63_GLO	Brassica napus	Illumina RNASeq
D69_HAN	Brassica napus	Illumina RNASeq
D6_APE	Brassica napus	Illumina RNASeq
D71_HEL	Brassica napus	Illumina RNASeq

D74_HURxNAV	Brassica napus	Illumina RNASeq
D75_INCxCON	Brassica napus	Illumina RNASeq
D76_JANS	Brassica napus	Illumina RNASeq
D79_JETN	Brassica napus	Illumina RNASeq
D7_APExGIN	Brassica napus	Illumina RNASeq
D83_KAR	Brassica napus	Illumina RNASeq
D85_KRO	Brassica napus	Illumina RNASeq
D8_AphRR	Brassica napus	Illumina RNASeq
DH12075	Brassica napus	Roche 454
DH12075_chilean	Brassica napus	Roche 454
DH12075_illumina_chilean	Brassica napus	Illumina
93-50532_chilean	Brassica napus	Roche 454
93-50537_chilean	Brassica napus	Roche 454
Express	Brassica napus	Roche 454
Express_chilean	Brassica napus	Roche 454
Express_illumina_chilean	Brassica napus	Illumina
Express	Brassica napus	Illumina
Express_rnaseq	Brassica napus	Illumina RNASeq
Hamburger	Brassica napus	Roche 454
Ningyou_chilean	Brassica napus	Roche 454
Ningyou_illumina	Brassica napus	Illumina
Ningyou_rnaseq	Brassica napus	Illumina RNASeq
Ningyou	Brassica napus	Illumina
PSA12	Brassica napus	Roche 454
PSA12_chilean	Brassica napus	Roche 454
Paroll	Brassica napus	Roche 454
Quantum	Brassica napus	Roche 454
Rainbow_chilean	Brassica napus	Roche 454
Surpass	Brassica napus	Illumina
Tapidor	Brassica napus	Illumina
Tapidor_rnaseq	Brassica napus	Illumina RNASeq
Tapidor_chilean	Brassica napus	Roche 454
Tapidor_illumina	Brassica napus	Roche 454
Yudal	Brassica napus	Illumina
Topas	Brassica napus	Illumina
Polo	Brassica napus	Illumina
Glacier	Brassica napus	Illumina
Glacier_rnaseq	Brassica napus	Illumina RNASeq
Quinta	Brassica napus	Illumina
Quinta_rnaseq	Brassica napus	Illumina RNASeq
Stellar	Brassica napus	Illumina
Scoop	Brassica napus	Illumina
Samourai	Brassica napus	Illumina
Samourai_rnaseq	Brassica napus	Illumina RNASeq
Mendel	Brassica napus	Illumina
Mendel_rnaseq	Brassica napus	Illumina RNASeq

V8	Brassica napus	Roche 454
V8_chilean	Brassica napus	Roche 454
YN429_chilean	Brassica napus	Roche 454

Sequenced reads	Unique Mapped Reads	Gb
93537184	83984515	
87149532	74804454	
157057996	135197219	
159402046	137036735	
166874868	148539034	
50572178	48084783	
53856212	50514136	
59102774	55559595	
53966062	50339970	
84838482	80934190	
36158322	32721700	
38162349	34671780	
29445977	27136213	
36110949	28521798	
38988726	33695055	
39594773	31751072	
35776273	28014190	
39069961	34269642	
39328918	35326870	
38813531	35290987	
37940783	34152978	
38188002	34582859	
33705961	29248689	
29974230	27772249	
38990423	35980986	
38492195	34387311	
37719204	34056466	
34807008	29664922	
30955744	28830790	
22935657	20151056	
28036702	26354686	
18052349	16504946	
29403131	27555540	
29080184	27307001	
30309874	27307452	
26575511	23668740	
28908899	26902006	
30060091	28271702	
27999290	25478403	
30199833	27087428	
31220585	28178041	
32452527	29894517	
32815906	30713186	
33117600	29716337	

25438243	22797851
32729048	29489839
30647477	26254893
18441603	16720530
29760647	27758030
38039017	33932578
36534976	33895104
28334337	25878110
887769	996364
1289496	996364
167215494	156161565
742361	544211
717016	524730
636405	507686
827074	606370
184559482	175195107
182678822	155549603
57841114	54865304
404192	318272
803553	606835
533612540	495241504
56524574	53553743
154359284	135974829
866710	655021
826680	630083
286450	196676
251531	178647
742283	563478
190635716	168176334
150206516	128941204
53139260	50419189
778116	583084
523298850	445443413
191318120	168845353
195843394	172199886
190482262	167653011
152810638	134241959
53961478	51304667
146150116	128117608
55783212	52587168
198474328	174607069
185982920	164160105
181394104	158282296
58288548	55143555
160141118	137431417
55600134	52584420

459158	365304
711244	524728
735005	540308
