# Supplementary material for: A high-density SNP genotyping array for Brassica napus and its ancestral diploid species based on optimised selection of single-locus markers in the allotetraploid genome
Source: Theor Appl Genet. 2016 Jun 30;129(10):1887–99. doi: 10.1007/s00122-016-2746-7 (PMC5025514; doi:10.1007/s00122-016-2746-7)
Supplement: Supplementary file 5 — List of SNP loci which fall within annotated B. napus genes. (PDF 1071 kb) [file 122_2016_2746_MOESM5_ESM.pdf]

Table 4: List of SNP loci which fall within annotated *B. napus* (DH12075v3.1) genes

SNP_ID	DH12075v3.1_ID		
Bn-A01-p10000230	Bn-N1-p9549887	gene	
Bn-A01-p1000115	Bn-N1-p1030519	gene	exon
Bn-A01-p1001022	Bn-N1-p1031427	gene	exon
Bn-A01-p10069493	Bn-N1-p9620973	gene	
Bn-A01-p10071456	Bn-N1-p9622923	gene	exon
Bn-A01-p10071903	Bn-N1-p9623369	gene	
Bn-A01-p10105499	Bn-N1-p9656392	gene	exon
Bn-A01-p1012990	Bn-N1-p1038774	gene	exon
Bn-A01-p10234366	Bn-N1-p9784407	gene	exon
Bn-A01-p10246312	Bn-N1-p9794780	gene	
Bn-A01-p10251052	Bn-N1-p9799309	gene	
Bn-A01-p10257919	Bn-N1-p9805260	gene	exon
Bn-A01-p1026631	Bn-N1-p1053515	gene	exon
Bn-A01-p10278752	Bn-N1-p9830448	gene	
Bn-A01-p10285074	Bn-N1-p9839493	gene	exon
Bn-A01-p10297732	Bn-N1-p9847473	gene	exon
Bn-A01-p10299187	Bn-N1-p9848920	gene	
Bn-A01-p10340159	Bn-N1-p9907693	gene	exon
Bn-A01-p10340283	Bn-N1-p9907816	gene	exon
Bn-A01-p10354645	Bn-N1-p9922762	gene	
Bn-A01-p10410665	Bn-N1-p9970782	gene	exon
Bn-A01-p10426647	Bn-N1-p9982395	gene	exon
Bn-A01-p10426714	Bn-N1-p9982461	gene	
Bn-A01-p10493092	Bn-N1-p10087131	gene	exon
Bn-A01-p10499882	Bn-N1-p10100505	gene	exon
Bn-A01-p10513529	Bn-N7-p7136845	gene	
Bn-A01-p10559083	Bn-N7-p7192886	gene	exon
Bn-A01-p1056386	Bn-N1-p1084599	gene	
Bn-A01-p10590357	Bn-N1-p10129316	gene	
Bn-A01-p10611800	Bn-N1-p10147556	gene	
Bn-A01-p10636637	Bn-N1-p10175252	gene	exon
Bn-A01-p10636994	Bn-N1-p10175609	gene	
Bn-A01-p10638747	Bn-N1-p10177362	gene	
Bn-A01-p10639421	Bn-N1-p10178036	gene	
Bn-A01-p10640881	Bn-N1-p10179496	gene	exon
Bn-A01-p10641656	Bn-N1-p10189977	gene	
Bn-A01-p1067371	Bn-N1-p1099188	gene	
Bn-A01-p1073387	Bn-N1-p1104160	gene	
Bn-A01-p10835605	Bn-N1-p10379201	gene	exon
Bn-A01-p10859527	Bn-N1-p10401833	gene	
Bn-A01-p10889788	Bn-N1-p10427892	gene	
Bn-A01-p1091610	Bn-N1-p1127323	gene	exon
Bn-A01-p10933947	Bn-N1-p10489280	gene	
Bn-A01-p10934377	Bn-N1-p10489679	gene	
Bn-A01-p1104187	Bn-N1-p1140701	gene	
Bn-A01-p11108443	Bn-N1-p10623142	gene	
Bn-A01-p11119985	Bn-N1-p10628286	gene	
Bn-A01-p11163785	Bn-N1-p10671550	gene	exon
Bn-A01-p11174997	Bn-N11-p16368364	gene	exon
Bn-A01-p11206467	Bn-N11-p16393184	gene	exon

Bn-A01-p11233207	Bn-N1-p10742834	gene	
Bn-A01-p11234159	Bn-N1-p10743860	gene	
Bn-A01-p11238778	Bn-N5-p15435464	gene	exon
Bn-A01-p11249993	Bn-N1-p10758006	gene	
Bn-A01-p11253272	Bn-N1-p10762480	gene	
Bn-A01-p11306490	Bn-N1-p10842165	gene	
Bn-A01-p11309186	Bn-N1-p10844861	gene	
Bn-A01-p1132885	Bn-N1-p1169679	gene	exon
Bn-A01-p11330661	Bn-N1-p10860453	gene	
Bn-A01-p11441851	Bn-N1-p10971971	gene	
Bn-A01-p11471489	Bn-N1-p11002226	gene	exon
Bn-A01-p11479565	Bn-N1-p11009759	gene	exon
Bn-A01-p11480814	Bn-N1-p11014535	gene	exon
Bn-A01-p11483435	Bn-N1-p11017255	gene	exon
Bn-A01-p1148760	Bn-N1-p1196607	gene	
Bn-A01-p1149351	Bn-N1-p1197198	gene	
Bn-A01-p1149698	Bn-N1-p1197545	gene	exon
Bn-A01-p1151961	Bn-N1-p1199814	gene	exon
Bn-A01-p1152123	Bn-N1-p1199976	gene	exon
Bn-A01-p1152359	Bn-N1-p1200214	gene	exon
Bn-A01-p1152369	Bn-N1-p1200224	gene	exon
Bn-A01-p11543126	Bn-N1-p11101231	gene	
Bn-A01-p11543191	Bn-N1-p11101339	gene	
Bn-A01-p11559064	Bn-N1-p11118034	gene	exon
Bn-A01-p1156081	Bn-N1-p1203925	gene	exon
Bn-A01-p1156979	Bn-N1-p1204822	gene	
Bn-A01-p11652789	Bn-N4-p4891849	gene	
Bn-A01-p11653461	Bn-N4-p4891177	gene	
Bn-A01-p11702957	Bn-N1-p11187449	gene	
Bn-A01-p11741112	Bn-N8-p556921	gene	
Bn-A01-p1187868	Bn-N1-p1237997	gene	exon
Bn-A01-p1188004	Bn-N1-p1238133	gene	exon
Bn-A01-p1192928	Bn-N1-p1242752	gene	exon
Bn-A01-p11938379	Bn-N1-p11446368	gene	
Bn-A01-p1194009	Bn-N1-p1243827	gene	exon
Bn-A01-p11947255	Bn-N1-p11456056	gene	exon
Bn-A01-p120225	Bn-N1-p131604	gene	exon
Bn-A01-p12066369	Bn-N1-p11497027	gene	exon
Bn-A01-p1208515	Bn-N1-p1255249	gene	
Bn-A01-p12145148	Bn-N1-p11567265	gene	
Bn-A01-p12158810	Bn-N1-p11580929	gene	exon
Bn-A01-p12178646	Bn-N1-p11606752	gene	exon
Bn-A01-p12179784	Bn-N1-p11607855	gene	
Bn-A01-p12192020	Bn-N1-p11617868	gene	
Bn-A01-p1220904	Bn-Scaffold01553-p1629	gene	exon
Bn-A01-p12233209	Bn-N1-p11635701	gene	exon
Bn-A01-p1226475	Bn-N1-p1271278	gene	
Bn-A01-p1227000	Bn-N1-p1271805	gene	
Bn-A01-p1227023	Bn-N1-p1271828	gene	
Bn-A01-p1232064	Bn-N1-p1276870	gene	exon
Bn-A01-p1248141	Bn-N1-p1290257	gene	
Bn-A01-p12484368	Bn-N1-p11892376	gene	exon

Bn-A01-p1250595	Bn-N1-p1292716	gene	
Bn-A01-p12516944	Bn-N1-p11891352	gene	exon
Bn-A01-p12522369	Bn-N1-p11939222	gene	
Bn-A01-p1252553	Bn-N11-p1604240	gene	exon
Bn-A01-p12577268	Bn-N1-p11976112	gene	
Bn-A01-p12580778	Bn-N1-p11989462	gene	exon
Bn-A01-p12593173	Bn-N1-p12015522	gene	
Bn-A01-p12593802	Bn-N11-p18444093	gene	
Bn-A01-p12625892	Bn-N1-p12103289	gene	
Bn-A01-p12665045	Bn-N1-p12143466	gene	
Bn-A01-p12665250	Bn-N1-p12143671	gene	
Bn-A01-p12672899	Bn-N1-p12154035	gene	exon
Bn-A01-p12774270	Bn-N1-p12261697	gene	
Bn-A01-p1296503	Bn-N11-p1708856	gene	exon
Bn-A01-p1314194	Bn-N1-p1355312	gene	exon
Bn-A01-p1314440	Bn-N1-p1355558	gene	exon
Bn-A01-p1320368	Bn-N11-p1735373	gene	
Bn-A01-p13622238	Bn-N2-p181357	gene	exon
Bn-A01-p13709801	Bn-N1-p13197936	gene	exon
Bn-A01-p1372430	Bn-N1-p1418752	gene	exon
Bn-A01-p1373104	Bn-N1-p1419426	gene	
Bn-A01-p1373239	Bn-N1-p1419561	gene	exon
Bn-A01-p1373827	Bn-N1-p1420152	gene	
Bn-A01-p1373838	Bn-N1-p1420163	gene	
Bn-A01-p1373965	Bn-N1-p1420294	gene	exon
Bn-A01-p13775817	Bn-N1-p13254584	gene	exon
Bn-A01-p1387174	Bn-N1-p1432886	gene	exon
Bn-A01-p14001039	Bn-N1-p13554075	gene	exon
Bn-A01-p1409723	Bn-N1-p1464686	gene	exon
Bn-A01-p1414695	Bn-N1-p1469639	gene	exon
Bn-A01-p14169412	Bn-N1-p13748387	gene	exon
Bn-A01-p14181463	Bn-N14-p49684496	gene	
Bn-A01-p14181651	Bn-N14-p49684684	gene	
Bn-A01-p14181829	Bn-N14-p49684862	gene	exon
Bn-A01-p14197545	Bn-N1-p13766078	gene	
Bn-A01-p14223709	Bn-N1-p13797566	gene	exon
Bn-A01-p1422384	Bn-N11-p1861046	gene	exon
Bn-A01-p1423163	Bn-N1-p1477507	gene	exon
Bn-A01-p14256104	Bn-N1-p13857145	gene	
Bn-A01-p14339825	Bn-N1-p13940499	gene	exon
Bn-A01-p1439621	Bn-N1-p1495430	gene	exon
Bn-A01-p1440009	Bn-N1-p1495817	gene	
Bn-A01-p14402434	Bn-N17-p12307568	gene	exon
Bn-A01-p14415386	Bn-N1-p13989587	gene	exon
Bn-A01-p1448811	Bn-N1-p1504639	gene	exon
Bn-A01-p1449865	Bn-N1-p1505698	gene	exon
Bn-A01-p14559770	Bn-N1-p1722929	gene	
Bn-A01-p1456256	Bn-N1-p1512420	gene	exon
Bn-A01-p146963	Bn-N11-p209233	gene	exon
Bn-A01-p1473188	Bn-N1-p1530325	gene	exon
Bn-A01-p1476216	Bn-N1-p1533136	gene	exon
Bn-A01-p14901403	Bn-N1-p14398108	gene	

Bn-A01-p14948237	Bn-N1-p14446586	gene	
Bn-A01-p14948379	Bn-N1-p14446728	gene	
Bn-A01-p14948614	Bn-N1-p14446963	gene	
Bn-A01-p14948748	Bn-N1-p14447097	gene	exon
Bn-A01-p14949030	Bn-N1-p14447379	gene	exon
Bn-A01-p14949121	Bn-N1-p14447470	gene	exon
Bn-A01-p14949256	Bn-N1-p14447605	gene	exon
Bn-A01-p14955594	Bn-N1-p14453956	gene	exon
Bn-A01-p14960233	Bn-N1-p14459360	gene	exon
Bn-A01-p14965172	Bn-N1-p14464498	gene	exon
Bn-A01-p14965596	Bn-N1-p14464922	gene	exon
Bn-A01-p150028	Bn-N1-p160751	gene	exon
Bn-A01-p15043967	Bn-N11-p24000093	gene	exon
Bn-A01-p15086787	Bn-N1-p14616211	gene	exon
Bn-A01-p15087045	Bn-N1-p14616469	gene	
Bn-A01-p15087171	Bn-N1-p14616595	gene	exon
Bn-A01-p15233095	Bn-N1-p14770954	gene	exon
Bn-A01-p1530160	Bn-N1-p1571664	gene	exon
Bn-A01-p1531829	Bn-N1-p1573337	gene	exon
Bn-A01-p15446864	Bn-N1-p14978764	gene	exon
Bn-A01-p15468033	Bn-N1-p15006427	gene	exon
Bn-A01-p15476640	Bn-N1-p15013837	gene	exon
Bn-A01-p15502589	Bn-N1-p15042884	gene	
Bn-A01-p15519090	Bn-N1-p15059254	gene	
Bn-A01-p1556145	Bn-N1-p1596831	gene	
Bn-A01-p15568028	Bn-N1-p15097995	gene	exon
Bn-A01-p1578682	Bn-N1-p1627035	gene	
Bn-A01-p16015382	Bn-N1-p16585172	gene	exon
Bn-A01-p1606312	Bn-N1-p1648516	gene	exon
Bn-A01-p16079153	Bn-N11-p26877557	gene	
Bn-A01-p16079370	Bn-N11-p26877781	gene	exon
Bn-A01-p16086381	Bn-N1-p16669166	gene	
Bn-A01-p16088721	Bn-Scaffold05442-p451	gene	exon
Bn-A01-p16106314	Bn-N1-p16697490	gene	exon
Bn-A01-p16106372	Bn-N1-p16697548	gene	
Bn-A01-p16111263	Bn-N1-p16698816	gene	
Bn-A01-p16217917	Bn-N1-p16804153	gene	
Bn-A01-p16253463	Bn-Scaffold04479-p1344	gene	exon
Bn-A01-p16257043	Bn-N1-p16830009	gene	
Bn-A01-p16276961	Bn-N1-p16849435	gene	exon
Bn-A01-p16303134	Bn-N1-p16877802	gene	
Bn-A01-p16323375	Bn-N1-p16897385	gene	
Bn-A01-p16323503	Bn-N1-p16897513	gene	
Bn-A01-p16324010	Bn-N1-p16898020	gene	
Bn-A01-p1632686	Bn-N1-p1674784	gene	exon
Bn-A01-p16331424	Bn-N1-p16906784	gene	exon
Bn-A01-p1637234	Bn-N1-p1679347	gene	
Bn-A01-p16374068	Bn-N1-p16958282	gene	exon
Bn-A01-p16389291	Bn-N1-p16976972	gene	
Bn-A01-p16393582	Bn-N1-p16981289	gene	
Bn-A01-p16393856	Bn-N1-p16981543	gene	exon
Bn-A01-p16393939	Bn-N1-p16981626	gene	

Bn-A01-p16394111	Bn-N1-p16981800	gene	exon
Bn-A01-p1639922	Bn-N11-p2083431	gene	
Bn-A01-p1641033	Bn-N1-p1683157	gene	exon
Bn-A01-p16432946	Bn-N1-p17035841	gene	
Bn-A01-p16476326	Bn-N1-p17076799	gene	exon
Bn-A01-p16580971	Bn-N1-p17178201	gene	exon
Bn-A01-p16598652	Bn-N1-p17196443	gene	
Bn-A01-p1661180	Bn-N1-p1703757	gene	
Bn-A01-p16669142	Bn-N1-p17236570	gene	
Bn-A01-p16705936	Bn-N1-p17291479	gene	
Bn-A01-p16715637	Bn-N1-p17302028	gene	exon
Bn-A01-p16730334	Bn-N1-p17333950	gene	
Bn-A01-p16749946	Bn-N1-p17349538	gene	
Bn-A01-p16749951	Bn-N1-p17349543	gene	
Bn-A01-p16774157	Bn-N1-p17393353	gene	exon
Bn-A01-p16829363	Bn-N1-p17412271	gene	
Bn-A01-p16847535	Bn-N1-p17428973	gene	exon
Bn-A01-p16857183	Bn-N1-p17446015	gene	exon
Bn-A01-p16857466	Bn-N1-p17446297	gene	
Bn-A01-p16869474	Bn-N1-p17459802	gene	
Bn-A01-p16893578	Bn-N1-p17482527	gene	
Bn-A01-p16997658	Bn-N1-p17595887	gene	exon
Bn-A01-p17015383	Bn-N1-p17625591	gene	
Bn-A01-p17036109	Bn-N1-p17653794	gene	exon
Bn-A01-p17081120	Bn-N1-p17714097	gene	
Bn-A01-p17081703	Bn-N1-p17714680	gene	
Bn-A01-p17110860	Bn-N1-p17742019	gene	
Bn-A01-p17118647	Bn-N1-p17745092	gene	exon
Bn-A01-p17129906	Bn-N1-p17753257	gene	
Bn-A01-p17160355	Bn-N1-p17776308	gene	
Bn-A01-p1719658	Bn-N1-p1781685	gene	exon
Bn-A01-p17216088	Bn-N1-p17856909	gene	
Bn-A01-p17216237	Bn-N1-p17857059	gene	
Bn-A01-p17242218	Bn-N1-p17887632	gene	
Bn-A01-p17258931	Bn-N1-p17910900	gene	exon
Bn-A01-p17283208	Bn-N1-p17936190	gene	exon
Bn-A01-p17315519	Bn-N1-p17973483	gene	exon
Bn-A01-p17377721	Bn-N1-p18040148	gene	
Bn-A01-p1742846	Bn-N1-p1806273	gene	
Bn-A01-p1746226	Bn-N1-p1812677	gene	
Bn-A01-p17481804	Bn-N1-p18140345	gene	exon
Bn-A01-p17482037	Bn-N1-p18140578	gene	
Bn-A01-p17483006	Bn-N1-p18141526	gene	
Bn-A01-p17492348	Bn-N1-p18150937	gene	exon
Bn-A01-p17518468	Bn-N1-p18167006	gene	
Bn-A01-p17535899	Bn-N1-p18183385	gene	exon
Bn-A01-p17536754	Bn-N1-p18184240	gene	
Bn-A01-p17548021	Bn-N1-p18188383	gene	exon
Bn-A01-p17548227	Bn-N1-p18188590	gene	
Bn-A01-p17549137	Bn-N11-p30196980	gene	exon
Bn-A01-p17550125	Bn-N1-p18190500	gene	
Bn-A01-p17550588	Bn-N1-p18190963	gene	

Bn-A01-p17552389	Bn-N1-p18192762	gene	exon
Bn-A01-p1768443	Bn-N1-p1838760	gene	
Bn-A01-p17696068	Bn-N1-p18378510	gene	exon
Bn-A01-p17696358	Bn-N1-p18378797	gene	
Bn-A01-p17751647	Bn-N1-p18434525	gene	exon
Bn-A01-p17755236	Bn-N1-p18438320	gene	exon
Bn-A01-p17771411	Bn-N1-p18443557	gene	
Bn-A01-p17899781	Bn-N1-p18550465	gene	
Bn-A01-p17905950	Bn-N1-p18559022	gene	
Bn-A01-p17906322	Bn-N1-p18559394	gene	exon
Bn-A01-p1793928	Bn-N1-p1864630	gene	
Bn-A01-p17997441	Bn-N1-p18656229	gene	exon
Bn-A01-p17998573	Bn-N1-p18657361	gene	
Bn-A01-p18006188	Bn-N1-p18659664	gene	
Bn-A01-p18008659	Bn-N1-p18662134	gene	
Bn-A01-p18032386	Bn-N1-p18688384	gene	
Bn-A01-p18119989	Bn-N1-p18779446	gene	
Bn-A01-p18121827	Bn-N11-p31100693	gene	exon
Bn-A01-p18178802	Bn-N1-p18826458	gene	exon
Bn-A01-p18197352	Bn-N1-p18850715	gene	exon
Bn-A01-p18200921	Bn-N1-p18852932	gene	
Bn-A01-p1823705	Bn-N1-p1897288	gene	exon
Bn-A01-p1831507	Bn-N1-p1905152	gene	
Bn-A01-p18327940	Bn-N1-p18982111	gene	exon
Bn-A01-p18329609	Bn-N1-p18984031	gene	exon
Bn-A01-p18367467	Bn-N1-p19021045	gene	
Bn-A01-p18383994	Bn-N1-p19034080	gene	
Bn-A01-p1842079	Bn-N1-p1914802	gene	
Bn-A01-p1842658	Bn-N11-p2404208	gene	exon
Bn-A01-p18435876	Bn-N1-p19082054	gene	exon
Bn-A01-p18438154	Bn-N1-p19084332	gene	
Bn-A01-p1855903	Bn-N1-p1931677	gene	exon
Bn-A01-p1858279	Bn-N1-p1934009	gene	
Bn-A01-p1864705	Bn-N11-p2433444	gene	
Bn-A01-p1879401	Bn-N1-p1955958	gene	exon
Bn-A01-p18813615	Bn-N10-p5489130	gene	exon
Bn-A01-p18813849	Bn-N10-p5488896	gene	exon
Bn-A01-p18837660	Bn-N10-p5469414	gene	exon
Bn-A01-p18850969	Bn-N10-p5455380	gene	
Bn-A01-p18881723	Bn-N10-p5423154	gene	
Bn-A01-p18882626	Bn-N10-p5422236	gene	
Bn-A01-p18917698	Bn-N10-p5395574	gene	
Bn-A01-p19097875	Bn-N10-p5191978	gene	exon
Bn-A01-p19098195	Bn-N10-p5191658	gene	exon
Bn-A01-p19107773	Bn-N10-p5182232	gene	
Bn-A01-p19125668	Bn-N10-p5164097	gene	
Bn-A01-p19132144	Bn-N10-p5157599	gene	
Bn-A01-p19150923	Bn-N10-p5131193	gene	
Bn-A01-p19170760	Bn-N10-p5108815	gene	exon
Bn-A01-p1917414	Bn-N11-p2484486	gene	
Bn-A01-p1929982	Bn-N1-p1996291	gene	exon
Bn-A01-p1930111	Bn-N1-p1996420	gene	exon

Bn-A01-p19355180	Bn-N1-p19347053	gene	exon
Bn-A01-p19446599	Bn-N1-p19441811	gene	
Bn-A01-p19447794	Bn-N1-p19443005	gene	
Bn-A01-p19512499	Bn-N1-p19508482	gene	exon
Bn-A01-p1951988	Bn-N1-p2007372	gene	exon
Bn-A01-p195253	Bn-N1-p208162	gene	
Bn-A01-p19633815	Bn-N1-p19597950	gene	
Bn-A01-p1966955	Bn-N1-p2016917	gene	exon
Bn-A01-p1975231	Bn-N1-p2025297	gene	
Bn-A01-p19762477	Bn-N1-p19691017	gene	
Bn-A01-p19764240	Bn-N1-p19692776	gene	
Bn-A01-p1977270	Bn-N1-p2027327	gene	exon
Bn-A01-p1980181	Bn-N1-p2030241	gene	exon
Bn-A01-p19855343	Bn-N11-p44526660	gene	exon
Bn-A01-p19929280	Bn-N1-p19844885	gene	
Bn-A01-p19961176	Bn-N1-p19884679	gene	exon
Bn-A01-p19993388	Bn-N1-p19915857	gene	
Bn-A01-p20133687	Bn-N1-p20042069	gene	exon
Bn-A01-p20181046	Bn-N1-p20096054	gene	
Bn-A01-p20181346	Bn-N1-p20096357	gene	exon
Bn-A01-p2025400	Bn-N1-p2079512	gene	
Bn-A01-p20323059	Bn-N1-p20216069	gene	
Bn-A01-p20393111	Bn-N11-p33747321	gene	exon
Bn-A01-p20433111	Bn-N11-p33891719	gene	
Bn-A01-p2046061	Bn-N1-p2097038	gene	
Bn-A01-p2069218	Bn-N1-p2120534	gene	
Bn-A01-p2069488	Bn-N1-p2120803	gene	
Bn-A01-p207098	Bn-N1-p219745	gene	exon
Bn-A01-p207363	Bn-N1-p220010	gene	
Bn-A01-p20782963	Bn-N18-p19189424	gene	
Bn-A01-p20784377	Bn-N13-p54157433	gene	
Bn-A01-p20899472	Bn-N1-p20797508	gene	
Bn-A01-p20909649	Bn-N1-p20809797	gene	exon
Bn-A01-p20909816	Bn-N1-p20809964	gene	exon
Bn-A01-p2091322	Bn-N1-p2141306	gene	
Bn-A01-p20924050	Bn-N1-p20816306	gene	exon
Bn-A01-p20933189	Bn-Scaffold07211-p725	gene	
Bn-A01-p20990218	Bn-N1-p20874310	gene	
Bn-A01-p2100996	Bn-N1-p2151261	gene	exon
Bn-A01-p21035670	Bn-N1-p20906257	gene	
Bn-A01-p21075183	Bn-N1-p20952653	gene	
Bn-A01-p2111978	Bn-N1-p2158333	gene	
Bn-A01-p2114552	Bn-N1-p2160898	gene	
Bn-A01-p2114803	Bn-N1-p2161149	gene	exon
Bn-A01-p2121224	Bn-N11-p2744541	gene	
Bn-A01-p21236322	Bn-N1-p21104169	gene	exon
Bn-A01-p21277841	Bn-N11-p35608801	gene	
Bn-A01-p21444041	Bn-N1-p21434496	gene	
Bn-A01-p21473161	Bn-N1-p21471174	gene	exon
Bn-A01-p2148059	Bn-N1-p2186745	gene	
Bn-A01-p21519780	Bn-N1-p21514264	gene	exon
Bn-A01-p21527187	Bn-N1-p21522465	gene	

Bn-A01-p2158511	Bn-N1-p2198901	gene	exon
Bn-A01-p21638516	Bn-N1-p21622853	gene	
Bn-A01-p21708353	Bn-N1-p21688436	gene	exon
Bn-A01-p21715162	Bn-N1-p21695724	gene	
Bn-A01-p21724511	Bn-N1-p21704132	gene	exon
Bn-A01-p21758046	Bn-N1-p21744520	gene	
Bn-A01-p21778610	Bn-N1-p21766939	gene	exon
Bn-A01-p2177864	Bn-N11-p2795251	gene	exon
Bn-A01-p21846342	Bn-N1-p21821902	gene	exon
Bn-A01-p21860162	Bn-N1-p21837377	gene	
Bn-A01-p2187099	Bn-N1-p2227901	gene	
Bn-A01-p21938751	Bn-N1-p21915672	gene	
Bn-A01-p21960901	Bn-N11-p36735444	gene	
Bn-A01-p21969405	Bn-N1-p21946464	gene	
Bn-A01-p2197707	Bn-N1-p2235975	gene	exon
Bn-A01-p2197976	Bn-N1-p2236253	gene	
Bn-A01-p21980567	Bn-N1-p21959787	gene	exon
Bn-A01-p22014179	Bn-N1-p22001815	gene	exon
Bn-A01-p22014707	Bn-N1-p22002341	gene	exon
Bn-A01-p22016353	Bn-N1-p22003987	gene	exon
Bn-A01-p22018616	Bn-N1-p22010652	gene	exon
Bn-A01-p22022023	Bn-N1-p22014059	gene	exon
Bn-A01-p22038749	Bn-N1-p22027144	gene	
Bn-A01-p22038776	Bn-N1-p22027171	gene	
Bn-A01-p22051021	Bn-N1-p22039403	gene	exon
Bn-A01-p22051502	Bn-N1-p22039884	gene	
Bn-A01-p22051510	Bn-N1-p22039892	gene	
Bn-A01-p220693	Bn-N1-p233449	gene	exon
Bn-A01-p2207452	Bn-N1-p2246891	gene	
Bn-A01-p22085117	Bn-N1-p22087529	gene	exon
Bn-A01-p22086244	Bn-N1-p22088656	gene	
Bn-A01-p22087364	Bn-N1-p22089776	gene	exon
Bn-A01-p22087629	Bn-N1-p22090041	gene	
Bn-A01-p22156169	Bn-N15-p38746904	gene	
Bn-A01-p22182442	Bn-N1-p22215004	gene	exon
Bn-A01-p2230371	Bn-N1-p2270625	gene	exon
Bn-A01-p2245780	Bn-N1-p2291747	gene	exon
Bn-A01-p2246317	Bn-N1-p2292283	gene	
Bn-A01-p2246373	Bn-N1-p2292339	gene	exon
Bn-A01-p2251106	Bn-N1-p2297029	gene	exon
Bn-A01-p22602330	Bn-N3-p34356507	gene	exon
Bn-A01-p2260859	Bn-N1-p2308181	gene	exon
Bn-A01-p22684301	Bn-N1-p22245609	gene	exon
Bn-A01-p22741943	Bn-N1-p22296467	gene	
Bn-A01-p22743403	Bn-N11-p37295377	gene	exon
Bn-A01-p22768523	Bn-N1-p22311096	gene	exon
Bn-A01-p2277290	Bn-N1-p2319617	gene	exon
Bn-A01-p22841111	Bn-N1-p22377873	gene	exon
Bn-A01-p22841314	Bn-N1-p22378076	gene	exon
Bn-A01-p2288902	Bn-N1-p2331102	gene	exon
Bn-A01-p2292174	Bn-N1-p2334618	gene	exon
Bn-A01-p22948925	Bn-N1-p22452695	gene	

Bn-A01-p22949106	Bn-N1-p22452876	gene	
Bn-A01-p22982065	Bn-N11-p37708628	gene	exon
Bn-A01-p2306208	Bn-N1-p2344506	gene	exon
Bn-A01-p2306938	Bn-N1-p2345236	gene	
Bn-A01-p2307144	Bn-N1-p2345442	gene	
Bn-A01-p23078554	Bn-N1-p22579592	gene	
Bn-A01-p23080525	Bn-N1-p22581568	gene	exon
Bn-A01-p2311015	Bn-N1-p2349273	gene	
Bn-A01-p23169607	Bn-N1-p22645941	gene	
Bn-A01-p23175111	Bn-N1-p22651446	gene	
Bn-A01-p2318972	Bn-N1-p2361058	gene	exon
Bn-A01-p23195492	Bn-N1-p22672365	gene	
Bn-A01-p23196022	Bn-N1-p22672895	gene	
Bn-A01-p23330944	Bn-N1-p22823086	gene	
Bn-A01-p2340048	Bn-N1-p2375360	gene	
Bn-A01-p2340359	Bn-N1-p2375614	gene	exon
Bn-A01-p2345255	Bn-N11-p3025954	gene	exon
Bn-A01-p23472380	Bn-N1-p22980942	gene	
Bn-A01-p23472646	Bn-N1-p22981209	gene	exon
Bn-A01-p23477194	Bn-N1-p22984780	gene	
Bn-A01-p23478530	Bn-N1-p22986116	gene	
Bn-A01-p23479473	Bn-N1-p22987059	gene	exon
Bn-A01-p234898	Bn-N1-p240997	gene	exon
Bn-A01-p235115	Bn-N1-p241218	gene	exon
Bn-A01-p23536634	Bn-N1-p23036989	gene	
Bn-A01-p23538267	Bn-N1-p23038622	gene	exon
Bn-A01-p23582571	Bn-N1-p23086314	gene	exon
Bn-A01-p23586266	Bn-N1-p23089995	gene	
Bn-A01-p23613283	Bn-N1-p23130723	gene	exon
Bn-A01-p23627526	Bn-N1-p23147138	gene	
Bn-A01-p23634753	Bn-N1-p23155057	gene	exon
Bn-A01-p23640103	Bn-N1-p23161915	gene	exon
Bn-A01-p23658023	Bn-N1-p23176240	gene	
Bn-A01-p23672301	Bn-N1-p23189858	gene	exon
Bn-A01-p23685978	Bn-N1-p23199810	gene	
Bn-A01-p23718726	Bn-N11-p38924538	gene	exon
Bn-A01-p23719321	Bn-N1-p23232558	gene	
Bn-A01-p23730610	Bn-N1-p23241673	gene	
Bn-A01-p23736208	Bn-N1-p23247097	gene	exon
Bn-A01-p23739022	Bn-N1-p23249921	gene	exon
Bn-A01-p23785561	Bn-N1-p23307739	gene	
Bn-A01-p23810423	Bn-N1-p23326638	gene	exon
Bn-A01-p23813837	Bn-N1-p23330051	gene	exon
Bn-A01-p23842865	Bn-N1-p23361899	gene	exon
Bn-A01-p23843030	Bn-N1-p23362064	gene	exon
Bn-A01-p23843222	Bn-N1-p23362256	gene	exon
Bn-A01-p23859890	Bn-N11-p39959507	gene	exon
Bn-A01-p23905191	Bn-N1-p23420845	gene	
Bn-A01-p23938571	Bn-N1-p23451987	gene	exon
Bn-A01-p23950559	Bn-N1-p23460955	gene	
Bn-A01-p23961368	Bn-N1-p23476953	gene	
Bn-A01-p23965220	Bn-N1-p23480933	gene	

Bn-A01-p23966537	Bn-N1-p23482250	gene	exon
Bn-A01-p23972100	Bn-N1-p23487811	gene	
Bn-A01-p2403471	Bn-N1-p2439812	gene	exon
Bn-A01-p24107515	Bn-N6-p16491047	gene	exon
Bn-A01-p24145113	Bn-N17-p7038322	gene	exon
Bn-A01-p24226107	Bn-N6-p16586359	gene	exon
Bn-A01-p2431503	Bn-N1-p2465850	gene	exon
Bn-A01-p24360206	Bn-N1-p23592016	gene	
Bn-A01-p24390991	Bn-N1-p23626240	gene	
Bn-A01-p24394537	Bn-N1-p23629804	gene	exon
Bn-A01-p24395406	Bn-N1-p23631005	gene	
Bn-A01-p24425211	Bn-N1-p23670004	gene	
Bn-A01-p24425829	Bn-N1-p23670623	gene	exon
Bn-A01-p24429653	Bn-N1-p23674556	gene	exon
Bn-A01-p24435452	Bn-N1-p23680494	gene	exon
Bn-A01-p2445274	Bn-N1-p2603063	gene	exon
Bn-A01-p2445468	Bn-N1-p2602869	gene	exon
Bn-A01-p2445602	Bn-N1-p2602735	gene	exon
Bn-A01-p24525303	Bn-N1-p23773086	gene	exon
Bn-A01-p2453633	Bn-N1-p2599772	gene	exon
Bn-A01-p2453758	Bn-N1-p2599647	gene	exon
Bn-A01-p24541095	Bn-N1-p23786611	gene	exon
Bn-A01-p2455219	Bn-N1-p2598186	gene	exon
Bn-A01-p2457750	Bn-N1-p2595584	gene	
Bn-A01-p24646334	Bn-N1-p23890376	gene	
Bn-A01-p24664331	Bn-N1-p23910662	gene	exon
Bn-A01-p24696367	Bn-N1-p23943419	gene	
Bn-A01-p24697185	Bn-N1-p23944238	gene	
Bn-A01-p24701885	Bn-N1-p23949100	gene	
Bn-A01-p24703150	Bn-N1-p23950352	gene	exon
Bn-A01-p24706378	Bn-N1-p23953508	gene	
Bn-A01-p24707425	Bn-N1-p23954561	gene	exon
Bn-A01-p24707727	Bn-N1-p23954863	gene	
Bn-A01-p24737418	Bn-N1-p23977024	gene	exon
Bn-A01-p24756888	Bn-N1-p23996711	gene	exon
Bn-A01-p24811222	Bn-N1-p24028871	gene	
Bn-A01-p24812396	Bn-N13-p26104669	gene	exon
Bn-A01-p24848629	Bn-N1-p24056843	gene	exon
Bn-A01-p24858036	Bn-N11-p40460287	gene	exon
Bn-A01-p24862401	Bn-N1-p24070838	gene	
Bn-A01-p2491976	Bn-N1-p2562581	gene	
Bn-A01-p24974533	Bn-N1-p24186494	gene	exon
Bn-A01-p2501423	Bn-N1-p2552952	gene	
Bn-A01-p2504370	Bn-N1-p2550026	gene	exon
Bn-A01-p25079505	Bn-N1-p26979908	gene	exon
Bn-A01-p2509163	Bn-N1-p2543483	gene	exon
Bn-A01-p25171870	Bn-N4-p21066278	gene	
Bn-A01-p25175675	Bn-N4-p21062470	gene	
Bn-A01-p2517658	Bn-N1-p2535164	gene	
Bn-A01-p25183707	Bn-N4-p21054418	gene	exon
Bn-A01-p25235998	Bn-N1-p26886005	gene	exon
Bn-A01-p25255567	Bn-N1-p26866822	gene	exon

Bn-A01-p25259103	Bn-N1-p26863282	gene	exon
Bn-A01-p25259292	Bn-N1-p26863067	gene	
Bn-A01-p25260327	Bn-N1-p26862107	gene	exon
Bn-A01-p25260589	Bn-N1-p26861843	gene	
Bn-A01-p25267377	Bn-N1-p26854811	gene	exon
Bn-A01-p25279161	Bn-N1-p26840028	gene	
Bn-A01-p25326722	Bn-N1-p26804360	gene	exon
Bn-A01-p25327241	Bn-N1-p26803842	gene	exon
Bn-A01-p2533727	Bn-N1-p2516058	gene	
Bn-A01-p25377739	Bn-Scaffold00895-p62181	gene	exon
Bn-A01-p25405698	Bn-Scaffold00895-p89635	gene	exon
Bn-A01-p25453874	Bn-N1-p26770166	gene	exon
Bn-A01-p25509783	Bn-N1-p26711185	gene	
Bn-A01-p25518745	Bn-N1-p26696571	gene	exon
Bn-A01-p25555508	Bn-N1-p26660503	gene	
Bn-A01-p25561204	Bn-N1-p26655514	gene	
Bn-A01-p25570701	Bn-N1-p26644766	gene	
Bn-A01-p25572254	Bn-N1-p26643152	gene	
Bn-A01-p25577348	Bn-N1-p26638166	gene	
Bn-A01-p25580651	Bn-N1-p26634866	gene	
Bn-A01-p2574969	Bn-N1-p2616127	gene	exon
Bn-A01-p25898606	Bn-N16-p17821360	gene	exon
Bn-A01-p26064479	Bn-N1-p4486639	gene	
Bn-A01-p2608660	Bn-N1-p2657233	gene	exon
Bn-A01-p2624222	Bn-N1-p2671811	gene	exon
Bn-A01-p2626270	Bn-N1-p2675493	gene	
Bn-A01-p2634563	Bn-N1-p2683740	gene	exon
Bn-A01-p26369426	Bn-N11-p41476941	gene	exon
Bn-A01-p26369651	Bn-N1-p24501624	gene	exon
Bn-A01-p2637699	Bn-N1-p2686959	gene	
Bn-A01-p26400154	Bn-N1-p24527640	gene	exon
Bn-A01-p26423587	Bn-N1-p24541048	gene	
Bn-A01-p26425810	Bn-N1-p24543088	gene	
Bn-A01-p26426004	Bn-N11-p41571399	gene	exon
Bn-A01-p2647221	Bn-N1-p2697173	gene	exon
Bn-A01-p26519047	Bn-N1-p24640051	gene	exon
Bn-A01-p26568738	Bn-N1-p24698643	gene	exon
Bn-A01-p26571596	Bn-N5-p22509511	gene	
Bn-A01-p26572331	Bn-N1-p24702848	gene	
Bn-A01-p26575652	Bn-N1-p24707206	gene	exon
Bn-A01-p26589959	Bn-N1-p24718839	gene	exon
Bn-A01-p26592021	Bn-N1-p24721846	gene	
Bn-A01-p26594603	Bn-N1-p24724459	gene	
Bn-A01-p26604864	Bn-N1-p24735170	gene	
Bn-A01-p26624315	Bn-N1-p24754935	gene	
Bn-A01-p26627091	Bn-N1-p24757734	gene	
Bn-A01-p26688333	Bn-N1-p24829825	gene	
Bn-A01-p2668951	Bn-N1-p2721895	gene	exon
Bn-A01-p26715665	Bn-N1-p24855239	gene	exon
Bn-A01-p26759778	Bn-N9-p1481062	gene	exon
Bn-A01-p26760158	Bn-N9-p1480682	gene	exon
Bn-A01-p26829665	Bn-N19-p1641358	gene	exon

Bn-A01-p26837207	Bn-N9-p1407285	gene	
Bn-A01-p26866541	Bn-N9-p1378090	gene	
Bn-A01-p2688662	Bn-N1-p2742283	gene	exon
Bn-A01-p26910983	Bn-N9-p1350499	gene	exon
Bn-A01-p26914512	Bn-N9-p1346927	gene	exon
Bn-A01-p26914851	Bn-N9-p1346589	gene	
Bn-A01-p26921219	Bn-N9-p1339122	gene	exon
Bn-A01-p26921609	Bn-N9-p1338732	gene	exon
Bn-A01-p26940404	Bn-N9-p1316930	gene	
Bn-A01-p2694323	Bn-N1-p2747936	gene	exon
Bn-A01-p26945726	Bn-N9-p1310708	gene	
Bn-A01-p26946254	Bn-N9-p1310180	gene	exon
Bn-A01-p26965229	Bn-N9-p1275641	gene	exon
Bn-A01-p26968952	Bn-N9-p1271915	gene	
Bn-A01-p26969210	Bn-N9-p1271660	gene	
Bn-A01-p26996900	Bn-N9-p1242431	gene	exon
Bn-A01-p2699907	Bn-N1-p2753537	gene	exon
Bn-A01-p27017324	Bn-N9-p1211774	gene	
Bn-A01-p27017651	Bn-N9-p1211447	gene	
Bn-A01-p27019745	Bn-N9-p1209298	gene	exon
Bn-A01-p27029107	Bn-N9-p1200086	gene	exon
Bn-A01-p27029254	Bn-N9-p1199939	gene	exon
Bn-A01-p27037472	Bn-N9-p1191682	gene	
Bn-A01-p27050083	Bn-N9-p1179090	gene	exon
Bn-A01-p27060364	Bn-N9-p1169123	gene	
Bn-A01-p27061075	Bn-N9-p1168411	gene	
Bn-A01-p27101289	Bn-N1-p24969557	gene	exon
Bn-A01-p27107081	Bn-N1-p24973721	gene	exon
Bn-A01-p27115455	Bn-N1-p24985491	gene	
Bn-A01-p27117954	Bn-N1-p24987974	gene	
Bn-A01-p27119250	Bn-N1-p24989268	gene	
Bn-A01-p27125649	Bn-N11-p42647301	gene	exon
Bn-A01-p2713439	Bn-N8-p12837754	gene	
Bn-A01-p2713528	Bn-N1-p2770797	gene	
Bn-A01-p2713583	Bn-N1-p2770852	gene	
Bn-A01-p27147782	Bn-N11-p42602531	gene	exon
Bn-A01-p2715156	Bn-N1-p2772407	gene	
Bn-A01-p2718126	Bn-N1-p2775946	gene	
Bn-A01-p27202461	Bn-N11-p42336133	gene	exon
Bn-A01-p27225628	Bn-N11-p42349033	gene	exon
Bn-A01-p27272066	Bn-N1-p25157507	gene	exon
Bn-A01-p27299339	Bn-N1-p25185733	gene	
Bn-A01-p27301637	Bn-N1-p25190903	gene	
Bn-A01-p27301980	Bn-N1-p25191246	gene	
Bn-A01-p27302144	Bn-N1-p25191410	gene	
Bn-A01-p27361855	Bn-N1-p25237072	gene	exon
Bn-A01-p27381111	Bn-N1-p25257937	gene	exon
Bn-A01-p27389703	Bn-N1-p25263989	gene	exon
Bn-A01-p27392534	Bn-Scaffold03168-p766	gene	exon
Bn-A01-p27417163	Bn-N1-p25308592	gene	exon
Bn-A01-p2746862	Bn-N1-p2808336	gene	
Bn-A01-p27522299	Bn-N1-p25411408	gene	exon

Bn-A01-p27573404	Bn-N1-p25459315	gene	exon
Bn-A01-p27580681	Bn-N1-p25466344	gene	
Bn-A01-p27581056	Bn-N1-p25466719	gene	
Bn-A01-p27668070	Bn-N1-p25547775	gene	exon
Bn-A01-p27673018	Bn-N1-p25558340	gene	
Bn-A01-p27722989	Bn-N1-p25619480	gene	
Bn-A01-p2775635	Bn-N1-p2829094	gene	exon
Bn-A01-p27774124	Bn-N1-p25700994	gene	exon
Bn-A01-p27774386	Bn-N1-p25701256	gene	
Bn-A01-p27774666	Bn-N1-p25701534	gene	exon
Bn-A01-p27796877	Bn-N1-p25722543	gene	
Bn-A01-p27797076	Bn-N1-p25722742	gene	exon
Bn-A01-p27822000	Bn-N1-p25755518	gene	exon
Bn-A01-p27822790	Bn-N1-p25756308	gene	
Bn-A01-p27830372	Bn-N1-p25763891	gene	
Bn-A01-p27834392	Bn-N1-p25768895	gene	
Bn-A01-p27834452	Bn-N1-p25768956	gene	
Bn-A01-p27843242	Bn-N1-p25778161	gene	
Bn-A01-p27845673	Bn-N1-p25779974	gene	
Bn-A01-p27846002	Bn-N1-p25780303	gene	
Bn-A01-p27846227	Bn-N1-p25780528	gene	
Bn-A01-p27846544	Bn-N1-p25780845	gene	
Bn-A01-p27854949	Bn-N11-p43517628	gene	exon
Bn-A01-p27866419	Bn-N1-p25801701	gene	
Bn-A01-p27877194	Bn-N1-p25814170	gene	exon
Bn-A01-p27907325	Bn-N1-p25844436	gene	
Bn-A01-p27907422	Bn-N1-p25844533	gene	exon
Bn-A01-p27926574	Bn-N1-p25873087	gene	exon
Bn-A01-p27926866	Bn-N1-p25873379	gene	exon
Bn-A01-p2792719	Bn-N1-p2850328	gene	exon
Bn-A01-p27965742	Bn-N1-p25938114	gene	exon
Bn-A01-p27968584	Bn-N1-p25940954	gene	exon
Bn-A01-p27988223	Bn-N1-p25965645	gene	
Bn-A01-p27989439	Bn-N11-p43703019	gene	exon
Bn-A01-p28049872	Bn-N1-p26030653	gene	exon
Bn-A01-p28089456	Bn-N11-p43830963	gene	
Bn-A01-p28110393	Bn-N1-p26103627	gene	
Bn-A01-p28186318	Bn-Scaffold01504-p6253	gene	
Bn-A01-p28193774	Bn-Scaffold01107-p34210	gene	exon
Bn-A01-p28217427	Bn-Scaffold01107-p12950	gene	
Bn-A01-p28220430	Bn-Scaffold01107-p9803	gene	
Bn-A01-p28222170	Bn-Scaffold01107-p8092	gene	
Bn-A01-p28261145	Bn-N11-p44124748	gene	exon
Bn-A01-p28285580	Bn-N11-p44154514	gene	exon
Bn-A01-p28326025	Bn-N11-p44194806	gene	exon
Bn-A01-p28346931	Bn-N1-p26178309	gene	exon
Bn-A01-p28360889	Bn-N1-p26191337	gene	exon
Bn-A01-p28364600	Bn-N1-p26195825	gene	exon
Bn-A01-p28385501	Bn-N1-p26215591	gene	
Bn-A01-p28426820	Bn-N1-p26248753	gene	exon
Bn-A01-p28462071	Bn-N1-p26290806	gene	exon
Bn-A01-p2846364	Bn-N14-p31172883	gene	

Bn-A01-p2848253	Bn-N14-p31171076	gene	
Bn-A01-p2849034	Bn-N14-p31170295	gene	
Bn-A01-p2849540	Bn-N14-p31169787	gene	
Bn-A01-p28497362	Bn-N1-p26332052	gene	
Bn-A01-p28497900	Bn-N1-p26332590	gene	
Bn-A01-p28500378	Bn-N1-p26335066	gene	
Bn-A01-p28551714	Bn-Scaffold00891-p28220	gene	
Bn-A01-p28571117	Bn-Scaffold00891-p75320	gene	
Bn-A01-p28577026	Bn-Scaffold00891-p81308	gene	
Bn-A01-p2864193	Bn-N1-p2918757	gene	exon
Bn-A01-p2867284	Bn-N1-p2921846	gene	exon
Bn-A01-p2882270	Bn-N1-p2938627	gene	
Bn-A01-p2884934	Bn-N1-p2941286	gene	
Bn-A01-p2897071	Bn-N1-p2953448	gene	exon
Bn-A01-p2915231	Bn-N1-p2981735	gene	exon
Bn-A01-p2928824	Bn-N1-p2997150	gene	
Bn-A01-p2934166	Bn-N1-p3002527	gene	exon
Bn-A01-p2956393	Bn-N1-p3018205	gene	exon
Bn-A01-p3000989	Bn-N1-p3061785	gene	exon
Bn-A01-p3005310	Bn-N1-p3065971	gene	
Bn-A01-p3005793	Bn-N1-p3066454	gene	
Bn-A01-p3061223	Bn-N1-p3119720	gene	
Bn-A01-p3069312	Bn-N1-p3127809	gene	exon
Bn-A01-p3070657	Bn-N1-p3129154	gene	
Bn-A01-p3077309	Bn-N1-p3135680	gene	exon
Bn-A01-p3078457	Bn-N1-p3136822	gene	exon
Bn-A01-p3097105	Bn-N1-p3154795	gene	exon
Bn-A01-p3111607	Bn-N1-p3174757	gene	
Bn-A01-p3136468	Bn-N1-p3199609	gene	
Bn-A01-p3179210	Bn-Scaffold08692-p250	gene	exon
Bn-A01-p3179292	Bn-Scaffold08692-p169	gene	exon
Bn-A01-p3223005	Bn-N1-p3281849	gene	
Bn-A01-p324562	Bn-N1-p325704	gene	exon
Bn-A01-p3279678	Bn-N1-p3328131	gene	
Bn-A01-p3306526	Bn-N1-p3356185	gene	
Bn-A01-p3307173	Bn-N1-p3356763	gene	exon
Bn-A01-p3378332	Bn-N1-p3408988	gene	exon
Bn-A01-p3567127	Bn-N1-p3612730	gene	
Bn-A01-p3579887	Bn-N1-p3626398	gene	
Bn-A01-p3599097	Bn-N1-p3656214	gene	exon
Bn-A01-p3610530	Bn-N1-p3665703	gene	exon
Bn-A01-p3619975	Bn-N1-p3672616	gene	
Bn-A01-p3626821	Bn-N1-p3679483	gene	exon
Bn-A01-p3631990	Bn-N1-p3690151	gene	exon
Bn-A01-p3643352	Bn-N1-p3700085	gene	exon
Bn-A01-p3664698	Bn-N1-p3718064	gene	exon
Bn-A01-p3666241	Bn-N1-p3719607	gene	exon
Bn-A01-p3703676	Bn-N1-p3749245	gene	exon
Bn-A01-p3707366	Bn-N1-p3752956	gene	exon
Bn-A01-p3713266	Bn-N1-p3759089	gene	exon
Bn-A01-p3737522	Bn-N1-p3782540	gene	exon
Bn-A01-p3744726	Bn-N1-p3789910	gene	exon

Bn-A01-p3791028	Bn-N1-p3835643	gene	
Bn-A01-p3829226	Bn-N11-p5311688	gene	exon
Bn-A01-p3829785	Bn-N1-p3869223	gene	exon
Bn-A01-p3858902	Bn-N1-p3896021	gene	exon
Bn-A01-p3859106	Bn-N11-p5352241	gene	exon
Bn-A01-p3859610	Bn-N1-p3896729	gene	exon
Bn-A01-p3906965	Bn-N1-p3955040	gene	
Bn-A01-p3938241	Bn-N1-p3986378	gene	exon
Bn-A01-p3939508	Bn-N1-p3987645	gene	exon
Bn-A01-p4070059	Bn-N1-p4129839	gene	exon
Bn-A01-p4077105	Bn-N1-p4136147	gene	
Bn-A01-p4084366	Bn-N1-p4143665	gene	
Bn-A01-p4084739	Bn-N1-p4144038	gene	exon
Bn-A01-p4087533	Bn-N1-p4147074	gene	
Bn-A01-p4109157	Bn-N1-p4164079	gene	exon
Bn-A01-p4113202	Bn-N1-p4168098	gene	exon
Bn-A01-p4116110	Bn-N1-p4171006	gene	exon
Bn-A01-p4117153	Bn-N1-p4172049	gene	exon
Bn-A01-p4167795	Bn-N1-p4219923	gene	
Bn-A01-p4173668	Bn-N1-p4226689	gene	
Bn-A01-p4220987	Bn-N11-p5913888	gene	
Bn-A01-p4227703	Bn-N1-p4274490	gene	
Bn-A01-p4230829	Bn-N1-p4277619	gene	exon
Bn-A01-p4242269	Bn-N1-p4293684	gene	exon
Bn-A01-p4246586	Bn-N1-p4297042	gene	
Bn-A01-p4258195	Bn-N1-p4304559	gene	exon
Bn-A01-p4293847	Bn-N1-p4333914	gene	
Bn-A01-p4293878	Bn-N1-p4333945	gene	
Bn-A01-p4322340	Bn-N1-p4367773	gene	
Bn-A01-p4325162	Bn-N1-p4372440	gene	
Bn-A01-p4335144	Bn-N1-p4380902	gene	
Bn-A01-p4353657	Bn-N1-p4399471	gene	exon
Bn-A01-p436141	Bn-N1-p435708	gene	exon
Bn-A01-p4390611	Bn-N1-p4436209	gene	
Bn-A01-p4394660	Bn-N11-p6130132	gene	exon
Bn-A01-p442492	Bn-N1-p442085	gene	exon
Bn-A01-p4452779	Bn-N1-p4488299	gene	exon
Bn-A01-p4519762	Bn-N1-p4549496	gene	exon
Bn-A01-p460072	Bn-N1-p455627	gene	
Bn-A01-p4612881	Bn-N1-p4651489	gene	exon
Bn-A01-p4641747	Bn-N1-p4679669	gene	
Bn-A01-p4641802	Bn-N1-p4679724	gene	
Bn-A01-p4734188	Bn-N1-p4744328	gene	exon
Bn-A01-p4736924	Bn-N1-p4747053	gene	
Bn-A01-p4773772	Bn-N1-p4785853	gene	
Bn-A01-p4774065	Bn-N1-p4786146	gene	
Bn-A01-p4786653	Bn-N1-p4802652	gene	exon
Bn-A01-p4786754	Bn-N1-p4802753	gene	
Bn-A01-p4786787	Bn-N1-p4802786	gene	
Bn-A01-p4787515	Bn-N1-p4803559	gene	exon
Bn-A01-p4812269	Bn-N1-p4828077	gene	exon
Bn-A01-p4820187	Bn-N1-p4830975	gene	

Bn-A01-p4883188	Bn-N1-p4892640	gene	exon
Bn-A01-p4920622	Bn-N1-p4922079	gene	
Bn-A01-p4922557	Bn-N1-p4924007	gene	
Bn-A01-p4925004	Bn-N1-p4926465	gene	exon
Bn-A01-p4940549	Bn-N1-p4944359	gene	
Bn-A01-p4947577	Bn-N1-p4952041	gene	exon
Bn-A01-p4948316	Bn-N1-p4952735	gene	exon
Bn-A01-p5017444	Bn-N1-p5004859	gene	exon
Bn-A01-p5017446	Bn-N1-p5004861	gene	exon
Bn-A01-p5020890	Bn-N1-p5008304	gene	
Bn-A01-p5106042	Bn-N1-p5078759	gene	exon
Bn-A01-p511682	Bn-N1-p499736	gene	exon
Bn-A01-p5215209	Bn-N1-p5181745	gene	
Bn-A01-p5238564	Bn-N1-p5202234	gene	
Bn-A01-p5239959	Bn-N1-p5203630	gene	
Bn-A01-p5245778	Bn-N11-p7408118	gene	exon
Bn-A01-p5246582	Bn-N11-p7408943	gene	
Bn-A01-p5259856	Bn-N1-p5226110	gene	exon
Bn-A01-p5268871	Bn-N1-p5239470	gene	
Bn-A01-p5269368	Bn-N1-p5239967	gene	exon
Bn-A01-p5284702	Bn-N1-p5260212	gene	exon
Bn-A01-p5296564	Bn-N1-p5270686	gene	
Bn-A01-p5298283	Bn-N1-p5272405	gene	
Bn-A01-p5357589	Bn-N1-p5336362	gene	exon
Bn-A01-p5378430	Bn-N1-p5359089	gene	exon
Bn-A01-p5391551	Bn-N1-p5370787	gene	
Bn-A01-p5469727	Bn-N1-p5442893	gene	
Bn-A01-p5506421	Bn-N1-p5479028	gene	exon
Bn-A01-p5508840	Bn-N1-p5481447	gene	exon
Bn-A01-p5508998	Bn-N1-p5481605	gene	
Bn-A01-p5512	Bn-Scaffold01043-p18665	gene	exon
Bn-A01-p5579695	Bn-N1-p5576501	gene	
Bn-A01-p5599369	Bn-N1-p5594557	gene	exon
Bn-A01-p5601232	Bn-N1-p5596422	gene	
Bn-A01-p5621392	Bn-N1-p5613276	gene	exon
Bn-A01-p5647795	Bn-N1-p5631458	gene	exon
Bn-A01-p5659083	Bn-N1-p5644004	gene	exon
Bn-A01-p5660238	Bn-N1-p5645159	gene	exon
Bn-A01-p5661294	Bn-N1-p5646214	gene	exon
Bn-A01-p5678678	Bn-N1-p5668444	gene	
Bn-A01-p5679389	Bn-N1-p5669155	gene	
Bn-A01-p5688168	Bn-N1-p5679756	gene	
Bn-A01-p5697529	Bn-N1-p5687445	gene	
Bn-A01-p5697706	Bn-N1-p5687622	gene	exon
Bn-A01-p5715141	Bn-N1-p5703321	gene	
Bn-A01-p5718049	Bn-N11-p8176749	gene	exon
Bn-A01-p5730640	Bn-N1-p5719044	gene	
Bn-A01-p5731429	Bn-N1-p5719833	gene	
Bn-A01-p5731932	Bn-N1-p5720336	gene	exon
Bn-A01-p5732226	Bn-N1-p5720630	gene	
Bn-A01-p5752342	Bn-N1-p5743225	gene	exon
Bn-A01-p5762190	Bn-N1-p5759503	gene	

Bn-A01-p5795016	Bn-N1-p5795297	gene	
Bn-A01-p5796314	Bn-N1-p5796499	gene	exon
Bn-A01-p5796425	Bn-N1-p5796610	gene	
Bn-A01-p5797296	Bn-N1-p5797482	gene	exon
Bn-A01-p5797810	Bn-N1-p5797996	gene	
Bn-A01-p5805154	Bn-N1-p5805475	gene	exon
Bn-A01-p5806635	Bn-N1-p5806986	gene	
Bn-A01-p5821651	Bn-N1-p5822192	gene	
Bn-A01-p5822015	Bn-N1-p5822556	gene	
Bn-A01-p5835558	Bn-N1-p5839444	gene	exon
Bn-A01-p5868598	Bn-N1-p5878881	gene	exon
Bn-A01-p5873504	Bn-N1-p5883788	gene	
Bn-A01-p5886182	Bn-N1-p5896146	gene	exon
Bn-A01-p5899092	Bn-N1-p5914797	gene	
Bn-A01-p5917688	Bn-N1-p5927079	gene	exon
Bn-A01-p5918340	Bn-N11-p8475688	gene	
Bn-A01-p5921497	Bn-N1-p5930921	gene	exon
Bn-A01-p5930704	Bn-N1-p5940336	gene	exon
Bn-A01-p5943392	Bn-N1-p5953742	gene	exon
Bn-A01-p5949498	Bn-N1-p5959596	gene	exon
Bn-A01-p597363	Bn-N1-p601625	gene	exon
Bn-A01-p598242	Bn-N1-p602504	gene	
Bn-A01-p5990203	Bn-N1-p6006584	gene	exon
Bn-A01-p5994840	Bn-N1-p6011242	gene	exon
Bn-A01-p5994980	Bn-N1-p6011382	gene	exon
Bn-A01-p6035491	Bn-N1-p6057148	gene	
Bn-A01-p6038249	Bn-N1-p6059919	gene	exon
Bn-A01-p6040667	Bn-N1-p6062283	gene	
Bn-A01-p6083015	Bn-N1-p6102164	gene	exon
Bn-A01-p6111016	Bn-N1-p6134186	gene	
Bn-A01-p6180257	Bn-N1-p6204502	gene	
Bn-A01-p6182710	Bn-N1-p6206809	gene	
Bn-A01-p6188347	Bn-N1-p6212733	gene	exon
Bn-A01-p6215196	Bn-N1-p6237449	gene	exon
Bn-A01-p6216424	Bn-N11-p8996200	gene	
Bn-A01-p6217011	Bn-N1-p6240730	gene	exon
Bn-A01-p6220667	Bn-N1-p6244388	gene	
Bn-A01-p622550	Bn-N1-p627350	gene	
Bn-A01-p6232061	Bn-N1-p6255985	gene	exon
Bn-A01-p623568	Bn-N1-p628366	gene	exon
Bn-A01-p6270054	Bn-N1-p6282220	gene	exon
Bn-A01-p6271245	Bn-N1-p6283411	gene	exon
Bn-A01-p6290508	Bn-N1-p6303043	gene	exon
Bn-A01-p6349933	Bn-N1-p6348505	gene	
Bn-A01-p637586	Bn-N1-p642087	gene	exon
Bn-A01-p644410	Bn-N1-p648675	gene	
Bn-A01-p645951	Bn-N1-p650216	gene	
Bn-A01-p648196	Bn-N1-p652446	gene	
Bn-A01-p6482543	Bn-N1-p6482538	gene	
Bn-A01-p6487075	Bn-N1-p6489048	gene	
Bn-A01-p6546245	Bn-N1-p6542526	gene	exon
Bn-A01-p6547052	Bn-N1-p6543334	gene	

Bn-A01-p6552596	Bn-N1-p6548875	gene	exon
Bn-A01-p6558843	Bn-N1-p6555124	gene	exon
Bn-A01-p6560270	Bn-N1-p6556551	gene	
Bn-A01-p657852	Bn-N1-p657755	gene	
Bn-A01-p6643749	Bn-N1-p6624569	gene	
Bn-A01-p6678914	Bn-N1-p6660632	gene	exon
Bn-A01-p6679394	Bn-N1-p6661113	gene	
Bn-A01-p6747706	Bn-N1-p6723020	gene	
Bn-A01-p6776634	Bn-N1-p6755980	gene	exon
Bn-A01-p6776950	Bn-N1-p6756296	gene	exon
Bn-A01-p678018	Bn-N1-p678856	gene	exon
Bn-A01-p6787691	Bn-N1-p6778779	gene	
Bn-A01-p6787930	Bn-N1-p6779018	gene	
Bn-A01-p6788044	Bn-N1-p6779131	gene	
Bn-A01-p6801449	Bn-N1-p6771861	gene	
Bn-A01-p6826327	Bn-N1-p6810755	gene	
Bn-A01-p6838738	Bn-N1-p6828366	gene	
Bn-A01-p6841235	Bn-N1-p6830489	gene	exon
Bn-A01-p6877281	Bn-N11-p10119319	gene	exon
Bn-A01-p688355	Bn-N1-p687818	gene	
Bn-A01-p6899980	Bn-N13-p60803183	gene	exon
Bn-A01-p6907522	Bn-N1-p6896133	gene	
Bn-A01-p6920456	Bn-N1-p6908046	gene	
Bn-A01-p6925167	Bn-N1-p6911249	gene	exon
Bn-A01-p6925436	Bn-N1-p6911513	gene	exon
Bn-A01-p6950868	Bn-N11-p10237431	gene	exon
Bn-A01-p6962542	Bn-N1-p6949268	gene	
Bn-A01-p6972779	Bn-N1-p6961785	gene	exon
Bn-A01-p6976938	Bn-N1-p6971482	gene	exon
Bn-A01-p6986405	Bn-N1-p6981704	gene	exon
Bn-A01-p7068752	Bn-N1-p7060343	gene	
Bn-A01-p7069263	Bn-N1-p7060854	gene	exon
Bn-A01-p710239	Bn-N11-p910546	gene	
Bn-A01-p71129	Bn-N1-p68429	gene	exon
Bn-A01-p7121832	Bn-N1-p7130180	gene	
Bn-A01-p7122036	Bn-N1-p7130384	gene	exon
Bn-A01-p7132729	Bn-N1-p7146539	gene	exon
Bn-A01-p713562	Bn-N1-p728364	gene	exon
Bn-A01-p7137897	Bn-N1-p7151712	gene	
Bn-A01-p7150748	Bn-N1-p7159741	gene	
Bn-A01-p7151096	Bn-N1-p7160087	gene	
Bn-A01-p733483	Bn-N11-p967160	gene	
Bn-A01-p7363480	Bn-N1-p7373856	gene	
Bn-A01-p7367598	Bn-N1-p7377974	gene	exon
Bn-A01-p7372570	Bn-N1-p7383038	gene	
Bn-A01-p7372653	Bn-N1-p7383121	gene	exon
Bn-A01-p7373427	Bn-N1-p7383894	gene	
Bn-A01-p7374290	Bn-N1-p7384740	gene	exon
Bn-A01-p7374589	Bn-N1-p7385039	gene	exon
Bn-A01-p737560	Bn-N1-p752694	gene	
Bn-A01-p7383179	Bn-N1-p7391780	gene	
Bn-A01-p739098	Bn-N1-p754255	gene	exon

Bn-A01-p7455231	Bn-N1-p7488564	gene	
Bn-A01-p7455852	Bn-N1-p7489185	gene	
Bn-A01-p7479483	Bn-N1-p7515195	gene	
Bn-A01-p7493373	Bn-N1-p7527425	gene	exon
Bn-A01-p7530968	Bn-N1-p7557701	gene	
Bn-A01-p7548806	Bn-N1-p7577062	gene	
Bn-A01-p7551163	Bn-N1-p7579411	gene	
Bn-A01-p7554673	Bn-N1-p7582871	gene	
Bn-A01-p7573871	Bn-N1-p7606082	gene	
Bn-A01-p7574355	Bn-N1-p7606566	gene	
Bn-A01-p7603030	Bn-N1-p7632937	gene	exon
Bn-A01-p7615707	Bn-N1-p7645721	gene	
Bn-A01-p7643514	Bn-N1-p7681242	gene	
Bn-A01-p7643571	Bn-N1-p7681299	gene	exon
Bn-A01-p7702471	Bn-N1-p7744599	gene	exon
Bn-A01-p7714470	Bn-N1-p7759078	gene	
Bn-A01-p7717618	Bn-N11-p11698999	gene	exon
Bn-A01-p7721497	Bn-N1-p7767388	gene	exon
Bn-A01-p7724859	Bn-N1-p7770754	gene	exon
Bn-A01-p7771617	Bn-N1-p7849593	gene	
Bn-A01-p7772345	Bn-N1-p7850321	gene	exon
Bn-A01-p7787153	Bn-N1-p7857203	gene	exon
Bn-A01-p7825044	Bn-N1-p7886156	gene	
Bn-A01-p7827260	Bn-N1-p7888408	gene	
Bn-A01-p7831289	Bn-N1-p7892564	gene	
Bn-A01-p7834871	Bn-N1-p7896286	gene	exon
Bn-A01-p7850092	Bn-N11-p11910851	gene	exon
Bn-A01-p7852440	Bn-N1-p7911511	gene	exon
Bn-A01-p7861283	Bn-N1-p7920296	gene	exon
Bn-A01-p7875358	Bn-N1-p7934380	gene	
Bn-A01-p7889385	Bn-N1-p7947519	gene	
Bn-A01-p7890174	Bn-N1-p7948308	gene	
Bn-A01-p7926287	Bn-N1-p7973148	gene	exon
Bn-A01-p7953001	Bn-N1-p7998583	gene	
Bn-A01-p7961620	Bn-N1-p8008195	gene	exon
Bn-A01-p7973418	Bn-N1-p8025818	gene	
Bn-A01-p7974551	Bn-N1-p8027772	gene	exon
Bn-A01-p7979458	Bn-N1-p8033912	gene	
Bn-A01-p7982220	Bn-N11-p12094492	gene	
Bn-A01-p7983241	Bn-N1-p8037687	gene	exon
Bn-A01-p7987687	Bn-N1-p8044316	gene	
Bn-A01-p8016433	Bn-N1-p8071288	gene	
Bn-A01-p8017380	Bn-N1-p8072235	gene	
Bn-A01-p80385	Bn-N1-p77986	gene	
Bn-A01-p8043776	Bn-N1-p8093914	gene	exon
Bn-A01-p8051029	Bn-N1-p8105843	gene	
Bn-A01-p8051804	Bn-N1-p8106618	gene	
Bn-A01-p8063652	Bn-N1-p8117459	gene	
Bn-A01-p8076080	Bn-N1-p8129897	gene	exon
Bn-A01-p8108178	Bn-N1-p8150073	gene	
Bn-A01-p8115849	Bn-N1-p8154622	gene	
Bn-A01-p8116314	Bn-N1-p8155064	gene	

Bn-A01-p8121877	Bn-N1-p8160621	gene	
Bn-A01-p8122779	Bn-N1-p8161525	gene	
Bn-A01-p816488	Bn-N1-p836147	gene	exon
Bn-A01-p820317	Bn-N1-p839949	gene	exon
Bn-A01-p821328	Bn-N1-p840960	gene	
Bn-A01-p8221245	Bn-N1-p8246043	gene	
Bn-A01-p8242690	Bn-Scaffold01941-p1422	gene	
Bn-A01-p8246641	Bn-N1-p8273622	gene	exon
Bn-A01-p830221	Bn-N1-p849820	gene	exon
Bn-A01-p8335308	Bn-N1-p8372080	gene	exon
Bn-A01-p8353426	Bn-N1-p8396150	gene	exon
Bn-A01-p8354196	Bn-N11-p12707834	gene	
Bn-A01-p8378967	Bn-N1-p8419568	gene	
Bn-A01-p8404588	Bn-N1-p8448286	gene	exon
Bn-A01-p8417520	Bn-N1-p8460647	gene	
Bn-A01-p8418102	Bn-N1-p8461229	gene	exon
Bn-A01-p8439363	Bn-N1-p8481766	gene	exon
Bn-A01-p8451192	Bn-N11-p12860731	gene	
Bn-A01-p8501753	Bn-N1-p8547388	gene	
Bn-A01-p8504134	Bn-N1-p8549776	gene	exon
Bn-A01-p8543512	Bn-N1-p8581598	gene	exon
Bn-A01-p857771	Bn-N1-p882928	gene	
Bn-A01-p8647625	Bn-N1-p8687484	gene	exon
Bn-A01-p866260	Bn-N1-p891435	gene	exon
Bn-A01-p8683487	Bn-N1-p8727492	gene	
Bn-A01-p872436	Bn-N11-p1137301	gene	exon
Bn-A01-p877322	Bn-N1-p899365	gene	exon
Bn-A01-p878256	Bn-N1-p900297	gene	exon
Bn-A01-p8795719	Bn-N9-p18852883	gene	exon
Bn-A01-p879755	Bn-N1-p902872	gene	
Bn-A01-p8821722	Bn-N9-p18882233	gene	
Bn-A01-p8836783	Bn-N9-p18897377	gene	
Bn-A01-p8859217	Bn-N9-p18918063	gene	exon
Bn-A01-p887815	Bn-N1-p910193	gene	exon
Bn-A01-p8901327	Bn-N9-p18985716	gene	
Bn-A01-p8901490	Bn-N9-p18985879	gene	exon
Bn-A01-p8960719	Bn-N9-p3365113	gene	
Bn-A01-p8982996	Bn-N9-p3345599	gene	exon
Bn-A01-p89956	Bn-N1-p88996	gene	
Bn-A01-p9030262	Bn-N9-p3301660	gene	
Bn-A01-p9065564	Bn-Scaffold06532-p1209	gene	exon
Bn-A01-p9075803	Bn-N9-p3248371	gene	
Bn-A01-p9075931	Bn-N9-p3248243	gene	
Bn-A01-p9080359	Bn-N9-p3243748	gene	
Bn-A01-p9080573	Bn-N9-p3243534	gene	exon
Bn-A01-p9129693	Bn-N9-p3184444	gene	exon
Bn-A01-p9130391	Bn-N9-p3183735	gene	
Bn-A01-p9138209	Bn-N9-p3175914	gene	
Bn-A01-p9145211	Bn-N9-p3170011	gene	
Bn-A01-p9145630	Bn-N9-p3169592	gene	
Bn-A01-p9146490	Bn-N9-p3168728	gene	
Bn-A01-p9147096	Bn-N9-p3168122	gene	

Bn-A01-p916774	Bn-N1-p936377	gene	
Bn-A01-p916852	Bn-N1-p936455	gene	
Bn-A01-p9170858	Bn-N1-p8737520	gene	exon
Bn-A01-p9176104	Bn-N1-p8743891	gene	exon
Bn-A01-p9180340	Bn-N1-p8751591	gene	exon
Bn-A01-p9180828	Bn-N1-p8752082	gene	
Bn-A01-p9259611	Bn-N1-p8812569	gene	
Bn-A01-p9260073	Bn-N1-p8813031	gene	
Bn-A01-p9269627	Bn-N1-p8841697	gene	exon
Bn-A01-p9326695	Bn-N1-p8879780	gene	exon
Bn-A01-p9331463	Bn-N1-p8884533	gene	exon
Bn-A01-p933795	Bn-N11-p1196494	gene	exon
Bn-A01-p9360043	Bn-N1-p8904052	gene	
Bn-A01-p9360971	Bn-N1-p8904980	gene	
Bn-A01-p9369257	Bn-N1-p8913447	gene	
Bn-A01-p9371414	Bn-N11-p13619579	gene	
Bn-A01-p938887	Bn-N1-p958851	gene	exon
Bn-A01-p939202	Bn-N1-p959173	gene	
Bn-A01-p9408018	Bn-N1-p8952049	gene	exon
Bn-A01-p941792	Bn-N1-p966110	gene	
Bn-A01-p9469933	Bn-N1-p9007626	gene	exon
Bn-A01-p9476520	Bn-N1-p9013686	gene	exon
Bn-A01-p9499100	Bn-N1-p9029564	gene	exon
Bn-A01-p9523572	Bn-N1-p9051751	gene	exon
Bn-A01-p9565804	Bn-N1-p9081251	gene	exon
Bn-A01-p95718	Bn-N1-p101308	gene	
Bn-A01-p9599644	Bn-N1-p9110966	gene	
Bn-A01-p9614730	Bn-N1-p9123417	gene	exon
Bn-A01-p9619932	Bn-N1-p9128645	gene	exon
Bn-A01-p9621623	Bn-N1-p9130259	gene	exon
Bn-A01-p977861	Bn-N11-p1264828	gene	
Bn-A01-p978860	Bn-N1-p1005541	gene	exon
Bn-A01-p9810552	Bn-N1-p9320614	gene	exon
Bn-A01-p9853365	Bn-N1-p9366090	gene	exon
Bn-A01-p9863114	Bn-N1-p9375414	gene	exon
Bn-A01-p994722	Bn-N1-p1025240	gene	exon
Bn-A01-p9961425	Bn-N1-p9496705	gene	exon
Bn-A01-p9961553	Bn-N1-p9496833	gene	exon
Bn-A01-p9982115	Bn-N1-p9522795	gene	
Bn-A01-p9989240	Bn-N1-p9529910	gene	
Bn-A01-p9999485	Bn-N1-p9549138	gene	
Bn-A02-p10019636	Bn-N2-p9430743	gene	exon
Bn-A02-p10020231	Bn-N2-p9431338	gene	exon
Bn-A02-p10096185	Bn-N2-p9499093	gene	
Bn-A02-p10126530	Bn-N2-p9546173	gene	exon
Bn-A02-p10138982	Bn-N2-p9559614	gene	
Bn-A02-p10140065	Bn-N2-p9560595	gene	
Bn-A02-p10153359	Bn-N2-p9575002	gene	exon
Bn-A02-p10156025	Bn-N2-p9577668	gene	
Bn-A02-p10193709	Bn-N2-p9620480	gene	exon
Bn-A02-p1020806	Bn-N7-p13945724	gene	
Bn-A02-p10231298	Bn-N2-p9655300	gene	exon

Bn-A02-p10247756	Bn-N2-p9675879	gene	exon
Bn-A02-p1025518	Bn-N7-p13950938	gene	
Bn-A02-p10255955	Bn-N2-p9684278	gene	exon
Bn-A02-p10259746	Bn-N2-p9688844	gene	
Bn-A02-p1028349	Bn-N7-p13953747	gene	exon
Bn-A02-p10325260	Bn-N2-p9739682	gene	
Bn-A02-p10332863	Bn-N2-p9746334	gene	
Bn-A02-p10338350	Bn-N2-p9750165	gene	
Bn-A02-p10377179	Bn-N2-p9789893	gene	
Bn-A02-p10395108	Bn-N2-p9812760	gene	exon
Bn-A02-p10444138	Bn-N2-p9877373	gene	exon
Bn-A02-p10510996	Bn-N2-p9937231	gene	exon
Bn-A02-p10524842	Bn-N2-p9961771	gene	
Bn-A02-p10591779	Bn-Scaffold01435-p4966	gene	exon
Bn-A02-p10616871	Bn-N2-p10056794	gene	exon
Bn-A02-p10623838	Bn-N2-p10063922	gene	
Bn-A02-p10624373	Bn-N2-p10064457	gene	exon
Bn-A02-p10625720	Bn-N2-p10065821	gene	
Bn-A02-p10626782	Bn-N2-p10066883	gene	
Bn-A02-p10629886	Bn-N2-p10069986	gene	exon
Bn-A02-p10711353	Bn-N2-p10136517	gene	exon
Bn-A02-p10711442	Bn-N2-p10136606	gene	exon
Bn-A02-p10711565	Bn-N2-p10136729	gene	exon
Bn-A02-p1075920	Bn-N2-p1806142	gene	
Bn-A02-p10775283	Bn-N12-p15120898	gene	exon
Bn-A02-p10850012	Bn-N2-p10364056	gene	exon
Bn-A02-p10850323	Bn-N2-p10364367	gene	
Bn-A02-p1085455	Bn-N12-p1811265	gene	
Bn-A02-p1091779	Bn-N2-p1790897	gene	
Bn-A02-p1092146	Bn-N2-p1790534	gene	
Bn-A02-p10949322	Bn-N2-p10473667	gene	
Bn-A02-p1096082	Bn-N2-p1786578	gene	
Bn-A02-p10983922	Bn-N2-p10505168	gene	exon
Bn-A02-p10996151	Bn-N2-p10519407	gene	exon
Bn-A02-p11038401	Bn-N2-p10576393	gene	
Bn-A02-p11039119	Bn-N2-p10577070	gene	exon
Bn-A02-p11041638	Bn-N2-p10579589	gene	exon
Bn-A02-p11041726	Bn-N2-p10579677	gene	exon
Bn-A02-p11086855	Bn-N2-p10621790	gene	
Bn-A02-p11087046	Bn-N2-p10621981	gene	
Bn-A02-p11087388	Bn-N2-p10622333	gene	exon
Bn-A02-p11155686	Bn-N2-p10688424	gene	
Bn-A02-p11156474	Bn-N2-p10689212	gene	exon
Bn-A02-p11159353	Bn-N2-p10692160	gene	
Bn-A02-p11160726	Bn-N2-p10693526	gene	
Bn-A02-p11161716	Bn-N2-p10694521	gene	exon
Bn-A02-p11182703	Bn-N2-p10715981	gene	
Bn-A02-p11235969	Bn-N2-p10796763	gene	
Bn-A02-p11291606	Bn-N2-p10856085	gene	exon
Bn-A02-p11293986	Bn-N2-p10858464	gene	exon
Bn-A02-p11346389	Bn-N2-p10907135	gene	
Bn-A02-p11355276	Bn-N2-p10915864	gene	exon

Bn-A02-p11356944	Bn-N2-p10917650	gene	
Bn-A02-p11365167	Bn-N2-p10926253	gene	
Bn-A02-p11449348	Bn-N2-p11155737	gene	exon
Bn-A02-p11454658	Bn-N2-p11167023	gene	exon
Bn-A02-p11454746	Bn-N2-p11167111	gene	exon
Bn-A02-p11455068	Bn-N2-p11167436	gene	exon
Bn-A02-p11455310	Bn-N2-p11167691	gene	
Bn-A02-p11455369	Bn-N2-p11167750	gene	exon
Bn-A02-p11455659	Bn-N2-p11168040	gene	
Bn-A02-p11535570	Bn-N2-p11254616	gene	exon
Bn-A02-p1155656	Bn-N2-p1716507	gene	exon
Bn-A02-p1157373	Bn-N2-p1714788	gene	
Bn-A02-p11616676	Bn-N2-p11344990	gene	
Bn-A02-p11713499	Bn-N2-p11442395	gene	exon
Bn-A02-p11723698	Bn-N2-p11453116	gene	
Bn-A02-p11759281	Bn-N2-p11485420	gene	exon
Bn-A02-p11772983	Bn-N12-p16895215	gene	
Bn-A02-p11786253	Bn-N12-p16904124	gene	
Bn-A02-p11786572	Bn-N2-p11519176	gene	
Bn-A02-p11842725	Bn-N2-p11585881	gene	
Bn-A02-p11859430	Bn-N2-p11603565	gene	
Bn-A02-p11918483	Bn-N2-p11657722	gene	
Bn-A02-p11929538	Bn-N2-p11673849	gene	
Bn-A02-p11936177	Bn-N2-p11680540	gene	
Bn-A02-p11937221	Bn-N2-p11681573	gene	
Bn-A02-p12044265	Bn-N2-p11820483	gene	
Bn-A02-p12061350	Bn-N2-p11825220	gene	
Bn-A02-p12105374	Bn-N12-p17401558	gene	exon
Bn-A02-p12106636	Bn-N2-p11875156	gene	exon
Bn-A02-p12107746	Bn-N2-p11876266	gene	exon
Bn-A02-p12107868	Bn-N2-p11876388	gene	
Bn-A02-p12145607	Bn-N2-p11905764	gene	
Bn-A02-p12150733	Bn-N2-p11914823	gene	
Bn-A02-p12157799	Bn-N2-p11921905	gene	exon
Bn-A02-p12198897	Bn-N2-p11962322	gene	
Bn-A02-p12269431	Bn-N2-p12021186	gene	
Bn-A02-p12278577	Bn-N2-p12031940	gene	exon
Bn-A02-p12284078	Bn-N2-p12037386	gene	
Bn-A02-p12284671	Bn-N2-p12037981	gene	exon
Bn-A02-p12284882	Bn-N2-p12038192	gene	
Bn-A02-p12295281	Bn-N2-p12048480	gene	
Bn-A02-p12295682	Bn-N2-p12048881	gene	exon
Bn-A02-p1232964	Bn-N2-p1648567	gene	
Bn-A02-p12393617	Bn-N2-p12223294	gene	exon
Bn-A02-p12397326	Bn-N2-p12226976	gene	
Bn-A02-p12397585	Bn-N2-p12227235	gene	
Bn-A02-p1250673	Bn-N2-p1628726	gene	
Bn-A02-p12520861	Bn-N2-p12364780	gene	
Bn-A02-p12521545	Bn-N2-p12365464	gene	exon
Bn-A02-p12589304	Bn-N2-p12445215	gene	
Bn-A02-p1259929	Bn-N2-p1619108	gene	exon
Bn-A02-p126006	Bn-N7-p13000679	gene	exon

Bn-A02-p12608089	Bn-N2-p12466634	gene	exon
Bn-A02-p12680940	Bn-N2-p12555310	gene	exon
Bn-A02-p12681201	Bn-N2-p12555566	gene	
Bn-A02-p12681247	Bn-N2-p12555614	gene	
Bn-A02-p12688495	Bn-N2-p12562841	gene	exon
Bn-A02-p12688906	Bn-N2-p12563252	gene	exon
Bn-A02-p12693516	Bn-N2-p12569885	gene	
Bn-A02-p12739678	Bn-N2-p12597648	gene	exon
Bn-A02-p12752915	Bn-N2-p12636981	gene	exon
Bn-A02-p12815817	Bn-N2-p12709133	gene	exon
Bn-A02-p12819772	Bn-N12-p18227776	gene	
Bn-A02-p12821038	Bn-N2-p12728874	gene	exon
Bn-A02-p12822311	Bn-N12-p18230297	gene	
Bn-A02-p12822315	Bn-N2-p12730149	gene	
Bn-A02-p1290847	Bn-N2-p1587146	gene	exon
Bn-A02-p1292023	Bn-N2-p1585968	gene	
Bn-A02-p12939509	Bn-N2-p12812391	gene	
Bn-A02-p12999856	Bn-N2-p12861351	gene	
Bn-A02-p13000494	Bn-N2-p12861986	gene	exon
Bn-A02-p13019236	Bn-N2-p12877301	gene	exon
Bn-A02-p13067449	Bn-N2-p12914411	gene	
Bn-A02-p13068355	Bn-N2-p12912891	gene	
Bn-A02-p13118529	Bn-N2-p12981704	gene	
Bn-A02-p13167989	Bn-N12-p18773573	gene	exon
Bn-A02-p1324368	Bn-N2-p1553708	gene	exon
Bn-A02-p13252027	Bn-N1-p22788462	gene	
Bn-A02-p13252137	Bn-N1-p22788352	gene	exon
Bn-A02-p13252354	Bn-N1-p22788137	gene	
Bn-A02-p13252652	Bn-N1-p22787838	gene	
Bn-A02-p1327787	Bn-N2-p1550342	gene	
Bn-A02-p13382591	Bn-N2-p13258690	gene	exon
Bn-A02-p1344870	Bn-N12-p1467532	gene	exon
Bn-A02-p13572191	Bn-N2-p13366263	gene	exon
Bn-A02-p13627045	Bn-N2-p13426522	gene	exon
Bn-A02-p13637436	Bn-N2-p13438528	gene	
Bn-A02-p13662225	Bn-N2-p13470531	gene	
Bn-A02-p1370581	Bn-N2-p1508906	gene	exon
Bn-A02-p1372727	Bn-N2-p1506659	gene	
Bn-A02-p13874627	Bn-N2-p13723961	gene	exon
Bn-A02-p13875459	Bn-N2-p13724898	gene	
Bn-A02-p14040220	Bn-N2-p13879570	gene	
Bn-A02-p14078497	Bn-N2-p13922267	gene	exon
Bn-A02-p14134708	Bn-N2-p13977379	gene	
Bn-A02-p14170476	Bn-N2-p14009194	gene	
Bn-A02-p14215327	Bn-Scaffold21546-p444	gene	exon
Bn-A02-p14287347	Bn-N2-p14133767	gene	exon
Bn-A02-p14287428	Bn-N2-p14133848	gene	exon
Bn-A02-p14328410	Bn-N2-p14160230	gene	
Bn-A02-p14527781	Bn-N2-p14409203	gene	exon
Bn-A02-p14529778	Bn-N2-p14411210	gene	exon
Bn-A02-p14592602	Bn-N2-p14456030	gene	exon
Bn-A02-p14605944	Bn-N2-p14466194	gene	exon

Bn-A02-p14612382	Bn-N2-p14473251	gene	exon
Bn-A02-p14615703	Bn-N2-p14476576	gene	exon
Bn-A02-p14781371	Bn-N2-p14671951	gene	exon
Bn-A02-p14956864	Bn-N2-p14875554	gene	
Bn-A02-p14992706	Bn-N2-p14911092	gene	
Bn-A02-p14992781	Bn-N2-p14911167	gene	
Bn-A02-p14992802	Bn-N2-p14911188	gene	
Bn-A02-p1506579	Bn-N2-p169311	gene	
Bn-A02-p1530996	Bn-N2-p207019	gene	
Bn-A02-p15457681	Bn-N18-p23083283	gene	
Bn-A02-p15569271	Bn-N2-p15445555	gene	exon
Bn-A02-p15693192	Bn-N2-p15585036	gene	
Bn-A02-p15745628	Bn-N2-p15638511	gene	exon
Bn-A02-p15758928	Bn-N2-p15651816	gene	exon
Bn-A02-p1576350	Bn-N2-p249451	gene	
Bn-A02-p15764198	Bn-N2-p15655290	gene	exon
Bn-A02-p15766649	Bn-N2-p15656768	gene	exon
Bn-A02-p15789088	Bn-N2-p15667274	gene	exon
Bn-A02-p15832342	Bn-N2-p15718293	gene	exon
Bn-A02-p15870327	Bn-N2-p15740491	gene	
Bn-A02-p15912199	Bn-N2-p15783884	gene	
Bn-A02-p16058769	Bn-N2-p15945767	gene	
Bn-A02-p16083197	Bn-N2-p15961807	gene	exon
Bn-A02-p16209439	Bn-N2-p16108396	gene	exon
Bn-A02-p16223740	Bn-N2-p16125170	gene	
Bn-A02-p1629448	Bn-N2-p304853	gene	
Bn-A02-p16339698	Bn-N2-p16248137	gene	exon
Bn-A02-p16528486	Bn-N2-p16440230	gene	
Bn-A02-p16529092	Bn-N2-p16440836	gene	
Bn-A02-p16571709	Bn-N2-p16481172	gene	
Bn-A02-p16666866	Bn-N2-p17563229	gene	
Bn-A02-p1684625	Bn-N2-p368153	gene	
Bn-A02-p1705187	Bn-N12-p22425	gene	
Bn-A02-p1707420	Bn-N2-p392255	gene	
Bn-A02-p17156661	Bn-N2-p18059115	gene	exon
Bn-A02-p174249	Bn-N7-p13043612	gene	
Bn-A02-p175605	Bn-N7-p13044968	gene	
Bn-A02-p17938666	Bn-N2-p7355591	gene	
Bn-A02-p179557	Bn-N7-p13049089	gene	
Bn-A02-p182447	Bn-N7-p13051977	gene	exon
Bn-A02-p182618	Bn-N7-p13052148	gene	exon
Bn-A02-p1890913	Bn-N2-p590652	gene	
Bn-A02-p18917772	Bn-N2-p21457111	gene	exon
Bn-A02-p18966918	Bn-N2-p21499463	gene	exon
Bn-A02-p18974322	Bn-N2-p21506168	gene	exon
Bn-A02-p18984484	Bn-N2-p21515507	gene	
Bn-A02-p19003944	Bn-N2-p21534227	gene	
Bn-A02-p19058136	Bn-N2-p21596996	gene	exon
Bn-A02-p19207431	Bn-N2-p21760905	gene	
Bn-A02-p19212766	Bn-N2-p21773179	gene	
Bn-A02-p19316657	Bn-N2-p21856271	gene	exon
Bn-A02-p19317643	Bn-N2-p21857257	gene	exon

Bn-A02-p19395795	Bn-N2-p21920826	gene	exon
Bn-A02-p19573867	Bn-N2-p22285981	gene	
Bn-A02-p19575587	Bn-N2-p22287665	gene	exon
Bn-A02-p19587474	Bn-N2-p22291612	gene	exon
Bn-A02-p19587812	Bn-N2-p22291950	gene	exon
Bn-A02-p19645773	Bn-N2-p22331062	gene	exon
Bn-A02-p19682273	Bn-N2-p22376166	gene	exon
Bn-A02-p19682984	Bn-N2-p22376878	gene	
Bn-A02-p19683093	Bn-N2-p22376987	gene	exon
Bn-A02-p19702955	Bn-N2-p22414997	gene	
Bn-A02-p19704677	Bn-N2-p22416723	gene	exon
Bn-A02-p19731316	Bn-N2-p24147282	gene	exon
Bn-A02-p19872956	Bn-N2-p24032897	gene	
Bn-A02-p19889192	Bn-N2-p24017850	gene	
Bn-A02-p19892632	Bn-N2-p24011891	gene	
Bn-A02-p19892839	Bn-N2-p24011684	gene	
Bn-A02-p19927952	Bn-N2-p23977051	gene	
Bn-A02-p1997812	Bn-N2-p705902	gene	
Bn-A02-p20200723	Bn-N2-p23729895	gene	
Bn-A02-p20242753	Bn-N2-p23685668	gene	exon
Bn-A02-p202819	Bn-N7-p13063423	gene	
Bn-A02-p20371774	Bn-N2-p23584531	gene	exon
Bn-A02-p20396880	Bn-N2-p23560271	gene	exon
Bn-A02-p20461477	Bn-N2-p23499487	gene	exon
Bn-A02-p20536885	Bn-N2-p23428442	gene	
Bn-A02-p20561132	Bn-N2-p23404761	gene	exon
Bn-A02-p20735027	Bn-N2-p23238672	gene	
Bn-A02-p2075762	Bn-N2-p775778	gene	exon
Bn-A02-p21009288	Bn-N12-p36496167	gene	
Bn-A02-p21023727	Bn-N2-p22923264	gene	
Bn-A02-p2105937	Bn-N2-p808758	gene	
Bn-A02-p21073755	Bn-N2-p22873663	gene	
Bn-A02-p21207148	Bn-N2-p22699171	gene	
Bn-A02-p21211198	Bn-N2-p22695143	gene	exon
Bn-A02-p2128775	Bn-N2-p828865	gene	exon
Bn-A02-p212959	Bn-N7-p13072225	gene	exon
Bn-A02-p21325110	Bn-N2-p22602967	gene	exon
Bn-A02-p2134112	Bn-N2-p834279	gene	exon
Bn-A02-p2135053	Bn-N2-p835204	gene	
Bn-A02-p21379810	Bn-N2-p25490754	gene	
Bn-A02-p2138408	Bn-N2-p839451	gene	
Bn-A02-p2146210	Bn-N2-p847432	gene	exon
Bn-A02-p21467101	Bn-N2-p25412797	gene	exon
Bn-A02-p21468	Bn-N7-p12887903	gene	exon
Bn-A02-p21489620	Bn-N2-p25392820	gene	
Bn-A02-p21491139	Bn-N2-p25391305	gene	exon
Bn-A02-p21510968	Bn-N2-p25366085	gene	
Bn-A02-p21544735	Bn-N2-p25325581	gene	exon
Bn-A02-p21570784	Bn-N12-p41064633	gene	exon
Bn-A02-p21595382	Bn-N2-p25269719	gene	
Bn-A02-p21616494	Bn-N2-p25248893	gene	exon
Bn-A02-p21625125	Bn-N2-p25238071	gene	exon

Bn-A02-p21639869	Bn-N2-p25226448	gene	exon
Bn-A02-p21648504	Bn-N2-p25217692	gene	exon
Bn-A02-p21713756	Bn-N12-p40864854	gene	
Bn-A02-p21713818	Bn-N12-p40864792	gene	
Bn-A02-p21891973	Bn-N2-p24954183	gene	exon
Bn-A02-p21930156	Bn-N2-p24931155	gene	
Bn-A02-p22296426	Bn-N2-p24561969	gene	
Bn-A02-p22357265	Bn-N2-p24503527	gene	
Bn-A02-p22375400	Bn-N2-p24489078	gene	
Bn-A02-p22394632	Bn-N2-p24463173	gene	exon
Bn-A02-p22395060	Bn-N2-p24462745	gene	exon
Bn-A02-p22478377	Bn-N10-p7103511	gene	
Bn-A02-p22479272	Bn-N10-p7102617	gene	exon
Bn-A02-p22493338	Bn-N10-p7087848	gene	
Bn-A02-p22550542	Bn-N10-p7034603	gene	exon
Bn-A02-p2258714	Bn-N2-p952606	gene	
Bn-A02-p22599833	Bn-N10-p6996400	gene	
Bn-A02-p22601555	Bn-N10-p6994289	gene	
Bn-A02-p22602317	Bn-N10-p6993526	gene	
Bn-A02-p22650896	Bn-N10-p6960950	gene	
Bn-A02-p22651355	Bn-N10-p6960491	gene	
Bn-A02-p22741797	Bn-N2-p24315106	gene	
Bn-A02-p2274901	Bn-N2-p974551	gene	
Bn-A02-p22847472	Bn-N2-p24218337	gene	exon
Bn-A02-p2287712	Bn-N2-p991484	gene	exon
Bn-A02-p2288207	Bn-N2-p991976	gene	exon
Bn-A02-p2288216	Bn-N2-p991985	gene	exon
Bn-A02-p2292871	Bn-N2-p1000576	gene	
Bn-A02-p23095572	Bn-N5-p24533762	gene	
Bn-A02-p23108517	Bn-N5-p24518497	gene	
Bn-A02-p23112746	Bn-N5-p24514024	gene	exon
Bn-A02-p23277919	Bn-N5-p24340494	gene	exon
Bn-A02-p23279287	Bn-N5-p24339131	gene	exon
Bn-A02-p23280472	Bn-N5-p24337931	gene	exon
Bn-A02-p23280665	Bn-N5-p24337738	gene	exon
Bn-A02-p23280869	Bn-N5-p24337534	gene	
Bn-A02-p23295254	Bn-N5-p24327677	gene	
Bn-A02-p23297690	Bn-N15-p46318587	gene	
Bn-A02-p23297789	Bn-N15-p46318493	gene	exon
Bn-A02-p23408870	Bn-N2-p25826230	gene	
Bn-A02-p2341934	Bn-N2-p1046388	gene	exon
Bn-A02-p23454935	Bn-N2-p25872381	gene	exon
Bn-A02-p23456103	Bn-N2-p25873549	gene	
Bn-A02-p23472944	Bn-N2-p25888075	gene	exon
Bn-A02-p23477774	Bn-N2-p25895492	gene	exon
Bn-A02-p23477968	Bn-N2-p25895693	gene	exon
Bn-A02-p23479432	Bn-N12-p41767720	gene	exon
Bn-A02-p23482499	Bn-N2-p25900190	gene	
Bn-A02-p23486694	Bn-N2-p25904143	gene	
Bn-A02-p23488731	Bn-N2-p25906177	gene	
Bn-A02-p23495061	Bn-N2-p25912435	gene	
Bn-A02-p23523709	Bn-N2-p25944732	gene	exon

Bn-A02-p2364069	Bn-N2-p1068047	gene	
Bn-A02-p2364479	Bn-N2-p1068443	gene	exon
Bn-A02-p23685036	Bn-N2-p26099373	gene	exon
Bn-A02-p23686560	Bn-N2-p26100891	gene	
Bn-A02-p23708299	Bn-N17-p35241902	gene	
Bn-A02-p23708516	Bn-N6-p23435721	gene	exon
Bn-A02-p23726200	Bn-N12-p42159516	gene	
Bn-A02-p23729046	Bn-N11-p17266003	gene	exon
Bn-A02-p23743267	Bn-N2-p26126049	gene	exon
Bn-A02-p23757876	Bn-N2-p26139876	gene	
Bn-A02-p23824343	Bn-N2-p26213639	gene	
Bn-A02-p23826324	Bn-N2-p26215628	gene	
Bn-A02-p23836232	Bn-N2-p26224099	gene	
Bn-A02-p23856500	Bn-N2-p26249411	gene	
Bn-A02-p23857384	Bn-N2-p26250302	gene	
Bn-A02-p23875682	Bn-N2-p26268225	gene	exon
Bn-A02-p23889188	Bn-N2-p26279361	gene	
Bn-A02-p2389418	Bn-N12-p988560	gene	
Bn-A02-p23920155	Bn-N10-p2032226	gene	exon
Bn-A02-p23925334	Bn-N10-p2027091	gene	exon
Bn-A02-p24216905	Bn-N2-p26572947	gene	exon
Bn-A02-p24237359	Bn-N2-p26593264	gene	exon
Bn-A02-p24268496	Bn-N2-p26620206	gene	
Bn-A02-p24269435	Bn-N2-p26621146	gene	
Bn-A02-p24359370	Bn-N2-p26707970	gene	
Bn-A02-p24378297	Bn-N2-p26731687	gene	
Bn-A02-p24415454	Bn-N2-p26761096	gene	exon
Bn-A02-p24438115	Bn-N2-p26785340	gene	exon
Bn-A02-p24449690	Bn-N2-p26799071	gene	exon
Bn-A02-p24462762	Bn-N2-p26817344	gene	exon
Bn-A02-p24522983	Bn-N2-p26882288	gene	
Bn-A02-p24584058	Bn-N2-p26950533	gene	exon
Bn-A02-p2466355	Bn-N2-p1163220	gene	
Bn-A02-p24675182	Bn-N2-p27027234	gene	exon
Bn-A02-p24688870	Bn-N2-p27041388	gene	
Bn-A02-p24706646	Bn-N2-p27057373	gene	
Bn-A02-p24722684	Bn-N2-p27070634	gene	
Bn-A02-p24809526	Bn-N2-p27155988	gene	exon
Bn-A02-p24810609	Bn-N2-p27157071	gene	
Bn-A02-p24844291	Bn-N2-p27195801	gene	
Bn-A02-p24851624	Bn-N2-p27203225	gene	
Bn-A02-p24878657	Bn-N2-p27227551	gene	exon
Bn-A02-p2489530	Bn-N2-p1187700	gene	
Bn-A02-p2490293	Bn-N2-p1188463	gene	
Bn-A02-p24911505	Bn-N2-p27262414	gene	
Bn-A02-p24914083	Bn-N2-p27264991	gene	
Bn-A02-p24919366	Bn-N2-p27271338	gene	
Bn-A02-p24945785	Bn-N2-p27306231	gene	exon
Bn-A02-p24967958	Bn-N2-p27323065	gene	exon
Bn-A02-p25022333	Bn-N12-p44340035	gene	
Bn-A02-p25022921	Bn-N2-p27354623	gene	exon
Bn-A02-p25027576	Bn-N12-p44346012	gene	exon

Bn-A02-p2502950	Bn-N2-p1201098	gene	
Bn-A02-p25081911	Bn-N2-p27417291	gene	
Bn-A02-p25081916	Bn-N2-p27417296	gene	
Bn-A02-p25100451	Bn-N2-p27435756	gene	
Bn-A02-p25105527	Bn-N2-p27441225	gene	
Bn-A02-p25174583	Bn-N6-p19264943	gene	
Bn-A02-p25181474	Bn-N2-p27515640	gene	exon
Bn-A02-p25181726	Bn-N2-p27515892	gene	exon
Bn-A02-p25190854	Bn-N2-p27521196	gene	exon
Bn-A02-p25199281	Bn-N12-p44617912	gene	exon
Bn-A02-p25201372	Bn-N2-p27529744	gene	
Bn-A02-p2520421	Bn-N2-p1218503	gene	exon
Bn-A02-p25205468	Bn-N2-p27537494	gene	
Bn-A02-p25260066	Bn-N2-p27602638	gene	
Bn-A02-p25334100	Bn-N2-p27636469	gene	exon
Bn-A02-p25334266	Bn-N2-p27636635	gene	
Bn-A02-p25334271	Bn-N2-p27636640	gene	
Bn-A02-p2536779	Bn-N2-p1239345	gene	exon
Bn-A02-p25375825	Bn-N2-p27670381	gene	exon
Bn-A02-p25400515	Bn-N2-p27686740	gene	
Bn-A02-p25400591	Bn-N2-p27686816	gene	
Bn-A02-p25401060	Bn-N2-p27687274	gene	
Bn-A02-p25402286	Bn-N2-p27687764	gene	
Bn-A02-p25402971	Bn-N2-p27689228	gene	exon
Bn-A02-p25419536	Bn-N2-p27704681	gene	
Bn-A02-p2542065	Bn-N2-p1244624	gene	
Bn-A02-p25429624	Bn-N2-p27705618	gene	
Bn-A02-p25429837	Bn-N2-p27705832	gene	exon
Bn-A02-p2544070	Bn-N2-p1246634	gene	
Bn-A02-p25446521	Bn-N2-p27724158	gene	
Bn-A02-p25448092	Bn-N2-p27725728	gene	
Bn-A02-p25453070	Bn-N2-p27730776	gene	
Bn-A02-p25453447	Bn-N2-p27731154	gene	
Bn-A02-p25454242	Bn-N2-p27732531	gene	
Bn-A02-p25454499	Bn-N2-p27732788	gene	
Bn-A02-p25454882	Bn-N2-p27733184	gene	
Bn-A02-p25456985	Bn-N2-p27735438	gene	
Bn-A02-p25457045	Bn-N2-p27735498	gene	
Bn-A02-p25457843	Bn-N2-p27735941	gene	
Bn-A02-p25479893	Bn-N2-p27760434	gene	
Bn-A02-p25485627	Bn-N2-p27765939	gene	
Bn-A02-p25488435	Bn-N2-p27768345	gene	exon
Bn-A02-p2549141	Bn-N2-p1250691	gene	
Bn-A02-p2551178	Bn-N2-p1252896	gene	exon
Bn-A02-p25530111	Bn-N2-p27786711	gene	
Bn-A02-p25530994	Bn-N2-p27787594	gene	exon
Bn-A02-p25564792	Bn-N2-p27816660	gene	exon
Bn-A02-p25572877	Bn-N2-p27824731	gene	exon
Bn-A02-p25612808	Bn-N2-p27880540	gene	exon
Bn-A02-p25644999	Bn-N2-p27912524	gene	exon
Bn-A02-p25670193	Bn-N2-p27937138	gene	
Bn-A02-p2568989	Bn-N2-p1270380	gene	exon

Bn-A02-p25735700	Bn-N2-p28014498	gene	
Bn-A02-p25736307	Bn-N2-p28015128	gene	
Bn-A02-p25746504	Bn-N2-p28033328	gene	
Bn-A02-p25747937	Bn-N12-p45461697	gene	exon
Bn-A02-p25762081	Bn-N2-p28047144	gene	exon
Bn-A02-p25795588	Bn-N2-p28058120	gene	exon
Bn-A02-p25814719	Bn-N12-p45535436	gene	exon
Bn-A02-p25820450	Bn-N2-p28086130	gene	
Bn-A02-p25821750	Bn-N2-p28087430	gene	
Bn-A02-p25826690	Bn-N2-p28092398	gene	exon
Bn-A02-p25827208	Bn-N2-p28092916	gene	
Bn-A02-p25841382	Bn-N2-p28101760	gene	exon
Bn-A02-p25841722	Bn-N2-p28102100	gene	
Bn-A02-p25864853	Bn-N2-p28124925	gene	exon
Bn-A02-p25865360	Bn-N2-p28125432	gene	exon
Bn-A02-p25876712	Bn-N2-p28136615	gene	exon
Bn-A02-p25881408	Bn-N2-p28144982	gene	exon
Bn-A02-p25883567	Bn-N2-p28147165	gene	
Bn-A02-p25966536	Bn-Scaffold00372b-p49885	gene	exon
Bn-A02-p26013503	Bn-N2-p28212500	gene	
Bn-A02-p2608125	Bn-N2-p1305765	gene	
Bn-A02-p2615923	Bn-N2-p1313171	gene	
Bn-A02-p2617701	Bn-N2-p1314947	gene	
Bn-A02-p2617781	Bn-N2-p1315027	gene	
Bn-A02-p26193572	Bn-N2-p28398142	gene	
Bn-A02-p2620397	Bn-N2-p1317615	gene	exon
Bn-A02-p2624375	Bn-N2-p1321568	gene	
Bn-A02-p26304354	Bn-N2-p28494437	gene	
Bn-A02-p26322916	Bn-N2-p28512399	gene	
Bn-A02-p26411585	Bn-N2-p28604732	gene	exon
Bn-A02-p26421790	Bn-N2-p28617133	gene	
Bn-A02-p26425347	Bn-N2-p28620666	gene	
Bn-A02-p2646929	Bn-N12-p1289657	gene	exon
Bn-A02-p26514827	Bn-N2-p28696696	gene	exon
Bn-A02-p2654991	Bn-N2-p1355422	gene	
Bn-A02-p26554539	Bn-N12-p45964408	gene	exon
Bn-A02-p26567426	Bn-N2-p28741025	gene	
Bn-A02-p26571107	Bn-N2-p28744664	gene	
Bn-A02-p266121	Bn-N7-p13133586	gene	exon
Bn-A02-p26625153	Bn-N5-p25377236	gene	exon
Bn-A02-p26625696	Bn-N5-p25376687	gene	
Bn-A02-p26641996	Bn-N5-p25359883	gene	
Bn-A02-p26644297	Bn-N5-p25357590	gene	exon
Bn-A02-p26651278	Bn-N5-p25350622	gene	
Bn-A02-p26653626	Bn-N5-p25348367	gene	exon
Bn-A02-p26654006	Bn-N5-p25347987	gene	exon
Bn-A02-p26656981	Bn-N5-p25344966	gene	
Bn-A02-p26668289	Bn-N5-p25333670	gene	exon
Bn-A02-p26669888	Bn-N5-p25332080	gene	
Bn-A02-p26689319	Bn-N5-p25304193	gene	
Bn-A02-p26713316	Bn-N5-p25279974	gene	
Bn-A02-p26733511	Bn-N5-p25264790	gene	exon

Bn-A02-p2673795	Bn-N2-p1372334	gene	exon
Bn-A02-p26792851	Bn-Scaffold00893-p90555	gene	exon
Bn-A02-p26803902	Bn-Scaffold00893-p77934	gene	
Bn-A02-p26804091	Bn-Scaffold00893-p77746	gene	
Bn-A02-p26832454	Bn-Scaffold00893-p49364	gene	
Bn-A02-p26845132	Bn-Scaffold00893-p31826	gene	exon
Bn-A02-p2692116	Bn-N2-p1390902	gene	exon
Bn-A02-p26921384	Bn-N5-p25156493	gene	exon
Bn-A02-p26946005	Bn-N5-p25132913	gene	exon
Bn-A02-p26947254	Bn-N5-p25131677	gene	exon
Bn-A02-p2695870	Bn-N2-p1394706	gene	
Bn-A02-p2698217	Bn-N2-p1397046	gene	
Bn-A02-p26982864	Bn-N2-p28771949	gene	
Bn-A02-p26986319	Bn-N12-p46027967	gene	
Bn-A02-p26987864	Bn-N2-p28776996	gene	
Bn-A02-p27024443	Bn-N2-p28806279	gene	
Bn-A02-p27051969	Bn-N2-p28834782	gene	
Bn-A02-p27052532	Bn-N2-p28835599	gene	
Bn-A02-p27052742	Bn-N2-p28835810	gene	
Bn-A02-p27121126	Bn-N2-p28903046	gene	
Bn-A02-p27124090	Bn-N2-p28905991	gene	
Bn-A02-p27134251	Bn-N2-p28917373	gene	
Bn-A02-p27172338	Bn-N2-p28949788	gene	
Bn-A02-p27203061	Bn-N2-p28990408	gene	
Bn-A02-p27220702	Bn-N12-p46405278	gene	exon
Bn-A02-p27255197	Bn-N12-p46472742	gene	exon
Bn-A02-p2727560	Bn-N12-p1375621	gene	exon
Bn-A02-p27286923	Bn-N2-p29080099	gene	
Bn-A02-p27288011	Bn-N2-p29084819	gene	
Bn-A02-p27288044	Bn-N2-p29084852	gene	
Bn-A02-p27288121	Bn-N2-p29084929	gene	
Bn-A02-p27321599	Bn-N2-p29101998	gene	exon
Bn-A02-p27327804	Bn-N2-p29103428	gene	exon
Bn-A02-p2738923	Bn-N2-p1452300	gene	
Bn-A02-p27405658	Bn-N2-p29191169	gene	exon
Bn-A02-p27420743	Bn-N2-p29206182	gene	
Bn-A02-p27420799	Bn-N2-p29206238	gene	exon
Bn-A02-p27450764	Bn-N2-p29238960	gene	exon
Bn-A02-p27503846	Bn-N2-p29291919	gene	
Bn-A02-p27504018	Bn-N2-p29292091	gene	exon
Bn-A02-p27613710	Bn-N2-p29402065	gene	exon
Bn-A02-p276572	Bn-Scaffold12647-p249	gene	exon
Bn-A02-p27680472	Bn-N2-p29466488	gene	exon
Bn-A02-p27831174	Bn-N2-p29609872	gene	
Bn-A02-p27833071	Bn-N2-p29611851	gene	
Bn-A02-p27834072	Bn-N2-p29612855	gene	
Bn-A02-p2784335	Bn-N2-p1858150	gene	
Bn-A02-p27845279	Bn-N2-p29621344	gene	
Bn-A02-p2785185	Bn-N2-p1859025	gene	exon
Bn-A02-p2788144	Bn-N2-p1865260	gene	
Bn-A02-p2799146	Bn-N2-p1874242	gene	
Bn-A02-p2800227	Bn-N2-p1875323	gene	exon

Bn-A02-p2800579	Bn-N2-p1875675	gene	
Bn-A02-p2800698	Bn-N2-p1875794	gene	exon
Bn-A02-p284968	Bn-N7-p13154675	gene	
Bn-A02-p2867545	Bn-N2-p1943312	gene	exon
Bn-A02-p2877222	Bn-N2-p1952858	gene	
Bn-A02-p2923889	Bn-N2-p1983351	gene	exon
Bn-A02-p293860	Bn-N7-p13165342	gene	
Bn-A02-p294984	Bn-N7-p13166456	gene	exon
Bn-A02-p2962298	Bn-N2-p2021013	gene	exon
Bn-A02-p300727	Bn-N7-p13184963	gene	
Bn-A02-p3011103	Bn-N2-p2069982	gene	
Bn-A02-p3033620	Bn-N2-p2091947	gene	
Bn-A02-p307342	Bn-N7-p13196459	gene	exon
Bn-A02-p3078017	Bn-N2-p2155233	gene	
Bn-A02-p3080150	Bn-N2-p2157365	gene	exon
Bn-A02-p3096890	Bn-N2-p2173479	gene	exon
Bn-A02-p3121742	Bn-N2-p2196718	gene	exon
Bn-A02-p3142416	Bn-N2-p2220109	gene	
Bn-A02-p3145923	Bn-N2-p2223558	gene	exon
Bn-A02-p3148346	Bn-N2-p2225846	gene	
Bn-A02-p3160461	Bn-N2-p2238075	gene	
Bn-A02-p3281338	Bn-N2-p2367290	gene	
Bn-A02-p3281452	Bn-N2-p2367418	gene	
Bn-A02-p3294682	Bn-N2-p2380926	gene	
Bn-A02-p3295898	Bn-N2-p2382134	gene	
Bn-A02-p3299206	Bn-N2-p2385418	gene	
Bn-A02-p3300731	Bn-N2-p2387091	gene	
Bn-A02-p3344176	Bn-N2-p2434012	gene	
Bn-A02-p3352863	Bn-N2-p2442666	gene	
Bn-A02-p3354301	Bn-N12-p2686843	gene	exon
Bn-A02-p3361391	Bn-N2-p2445850	gene	
Bn-A02-p3398506	Bn-N2-p2482742	gene	exon
Bn-A02-p3398710	Bn-N2-p2482946	gene	exon
Bn-A02-p3430069	Bn-N2-p2527335	gene	exon
Bn-A02-p3478450	Bn-N12-p2849930	gene	
Bn-A02-p3503647	Bn-N2-p2580923	gene	exon
Bn-A02-p3507057	Bn-N12-p2889730	gene	exon
Bn-A02-p3558502	Bn-N2-p2633436	gene	
Bn-A02-p3597721	Bn-N2-p2670223	gene	
Bn-A02-p3606635	Bn-N2-p2679040	gene	
Bn-A02-p3607564	Bn-N2-p2679970	gene	exon
Bn-A02-p3627856	Bn-N2-p2699130	gene	
Bn-A02-p3633790	Bn-N2-p2708860	gene	
Bn-A02-p3635114	Bn-N2-p2712160	gene	exon
Bn-A02-p3640490	Bn-N2-p2717536	gene	exon
Bn-A02-p3730794	Bn-N2-p2801052	gene	exon
Bn-A02-p3735101	Bn-N2-p2805334	gene	
Bn-A02-p3742509	Bn-N2-p2812081	gene	
Bn-A02-p3759462	Bn-N2-p2826887	gene	
Bn-A02-p3760177	Bn-N2-p2827602	gene	
Bn-A02-p3760247	Bn-N2-p2827672	gene	
Bn-A02-p3792136	Bn-N2-p2859571	gene	

Bn-A02-p3801428	Bn-N2-p2866053	gene	exon
Bn-A02-p3807577	Bn-N2-p2875058	gene	
Bn-A02-p3809970	Bn-N2-p2877458	gene	exon
Bn-A02-p3810028	Bn-N2-p2877516	gene	exon
Bn-A02-p3810078	Bn-N2-p2877566	gene	exon
Bn-A02-p3821814	Bn-N2-p2890915	gene	
Bn-A02-p3821944	Bn-N2-p2891045	gene	
Bn-A02-p3822985	Bn-N2-p2892084	gene	
Bn-A02-p3860483	Bn-N2-p2926014	gene	exon
Bn-A02-p3897213	Bn-N2-p2960517	gene	
Bn-A02-p3897546	Bn-N2-p2960857	gene	
Bn-A02-p3898766	Bn-N2-p2962079	gene	exon
Bn-A02-p3914269	Bn-N2-p2975056	gene	exon
Bn-A02-p3934729	Bn-Scaffold03677-p1641	gene	
Bn-A02-p3950581	Bn-N2-p3016075	gene	exon
Bn-A02-p3965286	Bn-N2-p3046915	gene	
Bn-A02-p4035808	Bn-N2-p3119297	gene	
Bn-A02-p4037028	Bn-N2-p3120499	gene	
Bn-A02-p4038224	Bn-N2-p3121692	gene	
Bn-A02-p4065755	Bn-N2-p3142609	gene	exon
Bn-A02-p4102727	Bn-N2-p3171759	gene	exon
Bn-A02-p4103096	Bn-N2-p3172123	gene	exon
Bn-A02-p4103158	Bn-N2-p3172185	gene	exon
Bn-A02-p4104054	Bn-N2-p3173081	gene	exon
Bn-A02-p4110181	Bn-N2-p3178749	gene	
Bn-A02-p4118946	Bn-N12-p3809918	gene	
Bn-A02-p4174594	Bn-N2-p3240276	gene	exon
Bn-A02-p4183553	Bn-N2-p3249801	gene	
Bn-A02-p4199792	Bn-N2-p3261807	gene	
Bn-A02-p4210810	Bn-N2-p3283944	gene	exon
Bn-A02-p4220181	Bn-N2-p3292631	gene	
Bn-A02-p4232093	Bn-N2-p3301771	gene	exon
Bn-A02-p4237047	Bn-N2-p3309912	gene	
Bn-A02-p4241100	Bn-N2-p3314254	gene	
Bn-A02-p4243717	Bn-N2-p3316876	gene	exon
Bn-A02-p4262796	Bn-N2-p3349263	gene	
Bn-A02-p4262907	Bn-N2-p3349374	gene	
Bn-A02-p4277649	Bn-N2-p3355557	gene	
Bn-A02-p4295967	Bn-N2-p3377254	gene	exon
Bn-A02-p4306196	Bn-N2-p3387309	gene	
Bn-A02-p4351893	Bn-N12-p4180934	gene	exon
Bn-A02-p4374253	Bn-N2-p3456000	gene	
Bn-A02-p441751	Bn-N7-p13316291	gene	exon
Bn-A02-p4568023	Bn-N2-p3649128	gene	
Bn-A02-p459981	Bn-N7-p13338835	gene	
Bn-A02-p4611221	Bn-N12-p4596088	gene	exon
Bn-A02-p4681731	Bn-N2-p3759227	gene	exon
Bn-A02-p4698141	Bn-N2-p3778378	gene	
Bn-A02-p4721726	Bn-N12-p4796406	gene	exon
Bn-A02-p4744168	Bn-N2-p3833121	gene	exon
Bn-A02-p474724	Bn-N7-p13356720	gene	exon
Bn-A02-p476011	Bn-N7-p13358348	gene	exon

Bn-A02-p4761483	Bn-N2-p3853545	gene	exon
Bn-A02-p4773307	Bn-N2-p3865572	gene	
Bn-A02-p4774095	Bn-N2-p3866360	gene	exon
Bn-A02-p4774099	Bn-N2-p3866363	gene	exon
Bn-A02-p4777053	Bn-N2-p3872871	gene	
Bn-A02-p4779271	Bn-N2-p3875092	gene	exon
Bn-A02-p4783596	Bn-N2-p3879029	gene	
Bn-A02-p4881323	Bn-N2-p3980652	gene	
Bn-A02-p488160	Bn-N7-p13371113	gene	exon
Bn-A02-p490065	Bn-N7-p13373789	gene	exon
Bn-A02-p4928982	Bn-N2-p4014476	gene	exon
Bn-A02-p5040691	Bn-N2-p4122354	gene	exon
Bn-A02-p5063790	Bn-N12-p5375855	gene	exon
Bn-A02-p5166772	Bn-N2-p4257512	gene	exon
Bn-A02-p5201568	Bn-N2-p4286646	gene	exon
Bn-A02-p5242706	Bn-Scaffold25363-p252	gene	exon
Bn-A02-p5271340	Bn-N12-p5815167	gene	exon
Bn-A02-p5304300	Bn-N2-p4438281	gene	
Bn-A02-p5326222	Bn-N2-p4461220	gene	exon
Bn-A02-p5327209	Bn-N2-p4462207	gene	exon
Bn-A02-p5429003	Bn-N2-p4567982	gene	exon
Bn-A02-p5439746	Bn-N2-p4582902	gene	
Bn-A02-p5442618	Bn-N2-p4585736	gene	exon
Bn-A02-p545783	Bn-N7-p13444606	gene	
Bn-A02-p5485978	Bn-N2-p4642849	gene	exon
Bn-A02-p5500107	Bn-N2-p4652864	gene	
Bn-A02-p5501733	Bn-N2-p4654749	gene	
Bn-A02-p5570144	Bn-N2-p4720861	gene	exon
Bn-A02-p5571981	Bn-N2-p4722669	gene	
Bn-A02-p5582861	Bn-N2-p4733516	gene	
Bn-A02-p5592678	Bn-N2-p4755097	gene	exon
Bn-A02-p5601991	Bn-N2-p4769587	gene	exon
Bn-A02-p5602271	Bn-N2-p4769867	gene	exon
Bn-A02-p5604926	Bn-N2-p4778603	gene	
Bn-A02-p5612147	Bn-N2-p4789072	gene	exon
Bn-A02-p5628094	Bn-N2-p4807150	gene	
Bn-A02-p5628473	Bn-N2-p4807529	gene	
Bn-A02-p5628535	Bn-N2-p4807591	gene	
Bn-A02-p5640408	Bn-Scaffold01856-p3603	gene	
Bn-A02-p5664842	Bn-N2-p4841926	gene	exon
Bn-A02-p5722550	Bn-N2-p4911936	gene	
Bn-A02-p5731499	Bn-N2-p4924739	gene	
Bn-A02-p5797724	Bn-N2-p4977779	gene	exon
Bn-A02-p5812506	Bn-N2-p4989347	gene	exon
Bn-A02-p5826306	Bn-N2-p5006941	gene	
Bn-A02-p5826505	Bn-Scaffold16162-p544	gene	exon
Bn-A02-p5826541	Bn-Scaffold16162-p508	gene	exon
Bn-A02-p5832870	Bn-N2-p5019782	gene	exon
Bn-A02-p584200	Bn-N7-p13489571	gene	
Bn-A02-p5866325	Bn-N2-p5045512	gene	exon
Bn-A02-p5882799	Bn-N2-p5065792	gene	
Bn-A02-p5924483	Bn-N2-p5120600	gene	

Bn-A02-p5938385	Bn-N2-p5134791	gene	
Bn-A02-p6097441	Bn-N2-p5303980	gene	exon
Bn-A02-p6105931	Bn-N2-p5337505	gene	exon
Bn-A02-p6149358	Bn-N2-p5357074	gene	exon
Bn-A02-p615312	Bn-N7-p13513016	gene	exon
Bn-A02-p6156818	Bn-N2-p5359276	gene	
Bn-A02-p6164030	Bn-N12-p7393997	gene	exon
Bn-A02-p6233	Bn-N7-p12871398	gene	exon
Bn-A02-p6244013	Bn-N2-p5458662	gene	
Bn-A02-p624595	Bn-N7-p13523552	gene	exon
Bn-A02-p6252196	Bn-N2-p5465834	gene	exon
Bn-A02-p6265274	Bn-N2-p5483616	gene	
Bn-A02-p6267188	Bn-N2-p5485530	gene	
Bn-A02-p6267300	Bn-N2-p5485642	gene	
Bn-A02-p6277812	Bn-N2-p5496117	gene	
Bn-A02-p6285260	Bn-N12-p7645920	gene	exon
Bn-A02-p631297	Bn-N7-p13530951	gene	
Bn-A02-p6315194	Bn-N2-p5530606	gene	
Bn-A02-p636428	Bn-N7-p13536098	gene	exon
Bn-A02-p6366262	Bn-N2-p5578109	gene	exon
Bn-A02-p6373647	Bn-N2-p5592862	gene	
Bn-A02-p6375486	Bn-N2-p5598295	gene	exon
Bn-A02-p6384663	Bn-N2-p5610536	gene	exon
Bn-A02-p6391751	Bn-N2-p5618792	gene	
Bn-A02-p6392450	Bn-N2-p5619470	gene	
Bn-A02-p6399820	Bn-N2-p5627536	gene	
Bn-A02-p647024	Bn-N7-p13548076	gene	
Bn-A02-p6476748	Bn-N12-p8063604	gene	exon
Bn-A02-p647706	Bn-N16-p17927716	gene	exon
Bn-A02-p6478309	Bn-N2-p5711970	gene	
Bn-A02-p6483805	Bn-N2-p5717447	gene	exon
Bn-A02-p6500009	Bn-N12-p8122400	gene	exon
Bn-A02-p6523411	Bn-N2-p5774235	gene	exon
Bn-A02-p6523609	Bn-N2-p5774433	gene	exon
Bn-A02-p652623	Bn-N7-p13551729	gene	exon
Bn-A02-p655765	Bn-N7-p13556789	gene	
Bn-A02-p6564856	Bn-N2-p5818153	gene	exon
Bn-A02-p6564861	Bn-N2-p5818158	gene	exon
Bn-A02-p6576389	Bn-N2-p5830462	gene	
Bn-A02-p6578877	Bn-N2-p5832950	gene	
Bn-A02-p6584506	Bn-N2-p5838571	gene	exon
Bn-A02-p6627382	Bn-N2-p5881656	gene	
Bn-A02-p6633	Bn-N7-p12871798	gene	
Bn-A02-p6653554	Bn-N2-p5914507	gene	exon
Bn-A02-p6658392	Bn-N2-p5919336	gene	
Bn-A02-p6692347	Bn-N2-p5948780	gene	
Bn-A02-p6711579	Bn-N2-p5970390	gene	exon
Bn-A02-p677501	Bn-N7-p13572791	gene	exon
Bn-A02-p6794013	Bn-N2-p6042570	gene	
Bn-A02-p6813	Bn-N7-p12871978	gene	exon
Bn-A02-p6859259	Bn-N2-p6114654	gene	exon
Bn-A02-p6862619	Bn-N2-p6118405	gene	exon

Bn-A02-p6871477	Bn-N2-p6126225	gene	exon
Bn-A02-p6917044	Bn-Scaffold10158-p209	gene	exon
Bn-A02-p7029950	Bn-N2-p6305274	gene	exon
Bn-A02-p7043901	Bn-N2-p6317407	gene	exon
Bn-A02-p7147282	Bn-N2-p6425973	gene	exon
Bn-A02-p714791	Bn-N7-p13615770	gene	exon
Bn-A02-p7245587	Bn-N2-p6521847	gene	exon
Bn-A02-p7245763	Bn-N2-p6522023	gene	
Bn-A02-p7306278	Bn-N2-p6581856	gene	exon
Bn-A02-p7361744	Bn-N2-p6641973	gene	
Bn-A02-p7363071	Bn-N2-p6643287	gene	
Bn-A02-p7368167	Bn-N2-p6648450	gene	
Bn-A02-p7396400	Bn-N2-p6679169	gene	exon
Bn-A02-p7409699	Bn-N12-p9640107	gene	exon
Bn-A02-p7439205	Bn-N12-p9736465	gene	exon
Bn-A02-p7463965	Bn-N2-p6762423	gene	
Bn-A02-p7464840	Bn-N2-p6763298	gene	
Bn-A02-p7464997	Bn-N2-p6763455	gene	
Bn-A02-p7476709	Bn-N2-p6775277	gene	exon
Bn-A02-p7498192	Bn-N2-p6796960	gene	
Bn-A02-p7514338	Bn-N2-p6808561	gene	exon
Bn-A02-p7516226	Bn-N2-p6810596	gene	exon
Bn-A02-p7516732	Bn-N2-p6811103	gene	exon
Bn-A02-p7520783	Bn-N2-p6815147	gene	exon
Bn-A02-p7525472	Bn-N2-p6819958	gene	
Bn-A02-p7595911	Bn-N2-p6885350	gene	exon
Bn-A02-p7597314	Bn-N2-p6886805	gene	exon
Bn-A02-p761524	Bn-N16-p18500268	gene	
Bn-A02-p770821	Bn-N7-p13675563	gene	exon
Bn-A02-p771019	Bn-N7-p13675761	gene	exon
Bn-A02-p771313	Bn-N7-p13676055	gene	exon
Bn-A02-p771951	Bn-N7-p13676693	gene	exon
Bn-A02-p7784914	Bn-N12-p10410491	gene	
Bn-A02-p7804291	Bn-N2-p7107216	gene	
Bn-A02-p7812681	Bn-N2-p7115449	gene	
Bn-A02-p7831145	Bn-N2-p7134497	gene	exon
Bn-A02-p7840077	Bn-N2-p7145230	gene	exon
Bn-A02-p7885731	Bn-N12-p10659504	gene	exon
Bn-A02-p7886882	Bn-N2-p7192865	gene	
Bn-A02-p7970283	Bn-N2-p7269635	gene	
Bn-A02-p7976278	Bn-N2-p7275411	gene	exon
Bn-A02-p7978230	Bn-N2-p7277351	gene	exon
Bn-A02-p799930	Bn-N7-p13707097	gene	exon
Bn-A02-p806025	Bn-N7-p13712824	gene	
Bn-A02-p806949	Bn-N7-p13713751	gene	
Bn-A02-p8084362	Bn-N2-p7416013	gene	exon
Bn-A02-p8084605	Bn-N2-p7416256	gene	exon
Bn-A02-p808711	Bn-N7-p13715521	gene	
Bn-A02-p809627	Bn-N7-p13718122	gene	
Bn-A02-p8140997	Bn-N2-p7467376	gene	exon
Bn-A02-p8141909	Bn-N2-p7468288	gene	exon
Bn-A02-p8160522	Bn-N2-p7486923	gene	exon

Bn-A02-p8191099	Bn-N2-p7510888	gene	exon
Bn-A02-p8284992	Bn-N2-p7603576	gene	
Bn-A02-p8285151	Bn-N2-p7603734	gene	exon
Bn-A02-p8289331	Bn-N2-p7610024	gene	exon
Bn-A02-p8335312	Bn-N2-p7654564	gene	
Bn-A02-p839171	Bn-N7-p13760257	gene	exon
Bn-A02-p8396864	Bn-N10-p6196801	gene	
Bn-A02-p8412911	Bn-N2-p7748607	gene	
Bn-A02-p8413531	Bn-N2-p7749409	gene	
Bn-A02-p8440451	Bn-N2-p7767730	gene	
Bn-A02-p8443355	Bn-N2-p7770939	gene	
Bn-A02-p8444558	Bn-N2-p7771866	gene	
Bn-A02-p8453414	Bn-N2-p7787059	gene	exon
Bn-A02-p8453695	Bn-N2-p7787295	gene	
Bn-A02-p847638	Bn-N7-p13768733	gene	exon
Bn-A02-p8509407	Bn-N2-p7872796	gene	
Bn-A02-p8509694	Bn-N2-p7873083	gene	
Bn-A02-p852096	Bn-N7-p13776977	gene	
Bn-A02-p8527757	Bn-N2-p7894091	gene	exon
Bn-A02-p8549867	Bn-N12-p11843110	gene	
Bn-A02-p8554870	Bn-N2-p7927221	gene	exon
Bn-A02-p8567245	Bn-N2-p7932553	gene	exon
Bn-A02-p8575325	Bn-N2-p7943615	gene	
Bn-A02-p859079	Bn-N7-p13781552	gene	exon
Bn-A02-p8621264	Bn-N2-p7979682	gene	exon
Bn-A02-p8633119	Bn-N2-p7991268	gene	
Bn-A02-p8663297	Bn-N2-p8017864	gene	
Bn-A02-p8676211	Bn-N2-p8031461	gene	exon
Bn-A02-p8682290	Bn-N2-p8036705	gene	
Bn-A02-p8724443	Bn-N2-p8079259	gene	
Bn-A02-p8735305	Bn-N2-p8089962	gene	exon
Bn-A02-p8849037	Bn-N2-p8196394	gene	
Bn-A02-p8934314	Bn-N2-p8277914	gene	exon
Bn-A02-p8934537	Bn-N2-p8278210	gene	
Bn-A02-p8949465	Bn-N2-p8293065	gene	exon
Bn-A02-p8950348	Bn-N2-p8293937	gene	
Bn-A02-p8954851	Bn-N2-p8300842	gene	
Bn-A02-p8955992	Bn-N2-p8301981	gene	
Bn-A02-p8967830	Bn-N14-p3732818	gene	
Bn-A02-p9051321	Bn-N2-p8404290	gene	exon
Bn-A02-p9108719	Bn-Scaffold16068-p488	gene	
Bn-A02-p9140734	Bn-N2-p8490773	gene	exon
Bn-A02-p9143216	Bn-N2-p8493277	gene	exon
Bn-A02-p9195997	Bn-N2-p8558461	gene	exon
Bn-A02-p9206548	Bn-N8-p17886683	gene	
Bn-A02-p9225798	Bn-N2-p8575828	gene	
Bn-A02-p9231115	Bn-N2-p8581134	gene	
Bn-A02-p9254995	Bn-N2-p8600433	gene	exon
Bn-A02-p9274719	Bn-N2-p8622494	gene	
Bn-A02-p9281731	Bn-N2-p8629774	gene	
Bn-A02-p9319210	Bn-N12-p12993859	gene	exon
Bn-A02-p9341487	Bn-N2-p8691192	gene	exon

Bn-A02-p9351376	Bn-N12-p4270460	gene	
Bn-A02-p9353942	Bn-N2-p8701320	gene	exon
Bn-A02-p9361967	Bn-N2-p8709878	gene	
Bn-A02-p9415380	Bn-N2-p8777642	gene	exon
Bn-A02-p9415636	Bn-N2-p8777898	gene	exon
Bn-A02-p9415901	Bn-N2-p8778163	gene	exon
Bn-A02-p9416095	Bn-N2-p8778357	gene	exon
Bn-A02-p9420837	Bn-N2-p8785290	gene	
Bn-A02-p9422112	Bn-N2-p8786433	gene	exon
Bn-A02-p9425125	Bn-N2-p8789447	gene	exon
Bn-A02-p9434869	Bn-N12-p13164607	gene	
Bn-A02-p9464293	Bn-N2-p8815383	gene	exon
Bn-A02-p9465668	Bn-N2-p8816758	gene	exon
Bn-A02-p9480465	Bn-N2-p8831634	gene	
Bn-A02-p9513250	Bn-N2-p8867444	gene	
Bn-A02-p9526960	Bn-N2-p8884509	gene	
Bn-A02-p9607043	Bn-N2-p8936030	gene	
Bn-A02-p9622161	Bn-N2-p8955292	gene	
Bn-A02-p9643734	Bn-N2-p8982954	gene	exon
Bn-A02-p9646068	Bn-N2-p8985287	gene	
Bn-A02-p9648484	Bn-N2-p8987694	gene	
Bn-A02-p9664226	Bn-N2-p9002188	gene	exon
Bn-A02-p9719196	Bn-N12-p13555184	gene	exon
Bn-A02-p9722276	Bn-N2-p9059358	gene	exon
Bn-A02-p9829719	Bn-N2-p9203173	gene	exon
Bn-A02-p985366	Bn-N7-p13907779	gene	
Bn-A02-p9888208	Bn-N2-p9300950	gene	exon
Bn-A02-p9970625	Bn-N2-p9391132	gene	
Bn-A02-p9970848	Bn-N2-p9391355	gene	
Bn-A02-p9973775	Bn-N12-p13996795	gene	exon
Bn-A02-p9973927	Bn-N12-p13996958	gene	exon
Bn-A02-p99883	Bn-N7-p12969397	gene	exon
Bn-A02-p9990613	Bn-N2-p9406491	gene	exon
Bn-A02-p9996703	Bn-N2-p9413235	gene	
Bn-A02-p9997237	Bn-N2-p9413769	gene	exon
Bn-A03-p10008905	Bn-N13-p14663572	gene	exon
Bn-A03-p10011955	Bn-N3-p10204804	gene	
Bn-A03-p10047160	Bn-N3-p10235963	gene	exon
Bn-A03-p1004918	Bn-N3-p1022213	gene	exon
Bn-A03-p10062838	Bn-N3-p10256288	gene	exon
Bn-A03-p10076616	Bn-N3-p10270934	gene	
Bn-A03-p10081359	Bn-N13-p14764453	gene	
Bn-A03-p1008445	Bn-N3-p1025736	gene	
Bn-A03-p10084501	Bn-N3-p10278812	gene	exon
Bn-A03-p10087723	Bn-N3-p10282080	gene	
Bn-A03-p10092390	Bn-N3-p10286701	gene	
Bn-A03-p10092885	Bn-N3-p10287196	gene	
Bn-A03-p10129836	Bn-N3-p10327525	gene	exon
Bn-A03-p10130816	Bn-N3-p10328499	gene	exon
Bn-A03-p1013445	Bn-N3-p1032818	gene	exon
Bn-A03-p10218040	Bn-N13-p14933641	gene	exon
Bn-A03-p10321263	Bn-N3-p10555810	gene	

Bn-A03-p10321416	Bn-N3-p10555964	gene	exon
Bn-A03-p10350207	Bn-N3-p10585533	gene	exon
Bn-A03-p10388827	Bn-N3-p10628159	gene	
Bn-A03-p10393461	Bn-N3-p10632845	gene	exon
Bn-A03-p10400341	Bn-N3-p10639501	gene	exon
Bn-A03-p10402639	Bn-N3-p10641727	gene	exon
Bn-A03-p10464551	Bn-N3-p10704304	gene	exon
Bn-A03-p10466633	Bn-N3-p10705333	gene	
Bn-A03-p1050437	Bn-N3-p1080752	gene	exon
Bn-A03-p1050893	Bn-N3-p1081207	gene	
Bn-A03-p1051774	Bn-N3-p1082089	gene	
Bn-A03-p10533775	Bn-N3-p10779956	gene	exon
Bn-A03-p10547280	Bn-N3-p10795852	gene	exon
Bn-A03-p10562274	Bn-N3-p10812473	gene	exon
Bn-A03-p10587547	Bn-N3-p10840760	gene	
Bn-A03-p10600103	Bn-N3-p10850182	gene	
Bn-A03-p10600567	Bn-N3-p10850644	gene	
Bn-A03-p10607288	Bn-N3-p10864571	gene	
Bn-A03-p10608519	Bn-N3-p10866539	gene	
Bn-A03-p10623167	Bn-N3-p10883047	gene	exon
Bn-A03-p10649410	Bn-N3-p10913353	gene	
Bn-A03-p10664726	Bn-N3-p10936357	gene	
Bn-A03-p10676505	Bn-N3-p10945231	gene	
Bn-A03-p10692514	Bn-N3-p10961822	gene	
Bn-A03-p10714724	Bn-N3-p10980676	gene	
Bn-A03-p10732934	Bn-N3-p10999696	gene	
Bn-A03-p10749391	Bn-N3-p11020846	gene	
Bn-A03-p10761386	Bn-N3-p11032703	gene	exon
Bn-A03-p10761460	Bn-N3-p11032777	gene	exon
Bn-A03-p10794513	Bn-N3-p11072359	gene	exon
Bn-A03-p10802728	Bn-N3-p11080901	gene	
Bn-A03-p10803927	Bn-N3-p11082099	gene	exon
Bn-A03-p10823720	Bn-N3-p11103919	gene	
Bn-A03-p1084819	Bn-N3-p1107969	gene	exon
Bn-A03-p10855422	Bn-N3-p11138274	gene	exon
Bn-A03-p10862251	Bn-N3-p11145180	gene	
Bn-A03-p10883930	Bn-N3-p11171589	gene	
Bn-A03-p10900301	Bn-N3-p11185817	gene	
Bn-A03-p10900813	Bn-N3-p11186329	gene	
Bn-A03-p10912453	Bn-N3-p11195905	gene	
Bn-A03-p10912815	Bn-N3-p11197689	gene	
Bn-A03-p10928669	Bn-N3-p11206326	gene	
Bn-A03-p10928990	Bn-N3-p11206647	gene	
Bn-A03-p10958194	Bn-N3-p11239704	gene	
Bn-A03-p10967268	Bn-N3-p11247217	gene	
Bn-A03-p10993904	Bn-N3-p11276586	gene	
Bn-A03-p11051970	Bn-N3-p11346446	gene	exon
Bn-A03-p11053758	Bn-N3-p11348227	gene	exon
Bn-A03-p11053857	Bn-N3-p11348345	gene	exon
Bn-A03-p11087692	Bn-N3-p11376031	gene	exon
Bn-A03-p11138137	Bn-N13-p16498143	gene	exon
Bn-A03-p11156293	Bn-N3-p11438941	gene	

Bn-A03-p11171067	Bn-N3-p11447645	gene	exon
Bn-A03-p11177892	Bn-N3-p11458348	gene	exon
Bn-A03-p11191805	Bn-N3-p11472341	gene	
Bn-A03-p11202719	Bn-N3-p11485348	gene	exon
Bn-A03-p11217606	Bn-N3-p11500395	gene	
Bn-A03-p11218468	Bn-N3-p11501278	gene	
Bn-A03-p11232586	Bn-N3-p11515431	gene	exon
Bn-A03-p11294370	Bn-N3-p11581195	gene	exon
Bn-A03-p11294858	Bn-N3-p11581684	gene	exon
Bn-A03-p11399915	Bn-N3-p11685041	gene	
Bn-A03-p11402714	Bn-N3-p11687825	gene	exon
Bn-A03-p11418849	Bn-N3-p11699513	gene	exon
Bn-A03-p11456115	Bn-N3-p11746601	gene	
Bn-A03-p11468741	Bn-N3-p11761527	gene	exon
Bn-A03-p11505658	Bn-N3-p11789875	gene	
Bn-A03-p11517597	Bn-N3-p11803507	gene	
Bn-A03-p11520156	Bn-N3-p11806708	gene	
Bn-A03-p11578563	Bn-N3-p11866652	gene	
Bn-A03-p11613167	Bn-N3-p11925893	gene	exon
Bn-A03-p11613865	Bn-N3-p11926590	gene	
Bn-A03-p11618241	Bn-N3-p11934741	gene	exon
Bn-A03-p11621375	Bn-N3-p11937837	gene	
Bn-A03-p11623083	Bn-N3-p11939547	gene	
Bn-A03-p11624578	Bn-N3-p11941042	gene	
Bn-A03-p11626015	Bn-N3-p11942485	gene	exon
Bn-A03-p11626279	Bn-N3-p11942749	gene	
Bn-A03-p11633959	Bn-N7-p9599253	gene	
Bn-A03-p11683045	Bn-N13-p17776730	gene	
Bn-A03-p11704711	Bn-N3-p12015363	gene	exon
Bn-A03-p11708366	Bn-N3-p12019703	gene	exon
Bn-A03-p11739768	Bn-N3-p12055781	gene	exon
Bn-A03-p11744082	Bn-N3-p12061079	gene	
Bn-A03-p11751694	Bn-N3-p12075423	gene	exon
Bn-A03-p11771139	Bn-N3-p12093780	gene	
Bn-A03-p11772387	Bn-N3-p12095031	gene	exon
Bn-A03-p11785159	Bn-N3-p12115162	gene	exon
Bn-A03-p11792328	Bn-N3-p12122804	gene	exon
Bn-A03-p11798309	Bn-N3-p12129486	gene	
Bn-A03-p11822847	Bn-N3-p12151785	gene	
Bn-A03-p1194072	Bn-N3-p1196191	gene	exon
Bn-A03-p1195712	Bn-N13-p1532292	gene	exon
Bn-A03-p12008511	Bn-N3-p12346608	gene	exon
Bn-A03-p12065947	Bn-N3-p12412283	gene	
Bn-A03-p12076661	Bn-N3-p12426160	gene	exon
Bn-A03-p1218291	Bn-N3-p1220578	gene	
Bn-A03-p12186958	Bn-N3-p12537961	gene	
Bn-A03-p12219523	Bn-N3-p12575097	gene	
Bn-A03-p12279241	Bn-N3-p12629150	gene	
Bn-A03-p12279706	Bn-N3-p12629615	gene	
Bn-A03-p12296442	Bn-N3-p12643784	gene	exon
Bn-A03-p12319647	Bn-N3-p12668118	gene	
Bn-A03-p12359936	Bn-N3-p12716589	gene	

Bn-A03-p12393626	Bn-N3-p12750656	gene	
Bn-A03-p12393711	Bn-N3-p12750742	gene	
Bn-A03-p12402349	Bn-N3-p12759891	gene	exon
Bn-A03-p12411362	Bn-N3-p12781741	gene	
Bn-A03-p12426461	Bn-N3-p12802514	gene	
Bn-A03-p12453877	Bn-N3-p12830096	gene	exon
Bn-A03-p12457385	Bn-N3-p12833459	gene	exon
Bn-A03-p12457751	Bn-N3-p12833828	gene	exon
Bn-A03-p12471513	Bn-N3-p12849878	gene	exon
Bn-A03-p12477334	Bn-N3-p12856222	gene	
Bn-A03-p12518314	Bn-N3-p12898989	gene	
Bn-A03-p12541037	Bn-N3-p12920844	gene	
Bn-A03-p12541207	Bn-N3-p12921014	gene	
Bn-A03-p12541337	Bn-N3-p12921144	gene	
Bn-A03-p12623286	Bn-N3-p13010524	gene	
Bn-A03-p12631124	Bn-N3-p13019250	gene	exon
Bn-A03-p12737336	Bn-N13-p20002801	gene	exon
Bn-A03-p12820905	Bn-N3-p13217701	gene	
Bn-A03-p12826949	Bn-Scaffold01631-p5853	gene	
Bn-A03-p12859516	Bn-N3-p13257421	gene	exon
Bn-A03-p12863264	Bn-N3-p13260650	gene	exon
Bn-A03-p12863642	Bn-N3-p13261028	gene	exon
Bn-A03-p12863684	Bn-N3-p13261070	gene	exon
Bn-A03-p12869911	Bn-N3-p13261481	gene	
Bn-A03-p12932399	Bn-N3-p13323285	gene	
Bn-A03-p12932760	Bn-N3-p13323628	gene	exon
Bn-A03-p12945091	Bn-N3-p13340397	gene	
Bn-A03-p12993600	Bn-N3-p13388486	gene	
Bn-A03-p12994885	Bn-N3-p13389764	gene	
Bn-A03-p13061709	Bn-N3-p13458036	gene	exon
Bn-A03-p1308820	Bn-N3-p1322771	gene	
Bn-A03-p13107268	Bn-N3-p13501411	gene	exon
Bn-A03-p13145923	Bn-N3-p13529675	gene	exon
Bn-A03-p13169138	Bn-N3-p13558157	gene	
Bn-A03-p13169346	Bn-N3-p13558365	gene	
Bn-A03-p13216785	Bn-N3-p13605162	gene	exon
Bn-A03-p13218451	Bn-N3-p13606828	gene	
Bn-A03-p13236840	Bn-N13-p20803975	gene	
Bn-A03-p13253155	Bn-N13-p20830258	gene	exon
Bn-A03-p13254564	Bn-N13-p20831776	gene	
Bn-A03-p13286574	Bn-N3-p13645159	gene	
Bn-A03-p13287670	Bn-N3-p13646257	gene	
Bn-A03-p13288429	Bn-N3-p13647015	gene	
Bn-A03-p13291935	Bn-N3-p13650548	gene	
Bn-A03-p13302882	Bn-N13-p20897815	gene	exon
Bn-A03-p13335275	Bn-N3-p13687875	gene	
Bn-A03-p13335946	Bn-N13-p20967497	gene	exon
Bn-A03-p13365712	Bn-N3-p13707451	gene	exon
Bn-A03-p13469770	Bn-N3-p13802351	gene	
Bn-A03-p13492986	Bn-N3-p13823753	gene	exon
Bn-A03-p13502336	Bn-N3-p13834517	gene	exon
Bn-A03-p13514947	Bn-N13-p21438071	gene	exon

Bn-A03-p13523343	Bn-N3-p13853217	gene	exon
Bn-A03-p1352996	Bn-N3-p1362960	gene	
Bn-A03-p1353309	Bn-N3-p1363269	gene	
Bn-A03-p13546174	Bn-N3-p13887442	gene	exon
Bn-A03-p13567559	Bn-N3-p13920556	gene	
Bn-A03-p13605344	Bn-N3-p13961114	gene	
Bn-A03-p13610858	Bn-N3-p13966548	gene	exon
Bn-A03-p13624310	Bn-N3-p13985853	gene	
Bn-A03-p13630048	Bn-N3-p13991377	gene	
Bn-A03-p13658460	Bn-N3-p14017333	gene	exon
Bn-A03-p13678735	Bn-N13-p21744521	gene	exon
Bn-A03-p13679854	Bn-N3-p14037826	gene	exon
Bn-A03-p1373885	Bn-N13-p1752270	gene	
Bn-A03-p13773583	Bn-N3-p14130625	gene	
Bn-A03-p13777203	Bn-N3-p14134241	gene	exon
Bn-A03-p13778786	Bn-N3-p14135815	gene	
Bn-A03-p13783263	Bn-N3-p14140217	gene	exon
Bn-A03-p13802162	Bn-N3-p14145741	gene	
Bn-A03-p13810458	Bn-N13-p21976769	gene	
Bn-A03-p13814543	Bn-N13-p21981294	gene	exon
Bn-A03-p13861679	Bn-N3-p14182950	gene	
Bn-A03-p13861749	Bn-N3-p14183025	gene	
Bn-A03-p13863828	Bn-N13-p22045080	gene	
Bn-A03-p13912476	Bn-N3-p14236228	gene	exon
Bn-A03-p13996014	Bn-N3-p14323705	gene	exon
Bn-A03-p14003701	Bn-N3-p14331357	gene	exon
Bn-A03-p14009871	Bn-N3-p14338096	gene	exon
Bn-A03-p14015779	Bn-N3-p14341517	gene	
Bn-A03-p14023392	Bn-N3-p14348717	gene	exon
Bn-A03-p14024778	Bn-N3-p14350104	gene	exon
Bn-A03-p14037892	Bn-N3-p14366149	gene	exon
Bn-A03-p14041931	Bn-N3-p14371157	gene	exon
Bn-A03-p14092692	Bn-N3-p14418412	gene	
Bn-A03-p14095948	Bn-N3-p14422444	gene	
Bn-A03-p14112910	Bn-N3-p14438774	gene	
Bn-A03-p14121492	Bn-N3-p14447394	gene	exon
Bn-A03-p14127649	Bn-N3-p14453580	gene	exon
Bn-A03-p14128333	Bn-N3-p14454264	gene	exon
Bn-A03-p141681	Bn-N3-p152180	gene	exon
Bn-A03-p14201146	Bn-N3-p14535159	gene	exon
Bn-A03-p14276328	Bn-N3-p14611514	gene	
Bn-A03-p14282460	Bn-N3-p14617650	gene	exon
Bn-A03-p14290741	Bn-N3-p14626122	gene	
Bn-A03-p14291952	Bn-N3-p14627320	gene	exon
Bn-A03-p14306440	Bn-N3-p14641501	gene	exon
Bn-A03-p14310805	Bn-N3-p14645865	gene	
Bn-A03-p14353080	Bn-N3-p14687684	gene	
Bn-A03-p14355646	Bn-N3-p14688286	gene	exon
Bn-A03-p14385006	Bn-N3-p14711636	gene	
Bn-A03-p14398744	Bn-N3-p14728197	gene	
Bn-A03-p14399972	Bn-N3-p14729444	gene	
Bn-A03-p14400020	Bn-N3-p14729492	gene	

Bn-A03-p14413451	Bn-N3-p14738917	gene	
Bn-A03-p14418556	Bn-N13-p22939644	gene	
Bn-A03-p14418650	Bn-N3-p14747284	gene	exon
Bn-A03-p14423477	Bn-N3-p14752111	gene	exon
Bn-A03-p14434665	Bn-N3-p14764950	gene	
Bn-A03-p14462570	Bn-N3-p14794327	gene	exon
Bn-A03-p14472406	Bn-N3-p14804413	gene	
Bn-A03-p14473184	Bn-N3-p14805191	gene	exon
Bn-A03-p14473296	Bn-N3-p14805303	gene	
Bn-A03-p14474478	Bn-N3-p14806463	gene	
Bn-A03-p14552086	Bn-N3-p14890862	gene	
Bn-A03-p14557663	Bn-N3-p14896577	gene	exon
Bn-A03-p14577690	Bn-N3-p14922691	gene	exon
Bn-A03-p14585799	Bn-N3-p14930339	gene	exon
Bn-A03-p14586392	Bn-N3-p14931181	gene	exon
Bn-A03-p14611641	Bn-N3-p14954238	gene	
Bn-A03-p1461633	Bn-N3-p1475985	gene	
Bn-A03-p1466010	Bn-N3-p1480002	gene	
Bn-A03-p14684924	Bn-N3-p15060573	gene	exon
Bn-A03-p14685500	Bn-N3-p15061007	gene	
Bn-A03-p14710981	Bn-N3-p15077833	gene	exon
Bn-A03-p14758285	Bn-N3-p15112267	gene	exon
Bn-A03-p14811204	Bn-Scaffold01345-p10364	gene	exon
Bn-A03-p14811653	Bn-N3-p15168702	gene	exon
Bn-A03-p14848117	Bn-N3-p15196753	gene	exon
Bn-A03-p14852785	Bn-N3-p15208068	gene	
Bn-A03-p14870270	Bn-N3-p15234864	gene	exon
Bn-A03-p14870763	Bn-N3-p15235357	gene	
Bn-A03-p14871005	Bn-N13-p23681566	gene	
Bn-A03-p1488306	Bn-N3-p1499294	gene	exon
Bn-A03-p14888403	Bn-N3-p15253419	gene	exon
Bn-A03-p14903814	Bn-N3-p15262450	gene	
Bn-A03-p14949812	Bn-N3-p15309330	gene	
Bn-A03-p14962288	Bn-N3-p15323141	gene	
Bn-A03-p15002477	Bn-N3-p15359664	gene	
Bn-A03-p15004059	Bn-N3-p15361204	gene	
Bn-A03-p15015706	Bn-N3-p15373470	gene	exon
Bn-A03-p1503462	Bn-N3-p1513506	gene	exon
Bn-A03-p15038774	Bn-N3-p15403139	gene	
Bn-A03-p15044158	Bn-N3-p15409110	gene	exon
Bn-A03-p1507769	Bn-N3-p1517990	gene	
Bn-A03-p15103575	Bn-N3-p15456683	gene	exon
Bn-A03-p15139190	Bn-N3-p15493124	gene	
Bn-A03-p15139484	Bn-N3-p15493418	gene	exon
Bn-A03-p15141821	Bn-N3-p15495724	gene	exon
Bn-A03-p15237693	Bn-N3-p15595799	gene	
Bn-A03-p15240794	Bn-N3-p15598875	gene	exon
Bn-A03-p15240992	Bn-N3-p15599073	gene	exon
Bn-A03-p15245600	Bn-Scaffold01107-p16315	gene	
Bn-A03-p15300653	Bn-N3-p15667081	gene	
Bn-A03-p1532114	Bn-N3-p1543888	gene	exon
Bn-A03-p15326805	Bn-N3-p15677762	gene	

Bn-A03-p15327817	Bn-N3-p15678774	gene	exon
Bn-A03-p15327921	Bn-N3-p15678878	gene	exon
Bn-A03-p15347967	Bn-N3-p15694477	gene	exon
Bn-A03-p15349925	Bn-N3-p15696427	gene	exon
Bn-A03-p15359867	Bn-N3-p15710902	gene	
Bn-A03-p15363919	Bn-N3-p15714953	gene	
Bn-A03-p1538555	Bn-N3-p1550326	gene	
Bn-A03-p15397187	Bn-N3-p15746620	gene	exon
Bn-A03-p15404736	Bn-N3-p15754783	gene	
Bn-A03-p15406047	Bn-N3-p15759493	gene	exon
Bn-A03-p15416728	Bn-N3-p15771428	gene	exon
Bn-A03-p15417676	Bn-N13-p24436448	gene	exon
Bn-A03-p15424536	Bn-N3-p15779216	gene	
Bn-A03-p15435114	Bn-N3-p15791833	gene	exon
Bn-A03-p15435174	Bn-N3-p15791893	gene	exon
Bn-A03-p15435442	Bn-N3-p15792170	gene	
Bn-A03-p15481319	Bn-N3-p15854768	gene	exon
Bn-A03-p15481682	Bn-N3-p15855146	gene	
Bn-A03-p15496608	Bn-N3-p15876140	gene	
Bn-A03-p15496962	Bn-N3-p15876498	gene	
Bn-A03-p15546590	Bn-N3-p15918295	gene	
Bn-A03-p15576074	Bn-N3-p15954172	gene	exon
Bn-A03-p15577849	Bn-N3-p15957400	gene	
Bn-A03-p15593483	Bn-N3-p15973724	gene	exon
Bn-A03-p15605355	Bn-N3-p15980808	gene	exon
Bn-A03-p15634432	Bn-N3-p16009808	gene	exon
Bn-A03-p15675871	Bn-N13-p24799629	gene	exon
Bn-A03-p15689225	Bn-N3-p16036365	gene	
Bn-A03-p15702943	Bn-N3-p16050732	gene	exon
Bn-A03-p15704793	Bn-N3-p16052583	gene	
Bn-A03-p15704830	Bn-N3-p16052620	gene	
Bn-A03-p15705021	Bn-N3-p16052811	gene	exon
Bn-A03-p15744983	Bn-N3-p16097061	gene	exon
Bn-A03-p15766386	Bn-N3-p16118711	gene	exon
Bn-A03-p1577561	Bn-N3-p1585403	gene	exon
Bn-A03-p15776521	Bn-N3-p16129121	gene	
Bn-A03-p15776756	Bn-N3-p16129355	gene	
Bn-A03-p15818896	Bn-N3-p16176575	gene	exon
Bn-A03-p15852439	Bn-N3-p16210478	gene	exon
Bn-A03-p15871284	Bn-N3-p16228427	gene	
Bn-A03-p15875404	Bn-N3-p16232548	gene	
Bn-A03-p1588735	Bn-N3-p1596648	gene	exon
Bn-A03-p15892296	Bn-N3-p16242827	gene	
Bn-A03-p15900189	Bn-N3-p16247749	gene	
Bn-A03-p15906810	Bn-N3-p16254855	gene	
Bn-A03-p15916054	Bn-N3-p16264170	gene	exon
Bn-A03-p15923626	Bn-N13-p25175440	gene	
Bn-A03-p15954297	Bn-N3-p16309095	gene	exon
Bn-A03-p1596091	Bn-N3-p1607870	gene	exon
Bn-A03-p15979252	Bn-N3-p16334534	gene	
Bn-A03-p16000194	Bn-N13-p25301116	gene	
Bn-A03-p16017186	Bn-N3-p16376636	gene	

Bn-A03-p16038553	Bn-N3-p16401172	gene	exon
Bn-A03-p16051661	Bn-N3-p16417695	gene	exon
Bn-A03-p16055930	Bn-N3-p16422048	gene	
Bn-A03-p16056039	Bn-N13-p25412548	gene	
Bn-A03-p16056249	Bn-N3-p16422373	gene	exon
Bn-A03-p16056460	Bn-N3-p16422587	gene	exon
Bn-A03-p16056621	Bn-N3-p16422754	gene	exon
Bn-A03-p16081434	Bn-N3-p16447196	gene	
Bn-A03-p16084302	Bn-N3-p16450065	gene	
Bn-A03-p16123758	Bn-N3-p16484258	gene	
Bn-A03-p16126013	Bn-N3-p16486504	gene	
Bn-A03-p16162908	Bn-N3-p16530314	gene	exon
Bn-A03-p16167780	Bn-N11-p26752851	gene	
Bn-A03-p16170389	Bn-N7-p21285723	gene	exon
Bn-A03-p16185537	Bn-N3-p16558996	gene	
Bn-A03-p162225	Bn-N3-p173341	gene	exon
Bn-A03-p16229294	Bn-N3-p16606777	gene	exon
Bn-A03-p16269745	Bn-N3-p16649156	gene	
Bn-A03-p16275650	Bn-N3-p16651582	gene	
Bn-A03-p16278251	Bn-N3-p16653377	gene	
Bn-A03-p16284595	Bn-N3-p16660095	gene	
Bn-A03-p16303780	Bn-N3-p16680815	gene	
Bn-A03-p16342771	Bn-N3-p16718081	gene	
Bn-A03-p16371467	Bn-N3-p16745660	gene	
Bn-A03-p16378233	Bn-N3-p16753163	gene	
Bn-A03-p16379045	Bn-N3-p16753948	gene	
Bn-A03-p16380304	Bn-N3-p16755208	gene	
Bn-A03-p1638326	Bn-N3-p1664185	gene	
Bn-A03-p1639446	Bn-N3-p1665303	gene	exon
Bn-A03-p16404871	Bn-N3-p16774587	gene	exon
Bn-A03-p16405269	Bn-N3-p16774984	gene	
Bn-A03-p16409353	Bn-N3-p16781840	gene	
Bn-A03-p16422604	Bn-N3-p16798020	gene	exon
Bn-A03-p16423239	Bn-N3-p16798655	gene	
Bn-A03-p16431100	Bn-N3-p16801093	gene	exon
Bn-A03-p16437979	Bn-N3-p16805278	gene	
Bn-A03-p16444510	Bn-N3-p16812048	gene	
Bn-A03-p16481995	Bn-N3-p16847571	gene	exon
Bn-A03-p16482731	Bn-N3-p16848307	gene	exon
Bn-A03-p1652490	Bn-N3-p1683017	gene	
Bn-A03-p16530135	Bn-N3-p16901349	gene	exon
Bn-A03-p16565487	Bn-N3-p16933963	gene	exon
Bn-A03-p16607714	Bn-N3-p16961243	gene	exon
Bn-A03-p16610235	Bn-N3-p16963783	gene	exon
Bn-A03-p16611349	Bn-N3-p16964899	gene	
Bn-A03-p16613382	Bn-N3-p16966918	gene	exon
Bn-A03-p16742011	Bn-N3-p17112362	gene	
Bn-A03-p16758311	Bn-N13-p26698823	gene	exon
Bn-A03-p16759411	Bn-N3-p17131381	gene	exon
Bn-A03-p16769908	Bn-N3-p17141747	gene	exon
Bn-A03-p16811562	Bn-N3-p17184568	gene	
Bn-A03-p16812953	Bn-N3-p17185957	gene	exon

Bn-A03-p16852209	Bn-N3-p17230620	gene	
Bn-A03-p16955732	Bn-N3-p17352306	gene	exon
Bn-A03-p1698016	Bn-N3-p1740688	gene	
Bn-A03-p16981919	Bn-N3-p17381095	gene	exon
Bn-A03-p16989353	Bn-N3-p17388681	gene	
Bn-A03-p16990947	Bn-N3-p17390270	gene	
Bn-A03-p17038064	Bn-N3-p17438283	gene	
Bn-A03-p1711856	Bn-N3-p1755477	gene	exon
Bn-A03-p17125715	Bn-N13-p27358796	gene	
Bn-A03-p17139931	Bn-N3-p17542662	gene	exon
Bn-A03-p17182494	Bn-N3-p17584688	gene	
Bn-A03-p17194139	Bn-N3-p17590948	gene	exon
Bn-A03-p17207547	Bn-N3-p17607592	gene	
Bn-A03-p17213825	Bn-N3-p17614336	gene	exon
Bn-A03-p17281617	Bn-N3-p17683591	gene	
Bn-A03-p1741556	Bn-N3-p1787910	gene	exon
Bn-A03-p17532322	Bn-N3-p17889546	gene	exon
Bn-A03-p1762980	Bn-N3-p1802282	gene	exon
Bn-A03-p1777701	Bn-N3-p1824146	gene	exon
Bn-A03-p17782292	Bn-N3-p18120292	gene	exon
Bn-A03-p17790084	Bn-N3-p18124358	gene	exon
Bn-A03-p17921239	Bn-N3-p18249832	gene	exon
Bn-A03-p17926123	Bn-N3-p18254716	gene	
Bn-A03-p18014273	Bn-N13-p28957446	gene	exon
Bn-A03-p18019822	Bn-N3-p18370833	gene	exon
Bn-A03-p1805828	Bn-N3-p1857444	gene	
Bn-A03-p1813564	Bn-N3-p1869293	gene	exon
Bn-A03-p1817556	Bn-N3-p1873254	gene	exon
Bn-A03-p18183550	Bn-N3-p18538515	gene	
Bn-A03-p18198495	Bn-N3-p18559964	gene	exon
Bn-A03-p18376493	Bn-N3-p18740982	gene	exon
Bn-A03-p1839932	Bn-N13-p2372489	gene	
Bn-A03-p18405869	Bn-N3-p18771675	gene	
Bn-A03-p18461597	Bn-N3-p18828574	gene	
Bn-A03-p18461778	Bn-N3-p18828755	gene	
Bn-A03-p18462261	Bn-N3-p18829258	gene	
Bn-A03-p18468633	Bn-N3-p18835812	gene	
Bn-A03-p18471820	Bn-N3-p18839043	gene	
Bn-A03-p18570046	Bn-N3-p18949264	gene	
Bn-A03-p18594273	Bn-N3-p18978824	gene	exon
Bn-A03-p18595122	Bn-N3-p18979675	gene	exon
Bn-A03-p18606297	Bn-N3-p18990492	gene	
Bn-A03-p18618117	Bn-N3-p19000422	gene	exon
Bn-A03-p18618990	Bn-N3-p19001295	gene	exon
Bn-A03-p18638146	Bn-N3-p19022428	gene	
Bn-A03-p18692928	Bn-N3-p19078178	gene	
Bn-A03-p187017	Bn-N3-p198287	gene	exon
Bn-A03-p1871012	Bn-N3-p1958934	gene	exon
Bn-A03-p18762440	Bn-N3-p19125889	gene	exon
Bn-A03-p18762453	Bn-N3-p19125902	gene	exon
Bn-A03-p18762984	Bn-N3-p19126433	gene	exon
Bn-A03-p18765971	Bn-N3-p19135030	gene	

Bn-A03-p18766028	Bn-N3-p19135086	gene	
Bn-A03-p18768321	Bn-N3-p19142067	gene	
Bn-A03-p18770982	Bn-N3-p19144502	gene	
Bn-A03-p18775919	Bn-N3-p19147826	gene	exon
Bn-A03-p18776071	Bn-N3-p19147979	gene	
Bn-A03-p187972	Bn-N3-p199219	gene	
Bn-A03-p18807150	Bn-N3-p19184017	gene	
Bn-A03-p18824580	Bn-N3-p19198936	gene	
Bn-A03-p18834464	Bn-N3-p19214241	gene	exon
Bn-A03-p18836509	Bn-N3-p19216285	gene	exon
Bn-A03-p18848732	Bn-N3-p19225129	gene	exon
Bn-A03-p18899251	Bn-N3-p19265231	gene	
Bn-A03-p18899284	Bn-N3-p19265264	gene	
Bn-A03-p1894119	Bn-N3-p1980524	gene	
Bn-A03-p1899032	Bn-N3-p1989483	gene	
Bn-A03-p19045801	Bn-N3-p19315281	gene	
Bn-A03-p19078721	Bn-N3-p19349248	gene	
Bn-A03-p19129006	Bn-N3-p19402033	gene	exon
Bn-A03-p19132431	Bn-N3-p19405475	gene	
Bn-A03-p19147644	Bn-N3-p19431402	gene	exon
Bn-A03-p19191616	Bn-N3-p19482961	gene	
Bn-A03-p1922712	Bn-N3-p2016565	gene	
Bn-A03-p1923025	Bn-N3-p2016876	gene	
Bn-A03-p19333794	Bn-N3-p19627079	gene	
Bn-A03-p19344007	Bn-N3-p19638274	gene	exon
Bn-A03-p19389076	Bn-N3-p19683020	gene	
Bn-A03-p19441544	Bn-N3-p19736425	gene	
Bn-A03-p19441620	Bn-N3-p19736501	gene	
Bn-A03-p19442945	Bn-N3-p19737829	gene	
Bn-A03-p19462036	Bn-N3-p19752040	gene	exon
Bn-A03-p19501529	Bn-N3-p19795423	gene	
Bn-A03-p19510486	Bn-N3-p19814265	gene	
Bn-A03-p1951453	Bn-N3-p2051958	gene	
Bn-A03-p19526788	Bn-N3-p19828634	gene	
Bn-A03-p19527199	Bn-N3-p19829045	gene	
Bn-A03-p19573902	Bn-N3-p19870417	gene	
Bn-A03-p1960313	Bn-N3-p2060586	gene	exon
Bn-A03-p1960694	Bn-N3-p2060923	gene	exon
Bn-A03-p19740654	Bn-N3-p20028569	gene	exon
Bn-A03-p19751864	Bn-N13-p32798196	gene	exon
Bn-A03-p19753364	Bn-N3-p20058074	gene	
Bn-A03-p19753436	Bn-N3-p20058146	gene	exon
Bn-A03-p19753470	Bn-N3-p20058180	gene	exon
Bn-A03-p19768447	Bn-N3-p20073818	gene	
Bn-A03-p19772056	Bn-N3-p20078266	gene	
Bn-A03-p19793770	Bn-N3-p20092686	gene	exon
Bn-A03-p19798208	Bn-N3-p20097172	gene	exon
Bn-A03-p19805933	Bn-N3-p20104897	gene	exon
Bn-A03-p19806018	Bn-N3-p20104982	gene	
Bn-A03-p19832980	Bn-N3-p20133234	gene	
Bn-A03-p19850198	Bn-N3-p20157427	gene	
Bn-A03-p19868485	Bn-N3-p20178063	gene	exon

Bn-A03-p19880918	Bn-N3-p20192016	gene	exon
Bn-A03-p19896843	Bn-N13-p33240254	gene	
Bn-A03-p19897251	Bn-N3-p20214153	gene	exon
Bn-A03-p1990974	Bn-N13-p2605446	gene	exon
Bn-A03-p19925593	Bn-N3-p20240617	gene	
Bn-A03-p19928269	Bn-N3-p20244850	gene	
Bn-A03-p19953514	Bn-N3-p20259977	gene	exon
Bn-A03-p19956358	Bn-N3-p20262720	gene	
Bn-A03-p19973423	Bn-N3-p20283079	gene	exon
Bn-A03-p19974784	Bn-N3-p20284442	gene	exon
Bn-A03-p19975539	Bn-N3-p20285197	gene	exon
Bn-A03-p20000930	Bn-N3-p20301366	gene	exon
Bn-A03-p20005216	Bn-N3-p20304899	gene	exon
Bn-A03-p20007146	Bn-N3-p20306827	gene	exon
Bn-A03-p20008868	Bn-N3-p20308557	gene	exon
Bn-A03-p2003715	Bn-N3-p2098853	gene	
Bn-A03-p20044499	Bn-N3-p20342349	gene	
Bn-A03-p2004585	Bn-N3-p2099723	gene	exon
Bn-A03-p2005352	Bn-N3-p2100490	gene	exon
Bn-A03-p20080800	Bn-N3-p20374281	gene	exon
Bn-A03-p20086767	Bn-N3-p20380413	gene	exon
Bn-A03-p20100490	Bn-N3-p20394143	gene	exon
Bn-A03-p201146	Bn-N3-p213799	gene	
Bn-A03-p20114910	Bn-N3-p20408835	gene	exon
Bn-A03-p20122616	Bn-N3-p20417490	gene	
Bn-A03-p20125361	Bn-N3-p20420513	gene	
Bn-A03-p20158806	Bn-N3-p20453522	gene	exon
Bn-A03-p20180463	Bn-N3-p20469807	gene	exon
Bn-A03-p20213689	Bn-N3-p20500455	gene	exon
Bn-A03-p20225335	Bn-N3-p20518496	gene	exon
Bn-A03-p20262635	Bn-N3-p20559209	gene	
Bn-A03-p20286113	Bn-N3-p20584427	gene	
Bn-A03-p203000	Bn-N3-p215656	gene	
Bn-A03-p2030676	Bn-Scaffold10148-p525	gene	exon
Bn-A03-p20350690	Bn-N3-p20662632	gene	exon
Bn-A03-p20375108	Bn-N13-p34174138	gene	exon
Bn-A03-p20375514	Bn-N3-p20690914	gene	
Bn-A03-p20409233	Bn-N3-p20717493	gene	exon
Bn-A03-p20429794	Bn-N3-p20746570	gene	exon
Bn-A03-p20495253	Bn-N3-p20805694	gene	
Bn-A03-p20495700	Bn-N3-p20806147	gene	
Bn-A03-p20523474	Bn-N3-p20834069	gene	exon
Bn-A03-p20523585	Bn-N3-p20834180	gene	
Bn-A03-p20524035	Bn-N3-p20834644	gene	exon
Bn-A03-p20525474	Bn-N3-p20836118	gene	exon
Bn-A03-p20525591	Bn-N3-p20836235	gene	
Bn-A03-p20560569	Bn-N3-p20885422	gene	exon
Bn-A03-p20568764	Bn-N3-p20893626	gene	exon
Bn-A03-p20584351	Bn-N3-p20899843	gene	
Bn-A03-p20604949	Bn-N3-p20913968	gene	exon
Bn-A03-p20609910	Bn-N3-p20919598	gene	
Bn-A03-p20664670	Bn-N3-p20953283	gene	

Bn-A03-p20670561	Bn-N3-p20960556	gene	
Bn-A03-p20673486	Bn-N3-p20963481	gene	
Bn-A03-p2071494	Bn-N3-p2173818	gene	exon
Bn-A03-p20730830	Bn-N3-p21008614	gene	exon
Bn-A03-p2073180	Bn-N3-p2175513	gene	exon
Bn-A03-p20762376	Bn-N3-p21035723	gene	exon
Bn-A03-p207748	Bn-N3-p226168	gene	
Bn-A03-p20909567	Bn-N3-p21159284	gene	
Bn-A03-p20983297	Bn-N3-p21231831	gene	
Bn-A03-p20983398	Bn-N3-p21231932	gene	
Bn-A03-p2100226	Bn-N3-p2202584	gene	
Bn-A03-p21076805	Bn-N17-p38456629	gene	exon
Bn-A03-p21155851	Bn-N17-p38567519	gene	
Bn-A03-p21199018	Bn-N17-p38615424	gene	exon
Bn-A03-p21205471	Bn-N3-p21448155	gene	exon
Bn-A03-p21210189	Bn-N3-p21451794	gene	exon
Bn-A03-p21226758	Bn-N3-p21486750	gene	
Bn-A03-p21237671	Bn-N3-p21499493	gene	exon
Bn-A03-p21238466	Bn-N3-p21500288	gene	exon
Bn-A03-p21241827	Bn-N3-p21503657	gene	exon
Bn-A03-p21252366	Bn-N3-p21524087	gene	
Bn-A03-p21271941	Bn-N3-p21543364	gene	exon
Bn-A03-p21292773	Bn-N3-p21568955	gene	exon
Bn-A03-p2130281	Bn-N3-p2226318	gene	exon
Bn-A03-p21344477	Bn-N3-p21637085	gene	exon
Bn-A03-p21353686	Bn-N3-p21646759	gene	exon
Bn-A03-p21375969	Bn-N3-p21667099	gene	
Bn-A03-p2140542	Bn-N3-p2233968	gene	exon
Bn-A03-p21414316	Bn-N3-p21705306	gene	exon
Bn-A03-p21436299	Bn-N3-p21728055	gene	exon
Bn-A03-p21478889	Bn-N3-p21745571	gene	
Bn-A03-p21487106	Bn-N3-p21756700	gene	exon
Bn-A03-p21507834	Bn-N3-p21776664	gene	
Bn-A03-p21507915	Bn-N3-p21776583	gene	
Bn-A03-p21508594	Bn-N3-p21775904	gene	
Bn-A03-p21632587	Bn-N3-p21900335	gene	exon
Bn-A03-p21685480	Bn-N3-p21947903	gene	exon
Bn-A03-p21744993	Bn-N3-p22019268	gene	exon
Bn-A03-p21813117	Bn-N3-p22063994	gene	
Bn-A03-p21839200	Bn-N3-p22091569	gene	
Bn-A03-p21860400	Bn-N3-p22117895	gene	
Bn-A03-p21862988	Bn-N3-p22120476	gene	
Bn-A03-p21864294	Bn-N3-p22121774	gene	exon
Bn-A03-p21904842	Bn-N3-p22166287	gene	exon
Bn-A03-p21909917	Bn-N3-p22173162	gene	exon
Bn-A03-p21921653	Bn-N3-p22190031	gene	exon
Bn-A03-p21927724	Bn-N3-p22195846	gene	
Bn-A03-p21937030	Bn-N3-p22206595	gene	
Bn-A03-p21979477	Bn-N3-p22258835	gene	exon
Bn-A03-p21980336	Bn-N3-p22259691	gene	exon
Bn-A03-p22014622	Bn-N3-p22297575	gene	
Bn-A03-p2206256	Bn-N13-p2851955	gene	exon

Bn-A03-p22065637	Bn-N3-p22341029	gene	exon
Bn-A03-p22072036	Bn-N3-p22348081	gene	exon
Bn-A03-p22088201	Bn-N3-p22360867	gene	exon
Bn-A03-p22090414	Bn-N3-p22363065	gene	exon
Bn-A03-p22098429	Bn-N3-p22371208	gene	exon
Bn-A03-p22102124	Bn-N3-p22374903	gene	
Bn-A03-p22112518	Bn-N3-p22385031	gene	
Bn-A03-p22132164	Bn-N3-p22403764	gene	
Bn-A03-p22132555	Bn-N3-p22404155	gene	
Bn-A03-p22132662	Bn-N3-p22404262	gene	
Bn-A03-p22237846	Bn-N3-p22526046	gene	
Bn-A03-p22238801	Bn-N3-p22527001	gene	
Bn-A03-p22285105	Bn-N3-p22561791	gene	exon
Bn-A03-p2228813	Bn-N3-p2307358	gene	exon
Bn-A03-p22300372	Bn-N3-p22577562	gene	exon
Bn-A03-p22304555	Bn-N3-p22580370	gene	
Bn-A03-p22313737	Bn-N3-p22591655	gene	exon
Bn-A03-p22378361	Bn-N3-p22664130	gene	
Bn-A03-p22382259	Bn-N3-p22667980	gene	
Bn-A03-p22382828	Bn-N3-p22668550	gene	exon
Bn-A03-p22385937	Bn-N3-p22671584	gene	exon
Bn-A03-p22398141	Bn-N3-p22691366	gene	exon
Bn-A03-p22588041	Bn-N3-p22886076	gene	exon
Bn-A03-p22610222	Bn-N3-p22902373	gene	exon
Bn-A03-p22619997	Bn-N3-p22908167	gene	
Bn-A03-p2262214	Bn-N3-p2332621	gene	
Bn-A03-p22626240	Bn-N3-p22918251	gene	
Bn-A03-p22643433	Bn-N3-p22936404	gene	
Bn-A03-p22655575	Bn-N3-p22944155	gene	
Bn-A03-p22679507	Bn-N3-p22977266	gene	exon
Bn-A03-p2271873	Bn-N3-p2339537	gene	
Bn-A03-p22739253	Bn-N3-p23049133	gene	exon
Bn-A03-p22744766	Bn-N3-p23054682	gene	
Bn-A03-p22750972	Bn-N3-p23059076	gene	
Bn-A03-p22752812	Bn-N3-p23060917	gene	
Bn-A03-p22752871	Bn-N3-p23060976	gene	
Bn-A03-p22776355	Bn-N3-p23085025	gene	
Bn-A03-p22779259	Bn-N3-p23087925	gene	
Bn-A03-p22838495	Bn-N3-p23140811	gene	
Bn-A03-p22841929	Bn-N3-p23144235	gene	exon
Bn-A03-p22886478	Bn-N3-p23187226	gene	exon
Bn-A03-p22974798	Bn-N3-p23289690	gene	exon
Bn-A03-p22975377	Bn-N3-p23290269	gene	exon
Bn-A03-p22976259	Bn-N3-p23291222	gene	exon
Bn-A03-p22976648	Bn-N3-p23291624	gene	exon
Bn-A03-p23017354	Bn-N3-p23342059	gene	
Bn-A03-p23020431	Bn-N3-p23345136	gene	
Bn-A03-p23046592	Bn-N3-p23369171	gene	
Bn-A03-p23080487	Bn-N3-p23402061	gene	exon
Bn-A03-p23088716	Bn-N3-p23411579	gene	exon
Bn-A03-p2313208	Bn-N3-p2388820	gene	
Bn-A03-p23143134	Bn-N3-p23445947	gene	

Bn-A03-p23170401	Bn-N3-p23473792	gene	exon
Bn-A03-p23185327	Bn-N17-p41198220	gene	exon
Bn-A03-p23193342	Bn-N3-p23495929	gene	
Bn-A03-p23222657	Bn-N3-p23523310	gene	exon
Bn-A03-p23240923	Bn-N3-p23545175	gene	
Bn-A03-p23243420	Bn-N3-p23547636	gene	
Bn-A03-p23253636	Bn-N3-p23557909	gene	
Bn-A03-p23286977	Bn-N3-p23594045	gene	
Bn-A03-p23318910	Bn-N3-p23628736	gene	
Bn-A03-p23319135	Bn-N3-p23628961	gene	
Bn-A03-p2342965	Bn-N3-p2425829	gene	exon
Bn-A03-p2344066	Bn-N13-p3023239	gene	exon
Bn-A03-p235039	Bn-N3-p253211	gene	
Bn-A03-p23526967	Bn-N3-p23815659	gene	exon
Bn-A03-p23528989	Bn-N3-p23817639	gene	
Bn-A03-p23529225	Bn-N3-p23817882	gene	
Bn-A03-p23550322	Bn-N3-p23845219	gene	
Bn-A03-p23552464	Bn-N3-p23847416	gene	
Bn-A03-p23552601	Bn-N3-p23847553	gene	
Bn-A03-p23562008	Bn-N3-p23857806	gene	exon
Bn-A03-p23674889	Bn-N3-p23974210	gene	exon
Bn-A03-p23704255	Bn-N17-p41798847	gene	
Bn-A03-p23708487	Bn-N3-p24009643	gene	
Bn-A03-p23719771	Bn-N3-p24019665	gene	
Bn-A03-p23798235	Bn-N3-p24090508	gene	
Bn-A03-p23799968	Bn-N3-p24092242	gene	
Bn-A03-p23801324	Bn-N3-p24093598	gene	
Bn-A03-p23862018	Bn-N3-p24127685	gene	
Bn-A03-p23862683	Bn-N3-p24128350	gene	exon
Bn-A03-p23899	Bn-N3-p6712	gene	
Bn-A03-p23917701	Bn-N3-p24186212	gene	
Bn-A03-p23973475	Bn-N3-p24237586	gene	
Bn-A03-p23984129	Bn-N3-p24247463	gene	
Bn-A03-p23992075	Bn-N3-p24259165	gene	
Bn-A03-p24002443	Bn-N3-p24269601	gene	
Bn-A03-p24016551	Bn-N3-p24283710	gene	exon
Bn-A03-p24017311	Bn-N3-p24284469	gene	
Bn-A03-p24017859	Bn-N3-p24285016	gene	exon
Bn-A03-p24059470	Bn-N3-p24342623	gene	exon
Bn-A03-p24092986	Bn-N3-p24362517	gene	
Bn-A03-p24104930	Bn-N3-p24373519	gene	
Bn-A03-p24117554	Bn-N3-p24385535	gene	
Bn-A03-p2412263	Bn-N3-p2505310	gene	exon
Bn-A03-p24134454	Bn-N17-p42241788	gene	exon
Bn-A03-p24249333	Bn-N3-p24515175	gene	
Bn-A03-p24294571	Bn-N3-p24553962	gene	
Bn-A03-p24298999	Bn-N3-p24559224	gene	
Bn-A03-p24349898	Bn-N3-p24608085	gene	exon
Bn-A03-p24396004	Bn-N3-p24632888	gene	exon
Bn-A03-p24410521	Bn-N3-p24637392	gene	exon
Bn-A03-p24420842	Bn-N3-p24651687	gene	
Bn-A03-p24422801	Bn-N4-p19627187	gene	exon

Bn-A03-p24497262	Bn-N3-p24731628	gene	
Bn-A03-p24527313	Bn-N3-p24771219	gene	exon
Bn-A03-p24537588	Bn-N3-p24774096	gene	
Bn-A03-p2453901	Bn-N3-p2552556	gene	exon
Bn-A03-p24565421	Bn-N3-p24794707	gene	exon
Bn-A03-p2457869	Bn-N3-p2556456	gene	
Bn-A03-p2457934	Bn-N3-p2556520	gene	
Bn-A03-p24581655	Bn-N3-p24810883	gene	
Bn-A03-p24650359	Bn-N3-p24866051	gene	
Bn-A03-p2468983	Bn-N3-p2567726	gene	
Bn-A03-p24690789	Bn-N3-p24912822	gene	
Bn-A03-p24704126	Bn-N3-p24919591	gene	
Bn-A03-p24710055	Bn-N3-p24925103	gene	
Bn-A03-p24745951	Bn-N3-p24956620	gene	
Bn-A03-p24746007	Bn-N3-p24956676	gene	
Bn-A03-p24748004	Bn-N3-p24957877	gene	
Bn-A03-p24773584	Bn-N3-p24984578	gene	
Bn-A03-p24780067	Bn-N17-p42904503	gene	exon
Bn-A03-p24800655	Bn-N3-p25012870	gene	exon
Bn-A03-p24809290	Bn-N3-p25023698	gene	
Bn-A03-p24811187	Bn-N3-p25025579	gene	exon
Bn-A03-p24827646	Bn-N3-p25048900	gene	exon
Bn-A03-p24829281	Bn-N17-p42963272	gene	
Bn-A03-p2483644	Bn-N3-p2583835	gene	
Bn-A03-p24906226	Bn-N3-p25124773	gene	exon
Bn-A03-p2491346	Bn-N3-p2590934	gene	
Bn-A03-p2493541	Bn-N13-p3174052	gene	
Bn-A03-p24990059	Bn-N3-p25201832	gene	
Bn-A03-p24998976	Bn-N17-p43120499	gene	
Bn-A03-p24999716	Bn-N17-p43120844	gene	
Bn-A03-p25008908	Bn-Scaffold01419-p7069	gene	
Bn-A03-p25009736	Bn-N3-p25207170	gene	
Bn-A03-p25009919	Bn-N3-p25207353	gene	
Bn-A03-p25009925	Bn-N3-p25207359	gene	
Bn-A03-p25024647	Bn-N3-p25214769	gene	
Bn-A03-p25024749	Bn-N3-p25214871	gene	
Bn-A03-p25024928	Bn-N3-p25215047	gene	
Bn-A03-p25025360	Bn-N3-p25215488	gene	exon
Bn-A03-p25033468	Bn-N17-p43145031	gene	
Bn-A03-p25046120	Bn-N3-p25230750	gene	
Bn-A03-p25047750	Bn-N3-p25232380	gene	exon
Bn-A03-p25071259	Bn-N3-p25256225	gene	
Bn-A03-p25073363	Bn-N3-p25258404	gene	exon
Bn-A03-p25082044	Bn-N3-p25266974	gene	
Bn-A03-p2511868	Bn-N3-p2621627	gene	exon
Bn-A03-p25119771	Bn-N3-p25323805	gene	exon
Bn-A03-p2520008	Bn-N3-p2632049	gene	
Bn-A03-p25265364	Bn-N3-p25434700	gene	exon
Bn-A03-p25296332	Bn-N3-p25475054	gene	exon
Bn-A03-p25314490	Bn-N3-p25499702	gene	
Bn-A03-p25314570	Bn-N3-p25499782	gene	
Bn-A03-p2532699	Bn-N3-p2645979	gene	exon

Bn-A03-p25342507	Bn-N3-p25529747	gene	exon
Bn-A03-p25471848	Bn-N3-p25668602	gene	exon
Bn-A03-p2548187	Bn-N3-p2657992	gene	
Bn-A03-p25489551	Bn-N3-p25688788	gene	exon
Bn-A03-p25521989	Bn-N3-p25721078	gene	exon
Bn-A03-p25582691	Bn-N3-p25795613	gene	exon
Bn-A03-p25584526	Bn-N3-p25797448	gene	exon
Bn-A03-p25591818	Bn-N3-p25804790	gene	
Bn-A03-p25608893	Bn-N3-p25822853	gene	
Bn-A03-p25625732	Bn-N3-p25839627	gene	
Bn-A03-p25631316	Bn-N3-p25845246	gene	
Bn-A03-p25636300	Bn-N3-p25849712	gene	exon
Bn-A03-p25637279	Bn-N3-p25850691	gene	
Bn-A03-p25640844	Bn-N3-p25855762	gene	
Bn-A03-p25668597	Bn-N3-p25885248	gene	
Bn-A03-p25671744	Bn-Scaffold04006-p242	gene	exon
Bn-A03-p25679775	Bn-N3-p25904465	gene	
Bn-A03-p25683964	Bn-N17-p43759613	gene	
Bn-A03-p25704793	Bn-N3-p25936050	gene	exon
Bn-A03-p25708944	Bn-N3-p25951642	gene	
Bn-A03-p25718518	Bn-N3-p25965615	gene	exon
Bn-A03-p25718716	Bn-N3-p25965813	gene	exon
Bn-A03-p25751759	Bn-N3-p26001616	gene	
Bn-A03-p25756310	Bn-N3-p26003524	gene	
Bn-A03-p25779411	Bn-N3-p26020241	gene	exon
Bn-A03-p25785875	Bn-N3-p26026651	gene	exon
Bn-A03-p2579866	Bn-N3-p2679025	gene	exon
Bn-A03-p25801783	Bn-N3-p26044405	gene	
Bn-A03-p25810585	Bn-N3-p26055225	gene	exon
Bn-A03-p25819955	Bn-N3-p26062430	gene	
Bn-A03-p25826578	Bn-N3-p26072457	gene	
Bn-A03-p25827210	Bn-N3-p26073088	gene	
Bn-A03-p25827833	Bn-N3-p26073710	gene	
Bn-A03-p25828479	Bn-N3-p26074364	gene	
Bn-A03-p25841013	Bn-N3-p26086965	gene	exon
Bn-A03-p25843937	Bn-N3-p26089889	gene	exon
Bn-A03-p25849429	Bn-N3-p26092619	gene	exon
Bn-A03-p25849463	Bn-N3-p26092653	gene	
Bn-A03-p25850146	Bn-N3-p26093336	gene	exon
Bn-A03-p25851789	Bn-N3-p26094977	gene	
Bn-A03-p2596410	Bn-N3-p2695450	gene	
Bn-A03-p26001258	Bn-N3-p26239055	gene	exon
Bn-A03-p26013651	Bn-N17-p44067603	gene	exon
Bn-A03-p26014745	Bn-N3-p26249043	gene	
Bn-A03-p26021585	Bn-N3-p26255287	gene	exon
Bn-A03-p2606192	Bn-N3-p2705395	gene	
Bn-A03-p26066930	Bn-N3-p26287335	gene	
Bn-A03-p26081548	Bn-N3-p26301236	gene	
Bn-A03-p26116027	Bn-N3-p26344598	gene	exon
Bn-A03-p26117920	Bn-N3-p26346507	gene	
Bn-A03-p26131473	Bn-N3-p26354110	gene	
Bn-A03-p26132481	Bn-N3-p26355096	gene	

Bn-A03-p2616068	Bn-N3-p2715484	gene	exon
Bn-A03-p26167847	Bn-N3-p26383967	gene	exon
Bn-A03-p26167992	Bn-N3-p26384112	gene	
Bn-A03-p26168881	Bn-N3-p26385056	gene	exon
Bn-A03-p26232681	Bn-N3-p26447546	gene	exon
Bn-A03-p26272560	Bn-N3-p26505748	gene	exon
Bn-A03-p2627552	Bn-N3-p2724321	gene	
Bn-A03-p262793	Bn-N3-p288657	gene	exon
Bn-A03-p26284129	Bn-N3-p26516781	gene	exon
Bn-A03-p26284449	Bn-N3-p26517101	gene	exon
Bn-A03-p26284882	Bn-N3-p26517534	gene	
Bn-A03-p26286301	Bn-N3-p26518958	gene	
Bn-A03-p26288769	Bn-N3-p26521343	gene	exon
Bn-A03-p26319168	Bn-N3-p26584329	gene	
Bn-A03-p26359851	Bn-N3-p26621508	gene	
Bn-A03-p2638510	Bn-N3-p2735170	gene	
Bn-A03-p26405367	Bn-N3-p26663953	gene	
Bn-A03-p26406372	Bn-N3-p26664961	gene	
Bn-A03-p26437565	Bn-N3-p26692066	gene	
Bn-A03-p26437680	Bn-N3-p26692181	gene	exon
Bn-A03-p26437883	Bn-N3-p26692384	gene	
Bn-A03-p26444464	Bn-N3-p26702481	gene	exon
Bn-A03-p26546744	Bn-N17-p44634091	gene	exon
Bn-A03-p26567111	Bn-N3-p26835139	gene	exon
Bn-A03-p26603077	Bn-N3-p26873097	gene	
Bn-A03-p26604097	Bn-N3-p26874117	gene	
Bn-A03-p26604578	Bn-N3-p26874599	gene	
Bn-A03-p2662675	Bn-N13-p3373305	gene	
Bn-A03-p2665731	Bn-N3-p2765209	gene	exon
Bn-A03-p2666271	Bn-N3-p2765749	gene	exon
Bn-A03-p26740723	Bn-N3-p27032084	gene	
Bn-A03-p26756701	Bn-N17-p44816015	gene	exon
Bn-A03-p26763577	Bn-N3-p27047775	gene	
Bn-A03-p26764606	Bn-N3-p27048805	gene	
Bn-A03-p26786549	Bn-N3-p27070755	gene	
Bn-A03-p26788875	Bn-N3-p27073110	gene	
Bn-A03-p26789755	Bn-N3-p27073988	gene	
Bn-A03-p26798652	Bn-N3-p27083185	gene	exon
Bn-A03-p2683534	Bn-N3-p2782003	gene	
Bn-A03-p26848326	Bn-N3-p27158857	gene	exon
Bn-A03-p26852795	Bn-N3-p27163563	gene	exon
Bn-A03-p26931199	Bn-N3-p27246627	gene	exon
Bn-A03-p27028300	Bn-N3-p27340263	gene	
Bn-A03-p2704795	Bn-N3-p2807568	gene	
Bn-A03-p27066000	Bn-N3-p27367099	gene	exon
Bn-A03-p27101177	Bn-N3-p27401411	gene	
Bn-A03-p27104733	Bn-N3-p27404993	gene	exon
Bn-A03-p27112789	Bn-N3-p27412155	gene	exon
Bn-A03-p27156983	Bn-N3-p27503944	gene	exon
Bn-A03-p2719384	Bn-N3-p2823603	gene	
Bn-A03-p27253035	Bn-N3-p27602495	gene	exon
Bn-A03-p27253678	Bn-N3-p27603138	gene	

Bn-A03-p27269401	Bn-N3-p27642984	gene	
Bn-A03-p27288521	Bn-N3-p27649606	gene	exon
Bn-A03-p27298810	Bn-N3-p27661207	gene	
Bn-A03-p2731426	Bn-N3-p2835990	gene	exon
Bn-A03-p27337536	Bn-N3-p27696860	gene	exon
Bn-A03-p27339890	Bn-N3-p27699176	gene	
Bn-A03-p2736960	Bn-N3-p2839213	gene	exon
Bn-A03-p2737116	Bn-N3-p2839369	gene	
Bn-A03-p2737190	Bn-N3-p2839443	gene	exon
Bn-A03-p2737338	Bn-N3-p2839591	gene	exon
Bn-A03-p27385155	Bn-N3-p27755002	gene	exon
Bn-A03-p27423379	Bn-N3-p27796507	gene	exon
Bn-A03-p27438081	Bn-N3-p27810899	gene	exon
Bn-A03-p27445819	Bn-N3-p27818670	gene	exon
Bn-A03-p27454930	Bn-N3-p27826872	gene	exon
Bn-A03-p27455156	Bn-N3-p27827095	gene	exon
Bn-A03-p27489686	Bn-N3-p27861973	gene	
Bn-A03-p27541115	Bn-N3-p27914811	gene	exon
Bn-A03-p27546971	Bn-N3-p27923490	gene	
Bn-A03-p27547270	Bn-N3-p27923776	gene	
Bn-A03-p27564692	Bn-N3-p27940257	gene	
Bn-A03-p27581773	Bn-N3-p27957944	gene	
Bn-A03-p27582705	Bn-N3-p27958875	gene	
Bn-A03-p27629415	Bn-N3-p28022921	gene	
Bn-A03-p27650553	Bn-N3-p28035188	gene	exon
Bn-A03-p27665971	Bn-Scaffold23998-p141	gene	exon
Bn-A03-p27719757	Bn-N3-p28143273	gene	
Bn-A03-p27720045	Bn-N3-p28143561	gene	
Bn-A03-p27747178	Bn-N3-p28164136	gene	exon
Bn-A03-p27747229	Bn-N3-p28164187	gene	exon
Bn-A03-p27793125	Bn-N3-p28201195	gene	exon
Bn-A03-p27794041	Bn-N3-p28202111	gene	exon
Bn-A03-p27825739	Bn-N3-p28233409	gene	exon
Bn-A03-p27838177	Bn-N3-p28247743	gene	
Bn-A03-p27841728	Bn-N3-p28251286	gene	exon
Bn-A03-p27853312	Bn-N3-p28262385	gene	
Bn-A03-p27866782	Bn-N3-p28280474	gene	
Bn-A03-p2789113	Bn-N13-p3541265	gene	exon
Bn-A03-p27917455	Bn-N3-p28356341	gene	exon
Bn-A03-p2793288	Bn-N3-p2892489	gene	
Bn-A03-p27951283	Bn-N3-p28379354	gene	exon
Bn-A03-p27972408	Bn-N3-p28386690	gene	exon
Bn-A03-p27972738	Bn-N3-p28387020	gene	
Bn-A03-p28036367	Bn-N3-p28465332	gene	
Bn-A03-p28043216	Bn-N3-p28478610	gene	
Bn-A03-p28046101	Bn-N3-p28480905	gene	
Bn-A03-p28117836	Bn-N3-p28558388	gene	exon
Bn-A03-p28126287	Bn-N3-p28566839	gene	
Bn-A03-p28153736	Bn-N3-p28574150	gene	exon
Bn-A03-p28153797	Bn-N3-p28574211	gene	exon
Bn-A03-p28154053	Bn-N3-p28574467	gene	exon
Bn-A03-p28154830	Bn-N3-p28575244	gene	exon

Bn-A03-p28154970	Bn-N17-p46115361	gene	exon
Bn-A03-p28184972	Bn-N3-p28609414	gene	exon
Bn-A03-p28202050	Bn-N3-p28631422	gene	
Bn-A03-p28227026	Bn-N3-p28656504	gene	
Bn-A03-p28229360	Bn-N3-p28658838	gene	
Bn-A03-p28256169	Bn-N3-p28692893	gene	exon
Bn-A03-p28265602	Bn-N3-p28713559	gene	
Bn-A03-p28297407	Bn-N3-p28747350	gene	
Bn-A03-p28302949	Bn-N3-p28752921	gene	
Bn-A03-p2834684	Bn-N3-p2918902	gene	
Bn-A03-p28360281	Bn-N3-p28798265	gene	
Bn-A03-p28389539	Bn-Scaffold12025-p327	gene	exon
Bn-A03-p28464995	Bn-N3-p28886686	gene	exon
Bn-A03-p28469194	Bn-N3-p28890890	gene	exon
Bn-A03-p28498617	Bn-N3-p28933444	gene	exon
Bn-A03-p28498755	Bn-N3-p28933582	gene	
Bn-A03-p28499049	Bn-N3-p28933876	gene	exon
Bn-A03-p28499141	Bn-N3-p28933967	gene	
Bn-A03-p28535638	Bn-N3-p28971341	gene	
Bn-A03-p28560125	Bn-N3-p29014512	gene	exon
Bn-A03-p28560659	Bn-N17-p46458977	gene	exon
Bn-A03-p28583587	Bn-N3-p29038514	gene	exon
Bn-A03-p28583685	Bn-N3-p29038612	gene	
Bn-A03-p2858882	Bn-N3-p2940916	gene	
Bn-A03-p2858972	Bn-N3-p2941090	gene	
Bn-A03-p28698732	Bn-N17-p46544136	gene	exon
Bn-A03-p2879773	Bn-N3-p2954702	gene	exon
Bn-A03-p28808705	Bn-N3-p29254604	gene	
Bn-A03-p28847903	Bn-N3-p29286039	gene	
Bn-A03-p28873781	Bn-N1-p7410314	gene	exon
Bn-A03-p28877626	Bn-N3-p29314459	gene	
Bn-A03-p28877719	Bn-N3-p29314552	gene	
Bn-A03-p28878501	Bn-N3-p29315338	gene	
Bn-A03-p28878698	Bn-N3-p29315535	gene	
Bn-A03-p28878703	Bn-N3-p29315540	gene	
Bn-A03-p28879610	Bn-N3-p29316444	gene	exon
Bn-A03-p28882585	Bn-N3-p29319418	gene	
Bn-A03-p28883986	Bn-N3-p29320816	gene	
Bn-A03-p28924123	Bn-N3-p29356139	gene	
Bn-A03-p28926445	Bn-N3-p29358461	gene	
Bn-A03-p28928570	Bn-N3-p29360580	gene	
Bn-A03-p28948799	Bn-N3-p29379477	gene	exon
Bn-A03-p28963072	Bn-N3-p29389977	gene	exon
Bn-A03-p28977058	Bn-N3-p29404545	gene	exon
Bn-A03-p2898043	Bn-N3-p2973544	gene	exon
Bn-A03-p28982546	Bn-N3-p29410176	gene	
Bn-A03-p28995612	Bn-N17-p46747013	gene	exon
Bn-A03-p28996082	Bn-N3-p29425146	gene	
Bn-A03-p29005979	Bn-N3-p29441263	gene	
Bn-A03-p29046284	Bn-N3-p29475909	gene	
Bn-A03-p29053385	Bn-N3-p29480332	gene	
Bn-A03-p29081614	Bn-N3-p29513622	gene	exon

Bn-A03-p29198982	Bn-N3-p29679770	gene	
Bn-A03-p29269385	Bn-N3-p29720055	gene	
Bn-A03-p29271424	Bn-N3-p29722078	gene	
Bn-A03-p29271737	Bn-N3-p29722391	gene	
Bn-A03-p29274532	Bn-N3-p29726484	gene	exon
Bn-A03-p29279909	Bn-N3-p29731867	gene	
Bn-A03-p2929711	Bn-N3-p3005203	gene	
Bn-A03-p29346125	Bn-N3-p29808039	gene	exon
Bn-A03-p29364596	Bn-N3-p29824338	gene	exon
Bn-A03-p29391849	Bn-N3-p29844867	gene	
Bn-A03-p2946358	Bn-N3-p3023164	gene	exon
Bn-A03-p2948394	Bn-N3-p3025293	gene	exon
Bn-A03-p2948695	Bn-N3-p3025594	gene	exon
Bn-A03-p29564970	Bn-N3-p30007309	gene	
Bn-A03-p29565087	Bn-N3-p30007426	gene	
Bn-A03-p29566168	Bn-N3-p30008507	gene	exon
Bn-A03-p29571938	Bn-N3-p23667615	gene	
Bn-A03-p29583640	Bn-N3-p30022628	gene	
Bn-A03-p29590349	Bn-N3-p30028438	gene	exon
Bn-A03-p29598801	Bn-N3-p30037033	gene	
Bn-A03-p29629982	Bn-N3-p30096660	gene	
Bn-A03-p29632526	Bn-N3-p30099193	gene	
Bn-A03-p29656801	Bn-N3-p30118965	gene	
Bn-A03-p2971192	Bn-N3-p3047863	gene	exon
Bn-A03-p2976496	Bn-N3-p3053166	gene	
Bn-A03-p29776900	Bn-N3-p30223749	gene	
Bn-A03-p29780293	Bn-N3-p30227139	gene	
Bn-A03-p29781065	Bn-N3-p30227911	gene	
Bn-A03-p2978588	Bn-N3-p3055256	gene	
Bn-A03-p29899721	Bn-N3-p30358187	gene	
Bn-A03-p29926117	Bn-N3-p30377785	gene	
Bn-A03-p29938	Bn-N3-p14251	gene	exon
Bn-A03-p2997270	Bn-N3-p3074368	gene	
Bn-A03-p29998247	Bn-N3-p30445825	gene	
Bn-A03-p29999738	Bn-N3-p30447312	gene	
Bn-A03-p3001467	Bn-N3-p3078563	gene	
Bn-A03-p30170124	Bn-N3-p30585268	gene	exon
Bn-A03-p30243799	Bn-N3-p30665384	gene	exon
Bn-A03-p30358487	Bn-N3-p30833534	gene	exon
Bn-A03-p3036102	Bn-N3-p3110843	gene	
Bn-A03-p3036446	Bn-N3-p3111187	gene	exon
Bn-A03-p3052882	Bn-N3-p3129308	gene	exon
Bn-A03-p3053702	Bn-N3-p3130128	gene	exon
Bn-A03-p30564852	Bn-N3-p31074167	gene	
Bn-A03-p306814	Bn-N3-p301911	gene	
Bn-A03-p30693412	Bn-N3-p31246596	gene	
Bn-A03-p3073797	Bn-N3-p3156265	gene	
Bn-A03-p3084593	Bn-N3-p3167245	gene	
Bn-A03-p3084866	Bn-N3-p3167518	gene	
Bn-A03-p30873818	Bn-N3-p31430489	gene	
Bn-A03-p30873956	Bn-N3-p31430627	gene	exon
Bn-A03-p30874086	Bn-N3-p31430757	gene	

Bn-A03-p30876909	Bn-N3-p31433263	gene	
Bn-A03-p30905429	Bn-N3-p31465405	gene	
Bn-A03-p30951542	Bn-N3-p31523807	gene	
Bn-A03-p3097703	Bn-N3-p3181533	gene	
Bn-A03-p3098147	Bn-N3-p3181977	gene	exon
Bn-A03-p31078490	Bn-N3-p31716605	gene	exon
Bn-A03-p31093169	Bn-N3-p31730813	gene	
Bn-A03-p31095221	Bn-N3-p31733173	gene	
Bn-A03-p31147953	Bn-N3-p32213844	gene	
Bn-A03-p3116829	Bn-N3-p3207213	gene	
Bn-A03-p31179815	Bn-N3-p31785347	gene	
Bn-A03-p3131450	Bn-N3-p3221824	gene	exon
Bn-A03-p31546901	Bn-N1-p16898334	gene	
Bn-A03-p3156872	Bn-N13-p4106390	gene	exon
Bn-A03-p3189093	Bn-N3-p3292006	gene	
Bn-A03-p3189333	Bn-N3-p3292246	gene	
Bn-A03-p319304	Bn-N3-p336597	gene	exon
Bn-A03-p3219484	Bn-N3-p3317486	gene	exon
Bn-A03-p3272346	Bn-N3-p3357744	gene	
Bn-A03-p3273629	Bn-N3-p3359027	gene	
Bn-A03-p3319357	Bn-N3-p3406926	gene	
Bn-A03-p3381122	Bn-N13-p4396027	gene	
Bn-A03-p3392103	Bn-N3-p3477614	gene	
Bn-A03-p3507222	Bn-N3-p3596471	gene	exon
Bn-A03-p3517306	Bn-N3-p3606622	gene	
Bn-A03-p3544165	Bn-N3-p3632093	gene	
Bn-A03-p3544245	Bn-N3-p3632173	gene	
Bn-A03-p3561270	Bn-N3-p3648874	gene	exon
Bn-A03-p3571622	Bn-N13-p4633364	gene	
Bn-A03-p3571859	Bn-N13-p4633600	gene	
Bn-A03-p360880	Bn-N3-p447326	gene	exon
Bn-A03-p3698620	Bn-N3-p3778772	gene	exon
Bn-A03-p3795299	Bn-N3-p3885826	gene	
Bn-A03-p3795523	Bn-N3-p3886050	gene	
Bn-A03-p3823071	Bn-N3-p3913548	gene	
Bn-A03-p3839084	Bn-N3-p3931501	gene	
Bn-A03-p3851195	Bn-N3-p3941404	gene	
Bn-A03-p3857752	Bn-N3-p3948397	gene	exon
Bn-A03-p3862928	Bn-N3-p3954498	gene	
Bn-A03-p386542	Bn-N3-p431647	gene	exon
Bn-A03-p3969037	Bn-N3-p4057117	gene	
Bn-A03-p3969278	Bn-N3-p4065533	gene	
Bn-A03-p3984795	Bn-N3-p4079139	gene	exon
Bn-A03-p3994857	Bn-N3-p4088002	gene	exon
Bn-A03-p4008078	Bn-N3-p4100924	gene	
Bn-A03-p403559	Bn-N3-p417823	gene	exon
Bn-A03-p4100027	Bn-N3-p4188260	gene	
Bn-A03-p4116886	Bn-N3-p4210706	gene	
Bn-A03-p4138735	Bn-N3-p4244632	gene	
Bn-A03-p4167644	Bn-N3-p4287809	gene	
Bn-A03-p4180932	Bn-N13-p5989045	gene	
Bn-A03-p4183056	Bn-N3-p4303170	gene	exon

Bn-A03-p419250	Bn-N3-p397357	gene	exon
Bn-A03-p4202242	Bn-N3-p4321291	gene	exon
Bn-A03-p420642	Bn-N3-p395965	gene	exon
Bn-A03-p4232493	Bn-N3-p4352276	gene	
Bn-A03-p4235658	Bn-N3-p4355441	gene	exon
Bn-A03-p4309562	Bn-N3-p4421100	gene	
Bn-A03-p4339301	Bn-N3-p4448837	gene	
Bn-A03-p4348997	Bn-N3-p4458513	gene	
Bn-A03-p4378124	Bn-N3-p4480524	gene	exon
Bn-A03-p4382093	Bn-N3-p4484494	gene	
Bn-A03-p4399285	Bn-N3-p4500151	gene	
Bn-A03-p4407987	Bn-N3-p4510032	gene	exon
Bn-A03-p4408006	Bn-N3-p4510051	gene	exon
Bn-A03-p4408895	Bn-N3-p4510940	gene	
Bn-A03-p4409606	Bn-N3-p4511651	gene	
Bn-A03-p4409970	Bn-N3-p4512015	gene	exon
Bn-A03-p4416817	Bn-N3-p4518862	gene	exon
Bn-A03-p4425566	Bn-N3-p4531826	gene	
Bn-A03-p4425819	Bn-N3-p4532079	gene	
Bn-A03-p4498766	Bn-N3-p4593459	gene	
Bn-A03-p4548924	Bn-Scaffold20404-p489	gene	exon
Bn-A03-p4549344	Bn-N3-p4647289	gene	exon
Bn-A03-p4550270	Bn-N3-p4648215	gene	
Bn-A03-p4560576	Bn-N3-p4663300	gene	
Bn-A03-p4567578	Bn-N3-p4670615	gene	exon
Bn-A03-p4575317	Bn-N3-p4678354	gene	
Bn-A03-p4590174	Bn-N3-p4684505	gene	
Bn-A03-p4593298	Bn-N3-p4687569	gene	
Bn-A03-p4593331	Bn-N3-p4687602	gene	
Bn-A03-p4594763	Bn-N3-p4689034	gene	exon
Bn-A03-p4620459	Bn-N3-p4715690	gene	exon
Bn-A03-p4659068	Bn-N3-p4744028	gene	
Bn-A03-p4682207	Bn-N13-p6630129	gene	exon
Bn-A03-p4753001	Bn-N3-p4805875	gene	
Bn-A03-p4769653	Bn-N3-p4817421	gene	exon
Bn-A03-p4783485	Bn-N3-p4831677	gene	
Bn-A03-p478702	Bn-N3-p479111	gene	
Bn-A03-p4798485	Bn-N3-p4851110	gene	exon
Bn-A03-p4827846	Bn-N3-p4880681	gene	
Bn-A03-p4871103	Bn-N3-p4919913	gene	exon
Bn-A03-p487666	Bn-N4-p19088456	gene	
Bn-A03-p4879812	Bn-N3-p4928778	gene	exon
Bn-A03-p4909313	Bn-N3-p4959008	gene	
Bn-A03-p4909522	Bn-N3-p4959215	gene	
Bn-A03-p4916224	Bn-N3-p4964782	gene	
Bn-A03-p493766	Bn-N3-p489352	gene	
Bn-A03-p4944913	Bn-N3-p4985911	gene	exon
Bn-A03-p4989769	Bn-N3-p5023410	gene	
Bn-A03-p4992039	Bn-N3-p5025754	gene	
Bn-A03-p4997351	Bn-N3-p5030697	gene	exon
Bn-A03-p5006544	Bn-N3-p5040509	gene	exon
Bn-A03-p5025156	Bn-N3-p5059196	gene	exon

Bn-A03-p5038403	Bn-N10-p9907929	gene	
Bn-A03-p5042719	Bn-N3-p5076308	gene	exon
Bn-A03-p5072729	Bn-N3-p5106348	gene	exon
Bn-A03-p5083198	Bn-N3-p5115640	gene	
Bn-A03-p5083386	Bn-N3-p5115828	gene	
Bn-A03-p5083430	Bn-N3-p5115872	gene	
Bn-A03-p5111446	Bn-N3-p5149867	gene	exon
Bn-A03-p5115321	Bn-N3-p5153991	gene	
Bn-A03-p5153735	Bn-N3-p5185451	gene	exon
Bn-A03-p5159930	Bn-N3-p5191753	gene	exon
Bn-A03-p5160088	Bn-N3-p5191911	gene	
Bn-A03-p5163056	Bn-N3-p5194879	gene	
Bn-A03-p5182850	Bn-N3-p5216734	gene	exon
Bn-A03-p5227700	Bn-N3-p5261164	gene	exon
Bn-A03-p5230726	Bn-N3-p5264185	gene	exon
Bn-A03-p5268272	Bn-N3-p5300453	gene	exon
Bn-A03-p5273046	Bn-N3-p5306760	gene	exon
Bn-A03-p5333699	Bn-N3-p5352536	gene	
Bn-A03-p5357737	Bn-N3-p5379420	gene	exon
Bn-A03-p5374417	Bn-N3-p5394308	gene	
Bn-A03-p5380857	Bn-N3-p5400514	gene	
Bn-A03-p5408678	Bn-N13-p7615820	gene	
Bn-A03-p5423318	Bn-N3-p5447066	gene	exon
Bn-A03-p5439400	Bn-N3-p5461030	gene	exon
Bn-A03-p5455069	Bn-N3-p5476226	gene	
Bn-A03-p5471743	Bn-N3-p5493961	gene	exon
Bn-A03-p5502308	Bn-N3-p5535586	gene	exon
Bn-A03-p5503857	Bn-N3-p5537135	gene	
Bn-A03-p5523239	Bn-N3-p5558176	gene	exon
Bn-A03-p5523813	Bn-N3-p5558750	gene	exon
Bn-A03-p5523943	Bn-N3-p5558880	gene	exon
Bn-A03-p5532043	Bn-N3-p5567045	gene	exon
Bn-A03-p5564849	Bn-N3-p5599272	gene	
Bn-A03-p5578777	Bn-N3-p5607743	gene	exon
Bn-A03-p5597551	Bn-N3-p5628250	gene	
Bn-A03-p5608927	Bn-N3-p5639957	gene	
Bn-A03-p5626575	Bn-N3-p5668974	gene	exon
Bn-A03-p5626694	Bn-N3-p5669092	gene	exon
Bn-A03-p5627074	Bn-N3-p5669472	gene	exon
Bn-A03-p5627585	Bn-N3-p5669983	gene	exon
Bn-A03-p563787	Bn-N3-p548319	gene	
Bn-A03-p5649178	Bn-N3-p5682437	gene	exon
Bn-A03-p565187	Bn-N3-p549719	gene	
Bn-A03-p565363	Bn-N3-p549895	gene	
Bn-A03-p565637	Bn-N3-p550169	gene	exon
Bn-A03-p5682911	Bn-N3-p5710737	gene	exon
Bn-A03-p5694401	Bn-N3-p5723046	gene	exon
Bn-A03-p5696470	Bn-N3-p5727160	gene	
Bn-A03-p5711554	Bn-N3-p5740667	gene	exon
Bn-A03-p5746114	Bn-N3-p5784019	gene	exon
Bn-A03-p5762915	Bn-N3-p5800914	gene	exon
Bn-A03-p5770833	Bn-N13-p8100180	gene	exon

Bn-A03-p5796033	Bn-N3-p5834914	gene	
Bn-A03-p5800566	Bn-N3-p5839462	gene	exon
Bn-A03-p5814447	Bn-N3-p5854130	gene	exon
Bn-A03-p5866896	Bn-N3-p5900253	gene	exon
Bn-A03-p5903435	Bn-N3-p5931743	gene	exon
Bn-A03-p5952923	Bn-N3-p5980466	gene	exon
Bn-A03-p5984123	Bn-N3-p6020429	gene	
Bn-A03-p6036521	Bn-N3-p6077412	gene	
Bn-A03-p6039029	Bn-N3-p6079930	gene	
Bn-A03-p6057369	Bn-N3-p6097929	gene	exon
Bn-A03-p6126012	Bn-N3-p6172559	gene	exon
Bn-A03-p6157864	Bn-N3-p6205135	gene	
Bn-A03-p6158211	Bn-N3-p6205483	gene	exon
Bn-A03-p6204498	Bn-N3-p6249247	gene	
Bn-A03-p6274828	Bn-N3-p6308534	gene	
Bn-A03-p6276385	Bn-N3-p6310134	gene	exon
Bn-A03-p630979	Bn-N3-p624785	gene	
Bn-A03-p6317002	Bn-N3-p6356236	gene	
Bn-A03-p6332797	Bn-N3-p6374184	gene	exon
Bn-A03-p6404659	Bn-N3-p6461195	gene	exon
Bn-A03-p6404903	Bn-N3-p6461439	gene	
Bn-A03-p641927	Bn-N3-p643986	gene	
Bn-A03-p6423795	Bn-N3-p6478911	gene	
Bn-A03-p6424980	Bn-N3-p6480096	gene	exon
Bn-A03-p6425315	Bn-N3-p6480430	gene	
Bn-A03-p6425858	Bn-N3-p6480974	gene	
Bn-A03-p6427905	Bn-N3-p6487560	gene	exon
Bn-A03-p6428220	Bn-N3-p6487875	gene	
Bn-A03-p6439432	Bn-N3-p6497920	gene	
Bn-A03-p6484137	Bn-N3-p6522588	gene	
Bn-A03-p6498305	Bn-N3-p6535515	gene	exon
Bn-A03-p6502463	Bn-N3-p6539671	gene	exon
Bn-A03-p6514858	Bn-N3-p6554535	gene	
Bn-A03-p6522454	Bn-N3-p6560772	gene	
Bn-A03-p6528914	Bn-N3-p6568754	gene	
Bn-A03-p654363	Bn-N3-p658962	gene	
Bn-A03-p6556647	Bn-N3-p6593693	gene	exon
Bn-A03-p657593	Bn-N3-p662130	gene	
Bn-A03-p6576575	Bn-N3-p6618284	gene	
Bn-A03-p6577213	Bn-N3-p6618922	gene	
Bn-A03-p6585645	Bn-N3-p6629360	gene	
Bn-A03-p6586436	Bn-N3-p6630151	gene	
Bn-A03-p6590051	Bn-N3-p6633766	gene	exon
Bn-A03-p6646094	Bn-N3-p6682601	gene	exon
Bn-A03-p6668218	Bn-N3-p6704487	gene	exon
Bn-A03-p6668259	Bn-N3-p6704528	gene	exon
Bn-A03-p6672024	Bn-N3-p6708373	gene	exon
Bn-A03-p6677819	Bn-N3-p6710985	gene	exon
Bn-A03-p6681953	Bn-N3-p6715108	gene	exon
Bn-A03-p6708217	Bn-N3-p6736061	gene	exon
Bn-A03-p671279	Bn-N3-p671635	gene	exon
Bn-A03-p6717546	Bn-N3-p6745534	gene	exon

Bn-A03-p6717785	Bn-N3-p6745773	gene	exon
Bn-A03-p6771876	Bn-N3-p6803630	gene	
Bn-A03-p6772326	Bn-N3-p6804089	gene	
Bn-A03-p6781156	Bn-N3-p6805943	gene	
Bn-A03-p6783685	Bn-N3-p6809695	gene	
Bn-A03-p6804536	Bn-N3-p6834962	gene	
Bn-A03-p6804857	Bn-N3-p6835283	gene	
Bn-A03-p6845546	Bn-N3-p6875205	gene	exon
Bn-A03-p6907743	Bn-N18-p17531568	gene	
Bn-A03-p6984742	Bn-N3-p7005668	gene	
Bn-A03-p7007376	Bn-N3-p7039005	gene	exon
Bn-A03-p7007377	Bn-N3-p7039006	gene	exon
Bn-A03-p7011698	Bn-N3-p7044416	gene	
Bn-A03-p7011746	Bn-N3-p7044464	gene	
Bn-A03-p7012232	Bn-N3-p7044950	gene	exon
Bn-A03-p7049396	Bn-N3-p7100631	gene	
Bn-A03-p7058001	Bn-N3-p7112725	gene	
Bn-A03-p7074078	Bn-N3-p7124947	gene	exon
Bn-A03-p7075595	Bn-N3-p7126465	gene	
Bn-A03-p7079712	Bn-N3-p7130834	gene	exon
Bn-A03-p7088375	Bn-N3-p7138714	gene	
Bn-A03-p709332	Bn-N3-p713087	gene	
Bn-A03-p711408	Bn-N3-p715159	gene	
Bn-A03-p712308	Bn-N3-p716059	gene	exon
Bn-A03-p7178917	Bn-N3-p7235721	gene	exon
Bn-A03-p7180288	Bn-N3-p7237090	gene	
Bn-A03-p7186958	Bn-N3-p7242704	gene	exon
Bn-A03-p7224854	Bn-N3-p7284957	gene	
Bn-A03-p7235826	Bn-N3-p7300243	gene	
Bn-A03-p7236103	Bn-N3-p7300520	gene	
Bn-A03-p7239205	Bn-N3-p7307021	gene	exon
Bn-A03-p7239260	Bn-N3-p7307076	gene	
Bn-A03-p726812	Bn-N13-p892838	gene	exon
Bn-A03-p7281096	Bn-N3-p7343067	gene	exon
Bn-A03-p7284090	Bn-N3-p7346059	gene	exon
Bn-A03-p72933	Bn-N3-p72904	gene	
Bn-A03-p7295912	Bn-N3-p7359855	gene	exon
Bn-A03-p731558	Bn-N3-p738745	gene	exon
Bn-A03-p7366616	Bn-N3-p7435689	gene	exon
Bn-A03-p7366783	Bn-N3-p7435856	gene	exon
Bn-A03-p7382887	Bn-N3-p7452614	gene	exon
Bn-A03-p7428803	Bn-N13-p10438134	gene	exon
Bn-A03-p7445206	Bn-N3-p7511807	gene	
Bn-A03-p7460244	Bn-N3-p7525249	gene	
Bn-A03-p7467515	Bn-N3-p7531661	gene	
Bn-A03-p7482812	Bn-N3-p7546148	gene	exon
Bn-A03-p7485450	Bn-N3-p7548786	gene	exon
Bn-A03-p7491211	Bn-N3-p7550312	gene	
Bn-A03-p7493664	Bn-N3-p7552746	gene	exon
Bn-A03-p7501352	Bn-N3-p7560441	gene	
Bn-A03-p7506855	Bn-N3-p7565998	gene	
Bn-A03-p7528981	Bn-N3-p7587681	gene	exon

Bn-A03-p7543362	Bn-N3-p7601180	gene	exon
Bn-A03-p7551518	Bn-N3-p7608265	gene	exon
Bn-A03-p7555264	Bn-N3-p7612737	gene	exon
Bn-A03-p7571364	Bn-N3-p7630005	gene	exon
Bn-A03-p7575620	Bn-N3-p7634144	gene	
Bn-A03-p757592	Bn-N3-p764664	gene	
Bn-A03-p7583703	Bn-N3-p7642964	gene	exon
Bn-A03-p7613494	Bn-N3-p7669799	gene	exon
Bn-A03-p7614596	Bn-N3-p7670901	gene	
Bn-A03-p7628749	Bn-N3-p7685208	gene	
Bn-A03-p7628905	Bn-N3-p7685364	gene	
Bn-A03-p7629219	Bn-N13-p10704969	gene	
Bn-A03-p7630376	Bn-N13-p10706102	gene	exon
Bn-A03-p7630628	Bn-N3-p7687041	gene	
Bn-A03-p7631083	Bn-N3-p7687496	gene	
Bn-A03-p7631177	Bn-N3-p7687592	gene	
Bn-A03-p764274	Bn-N3-p771325	gene	
Bn-A03-p7643795	Bn-N3-p7699902	gene	exon
Bn-A03-p765077	Bn-N3-p772129	gene	exon
Bn-A03-p7652326	Bn-N3-p7708687	gene	
Bn-A03-p766322	Bn-N3-p773472	gene	exon
Bn-A03-p7675340	Bn-N3-p7731832	gene	exon
Bn-A03-p7688578	Bn-N3-p7746543	gene	exon
Bn-A03-p77099	Bn-N3-p80627	gene	
Bn-A03-p7788800	Bn-N3-p7843561	gene	
Bn-A03-p7793076	Bn-N3-p7847636	gene	exon
Bn-A03-p7799483	Bn-N3-p7854102	gene	exon
Bn-A03-p7814324	Bn-N3-p7866417	gene	
Bn-A03-p7818081	Bn-N3-p7870422	gene	exon
Bn-A03-p7818503	Bn-N3-p7870844	gene	exon
Bn-A03-p7835836	Bn-N3-p7891520	gene	
Bn-A03-p7838070	Bn-N13-p10974843	gene	
Bn-A03-p7851577	Bn-N3-p7907076	gene	exon
Bn-A03-p78559	Bn-N3-p82106	gene	
Bn-A03-p7857447	Bn-N3-p7917659	gene	exon
Bn-A03-p7870307	Bn-N3-p7929566	gene	exon
Bn-A03-p7870541	Bn-N3-p7929800	gene	exon
Bn-A03-p7873426	Bn-N3-p7932668	gene	exon
Bn-A03-p7878888	Bn-N3-p7939377	gene	
Bn-A03-p7883846	Bn-N3-p7944789	gene	exon
Bn-A03-p7897822	Bn-N3-p7961090	gene	exon
Bn-A03-p7902457	Bn-N3-p7965564	gene	
Bn-A03-p7920650	Bn-N3-p7977117	gene	exon
Bn-A03-p7943450	Bn-N3-p8003151	gene	
Bn-A03-p7983071	Bn-N13-p11170486	gene	exon
Bn-A03-p7985158	Bn-N3-p8045443	gene	exon
Bn-A03-p8008867	Bn-N3-p8069505	gene	
Bn-A03-p8038504	Bn-N3-p8102537	gene	exon
Bn-A03-p8039401	Bn-N3-p8103434	gene	exon
Bn-A03-p8043620	Bn-N3-p8107615	gene	
Bn-A03-p8070883	Bn-N3-p8129882	gene	
Bn-A03-p8080680	Bn-N3-p8137197	gene	exon

Bn-A03-p8083083	Bn-N3-p8139630	gene	exon
Bn-A03-p8095796	Bn-N3-p8156219	gene	exon
Bn-A03-p8109045	Bn-N13-p11370963	gene	exon
Bn-A03-p8112016	Bn-N13-p11373962	gene	
Bn-A03-p8122222	Bn-N3-p8178415	gene	exon
Bn-A03-p8148926	Bn-N17-p19243259	gene	exon
Bn-A03-p8158265	Bn-N3-p8201678	gene	exon
Bn-A03-p8218946	Bn-N3-p8261863	gene	exon
Bn-A03-p8279849	Bn-N3-p8324319	gene	exon
Bn-A03-p8285385	Bn-N3-p8330205	gene	
Bn-A03-p8298336	Bn-N3-p8341863	gene	exon
Bn-A03-p830116	Bn-N3-p837687	gene	exon
Bn-A03-p830487	Bn-N3-p838058	gene	exon
Bn-A03-p8308938	Bn-N3-p8353930	gene	
Bn-A03-p8314005	Bn-N3-p8359018	gene	
Bn-A03-p8347370	Bn-N3-p8402995	gene	exon
Bn-A03-p8359092	Bn-N3-p8414225	gene	exon
Bn-A03-p8360894	Bn-N13-p41521362	gene	
Bn-A03-p8372559	Bn-N3-p8424764	gene	exon
Bn-A03-p8394184	Bn-N3-p8446939	gene	
Bn-A03-p8396525	Bn-N3-p8454576	gene	
Bn-A03-p8398790	Bn-N3-p8456836	gene	
Bn-A03-p8408815	Bn-N3-p8465479	gene	exon
Bn-A03-p8467104	Bn-N3-p8551517	gene	exon
Bn-A03-p8475614	Bn-N3-p8559885	gene	exon
Bn-A03-p8479803	Bn-N3-p8564064	gene	exon
Bn-A03-p8480669	Bn-N3-p8564933	gene	exon
Bn-A03-p8497543	Bn-N3-p8582113	gene	
Bn-A03-p8559617	Bn-N3-p8645677	gene	exon
Bn-A03-p8559794	Bn-N3-p8645854	gene	exon
Bn-A03-p858910	Bn-N3-p873123	gene	
Bn-A03-p8631833	Bn-N3-p8713343	gene	
Bn-A03-p8645654	Bn-N3-p8728661	gene	exon
Bn-A03-p8663803	Bn-N3-p8747803	gene	
Bn-A03-p8681690	Bn-N3-p8762847	gene	
Bn-A03-p8733974	Bn-N3-p8831928	gene	exon
Bn-A03-p8735039	Bn-N3-p8832993	gene	exon
Bn-A03-p8745839	Bn-N3-p8846748	gene	
Bn-A03-p8823042	Bn-N3-p8950062	gene	exon
Bn-A03-p8836751	Bn-N3-p8965635	gene	exon
Bn-A03-p8842546	Bn-N3-p8971444	gene	
Bn-A03-p8881377	Bn-N3-p9011072	gene	exon
Bn-A03-p8881585	Bn-N3-p9011283	gene	
Bn-A03-p8883782	Bn-N3-p9013572	gene	
Bn-A03-p8883841	Bn-N3-p9013631	gene	
Bn-A03-p8924852	Bn-N3-p9057017	gene	
Bn-A03-p8973137	Bn-N3-p9103360	gene	
Bn-A03-p898067	Bn-N3-p910599	gene	exon
Bn-A03-p90436	Bn-N3-p102637	gene	
Bn-A03-p9093699	Bn-N3-p9226323	gene	
Bn-A03-p9098773	Bn-N3-p9230818	gene	exon
Bn-A03-p9115020	Bn-N3-p9247475	gene	

Bn-A03-p912314	Bn-N3-p924972	gene	exon
Bn-A03-p9123542	Bn-N3-p9264008	gene	exon
Bn-A03-p9148822	Bn-N3-p9302770	gene	exon
Bn-A03-p9191787	Bn-N13-p13262973	gene	
Bn-A03-p9203536	Bn-N3-p9356933	gene	exon
Bn-A03-p9203834	Bn-N3-p9357231	gene	exon
Bn-A03-p9203992	Bn-N3-p9357389	gene	exon
Bn-A03-p9203999	Bn-N3-p9357396	gene	exon
Bn-A03-p9208735	Bn-N3-p9362802	gene	
Bn-A03-p9233645	Bn-N3-p9386412	gene	exon
Bn-A03-p9243701	Bn-N3-p9397787	gene	
Bn-A03-p9244195	Bn-N3-p9398282	gene	
Bn-A03-p9250832	Bn-N3-p9405991	gene	exon
Bn-A03-p9255139	Bn-N3-p9410111	gene	
Bn-A03-p9265554	Bn-N3-p9422013	gene	exon
Bn-A03-p927517	Bn-N3-p937636	gene	exon
Bn-A03-p9304885	Bn-N13-p13508556	gene	exon
Bn-A03-p9324653	Bn-N3-p9485997	gene	
Bn-A03-p9328470	Bn-N3-p9496324	gene	exon
Bn-A03-p9338698	Bn-N3-p9507021	gene	
Bn-A03-p9383602	Bn-N13-p13601842	gene	exon
Bn-A03-p9439354	Bn-N3-p9621591	gene	
Bn-A03-p9439893	Bn-N3-p9622130	gene	
Bn-A03-p9448206	Bn-N3-p9630571	gene	exon
Bn-A03-p9449316	Bn-N3-p9631681	gene	exon
Bn-A03-p9476053	Bn-N3-p9659236	gene	exon
Bn-A03-p9485365	Bn-N3-p9671897	gene	
Bn-A03-p9497697	Bn-N3-p9679562	gene	exon
Bn-A03-p9497821	Bn-N3-p9679686	gene	exon
Bn-A03-p950999	Bn-N3-p968276	gene	
Bn-A03-p951365	Bn-N3-p968642	gene	
Bn-A03-p952091	Bn-N3-p969367	gene	
Bn-A03-p9522072	Bn-N13-p13808871	gene	exon
Bn-A03-p9537618	Bn-N3-p9707126	gene	exon
Bn-A03-p9564515	Bn-N13-p13849965	gene	
Bn-A03-p9601581	Bn-N3-p9768372	gene	
Bn-A03-p9605319	Bn-N3-p9772136	gene	exon
Bn-A03-p9613933	Bn-N3-p9778512	gene	exon
Bn-A03-p9615126	Bn-N3-p9779701	gene	exon
Bn-A03-p9638708	Bn-N3-p9810417	gene	
Bn-A03-p9638726	Bn-N3-p9810435	gene	
Bn-A03-p9651526	Bn-N3-p9829080	gene	
Bn-A03-p9657864	Bn-N3-p9835434	gene	exon
Bn-A03-p9677969	Bn-N3-p9845555	gene	
Bn-A03-p9705481	Bn-N3-p9870689	gene	exon
Bn-A03-p9706640	Bn-N3-p9871849	gene	exon
Bn-A03-p9764680	Bn-N3-p9956468	gene	
Bn-A03-p9765420	Bn-N3-p9957200	gene	
Bn-A03-p9765623	Bn-N3-p9957420	gene	
Bn-A03-p9823618	Bn-N3-p10018687	gene	exon
Bn-A03-p9825032	Bn-N3-p10020110	gene	exon
Bn-A03-p9833648	Bn-N3-p10026056	gene	exon

Bn-A03-p9839212	Bn-N3-p10031520	gene	exon
Bn-A03-p9847680	Bn-N3-p10040187	gene	exon
Bn-A03-p9863138	Bn-N3-p10056625	gene	
Bn-A03-p9863553	Bn-N3-p10057039	gene	exon
Bn-A03-p9863600	Bn-N3-p10057086	gene	
Bn-A03-p9873327	Bn-N3-p10066296	gene	exon
Bn-A03-p9873384	Bn-N3-p10066353	gene	exon
Bn-A03-p9876378	Bn-N3-p10069357	gene	exon
Bn-A03-p9883544	Bn-N3-p10076547	gene	
Bn-A03-p9920847	Bn-N3-p10103147	gene	exon
Bn-A04-p1001237	Bn-N4-p932584	gene	
Bn-A04-p1007534	Bn-N4-p942388	gene	
Bn-A04-p10099096	Bn-N4-p12112331	gene	exon
Bn-A04-p10129538	Bn-N4-p12141458	gene	exon
Bn-A04-p10154400	Bn-N4-p12164658	gene	exon
Bn-A04-p10155907	Bn-N4-p12166165	gene	exon
Bn-A04-p10176387	Bn-N4-p12196262	gene	
Bn-A04-p1018807	Bn-N4-p954367	gene	
Bn-A04-p10196289	Bn-N4-p12216738	gene	exon
Bn-A04-p1021879	Bn-N4-p957845	gene	exon
Bn-A04-p10261874	Bn-N4-p12283635	gene	exon
Bn-A04-p1032336	Bn-N4-p965926	gene	
Bn-A04-p10383434	Bn-N4-p12637478	gene	exon
Bn-A04-p1040916	Bn-N4-p975439	gene	exon
Bn-A04-p1042273	Bn-N4-p975963	gene	exon
Bn-A04-p1043111	Bn-N4-p976801	gene	exon
Bn-A04-p10434805	Bn-N4-p12697821	gene	
Bn-A04-p10458946	Bn-N4-p12731491	gene	
Bn-A04-p10464942	Bn-N4-p12728711	gene	exon
Bn-A04-p10494765	Bn-N4-p12772709	gene	exon
Bn-A04-p10541268	Bn-N4-p12819154	gene	
Bn-A04-p10566018	Bn-N4-p12842304	gene	exon
Bn-A04-p10600455	Bn-N4-p12872786	gene	exon
Bn-A04-p10602815	Bn-N14-p43786251	gene	exon
Bn-A04-p1065480	Bn-N4-p1006422	gene	exon
Bn-A04-p10661890	Bn-N4-p12928183	gene	
Bn-A04-p10661931	Bn-N4-p12928224	gene	
Bn-A04-p10662404	Bn-N4-p12928696	gene	
Bn-A04-p10744003	Bn-N4-p13003736	gene	
Bn-A04-p10760352	Bn-N4-p13017710	gene	exon
Bn-A04-p10769811	Bn-N4-p13027215	gene	
Bn-A04-p10774641	Bn-N4-p13032331	gene	exon
Bn-A04-p10786704	Bn-N4-p13035554	gene	
Bn-A04-p10808805	Bn-N4-p13064596	gene	exon
Bn-A04-p10809637	Bn-N4-p13065428	gene	
Bn-A04-p10852032	Bn-N4-p13137303	gene	
Bn-A04-p10902724	Bn-N4-p13187409	gene	exon
Bn-A04-p10903353	Bn-N14-p44379751	gene	
Bn-A04-p10914108	Bn-N4-p13200712	gene	
Bn-A04-p1091727	Bn-N4-p1036358	gene	
Bn-A04-p10975832	Bn-N4-p13271446	gene	
Bn-A04-p11015443	Bn-N4-p13326516	gene	

Bn-A04-p11020444	Bn-N4-p13331211	gene	
Bn-A04-p1104385	Bn-N4-p1053356	gene	
Bn-A04-p11058870	Bn-N4-p13372031	gene	
Bn-A04-p11119216	Bn-N4-p13412903	gene	
Bn-A04-p11119582	Bn-N4-p13413271	gene	
Bn-A04-p11164883	Bn-N4-p13449277	gene	
Bn-A04-p11197346	Bn-N4-p13491897	gene	
Bn-A04-p11197707	Bn-N4-p13492258	gene	exon
Bn-A04-p11209663	Bn-N4-p13515177	gene	exon
Bn-A04-p11209773	Bn-N4-p13515286	gene	
Bn-A04-p11209827	Bn-N4-p13515340	gene	exon
Bn-A04-p11213108	Bn-N4-p13518558	gene	
Bn-A04-p11243983	Bn-N4-p13553512	gene	
Bn-A04-p11243994	Bn-N4-p13553523	gene	
Bn-A04-p11244643	Bn-N4-p13554202	gene	exon
Bn-A04-p11244673	Bn-N4-p13554232	gene	exon
Bn-A04-p11245906	Bn-N4-p13555465	gene	exon
Bn-A04-p11250035	Bn-N4-p13559742	gene	exon
Bn-A04-p11250141	Bn-N4-p13559848	gene	
Bn-A04-p11274255	Bn-N4-p13575212	gene	
Bn-A04-p11291596	Bn-N4-p13584873	gene	
Bn-A04-p11319703	Bn-N4-p13621425	gene	
Bn-A04-p11320045	Bn-N4-p13621735	gene	exon
Bn-A04-p11329756	Bn-N4-p13636377	gene	
Bn-A04-p11376488	Bn-N4-p13688766	gene	exon
Bn-A04-p11377702	Bn-N4-p13690012	gene	exon
Bn-A04-p11377775	Bn-N4-p13690084	gene	exon
Bn-A04-p11402649	Bn-N4-p13716359	gene	
Bn-A04-p11420466	Bn-N4-p13744208	gene	
Bn-A04-p11425304	Bn-N4-p13749037	gene	exon
Bn-A04-p11449464	Bn-N4-p13775526	gene	exon
Bn-A04-p11450339	Bn-N4-p13776401	gene	exon
Bn-A04-p11503303	Bn-N4-p13828317	gene	exon
Bn-A04-p11509775	Bn-N4-p13835033	gene	
Bn-A04-p11510797	Bn-N4-p13836054	gene	
Bn-A04-p11512379	Bn-N4-p13837635	gene	exon
Bn-A04-p11513352	Bn-N4-p13838707	gene	exon
Bn-A04-p11595428	Bn-N14-p45517823	gene	exon
Bn-A04-p1159662	Bn-N4-p1093634	gene	exon
Bn-A04-p1161563	Bn-N4-p1095696	gene	exon
Bn-A04-p11633209	Bn-N4-p13967615	gene	
Bn-A04-p11633439	Bn-N4-p13967845	gene	
Bn-A04-p11791366	Bn-N4-p14083506	gene	exon
Bn-A04-p11810574	Bn-N4-p14106202	gene	exon
Bn-A04-p11818064	Bn-N4-p14114079	gene	
Bn-A04-p11818085	Bn-N4-p14114100	gene	
Bn-A04-p11865854	Bn-N4-p14170993	gene	exon
Bn-A04-p11888044	Bn-N4-p14211428	gene	
Bn-A04-p11896581	Bn-N4-p14225042	gene	exon
Bn-A04-p11896696	Bn-N4-p14225154	gene	exon
Bn-A04-p11897079	Bn-N4-p14225546	gene	exon
Bn-A04-p11900571	Bn-N4-p14229076	gene	

Bn-A04-p11907565	Bn-N4-p14236714	gene	
Bn-A04-p11922910	Bn-N4-p14244731	gene	
Bn-A04-p11923310	Bn-N4-p14245131	gene	exon
Bn-A04-p11923511	Bn-N4-p14245332	gene	
Bn-A04-p11924219	Bn-N4-p14246038	gene	
Bn-A04-p11957518	Bn-N4-p14285273	gene	exon
Bn-A04-p11958047	Bn-N4-p14285802	gene	exon
Bn-A04-p11961175	Bn-N4-p14293805	gene	exon
Bn-A04-p12051277	Bn-N4-p14385352	gene	
Bn-A04-p12084855	Bn-N4-p14424587	gene	exon
Bn-A04-p12087644	Bn-N4-p14427376	gene	exon
Bn-A04-p1212589	Bn-N4-p1147464	gene	exon
Bn-A04-p1213196	Bn-N4-p1148070	gene	
Bn-A04-p1215863	Bn-N4-p1150735	gene	exon
Bn-A04-p12163578	Bn-N4-p14496571	gene	
Bn-A04-p12163967	Bn-N4-p14496966	gene	exon
Bn-A04-p12204775	Bn-N4-p14540890	gene	
Bn-A04-p12205378	Bn-N4-p14541493	gene	
Bn-A04-p12210199	Bn-N4-p14546314	gene	exon
Bn-A04-p12211993	Bn-N4-p14548108	gene	
Bn-A04-p122280	Bn-N4-p193375	gene	exon
Bn-A04-p12243311	Bn-N4-p14580040	gene	
Bn-A04-p12259499	Bn-N4-p14596496	gene	exon
Bn-A04-p12263920	Bn-N4-p14600964	gene	exon
Bn-A04-p1231047	Bn-N4-p1164595	gene	exon
Bn-A04-p1231127	Bn-N4-p1164675	gene	
Bn-A04-p12314901	Bn-N4-p14652564	gene	
Bn-A04-p12314909	Bn-N4-p14652572	gene	
Bn-A04-p12509027	Bn-N10-p4733803	gene	
Bn-A04-p12526741	Bn-N10-p4716216	gene	exon
Bn-A04-p12541660	Bn-N10-p4688367	gene	
Bn-A04-p12542184	Bn-N10-p4687831	gene	
Bn-A04-p12547910	Bn-N10-p4676521	gene	
Bn-A04-p12550756	Bn-N10-p4673693	gene	
Bn-A04-p12551118	Bn-N10-p4673331	gene	
Bn-A04-p12599627	Bn-N10-p4608812	gene	
Bn-A04-p12599843	Bn-N10-p4608596	gene	
Bn-A04-p12635986	Bn-N10-p4575735	gene	
Bn-A04-p12659254	Bn-N4-p14726255	gene	
Bn-A04-p12659402	Bn-N4-p14726403	gene	exon
Bn-A04-p12671369	Bn-N4-p14747087	gene	
Bn-A04-p12675088	Bn-N4-p14751019	gene	
Bn-A04-p12677961	Bn-N4-p14753696	gene	
Bn-A04-p12680156	Bn-N4-p14758361	gene	
Bn-A04-p1276308	Bn-N4-p1215871	gene	
Bn-A04-p12793333	Bn-N4-p14849520	gene	
Bn-A04-p12793456	Bn-N4-p14849643	gene	exon
Bn-A04-p12793487	Bn-N4-p14849674	gene	exon
Bn-A04-p1279752	Bn-N4-p1219316	gene	
Bn-A04-p12798976	Bn-N4-p14855183	gene	
Bn-A04-p12840183	Bn-N4-p14892410	gene	
Bn-A04-p12877134	Bn-N4-p14930735	gene	

Bn-A04-p12888388	Bn-N4-p14942933	gene	exon
Bn-A04-p12917949	Bn-N4-p14969070	gene	exon
Bn-A04-p12953418	Bn-N4-p14998960	gene	exon
Bn-A04-p12959668	Bn-N4-p15005514	gene	
Bn-A04-p12994676	Bn-N4-p15036643	gene	exon
Bn-A04-p13009893	Bn-N4-p15054322	gene	exon
Bn-A04-p13050240	Bn-N4-p15092086	gene	
Bn-A04-p13052013	Bn-N4-p15093867	gene	
Bn-A04-p13056296	Bn-N4-p15102871	gene	
Bn-A04-p13056382	Bn-N4-p15102956	gene	
Bn-A04-p13056438	Bn-N4-p15103012	gene	
Bn-A04-p13059485	Bn-N4-p15106045	gene	
Bn-A04-p13069741	Bn-N4-p15116045	gene	exon
Bn-A04-p13078078	Bn-N4-p15122228	gene	
Bn-A04-p1311487	Bn-N4-p1247303	gene	
Bn-A04-p1311567	Bn-N4-p1247383	gene	
Bn-A04-p13116453	Bn-N4-p15199773	gene	exon
Bn-A04-p13122589	Bn-N4-p15205829	gene	
Bn-A04-p13129306	Bn-N4-p15213405	gene	
Bn-A04-p13129601	Bn-N4-p15213699	gene	
Bn-A04-p13129741	Bn-N4-p15213841	gene	
Bn-A04-p13145539	Bn-N4-p15233023	gene	exon
Bn-A04-p1317446	Bn-N4-p1252454	gene	exon
Bn-A04-p1318613	Bn-N4-p1253627	gene	
Bn-A04-p13206432	Bn-N4-p15287831	gene	
Bn-A04-p13207715	Bn-N4-p15289091	gene	exon
Bn-A04-p13208442	Bn-N4-p15289638	gene	exon
Bn-A04-p13235708	Bn-N4-p15316208	gene	
Bn-A04-p13241959	Bn-N4-p15322956	gene	exon
Bn-A04-p13247108	Bn-N4-p15329791	gene	
Bn-A04-p13293545	Bn-N4-p15352692	gene	exon
Bn-A04-p13293640	Bn-N4-p15352787	gene	exon
Bn-A04-p13294322	Bn-N4-p15353469	gene	exon
Bn-A04-p1330321	Bn-N4-p1271987	gene	exon
Bn-A04-p13306369	Bn-N4-p15377894	gene	
Bn-A04-p13336924	Bn-N4-p15405333	gene	
Bn-A04-p13370433	Bn-N4-p15437344	gene	
Bn-A04-p13384606	Bn-N4-p15444121	gene	exon
Bn-A04-p13389366	Bn-N4-p15444250	gene	exon
Bn-A04-p13389622	Bn-N4-p15444506	gene	exon
Bn-A04-p13394479	Bn-N4-p15449389	gene	exon
Bn-A04-p13418563	Bn-N4-p15477342	gene	
Bn-A04-p1346738	Bn-N4-p1274801	gene	
Bn-A04-p13490747	Bn-N4-p15520771	gene	exon
Bn-A04-p1349255	Bn-N4-p1277334	gene	exon
Bn-A04-p13497608	Bn-N4-p15527167	gene	
Bn-A04-p13521405	Bn-N4-p15548838	gene	exon
Bn-A04-p1353175	Bn-N4-p1282151	gene	
Bn-A04-p1353238	Bn-N4-p1282214	gene	
Bn-A04-p13532829	Bn-N4-p15554194	gene	
Bn-A04-p13552122	Bn-N4-p15580400	gene	exon
Bn-A04-p1355502	Bn-N4-p1284481	gene	exon

Bn-A04-p13562332	Bn-N4-p15592185	gene	
Bn-A04-p13583325	Bn-N4-p15605130	gene	exon
Bn-A04-p13589498	Bn-N4-p15608137	gene	
Bn-A04-p13600415	Bn-N4-p15622445	gene	exon
Bn-A04-p13655958	Bn-N4-p15674600	gene	
Bn-A04-p13665489	Bn-N4-p15684044	gene	exon
Bn-A04-p13685561	Bn-N4-p15701910	gene	
Bn-A04-p13689816	Bn-N4-p15707430	gene	
Bn-A04-p13705410	Bn-N4-p15714656	gene	
Bn-A04-p13705548	Bn-N4-p15714794	gene	
Bn-A04-p13705636	Bn-N4-p15714880	gene	
Bn-A04-p13705760	Bn-N4-p15715005	gene	
Bn-A04-p13726056	Bn-N4-p15738093	gene	exon
Bn-A04-p13856134	Bn-N4-p15881188	gene	exon
Bn-A04-p1390449	Bn-N4-p1324713	gene	exon
Bn-A04-p13992889	Bn-N4-p15997563	gene	
Bn-A04-p14020738	Bn-N4-p16025942	gene	
Bn-A04-p14020855	Bn-N4-p16026059	gene	
Bn-A04-p14023419	Bn-N4-p16028620	gene	exon
Bn-A04-p14026143	Bn-N4-p16031244	gene	
Bn-A04-p14060096	Bn-N4-p16068662	gene	exon
Bn-A04-p14092385	Bn-N4-p16096135	gene	
Bn-A04-p14156506	Bn-N14-p49045771	gene	
Bn-A04-p14159293	Bn-N4-p16160739	gene	exon
Bn-A04-p14159494	Bn-N4-p16160940	gene	exon
Bn-A04-p1416287	Bn-N4-p1354082	gene	
Bn-A04-p1417481	Bn-N4-p1355252	gene	
Bn-A04-p14289058	Bn-N4-p16277785	gene	exon
Bn-A04-p1429850	Bn-N4-p1370880	gene	
Bn-A04-p14312019	Bn-N4-p16304417	gene	exon
Bn-A04-p14315336	Bn-N4-p16307392	gene	
Bn-A04-p14332759	Bn-N4-p16322233	gene	exon
Bn-A04-p14353463	Bn-N4-p16341393	gene	exon
Bn-A04-p14360385	Bn-N4-p16350269	gene	
Bn-A04-p14360799	Bn-N4-p16350682	gene	
Bn-A04-p14380140	Bn-N4-p16378096	gene	
Bn-A04-p14397957	Bn-N4-p16401198	gene	
Bn-A04-p14398335	Bn-N4-p16401576	gene	exon
Bn-A04-p14478925	Bn-N4-p16451168	gene	
Bn-A04-p14479867	Bn-N4-p16452111	gene	exon
Bn-A04-p1451594	Bn-N4-p1389366	gene	
Bn-A04-p14532353	Bn-N4-p16495684	gene	
Bn-A04-p1454434	Bn-N4-p1392198	gene	
Bn-A04-p14563007	Bn-N14-p49700767	gene	exon
Bn-A04-p14572391	Bn-N14-p49710064	gene	
Bn-A04-p14582431	Bn-N4-p16548395	gene	exon
Bn-A04-p14582584	Bn-N4-p16548548	gene	exon
Bn-A04-p14588183	Bn-N4-p16554984	gene	exon
Bn-A04-p14622218	Bn-N4-p16573987	gene	exon
Bn-A04-p14630533	Bn-N14-p49822368	gene	exon
Bn-A04-p14630759	Bn-N14-p49822587	gene	exon
Bn-A04-p14631343	Bn-N14-p49823213	gene	

Bn-A04-p14631586	Bn-N4-p16585003	gene	
Bn-A04-p14640007	Bn-N4-p16594072	gene	exon
Bn-A04-p14642152	Bn-N4-p16596219	gene	exon
Bn-A04-p14661285	Bn-N4-p16617010	gene	exon
Bn-A04-p14662924	Bn-N4-p16618649	gene	
Bn-A04-p14670992	Bn-N4-p16626708	gene	exon
Bn-A04-p14673783	Bn-N4-p16627428	gene	exon
Bn-A04-p14687930	Bn-N4-p16642399	gene	
Bn-A04-p1471607	Bn-N4-p1412969	gene	exon
Bn-A04-p1471982	Bn-N4-p1413344	gene	exon
Bn-A04-p1472416	Bn-N4-p1413778	gene	exon
Bn-A04-p1472739	Bn-N4-p1414101	gene	exon
Bn-A04-p14742779	Bn-N4-p16697370	gene	
Bn-A04-p14807002	Bn-N14-p50171536	gene	
Bn-A04-p14807150	Bn-N14-p50171714	gene	exon
Bn-A04-p14807561	Bn-N4-p16760524	gene	exon
Bn-A04-p14815251	Bn-N4-p16768147	gene	exon
Bn-A04-p14822521	Bn-N4-p16777601	gene	exon
Bn-A04-p14829686	Bn-N4-p16792438	gene	exon
Bn-A04-p14877869	Bn-N4-p16852137	gene	exon
Bn-A04-p1490485	Bn-N4-p1439997	gene	
Bn-A04-p14917114	Bn-N4-p16884111	gene	
Bn-A04-p14922562	Bn-N4-p16889546	gene	exon
Bn-A04-p14923168	Bn-N4-p16890152	gene	exon
Bn-A04-p14972825	Bn-N4-p16942573	gene	
Bn-A04-p149787	Bn-N14-p25516698	gene	exon
Bn-A04-p14986920	Bn-N14-p50454869	gene	
Bn-A04-p14987934	Bn-N4-p16954493	gene	exon
Bn-A04-p14988304	Bn-N4-p16954861	gene	exon
Bn-A04-p14999714	Bn-N4-p16963218	gene	
Bn-A04-p14999768	Bn-N4-p16963272	gene	
Bn-A04-p15001710	Bn-N4-p16963400	gene	
Bn-A04-p15030355	Bn-N4-p16985302	gene	exon
Bn-A04-p15030899	Bn-N4-p16985846	gene	exon
Bn-A04-p15030963	Bn-N4-p16985910	gene	exon
Bn-A04-p15048204	Bn-N4-p17002317	gene	exon
Bn-A04-p15108913	Bn-N4-p17051397	gene	exon
Bn-A04-p15122412	Bn-Scaffold00208b-p36326	gene	
Bn-A04-p15150564	Bn-Scaffold00208b-p15947	gene	exon
Bn-A04-p15152016	Bn-N4-p17082281	gene	exon
Bn-A04-p15164920	Bn-N4-p17088020	gene	
Bn-A04-p15168889	Bn-Scaffold03862-p1699	gene	exon
Bn-A04-p15195920	Bn-N4-p17137351	gene	
Bn-A04-p15310116	Bn-Scaffold12290-p631	gene	
Bn-A04-p15312437	Bn-N4-p17253809	gene	
Bn-A04-p15399118	Bn-N4-p17361369	gene	exon
Bn-A04-p15404568	Bn-N4-p17369430	gene	
Bn-A04-p15409836	Bn-N4-p17378738	gene	
Bn-A04-p15411699	Bn-N4-p17380605	gene	
Bn-A04-p15420126	Bn-N4-p17387416	gene	
Bn-A04-p15436139	Bn-N4-p17402396	gene	exon
Bn-A04-p15456188	Bn-N14-p50670440	gene	

Bn-A04-p15485629	Bn-N4-p17459433	gene	
Bn-A04-p15492182	Bn-N4-p17465788	gene	
Bn-A04-p15498393	Bn-N4-p17476425	gene	exon
Bn-A04-p15514869	Bn-N4-p17499224	gene	
Bn-A04-p15519877	Bn-N4-p17504210	gene	exon
Bn-A04-p15530059	Bn-N4-p17513547	gene	exon
Bn-A04-p15530609	Bn-N4-p17514102	gene	exon
Bn-A04-p15530625	Bn-N4-p17514118	gene	exon
Bn-A04-p15555443	Bn-N4-p17547137	gene	exon
Bn-A04-p15631388	Bn-N4-p17617446	gene	exon
Bn-A04-p15655213	Bn-N4-p17648503	gene	exon
Bn-A04-p15707649	Bn-N4-p17703045	gene	
Bn-A04-p1576564	Bn-N4-p1537124	gene	exon
Bn-A04-p1589006	Bn-N4-p1553265	gene	
Bn-A04-p15893499	Bn-Scaffold00305b-p65905	gene	
Bn-A04-p1590669	Bn-N4-p1554928	gene	
Bn-A04-p1590931	Bn-N4-p1555193	gene	
Bn-A04-p15951138	Bn-N5-p4477991	gene	exon
Bn-A04-p16056186	Bn-N4-p17983696	gene	exon
Bn-A04-p16090875	Bn-N4-p18045765	gene	exon
Bn-A04-p16107844	Bn-N14-p51173063	gene	exon
Bn-A04-p16109558	Bn-N14-p51174766	gene	
Bn-A04-p16119082	Bn-N14-p51189070	gene	
Bn-A04-p16120092	Bn-N14-p51190072	gene	exon
Bn-A04-p16166839	Bn-N4-p18132806	gene	
Bn-A04-p16268792	Bn-N4-p18239986	gene	
Bn-A04-p16313477	Bn-N4-p18286010	gene	
Bn-A04-p16320671	Bn-N14-p51452393	gene	exon
Bn-A04-p16365226	Bn-N4-p18338936	gene	exon
Bn-A04-p16368852	Bn-N4-p18343289	gene	
Bn-A04-p16394755	Bn-N4-p18368736	gene	
Bn-A04-p16427350	Bn-N4-p18408150	gene	
Bn-A04-p16446976	Bn-N4-p18422343	gene	
Bn-A04-p16447217	Bn-N4-p18422584	gene	
Bn-A04-p16459969	Bn-N4-p18435319	gene	
Bn-A04-p16477853	Bn-N4-p18442566	gene	
Bn-A04-p16508914	Bn-N4-p18466378	gene	exon
Bn-A04-p16514488	Bn-N4-p18470743	gene	
Bn-A04-p16523010	Bn-N4-p18477898	gene	exon
Bn-A04-p16523116	Bn-N4-p18478004	gene	exon
Bn-A04-p16528010	Bn-N4-p18485408	gene	
Bn-A04-p16528107	Bn-N4-p18485498	gene	
Bn-A04-p1655416	Bn-N4-p1589334	gene	exon
Bn-A04-p16555982	Bn-N4-p18508172	gene	
Bn-A04-p16563543	Bn-N4-p18512244	gene	exon
Bn-A04-p16584084	Bn-N14-p51777752	gene	exon
Bn-A04-p16595064	Bn-N4-p18534997	gene	
Bn-A04-p16598514	Bn-N4-p18538501	gene	exon
Bn-A04-p16605277	Bn-N4-p18544659	gene	exon
Bn-A04-p16607433	Bn-N4-p18547436	gene	
Bn-A04-p16608584	Bn-N4-p18548589	gene	
Bn-A04-p16621448	Bn-N4-p18562429	gene	exon

Bn-A04-p16627363	Bn-N4-p18566978	gene	
Bn-A04-p16743976	Bn-N4-p19028528	gene	
Bn-A04-p16744035	Bn-N4-p19028469	gene	
Bn-A04-p16745356	Bn-N4-p19027130	gene	
Bn-A04-p1674763	Bn-N4-p1602173	gene	
Bn-A04-p16748162	Bn-N4-p19024648	gene	
Bn-A04-p16791082	Bn-N4-p18980996	gene	exon
Bn-A04-p16798852	Bn-N4-p18973231	gene	exon
Bn-A04-p1680506	Bn-N4-p1607740	gene	
Bn-A04-p1680518	Bn-N4-p1607752	gene	
Bn-A04-p1680673	Bn-N4-p1607903	gene	
Bn-A04-p16819445	Bn-N4-p18957489	gene	
Bn-A04-p16830389	Bn-N4-p18946420	gene	exon
Bn-A04-p16832545	Bn-N4-p18943797	gene	exon
Bn-A04-p16841462	Bn-N4-p18935470	gene	
Bn-A04-p16892915	Bn-N4-p18888967	gene	
Bn-A04-p16919648	Bn-N4-p18859650	gene	
Bn-A04-p16919672	Bn-N4-p18859626	gene	
Bn-A04-p16998111	Bn-N4-p18791320	gene	exon
Bn-A04-p17019529	Bn-N4-p18775521	gene	
Bn-A04-p17044228	Bn-N4-p18752848	gene	
Bn-A04-p17052071	Bn-N4-p18743497	gene	
Bn-A04-p17105440	Bn-N4-p18698782	gene	exon
Bn-A04-p17106064	Bn-N4-p18698148	gene	
Bn-A04-p17107045	Bn-N4-p18697146	gene	exon
Bn-A04-p17125513	Bn-N4-p19050827	gene	exon
Bn-A04-p17155901	Bn-N4-p19083440	gene	
Bn-A04-p17157304	Bn-N4-p19084843	gene	
Bn-A04-p17170974	Bn-N4-p19106540	gene	exon
Bn-A04-p17171349	Bn-N4-p19106930	gene	
Bn-A04-p17179870	Bn-N4-p19115278	gene	exon
Bn-A04-p1718231	Bn-N4-p1659171	gene	exon
Bn-A04-p1718248	Bn-N4-p1659191	gene	exon
Bn-A04-p17212731	Bn-N4-p19152134	gene	exon
Bn-A04-p17239509	Bn-N4-p19180742	gene	
Bn-A04-p17277010	Bn-N14-p52562849	gene	exon
Bn-A04-p17278229	Bn-N4-p19217525	gene	
Bn-A04-p17281577	Bn-N4-p19221228	gene	
Bn-A04-p17295665	Bn-N4-p19237849	gene	exon
Bn-A04-p17300068	Bn-N4-p19242580	gene	exon
Bn-A04-p17322241	Bn-N4-p19262174	gene	exon
Bn-A04-p17331915	Bn-N4-p19271681	gene	exon
Bn-A04-p17372718	Bn-N4-p19322488	gene	exon
Bn-A04-p17391232	Bn-N4-p19341607	gene	exon
Bn-A04-p17391802	Bn-N4-p19342177	gene	exon
Bn-A04-p17394552	Bn-N4-p19346776	gene	exon
Bn-A04-p17394736	Bn-N4-p19346960	gene	exon
Bn-A04-p17412823	Bn-N4-p19364932	gene	exon
Bn-A04-p17416243	Bn-N4-p19368191	gene	exon
Bn-A04-p17422800	Bn-N4-p19374651	gene	
Bn-A04-p17487757	Bn-N4-p19437505	gene	exon
Bn-A04-p17489063	Bn-N4-p19438815	gene	

Bn-A04-p17530621	Bn-N4-p19479475	gene	exon
Bn-A04-p17541500	Bn-N4-p19497129	gene	exon
Bn-A04-p17541623	Bn-N4-p19497252	gene	
Bn-A04-p17576444	Bn-N4-p19544316	gene	exon
Bn-A04-p17577700	Bn-N4-p19545572	gene	
Bn-A04-p17578444	Bn-N4-p19546312	gene	
Bn-A04-p17614504	Bn-N4-p19580311	gene	
Bn-A04-p17615088	Bn-N4-p19580895	gene	exon
Bn-A04-p17623914	Bn-N4-p19587713	gene	exon
Bn-A04-p17624469	Bn-N4-p19588268	gene	exon
Bn-A04-p17624526	Bn-N4-p19588325	gene	exon
Bn-A04-p17632009	Bn-N4-p19595954	gene	
Bn-A04-p17634469	Bn-N4-p19598402	gene	
Bn-A04-p17685210	Bn-N4-p19648488	gene	
Bn-A04-p17686228	Bn-N4-p19649505	gene	
Bn-A04-p17689002	Bn-N4-p19652081	gene	exon
Bn-A04-p17689324	Bn-N4-p19652403	gene	exon
Bn-A04-p17704458	Bn-N4-p19668024	gene	
Bn-A04-p17712184	Bn-N4-p19675646	gene	
Bn-A04-p1773333	Bn-N4-p1721944	gene	
Bn-A04-p17736387	Bn-N4-p19701055	gene	
Bn-A04-p17736770	Bn-N4-p19701438	gene	
Bn-A04-p1774031	Bn-N4-p1722642	gene	exon
Bn-A04-p17740663	Bn-N4-p19705522	gene	
Bn-A04-p17742799	Bn-N4-p19707735	gene	exon
Bn-A04-p1774926	Bn-N4-p1723549	gene	
Bn-A04-p1775155	Bn-N4-p1723774	gene	exon
Bn-A04-p17756059	Bn-N4-p19720981	gene	exon
Bn-A04-p17760280	Bn-N4-p19725124	gene	
Bn-A04-p17766173	Bn-N4-p19730522	gene	exon
Bn-A04-p17768404	Bn-N4-p19732754	gene	exon
Bn-A04-p17770281	Bn-N4-p19734627	gene	exon
Bn-A04-p1780149	Bn-N4-p1728952	gene	
Bn-A04-p17811740	Bn-N4-p19775221	gene	
Bn-A04-p17816796	Bn-N4-p19780403	gene	
Bn-A04-p17816969	Bn-N4-p19780571	gene	exon
Bn-A04-p17820683	Bn-N4-p19784349	gene	exon
Bn-A04-p17832455	Bn-N4-p19794435	gene	exon
Bn-A04-p17858149	Bn-N4-p19821474	gene	
Bn-A04-p17864572	Bn-N4-p19827880	gene	exon
Bn-A04-p17864879	Bn-N4-p19828476	gene	
Bn-A04-p17870056	Bn-N4-p19832166	gene	
Bn-A04-p17871505	Bn-N4-p19834650	gene	exon
Bn-A04-p17910578	Bn-N4-p19885124	gene	exon
Bn-A04-p17969838	Bn-N4-p19932331	gene	
Bn-A04-p17976746	Bn-N4-p19941543	gene	exon
Bn-A04-p18027570	Bn-N4-p19995455	gene	exon
Bn-A04-p18031439	Bn-N4-p20005862	gene	
Bn-A04-p18047327	Bn-N4-p20021655	gene	exon
Bn-A04-p18047606	Bn-N4-p20021934	gene	exon
Bn-A04-p18055770	Bn-N4-p20029924	gene	exon
Bn-A04-p18062365	Bn-N4-p20036288	gene	

Bn-A04-p18071582	Bn-N4-p20047890	gene	exon
Bn-A04-p18075680	Bn-N4-p20051903	gene	
Bn-A04-p18083568	Bn-N4-p20063171	gene	exon
Bn-A04-p18097103	Bn-N4-p20080551	gene	exon
Bn-A04-p18097421	Bn-N4-p20080869	gene	
Bn-A04-p18125528	Bn-N4-p20114547	gene	exon
Bn-A04-p18129552	Bn-N4-p20119480	gene	
Bn-A04-p18152854	Bn-N4-p20141390	gene	exon
Bn-A04-p1815404	Bn-N4-p1753561	gene	
Bn-A04-p18156458	Bn-N4-p20144992	gene	
Bn-A04-p18167535	Bn-N4-p20156162	gene	
Bn-A04-p18167614	Bn-N4-p20156241	gene	
Bn-A04-p18168733	Bn-N14-p53772914	gene	
Bn-A04-p18180777	Bn-N4-p20169038	gene	exon
Bn-A04-p1824456	Bn-N4-p1770094	gene	
Bn-A04-p1837535	Bn-N4-p1787089	gene	exon
Bn-A04-p18457550	Bn-N14-p54136125	gene	
Bn-A04-p18458146	Bn-N4-p20450574	gene	
Bn-A04-p18487715	Bn-N4-p20483625	gene	
Bn-A04-p18502854	Bn-N4-p20498001	gene	
Bn-A04-p18562244	Bn-N4-p20563734	gene	exon
Bn-A04-p18572841	Bn-N4-p20574835	gene	exon
Bn-A04-p18577466	Bn-N4-p20579460	gene	exon
Bn-A04-p18585603	Bn-N4-p20585798	gene	
Bn-A04-p18595858	Bn-N4-p20602010	gene	
Bn-A04-p18606660	Bn-N4-p20612582	gene	
Bn-A04-p18613344	Bn-N4-p20621135	gene	exon
Bn-A04-p18620226	Bn-N4-p20626993	gene	exon
Bn-A04-p18621492	Bn-N4-p20628250	gene	
Bn-A04-p18639840	Bn-N4-p20641939	gene	exon
Bn-A04-p18639922	Bn-N14-p54400429	gene	exon
Bn-A04-p1865434	Bn-N4-p1805109	gene	
Bn-A04-p18693211	Bn-N4-p20687712	gene	
Bn-A04-p18702920	Bn-N4-p20696885	gene	
Bn-A04-p18731541	Bn-N4-p20731935	gene	exon
Bn-A04-p18789871	Bn-N4-p20817952	gene	
Bn-A04-p1880654	Bn-N4-p1836032	gene	
Bn-A04-p18806834	Bn-N4-p20835363	gene	
Bn-A04-p1882240	Bn-N4-p1837609	gene	
Bn-A04-p18913495	Bn-N4-p20955081	gene	exon
Bn-A04-p18918997	Bn-N14-p54728683	gene	exon
Bn-A04-p18946513	Bn-N4-p20990324	gene	exon
Bn-A04-p18961456	Bn-N4-p21004100	gene	exon
Bn-A04-p1900422	Bn-N4-p1855279	gene	exon
Bn-A04-p1948034	Bn-N4-p2246771	gene	
Bn-A04-p1962888	Bn-N4-p2230820	gene	exon
Bn-A04-p1979834	Bn-N4-p2220145	gene	
Bn-A04-p1991843	Bn-N4-p2207545	gene	exon
Bn-A04-p2029030	Bn-N4-p2172423	gene	
Bn-A04-p2037077	Bn-N4-p2161110	gene	
Bn-A04-p2048587	Bn-N4-p2149677	gene	
Bn-A04-p2111647	Bn-N4-p2089154	gene	

Bn-A04-p2122324	Bn-N4-p2075647	gene	exon
Bn-A04-p2124879	Bn-N4-p2070320	gene	
Bn-A04-p2146459	Bn-N4-p2059248	gene	
Bn-A04-p2149134	Bn-N4-p2056362	gene	exon
Bn-A04-p2149214	Bn-N4-p2056282	gene	exon
Bn-A04-p2178146	Bn-N4-p2030254	gene	
Bn-A04-p2191612	Bn-N4-p2016910	gene	exon
Bn-A04-p2192192	Bn-N4-p2016330	gene	exon
Bn-A04-p2215376	Bn-N4-p1987197	gene	exon
Bn-A04-p2215559	Bn-N4-p1987014	gene	exon
Bn-A04-p2222852	Bn-N4-p1977753	gene	
Bn-A04-p2288114	Bn-N4-p1930836	gene	exon
Bn-A04-p2309625	Bn-N4-p1905792	gene	exon
Bn-A04-p2330084	Bn-N4-p1892856	gene	
Bn-A04-p233588	Bn-N4-p239356	gene	exon
Bn-A04-p2401413	Bn-N4-p2305853	gene	
Bn-A04-p24368	Bn-N4-p133409	gene	exon
Bn-A04-p2439163	Bn-N4-p2340231	gene	
Bn-A04-p24411	Bn-N4-p133366	gene	exon
Bn-A04-p2456200	Bn-N4-p2354532	gene	exon
Bn-A04-p2498254	Bn-N4-p2415593	gene	
Bn-A04-p251383	Bn-N4-p252312	gene	exon
Bn-A04-p261340	Bn-N4-p263953	gene	
Bn-A04-p2650427	Bn-N4-p2564526	gene	exon
Bn-A04-p2651171	Bn-N4-p2565270	gene	
Bn-A04-p2680732	Bn-N4-p2593065	gene	exon
Bn-A04-p2723787	Bn-N4-p2632977	gene	
Bn-A04-p2748876	Bn-N14-p30998304	gene	
Bn-A04-p2802411	Bn-N4-p2705775	gene	exon
Bn-A04-p2825294	Bn-N4-p2721433	gene	exon
Bn-A04-p2825362	Bn-N4-p2721501	gene	
Bn-A04-p2833736	Bn-N4-p2729937	gene	
Bn-A04-p2841433	Bn-N4-p2737553	gene	
Bn-A04-p2843802	Bn-N4-p2740497	gene	
Bn-A04-p2889239	Bn-N4-p2767110	gene	exon
Bn-A04-p2892557	Bn-N4-p2770374	gene	
Bn-A04-p2892722	Bn-N4-p2770540	gene	exon
Bn-A04-p2892985	Bn-N4-p2770803	gene	exon
Bn-A04-p2893053	Bn-N4-p2770871	gene	exon
Bn-A04-p289974	Bn-N4-p299261	gene	
Bn-A04-p2903530	Bn-N4-p2778620	gene	
Bn-A04-p2941201	Bn-N4-p2813079	gene	exon
Bn-A04-p2969081	Bn-N4-p2879542	gene	exon
Bn-A04-p2983187	Bn-N4-p2887116	gene	exon
Bn-A04-p3002670	Bn-N4-p2905453	gene	exon
Bn-A04-p3004068	Bn-N4-p2906851	gene	exon
Bn-A04-p3021710	Bn-N4-p2914174	gene	exon
Bn-A04-p3022681	Bn-N4-p2915145	gene	exon
Bn-A04-p3126855	Bn-N4-p3021601	gene	
Bn-A04-p3144839	Bn-N4-p3045094	gene	
Bn-A04-p3181336	Bn-N4-p3088237	gene	
Bn-A04-p3182956	Bn-N4-p3089856	gene	

Bn-A04-p320972	Bn-N4-p334926	gene	exon
Bn-A04-p3220424	Bn-N4-p3124308	gene	
Bn-A04-p3220819	Bn-N4-p3124704	gene	exon
Bn-A04-p3225119	Bn-N4-p3129508	gene	exon
Bn-A04-p322820	Bn-N4-p336773	gene	
Bn-A04-p3235336	Bn-N4-p3138888	gene	exon
Bn-A04-p324144	Bn-N4-p338158	gene	
Bn-A04-p325019	Bn-N4-p338730	gene	exon
Bn-A04-p326007	Bn-N4-p339717	gene	
Bn-A04-p3262432	Bn-N4-p3170115	gene	
Bn-A04-p3263085	Bn-N4-p3170762	gene	exon
Bn-A04-p3267803	Bn-N4-p3175665	gene	exon
Bn-A04-p3298487	Bn-N4-p3206903	gene	exon
Bn-A04-p3345530	Bn-N4-p3261325	gene	exon
Bn-A04-p3357098	Bn-N4-p3272380	gene	exon
Bn-A04-p3357519	Bn-N4-p3272801	gene	
Bn-A04-p3357700	Bn-N4-p3272981	gene	
Bn-A04-p3358441	Bn-N4-p3273734	gene	
Bn-A04-p3363190	Bn-N4-p3278771	gene	exon
Bn-A04-p3395103	Bn-N4-p3317226	gene	
Bn-A04-p3413548	Bn-N4-p3332043	gene	exon
Bn-A04-p3414140	Bn-N4-p3332635	gene	
Bn-A04-p3414232	Bn-N4-p3332727	gene	
Bn-A04-p3414449	Bn-N4-p3332942	gene	
Bn-A04-p3528213	Bn-N4-p3440624	gene	exon
Bn-A04-p354688	Bn-N4-p367121	gene	
Bn-A04-p3571259	Bn-N4-p3486900	gene	
Bn-A04-p3572717	Bn-N4-p3488326	gene	
Bn-A04-p357272	Bn-N4-p369707	gene	exon
Bn-A04-p3572777	Bn-N4-p3488386	gene	
Bn-A04-p3573078	Bn-N4-p3488718	gene	
Bn-A04-p3583216	Bn-N4-p3498786	gene	
Bn-A04-p3589494	Bn-N4-p3505048	gene	
Bn-A04-p3628682	Bn-N4-p3544790	gene	
Bn-A04-p3630793	Bn-N4-p3546895	gene	exon
Bn-A04-p3631563	Bn-N4-p3547667	gene	
Bn-A04-p3632226	Bn-N4-p3548333	gene	
Bn-A04-p3632611	Bn-N4-p3548718	gene	
Bn-A04-p3651589	Bn-N4-p3565731	gene	exon
Bn-A04-p3702896	Bn-N4-p3609768	gene	
Bn-A04-p3721587	Bn-N4-p3624036	gene	
Bn-A04-p3724573	Bn-N4-p3627001	gene	exon
Bn-A04-p3743549	Bn-N4-p3642885	gene	
Bn-A04-p3761523	Bn-N4-p3662467	gene	exon
Bn-A04-p3875034	Bn-N4-p3744805	gene	exon
Bn-A04-p3915978	Bn-N4-p3773355	gene	exon
Bn-A04-p3923982	Bn-N12-p39746008	gene	
Bn-A04-p397523	Bn-N4-p397170	gene	
Bn-A04-p398807	Bn-N4-p398455	gene	exon
Bn-A04-p4002585	Bn-N14-p33047165	gene	
Bn-A04-p404042	Bn-N1-p3955833	gene	
Bn-A04-p405060	Bn-N4-p404783	gene	

Bn-A04-p4221603	Bn-N4-p4491938	gene	exon
Bn-A04-p4222491	Bn-N4-p4492827	gene	exon
Bn-A04-p4223859	Bn-N4-p4494197	gene	
Bn-A04-p4224646	Bn-N4-p4494988	gene	
Bn-A04-p4224705	Bn-N4-p4495047	gene	
Bn-A04-p4249132	Bn-N4-p4523660	gene	exon
Bn-A04-p4265054	Bn-N4-p4532069	gene	exon
Bn-A04-p4265606	Bn-N4-p4532621	gene	exon
Bn-A04-p4276334	Bn-N4-p4547759	gene	
Bn-A04-p4286343	Bn-N4-p4577694	gene	exon
Bn-A04-p4294569	Bn-N4-p4585331	gene	exon
Bn-A04-p4307559	Bn-N4-p4600850	gene	exon
Bn-A04-p4319736	Bn-N4-p4613586	gene	exon
Bn-A04-p4330419	Bn-N4-p4625457	gene	
Bn-A04-p4346492	Bn-N4-p4641714	gene	
Bn-A04-p461679	Bn-N14-p23453552	gene	exon
Bn-A04-p4717909	Bn-N4-p5041861	gene	
Bn-A04-p4736023	Bn-N4-p5076254	gene	
Bn-A04-p4742377	Bn-N4-p5091187	gene	
Bn-A04-p4742432	Bn-N4-p5091242	gene	
Bn-A04-p4823556	Bn-N4-p5186650	gene	
Bn-A04-p4825669	Bn-N4-p5188763	gene	
Bn-A04-p4874840	Bn-N4-p5232333	gene	
Bn-A04-p4881168	Bn-N4-p5238741	gene	
Bn-A04-p4881807	Bn-N14-p34700808	gene	exon
Bn-A04-p4897489	Bn-N4-p5314588	gene	
Bn-A04-p4976932	Bn-N4-p5380466	gene	exon
Bn-A04-p4977488	Bn-N4-p5381022	gene	exon
Bn-A04-p4978478	Bn-N4-p5382012	gene	exon
Bn-A04-p4978492	Bn-N4-p5382026	gene	exon
Bn-A04-p4978780	Bn-N4-p5382314	gene	exon
Bn-A04-p5183306	Bn-N4-p5614929	gene	exon
Bn-A04-p5270881	Bn-N4-p5713624	gene	exon
Bn-A04-p5273199	Bn-N4-p5715924	gene	
Bn-A04-p5378408	Bn-N4-p5827825	gene	
Bn-A04-p560622	Bn-N4-p554487	gene	
Bn-A04-p570250	Bn-N4-p564486	gene	exon
Bn-A04-p5715780	Bn-N4-p6269396	gene	
Bn-A04-p5788908	Bn-N4-p7409965	gene	
Bn-A04-p5852413	Bn-N4-p7460179	gene	
Bn-A04-p5853514	Bn-N4-p7457881	gene	exon
Bn-A04-p5872564	Bn-N4-p7480361	gene	
Bn-A04-p5938125	Bn-N4-p7586749	gene	exon
Bn-A04-p5938245	Bn-N4-p7586869	gene	exon
Bn-A04-p5982123	Bn-N4-p7640512	gene	exon
Bn-A04-p5985513	Bn-N4-p7643273	gene	
Bn-A04-p5987463	Bn-N4-p7645250	gene	
Bn-A04-p6023914	Bn-N4-p7681393	gene	exon
Bn-A04-p6228211	Bn-N14-p37289690	gene	exon
Bn-A04-p6240855	Bn-N4-p7917200	gene	exon
Bn-A04-p6277634	Bn-N4-p7970235	gene	exon
Bn-A04-p6284377	Bn-N4-p7977624	gene	exon

Bn-A04-p6319430	Bn-N4-p8014387	gene	exon
Bn-A04-p6376472	Bn-N14-p37577067	gene	exon
Bn-A04-p637683	Bn-N4-p648894	gene	exon
Bn-A04-p6402808	Bn-N4-p8099707	gene	exon
Bn-A04-p6445083	Bn-Scaffold01456-p1233	gene	
Bn-A04-p646064	Bn-N4-p657427	gene	exon
Bn-A04-p646105	Bn-N4-p657447	gene	exon
Bn-A04-p6465442	Bn-N14-p37632125	gene	
Bn-A04-p648152	Bn-N4-p659515	gene	exon
Bn-A04-p6555913	Bn-N4-p8247861	gene	
Bn-A04-p6563107	Bn-N4-p8255950	gene	exon
Bn-A04-p6563200	Bn-N4-p8256043	gene	exon
Bn-A04-p6588935	Bn-N4-p8296575	gene	exon
Bn-A04-p6632126	Bn-N4-p8348683	gene	exon
Bn-A04-p6640990	Bn-N4-p8360809	gene	exon
Bn-A04-p665754	Bn-N4-p671632	gene	exon
Bn-A04-p665932	Bn-N4-p671810	gene	exon
Bn-A04-p665967	Bn-N4-p671845	gene	exon
Bn-A04-p6673120	Bn-N4-p8391311	gene	exon
Bn-A04-p6678797	Bn-N4-p8396236	gene	exon
Bn-A04-p6679263	Bn-N4-p8396702	gene	exon
Bn-A04-p6679568	Bn-N4-p8397006	gene	exon
Bn-A04-p669182	Bn-N4-p675443	gene	exon
Bn-A04-p6719949	Bn-N4-p8439218	gene	exon
Bn-A04-p6764233	Bn-N4-p8500149	gene	
Bn-A04-p6765045	Bn-N4-p8500961	gene	
Bn-A04-p6773984	Bn-N4-p8509867	gene	
Bn-A04-p6775008	Bn-N4-p8510915	gene	
Bn-A04-p6776597	Bn-N4-p8512504	gene	exon
Bn-A04-p6776786	Bn-N4-p8512693	gene	
Bn-A04-p6790373	Bn-N4-p8525888	gene	exon
Bn-A04-p6929056	Bn-N4-p8682719	gene	exon
Bn-A04-p6953674	Bn-N4-p8706711	gene	exon
Bn-A04-p6958157	Bn-N4-p8711151	gene	exon
Bn-A04-p7003715	Bn-N4-p8751132	gene	exon
Bn-A04-p7044446	Bn-N14-p38203369	gene	exon
Bn-A04-p7077586	Bn-N4-p8807525	gene	exon
Bn-A04-p7093491	Bn-N4-p8824849	gene	exon
Bn-A04-p7141171	Bn-N4-p8866013	gene	
Bn-A04-p7142597	Bn-N4-p8867424	gene	
Bn-A04-p7157859	Bn-N4-p8893629	gene	
Bn-A04-p7161326	Bn-N4-p8897087	gene	
Bn-A04-p7162690	Bn-N4-p8898451	gene	
Bn-A04-p7233978	Bn-N4-p8978598	gene	exon
Bn-A04-p7322000	Bn-N4-p9083388	gene	exon
Bn-A04-p7322497	Bn-N4-p9083885	gene	exon
Bn-A04-p7349115	Bn-N4-p9160821	gene	exon
Bn-A04-p7349794	Bn-N4-p9161498	gene	exon
Bn-A04-p7350870	Bn-N4-p9162577	gene	
Bn-A04-p7389149	Bn-N4-p9203787	gene	
Bn-A04-p7407657	Bn-N4-p9228932	gene	exon
Bn-A04-p741528	Bn-N4-p745025	gene	

Bn-A04-p7431245	Bn-N4-p9252792	gene	
Bn-A04-p7431816	Bn-N4-p9253344	gene	
Bn-A04-p745024	Bn-N4-p748522	gene	exon
Bn-A04-p7450678	Bn-N4-p9273747	gene	exon
Bn-A04-p7471870	Bn-N4-p9311436	gene	
Bn-A04-p7474589	Bn-N4-p9314153	gene	
Bn-A04-p7488675	Bn-N4-p9348797	gene	
Bn-A04-p7490390	Bn-N14-p38833708	gene	
Bn-A04-p7520330	Bn-N4-p9383429	gene	exon
Bn-A04-p7541325	Bn-N4-p9414441	gene	
Bn-A04-p7541762	Bn-N4-p9414878	gene	exon
Bn-A04-p7542594	Bn-N4-p9415709	gene	exon
Bn-A04-p7582986	Bn-N4-p9451011	gene	exon
Bn-A04-p7584788	Bn-N4-p9452812	gene	
Bn-A04-p7586476	Bn-N4-p9454517	gene	exon
Bn-A04-p7588820	Bn-N4-p9456872	gene	
Bn-A04-p761383	Bn-N4-p759440	gene	exon
Bn-A04-p7625503	Bn-N4-p9498896	gene	
Bn-A04-p7629926	Bn-N4-p9503381	gene	
Bn-A04-p7635112	Bn-N4-p9510178	gene	
Bn-A04-p7666812	Bn-N4-p9540723	gene	
Bn-A04-p7666981	Bn-N4-p9540892	gene	
Bn-A04-p7694288	Bn-N4-p9562834	gene	exon
Bn-A04-p7819391	Bn-N4-p9710288	gene	
Bn-A04-p7819525	Bn-N4-p9710410	gene	
Bn-A04-p783339	Bn-N4-p781449	gene	exon
Bn-A04-p784708	Bn-N4-p782818	gene	exon
Bn-A04-p784894	Bn-N4-p783004	gene	exon
Bn-A04-p785628	Bn-N4-p783742	gene	exon
Bn-A04-p786588	Bn-N4-p784701	gene	
Bn-A04-p792399	Bn-N4-p790541	gene	exon
Bn-A04-p803315	Bn-N4-p801225	gene	exon
Bn-A04-p810935	Bn-N4-p811294	gene	exon
Bn-A04-p813173	Bn-N4-p813484	gene	exon
Bn-A04-p8178053	Bn-N14-p39909486	gene	exon
Bn-A04-p8178997	Bn-N4-p10061305	gene	exon
Bn-A04-p8348750	Bn-N4-p10238804	gene	exon
Bn-A04-p8348806	Bn-N4-p10238860	gene	exon
Bn-A04-p8441932	Bn-N4-p10359241	gene	
Bn-A04-p8555881	Bn-N4-p10457792	gene	exon
Bn-A04-p8559580	Bn-N4-p10461522	gene	exon
Bn-A04-p8564072	Bn-N4-p10466045	gene	
Bn-A04-p8595035	Bn-N7-p13676973	gene	exon
Bn-A04-p860212	Bn-N4-p855702	gene	exon
Bn-A04-p8609799	Bn-N14-p40537353	gene	exon
Bn-A04-p8612311	Bn-N4-p10512694	gene	
Bn-A04-p8615093	Bn-N14-p40543502	gene	
Bn-A04-p8790433	Bn-N4-p10723288	gene	exon
Bn-A04-p8803630	Bn-N4-p10744229	gene	exon
Bn-A04-p8817223	Bn-N4-p10773879	gene	
Bn-A04-p8828395	Bn-N4-p10779021	gene	exon
Bn-A04-p8828462	Bn-N4-p10779088	gene	exon

Bn-A04-p8892416	Bn-N4-p10846981	gene	
Bn-A04-p8906594	Bn-N4-p10861544	gene	exon
Bn-A04-p8974361	Bn-N4-p10922129	gene	
Bn-A04-p9024009	Bn-N4-p10964749	gene	exon
Bn-A04-p9086923	Bn-N4-p11036134	gene	
Bn-A04-p9088043	Bn-N4-p11037233	gene	exon
Bn-A04-p9088760	Bn-N4-p11037947	gene	
Bn-A04-p9097344	Bn-N4-p11046720	gene	exon
Bn-A04-p9098709	Bn-N4-p11048084	gene	exon
Bn-A04-p9099209	Bn-N4-p11048585	gene	exon
Bn-A04-p9107147	Bn-N4-p11056629	gene	
Bn-A04-p9119079	Bn-N4-p11071258	gene	exon
Bn-A04-p9119142	Bn-N4-p11071321	gene	exon
Bn-A04-p922828	Bn-N4-p912024	gene	
Bn-A04-p9231842	Bn-N4-p11168852	gene	exon
Bn-A04-p9232024	Bn-N4-p11169034	gene	
Bn-A04-p9232365	Bn-N4-p11169375	gene	exon
Bn-A04-p9232828	Bn-N4-p11169849	gene	
Bn-A04-p928350	Bn-N4-p917323	gene	exon
Bn-A04-p9340809	Bn-N6-p5084813	gene	exon
Bn-A04-p934150	Bn-N4-p923006	gene	exon
Bn-A04-p9355248	Bn-N4-p11290169	gene	
Bn-A04-p9363550	Bn-N4-p11302129	gene	
Bn-A04-p9379937	Bn-N4-p11330582	gene	
Bn-A04-p9484747	Bn-N4-p11472813	gene	
Bn-A04-p9505012	Bn-N4-p11485501	gene	exon
Bn-A04-p9524800	Bn-N4-p11513641	gene	
Bn-A04-p9543062	Bn-N4-p11529941	gene	
Bn-A04-p9566708	Bn-N4-p11552797	gene	exon
Bn-A04-p9606190	Bn-N4-p11603803	gene	exon
Bn-A04-p9623505	Bn-N4-p11612168	gene	exon
Bn-A04-p9623913	Bn-N4-p11612576	gene	exon
Bn-A04-p9639648	Bn-N4-p11631956	gene	exon
Bn-A04-p9673337	Bn-N4-p11667162	gene	
Bn-A04-p9675790	Bn-N4-p11669634	gene	exon
Bn-A04-p9746407	Bn-N4-p11733588	gene	exon
Bn-A04-p980952	Bn-N4-p930085	gene	
Bn-A04-p9811506	Bn-N4-p11792965	gene	exon
Bn-A04-p9814236	Bn-N4-p11795696	gene	exon
Bn-A04-p9814284	Bn-N4-p11795744	gene	exon
Bn-A04-p9815052	Bn-N4-p11796512	gene	exon
Bn-A04-p9819724	Bn-N4-p11801084	gene	exon
Bn-A04-p9819885	Bn-N4-p11801243	gene	
Bn-A04-p982068	Bn-N4-p931248	gene	
Bn-A04-p9830766	Bn-N4-p11814273	gene	
Bn-A04-p9844020	Bn-N4-p11826893	gene	exon
Bn-A04-p9847875	Bn-N4-p11830458	gene	
Bn-A04-p9848849	Bn-N4-p11831435	gene	
Bn-A04-p9868258	Bn-N4-p11856220	gene	
Bn-A04-p9870167	Bn-N4-p11858133	gene	
Bn-A04-p9920827	Bn-N4-p11910916	gene	
Bn-A04-p9920873	Bn-N4-p11910962	gene	

Bn-A04-p9921876	Bn-N4-p11911966	gene	
Bn-A04-p9983165	Bn-N4-p11965185	gene	exon
Bn-A05-p1006754	Bn-N5-p1117066	gene	exon
Bn-A05-p10179640	Bn-N5-p9731217	gene	
Bn-A05-p10181096	Bn-N5-p9732665	gene	
Bn-A05-p10241814	Bn-N5-p9805761	gene	
Bn-A05-p10278245	Bn-N5-p9842754	gene	
Bn-A05-p10305352	Bn-N5-p9880048	gene	exon
Bn-A05-p10320143	Bn-N5-p9895872	gene	exon
Bn-A05-p10320501	Bn-N5-p9896230	gene	exon
Bn-A05-p10321304	Bn-N5-p9897033	gene	
Bn-A05-p10321580	Bn-N5-p9897309	gene	exon
Bn-A05-p1035167	Bn-N5-p1142967	gene	exon
Bn-A05-p103817	Bn-N5-p123677	gene	exon
Bn-A05-p10423564	Bn-N5-p10023951	gene	exon
Bn-A05-p10426347	Bn-N5-p10026726	gene	exon
Bn-A05-p10426618	Bn-N5-p10027013	gene	exon
Bn-A05-p10622526	Bn-N5-p10251993	gene	
Bn-A05-p10697862	Bn-N5-p10321363	gene	exon
Bn-A05-p10719967	Bn-N5-p10361634	gene	exon
Bn-A05-p10760570	Bn-N5-p10401975	gene	
Bn-A05-p10783057	Bn-N5-p10419340	gene	
Bn-A05-p108510	Bn-N5-p127460	gene	
Bn-A05-p10922835	Bn-N5-p10577272	gene	
Bn-A05-p10939686	Bn-N5-p10599417	gene	exon
Bn-A05-p10939740	Bn-N5-p10599471	gene	exon
Bn-A05-p10940401	Bn-N5-p10600132	gene	exon
Bn-A05-p109998	Bn-N5-p128987	gene	
Bn-A05-p10999977	Bn-N5-p10656438	gene	exon
Bn-A05-p11000036	Bn-N5-p10656497	gene	exon
Bn-A05-p11000261	Bn-N5-p10656719	gene	
Bn-A05-p11012409	Bn-N5-p10662213	gene	
Bn-A05-p11013933	Bn-N5-p10663727	gene	
Bn-A05-p11013981	Bn-N5-p10663775	gene	exon
Bn-A05-p11044537	Bn-N5-p10674781	gene	
Bn-A05-p11056573	Bn-N5-p10686874	gene	exon
Bn-A05-p11057820	Bn-N5-p10688058	gene	
Bn-A05-p1107888	Bn-N5-p1216224	gene	
Bn-A05-p11084469	Bn-N5-p10708377	gene	exon
Bn-A05-p11225095	Bn-N5-p10868110	gene	
Bn-A05-p11279325	Bn-N5-p10912453	gene	
Bn-A05-p11279669	Bn-N5-p10912797	gene	
Bn-A05-p1134104	Bn-N5-p1245455	gene	
Bn-A05-p11377111	Bn-N5-p11021231	gene	
Bn-A05-p11384651	Bn-N5-p11029410	gene	exon
Bn-A05-p11429310	Bn-N5-p11076942	gene	
Bn-A05-p114598	Bn-N5-p133603	gene	exon
Bn-A05-p11514604	Bn-N5-p11144951	gene	exon
Bn-A05-p11593933	Bn-N15-p25890644	gene	exon
Bn-A05-p11606554	Bn-N5-p11228759	gene	exon
Bn-A05-p11607344	Bn-N5-p11229549	gene	exon
Bn-A05-p11785796	Bn-N5-p11394645	gene	

Bn-A05-p11786537	Bn-N5-p11395386	gene	
Bn-A05-p11840240	Bn-N5-p11445225	gene	
Bn-A05-p11856345	Bn-N5-p11464718	gene	
Bn-A05-p11966222	Bn-N5-p11902010	gene	
Bn-A05-p11975840	Bn-N5-p11911662	gene	
Bn-A05-p11990369	Bn-N5-p11929939	gene	
Bn-A05-p1204987	Bn-N5-p1293109	gene	exon
Bn-A05-p12056452	Bn-N5-p11994559	gene	exon
Bn-A05-p12158169	Bn-N5-p12100389	gene	exon
Bn-A05-p1216828	Bn-N5-p1309989	gene	exon
Bn-A05-p1220485	Bn-N5-p1313649	gene	exon
Bn-A05-p12208604	Bn-N5-p12152813	gene	exon
Bn-A05-p12208728	Bn-N5-p12152937	gene	exon
Bn-A05-p12208840	Bn-N5-p12153049	gene	exon
Bn-A05-p12209129	Bn-N5-p12153338	gene	
Bn-A05-p12209466	Bn-N5-p12153675	gene	
Bn-A05-p1237296	Bn-N5-p1331338	gene	exon
Bn-A05-p12378382	Bn-N5-p12329634	gene	
Bn-A05-p12408504	Bn-N5-p12360876	gene	
Bn-A05-p12597194	Bn-N5-p12521345	gene	exon
Bn-A05-p1279304	Bn-N5-p1383086	gene	exon
Bn-A05-p12845300	Bn-N5-p12782155	gene	
Bn-A05-p12952248	Bn-N15-p22372553	gene	exon
Bn-A05-p1299570	Bn-N5-p1401301	gene	exon
Bn-A05-p13013349	Bn-N5-p12952826	gene	
Bn-A05-p1308471	Bn-N5-p1410753	gene	exon
Bn-A05-p13119450	Bn-N5-p11820809	gene	exon
Bn-A05-p13126937	Bn-N5-p11813560	gene	exon
Bn-A05-p13165406	Bn-N5-p11787351	gene	exon
Bn-A05-p13190945	Bn-N5-p17871658	gene	exon
Bn-A05-p13224690	Bn-N5-p17912096	gene	exon
Bn-A05-p13227178	Bn-N5-p17914574	gene	exon
Bn-A05-p1335771	Bn-N5-p1436231	gene	exon
Bn-A05-p1349520	Bn-N5-p1453955	gene	
Bn-A05-p1350976	Bn-N5-p1455247	gene	exon
Bn-A05-p1353104	Bn-N14-p2211245	gene	exon
Bn-A05-p1356378	Bn-N5-p1459257	gene	exon
Bn-A05-p13565564	Bn-N5-p13247076	gene	
Bn-A05-p13687507	Bn-Scaffold03512-p2287	gene	
Bn-A05-p13688426	Bn-N5-p13343674	gene	
Bn-A05-p13688982	Bn-N5-p13344230	gene	exon
Bn-A05-p13690413	Bn-N5-p13345661	gene	
Bn-A05-p1372057	Bn-N5-p1480737	gene	exon
Bn-A05-p13778123	Bn-N13-p36788938	gene	
Bn-A05-p13813146	Bn-N5-p13489952	gene	
Bn-A05-p13822414	Bn-N5-p13499507	gene	exon
Bn-A05-p13822616	Bn-N5-p13499709	gene	
Bn-A05-p13823408	Bn-N5-p13500501	gene	exon
Bn-A05-p13825402	Bn-N5-p13502511	gene	
Bn-A05-p1384000	Bn-N5-p1493281	gene	exon
Bn-A05-p1384677	Bn-N5-p1493971	gene	
Bn-A05-p13897888	Bn-N5-p13579644	gene	

Bn-A05-p13948854	Bn-N5-p13668111	gene	
Bn-A05-p1396162	Bn-N5-p1505543	gene	exon
Bn-A05-p1396567	Bn-N5-p1505947	gene	exon
Bn-A05-p14040907	Bn-N5-p13760234	gene	exon
Bn-A05-p14042687	Bn-N5-p13761427	gene	exon
Bn-A05-p140798	Bn-N5-p173295	gene	exon
Bn-A05-p14104827	Bn-N5-p13828339	gene	exon
Bn-A05-p14106384	Bn-N5-p13829896	gene	exon
Bn-A05-p14146305	Bn-N5-p13891987	gene	exon
Bn-A05-p14198626	Bn-N5-p13959974	gene	exon
Bn-A05-p14213049	Bn-N5-p13982304	gene	exon
Bn-A05-p14325043	Bn-N5-p14842237	gene	exon
Bn-A05-p1436011	Bn-N5-p1568006	gene	exon
Bn-A05-p14374199	Bn-N5-p14911377	gene	
Bn-A05-p14426042	Bn-N5-p14948520	gene	
Bn-A05-p14426121	Bn-N5-p14948599	gene	
Bn-A05-p14443915	Bn-N5-p14967024	gene	exon
Bn-A05-p14444179	Bn-N5-p14967288	gene	exon
Bn-A05-p14447476	Bn-N5-p14970698	gene	
Bn-A05-p14477462	Bn-N5-p14992271	gene	exon
Bn-A05-p14483962	Bn-N5-p15020078	gene	
Bn-A05-p1451750	Bn-N5-p1577981	gene	exon
Bn-A05-p14521315	Bn-N5-p15036982	gene	exon
Bn-A05-p1457058	Bn-N5-p1583214	gene	exon
Bn-A05-p14580151	Bn-N5-p15080209	gene	
Bn-A05-p14600386	Bn-N5-p15107862	gene	exon
Bn-A05-p14612446	Bn-N5-p15115218	gene	
Bn-A05-p14662656	Bn-N5-p15173819	gene	exon
Bn-A05-p14663154	Bn-N15-p31441363	gene	
Bn-A05-p14673407	Bn-N5-p15189140	gene	
Bn-A05-p1475369	Bn-N5-p1602173	gene	exon
Bn-A05-p1492327	Bn-N5-p1619320	gene	exon
Bn-A05-p1496037	Bn-N5-p1623592	gene	exon
Bn-A05-p1499192	Bn-N5-p1626797	gene	
Bn-A05-p15004033	Bn-N5-p15551040	gene	exon
Bn-A05-p15107406	Bn-N5-p15642723	gene	
Bn-A05-p15126127	Bn-N5-p15684555	gene	
Bn-A05-p15224458	Bn-N5-p15731705	gene	exon
Bn-A05-p15290524	Bn-N5-p15778804	gene	
Bn-A05-p15297460	Bn-N5-p15782664	gene	exon
Bn-A05-p15320768	Bn-N5-p15801621	gene	
Bn-A05-p15322236	Bn-N5-p15803039	gene	
Bn-A05-p15491528	Bn-N5-p15975442	gene	
Bn-A05-p1554943	Bn-N5-p1722333	gene	
Bn-A05-p15551715	Bn-N9-p27467116	gene	exon
Bn-A05-p1561518	Bn-N5-p1728875	gene	exon
Bn-A05-p15663987	Bn-N9-p27582128	gene	
Bn-A05-p15702744	Bn-N9-p27627392	gene	
Bn-A05-p1578074	Bn-N5-p1753871	gene	
Bn-A05-p15780983	Bn-N9-p27718465	gene	exon
Bn-A05-p1579155	Bn-N5-p1754900	gene	
Bn-A05-p1579208	Bn-N5-p1754953	gene	exon

Bn-A05-p1580246	Bn-N5-p1755984	gene	exon
Bn-A05-p15812933	Bn-N9-p27767000	gene	exon
Bn-A05-p15859690	Bn-N5-p16065172	gene	exon
Bn-A05-p15906659	Bn-N5-p16124527	gene	exon
Bn-A05-p15938967	Bn-N5-p16147503	gene	
Bn-A05-p15940232	Bn-N5-p16148773	gene	
Bn-A05-p15948690	Bn-N5-p16159482	gene	
Bn-A05-p16195531	Bn-N5-p16479473	gene	
Bn-A05-p16272686	Bn-N5-p16556180	gene	exon
Bn-A05-p1630204	Bn-N5-p1805167	gene	exon
Bn-A05-p16359545	Bn-N5-p16652852	gene	
Bn-A05-p16392699	Bn-N5-p16690718	gene	
Bn-A05-p16460167	Bn-N5-p16762643	gene	
Bn-A05-p16468152	Bn-N4-p1024552	gene	
Bn-A05-p16538605	Bn-N5-p16825020	gene	
Bn-A05-p16590105	Bn-N5-p16888750	gene	exon
Bn-A05-p16625783	Bn-N5-p16955195	gene	exon
Bn-A05-p16664411	Bn-N5-p17005160	gene	
Bn-A05-p16735186	Bn-N5-p17072997	gene	
Bn-A05-p16738292	Bn-N5-p17074249	gene	
Bn-A05-p16738871	Bn-N5-p17074827	gene	
Bn-A05-p1679589	Bn-N5-p1885398	gene	exon
Bn-A05-p16892759	Bn-N5-p17224711	gene	
Bn-A05-p1689277	Bn-N5-p1890245	gene	
Bn-A05-p16897600	Bn-N5-p17229653	gene	
Bn-A05-p16898305	Bn-N5-p17230322	gene	
Bn-A05-p16900864	Bn-N5-p17232898	gene	
Bn-A05-p1690598	Bn-N5-p1891553	gene	exon
Bn-A05-p1697401	Bn-N5-p1896787	gene	exon
Bn-A05-p1701030	Bn-N5-p1901300	gene	
Bn-A05-p17108506	Bn-N5-p17595916	gene	exon
Bn-A05-p17215153	Bn-N5-p18137448	gene	exon
Bn-A05-p17241091	Bn-N5-p18160629	gene	
Bn-A05-p17243214	Bn-N5-p18167468	gene	
Bn-A05-p17262653	Bn-N5-p18175902	gene	exon
Bn-A05-p17292224	Bn-N5-p18232665	gene	exon
Bn-A05-p17366544	Bn-N15-p36454254	gene	
Bn-A05-p17368155	Bn-N5-p18290404	gene	exon
Bn-A05-p17417422	Bn-N15-p36517662	gene	exon
Bn-A05-p17419591	Bn-N5-p18337436	gene	
Bn-A05-p17420484	Bn-N5-p18338329	gene	exon
Bn-A05-p17420613	Bn-N5-p18338458	gene	exon
Bn-A05-p17442162	Bn-N15-p36557068	gene	
Bn-A05-p17544675	Bn-N5-p18437903	gene	exon
Bn-A05-p17566846	Bn-N5-p18574806	gene	exon
Bn-A05-p17567179	Bn-N5-p18575139	gene	exon
Bn-A05-p17568606	Bn-N5-p18576572	gene	
Bn-A05-p1761473	Bn-N5-p1949635	gene	
Bn-A05-p17617527	Bn-N5-p18610123	gene	
Bn-A05-p17617724	Bn-N5-p18740415	gene	exon
Bn-A05-p1762229	Bn-N5-p1949899	gene	
Bn-A05-p1764530	Bn-N5-p1953359	gene	

Bn-A05-p17675829	Bn-N5-p18660925	gene	exon
Bn-A05-p17739455	Bn-N5-p18759377	gene	exon
Bn-A05-p17758084	Bn-N5-p18779379	gene	exon
Bn-A05-p17774694	Bn-N5-p18803472	gene	exon
Bn-A05-p17822322	Bn-N5-p18839244	gene	
Bn-A05-p17894045	Bn-N5-p18900369	gene	exon
Bn-A05-p17895197	Bn-N5-p18901501	gene	exon
Bn-A05-p17928363	Bn-N5-p18933887	gene	
Bn-A05-p17931505	Bn-N5-p18936830	gene	
Bn-A05-p1806574	Bn-N5-p1985171	gene	exon
Bn-A05-p18066693	Bn-N5-p19035144	gene	exon
Bn-A05-p18088421	Bn-N5-p19057410	gene	
Bn-A05-p18112139	Bn-N5-p19080269	gene	
Bn-A05-p18125663	Bn-N5-p19101504	gene	
Bn-A05-p18139526	Bn-N5-p19115298	gene	
Bn-A05-p18147040	Bn-Scaffold01376-p6621	gene	
Bn-A05-p18161591	Bn-N5-p19136523	gene	exon
Bn-A05-p18191062	Bn-N5-p19155169	gene	
Bn-A05-p18196942	Bn-N5-p19164488	gene	exon
Bn-A05-p18229790	Bn-N5-p19205661	gene	
Bn-A05-p182664	Bn-N5-p215149	gene	
Bn-A05-p18309576	Bn-N5-p19266967	gene	exon
Bn-A05-p18310658	Bn-N5-p19268047	gene	
Bn-A05-p18310838	Bn-N5-p19268227	gene	
Bn-A05-p18391243	Bn-N5-p19350612	gene	
Bn-A05-p18391581	Bn-N5-p19350950	gene	
Bn-A05-p18452462	Bn-N5-p19412722	gene	
Bn-A05-p18452563	Bn-N5-p19412824	gene	
Bn-A05-p18453140	Bn-N5-p19413401	gene	
Bn-A05-p18458181	Bn-N5-p19422118	gene	exon
Bn-A05-p18490530	Bn-N5-p19458814	gene	
Bn-A05-p18505935	Bn-N5-p19477927	gene	exon
Bn-A05-p18543237	Bn-N5-p19498317	gene	
Bn-A05-p18543933	Bn-N5-p19498971	gene	
Bn-A05-p18547040	Bn-N5-p19501941	gene	
Bn-A05-p1857946	Bn-N5-p2039192	gene	exon
Bn-A05-p18598001	Bn-N5-p19546408	gene	
Bn-A05-p18642842	Bn-N5-p19591839	gene	
Bn-A05-p18643459	Bn-N5-p19592456	gene	
Bn-A05-p18689306	Bn-N5-p19642096	gene	
Bn-A05-p18730286	Bn-N5-p19673681	gene	
Bn-A05-p18768801	Bn-N5-p19730837	gene	
Bn-A05-p18770393	Bn-N5-p19732402	gene	exon
Bn-A05-p18773930	Bn-N5-p19736037	gene	
Bn-A05-p18793322	Bn-N5-p19752600	gene	exon
Bn-A05-p18807426	Bn-N5-p19772031	gene	exon
Bn-A05-p18828578	Bn-N5-p19793031	gene	exon
Bn-A05-p18828589	Bn-N5-p19793042	gene	exon
Bn-A05-p18905070	Bn-N5-p19863530	gene	
Bn-A05-p18922154	Bn-N5-p19878612	gene	
Bn-A05-p18958059	Bn-N5-p19908496	gene	
Bn-A05-p18958083	Bn-N5-p19908520	gene	

Bn-A05-p1898273	Bn-N5-p2075840	gene	
Bn-A05-p19001293	Bn-N5-p19946962	gene	
Bn-A05-p19017469	Bn-N5-p19964602	gene	exon
Bn-A05-p19031269	Bn-N5-p19975569	gene	
Bn-A05-p19041695	Bn-N5-p19986389	gene	exon
Bn-A05-p19048941	Bn-N5-p19993850	gene	exon
Bn-A05-p19064234	Bn-N5-p20005163	gene	
Bn-A05-p19067421	Bn-N5-p20008350	gene	
Bn-A05-p19071118	Bn-N5-p20016769	gene	
Bn-A05-p19096242	Bn-N5-p20037433	gene	exon
Bn-A05-p19128595	Bn-N5-p20074142	gene	
Bn-A05-p19130774	Bn-N5-p20076321	gene	
Bn-A05-p19187149	Bn-N5-p20135232	gene	
Bn-A05-p19195181	Bn-N5-p20142943	gene	
Bn-A05-p19209866	Bn-N5-p20155541	gene	exon
Bn-A05-p19224833	Bn-N5-p20178234	gene	exon
Bn-A05-p19238078	Bn-N5-p20191672	gene	
Bn-A05-p19238134	Bn-N5-p20191728	gene	
Bn-A05-p19238534	Bn-N5-p20192126	gene	
Bn-A05-p19268141	Bn-N5-p20211837	gene	exon
Bn-A05-p19278249	Bn-N5-p20230377	gene	
Bn-A05-p19278438	Bn-N5-p20230566	gene	
Bn-A05-p19359433	Bn-N5-p20308057	gene	
Bn-A05-p19359443	Bn-N5-p20308067	gene	
Bn-A05-p1942242	Bn-N5-p2132498	gene	
Bn-A05-p1943093	Bn-N5-p2132766	gene	
Bn-A05-p19473882	Bn-N5-p20426549	gene	exon
Bn-A05-p19530568	Bn-N5-p20484395	gene	
Bn-A05-p19532660	Bn-N5-p20486481	gene	
Bn-A05-p19550645	Bn-N5-p20506085	gene	exon
Bn-A05-p19554281	Bn-N5-p20509798	gene	exon
Bn-A05-p19571798	Bn-N5-p20531407	gene	
Bn-A05-p19583714	Bn-N5-p20543965	gene	
Bn-A05-p19622826	Bn-N5-p20590239	gene	exon
Bn-A05-p19650213	Bn-N5-p20653161	gene	
Bn-A05-p19650278	Bn-N5-p20653226	gene	
Bn-A05-p19653084	Bn-N5-p20656318	gene	
Bn-A05-p19728614	Bn-N15-p40940767	gene	exon
Bn-A05-p19745674	Bn-N5-p20746277	gene	
Bn-A05-p1975785	Bn-N5-p2163761	gene	
Bn-A05-p19777231	Bn-N5-p20764810	gene	
Bn-A05-p19777547	Bn-N5-p20765142	gene	
Bn-A05-p19802577	Bn-N5-p20796236	gene	exon
Bn-A05-p19857351	Bn-N5-p20863496	gene	
Bn-A05-p19857532	Bn-N15-p41170678	gene	exon
Bn-A05-p19919111	Bn-N5-p20921026	gene	
Bn-A05-p19919179	Bn-N5-p20921094	gene	
Bn-A05-p19930910	Bn-N5-p20933033	gene	
Bn-A05-p19943528	Bn-N15-p41305004	gene	
Bn-A05-p19946413	Bn-N5-p20947845	gene	
Bn-A05-p19967151	Bn-N5-p20959304	gene	exon
Bn-A05-p19973413	Bn-N5-p20966234	gene	exon

Bn-A05-p20009925	Bn-N5-p20990105	gene	exon
Bn-A05-p20019130	Bn-N5-p20999054	gene	
Bn-A05-p20075053	Bn-N5-p21047945	gene	exon
Bn-A05-p20100234	Bn-N15-p41508343	gene	exon
Bn-A05-p20124425	Bn-N5-p21103930	gene	
Bn-A05-p20128796	Bn-N5-p21111137	gene	exon
Bn-A05-p20139872	Bn-N5-p21124163	gene	
Bn-A05-p20161210	Bn-N5-p21148095	gene	exon
Bn-A05-p20163118	Bn-N5-p21150003	gene	
Bn-A05-p20168806	Bn-N5-p21155697	gene	exon
Bn-A05-p20168909	Bn-N5-p21155799	gene	exon
Bn-A05-p20174313	Bn-N5-p21161214	gene	exon
Bn-A05-p20178007	Bn-N5-p21164918	gene	
Bn-A05-p20232989	Bn-N5-p21229250	gene	exon
Bn-A05-p2024975	Bn-N5-p2214915	gene	
Bn-A05-p20292751	Bn-N5-p21285273	gene	exon
Bn-A05-p20302128	Bn-N5-p21301220	gene	exon
Bn-A05-p20314128	Bn-N5-p21314748	gene	
Bn-A05-p20316923	Bn-N5-p21317537	gene	exon
Bn-A05-p20319622	Bn-N5-p21320240	gene	
Bn-A05-p20328249	Bn-N5-p21328859	gene	exon
Bn-A05-p20328443	Bn-N6-p12740623	gene	
Bn-A05-p20337405	Bn-N15-p41803731	gene	exon
Bn-A05-p20359553	Bn-N5-p21367608	gene	exon
Bn-A05-p20365953	Bn-N15-p41914828	gene	exon
Bn-A05-p20372600	Bn-N5-p21377099	gene	exon
Bn-A05-p20375812	Bn-N5-p21380281	gene	exon
Bn-A05-p20386700	Bn-N5-p21394148	gene	
Bn-A05-p2039690	Bn-N5-p2230561	gene	
Bn-A05-p20399452	Bn-N5-p21409222	gene	exon
Bn-A05-p20422081	Bn-N15-p42032342	gene	exon
Bn-A05-p20425452	Bn-N5-p21430652	gene	
Bn-A05-p20425648	Bn-N5-p21430848	gene	exon
Bn-A05-p20461416	Bn-N5-p21457459	gene	
Bn-A05-p20461727	Bn-N5-p21457770	gene	
Bn-A05-p20478486	Bn-N5-p21473270	gene	exon
Bn-A05-p20492525	Bn-N5-p21490939	gene	
Bn-A05-p20550694	Bn-N5-p21552451	gene	exon
Bn-A05-p20555996	Bn-N5-p21557718	gene	
Bn-A05-p20563653	Bn-N5-p21565316	gene	exon
Bn-A05-p20564729	Bn-N5-p21566392	gene	exon
Bn-A05-p20594196	Bn-N5-p21593116	gene	exon
Bn-A05-p20601139	Bn-N5-p21609623	gene	
Bn-A05-p20673293	Bn-N5-p21686601	gene	
Bn-A05-p20681871	Bn-N5-p21694862	gene	
Bn-A05-p20690058	Bn-N5-p21700221	gene	
Bn-A05-p20698928	Bn-N5-p21707505	gene	exon
Bn-A05-p20699671	Bn-N5-p21707895	gene	
Bn-A05-p20712388	Bn-N5-p21721917	gene	
Bn-A05-p20714207	Bn-N5-p21723709	gene	exon
Bn-A05-p20714302	Bn-N5-p21723804	gene	exon
Bn-A05-p20714581	Bn-N5-p21724083	gene	exon

Bn-A05-p20716500	Bn-N5-p21726006	gene	exon
Bn-A05-p20718776	Bn-N5-p21728375	gene	exon
Bn-A05-p20728232	Bn-N5-p21737810	gene	
Bn-A05-p20728284	Bn-N15-p42678814	gene	
Bn-A05-p20811510	Bn-N5-p21835724	gene	exon
Bn-A05-p20824936	Bn-N5-p21843939	gene	
Bn-A05-p20828693	Bn-N5-p21848115	gene	exon
Bn-A05-p20888695	Bn-N5-p21899709	gene	
Bn-A05-p20894465	Bn-N15-p42927017	gene	
Bn-A05-p20903214	Bn-N5-p21916523	gene	
Bn-A05-p20903608	Bn-N5-p21916921	gene	
Bn-A05-p21000614	Bn-N5-p22021619	gene	
Bn-A05-p2102634	Bn-N5-p2295512	gene	exon
Bn-A05-p21036828	Bn-N5-p22058965	gene	
Bn-A05-p21064254	Bn-N5-p22086864	gene	exon
Bn-A05-p21065087	Bn-N5-p22087896	gene	
Bn-A05-p21070302	Bn-N5-p22092218	gene	exon
Bn-A05-p21070991	Bn-N5-p22092907	gene	exon
Bn-A05-p2107808	Bn-N5-p2299339	gene	exon
Bn-A05-p21081145	Bn-N5-p22097194	gene	
Bn-A05-p2108713	Bn-N5-p2300244	gene	exon
Bn-A05-p21093954	Bn-N5-p22109604	gene	exon
Bn-A05-p2109546	Bn-N5-p2301077	gene	
Bn-A05-p2110255	Bn-N5-p2302923	gene	
Bn-A05-p2110344	Bn-N5-p2303012	gene	
Bn-A05-p2111165	Bn-N5-p2303825	gene	exon
Bn-A05-p21163226	Bn-N5-p22198721	gene	
Bn-A05-p21182276	Bn-N5-p22216406	gene	
Bn-A05-p21188442	Bn-N5-p22222880	gene	
Bn-A05-p21193491	Bn-N5-p22228410	gene	
Bn-A05-p21201908	Bn-N5-p22236093	gene	exon
Bn-A05-p21201956	Bn-N5-p22236141	gene	exon
Bn-A05-p21210078	Bn-N5-p22244271	gene	exon
Bn-A05-p21233666	Bn-N5-p22267180	gene	exon
Bn-A05-p21243931	Bn-N5-p22284184	gene	exon
Bn-A05-p21248382	Bn-N5-p22288664	gene	
Bn-A05-p21248720	Bn-N5-p22288997	gene	exon
Bn-A05-p21267598	Bn-N5-p22308940	gene	
Bn-A05-p21299910	Bn-N5-p22345549	gene	
Bn-A05-p21319243	Bn-N5-p22361875	gene	exon
Bn-A05-p21326091	Bn-N5-p22365600	gene	
Bn-A05-p21326775	Bn-N5-p22366288	gene	exon
Bn-A05-p21345579	Bn-N15-p43570276	gene	
Bn-A05-p21347821	Bn-N5-p22392693	gene	
Bn-A05-p21370435	Bn-N5-p22475280	gene	exon
Bn-A05-p21370618	Bn-N5-p22475463	gene	exon
Bn-A05-p21375513	Bn-N5-p22477845	gene	
Bn-A05-p21375514	Bn-N5-p22477845	gene	
Bn-A05-p21377682	Bn-N5-p22480062	gene	exon
Bn-A05-p21381782	Bn-N5-p22483019	gene	
Bn-A05-p2138342	Bn-N5-p2335324	gene	
Bn-A05-p21385524	Bn-N5-p22489513	gene	

Bn-A05-p2139529	Bn-N5-p2336512	gene	exon
Bn-A05-p2143241	Bn-N5-p2340764	gene	exon
Bn-A05-p2143292	Bn-N5-p2340815	gene	exon
Bn-A05-p21463902	Bn-N15-p43799232	gene	exon
Bn-A05-p21470614	Bn-N5-p22571580	gene	
Bn-A05-p21480994	Bn-N5-p22581296	gene	
Bn-A05-p21484113	Bn-N5-p22584410	gene	
Bn-A05-p21532839	Bn-N5-p22635384	gene	exon
Bn-A05-p2155466	Bn-N5-p2357631	gene	exon
Bn-A05-p21633055	Bn-N5-p22738083	gene	
Bn-A05-p21668872	Bn-N5-p22769764	gene	exon
Bn-A05-p21806801	Bn-N5-p22896160	gene	exon
Bn-A05-p21848003	Bn-N5-p22904785	gene	exon
Bn-A05-p21852014	Bn-N5-p22908803	gene	exon
Bn-A05-p21865451	Bn-N5-p22920042	gene	exon
Bn-A05-p21886422	Bn-N5-p22949280	gene	exon
Bn-A05-p21915455	Bn-Scaffold03647-p2401	gene	exon
Bn-A05-p21973951	Bn-N5-p23033256	gene	
Bn-A05-p22015412	Bn-N5-p23079653	gene	
Bn-A05-p22028401	Bn-N5-p23094897	gene	
Bn-A05-p22060825	Bn-N5-p23110211	gene	
Bn-A05-p22089650	Bn-N5-p23135358	gene	
Bn-A05-p22101592	Bn-N5-p23143905	gene	
Bn-A05-p22111286	Bn-N5-p23146885	gene	exon
Bn-A05-p22111345	Bn-N5-p23146944	gene	exon
Bn-A05-p22114137	Bn-N5-p23149741	gene	
Bn-A05-p22132297	Bn-N5-p23167931	gene	
Bn-A05-p22135265	Bn-N5-p23170850	gene	exon
Bn-A05-p22141046	Bn-N5-p23176773	gene	exon
Bn-A05-p22142609	Bn-N5-p23178285	gene	exon
Bn-A05-p22153383	Bn-N5-p23204638	gene	exon
Bn-A05-p22159613	Bn-N5-p23215538	gene	
Bn-A05-p22169560	Bn-N5-p23229552	gene	
Bn-A05-p22221563	Bn-N5-p23283281	gene	
Bn-A05-p22269511	Bn-N5-p23332175	gene	exon
Bn-A05-p22320477	Bn-N5-p23383035	gene	exon
Bn-A05-p22324761	Bn-N5-p23387315	gene	exon
Bn-A05-p22392750	Bn-N5-p23463991	gene	
Bn-A05-p22406985	Bn-N5-p23484017	gene	
Bn-A05-p22427412	Bn-N5-p23503111	gene	exon
Bn-A05-p22459438	Bn-N5-p23534788	gene	
Bn-A05-p22463256	Bn-N5-p23538588	gene	exon
Bn-A05-p22476414	Bn-N5-p23551706	gene	exon
Bn-A05-p22478538	Bn-N5-p23553830	gene	exon
Bn-A05-p22506006	Bn-N5-p23578251	gene	exon
Bn-A05-p22513075	Bn-N5-p23587400	gene	exon
Bn-A05-p22519751	Bn-N5-p23601213	gene	
Bn-A05-p22519836	Bn-N5-p23601298	gene	
Bn-A05-p2254100	Bn-N5-p2449849	gene	
Bn-A05-p22553631	Bn-N5-p23621952	gene	exon
Bn-A05-p22563719	Bn-N5-p23632498	gene	
Bn-A05-p22589874	Bn-N5-p23651326	gene	exon

Bn-A05-p22591072	Bn-N5-p23652525	gene	
Bn-A05-p22591626	Bn-N5-p23653076	gene	
Bn-A05-p22599241	Bn-N5-p23662711	gene	exon
Bn-A05-p22636722	Bn-N5-p23705220	gene	exon
Bn-A05-p22650582	Bn-N5-p23716430	gene	
Bn-A05-p22651145	Bn-N5-p23716993	gene	exon
Bn-A05-p22660561	Bn-N5-p23726662	gene	exon
Bn-A05-p22677701	Bn-N5-p23742924	gene	
Bn-A05-p22677829	Bn-N5-p23743052	gene	
Bn-A05-p22677937	Bn-N5-p23743160	gene	
Bn-A05-p22689848	Bn-N5-p23757583	gene	exon
Bn-A05-p22712783	Bn-N5-p23775665	gene	exon
Bn-A05-p2272518	Bn-N5-p2468787	gene	
Bn-A05-p2273196	Bn-N5-p2469464	gene	
Bn-A05-p2274301	Bn-N5-p2470569	gene	exon
Bn-A05-p22780601	Bn-N5-p23850449	gene	
Bn-A05-p22780670	Bn-N5-p23850516	gene	
Bn-A05-p22789344	Bn-N5-p23859137	gene	exon
Bn-A05-p2280549	Bn-N5-p2476665	gene	
Bn-A05-p22812957	Bn-N5-p23882504	gene	exon
Bn-A05-p22813120	Bn-N5-p23882667	gene	exon
Bn-A05-p22813805	Bn-N5-p23883352	gene	exon
Bn-A05-p2285903	Bn-N5-p2481355	gene	
Bn-A05-p22863641	Bn-N5-p23926491	gene	
Bn-A05-p2287815	Bn-N5-p2483134	gene	
Bn-A05-p22889788	Bn-N5-p23989852	gene	
Bn-A05-p22899595	Bn-N5-p23997708	gene	
Bn-A05-p22911906	Bn-N5-p24009871	gene	
Bn-A05-p22912544	Bn-N5-p24010509	gene	
Bn-A05-p22913147	Bn-N5-p24011109	gene	
Bn-A05-p22913571	Bn-N5-p24011532	gene	
Bn-A05-p22913874	Bn-N5-p24011823	gene	
Bn-A05-p22915357	Bn-N5-p24013306	gene	
Bn-A05-p22917436	Bn-N5-p24015388	gene	
Bn-A05-p22920228	Bn-N5-p24018180	gene	
Bn-A05-p22934224	Bn-N5-p24027080	gene	exon
Bn-A05-p2293577	Bn-N5-p2490392	gene	
Bn-A05-p22938228	Bn-N5-p24032689	gene	exon
Bn-A05-p22943215	Bn-N5-p24038161	gene	
Bn-A05-p22946715	Bn-N5-p24041620	gene	
Bn-A05-p22946858	Bn-N5-p24041763	gene	
Bn-A05-p22948751	Bn-N5-p24043719	gene	exon
Bn-A05-p2296307	Bn-N5-p2493077	gene	exon
Bn-A05-p22969236	Bn-N15-p45959527	gene	exon
Bn-A05-p22973324	Bn-N5-p24063948	gene	
Bn-A05-p22976431	Bn-N5-p24070006	gene	
Bn-A05-p22977061	Bn-N5-p24070632	gene	
Bn-A05-p23019620	Bn-N5-p24114691	gene	
Bn-A05-p23020463	Bn-N5-p24114724	gene	
Bn-A05-p23020834	Bn-N5-p24115095	gene	
Bn-A05-p23027881	Bn-N5-p24122493	gene	exon
Bn-A05-p23030143	Bn-N5-p24126846	gene	exon

Bn-A05-p2303112	Bn-N5-p2509507	gene	exon
Bn-A05-p23032803	Bn-N5-p24129511	gene	
Bn-A05-p23079485	Bn-N5-p24168396	gene	exon
Bn-A05-p23102053	Bn-N5-p24193947	gene	exon
Bn-A05-p23126108	Bn-N5-p24227096	gene	
Bn-A05-p23140205	Bn-N5-p24252004	gene	
Bn-A05-p23141080	Bn-N5-p24252879	gene	
Bn-A05-p23143046	Bn-N5-p24254846	gene	exon
Bn-A05-p23145678	Bn-N5-p24257477	gene	exon
Bn-A05-p23146882	Bn-N5-p24258683	gene	
Bn-A05-p23188406	Bn-N5-p24299292	gene	exon
Bn-A05-p23253826	Bn-N5-p24618267	gene	
Bn-A05-p23276053	Bn-N5-p24638929	gene	
Bn-A05-p23285926	Bn-N5-p24655514	gene	
Bn-A05-p23288913	Bn-N5-p24657940	gene	exon
Bn-A05-p23290863	Bn-N5-p24659891	gene	
Bn-A05-p23314738	Bn-N5-p24678264	gene	exon
Bn-A05-p23340419	Bn-N5-p24703204	gene	
Bn-A05-p23358283	Bn-N5-p24720294	gene	
Bn-A05-p2337684	Bn-N5-p2530906	gene	
Bn-A05-p23377419	Bn-N5-p24738956	gene	
Bn-A05-p23424482	Bn-N5-p24780757	gene	
Bn-A05-p23445454	Bn-N5-p24808015	gene	exon
Bn-A05-p2345233	Bn-N14-p3987756	gene	
Bn-A05-p2345849	Bn-N14-p3988373	gene	exon
Bn-A05-p23498540	Bn-N5-p24861878	gene	
Bn-A05-p2350131	Bn-N5-p2546124	gene	
Bn-A05-p23502993	Bn-N5-p24865653	gene	exon
Bn-A05-p23512396	Bn-N5-p24875054	gene	
Bn-A05-p23512931	Bn-N5-p24875589	gene	exon
Bn-A05-p2351545	Bn-N5-p2547531	gene	exon
Bn-A05-p23543751	Bn-N5-p25412615	gene	exon
Bn-A05-p23547791	Bn-N5-p25416667	gene	exon
Bn-A05-p23588992	Bn-N5-p25460397	gene	exon
Bn-A05-p23593836	Bn-N5-p25465340	gene	
Bn-A05-p23607588	Bn-N5-p25483004	gene	exon
Bn-A05-p2367876	Bn-N5-p2562704	gene	exon
Bn-A05-p23692249	Bn-N5-p25561438	gene	
Bn-A05-p23693572	Bn-N5-p25562767	gene	exon
Bn-A05-p23745136	Bn-N5-p25622925	gene	exon
Bn-A05-p2376801	Bn-N5-p2572299	gene	
Bn-A05-p23776343	Bn-N5-p25659292	gene	
Bn-A05-p23776869	Bn-N5-p25659796	gene	
Bn-A05-p23802326	Bn-N5-p25684089	gene	exon
Bn-A05-p23844016	Bn-Scaffold01069-p22378	gene	
Bn-A05-p23844225	Bn-Scaffold01069-p22210	gene	
Bn-A05-p23855991	Bn-Scaffold01727-p1531	gene	
Bn-A05-p2416806	Bn-N5-p2612057	gene	exon
Bn-A05-p244051	Bn-N5-p272544	gene	
Bn-A05-p2460244	Bn-N5-p2649995	gene	
Bn-A05-p2472050	Bn-N5-p2661410	gene	exon
Bn-A05-p2472584	Bn-N5-p2661943	gene	

Bn-A05-p2488952	Bn-N5-p2675301	gene	
Bn-A05-p2489101	Bn-N5-p2675450	gene	
Bn-A05-p2492451	Bn-N5-p2676866	gene	
Bn-A05-p2494204	Bn-N5-p2678611	gene	exon
Bn-A05-p2495066	Bn-N5-p2679473	gene	
Bn-A05-p2495419	Bn-N5-p2679827	gene	
Bn-A05-p2495697	Bn-N5-p2680108	gene	
Bn-A05-p2497466	Bn-N5-p2681879	gene	
Bn-A05-p253584	Bn-N5-p281613	gene	exon
Bn-A05-p2563320	Bn-N5-p2745504	gene	
Bn-A05-p2565185	Bn-N5-p2748760	gene	exon
Bn-A05-p2565586	Bn-N5-p2749161	gene	exon
Bn-A05-p2582423	Bn-N5-p2769101	gene	exon
Bn-A05-p2592847	Bn-N5-p2778500	gene	exon
Bn-A05-p2604531	Bn-N5-p2794879	gene	exon
Bn-A05-p2604957	Bn-N5-p2795396	gene	
Bn-A05-p2610006	Bn-N5-p2816986	gene	
Bn-A05-p262934	Bn-N5-p291486	gene	exon
Bn-A05-p2645307	Bn-N5-p2830757	gene	exon
Bn-A05-p2645365	Bn-N5-p2830821	gene	
Bn-A05-p2648679	Bn-N5-p2843690	gene	exon
Bn-A05-p2653139	Bn-N5-p2848406	gene	exon
Bn-A05-p2656042	Bn-N5-p2851329	gene	exon
Bn-A05-p2671921	Bn-N5-p2870666	gene	exon
Bn-A05-p2711007	Bn-N5-p2902787	gene	
Bn-A05-p2711208	Bn-N5-p2902988	gene	exon
Bn-A05-p2718015	Bn-N5-p2912485	gene	exon
Bn-A05-p2732409	Bn-N5-p2927711	gene	exon
Bn-A05-p2777824	Bn-N5-p2974613	gene	
Bn-A05-p2777912	Bn-N5-p2974701	gene	
Bn-A05-p2778040	Bn-N5-p2974829	gene	exon
Bn-A05-p2778230	Bn-N5-p2975016	gene	
Bn-A05-p2778577	Bn-N5-p2975363	gene	
Bn-A05-p2778679	Bn-N5-p2975465	gene	
Bn-A05-p2778737	Bn-N5-p2975523	gene	
Bn-A05-p2779142	Bn-N5-p2975937	gene	
Bn-A05-p2804703	Bn-N5-p2992931	gene	exon
Bn-A05-p2830103	Bn-N5-p3017274	gene	exon
Bn-A05-p2839985	Bn-N5-p3028806	gene	
Bn-A05-p2840537	Bn-N5-p3029498	gene	
Bn-A05-p2840734	Bn-N5-p3029695	gene	
Bn-A05-p2841024	Bn-N5-p3029985	gene	exon
Bn-A05-p2843347	Bn-N5-p3032379	gene	
Bn-A05-p2844059	Bn-N5-p3033090	gene	
Bn-A05-p2898061	Bn-N14-p4843841	gene	exon
Bn-A05-p2937826	Bn-N5-p3162058	gene	exon
Bn-A05-p2937829	Bn-N5-p3162061	gene	exon
Bn-A05-p2984656	Bn-N5-p3208071	gene	exon
Bn-A05-p3043365	Bn-N5-p3275694	gene	
Bn-A05-p3099878	Bn-N5-p3333556	gene	
Bn-A05-p3117520	Bn-N5-p3361502	gene	
Bn-A05-p3188172	Bn-N5-p3436050	gene	exon

Bn-A05-p3191390	Bn-N5-p3439087	gene	
Bn-A05-p3198955	Bn-N5-p3445947	gene	exon
Bn-A05-p3242299	Bn-N5-p3492083	gene	exon
Bn-A05-p3246311	Bn-N5-p3496090	gene	
Bn-A05-p3248878	Bn-N5-p3498660	gene	
Bn-A05-p3264129	Bn-N5-p3515043	gene	
Bn-A05-p3278023	Bn-N5-p3523252	gene	
Bn-A05-p3280592	Bn-N5-p3525724	gene	
Bn-A05-p3312679	Bn-N5-p3558019	gene	exon
Bn-A05-p3312786	Bn-N5-p3558126	gene	exon
Bn-A05-p3320783	Bn-N5-p3569934	gene	exon
Bn-A05-p3323878	Bn-N14-p5658458	gene	exon
Bn-A05-p3361741	Bn-N5-p3602767	gene	
Bn-A05-p3369681	Bn-N5-p3609983	gene	
Bn-A05-p3394311	Bn-N5-p3631412	gene	
Bn-A05-p3406573	Bn-N5-p3644816	gene	
Bn-A05-p3467036	Bn-N5-p3707907	gene	exon
Bn-A05-p346822	Bn-N5-p384683	gene	exon
Bn-A05-p347486	Bn-N5-p385347	gene	
Bn-A05-p3482813	Bn-N5-p3729580	gene	exon
Bn-A05-p3516602	Bn-N5-p3752902	gene	exon
Bn-A05-p3548736	Bn-N5-p3785693	gene	exon
Bn-A05-p3610111	Bn-N5-p3853841	gene	
Bn-A05-p3611878	Bn-N5-p3857996	gene	exon
Bn-A05-p3618356	Bn-N5-p3873260	gene	
Bn-A05-p363138	Bn-N5-p419017	gene	
Bn-A05-p3681637	Bn-N5-p3957300	gene	
Bn-A05-p3719423	Bn-N5-p3984819	gene	exon
Bn-A05-p3833308	Bn-N14-p6639070	gene	exon
Bn-A05-p3834445	Bn-N5-p4104667	gene	
Bn-A05-p3834639	Bn-N5-p4104862	gene	
Bn-A05-p3882826	Bn-N5-p4150021	gene	exon
Bn-A05-p3901565	Bn-N5-p4166065	gene	
Bn-A05-p3939102	Bn-N5-p4203438	gene	
Bn-A05-p3966848	Bn-N5-p4230168	gene	exon
Bn-A05-p3988218	Bn-N5-p4249789	gene	
Bn-A05-p3988279	Bn-N5-p4249850	gene	
Bn-A05-p3988958	Bn-N5-p4250529	gene	exon
Bn-A05-p3989555	Bn-N5-p4251126	gene	
Bn-A05-p4006770	Bn-N5-p4268889	gene	exon
Bn-A05-p4016172	Bn-N5-p4278430	gene	exon
Bn-A05-p4017270	Bn-N5-p4279532	gene	
Bn-A05-p4022440	Bn-N5-p4284420	gene	
Bn-A05-p4023464	Bn-N5-p4285459	gene	
Bn-A05-p4036806	Bn-N5-p4292309	gene	
Bn-A05-p4043474	Bn-N5-p4302708	gene	exon
Bn-A05-p4054823	Bn-N5-p4314001	gene	
Bn-A05-p4055656	Bn-N5-p4314837	gene	
Bn-A05-p4055839	Bn-N5-p4315020	gene	
Bn-A05-p4078496	Bn-N5-p4338418	gene	
Bn-A05-p4086144	Bn-N5-p4346940	gene	
Bn-A05-p4104070	Bn-N5-p4366930	gene	exon

Bn-A05-p4108818	Bn-N5-p4372981	gene	exon
Bn-A05-p4110015	Bn-N5-p4374186	gene	exon
Bn-A05-p4121158	Bn-N5-p4386273	gene	exon
Bn-A05-p4176921	Bn-N5-p4433574	gene	exon
Bn-A05-p4197697	Bn-N5-p4451869	gene	
Bn-A05-p4230422	Bn-N5-p4488675	gene	
Bn-A05-p4316943	Bn-N5-p4565962	gene	
Bn-A05-p4336667	Bn-N5-p4597112	gene	exon
Bn-A05-p4353454	Bn-N5-p4607614	gene	
Bn-A05-p4354442	Bn-N5-p4608601	gene	
Bn-A05-p4373694	Bn-N5-p4623279	gene	exon
Bn-A05-p4376029	Bn-N5-p4625600	gene	
Bn-A05-p4399949	Bn-N5-p4641889	gene	
Bn-A05-p4422786	Bn-N5-p4663810	gene	
Bn-A05-p44690	Bn-N5-p49148	gene	
Bn-A05-p449086	Bn-N5-p524531	gene	
Bn-A05-p4498290	Bn-N5-p4720302	gene	exon
Bn-A05-p4498398	Bn-N5-p4720410	gene	exon
Bn-A05-p4516526	Bn-N5-p4775030	gene	exon
Bn-A05-p4516748	Bn-N14-p7707113	gene	exon
Bn-A05-p4520844	Bn-N14-p7710650	gene	exon
Bn-A05-p452939	Bn-N5-p528432	gene	exon
Bn-A05-p4569670	Bn-N5-p4831145	gene	
Bn-A05-p458357	Bn-N5-p533849	gene	
Bn-A05-p4593001	Bn-N5-p4848123	gene	exon
Bn-A05-p4625019	Bn-N5-p4875635	gene	
Bn-A05-p4644984	Bn-N5-p4902267	gene	
Bn-A05-p4679663	Bn-N5-p4945932	gene	exon
Bn-A05-p472271	Bn-N5-p547781	gene	exon
Bn-A05-p4820365	Bn-N5-p5097227	gene	
Bn-A05-p482473	Bn-N5-p561498	gene	
Bn-A05-p484591	Bn-N5-p564912	gene	exon
Bn-A05-p4919899	Bn-N5-p5176166	gene	
Bn-A05-p4924856	Bn-N5-p5181567	gene	exon
Bn-A05-p4959282	Bn-N5-p5206299	gene	
Bn-A05-p4961402	Bn-N5-p5216947	gene	exon
Bn-A05-p5026139	Bn-N5-p5284473	gene	exon
Bn-A05-p5037839	Bn-N5-p5295656	gene	exon
Bn-A05-p5043499	Bn-N5-p5301311	gene	exon
Bn-A05-p5057096	Bn-N5-p5316568	gene	exon
Bn-A05-p5057141	Bn-N5-p5316613	gene	exon
Bn-A05-p5058143	Bn-N5-p5317614	gene	exon
Bn-A05-p5093548	Bn-N5-p5361751	gene	
Bn-A05-p5100352	Bn-N5-p5368829	gene	exon
Bn-A05-p5104821	Bn-N5-p5376501	gene	exon
Bn-A05-p5135426	Bn-N5-p5403580	gene	
Bn-A05-p518923	Bn-N5-p593059	gene	exon
Bn-A05-p5251613	Bn-N5-p5501272	gene	exon
Bn-A05-p5252542	Bn-N5-p5502201	gene	
Bn-A05-p5279229	Bn-N5-p5552874	gene	exon
Bn-A05-p5280016	Bn-N5-p5553661	gene	
Bn-A05-p5281478	Bn-N5-p5555132	gene	

Bn-A05-p5283304	Bn-N14-p9197951	gene	exon
Bn-A05-p5328959	Bn-N5-p5602569	gene	exon
Bn-A05-p5337620	Bn-N5-p5611393	gene	exon
Bn-A05-p5391937	Bn-N5-p5672976	gene	
Bn-A05-p5437257	Bn-N5-p5711890	gene	exon
Bn-A05-p5445252	Bn-N5-p5722369	gene	
Bn-A05-p5446122	Bn-N5-p5722728	gene	
Bn-A05-p5457877	Bn-N5-p5731679	gene	
Bn-A05-p547582	Bn-N5-p622657	gene	
Bn-A05-p5521242	Bn-N5-p5789631	gene	exon
Bn-A05-p5522210	Bn-N5-p5790600	gene	exon
Bn-A05-p5541232	Bn-N5-p5806607	gene	exon
Bn-A05-p5542434	Bn-N5-p5807936	gene	exon
Bn-A05-p5552246	Bn-N5-p5817607	gene	exon
Bn-A05-p5565945	Bn-N5-p5831310	gene	exon
Bn-A05-p5614651	Bn-N5-p5885217	gene	
Bn-A05-p5657708	Bn-N5-p5929818	gene	
Bn-A05-p5661136	Bn-N5-p5933631	gene	exon
Bn-A05-p5662601	Bn-N5-p5935093	gene	exon
Bn-A05-p5714702	Bn-N5-p5978793	gene	exon
Bn-A05-p5714756	Bn-N5-p5978847	gene	
Bn-A05-p5714836	Bn-N5-p5978927	gene	
Bn-A05-p5715403	Bn-N5-p5979494	gene	exon
Bn-A05-p5778158	Bn-N5-p6046199	gene	exon
Bn-A05-p5790313	Bn-N5-p6064158	gene	exon
Bn-A05-p5796678	Bn-N5-p6070566	gene	
Bn-A05-p5805445	Bn-N5-p6077945	gene	exon
Bn-A05-p5808654	Bn-N5-p6081172	gene	
Bn-A05-p5808895	Bn-N5-p6081413	gene	
Bn-A05-p5809043	Bn-N5-p6081561	gene	
Bn-A05-p5817231	Bn-N5-p6087991	gene	
Bn-A05-p5831673	Bn-N5-p6099375	gene	exon
Bn-A05-p5878329	Bn-N5-p6148874	gene	
Bn-A05-p58992	Bn-N5-p78052	gene	
Bn-A05-p59055	Bn-N5-p78115	gene	exon
Bn-A05-p59178	Bn-N5-p78238	gene	
Bn-A05-p5924319	Bn-N5-p6195750	gene	
Bn-A05-p5992612	Bn-N5-p6282683	gene	exon
Bn-A05-p600196	Bn-N5-p684459	gene	
Bn-A05-p6004356	Bn-N5-p6293193	gene	exon
Bn-A05-p6020205	Bn-N5-p6308665	gene	exon
Bn-A05-p6021593	Bn-N5-p6310052	gene	
Bn-A05-p6074427	Bn-N5-p6347876	gene	exon
Bn-A05-p6154871	Bn-N5-p6434195	gene	exon
Bn-A05-p619336	Bn-N5-p701378	gene	
Bn-A05-p6236807	Bn-N5-p6514458	gene	exon
Bn-A05-p6261796	Bn-N5-p6540668	gene	
Bn-A05-p6269991	Bn-N5-p6552395	gene	exon
Bn-A05-p6271443	Bn-N5-p6553847	gene	
Bn-A05-p6288330	Bn-N5-p6575827	gene	
Bn-A05-p6308471	Bn-N5-p6599347	gene	
Bn-A05-p6308543	Bn-N5-p6599419	gene	

Bn-A05-p6402777	Bn-N5-p6686533	gene	
Bn-A05-p6404424	Bn-N5-p6688182	gene	
Bn-A05-p6404595	Bn-N5-p6688353	gene	
Bn-A05-p6404775	Bn-N5-p6688533	gene	exon
Bn-A05-p6405021	Bn-N5-p6688779	gene	exon
Bn-A05-p6405095	Bn-N5-p6688853	gene	exon
Bn-A05-p6405415	Bn-N5-p6689173	gene	exon
Bn-A05-p6412530	Bn-N5-p6696504	gene	
Bn-A05-p6441813	Bn-N5-p6721334	gene	exon
Bn-A05-p6473296	Bn-N5-p6767318	gene	exon
Bn-A05-p6479603	Bn-N5-p6773398	gene	exon
Bn-A05-p6480168	Bn-N5-p6773964	gene	
Bn-A05-p6480907	Bn-N5-p6774703	gene	exon
Bn-A05-p6480981	Bn-N5-p6774777	gene	
Bn-A05-p6494313	Bn-N5-p6784054	gene	
Bn-A05-p652475	Bn-N14-p936241	gene	exon
Bn-A05-p6535233	Bn-N5-p6831295	gene	exon
Bn-A05-p653606	Bn-N5-p738760	gene	
Bn-A05-p6541429	Bn-N5-p6966977	gene	exon
Bn-A05-p6542040	Bn-N5-p6966372	gene	
Bn-A05-p6562415	Bn-N5-p6940989	gene	
Bn-A05-p6571327	Bn-N5-p6932082	gene	
Bn-A05-p6571330	Bn-N5-p6932079	gene	
Bn-A05-p6672638	Bn-N8-p18042252	gene	
Bn-A05-p6674758	Bn-N5-p6976079	gene	exon
Bn-A05-p6682796	Bn-N5-p6984110	gene	
Bn-A05-p671910	Bn-N5-p748023	gene	exon
Bn-A05-p6774249	Bn-N5-p7074505	gene	exon
Bn-A05-p6775737	Bn-N5-p7075993	gene	
Bn-A05-p677619	Bn-N5-p757125	gene	exon
Bn-A05-p6776954	Bn-N5-p7077214	gene	
Bn-A05-p6786862	Bn-N5-p7081718	gene	
Bn-A05-p6787164	Bn-N5-p7082020	gene	
Bn-A05-p6867531	Bn-N5-p7163028	gene	exon
Bn-A05-p6877058	Bn-N5-p7170758	gene	
Bn-A05-p687790	Bn-N5-p767412	gene	
Bn-A05-p6878538	Bn-N5-p7172426	gene	exon
Bn-A05-p6898696	Bn-N14-p12382962	gene	
Bn-A05-p6910402	Bn-N5-p7201619	gene	exon
Bn-A05-p6951707	Bn-N5-p7239361	gene	exon
Bn-A05-p6955996	Bn-Scaffold09921-p280	gene	exon
Bn-A05-p6975353	Bn-N5-p7267602	gene	exon
Bn-A05-p6984362	Bn-N5-p7276887	gene	exon
Bn-A05-p7062575	Bn-N5-p7378703	gene	exon
Bn-A05-p7062784	Bn-N5-p7378912	gene	
Bn-A05-p7076442	Bn-N5-p7398390	gene	
Bn-A05-p7098949	Bn-N5-p7418100	gene	
Bn-A05-p7154689	Bn-N5-p7457067	gene	exon
Bn-A05-p7222795	Bn-N5-p7509876	gene	exon
Bn-A05-p7231369	Bn-N5-p7525358	gene	
Bn-A05-p726481	Bn-N5-p802595	gene	
Bn-A05-p7352554	Bn-N5-p7638503	gene	exon

Bn-A05-p7496554	Bn-N5-p7850202	gene	exon
Bn-A05-p7558647	Bn-N14-p13675046	gene	exon
Bn-A05-p7589125	Bn-N5-p7948967	gene	exon
Bn-A05-p7589257	Bn-N5-p7949099	gene	exon
Bn-A05-p771943	Bn-N5-p871128	gene	
Bn-A05-p772777	Bn-N5-p871952	gene	
Bn-A05-p7729367	Bn-N5-p8083716	gene	exon
Bn-A05-p7741244	Bn-N5-p8094580	gene	exon
Bn-A05-p7781811	Bn-N5-p8137605	gene	exon
Bn-A05-p780844	Bn-N5-p880252	gene	exon
Bn-A05-p7809870	Bn-N5-p8178085	gene	exon
Bn-A05-p784331	Bn-N5-p883877	gene	exon
Bn-A05-p7844876	Bn-N5-p8207484	gene	
Bn-A05-p7855766	Bn-N5-p8218313	gene	exon
Bn-A05-p7903619	Bn-N5-p8251744	gene	
Bn-A05-p7911054	Bn-N5-p8260953	gene	
Bn-A05-p802111	Bn-N5-p903818	gene	
Bn-A05-p8120795	Bn-N8-p20095914	gene	exon
Bn-A05-p8120997	Bn-N8-p20096116	gene	
Bn-A05-p816963	Bn-N5-p916761	gene	exon
Bn-A05-p817036	Bn-N5-p916834	gene	exon
Bn-A05-p8206349	Bn-N8-p20173578	gene	exon
Bn-A05-p8210067	Bn-N18-p21801715	gene	exon
Bn-A05-p822087	Bn-N5-p923155	gene	exon
Bn-A05-p822597	Bn-N5-p923666	gene	
Bn-A05-p8245454	Bn-N8-p20224691	gene	exon
Bn-A05-p8261872	Bn-N8-p20236998	gene	
Bn-A05-p8297352	Bn-N8-p20277216	gene	exon
Bn-A05-p8297563	Bn-N8-p20277427	gene	exon
Bn-A05-p8320167	Bn-N8-p20301042	gene	exon
Bn-A05-p8322435	Bn-N8-p20303309	gene	exon
Bn-A05-p8327216	Bn-N8-p20308092	gene	exon
Bn-A05-p8542569	Bn-N2-p17286059	gene	exon
Bn-A05-p8786891	Bn-N2-p17049997	gene	
Bn-A05-p8798628	Bn-N2-p17038049	gene	exon
Bn-A05-p889343	Bn-N5-p997791	gene	exon
Bn-A05-p894768	Bn-N14-p1335565	gene	exon
Bn-A05-p895276	Bn-N5-p1003703	gene	
Bn-A05-p895522	Bn-N5-p1003937	gene	exon
Bn-A05-p9058906	Bn-N2-p16771429	gene	
Bn-A05-p9063726	Bn-N2-p16762042	gene	exon
Bn-A05-p907258	Bn-N5-p1015308	gene	exon
Bn-A05-p9116236	Bn-N2-p16703689	gene	exon
Bn-A05-p9118240	Bn-N2-p16701742	gene	exon
Bn-A05-p917769	Bn-N5-p1025845	gene	
Bn-A05-p9209378	Bn-N2-p16611823	gene	exon
Bn-A05-p9225459	Bn-N2-p16589487	gene	
Bn-A05-p922813	Bn-N5-p1030016	gene	
Bn-A05-p9247748	Bn-N12-p23584328	gene	
Bn-A05-p926361	Bn-N5-p1033381	gene	
Bn-A05-p9277011	Bn-Scaffold02521-p2192	gene	
Bn-A05-p9319851	Bn-N5-p8702877	gene	

Bn-A05-p9347113	Bn-N5-p8672485	gene	exon
Bn-A05-p9364170	Bn-N5-p8656664	gene	exon
Bn-A05-p9376879	Bn-N5-p8652776	gene	
Bn-A05-p9409136	Bn-N5-p8624398	gene	
Bn-A05-p9455372	Bn-N5-p8584357	gene	
Bn-A05-p9472646	Bn-N5-p8564641	gene	
Bn-A05-p9472820	Bn-N5-p8564467	gene	
Bn-A05-p9495579	Bn-N5-p8545241	gene	exon
Bn-A05-p9555189	Bn-N5-p8494946	gene	
Bn-A05-p9623131	Bn-N5-p8791583	gene	exon
Bn-A05-p966483	Bn-N5-p1076071	gene	exon
Bn-A05-p9668222	Bn-N5-p8856168	gene	
Bn-A05-p967131	Bn-N5-p1076788	gene	
Bn-A05-p967575	Bn-N5-p1077255	gene	
Bn-A05-p9758517	Bn-N5-p8936796	gene	
Bn-A05-p9849049	Bn-N5-p9030784	gene	
Bn-A05-p9937099	Bn-N5-p9135367	gene	
Bn-A05-p996188	Bn-N5-p1105629	gene	
Bn-A06-p10040506	Bn-N6-p9798452	gene	
Bn-A06-p10046575	Bn-N6-p9804523	gene	exon
Bn-A06-p10046691	Bn-N6-p9804639	gene	exon
Bn-A06-p10053057	Bn-N6-p9811002	gene	
Bn-A06-p10053801	Bn-N6-p9811745	gene	
Bn-A06-p10124135	Bn-N6-p9886369	gene	exon
Bn-A06-p10129250	Bn-N6-p9894522	gene	
Bn-A06-p10135140	Bn-N6-p9900416	gene	exon
Bn-A06-p1015217	Bn-N6-p1056120	gene	
Bn-A06-p1016417	Bn-N6-p1057320	gene	
Bn-A06-p10240329	Bn-N6-p10002027	gene	
Bn-A06-p10251899	Bn-N6-p10013601	gene	exon
Bn-A06-p10300664	Bn-N6-p10051722	gene	
Bn-A06-p10320803	Bn-N6-p10070686	gene	
Bn-A06-p10327909	Bn-N6-p10079243	gene	exon
Bn-A06-p10391346	Bn-N6-p10140172	gene	
Bn-A06-p10401376	Bn-N13-p51376456	gene	
Bn-A06-p10499003	Bn-N6-p10265386	gene	exon
Bn-A06-p10594170	Bn-N6-p10363440	gene	
Bn-A06-p10609574	Bn-N6-p10379031	gene	
Bn-A06-p10609641	Bn-N6-p10379098	gene	
Bn-A06-p10642761	Bn-N6-p10407153	gene	exon
Bn-A06-p1071392	Bn-N6-p1106111	gene	
Bn-A06-p10781335	Bn-N6-p10551637	gene	exon
Bn-A06-p10795156	Bn-N6-p10567138	gene	exon
Bn-A06-p10833219	Bn-N6-p10602504	gene	
Bn-A06-p10836353	Bn-N6-p10605729	gene	exon
Bn-A06-p10854312	Bn-N6-p10616850	gene	
Bn-A06-p10859915	Bn-N6-p10624189	gene	
Bn-A06-p10863673	Bn-N6-p10628123	gene	
Bn-A06-p10873048	Bn-N6-p10638902	gene	exon
Bn-A06-p11021085	Bn-N6-p10764131	gene	
Bn-A06-p11024576	Bn-N6-p10767902	gene	
Bn-A06-p11165324	Bn-N6-p10948454	gene	

Bn-A06-p11165375	Bn-N6-p10948505	gene	
Bn-A06-p1120102	Bn-N6-p1146457	gene	
Bn-A06-p1120107	Bn-N6-p1146462	gene	
Bn-A06-p112339	Bn-N6-p100294	gene	exon
Bn-A06-p11301532	Bn-N6-p11097112	gene	
Bn-A06-p11362290	Bn-N6-p11169205	gene	
Bn-A06-p113900	Bn-N6-p101855	gene	
Bn-A06-p11415584	Bn-N6-p11203715	gene	
Bn-A06-p1142557	Bn-N8-p1704686	gene	
Bn-A06-p114634	Bn-N6-p102589	gene	
Bn-A06-p1149034	Bn-N6-p1179470	gene	
Bn-A06-p1154840	Bn-N6-p1187003	gene	exon
Bn-A06-p11588495	Bn-N6-p11367282	gene	exon
Bn-A06-p1160984	Bn-N6-p1195652	gene	exon
Bn-A06-p1168447	Bn-N6-p1204309	gene	
Bn-A06-p1173126	Bn-N6-p1205246	gene	
Bn-A06-p1176062	Bn-N6-p1209543	gene	exon
Bn-A06-p11776560	Bn-N6-p14530687	gene	
Bn-A06-p11819954	Bn-N6-p14471323	gene	
Bn-A06-p1184282	Bn-N6-p1217343	gene	exon
Bn-A06-p1185371	Bn-N6-p1218432	gene	exon
Bn-A06-p1187915	Bn-N6-p1220973	gene	exon
Bn-A06-p11919396	Bn-N6-p14359487	gene	
Bn-A06-p12078648	Bn-N11-p43130600	gene	exon
Bn-A06-p12093192	Bn-N6-p14162119	gene	
Bn-A06-p12135104	Bn-N6-p14118028	gene	exon
Bn-A06-p1217543	Bn-N6-p1239128	gene	
Bn-A06-p1225010	Bn-N16-p5520725	gene	
Bn-A06-p12337022	Bn-N6-p13901223	gene	
Bn-A06-p12337596	Bn-N6-p13900655	gene	
Bn-A06-p12337598	Bn-N6-p13900653	gene	
Bn-A06-p12357497	Bn-N6-p13872656	gene	
Bn-A06-p12358748	Bn-N6-p13871404	gene	
Bn-A06-p12436092	Bn-N6-p13777306	gene	
Bn-A06-p12517420	Bn-N6-p13700663	gene	
Bn-A06-p12645785	Bn-N6-p13596651	gene	exon
Bn-A06-p12658161	Bn-N6-p13590523	gene	
Bn-A06-p12696157	Bn-N6-p13572718	gene	exon
Bn-A06-p12732487	Bn-N6-p13542781	gene	exon
Bn-A06-p12768136	Bn-N6-p13500684	gene	
Bn-A06-p1278419	Bn-N6-p1290586	gene	
Bn-A06-p12846574	Bn-N6-p13448885	gene	
Bn-A06-p12868864	Bn-N6-p13424479	gene	exon
Bn-A06-p12884843	Bn-N6-p13378376	gene	exon
Bn-A06-p12911577	Bn-N6-p13339902	gene	
Bn-A06-p1291667	Bn-N6-p1303631	gene	exon
Bn-A06-p12986192	Bn-N6-p13275473	gene	
Bn-A06-p12995133	Bn-N6-p13252043	gene	
Bn-A06-p1312619	Bn-N6-p1327589	gene	exon
Bn-A06-p13162770	Bn-N6-p13143010	gene	exon
Bn-A06-p13230875	Bn-N6-p13098469	gene	exon
Bn-A06-p1330567	Bn-N6-p1345326	gene	

Bn-A06-p1345642	Bn-N6-p1360070	gene	
Bn-A06-p1358955	Bn-N6-p1373174	gene	exon
Bn-A06-p13610210	Bn-N6-p12734548	gene	
Bn-A06-p1366713	Bn-N6-p1379872	gene	
Bn-A06-p13726921	Bn-N13-p47843647	gene	exon
Bn-A06-p13728321	Bn-N6-p11835582	gene	
Bn-A06-p1383275	Bn-N6-p1396905	gene	exon
Bn-A06-p13859891	Bn-N6-p11668711	gene	exon
Bn-A06-p1386474	Bn-N6-p1400036	gene	
Bn-A06-p139033	Bn-N6-p129567	gene	
Bn-A06-p1403969	Bn-N6-p1417507	gene	
Bn-A06-p1408273	Bn-N6-p1424022	gene	exon
Bn-A06-p14192915	Bn-N6-p15617622	gene	
Bn-A06-p14193381	Bn-N6-p15618086	gene	
Bn-A06-p14198240	Bn-N6-p15623590	gene	exon
Bn-A06-p14232702	Bn-Scaffold00776-p6669	gene	exon
Bn-A06-p14232703	Bn-Scaffold00776-p6668	gene	exon
Bn-A06-p14266102	Bn-N6-p15671764	gene	exon
Bn-A06-p14269763	Bn-N19-p5165096	gene	exon
Bn-A06-p1427206	Bn-N6-p1448225	gene	
Bn-A06-p14316307	Bn-N6-p15724343	gene	
Bn-A06-p1432446	Bn-N6-p1453449	gene	
Bn-A06-p1433021	Bn-N6-p1454023	gene	
Bn-A06-p14369166	Bn-N6-p15794210	gene	
Bn-A06-p14373935	Bn-N6-p15799654	gene	
Bn-A06-p14389679	Bn-N6-p15810337	gene	
Bn-A06-p14417437	Bn-N6-p15852103	gene	exon
Bn-A06-p14428186	Bn-N6-p15863068	gene	exon
Bn-A06-p14428293	Bn-N6-p15863175	gene	exon
Bn-A06-p1443684	Bn-N6-p1465234	gene	exon
Bn-A06-p14441081	Bn-N13-p38763344	gene	exon
Bn-A06-p14457099	Bn-N6-p15895022	gene	exon
Bn-A06-p14474461	Bn-N6-p15908274	gene	
Bn-A06-p14495889	Bn-N6-p15932979	gene	
Bn-A06-p1450741	Bn-N6-p1481418	gene	
Bn-A06-p14546946	Bn-N6-p15978460	gene	
Bn-A06-p1456521	Bn-N6-p1487539	gene	exon
Bn-A06-p14576278	Bn-N6-p16000536	gene	exon
Bn-A06-p14578177	Bn-N6-p16002426	gene	
Bn-A06-p14578303	Bn-N6-p16002552	gene	
Bn-A06-p14594965	Bn-N6-p16625034	gene	exon
Bn-A06-p1460039	Bn-N6-p1491080	gene	
Bn-A06-p14621640	Bn-N2-p11813392	gene	
Bn-A06-p14664213	Bn-N6-p16688581	gene	
Bn-A06-p14676716	Bn-N6-p16701089	gene	exon
Bn-A06-p14737621	Bn-N6-p16748548	gene	exon
Bn-A06-p14773179	Bn-N6-p16779217	gene	exon
Bn-A06-p14809503	Bn-N6-p16823168	gene	
Bn-A06-p14816143	Bn-N6-p16829860	gene	
Bn-A06-p14819465	Bn-N6-p16835023	gene	exon
Bn-A06-p14843109	Bn-N6-p16852047	gene	exon
Bn-A06-p14848662	Bn-N6-p16857016	gene	

Bn-A06-p14850300	Bn-N6-p16859278	gene	exon
Bn-A06-p14852091	Bn-N6-p16861058	gene	
Bn-A06-p14852157	Bn-N6-p16861124	gene	
Bn-A06-p1485525	Bn-N6-p1507277	gene	exon
Bn-A06-p14878721	Bn-N6-p16886918	gene	exon
Bn-A06-p14880259	Bn-N6-p16888452	gene	exon
Bn-A06-p14880848	Bn-N6-p16889039	gene	exon
Bn-A06-p1490969	Bn-N6-p1510313	gene	exon
Bn-A06-p15013369	Bn-N6-p17022469	gene	exon
Bn-A06-p15048	Bn-N6-p19860	gene	exon
Bn-A06-p15059166	Bn-N6-p17060917	gene	exon
Bn-A06-p15065078	Bn-N6-p17068592	gene	exon
Bn-A06-p1507197	Bn-N16-p4752677	gene	
Bn-A06-p15262271	Bn-N6-p17270352	gene	exon
Bn-A06-p15285770	Bn-N6-p17291886	gene	
Bn-A06-p1530126	Bn-N6-p1556549	gene	exon
Bn-A06-p15375170	Bn-N6-p17381086	gene	
Bn-A06-p15375395	Bn-N6-p17381311	gene	
Bn-A06-p15406244	Bn-N6-p17409318	gene	
Bn-A06-p15415078	Bn-N6-p17418106	gene	exon
Bn-A06-p15420581	Bn-N6-p17423599	gene	
Bn-A06-p15421020	Bn-N6-p17424038	gene	
Bn-A06-p15489475	Bn-N6-p17497852	gene	
Bn-A06-p15515345	Bn-N6-p17520327	gene	exon
Bn-A06-p15581849	Bn-N6-p17585308	gene	
Bn-A06-p15582577	Bn-N6-p17586036	gene	exon
Bn-A06-p15582617	Bn-N6-p17586075	gene	exon
Bn-A06-p15632434	Bn-N6-p17634501	gene	
Bn-A06-p15661345	Bn-N6-p17666374	gene	
Bn-A06-p15709579	Bn-N6-p17719839	gene	exon
Bn-A06-p15749428	Bn-N6-p17759688	gene	
Bn-A06-p15760165	Bn-N6-p17772993	gene	exon
Bn-A06-p15775625	Bn-N6-p17787686	gene	exon
Bn-A06-p15778397	Bn-N6-p17790463	gene	exon
Bn-A06-p15791244	Bn-N6-p17805002	gene	
Bn-A06-p15801373	Bn-N6-p17814208	gene	
Bn-A06-p15893221	Bn-N6-p17911556	gene	exon
Bn-A06-p15966650	Bn-N6-p17991412	gene	
Bn-A06-p15967529	Bn-N6-p17992288	gene	
Bn-A06-p15967682	Bn-N6-p17992441	gene	exon
Bn-A06-p16009894	Bn-N6-p18030366	gene	exon
Bn-A06-p16014870	Bn-N6-p18041152	gene	
Bn-A06-p16014941	Bn-N6-p18041223	gene	
Bn-A06-p16015026	Bn-N6-p18041308	gene	
Bn-A06-p16015727	Bn-N6-p18041965	gene	
Bn-A06-p16015795	Bn-N6-p18042033	gene	
Bn-A06-p16064239	Bn-N6-p18087846	gene	exon
Bn-A06-p16106230	Bn-N6-p18126580	gene	exon
Bn-A06-p1612900	Bn-N6-p1649426	gene	
Bn-A06-p16148736	Bn-N6-p18169290	gene	
Bn-A06-p16149093	Bn-N6-p18169647	gene	exon
Bn-A06-p16150365	Bn-N6-p18170919	gene	

Bn-A06-p16150919	Bn-N6-p18171473	gene	exon
Bn-A06-p16181242	Bn-N6-p18232789	gene	
Bn-A06-p16188512	Bn-N6-p18240129	gene	
Bn-A06-p16237929	Bn-N6-p18284170	gene	
Bn-A06-p1626020	Bn-N6-p1663246	gene	exon
Bn-A06-p16283909	Bn-N6-p18303343	gene	
Bn-A06-p16284324	Bn-N6-p18303758	gene	
Bn-A06-p16284610	Bn-N6-p18304044	gene	
Bn-A06-p16303805	Bn-N6-p18328984	gene	exon
Bn-A06-p16303860	Bn-N6-p18329039	gene	exon
Bn-A06-p16330377	Bn-N6-p18350463	gene	
Bn-A06-p1636449	Bn-N16-p4442046	gene	exon
Bn-A06-p16386782	Bn-N6-p18433771	gene	
Bn-A06-p16386992	Bn-N6-p18433981	gene	
Bn-A06-p16449642	Bn-N6-p18500196	gene	
Bn-A06-p16460575	Bn-N6-p18510588	gene	
Bn-A06-p16461885	Bn-N6-p18511901	gene	exon
Bn-A06-p1650453	Bn-N6-p1687064	gene	
Bn-A06-p1650457	Bn-N6-p1687068	gene	
Bn-A06-p1650529	Bn-N6-p1687140	gene	
Bn-A06-p1650786	Bn-N6-p1687397	gene	
Bn-A06-p1652088	Bn-N6-p1688698	gene	
Bn-A06-p16553412	Bn-N6-p18592834	gene	
Bn-A06-p16553640	Bn-N6-p18593062	gene	
Bn-A06-p16554297	Bn-N6-p18593718	gene	
Bn-A06-p16613538	Bn-N6-p18656124	gene	
Bn-A06-p1661477	Bn-N6-p1702037	gene	
Bn-A06-p16620858	Bn-N6-p18662738	gene	exon
Bn-A06-p16620984	Bn-N6-p18662864	gene	exon
Bn-A06-p16624574	Bn-N6-p18665999	gene	exon
Bn-A06-p16645035	Bn-N6-p18693881	gene	
Bn-A06-p16645140	Bn-N6-p18693986	gene	exon
Bn-A06-p16656745	Bn-N6-p18704283	gene	exon
Bn-A06-p16657894	Bn-N6-p18705429	gene	
Bn-A06-p16660065	Bn-N6-p18707593	gene	exon
Bn-A06-p16689717	Bn-N6-p18733711	gene	
Bn-A06-p16701773	Bn-N6-p18742415	gene	
Bn-A06-p16702471	Bn-N6-p18743110	gene	
Bn-A06-p16703929	Bn-N6-p18744562	gene	exon
Bn-A06-p16718752	Bn-N6-p18762305	gene	exon
Bn-A06-p16728885	Bn-N6-p18766418	gene	
Bn-A06-p16829292	Bn-N6-p18786178	gene	exon
Bn-A06-p16847178	Bn-N6-p18799664	gene	exon
Bn-A06-p16847229	Bn-N6-p18799715	gene	exon
Bn-A06-p16933880	Bn-N6-p19067826	gene	
Bn-A06-p16985788	Bn-N6-p18942604	gene	
Bn-A06-p16992951	Bn-N6-p18954240	gene	exon
Bn-A06-p16993923	Bn-N6-p18955214	gene	
Bn-A06-p16994210	Bn-N6-p18955501	gene	exon
Bn-A06-p17003685	Bn-N6-p18964995	gene	exon
Bn-A06-p17006799	Bn-N6-p18968109	gene	exon
Bn-A06-p17007775	Bn-N6-p18969085	gene	

Bn-A06-p17010360	Bn-N6-p18971703	gene	exon
Bn-A06-p17011382	Bn-N6-p18972716	gene	exon
Bn-A06-p17021956	Bn-N6-p18982111	gene	
Bn-A06-p17024179	Bn-N6-p18984139	gene	
Bn-A06-p17026294	Bn-N6-p18986238	gene	
Bn-A06-p17026843	Bn-N6-p18986787	gene	exon
Bn-A06-p17037739	Bn-N6-p18997745	gene	exon
Bn-A06-p17037912	Bn-N6-p18997918	gene	exon
Bn-A06-p17097734	Bn-N6-p19055319	gene	
Bn-A06-p17109583	Bn-N6-p19069640	gene	
Bn-A06-p17143376	Bn-N6-p19092537	gene	
Bn-A06-p17175388	Bn-Scaffold08133-p758	gene	exon
Bn-A06-p17176086	Bn-N6-p19142066	gene	exon
Bn-A06-p17208027	Bn-N6-p19173275	gene	
Bn-A06-p17242631	Bn-N3-p2167500	gene	exon
Bn-A06-p17262265	Bn-N6-p19212690	gene	
Bn-A06-p17289947	Bn-N6-p19234621	gene	exon
Bn-A06-p17291868	Bn-N6-p19238162	gene	
Bn-A06-p17301456	Bn-N6-p19247744	gene	
Bn-A06-p17313345	Bn-N6-p19259582	gene	exon
Bn-A06-p17332795	Bn-N6-p19278754	gene	exon
Bn-A06-p17337842	Bn-N6-p19283769	gene	exon
Bn-A06-p17375407	Bn-N6-p19322585	gene	exon
Bn-A06-p17375882	Bn-N6-p19323060	gene	exon
Bn-A06-p1743446	Bn-N6-p1781188	gene	
Bn-A06-p17452087	Bn-N6-p19399601	gene	exon
Bn-A06-p17548258	Bn-N6-p19500042	gene	
Bn-A06-p17548894	Bn-N6-p19500680	gene	
Bn-A06-p17549347	Bn-N6-p19501133	gene	exon
Bn-A06-p17549603	Bn-N6-p19501390	gene	
Bn-A06-p17671185	Bn-N6-p19646596	gene	exon
Bn-A06-p17710201	Bn-N6-p19685508	gene	
Bn-A06-p17721678	Bn-N6-p19697278	gene	
Bn-A06-p17729064	Bn-N6-p19704663	gene	
Bn-A06-p17734402	Bn-N6-p19710973	gene	
Bn-A06-p17734904	Bn-N6-p19711475	gene	exon
Bn-A06-p17741868	Bn-N6-p19721994	gene	exon
Bn-A06-p17741952	Bn-N6-p19722078	gene	exon
Bn-A06-p1776668	Bn-N6-p1815642	gene	exon
Bn-A06-p1777127	Bn-N6-p1816104	gene	exon
Bn-A06-p17778079	Bn-N6-p19757990	gene	
Bn-A06-p17778210	Bn-N6-p19758120	gene	
Bn-A06-p17785331	Bn-N6-p19764946	gene	
Bn-A06-p17791496	Bn-N6-p19771178	gene	exon
Bn-A06-p17791746	Bn-N6-p19771428	gene	exon
Bn-A06-p17823115	Bn-N6-p19786851	gene	exon
Bn-A06-p17831661	Bn-N6-p19795172	gene	exon
Bn-A06-p17887060	Bn-N6-p19840998	gene	exon
Bn-A06-p17888567	Bn-N6-p19842506	gene	exon
Bn-A06-p17929532	Bn-N6-p19882884	gene	exon
Bn-A06-p17932959	Bn-N6-p19886316	gene	exon
Bn-A06-p1793586	Bn-N6-p1831047	gene	

Bn-A06-p17938497	Bn-N6-p19890604	gene	exon
Bn-A06-p17956497	Bn-N9-p35866953	gene	
Bn-A06-p18000403	Bn-N6-p19964006	gene	exon
Bn-A06-p18000461	Bn-N6-p19964064	gene	
Bn-A06-p1800467	Bn-N6-p1837502	gene	
Bn-A06-p18006686	Bn-N6-p19974379	gene	
Bn-A06-p18040363	Bn-N6-p20017987	gene	
Bn-A06-p18040423	Bn-N6-p20018047	gene	
Bn-A06-p18058671	Bn-N6-p20031446	gene	
Bn-A06-p18059278	Bn-N6-p20032092	gene	
Bn-A06-p18059565	Bn-N6-p20032380	gene	exon
Bn-A06-p18059678	Bn-N6-p20032493	gene	
Bn-A06-p18102164	Bn-N6-p20094346	gene	
Bn-A06-p18115817	Bn-N6-p20107852	gene	
Bn-A06-p18129979	Bn-N6-p20127975	gene	
Bn-A06-p18141167	Bn-N6-p20144469	gene	
Bn-A06-p18144127	Bn-N6-p20147429	gene	
Bn-A06-p18220327	Bn-N6-p20218146	gene	
Bn-A06-p18246821	Bn-N6-p20245047	gene	
Bn-A06-p18248917	Bn-N6-p20247145	gene	
Bn-A06-p18249054	Bn-N6-p20247282	gene	
Bn-A06-p18252711	Bn-N6-p20250985	gene	
Bn-A06-p18254877	Bn-N6-p20253151	gene	exon
Bn-A06-p18260387	Bn-N6-p20258633	gene	
Bn-A06-p18277724	Bn-N6-p20273817	gene	exon
Bn-A06-p18277802	Bn-N6-p20273964	gene	exon
Bn-A06-p183811	Bn-N6-p176405	gene	exon
Bn-A06-p18388041	Bn-N6-p20381692	gene	
Bn-A06-p18394892	Bn-N6-p20388629	gene	
Bn-A06-p18397592	Bn-N6-p20391329	gene	
Bn-A06-p18402713	Bn-N6-p20396437	gene	exon
Bn-A06-p18403451	Bn-N6-p20397175	gene	
Bn-A06-p18408257	Bn-N6-p20402074	gene	exon
Bn-A06-p18430792	Bn-N6-p20421348	gene	exon
Bn-A06-p18432812	Bn-N6-p20423354	gene	
Bn-A06-p18454864	Bn-N6-p20445389	gene	exon
Bn-A06-p18455125	Bn-N6-p20445650	gene	
Bn-A06-p18500429	Bn-N6-p20485900	gene	exon
Bn-A06-p18500596	Bn-N6-p20486067	gene	exon
Bn-A06-p18500625	Bn-N6-p20486096	gene	exon
Bn-A06-p18563686	Bn-N6-p20547118	gene	exon
Bn-A06-p18624741	Bn-N6-p20614321	gene	
Bn-A06-p18634396	Bn-N6-p20623983	gene	exon
Bn-A06-p18642085	Bn-N6-p20631670	gene	
Bn-A06-p18706102	Bn-N6-p20685634	gene	
Bn-A06-p18709596	Bn-N6-p20689130	gene	
Bn-A06-p18717323	Bn-N6-p20697012	gene	exon
Bn-A06-p18718829	Bn-N6-p20698355	gene	exon
Bn-A06-p1872484	Bn-N6-p1918225	gene	
Bn-A06-p1872597	Bn-N6-p1918338	gene	
Bn-A06-p18755859	Bn-N6-p20732196	gene	
Bn-A06-p18783	Bn-N6-p23558	gene	

Bn-A06-p18794208	Bn-N6-p20776944	gene	
Bn-A06-p18813274	Bn-N6-p14648727	gene	
Bn-A06-p18872147	Bn-N6-p14708135	gene	exon
Bn-A06-p18898902	Bn-N6-p14740468	gene	
Bn-A06-p1892397	Bn-N6-p1927804	gene	exon
Bn-A06-p1892464	Bn-N6-p1927871	gene	exon
Bn-A06-p18998806	Bn-N6-p14828458	gene	
Bn-A06-p1903170	Bn-N6-p1940469	gene	
Bn-A06-p1903532	Bn-N6-p1940831	gene	
Bn-A06-p19151996	Bn-N6-p14972121	gene	
Bn-A06-p19153385	Bn-N6-p14973511	gene	
Bn-A06-p19153719	Bn-N6-p14973844	gene	
Bn-A06-p19163919	Bn-N6-p14984047	gene	
Bn-A06-p19184868	Bn-N6-p15006419	gene	
Bn-A06-p19185585	Bn-N6-p15007134	gene	exon
Bn-A06-p19245025	Bn-N6-p15082715	gene	exon
Bn-A06-p19265815	Bn-N6-p15104807	gene	
Bn-A06-p19333055	Bn-N6-p15166699	gene	
Bn-A06-p19333117	Bn-N6-p15166762	gene	
Bn-A06-p19333349	Bn-N6-p15166993	gene	
Bn-A06-p19392651	Bn-N6-p15226492	gene	exon
Bn-A06-p19405765	Bn-N6-p15235318	gene	exon
Bn-A06-p19411124	Bn-N6-p15240234	gene	
Bn-A06-p19416292	Bn-N6-p15245415	gene	
Bn-A06-p19418423	Bn-N6-p15247555	gene	
Bn-A06-p19419215	Bn-N6-p15248345	gene	
Bn-A06-p19434905	Bn-N6-p15264691	gene	
Bn-A06-p19437625	Bn-N6-p15267450	gene	
Bn-A06-p1947247	Bn-N6-p1991978	gene	exon
Bn-A06-p195449	Bn-N6-p188974	gene	exon
Bn-A06-p19555116	Bn-N6-p15378380	gene	
Bn-A06-p19574863	Bn-N6-p15402830	gene	exon
Bn-A06-p19579765	Bn-N6-p15404645	gene	exon
Bn-A06-p19593266	Bn-N6-p15413071	gene	
Bn-A06-p1961013	Bn-N6-p2006985	gene	exon
Bn-A06-p19635380	Bn-N6-p15453006	gene	exon
Bn-A06-p19646183	Bn-N6-p15467377	gene	exon
Bn-A06-p19656363	Bn-N6-p15479154	gene	exon
Bn-A06-p19715332	Bn-Scaffold00926-p20936	gene	
Bn-A06-p1973105	Bn-N6-p2022757	gene	
Bn-A06-p19739501	Bn-Scaffold00926-p3388	gene	exon
Bn-A06-p197604	Bn-N6-p191127	gene	
Bn-A06-p19792160	Bn-N9-p18529473	gene	
Bn-A06-p19968113	Bn-N9-p18718935	gene	exon
Bn-A06-p20320209	Bn-N5-p14653039	gene	
Bn-A06-p20320258	Bn-N5-p14652990	gene	
Bn-A06-p2060583	Bn-N6-p2116721	gene	exon
Bn-A06-p21045315	Bn-N6-p21295598	gene	
Bn-A06-p21060490	Bn-N17-p34995753	gene	exon
Bn-A06-p21064129	Bn-N6-p21276666	gene	
Bn-A06-p21064168	Bn-N6-p21276627	gene	
Bn-A06-p21064290	Bn-N6-p21276505	gene	exon

Bn-A06-p21064341	Bn-N17-p35005435	gene	
Bn-A06-p21065019	Bn-N6-p21275774	gene	
Bn-A06-p2107176	Bn-N6-p2171415	gene	exon
Bn-A06-p21072392	Bn-N6-p21268255	gene	
Bn-A06-p2107610	Bn-N6-p2171861	gene	exon
Bn-A06-p21080778	Bn-N6-p21260524	gene	
Bn-A06-p21080915	Bn-N6-p21260159	gene	
Bn-A06-p21098677	Bn-N6-p21245347	gene	exon
Bn-A06-p21101957	Bn-N6-p21242087	gene	exon
Bn-A06-p21105437	Bn-N6-p21239160	gene	exon
Bn-A06-p21116438	Bn-N6-p21226214	gene	
Bn-A06-p21126329	Bn-N6-p21217733	gene	exon
Bn-A06-p21127138	Bn-N6-p21217121	gene	
Bn-A06-p21127779	Bn-N6-p21216470	gene	exon
Bn-A06-p21127805	Bn-N6-p21216444	gene	
Bn-A06-p21127973	Bn-N6-p21216276	gene	exon
Bn-A06-p21132765	Bn-N6-p21210684	gene	
Bn-A06-p21134315	Bn-N6-p21209138	gene	exon
Bn-A06-p21150459	Bn-N6-p21195020	gene	
Bn-A06-p21150622	Bn-N6-p21194846	gene	
Bn-A06-p21162770	Bn-N6-p21186860	gene	
Bn-A06-p21163950	Bn-N6-p21185677	gene	
Bn-A06-p21163993	Bn-N6-p21185634	gene	
Bn-A06-p21164043	Bn-N6-p21185584	gene	exon
Bn-A06-p21164284	Bn-N6-p21185343	gene	exon
Bn-A06-p21164289	Bn-N6-p21185338	gene	exon
Bn-A06-p21183207	Bn-N6-p21165944	gene	exon
Bn-A06-p21187999	Bn-N6-p21161093	gene	
Bn-A06-p21191463	Bn-N6-p21157674	gene	
Bn-A06-p21194827	Bn-N6-p21149611	gene	exon
Bn-A06-p21195115	Bn-N6-p21149323	gene	exon
Bn-A06-p21196739	Bn-N6-p21147702	gene	exon
Bn-A06-p21221869	Bn-N6-p21120213	gene	
Bn-A06-p21247593	Bn-N6-p21094107	gene	
Bn-A06-p21252883	Bn-N6-p21088817	gene	
Bn-A06-p21268699	Bn-N17-p35259636	gene	exon
Bn-A06-p21268967	Bn-N6-p21072654	gene	
Bn-A06-p21269830	Bn-N6-p21071791	gene	
Bn-A06-p21270132	Bn-N6-p21071489	gene	exon
Bn-A06-p21273211	Bn-N6-p21068336	gene	exon
Bn-A06-p21278243	Bn-N6-p21062261	gene	
Bn-A06-p21301745	Bn-N9-p5942255	gene	exon
Bn-A06-p21302070	Bn-N9-p5941930	gene	exon
Bn-A06-p21308348	Bn-N6-p21038101	gene	exon
Bn-A06-p21310030	Bn-N6-p21031628	gene	
Bn-A06-p21315016	Bn-N6-p21026631	gene	
Bn-A06-p21318925	Bn-N17-p35308010	gene	exon
Bn-A06-p21321797	Bn-N6-p21019844	gene	exon
Bn-A06-p21438687	Bn-N6-p20903951	gene	exon
Bn-A06-p21462006	Bn-N6-p20879596	gene	
Bn-A06-p21463076	Bn-N6-p20878529	gene	
Bn-A06-p21475556	Bn-N6-p20866349	gene	exon

Bn-A06-p21490506	Bn-N6-p20850567	gene	exon
Bn-A06-p21491454	Bn-N6-p20849620	gene	
Bn-A06-p21501350	Bn-N6-p20843710	gene	exon
Bn-A06-p21509934	Bn-N6-p20833612	gene	exon
Bn-A06-p21525576	Bn-N6-p20817476	gene	exon
Bn-A06-p21550710	Bn-N6-p20800759	gene	exon
Bn-A06-p21572310	Bn-N6-p20783353	gene	exon
Bn-A06-p21573640	Bn-N6-p20781750	gene	
Bn-A06-p21574157	Bn-N6-p20781234	gene	
Bn-A06-p21589512	Bn-N6-p21310132	gene	
Bn-A06-p21591920	Bn-N6-p21312540	gene	
Bn-A06-p21600894	Bn-N6-p21321514	gene	
Bn-A06-p21602381	Bn-N6-p21323001	gene	
Bn-A06-p21613020	Bn-N6-p21333702	gene	
Bn-A06-p2165351	Bn-N6-p2225291	gene	exon
Bn-A06-p21653611	Bn-N6-p21376133	gene	
Bn-A06-p21663690	Bn-N6-p21385957	gene	exon
Bn-A06-p21677028	Bn-N6-p21389385	gene	
Bn-A06-p21797963	Bn-N6-p21515905	gene	
Bn-A06-p21800812	Bn-N6-p21518841	gene	exon
Bn-A06-p21800927	Bn-N6-p21518956	gene	exon
Bn-A06-p21801638	Bn-N6-p21519667	gene	exon
Bn-A06-p21826106	Bn-N6-p21564067	gene	
Bn-A06-p21846491	Bn-N6-p21580042	gene	exon
Bn-A06-p21848747	Bn-N6-p21582195	gene	exon
Bn-A06-p2185099	Bn-N6-p2247063	gene	exon
Bn-A06-p21852261	Bn-N6-p21585709	gene	
Bn-A06-p21902421	Bn-N6-p21647204	gene	
Bn-A06-p21902773	Bn-N6-p21647556	gene	
Bn-A06-p21917612	Bn-N6-p21657109	gene	
Bn-A06-p21926982	Bn-N6-p21665633	gene	exon
Bn-A06-p21930942	Bn-N6-p21669754	gene	
Bn-A06-p21933122	Bn-N6-p21671942	gene	exon
Bn-A06-p21933776	Bn-N6-p21672596	gene	
Bn-A06-p21933808	Bn-N6-p21672628	gene	
Bn-A06-p21933870	Bn-N6-p21672690	gene	exon
Bn-A06-p21980483	Bn-N6-p21729330	gene	exon
Bn-A06-p21980699	Bn-N17-p34313338	gene	exon
Bn-A06-p22051862	Bn-N6-p21797366	gene	
Bn-A06-p22054532	Bn-N6-p21800071	gene	
Bn-A06-p22056809	Bn-N6-p21802385	gene	exon
Bn-A06-p22063657	Bn-N6-p21810521	gene	
Bn-A06-p22074841	Bn-N6-p21821704	gene	
Bn-A06-p22077748	Bn-N6-p21824609	gene	
Bn-A06-p22088550	Bn-N6-p21834891	gene	exon
Bn-A06-p22096615	Bn-N6-p21842565	gene	exon
Bn-A06-p22105035	Bn-N6-p21850966	gene	exon
Bn-A06-p22121666	Bn-N6-p21868896	gene	exon
Bn-A06-p22160452	Bn-N6-p21908673	gene	exon
Bn-A06-p22163702	Bn-N6-p21911923	gene	exon
Bn-A06-p2217578	Bn-N6-p2284554	gene	exon
Bn-A06-p2218956	Bn-N6-p2285927	gene	exon

Bn-A06-p22209347	Bn-N6-p21961235	gene	exon
Bn-A06-p2222872	Bn-N6-p2289854	gene	
Bn-A06-p22262511	Bn-N6-p22018480	gene	
Bn-A06-p22267850	Bn-N6-p22023819	gene	
Bn-A06-p22268128	Bn-N6-p22024097	gene	exon
Bn-A06-p22271048	Bn-N6-p22027017	gene	exon
Bn-A06-p22278468	Bn-N6-p22032653	gene	exon
Bn-A06-p22279513	Bn-N6-p22033707	gene	exon
Bn-A06-p22329026	Bn-N6-p22083891	gene	exon
Bn-A06-p22329819	Bn-N6-p22084684	gene	exon
Bn-A06-p22330861	Bn-N6-p22085726	gene	exon
Bn-A06-p22331680	Bn-N6-p22086545	gene	exon
Bn-A06-p2234696	Bn-N6-p2310593	gene	
Bn-A06-p2234718	Bn-N6-p2310615	gene	
Bn-A06-p22391357	Bn-N17-p33472677	gene	exon
Bn-A06-p22397061	Bn-N6-p22169567	gene	exon
Bn-A06-p22503987	Bn-N6-p22274288	gene	
Bn-A06-p22505810	Bn-N6-p22276050	gene	exon
Bn-A06-p2251910	Bn-N6-p2323861	gene	exon
Bn-A06-p2252815	Bn-N6-p2324749	gene	
Bn-A06-p22554741	Bn-N6-p22355457	gene	
Bn-A06-p22555168	Bn-N6-p22355884	gene	
Bn-A06-p22557239	Bn-N6-p22357954	gene	exon
Bn-A06-p22559895	Bn-N6-p22360620	gene	exon
Bn-A06-p22577587	Bn-N6-p22382558	gene	exon
Bn-A06-p22582397	Bn-Scaffold05279-p349	gene	exon
Bn-A06-p22594488	Bn-N6-p22400553	gene	
Bn-A06-p22605217	Bn-N6-p22410943	gene	
Bn-A06-p22644580	Bn-N6-p22456205	gene	
Bn-A06-p22647955	Bn-N6-p22458486	gene	
Bn-A06-p2268393	Bn-N6-p2342201	gene	exon
Bn-A06-p22691250	Bn-N6-p22488724	gene	
Bn-A06-p22709184	Bn-N17-p32930419	gene	exon
Bn-A06-p22709872	Bn-N6-p22511393	gene	
Bn-A06-p2274318	Bn-N6-p2348122	gene	exon
Bn-A06-p2275754	Bn-N16-p3090573	gene	
Bn-A06-p22784485	Bn-N2-p27875310	gene	
Bn-A06-p22796718	Bn-N6-p22587749	gene	exon
Bn-A06-p22806976	Bn-N6-p22597910	gene	
Bn-A06-p22808095	Bn-N6-p22599025	gene	exon
Bn-A06-p22835281	Bn-N6-p22631670	gene	
Bn-A06-p22847296	Bn-N6-p22644867	gene	exon
Bn-A06-p22882207	Bn-N6-p22678173	gene	
Bn-A06-p22894392	Bn-N17-p32608696	gene	exon
Bn-A06-p22910339	Bn-N6-p22705689	gene	exon
Bn-A06-p22917369	Bn-N6-p22712955	gene	
Bn-A06-p22938934	Bn-N17-p32545000	gene	exon
Bn-A06-p2300342	Bn-N6-p2380085	gene	
Bn-A06-p23041811	Bn-N6-p22850310	gene	
Bn-A06-p23042397	Bn-N6-p22850896	gene	
Bn-A06-p23042849	Bn-N6-p22851348	gene	
Bn-A06-p23043135	Bn-N6-p22851631	gene	

Bn-A06-p2306499	Bn-N6-p2386953	gene	exon
Bn-A06-p23258948	Bn-N6-p23078241	gene	
Bn-A06-p23259546	Bn-N6-p23078841	gene	
Bn-A06-p23268021	Bn-N6-p23087867	gene	
Bn-A06-p23295405	Bn-N6-p23105851	gene	
Bn-A06-p23341715	Bn-N6-p23198691	gene	
Bn-A06-p23347767	Bn-N6-p23205777	gene	exon
Bn-A06-p23406582	Bn-N6-p23286174	gene	exon
Bn-A06-p23442751	Bn-N17-p31512414	gene	exon
Bn-A06-p23468803	Bn-N6-p23351373	gene	exon
Bn-A06-p2348428	Bn-N6-p2430074	gene	exon
Bn-A06-p2357647	Bn-N6-p2439412	gene	
Bn-A06-p23585772	Bn-N6-p23480882	gene	
Bn-A06-p23586019	Bn-N6-p23481129	gene	
Bn-A06-p23612730	Bn-N6-p23506709	gene	
Bn-A06-p23661679	Bn-N6-p23549359	gene	exon
Bn-A06-p23665384	Bn-N6-p23553077	gene	
Bn-A06-p23665564	Bn-N6-p23553257	gene	
Bn-A06-p23666378	Bn-N6-p23554072	gene	
Bn-A06-p23744370	Bn-N18-p22925019	gene	exon
Bn-A06-p23795599	Bn-N6-p23686370	gene	
Bn-A06-p23795763	Bn-N6-p23686534	gene	
Bn-A06-p23796020	Bn-N6-p23686792	gene	
Bn-A06-p23857345	Bn-N6-p23753179	gene	
Bn-A06-p23865356	Bn-N6-p23761177	gene	
Bn-A06-p23983648	Bn-N6-p23877592	gene	exon
Bn-A06-p24032427	Bn-N6-p23921418	gene	exon
Bn-A06-p24046488	Bn-N6-p23936898	gene	
Bn-A06-p2405022	Bn-N16-p2735237	gene	
Bn-A06-p2412998	Bn-N6-p2493840	gene	exon
Bn-A06-p24167592	Bn-N6-p24064554	gene	exon
Bn-A06-p24168470	Bn-N6-p24065432	gene	exon
Bn-A06-p24178982	Bn-N6-p24071269	gene	
Bn-A06-p24204030	Bn-N6-p24104375	gene	
Bn-A06-p24206019	Bn-N6-p24106364	gene	
Bn-A06-p24206556	Bn-N6-p24106901	gene	
Bn-A06-p24207308	Bn-N6-p24107654	gene	
Bn-A06-p24210014	Bn-N6-p24110360	gene	
Bn-A06-p24218304	Bn-N6-p24118837	gene	exon
Bn-A06-p24220121	Bn-N6-p24120811	gene	
Bn-A06-p24222064	Bn-N6-p24122752	gene	
Bn-A06-p24236385	Bn-N6-p24137157	gene	
Bn-A06-p24267492	Bn-N6-p24168953	gene	
Bn-A06-p24267499	Bn-N6-p24168960	gene	
Bn-A06-p24267745	Bn-N6-p24169206	gene	
Bn-A06-p24270718	Bn-N6-p24172179	gene	
Bn-A06-p24284014	Bn-N6-p24185514	gene	exon
Bn-A06-p24297216	Bn-N6-p24203879	gene	
Bn-A06-p24307250	Bn-N6-p24216449	gene	exon
Bn-A06-p24319329	Bn-N6-p24231951	gene	
Bn-A06-p24327317	Bn-N6-p24240336	gene	exon
Bn-A06-p24340685	Bn-N6-p24253950	gene	

Bn-A06-p24340830	Bn-N6-p24254096	gene	exon
Bn-A06-p24370009	Bn-N6-p24290033	gene	
Bn-A06-p2437284	Bn-N6-p2519721	gene	exon
Bn-A06-p24386467	Bn-N6-p24305347	gene	exon
Bn-A06-p24395608	Bn-N6-p24314023	gene	
Bn-A06-p24401543	Bn-N6-p24318789	gene	exon
Bn-A06-p24404374	Bn-N6-p24321461	gene	
Bn-A06-p24404996	Bn-N6-p24322081	gene	
Bn-A06-p24405065	Bn-N6-p24322150	gene	
Bn-A06-p24407165	Bn-N6-p24324247	gene	
Bn-A06-p24407535	Bn-N6-p24324617	gene	
Bn-A06-p24407609	Bn-N6-p24324691	gene	
Bn-A06-p24410007	Bn-N6-p24327124	gene	
Bn-A06-p24428073	Bn-N6-p24343088	gene	
Bn-A06-p24465494	Bn-N6-p24388412	gene	
Bn-A06-p24466136	Bn-N6-p24389054	gene	
Bn-A06-p24467652	Bn-N6-p24390570	gene	
Bn-A06-p24501315	Bn-N6-p24426203	gene	
Bn-A06-p24501540	Bn-N6-p24426433	gene	
Bn-A06-p24508464	Bn-N6-p24434163	gene	exon
Bn-A06-p24557108	Bn-N6-p24483260	gene	exon
Bn-A06-p24557193	Bn-N6-p24483345	gene	exon
Bn-A06-p24602106	Bn-N6-p24528502	gene	exon
Bn-A06-p24607556	Bn-N6-p24531745	gene	exon
Bn-A06-p24618096	Bn-N6-p24541836	gene	exon
Bn-A06-p24678077	Bn-N6-p24609360	gene	exon
Bn-A06-p24695860	Bn-N6-p24629879	gene	exon
Bn-A06-p24709822	Bn-N6-p24641624	gene	
Bn-A06-p24713872	Bn-N6-p24648594	gene	
Bn-A06-p24717767	Bn-N6-p24652460	gene	
Bn-A06-p24726203	Bn-N6-p24660985	gene	exon
Bn-A06-p24730935	Bn-N6-p24666030	gene	exon
Bn-A06-p24744523	Bn-N6-p24680736	gene	exon
Bn-A06-p24814906	Bn-N6-p24748982	gene	
Bn-A06-p24822573	Bn-N6-p24756372	gene	exon
Bn-A06-p24836495	Bn-N17-p27245347	gene	exon
Bn-A06-p24886436	Bn-N6-p24818237	gene	exon
Bn-A06-p24913140	Bn-N6-p24828591	gene	exon
Bn-A06-p24922072	Bn-N6-p24838130	gene	
Bn-A06-p24929938	Bn-N6-p24845946	gene	exon
Bn-A06-p24934604	Bn-N6-p24853461	gene	
Bn-A06-p24973824	Bn-N6-p24897402	gene	exon
Bn-A06-p24976434	Bn-N6-p24900064	gene	exon
Bn-A06-p2514723	Bn-N6-p2549445	gene	exon
Bn-A06-p2521454	Bn-N6-p2556154	gene	exon
Bn-A06-p2521668	Bn-N6-p2556368	gene	exon
Bn-A06-p2525037	Bn-N6-p2559757	gene	exon
Bn-A06-p2526552	Bn-N6-p2561249	gene	
Bn-A06-p25329425	Bn-N6-p25237706	gene	exon
Bn-A06-p25343454	Bn-N6-p25251925	gene	exon
Bn-A06-p25350033	Bn-N6-p25259115	gene	
Bn-A06-p25351980	Bn-N6-p25261078	gene	exon

Bn-A06-p25377321	Bn-N6-p25287774	gene	
Bn-A06-p25409852	Bn-N6-p25304379	gene	exon
Bn-A06-p25471463	Bn-N6-p25360464	gene	
Bn-A06-p2563025	Bn-N6-p2593860	gene	exon
Bn-A06-p2582613	Bn-N6-p2620055	gene	
Bn-A06-p25840740	Bn-N6-p25719735	gene	exon
Bn-A06-p25878721	Bn-N6-p25758868	gene	exon
Bn-A06-p26023391	Bn-N6-p25901016	gene	exon
Bn-A06-p26039465	Bn-N6-p25913173	gene	exon
Bn-A06-p26045429	Bn-N6-p25920137	gene	exon
Bn-A06-p26066533	Bn-N17-p48616786	gene	
Bn-A06-p26080915	Bn-N6-p25952938	gene	
Bn-A06-p26096667	Bn-N6-p25972810	gene	exon
Bn-A06-p26096963	Bn-N6-p25973106	gene	
Bn-A06-p26097037	Bn-N6-p25973180	gene	
Bn-A06-p26108669	Bn-N6-p25984695	gene	exon
Bn-A06-p26113448	Bn-N6-p25989421	gene	exon
Bn-A06-p26144134	Bn-N6-p26014839	gene	
Bn-A06-p26144417	Bn-N6-p26015122	gene	
Bn-A06-p26144731	Bn-N6-p26015436	gene	
Bn-A06-p26152237	Bn-N6-p26022941	gene	
Bn-A06-p26154413	Bn-N6-p26025052	gene	
Bn-A06-p26155068	Bn-N6-p26025707	gene	
Bn-A06-p26173206	Bn-N6-p26045574	gene	
Bn-A06-p26186229	Bn-N6-p26052502	gene	exon
Bn-A06-p2619089	Bn-N6-p2651143	gene	exon
Bn-A06-p26212942	Bn-N6-p26083374	gene	
Bn-A06-p26213014	Bn-N6-p26083446	gene	
Bn-A06-p26213205	Bn-N6-p26083637	gene	exon
Bn-A06-p26213281	Bn-N6-p26083713	gene	
Bn-A06-p26219274	Bn-N6-p26088740	gene	exon
Bn-A06-p26219617	Bn-N6-p26089080	gene	exon
Bn-A06-p26248182	Bn-N6-p26119667	gene	exon
Bn-A06-p2630468	Bn-N6-p2668296	gene	exon
Bn-A06-p2633224	Bn-N6-p2671059	gene	exon
Bn-A06-p2633764	Bn-N6-p2671598	gene	exon
Bn-A06-p2646572	Bn-N6-p2682622	gene	exon
Bn-A06-p2650442	Bn-N6-p2686460	gene	
Bn-A06-p2651708	Bn-N6-p2687756	gene	exon
Bn-A06-p2682929	Bn-N15-p2990291	gene	exon
Bn-A06-p2698067	Bn-N6-p2727686	gene	exon
Bn-A06-p2709177	Bn-N6-p2732304	gene	exon
Bn-A06-p2714610	Bn-N6-p2737753	gene	
Bn-A06-p2717967	Bn-N6-p2741108	gene	exon
Bn-A06-p2718852	Bn-N6-p2741979	gene	exon
Bn-A06-p2729577	Bn-N6-p2752302	gene	
Bn-A06-p2743076	Bn-N6-p2757743	gene	
Bn-A06-p2756247	Bn-N6-p2768293	gene	
Bn-A06-p2756792	Bn-N6-p2768838	gene	
Bn-A06-p2758495	Bn-N6-p2770541	gene	
Bn-A06-p2778072	Bn-N6-p2786608	gene	
Bn-A06-p2778829	Bn-N6-p2787365	gene	

Bn-A06-p2778837	Bn-N6-p2787373	gene	
Bn-A06-p2779243	Bn-N6-p2787778	gene	
Bn-A06-p2788585	Bn-N6-p2797188	gene	exon
Bn-A06-p2801792	Bn-N6-p2810106	gene	
Bn-A06-p2807759	Bn-N6-p2816073	gene	exon
Bn-A06-p2823066	Bn-N6-p2826199	gene	
Bn-A06-p2824500	Bn-N6-p2827633	gene	
Bn-A06-p2826315	Bn-N6-p2829463	gene	exon
Bn-A06-p2826630	Bn-N6-p2829778	gene	
Bn-A06-p2842757	Bn-N6-p2842298	gene	
Bn-A06-p2851834	Bn-N6-p2851376	gene	
Bn-A06-p2870413	Bn-N6-p2866315	gene	exon
Bn-A06-p287053	Bn-N6-p282831	gene	exon
Bn-A06-p2874216	Bn-N6-p2870151	gene	exon
Bn-A06-p288377	Bn-N6-p284723	gene	
Bn-A06-p2890703	Bn-N6-p2896493	gene	exon
Bn-A06-p2891175	Bn-N6-p2897013	gene	
Bn-A06-p2891290	Bn-N6-p2897128	gene	
Bn-A06-p2903017	Bn-N6-p2903792	gene	exon
Bn-A06-p2929852	Bn-N6-p2935540	gene	exon
Bn-A06-p2930156	Bn-N6-p2935844	gene	
Bn-A06-p2938450	Bn-N6-p2943858	gene	exon
Bn-A06-p2938890	Bn-N6-p2944300	gene	exon
Bn-A06-p2949781	Bn-N6-p2953219	gene	
Bn-A06-p2949858	Bn-N6-p2953296	gene	
Bn-A06-p2954752	Bn-N6-p2957078	gene	
Bn-A06-p2956891	Bn-N6-p2959219	gene	exon
Bn-A06-p2958095	Bn-N6-p2960373	gene	exon
Bn-A06-p2973074	Bn-N6-p2973146	gene	
Bn-A06-p2981395	Bn-N6-p2981638	gene	exon
Bn-A06-p2984184	Bn-N15-p3364850	gene	exon
Bn-A06-p2993800	Bn-N15-p3374204	gene	exon
Bn-A06-p3009854	Bn-N6-p3006589	gene	
Bn-A06-p3012876	Bn-N6-p3010680	gene	
Bn-A06-p3013547	Bn-N6-p3011370	gene	
Bn-A06-p3016186	Bn-N6-p3013918	gene	
Bn-A06-p3016356	Bn-N6-p3014089	gene	
Bn-A06-p3031327	Bn-N6-p3030154	gene	exon
Bn-A06-p3049884	Bn-N6-p3048326	gene	exon
Bn-A06-p3050205	Bn-N6-p3048647	gene	
Bn-A06-p3050747	Bn-N6-p3049189	gene	
Bn-A06-p3051257	Bn-N6-p3049699	gene	
Bn-A06-p3065773	Bn-N6-p3065004	gene	
Bn-A06-p3066038	Bn-N6-p3065269	gene	
Bn-A06-p3066372	Bn-N6-p3065603	gene	
Bn-A06-p307436	Bn-N6-p302033	gene	exon
Bn-A06-p3088460	Bn-N6-p3088020	gene	
Bn-A06-p3090078	Bn-N6-p3089638	gene	exon
Bn-A06-p3096813	Bn-N6-p3104738	gene	
Bn-A06-p3125473	Bn-N6-p3140846	gene	exon
Bn-A06-p3132058	Bn-N6-p3147710	gene	
Bn-A06-p3132860	Bn-N6-p3148516	gene	

Bn-A06-p3146367	Bn-N6-p3162102	gene	
Bn-A06-p3146720	Bn-N6-p3162460	gene	
Bn-A06-p3184395	Bn-N6-p3195839	gene	
Bn-A06-p3202098	Bn-N6-p3213931	gene	exon
Bn-A06-p3202615	Bn-N6-p3214421	gene	
Bn-A06-p3206017	Bn-N6-p3219017	gene	exon
Bn-A06-p3207623	Bn-N6-p3220626	gene	
Bn-A06-p3226693	Bn-N6-p3230970	gene	exon
Bn-A06-p3257163	Bn-N6-p3249953	gene	exon
Bn-A06-p3278682	Bn-N6-p3270169	gene	exon
Bn-A06-p3287018	Bn-N16-p37184565	gene	exon
Bn-A06-p329217	Bn-N6-p330032	gene	
Bn-A06-p3295431	Bn-N6-p3286024	gene	
Bn-A06-p3302657	Bn-N6-p3292678	gene	
Bn-A06-p330826	Bn-N6-p331683	gene	
Bn-A06-p3308398	Bn-N6-p3300591	gene	
Bn-A06-p3318611	Bn-N6-p3310703	gene	exon
Bn-A06-p3325502	Bn-N6-p3317535	gene	exon
Bn-A06-p3325566	Bn-N6-p3317599	gene	exon
Bn-A06-p3329211	Bn-N6-p3322607	gene	exon
Bn-A06-p3353405	Bn-N6-p3347064	gene	
Bn-A06-p3354754	Bn-N6-p3349067	gene	exon
Bn-A06-p3363188	Bn-N6-p3356903	gene	
Bn-A06-p3380495	Bn-N6-p3374808	gene	
Bn-A06-p3399869	Bn-N6-p3392743	gene	
Bn-A06-p3399973	Bn-N6-p3392847	gene	exon
Bn-A06-p3405779	Bn-N6-p3398917	gene	exon
Bn-A06-p3406041	Bn-N6-p3399178	gene	
Bn-A06-p3414128	Bn-N6-p3407281	gene	
Bn-A06-p3416803	Bn-N6-p3409871	gene	
Bn-A06-p3428214	Bn-N6-p3424307	gene	exon
Bn-A06-p3444868	Bn-N6-p3444530	gene	
Bn-A06-p3446956	Bn-N6-p3446613	gene	
Bn-A06-p3452904	Bn-N6-p3449964	gene	
Bn-A06-p3468374	Bn-N6-p3465457	gene	
Bn-A06-p3483249	Bn-N6-p3479969	gene	
Bn-A06-p3483382	Bn-N6-p3480103	gene	
Bn-A06-p3496759	Bn-N6-p3499375	gene	
Bn-A06-p3528559	Bn-N6-p3533772	gene	
Bn-A06-p3528628	Bn-N6-p3533841	gene	
Bn-A06-p3584489	Bn-N6-p3596674	gene	
Bn-A06-p3586728	Bn-N6-p3598871	gene	exon
Bn-A06-p3587032	Bn-N6-p3599168	gene	
Bn-A06-p3611806	Bn-N6-p3615094	gene	exon
Bn-A06-p3612397	Bn-N6-p3615685	gene	
Bn-A06-p362402	Bn-N6-p375479	gene	
Bn-A06-p3625328	Bn-N6-p3627477	gene	exon
Bn-A06-p3628159	Bn-N6-p3630437	gene	exon
Bn-A06-p3651074	Bn-N15-p4343868	gene	
Bn-A06-p3651473	Bn-N6-p3658326	gene	exon
Bn-A06-p3661342	Bn-N6-p3662706	gene	exon
Bn-A06-p3691343	Bn-N6-p3681470	gene	exon

Bn-A06-p3712646	Bn-N6-p3695960	gene	
Bn-A06-p3731147	Bn-N6-p3717253	gene	exon
Bn-A06-p3805018	Bn-N6-p3788444	gene	exon
Bn-A06-p3809199	Bn-N6-p3792731	gene	
Bn-A06-p3825492	Bn-N6-p3807329	gene	exon
Bn-A06-p3839293	Bn-N6-p3825033	gene	
Bn-A06-p3867353	Bn-N6-p3855138	gene	
Bn-A06-p3871984	Bn-N6-p3859821	gene	exon
Bn-A06-p3900404	Bn-N6-p3890089	gene	
Bn-A06-p3913280	Bn-N6-p3903064	gene	
Bn-A06-p393328	Bn-N6-p408495	gene	
Bn-A06-p3953661	Bn-N6-p3951758	gene	exon
Bn-A06-p3970972	Bn-N6-p3973819	gene	
Bn-A06-p3976759	Bn-N6-p3981557	gene	
Bn-A06-p3986320	Bn-N6-p3991299	gene	exon
Bn-A06-p3987067	Bn-N6-p3992372	gene	
Bn-A06-p3992289	Bn-N6-p4001869	gene	
Bn-A06-p3997307	Bn-N6-p4006737	gene	
Bn-A06-p3998593	Bn-N6-p4008023	gene	
Bn-A06-p3999986	Bn-N6-p4009416	gene	exon
Bn-A06-p4001378	Bn-N6-p4010808	gene	exon
Bn-A06-p4013010	Bn-N6-p4030632	gene	exon
Bn-A06-p4016422	Bn-N15-p4844920	gene	
Bn-A06-p4017460	Bn-N6-p4034974	gene	exon
Bn-A06-p4060202	Bn-N6-p4091519	gene	exon
Bn-A06-p4060310	Bn-N6-p4091627	gene	exon
Bn-A06-p4315934	Bn-N6-p4334089	gene	
Bn-A06-p4316905	Bn-N6-p4335061	gene	
Bn-A06-p4362245	Bn-N6-p4370566	gene	
Bn-A06-p4362386	Bn-N6-p4370707	gene	exon
Bn-A06-p4362700	Bn-N6-p4371129	gene	exon
Bn-A06-p4409355	Bn-N6-p4415508	gene	exon
Bn-A06-p4418231	Bn-N6-p4422646	gene	exon
Bn-A06-p4419571	Bn-N6-p4424002	gene	
Bn-A06-p4420263	Bn-N6-p4424692	gene	exon
Bn-A06-p4421214	Bn-N6-p4425647	gene	
Bn-A06-p4436168	Bn-N6-p4442587	gene	exon
Bn-A06-p4452778	Bn-N6-p4457051	gene	exon
Bn-A06-p4508774	Bn-N6-p4499520	gene	exon
Bn-A06-p4517457	Bn-N6-p4514328	gene	
Bn-A06-p4597746	Bn-N6-p4591680	gene	exon
Bn-A06-p4623401	Bn-N6-p4609266	gene	exon
Bn-A06-p4703366	Bn-N6-p4654719	gene	
Bn-A06-p4874556	Bn-N6-p4876095	gene	
Bn-A06-p4881785	Bn-N6-p4883065	gene	exon
Bn-A06-p4896084	Bn-N6-p4913674	gene	
Bn-A06-p4901091	Bn-N6-p4918601	gene	
Bn-A06-p4927344	Bn-N15-p6144435	gene	exon
Bn-A06-p4929018	Bn-N6-p4954842	gene	exon
Bn-A06-p5040441	Bn-N6-p5030955	gene	
Bn-A06-p5047871	Bn-N6-p5038147	gene	
Bn-A06-p5094790	Bn-N6-p5100993	gene	

Bn-A06-p5103824	Bn-N6-p5109958	gene	exon
Bn-A06-p5114238	Bn-N6-p5121273	gene	exon
Bn-A06-p5147575	Bn-N6-p5158557	gene	exon
Bn-A06-p5151254	Bn-N6-p5162258	gene	
Bn-A06-p5151337	Bn-N6-p5162341	gene	
Bn-A06-p5276150	Bn-N4-p4098388	gene	exon
Bn-A06-p5276251	Bn-N4-p4098287	gene	exon
Bn-A06-p5276641	Bn-N4-p4097897	gene	exon
Bn-A06-p5276713	Bn-N4-p4097825	gene	exon
Bn-A06-p5276751	Bn-N4-p4097787	gene	exon
Bn-A06-p5284260	Bn-N4-p4090036	gene	
Bn-A06-p5346654	Bn-N14-p33160018	gene	
Bn-A06-p5384725	Bn-N4-p3995321	gene	
Bn-A06-p5386579	Bn-N4-p3993216	gene	
Bn-A06-p5427889	Bn-N4-p3947979	gene	
Bn-A06-p54687	Bn-N6-p49393	gene	exon
Bn-A06-p5535537	Bn-N6-p5208306	gene	
Bn-A06-p5546411	Bn-N6-p5216807	gene	exon
Bn-A06-p5577934	Bn-N6-p5241155	gene	exon
Bn-A06-p5600636	Bn-N6-p5269329	gene	
Bn-A06-p5616164	Bn-N6-p5281086	gene	
Bn-A06-p5618302	Bn-N6-p5283223	gene	
Bn-A06-p5669960	Bn-N6-p5336105	gene	
Bn-A06-p568101	Bn-N6-p609797	gene	
Bn-A06-p5693735	Bn-N6-p5493084	gene	exon
Bn-A06-p569582	Bn-N6-p611282	gene	exon
Bn-A06-p5747694	Bn-N6-p5422622	gene	exon
Bn-A06-p5767097	Bn-N6-p5442981	gene	
Bn-A06-p576740	Bn-N6-p618512	gene	
Bn-A06-p5830298	Bn-N6-p5501201	gene	
Bn-A06-p5844403	Bn-N6-p5509500	gene	
Bn-A06-p5878990	Bn-N6-p5546435	gene	
Bn-A06-p5880980	Bn-N6-p5548424	gene	
Bn-A06-p5888779	Bn-N6-p5556316	gene	exon
Bn-A06-p5922951	Bn-N6-p5593618	gene	exon
Bn-A06-p5930501	Bn-N6-p5605488	gene	
Bn-A06-p5931022	Bn-N6-p5606794	gene	exon
Bn-A06-p5931130	Bn-N6-p5606902	gene	exon
Bn-A06-p5945068	Bn-N6-p5625931	gene	
Bn-A06-p5945471	Bn-N6-p5626333	gene	exon
Bn-A06-p5947781	Bn-N6-p5628643	gene	
Bn-A06-p5956274	Bn-N6-p5646319	gene	exon
Bn-A06-p5957378	Bn-N6-p5647423	gene	exon
Bn-A06-p5995778	Bn-N6-p5693826	gene	
Bn-A06-p600029	Bn-N6-p633463	gene	
Bn-A06-p6009064	Bn-N6-p5700145	gene	exon
Bn-A06-p6059527	Bn-N6-p5744726	gene	exon
Bn-A06-p6066073	Bn-N6-p5752754	gene	exon
Bn-A06-p608554	Bn-N6-p641913	gene	
Bn-A06-p608570	Bn-N6-p641929	gene	exon
Bn-A06-p6091453	Bn-N6-p5782313	gene	exon
Bn-A06-p611589	Bn-N6-p644948	gene	

Bn-A06-p6129072	Bn-N6-p5809181	gene	
Bn-A06-p613789	Bn-N6-p647148	gene	
Bn-A06-p6154470	Bn-N6-p5820705	gene	exon
Bn-A06-p6182046	Bn-N6-p5841280	gene	exon
Bn-A06-p6182593	Bn-N6-p5841830	gene	
Bn-A06-p6183859	Bn-N6-p5843097	gene	exon
Bn-A06-p6184455	Bn-N6-p5843693	gene	exon
Bn-A06-p6198128	Bn-N6-p5863331	gene	exon
Bn-A06-p6204652	Bn-N15-p7700309	gene	exon
Bn-A06-p621506	Bn-N6-p659207	gene	
Bn-A06-p621695	Bn-N6-p646998	gene	exon
Bn-A06-p6270772	Bn-N6-p5935194	gene	
Bn-A06-p6311231	Bn-N6-p5979291	gene	
Bn-A06-p6311289	Bn-N6-p5979349	gene	
Bn-A06-p6319212	Bn-N6-p5989236	gene	
Bn-A06-p6358974	Bn-N6-p6030067	gene	
Bn-A06-p6359207	Bn-N6-p6030300	gene	
Bn-A06-p6382773	Bn-N6-p6055728	gene	
Bn-A06-p6388760	Bn-N6-p6062781	gene	exon
Bn-A06-p6389318	Bn-N6-p6063346	gene	
Bn-A06-p6403382	Bn-N6-p6077465	gene	
Bn-A06-p6437068	Bn-N6-p6105649	gene	exon
Bn-A06-p6439396	Bn-N6-p6107952	gene	
Bn-A06-p6451055	Bn-N6-p6120744	gene	
Bn-A06-p6457529	Bn-N6-p6127519	gene	exon
Bn-A06-p6464476	Bn-N6-p6134437	gene	exon
Bn-A06-p6477943	Bn-N6-p6147723	gene	
Bn-A06-p6482307	Bn-N6-p6151909	gene	exon
Bn-A06-p6483553	Bn-N6-p6153153	gene	exon
Bn-A06-p6486764	Bn-N6-p6155437	gene	
Bn-A06-p649348	Bn-N6-p689284	gene	exon
Bn-A06-p650072	Bn-N16-p7142200	gene	exon
Bn-A06-p650790	Bn-N16-p7141516	gene	exon
Bn-A06-p6520301	Bn-N6-p6182653	gene	
Bn-A06-p6532740	Bn-N6-p6198691	gene	
Bn-A06-p6563295	Bn-N6-p6240258	gene	exon
Bn-A06-p6569468	Bn-N15-p8359521	gene	exon
Bn-A06-p659391	Bn-N6-p694734	gene	
Bn-A06-p6617274	Bn-N6-p6297113	gene	
Bn-A06-p6622319	Bn-N6-p6305165	gene	exon
Bn-A06-p6624071	Bn-N6-p6306917	gene	
Bn-A06-p6636202	Bn-N6-p6319977	gene	
Bn-A06-p6647323	Bn-N6-p6327396	gene	exon
Bn-A06-p6655047	Bn-N15-p8481600	gene	exon
Bn-A06-p6689858	Bn-N6-p6374095	gene	exon
Bn-A06-p6691628	Bn-N6-p6375814	gene	exon
Bn-A06-p6692851	Bn-N6-p6377031	gene	exon
Bn-A06-p6699971	Bn-N6-p6380905	gene	exon
Bn-A06-p6705911	Bn-N6-p6386967	gene	exon
Bn-A06-p6706067	Bn-N6-p6387123	gene	
Bn-A06-p6723804	Bn-N6-p6400593	gene	exon
Bn-A06-p6744737	Bn-N6-p6420813	gene	exon

Bn-A06-p6747460	Bn-N6-p6423259	gene	
Bn-A06-p6847261	Bn-N6-p6507267	gene	exon
Bn-A06-p6847367	Bn-N6-p6507373	gene	exon
Bn-A06-p6864079	Bn-N6-p6524011	gene	
Bn-A06-p6875144	Bn-N6-p6536016	gene	
Bn-A06-p6878792	Bn-N6-p6539667	gene	exon
Bn-A06-p6879017	Bn-N6-p6539892	gene	exon
Bn-A06-p6895399	Bn-N6-p6574346	gene	exon
Bn-A06-p6913483	Bn-N6-p6629503	gene	
Bn-A06-p6919765	Bn-N6-p6633849	gene	
Bn-A06-p6920925	Bn-N6-p6635009	gene	exon
Bn-A06-p69515	Bn-N6-p56922	gene	exon
Bn-A06-p6955437	Bn-N6-p6668110	gene	
Bn-A06-p6966525	Bn-N6-p6678413	gene	
Bn-A06-p6971737	Bn-N6-p6683545	gene	
Bn-A06-p6971900	Bn-N6-p6683803	gene	
Bn-A06-p6972345	Bn-N6-p6684244	gene	
Bn-A06-p6985717	Bn-N6-p6701684	gene	
Bn-A06-p6990496	Bn-N6-p6706458	gene	
Bn-A06-p6993862	Bn-N6-p6709824	gene	
Bn-A06-p6995492	Bn-N6-p6711454	gene	
Bn-A06-p7083415	Bn-N6-p6792473	gene	exon
Bn-A06-p709547	Bn-N6-p749143	gene	exon
Bn-A06-p7179419	Bn-N6-p6921708	gene	
Bn-A06-p7187455	Bn-N6-p6929922	gene	exon
Bn-A06-p72383	Bn-N6-p59864	gene	exon
Bn-A06-p7266200	Bn-N6-p7005622	gene	
Bn-A06-p7279909	Bn-N6-p7023374	gene	
Bn-A06-p72878	Bn-N6-p60359	gene	exon
Bn-A06-p7288881	Bn-N6-p7048578	gene	exon
Bn-A06-p7289388	Bn-N6-p7049085	gene	
Bn-A06-p7355866	Bn-N6-p7112881	gene	exon
Bn-A06-p7384367	Bn-N15-p9456872	gene	
Bn-A06-p73924	Bn-N6-p61405	gene	exon
Bn-A06-p7420466	Bn-N6-p7191794	gene	exon
Bn-A06-p7444483	Bn-N6-p7219460	gene	exon
Bn-A06-p7553160	Bn-N6-p7331811	gene	
Bn-A06-p7583628	Bn-N6-p7371216	gene	exon
Bn-A06-p7584691	Bn-N6-p7372279	gene	
Bn-A06-p7589883	Bn-N15-p9848110	gene	exon
Bn-A06-p7658535	Bn-N6-p7449964	gene	exon
Bn-A06-p7665759	Bn-N6-p7457260	gene	
Bn-A06-p7701069	Bn-N6-p7494036	gene	
Bn-A06-p7726350	Bn-N6-p7520076	gene	exon
Bn-A06-p7771695	Bn-N6-p7563220	gene	exon
Bn-A06-p7927622	Bn-N6-p7719763	gene	
Bn-A06-p7927767	Bn-N6-p7719908	gene	
Bn-A06-p7931177	Bn-N6-p7724897	gene	
Bn-A06-p7959476	Bn-N6-p7750782	gene	exon
Bn-A06-p7973826	Bn-N6-p7763966	gene	
Bn-A06-p798821	Bn-N6-p838127	gene	
Bn-A06-p8021875	Bn-N6-p7800280	gene	

Bn-A06-p8022058	Bn-N6-p7800462	gene	
Bn-A06-p8042439	Bn-N6-p7818608	gene	
Bn-A06-p8042901	Bn-N6-p7819062	gene	
Bn-A06-p8043214	Bn-N6-p7819375	gene	
Bn-A06-p8047893	Bn-N6-p7824149	gene	
Bn-A06-p8080390	Bn-N6-p7859729	gene	
Bn-A06-p8080666	Bn-N6-p7860005	gene	
Bn-A06-p8090520	Bn-N6-p7869882	gene	
Bn-A06-p8109713	Bn-N6-p7882207	gene	exon
Bn-A06-p8200169	Bn-N6-p7967911	gene	
Bn-A06-p8201495	Bn-N6-p7969229	gene	
Bn-A06-p8201581	Bn-N6-p7969315	gene	exon
Bn-A06-p8263325	Bn-N6-p8037352	gene	
Bn-A06-p8356542	Bn-N6-p8114394	gene	exon
Bn-A06-p836912	Bn-N6-p876249	gene	exon
Bn-A06-p8416265	Bn-N6-p8178953	gene	
Bn-A06-p8430642	Bn-N6-p8192891	gene	
Bn-A06-p8434088	Bn-N6-p8196332	gene	exon
Bn-A06-p8472457	Bn-N6-p8230082	gene	
Bn-A06-p8536023	Bn-N6-p8277780	gene	
Bn-A06-p853722	Bn-N6-p892885	gene	exon
Bn-A06-p853974	Bn-N6-p893137	gene	exon
Bn-A06-p8573607	Bn-N6-p8331335	gene	exon
Bn-A06-p860631	Bn-N6-p901756	gene	exon
Bn-A06-p8615815	Bn-N6-p8374632	gene	exon
Bn-A06-p8633786	Bn-N6-p8397148	gene	
Bn-A06-p8634166	Bn-N6-p8397528	gene	
Bn-A06-p8634648	Bn-N6-p8398010	gene	
Bn-A06-p8643665	Bn-N14-p25133590	gene	exon
Bn-A06-p8697187	Bn-N6-p8462318	gene	
Bn-A06-p8697193	Bn-N6-p8462324	gene	
Bn-A06-p8792429	Bn-N6-p8551424	gene	exon
Bn-A06-p8792843	Bn-N6-p8551850	gene	
Bn-A06-p8809314	Bn-N6-p8575504	gene	exon
Bn-A06-p8837251	Bn-N6-p8604210	gene	exon
Bn-A06-p8837290	Bn-N6-p8604249	gene	exon
Bn-A06-p8907559	Bn-N6-p8674294	gene	
Bn-A06-p89131	Bn-Scaffold10358-p220	gene	exon
Bn-A06-p8923160	Bn-N6-p8689728	gene	exon
Bn-A06-p8948420	Bn-N6-p8715557	gene	
Bn-A06-p8968750	Bn-N6-p8733481	gene	
Bn-A06-p8972533	Bn-N6-p8737187	gene	exon
Bn-A06-p8998572	Bn-N6-p8767207	gene	
Bn-A06-p9009861	Bn-N6-p8776269	gene	
Bn-A06-p9064537	Bn-N6-p8802966	gene	
Bn-A06-p9074653	Bn-N6-p8820097	gene	exon
Bn-A06-p9076011	Bn-N6-p8821455	gene	
Bn-A06-p9087487	Bn-N6-p8833369	gene	exon
Bn-A06-p9135772	Bn-N6-p8876786	gene	exon
Bn-A06-p918871	Bn-N6-p965609	gene	
Bn-A06-p9287101	Bn-N6-p9033717	gene	
Bn-A06-p9299877	Bn-N6-p9047406	gene	exon

Bn-A06-p9371495	Bn-N1-p23082579	gene	exon
Bn-A06-p938677	Bn-N6-p975926	gene	exon
Bn-A06-p9399854	Bn-N18-p28491976	gene	exon
Bn-A06-p947740	Bn-N6-p985341	gene	exon
Bn-A06-p9495829	Bn-N6-p9270854	gene	
Bn-A06-p9517147	Bn-N6-p9281645	gene	
Bn-A06-p9562200	Bn-N6-p9345827	gene	
Bn-A06-p9593892	Bn-N6-p9366702	gene	exon
Bn-A06-p9630478	Bn-N6-p9394235	gene	
Bn-A06-p9684583	Bn-N6-p9446970	gene	
Bn-A06-p9721031	Bn-N6-p9479849	gene	
Bn-A06-p999044	Bn-N6-p1041874	gene	exon
Bn-A07-p10003378	Bn-N7-p11491755	gene	exon
Bn-A07-p10004245	Bn-N7-p11492622	gene	exon
Bn-A07-p10010327	Bn-N7-p11498680	gene	exon
Bn-A07-p10033055	Bn-N7-p11508010	gene	
Bn-A07-p10037462	Bn-N7-p11512955	gene	exon
Bn-A07-p10099786	Bn-N7-p11584293	gene	exon
Bn-A07-p10100811	Bn-N7-p11585318	gene	exon
Bn-A07-p10101125	Bn-N7-p11585635	gene	exon
Bn-A07-p10169211	Bn-N7-p11645303	gene	exon
Bn-A07-p10209077	Bn-N7-p11678835	gene	exon
Bn-A07-p10209154	Bn-N7-p11678912	gene	exon
Bn-A07-p10233337	Bn-N7-p11710832	gene	exon
Bn-A07-p10281077	Bn-N7-p11760233	gene	exon
Bn-A07-p10282316	Bn-N7-p11761471	gene	
Bn-A07-p10282792	Bn-N7-p11761947	gene	
Bn-A07-p10284031	Bn-N7-p11763083	gene	
Bn-A07-p10285778	Bn-N7-p11764829	gene	
Bn-A07-p10289778	Bn-N7-p11768840	gene	
Bn-A07-p10291035	Bn-N7-p11770094	gene	exon
Bn-A07-p10340211	Bn-N7-p11813359	gene	
Bn-A07-p10379059	Bn-N7-p11858814	gene	
Bn-A07-p10398999	Bn-N7-p11879724	gene	
Bn-A07-p10399235	Bn-N7-p11879960	gene	
Bn-A07-p10421977	Bn-N7-p11910872	gene	
Bn-A07-p10430301	Bn-N7-p11918244	gene	
Bn-A07-p10437749	Bn-N7-p11925680	gene	exon
Bn-A07-p10437970	Bn-N7-p11925901	gene	
Bn-A07-p10451411	Bn-N7-p11937147	gene	
Bn-A07-p10455192	Bn-N7-p11940967	gene	exon
Bn-A07-p10471024	Bn-N7-p11945984	gene	exon
Bn-A07-p10473511	Bn-N7-p11948511	gene	exon
Bn-A07-p10483339	Bn-N7-p11958317	gene	exon
Bn-A07-p10486045	Bn-N7-p11961032	gene	exon
Bn-A07-p10494571	Bn-N7-p11969210	gene	
Bn-A07-p10554944	Bn-N7-p12026837	gene	exon
Bn-A07-p10584018	Bn-N7-p12061103	gene	exon
Bn-A07-p10650508	Bn-N7-p12121407	gene	exon
Bn-A07-p10651899	Bn-N7-p12122798	gene	exon
Bn-A07-p10667459	Bn-N7-p12151327	gene	
Bn-A07-p10672691	Bn-N7-p12171039	gene	exon

Bn-A07-p10673477	Bn-N7-p12171825	gene	exon
Bn-A07-p10673700	Bn-N7-p12172048	gene	exon
Bn-A07-p10675102	Bn-N7-p12173458	gene	exon
Bn-A07-p10733528	Bn-N7-p12222357	gene	
Bn-A07-p10750190	Bn-N7-p12236021	gene	
Bn-A07-p10755129	Bn-N7-p12240892	gene	
Bn-A07-p10780571	Bn-N7-p12269959	gene	
Bn-A07-p107832	Bn-N7-p122997	gene	exon
Bn-A07-p10847704	Bn-N14-p16051073	gene	
Bn-A07-p10848104	Bn-N7-p12322177	gene	
Bn-A07-p10848190	Bn-N7-p12322263	gene	
Bn-A07-p10890061	Bn-N7-p12364144	gene	exon
Bn-A07-p10891272	Bn-N7-p12365945	gene	exon
Bn-A07-p1093997	Bn-N7-p1112292	gene	
Bn-A07-p10968184	Bn-N7-p12441442	gene	
Bn-A07-p11021668	Bn-N7-p12509207	gene	
Bn-A07-p11068231	Bn-N7-p12556265	gene	exon
Bn-A07-p11068735	Bn-N7-p12556769	gene	exon
Bn-A07-p11114150	Bn-N7-p12596579	gene	
Bn-A07-p11152131	Bn-N7-p12641457	gene	
Bn-A07-p11156840	Bn-N7-p12646164	gene	exon
Bn-A07-p11178807	Bn-N7-p12663707	gene	
Bn-A07-p11196478	Bn-N7-p12684358	gene	exon
Bn-A07-p11196510	Bn-N7-p12684390	gene	exon
Bn-A07-p11204177	Bn-N7-p12692110	gene	exon
Bn-A07-p11255851	Bn-N7-p12728638	gene	
Bn-A07-p11323366	Bn-N7-p12802492	gene	exon
Bn-A07-p11324360	Bn-N7-p12803486	gene	exon
Bn-A07-p1135440	Bn-N7-p1164517	gene	
Bn-A07-p11390804	Bn-N5-p9409156	gene	exon
Bn-A07-p11477634	Bn-N7-p13989123	gene	
Bn-A07-p11542386	Bn-N7-p14051166	gene	exon
Bn-A07-p11542638	Bn-N7-p14051418	gene	exon
Bn-A07-p11570097	Bn-N7-p14083332	gene	exon
Bn-A07-p11572985	Bn-N7-p14086176	gene	
Bn-A07-p11599915	Bn-N7-p14106059	gene	
Bn-A07-p11698185	Bn-N16-p19893589	gene	exon
Bn-A07-p11715576	Bn-N7-p14261264	gene	exon
Bn-A07-p11724009	Bn-N7-p14269743	gene	
Bn-A07-p11764922	Bn-N7-p14316847	gene	
Bn-A07-p11765027	Bn-N7-p14316952	gene	
Bn-A07-p11765232	Bn-N7-p14317158	gene	
Bn-A07-p11832389	Bn-N7-p14389577	gene	exon
Bn-A07-p11846645	Bn-N7-p14403862	gene	
Bn-A07-p11861194	Bn-N7-p14417147	gene	
Bn-A07-p11864371	Bn-N7-p14420451	gene	
Bn-A07-p11870301	Bn-N7-p14429056	gene	exon
Bn-A07-p11870468	Bn-N7-p14429223	gene	exon
Bn-A07-p11870852	Bn-N7-p14429607	gene	exon
Bn-A07-p1196596	Bn-N7-p1235148	gene	exon
Bn-A07-p12017702	Bn-N7-p14569170	gene	
Bn-A07-p12066704	Bn-N7-p14614851	gene	

Bn-A07-p12115911	Bn-N7-p14664185	gene	
Bn-A07-p12131198	Bn-N7-p14680023	gene	
Bn-A07-p1217535	Bn-N7-p1251465	gene	
Bn-A07-p1217876	Bn-N7-p1251806	gene	exon
Bn-A07-p12193031	Bn-N7-p14709044	gene	
Bn-A07-p1227140	Bn-N7-p1262567	gene	
Bn-A07-p12297809	Bn-N7-p14833511	gene	exon
Bn-A07-p12318667	Bn-N16-p21611826	gene	exon
Bn-A07-p12328863	Bn-N7-p14864972	gene	
Bn-A07-p12333270	Bn-N7-p14869394	gene	exon
Bn-A07-p12385490	Bn-N7-p14909637	gene	exon
Bn-A07-p12412116	Bn-N7-p14957010	gene	exon
Bn-A07-p12415996	Bn-N7-p14967498	gene	exon
Bn-A07-p12424938	Bn-N7-p14976509	gene	exon
Bn-A07-p12425115	Bn-N7-p14976686	gene	
Bn-A07-p12425319	Bn-N7-p14976890	gene	
Bn-A07-p12425566	Bn-N7-p14977137	gene	
Bn-A07-p12426500	Bn-N7-p14978071	gene	
Bn-A07-p12426652	Bn-N7-p14978223	gene	
Bn-A07-p12426907	Bn-N7-p14978478	gene	
Bn-A07-p12429159	Bn-N7-p14980865	gene	
Bn-A07-p12443929	Bn-N7-p14998858	gene	
Bn-A07-p12470367	Bn-N16-p21977371	gene	exon
Bn-A07-p12487549	Bn-N7-p15041071	gene	
Bn-A07-p12487688	Bn-N7-p15041210	gene	
Bn-A07-p12487765	Bn-N7-p15041287	gene	exon
Bn-A07-p1254564	Bn-N7-p1298977	gene	
Bn-A07-p12546889	Bn-N7-p15109085	gene	exon
Bn-A07-p12547012	Bn-N7-p15109208	gene	exon
Bn-A07-p12547288	Bn-N7-p15109484	gene	exon
Bn-A07-p1255437	Bn-N7-p1299849	gene	
Bn-A07-p12554962	Bn-N7-p15115102	gene	
Bn-A07-p12583860	Bn-N7-p15153157	gene	exon
Bn-A07-p12602102	Bn-N7-p15181717	gene	exon
Bn-A07-p12605125	Bn-N7-p15186297	gene	
Bn-A07-p12609078	Bn-N7-p15189566	gene	
Bn-A07-p12609202	Bn-N7-p15189690	gene	
Bn-A07-p12631921	Bn-N16-p22325394	gene	
Bn-A07-p12685266	Bn-N7-p15276004	gene	exon
Bn-A07-p12688545	Bn-N7-p15278966	gene	exon
Bn-A07-p12693030	Bn-N7-p15282841	gene	exon
Bn-A07-p12705404	Bn-N7-p15297868	gene	
Bn-A07-p12708718	Bn-N7-p15301183	gene	exon
Bn-A07-p12716261	Bn-N7-p15308770	gene	exon
Bn-A07-p12732142	Bn-N7-p15319071	gene	
Bn-A07-p12751218	Bn-N7-p15329565	gene	
Bn-A07-p12754144	Bn-N7-p15332485	gene	exon
Bn-A07-p12787260	Bn-N7-p15363154	gene	
Bn-A07-p12827973	Bn-N7-p15408930	gene	exon
Bn-A07-p12828848	Bn-N7-p15409806	gene	exon
Bn-A07-p12840368	Bn-N16-p22717731	gene	
Bn-A07-p12841026	Bn-N7-p15425789	gene	exon

Bn-A07-p12874349	Bn-N7-p15458367	gene	exon
Bn-A07-p12876226	Bn-N16-p22781030	gene	
Bn-A07-p12878986	Bn-N7-p15463003	gene	exon
Bn-A07-p12904129	Bn-N7-p15489905	gene	exon
Bn-A07-p12918175	Bn-N7-p15507530	gene	
Bn-A07-p12926496	Bn-N7-p15515854	gene	
Bn-A07-p1292866	Bn-N7-p1320908	gene	
Bn-A07-p1293366	Bn-N17-p3287749	gene	exon
Bn-A07-p12935086	Bn-N16-p22981025	gene	exon
Bn-A07-p12935190	Bn-N16-p22981129	gene	
Bn-A07-p12963622	Bn-N7-p15559143	gene	exon
Bn-A07-p1300457	Bn-N7-p1329349	gene	
Bn-A07-p13007323	Bn-N7-p15598298	gene	
Bn-A07-p1301127	Bn-N7-p1330026	gene	exon
Bn-A07-p13017844	Bn-N7-p15607323	gene	
Bn-A07-p1303513	Bn-N7-p1333816	gene	exon
Bn-A07-p13037426	Bn-N7-p15631631	gene	exon
Bn-A07-p1306286	Bn-N7-p1336584	gene	
Bn-A07-p13090054	Bn-N7-p15689204	gene	
Bn-A07-p13154645	Bn-N7-p15762727	gene	
Bn-A07-p13167740	Bn-N7-p15781239	gene	
Bn-A07-p13167844	Bn-N7-p15781366	gene	
Bn-A07-p13171850	Bn-N7-p15785405	gene	
Bn-A07-p13172047	Bn-N7-p15785601	gene	exon
Bn-A07-p13184664	Bn-N7-p15800345	gene	exon
Bn-A07-p13186090	Bn-N7-p15801770	gene	exon
Bn-A07-p13259825	Bn-N7-p15876660	gene	exon
Bn-A07-p13273073	Bn-N7-p15888530	gene	exon
Bn-A07-p1327427	Bn-N7-p1356597	gene	
Bn-A07-p13283299	Bn-N7-p15899977	gene	
Bn-A07-p13324733	Bn-N7-p15945033	gene	
Bn-A07-p13334430	Bn-N7-p15960245	gene	
Bn-A07-p13347715	Bn-N7-p15972076	gene	
Bn-A07-p13350234	Bn-N7-p15974592	gene	exon
Bn-A07-p13358954	Bn-N7-p15983477	gene	exon
Bn-A07-p13359413	Bn-N7-p15983933	gene	
Bn-A07-p13391170	Bn-N7-p16013400	gene	exon
Bn-A07-p13391724	Bn-N7-p16013955	gene	
Bn-A07-p13425409	Bn-N7-p16041939	gene	exon
Bn-A07-p13481981	Bn-N7-p16102859	gene	exon
Bn-A07-p13482160	Bn-N7-p16103038	gene	exon
Bn-A07-p13485321	Bn-N7-p16106200	gene	
Bn-A07-p13485413	Bn-N7-p16106292	gene	
Bn-A07-p13485722	Bn-N7-p16106600	gene	
Bn-A07-p13488332	Bn-N7-p16109178	gene	
Bn-A07-p13488433	Bn-N7-p16109279	gene	
Bn-A07-p13497516	Bn-N7-p16130688	gene	exon
Bn-A07-p13497654	Bn-N7-p16130826	gene	exon
Bn-A07-p13512124	Bn-N7-p16149816	gene	exon
Bn-A07-p13513850	Bn-N7-p16154516	gene	exon
Bn-A07-p13537021	Bn-N7-p16184503	gene	exon
Bn-A07-p13543954	Bn-N7-p16191418	gene	

Bn-A07-p13587753	Bn-N7-p16230522	gene	
Bn-A07-p13599579	Bn-N7-p16236383	gene	
Bn-A07-p13605650	Bn-N7-p16242457	gene	exon
Bn-A07-p13605726	Bn-N7-p16242533	gene	
Bn-A07-p13615643	Bn-N7-p16250566	gene	exon
Bn-A07-p13639374	Bn-N7-p16271735	gene	exon
Bn-A07-p13650653	Bn-N7-p16283115	gene	
Bn-A07-p13653958	Bn-N7-p16286422	gene	
Bn-A07-p13654191	Bn-N7-p16286654	gene	
Bn-A07-p13657646	Bn-N7-p16290109	gene	
Bn-A07-p13659939	Bn-N7-p16292466	gene	
Bn-A07-p13662635	Bn-N7-p16295171	gene	exon
Bn-A07-p13665031	Bn-N7-p16297590	gene	exon
Bn-A07-p13693377	Bn-N7-p16328594	gene	
Bn-A07-p13705188	Bn-N7-p16339498	gene	
Bn-A07-p13712520	Bn-N7-p16346921	gene	
Bn-A07-p13712881	Bn-N7-p16347282	gene	
Bn-A07-p13715621	Bn-N7-p16350064	gene	exon
Bn-A07-p13747722	Bn-N7-p16379790	gene	
Bn-A07-p13767766	Bn-N7-p16391750	gene	exon
Bn-A07-p13828973	Bn-N7-p16440233	gene	exon
Bn-A07-p13830862	Bn-N7-p16442121	gene	
Bn-A07-p13855997	Bn-N7-p16469862	gene	
Bn-A07-p13857064	Bn-N7-p16470929	gene	
Bn-A07-p13868960	Bn-N7-p16480181	gene	exon
Bn-A07-p13873942	Bn-N7-p16485186	gene	
Bn-A07-p13902763	Bn-N7-p16514874	gene	exon
Bn-A07-p13926029	Bn-N7-p16533634	gene	exon
Bn-A07-p13930308	Bn-N7-p16537885	gene	exon
Bn-A07-p13931176	Bn-N7-p16538753	gene	
Bn-A07-p13963849	Bn-N7-p16570707	gene	exon
Bn-A07-p13971525	Bn-N7-p16587947	gene	
Bn-A07-p14002586	Bn-N7-p16631479	gene	
Bn-A07-p14002656	Bn-N7-p16631549	gene	
Bn-A07-p14005263	Bn-N7-p16634145	gene	
Bn-A07-p14016405	Bn-N7-p16643860	gene	
Bn-A07-p14024559	Bn-N7-p16652499	gene	exon
Bn-A07-p14024677	Bn-N7-p16652617	gene	
Bn-A07-p14024930	Bn-N7-p16652870	gene	exon
Bn-A07-p14026612	Bn-N7-p16654556	gene	exon
Bn-A07-p14029820	Bn-N7-p16657716	gene	exon
Bn-A07-p14034468	Bn-N7-p16662443	gene	
Bn-A07-p14045156	Bn-N7-p16672532	gene	exon
Bn-A07-p14059112	Bn-N7-p16686621	gene	exon
Bn-A07-p14092905	Bn-N7-p16724230	gene	
Bn-A07-p14103536	Bn-N7-p16735378	gene	
Bn-A07-p14124058	Bn-N7-p16758959	gene	
Bn-A07-p14129550	Bn-N7-p16764574	gene	
Bn-A07-p14129566	Bn-N7-p16764589	gene	
Bn-A07-p14144205	Bn-N7-p16778327	gene	
Bn-A07-p14146406	Bn-N16-p25708370	gene	
Bn-A07-p14162450	Bn-N7-p16796388	gene	

Bn-A07-p14162566	Bn-N7-p16796504	gene	
Bn-A07-p14162622	Bn-N7-p16796560	gene	
Bn-A07-p14164068	Bn-N7-p16798007	gene	
Bn-A07-p14193108	Bn-N7-p16833259	gene	exon
Bn-A07-p14199690	Bn-N7-p16839482	gene	
Bn-A07-p14225102	Bn-N7-p16864057	gene	
Bn-A07-p14225642	Bn-N7-p16864597	gene	exon
Bn-A07-p14263288	Bn-N7-p16901581	gene	
Bn-A07-p14325380	Bn-N7-p16962092	gene	
Bn-A07-p1437777	Bn-N7-p1574926	gene	
Bn-A07-p14382008	Bn-N7-p17019859	gene	exon
Bn-A07-p14390667	Bn-N7-p17028212	gene	
Bn-A07-p14437313	Bn-N7-p17070776	gene	
Bn-A07-p14453848	Bn-N7-p17091758	gene	
Bn-A07-p14523713	Bn-N7-p17164096	gene	exon
Bn-A07-p14540334	Bn-N7-p17179658	gene	
Bn-A07-p14562613	Bn-N7-p17201575	gene	
Bn-A07-p14585610	Bn-N11-p43890733	gene	exon
Bn-A07-p14598430	Bn-N7-p17232223	gene	
Bn-A07-p14611227	Bn-N7-p17247704	gene	
Bn-A07-p14623449	Bn-N7-p17257181	gene	
Bn-A07-p14627135	Bn-N7-p17260873	gene	exon
Bn-A07-p14666868	Bn-N7-p17294374	gene	
Bn-A07-p14735920	Bn-N16-p26969672	gene	
Bn-A07-p14736696	Bn-N16-p26970446	gene	exon
Bn-A07-p14759734	Bn-N7-p17387372	gene	
Bn-A07-p14759792	Bn-N7-p17387430	gene	exon
Bn-A07-p14759961	Bn-N7-p17387599	gene	
Bn-A07-p14759962	Bn-N7-p17387599	gene	
Bn-A07-p14760232	Bn-N7-p17387872	gene	
Bn-A07-p14766907	Bn-N7-p17394595	gene	exon
Bn-A07-p14770619	Bn-N7-p17398077	gene	
Bn-A07-p14792267	Bn-N7-p17416205	gene	
Bn-A07-p14792772	Bn-N7-p17416711	gene	
Bn-A07-p14804953	Bn-N7-p17428408	gene	exon
Bn-A07-p14812317	Bn-N7-p17434977	gene	
Bn-A07-p14814393	Bn-N7-p17437078	gene	exon
Bn-A07-p14820942	Bn-N7-p17440792	gene	exon
Bn-A07-p14865976	Bn-N7-p17490629	gene	exon
Bn-A07-p14895551	Bn-N7-p17534137	gene	
Bn-A07-p14895675	Bn-N16-p27297695	gene	
Bn-A07-p14920018	Bn-N7-p17559082	gene	
Bn-A07-p14922137	Bn-N7-p17561201	gene	exon
Bn-A07-p14934271	Bn-N7-p17572824	gene	exon
Bn-A07-p14958606	Bn-N7-p17595363	gene	
Bn-A07-p14969298	Bn-N16-p27408804	gene	exon
Bn-A07-p14970273	Bn-N7-p17609212	gene	exon
Bn-A07-p14975152	Bn-N7-p17614051	gene	exon
Bn-A07-p15006983	Bn-N7-p17648991	gene	exon
Bn-A07-p15015434	Bn-N7-p17656934	gene	
Bn-A07-p15033167	Bn-N7-p17670224	gene	exon
Bn-A07-p15033648	Bn-N7-p17670705	gene	

Bn-A07-p15035369	Bn-N7-p17672425	gene	exon
Bn-A07-p15043209	Bn-N7-p17682010	gene	exon
Bn-A07-p15050270	Bn-N7-p17688042	gene	exon
Bn-A07-p15054860	Bn-N7-p17692604	gene	exon
Bn-A07-p15055528	Bn-N7-p17693272	gene	exon
Bn-A07-p15059054	Bn-N7-p17696669	gene	exon
Bn-A07-p15064528	Bn-N7-p17703725	gene	
Bn-A07-p15107656	Bn-N7-p17764265	gene	exon
Bn-A07-p15129531	Bn-N7-p17791210	gene	exon
Bn-A07-p15162548	Bn-N7-p17830159	gene	
Bn-A07-p15162767	Bn-N7-p17830380	gene	exon
Bn-A07-p15162837	Bn-N7-p17830450	gene	exon
Bn-A07-p15165145	Bn-N7-p17833148	gene	exon
Bn-A07-p15175027	Bn-N7-p17842911	gene	exon
Bn-A07-p15175141	Bn-N7-p17843024	gene	exon
Bn-A07-p15183066	Bn-N16-p27909405	gene	exon
Bn-A07-p15220175	Bn-N7-p17895417	gene	exon
Bn-A07-p15233647	Bn-N7-p17910141	gene	
Bn-A07-p15239170	Bn-N7-p17916052	gene	exon
Bn-A07-p15245952	Bn-N7-p17921873	gene	exon
Bn-A07-p15247793	Bn-N7-p17923677	gene	
Bn-A07-p15248927	Bn-N7-p17924813	gene	exon
Bn-A07-p15262851	Bn-N7-p17940636	gene	
Bn-A07-p15273529	Bn-N7-p17956020	gene	
Bn-A07-p15298239	Bn-N7-p17979504	gene	exon
Bn-A07-p15298338	Bn-N7-p17979603	gene	exon
Bn-A07-p15299046	Bn-N7-p17980311	gene	exon
Bn-A07-p15303340	Bn-N7-p17984598	gene	
Bn-A07-p15331892	Bn-N7-p18016065	gene	
Bn-A07-p15332201	Bn-N7-p18016379	gene	
Bn-A07-p15352802	Bn-N7-p18034004	gene	
Bn-A07-p15354488	Bn-N7-p18037237	gene	exon
Bn-A07-p15357890	Bn-N16-p28385249	gene	
Bn-A07-p15364409	Bn-N7-p18048568	gene	
Bn-A07-p15369326	Bn-N7-p18053484	gene	exon
Bn-A07-p15372787	Bn-N12-p18557545	gene	exon
Bn-A07-p15404895	Bn-N7-p18087198	gene	exon
Bn-A07-p15405942	Bn-N7-p18088245	gene	exon
Bn-A07-p15410837	Bn-N7-p18093108	gene	
Bn-A07-p15415388	Bn-N7-p18097525	gene	exon
Bn-A07-p15417895	Bn-N7-p18100041	gene	exon
Bn-A07-p15425959	Bn-N7-p18108303	gene	
Bn-A07-p15426090	Bn-N7-p18108434	gene	
Bn-A07-p15428458	Bn-N7-p18112046	gene	exon
Bn-A07-p15453076	Bn-N7-p18139396	gene	exon
Bn-A07-p15459314	Bn-N7-p18149018	gene	exon
Bn-A07-p15545260	Bn-N7-p18227733	gene	exon
Bn-A07-p15546893	Bn-N7-p18229666	gene	exon
Bn-A07-p15549943	Bn-N7-p18232812	gene	exon
Bn-A07-p1557675	Bn-N7-p1675377	gene	
Bn-A07-p15578269	Bn-N7-p18257843	gene	exon
Bn-A07-p15591535	Bn-N7-p18267292	gene	

Bn-A07-p15591699	Bn-N7-p18267456	gene	
Bn-A07-p15606996	Bn-N7-p18282644	gene	
Bn-A07-p15612140	Bn-N7-p18287694	gene	
Bn-A07-p15622870	Bn-N7-p18297859	gene	exon
Bn-A07-p1564369	Bn-N7-p1682483	gene	exon
Bn-A07-p15648620	Bn-N7-p18322939	gene	exon
Bn-A07-p15650680	Bn-N7-p18324891	gene	
Bn-A07-p15652974	Bn-N7-p18326907	gene	
Bn-A07-p15695422	Bn-N7-p18373032	gene	
Bn-A07-p15698133	Bn-N7-p18375871	gene	exon
Bn-A07-p15768026	Bn-N7-p18449193	gene	exon
Bn-A07-p15771342	Bn-N7-p18452509	gene	
Bn-A07-p15791885	Bn-N7-p18467443	gene	exon
Bn-A07-p15801291	Bn-N7-p18476233	gene	
Bn-A07-p15802174	Bn-N7-p18477116	gene	
Bn-A07-p15802229	Bn-N7-p18477171	gene	
Bn-A07-p15809590	Bn-N7-p18485768	gene	
Bn-A07-p15812852	Bn-N7-p18488979	gene	exon
Bn-A07-p15814161	Bn-N7-p18490278	gene	exon
Bn-A07-p15816246	Bn-N7-p18491537	gene	
Bn-A07-p15821030	Bn-N7-p18496325	gene	exon
Bn-A07-p15829114	Bn-N7-p18500972	gene	
Bn-A07-p15829285	Bn-N7-p18501143	gene	exon
Bn-A07-p15831043	Bn-N7-p18502907	gene	exon
Bn-A07-p15833211	Bn-N7-p18505098	gene	exon
Bn-A07-p15856545	Bn-N7-p18531264	gene	exon
Bn-A07-p15856978	Bn-N7-p18531697	gene	
Bn-A07-p15871261	Bn-N7-p18541543	gene	
Bn-A07-p15872578	Bn-N7-p18542858	gene	
Bn-A07-p15885711	Bn-N7-p18559070	gene	
Bn-A07-p158888	Bn-N7-p172010	gene	exon
Bn-A07-p15931750	Bn-N7-p18594936	gene	exon
Bn-A07-p15938534	Bn-N7-p18601865	gene	
Bn-A07-p15948472	Bn-N7-p18606472	gene	
Bn-A07-p15957061	Bn-N7-p18631534	gene	
Bn-A07-p15986673	Bn-N7-p18657326	gene	
Bn-A07-p16009799	Bn-N7-p18688438	gene	exon
Bn-A07-p16013075	Bn-N7-p18691714	gene	
Bn-A07-p16020955	Bn-N7-p18696705	gene	
Bn-A07-p16021617	Bn-N7-p18697367	gene	exon
Bn-A07-p16050076	Bn-N7-p18729717	gene	exon
Bn-A07-p160648	Bn-N17-p394283	gene	
Bn-A07-p16146576	Bn-N7-p18830062	gene	
Bn-A07-p16146639	Bn-N7-p18830125	gene	
Bn-A07-p16183869	Bn-N7-p18872267	gene	exon
Bn-A07-p16395444	Bn-N7-p19097039	gene	
Bn-A07-p16406072	Bn-N16-p30776587	gene	
Bn-A07-p16423207	Bn-N7-p19132231	gene	exon
Bn-A07-p16449661	Bn-N7-p19159713	gene	exon
Bn-A07-p16449988	Bn-N7-p19160040	gene	
Bn-A07-p16469173	Bn-N7-p19177559	gene	exon
Bn-A07-p16519850	Bn-N7-p19231704	gene	exon

Bn-A07-p16527225	Bn-N7-p19239077	gene	exon
Bn-A07-p16553147	Bn-N7-p19263682	gene	
Bn-A07-p16557893	Bn-N7-p19268240	gene	exon
Bn-A07-p16559043	Bn-N7-p19269390	gene	exon
Bn-A07-p16566633	Bn-N7-p19277009	gene	
Bn-A07-p16567234	Bn-N7-p19277609	gene	
Bn-A07-p16595704	Bn-N7-p19308425	gene	
Bn-A07-p16636687	Bn-N7-p19358087	gene	
Bn-A07-p16659232	Bn-N7-p19383325	gene	
Bn-A07-p16659396	Bn-N7-p19383489	gene	
Bn-A07-p16709303	Bn-N7-p19433945	gene	exon
Bn-A07-p16721483	Bn-N7-p19450162	gene	exon
Bn-A07-p16722346	Bn-N7-p19451034	gene	
Bn-A07-p16722383	Bn-N7-p19451071	gene	
Bn-A07-p16722623	Bn-N7-p19451310	gene	
Bn-A07-p16745833	Bn-N7-p19465227	gene	exon
Bn-A07-p16749101	Bn-N7-p19468523	gene	
Bn-A07-p16755801	Bn-N7-p19475138	gene	
Bn-A07-p16756509	Bn-N7-p19475846	gene	
Bn-A07-p16765596	Bn-N7-p19484933	gene	exon
Bn-A07-p16792991	Bn-N7-p19499588	gene	exon
Bn-A07-p16831414	Bn-N7-p19541188	gene	
Bn-A07-p16833174	Bn-N7-p19542578	gene	exon
Bn-A07-p16872148	Bn-N7-p19574677	gene	exon
Bn-A07-p1689072	Bn-N7-p1805953	gene	exon
Bn-A07-p16895848	Bn-N7-p19595432	gene	
Bn-A07-p16896370	Bn-N7-p19595976	gene	
Bn-A07-p16898207	Bn-N7-p19597887	gene	
Bn-A07-p16909798	Bn-N7-p19610807	gene	
Bn-A07-p16912963	Bn-N7-p19613978	gene	exon
Bn-A07-p16919795	Bn-N7-p19620923	gene	exon
Bn-A07-p16924814	Bn-N7-p19626423	gene	exon
Bn-A07-p16925201	Bn-N7-p19626810	gene	exon
Bn-A07-p16936529	Bn-N7-p19638052	gene	
Bn-A07-p16946173	Bn-N7-p19647643	gene	
Bn-A07-p16965943	Bn-N7-p19667355	gene	
Bn-A07-p16967567	Bn-N7-p19668971	gene	exon
Bn-A07-p16970000	Bn-N7-p19671405	gene	
Bn-A07-p16987468	Bn-N7-p19689583	gene	exon
Bn-A07-p16996070	Bn-N7-p19698250	gene	
Bn-A07-p17001912	Bn-N7-p19705950	gene	
Bn-A07-p17026444	Bn-N7-p19728421	gene	
Bn-A07-p17047263	Bn-N7-p19743562	gene	
Bn-A07-p17053061	Bn-N7-p19749483	gene	exon
Bn-A07-p17085728	Bn-N7-p19781419	gene	exon
Bn-A07-p17100649	Bn-N7-p19800272	gene	
Bn-A07-p17104148	Bn-N7-p19803770	gene	exon
Bn-A07-p17123712	Bn-N7-p19825901	gene	exon
Bn-A07-p17142058	Bn-N16-p32174578	gene	
Bn-A07-p17148373	Bn-N7-p19873366	gene	
Bn-A07-p17177279	Bn-N7-p19900751	gene	exon
Bn-A07-p17186393	Bn-N7-p19913925	gene	exon

Bn-A07-p17225853	Bn-N7-p19950600	gene	
Bn-A07-p17260356	Bn-N16-p32479391	gene	exon
Bn-A07-p1729364	Bn-N7-p1849388	gene	exon
Bn-A07-p17298132	Bn-N7-p19989191	gene	exon
Bn-A07-p17353806	Bn-N16-p32765125	gene	
Bn-A07-p17400106	Bn-N7-p20100304	gene	exon
Bn-A07-p17409328	Bn-N7-p20107667	gene	exon
Bn-A07-p17411297	Bn-N7-p20109636	gene	exon
Bn-A07-p17411599	Bn-N7-p20109938	gene	
Bn-A07-p17412755	Bn-N7-p20111094	gene	exon
Bn-A07-p17418824	Bn-N7-p20117163	gene	
Bn-A07-p17419179	Bn-N7-p20117518	gene	
Bn-A07-p17421675	Bn-N7-p20120014	gene	
Bn-A07-p17435418	Bn-N7-p20133134	gene	
Bn-A07-p17450697	Bn-N7-p20146850	gene	exon
Bn-A07-p17465762	Bn-N7-p20160318	gene	
Bn-A07-p17470966	Bn-N7-p20167404	gene	exon
Bn-A07-p17477080	Bn-N7-p20173519	gene	
Bn-A07-p17483655	Bn-N7-p20180334	gene	exon
Bn-A07-p17520539	Bn-N7-p20220294	gene	exon
Bn-A07-p17523277	Bn-N16-p33053742	gene	
Bn-A07-p17583998	Bn-N7-p20276166	gene	
Bn-A07-p17587315	Bn-N7-p20279530	gene	
Bn-A07-p17588031	Bn-N7-p20280260	gene	exon
Bn-A07-p17598687	Bn-N7-p20294291	gene	exon
Bn-A07-p17605668	Bn-N7-p20301104	gene	exon
Bn-A07-p17607958	Bn-N7-p20305544	gene	
Bn-A07-p17628920	Bn-N7-p20326009	gene	
Bn-A07-p17636141	Bn-N7-p20335931	gene	exon
Bn-A07-p17679589	Bn-N7-p20396156	gene	exon
Bn-A07-p17814268	Bn-N7-p20528512	gene	exon
Bn-A07-p17823544	Bn-N7-p20537658	gene	
Bn-A07-p17827124	Bn-N7-p20541231	gene	
Bn-A07-p178437	Bn-N7-p195940	gene	
Bn-A07-p17860745	Bn-N7-p20582995	gene	
Bn-A07-p17959118	Bn-N7-p20681326	gene	
Bn-A07-p18035698	Bn-N7-p20760589	gene	exon
Bn-A07-p18103464	Bn-N7-p20844230	gene	exon
Bn-A07-p18103505	Bn-N7-p20844271	gene	exon
Bn-A07-p18104828	Bn-N7-p20845604	gene	
Bn-A07-p18106007	Bn-N7-p20846772	gene	
Bn-A07-p18134446	Bn-N7-p20874908	gene	exon
Bn-A07-p18157564	Bn-N7-p20910957	gene	
Bn-A07-p18167424	Bn-N7-p20920433	gene	exon
Bn-A07-p18187317	Bn-N16-p34443093	gene	exon
Bn-A07-p18204966	Bn-N7-p20966934	gene	exon
Bn-A07-p18210547	Bn-N7-p20972516	gene	exon
Bn-A07-p18216943	Bn-N7-p20978354	gene	exon
Bn-A07-p18226326	Bn-N7-p20987309	gene	
Bn-A07-p18227328	Bn-N7-p20988316	gene	
Bn-A07-p18262143	Bn-N7-p21028191	gene	
Bn-A07-p18265668	Bn-N7-p21031716	gene	exon

Bn-A07-p18265772	Bn-N7-p21031821	gene	
Bn-A07-p18274656	Bn-N7-p21042702	gene	exon
Bn-A07-p18308378	Bn-N7-p21081019	gene	exon
Bn-A07-p1832580	Bn-N7-p2013193	gene	
Bn-A07-p18338860	Bn-N16-p34681807	gene	
Bn-A07-p18346973	Bn-N7-p21120187	gene	
Bn-A07-p18812613	Bn-N7-p21658703	gene	exon
Bn-A07-p18822708	Bn-N7-p21671143	gene	exon
Bn-A07-p18832178	Bn-N7-p21681303	gene	exon
Bn-A07-p18947073	Bn-N7-p21820922	gene	exon
Bn-A07-p18955989	Bn-N7-p21829421	gene	
Bn-A07-p18956207	Bn-N7-p21829639	gene	
Bn-A07-p18957162	Bn-N7-p21830585	gene	
Bn-A07-p18985169	Bn-N7-p21860887	gene	exon
Bn-A07-p19001556	Bn-N7-p21876445	gene	exon
Bn-A07-p19001778	Bn-N7-p21876667	gene	exon
Bn-A07-p19009397	Bn-N7-p21885980	gene	
Bn-A07-p19090612	Bn-N7-p21968936	gene	exon
Bn-A07-p19093712	Bn-N7-p21972098	gene	exon
Bn-A07-p19113444	Bn-N7-p21993479	gene	
Bn-A07-p19118066	Bn-N7-p21997596	gene	
Bn-A07-p19129406	Bn-N7-p22009465	gene	
Bn-A07-p19142808	Bn-N7-p22015948	gene	exon
Bn-A07-p19163856	Bn-N7-p22035316	gene	
Bn-A07-p19166676	Bn-N7-p22038136	gene	
Bn-A07-p19175358	Bn-N7-p22048526	gene	exon
Bn-A07-p19176340	Bn-N16-p36266862	gene	exon
Bn-A07-p19186300	Bn-N7-p22059448	gene	exon
Bn-A07-p19190510	Bn-N7-p22063739	gene	
Bn-A07-p19512603	Bn-N7-p22378556	gene	
Bn-A07-p19512916	Bn-N7-p22378869	gene	exon
Bn-A07-p19824864	Bn-N7-p22725156	gene	
Bn-A07-p19825063	Bn-N7-p22725356	gene	
Bn-A07-p19865918	Bn-N7-p22777396	gene	
Bn-A07-p19865996	Bn-N7-p22777474	gene	
Bn-A07-p19871923	Bn-N7-p22783472	gene	
Bn-A07-p19900592	Bn-N7-p22818133	gene	exon
Bn-A07-p19901175	Bn-N16-p28333077	gene	
Bn-A07-p19915062	Bn-N7-p22833926	gene	exon
Bn-A07-p19924753	Bn-N7-p22846933	gene	
Bn-A07-p199589	Bn-N7-p221507	gene	
Bn-A07-p19965401	Bn-N7-p22889167	gene	exon
Bn-A07-p19967695	Bn-N7-p22892309	gene	
Bn-A07-p19975808	Bn-N16-p37540850	gene	exon
Bn-A07-p19987483	Bn-N7-p22914333	gene	exon
Bn-A07-p19987485	Bn-N7-p22914335	gene	exon
Bn-A07-p19999651	Bn-N7-p22927363	gene	exon
Bn-A07-p20000340	Bn-N7-p22928052	gene	exon
Bn-A07-p20003438	Bn-N7-p22931174	gene	exon
Bn-A07-p20006245	Bn-N7-p22934417	gene	
Bn-A07-p20029449	Bn-N7-p22957030	gene	exon
Bn-A07-p20044413	Bn-N7-p22973485	gene	exon

Bn-A07-p20090726	Bn-N7-p23020123	gene	
Bn-A07-p20144687	Bn-N7-p23083318	gene	exon
Bn-A07-p20190653	Bn-N7-p23121055	gene	
Bn-A07-p20193221	Bn-N7-p23123586	gene	
Bn-A07-p20209068	Bn-N7-p23139133	gene	exon
Bn-A07-p20221692	Bn-N7-p23146689	gene	exon
Bn-A07-p20230189	Bn-N7-p23153248	gene	exon
Bn-A07-p20231605	Bn-N7-p23154664	gene	exon
Bn-A07-p20369599	Bn-N16-p38160484	gene	exon
Bn-A07-p20467347	Bn-N7-p23418291	gene	exon
Bn-A07-p20471091	Bn-N7-p23422028	gene	
Bn-A07-p20471143	Bn-N7-p23422080	gene	
Bn-A07-p20511320	Bn-N7-p23459139	gene	exon
Bn-A07-p20554194	Bn-N7-p23514296	gene	
Bn-A07-p20558164	Bn-N7-p23518260	gene	
Bn-A07-p20561173	Bn-N7-p23521267	gene	exon
Bn-A07-p20646388	Bn-N7-p23615357	gene	exon
Bn-A07-p20661885	Bn-N7-p23622894	gene	exon
Bn-A07-p20662200	Bn-N7-p23623208	gene	exon
Bn-A07-p20675049	Bn-N7-p23636157	gene	exon
Bn-A07-p20771568	Bn-N7-p23721465	gene	
Bn-A07-p20797755	Bn-N7-p23748355	gene	
Bn-A07-p20871163	Bn-N7-p23811865	gene	exon
Bn-A07-p20887568	Bn-N7-p23826853	gene	
Bn-A07-p20935217	Bn-N7-p23869260	gene	exon
Bn-A07-p20941237	Bn-N16-p38817271	gene	
Bn-A07-p20941338	Bn-N16-p38817372	gene	
Bn-A07-p20951340	Bn-N16-p38850702	gene	
Bn-A07-p20981410	Bn-N7-p23882909	gene	exon
Bn-A07-p20986876	Bn-N7-p23888405	gene	exon
Bn-A07-p20999615	Bn-N7-p23898726	gene	exon
Bn-A07-p21019499	Bn-N7-p23918790	gene	exon
Bn-A07-p21160078	Bn-N7-p24081454	gene	
Bn-A07-p21173896	Bn-N7-p24091608	gene	
Bn-A07-p21280027	Bn-N7-p24201869	gene	exon
Bn-A07-p21328168	Bn-N7-p24246510	gene	
Bn-A07-p21332273	Bn-N7-p24250622	gene	
Bn-A07-p21395576	Bn-N16-p39359806	gene	exon
Bn-A07-p21400508	Bn-N16-p39365019	gene	
Bn-A07-p21404492	Bn-N16-p39369178	gene	exon
Bn-A07-p21410292	Bn-N16-p39376975	gene	
Bn-A07-p21413042	Bn-N16-p39379780	gene	
Bn-A07-p21449796	Bn-N16-p39420044	gene	
Bn-A07-p21496070	Bn-N7-p24345207	gene	exon
Bn-A07-p21546078	Bn-N16-p39534992	gene	
Bn-A07-p21567127	Bn-N7-p24424123	gene	
Bn-A07-p21568086	Bn-N7-p24425050	gene	
Bn-A07-p21569289	Bn-N7-p24426258	gene	
Bn-A07-p21577164	Bn-N7-p24433733	gene	
Bn-A07-p21586613	Bn-N7-p24443471	gene	
Bn-A07-p21587819	Bn-N7-p24444677	gene	
Bn-A07-p21600829	Bn-N7-p24457796	gene	exon

Bn-A07-p216152	Bn-N7-p231855	gene	
Bn-A07-p21631043	Bn-N7-p24486028	gene	
Bn-A07-p21643815	Bn-N7-p24498837	gene	
Bn-A07-p21678440	Bn-N7-p24535423	gene	exon
Bn-A07-p21679566	Bn-N7-p24536529	gene	exon
Bn-A07-p21792030	Bn-N7-p24574746	gene	exon
Bn-A07-p21808739	Bn-N7-p24590257	gene	exon
Bn-A07-p21820686	Bn-N7-p24600079	gene	exon
Bn-A07-p21835729	Bn-N7-p24613096	gene	
Bn-A07-p21837742	Bn-N7-p24619288	gene	exon
Bn-A07-p21844673	Bn-N7-p24626217	gene	exon
Bn-A07-p21875901	Bn-N7-p24662708	gene	exon
Bn-A07-p21962752	Bn-N7-p24768075	gene	exon
Bn-A07-p21965076	Bn-N7-p24770398	gene	exon
Bn-A07-p21979205	Bn-N7-p24784384	gene	
Bn-A07-p21993375	Bn-N7-p24799309	gene	
Bn-A07-p21994050	Bn-N7-p24800048	gene	
Bn-A07-p21995054	Bn-N7-p24801058	gene	
Bn-A07-p22005058	Bn-N7-p24811031	gene	exon
Bn-A07-p22010671	Bn-N7-p24814522	gene	exon
Bn-A07-p22034462	Bn-N7-p24839696	gene	exon
Bn-A07-p22050904	Bn-N7-p24856389	gene	exon
Bn-A07-p22092610	Bn-N7-p24897405	gene	exon
Bn-A07-p22092775	Bn-N7-p24897570	gene	exon
Bn-A07-p22095943	Bn-N7-p24900757	gene	
Bn-A07-p22103902	Bn-N7-p24910676	gene	exon
Bn-A07-p22119502	Bn-N7-p24927237	gene	
Bn-A07-p22139480	Bn-N7-p24947975	gene	
Bn-A07-p22140320	Bn-N7-p24948807	gene	
Bn-A07-p22156609	Bn-N7-p24967569	gene	exon
Bn-A07-p22168947	Bn-N7-p24983502	gene	exon
Bn-A07-p22169573	Bn-N7-p24984126	gene	
Bn-A07-p22176569	Bn-N7-p24991322	gene	exon
Bn-A07-p22198393	Bn-N7-p25015613	gene	
Bn-A07-p22209952	Bn-N7-p25028678	gene	
Bn-A07-p22231852	Bn-N16-p40327922	gene	exon
Bn-A07-p22250567	Bn-N7-p25075523	gene	exon
Bn-A07-p22251229	Bn-N7-p25076175	gene	
Bn-A07-p22251906	Bn-N7-p25076853	gene	
Bn-A07-p22253687	Bn-N7-p25078634	gene	
Bn-A07-p22282923	Bn-N16-p40401185	gene	exon
Bn-A07-p22302158	Bn-N7-p25126505	gene	exon
Bn-A07-p22308030	Bn-N7-p25133285	gene	
Bn-A07-p22309060	Bn-N7-p25134315	gene	
Bn-A07-p22309183	Bn-N7-p25134438	gene	exon
Bn-A07-p22310107	Bn-N7-p25135147	gene	exon
Bn-A07-p22329359	Bn-N7-p25157288	gene	
Bn-A07-p22382675	Bn-N7-p25210618	gene	
Bn-A07-p22412915	Bn-N7-p25247687	gene	exon
Bn-A07-p22440144	Bn-N7-p25284734	gene	
Bn-A07-p22488507	Bn-N7-p25334331	gene	
Bn-A07-p22510199	Bn-N7-p25356625	gene	

Bn-A07-p22575730	Bn-N7-p25437729	gene	
Bn-A07-p2264217	Bn-N7-p4001170	gene	exon
Bn-A07-p241997	Bn-N17-p652619	gene	exon
Bn-A07-p244051	Bn-N7-p269475	gene	
Bn-A07-p2524777	Bn-N7-p4182170	gene	
Bn-A07-p2575548	Bn-N7-p4236411	gene	
Bn-A07-p267110	Bn-N7-p296070	gene	
Bn-A07-p276584	Bn-N7-p305580	gene	
Bn-A07-p3041012	Bn-N7-p4632441	gene	exon
Bn-A07-p3081602	Bn-N7-p4691361	gene	exon
Bn-A07-p3093306	Bn-N7-p4698981	gene	
Bn-A07-p3102715	Bn-N19-p36899879	gene	
Bn-A07-p3132316	Bn-N7-p4713420	gene	
Bn-A07-p3202245	Bn-N7-p4780498	gene	
Bn-A07-p3262688	Bn-N7-p4845203	gene	
Bn-A07-p3262752	Bn-N7-p4845267	gene	
Bn-A07-p3262864	Bn-N7-p4845380	gene	exon
Bn-A07-p3275876	Bn-N7-p4866520	gene	exon
Bn-A07-p3287486	Bn-N7-p4875256	gene	exon
Bn-A07-p3309722	Bn-N7-p4897882	gene	
Bn-A07-p3467400	Bn-N17-p12181043	gene	exon
Bn-A07-p3482570	Bn-N7-p5116632	gene	exon
Bn-A07-p3482967	Bn-N7-p5117026	gene	
Bn-A07-p3517211	Bn-N7-p5153323	gene	exon
Bn-A07-p3646853	Bn-N7-p5260334	gene	
Bn-A07-p3648246	Bn-N7-p5261722	gene	
Bn-A07-p3649647	Bn-N7-p5263276	gene	
Bn-A07-p367569	Bn-N7-p392915	gene	
Bn-A07-p3746131	Bn-N7-p5345350	gene	
Bn-A07-p3796068	Bn-N7-p5390231	gene	
Bn-A07-p3796474	Bn-N7-p5390636	gene	exon
Bn-A07-p3796859	Bn-N7-p5391030	gene	exon
Bn-A07-p3826249	Bn-N7-p5421931	gene	
Bn-A07-p3862192	Bn-N7-p5449697	gene	exon
Bn-A07-p3899866	Bn-N7-p5472422	gene	exon
Bn-A07-p3900934	Bn-N7-p5473488	gene	exon
Bn-A07-p3916567	Bn-N7-p5478132	gene	exon
Bn-A07-p3918449	Bn-N7-p5480005	gene	exon
Bn-A07-p3934301	Bn-N7-p5499638	gene	
Bn-A07-p3995605	Bn-N7-p5563726	gene	
Bn-A07-p4095559	Bn-N7-p5649088	gene	
Bn-A07-p4097388	Bn-N7-p5650918	gene	
Bn-A07-p4097690	Bn-N7-p5651220	gene	
Bn-A07-p4173116	Bn-N7-p5732895	gene	exon
Bn-A07-p4173583	Bn-N7-p5733362	gene	exon
Bn-A07-p4289732	Bn-N7-p5861096	gene	
Bn-A07-p4309577	Bn-N7-p5878177	gene	exon
Bn-A07-p4387388	Bn-N7-p5955748	gene	exon
Bn-A07-p4414432	Bn-N7-p5979312	gene	
Bn-A07-p4477010	Bn-N7-p6044743	gene	
Bn-A07-p4483935	Bn-N7-p6050372	gene	exon
Bn-A07-p4536488	Bn-N7-p6087716	gene	exon

Bn-A07-p4536608	Bn-N7-p6087836	gene	exon
Bn-A07-p4582463	Bn-N7-p6132922	gene	
Bn-A07-p4587108	Bn-N17-p14444200	gene	
Bn-A07-p4607332	Bn-N7-p6164763	gene	
Bn-A07-p4639103	Bn-N18-p6352800	gene	
Bn-A07-p4644371	Bn-N7-p6189089	gene	
Bn-A07-p4678510	Bn-N7-p6243950	gene	exon
Bn-A07-p4704042	Bn-N7-p6267952	gene	exon
Bn-A07-p4802709	Bn-N7-p6366961	gene	
Bn-A07-p4815239	Bn-N7-p6379719	gene	
Bn-A07-p4815910	Bn-N7-p6380390	gene	
Bn-A07-p4817318	Bn-N7-p6381799	gene	
Bn-A07-p4825210	Bn-N7-p6393903	gene	exon
Bn-A07-p483451	Bn-N3-p2329168	gene	
Bn-A07-p483541	Bn-Scaffold17351-p645	gene	exon
Bn-A07-p4838826	Bn-N7-p6407686	gene	exon
Bn-A07-p4912587	Bn-N7-p6483683	gene	exon
Bn-A07-p4912709	Bn-N7-p6483805	gene	
Bn-A07-p4937244	Bn-N7-p6506698	gene	
Bn-A07-p4947273	Bn-N7-p6514933	gene	
Bn-A07-p4954667	Bn-N7-p6520825	gene	
Bn-A07-p4958794	Bn-N7-p6524952	gene	
Bn-A07-p4958868	Bn-N7-p6525026	gene	
Bn-A07-p4959975	Bn-N7-p6526166	gene	exon
Bn-A07-p5008797	Bn-N7-p6573394	gene	
Bn-A07-p5016523	Bn-N7-p6580894	gene	
Bn-A07-p5050160	Bn-N7-p6632110	gene	exon
Bn-A07-p5072420	Bn-N7-p6659468	gene	exon
Bn-A07-p5118743	Bn-N7-p6710788	gene	
Bn-A07-p5119149	Bn-N7-p6711189	gene	
Bn-A07-p5126106	Bn-N7-p6718144	gene	exon
Bn-A07-p5145409	Bn-N7-p6730359	gene	exon
Bn-A07-p5211320	Bn-N7-p6774120	gene	exon
Bn-A07-p5228632	Bn-N7-p6781198	gene	
Bn-A07-p5229070	Bn-N7-p6781636	gene	
Bn-A07-p5234114	Bn-N7-p6798260	gene	exon
Bn-A07-p5243525	Bn-N7-p6805566	gene	
Bn-A07-p5256311	Bn-N7-p6823370	gene	exon
Bn-A07-p5365136	Bn-N7-p6921020	gene	exon
Bn-A07-p53742	Bn-N7-p47215	gene	
Bn-A07-p5401960	Bn-N7-p6949695	gene	
Bn-A07-p5402372	Bn-N7-p6950841	gene	
Bn-A07-p5402451	Bn-N7-p6950920	gene	
Bn-A07-p5402605	Bn-N7-p6951074	gene	
Bn-A07-p5402835	Bn-N7-p6951305	gene	
Bn-A07-p5403065	Bn-N7-p6951535	gene	
Bn-A07-p5407029	Bn-N7-p6955556	gene	
Bn-A07-p5407143	Bn-N7-p6955670	gene	
Bn-A07-p5407683	Bn-N7-p6956210	gene	exon
Bn-A07-p5408025	Bn-N7-p6963842	gene	
Bn-A07-p541617	Bn-N9-p29257429	gene	exon
Bn-A07-p5431144	Bn-N7-p6985800	gene	

Bn-A07-p5455024	Bn-N7-p7010907	gene	exon
Bn-A07-p5482599	Bn-N7-p7047312	gene	
Bn-A07-p5539902	Bn-N17-p15997447	gene	exon
Bn-A07-p5568504	Bn-N7-p7108520	gene	
Bn-A07-p5591930	Bn-N7-p7124905	gene	
Bn-A07-p5618903	Bn-N7-p7215114	gene	
Bn-A07-p5619095	Bn-N7-p7215306	gene	
Bn-A07-p5619285	Bn-N7-p7215496	gene	
Bn-A07-p5620067	Bn-N7-p7216278	gene	
Bn-A07-p5633352	Bn-N7-p7229050	gene	
Bn-A07-p5733051	Bn-N9-p17767212	gene	
Bn-A07-p5758215	Bn-N9-p17736080	gene	exon
Bn-A07-p5768726	Bn-N7-p7332148	gene	exon
Bn-A07-p5810641	Bn-N7-p7375207	gene	
Bn-A07-p5871275	Bn-N7-p7434664	gene	exon
Bn-A07-p5895170	Bn-N7-p7471626	gene	exon
Bn-A07-p5905223	Bn-N7-p7479495	gene	
Bn-A07-p5983825	Bn-N7-p7565383	gene	
Bn-A07-p6055505	Bn-N3-p7073378	gene	exon
Bn-A07-p6197889	Bn-N7-p7757299	gene	
Bn-A07-p6207835	Bn-N7-p7775654	gene	exon
Bn-A07-p6215131	Bn-N7-p7782954	gene	
Bn-A07-p6217329	Bn-N7-p7785152	gene	
Bn-A07-p6217365	Bn-N7-p7785188	gene	
Bn-A07-p6238777	Bn-N7-p7808729	gene	exon
Bn-A07-p6259787	Bn-N7-p7826390	gene	exon
Bn-A07-p6262438	Bn-N7-p7829039	gene	
Bn-A07-p6276115	Bn-N7-p7851921	gene	exon
Bn-A07-p6281546	Bn-N7-p7854896	gene	
Bn-A07-p6282717	Bn-N7-p7856070	gene	exon
Bn-A07-p6283126	Bn-N7-p7856478	gene	
Bn-A07-p6309969	Bn-N7-p7879982	gene	
Bn-A07-p6346472	Bn-N7-p7931431	gene	exon
Bn-A07-p6428365	Bn-N7-p8019506	gene	
Bn-A07-p6432326	Bn-N7-p8023341	gene	
Bn-A07-p6432331	Bn-N7-p8023346	gene	
Bn-A07-p6460270	Bn-N7-p8037884	gene	
Bn-A07-p6494007	Bn-N7-p8076082	gene	
Bn-A07-p6501207	Bn-N17-p17601417	gene	
Bn-A07-p6516437	Bn-N7-p8094964	gene	
Bn-A07-p6518480	Bn-N7-p8097006	gene	exon
Bn-A07-p6523973	Bn-N17-p17644049	gene	
Bn-A07-p6524220	Bn-N7-p8102738	gene	exon
Bn-A07-p653283	Bn-N7-p594197	gene	exon
Bn-A07-p6534557	Bn-N7-p8114866	gene	exon
Bn-A07-p6542254	Bn-N7-p8122646	gene	
Bn-A07-p663772	Bn-N7-p607970	gene	
Bn-A07-p6641473	Bn-N7-p8218371	gene	exon
Bn-A07-p6719583	Bn-N7-p8299963	gene	exon
Bn-A07-p6722244	Bn-N7-p8302625	gene	
Bn-A07-p6735264	Bn-N7-p8323574	gene	
Bn-A07-p6841662	Bn-N17-p18288854	gene	exon

Bn-A07-p6850383	Bn-N7-p8426478	gene	exon
Bn-A07-p6898363	Bn-N7-p8483939	gene	
Bn-A07-p6906296	Bn-N13-p53096085	gene	exon
Bn-A07-p6926020	Bn-N7-p8510444	gene	
Bn-A07-p6942882	Bn-N7-p8523017	gene	exon
Bn-A07-p6943725	Bn-N7-p8523858	gene	
Bn-A07-p6947953	Bn-N7-p8528691	gene	
Bn-A07-p6952910	Bn-N7-p8532856	gene	exon
Bn-A07-p6981435	Bn-N7-p8565060	gene	
Bn-A07-p7009935	Bn-N7-p8592644	gene	
Bn-A07-p7027400	Bn-N7-p8611224	gene	exon
Bn-A07-p7055420	Bn-N7-p8632712	gene	exon
Bn-A07-p7069624	Bn-N7-p8643592	gene	
Bn-A07-p7078464	Bn-N7-p8657424	gene	exon
Bn-A07-p7079099	Bn-N7-p8658089	gene	
Bn-A07-p7082333	Bn-N7-p8661352	gene	
Bn-A07-p7099002	Bn-N17-p18732130	gene	exon
Bn-A07-p7117228	Bn-N7-p8706303	gene	
Bn-A07-p7117229	Bn-N7-p8706304	gene	
Bn-A07-p7118584	Bn-N7-p8708759	gene	exon
Bn-A07-p7119962	Bn-N7-p8710138	gene	exon
Bn-A07-p7124154	Bn-N7-p8714434	gene	
Bn-A07-p7215862	Bn-N7-p8826403	gene	
Bn-A07-p7230543	Bn-N7-p8834828	gene	exon
Bn-A07-p7232691	Bn-N7-p8840367	gene	exon
Bn-A07-p7237742	Bn-N7-p8845478	gene	
Bn-A07-p7261357	Bn-N7-p8864825	gene	
Bn-A07-p7261508	Bn-N7-p8864976	gene	
Bn-A07-p7268294	Bn-N7-p8874774	gene	exon
Bn-A07-p7287398	Bn-N7-p8896085	gene	
Bn-A07-p7298603	Bn-N7-p8909681	gene	exon
Bn-A07-p7309287	Bn-N7-p8916937	gene	exon
Bn-A07-p7312075	Bn-N7-p8922644	gene	exon
Bn-A07-p73440	Bn-N7-p72884	gene	exon
Bn-A07-p7352643	Bn-N7-p8951323	gene	
Bn-A07-p7367247	Bn-N7-p8962238	gene	
Bn-A07-p7380906	Bn-N7-p8977570	gene	
Bn-A07-p7391449	Bn-N7-p8988114	gene	exon
Bn-A07-p7392394	Bn-N7-p8989059	gene	exon
Bn-A07-p7392454	Bn-N7-p8989119	gene	exon
Bn-A07-p7392457	Bn-N7-p8989122	gene	exon
Bn-A07-p7392753	Bn-N7-p8989418	gene	
Bn-A07-p7392879	Bn-N7-p8989544	gene	
Bn-A07-p7393007	Bn-N7-p8989673	gene	exon
Bn-A07-p7395038	Bn-N7-p8991585	gene	exon
Bn-A07-p7400268	Bn-N7-p8997082	gene	exon
Bn-A07-p7400432	Bn-N7-p8997246	gene	
Bn-A07-p7433228	Bn-N7-p9011407	gene	exon
Bn-A07-p7433707	Bn-N7-p9011886	gene	
Bn-A07-p7434126	Bn-N7-p9012305	gene	
Bn-A07-p7436573	Bn-N7-p9014762	gene	
Bn-A07-p7499400	Bn-N7-p9095651	gene	exon

Bn-A07-p7509139	Bn-N7-p9105871	gene	
Bn-A07-p7519375	Bn-N7-p9116100	gene	exon
Bn-A07-p7528011	Bn-N7-p9125239	gene	exon
Bn-A07-p7528095	Bn-N7-p9125323	gene	
Bn-A07-p7542335	Bn-N7-p9135461	gene	exon
Bn-A07-p7551371	Bn-N17-p19834270	gene	
Bn-A07-p7584364	Bn-N7-p9184566	gene	
Bn-A07-p7678712	Bn-N7-p9258227	gene	exon
Bn-A07-p7679469	Bn-N7-p9258984	gene	
Bn-A07-p7717365	Bn-Scaffold11459-p506	gene	
Bn-A07-p7738577	Bn-N7-p9300146	gene	
Bn-A07-p7740173	Bn-N7-p9301388	gene	
Bn-A07-p7773921	Bn-N7-p9336719	gene	
Bn-A07-p7796025	Bn-N7-p9360955	gene	exon
Bn-A07-p7872194	Bn-N7-p9420763	gene	
Bn-A07-p7931519	Bn-N7-p9298824	gene	exon
Bn-A07-p8037081	Bn-N7-p9618283	gene	
Bn-A07-p8046121	Bn-N7-p9627350	gene	exon
Bn-A07-p8084624	Bn-N7-p9659378	gene	exon
Bn-A07-p8096658	Bn-N7-p9667551	gene	exon
Bn-A07-p8122640	Bn-N7-p9685775	gene	exon
Bn-A07-p8134950	Bn-N7-p9697098	gene	exon
Bn-A07-p8167856	Bn-N7-p9734603	gene	exon
Bn-A07-p8178839	Bn-N7-p9744672	gene	exon
Bn-A07-p8181654	Bn-N7-p9747476	gene	exon
Bn-A07-p8183365	Bn-N7-p9750272	gene	
Bn-A07-p8184393	Bn-N7-p9751304	gene	exon
Bn-A07-p8207791	Bn-N7-p9764669	gene	exon
Bn-A07-p8258521	Bn-N7-p9809726	gene	exon
Bn-A07-p8259212	Bn-N7-p9810415	gene	exon
Bn-A07-p8259218	Bn-N7-p9810421	gene	exon
Bn-A07-p828139	Bn-N7-p751984	gene	exon
Bn-A07-p829095	Bn-N7-p752939	gene	
Bn-A07-p8314613	Bn-N7-p9874342	gene	exon
Bn-A07-p8359729	Bn-N7-p9921479	gene	
Bn-A07-p8530938	Bn-N7-p10088538	gene	
Bn-A07-p8531068	Bn-N17-p21518315	gene	
Bn-A07-p8545569	Bn-N7-p10101866	gene	
Bn-A07-p8558330	Bn-N7-p10114125	gene	exon
Bn-A07-p8560948	Bn-N7-p10116766	gene	
Bn-A07-p8572785	Bn-N7-p10129706	gene	
Bn-A07-p8610828	Bn-N7-p10167179	gene	
Bn-A07-p8675164	Bn-N7-p10229408	gene	
Bn-A07-p8675574	Bn-N7-p10229818	gene	exon
Bn-A07-p8701644	Bn-N7-p10248306	gene	
Bn-A07-p8745024	Bn-N7-p10295054	gene	
Bn-A07-p8775069	Bn-N7-p10328682	gene	
Bn-A07-p8790021	Bn-N7-p10341860	gene	exon
Bn-A07-p8796823	Bn-N7-p10348699	gene	exon
Bn-A07-p8851644	Bn-N7-p10432761	gene	exon
Bn-A07-p8852716	Bn-N17-p21908599	gene	
Bn-A07-p8857433	Bn-N7-p10438066	gene	

Bn-A07-p8888056	Bn-N17-p42441570	gene	
Bn-A07-p8929154	Bn-N7-p10527946	gene	
Bn-A07-p8947431	Bn-N7-p10546669	gene	
Bn-A07-p9034194	Bn-N17-p22687236	gene	exon
Bn-A07-p9044585	Bn-N13-p41261292	gene	exon
Bn-A07-p9046330	Bn-N7-p10639867	gene	
Bn-A07-p9071682	Bn-N7-p10663266	gene	exon
Bn-A07-p9079474	Bn-N7-p10670871	gene	exon
Bn-A07-p9089710	Bn-N7-p10681336	gene	exon
Bn-A07-p9091839	Bn-N7-p10683458	gene	
Bn-A07-p9095204	Bn-N7-p10686823	gene	
Bn-A07-p9095357	Bn-N7-p10686976	gene	exon
Bn-A07-p9095557	Bn-N7-p10687176	gene	
Bn-A07-p9097136	Bn-N7-p10688753	gene	
Bn-A07-p9115370	Bn-N7-p10704581	gene	
Bn-A07-p9120043	Bn-N3-p24974954	gene	
Bn-A07-p930068	Bn-N7-p929818	gene	exon
Bn-A07-p9320476	Bn-N7-p10880110	gene	
Bn-A07-p9321347	Bn-N7-p10880981	gene	exon
Bn-A07-p9324529	Bn-N7-p10884131	gene	
Bn-A07-p9402974	Bn-N7-p10952981	gene	exon
Bn-A07-p9462417	Bn-N7-p10990897	gene	
Bn-A07-p9493282	Bn-N7-p11018459	gene	
Bn-A07-p9509100	Bn-N7-p11033575	gene	
Bn-A07-p9512042	Bn-N7-p11039410	gene	
Bn-A07-p9512256	Bn-N7-p11039624	gene	exon
Bn-A07-p9516564	Bn-N12-p34412387	gene	
Bn-A07-p9523515	Bn-N7-p11047560	gene	
Bn-A07-p9525348	Bn-N7-p11049392	gene	exon
Bn-A07-p9565657	Bn-N7-p11072298	gene	exon
Bn-A07-p9583355	Bn-N7-p11089964	gene	
Bn-A07-p9583457	Bn-N7-p11090066	gene	
Bn-A07-p959523	Bn-N7-p974049	gene	exon
Bn-A07-p9617534	Bn-N7-p11120243	gene	exon
Bn-A07-p9637867	Bn-N7-p11138451	gene	
Bn-A07-p9661444	Bn-N7-p11159340	gene	exon
Bn-A07-p9670298	Bn-N7-p11177134	gene	exon
Bn-A07-p9684829	Bn-N7-p11191362	gene	
Bn-A07-p9724274	Bn-N17-p24104461	gene	exon
Bn-A07-p9831545	Bn-N7-p11323969	gene	
Bn-A07-p9841369	Bn-N7-p11333958	gene	
Bn-A07-p9849702	Bn-N7-p11347009	gene	
Bn-A07-p9897363	Bn-N7-p11393929	gene	exon
Bn-A07-p9908315	Bn-N7-p11406142	gene	
Bn-A07-p9921856	Bn-N7-p11421553	gene	exon
Bn-A07-p9923514	Bn-N7-p11423214	gene	
Bn-A07-p9924604	Bn-N7-p11424323	gene	
Bn-A07-p9924929	Bn-N7-p11424648	gene	
Bn-A07-p9985625	Bn-N7-p11476372	gene	
Bn-A07-p9987682	Bn-N7-p11478420	gene	
Bn-A08-p10026421	Bn-N8-p9593043	gene	exon
Bn-A08-p10067927	Bn-N8-p9634172	gene	exon

Bn-A08-p1007687	Bn-N8-p1217690	gene	exon
Bn-A08-p10106723	Bn-N8-p9686586	gene	exon
Bn-A08-p10115835	Bn-N8-p9694008	gene	exon
Bn-A08-p10118532	Bn-N18-p17777275	gene	exon
Bn-A08-p10128352	Bn-N8-p9705188	gene	
Bn-A08-p10136371	Bn-N8-p9724412	gene	
Bn-A08-p10190275	Bn-N8-p9790118	gene	
Bn-A08-p10214814	Bn-N8-p9810999	gene	
Bn-A08-p10215334	Bn-N8-p9811598	gene	exon
Bn-A08-p10215623	Bn-N8-p9811890	gene	exon
Bn-A08-p10281337	Bn-N8-p9946893	gene	exon
Bn-A08-p10528898	Bn-N8-p10210186	gene	
Bn-A08-p10615012	Bn-N8-p10291520	gene	exon
Bn-A08-p10663544	Bn-N8-p10331185	gene	exon
Bn-A08-p10716777	Bn-N8-p10375220	gene	exon
Bn-A08-p1077419	Bn-N8-p1277816	gene	exon
Bn-A08-p1077573	Bn-N8-p1277970	gene	exon
Bn-A08-p10795996	Bn-N8-p10449072	gene	exon
Bn-A08-p10798067	Bn-N8-p10454779	gene	
Bn-A08-p10809139	Bn-N8-p10465446	gene	exon
Bn-A08-p10822662	Bn-N8-p10475491	gene	exon
Bn-A08-p10824964	Bn-N8-p10477792	gene	
Bn-A08-p1084991	Bn-N3-p34880873	gene	
Bn-A08-p1088499	Bn-N8-p1284504	gene	
Bn-A08-p1088701	Bn-N8-p1284706	gene	
Bn-A08-p10887204	Bn-N8-p10534243	gene	exon
Bn-A08-p1089123	Bn-N8-p1285128	gene	exon
Bn-A08-p11308315	Bn-N13-p59642218	gene	
Bn-A08-p11310535	Bn-N8-p10992150	gene	
Bn-A08-p11329864	Bn-N8-p11010771	gene	
Bn-A08-p11352069	Bn-N8-p11022045	gene	exon
Bn-A08-p11458466	Bn-N8-p11129532	gene	
Bn-A08-p11459293	Bn-N8-p11131297	gene	exon
Bn-A08-p11667191	Bn-N8-p11338956	gene	exon
Bn-A08-p11679083	Bn-N8-p11352102	gene	
Bn-A08-p12054745	Bn-N3-p26994721	gene	exon
Bn-A08-p12058242	Bn-N3-p26998101	gene	exon
Bn-A08-p12555227	Bn-N8-p12138124	gene	
Bn-A08-p12556455	Bn-N8-p12139346	gene	exon
Bn-A08-p12599446	Bn-N8-p12191131	gene	
Bn-A08-p12638473	Bn-N8-p12235835	gene	exon
Bn-A08-p12752628	Bn-N8-p12372214	gene	
Bn-A08-p12761022	Bn-N8-p12380623	gene	exon
Bn-A08-p12767403	Bn-N8-p12386789	gene	
Bn-A08-p12781426	Bn-N8-p12398027	gene	exon
Bn-A08-p12794893	Bn-N8-p12401667	gene	exon
Bn-A08-p12812977	Bn-N8-p12415810	gene	exon
Bn-A08-p12820786	Bn-N8-p12430957	gene	
Bn-A08-p12828472	Bn-N8-p12438569	gene	
Bn-A08-p12923799	Bn-N8-p12556531	gene	
Bn-A08-p13009633	Bn-N8-p12643613	gene	
Bn-A08-p13011710	Bn-N8-p12646066	gene	

Bn-A08-p13019549	Bn-N13-p63010986	gene	exon
Bn-A08-p13030362	Bn-N8-p12708538	gene	exon
Bn-A08-p13030439	Bn-N8-p12708615	gene	
Bn-A08-p13076304	Bn-N8-p12763413	gene	
Bn-A08-p13076305	Bn-N8-p12763413	gene	
Bn-A08-p13091365	Bn-N8-p12779087	gene	exon
Bn-A08-p1319548	Bn-N5-p9587568	gene	
Bn-A08-p1319949	Bn-N5-p9587167	gene	exon
Bn-A08-p13200580	Bn-N8-p12863686	gene	
Bn-A08-p13207923	Bn-N13-p63308773	gene	
Bn-A08-p13221380	Bn-N8-p12891668	gene	exon
Bn-A08-p13239621	Bn-N8-p12910240	gene	exon
Bn-A08-p13239816	Bn-N8-p12910435	gene	exon
Bn-A08-p13269999	Bn-N2-p25048579	gene	
Bn-A08-p13270793	Bn-N8-p12940453	gene	
Bn-A08-p13271519	Bn-N8-p12941278	gene	
Bn-A08-p13271950	Bn-N8-p12941709	gene	exon
Bn-A08-p13284369	Bn-N8-p12952657	gene	
Bn-A08-p13327070	Bn-N8-p12999889	gene	exon
Bn-A08-p13328135	Bn-N8-p13000954	gene	exon
Bn-A08-p13342419	Bn-N8-p13014295	gene	exon
Bn-A08-p13352430	Bn-N8-p13023342	gene	
Bn-A08-p13354792	Bn-N8-p13027358	gene	
Bn-A08-p13357594	Bn-N8-p13030141	gene	
Bn-A08-p13363830	Bn-N8-p13038993	gene	exon
Bn-A08-p13376746	Bn-N8-p13049362	gene	exon
Bn-A08-p13379983	Bn-N8-p13052563	gene	
Bn-A08-p13409843	Bn-N8-p13070885	gene	exon
Bn-A08-p13427247	Bn-N8-p13081679	gene	exon
Bn-A08-p13427290	Bn-N8-p13081722	gene	exon
Bn-A08-p13428079	Bn-N8-p13082114	gene	exon
Bn-A08-p13428547	Bn-N8-p13082578	gene	exon
Bn-A08-p13484039	Bn-N8-p13158827	gene	exon
Bn-A08-p13485948	Bn-N8-p13160734	gene	exon
Bn-A08-p13486650	Bn-N8-p13161435	gene	
Bn-A08-p13544679	Bn-N8-p13225619	gene	exon
Bn-A08-p13559192	Bn-N8-p13239149	gene	
Bn-A08-p13573114	Bn-N8-p13253873	gene	exon
Bn-A08-p13577914	Bn-N8-p13258045	gene	
Bn-A08-p13580050	Bn-N8-p13260187	gene	
Bn-A08-p1364252	Bn-N5-p9541711	gene	exon
Bn-A08-p1365508	Bn-N5-p9540466	gene	exon
Bn-A08-p1365676	Bn-N5-p9540298	gene	
Bn-A08-p13670107	Bn-N8-p13329479	gene	
Bn-A08-p1369206	Bn-N5-p9527984	gene	exon
Bn-A08-p13692951	Bn-N8-p13376528	gene	
Bn-A08-p13707991	Bn-N8-p13387562	gene	exon
Bn-A08-p13785392	Bn-N8-p13468253	gene	exon
Bn-A08-p13785675	Bn-N8-p13468536	gene	exon
Bn-A08-p1379662	Bn-N5-p9515624	gene	
Bn-A08-p1379663	Bn-N5-p9515623	gene	
Bn-A08-p1379996	Bn-N5-p9515292	gene	

Bn-A08-p13856852	Bn-N8-p13544730	gene	
Bn-A08-p13860913	Bn-N8-p13548962	gene	
Bn-A08-p13876356	Bn-N8-p13565760	gene	exon
Bn-A08-p13892926	Bn-N8-p13577434	gene	
Bn-A08-p13897110	Bn-N8-p13586404	gene	exon
Bn-A08-p13901348	Bn-N8-p13590881	gene	
Bn-A08-p13964091	Bn-N8-p13664714	gene	
Bn-A08-p13966241	Bn-N8-p13668355	gene	exon
Bn-A08-p1400360	Bn-N5-p9491353	gene	
Bn-A08-p14011808	Bn-N8-p13732712	gene	exon
Bn-A08-p14014365	Bn-N8-p13735227	gene	exon
Bn-A08-p1404321	Bn-N5-p9487392	gene	exon
Bn-A08-p1404439	Bn-N5-p9487275	gene	exon
Bn-A08-p14067094	Bn-N8-p13785515	gene	exon
Bn-A08-p14067361	Bn-N8-p13785782	gene	exon
Bn-A08-p14086982	Bn-N8-p13804894	gene	exon
Bn-A08-p14133917	Bn-N8-p13842510	gene	exon
Bn-A08-p14156050	Bn-N8-p13860004	gene	
Bn-A08-p14163751	Bn-N18-p20069358	gene	
Bn-A08-p14167389	Bn-N8-p13866389	gene	exon
Bn-A08-p14218543	Bn-N8-p13911141	gene	
Bn-A08-p14218712	Bn-N8-p13910604	gene	exon
Bn-A08-p14351609	Bn-N8-p14059113	gene	
Bn-A08-p14351709	Bn-N8-p14059213	gene	exon
Bn-A08-p14369220	Bn-N8-p14071010	gene	
Bn-A08-p14375712	Bn-N8-p14071271	gene	
Bn-A08-p14447288	Bn-N8-p14161558	gene	exon
Bn-A08-p14493385	Bn-N8-p14218449	gene	exon
Bn-A08-p14496135	Bn-N18-p19427490	gene	exon
Bn-A08-p14498301	Bn-N8-p14223379	gene	exon
Bn-A08-p14538807	Bn-N8-p14264539	gene	exon
Bn-A08-p14597378	Bn-N8-p14303241	gene	
Bn-A08-p14597858	Bn-N8-p14303721	gene	
Bn-A08-p14658507	Bn-N8-p14358001	gene	
Bn-A08-p14673671	Bn-N8-p14373101	gene	exon
Bn-A08-p14692156	Bn-N8-p14400848	gene	
Bn-A08-p14695487	Bn-N8-p14404179	gene	
Bn-A08-p14695762	Bn-N8-p14404456	gene	
Bn-A08-p14697337	Bn-N8-p14406206	gene	
Bn-A08-p14704782	Bn-N8-p14413106	gene	exon
Bn-A08-p14713741	Bn-N8-p14426462	gene	
Bn-A08-p14715630	Bn-N8-p14428351	gene	
Bn-A08-p14718347	Bn-N8-p14431283	gene	
Bn-A08-p14739774	Bn-N8-p14453064	gene	
Bn-A08-p14740175	Bn-N8-p14453465	gene	
Bn-A08-p14740662	Bn-N8-p14453952	gene	
Bn-A08-p14759185	Bn-N8-p14479446	gene	
Bn-A08-p14766663	Bn-N8-p14487515	gene	exon
Bn-A08-p14784965	Bn-N8-p14506665	gene	
Bn-A08-p14790412	Bn-N8-p14512675	gene	exon
Bn-A08-p14813540	Bn-N8-p14537632	gene	exon
Bn-A08-p14846467	Bn-N8-p14569947	gene	exon

Bn-A08-p14906652	Bn-N8-p14630847	gene	
Bn-A08-p14955455	Bn-N8-p14684717	gene	
Bn-A08-p14970925	Bn-N8-p14700421	gene	exon
Bn-A08-p14976685	Bn-N8-p14706183	gene	
Bn-A08-p14980805	Bn-N8-p14710303	gene	exon
Bn-A08-p15027690	Bn-N8-p1516253	gene	
Bn-A08-p15067659	Bn-N8-p14800175	gene	exon
Bn-A08-p15067721	Bn-N8-p14800237	gene	exon
Bn-A08-p15122707	Bn-N8-p14852936	gene	exon
Bn-A08-p15127926	Bn-N8-p14858153	gene	exon
Bn-A08-p15213340	Bn-N13-p57224861	gene	exon
Bn-A08-p15239295	Bn-N8-p14975889	gene	
Bn-A08-p15239790	Bn-N8-p14976383	gene	
Bn-A08-p15243115	Bn-N8-p14979752	gene	exon
Bn-A08-p15273100	Bn-N8-p15008502	gene	exon
Bn-A08-p15322011	Bn-N8-p15068724	gene	exon
Bn-A08-p15326882	Bn-N13-p56974805	gene	exon
Bn-A08-p15341166	Bn-N8-p15080346	gene	exon
Bn-A08-p15342008	Bn-N8-p15081192	gene	exon
Bn-A08-p15353881	Bn-N8-p15090232	gene	exon
Bn-A08-p15362382	Bn-N8-p15099462	gene	
Bn-A08-p15448484	Bn-N8-p15181502	gene	exon
Bn-A08-p15489686	Bn-N8-p15224551	gene	exon
Bn-A08-p15594956	Bn-N8-p15335573	gene	exon
Bn-A08-p15614498	Bn-N13-p56349992	gene	exon
Bn-A08-p15615914	Bn-N8-p15354868	gene	exon
Bn-A08-p15616595	Bn-N8-p15355549	gene	exon
Bn-A08-p15619709	Bn-N8-p15358663	gene	exon
Bn-A08-p15620062	Bn-N8-p15359016	gene	exon
Bn-A08-p15620471	Bn-N8-p15359431	gene	
Bn-A08-p15620702	Bn-N8-p15359662	gene	
Bn-A08-p15700195	Bn-N8-p15416748	gene	exon
Bn-A08-p15710460	Bn-N8-p15426114	gene	exon
Bn-A08-p15710698	Bn-N8-p15426352	gene	
Bn-A08-p15740524	Bn-N8-p15455358	gene	
Bn-A08-p15767590	Bn-N8-p15492485	gene	
Bn-A08-p15782077	Bn-N8-p15503840	gene	exon
Bn-A08-p15782130	Bn-N8-p15503893	gene	exon
Bn-A08-p15782229	Bn-N8-p15503992	gene	exon
Bn-A08-p15782241	Bn-N8-p15504004	gene	exon
Bn-A08-p15782307	Bn-N8-p15504070	gene	exon
Bn-A08-p15790373	Bn-N8-p15512424	gene	
Bn-A08-p15792069	Bn-N8-p15514120	gene	
Bn-A08-p15792369	Bn-N8-p15514420	gene	
Bn-A08-p15792684	Bn-N8-p15514735	gene	
Bn-A08-p15792942	Bn-N8-p15514993	gene	
Bn-A08-p15802943	Bn-N8-p15526236	gene	
Bn-A08-p15804301	Bn-N8-p15527596	gene	
Bn-A08-p1581891	Bn-N2-p22571764	gene	
Bn-A08-p1584454	Bn-N2-p22574322	gene	exon
Bn-A08-p1584455	Bn-N2-p22574322	gene	exon
Bn-A08-p15945454	Bn-N8-p15640322	gene	exon

Bn-A08-p15952558	Bn-N8-p15649647	gene	exon
Bn-A08-p15961655	Bn-N19-p54773025	gene	
Bn-A08-p15967287	Bn-N8-p15659901	gene	
Bn-A08-p15991441	Bn-N8-p15686563	gene	exon
Bn-A08-p15994149	Bn-N8-p15687942	gene	exon
Bn-A08-p16028375	Bn-N8-p15714872	gene	
Bn-A08-p16030898	Bn-N8-p15717300	gene	
Bn-A08-p16039443	Bn-N8-p15726713	gene	exon
Bn-A08-p16039755	Bn-N8-p15727025	gene	
Bn-A08-p16061843	Bn-N8-p15748397	gene	
Bn-A08-p16141425	Bn-N8-p15834199	gene	exon
Bn-A08-p16171460	Bn-N8-p15863075	gene	exon
Bn-A08-p16201029	Bn-N8-p15898446	gene	
Bn-A08-p16216893	Bn-N8-p15914242	gene	exon
Bn-A08-p16259976	Bn-N8-p15960786	gene	
Bn-A08-p16260215	Bn-N8-p15961025	gene	exon
Bn-A08-p16267662	Bn-N8-p15969400	gene	exon
Bn-A08-p16286008	Bn-N8-p15985095	gene	exon
Bn-A08-p16286082	Bn-N8-p15985169	gene	
Bn-A08-p16293275	Bn-N8-p15992471	gene	exon
Bn-A08-p16299351	Bn-N8-p15998537	gene	exon
Bn-A08-p16306425	Bn-N8-p16006346	gene	
Bn-A08-p16313016	Bn-N8-p16012975	gene	
Bn-A08-p16314818	Bn-N8-p16014776	gene	
Bn-A08-p16317873	Bn-N8-p16017715	gene	
Bn-A08-p16318709	Bn-N8-p16018551	gene	exon
Bn-A08-p16318890	Bn-N8-p16018732	gene	
Bn-A08-p16325858	Bn-N8-p16025918	gene	
Bn-A08-p16328281	Bn-N8-p16028989	gene	
Bn-A08-p16344396	Bn-N8-p16042964	gene	
Bn-A08-p16349875	Bn-N8-p16048457	gene	exon
Bn-A08-p16392743	Bn-N8-p16096353	gene	
Bn-A08-p16392957	Bn-N8-p16096567	gene	
Bn-A08-p16403550	Bn-N8-p16111116	gene	exon
Bn-A08-p16457656	Bn-N8-p16162924	gene	exon
Bn-A08-p165219	Bn-N8-p295324	gene	exon
Bn-A08-p16572658	Bn-N8-p16282351	gene	exon
Bn-A08-p16575548	Bn-N8-p16284975	gene	exon
Bn-A08-p16575796	Bn-N8-p16285223	gene	exon
Bn-A08-p16582191	Bn-N8-p16291688	gene	exon
Bn-A08-p16673732	Bn-N8-p16382244	gene	exon
Bn-A08-p16676967	Bn-N8-p16385477	gene	exon
Bn-A08-p16770669	Bn-N8-p16480922	gene	exon
Bn-A08-p16770923	Bn-N8-p16481176	gene	
Bn-A08-p16771030	Bn-N8-p16481283	gene	
Bn-A08-p16771161	Bn-N8-p16481414	gene	
Bn-A08-p16829757	Bn-N8-p16534122	gene	exon
Bn-A08-p16842687	Bn-N8-p16547530	gene	
Bn-A08-p16842690	Bn-N8-p16547533	gene	
Bn-A08-p16870021	Bn-N13-p53640332	gene	exon
Bn-A08-p16943139	Bn-N8-p16642216	gene	
Bn-A08-p16943263	Bn-N8-p16642340	gene	exon

Bn-A08-p16965932	Bn-N8-p16671094	gene	exon
Bn-A08-p16966023	Bn-N8-p16671185	gene	exon
Bn-A08-p16980526	Bn-N8-p16685917	gene	
Bn-A08-p17062689	Bn-N8-p16760643	gene	
Bn-A08-p17070496	Bn-N8-p16768413	gene	
Bn-A08-p17088758	Bn-N8-p16786044	gene	
Bn-A08-p17094421	Bn-N8-p16791557	gene	exon
Bn-A08-p17105262	Bn-N8-p16802291	gene	exon
Bn-A08-p17111194	Bn-N8-p16814168	gene	exon
Bn-A08-p17130151	Bn-N8-p16834676	gene	exon
Bn-A08-p17152372	Bn-N8-p16858120	gene	
Bn-A08-p17186790	Bn-N8-p16894646	gene	
Bn-A08-p17187064	Bn-N8-p16894920	gene	exon
Bn-A08-p17190098	Bn-N8-p16897922	gene	
Bn-A08-p17191196	Bn-N13-p52891907	gene	exon
Bn-A08-p17191740	Bn-N8-p16899564	gene	
Bn-A08-p17193797	Bn-N8-p16901618	gene	exon
Bn-A08-p17202184	Bn-N8-p16911036	gene	
Bn-A08-p17202527	Bn-N8-p16911379	gene	
Bn-A08-p17202584	Bn-N8-p16911436	gene	
Bn-A08-p17202745	Bn-N8-p16911597	gene	
Bn-A08-p1720366	Bn-N8-p1579503	gene	
Bn-A08-p17217296	Bn-N8-p16923386	gene	exon
Bn-A08-p17221534	Bn-N8-p16927699	gene	
Bn-A08-p17235030	Bn-N8-p16945126	gene	
Bn-A08-p17238995	Bn-N13-p52787768	gene	
Bn-A08-p17249190	Bn-N8-p16959265	gene	
Bn-A08-p17255997	Bn-N8-p16968469	gene	
Bn-A08-p17256848	Bn-N8-p16969322	gene	exon
Bn-A08-p17291598	Bn-N8-p17004603	gene	exon
Bn-A08-p17291679	Bn-N8-p17004684	gene	exon
Bn-A08-p17293594	Bn-N8-p17006572	gene	
Bn-A08-p17306802	Bn-N8-p17019485	gene	exon
Bn-A08-p17307247	Bn-N8-p17019930	gene	
Bn-A08-p17363588	Bn-N8-p17094950	gene	
Bn-A08-p17366299	Bn-N8-p17097654	gene	
Bn-A08-p17381323	Bn-N13-p52431923	gene	
Bn-A08-p17382135	Bn-N8-p17115644	gene	
Bn-A08-p17382231	Bn-N8-p17115740	gene	
Bn-A08-p17402165	Bn-N8-p17136992	gene	
Bn-A08-p17403283	Bn-N8-p17138121	gene	exon
Bn-A08-p17434041	Bn-N8-p17157955	gene	exon
Bn-A08-p17437898	Bn-N8-p17161812	gene	
Bn-A08-p17449031	Bn-N3-p31715748	gene	exon
Bn-A08-p17449089	Bn-N3-p31715690	gene	exon
Bn-A08-p17449196	Bn-N8-p17166986	gene	exon
Bn-A08-p17449410	Bn-N8-p17167199	gene	exon
Bn-A08-p17472208	Bn-N8-p17190258	gene	exon
Bn-A08-p17530580	Bn-N8-p17245366	gene	exon
Bn-A08-p17530617	Bn-N8-p17245403	gene	
Bn-A08-p17534858	Bn-N8-p17249326	gene	exon
Bn-A08-p17559236	Bn-N8-p17270027	gene	exon

Bn-A08-p17565873	Bn-N8-p17275506	gene	exon
Bn-A08-p17570589	Bn-N8-p17282600	gene	
Bn-A08-p17578990	Bn-N8-p17290924	gene	
Bn-A08-p17612777	Bn-N8-p17333121	gene	exon
Bn-A08-p17612865	Bn-N8-p17333209	gene	
Bn-A08-p17639419	Bn-N8-p17373422	gene	exon
Bn-A08-p17648611	Bn-N8-p17386226	gene	
Bn-A08-p17649841	Bn-N8-p17387381	gene	
Bn-A08-p17654367	Bn-N8-p17391268	gene	exon
Bn-A08-p17654459	Bn-N8-p17391360	gene	
Bn-A08-p17696796	Bn-N8-p17425347	gene	
Bn-A08-p17710867	Bn-N8-p17444751	gene	
Bn-A08-p17721455	Bn-N8-p17457023	gene	
Bn-A08-p17735673	Bn-N8-p17469786	gene	
Bn-A08-p17748884	Bn-N8-p17488093	gene	exon
Bn-A08-p17813465	Bn-N8-p17543400	gene	
Bn-A08-p17848907	Bn-N18-p27148653	gene	exon
Bn-A08-p17849621	Bn-N8-p17573103	gene	exon
Bn-A08-p17851106	Bn-N8-p17574579	gene	exon
Bn-A08-p17857870	Bn-N8-p17579698	gene	
Bn-A08-p17859330	Bn-N8-p17581149	gene	exon
Bn-A08-p17938347	Bn-N5-p24244068	gene	
Bn-A08-p17981717	Bn-N8-p17711290	gene	
Bn-A08-p18000876	Bn-N8-p17732398	gene	
Bn-A08-p18045254	Bn-N8-p17778288	gene	
Bn-A08-p1809204	Bn-N8-p1682130	gene	
Bn-A08-p18112619	Bn-N18-p26552559	gene	
Bn-A08-p18144819	Bn-N8-p17878011	gene	
Bn-A08-p18153504	Bn-N8-p17884807	gene	exon
Bn-A08-p18200896	Bn-N8-p17928683	gene	
Bn-A08-p18234758	Bn-N8-p17964906	gene	exon
Bn-A08-p18286374	Bn-N8-p18021601	gene	
Bn-A08-p18290748	Bn-N8-p18030952	gene	exon
Bn-A08-p18291268	Bn-N8-p18031474	gene	exon
Bn-A08-p18321643	Bn-N8-p18067482	gene	exon
Bn-A08-p18331012	Bn-N8-p18076240	gene	exon
Bn-A08-p18353208	Bn-N8-p18098656	gene	exon
Bn-A08-p18356257	Bn-N8-p18101702	gene	exon
Bn-A08-p18409864	Bn-N8-p18146353	gene	
Bn-A08-p18432711	Bn-N8-p18182156	gene	exon
Bn-A08-p18432800	Bn-N8-p18182245	gene	exon
Bn-A08-p18432919	Bn-N8-p18182364	gene	exon
Bn-A08-p18433093	Bn-N8-p18182538	gene	exon
Bn-A08-p18433672	Bn-N8-p18183114	gene	exon
Bn-A08-p18462668	Bn-N8-p18208682	gene	exon
Bn-A08-p18463273	Bn-N8-p18209287	gene	
Bn-A08-p1847328	Bn-N8-p1726629	gene	
Bn-A08-p18526873	Bn-N8-p18279878	gene	exon
Bn-A08-p18538166	Bn-N8-p18290523	gene	
Bn-A08-p18592850	Bn-N8-p18351042	gene	exon
Bn-A08-p18597209	Bn-N8-p18355625	gene	exon
Bn-A08-p18605816	Bn-N8-p18359822	gene	exon

Bn-A08-p18630178	Bn-N18-p25494078	gene	exon
Bn-A08-p18631047	Bn-N18-p25490266	gene	
Bn-A08-p18680503	Bn-N8-p18436703	gene	exon
Bn-A08-p18704383	Bn-N8-p18453392	gene	exon
Bn-A08-p18750116	Bn-N8-p18503671	gene	exon
Bn-A08-p18750253	Bn-N8-p18503808	gene	exon
Bn-A08-p18754717	Bn-N8-p18507294	gene	
Bn-A08-p18784592	Bn-Scaffold02717-p1832	gene	
Bn-A08-p18789560	Bn-N8-p18545244	gene	exon
Bn-A08-p18798063	Bn-N8-p18564132	gene	exon
Bn-A08-p1880862	Bn-N8-p1772680	gene	exon
Bn-A08-p18827698	Bn-N8-p18593363	gene	
Bn-A08-p18953939	Bn-N8-p18721579	gene	exon
Bn-A08-p18954204	Bn-N8-p18721844	gene	exon
Bn-A08-p18966828	Bn-N8-p18734465	gene	exon
Bn-A08-p19005550	Bn-N8-p18773926	gene	exon
Bn-A08-p19007302	Bn-N8-p18775647	gene	exon
Bn-A08-p19008575	Bn-N8-p18776922	gene	exon
Bn-A08-p19008671	Bn-N8-p18777018	gene	exon
Bn-A08-p19009254	Bn-N8-p18777760	gene	exon
Bn-A08-p19017689	Bn-N8-p18786566	gene	exon
Bn-A08-p19035945	Bn-N8-p18799600	gene	exon
Bn-A08-p19052340	Bn-N8-p18819257	gene	
Bn-A08-p19052519	Bn-N8-p18819436	gene	exon
Bn-A08-p19056545	Bn-N8-p18823461	gene	exon
Bn-A08-p19084386	Bn-N8-p18848906	gene	
Bn-A08-p19122274	Bn-N8-p18888323	gene	exon
Bn-A08-p19173201	Bn-N8-p18941616	gene	exon
Bn-A08-p19176979	Bn-N8-p18945362	gene	exon
Bn-A08-p19191183	Bn-N8-p18960253	gene	exon
Bn-A08-p19191695	Bn-N6-p6446926	gene	
Bn-A08-p19197746	Bn-N8-p18966855	gene	exon
Bn-A08-p19217869	Bn-N8-p18989146	gene	
Bn-A08-p19284258	Bn-N8-p19053908	gene	exon
Bn-A08-p19287037	Bn-N8-p19056687	gene	exon
Bn-A08-p19346186	Bn-N18-p24079296	gene	exon
Bn-A08-p19346369	Bn-N8-p19133093	gene	
Bn-A08-p19363491	Bn-N8-p19150840	gene	exon
Bn-A08-p19365594	Bn-N8-p19152943	gene	
Bn-A08-p19407950	Bn-N8-p19208221	gene	exon
Bn-A08-p19410224	Bn-N8-p19210493	gene	
Bn-A08-p19428204	Bn-N8-p19229698	gene	
Bn-A08-p19454534	Bn-N8-p19251282	gene	
Bn-A08-p19454590	Bn-N8-p19251339	gene	
Bn-A08-p19460659	Bn-N8-p19258338	gene	exon
Bn-A08-p19483726	Bn-N18-p23810373	gene	exon
Bn-A08-p19487830	Bn-N8-p19289995	gene	exon
Bn-A08-p19504101	Bn-N8-p19303607	gene	exon
Bn-A08-p19538361	Bn-N8-p19341873	gene	
Bn-A08-p19553756	Bn-N8-p19357480	gene	
Bn-A08-p19573947	Bn-N8-p19378150	gene	exon
Bn-A08-p19578180	Bn-N18-p23545795	gene	exon

Bn-A08-p19599590	Bn-N8-p19399357	gene	exon
Bn-A08-p19619504	Bn-N8-p19420061	gene	exon
Bn-A08-p19626269	Bn-N8-p19426783	gene	
Bn-A08-p19642040	Bn-N8-p19441441	gene	
Bn-A08-p19675091	Bn-N8-p19477234	gene	
Bn-A08-p19676653	Bn-N18-p23318644	gene	exon
Bn-A08-p19676751	Bn-N18-p23318546	gene	exon
Bn-A08-p19690298	Bn-N8-p19490115	gene	
Bn-A08-p19690393	Bn-N8-p19490210	gene	
Bn-A08-p19736692	Bn-N8-p19543607	gene	exon
Bn-A08-p19736774	Bn-N18-p23160340	gene	
Bn-A08-p19782306	Bn-N8-p19586029	gene	exon
Bn-A08-p19782433	Bn-N8-p19586156	gene	exon
Bn-A08-p19834705	Bn-N8-p19651757	gene	
Bn-A08-p19879173	Bn-N8-p19696516	gene	exon
Bn-A08-p19879285	Bn-N8-p19696628	gene	
Bn-A08-p19879343	Bn-N8-p19696686	gene	
Bn-A08-p19879488	Bn-N8-p19696831	gene	exon
Bn-A08-p19879499	Bn-N8-p19696842	gene	exon
Bn-A08-p19888431	Bn-N8-p19707140	gene	
Bn-A08-p19903284	Bn-N8-p19719543	gene	
Bn-A08-p19907560	Bn-N8-p19723958	gene	
Bn-A08-p19919658	Bn-N8-p19737576	gene	
Bn-A08-p19919901	Bn-N8-p19737820	gene	exon
Bn-A08-p19921292	Bn-N8-p19739247	gene	exon
Bn-A08-p19924214	Bn-N8-p19741701	gene	exon
Bn-A08-p19983972	Bn-N8-p19785184	gene	exon
Bn-A08-p20025646	Bn-N8-p19824393	gene	
Bn-A08-p20037426	Bn-N8-p19835979	gene	exon
Bn-A08-p20042299	Bn-N8-p19837252	gene	exon
Bn-A08-p20068678	Bn-N8-p19858500	gene	
Bn-A08-p20068977	Bn-N8-p19858799	gene	exon
Bn-A08-p20069265	Bn-N8-p19859087	gene	exon
Bn-A08-p20072457	Bn-N8-p19862815	gene	
Bn-A08-p20075062	Bn-N8-p19865386	gene	exon
Bn-A08-p20085504	Bn-N8-p19875990	gene	exon
Bn-A08-p20091818	Bn-N8-p19882294	gene	
Bn-A08-p20092115	Bn-N8-p19882591	gene	
Bn-A08-p20096558	Bn-N8-p19886966	gene	exon
Bn-A08-p20106529	Bn-N8-p19896514	gene	exon
Bn-A08-p20169768	Bn-N8-p19933880	gene	
Bn-A08-p20170453	Bn-N8-p19934563	gene	exon
Bn-A08-p20189027	Bn-N8-p19957910	gene	
Bn-A08-p20244218	Bn-N8-p20001777	gene	
Bn-A08-p20286379	Bn-N8-p20051389	gene	
Bn-A08-p20305036	Bn-N8-p20348461	gene	
Bn-A08-p20306059	Bn-N8-p20351252	gene	
Bn-A08-p20306463	Bn-N8-p20351656	gene	exon
Bn-A08-p20306567	Bn-N8-p20351760	gene	
Bn-A08-p20319696	Bn-N8-p20361964	gene	exon
Bn-A08-p20319815	Bn-N8-p20362083	gene	
Bn-A08-p20336178	Bn-N8-p20379990	gene	exon

Bn-A08-p20343735	Bn-N18-p21457522	gene	exon
Bn-A08-p20347040	Bn-N8-p20392010	gene	
Bn-A08-p20354609	Bn-N8-p20398894	gene	
Bn-A08-p20355213	Bn-N8-p20399499	gene	
Bn-A08-p20394042	Bn-N8-p20432212	gene	exon
Bn-A08-p20395388	Bn-N8-p20433558	gene	exon
Bn-A08-p20416050	Bn-N8-p20463692	gene	
Bn-A08-p20441889	Bn-N8-p20495674	gene	
Bn-A08-p20474660	Bn-N8-p20534215	gene	exon
Bn-A08-p20476359	Bn-N8-p20535921	gene	
Bn-A08-p20480348	Bn-N8-p20539877	gene	exon
Bn-A08-p20485374	Bn-N8-p20547159	gene	
Bn-A08-p20490539	Bn-N8-p20552338	gene	exon
Bn-A08-p20490691	Bn-N8-p20552490	gene	exon
Bn-A08-p205262	Bn-N8-p330395	gene	exon
Bn-A08-p20529396	Bn-N8-p20593300	gene	
Bn-A08-p20530487	Bn-N8-p20594384	gene	
Bn-A08-p20537757	Bn-N18-p21006384	gene	
Bn-A08-p20538517	Bn-N8-p20600222	gene	
Bn-A08-p20546003	Bn-N8-p20606125	gene	exon
Bn-A08-p20546044	Bn-N8-p20606166	gene	exon
Bn-A08-p20546110	Bn-N8-p20606232	gene	exon
Bn-A08-p20547091	Bn-N8-p20606688	gene	exon
Bn-A08-p20551301	Bn-N8-p20610895	gene	exon
Bn-A08-p20554540	Bn-N8-p20614128	gene	
Bn-A08-p20555629	Bn-N8-p20615247	gene	exon
Bn-A08-p20587556	Bn-N8-p20641784	gene	
Bn-A08-p20692045	Bn-N8-p20760472	gene	exon
Bn-A08-p20717142	Bn-N18-p2025883	gene	exon
Bn-A08-p20778097	Bn-N8-p20847011	gene	exon
Bn-A08-p20801363	Bn-N8-p20869624	gene	exon
Bn-A08-p20820768	Bn-N8-p20879154	gene	exon
Bn-A08-p20838949	Bn-N8-p20900858	gene	exon
Bn-A08-p20839304	Bn-N8-p20901235	gene	
Bn-A08-p20846182	Bn-N8-p20908110	gene	exon
Bn-A08-p20904948	Bn-N8-p20968196	gene	
Bn-A08-p20913891	Bn-N8-p20977267	gene	
Bn-A08-p20927339	Bn-N8-p20989516	gene	exon
Bn-A08-p20931438	Bn-N8-p20993661	gene	
Bn-A08-p20931690	Bn-N8-p20994132	gene	
Bn-A08-p20934184	Bn-N8-p20996625	gene	
Bn-A08-p20950199	Bn-N8-p21010676	gene	exon
Bn-A08-p20967165	Bn-N8-p21031047	gene	exon
Bn-A08-p20968239	Bn-N18-p1551819	gene	exon
Bn-A08-p20971092	Bn-N8-p21035086	gene	
Bn-A08-p20993982	Bn-N8-p21055202	gene	
Bn-A08-p21036778	Bn-N8-p21100372	gene	exon
Bn-A08-p21062076	Bn-N18-p1305030	gene	exon
Bn-A08-p211090	Bn-N13-p67517978	gene	exon
Bn-A08-p21132027	Bn-N18-p1122222	gene	exon
Bn-A08-p21149542	Bn-N8-p21210689	gene	exon
Bn-A08-p21168203	Bn-N8-p21226954	gene	exon

Bn-A08-p21189645	Bn-N8-p21264787	gene	exon
Bn-A08-p21227076	Bn-N8-p21302699	gene	
Bn-A08-p21228678	Bn-N8-p21304221	gene	exon
Bn-A08-p21282852	Bn-N8-p21361253	gene	exon
Bn-A08-p21283107	Bn-N18-p774166	gene	exon
Bn-A08-p21283562	Bn-N8-p21361961	gene	exon
Bn-A08-p21330967	Bn-N8-p21408616	gene	
Bn-A08-p21348310	Bn-N8-p21425973	gene	exon
Bn-A08-p21352716	Bn-N8-p21430377	gene	exon
Bn-A08-p21385759	Bn-N8-p21460702	gene	
Bn-A08-p21417592	Bn-N8-p21495935	gene	
Bn-A08-p21434747	Bn-N8-p21511255	gene	exon
Bn-A08-p21463036	Bn-N8-p21536587	gene	exon
Bn-A08-p2275410	Bn-N8-p2135016	gene	
Bn-A08-p2371175	Bn-N8-p2230272	gene	
Bn-A08-p2397885	Bn-N8-p2257095	gene	
Bn-A08-p2406380	Bn-N8-p2266693	gene	
Bn-A08-p2425488	Bn-N8-p2286556	gene	
Bn-A08-p2461697	Bn-N8-p2321801	gene	exon
Bn-A08-p2533244	Bn-N8-p2377256	gene	
Bn-A08-p2536015	Bn-N8-p2380024	gene	exon
Bn-A08-p2564131	Bn-N8-p2407710	gene	exon
Bn-A08-p2574453	Bn-N8-p2428978	gene	
Bn-A08-p2589956	Bn-N8-p2442184	gene	
Bn-A08-p2590415	Bn-N8-p2442643	gene	
Bn-A08-p2660414	Bn-N8-p2507725	gene	exon
Bn-A08-p2711497	Bn-N8-p2573766	gene	
Bn-A08-p2715086	Bn-N8-p2577309	gene	
Bn-A08-p2728670	Bn-N8-p2600395	gene	
Bn-A08-p2740174	Bn-N8-p2610879	gene	
Bn-A08-p2771143	Bn-N8-p2641528	gene	
Bn-A08-p2885855	Bn-N8-p2777219	gene	
Bn-A08-p2897827	Bn-N8-p2784310	gene	exon
Bn-A08-p2898446	Bn-N8-p2784929	gene	
Bn-A08-p293480	Bn-N13-p67434994	gene	exon
Bn-A08-p3031902	Bn-N8-p2911497	gene	
Bn-A08-p3120976	Bn-N8-p3000167	gene	
Bn-A08-p3120986	Bn-N8-p3000176	gene	
Bn-A08-p3121492	Bn-N8-p3000683	gene	exon
Bn-A08-p3182594	Bn-N8-p3069193	gene	
Bn-A08-p3182798	Bn-N8-p3069397	gene	
Bn-A08-p3184233	Bn-N8-p3070767	gene	
Bn-A08-p3193662	Bn-N8-p3085952	gene	
Bn-A08-p3195277	Bn-N18-p3962969	gene	
Bn-A08-p3223002	Bn-N8-p3102300	gene	
Bn-A08-p3269802	Bn-N8-p3151120	gene	exon
Bn-A08-p3302700	Bn-N8-p3186724	gene	
Bn-A08-p3388906	Bn-N8-p3234254	gene	
Bn-A08-p345270	Bn-N8-p513642	gene	
Bn-A08-p350287	Bn-N8-p518715	gene	
Bn-A08-p3546013	Bn-N8-p3357184	gene	exon
Bn-A08-p355936	Bn-N8-p523067	gene	

Bn-A08-p361637	Bn-N8-p528768	gene	exon
Bn-A08-p361910	Bn-N8-p529041	gene	exon
Bn-A08-p3628921	Bn-N8-p3465024	gene	exon
Bn-A08-p3737287	Bn-N8-p3558110	gene	exon
Bn-A08-p3840267	Bn-N8-p3739128	gene	exon
Bn-A08-p3846272	Bn-N8-p3749216	gene	exon
Bn-A08-p3883962	Bn-N19-p18840612	gene	
Bn-A08-p4152083	Bn-N8-p3988274	gene	
Bn-A08-p4182726	Bn-N8-p4031637	gene	exon
Bn-A08-p4207231	Bn-N8-p4052341	gene	
Bn-A08-p4209834	Bn-N8-p4054898	gene	exon
Bn-A08-p4253915	Bn-N8-p4086978	gene	exon
Bn-A08-p4254041	Bn-N8-p4087104	gene	
Bn-A08-p4271010	Bn-N8-p4104241	gene	exon
Bn-A08-p4271262	Bn-N8-p4104493	gene	exon
Bn-A08-p4273091	Bn-N8-p4106457	gene	
Bn-A08-p4293226	Bn-N8-p4127375	gene	exon
Bn-A08-p4342485	Bn-N8-p4176136	gene	
Bn-A08-p4483232	Bn-N8-p4320169	gene	
Bn-A08-p4521258	Bn-N8-p4365967	gene	
Bn-A08-p4528943	Bn-N8-p4373662	gene	
Bn-A08-p4598270	Bn-N8-p4441754	gene	
Bn-A08-p4668561	Bn-N8-p4507005	gene	
Bn-A08-p4670469	Bn-N8-p4508913	gene	
Bn-A08-p482401	Bn-N8-p658897	gene	
Bn-A08-p482505	Bn-N8-p659001	gene	exon
Bn-A08-p4837890	Bn-N8-p4638527	gene	exon
Bn-A08-p4876592	Bn-N10-p13185109	gene	exon
Bn-A08-p4902282	Bn-N10-p13161705	gene	
Bn-A08-p4936436	Bn-N10-p13122698	gene	exon
Bn-A08-p5017136	Bn-N8-p4806558	gene	exon
Bn-A08-p5026482	Bn-N8-p4815377	gene	exon
Bn-A08-p5096382	Bn-N8-p4876764	gene	
Bn-A08-p5146710	Bn-N8-p4908806	gene	
Bn-A08-p5174518	Bn-N8-p4925818	gene	exon
Bn-A08-p5174818	Bn-N8-p4926118	gene	exon
Bn-A08-p5249573	Bn-N8-p5021473	gene	exon
Bn-A08-p537474	Bn-N8-p711266	gene	
Bn-A08-p5420503	Bn-N8-p5216482	gene	
Bn-A08-p5490050	Bn-N8-p5280916	gene	
Bn-A08-p5591333	Bn-N8-p5464803	gene	exon
Bn-A08-p5636755	Bn-N8-p5499242	gene	
Bn-A08-p5636764	Bn-N8-p5499251	gene	
Bn-A08-p5638757	Bn-N8-p5501244	gene	
Bn-A08-p5640946	Bn-N8-p5503440	gene	
Bn-A08-p5698262	Bn-N8-p5571566	gene	exon
Bn-A08-p5698705	Bn-N8-p5572009	gene	exon
Bn-A08-p5699040	Bn-N8-p5572344	gene	exon
Bn-A08-p570304	Bn-N8-p758575	gene	exon
Bn-A08-p5705625	Bn-N8-p5578918	gene	
Bn-A08-p5730628	Bn-N8-p5603234	gene	exon
Bn-A08-p5924891	Bn-N8-p5785368	gene	

Bn-A08-p6029041	Bn-N8-p4690333	gene	
Bn-A08-p6101880	Bn-N8-p8407250	gene	exon
Bn-A08-p6114046	Bn-N8-p8419492	gene	exon
Bn-A08-p6133005	Bn-N8-p8423399	gene	
Bn-A08-p6133601	Bn-N8-p8423995	gene	
Bn-A08-p6157861	Bn-N8-p8439711	gene	exon
Bn-A08-p6164968	Bn-N8-p8454598	gene	
Bn-A08-p6165340	Bn-N8-p8454970	gene	
Bn-A08-p6165665	Bn-N8-p8455295	gene	
Bn-A08-p6257665	Bn-N8-p8545244	gene	exon
Bn-A08-p626649	Bn-N13-p66924325	gene	
Bn-A08-p6268593	Bn-N8-p8553043	gene	exon
Bn-A08-p6332878	Bn-N8-p8593283	gene	exon
Bn-A08-p6343431	Bn-N8-p8604736	gene	exon
Bn-A08-p6394232	Bn-N8-p8651448	gene	exon
Bn-A08-p6400378	Bn-N8-p8656368	gene	
Bn-A08-p6523926	Bn-N8-p8769174	gene	
Bn-A08-p6523952	Bn-N8-p8769200	gene	
Bn-A08-p6828854	Bn-N8-p9121537	gene	exon
Bn-A08-p6828857	Bn-N8-p9121540	gene	exon
Bn-A08-p6829643	Bn-N8-p9122324	gene	exon
Bn-A08-p6887868	Bn-N8-p9180282	gene	
Bn-A08-p6889067	Bn-N8-p9181481	gene	exon
Bn-A08-p6937293	Bn-N8-p9428077	gene	exon
Bn-A08-p6943246	Bn-N8-p9427004	gene	exon
Bn-A08-p6971574	Bn-N8-p9402200	gene	exon
Bn-A08-p7099327	Bn-N8-p5998133	gene	exon
Bn-A08-p7151447	Bn-N8-p6036123	gene	
Bn-A08-p7152271	Bn-N8-p6036959	gene	
Bn-A08-p7303995	Bn-N18-p8810165	gene	
Bn-A08-p7318043	Bn-N8-p6317339	gene	exon
Bn-A08-p7355372	Bn-N18-p8875292	gene	
Bn-A08-p743985	Bn-N8-p971806	gene	exon
Bn-A08-p7444336	Bn-N8-p6413426	gene	exon
Bn-A08-p744725	Bn-N8-p972494	gene	
Bn-A08-p744837	Bn-N8-p972606	gene	exon
Bn-A08-p7458611	Bn-N8-p6430504	gene	
Bn-A08-p7563202	Bn-N8-p6550967	gene	
Bn-A08-p758590	Bn-N8-p986604	gene	exon
Bn-A08-p758977	Bn-N8-p986992	gene	
Bn-A08-p7596192	Bn-N18-p9364917	gene	exon
Bn-A08-p7698145	Bn-N8-p6764076	gene	exon
Bn-A08-p7791369	Bn-N8-p6895245	gene	exon
Bn-A08-p7814328	Bn-N8-p6907707	gene	
Bn-A08-p7814432	Bn-N8-p6907811	gene	
Bn-A08-p782784	Bn-N8-p1011139	gene	exon
Bn-A08-p7835995	Bn-N8-p6931659	gene	exon
Bn-A08-p7856305	Bn-N18-p10249395	gene	
Bn-A08-p7856849	Bn-N8-p6948450	gene	
Bn-A08-p797453	Bn-N8-p1026397	gene	
Bn-A08-p7984584	Bn-N8-p7113378	gene	
Bn-A08-p798867	Bn-N8-p1027811	gene	

Bn-A08-p798970	Bn-N8-p1027914	gene	
Bn-A08-p8031289	Bn-N8-p7175077	gene	
Bn-A08-p8091811	Bn-N8-p7216514	gene	
Bn-A08-p813316	Bn-N8-p1047794	gene	
Bn-A08-p813510	Bn-N8-p1047988	gene	exon
Bn-A08-p814234	Bn-N8-p1048712	gene	exon
Bn-A08-p8161845	Bn-N8-p7278568	gene	
Bn-A08-p8162230	Bn-N8-p7278965	gene	
Bn-A08-p8179629	Bn-N8-p7643523	gene	exon
Bn-A08-p8320380	Bn-N18-p11290982	gene	exon
Bn-A08-p8385137	Bn-N8-p7351381	gene	
Bn-A08-p8386874	Bn-N8-p7349644	gene	
Bn-A08-p8396508	Bn-N8-p7336453	gene	
Bn-A08-p8397266	Bn-N8-p7335695	gene	exon
Bn-A08-p8434044	Bn-N8-p7655528	gene	
Bn-A08-p8479611	Bn-N8-p7698170	gene	exon
Bn-A08-p8479932	Bn-N8-p7698491	gene	exon
Bn-A08-p8480536	Bn-N8-p7699095	gene	exon
Bn-A08-p8480734	Bn-N8-p7699293	gene	exon
Bn-A08-p8493076	Bn-N8-p7710546	gene	
Bn-A08-p8494172	Bn-N18-p11577952	gene	exon
Bn-A08-p8581123	Bn-N8-p7834013	gene	exon
Bn-A08-p8581899	Bn-N8-p7834794	gene	
Bn-A08-p8644010	Bn-N8-p7884860	gene	
Bn-A08-p8644734	Bn-N8-p7885583	gene	
Bn-A08-p8672999	Bn-N8-p7915843	gene	exon
Bn-A08-p8710166	Bn-N8-p7940018	gene	
Bn-A08-p8727608	Bn-N8-p7972048	gene	exon
Bn-A08-p8740002	Bn-N8-p7987878	gene	exon
Bn-A08-p8755323	Bn-N8-p8005536	gene	
Bn-A08-p8783951	Bn-N8-p8031999	gene	
Bn-A08-p8784663	Bn-N8-p8032711	gene	
Bn-A08-p8869180	Bn-N8-p8129492	gene	exon
Bn-A08-p8879495	Bn-N8-p8137151	gene	
Bn-A08-p9172893	Bn-N9-p17586800	gene	exon
Bn-A08-p9216064	Bn-N9-p17530291	gene	exon
Bn-A08-p9216239	Bn-N9-p17530116	gene	exon
Bn-A08-p9216723	Bn-N9-p17529632	gene	
Bn-A08-p9217601	Bn-N9-p17528742	gene	exon
Bn-A08-p9217703	Bn-N9-p17528640	gene	exon
Bn-A08-p9218159	Bn-N9-p17528184	gene	exon
Bn-A08-p9221373	Bn-N9-p17519787	gene	exon
Bn-A08-p9230750	Bn-N9-p17504276	gene	
Bn-A08-p9247445	Bn-N9-p17499010	gene	exon
Bn-A08-p9341774	Bn-N9-p17403898	gene	exon
Bn-A08-p9341953	Bn-N9-p17403719	gene	exon
Bn-A08-p9353221	Bn-N19-p26431260	gene	
Bn-A08-p937299	Bn-N8-p1138446	gene	exon
Bn-A08-p9388258	Bn-N9-p17363103	gene	
Bn-A08-p940619	Bn-N8-p1141887	gene	
Bn-A08-p9429379	Bn-N9-p17306796	gene	exon
Bn-A08-p9433046	Bn-N9-p17303142	gene	exon

Bn-A08-p9503231	Bn-N19-p26682267	gene	exon
Bn-A08-p9505581	Bn-N9-p17247889	gene	
Bn-A08-p9555028	Bn-N9-p17229664	gene	exon
Bn-A08-p9555984	Bn-N9-p17228714	gene	exon
Bn-A08-p9582412	Bn-N9-p17196517	gene	exon
Bn-A08-p9715574	Bn-N9-p17067191	gene	
Bn-A08-p9795033	Bn-N9-p16978041	gene	
Bn-A08-p9795423	Bn-N9-p16977651	gene	exon
Bn-A08-p980768	Bn-N8-p1189412	gene	
Bn-A08-p9917420	Bn-N7-p4007819	gene	exon
Bn-A08-p9918228	Bn-N7-p4008627	gene	exon
Bn-A09-p10019827	Bn-N9-p10788455	gene	exon
Bn-A09-p10023881	Bn-N9-p10790825	gene	
Bn-A09-p100554	Bn-N9-p1037437	gene	exon
Bn-A09-p100631	Bn-N9-p1037360	gene	exon
Bn-A09-p100643	Bn-N9-p1037348	gene	exon
Bn-A09-p10092753	Bn-N9-p10866671	gene	
Bn-A09-p1011107	Bn-N9-p147637	gene	
Bn-A09-p10212596	Bn-N9-p10978908	gene	
Bn-A09-p10212674	Bn-N9-p10978986	gene	
Bn-A09-p10212726	Bn-N9-p10979038	gene	
Bn-A09-p10247512	Bn-N9-p11010077	gene	
Bn-A09-p10296510	Bn-N9-p11060546	gene	
Bn-A09-p10297982	Bn-N9-p11062656	gene	
Bn-A09-p1032996	Bn-N9-p126850	gene	
Bn-A09-p10336331	Bn-N9-p11108405	gene	
Bn-A09-p10495974	Bn-N9-p18376183	gene	
Bn-A09-p10504507	Bn-N9-p18367623	gene	
Bn-A09-p10536531	Bn-N9-p18332961	gene	
Bn-A09-p10536718	Bn-N9-p18332777	gene	
Bn-A09-p10544697	Bn-N9-p18324503	gene	
Bn-A09-p10759604	Bn-N9-p15825988	gene	
Bn-A09-p10836683	Bn-N9-p15942153	gene	
Bn-A09-p10900375	Bn-N9-p16005270	gene	
Bn-A09-p10901534	Bn-N9-p16006426	gene	
Bn-A09-p109917	Bn-N19-p1200873	gene	exon
Bn-A09-p11110730	Bn-N9-p16220290	gene	exon
Bn-A09-p11126016	Bn-N9-p16227060	gene	
Bn-A09-p11126583	Bn-N9-p16227644	gene	
Bn-A09-p11164079	Bn-N9-p37292480	gene	exon
Bn-A09-p11180241	Bn-N9-p16275825	gene	exon
Bn-A09-p11181581	Bn-N9-p16277162	gene	
Bn-A09-p11210109	Bn-N9-p16311666	gene	exon
Bn-A09-p112102	Bn-N9-p1017201	gene	exon
Bn-A09-p11297498	Bn-N9-p16373161	gene	
Bn-A09-p11297772	Bn-N9-p16373436	gene	
Bn-A09-p11393003	Bn-N9-p16502384	gene	
Bn-A09-p11461510	Bn-N9-p16556704	gene	
Bn-A09-p11476056	Bn-N9-p16585244	gene	
Bn-A09-p11491871	Bn-N19-p27886982	gene	
Bn-A09-p11589729	Bn-N9-p16716268	gene	
Bn-A09-p1165212	Bn-N9-p1534774	gene	

Bn-A09-p11727553	Bn-N9-p16858337	gene	exon
Bn-A09-p1188091	Bn-N9-p1554817	gene	
Bn-A09-p1227639	Bn-N9-p1605889	gene	exon
Bn-A09-p1243032	Bn-N9-p1617114	gene	
Bn-A09-p1252014	Bn-N9-p1626111	gene	exon
Bn-A09-p1252150	Bn-N9-p1626247	gene	exon
Bn-A09-p12557952	Bn-N9-p25202397	gene	exon
Bn-A09-p1272298	Bn-N9-p1649433	gene	exon
Bn-A09-p12929078	Bn-N9-p12432502	gene	
Bn-A09-p13251498	Bn-N9-p12159209	gene	exon
Bn-A09-p13253434	Bn-N19-p19760365	gene	exon
Bn-A09-p13384525	Bn-N19-p18863999	gene	exon
Bn-A09-p13482934	Bn-N19-p18685776	gene	exon
Bn-A09-p13581136	Bn-N9-p11894655	gene	
Bn-A09-p13582084	Bn-N9-p11893701	gene	
Bn-A09-p13584189	Bn-N9-p11891574	gene	
Bn-A09-p13585262	Bn-N9-p11890508	gene	
Bn-A09-p13587429	Bn-N9-p11888156	gene	
Bn-A09-p135892	Bn-N9-p994517	gene	
Bn-A09-p13616262	Bn-N19-p18424309	gene	
Bn-A09-p13641153	Bn-N9-p11837130	gene	
Bn-A09-p13666209	Bn-N9-p11825208	gene	exon
Bn-A09-p13667207	Bn-N9-p11824216	gene	exon
Bn-A09-p13667337	Bn-N9-p11824086	gene	exon
Bn-A09-p13682690	Bn-N9-p11815277	gene	
Bn-A09-p13692953	Bn-N9-p11804858	gene	
Bn-A09-p13693010	Bn-N9-p11804802	gene	
Bn-A09-p13693499	Bn-N9-p11804324	gene	
Bn-A09-p13696043	Bn-N19-p18326124	gene	
Bn-A09-p13737711	Bn-N9-p11770134	gene	exon
Bn-A09-p13776245	Bn-N9-p11737276	gene	
Bn-A09-p13827265	Bn-N9-p11671670	gene	exon
Bn-A09-p13836909	Bn-N9-p11668982	gene	
Bn-A09-p13952461	Bn-N19-p17992612	gene	
Bn-A09-p13957074	Bn-N19-p17988021	gene	
Bn-A09-p13983057	Bn-N9-p11513574	gene	
Bn-A09-p13983626	Bn-N9-p11513006	gene	
Bn-A09-p14017321	Bn-N9-p11479174	gene	
Bn-A09-p1404415	Bn-N9-p1774736	gene	
Bn-A09-p1405207	Bn-N9-p1775528	gene	
Bn-A09-p14105923	Bn-N19-p17836522	gene	exon
Bn-A09-p14106312	Bn-N9-p11383227	gene	exon
Bn-A09-p14115991	Bn-N9-p11368203	gene	exon
Bn-A09-p14166446	Bn-N9-p11284473	gene	exon
Bn-A09-p14167293	Bn-N19-p17754029	gene	exon
Bn-A09-p14199197	Bn-N9-p11253207	gene	exon
Bn-A09-p14223034	Bn-N9-p11225868	gene	exon
Bn-A09-p14282683	Bn-N9-p11148022	gene	
Bn-A09-p14285217	Bn-N9-p11145480	gene	exon
Bn-A09-p14287005	Bn-N19-p17533617	gene	
Bn-A09-p14287061	Bn-N9-p11143635	gene	exon
Bn-A09-p14432426	Bn-N9-p14596462	gene	exon

Bn-A09-p14468231	Bn-N9-p14563728	gene	
Bn-A09-p14630762	Bn-N9-p14382789	gene	
Bn-A09-p14647697	Bn-N9-p27672263	gene	exon
Bn-A09-p146640	Bn-N9-p983758	gene	exon
Bn-A09-p149032	Bn-N19-p1132101	gene	exon
Bn-A09-p149317	Bn-N9-p979003	gene	exon
Bn-A09-p14987165	Bn-N9-p14172767	gene	exon
Bn-A09-p15042985	Bn-N9-p14128345	gene	exon
Bn-A09-p15090180	Bn-N9-p14086987	gene	exon
Bn-A09-p15105347	Bn-N9-p14069887	gene	
Bn-A09-p15112850	Bn-N5-p13759318	gene	exon
Bn-A09-p15262	Bn-N9-p1146859	gene	exon
Bn-A09-p15353026	Bn-N9-p13780787	gene	
Bn-A09-p1541696	Bn-N9-p3097254	gene	exon
Bn-A09-p15556325	Bn-N19-p7430775	gene	exon
Bn-A09-p1558529	Bn-N9-p3082660	gene	exon
Bn-A09-p15648941	Bn-N9-p13548099	gene	exon
Bn-A09-p1567071	Bn-N9-p3070052	gene	exon
Bn-A09-p1570558	Bn-N9-p3066467	gene	exon
Bn-A09-p15826536	Bn-N9-p13362920	gene	exon
Bn-A09-p15984968	Bn-N9-p13173027	gene	exon
Bn-A09-p15986311	Bn-N9-p13171686	gene	
Bn-A09-p160775	Bn-N9-p966527	gene	
Bn-A09-p1608051	Bn-N9-p3026742	gene	
Bn-A09-p1610631	Bn-N9-p3024118	gene	
Bn-A09-p16157117	Bn-N9-p12967181	gene	exon
Bn-A09-p16172780	Bn-N9-p12956159	gene	exon
Bn-A09-p16187931	Bn-N9-p12933962	gene	
Bn-A09-p1623725	Bn-N9-p3010191	gene	
Bn-A09-p16322417	Bn-N16-p14945984	gene	exon
Bn-A09-p16479468	Bn-N1-p9572063	gene	
Bn-A09-p16479915	Bn-Scaffold00929-p45360	gene	exon
Bn-A09-p16519073	Bn-N9-p12849695	gene	
Bn-A09-p16540542	Bn-N9-p12820481	gene	
Bn-A09-p16600344	Bn-N9-p12761886	gene	
Bn-A09-p16620672	Bn-N9-p12748362	gene	
Bn-A09-p16639867	Bn-N9-p12729687	gene	
Bn-A09-p16640011	Bn-N9-p12729543	gene	
Bn-A09-p16722238	Bn-N9-p12636144	gene	
Bn-A09-p16722618	Bn-N9-p12635768	gene	
Bn-A09-p16722814	Bn-N9-p12635567	gene	
Bn-A09-p16766275	Bn-N9-p12596604	gene	exon
Bn-A09-p16771727	Bn-N9-p12592843	gene	exon
Bn-A09-p16805527	Bn-N9-p12554979	gene	
Bn-A09-p1680594	Bn-N9-p2954788	gene	
Bn-A09-p1681240	Bn-N9-p2954142	gene	exon
Bn-A09-p16833397	Bn-N9-p12526525	gene	exon
Bn-A09-p16835122	Bn-N9-p12524782	gene	
Bn-A09-p16846786	Bn-N9-p12514031	gene	
Bn-A09-p1690633	Bn-N9-p2934202	gene	
Bn-A09-p1691589	Bn-N9-p2933246	gene	exon
Bn-A09-p16998291	Bn-N9-p14761259	gene	exon

Bn-A09-p1721492	Bn-N9-p2894989	gene	exon
Bn-A09-p1721507	Bn-N9-p2894974	gene	exon
Bn-A09-p17332686	Bn-N9-p15065639	gene	
Bn-A09-p17415909	Bn-N19-p24370712	gene	
Bn-A09-p174327	Bn-N9-p954671	gene	
Bn-A09-p17483853	Bn-N9-p15217795	gene	exon
Bn-A09-p17484188	Bn-N9-p15218143	gene	exon
Bn-A09-p175303	Bn-N9-p948283	gene	
Bn-A09-p17538848	Bn-N9-p15271543	gene	
Bn-A09-p1756759	Bn-N9-p2853754	gene	
Bn-A09-p17582590	Bn-N9-p15315290	gene	exon
Bn-A09-p17600384	Bn-N13-p41406354	gene	exon
Bn-A09-p17600725	Bn-N13-p41406016	gene	
Bn-A09-p17609859	Bn-N9-p15340926	gene	
Bn-A09-p1765907	Bn-N9-p2844896	gene	
Bn-A09-p17686638	Bn-N9-p15442766	gene	exon
Bn-A09-p17723940	Bn-N9-p15490906	gene	exon
Bn-A09-p17739465	Bn-N9-p15500666	gene	
Bn-A09-p1786726	Bn-N9-p2821962	gene	exon
Bn-A09-p17869345	Bn-N9-p18209625	gene	
Bn-A09-p17955698	Bn-N9-p18105082	gene	
Bn-A09-p1796337	Bn-N9-p2816747	gene	exon
Bn-A09-p18132317	Bn-N9-p17941873	gene	
Bn-A09-p18156994	Bn-N9-p18110651	gene	exon
Bn-A09-p1821136	Bn-N9-p2785259	gene	exon
Bn-A09-p18258259	Bn-N19-p25877131	gene	
Bn-A09-p18258443	Bn-N9-p17788208	gene	
Bn-A09-p18283186	Bn-N7-p1493424	gene	exon
Bn-A09-p1829952	Bn-N9-p2777065	gene	
Bn-A09-p18316084	Bn-N7-p1458970	gene	exon
Bn-A09-p18324038	Bn-N7-p1450968	gene	
Bn-A09-p1841381	Bn-N9-p2764087	gene	
Bn-A09-p184319	Bn-N9-p935677	gene	exon
Bn-A09-p18588652	Bn-N6-p19223438	gene	exon
Bn-A09-p1860063	Bn-N9-p2742026	gene	
Bn-A09-p18922925	Bn-N9-p20053677	gene	
Bn-A09-p18923026	Bn-N9-p20053778	gene	exon
Bn-A09-p19040325	Bn-N9-p20147639	gene	
Bn-A09-p19043442	Bn-N9-p20150756	gene	exon
Bn-A09-p19113008	Bn-N9-p20239579	gene	
Bn-A09-p19181075	Bn-N9-p20293471	gene	exon
Bn-A09-p19198146	Bn-N9-p20308599	gene	exon
Bn-A09-p19227824	Bn-N9-p20339917	gene	exon
Bn-A09-p19244125	Bn-N9-p20364561	gene	exon
Bn-A09-p19244566	Bn-N9-p20365002	gene	
Bn-A09-p19267307	Bn-N9-p20371867	gene	
Bn-A09-p19268137	Bn-N9-p20372719	gene	
Bn-A09-p19268663	Bn-N9-p20373245	gene	
Bn-A09-p19282217	Bn-N9-p20391806	gene	
Bn-A09-p19283001	Bn-N9-p20392590	gene	
Bn-A09-p1929245	Bn-N9-p2676238	gene	
Bn-A09-p19297523	Bn-N9-p20406812	gene	

Bn-A09-p19316704	Bn-N9-p20419922	gene	
Bn-A09-p19347448	Bn-N16-p11601742	gene	
Bn-A09-p19348447	Bn-N9-p20470408	gene	
Bn-A09-p19364150	Bn-N9-p20491881	gene	
Bn-A09-p19400882	Bn-N12-p31281870	gene	
Bn-A09-p19413084	Bn-N15-p17860803	gene	
Bn-A09-p19462041	Bn-N9-p20578579	gene	exon
Bn-A09-p19489065	Bn-N9-p20600856	gene	exon
Bn-A09-p19494485	Bn-N9-p20604964	gene	exon
Bn-A09-p19539215	Bn-N9-p20667445	gene	
Bn-A09-p19542797	Bn-N9-p20669583	gene	exon
Bn-A09-p19545956	Bn-N9-p20673400	gene	
Bn-A09-p19583295	Bn-N9-p20728115	gene	
Bn-A09-p19589751	Bn-N9-p20730988	gene	exon
Bn-A09-p19619009	Bn-N9-p20767013	gene	exon
Bn-A09-p19689884	Bn-N9-p21012291	gene	
Bn-A09-p19690311	Bn-N9-p21012776	gene	
Bn-A09-p19690392	Bn-N9-p21012857	gene	
Bn-A09-p19690521	Bn-N9-p21012986	gene	
Bn-A09-p19690574	Bn-N9-p21013039	gene	
Bn-A09-p19690777	Bn-N9-p21013245	gene	
Bn-A09-p19766078	Bn-N9-p21072022	gene	exon
Bn-A09-p19766741	Bn-N9-p21072685	gene	
Bn-A09-p19766911	Bn-N9-p21072861	gene	exon
Bn-A09-p19771174	Bn-N9-p21078816	gene	exon
Bn-A09-p19805361	Bn-N9-p21109062	gene	
Bn-A09-p19835714	Bn-N9-p21139004	gene	
Bn-A09-p19939967	Bn-N9-p21252591	gene	exon
Bn-A09-p20061287	Bn-N9-p21409790	gene	
Bn-A09-p20113369	Bn-N9-p21456587	gene	exon
Bn-A09-p20115072	Bn-N9-p21458259	gene	
Bn-A09-p20124340	Bn-N9-p21485661	gene	
Bn-A09-p20129620	Bn-N9-p21490082	gene	
Bn-A09-p20145310	Bn-N9-p21522397	gene	
Bn-A09-p20149594	Bn-N9-p21526611	gene	exon
Bn-A09-p20214059	Bn-N9-p21590661	gene	
Bn-A09-p20336623	Bn-N9-p21743462	gene	
Bn-A09-p2044817	Bn-N9-p2550455	gene	exon
Bn-A09-p20523068	Bn-N9-p21910204	gene	exon
Bn-A09-p20554631	Bn-N9-p21936966	gene	
Bn-A09-p20569783	Bn-N9-p21944496	gene	exon
Bn-A09-p20569958	Bn-N9-p21944669	gene	
Bn-A09-p20649745	Bn-N9-p22007527	gene	
Bn-A09-p2065608	Bn-N9-p2532471	gene	
Bn-A09-p20720084	Bn-N9-p22091373	gene	exon
Bn-A09-p2081648	Bn-N9-p2523586	gene	exon
Bn-A09-p2082298	Bn-N9-p2522938	gene	exon
Bn-A09-p20856553	Bn-N6-p15584982	gene	
Bn-A09-p21224256	Bn-N9-p22646736	gene	
Bn-A09-p2131637	Bn-N19-p2220394	gene	exon
Bn-A09-p2131851	Bn-N9-p1892403	gene	
Bn-A09-p2160040	Bn-N9-p1915929	gene	

Bn-A09-p21795879	Bn-N9-p23251195	gene	exon
Bn-A09-p21821779	Bn-N6-p23920971	gene	
Bn-A09-p21865966	Bn-N9-p23314517	gene	
Bn-A09-p219538	Bn-N9-p915609	gene	exon
Bn-A09-p21969014	Bn-N9-p23415055	gene	
Bn-A09-p22003936	Bn-N9-p23462924	gene	exon
Bn-A09-p2208929	Bn-N9-p1959990	gene	
Bn-A09-p2209142	Bn-N9-p1960203	gene	
Bn-A09-p22344523	Bn-N9-p23842926	gene	exon
Bn-A09-p22345800	Bn-N9-p23844203	gene	exon
Bn-A09-p2241625	Bn-N9-p1990313	gene	
Bn-A09-p22462771	Bn-N9-p23950174	gene	exon
Bn-A09-p22495700	Bn-N9-p23985383	gene	
Bn-A09-p22612980	Bn-N6-p23795660	gene	exon
Bn-A09-p2280336	Bn-N9-p2040201	gene	exon
Bn-A09-p22965277	Bn-N9-p25657346	gene	
Bn-A09-p23089160	Bn-N9-p25792057	gene	exon
Bn-A09-p23107846	Bn-N9-p25809995	gene	
Bn-A09-p23107847	Bn-N9-p25809995	gene	
Bn-A09-p23144404	Bn-N9-p25845832	gene	
Bn-A09-p23144931	Bn-N9-p25846359	gene	exon
Bn-A09-p2323366	Bn-N9-p2122479	gene	exon
Bn-A09-p23250129	Bn-N9-p25936796	gene	exon
Bn-A09-p23254203	Bn-N9-p25940347	gene	
Bn-A09-p23269680	Bn-N9-p25954329	gene	
Bn-A09-p23325795	Bn-N9-p26028979	gene	exon
Bn-A09-p23334889	Bn-N9-p26038289	gene	
Bn-A09-p23345719	Bn-N9-p26047448	gene	exon
Bn-A09-p23382659	Bn-N9-p26090655	gene	exon
Bn-A09-p23410187	Bn-N9-p26118153	gene	
Bn-A09-p23416800	Bn-N9-p26124779	gene	exon
Bn-A09-p23436500	Bn-N9-p26147168	gene	
Bn-A09-p234493	Bn-N9-p905188	gene	exon
Bn-A09-p23489920	Bn-N9-p26201599	gene	
Bn-A09-p23545925	Bn-N9-p26252968	gene	
Bn-A09-p23546242	Bn-N9-p26253285	gene	exon
Bn-A09-p23552530	Bn-N9-p26259106	gene	
Bn-A09-p23553955	Bn-N9-p26260531	gene	
Bn-A09-p23554212	Bn-N9-p26260786	gene	
Bn-A09-p23554351	Bn-N9-p26260925	gene	
Bn-A09-p23554402	Bn-N9-p26260976	gene	
Bn-A09-p23693948	Bn-N9-p26406113	gene	exon
Bn-A09-p23707462	Bn-N9-p26415577	gene	
Bn-A09-p23708692	Bn-N9-p26416798	gene	exon
Bn-A09-p23713670	Bn-N9-p26421777	gene	exon
Bn-A09-p23717291	Bn-N9-p26425397	gene	exon
Bn-A09-p23722611	Bn-N9-p26430738	gene	exon
Bn-A09-p23724102	Bn-N9-p26432203	gene	
Bn-A09-p23724105	Bn-N9-p26432206	gene	
Bn-A09-p23724303	Bn-N9-p26432401	gene	
Bn-A09-p23790907	Bn-N6-p2643129	gene	
Bn-A09-p23802586	Bn-N9-p26512622	gene	exon

Bn-A09-p23802640	Bn-N9-p26512677	gene	exon
Bn-A09-p23802762	Bn-N9-p26512799	gene	exon
Bn-A09-p23802813	Bn-N9-p26512850	gene	exon
Bn-A09-p23808067	Bn-N15-p14289776	gene	exon
Bn-A09-p23861045	Bn-N9-p26570880	gene	
Bn-A09-p23868070	Bn-N9-p26577901	gene	exon
Bn-A09-p23904657	Bn-N9-p26611721	gene	
Bn-A09-p23904905	Bn-N9-p26611969	gene	
Bn-A09-p23904964	Bn-N9-p26612028	gene	
Bn-A09-p23913406	Bn-N9-p26621379	gene	
Bn-A09-p23921036	Bn-N9-p26636261	gene	exon
Bn-A09-p23940132	Bn-N9-p26652313	gene	
Bn-A09-p23948738	Bn-N9-p26660928	gene	
Bn-A09-p239851	Bn-N9-p899826	gene	
Bn-A09-p23993823	Bn-N9-p26702498	gene	
Bn-A09-p24003401	Bn-N9-p26711518	gene	
Bn-A09-p24003969	Bn-N9-p26712086	gene	
Bn-A09-p24086990	Bn-N9-p26787014	gene	exon
Bn-A09-p24104666	Bn-N9-p26823650	gene	
Bn-A09-p24105838	Bn-N9-p26824825	gene	exon
Bn-A09-p24106965	Bn-N9-p26825952	gene	exon
Bn-A09-p24113289	Bn-N15-p13633484	gene	exon
Bn-A09-p24113399	Bn-N15-p13633374	gene	exon
Bn-A09-p24216715	Bn-N9-p26924860	gene	exon
Bn-A09-p2429402	Bn-N9-p2226143	gene	exon
Bn-A09-p24506395	Bn-N9-p27218676	gene	exon
Bn-A09-p24556003	Bn-N15-p12912611	gene	
Bn-A09-p25004061	Bn-Scaffold06475-p624	gene	exon
Bn-A09-p25018422	Bn-N9-p28006407	gene	exon
Bn-A09-p25050662	Bn-N9-p28033765	gene	
Bn-A09-p25076813	Bn-N9-p28059219	gene	exon
Bn-A09-p25133968	Bn-N9-p28127755	gene	exon
Bn-A09-p25139597	Bn-N9-p28131332	gene	
Bn-A09-p25141537	Bn-N9-p28125648	gene	exon
Bn-A09-p25166951	Bn-N9-p28152900	gene	
Bn-A09-p25224568	Bn-N9-p28210747	gene	
Bn-A09-p25236454	Bn-N9-p28226168	gene	
Bn-A09-p25236781	Bn-N9-p28226496	gene	
Bn-A09-p25237569	Bn-N9-p28227284	gene	exon
Bn-A09-p25238294	Bn-N9-p28228008	gene	
Bn-A09-p25372544	Bn-N9-p28382633	gene	exon
Bn-A09-p25376723	Bn-N9-p28386821	gene	
Bn-A09-p25440365	Bn-N9-p28446824	gene	exon
Bn-A09-p25443253	Bn-N9-p28448463	gene	
Bn-A09-p25443581	Bn-N9-p28448902	gene	
Bn-A09-p25455888	Bn-N9-p28461360	gene	
Bn-A09-p25523186	Bn-N9-p28537650	gene	
Bn-A09-p25532977	Bn-N9-p28547711	gene	exon
Bn-A09-p2555630	Bn-N9-p2348684	gene	exon
Bn-A09-p2557243	Bn-N9-p2350301	gene	
Bn-A09-p25575397	Bn-N9-p28590392	gene	
Bn-A09-p25618420	Bn-N9-p28613017	gene	

Bn-A09-p25635243	Bn-N9-p28627112	gene	exon
Bn-A09-p25636375	Bn-N9-p28628244	gene	exon
Bn-A09-p25638579	Bn-N9-p28630422	gene	
Bn-A09-p25652958	Bn-N9-p28650773	gene	
Bn-A09-p25652968	Bn-N9-p28650782	gene	
Bn-A09-p25653162	Bn-N9-p28650977	gene	
Bn-A09-p25654791	Bn-N9-p28652633	gene	exon
Bn-A09-p25670603	Bn-N9-p28673764	gene	
Bn-A09-p25736905	Bn-N9-p28730894	gene	exon
Bn-A09-p25738255	Bn-N9-p28732244	gene	exon
Bn-A09-p25738298	Bn-N9-p28732287	gene	exon
Bn-A09-p25739064	Bn-N9-p28732881	gene	exon
Bn-A09-p25740281	Bn-N9-p28734131	gene	exon
Bn-A09-p25747213	Bn-N9-p28741431	gene	
Bn-A09-p25747305	Bn-N9-p28741523	gene	
Bn-A09-p25777776	Bn-N9-p28779033	gene	
Bn-A09-p25779397	Bn-N9-p28781371	gene	exon
Bn-A09-p25793645	Bn-N9-p28804235	gene	exon
Bn-A09-p25823689	Bn-N9-p28830751	gene	
Bn-A09-p25832607	Bn-N9-p28835826	gene	exon
Bn-A09-p25872184	Bn-N9-p28879132	gene	exon
Bn-A09-p25886596	Bn-N9-p28909547	gene	exon
Bn-A09-p25923686	Bn-N9-p28946507	gene	exon
Bn-A09-p25942252	Bn-N9-p28963398	gene	
Bn-A09-p25960397	Bn-N9-p28977307	gene	
Bn-A09-p26054532	Bn-N9-p29069367	gene	exon
Bn-A09-p26062484	Bn-N9-p29077267	gene	
Bn-A09-p26101323	Bn-N9-p29097891	gene	
Bn-A09-p26133745	Bn-N9-p29124155	gene	exon
Bn-A09-p26136023	Bn-N18-p30854660	gene	exon
Bn-A09-p26138669	Bn-N9-p29129081	gene	exon
Bn-A09-p26142571	Bn-N9-p29134890	gene	exon
Bn-A09-p26244223	Bn-N9-p29268040	gene	exon
Bn-A09-p26301343	Bn-N9-p29304611	gene	exon
Bn-A09-p26323563	Bn-N9-p29324396	gene	exon
Bn-A09-p26347498	Bn-N9-p29340919	gene	
Bn-A09-p26347709	Bn-N9-p29341135	gene	
Bn-A09-p26353266	Bn-N9-p29346686	gene	exon
Bn-A09-p26355161	Bn-N9-p29348575	gene	
Bn-A09-p26397574	Bn-N9-p29391658	gene	exon
Bn-A09-p26400930	Bn-N9-p29395393	gene	
Bn-A09-p26451184	Bn-N9-p29433289	gene	exon
Bn-A09-p26456605	Bn-N12-p20836411	gene	exon
Bn-A09-p26461272	Bn-N9-p29443692	gene	
Bn-A09-p26477673	Bn-N9-p29458274	gene	exon
Bn-A09-p26481517	Bn-N9-p29462118	gene	exon
Bn-A09-p26492309	Bn-N19-p39309662	gene	exon
Bn-A09-p26524436	Bn-N9-p29491686	gene	
Bn-A09-p26577478	Bn-N9-p29538507	gene	exon
Bn-A09-p26606418	Bn-N9-p29578431	gene	
Bn-A09-p26625665	Bn-N9-p29596766	gene	
Bn-A09-p26637002	Bn-N9-p29609679	gene	exon

Bn-A09-p26727566	Bn-N18-p31610682	gene	
Bn-A09-p26825500	Bn-N9-p29793299	gene	
Bn-A09-p26831710	Bn-N9-p29799428	gene	exon
Bn-A09-p26873627	Bn-N9-p29829829	gene	exon
Bn-A09-p26874249	Bn-N9-p29830527	gene	
Bn-A09-p26877444	Bn-N9-p29833881	gene	exon
Bn-A09-p26881902	Bn-N9-p29850434	gene	
Bn-A09-p27036273	Bn-N9-p30042560	gene	exon
Bn-A09-p27103683	Bn-N9-p30112538	gene	
Bn-A09-p27108615	Bn-N9-p30116248	gene	
Bn-A09-p27109839	Bn-N9-p30117483	gene	exon
Bn-A09-p2730673	Bn-N9-p3411364	gene	
Bn-A09-p27351028	Bn-N9-p30396553	gene	exon
Bn-A09-p27351047	Bn-N9-p30396572	gene	exon
Bn-A09-p27370873	Bn-N9-p30414792	gene	exon
Bn-A09-p27436536	Bn-N9-p30488192	gene	exon
Bn-A09-p27531548	Bn-N9-p30562995	gene	exon
Bn-A09-p27642687	Bn-N9-p30666401	gene	
Bn-A09-p27642788	Bn-N9-p30666485	gene	
Bn-A09-p27642842	Bn-N9-p30666538	gene	
Bn-A09-p27673543	Bn-N9-p30709586	gene	
Bn-A09-p27678823	Bn-N9-p30715016	gene	exon
Bn-A09-p27688300	Bn-N9-p30726093	gene	
Bn-A09-p27787013	Bn-N9-p30817307	gene	
Bn-A09-p27864717	Bn-N9-p30908247	gene	
Bn-A09-p27869414	Bn-N9-p30914287	gene	
Bn-A09-p27887024	Bn-N9-p30927905	gene	
Bn-A09-p27918462	Bn-N9-p30957622	gene	exon
Bn-A09-p27939579	Bn-N9-p30975876	gene	exon
Bn-A09-p28003463	Bn-N9-p31058170	gene	exon
Bn-A09-p28017415	Bn-N9-p31079966	gene	
Bn-A09-p28025169	Bn-N9-p31083527	gene	
Bn-A09-p28026997	Bn-N9-p31085355	gene	
Bn-A09-p28027675	Bn-N9-p31086038	gene	
Bn-A09-p28027779	Bn-N9-p31086142	gene	
Bn-A09-p28044674	Bn-N18-p33429704	gene	
Bn-A09-p28060237	Bn-N9-p31131118	gene	
Bn-A09-p28070330	Bn-N9-p31138236	gene	
Bn-A09-p28074719	Bn-N9-p31142638	gene	
Bn-A09-p28082973	Bn-N9-p31149111	gene	
Bn-A09-p28083029	Bn-N9-p31149167	gene	
Bn-A09-p28142154	Bn-N9-p31211549	gene	exon
Bn-A09-p28142685	Bn-N9-p31212080	gene	exon
Bn-A09-p28149545	Bn-N9-p31218936	gene	exon
Bn-A09-p281844	Bn-N9-p867634	gene	exon
Bn-A09-p28191601	Bn-N9-p31262552	gene	exon
Bn-A09-p28209274	Bn-Scaffold12533-p573	gene	exon
Bn-A09-p28212233	Bn-N9-p31286465	gene	exon
Bn-A09-p28225347	Bn-N9-p31299424	gene	exon
Bn-A09-p28259332	Bn-N9-p31335679	gene	exon
Bn-A09-p28290848	Bn-N9-p31372875	gene	
Bn-A09-p28291555	Bn-N9-p31373585	gene	

Bn-A09-p28302787	Bn-N9-p31388617	gene	
Bn-A09-p28322032	Bn-N9-p31415979	gene	exon
Bn-A09-p28322383	Bn-N9-p31416330	gene	exon
Bn-A09-p28466750	Bn-N9-p31553347	gene	
Bn-A09-p28510409	Bn-N9-p31592124	gene	exon
Bn-A09-p28511369	Bn-N9-p31593084	gene	exon
Bn-A09-p285148	Bn-N9-p864331	gene	
Bn-A09-p28518619	Bn-N9-p31600329	gene	exon
Bn-A09-p28565181	Bn-N9-p31647170	gene	exon
Bn-A09-p28565331	Bn-N9-p31647322	gene	exon
Bn-A09-p28566621	Bn-N9-p31648612	gene	exon
Bn-A09-p28580489	Bn-N9-p31665586	gene	exon
Bn-A09-p28602135	Bn-N9-p31688305	gene	
Bn-A09-p28603202	Bn-N9-p31689372	gene	
Bn-A09-p28603388	Bn-N9-p31689556	gene	exon
Bn-A09-p28654058	Bn-N9-p31742669	gene	
Bn-A09-p28695042	Bn-N9-p31788810	gene	exon
Bn-A09-p28699275	Bn-N9-p31792823	gene	
Bn-A09-p28705871	Bn-N9-p31797994	gene	exon
Bn-A09-p28712265	Bn-N9-p31804625	gene	exon
Bn-A09-p28717690	Bn-N9-p31814388	gene	
Bn-A09-p28721875	Bn-N18-p34373489	gene	
Bn-A09-p28722989	Bn-N9-p31819589	gene	
Bn-A09-p28723813	Bn-N9-p31820413	gene	
Bn-A09-p28726474	Bn-N9-p31823074	gene	exon
Bn-A09-p28779232	Bn-N2-p9384090	gene	
Bn-A09-p28822639	Bn-N9-p31918034	gene	
Bn-A09-p28865744	Bn-N9-p31946357	gene	exon
Bn-A09-p28881091	Bn-N9-p31961966	gene	exon
Bn-A09-p28885939	Bn-N9-p31965342	gene	exon
Bn-A09-p28914189	Bn-N9-p31997772	gene	exon
Bn-A09-p28925363	Bn-N9-p32008873	gene	
Bn-A09-p28966938	Bn-N18-p34658354	gene	
Bn-A09-p28975119	Bn-N9-p32055497	gene	
Bn-A09-p28985128	Bn-N18-p34672855	gene	exon
Bn-A09-p28987525	Bn-N18-p34675526	gene	exon
Bn-A09-p28988322	Bn-N9-p32067950	gene	
Bn-A09-p28988381	Bn-N9-p32068018	gene	
Bn-A09-p28990648	Bn-N9-p32070470	gene	exon
Bn-A09-p28996871	Bn-N9-p32076649	gene	
Bn-A09-p29000054	Bn-N9-p32079842	gene	
Bn-A09-p29035265	Bn-N9-p32117153	gene	
Bn-A09-p29048790	Bn-N9-p32123622	gene	
Bn-A09-p29052515	Bn-N9-p32127354	gene	
Bn-A09-p29052616	Bn-N9-p32127455	gene	
Bn-A09-p29070287	Bn-N9-p32150935	gene	exon
Bn-A09-p29084281	Bn-N9-p32167209	gene	exon
Bn-A09-p29086743	Bn-N18-p34816481	gene	exon
Bn-A09-p29092594	Bn-N9-p32175501	gene	exon
Bn-A09-p29106108	Bn-N9-p32189368	gene	
Bn-A09-p29134633	Bn-N9-p32221026	gene	exon
Bn-A09-p29163307	Bn-N9-p32242477	gene	exon

Bn-A09-p29186255	Bn-N9-p32264891	gene	
Bn-A09-p29245415	Bn-N9-p32322014	gene	
Bn-A09-p29265453	Bn-N9-p32352864	gene	exon
Bn-A09-p29282189	Bn-N9-p32368035	gene	exon
Bn-A09-p29310462	Bn-N9-p32399459	gene	exon
Bn-A09-p29321510	Bn-N9-p32411874	gene	exon
Bn-A09-p29400976	Bn-N9-p32512470	gene	
Bn-A09-p29416708	Bn-N9-p32528115	gene	
Bn-A09-p29420099	Bn-N9-p32531531	gene	
Bn-A09-p29449180	Bn-N9-p32557398	gene	
Bn-A09-p29454619	Bn-N9-p32558190	gene	exon
Bn-A09-p29494065	Bn-N9-p32584007	gene	exon
Bn-A09-p29496129	Bn-N9-p32586060	gene	
Bn-A09-p29496571	Bn-N9-p32586503	gene	
Bn-A09-p29496588	Bn-N9-p32586520	gene	
Bn-A09-p29507416	Bn-N9-p32593460	gene	
Bn-A09-p29508036	Bn-N9-p32594074	gene	
Bn-A09-p29617756	Bn-N9-p32727243	gene	exon
Bn-A09-p29617813	Bn-N9-p32727300	gene	
Bn-A09-p29628819	Bn-N9-p32738483	gene	exon
Bn-A09-p29629155	Bn-N9-p32738819	gene	exon
Bn-A09-p29630906	Bn-N9-p32740570	gene	exon
Bn-A09-p29631122	Bn-N9-p32740786	gene	exon
Bn-A09-p29676939	Bn-N9-p32779190	gene	
Bn-A09-p297029	Bn-N9-p848747	gene	exon
Bn-A09-p29859019	Bn-N9-p32953844	gene	
Bn-A09-p29944855	Bn-N9-p33051403	gene	exon
Bn-A09-p29951785	Bn-N18-p35851104	gene	exon
Bn-A09-p29953651	Bn-N9-p33061844	gene	exon
Bn-A09-p29968210	Bn-N9-p33074073	gene	
Bn-A09-p29968302	Bn-N9-p33074165	gene	exon
Bn-A09-p30007298	Bn-N9-p33118727	gene	
Bn-A09-p30010889	Bn-N9-p33122318	gene	exon
Bn-A09-p30032125	Bn-N9-p33138568	gene	exon
Bn-A09-p30073403	Bn-N9-p33178686	gene	exon
Bn-A09-p30102296	Bn-N9-p33206446	gene	exon
Bn-A09-p30105255	Bn-N9-p33209402	gene	
Bn-A09-p30105467	Bn-N9-p33209842	gene	exon
Bn-A09-p30108245	Bn-N9-p33212673	gene	
Bn-A09-p30108430	Bn-N9-p33212858	gene	exon
Bn-A09-p30112316	Bn-N9-p33219152	gene	exon
Bn-A09-p30115693	Bn-N9-p33222528	gene	
Bn-A09-p30116087	Bn-N9-p33222921	gene	
Bn-A09-p30117412	Bn-N9-p33226477	gene	
Bn-A09-p30117469	Bn-N9-p33226533	gene	
Bn-A09-p30129045	Bn-N9-p33233059	gene	exon
Bn-A09-p301754	Bn-N9-p843863	gene	
Bn-A09-p301830	Bn-N9-p843787	gene	exon
Bn-A09-p302062	Bn-N19-p965061	gene	
Bn-A09-p30280052	Bn-N9-p33397646	gene	
Bn-A09-p30280973	Bn-N9-p33398567	gene	
Bn-A09-p30282007	Bn-N9-p33399601	gene	

Bn-A09-p30284632	Bn-N9-p33402226	gene	
Bn-A09-p30284813	Bn-N9-p33402407	gene	exon
Bn-A09-p30292279	Bn-N9-p33408783	gene	exon
Bn-A09-p30344978	Bn-N9-p33472614	gene	
Bn-A09-p30348281	Bn-N9-p33475898	gene	
Bn-A09-p30358198	Bn-N9-p33485793	gene	exon
Bn-A09-p30371768	Bn-N9-p33502593	gene	exon
Bn-A09-p30373180	Bn-N9-p33504078	gene	
Bn-A09-p30391674	Bn-N9-p33515525	gene	exon
Bn-A09-p30404228	Bn-N9-p33526161	gene	exon
Bn-A09-p30406985	Bn-N9-p33528909	gene	exon
Bn-A09-p30424373	Bn-N9-p33558503	gene	exon
Bn-A09-p30437673	Bn-N9-p33571425	gene	exon
Bn-A09-p30463234	Bn-N9-p33607106	gene	
Bn-A09-p30464712	Bn-N18-p36549521	gene	
Bn-A09-p30466030	Bn-N9-p33610037	gene	
Bn-A09-p30488258	Bn-N18-p36861419	gene	
Bn-A09-p30494224	Bn-N9-p33637337	gene	exon
Bn-A09-p30495797	Bn-N9-p33638910	gene	exon
Bn-A09-p30506895	Bn-N9-p33652890	gene	
Bn-A09-p30559416	Bn-N9-p33696438	gene	exon
Bn-A09-p30570466	Bn-N9-p33705142	gene	exon
Bn-A09-p30587291	Bn-N9-p33717957	gene	
Bn-A09-p30687979	Bn-N9-p33823886	gene	
Bn-A09-p30697212	Bn-N18-p36808723	gene	exon
Bn-A09-p30710881	Bn-N9-p33846656	gene	exon
Bn-A09-p30711004	Bn-N9-p33846779	gene	exon
Bn-A09-p30718480	Bn-N9-p33854257	gene	
Bn-A09-p30733739	Bn-N9-p33870456	gene	exon
Bn-A09-p30740855	Bn-N9-p33877684	gene	exon
Bn-A09-p30755882	Bn-N9-p33901968	gene	
Bn-A09-p30771886	Bn-N9-p33916963	gene	
Bn-A09-p30780421	Bn-N9-p33929638	gene	exon
Bn-A09-p30780679	Bn-N9-p33929911	gene	
Bn-A09-p30780890	Bn-N9-p33930122	gene	
Bn-A09-p30788157	Bn-N9-p33933296	gene	
Bn-A09-p30805314	Bn-N9-p33947529	gene	
Bn-A09-p30805387	Bn-N9-p33947602	gene	
Bn-A09-p30835062	Bn-N9-p33992893	gene	
Bn-A09-p30846003	Bn-N9-p34008029	gene	exon
Bn-A09-p30847721	Bn-N9-p34009664	gene	exon
Bn-A09-p30869609	Bn-N9-p34032434	gene	
Bn-A09-p30877022	Bn-N9-p34039910	gene	exon
Bn-A09-p30877241	Bn-N9-p34040129	gene	exon
Bn-A09-p30877372	Bn-N9-p34040260	gene	exon
Bn-A09-p30900431	Bn-N9-p34067275	gene	exon
Bn-A09-p30901490	Bn-N18-p37070442	gene	
Bn-A09-p30908648	Bn-N9-p34074998	gene	exon
Bn-A09-p30909393	Bn-N9-p34075742	gene	exon
Bn-A09-p30921980	Bn-N9-p34083100	gene	
Bn-A09-p30954374	Bn-N9-p34116489	gene	exon
Bn-A09-p30965242	Bn-N9-p34123024	gene	

Bn-A09-p31013896	Bn-N9-p34181393	gene	
Bn-A09-p31152287	Bn-N9-p34320872	gene	
Bn-A09-p31204477	Bn-N9-p34366655	gene	exon
Bn-A09-p31204602	Bn-N9-p34366780	gene	exon
Bn-A09-p31231616	Bn-N9-p34393086	gene	exon
Bn-A09-p31270539	Bn-N9-p34427517	gene	exon
Bn-A09-p31388253	Bn-N9-p34576071	gene	exon
Bn-A09-p31402193	Bn-N9-p34590601	gene	
Bn-A09-p31412669	Bn-N18-p37714208	gene	exon
Bn-A09-p31413712	Bn-N9-p34601580	gene	exon
Bn-A09-p31416695	Bn-N9-p34604575	gene	
Bn-A09-p31492693	Bn-N9-p34676656	gene	exon
Bn-A09-p31493152	Bn-N18-p37805730	gene	exon
Bn-A09-p31494058	Bn-N9-p34677629	gene	exon
Bn-A09-p31576965	Bn-N9-p34765864	gene	exon
Bn-A09-p31578167	Bn-N9-p34767066	gene	exon
Bn-A09-p31582088	Bn-N9-p34770977	gene	
Bn-A09-p31587667	Bn-N9-p34780274	gene	exon
Bn-A09-p31610860	Bn-N9-p34805405	gene	
Bn-A09-p31611193	Bn-N9-p34805737	gene	
Bn-A09-p31611279	Bn-N9-p34805823	gene	exon
Bn-A09-p31611436	Bn-N9-p34805980	gene	exon
Bn-A09-p31611521	Bn-N9-p34806065	gene	exon
Bn-A09-p31611638	Bn-N9-p34806182	gene	
Bn-A09-p31612154	Bn-N9-p34806698	gene	
Bn-A09-p31613718	Bn-N9-p34808955	gene	
Bn-A09-p31614055	Bn-N9-p34809292	gene	
Bn-A09-p31614130	Bn-N9-p34809367	gene	
Bn-A09-p31614376	Bn-N9-p34809613	gene	
Bn-A09-p31615419	Bn-N9-p34810763	gene	exon
Bn-A09-p31635684	Bn-N9-p34832111	gene	exon
Bn-A09-p31663954	Bn-N9-p34891067	gene	
Bn-A09-p3173488	Bn-N19-p4919686	gene	exon
Bn-A09-p31741551	Bn-N9-p34968983	gene	exon
Bn-A09-p31979689	Bn-N18-p38701686	gene	
Bn-A09-p31982487	Bn-N9-p35436621	gene	
Bn-A09-p32027483	Bn-N9-p35492302	gene	exon
Bn-A09-p32066349	Bn-N9-p35530225	gene	
Bn-A09-p32104929	Bn-N9-p35573213	gene	exon
Bn-A09-p32104974	Bn-N9-p35573257	gene	exon
Bn-A09-p32105188	Bn-N9-p35573472	gene	exon
Bn-A09-p321430	Bn-N9-p812277	gene	exon
Bn-A09-p32223480	Bn-N9-p35667486	gene	
Bn-A09-p32225646	Bn-N9-p35669625	gene	
Bn-A09-p3225300	Bn-N9-p3866498	gene	
Bn-A09-p32254190	Bn-N9-p35689490	gene	
Bn-A09-p32270596	Bn-N9-p35705227	gene	exon
Bn-A09-p32275168	Bn-N9-p35708033	gene	exon
Bn-A09-p32321534	Bn-N9-p35739529	gene	
Bn-A09-p3233129	Bn-N19-p5017427	gene	exon
Bn-A09-p3234323	Bn-N9-p3875594	gene	
Bn-A09-p3251545	Bn-N9-p3890593	gene	

Bn-A09-p32553111	Bn-N9-p35973804	gene	
Bn-A09-p3257371	Bn-N9-p3897790	gene	exon
Bn-A09-p32624472	Bn-N8-p5130068	gene	
Bn-A09-p32656218	Bn-N18-p13436786	gene	exon
Bn-A09-p32665710	Bn-N9-p36061302	gene	
Bn-A09-p32666778	Bn-N9-p36062370	gene	exon
Bn-A09-p32713083	Bn-N9-p36111319	gene	
Bn-A09-p32717048	Bn-N9-p36115269	gene	
Bn-A09-p32787589	Bn-N9-p36173180	gene	
Bn-A09-p32811261	Bn-N9-p36193076	gene	
Bn-A09-p32849664	Bn-N18-p39671307	gene	exon
Bn-A09-p32862905	Bn-N9-p36243125	gene	
Bn-A09-p32864411	Bn-N9-p36244647	gene	
Bn-A09-p32884256	Bn-N9-p36265098	gene	exon
Bn-A09-p32903518	Bn-N9-p36284874	gene	
Bn-A09-p329151	Bn-N9-p820196	gene	
Bn-A09-p32937856	Bn-N9-p36311368	gene	
Bn-A09-p32944206	Bn-N9-p36317718	gene	
Bn-A09-p32945202	Bn-N9-p36318714	gene	
Bn-A09-p32980100	Bn-N9-p36353569	gene	
Bn-A09-p32980153	Bn-N9-p36353603	gene	
Bn-A09-p33009761	Bn-N9-p36389886	gene	
Bn-A09-p33025970	Bn-N9-p36404730	gene	exon
Bn-A09-p33029336	Bn-N9-p36413463	gene	exon
Bn-A09-p33037403	Bn-N9-p36422919	gene	
Bn-A09-p33040441	Bn-N9-p36426733	gene	
Bn-A09-p33061472	Bn-N9-p36435494	gene	exon
Bn-A09-p33105121	Bn-N9-p36486000	gene	exon
Bn-A09-p33280	Bn-N9-p1129797	gene	
Bn-A09-p33349373	Bn-N9-p36688936	gene	exon
Bn-A09-p33349948	Bn-N9-p36689515	gene	
Bn-A09-p33358168	Bn-N9-p36701856	gene	exon
Bn-A09-p33358298	Bn-N9-p36701986	gene	exon
Bn-A09-p33358356	Bn-N9-p36702043	gene	
Bn-A09-p33398481	Bn-N9-p36741687	gene	exon
Bn-A09-p33406899	Bn-N9-p36749552	gene	
Bn-A09-p33419515	Bn-N9-p36762500	gene	
Bn-A09-p33427256	Bn-N9-p36780736	gene	
Bn-A09-p33428597	Bn-N9-p36782086	gene	
Bn-A09-p33434187	Bn-N9-p36788333	gene	exon
Bn-A09-p33456655	Bn-N9-p36822177	gene	exon
Bn-A09-p33458611	Bn-N9-p36824202	gene	
Bn-A09-p33459299	Bn-N9-p36824891	gene	exon
Bn-A09-p33464355	Bn-N9-p36829929	gene	
Bn-A09-p33473254	Bn-N9-p36838946	gene	exon
Bn-A09-p33475518	Bn-N9-p36840288	gene	
Bn-A09-p33487356	Bn-N9-p36847011	gene	exon
Bn-A09-p33491501	Bn-N9-p36851155	gene	
Bn-A09-p33499505	Bn-N9-p36859886	gene	exon
Bn-A09-p33535361	Bn-N9-p36883548	gene	
Bn-A09-p33544191	Bn-N9-p36895783	gene	
Bn-A09-p33587432	Bn-N9-p36934279	gene	exon

Bn-A09-p33641390	Bn-N9-p36997498	gene	exon
Bn-A09-p33650317	Bn-N9-p37009406	gene	exon
Bn-A09-p33660289	Bn-N9-p37021528	gene	exon
Bn-A09-p33694154	Bn-N9-p37061860	gene	
Bn-A09-p33694194	Bn-N9-p37061900	gene	exon
Bn-A09-p33726811	Bn-N9-p37089844	gene	
Bn-A09-p33777546	Bn-N9-p37125288	gene	
Bn-A09-p33777987	Bn-N9-p37125729	gene	
Bn-A09-p33815163	Bn-N9-p37157555	gene	exon
Bn-A09-p33822952	Bn-N9-p37166762	gene	
Bn-A09-p33894393	Bn-N18-p40907881	gene	exon
Bn-A09-p33927099	Bn-N9-p37303753	gene	
Bn-A09-p33942716	Bn-N9-p37319590	gene	exon
Bn-A09-p33948825	Bn-N9-p37325699	gene	exon
Bn-A09-p33973275	Bn-N9-p37349418	gene	
Bn-A09-p33975396	Bn-N9-p37351983	gene	
Bn-A09-p33975959	Bn-N9-p37355176	gene	
Bn-A09-p33976066	Bn-N9-p37355283	gene	exon
Bn-A09-p33976347	Bn-N9-p37355564	gene	
Bn-A09-p33976464	Bn-N9-p37355681	gene	
Bn-A09-p33976515	Bn-N9-p37355732	gene	
Bn-A09-p33981445	Bn-N9-p37358958	gene	
Bn-A09-p33983999	Bn-N18-p41042789	gene	exon
Bn-A09-p34022183	Bn-N9-p37399070	gene	exon
Bn-A09-p34028225	Bn-N9-p37405084	gene	
Bn-A09-p34029934	Bn-N9-p37406793	gene	
Bn-A09-p34044914	Bn-N9-p37421869	gene	exon
Bn-A09-p34053851	Bn-N9-p37430821	gene	exon
Bn-A09-p34067269	Bn-N9-p37445603	gene	exon
Bn-A09-p34331338	Bn-N18-p41429158	gene	
Bn-A09-p34373261	Bn-N9-p37751407	gene	
Bn-A09-p34390255	Bn-N9-p37770036	gene	exon
Bn-A09-p34393068	Bn-N9-p37773158	gene	exon
Bn-A09-p34437367	Bn-N9-p37816830	gene	exon
Bn-A09-p34469671	Bn-N9-p37851437	gene	exon
Bn-A09-p34482075	Bn-N9-p37864555	gene	exon
Bn-A09-p3452203	Bn-N9-p4101256	gene	exon
Bn-A09-p34591600	Bn-N18-p41737490	gene	exon
Bn-A09-p34779068	Bn-N9-p38152140	gene	exon
Bn-A09-p34794738	Bn-N9-p38177898	gene	exon
Bn-A09-p34830	Bn-N9-p1128216	gene	exon
Bn-A09-p34995048	Bn-N9-p38402439	gene	
Bn-A09-p34995922	Bn-N9-p38403313	gene	
Bn-A09-p35278018	Bn-N18-p42699168	gene	exon
Bn-A09-p35279845	Bn-N9-p38700381	gene	
Bn-A09-p35292945	Bn-N9-p38715294	gene	exon
Bn-A09-p35299111	Bn-N9-p38723451	gene	exon
Bn-A09-p35303838	Bn-N9-p38733006	gene	exon
Bn-A09-p35306248	Bn-N9-p38735420	gene	exon
Bn-A09-p35321301	Bn-N9-p38752121	gene	exon
Bn-A09-p35368467	Bn-N9-p38801419	gene	exon
Bn-A09-p35372226	Bn-N9-p38805268	gene	exon

Bn-A09-p35389045	Bn-N9-p38828320	gene	exon
Bn-A09-p35404670	Bn-N9-p38845336	gene	exon
Bn-A09-p35425963	Bn-N9-p38864755	gene	exon
Bn-A09-p35426888	Bn-N9-p38865680	gene	
Bn-A09-p35437924	Bn-N9-p38876666	gene	exon
Bn-A09-p35466517	Bn-N9-p38908464	gene	exon
Bn-A09-p35476619	Bn-N9-p38919112	gene	exon
Bn-A09-p35479723	Bn-N9-p38922217	gene	
Bn-A09-p35480425	Bn-N9-p38922946	gene	
Bn-A09-p35480484	Bn-N9-p38923005	gene	
Bn-A09-p35481880	Bn-N9-p38924401	gene	exon
Bn-A09-p35484843	Bn-N9-p38927365	gene	exon
Bn-A09-p35485049	Bn-N9-p38927571	gene	
Bn-A09-p35485458	Bn-N9-p38927980	gene	exon
Bn-A09-p35505057	Bn-N9-p38945900	gene	
Bn-A09-p35505120	Bn-N9-p38945963	gene	
Bn-A09-p35508720	Bn-N9-p38949671	gene	exon
Bn-A09-p35508955	Bn-N9-p38949905	gene	
Bn-A09-p35509044	Bn-N9-p38949994	gene	exon
Bn-A09-p35510262	Bn-N9-p38951200	gene	
Bn-A09-p35510324	Bn-N9-p38951262	gene	
Bn-A09-p35519543	Bn-N9-p38954628	gene	
Bn-A09-p35520884	Bn-N9-p38955969	gene	
Bn-A09-p35520944	Bn-N9-p38956029	gene	
Bn-A09-p35529865	Bn-N9-p38965296	gene	
Bn-A09-p35531421	Bn-N9-p38966852	gene	
Bn-A09-p35559972	Bn-N9-p38995774	gene	exon
Bn-A09-p35563022	Bn-N9-p39002787	gene	exon
Bn-A09-p35571119	Bn-N9-p39010870	gene	exon
Bn-A09-p35594338	Bn-N9-p39028751	gene	
Bn-A09-p35604358	Bn-N9-p39038217	gene	
Bn-A09-p35609789	Bn-N9-p39045025	gene	
Bn-A09-p35611451	Bn-N9-p39046687	gene	exon
Bn-A09-p35624679	Bn-N9-p39061009	gene	
Bn-A09-p35624767	Bn-N9-p39061102	gene	
Bn-A09-p35647477	Bn-N9-p39081551	gene	exon
Bn-A09-p35661872	Bn-N9-p39097820	gene	exon
Bn-A09-p35672030	Bn-Scaffold01321-p9761	gene	
Bn-A09-p35691478	Bn-N9-p39121587	gene	exon
Bn-A09-p35703402	Bn-N9-p39131741	gene	exon
Bn-A09-p35707505	Bn-N18-p43252029	gene	
Bn-A09-p35724973	Bn-N9-p39150989	gene	exon
Bn-A09-p35728172	Bn-N9-p39154189	gene	exon
Bn-A09-p35790555	Bn-N9-p39206858	gene	exon
Bn-A09-p35848630	Bn-N18-p43381931	gene	exon
Bn-A09-p35866195	Bn-N18-p43399598	gene	
Bn-A09-p35867904	Bn-N18-p43401280	gene	
Bn-A09-p35890304	Bn-N9-p39286943	gene	
Bn-A09-p35901593	Bn-N9-p39300694	gene	
Bn-A09-p35964883	Bn-N9-p39360714	gene	
Bn-A09-p35979119	Bn-N9-p39375212	gene	exon
Bn-A09-p35981493	Bn-N9-p39377586	gene	

Bn-A09-p35981539	Bn-N9-p39377632	gene	
Bn-A09-p36019854	Bn-N9-p39396878	gene	exon
Bn-A09-p36033827	Bn-N9-p39417090	gene	
Bn-A09-p36062760	Bn-N9-p39444862	gene	
Bn-A09-p36062825	Bn-N9-p39444927	gene	
Bn-A09-p36112515	Bn-N9-p39502154	gene	
Bn-A09-p36120469	Bn-N9-p39522329	gene	exon
Bn-A09-p36136583	Bn-N9-p39538036	gene	
Bn-A09-p36172635	Bn-N18-p43731092	gene	
Bn-A09-p36173579	Bn-N9-p39576329	gene	
Bn-A09-p36200543	Bn-N9-p39608592	gene	
Bn-A09-p36201113	Bn-N9-p39609165	gene	exon
Bn-A09-p36204736	Bn-N9-p39612788	gene	exon
Bn-A09-p36210166	Bn-N9-p39618274	gene	
Bn-A09-p36210247	Bn-N9-p39618355	gene	
Bn-A09-p36211880	Bn-N9-p39620502	gene	
Bn-A09-p36221958	Bn-N9-p39637035	gene	
Bn-A09-p36268830	Bn-N18-p43850890	gene	
Bn-A09-p36290340	Bn-N9-p39706035	gene	exon
Bn-A09-p36298053	Bn-N9-p39714638	gene	
Bn-A09-p36330477	Bn-N18-p43997860	gene	exon
Bn-A09-p36363739	Bn-N9-p39783836	gene	exon
Bn-A09-p36434436	Bn-N9-p39856164	gene	exon
Bn-A09-p36455112	Bn-N9-p39876911	gene	
Bn-A09-p36456115	Bn-N9-p39877914	gene	
Bn-A09-p36456505	Bn-N9-p39878304	gene	
Bn-A09-p36462450	Bn-N9-p39883918	gene	exon
Bn-A09-p36467520	Bn-N9-p39888992	gene	exon
Bn-A09-p36550750	Bn-N9-p39979652	gene	
Bn-A09-p36551108	Bn-N9-p39980235	gene	
Bn-A09-p36558536	Bn-N9-p39987599	gene	exon
Bn-A09-p36619537	Bn-N9-p40049828	gene	exon
Bn-A09-p36727719	Bn-N18-p44485147	gene	exon
Bn-A09-p36803825	Bn-N13-p55061222	gene	exon
Bn-A09-p36812148	Bn-N9-p40297556	gene	exon
Bn-A09-p36815767	Bn-N9-p40192302	gene	
Bn-A09-p36915816	Bn-Scaffold06015-p615	gene	exon
Bn-A09-p36939006	Bn-N9-p40375145	gene	exon
Bn-A09-p36969978	Bn-Scaffold03369-p1628	gene	exon
Bn-A09-p36991758	Bn-N9-p40431805	gene	
Bn-A09-p37019231	Bn-N9-p40466380	gene	
Bn-A09-p37024383	Bn-N18-p44587258	gene	
Bn-A09-p37029924	Bn-N9-p40512392	gene	
Bn-A09-p37031129	Bn-N9-p40514752	gene	
Bn-A09-p37034993	Bn-N18-p44622559	gene	exon
Bn-A09-p37048611	Bn-N18-p44637869	gene	exon
Bn-A09-p381059	Bn-N12-p14270242	gene	exon
Bn-A09-p384043	Bn-N16-p31486339	gene	
Bn-A09-p388989	Bn-N9-p747002	gene	
Bn-A09-p3955243	Bn-N9-p5005272	gene	exon
Bn-A09-p403519	Bn-N19-p849968	gene	
Bn-A09-p408412	Bn-N9-p724239	gene	exon

Bn-A09-p4163474	Bn-N9-p4792753	gene	exon
Bn-A09-p4163989	Bn-N9-p4792238	gene	exon
Bn-A09-p4164576	Bn-N9-p4791651	gene	exon
Bn-A09-p4168039	Bn-N9-p4788032	gene	exon
Bn-A09-p417110	Bn-N9-p719287	gene	exon
Bn-A09-p4207679	Bn-N9-p4742916	gene	exon
Bn-A09-p4282686	Bn-N9-p4664120	gene	exon
Bn-A09-p4288843	Bn-N9-p4658006	gene	exon
Bn-A09-p4300103	Bn-N9-p4649474	gene	exon
Bn-A09-p4347835	Bn-N9-p4608933	gene	exon
Bn-A09-p4356331	Bn-N9-p4605185	gene	
Bn-A09-p4384911	Bn-N9-p4580472	gene	
Bn-A09-p4416731	Bn-N9-p4544875	gene	exon
Bn-A09-p4433127	Bn-N9-p5340674	gene	exon
Bn-A09-p4433252	Bn-N9-p5340799	gene	exon
Bn-A09-p4459348	Bn-N9-p5366750	gene	
Bn-A09-p4465163	Bn-N9-p5374076	gene	
Bn-A09-p4509627	Bn-N9-p5419243	gene	
Bn-A09-p4652268	Bn-N19-p7859662	gene	
Bn-A09-p4655322	Bn-N9-p5569467	gene	exon
Bn-A09-p4671467	Bn-N9-p5587113	gene	exon
Bn-A09-p469758	Bn-N9-p675523	gene	exon
Bn-A09-p4735640	Bn-N9-p5627772	gene	
Bn-A09-p4835780	Bn-N9-p5734320	gene	exon
Bn-A09-p4838767	Bn-N19-p8115030	gene	exon
Bn-A09-p4850116	Bn-N9-p5748041	gene	exon
Bn-A09-p4850180	Bn-N19-p8127893	gene	exon
Bn-A09-p4863472	Bn-N9-p5761647	gene	exon
Bn-A09-p487360	Bn-N9-p662145	gene	exon
Bn-A09-p487432	Bn-N9-p662073	gene	exon
Bn-A09-p4905706	Bn-N9-p5829540	gene	
Bn-A09-p4905766	Bn-N9-p5829600	gene	
Bn-A09-p4990811	Bn-N9-p35114498	gene	exon
Bn-A09-p4996710	Bn-N9-p35120366	gene	exon
Bn-A09-p4997670	Bn-N9-p35121321	gene	exon
Bn-A09-p5006353	Bn-N9-p35134896	gene	
Bn-A09-p5031583	Bn-N9-p35161613	gene	exon
Bn-A09-p5054103	Bn-N18-p38400259	gene	
Bn-A09-p5062101	Bn-N9-p35194528	gene	exon
Bn-A09-p5118197	Bn-N9-p35237446	gene	exon
Bn-A09-p5157169	Bn-N9-p35266060	gene	exon
Bn-A09-p5200256	Bn-N9-p5953243	gene	exon
Bn-A09-p5215814	Bn-N9-p5966760	gene	exon
Bn-A09-p5244764	Bn-N9-p5990474	gene	
Bn-A09-p5246171	Bn-N9-p5991884	gene	exon
Bn-A09-p5246229	Bn-N9-p5991942	gene	exon
Bn-A09-p5255753	Bn-N9-p6004794	gene	
Bn-A09-p5285034	Bn-N9-p6034586	gene	exon
Bn-A09-p5322252	Bn-N9-p6068414	gene	exon
Bn-A09-p5348717	Bn-N9-p6095587	gene	exon
Bn-A09-p5406597	Bn-N9-p6139920	gene	exon
Bn-A09-p5406959	Bn-N9-p6140282	gene	exon

Bn-A09-p5416801	Bn-N9-p6149418	gene	exon
Bn-A09-p5418207	Bn-N9-p6150830	gene	exon
Bn-A09-p5418342	Bn-N9-p6150965	gene	exon
Bn-A09-p5431352	Bn-N9-p6163934	gene	exon
Bn-A09-p5442608	Bn-N9-p6172279	gene	
Bn-A09-p5455608	Bn-N9-p6187862	gene	exon
Bn-A09-p5601358	Bn-N9-p6328235	gene	exon
Bn-A09-p5603209	Bn-N9-p6330076	gene	exon
Bn-A09-p5678953	Bn-N9-p6405447	gene	exon
Bn-A09-p5680812	Bn-N9-p6407307	gene	
Bn-A09-p574205	Bn-N9-p567757	gene	exon
Bn-A09-p5781490	Bn-N9-p6522956	gene	exon
Bn-A09-p584756	Bn-N9-p558672	gene	
Bn-A09-p586357	Bn-N9-p557067	gene	
Bn-A09-p5958009	Bn-N9-p6697872	gene	
Bn-A09-p5958764	Bn-N9-p6698627	gene	
Bn-A09-p5966342	Bn-N1-p1112754	gene	exon
Bn-A09-p5966858	Bn-N1-p1112176	gene	exon
Bn-A09-p5975583	Bn-N18-p38563178	gene	exon
Bn-A09-p5977032	Bn-N9-p35302828	gene	
Bn-A09-p5992	Bn-N9-p1160219	gene	exon
Bn-A09-p6021939	Bn-N9-p8045087	gene	
Bn-A09-p603278	Bn-N9-p534104	gene	
Bn-A09-p6133524	Bn-N9-p7905525	gene	
Bn-A09-p6140805	Bn-N9-p7898169	gene	exon
Bn-A09-p616919	Bn-N9-p508992	gene	
Bn-A09-p6459935	Bn-N9-p7635565	gene	
Bn-A09-p660753	Bn-N9-p464464	gene	
Bn-A09-p660775	Bn-N9-p464442	gene	
Bn-A09-p6615722	Bn-N19-p11198841	gene	exon
Bn-A09-p6616042	Bn-N9-p7456587	gene	exon
Bn-A09-p6616405	Bn-N9-p7456223	gene	
Bn-A09-p6630867	Bn-N9-p7443977	gene	exon
Bn-A09-p6632506	Bn-N19-p11176935	gene	exon
Bn-A09-p664845	Bn-N9-p459340	gene	
Bn-A09-p6783843	Bn-N9-p7329180	gene	exon
Bn-A09-p679276	Bn-N9-p448494	gene	
Bn-A09-p6922467	Bn-N9-p7181761	gene	exon
Bn-A09-p694190	Bn-N19-p417038	gene	exon
Bn-A09-p702173	Bn-N9-p430601	gene	
Bn-A09-p704028	Bn-N9-p428711	gene	
Bn-A09-p7047022	Bn-N9-p7063456	gene	exon
Bn-A09-p7086863	Bn-N9-p6995161	gene	
Bn-A09-p7087772	Bn-N9-p6994252	gene	exon
Bn-A09-p7174944	Bn-N19-p10270316	gene	
Bn-A09-p7176234	Bn-N9-p6908092	gene	
Bn-A09-p7191362	Bn-N9-p6896173	gene	exon
Bn-A09-p7191369	Bn-N9-p6896166	gene	exon
Bn-A09-p7199203	Bn-N9-p6888125	gene	
Bn-A09-p7287912	Bn-N9-p6806829	gene	exon
Bn-A09-p7327691	Bn-N9-p6777654	gene	exon
Bn-A09-p733947	Bn-N9-p396353	gene	

Bn-A09-p7397116	Bn-N9-p6714104	gene	
Bn-A09-p7490528	Bn-N9-p8183157	gene	
Bn-A09-p7490626	Bn-N9-p8183255	gene	exon
Bn-A09-p7491672	Bn-N9-p8184301	gene	exon
Bn-A09-p7503009	Bn-N9-p8204408	gene	exon
Bn-A09-p750455	Bn-N19-p320463	gene	exon
Bn-A09-p7510345	Bn-N9-p8210913	gene	
Bn-A09-p751817	Bn-N9-p382442	gene	
Bn-A09-p7560188	Bn-N9-p8266261	gene	
Bn-A09-p757653	Bn-N9-p379694	gene	exon
Bn-A09-p788109	Bn-N9-p348078	gene	exon
Bn-A09-p788595	Bn-N9-p347592	gene	exon
Bn-A09-p7892057	Bn-N7-p9784621	gene	
Bn-A09-p8044910	Bn-N9-p8790084	gene	
Bn-A09-p8239411	Bn-N9-p8968316	gene	
Bn-A09-p8598652	Bn-N9-p9304419	gene	exon
Bn-A09-p8611536	Bn-N9-p9320169	gene	exon
Bn-A09-p86293	Bn-N9-p1053679	gene	exon
Bn-A09-p8634971	Bn-N9-p9339112	gene	exon
Bn-A09-p8635608	Bn-N9-p9339748	gene	
Bn-A09-p8635777	Bn-N19-p14542553	gene	
Bn-A09-p869372	Bn-N9-p306757	gene	
Bn-A09-p8798692	Bn-N9-p9506415	gene	
Bn-A09-p8798760	Bn-N9-p9506483	gene	
Bn-A09-p8800446	Bn-N9-p9508156	gene	exon
Bn-A09-p8810292	Bn-N9-p9528241	gene	
Bn-A09-p8877444	Bn-N9-p9612935	gene	exon
Bn-A09-p8891277	Bn-N9-p9617045	gene	
Bn-A09-p9040117	Bn-N9-p9778514	gene	
Bn-A09-p9083090	Bn-N9-p9820124	gene	
Bn-A09-p9083366	Bn-N9-p9820348	gene	exon
Bn-A09-p9083445	Bn-N9-p9820428	gene	exon
Bn-A09-p9206874	Bn-N9-p9964908	gene	
Bn-A09-p9317714	Bn-N9-p10088321	gene	
Bn-A09-p9371106	Bn-N19-p15847047	gene	
Bn-A09-p9379347	Bn-N9-p10155921	gene	
Bn-A09-p941202	Bn-N9-p227026	gene	
Bn-A09-p9421306	Bn-N9-p10198203	gene	exon
Bn-A09-p9497384	Bn-N9-p10276370	gene	
Bn-A09-p951202	Bn-N9-p216968	gene	
Bn-A09-p9512316	Bn-N9-p10292141	gene	
Bn-A09-p9558015	Bn-N9-p10331661	gene	
Bn-A09-p9558692	Bn-N9-p10332338	gene	
Bn-A09-p9558834	Bn-N9-p10333379	gene	
Bn-A09-p9559997	Bn-N19-p16199574	gene	
Bn-A09-p9624934	Bn-N19-p16287034	gene	
Bn-A09-p9667631	Bn-N9-p10471544	gene	exon
Bn-A09-p9671359	Bn-N9-p10475250	gene	exon
Bn-A09-p9702226	Bn-N13-p39819389	gene	
Bn-A09-p972043	Bn-N9-p197070	gene	exon
Bn-A09-p9728715	Bn-N3-p19023544	gene	exon
Bn-A09-p9747402	Bn-N9-p10543852	gene	exon

Bn-A09-p9789590	Bn-N9-p10590554	gene	
Bn-A09-p9789898	Bn-N9-p10590863	gene	
Bn-A09-p9789943	Bn-N9-p10590908	gene	exon
Bn-A09-p9845028	Bn-N9-p10675202	gene	exon
Bn-A09-p985671	Bn-N9-p180078	gene	
Bn-A09-p9862616	Bn-N9-p10692415	gene	
Bn-A09-p9863993	Bn-N9-p10693791	gene	
Bn-A09-p996577	Bn-N9-p169841	gene	
Bn-A09-p9980036	Bn-N9-p10761224	gene	exon
Bn-A09-p9987709	Bn-N9-p10768890	gene	exon
Bn-A09-p999098	Bn-N9-p166061	gene	exon
Bn-A10-p10020060	Bn-N10-p11657804	gene	
Bn-A10-p10026077	Bn-N19-p46930573	gene	
Bn-A10-p10083397	Bn-N10-p11698546	gene	
Bn-A10-p10083483	Bn-N10-p11698632	gene	exon
Bn-A10-p10108321	Bn-N19-p47019905	gene	
Bn-A10-p1010963	Bn-N10-p2994980	gene	exon
Bn-A10-p10120142	Bn-N10-p11746996	gene	exon
Bn-A10-p10132309	Bn-N10-p11760675	gene	exon
Bn-A10-p10133034	Bn-N10-p11761400	gene	
Bn-A10-p10134254	Bn-N10-p11762640	gene	
Bn-A10-p10134322	Bn-N10-p11762708	gene	
Bn-A10-p10141249	Bn-N10-p11769661	gene	
Bn-A10-p10149557	Bn-N10-p11778321	gene	exon
Bn-A10-p10155781	Bn-N10-p11784531	gene	exon
Bn-A10-p10156893	Bn-N10-p11785618	gene	
Bn-A10-p10186330	Bn-N10-p11818254	gene	
Bn-A10-p10244709	Bn-N10-p11868078	gene	
Bn-A10-p10245283	Bn-N11-p43901894	gene	
Bn-A10-p10251191	Bn-N10-p11873821	gene	
Bn-A10-p10252741	Bn-N10-p11875373	gene	
Bn-A10-p10298013	Bn-N10-p11912249	gene	exon
Bn-A10-p10361031	Bn-N10-p11964992	gene	exon
Bn-A10-p10374644	Bn-N10-p11985610	gene	exon
Bn-A10-p10387954	Bn-N10-p11999583	gene	
Bn-A10-p10392191	Bn-N10-p12003829	gene	
Bn-A10-p10395508	Bn-N19-p47520526	gene	exon
Bn-A10-p10395990	Bn-N10-p12009044	gene	exon
Bn-A10-p10402358	Bn-Scaffold02259-p694	gene	exon
Bn-A10-p10423500	Bn-N10-p12039050	gene	
Bn-A10-p10429303	Bn-N10-p12045392	gene	
Bn-A10-p10454385	Bn-N10-p12068595	gene	
Bn-A10-p10460400	Bn-N10-p12074615	gene	exon
Bn-A10-p10469649	Bn-N10-p12083813	gene	exon
Bn-A10-p10485888	Bn-N19-p47652419	gene	
Bn-A10-p10509825	Bn-N10-p12122863	gene	exon
Bn-A10-p10516377	Bn-N10-p12127339	gene	exon
Bn-A10-p10540729	Bn-N10-p12150832	gene	
Bn-A10-p10555057	Bn-N10-p12164282	gene	exon
Bn-A10-p10575106	Bn-N10-p12184538	gene	
Bn-A10-p10576878	Bn-N10-p12186310	gene	
Bn-A10-p10613361	Bn-Scaffold01263-p707	gene	exon

Bn-A10-p10651432	Bn-N10-p12247935	gene	
Bn-A10-p10651872	Bn-N10-p12248375	gene	exon
Bn-A10-p10652964	Bn-N10-p12249467	gene	exon
Bn-A10-p10654061	Bn-N10-p12250552	gene	
Bn-A10-p10660780	Bn-N10-p12255516	gene	exon
Bn-A10-p10668111	Bn-N10-p12265600	gene	
Bn-A10-p10669303	Bn-N10-p12266804	gene	
Bn-A10-p10677237	Bn-N10-p12274787	gene	exon
Bn-A10-p10691761	Bn-N10-p12290489	gene	
Bn-A10-p10714593	Bn-N10-p12315331	gene	
Bn-A10-p10717595	Bn-N10-p12318665	gene	
Bn-A10-p10725779	Bn-N10-p12326872	gene	
Bn-A10-p10726590	Bn-N10-p12327683	gene	
Bn-A10-p10728078	Bn-N10-p12328932	gene	exon
Bn-A10-p10748653	Bn-N10-p12344167	gene	
Bn-A10-p10751752	Bn-N10-p12347251	gene	exon
Bn-A10-p10755216	Bn-N10-p12352772	gene	
Bn-A10-p10764928	Bn-N10-p12365473	gene	exon
Bn-A10-p10777142	Bn-N10-p12385408	gene	
Bn-A10-p10830979	Bn-N10-p12432749	gene	
Bn-A10-p10840792	Bn-N10-p12439657	gene	exon
Bn-A10-p10882669	Bn-N10-p12473971	gene	exon
Bn-A10-p10902810	Bn-N10-p12500338	gene	exon
Bn-A10-p10936233	Bn-N10-p12537487	gene	exon
Bn-A10-p10936559	Bn-N10-p12537825	gene	
Bn-A10-p10949218	Bn-N10-p12557367	gene	exon
Bn-A10-p10954176	Bn-N10-p12563640	gene	exon
Bn-A10-p10954641	Bn-N10-p12564099	gene	exon
Bn-A10-p10982591	Bn-N10-p12613401	gene	
Bn-A10-p10997884	Bn-N10-p12627689	gene	exon
Bn-A10-p11038926	Bn-N10-p12638872	gene	exon
Bn-A10-p11043549	Bn-N19-p48776688	gene	exon
Bn-A10-p11043924	Bn-N10-p12643027	gene	exon
Bn-A10-p11048886	Bn-N10-p12648161	gene	exon
Bn-A10-p11052814	Bn-N19-p48788308	gene	
Bn-A10-p11052877	Bn-N10-p12653364	gene	
Bn-A10-p11064857	Bn-N10-p12663925	gene	exon
Bn-A10-p11075240	Bn-N10-p12675560	gene	exon
Bn-A10-p11082862	Bn-N10-p12689085	gene	
Bn-A10-p1108975	Bn-N10-p2886382	gene	exon
Bn-A10-p11138874	Bn-N10-p12755352	gene	exon
Bn-A10-p11169419	Bn-N10-p12766108	gene	exon
Bn-A10-p11183840	Bn-N10-p12783717	gene	exon
Bn-A10-p11192168	Bn-N10-p12791907	gene	exon
Bn-A10-p11201481	Bn-N10-p12800841	gene	
Bn-A10-p11221861	Bn-N19-p49104875	gene	exon
Bn-A10-p1123189	Bn-N10-p2868701	gene	
Bn-A10-p11238287	Bn-N19-p49137317	gene	
Bn-A10-p1125100	Bn-N10-p2866782	gene	exon
Bn-A10-p1125156	Bn-N10-p2866726	gene	
Bn-A10-p11268601	Bn-N10-p12875513	gene	
Bn-A10-p11299415	Bn-N10-p12912983	gene	exon

Bn-A10-p11302032	Bn-N10-p12917622	gene	exon
Bn-A10-p11312705	Bn-N10-p12928294	gene	exon
Bn-A10-p1131424	Bn-N10-p2860391	gene	
Bn-A10-p11321778	Bn-N10-p12939316	gene	
Bn-A10-p11330183	Bn-N10-p12943850	gene	
Bn-A10-p11332256	Bn-N10-p12945925	gene	exon
Bn-A10-p11358033	Bn-N10-p12973917	gene	exon
Bn-A10-p11369880	Bn-N10-p12987970	gene	exon
Bn-A10-p11394407	Bn-N10-p13022743	gene	exon
Bn-A10-p113985	Bn-N10-p4482689	gene	
Bn-A10-p11411280	Bn-N10-p13042901	gene	
Bn-A10-p11420261	Bn-N10-p13050450	gene	exon
Bn-A10-p11448249	Bn-N10-p13072679	gene	
Bn-A10-p114515	Bn-N10-p4482159	gene	
Bn-A10-p11464311	Bn-N7-p2032449	gene	
Bn-A10-p11466367	Bn-N7-p2034506	gene	
Bn-A10-p11466727	Bn-N7-p2034866	gene	
Bn-A10-p1149411	Bn-N10-p2837553	gene	
Bn-A10-p11527601	Bn-N7-p2093868	gene	exon
Bn-A10-p11541544	Bn-N7-p2111403	gene	
Bn-A10-p11601681	Bn-N7-p2180386	gene	exon
Bn-A10-p11637678	Bn-N7-p2213671	gene	exon
Bn-A10-p11637847	Bn-N7-p2213840	gene	
Bn-A10-p11639214	Bn-N7-p2215189	gene	exon
Bn-A10-p11649398	Bn-N7-p2222212	gene	exon
Bn-A10-p11723249	Bn-N7-p2294109	gene	exon
Bn-A10-p11723882	Bn-N7-p2294742	gene	exon
Bn-A10-p11744746	Bn-N7-p2306631	gene	
Bn-A10-p11745226	Bn-N7-p2307111	gene	exon
Bn-A10-p11814912	Bn-N7-p2337784	gene	
Bn-A10-p11826776	Bn-N7-p2357158	gene	
Bn-A10-p11871893	Bn-N7-p2388946	gene	
Bn-A10-p11874688	Bn-N7-p2391746	gene	exon
Bn-A10-p1187541	Bn-N10-p2795757	gene	
Bn-A10-p11888837	Bn-N7-p2403156	gene	exon
Bn-A10-p1193336	Bn-N10-p2790114	gene	
Bn-A10-p1196851	Bn-N10-p2786588	gene	
Bn-A10-p12000106	Bn-N7-p2482675	gene	exon
Bn-A10-p1215039	Bn-N10-p2759191	gene	
Bn-A10-p1215054	Bn-N10-p2759176	gene	
Bn-A10-p12198293	Bn-N7-p16086234	gene	
Bn-A10-p12201411	Bn-N6-p15273363	gene	
Bn-A10-p12214779	Bn-N7-p2722848	gene	exon
Bn-A10-p12231523	Bn-N7-p2737927	gene	
Bn-A10-p12297280	Bn-N7-p2793248	gene	
Bn-A10-p12307115	Bn-N7-p2809465	gene	exon
Bn-A10-p12316390	Bn-N7-p2821655	gene	
Bn-A10-p12316420	Bn-N7-p2821685	gene	
Bn-A10-p12316487	Bn-N7-p2821752	gene	
Bn-A10-p12353464	Bn-N7-p2854426	gene	exon
Bn-A10-p12439754	Bn-Scaffold15041-p533	gene	
Bn-A10-p12463322	Bn-N7-p2970827	gene	exon

Bn-A10-p12485323	Bn-N7-p2992326	gene	
Bn-A10-p12501815	Bn-N7-p3009536	gene	
Bn-A10-p12597952	Bn-N7-p3080446	gene	
Bn-A10-p12611994	Bn-N7-p3093734	gene	exon
Bn-A10-p126180	Bn-N19-p32582381	gene	
Bn-A10-p12627247	Bn-N7-p3114500	gene	
Bn-A10-p12632289	Bn-N7-p3119690	gene	exon
Bn-A10-p12633352	Bn-N7-p3120753	gene	exon
Bn-A10-p1264986	Bn-N15-p2523137	gene	
Bn-A10-p1268549	Bn-N10-p2691862	gene	exon
Bn-A10-p1269116	Bn-N15-p2518877	gene	exon
Bn-A10-p12727662	Bn-N7-p3222825	gene	exon
Bn-A10-p1273491	Bn-N10-p2686920	gene	
Bn-A10-p12828161	Bn-N10-p13226976	gene	exon
Bn-A10-p12842513	Bn-N10-p13241309	gene	
Bn-A10-p12885978	Bn-N10-p13286395	gene	
Bn-A10-p12900258	Bn-N10-p13307673	gene	exon
Bn-A10-p12901822	Bn-N19-p49954038	gene	
Bn-A10-p12928142	Bn-N10-p13336279	gene	exon
Bn-A10-p12933288	Bn-N10-p13341433	gene	exon
Bn-A10-p12938355	Bn-N10-p13346427	gene	exon
Bn-A10-p12968361	Bn-N10-p13364991	gene	exon
Bn-A10-p12984425	Bn-N10-p13374408	gene	exon
Bn-A10-p12990499	Bn-N10-p13380117	gene	
Bn-A10-p12999930	Bn-N10-p13392000	gene	exon
Bn-A10-p13008713	Bn-N19-p50056272	gene	exon
Bn-A10-p13009957	Bn-N10-p13404021	gene	
Bn-A10-p13019909	Bn-N10-p13415737	gene	exon
Bn-A10-p13028260	Bn-N10-p13424174	gene	
Bn-A10-p1303093	Bn-N10-p2679239	gene	exon
Bn-A10-p13038966	Bn-N10-p13436855	gene	
Bn-A10-p13049564	Bn-N10-p13447390	gene	
Bn-A10-p13094022	Bn-N10-p13496580	gene	
Bn-A10-p13130002	Bn-N10-p13530217	gene	exon
Bn-A10-p1313220	Bn-N10-p2672199	gene	
Bn-A10-p13137531	Bn-N10-p13558959	gene	exon
Bn-A10-p13137658	Bn-N10-p13559086	gene	
Bn-A10-p13138159	Bn-N10-p13559594	gene	
Bn-A10-p13138769	Bn-N10-p13560204	gene	exon
Bn-A10-p13139635	Bn-N10-p13561070	gene	
Bn-A10-p13142215	Bn-N10-p13563545	gene	
Bn-A10-p13142579	Bn-N10-p13563909	gene	exon
Bn-A10-p13162021	Bn-N10-p13584582	gene	exon
Bn-A10-p13164379	Bn-N10-p13586951	gene	
Bn-A10-p13164536	Bn-N10-p13587108	gene	
Bn-A10-p13165001	Bn-N10-p13587574	gene	
Bn-A10-p13165101	Bn-N10-p13587674	gene	
Bn-A10-p13169213	Bn-N10-p13591796	gene	
Bn-A10-p1318385	Bn-N10-p2667034	gene	
Bn-A10-p13195966	Bn-N10-p13628985	gene	exon
Bn-A10-p13205889	Bn-N10-p13634244	gene	
Bn-A10-p13207985	Bn-N10-p13636114	gene	

Bn-A10-p13230002	Bn-N10-p13654405	gene	
Bn-A10-p13245203	Bn-N10-p13669486	gene	
Bn-A10-p13263227	Bn-N10-p13696841	gene	exon
Bn-A10-p13267698	Bn-N19-p50510017	gene	
Bn-A10-p13283073	Bn-N10-p13724909	gene	exon
Bn-A10-p13289368	Bn-N10-p13729447	gene	exon
Bn-A10-p13299250	Bn-N10-p13739986	gene	exon
Bn-A10-p13327024	Bn-N10-p13775604	gene	exon
Bn-A10-p13330418	Bn-N10-p13778955	gene	exon
Bn-A10-p13358856	Bn-N10-p13806544	gene	exon
Bn-A10-p13364232	Bn-N10-p13811605	gene	
Bn-A10-p13375342	Bn-N10-p13820657	gene	
Bn-A10-p13384066	Bn-N10-p13827222	gene	exon
Bn-A10-p13387522	Bn-N10-p13836637	gene	
Bn-A10-p13390065	Bn-N10-p13837385	gene	
Bn-A10-p13401328	Bn-N10-p13850989	gene	exon
Bn-A10-p13410287	Bn-N10-p13860320	gene	exon
Bn-A10-p13410414	Bn-N10-p13860440	gene	exon
Bn-A10-p13423663	Bn-N10-p13875193	gene	exon
Bn-A10-p13435288	Bn-N19-p50796641	gene	exon
Bn-A10-p1345255	Bn-N10-p2631797	gene	exon
Bn-A10-p13455391	Bn-N10-p13907941	gene	
Bn-A10-p13471114	Bn-N10-p13924651	gene	
Bn-A10-p13473460	Bn-N10-p13926768	gene	
Bn-A10-p13473595	Bn-N10-p13926902	gene	
Bn-A10-p13495547	Bn-N10-p13938984	gene	exon
Bn-A10-p13508529	Bn-N10-p13951994	gene	exon
Bn-A10-p13508970	Bn-N10-p13952435	gene	
Bn-A10-p13509116	Bn-N10-p13952581	gene	exon
Bn-A10-p135462	Bn-N10-p4452891	gene	
Bn-A10-p1354965	Bn-N15-p2451181	gene	exon
Bn-A10-p13577876	Bn-N10-p14028553	gene	
Bn-A10-p13590061	Bn-N10-p14041793	gene	
Bn-A10-p13590367	Bn-N10-p14042099	gene	exon
Bn-A10-p1359047	Bn-N15-p2448948	gene	
Bn-A10-p13606837	Bn-N10-p14062201	gene	exon
Bn-A10-p13608040	Bn-N10-p14063421	gene	
Bn-A10-p13618703	Bn-N10-p14079656	gene	
Bn-A10-p13638222	Bn-N10-p14099908	gene	exon
Bn-A10-p13638489	Bn-N10-p14100175	gene	exon
Bn-A10-p13640346	Bn-N10-p14102032	gene	exon
Bn-A10-p13659996	Bn-N10-p14117563	gene	
Bn-A10-p13669875	Bn-N10-p14128485	gene	
Bn-A10-p13680808	Bn-N10-p14144802	gene	
Bn-A10-p13685654	Bn-N10-p14149654	gene	exon
Bn-A10-p13701342	Bn-N10-p14162042	gene	
Bn-A10-p1370599	Bn-N10-p2610745	gene	
Bn-A10-p13710321	Bn-N10-p14175760	gene	
Bn-A10-p13723140	Bn-N10-p14189516	gene	
Bn-A10-p13734245	Bn-N10-p14199560	gene	
Bn-A10-p13739326	Bn-N10-p14204498	gene	
Bn-A10-p13747995	Bn-N10-p14213173	gene	exon

Bn-A10-p13778875	Bn-N10-p14243624	gene	exon
Bn-A10-p13779014	Bn-N10-p14243763	gene	exon
Bn-A10-p13786294	Bn-N10-p14251001	gene	
Bn-A10-p13786487	Bn-N19-p51548397	gene	exon
Bn-A10-p13787477	Bn-N10-p14252185	gene	
Bn-A10-p13788629	Bn-N10-p14253316	gene	
Bn-A10-p13789579	Bn-N10-p14254267	gene	
Bn-A10-p13796417	Bn-N10-p14260941	gene	
Bn-A10-p13796426	Bn-N10-p14260950	gene	
Bn-A10-p13798602	Bn-N10-p14263300	gene	exon
Bn-A10-p13815263	Bn-N10-p14280519	gene	exon
Bn-A10-p13827678	Bn-N10-p14293030	gene	
Bn-A10-p13841213	Bn-N10-p14311399	gene	exon
Bn-A10-p13845694	Bn-N10-p14315829	gene	exon
Bn-A10-p13846108	Bn-N10-p14316243	gene	exon
Bn-A10-p13849675	Bn-N10-p14319805	gene	exon
Bn-A10-p13875041	Bn-N10-p14342649	gene	
Bn-A10-p13882091	Bn-N10-p14348550	gene	exon
Bn-A10-p13882718	Bn-N10-p14349168	gene	
Bn-A10-p13932541	Bn-N10-p14390423	gene	exon
Bn-A10-p13938504	Bn-N10-p14396084	gene	exon
Bn-A10-p13941742	Bn-N10-p14399277	gene	exon
Bn-A10-p13965313	Bn-N10-p14423705	gene	exon
Bn-A10-p13973182	Bn-N10-p14432266	gene	
Bn-A10-p13973954	Bn-N10-p14433038	gene	
Bn-A10-p13985926	Bn-N10-p14445060	gene	
Bn-A10-p13995725	Bn-N10-p14454462	gene	
Bn-A10-p13997359	Bn-N10-p14456091	gene	exon
Bn-A10-p14012013	Bn-N10-p14461495	gene	exon
Bn-A10-p14042374	Bn-N10-p14488682	gene	
Bn-A10-p14049684	Bn-N10-p14497144	gene	exon
Bn-A10-p14058257	Bn-N10-p14505713	gene	exon
Bn-A10-p1406327	Bn-N15-p2409529	gene	
Bn-A10-p14064793	Bn-N19-p51994809	gene	exon
Bn-A10-p14075685	Bn-N10-p14522217	gene	
Bn-A10-p14092948	Bn-N10-p14543149	gene	
Bn-A10-p14097282	Bn-N10-p14549323	gene	exon
Bn-A10-p14142060	Bn-N10-p14593603	gene	exon
Bn-A10-p14143198	Bn-N19-p52096410	gene	exon
Bn-A10-p14143770	Bn-N10-p14595313	gene	exon
Bn-A10-p14150442	Bn-N10-p14601992	gene	exon
Bn-A10-p14160561	Bn-N10-p14612957	gene	exon
Bn-A10-p1416938	Bn-N10-p2567262	gene	exon
Bn-A10-p14198466	Bn-N10-p14651413	gene	
Bn-A10-p14254487	Bn-N10-p14715060	gene	exon
Bn-A10-p14297432	Bn-N10-p14753438	gene	exon
Bn-A10-p14297749	Bn-N10-p14753755	gene	exon
Bn-A10-p14299800	Bn-N10-p14755806	gene	
Bn-A10-p14300320	Bn-N10-p14756326	gene	
Bn-A10-p14307417	Bn-N10-p14765541	gene	
Bn-A10-p14313275	Bn-N10-p14771350	gene	exon
Bn-A10-p14327285	Bn-N10-p14784956	gene	

Bn-A10-p14334390	Bn-N10-p14792079	gene	
Bn-A10-p14353770	Bn-N10-p14810503	gene	exon
Bn-A10-p14354612	Bn-N10-p14811340	gene	exon
Bn-A10-p14365581	Bn-N10-p14823751	gene	
Bn-A10-p14373433	Bn-N10-p14831501	gene	
Bn-A10-p14384277	Bn-N10-p14842393	gene	exon
Bn-A10-p14385282	Bn-N10-p14843399	gene	
Bn-A10-p14394392	Bn-N10-p14852555	gene	exon
Bn-A10-p14408281	Bn-N10-p14866420	gene	
Bn-A10-p14431375	Bn-N10-p14890034	gene	
Bn-A10-p14464434	Bn-N10-p14908626	gene	
Bn-A10-p14475546	Bn-N10-p14923063	gene	exon
Bn-A10-p14488358	Bn-N10-p14935901	gene	exon
Bn-A10-p14490038	Bn-N10-p14937581	gene	
Bn-A10-p14528560	Bn-N10-p14983813	gene	exon
Bn-A10-p14545046	Bn-N10-p14998321	gene	
Bn-A10-p14547767	Bn-N10-p15001063	gene	exon
Bn-A10-p14554456	Bn-N10-p15007769	gene	exon
Bn-A10-p14574100	Bn-N10-p15026776	gene	exon
Bn-A10-p14618311	Bn-N10-p15060399	gene	
Bn-A10-p14620951	Bn-N10-p15062988	gene	
Bn-A10-p14636670	Bn-N10-p15079362	gene	exon
Bn-A10-p14646222	Bn-N10-p15090804	gene	exon
Bn-A10-p14653123	Bn-N10-p15097630	gene	
Bn-A10-p14658742	Bn-N10-p15103352	gene	exon
Bn-A10-p14664967	Bn-N10-p15109709	gene	exon
Bn-A10-p14665064	Bn-N10-p15109806	gene	exon
Bn-A10-p14681027	Bn-N10-p15124145	gene	exon
Bn-A10-p14687193	Bn-N10-p15130797	gene	exon
Bn-A10-p14687699	Bn-N10-p15131303	gene	exon
Bn-A10-p14693723	Bn-N10-p15137303	gene	exon
Bn-A10-p14694580	Bn-N10-p15138161	gene	exon
Bn-A10-p14728923	Bn-N10-p15186752	gene	exon
Bn-A10-p14734107	Bn-N10-p15193088	gene	exon
Bn-A10-p1474859	Bn-N7-p1893662	gene	exon
Bn-A10-p14770174	Bn-N10-p15226589	gene	exon
Bn-A10-p14772012	Bn-N10-p15228428	gene	
Bn-A10-p14809502	Bn-N10-p15275509	gene	exon
Bn-A10-p14814896	Bn-N10-p15280900	gene	exon
Bn-A10-p14820463	Bn-N10-p15286509	gene	
Bn-A10-p14821861	Bn-N10-p15287902	gene	exon
Bn-A10-p14823906	Bn-N10-p15289945	gene	
Bn-A10-p14834518	Bn-N10-p15307795	gene	exon
Bn-A10-p14835887	Bn-N10-p15309164	gene	exon
Bn-A10-p14844217	Bn-N10-p15322850	gene	exon
Bn-A10-p1486296	Bn-N10-p2483504	gene	exon
Bn-A10-p14883536	Bn-N10-p15359194	gene	
Bn-A10-p14914898	Bn-N10-p15401933	gene	
Bn-A10-p14918537	Bn-N10-p15404656	gene	
Bn-A10-p14928362	Bn-N19-p53371026	gene	exon
Bn-A10-p14950115	Bn-N10-p15438914	gene	exon
Bn-A10-p14958522	Bn-N10-p15446707	gene	

Bn-A10-p14962307	Bn-N10-p15449313	gene	
Bn-A10-p14971063	Bn-N10-p15457676	gene	
Bn-A10-p14994086	Bn-N10-p15482403	gene	exon
Bn-A10-p14994341	Bn-N10-p15482658	gene	
Bn-A10-p15020436	Bn-N10-p15511387	gene	exon
Bn-A10-p15021776	Bn-N10-p15512727	gene	exon
Bn-A10-p15031810	Bn-N10-p15522899	gene	exon
Bn-A10-p15069552	Bn-N19-p53661499	gene	exon
Bn-A10-p15083548	Bn-N10-p15577790	gene	exon
Bn-A10-p15086931	Bn-N10-p15581153	gene	
Bn-A10-p15106056	Bn-N10-p15599277	gene	
Bn-A10-p15108947	Bn-N10-p15602672	gene	
Bn-A10-p15117055	Bn-N10-p15610190	gene	exon
Bn-A10-p1513127	Bn-N10-p2450672	gene	exon
Bn-A10-p15156277	Bn-N10-p15639341	gene	exon
Bn-A10-p15160966	Bn-N10-p15644031	gene	exon
Bn-A10-p15227741	Bn-N10-p16983649	gene	exon
Bn-A10-p15237864	Bn-N10-p16973367	gene	exon
Bn-A10-p15237975	Bn-N10-p16973256	gene	exon
Bn-A10-p15265102	Bn-N10-p16945504	gene	exon
Bn-A10-p15276369	Bn-N19-p55393882	gene	
Bn-A10-p15276444	Bn-N19-p55393806	gene	
Bn-A10-p15296322	Bn-N10-p16904775	gene	
Bn-A10-p15299272	Bn-N10-p16901840	gene	
Bn-A10-p15300203	Bn-N10-p16900889	gene	exon
Bn-A10-p15310323	Bn-N10-p16891673	gene	exon
Bn-A10-p15312311	Bn-N10-p16889689	gene	
Bn-A10-p15327377	Bn-N10-p16878297	gene	
Bn-A10-p15327814	Bn-N10-p16877860	gene	
Bn-A10-p15327850	Bn-N10-p16877824	gene	
Bn-A10-p15328039	Bn-N19-p55342118	gene	
Bn-A10-p15355521	Bn-N10-p16845539	gene	exon
Bn-A10-p15361519	Bn-N10-p16838219	gene	exon
Bn-A10-p15401999	Bn-N10-p16794775	gene	exon
Bn-A10-p15405149	Bn-N10-p16791314	gene	exon
Bn-A10-p15408188	Bn-N19-p55220975	gene	
Bn-A10-p15412233	Bn-N10-p16786323	gene	
Bn-A10-p15433413	Bn-N19-p55179397	gene	exon
Bn-A10-p15442975	Bn-N10-p16749269	gene	
Bn-A10-p15444223	Bn-N10-p16748021	gene	
Bn-A10-p15458720	Bn-N10-p16732070	gene	
Bn-A10-p15485139	Bn-N10-p16703595	gene	
Bn-A10-p15488923	Bn-N10-p16700077	gene	
Bn-A10-p15496817	Bn-N10-p16692507	gene	
Bn-A10-p15507350	Bn-N10-p16681932	gene	exon
Bn-A10-p15591443	Bn-N10-p16576269	gene	exon
Bn-A10-p15597364	Bn-N10-p16570303	gene	exon
Bn-A10-p15601027	Bn-N10-p16566578	gene	exon
Bn-A10-p15606935	Bn-N10-p16560438	gene	exon
Bn-A10-p15607468	Bn-N10-p16559905	gene	
Bn-A10-p15633321	Bn-N10-p16533851	gene	exon
Bn-A10-p15637848	Bn-N10-p16529373	gene	exon

Bn-A10-p15651383	Bn-N10-p16515942	gene	
Bn-A10-p15657263	Bn-N10-p16510169	gene	
Bn-A10-p15660472	Bn-N19-p54843341	gene	exon
Bn-A10-p15668415	Bn-N10-p16498167	gene	
Bn-A10-p15672303	Bn-N10-p16495323	gene	
Bn-A10-p15711469	Bn-N10-p16457656	gene	exon
Bn-A10-p15719803	Bn-N19-p54792481	gene	exon
Bn-A10-p15729205	Bn-N10-p16437711	gene	exon
Bn-A10-p15737116	Bn-N10-p16429319	gene	exon
Bn-A10-p15737423	Bn-N10-p16429012	gene	exon
Bn-A10-p15738808	Bn-N10-p16427579	gene	
Bn-A10-p15742689	Bn-N10-p16423703	gene	exon
Bn-A10-p15746238	Bn-N10-p16420172	gene	
Bn-A10-p15760933	Bn-N10-p16402700	gene	
Bn-A10-p15786012	Bn-N10-p16373748	gene	exon
Bn-A10-p15817344	Bn-N10-p16340492	gene	exon
Bn-A10-p15824883	Bn-N19-p54662336	gene	exon
Bn-A10-p15838863	Bn-N10-p16318461	gene	
Bn-A10-p15838932	Bn-N10-p16318392	gene	
Bn-A10-p15863143	Bn-N10-p16296245	gene	
Bn-A10-p15863972	Bn-N10-p16295415	gene	
Bn-A10-p15864056	Bn-N10-p16295331	gene	
Bn-A10-p15868115	Bn-N10-p16291242	gene	exon
Bn-A10-p15868364	Bn-N19-p54618907	gene	exon
Bn-A10-p15884544	Bn-N10-p16282653	gene	
Bn-A10-p15885356	Bn-N10-p16281841	gene	exon
Bn-A10-p15894309	Bn-N10-p16271426	gene	
Bn-A10-p15894501	Bn-N10-p16271234	gene	
Bn-A10-p15908393	Bn-N10-p16258531	gene	
Bn-A10-p15924608	Bn-N10-p16242092	gene	exon
Bn-A10-p15924755	Bn-N10-p16241945	gene	
Bn-A10-p15925135	Bn-N10-p16241644	gene	exon
Bn-A10-p1595235	Bn-N10-p2354972	gene	
Bn-A10-p15955591	Bn-N10-p16204253	gene	
Bn-A10-p15971312	Bn-N10-p16187749	gene	exon
Bn-A10-p15997363	Bn-N10-p16166145	gene	
Bn-A10-p15997433	Bn-N10-p16166075	gene	
Bn-A10-p15997448	Bn-N10-p16166060	gene	
Bn-A10-p16005880	Bn-N10-p16158767	gene	
Bn-A10-p16017024	Bn-N10-p16147283	gene	exon
Bn-A10-p16029222	Bn-N10-p16135174	gene	
Bn-A10-p16030162	Bn-N10-p16134232	gene	exon
Bn-A10-p16032125	Bn-N10-p16132296	gene	
Bn-A10-p16036488	Bn-N10-p16126711	gene	
Bn-A10-p16037268	Bn-N10-p16125930	gene	
Bn-A10-p1606279	Bn-N10-p2343996	gene	
Bn-A10-p16067573	Bn-N10-p16090488	gene	exon
Bn-A10-p16071525	Bn-N10-p16086536	gene	
Bn-A10-p16074706	Bn-N10-p16083357	gene	
Bn-A10-p16109898	Bn-N10-p16046319	gene	
Bn-A10-p1611838	Bn-N10-p2336662	gene	exon
Bn-A10-p1611973	Bn-N10-p2336527	gene	

Bn-A10-p16142232	Bn-N10-p16013752	gene	exon
Bn-A10-p16152327	Bn-N10-p16003684	gene	exon
Bn-A10-p16158247	Bn-N10-p16000605	gene	exon
Bn-A10-p16158770	Bn-N10-p16000082	gene	exon
Bn-A10-p16162575	Bn-N10-p15996235	gene	
Bn-A10-p16164252	Bn-N10-p15994574	gene	
Bn-A10-p16184233	Bn-N10-p15975946	gene	
Bn-A10-p16185535	Bn-N10-p15974619	gene	
Bn-A10-p16187017	Bn-N10-p15973136	gene	
Bn-A10-p16187357	Bn-N10-p15972796	gene	
Bn-A10-p16190708	Bn-N10-p15969233	gene	exon
Bn-A10-p16207908	Bn-N10-p15957847	gene	exon
Bn-A10-p16210702	Bn-N10-p15955037	gene	exon
Bn-A10-p16213045	Bn-N10-p15952698	gene	
Bn-A10-p16224024	Bn-N10-p15939293	gene	
Bn-A10-p16240958	Bn-N10-p15927241	gene	
Bn-A10-p16249484	Bn-N10-p15918848	gene	
Bn-A10-p16285282	Bn-N10-p15878279	gene	exon
Bn-A10-p16287574	Bn-N10-p15876004	gene	
Bn-A10-p16296069	Bn-N10-p15867500	gene	exon
Bn-A10-p16312405	Bn-N10-p15851140	gene	
Bn-A10-p16317798	Bn-N10-p15847683	gene	exon
Bn-A10-p16344917	Bn-N10-p15825188	gene	
Bn-A10-p16354622	Bn-N10-p15813657	gene	exon
Bn-A10-p16357479	Bn-N10-p15811331	gene	exon
Bn-A10-p16360911	Bn-N10-p15807897	gene	exon
Bn-A10-p16363196	Bn-N19-p53976276	gene	exon
Bn-A10-p16396586	Bn-N10-p15768881	gene	exon
Bn-A10-p16439150	Bn-N10-p15720562	gene	exon
Bn-A10-p16442531	Bn-N10-p15717099	gene	exon
Bn-A10-p16451834	Bn-N10-p15707679	gene	exon
Bn-A10-p16516985	Bn-Scaffold09297-p138	gene	exon
Bn-A10-p16522658	Bn-N10-p17075724	gene	
Bn-A10-p16595840	Bn-N10-p17146693	gene	
Bn-A10-p16626615	Bn-N10-p17179621	gene	
Bn-A10-p16640315	Bn-Scaffold02219-p1504	gene	exon
Bn-A10-p16672063	Bn-N10-p17216045	gene	
Bn-A10-p16673324	Bn-N2-p771574	gene	
Bn-A10-p16673808	Bn-N10-p17217790	gene	
Bn-A10-p16674476	Bn-N10-p17218458	gene	
Bn-A10-p16694218	Bn-N10-p17237348	gene	
Bn-A10-p16694564	Bn-N10-p17237695	gene	
Bn-A10-p16695705	Bn-N10-p17238838	gene	exon
Bn-A10-p16696977	Bn-N10-p17240110	gene	exon
Bn-A10-p16706289	Bn-N10-p17257027	gene	
Bn-A10-p16712853	Bn-N10-p17263462	gene	
Bn-A10-p16718558	Bn-N10-p17268771	gene	
Bn-A10-p16737974	Bn-N10-p17296870	gene	exon
Bn-A10-p16752445	Bn-Scaffold02476-p1343	gene	
Bn-A10-p16754116	Bn-Scaffold02476-p3014	gene	
Bn-A10-p16816027	Bn-N10-p17374907	gene	
Bn-A10-p16821888	Bn-N10-p17382668	gene	

Bn-A10-p16824950	Bn-N10-p17385723	gene	
Bn-A10-p16836688	Bn-N10-p17398551	gene	exon
Bn-A10-p16837056	Bn-N10-p17398918	gene	exon
Bn-A10-p16871085	Bn-N10-p17431885	gene	exon
Bn-A10-p16924111	Bn-N10-p17489488	gene	exon
Bn-A10-p16932737	Bn-N10-p17498118	gene	exon
Bn-A10-p16935603	Bn-N12-p352125	gene	
Bn-A10-p1698746	Bn-N10-p2222171	gene	
Bn-A10-p17146557	Bn-N10-p17715579	gene	
Bn-A10-p17160136	Bn-N10-p17729715	gene	exon
Bn-A10-p17169081	Bn-N10-p17739220	gene	
Bn-A10-p17262954	Bn-N10-p17829335	gene	
Bn-A10-p17302908	Bn-N10-p17867565	gene	
Bn-A10-p17367157	Bn-N19-p55680548	gene	
Bn-A10-p17434951	Bn-N19-p55778823	gene	
Bn-A10-p1746455	Bn-N10-p2160505	gene	
Bn-A10-p17470876	Bn-N19-p55832045	gene	exon
Bn-A10-p17500897	Bn-N19-p55866113	gene	exon
Bn-A10-p17541781	Bn-N19-p55895218	gene	
Bn-A10-p17549759	Bn-N19-p55902977	gene	exon
Bn-A10-p17565344	Bn-N19-p55954117	gene	
Bn-A10-p17565347	Bn-N19-p55954120	gene	
Bn-A10-p17566833	Bn-N19-p55962124	gene	
Bn-A10-p17568393	Bn-N19-p55967560	gene	exon
Bn-A10-p17575474	Bn-N19-p55973742	gene	exon
Bn-A10-p1782825	Bn-N10-p2132362	gene	
Bn-A10-p1787348	Bn-N15-p2061201	gene	
Bn-A10-p1791974	Bn-N10-p2123204	gene	exon
Bn-A10-p180934	Bn-N10-p4362516	gene	
Bn-A10-p182060	Bn-N10-p4361353	gene	
Bn-A10-p2034005	Bn-N10-p1844715	gene	exon
Bn-A10-p2034006	Bn-N10-p1844714	gene	exon
Bn-A10-p2035810	Bn-N15-p1843710	gene	
Bn-A10-p2048923	Bn-N10-p1827080	gene	
Bn-A10-p2056053	Bn-N10-p1819797	gene	
Bn-A10-p2059382	Bn-N10-p1816479	gene	
Bn-A10-p2074000	Bn-N10-p1798135	gene	
Bn-A10-p2084182	Bn-N10-p1789509	gene	
Bn-A10-p2096179	Bn-N10-p1783003	gene	
Bn-A10-p2096482	Bn-N10-p1782700	gene	
Bn-A10-p2104477	Bn-N10-p1773677	gene	
Bn-A10-p2106781	Bn-N10-p1771367	gene	exon
Bn-A10-p2107649	Bn-N10-p1770499	gene	
Bn-A10-p2107673	Bn-N10-p1770475	gene	
Bn-A10-p2171166	Bn-N10-p1704397	gene	exon
Bn-A10-p2176252	Bn-N10-p1699311	gene	exon
Bn-A10-p2196623	Bn-N10-p1674904	gene	
Bn-A10-p2240635	Bn-N10-p1629949	gene	
Bn-A10-p2247699	Bn-N10-p1625365	gene	
Bn-A10-p2260170	Bn-N15-p1632525	gene	exon
Bn-A10-p2266029	Bn-N10-p1610466	gene	
Bn-A10-p2268467	Bn-N10-p1608071	gene	

Bn-A10-p2268881	Bn-N10-p1607657	gene	
Bn-A10-p2279962	Bn-N10-p1603334	gene	exon
Bn-A10-p2292633	Bn-N10-p1597356	gene	exon
Bn-A10-p2294849	Bn-N10-p1594492	gene	
Bn-A10-p2295027	Bn-N10-p1594314	gene	
Bn-A10-p2309307	Bn-N10-p1580565	gene	exon
Bn-A10-p2309584	Bn-N10-p1580288	gene	exon
Bn-A10-p2315491	Bn-N10-p5685437	gene	
Bn-A10-p2315612	Bn-N10-p5685558	gene	
Bn-A10-p2337558	Bn-N10-p1554391	gene	exon
Bn-A10-p2356781	Bn-N10-p1536384	gene	exon
Bn-A10-p2357401	Bn-N10-p1535766	gene	exon
Bn-A10-p2369024	Bn-N10-p1532307	gene	exon
Bn-A10-p2369622	Bn-N10-p1531709	gene	
Bn-A10-p2374648	Bn-N10-p1526705	gene	
Bn-A10-p2415609	Bn-N10-p1497520	gene	exon
Bn-A10-p2416648	Bn-N10-p1496521	gene	exon
Bn-A10-p2425437	Bn-N15-p1517237	gene	
Bn-A10-p2449120	Bn-N10-p1467040	gene	
Bn-A10-p2500393	Bn-N10-p1420348	gene	
Bn-A10-p2500523	Bn-N10-p1419901	gene	
Bn-A10-p2523110	Bn-N10-p1398053	gene	
Bn-A10-p2523239	Bn-N10-p1397924	gene	
Bn-A10-p2529144	Bn-N10-p1388373	gene	
Bn-A10-p2535072	Bn-N10-p1379545	gene	
Bn-A10-p2580811	Bn-N10-p1337003	gene	exon
Bn-A10-p2589024	Bn-N10-p1328701	gene	exon
Bn-A10-p2687844	Bn-N10-p1233095	gene	
Bn-A10-p2710961	Bn-N10-p1213968	gene	
Bn-A10-p2728481	Bn-N10-p1200010	gene	
Bn-A10-p2747164	Bn-N10-p1181109	gene	
Bn-A10-p2764779	Bn-N10-p1168468	gene	exon
Bn-A10-p2805505	Bn-N10-p1125026	gene	exon
Bn-A10-p2890008	Bn-N7-p3299226	gene	exon
Bn-A10-p2899850	Bn-N7-p3305498	gene	exon
Bn-A10-p2937911	Bn-N7-p3325254	gene	exon
Bn-A10-p2939364	Bn-N7-p3326707	gene	
Bn-A10-p2941216	Bn-N7-p3328567	gene	
Bn-A10-p2997098	Bn-N7-p3371486	gene	exon
Bn-A10-p2997237	Bn-N7-p3371625	gene	
Bn-A10-p302099	Bn-N10-p4247550	gene	
Bn-A10-p3042880	Bn-N7-p3415813	gene	exon
Bn-A10-p306851	Bn-N10-p4242768	gene	
Bn-A10-p3235646	Bn-N7-p3620878	gene	
Bn-A10-p353725	Bn-N10-p4218844	gene	
Bn-A10-p3605582	Bn-N10-p4791358	gene	
Bn-A10-p3605726	Bn-N10-p4791502	gene	
Bn-A10-p3605865	Bn-N10-p4791641	gene	
Bn-A10-p3652309	Bn-N10-p4841394	gene	exon
Bn-A10-p3652425	Bn-N10-p4841510	gene	exon
Bn-A10-p3728041	Bn-N10-p4933619	gene	exon
Bn-A10-p3745973	Bn-N10-p4947658	gene	exon

Bn-A10-p3782582	Bn-N10-p1041846	gene	exon
Bn-A10-p3782643	Bn-N10-p1041785	gene	exon
Bn-A10-p378724	Bn-N10-p4195288	gene	
Bn-A10-p3809773	Bn-N10-p1015618	gene	exon
Bn-A10-p3845546	Bn-N10-p984144	gene	exon
Bn-A10-p3903845	Bn-N10-p932923	gene	
Bn-A10-p3905100	Bn-N10-p931657	gene	
Bn-A10-p3905295	Bn-N10-p931462	gene	
Bn-A10-p3909275	Bn-N10-p927565	gene	exon
Bn-A10-p3944803	Bn-Scaffold03150-p963	gene	exon
Bn-A10-p3955901	Bn-N10-p912738	gene	exon
Bn-A10-p3966740	Bn-N10-p898703	gene	
Bn-A10-p3978242	Bn-N10-p886752	gene	
Bn-A10-p3988046	Bn-N10-p866437	gene	exon
Bn-A10-p4038884	Bn-N10-p831013	gene	
Bn-A10-p4040298	Bn-N10-p829577	gene	exon
Bn-A10-p4076884	Bn-N10-p795050	gene	
Bn-A10-p4078158	Bn-N10-p793644	gene	
Bn-A10-p4079529	Bn-N10-p792273	gene	
Bn-A10-p4080011	Bn-N10-p791792	gene	
Bn-A10-p4088438	Bn-N10-p782670	gene	
Bn-A10-p4089670	Bn-N10-p781432	gene	
Bn-A10-p4112636	Bn-N10-p763440	gene	
Bn-A10-p4115062	Bn-N10-p761222	gene	
Bn-A10-p4120782	Bn-N10-p756176	gene	
Bn-A10-p4127274	Bn-N10-p749727	gene	exon
Bn-A10-p4134125	Bn-N15-p791259	gene	exon
Bn-A10-p4138782	Bn-N10-p738423	gene	
Bn-A10-p4138801	Bn-N10-p738404	gene	exon
Bn-A10-p4214836	Bn-N10-p647918	gene	exon
Bn-A10-p4311291	Bn-N10-p556861	gene	
Bn-A10-p4347862	Bn-N10-p518121	gene	exon
Bn-A10-p4425066	Bn-N10-p457441	gene	
Bn-A10-p4425312	Bn-N10-p457194	gene	
Bn-A10-p4460099	Bn-N10-p411194	gene	
Bn-A10-p4506059	Bn-N10-p379016	gene	exon
Bn-A10-p4506119	Bn-N10-p378956	gene	exon
Bn-A10-p4553957	Bn-N10-p341281	gene	
Bn-A10-p4558688	Bn-N10-p334970	gene	exon
Bn-A10-p4574423	Bn-N10-p322875	gene	exon
Bn-A10-p4575458	Bn-N10-p321834	gene	
Bn-A10-p4577455	Bn-N10-p319837	gene	exon
Bn-A10-p4605721	Bn-N10-p298208	gene	exon
Bn-A10-p4606815	Bn-N10-p297188	gene	
Bn-A10-p4624712	Bn-N10-p280829	gene	
Bn-A10-p4625350	Bn-N10-p280191	gene	
Bn-A10-p4639202	Bn-N10-p260628	gene	exon
Bn-A10-p4642637	Bn-N10-p257232	gene	
Bn-A10-p466849	Bn-N10-p4150990	gene	exon
Bn-A10-p4701756	Bn-N10-p195559	gene	exon
Bn-A10-p4725713	Bn-N10-p176298	gene	exon
Bn-A10-p4727374	Bn-N10-p174643	gene	

Bn-A10-p4731968	Bn-N10-p172505	gene	exon
Bn-A10-p4742940	Bn-N10-p165000	gene	exon
Bn-A10-p4755899	Bn-N10-p146549	gene	exon
Bn-A10-p4757850	Bn-N10-p144599	gene	
Bn-A10-p4786596	Bn-N10-p109572	gene	exon
Bn-A10-p4792793	Bn-N10-p100701	gene	exon
Bn-A10-p4802341	Bn-N10-p88239	gene	exon
Bn-A10-p4813171	Bn-N10-p64218	gene	
Bn-A10-p4840892	Bn-N10-p41211	gene	exon
Bn-A10-p4985950	Bn-N10-p5764268	gene	
Bn-A10-p4986275	Bn-N10-p5764593	gene	
Bn-A10-p510846	Bn-N10-p6506271	gene	
Bn-A10-p5128067	Bn-N19-p36422057	gene	exon
Bn-A10-p5250586	Bn-N10-p6068974	gene	
Bn-A10-p5299784	Bn-N10-p6114631	gene	exon
Bn-A10-p5317804	Bn-N10-p6132376	gene	
Bn-A10-p5635816	Bn-N10-p7187678	gene	
Bn-A10-p5730775	Bn-N10-p7287797	gene	exon
Bn-A10-p5799347	Bn-N10-p7364123	gene	
Bn-A10-p5819027	Bn-N10-p7382826	gene	
Bn-A10-p5869175	Bn-N10-p7430860	gene	
Bn-A10-p600021	Bn-N10-p3397365	gene	
Bn-A10-p6120400	Bn-N10-p7704661	gene	exon
Bn-A10-p6126590	Bn-N10-p7711290	gene	
Bn-A10-p6137434	Bn-N10-p7723073	gene	
Bn-A10-p6159693	Bn-N10-p7762914	gene	exon
Bn-A10-p6160352	Bn-N10-p7763571	gene	
Bn-A10-p6162838	Bn-N10-p7765879	gene	exon
Bn-A10-p6180693	Bn-N10-p7783581	gene	exon
Bn-A10-p6192971	Bn-N10-p7796722	gene	
Bn-A10-p6204788	Bn-N10-p7808581	gene	exon
Bn-A10-p6228003	Bn-N10-p7833539	gene	exon
Bn-A10-p6233556	Bn-N10-p7839095	gene	
Bn-A10-p6247519	Bn-N10-p7856544	gene	exon
Bn-A10-p6271467	Bn-N10-p7880985	gene	
Bn-A10-p6271554	Bn-N10-p7881072	gene	
Bn-A10-p6271776	Bn-N10-p7881288	gene	
Bn-A10-p6280436	Bn-N10-p7890668	gene	exon
Bn-A10-p6405472	Bn-N10-p8026286	gene	
Bn-A10-p6457220	Bn-N10-p8076350	gene	
Bn-A10-p6459258	Bn-N10-p8078561	gene	exon
Bn-A10-p6459782	Bn-N10-p8079085	gene	exon
Bn-A10-p6600373	Bn-N10-p8197048	gene	exon
Bn-A10-p6600844	Bn-N10-p8197519	gene	
Bn-A10-p6632002	Bn-N10-p8228294	gene	
Bn-A10-p6643953	Bn-N10-p8242779	gene	
Bn-A10-p6724918	Bn-N10-p8317394	gene	
Bn-A10-p6730588	Bn-N10-p8322916	gene	
Bn-A10-p6743896	Bn-N10-p8345249	gene	
Bn-A10-p6760255	Bn-N10-p8361662	gene	exon
Bn-A10-p6760664	Bn-N10-p8362071	gene	exon
Bn-A10-p6765523	Bn-N10-p8367620	gene	exon

Bn-A10-p6792798	Bn-N19-p39887885	gene	
Bn-A10-p6796120	Bn-N10-p8395757	gene	
Bn-A10-p6838885	Bn-N10-p8410308	gene	exon
Bn-A10-p6851091	Bn-N10-p8427566	gene	exon
Bn-A10-p6851375	Bn-N10-p8427848	gene	exon
Bn-A10-p6880184	Bn-N10-p8458434	gene	exon
Bn-A10-p6881287	Bn-N10-p8459537	gene	exon
Bn-A10-p6896063	Bn-N10-p8472459	gene	exon
Bn-A10-p6915231	Bn-N10-p8499914	gene	
Bn-A10-p6967288	Bn-N19-p40237283	gene	
Bn-A10-p7065016	Bn-N10-p8633594	gene	exon
Bn-A10-p7076810	Bn-N10-p8648033	gene	exon
Bn-A10-p7109137	Bn-N10-p8674036	gene	exon
Bn-A10-p718048	Bn-N10-p3290241	gene	exon
Bn-A10-p7186465	Bn-N19-p40656394	gene	exon
Bn-A10-p7247322	Bn-N10-p8810546	gene	exon
Bn-A10-p7250703	Bn-N10-p8818594	gene	exon
Bn-A10-p7252424	Bn-N10-p8820235	gene	exon
Bn-A10-p7283605	Bn-N10-p8846180	gene	exon
Bn-A10-p7309029	Bn-N10-p8869981	gene	exon
Bn-A10-p73255	Bn-N5-p12497761	gene	
Bn-A10-p7326339	Bn-N10-p8881369	gene	
Bn-A10-p7341107	Bn-Scaffold17729-p319	gene	exon
Bn-A10-p7349925	Bn-N10-p8924295	gene	exon
Bn-A10-p7357100	Bn-N10-p8938488	gene	
Bn-A10-p7357442	Bn-N10-p8938830	gene	
Bn-A10-p7357555	Bn-N10-p8938943	gene	exon
Bn-A10-p7360229	Bn-N10-p8940805	gene	exon
Bn-A10-p7365704	Bn-N19-p41150470	gene	exon
Bn-A10-p7410271	Bn-N10-p8986806	gene	exon
Bn-A10-p7480918	Bn-N19-p41478141	gene	
Bn-A10-p7482568	Bn-N19-p41479811	gene	exon
Bn-A10-p749870	Bn-N10-p3250971	gene	exon
Bn-A10-p7502551	Bn-N10-p9063787	gene	
Bn-A10-p7502935	Bn-N10-p9064197	gene	exon
Bn-A10-p7504317	Bn-N10-p9065708	gene	exon
Bn-A10-p7510542	Bn-N10-p9071873	gene	
Bn-A10-p7513409	Bn-N10-p9074740	gene	
Bn-A10-p758707	Bn-N10-p3233916	gene	exon
Bn-A10-p7599250	Bn-N10-p9167144	gene	exon
Bn-A10-p7600250	Bn-N10-p9168144	gene	
Bn-A10-p7636136	Bn-N10-p9210764	gene	
Bn-A10-p7704373	Bn-N10-p9279196	gene	exon
Bn-A10-p7720931	Bn-N10-p9299579	gene	
Bn-A10-p7767853	Bn-N10-p9352782	gene	
Bn-A10-p7830321	Bn-N10-p9426238	gene	exon
Bn-A10-p7835472	Bn-N10-p9431923	gene	exon
Bn-A10-p78356	Bn-N6-p16371981	gene	
Bn-A10-p7841942	Bn-N10-p9438122	gene	exon
Bn-A10-p7850480	Bn-N10-p9066465	gene	
Bn-A10-p7866191	Bn-N10-p9462270	gene	exon
Bn-A10-p7873560	Bn-N10-p9471356	gene	exon

Bn-A10-p788337	Bn-N10-p3206951	gene	exon
Bn-A10-p7904737	Bn-N10-p9510368	gene	exon
Bn-A10-p7905059	Bn-N10-p9510690	gene	exon
Bn-A10-p7906281	Bn-N10-p9511912	gene	exon
Bn-A10-p7913022	Bn-N10-p9518968	gene	exon
Bn-A10-p7932013	Bn-N10-p9533415	gene	
Bn-A10-p7975206	Bn-N19-p42508285	gene	exon
Bn-A10-p7979799	Bn-N10-p9586416	gene	exon
Bn-A10-p8025520	Bn-Scaffold15780-p179	gene	exon
Bn-A10-p8027866	Bn-N10-p9656550	gene	
Bn-A10-p8040898	Bn-N10-p9674931	gene	exon
Bn-A10-p8213398	Bn-N10-p9828138	gene	exon
Bn-A10-p8237821	Bn-N10-p9842422	gene	
Bn-A10-p8257748	Bn-N10-p9857551	gene	exon
Bn-A10-p8285049	Bn-N19-p43117189	gene	exon
Bn-A10-p8290065	Bn-N10-p9880744	gene	
Bn-A10-p8290448	Bn-N10-p9881123	gene	
Bn-A10-p8292854	Bn-N10-p9883529	gene	exon
Bn-A10-p8304815	Bn-N10-p9897698	gene	
Bn-A10-p8308769	Bn-N10-p9901618	gene	
Bn-A10-p8309983	Bn-N19-p43212085	gene	exon
Bn-A10-p8315549	Bn-N10-p9908248	gene	exon
Bn-A10-p8320886	Bn-N10-p9913730	gene	
Bn-A10-p8322965	Bn-N10-p9915810	gene	
Bn-A10-p8328622	Bn-N10-p9921708	gene	
Bn-A10-p8329751	Bn-N10-p9922837	gene	exon
Bn-A10-p8330489	Bn-N10-p9923575	gene	
Bn-A10-p8340529	Bn-N10-p9934261	gene	
Bn-A10-p8359990	Bn-N10-p9953103	gene	exon
Bn-A10-p8379385	Bn-N19-p43408538	gene	exon
Bn-A10-p8383686	Bn-N10-p9976868	gene	
Bn-A10-p8383995	Bn-N10-p9977168	gene	
Bn-A10-p8387158	Bn-N10-p9981252	gene	exon
Bn-A10-p8408212	Bn-Scaffold11791-p859	gene	
Bn-A10-p8423162	Bn-N19-p43495014	gene	
Bn-A10-p8443505	Bn-N10-p10057518	gene	
Bn-A10-p8444450	Bn-N19-p43560319	gene	exon
Bn-A10-p8448368	Bn-N19-p43567472	gene	exon
Bn-A10-p8456410	Bn-N10-p10070492	gene	
Bn-A10-p8456551	Bn-N10-p10070632	gene	
Bn-A10-p8470821	Bn-N10-p10085107	gene	
Bn-A10-p8487448	Bn-N19-p43674787	gene	
Bn-A10-p8503752	Bn-N10-p10121257	gene	exon
Bn-A10-p8529364	Bn-N10-p10140677	gene	exon
Bn-A10-p8559413	Bn-N10-p10187618	gene	exon
Bn-A10-p8560686	Bn-N10-p10188889	gene	exon
Bn-A10-p8564698	Bn-N10-p10192915	gene	exon
Bn-A10-p8572267	Bn-N19-p43940819	gene	exon
Bn-A10-p8577924	Bn-N10-p10210551	gene	exon
Bn-A10-p8582852	Bn-N19-p43963228	gene	
Bn-A10-p8583543	Bn-N10-p10219233	gene	exon
Bn-A10-p8589353	Bn-N10-p10224379	gene	exon

Bn-A10-p8608729	Bn-N10-p10236420	gene	exon
Bn-A10-p8620702	Bn-N10-p10246201	gene	exon
Bn-A10-p8646816	Bn-N10-p10277442	gene	exon
Bn-A10-p8655897	Bn-N10-p10287127	gene	exon
Bn-A10-p8656908	Bn-N10-p10288138	gene	
Bn-A10-p8701134	Bn-N10-p10327963	gene	exon
Bn-A10-p8722713	Bn-N10-p10343369	gene	
Bn-A10-p8723171	Bn-N10-p10343794	gene	
Bn-A10-p8729146	Bn-N10-p10349100	gene	
Bn-A10-p8741886	Bn-N10-p10371085	gene	
Bn-A10-p8766754	Bn-N6-p465259	gene	
Bn-A10-p8767803	Bn-N10-p10399961	gene	exon
Bn-A10-p8822075	Bn-N19-p44525880	gene	exon
Bn-A10-p8826205	Bn-N10-p10462821	gene	exon
Bn-A10-p8832970	Bn-N19-p44533303	gene	
Bn-A10-p8834914	Bn-N10-p10470766	gene	exon
Bn-A10-p8867175	Bn-N10-p10502521	gene	
Bn-A10-p8890067	Bn-N10-p10520273	gene	
Bn-A10-p8917543	Bn-N10-p10553880	gene	exon
Bn-A10-p8937723	Bn-N10-p10566873	gene	
Bn-A10-p8951109	Bn-N19-p44718829	gene	
Bn-A10-p8957186	Bn-N19-p44732365	gene	
Bn-A10-p8961320	Bn-N12-p21998589	gene	exon
Bn-A10-p8963952	Bn-N3-p22188985	gene	exon
Bn-A10-p8964484	Bn-N3-p22188456	gene	exon
Bn-A10-p8965800	Bn-N19-p44763440	gene	
Bn-A10-p8981561	Bn-N10-p10601788	gene	
Bn-A10-p8984096	Bn-N10-p10604323	gene	
Bn-A10-p8996669	Bn-N10-p10620518	gene	exon
Bn-A10-p902245	Bn-N10-p3094856	gene	
Bn-A10-p9039610	Bn-N10-p10661696	gene	exon
Bn-A10-p9050329	Bn-N10-p10668987	gene	exon
Bn-A10-p9065520	Bn-N10-p10687034	gene	
Bn-A10-p9079327	Bn-N10-p10702554	gene	exon
Bn-A10-p9084421	Bn-N10-p10709112	gene	
Bn-A10-p9118672	Bn-N10-p10749461	gene	exon
Bn-A10-p9118675	Bn-N10-p10749464	gene	exon
Bn-A10-p9120500	Bn-N10-p10751271	gene	
Bn-A10-p9121306	Bn-N10-p10752076	gene	exon
Bn-A10-p9124552	Bn-N10-p10755322	gene	exon
Bn-A10-p9128660	Bn-N10-p10759514	gene	exon
Bn-A10-p9150150	Bn-N10-p10771171	gene	
Bn-A10-p9150359	Bn-N10-p10771380	gene	
Bn-A10-p9160244	Bn-N10-p10780276	gene	
Bn-A10-p9181902	Bn-N10-p10810668	gene	exon
Bn-A10-p9184064	Bn-N10-p10813292	gene	exon
Bn-A10-p9186877	Bn-N10-p10816121	gene	
Bn-A10-p9193298	Bn-N10-p10823160	gene	
Bn-A10-p9209872	Bn-N10-p10841185	gene	exon
Bn-A10-p9218381	Bn-N10-p10844684	gene	exon
Bn-A10-p9241008	Bn-N10-p10863692	gene	
Bn-A10-p9255041	Bn-N10-p10875719	gene	

Bn-A10-p9259792	Bn-N10-p10880421	gene	exon
Bn-A10-p926733	Bn-N10-p3083156	gene	exon
Bn-A10-p9270767	Bn-N10-p10893055	gene	exon
Bn-A10-p9271334	Bn-N13-p6453628	gene	
Bn-A10-p9271807	Bn-N19-p45447749	gene	exon
Bn-A10-p9272156	Bn-N10-p10894406	gene	
Bn-A10-p9287837	Bn-N10-p10914532	gene	
Bn-A10-p9318940	Bn-N10-p10948364	gene	
Bn-A10-p9327364	Bn-N10-p10959596	gene	
Bn-A10-p9327975	Bn-N10-p10960209	gene	exon
Bn-A10-p9329804	Bn-N10-p10963914	gene	
Bn-A10-p9365540	Bn-N19-p45645175	gene	
Bn-A10-p9370046	Bn-N10-p11000973	gene	exon
Bn-A10-p9374169	Bn-N10-p11004419	gene	exon
Bn-A10-p9378974	Bn-N10-p11005360	gene	
Bn-A10-p9381557	Bn-N10-p11007913	gene	
Bn-A10-p9436205	Bn-N19-p45741636	gene	exon
Bn-A10-p9470646	Bn-N10-p11098941	gene	
Bn-A10-p9473804	Bn-N10-p11108265	gene	
Bn-A10-p9473886	Bn-N10-p11108347	gene	
Bn-A10-p9496981	Bn-N10-p11129479	gene	
Bn-A10-p9561249	Bn-N10-p11212698	gene	
Bn-A10-p9579123	Bn-N10-p11215987	gene	exon
Bn-A10-p9581812	Bn-N10-p11226589	gene	exon
Bn-A10-p9605069	Bn-N10-p11253656	gene	exon
Bn-A10-p9605153	Bn-N10-p11253740	gene	exon
Bn-A10-p9607076	Bn-N10-p11255699	gene	exon
Bn-A10-p9607474	Bn-N10-p11256096	gene	
Bn-A10-p9615291	Bn-N10-p11263782	gene	exon
Bn-A10-p9615362	Bn-N10-p11263853	gene	exon
Bn-A10-p9624125	Bn-N10-p11279332	gene	exon
Bn-A10-p9630194	Bn-N10-p11283552	gene	
Bn-A10-p9631366	Bn-N10-p11284647	gene	
Bn-A10-p9648940	Bn-N10-p11302212	gene	
Bn-A10-p9694236	Bn-N19-p46194919	gene	
Bn-A10-p971793	Bn-N10-p3037089	gene	
Bn-A10-p9722784	Bn-N10-p11356708	gene	
Bn-A10-p973131	Bn-N10-p3032751	gene	exon
Bn-A10-p9732666	Bn-N10-p11368340	gene	
Bn-A10-p9738703	Bn-N10-p11375913	gene	exon
Bn-A10-p9746391	Bn-N10-p11381704	gene	
Bn-A10-p9774980	Bn-N10-p11403795	gene	
Bn-A10-p9845811	Bn-N10-p11476015	gene	
Bn-A10-p9846287	Bn-N10-p11476491	gene	exon
Bn-A10-p9868026	Bn-N10-p11495432	gene	exon
Bn-A10-p9868333	Bn-N10-p11495739	gene	
Bn-A10-p9892016	Bn-N10-p11519023	gene	exon
Bn-A10-p992281	Bn-N10-p3016644	gene	exon
Bn-A10-p9979372	Bn-N10-p11610439	gene	
Bn-A10-p9981528	Bn-N10-p11612535	gene	
Bn-A10-p9981957	Bn-N10-p11612967	gene	
Bn-C13488683-p154	Bn-N14-p1310660	gene	exon

Bn-C13706737-p102	Bn-N8-p20719937	gene	exon
Bn-C13729753-p243	Bn-N15-p11973629	gene	
Bn-C13734643-p116	Bn-N14-p16289078	gene	exon
Bn-C13793433-p23	Bn-Scaffold01365-p5705	gene	
Bn-C13803703-p146	Bn-N17-p4962958	gene	
Bn-C13824437-p146	Bn-N4-p15324215	gene	
Bn-C13873857-p268	Bn-N14-p50637620	gene	
Bn-C14160250-p3687	Bn-N15-p48510589	gene	
Bn-Scaffold000096-p454538	Bn-N3-p33815047	gene	exon
Bn-Scaffold000096-p513249	Bn-N3-p33777294	gene	
Bn-Scaffold000096-p533173	Bn-N3-p33767642	gene	exon
Bn-Scaffold000096-p533387	Bn-N3-p33767428	gene	exon
Bn-Scaffold000096-p533916	Bn-N3-p33766899	gene	exon
Bn-Scaffold000096-p588135	Bn-N3-p33731872	gene	exon
Bn-Scaffold000096-p598260	Bn-N3-p33721988	gene	
Bn-Scaffold000096-p598820	Bn-N3-p33721428	gene	
Bn-Scaffold000096-p603806	Bn-N3-p33716469	gene	exon
Bn-Scaffold000096-p624954	Bn-N2-p20210932	gene	exon
Bn-Scaffold000096-p625022	Bn-N2-p20211000	gene	exon
Bn-Scaffold000096-p693657	Bn-N9-p21798240	gene	
Bn-Scaffold000096-p762429	Bn-N3-p33598434	gene	exon
Bn-Scaffold000096-p762911	Bn-N3-p33597953	gene	exon
Bn-Scaffold000096-p763000	Bn-N3-p33597864	gene	exon
Bn-Scaffold000096-p763301	Bn-N3-p33597563	gene	exon
Bn-Scaffold000100-p138485	Bn-N10-p10945393	gene	
Bn-Scaffold000100-p631465	Bn-N6-p12148629	gene	exon
Bn-Scaffold000100-p873567	Bn-N6-p11891154	gene	exon
Bn-Scaffold000100-p9450	Bn-N4-p9962350	gene	exon
Bn-Scaffold000103-p494957	Bn-N2-p18718259	gene	exon
Bn-Scaffold000103-p527452	Bn-N12-p29607207	gene	exon
Bn-Scaffold000103-p85526	Bn-N2-p19133570	gene	exon
Bn-Scaffold000104-p143432	Bn-N4-p7171316	gene	
Bn-Scaffold000104-p143849	Bn-N4-p7170917	gene	
Bn-Scaffold000104-p158234	Bn-N4-p7151662	gene	exon
Bn-Scaffold000104-p158481	Bn-N4-p7151412	gene	
Bn-Scaffold000104-p193967	Bn-N4-p7110281	gene	
Bn-Scaffold000104-p199071	Bn-N4-p7105171	gene	
Bn-Scaffold000104-p199126	Bn-N4-p7105150	gene	
Bn-Scaffold000104-p33765	Bn-N4-p7271031	gene	
Bn-Scaffold000104-p404978	Bn-N19-p36899078	gene	
Bn-Scaffold000104-p502698	Bn-N4-p6799111	gene	exon
Bn-Scaffold000104-p546329	Bn-N4-p6753284	gene	
Bn-Scaffold000104-p613533	Bn-N4-p6682724	gene	
Bn-Scaffold000104-p622483	Bn-N4-p6674800	gene	exon
Bn-Scaffold000104-p628225	Bn-N4-p6665782	gene	
Bn-Scaffold000111-p125047	Bn-N2-p19453800	gene	
Bn-Scaffold000111-p50108	Bn-N2-p19378006	gene	exon
Bn-Scaffold000123-p155648	Bn-N10-p3945997	gene	
Bn-Scaffold000123-p156913	Bn-N10-p3944731	gene	
Bn-Scaffold000123-p226882	Bn-N10-p3872255	gene	exon
Bn-Scaffold000123-p238360	Bn-N10-p3863424	gene	
Bn-Scaffold000123-p295468	Bn-N10-p3809500	gene	

Bn-Scaffold000123-p387774	Bn-N10-p3727066	gene	exon
Bn-Scaffold000123-p403102	Bn-N10-p3710933	gene	exon
Bn-Scaffold000123-p426461	Bn-N10-p3677827	gene	
Bn-Scaffold000123-p511169	Bn-N10-p3619357	gene	exon
Bn-Scaffold000123-p5714	Bn-N10-p4089175	gene	
Bn-Scaffold000123-p86645	Bn-N10-p3999437	gene	
Bn-Scaffold000123-p86967	Bn-N10-p3999106	gene	
Bn-Scaffold000123-p94132	Bn-N10-p3991789	gene	exon
Bn-Scaffold000140-p310787	Bn-N1-p15753553	gene	
Bn-Scaffold000149-p131079	Bn-N3-p35267553	gene	
Bn-Scaffold000149-p171606	Bn-N18-p22315132	gene	
Bn-Scaffold000149-p188680	Bn-N3-p35179554	gene	exon
Bn-Scaffold000155-p264365	Bn-N15-p34145216	gene	
Bn-Scaffold000162-p117310	Bn-N3-p26144147	gene	exon
Bn-Scaffold000162-p320522	Bn-N3-p33233203	gene	
Bn-Scaffold000164-p11112	Bn-N1-p24455809	gene	
Bn-Scaffold000164-p120459	Bn-N1-p24339405	gene	exon
Bn-Scaffold000164-p123574	Bn-N7-p14449439	gene	exon
Bn-Scaffold000164-p15854	Bn-N1-p24449515	gene	exon
Bn-Scaffold000164-p160466	Bn-N1-p24309879	gene	exon
Bn-Scaffold000164-p174512	Bn-N1-p24295887	gene	exon
Bn-Scaffold000164-p17878	Bn-N1-p24447499	gene	exon
Bn-Scaffold000164-p192732	Bn-N1-p24280824	gene	
Bn-Scaffold000164-p195645	Bn-N1-p24277939	gene	exon
Bn-Scaffold000164-p196286	Bn-N1-p24277298	gene	
Bn-Scaffold000164-p196290	Bn-N1-p24277294	gene	
Bn-Scaffold000164-p196553	Bn-N1-p24277031	gene	exon
Bn-Scaffold000164-p242428	Bn-N11-p40943214	gene	
Bn-Scaffold000164-p261392	Bn-Scaffold01007-p52117	gene	
Bn-Scaffold000164-p271091	Bn-Scaffold01007-p42419	gene	
Bn-Scaffold000164-p46170	Bn-N1-p24418714	gene	
Bn-Scaffold000164-p55747	Bn-N1-p24408926	gene	
Bn-Scaffold000167-p105601	Bn-N3-p11573697	gene	exon
Bn-Scaffold000167-p106164	Bn-N3-p11574253	gene	
Bn-Scaffold000172-p110842	Bn-N5-p11530724	gene	
Bn-Scaffold000172-p135389	Bn-N5-p11553008	gene	
Bn-Scaffold000172-p243096	Bn-N5-p11683983	gene	
Bn-Scaffold000172-p98111	Bn-N15-p24930106	gene	exon
Bn-Scaffold000178-p114893	Bn-N9-p5200515	gene	
Bn-Scaffold000178-p33587	Bn-N9-p5112709	gene	exon
Bn-Scaffold000191-p54987	Bn-N5-p25032882	gene	
Bn-Scaffold000191-p65525	Bn-N5-p25043351	gene	
Bn-Scaffold000191-p70336	Bn-N15-p47417549	gene	exon
Bn-Scaffold000191-p89779	Bn-N5-p25066726	gene	
Bn-Scaffold000193-p93659	Bn-N10-p6859125	gene	
Bn-Scaffold000196-p129412	Bn-N10-p3513046	gene	exon
Bn-Scaffold000196-p132185	Bn-N10-p3515829	gene	
Bn-Scaffold000196-p132202	Bn-N10-p3515846	gene	
Bn-Scaffold000196-p132328	Bn-N10-p3515972	gene	exon
Bn-Scaffold000196-p67283	Bn-Scaffold00912-p67106	gene	
Bn-Scaffold000203-p24382	Bn-N5-p24958325	gene	
Bn-Scaffold000203-p9485	Bn-N5-p24969336	gene	exon

Bn-Scaffold000203-p9544	Bn-N5-p24969277	gene	exon
Bn-Scaffold000212-p66182	Bn-N10-p4115981	gene	
Bn-Scaffold000213-p30061	Bn-N2-p18257798	gene	
Bn-Scaffold000215-p13136	Bn-N11-p44225821	gene	
Bn-Scaffold000215-p42890	Bn-N11-p44260579	gene	exon
Bn-Scaffold000215-p4493	Bn-N11-p44217186	gene	exon
Bn-Scaffold000215-p60955	Bn-N3-p15363807	gene	exon
Bn-Scaffold000215-p6257	Bn-N11-p44218469	gene	exon
Bn-Scaffold000215-p67874	Bn-N11-p44278826	gene	
Bn-Scaffold000216-p13511	Bn-N3-p34881763	gene	
Bn-Scaffold000217-p20168	Bn-Scaffold01003-p20106	gene	
Bn-Scaffold000217-p6025	Bn-Scaffold01003-p5965	gene	
Bn-Scaffold000220-p29775	Bn-N3-p35607330	gene	
Bn-Scaffold000224-p32291	Bn-N10-p3602078	gene	
Bn-Scaffold000229-p11330	Bn-Scaffold00900-p16800	gene	
Bn-Scaffold000229-p12311	Bn-Scaffold00900-p17779	gene	
Bn-Scaffold000229-p12556	Bn-Scaffold00900-p18024	gene	
Bn-Scaffold000229-p47997	Bn-Scaffold00900-p52259	gene	
Bn-Scaffold000232-p38316	Bn-N7-p825617	gene	
Bn-Scaffold000237-p12082	Bn-N1-p27064751	gene	
Bn-Scaffold000237-p24404	Bn-N1-p27074965	gene	
Bn-Scaffold000240-p44866	Bn-N6-p4820425	gene	exon
Bn-Scaffold000247-p34082	Bn-N8-p9505869	gene	
Bn-Scaffold000251-p33332	Bn-N4-p15152705	gene	
Bn-Scaffold000251-p34051	Bn-N4-p15151940	gene	
Bn-Scaffold000257-p33259	Bn-Scaffold01089-p5089	gene	exon
Bn-Scaffold000259-p37148	Bn-N8-p3797614	gene	exon
Bn-Scaffold000264-p20309	Bn-Scaffold01017-p7354	gene	exon
Bn-Scaffold000270-p33166	Bn-N2-p24333529	gene	
Bn-Scaffold000280-p5190	Bn-Scaffold01040-p31149	gene	exon
Bn-Scaffold000280-p5271	Bn-Scaffold01040-p31230	gene	exon
Bn-Scaffold000281-p25625	Bn-N4-p9127651	gene	exon
Bn-Scaffold000281-p4606	Bn-N4-p9101667	gene	
Bn-Scaffold000282-p6257	Bn-N9-p27422574	gene	exon
Bn-Scaffold000283-p1502	Bn-N8-p3683769	gene	exon
Bn-Scaffold000283-p24053	Bn-N6-p3216621	gene	
Bn-Scaffold000285-p18278	Bn-Scaffold01222-p2349	gene	exon
Bn-Scaffold000286-p11806	Bn-Scaffold00637-p33390	gene	exon
Bn-Scaffold000289-p27459	Bn-N6-p18207764	gene	
Bn-Scaffold000291-p13489	Bn-N10-p3550653	gene	
Bn-Scaffold000291-p13580	Bn-N10-p3550562	gene	
Bn-Scaffold000295-p23235	Bn-N9-p17690939	gene	
Bn-Scaffold000296-p9362	Bn-N12-p16960102	gene	
Bn-Scaffold000300-p15526	Bn-N1-p12057480	gene	exon
Bn-Scaffold000300-p17011	Bn-N1-p12058978	gene	
Bn-Scaffold000300-p4564	Bn-N1-p12046264	gene	
Bn-Scaffold000303-p7649	Bn-N1-p21137586	gene	
Bn-Scaffold000306-p22417	Bn-N10-p16285653	gene	
Bn-Scaffold000316-p9840	Bn-N8-p10128229	gene	
Bn-Scaffold000317-p23246	Bn-N7-p12857776	gene	
Bn-Scaffold000320-p11480	Bn-N8-p12686182	gene	
Bn-Scaffold000324-p11634	Bn-N5-p11834989	gene	

Bn-Scaffold000324-p11905	Bn-N5-p11835261	gene	exon
Bn-Scaffold000337-p6012	Bn-N10-p6470476	gene	
Bn-Scaffold000344-p1622	Bn-N9-p17661188	gene	
Bn-Scaffold000355-p19729	Bn-N4-p8886866	gene	
Bn-Scaffold000366-p2717	Bn-N5-p9963039	gene	exon
Bn-Scaffold000366-p4685	Bn-N5-p9961073	gene	exon
Bn-Scaffold000366-p4803	Bn-N5-p9960955	gene	exon
Bn-Scaffold000366-p5068	Bn-N5-p9960690	gene	exon
Bn-Scaffold000367-p2871	Bn-N9-p20910355	gene	
Bn-Scaffold000369-p1902	Bn-N10-p7139263	gene	
Bn-Scaffold000379-p15742	Bn-N3-p26500219	gene	exon
Bn-Scaffold000386-p10437	Bn-N1-p24881125	gene	
Bn-Scaffold000397-p6640	Bn-N5-p7356985	gene	
Bn-Scaffold000397-p6778	Bn-N5-p7357123	gene	exon
Bn-Scaffold000397-p7197	Bn-N5-p7357542	gene	
Bn-Scaffold000403-p12455	Bn-N5-p407617	gene	exon
Bn-Scaffold000411-p10252	Bn-N16-p33563993	gene	
Bn-Scaffold000512-p1602	Bn-N8-p5279596	gene	
Bn-Scaffold000521-p2857	Bn-N6-p23249963	gene	exon
Bn-Scaffold000565-p4438	Bn-Scaffold01017-p5337	gene	exon
Bn-Scaffold000684-p2321	Bn-N7-p11697120	gene	exon
Bn-Scaffold000783-p133	Bn-N9-p803310	gene	
Bn-Scaffold000796-p1119	Bn-N6-p12056354	gene	exon
Bn-Scaffold000892-p1200	Bn-Scaffold00821-p39712	gene	
Bn-Scaffold000932-p575	Bn-N18-p21748724	gene	
Bn-Scaffold000996-p195	Bn-N7-p576571	gene	exon
Bn-Scaffold001055-p797	Bn-N3-p34880837	gene	
Bn-Scaffold001110-p441	Bn-N7-p6834202	gene	exon
Bn-Scaffold001128-p1186	Bn-N3-p11450935	gene	
Bn-Scaffold001298-p970	Bn-N6-p18405797	gene	exon
Bn-Scaffold001320-p775	Bn-N9-p16129231	gene	
Bn-Scaffold001332-p452	Bn-N7-p579773	gene	exon
Bn-Scaffold001532-p334	Bn-N4-p16960038	gene	exon
Bn-Scaffold001557-p226	Bn-N1-p9922090	gene	
Bn-Scaffold001770-p612	Bn-N9-p25011853	gene	
Bn-Scaffold001988-p613	Bn-N7-p555405	gene	exon
Bn-Scaffold002092-p583	Bn-N5-p21310204	gene	exon
Bn-Scaffold002315-p80	Bn-N2-p1998781	gene	exon
Bn-Scaffold002531-p122	Bn-N7-p573092	gene	
Bn-Scaffold002856-p361	Bn-N7-p578561	gene	exon
Bn-Scaffold003510-p152	Bn-N8-p20395555	gene	exon
Bn-Scaffold003510-p371	Bn-N18-p21450319	gene	exon
Bn-Scaffold004298-p436	Bn-N7-p573280	gene	exon
Bn-Scaffold004542-p45	Bn-N4-p14466779	gene	exon
Bn-Scaffold004639-p267	Bn-N7-p22373517	gene	
Bn-Scaffold005215-p31	Bn-N9-p32994996	gene	exon
Bn-Scaffold005215-p317	Bn-N9-p32995282	gene	exon
Bn-Scaffold005444-p94	Bn-N17-p24943777	gene	exon
Bn-Scaffold006179-p319	Bn-N1-p3353916	gene	exon
Bn-Scaffold006825-p88	Bn-N18-p20996128	gene	exon
Bn-Scaffold006833-p103	Bn-N18-p11804382	gene	
Bn-Scaffold009013-p209	Bn-N2-p6071971	gene	

Bn-Scaffold009788-p170	Bn-N6-p4990542	gene	exon
Bn-Scaffold010519-p163	Bn-N3-p11448216	gene	
Bn-Scaffold011309-p72	Bn-N17-p7038114	gene	exon
Bn-Scaffold011425-p94	Bn-N3-p28889560	gene	exon
Bn-Scaffold012150-p185	Bn-N2-p8481277	gene	exon
Bn-Scaffold012206-p170	Bn-N13-p39017405	gene	exon
Bn-Scaffold012485-p58	Bn-N6-p5794487	gene	
Bn-Scaffold012703-p114	Bn-N5-p17840334	gene	
Bn-Scaffold012966-p76	Bn-N15-p37168947	gene	exon
Bn-Scaffold013089-p53	Bn-N6-p2539908	gene	
Bn-Scaffold014583-p78	Bn-N3-p6900247	gene	exon
Bn-Scaffold014586-p83	Bn-N7-p573769	gene	
Bn-Scaffold014880-p22	Bn-N1-p1112141	gene	exon
Bn-Scaffold014949-p111	Bn-N7-p15876337	gene	exon
Bn-Scaffold015028-p146	Bn-N4-p15823270	gene	
Bn-Scaffold015689-p116	Bn-N5-p17839871	gene	exon
Bn-Scaffold016308-p77	Bn-N1-p22156466	gene	exon
Bn-Scaffold016479-p127	Bn-N3-p11448016	gene	exon
Bn-Scaffold017668-p98	Bn-N7-p9298822	gene	exon
Bn-Scaffold018996-p33	Bn-N16-p16868647	gene	exon
Bn-Scaffold019560-p58	Bn-N3-p25418964	gene	
Bn-Scaffold019655-p1	Bn-N12-p35225741	gene	exon
Bn-Scaffold020953-p21	Bn-N1-p18623	gene	exon
Bn-Scaffold024114-p71	Bn-N19-p45102099	gene	exon
Bn-Scaffold026273-p6	Bn-N5-p17839672	gene	exon
Bn-Scaffold030702-p88	Bn-N7-p12744107	gene	
Bn-Scaffold032460-p85	Bn-N15-p2384036	gene	exon
Bn-Scaffold035376-p42	Bn-N11-p14135111	gene	exon
Bn-Scaffold037025-p103	Bn-N3-p3109274	gene	exon
Bn-scaff_15576_1-p111352	Bn-N19-p48107009	gene	
Bn-scaff_15576_1-p159597	Bn-N10-p12364300	gene	exon
Bn-scaff_15576_1-p162267	Bn-N19-p48155198	gene	exon
Bn-scaff_15576_1-p201715	Bn-N19-p48188259	gene	exon
Bn-scaff_15576_1-p240863	Bn-N19-p48227200	gene	
Bn-scaff_15576_1-p323211	Bn-N19-p48318735	gene	
Bn-scaff_15576_1-p335372	Bn-N19-p48338862	gene	exon
Bn-scaff_15576_1-p335942	Bn-N19-p48339431	gene	
Bn-scaff_15576_1-p335950	Bn-N19-p48339439	gene	
Bn-scaff_15576_1-p374078	Bn-N10-p12474103	gene	exon
Bn-scaff_15576_1-p375237	Bn-N19-p48378629	gene	exon
Bn-scaff_15576_1-p437413	Bn-N19-p48443932	gene	
Bn-scaff_15576_1-p455734	Bn-N19-p48477749	gene	
Bn-scaff_15576_1-p540624	Bn-N19-p48552942	gene	
Bn-scaff_15576_1-p579069	Bn-N19-p48600957	gene	exon
Bn-scaff_15576_1-p634652	Bn-N19-p48654996	gene	exon
Bn-scaff_15576_1-p637717	Bn-N19-p48657054	gene	
Bn-scaff_15576_1-p639470	Bn-N16-p25997897	gene	
Bn-scaff_15576_1-p653601	Bn-N19-p48730533	gene	
Bn-scaff_15576_1-p654663	Bn-N19-p48731604	gene	
Bn-scaff_15576_1-p663373	Bn-N10-p12629368	gene	exon
Bn-scaff_15576_1-p663482	Bn-N19-p48744959	gene	
Bn-scaff_15576_1-p697886	Bn-N10-p12639875	gene	exon

Bn-scaff_15576_1-p727242	Bn-N19-p48788170	gene	
Bn-scaff_15576_1-p728114	Bn-N10-p12654041	gene	exon
Bn-scaff_15576_1-p739936	Bn-N19-p48800264	gene	
Bn-scaff_15576_1-p761764	Bn-N19-p48822467	gene	
Bn-scaff_15576_1-p772503	Bn-N19-p48833620	gene	
Bn-scaff_15585_1-p1020764	Bn-N14-p50517443	gene	exon
Bn-scaff_15585_1-p1028759	Bn-N14-p50525438	gene	exon
Bn-scaff_15585_1-p1029921	Bn-N14-p50526600	gene	
Bn-scaff_15585_1-p1030484	Bn-N7-p8648385	gene	
Bn-scaff_15585_1-p1034252	Bn-N14-p50530931	gene	
Bn-scaff_15585_1-p1046789	Bn-N14-p50542537	gene	
Bn-scaff_15585_1-p1047449	Bn-N14-p50543198	gene	
Bn-scaff_15585_1-p1048094	Bn-N14-p50543842	gene	
Bn-scaff_15585_1-p1060500	Bn-N4-p17402347	gene	
Bn-scaff_15585_1-p1060548	Bn-N14-p50559020	gene	exon
Bn-scaff_15585_1-p1060700	Bn-N4-p17402546	gene	
Bn-scaff_15585_1-p1060833	Bn-N14-p50559304	gene	
Bn-scaff_15585_1-p1065032	Bn-N14-p50561854	gene	
Bn-scaff_15585_1-p1069334	Bn-N14-p50566156	gene	exon
Bn-scaff_15585_1-p1102807	Bn-N14-p50608258	gene	
Bn-scaff_15585_1-p1109141	Bn-N14-p50622477	gene	exon
Bn-scaff_15585_1-p121993	Bn-N4-p16792152	gene	
Bn-scaff_15585_1-p127973	Bn-N14-p50225438	gene	
Bn-scaff_15585_1-p18623	Bn-N14-p50062743	gene	exon
Bn-scaff_15585_1-p265441	Bn-N14-p50363030	gene	
Bn-scaff_15585_1-p266123	Bn-N14-p50363707	gene	
Bn-scaff_15585_1-p291968	Bn-N14-p50390541	gene	exon
Bn-scaff_15585_1-p292538	Bn-N14-p50391111	gene	exon
Bn-scaff_15585_1-p292549	Bn-N14-p50391122	gene	exon
Bn-scaff_15585_1-p436385	Bn-Scaffold00208c-p9658	gene	exon
Bn-scaff_15585_1-p456291	Bn-Scaffold00208b-p101236	gene	exon
Bn-scaff_15585_1-p456596	Bn-Scaffold00208b-p100931	gene	exon
Bn-scaff_15585_1-p56427	Bn-N14-p50108832	gene	
Bn-scaff_15585_1-p567560	Bn-Scaffold02372-p1412	gene	exon
Bn-scaff_15585_1-p590757	Bn-Scaffold00208b-p3454	gene	exon
Bn-scaff_15585_1-p666861	Bn-N5-p5621542	gene	
Bn-scaff_15585_1-p763077	Bn-N5-p5454604	gene	exon
Bn-scaff_15585_1-p82510	Bn-N4-p16756646	gene	exon
Bn-scaff_15585_1-p904564	Bn-N4-p17311855	gene	exon
Bn-scaff_15585_1-p929360	Bn-Scaffold00883b-p18809	gene	exon
Bn-scaff_15585_1-p978062	Bn-N5-p5224670	gene	exon
Bn-scaff_15585_1-p978781	Bn-N5-p5223997	gene	exon
Bn-scaff_15585_1-p9965	Bn-N14-p50058232	gene	
Bn-scaff_15586_1-p3523	Bn-N17-p29142426	gene	
Bn-scaff_15607_1-p13090	Bn-N14-p14512485	gene	
Bn-scaff_15611_1-p500444	Bn-N17-p32319296	gene	exon
Bn-scaff_15611_1-p626686	Bn-N6-p11105198	gene	exon
Bn-scaff_15626_1-p1072079	Bn-N7-p6421901	gene	
Bn-scaff_15626_1-p1084223	Bn-N17-p14892900	gene	
Bn-scaff_15626_1-p548743	Bn-N17-p15440338	gene	
Bn-scaff_15626_1-p651986	Bn-N17-p15344777	gene	
Bn-scaff_15626_1-p673079	Bn-N17-p15320481	gene	

Bn-scaff_15626_1-p729257	Bn-N17-p15265903	gene	
Bn-scaff_15626_1-p743167	Bn-N17-p15245657	gene	exon
Bn-scaff_15626_1-p787026	Bn-N17-p15187885	gene	
Bn-scaff_15627_1-p14438	Bn-N17-p936603	gene	
Bn-scaff_15627_1-p346925	Bn-N7-p270655	gene	
Bn-scaff_15627_1-p472369	Bn-N17-p411358	gene	
Bn-scaff_15627_1-p481878	Bn-N17-p400174	gene	exon
Bn-scaff_15627_1-p482235	Bn-N17-p399808	gene	exon
Bn-scaff_15627_1-p485914	Bn-N7-p176414	gene	
Bn-scaff_15648_1-p468518	Bn-N5-p13423832	gene	
Bn-scaff_15648_1-p468537	Bn-N15-p28477108	gene	
Bn-scaff_15648_1-p538568	Bn-N5-p13344625	gene	
Bn-scaff_15648_1-p541272	Bn-Scaffold03512-p1371	gene	exon
Bn-scaff_15648_1-p541334	Bn-Scaffold03512-p1309	gene	exon
Bn-scaff_15648_1-p541620	Bn-Scaffold03512-p1023	gene	exon
Bn-scaff_15650_1-p107513	Bn-N16-p14480409	gene	
Bn-scaff_15650_1-p256179	Bn-Scaffold20455-p294	gene	exon
Bn-scaff_15650_1-p643013	Bn-N19-p21651158	gene	exon
Bn-scaff_15650_1-p655781	Bn-N19-p21638623	gene	exon
Bn-scaff_15650_1-p683404	Bn-N19-p21603035	gene	
Bn-scaff_15650_1-p707618	Bn-N2-p22331923	gene	exon
Bn-scaff_15650_1-p714363	Bn-N19-p21571246	gene	
Bn-scaff_15650_1-p754414	Bn-N19-p21528211	gene	
Bn-scaff_15650_1-p886508	Bn-N19-p21361546	gene	exon
Bn-scaff_15650_1-p908638	Bn-N19-p21338800	gene	exon
Bn-scaff_15676_1-p298679	Bn-N15-p47567740	gene	exon
Bn-scaff_15676_1-p343087	Bn-N15-p47608607	gene	exon
Bn-scaff_15676_1-p361507	Bn-N15-p47653500	gene	
Bn-scaff_15676_1-p471570	Bn-Scaffold00893-p25886	gene	exon
Bn-scaff_15690_1-p372287	Bn-N15-p39132084	gene	exon
Bn-scaff_15690_1-p406371	Bn-N15-p39168145	gene	exon
Bn-scaff_15695_1-p281125	Bn-N15-p34609173	gene	
Bn-scaff_15695_1-p288239	Bn-N15-p34616287	gene	
Bn-scaff_15695_1-p288827	Bn-N5-p16921456	gene	exon
Bn-scaff_15695_1-p294894	Bn-N15-p34623387	gene	
Bn-scaff_15695_1-p382951	Bn-N13-p34416175	gene	
Bn-scaff_15695_1-p383012	Bn-N13-p34416114	gene	exon
Bn-scaff_15695_1-p611509	Bn-N13-p34176483	gene	exon
Bn-scaff_15695_2-p265894	Bn-N15-p34954015	gene	exon
Bn-scaff_15695_2-p288544	Bn-N15-p34976295	gene	
Bn-scaff_15695_2-p413042	Bn-N15-p35097660	gene	
Bn-scaff_15695_2-p413159	Bn-N15-p35097777	gene	
Bn-scaff_15695_2-p413223	Bn-N15-p35097841	gene	
Bn-scaff_15695_2-p413633	Bn-N5-p17072340	gene	
Bn-scaff_15695_2-p423869	Bn-N9-p35660900	gene	exon
Bn-scaff_15699_1-p340486	Bn-N8-p14644251	gene	exon
Bn-scaff_15699_1-p577914	Bn-N18-p18531864	gene	exon
Bn-scaff_15699_1-p578121	Bn-N18-p18532072	gene	
Bn-scaff_15699_1-p696260	Bn-N8-p14551041	gene	exon
Bn-scaff_15701_1-p1015232	Bn-N19-p12291662	gene	exon
Bn-scaff_15701_1-p152026	Bn-N15-p26047661	gene	
Bn-scaff_15701_1-p703352	Bn-N19-p12620307	gene	

Bn-scaff_15701_1-p994892	Bn-N19-p12311241	gene	exon
Bn-scaff_15703_1-p186311	Bn-N13-p58561976	gene	exon
Bn-scaff_15703_1-p186424	Bn-N8-p10545891	gene	exon
Bn-scaff_15705_1-p1090470	Bn-N2-p27510258	gene	exon
Bn-scaff_15705_1-p1214538	Bn-N17-p37614360	gene	
Bn-scaff_15705_1-p1214955	Bn-N17-p37614576	gene	
Bn-scaff_15705_1-p123831	Bn-N17-p36479753	gene	
Bn-scaff_15705_1-p1308702	Bn-N17-p37701535	gene	exon
Bn-scaff_15705_1-p1324812	Bn-N17-p37722774	gene	exon
Bn-scaff_15705_1-p1350770	Bn-N17-p37736589	gene	exon
Bn-scaff_15705_1-p1393933	Bn-N17-p37782798	gene	
Bn-scaff_15705_1-p1394205	Bn-N17-p37783067	gene	
Bn-scaff_15705_1-p1415123	Bn-N17-p37803986	gene	exon
Bn-scaff_15705_1-p1433099	Bn-N17-p37815441	gene	exon
Bn-scaff_15705_1-p1433154	Bn-N17-p37815496	gene	exon
Bn-scaff_15705_1-p1433369	Bn-N17-p37815711	gene	
Bn-scaff_15705_1-p1435562	Bn-N6-p18969658	gene	exon
Bn-scaff_15705_1-p1479071	Bn-N17-p37851445	gene	
Bn-scaff_15705_1-p1480198	Bn-N17-p37852572	gene	
Bn-scaff_15705_1-p1481509	Bn-N17-p37853883	gene	
Bn-scaff_15705_1-p1481530	Bn-N17-p37853904	gene	
Bn-scaff_15705_1-p1524031	Bn-N17-p37900750	gene	
Bn-scaff_15705_1-p1553993	Bn-N17-p37934475	gene	exon
Bn-scaff_15705_1-p156530	Bn-N6-p20103217	gene	
Bn-scaff_15705_1-p157289	Bn-N6-p20102454	gene	exon
Bn-scaff_15705_1-p1619245	Bn-N17-p38013010	gene	exon
Bn-scaff_15705_1-p1673044	Bn-N17-p38052647	gene	
Bn-scaff_15705_1-p1710163	Bn-N17-p38090703	gene	
Bn-scaff_15705_1-p1719177	Bn-N17-p38099717	gene	
Bn-scaff_15705_1-p1719589	Bn-N17-p38100129	gene	
Bn-scaff_15705_1-p1719850	Bn-N17-p38100390	gene	
Bn-scaff_15705_1-p1723798	Bn-N17-p38103815	gene	
Bn-scaff_15705_1-p1724041	Bn-N17-p38104058	gene	
Bn-scaff_15705_1-p1730509	Bn-N17-p38110714	gene	
Bn-scaff_15705_1-p1812021	Bn-N17-p38195317	gene	
Bn-scaff_15705_1-p1818177	Bn-N17-p38201502	gene	exon
Bn-scaff_15705_1-p2195474	Bn-N17-p38566411	gene	
Bn-scaff_15705_1-p2195647	Bn-N17-p38566585	gene	
Bn-scaff_15705_1-p2244459	Bn-N17-p38615911	gene	exon
Bn-scaff_15705_1-p2247844	Bn-N17-p38619375	gene	exon
Bn-scaff_15705_1-p2248267	Bn-N17-p38619798	gene	exon
Bn-scaff_15705_1-p2249211	Bn-N17-p38620761	gene	exon
Bn-scaff_15705_1-p2249214	Bn-N17-p38620768	gene	exon
Bn-scaff_15705_1-p2274493	Bn-N17-p38658044	gene	exon
Bn-scaff_15705_1-p2279820	Bn-N17-p38664474	gene	
Bn-scaff_15705_1-p2282942	Bn-N17-p38666703	gene	exon
Bn-scaff_15705_1-p2283255	Bn-N17-p38667953	gene	
Bn-scaff_15705_1-p2317575	Bn-N17-p38706970	gene	
Bn-scaff_15705_1-p2504173	Bn-N17-p38887023	gene	
Bn-scaff_15705_1-p2504492	Bn-N17-p38887343	gene	
Bn-scaff_15705_1-p2590289	Bn-N17-p38967842	gene	
Bn-scaff_15705_1-p2740790	Bn-N3-p21862451	gene	

Bn-scaff_15705_1-p2758961	Bn-N17-p39151354	gene	
Bn-scaff_15705_1-p2971358	Bn-N18-p1782299	gene	exon
Bn-scaff_15705_1-p329173	Bn-N17-p36730215	gene	
Bn-scaff_15705_1-p374398	Bn-N17-p36777124	gene	exon
Bn-scaff_15705_1-p379864	Bn-N17-p36785071	gene	
Bn-scaff_15705_1-p392673	Bn-N17-p36797874	gene	
Bn-scaff_15705_1-p394094	Bn-N17-p36799295	gene	
Bn-scaff_15705_1-p394867	Bn-N17-p36800068	gene	
Bn-scaff_15705_1-p431621	Bn-N6-p19844476	gene	exon
Bn-scaff_15705_1-p432224	Bn-N17-p36829930	gene	
Bn-scaff_15705_1-p459906	Bn-N17-p36857699	gene	
Bn-scaff_15705_1-p460083	Bn-N17-p36857876	gene	exon
Bn-scaff_15705_1-p477053	Bn-N17-p36868413	gene	
Bn-scaff_15705_1-p478465	Bn-N17-p36869841	gene	
Bn-scaff_15705_1-p480964	Bn-N17-p36872295	gene	exon
Bn-scaff_15705_1-p523721	Bn-N6-p19765996	gene	exon
Bn-scaff_15705_1-p562818	Bn-N17-p36942241	gene	exon
Bn-scaff_15705_1-p605605	Bn-N17-p36986539	gene	exon
Bn-scaff_15705_1-p773910	Bn-N17-p37149977	gene	
Bn-scaff_15705_1-p780228	Bn-N6-p19551466	gene	exon
Bn-scaff_15705_1-p841235	Bn-N6-p19500355	gene	
Bn-scaff_15705_2-p766564	Bn-N15-p29534218	gene	exon
Bn-scaff_15705_2-p797337	Bn-N15-p29502817	gene	
Bn-scaff_15705_2-p798638	Bn-N15-p29501516	gene	
Bn-scaff_15705_2-p92026	Bn-N15-p30229660	gene	exon
Bn-scaff_15705_2-p931546	Bn-N15-p29271026	gene	exon
Bn-scaff_15705_2-p962347	Bn-N9-p20372858	gene	exon
Bn-scaff_15705_3-p129321	Bn-N17-p39419198	gene	
Bn-scaff_15705_3-p131881	Bn-N17-p39425879	gene	
Bn-scaff_15705_3-p234116	Bn-N17-p39529839	gene	
Bn-scaff_15705_3-p241821	Bn-N17-p39537341	gene	exon
Bn-scaff_15705_3-p253023	Bn-N17-p39542139	gene	
Bn-scaff_15705_3-p261350	Bn-N17-p39558857	gene	exon
Bn-scaff_15705_3-p262027	Bn-N17-p39559532	gene	exon
Bn-scaff_15705_3-p308195	Bn-N3-p22258665	gene	
Bn-scaff_15705_3-p351405	Bn-N17-p39660615	gene	exon
Bn-scaff_15705_3-p436841	Bn-N17-p39754792	gene	exon
Bn-scaff_15705_3-p440761	Bn-N17-p39758705	gene	exon
Bn-scaff_15705_3-p450433	Bn-N17-p39768456	gene	exon
Bn-scaff_15705_3-p455031	Bn-N17-p39773053	gene	
Bn-scaff_15705_3-p582863	Bn-N17-p39875700	gene	exon
Bn-scaff_15705_3-p601831	Bn-N17-p39899760	gene	
Bn-scaff_15705_3-p616618	Bn-N17-p39912815	gene	exon
Bn-scaff_15705_3-p620597	Bn-N17-p39916125	gene	exon
Bn-scaff_15709_1-p58075	Bn-N1-p26854340	gene	exon
Bn-scaff_15709_1-p67334	Bn-N1-p26848911	gene	exon
Bn-scaff_15709_1-p74248	Bn-N11-p45166914	gene	
Bn-scaff_15712_1-p816541	Bn-N6-p25237115	gene	exon
Bn-scaff_15712_1-p841920	Bn-N17-p26695844	gene	exon
Bn-scaff_15712_10-p103802	Bn-N15-p5289572	gene	exon
Bn-scaff_15712_10-p1182534	Bn-N15-p6634826	gene	exon
Bn-scaff_15712_10-p14121	Bn-N15-p5224172	gene	

Bn-scaff_15712_10-p377155	Bn-N15-p5531125	gene	
Bn-scaff_15712_10-p378255	Bn-N15-p5533141	gene	
Bn-scaff_15712_10-p443730	Bn-N15-p5582619	gene	exon
Bn-scaff_15712_10-p528204	Bn-N15-p5660268	gene	exon
Bn-scaff_15712_10-p598383	Bn-N15-p5743185	gene	exon
Bn-scaff_15712_10-p669486	Bn-N15-p5823015	gene	exon
Bn-scaff_15712_10-p717090	Bn-N6-p4727217	gene	
Bn-scaff_15712_10-p927257	Bn-N15-p6076670	gene	
Bn-scaff_15712_11-p40223	Bn-N18-p6869024	gene	
Bn-scaff_15712_11-p46467	Bn-N18-p6866452	gene	exon
Bn-scaff_15712_11-p60195	Bn-N18-p6847616	gene	
Bn-scaff_15712_11-p63788	Bn-N18-p6844023	gene	
Bn-scaff_15712_11-p64730	Bn-N18-p6843081	gene	
Bn-scaff_15712_11-p64879	Bn-N18-p6842932	gene	
Bn-scaff_15712_12-p21418	Bn-N12-p42499224	gene	
Bn-scaff_15712_12-p5675	Bn-N12-p42486098	gene	exon
Bn-scaff_15712_12-p5737	Bn-N12-p42486160	gene	
Bn-scaff_15712_12-p5921	Bn-N12-p42486342	gene	
Bn-scaff_15712_13-p33062	Bn-N12-p9756996	gene	exon
Bn-scaff_15712_13-p43168	Bn-N12-p9767491	gene	
Bn-scaff_15712_13-p61367	Bn-N12-p9789812	gene	
Bn-scaff_15712_13-p63324	Bn-Scaffold01917-p4110	gene	
Bn-scaff_15712_2-p14588	Bn-N17-p21262009	gene	
Bn-scaff_15712_2-p15006	Bn-N17-p21261593	gene	
Bn-scaff_15712_2-p275897	Bn-N12-p38769161	gene	exon
Bn-scaff_15712_2-p800802	Bn-N12-p39380771	gene	
Bn-scaff_15712_2-p800968	Bn-N12-p39380937	gene	
Bn-scaff_15712_2-p810721	Bn-N12-p39391625	gene	
Bn-scaff_15712_2-p810782	Bn-N12-p39391686	gene	
Bn-scaff_15712_2-p811032	Bn-N12-p39391936	gene	
Bn-scaff_15712_2-p817397	Bn-N12-p39396046	gene	
Bn-scaff_15712_2-p86441	Bn-N12-p38597827	gene	exon
Bn-scaff_15712_3-p203186	Bn-N11-p43246088	gene	
Bn-scaff_15712_3-p205773	Bn-N11-p43243501	gene	exon
Bn-scaff_15712_3-p205825	Bn-N11-p43243449	gene	exon
Bn-scaff_15712_3-p251179	Bn-N11-p43197921	gene	
Bn-scaff_15712_3-p353404	Bn-N11-p43073005	gene	
Bn-scaff_15712_3-p46743	Bn-N11-p43420313	gene	
Bn-scaff_15712_3-p480089	Bn-N11-p42961129	gene	exon
Bn-scaff_15712_3-p489739	Bn-N11-p42951680	gene	exon
Bn-scaff_15712_3-p493370	Bn-N11-p42938722	gene	
Bn-scaff_15712_3-p507666	Bn-N1-p25411486	gene	exon
Bn-scaff_15712_3-p508068	Bn-N1-p25411084	gene	exon
Bn-scaff_15712_3-p516388	Bn-N11-p42921474	gene	
Bn-scaff_15712_3-p543124	Bn-N11-p42896042	gene	
Bn-scaff_15712_3-p562225	Bn-Scaffold01128-p3952	gene	exon
Bn-scaff_15712_3-p627192	Bn-N11-p42825633	gene	
Bn-scaff_15712_3-p627461	Bn-N11-p42825368	gene	
Bn-scaff_15712_3-p627936	Bn-N11-p42824903	gene	
Bn-scaff_15712_3-p636695	Bn-N11-p42815944	gene	exon
Bn-scaff_15712_3-p653523	Bn-N11-p42798163	gene	
Bn-scaff_15712_3-p703293	Bn-N11-p42744035	gene	

Bn-scaff_15712_3-p804205	Bn-N11-p42480309	gene	exon
Bn-scaff_15712_3-p804356	Bn-N11-p42480158	gene	exon
Bn-scaff_15712_3-p807914	Bn-N11-p42476600	gene	
Bn-scaff_15712_3-p808416	Bn-N11-p42476098	gene	
Bn-scaff_15712_3-p818174	Bn-N11-p42466501	gene	exon
Bn-scaff_15712_5-p1021105	Bn-N12-p9934997	gene	
Bn-scaff_15712_5-p104472	Bn-N12-p10714800	gene	exon
Bn-scaff_15712_5-p107128	Bn-N12-p10712144	gene	
Bn-scaff_15712_5-p107189	Bn-N12-p10712083	gene	
Bn-scaff_15712_5-p1123924	Bn-N15-p8772295	gene	exon
Bn-scaff_15712_5-p1124972	Bn-N15-p8771247	gene	exon
Bn-scaff_15712_5-p124174	Bn-N12-p10693196	gene	
Bn-scaff_15712_5-p17640	Bn-N12-p10802780	gene	
Bn-scaff_15712_5-p239953	Bn-N12-p10583390	gene	exon
Bn-scaff_15712_5-p263761	Bn-N12-p10556939	gene	
Bn-scaff_15712_5-p266747	Bn-N12-p10553953	gene	exon
Bn-scaff_15712_5-p266971	Bn-N12-p10553729	gene	exon
Bn-scaff_15712_5-p315320	Bn-N12-p10504926	gene	
Bn-scaff_15712_5-p355840	Bn-N2-p7134265	gene	exon
Bn-scaff_15712_5-p384769	Bn-N12-p10452849	gene	exon
Bn-scaff_15712_5-p389200	Bn-N12-p10448432	gene	
Bn-scaff_15712_5-p40206	Bn-N12-p10776432	gene	
Bn-scaff_15712_5-p427179	Bn-N12-p10410784	gene	
Bn-scaff_15712_5-p442786	Bn-N12-p10394988	gene	exon
Bn-scaff_15712_5-p442792	Bn-N12-p10394982	gene	exon
Bn-scaff_15712_5-p443017	Bn-N12-p10394757	gene	exon
Bn-scaff_15712_5-p50347	Bn-N12-p10766514	gene	
Bn-scaff_15712_5-p53061	Bn-N12-p10763598	gene	
Bn-scaff_15712_5-p581641	Bn-N12-p10296647	gene	
Bn-scaff_15712_5-p581712	Bn-N12-p10296576	gene	
Bn-scaff_15712_5-p581943	Bn-N12-p10296345	gene	
Bn-scaff_15712_5-p582188	Bn-N2-p7016815	gene	exon
Bn-scaff_15712_5-p647318	Bn-N2-p6984095	gene	exon
Bn-scaff_15712_5-p652960	Bn-N12-p10233924	gene	exon
Bn-scaff_15712_5-p755073	Bn-N12-p10136477	gene	
Bn-scaff_15712_5-p795461	Bn-N12-p10105970	gene	exon
Bn-scaff_15712_5-p823875	Bn-N12-p10079335	gene	
Bn-scaff_15712_5-p941560	Bn-N12-p9983483	gene	
Bn-scaff_15712_5-p942881	Bn-N12-p9982019	gene	
Bn-scaff_15712_6-p1170156	Bn-N12-p38305459	gene	exon
Bn-scaff_15712_6-p1170308	Bn-N12-p38305611	gene	exon
Bn-scaff_15712_6-p1182178	Bn-N12-p38317508	gene	
Bn-scaff_15712_6-p1227268	Bn-N12-p38356579	gene	exon
Bn-scaff_15712_6-p1254900	Bn-N12-p38380144	gene	exon
Bn-scaff_15712_6-p1356799	Bn-N12-p38486441	gene	
Bn-scaff_15712_6-p157246	Bn-N12-p37245848	gene	exon
Bn-scaff_15712_6-p158134	Bn-N12-p37246723	gene	exon
Bn-scaff_15712_6-p207205	Bn-N12-p37290118	gene	exon
Bn-scaff_15712_6-p214229	Bn-N12-p37297028	gene	
Bn-scaff_15712_6-p357924	Bn-N12-p37431610	gene	exon
Bn-scaff_15712_6-p470292	Bn-N2-p23510539	gene	
Bn-scaff_15712_6-p470462	Bn-N2-p23510709	gene	

Bn-scaff_15712_6-p561242	Bn-N2-p23560531	gene	exon
Bn-scaff_15712_6-p561498	Bn-N12-p37678466	gene	exon
Bn-scaff_15712_6-p585395	Bn-N12-p37706849	gene	exon
Bn-scaff_15712_6-p590850	Bn-N12-p37712319	gene	
Bn-scaff_15712_6-p592438	Bn-N2-p23587175	gene	
Bn-scaff_15712_6-p623654	Bn-N2-p23607818	gene	
Bn-scaff_15712_6-p648969	Bn-N12-p37771467	gene	exon
Bn-scaff_15712_6-p801970	Bn-N12-p37924482	gene	
Bn-scaff_15712_6-p802708	Bn-N12-p37925221	gene	
Bn-scaff_15712_6-p803175	Bn-N12-p37925687	gene	
Bn-scaff_15712_6-p803381	Bn-N12-p37925893	gene	
Bn-scaff_15712_6-p803807	Bn-N12-p37926318	gene	exon
Bn-scaff_15712_6-p836450	Bn-N12-p37983843	gene	
Bn-scaff_15712_6-p932230	Bn-N12-p38089110	gene	
Bn-scaff_15712_8-p359006	Bn-N14-p32786312	gene	
Bn-scaff_15712_9-p518410	Bn-N12-p39747214	gene	
Bn-scaff_15712_9-p528882	Bn-N12-p39758584	gene	exon
Bn-scaff_15712_9-p530201	Bn-N12-p39759903	gene	exon
Bn-scaff_15712_9-p618165	Bn-N12-p39845573	gene	exon
Bn-scaff_15712_9-p621118	Bn-N12-p39848526	gene	
Bn-scaff_15712_9-p621830	Bn-N12-p39849238	gene	
Bn-scaff_15712_9-p636935	Bn-N12-p39869049	gene	
Bn-scaff_15712_9-p801464	Bn-N12-p40040212	gene	
Bn-scaff_15712_9-p822331	Bn-N12-p40059391	gene	exon
Bn-scaff_15713_1-p3776	Bn-N11-p33938827	gene	
Bn-scaff_15714_1-p1026389	Bn-N12-p3310310	gene	
Bn-scaff_15714_1-p1063274	Bn-N12-p3273126	gene	exon
Bn-scaff_15714_1-p1080911	Bn-N12-p3257054	gene	
Bn-scaff_15714_1-p1091353	Bn-N12-p3244236	gene	exon
Bn-scaff_15714_1-p1099018	Bn-N12-p3231957	gene	exon
Bn-scaff_15714_1-p1210756	Bn-Scaffold05044-p1458	gene	exon
Bn-scaff_15714_1-p1767499	Bn-N12-p2566972	gene	exon
Bn-scaff_15714_1-p1787439	Bn-N12-p2532902	gene	
Bn-scaff_15714_1-p1890215	Bn-N12-p2431198	gene	
Bn-scaff_15714_1-p1892918	Bn-N12-p2428493	gene	
Bn-scaff_15714_1-p1934829	Bn-N2-p2196579	gene	exon
Bn-scaff_15714_1-p1949380	Bn-N12-p2360318	gene	exon
Bn-scaff_15714_1-p1949467	Bn-N12-p2360232	gene	exon
Bn-scaff_15714_1-p1949689	Bn-N12-p2360049	gene	
Bn-scaff_15714_1-p1983642	Bn-N12-p2332826	gene	exon
Bn-scaff_15714_1-p2048498	Bn-N2-p2086382	gene	
Bn-scaff_15714_1-p2071571	Bn-N2-p2069493	gene	exon
Bn-scaff_15714_1-p210487	Bn-N12-p4181914	gene	exon
Bn-scaff_15714_1-p2113009	Bn-N12-p2196256	gene	
Bn-scaff_15714_1-p2299285	Bn-N12-p1985719	gene	exon
Bn-scaff_15714_1-p2368497	Bn-N2-p1867396	gene	
Bn-scaff_15714_1-p2383620	Bn-N12-p1897640	gene	exon
Bn-scaff_15714_1-p2383694	Bn-N12-p1897566	gene	exon
Bn-scaff_15714_1-p2383776	Bn-N2-p1848365	gene	exon
Bn-scaff_15714_1-p2481342	Bn-N12-p1816687	gene	
Bn-scaff_15714_1-p2489495	Bn-N12-p1809430	gene	exon
Bn-scaff_15714_1-p2489824	Bn-N12-p1809100	gene	exon

Bn-scaff_15714_1-p2492549	Bn-N2-p1792238	gene	exon
Bn-scaff_15714_1-p2555033	Bn-N12-p1739717	gene	exon
Bn-scaff_15714_1-p2557722	Bn-N2-p1730209	gene	exon
Bn-scaff_15714_1-p2567188	Bn-N2-p1716440	gene	exon
Bn-scaff_15714_1-p2569623	Bn-N2-p1714008	gene	exon
Bn-scaff_15714_1-p2723978	Bn-N2-p1586810	gene	exon
Bn-scaff_15714_1-p2794889	Bn-N12-p1466581	gene	exon
Bn-scaff_15714_1-p2817933	Bn-N12-p1447634	gene	exon
Bn-scaff_15714_1-p2894962	Bn-N12-p1375815	gene	exon
Bn-scaff_15714_1-p2895029	Bn-N12-p1375748	gene	
Bn-scaff_15714_1-p2895311	Bn-N2-p1444923	gene	exon
Bn-scaff_15714_1-p2978071	Bn-N12-p1294128	gene	exon
Bn-scaff_15714_1-p2989937	Bn-N12-p1282905	gene	exon
Bn-scaff_15714_1-p2995270	Bn-N12-p1277324	gene	
Bn-scaff_15714_1-p2995346	Bn-N12-p1277247	gene	
Bn-scaff_15714_1-p3001398	Bn-N12-p1271195	gene	exon
Bn-scaff_15714_1-p3007686	Bn-N12-p1264901	gene	
Bn-scaff_15714_1-p3007703	Bn-N12-p1264884	gene	
Bn-scaff_15714_1-p3110986	Bn-N12-p1175456	gene	exon
Bn-scaff_15714_1-p3216292	Bn-N18-p12134482	gene	
Bn-scaff_15714_1-p3216346	Bn-N18-p12134428	gene	
Bn-scaff_15714_1-p328756	Bn-N12-p4085502	gene	exon
Bn-scaff_15714_1-p342901	Bn-N12-p4076106	gene	
Bn-scaff_15714_1-p483395	Bn-N2-p3262071	gene	exon
Bn-scaff_15714_1-p517527	Bn-N12-p3895633	gene	
Bn-scaff_15714_1-p519285	Bn-N2-p3246461	gene	
Bn-scaff_15714_1-p585548	Bn-N12-p3818883	gene	
Bn-scaff_15714_1-p585874	Bn-N12-p3818556	gene	exon
Bn-scaff_15714_1-p592895	Bn-N2-p3193027	gene	exon
Bn-scaff_15714_1-p593159	Bn-N2-p3192762	gene	exon
Bn-scaff_15714_1-p594799	Bn-N12-p3809749	gene	exon
Bn-scaff_15714_1-p615978	Bn-N2-p3174149	gene	exon
Bn-scaff_15714_1-p633772	Bn-N12-p3769303	gene	exon
Bn-scaff_15714_1-p633944	Bn-N12-p3769131	gene	
Bn-scaff_15714_1-p642855	Bn-N2-p3150661	gene	exon
Bn-scaff_15714_1-p692582	Bn-N12-p3709324	gene	
Bn-scaff_15714_1-p696326	Bn-N12-p3705521	gene	exon
Bn-scaff_15714_1-p696585	Bn-N12-p3705261	gene	
Bn-scaff_15714_1-p696586	Bn-N12-p3705260	gene	
Bn-scaff_15714_1-p723481	Bn-N12-p3677074	gene	
Bn-scaff_15714_1-p723890	Bn-N2-p3083954	gene	exon
Bn-scaff_15714_1-p773455	Bn-N12-p3618105	gene	
Bn-scaff_15714_1-p835459	Bn-N12-p3551736	gene	exon
Bn-scaff_15714_1-p865598	Bn-N12-p3517585	gene	exon
Bn-scaff_15714_1-p881090	Bn-N12-p3501852	gene	
Bn-scaff_15714_1-p966029	Bn-N12-p3371482	gene	
Bn-scaff_15714_1-p977841	Bn-N2-p2891505	gene	exon
Bn-scaff_15714_1-p990729	Bn-N2-p2877737	gene	exon
Bn-scaff_15715_1-p202940	Bn-N17-p27277424	gene	exon
Bn-scaff_15743_1-p13056	Bn-N19-p42328064	gene	exon
Bn-scaff_15743_1-p224179	Bn-N19-p42129008	gene	exon
Bn-scaff_15743_1-p232722	Bn-N19-p42120466	gene	

Bn-scaff_15743_1-p233012	Bn-N19-p42120176	gene	exon
Bn-scaff_15743_1-p318571	Bn-N19-p42029446	gene	
Bn-scaff_15743_1-p535306	Bn-N16-p31031744	gene	
Bn-scaff_15743_1-p536811	Bn-N16-p31033247	gene	exon
Bn-scaff_15743_1-p538763	Bn-N16-p31035199	gene	
Bn-scaff_15743_1-p549066	Bn-N16-p31055385	gene	exon
Bn-scaff_15743_1-p635780	Bn-N16-p31117926	gene	
Bn-scaff_15743_1-p635943	Bn-N16-p31118089	gene	exon
Bn-scaff_15743_1-p638252	Bn-N16-p31120415	gene	
Bn-scaff_15743_1-p638555	Bn-N16-p31120704	gene	
Bn-scaff_15743_1-p644117	Bn-N7-p19267203	gene	exon
Bn-scaff_15743_1-p660655	Bn-N16-p31149996	gene	exon
Bn-scaff_15743_1-p660665	Bn-N16-p31150006	gene	
Bn-scaff_15743_1-p75010	Bn-N19-p42267640	gene	exon
Bn-scaff_15746_1-p128358	Bn-N16-p26632870	gene	
Bn-scaff_15746_1-p152007	Bn-N16-p26608539	gene	exon
Bn-scaff_15746_1-p241457	Bn-N16-p26503290	gene	exon
Bn-scaff_15746_1-p403163	Bn-N7-p17076360	gene	exon
Bn-scaff_15746_1-p428738	Bn-N7-p17063577	gene	
Bn-scaff_15747_1-p105633	Bn-N11-p14577555	gene	
Bn-scaff_15747_1-p106047	Bn-N11-p14577969	gene	
Bn-scaff_15747_1-p339862	Bn-N11-p14828607	gene	
Bn-scaff_15747_1-p340806	Bn-N11-p14829557	gene	exon
Bn-scaff_15747_1-p397042	Bn-N11-p14876362	gene	exon
Bn-scaff_15747_1-p397566	Bn-N1-p9805323	gene	exon
Bn-scaff_15747_1-p443186	Bn-N11-p14918285	gene	
Bn-scaff_15747_1-p544031	Bn-N1-p9907465	gene	exon
Bn-scaff_15747_1-p544523	Bn-N11-p14995262	gene	exon
Bn-scaff_15747_1-p643342	Bn-N11-p15121104	gene	
Bn-scaff_15747_1-p649039	Bn-N1-p9970250	gene	exon
Bn-scaff_15747_1-p649424	Bn-N1-p9970635	gene	exon
Bn-scaff_15747_1-p750280	Bn-Scaffold05944-p1025	gene	exon
Bn-scaff_15747_1-p750297	Bn-Scaffold05944-p1008	gene	exon
Bn-scaff_15747_1-p772985	Bn-N11-p15265044	gene	
Bn-scaff_15747_1-p776112	Bn-N11-p15268172	gene	exon
Bn-scaff_15747_1-p777863	Bn-N11-p15269922	gene	
Bn-scaff_15747_1-p784675	Bn-N11-p15276734	gene	
Bn-scaff_15747_1-p785543	Bn-N11-p15277602	gene	
Bn-scaff_15747_1-p94111	Bn-N11-p14564335	gene	exon
Bn-scaff_15747_2-p217901	Bn-N13-p36902929	gene	
Bn-scaff_15747_2-p259749	Bn-N13-p36859670	gene	
Bn-scaff_15747_2-p387345	Bn-N13-p36718167	gene	exon
Bn-scaff_15747_2-p387704	Bn-N13-p36717808	gene	
Bn-scaff_15749_1-p282242	Bn-N1-p16944932	gene	
Bn-scaff_15749_1-p332783	Bn-N11-p27985220	gene	
Bn-scaff_15749_1-p333180	Bn-N11-p27984829	gene	
Bn-scaff_15749_1-p404917	Bn-N11-p27915576	gene	
Bn-scaff_15749_1-p405353	Bn-N11-p27915139	gene	exon
Bn-scaff_15749_1-p406036	Bn-N11-p27914456	gene	
Bn-scaff_15749_1-p406268	Bn-N11-p27914228	gene	
Bn-scaff_15749_1-p406376	Bn-N11-p27914120	gene	
Bn-scaff_15749_1-p409261	Bn-N11-p27911236	gene	

Bn-scaff_15754_1-p1212459	Bn-N17-p30454636	gene	exon
Bn-scaff_15754_1-p1214097	Bn-N17-p30456274	gene	
Bn-scaff_15754_1-p1215465	Bn-N17-p30457641	gene	
Bn-scaff_15754_1-p1225357	Bn-N17-p30467558	gene	
Bn-scaff_15754_1-p12686	Bn-N17-p29192826	gene	exon
Bn-scaff_15754_1-p1403758	Bn-N17-p30654212	gene	exon
Bn-scaff_15754_1-p1425457	Bn-N17-p30632168	gene	exon
Bn-scaff_15754_1-p1426899	Bn-N17-p30630717	gene	exon
Bn-scaff_15754_1-p294973	Bn-N6-p24320035	gene	
Bn-scaff_15754_1-p44190	Bn-N17-p29227847	gene	exon
Bn-scaff_15754_1-p465154	Bn-N17-p29606684	gene	exon
Bn-scaff_15754_1-p477663	Bn-N17-p29620442	gene	exon
Bn-scaff_15754_1-p478730	Bn-N17-p29621509	gene	exon
Bn-scaff_15754_1-p478990	Bn-N17-p29621770	gene	
Bn-scaff_15754_1-p555074	Bn-N17-p29713676	gene	
Bn-scaff_15754_1-p688470	Bn-N17-p29860763	gene	
Bn-scaff_15754_1-p691892	Bn-N17-p29883169	gene	
Bn-scaff_15754_1-p692148	Bn-N17-p29883425	gene	
Bn-scaff_15754_1-p692916	Bn-N17-p29884193	gene	exon
Bn-scaff_15754_1-p730879	Bn-N17-p29931236	gene	exon
Bn-scaff_15754_1-p827585	Bn-N17-p30023704	gene	exon
Bn-scaff_15754_1-p827708	Bn-N17-p30023827	gene	exon
Bn-scaff_15754_1-p827981	Bn-N6-p24021965	gene	exon
Bn-scaff_15758_1-p36244	Bn-N13-p45008685	gene	exon
Bn-scaff_15758_3-p1324936	Bn-N17-p9145611	gene	exon
Bn-scaff_15758_3-p358181	Bn-N6-p12992142	gene	
Bn-scaff_15762_1-p1096634	Bn-N7-p955943	gene	exon
Bn-scaff_15762_1-p1131894	Bn-N17-p2252247	gene	exon
Bn-scaff_15762_1-p1132736	Bn-N7-p1380806	gene	
Bn-scaff_15762_1-p995371	Bn-N7-p869488	gene	
Bn-scaff_15763_1-p1029560	Bn-N7-p15764803	gene	
Bn-scaff_15763_1-p1048413	Bn-N7-p15783785	gene	exon
Bn-scaff_15763_1-p1089975	Bn-N7-p15800681	gene	exon
Bn-scaff_15763_1-p1090874	Bn-N7-p15801668	gene	exon
Bn-scaff_15763_1-p1090987	Bn-N7-p15801781	gene	exon
Bn-scaff_15763_1-p123436	Bn-N7-p15355618	gene	
Bn-scaff_15763_1-p1332687	Bn-N16-p23748643	gene	exon
Bn-scaff_15763_1-p1382176	Bn-N16-p23767263	gene	exon
Bn-scaff_15763_1-p1403988	Bn-N16-p23785191	gene	
Bn-scaff_15763_1-p1411892	Bn-N16-p23791305	gene	
Bn-scaff_15763_1-p1413613	Bn-N16-p23793027	gene	exon
Bn-scaff_15763_1-p1413941	Bn-N16-p23793355	gene	
Bn-scaff_15763_1-p1418783	Bn-N16-p23798819	gene	
Bn-scaff_15763_1-p1419543	Bn-N16-p23799596	gene	
Bn-scaff_15763_1-p1481622	Bn-N16-p23863227	gene	
Bn-scaff_15763_1-p1492349	Bn-N16-p23873923	gene	exon
Bn-scaff_15763_1-p1557780	Bn-N7-p15983519	gene	exon
Bn-scaff_15763_1-p188023	Bn-N16-p22690252	gene	
Bn-scaff_15763_1-p189152	Bn-N16-p22691369	gene	
Bn-scaff_15763_1-p221789	Bn-N16-p22718536	gene	exon
Bn-scaff_15763_1-p233149	Bn-N16-p22741620	gene	
Bn-scaff_15763_1-p233999	Bn-N16-p22742470	gene	exon

Bn-scaff_15763_1-p241894	Bn-N16-p22750490	gene	exon
Bn-scaff_15763_1-p255875	Bn-N16-p22763105	gene	
Bn-scaff_15763_1-p262418	Bn-N16-p22770059	gene	
Bn-scaff_15763_1-p282019	Bn-N16-p22779031	gene	exon
Bn-scaff_15763_1-p285424	Bn-N16-p22782141	gene	exon
Bn-scaff_15763_1-p287565	Bn-N16-p22784281	gene	exon
Bn-scaff_15763_1-p313075	Bn-N16-p22803057	gene	
Bn-scaff_15763_1-p313425	Bn-N16-p22803407	gene	
Bn-scaff_15763_1-p320413	Bn-N16-p22810318	gene	exon
Bn-scaff_15763_1-p506136	Bn-N16-p22981280	gene	
Bn-scaff_15763_1-p506301	Bn-N16-p22981445	gene	
Bn-scaff_15763_1-p514953	Bn-N16-p22990094	gene	exon
Bn-scaff_15763_1-p522845	Bn-N16-p22998009	gene	
Bn-scaff_15763_1-p553985	Bn-N16-p23025749	gene	exon
Bn-scaff_15763_1-p572494	Bn-N7-p15556233	gene	
Bn-scaff_15763_1-p573751	Bn-N16-p23045027	gene	exon
Bn-scaff_15763_1-p575274	Bn-N16-p23046550	gene	
Bn-scaff_15763_1-p637496	Bn-N7-p15585204	gene	
Bn-scaff_15763_1-p648473	Bn-N16-p23094429	gene	
Bn-scaff_15763_1-p648869	Bn-N16-p23094825	gene	
Bn-scaff_15763_1-p648971	Bn-N16-p23094927	gene	
Bn-scaff_15763_1-p657663	Bn-N16-p23097770	gene	
Bn-scaff_15763_1-p662057	Bn-N16-p23102163	gene	
Bn-scaff_15763_1-p675547	Bn-N16-p23115663	gene	exon
Bn-scaff_15763_1-p677594	Bn-N16-p23117710	gene	
Bn-scaff_15763_1-p681318	Bn-N16-p23121434	gene	exon
Bn-scaff_15763_1-p681523	Bn-N16-p23121639	gene	exon
Bn-scaff_15763_1-p682251	Bn-N16-p23122367	gene	
Bn-scaff_15763_1-p87690	Bn-N16-p22570082	gene	exon
Bn-scaff_15765_1-p145525	Bn-N14-p12059033	gene	
Bn-scaff_15766_1-p103722	Bn-N17-p10799935	gene	exon
Bn-scaff_15766_1-p103742	Bn-N17-p10799956	gene	exon
Bn-scaff_15766_1-p103851	Bn-N17-p10800064	gene	exon
Bn-scaff_15766_1-p117523	Bn-N17-p10810558	gene	exon
Bn-scaff_15766_1-p117700	Bn-N17-p10810735	gene	exon
Bn-scaff_15766_1-p141085	Bn-N17-p10838548	gene	
Bn-scaff_15766_1-p141160	Bn-N17-p10838623	gene	
Bn-scaff_15766_1-p20339	Bn-N17-p10707305	gene	
Bn-scaff_15766_1-p20537	Bn-N17-p10707503	gene	
Bn-scaff_15766_1-p21067	Bn-N17-p10708033	gene	
Bn-scaff_15766_1-p21074	Bn-N17-p10708040	gene	
Bn-scaff_15766_1-p21706	Bn-N17-p10708325	gene	
Bn-scaff_15766_1-p237467	Bn-N14-p21562245	gene	exon
Bn-scaff_15766_1-p34395	Bn-N17-p10717828	gene	
Bn-scaff_15766_1-p34398	Bn-N17-p10717831	gene	
Bn-scaff_15767_1-p104948	Bn-N12-p28736092	gene	
Bn-scaff_15767_1-p108658	Bn-N12-p28739802	gene	
Bn-scaff_15767_1-p110471	Bn-N12-p28741615	gene	
Bn-scaff_15767_1-p113288	Bn-N12-p28744432	gene	
Bn-scaff_15767_1-p174572	Bn-N12-p28799664	gene	exon
Bn-scaff_15767_1-p174671	Bn-N12-p28799763	gene	exon
Bn-scaff_15779_1-p400004	Bn-N14-p34636471	gene	

Bn-scaff_15779_1-p41353	Bn-N14-p34336145	gene	
Bn-scaff_15779_1-p94004	Bn-N14-p34388709	gene	exon
Bn-scaff_15779_1-p94543	Bn-N14-p34389254	gene	exon
Bn-scaff_15782_1-p220040	Bn-N3-p12663515	gene	exon
Bn-scaff_15782_1-p37811	Bn-N13-p19249996	gene	
Bn-scaff_15782_1-p71063	Bn-N13-p19214993	gene	exon
Bn-scaff_15782_1-p72137	Bn-N3-p12728298	gene	
Bn-scaff_15783_1-p178333	Bn-N9-p1128019	gene	
Bn-scaff_15783_1-p262930	Bn-N9-p1037793	gene	exon
Bn-scaff_15783_1-p273092	Bn-N9-p1018784	gene	exon
Bn-scaff_15783_1-p346149	Bn-N9-p984087	gene	
Bn-scaff_15783_1-p367803	Bn-N19-p1110097	gene	
Bn-scaff_15783_1-p368397	Bn-N19-p1109503	gene	
Bn-scaff_15783_1-p379962	Bn-N19-p945787	gene	exon
Bn-scaff_15783_1-p381165	Bn-N19-p1087400	gene	exon
Bn-scaff_15783_1-p500695	Bn-N19-p965542	gene	
Bn-scaff_15783_1-p501174	Bn-N19-p965062	gene	
Bn-scaff_15783_1-p501489	Bn-N19-p964748	gene	
Bn-scaff_15783_1-p519816	Bn-N19-p1087618	gene	exon
Bn-scaff_15783_1-p520465	Bn-N19-p946120	gene	exon
Bn-scaff_15794_1-p157188	Bn-N13-p62353372	gene	
Bn-scaff_15794_1-p236621	Bn-N13-p62236523	gene	exon
Bn-scaff_15794_1-p236720	Bn-N13-p62236423	gene	exon
Bn-scaff_15794_1-p240208	Bn-N13-p62232165	gene	
Bn-scaff_15794_1-p354725	Bn-N13-p62097166	gene	
Bn-scaff_15794_1-p437864	Bn-N13-p62015568	gene	
Bn-scaff_15794_1-p84457	Bn-N13-p62429054	gene	exon
Bn-scaff_15794_2-p214881	Bn-N11-p36341960	gene	
Bn-scaff_15794_2-p251230	Bn-N1-p21706353	gene	exon
Bn-scaff_15794_3-p137832	Bn-N13-p61741280	gene	exon
Bn-scaff_15794_3-p154541	Bn-N13-p61712262	gene	exon
Bn-scaff_15794_3-p256042	Bn-N13-p61643005	gene	exon
Bn-scaff_15794_3-p62430	Bn-N2-p9008665	gene	exon
Bn-scaff_15798_1-p1057546	Bn-N4-p12874728	gene	exon
Bn-scaff_15798_1-p1058384	Bn-N14-p43786578	gene	
Bn-scaff_15798_1-p135614	Bn-N14-p42789267	gene	
Bn-scaff_15798_1-p194900	Bn-N4-p12011049	gene	exon
Bn-scaff_15798_1-p289396	Bn-N4-p12111810	gene	exon
Bn-scaff_15798_1-p290204	Bn-N4-p12112618	gene	exon
Bn-scaff_15798_1-p310424	Bn-N18-p32082324	gene	
Bn-scaff_15798_1-p419058	Bn-N4-p12216719	gene	exon
Bn-scaff_15798_1-p699979	Bn-N14-p43424560	gene	exon
Bn-scaff_15798_1-p711199	Bn-N14-p43436831	gene	exon
Bn-scaff_15798_1-p712022	Bn-N14-p43439216	gene	exon
Bn-scaff_15798_1-p725659	Bn-N14-p43451574	gene	exon
Bn-scaff_15798_1-p729336	Bn-N14-p43455251	gene	
Bn-scaff_15798_1-p731733	Bn-N14-p43457650	gene	
Bn-scaff_15798_1-p732529	Bn-N14-p43458447	gene	
Bn-scaff_15798_1-p982019	Bn-N14-p43693473	gene	exon
Bn-scaff_15803_1-p452288	Bn-N11-p15721007	gene	
Bn-scaff_15803_1-p452639	Bn-N11-p15720656	gene	
Bn-scaff_15803_1-p558476	Bn-N11-p15612184	gene	

Bn-scaff_15803_1-p668267	Bn-N11-p15505848	gene	
Bn-scaff_15803_1-p677518	Bn-N11-p15497251	gene	
Bn-scaff_15803_1-p729620	Bn-N11-p15422454	gene	exon
Bn-scaff_15803_1-p802390	Bn-N11-p15346780	gene	
Bn-scaff_15803_1-p811739	Bn-N1-p10087033	gene	exon
Bn-scaff_15803_1-p813514	Bn-N1-p10085287	gene	exon
Bn-scaff_15803_1-p855436	Bn-N11-p15294448	gene	
Bn-scaff_15808_1-p236986	Bn-N19-p43408065	gene	exon
Bn-scaff_15808_1-p240156	Bn-N19-p43411238	gene	exon
Bn-scaff_15808_1-p240644	Bn-N19-p43411724	gene	exon
Bn-scaff_15808_1-p247006	Bn-N19-p43420231	gene	exon
Bn-scaff_15808_1-p337084	Bn-N10-p10024667	gene	
Bn-scaff_15808_1-p337452	Bn-N19-p43496389	gene	exon
Bn-scaff_15808_1-p388294	Bn-N19-p43560390	gene	exon
Bn-scaff_15808_1-p393449	Bn-N19-p43567591	gene	exon
Bn-scaff_15808_1-p405938	Bn-N19-p43583099	gene	
Bn-scaff_15808_1-p432946	Bn-N19-p43614257	gene	exon
Bn-scaff_15808_1-p433123	Bn-N19-p43614433	gene	exon
Bn-scaff_15808_1-p433195	Bn-N19-p43614505	gene	exon
Bn-scaff_15808_1-p433496	Bn-N19-p43614806	gene	
Bn-scaff_15808_1-p433945	Bn-N19-p43615255	gene	
Bn-scaff_15808_1-p434342	Bn-N19-p43615653	gene	
Bn-scaff_15808_1-p445999	Bn-N19-p43635224	gene	
Bn-scaff_15808_1-p446050	Bn-N19-p43635275	gene	
Bn-scaff_15808_1-p446701	Bn-N19-p43636655	gene	
Bn-scaff_15808_1-p487486	Bn-N19-p43674351	gene	exon
Bn-scaff_15808_1-p50319	Bn-N19-p43213003	gene	exon
Bn-scaff_15808_1-p519223	Bn-N10-p10121072	gene	
Bn-scaff_15808_1-p519665	Bn-N19-p43704435	gene	exon
Bn-scaff_15808_1-p525268	Bn-N19-p43710040	gene	exon
Bn-scaff_15808_1-p56362	Bn-N10-p9909941	gene	exon
Bn-scaff_15808_1-p583082	Bn-N19-p43768862	gene	
Bn-scaff_15808_1-p644839	Bn-N19-p43876948	gene	exon
Bn-scaff_15808_1-p645700	Bn-N19-p43877881	gene	
Bn-scaff_15808_1-p645707	Bn-N19-p43877887	gene	
Bn-scaff_15808_1-p645798	Bn-N19-p43877978	gene	
Bn-scaff_15808_1-p646346	Bn-N19-p43878526	gene	exon
Bn-scaff_15808_1-p646435	Bn-N19-p43878615	gene	
Bn-scaff_15808_1-p646761	Bn-N19-p43878942	gene	
Bn-scaff_15808_1-p647845	Bn-N19-p43880025	gene	
Bn-scaff_15808_1-p647937	Bn-N19-p43880117	gene	exon
Bn-scaff_15808_1-p680975	Bn-N10-p10192353	gene	exon
Bn-scaff_15808_1-p742572	Bn-N19-p43963712	gene	exon
Bn-scaff_15808_1-p742673	Bn-N19-p43963812	gene	exon
Bn-scaff_15808_1-p743841	Bn-N19-p43964981	gene	exon
Bn-scaff_15808_1-p784093	Bn-N19-p43997828	gene	
Bn-scaff_15808_1-p786011	Bn-N19-p43999734	gene	
Bn-scaff_15808_1-p92071	Bn-N19-p43251404	gene	exon
Bn-scaff_15808_1-p92391	Bn-N19-p43251724	gene	
Bn-scaff_15809_1-p110149	Bn-N14-p19195326	gene	
Bn-scaff_15809_1-p207828	Bn-N14-p19287829	gene	
Bn-scaff_15809_1-p209065	Bn-N14-p19289078	gene	

Bn-scaff_15809_1-p214708	Bn-N14-p19294587	gene	
Bn-scaff_15809_1-p219058	Bn-N15-p3218959	gene	exon
Bn-scaff_15809_1-p81364	Bn-N14-p19164997	gene	
Bn-scaff_15818_1-p1579953	Bn-N7-p16190523	gene	exon
Bn-scaff_15818_1-p1871998	Bn-N7-p16308315	gene	
Bn-scaff_15818_1-p2029150	Bn-N16-p24860781	gene	exon
Bn-scaff_15818_1-p2029912	Bn-N16-p24861544	gene	
Bn-scaff_15818_1-p2103793	Bn-N7-p16405417	gene	exon
Bn-scaff_15818_1-p2165992	Bn-N7-p16448551	gene	
Bn-scaff_15818_1-p2331085	Bn-N7-p16538251	gene	exon
Bn-scaff_15818_1-p2399771	Bn-N16-p25235002	gene	
Bn-scaff_15818_1-p2399825	Bn-N16-p25235044	gene	
Bn-scaff_15818_1-p2515033	Bn-N16-p25327888	gene	
Bn-scaff_15818_1-p2516251	Bn-N16-p25329106	gene	exon
Bn-scaff_15818_1-p2532249	Bn-N16-p25345988	gene	
Bn-scaff_15818_1-p2543219	Bn-N7-p16672343	gene	exon
Bn-scaff_15818_1-p2633791	Bn-N7-p16702198	gene	exon
Bn-scaff_15818_1-p277309	Bn-N16-p17319523	gene	
Bn-scaff_15818_1-p2876784	Bn-N16-p25691260	gene	exon
Bn-scaff_15818_1-p2889771	Bn-N16-p25704426	gene	
Bn-scaff_15818_1-p2890837	Bn-N16-p25705492	gene	
Bn-scaff_15818_1-p2891179	Bn-N16-p25705835	gene	
Bn-scaff_15818_1-p2891900	Bn-N16-p25706555	gene	
Bn-scaff_15818_1-p2921633	Bn-N16-p25737438	gene	
Bn-scaff_15818_1-p292872	Bn-N16-p17336642	gene	exon
Bn-scaff_15818_1-p297955	Bn-N16-p17341747	gene	exon
Bn-scaff_15818_1-p3109988	Bn-N16-p25925645	gene	
Bn-scaff_15818_1-p3172268	Bn-N16-p25975890	gene	
Bn-scaff_15818_1-p3308182	Bn-N7-p16960659	gene	exon
Bn-scaff_15818_1-p3310016	Bn-N16-p26110933	gene	
Bn-scaff_15818_1-p3310794	Bn-N7-p16963235	gene	exon
Bn-scaff_15818_1-p352205	Bn-N16-p17406504	gene	exon
Bn-scaff_15818_1-p471106	Bn-N16-p17484947	gene	
Bn-scaff_15818_1-p493044	Bn-N7-p13383887	gene	
Bn-scaff_15818_1-p832468	Bn-N7-p13511104	gene	exon
Bn-scaff_15818_1-p858733	Bn-N7-p13523262	gene	exon
Bn-scaff_15818_1-p858798	Bn-N7-p13523326	gene	
Bn-scaff_15818_2-p1142391	Bn-N16-p19451304	gene	
Bn-scaff_15818_2-p1144939	Bn-N16-p19453915	gene	exon
Bn-scaff_15818_2-p1146207	Bn-N16-p19455199	gene	exon
Bn-scaff_15818_2-p1209251	Bn-N16-p19534252	gene	
Bn-scaff_15818_2-p1235556	Bn-N16-p19559397	gene	
Bn-scaff_15818_2-p1236404	Bn-N16-p19560245	gene	
Bn-scaff_15818_2-p1238506	Bn-N16-p19562347	gene	exon
Bn-scaff_15818_2-p1430036	Bn-N16-p19778089	gene	exon
Bn-scaff_15818_2-p1534553	Bn-N16-p19895770	gene	exon
Bn-scaff_15818_2-p1554832	Bn-N16-p19917101	gene	
Bn-scaff_15818_2-p1554957	Bn-N16-p19917226	gene	
Bn-scaff_15818_2-p1555112	Bn-N16-p19917381	gene	
Bn-scaff_15818_2-p1555114	Bn-N16-p19917383	gene	
Bn-scaff_15818_2-p1566134	Bn-N16-p19923807	gene	exon
Bn-scaff_15818_2-p1567500	Bn-N16-p19922441	gene	exon

Bn-scaff_15818_2-p172987	Bn-N16-p18523560	gene	
Bn-scaff_15818_2-p175898	Bn-N16-p18525943	gene	exon
Bn-scaff_15818_2-p175935	Bn-N16-p18525980	gene	exon
Bn-scaff_15818_2-p176007	Bn-N16-p18526052	gene	exon
Bn-scaff_15818_2-p1943374	Bn-N16-p20266466	gene	
Bn-scaff_15818_2-p1945022	Bn-N16-p20268414	gene	
Bn-scaff_15818_2-p1945232	Bn-N16-p20268603	gene	
Bn-scaff_15818_2-p1945498	Bn-N16-p20268871	gene	exon
Bn-scaff_15818_2-p1945612	Bn-N16-p20268985	gene	exon
Bn-scaff_15818_2-p2060720	Bn-N16-p20378717	gene	
Bn-scaff_15818_2-p2080735	Bn-N16-p20397189	gene	exon
Bn-scaff_15818_2-p485119	Bn-N16-p18852696	gene	exon
Bn-scaff_15818_2-p502870	Bn-N16-p18870481	gene	exon
Bn-scaff_15818_2-p503599	Bn-N16-p18871211	gene	exon
Bn-scaff_15818_2-p504385	Bn-N7-p13818120	gene	
Bn-scaff_15818_2-p505807	Bn-N7-p13819541	gene	exon
Bn-scaff_15818_2-p693286	Bn-N16-p19061654	gene	exon
Bn-scaff_15832_1-p74342	Bn-N14-p505347	gene	exon
Bn-scaff_15838_1-p1045102	Bn-N11-p1646614	gene	exon
Bn-scaff_15838_1-p1065318	Bn-N11-p1669416	gene	
Bn-scaff_15838_1-p1126349	Bn-N11-p1736348	gene	
Bn-scaff_15838_1-p1218165	Bn-N11-p1833879	gene	
Bn-scaff_15838_1-p1347132	Bn-N1-p1574222	gene	exon
Bn-scaff_15838_1-p1347433	Bn-N1-p1574535	gene	exon
Bn-scaff_15838_1-p1347959	Bn-N1-p1575049	gene	exon
Bn-scaff_15838_1-p1372150	Bn-N11-p1992241	gene	
Bn-scaff_15838_1-p1403775	Bn-N11-p2029142	gene	
Bn-scaff_15838_1-p1430057	Bn-N11-p2059634	gene	
Bn-scaff_15838_1-p1451211	Bn-N11-p2083358	gene	exon
Bn-scaff_15838_1-p1452641	Bn-N1-p1683410	gene	exon
Bn-scaff_15838_1-p1467858	Bn-N1-p1697189	gene	exon
Bn-scaff_15838_1-p1476644	Bn-N11-p2110760	gene	
Bn-scaff_15838_1-p1572850	Bn-N1-p1769679	gene	exon
Bn-scaff_15838_1-p1573954	Bn-N11-p2206997	gene	exon
Bn-scaff_15838_1-p1585380	Bn-N11-p2221095	gene	
Bn-scaff_15838_1-p1680792	Bn-N11-p2325230	gene	
Bn-scaff_15838_1-p1684617	Bn-N11-p2329121	gene	exon
Bn-scaff_15838_1-p1810027	Bn-N11-p2452848	gene	
Bn-scaff_15838_1-p1917196	Bn-N11-p2565721	gene	
Bn-scaff_15838_1-p1942196	Bn-N11-p2594884	gene	
Bn-scaff_15838_1-p2018342	Bn-N11-p2682441	gene	exon
Bn-scaff_15838_1-p2033116	Bn-N1-p2128324	gene	
Bn-scaff_15838_1-p2035980	Bn-N11-p2700993	gene	
Bn-scaff_15838_1-p2039607	Bn-N11-p2704674	gene	
Bn-scaff_15838_1-p2103661	Bn-N11-p2765238	gene	
Bn-scaff_15838_1-p2104239	Bn-N11-p2765816	gene	exon
Bn-scaff_15838_1-p2144986	Bn-N11-p2803667	gene	exon
Bn-scaff_15838_1-p2212925	Bn-N11-p2874100	gene	exon
Bn-scaff_15838_1-p2253503	Bn-N11-p2924028	gene	
Bn-scaff_15838_1-p2261053	Bn-N11-p2931702	gene	exon
Bn-scaff_15838_1-p2268600	Bn-N11-p2939439	gene	exon
Bn-scaff_15838_1-p2363391	Bn-N11-p3039587	gene	exon

Bn-scaff_15838_1-p2366465	Bn-N1-p2399522	gene	exon
Bn-scaff_15838_1-p3224	Bn-N14-p382427	gene	exon
Bn-scaff_15838_1-p431092	Bn-N11-p1100614	gene	exon
Bn-scaff_15838_1-p431266	Bn-N11-p1100788	gene	exon
Bn-scaff_15838_1-p517875	Bn-N1-p894187	gene	exon
Bn-scaff_15838_1-p533029	Bn-N11-p1150870	gene	exon
Bn-scaff_15838_1-p536524	Bn-N11-p1154327	gene	exon
Bn-scaff_15838_1-p554584	Bn-N11-p1169242	gene	exon
Bn-scaff_15838_1-p586436	Bn-N11-p1201469	gene	
Bn-scaff_15838_1-p614550	Bn-N11-p1232917	gene	
Bn-scaff_15838_1-p693814	Bn-N11-p1327164	gene	exon
Bn-scaff_15838_1-p694016	Bn-N1-p1048604	gene	
Bn-scaff_15838_1-p714484	Bn-N11-p1346032	gene	
Bn-scaff_15838_1-p740881	Bn-N11-p1373990	gene	
Bn-scaff_15838_1-p741071	Bn-N11-p1374194	gene	
Bn-scaff_15838_1-p747634	Bn-N11-p1380587	gene	
Bn-scaff_15838_1-p767750	Bn-N11-p1407403	gene	
Bn-scaff_15838_1-p775566	Bn-N11-p1418958	gene	exon
Bn-scaff_15838_1-p787184	Bn-N11-p1430592	gene	
Bn-scaff_15838_1-p805646	Bn-N11-p1453637	gene	exon
Bn-scaff_15838_1-p830622	Bn-N11-p1475601	gene	
Bn-scaff_15838_1-p842481	Bn-N11-p1486285	gene	
Bn-scaff_15838_1-p867997	Bn-N11-p1513650	gene	exon
Bn-scaff_15838_1-p868199	Bn-N11-p1513851	gene	
Bn-scaff_15838_1-p868461	Bn-N11-p1514103	gene	
Bn-scaff_15838_1-p868592	Bn-N11-p1514242	gene	exon
Bn-scaff_15838_1-p943061	Bn-N11-p1558871	gene	exon
Bn-scaff_15838_2-p107992	Bn-N15-p4449140	gene	
Bn-scaff_15838_2-p115812	Bn-N15-p4436641	gene	exon
Bn-scaff_15838_2-p126220	Bn-N15-p4430761	gene	
Bn-scaff_15838_2-p135868	Bn-N15-p4426226	gene	exon
Bn-scaff_15838_2-p136603	Bn-N15-p4425491	gene	exon
Bn-scaff_15838_2-p142579	Bn-N15-p4419019	gene	
Bn-scaff_15838_2-p143070	Bn-N15-p4418528	gene	
Bn-scaff_15838_3-p220348	Bn-N2-p10883664	gene	exon
Bn-scaff_15838_3-p227340	Bn-N2-p10876389	gene	exon
Bn-scaff_15838_3-p256125	Bn-N2-p10854695	gene	
Bn-scaff_15838_3-p612728	Bn-N2-p10717080	gene	exon
Bn-scaff_15838_3-p613154	Bn-N2-p10716672	gene	exon
Bn-scaff_15838_5-p1004507	Bn-N11-p4210580	gene	exon
Bn-scaff_15838_5-p1092811	Bn-N11-p4319272	gene	
Bn-scaff_15838_5-p1093365	Bn-N11-p4319826	gene	exon
Bn-scaff_15838_5-p1158185	Bn-N11-p4379087	gene	
Bn-scaff_15838_5-p1162338	Bn-N11-p4383249	gene	
Bn-scaff_15838_5-p1167562	Bn-N11-p4388971	gene	exon
Bn-scaff_15838_5-p1170066	Bn-N11-p4391477	gene	exon
Bn-scaff_15838_5-p1182961	Bn-N11-p4400975	gene	exon
Bn-scaff_15838_5-p1208686	Bn-N11-p4410676	gene	exon
Bn-scaff_15838_5-p1208759	Bn-N11-p4410749	gene	exon
Bn-scaff_15838_5-p1208892	Bn-N11-p4410882	gene	exon
Bn-scaff_15838_5-p1209957	Bn-N11-p4417387	gene	
Bn-scaff_15838_5-p1210444	Bn-N11-p4417868	gene	

Bn-scaff_15838_5-p1215697	Bn-N11-p4422754	gene	exon
Bn-scaff_15838_5-p123944	Bn-N11-p3245136	gene	exon
Bn-scaff_15838_5-p151738	Bn-N11-p3280931	gene	
Bn-scaff_15838_5-p152076	Bn-N11-p3281268	gene	
Bn-scaff_15838_5-p164551	Bn-N11-p3293391	gene	
Bn-scaff_15838_5-p165094	Bn-N11-p3293934	gene	
Bn-scaff_15838_5-p202432	Bn-N11-p3315863	gene	exon
Bn-scaff_15838_5-p217291	Bn-N11-p3329956	gene	exon
Bn-scaff_15838_5-p224495	Bn-N11-p3340342	gene	exon
Bn-scaff_15838_5-p227630	Bn-N11-p3343538	gene	
Bn-scaff_15838_5-p236972	Bn-N11-p3355446	gene	
Bn-scaff_15838_5-p241183	Bn-N11-p3359669	gene	
Bn-scaff_15838_5-p304302	Bn-N11-p3419694	gene	
Bn-scaff_15838_5-p329539	Bn-N11-p3445612	gene	
Bn-scaff_15838_5-p330543	Bn-N11-p3446628	gene	
Bn-scaff_15838_5-p331638	Bn-N11-p3450237	gene	
Bn-scaff_15838_5-p335984	Bn-N11-p3455578	gene	
Bn-scaff_15838_5-p382969	Bn-N11-p3496067	gene	exon
Bn-scaff_15838_5-p441651	Bn-N11-p3563780	gene	exon
Bn-scaff_15838_5-p444851	Bn-N11-p3567003	gene	
Bn-scaff_15838_5-p444873	Bn-N11-p3567025	gene	exon
Bn-scaff_15838_5-p468773	Bn-N1-p2772283	gene	exon
Bn-scaff_15838_5-p478688	Bn-N11-p3601869	gene	
Bn-scaff_15838_5-p479433	Bn-N11-p3602607	gene	
Bn-scaff_15838_5-p480491	Bn-N11-p3603661	gene	
Bn-scaff_15838_5-p480567	Bn-N11-p3603737	gene	
Bn-scaff_15838_5-p480957	Bn-N11-p3604118	gene	
Bn-scaff_15838_5-p483184	Bn-N11-p3607555	gene	exon
Bn-scaff_15838_5-p484014	Bn-N1-p2788020	gene	
Bn-scaff_15838_5-p513730	Bn-N1-p2815315	gene	exon
Bn-scaff_15838_5-p568283	Bn-N1-p2854509	gene	
Bn-scaff_15838_5-p603655	Bn-N11-p3801909	gene	
Bn-scaff_15838_5-p625786	Bn-N11-p3819489	gene	
Bn-scaff_15838_5-p626078	Bn-N11-p3819781	gene	exon
Bn-scaff_15838_5-p648080	Bn-N1-p2917518	gene	
Bn-scaff_15838_5-p672971	Bn-N1-p2930747	gene	
Bn-scaff_15838_5-p706717	Bn-N11-p3882758	gene	
Bn-scaff_15838_5-p779070	Bn-N11-p3953787	gene	exon
Bn-scaff_15838_5-p850445	Bn-N11-p4038071	gene	exon
Bn-scaff_15838_5-p850964	Bn-N11-p4038590	gene	
Bn-scaff_15838_5-p884827	Bn-N11-p4073422	gene	exon
Bn-scaff_15838_5-p885166	Bn-N11-p4073761	gene	exon
Bn-scaff_15838_5-p886564	Bn-N11-p4081169	gene	
Bn-scaff_15838_5-p929569	Bn-N11-p4135334	gene	exon
Bn-scaff_15838_5-p944359	Bn-N1-p3140026	gene	exon
Bn-scaff_15838_5-p954588	Bn-N11-p4161289	gene	
Bn-scaff_15844_1-p111925	Bn-N11-p36982647	gene	
Bn-scaff_15844_1-p119216	Bn-N11-p36990184	gene	exon
Bn-scaff_15844_1-p163992	Bn-N1-p22154564	gene	exon
Bn-scaff_15844_1-p184165	Bn-N11-p37069869	gene	
Bn-scaff_15844_1-p222290	Bn-N11-p37108036	gene	
Bn-scaff_15844_1-p6065	Bn-N11-p36876178	gene	exon

Bn-scaff_15844_1-p6285	Bn-N11-p36876398	gene	exon
Bn-scaff_15844_1-p9633	Bn-N11-p36879747	gene	
Bn-scaff_15847_1-p121544	Bn-N17-p13875407	gene	
Bn-scaff_15847_1-p264646	Bn-N19-p17339897	gene	exon
Bn-scaff_15847_1-p265134	Bn-N19-p17339410	gene	exon
Bn-scaff_15852_1-p234569	Bn-N14-p44390234	gene	
Bn-scaff_15852_1-p263989	Bn-N14-p44370575	gene	exon
Bn-scaff_15852_1-p435102	Bn-N4-p13065377	gene	exon
Bn-scaff_15852_1-p483817	Bn-N14-p44114987	gene	exon
Bn-scaff_15852_1-p526799	Bn-N14-p44070049	gene	exon
Bn-scaff_15852_1-p549809	Bn-N14-p44047060	gene	exon
Bn-scaff_15852_1-p593067	Bn-N14-p44002091	gene	
Bn-scaff_15852_1-p622579	Bn-N15-p21427152	gene	
Bn-scaff_15856_1-p119395	Bn-N15-p48268203	gene	
Bn-scaff_15856_1-p50968	Bn-N1-p26927669	gene	
Bn-scaff_15856_1-p55934	Bn-N15-p48200037	gene	
Bn-scaff_15856_1-p78904	Bn-N5-p25534920	gene	exon
Bn-scaff_15856_1-p79207	Bn-N5-p25535222	gene	exon
Bn-scaff_15856_1-p80690	Bn-N5-p25536705	gene	exon
Bn-scaff_15877_1-p1015445	Bn-N13-p5672849	gene	exon
Bn-scaff_15877_1-p1067997	Bn-N13-p5725424	gene	exon
Bn-scaff_15877_1-p152569	Bn-N13-p4209655	gene	exon
Bn-scaff_15877_1-p173651	Bn-N13-p4239190	gene	exon
Bn-scaff_15877_1-p173818	Bn-N13-p4239357	gene	exon
Bn-scaff_15877_1-p184815	Bn-N13-p4250640	gene	exon
Bn-scaff_15877_1-p184866	Bn-N13-p4250691	gene	exon
Bn-scaff_15877_1-p184927	Bn-N13-p4250751	gene	exon
Bn-scaff_15877_1-p185777	Bn-N3-p3351352	gene	exon
Bn-scaff_15877_1-p186528	Bn-N13-p4252360	gene	
Bn-scaff_15877_1-p205102	Bn-N3-p3363208	gene	exon
Bn-scaff_15877_1-p273957	Bn-N13-p4312899	gene	exon
Bn-scaff_15877_1-p282014	Bn-N13-p4321147	gene	exon
Bn-scaff_15877_1-p289796	Bn-N13-p4329042	gene	
Bn-scaff_15877_1-p292927	Bn-N13-p4331385	gene	exon
Bn-scaff_15877_1-p293326	Bn-N13-p4331784	gene	exon
Bn-scaff_15877_1-p293578	Bn-N13-p4332036	gene	exon
Bn-scaff_15877_1-p293599	Bn-N13-p4332057	gene	exon
Bn-scaff_15877_1-p294800	Bn-N13-p4333259	gene	
Bn-scaff_15877_1-p294806	Bn-N13-p4333265	gene	
Bn-scaff_15877_1-p295197	Bn-N13-p4333652	gene	exon
Bn-scaff_15877_1-p295224	Bn-N13-p4333679	gene	exon
Bn-scaff_15877_1-p306167	Bn-N13-p4343820	gene	
Bn-scaff_15877_1-p349800	Bn-N13-p4389906	gene	
Bn-scaff_15877_1-p353421	Bn-N13-p4393527	gene	
Bn-scaff_15877_1-p363914	Bn-N13-p4404019	gene	
Bn-scaff_15877_1-p364706	Bn-N13-p4404811	gene	
Bn-scaff_15877_1-p420181	Bn-N13-p4453122	gene	
Bn-scaff_15877_1-p423112	Bn-N13-p4456065	gene	exon
Bn-scaff_15877_1-p4822	Bn-N3-p3215603	gene	
Bn-scaff_15877_1-p555712	Bn-N13-p4592351	gene	
Bn-scaff_15877_1-p617921	Bn-N13-p4662761	gene	
Bn-scaff_15877_1-p655934	Bn-N13-p4700688	gene	

Bn-scaff_15877_1-p69630	Bn-N13-p4120535	gene	exon
Bn-scaff_15877_1-p69781	Bn-N13-p4120686	gene	
Bn-scaff_15877_1-p715686	Bn-N3-p3752600	gene	exon
Bn-scaff_15877_1-p717745	Bn-N19-p48785869	gene	exon
Bn-scaff_15877_1-p718090	Bn-N13-p5299943	gene	exon
Bn-scaff_15877_1-p719944	Bn-N13-p5302465	gene	
Bn-scaff_15877_1-p730349	Bn-N13-p5313276	gene	
Bn-scaff_15877_1-p730358	Bn-N13-p5313285	gene	
Bn-scaff_15877_1-p730440	Bn-N13-p5313367	gene	
Bn-scaff_15877_1-p730579	Bn-N13-p5313506	gene	
Bn-scaff_15877_1-p730758	Bn-N13-p5313735	gene	
Bn-scaff_15877_1-p730826	Bn-N13-p5313803	gene	
Bn-scaff_15877_1-p731133	Bn-N13-p5314110	gene	
Bn-scaff_15877_1-p731158	Bn-N13-p5314135	gene	
Bn-scaff_15877_1-p731212	Bn-N13-p5314189	gene	
Bn-scaff_15877_1-p732473	Bn-N13-p5315450	gene	exon
Bn-scaff_15877_1-p732865	Bn-N13-p5315842	gene	
Bn-scaff_15877_1-p741674	Bn-N13-p5324207	gene	
Bn-scaff_15877_1-p742470	Bn-N13-p5325003	gene	exon
Bn-scaff_15877_1-p743362	Bn-N13-p5325895	gene	
Bn-scaff_15877_1-p744228	Bn-N13-p5326762	gene	exon
Bn-scaff_15877_1-p744483	Bn-N13-p5327017	gene	
Bn-scaff_15877_1-p744489	Bn-N13-p5327023	gene	
Bn-scaff_15877_1-p744560	Bn-N13-p5327094	gene	exon
Bn-scaff_15877_1-p746992	Bn-N13-p5328849	gene	exon
Bn-scaff_15877_1-p754954	Bn-N13-p5346713	gene	
Bn-scaff_15877_1-p802484	Bn-N13-p5403885	gene	
Bn-scaff_15877_1-p835991	Bn-N13-p5435371	gene	
Bn-scaff_15877_1-p901317	Bn-N3-p3918675	gene	
Bn-scaff_15877_1-p907858	Bn-N13-p5563875	gene	
Bn-scaff_15877_1-p908058	Bn-N13-p5564075	gene	
Bn-scaff_15877_1-p921076	Bn-N13-p5579592	gene	exon
Bn-scaff_15877_1-p922952	Bn-N13-p5581467	gene	
Bn-scaff_15877_1-p923112	Bn-N13-p5581627	gene	
Bn-scaff_15877_1-p926737	Bn-N13-p5585256	gene	
Bn-scaff_15877_1-p92833	Bn-N13-p4142604	gene	
Bn-scaff_15877_1-p936945	Bn-N3-p3948603	gene	exon
Bn-scaff_15877_1-p985222	Bn-N13-p5650134	gene	exon
Bn-scaff_15879_1-p21018	Bn-N11-p34818028	gene	
Bn-scaff_15879_1-p22763	Bn-N11-p34819773	gene	
Bn-scaff_15879_1-p29129	Bn-N11-p34826133	gene	exon
Bn-scaff_15879_1-p29379	Bn-N11-p34826382	gene	exon
Bn-scaff_15879_1-p467229	Bn-N11-p35239153	gene	
Bn-scaff_15879_1-p690531	Bn-N11-p35454733	gene	exon
Bn-scaff_15879_1-p715384	Bn-N11-p35480242	gene	
Bn-scaff_15881_1-p123524	Bn-N19-p45838088	gene	
Bn-scaff_15881_1-p181813	Bn-N19-p45906521	gene	
Bn-scaff_15881_1-p92161	Bn-N14-p41903684	gene	exon
Bn-scaff_15892_1-p1113105	Bn-N16-p30379753	gene	exon
Bn-scaff_15892_1-p1115024	Bn-N16-p30381666	gene	exon
Bn-scaff_15892_1-p112551	Bn-N16-p29427599	gene	exon
Bn-scaff_15892_1-p1141154	Bn-N16-p30409424	gene	exon

Bn-scaff_15892_1-p114893	Bn-N16-p29429917	gene	
Bn-scaff_15892_1-p114985	Bn-N16-p29430009	gene	
Bn-scaff_15892_1-p115171	Bn-N16-p29430172	gene	
Bn-scaff_15892_1-p1170714	Bn-N7-p18936843	gene	exon
Bn-scaff_15892_1-p1170828	Bn-N16-p30437555	gene	exon
Bn-scaff_15892_1-p1298704	Bn-N16-p30500549	gene	
Bn-scaff_15892_1-p1300181	Bn-N16-p30502025	gene	
Bn-scaff_15892_1-p1481274	Bn-N7-p19035471	gene	exon
Bn-scaff_15892_1-p1482014	Bn-N16-p30628435	gene	exon
Bn-scaff_15892_1-p156854	Bn-N16-p29468217	gene	
Bn-scaff_15892_1-p1682924	Bn-N16-p30814307	gene	
Bn-scaff_15892_1-p169731	Bn-N7-p18501062	gene	exon
Bn-scaff_15892_1-p191252	Bn-N7-p18511142	gene	exon
Bn-scaff_15892_1-p446753	Bn-N16-p29717587	gene	
Bn-scaff_15892_1-p544810	Bn-N16-p29821909	gene	exon
Bn-scaff_15892_1-p561526	Bn-N16-p29838403	gene	exon
Bn-scaff_15892_1-p563981	Bn-N16-p29840858	gene	exon
Bn-scaff_15892_1-p623966	Bn-N16-p29896610	gene	exon
Bn-scaff_15892_1-p959005	Bn-N11-p36604602	gene	
Bn-scaff_15892_1-p993042	Bn-N7-p18850477	gene	exon
Bn-scaff_15895_1-p167099	Bn-N10-p9001680	gene	exon
Bn-scaff_15896_1-p550030	Bn-N15-p5111205	gene	
Bn-scaff_15896_1-p613370	Bn-N8-p4929175	gene	
Bn-scaff_15896_1-p636698	Bn-N18-p14465750	gene	
Bn-scaff_15896_1-p64515	Bn-N18-p7924734	gene	
Bn-scaff_15906_1-p269015	Bn-N1-p15221054	gene	
Bn-scaff_15906_1-p285153	Bn-N11-p25958520	gene	exon
Bn-scaff_15908_1-p1128507	Bn-N14-p7266863	gene	exon
Bn-scaff_15908_1-p1136151	Bn-N14-p7275265	gene	exon
Bn-scaff_15908_1-p1136503	Bn-N14-p7275617	gene	
Bn-scaff_15908_1-p1179977	Bn-N14-p7319370	gene	
Bn-scaff_15908_1-p1181021	Bn-N14-p7320414	gene	
Bn-scaff_15908_1-p1188324	Bn-N14-p7327717	gene	exon
Bn-scaff_15908_1-p138105	Bn-N14-p6198738	gene	exon
Bn-scaff_15908_1-p140050	Bn-N5-p3870304	gene	exon
Bn-scaff_15908_1-p280031	Bn-N14-p6337948	gene	exon
Bn-scaff_15908_1-p289000	Bn-N14-p6347876	gene	
Bn-scaff_15908_1-p290321	Bn-N14-p6349194	gene	
Bn-scaff_15908_1-p432859	Bn-N5-p4015010	gene	exon
Bn-scaff_15908_1-p472716	Bn-N14-p6546116	gene	
Bn-scaff_15908_1-p557100	Bn-N14-p6639696	gene	
Bn-scaff_15908_1-p612641	Bn-N14-p6700088	gene	exon
Bn-scaff_15908_1-p651765	Bn-N14-p6743480	gene	exon
Bn-scaff_15908_1-p755460	Bn-N14-p6857602	gene	exon
Bn-scaff_15908_1-p770939	Bn-N14-p6874479	gene	exon
Bn-scaff_15908_1-p798787	Bn-N14-p6910887	gene	
Bn-scaff_15908_1-p833150	Bn-N14-p6942123	gene	
Bn-scaff_15908_1-p835545	Bn-N14-p6944553	gene	
Bn-scaff_15908_1-p850054	Bn-N14-p6960431	gene	
Bn-scaff_15908_1-p854571	Bn-N14-p6965278	gene	exon
Bn-scaff_15908_1-p875945	Bn-N14-p6988164	gene	exon
Bn-scaff_15908_1-p923102	Bn-N14-p7031797	gene	

Bn-scaff_15908_1-p938821	Bn-N14-p7043134	gene	exon
Bn-scaff_15911_1-p15553	Bn-N14-p23691013	gene	
Bn-scaff_15911_1-p290639	Bn-N14-p23453649	gene	exon
Bn-scaff_15911_1-p331958	Bn-N14-p23395562	gene	
Bn-scaff_15911_1-p360033	Bn-N14-p23365170	gene	
Bn-scaff_15911_1-p360381	Bn-N14-p23364822	gene	
Bn-scaff_15911_1-p360436	Bn-N14-p23364767	gene	
Bn-scaff_15911_1-p510887	Bn-N14-p24290028	gene	exon
Bn-scaff_15911_1-p516900	Bn-N14-p24284533	gene	exon
Bn-scaff_15911_1-p539489	Bn-N3-p33810490	gene	
Bn-scaff_15911_1-p559862	Bn-N14-p24242191	gene	
Bn-scaff_15911_1-p571243	Bn-N14-p24231232	gene	
Bn-scaff_15911_1-p571842	Bn-N14-p24230632	gene	
Bn-scaff_15911_1-p584543	Bn-N14-p24216216	gene	exon
Bn-scaff_15911_1-p590682	Bn-N14-p24210070	gene	
Bn-scaff_15911_1-p600329	Bn-N14-p24200383	gene	
Bn-scaff_15911_1-p611941	Bn-N14-p24186203	gene	
Bn-scaff_15911_1-p623766	Bn-N14-p24174953	gene	
Bn-scaff_15911_1-p624117	Bn-N14-p24174602	gene	exon
Bn-scaff_15918_1-p227752	Bn-N12-p42704138	gene	exon
Bn-scaff_15918_1-p303294	Bn-N12-p42780913	gene	exon
Bn-scaff_15918_1-p303816	Bn-N12-p42781436	gene	exon
Bn-scaff_15918_1-p77389	Bn-N2-p26401841	gene	exon
Bn-scaff_15918_1-p77407	Bn-N2-p26401822	gene	exon
Bn-scaff_15918_1-p77494	Bn-N2-p26401735	gene	
Bn-scaff_15918_1-p77704	Bn-N12-p42603176	gene	
Bn-scaff_15918_1-p82985	Bn-N12-p42618481	gene	exon
Bn-scaff_15918_1-p83785	Bn-N12-p42619281	gene	exon
Bn-scaff_15923_1-p1096132	Bn-N8-p18393020	gene	exon
Bn-scaff_15923_1-p1269397	Bn-Scaffold25034-p422	gene	exon
Bn-scaff_15923_1-p635095	Bn-N18-p25932440	gene	exon
Bn-scaff_15923_1-p785679	Bn-N8-p18237372	gene	
Bn-scaff_15930_1-p104962	Bn-N10-p7602231	gene	exon
Bn-scaff_15930_1-p105169	Bn-N10-p7602024	gene	exon
Bn-scaff_15930_1-p202997	Bn-N19-p38163044	gene	
Bn-scaff_15930_1-p72371	Bn-N19-p38305414	gene	
Bn-scaff_15936_1-p159213	Bn-N11-p40112374	gene	exon
Bn-scaff_15936_1-p202187	Bn-N1-p23909704	gene	
Bn-scaff_15936_1-p245327	Bn-N11-p40253445	gene	exon
Bn-scaff_15936_1-p264595	Bn-N11-p40272759	gene	exon
Bn-scaff_15936_1-p358800	Bn-N11-p40361351	gene	
Bn-scaff_15936_1-p383132	Bn-N11-p40389202	gene	
Bn-scaff_15936_1-p441603	Bn-N1-p24066438	gene	exon
Bn-scaff_15936_1-p444875	Bn-N11-p40461971	gene	
Bn-scaff_15936_1-p444993	Bn-N11-p40462089	gene	
Bn-scaff_15959_1-p37450	Bn-N9-p21111927	gene	exon
Bn-scaff_15959_1-p657425	Bn-N15-p27479033	gene	exon
Bn-scaff_15969_1-p191154	Bn-N15-p24967534	gene	
Bn-scaff_15969_1-p205432	Bn-N15-p24950944	gene	
Bn-scaff_15969_1-p276159	Bn-N15-p24886067	gene	exon
Bn-scaff_15969_1-p276286	Bn-N15-p24885940	gene	
Bn-scaff_15983_1-p584698	Bn-N9-p16551507	gene	exon

Bn-scaff_15993_1-p3906	Bn-N14-p14408926	gene	exon
Bn-scaff_16002_1-p1002120	Bn-N13-p15070603	gene	exon
Bn-scaff_16002_1-p1014503	Bn-N13-p15083069	gene	exon
Bn-scaff_16002_1-p1224962	Bn-N13-p14875439	gene	exon
Bn-scaff_16002_1-p1227296	Bn-N13-p14871228	gene	exon
Bn-scaff_16002_1-p1235661	Bn-N13-p14863073	gene	
Bn-scaff_16002_1-p1270726	Bn-N13-p14834487	gene	
Bn-scaff_16002_1-p1270822	Bn-N13-p14834392	gene	exon
Bn-scaff_16002_1-p1274791	Bn-N13-p14830352	gene	
Bn-scaff_16002_1-p1334406	Bn-N3-p10285674	gene	exon
Bn-scaff_16002_1-p1350172	Bn-N13-p14764021	gene	
Bn-scaff_16002_1-p1354832	Bn-N13-p14759341	gene	
Bn-scaff_16002_1-p1392678	Bn-N13-p14723568	gene	exon
Bn-scaff_16002_1-p1400264	Bn-N3-p10236351	gene	exon
Bn-scaff_16002_1-p1400602	Bn-N13-p14712275	gene	exon
Bn-scaff_16002_1-p1537079	Bn-N3-p10146687	gene	exon
Bn-scaff_16002_1-p1537435	Bn-N13-p14580172	gene	
Bn-scaff_16002_1-p1538744	Bn-N3-p10144981	gene	exon
Bn-scaff_16002_1-p1554395	Bn-N13-p14564572	gene	
Bn-scaff_16002_1-p1569056	Bn-N13-p14545154	gene	
Bn-scaff_16002_1-p1569070	Bn-N13-p14545142	gene	
Bn-scaff_16002_1-p1595096	Bn-N13-p14522881	gene	exon
Bn-scaff_16002_1-p1595246	Bn-N13-p14522731	gene	exon
Bn-scaff_16002_1-p1602193	Bn-N13-p14506131	gene	exon
Bn-scaff_16002_1-p1603062	Bn-N13-p14505262	gene	
Bn-scaff_16002_1-p1627403	Bn-N13-p14478668	gene	exon
Bn-scaff_16002_1-p1627629	Bn-N13-p14478442	gene	exon
Bn-scaff_16002_1-p1628134	Bn-N13-p14477937	gene	
Bn-scaff_16002_1-p1628209	Bn-N13-p14477862	gene	exon
Bn-scaff_16002_1-p1660852	Bn-N13-p14441324	gene	
Bn-scaff_16002_1-p1666051	Bn-N13-p14431397	gene	exon
Bn-scaff_16002_1-p1676442	Bn-N13-p14421104	gene	
Bn-scaff_16002_1-p1677804	Bn-N13-p14419738	gene	
Bn-scaff_16002_1-p1717929	Bn-N13-p14360891	gene	exon
Bn-scaff_16002_1-p1725846	Bn-N13-p14352461	gene	exon
Bn-scaff_16002_1-p1731397	Bn-N13-p14346911	gene	exon
Bn-scaff_16002_1-p1735395	Bn-N13-p14342920	gene	
Bn-scaff_16002_1-p1762342	Bn-N13-p14322905	gene	
Bn-scaff_16002_1-p1762359	Bn-N13-p14322888	gene	
Bn-scaff_16002_1-p1762565	Bn-N13-p14322682	gene	
Bn-scaff_16002_1-p1766250	Bn-N3-p10020562	gene	
Bn-scaff_16002_1-p178243	Bn-N13-p15899106	gene	exon
Bn-scaff_16002_1-p1842049	Bn-N13-p14242856	gene	
Bn-scaff_16002_1-p1916889	Bn-N3-p9945268	gene	exon
Bn-scaff_16002_1-p1992812	Bn-N3-p9882221	gene	
Bn-scaff_16002_1-p2012656	Bn-N13-p14082682	gene	
Bn-scaff_16002_1-p2036813	Bn-N13-p14055368	gene	exon
Bn-scaff_16002_1-p2037221	Bn-N13-p14054964	gene	exon
Bn-scaff_16002_1-p2037999	Bn-N13-p14052434	gene	exon
Bn-scaff_16002_1-p2055593	Bn-N13-p14029722	gene	exon
Bn-scaff_16002_1-p2066074	Bn-N13-p14019525	gene	exon
Bn-scaff_16002_1-p2076397	Bn-N13-p14008475	gene	

Bn-scaff_16002_1-p2126973	Bn-N13-p13958424	gene	exon
Bn-scaff_16002_1-p2131870	Bn-N13-p13953302	gene	
Bn-scaff_16002_1-p2144304	Bn-N13-p13940881	gene	
Bn-scaff_16002_1-p2172421	Bn-N13-p13909588	gene	exon
Bn-scaff_16002_1-p2172850	Bn-N13-p13909159	gene	exon
Bn-scaff_16002_1-p2201513	Bn-N13-p13865522	gene	
Bn-scaff_16002_1-p2215859	Bn-N13-p13851151	gene	
Bn-scaff_16002_1-p2217361	Bn-N13-p13849646	gene	
Bn-scaff_16002_1-p2221754	Bn-N13-p13845543	gene	
Bn-scaff_16002_1-p2233131	Bn-N13-p13837631	gene	
Bn-scaff_16002_1-p2243475	Bn-N13-p13827794	gene	
Bn-scaff_16002_1-p2244145	Bn-N13-p13827124	gene	
Bn-scaff_16002_1-p2245949	Bn-N13-p13822957	gene	
Bn-scaff_16002_1-p2247639	Bn-N13-p13821267	gene	
Bn-scaff_16002_1-p2285097	Bn-N13-p13783610	gene	exon
Bn-scaff_16002_1-p2291915	Bn-N13-p13776017	gene	
Bn-scaff_16002_1-p2292068	Bn-N13-p13775864	gene	
Bn-scaff_16002_1-p2299056	Bn-N13-p13768672	gene	
Bn-scaff_16002_1-p2307265	Bn-N13-p13759779	gene	
Bn-scaff_16002_1-p2307317	Bn-N13-p13759727	gene	
Bn-scaff_16002_1-p2307932	Bn-N13-p13759112	gene	
Bn-scaff_16002_1-p2320915	Bn-N13-p13746504	gene	exon
Bn-scaff_16002_1-p2321222	Bn-N13-p13746205	gene	
Bn-scaff_16002_1-p2321291	Bn-N13-p13746136	gene	
Bn-scaff_16002_1-p2334646	Bn-N13-p13732920	gene	exon
Bn-scaff_16002_1-p2441323	Bn-N13-p13602488	gene	
Bn-scaff_16002_1-p2511119	Bn-N13-p13531578	gene	
Bn-scaff_16002_1-p2512925	Bn-N13-p13529779	gene	
Bn-scaff_16002_1-p2517762	Bn-N13-p13524887	gene	exon
Bn-scaff_16002_1-p309640	Bn-N13-p15781094	gene	exon
Bn-scaff_16002_1-p329748	Bn-N3-p10981968	gene	exon
Bn-scaff_16002_1-p341408	Bn-N13-p15751427	gene	exon
Bn-scaff_16002_1-p388314	Bn-N3-p10944658	gene	
Bn-scaff_16002_1-p406108	Bn-N13-p15670842	gene	
Bn-scaff_16002_1-p482742	Bn-N13-p15596827	gene	exon
Bn-scaff_16002_1-p580789	Bn-N3-p10817473	gene	exon
Bn-scaff_16002_1-p602768	Bn-N13-p15468841	gene	exon
Bn-scaff_16002_1-p650113	Bn-N13-p15422636	gene	
Bn-scaff_16002_1-p727475	Bn-N13-p15345380	gene	
Bn-scaff_16002_1-p765237	Bn-N13-p15317849	gene	
Bn-scaff_16002_1-p778601	Bn-N13-p15306409	gene	
Bn-scaff_16002_1-p785360	Bn-N13-p15299701	gene	
Bn-scaff_16002_1-p785370	Bn-N13-p15299691	gene	
Bn-scaff_16002_1-p785986	Bn-N3-p10670430	gene	
Bn-scaff_16002_1-p842967	Bn-N13-p15237191	gene	exon
Bn-scaff_16002_1-p843894	Bn-N13-p15236264	gene	
Bn-scaff_16002_1-p980192	Bn-N3-p10566907	gene	exon
Bn-scaff_16002_1-p980195	Bn-N3-p10566904	gene	exon
Bn-scaff_16002_1-p998658	Bn-N3-p10556684	gene	
Bn-scaff_16021_1-p115430	Bn-N18-p44635485	gene	exon
Bn-scaff_16021_1-p167469	Bn-N18-p44587485	gene	
Bn-scaff_16021_1-p167771	Bn-N18-p44587182	gene	exon

Bn-scaff_16021_1-p167843	Bn-N9-p40471446	gene	exon
Bn-scaff_16021_1-p167885	Bn-N9-p40471404	gene	exon
Bn-scaff_16021_1-p175579	Bn-N9-p40465724	gene	
Bn-scaff_16021_1-p225665	Bn-N9-p40431754	gene	
Bn-scaff_16021_1-p225933	Bn-N9-p40431470	gene	exon
Bn-scaff_16021_1-p231138	Bn-Scaffold02064-p3633	gene	
Bn-scaff_16021_1-p585766	Bn-N18-p44455906	gene	exon
Bn-scaff_16021_1-p588868	Bn-N18-p44452836	gene	
Bn-scaff_16021_1-p63795	Bn-N10-p62209	gene	
Bn-scaff_16021_1-p64439	Bn-N15-p66148	gene	
Bn-scaff_16021_1-p65088	Bn-N15-p65356	gene	
Bn-scaff_16021_1-p661548	Bn-N18-p44373070	gene	
Bn-scaff_16021_1-p75322	Bn-N18-p44672210	gene	exon
Bn-scaff_16022_1-p413000	Bn-N18-p2339914	gene	
Bn-scaff_16022_1-p449389	Bn-N18-p2300909	gene	
Bn-scaff_16022_1-p502304	Bn-N18-p2180635	gene	
Bn-scaff_16022_1-p502660	Bn-N18-p2180278	gene	exon
Bn-scaff_16022_1-p596005	Bn-N18-p2105542	gene	
Bn-scaff_16022_1-p849431	Bn-Scaffold01052-p44529	gene	exon
Bn-scaff_16027_1-p319312	Bn-N14-p1470020	gene	
Bn-scaff_16027_1-p319363	Bn-N14-p1470071	gene	
Bn-scaff_16027_1-p319454	Bn-N14-p1470162	gene	
Bn-scaff_16027_1-p364820	Bn-N14-p1515985	gene	exon
Bn-scaff_16027_1-p367097	Bn-N14-p1518262	gene	
Bn-scaff_16027_1-p397922	Bn-N14-p1543989	gene	
Bn-scaff_16027_1-p63045	Bn-N14-p1234398	gene	exon
Bn-scaff_16027_1-p668374	Bn-N5-p1310356	gene	exon
Bn-scaff_16027_1-p70704	Bn-N14-p1242221	gene	
Bn-scaff_16042_1-p126540	Bn-N10-p8782272	gene	exon
Bn-scaff_16042_1-p331317	Bn-N10-p8870416	gene	exon
Bn-scaff_16042_1-p98299	Bn-N10-p8756392	gene	exon
Bn-scaff_16045_1-p147607	Bn-N15-p8446814	gene	
Bn-scaff_16045_1-p326419	Bn-N6-p6427954	gene	
Bn-scaff_16055_1-p1055037	Bn-N11-p32234337	gene	exon
Bn-scaff_16055_1-p1060688	Bn-N11-p32240182	gene	
Bn-scaff_16055_1-p1060937	Bn-N11-p32240431	gene	exon
Bn-scaff_16055_1-p1062293	Bn-N11-p32241787	gene	
Bn-scaff_16055_1-p1117736	Bn-N11-p32293393	gene	
Bn-scaff_16055_1-p1117843	Bn-N11-p32293500	gene	exon
Bn-scaff_16055_1-p1187264	Bn-N11-p32356412	gene	
Bn-scaff_16055_1-p119822	Bn-N11-p31319343	gene	exon
Bn-scaff_16055_1-p1261857	Bn-N11-p32434882	gene	
Bn-scaff_16055_1-p1317053	Bn-N11-p32484669	gene	exon
Bn-scaff_16055_1-p1320481	Bn-N11-p32488252	gene	
Bn-scaff_16055_1-p1543652	Bn-N11-p32692534	gene	
Bn-scaff_16055_1-p16059	Bn-N11-p31207547	gene	
Bn-scaff_16055_1-p448968	Bn-N11-p31684435	gene	
Bn-scaff_16055_1-p449019	Bn-N11-p31684486	gene	
Bn-scaff_16055_1-p451428	Bn-N11-p31686896	gene	
Bn-scaff_16055_1-p475420	Bn-N11-p31708129	gene	
Bn-scaff_16055_1-p613375	Bn-N11-p31849159	gene	exon
Bn-scaff_16055_1-p742258	Bn-N11-p31980537	gene	

Bn-scaff_16055_1-p777673	Bn-N11-p32010330	gene	
Bn-scaff_16055_1-p777908	Bn-N11-p32010565	gene	
Bn-scaff_16062_1-p289885	Bn-N15-p17650426	gene	
Bn-scaff_16062_1-p295083	Bn-N15-p17655644	gene	exon
Bn-scaff_16062_1-p295374	Bn-N15-p17655934	gene	exon
Bn-scaff_16062_1-p295518	Bn-N15-p17656078	gene	exon
Bn-scaff_16062_1-p295690	Bn-N15-p17656250	gene	exon
Bn-scaff_16062_1-p345501	Bn-N15-p17718171	gene	exon
Bn-scaff_16062_1-p345601	Bn-N15-p17718271	gene	exon
Bn-scaff_16064_1-p1009773	Bn-N7-p16343646	gene	
Bn-scaff_16064_1-p1046468	Bn-N7-p17637012	gene	exon
Bn-scaff_16064_1-p1047774	Bn-N16-p27459269	gene	exon
Bn-scaff_16064_1-p1086474	Bn-N16-p27418279	gene	exon
Bn-scaff_16064_1-p1137134	Bn-N16-p27349553	gene	exon
Bn-scaff_16064_1-p1146984	Bn-N7-p17566875	gene	
Bn-scaff_16064_1-p1154213	Bn-N16-p27334821	gene	exon
Bn-scaff_16064_1-p13237	Bn-N7-p18108481	gene	exon
Bn-scaff_16064_1-p1373906	Bn-N16-p27142786	gene	
Bn-scaff_16064_1-p1418053	Bn-N16-p27090910	gene	exon
Bn-scaff_16064_1-p1442092	Bn-N16-p27073909	gene	exon
Bn-scaff_16064_1-p1442106	Bn-N16-p27073895	gene	exon
Bn-scaff_16064_1-p144717	Bn-N16-p28374351	gene	
Bn-scaff_16064_1-p145597	Bn-N16-p28373471	gene	
Bn-scaff_16064_1-p151843	Bn-N16-p28366853	gene	
Bn-scaff_16064_1-p152930	Bn-N7-p18036846	gene	exon
Bn-scaff_16064_1-p158730	Bn-N7-p18033067	gene	exon
Bn-scaff_16064_1-p249990	Bn-N16-p28234104	gene	
Bn-scaff_16064_1-p26703	Bn-N16-p28510878	gene	exon
Bn-scaff_16064_1-p322157	Bn-N16-p28191288	gene	
Bn-scaff_16064_1-p360023	Bn-N16-p28158935	gene	
Bn-scaff_16064_1-p375131	Bn-N16-p28139421	gene	
Bn-scaff_16064_1-p380045	Bn-N16-p28133813	gene	
Bn-scaff_16064_1-p380597	Bn-N16-p28133263	gene	
Bn-scaff_16064_1-p380874	Bn-N16-p28132986	gene	
Bn-scaff_16064_1-p550807	Bn-N16-p27965794	gene	exon
Bn-scaff_16064_1-p615620	Bn-N7-p17842726	gene	exon
Bn-scaff_16064_1-p7157	Bn-N16-p28530524	gene	exon
Bn-scaff_16064_1-p9281	Bn-N16-p28528402	gene	
Bn-scaff_16064_1-p938130	Bn-N16-p27574086	gene	
Bn-scaff_16064_1-p938404	Bn-N16-p27573812	gene	
Bn-scaff_16064_1-p948906	Bn-N16-p27563136	gene	exon
Bn-scaff_16069_1-p1050288	Bn-N17-p41047184	gene	
Bn-scaff_16069_1-p1101589	Bn-N17-p41097896	gene	exon
Bn-scaff_16069_1-p1150783	Bn-N3-p23476924	gene	exon
Bn-scaff_16069_1-p1151520	Bn-N17-p41187436	gene	exon
Bn-scaff_16069_1-p1152134	Bn-N17-p41188052	gene	exon
Bn-scaff_16069_1-p1162324	Bn-N17-p41198251	gene	
Bn-scaff_16069_1-p1164388	Bn-N17-p41200308	gene	
Bn-scaff_16069_1-p1184092	Bn-N9-p22854028	gene	exon
Bn-scaff_16069_1-p1199961	Bn-N17-p41245352	gene	
Bn-scaff_16069_1-p1202176	Bn-N17-p41247582	gene	exon
Bn-scaff_16069_1-p1202826	Bn-N17-p41248233	gene	exon

Bn-scaff_16069_1-p1204476	Bn-N17-p41249846	gene	
Bn-scaff_16069_1-p1204477	Bn-N17-p41249847	gene	
Bn-scaff_16069_1-p1268741	Bn-N17-p41311087	gene	
Bn-scaff_16069_1-p1269936	Bn-N17-p41312282	gene	
Bn-scaff_16069_1-p1456269	Bn-N17-p41489839	gene	
Bn-scaff_16069_1-p1464840	Bn-N17-p41493500	gene	
Bn-scaff_16069_1-p1469430	Bn-N17-p41498245	gene	exon
Bn-scaff_16069_1-p1552377	Bn-N3-p23817221	gene	
Bn-scaff_16069_1-p1708862	Bn-N17-p41738273	gene	
Bn-scaff_16069_1-p17322	Bn-N17-p39944819	gene	exon
Bn-scaff_16069_1-p1776014	Bn-N3-p23996914	gene	exon
Bn-scaff_16069_1-p1781070	Bn-N17-p41795436	gene	
Bn-scaff_16069_1-p1786996	Bn-N3-p24008019	gene	
Bn-scaff_16069_1-p1789002	Bn-N17-p41803223	gene	
Bn-scaff_16069_1-p1815891	Bn-N17-p41847698	gene	exon
Bn-scaff_16069_1-p1916253	Bn-N17-p41949267	gene	exon
Bn-scaff_16069_1-p1957984	Bn-N17-p41992633	gene	exon
Bn-scaff_16069_1-p198607	Bn-N3-p22663303	gene	exon
Bn-scaff_16069_1-p2142538	Bn-N3-p24244791	gene	exon
Bn-scaff_16069_1-p2174360	Bn-N17-p42115777	gene	
Bn-scaff_16069_1-p2193948	Bn-N3-p24284075	gene	exon
Bn-scaff_16069_1-p2232174	Bn-Scaffold04490-p191	gene	
Bn-scaff_16069_1-p2323317	Bn-N17-p42265894	gene	
Bn-scaff_16069_1-p2408540	Bn-N17-p42360747	gene	
Bn-scaff_16069_1-p2577660	Bn-N17-p42530127	gene	exon
Bn-scaff_16069_1-p2584610	Bn-N17-p42537537	gene	
Bn-scaff_16069_1-p2607295	Bn-N17-p42621836	gene	
Bn-scaff_16069_1-p2611780	Bn-N17-p42622579	gene	
Bn-scaff_16069_1-p2666161	Bn-N17-p42680766	gene	
Bn-scaff_16069_1-p270593	Bn-N17-p40175403	gene	
Bn-scaff_16069_1-p2764934	Bn-N17-p42769944	gene	
Bn-scaff_16069_1-p2764992	Bn-N17-p42770003	gene	
Bn-scaff_16069_1-p2765187	Bn-N17-p42770197	gene	exon
Bn-scaff_16069_1-p2820796	Bn-N17-p42839390	gene	
Bn-scaff_16069_1-p2821021	Bn-N17-p42839615	gene	exon
Bn-scaff_16069_1-p2821319	Bn-N17-p42839914	gene	exon
Bn-scaff_16069_1-p2866381	Bn-N3-p24984422	gene	
Bn-scaff_16069_1-p2867813	Bn-N17-p42894320	gene	
Bn-scaff_16069_1-p2897181	Bn-N17-p42928058	gene	
Bn-scaff_16069_1-p2946061	Bn-N17-p42975451	gene	exon
Bn-scaff_16069_1-p2974287	Bn-N17-p43021305	gene	
Bn-scaff_16069_1-p2993184	Bn-Scaffold03128-p2586	gene	
Bn-scaff_16069_1-p2996601	Bn-N17-p43032301	gene	
Bn-scaff_16069_1-p3024157	Bn-N17-p43059081	gene	exon
Bn-scaff_16069_1-p306662	Bn-N17-p40297671	gene	
Bn-scaff_16069_1-p3093804	Bn-N17-p43120635	gene	
Bn-scaff_16069_1-p3098868	Bn-N11-p10436292	gene	
Bn-scaff_16069_1-p3099631	Bn-N17-p43123318	gene	
Bn-scaff_16069_1-p3117793	Bn-N17-p43144989	gene	
Bn-scaff_16069_1-p3131384	Bn-N17-p43154339	gene	exon
Bn-scaff_16069_1-p3135269	Bn-N17-p43158306	gene	
Bn-scaff_16069_1-p3138539	Bn-N17-p43161574	gene	

Bn-scaff_16069_1-p3154456	Bn-N17-p43181417	gene	
Bn-scaff_16069_1-p3210071	Bn-N17-p43237130	gene	exon
Bn-scaff_16069_1-p3210581	Bn-N17-p43237640	gene	
Bn-scaff_16069_1-p3217156	Bn-N17-p43244196	gene	exon
Bn-scaff_16069_1-p3217193	Bn-N17-p43244233	gene	exon
Bn-scaff_16069_1-p3263731	Bn-N17-p43290999	gene	exon
Bn-scaff_16069_1-p3275296	Bn-N17-p43301300	gene	
Bn-scaff_16069_1-p3311150	Bn-N17-p43326846	gene	
Bn-scaff_16069_1-p3311744	Bn-N17-p43327165	gene	exon
Bn-scaff_16069_1-p3375579	Bn-N17-p43386199	gene	exon
Bn-scaff_16069_1-p342150	Bn-N17-p40329509	gene	
Bn-scaff_16069_1-p342188	Bn-N17-p40329547	gene	
Bn-scaff_16069_1-p3435850	Bn-N17-p43447093	gene	
Bn-scaff_16069_1-p3455597	Bn-N17-p43467262	gene	exon
Bn-scaff_16069_1-p3463763	Bn-N17-p43475775	gene	
Bn-scaff_16069_1-p3470600	Bn-N17-p43478738	gene	exon
Bn-scaff_16069_1-p3486333	Bn-N17-p43494874	gene	
Bn-scaff_16069_1-p3583547	Bn-N3-p25706558	gene	exon
Bn-scaff_16069_1-p3596469	Bn-N17-p43606800	gene	exon
Bn-scaff_16069_1-p3596576	Bn-N17-p43606907	gene	exon
Bn-scaff_16069_1-p3602641	Bn-N17-p43612998	gene	exon
Bn-scaff_16069_1-p3608406	Bn-N17-p43618094	gene	exon
Bn-scaff_16069_1-p361062	Bn-N17-p40348972	gene	
Bn-scaff_16069_1-p3611263	Bn-N17-p43620938	gene	
Bn-scaff_16069_1-p3611621	Bn-N17-p43621296	gene	
Bn-scaff_16069_1-p3623226	Bn-N17-p43631978	gene	exon
Bn-scaff_16069_1-p3631187	Bn-N17-p43639910	gene	exon
Bn-scaff_16069_1-p3636672	Bn-N17-p43645650	gene	exon
Bn-scaff_16069_1-p3663377	Bn-N17-p43672300	gene	exon
Bn-scaff_16069_1-p3664108	Bn-N17-p43673031	gene	exon
Bn-scaff_16069_1-p3674000	Bn-N17-p43683771	gene	
Bn-scaff_16069_1-p3675332	Bn-N17-p43685103	gene	
Bn-scaff_16069_1-p3675519	Bn-N17-p43685291	gene	
Bn-scaff_16069_1-p3676607	Bn-N17-p43686378	gene	
Bn-scaff_16069_1-p3689610	Bn-N17-p43699329	gene	exon
Bn-scaff_16069_1-p3724227	Bn-N3-p25868620	gene	
Bn-scaff_16069_1-p3731985	Bn-N17-p43724035	gene	exon
Bn-scaff_16069_1-p3732050	Bn-N17-p43724100	gene	exon
Bn-scaff_16069_1-p3732051	Bn-N17-p43724101	gene	exon
Bn-scaff_16069_1-p3748310	Bn-N17-p43734683	gene	
Bn-scaff_16069_1-p3749614	Bn-N17-p43735984	gene	exon
Bn-scaff_16069_1-p3750766	Bn-Scaffold04006-p90	gene	exon
Bn-scaff_16069_1-p3780494	Bn-N17-p43775738	gene	
Bn-scaff_16069_1-p3861034	Bn-N17-p43880846	gene	
Bn-scaff_16069_1-p3864928	Bn-N17-p43882490	gene	exon
Bn-scaff_16069_1-p3887367	Bn-N17-p43906140	gene	
Bn-scaff_16069_1-p3906310	Bn-N17-p43924735	gene	
Bn-scaff_16069_1-p3909013	Bn-N17-p43927473	gene	
Bn-scaff_16069_1-p3926701	Bn-N17-p43942226	gene	exon
Bn-scaff_16069_1-p3937772	Bn-N17-p43953157	gene	exon
Bn-scaff_16069_1-p3940746	Bn-N17-p43956129	gene	
Bn-scaff_16069_1-p3942923	Bn-N17-p43958306	gene	

Bn-scaff_16069_1-p3945232	Bn-N17-p43960610	gene	
Bn-scaff_16069_1-p4039727	Bn-N17-p44054980	gene	exon
Bn-scaff_16069_1-p4045434	Bn-N17-p44060606	gene	
Bn-scaff_16069_1-p4071609	Bn-N17-p44086938	gene	exon
Bn-scaff_16069_1-p4074074	Bn-N17-p44089405	gene	
Bn-scaff_16069_1-p4240632	Bn-N17-p44256048	gene	
Bn-scaff_16069_1-p4246262	Bn-N17-p44262010	gene	
Bn-scaff_16069_1-p4252709	Bn-N17-p44268521	gene	
Bn-scaff_16069_1-p4306415	Bn-N17-p44318352	gene	
Bn-scaff_16069_1-p4306874	Bn-N17-p44318806	gene	
Bn-scaff_16069_1-p4327327	Bn-N17-p44340801	gene	
Bn-scaff_16069_1-p4332977	Bn-N17-p44349961	gene	exon
Bn-scaff_16069_1-p4333464	Bn-N17-p44350448	gene	exon
Bn-scaff_16069_1-p4333601	Bn-N17-p44350585	gene	exon
Bn-scaff_16069_1-p4341105	Bn-N17-p44353390	gene	exon
Bn-scaff_16069_1-p4358397	Bn-N17-p44374440	gene	
Bn-scaff_16069_1-p4382026	Bn-N17-p44395570	gene	exon
Bn-scaff_16069_1-p439239	Bn-N17-p40422106	gene	exon
Bn-scaff_16069_1-p4396361	Bn-N3-p26623905	gene	
Bn-scaff_16069_1-p4464312	Bn-N17-p44505279	gene	
Bn-scaff_16069_1-p4479258	Bn-N3-p26685356	gene	exon
Bn-scaff_16069_1-p4482899	Bn-N17-p44530761	gene	
Bn-scaff_16069_1-p4484876	Bn-N17-p44533688	gene	
Bn-scaff_16069_1-p4489254	Bn-N17-p44540283	gene	
Bn-scaff_16069_1-p4510578	Bn-N17-p44562731	gene	
Bn-scaff_16069_1-p4528345	Bn-N17-p44579386	gene	exon
Bn-scaff_16069_1-p4530987	Bn-N17-p44582055	gene	
Bn-scaff_16069_1-p4546846	Bn-N17-p44595392	gene	
Bn-scaff_16069_1-p4605280	Bn-N17-p44650324	gene	
Bn-scaff_16069_1-p4605330	Bn-N17-p44650374	gene	
Bn-scaff_16069_1-p4605588	Bn-N17-p44650632	gene	exon
Bn-scaff_16069_1-p4605776	Bn-N17-p44650820	gene	
Bn-scaff_16069_1-p4642584	Bn-N17-p44686294	gene	
Bn-scaff_16069_1-p4646214	Bn-N17-p44689015	gene	
Bn-scaff_16069_1-p4646255	Bn-N17-p44689056	gene	
Bn-scaff_16069_1-p4646457	Bn-N17-p44689272	gene	
Bn-scaff_16069_1-p4648828	Bn-N17-p44691641	gene	
Bn-scaff_16069_1-p4666209	Bn-N17-p44710279	gene	
Bn-scaff_16069_1-p4686169	Bn-N17-p44732624	gene	
Bn-scaff_16069_1-p473539	Bn-N17-p40455696	gene	
Bn-scaff_16069_1-p4741693	Bn-N17-p44761961	gene	
Bn-scaff_16069_1-p4752630	Bn-N17-p44771875	gene	exon
Bn-scaff_16069_1-p4802994	Bn-N17-p44822991	gene	
Bn-scaff_16069_1-p4803141	Bn-N17-p44823139	gene	exon
Bn-scaff_16069_1-p4803203	Bn-N17-p44823201	gene	exon
Bn-scaff_16069_1-p4805298	Bn-N17-p44825295	gene	
Bn-scaff_16069_1-p4805351	Bn-N17-p44825348	gene	exon
Bn-scaff_16069_1-p4805385	Bn-N17-p44825382	gene	
Bn-scaff_16069_1-p481921	Bn-N3-p22911707	gene	exon
Bn-scaff_16069_1-p4819823	Bn-N3-p27098073	gene	exon
Bn-scaff_16069_1-p482315	Bn-N3-p22906612	gene	
Bn-scaff_16069_1-p4828119	Bn-N17-p44849908	gene	exon

Bn-scaff_16069_1-p4849379	Bn-N17-p44867058	gene	exon
Bn-scaff_16069_1-p4854537	Bn-N17-p44876223	gene	exon
Bn-scaff_16069_1-p4896946	Bn-N17-p44921045	gene	exon
Bn-scaff_16069_1-p496534	Bn-N17-p40511220	gene	exon
Bn-scaff_16069_1-p541003	Bn-N3-p22989923	gene	
Bn-scaff_16069_1-p550328	Bn-N17-p40541999	gene	exon
Bn-scaff_16069_1-p557756	Bn-N17-p40549510	gene	
Bn-scaff_16069_1-p557815	Bn-N17-p40549569	gene	
Bn-scaff_16069_1-p557875	Bn-N17-p40549629	gene	
Bn-scaff_16069_1-p607537	Bn-N17-p40599650	gene	
Bn-scaff_16069_1-p625992	Bn-N17-p40625710	gene	
Bn-scaff_16069_1-p628565	Bn-N17-p40628259	gene	
Bn-scaff_16069_1-p652895	Bn-N3-p23060981	gene	
Bn-scaff_16069_1-p685101	Bn-N17-p40667082	gene	exon
Bn-scaff_16069_1-p695279	Bn-N17-p40675312	gene	exon
Bn-scaff_16069_1-p695336	Bn-N17-p40675369	gene	
Bn-scaff_16069_1-p695414	Bn-N17-p40675447	gene	
Bn-scaff_16069_1-p700577	Bn-N17-p40679688	gene	exon
Bn-scaff_16069_1-p700623	Bn-N17-p40679734	gene	exon
Bn-scaff_16069_1-p726538	Bn-N17-p40699735	gene	
Bn-scaff_16069_1-p754180	Bn-N17-p40730683	gene	
Bn-scaff_16069_1-p7747	Bn-N17-p39934947	gene	
Bn-scaff_16069_1-p801743	Bn-N17-p40787811	gene	
Bn-scaff_16069_1-p810060	Bn-N17-p40796148	gene	
Bn-scaff_16069_1-p812381	Bn-N17-p40798468	gene	exon
Bn-scaff_16069_1-p850880	Bn-N17-p40836508	gene	exon
Bn-scaff_16069_1-p942685	Bn-N17-p40934729	gene	
Bn-scaff_16069_1-p943494	Bn-N3-p23289552	gene	exon
Bn-scaff_16069_1-p944313	Bn-N3-p23290371	gene	exon
Bn-scaff_16069_1-p944589	Bn-N3-p23290647	gene	exon
Bn-scaff_16082_1-p17710	Bn-N15-p9413536	gene	
Bn-scaff_16082_1-p237359	Bn-N6-p6972790	gene	exon
Bn-scaff_16082_1-p33791	Bn-N15-p9406635	gene	exon
Bn-scaff_16082_1-p454878	Bn-N15-p8958496	gene	exon
Bn-scaff_16082_1-p466994	Bn-N15-p8936192	gene	
Bn-scaff_16082_1-p503495	Bn-N6-p6721063	gene	exon
Bn-scaff_16082_1-p518060	Bn-N15-p8898796	gene	
Bn-scaff_16082_1-p543841	Bn-N15-p8869053	gene	exon
Bn-scaff_16082_1-p543947	Bn-N6-p6685454	gene	exon
Bn-scaff_16082_1-p551611	Bn-N6-p6678266	gene	
Bn-scaff_16082_1-p566308	Bn-N15-p8844240	gene	
Bn-scaff_16082_1-p579183	Bn-N15-p8816261	gene	
Bn-scaff_16092_1-p10969	Bn-N13-p31101404	gene	
Bn-scaff_16092_1-p11235	Bn-N13-p31101670	gene	
Bn-scaff_16092_1-p129830	Bn-N13-p31278105	gene	
Bn-scaff_16092_1-p147262	Bn-N10-p3712944	gene	
Bn-scaff_16092_1-p370286	Bn-N3-p4722127	gene	
Bn-scaff_16092_1-p476550	Bn-N13-p31642393	gene	exon
Bn-scaff_16092_1-p671871	Bn-N3-p19639126	gene	exon
Bn-scaff_16092_1-p671925	Bn-N3-p19639180	gene	
Bn-scaff_16092_1-p677092	Bn-N13-p31843063	gene	exon
Bn-scaff_16092_1-p681859	Bn-N13-p31848052	gene	

Bn-scaff_16092_1-p703657	Bn-N13-p31867440	gene	exon
Bn-scaff_16092_1-p753442	Bn-N13-p31917295	gene	
Bn-scaff_16092_1-p846667	Bn-N13-p32019774	gene	exon
Bn-scaff_16092_1-p848001	Bn-N13-p32021111	gene	
Bn-scaff_16092_1-p866917	Bn-N3-p19734381	gene	exon
Bn-scaff_16092_1-p870157	Bn-N13-p32038310	gene	
Bn-scaff_16092_1-p889505	Bn-N13-p32057983	gene	
Bn-scaff_16092_1-p924766	Bn-N13-p32089397	gene	exon
Bn-scaff_16093_1-p44383	Bn-N15-p11405952	gene	exon
Bn-scaff_16095_1-p120222	Bn-N14-p11725431	gene	
Bn-scaff_16095_1-p22585	Bn-N14-p11628874	gene	
Bn-scaff_16095_1-p24601	Bn-N14-p11630926	gene	
Bn-scaff_16095_1-p28021	Bn-N14-p11634346	gene	
Bn-scaff_16095_1-p43615	Bn-N14-p11650614	gene	
Bn-scaff_16095_1-p82767	Bn-N14-p11690719	gene	exon
Bn-scaff_16100_1-p617160	Bn-N17-p21604862	gene	
Bn-scaff_16110_1-p1049197	Bn-N3-p30805159	gene	exon
Bn-scaff_16110_1-p1076947	Bn-N3-p30739869	gene	exon
Bn-scaff_16110_1-p111601	Bn-N17-p48719259	gene	exon
Bn-scaff_16110_1-p111711	Bn-N6-p26026798	gene	exon
Bn-scaff_16110_1-p1139348	Bn-N17-p47682165	gene	
Bn-scaff_16110_1-p1181825	Bn-N17-p47613654	gene	
Bn-scaff_16110_1-p1185491	Bn-N17-p47609993	gene	
Bn-scaff_16110_1-p1204999	Bn-N17-p47594347	gene	
Bn-scaff_16110_1-p1230955	Bn-N17-p47568578	gene	
Bn-scaff_16110_1-p1315734	Bn-N17-p47482705	gene	
Bn-scaff_16110_1-p1324212	Bn-N17-p47474221	gene	
Bn-scaff_16110_1-p1329125	Bn-N17-p47469311	gene	
Bn-scaff_16110_1-p1329246	Bn-N17-p47469190	gene	exon
Bn-scaff_16110_1-p1330898	Bn-N17-p47467532	gene	
Bn-scaff_16110_1-p135610	Bn-N17-p48696724	gene	exon
Bn-scaff_16110_1-p1389044	Bn-N17-p47408849	gene	exon
Bn-scaff_16110_1-p1389443	Bn-N17-p47408450	gene	
Bn-scaff_16110_1-p1451801	Bn-N17-p47352443	gene	
Bn-scaff_16110_1-p1533906	Bn-N15-p45246777	gene	
Bn-scaff_16110_1-p1582977	Bn-N17-p47242861	gene	
Bn-scaff_16110_1-p17868	Bn-N6-p26083624	gene	exon
Bn-scaff_16110_1-p18020	Bn-N6-p26083472	gene	exon
Bn-scaff_16110_1-p1891578	Bn-N17-p46855285	gene	
Bn-scaff_16110_1-p1939090	Bn-N17-p46811171	gene	
Bn-scaff_16110_1-p1948461	Bn-N17-p46790427	gene	
Bn-scaff_16110_1-p1986889	Bn-N17-p46746858	gene	exon
Bn-scaff_16110_1-p1987048	Bn-N17-p46746699	gene	exon
Bn-scaff_16110_1-p2028032	Bn-N17-p46702285	gene	
Bn-scaff_16110_1-p2029853	Bn-N3-p29361143	gene	
Bn-scaff_16110_1-p2030050	Bn-N17-p46700265	gene	
Bn-scaff_16110_1-p204649	Bn-N6-p25953086	gene	exon
Bn-scaff_16110_1-p2168026	Bn-N17-p46544240	gene	exon
Bn-scaff_16110_1-p2168404	Bn-N17-p46543863	gene	exon
Bn-scaff_16110_1-p2281371	Bn-N17-p46435741	gene	
Bn-scaff_16110_1-p2281432	Bn-N17-p46435680	gene	
Bn-scaff_16110_1-p2336219	Bn-N17-p46379593	gene	exon

Bn-scaff_16110_1-p2403789	Bn-N17-p46315458	gene	exon
Bn-scaff_16110_1-p2416207	Bn-N17-p46302881	gene	
Bn-scaff_16110_1-p2439331	Bn-N17-p46279913	gene	
Bn-scaff_16110_1-p2532535	Bn-N17-p46171749	gene	
Bn-scaff_16110_1-p2540852	Bn-N17-p46158475	gene	
Bn-scaff_16110_1-p2554704	Bn-N17-p46144687	gene	exon
Bn-scaff_16110_1-p2556157	Bn-N17-p46143233	gene	exon
Bn-scaff_16110_1-p2557297	Bn-N3-p28606805	gene	exon
Bn-scaff_16110_1-p2570414	Bn-N17-p46133260	gene	exon
Bn-scaff_16110_1-p2571584	Bn-N17-p46132089	gene	
Bn-scaff_16110_1-p2598394	Bn-N17-p46100692	gene	exon
Bn-scaff_16110_1-p2721568	Bn-N3-p28376955	gene	exon
Bn-scaff_16110_1-p2793943	Bn-N17-p45884797	gene	
Bn-scaff_16110_1-p2890287	Bn-N17-p45782916	gene	exon
Bn-scaff_16110_1-p2891118	Bn-N17-p45782085	gene	exon
Bn-scaff_16110_1-p2971780	Bn-N3-p28047133	gene	exon
Bn-scaff_16110_1-p2986286	Bn-N3-p28020434	gene	exon
Bn-scaff_16110_1-p2986510	Bn-N3-p28020210	gene	exon
Bn-scaff_16110_1-p2986546	Bn-N17-p45671127	gene	exon
Bn-scaff_16110_1-p2990045	Bn-N17-p45667629	gene	exon
Bn-scaff_16110_1-p2992015	Bn-N17-p45664990	gene	
Bn-scaff_16110_1-p3015887	Bn-N17-p45639424	gene	exon
Bn-scaff_16110_1-p3016282	Bn-N17-p45639021	gene	exon
Bn-scaff_16110_1-p3016602	Bn-N17-p45638701	gene	
Bn-scaff_16110_1-p3075966	Bn-N17-p45568368	gene	
Bn-scaff_16110_1-p3077130	Bn-N17-p45567203	gene	
Bn-scaff_16110_1-p3092217	Bn-N17-p45556463	gene	exon
Bn-scaff_16110_1-p3168173	Bn-N17-p45472597	gene	
Bn-scaff_16110_1-p3168236	Bn-N17-p45472534	gene	
Bn-scaff_16110_1-p3246958	Bn-N17-p45461078	gene	
Bn-scaff_16110_1-p3277472	Bn-N17-p45361594	gene	
Bn-scaff_16110_1-p3503616	Bn-N17-p45160580	gene	
Bn-scaff_16110_1-p3504170	Bn-N17-p45160012	gene	
Bn-scaff_16110_1-p3504222	Bn-N17-p45159960	gene	
Bn-scaff_16110_1-p3512482	Bn-N17-p45144008	gene	
Bn-scaff_16110_1-p3563652	Bn-N17-p45080325	gene	exon
Bn-scaff_16110_1-p3660759	Bn-N17-p44982614	gene	
Bn-scaff_16110_1-p3660807	Bn-N17-p44982566	gene	
Bn-scaff_16110_1-p3665531	Bn-N17-p44977842	gene	
Bn-scaff_16110_1-p3665685	Bn-N17-p44977688	gene	
Bn-scaff_16110_1-p3665779	Bn-N17-p44977594	gene	
Bn-scaff_16110_1-p3674923	Bn-N17-p44967898	gene	
Bn-scaff_16110_1-p410525	Bn-N6-p25783615	gene	exon
Bn-scaff_16110_1-p426547	Bn-N17-p48428449	gene	
Bn-scaff_16110_1-p436278	Bn-N17-p48419347	gene	exon
Bn-scaff_16110_1-p527111	Bn-N17-p48343837	gene	exon
Bn-scaff_16110_1-p550475	Bn-N17-p48312502	gene	exon
Bn-scaff_16110_1-p554783	Bn-N17-p48308176	gene	
Bn-scaff_16110_1-p594253	Bn-N17-p48264289	gene	exon
Bn-scaff_16110_1-p931172	Bn-N17-p47906587	gene	
Bn-scaff_16110_1-p947765	Bn-N17-p47889885	gene	exon
Bn-scaff_16116_1-p264169	Bn-N7-p19427144	gene	exon

Bn-scaff_16116_1-p265025	Bn-N16-p31464126	gene	exon
Bn-scaff_16116_1-p367607	Bn-N7-p19468658	gene	exon
Bn-scaff_16116_1-p373851	Bn-N7-p19474459	gene	exon
Bn-scaff_16116_1-p409531	Bn-N16-p31575631	gene	
Bn-scaff_16116_1-p50186	Bn-N16-p31292680	gene	
Bn-scaff_16116_1-p545075	Bn-N16-p31713864	gene	exon
Bn-scaff_16116_1-p7960	Bn-N16-p31251374	gene	
Bn-scaff_16116_1-p840566	Bn-N7-p19729843	gene	exon
Bn-scaff_16128_1-p115117	Bn-N14-p31608300	gene	exon
Bn-scaff_16128_1-p471514	Bn-N11-p9250775	gene	
Bn-scaff_16128_1-p499556	Bn-N1-p6377216	gene	exon
Bn-scaff_16128_1-p505089	Bn-N11-p9215794	gene	exon
Bn-scaff_16128_2-p106250	Bn-N11-p9588002	gene	
Bn-scaff_16128_2-p121684	Bn-N11-p9563586	gene	
Bn-scaff_16128_2-p123105	Bn-N11-p9562167	gene	
Bn-scaff_16128_2-p124279	Bn-N11-p9560992	gene	
Bn-scaff_16128_2-p149602	Bn-N11-p9532277	gene	
Bn-scaff_16128_2-p150911	Bn-N11-p9530956	gene	
Bn-scaff_16130_1-p1042526	Bn-N17-p31624844	gene	
Bn-scaff_16130_1-p1050666	Bn-N17-p31632419	gene	
Bn-scaff_16130_1-p122971	Bn-N17-p30760438	gene	exon
Bn-scaff_16130_1-p1313793	Bn-N6-p23193164	gene	exon
Bn-scaff_16130_1-p1623241	Bn-N6-p23018378	gene	exon
Bn-scaff_16130_1-p1997926	Bn-N17-p32310007	gene	exon
Bn-scaff_16130_1-p2006227	Bn-N17-p32319280	gene	exon
Bn-scaff_16130_1-p2007326	Bn-N17-p32320379	gene	exon
Bn-scaff_16130_1-p2105546	Bn-N17-p32422720	gene	exon
Bn-scaff_16130_1-p2106542	Bn-N17-p32423726	gene	
Bn-scaff_16130_1-p2106991	Bn-N17-p32424175	gene	
Bn-scaff_16130_1-p216749	Bn-N17-p30833449	gene	
Bn-scaff_16130_1-p2248228	Bn-N17-p32567869	gene	
Bn-scaff_16130_1-p2251656	Bn-N17-p32571279	gene	
Bn-scaff_16130_1-p2259290	Bn-N2-p2975407	gene	
Bn-scaff_16130_1-p2264713	Bn-N17-p32593754	gene	
Bn-scaff_16130_1-p2282903	Bn-N6-p22690942	gene	
Bn-scaff_16130_1-p2331239	Bn-N17-p32657823	gene	
Bn-scaff_16130_1-p2426412	Bn-N17-p32756610	gene	exon
Bn-scaff_16130_1-p2433175	Bn-N17-p32763644	gene	exon
Bn-scaff_16130_1-p2525629	Bn-N17-p32855427	gene	
Bn-scaff_16130_1-p258535	Bn-N17-p30881825	gene	exon
Bn-scaff_16130_1-p260449	Bn-N17-p30882700	gene	
Bn-scaff_16130_1-p260865	Bn-N17-p30883115	gene	exon
Bn-scaff_16130_1-p280712	Bn-N17-p30900473	gene	
Bn-scaff_16130_1-p280914	Bn-N17-p30900678	gene	
Bn-scaff_16130_1-p280940	Bn-N17-p30900704	gene	
Bn-scaff_16130_1-p286182	Bn-N17-p30910178	gene	
Bn-scaff_16130_1-p287427	Bn-N17-p30911424	gene	
Bn-scaff_16130_1-p293403	Bn-N17-p30917405	gene	
Bn-scaff_16130_1-p293649	Bn-N17-p30917651	gene	
Bn-scaff_16130_1-p447718	Bn-N17-p31076973	gene	
Bn-scaff_16130_1-p542753	Bn-N17-p31158770	gene	
Bn-scaff_16130_1-p646330	Bn-N2-p23542041	gene	exon

Bn-scaff_16130_1-p659049	Bn-N17-p31283084	gene	
Bn-scaff_16130_1-p659599	Bn-N17-p31283633	gene	exon
Bn-scaff_16130_1-p733651	Bn-N4-p12541782	gene	exon
Bn-scaff_16130_1-p763633	Bn-N17-p31307076	gene	exon
Bn-scaff_16130_1-p764056	Bn-N17-p31307499	gene	
Bn-scaff_16130_1-p799679	Bn-N17-p31344011	gene	exon
Bn-scaff_16130_1-p800124	Bn-N17-p31344456	gene	exon
Bn-scaff_16130_1-p800527	Bn-N17-p31344859	gene	
Bn-scaff_16130_2-p374682	Bn-N13-p33939811	gene	exon
Bn-scaff_16130_2-p374990	Bn-N3-p20576628	gene	exon
Bn-scaff_16130_2-p375201	Bn-N13-p33939292	gene	exon
Bn-scaff_16130_2-p403395	Bn-N13-p33912030	gene	
Bn-scaff_16130_2-p524217	Bn-N13-p33781131	gene	exon
Bn-scaff_16130_2-p525577	Bn-N13-p33779770	gene	exon
Bn-scaff_16130_2-p546231	Bn-N13-p33766847	gene	
Bn-scaff_16130_2-p713723	Bn-N13-p33591961	gene	exon
Bn-scaff_16130_2-p714662	Bn-N13-p33591022	gene	exon
Bn-scaff_16130_2-p718383	Bn-N13-p33588319	gene	exon
Bn-scaff_16130_5-p129193	Bn-N13-p33376773	gene	exon
Bn-scaff_16130_5-p92194	Bn-N13-p33413151	gene	exon
Bn-scaff_16130_5-p92290	Bn-N3-p20307270	gene	exon
Bn-scaff_16135_1-p183824	Bn-N13-p52085239	gene	
Bn-scaff_16135_1-p184280	Bn-N13-p52085695	gene	
Bn-scaff_16135_1-p195975	Bn-N13-p52098514	gene	exon
Bn-scaff_16135_1-p196922	Bn-N8-p17275444	gene	exon
Bn-scaff_16135_1-p230416	Bn-N13-p52124627	gene	
Bn-scaff_16135_1-p257329	Bn-N13-p52152019	gene	exon
Bn-scaff_16135_1-p290412	Bn-N13-p52183172	gene	
Bn-scaff_16135_1-p309515	Bn-N13-p52195526	gene	
Bn-scaff_16135_1-p309577	Bn-N13-p52195586	gene	
Bn-scaff_16135_1-p315469	Bn-N13-p52207197	gene	exon
Bn-scaff_16139_1-p1027358	Bn-N12-p46309771	gene	exon
Bn-scaff_16139_1-p1072066	Bn-N12-p46260370	gene	
Bn-scaff_16139_1-p1074318	Bn-N2-p28890715	gene	exon
Bn-scaff_16139_1-p1090344	Bn-N12-p46245892	gene	exon
Bn-scaff_16139_1-p1266852	Bn-N12-p46067022	gene	exon
Bn-scaff_16139_1-p1275385	Bn-N12-p46054802	gene	
Bn-scaff_16139_1-p1275964	Bn-N12-p46054223	gene	
Bn-scaff_16139_1-p1276670	Bn-N12-p46053331	gene	
Bn-scaff_16139_1-p1277806	Bn-N12-p46046906	gene	
Bn-scaff_16139_1-p1279588	Bn-N12-p46045123	gene	exon
Bn-scaff_16139_1-p1291126	Bn-N12-p46033601	gene	exon
Bn-scaff_16139_1-p1295124	Bn-N12-p46029446	gene	exon
Bn-scaff_16139_1-p1337221	Bn-N2-p28735992	gene	exon
Bn-scaff_16139_1-p1496638	Bn-N17-p48345883	gene	
Bn-scaff_16139_1-p259289	Bn-N12-p46958995	gene	
Bn-scaff_16139_1-p28203	Bn-N12-p46774538	gene	exon
Bn-scaff_16139_1-p41217	Bn-N12-p46786046	gene	
Bn-scaff_16139_1-p4995	Bn-N12-p46751624	gene	
Bn-scaff_16139_1-p660500	Bn-N12-p46660190	gene	exon
Bn-scaff_16139_1-p662939	Bn-N12-p46654390	gene	exon
Bn-scaff_16139_1-p721705	Bn-N12-p46601255	gene	exon

Bn-scaff_16139_1-p921208	Bn-N12-p46410950	gene	
Bn-scaff_16139_1-p921558	Bn-N12-p46410600	gene	exon
Bn-scaff_16139_1-p928671	Bn-N12-p46401639	gene	
Bn-scaff_16139_1-p959373	Bn-N12-p46370308	gene	
Bn-scaff_16139_1-p978370	Bn-N12-p46351331	gene	exon
Bn-scaff_16141_1-p131942	Bn-N17-p13596292	gene	exon
Bn-scaff_16141_1-p55687	Bn-N3-p19680763	gene	exon
Bn-scaff_16142_1-p166962	Bn-N18-p11577254	gene	
Bn-scaff_16142_1-p284811	Bn-N18-p11464139	gene	exon
Bn-scaff_16142_1-p295868	Bn-N18-p11452153	gene	
Bn-scaff_16142_1-p411484	Bn-N8-p7374438	gene	exon
Bn-scaff_16142_1-p418818	Bn-N18-p11326372	gene	
Bn-scaff_16142_1-p711070	Bn-N2-p10261029	gene	
Bn-scaff_16142_1-p723944	Bn-N9-p21096259	gene	exon
Bn-scaff_16142_1-p724998	Bn-N9-p21097313	gene	exon
Bn-scaff_16142_1-p726414	Bn-N14-p4044671	gene	
Bn-scaff_16142_1-p97803	Bn-N8-p7743305	gene	exon
Bn-scaff_16148_1-p121322	Bn-N8-p1558964	gene	
Bn-scaff_16148_1-p121470	Bn-N8-p1559113	gene	exon
Bn-scaff_16148_1-p21352	Bn-N13-p65375656	gene	exon
Bn-scaff_16148_1-p23001	Bn-N13-p65377297	gene	
Bn-scaff_16148_1-p294989	Bn-N13-p65737377	gene	
Bn-scaff_16148_1-p334833	Bn-N3-p35605083	gene	
Bn-scaff_16148_1-p365481	Bn-N13-p65817586	gene	exon
Bn-scaff_16148_1-p61488	Bn-N13-p65432143	gene	
Bn-scaff_16148_1-p63709	Bn-N13-p65434365	gene	exon
Bn-scaff_16148_1-p93513	Bn-N13-p65507397	gene	
Bn-scaff_16148_1-p94877	Bn-N13-p65508748	gene	exon
Bn-scaff_16148_1-p94937	Bn-N8-p1517049	gene	
Bn-scaff_16158_1-p284205	Bn-N18-p6091100	gene	exon
Bn-scaff_16158_1-p284923	Bn-N18-p6091856	gene	exon
Bn-scaff_16158_1-p377774	Bn-N18-p6179988	gene	
Bn-scaff_16166_1-p407410	Bn-N5-p18901492	gene	exon
Bn-scaff_16166_1-p44558	Bn-N5-p19038336	gene	exon
Bn-scaff_16174_1-p1033507	Bn-N6-p9393311	gene	
Bn-scaff_16174_1-p1163637	Bn-N18-p28358352	gene	
Bn-scaff_16174_1-p1340704	Bn-N18-p28527471	gene	
Bn-scaff_16174_1-p1342264	Bn-N18-p28529029	gene	exon
Bn-scaff_16174_1-p1505515	Bn-N18-p28707191	gene	
Bn-scaff_16174_1-p19311	Bn-N7-p9421680	gene	
Bn-scaff_16174_1-p6960	Bn-N18-p27176468	gene	exon
Bn-scaff_16181_1-p145919	Bn-N15-p18514292	gene	
Bn-scaff_16181_1-p15961	Bn-N15-p18650058	gene	exon
Bn-scaff_16181_1-p44437	Bn-N15-p18620814	gene	exon
Bn-scaff_16181_1-p45725	Bn-N15-p18619526	gene	exon
Bn-scaff_16182_1-p319123	Bn-N13-p58054641	gene	
Bn-scaff_16182_1-p481707	Bn-N5-p13220675	gene	exon
Bn-scaff_16182_1-p542363	Bn-N13-p57828272	gene	
Bn-scaff_16182_1-p571563	Bn-Scaffold01185-p24823	gene	
Bn-scaff_16182_1-p629084	Bn-N11-p2301442	gene	
Bn-scaff_16197_1-p1008308	Bn-N18-p38416954	gene	
Bn-scaff_16197_1-p1039642	Bn-N9-p35171964	gene	exon

Bn-scaff_16197_1-p1039692	Bn-N9-p35171914	gene	exon
Bn-scaff_16197_1-p1063639	Bn-N18-p38376281	gene	
Bn-scaff_16197_1-p1087526	Bn-N18-p38347170	gene	
Bn-scaff_16197_1-p1096399	Bn-N18-p38336612	gene	
Bn-scaff_16197_1-p1098469	Bn-N18-p38334542	gene	exon
Bn-scaff_16197_1-p1098747	Bn-N18-p38334263	gene	
Bn-scaff_16197_1-p1120033	Bn-N18-p38310987	gene	
Bn-scaff_16197_1-p1156345	Bn-N18-p38275203	gene	
Bn-scaff_16197_1-p1160568	Bn-N18-p38270960	gene	
Bn-scaff_16197_1-p1192886	Bn-N18-p38236074	gene	
Bn-scaff_16197_1-p1193122	Bn-N18-p38235838	gene	
Bn-scaff_16197_1-p1193512	Bn-N18-p38235447	gene	exon
Bn-scaff_16197_1-p1223841	Bn-N18-p38188114	gene	exon
Bn-scaff_16197_1-p1249438	Bn-N18-p38170754	gene	exon
Bn-scaff_16197_1-p1257731	Bn-N9-p35002894	gene	
Bn-scaff_16197_1-p1333425	Bn-N18-p38077716	gene	
Bn-scaff_16197_1-p1348777	Bn-N18-p38060280	gene	
Bn-scaff_16197_1-p135078	Bn-N18-p39334616	gene	exon
Bn-scaff_16197_1-p1382140	Bn-N18-p38030050	gene	
Bn-scaff_16197_1-p1388213	Bn-N9-p34891871	gene	exon
Bn-scaff_16197_1-p1404163	Bn-N18-p38008950	gene	exon
Bn-scaff_16197_1-p1486027	Bn-N18-p37922559	gene	exon
Bn-scaff_16197_1-p1635223	Bn-N18-p37787284	gene	
Bn-scaff_16197_1-p1725558	Bn-N18-p37701779	gene	
Bn-scaff_16197_1-p1745860	Bn-N18-p37673174	gene	exon
Bn-scaff_16197_1-p1758608	Bn-N18-p37666699	gene	
Bn-scaff_16197_1-p1760380	Bn-N18-p37664925	gene	exon
Bn-scaff_16197_1-p1761489	Bn-N18-p37663811	gene	exon
Bn-scaff_16197_1-p177403	Bn-N18-p39279720	gene	exon
Bn-scaff_16197_1-p177710	Bn-N18-p39279413	gene	
Bn-scaff_16197_1-p178006	Bn-N18-p39279119	gene	
Bn-scaff_16197_1-p1787278	Bn-N18-p37637096	gene	
Bn-scaff_16197_1-p1794176	Bn-N18-p37630210	gene	
Bn-scaff_16197_1-p1808027	Bn-N18-p37619057	gene	exon
Bn-scaff_16197_1-p1808165	Bn-N18-p37618919	gene	exon
Bn-scaff_16197_1-p1822297	Bn-N18-p37604727	gene	exon
Bn-scaff_16197_1-p1849406	Bn-N14-p38142057	gene	exon
Bn-scaff_16197_1-p1855471	Bn-N18-p37570726	gene	exon
Bn-scaff_16197_1-p1888759	Bn-N18-p37539300	gene	
Bn-scaff_16197_1-p1901158	Bn-N18-p37527241	gene	
Bn-scaff_16197_1-p1906605	Bn-N18-p37521794	gene	
Bn-scaff_16197_1-p1944534	Bn-N18-p37483413	gene	exon
Bn-scaff_16197_1-p1980881	Bn-N18-p37446103	gene	exon
Bn-scaff_16197_1-p1981260	Bn-N18-p37445724	gene	exon
Bn-scaff_16197_1-p1981549	Bn-N18-p37445434	gene	
Bn-scaff_16197_1-p2042922	Bn-N18-p37383018	gene	
Bn-scaff_16197_1-p2048929	Bn-N18-p37377078	gene	exon
Bn-scaff_16197_1-p2096622	Bn-N18-p37322645	gene	
Bn-scaff_16197_1-p2117067	Bn-N18-p37311186	gene	
Bn-scaff_16197_1-p2117522	Bn-N18-p37310731	gene	
Bn-scaff_16197_1-p2118928	Bn-N18-p37309705	gene	
Bn-scaff_16197_1-p2119076	Bn-N18-p37309556	gene	

Bn-scaff_16197_1-p2119334	Bn-N18-p37309297	gene	
Bn-scaff_16197_1-p2119424	Bn-N18-p37309207	gene	
Bn-scaff_16197_1-p2149887	Bn-N18-p37274397	gene	
Bn-scaff_16197_1-p2150128	Bn-N18-p37274170	gene	
Bn-scaff_16197_1-p222159	Bn-N18-p39241000	gene	
Bn-scaff_16197_1-p222284	Bn-N18-p39240875	gene	exon
Bn-scaff_16197_1-p225455	Bn-N18-p39237702	gene	
Bn-scaff_16197_1-p225468	Bn-N18-p39237690	gene	
Bn-scaff_16197_1-p2257972	Bn-N18-p37161528	gene	
Bn-scaff_16197_1-p225901	Bn-N18-p39237257	gene	
Bn-scaff_16197_1-p2313484	Bn-N18-p37099393	gene	
Bn-scaff_16197_1-p2329864	Bn-N18-p37084121	gene	exon
Bn-scaff_16197_1-p2337949	Bn-N9-p34068690	gene	exon
Bn-scaff_16197_1-p2356427	Bn-N18-p37044597	gene	exon
Bn-scaff_16197_1-p2356518	Bn-N18-p37044506	gene	exon
Bn-scaff_16197_1-p2365578	Bn-N18-p37027751	gene	exon
Bn-scaff_16197_1-p2366422	Bn-N18-p37026923	gene	
Bn-scaff_16197_1-p2378207	Bn-N18-p37637739	gene	
Bn-scaff_16197_1-p2398598	Bn-N18-p36985888	gene	exon
Bn-scaff_16197_1-p2433378	Bn-N18-p36963450	gene	
Bn-scaff_16197_1-p2476076	Bn-N18-p36926828	gene	
Bn-scaff_16197_1-p2481235	Bn-N18-p36921575	gene	
Bn-scaff_16197_1-p2488559	Bn-N18-p36913933	gene	
Bn-scaff_16197_1-p2491827	Bn-N18-p36910665	gene	
Bn-scaff_16197_1-p2493450	Bn-N18-p36909041	gene	
Bn-scaff_16197_1-p2495403	Bn-N18-p36907089	gene	
Bn-scaff_16197_1-p2520910	Bn-N18-p36872112	gene	exon
Bn-scaff_16197_1-p2521569	Bn-N18-p36871452	gene	exon
Bn-scaff_16197_1-p2521682	Bn-N18-p36871339	gene	
Bn-scaff_16197_1-p2521968	Bn-N18-p36871060	gene	exon
Bn-scaff_16197_1-p2522155	Bn-N18-p36870874	gene	
Bn-scaff_16197_1-p2522210	Bn-N18-p36870807	gene	
Bn-scaff_16197_1-p2523378	Bn-N18-p36869632	gene	exon
Bn-scaff_16197_1-p2525226	Bn-N18-p36867777	gene	
Bn-scaff_16197_1-p2540923	Bn-N18-p36851565	gene	
Bn-scaff_16197_1-p2541363	Bn-N18-p36851124	gene	
Bn-scaff_16197_1-p2581072	Bn-N18-p36809130	gene	exon
Bn-scaff_16197_1-p2581685	Bn-N18-p36808517	gene	
Bn-scaff_16197_1-p2581920	Bn-N18-p36808283	gene	
Bn-scaff_16197_1-p2582618	Bn-N18-p36807575	gene	exon
Bn-scaff_16197_1-p2593050	Bn-N18-p36800943	gene	exon
Bn-scaff_16197_1-p2597305	Bn-N18-p36796541	gene	exon
Bn-scaff_16197_1-p2626407	Bn-N18-p36767461	gene	
Bn-scaff_16197_1-p2640097	Bn-N18-p36753902	gene	exon
Bn-scaff_16197_1-p2836132	Bn-Scaffold04535-p777	gene	
Bn-scaff_16197_1-p2852488	Bn-N18-p36548316	gene	
Bn-scaff_16197_1-p2853374	Bn-N18-p36547424	gene	exon
Bn-scaff_16197_1-p2883093	Bn-N18-p36524368	gene	
Bn-scaff_16197_1-p2891250	Bn-N18-p36519429	gene	exon
Bn-scaff_16197_1-p2897439	Bn-N18-p36513244	gene	exon
Bn-scaff_16197_1-p293459	Bn-N10-p16607096	gene	
Bn-scaff_16197_1-p2958011	Bn-N9-p33529630	gene	exon

Bn-scaff_16197_1-p2958264	Bn-N18-p36464614	gene	exon
Bn-scaff_16197_1-p2958549	Bn-N18-p36464329	gene	
Bn-scaff_16197_1-p2958698	Bn-N18-p36464179	gene	exon
Bn-scaff_16197_1-p2962341	Bn-N18-p36460535	gene	
Bn-scaff_16197_1-p2963497	Bn-N18-p36459392	gene	exon
Bn-scaff_16197_1-p2968446	Bn-N18-p36454184	gene	exon
Bn-scaff_16197_1-p3006933	Bn-N18-p36431395	gene	exon
Bn-scaff_16197_1-p3019762	Bn-N18-p36418297	gene	exon
Bn-scaff_16197_1-p3019951	Bn-N18-p36418108	gene	
Bn-scaff_16197_1-p3020219	Bn-N9-p33485812	gene	exon
Bn-scaff_16197_1-p3020399	Bn-N9-p33485633	gene	exon
Bn-scaff_16197_1-p3020713	Bn-N9-p33485319	gene	exon
Bn-scaff_16197_1-p3021149	Bn-N18-p36416695	gene	exon
Bn-scaff_16197_1-p3021158	Bn-N18-p36416686	gene	exon
Bn-scaff_16197_1-p3023882	Bn-N18-p36413917	gene	exon
Bn-scaff_16197_1-p3054920	Bn-N18-p36386183	gene	exon
Bn-scaff_16197_1-p3062579	Bn-N18-p36378562	gene	exon
Bn-scaff_16197_1-p3070176	Bn-N18-p36370965	gene	
Bn-scaff_16197_1-p3111917	Bn-N18-p36314955	gene	
Bn-scaff_16197_1-p3112286	Bn-N18-p36314585	gene	exon
Bn-scaff_16197_1-p3112359	Bn-N18-p36314513	gene	exon
Bn-scaff_16197_1-p3112707	Bn-N18-p36314165	gene	exon
Bn-scaff_16197_1-p3148021	Bn-N9-p33380515	gene	exon
Bn-scaff_16197_1-p325607	Bn-N18-p39135243	gene	
Bn-scaff_16197_1-p3294723	Bn-N18-p36117831	gene	
Bn-scaff_16197_1-p362815	Bn-N9-p35795927	gene	exon
Bn-scaff_16197_1-p394169	Bn-N18-p39075893	gene	exon
Bn-scaff_16197_1-p453838	Bn-N18-p39000256	gene	exon
Bn-scaff_16197_1-p47391	Bn-N18-p39441240	gene	exon
Bn-scaff_16197_1-p489638	Bn-N18-p38965167	gene	exon
Bn-scaff_16197_1-p489972	Bn-N18-p38964833	gene	exon
Bn-scaff_16197_1-p490092	Bn-N18-p38964713	gene	
Bn-scaff_16197_1-p490649	Bn-N9-p35681679	gene	exon
Bn-scaff_16197_1-p504820	Bn-N18-p38948144	gene	
Bn-scaff_16197_1-p506765	Bn-N18-p38946203	gene	
Bn-scaff_16197_1-p508785	Bn-N9-p35661024	gene	exon
Bn-scaff_16197_1-p595290	Bn-N18-p38868460	gene	
Bn-scaff_16197_1-p630099	Bn-N18-p38836466	gene	
Bn-scaff_16197_1-p634285	Bn-N18-p38831112	gene	
Bn-scaff_16197_1-p63640	Bn-N9-p36100262	gene	exon
Bn-scaff_16197_1-p637120	Bn-N18-p38827089	gene	
Bn-scaff_16197_1-p652619	Bn-N18-p38808093	gene	exon
Bn-scaff_16197_1-p679283	Bn-N18-p38787368	gene	exon
Bn-scaff_16197_1-p689617	Bn-N18-p38774778	gene	
Bn-scaff_16197_1-p697008	Bn-N18-p38767644	gene	exon
Bn-scaff_16197_1-p732813	Bn-N18-p38712876	gene	exon
Bn-scaff_16197_1-p739645	Bn-N18-p38705973	gene	
Bn-scaff_16197_1-p739973	Bn-N18-p38705645	gene	
Bn-scaff_16197_1-p740084	Bn-N18-p38705533	gene	
Bn-scaff_16197_1-p74067	Bn-N18-p39416263	gene	
Bn-scaff_16197_1-p870393	Bn-N9-p35302873	gene	
Bn-scaff_16197_1-p974284	Bn-N18-p38459102	gene	exon

Bn-scaff_16200_1-p105426	Bn-N18-p32473648	gene	exon
Bn-scaff_16200_1-p111092	Bn-N18-p32467969	gene	
Bn-scaff_16200_1-p122106	Bn-N18-p32456956	gene	
Bn-scaff_16200_1-p280959	Bn-N17-p12862972	gene	
Bn-scaff_16200_1-p338436	Bn-N17-p12905730	gene	
Bn-scaff_16200_1-p339334	Bn-N7-p5500554	gene	exon
Bn-scaff_16200_1-p388097	Bn-N17-p12969459	gene	exon
Bn-scaff_16200_1-p388152	Bn-N17-p12969514	gene	
Bn-scaff_16200_1-p388253	Bn-N17-p12969615	gene	
Bn-scaff_16200_1-p388352	Bn-N17-p12969714	gene	exon
Bn-scaff_16200_1-p536263	Bn-N17-p13143312	gene	exon
Bn-scaff_16200_1-p608563	Bn-N17-p13223587	gene	
Bn-scaff_16200_1-p609532	Bn-N17-p13224555	gene	exon
Bn-scaff_16200_1-p616097	Bn-N17-p13230415	gene	
Bn-scaff_16200_1-p616394	Bn-N17-p13230711	gene	
Bn-scaff_16200_1-p616552	Bn-N17-p13230871	gene	
Bn-scaff_16200_1-p616708	Bn-N17-p13231027	gene	
Bn-scaff_16200_1-p616843	Bn-N17-p13231162	gene	
Bn-scaff_16200_1-p617013	Bn-N17-p13231332	gene	
Bn-scaff_16200_1-p617473	Bn-N17-p13231792	gene	
Bn-scaff_16200_1-p618169	Bn-N17-p13232488	gene	
Bn-scaff_16200_1-p622179	Bn-N17-p13236594	gene	
Bn-scaff_16200_1-p65647	Bn-N18-p32515926	gene	exon
Bn-scaff_16200_1-p91053	Bn-N18-p32488398	gene	exon
Bn-scaff_16200_1-p95910	Bn-N18-p32484549	gene	exon
Bn-scaff_16209_1-p117013	Bn-N12-p40587598	gene	
Bn-scaff_16209_1-p117145	Bn-N12-p40587466	gene	
Bn-scaff_16214_1-p172721	Bn-N14-p3043034	gene	
Bn-scaff_16214_1-p195959	Bn-N14-p3052920	gene	exon
Bn-scaff_16214_1-p263853	Bn-N14-p3144754	gene	
Bn-scaff_16214_1-p265043	Bn-N14-p3145948	gene	exon
Bn-scaff_16214_1-p265231	Bn-N5-p2096496	gene	exon
Bn-scaff_16214_1-p300788	Bn-N14-p3187446	gene	
Bn-scaff_16214_1-p301269	Bn-N14-p3187927	gene	exon
Bn-scaff_16214_1-p347223	Bn-N14-p3226772	gene	
Bn-scaff_16214_1-p347579	Bn-N14-p3227127	gene	
Bn-scaff_16214_1-p347758	Bn-N14-p3227306	gene	
Bn-scaff_16214_1-p349072	Bn-N14-p3228621	gene	
Bn-scaff_16214_1-p40152	Bn-N14-p2890268	gene	
Bn-scaff_16214_1-p40230	Bn-N14-p2890346	gene	
Bn-scaff_16214_1-p477058	Bn-N5-p2179039	gene	exon
Bn-scaff_16214_1-p562727	Bn-N5-p2211258	gene	
Bn-scaff_16214_1-p69186	Bn-N14-p2915439	gene	exon
Bn-scaff_16217_1-p116824	Bn-N14-p26013556	gene	
Bn-scaff_16217_1-p121250	Bn-N14-p26009127	gene	
Bn-scaff_16217_1-p123037	Bn-N14-p26007340	gene	exon
Bn-scaff_16217_1-p132134	Bn-N14-p25998464	gene	exon
Bn-scaff_16217_1-p181427	Bn-N14-p25948672	gene	
Bn-scaff_16217_1-p270268	Bn-N14-p25857550	gene	exon
Bn-scaff_16217_1-p428402	Bn-N14-p25702349	gene	exon
Bn-scaff_16217_1-p457905	Bn-N14-p25656254	gene	exon
Bn-scaff_16217_1-p473162	Bn-N14-p25639298	gene	

Bn-scaff_16217_1-p87998	Bn-N14-p26045300	gene	exon
Bn-scaff_16228_1-p171719	Bn-N5-p15101132	gene	exon
Bn-scaff_16231_1-p1011395	Bn-N18-p24196802	gene	exon
Bn-scaff_16231_1-p1014215	Bn-N18-p24193993	gene	
Bn-scaff_16231_1-p1015173	Bn-N18-p24193032	gene	
Bn-scaff_16231_1-p1024600	Bn-N18-p24183605	gene	exon
Bn-scaff_16231_1-p1026375	Bn-N18-p24181807	gene	exon
Bn-scaff_16231_1-p1036610	Bn-N8-p19062530	gene	exon
Bn-scaff_16231_1-p1093410	Bn-N18-p24104545	gene	exon
Bn-scaff_16231_1-p1120106	Bn-N18-p24076764	gene	
Bn-scaff_16231_1-p1176514	Bn-N18-p24011698	gene	exon
Bn-scaff_16231_1-p1176794	Bn-N18-p24011417	gene	exon
Bn-scaff_16231_1-p1176976	Bn-N18-p24011236	gene	exon
Bn-scaff_16231_1-p1177104	Bn-N18-p24011108	gene	exon
Bn-scaff_16231_1-p1180991	Bn-N18-p24007532	gene	
Bn-scaff_16231_1-p1188910	Bn-N18-p23995226	gene	
Bn-scaff_16231_1-p1189052	Bn-N18-p23995115	gene	
Bn-scaff_16231_1-p1209255	Bn-N18-p23972799	gene	
Bn-scaff_16231_1-p1231804	Bn-N18-p23952842	gene	
Bn-scaff_16231_1-p1235878	Bn-N18-p23948767	gene	exon
Bn-scaff_16231_1-p124584	Bn-N18-p25136170	gene	
Bn-scaff_16231_1-p1256187	Bn-N18-p23928309	gene	
Bn-scaff_16231_1-p1256308	Bn-N18-p23928188	gene	
Bn-scaff_16231_1-p1256858	Bn-N18-p23927638	gene	exon
Bn-scaff_16231_1-p1484437	Bn-N18-p23614763	gene	exon
Bn-scaff_16231_1-p1484816	Bn-N18-p23614384	gene	
Bn-scaff_16231_1-p1484914	Bn-N18-p23614287	gene	exon
Bn-scaff_16231_1-p1506772	Bn-N18-p23594905	gene	
Bn-scaff_16231_1-p1629494	Bn-N8-p19416572	gene	exon
Bn-scaff_16231_1-p1643097	Bn-N18-p23438785	gene	exon
Bn-scaff_16231_1-p1778148	Bn-N18-p23321376	gene	exon
Bn-scaff_16231_1-p1778471	Bn-N18-p23321052	gene	exon
Bn-scaff_16231_1-p1841458	Bn-N8-p19488518	gene	exon
Bn-scaff_16231_1-p203872	Bn-N18-p25076370	gene	exon
Bn-scaff_16231_1-p2042129	Bn-N18-p23042268	gene	
Bn-scaff_16231_1-p204841	Bn-N18-p25074822	gene	exon
Bn-scaff_16231_1-p205060	Bn-N18-p25074603	gene	exon
Bn-scaff_16231_1-p2213239	Bn-N18-p22891395	gene	
Bn-scaff_16231_1-p2213779	Bn-N18-p22890855	gene	
Bn-scaff_16231_1-p2274371	Bn-N8-p19696722	gene	exon
Bn-scaff_16231_1-p2274454	Bn-N18-p22796016	gene	exon
Bn-scaff_16231_1-p2287394	Bn-N8-p19706556	gene	exon
Bn-scaff_16231_1-p296794	Bn-N18-p24988414	gene	
Bn-scaff_16231_1-p490553	Bn-N18-p24772431	gene	
Bn-scaff_16231_1-p563765	Bn-N17-p42400764	gene	
Bn-scaff_16231_1-p576383	Bn-N18-p24679977	gene	
Bn-scaff_16231_1-p576632	Bn-N18-p24679727	gene	exon
Bn-scaff_16231_1-p577104	Bn-N18-p24679255	gene	exon
Bn-scaff_16231_1-p662722	Bn-N18-p24582718	gene	
Bn-scaff_16231_1-p678599	Bn-N18-p24561072	gene	
Bn-scaff_16231_1-p715762	Bn-N18-p24521394	gene	
Bn-scaff_16231_1-p734971	Bn-N5-p16486656	gene	

Bn-scaff_16231_1-p745514	Bn-N18-p24483166	gene	
Bn-scaff_16231_1-p745813	Bn-N18-p24482874	gene	
Bn-scaff_16231_1-p746922	Bn-N18-p24481717	gene	exon
Bn-scaff_16231_1-p747021	Bn-N18-p24481618	gene	exon
Bn-scaff_16231_1-p780465	Bn-N18-p24453181	gene	exon
Bn-scaff_16231_1-p780489	Bn-N18-p24453157	gene	
Bn-scaff_16231_1-p780750	Bn-N8-p18940986	gene	
Bn-scaff_16231_1-p786260	Bn-N18-p24447388	gene	exon
Bn-scaff_16231_1-p795075	Bn-N18-p24433888	gene	
Bn-scaff_16231_1-p795462	Bn-N18-p24433501	gene	
Bn-scaff_16231_1-p795518	Bn-N18-p24433445	gene	
Bn-scaff_16231_1-p795641	Bn-N18-p24433335	gene	
Bn-scaff_16231_1-p801359	Bn-N8-p18961142	gene	exon
Bn-scaff_16231_1-p847032	Bn-N18-p24405897	gene	
Bn-scaff_16231_1-p850157	Bn-N18-p24402776	gene	exon
Bn-scaff_16231_1-p853799	Bn-N8-p18989114	gene	
Bn-scaff_16231_1-p903042	Bn-N18-p24309959	gene	
Bn-scaff_16231_1-p905549	Bn-N18-p24307453	gene	exon
Bn-scaff_16231_1-p905567	Bn-N18-p24307436	gene	exon
Bn-scaff_16240_1-p13277	Bn-N14-p49708079	gene	
Bn-scaff_16240_1-p15203	Bn-N14-p49706152	gene	exon
Bn-scaff_16240_1-p246238	Bn-N4-p16394061	gene	
Bn-scaff_16240_1-p310848	Bn-N4-p16342115	gene	exon
Bn-scaff_16240_1-p310915	Bn-N14-p49354622	gene	exon
Bn-scaff_16240_1-p311457	Bn-N14-p49354082	gene	exon
Bn-scaff_16244_1-p25214	Bn-N11-p33701989	gene	
Bn-scaff_16244_1-p28539	Bn-N11-p33698641	gene	exon
Bn-scaff_16246_2-p21963	Bn-N19-p36750761	gene	
Bn-scaff_16246_2-p75351	Bn-N19-p36790794	gene	
Bn-scaff_16263_1-p445748	Bn-N5-p18589874	gene	exon
Bn-scaff_16263_1-p446017	Bn-N5-p18589605	gene	
Bn-scaff_16263_1-p446092	Bn-N5-p18589546	gene	
Bn-scaff_16263_1-p446224	Bn-N5-p18589414	gene	exon
Bn-scaff_16263_1-p459593	Bn-N5-p18574406	gene	exon
Bn-scaff_16263_1-p459656	Bn-N5-p18574343	gene	exon
Bn-scaff_16263_1-p459932	Bn-N5-p18574067	gene	exon
Bn-scaff_16268_1-p262996	Bn-N15-p14301787	gene	
Bn-scaff_16268_1-p533546	Bn-N15-p14019003	gene	exon
Bn-scaff_16268_1-p543187	Bn-N15-p14002109	gene	
Bn-scaff_16268_1-p564846	Bn-N15-p13978014	gene	exon
Bn-scaff_16268_1-p600077	Bn-N15-p13958409	gene	exon
Bn-scaff_16268_1-p600528	Bn-N15-p13957794	gene	exon
Bn-scaff_16268_1-p601866	Bn-N15-p13956454	gene	exon
Bn-scaff_16268_1-p87361	Bn-N9-p26405945	gene	exon
Bn-scaff_16269_1-p192431	Bn-N12-p8218856	gene	exon
Bn-scaff_16269_1-p197772	Bn-N2-p5818341	gene	exon
Bn-scaff_16269_1-p242401	Bn-N2-p5780519	gene	exon
Bn-scaff_16269_1-p296261	Bn-N2-p5732602	gene	exon
Bn-scaff_16269_1-p301771	Bn-N12-p8074757	gene	exon
Bn-scaff_16269_1-p304514	Bn-N2-p5717244	gene	
Bn-scaff_16269_1-p484840	Bn-N2-p5627603	gene	
Bn-scaff_16269_1-p529343	Bn-N12-p7835866	gene	

Bn-scaff_16269_1-p529695	Bn-N19-p43768379	gene	exon
Bn-scaff_16269_1-p533948	Bn-N12-p7830661	gene	
Bn-scaff_16269_1-p534639	Bn-N2-p5568974	gene	exon
Bn-scaff_16269_1-p599575	Bn-N12-p7763823	gene	
Bn-scaff_16269_1-p600151	Bn-N12-p21335454	gene	
Bn-scaff_16269_1-p609618	Bn-N12-p7758251	gene	exon
Bn-scaff_16269_1-p98735	Bn-N12-p8304924	gene	
Bn-scaff_16273_1-p227959	Bn-N8-p5959818	gene	exon
Bn-scaff_16273_1-p290254	Bn-N8-p6002800	gene	
Bn-scaff_16273_1-p291283	Bn-N8-p6003831	gene	
Bn-scaff_16273_1-p291496	Bn-N18-p8392653	gene	exon
Bn-scaff_16273_1-p340678	Bn-N18-p8436994	gene	exon
Bn-scaff_16273_1-p340796	Bn-N18-p8437112	gene	exon
Bn-scaff_16275_1-p819	Bn-N16-p2003770	gene	
Bn-scaff_16287_1-p21083	Bn-N18-p12154172	gene	
Bn-scaff_16287_1-p248964	Bn-N18-p12397041	gene	exon
Bn-scaff_16287_1-p28498	Bn-N18-p12161537	gene	
Bn-scaff_16287_1-p294064	Bn-N18-p12421848	gene	exon
Bn-scaff_16287_1-p294080	Bn-N18-p12421864	gene	exon
Bn-scaff_16287_1-p294213	Bn-N18-p12421997	gene	exon
Bn-scaff_16287_1-p343693	Bn-N18-p12442346	gene	exon
Bn-scaff_16287_1-p366585	Bn-N18-p12462117	gene	
Bn-scaff_16287_1-p367970	Bn-N18-p12463502	gene	
Bn-scaff_16287_1-p368299	Bn-N18-p12463832	gene	
Bn-scaff_16287_1-p368373	Bn-N18-p12463905	gene	
Bn-scaff_16287_1-p395887	Bn-N8-p8134585	gene	exon
Bn-scaff_16287_1-p416151	Bn-N18-p12514226	gene	
Bn-scaff_16287_1-p441896	Bn-N18-p12544944	gene	
Bn-scaff_16287_1-p441940	Bn-N18-p12544988	gene	
Bn-scaff_16297_1-p259650	Bn-N9-p18853705	gene	exon
Bn-scaff_16297_2-p34182	Bn-N16-p33563951	gene	
Bn-scaff_16300_1-p1233910	Bn-N12-p21266657	gene	
Bn-scaff_16300_1-p1234612	Bn-N12-p21265955	gene	
Bn-scaff_16300_1-p186784	Bn-N2-p15718224	gene	exon
Bn-scaff_16300_1-p214856	Bn-N12-p22183663	gene	
Bn-scaff_16300_1-p250259	Bn-N12-p22151766	gene	exon
Bn-scaff_16300_1-p284543	Bn-N12-p22118094	gene	exon
Bn-scaff_16300_1-p31299	Bn-N12-p22383154	gene	exon
Bn-scaff_16300_1-p33977	Bn-N12-p22382107	gene	exon
Bn-scaff_16300_1-p561129	Bn-N12-p21903253	gene	
Bn-scaff_16300_1-p614082	Bn-N12-p21828760	gene	
Bn-scaff_16300_1-p625676	Bn-N12-p21824016	gene	exon
Bn-scaff_16300_1-p625805	Bn-N12-p21823890	gene	exon
Bn-scaff_16300_1-p831382	Bn-Scaffold13282-p151	gene	exon
Bn-scaff_16300_1-p831651	Bn-N10-p5926189	gene	
Bn-scaff_16300_1-p890245	Bn-N12-p21536663	gene	
Bn-scaff_16309_1-p116361	Bn-N14-p27208002	gene	exon
Bn-scaff_16309_1-p144811	Bn-N14-p27173265	gene	
Bn-scaff_16309_1-p164550	Bn-N14-p27149568	gene	
Bn-scaff_16309_1-p187260	Bn-N14-p27120264	gene	exon
Bn-scaff_16309_1-p187425	Bn-N14-p27120099	gene	
Bn-scaff_16309_1-p260352	Bn-N14-p27072112	gene	

Bn-scaff_16309_1-p62538	Bn-N14-p27265481	gene	
Bn-scaff_16310_1-p239317	Bn-N3-p18539921	gene	exon
Bn-scaff_16310_1-p239386	Bn-N3-p18539852	gene	exon
Bn-scaff_16310_1-p279083	Bn-N13-p29383446	gene	
Bn-scaff_16312_1-p126580	Bn-N14-p16873438	gene	exon
Bn-scaff_16312_1-p126687	Bn-N14-p16873588	gene	exon
Bn-scaff_16312_1-p33849	Bn-N14-p16727903	gene	
Bn-scaff_16312_1-p34040	Bn-N14-p16728085	gene	
Bn-scaff_16312_1-p34201	Bn-N14-p16728246	gene	
Bn-scaff_16312_1-p49769	Bn-N14-p16740426	gene	
Bn-scaff_16323_1-p171333	Bn-N1-p7149541	gene	
Bn-scaff_16323_1-p226708	Bn-N11-p10538278	gene	exon
Bn-scaff_16328_1-p184165	Bn-N12-p26681067	gene	exon
Bn-scaff_16328_1-p200394	Bn-N12-p26699227	gene	exon
Bn-scaff_16328_1-p25550	Bn-N12-p26503092	gene	exon
Bn-scaff_16328_1-p334896	Bn-N12-p26819565	gene	exon
Bn-scaff_16328_1-p751831	Bn-N12-p27210030	gene	exon
Bn-scaff_16328_1-p971718	Bn-N15-p48513661	gene	exon
Bn-scaff_16328_1-p971878	Bn-N15-p48513500	gene	exon
Bn-scaff_16342_1-p104963	Bn-N11-p44211887	gene	exon
Bn-scaff_16342_1-p28765	Bn-N11-p44275475	gene	exon
Bn-scaff_16342_1-p37982	Bn-N11-p44265748	gene	exon
Bn-scaff_16350_1-p172744	Bn-N13-p41971769	gene	
Bn-scaff_16352_1-p100761	Bn-N3-p11926582	gene	
Bn-scaff_16352_1-p1204075	Bn-N13-p18806772	gene	
Bn-scaff_16352_1-p1287850	Bn-N13-p18896653	gene	
Bn-scaff_16352_1-p17155	Bn-N3-p11884861	gene	exon
Bn-scaff_16352_1-p190051	Bn-N13-p17758501	gene	exon
Bn-scaff_16352_1-p2104	Bn-N13-p17574621	gene	exon
Bn-scaff_16352_1-p255957	Bn-N13-p17838976	gene	exon
Bn-scaff_16352_1-p259954	Bn-N13-p17844117	gene	exon
Bn-scaff_16352_1-p335290	Bn-N13-p17932153	gene	
Bn-scaff_16352_1-p359772	Bn-N13-p17958699	gene	exon
Bn-scaff_16352_1-p367247	Bn-N13-p17966579	gene	exon
Bn-scaff_16352_1-p377620	Bn-N3-p12094665	gene	exon
Bn-scaff_16352_1-p46887	Bn-N3-p11895719	gene	exon
Bn-scaff_16352_1-p496916	Bn-N3-p12169254	gene	exon
Bn-scaff_16352_1-p5784	Bn-N13-p17578301	gene	exon
Bn-scaff_16352_1-p5909	Bn-N3-p11867076	gene	exon
Bn-scaff_16352_1-p720619	Bn-N13-p18371385	gene	exon
Bn-scaff_16352_1-p83169	Bn-N13-p17646915	gene	
Bn-scaff_16352_1-p838453	Bn-N13-p18473637	gene	exon
Bn-scaff_16353_1-p41335	Bn-N11-p10916321	gene	
Bn-scaff_16353_1-p41558	Bn-N11-p10916098	gene	
Bn-scaff_16356_1-p17864	Bn-N14-p2603825	gene	
Bn-scaff_16356_1-p41349	Bn-N14-p2626996	gene	
Bn-scaff_16356_1-p41354	Bn-N14-p2627001	gene	
Bn-scaff_16356_1-p42154	Bn-N14-p2627801	gene	exon
Bn-scaff_16356_1-p42317	Bn-N14-p2627964	gene	exon
Bn-scaff_16356_1-p42628	Bn-N14-p2628405	gene	exon
Bn-scaff_16356_1-p42659	Bn-N14-p2628437	gene	exon
Bn-scaff_16356_1-p60006	Bn-N14-p2642891	gene	exon

Bn-scaff_16356_1-p60107	Bn-N14-p2642991	gene	exon
Bn-scaff_16356_1-p60133	Bn-N14-p2643017	gene	exon
Bn-scaff_16356_1-p61309	Bn-N5-p1764461	gene	exon
Bn-scaff_16356_1-p69775	Bn-N5-p1773582	gene	exon
Bn-scaff_16356_1-p69920	Bn-N5-p1773727	gene	exon
Bn-scaff_16361_1-p100399	Bn-N18-p32758741	gene	exon
Bn-scaff_16361_1-p1007904	Bn-N18-p33709219	gene	exon
Bn-scaff_16361_1-p1052698	Bn-N9-p31373329	gene	exon
Bn-scaff_16361_1-p1056305	Bn-N18-p33748571	gene	exon
Bn-scaff_16361_1-p1056657	Bn-N18-p33748922	gene	
Bn-scaff_16361_1-p1072715	Bn-N18-p33767888	gene	exon
Bn-scaff_16361_1-p1127803	Bn-N18-p33836942	gene	exon
Bn-scaff_16361_1-p1205638	Bn-N18-p33915303	gene	exon
Bn-scaff_16361_1-p1205826	Bn-N18-p33915459	gene	exon
Bn-scaff_16361_1-p1227636	Bn-N9-p31486334	gene	exon
Bn-scaff_16361_1-p1277572	Bn-N18-p33987884	gene	exon
Bn-scaff_16361_1-p1379846	Bn-N18-p34090563	gene	
Bn-scaff_16361_1-p1380155	Bn-N18-p34090875	gene	
Bn-scaff_16361_1-p1380357	Bn-N18-p34091077	gene	exon
Bn-scaff_16361_1-p1387122	Bn-N18-p34098757	gene	
Bn-scaff_16361_1-p1389686	Bn-N18-p34101345	gene	exon
Bn-scaff_16361_1-p1390712	Bn-N18-p34102368	gene	exon
Bn-scaff_16361_1-p1398166	Bn-N18-p34107972	gene	
Bn-scaff_16361_1-p1443077	Bn-N18-p34139782	gene	
Bn-scaff_16361_1-p1466598	Bn-N18-p34167166	gene	exon
Bn-scaff_16361_1-p1467945	Bn-N18-p34168513	gene	exon
Bn-scaff_16361_1-p1496856	Bn-N18-p34197768	gene	exon
Bn-scaff_16361_1-p1497208	Bn-N18-p34198120	gene	exon
Bn-scaff_16361_1-p1497666	Bn-N18-p34198577	gene	
Bn-scaff_16361_1-p1500008	Bn-N9-p31690570	gene	
Bn-scaff_16361_1-p1500169	Bn-N9-p31690730	gene	
Bn-scaff_16361_1-p1505687	Bn-N18-p34206598	gene	exon
Bn-scaff_16361_1-p1569143	Bn-N18-p34266593	gene	exon
Bn-scaff_16361_1-p1569363	Bn-N18-p34266812	gene	exon
Bn-scaff_16361_1-p1569804	Bn-N18-p34267253	gene	
Bn-scaff_16361_1-p1570938	Bn-N18-p34268387	gene	exon
Bn-scaff_16361_1-p1656248	Bn-N18-p34369176	gene	exon
Bn-scaff_16361_1-p1658395	Bn-N18-p34371203	gene	
Bn-scaff_16361_1-p1669677	Bn-N18-p34378648	gene	
Bn-scaff_16361_1-p1690357	Bn-N9-p31836658	gene	exon
Bn-scaff_16361_1-p1713891	Bn-N18-p34420071	gene	exon
Bn-scaff_16361_1-p1715022	Bn-N18-p34421191	gene	exon
Bn-scaff_16361_1-p1749058	Bn-N18-p34457232	gene	exon
Bn-scaff_16361_1-p1974251	Bn-N18-p34657338	gene	
Bn-scaff_16361_1-p2016133	Bn-N18-p34699643	gene	
Bn-scaff_16361_1-p2037720	Bn-N18-p34716326	gene	
Bn-scaff_16361_1-p2038122	Bn-N18-p34716743	gene	
Bn-scaff_16361_1-p2052324	Bn-N18-p34734011	gene	
Bn-scaff_16361_1-p2064780	Bn-N18-p34746418	gene	exon
Bn-scaff_16361_1-p2080257	Bn-N18-p34762202	gene	exon
Bn-scaff_16361_1-p2138712	Bn-N9-p32168758	gene	
Bn-scaff_16361_1-p2256702	Bn-N18-p34936297	gene	exon

Bn-scaff_16361_1-p2262641	Bn-N18-p34942236	gene	exon
Bn-scaff_16361_1-p2263072	Bn-N18-p34942668	gene	exon
Bn-scaff_16361_1-p2273130	Bn-N18-p34952204	gene	
Bn-scaff_16361_1-p2291028	Bn-N18-p34972083	gene	
Bn-scaff_16361_1-p2291795	Bn-N18-p34972849	gene	
Bn-scaff_16361_1-p2342831	Bn-N9-p32349114	gene	
Bn-scaff_16361_1-p2350469	Bn-N18-p35040928	gene	
Bn-scaff_16361_1-p2357373	Bn-N18-p35053164	gene	exon
Bn-scaff_16361_1-p2400621	Bn-N18-p35079769	gene	
Bn-scaff_16361_1-p2411581	Bn-N18-p35090882	gene	exon
Bn-scaff_16361_1-p2493469	Bn-N18-p35146481	gene	
Bn-scaff_16361_1-p2518617	Bn-N18-p35166068	gene	
Bn-scaff_16361_1-p2529346	Bn-N18-p35178875	gene	exon
Bn-scaff_16361_1-p2532318	Bn-N18-p35181848	gene	exon
Bn-scaff_16361_1-p2532823	Bn-N18-p35182353	gene	
Bn-scaff_16361_1-p2532927	Bn-N18-p35182456	gene	
Bn-scaff_16361_1-p2533115	Bn-N18-p35182645	gene	exon
Bn-scaff_16361_1-p2545402	Bn-N18-p35190298	gene	
Bn-scaff_16361_1-p2545776	Bn-N18-p35190672	gene	
Bn-scaff_16361_1-p2563720	Bn-N18-p35208760	gene	exon
Bn-scaff_16361_1-p2730705	Bn-N18-p35377574	gene	
Bn-scaff_16361_1-p2750042	Bn-N18-p35398843	gene	
Bn-scaff_16361_1-p2750581	Bn-N18-p35399382	gene	
Bn-scaff_16361_1-p2793280	Bn-N18-p35442085	gene	exon
Bn-scaff_16361_1-p2793418	Bn-N18-p35442223	gene	
Bn-scaff_16361_1-p2793822	Bn-N18-p35442628	gene	exon
Bn-scaff_16361_1-p2813910	Bn-N9-p32741105	gene	exon
Bn-scaff_16361_1-p2818711	Bn-N9-p32745901	gene	exon
Bn-scaff_16361_1-p2911258	Bn-N9-p32797763	gene	exon
Bn-scaff_16361_1-p3094172	Bn-N9-p32919079	gene	exon
Bn-scaff_16361_1-p3120761	Bn-N18-p35707246	gene	
Bn-scaff_16361_1-p3250247	Bn-N18-p35851106	gene	exon
Bn-scaff_16361_1-p3265282	Bn-N18-p35865354	gene	
Bn-scaff_16361_1-p330276	Bn-N18-p32966327	gene	
Bn-scaff_16361_1-p3325801	Bn-N18-p35932322	gene	
Bn-scaff_16361_1-p3328159	Bn-N9-p33118044	gene	exon
Bn-scaff_16361_1-p3354199	Bn-N18-p35967462	gene	
Bn-scaff_16361_1-p3354234	Bn-N18-p35967497	gene	
Bn-scaff_16361_1-p3354265	Bn-N9-p33154616	gene	
Bn-scaff_16361_1-p3354701	Bn-N18-p35967970	gene	exon
Bn-scaff_16361_1-p3354942	Bn-N9-p33155300	gene	exon
Bn-scaff_16361_1-p3355395	Bn-N18-p35968663	gene	exon
Bn-scaff_16361_1-p3397399	Bn-N18-p36010220	gene	exon
Bn-scaff_16361_1-p3397563	Bn-N18-p36010384	gene	
Bn-scaff_16361_1-p423090	Bn-N18-p33042017	gene	
Bn-scaff_16361_1-p456356	Bn-N18-p33106233	gene	exon
Bn-scaff_16361_1-p457936	Bn-N18-p33107813	gene	
Bn-scaff_16361_1-p461213	Bn-N18-p33111090	gene	exon
Bn-scaff_16361_1-p508319	Bn-N9-p30870656	gene	
Bn-scaff_16361_1-p563785	Bn-N9-p30929785	gene	exon
Bn-scaff_16361_1-p635435	Bn-N9-p30993981	gene	exon
Bn-scaff_16361_1-p686911	Bn-N18-p33372017	gene	exon

Bn-scaff_16361_1-p794887	Bn-N18-p33471666	gene	
Bn-scaff_16361_1-p888733	Bn-N18-p33590255	gene	exon
Bn-scaff_16361_1-p889103	Bn-N18-p33590625	gene	
Bn-scaff_16361_1-p889127	Bn-N18-p33590649	gene	
Bn-scaff_16361_1-p930064	Bn-N18-p33631903	gene	
Bn-scaff_16361_1-p930550	Bn-N18-p33632414	gene	
Bn-scaff_16361_1-p930618	Bn-N18-p33632491	gene	
Bn-scaff_16361_1-p930719	Bn-N18-p33632592	gene	
Bn-scaff_16361_1-p930857	Bn-N18-p33632730	gene	
Bn-scaff_16361_1-p933392	Bn-N18-p33635254	gene	
Bn-scaff_16361_1-p941757	Bn-N18-p33643656	gene	
Bn-scaff_16361_1-p952834	Bn-N18-p33659461	gene	exon
Bn-scaff_16361_1-p953048	Bn-N18-p33659675	gene	exon
Bn-scaff_16362_1-p124177	Bn-N10-p13890645	gene	exon
Bn-scaff_16362_1-p143283	Bn-N19-p50777916	gene	exon
Bn-scaff_16362_1-p381462	Bn-N19-p50557941	gene	exon
Bn-scaff_16362_1-p396617	Bn-N19-p50544180	gene	exon
Bn-scaff_16362_1-p490371	Bn-N19-p50461247	gene	
Bn-scaff_16362_1-p568462	Bn-N19-p50382502	gene	
Bn-scaff_16362_1-p572387	Bn-N19-p50378596	gene	exon
Bn-scaff_16362_1-p572972	Bn-N19-p50378008	gene	
Bn-scaff_16362_1-p616006	Bn-N19-p50337104	gene	
Bn-scaff_16362_1-p616122	Bn-N19-p50336988	gene	
Bn-scaff_16362_1-p621813	Bn-N19-p50334243	gene	
Bn-scaff_16362_1-p632561	Bn-N19-p50323780	gene	
Bn-scaff_16362_1-p633987	Bn-N19-p50322354	gene	exon
Bn-scaff_16362_1-p650685	Bn-N19-p50308208	gene	exon
Bn-scaff_16362_1-p655565	Bn-N19-p50302870	gene	exon
Bn-scaff_16362_1-p713218	Bn-N19-p50272436	gene	exon
Bn-scaff_16362_1-p815199	Bn-N19-p50166559	gene	exon
Bn-scaff_16362_1-p815264	Bn-N19-p50166494	gene	exon
Bn-scaff_16362_1-p842973	Bn-N19-p50128276	gene	
Bn-scaff_16362_1-p847467	Bn-N10-p13420831	gene	
Bn-scaff_16362_1-p91070	Bn-N10-p13921386	gene	exon
Bn-scaff_16369_1-p173601	Bn-N2-p13307559	gene	exon
Bn-scaff_16369_1-p181692	Bn-N12-p18362814	gene	exon
Bn-scaff_16369_1-p181890	Bn-N12-p18362617	gene	exon
Bn-scaff_16369_1-p182886	Bn-N12-p18361610	gene	
Bn-scaff_16369_1-p185577	Bn-N12-p18358534	gene	
Bn-scaff_16369_1-p82394	Bn-N12-p18444315	gene	
Bn-scaff_16371_1-p110691	Bn-N7-p5777093	gene	exon
Bn-scaff_16371_1-p99818	Bn-N17-p13425846	gene	
Bn-scaff_16372_1-p106914	Bn-N13-p54187721	gene	exon
Bn-scaff_16372_1-p78027	Bn-N13-p54163010	gene	
Bn-scaff_16372_1-p93422	Bn-N13-p54178298	gene	
Bn-scaff_16378_1-p20179	Bn-N3-p12336721	gene	exon
Bn-scaff_16378_1-p20798	Bn-N13-p18428451	gene	exon
Bn-scaff_16389_1-p142328	Bn-N18-p43382239	gene	
Bn-scaff_16389_1-p152168	Bn-N18-p43400861	gene	
Bn-scaff_16389_1-p152360	Bn-N18-p43401052	gene	
Bn-scaff_16389_1-p214204	Bn-N18-p43462116	gene	exon
Bn-scaff_16389_1-p215602	Bn-N18-p43463516	gene	exon

Bn-scaff_16389_1-p374339	Bn-N9-p39481881	gene	exon
Bn-scaff_16389_1-p394420	Bn-N18-p43652457	gene	
Bn-scaff_16389_1-p403394	Bn-N9-p39501393	gene	exon
Bn-scaff_16389_1-p407508	Bn-N9-p39505465	gene	exon
Bn-scaff_16389_1-p410098	Bn-N9-p39520346	gene	exon
Bn-scaff_16389_1-p411729	Bn-N18-p43668549	gene	
Bn-scaff_16389_1-p425903	Bn-N18-p43683893	gene	
Bn-scaff_16389_1-p426263	Bn-N18-p43684252	gene	
Bn-scaff_16389_1-p426336	Bn-N18-p43684324	gene	exon
Bn-scaff_16389_1-p426792	Bn-N18-p43684781	gene	exon
Bn-scaff_16389_1-p427823	Bn-N9-p39536234	gene	exon
Bn-scaff_16389_1-p428067	Bn-N9-p39536479	gene	
Bn-scaff_16389_1-p436578	Bn-N18-p43694861	gene	
Bn-scaff_16389_1-p468392	Bn-N18-p43725683	gene	
Bn-scaff_16389_1-p513392	Bn-N18-p43779352	gene	
Bn-scaff_16389_1-p539974	Bn-N18-p43805143	gene	
Bn-scaff_16389_1-p577783	Bn-N18-p43829874	gene	exon
Bn-scaff_16389_1-p578073	Bn-N18-p43830164	gene	exon
Bn-scaff_16389_1-p610756	Bn-N18-p43882051	gene	
Bn-scaff_16389_1-p633959	Bn-N9-p39720791	gene	exon
Bn-scaff_16389_1-p660552	Bn-N18-p43996802	gene	exon
Bn-scaff_16389_1-p661491	Bn-N18-p43997776	gene	exon
Bn-scaff_16389_1-p679212	Bn-N18-p44009357	gene	
Bn-scaff_16389_1-p688011	Bn-N18-p44018156	gene	
Bn-scaff_16389_1-p688405	Bn-N18-p44018550	gene	
Bn-scaff_16389_1-p740326	Bn-N18-p44070544	gene	exon
Bn-scaff_16389_1-p74961	Bn-N18-p43313169	gene	
Bn-scaff_16389_1-p751453	Bn-N18-p44095166	gene	
Bn-scaff_16389_1-p767175	Bn-N18-p44094835	gene	
Bn-scaff_16389_1-p776698	Bn-N18-p44107160	gene	
Bn-scaff_16389_1-p820513	Bn-N18-p44152304	gene	
Bn-scaff_16389_1-p842667	Bn-N18-p44181155	gene	exon
Bn-scaff_16391_1-p142	Bn-N19-p33321646	gene	
Bn-scaff_16394_1-p1002503	Bn-N14-p37170308	gene	exon
Bn-scaff_16394_1-p1121499	Bn-N14-p37319459	gene	
Bn-scaff_16394_1-p1129497	Bn-N14-p37323263	gene	exon
Bn-scaff_16394_1-p1195388	Bn-N4-p8003046	gene	exon
Bn-scaff_16394_1-p1342083	Bn-N14-p37593537	gene	exon
Bn-scaff_16394_1-p1386812	Bn-N14-p37638439	gene	
Bn-scaff_16394_1-p1442938	Bn-N14-p37691491	gene	
Bn-scaff_16394_1-p1502876	Bn-N4-p8253031	gene	exon
Bn-scaff_16394_1-p1511035	Bn-N14-p37761797	gene	exon
Bn-scaff_16394_1-p1516390	Bn-N14-p37768183	gene	
Bn-scaff_16394_1-p1516423	Bn-N14-p37768216	gene	exon
Bn-scaff_16394_1-p1875336	Bn-N4-p8682010	gene	exon
Bn-scaff_16394_1-p1945222	Bn-N14-p38179966	gene	exon
Bn-scaff_16394_1-p2229812	Bn-N13-p55728682	gene	exon
Bn-scaff_16394_1-p2335614	Bn-N13-p55630761	gene	exon
Bn-scaff_16394_1-p236787	Bn-N14-p36325853	gene	
Bn-scaff_16394_1-p237041	Bn-N14-p36326107	gene	
Bn-scaff_16394_1-p237325	Bn-N14-p36326391	gene	
Bn-scaff_16394_1-p308897	Bn-N14-p36402759	gene	

Bn-scaff_16394_1-p363570	Bn-N14-p16505270	gene	
Bn-scaff_16394_1-p401584	Bn-N14-p36500797	gene	exon
Bn-scaff_16394_1-p401893	Bn-N14-p36501106	gene	exon
Bn-scaff_16394_1-p454360	Bn-N14-p36578991	gene	
Bn-scaff_16394_1-p454523	Bn-N14-p36579154	gene	
Bn-scaff_16394_1-p474226	Bn-N14-p36599918	gene	
Bn-scaff_16394_1-p531248	Bn-N14-p36649450	gene	exon
Bn-scaff_16394_1-p551690	Bn-N4-p7421519	gene	
Bn-scaff_16394_1-p551903	Bn-N4-p7421732	gene	exon
Bn-scaff_16394_1-p623306	Bn-N14-p36739167	gene	
Bn-scaff_16394_1-p722315	Bn-N4-p7587022	gene	
Bn-scaff_16394_1-p722580	Bn-N4-p7587287	gene	exon
Bn-scaff_16394_1-p842382	Bn-N14-p37008314	gene	
Bn-scaff_16394_2-p101403	Bn-N8-p14835049	gene	exon
Bn-scaff_16394_2-p1061499	Bn-N13-p56363786	gene	exon
Bn-scaff_16394_2-p1061987	Bn-N13-p56363298	gene	exon
Bn-scaff_16394_2-p1075592	Bn-N13-p56349432	gene	exon
Bn-scaff_16394_2-p1075976	Bn-N13-p56349048	gene	exon
Bn-scaff_16394_2-p1084167	Bn-N13-p56340838	gene	
Bn-scaff_16394_2-p1084424	Bn-N8-p15359425	gene	
Bn-scaff_16394_2-p1085415	Bn-N8-p15360167	gene	exon
Bn-scaff_16394_2-p1086630	Bn-N13-p56338358	gene	
Bn-scaff_16394_2-p1165251	Bn-N13-p56255970	gene	
Bn-scaff_16394_2-p1178077	Bn-N13-p56245794	gene	exon
Bn-scaff_16394_2-p1198592	Bn-N8-p15443540	gene	exon
Bn-scaff_16394_2-p120638	Bn-N1-p1239507	gene	exon
Bn-scaff_16394_2-p1263079	Bn-N13-p56166043	gene	
Bn-scaff_16394_2-p1263387	Bn-N8-p15473666	gene	
Bn-scaff_16394_2-p1364720	Bn-Scaffold02005-p4060	gene	exon
Bn-scaff_16394_2-p1431681	Bn-N13-p55961449	gene	exon
Bn-scaff_16394_2-p1498560	Bn-N13-p55880297	gene	
Bn-scaff_16394_2-p174513	Bn-N8-p14896931	gene	exon
Bn-scaff_16394_2-p178524	Bn-N13-p57284933	gene	
Bn-scaff_16394_2-p178759	Bn-N8-p14900658	gene	exon
Bn-scaff_16394_2-p178827	Bn-N13-p57284630	gene	
Bn-scaff_16394_2-p203450	Bn-N13-p57259748	gene	exon
Bn-scaff_16394_2-p207187	Bn-N13-p57255997	gene	
Bn-scaff_16394_2-p243183	Bn-N13-p57227447	gene	
Bn-scaff_16394_2-p247786	Bn-N13-p57222895	gene	exon
Bn-scaff_16394_2-p252811	Bn-N13-p57209345	gene	exon
Bn-scaff_16394_2-p347960	Bn-N13-p57080525	gene	
Bn-scaff_16394_2-p348198	Bn-N13-p57080292	gene	
Bn-scaff_16394_2-p40571	Bn-N13-p57436782	gene	exon
Bn-scaff_16394_2-p449512	Bn-N13-p56980124	gene	exon
Bn-scaff_16394_2-p471655	Bn-N13-p56956424	gene	exon
Bn-scaff_16394_2-p482093	Bn-N8-p15081024	gene	exon
Bn-scaff_16394_2-p510062	Bn-N13-p56913833	gene	exon
Bn-scaff_16394_2-p525073	Bn-N13-p56899215	gene	
Bn-scaff_16394_2-p525169	Bn-N13-p56899119	gene	
Bn-scaff_16394_2-p526954	Bn-N13-p56897408	gene	exon
Bn-scaff_16394_2-p527071	Bn-N13-p56897291	gene	exon
Bn-scaff_16394_2-p527689	Bn-N13-p56896673	gene	

Bn-scaff_16394_2-p527944	Bn-N13-p56896419	gene	exon
Bn-scaff_16394_2-p553789	Bn-N13-p56863804	gene	exon
Bn-scaff_16394_2-p553815	Bn-N13-p56863778	gene	exon
Bn-scaff_16394_2-p672613	Bn-N13-p56780988	gene	exon
Bn-scaff_16394_2-p672690	Bn-N13-p56780911	gene	exon
Bn-scaff_16394_2-p673824	Bn-N13-p56779756	gene	exon
Bn-scaff_16394_2-p776520	Bn-N13-p56698687	gene	exon
Bn-scaff_16394_2-p781694	Bn-N13-p56693678	gene	
Bn-scaff_16397_1-p114405	Bn-N16-p36419944	gene	exon
Bn-scaff_16397_1-p181125	Bn-N16-p36342187	gene	
Bn-scaff_16397_1-p21413	Bn-N16-p36519805	gene	exon
Bn-scaff_16397_1-p21961	Bn-N16-p36519257	gene	
Bn-scaff_16397_1-p22776	Bn-N16-p36518442	gene	
Bn-scaff_16397_1-p22778	Bn-N16-p36518440	gene	
Bn-scaff_16397_1-p32614	Bn-N16-p36504309	gene	
Bn-scaff_16397_1-p360292	Bn-N16-p36217827	gene	exon
Bn-scaff_16397_1-p360296	Bn-N16-p36217822	gene	exon
Bn-scaff_16397_1-p360372	Bn-N16-p36217747	gene	exon
Bn-scaff_16397_1-p424878	Bn-N16-p36140448	gene	exon
Bn-scaff_16397_1-p561129	Bn-N16-p36006161	gene	exon
Bn-scaff_16397_1-p568133	Bn-N16-p35992409	gene	exon
Bn-scaff_16397_1-p568202	Bn-N16-p35992340	gene	
Bn-scaff_16397_1-p568238	Bn-N16-p35992304	gene	
Bn-scaff_16397_1-p568303	Bn-N16-p35990400	gene	
Bn-scaff_16397_1-p568389	Bn-N16-p35990314	gene	
Bn-scaff_16397_1-p584351	Bn-N16-p35951834	gene	
Bn-scaff_16397_1-p597210	Bn-N16-p35948853	gene	
Bn-scaff_16397_1-p597986	Bn-N16-p35948077	gene	
Bn-scaff_16397_1-p598096	Bn-N16-p35947968	gene	
Bn-scaff_16397_1-p602190	Bn-N7-p21871184	gene	
Bn-scaff_16397_1-p716613	Bn-N7-p21804394	gene	
Bn-scaff_16397_1-p735665	Bn-N16-p35787732	gene	exon
Bn-scaff_16397_1-p805714	Bn-N16-p35707507	gene	
Bn-scaff_16397_1-p809070	Bn-N16-p35704582	gene	
Bn-scaff_16397_1-p809142	Bn-N16-p35704510	gene	
Bn-scaff_16397_1-p813786	Bn-N16-p35699867	gene	
Bn-scaff_16397_1-p818901	Bn-N16-p35694768	gene	exon
Bn-scaff_16402_1-p16325	Bn-N7-p12805206	gene	exon
Bn-scaff_16402_1-p16404	Bn-N7-p12805127	gene	exon
Bn-scaff_16402_1-p76139	Bn-N7-p12781998	gene	exon
Bn-scaff_16414_1-p1031719	Bn-N10-p1014379	gene	exon
Bn-scaff_16414_1-p1047975	Bn-N15-p1081391	gene	
Bn-scaff_16414_1-p1072066	Bn-N10-p936330	gene	exon
Bn-scaff_16414_1-p1086180	Bn-N15-p1034501	gene	exon
Bn-scaff_16414_1-p1087384	Bn-N15-p1033299	gene	
Bn-scaff_16414_1-p1087443	Bn-N15-p1033240	gene	
Bn-scaff_16414_1-p1087497	Bn-N15-p1033186	gene	
Bn-scaff_16414_1-p1114696	Bn-N15-p1004798	gene	exon
Bn-scaff_16414_1-p1186210	Bn-N10-p808551	gene	exon
Bn-scaff_16414_1-p1186599	Bn-N10-p808106	gene	exon
Bn-scaff_16414_1-p1208159	Bn-N15-p910820	gene	exon
Bn-scaff_16414_1-p1332150	Bn-N15-p757030	gene	exon

Bn-scaff_16414_1-p1421347	Bn-N15-p663780	gene	exon
Bn-scaff_16414_1-p1469798	Bn-N15-p631168	gene	exon
Bn-scaff_16414_1-p1496608	Bn-N15-p600737	gene	exon
Bn-scaff_16414_1-p1499983	Bn-N10-p484168	gene	exon
Bn-scaff_16414_1-p1602060	Bn-N15-p492352	gene	
Bn-scaff_16414_1-p1610723	Bn-N15-p484356	gene	
Bn-scaff_16414_1-p1610776	Bn-N15-p484303	gene	
Bn-scaff_16414_1-p1627334	Bn-N15-p464506	gene	
Bn-scaff_16414_1-p1627393	Bn-N15-p464447	gene	
Bn-scaff_16414_1-p1631188	Bn-N15-p460659	gene	exon
Bn-scaff_16414_1-p1635975	Bn-N15-p455905	gene	
Bn-scaff_16414_1-p1636351	Bn-N15-p455529	gene	
Bn-scaff_16414_1-p1652972	Bn-N15-p436739	gene	exon
Bn-scaff_16414_1-p1679802	Bn-N10-p334540	gene	exon
Bn-scaff_16414_1-p1726455	Bn-N15-p348225	gene	exon
Bn-scaff_16414_1-p1747803	Bn-N15-p328893	gene	
Bn-scaff_16414_1-p175360	Bn-N15-p1928788	gene	exon
Bn-scaff_16414_1-p309442	Bn-N15-p1788751	gene	exon
Bn-scaff_16414_1-p309753	Bn-N15-p1788428	gene	
Bn-scaff_16414_1-p309830	Bn-N15-p1788350	gene	exon
Bn-scaff_16414_1-p310240	Bn-N15-p1787941	gene	
Bn-scaff_16414_1-p428111	Bn-N10-p1644361	gene	exon
Bn-scaff_16414_1-p50604	Bn-N15-p2055863	gene	exon
Bn-scaff_16414_1-p539328	Bn-N15-p1581612	gene	
Bn-scaff_16414_1-p539478	Bn-N15-p1581462	gene	exon
Bn-scaff_16414_1-p620295	Bn-N15-p1501446	gene	exon
Bn-scaff_16414_1-p623673	Bn-N15-p1498537	gene	
Bn-scaff_16414_1-p624848	Bn-N15-p1497363	gene	
Bn-scaff_16414_1-p650418	Bn-N15-p1474088	gene	exon
Bn-scaff_16414_1-p740570	Bn-N15-p1372661	gene	
Bn-scaff_16414_1-p772316	Bn-N15-p1350052	gene	
Bn-scaff_16414_1-p774881	Bn-N15-p1344027	gene	
Bn-scaff_16414_1-p777277	Bn-N15-p1342122	gene	
Bn-scaff_16414_1-p798013	Bn-N15-p1327090	gene	exon
Bn-scaff_16414_1-p834366	Bn-N15-p1291936	gene	
Bn-scaff_16414_1-p836176	Bn-N15-p1290119	gene	exon
Bn-scaff_16414_1-p863592	Bn-N15-p1261974	gene	
Bn-scaff_16414_1-p863783	Bn-N15-p1261783	gene	exon
Bn-scaff_16414_1-p864591	Bn-N15-p1260975	gene	exon
Bn-scaff_16414_1-p874497	Bn-N15-p1251075	gene	
Bn-scaff_16414_1-p879412	Bn-N10-p1230343	gene	
Bn-scaff_16414_1-p884592	Bn-N15-p1240984	gene	
Bn-scaff_16414_1-p91571	Bn-N15-p2012720	gene	exon
Bn-scaff_16414_1-p917631	Bn-N15-p1208899	gene	
Bn-scaff_16414_1-p932272	Bn-N15-p1197638	gene	exon
Bn-scaff_16414_1-p932352	Bn-N15-p1197559	gene	exon
Bn-scaff_16437_1-p36113	Bn-N12-p18113655	gene	exon
Bn-scaff_16437_1-p53425	Bn-N12-p18093676	gene	
Bn-scaff_16445_1-p1076112	Bn-N18-p41423647	gene	
Bn-scaff_16445_1-p1291783	Bn-N18-p41190194	gene	
Bn-scaff_16445_1-p1325537	Bn-N18-p41156279	gene	exon
Bn-scaff_16445_1-p1355599	Bn-N9-p37430430	gene	exon

Bn-scaff_16445_1-p1369864	Bn-N18-p41113256	gene	exon
Bn-scaff_16445_1-p1405783	Bn-N18-p41075300	gene	
Bn-scaff_16445_1-p1419218	Bn-N18-p41063612	gene	
Bn-scaff_16445_1-p1441125	Bn-N18-p41042904	gene	exon
Bn-scaff_16445_1-p1441308	Bn-N18-p41042781	gene	exon
Bn-scaff_16445_1-p1445958	Bn-N18-p41038071	gene	
Bn-scaff_16445_1-p1447054	Bn-N18-p41037041	gene	
Bn-scaff_16445_1-p1459241	Bn-N18-p41024854	gene	
Bn-scaff_16445_1-p1460133	Bn-N18-p41023962	gene	
Bn-scaff_16445_1-p1460618	Bn-N18-p41023477	gene	
Bn-scaff_16445_1-p1530516	Bn-N18-p40947014	gene	
Bn-scaff_16445_1-p1548492	Bn-N18-p40899575	gene	
Bn-scaff_16445_1-p1548555	Bn-N18-p40899512	gene	
Bn-scaff_16445_1-p1602173	Bn-N18-p40836028	gene	
Bn-scaff_16445_1-p1603116	Bn-N18-p40835086	gene	
Bn-scaff_16445_1-p1603383	Bn-N18-p40834819	gene	
Bn-scaff_16445_1-p1607170	Bn-N18-p40830994	gene	
Bn-scaff_16445_1-p1607426	Bn-N18-p40830738	gene	
Bn-scaff_16445_1-p1607691	Bn-N18-p40830472	gene	exon
Bn-scaff_16445_1-p1609147	Bn-N18-p40829021	gene	
Bn-scaff_16445_1-p1628220	Bn-N18-p40809911	gene	exon
Bn-scaff_16445_1-p1639969	Bn-N18-p40797253	gene	
Bn-scaff_16445_1-p1656512	Bn-N18-p40780386	gene	
Bn-scaff_16445_1-p1656718	Bn-N18-p40780180	gene	exon
Bn-scaff_16445_1-p1673090	Bn-N18-p40763754	gene	
Bn-scaff_16445_1-p1673540	Bn-N18-p40763304	gene	
Bn-scaff_16445_1-p1673779	Bn-N18-p40763065	gene	
Bn-scaff_16445_1-p1674687	Bn-N18-p40762154	gene	
Bn-scaff_16445_1-p1679519	Bn-N18-p40756291	gene	
Bn-scaff_16445_1-p178795	Bn-N18-p42326720	gene	exon
Bn-scaff_16445_1-p179795	Bn-N18-p42325720	gene	
Bn-scaff_16445_1-p1807153	Bn-N18-p40638607	gene	
Bn-scaff_16445_1-p1815534	Bn-N18-p40629626	gene	exon
Bn-scaff_16445_1-p1823431	Bn-N18-p40624849	gene	
Bn-scaff_16445_1-p1834116	Bn-N18-p40616505	gene	exon
Bn-scaff_16445_1-p1834644	Bn-N9-p37021234	gene	
Bn-scaff_16445_1-p1836681	Bn-N18-p40612335	gene	
Bn-scaff_16445_1-p1836752	Bn-N18-p40612264	gene	
Bn-scaff_16445_1-p1839966	Bn-N9-p37015753	gene	exon
Bn-scaff_16445_1-p194744	Bn-N18-p42316717	gene	exon
Bn-scaff_16445_1-p197721	Bn-N18-p42313740	gene	
Bn-scaff_16445_1-p2011296	Bn-N18-p40422126	gene	
Bn-scaff_16445_1-p2011490	Bn-N18-p40421931	gene	exon
Bn-scaff_16445_1-p2012444	Bn-N18-p40420978	gene	exon
Bn-scaff_16445_1-p2013455	Bn-N18-p40419966	gene	exon
Bn-scaff_16445_1-p2022692	Bn-N18-p40410730	gene	
Bn-scaff_16445_1-p202619	Bn-N18-p42308849	gene	
Bn-scaff_16445_1-p2106869	Bn-N9-p36795604	gene	exon
Bn-scaff_16445_1-p2114822	Bn-N18-p40320031	gene	
Bn-scaff_16445_1-p2117906	Bn-N18-p40316962	gene	
Bn-scaff_16445_1-p2118684	Bn-N18-p40316184	gene	exon
Bn-scaff_16445_1-p2118822	Bn-N18-p40316046	gene	exon

Bn-scaff_16445_1-p2118895	Bn-N18-p40315973	gene	exon
Bn-scaff_16445_1-p2119017	Bn-N18-p40315851	gene	exon
Bn-scaff_16445_1-p2119496	Bn-N18-p40315371	gene	
Bn-scaff_16445_1-p2120560	Bn-N18-p40314313	gene	
Bn-scaff_16445_1-p2132184	Bn-N18-p40303413	gene	
Bn-scaff_16445_1-p2133229	Bn-N18-p40302377	gene	exon
Bn-scaff_16445_1-p2133282	Bn-N18-p40302324	gene	exon
Bn-scaff_16445_1-p2133586	Bn-N18-p40302009	gene	exon
Bn-scaff_16445_1-p214654	Bn-N18-p42296822	gene	exon
Bn-scaff_16445_1-p2227124	Bn-N18-p40232028	gene	
Bn-scaff_16445_1-p2262279	Bn-N18-p40195326	gene	exon
Bn-scaff_16445_1-p228498	Bn-N18-p42282987	gene	exon
Bn-scaff_16445_1-p2444795	Bn-N18-p40019031	gene	exon
Bn-scaff_16445_1-p2549752	Bn-N9-p36437629	gene	exon
Bn-scaff_16445_1-p2601107	Bn-N18-p39869059	gene	
Bn-scaff_16445_1-p2613244	Bn-N18-p39854595	gene	
Bn-scaff_16445_1-p2624353	Bn-N18-p39834588	gene	exon
Bn-scaff_16445_1-p263067	Bn-N18-p42249036	gene	exon
Bn-scaff_16445_1-p2639425	Bn-N18-p39815744	gene	
Bn-scaff_16445_1-p2656698	Bn-N18-p39796465	gene	exon
Bn-scaff_16445_1-p2665772	Bn-N18-p39783314	gene	exon
Bn-scaff_16445_1-p268218	Bn-N6-p3798284	gene	
Bn-scaff_16445_1-p2929527	Bn-N18-p39528392	gene	exon
Bn-scaff_16445_1-p311546	Bn-N18-p42197192	gene	exon
Bn-scaff_16445_1-p31451	Bn-N18-p42467540	gene	exon
Bn-scaff_16445_1-p351646	Bn-N18-p42163711	gene	
Bn-scaff_16445_1-p355779	Bn-N18-p42159586	gene	
Bn-scaff_16445_1-p358643	Bn-N18-p42156783	gene	
Bn-scaff_16445_1-p364847	Bn-N18-p42150534	gene	
Bn-scaff_16445_1-p365999	Bn-N18-p42149382	gene	exon
Bn-scaff_16445_1-p366238	Bn-N18-p42149130	gene	exon
Bn-scaff_16445_1-p375092	Bn-N18-p42140269	gene	
Bn-scaff_16445_1-p414675	Bn-N18-p42110361	gene	exon
Bn-scaff_16445_1-p417888	Bn-N18-p42107125	gene	exon
Bn-scaff_16445_1-p418945	Bn-N18-p42106103	gene	exon
Bn-scaff_16445_1-p418957	Bn-N18-p42106091	gene	exon
Bn-scaff_16445_1-p437285	Bn-N18-p42080488	gene	
Bn-scaff_16445_1-p460948	Bn-N18-p42051904	gene	
Bn-scaff_16445_1-p478398	Bn-N18-p42033240	gene	
Bn-scaff_16445_1-p491592	Bn-N18-p42020425	gene	exon
Bn-scaff_16445_1-p492739	Bn-N18-p42019313	gene	
Bn-scaff_16445_1-p566534	Bn-N18-p41934206	gene	
Bn-scaff_16445_1-p584652	Bn-N18-p41915995	gene	
Bn-scaff_16445_1-p588681	Bn-N18-p41911762	gene	
Bn-scaff_16445_1-p591427	Bn-N18-p41908982	gene	
Bn-scaff_16445_1-p594145	Bn-N18-p41906145	gene	exon
Bn-scaff_16445_1-p611520	Bn-N18-p41892601	gene	
Bn-scaff_16445_1-p615520	Bn-N18-p41883614	gene	
Bn-scaff_16445_1-p621269	Bn-N18-p41877595	gene	
Bn-scaff_16445_1-p632372	Bn-N18-p41866778	gene	exon
Bn-scaff_16445_1-p723609	Bn-Scaffold01015-p3952	gene	exon
Bn-scaff_16445_1-p724011	Bn-N18-p41774612	gene	exon

Bn-scaff_16445_1-p727477	Bn-N18-p41771120	gene	
Bn-scaff_16445_1-p727690	Bn-N18-p41770907	gene	
Bn-scaff_16445_1-p728844	Bn-N18-p41769724	gene	
Bn-scaff_16445_1-p728936	Bn-N18-p41769633	gene	
Bn-scaff_16445_1-p743559	Bn-N18-p41757106	gene	exon
Bn-scaff_16445_1-p746383	Bn-N18-p41755497	gene	
Bn-scaff_16445_1-p749430	Bn-N18-p41752394	gene	exon
Bn-scaff_16445_1-p766444	Bn-N18-p41737747	gene	exon
Bn-scaff_16445_1-p766648	Bn-N18-p41737543	gene	exon
Bn-scaff_16445_1-p766708	Bn-N18-p41737484	gene	exon
Bn-scaff_16445_1-p766826	Bn-N18-p41737365	gene	exon
Bn-scaff_16445_1-p782939	Bn-N18-p41722181	gene	
Bn-scaff_16445_1-p783410	Bn-N18-p41721710	gene	
Bn-scaff_16445_1-p800267	Bn-N18-p41704383	gene	
Bn-scaff_16445_1-p800334	Bn-N18-p41704316	gene	
Bn-scaff_16445_1-p809862	Bn-N18-p41696040	gene	
Bn-scaff_16445_1-p815008	Bn-N18-p41689264	gene	exon
Bn-scaff_16445_1-p825012	Bn-N18-p41682300	gene	exon
Bn-scaff_16445_1-p82664	Bn-N18-p42401622	gene	exon
Bn-scaff_16445_1-p856678	Bn-N18-p41663055	gene	exon
Bn-scaff_16445_1-p894350	Bn-N18-p41612841	gene	exon
Bn-scaff_16445_1-p925530	Bn-N18-p41563606	gene	exon
Bn-scaff_16445_1-p932699	Bn-N18-p41557308	gene	
Bn-scaff_16445_1-p933254	Bn-N18-p41556749	gene	exon
Bn-scaff_16445_1-p968765	Bn-N18-p41517888	gene	exon
Bn-scaff_16445_1-p973672	Bn-N18-p41512750	gene	
Bn-scaff_16445_1-p992590	Bn-N9-p37770214	gene	exon
Bn-scaff_16447_1-p393381	Bn-N5-p6132265	gene	exon
Bn-scaff_16447_1-p403240	Bn-N14-p10320518	gene	
Bn-scaff_16447_1-p446856	Bn-N3-p7909566	gene	exon
Bn-scaff_16449_1-p1020504	Bn-N12-p33132303	gene	
Bn-scaff_16449_1-p1020573	Bn-N12-p33132234	gene	
Bn-scaff_16449_1-p1020659	Bn-N12-p33132148	gene	
Bn-scaff_16449_1-p13856	Bn-N12-p34212409	gene	
Bn-scaff_16449_1-p13914	Bn-N12-p34212351	gene	
Bn-scaff_16449_1-p14328	Bn-N12-p34211937	gene	
Bn-scaff_16449_1-p17755	Bn-N12-p34208509	gene	
Bn-scaff_16449_1-p18120	Bn-N12-p34208144	gene	
Bn-scaff_16449_1-p248131	Bn-N12-p33982035	gene	exon
Bn-scaff_16449_1-p251526	Bn-N12-p33978639	gene	exon
Bn-scaff_16449_1-p350893	Bn-N12-p33831807	gene	
Bn-scaff_16449_1-p555329	Bn-N12-p33626168	gene	exon
Bn-scaff_16449_1-p555639	Bn-Scaffold29624-p373	gene	exon
Bn-scaff_16449_1-p688961	Bn-N12-p33459424	gene	exon
Bn-scaff_16449_1-p69605	Bn-N12-p34152061	gene	
Bn-scaff_16449_1-p762227	Bn-N2-p20550310	gene	exon
Bn-scaff_16449_1-p850527	Bn-N12-p33285521	gene	exon
Bn-scaff_16449_1-p867180	Bn-N12-p33278545	gene	
Bn-scaff_16449_1-p875975	Bn-N12-p33270786	gene	exon
Bn-scaff_16449_1-p878143	Bn-N12-p33268615	gene	exon
Bn-scaff_16449_1-p92326	Bn-N12-p34130290	gene	exon
Bn-scaff_16449_1-p92407	Bn-N12-p34130209	gene	exon

Bn-scaff_16450_1-p368974	Bn-N17-p20056001	gene	
Bn-scaff_16450_1-p406085	Bn-N17-p20019535	gene	
Bn-scaff_16454_1-p393468	Bn-N5-p12252669	gene	exon
Bn-scaff_16454_1-p393741	Bn-N5-p12252394	gene	exon
Bn-scaff_16454_1-p459442	Bn-N15-p23537373	gene	exon
Bn-scaff_16454_1-p564668	Bn-N5-p12144860	gene	exon
Bn-scaff_16454_1-p73573	Bn-N19-p9093845	gene	
Bn-scaff_16454_1-p880400	Bn-N15-p23839011	gene	
Bn-scaff_16454_1-p943860	Bn-N15-p23886715	gene	
Bn-scaff_16456_1-p121347	Bn-N19-p40347689	gene	exon
Bn-scaff_16456_1-p230098	Bn-N19-p40250621	gene	
Bn-scaff_16456_1-p453404	Bn-N19-p40065238	gene	exon
Bn-scaff_16456_1-p485647	Bn-N19-p40038051	gene	exon
Bn-scaff_16456_1-p570339	Bn-N10-p8410591	gene	exon
Bn-scaff_16456_1-p734822	Bn-N10-p10135868	gene	
Bn-scaff_16456_1-p735437	Bn-N10-p10136496	gene	
Bn-scaff_16456_1-p735749	Bn-N10-p10136803	gene	exon
Bn-scaff_16456_1-p832346	Bn-N19-p39701784	gene	exon
Bn-scaff_16468_1-p241032	Bn-N18-p7124802	gene	exon
Bn-scaff_16468_1-p242072	Bn-N18-p7125842	gene	
Bn-scaff_16468_1-p281212	Bn-N18-p7167100	gene	
Bn-scaff_16468_1-p290029	Bn-N18-p7175786	gene	
Bn-scaff_16468_1-p308201	Bn-N18-p7192254	gene	exon
Bn-scaff_16468_1-p308268	Bn-N18-p7192321	gene	exon
Bn-scaff_16468_1-p311691	Bn-N18-p7195744	gene	
Bn-scaff_16478_1-p1015336	Bn-N15-p19697921	gene	exon
Bn-scaff_16478_1-p1051523	Bn-N9-p23216151	gene	
Bn-scaff_16478_1-p756672	Bn-N15-p19443074	gene	exon
Bn-scaff_16485_1-p1400193	Bn-N2-p18441355	gene	exon
Bn-scaff_16485_1-p1561149	Bn-N12-p28177914	gene	
Bn-scaff_16485_1-p1693762	Bn-N16-p17821692	gene	
Bn-scaff_16485_1-p46055	Bn-N16-p3688412	gene	
Bn-scaff_16485_1-p53130	Bn-N16-p3696796	gene	
Bn-scaff_16485_1-p647035	Bn-N16-p4277496	gene	exon
Bn-scaff_16485_1-p676613	Bn-N16-p4300629	gene	
Bn-scaff_16485_1-p682420	Bn-N16-p4310556	gene	exon
Bn-scaff_16485_1-p691422	Bn-N6-p1830748	gene	
Bn-scaff_16485_1-p747170	Bn-N16-p4375847	gene	exon
Bn-scaff_16486_1-p22444	Bn-N9-p242192	gene	
Bn-scaff_16486_1-p39141	Bn-N19-p137645	gene	
Bn-scaff_16486_1-p77468	Bn-N19-p92689	gene	exon
Bn-scaff_16486_1-p88236	Bn-N19-p80930	gene	exon
Bn-scaff_16486_1-p88463	Bn-N19-p80703	gene	exon
Bn-scaff_16503_1-p152489	Bn-N14-p20771166	gene	exon
Bn-scaff_16503_1-p152805	Bn-N14-p20770850	gene	
Bn-scaff_16503_1-p154948	Bn-N14-p20768710	gene	
Bn-scaff_16503_1-p178769	Bn-N14-p20753444	gene	exon
Bn-scaff_16503_1-p179067	Bn-N14-p20753141	gene	
Bn-scaff_16503_1-p180312	Bn-N14-p20752643	gene	
Bn-scaff_16503_1-p182207	Bn-N14-p20750746	gene	
Bn-scaff_16503_1-p191084	Bn-N14-p20749834	gene	exon
Bn-scaff_16503_1-p191126	Bn-N14-p20749792	gene	exon

Bn-scaff_16503_1-p201761	Bn-N14-p20697166	gene	
Bn-scaff_16503_1-p222903	Bn-N14-p20670089	gene	exon
Bn-scaff_16503_1-p332592	Bn-N14-p20579804	gene	
Bn-scaff_16503_1-p332687	Bn-N14-p20579709	gene	exon
Bn-scaff_16503_1-p336686	Bn-N14-p20576539	gene	exon
Bn-scaff_16503_1-p337729	Bn-N14-p20575544	gene	
Bn-scaff_16503_1-p337950	Bn-N14-p20575323	gene	
Bn-scaff_16503_1-p341200	Bn-N14-p20572057	gene	
Bn-scaff_16503_1-p341409	Bn-N14-p20571848	gene	
Bn-scaff_16503_1-p345397	Bn-N14-p20567183	gene	
Bn-scaff_16503_1-p53371	Bn-N14-p20864882	gene	exon
Bn-scaff_16503_1-p53448	Bn-N14-p20864805	gene	exon
Bn-scaff_16503_1-p53669	Bn-N14-p20864584	gene	
Bn-scaff_16503_1-p53912	Bn-N14-p20864341	gene	
Bn-scaff_16503_1-p53965	Bn-N14-p20864288	gene	exon
Bn-scaff_16503_1-p54138	Bn-N14-p20864115	gene	exon
Bn-scaff_16503_1-p54145	Bn-N14-p20864108	gene	exon
Bn-scaff_16503_1-p54559	Bn-N14-p20863694	gene	exon
Bn-scaff_16510_1-p234240	Bn-N16-p15599318	gene	exon
Bn-scaff_16510_1-p279307	Bn-N16-p15548400	gene	exon
Bn-scaff_16510_1-p279825	Bn-N16-p15547882	gene	exon
Bn-scaff_16511_1-p1103171	Bn-N14-p18175785	gene	
Bn-scaff_16511_1-p1122889	Bn-N14-p18156005	gene	
Bn-scaff_16511_1-p1123416	Bn-N14-p18155478	gene	
Bn-scaff_16511_1-p1123619	Bn-N14-p18155275	gene	
Bn-scaff_16511_1-p1123940	Bn-N14-p18154954	gene	
Bn-scaff_16511_1-p1124212	Bn-N14-p18154702	gene	
Bn-scaff_16511_1-p1124636	Bn-N14-p18154278	gene	
Bn-scaff_16511_1-p1125753	Bn-N14-p18153160	gene	
Bn-scaff_16511_1-p1201777	Bn-N14-p18076008	gene	
Bn-scaff_16511_1-p1202164	Bn-N14-p18075690	gene	
Bn-scaff_16511_1-p1204394	Bn-N14-p18073458	gene	exon
Bn-scaff_16511_1-p1238622	Bn-N14-p18039424	gene	exon
Bn-scaff_16511_1-p1238976	Bn-N14-p18039061	gene	
Bn-scaff_16511_1-p1239108	Bn-N14-p18038929	gene	
Bn-scaff_16511_1-p1239857	Bn-N14-p18038180	gene	exon
Bn-scaff_16511_1-p1239859	Bn-N14-p18038178	gene	exon
Bn-scaff_16511_1-p1241573	Bn-N14-p18036463	gene	
Bn-scaff_16511_1-p1256064	Bn-N14-p18016720	gene	
Bn-scaff_16511_1-p1256652	Bn-N14-p18016133	gene	
Bn-scaff_16511_1-p1256780	Bn-N14-p18016005	gene	
Bn-scaff_16511_1-p529904	Bn-N14-p18916942	gene	exon
Bn-scaff_16511_1-p539769	Bn-N14-p18926841	gene	exon
Bn-scaff_16511_1-p540146	Bn-N14-p18927220	gene	
Bn-scaff_16511_1-p540559	Bn-N14-p18927633	gene	
Bn-scaff_16511_1-p550592	Bn-N14-p18939039	gene	exon
Bn-scaff_16511_1-p551625	Bn-N14-p18940071	gene	
Bn-scaff_16511_1-p617438	Bn-N14-p19006672	gene	
Bn-scaff_16511_1-p617819	Bn-N14-p19007053	gene	exon
Bn-scaff_16511_1-p623035	Bn-N14-p19010601	gene	
Bn-scaff_16511_1-p6787	Bn-N14-p18545244	gene	
Bn-scaff_16511_1-p703856	Bn-N14-p19095010	gene	exon

Bn-scaff_16511_1-p703878	Bn-N14-p19095032	gene	exon
Bn-scaff_16511_1-p711537	Bn-N14-p19102622	gene	exon
Bn-scaff_16511_1-p717330	Bn-N14-p19108412	gene	exon
Bn-scaff_16511_1-p719704	Bn-N14-p19109807	gene	exon
Bn-scaff_16511_1-p721075	Bn-N14-p19111177	gene	
Bn-scaff_16511_1-p722134	Bn-N14-p19112400	gene	
Bn-scaff_16511_1-p722191	Bn-N14-p19112457	gene	
Bn-scaff_16511_1-p722442	Bn-N14-p19112708	gene	
Bn-scaff_16511_1-p722453	Bn-N14-p19112719	gene	
Bn-scaff_16511_1-p722634	Bn-N14-p19112900	gene	
Bn-scaff_16511_1-p722642	Bn-N14-p19112908	gene	
Bn-scaff_16511_1-p722842	Bn-N14-p19113108	gene	
Bn-scaff_16511_1-p729125	Bn-N14-p19119747	gene	
Bn-scaff_16511_1-p7404	Bn-N14-p18545861	gene	
Bn-scaff_16511_1-p824090	Bn-N14-p18468156	gene	
Bn-scaff_16511_1-p836262	Bn-N14-p18454881	gene	exon
Bn-scaff_16511_1-p836849	Bn-N14-p18454294	gene	
Bn-scaff_16511_1-p837471	Bn-N14-p18453672	gene	
Bn-scaff_16511_1-p837536	Bn-N14-p18453607	gene	
Bn-scaff_16511_1-p84994	Bn-N14-p18622462	gene	
Bn-scaff_16511_1-p991704	Bn-N14-p18287674	gene	
Bn-scaff_16511_1-p994613	Bn-N14-p18284764	gene	
Bn-scaff_16514_1-p159695	Bn-N17-p33558701	gene	exon
Bn-scaff_16514_1-p159841	Bn-N17-p33558847	gene	exon
Bn-scaff_16514_1-p161190	Bn-N17-p33560200	gene	exon
Bn-scaff_16514_1-p41089	Bn-N17-p33472102	gene	exon
Bn-scaff_16514_1-p50048	Bn-N17-p33481196	gene	exon
Bn-scaff_16514_1-p65334	Bn-N17-p33496913	gene	
Bn-scaff_16514_1-p73994	Bn-N8-p10784751	gene	
Bn-scaff_16516_1-p57960	Bn-N5-p20349142	gene	exon
Bn-scaff_16517_1-p105105	Bn-N14-p48097987	gene	
Bn-scaff_16517_1-p133250	Bn-N14-p48071420	gene	
Bn-scaff_16517_1-p192158	Bn-N4-p15657492	gene	exon
Bn-scaff_16517_1-p243867	Bn-N14-p48147453	gene	exon
Bn-scaff_16517_1-p244040	Bn-N14-p48147599	gene	exon
Bn-scaff_16517_1-p258949	Bn-N14-p48167350	gene	
Bn-scaff_16517_1-p354305	Bn-N14-p48252486	gene	exon
Bn-scaff_16517_1-p46045	Bn-N4-p15561036	gene	exon
Bn-scaff_16517_1-p561513	Bn-N14-p48429850	gene	exon
Bn-scaff_16517_1-p561821	Bn-N4-p15824941	gene	
Bn-scaff_16517_1-p561851	Bn-N14-p48430188	gene	exon
Bn-scaff_16517_1-p584031	Bn-N14-p48446731	gene	exon
Bn-scaff_16517_1-p630053	Bn-N14-p48485375	gene	exon
Bn-scaff_16517_1-p630137	Bn-N14-p48485459	gene	exon
Bn-scaff_16517_1-p634132	Bn-N4-p15864885	gene	exon
Bn-scaff_16517_1-p656332	Bn-N12-p2795089	gene	
Bn-scaff_16517_1-p679954	Bn-N14-p48531091	gene	exon
Bn-scaff_16517_1-p680544	Bn-N14-p48531754	gene	
Bn-scaff_16517_1-p681400	Bn-N14-p48532610	gene	exon
Bn-scaff_16517_1-p682628	Bn-N14-p48533839	gene	
Bn-scaff_16517_1-p682660	Bn-N14-p48533872	gene	
Bn-scaff_16517_1-p683951	Bn-N14-p48535162	gene	exon

Bn-scaff_16517_1-p91597	Bn-N4-p15595126	gene	
Bn-scaff_16526_1-p28769	Bn-N15-p46465629	gene	
Bn-scaff_16526_1-p28879	Bn-N15-p46465519	gene	
Bn-scaff_16531_1-p228553	Bn-N14-p24960291	gene	
Bn-scaff_16531_1-p231683	Bn-N14-p24972754	gene	
Bn-scaff_16531_1-p339446	Bn-N14-p25038773	gene	exon
Bn-scaff_16531_1-p379304	Bn-N14-p25081438	gene	exon
Bn-scaff_16531_1-p42657	Bn-N14-p24821016	gene	exon
Bn-scaff_16531_1-p42774	Bn-N14-p24821133	gene	exon
Bn-scaff_16531_1-p428890	Bn-N14-p25197413	gene	exon
Bn-scaff_16531_1-p435324	Bn-N14-p25204224	gene	
Bn-scaff_16531_1-p521807	Bn-N14-p25253684	gene	
Bn-scaff_16531_1-p521908	Bn-N14-p25253785	gene	exon
Bn-scaff_16531_1-p525553	Bn-N14-p25257433	gene	
Bn-scaff_16531_1-p526136	Bn-N14-p25258016	gene	
Bn-scaff_16531_1-p526222	Bn-N14-p25258102	gene	
Bn-scaff_16531_1-p526677	Bn-N14-p25258554	gene	
Bn-scaff_16531_1-p629084	Bn-N14-p25358389	gene	exon
Bn-scaff_16531_1-p630917	Bn-N14-p25360897	gene	
Bn-scaff_16531_1-p630968	Bn-N14-p25360947	gene	
Bn-scaff_16531_1-p640411	Bn-N14-p25369421	gene	exon
Bn-scaff_16531_1-p640933	Bn-N14-p25369942	gene	exon
Bn-scaff_16531_1-p642247	Bn-N14-p25371241	gene	
Bn-scaff_16531_1-p642716	Bn-N14-p25371710	gene	
Bn-scaff_16531_1-p643592	Bn-N14-p25372586	gene	exon
Bn-scaff_16531_1-p684695	Bn-N14-p25406165	gene	exon
Bn-scaff_16531_1-p88934	Bn-N14-p24858759	gene	exon
Bn-scaff_16532_1-p4546	Bn-N11-p26668349	gene	
Bn-scaff_16534_1-p1112687	Bn-N14-p4678584	gene	
Bn-scaff_16534_1-p1146423	Bn-N14-p4708067	gene	
Bn-scaff_16534_1-p1161390	Bn-N14-p4723352	gene	exon
Bn-scaff_16534_1-p1162866	Bn-N14-p4724824	gene	exon
Bn-scaff_16534_1-p1181744	Bn-N14-p4742094	gene	exon
Bn-scaff_16534_1-p1255498	Bn-N14-p4843633	gene	
Bn-scaff_16534_1-p1263272	Bn-N14-p4852004	gene	
Bn-scaff_16534_1-p1267478	Bn-N14-p4856291	gene	
Bn-scaff_16534_1-p1280130	Bn-N14-p4865081	gene	
Bn-scaff_16534_1-p1339750	Bn-N14-p4930735	gene	
Bn-scaff_16534_1-p1340189	Bn-N14-p4931174	gene	
Bn-scaff_16534_1-p1340741	Bn-N14-p4931726	gene	
Bn-scaff_16534_1-p137258	Bn-N14-p3583549	gene	exon
Bn-scaff_16534_1-p138772	Bn-N14-p3585063	gene	
Bn-scaff_16534_1-p1468318	Bn-N14-p5059985	gene	
Bn-scaff_16534_1-p1474844	Bn-N14-p5066499	gene	exon
Bn-scaff_16534_1-p1500199	Bn-N14-p5089651	gene	exon
Bn-scaff_16534_1-p1507780	Bn-N5-p3300147	gene	
Bn-scaff_16534_1-p154616	Bn-N5-p2368002	gene	
Bn-scaff_16534_1-p1770989	Bn-N14-p5361259	gene	exon
Bn-scaff_16534_1-p1804261	Bn-N14-p16476636	gene	
Bn-scaff_16534_1-p1846740	Bn-N5-p3458338	gene	
Bn-scaff_16534_1-p1891486	Bn-N14-p5476708	gene	
Bn-scaff_16534_1-p1897963	Bn-N14-p5483009	gene	

Bn-scaff_16534_1-p1900588	Bn-N5-p3498024	gene	
Bn-scaff_16534_1-p1901223	Bn-N14-p5486002	gene	
Bn-scaff_16534_1-p1987292	Bn-N14-p5573181	gene	exon
Bn-scaff_16534_1-p2007183	Bn-N14-p5587018	gene	exon
Bn-scaff_16534_1-p2222544	Bn-N14-p5699850	gene	
Bn-scaff_16534_1-p2222998	Bn-N14-p5700304	gene	
Bn-scaff_16534_1-p22373	Bn-N14-p3457730	gene	exon
Bn-scaff_16534_1-p2248344	Bn-N14-p5729690	gene	exon
Bn-scaff_16534_1-p2328268	Bn-N14-p5817374	gene	
Bn-scaff_16534_1-p2342844	Bn-N14-p5835752	gene	
Bn-scaff_16534_1-p2424886	Bn-N14-p5913307	gene	exon
Bn-scaff_16534_1-p2424934	Bn-N14-p5913354	gene	exon
Bn-scaff_16534_1-p2434398	Bn-N5-p3722780	gene	exon
Bn-scaff_16534_1-p2435936	Bn-N14-p5925076	gene	
Bn-scaff_16534_1-p2435960	Bn-N14-p5925100	gene	
Bn-scaff_16534_1-p2580555	Bn-N14-p6047697	gene	exon
Bn-scaff_16534_1-p2581985	Bn-N14-p6049127	gene	
Bn-scaff_16534_1-p288565	Bn-N14-p3771563	gene	
Bn-scaff_16534_1-p288682	Bn-N14-p3771680	gene	
Bn-scaff_16534_1-p332911	Bn-N14-p3829660	gene	exon
Bn-scaff_16534_1-p334066	Bn-N14-p3830820	gene	
Bn-scaff_16534_1-p500718	Bn-N14-p4071215	gene	exon
Bn-scaff_16534_1-p500900	Bn-N5-p2594467	gene	exon
Bn-scaff_16534_1-p509606	Bn-N14-p4080100	gene	
Bn-scaff_16534_1-p514275	Bn-N14-p4084770	gene	exon
Bn-scaff_16534_1-p514720	Bn-N14-p4085214	gene	
Bn-scaff_16534_1-p584066	Bn-N14-p4154410	gene	exon
Bn-scaff_16534_1-p648790	Bn-N5-p2673519	gene	exon
Bn-scaff_16534_1-p650838	Bn-N5-p2675497	gene	
Bn-scaff_16534_1-p666334	Bn-N14-p4231239	gene	exon
Bn-scaff_16534_1-p679107	Bn-N14-p4244895	gene	
Bn-scaff_16534_1-p729809	Bn-N14-p4298504	gene	exon
Bn-scaff_16534_1-p801233	Bn-N14-p4357824	gene	
Bn-scaff_16534_1-p808074	Bn-N14-p4361639	gene	
Bn-scaff_16534_1-p827888	Bn-N14-p4381239	gene	exon
Bn-scaff_16534_2-p307964	Bn-N1-p20777150	gene	exon
Bn-scaff_16534_2-p310612	Bn-N11-p34774339	gene	exon
Bn-scaff_16534_2-p40563	Bn-N11-p34481372	gene	
Bn-scaff_16534_2-p78431	Bn-N11-p34503137	gene	
Bn-scaff_16534_2-p79059	Bn-N11-p34503765	gene	
Bn-scaff_16534_2-p79383	Bn-N11-p34504089	gene	
Bn-scaff_16534_2-p80260	Bn-N11-p34504966	gene	
Bn-scaff_16534_2-p80512	Bn-N11-p34505218	gene	
Bn-scaff_16534_2-p86956	Bn-N11-p34511592	gene	exon
Bn-scaff_16534_2-p88292	Bn-N11-p34512927	gene	exon
Bn-scaff_16534_2-p89468	Bn-N11-p34513768	gene	
Bn-scaff_16541_1-p238205	Bn-N7-p8631232	gene	
Bn-scaff_16541_1-p526325	Bn-N17-p18926136	gene	
Bn-scaff_16541_1-p72003	Bn-N7-p8532467	gene	exon
Bn-scaff_16545_1-p110342	Bn-N8-p8342847	gene	
Bn-scaff_16545_1-p236222	Bn-N18-p14959257	gene	exon
Bn-scaff_16545_1-p256454	Bn-N18-p14980417	gene	exon

Bn-scaff_16545_1-p351117	Bn-N12-p31927426	gene	
Bn-scaff_16545_1-p855589	Bn-N12-p31364551	gene	
Bn-scaff_16547_1-p179544	Bn-N16-p32967926	gene	
Bn-scaff_16547_1-p255118	Bn-N16-p33050789	gene	
Bn-scaff_16547_1-p290781	Bn-N16-p33087104	gene	exon
Bn-scaff_16547_1-p290890	Bn-N16-p33087213	gene	
Bn-scaff_16547_1-p292593	Bn-N16-p33088917	gene	
Bn-scaff_16547_1-p310844	Bn-N16-p33178930	gene	exon
Bn-scaff_16547_1-p331785	Bn-N7-p20104021	gene	exon
Bn-scaff_16547_1-p34318	Bn-N16-p32823644	gene	
Bn-scaff_16547_1-p345176	Bn-N18-p21042421	gene	exon
Bn-scaff_16547_1-p59615	Bn-N16-p32838815	gene	exon
Bn-scaff_16547_1-p95612	Bn-N16-p32870419	gene	
Bn-scaff_16553_1-p15698	Bn-N16-p3050081	gene	exon
Bn-scaff_16553_1-p33772	Bn-N16-p3092008	gene	exon
Bn-scaff_16553_1-p34179	Bn-N16-p3092416	gene	
Bn-scaff_16553_1-p34303	Bn-N16-p3092539	gene	
Bn-scaff_16553_1-p36608	Bn-N16-p3094849	gene	
Bn-scaff_16553_1-p78455	Bn-N16-p3136944	gene	exon
Bn-scaff_16553_1-p88896	Bn-N16-p3148514	gene	
Bn-scaff_16554_1-p133285	Bn-N11-p44831407	gene	exon
Bn-scaff_16554_1-p160009	Bn-N1-p26464829	gene	
Bn-scaff_16554_1-p160063	Bn-N1-p26464883	gene	
Bn-scaff_16554_1-p160094	Bn-N11-p44855558	gene	
Bn-scaff_16554_1-p160189	Bn-N11-p44855653	gene	
Bn-scaff_16554_1-p160287	Bn-N11-p44855751	gene	
Bn-scaff_16554_1-p160463	Bn-N11-p44855927	gene	
Bn-scaff_16554_1-p160955	Bn-N1-p26465911	gene	
Bn-scaff_16554_1-p161479	Bn-N11-p44856889	gene	exon
Bn-scaff_16565_1-p1062007	Bn-N2-p9500340	gene	exon
Bn-scaff_16565_1-p1065413	Bn-N2-p9493071	gene	
Bn-scaff_16565_1-p414573	Bn-N12-p14618582	gene	exon
Bn-scaff_16565_1-p860022	Bn-N12-p14328459	gene	exon
Bn-scaff_16565_1-p937414	Bn-N2-p9561430	gene	
Bn-scaff_16565_1-p977015	Bn-N2-p9525819	gene	exon
Bn-scaff_16567_1-p40709	Bn-N11-p40692838	gene	exon
Bn-scaff_16567_1-p97437	Bn-N11-p40748415	gene	
Bn-scaff_16569_1-p411229	Bn-N19-p26731550	gene	
Bn-scaff_16569_1-p567777	Bn-N9-p17314152	gene	exon
Bn-scaff_16569_1-p593723	Bn-N19-p26573216	gene	exon
Bn-scaff_16576_1-p165518	Bn-N14-p13522686	gene	
Bn-scaff_16576_1-p166604	Bn-N14-p13523772	gene	
Bn-scaff_16576_1-p262132	Bn-N14-p13681638	gene	exon
Bn-scaff_16576_1-p262230	Bn-N14-p13681736	gene	exon
Bn-scaff_16576_1-p262357	Bn-N14-p13681863	gene	
Bn-scaff_16576_1-p262623	Bn-N14-p13682129	gene	exon
Bn-scaff_16576_1-p282069	Bn-N14-p13723021	gene	
Bn-scaff_16576_1-p289277	Bn-N14-p13732022	gene	exon
Bn-scaff_16576_1-p318294	Bn-N14-p13769400	gene	
Bn-scaff_16576_1-p385377	Bn-N5-p7993175	gene	
Bn-scaff_16576_1-p582535	Bn-N14-p14026065	gene	exon
Bn-scaff_16576_1-p95164	Bn-N14-p13455433	gene	

Bn-scaff_16614_1-p1013432	Bn-N13-p1238988	gene	exon
Bn-scaff_16614_1-p1059872	Bn-N13-p1191026	gene	exon
Bn-scaff_16614_1-p1083236	Bn-N13-p1168401	gene	exon
Bn-scaff_16614_1-p1122477	Bn-N3-p918098	gene	exon
Bn-scaff_16614_1-p1167537	Bn-N13-p1101910	gene	
Bn-scaff_16614_1-p1167942	Bn-N13-p1101502	gene	
Bn-scaff_16614_1-p1171440	Bn-N13-p1097957	gene	exon
Bn-scaff_16614_1-p1189027	Bn-N3-p869421	gene	exon
Bn-scaff_16614_1-p119144	Bn-N3-p1792209	gene	
Bn-scaff_16614_1-p1194872	Bn-N13-p1072053	gene	
Bn-scaff_16614_1-p1195406	Bn-N13-p1071518	gene	exon
Bn-scaff_16614_1-p1203725	Bn-N13-p1068696	gene	exon
Bn-scaff_16614_1-p1326652	Bn-N13-p929943	gene	exon
Bn-scaff_16614_1-p1361646	Bn-N13-p892897	gene	exon
Bn-scaff_16614_1-p1424702	Bn-N13-p835441	gene	
Bn-scaff_16614_1-p1480092	Bn-N13-p755261	gene	
Bn-scaff_16614_1-p1649933	Bn-N13-p606295	gene	
Bn-scaff_16614_1-p174856	Bn-N13-p2167540	gene	exon
Bn-scaff_16614_1-p1804868	Bn-Scaffold13202-p324	gene	exon
Bn-scaff_16614_1-p1892380	Bn-N3-p227875	gene	exon
Bn-scaff_16614_1-p1893345	Bn-N3-p226267	gene	exon
Bn-scaff_16614_1-p1905215	Bn-N13-p344707	gene	
Bn-scaff_16614_1-p1995086	Bn-N13-p260564	gene	exon
Bn-scaff_16614_1-p1995102	Bn-N13-p260548	gene	exon
Bn-scaff_16614_1-p1999158	Bn-N13-p256492	gene	
Bn-scaff_16614_1-p1999433	Bn-N13-p256217	gene	exon
Bn-scaff_16614_1-p2013370	Bn-N13-p233795	gene	
Bn-scaff_16614_1-p2014049	Bn-N13-p233116	gene	exon
Bn-scaff_16614_1-p2035305	Bn-N3-p129577	gene	exon
Bn-scaff_16614_1-p2138906	Bn-N13-p87066	gene	exon
Bn-scaff_16614_1-p2191153	Bn-N13-p44229	gene	exon
Bn-scaff_16614_1-p2218769	Bn-N13-p9834	gene	
Bn-scaff_16614_1-p226165	Bn-N3-p1696978	gene	
Bn-scaff_16614_1-p281452	Bn-N13-p2043648	gene	exon
Bn-scaff_16614_1-p301846	Bn-N13-p2022937	gene	
Bn-scaff_16614_1-p358098	Bn-N13-p1951861	gene	exon
Bn-scaff_16614_1-p358276	Bn-N13-p1951683	gene	exon
Bn-scaff_16614_1-p369346	Bn-N3-p1519413	gene	
Bn-scaff_16614_1-p373513	Bn-N3-p1515411	gene	exon
Bn-scaff_16614_1-p400790	Bn-N13-p1910299	gene	exon
Bn-scaff_16614_1-p432138	Bn-N13-p1871532	gene	
Bn-scaff_16614_1-p534173	Bn-N13-p1766310	gene	
Bn-scaff_16614_1-p546020	Bn-N13-p1753320	gene	
Bn-scaff_16614_1-p56536	Bn-N13-p2275896	gene	exon
Bn-scaff_16614_1-p571357	Bn-N13-p1734011	gene	exon
Bn-scaff_16614_1-p578598	Bn-N13-p1724394	gene	
Bn-scaff_16614_1-p611916	Bn-N3-p1335072	gene	exon
Bn-scaff_16614_1-p636297	Bn-N13-p1662465	gene	
Bn-scaff_16614_1-p636358	Bn-N13-p1662457	gene	
Bn-scaff_16614_1-p636420	Bn-N13-p1662395	gene	exon
Bn-scaff_16614_1-p636428	Bn-N13-p1662387	gene	exon
Bn-scaff_16614_1-p642560	Bn-N13-p1656267	gene	

Bn-scaff_16614_1-p642888	Bn-N13-p1655896	gene	exon
Bn-scaff_16614_1-p643298	Bn-N13-p1655529	gene	
Bn-scaff_16614_1-p643378	Bn-N13-p1655449	gene	exon
Bn-scaff_16614_1-p643845	Bn-N13-p1654982	gene	exon
Bn-scaff_16614_1-p661193	Bn-N3-p1289877	gene	
Bn-scaff_16614_1-p677445	Bn-N3-p1278304	gene	
Bn-scaff_16614_1-p702187	Bn-N13-p1588380	gene	
Bn-scaff_16614_1-p717017	Bn-N13-p1573556	gene	
Bn-scaff_16614_1-p722822	Bn-N13-p1567736	gene	
Bn-scaff_16614_1-p731075	Bn-N13-p1561460	gene	
Bn-scaff_16614_1-p731938	Bn-N13-p1555052	gene	
Bn-scaff_16614_1-p750688	Bn-N3-p1197574	gene	
Bn-scaff_16614_1-p889881	Bn-N13-p1354589	gene	exon
Bn-scaff_16619_1-p208263	Bn-N19-p34952973	gene	
Bn-scaff_16619_1-p208935	Bn-N19-p34953645	gene	exon
Bn-scaff_16619_1-p228456	Bn-N19-p34973447	gene	
Bn-scaff_16619_1-p229074	Bn-N19-p34974217	gene	
Bn-scaff_16625_1-p41761	Bn-N14-p26758658	gene	exon
Bn-scaff_16625_1-p60807	Bn-N4-p812278	gene	exon
Bn-scaff_16625_1-p64201	Bn-N14-p26780967	gene	
Bn-scaff_16647_1-p162647	Bn-N16-p6317069	gene	
Bn-scaff_16647_1-p287095	Bn-N16-p6126513	gene	
Bn-scaff_16647_1-p288006	Bn-N16-p6125602	gene	
Bn-scaff_16647_1-p294824	Bn-N16-p6118700	gene	exon
Bn-scaff_16647_1-p296976	Bn-N16-p6116548	gene	exon
Bn-scaff_16647_1-p297173	Bn-N16-p6116351	gene	
Bn-scaff_16647_1-p297315	Bn-N16-p6116209	gene	
Bn-scaff_16647_1-p297412	Bn-N16-p6116112	gene	
Bn-scaff_16647_1-p297560	Bn-N16-p6115964	gene	
Bn-scaff_16647_1-p347369	Bn-N16-p6081277	gene	
Bn-scaff_16647_1-p398429	Bn-N6-p1071631	gene	exon
Bn-scaff_16647_1-p462650	Bn-N16-p5992011	gene	
Bn-scaff_16647_1-p464272	Bn-N16-p5990387	gene	
Bn-scaff_16647_1-p486000	Bn-N16-p5973313	gene	
Bn-scaff_16647_1-p499920	Bn-N16-p5961851	gene	
Bn-scaff_16656_1-p171648	Bn-Scaffold04219-p738	gene	exon
Bn-scaff_16665_1-p127087	Bn-N8-p16186531	gene	exon
Bn-scaff_16665_1-p127450	Bn-N8-p16186171	gene	
Bn-scaff_16665_1-p141052	Bn-N13-p54488235	gene	exon
Bn-scaff_16665_1-p158336	Bn-N13-p54498507	gene	exon
Bn-scaff_16665_1-p158462	Bn-N13-p54498633	gene	exon
Bn-scaff_16670_1-p51961	Bn-N16-p1965975	gene	
Bn-scaff_16676_1-p6894	Bn-N13-p64649039	gene	
Bn-scaff_16677_1-p101641	Bn-N6-p1460278	gene	
Bn-scaff_16677_1-p102715	Bn-N6-p1459207	gene	
Bn-scaff_16677_1-p1237	Bn-N16-p4921864	gene	exon
Bn-scaff_16677_1-p208279	Bn-N18-p5442895	gene	exon
Bn-scaff_16677_1-p310736	Bn-N18-p5324199	gene	exon
Bn-scaff_16691_1-p1332173	Bn-N1-p24998977	gene	exon
Bn-scaff_16691_1-p1350013	Bn-N11-p42276032	gene	exon
Bn-scaff_16691_1-p1402284	Bn-N1-p25086811	gene	exon
Bn-scaff_16691_1-p1441263	Bn-N13-p38449752	gene	

Bn-scaff_16691_1-p144715	Bn-N11-p24368676	gene	
Bn-scaff_16691_1-p1459639	Bn-N11-p42394452	gene	exon
Bn-scaff_16691_1-p1479528	Bn-N17-p47101815	gene	exon
Bn-scaff_16691_1-p278826	Bn-N11-p24206076	gene	exon
Bn-scaff_16691_1-p483096	Bn-N11-p24013028	gene	exon
Bn-scaff_16691_1-p50579	Bn-N1-p14800251	gene	exon
Bn-scaff_16691_1-p565328	Bn-N11-p23923592	gene	exon
Bn-scaff_16691_1-p61463	Bn-N11-p24447418	gene	exon
Bn-scaff_16691_1-p637815	Bn-N11-p23841636	gene	
Bn-scaff_16691_1-p638223	Bn-N11-p23841113	gene	
Bn-scaff_16691_1-p639122	Bn-N11-p23840213	gene	
Bn-scaff_16691_1-p639227	Bn-N11-p23840108	gene	exon
Bn-scaff_16691_1-p640408	Bn-N11-p23838935	gene	
Bn-scaff_16691_1-p650869	Bn-N11-p23828326	gene	
Bn-scaff_16691_1-p657224	Bn-N11-p23822039	gene	
Bn-scaff_16691_1-p658442	Bn-N11-p23820825	gene	exon
Bn-scaff_16691_1-p658665	Bn-N11-p23820602	gene	
Bn-scaff_16691_1-p658917	Bn-N11-p23820350	gene	
Bn-scaff_16691_1-p675741	Bn-N1-p14413420	gene	exon
Bn-scaff_16691_1-p697108	Bn-N11-p23777494	gene	
Bn-scaff_16691_1-p697516	Bn-N11-p23777085	gene	
Bn-scaff_16691_1-p792721	Bn-N11-p23679845	gene	exon
Bn-scaff_16691_1-p792979	Bn-N11-p23679587	gene	
Bn-scaff_16691_1-p793078	Bn-N11-p23679488	gene	
Bn-scaff_16691_1-p793355	Bn-N11-p23675725	gene	
Bn-scaff_16691_1-p881093	Bn-N11-p23587861	gene	
Bn-scaff_16691_1-p881477	Bn-N1-p14338784	gene	
Bn-scaff_16691_1-p913942	Bn-N11-p23540225	gene	
Bn-scaff_16691_1-p914035	Bn-N11-p23540132	gene	
Bn-scaff_16691_1-p954339	Bn-N11-p23547890	gene	
Bn-scaff_16693_1-p128559	Bn-N2-p396080	gene	exon
Bn-scaff_16693_1-p144993	Bn-N10-p17802637	gene	exon
Bn-scaff_16693_1-p145372	Bn-N10-p17802258	gene	
Bn-scaff_16693_1-p199016	Bn-N2-p424769	gene	exon
Bn-scaff_16693_1-p300736	Bn-N10-p17739702	gene	
Bn-scaff_16693_1-p46449	Bn-N10-p17858492	gene	
Bn-scaff_16694_1-p305039	Bn-N14-p40732336	gene	
Bn-scaff_16700_1-p126138	Bn-N14-p44802568	gene	
Bn-scaff_16700_1-p126438	Bn-N4-p13452964	gene	
Bn-scaff_16700_1-p164216	Bn-N14-p44889927	gene	exon
Bn-scaff_16700_1-p60575	Bn-N14-p44700201	gene	exon
Bn-scaff_16700_1-p73552	Bn-N14-p44738247	gene	exon
Bn-scaff_16700_1-p73598	Bn-N14-p44738292	gene	exon
Bn-scaff_16700_1-p75339	Bn-N14-p44738624	gene	
Bn-scaff_16700_1-p75524	Bn-N14-p44738809	gene	
Bn-scaff_16700_1-p75804	Bn-N14-p44739084	gene	
Bn-scaff_16704_1-p119074	Bn-N12-p19060123	gene	
Bn-scaff_16704_1-p121608	Bn-N12-p19057578	gene	exon
Bn-scaff_16704_1-p141970	Bn-N12-p19020827	gene	exon
Bn-scaff_16704_1-p173789	Bn-N12-p18986246	gene	
Bn-scaff_16704_1-p174270	Bn-N12-p18985760	gene	
Bn-scaff_16704_1-p349056	Bn-N12-p18867322	gene	

Bn-scaff_16704_1-p454876	Bn-N12-p18773054	gene	exon
Bn-scaff_16712_1-p33885	Bn-N11-p3590029	gene	exon
Bn-scaff_16712_1-p33920	Bn-N13-p63286665	gene	
Bn-scaff_16712_1-p53301	Bn-N13-p63305448	gene	exon
Bn-scaff_16714_1-p104848	Bn-N14-p26493065	gene	
Bn-scaff_16714_1-p111822	Bn-N14-p26486327	gene	
Bn-scaff_16714_1-p112449	Bn-N4-p671668	gene	
Bn-scaff_16714_1-p172282	Bn-N14-p26418743	gene	exon
Bn-scaff_16714_1-p215248	Bn-N14-p26362564	gene	exon
Bn-scaff_16714_1-p229922	Bn-N14-p26346285	gene	exon
Bn-scaff_16714_1-p248892	Bn-N14-p26325586	gene	exon
Bn-scaff_16714_1-p249437	Bn-N14-p26325040	gene	exon
Bn-scaff_16714_1-p329155	Bn-N14-p26252074	gene	exon
Bn-scaff_16714_1-p329461	Bn-N14-p26251768	gene	exon
Bn-scaff_16714_1-p342976	Bn-N14-p26234058	gene	exon
Bn-scaff_16714_1-p343537	Bn-N4-p618611	gene	exon
Bn-scaff_16714_1-p346340	Bn-N14-p26230691	gene	
Bn-scaff_16714_1-p348941	Bn-N14-p26228090	gene	
Bn-scaff_16714_1-p375535	Bn-N14-p26203507	gene	
Bn-scaff_16714_1-p406026	Bn-N14-p26174106	gene	
Bn-scaff_16714_1-p407589	Bn-N14-p26172543	gene	exon
Bn-scaff_16714_1-p98921	Bn-N14-p26498993	gene	
Bn-scaff_16721_1-p1173038	Bn-N17-p11827490	gene	
Bn-scaff_16721_1-p1254647	Bn-N17-p11900790	gene	exon
Bn-scaff_16721_1-p1263674	Bn-N17-p11909868	gene	exon
Bn-scaff_16721_1-p1264229	Bn-N17-p11910515	gene	
Bn-scaff_16721_1-p1335429	Bn-N17-p11995124	gene	
Bn-scaff_16721_1-p1335851	Bn-N17-p11995546	gene	
Bn-scaff_16721_1-p1347752	Bn-N17-p12001901	gene	exon
Bn-scaff_16721_1-p1521206	Bn-N17-p12177885	gene	exon
Bn-scaff_16721_1-p1532675	Bn-N7-p5111309	gene	exon
Bn-scaff_16721_1-p158260	Bn-N17-p11263780	gene	
Bn-scaff_16721_1-p158546	Bn-N7-p4580094	gene	
Bn-scaff_16721_1-p161261	Bn-N17-p11266824	gene	
Bn-scaff_16721_1-p169588	Bn-N17-p11276011	gene	
Bn-scaff_16721_1-p170488	Bn-N17-p11276911	gene	exon
Bn-scaff_16721_1-p1775159	Bn-N17-p12370670	gene	
Bn-scaff_16721_1-p1783089	Bn-N17-p12378337	gene	
Bn-scaff_16721_1-p1805920	Bn-N7-p5208939	gene	
Bn-scaff_16721_1-p1901248	Bn-N17-p12464968	gene	
Bn-scaff_16721_1-p1901401	Bn-N17-p12465121	gene	
Bn-scaff_16721_1-p2118530	Bn-N17-p12643865	gene	
Bn-scaff_16721_1-p2136567	Bn-N7-p5390406	gene	exon
Bn-scaff_16721_1-p2171161	Bn-N7-p5422323	gene	
Bn-scaff_16721_1-p24679	Bn-N17-p11094465	gene	exon
Bn-scaff_16721_1-p25103	Bn-N17-p11094889	gene	
Bn-scaff_16721_1-p25525	Bn-N17-p11095311	gene	exon
Bn-scaff_16721_1-p360637	Bn-Scaffold00929-p44810	gene	exon
Bn-scaff_16721_1-p360689	Bn-N17-p11449356	gene	exon
Bn-scaff_16721_1-p361967	Bn-N19-p3992906	gene	exon
Bn-scaff_16721_1-p377743	Bn-N17-p11461087	gene	
Bn-scaff_16721_1-p4674	Bn-N17-p10621846	gene	

Bn-scaff_16721_1-p4809	Bn-N17-p10621710	gene	exon
Bn-scaff_16721_1-p673	Bn-N17-p10623387	gene	
Bn-scaff_16721_1-p744	Bn-N17-p10623317	gene	
Bn-scaff_16721_1-p7795	Bn-N17-p10620061	gene	
Bn-scaff_16721_1-p825	Bn-N17-p10623235	gene	
Bn-scaff_16721_1-p827	Bn-N17-p10623233	gene	
Bn-scaff_16755_1-p1008572	Bn-N13-p66818565	gene	
Bn-scaff_16755_1-p1112413	Bn-N8-p987367	gene	exon
Bn-scaff_16755_1-p1112518	Bn-N8-p987472	gene	exon
Bn-scaff_16755_1-p116429	Bn-N13-p67667405	gene	exon
Bn-scaff_16755_1-p117421	Bn-N13-p67666172	gene	exon
Bn-scaff_16755_1-p1191145	Bn-N13-p66653654	gene	
Bn-scaff_16755_1-p1201419	Bn-N13-p66641183	gene	
Bn-scaff_16755_1-p1208174	Bn-N13-p66634448	gene	exon
Bn-scaff_16755_1-p12541	Bn-N13-p67764811	gene	
Bn-scaff_16755_1-p1290310	Bn-N13-p66541393	gene	
Bn-scaff_16755_1-p1426914	Bn-N2-p10213564	gene	exon
Bn-scaff_16755_1-p1427195	Bn-N2-p10213284	gene	exon
Bn-scaff_16755_1-p1427621	Bn-N2-p10212859	gene	exon
Bn-scaff_16755_1-p1428088	Bn-N2-p10212435	gene	
Bn-scaff_16755_1-p1494849	Bn-N13-p66317548	gene	exon
Bn-scaff_16755_1-p1497175	Bn-N13-p66315241	gene	exon
Bn-scaff_16755_1-p150355	Bn-N13-p67617970	gene	exon
Bn-scaff_16755_1-p150462	Bn-N13-p67617863	gene	exon
Bn-scaff_16755_1-p325875	Bn-N13-p67436102	gene	exon
Bn-scaff_16755_1-p327067	Bn-N13-p67434911	gene	exon
Bn-scaff_16755_1-p352874	Bn-N13-p67404934	gene	
Bn-scaff_16755_1-p411912	Bn-N13-p67341586	gene	exon
Bn-scaff_16755_1-p436280	Bn-N13-p67315054	gene	exon
Bn-scaff_16755_1-p439395	Bn-N13-p67313411	gene	exon
Bn-scaff_16755_1-p502073	Bn-N8-p1285212	gene	exon
Bn-scaff_16755_1-p515388	Bn-N9-p9320121	gene	
Bn-scaff_16755_1-p515807	Bn-N9-p9319701	gene	exon
Bn-scaff_16755_1-p614641	Bn-N8-p581805	gene	exon
Bn-scaff_16755_1-p970375	Bn-N13-p66857529	gene	
Bn-scaff_16755_1-p970466	Bn-N13-p66857438	gene	
Bn-scaff_16755_1-p982092	Bn-N16-p14474806	gene	exon
Bn-scaff_16766_1-p119819	Bn-N3-p5799995	gene	
Bn-scaff_16766_1-p289124	Bn-N8-p6331205	gene	exon
Bn-scaff_16770_1-p107862	Bn-N15-p40139455	gene	
Bn-scaff_16770_1-p1746549	Bn-N18-p29413393	gene	exon
Bn-scaff_16770_1-p1969319	Bn-N9-p28210405	gene	
Bn-scaff_16770_1-p2082683	Bn-N18-p29793921	gene	
Bn-scaff_16770_1-p2217095	Bn-N9-p28364458	gene	
Bn-scaff_16770_1-p2577945	Bn-N9-p29140595	gene	exon
Bn-scaff_16770_1-p2803733	Bn-N9-p28957647	gene	
Bn-scaff_16770_1-p2818688	Bn-N18-p30615117	gene	exon
Bn-scaff_16770_1-p2818770	Bn-N18-p30615036	gene	
Bn-scaff_16770_1-p2818885	Bn-N18-p30614921	gene	exon
Bn-scaff_16770_1-p2861442	Bn-N18-p30588955	gene	exon
Bn-scaff_16770_1-p2909313	Bn-N18-p30539869	gene	exon
Bn-scaff_16770_1-p2946678	Bn-N18-p30502022	gene	

Bn-scaff_16770_1-p2948588	Bn-N18-p30500155	gene	
Bn-scaff_16770_1-p298311	Bn-N15-p39942722	gene	exon
Bn-scaff_16770_1-p2988236	Bn-N9-p28657576	gene	exon
Bn-scaff_16770_1-p298928	Bn-N15-p39942105	gene	exon
Bn-scaff_16770_1-p3013229	Bn-N18-p30430944	gene	
Bn-scaff_16770_1-p3013339	Bn-N18-p30430834	gene	
Bn-scaff_16770_1-p3050704	Bn-N9-p28610378	gene	exon
Bn-scaff_16770_1-p3052263	Bn-N9-p28608755	gene	exon
Bn-scaff_16770_1-p3121600	Bn-N9-p28545552	gene	exon
Bn-scaff_16770_1-p3142138	Bn-N9-p28517012	gene	exon
Bn-scaff_16770_1-p317634	Bn-N15-p39923313	gene	
Bn-scaff_16770_1-p332122	Bn-N5-p20136481	gene	exon
Bn-scaff_16770_1-p3930920	Bn-N18-p31719694	gene	exon
Bn-scaff_16770_1-p3943733	Bn-N18-p31736733	gene	exon
Bn-scaff_16770_1-p4054911	Bn-N18-p31864375	gene	exon
Bn-scaff_16770_1-p4054998	Bn-N18-p31864462	gene	
Bn-scaff_16770_1-p4068629	Bn-N18-p31875046	gene	
Bn-scaff_16770_1-p4071245	Bn-N18-p31877221	gene	
Bn-scaff_16770_1-p4204848	Bn-N18-p31996276	gene	
Bn-scaff_16770_1-p4219765	Bn-N18-p32012310	gene	
Bn-scaff_16770_1-p4231526	Bn-N18-p32021176	gene	
Bn-scaff_16770_1-p4235967	Bn-N18-p32028258	gene	
Bn-scaff_16770_1-p4246969	Bn-N18-p32039448	gene	exon
Bn-scaff_16770_1-p4247182	Bn-N18-p32039661	gene	exon
Bn-scaff_16770_1-p4253992	Bn-N18-p32046472	gene	
Bn-scaff_16770_1-p4261177	Bn-N18-p32053675	gene	
Bn-scaff_16770_1-p4273193	Bn-N18-p32059058	gene	
Bn-scaff_16770_1-p4273299	Bn-N18-p32059164	gene	
Bn-scaff_16770_1-p4273315	Bn-N18-p32059180	gene	
Bn-scaff_16770_1-p4273479	Bn-N18-p32059344	gene	
Bn-scaff_16770_1-p4273654	Bn-N18-p32059519	gene	
Bn-scaff_16770_1-p4279495	Bn-N18-p32066808	gene	
Bn-scaff_16770_1-p4283902	Bn-N18-p32071215	gene	
Bn-scaff_16770_1-p4296535	Bn-N18-p32083896	gene	
Bn-scaff_16770_1-p4331835	Bn-N18-p32115345	gene	
Bn-scaff_16770_1-p4338179	Bn-N18-p32130575	gene	exon
Bn-scaff_16770_1-p4352929	Bn-N18-p32150182	gene	
Bn-scaff_16770_1-p4353199	Bn-N18-p32150422	gene	
Bn-scaff_16770_1-p4353457	Bn-N18-p32150596	gene	
Bn-scaff_16770_1-p4374851	Bn-N18-p32167058	gene	
Bn-scaff_16770_1-p65546	Bn-N15-p40190660	gene	
Bn-scaff_16770_1-p65799	Bn-N15-p40190406	gene	
Bn-scaff_16770_1-p677152	Bn-N5-p19987638	gene	exon
Bn-scaff_16770_1-p684639	Bn-N15-p39554368	gene	
Bn-scaff_16770_1-p982799	Bn-N4-p407412	gene	exon
Bn-scaff_16792_1-p21096	Bn-N15-p12505820	gene	
Bn-scaff_16792_1-p21478	Bn-N15-p12506202	gene	
Bn-scaff_16792_1-p21595	Bn-N15-p12506319	gene	
Bn-scaff_16792_1-p21956	Bn-N15-p12506680	gene	
Bn-scaff_16793_1-p821453	Bn-N19-p29509411	gene	
Bn-scaff_16804_1-p383275	Bn-N2-p6234189	gene	
Bn-scaff_16804_1-p64080	Bn-N2-p6062784	gene	

Bn-scaff_16804_1-p653237	Bn-N12-p9069045	gene	exon
Bn-scaff_16804_1-p670370	Bn-N14-p47143243	gene	
Bn-scaff_16804_1-p695166	Bn-N14-p47179219	gene	
Bn-scaff_16804_1-p704013	Bn-N14-p47187128	gene	
Bn-scaff_16804_1-p704907	Bn-N14-p47188023	gene	exon
Bn-scaff_16804_1-p755719	Bn-N14-p47233222	gene	
Bn-scaff_16804_1-p773033	Bn-N14-p47250018	gene	exon
Bn-scaff_16804_1-p794568	Bn-N14-p47276519	gene	
Bn-scaff_16804_1-p796198	Bn-N4-p15123163	gene	exon
Bn-scaff_16804_2-p156230	Bn-N14-p48951207	gene	
Bn-scaff_16804_2-p157923	Bn-N4-p16108021	gene	
Bn-scaff_16804_2-p165752	Bn-N4-p16097093	gene	exon
Bn-scaff_16804_2-p166441	Bn-N4-p16096442	gene	
Bn-scaff_16804_2-p167019	Bn-N14-p48938815	gene	
Bn-scaff_16804_2-p195554	Bn-N14-p48907212	gene	exon
Bn-scaff_16804_2-p210553	Bn-N14-p48893266	gene	exon
Bn-scaff_16804_2-p211134	Bn-N14-p48892685	gene	exon
Bn-scaff_16804_2-p255570	Bn-N14-p48848042	gene	
Bn-scaff_16804_2-p283580	Bn-N14-p48818989	gene	
Bn-scaff_16804_2-p284402	Bn-N14-p48818167	gene	
Bn-scaff_16804_2-p298602	Bn-N14-p48805465	gene	
Bn-scaff_16804_4-p107537	Bn-N12-p9136673	gene	exon
Bn-scaff_16804_4-p112136	Bn-N12-p9141287	gene	
Bn-scaff_16804_4-p272285	Bn-N2-p6512012	gene	exon
Bn-scaff_16809_1-p21714	Bn-Scaffold00952-p74230	gene	exon
Bn-scaff_16809_1-p46229	Bn-N11-p20128849	gene	
Bn-scaff_16809_1-p54602	Bn-N11-p20108993	gene	exon
Bn-scaff_16809_1-p55782	Bn-N11-p20099934	gene	exon
Bn-scaff_16809_1-p56352	Bn-N11-p20099362	gene	
Bn-scaff_16818_1-p222830	Bn-N15-p33407046	gene	exon
Bn-scaff_16818_3-p10386	Bn-Scaffold03234-p2275	gene	exon
Bn-scaff_16822_1-p110220	Bn-N13-p30886079	gene	exon
Bn-scaff_16822_1-p137478	Bn-N13-p30866763	gene	exon
Bn-scaff_16822_1-p139438	Bn-N13-p30864833	gene	
Bn-scaff_16822_1-p56747	Bn-N13-p31030768	gene	exon
Bn-scaff_16822_1-p96591	Bn-N13-p30900756	gene	
Bn-scaff_16822_1-p96861	Bn-N13-p30900486	gene	
Bn-scaff_16825_1-p103398	Bn-N12-p4472399	gene	exon
Bn-scaff_16825_1-p63375	Bn-N12-p4409413	gene	exon
Bn-scaff_16825_1-p94370	Bn-N12-p4460737	gene	exon
Bn-scaff_16825_1-p95545	Bn-N6-p8606002	gene	
Bn-scaff_16864_1-p113683	Bn-N14-p2276187	gene	exon
Bn-scaff_16864_1-p22602	Bn-N14-p2171696	gene	exon
Bn-scaff_16864_1-p22764	Bn-N14-p2171858	gene	exon
Bn-scaff_16865_1-p221176	Bn-N4-p21071745	gene	
Bn-scaff_16865_1-p223681	Bn-N4-p21076005	gene	exon
Bn-scaff_16865_1-p40832	Bn-N16-p28602368	gene	exon
Bn-scaff_16874_1-p172441	Bn-N16-p35142307	gene	
Bn-scaff_16874_1-p172867	Bn-N16-p35142733	gene	exon
Bn-scaff_16874_1-p173312	Bn-N16-p35143178	gene	
Bn-scaff_16874_1-p173544	Bn-N16-p35143410	gene	
Bn-scaff_16874_1-p285169	Bn-N16-p35261709	gene	exon

Bn-scaff_16874_1-p325339	Bn-N16-p35304721	gene	
Bn-scaff_16874_1-p325632	Bn-N16-p35305010	gene	
Bn-scaff_16874_1-p326376	Bn-N16-p35305774	gene	
Bn-scaff_16874_1-p327692	Bn-N16-p35307090	gene	exon
Bn-scaff_16874_1-p342999	Bn-N16-p35330834	gene	exon
Bn-scaff_16874_1-p425401	Bn-N16-p35417857	gene	
Bn-scaff_16874_1-p616918	Bn-N16-p35599783	gene	exon
Bn-scaff_16874_1-p617228	Bn-N16-p35600092	gene	exon
Bn-scaff_16874_1-p642411	Bn-N16-p35628223	gene	
Bn-scaff_16874_1-p642780	Bn-N16-p35628592	gene	exon
Bn-scaff_16874_1-p644530	Bn-N16-p35630340	gene	
Bn-scaff_16874_1-p644836	Bn-N16-p35630646	gene	
Bn-scaff_16874_2-p156435	Bn-N11-p13331984	gene	exon
Bn-scaff_16874_2-p156587	Bn-N11-p13331833	gene	exon
Bn-scaff_16874_2-p157688	Bn-N1-p8750275	gene	exon
Bn-scaff_16874_2-p217681	Bn-N1-p8711177	gene	
Bn-scaff_16874_2-p85853	Bn-N8-p14248847	gene	exon
Bn-scaff_16876_1-p1013994	Bn-N14-p38900834	gene	exon
Bn-scaff_16876_1-p1016989	Bn-N14-p38897836	gene	exon
Bn-scaff_16876_1-p1017087	Bn-N14-p38897738	gene	exon
Bn-scaff_16876_1-p1017291	Bn-N14-p38897534	gene	exon
Bn-scaff_16876_1-p1038199	Bn-N14-p38879446	gene	exon
Bn-scaff_16876_1-p1066302	Bn-N14-p38851491	gene	
Bn-scaff_16876_1-p1092787	Bn-N14-p38832572	gene	
Bn-scaff_16876_1-p1093245	Bn-N14-p38832113	gene	exon
Bn-scaff_16876_1-p110047	Bn-N14-p39791703	gene	
Bn-scaff_16876_1-p1116580	Bn-N14-p38808273	gene	exon
Bn-scaff_16876_1-p1116894	Bn-N14-p38807959	gene	
Bn-scaff_16876_1-p1228854	Bn-N14-p38679704	gene	exon
Bn-scaff_16876_1-p257162	Bn-N14-p39665542	gene	
Bn-scaff_16876_1-p287148	Bn-N14-p39630648	gene	exon
Bn-scaff_16876_1-p390080	Bn-N14-p39551702	gene	exon
Bn-scaff_16876_1-p574590	Bn-N14-p39346902	gene	exon
Bn-scaff_16876_1-p732973	Bn-N14-p39178511	gene	
Bn-scaff_16876_1-p733124	Bn-N14-p39178360	gene	
Bn-scaff_16876_1-p7359	Bn-N14-p39911026	gene	
Bn-scaff_16876_1-p851344	Bn-N14-p39061279	gene	
Bn-scaff_16876_1-p863842	Bn-N4-p9501537	gene	
Bn-scaff_16876_1-p865850	Bn-N14-p39045757	gene	
Bn-scaff_16876_1-p873216	Bn-N14-p39038391	gene	exon
Bn-scaff_16876_1-p8795	Bn-N14-p39909613	gene	exon
Bn-scaff_16876_1-p908602	Bn-N14-p39005334	gene	
Bn-scaff_16876_1-p93444	Bn-N14-p39820130	gene	exon
Bn-scaff_16876_1-p948070	Bn-N14-p38965582	gene	
Bn-scaff_16876_1-p963956	Bn-N14-p38949692	gene	exon
Bn-scaff_16876_1-p964636	Bn-N14-p38949012	gene	exon
Bn-scaff_16888_1-p1023505	Bn-N4-p18295066	gene	
Bn-scaff_16888_1-p1031305	Bn-N4-p18302479	gene	exon
Bn-scaff_16888_1-p1036219	Bn-N4-p18307337	gene	
Bn-scaff_16888_1-p1037072	Bn-N14-p51467109	gene	exon
Bn-scaff_16888_1-p1070420	Bn-N4-p18345359	gene	exon
Bn-scaff_16888_1-p1085702	Bn-N14-p51517536	gene	

Bn-scaff_16888_1-p1140119	Bn-N14-p51573492	gene	
Bn-scaff_16888_1-p1193844	Bn-N14-p51627798	gene	exon
Bn-scaff_16888_1-p1194989	Bn-N14-p51628944	gene	exon
Bn-scaff_16888_1-p1206146	Bn-N14-p51640559	gene	
Bn-scaff_16888_1-p1221383	Bn-N14-p51655635	gene	
Bn-scaff_16888_1-p1221973	Bn-N14-p51656227	gene	
Bn-scaff_16888_1-p1222121	Bn-N14-p51656375	gene	
Bn-scaff_16888_1-p1222131	Bn-N14-p51656384	gene	
Bn-scaff_16888_1-p1222789	Bn-N14-p51657042	gene	exon
Bn-scaff_16888_1-p1273637	Bn-N4-p18475690	gene	exon
Bn-scaff_16888_1-p1306317	Bn-N14-p51747316	gene	
Bn-scaff_16888_1-p1337706	Bn-N14-p51767998	gene	exon
Bn-scaff_16888_1-p1341139	Bn-N14-p51771430	gene	
Bn-scaff_16888_1-p1347380	Bn-N14-p51776604	gene	
Bn-scaff_16888_1-p1347381	Bn-N14-p51776605	gene	
Bn-scaff_16888_1-p1364784	Bn-N14-p51797996	gene	
Bn-scaff_16888_1-p1400424	Bn-N14-p51834778	gene	exon
Bn-scaff_16888_1-p1419203	Bn-N14-p51872310	gene	
Bn-scaff_16888_1-p1426135	Bn-N14-p51877587	gene	exon
Bn-scaff_16888_1-p1431260	Bn-N4-p18618578	gene	
Bn-scaff_16888_1-p1435412	Bn-N14-p51884510	gene	exon
Bn-scaff_16888_1-p1458728	Bn-N4-p18652399	gene	exon
Bn-scaff_16888_1-p1484731	Bn-N14-p51926975	gene	
Bn-scaff_16888_1-p1538459	Bn-N14-p51986211	gene	exon
Bn-scaff_16888_1-p1587885	Bn-N4-p18963932	gene	exon
Bn-scaff_16888_1-p163778	Bn-N14-p51029968	gene	
Bn-scaff_16888_1-p173815	Bn-N14-p51035913	gene	
Bn-scaff_16888_1-p177029	Bn-N14-p51039887	gene	exon
Bn-scaff_16888_1-p1786111	Bn-N14-p52189935	gene	
Bn-scaff_16888_1-p1786468	Bn-N14-p52190292	gene	
Bn-scaff_16888_1-p1786897	Bn-N14-p52190722	gene	
Bn-scaff_16888_1-p1790902	Bn-N14-p52193999	gene	
Bn-scaff_16888_1-p1796428	Bn-N14-p52200006	gene	exon
Bn-scaff_16888_1-p1796793	Bn-N14-p52200371	gene	exon
Bn-scaff_16888_1-p1803946	Bn-N14-p52207524	gene	exon
Bn-scaff_16888_1-p1804195	Bn-N14-p52207773	gene	exon
Bn-scaff_16888_1-p1804460	Bn-N14-p52208038	gene	exon
Bn-scaff_16888_1-p1805449	Bn-N14-p52209027	gene	
Bn-scaff_16888_1-p1805506	Bn-N14-p52209085	gene	
Bn-scaff_16888_1-p1815678	Bn-N14-p52216124	gene	exon
Bn-scaff_16888_1-p1816216	Bn-N14-p52216672	gene	
Bn-scaff_16888_1-p1817414	Bn-N14-p52217881	gene	exon
Bn-scaff_16888_1-p1855862	Bn-N14-p52262832	gene	exon
Bn-scaff_16888_1-p1866577	Bn-N14-p52267076	gene	exon
Bn-scaff_16888_1-p1867396	Bn-N14-p52267888	gene	
Bn-scaff_16888_1-p1867573	Bn-N14-p52268065	gene	exon
Bn-scaff_16888_1-p189267	Bn-N14-p51053084	gene	exon
Bn-scaff_16888_1-p189582	Bn-N14-p51053399	gene	exon
Bn-scaff_16888_1-p193279	Bn-N14-p51057431	gene	
Bn-scaff_16888_1-p242818	Bn-N14-p51118397	gene	
Bn-scaff_16888_1-p384418	Bn-Scaffold00305b-p101621	gene	
Bn-scaff_16888_1-p384581	Bn-Scaffold00305b-p101449	gene	

Bn-scaff_16888_1-p469136	Bn-Scaffold00305b-p38000	gene	exon
Bn-scaff_16888_1-p516727	Bn-N4-p17893486	gene	exon
Bn-scaff_16888_1-p562258	Bn-N4-p17923761	gene	
Bn-scaff_16888_1-p569403	Bn-N10-p13830859	gene	exon
Bn-scaff_16888_1-p569748	Bn-N17-p43617607	gene	exon
Bn-scaff_16888_1-p570617	Bn-N1-p243904	gene	exon
Bn-scaff_16888_1-p59297	Bn-N14-p50917236	gene	exon
Bn-scaff_16888_1-p637751	Bn-N4-p17976885	gene	exon
Bn-scaff_16888_1-p746439	Bn-N14-p51174389	gene	
Bn-scaff_16888_1-p747827	Bn-N14-p51175775	gene	exon
Bn-scaff_16888_1-p748045	Bn-N14-p51175992	gene	
Bn-scaff_16888_1-p748106	Bn-N14-p51176053	gene	exon
Bn-scaff_16888_1-p763522	Bn-N14-p51189659	gene	exon
Bn-scaff_16888_1-p764043	Bn-N14-p51190178	gene	
Bn-scaff_16888_1-p80216	Bn-N14-p50931701	gene	
Bn-scaff_16888_1-p80455	Bn-N14-p50931940	gene	
Bn-scaff_16888_1-p830529	Bn-N14-p51257595	gene	
Bn-scaff_16888_1-p830636	Bn-N14-p51257702	gene	exon
Bn-scaff_16888_1-p830750	Bn-N14-p51257816	gene	exon
Bn-scaff_16888_1-p88359	Bn-N14-p50940118	gene	
Bn-scaff_16888_1-p894418	Bn-N14-p51329477	gene	exon
Bn-scaff_16888_1-p899785	Bn-N14-p51334335	gene	exon
Bn-scaff_16903_1-p168859	Bn-N16-p16868686	gene	exon
Bn-scaff_16903_1-p168943	Bn-N16-p16868770	gene	exon
Bn-scaff_16903_1-p230137	Bn-N16-p16931563	gene	exon
Bn-scaff_16903_1-p332611	Bn-N16-p16669389	gene	exon
Bn-scaff_16903_1-p34282	Bn-N7-p13206730	gene	exon
Bn-scaff_16903_1-p49977	Bn-N16-p16732780	gene	exon
Bn-scaff_16903_2-p305070	Bn-N12-p20074167	gene	exon
Bn-scaff_16903_3-p65171	Bn-N9-p14231177	gene	
Bn-scaff_16912_1-p112569	Bn-N19-p16504495	gene	
Bn-scaff_16912_1-p182324	Bn-N19-p16554732	gene	
Bn-scaff_16912_1-p187495	Bn-N19-p16569159	gene	
Bn-scaff_16912_1-p190291	Bn-N19-p16571976	gene	
Bn-scaff_16912_1-p201151	Bn-N19-p16592624	gene	
Bn-scaff_16925_1-p168760	Bn-N13-p29946745	gene	
Bn-scaff_16925_1-p183625	Bn-N13-p29943421	gene	exon
Bn-scaff_16925_1-p216039	Bn-N13-p29903419	gene	
Bn-scaff_16925_1-p227550	Bn-N13-p29884060	gene	
Bn-scaff_16925_1-p247087	Bn-N13-p29864198	gene	
Bn-scaff_16925_1-p87032	Bn-N13-p30046234	gene	exon
Bn-scaff_16926_1-p9295	Bn-N12-p29027779	gene	exon
Bn-scaff_16929_1-p154513	Bn-N1-p18655777	gene	exon
Bn-scaff_16929_1-p155104	Bn-N11-p30869026	gene	
Bn-scaff_16929_1-p175989	Bn-N11-p30847102	gene	exon
Bn-scaff_16929_1-p378424	Bn-N1-p18497212	gene	exon
Bn-scaff_16929_1-p383176	Bn-N1-p18471102	gene	
Bn-scaff_16929_1-p495739	Bn-N11-p30574635	gene	
Bn-scaff_16929_1-p497165	Bn-N11-p30573208	gene	exon
Bn-scaff_16929_1-p797092	Bn-N11-p30281752	gene	exon
Bn-scaff_16929_1-p841653	Bn-N1-p18188897	gene	exon
Bn-scaff_16929_1-p842076	Bn-N1-p18188474	gene	exon

Bn-scaff_16929_1-p886262	Bn-N11-p30185925	gene	
Bn-scaff_16929_1-p886405	Bn-N11-p30185782	gene	exon
Bn-scaff_16929_1-p887518	Bn-N1-p18183475	gene	
Bn-scaff_16929_1-p887720	Bn-N11-p30184416	gene	exon
Bn-scaff_16934_1-p145998	Bn-N12-p7629494	gene	
Bn-scaff_16934_1-p146521	Bn-N12-p7628955	gene	
Bn-scaff_16934_1-p148328	Bn-N12-p7627148	gene	
Bn-scaff_16934_1-p148330	Bn-N12-p7627148	gene	
Bn-scaff_16934_1-p167048	Bn-N12-p7607907	gene	
Bn-scaff_16934_1-p409102	Bn-N12-p7388807	gene	
Bn-scaff_16934_1-p411362	Bn-N12-p7386557	gene	
Bn-scaff_16934_1-p425999	Bn-N12-p7371080	gene	
Bn-scaff_16934_1-p426000	Bn-N12-p7371079	gene	
Bn-scaff_16934_1-p432017	Bn-N12-p7365092	gene	
Bn-scaff_16934_1-p98107	Bn-Scaffold01300-p8957	gene	
Bn-scaff_16935_1-p102075	Bn-N14-p677430	gene	
Bn-scaff_16935_1-p116139	Bn-N14-p691582	gene	
Bn-scaff_16935_1-p116701	Bn-N14-p692149	gene	
Bn-scaff_16935_1-p220838	Bn-N14-p785230	gene	
Bn-scaff_16935_1-p222793	Bn-N14-p787197	gene	
Bn-scaff_16935_1-p69744	Bn-N14-p647832	gene	
Bn-scaff_16935_1-p93509	Bn-N14-p666337	gene	
Bn-scaff_16935_1-p98166	Bn-N14-p671258	gene	
Bn-scaff_16935_1-p99442	Bn-N14-p672526	gene	
Bn-scaff_16962_1-p359041	Bn-N18-p22715061	gene	exon
Bn-scaff_16962_1-p376380	Bn-N18-p22712824	gene	exon
Bn-scaff_16962_1-p449385	Bn-N18-p22668381	gene	exon
Bn-scaff_16962_1-p454442	Bn-N18-p22663905	gene	
Bn-scaff_16962_1-p555042	Bn-N18-p22570835	gene	
Bn-scaff_16962_1-p565075	Bn-N18-p22559135	gene	
Bn-scaff_16962_1-p578438	Bn-N18-p22552608	gene	exon
Bn-scaff_16975_1-p129887	Bn-N17-p9285376	gene	exon
Bn-scaff_16975_1-p177167	Bn-N17-p9236970	gene	
Bn-scaff_16975_1-p6371	Bn-N17-p9391971	gene	
Bn-scaff_16975_1-p95169	Bn-N17-p9320151	gene	
Bn-scaff_16976_1-p12018	Bn-N14-p47099344	gene	
Bn-scaff_16984_1-p129923	Bn-N16-p3382589	gene	
Bn-scaff_16984_1-p130673	Bn-N16-p3381841	gene	exon
Bn-scaff_16984_1-p130702	Bn-N16-p3381812	gene	exon
Bn-scaff_16984_1-p200546	Bn-N16-p3309877	gene	
Bn-scaff_16984_1-p204543	Bn-N16-p3305881	gene	exon
Bn-scaff_16984_1-p21869	Bn-N16-p3491913	gene	
Bn-scaff_16984_1-p22326	Bn-N16-p3491456	gene	
Bn-scaff_16984_1-p22381	Bn-N16-p3491401	gene	
Bn-scaff_16984_1-p22409	Bn-N16-p3491373	gene	
Bn-scaff_16984_1-p22475	Bn-N16-p3491307	gene	
Bn-scaff_16984_1-p287791	Bn-N16-p3235572	gene	exon
Bn-scaff_16984_1-p312166	Bn-N16-p3212298	gene	
Bn-scaff_16984_1-p324673	Bn-N16-p3196094	gene	exon
Bn-scaff_16984_1-p353017	Bn-N16-p3155077	gene	exon
Bn-scaff_16984_1-p353514	Bn-N16-p3154580	gene	exon
Bn-scaff_16984_1-p353573	Bn-N6-p2313672	gene	exon

Bn-scaff_16984_1-p55002	Bn-N16-p3458189	gene	
Bn-scaff_16996_1-p283285	Bn-N13-p62743952	gene	exon
Bn-scaff_16996_1-p335143	Bn-N8-p12415213	gene	exon
Bn-scaff_17028_1-p134055	Bn-N19-p49462799	gene	
Bn-scaff_17028_1-p166275	Bn-N19-p49511084	gene	exon
Bn-scaff_17028_1-p1940	Bn-N10-p12975091	gene	exon
Bn-scaff_17028_1-p431988	Bn-N19-p49750038	gene	
Bn-scaff_17028_1-p432005	Bn-N19-p49750055	gene	
Bn-scaff_17028_1-p432707	Bn-N19-p49750758	gene	
Bn-scaff_17028_1-p444352	Bn-N19-p49761914	gene	exon
Bn-scaff_17028_1-p444459	Bn-N19-p49762022	gene	exon
Bn-scaff_17036_1-p2905	Bn-N11-p36045115	gene	exon
Bn-scaff_17036_1-p4953	Bn-N11-p36043057	gene	exon
Bn-scaff_17036_1-p91726	Bn-N11-p35955666	gene	
Bn-scaff_17036_1-p94786	Bn-N1-p21421992	gene	exon
Bn-scaff_17042_1-p14824	Bn-N13-p59278951	gene	exon
Bn-scaff_17042_1-p14943	Bn-N13-p59279070	gene	exon
Bn-scaff_17042_1-p15113	Bn-N13-p59279240	gene	exon
Bn-scaff_17042_1-p298610	Bn-N13-p59561211	gene	
Bn-scaff_17042_1-p319410	Bn-N13-p59579439	gene	exon
Bn-scaff_17042_1-p359732	Bn-N8-p10979512	gene	
Bn-scaff_17042_1-p359779	Bn-N8-p10979559	gene	exon
Bn-scaff_17042_1-p361073	Bn-N13-p59625974	gene	exon
Bn-scaff_17042_1-p456580	Bn-N13-p59729923	gene	exon
Bn-scaff_17042_1-p458609	Bn-N13-p59732075	gene	
Bn-scaff_17042_1-p458664	Bn-N13-p59732130	gene	
Bn-scaff_17042_1-p516850	Bn-N13-p59792507	gene	exon
Bn-scaff_17042_1-p517224	Bn-N13-p59792881	gene	
Bn-scaff_17042_1-p52695	Bn-N13-p59312403	gene	exon
Bn-scaff_17042_1-p642311	Bn-N13-p59908230	gene	
Bn-scaff_17042_1-p694383	Bn-N13-p60001509	gene	
Bn-scaff_17042_1-p701551	Bn-N13-p60008192	gene	exon
Bn-scaff_17044_1-p310881	Bn-N9-p20669226	gene	exon
Bn-scaff_17044_1-p521346	Bn-N6-p17791054	gene	exon
Bn-scaff_17044_1-p522803	Bn-N13-p36668857	gene	
Bn-scaff_17044_1-p529538	Bn-N13-p36662124	gene	
Bn-scaff_17044_1-p545336	Bn-N13-p36646684	gene	
Bn-scaff_17044_1-p612759	Bn-N13-p36580375	gene	
Bn-scaff_17044_1-p614454	Bn-N13-p36578680	gene	
Bn-scaff_17044_1-p614478	Bn-N13-p36578656	gene	
Bn-scaff_17044_1-p614535	Bn-N13-p36578599	gene	
Bn-scaff_17044_1-p630648	Bn-N13-p36558598	gene	exon
Bn-scaff_17044_1-p631059	Bn-N13-p36558187	gene	exon
Bn-scaff_17048_1-p105215	Bn-N19-p26296562	gene	
Bn-scaff_17048_1-p206502	Bn-N19-p26189233	gene	
Bn-scaff_17067_1-p1117534	Bn-N18-p3090365	gene	exon
Bn-scaff_17067_1-p507838	Bn-N12-p29943968	gene	
Bn-scaff_17067_1-p716724	Bn-N12-p30142152	gene	
Bn-scaff_17077_1-p329966	Bn-N2-p12363988	gene	
Bn-scaff_17077_1-p588579	Bn-N2-p12469235	gene	exon
Bn-scaff_17077_1-p589085	Bn-N2-p12469741	gene	exon
Bn-scaff_17079_1-p102621	Bn-N2-p11986827	gene	exon

Bn-scaff_17079_1-p28593	Bn-N4-p12479382	gene	
Bn-scaff_17079_1-p79663	Bn-N4-p12455274	gene	exon
Bn-scaff_17084_1-p234852	Bn-N17-p4947848	gene	
Bn-scaff_17084_1-p256953	Bn-N17-p4963722	gene	
Bn-scaff_17084_1-p257435	Bn-N17-p4964204	gene	
Bn-scaff_17084_1-p341281	Bn-N7-p2097330	gene	exon
Bn-scaff_17084_1-p850947	Bn-N9-p5350421	gene	exon
Bn-scaff_17084_1-p851121	Bn-N9-p5350247	gene	exon
Bn-scaff_17084_1-p896728	Bn-N9-p5340759	gene	exon
Bn-scaff_17088_1-p239732	Bn-N19-p46866964	gene	
Bn-scaff_17088_1-p240030	Bn-N10-p11610150	gene	
Bn-scaff_17088_1-p245502	Bn-N10-p11615606	gene	exon
Bn-scaff_17088_1-p327069	Bn-N19-p46949448	gene	
Bn-scaff_17088_1-p337567	Bn-N10-p11698760	gene	exon
Bn-scaff_17088_1-p353765	Bn-N10-p11718757	gene	exon
Bn-scaff_17088_1-p444918	Bn-N19-p47069927	gene	
Bn-scaff_17088_1-p445721	Bn-N10-p11778172	gene	exon
Bn-scaff_17088_1-p455184	Bn-N19-p47080269	gene	
Bn-scaff_17088_2-p124580	Bn-N16-p14103068	gene	
Bn-scaff_17088_2-p124900	Bn-N16-p14103387	gene	
Bn-scaff_17088_2-p126958	Bn-N16-p14105439	gene	
Bn-scaff_17088_2-p163374	Bn-N16-p14136006	gene	
Bn-scaff_17088_2-p236616	Bn-N5-p9929814	gene	exon
Bn-scaff_17088_3-p117282	Bn-N5-p10023837	gene	exon
Bn-scaff_17088_3-p123625	Bn-N5-p10027591	gene	exon
Bn-scaff_17088_3-p242403	Bn-N16-p13543105	gene	
Bn-scaff_17088_3-p275459	Bn-N16-p13481351	gene	
Bn-scaff_17088_3-p61230	Bn-N16-p13710878	gene	exon
Bn-scaff_17092_1-p426203	Bn-N5-p11876624	gene	exon
Bn-scaff_17109_1-p1044330	Bn-N12-p41825672	gene	
Bn-scaff_17109_1-p1044350	Bn-N12-p41825651	gene	exon
Bn-scaff_17109_1-p1044411	Bn-N12-p41825591	gene	exon
Bn-scaff_17109_1-p1058256	Bn-N12-p41811421	gene	
Bn-scaff_17109_1-p1096035	Bn-N12-p41767921	gene	exon
Bn-scaff_17109_1-p1659373	Bn-N10-p14962795	gene	
Bn-scaff_17109_1-p1694927	Bn-N19-p52591053	gene	
Bn-scaff_17109_1-p172384	Bn-N19-p22829826	gene	
Bn-scaff_17109_1-p1868715	Bn-N10-p14816420	gene	exon
Bn-scaff_17109_1-p1959932	Bn-N19-p52346260	gene	
Bn-scaff_17109_1-p2172912	Bn-N15-p25601259	gene	
Bn-scaff_17109_1-p2174572	Bn-N10-p14595133	gene	exon
Bn-scaff_17109_1-p280020	Bn-N19-p22723404	gene	exon
Bn-scaff_17109_1-p387181	Bn-N9-p14087191	gene	
Bn-scaff_17109_1-p498072	Bn-N12-p42405769	gene	exon
Bn-scaff_17109_1-p508730	Bn-N12-p42395018	gene	
Bn-scaff_17109_1-p509718	Bn-N12-p42394030	gene	
Bn-scaff_17109_1-p548640	Bn-N12-p42356118	gene	exon
Bn-scaff_17109_1-p548999	Bn-N12-p42355759	gene	
Bn-scaff_17109_1-p556641	Bn-N12-p42348325	gene	
Bn-scaff_17109_1-p557169	Bn-N12-p42347797	gene	exon
Bn-scaff_17109_1-p569458	Bn-N2-p26212825	gene	
Bn-scaff_17109_1-p626573	Bn-N12-p42268794	gene	

Bn-scaff_17109_1-p687016	Bn-N12-p42196603	gene	exon
Bn-scaff_17109_1-p690819	Bn-N12-p42192904	gene	exon
Bn-scaff_17109_1-p691274	Bn-N12-p42192449	gene	exon
Bn-scaff_17109_1-p715785	Bn-N12-p42172033	gene	
Bn-scaff_17109_1-p796852	Bn-N2-p26113992	gene	exon
Bn-scaff_17109_1-p827316	Bn-N12-p42070274	gene	
Bn-scaff_17109_1-p832774	Bn-N12-p42064838	gene	
Bn-scaff_17109_1-p833859	Bn-N12-p42063752	gene	
Bn-scaff_17109_1-p857649	Bn-N12-p42038852	gene	
Bn-scaff_17109_1-p859844	Bn-N12-p42036627	gene	
Bn-scaff_17109_1-p868072	Bn-N12-p42027555	gene	
Bn-scaff_17109_1-p868668	Bn-N2-p26054274	gene	exon
Bn-scaff_17109_1-p889768	Bn-N12-p42005074	gene	
Bn-scaff_17109_1-p899388	Bn-N12-p41995560	gene	exon
Bn-scaff_17109_1-p910796	Bn-N12-p41985546	gene	exon
Bn-scaff_17109_1-p910811	Bn-N12-p41985531	gene	exon
Bn-scaff_17109_1-p982274	Bn-N12-p41904103	gene	exon
Bn-scaff_17109_1-p982337	Bn-N12-p41904040	gene	exon
Bn-scaff_17109_1-p997171	Bn-N12-p41888813	gene	
Bn-scaff_17109_2-p150259	Bn-N10-p15439225	gene	
Bn-scaff_17109_2-p150298	Bn-N10-p15439185	gene	exon
Bn-scaff_17109_2-p188015	Bn-N19-p53374219	gene	exon
Bn-scaff_17109_2-p190946	Bn-N19-p53371260	gene	exon
Bn-scaff_17109_2-p205003	Bn-N19-p53357982	gene	exon
Bn-scaff_17109_2-p206902	Bn-N10-p15400129	gene	exon
Bn-scaff_17109_2-p207004	Bn-N10-p15400027	gene	
Bn-scaff_17109_2-p315902	Bn-N3-p18897762	gene	exon
Bn-scaff_17109_2-p361161	Bn-N12-p1826636	gene	
Bn-scaff_17109_2-p364002	Bn-N19-p53094461	gene	exon
Bn-scaff_17109_2-p370345	Bn-N19-p53090532	gene	
Bn-scaff_17109_2-p392938	Bn-N10-p15256965	gene	exon
Bn-scaff_17109_2-p405700	Bn-N19-p53059810	gene	exon
Bn-scaff_17109_2-p406679	Bn-N19-p53058830	gene	exon
Bn-scaff_17109_2-p413792	Bn-N19-p53051757	gene	
Bn-scaff_17109_2-p79906	Bn-N10-p15465109	gene	
Bn-scaff_17109_4-p114755	Bn-N12-p41266997	gene	
Bn-scaff_17109_4-p114822	Bn-N12-p41266930	gene	
Bn-scaff_17109_4-p159217	Bn-N12-p41230648	gene	exon
Bn-scaff_17109_4-p283045	Bn-N12-p41191535	gene	
Bn-scaff_17109_4-p95949	Bn-N12-p41285545	gene	exon
Bn-scaff_17109_4-p96390	Bn-N12-p41285105	gene	exon
Bn-scaff_17109_4-p97410	Bn-N12-p41284084	gene	exon
Bn-scaff_17119_1-p114444	Bn-N8-p12891309	gene	exon
Bn-scaff_17119_1-p114898	Bn-N8-p12891764	gene	exon
Bn-scaff_17119_1-p115218	Bn-N8-p12892084	gene	
Bn-scaff_17119_1-p172528	Bn-N8-p12909861	gene	exon
Bn-scaff_17119_1-p183714	Bn-N13-p63388579	gene	
Bn-scaff_17119_1-p247998	Bn-N13-p63470097	gene	exon
Bn-scaff_17119_1-p273017	Bn-N13-p63483753	gene	exon
Bn-scaff_17119_1-p298555	Bn-N13-p63505621	gene	exon
Bn-scaff_17119_1-p298861	Bn-N13-p63505927	gene	
Bn-scaff_17119_1-p299060	Bn-N13-p63506126	gene	exon

Bn-scaff_17119_1-p300606	Bn-N13-p63506858	gene	
Bn-scaff_17119_1-p410578	Bn-N13-p63620459	gene	
Bn-scaff_17119_1-p410774	Bn-N13-p63620655	gene	exon
Bn-scaff_17119_1-p69237	Bn-N13-p63308406	gene	exon
Bn-scaff_17119_1-p84986	Bn-N8-p12877341	gene	
Bn-scaff_17119_1-p85292	Bn-N8-p12877637	gene	exon
Bn-scaff_17163_1-p221326	Bn-N11-p44946293	gene	
Bn-scaff_17163_1-p42534	Bn-N1-p26793695	gene	
Bn-scaff_17174_1-p114224	Bn-N19-p17203746	gene	exon
Bn-scaff_17174_1-p216229	Bn-N19-p17082722	gene	
Bn-scaff_17174_1-p291582	Bn-N19-p17010571	gene	
Bn-scaff_17174_1-p37586	Bn-N19-p17272421	gene	exon
Bn-scaff_17174_1-p38610	Bn-N19-p17271414	gene	exon
Bn-scaff_17174_1-p400192	Bn-N19-p16959792	gene	
Bn-scaff_17174_1-p41873	Bn-N19-p17268150	gene	
Bn-scaff_17174_1-p88911	Bn-N19-p17203737	gene	exon
Bn-scaff_17177_1-p105819	Bn-N2-p28212988	gene	exon
Bn-scaff_17177_1-p131333	Bn-N2-p28191825	gene	
Bn-scaff_17177_1-p161160	Bn-Scaffold00372b-p50350	gene	exon
Bn-scaff_17177_1-p381225	Bn-N12-p45544093	gene	
Bn-scaff_17177_1-p385429	Bn-N12-p45539903	gene	
Bn-scaff_17177_1-p409992	Bn-N12-p45515087	gene	exon
Bn-scaff_17177_1-p462784	Bn-N12-p45461870	gene	exon
Bn-scaff_17177_1-p463843	Bn-N2-p28033916	gene	
Bn-scaff_17177_1-p504084	Bn-N12-p45406485	gene	exon
Bn-scaff_17177_1-p593541	Bn-N2-p27936056	gene	
Bn-scaff_17177_1-p667432	Bn-N12-p45200837	gene	exon
Bn-scaff_17177_1-p667932	Bn-N2-p27868077	gene	
Bn-scaff_17177_1-p731457	Bn-N12-p45137315	gene	exon
Bn-scaff_17180_1-p153332	Bn-N14-p30536203	gene	exon
Bn-scaff_17180_1-p253719	Bn-N14-p30628272	gene	exon
Bn-scaff_17180_1-p263031	Bn-N14-p30637109	gene	
Bn-scaff_17180_1-p263223	Bn-N14-p30637301	gene	exon
Bn-scaff_17180_1-p471429	Bn-N2-p24295289	gene	exon
Bn-scaff_17180_1-p59560	Bn-N14-p30328864	gene	exon
Bn-scaff_17180_1-p72890	Bn-N14-p30314792	gene	
Bn-scaff_17190_1-p1116406	Bn-N19-p6038475	gene	exon
Bn-scaff_17190_1-p1119408	Bn-N19-p6041483	gene	
Bn-scaff_17190_1-p1119825	Bn-N19-p6041900	gene	
Bn-scaff_17190_1-p1134270	Bn-N19-p6044429	gene	
Bn-scaff_17190_1-p18409	Bn-N9-p3875259	gene	exon
Bn-scaff_17190_1-p193506	Bn-N19-p5182418	gene	exon
Bn-scaff_17190_1-p237474	Bn-N9-p4020145	gene	
Bn-scaff_17190_1-p237949	Bn-N9-p4020612	gene	exon
Bn-scaff_17190_1-p238452	Bn-N9-p4021117	gene	exon
Bn-scaff_17190_1-p495674	Bn-N12-p46320791	gene	exon
Bn-scaff_17190_1-p660881	Bn-N19-p5638360	gene	exon
Bn-scaff_17190_1-p9206	Bn-N19-p5009107	gene	exon
Bn-scaff_17203_1-p315290	Bn-N11-p16553834	gene	exon
Bn-scaff_17203_1-p318274	Bn-N11-p16550844	gene	
Bn-scaff_17203_1-p534613	Bn-N1-p10670256	gene	exon
Bn-scaff_17203_1-p544482	Bn-N11-p16328950	gene	

Bn-scaff_17227_1-p1101239	Bn-N8-p20113458	gene	
Bn-scaff_17227_1-p1380795	Bn-N18-p22264975	gene	exon
Bn-scaff_17227_1-p253510	Bn-N18-p21154511	gene	
Bn-scaff_17227_1-p626934	Bn-N18-p21530494	gene	
Bn-scaff_17227_1-p697412	Bn-N8-p20308029	gene	exon
Bn-scaff_17227_1-p697710	Bn-N8-p20307730	gene	
Bn-scaff_17227_1-p699721	Bn-N8-p20305759	gene	
Bn-scaff_17227_1-p699815	Bn-N8-p20305665	gene	
Bn-scaff_17227_1-p700248	Bn-N8-p20305257	gene	
Bn-scaff_17227_1-p701651	Bn-N6-p3486262	gene	exon
Bn-scaff_17227_1-p895880	Bn-N8-p20191647	gene	
Bn-scaff_17227_1-p909256	Bn-N18-p21792423	gene	
Bn-scaff_17227_1-p991724	Bn-N18-p21877741	gene	exon
Bn-scaff_17233_1-p35444	Bn-N11-p36091941	gene	
Bn-scaff_17233_1-p9290	Bn-N11-p36057329	gene	
Bn-scaff_17249_1-p1042660	Bn-N6-p15368915	gene	exon
Bn-scaff_17249_1-p1054417	Bn-N6-p15378213	gene	
Bn-scaff_17249_1-p1094761	Bn-N13-p39918456	gene	
Bn-scaff_17249_1-p1295955	Bn-N13-p39818348	gene	
Bn-scaff_17249_1-p749288	Bn-N13-p40369251	gene	
Bn-scaff_17249_1-p985993	Bn-N13-p40093373	gene	exon
Bn-scaff_17257_1-p97933	Bn-N8-p9121603	gene	exon
Bn-scaff_17257_1-p98421	Bn-N8-p9122111	gene	exon
Bn-scaff_17257_1-p98652	Bn-N18-p16582401	gene	exon
Bn-scaff_17275_1-p34341	Bn-N19-p46571823	gene	exon
Bn-scaff_17289_1-p18657	Bn-N12-p35641314	gene	exon
Bn-scaff_17289_1-p460845	Bn-N2-p21761539	gene	exon
Bn-scaff_17289_1-p461538	Bn-N2-p21760848	gene	
Bn-scaff_17289_1-p627205	Bn-N2-p21597671	gene	exon
Bn-scaff_17289_1-p674943	Bn-N12-p35058761	gene	
Bn-scaff_17289_1-p807830	Bn-N12-p34919305	gene	
Bn-scaff_17289_1-p808294	Bn-N12-p34918841	gene	exon
Bn-scaff_17289_1-p808429	Bn-N12-p34918706	gene	exon
Bn-scaff_17289_1-p808558	Bn-N12-p34918577	gene	
Bn-scaff_17289_1-p882945	Bn-N9-p26499791	gene	exon
Bn-scaff_17291_1-p131907	Bn-N17-p36209498	gene	
Bn-scaff_17291_1-p188013	Bn-N17-p36132457	gene	exon
Bn-scaff_17291_1-p216152	Bn-N17-p36103147	gene	exon
Bn-scaff_17291_1-p22307	Bn-N6-p20279179	gene	exon
Bn-scaff_17291_1-p226218	Bn-N17-p36093081	gene	exon
Bn-scaff_17291_1-p239559	Bn-N13-p6404488	gene	
Bn-scaff_17291_1-p249622	Bn-N17-p36064701	gene	
Bn-scaff_17291_1-p250443	Bn-N17-p36063881	gene	
Bn-scaff_17291_1-p305279	Bn-N17-p36004078	gene	
Bn-scaff_17291_1-p305613	Bn-N6-p20470187	gene	exon
Bn-scaff_17291_1-p340775	Bn-N17-p35959762	gene	exon
Bn-scaff_17291_1-p360274	Bn-N17-p35939145	gene	
Bn-scaff_17291_1-p360422	Bn-N17-p35938997	gene	
Bn-scaff_17291_1-p398311	Bn-N17-p35900093	gene	
Bn-scaff_17291_1-p398501	Bn-N17-p35899903	gene	
Bn-scaff_17291_1-p409375	Bn-N17-p35888579	gene	
Bn-scaff_17291_1-p409673	Bn-N17-p35888281	gene	exon

Bn-scaff_17291_1-p409933	Bn-N17-p35888021	gene	
Bn-scaff_17291_1-p423933	Bn-N17-p35864789	gene	
Bn-scaff_17291_1-p467455	Bn-N17-p35814515	gene	exon
Bn-scaff_17291_1-p467671	Bn-N17-p35814298	gene	exon
Bn-scaff_17291_1-p475978	Bn-N6-p20607579	gene	exon
Bn-scaff_17291_1-p476220	Bn-N17-p35806298	gene	
Bn-scaff_17291_1-p722344	Bn-N17-p35630374	gene	
Bn-scaff_17291_1-p732305	Bn-N6-p20751653	gene	exon
Bn-scaff_17291_1-p732388	Bn-N6-p20751736	gene	exon
Bn-scaff_17291_1-p779676	Bn-N17-p35573134	gene	exon
Bn-scaff_17291_1-p844298	Bn-N17-p35499700	gene	
Bn-scaff_17291_1-p867646	Bn-N17-p35476841	gene	
Bn-scaff_17297_1-p214513	Bn-N19-p6321757	gene	exon
Bn-scaff_17298_1-p1055071	Bn-N13-p26575324	gene	
Bn-scaff_17298_1-p1058052	Bn-N13-p26578315	gene	
Bn-scaff_17298_1-p1064361	Bn-N13-p26584639	gene	exon
Bn-scaff_17298_1-p1086876	Bn-N13-p26602110	gene	exon
Bn-scaff_17298_1-p1092986	Bn-N13-p26608211	gene	
Bn-scaff_17298_1-p1136461	Bn-Scaffold01203-p8906	gene	
Bn-scaff_17298_1-p1170798	Bn-N13-p26697986	gene	
Bn-scaff_17298_1-p1173132	Bn-N13-p26700341	gene	
Bn-scaff_17298_1-p1173360	Bn-N3-p17132038	gene	exon
Bn-scaff_17298_1-p1173974	Bn-N3-p17132683	gene	exon
Bn-scaff_17298_1-p1174018	Bn-N3-p17132728	gene	exon
Bn-scaff_17298_1-p1174337	Bn-N13-p26701563	gene	
Bn-scaff_17298_1-p119718	Bn-N13-p25591525	gene	exon
Bn-scaff_17298_1-p1249353	Bn-N13-p26783811	gene	
Bn-scaff_17298_1-p1249839	Bn-N13-p26784330	gene	
Bn-scaff_17298_1-p1251153	Bn-N13-p26785623	gene	exon
Bn-scaff_17298_1-p1302996	Bn-N13-p26834187	gene	
Bn-scaff_17298_1-p1320886	Bn-N13-p26848042	gene	
Bn-scaff_17298_1-p1321549	Bn-N13-p26848688	gene	exon
Bn-scaff_17298_1-p134814	Bn-N13-p25605150	gene	exon
Bn-scaff_17298_1-p1370828	Bn-N13-p26902100	gene	exon
Bn-scaff_17298_1-p138775	Bn-N13-p25609110	gene	
Bn-scaff_17298_1-p139159	Bn-N13-p25609494	gene	
Bn-scaff_17298_1-p140847	Bn-N13-p25611164	gene	
Bn-scaff_17298_1-p1471882	Bn-N13-p26998039	gene	exon
Bn-scaff_17298_1-p1558470	Bn-N11-p39924400	gene	exon
Bn-scaff_17298_1-p1744778	Bn-N3-p17471016	gene	
Bn-scaff_17298_1-p1758734	Bn-N3-p27246678	gene	exon
Bn-scaff_17298_1-p1964250	Bn-N13-p27488482	gene	exon
Bn-scaff_17298_1-p1972479	Bn-N5-p20306526	gene	exon
Bn-scaff_17298_1-p2045159	Bn-N13-p27509870	gene	
Bn-scaff_17298_1-p2049303	Bn-N13-p27515019	gene	exon
Bn-scaff_17298_1-p2096293	Bn-N13-p27568024	gene	
Bn-scaff_17298_1-p2097436	Bn-N13-p27569145	gene	
Bn-scaff_17298_1-p2114220	Bn-N5-p20945128	gene	exon
Bn-scaff_17298_1-p2114326	Bn-N13-p27581498	gene	
Bn-scaff_17298_1-p220381	Bn-N13-p25693527	gene	exon
Bn-scaff_17298_1-p233298	Bn-N13-p25705063	gene	exon
Bn-scaff_17298_1-p250036	Bn-N13-p25722744	gene	exon

Bn-scaff_17298_1-p290841	Bn-N13-p25764799	gene	exon
Bn-scaff_17298_1-p302442	Bn-N13-p25777742	gene	
Bn-scaff_17298_1-p302930	Bn-N13-p25778578	gene	exon
Bn-scaff_17298_1-p307943	Bn-N13-p25785157	gene	
Bn-scaff_17298_1-p504210	Bn-N3-p16676434	gene	
Bn-scaff_17298_1-p586528	Bn-N13-p26047571	gene	
Bn-scaff_17298_1-p61168	Bn-N13-p25554878	gene	
Bn-scaff_17298_1-p61238	Bn-N13-p25554948	gene	exon
Bn-scaff_17298_1-p627166	Bn-N13-p26100579	gene	
Bn-scaff_17298_1-p649558	Bn-N13-p26118165	gene	exon
Bn-scaff_17298_1-p663900	Bn-N3-p16755441	gene	exon
Bn-scaff_17298_1-p679830	Bn-N13-p26157491	gene	
Bn-scaff_17298_1-p685381	Bn-N13-p26163067	gene	
Bn-scaff_17298_1-p699493	Bn-N13-p26177116	gene	
Bn-scaff_17298_1-p705887	Bn-N13-p26183664	gene	
Bn-scaff_17298_1-p720480	Bn-N12-p36105673	gene	
Bn-scaff_17298_1-p775436	Bn-N13-p26283261	gene	exon
Bn-scaff_17298_1-p779577	Bn-N13-p26287402	gene	exon
Bn-scaff_17298_1-p795098	Bn-N3-p16864326	gene	
Bn-scaff_17298_1-p928353	Bn-N3-p16966820	gene	exon
Bn-scaff_17298_1-p928410	Bn-N3-p16966877	gene	exon
Bn-scaff_17298_1-p936345	Bn-N13-p26464329	gene	
Bn-scaff_17316_1-p35218	Bn-N14-p20083377	gene	
Bn-scaff_17316_1-p36051	Bn-N14-p20084210	gene	
Bn-scaff_17326_1-p243497	Bn-N7-p7512911	gene	exon
Bn-scaff_17326_1-p254820	Bn-N7-p7565160	gene	exon
Bn-scaff_17326_1-p536514	Bn-N17-p17061241	gene	
Bn-scaff_17326_1-p718434	Bn-N7-p7828290	gene	
Bn-scaff_17326_1-p911613	Bn-N7-p7940185	gene	exon
Bn-scaff_17339_1-p23654	Bn-N19-p25945623	gene	exon
Bn-scaff_17339_1-p262780	Bn-N19-p25727119	gene	
Bn-scaff_17339_1-p292992	Bn-N19-p25689081	gene	
Bn-scaff_17339_1-p393814	Bn-N19-p25595655	gene	
Bn-scaff_17346_1-p103209	Bn-N14-p13211105	gene	
Bn-scaff_17367_1-p231214	Bn-N3-p6285935	gene	exon
Bn-scaff_17367_1-p231614	Bn-N19-p37218201	gene	exon
Bn-scaff_17367_1-p309816	Bn-N19-p37284711	gene	
Bn-scaff_17367_1-p310247	Bn-N19-p37285142	gene	
Bn-scaff_17367_1-p310274	Bn-N19-p37285169	gene	
Bn-scaff_17367_1-p345554	Bn-N19-p37312410	gene	
Bn-scaff_17367_1-p345745	Bn-N19-p37312602	gene	
Bn-scaff_17367_1-p346946	Bn-N19-p37313804	gene	
Bn-scaff_17367_1-p347096	Bn-N19-p37313953	gene	
Bn-scaff_17367_1-p575654	Bn-N19-p37524108	gene	exon
Bn-scaff_17367_1-p576145	Bn-N19-p37524553	gene	
Bn-scaff_17367_1-p576489	Bn-N19-p37524898	gene	exon
Bn-scaff_17367_1-p590827	Bn-N19-p37538128	gene	exon
Bn-scaff_17367_1-p590933	Bn-N19-p37538234	gene	exon
Bn-scaff_17367_1-p590982	Bn-N19-p37538284	gene	exon
Bn-scaff_17367_1-p791787	Bn-N19-p37728047	gene	exon
Bn-scaff_17367_1-p864352	Bn-N10-p7361314	gene	exon
Bn-scaff_17367_1-p864566	Bn-N10-p7361528	gene	exon

Bn-scaff_17367_1-p866103	Bn-N10-p7364351	gene	exon
Bn-scaff_17367_1-p9887	Bn-N19-p36942034	gene	
Bn-scaff_17369_1-p1016703	Bn-N11-p12029642	gene	
Bn-scaff_17369_1-p1017003	Bn-N11-p12029342	gene	
Bn-scaff_17369_1-p1017156	Bn-N11-p12029189	gene	
Bn-scaff_17369_1-p1041740	Bn-N11-p12010529	gene	
Bn-scaff_17369_1-p1041795	Bn-N11-p12010474	gene	
Bn-scaff_17369_1-p1062823	Bn-N11-p11982850	gene	
Bn-scaff_17369_1-p1111724	Bn-N11-p11929026	gene	
Bn-scaff_17369_1-p1112630	Bn-N11-p11928120	gene	
Bn-scaff_17369_1-p1150177	Bn-N11-p11890678	gene	exon
Bn-scaff_17369_1-p1151937	Bn-N1-p7893974	gene	
Bn-scaff_17369_1-p1152675	Bn-N1-p7893243	gene	exon
Bn-scaff_17369_1-p116095	Bn-N11-p12936658	gene	
Bn-scaff_17369_1-p1199108	Bn-N11-p11830343	gene	
Bn-scaff_17369_1-p1199959	Bn-N11-p11829490	gene	exon
Bn-scaff_17369_1-p1207575	Bn-N11-p11823038	gene	
Bn-scaff_17369_1-p1208179	Bn-N11-p11822434	gene	exon
Bn-scaff_17369_1-p1235509	Bn-N11-p11796613	gene	exon
Bn-scaff_17369_1-p1244703	Bn-N11-p11786232	gene	
Bn-scaff_17369_1-p1244776	Bn-N11-p11786159	gene	
Bn-scaff_17369_1-p318635	Bn-N11-p12708904	gene	exon
Bn-scaff_17369_1-p319471	Bn-N11-p12708075	gene	
Bn-scaff_17369_1-p32803	Bn-N11-p13056472	gene	exon
Bn-scaff_17369_1-p366584	Bn-N11-p12678603	gene	
Bn-scaff_17369_1-p374923	Bn-N11-p12670266	gene	exon
Bn-scaff_17369_1-p599615	Bn-N11-p12450690	gene	exon
Bn-scaff_17369_1-p617359	Bn-N11-p12439574	gene	
Bn-scaff_17369_1-p617726	Bn-N13-p22556797	gene	
Bn-scaff_17369_1-p703501	Bn-N11-p12354149	gene	
Bn-scaff_17369_1-p739904	Bn-N1-p8153722	gene	exon
Bn-scaff_17369_1-p75743	Bn-N11-p12986471	gene	
Bn-scaff_17369_1-p794147	Bn-N11-p12271959	gene	exon
Bn-scaff_17369_1-p802254	Bn-N11-p12263895	gene	exon
Bn-scaff_17369_1-p805277	Bn-N11-p12260876	gene	exon
Bn-scaff_17369_1-p83617	Bn-N11-p12979281	gene	
Bn-scaff_17369_1-p837839	Bn-N11-p12227469	gene	
Bn-scaff_17369_1-p843089	Bn-N11-p12224260	gene	
Bn-scaff_17369_1-p850330	Bn-N11-p12217285	gene	
Bn-scaff_17369_1-p851091	Bn-N11-p12216524	gene	
Bn-scaff_17369_1-p855743	Bn-N11-p12211408	gene	exon
Bn-scaff_17369_1-p858259	Bn-N11-p12208866	gene	
Bn-scaff_17369_1-p884077	Bn-N11-p12182854	gene	exon
Bn-scaff_17369_1-p917192	Bn-N11-p12147720	gene	exon
Bn-scaff_17369_1-p965580	Bn-N11-p12085665	gene	
Bn-scaff_17369_1-p974691	Bn-N11-p12076548	gene	
Bn-scaff_17369_1-p992249	Bn-N11-p12056702	gene	
Bn-scaff_17371_1-p462701	Bn-N9-p16221065	gene	exon
Bn-scaff_17395_1-p20236	Bn-N13-p30250915	gene	exon
Bn-scaff_17395_1-p32420	Bn-N13-p30244250	gene	
Bn-scaff_17413_1-p76511	Bn-N15-p37167817	gene	exon
Bn-scaff_17423_1-p100318	Bn-N9-p4692194	gene	

Bn-scaff_17423_1-p376558	Bn-N19-p6771134	gene	
Bn-scaff_17423_1-p549385	Bn-N9-p4971024	gene	exon
Bn-scaff_17423_1-p737034	Bn-N9-p5098325	gene	
Bn-scaff_17423_1-p99662	Bn-N9-p4691477	gene	
Bn-scaff_17436_1-p239369	Bn-N17-p7028436	gene	exon
Bn-scaff_17436_1-p260772	Bn-N16-p32308186	gene	
Bn-scaff_17436_1-p261063	Bn-N7-p19896215	gene	exon
Bn-scaff_17436_1-p26325	Bn-N16-p32730713	gene	exon
Bn-scaff_17436_1-p76050	Bn-N7-p19990324	gene	exon
Bn-scaff_17440_1-p107030	Bn-N13-p53984842	gene	
Bn-scaff_17440_1-p25854	Bn-N13-p54063279	gene	exon
Bn-scaff_17440_1-p268977	Bn-N13-p53820816	gene	exon
Bn-scaff_17440_1-p306539	Bn-N13-p53785208	gene	
Bn-scaff_17440_1-p311228	Bn-N13-p53780464	gene	
Bn-scaff_17440_1-p364383	Bn-N13-p53700581	gene	
Bn-scaff_17440_1-p364477	Bn-N13-p53700526	gene	
Bn-scaff_17440_1-p411613	Bn-N13-p53651160	gene	exon
Bn-scaff_17440_1-p523867	Bn-N13-p53527538	gene	exon
Bn-scaff_17440_1-p590665	Bn-N13-p53445599	gene	
Bn-scaff_17440_1-p592842	Bn-N13-p53443386	gene	
Bn-scaff_17440_1-p593576	Bn-N13-p53442652	gene	
Bn-scaff_17440_1-p761980	Bn-N13-p53270627	gene	exon
Bn-scaff_17440_1-p803525	Bn-N13-p53229934	gene	exon
Bn-scaff_17440_1-p829293	Bn-N13-p53204129	gene	
Bn-scaff_17440_1-p832657	Bn-N13-p53200707	gene	exon
Bn-scaff_17440_1-p868006	Bn-N13-p53167783	gene	
Bn-scaff_17440_1-p871646	Bn-N8-p16789132	gene	exon
Bn-scaff_17440_1-p958665	Bn-N13-p53081957	gene	
Bn-scaff_17441_1-p1037899	Bn-N15-p44948322	gene	
Bn-scaff_17441_1-p1039913	Bn-N15-p44950332	gene	
Bn-scaff_17441_1-p1045065	Bn-N15-p44959556	gene	
Bn-scaff_17441_1-p1140035	Bn-N15-p45045385	gene	
Bn-scaff_17441_1-p1142314	Bn-N15-p45047665	gene	exon
Bn-scaff_17441_1-p1182660	Bn-N5-p23357519	gene	exon
Bn-scaff_17441_1-p1211653	Bn-N15-p45110303	gene	
Bn-scaff_17441_1-p348811	Bn-N15-p44234543	gene	exon
Bn-scaff_17441_1-p349027	Bn-N5-p22822682	gene	exon
Bn-scaff_17441_1-p349187	Bn-N15-p44234955	gene	exon
Bn-scaff_17441_1-p357570	Bn-N19-p3784583	gene	
Bn-scaff_17441_1-p369136	Bn-N5-p22836261	gene	exon
Bn-scaff_17441_1-p369176	Bn-N5-p22836301	gene	exon
Bn-scaff_17441_1-p388434	Bn-N15-p44269414	gene	
Bn-scaff_17441_1-p388678	Bn-N15-p44269658	gene	
Bn-scaff_17441_1-p569870	Bn-N15-p44451287	gene	
Bn-scaff_17441_1-p575038	Bn-N15-p44462703	gene	exon
Bn-scaff_17441_1-p671270	Bn-N5-p23025194	gene	
Bn-scaff_17441_1-p671782	Bn-N5-p23025706	gene	
Bn-scaff_17441_1-p693032	Bn-N15-p44587703	gene	
Bn-scaff_17441_1-p693042	Bn-N15-p44587713	gene	
Bn-scaff_17441_1-p720235	Bn-N15-p44613455	gene	exon
Bn-scaff_17441_1-p720405	Bn-N15-p44613624	gene	exon
Bn-scaff_17441_1-p731079	Bn-N15-p44623922	gene	

Bn-scaff_17441_1-p900689	Bn-N15-p44796085	gene	
Bn-scaff_17441_1-p949135	Bn-N5-p23232599	gene	exon
Bn-scaff_17441_1-p950045	Bn-N15-p44858437	gene	exon
Bn-scaff_17441_1-p950341	Bn-N5-p23233877	gene	exon
Bn-scaff_17441_1-p958342	Bn-N15-p44871455	gene	
Bn-scaff_17441_1-p958418	Bn-N15-p44871531	gene	exon
Bn-scaff_17441_1-p974195	Bn-N15-p44884264	gene	
Bn-scaff_17441_1-p974705	Bn-N15-p44884773	gene	
Bn-scaff_17441_1-p974871	Bn-N15-p44884939	gene	exon
Bn-scaff_17441_1-p977042	Bn-N15-p44887111	gene	exon
Bn-scaff_17441_1-p986906	Bn-N15-p44897065	gene	
Bn-scaff_17441_1-p986974	Bn-N15-p44897133	gene	
Bn-scaff_17441_1-p987771	Bn-N15-p44898205	gene	
Bn-scaff_17441_3-p147254	Bn-N15-p45267488	gene	exon
Bn-scaff_17441_3-p161657	Bn-N15-p45281868	gene	
Bn-scaff_17441_3-p165957	Bn-N15-p45286728	gene	
Bn-scaff_17441_3-p174529	Bn-N5-p23545128	gene	
Bn-scaff_17441_3-p183168	Bn-N15-p45298286	gene	exon
Bn-scaff_17441_3-p213241	Bn-N15-p45333439	gene	
Bn-scaff_17441_3-p56937	Bn-N15-p45181301	gene	
Bn-scaff_17454_1-p416530	Bn-N18-p131583	gene	
Bn-scaff_17454_1-p87022	Bn-N16-p11741573	gene	
Bn-scaff_17457_1-p114659	Bn-N13-p60507954	gene	exon
Bn-scaff_17457_1-p176662	Bn-N13-p60460820	gene	
Bn-scaff_17457_1-p177567	Bn-N13-p60459918	gene	exon
Bn-scaff_17457_1-p178292	Bn-N13-p60459171	gene	exon
Bn-scaff_17457_1-p178355	Bn-N13-p60459108	gene	exon
Bn-scaff_17457_1-p178466	Bn-N13-p60458997	gene	exon
Bn-scaff_17457_1-p250253	Bn-N13-p60395711	gene	
Bn-scaff_17457_1-p253824	Bn-N13-p60392106	gene	exon
Bn-scaff_17457_1-p290434	Bn-N13-p60348841	gene	exon
Bn-scaff_17457_1-p505494	Bn-N13-p60047795	gene	
Bn-scaff_17457_1-p81967	Bn-N13-p60541088	gene	
Bn-scaff_17457_1-p82363	Bn-N13-p60540692	gene	
Bn-scaff_17457_1-p82523	Bn-N13-p60540532	gene	
Bn-scaff_17457_1-p82664	Bn-N13-p60540391	gene	
Bn-scaff_17457_1-p85083	Bn-N13-p60537972	gene	
Bn-scaff_17457_1-p85663	Bn-N13-p60537392	gene	
Bn-scaff_17461_1-p1039768	Bn-N17-p6202428	gene	exon
Bn-scaff_17461_1-p1185821	Bn-N16-p5380133	gene	exon
Bn-scaff_17461_1-p15221	Bn-N7-p2119589	gene	exon
Bn-scaff_17461_1-p15516	Bn-N7-p2119884	gene	exon
Bn-scaff_17461_1-p177714	Bn-N17-p5270230	gene	exon
Bn-scaff_17461_1-p199794	Bn-N7-p2210827	gene	
Bn-scaff_17461_1-p209013	Bn-N17-p5295515	gene	
Bn-scaff_17461_1-p369095	Bn-N7-p2298827	gene	
Bn-scaff_17480_1-p585	Bn-N9-p36392522	gene	
Bn-scaff_17482_1-p6632	Bn-N14-p43890545	gene	
Bn-scaff_17482_1-p6769	Bn-N14-p43890408	gene	
Bn-scaff_17482_1-p7299	Bn-N14-p43889871	gene	exon
Bn-scaff_17482_1-p9837	Bn-N6-p4390740	gene	exon
Bn-scaff_17487_1-p1244940	Bn-N9-p6697596	gene	exon

Bn-scaff_17487_1-p1371187	Bn-N9-p6777819	gene	exon
Bn-scaff_17487_1-p1371321	Bn-N9-p6777951	gene	exon
Bn-scaff_17487_1-p1573204	Bn-N9-p6876029	gene	exon
Bn-scaff_17487_1-p160800	Bn-N19-p8685501	gene	
Bn-scaff_17487_1-p160927	Bn-N19-p8685628	gene	
Bn-scaff_17487_1-p160966	Bn-N9-p6066863	gene	
Bn-scaff_17487_1-p161463	Bn-N9-p6067360	gene	exon
Bn-scaff_17487_1-p1782018	Bn-N19-p10437194	gene	exon
Bn-scaff_17487_1-p1782181	Bn-N19-p10437358	gene	exon
Bn-scaff_17487_1-p1888000	Bn-N19-p10533617	gene	exon
Bn-scaff_17487_1-p1890044	Bn-N19-p10535660	gene	exon
Bn-scaff_17487_1-p1909011	Bn-N19-p10546168	gene	exon
Bn-scaff_17487_1-p1959291	Bn-N19-p10615373	gene	exon
Bn-scaff_17487_1-p1959431	Bn-N19-p10615513	gene	
Bn-scaff_17487_1-p235174	Bn-N19-p8760197	gene	exon
Bn-scaff_17487_1-p303109	Bn-N9-p6139965	gene	exon
Bn-scaff_17487_1-p376857	Bn-N19-p8926497	gene	
Bn-scaff_17487_1-p415797	Bn-N19-p8959048	gene	
Bn-scaff_17487_1-p416211	Bn-N19-p8959462	gene	exon
Bn-scaff_17487_1-p441639	Bn-N19-p9000256	gene	
Bn-scaff_17487_1-p491031	Bn-N19-p9114750	gene	
Bn-scaff_17487_1-p527387	Bn-N19-p9161418	gene	
Bn-scaff_17487_1-p545307	Bn-N19-p9173532	gene	
Bn-scaff_17487_1-p611346	Bn-N19-p9233935	gene	exon
Bn-scaff_17487_1-p615064	Bn-N19-p9237653	gene	exon
Bn-scaff_17487_1-p616916	Bn-N19-p9239505	gene	
Bn-scaff_17487_1-p682697	Bn-N19-p9302556	gene	exon
Bn-scaff_17487_1-p723169	Bn-N19-p9345211	gene	exon
Bn-scaff_17487_1-p723256	Bn-N19-p9345298	gene	
Bn-scaff_17487_1-p74837	Bn-N12-p21213971	gene	
Bn-scaff_17487_1-p781216	Bn-N19-p9400513	gene	
Bn-scaff_17515_1-p217213	Bn-N11-p38612367	gene	exon
Bn-scaff_17515_1-p220339	Bn-N11-p38609240	gene	
Bn-scaff_17515_1-p243685	Bn-N11-p38585798	gene	
Bn-scaff_17515_1-p243778	Bn-N11-p38585705	gene	
Bn-scaff_17515_1-p243869	Bn-N11-p38585614	gene	
Bn-scaff_17515_1-p244504	Bn-N11-p38584979	gene	exon
Bn-scaff_17515_1-p247763	Bn-N1-p23036826	gene	exon
Bn-scaff_17515_1-p257014	Bn-N1-p23026364	gene	exon
Bn-scaff_17515_1-p27159	Bn-N11-p38786121	gene	exon
Bn-scaff_17515_1-p301057	Bn-N11-p38528118	gene	exon
Bn-scaff_17515_1-p302194	Bn-N11-p38526981	gene	
Bn-scaff_17515_1-p302518	Bn-N11-p38526657	gene	
Bn-scaff_17515_1-p302615	Bn-N11-p38526560	gene	
Bn-scaff_17515_1-p304774	Bn-N11-p38524401	gene	
Bn-scaff_17515_1-p311556	Bn-N11-p38517614	gene	
Bn-scaff_17515_1-p31995	Bn-N11-p38781306	gene	exon
Bn-scaff_17515_1-p376922	Bn-N11-p38451601	gene	exon
Bn-scaff_17515_1-p388362	Bn-N11-p38440232	gene	exon
Bn-scaff_17515_1-p388588	Bn-N11-p38440006	gene	exon
Bn-scaff_17515_1-p388701	Bn-N11-p38439893	gene	exon
Bn-scaff_17515_1-p396838	Bn-N11-p38432867	gene	

Bn-scaff_17515_1-p407792	Bn-N11-p38421917	gene	
Bn-scaff_17515_1-p51302	Bn-N1-p23129730	gene	exon
Bn-scaff_17515_1-p533140	Bn-N11-p38284920	gene	
Bn-scaff_17515_1-p533532	Bn-N11-p38284529	gene	
Bn-scaff_17515_1-p557620	Bn-N11-p38261768	gene	exon
Bn-scaff_17515_1-p563479	Bn-N11-p38256652	gene	exon
Bn-scaff_17515_1-p563548	Bn-N1-p22839689	gene	exon
Bn-scaff_17515_1-p584565	Bn-N11-p38235846	gene	exon
Bn-scaff_17515_1-p588895	Bn-N11-p38231512	gene	
Bn-scaff_17515_1-p749666	Bn-N11-p38058541	gene	exon
Bn-scaff_17515_1-p780999	Bn-N9-p38677110	gene	exon
Bn-scaff_17517_1-p11108	Bn-N11-p33304619	gene	exon
Bn-scaff_17517_1-p321895	Bn-N11-p33682951	gene	
Bn-scaff_17517_1-p324434	Bn-N11-p33686536	gene	
Bn-scaff_17517_1-p361588	Bn-N11-p33742799	gene	exon
Bn-scaff_17517_1-p361630	Bn-N11-p33742840	gene	exon
Bn-scaff_17517_1-p377821	Bn-N2-p24295397	gene	
Bn-scaff_17517_1-p377929	Bn-N11-p33753964	gene	
Bn-scaff_17517_1-p418933	Bn-N11-p33794669	gene	exon
Bn-scaff_17517_1-p419997	Bn-N11-p33795733	gene	
Bn-scaff_17517_1-p420485	Bn-N11-p33796222	gene	
Bn-scaff_17517_1-p420638	Bn-N1-p20290608	gene	exon
Bn-scaff_17517_1-p564869	Bn-N11-p33964545	gene	
Bn-scaff_17517_1-p600097	Bn-N11-p34003494	gene	
Bn-scaff_17517_1-p600876	Bn-N11-p34004273	gene	
Bn-scaff_17517_1-p642529	Bn-N11-p34050312	gene	exon
Bn-scaff_17517_1-p78363	Bn-N11-p33378200	gene	
Bn-scaff_17517_1-p816801	Bn-N11-p34233601	gene	
Bn-scaff_17517_1-p817376	Bn-N11-p34234201	gene	
Bn-scaff_17517_1-p859669	Bn-N11-p34277584	gene	
Bn-scaff_17517_1-p980938	Bn-N11-p34407498	gene	exon
Bn-scaff_17521_1-p1050704	Bn-N13-p24373712	gene	exon
Bn-scaff_17521_1-p1052439	Bn-N13-p24371985	gene	exon
Bn-scaff_17521_1-p1052808	Bn-N13-p24371616	gene	exon
Bn-scaff_17521_1-p1054284	Bn-N3-p15720729	gene	
Bn-scaff_17521_1-p1166900	Bn-N3-p15629676	gene	exon
Bn-scaff_17521_1-p1167341	Bn-N3-p15629235	gene	exon
Bn-scaff_17521_1-p1214522	Bn-N3-p15599465	gene	exon
Bn-scaff_17521_1-p125175	Bn-N13-p25295898	gene	exon
Bn-scaff_17521_1-p1327223	Bn-N3-p15493388	gene	exon
Bn-scaff_17521_1-p1338444	Bn-N3-p15485935	gene	
Bn-scaff_17521_1-p1354785	Bn-N13-p24022685	gene	
Bn-scaff_17521_1-p1354850	Bn-N13-p24022620	gene	
Bn-scaff_17521_1-p1355354	Bn-N13-p24022116	gene	
Bn-scaff_17521_1-p1355419	Bn-N13-p24022051	gene	
Bn-scaff_17521_1-p137019	Bn-N13-p25279346	gene	
Bn-scaff_17521_1-p1385225	Bn-N13-p23995974	gene	
Bn-scaff_17521_1-p1385470	Bn-N13-p23995729	gene	exon
Bn-scaff_17521_1-p1400234	Bn-N3-p15445011	gene	exon
Bn-scaff_17521_1-p194710	Bn-N19-p37376956	gene	
Bn-scaff_17521_1-p246991	Bn-N3-p16282331	gene	
Bn-scaff_17521_1-p286106	Bn-N13-p25151830	gene	

Bn-scaff_17521_1-p290063	Bn-N13-p25148157	gene	
Bn-scaff_17521_1-p353123	Bn-N13-p25090001	gene	exon
Bn-scaff_17521_1-p37024	Bn-N13-p25396034	gene	exon
Bn-scaff_17521_1-p38766	Bn-N13-p25394329	gene	
Bn-scaff_17521_1-p403946	Bn-N13-p25044606	gene	exon
Bn-scaff_17521_1-p404270	Bn-N13-p25044282	gene	
Bn-scaff_17521_1-p404658	Bn-N13-p25043894	gene	
Bn-scaff_17521_1-p405038	Bn-N13-p25043514	gene	exon
Bn-scaff_17521_1-p415393	Bn-N13-p25032997	gene	exon
Bn-scaff_17521_1-p460875	Bn-N3-p16129428	gene	exon
Bn-scaff_17521_1-p460933	Bn-N13-p24985969	gene	
Bn-scaff_17521_1-p464847	Bn-N3-p16124912	gene	exon
Bn-scaff_17521_1-p508913	Bn-N13-p24929801	gene	exon
Bn-scaff_17521_1-p511054	Bn-N13-p24927635	gene	exon
Bn-scaff_17521_1-p550417	Bn-N3-p16061572	gene	exon
Bn-scaff_17521_1-p561565	Bn-N13-p24854055	gene	
Bn-scaff_17521_1-p579834	Bn-N13-p24839164	gene	
Bn-scaff_17521_1-p584297	Bn-N13-p24834701	gene	
Bn-scaff_17521_1-p585002	Bn-N13-p24833989	gene	
Bn-scaff_17521_1-p596578	Bn-N3-p16034322	gene	exon
Bn-scaff_17521_1-p596662	Bn-N3-p16034239	gene	
Bn-scaff_17521_1-p644797	Bn-N13-p24765862	gene	
Bn-scaff_17521_1-p647350	Bn-N13-p24763309	gene	exon
Bn-scaff_17521_1-p663497	Bn-N13-p24746200	gene	exon
Bn-scaff_17521_1-p682662	Bn-N13-p24726130	gene	
Bn-scaff_17521_1-p742161	Bn-N13-p24665592	gene	exon
Bn-scaff_17521_1-p749297	Bn-N13-p24658268	gene	
Bn-scaff_17521_1-p749319	Bn-N13-p24658246	gene	
Bn-scaff_17521_1-p759119	Bn-N13-p24648462	gene	exon
Bn-scaff_17521_1-p807610	Bn-N13-p24590915	gene	exon
Bn-scaff_17521_1-p837796	Bn-N13-p24562269	gene	exon
Bn-scaff_17521_1-p842968	Bn-N13-p24554201	gene	
Bn-scaff_17521_1-p843450	Bn-N13-p24553723	gene	exon
Bn-scaff_17521_1-p869381	Bn-N13-p24528039	gene	
Bn-scaff_17521_1-p88281	Bn-N13-p25341694	gene	exon
Bn-scaff_17521_1-p952802	Bn-N13-p24454079	gene	exon
Bn-scaff_17521_1-p969711	Bn-N13-p24440833	gene	
Bn-scaff_17521_1-p988210	Bn-N13-p24425628	gene	
Bn-scaff_17521_1-p992171	Bn-N13-p24421667	gene	exon
Bn-scaff_17521_1-p997498	Bn-N13-p24416336	gene	exon
Bn-scaff_17522_1-p1234413	Bn-N2-p4687278	gene	exon
Bn-scaff_17522_1-p1361919	Bn-N2-p4653612	gene	exon
Bn-scaff_17522_1-p1394030	Bn-N12-p6138926	gene	exon
Bn-scaff_17522_1-p144926	Bn-N12-p7245498	gene	
Bn-scaff_17522_1-p1454520	Bn-N12-p6081749	gene	
Bn-scaff_17522_1-p1469252	Bn-N2-p4585093	gene	exon
Bn-scaff_17522_1-p1469455	Bn-N2-p4584890	gene	exon
Bn-scaff_17522_1-p1512412	Bn-N2-p4558829	gene	exon
Bn-scaff_17522_1-p1580565	Bn-N12-p5956085	gene	exon
Bn-scaff_17522_1-p1607150	Bn-N12-p5937746	gene	
Bn-scaff_17522_1-p1702091	Bn-N12-p5850892	gene	
Bn-scaff_17522_1-p1724143	Bn-N12-p5833650	gene	exon

Bn-scaff_17522_1-p1818546	Bn-N2-p4313592	gene	exon
Bn-scaff_17522_1-p1837209	Bn-N2-p4314105	gene	exon
Bn-scaff_17522_1-p1837786	Bn-N2-p4313528	gene	exon
Bn-scaff_17522_1-p234775	Bn-N12-p7195871	gene	
Bn-scaff_17522_1-p258646	Bn-N12-p7177286	gene	
Bn-scaff_17522_1-p275002	Bn-N15-p13327289	gene	
Bn-scaff_17522_1-p371170	Bn-N12-p7082817	gene	
Bn-scaff_17522_1-p479182	Bn-N12-p6986605	gene	exon
Bn-scaff_17522_1-p481078	Bn-N12-p6984710	gene	
Bn-scaff_17522_1-p490835	Bn-N12-p6974515	gene	
Bn-scaff_17522_1-p60662	Bn-N12-p7328399	gene	
Bn-scaff_17522_1-p60776	Bn-N12-p7328285	gene	
Bn-scaff_17522_1-p61884	Bn-N12-p7327177	gene	exon
Bn-scaff_17522_1-p62530	Bn-N12-p7326531	gene	exon
Bn-scaff_17522_1-p653541	Bn-N12-p6814181	gene	exon
Bn-scaff_17522_1-p67815	Bn-N12-p7324876	gene	exon
Bn-scaff_17522_1-p71448	Bn-N2-p5284351	gene	exon
Bn-scaff_17522_1-p774153	Bn-N12-p6723806	gene	exon
Bn-scaff_17522_1-p88139	Bn-N12-p7306914	gene	
Bn-scaff_17522_1-p908204	Bn-N12-p6599168	gene	
Bn-scaff_17525_1-p117065	Bn-N14-p40532786	gene	
Bn-scaff_17525_1-p125978	Bn-N4-p10499891	gene	exon
Bn-scaff_17525_1-p126933	Bn-N4-p10498935	gene	exon
Bn-scaff_17525_1-p267589	Bn-N14-p40387568	gene	exon
Bn-scaff_17525_1-p272430	Bn-N4-p10459172	gene	exon
Bn-scaff_17525_1-p272544	Bn-N4-p10459058	gene	exon
Bn-scaff_17525_1-p302132	Bn-Scaffold02570-p2976	gene	exon
Bn-scaff_17525_1-p503094	Bn-N4-p10351731	gene	exon
Bn-scaff_17525_1-p548959	Bn-N13-p61294947	gene	
Bn-scaff_17525_1-p665258	Bn-N14-p40014542	gene	
Bn-scaff_17525_1-p665421	Bn-N4-p10195479	gene	exon
Bn-scaff_17525_1-p665586	Bn-N14-p40014215	gene	
Bn-scaff_17525_1-p665622	Bn-N14-p40014178	gene	
Bn-scaff_17525_1-p666433	Bn-N14-p40013368	gene	
Bn-scaff_17525_1-p666500	Bn-N14-p40013301	gene	
Bn-scaff_17526_1-p1005324	Bn-N19-p2736985	gene	exon
Bn-scaff_17526_1-p1063974	Bn-N19-p2675298	gene	exon
Bn-scaff_17526_1-p1143773	Bn-N19-p2579440	gene	
Bn-scaff_17526_1-p1147911	Bn-N19-p2575296	gene	exon
Bn-scaff_17526_1-p1264483	Bn-N19-p2462824	gene	
Bn-scaff_17526_1-p1279502	Bn-N9-p2049610	gene	exon
Bn-scaff_17526_1-p1348890	Bn-N19-p2377293	gene	
Bn-scaff_17526_1-p1348970	Bn-N19-p2377212	gene	exon
Bn-scaff_17526_1-p1349476	Bn-N19-p2376706	gene	exon
Bn-scaff_17526_1-p1469380	Bn-N19-p2267198	gene	
Bn-scaff_17526_1-p1474906	Bn-N19-p2260255	gene	exon
Bn-scaff_17526_1-p1504264	Bn-N9-p1900911	gene	exon
Bn-scaff_17526_1-p1649875	Bn-N19-p2092209	gene	
Bn-scaff_17526_1-p1667260	Bn-N19-p2074812	gene	exon
Bn-scaff_17526_1-p1718326	Bn-N9-p1727887	gene	
Bn-scaff_17526_1-p1834362	Bn-N19-p1924999	gene	
Bn-scaff_17526_1-p1910152	Bn-N19-p1840358	gene	exon

Bn-scaff_17526_1-p1912248	Bn-N19-p1838258	gene	exon
Bn-scaff_17526_1-p2177941	Bn-N19-p1540726	gene	exon
Bn-scaff_17526_1-p2178654	Bn-N19-p1540012	gene	exon
Bn-scaff_17526_1-p2179198	Bn-N9-p1338662	gene	exon
Bn-scaff_17526_1-p220057	Bn-N19-p3578306	gene	
Bn-scaff_17526_1-p2206429	Bn-N9-p1310315	gene	exon
Bn-scaff_17526_1-p220813	Bn-N9-p2890933	gene	exon
Bn-scaff_17526_1-p220816	Bn-N19-p3577521	gene	exon
Bn-scaff_17526_1-p221576	Bn-N9-p2890148	gene	
Bn-scaff_17526_1-p256458	Bn-N17-p43902457	gene	exon
Bn-scaff_17526_1-p300781	Bn-N9-p2832960	gene	exon
Bn-scaff_17526_1-p301127	Bn-N9-p2832606	gene	exon
Bn-scaff_17526_1-p412410	Bn-N9-p2739289	gene	
Bn-scaff_17526_1-p463391	Bn-N19-p3322304	gene	
Bn-scaff_17526_1-p480659	Bn-N19-p3301357	gene	exon
Bn-scaff_17526_1-p729024	Bn-N19-p3014547	gene	exon
Bn-scaff_17526_1-p776163	Bn-N19-p2966703	gene	
Bn-scaff_17526_1-p860459	Bn-N19-p2887140	gene	
Bn-scaff_17526_1-p860504	Bn-N19-p2887095	gene	
Bn-scaff_17526_1-p879036	Bn-N19-p2867698	gene	exon
Bn-scaff_17526_1-p951929	Bn-N19-p2784980	gene	
Bn-scaff_17526_1-p969477	Bn-N19-p2767307	gene	
Bn-scaff_17566_1-p21523	Bn-Scaffold01156-p5994	gene	exon
Bn-scaff_17566_1-p76755	Bn-N12-p17324877	gene	
Bn-scaff_17573_1-p41354	Bn-N13-p41122140	gene	exon
Bn-scaff_17573_1-p95384	Bn-N13-p41187292	gene	exon
Bn-scaff_17580_1-p160243	Bn-N7-p7242166	gene	exon
Bn-scaff_17580_1-p194288	Bn-N7-p7217932	gene	
Bn-scaff_17580_1-p70475	Bn-N7-p7306922	gene	exon
Bn-scaff_17584_1-p109173	Bn-N1-p19081517	gene	exon
Bn-scaff_17584_1-p223738	Bn-N7-p6203463	gene	
Bn-scaff_17584_1-p268654	Bn-N17-p14483219	gene	
Bn-scaff_17584_1-p682870	Bn-N17-p14099603	gene	exon
Bn-scaff_17584_1-p683262	Bn-N17-p14099211	gene	exon
Bn-scaff_17584_1-p683277	Bn-N17-p14099196	gene	exon
Bn-scaff_17591_1-p578277	Bn-N18-p9991776	gene	exon
Bn-scaff_17591_1-p75901	Bn-N18-p10444900	gene	exon
Bn-scaff_17592_1-p267740	Bn-N1-p7450717	gene	exon
Bn-scaff_17592_1-p295310	Bn-N11-p11237433	gene	exon
Bn-scaff_17592_1-p485859	Bn-N11-p11432081	gene	exon
Bn-scaff_17592_1-p535117	Bn-N11-p11475975	gene	exon
Bn-scaff_17592_1-p535173	Bn-N11-p11476032	gene	exon
Bn-scaff_17592_1-p546118	Bn-N11-p11487633	gene	exon
Bn-scaff_17592_1-p603755	Bn-N1-p7683662	gene	
Bn-scaff_17592_1-p642504	Bn-N11-p11600571	gene	exon
Bn-scaff_17592_1-p685168	Bn-N11-p11641619	gene	exon
Bn-scaff_17592_1-p714613	Bn-N11-p11674465	gene	exon
Bn-scaff_17592_1-p725692	Bn-N11-p11700517	gene	exon
Bn-scaff_17592_1-p727914	Bn-N11-p11702731	gene	exon
Bn-scaff_17592_1-p735049	Bn-N11-p11709906	gene	
Bn-scaff_17592_1-p739203	Bn-N11-p11714060	gene	exon
Bn-scaff_17592_1-p764187	Bn-N11-p11743002	gene	exon

Bn-scaff_17592_1-p775877	Bn-N11-p11757806	gene	
Bn-scaff_17592_1-p791809	Bn-N11-p11774220	gene	
Bn-scaff_17592_1-p793269	Bn-N11-p11775681	gene	exon
Bn-scaff_17592_1-p794766	Bn-N11-p11777177	gene	
Bn-scaff_17592_1-p794801	Bn-N11-p11777212	gene	
Bn-scaff_17592_1-p795171	Bn-N11-p11777582	gene	
Bn-scaff_17592_1-p795494	Bn-N11-p11777905	gene	
Bn-scaff_17592_1-p795582	Bn-N11-p11777993	gene	
Bn-scaff_17592_1-p795799	Bn-N11-p11778210	gene	
Bn-scaff_17592_1-p795882	Bn-N11-p11778293	gene	
Bn-scaff_17592_1-p796290	Bn-N11-p11778701	gene	
Bn-scaff_17592_1-p796881	Bn-N11-p11779292	gene	
Bn-scaff_17592_1-p798798	Bn-N11-p11781209	gene	exon
Bn-scaff_17592_1-p799061	Bn-N11-p11781472	gene	
Bn-scaff_17592_1-p857899	Bn-N14-p14732598	gene	
Bn-scaff_17592_1-p869743	Bn-N14-p14730135	gene	exon
Bn-scaff_17592_1-p888125	Bn-N14-p14707497	gene	exon
Bn-scaff_17623_1-p1006646	Bn-N12-p43041867	gene	
Bn-scaff_17623_1-p120681	Bn-N2-p27068249	gene	exon
Bn-scaff_17623_1-p120748	Bn-N2-p27068181	gene	
Bn-scaff_17623_1-p172641	Bn-N12-p43782961	gene	
Bn-scaff_17623_1-p472440	Bn-N2-p26947248	gene	
Bn-scaff_17623_1-p546659	Bn-N12-p43462429	gene	exon
Bn-scaff_17623_1-p648231	Bn-N2-p26836930	gene	exon
Bn-scaff_17623_1-p688369	Bn-N12-p43332654	gene	
Bn-scaff_17623_1-p706105	Bn-N12-p43321758	gene	
Bn-scaff_17623_1-p714325	Bn-N12-p43314558	gene	
Bn-scaff_17623_1-p715915	Bn-N12-p43312968	gene	
Bn-scaff_17623_1-p73661	Bn-N12-p43878631	gene	
Bn-scaff_17623_1-p738869	Bn-N12-p43289264	gene	
Bn-scaff_17623_1-p990301	Bn-N9-p7425252	gene	exon
Bn-scaff_17623_1-p990628	Bn-N9-p7425592	gene	exon
Bn-scaff_17637_1-p114798	Bn-N18-p11946344	gene	
Bn-scaff_17637_1-p84569	Bn-N18-p11978697	gene	
Bn-scaff_17637_1-p84671	Bn-N18-p11978595	gene	
Bn-scaff_17647_1-p6629	Bn-N14-p13228686	gene	exon
Bn-scaff_17658_1-p25183	Bn-N13-p17554706	gene	
Bn-scaff_17658_1-p25628	Bn-N13-p17555150	gene	exon
Bn-scaff_17658_1-p25655	Bn-N13-p17555177	gene	exon
Bn-scaff_17683_1-p280237	Bn-N9-p7467105	gene	
Bn-scaff_17683_1-p280296	Bn-N9-p7467046	gene	
Bn-scaff_17683_1-p286463	Bn-N19-p11201336	gene	
Bn-scaff_17683_1-p28746	Bn-N19-p11436491	gene	exon
Bn-scaff_17683_1-p311331	Bn-N19-p11180562	gene	exon
Bn-scaff_17683_1-p313031	Bn-N19-p11178842	gene	exon
Bn-scaff_17683_1-p313826	Bn-N19-p11178045	gene	
Bn-scaff_17685_1-p158320	Bn-N11-p20962571	gene	exon
Bn-scaff_17685_1-p158870	Bn-N11-p20963121	gene	
Bn-scaff_17685_1-p159088	Bn-N11-p20963340	gene	exon
Bn-scaff_17685_1-p163978	Bn-N11-p20968243	gene	exon
Bn-scaff_17685_1-p165553	Bn-N11-p20969818	gene	exon
Bn-scaff_17685_1-p176536	Bn-N11-p20985598	gene	

Bn-scaff_17685_1-p176713	Bn-N11-p20985775	gene	
Bn-scaff_17685_1-p176850	Bn-N11-p20985912	gene	
Bn-scaff_17686_1-p105094	Bn-N14-p15460932	gene	exon
Bn-scaff_17708_1-p14790	Bn-N17-p3124164	gene	
Bn-scaff_17708_1-p14926	Bn-N17-p3124300	gene	
Bn-scaff_17708_1-p15115	Bn-N17-p3124489	gene	
Bn-scaff_17708_1-p169681	Bn-N7-p1321060	gene	exon
Bn-scaff_17708_1-p650212	Bn-N17-p3741010	gene	exon
Bn-scaff_17721_1-p175463	Bn-N12-p44711602	gene	
Bn-scaff_17721_1-p175936	Bn-N12-p44711129	gene	
Bn-scaff_17721_1-p212032	Bn-N12-p44674262	gene	
Bn-scaff_17721_1-p221548	Bn-N12-p44664761	gene	exon
Bn-scaff_17721_1-p263958	Bn-N12-p44620172	gene	exon
Bn-scaff_17721_1-p273764	Bn-N12-p44609296	gene	
Bn-scaff_17721_1-p297336	Bn-N2-p27511161	gene	exon
Bn-scaff_17721_1-p299314	Bn-N12-p44584944	gene	exon
Bn-scaff_17721_1-p347555	Bn-N12-p44511427	gene	exon
Bn-scaff_17721_1-p381227	Bn-N12-p44468918	gene	
Bn-scaff_17721_1-p39186	Bn-N2-p27679843	gene	exon
Bn-scaff_17721_1-p404462	Bn-N12-p44446154	gene	exon
Bn-scaff_17721_1-p644860	Bn-N12-p44239709	gene	
Bn-scaff_17721_1-p648583	Bn-N2-p27323063	gene	exon
Bn-scaff_17721_1-p676081	Bn-N12-p44212766	gene	
Bn-scaff_17721_1-p676383	Bn-N12-p44212464	gene	exon
Bn-scaff_17721_1-p677326	Bn-N12-p44209216	gene	exon
Bn-scaff_17721_1-p811886	Bn-N12-p44091960	gene	exon
Bn-scaff_17721_1-p884087	Bn-N2-p27196717	gene	
Bn-scaff_17725_1-p115788	Bn-N12-p11248166	gene	
Bn-scaff_17725_1-p115992	Bn-N12-p11248370	gene	
Bn-scaff_17725_1-p116642	Bn-N12-p11249029	gene	
Bn-scaff_17725_1-p199859	Bn-N12-p11357101	gene	
Bn-scaff_17725_1-p430981	Bn-N12-p11555365	gene	
Bn-scaff_17725_1-p431238	Bn-N12-p11555622	gene	
Bn-scaff_17725_1-p431566	Bn-N12-p11555950	gene	
Bn-scaff_17725_1-p505212	Bn-N2-p7776012	gene	
Bn-scaff_17725_1-p510392	Bn-N2-p7778581	gene	
Bn-scaff_17725_1-p514557	Bn-N12-p11636658	gene	exon
Bn-scaff_17725_1-p514758	Bn-N2-p7781368	gene	
Bn-scaff_17726_1-p287320	Bn-N14-p31908104	gene	
Bn-scaff_17726_1-p329807	Bn-N2-p18257173	gene	
Bn-scaff_17731_1-p154621	Bn-N11-p6674524	gene	exon
Bn-scaff_17731_1-p155870	Bn-N1-p4747599	gene	exon
Bn-scaff_17731_1-p166950	Bn-N11-p6684280	gene	
Bn-scaff_17731_1-p231809	Bn-N11-p6752025	gene	
Bn-scaff_17731_1-p241423	Bn-N11-p6763064	gene	exon
Bn-scaff_17731_1-p258929	Bn-N11-p6776210	gene	exon
Bn-scaff_17731_1-p262448	Bn-N11-p6783414	gene	
Bn-scaff_17731_1-p262543	Bn-N11-p6783509	gene	exon
Bn-scaff_17731_1-p275059	Bn-N11-p6791116	gene	
Bn-scaff_17731_1-p385358	Bn-N11-p6934402	gene	exon
Bn-scaff_17731_1-p429660	Bn-N11-p6981749	gene	
Bn-scaff_17731_1-p478972	Bn-N11-p7014690	gene	exon

Bn-scaff_17731_1-p482821	Bn-N11-p7017265	gene	exon
Bn-scaff_17731_1-p538617	Bn-N11-p7067204	gene	
Bn-scaff_17731_1-p569481	Bn-N11-p7094237	gene	
Bn-scaff_17731_1-p569486	Bn-N11-p7094242	gene	
Bn-scaff_17731_1-p746793	Bn-N11-p7290255	gene	
Bn-scaff_17731_1-p748416	Bn-N11-p7292145	gene	
Bn-scaff_17731_1-p748518	Bn-N11-p7292250	gene	exon
Bn-scaff_17731_1-p748522	Bn-N11-p7292254	gene	exon
Bn-scaff_17731_1-p824980	Bn-N11-p7361579	gene	
Bn-scaff_17731_1-p842133	Bn-N1-p5198058	gene	exon
Bn-scaff_17731_1-p863967	Bn-N11-p7408370	gene	exon
Bn-scaff_17731_1-p892885	Bn-N1-p5239670	gene	exon
Bn-scaff_17731_1-p979512	Bn-N11-p7536587	gene	
Bn-scaff_17740_1-p489699	Bn-N17-p33419128	gene	exon
Bn-scaff_17740_1-p676503	Bn-N6-p22325909	gene	exon
Bn-scaff_17740_1-p790888	Bn-N17-p33139902	gene	
Bn-scaff_17740_1-p793301	Bn-N17-p33137490	gene	exon
Bn-scaff_17740_1-p799766	Bn-N17-p33131009	gene	
Bn-scaff_17740_1-p814634	Bn-N17-p33097899	gene	
Bn-scaff_17740_1-p814830	Bn-N17-p33097703	gene	
Bn-scaff_17740_1-p815135	Bn-N17-p33097398	gene	exon
Bn-scaff_17740_1-p816267	Bn-N17-p33096283	gene	
Bn-scaff_17740_1-p816319	Bn-N17-p33096230	gene	
Bn-scaff_17740_1-p827481	Bn-N17-p33095122	gene	
Bn-scaff_17740_1-p828759	Bn-N17-p33093832	gene	exon
Bn-scaff_17740_1-p829172	Bn-N17-p33093419	gene	exon
Bn-scaff_17740_1-p830996	Bn-N17-p33091624	gene	
Bn-scaff_17740_1-p834125	Bn-N17-p33088497	gene	
Bn-scaff_17740_1-p839014	Bn-N17-p33083608	gene	
Bn-scaff_17740_1-p839294	Bn-N17-p33083327	gene	exon
Bn-scaff_17740_1-p845263	Bn-N17-p33077360	gene	exon
Bn-scaff_17740_1-p85367	Bn-N19-p35542619	gene	exon
Bn-scaff_17740_1-p955896	Bn-N17-p32964494	gene	exon
Bn-scaff_17740_1-p987288	Bn-N17-p32931939	gene	
Bn-scaff_17740_1-p988132	Bn-N17-p32931094	gene	exon
Bn-scaff_17750_1-p1105057	Bn-N19-p54667995	gene	exon
Bn-scaff_17750_1-p1165913	Bn-N19-p54610662	gene	
Bn-scaff_17750_1-p1165935	Bn-N19-p54610640	gene	
Bn-scaff_17750_1-p1166353	Bn-N19-p54610222	gene	
Bn-scaff_17750_1-p1167557	Bn-N19-p54608921	gene	
Bn-scaff_17750_1-p1168839	Bn-N10-p16281841	gene	exon
Bn-scaff_17750_1-p117385	Bn-N19-p55562109	gene	exon
Bn-scaff_17750_1-p1193301	Bn-N10-p16263606	gene	exon
Bn-scaff_17750_1-p1243511	Bn-N19-p54525486	gene	exon
Bn-scaff_17750_1-p1252439	Bn-N19-p54520183	gene	
Bn-scaff_17750_1-p1257734	Bn-N10-p16196927	gene	exon
Bn-scaff_17750_1-p1317267	Bn-N19-p54454858	gene	
Bn-scaff_17750_1-p1317272	Bn-N19-p54454855	gene	
Bn-scaff_17750_1-p132154	Bn-N10-p17056039	gene	exon
Bn-scaff_17750_1-p1326856	Bn-N19-p54445865	gene	
Bn-scaff_17750_1-p1506896	Bn-N10-p16002782	gene	exon
Bn-scaff_17750_1-p1576968	Bn-N19-p46962596	gene	exon

Bn-scaff_17750_1-p1577506	Bn-N19-p46963209	gene	
Bn-scaff_17750_1-p161563	Bn-N19-p55512276	gene	
Bn-scaff_17750_1-p1620719	Bn-N19-p54119207	gene	
Bn-scaff_17750_1-p1621241	Bn-N10-p15911145	gene	exon
Bn-scaff_17750_1-p1621294	Bn-N19-p54118626	gene	exon
Bn-scaff_17750_1-p166173	Bn-N19-p55507666	gene	exon
Bn-scaff_17750_1-p1692443	Bn-N19-p54057838	gene	exon
Bn-scaff_17750_1-p1692483	Bn-N19-p54057799	gene	exon
Bn-scaff_17750_1-p1801981	Bn-N19-p53945924	gene	
Bn-scaff_17750_1-p1803210	Bn-N19-p53944702	gene	
Bn-scaff_17750_1-p1839429	Bn-N19-p53910139	gene	
Bn-scaff_17750_1-p1871862	Bn-N10-p15708239	gene	exon
Bn-scaff_17750_1-p1905208	Bn-N19-p53849214	gene	
Bn-scaff_17750_1-p1981563	Bn-N10-p15646007	gene	exon
Bn-scaff_17750_1-p2007623	Bn-N10-p15617354	gene	exon
Bn-scaff_17750_1-p2082478	Bn-N19-p53678595	gene	
Bn-scaff_17750_1-p261491	Bn-N19-p55467168	gene	exon
Bn-scaff_17750_1-p274778	Bn-N19-p55453837	gene	exon
Bn-scaff_17750_1-p274914	Bn-N19-p55453701	gene	
Bn-scaff_17750_1-p409857	Bn-N10-p16874115	gene	exon
Bn-scaff_17750_1-p413107	Bn-N19-p55333334	gene	
Bn-scaff_17750_1-p435403	Bn-N19-p55310596	gene	
Bn-scaff_17750_1-p436527	Bn-N19-p55309293	gene	exon
Bn-scaff_17750_1-p555679	Bn-N19-p55203164	gene	exon
Bn-scaff_17750_1-p597135	Bn-N19-p55161212	gene	
Bn-scaff_17750_1-p602923	Bn-N19-p55154352	gene	
Bn-scaff_17750_1-p610438	Bn-N19-p55147912	gene	
Bn-scaff_17750_1-p621298	Bn-N19-p55137923	gene	
Bn-scaff_17750_1-p666634	Bn-N19-p55095413	gene	
Bn-scaff_17750_1-p669492	Bn-N19-p55092556	gene	
Bn-scaff_17750_1-p684757	Bn-N19-p55072003	gene	
Bn-scaff_17750_1-p770456	Bn-N19-p54993207	gene	
Bn-scaff_17750_1-p823170	Bn-N19-p54939364	gene	exon
Bn-scaff_17750_1-p892716	Bn-N19-p54879239	gene	
Bn-scaff_17750_1-p932637	Bn-N19-p54839328	gene	
Bn-scaff_17752_1-p73834	Bn-N12-p398718	gene	
Bn-scaff_17799_1-p1053450	Bn-N7-p24106556	gene	exon
Bn-scaff_17799_1-p1070542	Bn-N16-p39104677	gene	
Bn-scaff_17799_1-p1178394	Bn-N16-p39008227	gene	
Bn-scaff_17799_1-p1179110	Bn-N16-p39007511	gene	
Bn-scaff_17799_1-p1214222	Bn-N16-p38970697	gene	exon
Bn-scaff_17799_1-p122335	Bn-N16-p25690919	gene	exon
Bn-scaff_17799_1-p123518	Bn-N7-p16773046	gene	exon
Bn-scaff_17799_1-p1278642	Bn-N16-p38904676	gene	
Bn-scaff_17799_1-p1293186	Bn-N16-p38891493	gene	exon
Bn-scaff_17799_1-p1308909	Bn-N16-p38875406	gene	
Bn-scaff_17799_1-p1322660	Bn-N16-p38860629	gene	
Bn-scaff_17799_1-p1399131	Bn-N16-p38744500	gene	exon
Bn-scaff_17799_1-p1428594	Bn-N16-p38714052	gene	exon
Bn-scaff_17799_1-p150488	Bn-N7-p24856683	gene	exon
Bn-scaff_17799_1-p1564366	Bn-N16-p38575084	gene	exon
Bn-scaff_17799_1-p1577464	Bn-N7-p23674713	gene	exon

Bn-scaff_17799_1-p1627022	Bn-N7-p23629541	gene	
Bn-scaff_17799_1-p1629892	Bn-N16-p38519009	gene	
Bn-scaff_17799_1-p1916350	Bn-N7-p23409205	gene	
Bn-scaff_17799_1-p1917487	Bn-N7-p23408003	gene	exon
Bn-scaff_17799_1-p1917543	Bn-N7-p23407947	gene	exon
Bn-scaff_17799_1-p1951179	Bn-N16-p38206123	gene	
Bn-scaff_17799_1-p1952920	Bn-N16-p38204361	gene	exon
Bn-scaff_17799_1-p1953043	Bn-N16-p38204239	gene	
Bn-scaff_17799_1-p1954076	Bn-N16-p38203205	gene	
Bn-scaff_17799_1-p1960877	Bn-N16-p38198398	gene	
Bn-scaff_17799_1-p1960995	Bn-N16-p38198281	gene	
Bn-scaff_17799_1-p1991849	Bn-N16-p38166649	gene	exon
Bn-scaff_17799_1-p2051989	Bn-N7-p23264448	gene	exon
Bn-scaff_17799_1-p2054776	Bn-N16-p38108424	gene	exon
Bn-scaff_17799_1-p2065343	Bn-N16-p38096354	gene	
Bn-scaff_17799_1-p2065835	Bn-N16-p38095925	gene	
Bn-scaff_17799_1-p2066439	Bn-N16-p38095321	gene	
Bn-scaff_17799_1-p2111711	Bn-N16-p38035827	gene	exon
Bn-scaff_17799_1-p2161244	Bn-N16-p38002862	gene	
Bn-scaff_17799_1-p2233442	Bn-N7-p23154757	gene	exon
Bn-scaff_17799_1-p2273825	Bn-N16-p37881459	gene	
Bn-scaff_17799_1-p2278557	Bn-N16-p37834805	gene	exon
Bn-scaff_17799_1-p2278846	Bn-N16-p37834516	gene	exon
Bn-scaff_17799_1-p2280106	Bn-N16-p37833468	gene	
Bn-scaff_17799_1-p2284083	Bn-N16-p37831922	gene	exon
Bn-scaff_17799_1-p2284135	Bn-N16-p37831870	gene	exon
Bn-scaff_17799_1-p2284230	Bn-N16-p37831775	gene	
Bn-scaff_17799_1-p2293785	Bn-N16-p37812451	gene	exon
Bn-scaff_17799_1-p2300742	Bn-N16-p37806382	gene	
Bn-scaff_17799_1-p2301106	Bn-N16-p37806017	gene	
Bn-scaff_17799_1-p2334952	Bn-N16-p37783613	gene	exon
Bn-scaff_17799_1-p2391172	Bn-N16-p37714922	gene	
Bn-scaff_17799_1-p2391248	Bn-N16-p37714847	gene	
Bn-scaff_17799_1-p2426803	Bn-N16-p37684590	gene	
Bn-scaff_17799_1-p2426871	Bn-N16-p37684523	gene	
Bn-scaff_17799_1-p2495638	Bn-N16-p37607942	gene	exon
Bn-scaff_17799_1-p2548952	Bn-N16-p37570921	gene	exon
Bn-scaff_17799_1-p2577368	Bn-N7-p22898719	gene	exon
Bn-scaff_17799_1-p264732	Bn-N16-p39955379	gene	
Bn-scaff_17799_1-p2747856	Bn-N19-p46387273	gene	
Bn-scaff_17799_1-p2764738	Bn-N19-p46367929	gene	
Bn-scaff_17799_1-p2821394	Bn-N19-p46315260	gene	
Bn-scaff_17799_1-p2838665	Bn-N19-p46297441	gene	
Bn-scaff_17799_1-p2885267	Bn-N19-p46247286	gene	exon
Bn-scaff_17799_1-p2937790	Bn-N19-p46154952	gene	exon
Bn-scaff_17799_1-p2990222	Bn-N19-p46092475	gene	
Bn-scaff_17799_1-p2993406	Bn-N19-p46088549	gene	
Bn-scaff_17799_1-p2993905	Bn-N19-p46088051	gene	
Bn-scaff_17799_1-p2994637	Bn-N19-p46087319	gene	
Bn-scaff_17799_1-p2994660	Bn-N19-p46087296	gene	
Bn-scaff_17799_1-p300944	Bn-N16-p39921733	gene	
Bn-scaff_17799_1-p3010894	Bn-N19-p46070104	gene	

Bn-scaff_17799_1-p3014813	Bn-N19-p46066885	gene	
Bn-scaff_17799_1-p3015559	Bn-N10-p11272495	gene	exon
Bn-scaff_17799_1-p3015777	Bn-N10-p11272265	gene	exon
Bn-scaff_17799_1-p3016075	Bn-N19-p46065748	gene	exon
Bn-scaff_17799_1-p393729	Bn-N7-p24627680	gene	
Bn-scaff_17799_1-p397275	Bn-N7-p24624123	gene	exon
Bn-scaff_17799_1-p401453	Bn-N16-p39816758	gene	
Bn-scaff_17799_1-p462622	Bn-N16-p39745453	gene	
Bn-scaff_17799_1-p462623	Bn-N16-p39745452	gene	
Bn-scaff_17799_1-p668789	Bn-N16-p39522760	gene	exon
Bn-scaff_17799_1-p670065	Bn-N16-p39521484	gene	exon
Bn-scaff_17799_1-p678129	Bn-N16-p26174419	gene	exon
Bn-scaff_17799_1-p743957	Bn-N7-p24320722	gene	exon
Bn-scaff_17799_1-p744199	Bn-N7-p24320481	gene	
Bn-scaff_17799_1-p836919	Bn-N16-p39349749	gene	
Bn-scaff_17799_1-p840352	Bn-N16-p39346156	gene	
Bn-scaff_17799_1-p841812	Bn-N16-p39344538	gene	
Bn-scaff_17799_1-p925281	Bn-N16-p39266297	gene	
Bn-scaff_17799_1-p945289	Bn-N16-p39244338	gene	
Bn-scaff_17801_1-p179884	Bn-N19-p19300295	gene	
Bn-scaff_17801_1-p180565	Bn-Scaffold10980-p367	gene	
Bn-scaff_17801_1-p185077	Bn-N19-p19294893	gene	
Bn-scaff_17801_1-p203055	Bn-N19-p19272234	gene	exon
Bn-scaff_17801_1-p314822	Bn-N9-p12576733	gene	exon
Bn-scaff_17807_1-p192053	Bn-N18-p26913742	gene	
Bn-scaff_17807_1-p364572	Bn-N18-p4039298	gene	
Bn-scaff_17807_1-p461276	Bn-N8-p3085739	gene	
Bn-scaff_17807_1-p461826	Bn-N18-p3964246	gene	exon
Bn-scaff_17807_1-p462009	Bn-N18-p3964064	gene	
Bn-scaff_17807_1-p462854	Bn-N18-p3963219	gene	exon
Bn-scaff_17821_1-p119310	Bn-N8-p12762599	gene	
Bn-scaff_17821_1-p127254	Bn-N13-p63142532	gene	exon
Bn-scaff_17821_1-p21053	Bn-N13-p63041416	gene	exon
Bn-scaff_17827_1-p1028452	Bn-N11-p8544945	gene	exon
Bn-scaff_17827_1-p111920	Bn-N11-p7700606	gene	exon
Bn-scaff_17827_1-p197955	Bn-N7-p16568832	gene	
Bn-scaff_17827_1-p20213	Bn-N11-p7616076	gene	
Bn-scaff_17827_1-p271972	Bn-N11-p7823186	gene	exon
Bn-scaff_17827_1-p329960	Bn-N1-p5488275	gene	exon
Bn-scaff_17827_1-p523112	Bn-N11-p8031171	gene	exon
Bn-scaff_17827_1-p600033	Bn-N11-p8106740	gene	exon
Bn-scaff_17827_1-p628709	Bn-N11-p8138086	gene	
Bn-scaff_17827_1-p629081	Bn-N11-p8138458	gene	
Bn-scaff_17827_1-p629351	Bn-N1-p5680273	gene	exon
Bn-scaff_17827_1-p646146	Bn-N11-p8148266	gene	
Bn-scaff_17827_1-p653879	Bn-N11-p8156000	gene	exon
Bn-scaff_17827_1-p654250	Bn-N11-p8156369	gene	exon
Bn-scaff_17827_1-p747924	Bn-N11-p8252973	gene	exon
Bn-scaff_17827_1-p836925	Bn-N11-p8342276	gene	exon
Bn-scaff_17827_1-p897345	Bn-N11-p8423772	gene	
Bn-scaff_17827_1-p936522	Bn-N11-p8452106	gene	
Bn-scaff_17827_1-p953712	Bn-N11-p8479761	gene	exon

Bn-scaff_17827_1-p953907	Bn-N11-p8479956	gene	exon
Bn-scaff_17831_1-p137251	Bn-N12-p40806307	gene	
Bn-scaff_17831_1-p138012	Bn-N12-p40807068	gene	
Bn-scaff_17831_1-p167412	Bn-N12-p40838046	gene	exon
Bn-scaff_17831_1-p173520	Bn-Scaffold01566-p4791	gene	exon
Bn-scaff_17831_1-p198992	Bn-N12-p40864279	gene	
Bn-scaff_17831_1-p272649	Bn-N12-p40946972	gene	
Bn-scaff_17831_1-p272703	Bn-N12-p40947026	gene	
Bn-scaff_17831_1-p273579	Bn-N12-p40947902	gene	
Bn-scaff_17831_1-p273961	Bn-N12-p40948284	gene	
Bn-scaff_17831_1-p274241	Bn-N12-p40948564	gene	
Bn-scaff_17831_1-p274650	Bn-N12-p40948964	gene	
Bn-scaff_17831_1-p288494	Bn-N12-p40963828	gene	exon
Bn-scaff_17831_1-p291371	Bn-N12-p40966705	gene	exon
Bn-scaff_17831_1-p291865	Bn-N12-p40967199	gene	exon
Bn-scaff_17831_1-p292580	Bn-N12-p40967914	gene	exon
Bn-scaff_17867_1-p65627	Bn-N16-p16361408	gene	
Bn-scaff_17867_1-p69453	Bn-N16-p16357628	gene	exon
Bn-scaff_17867_1-p70307	Bn-N16-p16356805	gene	
Bn-scaff_17869_1-p1056525	Bn-N15-p16510311	gene	
Bn-scaff_17869_1-p1067608	Bn-N15-p16524114	gene	
Bn-scaff_17869_1-p115726	Bn-N14-p10840151	gene	
Bn-scaff_17869_1-p115797	Bn-N14-p10840222	gene	
Bn-scaff_17869_1-p1271417	Bn-N15-p16735860	gene	exon
Bn-scaff_17869_1-p1272586	Bn-N15-p16737031	gene	exon
Bn-scaff_17869_1-p1272783	Bn-N15-p16737228	gene	exon
Bn-scaff_17869_1-p135956	Bn-N14-p10860683	gene	exon
Bn-scaff_17869_1-p174416	Bn-N14-p10897686	gene	
Bn-scaff_17869_1-p174480	Bn-N14-p10897743	gene	
Bn-scaff_17869_1-p207523	Bn-N14-p10930559	gene	
Bn-scaff_17869_1-p331838	Bn-N14-p48525363	gene	exon
Bn-scaff_17869_1-p332267	Bn-N19-p49512320	gene	exon
Bn-scaff_17869_1-p411256	Bn-N5-p6538816	gene	exon
Bn-scaff_17869_1-p592847	Bn-N14-p11340141	gene	
Bn-scaff_17869_1-p593516	Bn-N5-p6689274	gene	
Bn-scaff_17869_1-p593823	Bn-N14-p11339151	gene	exon
Bn-scaff_17869_1-p624110	Bn-N14-p11302004	gene	
Bn-scaff_17869_1-p709145	Bn-N5-p6774794	gene	
Bn-scaff_17869_1-p814323	Bn-N14-p11571068	gene	exon
Bn-scaff_17869_1-p814402	Bn-N14-p11571147	gene	exon
Bn-scaff_17869_1-p815096	Bn-N14-p11571841	gene	
Bn-scaff_17869_1-p820117	Bn-N14-p11576785	gene	
Bn-scaff_17869_1-p947940	Bn-N15-p16397450	gene	
Bn-scaff_17869_1-p972743	Bn-N15-p16428702	gene	
Bn-scaff_17869_1-p975256	Bn-N15-p16431209	gene	
Bn-scaff_17882_1-p20385	Bn-N17-p7132913	gene	exon
Bn-scaff_17882_1-p21938	Bn-N17-p7131360	gene	
Bn-scaff_17882_1-p22070	Bn-N17-p7131228	gene	exon
Bn-scaff_17882_1-p74497	Bn-N7-p3009263	gene	
Bn-scaff_17882_1-p75280	Bn-N17-p7072533	gene	exon
Bn-scaff_17882_1-p91047	Bn-N17-p7049566	gene	
Bn-scaff_17882_1-p91274	Bn-N17-p7049339	gene	

Bn-scaff_17882_1-p91894	Bn-N17-p7048719	gene	exon
Bn-scaff_17888_1-p156678	Bn-N17-p47610693	gene	exon
Bn-scaff_17888_1-p183402	Bn-N19-p20139547	gene	exon
Bn-scaff_17888_1-p199868	Bn-N19-p20160224	gene	exon
Bn-scaff_17888_1-p201117	Bn-N19-p20161470	gene	exon
Bn-scaff_17888_1-p234601	Bn-N19-p20198436	gene	exon
Bn-scaff_17888_1-p435307	Bn-N9-p12515313	gene	
Bn-scaff_17888_1-p65372	Bn-N19-p20049810	gene	exon
Bn-scaff_17910_1-p126780	Bn-N19-p38533778	gene	
Bn-scaff_17910_1-p134686	Bn-N10-p7711371	gene	
Bn-scaff_17910_1-p135761	Bn-N10-p7712561	gene	exon
Bn-scaff_17910_1-p135864	Bn-N19-p38541697	gene	exon
Bn-scaff_17917_1-p171340	Bn-N16-p8302589	gene	exon
Bn-scaff_17917_1-p187293	Bn-N16-p8285149	gene	
Bn-scaff_17917_1-p190454	Bn-N16-p8282258	gene	
Bn-scaff_17917_1-p191484	Bn-N16-p8281226	gene	
Bn-scaff_17917_1-p93511	Bn-N16-p8387232	gene	
Bn-scaff_17919_1-p174590	Bn-N19-p39531304	gene	exon
Bn-scaff_17919_1-p175012	Bn-N19-p39530881	gene	
Bn-scaff_17919_1-p177730	Bn-N19-p39528164	gene	
Bn-scaff_17919_1-p282066	Bn-N19-p39404555	gene	
Bn-scaff_17959_1-p284214	Bn-N11-p21461673	gene	
Bn-scaff_17959_1-p340854	Bn-N11-p21403051	gene	exon
Bn-scaff_17959_1-p346956	Bn-N11-p21396845	gene	
Bn-scaff_17959_1-p75322	Bn-N11-p21665069	gene	
Bn-scaff_17959_1-p75903	Bn-N11-p21664488	gene	
Bn-scaff_17959_1-p76427	Bn-N11-p21663964	gene	
Bn-scaff_17959_1-p76589	Bn-N11-p21663802	gene	
Bn-scaff_17959_1-p95559	Bn-N11-p21645046	gene	
Bn-scaff_17959_1-p95580	Bn-N11-p21645025	gene	
Bn-scaff_17969_1-p2047	Bn-N12-p4355401	gene	
Bn-scaff_17972_1-p113066	Bn-N1-p26161144	gene	exon
Bn-scaff_17972_1-p146234	Bn-N17-p34193482	gene	
Bn-scaff_17972_1-p146235	Bn-N17-p34193481	gene	
Bn-scaff_17972_1-p172077	Bn-N17-p34165706	gene	exon
Bn-scaff_17972_1-p183765	Bn-N6-p21821758	gene	
Bn-scaff_17972_1-p279228	Bn-N17-p34061229	gene	
Bn-scaff_17972_1-p299054	Bn-N6-p21873030	gene	exon
Bn-scaff_17972_1-p361917	Bn-N17-p33939774	gene	exon
Bn-scaff_17972_1-p421559	Bn-N17-p33882155	gene	
Bn-scaff_17972_1-p424356	Bn-N17-p33879357	gene	exon
Bn-scaff_17972_1-p424678	Bn-N17-p33879035	gene	exon
Bn-scaff_17972_1-p424888	Bn-N17-p33878826	gene	
Bn-scaff_17972_1-p425171	Bn-N17-p33878543	gene	
Bn-scaff_17972_1-p425274	Bn-N17-p33878440	gene	
Bn-scaff_17972_1-p425581	Bn-N17-p33878132	gene	
Bn-scaff_17972_1-p425760	Bn-N17-p33877954	gene	
Bn-scaff_17972_1-p426067	Bn-N17-p33877647	gene	
Bn-scaff_17972_1-p426268	Bn-N17-p33877446	gene	exon
Bn-scaff_17972_1-p426297	Bn-N17-p33877417	gene	exon
Bn-scaff_17972_1-p526216	Bn-N17-p33785592	gene	
Bn-scaff_17972_1-p581578	Bn-N17-p33738524	gene	exon

Bn-scaff_17972_1-p68614	Bn-N11-p44025629	gene	
Bn-scaff_17972_1-p68665	Bn-N11-p44025578	gene	
Bn-scaff_17972_1-p69160	Bn-Scaffold01504-p5604	gene	
Bn-scaff_17977_1-p146911	Bn-N13-p50449853	gene	exon
Bn-scaff_17978_1-p22148	Bn-N11-p4640656	gene	exon
Bn-scaff_17978_1-p225133	Bn-N11-p4852289	gene	
Bn-scaff_17978_1-p23608	Bn-N11-p4655523	gene	exon
Bn-scaff_17978_1-p23955	Bn-N11-p4655870	gene	
Bn-scaff_17978_1-p329567	Bn-N1-p3688925	gene	exon
Bn-scaff_17978_1-p47592	Bn-N11-p4679189	gene	
Bn-scaff_17983_1-p52117	Bn-N14-p7467945	gene	exon
Bn-scaff_17983_1-p52946	Bn-N14-p7468767	gene	
Bn-scaff_17984_1-p1228297	Bn-N9-p19647213	gene	exon
Bn-scaff_17984_1-p222271	Bn-N16-p10971267	gene	exon
Bn-scaff_17984_1-p222990	Bn-N16-p10970549	gene	exon
Bn-scaff_17984_1-p317879	Bn-N16-p10862059	gene	exon
Bn-scaff_17984_1-p792511	Bn-N18-p8944223	gene	exon
Bn-scaff_17996_1-p229883	Bn-N19-p20519747	gene	
Bn-scaff_17996_1-p32471	Bn-N15-p26047554	gene	
Bn-scaff_18002_1-p156755	Bn-N16-p9142685	gene	exon
Bn-scaff_18002_1-p240843	Bn-N16-p9229439	gene	
Bn-scaff_18013_1-p194773	Bn-N13-p48108178	gene	exon
Bn-scaff_18022_1-p991	Bn-N15-p45592595	gene	exon
Bn-scaff_18034_1-p365	Bn-N14-p44914645	gene	
Bn-scaff_18039_1-p166807	Bn-N11-p36586000	gene	
Bn-scaff_18039_1-p183225	Bn-N11-p36602391	gene	
Bn-scaff_18039_1-p183233	Bn-N11-p36602399	gene	
Bn-scaff_18039_1-p189309	Bn-N11-p36608515	gene	exon
Bn-scaff_18062_1-p121756	Bn-N14-p35983012	gene	
Bn-scaff_18062_1-p139457	Bn-N14-p35972577	gene	exon
Bn-scaff_18062_1-p219340	Bn-N14-p35882033	gene	exon
Bn-scaff_18062_1-p219780	Bn-N14-p35881591	gene	
Bn-scaff_18062_1-p220233	Bn-N14-p35881138	gene	
Bn-scaff_18062_1-p220299	Bn-N14-p35881072	gene	
Bn-scaff_18062_1-p220320	Bn-N14-p35881051	gene	
Bn-scaff_18062_1-p220375	Bn-N14-p35880996	gene	exon
Bn-scaff_18062_1-p242986	Bn-N14-p35855606	gene	
Bn-scaff_18062_1-p281711	Bn-N14-p35817270	gene	
Bn-scaff_18062_1-p314727	Bn-N14-p35792556	gene	
Bn-scaff_18062_1-p315025	Bn-N14-p35792258	gene	
Bn-scaff_18062_1-p323927	Bn-N14-p35783356	gene	exon
Bn-scaff_18062_1-p45782	Bn-N14-p36071896	gene	
Bn-scaff_18062_1-p68600	Bn-N14-p36048027	gene	
Bn-scaff_18095_1-p758	Bn-N4-p3083641	gene	exon
Bn-scaff_18096_1-p30025	Bn-N11-p39896739	gene	
Bn-scaff_18100_1-p1123718	Bn-N19-p18498106	gene	
Bn-scaff_18100_1-p1124889	Bn-N9-p11895221	gene	exon
Bn-scaff_18100_1-p1166204	Bn-N19-p18537507	gene	
Bn-scaff_18100_1-p1168487	Bn-N19-p18539789	gene	exon
Bn-scaff_18100_1-p1229515	Bn-N19-p18601945	gene	
Bn-scaff_18100_1-p1255808	Bn-N19-p18634831	gene	exon
Bn-scaff_18100_1-p1255862	Bn-N19-p18634885	gene	exon

Bn-scaff_18100_1-p1311597	Bn-N19-p18685207	gene	exon
Bn-scaff_18100_1-p1312267	Bn-N19-p18685877	gene	
Bn-scaff_18100_1-p1335869	Bn-N19-p18728249	gene	
Bn-scaff_18100_1-p1335875	Bn-N19-p18728254	gene	
Bn-scaff_18100_1-p1335954	Bn-N19-p18728334	gene	
Bn-scaff_18100_1-p1345991	Bn-N19-p18742248	gene	exon
Bn-scaff_18100_1-p1364099	Bn-N19-p18756344	gene	
Bn-scaff_18100_1-p1376382	Bn-N16-p24916897	gene	exon
Bn-scaff_18100_1-p142231	Bn-N19-p17534089	gene	
Bn-scaff_18100_1-p232146	Bn-N19-p17629028	gene	exon
Bn-scaff_18100_1-p232785	Bn-N19-p17629668	gene	exon
Bn-scaff_18100_1-p234842	Bn-N19-p17631725	gene	exon
Bn-scaff_18100_1-p275291	Bn-N19-p17668520	gene	exon
Bn-scaff_18100_1-p275630	Bn-N19-p17668859	gene	exon
Bn-scaff_18100_1-p31176	Bn-N19-p17386852	gene	exon
Bn-scaff_18100_1-p446182	Bn-N9-p11383376	gene	exon
Bn-scaff_18100_1-p54657	Bn-N19-p17410395	gene	
Bn-scaff_18100_1-p54706	Bn-N19-p17410444	gene	exon
Bn-scaff_18100_1-p559555	Bn-N12-p9480427	gene	exon
Bn-scaff_18100_1-p568863	Bn-N19-p17949506	gene	exon
Bn-scaff_18100_1-p593993	Bn-N9-p11525005	gene	exon
Bn-scaff_18100_1-p68477	Bn-N9-p11074977	gene	exon
Bn-scaff_18100_1-p69205	Bn-N9-p11075701	gene	exon
Bn-scaff_18100_1-p762388	Bn-N19-p18177911	gene	exon
Bn-scaff_18100_1-p762611	Bn-N19-p18178135	gene	exon
Bn-scaff_18100_1-p763088	Bn-N9-p11667951	gene	
Bn-scaff_18100_1-p785785	Bn-N9-p11676105	gene	exon
Bn-scaff_18125_1-p33315	Bn-N16-p32805298	gene	
Bn-scaff_18125_1-p37317	Bn-N7-p20073375	gene	exon
Bn-scaff_18140_1-p183860	Bn-N16-p2731042	gene	exon
Bn-scaff_18140_1-p536327	Bn-N10-p3251139	gene	exon
Bn-scaff_18140_1-p771443	Bn-N16-p2093907	gene	
Bn-scaff_18140_1-p94701	Bn-N16-p2772361	gene	
Bn-scaff_18181_1-p113928	Bn-N15-p8225544	gene	
Bn-scaff_18181_1-p1150001	Bn-N6-p5461586	gene	exon
Bn-scaff_18181_1-p1150890	Bn-N15-p7101313	gene	exon
Bn-scaff_18181_1-p1175296	Bn-N19-p54239304	gene	exon
Bn-scaff_18181_1-p1265722	Bn-N15-p6956802	gene	
Bn-scaff_18181_1-p1266768	Bn-N15-p6955756	gene	exon
Bn-scaff_18181_1-p1289692	Bn-N15-p6935723	gene	
Bn-scaff_18181_1-p1341113	Bn-N6-p5375333	gene	exon
Bn-scaff_18181_1-p1342505	Bn-N6-p5373954	gene	exon
Bn-scaff_18181_1-p140221	Bn-N15-p8187090	gene	
Bn-scaff_18181_1-p141261	Bn-N6-p6152761	gene	exon
Bn-scaff_18181_1-p1481102	Bn-N17-p32066173	gene	
Bn-scaff_18181_1-p1590136	Bn-N15-p6313834	gene	
Bn-scaff_18181_1-p1603841	Bn-N15-p6339812	gene	
Bn-scaff_18181_1-p1664330	Bn-N15-p6436743	gene	
Bn-scaff_18181_1-p1691104	Bn-N15-p6459843	gene	exon
Bn-scaff_18181_1-p1691208	Bn-N15-p6459947	gene	exon
Bn-scaff_18181_1-p1691801	Bn-N15-p6460541	gene	exon
Bn-scaff_18181_1-p1707481	Bn-N15-p6475053	gene	

Bn-scaff_18181_1-p176182	Bn-N15-p8151214	gene	
Bn-scaff_18181_1-p1774469	Bn-N15-p6549784	gene	
Bn-scaff_18181_1-p1849246	Bn-N8-p12331547	gene	exon
Bn-scaff_18181_1-p195543	Bn-N6-p6098271	gene	exon
Bn-scaff_18181_1-p217804	Bn-N6-p6086604	gene	exon
Bn-scaff_18181_1-p522178	Bn-N15-p7766993	gene	exon
Bn-scaff_18181_1-p522373	Bn-N15-p7766798	gene	exon
Bn-scaff_18181_1-p522529	Bn-N15-p7766642	gene	exon
Bn-scaff_18181_1-p522702	Bn-N15-p7766469	gene	
Bn-scaff_18181_1-p529589	Bn-N15-p7760272	gene	exon
Bn-scaff_18181_1-p616899	Bn-N15-p7655747	gene	exon
Bn-scaff_18181_1-p620712	Bn-N15-p7653550	gene	
Bn-scaff_18181_1-p622258	Bn-N15-p7652030	gene	
Bn-scaff_18181_1-p626037	Bn-N15-p7646696	gene	exon
Bn-scaff_18181_1-p630071	Bn-N6-p5817465	gene	
Bn-scaff_18181_1-p674948	Bn-N15-p7586759	gene	
Bn-scaff_18181_1-p917633	Bn-N15-p7348802	gene	exon
Bn-scaff_18181_1-p948888	Bn-N15-p7317796	gene	
Bn-scaff_18181_1-p952071	Bn-N6-p5625803	gene	
Bn-scaff_18181_1-p953276	Bn-N15-p7313800	gene	exon
Bn-scaff_18181_1-p960226	Bn-N15-p7306859	gene	exon
Bn-scaff_18181_1-p962029	Bn-N15-p7304949	gene	
Bn-scaff_18189_1-p191178	Bn-N5-p19212621	gene	exon
Bn-scaff_18189_1-p301812	Bn-N5-p19133883	gene	exon
Bn-scaff_18199_1-p108926	Bn-N12-p24332679	gene	
Bn-scaff_18199_1-p121854	Bn-N12-p24320819	gene	exon
Bn-scaff_18199_1-p190902	Bn-N12-p24257615	gene	
Bn-scaff_18199_1-p234489	Bn-N12-p24216983	gene	
Bn-scaff_18199_1-p315029	Bn-N12-p24148282	gene	
Bn-scaff_18199_1-p315112	Bn-N12-p24148199	gene	exon
Bn-scaff_18199_1-p315242	Bn-N12-p24148069	gene	
Bn-scaff_18199_1-p524720	Bn-N2-p16703689	gene	exon
Bn-scaff_18202_1-p1088626	Bn-N17-p24742496	gene	exon
Bn-scaff_18202_1-p1088668	Bn-N17-p24742395	gene	exon
Bn-scaff_18202_1-p1481243	Bn-N17-p24400119	gene	exon
Bn-scaff_18202_1-p149190	Bn-N17-p25647141	gene	
Bn-scaff_18202_1-p1503040	Bn-N17-p24384574	gene	exon
Bn-scaff_18202_1-p1631779	Bn-N17-p24254633	gene	exon
Bn-scaff_18202_1-p182631	Bn-N17-p25621099	gene	
Bn-scaff_18202_1-p276140	Bn-N17-p25552773	gene	exon
Bn-scaff_18202_1-p280150	Bn-N17-p25548739	gene	exon
Bn-scaff_18202_1-p356089	Bn-N17-p25469565	gene	exon
Bn-scaff_18202_1-p407315	Bn-N17-p25425045	gene	
Bn-scaff_18202_1-p505132	Bn-N17-p25327371	gene	exon
Bn-scaff_18206_1-p142860	Bn-N15-p3458353	gene	
Bn-scaff_18206_1-p14924	Bn-N16-p20438919	gene	
Bn-scaff_18206_1-p457621	Bn-Scaffold01272-p6939	gene	exon
Bn-scaff_18206_1-p62235	Bn-N16-p20469204	gene	exon
Bn-scaff_18206_2-p114119	Bn-N16-p20966774	gene	
Bn-scaff_18206_2-p114197	Bn-N16-p20966853	gene	
Bn-scaff_18206_2-p33548	Bn-N16-p20866098	gene	
Bn-scaff_18206_3-p1040029	Bn-N17-p32657901	gene	

Bn-scaff_18206_3-p536950	Bn-N7-p14869730	gene	exon
Bn-scaff_18206_3-p923334	Bn-N16-p21979260	gene	exon
Bn-scaff_18206_3-p995621	Bn-N16-p22055835	gene	
Bn-scaff_18206_3-p996477	Bn-N16-p22056691	gene	exon
Bn-scaff_18206_3-p996964	Bn-N16-p22057178	gene	
Bn-scaff_18275_1-p1067723	Bn-N18-p17256451	gene	
Bn-scaff_18275_1-p1210524	Bn-N18-p17490884	gene	
Bn-scaff_18275_1-p1285880	Bn-N8-p9613284	gene	exon
Bn-scaff_18275_1-p1358799	Bn-N18-p17666886	gene	exon
Bn-scaff_18275_1-p269456	Bn-N14-p28502139	gene	exon
Bn-scaff_18275_1-p269623	Bn-N14-p28501972	gene	exon
Bn-scaff_18275_1-p477077	Bn-N4-p1294668	gene	
Bn-scaff_18279_1-p10124	Bn-N16-p4891006	gene	
Bn-scaff_18310_1-p1266233	Bn-N18-p14104849	gene	exon
Bn-scaff_18310_1-p151869	Bn-N18-p12997662	gene	
Bn-scaff_18310_1-p151946	Bn-N18-p12997739	gene	
Bn-scaff_18310_1-p152041	Bn-N18-p12997834	gene	
Bn-scaff_18310_1-p520653	Bn-N18-p13340272	gene	exon
Bn-scaff_18310_1-p577933	Bn-N18-p13407331	gene	
Bn-scaff_18310_1-p868154	Bn-N18-p13689515	gene	exon
Bn-scaff_18310_1-p868285	Bn-N18-p13689646	gene	exon
Bn-scaff_18310_1-p961622	Bn-N18-p13783672	gene	
Bn-scaff_18310_1-p961688	Bn-N18-p13783738	gene	
Bn-scaff_18310_1-p964552	Bn-N18-p13786603	gene	exon
Bn-scaff_18310_1-p964858	Bn-N18-p13786909	gene	
Bn-scaff_18310_1-p98775	Bn-N18-p12947528	gene	exon
Bn-scaff_18310_1-p99113	Bn-N18-p12947866	gene	exon
Bn-scaff_18310_1-p9997	Bn-N18-p12882687	gene	exon
Bn-scaff_18321_1-p116626	Bn-N12-p208129	gene	
Bn-scaff_18321_1-p117903	Bn-N12-p209374	gene	exon
Bn-scaff_18321_1-p122698	Bn-N12-p213849	gene	
Bn-scaff_18321_1-p129176	Bn-N12-p227245	gene	exon
Bn-scaff_18321_1-p169542	Bn-N2-p574808	gene	exon
Bn-scaff_18321_1-p170290	Bn-N12-p264829	gene	exon
Bn-scaff_18321_1-p203219	Bn-N12-p363529	gene	exon
Bn-scaff_18321_1-p29016	Bn-N2-p461838	gene	exon
Bn-scaff_18321_1-p30360	Bn-N12-p126030	gene	exon
Bn-scaff_18322_1-p1036896	Bn-N13-p9312883	gene	
Bn-scaff_18322_1-p1043643	Bn-N13-p9304824	gene	
Bn-scaff_18322_1-p1043993	Bn-N13-p9304474	gene	
Bn-scaff_18322_1-p1044275	Bn-N13-p9304195	gene	
Bn-scaff_18322_1-p1044730	Bn-N13-p9303740	gene	
Bn-scaff_18322_1-p1122025	Bn-N13-p9209242	gene	
Bn-scaff_18322_1-p1123656	Bn-N13-p9207611	gene	
Bn-scaff_18322_1-p1219747	Bn-N13-p9121392	gene	
Bn-scaff_18322_1-p1240715	Bn-N13-p9105352	gene	exon
Bn-scaff_18322_1-p1315547	Bn-N13-p9055329	gene	exon
Bn-scaff_18322_1-p1351673	Bn-N13-p9017502	gene	
Bn-scaff_18322_1-p1361529	Bn-N13-p9008505	gene	
Bn-scaff_18322_1-p1368804	Bn-N3-p6460568	gene	
Bn-scaff_18322_1-p1390788	Bn-N13-p8978895	gene	
Bn-scaff_18322_1-p1390926	Bn-N13-p8978757	gene	

Bn-scaff_18322_1-p1391958	Bn-N3-p6452388	gene	
Bn-scaff_18322_1-p1417497	Bn-N13-p8956827	gene	exon
Bn-scaff_18322_1-p1476976	Bn-N13-p8892370	gene	exon
Bn-scaff_18322_1-p1477883	Bn-N13-p8891463	gene	
Bn-scaff_18322_1-p1478173	Bn-N13-p8891173	gene	
Bn-scaff_18322_1-p1478389	Bn-N13-p8890968	gene	
Bn-scaff_18322_1-p1478526	Bn-N13-p8890831	gene	
Bn-scaff_18322_1-p1478842	Bn-N13-p8890513	gene	
Bn-scaff_18322_1-p1479449	Bn-N13-p8889897	gene	
Bn-scaff_18322_1-p1481186	Bn-N13-p8888117	gene	
Bn-scaff_18322_1-p1481418	Bn-N13-p8887885	gene	
Bn-scaff_18322_1-p1481509	Bn-N13-p8887794	gene	
Bn-scaff_18322_1-p1481654	Bn-N13-p8887649	gene	exon
Bn-scaff_18322_1-p1482713	Bn-N13-p8886591	gene	
Bn-scaff_18322_1-p1486915	Bn-N13-p8881146	gene	exon
Bn-scaff_18322_1-p1487173	Bn-N13-p8880888	gene	
Bn-scaff_18322_1-p1487354	Bn-N13-p8880707	gene	exon
Bn-scaff_18322_1-p1487593	Bn-N13-p8880468	gene	
Bn-scaff_18322_1-p1490180	Bn-N13-p8877879	gene	
Bn-scaff_18322_1-p1490792	Bn-N13-p8877266	gene	exon
Bn-scaff_18322_1-p1490880	Bn-N13-p8877175	gene	
Bn-scaff_18322_1-p1490938	Bn-N13-p8877117	gene	exon
Bn-scaff_18322_1-p1516256	Bn-N3-p6389292	gene	exon
Bn-scaff_18322_1-p1552078	Bn-N13-p8824071	gene	
Bn-scaff_18322_1-p1552930	Bn-N13-p8823218	gene	
Bn-scaff_18322_1-p1553314	Bn-N13-p8822834	gene	
Bn-scaff_18322_1-p1680489	Bn-N13-p8698769	gene	
Bn-scaff_18322_1-p1716237	Bn-N13-p8663161	gene	
Bn-scaff_18322_1-p1717349	Bn-N13-p8662055	gene	
Bn-scaff_18322_1-p1751672	Bn-N13-p8630968	gene	
Bn-scaff_18322_1-p1763220	Bn-N13-p8619423	gene	exon
Bn-scaff_18322_1-p1794782	Bn-N13-p8588527	gene	exon
Bn-scaff_18322_1-p1846275	Bn-N13-p8543566	gene	exon
Bn-scaff_18322_1-p1866564	Bn-Scaffold03063-p2721	gene	
Bn-scaff_18322_1-p1910313	Bn-N3-p6086296	gene	
Bn-scaff_18322_1-p1914840	Bn-N13-p8487022	gene	exon
Bn-scaff_18322_1-p1963622	Bn-N3-p6060430	gene	exon
Bn-scaff_18322_1-p2015929	Bn-N13-p8377292	gene	
Bn-scaff_18322_1-p2018782	Bn-N3-p6006066	gene	exon
Bn-scaff_18322_1-p2061517	Bn-N13-p8328774	gene	
Bn-scaff_18322_1-p2083290	Bn-N3-p5951003	gene	exon
Bn-scaff_18322_1-p2121463	Bn-N13-p8262154	gene	
Bn-scaff_18322_1-p2121603	Bn-N13-p8262014	gene	
Bn-scaff_18322_1-p2121991	Bn-N13-p8260435	gene	
Bn-scaff_18322_1-p2132957	Bn-N13-p8256260	gene	
Bn-scaff_18322_1-p2133523	Bn-N13-p8255695	gene	
Bn-scaff_18322_1-p2155092	Bn-N13-p8237432	gene	
Bn-scaff_18322_1-p2155609	Bn-N13-p8236915	gene	
Bn-scaff_18322_1-p2194066	Bn-N13-p8197899	gene	exon
Bn-scaff_18322_1-p2194125	Bn-N13-p8197840	gene	exon
Bn-scaff_18322_1-p2205139	Bn-N13-p8188030	gene	
Bn-scaff_18322_1-p2206270	Bn-N13-p8186899	gene	exon

Bn-scaff_18322_1-p2207543	Bn-N13-p8185626	gene	
Bn-scaff_18322_1-p2208011	Bn-N13-p8185158	gene	exon
Bn-scaff_18322_1-p2227308	Bn-N13-p8165057	gene	exon
Bn-scaff_18322_1-p2250566	Bn-N13-p8134236	gene	exon
Bn-scaff_18322_1-p2266003	Bn-N13-p8118293	gene	
Bn-scaff_18322_1-p2266068	Bn-N13-p8118228	gene	exon
Bn-scaff_18322_1-p2322027	Bn-N3-p5768074	gene	exon
Bn-scaff_18322_1-p2327100	Bn-N13-p8050051	gene	
Bn-scaff_18322_1-p2357361	Bn-N13-p8015497	gene	
Bn-scaff_18322_1-p2378816	Bn-N13-p7994144	gene	
Bn-scaff_18322_1-p2382266	Bn-N13-p7990694	gene	
Bn-scaff_18322_1-p2382544	Bn-N13-p7990416	gene	exon
Bn-scaff_18322_1-p2385043	Bn-N13-p7987917	gene	
Bn-scaff_18322_1-p243213	Bn-N3-p8837920	gene	exon
Bn-scaff_18322_1-p2442862	Bn-N13-p7929180	gene	exon
Bn-scaff_18322_1-p2443171	Bn-N3-p5669420	gene	exon
Bn-scaff_18322_1-p2487448	Bn-N13-p7887964	gene	
Bn-scaff_18322_1-p2518158	Bn-N13-p7857286	gene	exon
Bn-scaff_18322_1-p2560823	Bn-Scaffold17879-p117	gene	exon
Bn-scaff_18322_1-p2633571	Bn-N13-p7746041	gene	
Bn-scaff_18322_1-p2647238	Bn-N13-p7732494	gene	
Bn-scaff_18322_1-p2731053	Bn-N3-p5455764	gene	
Bn-scaff_18322_1-p320439	Bn-N3-p7240873	gene	
Bn-scaff_18322_1-p475040	Bn-N13-p9919516	gene	exon
Bn-scaff_18322_1-p476799	Bn-N13-p9917506	gene	exon
Bn-scaff_18322_1-p47968	Bn-N13-p10370521	gene	exon
Bn-scaff_18322_1-p483834	Bn-N13-p9901359	gene	exon
Bn-scaff_18322_1-p511201	Bn-N3-p7100307	gene	
Bn-scaff_18322_1-p590148	Bn-N13-p9799827	gene	exon
Bn-scaff_18322_1-p625299	Bn-N13-p9752538	gene	exon
Bn-scaff_18322_1-p744117	Bn-N13-p9606789	gene	
Bn-scaff_18322_1-p758030	Bn-N13-p9592168	gene	
Bn-scaff_18322_1-p802643	Bn-N13-p9531967	gene	exon
Bn-scaff_18322_1-p804580	Bn-N13-p9530024	gene	
Bn-scaff_18322_1-p813458	Bn-N13-p9520029	gene	
Bn-scaff_18322_1-p817790	Bn-N13-p9515353	gene	exon
Bn-scaff_18322_1-p817900	Bn-N13-p9515244	gene	exon
Bn-scaff_18322_1-p818265	Bn-N13-p9514879	gene	
Bn-scaff_18322_1-p881842	Bn-N13-p9457422	gene	
Bn-scaff_18322_1-p903761	Bn-N13-p9432653	gene	exon
Bn-scaff_18322_1-p918037	Bn-N13-p9417386	gene	exon
Bn-scaff_18322_1-p918195	Bn-N3-p6774295	gene	exon
Bn-scaff_18322_1-p918715	Bn-N13-p9416709	gene	
Bn-scaff_18322_1-p978540	Bn-N13-p9369107	gene	exon
Bn-scaff_18326_1-p324525	Bn-N19-p31615611	gene	exon
Bn-scaff_18338_1-p1073237	Bn-N15-p15145336	gene	exon
Bn-scaff_18338_1-p115595	Bn-N15-p16144070	gene	
Bn-scaff_18338_1-p342516	Bn-N15-p15895383	gene	
Bn-scaff_18338_1-p807466	Bn-N9-p25957850	gene	
Bn-scaff_18338_1-p911753	Bn-N15-p15311138	gene	
Bn-scaff_18338_1-p912633	Bn-N15-p15310259	gene	exon
Bn-scaff_18338_1-p920459	Bn-N3-p24787485	gene	

Bn-scaff_18338_1-p984141	Bn-N15-p15236727	gene	
Bn-scaff_18338_1-p984735	Bn-N15-p15236134	gene	exon
Bn-scaff_18344_1-p147881	Bn-N13-p5206344	gene	
Bn-scaff_18344_1-p236941	Bn-N16-p4426311	gene	
Bn-scaff_18344_1-p237963	Bn-N6-p1678371	gene	
Bn-scaff_18344_1-p386545	Bn-N16-p4571324	gene	
Bn-scaff_18344_1-p386657	Bn-N16-p4571436	gene	
Bn-scaff_18344_1-p386725	Bn-N16-p4571504	gene	
Bn-scaff_18344_1-p398871	Bn-N6-p1605226	gene	exon
Bn-scaff_18344_1-p666462	Bn-N16-p4853402	gene	
Bn-scaff_18344_1-p89409	Bn-N13-p5154425	gene	
Bn-scaff_18356_1-p116223	Bn-N13-p38471092	gene	
Bn-scaff_18356_1-p181684	Bn-N14-p5350741	gene	exon
Bn-scaff_18356_1-p218731	Bn-N13-p38364922	gene	exon
Bn-scaff_18356_1-p218924	Bn-N13-p38364728	gene	exon
Bn-scaff_18356_1-p220316	Bn-N13-p38363334	gene	
Bn-scaff_18356_1-p272201	Bn-N6-p16818614	gene	
Bn-scaff_18356_1-p309880	Bn-N6-p16830980	gene	exon
Bn-scaff_18356_1-p311981	Bn-N13-p38258630	gene	exon
Bn-scaff_18356_1-p349741	Bn-N13-p38217511	gene	exon
Bn-scaff_18356_1-p39463	Bn-N13-p38524463	gene	exon
Bn-scaff_18360_1-p151712	Bn-N12-p35803119	gene	exon
Bn-scaff_18360_1-p151873	Bn-N12-p35803280	gene	
Bn-scaff_18360_1-p31167	Bn-N12-p35696854	gene	exon
Bn-scaff_18360_1-p382074	Bn-Scaffold11521-p333	gene	exon
Bn-scaff_18360_1-p45199	Bn-N12-p35708348	gene	
Bn-scaff_18360_1-p570258	Bn-N12-p36252747	gene	
Bn-scaff_18360_1-p723514	Bn-N12-p36412649	gene	exon
Bn-scaff_18360_1-p873769	Bn-N12-p36605333	gene	exon
Bn-scaff_18371_1-p300088	Bn-Scaffold01104-p19398	gene	
Bn-scaff_18371_1-p777081	Bn-N15-p22549562	gene	
Bn-scaff_18371_1-p962353	Bn-N8-p6729438	gene	
Bn-scaff_18406_1-p266026	Bn-N12-p30662796	gene	exon
Bn-scaff_18406_1-p69905	Bn-N12-p30466433	gene	
Bn-scaff_18412_1-p280164	Bn-N18-p594209	gene	
Bn-scaff_18412_1-p283656	Bn-N18-p590717	gene	exon
Bn-scaff_18412_1-p29779	Bn-N18-p831461	gene	
Bn-scaff_18412_1-p385710	Bn-N18-p486080	gene	
Bn-scaff_18412_1-p387438	Bn-N18-p484351	gene	exon
Bn-scaff_18412_1-p387658	Bn-N18-p484132	gene	
Bn-scaff_18424_1-p411190	Bn-N9-p14748472	gene	
Bn-scaff_18424_1-p672600	Bn-N9-p14607669	gene	exon
Bn-scaff_18439_1-p1014445	Bn-N16-p14895759	gene	exon
Bn-scaff_18439_1-p1062345	Bn-N5-p5788630	gene	exon
Bn-scaff_18439_1-p1062495	Bn-N14-p9755975	gene	exon
Bn-scaff_18439_1-p1063837	Bn-N5-p5790139	gene	exon
Bn-scaff_18439_1-p127216	Bn-N16-p26903117	gene	exon
Bn-scaff_18439_1-p237947	Bn-N16-p27004080	gene	exon
Bn-scaff_18439_1-p277248	Bn-N16-p14152896	gene	
Bn-scaff_18439_1-p277935	Bn-N16-p14153586	gene	
Bn-scaff_18439_1-p376735	Bn-N16-p14257983	gene	exon
Bn-scaff_18439_1-p515154	Bn-N16-p14381101	gene	

Bn-scaff_18439_1-p633449	Bn-N16-p14510721	gene	exon
Bn-scaff_18439_1-p639659	Bn-N16-p14513721	gene	
Bn-scaff_18439_1-p643793	Bn-N16-p14518284	gene	
Bn-scaff_18439_1-p658725	Bn-N16-p14532832	gene	exon
Bn-scaff_18439_1-p659075	Bn-N16-p14533182	gene	exon
Bn-scaff_18439_1-p659120	Bn-N16-p14533185	gene	exon
Bn-scaff_18439_1-p675307	Bn-N16-p14549236	gene	exon
Bn-scaff_18439_1-p706567	Bn-N16-p14580422	gene	exon
Bn-scaff_18439_1-p79427	Bn-N7-p17295637	gene	exon
Bn-scaff_18439_1-p798045	Bn-N16-p14683521	gene	
Bn-scaff_18439_1-p798756	Bn-N16-p14684818	gene	exon
Bn-scaff_18439_1-p799057	Bn-N16-p14685119	gene	exon
Bn-scaff_18439_1-p952226	Bn-N16-p14833023	gene	
Bn-scaff_18439_1-p953290	Bn-N16-p14834087	gene	
Bn-scaff_18439_1-p953602	Bn-N16-p14834399	gene	
Bn-scaff_18439_1-p954410	Bn-N16-p14835207	gene	
Bn-scaff_18439_1-p978904	Bn-N16-p14859035	gene	exon
Bn-scaff_18461_1-p250715	Bn-N15-p17769317	gene	
Bn-scaff_18464_1-p2370	Bn-N17-p12820981	gene	exon
Bn-scaff_18464_1-p2513	Bn-N17-p12820838	gene	exon
Bn-scaff_18482_1-p246408	Bn-N13-p23025757	gene	exon
Bn-scaff_18482_1-p246941	Bn-N13-p23025187	gene	exon
Bn-scaff_18482_1-p322283	Bn-N3-p14747648	gene	exon
Bn-scaff_18482_1-p437945	Bn-N13-p22843364	gene	
Bn-scaff_18482_1-p438118	Bn-N13-p22843191	gene	
Bn-scaff_18482_1-p445207	Bn-N13-p22836602	gene	
Bn-scaff_18482_1-p447039	Bn-N3-p14665052	gene	exon
Bn-scaff_18482_1-p449692	Bn-N13-p22832555	gene	exon
Bn-scaff_18482_1-p517204	Bn-N13-p22765153	gene	
Bn-scaff_18482_1-p517555	Bn-N13-p22764020	gene	exon
Bn-scaff_18482_1-p646881	Bn-N13-p22614439	gene	
Bn-scaff_18482_1-p663209	Bn-N13-p22585564	gene	
Bn-scaff_18482_1-p663758	Bn-N13-p22585007	gene	
Bn-scaff_18482_1-p663864	Bn-N13-p22584900	gene	
Bn-scaff_18482_1-p677287	Bn-N3-p14484901	gene	exon
Bn-scaff_18482_1-p698896	Bn-N13-p22539845	gene	exon
Bn-scaff_18482_1-p699135	Bn-N13-p22539406	gene	
Bn-scaff_18482_1-p699636	Bn-N13-p22538911	gene	
Bn-scaff_18482_1-p710052	Bn-N13-p22520273	gene	exon
Bn-scaff_18482_1-p739133	Bn-N13-p22496045	gene	
Bn-scaff_18482_1-p739195	Bn-N13-p22495983	gene	
Bn-scaff_18482_1-p90983	Bn-N13-p23184583	gene	exon
Bn-scaff_18490_1-p12404	Bn-N5-p889651	gene	
Bn-scaff_18501_1-p1007207	Bn-N17-p23755812	gene	exon
Bn-scaff_18501_1-p114150	Bn-N17-p22812520	gene	
Bn-scaff_18501_1-p114412	Bn-N7-p10680489	gene	exon
Bn-scaff_18501_1-p122257	Bn-N17-p22820651	gene	exon
Bn-scaff_18501_1-p169867	Bn-N7-p10704944	gene	exon
Bn-scaff_18501_1-p336612	Bn-N6-p7830346	gene	exon
Bn-scaff_18501_1-p345881	Bn-N7-p10825813	gene	
Bn-scaff_18501_1-p421712	Bn-N17-p23125248	gene	exon
Bn-scaff_18501_1-p424246	Bn-N17-p23126818	gene	exon

Bn-scaff_18501_1-p426392	Bn-N17-p23128970	gene	
Bn-scaff_18501_1-p462141	Bn-N17-p23155131	gene	
Bn-scaff_18501_1-p465658	Bn-N17-p23158464	gene	
Bn-scaff_18501_1-p471519	Bn-N7-p10890045	gene	exon
Bn-scaff_18501_1-p472321	Bn-N17-p23165084	gene	
Bn-scaff_18501_1-p530677	Bn-N17-p23208449	gene	
Bn-scaff_18501_1-p604558	Bn-N17-p23287902	gene	
Bn-scaff_18501_1-p656400	Bn-N17-p23335166	gene	
Bn-scaff_18501_1-p789797	Bn-N17-p23522278	gene	exon
Bn-scaff_18501_1-p795227	Bn-N17-p23532357	gene	
Bn-scaff_18501_1-p837918	Bn-N17-p23558606	gene	exon
Bn-scaff_18501_1-p837985	Bn-N17-p23558671	gene	
Bn-scaff_18501_1-p838299	Bn-N17-p23558984	gene	exon
Bn-scaff_18501_1-p939596	Bn-N17-p23681480	gene	
Bn-scaff_18501_1-p942638	Bn-N17-p23684490	gene	
Bn-scaff_18501_1-p942700	Bn-N17-p23684552	gene	
Bn-scaff_18501_1-p943346	Bn-N17-p23685228	gene	
Bn-scaff_18501_1-p980365	Bn-N17-p23733382	gene	
Bn-scaff_18501_1-p982494	Bn-N17-p23735385	gene	
Bn-scaff_18501_1-p983179	Bn-N17-p23736074	gene	exon
Bn-scaff_18503_1-p179825	Bn-N16-p38852719	gene	exon
Bn-scaff_18505_1-p133117	Bn-N7-p12122754	gene	exon
Bn-scaff_18505_1-p254578	Bn-N7-p12171622	gene	exon
Bn-scaff_18505_1-p295538	Bn-N16-p35615990	gene	exon
Bn-scaff_18505_1-p85179	Bn-N14-p16638887	gene	
Bn-scaff_18505_1-p85945	Bn-N14-p16638121	gene	
Bn-scaff_18505_1-p90828	Bn-N14-p16629492	gene	
Bn-scaff_18505_1-p91467	Bn-N14-p16628837	gene	
Bn-scaff_18507_1-p1063809	Bn-N12-p25582197	gene	
Bn-scaff_18507_1-p1063865	Bn-N12-p25582253	gene	
Bn-scaff_18507_1-p1190632	Bn-N12-p25692578	gene	exon
Bn-scaff_18507_1-p1280405	Bn-N12-p25785441	gene	exon
Bn-scaff_18507_1-p1280418	Bn-N12-p25785454	gene	exon
Bn-scaff_18507_1-p1280533	Bn-N12-p25785569	gene	exon
Bn-scaff_18507_1-p1280604	Bn-N12-p25785640	gene	exon
Bn-scaff_18507_1-p1281501	Bn-N12-p25786537	gene	
Bn-scaff_18507_1-p265244	Bn-N12-p24763762	gene	
Bn-scaff_18507_1-p6970	Bn-N12-p24521884	gene	
Bn-scaff_18507_1-p710307	Bn-N12-p25228733	gene	
Bn-scaff_18507_1-p783295	Bn-N12-p25304018	gene	exon
Bn-scaff_18507_1-p9039	Bn-N12-p24523953	gene	exon
Bn-scaff_18507_1-p9570	Bn-N12-p24524484	gene	exon
Bn-scaff_18507_1-p960146	Bn-N12-p25480582	gene	
Bn-scaff_18507_1-p981369	Bn-N12-p25502744	gene	
Bn-scaff_18514_1-p28001	Bn-N12-p9415333	gene	
Bn-scaff_18514_1-p74327	Bn-N12-p9370501	gene	exon
Bn-scaff_18520_1-p1922	Bn-N17-p34622129	gene	
Bn-scaff_18520_1-p198684	Bn-N17-p34819900	gene	exon
Bn-scaff_18520_1-p217435	Bn-N17-p34854138	gene	exon
Bn-scaff_18520_1-p301248	Bn-N17-p34948756	gene	exon
Bn-scaff_18520_1-p330575	Bn-N17-p34977895	gene	
Bn-scaff_18520_1-p347007	Bn-N12-p41768062	gene	

Bn-scaff_18520_1-p347665	Bn-N17-p34990613	gene	
Bn-scaff_18520_1-p348211	Bn-N17-p34991161	gene	
Bn-scaff_18520_1-p351091	Bn-N17-p34994041	gene	
Bn-scaff_18520_1-p362389	Bn-N17-p35005654	gene	exon
Bn-scaff_18520_1-p362416	Bn-N17-p35005681	gene	exon
Bn-scaff_18520_1-p363396	Bn-N17-p35006656	gene	exon
Bn-scaff_18520_1-p404968	Bn-N17-p35044257	gene	exon
Bn-scaff_18520_1-p405606	Bn-N17-p35044942	gene	
Bn-scaff_18520_1-p414565	Bn-N17-p35053487	gene	
Bn-scaff_18520_1-p414656	Bn-N17-p35053579	gene	exon
Bn-scaff_18520_1-p426018	Bn-N17-p35064276	gene	
Bn-scaff_18520_1-p486046	Bn-N6-p21190140	gene	
Bn-scaff_18520_1-p486331	Bn-N17-p35120304	gene	
Bn-scaff_18520_1-p486445	Bn-N17-p35120418	gene	exon
Bn-scaff_18520_1-p486567	Bn-N17-p35120540	gene	
Bn-scaff_18520_1-p488642	Bn-N6-p21187433	gene	exon
Bn-scaff_18520_1-p489562	Bn-N6-p21186511	gene	
Bn-scaff_18520_1-p492814	Bn-N17-p35128242	gene	
Bn-scaff_18520_1-p569076	Bn-N17-p35197236	gene	
Bn-scaff_18520_1-p603560	Bn-N17-p35240900	gene	
Bn-scaff_18520_1-p612557	Bn-N17-p35250422	gene	
Bn-scaff_18520_1-p621993	Bn-N17-p35259821	gene	exon
Bn-scaff_18520_1-p622295	Bn-N17-p35260123	gene	
Bn-scaff_18520_1-p630574	Bn-N17-p35268703	gene	
Bn-scaff_18520_1-p633499	Bn-N17-p35271567	gene	
Bn-scaff_18520_1-p675612	Bn-N17-p35303312	gene	
Bn-scaff_18520_1-p675968	Bn-N17-p35303678	gene	exon
Bn-scaff_18520_1-p688050	Bn-N17-p35314465	gene	exon
Bn-scaff_18520_1-p705102	Bn-N17-p35332960	gene	
Bn-scaff_18520_1-p720465	Bn-N17-p35348429	gene	
Bn-scaff_18520_1-p742015	Bn-N17-p35360870	gene	exon
Bn-scaff_18520_1-p795155	Bn-N17-p35415361	gene	
Bn-scaff_18520_1-p807494	Bn-N17-p35426338	gene	exon
Bn-scaff_18520_1-p813605	Bn-N6-p20885880	gene	exon
Bn-scaff_18520_1-p814291	Bn-N17-p35433082	gene	
Bn-scaff_18520_1-p817342	Bn-N17-p35435979	gene	exon
Bn-scaff_18520_1-p817618	Bn-N17-p35436255	gene	exon
Bn-scaff_18549_1-p121747	Bn-N16-p833785	gene	
Bn-scaff_18549_1-p14236	Bn-N16-p941924	gene	exon
Bn-scaff_18549_1-p181754	Bn-N16-p773735	gene	
Bn-scaff_18557_1-p42780	Bn-Scaffold01003-p25069	gene	exon
Bn-scaff_18559_1-p157785	Bn-N13-p64795790	gene	
Bn-scaff_18559_1-p161529	Bn-N13-p64792033	gene	exon
Bn-scaff_18559_1-p166394	Bn-N13-p64783224	gene	
Bn-scaff_18559_1-p175628	Bn-N13-p64764597	gene	
Bn-scaff_18559_1-p17754	Bn-N8-p2080560	gene	
Bn-scaff_18559_1-p23939	Bn-N13-p64883740	gene	exon
Bn-scaff_18559_1-p77372	Bn-N8-p2137845	gene	exon
Bn-scaff_18559_1-p77834	Bn-N13-p64939357	gene	
Bn-scaff_18559_2-p257147	Bn-N11-p20548046	gene	exon
Bn-scaff_18559_2-p39127	Bn-N11-p20763384	gene	exon
Bn-scaff_18589_1-p126670	Bn-N15-p34071863	gene	

Bn-scaff_18589_1-p127443	Bn-N15-p34072637	gene	exon
Bn-scaff_18602_1-p100151	Bn-N13-p57549964	gene	
Bn-scaff_18602_1-p24004	Bn-N13-p57491318	gene	
Bn-scaff_18602_1-p24122	Bn-N13-p57491436	gene	
Bn-scaff_18602_1-p24405	Bn-N13-p57491719	gene	exon
Bn-scaff_18602_1-p24410	Bn-N13-p57491725	gene	exon
Bn-scaff_18602_1-p244789	Bn-N8-p14670111	gene	
Bn-scaff_18602_1-p244845	Bn-N8-p14670055	gene	exon
Bn-scaff_18602_1-p276428	Bn-N8-p14645209	gene	exon
Bn-scaff_18602_1-p277012	Bn-N8-p14644626	gene	exon
Bn-scaff_18602_1-p278628	Bn-N13-p57731728	gene	exon
Bn-scaff_18602_1-p279696	Bn-N8-p14641940	gene	exon
Bn-scaff_18602_1-p280085	Bn-N8-p14641551	gene	exon
Bn-scaff_18602_1-p280096	Bn-N8-p14641540	gene	exon
Bn-scaff_18602_1-p289185	Bn-N13-p57743723	gene	
Bn-scaff_18602_1-p46083	Bn-N13-p57494719	gene	
Bn-scaff_18607_1-p349525	Bn-N10-p6446837	gene	
Bn-scaff_18621_1-p137223	Bn-N11-p44548229	gene	
Bn-scaff_18621_1-p137565	Bn-N11-p44547887	gene	
Bn-scaff_18621_1-p137934	Bn-N11-p44547518	gene	exon
Bn-scaff_18621_1-p183795	Bn-N1-p26214722	gene	exon
Bn-scaff_18621_1-p363772	Bn-N11-p44354501	gene	exon
Bn-scaff_18621_1-p426582	Bn-Scaffold00900-p79183	gene	exon
Bn-scaff_18621_1-p85309	Bn-N11-p44625904	gene	
Bn-scaff_18628_1-p15768	Bn-N7-p11556504	gene	exon
Bn-scaff_18636_1-p102186	Bn-N1-p9013677	gene	exon
Bn-scaff_18636_1-p11498	Bn-N11-p13679240	gene	exon
Bn-scaff_18636_1-p17733	Bn-N1-p8948927	gene	exon
Bn-scaff_18636_1-p34871	Bn-N11-p13705436	gene	
Bn-scaff_18636_1-p41731	Bn-N11-p13712446	gene	exon
Bn-scaff_18636_1-p51263	Bn-N11-p13721982	gene	
Bn-scaff_18645_1-p423	Bn-Scaffold15270-p437	gene	exon
Bn-scaff_18656_1-p12837	Bn-N14-p26670292	gene	
Bn-scaff_18656_1-p22362	Bn-N14-p26681000	gene	
Bn-scaff_18656_1-p22363	Bn-N14-p26681001	gene	
Bn-scaff_18656_1-p43235	Bn-N14-p26698670	gene	
Bn-scaff_18656_1-p44737	Bn-N14-p26700172	gene	
Bn-scaff_18656_1-p53047	Bn-N4-p783164	gene	exon
Bn-scaff_18656_1-p54366	Bn-N14-p26716452	gene	exon
Bn-scaff_18656_1-p54679	Bn-N4-p784797	gene	
Bn-scaff_18656_1-p8003	Bn-N14-p26665458	gene	
Bn-scaff_18672_1-p122715	Bn-N1-p23234168	gene	
Bn-scaff_18672_1-p139723	Bn-N11-p38946144	gene	
Bn-scaff_18672_1-p140782	Bn-N11-p38947191	gene	
Bn-scaff_18672_1-p175171	Bn-N11-p38958552	gene	
Bn-scaff_18672_1-p178765	Bn-N11-p38960173	gene	exon
Bn-scaff_18675_1-p1068457	Bn-N12-p20488873	gene	
Bn-scaff_18675_1-p109771	Bn-N15-p48497106	gene	
Bn-scaff_18675_1-p174256	Bn-N12-p21062254	gene	
Bn-scaff_18675_1-p347787	Bn-N12-p20926413	gene	exon
Bn-scaff_18675_1-p784779	Bn-N3-p10348866	gene	exon
Bn-scaff_18675_1-p786938	Bn-N12-p20684960	gene	

Bn-scaff_18675_1-p82128	Bn-N12-p21107585	gene	
Bn-scaff_18675_1-p8309	Bn-N12-p21178479	gene	exon
Bn-scaff_18675_1-p85245	Bn-N12-p21105708	gene	
Bn-scaff_18675_1-p919422	Bn-N12-p20574863	gene	exon
Bn-scaff_18675_1-p955936	Bn-N2-p14550513	gene	
Bn-scaff_18675_1-p958880	Bn-N12-p20554712	gene	
Bn-scaff_18675_1-p960242	Bn-N12-p20553311	gene	
Bn-scaff_18675_1-p980333	Bn-N14-p50225427	gene	
Bn-scaff_18702_1-p217650	Bn-N2-p11570035	gene	exon
Bn-scaff_18702_1-p224263	Bn-N7-p21684348	gene	exon
Bn-scaff_18702_1-p233858	Bn-N12-p17016171	gene	
Bn-scaff_18702_1-p270197	Bn-N12-p17053036	gene	exon
Bn-scaff_18702_1-p285210	Bn-N12-p17068049	gene	exon
Bn-scaff_18702_1-p289625	Bn-N12-p17072721	gene	exon
Bn-scaff_18702_1-p316851	Bn-N12-p17083690	gene	exon
Bn-scaff_18702_1-p384439	Bn-N12-p17143105	gene	exon
Bn-scaff_18702_1-p523869	Bn-N12-p17276095	gene	
Bn-scaff_18702_1-p586812	Bn-Scaffold01129-p10880	gene	
Bn-scaff_18702_1-p589589	Bn-Scaffold01129-p13652	gene	
Bn-scaff_18702_1-p603145	Bn-N14-p27340796	gene	exon
Bn-scaff_18712_1-p326442	Bn-N14-p15955010	gene	exon
Bn-scaff_18712_1-p326693	Bn-N7-p12364284	gene	exon
Bn-scaff_18712_1-p421039	Bn-N14-p16056265	gene	exon
Bn-scaff_18712_1-p431714	Bn-N14-p16080257	gene	
Bn-scaff_18712_1-p433569	Bn-N14-p16082120	gene	
Bn-scaff_18712_1-p529145	Bn-N14-p16171745	gene	
Bn-scaff_18712_1-p602155	Bn-N14-p16252731	gene	exon
Bn-scaff_18712_1-p602624	Bn-N14-p16253201	gene	
Bn-scaff_18712_1-p95379	Bn-N7-p12471476	gene	exon
Bn-scaff_18762_1-p73478	Bn-N18-p22865650	gene	
Bn-scaff_18776_1-p246454	Bn-N14-p33837910	gene	
Bn-scaff_18776_1-p32882	Bn-N14-p33628609	gene	
Bn-scaff_18776_1-p33105	Bn-N14-p33628823	gene	
Bn-scaff_18776_1-p331403	Bn-N4-p4526547	gene	
Bn-scaff_18776_1-p345564	Bn-N14-p33937585	gene	exon
Bn-scaff_18776_1-p508763	Bn-N14-p34091444	gene	
Bn-scaff_18776_1-p62815	Bn-N14-p33661272	gene	
Bn-scaff_18776_1-p62883	Bn-N14-p33661329	gene	
Bn-scaff_18784_1-p173126	Bn-N15-p20627958	gene	exon
Bn-scaff_18784_1-p385292	Bn-N9-p22646408	gene	
Bn-scaff_18806_1-p248783	Bn-N16-p22340328	gene	
Bn-scaff_18806_1-p323783	Bn-N18-p3042832	gene	exon
Bn-scaff_18806_1-p367722	Bn-N16-p22447183	gene	
Bn-scaff_18806_1-p387496	Bn-N7-p15308589	gene	exon
Bn-scaff_18807_1-p139558	Bn-N16-p34075492	gene	exon
Bn-scaff_18807_1-p151245	Bn-N16-p34062427	gene	exon
Bn-scaff_18807_1-p153480	Bn-N7-p20726888	gene	exon
Bn-scaff_18807_1-p154280	Bn-N16-p34059395	gene	exon
Bn-scaff_18807_1-p154796	Bn-N16-p34058706	gene	exon
Bn-scaff_18807_1-p154933	Bn-N16-p34058570	gene	exon
Bn-scaff_18807_1-p155143	Bn-N7-p20725214	gene	exon
Bn-scaff_18807_1-p155314	Bn-N16-p34058189	gene	

Bn-scaff_18807_1-p155524	Bn-N7-p20724840	gene	
Bn-scaff_18807_1-p169046	Bn-N16-p34043292	gene	
Bn-scaff_18807_1-p170611	Bn-N16-p34041727	gene	
Bn-scaff_18807_1-p196267	Bn-N16-p34019647	gene	exon
Bn-scaff_18807_1-p485563	Bn-N16-p33711799	gene	exon
Bn-scaff_18807_1-p498993	Bn-N16-p33698689	gene	exon
Bn-scaff_18807_1-p499464	Bn-N16-p33698235	gene	
Bn-scaff_18807_1-p504078	Bn-N16-p33693622	gene	exon
Bn-scaff_18807_1-p519628	Bn-N16-p33677940	gene	
Bn-scaff_18807_1-p646353	Bn-N16-p33534775	gene	exon
Bn-scaff_18807_1-p648317	Bn-N16-p33532811	gene	exon
Bn-scaff_18807_1-p658855	Bn-N16-p33515592	gene	exon
Bn-scaff_18807_1-p694694	Bn-N16-p33467443	gene	exon
Bn-scaff_18807_1-p694943	Bn-N16-p33467195	gene	exon
Bn-scaff_18807_1-p73328	Bn-N12-p10539949	gene	exon
Bn-scaff_18807_1-p753863	Bn-N16-p33447496	gene	
Bn-scaff_18817_1-p29976	Bn-N14-p38438650	gene	exon
Bn-scaff_18817_1-p31981	Bn-N14-p38436648	gene	
Bn-scaff_18826_1-p1000612	Bn-N15-p40606936	gene	
Bn-scaff_18826_1-p1046995	Bn-N5-p20449834	gene	exon
Bn-scaff_18826_1-p1111648	Bn-N14-p21562350	gene	
Bn-scaff_18826_1-p158606	Bn-N14-p11862559	gene	exon
Bn-scaff_18826_1-p17445	Bn-N15-p41635680	gene	
Bn-scaff_18826_1-p232573	Bn-N15-p41460871	gene	exon
Bn-scaff_18826_1-p324015	Bn-N15-p41364740	gene	
Bn-scaff_18826_1-p326874	Bn-N15-p41361882	gene	
Bn-scaff_18826_1-p326979	Bn-N5-p20966706	gene	exon
Bn-scaff_18826_1-p355653	Bn-N5-p20947193	gene	exon
Bn-scaff_18826_1-p357180	Bn-N5-p20945637	gene	exon
Bn-scaff_18826_1-p46301	Bn-N18-p23499040	gene	exon
Bn-scaff_18826_1-p472373	Bn-N15-p41171006	gene	
Bn-scaff_18826_1-p472785	Bn-N15-p41170593	gene	exon
Bn-scaff_18826_1-p548550	Bn-N16-p2390776	gene	exon
Bn-scaff_18826_1-p632914	Bn-N5-p20765698	gene	
Bn-scaff_18826_1-p633463	Bn-N15-p41008260	gene	exon
Bn-scaff_18826_1-p75232	Bn-N15-p41597514	gene	
Bn-scaff_18826_1-p75898	Bn-N15-p41596839	gene	exon
Bn-scaff_18826_1-p76018	Bn-N15-p41596718	gene	exon
Bn-scaff_18826_1-p764929	Bn-N15-p40879239	gene	
Bn-scaff_18826_1-p776957	Bn-N15-p40862517	gene	
Bn-scaff_18826_1-p837020	Bn-N15-p40769084	gene	exon
Bn-scaff_18826_1-p837750	Bn-N15-p40768326	gene	exon
Bn-scaff_18826_1-p837907	Bn-N15-p40768170	gene	exon
Bn-scaff_18826_1-p851258	Bn-N15-p40756846	gene	exon
Bn-scaff_18826_1-p857151	Bn-N15-p40755696	gene	exon
Bn-scaff_18826_1-p857416	Bn-N15-p40755433	gene	exon
Bn-scaff_18826_1-p862439	Bn-N15-p40754671	gene	exon
Bn-scaff_18826_1-p890208	Bn-N5-p20567389	gene	exon
Bn-scaff_18826_1-p96686	Bn-N5-p21156090	gene	exon
Bn-scaff_18826_1-p97796	Bn-N15-p41579668	gene	exon
Bn-scaff_18839_1-p1075302	Bn-N9-p10434568	gene	exon
Bn-scaff_18839_1-p1092839	Bn-N19-p16349214	gene	

Bn-scaff_18839_1-p1092979	Bn-N19-p16349352	gene	
Bn-scaff_18839_1-p138513	Bn-N5-p16931011	gene	
Bn-scaff_18839_1-p138616	Bn-N5-p16931066	gene	
Bn-scaff_18839_1-p2517	Bn-N13-p18473962	gene	
Bn-scaff_18839_1-p343327	Bn-N4-p3207023	gene	exon
Bn-scaff_18839_1-p503111	Bn-N17-p25348314	gene	
Bn-scaff_18839_1-p974219	Bn-N9-p10342956	gene	
Bn-scaff_18849_1-p136795	Bn-N13-p52904401	gene	
Bn-scaff_18849_1-p143695	Bn-N13-p52895056	gene	exon
Bn-scaff_18849_1-p145737	Bn-N8-p16897915	gene	
Bn-scaff_18849_1-p206436	Bn-N11-p14401642	gene	exon
Bn-scaff_18849_1-p209120	Bn-N2-p14552801	gene	exon
Bn-scaff_18849_1-p229964	Bn-N13-p52809260	gene	exon
Bn-scaff_18849_1-p230084	Bn-N8-p16918207	gene	exon
Bn-scaff_18849_1-p230136	Bn-N13-p52809088	gene	exon
Bn-scaff_18849_1-p230521	Bn-N13-p52808715	gene	exon
Bn-scaff_18849_1-p230541	Bn-N13-p52808696	gene	exon
Bn-scaff_18849_1-p233040	Bn-N15-p16510269	gene	exon
Bn-scaff_18849_1-p350498	Bn-N13-p52684406	gene	exon
Bn-scaff_18855_1-p184900	Bn-N13-p35493587	gene	exon
Bn-scaff_18855_1-p185593	Bn-N13-p35492946	gene	exon
Bn-scaff_18855_1-p309483	Bn-N13-p35364429	gene	exon
Bn-scaff_18855_1-p309735	Bn-N3-p21145552	gene	exon
Bn-scaff_18855_1-p404353	Bn-N13-p35265094	gene	
Bn-scaff_18855_1-p405082	Bn-N13-p35264365	gene	
Bn-scaff_18855_1-p405501	Bn-N13-p35263945	gene	
Bn-scaff_18855_1-p480400	Bn-N13-p35185141	gene	exon
Bn-scaff_18855_1-p481332	Bn-N3-p21094623	gene	exon
Bn-scaff_18855_1-p831044	Bn-N3-p20956058	gene	exon
Bn-scaff_18855_1-p840984	Bn-N17-p7820364	gene	
Bn-scaff_18901_1-p197391	Bn-N5-p22489174	gene	exon
Bn-scaff_18901_1-p86336	Bn-N15-p43799080	gene	exon
Bn-scaff_18903_1-p183876	Bn-N14-p53710497	gene	
Bn-scaff_18903_1-p211617	Bn-N14-p53747519	gene	exon
Bn-scaff_18903_1-p25620	Bn-N14-p53565662	gene	
Bn-scaff_18903_1-p265334	Bn-N14-p53787886	gene	exon
Bn-scaff_18903_1-p329111	Bn-N14-p53843999	gene	
Bn-scaff_18903_1-p371596	Bn-N14-p53883588	gene	exon
Bn-scaff_18903_1-p403008	Bn-N14-p53905954	gene	
Bn-scaff_18903_1-p404304	Bn-N14-p53907248	gene	
Bn-scaff_18903_1-p432481	Bn-N14-p53937531	gene	
Bn-scaff_18903_1-p460375	Bn-N14-p53958343	gene	
Bn-scaff_18903_1-p471938	Bn-N14-p53972936	gene	
Bn-scaff_18903_1-p489532	Bn-N14-p53989084	gene	exon
Bn-scaff_18903_1-p489764	Bn-N14-p53989316	gene	
Bn-scaff_18903_1-p491280	Bn-N14-p53990727	gene	
Bn-scaff_18903_1-p497319	Bn-N14-p53995031	gene	
Bn-scaff_18903_1-p497762	Bn-N14-p53995474	gene	exon
Bn-scaff_18903_1-p497804	Bn-N14-p53995518	gene	exon
Bn-scaff_18903_1-p535509	Bn-N4-p20354316	gene	exon
Bn-scaff_18903_1-p557077	Bn-N14-p54047458	gene	exon
Bn-scaff_18903_1-p690920	Bn-N14-p54157297	gene	exon

Bn-scaff_18903_1-p735820	Bn-N14-p54201442	gene	
Bn-scaff_18903_1-p748326	Bn-N14-p54215154	gene	exon
Bn-scaff_18903_1-p757633	Bn-N14-p54224677	gene	
Bn-scaff_18903_1-p82359	Bn-N4-p20009256	gene	exon
Bn-scaff_18903_1-p893924	Bn-N14-p54384902	gene	
Bn-scaff_18903_1-p897366	Bn-N14-p54387897	gene	
Bn-scaff_18903_1-p897378	Bn-N14-p54387909	gene	
Bn-scaff_18903_1-p994149	Bn-N4-p20732350	gene	exon
Bn-scaff_18903_1-p995626	Bn-N14-p54503459	gene	exon
Bn-scaff_18903_1-p998892	Bn-N14-p54505801	gene	exon
Bn-scaff_18917_1-p235384	Bn-N3-p20151246	gene	exon
Bn-scaff_18917_1-p236042	Bn-N3-p20150588	gene	exon
Bn-scaff_18917_1-p265790	Bn-N13-p32913081	gene	
Bn-scaff_18917_1-p267401	Bn-N13-p32911267	gene	
Bn-scaff_18917_1-p286596	Bn-N13-p32888632	gene	exon
Bn-scaff_18917_1-p286901	Bn-N3-p20105153	gene	exon
Bn-scaff_18917_1-p299930	Bn-N13-p32873899	gene	exon
Bn-scaff_18917_1-p320287	Bn-N13-p32873836	gene	
Bn-scaff_18917_1-p320503	Bn-N13-p32853545	gene	exon
Bn-scaff_18917_1-p492674	Bn-N13-p32657324	gene	
Bn-scaff_18917_1-p603698	Bn-N13-p32575299	gene	
Bn-scaff_18917_1-p603793	Bn-N13-p32575203	gene	
Bn-scaff_18917_1-p652748	Bn-N13-p32536680	gene	exon
Bn-scaff_18917_1-p657551	Bn-N13-p32527256	gene	exon
Bn-scaff_18917_1-p720029	Bn-N13-p32469555	gene	
Bn-scaff_18917_1-p807362	Bn-N13-p32391714	gene	exon
Bn-scaff_18917_1-p817255	Bn-N13-p32381614	gene	exon
Bn-scaff_18917_1-p857136	Bn-N13-p32344580	gene	exon
Bn-scaff_18917_1-p942272	Bn-N13-p32257752	gene	
Bn-scaff_18936_1-p102137	Bn-N13-p3017933	gene	exon
Bn-scaff_18936_1-p102755	Bn-N13-p3018551	gene	
Bn-scaff_18936_1-p1066008	Bn-N13-p4017874	gene	exon
Bn-scaff_18936_1-p1072527	Bn-N13-p4024367	gene	
Bn-scaff_18936_1-p1073995	Bn-N13-p4025845	gene	
Bn-scaff_18936_1-p1075001	Bn-N3-p3210426	gene	exon
Bn-scaff_18936_1-p112410	Bn-N3-p2431297	gene	exon
Bn-scaff_18936_1-p1128294	Bn-N16-p7497767	gene	
Bn-scaff_18936_1-p1349441	Bn-N14-p30536095	gene	exon
Bn-scaff_18936_1-p1403545	Bn-N6-p658403	gene	
Bn-scaff_18936_1-p1578780	Bn-N16-p7031121	gene	
Bn-scaff_18936_1-p1579348	Bn-N16-p7030553	gene	exon
Bn-scaff_18936_1-p1675459	Bn-N16-p6929192	gene	
Bn-scaff_18936_1-p1687554	Bn-N16-p6919339	gene	
Bn-scaff_18936_1-p1759991	Bn-N16-p6849884	gene	
Bn-scaff_18936_1-p2020755	Bn-N16-p6574378	gene	exon
Bn-scaff_18936_1-p2027486	Bn-N16-p6568241	gene	
Bn-scaff_18936_1-p240670	Bn-N13-p3161750	gene	
Bn-scaff_18936_1-p269153	Bn-N13-p3186352	gene	
Bn-scaff_18936_1-p274133	Bn-N13-p3191332	gene	
Bn-scaff_18936_1-p277517	Bn-N13-p3194716	gene	
Bn-scaff_18936_1-p297756	Bn-N13-p3202838	gene	exon
Bn-scaff_18936_1-p297918	Bn-N13-p3203005	gene	exon

Bn-scaff_18936_1-p298551	Bn-N13-p3203431	gene	
Bn-scaff_18936_1-p325272	Bn-N13-p3231257	gene	
Bn-scaff_18936_1-p327129	Bn-N13-p3233114	gene	
Bn-scaff_18936_1-p333540	Bn-N13-p3239600	gene	exon
Bn-scaff_18936_1-p358822	Bn-N3-p2682880	gene	exon
Bn-scaff_18936_1-p384730	Bn-N13-p3299068	gene	
Bn-scaff_18936_1-p439378	Bn-N13-p3341525	gene	
Bn-scaff_18936_1-p440619	Bn-N13-p3342907	gene	
Bn-scaff_18936_1-p472353	Bn-N13-p3375183	gene	
Bn-scaff_18936_1-p540327	Bn-N3-p2810303	gene	
Bn-scaff_18936_1-p550795	Bn-N3-p2823504	gene	
Bn-scaff_18936_1-p559490	Bn-N13-p3464602	gene	
Bn-scaff_18936_1-p563025	Bn-N13-p3468142	gene	exon
Bn-scaff_18936_1-p582850	Bn-N3-p2852260	gene	exon
Bn-scaff_18936_1-p611810	Bn-N13-p3523997	gene	
Bn-scaff_18936_1-p615525	Bn-N13-p3528228	gene	
Bn-scaff_18936_1-p618378	Bn-N13-p3531109	gene	
Bn-scaff_18936_1-p643990	Bn-N13-p3557440	gene	exon
Bn-scaff_18936_1-p72585	Bn-N3-p2389903	gene	
Bn-scaff_18936_1-p725932	Bn-N13-p3657864	gene	
Bn-scaff_18936_1-p744477	Bn-N13-p3679709	gene	
Bn-scaff_18936_1-p744540	Bn-N13-p3679772	gene	
Bn-scaff_18936_1-p751329	Bn-N3-p3012458	gene	
Bn-scaff_18936_1-p757305	Bn-N13-p3692320	gene	exon
Bn-scaff_18936_1-p758290	Bn-N13-p3693305	gene	exon
Bn-scaff_18936_1-p808967	Bn-N13-p3747203	gene	exon
Bn-scaff_18936_1-p861428	Bn-N13-p3762152	gene	exon
Bn-scaff_18936_1-p86415	Bn-N13-p3001133	gene	exon
Bn-scaff_18936_1-p867397	Bn-N13-p3768302	gene	
Bn-scaff_18936_1-p933467	Bn-N13-p3840730	gene	
Bn-scaff_18936_1-p93643	Bn-N13-p3008135	gene	
Bn-scaff_18944_1-p358968	Bn-N9-p15347430	gene	exon
Bn-scaff_18944_1-p394269	Bn-N9-p15330023	gene	
Bn-scaff_18944_1-p536342	Bn-N15-p12641548	gene	exon
Bn-scaff_19026_1-p101219	Bn-N8-p5615925	gene	
Bn-scaff_19026_1-p105795	Bn-N13-p30634252	gene	
Bn-scaff_19026_1-p184842	Bn-N13-p30555341	gene	
Bn-scaff_19026_1-p187828	Bn-N13-p30552079	gene	
Bn-scaff_19026_1-p187921	Bn-N13-p30551986	gene	
Bn-scaff_19026_1-p188341	Bn-N13-p30551546	gene	
Bn-scaff_19026_1-p191325	Bn-N13-p30548960	gene	exon
Bn-scaff_19026_1-p191536	Bn-N13-p30548752	gene	
Bn-scaff_19026_1-p192280	Bn-N13-p30548002	gene	
Bn-scaff_19026_1-p192351	Bn-N13-p30547931	gene	
Bn-scaff_19026_1-p192416	Bn-N13-p30547866	gene	
Bn-scaff_19026_1-p194582	Bn-N3-p19142981	gene	
Bn-scaff_19026_1-p200897	Bn-N13-p30539366	gene	
Bn-scaff_19026_1-p231124	Bn-N13-p30516857	gene	
Bn-scaff_19026_1-p232264	Bn-N13-p30515717	gene	
Bn-scaff_19026_1-p292357	Bn-N13-p30441403	gene	
Bn-scaff_19026_1-p45302	Bn-N13-p30703196	gene	
Bn-scaff_19043_1-p22356	Bn-N14-p42551050	gene	exon

Bn-scaff_19043_1-p24351	Bn-N14-p42548928	gene	
Bn-scaff_19043_1-p389754	Bn-N4-p11630934	gene	
Bn-scaff_19043_1-p50175	Bn-N14-p42529018	gene	
Bn-scaff_19043_1-p51461	Bn-N14-p42527729	gene	
Bn-scaff_19043_1-p51554	Bn-N14-p42527636	gene	
Bn-scaff_19043_1-p52253	Bn-N14-p42526937	gene	exon
Bn-scaff_19043_1-p53467	Bn-N14-p42524898	gene	exon
Bn-scaff_19043_1-p53629	Bn-N14-p42524735	gene	
Bn-scaff_19043_1-p53637	Bn-N14-p42524727	gene	
Bn-scaff_19043_1-p77359	Bn-N14-p42496178	gene	exon
Bn-scaff_19043_1-p83155	Bn-N14-p42488705	gene	exon
Bn-scaff_19043_1-p83566	Bn-N14-p42488296	gene	
Bn-scaff_19043_1-p97247	Bn-N14-p42473937	gene	exon
Bn-scaff_19044_1-p9802	Bn-N15-p32540593	gene	
Bn-scaff_19047_1-p159375	Bn-N13-p65157643	gene	
Bn-scaff_19047_1-p164520	Bn-N13-p65152308	gene	exon
Bn-scaff_19047_1-p169194	Bn-N13-p65147608	gene	exon
Bn-scaff_19047_1-p191638	Bn-N13-p65136153	gene	exon
Bn-scaff_19047_1-p196388	Bn-N13-p65133374	gene	
Bn-scaff_19047_1-p292986	Bn-N13-p65018002	gene	
Bn-scaff_19047_1-p293516	Bn-N13-p65017472	gene	
Bn-scaff_19047_1-p294145	Bn-N13-p65016841	gene	
Bn-scaff_19047_1-p294788	Bn-N13-p65016198	gene	
Bn-scaff_19047_1-p295106	Bn-N13-p65015880	gene	
Bn-scaff_19047_1-p295372	Bn-N13-p65015398	gene	
Bn-scaff_19047_1-p297198	Bn-N13-p65014421	gene	exon
Bn-scaff_19101_1-p10495	Bn-N13-p60982141	gene	exon
Bn-scaff_19104_1-p304889	Bn-N8-p8978414	gene	exon
Bn-scaff_19106_1-p307140	Bn-N16-p16508340	gene	
Bn-scaff_19106_1-p426627	Bn-N7-p9721973	gene	exon
Bn-scaff_19106_1-p427702	Bn-N7-p9720917	gene	exon
Bn-scaff_19106_1-p440312	Bn-N17-p20634441	gene	
Bn-scaff_19106_1-p605386	Bn-N17-p20446675	gene	
Bn-scaff_19111_1-p138623	Bn-N13-p12043290	gene	exon
Bn-scaff_19111_1-p140517	Bn-N3-p8561479	gene	
Bn-scaff_19111_1-p162577	Bn-N13-p12017700	gene	exon
Bn-scaff_19111_1-p217082	Bn-N13-p11967999	gene	
Bn-scaff_19111_1-p217129	Bn-N13-p11967954	gene	
Bn-scaff_19111_1-p258190	Bn-N13-p11924744	gene	exon
Bn-scaff_19111_1-p258531	Bn-N13-p11924404	gene	
Bn-scaff_19111_1-p363367	Bn-N13-p20932985	gene	exon
Bn-scaff_19168_1-p109723	Bn-N11-p39761603	gene	
Bn-scaff_19168_1-p15958	Bn-N11-p39847702	gene	
Bn-scaff_19168_1-p29925	Bn-N11-p39843040	gene	exon
Bn-scaff_19168_1-p30868	Bn-N11-p39842746	gene	
Bn-scaff_19168_1-p31612	Bn-N11-p39841999	gene	
Bn-scaff_19168_1-p85750	Bn-N11-p39773153	gene	exon
Bn-scaff_19170_1-p1028423	Bn-N14-p22453616	gene	
Bn-scaff_19170_1-p1028779	Bn-N14-p22453972	gene	
Bn-scaff_19170_1-p1034998	Bn-N14-p22460191	gene	
Bn-scaff_19170_1-p1118815	Bn-N14-p22544401	gene	
Bn-scaff_19170_1-p114391	Bn-N14-p21675001	gene	

Bn-scaff_19170_1-p114487	Bn-N14-p21675097	gene	
Bn-scaff_19170_1-p391172	Bn-N14-p21815095	gene	
Bn-scaff_19170_1-p391864	Bn-N14-p21815786	gene	
Bn-scaff_19170_1-p403539	Bn-N14-p21822614	gene	
Bn-scaff_19170_1-p405290	Bn-N14-p21824365	gene	
Bn-scaff_19170_1-p461594	Bn-N14-p21886362	gene	
Bn-scaff_19170_1-p464773	Bn-N14-p21898643	gene	
Bn-scaff_19170_1-p472715	Bn-N14-p21922684	gene	exon
Bn-scaff_19170_1-p473071	Bn-N14-p21923040	gene	exon
Bn-scaff_19170_1-p474592	Bn-N14-p21924561	gene	exon
Bn-scaff_19170_1-p51349	Bn-N14-p21620672	gene	
Bn-scaff_19170_1-p517947	Bn-N14-p21976537	gene	
Bn-scaff_19170_1-p532510	Bn-N14-p21992330	gene	exon
Bn-scaff_19170_1-p533180	Bn-N14-p21993000	gene	
Bn-scaff_19170_1-p550014	Bn-N14-p22010261	gene	exon
Bn-scaff_19170_1-p574456	Bn-N14-p22033771	gene	
Bn-scaff_19170_1-p586404	Bn-N14-p22051158	gene	exon
Bn-scaff_19170_1-p586539	Bn-N14-p22051293	gene	exon
Bn-scaff_19170_1-p590810	Bn-N14-p22055565	gene	
Bn-scaff_19170_1-p590894	Bn-N14-p22055649	gene	
Bn-scaff_19170_1-p829081	Bn-N14-p22259427	gene	exon
Bn-scaff_19183_1-p164144	Bn-N11-p23093895	gene	exon
Bn-scaff_19183_1-p164373	Bn-N11-p23094124	gene	exon
Bn-scaff_19183_1-p164485	Bn-N11-p23094236	gene	
Bn-scaff_19183_1-p249410	Bn-N11-p23186592	gene	
Bn-scaff_19183_1-p425206	Bn-N11-p22376276	gene	
Bn-scaff_19183_1-p465103	Bn-N11-p22331633	gene	
Bn-scaff_19183_1-p478485	Bn-N11-p22321019	gene	
Bn-scaff_19186_1-p466456	Bn-N6-p12377071	gene	exon
Bn-scaff_19193_1-p1189479	Bn-N11-p5310140	gene	
Bn-scaff_19193_1-p1191929	Bn-N11-p5307763	gene	
Bn-scaff_19193_1-p1250152	Bn-N11-p5243904	gene	
Bn-scaff_19193_1-p1252982	Bn-N11-p5241032	gene	exon
Bn-scaff_19193_1-p1288594	Bn-N11-p5198868	gene	
Bn-scaff_19193_1-p1290482	Bn-N11-p5196983	gene	
Bn-scaff_19193_1-p1347576	Bn-N1-p3787946	gene	
Bn-scaff_19193_1-p313981	Bn-N1-p4467673	gene	exon
Bn-scaff_19193_1-p345684	Bn-N11-p6135636	gene	exon
Bn-scaff_19193_1-p399060	Bn-N11-p6078846	gene	exon
Bn-scaff_19193_1-p399528	Bn-N11-p6078377	gene	
Bn-scaff_19193_1-p416656	Bn-N16-p16121031	gene	exon
Bn-scaff_19193_1-p491056	Bn-N11-p5983097	gene	exon
Bn-scaff_19193_1-p491853	Bn-N11-p5982300	gene	exon
Bn-scaff_19193_1-p527597	Bn-N11-p5948182	gene	
Bn-scaff_19193_1-p561943	Bn-N11-p5915505	gene	exon
Bn-scaff_19193_1-p565446	Bn-N11-p5912004	gene	
Bn-scaff_19193_1-p657373	Bn-N11-p5819997	gene	exon
Bn-scaff_19193_1-p6789	Bn-N11-p6552161	gene	
Bn-scaff_19193_1-p704018	Bn-N11-p5751693	gene	exon
Bn-scaff_19193_1-p704672	Bn-N11-p5751039	gene	exon
Bn-scaff_19193_1-p711927	Bn-N11-p5743795	gene	exon
Bn-scaff_19193_1-p725427	Bn-N1-p4153029	gene	exon

Bn-scaff_19193_1-p725739	Bn-N1-p4152717	gene	
Bn-scaff_19193_1-p86427	Bn-N11-p6461074	gene	exon
Bn-scaff_19208_1-p130237	Bn-N14-p41168253	gene	
Bn-scaff_19208_1-p242899	Bn-N4-p10960303	gene	exon
Bn-scaff_19208_1-p309287	Bn-N14-p41373952	gene	
Bn-scaff_19208_1-p431737	Bn-N18-p38964367	gene	exon
Bn-scaff_19224_1-p29613	Bn-N15-p33944976	gene	
Bn-scaff_19224_1-p29858	Bn-N15-p33944731	gene	exon
Bn-scaff_19224_1-p30859	Bn-N15-p33943730	gene	exon
Bn-scaff_19242_1-p421335	Bn-N18-p20024510	gene	exon
Bn-scaff_19244_1-p120852	Bn-N11-p205786	gene	
Bn-scaff_19244_1-p193513	Bn-N11-p283543	gene	exon
Bn-scaff_19244_1-p196631	Bn-N11-p286661	gene	
Bn-scaff_19244_1-p208209	Bn-N11-p298051	gene	exon
Bn-scaff_19244_1-p260990	Bn-N11-p348913	gene	exon
Bn-scaff_19244_1-p270599	Bn-N11-p358546	gene	exon
Bn-scaff_19244_1-p328817	Bn-N11-p413606	gene	exon
Bn-scaff_19244_1-p355868	Bn-N1-p338310	gene	exon
Bn-scaff_19244_1-p380858	Bn-N11-p462202	gene	
Bn-scaff_19244_1-p441730	Bn-N1-p405672	gene	
Bn-scaff_19244_1-p443382	Bn-N11-p521263	gene	exon
Bn-scaff_19244_1-p451742	Bn-N11-p529608	gene	
Bn-scaff_19244_1-p53159	Bn-N11-p137862	gene	
Bn-scaff_19244_1-p551168	Bn-N11-p614746	gene	
Bn-scaff_19244_1-p577643	Bn-N11-p640023	gene	exon
Bn-scaff_19244_1-p656563	Bn-N1-p6869501	gene	
Bn-scaff_19244_1-p698816	Bn-N11-p769136	gene	exon
Bn-scaff_19244_1-p699318	Bn-N11-p769638	gene	
Bn-scaff_19244_1-p738519	Bn-N11-p814744	gene	
Bn-scaff_19244_1-p755070	Bn-N11-p830406	gene	
Bn-scaff_19244_1-p816342	Bn-N11-p865603	gene	exon
Bn-scaff_19244_1-p843614	Bn-N3-p7620724	gene	
Bn-scaff_19248_1-p155179	Bn-N14-p7698700	gene	exon
Bn-scaff_19248_1-p166341	Bn-N14-p7707563	gene	exon
Bn-scaff_19248_1-p233324	Bn-N14-p7770440	gene	exon
Bn-scaff_19248_1-p235858	Bn-N14-p7770795	gene	exon
Bn-scaff_19248_1-p269648	Bn-N14-p7800499	gene	
Bn-scaff_19248_1-p357191	Bn-N3-p8465382	gene	exon
Bn-scaff_19248_1-p48952	Bn-N14-p7584354	gene	exon
Bn-scaff_19248_1-p523097	Bn-N3-p8455128	gene	exon
Bn-scaff_19248_1-p57521	Bn-N14-p7592933	gene	
Bn-scaff_19248_1-p57760	Bn-N14-p7593176	gene	exon
Bn-scaff_19253_1-p112082	Bn-N14-p17049286	gene	exon
Bn-scaff_19253_1-p112212	Bn-N14-p17049416	gene	exon
Bn-scaff_19253_1-p112702	Bn-N14-p17049879	gene	exon
Bn-scaff_19253_1-p112787	Bn-N14-p17049964	gene	
Bn-scaff_19253_1-p113020	Bn-N14-p17050197	gene	
Bn-scaff_19253_1-p113235	Bn-N14-p17050412	gene	exon
Bn-scaff_19253_1-p138537	Bn-N14-p17084705	gene	
Bn-scaff_19253_1-p167431	Bn-N14-p17119898	gene	
Bn-scaff_19253_1-p168128	Bn-N14-p17120595	gene	
Bn-scaff_19253_1-p170640	Bn-N14-p17123122	gene	exon

Bn-scaff_19253_1-p197419	Bn-N14-p17150158	gene	exon
Bn-scaff_19253_1-p228274	Bn-N14-p17188642	gene	exon
Bn-scaff_19253_1-p228569	Bn-N14-p17188938	gene	
Bn-scaff_19253_1-p228570	Bn-N14-p17188938	gene	
Bn-scaff_19253_1-p237811	Bn-N14-p17200413	gene	
Bn-scaff_19253_1-p24802	Bn-N14-p16965938	gene	
Bn-scaff_19253_1-p253157	Bn-N14-p17234620	gene	exon
Bn-scaff_19253_1-p25504	Bn-N14-p16966640	gene	
Bn-scaff_19253_1-p25939	Bn-N14-p16967144	gene	
Bn-scaff_19253_1-p26030	Bn-N14-p16967235	gene	exon
Bn-scaff_19253_1-p269230	Bn-N14-p17251163	gene	
Bn-scaff_19253_1-p285404	Bn-N14-p17268712	gene	
Bn-scaff_19253_1-p30153	Bn-N14-p16972719	gene	exon
Bn-scaff_19253_1-p30405	Bn-N14-p16972971	gene	
Bn-scaff_19253_1-p31161	Bn-N14-p16973727	gene	
Bn-scaff_19253_1-p31253	Bn-N14-p16973819	gene	
Bn-scaff_19253_1-p31721	Bn-N14-p16974287	gene	
Bn-scaff_19253_1-p535009	Bn-N14-p17610966	gene	
Bn-scaff_19253_1-p556898	Bn-N14-p17629542	gene	
Bn-scaff_19253_1-p600747	Bn-N14-p17673050	gene	exon
Bn-scaff_19253_1-p614151	Bn-N14-p17686362	gene	
Bn-scaff_19253_1-p75285	Bn-N14-p17004393	gene	
Bn-scaff_19253_1-p82507	Bn-N14-p17015415	gene	exon
Bn-scaff_19253_1-p93586	Bn-N14-p17030446	gene	exon
Bn-scaff_19271_1-p113773	Bn-N1-p14977117	gene	exon
Bn-scaff_19290_1-p70753	Bn-N11-p13619178	gene	
Bn-scaff_19310_1-p116554	Bn-N13-p38670958	gene	
Bn-scaff_19310_1-p209197	Bn-N13-p38756837	gene	
Bn-scaff_19310_1-p247331	Bn-N6-p15862828	gene	exon
Bn-scaff_19310_1-p276541	Bn-N13-p38812341	gene	exon
Bn-scaff_19310_1-p351188	Bn-N13-p38870659	gene	exon
Bn-scaff_19310_1-p364008	Bn-N13-p38880526	gene	exon
Bn-scaff_19310_1-p370263	Bn-N13-p38884692	gene	
Bn-scaff_19310_1-p37363	Bn-N13-p38606483	gene	exon
Bn-scaff_19310_1-p429319	Bn-N13-p38941733	gene	
Bn-scaff_19310_1-p432715	Bn-N13-p38945132	gene	
Bn-scaff_19310_1-p432770	Bn-N13-p38945187	gene	
Bn-scaff_19310_1-p432772	Bn-N13-p38945189	gene	
Bn-scaff_19310_1-p434081	Bn-N13-p38946491	gene	exon
Bn-scaff_19310_1-p509653	Bn-N13-p39442480	gene	
Bn-scaff_19310_1-p531191	Bn-N13-p39463755	gene	
Bn-scaff_19310_1-p531402	Bn-N13-p39463966	gene	
Bn-scaff_19310_1-p536472	Bn-N6-p15668445	gene	
Bn-scaff_19310_1-p626867	Bn-N13-p39567939	gene	exon
Bn-scaff_19310_1-p66830	Bn-N13-p38631850	gene	
Bn-scaff_19318_1-p167	Bn-N14-p48027478	gene	
Bn-scaff_19364_1-p362049	Bn-N11-p19707635	gene	
Bn-scaff_19364_1-p362066	Bn-N11-p19707618	gene	
Bn-scaff_19364_1-p362185	Bn-N11-p19707499	gene	
Bn-scaff_19364_1-p417674	Bn-N11-p19660358	gene	exon
Bn-scaff_19364_1-p461287	Bn-N11-p19601746	gene	
Bn-scaff_19364_1-p462138	Bn-N11-p19600907	gene	

Bn-scaff_19364_1-p462147	Bn-N11-p19600898	gene	
Bn-scaff_19364_1-p462199	Bn-N11-p19600846	gene	
Bn-scaff_19364_1-p468188	Bn-N11-p19594126	gene	exon
Bn-scaff_19364_1-p468644	Bn-N11-p19593670	gene	exon
Bn-scaff_19372_1-p7611	Bn-N2-p17327319	gene	exon
Bn-scaff_19458_1-p7894	Bn-N11-p26297640	gene	
Bn-scaff_19458_1-p8019	Bn-N11-p26297515	gene	exon
Bn-scaff_19483_1-p95805	Bn-N10-p8701886	gene	exon
Bn-scaff_19490_1-p119155	Bn-Scaffold04186-p1786	gene	
Bn-scaff_19523_1-p14392	Bn-N13-p21370240	gene	exon
Bn-scaff_19523_1-p188656	Bn-N13-p21545093	gene	exon
Bn-scaff_19523_1-p232023	Bn-N13-p21573788	gene	exon
Bn-scaff_19523_1-p261625	Bn-N13-p21606213	gene	
Bn-scaff_19523_1-p341606	Bn-N19-p1132010	gene	exon
Bn-scaff_19523_1-p366203	Bn-N13-p21713728	gene	
Bn-scaff_19523_1-p427343	Bn-N3-p14065808	gene	
Bn-scaff_19523_1-p428124	Bn-N3-p14066557	gene	exon
Bn-scaff_19523_1-p545907	Bn-N14-p53111005	gene	exon
Bn-scaff_19523_1-p622333	Bn-N13-p21999599	gene	exon
Bn-scaff_19523_1-p869340	Bn-N3-p14294755	gene	exon
Bn-scaff_19560_1-p50816	Bn-N19-p38645485	gene	
Bn-scaff_19560_1-p51121	Bn-N19-p38645789	gene	
Bn-scaff_19564_1-p17934	Bn-N11-p29100585	gene	
Bn-scaff_19569_1-p41924	Bn-N13-p4907742	gene	
Bn-scaff_19575_1-p165770	Bn-N14-p20304756	gene	
Bn-scaff_19575_1-p184301	Bn-N14-p20290150	gene	
Bn-scaff_19575_1-p237597	Bn-N14-p20234881	gene	
Bn-scaff_19575_1-p386837	Bn-N14-p20926497	gene	exon
Bn-scaff_19575_1-p540292	Bn-N14-p21060555	gene	exon
Bn-scaff_19575_1-p634889	Bn-N14-p21229689	gene	exon
Bn-scaff_19575_1-p723313	Bn-N14-p21319633	gene	
Bn-scaff_19575_1-p738860	Bn-N14-p21335435	gene	
Bn-scaff_19575_1-p740686	Bn-N14-p21337262	gene	
Bn-scaff_19575_1-p88445	Bn-N14-p20409427	gene	exon
Bn-scaff_19575_1-p88718	Bn-N14-p20409153	gene	exon
Bn-scaff_19575_1-p89370	Bn-N14-p20408501	gene	
Bn-scaff_19575_1-p90626	Bn-N14-p20407245	gene	
Bn-scaff_19614_1-p106170	Bn-N1-p9320598	gene	exon
Bn-scaff_19614_1-p119351	Bn-N17-p16148810	gene	exon
Bn-scaff_19614_1-p300765	Bn-N11-p13956787	gene	exon
Bn-scaff_19614_1-p305584	Bn-N11-p13952157	gene	exon
Bn-scaff_19614_1-p325704	Bn-N11-p13931879	gene	
Bn-scaff_19614_1-p36023	Bn-N11-p14181324	gene	exon
Bn-scaff_19614_1-p53484	Bn-N11-p14175577	gene	
Bn-scaff_19614_1-p98680	Bn-N11-p14150127	gene	exon
Bn-scaff_19619_1-p39510	Bn-N19-p45660289	gene	exon
Bn-scaff_19641_1-p100329	Bn-N6-p3895316	gene	
Bn-scaff_19641_1-p170202	Bn-N15-p4623425	gene	exon
Bn-scaff_19641_1-p206896	Bn-N15-p4588751	gene	
Bn-scaff_19641_1-p222124	Bn-N15-p4570733	gene	
Bn-scaff_19646_1-p410	Bn-N13-p5199414	gene	exon
Bn-scaff_19702_1-p204876	Bn-N13-p44314137	gene	

Bn-scaff_19702_1-p481302	Bn-N13-p42514966	gene	exon
Bn-scaff_19724_1-p202852	Bn-N17-p28975035	gene	exon
Bn-scaff_19724_1-p249787	Bn-N17-p28929164	gene	
Bn-scaff_19724_1-p462268	Bn-N17-p26837319	gene	exon
Bn-scaff_19724_1-p467818	Bn-N6-p24634391	gene	
Bn-scaff_19724_1-p522227	Bn-N17-p26909757	gene	
Bn-scaff_19724_1-p659910	Bn-N6-p24680432	gene	
Bn-scaff_19724_1-p749258	Bn-N6-p24725333	gene	exon
Bn-scaff_19740_1-p109782	Bn-N13-p51768695	gene	exon
Bn-scaff_19740_1-p114198	Bn-N13-p51764279	gene	
Bn-scaff_19740_1-p236040	Bn-N11-p9467842	gene	
Bn-scaff_19740_1-p35978	Bn-N13-p51845472	gene	
Bn-scaff_19740_1-p36568	Bn-N13-p51844882	gene	
Bn-scaff_19740_1-p45553	Bn-N13-p51835215	gene	exon
Bn-scaff_19740_1-p6264	Bn-N18-p40830649	gene	exon
Bn-scaff_19740_1-p6315	Bn-N18-p40830598	gene	exon
Bn-scaff_19740_1-p66841	Bn-N13-p51813749	gene	
Bn-scaff_19740_1-p67158	Bn-N13-p51813432	gene	
Bn-scaff_19740_1-p67802	Bn-N13-p51812788	gene	
Bn-scaff_19757_1-p22787	Bn-N10-p14515412	gene	exon
Bn-scaff_19757_1-p24032	Bn-N19-p51996756	gene	
Bn-scaff_19773_1-p137808	Bn-N16-p13928755	gene	
Bn-scaff_19783_1-p1055665	Bn-N19-p4917290	gene	exon
Bn-scaff_19783_1-p1060183	Bn-N9-p3825589	gene	
Bn-scaff_19783_1-p1061297	Bn-N9-p3819839	gene	exon
Bn-scaff_19783_1-p1061414	Bn-N19-p4913015	gene	exon
Bn-scaff_19783_1-p1062045	Bn-N9-p3827438	gene	exon
Bn-scaff_19783_1-p279292	Bn-N19-p4158931	gene	
Bn-scaff_19783_1-p379086	Bn-N19-p4266183	gene	
Bn-scaff_19783_1-p423234	Bn-N9-p3411466	gene	exon
Bn-scaff_19783_1-p472902	Bn-N2-p28015348	gene	
Bn-scaff_19783_1-p71479	Bn-N9-p3162962	gene	exon
Bn-scaff_19783_1-p922268	Bn-N19-p4788413	gene	
Bn-scaff_19783_1-p925928	Bn-N19-p4792046	gene	
Bn-scaff_19821_1-p218002	Bn-N14-p14246777	gene	exon
Bn-scaff_19821_1-p262184	Bn-N14-p14291632	gene	exon
Bn-scaff_19821_1-p268090	Bn-N14-p14297489	gene	
Bn-scaff_19821_1-p268347	Bn-N14-p14297746	gene	
Bn-scaff_19821_1-p388456	Bn-N14-p14333490	gene	exon
Bn-scaff_19821_1-p54826	Bn-N5-p8094550	gene	exon
Bn-scaff_19821_1-p96192	Bn-N14-p14121665	gene	
Bn-scaff_19892_1-p218532	Bn-N5-p10734186	gene	exon
Bn-scaff_19892_1-p278146	Bn-N5-p10690972	gene	exon
Bn-scaff_19899_1-p16013	Bn-N19-p47957574	gene	
Bn-scaff_19899_1-p17895	Bn-N19-p47955692	gene	
Bn-scaff_19899_1-p203618	Bn-N19-p47758421	gene	exon
Bn-scaff_19899_1-p303613	Bn-N19-p47658209	gene	exon
Bn-scaff_19899_1-p34734	Bn-N19-p47939658	gene	exon
Bn-scaff_19899_1-p356987	Bn-N10-p12070269	gene	exon
Bn-scaff_19899_1-p357191	Bn-N19-p47611912	gene	
Bn-scaff_19899_1-p357670	Bn-N10-p12069587	gene	exon
Bn-scaff_19899_1-p358095	Bn-N19-p47611007	gene	exon

Bn-scaff_19899_1-p465507	Bn-N10-p12009774	gene	exon
Bn-scaff_19899_1-p465611	Bn-N17-p12155579	gene	exon
Bn-scaff_19899_1-p466993	Bn-N19-p47520241	gene	exon
Bn-scaff_19899_1-p523832	Bn-N19-p47461911	gene	exon
Bn-scaff_19899_1-p559187	Bn-N19-p47451063	gene	
Bn-scaff_19899_1-p621547	Bn-N19-p47367335	gene	exon
Bn-scaff_19899_1-p621874	Bn-N19-p47367007	gene	
Bn-scaff_19899_1-p623253	Bn-N19-p47365638	gene	exon
Bn-scaff_19899_1-p639962	Bn-N19-p47346877	gene	
Bn-scaff_19899_1-p684603	Bn-N14-p26646615	gene	exon
Bn-scaff_19899_1-p709156	Bn-N19-p47288680	gene	exon
Bn-scaff_19899_1-p719729	Bn-N19-p47275220	gene	
Bn-scaff_19899_1-p719873	Bn-N19-p47275076	gene	
Bn-scaff_19904_1-p103831	Bn-Scaffold03812-p2020	gene	exon
Bn-scaff_19911_1-p54072	Bn-N16-p2920805	gene	exon
Bn-scaff_19911_1-p98995	Bn-N16-p2873079	gene	
Bn-scaff_19927_1-p24167	Bn-N17-p35228677	gene	exon
Bn-scaff_19927_1-p24375	Bn-N18-p26943592	gene	
Bn-scaff_19929_1-p1877	Bn-Scaffold02566-p3116	gene	
Bn-scaff_19937_1-p19550	Bn-N16-p36848274	gene	
Bn-scaff_19937_1-p20028	Bn-N7-p22429034	gene	exon
Bn-scaff_19975_1-p14174	Bn-Scaffold01814-p2311	gene	exon
Bn-scaff_19995_1-p11896	Bn-Scaffold01118-p7341	gene	
Bn-scaff_19995_1-p12647	Bn-Scaffold01118-p8069	gene	
Bn-scaff_19995_1-p20624	Bn-N5-p11922367	gene	exon
Bn-scaff_19995_1-p33414	Bn-Scaffold01118-p31325	gene	exon
Bn-scaff_20026_1-p359079	Bn-N11-p29876725	gene	
Bn-scaff_20042_1-p10474	Bn-N14-p23278101	gene	
Bn-scaff_20042_1-p12033	Bn-N14-p23279656	gene	exon
Bn-scaff_20042_1-p39005	Bn-N14-p23301810	gene	
Bn-scaff_20042_1-p74312	Bn-N14-p23336945	gene	
Bn-scaff_20042_1-p84111	Bn-N14-p23345899	gene	exon
Bn-scaff_20063_1-p25913	Bn-N14-p6398533	gene	
Bn-scaff_20079_1-p17049	Bn-N3-p19029227	gene	exon
Bn-scaff_20079_1-p20029	Bn-N13-p30342481	gene	
Bn-scaff_20079_1-p289504	Bn-N14-p46670100	gene	
Bn-scaff_20079_1-p465897	Bn-N14-p46494306	gene	exon
Bn-scaff_20079_1-p489358	Bn-N14-p46467861	gene	exon
Bn-scaff_20079_1-p494342	Bn-N14-p46461222	gene	exon
Bn-scaff_20079_1-p507355	Bn-N14-p46431734	gene	
Bn-scaff_20079_1-p58991	Bn-N13-p30280082	gene	exon
Bn-scaff_20079_1-p661258	Bn-N14-p46336661	gene	
Bn-scaff_20079_1-p721407	Bn-N4-p14458357	gene	
Bn-scaff_20084_1-p163373	Bn-N17-p6705572	gene	exon
Bn-scaff_20084_1-p176376	Bn-N7-p2807523	gene	exon
Bn-scaff_20084_1-p197706	Bn-N7-p2826090	gene	exon
Bn-scaff_20084_1-p325311	Bn-N17-p6840154	gene	exon
Bn-scaff_20084_1-p82863	Bn-N17-p6619795	gene	exon
Bn-scaff_20103_1-p142562	Bn-N13-p29131690	gene	exon
Bn-scaff_20103_1-p142790	Bn-N13-p29131461	gene	exon
Bn-scaff_20103_1-p298039	Bn-N13-p28955083	gene	exon
Bn-scaff_20103_1-p309490	Bn-N13-p28938029	gene	exon

Bn-scaff_20103_1-p379028	Bn-N13-p28877251	gene	
Bn-scaff_20103_1-p507458	Bn-N13-p28669333	gene	exon
Bn-scaff_20103_1-p564754	Bn-N3-p18519256	gene	exon
Bn-scaff_20125_1-p1071	Bn-N15-p12333408	gene	exon
Bn-scaff_20125_1-p150744	Bn-N15-p12176589	gene	
Bn-scaff_20125_1-p151394	Bn-N15-p12175939	gene	exon
Bn-scaff_20125_1-p152650	Bn-N15-p12174673	gene	
Bn-scaff_20125_1-p177173	Bn-N15-p12151683	gene	
Bn-scaff_20125_1-p18276	Bn-N15-p12315210	gene	
Bn-scaff_20125_1-p301419	Bn-N15-p12020198	gene	
Bn-scaff_20125_1-p378742	Bn-N15-p11923031	gene	
Bn-scaff_20125_1-p428266	Bn-N6-p8767352	gene	exon
Bn-scaff_20125_1-p522233	Bn-N15-p11761322	gene	exon
Bn-scaff_20125_1-p533196	Bn-N15-p11755153	gene	
Bn-scaff_20125_1-p566462	Bn-N15-p11731057	gene	
Bn-scaff_20125_1-p567498	Bn-N15-p11730021	gene	
Bn-scaff_20125_1-p63386	Bn-N15-p12258258	gene	
Bn-scaff_20125_1-p63532	Bn-N15-p12258112	gene	exon
Bn-scaff_20125_1-p663724	Bn-N15-p11643360	gene	exon
Bn-scaff_20125_1-p67021	Bn-N7-p10191283	gene	exon
Bn-scaff_20125_1-p863	Bn-N15-p12333616	gene	exon
Bn-scaff_20125_1-p86621	Bn-N15-p12238056	gene	
Bn-scaff_20125_1-p872243	Bn-N19-p20858476	gene	
Bn-scaff_20125_1-p88815	Bn-N15-p12236213	gene	exon
Bn-scaff_20131_1-p73163	Bn-N1-p4936980	gene	
Bn-scaff_20131_1-p76620	Bn-N4-p5589	gene	
Bn-scaff_20168_1-p6747	Bn-N19-p19937519	gene	
Bn-scaff_20191_1-p143344	Bn-N4-p5363976	gene	exon
Bn-scaff_20191_1-p146654	Bn-N14-p34845349	gene	
Bn-scaff_20191_1-p63120	Bn-N14-p34935820	gene	
Bn-scaff_20202_1-p138599	Bn-N19-p19835397	gene	exon
Bn-scaff_20210_1-p111101	Bn-N11-p8996544	gene	exon
Bn-scaff_20210_1-p111252	Bn-N11-p8996393	gene	exon
Bn-scaff_20210_1-p112010	Bn-N11-p8995634	gene	exon
Bn-scaff_20210_1-p112176	Bn-N11-p8995469	gene	exon
Bn-scaff_20210_1-p112767	Bn-N11-p8992465	gene	exon
Bn-scaff_20210_1-p136861	Bn-N11-p8967302	gene	exon
Bn-scaff_20210_1-p136943	Bn-N11-p8967220	gene	
Bn-scaff_20210_1-p161976	Bn-N11-p8940075	gene	
Bn-scaff_20210_1-p162115	Bn-N11-p8939936	gene	
Bn-scaff_20210_1-p166012	Bn-N11-p8936002	gene	exon
Bn-scaff_20210_1-p267408	Bn-N11-p8844681	gene	
Bn-scaff_20210_1-p346419	Bn-N11-p8743983	gene	
Bn-scaff_20210_1-p449263	Bn-N11-p8662570	gene	exon
Bn-scaff_20210_1-p457153	Bn-N11-p8445894	gene	exon
Bn-scaff_20210_1-p54473	Bn-N15-p15894819	gene	exon
Bn-scaff_20219_1-p148469	Bn-N15-p45711100	gene	
Bn-scaff_20219_1-p148573	Bn-N15-p45711204	gene	
Bn-scaff_20219_1-p148903	Bn-N15-p45711535	gene	exon
Bn-scaff_20219_1-p150787	Bn-N15-p45713405	gene	
Bn-scaff_20219_1-p205790	Bn-N15-p45768280	gene	
Bn-scaff_20219_1-p290323	Bn-N15-p45863364	gene	exon

Bn-scaff_20219_1-p290966	Bn-N15-p45864005	gene	
Bn-scaff_20219_1-p305658	Bn-N15-p45878877	gene	
Bn-scaff_20219_1-p308326	Bn-N5-p24009932	gene	exon
Bn-scaff_20219_1-p312051	Bn-N5-p24013714	gene	
Bn-scaff_20219_1-p343155	Bn-N15-p45916538	gene	
Bn-scaff_20219_1-p348628	Bn-N5-p24043483	gene	exon
Bn-scaff_20219_1-p361815	Bn-N15-p45935030	gene	exon
Bn-scaff_20219_1-p50731	Bn-N15-p45652782	gene	exon
Bn-scaff_20219_1-p50816	Bn-N15-p45652867	gene	
Bn-scaff_20219_1-p52642	Bn-N15-p45654698	gene	exon
Bn-scaff_20219_1-p53873	Bn-N15-p45655924	gene	
Bn-scaff_20221_1-p119381	Bn-N2-p16942193	gene	exon
Bn-scaff_20221_1-p119890	Bn-N2-p16941684	gene	
Bn-scaff_20221_1-p131095	Bn-N12-p24391258	gene	
Bn-scaff_20221_1-p132207	Bn-N12-p24390141	gene	
Bn-scaff_20221_1-p65440	Bn-N12-p24458135	gene	exon
Bn-scaff_20221_1-p67938	Bn-N12-p24455637	gene	exon
Bn-scaff_20221_1-p67945	Bn-N12-p24455630	gene	
Bn-scaff_20221_1-p75490	Bn-N12-p24448351	gene	
Bn-scaff_20250_1-p190243	Bn-N11-p18443406	gene	exon
Bn-scaff_20250_1-p190957	Bn-N11-p18444121	gene	
Bn-scaff_20250_1-p213668	Bn-N1-p12057902	gene	exon
Bn-scaff_20250_1-p252517	Bn-N11-p18512204	gene	exon
Bn-scaff_20250_1-p252691	Bn-N11-p18512378	gene	
Bn-scaff_20250_1-p252716	Bn-N11-p18512403	gene	
Bn-scaff_20250_1-p320616	Bn-N11-p18582219	gene	
Bn-scaff_20250_1-p329601	Bn-N11-p18590811	gene	
Bn-scaff_20250_1-p336475	Bn-N11-p18597683	gene	
Bn-scaff_20250_1-p395260	Bn-N11-p18664147	gene	exon
Bn-scaff_20250_1-p402102	Bn-N11-p18667636	gene	
Bn-scaff_20250_1-p402157	Bn-N11-p18667691	gene	
Bn-scaff_20250_1-p402491	Bn-N11-p18668026	gene	
Bn-scaff_20250_1-p436289	Bn-N11-p18725128	gene	exon
Bn-scaff_20250_2-p12010	Bn-N11-p19090680	gene	exon
Bn-scaff_20250_2-p12065	Bn-N11-p19090625	gene	exon
Bn-scaff_20250_2-p12470	Bn-N11-p19090220	gene	
Bn-scaff_20250_2-p12556	Bn-N11-p19090134	gene	
Bn-scaff_20250_2-p12794	Bn-N11-p19089896	gene	exon
Bn-scaff_20250_2-p129421	Bn-N11-p18971342	gene	
Bn-scaff_20250_2-p255304	Bn-N11-p18847629	gene	
Bn-scaff_20250_2-p255760	Bn-N11-p18847174	gene	
Bn-scaff_20270_1-p1061064	Bn-N15-p46530738	gene	
Bn-scaff_20270_1-p1070285	Bn-N15-p46540304	gene	
Bn-scaff_20270_1-p1092399	Bn-N15-p46560298	gene	exon
Bn-scaff_20270_1-p1095989	Bn-N15-p46563887	gene	
Bn-scaff_20270_1-p1172081	Bn-N15-p46632104	gene	exon
Bn-scaff_20270_1-p1199943	Bn-N15-p46655991	gene	exon
Bn-scaff_20270_1-p1293568	Bn-N15-p46749035	gene	exon
Bn-scaff_20270_1-p1299957	Bn-N15-p46755424	gene	exon
Bn-scaff_20270_1-p1300874	Bn-N15-p46756341	gene	
Bn-scaff_20270_1-p1314298	Bn-N15-p46771301	gene	
Bn-scaff_20270_1-p1323892	Bn-N15-p46774545	gene	exon

Bn-scaff_20270_1-p13443	Bn-N14-p52418293	gene	exon
Bn-scaff_20270_1-p138318	Bn-N4-p19184632	gene	exon
Bn-scaff_20270_1-p1440999	Bn-N15-p46890058	gene	exon
Bn-scaff_20270_1-p1446137	Bn-N15-p46895185	gene	exon
Bn-scaff_20270_1-p1477447	Bn-N15-p46926548	gene	exon
Bn-scaff_20270_1-p165323	Bn-N14-p52555769	gene	exon
Bn-scaff_20270_1-p166898	Bn-N4-p19211540	gene	exon
Bn-scaff_20270_1-p174459	Bn-N4-p19215844	gene	exon
Bn-scaff_20270_1-p210772	Bn-N14-p52591333	gene	exon
Bn-scaff_20270_1-p212224	Bn-N14-p52592986	gene	
Bn-scaff_20270_1-p224871	Bn-N4-p19251899	gene	
Bn-scaff_20270_1-p235987	Bn-N14-p52615840	gene	
Bn-scaff_20270_1-p295928	Bn-N14-p52676236	gene	exon
Bn-scaff_20270_1-p331696	Bn-N4-p19341283	gene	exon
Bn-scaff_20270_1-p332105	Bn-N14-p52713397	gene	
Bn-scaff_20270_1-p380914	Bn-N14-p52764451	gene	
Bn-scaff_20270_1-p456204	Bn-N14-p52836647	gene	exon
Bn-scaff_20270_1-p48716	Bn-N4-p19106207	gene	exon
Bn-scaff_20270_1-p48789	Bn-N14-p52446318	gene	exon
Bn-scaff_20270_1-p546450	Bn-N14-p52943567	gene	
Bn-scaff_20270_1-p619866	Bn-N14-p53025585	gene	exon
Bn-scaff_20270_1-p620404	Bn-N14-p53026140	gene	
Bn-scaff_20270_1-p627168	Bn-N14-p53042397	gene	exon
Bn-scaff_20270_1-p632286	Bn-N14-p53047526	gene	exon
Bn-scaff_20270_1-p639601	Bn-N4-p19599693	gene	
Bn-scaff_20270_1-p715501	Bn-N14-p53139128	gene	exon
Bn-scaff_20270_1-p721012	Bn-N14-p53146116	gene	
Bn-scaff_20270_1-p73558	Bn-N14-p52480958	gene	exon
Bn-scaff_20270_1-p781490	Bn-N14-p53217722	gene	exon
Bn-scaff_20270_1-p792475	Bn-N4-p19707997	gene	exon
Bn-scaff_20270_1-p849122	Bn-N14-p53269984	gene	exon
Bn-scaff_20270_1-p881395	Bn-N14-p53302363	gene	
Bn-scaff_20288_1-p281923	Bn-N17-p10108964	gene	
Bn-scaff_20288_1-p283766	Bn-N17-p10110806	gene	exon
Bn-scaff_20288_1-p483106	Bn-N17-p10287846	gene	exon
Bn-scaff_20288_1-p514714	Bn-N7-p4391875	gene	
Bn-scaff_20294_1-p235008	Bn-N16-p37281702	gene	exon
Bn-scaff_20294_1-p250512	Bn-N16-p37266026	gene	exon
Bn-scaff_20294_1-p250681	Bn-N16-p37265857	gene	exon
Bn-scaff_20294_1-p268948	Bn-N16-p37247044	gene	exon
Bn-scaff_20294_1-p269011	Bn-N16-p37246981	gene	
Bn-scaff_20294_1-p269248	Bn-N16-p37246743	gene	
Bn-scaff_20294_1-p269745	Bn-N7-p22680960	gene	
Bn-scaff_20294_1-p303417	Bn-N16-p37209378	gene	exon
Bn-scaff_20294_1-p378164	Bn-N16-p37150138	gene	
Bn-scaff_20294_1-p381496	Bn-N16-p37146640	gene	
Bn-scaff_20294_1-p421716	Bn-N16-p37107567	gene	exon
Bn-scaff_20294_1-p421721	Bn-N16-p37107567	gene	exon
Bn-scaff_20294_1-p422419	Bn-N16-p37106910	gene	
Bn-scaff_20294_1-p424857	Bn-N16-p37104470	gene	
Bn-scaff_20320_1-p52767	Bn-N11-p30326	gene	
Bn-scaff_20320_1-p82662	Bn-N11-p11522	gene	exon

Bn-scaff_20320_1-p83433	Bn-N11-p10756	gene	
Bn-scaff_20354_1-p179456	Bn-N19-p23209066	gene	exon
Bn-scaff_20354_1-p211850	Bn-N19-p23188406	gene	exon
Bn-scaff_20354_1-p53124	Bn-N9-p14374053	gene	exon
Bn-scaff_20376_1-p121796	Bn-N5-p24782347	gene	
Bn-scaff_20376_1-p168831	Bn-N15-p47091056	gene	
Bn-scaff_20376_1-p218414	Bn-N15-p47142137	gene	exon
Bn-scaff_20376_1-p281604	Bn-N15-p47190606	gene	exon
Bn-scaff_20428_1-p133089	Bn-N9-p7309234	gene	exon
Bn-scaff_20428_1-p282411	Bn-N3-p20559905	gene	
Bn-scaff_20428_1-p312666	Bn-N19-p10751765	gene	
Bn-scaff_20428_1-p333831	Bn-N9-p7144154	gene	exon
Bn-scaff_20428_1-p33886	Bn-N19-p11073861	gene	
Bn-scaff_20428_1-p360651	Bn-N19-p10697114	gene	exon
Bn-scaff_20428_1-p430947	Bn-N19-p10637780	gene	
Bn-scaff_20428_1-p431113	Bn-N19-p10637614	gene	
Bn-scaff_20452_1-p127374	Bn-N1-p17350625	gene	exon
Bn-scaff_20452_1-p306492	Bn-N13-p37596567	gene	exon
Bn-scaff_20452_1-p86850	Bn-N11-p28668831	gene	exon
Bn-scaff_20455_1-p42268	Bn-N18-p39028943	gene	
Bn-scaff_20459_1-p120552	Bn-N5-p18374898	gene	
Bn-scaff_20461_1-p110039	Bn-N12-p11144164	gene	
Bn-scaff_20461_1-p178421	Bn-N12-p11090200	gene	exon
Bn-scaff_20461_1-p290320	Bn-N2-p7449577	gene	exon
Bn-scaff_20461_1-p314199	Bn-N12-p10970256	gene	
Bn-scaff_20461_1-p321761	Bn-N12-p10962541	gene	
Bn-scaff_20461_1-p322463	Bn-N12-p10961860	gene	
Bn-scaff_20461_1-p324935	Bn-N12-p10959356	gene	exon
Bn-scaff_20462_1-p27589	Bn-Scaffold00938-p9622	gene	exon
Bn-scaff_20478_1-p149392	Bn-Scaffold09318-p156	gene	exon
Bn-scaff_20478_1-p334940	Bn-N9-p10494796	gene	exon
Bn-scaff_20478_1-p54432	Bn-N11-p25522556	gene	exon
Bn-scaff_20497_1-p71596	Bn-N18-p17173466	gene	exon
Bn-scaff_20497_1-p71756	Bn-N18-p17173626	gene	
Bn-scaff_20497_1-p95468	Bn-N18-p17197659	gene	
Bn-scaff_20567_1-p320309	Bn-N14-p21131303	gene	exon
Bn-scaff_20567_1-p336974	Bn-N14-p24386958	gene	exon
Bn-scaff_20567_1-p347036	Bn-N14-p24377241	gene	exon
Bn-scaff_20567_1-p64644	Bn-N14-p24654358	gene	exon
Bn-scaff_20619_1-p210474	Bn-N19-p36548416	gene	exon
Bn-scaff_20619_1-p210599	Bn-N10-p6006657	gene	
Bn-scaff_20619_1-p316049	Bn-N19-p36422167	gene	
Bn-scaff_20619_1-p451147	Bn-Scaffold01071-p27045	gene	exon
Bn-scaff_20619_1-p467200	Bn-N6-p14520754	gene	exon
Bn-scaff_20619_1-p58311	Bn-N10-p6068898	gene	exon
Bn-scaff_20619_1-p693773	Bn-N19-p36134261	gene	exon
Bn-scaff_20638_1-p142399	Bn-N1-p6663773	gene	exon
Bn-scaff_20646_1-p1065920	Bn-N13-p19519392	gene	
Bn-scaff_20646_1-p1066305	Bn-N13-p19519007	gene	
Bn-scaff_20646_1-p1111352	Bn-N3-p12859685	gene	exon
Bn-scaff_20646_1-p207297	Bn-N13-p20409850	gene	
Bn-scaff_20646_1-p208091	Bn-N13-p20409055	gene	

Bn-scaff_20646_1-p299775	Bn-N13-p20314379	gene	exon
Bn-scaff_20646_1-p299890	Bn-N13-p20314264	gene	
Bn-scaff_20646_1-p300084	Bn-N13-p20314070	gene	
Bn-scaff_20646_1-p300270	Bn-N13-p20313884	gene	
Bn-scaff_20646_1-p317660	Bn-N13-p20291245	gene	exon
Bn-scaff_20646_1-p348861	Bn-N13-p20263967	gene	
Bn-scaff_20646_1-p397340	Bn-N13-p20156123	gene	
Bn-scaff_20646_1-p463899	Bn-N13-p20094248	gene	
Bn-scaff_20646_1-p464029	Bn-N13-p20094118	gene	
Bn-scaff_20646_1-p495036	Bn-N13-p20062887	gene	exon
Bn-scaff_20646_1-p498394	Bn-N13-p20059530	gene	
Bn-scaff_20646_1-p564345	Bn-N13-p19998674	gene	
Bn-scaff_20646_1-p580318	Bn-N13-p51801267	gene	exon
Bn-scaff_20646_1-p583921	Bn-N13-p19981444	gene	exon
Bn-scaff_20646_1-p584496	Bn-N13-p19980881	gene	exon
Bn-scaff_20646_1-p586003	Bn-N13-p19979374	gene	exon
Bn-scaff_20646_1-p634009	Bn-N13-p19941602	gene	exon
Bn-scaff_20646_1-p634317	Bn-N13-p19941294	gene	
Bn-scaff_20646_1-p634413	Bn-N13-p19941198	gene	
Bn-scaff_20646_1-p635083	Bn-N19-p27002105	gene	
Bn-scaff_20646_1-p635267	Bn-N9-p17084790	gene	exon
Bn-scaff_20646_1-p639709	Bn-N13-p19936061	gene	exon
Bn-scaff_20646_1-p639773	Bn-N13-p19935997	gene	exon
Bn-scaff_20646_1-p639860	Bn-N13-p19935910	gene	exon
Bn-scaff_20646_1-p644913	Bn-N13-p19930875	gene	
Bn-scaff_20646_1-p675326	Bn-N13-p19900213	gene	
Bn-scaff_20646_1-p675506	Bn-N13-p19900033	gene	exon
Bn-scaff_20646_1-p677020	Bn-N13-p19898518	gene	exon
Bn-scaff_20646_1-p702004	Bn-N13-p19879529	gene	exon
Bn-scaff_20646_1-p714994	Bn-N13-p19867153	gene	
Bn-scaff_20646_1-p715363	Bn-N13-p19866786	gene	
Bn-scaff_20646_1-p730848	Bn-N13-p19850395	gene	
Bn-scaff_20646_1-p735337	Bn-N13-p19845918	gene	exon
Bn-scaff_20646_1-p748599	Bn-N13-p19832946	gene	exon
Bn-scaff_20646_1-p764847	Bn-N13-p19816227	gene	
Bn-scaff_20646_1-p926102	Bn-N13-p19656997	gene	
Bn-scaff_20646_1-p930628	Bn-N17-p7028343	gene	exon
Bn-scaff_20666_1-p54157	Bn-N19-p34549996	gene	exon
Bn-scaff_20675_1-p149133	Bn-N11-p10147936	gene	
Bn-scaff_20675_1-p156163	Bn-N11-p10139855	gene	
Bn-scaff_20675_1-p159903	Bn-N11-p10118131	gene	exon
Bn-scaff_20675_1-p160003	Bn-N11-p10118032	gene	exon
Bn-scaff_20675_1-p52417	Bn-N11-p10239767	gene	
Bn-scaff_20675_1-p61871	Bn-N11-p10230319	gene	exon
Bn-scaff_20675_1-p93956	Bn-N1-p6911520	gene	exon
Bn-scaff_20675_1-p94966	Bn-N1-p6910541	gene	exon
Bn-scaff_20675_1-p95063	Bn-N11-p10196641	gene	
Bn-scaff_20675_1-p98663	Bn-N1-p6907200	gene	exon
Bn-scaff_20676_1-p189015	Bn-Scaffold04835-p328	gene	exon
Bn-scaff_20676_1-p189097	Bn-Scaffold04835-p374	gene	
Bn-scaff_20676_1-p189507	Bn-Scaffold04835-p373	gene	
Bn-scaff_20676_1-p435529	Bn-N14-p29185922	gene	

Bn-scaff_20732_1-p9422	Bn-N12-p31100503	gene	
Bn-scaff_20732_1-p9483	Bn-N12-p31100564	gene	
Bn-scaff_20735_1-p4627	Bn-N2-p12708394	gene	exon
Bn-scaff_20741_1-p20543	Bn-N3-p13800648	gene	
Bn-scaff_20741_1-p20982	Bn-N3-p13801088	gene	exon
Bn-scaff_20773_1-p121051	Bn-N16-p5778184	gene	
Bn-scaff_20773_1-p123341	Bn-N16-p5775959	gene	
Bn-scaff_20773_1-p153100	Bn-N16-p5750855	gene	
Bn-scaff_20773_1-p155178	Bn-N6-p1213095	gene	exon
Bn-scaff_20773_1-p332944	Bn-N16-p5529879	gene	
Bn-scaff_20773_1-p339705	Bn-N16-p5518436	gene	
Bn-scaff_20773_1-p424697	Bn-N16-p5440718	gene	
Bn-scaff_20773_1-p472229	Bn-N16-p5368962	gene	
Bn-scaff_20773_1-p566178	Bn-N6-p1360692	gene	exon
Bn-scaff_20790_1-p102313	Bn-N13-p52573037	gene	exon
Bn-scaff_20790_1-p102558	Bn-N13-p52572792	gene	exon
Bn-scaff_20790_1-p103526	Bn-N13-p52571825	gene	
Bn-scaff_20790_1-p259929	Bn-N8-p17093491	gene	
Bn-scaff_20790_1-p259991	Bn-N8-p17093554	gene	
Bn-scaff_20790_1-p261248	Bn-N13-p52461375	gene	
Bn-scaff_20790_1-p264415	Bn-N8-p17098025	gene	
Bn-scaff_20790_1-p268409	Bn-N8-p17104166	gene	exon
Bn-scaff_20790_1-p3436	Bn-N13-p52659189	gene	
Bn-scaff_20796_1-p137393	Bn-N6-p23377863	gene	exon
Bn-scaff_20796_1-p148168	Bn-N18-p1550138	gene	exon
Bn-scaff_20796_1-p34573	Bn-N18-p1454470	gene	
Bn-scaff_20796_1-p69576	Bn-N8-p21071372	gene	
Bn-scaff_20804_1-p178251	Bn-N14-p50671270	gene	exon
Bn-scaff_20804_1-p180229	Bn-N14-p50669290	gene	
Bn-scaff_20804_1-p180721	Bn-N14-p50668798	gene	
Bn-scaff_20804_1-p180953	Bn-N14-p50668572	gene	
Bn-scaff_20804_1-p181022	Bn-N14-p50668503	gene	
Bn-scaff_20804_1-p60629	Bn-N14-p50793904	gene	exon
Bn-scaff_20804_1-p84419	Bn-N14-p50767485	gene	exon
Bn-scaff_20804_1-p84899	Bn-N14-p50767009	gene	exon
Bn-scaff_20804_1-p85446	Bn-N14-p50766462	gene	exon
Bn-scaff_20804_1-p9294	Bn-N14-p50850535	gene	
Bn-scaff_20807_1-p104549	Bn-N7-p18188389	gene	exon
Bn-scaff_20809_1-p116777	Bn-N2-p11021459	gene	
Bn-scaff_20809_1-p163800	Bn-N18-p1332183	gene	
Bn-scaff_20809_1-p164232	Bn-N18-p1332616	gene	
Bn-scaff_20809_1-p164254	Bn-N18-p1332638	gene	
Bn-scaff_20809_1-p164312	Bn-N18-p1332696	gene	
Bn-scaff_20809_1-p47064	Bn-N2-p10966418	gene	
Bn-scaff_20809_1-p48762	Bn-N2-p10968196	gene	exon
Bn-scaff_20809_1-p64287	Bn-Scaffold01727-p3326	gene	
Bn-scaff_20809_1-p74433	Bn-N15-p48526971	gene	exon
Bn-scaff_20814_1-p251867	Bn-N4-p6139904	gene	exon
Bn-scaff_20817_1-p50242	Bn-N14-p54860544	gene	exon
Bn-scaff_20817_1-p51382	Bn-N14-p54861691	gene	exon
Bn-scaff_20817_1-p60579	Bn-N14-p54875333	gene	
Bn-scaff_20819_1-p3631	Bn-N17-p20788038	gene	exon

Bn-scaff_20830_1-p183301	Bn-N11-p8191138	gene	exon
Bn-scaff_20830_1-p60025	Bn-N18-p7445903	gene	exon
Bn-scaff_20830_1-p61135	Bn-N18-p7447048	gene	exon
Bn-scaff_20835_1-p2147	Bn-N10-p12928517	gene	exon
Bn-scaff_20836_1-p134452	Bn-N19-p16713395	gene	exon
Bn-scaff_20836_1-p19306	Bn-N19-p16834690	gene	
Bn-scaff_20836_1-p30574	Bn-Scaffold00776-p85614	gene	exon
Bn-scaff_20838_1-p221095	Bn-N1-p26938766	gene	exon
Bn-scaff_20866_1-p108216	Bn-N16-p3539235	gene	exon
Bn-scaff_20866_1-p111789	Bn-N16-p3535662	gene	exon
Bn-scaff_20881_1-p11760	Bn-N13-p50056419	gene	exon
Bn-scaff_20901_1-p1088726	Bn-N6-p2850541	gene	exon
Bn-scaff_20901_1-p1103224	Bn-N15-p3192152	gene	exon
Bn-scaff_20901_1-p1110262	Bn-N15-p3182631	gene	
Bn-scaff_20901_1-p1210835	Bn-N15-p3041443	gene	exon
Bn-scaff_20901_1-p1220930	Bn-N15-p3037969	gene	
Bn-scaff_20901_1-p1246467	Bn-N15-p3018062	gene	
Bn-scaff_20901_1-p1247459	Bn-N15-p3017053	gene	
Bn-scaff_20901_1-p1311264	Bn-N15-p2960014	gene	
Bn-scaff_20901_1-p1505546	Bn-N15-p2779513	gene	exon
Bn-scaff_20901_1-p1506172	Bn-N15-p2778888	gene	exon
Bn-scaff_20901_1-p1509958	Bn-N15-p2775101	gene	
Bn-scaff_20901_1-p1510247	Bn-N15-p2774812	gene	
Bn-scaff_20901_1-p1510260	Bn-N15-p2774798	gene	
Bn-scaff_20901_1-p1537914	Bn-N15-p2750972	gene	
Bn-scaff_20901_1-p1577315	Bn-N10-p2995296	gene	exon
Bn-scaff_20901_1-p1587058	Bn-N10-p2984141	gene	exon
Bn-scaff_20901_1-p1613836	Bn-N15-p2654630	gene	
Bn-scaff_20901_1-p1621648	Bn-N15-p2645608	gene	
Bn-scaff_20901_1-p1641268	Bn-N15-p2625197	gene	
Bn-scaff_20901_1-p1674359	Bn-N15-p2591939	gene	
Bn-scaff_20901_1-p1681305	Bn-N15-p2578161	gene	
Bn-scaff_20901_1-p1705574	Bn-N15-p2559070	gene	
Bn-scaff_20901_1-p1719394	Bn-N15-p2543657	gene	exon
Bn-scaff_20901_1-p1736256	Bn-N15-p2529890	gene	exon
Bn-scaff_20901_1-p1743515	Bn-N15-p2522639	gene	exon
Bn-scaff_20901_1-p1761960	Bn-N15-p2503978	gene	
Bn-scaff_20901_1-p1765503	Bn-N15-p2500520	gene	
Bn-scaff_20901_1-p1783054	Bn-N15-p2484324	gene	
Bn-scaff_20901_1-p1783385	Bn-N15-p2483993	gene	exon
Bn-scaff_20901_1-p1783641	Bn-N15-p2483737	gene	
Bn-scaff_20901_1-p2013546	Bn-N15-p2257895	gene	
Bn-scaff_20901_1-p2018350	Bn-N10-p2344219	gene	exon
Bn-scaff_20901_1-p2019548	Bn-N18-p43666543	gene	exon
Bn-scaff_20901_1-p2029631	Bn-N15-p2241803	gene	
Bn-scaff_20901_1-p2081837	Bn-N15-p2189731	gene	exon
Bn-scaff_20901_1-p2096370	Bn-N15-p2176308	gene	
Bn-scaff_20901_1-p2125331	Bn-N15-p2143706	gene	
Bn-scaff_20901_1-p276384	Bn-N15-p4072410	gene	
Bn-scaff_20901_1-p345310	Bn-N15-p4007948	gene	
Bn-scaff_20901_1-p348242	Bn-N15-p4005029	gene	
Bn-scaff_20901_1-p365032	Bn-Scaffold09732-p367	gene	

Bn-scaff_20901_1-p380645	Bn-N15-p3967277	gene	
Bn-scaff_20901_1-p403764	Bn-N15-p3940028	gene	
Bn-scaff_20901_1-p449219	Bn-N15-p3895910	gene	exon
Bn-scaff_20901_1-p449884	Bn-N15-p3830247	gene	exon
Bn-scaff_20901_1-p566912	Bn-N6-p3241905	gene	
Bn-scaff_20901_1-p58099	Bn-N6-p3615757	gene	exon
Bn-scaff_20901_1-p58670	Bn-N15-p4275661	gene	exon
Bn-scaff_20901_1-p597476	Bn-N15-p3728694	gene	
Bn-scaff_20901_1-p653379	Bn-N15-p3671327	gene	exon
Bn-scaff_20901_1-p653492	Bn-N15-p3671214	gene	
Bn-scaff_20901_1-p656470	Bn-N15-p3668206	gene	
Bn-scaff_20901_1-p857332	Bn-N15-p3459894	gene	
Bn-scaff_20901_1-p870589	Bn-N18-p42922857	gene	exon
Bn-scaff_20901_1-p875264	Bn-N15-p3441990	gene	exon
Bn-scaff_20901_1-p879369	Bn-N15-p3436207	gene	
Bn-scaff_20901_1-p880825	Bn-N15-p3434747	gene	
Bn-scaff_20901_1-p882135	Bn-N15-p3433436	gene	
Bn-scaff_20901_1-p884877	Bn-N6-p3020188	gene	exon
Bn-scaff_20901_1-p890950	Bn-N15-p3419558	gene	
Bn-scaff_20901_1-p894349	Bn-N15-p3416186	gene	
Bn-scaff_20901_1-p900299	Bn-N15-p3409513	gene	
Bn-scaff_20901_1-p948057	Bn-N15-p3365432	gene	
Bn-scaff_20901_1-p948373	Bn-N15-p3365116	gene	
Bn-scaff_20901_1-p950532	Bn-N15-p3362946	gene	exon
Bn-scaff_20901_1-p950607	Bn-N15-p3362870	gene	exon
Bn-scaff_20901_1-p951018	Bn-N15-p3362460	gene	exon
Bn-scaff_20901_1-p951365	Bn-N6-p2981706	gene	exon
Bn-scaff_20901_1-p990137	Bn-N15-p3319836	gene	
Bn-scaff_20903_1-p264678	Bn-N19-p21035135	gene	
Bn-scaff_20903_1-p379070	Bn-N19-p21159907	gene	
Bn-scaff_20903_1-p380659	Bn-N19-p21161317	gene	
Bn-scaff_20903_1-p38574	Bn-N19-p20886726	gene	
Bn-scaff_20903_1-p398892	Bn-N9-p13270452	gene	exon
Bn-scaff_20903_1-p456556	Bn-N19-p21260985	gene	
Bn-scaff_20942_1-p1093417	Bn-Scaffold01396-p6634	gene	exon
Bn-scaff_20942_1-p149203	Bn-N2-p8241339	gene	exon
Bn-scaff_20942_1-p21122	Bn-N12-p12587564	gene	
Bn-scaff_20942_1-p246578	Bn-N12-p12397263	gene	exon
Bn-scaff_20942_1-p393532	Bn-N12-p12257251	gene	exon
Bn-scaff_20942_1-p440106	Bn-N12-p12209928	gene	exon
Bn-scaff_20942_1-p446103	Bn-N2-p8093488	gene	
Bn-scaff_20942_1-p453076	Bn-N18-p35724879	gene	
Bn-scaff_20942_1-p460319	Bn-N12-p12128747	gene	exon
Bn-scaff_20942_1-p526744	Bn-N2-p8076681	gene	exon
Bn-scaff_20942_1-p604955	Bn-N12-p12111703	gene	
Bn-scaff_20942_1-p607399	Bn-N2-p8034681	gene	exon
Bn-scaff_20942_1-p655677	Bn-N2-p8013649	gene	
Bn-scaff_20942_1-p672835	Bn-N2-p8007110	gene	
Bn-scaff_20942_1-p694535	Bn-N12-p12037899	gene	exon
Bn-scaff_20942_1-p695512	Bn-N2-p7996421	gene	exon
Bn-scaff_20942_1-p734832	Bn-N12-p12009928	gene	exon
Bn-scaff_20947_1-p127456	Bn-N18-p42693789	gene	exon

Bn-scaff_20947_1-p146378	Bn-N18-p42675828	gene	exon
Bn-scaff_20947_1-p146783	Bn-N18-p42675423	gene	exon
Bn-scaff_20947_1-p197588	Bn-N9-p38632400	gene	
Bn-scaff_20947_1-p327211	Bn-N16-p37460436	gene	exon
Bn-scaff_20947_1-p89554	Bn-N18-p42722177	gene	exon
Bn-scaff_20972_1-p21129	Bn-N19-p37834983	gene	exon
Bn-scaff_20979_1-p456432	Bn-N12-p16423990	gene	
Bn-scaff_20979_1-p624297	Bn-N12-p16334057	gene	exon
Bn-scaff_21003_1-p457127	Bn-N18-p4698077	gene	exon
Bn-scaff_21003_1-p457723	Bn-N18-p4697479	gene	exon
Bn-scaff_21003_1-p567379	Bn-N18-p4826429	gene	exon
Bn-scaff_21003_1-p647095	Bn-N18-p4552442	gene	
Bn-scaff_21003_1-p647723	Bn-N18-p4551814	gene	
Bn-scaff_21003_1-p655968	Bn-N18-p4535731	gene	
Bn-scaff_21003_1-p659725	Bn-N18-p4531763	gene	
Bn-scaff_21003_1-p703369	Bn-N18-p36861249	gene	exon
Bn-scaff_21015_1-p89425	Bn-N5-p19376008	gene	exon
Bn-scaff_21018_1-p138540	Bn-N16-p40670655	gene	
Bn-scaff_21018_1-p24333	Bn-N16-p40782147	gene	
Bn-scaff_21034_1-p41433	Bn-N12-p19884200	gene	
Bn-scaff_21124_1-p142833	Bn-N15-p41666095	gene	
Bn-scaff_21124_1-p174942	Bn-N15-p41694030	gene	
Bn-scaff_21124_1-p175003	Bn-N15-p41694092	gene	exon
Bn-scaff_21124_1-p212889	Bn-N15-p41721636	gene	exon
Bn-scaff_21124_1-p215984	Bn-N15-p41724790	gene	exon
Bn-scaff_21124_1-p216517	Bn-N15-p41725520	gene	exon
Bn-scaff_21124_1-p216889	Bn-N15-p41725892	gene	
Bn-scaff_21124_1-p242605	Bn-N15-p41749822	gene	exon
Bn-scaff_21124_1-p242718	Bn-N15-p41749935	gene	exon
Bn-scaff_21124_1-p298134	Bn-N15-p41807557	gene	
Bn-scaff_21124_1-p324211	Bn-N15-p41833428	gene	exon
Bn-scaff_21124_1-p375749	Bn-N15-p41876130	gene	exon
Bn-scaff_21131_1-p149854	Bn-N12-p32595538	gene	
Bn-scaff_21131_1-p245633	Bn-N16-p783965	gene	exon
Bn-scaff_21131_1-p266157	Bn-N12-p32780832	gene	exon
Bn-scaff_21131_1-p266236	Bn-N12-p32780911	gene	exon
Bn-scaff_21131_1-p284044	Bn-N12-p32786130	gene	exon
Bn-scaff_21131_1-p49530	Bn-N12-p32545937	gene	
Bn-scaff_21131_1-p95520	Bn-N2-p20227170	gene	
Bn-scaff_21138_1-p17277	Bn-N14-p23067688	gene	exon
Bn-scaff_21163_1-p3558	Bn-N11-p33872015	gene	exon
Bn-scaff_21163_1-p4255	Bn-N11-p33871318	gene	exon
Bn-scaff_21182_1-p42780	Bn-N18-p2799672	gene	exon
Bn-scaff_21186_1-p132310	Bn-N14-p49841274	gene	exon
Bn-scaff_21186_1-p153195	Bn-N14-p49823185	gene	
Bn-scaff_21186_1-p153520	Bn-N14-p49822860	gene	exon
Bn-scaff_21186_1-p153890	Bn-N14-p49822458	gene	exon
Bn-scaff_21186_1-p220377	Bn-N14-p49731821	gene	
Bn-scaff_21186_1-p220456	Bn-N14-p49731742	gene	exon
Bn-scaff_21186_1-p54820	Bn-N14-p49930428	gene	
Bn-scaff_21188_1-p10781	Bn-N8-p8747641	gene	exon
Bn-scaff_21188_1-p10848	Bn-N8-p8747574	gene	

Bn-scaff_21198_1-p161305	Bn-N4-p13975906	gene	exon
Bn-scaff_21198_1-p220051	Bn-N4-p14007604	gene	exon
Bn-scaff_21198_1-p220428	Bn-N14-p45632314	gene	exon
Bn-scaff_21198_1-p229395	Bn-N14-p45639815	gene	exon
Bn-scaff_21198_1-p231017	Bn-N14-p45640998	gene	
Bn-scaff_21198_1-p233297	Bn-N14-p45643592	gene	exon
Bn-scaff_21198_1-p233432	Bn-N14-p45643728	gene	
Bn-scaff_21198_1-p238719	Bn-N14-p45649019	gene	exon
Bn-scaff_21198_1-p238734	Bn-N14-p45649034	gene	exon
Bn-scaff_21198_1-p24520	Bn-N16-p15179014	gene	
Bn-scaff_21198_1-p34568	Bn-N16-p15166362	gene	
Bn-scaff_21198_1-p64964	Bn-N16-p15129517	gene	exon
Bn-scaff_21224_1-p114755	Bn-N12-p10940245	gene	exon
Bn-scaff_21224_1-p57955	Bn-N12-p10876408	gene	
Bn-scaff_21224_1-p61539	Bn-N12-p10879962	gene	
Bn-scaff_21225_1-p104810	Bn-N11-p35702743	gene	
Bn-scaff_21225_1-p53803	Bn-N1-p21306157	gene	exon
Bn-scaff_21225_1-p74961	Bn-N11-p35731746	gene	
Bn-scaff_21250_1-p212592	Bn-N2-p6658067	gene	
Bn-scaff_21250_1-p410732	Bn-N8-p8618284	gene	
Bn-scaff_21250_1-p452389	Bn-N18-p15474566	gene	
Bn-scaff_21261_1-p117005	Bn-N13-p31843078	gene	exon
Bn-scaff_21261_1-p98385	Bn-N12-p8470672	gene	exon
Bn-scaff_21268_1-p121804	Bn-N17-p27593979	gene	exon
Bn-scaff_21268_1-p122084	Bn-N17-p27593699	gene	exon
Bn-scaff_21268_1-p193486	Bn-N6-p24887152	gene	exon
Bn-scaff_21269_1-p118949	Bn-N9-p38917939	gene	
Bn-scaff_21269_1-p120803	Bn-N9-p38919535	gene	
Bn-scaff_21269_1-p121333	Bn-N18-p42958167	gene	exon
Bn-scaff_21269_1-p121394	Bn-N18-p42958228	gene	exon
Bn-scaff_21269_1-p123559	Bn-N18-p42960383	gene	
Bn-scaff_21269_1-p140454	Bn-N18-p42977027	gene	
Bn-scaff_21269_1-p143806	Bn-N18-p42980380	gene	
Bn-scaff_21269_1-p204828	Bn-N18-p43035968	gene	
Bn-scaff_21269_1-p285510	Bn-N9-p39033855	gene	exon
Bn-scaff_21269_1-p295000	Bn-N18-p43104061	gene	exon
Bn-scaff_21269_1-p295012	Bn-N18-p43104072	gene	exon
Bn-scaff_21269_1-p295511	Bn-N9-p39045207	gene	exon
Bn-scaff_21269_1-p306028	Bn-N18-p43114848	gene	exon
Bn-scaff_21269_1-p309569	Bn-N18-p43118388	gene	
Bn-scaff_21269_1-p311160	Bn-N18-p43120013	gene	
Bn-scaff_21269_1-p313587	Bn-N18-p43123724	gene	
Bn-scaff_21269_1-p355848	Bn-N9-p39140227	gene	exon
Bn-scaff_21269_1-p83142	Bn-N18-p42923160	gene	exon
Bn-scaff_21269_1-p83280	Bn-N18-p42923298	gene	exon
Bn-scaff_21271_1-p17516	Bn-N8-p9790938	gene	exon
Bn-scaff_21276_1-p302391	Bn-N19-p42708708	gene	exon
Bn-scaff_21276_1-p303670	Bn-N19-p42709986	gene	exon
Bn-scaff_21276_1-p378246	Bn-N19-p42792757	gene	exon
Bn-scaff_21276_1-p574134	Bn-N10-p9841852	gene	
Bn-scaff_21312_1-p102482	Bn-N13-p11712804	gene	
Bn-scaff_21312_1-p1111796	Bn-N3-p7744963	gene	

Bn-scaff_21312_1-p1156786	Bn-N3-p7706124	gene	exon
Bn-scaff_21312_1-p1206659	Bn-N13-p10664194	gene	exon
Bn-scaff_21312_1-p1341908	Bn-N3-p7567467	gene	exon
Bn-scaff_21312_1-p1343069	Bn-N3-p7566310	gene	
Bn-scaff_21312_1-p321858	Bn-N3-p8201584	gene	exon
Bn-scaff_21312_1-p323300	Bn-N3-p8199447	gene	exon
Bn-scaff_21312_1-p538444	Bn-N13-p11345362	gene	
Bn-scaff_21312_1-p546514	Bn-N13-p11337265	gene	
Bn-scaff_21312_1-p662797	Bn-N3-p8068851	gene	exon
Bn-scaff_21312_1-p694502	Bn-N13-p11190096	gene	exon
Bn-scaff_21312_1-p895326	Bn-N13-p11000650	gene	
Bn-scaff_21312_1-p90644	Bn-N3-p8359139	gene	exon
Bn-scaff_21312_1-p932709	Bn-N3-p7886666	gene	
Bn-scaff_21338_1-p1040635	Bn-N15-p13723909	gene	exon
Bn-scaff_21338_1-p1135741	Bn-N15-p13826354	gene	
Bn-scaff_21338_1-p1153932	Bn-N15-p13833175	gene	
Bn-scaff_21338_1-p1154261	Bn-N15-p13833504	gene	exon
Bn-scaff_21338_1-p1193458	Bn-N15-p13863790	gene	exon
Bn-scaff_21338_1-p1193513	Bn-N15-p13863845	gene	exon
Bn-scaff_21338_1-p1193631	Bn-N15-p13863925	gene	exon
Bn-scaff_21338_1-p1197350	Bn-N15-p13876547	gene	exon
Bn-scaff_21338_1-p1197474	Bn-N15-p13876671	gene	
Bn-scaff_21338_1-p1197568	Bn-N15-p13876765	gene	exon
Bn-scaff_21338_1-p1205110	Bn-N15-p13885042	gene	
Bn-scaff_21338_1-p151286	Bn-N15-p12739234	gene	
Bn-scaff_21338_1-p18825	Bn-N15-p12605534	gene	
Bn-scaff_21338_1-p215493	Bn-N15-p12808370	gene	exon
Bn-scaff_21338_1-p22459	Bn-N15-p12609502	gene	
Bn-scaff_21338_1-p22879	Bn-N15-p12609922	gene	
Bn-scaff_21338_1-p340741	Bn-N18-p31064837	gene	exon
Bn-scaff_21338_1-p359394	Bn-N9-p27254675	gene	exon
Bn-scaff_21338_1-p359709	Bn-N9-p27252711	gene	
Bn-scaff_21338_1-p363564	Bn-N15-p12967600	gene	exon
Bn-scaff_21338_1-p513997	Bn-N15-p13119574	gene	
Bn-scaff_21338_1-p514119	Bn-N15-p13119696	gene	
Bn-scaff_21338_1-p514135	Bn-N15-p13119712	gene	
Bn-scaff_21338_1-p514411	Bn-N15-p13119991	gene	
Bn-scaff_21338_1-p514512	Bn-N15-p13120078	gene	
Bn-scaff_21338_1-p567819	Bn-N15-p13204443	gene	
Bn-scaff_21338_1-p607624	Bn-N15-p13267341	gene	exon
Bn-scaff_21338_1-p669221	Bn-N15-p13339354	gene	exon
Bn-scaff_21338_1-p687350	Bn-N15-p13358719	gene	
Bn-scaff_21338_1-p760251	Bn-N15-p13420022	gene	
Bn-scaff_21338_1-p782134	Bn-N15-p13441585	gene	
Bn-scaff_21338_1-p832818	Bn-N15-p13496909	gene	
Bn-scaff_21338_1-p832899	Bn-N15-p13496990	gene	exon
Bn-scaff_21338_1-p921370	Bn-N15-p13603528	gene	
Bn-scaff_21338_1-p972059	Bn-N9-p26824762	gene	exon
Bn-scaff_21352_1-p110960	Bn-N16-p16206277	gene	
Bn-scaff_21352_1-p170063	Bn-N1-p13118186	gene	
Bn-scaff_21369_1-p1167373	Bn-N15-p42426081	gene	exon
Bn-scaff_21369_1-p1193882	Bn-N15-p42399398	gene	

Bn-scaff_21369_1-p1211715	Bn-N15-p42382656	gene	
Bn-scaff_21369_1-p1251615	Bn-N15-p42330001	gene	exon
Bn-scaff_21369_1-p190637	Bn-N15-p43392984	gene	
Bn-scaff_21369_1-p204692	Bn-N15-p43379026	gene	
Bn-scaff_21369_1-p205401	Bn-N15-p43378316	gene	exon
Bn-scaff_21369_1-p239290	Bn-N5-p22246291	gene	exon
Bn-scaff_21369_1-p260784	Bn-N15-p43322235	gene	exon
Bn-scaff_21369_1-p406725	Bn-N15-p43177706	gene	
Bn-scaff_21369_1-p456007	Bn-N15-p43126388	gene	
Bn-scaff_21369_1-p522214	Bn-N15-p43061512	gene	exon
Bn-scaff_21369_1-p628956	Bn-N15-p42952898	gene	exon
Bn-scaff_21369_1-p629372	Bn-N15-p42952482	gene	exon
Bn-scaff_21369_1-p653589	Bn-N5-p21908814	gene	exon
Bn-scaff_21369_1-p91227	Bn-N15-p43486391	gene	exon
Bn-scaff_21386_1-p35560	Bn-N13-p58900103	gene	exon
Bn-scaff_21386_1-p35636	Bn-N13-p58900027	gene	
Bn-scaff_21386_1-p35701	Bn-N13-p58899963	gene	
Bn-scaff_21386_1-p35918	Bn-N13-p58899746	gene	exon
Bn-scaff_21386_1-p36155	Bn-N13-p58899509	gene	exon
Bn-scaff_21386_1-p36891	Bn-N13-p58892102	gene	
Bn-scaff_21386_1-p37139	Bn-N13-p58891853	gene	
Bn-scaff_21386_1-p39383	Bn-N13-p58889429	gene	exon
Bn-scaff_21392_1-p175	Bn-N14-p50635357	gene	
Bn-scaff_21450_1-p68078	Bn-N17-p2783666	gene	exon
Bn-scaff_21450_1-p68242	Bn-N17-p2783830	gene	
Bn-scaff_21450_1-p77567	Bn-N7-p1171706	gene	exon
Bn-scaff_21453_1-p128447	Bn-N12-p13978112	gene	
Bn-scaff_21453_1-p130266	Bn-N12-p13979931	gene	
Bn-scaff_21453_1-p130962	Bn-N12-p13980627	gene	
Bn-scaff_21496_1-p140175	Bn-N15-p4919043	gene	exon
Bn-scaff_21496_1-p165566	Bn-N15-p4940949	gene	
Bn-scaff_21496_1-p185845	Bn-N15-p4962009	gene	exon
Bn-scaff_21496_1-p210320	Bn-N15-p4986125	gene	exon
Bn-scaff_21496_1-p320793	Bn-N15-p5078391	gene	
Bn-scaff_21496_1-p353654	Bn-N15-p5111592	gene	
Bn-scaff_21496_1-p359451	Bn-N15-p5116915	gene	exon
Bn-scaff_21496_1-p382380	Bn-N6-p4250338	gene	exon
Bn-scaff_21496_1-p419937	Bn-N15-p5173543	gene	exon
Bn-scaff_21496_1-p440091	Bn-N15-p5195547	gene	
Bn-scaff_21496_1-p440186	Bn-N15-p5195642	gene	
Bn-scaff_21496_1-p443487	Bn-N15-p5198529	gene	exon
Bn-scaff_21496_1-p444256	Bn-N15-p5201367	gene	
Bn-scaff_21496_1-p457336	Bn-N15-p5212596	gene	exon
Bn-scaff_21496_1-p91737	Bn-N15-p4849676	gene	exon
Bn-scaff_21542_1-p12789	Bn-N5-p11332136	gene	exon
Bn-scaff_21566_1-p109	Bn-N14-p50050491	gene	
Bn-scaff_21566_1-p181	Bn-N14-p50050419	gene	exon
Bn-scaff_21634_1-p62623	Bn-N15-p47853022	gene	exon
Bn-scaff_21636_1-p118433	Bn-N10-p17411781	gene	exon
Bn-scaff_21636_1-p118603	Bn-N10-p17411951	gene	exon
Bn-scaff_21636_1-p119179	Bn-N10-p17412523	gene	exon
Bn-scaff_21636_1-p124865	Bn-N10-p17418379	gene	exon

Bn-scaff_21636_1-p137260	Bn-N10-p17428951	gene	exon
Bn-scaff_21636_1-p137344	Bn-N10-p17429035	gene	exon
Bn-scaff_21636_1-p137408	Bn-N10-p17429101	gene	
Bn-scaff_21636_1-p137494	Bn-N10-p17429187	gene	exon
Bn-scaff_21636_1-p137951	Bn-N10-p17429638	gene	exon
Bn-scaff_21636_1-p137966	Bn-N10-p17429653	gene	exon
Bn-scaff_21636_1-p138951	Bn-N10-p17430622	gene	exon
Bn-scaff_21636_1-p15905	Bn-N10-p17313543	gene	
Bn-scaff_21636_1-p15908	Bn-N10-p17313546	gene	
Bn-scaff_21636_1-p226446	Bn-N10-p17483242	gene	exon
Bn-scaff_21636_1-p227057	Bn-N10-p17483861	gene	exon
Bn-scaff_21636_1-p238954	Bn-N10-p17493155	gene	exon
Bn-scaff_21636_1-p243640	Bn-N10-p17490863	gene	exon
Bn-scaff_21636_1-p278203	Bn-N10-p17522789	gene	exon
Bn-scaff_21636_1-p497038	Bn-Scaffold02659-p1681	gene	exon
Bn-scaff_21636_1-p7184	Bn-N5-p17840634	gene	exon
Bn-scaff_21656_1-p109389	Bn-N19-p11693818	gene	
Bn-scaff_21656_1-p312658	Bn-N19-p11475691	gene	exon
Bn-scaff_21705_1-p119297	Bn-N12-p34729436	gene	exon
Bn-scaff_21705_1-p119663	Bn-N12-p34729071	gene	exon
Bn-scaff_21705_1-p278164	Bn-N12-p34574580	gene	exon
Bn-scaff_21705_1-p289975	Bn-N12-p34554205	gene	exon
Bn-scaff_21705_1-p313590	Bn-N12-p34533998	gene	
Bn-scaff_21705_1-p315230	Bn-N12-p34532358	gene	
Bn-scaff_21705_1-p366615	Bn-N12-p34480702	gene	
Bn-scaff_21705_1-p470585	Bn-N12-p34378370	gene	
Bn-scaff_21705_1-p472001	Bn-N12-p34376948	gene	
Bn-scaff_21705_1-p488897	Bn-N12-p34361505	gene	exon
Bn-scaff_21705_1-p490306	Bn-N12-p34360097	gene	
Bn-scaff_21705_1-p497937	Bn-N12-p34352466	gene	
Bn-scaff_21705_1-p510001	Bn-N12-p34340413	gene	
Bn-scaff_21705_1-p51863	Bn-N12-p34788733	gene	
Bn-scaff_21705_1-p559758	Bn-N12-p34289632	gene	exon
Bn-scaff_21705_1-p608640	Bn-N12-p34242791	gene	exon
Bn-scaff_21705_1-p608952	Bn-N12-p34242479	gene	
Bn-scaff_21705_1-p609482	Bn-N12-p34241949	gene	
Bn-scaff_21705_1-p609934	Bn-N12-p34241497	gene	
Bn-scaff_21705_1-p609955	Bn-N12-p34241476	gene	
Bn-scaff_21705_1-p612632	Bn-N12-p34238796	gene	exon
Bn-scaff_21711_1-p12893	Bn-N17-p34583222	gene	
Bn-scaff_21711_1-p13060	Bn-N17-p34583055	gene	
Bn-scaff_21711_1-p18343	Bn-N17-p34577778	gene	exon
Bn-scaff_21711_1-p28431	Bn-N17-p34562507	gene	exon
Bn-scaff_21711_1-p34999	Bn-N17-p34556010	gene	
Bn-scaff_21711_1-p35589	Bn-N17-p34555421	gene	
Bn-scaff_21711_1-p39478	Bn-N17-p34551543	gene	
Bn-scaff_21711_1-p54607	Bn-N17-p34535913	gene	exon
Bn-scaff_21711_1-p75853	Bn-N6-p21595011	gene	
Bn-scaff_21711_1-p76752	Bn-N6-p21595897	gene	exon
Bn-scaff_21711_1-p79864	Bn-N17-p34516742	gene	exon
Bn-scaff_21744_1-p17282	Bn-N16-p1045794	gene	
Bn-scaff_21744_1-p25410	Bn-N14-p49822707	gene	

Bn-scaff_21778_1-p160881	Bn-N13-p5936201	gene	exon
Bn-scaff_21778_1-p163428	Bn-N13-p5938734	gene	
Bn-scaff_21778_1-p177121	Bn-N13-p5950883	gene	
Bn-scaff_21778_1-p184325	Bn-N13-p5958068	gene	exon
Bn-scaff_21778_1-p215559	Bn-N13-p5984689	gene	exon
Bn-scaff_21778_1-p217638	Bn-N13-p5986297	gene	exon
Bn-scaff_21778_1-p222165	Bn-N13-p5990681	gene	exon
Bn-scaff_21778_1-p222672	Bn-N13-p5991187	gene	
Bn-scaff_21778_1-p222993	Bn-N13-p5991507	gene	
Bn-scaff_21778_1-p223740	Bn-N13-p5992238	gene	
Bn-scaff_21778_1-p236024	Bn-N13-p31906538	gene	exon
Bn-scaff_21778_1-p236913	Bn-N13-p6002364	gene	exon
Bn-scaff_21778_1-p261833	Bn-N13-p6035825	gene	exon
Bn-scaff_21778_1-p262139	Bn-N13-p6036131	gene	exon
Bn-scaff_21778_1-p287068	Bn-N13-p6060543	gene	
Bn-scaff_21778_1-p287475	Bn-N13-p6060949	gene	
Bn-scaff_21778_1-p290583	Bn-N13-p6064076	gene	
Bn-scaff_21778_1-p312180	Bn-N13-p6079614	gene	
Bn-scaff_21778_1-p319366	Bn-N13-p6087379	gene	
Bn-scaff_21778_1-p364556	Bn-N13-p6133554	gene	
Bn-scaff_21778_1-p382522	Bn-N13-p6145467	gene	
Bn-scaff_21778_1-p43125	Bn-N13-p5780067	gene	
Bn-scaff_21778_1-p68265	Bn-N3-p4145513	gene	exon
Bn-scaff_21778_1-p80189	Bn-N13-p5828251	gene	
Bn-scaff_21778_1-p8688	Bn-N13-p5746094	gene	
Bn-scaff_21786_1-p127359	Bn-N18-p22441567	gene	
Bn-scaff_21786_1-p127974	Bn-N18-p22442183	gene	exon
Bn-scaff_21786_1-p128237	Bn-N18-p22442446	gene	
Bn-scaff_21786_1-p128461	Bn-N18-p22442670	gene	exon
Bn-scaff_21786_1-p128485	Bn-N18-p22442694	gene	exon
Bn-scaff_21786_1-p141890	Bn-N18-p22455980	gene	
Bn-scaff_21786_1-p149338	Bn-N18-p22462785	gene	
Bn-scaff_21786_1-p158103	Bn-N18-p22471550	gene	
Bn-scaff_21786_1-p160470	Bn-N18-p22473927	gene	
Bn-scaff_21786_1-p52446	Bn-N8-p19928494	gene	
Bn-scaff_21786_1-p52917	Bn-N8-p19928029	gene	
Bn-scaff_21820_1-p1028375	Bn-N11-p27840168	gene	
Bn-scaff_21820_1-p128894	Bn-N11-p26905119	gene	
Bn-scaff_21820_1-p129153	Bn-N11-p26904860	gene	exon
Bn-scaff_21820_1-p137002	Bn-N11-p26896866	gene	exon
Bn-scaff_21820_1-p137033	Bn-N11-p26896835	gene	exon
Bn-scaff_21820_1-p138221	Bn-N11-p26895647	gene	exon
Bn-scaff_21820_1-p143886	Bn-N1-p16690746	gene	exon
Bn-scaff_21820_1-p144148	Bn-N1-p16690463	gene	
Bn-scaff_21820_1-p169248	Bn-N1-p16639456	gene	exon
Bn-scaff_21820_1-p169785	Bn-N11-p26861253	gene	exon
Bn-scaff_21820_1-p169937	Bn-N11-p26861101	gene	
Bn-scaff_21820_1-p170850	Bn-N11-p26860189	gene	exon
Bn-scaff_21820_1-p173814	Bn-N11-p26857219	gene	
Bn-scaff_21820_1-p174491	Bn-N11-p26856541	gene	exon
Bn-scaff_21820_1-p209155	Bn-N11-p26829885	gene	exon
Bn-scaff_21820_1-p251742	Bn-N11-p26781422	gene	

Bn-scaff_21820_1-p270823	Bn-N1-p16546952	gene	exon
Bn-scaff_21820_1-p279193	Bn-N7-p21285755	gene	exon
Bn-scaff_21820_1-p279894	Bn-N11-p26754787	gene	exon
Bn-scaff_21820_1-p280779	Bn-N11-p26753900	gene	exon
Bn-scaff_21820_1-p323086	Bn-N11-p26707103	gene	exon
Bn-scaff_21820_1-p323485	Bn-N11-p26706709	gene	
Bn-scaff_21820_1-p336649	Bn-N11-p26695904	gene	exon
Bn-scaff_21820_1-p336987	Bn-N11-p26695566	gene	
Bn-scaff_21820_1-p338771	Bn-N11-p26693786	gene	
Bn-scaff_21820_1-p368142	Bn-N11-p27265013	gene	exon
Bn-scaff_21820_1-p42624	Bn-N11-p26968180	gene	
Bn-scaff_21820_1-p42746	Bn-N11-p26968058	gene	
Bn-scaff_21820_1-p490500	Bn-N11-p27371778	gene	
Bn-scaff_21820_1-p533341	Bn-N11-p27414833	gene	exon
Bn-scaff_21820_1-p543774	Bn-N11-p27432200	gene	
Bn-scaff_21820_1-p545415	Bn-N11-p27433853	gene	
Bn-scaff_21820_1-p611224	Bn-N1-p16115656	gene	
Bn-scaff_21820_1-p737794	Bn-N14-p21563058	gene	
Bn-scaff_21820_1-p956169	Bn-N11-p27759629	gene	
Bn-scaff_21820_1-p965485	Bn-N11-p27772917	gene	
Bn-scaff_21821_1-p75538	Bn-N15-p12400714	gene	
Bn-scaff_21841_1-p188116	Bn-N19-p49966534	gene	
Bn-scaff_21841_1-p230382	Bn-N19-p50003512	gene	exon
Bn-scaff_21841_1-p26836	Bn-N19-p49823533	gene	
Bn-scaff_21841_1-p297252	Bn-N10-p13396013	gene	exon
Bn-scaff_21841_1-p304850	Bn-N19-p50058248	gene	
Bn-scaff_21841_1-p329475	Bn-N19-p50090437	gene	exon
Bn-scaff_21841_1-p9955	Bn-N19-p49806198	gene	exon
Bn-scaff_21857_1-p293375	Bn-N11-p37440199	gene	
Bn-scaff_21857_1-p298054	Bn-N11-p37444866	gene	
Bn-scaff_21861_1-p146849	Bn-N2-p11905378	gene	exon
Bn-scaff_21861_1-p17903	Bn-Scaffold01330-p8167	gene	exon
Bn-scaff_21861_1-p20145	Bn-Scaffold01330-p10090	gene	exon
Bn-scaff_21861_1-p33827	Bn-Scaffold01376-p3913	gene	
Bn-scaff_21861_1-p33892	Bn-Scaffold01376-p3978	gene	
Bn-scaff_21861_1-p34223	Bn-Scaffold01376-p4309	gene	
Bn-scaff_21861_1-p37272	Bn-Scaffold01376-p7358	gene	exon
Bn-scaff_21861_1-p64738	Bn-N14-p2289026	gene	
Bn-scaff_21867_1-p69185	Bn-N13-p51241578	gene	exon
Bn-scaff_21867_1-p809384	Bn-N6-p10598107	gene	
Bn-scaff_21867_1-p872656	Bn-N6-p10615520	gene	
Bn-scaff_21867_1-p87764	Bn-N13-p51208823	gene	exon
Bn-scaff_21867_1-p879485	Bn-N6-p10624134	gene	
Bn-scaff_21884_1-p125365	Bn-N11-p40943706	gene	exon
Bn-scaff_21884_1-p153891	Bn-N11-p40969873	gene	exon
Bn-scaff_21884_1-p154442	Bn-N11-p40970424	gene	exon
Bn-scaff_21884_1-p154748	Bn-N11-p40970730	gene	exon
Bn-scaff_21884_1-p192677	Bn-N11-p41016725	gene	exon
Bn-scaff_21884_1-p219863	Bn-N11-p41039106	gene	
Bn-scaff_21884_1-p227529	Bn-N11-p41046800	gene	
Bn-scaff_21884_1-p28771	Bn-N11-p40846775	gene	exon
Bn-scaff_21884_1-p60083	Bn-N11-p40879023	gene	

Bn-scaff_21884_1-p638903	Bn-N11-p41575505	gene	
Bn-scaff_21884_1-p639289	Bn-N11-p41575868	gene	
Bn-scaff_21884_1-p650617	Bn-N11-p41583160	gene	
Bn-scaff_21884_1-p651359	Bn-N11-p41583903	gene	
Bn-scaff_21884_1-p664163	Bn-N1-p24563000	gene	exon
Bn-scaff_21884_1-p684281	Bn-N14-p3355676	gene	exon
Bn-scaff_21884_1-p720411	Bn-N1-p24602074	gene	exon
Bn-scaff_21884_1-p767376	Bn-N11-p41693934	gene	exon
Bn-scaff_21884_1-p768055	Bn-N1-p24640108	gene	exon
Bn-scaff_21884_1-p807260	Bn-N1-p24691988	gene	exon
Bn-scaff_21884_1-p819724	Bn-N11-p41778226	gene	
Bn-scaff_21884_1-p819958	Bn-N11-p41778460	gene	exon
Bn-scaff_21884_1-p819972	Bn-N11-p41778474	gene	exon
Bn-scaff_21884_1-p866739	Bn-N1-p24734752	gene	exon
Bn-scaff_21884_1-p914072	Bn-N6-p9808308	gene	
Bn-scaff_21884_1-p914724	Bn-N6-p9807656	gene	
Bn-scaff_21924_1-p88938	Bn-N10-p10493335	gene	exon
Bn-scaff_21925_1-p154909	Bn-N14-p2077247	gene	exon
Bn-scaff_21925_1-p200155	Bn-N14-p2126261	gene	exon
Bn-scaff_21925_1-p201015	Bn-N14-p2127122	gene	exon
Bn-scaff_21925_1-p55427	Bn-N14-p1975434	gene	
Bn-scaff_21925_1-p61702	Bn-N12-p46506765	gene	
Bn-scaff_21925_1-p88165	Bn-N14-p2008611	gene	
Bn-scaff_21956_1-p154372	Bn-N14-p45398869	gene	exon
Bn-scaff_21956_1-p160710	Bn-N14-p45405207	gene	exon
Bn-scaff_21956_1-p218752	Bn-N14-p45461565	gene	exon
Bn-scaff_21956_1-p219268	Bn-N14-p45462081	gene	exon
Bn-scaff_21956_1-p220255	Bn-N14-p45463066	gene	exon
Bn-scaff_21956_1-p253961	Bn-N14-p45500201	gene	
Bn-scaff_21956_1-p257039	Bn-N14-p45503329	gene	exon
Bn-scaff_21956_1-p262178	Bn-N14-p45514141	gene	
Bn-scaff_21956_1-p276706	Bn-N14-p45526701	gene	exon
Bn-scaff_21956_1-p289981	Bn-N14-p45540093	gene	
Bn-scaff_21956_1-p291369	Bn-N14-p45541481	gene	exon
Bn-scaff_21956_1-p50505	Bn-N14-p45255554	gene	exon
Bn-scaff_21956_1-p51220	Bn-N14-p45256273	gene	
Bn-scaff_21956_1-p95133	Bn-N4-p13781466	gene	exon
Bn-scaff_22002_1-p78733	Bn-N8-p4619176	gene	exon
Bn-scaff_22067_1-p139587	Bn-N13-p23770785	gene	
Bn-scaff_22067_1-p144570	Bn-N13-p23775118	gene	
Bn-scaff_22067_1-p149886	Bn-N13-p23780562	gene	exon
Bn-scaff_22067_1-p190904	Bn-N13-p23827143	gene	exon
Bn-scaff_22067_1-p200128	Bn-N13-p23836470	gene	
Bn-scaff_22067_1-p219321	Bn-N13-p23856396	gene	
Bn-scaff_22067_1-p225959	Bn-N11-p44285016	gene	exon
Bn-scaff_22067_1-p226242	Bn-N3-p15359516	gene	exon
Bn-scaff_22067_1-p70907	Bn-N13-p23680599	gene	
Bn-scaff_22067_1-p71973	Bn-N3-p15235704	gene	exon
Bn-scaff_22082_1-p115581	Bn-N19-p51827918	gene	exon
Bn-scaff_22082_1-p259621	Bn-N19-p51669549	gene	exon
Bn-scaff_22082_1-p26759	Bn-N19-p51926614	gene	exon
Bn-scaff_22082_1-p272003	Bn-N19-p51662834	gene	exon

Bn-scaff_22082_1-p272234	Bn-N19-p51662603	gene	exon
Bn-scaff_22082_1-p28114	Bn-N19-p51925259	gene	
Bn-scaff_22082_1-p301782	Bn-N19-p51634525	gene	exon
Bn-scaff_22082_1-p350	Bn-N15-p46069509	gene	exon
Bn-scaff_22082_1-p370189	Bn-N19-p51554161	gene	exon
Bn-scaff_22082_1-p372624	Bn-N19-p51551724	gene	exon
Bn-scaff_22082_1-p372841	Bn-N19-p51551508	gene	
Bn-scaff_22082_1-p373269	Bn-N19-p51551079	gene	
Bn-scaff_22082_1-p381196	Bn-N19-p51542741	gene	
Bn-scaff_22082_1-p381247	Bn-N19-p51542690	gene	
Bn-scaff_22082_1-p381255	Bn-N19-p51542682	gene	
Bn-scaff_22082_1-p383237	Bn-N19-p51540700	gene	exon
Bn-scaff_22082_1-p439197	Bn-N19-p51469262	gene	exon
Bn-scaff_22082_1-p444239	Bn-N19-p51464186	gene	
Bn-scaff_22082_1-p906323	Bn-N19-p50941833	gene	exon
Bn-scaff_22091_1-p59803	Bn-N13-p29679837	gene	
Bn-scaff_22091_1-p90799	Bn-N4-p15441018	gene	exon
Bn-scaff_22093_1-p101912	Bn-N12-p19770336	gene	exon
Bn-scaff_22093_1-p102888	Bn-N12-p19769360	gene	
Bn-scaff_22093_1-p103074	Bn-N12-p19769174	gene	
Bn-scaff_22093_1-p103093	Bn-N12-p19769155	gene	
Bn-scaff_22093_1-p103725	Bn-N12-p19768524	gene	
Bn-scaff_22093_1-p103799	Bn-N12-p19768450	gene	
Bn-scaff_22093_1-p53055	Bn-N12-p19948115	gene	exon
Bn-scaff_22093_1-p97166	Bn-N12-p19777735	gene	exon
Bn-scaff_22115_1-p120619	Bn-N11-p39522195	gene	exon
Bn-scaff_22115_1-p155376	Bn-N11-p39567545	gene	exon
Bn-scaff_22115_1-p182128	Bn-N11-p39601813	gene	
Bn-scaff_22115_1-p236953	Bn-N11-p39665719	gene	
Bn-scaff_22115_1-p238903	Bn-N1-p23629342	gene	exon
Bn-scaff_22115_1-p82010	Bn-N1-p23546207	gene	exon
Bn-scaff_22133_1-p31674	Bn-N16-p14927161	gene	exon
Bn-scaff_22133_1-p31920	Bn-N16-p14926915	gene	exon
Bn-scaff_22144_1-p193415	Bn-N12-p40235382	gene	
Bn-scaff_22144_1-p207843	Bn-N12-p40241593	gene	
Bn-scaff_22148_1-p251521	Bn-N14-p33416438	gene	
Bn-scaff_22148_1-p383901	Bn-N14-p33589085	gene	
Bn-scaff_22148_1-p5629	Bn-N14-p33177167	gene	
Bn-scaff_22183_1-p40909	Bn-N15-p10245234	gene	exon
Bn-scaff_22183_1-p41540	Bn-N15-p10244602	gene	exon
Bn-scaff_22183_1-p422126	Bn-N15-p9841752	gene	exon
Bn-scaff_22183_1-p61318	Bn-N15-p10225427	gene	
Bn-scaff_22183_1-p663781	Bn-N15-p9583241	gene	
Bn-scaff_22183_1-p670535	Bn-N15-p9570805	gene	
Bn-scaff_22183_1-p672707	Bn-N15-p9568632	gene	
Bn-scaff_22278_1-p136701	Bn-N4-p14211003	gene	exon
Bn-scaff_22278_1-p137755	Bn-N4-p14210018	gene	exon
Bn-scaff_22278_1-p137816	Bn-N4-p14209958	gene	exon
Bn-scaff_22278_1-p238329	Bn-N4-p14162714	gene	exon
Bn-scaff_22278_1-p304818	Bn-N14-p45747235	gene	exon
Bn-scaff_22278_1-p84630	Bn-N4-p14246038	gene	
Bn-scaff_22278_1-p95624	Bn-N17-p39892877	gene	

Bn-scaff_22278_1-p95949	Bn-N17-p39893202	gene	
Bn-scaff_22278_1-p96314	Bn-N17-p39893567	gene	
Bn-scaff_22301_1-p104455	Bn-N17-p7210457	gene	
Bn-scaff_22301_1-p105957	Bn-N17-p7208955	gene	
Bn-scaff_22301_1-p7514	Bn-N17-p7316254	gene	
Bn-scaff_22301_1-p84996	Bn-N17-p7230640	gene	
Bn-scaff_22310_1-p321188	Bn-N17-p8350002	gene	
Bn-scaff_22310_1-p329937	Bn-N7-p3705628	gene	exon
Bn-scaff_22310_1-p359751	Bn-N17-p8312132	gene	
Bn-scaff_22310_1-p359992	Bn-N17-p8311891	gene	
Bn-scaff_22310_1-p424210	Bn-N17-p8235998	gene	exon
Bn-scaff_22310_1-p436477	Bn-N17-p8222346	gene	exon
Bn-scaff_22310_1-p470575	Bn-N17-p8187090	gene	exon
Bn-scaff_22310_1-p557368	Bn-N7-p3556955	gene	exon
Bn-scaff_22310_1-p563505	Bn-N17-p8094208	gene	exon
Bn-scaff_22310_1-p568057	Bn-N17-p8089654	gene	exon
Bn-scaff_22310_1-p589151	Bn-N17-p8065857	gene	
Bn-scaff_22310_1-p653167	Bn-N17-p8000987	gene	exon
Bn-scaff_22310_1-p723646	Bn-N17-p7942321	gene	exon
Bn-scaff_22310_1-p779359	Bn-N17-p7884080	gene	
Bn-scaff_22310_1-p826319	Bn-N7-p3328802	gene	
Bn-scaff_22310_1-p835255	Bn-N17-p7820493	gene	
Bn-scaff_22310_1-p851873	Bn-N17-p7798171	gene	exon
Bn-scaff_22310_1-p852906	Bn-N17-p7797137	gene	exon
Bn-scaff_22310_1-p863593	Bn-N7-p3298796	gene	exon
Bn-scaff_22310_1-p895435	Bn-N17-p7755910	gene	exon
Bn-scaff_22321_1-p13103	Bn-N1-p26143514	gene	
Bn-scaff_22321_1-p247241	Bn-N11-p43675538	gene	exon
Bn-scaff_22321_1-p266340	Bn-N11-p43656394	gene	exon
Bn-scaff_22321_1-p45741	Bn-N11-p43861680	gene	
Bn-scaff_22395_1-p700	Bn-N12-p813067	gene	
Bn-scaff_22441_1-p108306	Bn-N17-p9075163	gene	
Bn-scaff_22441_1-p367333	Bn-N17-p8831048	gene	
Bn-scaff_22441_1-p381776	Bn-N17-p8822313	gene	
Bn-scaff_22441_1-p381973	Bn-N17-p8822116	gene	
Bn-scaff_22441_1-p382588	Bn-N17-p8821736	gene	
Bn-scaff_22451_1-p134565	Bn-N14-p26945363	gene	
Bn-scaff_22451_1-p193809	Bn-N12-p22524708	gene	
Bn-scaff_22451_2-p83262	Bn-N12-p22879782	gene	exon
Bn-scaff_22451_2-p85639	Bn-N12-p22882177	gene	
Bn-scaff_22451_2-p87375	Bn-N12-p22883992	gene	
Bn-scaff_22455_1-p12317	Bn-N11-p8389234	gene	exon
Bn-scaff_22466_1-p1427346	Bn-N3-p11816633	gene	
Bn-scaff_22466_1-p170754	Bn-N13-p16147925	gene	
Bn-scaff_22466_1-p182130	Bn-N13-p16159309	gene	exon
Bn-scaff_22466_1-p209190	Bn-N13-p16188929	gene	
Bn-scaff_22466_1-p209191	Bn-N13-p16188929	gene	
Bn-scaff_22466_1-p286093	Bn-N13-p16262071	gene	
Bn-scaff_22466_1-p381085	Bn-N13-p16365010	gene	
Bn-scaff_22466_1-p394461	Bn-N13-p16378372	gene	
Bn-scaff_22466_1-p618347	Bn-N3-p11447194	gene	
Bn-scaff_22466_1-p624476	Bn-N3-p11453373	gene	exon

Bn-scaff_22466_1-p667128	Bn-N13-p16667983	gene	exon
Bn-scaff_22466_1-p733665	Bn-N13-p16748300	gene	exon
Bn-scaff_22466_1-p746149	Bn-N13-p16752792	gene	exon
Bn-scaff_22466_1-p754489	Bn-N13-p16761369	gene	
Bn-scaff_22466_1-p803725	Bn-N13-p16811982	gene	exon
Bn-scaff_22466_1-p809447	Bn-N3-p11555917	gene	exon
Bn-scaff_22466_1-p847646	Bn-N3-p11581684	gene	exon
Bn-scaff_22466_1-p980721	Bn-N3-p11640806	gene	exon
Bn-scaff_22466_1-p986888	Bn-N13-p17003166	gene	exon
Bn-scaff_22466_1-p987521	Bn-N13-p17003799	gene	exon
Bn-scaff_22481_1-p143945	Bn-N19-p55806350	gene	
Bn-scaff_22481_1-p152101	Bn-N19-p55797728	gene	exon
Bn-scaff_22481_1-p167831	Bn-N19-p55782005	gene	
Bn-scaff_22481_1-p176508	Bn-N19-p55774267	gene	
Bn-scaff_22481_1-p177318	Bn-N19-p55773457	gene	
Bn-scaff_22481_1-p200007	Bn-N19-p55738631	gene	
Bn-scaff_22481_1-p200580	Bn-N19-p55738058	gene	
Bn-scaff_22481_1-p231024	Bn-N19-p55707406	gene	exon
Bn-scaff_22486_1-p7889	Bn-N18-p1570204	gene	exon
Bn-scaff_22527_1-p180660	Bn-N12-p5092817	gene	exon
Bn-scaff_22527_1-p180772	Bn-N2-p3983380	gene	exon
Bn-scaff_22527_1-p183208	Bn-N2-p3980899	gene	
Bn-scaff_22527_1-p338430	Bn-N2-p3880106	gene	exon
Bn-scaff_22527_1-p339764	Bn-N12-p4934944	gene	exon
Bn-scaff_22527_1-p350581	Bn-N2-p3865462	gene	exon
Bn-scaff_22527_1-p350640	Bn-N2-p3865403	gene	exon
Bn-scaff_22527_1-p412841	Bn-N12-p4872231	gene	
Bn-scaff_22527_1-p413147	Bn-N2-p3831363	gene	exon
Bn-scaff_22527_1-p613740	Bn-N19-p51976075	gene	exon
Bn-scaff_22536_1-p164551	Bn-N7-p8965510	gene	exon
Bn-scaff_22536_1-p319313	Bn-N17-p19627529	gene	
Bn-scaff_22536_1-p339132	Bn-N12-p41264836	gene	
Bn-scaff_22536_1-p7771	Bn-N17-p19256845	gene	exon
Bn-scaff_22563_1-p109636	Bn-N11-p22465175	gene	
Bn-scaff_22563_1-p111842	Bn-N11-p22462969	gene	exon
Bn-scaff_22563_1-p111921	Bn-N11-p22462890	gene	exon
Bn-scaff_22670_1-p170074	Bn-N12-p9672971	gene	exon
Bn-scaff_22728_1-p1012244	Bn-N13-p6643610	gene	exon
Bn-scaff_22728_1-p1012688	Bn-N13-p6643167	gene	
Bn-scaff_22728_1-p1013354	Bn-N13-p6642528	gene	exon
Bn-scaff_22728_1-p1018084	Bn-N13-p6638137	gene	
Bn-scaff_22728_1-p1024804	Bn-N13-p6634029	gene	exon
Bn-scaff_22728_1-p1080976	Bn-N13-p6593204	gene	
Bn-scaff_22728_1-p109366	Bn-N13-p7523555	gene	
Bn-scaff_22728_1-p1141898	Bn-N13-p6510945	gene	exon
Bn-scaff_22728_1-p1141914	Bn-N13-p6510928	gene	exon
Bn-scaff_22728_1-p1148454	Bn-N13-p6503417	gene	exon
Bn-scaff_22728_1-p1181721	Bn-N13-p6471383	gene	
Bn-scaff_22728_1-p1185330	Bn-N13-p6467443	gene	
Bn-scaff_22728_1-p1197316	Bn-N13-p6454206	gene	
Bn-scaff_22728_1-p1197524	Bn-N13-p6453998	gene	exon
Bn-scaff_22728_1-p1237743	Bn-N13-p6421333	gene	

Bn-scaff_22728_1-p1248875	Bn-N13-p6410202	gene	
Bn-scaff_22728_1-p1250616	Bn-N13-p6408467	gene	
Bn-scaff_22728_1-p1250818	Bn-N13-p6408264	gene	exon
Bn-scaff_22728_1-p1256248	Bn-N13-p6398173	gene	
Bn-scaff_22728_1-p1256416	Bn-N13-p6398005	gene	
Bn-scaff_22728_1-p1258206	Bn-N13-p6396242	gene	
Bn-scaff_22728_1-p1258510	Bn-N13-p6395938	gene	
Bn-scaff_22728_1-p1258526	Bn-N13-p6395922	gene	
Bn-scaff_22728_1-p1259166	Bn-N13-p6395291	gene	
Bn-scaff_22728_1-p1259217	Bn-N13-p6395241	gene	
Bn-scaff_22728_1-p1261707	Bn-N13-p6392723	gene	
Bn-scaff_22728_1-p1264219	Bn-N13-p6390202	gene	exon
Bn-scaff_22728_1-p1267084	Bn-N13-p6387284	gene	exon
Bn-scaff_22728_1-p1356495	Bn-N2-p4758977	gene	exon
Bn-scaff_22728_1-p1363526	Bn-N13-p6284917	gene	exon
Bn-scaff_22728_1-p1393497	Bn-N13-p6250100	gene	exon
Bn-scaff_22728_1-p1399313	Bn-N13-p6244288	gene	exon
Bn-scaff_22728_1-p263173	Bn-N13-p7371048	gene	exon
Bn-scaff_22728_1-p263319	Bn-N13-p7370901	gene	exon
Bn-scaff_22728_1-p285715	Bn-N13-p7347300	gene	exon
Bn-scaff_22728_1-p310071	Bn-N13-p7316777	gene	
Bn-scaff_22728_1-p344206	Bn-N3-p5184596	gene	exon
Bn-scaff_22728_1-p345946	Bn-N13-p7279510	gene	exon
Bn-scaff_22728_1-p356331	Bn-N13-p7269121	gene	exon
Bn-scaff_22728_1-p360792	Bn-N13-p7264894	gene	exon
Bn-scaff_22728_1-p400151	Bn-N13-p7223981	gene	
Bn-scaff_22728_1-p402134	Bn-N13-p7222251	gene	exon
Bn-scaff_22728_1-p458182	Bn-N13-p7168924	gene	
Bn-scaff_22728_1-p507391	Bn-N13-p7121994	gene	exon
Bn-scaff_22728_1-p513362	Bn-N13-p7116019	gene	exon
Bn-scaff_22728_1-p521385	Bn-N19-p10628505	gene	
Bn-scaff_22728_1-p526439	Bn-N13-p7112873	gene	
Bn-scaff_22728_1-p577795	Bn-N13-p7061079	gene	exon
Bn-scaff_22728_1-p600933	Bn-N13-p7037881	gene	exon
Bn-scaff_22728_1-p646719	Bn-N13-p6986158	gene	
Bn-scaff_22728_1-p737182	Bn-N13-p6898451	gene	exon
Bn-scaff_22728_1-p739263	Bn-N13-p6896425	gene	
Bn-scaff_22728_1-p739547	Bn-N13-p6896141	gene	exon
Bn-scaff_22728_1-p743128	Bn-N13-p6894078	gene	
Bn-scaff_22728_1-p749304	Bn-N13-p6889160	gene	exon
Bn-scaff_22728_1-p922794	Bn-N13-p6732192	gene	
Bn-scaff_22728_1-p924452	Bn-N13-p6730537	gene	
Bn-scaff_22728_1-p938985	Bn-N13-p6694313	gene	
Bn-scaff_22728_1-p967531	Bn-N13-p6670609	gene	
Bn-scaff_22728_1-p968340	Bn-N13-p6669797	gene	exon
Bn-scaff_22728_1-p968837	Bn-N13-p6669300	gene	
Bn-scaff_22728_1-p970076	Bn-N13-p6668303	gene	exon
Bn-scaff_22749_1-p144203	Bn-N12-p23513386	gene	
Bn-scaff_22749_1-p202280	Bn-N9-p843379	gene	exon
Bn-scaff_22749_1-p383075	Bn-N12-p23670211	gene	
Bn-scaff_22749_1-p436502	Bn-N12-p24001083	gene	
Bn-scaff_22749_1-p441257	Bn-N12-p23997910	gene	exon

Bn-scaff_22749_1-p443829	Bn-N12-p23995331	gene	exon
Bn-scaff_22749_1-p462312	Bn-N12-p23969432	gene	
Bn-scaff_22749_1-p462527	Bn-N12-p23969217	gene	exon
Bn-scaff_22749_1-p558264	Bn-N2-p16777070	gene	
Bn-scaff_22751_1-p137904	Bn-N3-p15090088	gene	exon
Bn-scaff_22765_1-p148520	Bn-N16-p1212865	gene	exon
Bn-scaff_22790_1-p1010980	Bn-N11-p17949280	gene	
Bn-scaff_22790_1-p1012274	Bn-N11-p17950577	gene	
Bn-scaff_22790_1-p1012275	Bn-N11-p17950577	gene	
Bn-scaff_22790_1-p10917	Bn-N11-p16959475	gene	exon
Bn-scaff_22790_1-p1155333	Bn-N11-p18088246	gene	
Bn-scaff_22790_1-p1271170	Bn-N11-p18191532	gene	
Bn-scaff_22790_1-p1273360	Bn-N11-p18193779	gene	
Bn-scaff_22790_1-p1274026	Bn-N11-p18194445	gene	exon
Bn-scaff_22790_1-p1317315	Bn-N11-p18234997	gene	exon
Bn-scaff_22790_1-p139306	Bn-N11-p17089917	gene	
Bn-scaff_22790_1-p207071	Bn-N11-p17178678	gene	
Bn-scaff_22790_1-p263811	Bn-Scaffold02684-p1834	gene	
Bn-scaff_22790_1-p323805	Bn-N11-p17258484	gene	exon
Bn-scaff_22790_1-p332765	Bn-N11-p17267254	gene	exon
Bn-scaff_22790_1-p370880	Bn-N11-p17307190	gene	exon
Bn-scaff_22790_1-p373418	Bn-N11-p17309728	gene	
Bn-scaff_22790_1-p457131	Bn-N11-p17395529	gene	
Bn-scaff_22790_1-p457517	Bn-N11-p17395915	gene	exon
Bn-scaff_22790_1-p550704	Bn-N19-p17759767	gene	
Bn-scaff_22790_1-p669477	Bn-N11-p17616355	gene	
Bn-scaff_22790_1-p717222	Bn-N11-p17650497	gene	exon
Bn-scaff_22790_1-p752821	Bn-N11-p17686725	gene	
Bn-scaff_22790_1-p771040	Bn-N11-p17718242	gene	exon
Bn-scaff_22790_1-p793565	Bn-N11-p17742121	gene	exon
Bn-scaff_22790_1-p793789	Bn-N11-p17742345	gene	
Bn-scaff_22790_1-p98222	Bn-N11-p17052554	gene	exon
Bn-scaff_22795_1-p195621	Bn-N1-p14032392	gene	
Bn-scaff_22814_1-p156349	Bn-N14-p33147241	gene	exon
Bn-scaff_22814_1-p166991	Bn-N14-p33159748	gene	
Bn-scaff_22814_1-p169781	Bn-N14-p33162535	gene	
Bn-scaff_22814_1-p58657	Bn-N14-p33045571	gene	exon
Bn-scaff_22821_1-p13976	Bn-N17-p25771233	gene	exon
Bn-scaff_22835_1-p534508	Bn-N19-p14655500	gene	
Bn-scaff_22835_1-p535692	Bn-N19-p14656684	gene	
Bn-scaff_22835_1-p537076	Bn-N19-p14658066	gene	
Bn-scaff_22835_1-p547549	Bn-N19-p14685129	gene	
Bn-scaff_22835_1-p716171	Bn-N19-p14457407	gene	
Bn-scaff_22933_1-p109175	Bn-N14-p80852	gene	
Bn-scaff_22933_1-p110027	Bn-N14-p80000	gene	
Bn-scaff_22933_1-p62015	Bn-N14-p121945	gene	
Bn-scaff_22933_1-p70880	Bn-N14-p113706	gene	exon
Bn-scaff_22970_1-p195963	Bn-N12-p689046	gene	
Bn-scaff_22970_1-p213807	Bn-N12-p705252	gene	
Bn-scaff_22970_1-p218555	Bn-N12-p709793	gene	exon
Bn-scaff_22970_1-p247775	Bn-N12-p739811	gene	exon
Bn-scaff_22970_1-p264018	Bn-N12-p756962	gene	

Bn-scaff_22970_1-p270779	Bn-N12-p763893	gene	
Bn-scaff_22970_1-p270891	Bn-N12-p764005	gene	
Bn-scaff_22970_1-p277300	Bn-N12-p770697	gene	
Bn-scaff_22970_1-p32371	Bn-N12-p523725	gene	
Bn-scaff_22970_1-p32963	Bn-N12-p524314	gene	exon
Bn-scaff_22970_1-p370489	Bn-N2-p974398	gene	
Bn-scaff_22970_1-p372634	Bn-N12-p850226	gene	
Bn-scaff_22970_1-p372788	Bn-N2-p976858	gene	exon
Bn-scaff_22970_1-p372931	Bn-N2-p977002	gene	
Bn-scaff_22970_1-p374054	Bn-N12-p851647	gene	
Bn-scaff_22970_1-p384574	Bn-N2-p991502	gene	exon
Bn-scaff_22970_1-p386413	Bn-N12-p866795	gene	exon
Bn-scaff_22970_1-p386559	Bn-N12-p866941	gene	exon
Bn-scaff_22970_1-p386610	Bn-N2-p993576	gene	exon
Bn-scaff_22970_1-p386682	Bn-N12-p867064	gene	
Bn-scaff_22970_1-p387055	Bn-N2-p994026	gene	exon
Bn-scaff_22970_1-p439123	Bn-N12-p911544	gene	
Bn-scaff_22970_1-p491664	Bn-N12-p956087	gene	
Bn-scaff_22970_1-p491692	Bn-N12-p956115	gene	
Bn-scaff_22970_1-p499830	Bn-N12-p964230	gene	exon
Bn-scaff_22970_1-p50892	Bn-N12-p541708	gene	exon
Bn-scaff_22970_1-p51056	Bn-N2-p771412	gene	
Bn-scaff_22970_1-p581710	Bn-N2-p1141057	gene	
Bn-scaff_22970_1-p72276	Bn-N12-p559533	gene	
Bn-scaff_23015_1-p2066	Bn-N6-p1899416	gene	exon
Bn-scaff_23066_1-p3540	Bn-N15-p10418325	gene	exon
Bn-scaff_23096_1-p447166	Bn-N10-p10752753	gene	exon
Bn-scaff_23096_1-p546726	Bn-N19-p45202855	gene	
Bn-scaff_23096_1-p548082	Bn-N19-p45204213	gene	exon
Bn-scaff_23098_1-p304246	Bn-N8-p1376618	gene	exon
Bn-scaff_23098_1-p319931	Bn-N8-p1387662	gene	
Bn-scaff_23098_1-p320181	Bn-Scaffold02512-p3189	gene	exon
Bn-scaff_23103_1-p2352	Bn-N19-p25919711	gene	exon
Bn-scaff_23104_1-p232139	Bn-N12-p32308178	gene	exon
Bn-scaff_23104_1-p29181	Bn-N12-p32489573	gene	exon
Bn-scaff_23107_1-p115633	Bn-N10-p111412	gene	exon
Bn-scaff_23107_1-p132163	Bn-N15-p145114	gene	
Bn-scaff_23107_1-p158176	Bn-N15-p170385	gene	
Bn-scaff_23107_1-p181951	Bn-N15-p199748	gene	
Bn-scaff_23108_1-p284977	Bn-N12-p19136456	gene	exon
Bn-scaff_23108_1-p362932	Bn-N2-p13429147	gene	exon
Bn-scaff_23140_1-p34812	Bn-N7-p7126313	gene	exon
Bn-scaff_23140_1-p456	Bn-N18-p23622812	gene	exon
Bn-scaff_23140_1-p68699	Bn-N7-p7117612	gene	exon
Bn-scaff_23167_1-p150107	Bn-N19-p55718471	gene	exon
Bn-scaff_23176_1-p55098	Bn-N12-p5382551	gene	exon
Bn-scaff_23176_1-p79652	Bn-N12-p5407807	gene	
Bn-scaff_23186_1-p102192	Bn-N15-p46330126	gene	exon
Bn-scaff_23186_1-p102352	Bn-N15-p46329966	gene	exon
Bn-scaff_23186_1-p18537	Bn-N15-p46434693	gene	exon
Bn-scaff_23186_1-p256752	Bn-N15-p46179480	gene	
Bn-scaff_23186_1-p303048	Bn-N15-p46131318	gene	exon

Bn-scaff_23186_1-p303208	Bn-N15-p46130595	gene	
Bn-scaff_23186_1-p331289	Bn-N5-p24167952	gene	
Bn-scaff_23186_1-p432591	Bn-N5-p24106491	gene	
Bn-scaff_23186_1-p52666	Bn-N15-p46394060	gene	exon
Bn-scaff_23186_1-p72105	Bn-N15-p46363653	gene	exon
Bn-scaff_23195_1-p9954	Bn-N15-p18309387	gene	
Bn-scaff_23200_1-p3350	Bn-N7-p22025097	gene	exon
Bn-scaff_23207_1-p284979	Bn-N19-p8223954	gene	exon
Bn-scaff_23207_1-p285998	Bn-N19-p8222927	gene	exon
Bn-scaff_23207_1-p384863	Bn-N19-p8114891	gene	exon
Bn-scaff_23207_1-p385010	Bn-N19-p8114744	gene	
Bn-scaff_23207_1-p385083	Bn-N19-p8114671	gene	
Bn-scaff_23207_1-p477296	Bn-N9-p5673010	gene	exon
Bn-scaff_23208_1-p22129	Bn-N19-p44019805	gene	exon
Bn-scaff_23229_1-p41596	Bn-N14-p3387730	gene	
Bn-scaff_23229_1-p55001	Bn-N5-p2219118	gene	
Bn-scaff_23286_1-p3025	Bn-N13-p36536860	gene	exon
Bn-scaff_23291_1-p150625	Bn-N12-p15556889	gene	
Bn-scaff_23293_1-p107816	Bn-N19-p22482081	gene	
Bn-scaff_23293_1-p175881	Bn-N19-p22425619	gene	
Bn-scaff_23293_1-p25406	Bn-N9-p14055857	gene	exon
Bn-scaff_23305_1-p46201	Bn-N15-p24099905	gene	exon
Bn-scaff_23347_1-p15430	Bn-N11-p39364344	gene	exon
Bn-scaff_23347_1-p39179	Bn-N11-p39345740	gene	exon
Bn-scaff_23347_1-p58373	Bn-N11-p39323391	gene	
Bn-scaff_23347_1-p58586	Bn-N11-p39323178	gene	
Bn-scaff_23347_1-p92439	Bn-N11-p39280696	gene	
Bn-scaff_23347_1-p92650	Bn-N11-p39280485	gene	
Bn-scaff_23350_1-p770686	Bn-N2-p26321027	gene	
Bn-scaff_23400_1-p101355	Bn-N17-p21558184	gene	
Bn-scaff_23400_1-p118029	Bn-N17-p21541510	gene	
Bn-scaff_23400_1-p249552	Bn-N17-p21402638	gene	exon
Bn-scaff_23400_1-p264065	Bn-N17-p21388125	gene	exon
Bn-scaff_23400_1-p265171	Bn-N17-p21387019	gene	exon
Bn-scaff_23400_1-p508657	Bn-N17-p21154167	gene	
Bn-scaff_23400_1-p57185	Bn-N17-p22576613	gene	
Bn-scaff_23400_1-p582590	Bn-N17-p21053844	gene	exon
Bn-scaff_23401_1-p222140	Bn-N12-p26274755	gene	exon
Bn-scaff_23401_1-p222743	Bn-N12-p26275358	gene	
Bn-scaff_23401_1-p222761	Bn-N12-p26275376	gene	
Bn-scaff_23401_1-p222826	Bn-N12-p26275441	gene	
Bn-scaff_23401_1-p301949	Bn-N12-p26342631	gene	
Bn-scaff_23401_1-p303746	Bn-N2-p17657106	gene	
Bn-scaff_23401_1-p317800	Bn-N12-p26358467	gene	
Bn-scaff_23407_1-p120583	Bn-N13-p51457114	gene	exon
Bn-scaff_23407_1-p125070	Bn-N13-p51452471	gene	exon
Bn-scaff_23407_1-p125338	Bn-N6-p10078134	gene	exon
Bn-scaff_23407_1-p128057	Bn-N13-p51450003	gene	exon
Bn-scaff_23407_1-p141198	Bn-N13-p51435797	gene	exon
Bn-scaff_23407_1-p141371	Bn-N13-p51435624	gene	exon
Bn-scaff_23407_1-p141430	Bn-N13-p51435565	gene	exon
Bn-scaff_23407_1-p141567	Bn-N13-p51435428	gene	

Bn-scaff_23407_1-p149870	Bn-N13-p51427052	gene	
Bn-scaff_23407_1-p26401	Bn-N6-p10023566	gene	
Bn-scaff_23407_1-p27013	Bn-N13-p51554299	gene	
Bn-scaff_23408_1-p104948	Bn-N15-p42033158	gene	
Bn-scaff_23408_1-p202762	Bn-N15-p42124264	gene	exon
Bn-scaff_23408_1-p243256	Bn-N15-p42154733	gene	
Bn-scaff_23408_1-p272922	Bn-N15-p42180350	gene	exon
Bn-scaff_23408_1-p273674	Bn-N15-p42181102	gene	
Bn-scaff_23432_1-p175763	Bn-N14-p22748412	gene	
Bn-scaff_23432_1-p175866	Bn-N14-p22748515	gene	
Bn-scaff_23432_1-p175867	Bn-N14-p22748516	gene	
Bn-scaff_23432_1-p176182	Bn-N14-p22748856	gene	exon
Bn-scaff_23432_1-p176886	Bn-N14-p22749536	gene	
Bn-scaff_23432_1-p176966	Bn-N14-p22749616	gene	exon
Bn-scaff_23432_1-p213377	Bn-N14-p22773939	gene	
Bn-scaff_23432_1-p254884	Bn-N17-p9131021	gene	exon
Bn-scaff_23472_1-p21637	Bn-N14-p42609392	gene	
Bn-scaff_23472_1-p42267	Bn-N14-p42626788	gene	exon
Bn-scaff_23534_1-p11593	Bn-N14-p213416	gene	
Bn-scaff_23534_1-p36688	Bn-N14-p187301	gene	exon
Bn-scaff_23534_1-p37312	Bn-N14-p186676	gene	
Bn-scaff_23534_1-p39080	Bn-N14-p184907	gene	
Bn-scaff_23546_1-p29147	Bn-N12-p13455739	gene	exon
Bn-scaff_23546_1-p29304	Bn-N12-p13455896	gene	exon
Bn-scaff_23546_1-p35707	Bn-N12-p13462338	gene	
Bn-scaff_23546_1-p35786	Bn-N12-p13462417	gene	
Bn-scaff_23546_1-p35962	Bn-N12-p13462593	gene	exon
Bn-scaff_23546_1-p416164	Bn-N2-p9199425	gene	exon
Bn-scaff_23546_1-p435046	Bn-N2-p9202142	gene	exon
Bn-scaff_23546_1-p44190	Bn-N12-p13470821	gene	
Bn-scaff_23546_1-p82197	Bn-N12-p13503977	gene	exon
Bn-scaff_23613_1-p26112	Bn-N5-p7720316	gene	exon
Bn-scaff_23613_1-p35766	Bn-N14-p13312005	gene	
Bn-scaff_23613_1-p51738	Bn-N14-p13299159	gene	
Bn-scaff_23622_1-p62088	Bn-N10-p10959317	gene	exon
Bn-scaff_23699_1-p106821	Bn-N14-p12452253	gene	exon
Bn-scaff_23699_1-p296012	Bn-N14-p12631929	gene	exon
Bn-scaff_23699_1-p296320	Bn-N14-p12632236	gene	exon
Bn-scaff_23699_1-p297000	Bn-N14-p12632916	gene	
Bn-scaff_23715_1-p109313	Bn-N8-p20705817	gene	exon
Bn-scaff_23761_1-p249628	Bn-N13-p64037256	gene	
Bn-scaff_23761_1-p27239	Bn-N13-p63750898	gene	
Bn-scaff_23761_1-p326272	Bn-N13-p64136202	gene	
Bn-scaff_23761_1-p326361	Bn-N8-p13285204	gene	exon
Bn-scaff_23761_1-p35445	Bn-N8-p13074265	gene	exon
Bn-scaff_23761_1-p392078	Bn-N13-p64197854	gene	exon
Bn-scaff_23761_1-p408137	Bn-N13-p64210511	gene	exon
Bn-scaff_23761_1-p434901	Bn-N13-p64237834	gene	exon
Bn-scaff_23761_1-p530135	Bn-N13-p64337717	gene	
Bn-scaff_23761_1-p581335	Bn-N13-p64389081	gene	exon
Bn-scaff_23761_1-p604319	Bn-N13-p64412087	gene	
Bn-scaff_23761_1-p604385	Bn-N13-p64412153	gene	

Bn-scaff_23761_1-p704264	Bn-N8-p13534677	gene	exon
Bn-scaff_23761_1-p704887	Bn-N13-p64521312	gene	
Bn-scaff_23761_1-p727007	Bn-N13-p64542355	gene	
Bn-scaff_23761_1-p728473	Bn-N13-p64543806	gene	
Bn-scaff_23761_1-p737998	Bn-N13-p64557277	gene	
Bn-scaff_23761_1-p738056	Bn-N13-p64557335	gene	
Bn-scaff_23761_1-p738582	Bn-N13-p64557861	gene	exon
Bn-scaff_23761_1-p738585	Bn-N13-p64557864	gene	exon
Bn-scaff_23761_1-p764152	Bn-N13-p64582781	gene	exon
Bn-scaff_23771_1-p149473	Bn-N17-p9505942	gene	
Bn-scaff_23771_1-p180581	Bn-N17-p9538692	gene	
Bn-scaff_23771_1-p195258	Bn-N17-p12465801	gene	
Bn-scaff_23795_1-p209345	Bn-N11-p33025383	gene	exon
Bn-scaff_23795_1-p227140	Bn-N11-p33007118	gene	
Bn-scaff_23795_1-p269807	Bn-N11-p32959163	gene	
Bn-scaff_23795_1-p82694	Bn-N11-p14770233	gene	
Bn-scaff_23795_1-p82795	Bn-N11-p14770132	gene	
Bn-scaff_23799_1-p21271	Bn-N11-p39046393	gene	exon
Bn-scaff_23799_1-p62076	Bn-N11-p39099716	gene	exon
Bn-scaff_23799_1-p89287	Bn-N11-p39126052	gene	
Bn-scaff_23813_1-p241423	Bn-N12-p12906232	gene	exon
Bn-scaff_23813_1-p396998	Bn-N2-p8678778	gene	exon
Bn-scaff_23813_1-p397311	Bn-N2-p8679091	gene	
Bn-scaff_23813_1-p495084	Bn-N2-p8744911	gene	exon
Bn-scaff_23813_1-p548372	Bn-N12-p13147079	gene	
Bn-scaff_23813_1-p581480	Bn-N12-p13176839	gene	exon
Bn-scaff_23813_1-p581761	Bn-N12-p13177120	gene	
Bn-scaff_23813_1-p582215	Bn-N12-p13177574	gene	
Bn-scaff_23813_1-p582286	Bn-N12-p13177645	gene	
Bn-scaff_23813_1-p582903	Bn-N12-p13178262	gene	exon
Bn-scaff_23813_1-p597970	Bn-N12-p13191805	gene	
Bn-scaff_23813_1-p603566	Bn-N12-p13197313	gene	
Bn-scaff_23813_1-p643415	Bn-N12-p13245846	gene	
Bn-scaff_23813_1-p665554	Bn-N12-p13267502	gene	
Bn-scaff_23813_1-p807747	Bn-N12-p13422477	gene	exon
Bn-scaff_23813_1-p807960	Bn-N12-p13422690	gene	exon
Bn-scaff_23817_1-p83	Bn-N13-p1472138	gene	exon
Bn-scaff_23821_1-p123235	Bn-N16-p33429030	gene	
Bn-scaff_23821_1-p21382	Bn-N7-p20309626	gene	
Bn-scaff_23821_1-p21725	Bn-N16-p33339684	gene	
Bn-scaff_23821_1-p22581	Bn-N16-p33340540	gene	
Bn-scaff_23821_1-p45516	Bn-N16-p33362757	gene	
Bn-scaff_23821_1-p45612	Bn-N16-p33362853	gene	exon
Bn-scaff_23821_1-p45657	Bn-N16-p33362898	gene	
Bn-scaff_23821_1-p61146	Bn-N16-p33374997	gene	exon
Bn-scaff_23821_1-p87496	Bn-N16-p33399998	gene	exon
Bn-scaff_23825_1-p530	Bn-N19-p1350584	gene	
Bn-scaff_23839_1-p144529	Bn-N17-p28546575	gene	
Bn-scaff_23839_1-p148899	Bn-N17-p28550890	gene	
Bn-scaff_23907_1-p1022401	Bn-N14-p9582758	gene	
Bn-scaff_23907_1-p1023804	Bn-N14-p9584160	gene	exon
Bn-scaff_23907_1-p1137072	Bn-N14-p9709463	gene	

Bn-scaff_23907_1-p195608	Bn-N14-p8488850	gene	exon
Bn-scaff_23907_1-p261237	Bn-N5-p5259588	gene	exon
Bn-scaff_23907_1-p29685	Bn-N5-p7082349	gene	
Bn-scaff_23907_1-p416774	Bn-N5-p5296794	gene	exon
Bn-scaff_23907_1-p433016	Bn-N14-p8919426	gene	
Bn-scaff_23907_1-p433223	Bn-N5-p5315855	gene	
Bn-scaff_23907_1-p434199	Bn-N5-p5316832	gene	exon
Bn-scaff_23907_1-p497391	Bn-N5-p5368766	gene	exon
Bn-scaff_23907_1-p542442	Bn-N14-p9025019	gene	
Bn-scaff_23907_1-p547717	Bn-N5-p5414304	gene	exon
Bn-scaff_23907_1-p793109	Bn-N14-p9289401	gene	
Bn-scaff_23907_1-p833956	Bn-N14-p9341108	gene	
Bn-scaff_23907_1-p930188	Bn-N14-p9470031	gene	
Bn-scaff_23907_1-p930514	Bn-N14-p9470357	gene	
Bn-scaff_23907_1-p930601	Bn-N5-p5646532	gene	exon
Bn-scaff_23907_1-p930754	Bn-N5-p5646685	gene	exon
Bn-scaff_23907_1-p931056	Bn-N14-p9471008	gene	
Bn-scaff_23907_1-p931535	Bn-N14-p9471503	gene	exon
Bn-scaff_23907_1-p931895	Bn-N14-p9471860	gene	exon
Bn-scaff_23907_1-p985972	Bn-N5-p5691195	gene	exon
Bn-scaff_23937_1-p26440	Bn-N18-p32625601	gene	exon
Bn-scaff_23937_1-p36794	Bn-N15-p15894800	gene	exon
Bn-scaff_23946_1-p35627	Bn-N14-p965975	gene	exon
Bn-scaff_23946_1-p38726	Bn-N14-p962885	gene	
Bn-scaff_23946_1-p65251	Bn-N14-p936533	gene	
Bn-scaff_23946_1-p69210	Bn-N14-p932574	gene	
Bn-scaff_23946_1-p74762	Bn-N5-p725211	gene	
Bn-scaff_23954_1-p1045541	Bn-N4-p17959348	gene	
Bn-scaff_23954_1-p1081348	Bn-N13-p12342526	gene	
Bn-scaff_23954_1-p1083780	Bn-N13-p12340049	gene	exon
Bn-scaff_23954_1-p1083875	Bn-N13-p12339955	gene	
Bn-scaff_23954_1-p1111123	Bn-N3-p8707588	gene	exon
Bn-scaff_23954_1-p1112688	Bn-N13-p12305427	gene	exon
Bn-scaff_23954_1-p119449	Bn-N14-p51795686	gene	exon
Bn-scaff_23954_1-p132570	Bn-N13-p13337168	gene	
Bn-scaff_23954_1-p13556	Bn-N14-p5582184	gene	exon
Bn-scaff_23954_1-p141044	Bn-N13-p13328692	gene	
Bn-scaff_23954_1-p196604	Bn-N13-p13212624	gene	exon
Bn-scaff_23954_1-p196953	Bn-N3-p9316350	gene	
Bn-scaff_23954_1-p209939	Bn-N13-p13201217	gene	exon
Bn-scaff_23954_1-p228756	Bn-N13-p13181989	gene	exon
Bn-scaff_23954_1-p239074	Bn-N3-p9290375	gene	exon
Bn-scaff_23954_1-p254934	Bn-N3-p9272497	gene	exon
Bn-scaff_23954_1-p269318	Bn-N13-p13140522	gene	
Bn-scaff_23954_1-p302686	Bn-N13-p13109867	gene	
Bn-scaff_23954_1-p41208	Bn-N3-p9421464	gene	
Bn-scaff_23954_1-p47363	Bn-N13-p13418695	gene	
Bn-scaff_23954_1-p622637	Bn-N3-p9069361	gene	
Bn-scaff_23954_1-p661198	Bn-N3-p9034675	gene	
Bn-scaff_23954_1-p768646	Bn-N13-p12659240	gene	
Bn-scaff_23954_1-p76992	Bn-N13-p13399581	gene	
Bn-scaff_23954_1-p870079	Bn-N3-p8926762	gene	exon

Bn-scaff_23954_1-p872722	Bn-N3-p8924092	gene	exon
Bn-scaff_23954_1-p882621	Bn-N13-p12563793	gene	
Bn-scaff_23954_1-p971859	Bn-N13-p12466319	gene	exon
Bn-scaff_23957_1-p127219	Bn-N7-p20887115	gene	exon
Bn-scaff_23957_1-p150263	Bn-N16-p34373829	gene	exon
Bn-scaff_23957_1-p150282	Bn-N16-p34373848	gene	exon
Bn-scaff_23957_1-p150459	Bn-N16-p34374025	gene	
Bn-scaff_23957_1-p150526	Bn-N16-p34374092	gene	exon
Bn-scaff_23957_1-p151052	Bn-N16-p34374618	gene	exon
Bn-scaff_23957_1-p163621	Bn-N16-p34392576	gene	exon
Bn-scaff_23957_1-p174718	Bn-N16-p34411232	gene	exon
Bn-scaff_23957_1-p175042	Bn-N16-p34411556	gene	exon
Bn-scaff_23957_1-p250533	Bn-N16-p34499856	gene	
Bn-scaff_23957_1-p252406	Bn-N16-p34501681	gene	exon
Bn-scaff_23957_1-p260266	Bn-N16-p34508295	gene	
Bn-scaff_23957_1-p36160	Bn-N16-p34276393	gene	exon
Bn-scaff_23957_1-p369426	Bn-N16-p34623151	gene	
Bn-scaff_23957_1-p37081	Bn-N16-p34277303	gene	
Bn-scaff_23957_1-p37633	Bn-N7-p20846821	gene	exon
Bn-scaff_23957_1-p393528	Bn-N16-p34642897	gene	
Bn-scaff_23957_1-p403566	Bn-N16-p34652877	gene	exon
Bn-scaff_23957_1-p419108	Bn-N16-p34667402	gene	exon
Bn-scaff_23957_1-p484991	Bn-N16-p34734914	gene	exon
Bn-scaff_23957_1-p484997	Bn-N16-p34734919	gene	exon
Bn-scaff_23957_1-p488858	Bn-N16-p34739912	gene	exon
Bn-scaff_23957_1-p676556	Bn-N16-p34935673	gene	exon
Bn-scaff_23957_1-p689992	Bn-N16-p34948994	gene	exon
Bn-scaff_23957_1-p704015	Bn-N16-p34963159	gene	
Bn-scaff_23974_1-p14812	Bn-N14-p989242	gene	exon
Bn-scaff_24047_1-p23400	Bn-N2-p12038332	gene	exon
Bn-scaff_24104_1-p111316	Bn-N16-p40524612	gene	
Bn-scaff_24104_1-p115364	Bn-N7-p25206725	gene	exon
Bn-scaff_24104_1-p153408	Bn-N16-p40476497	gene	
Bn-scaff_24104_1-p170165	Bn-N16-p40465775	gene	
Bn-scaff_24104_1-p19325	Bn-N7-p25309407	gene	exon
Bn-scaff_24104_1-p20590	Bn-N7-p25308105	gene	exon
Bn-scaff_24104_1-p344071	Bn-N7-p25024652	gene	
Bn-scaff_24104_1-p345097	Bn-N16-p40292325	gene	
Bn-scaff_24104_1-p359336	Bn-N16-p40274395	gene	
Bn-scaff_24104_1-p386708	Bn-N16-p40250112	gene	exon
Bn-scaff_24108_1-p456844	Bn-N19-p51918660	gene	
Bn-scaff_24119_1-p1067	Bn-N14-p14393284	gene	exon
Bn-scaff_24123_1-p35437	Bn-N14-p14590283	gene	exon
Bn-scaff_24126_1-p13903	Bn-N15-p26111409	gene	
Bn-scaff_24134_1-p25752	Bn-N18-p11711613	gene	
Bn-scaff_24208_1-p74641	Bn-N18-p15662944	gene	exon
Bn-scaff_24236_1-p107377	Bn-N11-p21904091	gene	
Bn-scaff_24236_1-p277754	Bn-N11-p22097234	gene	exon
Bn-scaff_24236_1-p297635	Bn-N11-p22127937	gene	
Bn-scaff_24236_1-p343935	Bn-N11-p22163436	gene	
Bn-scaff_24236_1-p346915	Bn-N11-p22166430	gene	
Bn-scaff_24236_1-p348123	Bn-N11-p22167637	gene	

Bn-scaff_24236_1-p425705	Bn-N11-p22259474	gene	exon
Bn-scaff_24236_1-p426034	Bn-N11-p22259806	gene	exon
Bn-scaff_24236_1-p473198	Bn-N11-p22303177	gene	exon
Bn-scaff_24236_1-p485959	Bn-N11-p22319804	gene	
Bn-scaff_24251_1-p56129	Bn-N15-p48109457	gene	exon
Bn-scaff_24251_1-p57967	Bn-N15-p48107618	gene	
Bn-scaff_24451_1-p4603	Bn-N19-p46257452	gene	exon
Bn-scaff_24457_1-p1741	Bn-N13-p58946328	gene	exon
Bn-scaff_24459_1-p11374	Bn-N13-p33973659	gene	exon
Bn-scaff_24459_1-p5391	Bn-N13-p33968181	gene	
Bn-scaff_24466_1-p53535	Bn-N11-p37961653	gene	
Bn-scaff_24466_1-p65988	Bn-N1-p22667184	gene	
Bn-scaff_24559_1-p418	Bn-N10-p2527567	gene	exon
Bn-scaff_24602_1-p69684	Bn-N14-p28925360	gene	
Bn-scaff_24608_1-p276823	Bn-N11-p29085158	gene	
Bn-scaff_24631_1-p150378	Bn-N15-p19696952	gene	exon
Bn-scaff_24631_1-p184986	Bn-N13-p54890638	gene	exon
Bn-scaff_24631_1-p187311	Bn-N13-p54892951	gene	
Bn-scaff_24631_1-p215956	Bn-N8-p15971865	gene	exon
Bn-scaff_24631_1-p251065	Bn-N13-p54990282	gene	exon
Bn-scaff_24631_1-p251133	Bn-N13-p54990350	gene	
Bn-scaff_24631_1-p276017	Bn-N13-p55012734	gene	exon
Bn-scaff_24631_1-p316671	Bn-N13-p55054599	gene	
Bn-scaff_24631_1-p372982	Bn-N13-p55109430	gene	
Bn-scaff_24631_1-p383340	Bn-N8-p15885084	gene	exon
Bn-scaff_24631_1-p383554	Bn-N8-p15884213	gene	exon
Bn-scaff_24631_1-p471093	Bn-N13-p55220190	gene	exon
Bn-scaff_24631_1-p501178	Bn-N8-p15820613	gene	exon
Bn-scaff_24631_1-p63534	Bn-N13-p54767699	gene	exon
Bn-scaff_24631_1-p745795	Bn-N8-p15696130	gene	
Bn-scaff_24631_1-p801855	Bn-N8-p15720124	gene	
Bn-scaff_24680_1-p45025	Bn-N16-p2986818	gene	
Bn-scaff_24680_1-p45076	Bn-N16-p2986768	gene	
Bn-scaff_24680_1-p46902	Bn-N16-p2984941	gene	exon
Bn-scaff_24705_1-p95	Bn-N19-p48555224	gene	exon
Bn-scaff_24721_1-p10525	Bn-N11-p20406726	gene	exon
Bn-scaff_24721_1-p257717	Bn-N11-p20169021	gene	exon
Bn-scaff_24721_1-p37669	Bn-N11-p20382982	gene	
Bn-scaff_24721_1-p38435	Bn-N11-p20382216	gene	exon
Bn-scaff_24721_1-p83101	Bn-N11-p20336263	gene	exon
Bn-scaff_24721_1-p83732	Bn-N11-p20335632	gene	exon
Bn-scaff_24721_1-p83843	Bn-N11-p20335521	gene	
Bn-scaff_24721_1-p83949	Bn-N11-p20335415	gene	exon
Bn-scaff_24726_1-p33555	Bn-N13-p54303531	gene	exon
Bn-scaff_24726_1-p33634	Bn-N13-p54303453	gene	exon
Bn-scaff_24726_1-p33823	Bn-N13-p54303264	gene	exon
Bn-scaff_24726_1-p39008	Bn-N8-p16275326	gene	exon
Bn-scaff_24859_1-p45913	Bn-N14-p1101599	gene	exon
Bn-scaff_24869_1-p119638	Bn-N11-p10424185	gene	
Bn-scaff_24869_1-p147699	Bn-N11-p10396879	gene	
Bn-scaff_24947_1-p27411	Bn-N11-p13224806	gene	
Bn-scaff_24947_1-p7191	Bn-N11-p13245581	gene	

Bn-scaff_24947_1-p79168	Bn-N11-p13166107	gene	exon
Bn-scaff_24947_1-p9756	Bn-N11-p13243006	gene	exon
Bn-scaff_24979_1-p13103	Bn-N14-p44929998	gene	
Bn-scaff_24979_1-p132892	Bn-N14-p45097950	gene	
Bn-scaff_24979_1-p134759	Bn-N4-p13660105	gene	exon
Bn-scaff_24979_1-p162534	Bn-N14-p45134860	gene	
Bn-scaff_24979_1-p163133	Bn-N14-p45135466	gene	
Bn-scaff_24979_1-p163668	Bn-N14-p45135989	gene	
Bn-scaff_24979_1-p169850	Bn-N14-p45151016	gene	
Bn-scaff_24979_1-p40341	Bn-N14-p44997527	gene	
Bn-scaff_24979_1-p40600	Bn-N14-p44997786	gene	
Bn-scaff_24979_1-p40845	Bn-N4-p13554052	gene	exon
Bn-scaff_24979_1-p41125	Bn-N14-p44998344	gene	
Bn-scaff_24979_1-p46544	Bn-N14-p45002642	gene	
Bn-scaff_24979_1-p46937	Bn-N14-p45003035	gene	
Bn-scaff_24979_1-p7134	Bn-N14-p44918337	gene	
Bn-scaff_24979_1-p73839	Bn-N14-p45036186	gene	
Bn-scaff_24979_1-p980	Bn-N14-p44915896	gene	
Bn-scaff_24999_1-p32039	Bn-N19-p14785578	gene	exon
Bn-scaff_25012_1-p10988	Bn-N14-p8274921	gene	exon
Bn-scaff_25040_1-p24261	Bn-N5-p4607613	gene	
Bn-scaff_25071_1-p14638	Bn-N12-p43936037	gene	exon
Bn-scaff_25094_1-p126581	Bn-N13-p35903137	gene	exon
Bn-scaff_25094_1-p13129	Bn-N13-p35717299	gene	
Bn-scaff_25094_1-p13184	Bn-N13-p35717355	gene	
Bn-scaff_25094_1-p131956	Bn-N13-p35906439	gene	
Bn-scaff_25094_1-p26060	Bn-N13-p35724962	gene	
Bn-scaff_25094_1-p26342	Bn-N13-p35725238	gene	exon
Bn-scaff_25094_1-p269207	Bn-N13-p36046561	gene	
Bn-scaff_25094_1-p336881	Bn-N6-p18171451	gene	exon
Bn-scaff_25094_1-p339638	Bn-N13-p36117613	gene	
Bn-scaff_25094_1-p345039	Bn-N13-p36122969	gene	
Bn-scaff_25094_1-p413818	Bn-N13-p36190936	gene	
Bn-scaff_25094_1-p497096	Bn-N13-p36278159	gene	exon
Bn-scaff_25094_1-p9143	Bn-N13-p35715570	gene	exon
Bn-scaff_25107_1-p22878	Bn-N16-p3439756	gene	
Bn-scaff_25107_1-p22897	Bn-N16-p3439775	gene	
Bn-scaff_25139_1-p2069	Bn-N14-p45733861	gene	
Bn-scaff_25139_1-p5705	Bn-N14-p45737496	gene	
Bn-scaff_25149_1-p76377	Bn-N11-p4592677	gene	
Bn-scaff_25149_1-p79962	Bn-N11-p4596273	gene	exon
Bn-scaff_25254_1-p499	Bn-N3-p10550968	gene	exon
Bn-scaff_25262_1-p114443	Bn-N12-p19855348	gene	exon
Bn-scaff_25311_1-p1307	Bn-N18-p30536578	gene	
Bn-scaff_25323_1-p33026	Bn-N13-p39756415	gene	
Bn-scaff_25323_1-p7019	Bn-N13-p39724930	gene	exon
Bn-scaff_25422_1-p95222	Bn-N1-p6813440	gene	exon
Bn-scaff_25427_1-p17459	Bn-N17-p39795270	gene	exon
Bn-scaff_25432_1-p24804	Bn-N14-p23772711	gene	exon
Bn-scaff_25432_1-p31881	Bn-N14-p23780006	gene	exon
Bn-scaff_25432_1-p33326	Bn-N14-p23781451	gene	exon
Bn-scaff_25457_1-p244144	Bn-N10-p10288025	gene	exon

Bn-scaff_25457_1-p244727	Bn-N19-p44133882	gene	exon
Bn-scaff_25457_1-p258094	Bn-N19-p44119976	gene	exon
Bn-scaff_25457_1-p279056	Bn-N19-p44105020	gene	
Bn-scaff_25457_1-p279291	Bn-N19-p44104785	gene	exon
Bn-scaff_25457_1-p279509	Bn-N19-p44104567	gene	
Bn-scaff_25457_1-p279510	Bn-N19-p44104566	gene	
Bn-scaff_25457_1-p297013	Bn-N19-p44063701	gene	exon
Bn-scaff_25457_1-p297098	Bn-N19-p44063615	gene	exon
Bn-scaff_25457_1-p297146	Bn-N19-p44063568	gene	
Bn-scaff_25457_1-p297885	Bn-N19-p44062830	gene	exon
Bn-scaff_25466_1-p11848	Bn-N16-p36827057	gene	
Bn-scaff_25466_1-p12042	Bn-N16-p36826863	gene	
Bn-scaff_25466_1-p12578	Bn-N16-p36826327	gene	
Bn-scaff_25466_1-p12628	Bn-N16-p36826277	gene	
Bn-scaff_25466_1-p12702	Bn-N16-p36826202	gene	
Bn-scaff_25466_1-p13102	Bn-N16-p36825796	gene	
Bn-scaff_25466_1-p13157	Bn-N16-p36825741	gene	
Bn-scaff_25466_1-p13426	Bn-N16-p36825472	gene	
Bn-scaff_25466_1-p14024	Bn-N16-p36824874	gene	
Bn-scaff_25466_1-p14416	Bn-N16-p36824482	gene	
Bn-scaff_25466_1-p15088	Bn-N16-p36823851	gene	
Bn-scaff_25466_1-p15362	Bn-N16-p36823577	gene	
Bn-scaff_25466_1-p15589	Bn-N16-p36823350	gene	
Bn-scaff_25466_1-p155973	Bn-N16-p36690681	gene	exon
Bn-scaff_25466_1-p192846	Bn-N7-p22299183	gene	exon
Bn-scaff_25466_1-p29121	Bn-N16-p36816062	gene	
Bn-scaff_25466_1-p29346	Bn-N16-p36815838	gene	
Bn-scaff_25466_1-p8800	Bn-N16-p36830106	gene	
Bn-scaff_25466_1-p9410	Bn-N16-p36829495	gene	exon
Bn-scaff_25491_1-p389	Bn-Scaffold02892-p2725	gene	exon
Bn-scaff_25491_1-p7540	Bn-N15-p36409108	gene	exon
Bn-scaff_25491_1-p81630	Bn-N15-p36486274	gene	
Bn-scaff_25543_1-p200758	Bn-N17-p7436496	gene	exon
Bn-scaff_25543_1-p56354	Bn-N2-p22105856	gene	
Bn-scaff_25545_1-p10968	Bn-Scaffold00952-p31257	gene	
Bn-scaff_25545_1-p11627	Bn-Scaffold00952-p30598	gene	
Bn-scaff_25595_1-p241671	Bn-N5-p15348794	gene	exon
Bn-scaff_25595_1-p331991	Bn-N5-p15406615	gene	exon
Bn-scaff_25595_1-p414663	Bn-N15-p31901716	gene	exon
Bn-scaff_25595_1-p414764	Bn-N15-p31901817	gene	
Bn-scaff_25628_1-p36781	Bn-N15-p32224239	gene	exon
Bn-scaff_25628_1-p37066	Bn-N15-p32224524	gene	exon
Bn-scaff_25628_1-p37260	Bn-N15-p32224719	gene	exon
Bn-scaff_25686_1-p7882	Bn-N14-p1921526	gene	exon
Bn-scaff_25686_1-p9156	Bn-N14-p1922800	gene	exon
Bn-scaff_25690_1-p287	Bn-Scaffold01365-p5237	gene	
Bn-scaff_25704_1-p19623	Bn-N12-p12432867	gene	exon
Bn-scaff_25705_1-p102800	Bn-N15-p10410031	gene	
Bn-scaff_25869_1-p1017	Bn-N16-p2005710	gene	
Bn-scaff_25869_1-p1568	Bn-N16-p2005164	gene	
Bn-scaff_25870_1-p48609	Bn-N14-p41769456	gene	
Bn-scaff_25870_1-p80252	Bn-N14-p41741508	gene	

Bn-scaff_25870_1-p81302	Bn-N14-p41740458	gene	
Bn-scaff_25960_1-p104315	Bn-N14-p41952984	gene	
Bn-scaff_25960_1-p104455	Bn-N14-p41953124	gene	
Bn-scaff_25960_1-p55555	Bn-N14-p41903699	gene	exon
Bn-scaff_25960_1-p76578	Bn-N4-p11416670	gene	
Bn-scaff_25960_1-p76841	Bn-N4-p11416929	gene	
Bn-scaff_25981_1-p191746	Bn-N8-p3797452	gene	exon
Bn-scaff_25991_1-p10385	Bn-N19-p9495974	gene	exon
Bn-scaff_25991_1-p10879	Bn-N19-p9495480	gene	exon
Bn-scaff_26023_1-p10596	Bn-N12-p32081113	gene	
Bn-scaff_26023_1-p19550	Bn-N12-p32072317	gene	
Bn-scaff_26033_1-p26555	Bn-N14-p50973719	gene	
Bn-scaff_26075_1-p534671	Bn-N15-p25615011	gene	
Bn-scaff_26075_1-p577183	Bn-N6-p8304848	gene	exon
Bn-scaff_26086_1-p11779	Bn-N12-p45658942	gene	
Bn-scaff_26139_1-p209686	Bn-N14-p35183507	gene	exon
Bn-scaff_26139_1-p268705	Bn-N14-p35134924	gene	exon
Bn-scaff_26139_1-p283121	Bn-N14-p35124802	gene	
Bn-scaff_26139_1-p283611	Bn-N14-p35124312	gene	
Bn-scaff_26139_1-p304734	Bn-N14-p35103174	gene	
Bn-scaff_26139_1-p305170	Bn-N14-p35102738	gene	exon
Bn-scaff_26139_1-p307625	Bn-N14-p35100385	gene	
Bn-scaff_26139_1-p313365	Bn-N4-p5564578	gene	
Bn-scaff_26139_1-p313572	Bn-N4-p5564369	gene	
Bn-scaff_26139_1-p342322	Bn-N14-p35058065	gene	
Bn-scaff_26139_1-p376956	Bn-N11-p13807196	gene	exon
Bn-scaff_26139_1-p54494	Bn-N14-p35315830	gene	
Bn-scaff_26139_1-p95647	Bn-N4-p5724551	gene	exon
Bn-scaff_26293_1-p117280	Bn-N18-p1633914	gene	
Bn-scaff_26293_1-p117439	Bn-N18-p1633794	gene	exon
Bn-scaff_26293_1-p133260	Bn-N8-p20993023	gene	
Bn-scaff_26293_1-p135449	Bn-N18-p1621343	gene	
Bn-scaff_26293_1-p141921	Bn-N18-p1616232	gene	
Bn-scaff_26293_1-p142747	Bn-N18-p1615521	gene	
Bn-scaff_26293_1-p14281	Bn-N18-p1744846	gene	
Bn-scaff_26293_1-p158489	Bn-N8-p21010352	gene	exon
Bn-scaff_26293_1-p89381	Bn-N18-p1665044	gene	
Bn-scaff_26293_1-p92022	Bn-N18-p1659195	gene	
Bn-scaff_26297_1-p1200	Bn-N17-p32880785	gene	exon
Bn-scaff_26320_1-p109124	Bn-N13-p34623036	gene	
Bn-scaff_26320_1-p156027	Bn-N13-p34574230	gene	exon
Bn-scaff_26320_1-p163494	Bn-N13-p34570495	gene	
Bn-scaff_26320_1-p235535	Bn-N13-p34500911	gene	
Bn-scaff_26320_1-p236701	Bn-N13-p34499745	gene	exon
Bn-scaff_26320_1-p242800	Bn-N8-p15558094	gene	exon
Bn-scaff_26320_1-p297674	Bn-N13-p34440251	gene	exon
Bn-scaff_26320_1-p298294	Bn-N13-p34439630	gene	exon
Bn-scaff_26320_1-p298590	Bn-N13-p34439334	gene	
Bn-scaff_26320_1-p35454	Bn-N13-p34698798	gene	exon
Bn-scaff_26320_1-p35819	Bn-N13-p34698432	gene	exon
Bn-scaff_26391_1-p10846	Bn-N14-p30055305	gene	
Bn-scaff_26391_1-p192467	Bn-N14-p29865387	gene	exon

Bn-scaff_26391_1-p292417	Bn-N4-p2135748	gene	exon
Bn-scaff_26391_1-p89273	Bn-N4-p1987134	gene	exon
Bn-scaff_26407_1-p187613	Bn-N15-p35073887	gene	exon
Bn-scaff_26407_1-p187668	Bn-N18-p20368116	gene	
Bn-scaff_26407_1-p209243	Bn-N12-p19690584	gene	
Bn-scaff_26505_1-p5590	Bn-N13-p32203145	gene	exon
Bn-scaff_26506_1-p42166	Bn-N18-p17060265	gene	
Bn-scaff_26642_1-p111569	Bn-N13-p59139337	gene	
Bn-scaff_26642_1-p111647	Bn-N13-p59139415	gene	
Bn-scaff_26642_1-p134911	Bn-N13-p59182158	gene	
Bn-scaff_26642_1-p135036	Bn-N13-p59182283	gene	
Bn-scaff_26642_1-p50926	Bn-N13-p59069525	gene	
Bn-scaff_26642_1-p51416	Bn-N13-p59070008	gene	exon
Bn-scaff_26642_1-p52834	Bn-N13-p59071429	gene	
Bn-scaff_26642_1-p52929	Bn-N13-p59071522	gene	
Bn-scaff_26712_1-p25842	Bn-N12-p41148444	gene	exon
Bn-scaff_26712_1-p26029	Bn-N12-p41148641	gene	
Bn-scaff_26712_1-p26624	Bn-N2-p25339339	gene	
Bn-scaff_26764_1-p779	Bn-N6-p22850930	gene	
Bn-scaff_26787_1-p102596	Bn-N14-p53518082	gene	
Bn-scaff_26787_1-p10517	Bn-N14-p53411349	gene	exon
Bn-scaff_26787_1-p20789	Bn-N14-p53422501	gene	
Bn-scaff_26787_1-p23954	Bn-N14-p53425949	gene	
Bn-scaff_26787_1-p58847	Bn-N4-p19897065	gene	exon
Bn-scaff_26846_1-p38699	Bn-N13-p58699520	gene	exon
Bn-scaff_26846_1-p40355	Bn-N13-p58701178	gene	exon
Bn-scaff_26859_1-p3097	Bn-N18-p20939796	gene	exon
Bn-scaff_26863_1-p685	Bn-N17-p13598060	gene	
Bn-scaff_26877_1-p31852	Bn-N14-p35952	gene	
Bn-scaff_26877_1-p40234	Bn-N14-p28402	gene	
Bn-scaff_26877_1-p64540	Bn-N5-p15900	gene	
Bn-scaff_26946_1-p175478	Bn-N4-p2308299	gene	
Bn-scaff_26946_1-p37150	Bn-N4-p1917705	gene	exon
Bn-scaff_26974_1-p8519	Bn-N17-p23580284	gene	exon
Bn-scaff_26997_1-p131520	Bn-N15-p35988056	gene	
Bn-scaff_26997_1-p86978	Bn-N15-p35927460	gene	exon
Bn-scaff_27039_1-p269523	Bn-N12-p15001910	gene	exon
Bn-scaff_27039_1-p483666	Bn-N2-p10167349	gene	exon
Bn-scaff_27039_1-p483799	Bn-Scaffold02199-p3036	gene	
Bn-scaff_27039_1-p485697	Bn-Scaffold02199-p1417	gene	exon
Bn-scaff_27039_1-p538644	Bn-N12-p15148139	gene	exon
Bn-scaff_27039_1-p647665	Bn-N14-p50493911	gene	
Bn-scaff_27039_1-p74093	Bn-N2-p10029465	gene	exon
Bn-scaff_27050_1-p101349	Bn-N5-p15270249	gene	exon
Bn-scaff_27050_1-p150244	Bn-N9-p22094465	gene	
Bn-scaff_27059_1-p12201	Bn-N17-p48820293	gene	exon
Bn-scaff_27059_1-p12272	Bn-N17-p48820364	gene	exon
Bn-scaff_27059_1-p12283	Bn-N6-p26112886	gene	exon
Bn-scaff_27059_1-p23114	Bn-Scaffold01198-p8135	gene	
Bn-scaff_27059_1-p23212	Bn-N6-p26125678	gene	
Bn-scaff_27076_1-p79989	Bn-N6-p15604921	gene	
Bn-scaff_27076_1-p80568	Bn-N13-p39647544	gene	

Bn-scaff_27129_1-p119948	Bn-N1-p9440919	gene	
Bn-scaff_27129_1-p129919	Bn-N1-p9458746	gene	exon
Bn-scaff_27129_1-p179619	Bn-N1-p9512913	gene	exon
Bn-scaff_27129_1-p200492	Bn-N11-p14427160	gene	
Bn-scaff_27180_1-p4389	Bn-N2-p6807343	gene	exon
Bn-scaff_27198_1-p101077	Bn-N13-p2429962	gene	
Bn-scaff_27198_1-p221778	Bn-N13-p2551115	gene	
Bn-scaff_27198_1-p254278	Bn-N13-p2601663	gene	
Bn-scaff_27198_1-p254314	Bn-N13-p2601699	gene	
Bn-scaff_27198_1-p263000	Bn-N13-p2610646	gene	
Bn-scaff_27198_1-p266270	Bn-N3-p2099214	gene	
Bn-scaff_27198_1-p300707	Bn-Scaffold10098-p454	gene	exon
Bn-scaff_27198_1-p421561	Bn-N13-p2754152	gene	
Bn-scaff_27198_1-p445589	Bn-N6-p15413329	gene	
Bn-scaff_27198_1-p574582	Bn-N13-p2896117	gene	
Bn-scaff_27198_1-p578320	Bn-N13-p2899855	gene	
Bn-scaff_27278_1-p1211	Bn-N1-p23796154	gene	exon
Bn-scaff_27313_1-p451	Bn-N13-p21632489	gene	
Bn-scaff_27313_1-p653	Bn-N13-p21632294	gene	
Bn-scaff_27421_1-p102892	Bn-N6-p1414544	gene	exon
Bn-scaff_27421_1-p106897	Bn-N16-p5090891	gene	
Bn-scaff_27421_1-p108926	Bn-N16-p5088862	gene	
Bn-scaff_27421_1-p11544	Bn-N11-p30023358	gene	exon
Bn-scaff_27421_1-p27395	Bn-N16-p5172613	gene	exon
Bn-scaff_27421_1-p7352	Bn-N16-p5188061	gene	
Bn-scaff_27421_1-p7596	Bn-N16-p5187817	gene	
Bn-scaff_27421_1-p7687	Bn-N16-p5187726	gene	
Bn-scaff_27421_1-p8399	Bn-N16-p5187014	gene	
Bn-scaff_27421_1-p9012	Bn-N16-p5186401	gene	
Bn-scaff_27421_1-p9941	Bn-N16-p5185472	gene	
Bn-scaff_27451_1-p7081	Bn-N14-p19146356	gene	
Bn-scaff_27469_1-p23521	Bn-N14-p2551829	gene	
Bn-scaff_27469_1-p28443	Bn-N14-p2541002	gene	
Bn-scaff_27469_1-p73595	Bn-N14-p2496913	gene	
Bn-scaff_27469_1-p73823	Bn-N5-p1644124	gene	
Bn-scaff_27469_1-p80430	Bn-N14-p2490076	gene	exon
Bn-scaff_27513_1-p8405	Bn-N14-p16291446	gene	exon
Bn-scaff_27554_1-p3448	Bn-N19-p42548736	gene	
Bn-scaff_27554_1-p3917	Bn-N19-p42548267	gene	
Bn-scaff_27567_1-p347	Bn-N9-p26641275	gene	exon
Bn-scaff_27585_1-p898	Bn-N1-p14798759	gene	exon
Bn-scaff_27630_1-p584	Bn-N16-p5213075	gene	exon
Bn-scaff_27634_1-p12586	Bn-N14-p20001609	gene	exon
Bn-scaff_27634_1-p129708	Bn-N14-p19865341	gene	
Bn-scaff_27634_1-p84941	Bn-N14-p19917307	gene	
Bn-scaff_27634_1-p85009	Bn-N14-p19917239	gene	
Bn-scaff_27634_1-p85133	Bn-N14-p19917115	gene	
Bn-scaff_27634_1-p90485	Bn-N14-p19911824	gene	exon
Bn-scaff_27650_1-p11659	Bn-N19-p3827541	gene	exon
Bn-scaff_27670_1-p347062	Bn-N12-p4549883	gene	exon
Bn-scaff_27670_1-p366718	Bn-N2-p3649033	gene	exon
Bn-scaff_27676_1-p175780	Bn-N14-p54715295	gene	

Bn-scaff_27676_1-p176043	Bn-N14-p54715554	gene	exon
Bn-scaff_27676_1-p191497	Bn-N14-p54729514	gene	
Bn-scaff_27676_1-p191772	Bn-N4-p20962060	gene	exon
Bn-scaff_27676_1-p33891	Bn-N4-p20817866	gene	exon
Bn-scaff_27677_1-p105296	Bn-N13-p20804689	gene	
Bn-scaff_27677_1-p118912	Bn-N13-p20797073	gene	exon
Bn-scaff_27677_1-p125947	Bn-Scaffold11358-p750	gene	exon
Bn-scaff_27677_1-p145105	Bn-N15-p36473267	gene	
Bn-scaff_27677_1-p196839	Bn-N2-p14964649	gene	
Bn-scaff_27677_1-p236486	Bn-N13-p20682700	gene	
Bn-scaff_27677_1-p246414	Bn-N3-p13529497	gene	exon
Bn-scaff_27677_1-p246472	Bn-N3-p13529439	gene	exon
Bn-scaff_27677_1-p247580	Bn-N3-p13528428	gene	exon
Bn-scaff_27677_1-p247804	Bn-N3-p13528204	gene	
Bn-scaff_27677_1-p249301	Bn-N13-p20673769	gene	
Bn-scaff_27677_1-p250638	Bn-N13-p20672417	gene	
Bn-scaff_27677_1-p301065	Bn-N3-p13479557	gene	exon
Bn-scaff_27677_1-p301097	Bn-N3-p13479526	gene	exon
Bn-scaff_27677_1-p330749	Bn-N3-p13469337	gene	exon
Bn-scaff_27705_1-p102681	Bn-N3-p3611112	gene	exon
Bn-scaff_27705_1-p111087	Bn-N19-p49176335	gene	
Bn-scaff_27705_1-p111279	Bn-N19-p49176144	gene	
Bn-scaff_27705_1-p21795	Bn-N10-p12940659	gene	
Bn-scaff_27747_1-p20694	Bn-N13-p52992671	gene	exon
Bn-scaff_27747_1-p20714	Bn-N13-p52992691	gene	exon
Bn-scaff_27747_1-p20852	Bn-N13-p52992829	gene	exon
Bn-scaff_27765_1-p332203	Bn-N8-p2706828	gene	
Bn-scaff_27765_1-p362839	Bn-N1-p22454032	gene	exon
Bn-scaff_27765_1-p58899	Bn-N18-p3783652	gene	
Bn-scaff_27815_1-p35807	Bn-N17-p4639605	gene	exon
Bn-scaff_27815_1-p367403	Bn-N7-p1700315	gene	exon
Bn-scaff_27870_1-p208671	Bn-N13-p21100276	gene	
Bn-scaff_27872_1-p39306	Bn-N13-p4814849	gene	
Bn-scaff_27872_1-p47050	Bn-N13-p4807089	gene	exon
Bn-scaff_27881_1-p14038	Bn-N13-p21009639	gene	exon
Bn-scaff_27881_1-p15080	Bn-N13-p21008597	gene	exon
Bn-scaff_27881_1-p15401	Bn-N13-p21008276	gene	exon
Bn-scaff_27914_1-p19775	Bn-N14-p32687869	gene	exon
Bn-scaff_27914_1-p40505	Bn-N4-p3625594	gene	exon
Bn-scaff_27914_1-p40676	Bn-N4-p3625765	gene	exon
Bn-scaff_27914_1-p74694	Bn-N14-p32736121	gene	
Bn-scaff_27946_1-p19861	Bn-N12-p42961199	gene	
Bn-scaff_27946_1-p6065	Bn-Scaffold01557-p4225	gene	
Bn-scaff_27955_1-p76	Bn-N7-p1087645	gene	
Bn-scaff_28053_1-p12340	Bn-N10-p17259635	gene	exon
Bn-scaff_28053_1-p60542	Bn-N10-p17228691	gene	exon
Bn-scaff_28179_1-p16904	Bn-N15-p3139942	gene	
Bn-scaff_28179_1-p21528	Bn-N15-p3135500	gene	exon
Bn-scaff_28179_1-p23683	Bn-N15-p3133348	gene	
Bn-scaff_28277_1-p5229	Bn-N14-p4000530	gene	
Bn-scaff_28371_1-p431	Bn-N11-p5942097	gene	exon
Bn-scaff_28382_1-p38738	Bn-N14-p2716875	gene	

Bn-scaff_28382_1-p39187	Bn-N14-p2717323	gene	exon
Bn-scaff_28382_1-p44654	Bn-N14-p2722831	gene	exon
Bn-scaff_28403_1-p116546	Bn-N17-p7436681	gene	exon
Bn-scaff_28403_1-p123434	Bn-N17-p7443564	gene	exon
Bn-scaff_28429_1-p325716	Bn-N7-p8365999	gene	exon
Bn-scaff_28429_1-p992606	Bn-N9-p8145546	gene	
Bn-scaff_28498_1-p107723	Bn-N14-p19686203	gene	
Bn-scaff_28498_1-p108458	Bn-N14-p19686926	gene	exon
Bn-scaff_28498_1-p163192	Bn-N14-p19699906	gene	
Bn-scaff_28498_1-p163305	Bn-N14-p19699793	gene	exon
Bn-scaff_28498_1-p163802	Bn-N14-p19699296	gene	exon
Bn-scaff_28498_1-p163884	Bn-N14-p19699214	gene	exon
Bn-scaff_28498_1-p164284	Bn-N14-p19698813	gene	
Bn-scaff_28498_1-p164307	Bn-N14-p19698790	gene	
Bn-scaff_28498_1-p165023	Bn-N14-p19698068	gene	
Bn-scaff_28498_1-p165148	Bn-N14-p19697945	gene	
Bn-scaff_28498_1-p165263	Bn-N14-p19697830	gene	exon
Bn-scaff_28498_1-p172641	Bn-Scaffold01576-p1116	gene	
Bn-scaff_28498_1-p245004	Bn-N14-p19744743	gene	
Bn-scaff_28498_1-p245518	Bn-N14-p19745257	gene	
Bn-scaff_28498_1-p246839	Bn-N14-p19746586	gene	
Bn-scaff_28498_1-p91470	Bn-N14-p19654365	gene	exon
Bn-scaff_28509_1-p33640	Bn-N13-p21636653	gene	exon
Bn-scaff_28509_1-p33710	Bn-N13-p21636583	gene	
Bn-scaff_28509_1-p33929	Bn-N13-p21636365	gene	
Bn-scaff_28509_1-p34128	Bn-N13-p21636166	gene	exon
Bn-scaff_28511_1-p19968	Bn-N19-p23618646	gene	exon
Bn-scaff_28522_1-p35475	Bn-N7-p13000790	gene	exon
Bn-scaff_28562_1-p10940	Bn-N13-p25462781	gene	
Bn-scaff_28637_1-p15131	Bn-N18-p32357196	gene	
Bn-scaff_28640_1-p43648	Bn-N13-p29314129	gene	
Bn-scaff_28666_1-p29889	Bn-N11-p9945397	gene	
Bn-scaff_28678_1-p272918	Bn-N9-p582355	gene	exon
Bn-scaff_28678_1-p34404	Bn-N9-p744727	gene	exon
Bn-scaff_28678_1-p441440	Bn-N19-p417144	gene	exon
Bn-scaff_28678_1-p442929	Bn-N19-p414554	gene	exon
Bn-scaff_28678_1-p443552	Bn-N19-p413534	gene	exon
Bn-scaff_28678_1-p591337	Bn-N19-p253766	gene	
Bn-scaff_28678_1-p591388	Bn-N19-p253766	gene	
Bn-scaff_28678_1-p591776	Bn-N19-p253390	gene	exon
Bn-scaff_28703_1-p31309	Bn-N14-p20494852	gene	exon
Bn-scaff_28703_1-p34884	Bn-N14-p20444850	gene	
Bn-scaff_28703_1-p49551	Bn-N14-p20518427	gene	
Bn-scaff_28703_1-p49734	Bn-N14-p20518610	gene	
Bn-scaff_28703_1-p50575	Bn-N14-p20519450	gene	exon
Bn-scaff_28703_1-p50822	Bn-N14-p20519697	gene	exon
Bn-scaff_28703_1-p50848	Bn-N14-p20519723	gene	exon
Bn-scaff_28703_1-p51095	Bn-N14-p20519970	gene	
Bn-scaff_28703_1-p51257	Bn-N14-p20520131	gene	
Bn-scaff_28703_1-p51978	Bn-N14-p20520852	gene	
Bn-scaff_28703_1-p53260	Bn-N14-p20522087	gene	
Bn-scaff_28703_1-p53619	Bn-N14-p20522446	gene	

Bn-scaff_28703_1-p53700	Bn-N14-p20522527	gene	exon
Bn-scaff_28703_1-p53783	Bn-N14-p20522610	gene	exon
Bn-scaff_28774_1-p13523	Bn-N13-p9257251	gene	
Bn-scaff_28774_1-p29882	Bn-N13-p9283447	gene	exon
Bn-scaff_28791_1-p5	Bn-N19-p46963129	gene	
Bn-scaff_28977_1-p7912	Bn-N2-p21350057	gene	
Bn-scaff_29028_1-p18552	Bn-N15-p45584381	gene	exon
Bn-scaff_29028_1-p9948	Bn-N15-p45574314	gene	exon
Bn-scaff_29061_1-p19327	Bn-N17-p43330956	gene	
Bn-scaffold12606-p486	Bn-N13-p4786482	gene	
Bn-scaffold12714-p261	Bn-Scaffold03435-p431	gene	
Bn-scaffold13646-p3	Bn-N16-p17821892	gene	exon
Bn-scaffold14071-p64	Bn-N14-p24388542	gene	exon
Bn-scaffold18039-p397	Bn-N14-p21035217	gene	exon
Bn-scaffold733-p492	Bn-Scaffold04835-p328	gene	exon
Bn-scaffold7442-p97	Bn-N15-p34454042	gene	
Bn-scaffold8358-p141	Bn-N2-p25495898	gene	
