# Supplementary material for: A high-density SNP genotyping array for Brassica napus and its ancestral diploid species based on optimised selection of single-locus markers in the allotetraploid genome
Source: Theor Appl Genet. 2016 Jun 30;129(10):1887–99. doi: 10.1007/s00122-016-2746-7 (PMC5025514; doi:10.1007/s00122-016-2746-7)
Supplement: Supplementary file 7 — Genetic map for Brassica napus based on SG DH population compressed into genetic bins according to recombination events. (PDF 1685 kb) [file 122_2016_2746_MOESM7_ESM.pdf]

Table 5: Genetic map for Brassica napus based on SG population compressed in

Order	DH12075v4_ID	New_name_rapa
1	Bn-N1-p148713	Bn-A01-p137814
13	Bn-N1-p455627	Bn-A01-p460072
31	Bn-N1-p819843	Bn-C2-p12033716
35	Bn-N1-p1001647	Bn-A01-p974934
60	Bn-N1-p1292716	Bn-A01-p1250595
65	Bn-N1-p1418752	Bn-A01-p1372430
76	Bn-N1-p1703757	Bn-A01-p1661180
79	Bn-N1-p1806273	Bn-A01-p1742846
82	Bn-N1-p1840607	Bn-A01-p1770291
94	Bn-N1-p2040832	Bn-A01-p1990755
101	Bn-N1-p2145056	Bn-A01-p2094793
104	Bn-N1-p2211868	Bn-A01-p2171217
106	Bn-N1-p2284563	Bn-A01-p2242890
123	Bn-N1-p2720419	Bn-A01-p2667471
130	Bn-N1-p2879286	Bn-A01-p2825565
137	Bn-N1-p3051730	Bn-A01-p2990104
151	Bn-N1-p3247018	Bn-A01-p3183888
152	Bn-N1-p3356185	Bn-A01-p3306526
170	Bn-N1-p3952570	Bn-A01-p3904495
199	Bn-N1-p4828077	Bn-A01-p4812269
215	Bn-N1-p5078759	Bn-A01-p5106042
216	Bn-N1-p5157847	Bn-A01-p5193639
254	Bn-N1-p5588331	Bn-A01-p5590998
286	Bn-N1-p6206809	Bn-A01-p6182710
292	Bn-N1-p6286302	Bn-A01-p6274135
300	Bn-N1-p6671771	Bn-A01-p6687707
309	Bn-N1-p6894209	Bn-A01-p6898291
324	Bn-N1-p7686806	Bn-A01-p7648929
329	Bn-N1-p7947519	Bn-A01-p7889385
335	Bn-N1-p8033912	Bn-A01-p7979458
342	Bn-N1-p8072235	Bn-A01-p8017380
388	Bn-N1-p8995481	Bn-A01-p9442471
400	Bn-N1-p9398357	Bn-A01-p9884449
404	Bn-N1-p9540618	Bn-A01-p9996458
405	Bn-N1-p9782678	Bn-A01-p10231780
423	Bn-N1-p10024494	Bn-C4-p24903660
471	Bn-N1-p11321978	Bn-A01-p11825880
678	Bn-N1-p19905997	Bn-A01-p19983739
711	Bn-N1-p21381690	Bn-A01-p21393260
724	Bn-N1-p21689831	Bn-A01-p21709916
727	Bn-N1-p21832963	Bn-A01-p21855770
746	Bn-N1-p22039892	Bn-A01-p22051510
748	Bn-N1-p22090041	Bn-A01-p22087629
750	Bn-N1-p22242804	Bn-A01-p22681496
781	Bn-N1-p23075763	Bn-A01-p23568963

783	Bn-N1-p23129730	Bn-C1-p37030858
784	Bn-N1-p23132864	Bn-A01-p23614494
791	Bn-N1-p23209338	Bn-A01-p23694782
805	Bn-N1-p23749821	Bn-A01-p24504575
819	Bn-N1-p23944238	Bn-A01-p24697185
830	Bn-N1-p24226858	Bn-Scaffold000164-p244631
844	Bn-N1-p24408926	Bn-Scaffold000164-p55747
859	Bn-N1-p24868250	Bn-Scaffold000386-p1325
865	Bn-N1-p25216255	Bn-A01-p27331467
870	Bn-N1-p25466344	Bn-A01-p27580681
873	Bn-N1-p25801701	Bn-A01-p27866419
880	Bn-N1-p26094193	Bn-A01-p28102080
888	Bn-N1-p26290806	Bn-A01-p28462071
895	Bn-N1-p26698258	Bn-A01-p25517197
909	Bn-N1-p27020484	Bn-A01-p25040577
922	Bn-N2-p392255	Bn-A02-p1707420
934	Bn-N2-p1000576	Bn-A02-p2292871
937	Bn-N2-p1244624	Bn-A02-p2542065
938	Bn-N2-p1274020	Bn-A02-p2572625
940	Bn-N2-p1314947	Bn-A02-p2617701
950	Bn-N2-p1453265	Bn-A02-p2739888
957	Bn-N2-p1786578	Bn-A02-p1096082
958	Bn-N2-p1790534	Bn-A02-p1092146
964	Bn-N2-p1952858	Bn-A02-p2877222
965	Bn-N2-p1983351	Bn-A02-p2923889
970	Bn-N2-p2151215	Bn-A02-p3074016
971	Bn-N2-p2196718	Bn-A02-p3121742
975	Bn-N2-p2256921	Bn-A02-p3175610
983	Bn-N2-p2345136	Bn-A02-p3260536
984	Bn-N2-p2380926	Bn-A02-p3294682
988	Bn-N2-p2434012	Bn-A02-p3344176
991	Bn-N2-p2458886	Bn-A02-p3374678
992	Bn-N2-p2482742	Bn-A02-p3398506
997	Bn-N2-p2616924	Bn-A02-p3542024
1009	Bn-N2-p3119297	Bn-A02-p4035808
1020	Bn-N2-p3355557	Bn-A02-p4277649
1029	Bn-N2-p3865572	Bn-A02-p4773307
1032	Bn-N2-p4461220	Bn-A02-p5326222
1033	Bn-N12-p6060989	Bn-A02-p5394958
1034	Bn-N2-p4567982	Bn-A02-p5429003
1042	Bn-N2-p4725469	Bn-A02-p5574727
1051	Bn-N2-p5035622	Bn-A02-p5859447
1052	Bn-N2-p5101425	Bn-A02-p5907701
1057	Bn-N2-p5291239	Bn-A02-p6084757
1083	Bn-N2-p5711970	Bn-A02-p6478309
1090	Bn-N2-p5919336	Bn-A02-p6658392
1117	Bn-N2-p6595573	Bn-A02-p7319800
1185	Bn-N2-p7984224	Bn-A02-p8625851
1194	Bn-N2-p8138197	Bn-A02-p8777846

1195	Bn-N2-p8215157	Bn-A02-p8865764
1232	Bn-N2-p8688243	Bn-A02-p9339288
1295	Bn-N2-p10579677	Bn-A02-p11041726
1356	Bn-N2-p10907135	Bn-A02-p11346389
1389	Bn-N2-p11561943	Bn-A02-p11817742
1407	Bn-N2-p11905764	Bn-A02-p12145607
1416	Bn-N2-p12045225	Bn-A02-p12292026
1489	Bn-N2-p13922267	Bn-A02-p14078497
1497	Bn-N2-p14075845	Bn-A02-p14227678
1503	Bn-N2-p14254515	Bn-Scaffold000262-p25395
1526	Bn-N2-p15525546	Bn-A02-p15637932
1551	Bn-N2-p16741741	Bn-A05-p9078281
1562	Bn-N2-p18281250	Bn-A02-p17381818
1568	Bn-N2-p21124875	Bn-A02-p18599005
1590	Bn-N2-p21822532	Bn-A02-p19261853
1636	Bn-N2-p22881947	Bn-A02-p21061002
1660	Bn-N2-p24122314	Bn-A02-p19756499
1662	Bn-N2-p24221591	Bn-A02-p22843446
1665	Bn-N2-p24299028	Bn-A02-p22757902
1673	Bn-N2-p24508335	Bn-A02-p22352518
1683	Bn-N2-p25829195	Bn-A02-p23411852
1698	Bn-N2-p25955982	Bn-Scaffold016439-p48
1705	Bn-N2-p26087020	Bn-A02-p23668607
1711	Bn-N12-p40545513	Bn-C2-p47337384
1713	Bn-N2-p26180452	Bn-A02-p23792703
1729	Bn-N2-p26534947	Bn-A02-p24178094
1744	Bn-N2-p26761096	Bn-A02-p24415454
1745	Bn-N2-p26799071	Bn-A02-p24449690
1752	Bn-N2-p26923902	Bn-A02-p24552542
1760	Bn-N2-p27068181	Bn-C2-p49029761
1765	Bn-N2-p27309412	Bn-A02-p24948938
1768	Bn-N2-p27435756	Bn-A02-p25100451
1772	Bn-N2-p27518281	Bn-A02-p25188041
1777	Bn-N2-p27686816	Bn-A02-p25400591
1814	Bn-N2-p27948371	Bn-A02-p25681480
1820	Bn-N2-p28092398	Bn-A02-p25826690
1821	Bn-N2-p28101502	Bn-A02-p25841130
1829	Bn-N2-p28125432	Bn-A02-p25865360
1835	Bn-N2-p28771949	Bn-A02-p26982864
1858	Bn-N2-p29609872	Bn-A02-p27831174
1860	Bn-N3-p330552	Bn-A03-p312762
1875	Bn-N3-p772129	Bn-A03-p765077
1879	Bn-N3-p1010731	Bn-A03-p991949
1882	Bn-N3-p1080752	Bn-A03-p1050437
1898	Bn-N3-p1499294	Bn-A03-p1488306
1908	Bn-N3-p1755477	Bn-A03-p1711856
1916	Bn-N3-p2019723	Bn-A03-p1926809
1919	Bn-N3-p2055094	Bn-A03-p1954639
1936	Bn-N3-p2679025	Bn-A03-p2579866

1941	Bn-N3-p2742051	Bn-A03-p2645391
1957	Bn-N3-p2966999	Bn-A03-p2891934
1961	Bn-N3-p3074368	Bn-A03-p2997270
1968	Bn-N3-p3183725	Bn-A03-p3099901
1975	Bn-N3-p3406926	Bn-A03-p3319357
1979	Bn-N3-p3772359	Bn-A03-p3692048
1980	Bn-N3-p3885826	Bn-A03-p3795299
2012	Bn-N3-p4831677	Bn-A03-p4783485
2016	Bn-N3-p4958930	Bn-A03-p4909233
2023	Bn-N3-p5115640	Bn-A03-p5083198
2037	Bn-N3-p5347595	Bn-Scaffold01280-p438
2062	Bn-N3-p5775835	Bn-A03-p5741340
2071	Bn-N3-p6077412	Bn-A03-p6036521
2077	Bn-N3-p6308534	Bn-A03-p6274828
2088	Bn-N3-p6633766	Bn-A03-p6590051
2092	Bn-N3-p6770990	Bn-A03-p6744344
2098	Bn-N3-p7039006	Bn-A03-p7007377
2102	Bn-N3-p7234314	Bn-A03-p7177504
2110	Bn-N3-p7435856	Bn-A03-p7366783
2114	Bn-N3-p7487460	Bn-A03-p7420835
2132	Bn-N3-p7994167	Bn-A03-p7939679
2140	Bn-N3-p8190537	Bn-A03-p8133096
2184	Bn-N3-p8872358	Bn-A03-p8764481
2224	Bn-N3-p9706667	Bn-A03-p9537155
2231	Bn-N3-p10026056	Bn-A03-p9833648
2258	Bn-N3-p10653298	Bn-A03-p10414176
2271	Bn-N3-p10915390	Bn-A03-p10651420
2276	Bn-N3-p11028842	Bn-A03-p10757525
2321	Bn-N3-p11599250	Bn-A03-p11319722
2333	Bn-N3-p11803507	Bn-A03-p11517597
2359	Bn-N3-p12537961	Bn-A03-p12186958
2379	Bn-N3-p13028750	Bn-A03-p12639200
2384	Bn-N3-p13385371	Bn-A03-p12990610
2402	Bn-N3-p13489967	Bn-A03-p13104283
2460	Bn-N3-p14438774	Bn-A03-p14112910
2463	Bn-N3-p14508983	Bn-A03-p14175904
2473	Bn-N3-p14641501	Bn-A03-p14306440
2489	Bn-N3-p14777622	Bn-A03-p14447238
2521	Bn-N3-p15710902	Bn-A03-p15359867
2535	Bn-N3-p15957400	Bn-A03-p15577849
2550	Bn-N3-p16312745	Bn-A03-p15957657
2568	Bn-N3-p16961243	Bn-A03-p16607714
2575	Bn-N3-p17185957	Bn-A03-p16812953
2576	Bn-N13-p26848688	Bn-C3-p25537230
2588	Bn-N3-p17584688	Bn-A03-p17182494
2591	Bn-N3-p18120292	Bn-A03-p17782292
2595	Bn-N3-p18475063	Bn-A03-p18120938
2618	Bn-N3-p19216285	Bn-A03-p18836509
2619	Bn-N3-p19222659	Bn-A03-p18846268

2655	Bn-N3-p20192016	Bn-A03-p19880918
2669	Bn-N3-p20329049	Bn-A03-p20033408
2670	Bn-N3-p20371571	Bn-A03-p20079452
2674	Bn-N3-p20445824	Bn-A03-p20151244
2675	Bn-N3-p20469807	Bn-A03-p20180463
2677	Bn-N3-p20532008	Bn-A03-p20237681
2698	Bn-N3-p20834180	Bn-A03-p20523585
2714	Bn-N3-p21231831	Bn-A03-p20983297
2719	Bn-N3-p21543364	Bn-A03-p21271941
2726	Bn-N3-p21713680	Bn-A03-p21422696
2733	Bn-N3-p21900335	Bn-A03-p21632587
2734	Bn-N3-p21947903	Bn-A03-p21685480
2751	Bn-N3-p22166287	Bn-A03-p21904842
2801	Bn-N3-p23119669	Bn-A03-p22820943
2807	Bn-N3-p23181434	Bn-A03-p22882585
2811	Bn-N3-p23342059	Bn-A03-p23017354
2818	Bn-N3-p23667615	Bn-A03-p29571938
2819	Bn-N3-p24031518	Bn-A03-p23735715
2835	Bn-N3-p24362517	Bn-A03-p24092986
2836	Bn-N3-p24431801	Bn-A03-p24176332
2848	Bn-N3-p11952122	Bn-A03-p24902631
2937	Bn-N3-p25600454	Bn-A03-p25402301
2943	Bn-N3-p25724701	Bn-A03-p25525612
2945	Bn-N3-p25828301	Bn-A03-p25614406
2946	Bn-N3-p25845246	Bn-A03-p25631316
2963	Bn-N3-p26231379	Bn-A03-p25993601
2967	Bn-N3-p26287335	Bn-A03-p26066930
2973	Bn-N3-p26517534	Bn-A03-p26284882
2974	Bn-N3-p26521343	Bn-A03-p26288769
2990	Bn-N3-p26736040	Bn-A03-p26477533
3001	Bn-N3-p27060036	Bn-A03-p26775994
3011	Bn-N3-p27367099	Bn-A03-p27066000
3047	Bn-N3-p28068549	Bn-A03-p27683723
3089	Bn-N3-p28971341	Bn-A03-p28535638
3149	Bn-N3-p33346228	Bn-Scaffold000096-p878406
3189	Bn-N3-p28566839	Bn-A03-p28126287
3190	Bn-N4-p193375	Bn-A04-p122280
3200	Bn-N4-p269934	Bn-A04-p267294
3254	Bn-N4-p811294	Bn-A04-p810935
3269	Bn-N4-p1148070	Bn-A04-p1213196
3277	Bn-N4-p1252454	Bn-A04-p1317446
3283	Bn-N4-p1324713	Bn-A04-p1390449
3288	Bn-N4-p1567651	Bn-A04-p1623151
3289	Bn-N4-p1589334	Bn-A04-p1655416
3290	Bn-N4-p1607740	Bn-A04-p1680506
3291	Bn-N4-p1686812	Bn-A04-p1745832
3313	Bn-N4-p1871339	Bn-A04-p1931502
3431	Bn-N4-p3129508	Bn-A04-p3225119
3456	Bn-N4-p3548333	Bn-A04-p3632226

3459	Bn-N4-p3642885	Bn-A04-p3743549
3492	Bn-N4-p7608030	Bn-A04-p5959753
3662	Bn-N4-p8850965	Bn-A04-p7109346
3754	Bn-N4-p10191316	Bn-A04-p8292499
3789	Bn-N4-p10512694	Bn-A04-p8612311
3826	Bn-N4-p11037233	Bn-A04-p9088043
3977	Bn-N4-p14285273	Bn-A04-p11957518
3985	Bn-N4-p14573838	Bn-A04-p12236240
4002	Bn-N4-p14726403	Bn-A04-p12659402
4075	Bn-N4-p15678260	Bn-A04-p13659690
4086	Bn-N4-p16108021	Bn-C4-p46736296
4088	Bn-N4-p16162014	Bn-C4-p46830608
4089	Bn-N4-p16255878	Bn-A04-p14255771
4101	Bn-N4-p16711540	Bn-A04-p14756001
4110	Bn-N4-p16965424	Bn-A04-p15007146
4114	Bn-N4-p17271998	Bn-A04-p15298740
4118	Bn-N4-p17369430	Bn-A04-p15404568
4126	Bn-N4-p17499224	Bn-A04-p15514869
4136	Bn-Scaffold31010-p309	Bn-A04-p15835310
4137	Bn-N7-p15002311	Bn-A04-p15895339
4142	Bn-N4-p17983696	Bn-A04-p16056186
4143	Bn-N4-p18198567	Bn-A04-p16233024
4155	Bn-N4-p18420558	Bn-A04-p16445225
4168	Bn-N4-p18620726	Bn-A04-p16663552
4173	Bn-N4-p18743497	Bn-A04-p17052071
4203	Bn-N4-p19262174	Bn-A04-p17322241
4218	Bn-N4-p19649505	Bn-A04-p17686228
4228	Bn-N4-p19775221	Bn-A04-p17811740
4230	Bn-N4-p19780403	Bn-A04-p17816796
4231	Bn-N4-p19827880	Bn-A04-p17864572
4241	Bn-N4-p20125262	Bn-A04-p18136301
4243	Bn-N4-p20169038	Bn-A04-p18180777
4244	Bn-N4-p20260734	Bn-A04-p18273048
4247	Bn-N4-p20419859	Bn-A04-p18429573
4248	Bn-N4-p20483625	Bn-A04-p18487715
4257	Bn-N4-p20625106	Bn-A04-p18617343
4268	Bn-N4-p21066278	Bn-A01-p25171870
4278	Bn-N5-p407617	Bn-Scaffold000403-p12455
4295	Bn-N5-p759307	Bn-A05-p679803
4314	Bn-N5-p1000999	Bn-A05-p892520
4324	Bn-N5-p1080492	Bn-A05-p970816
4325	Bn-N5-p1174032	Bn-A05-p1071371
4331	Bn-N5-p1410753	Bn-A05-p1308471
4333	Bn-N5-p1436231	Bn-A05-p1335771
4348	Bn-N5-p1619320	Bn-A05-p1492327
4360	Bn-N5-p1901300	Bn-A05-p1701030
4361	Bn-N5-p1949635	Bn-A05-p1761473
4362	Bn-N5-p2039192	Bn-A05-p1857946
4363	Bn-N5-p2075840	Bn-A05-p1898273

4371	Bn-N5-p2298341	Bn-A05-p2106680
4385	Bn-N5-p2509507	Bn-A05-p2303112
4386	Bn-N14-p3918239	Bn-A05-p2345233
4387	Bn-N5-p2562704	Bn-A05-p2367876
4392	Bn-N5-p2673519	Bn-C4-p3971988
4405	Bn-N5-p2836831	Bn-A05-p2641800
4412	Bn-N5-p2920992	Bn-A05-p2726301
4431	Bn-N5-p3236393	Bn-A05-p3005109
4451	Bn-N5-p3553066	Bn-A05-p3307736
4464	Bn-N5-p3749456	Bn-A05-p3507831
4472	Bn-N5-p4104667	Bn-A05-p3834445
4477	Bn-N5-p4182623	Bn-A05-p3923814
4478	Bn-N5-p4230168	Bn-A05-p3966848
4480	Bn-N5-p4250529	Bn-A05-p3988958
4494	Bn-N5-p4386273	Bn-A05-p4121158
4499	Bn-N5-p4597112	Bn-A05-p4336667
4523	Bn-N5-p5063788	Bn-C4-p7980371
4531	Bn-N5-p5194068	Bn-A05-p4940713
4538	Bn-N5-p5361751	Bn-A05-p5093548
4552	Bn-N5-p5652046	Bn-A05-p5372015
4557	Bn-N5-p5746359	Bn-A05-p5475992
4581	Bn-N5-p6077945	Bn-A05-p5805445
4592	Bn-N5-p6250576	Bn-A05-p5970916
4616	Bn-N5-p6966977	Bn-A05-p6541429
4642	Bn-N5-p7292023	Bn-A05-p6995896
4770	Bn-N5-p10708377	Bn-A05-p11084469
4967	Bn-N5-p16370464	Bn-A05-p16092993
5040	Bn-N5-p19205661	Bn-A05-p18229790
5062	Bn-N5-p19863530	Bn-A05-p18905070
5071	Bn-N5-p20015936	Bn-A05-p19070284
5072	Bn-N5-p20060451	Bn-A05-p19116891
5102	Bn-N5-p20653946	Bn-A05-p19650965
5110	Bn-N5-p20796236	Bn-A05-p19802577
5113	Bn-N5-p20921094	Bn-A05-p19919179
5144	Bn-N5-p21252649	Bn-A05-p20254809
5163	Bn-N5-p21557718	Bn-A05-p20555996
5197	Bn-N5-p21986809	Bn-A05-p20972649
5201	Bn-N5-p22049826	Bn-A05-p21028051
5213	Bn-N5-p22236141	Bn-A05-p21201956
5221	Bn-N5-p22288664	Bn-A05-p21248382
5222	Bn-N5-p22365600	Bn-A05-p21326091
5223	Bn-N5-p22392693	Bn-A05-p21347821
5228	Bn-N5-p22571580	Bn-A05-p21470614
5236	Bn-N5-p22695386	Bn-A05-p21587317
5247	Bn-N5-p22895992	Bn-A05-p21806633
5251	Bn-N5-p22951199	Bn-A05-p21889289
5252	Bn-N5-p22994971	Bn-A05-p21930978
5253	Bn-N5-p23203724	Bn-A05-p22152482
5256	Bn-N5-p23135358	Bn-A05-p22089650

5258	Bn-N5-p23280486	Bn-A05-p22218776
5263	Bn-N5-p23332175	Bn-A05-p22269511
5264	Bn-N5-p23348012	Bn-A05-p22282750
5273	Bn-N5-p23571097	Bn-A05-p22498838
5279	Bn-N5-p23652525	Bn-A05-p22591072
5282	Bn-N5-p23676910	Bn-A05-p22613926
5288	Bn-N5-p23759203	Bn-A05-p22691469
5290	Bn-N5-p23835201	Bn-A05-p22767106
5294	Bn-N5-p23859503	Bn-A05-p22789711
5299	Bn-N5-p23997708	Bn-A05-p22899595
5307	Bn-N5-p24063948	Bn-A05-p22973324
5335	Bn-N5-p24463861	Bn-A02-p23161914
5340	Bn-N5-p24618267	Bn-A05-p23253826
5357	Bn-N5-p25031207	Bn-Scaffold000191-p53301
5358	Bn-N5-p25180238	Bn-A02-p26901243
5375	Bn-N5-p25336681	Bn-A02-p26665282
5389	Bn-N5-p25659292	Bn-A05-p23776343
5403	Bn-N6-p108297	Bn-A06-p195449
5447	Bn-N6-p1011787	Bn-A06-p1054104
5458	Bn-N6-p1256849	Bn-A06-p1322724
5466	Bn-N6-p1337715	Bn-A06-p1404859
5479	Bn-N16-p4752677	Bn-A06-p1507197
5482	Bn-N6-p1700511	Bn-A06-p1743446
5483	Bn-N6-p1726972	Bn-A06-p1768254
5492	Bn-N6-p1911301	Bn-A06-p1947247
5499	Bn-N6-p2229938	Bn-A06-p2234718
5501	Bn-N6-p2243184	Bn-A06-p2251910
5505	Bn-N6-p2261524	Bn-A06-p2268393
5515	Bn-N6-p2407250	Bn-A06-p2407080
5516	Bn-N6-p2468768	Bn-A06-p2514723
5517	Bn-N6-p2492906	Bn-A06-p2542722
5520	Bn-N6-p2568453	Bn-A06-p2617079
5528	Bn-N6-p2616610	Bn-A06-p2676314
5546	Bn-N6-p2816451	Bn-A06-p2891290
5549	Bn-N6-p2861174	Bn-A06-p2936438
5552	Bn-N6-p2879696	Bn-A06-p2958095
5560	Bn-N6-p2968512	Bn-A06-p3050747
5585	Bn-N6-p3236858	Bn-A06-p3325502
5601	Bn-N6-p3411122	Bn-A06-p3491241
5607	Bn-N6-p3893142	Bn-A06-p3970972
5612	Bn-N6-p3958445	Bn-A06-p4019827
5613	Bn-N6-p3961778	Bn-A06-p4021930
5619	Bn-N6-p4046387	Bn-A06-p4098800
5620	Bn-N6-p4111353	Bn-A06-p4179318
5627	Bn-N6-p4304145	Bn-A06-p4371627
5637	Bn-N6-p4418843	Bn-A06-p4508774
5645	Bn-N6-p4795418	Bn-A06-p4874556
5661	Bn-N6-p5136130	Bn-A06-p5546411
5662	Bn-N6-p5200409	Bn-A06-p5616164

5668	Bn-N6-p5325363	Bn-A06-p5739682
5683	Bn-N6-p5547966	Bn-A06-p5947781
5684	Bn-N6-p5565642	Bn-A06-p5956274
5692	Bn-N6-p5707075	Bn-A06-p6103844
5695	Bn-N6-p5760603	Bn-A06-p6182046
5701	Bn-N6-p5898614	Bn-A06-p6311231
5705	Bn-N6-p5934735	Bn-A06-p6343931
5719	Bn-N6-p6226240	Bn-A06-p6624071
5722	Bn-N6-p6246719	Bn-A06-p6647323
5733	Bn-N6-p6349603	Bn-A06-p6761232
5780	Bn-N6-p7381938	Bn-A06-p7670696
5781	Bn-N6-p7413359	Bn-A06-p7701069
5800	Bn-N6-p7956675	Bn-A06-p8263325
5807	Bn-N6-p8125820	Bn-A06-p8446816
5871	Bn-N6-p10000718	Bn-A06-p10330066
6031	Bn-N6-p17186112	Bn-A06-p15258692
6084	Bn-N6-p18624752	Bn-A06-p16657894
6094	Bn-N6-p18887432	Bn-A06-p17006799
6110	Bn-N6-p19142761	Bn-A09-p18588652
6123	Bn-N6-p19420003	Bn-A06-p17548894
6130	Bn-N6-p19565919	Bn-A06-p17671185
6151	Bn-N6-p19927994	Bn-A06-p18029021
6165	Bn-N6-p20136498	Bn-A06-p18219362
6176	Bn-N6-p20277325	Bn-A06-p18364834
6184	Bn-N6-p20315760	Bn-A06-p18402713
6188	Bn-N6-p20405390	Bn-A06-p18500596
6202	Bn-N6-p20736799	Bn-A06-p21525576
6208	Bn-N6-p20905511	Bn-A06-p21349507
6218	Bn-N6-p21068646	Bn-A06-p21195115
6222	Bn-Scaffold74746-p122	Bn-A06-p21163037
6223	Bn-N6-p21106756	Bn-C7-p34444146
6224	Bn-N6-p21128461	Bn-A06-p21134315
6227	Bn-N6-p21161410	Bn-A06-p21101957
6239	Bn-N6-p21187578	Bn-A06-p21072392
6255	Bn-N6-p21432182	Bn-A06-p21796008
6256	Bn-N6-p21438164	Bn-A06-p21800812
6262	Bn-N6-p21463906	Bn-A06-p21826671
6268	Bn-N6-p21499365	Bn-A06-p21846491
6285	Bn-N6-p21827996	Bn-A06-p22160452
6295	Bn-N6-p22430716	Bn-A06-p22709872
6296	Bn-N6-p22667869	Bn-A06-p22947499
6312	Bn-N6-p23270696	Bn-A06-p23468803
6313	Bn-N6-p23406052	Bn-A06-p23591616
6318	Bn-N6-p23472580	Bn-A06-p23665564
6330	Bn-N6-p23666104	Bn-A06-p23852270
6331	Bn-N6-p23801589	Bn-A06-p23988702
6338	Bn-N6-p23909011	Bn-A06-p24099288
6354	Bn-N6-p24224670	Bn-A06-p24386467
6364	Bn-N6-p24309893	Bn-A06-p24467652

6366	Bn-N6-p24345526	Bn-A06-p24501315
6367	Bn-N6-p24347205	Bn-A06-p24503033
6379	Bn-N6-p24557399	Bn-A06-p24705718
6392	Bn-N6-p24806475	Bn-C7-p27578386
6393	Bn-N6-p25085426	Bn-C7-p26650096
6394	Bn-N6-p25171248	Bn-A06-p25343454
6401	Bn-N6-p25570516	Bn-A06-p25786733
6413	Bn-N6-p25934759	Bn-A06-p26144731
6498	Bn-N7-p1320908	Bn-A07-p1292866
6558	Bn-N7-p2187754	Bn-A10-p11608969
6579	Bn-N7-p2492673	Bn-A10-p12014652
6632	Bn-N7-p3119107	Bn-A10-p12631706
6432	Bn-N7-p6487300	Bn-A07-p4916203
6417	Bn-N7-p6968745	Bn-A07-p5412930
6787	Bn-N7-p10074654	Bn-A07-p8516667
7005	Bn-N7-p10832679	Bn-A07-p9272000
7023	Bn-N7-p11159340	Bn-A07-p9661444
7028	Bn-N7-p11291470	Bn-A07-p9791002
7091	Bn-N7-p12236021	Bn-A07-p10750190
7195	Bn-N7-p13676693	Bn-A02-p771951
7216	Bn-N7-p14143467	Bn-A07-p11629852
7226	Bn-N7-p14394583	Bn-A07-p11838401
7281	Bn-N7-p14888341	Bn-A07-p12362172
7282	Bn-N7-p15335414	Bn-A07-p12757009
7287	Bn-N7-p15417889	Bn-A07-p12833434
7303	Bn-N7-p15755571	Bn-A07-p13147502
7329	Bn-N7-p16230522	Bn-A07-p13587753
7332	Bn-N7-p16292466	Bn-A07-p13659939
7338	Bn-N7-p16373144	Bn-A07-p13739429
7363	Bn-N7-p16634145	Bn-A07-p14005263
7385	Bn-N7-p17091758	Bn-A07-p14453848
7392	Bn-N7-p17163479	Bn-A07-p14523096
7410	Bn-N7-p17387599	Bn-A07-p14759961
7451	Bn-N7-p18034004	Bn-A07-p15352802
7475	Bn-N7-p18257843	Bn-A07-p15578269
7498	Bn-N7-p18607472	Bn-A07-p15949460
7509	Bn-N7-p18746325	Bn-A07-p16074768
7512	Bn-N7-p18872267	Bn-A07-p16183869
7520	Bn-N7-p19176714	Bn-A07-p16468333
7564	Bn-N7-p19936860	Bn-A07-p17212130
7586	Bn-N7-p20518723	Bn-A07-p17804261
7594	Bn-N7-p20745840	Bn-A07-p18020732
7630	Bn-N7-p21793439	Bn-A07-p18922121
7642	Bn-N7-p22063739	Bn-A07-p19190510
7644	Bn-N7-p22327509	Bn-A07-p19458370
7651	Bn-N7-p22777474	Bn-A07-p19865996
7656	Bn-N7-p22885432	Bn-A07-p19961825
7664	Bn-N7-p23164955	Bn-A07-p20248654
7668	Bn-N7-p23434792	Bn-A07-p20481682

7672	Bn-N7-p23514296	Bn-A07-p20554194
7688	Bn-N7-p23918790	Bn-A07-p21019499
7697	Bn-N7-p24180943	Bn-A07-p21261518
7700	Bn-N7-p24325175	Bn-A07-p21476982
7745	Bn-N7-p25157288	Bn-A07-p22329359
7754	Bn-N8-p1020964	Bn-A08-p792162
7849	Bn-N8-p2854610	Bn-A08-p2973537
7818	Bn-N8-p7792310	Bn-A08-p8570810
8153	Bn-N8-p7859442	Bn-A08-p8601468
8219	Bn-N8-p9634172	Bn-A08-p10067927
8224	Bn-N8-p9946893	Bn-A08-p10281337
8226	Bn-N8-p10085528	Bn-A08-p10439396
8239	Bn-N8-p10979512	Bn-C3-p57295296
8248	Bn-N8-p12138124	Bn-A08-p12555227
8295	Bn-N8-p13023342	Bn-A08-p13352430
8299	Bn-N8-p13038993	Bn-A08-p13363830
8308	Bn-N8-p13128437	Bn-A08-p13454167
8322	Bn-N8-p13290340	Bn-A08-p13626189
8327	Bn-N8-p13391245	Bn-A08-p13708877
8363	Bn-N8-p14223379	Bn-A08-p14498301
8366	Bn-N8-p14328665	Bn-A08-p14616048
8379	Bn-N8-p14537632	Bn-A08-p14813540
8380	Bn-N8-p14670055	Bn-C3-p55722563
8399	Bn-N8-p15090232	Bn-A08-p15353881
8404	Bn-N8-p15416987	Bn-A08-p15701695
8423	Bn-N8-p15696130	Bn-C3-p53579689
8433	Bn-N8-p15876364	Bn-A08-p16183708
8436	Bn-N8-p15914242	Bn-A08-p16216893
8437	Bn-N8-p15952855	Bn-A08-p16248227
8442	Bn-N8-p16006346	Bn-A08-p16306425
8479	Bn-N8-p16552226	Bn-A08-p16846907
8484	Bn-N8-p16663666	Bn-A08-p16958537
8485	Bn-N8-p16685917	Bn-A08-p16980526
8503	Bn-N8-p16911036	Bn-A08-p17202184
8510	Bn-N8-p17004603	Bn-A08-p17291598
8515	Bn-N8-p17014513	Bn-A08-p17301557
8531	Bn-N8-p17227705	Bn-A08-p17513789
8532	Bn-N8-p17245403	Bn-A08-p17530617
8560	Bn-N8-p17556900	Bn-A08-p17824285
8565	Bn-N8-p17612784	Bn-A08-p17887796
8566	Bn-N8-p17732398	Bn-A08-p18000876
8569	Bn-N8-p17798612	Bn-A08-p18070928
8576	Bn-N8-p18042252	Bn-A05-p6672638
8579	Bn-N8-p18304756	Bn-A08-p18543984
8581	Bn-N8-p18359822	Bn-A08-p18605816
8586	Bn-N8-p18503671	Bn-A08-p18750116
8594	Bn-N8-p18776922	Bn-A08-p19008575
8604	Bn-N8-p19062530	Bn-C8-p21592037

8606	Bn-N8-p19229698	Bn-A08-p19428204
8611	Bn-N18-p23614287	Bn-C8-p21143733
8620	Bn-N8-p19448615	Bn-A08-p19646345
8621	Bn-N11-p34110475	Bn-A08-p19683899
8631	Bn-N8-p19651757	Bn-A08-p19834705
8634	Bn-N8-p19762815	Bn-A08-p19960214
8636	Bn-N8-p19858500	Bn-A08-p20068678
8653	Bn-N8-p20113458	Bn-C8-p19607543
8659	Bn-N8-p20348461	Bn-A08-p20305036
8684	Bn-N8-p20879154	Bn-A08-p20820768
8690	Bn-N8-p21005755	Bn-A08-p20945277
8700	Bn-N8-p21495935	Bn-A08-p21417592
8701	Bn-N9-p342104	Bn-A09-p957959
8711	Bn-N9-p560125	Bn-A09-p704028
8715	Bn-N9-p796829	Bn-A09-p86293
8717	Bn-N9-p1015065	Bn-A01-p26968952
8730	Bn-N9-p1150435	Bn-A01-p26837207
8732	Bn-N9-p1297967	Bn-A09-p1188091
8734	Bn-N9-p1613782	Bn-A09-p2116947
8736	Bn-N9-p1655795	Bn-A09-p2157775
8753	Bn-N9-p2419388	Bn-A09-p1929245
8755	Bn-N9-p2488499	Bn-A09-p1856828
8775	Bn-N9-p2809617	Bn-A09-p1570558
8776	Bn-N9-p2829986	Bn-A09-p1552993
8777	Bn-N9-p4609099	Bn-A09-p4096474
8788	Bn-N9-p4868714	Bn-Scaffold000178-p47905
8789	Bn-N9-p5116009	Bn-A09-p4463675
8792	Bn-N9-p5162393	Bn-A09-p4509627
8794	Bn-N9-p5312617	Bn-A09-p4655322
8834	Bn-N9-p5931012	Bn-A09-p5455608
8838	Bn-N9-p6202417	Bn-A07-p10188190
8844	Bn-N19-p10270316	Bn-A09-p7174944
8848	Bn-N9-p6806606	Bn-A09-p7047022
8849	Bn-N9-p6924911	Bn-A09-p6922467
8853	Bn-N9-p7052384	Bn-C9-p10793169
8858	Bn-N9-p7648675	Bn-A09-p6133524
8894	Bn-N9-p9899071	Bn-A09-p9379347
8904	Bn-N9-p10035291	Bn-A09-p9512316
8912	Bn-N9-p10441284	Bn-A09-p9865556
8937	Bn-N9-p10912967	Bn-A09-p14266174
8978	Bn-N9-p12472837	Bn-A09-p16639867
8994	Bn-N9-p13640142	Bn-C9-p21533473
8995	Bn-N9-p14240731	Bn-A09-p14535292
9004	Bn-N9-p14682425	Bn-A09-p17162263
9059	Bn-N9-p10197711	Bn-A09-p19272189
9398	Bn-N9-p23728533	Bn-A09-p22495700
9399	Bn-N13-p16057699	Bn-C3-p15381003
9402	Bn-N9-p25335523	Bn-A09-p22873685
9406	Bn-N9-p25588982	Bn-A09-p23144404

9410	Bn-N9-p25679946	Bn-A09-p23250129
9414	Bn-N9-p25797589	Bn-A09-p23354310
9421	Bn-N9-p25867929	Bn-A09-p23416800
9426	Bn-N9-p26001801	Bn-A09-p23552075
9432	Bn-N9-p26198959	Bn-A09-p23756115
9436	Bn-N9-p26355119	Bn-A09-p23904905
9443	Bn-N9-p26530164	Bn-A09-p24086990
9448	Bn-N9-p26823882	Bn-Scaffold003668-p282
9449	Bn-N9-p26997825	Bn-C5-p12386090
9451	Bn-N9-p27165724	Bn-Scaffold000282-p6257
9452	Bn-N9-p27491742	Bn-A05-p15802321
9453	Bn-N9-p27495421	Bn-A05-p15805806
9477	Bn-N9-p28204510	Bn-A09-p25455888
9516	Bn-N9-p28841041	Bn-A09-p26101323
9532	Bn-N9-p29283046	Bn-A09-p26578867
9541	Bn-N9-p29855688	Bn-A09-p27103683
9546	Bn-N9-p30306145	Bn-A09-p27531548
9584	Bn-N9-p31054476	Bn-C8-p30616202
9596	Bn-N9-p31562739	Bn-A09-p28722989
9620	Bn-N9-p32008041	Bn-A09-p29186255
9624	Bn-N9-p32102552	Bn-A09-p29272014
9628	Bn-N9-p32161397	Bn-A09-p29331470
9638	Bn-N9-p32326702	Bn-A09-p29493614
9646	Bn-N9-p32470450	Bn-A09-p29617813
9649	Bn-N9-p32550436	Bn-A09-p29699253
9660	Bn-N9-p32817315	Bn-A09-p29968302
9691	Bn-N9-p33307742	Bn-A09-p30431025
9708	Bn-N9-p33603704	Bn-A09-p30724789
9723	Bn-N9-p34324408	Bn-A09-p31393136
9738	Bn-N9-p34420779	Bn-A09-p31494058
9787	Bn-N9-p34992197	Bn-A09-p5132918
9815	Bn-N9-p35716954	Bn-A09-p32553111
9828	Bn-N9-p36096719	Bn-A09-p32980100
9832	Bn-N9-p36169883	Bn-A09-p33040441
9837	Bn-N9-p36445006	Bn-A09-p33358168
9855	Bn-N9-p36573079	Bn-A09-p33464355
9861	Bn-N9-p36603036	Bn-A09-p33499505
9889	Bn-N9-p37159171	Bn-A09-p34039082
9894	Bn-N9-p37607705	Bn-A09-p34482075
9895	Bn-N9-p37895290	Bn-A09-p34779068
9898	Bn-N9-p38146463	Bn-A09-p34995922
9899	Bn-N9-p38325101	Bn-A09-p35162641
9902	Bn-N9-p38427349	Bn-A09-p35262679
9909	Bn-N9-p38608830	Bn-A09-p35426888
9912	Bn-N9-p38663579	Bn-A09-p35477936
9927	Bn-N9-p38804159	Bn-A09-p35624679
9935	Bn-N9-p39021453	Bn-A09-p35879759
9942	Bn-N9-p39188012	Bn-A09-p36062760
9946	Bn-N9-p39351742	Bn-A09-p36200543

9956	Bn-N9-p39560080	Bn-A09-p36402398
9972	Bn-N9-p40060791	Bn-A09-p36886022
9978	Bn-N9-p40208874	Bn-C8-p41583107
9983	Bn-N10-p174643	Bn-A10-p4727374
9991	Bn-N10-p334540	Bn-C5-p335866
9993	Bn-N10-p297188	Bn-A10-p4606815
10019	Bn-N10-p912738	Bn-A10-p3955901
10022	Bn-N10-p1004835	Bn-A10-p3825331
10030	Bn-N10-p1108499	Bn-A10-p2819947
10034	Bn-N10-p1361370	Bn-A10-p2557960
10041	Bn-N10-p1398053	Bn-A10-p2523110
10090	Bn-N10-p2123204	Bn-A10-p1791974
10091	Bn-N10-p2222171	Bn-A10-p1698746
10096	Bn-N10-p2525099	Bn-A10-p1458849
10101	Bn-N10-p2631797	Bn-A10-p1345255
10123	Bn-N10-p3240452	Bn-A10-p754475
10288	Bn-N10-p6132376	Bn-A10-p5317804
10399	Bn-N10-p9518968	Bn-A10-p7913022
10422	Bn-N10-p10057518	Bn-A10-p8443505
10465	Bn-N10-p10343794	Bn-A10-p8723171
10474	Bn-N10-p10544389	Bn-A10-p8904827
10483	Bn-N10-p10790201	Bn-A10-p9166440
10495	Bn-N10-p10868858	Bn-A10-p9245966
10499	Bn-N10-p11000973	Bn-A10-p9370046
10506	Bn-N10-p11212698	Bn-A10-p9561249
10511	Bn-N10-p11235601	Bn-A10-p9594548
10523	Bn-N10-p11495739	Bn-A10-p9868333
10532	Bn-N10-p11718757	Bn-C9-p44593285
10541	Bn-N10-p12078376	Bn-A10-p10464159
10543	Bn-N10-p12243716	Bn-A10-p10647252
10560	Bn-N10-p12513083	Bn-A10-p10914713
10569	Bn-N10-p12717224	Bn-A10-p11105998
10573	Bn-N10-p12846026	Bn-A10-p11230418
10578	Bn-N10-p13024539	Bn-A10-p11396195
10579	Bn-N10-p13063530	Bn-A10-p11427179
10583	Bn-N19-p49762022	Bn-C9-p47439482
10585	Bn-N10-p13261881	Bn-A10-p12857310
10590	Bn-N10-p13341433	Bn-A10-p12933288
10596	Bn-N10-p13530217	Bn-A10-p13130002
10606	Bn-N10-p13642700	Bn-A10-p13218309
10613	Bn-N10-p13739986	Bn-A10-p13299250
10615	Bn-N10-p13792515	Bn-A10-p13343454
10622	Bn-N10-p13900036	Bn-A10-p13446086
10632	Bn-N10-p14102032	Bn-A10-p13640346
10634	Bn-N10-p14140344	Bn-A10-p13677412
10646	Bn-N10-p14283825	Bn-A10-p13818569
10664	Bn-N10-p14715060	Bn-A10-p14254487
10680	Bn-N10-p15065815	Bn-A10-p14623802
10707	Bn-N10-p15639341	Bn-A10-p15156277

10717	Bn-N10-p15738014	Bn-A10-p16421672
10726	Bn-N10-p15830303	Bn-A10-p16336708
10732	Bn-N10-p15939293	Bn-A10-p16224024
10738	Bn-N10-p16068992	Bn-A10-p16087066
10742	Bn-N10-p16296245	Bn-A10-p15863143
10745	Bn-N10-p16366292	Bn-A10-p15793623
10766	Bn-N10-p16559905	Bn-A10-p15607468
10771	Bn-N10-p16681932	Bn-A10-p15507350
10783	Bn-N10-p16798686	Bn-A10-p15398137
10786	Bn-N10-p16811029	Bn-A10-p15387894
10788	Bn-N10-p16832459	Bn-A10-p15367240
10792	Bn-N10-p16875078	Bn-A10-p15330596
10796	Bn-N10-p16945504	Bn-A10-p15265102
10802	Bn-N10-p17174060	Bn-A10-p16620627
10804	Bn-N14-p1859330	Bn-A10-p16862809
10805	Bn-N12-p421642	Bn-A10-p16935603
10809	Bn-N18-p44745193	Bn-A10-p17568393
10825	Bn-N11-p1459224	Bn-C1-p284003
10828	Bn-N11-p2038166	Bn-C1-p903714
10829	Bn-N11-p2335230	Bn-A01-p907721
10838	Bn-N11-p2972339	Bn-C1-p1914447
10841	Bn-N1-p1769679	Bn-C1-p2296948
10843	Bn-N11-p3873556	Bn-C1-p2760078
10848	Bn-N11-p4453494	Bn-C1-p3320931
10850	Bn-N11-p5429066	Bn-C1-p4212311
10896	Bn-N11-p7117911	Bn-Scaffold02362-p1538
10940	Bn-N11-p8709150	Bn-C1-p7446976
10956	Bn-N11-p9291289	Bn-C1-p8141825
10958	Bn-N11-p9303916	Bn-C1-p8150383
10983	Bn-N11-p10017244	Bn-C1-p8817529
10997	Bn-N11-p10852512	Bn-C1-p9647037
11001	Bn-N11-p11566204	Bn-C1-p10298103
11009	Bn-N11-p12954035	Bn-C1-p11604836
11015	Bn-N11-p13100683	Bn-C1-p11741511
11021	Bn-N11-p13229265	Bn-C1-p11861892
11027	Bn-N11-p13396823	Bn-C1-p12011052
11036	Bn-N11-p13604090	Bn-C1-p12228961
11041	Bn-N11-p13928723	Bn-C1-p12582975
11044	Bn-N11-p14229035	Bn-C1-p12821338
11072	Bn-N11-p15104442	Bn-C1-p13594012
11075	Bn-N11-p15348140	Bn-C1-p13866232
11086	Bn-N11-p15748495	Bn-C1-p14268880
11089	Bn-N11-p15812621	Bn-C1-p14332796
11091	Bn-N11-p16052156	Bn-C1-p14565806
11120	Bn-N11-p18914484	Bn-C1-p17234515
11500	Bn-N11-p23550723	Bn-C1-p22108831
11988	Bn-N11-p24506672	Bn-Scaffold01557-p399
12367	Bn-N11-p31551362	Bn-C1-p28860761
12432	Bn-N11-p35046298	Bn-Scaffold01540-p1838

12467	Bn-N11-p35998945	Bn-C1-p33165496
12476	Bn-N11-p36788702	Bn-Scaffold01385-p6273
12481	Bn-N11-p36904309	Bn-C1-p34090627
12516	Bn-N11-p38505222	Bn-C3-p21365850
12518	Bn-N11-p39029778	Bn-Scaffold00763-p50232
12538	Bn-N11-p39434331	Bn-C1-p36524540
12597	Bn-N11-p41026603	Bn-C1-p38334187
12613	Bn-N11-p42177018	Bn-C1-p39370506
12622	Bn-N11-p42748068	Bn-C1-p39827382
12639	Bn-N11-p43536774	Bn-C1-p40553550
12643	Bn-N11-p43652872	Bn-C1-p40710950
12647	Bn-N11-p43970726	Bn-C1-p40861632
12648	Bn-N11-p43984419	Bn-C1-p40875666
12702	Bn-N11-p44592876	Bn-C1-p41468412
12703	Bn-N12-p283366	Bn-C2-p671385
12735	Bn-N12-p936581	Bn-C2-p1296749
12742	Bn-N12-p1537049	Bn-A02-p1344870
12743	Bn-N12-p1640332	Bn-C2-p2054626
12747	Bn-N12-p1886204	Bn-C2-p2277308
12755	Bn-N12-p2411136	Bn-C2-p2791893
12758	Bn-N12-p2498010	Bn-C2-p2865732
12764	Bn-N12-p2959247	Bn-A02-p3507057
12766	Bn-N12-p3440999	Bn-C2-p3792621
12797	Bn-N12-p3912844	Bn-C2-p4204945
12802	Bn-N12-p4121985	Bn-C2-p4390372
12811	Bn-N12-p4336290	Bn-C2-p4623747
12833	Bn-N12-p6117636	Bn-C2-p6321978
12887	Bn-N12-p10151393	Bn-C2-p11298722
12961	Bn-N12-p12139142	Bn-C2-p13653557
13002	Bn-N12-p12889447	Bn-C2-p14539885
13063	Bn-N12-p16538763	Bn-C2-p19642551
13070	Bn-N12-p16729238	Bn-C2-p19861280
13126	Bn-N12-p18195253	Bn-C2-p22031369
13176	Bn-N12-p20496877	Bn-C2-p25538602
13257	Bn-N12-p21583340	Bn-C2-p26782405
13638	Bn-N12-p32987804	Bn-C2-p40369565
13657	Bn-N12-p34013662	Bn-C2-p41475606
13734	Bn-N12-p38135032	Bn-Scaffold00285-p269525
13748	Bn-N12-p39499870	Bn-Scaffold00992-p24644
13765	Bn-N12-p40164030	Bn-C2-p46969947
13766	Bn-N12-p40357683	Bn-C2-p47138435
13768	Bn-N12-p40391461	Bn-C2-p47170554
13772	Bn-N12-p40544490	Bn-C2-p47336361
13776	Bn-N12-p40838951	Bn-Scaffold00847-p5921
13779	Bn-N12-p40971090	Bn-C2-p47678596
13796	Bn-N12-p41334437	Bn-C2-p48091803
13797	Bn-N12-p41775492	Bn-C2-p48551446
13798	Bn-N12-p41815038	Bn-C2-p48603850
13799	Bn-N11-p10640405	Bn-C3-p49912971

13802	Bn-N12-p42046279	Bn-C2-p48894790
13823	Bn-N12-p42326091	Bn-C2-p49182188
13827	Bn-N12-p42598517	Bn-C2-p49449909
13874	Bn-N12-p43682190	Bn-C2-p50543794
13877	Bn-N12-p44011551	Bn-C2-p51343986
13878	Bn-N12-p44382055	Bn-C2-p51836264
13881	Bn-N12-p44392234	Bn-C2-p51846290
13899	Bn-N12-p44629331	Bn-C2-p52067557
13904	Bn-N12-p44732203	Bn-C2-p52179807
13910	Bn-N12-p44954857	Bn-Scaffold00615-p131853
13922	Bn-N12-p45453768	Bn-C2-p52680379
13939	Bn-N12-p45313388	Bn-C2-p52559929
13944	Bn-N13-p2992378	Bn-C3-p2915302
13947	Bn-N13-p3375183	Bn-C3-p3310082
13948	Bn-N13-p3557440	Bn-C3-p3481719
13950	Bn-N13-p3762152	Bn-C3-p3699157
13952	Bn-N13-p4085409	Bn-C3-p3953647
13955	Bn-N13-p4252360	Bn-C3-p4100659
13956	Bn-N13-p4633364	Bn-A03-p4682207
13959	Bn-N3-p4145513	Bn-C3-p5068957
13960	Bn-N13-p5986297	Bn-C3-p5218330
13965	Bn-N13-p6244288	Bn-C3-p5482090
13980	Bn-N13-p6465866	Bn-C3-p5694509
13982	Bn-N13-p6593204	Bn-C3-p5800427
13986	Bn-N13-p7464782	Bn-C4-p42681345
13987	Bn-N13-p8015497	Bn-C3-p7275851
13989	Bn-N13-p8245744	Bn-C3-p7486013
13992	Bn-N13-p8366065	Bn-C3-p7604728
13996	Bn-N13-p8761234	Bn-C3-p8017908
14005	Bn-N13-p9110162	Bn-C3-p8403185
14008	Bn-N13-p9886904	Bn-C3-p9135177
14012	Bn-N13-p11373962	Bn-A03-p8359092
14014	Bn-N13-p11865252	Bn-C3-p11172822
14019	Bn-N3-p9034675	Bn-C3-p12070773
14020	Bn-N13-p13544313	Bn-C3-p12808223
14028	Bn-N13-p14066106	Bn-C3-p13279392
14034	Bn-N13-p14759341	Bn-C3-p13950647
14037	Bn-N13-p15627092	Bn-C3-p14859675
14051	Bn-N13-p16484575	Bn-C3-p15798466
14158	Bn-N13-p19500387	Bn-C3-p18354125
14162	Bn-N13-p19751255	Bn-C3-p18596511
14168	Bn-N13-p19900213	Bn-C3-p18754012
14198	Bn-N13-p20649361	Bn-C3-p19981581
14206	Bn-N13-p21573788	Bn-C3-p20568555
14208	Bn-N13-p22495983	Bn-C3-p21443157
14215	Bn-N13-p23299576	Bn-C3-p22202335
14218	Bn-N13-p23741532	Bn-C3-p22562109
14221	Bn-N13-p23778608	Bn-C3-p22606381
14223	Bn-N13-p24102290	Bn-C3-p22945691

14224	Bn-N13-p24436448	Bn-A03-p15983019
14233	Bn-N13-p24927465	Bn-C3-p23704359
14245	Bn-N13-p25244880	Bn-C3-p24021907
14252	Bn-N13-p25461966	Bn-Scaffold01023-p11758
14292	Bn-N13-p25609110	Bn-C3-p24354456
14298	Bn-N13-p26100579	Bn-C3-p24842847
14299	Bn-N13-p26157491	Bn-C3-p24895511
14309	Bn-N13-p26575324	Bn-C3-p25270752
14318	Bn-N13-p26834187	Bn-C3-p25518677
14322	Bn-N13-p26945357	Bn-C3-p25627940
14335	Bn-N13-p28304014	Bn-C3-p27074199
14380	Bn-N13-p30096565	Bn-C3-p28808057
14494	Bn-N13-p32339346	Bn-C3-p30751017
14498	Bn-N13-p32444822	Bn-C3-p30872016
14503	Bn-N13-p32501325	Bn-C3-p30926907
14504	Bn-N13-p32520072	Bn-C3-p30949166
14525	Bn-N13-p32647170	Bn-C3-p31101837
14546	Bn-N13-p33816297	Bn-C3-p32159218
14627	Bn-N13-p36646684	Bn-C3-p34846496
14633	Bn-N13-p37011996	Bn-C3-p35168382
14636	Bn-N13-p38217511	Bn-C3-p36232123
14641	Bn-N13-p38520334	Bn-C3-p36538236
14645	Bn-N13-p35494025	Bn-C3-p33783470
14723	Bn-N13-p51208823	Bn-C3-p49533128
14729	Bn-N13-p51493654	Bn-C3-p49784531
14764	Bn-N13-p52659189	Bn-C3-p50876390
14776	Bn-N13-p52843875	Bn-C3-p51051765
14789	Bn-N13-p53059228	Bn-C3-p51220360
14799	Bn-N13-p53445599	Bn-C3-p51618353
14806	Bn-N13-p53775106	Bn-C3-p51892437
14828	Bn-N13-p54320108	Bn-C3-p52463498
14859	Bn-N13-p54890638	Bn-C3-p53018880
14862	Bn-N13-p55008263	Bn-C3-p53105434
14869	Bn-N13-p55065650	Bn-C3-p53172050
14877	Bn-N13-p55490354	Bn-C3-p53619703
14882	Bn-N13-p55880297	Bn-C3-p53979059
14886	Bn-N13-p56066278	Bn-C3-p54100388
14895	Bn-N13-p56483433	Bn-C3-p54484753
14949	Bn-N13-p56956424	Bn-C3-p55005964
14952	Bn-N13-p57080292	Bn-C3-p55129421
14956	Bn-N13-p57399905	Bn-C3-p55406195
14977	Bn-N13-p57494719	Bn-C3-p55523801
14978	Bn-N13-p57540903	Bn-C3-p55566792
14990	Bn-N13-p57793747	Bn-C3-p55817169
15009	Bn-N13-p58686520	Bn-Scaffold00889-p26261
15143	Bn-N13-p59403363	Bn-C3-p57082676
15149	Bn-N13-p59679488	Bn-C3-p57354163
15150	Bn-N13-p59742276	Bn-C3-p57404345
15162	Bn-N13-p60355266	Bn-C3-p57896585

15215	Bn-N13-p61395213	Bn-C3-p58884782
15228	Bn-N13-p61720359	Bn-C3-p59246536
15236	Bn-N13-p62353372	Bn-C3-p59803384
15241	Bn-N13-p63051506	Bn-C3-p60530925
15247	Bn-N13-p63388579	Bn-C3-p60849232
15262	Bn-N13-p63750898	Bn-C3-p61187054
15288	Bn-N13-p65014421	Bn-C3-p62366802
15294	Bn-N13-p65681849	Bn-C3-p62901691
15297	Bn-N13-p65434365	Bn-C3-p62727808
15333	Bn-N13-p66466754	Bn-C3-p63620341
15345	Bn-N13-p66892279	Bn-C3-p64050436
15351	Bn-N13-p66937567	Bn-C3-p64106691
15365	Bn-N13-p67764811	Bn-C3-p64972155
15366	Bn-N14-p10483	Bn-C4-p146560
15382	Bn-N14-p115390	Bn-C4-p264643
15388	Bn-N14-p381430	Bn-Scaffold00802-p78048
15391	Bn-N14-p607913	Bn-C4-p686637
15397	Bn-N14-p896458	Bn-C4-p983004
15402	Bn-N14-p1263905	Bn-C4-p1378830
15403	Bn-N14-p1400503	Bn-C4-p1516144
15422	Bn-N14-p1485582	Bn-C1-p31929709
15423	Bn-N14-p1704243	Bn-C4-p1782015
15428	Bn-N14-p2399700	Bn-C4-p2325430
15431	Bn-N14-p2638346	Bn-C4-p2539802
15439	Bn-N14-p2973517	Bn-C4-p2852349
15447	Bn-N14-p3510738	Bn-C4-p3457408
15459	Bn-N14-p3692891	Bn-C4-p3601694
15465	Bn-N14-p3828796	Bn-C4-p3722891
15466	Bn-N14-p3931013	Bn-Scaffold01730-p5229
15469	Bn-N14-p4209836	Bn-C4-p4030999
15473	Bn-N14-p4291107	Bn-C4-p4130322
15481	Bn-N14-p4781873	Bn-C4-p4585856
15484	Bn-N14-p4878672	Bn-C4-p4681049
15498	Bn-N14-p5357044	Bn-C4-p5169938
15505	Bn-N14-p5855559	Bn-C4-p5759134
15533	Bn-N14-p6788085	Bn-C4-p6677657
15540	Bn-N14-p6923580	Bn-C4-p6808204
15553	Bn-N14-p7388271	Bn-A05-p4304172
15570	Bn-N14-p7653870	Bn-C4-p7497891
15581	Bn-N14-p8042737	Bn-C4-p7925803
15590	Bn-N14-p8955502	Bn-C4-p8618530
15591	Bn-N14-p9067753	Bn-C4-p8723983
15600	Bn-N14-p9174618	Bn-C4-p8822093
15601	Bn-N14-p9401986	Bn-C4-p9007623
15604	Bn-N14-p10251001	Bn-C4-p9777719
15613	Bn-N14-p11501551	Bn-C4-p10995381
15628	Bn-N14-p12025135	Bn-C4-p11427976
15646	Bn-N14-p12698070	Bn-C4-p12096888
15714	Bn-N14-p15885493	Bn-C4-p15358035

15789	Bn-N14-p16960929	Bn-C4-p16825244
15887	Bn-N14-p23279842	Bn-C4-p22906600
16107	Bn-N14-p28855843	Bn-C4-p27836422
16115	Bn-N14-p29608726	Bn-Scaffold00866-p24484
16135	Bn-N14-p21949894	Bn-C4-p21291779
16209	Bn-N14-p34266628	Bn-C4-p32902510
16212	Bn-N14-p34552788	Bn-C4-p33179791
16224	Bn-N14-p34993877	Bn-C4-p33688222
16318	Bn-N14-p38673794	Bn-C4-p37078717
16323	Bn-N14-p38738442	Bn-C4-p37142354
16364	Bn-N14-p39683412	Bn-Scaffold04300-p95
16366	Bn-N14-p40463269	Bn-C4-p38898227
16379	Bn-N14-p41195848	Bn-C4-p39604628
16397	Bn-N14-p41883467	Bn-C4-p40183165
16526	Bn-N14-p43994865	Bn-C4-p42101528
16531	Bn-N14-p44301058	Bn-C4-p42370006
16534	Bn-N14-p44414316	Bn-C4-p42498181
16551	Bn-N14-p45065343	Bn-C4-p42981603
16572	Bn-N14-p46311853	Bn-C4-p44119685
16575	Bn-N14-p46389017	Bn-C4-p44247047
16581	Bn-N4-p14969070	Bn-A04-p12917949
16583	Bn-N14-p47057666	Bn-Scaffold01016-p29132
16585	Bn-N14-p48823749	Bn-C4-p46683666
16589	Bn-N14-p49662304	Bn-C4-p47419520
16595	Bn-N14-p50059672	Bn-Scaffold01527-p4691
16596	Bn-N2-p8682855	Bn-A04-p15287200
16600	Bn-N14-p50457083	Bn-C4-p48686430
16603	Bn-Scaffold01385-p601	Bn-C4-p48865332
16604	Bn-N14-p50751772	Bn-C4-p48943622
16606	Bn-N14-p50883109	Bn-C4-p49082294
16611	Bn-N14-p51259960	Bn-C4-p49871832
16612	Bn-N14-p51803213	Bn-C4-p50397026
16613	Bn-N14-p52137515	Bn-C4-p50780868
16615	Bn-N14-p52368863	Bn-C4-p51022991
16616	Bn-N14-p52488607	Bn-C4-p51151118
16617	Bn-N4-p19429574	Bn-A04-p17475728
16619	Bn-N14-p53148205	Bn-C4-p51764862
16621	Bn-N14-p53793394	Bn-C4-p52507724
16623	Bn-N14-p53977786	Bn-C4-p52713866
16625	Bn-N14-p54056004	Bn-C4-p52800188
16628	Bn-N14-p54436284	Bn-C4-p53155836
16630	Bn-N14-p54571000	Bn-C4-p53316094
16651	Bn-N15-p53524	Bn-C5-p160429
16643	Bn-N15-p1174899	Bn-C5-p1056773
16658	Bn-N15-p1342122	Bn-C5-p1238391
16662	Bn-N15-p1473353	Bn-C5-p1364481
16665	Bn-N15-p1578974	Bn-C5-p1474081
16670	Bn-N15-p1788350	Bn-C5-p1705838
16672	Bn-N15-p1999936	Bn-C5-p1917307

16676	Bn-N15-p2117649	Bn-C5-p2069364
16679	Bn-N15-p2193139	Bn-C5-p2151026
16680	Bn-N15-p2229669	Bn-C5-p2189242
16681	Bn-N15-p2241803	Bn-C5-p2199834
16685	Bn-N15-p2505335	Bn-C5-p2468868
16689	Bn-N15-p2578161	Bn-C5-p2548160
16690	Bn-N15-p2750972	Bn-C5-p2691551
16691	Bn-N15-p2960014	Bn-C5-p2918201
16692	Bn-N15-p2961100	Bn-C5-p2919330
16693	Bn-N15-p2970279	Bn-C5-p2928560
16695	Bn-N15-p3017053	Bn-C5-p2982006
16696	Bn-N15-p3192152	Bn-C5-p3126241
16698	Bn-N15-p3365432	Bn-C5-p3281408
16707	Bn-N15-p3663901	Bn-C5-p3568847
16714	Bn-N6-p3577649	Bn-A06-p3651473
16715	Bn-N15-p3730704	Bn-C5-p3633992
16726	Bn-N15-p4607710	Bn-C5-p4463720
16728	Bn-N15-p5195547	Bn-C5-p5091946
16738	Bn-N15-p5645612	Bn-C5-p5640950
16752	Bn-N15-p7309887	Bn-C5-p7266769
16756	Bn-N15-p7646696	Bn-C5-p7597925
16772	Bn-N15-p8958496	Bn-C5-p8762652
16773	Bn-N15-p8962076	Bn-C5-p8767284
16780	Bn-N15-p9595787	Bn-C5-p9370759
16790	Bn-N15-p10118146	Bn-C5-p9849982
16802	Bn-N15-p11363353	Bn-C5-p10853650
16960	Bn-N15-p13496909	Bn-C5-p12859514
17064	Bn-N15-p15332549	Bn-C5-p14153917
17065	Bn-N15-p15923332	Bn-C5-p14722118
17074	Bn-N16-p15485278	Bn-C6-p23268034
17075	Bn-N15-p17515855	Bn-C5-p18008781
17082	Bn-N15-p18376044	Bn-C5-p18812982
17087	Bn-N15-p19745473	Bn-C5-p20180980
17116	Bn-N5-p16808652	Bn-A05-p16509283
17117	Bn-N15-p34045905	Bn-C5-p32349790
17129	Bn-N15-p42180350	Bn-C5-p40472703
17134	Bn-N15-p40027801	Bn-C5-p38043152
17138	Bn-N15-p40606936	Bn-C5-p38756827
17139	Bn-N15-p40754671	Bn-C5-p38895000
17152	Bn-N15-p40858129	Bn-C5-p38976125
17206	Bn-N15-p43474159	Bn-C5-p41676458
17207	Bn-N5-p22366288	Bn-A05-p21326775
17208	Bn-N15-p44281647	Bn-C5-p42447050
17210	Bn-N15-p44461286	Bn-C5-p42619917
17212	Bn-N15-p44730765	Bn-C5-p42877318
17213	Bn-N15-p44959556	Bn-C5-p43091364
17214	Bn-N15-p45002883	Bn-C5-p43140924
17215	Bn-N15-p45051720	Bn-C5-p43192758
17223	Bn-N15-p45274243	Bn-C5-p43418714

17228	Bn-N15-p45401184	Bn-C5-p43590291
17242	Bn-N5-p24193947	Bn-A05-p23102053
17245	Bn-N15-p46329966	Bn-C5-p44548511
17252	Bn-N15-p46540304	Bn-C5-p44704155
17269	Bn-N15-p47239484	Bn-C5-p45479754
17271	Bn-N15-p47395762	Bn-Scaffold000191-p47497
17275	Bn-N15-p47606924	Bn-C5-p45853238
17279	Bn-N15-p48099448	Bn-C5-p46328665
17283	Bn-N15-p48278032	Bn-C5-p46523272
17289	Bn-N16-p37379113	Bn-C6-p1757213
17295	Bn-N16-p37278150	Bn-C6-p1854873
17296	Bn-N16-p37226193	Bn-C8-p138535
17301	Bn-N16-p37117974	Bn-C6-p1975362
17303	Bn-N16-p36961661	Bn-C6-p2132472
17306	Bn-N16-p36892483	Bn-C6-p2206123
17308	Bn-N16-p36841519	Bn-C6-p2259256
17311	Bn-N16-p36426332	Bn-C6-p2673885
17329	Bn-N16-p36396201	Bn-C6-p2701748
17332	Bn-N16-p36110171	Bn-C6-p2934075
17350	Bn-N16-p35980032	Bn-C6-p3073254
17373	Bn-N16-p35139350	Bn-C6-p3835667
17394	Bn-N16-p34266718	Bn-C6-p4683276
17426	Bn-N16-p33058389	Bn-C6-p5764339
17466	Bn-N16-p32870253	Bn-C6-p5949689
17479	Bn-N16-p32047782	Bn-C6-p6726844
17505	Bn-N16-p31856094	Bn-C6-p6875935
17516	Bn-N16-p31714014	Bn-C6-p7065345
17528	Bn-N16-p30681660	Bn-C6-p7863103
17534	Bn-N16-p28249935	Bn-C6-p10407391
17562	Bn-N16-p26901876	Bn-C6-p11765338
17597	Bn-N16-p26712994	Bn-C2-p41605604
17598	Bn-N16-p26506737	Bn-C6-p12136654
17607	Bn-N16-p24349364	Bn-C6-p14338971
17664	Bn-N16-p24223842	Bn-C6-p14478368
17671	Bn-N16-p23582092	Bn-C6-p15140213
17696	Bn-N16-p22247194	Bn-C6-p16403568
17706	Bn-N16-p21115094	Bn-C6-p17662536
17749	Bn-N16-p20370684	Bn-C6-p18415817
17752	Bn-N16-p17911957	Bn-C6-p20863048
17833	Bn-N16-p15871048	Bn-C6-p22863673
17858	Bn-N16-p12557485	Bn-C6-p26089476
17963	Bn-N16-p10094412	Bn-C6-p28787698
18003	Bn-N16-p5360756	Bn-C6-p34220372
18107	Bn-N16-p4535925	Bn-C6-p35031160
18143	Bn-N16-p4260159	Bn-C6-p35715999
18156	Bn-N16-p4216788	Bn-C6-p35758398
18158	Bn-N16-p3367847	Bn-C6-p36609795
18189	Bn-N16-p2735237	Bn-A06-p2405022
18229	Bn-N16-p1977431	Bn-C6-p38030144

18269	Bn-Scaffold27754-p161	Bn-Scaffold08858-p361
18271	Bn-N17-p394283	Bn-A07-p160648
18276	Bn-N17-p1329229	Bn-C7-p1192208
18285	Bn-N17-p4867251	Bn-C7-p4356314
18320	Bn-N17-p10157609	Bn-C7-p9654338
18427	Bn-N17-p11683264	Bn-C7-p11487715
18739	Bn-N17-p14892900	Bn-C7-p14676153
18769	Bn-N17-p16953516	Bn-C7-p16589778
18775	Bn-N13-p53096085	Bn-A07-p6906296
18781	Bn-N17-p20395324	Bn-C7-p20117250
18782	Bn-N17-p20446675	Bn-C7-p20307138
18800	Bn-N17-p20998852	Bn-C7-p20921325
18805	Bn-N17-p21264164	Bn-C7-p21135074
18812	Bn-N17-p21604862	Bn-C7-p21817897
18815	Bn-N17-p21712618	Bn-C7-p21544993
18827	Bn-N17-p23162790	Bn-C7-p22905190
18878	Bn-N17-p24193173	Bn-C7-p23829640
18883	Bn-N17-p24271722	Bn-C7-p23897491
18887	Bn-N17-p24742395	Bn-C7-p24424315
18890	Bn-N17-p24838256	Bn-C7-p24526764
18893	Bn-N17-p24950440	Bn-C7-p24641573
18898	Bn-N17-p25262220	Bn-C7-p24943849
18911	Bn-N17-p25771233	Bn-Scaffold00928-p13976
18915	Bn-N17-p26695844	Bn-C7-p26355002
18930	Bn-N17-p29426444	Bn-C7-p28973784
18943	Bn-N17-p29828465	Bn-C7-p29385521
18944	Bn-N17-p29860763	Bn-C7-p29422151
18998	Bn-N17-p31158770	Bn-C7-p30740034
18999	Bn-N17-p31283084	Bn-C7-p30856330
19031	Bn-N17-p31676667	Bn-C7-p31288691
19067	Bn-N17-p32855427	Bn-C7-p32722910
19118	Bn-N17-p33478895	Bn-C7-p33351378
19134	Bn-N17-p34061229	Bn-C7-p35151417
19137	Bn-N17-p34535913	Bn-C7-p33900798
19138	Bn-N17-p34551543	Bn-C7-p33915927
19140	Bn-N17-p34556010	Bn-C7-p33920406
19166	Bn-N17-p35120304	Bn-C7-p34441835
19170	Bn-N17-p35240900	Bn-C7-p34559064
19181	Bn-N17-p35433082	Bn-C7-p34769795
19244	Bn-N17-p36391568	Bn-C7-p36225591
19245	Bn-N17-p36829930	Bn-C7-p36618847
19254	Bn-N17-p36957581	Bn-C7-p36763950
19256	Bn-N17-p37614576	Bn-C7-p37401578
19261	Bn-N17-p37909489	Bn-C7-p37720330
19266	Bn-N17-p38619798	Bn-C7-p38434890
19268	Bn-N17-p38658044	Bn-C7-p38461116
19279	Bn-N17-p39425879	Bn-C7-p39218523
19280	Bn-N15-p41349137	Bn-C7-p39332602
19286	Bn-N17-p39754792	Bn-C7-p39523483

19295	Bn-N17-p40165190	Bn-C7-p39971347
19320	Bn-N17-p40699585	Bn-C7-p40436327
19323	Bn-N17-p40703416	Bn-C7-p40440151
19346	Bn-N15-p4027205	Bn-C8-p21229919
19409	Bn-N17-p42151468	Bn-C7-p41915528
19412	Bn-N17-p42170025	Bn-C7-p41932287
19415	Bn-N17-p42295766	Bn-C7-p42054639
19423	Bn-N17-p42975451	Bn-C7-p42655999
19437	Bn-N17-p43158306	Bn-C7-p42845207
19440	Bn-N17-p43260732	Bn-C7-p42942159
19451	Bn-N17-p43457031	Bn-C7-p43156370
19452	Bn-N17-p43466400	Bn-C7-p43164661
19468	Bn-N17-p43880846	Bn-C7-p43570972
19473	Bn-N17-p43960610	Bn-C7-p43655170
19478	Bn-N17-p44318806	Bn-C7-p44016812
19485	Bn-N17-p44595392	Bn-C7-p44256784
19488	Bn-N17-p44650324	Bn-C7-p44315218
19490	Bn-N17-p44654596	Bn-C7-p44319581
19556	Bn-N17-p45568856	Bn-C7-p45252338
19563	Bn-N17-p45707681	Bn-C7-p45377611
19565	Bn-N17-p45782916	Bn-C7-p45437529
19567	Bn-N17-p46039061	Bn-C7-p45671980
19569	Bn-N17-p46100692	Bn-C7-p45729422
19571	Bn-N17-p46122548	Bn-C7-p45751307
19588	Bn-N17-p46543863	Bn-C7-p46159412
19590	Bn-N17-p46700265	Bn-C7-p46297766
19594	Bn-N17-p46765619	Bn-C7-p46356119
19604	Bn-N3-p29480332	Bn-A03-p29053385
19605	Bn-N17-p47049196	Bn-C7-p46637900
19606	Bn-N17-p47352443	Bn-C7-p46876015
19610	Bn-N17-p47594347	Bn-C7-p47122817
19615	Bn-N17-p48184736	Bn-C7-p47656824
19627	Bn-N17-p48719259	Bn-C7-p48216215
19629	Bn-N18-p1454470	Bn-C8-p1210965
19634	Bn-N18-p5618557	Bn-C8-p4368350
19638	Bn-N18-p12616173	Bn-A01-p10059043
19685	Bn-N18-p1843932	Bn-C8-p1650985
19693	Bn-N18-p10112858	Bn-C8-p8635399
19857	Bn-N18-p15850482	Bn-C8-p13822836
19878	Bn-N18-p19251163	Bn-C8-p17004422
19881	Bn-N18-p20024510	Bn-C8-p17859005
19883	Bn-N18-p20934069	Bn-Scaffold01219-p8813
19886	Bn-N18-p21210849	Bn-C8-p18806260
19889	Bn-N8-p20305257	Bn-C8-p19206552
19891	Bn-N18-p22264975	Bn-C8-p19887099
19892	Bn-N18-p22441567	Bn-C8-p20069131
19894	Bn-N18-p22462785	Bn-C8-p20091110
19916	Bn-N18-p22891395	Bn-C8-p20415408
19917	Bn-N18-p23909536	Bn-C8-p21353685

19921	Bn-N18-p23952842	Bn-C8-p21396843
19922	Bn-N18-p23995115	Bn-C8-p21439595
19941	Bn-N18-p24433888	Bn-C8-p21833572
19946	Bn-N18-p24521394	Bn-C8-p21912885
19949	Bn-N18-p24988414	Bn-C8-p22331853
19952	Bn-N18-p26041888	Bn-Scaffold01074-p6212
19954	Bn-N18-p26132328	Bn-C8-p23430903
19955	Bn-N18-p26587389	Bn-C8-p23873394
19956	Bn-N18-p26913742	Bn-C8-p24127988
19960	Bn-N18-p28274042	Bn-C8-p25393055
19984	Bn-N18-p28819264	Bn-C8-p25926212
19987	Bn-N18-p30465380	Bn-C8-p27775136
19994	Bn-N18-p30754016	Bn-C3-p51141028
19995	Bn-N18-p31188241	Bn-C8-p28178287
19996	Bn-N18-p31877221	Bn-C8-p28860028
20012	Bn-N18-p32115345	Bn-C8-p29120618
20024	Bn-N18-p32357196	Bn-C8-p29299889
20050	Bn-N18-p33602937	Bn-C8-p30537649
20061	Bn-N18-p33767888	Bn-C8-p30708636
20063	Bn-N18-p34020085	Bn-C8-p30959960
20070	Bn-N18-p34098757	Bn-C8-p31023043
20072	Bn-N18-p34122556	Bn-C8-p31038829
20090	Bn-N18-p34647062	Bn-C8-p31579687
20120	Bn-N18-p35707246	Bn-C8-p32722330
20131	Bn-N18-p36370965	Bn-C8-p33331256
20199	Bn-N18-p37282326	Bn-C8-p34257170
20204	Bn-N18-p37515324	Bn-C8-p34488468
20248	Bn-N18-p39241000	Bn-C8-p36179273
20250	Bn-N18-p39869059	Bn-C8-p36742396
20257	Bn-N9-p36764678	Bn-A09-p33660289
20260	Bn-N18-p41556749	Bn-C8-p38410249
20264	Bn-N18-p41663055	Bn-C8-p38486825
20266	Bn-N18-p41737543	Bn-C8-p38576855
20269	Bn-N18-p41906145	Bn-C8-p38749358
20270	Bn-N18-p42467540	Bn-C8-p39312052
20271	Bn-N18-p43035968	Bn-C8-p39889339
20272	Bn-N18-p43996802	Bn-C8-p40757738
20274	Bn-N18-p44424911	Bn-C8-p41145335
20277	Bn-N4-p5589	Bn-C9-p7599
20278	Bn-N19-p55940966	Bn-A09-p281844
20281	Bn-N9-p1081882	Bn-A01-p26921609
20285	Bn-N19-p2092209	Bn-C9-p2129884
20289	Bn-N19-p2266494	Bn-C9-p2309674
20293	Bn-N19-p2637647	Bn-C9-p2688672
20300	Bn-N17-p11449356	Bn-C7-p10815743
20301	Bn-N19-p4207026	Bn-C9-p4177835
20305	Bn-N19-p5017304	Bn-A09-p3233005
20308	Bn-N9-p3763762	Bn-C9-p5218392
20309	Bn-N19-p5638360	Bn-C9-p5641324

20310	Bn-N19-p6041483	Bn-C9-p6099851
20312	Bn-N19-p6258583	Bn-C9-p6285792
20316	Bn-N19-p6771134	Bn-C9-p6763573
20318	Bn-N19-p7631927	Bn-C9-p7657260
20321	Bn-N19-p10533617	Bn-C9-p10411803
20351	Bn-N19-p10836063	Bn-C9-p10670207
20353	Bn-N19-p11436491	Bn-C9-p11335462
20361	Bn-N19-p13406984	Bn-C9-p13245838
20385	Bn-N19-p15841037	Bn-C9-p15329223
20389	Bn-N19-p16373749	Bn-C9-p15784039
20457	Bn-N19-p17203737	Bn-C9-p16683551
20476	Bn-N19-p17936845	Bn-C9-p17340499
20561	Bn-N19-p19118690	Bn-C9-p18692647
20648	Bn-N19-p25177594	Bn-C9-p24331999
20785	Bn-N19-p37974163	Bn-C9-p36438578
20791	Bn-N19-p38982545	Bn-C7-p13328709
20805	Bn-N19-p42314902	Bn-C9-p40440348
20904	Bn-N19-p45517324	Bn-C9-p43400331
20908	Bn-N19-p45851910	Bn-C9-p43720221
20925	Bn-N19-p46041580	Bn-C9-p43822424
20956	Bn-N19-p46754810	Bn-C9-p44386081
20959	Bn-N19-p47451063	Bn-C9-p45066216
20961	Bn-N19-p47678139	Bn-C9-p45342222
20962	Bn-N19-p47939658	Bn-C9-p45590669
20993	Bn-N19-p48639808	Bn-C9-p46165884
21008	Bn-N19-p48992662	Bn-C13791383-p286
21016	Bn-N19-p49157514	Bn-C9-p46830604
21019	Bn-N19-p49299421	Bn-C9-p47004610
21020	Bn-N19-p49725586	Bn-C9-p47391900
21032	Bn-N19-p50008223	Bn-A10-p12957851
21035	Bn-N19-p50302870	Bn-C9-p47989399
21050	Bn-N19-p50540424	Bn-C9-p48236162
21058	Bn-N2-p2679970	Bn-A02-p3607564
21060	Bn-N19-p51542682	Bn-C9-p49140588
21070	Bn-N19-p51918660	Bn-C3-p58636673
21071	Bn-N19-p52346260	Bn-C9-p49881005
21076	Bn-N19-p52423130	Bn-C9-p49978999
21080	Bn-N19-p53090532	Bn-C9-p50628723
21085	Bn-N19-p53678595	Bn-C9-p51113926
21087	Bn-N19-p53833236	Bn-C9-p51273237
21093	Bn-N19-p53945924	Bn-C9-p51394423
21096	Bn-N19-p54445865	Bn-C9-p51869548
21099	Bn-N19-p54525486	Bn-C9-p51952893
21100	Bn-N19-p54610222	Bn-C9-p52030051
21104	Bn-N19-p54800819	Bn-C9-p52227361
21105	Bn-N19-p54986241	Bn-C9-p52417079
21110	Bn-N10-p17259635	Bn-C9-p53208843

to bins according to recombination events

locus_name	LG	Bin	Distance	SG_152	SG_320	SG_65	SG_132	SG_221	SG_258	SG_9	SG_155	SG_324	SG_90	SG_137	SG_229	SG_259	SG_18	SG_159	SG_224	SG_103	SG_150	SG_232	SG_261	SG_39
Bn-A01-p137814	A1	1	0	B	A	0	A	B	B	A	0	B	A	B	B	B	B	B	B	B	A	A	A	B
Bn-A01-p460072	A1	2	1.9	B	A	0	A	B	B	A	0	B	A	B	B	B	B	B	B	B	A	A	A	B
Bn-scaff_20461_1-p169329	A1	3	1.9	B	A	0	A	B	B	A	0	B	A	B	B	B	B	B	B	B	A	A	A	B
Bn-A01-p974934	A1	4	3	B	A	0	A	B	B	A	0	B	0	B	B	B	B	B	B	B	A	A	A	B
Bn-A01-p1250595	A1	5	4.1	B	A	0	A	B	B	A	B	B	0	B	B	B	B	B	B	B	A	A	A	B
Bn-A01-p1372430	A1	6	6.3	B	A	0	A	B	B	A	0	B	0	B	B	B	B	B	B	B	A	A	A	B
Bn-A01-p1661180	A1	7	8.5	B	A	0	A	B	B	A	0	B	0	B	B	B	B	B	B	B	A	A	B	B
Bn-A01-p1742846	A1	8	9.6	B	A	0	A	B	B	A	0	B	0	B	B	B	B	B	B	B	A	A	B	B
Bn-A01-p1770291	A1	9	10.7	B	A	0	A	B	B	A	0	B	0	B	B	B	B	B	B	B	A	A	B	B
Bn-A01-p1990755	A1	10	11.8	B	A	0	A	B	B	A	0	B	0	B	B	B	B	B	B	B	A	A	B	B
Bn-A01-p2094793	A1	11	12.9	B	A	0	A	B	B	A	0	B	0	B	B	B	B	B	B	B	A	A	B	B
Bn-A01-p2171217	A1	12	14	B	A	0	A	B	B	A	0	B	0	B	B	B	B	B	B	B	A	A	B	B
Bn-A01-p2242890	A1	13	16.2	B	A	0	A	B	B	A	0	B	0	B	B	B	B	B	B	B	A	A	B	B
Bn-A01-p2667471	A1	14	19	B	A	0	A	B	B	A	0	B	0	B	B	B	B	B	B	B	A	A	B	B
Bn-A01-p2825565	A1	15	20.4	B	A	0	A	B	B	A	0	B	0	B	B	B	B	B	B	B	A	A	B	B
Bn-A01-p2990104	A1	16	21.8	B	A	0	A	B	B	A	0	B	0	B	B	B	B	B	B	B	A	A	B	B
Bn-A01-p3183888	A1	17	23.2	B	A	0	A	B	B	A	0	B	0	B	B	B	B	B	B	B	A	A	B	B
Bn-A01-p3306526	A1	18	24.4	B	A	0	A	B	B	A	0	B	0	B	B	B	B	B	B	A	A	A	B	B
Bn-A01-p3904495	A1	19	26.7	B	A	0	A	B	B	A	0	B	0	B	B	B	B	B	B	A	A	A	B	B
Bn-A01-p4812269	A1	20	26.7	B	A	0	A	B	B	A	0	B	0	B	B	B	B	B	B	A	A	A	B	B
Bn-A01-p5106042	A1	21	27.6	B	A	0	A	B	B	A	0	B	A	B	B	B	B	B	B	A	A	A	B	B
Bn-A01-p5193639	A1	22	28.5	B	A	0	A	B	B	A	0	B	A	B	B	B	B	B	B	A	A	A	B	B
Bn-A01-p5590998	A1	23	29.5	B	A	A	A	B	B	A	A	B	B	B	B	B	B	B	B	A	A	A	B	B
Bn-A01-p6182710	A1	24	30.4	B	A	A	A	B	B	A	A	B	B	B	B	B	B	B	B	A	A	A	B	B
Bn-A01-p6274135	A1	25	32.3	B	A	A	A	B	B	A	B	B	B	B	B	B	B	B	B	A	A	A	B	B
Bn-A01-p6687707	A1	26	34.2	B	A	A	A	B	B	A	B	B	B	B	B	B	B	B	B	A	A	A	B	B
Bn-A01-p6898291	A1	27	35.1	B	A	A	A	B	B	A	B	B	B	B	B	B	B	B	B	A	A	A	B	B
Bn-A01-p7648929	A1	28	36	B	A	A	A	B	B	A	B	B	B	B	B	B	B	B	B	A	A	A	B	B
Bn-A01-p7889385	A1	29	37.9	B	A	A	A	B	B	A	B	B	B	B	B	B	B	B	B	A	A	A	B	B
Bn-A01-p7979458	A1	30	38.8	B	A	A	A	B	B	A	B	B	B	B	B	B	B	B	B	A	A	A	B	B
Bn-A01-p8017380	A1	31	39.7	B	A	A	A	B	B	A	B	B	B	B	B	A	B	B	B	A	A	A	B	B
Bn-A01-p9442471	A1	32	40.6	B	A	A	A	B	B	A	B	B	B	B	B	A	B	B	B	A	A	A	B	B
Bn-A01-p9884449	A1	33	41.5	B	A	A	A	B	B	A	B	B	B	B	B	A	B	B	B	A	A	A	B	B
Bn-A01-p9996458	A1	34	42.4	B	A	A	A	B	B	A	B	B	B	B	B	A	B	B	B	A	A	A	B	B
Bn-A01-p10231780	A1	35	43.3	B	A	A	A	B	B	A	B	B	B	B	B	A	B	B	B	A	A	A	B	B
Bn-scaff_16217_1-p143344	A1	36	44.2	B	A	A	A	B	B	A	B	B	B	B	B	A	B	B	B	A	A	A	B	B
Bn-A01-p11825880	A1	37	45.1	B	A	A	A	B	B	A	B	B	B	B	B	A	B	B	B	A	A	A	B	B
Bn-A01-p19983739	A1	38	46	B	A	A	A	B	B	A	B	B	B	B	B	A	B	B	B	A	A	A	B	B
Bn-A01-p21393260	A1	39	46.9	B	A	A	A	B	B	A	B	B	B	B	B	A	B	B	B	A	A	A	B	B
Bn-A01-p21709916	A1	40	47.7	B	A	A	A	B	B	A	B	B	B	B	B	A	B	B	B	A	A	A	B	B
Bn-A01-p21855770	A1	41	48.5	B	A	A	A	B	B	A	B	B	B	B	B	A	B	B	B	A	A	A	B	B
Bn-A01-p22051510	A1	42	49.3	B	A	A	A	B	B	A	B	B	B	B	B	A	B	B	B	A	A	A	B	B
Bn-A01-p22087629	A1	43	50.1	B	A	A	A	B	B	A	B	B	B	B	B	A	B	B	B	A	A	A	B	B
Bn-A01-p22681496	A1	44	50.9	B	A	A	A	B	B	A	B	B	B	B	B	A	B	B	B	A	A	A	B	B
Bn-A01-p23568963	A1	45	55.2	A	A	A	A	B	B	A	B	B	B	B	B	A	B	A	B	A	A	A	B	B

Bn-scaff_17515_1-p51302	A1	46	56	A	A	B	A	B	B	A	B	B	B	B	B	A	B	A	B	A	A	A	B	B
Bn-A01-p23614494	A1	47	56.8	A	A	B	A	B	B	A	B	B	B	B	B	A	B	A	B	A	A	A	B	B
Bn-A01-p23694782	A1	48	57.6	A	A	B	A	B	B	A	B	B	B	B	B	A	B	A	B	A	A	A	B	B
Bn-A01-p24504575	A1	49	59.3	A	A	B	A	B	B	A	B	B	B	B	B	A	B	A	B	A	A	A	B	B
Bn-A01-p24697185	A1	50	61	A	A	B	A	B	B	A	B	B	B	A	B	A	B	A	B	A	A	A	B	B
Bn-Scaffold000164-p244631	A1	51	62.7	A	A	B	A	B	B	A	B	B	B	A	B	A	B	A	B	A	A	A	B	B
Bn-Scaffold000164-p55747	A1	52	65.2	A	A	B	A	B	B	A	B	A	B	A	B	A	B	A	B	A	A	A	B	B
Bn-Scaffold000386-p1325	A1	53	66	A	A	B	A	B	B	A	B	A	B	A	B	A	B	A	B	A	A	A	B	B
Bn-A01-p27331467	A1	54	66.8	A	A	B	A	B	B	A	B	A	B	A	B	A	B	A	A	A	A	A	B	B
Bn-A01-p27580681	A1	55	70.2	A	A	B	A	B	B	A	B	A	B	A	B	A	B	A	A	A	A	A	B	B
Bn-A01-p27866419	A1	56	71	A	A	B	A	B	B	A	B	A	B	A	B	A	B	A	A	A	A	A	B	B
Bn-A01-p28102080	A1	57	71.8	A	A	B	A	B	B	A	B	A	B	A	B	A	B	A	A	A	A	A	B	B
Bn-A01-p28462071	A1	58	74.3	A	A	B	A	B	B	A	B	A	B	A	B	A	B	A	A	A	A	A	B	B
Bn-A01-p25517197	A1	59	75.1	A	A	B	A	B	B	A	B	A	B	A	B	A	B	A	A	A	A	A	B	B
Bn-A01-p25040577	A1	60	75.9	A	A	B	A	B	B	A	B	A	B	A	B	A	B	A	A	A	A	A	B	B
Bn-A02-p1707420	A2	1	0	A	B	B	A	A	B	A	A	A	A	B	A	A	A	A	A	A	A	A	A	A
Bn-A02-p2292871	A2	2	1.8	A	B	B	A	A	B	A	B	A	A	B	A	A	A	A	A	A	A	A	A	A
Bn-A02-p2542065	A2	3	5.3	A	B	B	A	A	B	A	B	A	A	B	A	A	A	A	A	A	A	A	A	A
Bn-A02-p2572625	A2	4	6.2	A	B	B	A	A	B	A	B	A	A	B	A	A	A	A	A	A	A	A	A	A
Bn-A02-p2617701	A2	5	7.1	A	B	B	A	A	B	A	B	A	A	B	A	A	A	A	A	A	A	A	B	A
Bn-A02-p2739888	A2	6	8	A	B	B	A	A	B	A	B	A	A	B	A	A	A	A	A	A	A	A	B	A
Bn-A02-p1096082	A2	7	9.8	A	B	B	A	A	B	A	B	A	A	B	A	A	A	A	A	A	A	A	B	A
Bn-A02-p1092146	A2	8	10.6	A	B	B	A	A	B	A	B	A	A	B	A	A	A	A	A	A	A	A	B	A
Bn-A02-p2877222	A2	9	11.4	A	B	B	A	A	B	A	B	A	A	B	A	A	A	A	A	A	A	A	B	A
Bn-A02-p2923889	A2	10	12.2	A	B	B	A	A	B	A	B	A	A	B	B	A	A	A	A	A	A	A	B	A
Bn-A02-p3074016	A2	11	17.1	A	B	B	B	A	B	A	B	A	A	A	B	A	A	A	A	A	B	A	B	A
Bn-A02-p3121742	A2	12	17.9	A	B	B	B	A	B	A	B	A	A	A	B	A	B	A	A	A	B	A	B	A
Bn-A02-p3175610	A2	13	18.7	A	B	B	B	A	B	A	B	A	A	A	B	A	B	A	A	A	B	A	B	A
Bn-A02-p3260536	A2	14	19.5	A	B	B	B	A	B	A	B	A	A	A	B	A	B	A	A	A	B	A	B	A
Bn-A02-p3294682	A2	15	21.9	A	B	A	B	A	A	A	B	A	A	A	B	A	B	A	A	A	B	A	B	A
Bn-A02-p3344176	A2	16	22.7	A	B	A	B	A	A	A	B	A	A	A	B	A	B	A	A	A	B	A	B	A
Bn-A02-p3374678	A2	17	23.5	A	B	A	B	A	A	A	B	B	A	A	B	A	B	A	A	A	B	A	B	A
Bn-A02-p3398506	A2	18	24.3	A	B	A	B	A	A	A	B	B	A	A	B	A	B	A	A	A	B	A	B	A
Bn-A02-p3542024	A2	19	25.1	A	B	A	B	A	A	A	B	B	A	A	B	A	B	A	A	A	B	A	B	A
Bn-A02-p4035808	A2	20	25.9	A	B	A	B	A	A	A	B	B	A	A	B	A	B	A	A	A	B	A	B	A
Bn-A02-p4277649	A2	21	26.7	A	B	A	B	A	A	A	B	B	A	A	B	A	B	A	A	A	B	A	B	A
Bn-A02-p4773307	A2	22	28.3	A	B	A	B	A	A	A	B	B	A	A	B	A	B	A	A	A	B	A	B	A
Bn-A02-p5326222	A2	23	32.3	A	B	A	B	A	A	A	B	B	A	A	B	A	B	A	A	A	B	A	B	A
Bn-A02-p5394958	A2	24	33.1	A	B	A	B	A	A	A	B	B	A	A	B	A	B	A	A	A	B	A	B	A
Bn-A02-p5429003	A2	25	37.1	B	B	A	B	A	A	A	B	B	A	A	B	A	B	B	A	A	B	A	B	A
Bn-A02-p5574727	A2	26	37.9	B	B	A	B	A	A	A	B	B	A	A	B	A	B	B	A	A	B	A	B	A
Bn-A02-p5859447	A2	27	38.7	B	B	A	B	A	A	A	B	B	A	A	B	A	B	B	A	A	B	A	B	A
Bn-A02-p5907701	A2	28	40.3	B	B	A	B	A	A	A	B	B	A	A	B	A	B	B	A	A	B	A	B	A
Bn-A02-p6084757	A2	29	42.7	B	B	A	B	A	A	A	B	B	A	A	B	A	B	B	A	A	B	A	B	A
Bn-A02-p6478309	A2	30	43.5	B	B	A	B	A	A	A	B	B	A	A	B	A	B	B	A	A	B	A	B	A
Bn-A02-p6658392	A2	31	44.3	B	B	A	B	A	A	A	B	B	A	A	B	A	B	B	A	A	B	A	B	B
Bn-A02-p7319800	A2	32	45.1	B	B	A	B	A	A	A	B	B	A	A	B	A	B	B	A	A	B	A	B	B
Bn-A02-p8625851	A2	33	46.7	B	B	A	B	A	A	A	B	B	A	A	B	A	B	B	A	A	B	A	B	B
Bn-A02-p8777846	A2	34	47.5	B	B	A	B	A	A	A	B	B	A	A	B	A	B	B	A	A	B	A	B	B

Bn-A02-p8865764	A2	35	48.3	B	B	A	B	A	A	A	B	B	A	A	B	A	B	B	A	A	B	A	B	B
Bn-A02-p9339288	A2	36	49.1	B	B	A	B	A	A	A	B	B	A	A	B	A	B	B	A	A	B	A	B	B
Bn-A02-p11041726	A2	37	49.9	B	B	A	B	B	A	A	B	B	A	A	B	A	B	B	A	A	B	A	B	B
Bn-A02-p11346389	A2	38	50.7	B	B	A	B	B	A	B	B	B	A	A	B	A	B	B	A	A	B	A	B	B
Bn-A02-p11817742	A2	39	51.5	B	B	A	B	B	A	B	B	B	B	A	B	A	B	B	A	A	B	A	B	B
Bn-A02-p12145607	A2	40	53.1	B	B	A	B	B	A	B	B	B	B	A	A	B	B	B	A	A	B	A	B	B
Bn-A02-p12292026	A2	41	53.9	B	B	A	B	B	A	B	B	B	B	A	A	B	B	B	A	A	B	A	B	B
Bn-A02-p14078497	A2	42	54.7	B	B	A	B	B	A	B	B	B	B	A	A	B	B	B	A	A	B	A	B	B
Bn-A02-p14227678	A2	43	55.5	B	B	A	B	B	A	B	B	B	B	A	A	B	B	B	A	A	B	A	B	B
Bn-Scaffold000262-p25395	A2	44	56.3	B	B	A	B	B	A	B	B	B	B	A	A	B	B	B	A	A	B	A	B	B
Bn-A02-p15637932	A2	45	57.9	B	B	A	B	B	A	B	B	B	B	A	A	B	B	B	A	A	B	A	B	B
Bn-A05-p9078281	A2	46	58.7	B	B	A	B	B	A	B	B	B	B	A	A	B	B	B	A	A	B	A	B	B
Bn-A02-p17381818	A2	47	59.5	B	B	A	B	B	A	B	B	B	B	A	A	B	B	B	A	A	B	A	B	B
Bn-A02-p18599005	A2	48	62.7	B	B	A	A	B	A	B	B	B	B	A	A	B	B	B	A	A	A	A	B	B
Bn-A02-p19261853	A2	49	63.5	B	B	A	A	B	A	B	B	B	B	A	A	B	B	B	A	A	A	A	B	B
Bn-A02-p21061002	A2	50	64.3	B	B	A	A	B	A	B	B	B	B	A	A	B	B	B	A	A	A	A	B	B
Bn-A02-p19756499	A2	51	65.1	B	B	A	A	B	A	B	B	B	B	A	A	B	B	B	A	A	A	A	B	B
Bn-A02-p22843446	A2	52	67.5	B	B	A	A	B	A	B	B	B	B	A	A	B	B	B	A	A	A	A	B	B
Bn-A02-p22757902	A2	53	68.3	B	B	A	A	B	A	B	B	B	B	A	A	B	B	B	A	A	A	A	B	B
Bn-A02-p22352518	A2	54	69.1	B	B	A	A	B	A	B	B	B	B	A	A	B	B	B	A	A	A	A	B	B
Bn-A02-p23411852	A2	55	70.7	B	B	A	A	B	A	B	B	B	B	A	A	B	B	B	A	A	A	A	B	B
Bn-Scaffold016439-p48	A2	56	71.5	B	B	A	A	B	A	B	B	B	B	A	A	B	B	B	A	A	A	A	B	B
Bn-A02-p23668607	A2	57	73.1	B	B	A	A	B	A	B	B	B	B	B	A	B	B	B	A	A	A	A	B	B
Bn-scaff_17109_1-p690819	A2	58	74.2	B	B	A	A	B	A	B	B	B	B	B	A	B	B	B	A	A	A	A	B	B
Bn-A02-p23792703	A2	59	76.4	B	B	A	A	B	A	B	B	B	B	B	A	B	B	B	A	A	A	A	B	B
Bn-A02-p24178094	A2	60	77.2	B	B	A	A	B	A	B	B	B	B	B	A	B	B	B	A	A	A	A	B	B
Bn-A02-p24415454	A2	61	78.8	B	B	A	A	B	A	B	B	B	B	B	A	B	B	B	A	A	A	A	B	B
Bn-A02-p24449690	A2	62	79.6	B	B	A	A	B	A	B	B	B	B	B	A	B	B	B	A	A	A	A	B	B
Bn-A02-p24552542	A2	63	80.4	B	B	A	A	B	A	B	B	B	B	B	A	B	B	B	A	A	A	A	B	B
Bn-scaff_17623_1-p120748	A2	64	81.2	B	B	A	A	A	A	B	B	B	B	B	A	B	B	B	A	A	A	A	B	B
Bn-A02-p24948938	A2	65	83.6	B	B	A	A	A	A	B	B	B	B	B	A	B	B	B	A	A	A	A	B	B
Bn-A02-p25100451	A2	66	84.4	B	B	A	A	A	A	B	B	B	B	B	A	B	B	B	A	A	A	A	B	B
Bn-A02-p25188041	A2	67	85.2	B	B	A	A	A	A	B	B	B	B	B	A	B	B	B	A	A	A	A	B	B
Bn-A02-p25400591	A2	68	86	B	B	A	A	A	A	B	B	B	B	B	A	A	B	B	A	A	A	A	B	B
Bn-A02-p25681480	A2	69	87.6	B	B	A	A	A	A	B	B	B	B	B	A	A	B	B	A	A	A	A	B	B
Bn-A02-p25826690	A2	70	89.2	B	B	A	A	A	A	B	B	B	B	B	A	A	B	B	A	A	A	A	B	B
Bn-A02-p25841130	A2	71	90	B	B	A	A	A	A	B	B	B	B	B	A	A	B	B	A	A	A	A	B	B
Bn-A02-p25865360	A2	72	90.8	B	B	A	A	A	A	B	B	B	B	B	A	A	B	B	A	A	A	A	B	B
Bn-A02-p26982864	A2	73	97.4	B	B	A	B	A	A	B	B	A	B	B	A	A	B	B	A	A	B	A	A	B
Bn-A02-p27831174	A2	74	98.2	B	B	A	B	A	A	B	B	A	B	B	A	A	B	B	A	A	B	A	A	B
Bn-A03-p312762	A3	1	0	B	A	A	B	A	B	A	B	A	B	B	A	B	B	B	B	A	B	A	B	A
Bn-A03-p765077	A3	2	0.8	B	A	A	B	A	B	A	B	A	B	B	A	B	B	B	B	A	B	A	B	A
Bn-A03-p991949	A3	3	1.6	B	A	A	B	A	B	A	B	A	B	B	A	B	B	B	B	A	B	B	B	A
Bn-A03-p1050437	A3	4	3.2	B	A	A	B	A	B	A	B	A	B	B	A	B	B	B	B	A	B	B	B	A
Bn-A03-p1488306	A3	5	4.8	B	A	A	B	A	B	A	B	A	B	B	A	B	B	B	B	A	B	B	B	A
Bn-A03-p1711856	A3	6	5.6	B	A	A	B	A	B	A	B	A	B	B	A	B	B	B	B	A	B	B	B	A
Bn-A03-p1926809	A3	7	7.2	B	B	A	B	A	B	A	B	A	B	B	A	B	B	B	B	A	B	B	B	A
Bn-A03-p1954639	A3	8	8.8	B	B	A	B	A	B	A	B	A	B	A	A	B	B	B	B	A	B	B	B	A
Bn-A03-p2579866	A3	9	9.6	B	B	A	B	A	B	A	B	A	B	A	A	B	B	B	B	A	B	B	B	A

Bn-A03-p2645391	A3	10	10.4	B	B	A	B	B	B	A	B	A	B	A	A	B	B	B	B	A	B	B	B	A
Bn-A03-p2891934	A3	11	11.2	B	B	A	B	B	B	A	B	A	B	A	A	A	B	B	B	A	B	B	B	A
Bn-A03-p2997270	A3	12	13.6	B	B	A	B	B	B	A	B	A	B	A	A	A	B	B	B	A	B	B	B	A
Bn-A03-p3099901	A3	13	14.4	B	B	A	B	B	B	A	B	A	B	A	A	A	B	B	B	A	B	B	B	A
Bn-A03-p3319357	A3	14	15.2	B	B	A	B	B	B	A	B	A	B	A	A	A	B	B	B	A	B	B	B	A
Bn-A03-p3692048	A3	15	16.8	B	B	A	B	B	B	A	B	A	B	A	A	A	B	B	B	A	B	B	B	A
Bn-A03-p3795299	A3	16	17.6	B	B	A	B	B	B	A	B	A	B	A	A	A	B	B	B	A	B	B	B	A
Bn-A03-p4783485	A3	17	18.4	B	B	A	B	B	B	A	B	A	B	A	A	A	B	B	B	A	B	B	B	A
Bn-A03-p4909233	A3	18	19.2	B	B	A	B	B	B	A	B	A	B	A	A	A	B	B	B	A	B	B	B	A
Bn-A03-p5083198	A3	19	20.8	B	B	A	B	B	B	A	B	A	B	A	A	A	B	B	B	A	B	B	B	A
Bn-scaff_18523_1-p438	A3	20	24.9	A	B	A	B	B	B	A	B	A	B	A	A	A	B	A	B	A	B	B	B	A
Bn-A03-p5741340	A3	21	25.7	A	B	A	B	B	B	A	B	A	B	A	A	A	B	A	B	A	B	B	B	A
Bn-A03-p6036521	A3	22	26.5	A	B	A	B	B	B	A	B	A	B	A	A	A	B	A	0	A	B	B	B	A
Bn-A03-p6274828	A3	23	27.3	A	B	A	B	B	B	A	B	A	B	A	A	A	B	A	0	A	B	B	B	A
Bn-A03-p6590051	A3	24	28.9	A	B	A	B	B	B	A	B	A	B	A	A	A	B	A	0	A	B	B	B	A
Bn-A03-p6744344	A3	25	32.1	A	B	A	A	B	B	A	B	A	B	A	A	A	B	A	0	A	A	B	B	A
Bn-A03-p7007377	A3	26	32.9	A	B	A	A	B	B	A	B	A	B	A	A	A	B	A	0	A	A	B	B	A
Bn-A03-p7177504	A3	27	33.8	A	B	A	A	B	B	A	B	A	B	A	A	A	B	A	0	A	A	B	B	A
Bn-A03-p7366783	A3	28	35.5	A	B	A	A	B	B	A	B	A	B	A	A	A	B	A	0	A	A	B	B	A
Bn-A03-p7420835	A3	29	36.4	A	B	A	A	B	B	A	B	A	B	A	A	A	B	A	0	A	A	B	B	A
Bn-A03-p7939679	A3	30	37.3	A	B	A	A	B	B	A	B	A	B	A	A	A	B	A	0	A	A	B	B	A
Bn-A03-p8133096	A3	31	39	A	B	A	A	B	B	A	B	A	B	A	A	A	B	A	0	A	A	B	B	A
Bn-A03-p8764481	A3	32	41.6	A	B	A	A	B	B	A	B	A	A	A	A	A	B	A	0	A	A	B	B	A
Bn-A03-p9537155	A3	33	42.5	A	B	A	A	B	B	A	B	A	A	A	A	A	B	A	0	A	A	B	B	A
Bn-A03-p9833648	A3	34	43.4	A	B	A	A	B	B	A	B	A	A	A	A	A	B	A	0	A	A	B	B	A
Bn-A03-p10414176	A3	35	44.3	A	B	A	A	B	B	A	B	A	A	A	A	A	B	A	0	A	A	B	B	A
Bn-A03-p10651420	A3	36	45.2	A	B	A	A	B	B	A	B	A	A	A	A	A	B	A	0	B	A	B	B	A
Bn-A03-p10757525	A3	37	46.1	A	B	A	A	B	B	A	B	A	A	A	A	A	B	A	0	B	A	B	B	A
Bn-A03-p11319722	A3	38	47	A	B	A	A	B	B	A	B	A	A	A	A	A	B	A	0	B	A	B	B	A
Bn-A03-p11517597	A3	39	47.8	A	B	A	A	B	B	A	B	A	A	A	A	A	B	A	0	B	A	B	B	A
Bn-A03-p12186958	A3	40	49.4	A	B	B	A	B	B	A	B	A	A	A	A	A	B	A	0	B	A	B	B	A
Bn-A03-p12639200	A3	41	51	A	B	B	A	B	B	A	B	A	A	A	A	A	B	A	0	B	A	B	B	A
Bn-A03-p12990610	A3	42	51.8	A	B	B	A	B	B	A	B	A	A	A	A	A	B	A	0	B	A	B	B	A
Bn-A03-p13104283	A3	43	52.6	A	B	B	A	B	B	A	B	A	A	A	A	A	B	A	0	B	A	B	B	B
Bn-A03-p14112910	A3	44	56.7	A	B	B	A	B	B	A	B	A	A	A	A	A	B	A	B	B	A	B	B	B
Bn-A03-p14175904	A3	45	60.7	A	B	B	B	B	B	A	B	A	A	A	A	A	B	A	B	B	B	B	B	B
Bn-A03-p14306440	A3	46	62.3	A	B	B	B	B	B	A	B	A	A	B	A	A	B	A	B	B	B	B	B	B
Bn-A03-p14447238	A3	47	63.1	A	B	B	B	B	B	A	B	A	A	B	A	A	B	A	B	B	B	B	B	B
Bn-A03-p15359867	A3	48	64.7	A	B	B	B	B	B	A	B	A	A	B	A	A	B	A	B	B	B	B	B	B
Bn-A03-p15577849	A3	49	65.5	A	B	B	B	B	B	A	B	A	A	B	A	A	B	A	B	B	B	B	B	B
Bn-A03-p15957657	A3	50	67.1	A	B	B	B	B	B	A	B	A	A	B	A	A	B	A	B	B	B	B	B	B
Bn-A03-p16607714	A3	51	68.7	A	B	B	B	B	A	A	B	A	A	B	A	A	A	A	B	B	B	B	B	B
Bn-A03-p16812953	A3	52	69.5	A	B	B	B	B	A	A	B	A	A	B	A	A	A	A	B	B	B	B	B	B
Bn-scaff_17298_1-p1321549	A3	53	70.3	A	B	B	B	B	A	A	B	A	A	B	A	A	A	A	B	B	B	B	B	B
Bn-A03-p17182494	A3	54	71.1	A	B	B	B	B	A	A	B	A	A	B	A	A	A	A	B	B	B	B	B	B
Bn-A03-p17782292	A3	55	71.9	A	B	B	B	B	A	A	B	A	A	B	A	A	A	A	B	B	B	B	B	B
Bn-A03-p18120938	A3	56	72.7	A	B	B	B	B	A	A	B	A	A	B	A	A	A	A	B	B	B	B	B	B
Bn-A03-p18836509	A3	57	73.5	A	B	B	B	B	A	A	B	A	A	B	A	A	A	A	B	B	B	B	B	B
Bn-A03-p18846268	A3	58	74.3	A	B	B	B	B	A	A	B	A	A	B	A	A	A	A	B	B	B	B	B	B

Bn-A03-p19880918	A3	59	75.1	A	B	B	B	B	A	A	B	A	A	B	A	A	A	A	B	B	B	B	B	B
Bn-A03-p20033408	A3	60	75.9	A	B	B	B	B	A	A	B	A	A	B	B	A	A	A	B	B	B	B	B	B
Bn-A03-p20079452	A3	61	76.7	A	B	B	B	B	A	A	B	A	A	B	B	A	A	A	B	B	B	B	B	B
Bn-A03-p20151244	A3	62	77.5	A	B	B	B	B	A	A	B	A	A	B	B	A	A	A	B	B	B	B	B	B
Bn-A03-p20180463	A3	63	79.1	A	B	B	B	B	A	A	B	A	A	B	B	A	A	A	B	B	B	B	B	B
Bn-A03-p20237681	A3	64	79.9	A	B	B	B	B	A	A	B	A	A	B	B	A	A	A	B	B	B	B	B	B
Bn-A03-p20523585	A3	65	81.5	A	B	B	B	B	A	A	B	A	A	B	B	A	A	A	B	B	B	B	B	B
Bn-A03-p20983297	A3	66	83.9	A	B	B	B	B	A	B	B	A	A	B	B	B	A	A	B	B	B	B	B	B
Bn-A03-p21271941	A3	67	84.7	A	B	B	B	B	A	B	B	A	A	B	B	B	A	A	B	B	B	B	B	B
Bn-A03-p21422696	A3	68	85.5	A	B	B	B	B	A	B	B	A	A	B	B	B	A	A	B	B	B	B	B	B
Bn-A03-p21632587	A3	69	86.3	A	B	B	B	B	A	B	B	A	A	B	B	B	A	A	B	B	B	B	B	B
Bn-A03-p21685480	A3	70	87.1	A	B	B	B	B	A	B	B	A	A	B	B	B	A	A	B	B	B	B	B	B
Bn-A03-p21904842	A3	71	87.9	A	B	B	B	B	A	B	B	A	A	B	B	B	A	A	B	B	B	B	B	B
Bn-A03-p22820943	A3	72	88.7	A	B	B	B	B	A	B	B	A	A	B	B	B	A	A	B	B	B	B	B	B
Bn-A03-p22882585	A3	73	89.5	A	B	B	B	B	A	B	B	A	A	B	B	B	A	A	B	B	B	B	B	B
Bn-A03-p23017354	A3	74	90.3	A	B	B	B	B	A	B	B	A	A	B	B	B	A	A	B	B	B	B	B	B
Bn-A03-p29571938	A3	75	92.7	A	B	B	B	B	A	B	B	B	A	B	B	B	A	A	B	B	B	B	B	B
Bn-A03-p23735715	A3	76	93.5	A	B	B	B	B	A	B	B	B	A	B	B	B	A	A	B	B	B	B	B	A
Bn-A03-p24092986	A3	77	95.1	A	B	B	B	B	A	B	B	B	A	B	B	B	A	A	B	B	B	B	B	A
Bn-A03-p24176332	A3	78	95.9	A	B	B	B	B	A	B	B	B	A	B	B	B	A	A	B	B	B	B	B	A
Bn-A03-p24902631	A3	79	98.3	A	B	B	B	B	A	B	B	B	A	B	B	B	A	A	B	B	B	B	B	A
Bn-A03-p25402301	A3	80	99.1	A	B	B	B	B	A	B	B	B	A	B	B	B	A	A	B	B	B	B	B	A
Bn-A03-p25525612	A3	81	99.9	A	B	B	B	B	A	B	B	B	A	B	B	B	A	A	B	B	B	B	B	A
Bn-A03-p25614406	A3	82	100.7	A	B	B	B	B	A	B	B	B	A	B	B	B	A	A	B	B	B	B	B	A
Bn-A03-p25631316	A3	83	101.5	A	B	B	B	B	A	B	B	B	A	B	B	B	A	A	B	B	B	B	B	A
Bn-A03-p25993601	A3	84	102.3	A	B	B	B	B	A	B	B	B	A	B	B	B	A	A	B	B	B	B	B	A
Bn-A03-p26066930	A3	85	103.1	A	B	B	B	B	A	B	B	B	A	B	B	B	A	A	B	B	B	B	B	A
Bn-A03-p26284882	A3	86	103.9	A	B	B	B	B	A	B	B	B	A	B	B	B	A	A	B	B	B	B	B	A
Bn-A03-p26288769	A3	87	104.7	A	B	B	B	B	A	B	B	B	A	B	B	B	A	A	B	B	B	B	B	A
Bn-A03-p26477533	A3	88	105.5	A	B	B	B	B	A	B	B	B	A	B	B	B	A	A	B	B	B	B	B	A
Bn-A03-p26775994	A3	89	109.5	A	B	B	B	B	A	B	B	B	A	B	B	B	A	A	B	B	B	B	B	A
Bn-A03-p27066000	A3	90	110.3	A	B	B	B	B	A	B	B	B	A	B	B	B	A	A	B	B	B	B	B	A
Bn-A03-p27683723	A3	91	111.1	A	B	B	B	B	A	B	B	B	A	B	B	B	A	A	B	B	B	B	B	A
Bn-A03-p28535638	A3	92	111.9	A	B	B	B	B	A	B	B	B	A	B	B	B	A	A	B	B	B	B	B	A
Bn-Scaffold000096-p878406	A3	93	112.7	A	B	B	B	B	A	B	B	B	A	B	B	B	A	A	B	B	B	B	B	A
Bn-A03-p28126287	A3	94	114.3	A	B	B	B	B	A	B	B	B	A	B	B	B	A	A	B	B	B	B	B	A
Bn-A04-p122280	A4	1	0	A	B	A	A	A	A	A	B	A	B	A	B	B	B	A	B	A	A	A	B	B
Bn-A04-p267294	A4	2	0.8	A	B	A	A	A	A	A	B	A	B	A	B	B	B	A	B	A	A	A	B	B
Bn-A04-p810935	A4	3	3.2	A	B	A	A	A	A	A	B	A	B	A	B	B	B	A	B	A	A	A	B	B
Bn-A04-p1213196	A4	4	4	A	B	A	A	A	A	A	B	A	B	A	B	B	B	A	B	A	A	A	B	B
Bn-A04-p1317446	A4	5	4.8	A	B	A	A	A	A	A	B	A	B	A	B	B	B	A	B	A	A	A	B	B
Bn-A04-p1390449	A4	6	5.6	A	B	A	A	A	A	A	B	A	B	A	B	B	B	A	B	A	A	A	B	B
Bn-A04-p1623151	A4	7	6.4	A	B	A	A	A	A	A	B	A	B	A	B	B	B	A	B	A	A	A	B	B
Bn-A04-p1655416	A4	8	7.2	A	B	A	A	A	A	A	B	A	B	A	B	B	B	A	B	A	A	A	B	B
Bn-A04-p1680506	A4	9	8.8	A	B	A	A	A	B	A	B	A	B	A	B	B	B	A	B	A	A	A	B	B
Bn-A04-p1745832	A4	10	9.6	A	B	A	A	A	B	A	B	A	B	A	B	B	B	A	B	A	A	A	B	B
Bn-A04-p1931502	A4	11	10.4	A	B	A	A	A	B	A	B	A	B	A	B	B	B	A	B	A	A	A	B	B
Bn-A04-p3225119	A4	12	12	A	B	A	A	A	B	A	B	A	B	A	B	B	B	A	B	A	A	A	B	B
Bn-A04-p3632226	A4	13	13.6	A	B	A	A	A	B	A	B	A	B	A	B	B	B	A	B	A	A	A	B	B

Bn-A04-p3743549	A4	14	14.4	A	B	A	A	A	B	A	B	A	B	A	B	B	B	A	B	A	A	A	B	B
Bn-A04-p5959753	A4	15	16.8	A	B	A	A	A	B	A	B	A	B	A	B	B	B	A	B	B	A	A	B	B
Bn-A04-p7109346	A4	16	17.6	A	B	A	A	A	B	A	B	A	B	A	B	B	B	A	B	A	A	A	B	B
Bn-A04-p8292499	A4	17	18.4	A	B	A	A	A	B	A	B	A	B	A	B	B	B	A	B	A	A	A	B	B
Bn-A04-p8612311	A4	18	19.2	A	B	A	A	A	B	A	B	A	B	A	B	B	B	A	B	A	A	A	B	B
Bn-A04-p9088043	A4	19	20	A	B	A	A	A	B	B	B	A	B	A	B	B	B	A	B	A	A	A	B	B
Bn-A04-p11957518	A4	20	21.6	A	B	B	A	A	B	B	B	A	B	A	B	B	A	A	B	A	A	A	B	B
Bn-A04-p12236240	A4	21	23.2	A	B	B	A	A	B	B	A	A	B	A	B	B	A	A	B	A	A	A	B	B
Bn-A04-p12659402	A4	22	24.8	A	B	B	A	B	B	B	A	A	B	A	B	B	A	A	B	A	A	A	B	B
Bn-A04-p13659690	A4	23	25.6	A	B	B	A	B	B	B	A	A	B	A	B	B	A	A	B	A	A	B	B	B
Bn-scaff_16804_2-p157923	A4	24	26.4	A	B	B	A	B	B	B	A	A	B	A	B	B	A	A	B	A	A	B	B	B
Bn-scaff_16804_2-p63611	A4	25	28	A	B	B	A	B	B	B	A	A	B	A	B	B	A	A	B	A	A	B	B	B
Bn-A04-p14255771	A4	26	29.6	A	B	B	A	B	B	B	A	A	B	A	B	B	A	A	B	A	A	B	B	B
Bn-A04-p14756001	A4	27	32.8	A	B	B	B	B	B	B	A	A	B	A	B	B	A	A	B	A	B	B	B	B
Bn-A04-p15007146	A4	28	36.8	B	B	B	B	B	B	B	A	A	B	A	B	B	A	B	B	A	B	B	B	B
Bn-A04-p15298740	A4	29	37.6	B	B	B	B	B	B	B	A	A	B	A	B	B	A	B	B	B	B	B	B	B
Bn-A04-p15404568	A4	30	38.4	B	B	B	B	B	B	B	A	A	B	A	B	B	A	B	B	B	B	B	B	B
Bn-A04-p15514869	A4	31	39.2	B	B	B	B	B	B	B	A	A	B	A	B	B	A	B	B	B	B	B	B	B
Bn-A04-p15835310	A4	32	40	B	B	B	B	B	B	B	A	A	B	A	B	B	A	B	B	B	B	B	B	B
Bn-A04-p15895339	A4	33	41.6	B	B	B	B	B	B	B	A	A	B	A	B	B	A	B	B	B	B	B	B	B
Bn-A04-p16056186	A4	34	42.4	B	B	B	B	B	B	B	A	A	B	A	B	B	A	B	B	B	B	B	B	B
Bn-A04-p16233024	A4	35	43.4	B	B	B	B	B	B	B	A	A	B	A	B	B	A	B	B	B	B	B	A	B
Bn-A04-p16445225	A4	36	44.4	B	B	B	B	B	B	B	A	A	B	A	B	B	A	B	B	B	B	B	A	B
Bn-A04-p16663552	A4	37	45.4	B	B	B	B	B	B	B	A	A	B	A	B	B	A	B	B	B	B	B	A	B
Bn-A04-p17052071	A4	38	46.4	B	B	B	B	B	B	B	A	A	A	A	B	B	A	B	B	B	B	B	A	B
Bn-A04-p17322241	A4	39	47.2	B	B	B	B	B	B	B	A	A	A	A	B	B	A	B	B	B	B	B	A	B
Bn-A04-p17686228	A4	40	48	B	B	B	B	B	B	B	A	A	A	A	B	B	A	B	B	B	B	B	A	B
Bn-A04-p17811740	A4	41	48.8	B	B	B	B	B	B	B	A	A	A	A	B	B	A	B	B	B	B	B	A	B
Bn-A04-p17816796	A4	42	49.6	B	B	B	B	B	B	B	A	A	A	A	B	B	A	B	B	B	B	B	A	B
Bn-A04-p17864572	A4	43	50.4	B	B	B	B	B	B	B	A	A	A	A	B	B	A	B	B	B	B	B	A	B
Bn-A04-p18136301	A4	44	51.2	B	B	B	B	B	B	B	A	A	A	A	B	B	A	B	B	B	B	B	A	B
Bn-A04-p18180777	A4	45	52	B	B	B	B	B	B	B	A	A	A	A	B	B	A	B	B	B	B	B	A	B
Bn-A04-p18273048	A4	46	52.8	B	B	B	B	B	B	B	A	A	A	A	B	B	B	B	B	B	B	B	A	B
Bn-A04-p18429573	A4	47	55.2	B	B	B	B	B	B	B	A	A	A	A	B	B	B	B	B	B	B	B	A	B
Bn-A04-p18487715	A4	48	56	B	B	B	B	B	B	B	A	A	A	A	B	B	B	B	B	B	B	B	A	B
Bn-A04-p18617343	A4	49	56.8	B	B	B	B	B	B	B	A	A	A	A	B	B	B	B	B	B	B	B	A	B
Bn-A01-p25171870	A4	50	57.6	B	B	B	B	B	B	B	A	A	A	A	B	B	B	B	B	B	B	B	A	B
Bn-Scaffold000403-p12455	A5	1	0	A	B	A	A	B	B	0	A	A	B	A	B	A	A	A	A	A	A	A	A	A
Bn-A05-p679803	A5	2	0.8	A	B	A	A	B	B	0	A	A	B	A	B	A	A	A	A	A	A	A	A	A
Bn-A05-p892520	A5	3	2.4	A	B	A	A	B	B	0	A	A	B	A	B	A	A	A	A	A	A	A	A	A
Bn-A05-p970816	A5	4	2.4	A	B	A	A	B	B	0	A	A	B	A	B	A	A	A	A	A	A	A	A	A
Bn-A05-p1071371	A5	5	2.4	A	B	A	A	B	B	B	A	A	B	A	B	A	A	A	A	A	A	A	A	A
Bn-A05-p1308471	A5	6	4.8	A	B	A	A	B	B	B	B	A	B	A	B	A	A	A	A	A	A	A	A	A
Bn-A05-p1335771	A5	7	6.4	A	B	A	A	B	B	B	B	A	B	A	B	A	A	A	A	A	A	A	A	A
Bn-A05-p1492327	A5	8	7.2	A	B	A	A	B	B	B	B	A	B	A	B	A	A	A	A	A	A	A	A	A
Bn-A05-p1701030	A5	9	10.4	A	B	A	A	B	B	B	B	A	B	A	B	A	A	A	A	A	A	A	A	A
Bn-A05-p1761473	A5	10	11.2	A	B	A	A	B	B	B	B	A	B	A	B	A	A	A	A	A	A	A	A	A
Bn-A05-p1857946	A5	11	12	A	B	A	A	B	B	B	B	A	B	A	B	A	A	A	A	A	A	A	A	A
Bn-A05-p1898273	A5	12	14.4	A	B	A	A	B	B	B	B	A	B	A	B	A	A	A	A	A	A	A	A	A

Bn-A05-p2106680	A5	13	16.8	A	B	A	A	B	B	B	B	A	B	A	B	A	A	A	A	A	A	A	A	A
Bn-A05-p2303112	A5	14	17.6	A	B	A	A	B	B	B	B	A	B	A	B	A	A	A	A	A	A	A	A	A
Bn-A05-p2345233	A5	15	20	A	B	A	A	B	B	B	B	A	B	A	B	A	A	A	A	A	A	A	A	B
Bn-A05-p2367876	A5	16	22.4	A	B	A	A	B	B	B	B	A	B	A	B	A	A	A	A	A	A	A	A	A
Bn-scaff_16534_1-p648790	A5	17	23.2	A	B	A	A	B	B	B	B	A	B	A	B	A	A	A	A	A	A	A	A	A
Bn-A05-p2641800	A5	18	24	A	B	A	A	B	B	B	B	A	B	A	B	A	A	A	A	A	A	A	A	A
Bn-A05-p2726301	A5	19	26.4	A	B	A	A	B	B	B	B	A	B	A	B	A	A	A	A	A	A	A	A	A
Bn-A05-p3005109	A5	20	27.2	A	B	B	A	B	B	B	B	A	B	A	B	A	A	A	A	A	A	A	A	A
Bn-A05-p3307736	A5	21	28.8	A	B	B	A	B	B	B	B	A	B	A	B	A	A	A	A	A	A	A	A	A
Bn-A05-p3507831	A5	22	29.6	A	B	B	A	B	B	B	B	A	B	A	B	A	A	A	A	A	A	A	A	A
Bn-A05-p3834445	A5	23	31.2	A	B	B	A	B	B	B	B	A	B	A	B	A	A	A	A	A	A	A	A	A
Bn-A05-p3923814	A5	24	32	A	B	B	A	B	B	B	B	A	B	A	B	A	A	A	A	A	A	A	A	A
Bn-A05-p3966848	A5	25	32.8	A	B	B	A	B	A	B	B	A	B	A	B	A	A	A	A	A	A	A	A	A
Bn-A05-p3988958	A5	26	33.6	A	B	B	A	B	A	B	B	A	B	A	B	A	A	A	A	A	A	A	A	B
Bn-A05-p4121158	A5	27	34.4	A	B	B	A	B	A	B	B	A	B	A	B	A	A	A	A	A	A	A	A	B
Bn-A05-p4336667	A5	28	38.4	A	B	B	B	B	A	B	B	A	B	A	B	A	A	A	A	A	B	A	A	B
Bn-scaff_18440_1-p132335	A5	29	39.2	A	B	B	B	B	A	B	B	A	A	A	B	A	A	A	A	A	B	A	A	B
Bn-A05-p4940713	A5	30	40	A	B	B	B	B	A	B	B	A	A	A	B	A	A	A	A	A	B	A	A	B
Bn-A05-p5093548	A5	31	41.6	A	B	B	B	B	A	B	B	A	A	A	A	A	A	A	A	A	B	A	A	B
Bn-A05-p5372015	A5	32	43.2	A	A	B	B	B	A	B	B	A	A	A	A	A	A	A	A	A	B	A	A	B
Bn-A05-p5475992	A5	33	44	A	A	B	B	B	A	B	B	A	A	A	A	A	A	A	A	A	B	A	A	B
Bn-A05-p5805445	A5	34	44.8	A	A	B	B	B	A	B	B	A	A	A	A	A	A	A	A	A	B	A	A	B
Bn-A05-p5970916	A5	35	45.6	A	A	B	B	B	A	B	B	A	A	A	A	A	A	A	A	A	B	A	A	B
Bn-A05-p6541429	A5	36	47.2	A	A	B	B	B	A	B	B	A	A	A	A	A	A	A	A	A	B	A	A	B
Bn-A05-p6995896	A5	37	48	A	A	B	B	B	A	B	B	A	A	A	A	A	A	A	A	A	B	A	A	B
Bn-A05-p11084469	A5	38	49.6	A	A	B	B	B	A	B	B	A	A	B	A	A	A	A	A	A	B	A	A	B
Bn-A05-p16092993	A5	39	50.4	A	A	B	B	B	A	B	B	A	A	B	A	A	A	A	A	A	B	A	A	B
Bn-A05-p18229790	A5	40	51.2	A	A	B	B	B	A	B	B	A	A	B	A	A	A	A	A	A	B	A	A	B
Bn-A05-p18905070	A5	41	52	A	A	B	B	B	A	B	B	A	A	B	A	A	A	A	A	A	B	0	A	B
Bn-A05-p19070284	A5	42	54.5	A	A	B	B	B	A	B	B	A	A	A	A	A	A	A	A	A	B	0	A	B
Bn-A05-p19116891	A5	43	55.3	A	A	B	B	B	A	B	B	A	A	A	A	A	A	A	A	A	B	0	A	B
Bn-A05-p19650965	A5	44	56.1	A	A	B	B	B	A	B	B	A	A	A	A	A	A	A	A	A	B	0	A	B
Bn-A05-p19802577	A5	45	59.4	A	A	B	B	B	A	B	B	A	A	A	A	A	A	A	A	A	B	0	A	B
Bn-A05-p19919179	A5	46	60.2	A	A	B	B	B	A	B	B	A	A	A	A	A	A	A	A	A	B	0	A	B
Bn-A05-p20254809	A5	47	61	A	A	B	B	B	A	B	B	A	A	A	A	A	A	A	A	A	B	0	A	B
Bn-A05-p20555996	A5	48	62.7	A	A	B	B	B	A	B	B	A	A	A	A	A	A	A	A	A	B	0	A	B
Bn-A05-p20972649	A5	49	65.2	A	A	B	B	A	A	B	B	A	A	A	A	A	A	A	A	A	B	0	A	B
Bn-A05-p21028051	A5	50	66	A	A	B	B	A	A	B	B	A	A	A	A	A	A	A	A	A	B	0	A	B
Bn-A05-p21201956	A5	51	66.8	A	A	B	B	A	A	B	B	A	A	A	A	A	A	A	A	A	B	0	A	B
Bn-A05-p21248382	A5	52	67.6	A	A	B	B	A	A	B	B	A	A	A	A	A	A	A	A	A	B	0	A	B
Bn-A05-p21326091	A5	53	69.3	A	A	B	B	A	A	B	B	A	A	A	A	A	A	A	A	A	B	0	A	B
Bn-A05-p21347821	A5	54	70.1	A	A	B	B	A	A	B	B	A	A	A	A	A	A	A	A	A	B	0	A	B
Bn-A05-p21470614	A5	55	73.4	A	A	B	B	A	A	B	B	A	A	A	A	A	A	A	A	A	B	0	A	B
Bn-A05-p21587317	A5	56	74.2	A	A	B	B	A	A	B	B	A	A	A	A	A	A	A	A	A	B	0	A	B
Bn-A05-p21806633	A5	57	75	A	A	B	B	A	A	B	B	A	A	A	A	A	A	A	A	A	B	0	A	B
Bn-A05-p21889289	A5	58	79.2	A	A	B	B	A	A	B	B	A	A	A	A	A	A	A	A	A	B	0	A	B
Bn-A05-p21930978	A5	59	80	A	A	B	B	A	A	B	B	A	A	A	A	A	A	A	A	A	B	0	A	B
Bn-A05-p22152482	A5	60	83.3	A	A	B	A	A	A	B	B	A	A	A	A	A	A	A	A	A	A	0	A	B
Bn-A05-p22089650	A5	61	83.3	A	A	B	A	A	A	B	B	A	A	A	A	A	A	A	A	A	A	0	A	B

Bn-A05-p22218776	A5	62	85	A	A	B	A	A	A	B	B	A	A	A	A	A	A	A	A	A	A	0	A	B
Bn-A05-p22269511	A5	63	85.8	A	A	B	A	A	A	B	B	A	A	A	A	A	A	A	A	A	A	B	A	B
Bn-A05-p22282750	A5	64	86.6	A	A	B	A	A	A	B	B	A	A	A	A	A	A	A	A	A	A	B	A	B
Bn-A05-p22498838	A5	65	87.4	A	A	B	A	A	A	B	B	A	A	A	A	A	A	A	A	A	A	B	A	B
Bn-A05-p22591072	A5	66	88.2	A	A	0	A	A	A	B	B	A	A	A	A	A	A	A	A	B	A	B	A	B
Bn-A05-p22613926	A5	67	89	A	A	0	A	A	A	B	B	A	A	A	A	A	A	A	A	B	A	B	A	B
Bn-A05-p22691469	A5	68	89.8	A	A	0	A	A	A	B	B	A	A	A	A	A	A	A	A	B	A	B	A	B
Bn-A05-p22767106	A5	69	90.6	A	A	0	A	A	A	B	B	A	A	A	A	A	A	A	A	B	A	B	A	B
Bn-A05-p22789711	A5	70	91.4	A	A	0	A	A	A	B	B	A	A	A	A	A	A	A	A	B	A	B	A	B
Bn-A05-p22899595	A5	71	92.2	A	A	0	A	A	A	B	B	A	A	A	A	A	A	A	A	B	A	B	A	B
Bn-A05-p22973324	A5	72	93	A	A	0	A	A	A	B	B	A	A	A	A	A	A	A	A	B	A	B	A	B
Bn-A02-p23161914	A5	73	93.8	A	A	0	A	A	A	B	B	A	A	A	A	A	A	A	A	B	A	B	B	B
Bn-A05-p23253826	A5	74	94.6	A	A	0	A	A	A	B	B	A	A	A	A	A	A	A	A	B	A	B	B	B
Bn-Scaffold000191-p53301	A5	75	96.3	A	A	0	A	A	A	B	B	A	A	A	A	A	A	A	A	B	A	B	B	B
Bn-A02-p26901243	A5	76	98	A	A	0	A	A	A	B	B	A	A	A	A	A	A	A	A	B	A	B	B	B
Bn-A02-p26665282	A5	77	98.8	A	A	0	A	A	A	B	B	A	A	A	A	A	A	A	A	B	A	B	B	B
Bn-A05-p23776343	A5	78	99.6	A	A	0	A	A	A	B	B	A	A	A	A	A	A	A	A	B	A	B	B	B
Bn-A06-p195449	A6	1	0	B	B	B	A	B	B	A	B	B	B	A	A	B	B	B	A	A	A	B	B	A
Bn-A06-p1054104	A6	2	0.8	B	B	B	A	B	B	A	B	B	B	A	A	B	B	B	A	A	A	B	B	A
Bn-A06-p1322724	A6	3	1.6	B	B	B	A	B	B	A	B	B	B	A	A	B	B	B	A	A	A	B	B	A
Bn-A06-p1404859	A6	4	2.4	B	B	B	A	B	B	A	B	B	B	A	A	B	B	B	A	A	A	B	B	A
Bn-A06-p1507197	A6	5	3.2	B	B	B	A	B	B	A	B	B	B	A	A	B	B	B	A	A	A	B	B	A
Bn-A06-p1743446	A6	6	4	B	B	B	A	B	B	A	B	B	B	A	A	B	B	B	A	A	A	B	B	A
Bn-A06-p1768254	A6	7	4.8	B	B	B	A	B	B	A	B	A	B	A	A	B	B	B	A	A	A	B	B	A
Bn-A06-p1947247	A6	8	5.6	B	B	B	A	B	B	A	B	A	B	A	A	B	B	B	A	A	A	B	B	A
Bn-A06-p2234718	A6	9	6.4	B	B	B	A	B	B	A	B	A	B	A	A	B	B	B	A	A	A	B	B	A
Bn-A06-p2251910	A6	10	7.2	B	B	B	A	B	B	A	B	A	B	A	A	B	B	B	A	A	A	B	B	A
Bn-A06-p2268393	A6	11	11.2	B	B	B	A	B	B	A	B	A	B	A	A	B	B	B	A	A	A	B	B	A
Bn-A06-p2407080	A6	12	14.4	B	B	B	A	B	B	A	B	A	B	A	A	B	B	B	A	A	A	B	B	A
Bn-A06-p2514723	A6	13	16	B	B	B	A	B	B	A	B	A	B	A	A	B	B	B	A	A	A	B	B	A
Bn-A06-p2542722	A6	14	16.8	B	B	B	A	B	B	A	B	A	B	A	A	B	B	B	A	A	A	B	B	A
Bn-A06-p2617079	A6	15	18.4	B	B	B	A	B	B	A	A	A	B	A	A	B	B	B	A	A	A	B	B	A
Bn-A06-p2676314	A6	16	19.2	B	B	B	A	B	B	A	A	A	B	A	A	B	B	B	A	A	A	B	B	A
Bn-A06-p2891290	A6	17	21.6	B	B	B	A	B	B	A	A	A	B	A	A	B	B	B	A	A	A	B	B	A
Bn-A06-p2936438	A6	18	22.4	B	B	B	A	B	B	A	A	A	B	A	A	B	B	B	A	A	A	B	B	A
Bn-A06-p2958095	A6	19	23.2	B	B	B	A	B	B	A	A	A	B	A	A	B	B	B	A	A	A	B	B	A
Bn-A06-p3050747	A6	20	24.8	B	B	B	A	B	B	A	A	A	B	A	A	B	B	B	A	A	A	B	B	A
Bn-A06-p3325502	A6	21	25.6	B	B	B	A	B	B	A	A	A	B	A	A	B	B	B	A	A	A	B	B	A
Bn-A06-p3491241	A6	22	26.4	B	B	B	A	B	B	A	A	A	B	A	A	B	B	B	A	A	A	B	B	A
Bn-A06-p3970972	A6	23	28	B	B	B	A	B	B	A	A	A	B	A	A	B	B	B	A	A	A	B	B	A
Bn-A06-p4019827	A6	24	28.8	B	B	B	A	B	B	A	A	A	B	A	A	B	B	B	A	A	A	B	B	A
Bn-A06-p4021930	A6	25	29.6	B	B	B	A	B	B	A	A	A	B	A	A	B	B	B	A	A	A	B	B	A
Bn-A06-p4098800	A6	26	30.4	B	B	B	A	B	B	A	A	A	B	A	A	B	B	B	A	A	A	B	B	A
Bn-A06-p4179318	A6	27	31.2	B	B	B	A	B	B	A	A	A	B	A	A	B	B	B	A	A	A	B	B	A
Bn-A06-p4371627	A6	28	32.8	B	B	B	A	B	B	A	A	A	B	A	A	B	B	B	A	A	A	B	B	A
Bn-A06-p4508774	A6	29	33.6	B	B	B	A	B	B	A	A	A	B	A	A	B	B	B	A	A	A	B	B	A
Bn-A06-p4874556	A6	30	34.4	B	B	B	A	B	B	A	A	A	B	A	A	B	B	B	A	A	A	B	B	A
Bn-A06-p5546411	A6	31	35.2	B	B	B	A	B	B	A	A	A	B	A	A	B	B	B	A	A	A	B	B	A
Bn-A06-p5616164	A6	32	36.8	B	B	B	A	B	B	A	A	A	B	A	A	B	B	B	A	A	A	B	B	A

Bn-A06-p5739682	A6	33	37.6	B	B	B	A	B	B	A	A	A	B	A	A	B	B	B	A	B	A	B	B	A
Bn-A06-p5947781	A6	34	38.4	B	B	B	A	B	B	A	A	A	B	A	A	B	B	B	A	B	A	B	B	A
Bn-A06-p5956274	A6	35	39.2	B	B	B	A	B	B	A	A	A	B	A	A	B	B	B	A	B	A	B	B	A
Bn-A06-p6103844	A6	36	40	B	B	B	A	B	B	A	A	A	A	A	A	B	B	B	A	B	A	B	B	A
Bn-A06-p6182046	A6	37	40.8	B	B	B	A	B	B	A	A	A	A	A	A	B	B	B	A	B	A	B	B	A
Bn-A06-p6311231	A6	38	41.6	B	B	B	A	B	B	A	A	A	A	A	A	B	B	B	A	B	A	B	B	A
Bn-A06-p6343931	A6	39	42.4	B	B	B	A	B	B	A	A	A	A	A	A	B	B	B	A	B	A	B	B	A
Bn-A06-p6624071	A6	40	43.2	B	B	B	A	B	B	A	A	A	A	A	B	B	B	B	A	B	A	B	B	A
Bn-A06-p6647323	A6	41	44	B	B	B	A	B	B	A	A	A	A	A	B	A	B	B	A	B	A	B	B	A
Bn-A06-p6761232	A6	42	44.8	B	B	B	A	B	B	A	A	A	A	A	B	A	B	B	A	B	A	B	B	A
Bn-A06-p7670696	A6	43	45.6	B	B	B	A	A	B	A	A	A	A	A	B	A	B	B	A	B	A	B	B	A
Bn-A06-p7701069	A6	44	47.2	B	B	B	A	A	B	A	A	A	A	A	B	A	B	B	A	B	A	B	B	A
Bn-A06-p8263325	A6	45	48	B	B	B	A	A	B	A	A	A	A	A	B	A	B	B	A	B	A	B	B	A
Bn-A06-p8446816	A6	46	51.2	B	B	B	B	A	B	A	A	A	A	A	B	A	B	B	A	B	B	B	B	A
Bn-A06-p10330066	A6	47	52.8	B	B	B	B	A	B	A	A	A	A	A	B	A	B	B	A	B	B	B	B	A
Bn-A06-p15258692	A6	48	53.6	B	B	B	B	A	B	A	A	A	A	A	B	A	B	B	A	B	B	B	B	A
Bn-A06-p16657894	A6	49	54.4	B	B	B	B	A	B	A	A	A	A	A	B	A	B	B	A	B	B	B	B	A
Bn-A06-p17006799	A6	50	55.2	B	B	B	B	A	B	A	A	A	A	A	B	A	B	B	A	B	B	B	B	A
Bn-A09-p18588652	A6	51	56	B	B	B	B	A	B	A	A	A	B	A	B	A	B	B	A	B	B	B	B	A
Bn-A06-p17548894	A6	52	56.8	B	B	B	B	A	B	A	A	A	B	A	B	A	B	B	A	B	B	B	B	A
Bn-A06-p17671185	A6	53	57.6	B	B	B	B	A	B	A	A	A	B	A	B	A	B	B	A	B	B	B	B	A
Bn-A06-p18029021	A6	54	58.4	B	B	B	B	A	B	A	A	A	B	A	B	A	B	B	B	B	B	B	B	A
Bn-A06-p18219362	A6	55	59.2	B	B	B	B	A	B	A	A	A	B	A	B	A	B	B	B	B	B	B	B	A
Bn-A06-p18364834	A6	56	60	B	B	B	B	A	B	A	A	A	B	A	B	A	B	B	B	B	B	B	B	A
Bn-A06-p18402713	A6	57	61.6	B	B	B	B	A	B	A	A	A	B	A	B	A	B	B	B	B	B	B	B	A
Bn-A06-p18500596	A6	58	62.4	B	B	B	B	A	B	B	A	A	B	A	B	A	B	B	B	B	B	B	B	A
Bn-A06-p21525576	A6	59	63.2	B	B	B	B	A	B	B	A	A	B	A	B	A	B	B	B	B	B	B	B	A
Bn-A06-p21349507	A6	60	64	B	B	B	B	A	B	B	A	A	B	A	B	A	B	B	B	B	B	B	B	A
Bn-A06-p21195115	A6	61	64.8	B	B	B	B	A	B	B	A	A	B	A	B	A	B	B	B	B	B	B	B	A
Bn-A06-p21163037	A6	62	65.6	B	B	B	B	A	B	B	A	A	B	A	B	B	B	B	B	B	B	B	B	A
Bn-scaff_18520_1-p488642	A6	63	66.4	B	B	B	B	A	B	B	A	A	B	A	B	B	B	B	B	B	B	B	B	A
Bn-A06-p21134315	A6	64	67.2	B	B	B	B	A	B	B	A	A	B	A	B	B	B	B	B	B	B	B	B	B
Bn-A06-p21101957	A6	65	68	B	B	B	B	A	B	B	A	A	B	A	B	B	B	B	B	B	B	B	B	B
Bn-A06-p21072392	A6	66	68.8	B	B	B	B	A	B	B	A	A	B	A	B	B	B	B	B	B	B	A	B	B
Bn-A06-p21796008	A6	67	70.4	B	B	B	B	A	B	B	A	A	B	A	B	B	B	B	B	B	B	A	B	B
Bn-A06-p21800812	A6	68	71.2	B	B	B	B	A	B	B	A	A	B	A	B	B	B	B	B	B	B	A	B	B
Bn-A06-p21826671	A6	69	72	B	B	B	B	A	B	B	A	A	B	A	B	B	B	B	B	B	B	A	B	B
Bn-A06-p21846491	A6	70	73.6	B	B	B	B	A	B	B	A	A	B	A	B	B	B	B	B	B	B	A	B	B
Bn-A06-p22160452	A6	71	74.4	B	B	B	B	A	B	B	A	A	B	A	B	B	B	B	B	B	B	A	B	B
Bn-A06-p22709872	A6	72	76	B	B	B	B	A	B	B	A	A	B	A	B	B	B	B	B	B	B	A	B	B
Bn-A06-p22947499	A6	73	76.8	B	B	B	B	A	B	B	A	A	B	A	B	B	B	B	B	B	B	A	B	B
Bn-A06-p23468803	A6	74	78.4	B	B	B	B	A	B	B	A	A	B	A	B	B	B	B	B	B	B	A	B	B
Bn-A06-p23591616	A6	75	79.2	B	B	B	B	A	B	B	A	A	B	A	B	B	B	B	B	B	B	A	B	B
Bn-A06-p23665564	A6	76	80.8	B	B	B	B	A	B	B	A	A	B	A	B	B	B	B	B	B	B	A	B	B
Bn-A06-p23852270	A6	77	81.6	B	B	B	B	A	B	B	A	A	B	A	B	B	B	B	B	B	B	A	B	B
Bn-A06-p23988702	A6	78	83.2	B	B	B	B	A	B	B	A	A	B	A	B	B	B	B	A	B	B	A	B	B
Bn-A06-p24099288	A6	79	84	B	B	B	B	A	B	B	A	A	B	A	B	B	B	B	A	B	B	A	B	B
Bn-A06-p24386467	A6	80	84.8	B	B	B	B	A	B	B	A	A	A	A	B	B	B	B	A	B	B	A	B	B
Bn-A06-p24467652	A6	81	85.6	B	B	B	B	A	B	B	A	A	A	A	B	B	B	B	A	B	B	A	B	B

Bn-A06-p24501315	A6	82	87.2	B	B	A	B	A	B	B	A	A	A	A	B	B	B	B	A	B	B	A	B	B
Bn-A06-p24503033	A6	83	88	B	B	A	B	A	B	B	A	A	A	A	B	B	B	B	A	B	B	A	B	B
Bn-A06-p24705718	A6	84	90.4	B	B	A	B	A	B	B	A	A	A	A	B	B	B	B	A	B	B	A	B	B
Bn-scaff_21268_1-p193486	A6	85	91.2	B	B	A	B	A	B	B	A	A	A	A	B	B	B	B	A	B	B	A	B	B
Bn-scaff_15712_1-p1137014	A6	86	97.7	A	B	A	B	A	B	B	A	A	A	A	B	B	A	A	A	B	B	A	B	B
Bn-A06-p25343454	A6	87	98.5	A	B	A	B	A	B	B	A	A	A	A	B	B	A	A	A	B	B	A	B	B
Bn-A06-p25786733	A6	88	99.3	A	B	A	B	A	B	B	A	A	A	A	B	B	A	A	A	B	B	A	B	B
Bn-A06-p26144731	A6	89	100.1	A	B	A	B	A	B	B	A	A	A	A	B	B	A	A	A	B	B	A	B	B
Bn-A07-p1292866	A7	1	0	B	B	B	B	A	B	0	B	B	B	A	A	A	B	B	B	A	B	B	B	0
Bn-A10-p11608969	A7	2	0.8	B	B	B	B	A	B	0	B	B	B	A	A	A	B	B	B	A	B	B	B	0
Bn-A10-p12014652	A7	3	1.6	B	B	B	B	A	B	0	B	B	B	A	A	A	B	B	B	A	B	B	B	0
Bn-A10-p12631706	A7	4	2.4	B	B	B	B	A	B	0	B	B	B	A	A	A	B	B	B	A	B	A	B	0
Bn-A07-p4916203	A7	5	3.2	B	B	B	B	A	B	B	B	B	B	A	A	A	B	B	B	A	B	A	B	0
Bn-A07-p5412930	A7	6	4.1	B	B	B	B	A	B	B	B	B	B	A	A	B	B	B	B	A	B	A	B	0
Bn-A07-p8516667	A7	7	5	B	B	B	B	A	B	0	B	B	B	A	A	B	B	B	B	A	B	A	B	0
Bn-A07-p9272000	A7	8	6.6	B	B	B	B	A	B	0	B	B	B	A	A	B	B	B	B	A	B	A	A	0
Bn-A07-p9661444	A7	9	8.3	B	B	B	B	A	B	0	B	B	B	A	A	B	B	B	B	A	B	A	A	0
Bn-A07-p9791002	A7	10	9.1	B	B	A	B	A	B	0	B	B	B	A	A	B	B	B	B	A	B	A	A	0
Bn-A07-p10750190	A7	11	10	B	B	A	B	A	B	0	B	B	B	A	A	B	B	B	B	A	B	A	A	0
Bn-A02-p771951	A7	12	10.8	B	B	A	B	A	B	0	B	B	B	A	A	B	B	B	B	A	B	A	A	0
Bn-A07-p11629852	A7	13	11.6	B	B	A	B	A	B	0	B	B	B	A	A	B	B	B	B	A	B	A	A	0
Bn-A07-p11838401	A7	14	12.2	B	B	A	B	A	B	0	B	B	B	A	A	B	B	B	B	A	B	A	A	0
Bn-A07-p12362172	A7	15	13.1	B	B	B	B	A	B	0	B	B	B	A	A	B	B	B	B	A	B	A	A	0
Bn-A07-p12757009	A7	16	14	B	B	B	B	A	B	0	B	B	B	A	A	B	B	B	B	A	B	A	A	0
Bn-A07-p12833434	A7	17	17.5	B	B	B	A	A	B	0	B	B	B	A	A	B	B	B	B	A	A	A	A	0
Bn-A07-p13147502	A7	18	19.2	B	B	B	A	A	B	0	B	B	B	A	A	B	B	B	B	A	A	A	A	0
Bn-A07-p13587753	A7	19	20.1	B	B	B	A	A	B	0	B	B	B	A	A	B	B	B	A	A	A	A	A	0
Bn-A07-p13659939	A7	20	21	B	B	B	A	A	B	0	B	B	B	A	A	B	B	B	A	A	A	A	A	0
Bn-A07-p13739429	A7	21	21.9	B	B	B	A	A	B	0	B	B	B	A	A	B	B	B	A	A	A	A	A	0
Bn-A07-p14005263	A7	22	22.8	B	B	B	A	A	B	0	B	B	B	A	A	B	B	B	A	A	A	A	A	0
Bn-A07-p14453848	A7	23	23.7	B	B	B	A	A	A	0	B	B	B	A	A	B	B	B	A	A	A	A	A	0
Bn-A07-p14523096	A7	24	24.7	B	B	B	A	A	A	0	B	B	B	A	A	B	B	B	A	A	A	A	A	0
Bn-A07-p14759961	A7	25	25.6	B	B	B	A	A	A	0	B	B	B	A	A	B	B	B	A	B	A	A	A	0
Bn-A07-p15352802	A7	26	26.5	B	B	B	A	A	A	0	B	B	B	A	A	B	B	B	A	B	A	A	A	B
Bn-A07-p15578269	A7	27	27.4	B	B	B	A	A	A	0	B	B	B	A	A	B	B	B	A	B	A	A	A	B
Bn-A07-p15949460	A7	28	29.2	B	B	B	A	A	A	0	B	B	B	A	A	B	B	B	A	B	A	A	A	B
Bn-A07-p16074768	A7	29	30.1	B	B	B	A	A	A	0	B	B	B	A	A	B	B	B	A	B	A	A	B	B
Bn-A07-p16183869	A7	30	31	B	B	B	A	A	A	0	B	B	B	A	A	A	B	B	A	B	A	A	B	B
Bn-A07-p16468333	A7	31	31.9	B	B	B	A	A	A	0	B	B	B	A	A	A	B	B	A	B	A	A	B	B
Bn-A07-p17212130	A7	32	32.8	B	B	B	A	A	A	0	B	B	B	A	A	A	B	B	A	B	A	A	B	B
Bn-A07-p17804261	A7	33	33.7	B	B	B	A	A	A	0	B	B	B	A	A	A	B	B	A	B	A	A	B	B
Bn-A07-p18020732	A7	34	34.6	B	B	B	A	B	A	B	B	B	B	A	A	A	B	B	A	B	A	A	B	B
Bn-A07-p18922121	A7	35	35.5	B	B	B	A	B	A	B	B	B	B	A	A	A	B	B	A	B	A	A	B	B
Bn-A07-p19190510	A7	36	38.2	B	B	B	A	B	A	B	B	B	B	A	A	A	B	B	A	B	A	A	B	B
Bn-A07-p19458370	A7	37	39.1	B	B	B	A	B	A	B	B	B	B	A	A	A	B	B	A	B	A	A	B	B
Bn-A07-p19865996	A7	38	40	B	B	B	A	B	A	B	B	B	B	A	A	A	B	B	A	B	A	A	B	0
Bn-A07-p19961825	A7	39	42.5	B	B	B	A	B	A	B	B	B	B	A	A	A	B	B	A	0	A	A	B	0
Bn-A07-p20248654	A7	40	43.6	B	B	B	A	B	A	B	B	B	B	A	A	A	B	B	A	0	A	A	B	A
Bn-A07-p20481682	A7	41	44.5	B	B	B	A	B	A	B	B	B	A	B	B	A	B	B	A	0	A	A	B	A

Bn-A07-p20554194	A7	42	47.7	B	B	B	A	B	A	B	B	B	A	B	B	A	B	B	A	0	A	A	B	A
Bn-A07-p21019499	A7	43	55	B	B	B	A	B	A	B	B	B	A	B	B	A	B	B	A	0	A	A	B	A
Bn-A07-p21261518	A7	44	57.7	B	B	B	A	B	A	B	B	B	A	B	B	A	B	B	A	0	A	A	B	A
Bn-A07-p21476982	A7	45	58.7	B	B	B	A	B	A	B	B	B	A	B	B	A	B	B	A	0	A	A	B	A
Bn-A07-p22329359	A7	46	60	B	B	B	A	B	A	B	B	B	A	B	B	A	B	B	A	0	A	A	B	A
Bn-A08-p792162	A8	1	0	B	A	A	B	B	A	A	A	B	B	B	A	B	B	B	A	A	B	B	B	B
Bn-A08-p2973537	A8	2	0.8	B	A	A	B	B	A	A	A	B	B	B	A	B	B	B	A	A	B	B	B	B
Bn-A08-p8570810	A8	3	1.6	B	A	A	B	B	A	A	A	B	B	B	A	B	B	B	A	A	B	B	B	B
Bn-A08-p8601468	A8	4	4	B	A	A	B	B	A	A	A	B	B	B	A	B	B	B	A	A	B	B	B	B
Bn-A08-p10067927	A8	5	4.8	B	A	A	B	B	A	A	A	B	B	B	A	B	B	B	A	A	B	B	B	B
Bn-A08-p10281337	A8	6	5.6	B	A	A	B	B	B	A	A	B	B	B	A	B	B	B	A	A	B	B	B	B
Bn-A08-p10439396	A8	7	6.4	B	A	A	B	B	B	A	A	B	B	B	A	B	B	B	A	A	B	B	B	B
Bn-scaff_17042_1-p359732	A8	8	7.2	B	A	A	B	B	B	A	A	B	B	B	A	B	B	B	A	A	B	B	B	B
Bn-A08-p12555227	A8	9	9.6	B	A	A	B	B	B	A	A	B	B	B	A	B	B	B	A	A	B	B	B	B
Bn-A08-p13352430	A8	10	10.4	B	A	A	B	B	B	A	A	B	B	B	A	B	B	B	A	A	B	B	B	B
Bn-A08-p13363830	A8	11	12.8	B	A	A	B	B	B	A	A	B	B	B	A	B	B	B	A	A	B	B	B	B
Bn-A08-p13454167	A8	12	13.6	B	A	A	B	B	B	A	A	B	B	B	A	B	B	B	A	A	B	B	B	B
Bn-A08-p13626189	A8	13	16.8	B	A	A	A	B	B	A	A	B	B	B	A	B	B	B	A	A	A	B	B	B
Bn-A08-p13708877	A8	14	17.6	B	A	A	A	B	B	A	A	B	B	B	A	B	B	B	A	A	A	B	B	B
Bn-A08-p14498301	A8	15	18.4	B	A	A	A	B	B	A	A	B	B	B	A	B	B	B	A	A	A	B	B	B
Bn-A08-p14616048	A8	16	19.2	B	A	A	A	B	B	A	A	B	B	B	A	B	B	B	A	A	A	B	B	B
Bn-A08-p14813540	A8	17	20	B	A	A	A	B	B	A	A	B	B	B	A	B	B	B	A	A	A	B	B	B
Bn-scaff_18602_1-p244845	A8	18	20.8	B	A	A	A	B	B	A	A	B	B	B	A	B	B	B	A	A	A	B	B	B
Bn-A08-p15353881	A8	19	21.6	B	A	A	A	B	B	A	A	B	B	B	A	B	B	B	A	A	A	B	B	B
Bn-A08-p15701695	A8	20	22.4	B	A	A	A	B	B	A	A	B	B	B	A	B	B	B	A	A	A	B	B	B
Bn-scaff_24631_1-p745795	A8	21	23.2	B	A	A	A	B	B	A	A	B	B	B	A	B	B	B	A	A	A	B	B	B
Bn-A08-p16183708	A8	22	24	B	A	A	A	A	B	A	A	B	B	B	A	B	B	B	A	A	A	B	B	B
Bn-A08-p16216893	A8	23	24.8	B	A	A	A	A	B	A	A	B	B	B	A	B	B	B	A	A	A	B	B	B
Bn-A08-p16248227	A8	24	25.6	B	A	A	A	A	B	A	A	B	B	B	A	B	B	B	A	A	A	B	B	B
Bn-A08-p16306425	A8	25	26.4	B	A	A	A	A	B	A	A	B	B	B	A	B	B	B	A	A	A	B	B	B
Bn-A08-p16846907	A8	26	27.2	B	A	A	A	A	B	A	A	B	B	B	A	B	B	B	A	A	A	B	B	B
Bn-A08-p16958537	A8	27	28	B	A	B	A	A	B	A	A	B	B	B	A	B	B	B	A	A	A	B	B	B
Bn-A08-p16980526	A8	28	28.8	B	A	B	A	A	B	A	A	B	B	B	A	B	B	B	A	A	A	B	B	B
Bn-A08-p17202184	A8	29	31.2	B	A	B	A	A	B	A	A	B	B	B	A	B	B	B	A	A	A	B	B	B
Bn-A08-p17291598	A8	30	32	B	A	B	A	A	B	A	A	B	B	B	A	B	B	B	A	A	A	B	B	B
Bn-A08-p17301557	A8	31	32.8	B	A	B	A	A	B	A	A	B	B	B	A	B	B	B	A	A	A	B	B	B
Bn-A08-p17513789	A8	32	35.2	B	A	B	A	A	B	A	A	B	B	B	A	B	B	B	A	A	A	B	B	B
Bn-A08-p17530617	A8	33	39.2	B	A	B	A	A	B	A	A	B	B	B	A	B	B	B	A	A	A	B	B	B
Bn-A08-p17824285	A8	34	40	B	A	B	A	A	B	A	A	B	B	B	A	B	B	B	A	A	A	B	B	B
Bn-A08-p17887796	A8	35	41.6	B	A	B	A	A	B	A	A	B	B	B	A	B	B	B	A	A	A	B	B	B
Bn-A08-p18000876	A8	36	43.2	B	A	B	A	A	B	A	A	B	B	B	A	B	B	B	A	A	A	B	B	B
Bn-A08-p18070928	A8	37	44	B	A	B	A	A	B	A	A	B	B	B	A	B	B	B	A	A	A	B	B	B
Bn-A05-p6672638	A8	38	49.7	B	A	B	B	A	B	A	A	B	B	B	A	B	B	B	A	A	B	B	B	B
Bn-A08-p18543984	A8	39	52.9	B	A	B	B	A	A	B	A	B	B	B	A	B	B	B	A	A	B	B	B	B
Bn-A08-p18605816	A8	40	53.7	B	A	B	B	A	A	B	A	B	B	B	A	B	B	B	A	A	B	B	B	B
Bn-A08-p18750116	A8	41	54.5	B	A	B	B	A	A	B	A	B	B	B	B	B	B	B	A	A	B	B	B	B
Bn-A08-p19008575	A8	42	57.7	B	A	B	B	B	A	B	A	B	B	B	B	B	B	B	A	A	B	B	B	B
Bn-scaff_16231_1-p1036610	A8	43	60.1	B	A	B	B	B	A	B	A	B	B	B	B	B	B	B	A	A	B	B	B	B

Bn-A08-p19428204	A8	44	62.5	B	A	B	B	B	A	B	A	B	A	B	B	B	B	B	A	A	B	B	B	B
Bn-scaff_16231_1-p1484914	A8	45	64.1	B	A	B	B	B	A	B	A	B	A	B	B	B	B	B	A	A	B	B	B	B
Bn-A08-p19646345	A8	46	64.9	B	A	B	B	B	A	B	A	B	A	B	B	B	B	B	A	A	B	B	B	B
Bn-A08-p19683899	A8	47	68.1	B	A	B	B	B	A	B	A	B	A	B	B	B	B	B	A	A	B	B	B	B
Bn-A08-p19834705	A8	48	73	B	A	B	B	B	A	B	B	B	A	B	B	B	B	B	A	A	B	B	B	B
Bn-A08-p19960214	A8	49	73.9	B	A	B	B	B	A	B	B	B	A	B	B	B	A	B	A	A	B	B	B	B
Bn-A08-p20068678	A8	50	76.2	B	A	B	B	B	A	B	B	B	A	B	B	B	A	B	A	A	B	B	B	B
Bn-scaff_17227_1-p1101239	A8	51	77	B	A	B	B	B	A	B	B	B	A	B	B	B	A	B	A	A	B	B	B	B
Bn-A08-p20305036	A8	52	77.8	B	A	B	B	B	A	B	B	B	A	B	B	B	A	B	A	A	B	B	B	B
Bn-A08-p20820768	A8	53	81.8	B	A	B	A	B	A	B	B	B	A	B	B	B	A	B	A	A	A	B	B	B
Bn-A08-p20945277	A8	54	84.2	B	A	B	A	B	A	B	B	B	A	B	B	B	A	B	A	A	A	B	B	B
Bn-A08-p21417592	A8	55	85	B	A	B	A	B	A	B	B	B	A	B	B	B	A	B	A	A	A	B	B	B
Bn-A09-p957959	A9	1	0	A	A	A	A	A	B	A	B	A	A	B	A	A	A	A	B	A	A	B	A	A
Bn-A09-p704028	A9	2	0.8	A	A	A	A	A	B	A	B	A	A	B	A	A	A	A	B	A	A	B	A	A
Bn-A09-p86293	A9	3	2.1	A	A	A	A	A	B	A	B	A	A	B	A	A	A	A	B	A	A	B	A	A
Bn-A01-p26968952	A9	4	3.4	A	A	A	A	A	B	A	B	A	A	B	A	A	A	A	B	A	A	B	A	A
Bn-A01-p26837207	A9	5	4.3	A	A	A	A	A	B	A	B	A	A	B	A	A	A	A	B	A	A	B	B	A
Bn-A09-p1188091	A9	6	5.2	A	A	A	A	A	B	A	B	A	A	B	A	A	A	A	B	A	A	B	B	A
Bn-A09-p2116947	A9	7	7.9	A	A	A	A	A	B	A	B	A	A	B	A	A	A	A	B	A	A	B	B	A
Bn-A09-p2157775	A9	8	8.8	A	A	A	A	A	B	A	B	A	A	B	A	A	A	A	B	A	A	B	B	A
Bn-A09-p1929245	A9	9	9.7	A	A	A	A	A	B	A	B	A	A	B	A	A	A	A	B	A	A	B	B	A
Bn-A09-p1856828	A9	10	10.6	A	A	A	A	A	B	A	B	A	A	B	A	A	A	A	B	A	A	B	B	A
Bn-A09-p1570558	A9	11	12.3	A	A	A	A	A	B	A	B	A	A	B	A	A	A	A	B	A	A	B	B	A
Bn-A09-p1552993	A9	12	13.1	A	A	A	A	A	B	A	B	A	A	B	B	A	A	A	B	A	A	B	B	A
Bn-A09-p4096474	A9	13	24.6	A	A	A	A	A	B	A	B	A	A	A	B	A	A	A	B	A	A	B	B	A
Bn-Scaffold000178-p47905	A9	14	25.4	A	A	A	A	A	B	A	B	B	A	A	B	A	A	A	B	A	A	B	B	A
Bn-A09-p4463675	A9	15	26.2	A	A	A	A	A	B	A	B	B	A	A	B	A	A	A	B	A	A	B	B	A
Bn-A09-p4509627	A9	16	27	A	A	A	A	A	B	A	B	B	A	A	B	A	A	A	B	A	A	B	B	A
Bn-A09-p4655322	A9	17	28.6	A	A	A	A	A	B	A	B	B	A	A	B	A	A	A	B	A	A	B	B	A
Bn-A09-p5455608	A9	18	29.4	A	A	A	A	A	B	A	B	B	A	A	B	A	A	A	B	A	A	B	B	B
Bn-A07-p10188190	A9	19	30.2	A	A	A	A	A	B	A	B	B	A	A	B	A	A	A	B	A	A	B	B	B
Bn-A09-p7174944	A9	20	31	A	A	A	A	A	B	A	B	B	A	A	B	A	A	A	B	A	A	B	B	B
Bn-A09-p7047022	A9	21	34.2	A	A	A	A	A	B	B	B	B	A	A	B	A	A	A	A	A	A	B	B	B
Bn-A09-p6922467	A9	22	36.6	A	A	A	A	A	B	B	B	B	A	A	B	A	A	A	A	A	A	B	B	B
Bn-scaff_20428_1-p133089	A9	23	38.2	A	A	A	A	A	B	B	B	B	A	A	B	A	A	A	A	A	A	B	B	B
Bn-A09-p6133524	A9	24	39	A	A	A	A	A	B	B	B	B	A	A	B	A	A	A	A	A	A	B	B	B
Bn-A09-p9379347	A9	25	39.8	A	A	A	A	A	B	B	B	B	A	A	B	A	A	A	A	A	A	B	B	B
Bn-A09-p9512316	A9	26	40.6	A	A	A	A	A	B	B	B	B	A	A	B	A	A	A	A	A	A	B	A	B
Bn-A09-p9865556	A9	27	41.4	A	A	A	A	A	B	B	B	B	A	A	B	A	A	A	A	A	A	B	A	B
Bn-A09-p14266174	A9	28	43	A	A	A	A	A	B	B	B	B	A	A	B	A	A	A	A	A	A	B	A	B
Bn-A09-p16639867	A9	29	43.8	A	A	A	A	A	B	B	B	B	A	A	B	A	A	A	A	A	A	B	A	B
Bn-scaff_23293_1-p239190	A9	30	45.4	A	A	A	A	A	B	B	B	B	A	A	B	A	A	A	A	A	A	B	A	B
Bn-A09-p14535292	A9	31	47	A	A	A	A	A	B	B	B	B	A	A	B	A	A	A	A	A	A	B	A	B
Bn-A09-p17162263	A9	32	47.8	A	A	A	A	A	B	B	B	B	A	A	B	A	A	A	A	A	A	B	A	B
Bn-A09-p19272189	A9	33	49.4	A	A	A	A	A	B	B	B	B	A	A	B	A	A	A	A	A	A	B	A	B
Bn-A09-p22495700	A9	34	53.4	B	A	A	A	A	B	B	B	B	A	A	B	A	A	B	A	A	A	B	A	B
Bn-scaff_22466_1-p75425	A9	35	54.2	B	A	A	A	A	B	B	B	B	A	A	B	A	A	B	A	A	A	B	A	B
Bn-A09-p22873685	A9	36	55	B	A	A	A	A	B	B	B	B	A	A	B	A	A	B	A	A	A	B	A	B
Bn-A09-p23144404	A9	37	55.8	B	A	A	A	A	B	B	B	B	A	A	B	A	A	B	A	A	A	B	A	B

Bn-A09-p23250129	A9	38	56.6	B	A	A	A	A	B	B	B	B	A	A	B	A	A	B	A	A	A	B	A	B
Bn-A09-p23354310	A9	39	57.4	B	A	A	A	A	B	A	B	B	A	A	B	A	A	B	A	A	A	B	A	B
Bn-A09-p23416800	A9	40	59	B	A	A	A	A	B	A	B	B	A	A	B	A	A	B	A	A	A	B	A	B
Bn-A09-p23552075	A9	41	59.8	B	A	A	A	A	B	A	B	B	A	A	B	A	A	B	A	A	A	B	A	B
Bn-A09-p23756115	A9	42	61.4	B	A	A	A	A	B	A	B	B	A	A	B	A	A	B	A	A	A	B	A	B
Bn-A09-p23904905	A9	43	64.6	B	A	A	A	A	B	A	B	B	A	A	B	A	A	B	A	A	A	B	A	B
Bn-A09-p24086990	A9	44	65.4	B	A	A	A	A	B	A	B	B	A	A	B	A	A	B	A	A	A	B	A	B
Bn-Scaffold003668-p282	A9	45	67	B	A	A	A	A	B	A	B	B	A	A	B	A	A	B	A	A	A	B	A	B
Bn-scaff_21338_1-p359394	A9	46	69.4	B	B	A	A	A	B	A	B	B	A	A	B	A	A	B	A	A	A	B	A	B
Bn-Scaffold000282-p6257	A9	47	70.2	B	B	A	A	A	B	A	B	B	A	A	B	A	A	B	A	A	A	B	A	B
Bn-A05-p15802321	A9	48	71	B	B	A	A	A	B	A	B	B	A	A	B	A	A	B	A	A	A	B	A	B
Bn-A05-p15805806	A9	49	71.8	B	B	A	A	A	B	A	B	B	A	A	B	A	A	B	A	A	A	B	A	B
Bn-A09-p25455888	A9	50	72.6	B	B	A	A	A	B	A	B	B	A	A	B	A	A	B	A	A	A	B	A	B
Bn-A09-p26101323	A9	51	73.4	B	B	A	A	A	B	A	B	B	A	A	B	A	B	B	A	A	A	B	A	B
Bn-A09-p26578867	A9	52	74.2	B	B	A	A	A	B	A	B	B	A	A	B	A	B	B	A	A	A	B	A	A
Bn-A09-p27103683	A9	53	75.8	B	B	A	A	A	B	A	B	B	A	A	B	A	B	B	B	A	A	B	A	A
Bn-A09-p27531548	A9	54	76.6	B	B	A	A	A	B	A	B	B	A	A	B	A	B	B	B	A	A	B	A	A
Bn-scaff_16361_1-p980281	A9	55	77.4	B	B	A	A	A	A	A	B	B	A	A	B	A	B	B	B	A	A	B	A	A
Bn-A09-p28722989	A9	56	80.6	B	B	A	A	A	A	A	B	B	A	A	B	A	B	B	B	A	A	B	A	A
Bn-A09-p29186255	A9	57	82.2	B	B	A	A	A	A	A	A	B	A	A	B	A	B	B	B	A	A	B	A	A
Bn-A09-p29272014	A9	58	83.8	B	B	A	A	A	A	A	A	B	A	A	B	A	B	B	B	A	A	B	A	A
Bn-A09-p29331470	A9	59	84.6	B	B	A	A	A	A	A	A	B	A	A	B	A	B	B	B	A	A	B	A	A
Bn-A09-p29493614	A9	60	85.4	B	B	A	A	A	A	A	A	B	A	A	B	A	B	B	B	A	A	B	A	A
Bn-A09-p29617813	A9	61	86.2	B	B	A	A	A	A	A	A	B	A	A	B	A	B	B	B	A	A	B	A	A
Bn-A09-p29699253	A9	62	87	B	B	A	A	A	A	A	A	B	A	A	B	A	B	B	B	A	A	B	A	A
Bn-A09-p29968302	A9	63	87.8	B	B	A	A	A	A	A	A	B	A	A	B	A	B	B	B	A	A	B	A	A
Bn-A09-p30431025	A9	64	90.2	B	B	A	A	A	A	A	A	A	A	A	B	A	B	B	B	A	A	B	A	A
Bn-A09-p30724789	A9	65	91	B	B	A	A	A	A	A	A	A	A	A	B	A	B	B	B	B	A	B	A	A
Bn-A09-p31393136	A9	66	91.8	B	B	A	A	A	A	A	A	A	A	A	B	A	B	B	B	B	A	B	A	A
Bn-A09-p31494058	A9	67	92.6	B	B	A	A	A	A	A	A	A	A	A	B	A	B	B	B	B	A	B	A	A
Bn-A09-p5132918	A9	68	93.4	B	B	A	A	A	A	A	A	A	A	A	B	A	B	B	B	B	A	B	A	A
Bn-A09-p32553111	A9	69	95.8	B	B	A	A	A	A	A	A	A	A	A	B	A	B	B	B	B	A	B	A	A
Bn-A09-p32980100	A9	70	96.6	B	B	A	A	A	A	A	A	A	A	A	B	A	B	B	B	B	A	B	A	A
Bn-A09-p33040441	A9	71	97.4	B	B	A	A	A	A	A	A	A	A	A	B	A	B	B	B	B	A	B	A	A
Bn-A09-p33358168	A9	72	99	B	B	A	A	A	A	A	A	A	A	A	B	A	B	B	B	B	A	B	A	A
Bn-A09-p33464355	A9	73	99.8	B	B	A	A	A	A	A	A	A	A	A	B	A	B	B	B	B	A	B	A	A
Bn-A09-p33499505	A9	74	100.6	B	B	A	A	A	A	A	A	A	A	A	B	A	B	B	B	B	A	B	A	A
Bn-A09-p34039082	A9	75	101.4	B	B	A	A	A	A	A	A	A	A	A	B	A	B	B	B	B	A	B	A	A
Bn-A09-p34482075	A9	76	103.8	B	B	A	A	A	A	A	A	A	A	A	B	A	A	B	B	B	A	B	A	A
Bn-A09-p34779068	A9	77	105.4	B	B	A	A	A	A	A	A	A	A	A	B	A	A	B	B	B	A	B	A	A
Bn-A09-p34995922	A9	78	106.2	B	B	A	A	A	A	A	A	A	A	A	B	A	A	B	B	B	A	B	A	A
Bn-A09-p35162641	A9	79	107.8	B	B	B	A	A	A	A	A	A	A	A	B	A	A	B	B	B	A	B	A	A
Bn-A09-p35262679	A9	80	108.6	B	B	B	A	A	A	A	A	A	A	A	B	A	A	B	B	B	A	B	A	A
Bn-A09-p35426888	A9	81	109.4	B	B	B	A	A	A	A	A	A	A	A	B	A	A	B	B	B	A	B	A	B
Bn-A09-p35477936	A9	82	110.2	B	B	B	A	A	A	A	A	A	A	A	B	A	A	B	B	B	A	B	A	B
Bn-A09-p35624679	A9	83	111	B	B	B	A	A	A	A	A	A	A	A	B	A	A	B	B	B	A	B	A	B
Bn-A09-p35879759	A9	84	112.6	B	B	B	A	A	A	A	A	A	A	A	B	A	A	B	B	B	A	B	A	B
Bn-A09-p36062760	A9	85	113.4	B	B	B	A	A	A	A	A	A	A	A	B	A	A	B	B	B	A	B	A	B
Bn-A09-p36200543	A9	86	117.5	A	B	B	A	A	A	A	A	A	A	A	B	A	A	A	B	B	A	B	A	B

Bn-A09-p36402398	A9	87	118.3	A	B	B	A	A	A	A	A	A	A	A	B	A	A	A	B	B	A	B	A	B
Bn-A09-p36886022	A9	88	125	A	B	B	A	A	A	A	A	A	A	A	B	A	A	A	B	B	A	B	A	B
Bn-scaff_16021_1-p175579	A9	89	127.5	A	B	B	A	A	A	A	A	A	A	A	B	A	A	A	B	B	A	B	A	B
Bn-A10-p4727374	A10	1	0	A	A	A	B	A	B	B	A	A	A	A	A	B	A	A	B	A	B	B	A	A
Bn-scaff_16414_1-p1679802	A10	2	0.8	A	A	A	B	A	B	B	A	A	A	A	A	B	A	A	B	A	B	B	A	A
Bn-A10-p4606815	A10	3	0.8	A	A	A	B	A	B	B	A	A	A	A	A	B	A	A	B	A	B	B	A	A
Bn-A10-p3955901	A10	4	1.6	A	A	A	B	A	B	B	A	A	A	A	A	B	A	A	B	A	B	B	A	A
Bn-A10-p3825331	A10	5	2.4	A	A	A	B	A	B	B	A	A	A	A	A	B	A	A	B	A	B	B	A	A
Bn-A10-p2819947	A10	6	3.2	A	A	A	B	A	B	B	A	A	A	A	A	A	A	A	B	A	B	B	A	A
Bn-A10-p2557960	A10	7	4	A	A	A	B	A	B	B	A	A	A	A	A	A	A	A	B	A	B	B	A	A
Bn-A10-p2523110	A10	8	4.8	A	A	A	B	A	B	B	A	A	A	A	A	A	A	A	B	A	B	B	A	A
Bn-A10-p1791974	A10	9	5.6	A	A	A	B	A	B	B	A	B	A	A	A	A	A	A	B	A	B	B	A	A
Bn-A10-p1698746	A10	10	6.4	A	A	A	B	A	B	B	A	B	A	A	A	A	A	A	B	A	B	A	A	A
Bn-A10-p1458849	A10	11	7.2	A	A	A	B	A	B	B	A	B	A	A	A	A	A	A	B	A	B	A	A	A
Bn-A10-p1345255	A10	12	8	A	A	A	B	A	B	B	A	B	A	A	A	A	A	A	B	A	B	A	A	A
Bn-A10-p754475	A10	13	8.8	A	A	A	B	A	B	B	A	B	A	A	A	A	A	A	B	A	B	A	A	A
Bn-A10-p5317804	A10	14	9.6	A	A	A	B	A	B	B	A	B	A	A	A	A	A	A	B	A	B	A	B	A
Bn-A10-p7913022	A10	15	10.4	A	A	A	B	A	B	B	A	B	A	A	A	A	A	A	B	A	B	A	B	A
Bn-A10-p8443505	A10	16	11.2	A	A	A	B	A	B	B	A	B	A	A	A	A	A	A	B	A	B	A	B	A
Bn-A10-p8723171	A10	17	12	A	A	A	B	A	B	B	A	B	A	A	A	A	A	A	B	A	B	A	B	A
Bn-A10-p8904827	A10	18	13.6	A	A	A	B	A	B	B	A	B	A	B	A	A	A	A	B	A	B	A	B	A
Bn-A10-p9166440	A10	19	14.4	A	A	A	B	A	B	B	A	B	A	B	A	A	A	A	B	A	B	A	B	A
Bn-A10-p9245966	A10	20	15.2	A	A	A	B	A	B	B	A	B	A	B	A	A	A	A	B	A	B	A	B	A
Bn-A10-p9370046	A10	21	16	A	A	A	B	A	B	B	A	B	A	B	A	A	A	A	B	A	B	A	B	A
Bn-A10-p9561249	A10	22	17.6	A	A	A	B	A	B	B	A	B	A	B	A	A	A	A	B	A	B	A	B	A
Bn-A10-p9594548	A10	23	18.4	A	A	A	B	A	B	B	A	B	A	B	A	A	A	A	B	A	B	A	B	A
Bn-A10-p9868333	A10	24	19.2	A	A	A	B	A	B	B	A	B	A	B	A	A	A	A	B	A	B	A	B	A
Bn-scaff_17088_1-p353765	A10	25	20.8	A	A	A	B	A	B	B	A	B	A	B	A	A	A	A	B	A	B	A	B	A
Bn-A10-p10464159	A10	26	21.6	A	A	A	B	A	B	B	A	B	A	B	A	A	A	A	B	A	B	A	B	B
Bn-A10-p10647252	A10	27	26.5	B	A	A	B	A	B	B	A	B	A	B	A	A	A	B	B	A	B	A	B	B
Bn-A10-p10914713	A10	28	27.3	B	A	A	B	A	B	B	A	B	A	B	A	A	A	B	B	A	B	A	B	B
Bn-A10-p11105998	A10	29	28.1	B	A	A	B	A	B	B	A	B	A	B	B	A	A	B	B	A	B	A	B	B
Bn-A10-p11230418	A10	30	29.7	B	A	A	B	A	B	B	A	B	A	B	B	A	A	B	B	A	B	A	B	B
Bn-A10-p11396195	A10	31	30.5	B	A	A	B	A	B	B	A	B	A	B	B	A	A	B	B	A	B	A	B	B
Bn-A10-p11427179	A10	32	32.1	B	A	A	B	A	B	B	A	B	A	B	B	A	A	B	B	A	B	A	B	B
Bn-scaff_17028_1-p444459	A10	33	32.9	B	A	A	B	A	B	B	A	B	A	B	B	A	A	B	B	A	B	A	B	B
Bn-A10-p12857310	A10	34	34.5	B	A	A	B	A	B	B	A	B	A	B	B	A	A	B	B	A	B	A	B	B
Bn-A10-p12933288	A10	35	35.3	B	A	A	B	A	B	B	A	B	A	B	B	A	A	B	B	A	B	A	B	B
Bn-A10-p13130002	A10	36	36.1	B	A	A	B	A	B	B	A	B	A	B	B	A	A	B	B	A	B	A	B	B
Bn-A10-p13218309	A10	37	36.9	B	A	A	B	A	B	B	A	B	A	B	B	A	A	B	B	A	B	A	B	B
Bn-A10-p13299250	A10	38	37.7	B	A	A	B	A	B	B	A	B	A	B	B	A	A	B	B	A	B	A	B	B
Bn-A10-p13343454	A10	39	38.5	B	A	A	B	A	B	B	A	B	A	B	B	A	A	B	B	A	B	A	B	B
Bn-A10-p13446086	A10	40	39.3	B	A	A	B	A	B	B	A	B	A	B	B	A	A	B	B	A	B	A	B	B
Bn-A10-p13640346	A10	41	40.1	B	A	A	B	A	B	B	A	B	A	B	B	A	A	B	B	A	B	A	B	B
Bn-A10-p13677412	A10	42	41.7	B	A	A	B	A	B	B	A	B	A	B	B	A	A	B	B	A	B	A	B	B
Bn-A10-p13818569	A10	43	42.5	B	A	A	B	A	B	B	A	B	A	B	B	A	A	B	B	A	B	A	B	B
Bn-A10-p14254487	A10	44	43.3	B	A	A	B	A	B	B	A	B	A	B	B	A	A	B	B	A	B	A	B	B
Bn-A10-p14623802	A10	45	44.9	B	A	A	B	A	B	B	A	A	A	B	B	A	A	B	B	A	B	A	B	B
Bn-A10-p15156277	A10	46	45.8	B	A	A	B	A	B	B	A	A	A	B	B	A	A	B	B	A	B	A	B	B

Bn-A10-p16421672	A10	47	46.7	B	A	A	B	A	B	B	A	A	A	B	B	A	A	B	B	A	B	A	B	B
Bn-A10-p16336708	A10	48	51.8	B	A	A	B	A	B	B	B	A	A	B	B	A	A	B	B	A	B	A	B	B
Bn-A10-p16224024	A10	49	52.7	B	A	A	B	A	A	B	B	A	A	B	B	A	A	B	B	A	B	A	B	B
Bn-A10-p16087066	A10	50	53.6	B	A	A	B	A	A	B	B	A	A	B	B	A	A	B	B	A	B	A	B	B
Bn-A10-p15863143	A10	51	54.5	B	A	A	B	A	A	B	B	A	A	B	B	A	A	B	B	A	B	A	B	B
Bn-A10-p15793623	A10	52	56.2	B	A	A	B	A	A	B	B	A	A	B	B	A	A	B	B	A	B	A	B	B
Bn-A10-p15607468	A10	53	57.1	B	A	B	B	A	A	B	B	A	A	B	B	A	A	B	B	A	B	A	B	B
Bn-A10-p15507350	A10	54	58	B	A	B	B	A	A	B	B	A	A	B	B	A	A	B	A	A	B	A	B	B
Bn-A10-p15398137	A10	55	58.9	B	A	B	B	A	A	B	B	A	A	B	B	A	A	B	A	A	B	A	B	B
Bn-A10-p15387894	A10	56	59.8	B	A	B	B	A	A	B	B	A	A	B	B	A	A	B	A	A	B	A	B	B
Bn-A10-p15367240	A10	57	60.7	B	A	B	B	A	A	B	B	A	A	B	B	A	A	B	A	A	B	A	B	B
Bn-A10-p15330596	A10	58	61.6	B	A	B	B	A	A	B	B	A	A	B	B	A	A	B	A	A	B	A	B	B
Bn-A10-p15265102	A10	59	62.5	B	A	B	B	A	A	B	B	A	A	B	B	A	A	B	A	A	B	A	B	B
Bn-A10-p16620627	A10	60	63.4	B	A	B	B	A	A	B	B	A	A	B	B	A	A	B	A	A	B	A	B	B
Bn-A10-p16862809	A10	61	64.2	B	A	B	B	A	A	B	B	A	A	B	B	A	A	B	A	A	B	A	B	B
Bn-A10-p16935603	A10	62	73.2	B	A	B	B	A	A	B	B	A	A	B	B	A	A	B	A	A	B	A	B	B
Bn-A10-p17568393	A10	63	75.6	B	A	B	B	A	A	B	B	A	A	B	B	A	A	B	A	B	B	A	B	B
Bn-scaff_19244_1-p196631	C1	1	0	B	A	B	A	A	B	A	B	B	A	A	B	A	0	A	B	A	A	A	B	A
Bn-scaff_19244_1-p816342	C1	2	4.3	B	A	B	A	A	B	A	B	B	A	A	B	A	0	B	B	A	A	A	B	A
Bn-A01-p907721	C1	3	8.6	B	A	B	A	A	B	A	B	B	B	A	B	A	0	B	B	A	A	A	B	A
Bn-scaff_15838_1-p1190349	C1	4	10.3	B	A	B	A	A	B	A	B	B	B	A	B	A	0	B	B	A	A	A	B	A
Bn-scaff_15838_1-p1572850	C1	5	14	B	A	B	A	A	B	A	B	B	B	A	B	A	0	B	B	A	A	A	B	A
Bn-scaff_15838_1-p2035980	C1	6	19.6	B	A	B	A	A	B	A	B	B	B	A	B	A	A	B	B	A	A	B	B	A
Bn-scaff_15838_5-p151738	C1	7	21.3	B	A	B	A	B	B	A	B	B	B	A	B	A	A	B	B	A	A	B	B	A
Bn-scaff_15838_5-p1043118	C1	8	25.6	B	A	B	A	B	B	A	B	B	B	A	B	A	A	B	B	A	A	B	B	A
Bn-scaff_27215_1-p1538	C1	9	27.6	B	A	B	A	B	B	A	B	B	B	A	B	A	A	B	B	A	A	B	A	A
Bn-scaff_17731_1-p979512	C1	10	28.6	B	A	B	A	B	B	A	B	B	B	A	B	A	A	B	B	A	A	B	A	A
Bn-scaff_17827_1-p613418	C1	11	29.6	B	A	B	A	B	B	A	B	B	B	A	B	A	A	B	B	A	A	B	A	A
Bn-scaff_17827_1-p621976	C1	12	30.6	B	A	B	A	B	B	A	B	B	B	A	B	A	A	B	B	A	A	B	A	A
Bn-scaff_20210_1-p267408	C1	13	32.1	B	A	B	A	B	B	A	B	B	B	A	B	A	A	B	B	A	A	B	A	A
Bn-scaff_16128_2-p29287	C1	14	33.6	B	A	B	A	B	B	A	B	B	B	A	B	A	A	B	B	A	A	B	A	A
Bn-scaff_24869_1-p152736	C1	15	34.6	B	A	B	A	B	B	A	B	B	B	A	B	A	A	B	B	A	A	B	A	A
Bn-scaff_17592_1-p799061	C1	16	35.6	B	A	B	A	B	B	A	B	B	B	A	B	A	A	B	B	A	A	B	A	A
Bn-scaff_17369_1-p1112630	C1	17	36.6	B	A	B	A	B	B	A	B	B	B	A	B	A	A	B	B	A	A	B	A	A
Bn-scaff_17369_1-p992249	C1	18	37.6	B	A	B	A	B	B	A	B	B	B	A	B	A	A	B	B	A	A	B	A	A
Bn-scaff_17369_1-p843089	C1	19	39.6	B	A	B	A	B	B	A	B	B	B	A	B	A	A	B	B	A	A	B	A	A
Bn-scaff_17369_1-p625180	C1	20	40.6	B	A	B	A	B	B	A	B	B	B	A	B	A	A	B	B	A	A	B	A	A
Bn-scaff_17369_1-p271166	C1	21	41.6	B	A	B	A	B	B	A	B	B	B	A	B	A	A	B	B	A	A	B	A	A
Bn-scaff_17369_1-p32803	C1	22	43.6	B	A	B	A	B	B	A	B	B	B	A	B	A	A	B	B	A	A	A	A	A
Bn-scaff_19614_1-p325704	C1	23	46.6	B	A	B	A	B	B	A	B	B	B	A	B	A	A	B	B	A	A	A	A	A
Bn-scaff_19614_1-p53484	C1	24	48.6	B	A	B	A	A	B	A	B	B	B	A	B	A	A	B	B	A	A	A	A	A
Bn-scaff_15747_1-p104038	C1	25	49.6	B	A	B	A	A	B	A	B	B	B	A	B	A	A	B	B	A	A	A	A	A
Bn-scaff_15747_1-p167954	C1	26	50.5	B	A	B	A	A	B	A	B	B	B	A	B	A	A	B	B	A	A	A	A	A
Bn-scaff_15747_1-p400964	C1	27	52.4	B	A	B	A	A	B	A	B	B	B	A	B	A	A	B	B	A	A	A	A	A
Bn-scaff_22790_1-p793365	C1	28	54.3	B	A	B	A	A	B	A	B	B	B	A	B	A	A	B	B	A	A	A	A	A
Bn-scaff_19183_1-p423384	C1	29	54.3	B	A	B	A	A	B	A	B	B	B	A	B	A	A	B	B	A	A	A	A	A
Bn-scaff_25373_1-p399	C1	30	54.3	B	A	B	A	A	B	A	B	B	B	A	B	A	A	B	B	A	A	A	A	A
Bn-scaff_16929_1-p719482	C1	31	55.2	B	A	B	A	A	B	A	B	B	B	A	B	A	A	B	B	A	A	A	A	A
Bn-scaff_21163_1-p1838	C1	32	56.1	B	A	B	A	A	B	A	B	B	B	A	B	A	A	B	B	A	A	A	A	A

Bn-scaff_15879_1-p29379	C1	33	57	B	A	B	A	A	B	A	0	B	B	A	B	A	A	B	B	A	A	A	A	A
Bn-scaff_27823_1-p6273	C1	34	57.9	B	A	B	A	A	B	A	0	B	B	A	B	A	A	B	B	A	A	A	A	A
Bn-scaff_21225_1-p74961	C1	35	59.7	B	A	B	A	A	B	A	0	B	B	A	B	A	A	B	B	A	A	A	A	A
Bn-scaff_18482_1-p816502	C1	36	60.6	B	A	B	A	A	B	A	0	A	B	A	B	A	A	B	B	A	A	A	A	A
Bn-scaff_16962_1-p655721	C1	37	61.5	B	A	B	A	A	B	A	0	A	B	A	B	A	A	B	B	A	A	B	A	A
Bn-scaff_17515_1-p557620	C1	38	62.4	B	A	B	A	A	B	A	0	A	B	A	B	A	A	B	B	A	A	B	A	A
Bn-scaff_19168_1-p9620	C1	39	63.3	B	A	B	A	A	B	A	0	A	B	A	B	A	A	B	B	A	A	B	A	A
Bn-scaff_21884_1-p182027	C1	40	64.2	B	A	B	A	A	B	A	0	A	B	A	B	A	A	B	B	A	A	B	A	A
Bn-scaff_21884_1-p638903	C1	41	65.1	B	A	B	A	A	B	A	0	A	B	A	B	A	A	B	B	A	A	B	A	A
Bn-scaff_16691_1-p1434829	C1	42	67.7	B	A	A	A	A	B	A	0	A	B	A	B	A	A	B	B	A	A	B	A	A
Bn-scaff_15712_3-p804205	C1	43	68.6	B	A	A	A	A	B	A	0	A	A	A	B	A	A	B	B	A	A	B	A	A
Bn-scaff_15712_3-p653523	C1	44	69.5	B	A	A	A	A	B	A	0	A	A	A	B	A	A	B	B	A	A	B	A	A
Bn-scaff_15712_3-p639489	C1	45	70.4	B	A	A	A	A	B	A	0	A	A	A	B	A	A	B	B	A	A	B	A	A
Bn-scaff_15712_3-p46743	C1	46	71.3	B	A	A	A	A	B	A	0	A	A	A	B	A	A	B	B	A	A	B	A	A
Bn-scaff_18321_1-p122698	C2	1	0	A	B	A	A	A	B	A	A	0	A	A	B	B	A	A	B	A	A	A	A	A
Bn-scaff_22970_1-p386682	C2	2	1.7	A	B	A	A	A	B	A	A	0	A	A	B	B	A	A	B	A	A	A	A	A
Bn-A02-p1344870	C2	3	2.5	A	B	A	A	A	B	A	A	0	A	A	B	B	A	A	B	A	A	A	A	A
Bn-scaff_15714_1-p2704024	C2	4	6.7	A	A	A	A	A	B	A	A	0	A	A	B	B	A	A	B	A	A	A	A	A
Bn-scaff_15714_1-p2481342	C2	5	7.5	A	A	A	A	A	B	A	A	0	A	A	B	B	A	A	B	A	A	A	A	A
Bn-scaff_15714_1-p1966757	C2	6	9.6	A	A	A	A	A	B	A	A	0	A	A	B	B	A	A	B	A	A	A	A	A
Bn-scaff_15714_1-p1892918	C2	7	11.7	A	A	A	A	A	B	A	A	0	A	A	B	B	A	A	B	A	A	A	A	A
Bn-A02-p3507057	C2	8	13.4	A	A	A	A	A	B	A	A	0	A	A	A	B	A	A	B	A	A	A	A	A
Bn-scaff_15714_1-p966029	C2	9	14.2	A	A	A	A	A	A	A	A	0	A	A	A	B	A	A	B	A	A	A	A	A
Bn-scaff_15714_1-p553705	C2	10	15	A	A	A	A	A	A	A	A	B	A	A	A	B	A	A	B	A	A	A	A	A
Bn-scaff_15714_1-p368278	C2	11	15.8	A	A	A	A	A	A	A	A	B	A	A	A	B	A	A	B	A	A	A	A	A
Bn-scaff_15714_1-p134903	C2	12	18.3	A	A	A	A	A	A	A	A	B	A	A	A	B	A	A	B	A	A	A	A	A
Bn-scaff_17522_1-p1483337	C2	13	19.1	A	A	A	A	A	A	A	A	B	A	A	A	A	A	A	B	A	A	A	A	A
Bn-scaff_15712_5-p445598	C2	14	20.8	A	A	A	A	A	A	A	A	B	A	A	A	A	A	A	B	A	A	A	A	A
Bn-scaff_20942_1-p262998	C2	15	22.5	A	A	A	A	A	A	A	A	B	A	A	A	A	A	A	B	A	A	A	A	A
Bn-scaff_23813_1-p531505	C2	16	23.3	A	A	A	A	A	A	A	A	B	A	A	A	A	A	A	A	A	A	A	A	A
Bn-scaff_15839_1-p183517	C2	17	24.1	A	A	A	A	A	A	A	A	B	A	A	A	A	A	A	A	A	A	A	A	A
Bn-scaff_18702_1-p201508	C2	18	24.9	A	A	A	A	A	A	A	A	B	A	A	A	A	A	A	A	A	A	A	A	A
Bn-scaff_16369_1-p90302	C2	19	25.7	A	A	A	A	A	A	A	A	B	A	A	A	A	A	A	A	A	A	A	A	A
Bn-scaff_18675_1-p549332	C2	20	29	A	A	A	A	A	A	A	A	B	A	A	A	A	A	A	A	A	A	A	A	A
Bn-scaff_16300_1-p625676	C2	21	29.8	A	A	A	A	A	A	A	A	B	A	A	A	A	A	A	A	A	A	A	A	A
Bn-scaff_21705_1-p216607	C2	22	29.8	A	A	A	A	A	A	A	A	B	A	A	A	A	A	A	A	A	A	A	A	A
Bn-scaff_18360_1-p4195	C2	23	30.6	A	A	A	A	A	A	A	A	B	A	A	A	A	A	A	A	A	A	A	A	A
Bn-scaff_15712_9-p552692	C2	24	33.1	A	A	A	A	A	A	A	A	B	A	A	A	A	A	A	B	A	A	A	A	A
Bn-scaff_26712_1-p24644	C2	25	33.9	A	A	A	A	A	A	A	A	B	A	A	A	A	A	A	B	A	A	A	A	A
Bn-scaff_17109_1-p1058256	C2	26	35.5	A	A	A	A	A	A	A	A	B	A	A	A	A	A	A	B	A	A	A	A	A
Bn-scaff_17109_1-p889768	C2	27	37.1	A	A	A	A	A	A	A	A	B	A	A	B	A	A	A	B	A	A	A	A	A
Bn-scaff_17109_1-p857649	C2	28	37.9	A	A	A	A	A	A	A	A	B	A	A	B	A	A	A	B	A	A	A	B	A
Bn-scaff_17109_1-p691842	C2	29	38.7	A	A	A	A	A	A	A	A	B	A	A	B	A	A	A	B	A	A	A	B	A
Bn-scaff_15712_12-p5921	C2	30	39.5	A	A	A	A	A	A	A	A	B	A	A	B	A	A	A	B	A	A	A	B	A
Bn-scaff_15918_1-p82985	C2	31	41.1	A	A	A	A	A	A	A	A	B	A	A	B	A	A	A	B	A	A	A	B	A
Bn-scaff_27946_1-p1047	C2	32	42.7	A	A	A	A	A	A	A	A	B	A	A	B	A	A	A	B	A	A	A	B	A
Bn-scaff_17623_1-p599063	C2	33	45.2	A	A	A	A	A	A	A	A	B	A	A	B	A	A	A	B	A	A	A	B	A
Bn-scaff_17623_1-p546659	C2	34	50.2	A	A	A	A	A	A	A	A	B	B	A	B	A	A	A	B	A	A	A	B	A
Bn-scaff_19740_1-p236040	C2	35	51	A	A	A	A	B	A	A	A	B	B	A	B	A	A	A	B	A	A	A	B	A

Bn-scaff_17623_1-p255719	C2	36	51.8	A	A	A	A	B	A	A	A	B	B	A	B	A	A	A	B	B	A	A	B	A
Bn-scaff_17721_1-p906353	C2	37	53.4	A	A	B	A	B	A	A	A	B	B	A	B	A	A	A	B	B	A	A	B	A
Bn-scaff_17721_1-p638632	C2	38	54.2	A	A	B	A	B	A	A	A	A	B	A	B	A	A	A	B	B	A	A	B	A
Bn-scaff_17177_1-p546184	C2	39	56.7	A	A	B	A	B	A	A	A	A	B	A	B	A	A	A	B	B	A	A	B	A
Bn-scaff_26086_1-p11779	C2	40	59.2	A	A	B	A	B	A	A	A	A	B	A	B	A	A	A	B	B	A	A	B	A
Bn-scaff_16139_1-p1295124	C2	41	64.2	A	B	B	A	B	A	A	A	A	B	A	B	B	A	A	B	B	A	A	B	A
Bn-scaff_16139_1-p1285098	C2	42	65	A	B	B	A	B	A	A	A	A	B	A	B	B	A	A	B	B	A	A	B	A
Bn-scaff_16139_1-p1063831	C2	43	65.8	A	B	B	A	B	A	B	A	A	B	A	B	B	A	A	B	B	A	A	B	A
Bn-scaff_16139_1-p951581	C2	44	66.6	A	B	B	A	B	A	B	A	A	B	A	B	B	A	A	B	B	A	A	B	A
Bn-scaff_16139_1-p720716	C2	45	67.4	A	B	B	A	B	B	B	A	A	B	A	B	B	A	A	B	B	A	A	B	A
Bn-scaff_16139_1-p380924	C2	46	68.2	A	B	B	A	B	B	B	A	A	B	A	B	B	A	A	B	B	A	A	B	A
Bn-scaff_16139_1-p260474	C2	47	69.8	A	B	B	A	B	B	B	A	A	B	A	B	B	A	A	B	B	A	A	B	A
Bn-scaff_18936_1-p77573	C3	1	0	A	B	A	B	A	B	B	B	B	A	B	B	B	A	A	B	A	B	B	B	B
Bn-scaff_18936_1-p472353	C3	2	4	A	B	A	B	A	B	B	B	B	A	B	B	B	A	A	B	A	B	B	B	B
Bn-scaff_18936_1-p643990	C3	3	4.8	A	B	A	B	A	B	B	B	B	A	B	B	B	A	A	B	A	B	B	B	B
Bn-scaff_18936_1-p861428	C3	4	6.5	A	B	A	B	A	B	B	B	B	A	B	B	B	A	A	B	A	B	B	B	B
Bn-scaff_15877_1-p39516	C3	5	7.3	A	B	A	B	A	B	B	B	B	A	B	B	B	A	A	B	A	B	B	B	B
Bn-scaff_15877_1-p186528	C3	6	8.1	A	B	A	B	A	B	B	B	B	A	B	B	B	A	A	B	A	B	B	B	B
Bn-A03-p3571622	C3	7	12.3	A	B	B	B	A	B	B	B	B	A	B	B	B	A	A	B	A	B	B	B	B
Bn-scaff_21778_1-p68265	C3	8	15.7	A	B	B	B	A	B	B	B	B	A	B	B	B	A	A	B	A	B	B	B	B
Bn-scaff_21778_1-p217638	C3	9	16.5	A	B	B	B	A	B	B	B	B	A	B	B	B	A	A	B	A	B	B	B	B
Bn-scaff_22728_1-p1399313	C3	10	19	A	B	B	B	A	B	B	B	B	A	B	B	B	A	A	B	A	B	B	B	B
Bn-scaff_22728_1-p1186894	C3	11	19.8	A	B	B	B	A	B	B	B	B	A	B	B	B	A	A	B	A	B	B	B	B
Bn-scaff_22728_1-p1080976	C3	12	21.5	A	B	B	B	A	B	B	B	B	A	B	B	A	A	A	B	A	B	B	B	B
Bn-scaff_16700_1-p47251	C3	13	24	A	B	B	B	A	B	B	B	B	A	B	B	A	B	A	B	A	B	B	B	B
Bn-scaff_18322_1-p2357361	C3	14	24.8	A	B	B	B	A	B	B	B	B	A	B	B	A	B	A	B	A	B	B	B	B
Bn-scaff_18322_1-p2147199	C3	15	25.6	A	B	B	B	A	B	B	B	B	A	B	B	A	B	A	B	A	B	B	B	B
Bn-scaff_18322_1-p2028484	C3	16	26.4	A	B	B	B	A	B	B	B	B	A	B	B	A	B	A	A	A	B	B	B	B
Bn-scaff_18322_1-p1615304	C3	17	28.1	A	B	B	B	A	B	B	B	B	A	B	B	A	B	A	A	A	B	B	B	B
Bn-scaff_18322_1-p1230027	C3	18	28.9	A	B	B	B	A	B	B	B	B	A	B	B	A	B	A	A	A	B	B	B	B
Bn-scaff_18322_1-p498035	C3	19	29.7	A	B	B	B	A	B	B	B	B	A	B	B	A	B	A	A	A	B	B	B	B
Bn-A03-p8112016	C3	20	30.5	A	B	B	B	A	B	B	B	B	A	B	B	A	B	A	A	A	B	B	B	B
Bn-scaff_19111_1-p325137	C3	21	32.9	A	B	B	B	A	B	B	B	B	A	B	B	A	B	A	A	A	B	B	B	B
Bn-scaff_23954_1-p661198	C3	22	34.5	A	B	B	B	A	B	B	B	B	A	B	B	A	B	A	A	A	B	B	B	B
Bn-scaff_16002_1-p2497256	C3	23	35.3	A	B	B	B	A	B	B	B	B	A	B	B	A	B	A	0	A	B	B	B	B
Bn-scaff_16002_1-p2026087	C3	24	37	A	B	B	B	A	B	B	B	B	B	B	B	A	B	A	0	A	B	B	A	B
Bn-scaff_16002_1-p1354832	C3	25	37.8	A	B	B	B	A	B	B	B	B	B	B	B	A	B	A	0	A	B	B	A	B
Bn-scaff_16002_1-p445804	C3	26	39.5	A	B	B	B	A	B	B	B	B	B	B	B	A	B	A	0	A	B	B	A	B
Bn-scaff_22466_1-p492888	C3	27	41.3	A	B	B	B	A	B	B	B	B	B	B	B	A	B	A	0	A	B	B	A	A
Bn-scaff_20646_1-p1075213	C3	28	43.9	A	B	B	B	A	B	B	B	B	B	B	B	A	B	A	0	A	B	B	A	A
Bn-scaff_20646_1-p832827	C3	29	44.8	A	B	B	B	A	B	B	B	B	B	B	B	A	B	A	0	A	B	B	A	A
Bn-scaff_20646_1-p675326	C3	30	45.7	A	B	B	B	A	B	B	B	B	B	B	B	A	B	A	0	B	B	B	A	A
Bn-scaff_27677_1-p272923	C3	31	51	A	B	B	B	A	B	B	B	B	B	B	B	A	B	A	0	B	B	B	A	A
Bn-scaff_19523_1-p232023	C3	32	51.9	A	B	B	B	A	B	B	B	B	B	B	B	A	B	A	B	B	B	B	A	A
Bn-scaff_18482_1-p739195	C3	33	58.6	B	B	B	B	A	B	B	B	B	B	B	B	A	B	B	B	B	B	B	A	A
Bn-scaff_22751_1-p19884	C3	34	59.4	B	B	B	B	A	B	B	B	B	B	B	B	A	B	B	B	B	B	B	A	A
Bn-scaff_22067_1-p103662	C3	35	61.1	B	B	B	B	A	B	B	B	B	B	B	B	A	B	B	B	B	B	B	A	A
Bn-scaff_22067_1-p147934	C3	36	61.9	B	B	B	B	A	B	B	B	B	B	B	B	A	B	B	B	B	B	B	A	A
Bn-scaff_17521_1-p1269891	C3	37	66.8	B	B	B	B	A	B	B	B	B	B	B	B	A	B	B	B	B	B	B	A	A

Bn-A03-p15417676	C3	38	67.6	B	B	B	B	A	B	B	B	B	B	B	B	A	B	B	B	B	B	B	A	A
Bn-scaff_17521_1-p511223	C3	39	68.4	B	B	B	B	A	B	B	B	B	B	B	B	A	B	B	B	B	B	B	A	A
Bn-scaff_17521_1-p193675	C3	40	70	B	B	B	B	A	B	B	B	B	B	B	B	A	B	B	B	B	B	B	A	A
Bn-scaff_28562_1-p11758	C3	41	70.8	B	B	B	B	A	B	B	B	B	B	B	B	A	B	B	B	B	B	B	A	A
Bn-scaff_17298_1-p138775	C3	42	71.6	B	B	B	B	A	B	B	B	B	B	B	B	A	B	B	B	A	B	B	A	A
Bn-scaff_17298_1-p627166	C3	43	72.4	B	B	B	B	A	B	B	B	B	B	B	B	A	B	B	B	A	B	B	A	A
Bn-scaff_17298_1-p679830	C3	44	73.2	B	B	B	B	A	B	B	B	B	B	B	B	A	B	B	B	A	B	B	A	A
Bn-scaff_17298_1-p1055071	C3	45	75.6	B	B	B	B	A	B	B	B	B	B	B	B	A	B	B	B	A	B	B	A	A
Bn-scaff_17298_1-p1302996	C3	46	77.2	B	B	B	B	A	B	B	B	B	B	B	B	A	B	B	B	A	B	B	A	A
Bn-scaff_17298_1-p1412259	C3	47	78	B	B	B	B	A	B	B	B	B	B	B	B	A	B	B	B	A	B	B	A	A
Bn-scaff_23350_1-p643281	C3	48	78.8	B	B	B	B	A	B	B	B	B	B	B	B	A	B	B	B	A	B	B	A	A
Bn-scaff_17395_1-p191984	C3	49	79.6	B	B	B	B	A	B	B	B	B	B	B	B	A	B	B	B	A	B	B	A	A
Bn-scaff_18917_1-p865354	C3	50	81.2	B	B	B	B	A	B	B	B	B	B	B	B	A	B	B	B	A	B	B	A	A
Bn-scaff_18917_1-p744355	C3	51	82	B	B	B	B	A	B	B	B	B	B	B	B	A	B	B	B	A	B	B	A	A
Bn-scaff_18917_1-p689464	C3	52	82.8	B	B	B	B	A	B	B	B	B	B	B	B	A	A	B	B	A	B	B	A	A
Bn-scaff_18917_1-p667205	C3	53	84.4	B	B	B	B	A	B	B	B	B	B	B	B	A	A	B	B	A	B	B	A	A
Bn-scaff_18917_1-p514534	C3	54	85.2	B	B	B	B	B	B	B	B	B	B	B	B	A	A	B	B	A	B	B	A	A
Bn-scaff_16130_2-p484981	C3	55	86	B	B	B	B	B	B	B	B	B	B	B	B	A	A	B	B	A	B	B	A	A
Bn-scaff_17044_1-p545336	C3	56	86.8	B	B	B	B	B	B	B	B	B	B	B	B	A	A	B	B	A	B	B	A	A
Bn-scaff_15747_2-p127306	C3	57	89.2	B	B	B	B	B	B	B	B	B	B	B	B	A	A	B	B	A	B	B	A	A
Bn-scaff_18356_1-p349741	C3	58	92.4	B	B	B	B	B	B	B	B	B	B	B	B	B	A	B	B	A	B	B	A	A
Bn-scaff_18356_1-p43628	C3	59	93.2	B	B	B	B	B	B	B	B	B	B	B	B	B	A	B	B	A	B	B	A	A
Bn-scaff_18855_1-p179141	C3	60	94.8	B	B	B	B	B	B	B	B	B	B	B	B	B	A	B	B	A	B	B	A	A
Bn-scaff_21867_1-p87764	C3	61	95.6	B	B	B	B	B	B	B	B	B	B	B	B	B	A	B	B	A	B	B	A	A
Bn-scaff_23407_1-p87145	C3	62	96.4	B	B	B	B	B	B	B	B	B	B	B	B	B	A	B	B	A	B	B	A	A
Bn-scaff_20790_1-p3436	C3	63	98	B	B	B	B	B	B	B	B	B	B	A	B	B	A	B	B	A	B	B	A	A
Bn-scaff_18849_1-p191163	C3	64	102	B	B	B	B	B	B	B	B	B	B	A	B	B	A	B	B	A	B	B	A	A
Bn-scaff_18849_1-p22568	C3	65	103.6	B	B	B	B	B	B	B	B	B	B	A	B	B	A	B	B	B	B	B	A	A
Bn-scaff_17440_1-p590665	C3	66	104.4	B	B	B	B	B	B	B	B	B	B	A	B	B	A	B	B	B	B	B	A	A
Bn-scaff_17440_1-p316581	C3	67	105.2	B	B	B	B	B	B	B	B	B	B	A	B	B	A	B	B	B	B	B	A	A
Bn-scaff_24726_1-p16408	C3	68	106	B	B	B	B	B	B	B	B	B	B	A	B	B	A	B	B	B	B	B	A	A
Bn-scaff_24631_1-p184986	C3	69	106.8	B	B	B	B	B	B	B	B	B	B	A	B	B	A	B	B	B	B	B	A	A
Bn-scaff_24631_1-p271540	C3	70	108.4	B	B	B	B	B	B	B	B	B	B	A	B	B	A	B	B	B	B	B	A	A
Bn-scaff_24631_1-p338156	C3	71	109.2	B	B	B	B	B	B	B	B	B	B	A	B	B	A	B	B	B	B	B	A	A
Bn-scaff_24631_1-p785809	C3	72	110.8	B	B	B	B	B	B	B	B	B	B	A	B	B	A	B	B	B	B	B	A	A
Bn-scaff_16394_2-p1498560	C3	73	111.6	B	B	B	B	B	B	B	B	B	B	A	B	B	A	B	A	B	B	B	A	A
Bn-scaff_16394_2-p1377231	C3	74	112.4	B	B	B	B	B	B	B	B	B	B	A	B	B	A	B	A	B	B	B	A	A
Bn-scaff_16394_2-p992866	C3	75	113.2	B	B	B	B	B	B	A	B	B	B	A	B	B	A	B	A	B	B	B	A	A
Bn-scaff_16394_2-p471655	C3	76	117.2	A	B	B	B	B	B	A	B	B	B	A	B	B	A	A	A	B	B	B	A	A
Bn-scaff_16394_2-p348198	C3	77	118	A	B	B	B	B	B	A	B	B	B	A	B	B	A	A	A	B	B	B	A	A
Bn-scaff_16394_2-p71424	C3	78	118.8	A	B	B	B	B	B	A	B	B	B	A	B	B	A	A	A	B	B	B	A	A
Bn-scaff_18602_1-p46083	C3	79	119.6	A	B	B	B	B	B	A	B	B	B	A	B	B	A	A	A	B	B	B	A	A
Bn-scaff_18602_1-p89074	C3	80	120.4	A	B	B	B	B	B	A	B	B	B	A	B	B	A	A	A	B	B	B	A	A
Bn-scaff_16182_1-p632134	C3	81	121.2	A	B	B	B	B	B	A	B	B	B	A	B	B	A	A	A	B	B	B	B	A
Bn-scaff_26846_1-p26261	C3	82	122	A	B	B	B	B	B	A	B	B	B	A	B	B	A	A	A	B	B	B	B	A
Bn-scaff_17042_1-p147112	C3	83	122.8	A	B	B	B	B	B	A	B	B	B	A	B	B	A	A	A	B	B	B	B	A
Bn-scaff_17042_1-p418599	C3	84	123.6	A	B	B	B	B	B	A	B	B	B	A	B	B	A	A	A	B	B	B	B	A
Bn-scaff_17042_1-p468781	C3	85	124.4	A	B	B	B	B	B	A	B	B	B	A	B	B	A	A	A	B	B	B	B	A
Bn-scaff_17457_1-p283145	C3	86	127.6	A	B	B	B	B	B	A	B	B	B	A	B	B	A	A	A	B	B	B	B	A

Bn-scaff_15794_3-p508122	C3	87	129.2	A	B	B	B	B	B	A	B	B	B	A	B	B	A	A	A	B	B	B	B	A
Bn-scaff_15794_3-p146368	C3	88	130	A	B	B	B	B	B	A	B	B	B	A	B	B	A	A	A	B	B	B	B	A
Bn-scaff_15794_1-p157188	C3	89	130.8	A	B	B	B	B	B	A	B	B	B	A	B	B	A	A	A	B	B	B	B	A
Bn-scaff_17821_1-p30801	C3	90	134.8	A	B	B	B	B	B	A	B	B	B	A	B	B	A	A	A	B	B	B	B	A
Bn-scaff_17119_1-p183714	C3	91	136.4	A	B	B	B	B	B	A	B	B	B	A	B	B	A	A	A	B	B	B	B	A
Bn-scaff_23761_1-p27239	C3	92	137.2	A	B	B	B	B	B	A	B	B	B	A	B	B	A	A	A	B	B	B	B	A
Bn-scaff_19047_1-p297198	C3	93	140.4	A	B	B	B	B	B	A	B	B	B	A	B	B	A	A	A	A	B	B	B	A
Bn-scaff_16148_1-p237592	C3	94	141.2	A	B	B	B	B	B	A	B	B	B	A	B	B	A	A	A	A	B	B	B	A
Bn-scaff_16148_1-p63709	C3	95	142	A	B	B	B	B	B	A	B	B	B	A	B	B	A	A	A	A	B	B	B	A
Bn-scaff_16755_1-p1364355	C3	96	142.8	A	B	B	B	B	B	A	B	B	B	A	B	B	A	A	A	A	B	B	B	A
Bn-scaff_16755_1-p934260	C3	97	144.4	A	B	B	B	B	B	A	B	B	B	A	B	B	A	A	A	A	B	B	B	A
Bn-scaff_16755_1-p878005	C3	98	146	A	B	B	B	B	B	A	A	B	B	A	B	B	A	A	A	A	B	B	B	A
Bn-scaff_16755_1-p12541	C3	99	146.8	A	B	B	B	B	B	A	A	B	B	A	B	B	A	A	A	A	B	B	B	A
Bn-scaff_22933_1-p110027	C4	1	0	B	A	B	B	B	A	0	B	B	B	A	A	A	B	B	A	B	B	A	A	A
Bn-scaff_23534_1-p39080	C4	2	1.7	B	A	B	B	B	A	0	B	B	B	A	A	A	B	B	A	B	B	A	A	A
Bn-scaff_17712_1-p78048	C4	3	2.5	B	A	B	B	B	A	0	B	B	B	A	A	A	B	B	A	B	B	A	A	A
Bn-scaff_16935_1-p102075	C4	4	3.3	B	A	B	B	B	A	0	B	B	B	A	A	A	B	B	A	B	B	A	A	A
Bn-scaff_23946_1-p35627	C4	5	7.5	A	A	B	B	B	A	0	B	B	B	A	A	A	B	A	A	B	B	A	A	A
Bn-scaff_16027_1-p181998	C4	6	9.5	A	A	B	B	B	A	0	B	B	B	A	A	A	B	A	A	B	B	A	A	A
Bn-scaff_16027_1-p319312	C4	7	10.5	A	A	B	B	B	A	0	B	B	B	A	A	A	B	A	A	B	B	A	A	A
Bn-scaff_17517_1-p128364	C4	8	12.5	A	A	B	B	B	A	B	B	B	B	B	A	A	B	A	A	B	B	A	A	A
Bn-scaff_16027_1-p585183	C4	9	15.8	A	A	B	B	B	A	B	B	B	B	B	B	A	B	A	A	B	B	A	A	A
Bn-scaff_27469_1-p101056	C4	10	17.4	A	A	B	B	B	A	B	B	B	B	B	B	A	B	A	A	B	B	A	A	A
Bn-scaff_28382_1-p28399	C4	11	25.6	A	B	B	B	B	A	B	B	B	B	B	B	B	B	A	A	B	B	A	A	B
Bn-scaff_16214_1-p172721	C4	12	26.4	A	B	B	B	B	A	B	B	B	B	B	B	B	B	A	A	B	B	A	A	B
Bn-scaff_16534_1-p134210	C4	13	27.2	A	B	B	B	B	A	B	B	B	B	B	B	B	B	A	A	B	B	A	A	B
Bn-scaff_16534_1-p278496	C4	14	28	A	B	B	B	B	A	B	B	B	B	B	B	B	B	A	A	B	B	A	A	B
Bn-scaff_16534_1-p399693	C4	15	28.8	A	B	B	B	B	A	B	B	B	B	B	B	B	B	A	A	B	B	A	A	B
Bn-scaff_28277_1-p5229	C4	16	29.6	A	B	B	B	B	A	B	B	B	B	B	B	B	B	A	A	B	B	A	A	B
Bn-scaff_16534_1-p707801	C4	17	30.4	A	B	B	B	B	A	B	B	B	A	B	B	B	B	A	A	B	B	A	A	B
Bn-scaff_16534_1-p807124	C4	18	31.2	A	B	B	B	B	A	B	B	B	A	B	B	B	B	A	A	B	B	A	A	B
Bn-scaff_16534_1-p1262658	C4	19	32	A	B	B	B	B	A	B	B	B	A	B	B	B	B	A	A	B	B	A	A	B
Bn-scaff_16534_1-p1357851	C4	20	32.8	A	B	A	B	B	A	B	B	B	A	B	B	B	B	A	A	B	B	A	A	B
Bn-scaff_16534_1-p1846740	C4	21	33.6	A	B	A	B	B	A	B	B	B	A	B	B	B	B	A	A	B	B	A	A	B
Bn-scaff_16534_1-p2435936	C4	22	34.4	A	B	A	B	B	A	B	B	B	A	B	B	B	B	A	A	B	B	A	A	B
Bn-scaff_15908_1-p755460	C4	23	35.2	A	B	A	B	B	A	B	B	B	A	B	B	B	B	A	A	B	B	A	A	B
Bn-scaff_15908_1-p886007	C4	24	36	A	B	A	B	B	A	B	B	B	A	B	B	B	B	A	A	B	B	A	A	B
Bn-A05-p4304172	C4	25	37.6	A	B	A	B	B	A	B	B	B	A	B	B	B	B	A	A	B	B	A	A	B
Bn-scaff_19248_1-p187769	C4	26	39.2	A	B	A	B	B	A	B	B	B	A	B	B	B	B	A	A	B	B	A	B	B
Bn-scaff_18440_1-p77767	C4	27	40	A	B	A	B	B	A	B	B	B	A	B	B	B	B	A	A	B	B	A	B	B
Bn-scaff_23907_1-p542442	C4	28	44	A	B	A	B	B	A	B	B	B	A	B	B	B	B	A	A	B	B	A	B	B
Bn-scaff_23907_1-p647895	C4	29	44.8	A	B	A	B	B	A	B	B	B	A	B	B	B	B	A	A	B	B	A	B	B
Bn-scaff_23907_1-p746005	C4	30	45.6	A	B	A	B	B	A	B	B	B	A	B	B	B	B	A	A	B	B	A	B	B
Bn-scaff_23907_1-p931535	C4	31	46.4	A	B	A	B	B	A	B	B	B	A	B	B	B	B	A	A	B	B	A	B	B
Bn-scaff_16447_1-p403240	C4	32	48	A	B	A	B	B	A	B	B	B	A	B	B	B	B	A	A	B	B	A	B	B
Bn-scaff_17869_1-p814323	C4	33	48.8	A	B	A	B	B	A	B	B	B	A	B	A	B	B	A	A	B	B	A	B	B
Bn-scaff_18754_1-p233662	C4	34	51.2	A	B	A	B	A	A	B	B	B	A	B	A	B	B	A	A	B	B	A	B	B
Bn-scaff_23699_1-p435151	C4	35	52.8	A	B	A	B	A	A	B	B	B	A	B	A	B	B	A	A	B	B	A	B	B
Bn-scaff_18712_1-p326442	C4	36	54.4	A	B	A	B	A	A	B	B	B	A	B	A	B	B	A	A	B	B	A	B	B

Bn-scaff_19253_1-p93586	C4	37	55.2	A	B	A	B	A	A	A	B	B	A	B	A	B	B	A	A	B	B	A	B	B
Bn-scaff_15911_1-p376603	C4	38	56	A	B	A	B	A	A	A	B	B	A	B	A	B	B	A	A	B	B	A	B	B
Bn-scaff_24602_1-p69684	C4	39	62.5	A	B	A	B	A	A	A	B	B	A	B	A	A	B	A	B	B	B	A	B	B
Bn-scaff_19551_1-p24484	C4	40	63.3	A	B	A	B	B	A	A	B	B	A	B	A	A	B	A	B	B	B	A	B	B
Bn-scaff_19170_1-p559165	C4	41	64.9	A	B	A	B	B	A	A	B	B	A	B	A	A	B	A	B	B	B	A	B	B
Bn-scaff_15779_1-p41353	C4	42	68.1	A	B	A	B	B	A	A	B	B	A	B	A	A	B	A	B	B	B	B	B	B
Bn-scaff_15779_1-p318634	C4	43	68.9	A	B	A	B	B	A	A	B	B	A	B	A	A	B	A	B	B	B	B	B	B
Bn-scaff_26139_1-p333824	C4	44	69.7	A	B	A	B	B	A	A	B	B	A	B	A	A	B	A	B	B	B	B	B	B
Bn-scaff_16876_1-p1180531	C4	45	72.1	A	B	A	B	B	A	A	B	B	A	B	A	A	B	A	B	B	B	B	B	B
Bn-scaff_16876_1-p1116894	C4	46	72.9	A	B	A	B	B	A	B	B	B	A	B	A	A	B	A	B	B	B	B	B	B
Bn-scaff_22668_1-p95	C4	47	73.7	A	B	A	B	B	A	B	B	B	A	B	A	A	B	A	B	B	B	B	B	A
Bn-scaff_17525_1-p117065	C4	48	76.1	A	B	A	B	B	A	B	B	B	A	B	A	A	B	A	A	B	B	B	B	A
Bn-scaff_19208_1-p233040	C4	49	76.9	A	B	A	B	B	A	B	B	B	A	B	A	A	B	A	A	B	B	B	B	A
Bn-scaff_25960_1-p104315	C4	50	78.5	A	B	A	B	B	A	B	B	B	A	B	A	A	B	A	A	B	B	B	B	A
Bn-scaff_15852_1-p532467	C4	51	80.9	A	B	A	B	B	A	B	B	B	A	B	A	A	B	A	A	B	B	B	B	A
Bn-scaff_15852_1-p263989	C4	52	83.3	A	B	A	B	B	A	B	B	B	A	A	A	A	B	A	A	B	B	B	A	A
Bn-scaff_15852_1-p135814	C4	53	84.1	A	B	A	B	B	A	B	B	B	A	A	A	A	B	A	A	B	B	B	A	A
Bn-scaff_24979_1-p162534	C4	54	88.1	B	B	A	B	B	A	B	B	B	A	A	A	A	B	B	A	B	B	B	A	A
Bn-scaff_20079_1-p623803	C4	55	89.7	B	B	A	B	B	A	B	B	B	A	A	A	A	B	B	A	B	B	B	A	A
Bn-scaff_20079_1-p496441	C4	56	90.5	B	B	A	B	B	A	B	B	B	A	A	A	A	B	B	A	B	B	B	A	A
Bn-A04-p12917949	C4	57	93.7	B	B	A	A	B	A	B	B	B	A	A	A	A	B	B	A	B	A	B	A	A
Bn-scaff_16804_3-p29132	C4	58	94.5	B	B	A	A	B	A	B	B	B	A	A	A	A	B	B	A	B	A	B	A	A
Bn-scaff_16804_2-p210553	C4	59	97.7	B	B	A	A	B	A	B	B	B	A	A	A	A	B	B	A	B	A	B	A	A
Bn-scaff_21186_1-p220377	C4	60	100.1	B	B	A	A	B	A	B	B	B	A	A	A	A	B	B	A	B	A	B	A	A
Bn-scaff_17319_1-p4691	C4	61	100.9	B	B	A	A	B	A	B	B	B	A	A	B	A	B	B	A	B	A	B	A	A
Bn-A04-p15287200	C4	62	101.7	B	B	A	A	B	A	B	B	B	A	A	B	A	B	B	A	B	A	B	A	A
Bn-scaff_15585_1-p1029921	C4	63	102.5	B	B	A	A	B	A	B	B	B	A	A	B	A	B	B	A	B	A	B	A	A
Bn-scaff_20804_1-p111983	C4	64	103.3	B	B	A	A	B	A	B	B	B	A	A	B	A	B	B	A	B	A	B	A	A
Bn-scaff_20804_1-p33693	C4	65	104.1	B	B	A	A	B	A	B	B	B	A	A	B	A	B	B	A	B	A	B	A	A
Bn-scaff_16888_1-p104880	C4	66	104.9	B	B	A	A	B	A	B	B	B	A	A	B	A	B	B	A	B	A	B	A	A
Bn-scaff_16888_1-p894418	C4	67	109.8	B	B	A	A	B	A	B	B	B	A	A	B	A	B	B	A	B	A	B	A	A
Bn-scaff_16888_1-p1419612	C4	68	112.2	B	B	A	A	B	B	B	B	B	A	A	B	A	B	B	A	B	A	B	A	A
Bn-scaff_16888_1-p1803454	C4	69	116.2	B	B	A	A	B	B	B	B	B	A	A	B	A	B	B	A	B	A	B	A	A
Bn-scaff_20270_1-p39619	C4	70	117.8	B	B	A	A	B	B	B	B	B	A	A	B	A	B	B	A	B	A	B	A	A
Bn-scaff_20270_1-p167746	C4	71	118.6	B	B	A	A	B	B	B	B	B	A	A	B	B	B	B	A	B	A	B	A	A
Bn-A04-p17475728	C4	72	120.2	B	B	A	A	B	B	B	B	B	A	A	B	B	B	B	B	B	A	B	A	A
Bn-scaff_20270_1-p781490	C4	73	122.6	B	B	A	A	B	B	B	B	B	A	A	B	B	B	B	B	B	A	B	A	A
Bn-scaff_18903_1-p350780	C4	74	131.6	B	B	A	A	B	B	B	B	B	B	A	B	B	B	B	B	B	A	B	A	A
Bn-scaff_18903_1-p556922	C4	75	132.4	B	B	A	A	B	B	B	B	B	B	A	B	B	B	B	B	B	A	B	A	A
Bn-scaff_18903_1-p643244	C4	76	133.2	B	B	B	A	B	B	B	B	B	B	A	B	B	B	B	B	B	A	B	A	A
Bn-scaff_18903_1-p998892	C4	77	134.8	B	B	B	A	B	B	B	B	B	B	A	B	B	B	B	B	B	A	B	A	A
Bn-scaff_27676_1-p110403	C4	78	135.6	B	B	B	A	B	B	B	B	B	B	A	B	B	B	B	B	B	A	B	A	A
Bn-scaff_23107_1-p48188	C5	1	0	B	A	A	A	A	B	B	A	A	A	B	B	A	A	B	A	A	A	A	A	B
Bn-scaff_16414_1-p958895	C5	2	0.8	B	A	A	A	A	B	B	A	A	A	B	B	A	A	B	A	A	A	A	A	B
Bn-scaff_16414_1-p777277	C5	3	2.4	B	A	A	A	A	B	B	A	A	A	B	B	A	A	B	A	A	A	A	A	B
Bn-scaff_16414_1-p651187	C5	4	3.2	B	A	A	A	A	B	B	A	A	A	B	B	A	A	B	A	A	A	A	A	B
Bn-scaff_16414_1-p541587	C5	5	5.6	B	A	A	A	A	B	B	A	A	A	B	B	A	A	B	A	A	A	A	A	B
Bn-scaff_16414_1-p309830	C5	6	10.8	B	A	A	A	A	B	B	A	A	A	B	B	A	A	B	A	A	A	A	A	B
Bn-scaff_16414_1-p98361	C5	7	17.8	B	A	A	A	A	B	B	A	A	A	B	B	A	A	B	A	A	A	A	A	A

Bn-scaff_20901_1-p2160101	C5	8	18.6	B	A	A	A	A	B	B	A	A	A	B	B	A	A	B	A	A	A	A	A	A
Bn-scaff_20901_1-p2078439	C5	9	20.2	B	A	A	A	A	B	B	A	A	A	B	B	A	A	B	A	A	A	A	A	A
Bn-scaff_20901_1-p2040223	C5	10	21.8	B	A	A	A	A	B	B	A	A	B	B	B	A	A	B	A	A	A	A	A	A
Bn-scaff_20901_1-p2029631	C5	11	22.6	B	A	A	A	A	B	B	A	A	B	B	B	A	A	B	A	A	A	A	A	A
Bn-scaff_20901_1-p1760597	C5	12	23.4	B	A	A	A	A	B	B	A	A	B	B	B	A	A	B	A	A	A	A	A	A
Bn-scaff_20901_1-p1681305	C5	13	24.2	B	A	A	A	A	B	B	A	A	B	B	B	A	A	B	A	A	A	A	A	A
Bn-scaff_20901_1-p1537914	C5	14	29.1	B	A	A	A	A	B	A	A	A	B	B	B	A	A	B	A	A	A	A	A	A
Bn-scaff_20901_1-p1311264	C5	15	33.1	B	A	A	A	A	B	A	A	A	B	B	B	A	A	B	A	A	A	A	A	A
Bn-scaff_20901_1-p1310135	C5	16	33.9	B	A	A	A	A	A	A	A	A	B	B	B	A	A	B	A	A	A	A	A	A
Bn-scaff_20901_1-p1300905	C5	17	34.7	B	A	A	A	A	A	A	A	A	B	B	B	A	A	B	A	A	A	A	A	A
Bn-scaff_20901_1-p1247459	C5	18	36.3	B	A	A	A	A	A	A	A	A	B	B	B	A	A	B	A	A	A	A	A	A
Bn-scaff_20901_1-p1103224	C5	19	38.7	B	A	A	A	A	A	A	A	A	B	A	B	A	A	B	A	A	A	A	A	A
Bn-scaff_20901_1-p948057	C5	20	43.6	B	A	A	A	A	A	A	A	A	B	A	B	A	A	B	A	A	A	A	A	A
Bn-scaff_20901_1-p660618	C5	21	46	B	A	A	A	A	A	A	A	A	B	A	B	A	A	B	A	A	A	A	A	A
Bn-A06-p3651473	C5	22	50	A	A	A	A	B	A	A	A	A	B	A	B	A	A	B	A	A	A	A	A	A
Bn-scaff_20901_1-p595473	C5	23	50.8	A	A	A	A	B	A	A	A	A	B	A	B	A	A	A	A	A	A	A	A	A
Bn-scaff_19641_1-p188036	C5	24	51.6	A	A	A	A	B	A	A	A	A	B	A	B	A	A	A	A	A	A	A	A	A
Bn-scaff_21496_1-p440091	C5	25	55.6	A	A	A	A	B	A	A	A	A	B	A	B	A	A	A	A	A	A	A	A	A
Bn-scaff_15712_10-p520866	C5	26	56.4	A	A	A	A	B	A	A	A	A	B	A	A	A	A	A	A	A	A	A	A	A
Bn-scaff_18181_1-p957193	C5	27	57.2	A	A	A	A	B	A	A	A	A	B	A	A	A	A	A	A	A	A	A	A	A
Bn-scaff_18181_1-p626037	C5	28	58	A	A	A	A	B	A	A	A	A	B	A	A	A	A	A	A	A	A	A	A	A
Bn-scaff_16082_1-p454878	C5	29	58.8	A	A	A	A	B	A	A	A	A	B	A	A	A	A	A	A	A	A	A	A	A
Bn-scaff_16082_1-p450246	C5	30	59.6	A	A	A	A	B	A	A	A	A	B	A	A	A	A	A	B	A	A	A	A	A
Bn-scaff_22183_1-p644001	C5	31	60.4	A	A	A	A	B	A	A	A	A	B	A	A	A	A	A	B	A	A	A	A	A
Bn-scaff_22183_1-p164778	C5	32	62	A	A	A	A	B	A	A	A	A	B	A	A	A	A	A	B	A	A	A	A	A
Bn-scaff_16093_1-p5778	C5	33	64.4	A	A	A	A	B	A	A	A	A	B	A	A	A	A	A	B	A	A	A	A	A
Bn-scaff_21338_1-p832818	C5	34	65.2	A	A	A	A	B	A	A	A	A	B	A	A	A	A	A	B	A	A	A	A	A
Bn-scaff_18338_1-p891133	C5	35	66	A	A	A	A	B	A	A	A	A	B	A	A	A	A	A	B	A	A	A	A	A
Bn-scaff_18338_1-p322932	C5	36	70	A	A	A	B	B	A	A	A	A	A	A	A	A	A	A	B	A	B	A	A	A
Bn-scaff_15818_1-p74700	C5	37	70.8	A	A	A	B	B	A	B	A	A	A	A	A	A	A	A	B	A	B	A	A	A
Bn-scaff_16062_1-p148000	C5	38	71.6	A	A	A	B	B	A	B	A	A	A	A	A	A	A	A	B	A	B	A	A	A
Bn-scaff_23195_1-p70804	C5	39	74	A	A	A	B	B	A	B	A	A	A	B	A	A	A	A	A	A	B	A	A	A
Bn-scaff_16478_1-p1065763	C5	40	74.8	A	A	A	B	A	A	B	A	A	A	B	A	A	A	A	A	A	B	A	A	A
Bn-A05-p16509283	C5	41	75.6	A	A	A	B	A	A	B	A	A	A	B	A	A	A	A	A	A	B	A	A	A
Bn-scaff_18589_1-p94879	C5	42	76.4	A	A	A	B	A	A	B	A	A	A	B	A	A	A	A	A	A	B	A	A	A
Bn-scaff_23408_1-p272922	C5	43	77.2	A	A	A	B	A	A	B	A	A	A	B	A	A	A	A	B	A	B	A	A	A
Bn-scaff_16770_1-p217669	C5	44	78.9	A	A	A	B	A	A	B	A	A	A	B	A	A	A	A	B	A	B	0	A	A
Bn-scaff_18826_1-p1000612	C5	45	79.7	A	A	A	B	A	A	B	A	A	A	B	A	A	A	A	B	A	B	0	A	A
Bn-scaff_18826_1-p862439	C5	46	80.5	A	A	A	B	A	A	B	A	A	A	B	A	A	A	A	B	A	B	0	A	A
Bn-scaff_18826_1-p781314	C5	47	81.4	A	A	A	B	A	B	B	A	A	A	B	A	A	A	A	B	A	B	0	A	A
Bn-scaff_21369_1-p102870	C5	48	84	A	A	A	B	A	B	B	A	A	A	B	A	A	A	A	B	A	B	0	A	A
Bn-A05-p21326775	C5	49	85.7	A	A	A	B	A	B	B	A	A	A	B	A	A	A	A	B	A	B	0	A	A
Bn-scaff_17441_1-p400751	C5	50	90	A	A	A	B	A	B	B	A	A	A	B	A	A	A	A	B	A	B	0	A	A
Bn-scaff_17441_1-p573618	C5	51	91.7	A	A	A	B	A	B	B	A	A	A	B	A	A	A	A	B	A	B	0	A	A
Bn-scaff_17441_1-p831019	C5	52	93.4	A	A	A	B	A	B	B	A	A	A	B	A	A	A	A	B	A	B	0	A	A
Bn-scaff_17441_1-p1045065	C5	53	96	A	A	A	B	A	B	B	A	A	A	B	A	A	A	A	B	A	B	0	A	A
Bn-scaff_17441_1-p1094625	C5	54	96.9	A	A	A	B	A	B	B	A	A	A	B	A	A	A	A	B	A	B	0	A	A
Bn-scaff_17441_1-p1146459	C5	55	97.8	A	A	A	B	A	B	B	A	A	A	B	A	A	A	A	B	A	B	B	A	A
Bn-scaff_17441_3-p154031	C5	56	99.5	A	A	A	B	A	B	B	A	A	A	B	A	A	A	A	B	A	B	B	A	A

Bn-scaff_17441_3-p325608	C5	57	102	A	A	A	B	A	B	B	A	A	A	B	A	A	A	A	B	A	B	B	A	A
Bn-A05-p23102053	C5	58	106.1	A	A	A	B	A	B	A	A	A	A	A	A	A	A	A	B	A	B	B	A	A
Bn-scaff_23186_1-p102352	C5	59	108.6	A	A	A	B	A	B	A	A	A	A	A	A	A	A	A	B	A	B	B	A	A
Bn-scaff_20270_1-p1070285	C5	60	111.9	A	A	A	A	A	B	A	A	A	A	A	A	A	A	A	B	A	A	B	A	A
Bn-scaff_20376_1-p336606	C5	61	118.6	B	A	A	A	A	B	A	A	A	A	A	A	A	A	B	B	A	A	B	A	A
Bn-Scaffold000191-p47497	C5	62	119.4	B	A	A	A	A	B	A	A	A	A	A	A	A	A	B	B	A	A	B	A	A
Bn-scaff_15676_1-p341508	C5	63	121.9	B	A	A	A	A	B	A	A	A	A	A	A	A	B	B	B	A	A	B	A	A
Bn-scaff_24251_1-p65315	C5	64	123.6	B	A	A	A	A	B	A	A	A	A	A	A	A	B	B	B	A	A	B	A	A
Bn-scaff_15856_1-p129193	C5	65	124.4	B	A	A	A	A	B	A	A	A	A	A	A	A	B	B	B	A	A	B	A	A
Bn-scaff_17799_1-p1180982	C6	1	0	B	A	A	A	A	B	0	A	B	A	A	A	B	A	B	B	0	A	B	A	0
Bn-scaff_17799_1-p1278642	C6	2	1	B	A	A	A	A	B	0	A	B	A	A	A	B	A	B	B	0	A	B	A	0
Bn-scaff_18503_1-p179825	C6	3	2.9	B	A	A	A	A	B	0	A	B	A	A	A	B	A	B	B	0	A	B	A	0
Bn-scaff_17799_1-p1399131	C6	4	7.6	A	A	A	A	A	B	0	A	B	A	A	A	B	A	A	B	0	A	B	A	0
Bn-scaff_17799_1-p1556241	C6	5	9.5	A	A	A	A	A	B	0	A	B	A	A	A	B	A	A	B	0	A	B	A	0
Bn-scaff_17799_1-p1629892	C6	6	10.4	A	A	A	A	A	B	0	A	B	A	A	A	B	A	A	B	0	A	B	A	0
Bn-scaff_17799_1-p1683025	C6	7	12.3	A	A	A	A	A	B	0	A	B	A	A	A	B	A	A	B	0	A	B	A	0
Bn-scaff_17799_1-p2097654	C6	8	13.2	A	A	A	A	A	B	0	A	B	A	A	A	B	A	A	B	0	A	B	A	0
Bn-scaff_17799_1-p2125517	C6	9	14.1	A	A	A	A	A	B	0	A	B	A	A	A	B	A	A	B	0	A	B	A	0
Bn-scaff_17799_1-p2357844	C6	10	15	A	A	A	A	A	B	0	A	B	A	A	A	B	A	A	B	0	A	B	A	0
Bn-scaff_17799_1-p2497023	C6	11	15.9	A	A	A	A	A	A	0	A	B	A	A	A	B	A	A	B	0	A	B	A	0
Bn-scaff_25466_1-p74820	C6	12	18.4	A	A	A	A	A	A	A	A	B	A	A	A	B	A	A	B	A	A	B	A	A
Bn-scaff_16397_1-p636872	C6	13	20.4	A	A	A	A	A	A	A	A	B	A	A	A	B	A	A	B	A	A	B	A	A
Bn-scaff_23957_1-p432810	C6	14	21.3	A	A	A	A	A	A	A	A	B	A	A	A	B	A	A	B	A	A	B	A	A
Bn-scaff_23957_1-p247460	C6	15	22.2	A	A	A	A	A	A	A	A	B	A	A	A	B	A	A	B	A	A	B	A	A
Bn-scaff_18807_1-p529596	C6	16	23.1	A	A	A	A	A	A	A	A	B	A	A	A	B	A	A	B	A	A	B	A	A
Bn-scaff_18807_1-p678687	C6	17	23.1	A	A	A	A	A	A	A	A	B	A	A	A	B	A	A	B	A	A	B	A	A
Bn-scaff_23821_1-p22581	C6	18	23.1	A	A	A	A	A	A	A	A	B	A	A	A	B	A	A	B	A	A	B	A	A
Bn-scaff_17436_1-p260772	C6	19	23.1	A	A	A	A	A	A	A	A	B	A	A	A	B	A	A	B	A	A	B	A	A
Bn-scaff_15892_1-p610800	C6	20	24.8	A	A	A	A	A	A	A	A	B	A	A	A	B	A	A	B	A	A	B	A	A
Bn-scaff_16064_1-p9281	C6	21	25.6	A	A	A	A	A	A	A	A	B	A	A	A	B	A	A	B	A	A	B	B	A
Bn-scaff_18360_1-p134193	C6	22	26.4	A	A	A	A	A	A	A	A	B	A	A	A	B	A	A	B	A	A	B	B	A
Bn-scaff_16064_1-p380597	C6	23	27.2	A	A	A	A	A	A	A	A	B	A	A	A	B	A	A	B	A	A	B	B	A
Bn-scaff_15818_1-p3172268	C6	24	27.2	A	A	A	A	A	A	A	A	B	A	A	A	B	A	A	B	A	A	B	B	A
Bn-scaff_15818_1-p3032871	C6	25	28	A	A	A	A	A	A	A	A	B	A	A	A	A	A	A	B	A	A	B	B	A
Bn-scaff_15818_1-p2371026	C6	26	28.8	A	A	A	A	A	A	A	A	B	A	A	A	A	A	A	B	A	A	B	B	A
Bn-scaff_15763_1-p1492117	C6	27	29.6	A	A	A	A	A	A	A	A	B	A	A	A	A	A	A	B	A	A	B	B	A
Bn-scaff_15763_1-p233149	C6	28	30.4	A	A	A	A	A	A	A	A	B	A	A	A	A	A	A	B	A	A	B	B	A
Bn-scaff_18206_3-p934892	C6	29	31.2	A	A	A	A	A	A	A	A	B	A	A	A	A	A	A	B	A	A	B	B	A
Bn-scaff_15818_2-p1212586	C6	30	32	A	A	A	A	A	A	A	A	B	A	A	A	A	A	A	B	A	A	A	B	A
Bn-scaff_15818_1-p479061	C6	31	32.8	A	A	A	A	A	A	A	A	B	A	A	A	A	A	A	B	A	A	A	B	A
Bn-scaff_18439_1-p304224	C6	32	33.6	A	A	A	A	A	A	A	A	B	A	A	A	A	A	A	B	A	A	A	B	A
Bn-scaff_17984_1-p1076812	C6	33	34.4	A	A	A	A	A	A	A	A	B	A	A	A	A	A	A	B	A	A	A	B	A
Bn-scaff_20773_1-p481244	C6	34	35.2	A	A	A	A	A	A	A	A	B	A	A	A	A	A	A	B	A	A	A	B	A
Bn-scaff_18344_1-p341615	C6	35	36	A	A	A	A	A	A	A	A	B	A	A	A	A	A	A	A	A	A	A	B	A
Bn-scaff_16485_1-p630261	C6	36	36.8	A	A	A	A	A	A	A	A	B	A	A	A	A	A	A	A	A	A	A	B	A
Bn-scaff_16485_1-p587862	C6	37	38.5	A	A	A	A	A	A	A	A	B	A	A	A	A	A	A	A	A	A	A	B	A
Bn-scaff_16984_1-p145108	C6	38	39.3	A	A	A	A	A	A	A	A	B	A	A	A	A	A	A	A	A	A	A	B	A
Bn-A06-p2405022	C6	39	41.8	A	B	A	A	A	A	A	A	B	A	A	A	A	A	A	A	A	A	A	B	A
Bn-scaff_16670_1-p40390	C6	40	42.6	A	B	A	A	A	A	A	A	B	A	A	A	A	A	A	A	A	A	A	B	A

Bn-scaff_24413_1-p361	C7	1	0	A	B	A	A	A	A	B	B	A	B	A	A	A	A	A	A	B	A	A	A	A
Bn-A07-p160648	C7	2	0.8	A	B	A	A	A	A	B	B	A	B	A	A	A	A	A	A	B	A	A	A	A
Bn-scaff_15762_1-p258234	C7	3	2.4	A	B	A	A	A	A	B	B	A	B	A	A	A	A	A	A	B	A	A	A	A
Bn-scaff_17084_1-p161742	C7	4	4	A	B	A	A	A	A	B	B	A	B	A	A	A	A	A	A	B	A	A	A	A
Bn-scaff_20288_1-p344108	C7	5	4.8	A	B	A	A	A	A	B	B	A	B	A	A	A	A	A	A	B	A	A	A	A
Bn-scaff_16721_1-p1032661	C7	6	5.6	A	B	A	A	A	A	B	B	A	B	A	A	A	A	A	B	B	A	A	A	A
Bn-scaff_15626_1-p1084223	C7	7	6.4	A	B	A	A	A	A	B	B	A	B	A	A	A	A	A	B	B	A	A	A	A
Bn-scaff_17326_1-p357798	C7	8	7.2	A	B	A	A	A	A	A	B	A	B	A	A	A	A	A	B	B	A	A	A	A
Bn-A07-p6906296	C7	9	8	A	B	A	A	A	A	A	B	A	B	A	A	A	A	A	B	B	A	A	A	A
Bn-scaff_16450_1-p15270	C7	10	9.6	A	B	B	A	A	A	A	B	A	B	A	A	A	A	A	B	B	A	A	A	A
Bn-scaff_19106_1-p605386	C7	11	12.8	A	B	B	B	A	A	A	B	A	B	A	A	A	A	A	B	B	B	A	A	A
Bn-scaff_23400_1-p613560	C7	12	13.6	A	B	B	B	A	A	A	B	A	B	A	A	A	A	A	B	B	B	A	A	A
Bn-scaff_23400_1-p399811	C7	13	14.4	A	B	B	B	A	A	A	B	A	B	A	A	A	A	A	B	B	B	A	A	A
Bn-scaff_16100_1-p617160	C7	14	15.2	A	B	B	B	A	A	A	B	A	B	A	A	A	A	A	B	B	B	A	A	A
Bn-scaff_16100_1-p890064	C7	15	16	A	B	B	B	A	A	A	B	A	B	A	A	A	A	A	B	B	B	A	A	A
Bn-scaff_18501_1-p470034	C7	16	16.8	A	B	B	B	A	A	A	B	A	B	A	A	A	A	A	B	B	B	B	A	A
Bn-scaff_18202_1-p1683343	C7	17	20	A	B	B	B	A	A	A	B	B	B	A	A	A	A	A	B	B	B	B	A	A
Bn-scaff_18202_1-p1615492	C7	18	20.8	A	B	B	B	A	A	A	B	B	B	A	A	A	A	A	B	B	B	B	A	A
Bn-scaff_18202_1-p1088668	C7	19	24.8	A	B	B	B	A	A	A	B	B	B	A	A	A	A	A	B	B	B	B	A	A
Bn-scaff_18202_1-p986219	C7	20	25.6	A	B	B	B	A	A	A	B	B	B	A	A	A	A	A	B	B	B	B	A	A
Bn-scaff_18202_1-p871410	C7	21	26.4	A	B	B	B	A	A	A	B	B	B	A	A	A	A	A	B	B	B	B	A	A
Bn-scaff_18202_1-p569134	C7	22	28	A	B	B	B	A	A	A	B	B	B	A	A	A	A	A	B	B	B	B	A	A
Bn-scaff_22821_1-p13976	C7	23	28.8	A	B	B	B	A	A	A	B	B	B	A	A	A	A	A	B	B	B	B	A	A
Bn-scaff_15712_1-p841920	C7	24	29.6	A	B	B	B	A	A	A	B	B	B	A	A	A	A	A	B	B	B	B	A	A
Bn-scaff_15754_1-p240103	C7	25	30.4	A	B	B	B	A	A	A	B	B	B	A	A	A	A	A	B	B	B	B	A	A
Bn-scaff_15754_1-p651840	C7	26	32.8	A	B	B	B	A	A	A	B	B	B	A	A	A	A	A	B	B	B	B	A	A
Bn-scaff_15754_1-p688470	C7	27	33.6	A	B	B	B	A	A	A	B	B	B	A	A	A	A	A	B	B	B	B	A	A
Bn-scaff_16130_1-p542753	C7	28	34.4	A	B	B	B	A	A	A	B	B	B	A	A	A	A	A	B	B	B	B	A	A
Bn-scaff_16130_1-p659049	C7	29	35.2	A	B	B	B	A	A	A	B	B	B	A	A	A	A	A	B	B	B	B	A	A
Bn-scaff_16130_1-p1091410	C7	30	36	A	B	B	B	A	A	A	B	B	B	A	A	A	A	A	B	B	B	B	A	A
Bn-scaff_16130_1-p2525629	C7	31	36.8	A	B	B	B	A	A	A	B	B	B	A	A	A	A	A	B	B	B	B	A	A
Bn-scaff_16514_1-p47789	C7	32	40.8	A	B	B	A	A	A	A	B	B	B	A	A	A	A	A	B	B	A	B	A	A
Bn-scaff_17972_1-p279228	C7	33	42.4	A	B	B	A	A	A	A	A	B	B	A	A	A	A	A	B	B	A	B	A	A
Bn-scaff_21711_1-p54607	C7	34	43.2	A	B	B	A	A	A	A	A	B	B	A	A	A	A	A	B	B	A	B	A	A
Bn-scaff_21711_1-p39478	C7	35	45.6	A	B	B	A	A	A	A	A	B	B	A	A	A	A	A	B	B	A	B	A	A
Bn-scaff_21711_1-p34999	C7	36	46.4	A	B	B	A	A	A	A	A	B	B	A	A	A	A	A	B	B	A	B	A	A
Bn-scaff_18520_1-p486331	C7	37	47.2	A	B	B	A	A	A	A	A	B	B	A	A	A	A	A	B	B	A	B	A	A
Bn-scaff_18520_1-p603560	C7	38	48	A	B	B	A	A	A	A	A	B	A	A	A	A	A	A	B	B	A	B	A	A
Bn-scaff_18520_1-p814291	C7	39	48.8	A	B	B	A	A	A	A	A	B	A	A	B	A	A	A	B	B	A	B	A	A
Bn-scaff_15705_1-p38968	C7	40	49.6	A	B	B	A	A	A	A	A	B	A	A	B	B	A	A	B	B	A	B	A	A
Bn-scaff_15705_1-p432224	C7	41	50.4	A	B	B	A	A	A	A	A	B	A	A	B	B	A	A	B	B	A	B	A	A
Bn-scaff_15705_1-p577327	C7	42	51.2	A	B	B	A	A	A	A	A	B	A	A	B	B	A	A	B	B	A	B	A	A
Bn-scaff_15705_1-p1214955	C7	43	52	A	B	B	A	A	A	B	A	B	A	A	B	B	A	A	B	B	A	B	A	A
Bn-scaff_15705_1-p1533707	C7	44	52.8	A	B	B	A	A	A	B	A	B	A	A	B	B	A	A	B	B	A	B	A	A
Bn-scaff_15705_1-p2248267	C7	45	54.4	A	B	B	A	A	A	B	A	B	A	A	B	B	A	A	B	B	A	B	A	A
Bn-scaff_15705_1-p2274493	C7	46	56.8	A	B	B	A	A	A	B	A	B	A	B	B	B	A	A	B	B	A	B	A	A
Bn-scaff_15705_3-p131881	C7	47	58.4	A	B	B	A	A	A	B	A	B	A	B	B	B	A	A	B	B	A	B	A	A
Bn-scaff_15705_3-p245960	C7	48	59.2	A	B	B	A	A	A	B	A	B	A	B	B	B	A	A	B	B	A	B	A	A
Bn-scaff_15705_3-p436841	C7	49	60	A	B	B	A	A	A	B	A	B	A	B	B	B	A	A	B	B	A	B	A	A

Bn-scaff_16069_1-p261409	C7	50	60.8	A	B	B	A	A	A	B	A	B	A	B	B	B	A	A	B	B	A	B	A	A
Bn-scaff_16069_1-p726389	C7	51	61.6	A	B	B	A	A	A	B	A	B	A	B	B	B	A	A	B	B	A	B	A	A
Bn-scaff_16069_1-p730213	C7	52	62.4	A	B	B	A	A	A	B	A	B	A	B	B	B	A	A	B	B	A	B	A	A
Bn-scaff_16231_1-p1398728	C7	53	63.2	A	B	B	A	A	A	B	A	B	A	B	B	B	A	A	B	B	A	B	A	A
Bn-scaff_16069_1-p2205590	C7	54	66.4	A	B	B	A	A	A	B	A	B	A	B	B	B	A	A	B	B	A	B	A	A
Bn-scaff_16069_1-p2222349	C7	55	67.2	A	B	B	A	A	A	B	A	B	A	B	B	B	A	A	B	B	A	B	A	A
Bn-scaff_16069_1-p2344701	C7	56	68	A	B	B	A	A	A	B	A	B	A	B	B	B	A	A	B	B	A	B	A	A
Bn-scaff_16069_1-p2946061	C7	57	69.6	A	B	B	A	A	A	B	A	B	A	B	B	B	A	A	B	B	A	B	A	A
Bn-scaff_16069_1-p3135269	C7	58	71.2	A	B	B	A	A	A	B	B	B	A	B	B	B	A	A	B	B	A	B	A	A
Bn-scaff_16069_1-p3232221	C7	59	72	A	B	B	A	A	A	B	B	B	A	B	B	B	A	A	B	B	A	B	A	A
Bn-scaff_16069_1-p3446432	C7	60	72.8	A	B	B	A	A	A	B	B	B	A	B	B	B	A	A	A	B	A	B	A	A
Bn-scaff_16069_1-p3454723	C7	61	73.6	A	B	B	A	A	A	B	B	B	A	B	B	B	A	A	A	B	A	B	A	A
Bn-scaff_16069_1-p3861034	C7	62	76	A	B	B	A	A	A	B	B	B	A	B	B	B	A	A	A	B	A	B	A	A
Bn-scaff_16069_1-p3945232	C7	63	76.8	A	B	B	A	A	A	B	B	B	A	B	B	B	A	A	A	B	A	B	A	A
Bn-scaff_16069_1-p4306874	C7	64	77.6	A	B	B	A	A	A	B	B	B	A	B	B	B	A	A	A	B	A	B	A	A
Bn-scaff_16069_1-p4546846	C7	65	82.5	A	B	B	B	A	A	B	B	B	A	B	B	B	A	A	A	B	B	B	A	A
Bn-scaff_16069_1-p4605280	C7	66	84.1	A	B	B	B	A	A	B	B	B	A	B	B	B	A	A	A	B	B	B	A	A
Bn-scaff_16069_1-p4609643	C7	67	84.9	A	B	B	B	A	A	B	B	B	B	B	B	B	A	A	A	B	B	B	A	A
Bn-scaff_16110_1-p3075478	C7	68	86.5	A	B	A	B	A	A	B	B	B	B	B	B	B	A	A	A	B	B	B	A	A
Bn-scaff_16110_1-p2950205	C7	69	87.3	A	B	A	B	A	A	B	B	B	B	B	B	B	A	A	A	B	B	B	A	A
Bn-scaff_16110_1-p2890287	C7	70	88.1	A	B	A	B	A	A	B	B	B	B	B	B	B	A	A	A	B	B	B	A	A
Bn-scaff_16110_1-p2655836	C7	71	91.3	A	B	A	B	A	A	B	B	B	B	B	B	B	A	A	A	B	B	B	A	A
Bn-scaff_16110_1-p2598394	C7	72	92.1	A	B	A	B	A	A	B	B	B	B	B	B	B	A	A	A	B	B	B	A	A
Bn-scaff_16110_1-p2576509	C7	73	92.9	A	B	A	B	A	A	B	B	B	B	B	B	B	A	A	A	B	B	B	A	A
Bn-scaff_16110_1-p2168404	C7	74	92.9	A	B	A	B	A	A	B	B	B	B	B	B	B	A	A	A	B	B	B	A	A
Bn-scaff_16110_1-p2030050	C7	75	94.5	A	B	A	B	A	A	A	B	B	B	B	B	B	A	A	A	B	B	B	A	A
Bn-scaff_16110_1-p1971697	C7	76	95.3	A	B	A	B	A	A	A	B	B	B	B	B	B	A	A	A	B	B	B	A	A
Bn-A03-p29053385	C7	77	96.1	A	B	A	B	A	A	A	B	B	B	B	B	B	A	A	A	B	B	B	A	A
Bn-scaff_16110_1-p1689916	C7	78	96.9	A	B	A	B	A	A	A	B	B	B	B	B	A	A	A	A	B	B	B	A	A
Bn-scaff_16110_1-p1451801	C7	79	97.7	A	B	A	B	A	A	A	B	B	B	B	B	A	A	A	A	B	B	B	A	A
Bn-scaff_16110_1-p1204999	C7	80	98.5	A	B	A	B	A	A	A	B	B	B	B	B	A	B	A	A	B	B	B	A	A
Bn-scaff_16110_1-p670992	C7	81	100.9	A	B	A	B	A	A	A	B	B	B	B	B	A	B	A	A	B	B	B	A	A
Bn-scaff_16110_1-p111601	C7	82	101.7	A	B	A	B	A	A	A	B	B	A	B	B	A	B	A	A	B	B	B	A	A
Bn-scaff_20796_1-p34573	C8	1	0	B	B	A	B	A	B	A	A	A	A	B	A	A	A	B	A	A	B	A	A	A
Bn-scaff_25981_1-p240709	C8	2	1.6	B	B	A	B	A	B	A	A	A	A	B	A	A	A	B	A	A	B	A	A	A
Bn-A01-p10059043	C8	3	2.4	B	B	B	B	A	B	A	A	A	A	B	A	A	A	B	A	A	B	A	A	A
Bn-scaff_15705_1-p2909077	C8	4	3	B	B	B	B	A	B	A	A	A	A	B	A	A	A	B	A	A	B	A	A	A
Bn-scaff_17591_1-p461524	C8	5	4	B	B	B	B	A	B	A	A	A	A	B	A	A	A	B	A	A	B	A	A	A
Bn-scaff_19104_1-p662624	C8	6	5	B	B	B	B	A	B	A	A	A	A	B	A	A	A	B	A	A	B	A	A	A
Bn-scaff_15699_1-p1234178	C8	7	6	B	B	B	B	A	B	A	A	A	A	B	A	A	A	B	A	A	B	A	A	A
Bn-scaff_19242_1-p421335	C8	8	7	B	B	B	B	B	B	A	A	A	A	B	A	A	A	B	B	A	B	A	A	A
Bn-scaff_26859_1-p8813	C8	9	9	B	B	B	B	B	B	A	A	A	A	B	A	A	A	B	B	B	B	A	A	A
Bn-scaff_17227_1-p299956	C8	10	10	B	B	B	B	B	B	A	A	A	A	B	A	A	A	B	B	B	B	A	A	A
Bn-scaff_17227_1-p700248	C8	11	11	B	B	B	B	B	B	A	A	A	A	B	A	A	A	B	B	B	B	A	A	A
Bn-scaff_17227_1-p1380795	C8	12	15	B	B	B	B	B	B	A	A	A	A	B	A	A	A	B	B	B	B	A	A	A
Bn-scaff_21786_1-p127359	C8	13	16	B	B	B	B	B	B	A	A	A	A	B	A	A	A	B	B	B	B	A	A	A
Bn-scaff_21786_1-p149338	C8	14	18	B	B	B	B	B	B	A	A	A	A	B	A	A	A	B	B	B	B	A	A	A
Bn-scaff_16231_1-p2213239	C8	15	20	B	B	B	B	B	B	A	A	A	A	B	A	A	A	B	B	B	B	A	A	A
Bn-scaff_16231_1-p1274962	C8	16	25	B	A	B	B	B	B	A	A	A	A	B	A	A	A	B	B	B	B	A	A	A

Bn-scaff_16231_1-p1231804	C8	17	29	B	A	B	B	B	B	A	A	A	A	B	A	A	A	B	B	B	B	A	A	A
Bn-scaff_16231_1-p1189052	C8	18	31	B	A	B	B	B	B	A	A	A	A	A	A	A	A	B	B	B	B	A	A	A
Bn-scaff_16231_1-p795075	C8	19	32	B	A	B	B	B	B	A	A	A	A	A	A	A	A	B	B	B	B	A	A	A
Bn-scaff_16231_1-p715762	C8	20	33	B	A	B	B	B	B	B	A	A	A	A	A	A	A	B	B	B	B	A	A	A
Bn-scaff_16231_1-p296794	C8	21	35	B	A	B	B	B	B	B	A	B	A	A	A	A	A	B	B	B	B	A	A	A
Bn-scaff_17565_1-p6212	C8	22	38	B	A	B	B	B	B	B	A	B	A	A	A	A	A	B	B	B	B	A	A	A
Bn-scaff_15923_1-p489042	C8	23	40	B	A	B	B	B	B	B	A	B	A	A	A	A	A	B	B	B	B	A	A	A
Bn-scaff_15923_1-p46551	C8	24	43	B	A	B	B	B	B	B	A	B	A	A	A	A	A	B	B	B	B	A	A	A
Bn-scaff_17807_1-p192053	C8	25	43	B	A	B	B	B	B	B	A	B	A	A	A	A	A	B	B	B	B	A	A	A
Bn-scaff_16174_1-p1072915	C8	26	46	B	A	B	B	B	B	B	A	B	A	A	A	A	A	B	B	B	B	A	A	A
Bn-scaff_16770_1-p1137429	C8	27	47	B	A	B	B	B	B	B	A	B	A	A	A	A	B	B	B	B	B	A	A	A
Bn-scaff_16770_1-p2986353	C8	28	47	B	A	B	B	B	B	B	A	B	A	A	A	A	B	B	B	B	B	A	A	A
Bn-scaff_18849_1-p101900	C8	29	48	B	A	B	B	B	B	B	A	B	A	A	A	A	B	B	B	B	B	A	A	A
Bn-scaff_16770_1-p3389504	C8	30	49	B	A	B	B	B	B	B	A	B	A	A	A	A	B	B	B	B	B	A	A	A
Bn-scaff_16770_1-p4071245	C8	31	55	A	A	B	B	B	B	B	A	B	A	A	A	A	B	A	B	B	B	A	A	A
Bn-scaff_16770_1-p4331835	C8	32	55	A	A	B	B	B	B	B	A	B	A	A	A	A	B	A	B	B	B	A	A	A
Bn-scaff_28637_1-p15131	C8	33	56	A	A	B	B	B	B	B	A	B	A	A	A	A	B	A	B	B	B	A	A	A
Bn-scaff_16361_1-p901728	C8	34	58	A	A	B	B	B	B	B	A	B	A	A	A	A	B	A	B	B	B	A	A	A
Bn-scaff_16361_1-p1072715	C8	35	59	A	A	B	B	B	B	B	A	B	A	A	A	A	B	A	B	B	B	A	A	A
Bn-scaff_16361_1-p1324039	C8	36	59	A	A	B	B	B	B	B	A	B	A	A	A	A	B	A	B	B	B	A	A	A
Bn-scaff_16361_1-p1387122	C8	37	60	A	A	B	B	B	B	B	A	B	A	A	A	A	B	A	B	B	B	A	A	A
Bn-scaff_16361_1-p1402908	C8	38	61	A	A	B	B	B	B	B	A	B	A	A	A	A	B	A	B	B	B	A	A	A
Bn-scaff_16361_1-p1943766	C8	39	63	A	A	B	B	B	B	B	A	A	A	A	A	A	B	A	B	B	B	A	A	A
Bn-scaff_16361_1-p3120761	C8	40	66	A	A	B	A	B	B	B	A	A	A	A	A	A	B	A	B	B	A	A	A	A
Bn-scaff_16197_1-p3070176	C8	41	67	A	A	B	A	B	B	B	A	A	A	A	A	A	B	A	B	B	A	A	A	A
Bn-scaff_16197_1-p2144262	C8	42	67	A	A	B	A	B	B	B	A	A	A	A	A	A	B	A	B	B	A	A	A	A
Bn-scaff_16197_1-p1912964	C8	43	68	A	A	B	A	B	B	B	A	A	B	A	A	A	B	A	B	B	A	A	A	A
Bn-scaff_16197_1-p222159	C8	44	72	A	A	B	A	B	B	B	A	A	B	A	B	A	B	A	B	B	A	A	A	A
Bn-scaff_16445_1-p2601107	C8	45	73	A	A	B	A	B	B	B	A	A	B	A	B	A	B	A	B	B	A	A	A	A
Bn-A09-p33660289	C8	46	75	0	A	B	A	B	B	B	A	A	B	A	B	A	B	A	B	B	A	0	A	A
Bn-scaff_16445_1-p933254	C8	47	79	0	B	B	A	B	B	B	A	A	B	A	B	A	B	A	B	B	A	0	A	A
Bn-scaff_16445_1-p856678	C8	48	83	0	B	B	A	B	B	B	A	A	B	A	B	A	B	A	B	B	A	0	A	A
Bn-scaff_16445_1-p766648	C8	49	85	0	B	B	A	B	B	B	A	A	B	A	B	A	B	A	B	B	A	0	A	A
Bn-scaff_16445_1-p594145	C8	50	86	0	B	B	A	B	B	B	A	A	B	A	B	A	B	A	B	B	A	A	A	A
Bn-scaff_16445_1-p31451	C8	51	88	0	B	A	A	B	B	B	A	A	B	A	B	A	B	A	B	B	A	A	B	A
Bn-scaff_21269_1-p204828	C8	52	92	0	B	A	A	B	B	B	A	A	B	B	B	A	B	A	B	B	A	A	B	A
Bn-scaff_16389_1-p660552	C8	53	98	0	B	A	A	B	B	B	A	B	B	B	B	A	B	A	B	B	A	B	B	A
Bn-scaff_16021_1-p613351	C8	54	100	0	B	A	A	B	B	A	A	B	B	B	B	A	B	A	B	B	A	B	B	A
Bn-scaff_20131_1-p76620	C9	1	0	A	B	B	B	B	A	A	A	A	A	B	B	A	B	A	B	A	B	A	B	A
Bn-A09-p281844	C9	2	4.9	A	B	B	B	B	A	A	A	A	A	B	B	A	B	A	B	A	B	A	B	A
Bn-A01-p26921609	C9	3	6.5	A	B	B	B	B	A	A	A	A	A	B	B	A	B	A	B	A	B	A	B	A
Bn-scaff_17526_1-p1649875	C9	4	10.6	A	B	B	B	B	A	A	B	A	A	B	B	A	B	A	B	A	B	A	B	A
Bn-scaff_17526_1-p1470085	C9	5	12.2	A	B	B	B	B	A	A	B	A	A	B	B	A	B	A	B	A	B	A	B	A
Bn-scaff_17526_1-p1091087	C9	6	13.8	A	B	B	B	B	A	A	B	A	A	B	B	A	B	A	B	A	B	B	B	A
Bn-scaff_16721_1-p360689	C9	7	23.7	A	B	B	B	B	A	A	B	A	A	B	A	A	B	A	B	B	B	B	B	A
Bn-scaff_19783_1-p327125	C9	8	24.5	A	B	B	B	B	A	A	B	A	A	B	A	A	B	A	B	B	B	B	B	A
Bn-A09-p3233005	C9	9	25.3	A	B	B	B	B	A	A	B	A	A	B	A	A	B	A	B	B	B	B	B	A
Bn-scaff_17190_1-p237949	C9	10	26.1	A	B	B	B	B	A	A	B	A	B	B	A	A	B	A	B	B	B	B	B	A
Bn-scaff_17190_1-p660881	C9	11	27.7	A	B	B	B	B	A	A	B	B	B	B	A	A	B	A	B	B	B	B	B	A

Bn-scaff_17190_1-p1119408	C9	12	28.5	A	B	B	B	B	A	A	B	B	B	B	A	B	B	A	B	B	B	B	B	A
Bn-scaff_17297_1-p143112	C9	13	29.3	A	B	B	B	B	A	A	B	B	B	B	A	B	B	A	B	B	B	B	B	A
Bn-scaff_17423_1-p376558	C9	14	30.1	A	B	B	B	B	A	A	B	B	B	B	A	B	B	A	B	B	B	B	B	A
Bn-scaff_17084_1-p760594	C9	15	31.7	A	B	B	B	B	A	A	B	B	B	B	A	B	B	A	B	B	B	B	B	A
Bn-scaff_17487_1-p1888000	C9	16	37.4	A	A	B	B	B	A	A	B	B	B	B	A	B	B	A	B	B	B	B	B	A
Bn-scaff_20428_1-p256051	C9	17	39	A	A	B	B	B	A	A	B	B	B	B	A	B	B	A	B	B	B	B	B	A
Bn-scaff_17683_1-p28746	C9	18	39.8	A	A	B	B	A	A	A	B	B	B	B	A	B	B	A	B	B	B	B	B	A
Bn-scaff_28725_1-p72570	C9	19	40.6	A	A	B	B	A	A	A	B	B	B	B	A	B	B	A	B	B	B	B	B	A
Bn-scaff_23618_1-p120152	C9	20	41	A	A	B	B	A	A	A	B	B	B	B	A	B	B	A	B	B	B	B	B	A
Bn-scaff_16912_1-p13193	C9	21	43	A	A	B	B	A	A	A	B	B	B	B	A	B	B	A	B	B	B	B	B	A
Bn-scaff_17174_1-p88911	C9	22	44	A	A	B	B	A	A	A	B	B	B	B	A	B	B	A	B	B	B	B	B	A
Bn-scaff_18100_1-p541042	C9	23	45	A	A	B	B	A	A	A	B	B	B	B	A	B	B	A	B	B	B	B	B	A
Bn-scaff_17888_1-p378622	C9	24	46	A	A	B	B	A	A	A	B	B	B	B	A	B	B	A	B	B	B	B	B	A
Bn-scaff_19691_1-p105535	C9	25	49	A	A	B	B	A	A	A	B	B	B	B	A	B	B	A	B	B	B	B	B	A
Bn-scaff_20972_1-p163951	C9	26	50	A	A	B	B	A	A	A	B	B	B	B	A	B	B	A	B	A	B	B	B	A
Bn-scaff_15712_7-p58147	C9	27	56	B	A	B	B	A	A	A	B	B	B	B	A	B	B	B	B	A	B	B	B	A
Bn-scaff_15743_1-p26884	C9	28	57	B	A	B	B	A	A	A	B	B	B	B	A	B	B	B	B	A	B	B	B	A
Bn-scaff_23622_1-p3989	C9	29	58	B	A	B	B	A	A	A	B	B	B	B	A	B	B	B	B	A	B	B	B	A
Bn-scaff_15881_1-p139441	C9	30	59	B	A	B	B	A	A	A	B	B	B	B	A	B	B	B	B	A	B	B	B	A
Bn-scaff_17799_1-p3029724	C9	31	60	B	A	B	B	A	A	A	B	B	B	B	A	B	B	B	B	A	B	B	B	A
Bn-scaff_17088_1-p146561	C9	32	63	B	A	B	A	A	A	A	B	B	B	B	A	B	B	B	B	A	A	B	B	A
Bn-scaff_19899_1-p559187	C9	33	64	B	A	B	A	A	A	A	B	B	B	B	A	B	A	B	B	A	A	B	B	A
Bn-scaff_19899_1-p283181	C9	34	68	B	A	B	A	A	A	A	B	B	B	B	A	B	A	B	B	A	A	B	B	A
Bn-scaff_19899_1-p34734	C9	35	69	B	A	B	A	A	A	A	B	B	B	B	A	B	A	B	B	A	A	B	B	A
Bn-scaff_15576_1-p614226	C9	36	70	B	A	B	A	A	A	A	B	B	B	B	A	B	A	B	B	A	A	B	B	A
Bn-C13791383-p286	C9	37	71	B	A	B	A	A	A	A	B	B	B	B	A	B	A	B	B	A	A	B	B	A
Bn-scaff_27705_1-p129876	C9	38	72	B	A	B	A	A	A	A	B	B	B	B	A	B	A	B	B	A	A	B	B	A
Bn-scaff_17028_1-p9587	C9	39	73	B	A	B	A	A	A	A	B	B	B	B	A	B	A	B	B	A	A	B	B	A
Bn-scaff_17028_1-p396877	C9	40	76	B	A	B	A	A	A	A	B	B	B	B	A	B	A	B	B	A	A	B	B	A
Bn-A10-p12957851	C9	41	80	B	A	B	A	A	A	A	B	B	B	B	A	B	A	B	B	A	A	B	B	A
Bn-scaff_16362_1-p655565	C9	42	81	B	A	B	A	A	A	A	B	B	B	B	A	B	A	B	B	A	A	B	B	A
Bn-scaff_16362_1-p408802	C9	43	82	B	A	B	A	A	A	A	B	B	B	B	A	B	A	B	B	A	A	B	B	A
Bn-A02-p3607564	C9	44	85	B	A	B	A	A	A	A	B	B	B	B	A	B	A	B	B	A	A	B	A	A
Bn-scaff_22082_1-p381255	C9	45	86	B	A	A	A	A	A	A	B	B	B	B	A	B	A	B	B	A	A	B	A	A
Bn-scaff_24108_1-p456844	C9	46	86	B	A	A	A	A	A	A	B	B	B	B	A	A	A	B	B	A	A	B	A	A
Bn-scaff_17109_1-p1959932	C9	47	87	B	A	A	A	A	A	A	B	B	B	B	A	A	A	B	B	A	A	B	A	A
Bn-scaff_17109_1-p1861938	C9	48	88	B	A	A	A	A	A	A	B	B	B	B	A	A	A	B	B	A	A	A	A	A
Bn-scaff_17109_2-p370345	C9	49	90	B	A	A	A	A	A	A	B	B	B	B	A	A	A	B	B	A	A	A	A	A
Bn-scaff_17750_1-p2082478	C9	50	90	B	A	A	A	A	A	A	B	B	A	B	A	A	A	B	B	A	A	A	A	A
Bn-scaff_17750_1-p1923167	C9	51	93	B	A	A	A	A	A	A	B	B	A	A	A	A	A	B	B	A	A	A	A	A
Bn-scaff_17750_1-p1801981	C9	52	94	B	A	A	A	A	A	A	B	B	A	A	A	A	A	B	B	A	A	A	A	A
Bn-scaff_17750_1-p1326856	C9	53	95	B	A	A	A	A	A	A	B	B	A	A	A	A	A	B	B	A	A	A	A	A
Bn-scaff_17750_1-p1243511	C9	54	95	B	A	A	A	A	A	A	B	B	A	A	A	A	A	B	B	A	A	A	A	0
Bn-scaff_17750_1-p1166353	C9	55	96	B	A	A	A	A	A	A	B	B	A	A	A	A	A	B	B	A	A	A	A	0
Bn-scaff_17750_1-p969043	C9	56	97	B	A	A	A	A	A	A	B	B	A	A	A	A	A	B	B	A	A	A	A	0
Bn-scaff_17750_1-p779325	C9	57	99	B	A	A	A	A	A	A	B	B	A	A	A	A	A	B	B	A	A	A	A	0
Bn-scaff_28053_1-p12340	C9	58	106	B	A	A	A	A	A	A	B	A	A	A	A	A	A	B	B	A	A	A	A	0

SG_201	SG_29	SG_107	SG_166	SG_233	SG_262	SG_54	SG_215	SG_33	SG_108	SG_171	SG_241	SG_265	SG_67	SG_24	SG_42	SG_118	SG_172	SG_243	SG_267	SG_121	SG_290	SG_57	SG_119	SG_208	SG_246	SG_269	SG_146	SG_318	SG_64	SG_122	SG_216	SG_247	SG_271	SG_100	SG_115	SG_120	SG_130	SG_135	SG_165	SG_168	SG_175	SG_178	SG_19	SG_204	SG_209
B	B	A	B	B	A	A	B	B	A	B	B	A	B	B	0	B	B	A	0	B	B	B	B	B	B	B	0	0	A	B	B	A	B	B	A	B	A	0	0	B	0	B	B	B	A
B	B	A	B	B	A	A	B	B	A	B	B	B	B	B	0	B	B	A	0	B	B	B	B	B	B	B	0	0	A	B	B	A	B	B	A	B	A	0	0	B	0	B	B	B	A
B	B	A	B	B	A	0	B	B	A	B	B	B	B	B	0	B	B	A	B	B	B	B	B	B	B	B	0	A	A	B	B	A	B	B	A	B	A	0	0	B	0	B	B	B	A
B	B	A	B	B	A	0	B	B	A	B	B	B	B	B	0	B	B	A	B	B	B	B	B	B	B	B	0	A	A	B	B	A	B	B	A	B	A	0	0	B	0	B	B	B	A
B	B	A	B	B	A	0	B	B	A	B	B	B	B	B	0	B	B	A	B	B	B	B	B	B	B	B	0	A	A	B	B	A	B	B	A	B	A	0	0	B	0	B	B	B	A
B	B	A	B	B	A	0	B	B	A	B	B	B	B	B	0	B	B	B	B	B	B	B	B	B	B	B	0	A	A	B	B	B	B	B	A	B	A	0	0	B	0	B	B	B	A
B	B	A	B	B	A	0	B	B	A	B	B	B	B	B	0	B	B	B	B	B	B	B	B	B	B	B	0	A	A	B	B	B	B	B	A	B	A	0	0	B	0	B	B	B	A
B	B	A	B	B	A	0	B	B	A	B	B	B	B	B	0	B	B	B	B	B	B	B	B	B	B	B	0	A	A	B	B	B	B	B	A	B	A	0	0	B	0	B	B	B	A
B	B	A	B	B	B	0	B	B	A	B	B	B	B	B	0	B	B	B	B	B	B	B	B	B	B	B	0	A	A	B	B	B	B	B	A	B	A	0	0	B	0	B	B	B	A
B	B	A	B	B	B	0	B	B	A	B	B	B	B	B	0	B	B	B	B	B	B	B	B	B	B	B	0	A	A	B	B	B	B	B	A	B	A	0	0	B	0	B	B	B	A
B	B	A	B	B	B	0	B	B	A	B	A	B	B	B	0	B	B	B	B	B	B	B	B	B	B	B	0	A	A	B	B	B	B	B	A	B	A	0	0	B	0	B	B	B	A
B	B	A	B	B	B	0	B	B	A	B	A	B	B	B	0	B	B	B	B	B	B	B	B	B	B	B	0	A	A	B	B	B	B	B	A	B	A	0	0	B	0	B	B	B	A
B	B	A	B	B	B	0	B	B	A	B	A	B	B	B	0	B	B	B	B	B	B	B	B	B	B	B	0	A	B	B	B	B	B	B	A	B	A	0	0	B	0	B	B	B	A
B	B	0	B	B	B	0	B	B	0	B	A	B	B	B	0	B	B	B	B	B	B	B	B	B	B	B	0	B	B	B	B	B	B	B	A	B	A	0	0	B	0	B	B	B	A
B	B	0	B	B	B	0	B	B	0	B	A	B	B	B	0	B	B	B	B	A	B	B	B	B	B	B	0	B	B	B	B	B	B	B	A	B	A	0	0	B	0	B	B	B	A
B	B	0	B	B	B	0	A	B	0	B	A	B	B	B	0	B	B	B	B	A	B	B	B	B	B	B	0	B	B	B	B	B	B	B	A	B	A	0	0	B	0	B	B	B	A
B	B	0	B	B	B	B	A	B	0	B	A	B	B	B	0	B	B	B	B	A	B	B	B	B	B	B	0	B	B	B	B	B	B	B	A	B	A	0	0	B	0	B	A	B	A
B	B	0	B	B	B	B	A	B	0	B	A	B	B	B	0	B	B	B	B	A	B	B	B	B	B	B	0	B	B	B	B	B	B	B	A	B	A	0	0	B	0	B	A	B	A
B	B	0	B	B	B	B	A	B	0	B	A	B	B	B	0	B	B	B	B	A	B	B	B	B	B	B	0	B	B	B	B	B	B	B	A	B	A	0	A	B	0	B	A	B	A
B	B	B	B	B	B	B	A	B	B	B	A	B	B	B	A	B	B	B	B	A	B	B	B	B	B	B	A	B	B	B	B	B	B	B	A	B	A	A	A	B	A	B	A	B	A
B	B	B	B	B	B	B	A	B	B	B	A	B	B	B	A	B	B	B	B	A	B	B	B	B	B	B	A	B	B	B	B	B	B	B	A	B	A	A	A	B	A	B	A	A	A
B	B	B	B	B	B	B	A	B	B	B	A	B	A	B	A	B	B	B	B	A	B	B	B	B	B	B	A	B	B	B	B	B	B	B	A	B	A	A	A	B	A	B	A	A	A
B	B	B	B	B	B	B	A	B	B	B	A	B	A	B	A	B	B	B	B	A	B	B	B	B	B	B	A	B	B	B	B	B	B	B	A	B	A	A	A	B	A	0	A	A	A
B	B	B	B	B	B	B	A	B	B	B	A	B	A	B	A	B	B	B	B	A	B	B	B	B	B	B	A	B	B	B	B	B	B	B	A	B	A	A	A	B	A	0	A	A	A
B	B	B	B	B	B	B	A	B	B	B	A	B	A	B	A	B	B	B	B	A	B	B	B	B	B	B	A	B	B	B	B	B	B	B	A	B	A	B	A	B	A	0	A	A	A
B	B	B	B	B	B	B	A	B	B	B	A	B	A	B	A	B	B	B	B	A	B	B	B	B	B	B	A	B	B	B	B	B	B	B	A	B	A	B	A	B	A	0	A	A	A
B	B	B	B	B	B	B	A	B	B	B	A	B	A	B	A	B	B	B	B	A	B	B	B	B	B	B	A	B	B	B	B	B	B	B	A	B	A	B	A	B	A	0	A	A	A
B	B	B	B	B	B	B	A	B	B	B	A	B	A	B	A	B	B	B	B	A	B	B	B	B	B	B	A	B	B	B	B	B	B	B	A	B	A	B	A	B	A	0	A	A	A
B	B	B	B	B	B	B	A	B	B	A	A	B	A	B	A	B	B	B	B	A	B	B	B	B	B	B	A	B	B	B	B	B	B	B	A	B	A	B	A	A	A	0	A	A	A
B	B	B	B	B	B	B	A	B	B	A	A	B	A	B	A	B	B	B	B	A	B	B	B	B	B	B	B	B	B	B	B	B	B	B	A	B	A	B	A	A	A	0	A	A	A
B	B	B	B	B	B	B	A	B	B	A	A	B	A	B	A	B	B	B	B	A	B	B	B	B	B	B	B	B	B	B	B	B	B	B	A	B	A	B	A	A	A	0	A	A	A
B	B	B	B	B	B	B	A	0	B	A	A	B	A	B	A	B	B	B	B	A	B	B	B	B	B	B	B	B	B	B	B	B	B	B	A	B	A	B	A	A	A	0	A	A	A
B	B	B	B	B	B	B	A	0	B	A	A	B	A	B	A	B	B	B	B	A	B	B	B	B	B	B	B	B	B	B	B	B	B	B	B	B	A	B	A	A	A	0	A	A	A
B	B	B	B	B	B	B	A	0	B	A	A	B	A	B	A	B	B	B	B	A	B	B	B	B	B	B	B	B	B	B	B	B	B	B	B	B	A	B	A	A	A	0	A	A	A
B	B	B	B	B	B	B	A	0	B	A	A	B	A	B	B	B	B	B	B	A	B	B	B	B	B	B	B	B	B	B	B	B	B	B	B	B	A	B	A	A	A	0	A	A	A
B	B	B	B	B	B	B	A	0	B	A	A	B	A	B	B	B	B	B	B	A	B	B	B	B	A	B	B	B	B	B	B	B	B	B	B	B	A	B	A	A	A	0	A	A	A
B	B	B	B	B	B	B	A	0	B	A	A	B	A	B	B	B	B	B	B	A	B	B	B	B	A	B	B	B	B	B	B	B	B	B	B	B	A	B	A	A	A	0	A	A	A
B	B	B	B	B	B	B	A	B	B	A	A	B	A	B	B	B	B	B	B	A	B	B	B	B	A	B	B	B	B	B	B	B	B	B	B	B	A	B	A	A	A	0	A	A	A
B	B	B	B	B	B	B	A	B	B	A	A	B	A	B	B	B	B	B	B	A	B	B	B	B	A	B	B	B	B	B	B	B	B	B	B	B	A	B	A	A	A	0	A	A	A
B	B	B	B	B	B	B	A	B	B	A	A	B	A	B	B	B	B	B	B	A	B	B	B	B	A	B	B	B	B	B	B	B	B	B	B	B	A	B	A	A	A	0	A	A	A
B	B	B	B	B	B	B	A	B	B	A	A	B	A	B	B	B	B	B	B	A	B	B	B	B	A	B	B	B	B	B	B	B	B	B	B	B	A	B	A	A	A	0	A	A	A
B	B	B	B	B	B	B	A	B	B	A	A	B	A	B	B	B	B	B	B	A	B	B	B	B	A	B	B	B	B	B	B	B	B	B	B	B	A	B	A	A	A	0	A	A	A
B	B	B	B	B	B	B	A	B	B	A	A	B	A	B	B	B	B	B	B	A	B	B	B	B	A	B	B	B	B	B	B	B	B	B	B	B	A	B	A	A	A	0	A	A	A
B	B	B	B	B	B	B	A	B	B	A	A	B	A	B	B	B	B	B	B	A	B	B	B	B	A	B	B	B	B	B	B	B	B	B	B	B	A	B	A	A	A	0	A	A	A
B	B	B	B	B	B	B	A	B	B	A	A	B	A	B	B	B	B	B	B	A	B	B	B	B	A	B	B	B	B	B	B	B	B	B	B	B	A	B	A	A	A	0	A	A	A

B	B	B	B	B	B	B	A	B	B	A	A	B	A	B	B	B	B	B	B	A	B	B	B	B	A	B	B	B	B	B	B	B	B	B	B	B	A	B	A	A	A	0	A	A	A
B	B	B	B	B	B	B	A	B	B	A	A	B	A	B	B	B	B	B	B	A	B	B	B	B	A	B	B	B	B	B	B	B	B	B	B	B	A	B	A	A	A	0	A	A	A
B	B	B	B	B	B	B	A	B	B	A	A	B	A	B	B	B	B	B	B	A	B	B	B	B	A	B	B	B	A	B	B	B	B	B	B	B	A	B	A	A	A	0	A	A	A
A	B	B	B	B	B	B	A	B	B	A	A	B	A	B	B	B	B	B	B	A	B	B	B	B	A	B	B	B	A	B	B	B	B	B	B	B	A	B	A	A	A	0	A	A	A
A	B	B	B	B	B	B	A	B	B	A	A	B	A	A	B	B	B	B	B	A	B	B	B	B	A	B	B	B	A	B	B	B	B	B	B	B	A	B	A	A	A	0	A	A	A
A	B	B	B	B	B	B	A	B	B	A	A	B	A	A	B	B	B	B	B	A	B	B	B	B	A	B	B	B	A	B	B	B	B	B	B	B	A	B	A	A	A	0	A	A	A
A	B	B	B	B	B	B	A	B	B	A	A	B	A	A	B	B	B	B	B	A	B	B	B	B	A	B	B	B	A	B	B	B	B	B	B	B	A	B	A	A	A	0	A	A	A
A	B	B	B	B	B	B	A	B	B	A	A	B	A	A	B	B	B	B	B	A	B	B	B	B	A	B	B	B	A	B	B	B	B	B	B	B	A	B	A	A	A	0	A	A	A
A	B	B	B	B	B	B	A	B	B	A	A	B	A	A	B	B	B	B	B	A	B	B	B	B	A	B	B	B	A	B	B	B	B	B	B	B	A	B	A	A	A	0	A	A	A
A	B	B	B	B	B	B	A	B	B	A	A	B	A	A	B	B	B	B	B	A	B	B	B	B	A	B	B	B	A	B	B	B	B	B	B	B	A	B	A	A	A	0	A	A	A
A	B	B	B	B	B	B	A	B	B	A	A	B	A	A	B	B	B	B	B	A	A	B	B	B	A	B	B	B	A	B	B	B	B	B	B	B	A	B	A	A	A	0	A	A	A
A	B	B	B	B	B	B	A	B	B	A	A	B	A	A	B	B	B	B	B	A	A	B	B	B	A	B	B	B	A	B	B	B	B	B	B	B	A	B	A	A	A	0	A	B	A
A	B	B	B	A	B	B	A	B	B	A	A	B	A	A	B	B	B	B	B	A	A	B	B	B	A	A	B	B	A	B	B	B	B	B	B	B	A	B	A	A	A	0	A	B	A
A	B	B	B	A	B	B	A	B	B	A	A	B	A	A	B	B	B	B	B	A	A	B	B	B	A	A	B	B	A	B	B	B	B	B	B	B	A	B	A	A	A	0	A	B	A
A	B	B	B	A	B	B	A	B	B	A	A	B	A	A	B	B	B	B	B	A	A	B	B	B	A	A	B	B	A	B	B	B	B	B	B	B	A	B	A	A	A	0	A	B	A
B	B	A	B	A	A	B	B	B	A	B	A	B	B	B	B	B	B	A	B	B	A	B	B	A	B	B	A	B	B	B	B	A	B	B	A	B	A	A	B	B	A	B	B	A	A
B	B	A	B	A	A	B	B	B	A	B	A	B	B	B	B	B	B	A	B	B	A	B	B	A	B	B	A	B	B	B	B	A	B	B	A	B	A	B	B	B	A	B	B	A	A
B	B	A	B	A	A	B	B	B	A	B	A	B	B	B	B	B	B	A	B	B	A	B	B	A	B	B	A	B	A	B	B	A	B	B	A	B	A	B	B	B	A	B	B	A	A
B	B	A	B	A	A	B	B	B	A	B	A	A	B	B	B	B	B	A	B	B	A	B	B	A	B	B	A	B	A	B	B	A	B	B	A	B	A	B	B	B	A	B	B	A	A
B	B	A	B	A	A	B	B	B	A	B	A	A	B	B	B	B	B	A	B	B	A	B	B	A	B	B	A	B	A	B	B	A	B	B	A	B	A	B	B	B	A	B	B	A	A
B	B	A	B	A	A	B	B	B	A	B	A	A	B	B	B	B	B	A	B	B	A	B	B	A	B	B	A	B	A	B	B	A	B	B	A	B	A	B	B	B	A	B	B	A	A
B	B	A	B	A	A	B	B	B	A	B	A	A	B	B	B	B	B	A	B	B	A	B	B	B	B	B	A	B	A	B	B	A	B	B	A	B	A	B	B	B	A	B	B	A	A
B	B	A	B	A	A	B	B	B	A	B	A	A	B	B	B	B	B	A	B	B	A	B	B	B	A	B	A	B	A	B	B	A	B	B	A	B	A	B	B	B	A	B	B	A	A
B	B	A	B	A	A	B	B	B	A	B	A	A	B	B	B	B	B	A	B	B	A	B	B	B	A	B	A	B	A	B	B	A	B	B	A	B	A	B	B	B	A	B	B	A	A
B	B	A	B	A	A	B	B	B	A	B	A	A	B	B	B	B	B	A	B	B	A	B	B	B	A	B	A	B	A	B	B	A	B	B	A	B	A	B	B	B	A	B	B	A	A
B	B	A	B	A	A	B	B	B	A	B	A	A	B	A	B	B	B	A	B	B	A	B	B	B	A	B	A	B	A	B	B	A	B	B	A	B	B	B	B	B	A	B	B	A	A
B	B	A	B	A	A	B	B	B	A	B	A	A	B	A	B	B	B	A	B	B	A	B	B	B	A	B	A	B	A	B	B	A	B	B	A	B	B	B	B	B	A	B	B	A	A
B	B	A	B	A	A	B	B	B	A	B	A	A	B	A	B	B	B	A	B	B	A	B	B	B	A	B	A	B	A	B	B	A	B	B	A	B	B	B	B	B	A	B	B	A	A
B	B	A	B	A	B	B	B	B	A	B	A	A	B	A	B	B	B	A	B	B	A	B	B	B	A	B	A	B	A	B	B	A	B	B	A	B	B	B	B	B	A	B	B	A	A
B	B	A	B	A	B	B	B	B	A	B	A	A	B	A	B	B	B	A	B	B	A	B	B	B	A	B	A	B	A	B	B	A	B	B	A	B	B	B	B	B	A	B	B	A	A
B	B	A	B	A	B	B	B	B	A	B	A	A	B	A	B	B	B	A	B	B	A	B	B	B	A	B	B	B	A	B	B	A	B	B	A	B	B	B	B	B	A	B	B	A	A
B	B	A	B	A	B	B	B	B	A	B	A	A	B	A	B	B	B	A	B	B	A	B	B	B	A	B	B	B	A	B	B	A	B	B	A	B	B	B	B	B	A	B	B	A	A
B	B	A	B	A	B	B	B	B	A	B	A	A	B	A	B	B	B	A	B	B	A	B	B	B	A	B	B	B	A	B	B	A	B	B	A	B	B	B	B	B	A	B	B	A	A
B	B	A	B	A	B	B	B	B	A	B	A	A	B	A	B	B	B	A	B	B	A	B	B	B	A	B	B	B	A	B	B	A	B	B	A	B	B	B	B	B	A	B	B	B	A
B	B	A	B	A	B	B	B	B	A	B	A	A	B	A	B	B	B	A	B	B	A	B	B	B	A	B	B	B	A	B	B	A	B	B	B	B	B	B	B	B	A	B	B	B	A
B	B	A	B	A	B	B	B	B	A	B	A	A	B	A	B	B	B	A	B	B	A	B	B	B	A	B	B	B	A	B	B	A	B	B	B	B	B	B	B	B	A	B	B	B	A
B	B	A	B	A	B	B	B	B	A	B	A	A	B	A	B	B	B	A	B	B	A	B	B	B	A	B	B	B	A	B	B	A	B	B	B	B	B	B	B	B	A	B	B	B	A
B	B	A	B	A	B	B	B	B	A	B	A	A	B	A	B	B	B	A	B	B	A	B	B	B	A	B	B	B	A	B	B	A	B	B	B	B	B	B	A	B	A	B	B	B	A
B	B	A	B	A	B	B	B	B	A	B	A	A	B	A	B	B	B	A	B	B	A	B	B	B	A	B	B	B	A	B	B	A	B	B	B	B	B	B	A	B	A	B	B	B	A
B	B	A	B	A	B	B	B	B	A	B	A	A	B	A	B	B	B	A	B	B	A	B	B	B	A	B	B	B	A	B	B	A	B	B	B	B	B	B	A	B	A	B	B	B	A
B	B	A	B	A	B	B	B	B	A	B	A	A	B	A	B	B	B	A	B	B	A	B	B	B	A	B	B	B	A	B	B	A	B	B	B	B	B	B	A	B	A	B	B	B	A
B	B	A	B	A	B	B	B	B	A	B	A	A	B	A	B	B	B	A	B	B	A	B	B	B	A	B	B	B	A	B	B	A	B	B	B	B	B	B	A	B	A	B	B	B	A
B	B	B	B	A	B	B	B	B	B	B	A	A	B	A	B	B	B	A	B	B	A	B	B	B	A	B	B	B	A	B	B	A	B	B	B	B	B	B	A	B	A	B	B	B	A
B	B	B	B	A	B	B	B	B	B	B	A	A	B	A	B	B	B	A	B	B	A	B	B	B	A	B	B	B	A	B	B	A	B	B	B	B	B	B	A	B	A	B	A	B	B
B	B	B	B	A	B	B	B	B	B	B	A	A	B	A	B	B	B	A	B	B	A	B	B	B	A	B	B	B	A	B	B	A	B	B	B	B	B	B	A	B	A	B	A	B	B
B	B	B	B	A	B	B	B	B	B	B	A	A	B	A	B	B	B	A	B	B	A	B	B	B	A	B	B	B	A	B	B	A	B	B	B	B	B	B	A	B	A	B	A	B	B
B	B	B	B	A	B	B	B	B	B	B	A	A	B	A	B	B	B	A	B	B	A	B	B	B	A	B	B	B	A	B	B	A	B	B	B	B	B	B	A	B	A	B	A	B	B
B	B	B	B	A	B	B	B	B	B	B	A	A	B	A	B	B	B	B	B	B	A	B	B	B	A	B	B	B	A	B	B	B	B	B	B	B	B	B	A	B	A	B	A	B	B
B	B	B	B	A	B	B	B	B	B	B	A	A	B	A	B	B	B	B	B	B	A	B	B	B	A	B	B	B	A	B	B	B	B	B	B	B	B	B	A	B	A	A	A	B	B

B	B	B	B	A	B	B	B	B	B	B	A	A	B	A	B	B	B	B	B	B	A	B	B	B	A	B	B	B	A	B	B	B	B	B	B	B	B	B	A	B	A	A	A	B	B
B	B	B	B	A	B	B	A	B	B	B	A	A	B	A	B	B	B	B	B	B	A	B	B	B	A	B	B	B	A	B	B	B	B	B	B	B	B	B	A	B	A	A	A	B	B
B	B	B	B	A	B	B	A	B	B	B	A	A	B	A	B	B	B	B	B	B	A	B	B	B	A	B	B	B	A	B	B	B	B	B	B	B	B	B	A	B	A	A	A	B	B
B	B	B	B	A	B	B	A	B	B	B	A	A	B	A	B	B	B	B	B	B	A	B	B	B	A	B	B	B	A	B	B	B	B	B	B	B	B	B	A	B	A	A	A	B	B
B	B	B	B	A	B	B	A	B	B	B	A	A	B	A	B	B	B	B	B	B	A	B	B	B	A	B	B	B	A	B	B	B	B	B	B	B	B	B	A	B	A	A	A	B	B
B	B	B	B	A	B	B	A	B	B	B	A	A	B	A	B	B	B	B	B	B	A	B	B	B	A	B	B	B	A	B	B	B	B	B	B	B	B	B	A	B	A	A	A	B	B
B	B	B	B	A	B	B	A	B	B	B	A	A	B	A	B	B	B	B	B	B	A	B	B	B	A	B	B	B	A	B	B	B	B	B	B	B	B	B	A	B	A	A	A	B	B
B	B	B	B	A	B	B	A	B	B	B	A	A	B	A	B	B	B	B	B	B	A	B	B	B	A	B	B	B	A	B	B	B	B	B	B	B	B	B	A	B	A	A	A	B	B
B	B	B	B	A	B	B	A	B	B	B	A	A	B	A	B	B	B	B	B	B	A	B	B	B	A	B	B	B	A	B	B	B	B	B	B	B	B	B	A	B	A	A	A	B	B
B	B	B	B	A	B	B	A	B	B	B	A	A	B	A	B	B	B	B	B	B	A	B	B	B	A	B	B	B	A	B	B	B	B	B	B	B	B	B	A	B	A	A	A	B	B
B	B	B	A	A	B	B	A	B	B	B	A	A	B	A	B	B	B	B	B	B	A	B	B	B	A	B	B	B	A	B	B	B	B	B	B	B	B	B	A	B	A	A	A	B	B
B	B	B	A	A	B	B	A	B	B	B	A	A	B	A	B	B	B	B	B	B	A	B	B	B	A	B	B	B	A	B	B	B	B	B	B	B	B	B	A	B	A	A	A	B	B
B	B	B	A	A	B	B	A	B	B	B	A	A	B	A	B	B	B	B	B	B	A	B	B	B	A	B	B	B	A	B	B	B	B	B	B	B	B	B	A	B	A	A	A	B	B
B	B	B	A	A	B	B	A	B	B	B	A	A	B	A	B	B	B	B	B	B	A	B	B	B	A	B	B	B	A	B	B	B	B	B	B	B	A	B	A	B	A	A	A	B	B
B	B	B	A	A	B	B	A	B	B	B	A	A	B	A	B	B	B	B	B	B	A	B	B	B	A	B	B	B	A	B	B	B	B	B	B	B	A	B	A	B	A	A	A	B	B
B	B	B	A	A	B	B	A	B	B	B	A	A	B	A	B	B	B	B	B	B	A	B	B	B	A	B	B	B	A	B	B	B	B	B	B	B	A	B	A	B	A	A	A	B	B
B	B	B	A	A	B	B	A	B	B	B	A	A	B	A	B	B	B	B	B	B	A	B	B	B	A	B	B	B	A	B	B	B	B	B	B	B	A	B	B	B	A	A	A	B	B
B	B	B	A	A	B	B	A	B	B	B	A	A	B	A	B	B	B	B	B	A	A	B	B	B	A	B	B	A	A	B	B	B	B	B	B	B	A	B	B	B	A	A	A	B	0
B	B	B	A	A	B	B	A	B	B	B	A	A	B	A	B	B	B	B	B	A	A	B	B	B	A	B	B	A	A	B	B	B	B	B	B	B	A	B	B	B	A	A	A	A	0
B	B	B	A	A	B	B	A	B	B	B	A	A	B	A	A	B	B	B	B	A	A	B	B	B	A	B	B	A	A	B	B	B	B	B	B	B	A	B	B	B	A	A	A	A	0
B	B	B	A	A	B	B	A	B	B	B	A	A	B	A	A	B	B	B	B	A	A	B	B	B	A	B	B	A	A	B	B	B	B	B	B	B	A	B	B	B	A	A	A	A	0
B	B	B	A	A	B	B	A	B	B	B	A	A	B	A	A	B	B	B	B	A	A	B	B	B	A	B	B	A	A	B	B	B	B	B	B	B	A	B	B	B	A	A	A	A	0
B	B	B	A	A	B	B	A	B	B	B	A	A	B	B	A	B	B	B	B	A	A	B	B	B	A	B	B	A	A	B	B	B	B	B	B	B	A	B	B	B	A	A	A	A	0
B	B	B	A	A	B	B	A	B	B	B	A	A	B	B	A	B	B	B	B	A	A	B	B	B	A	B	B	A	A	B	B	B	B	B	B	B	A	B	B	0	A	A	A	A	0
B	B	B	A	A	B	B	A	B	B	A	A	A	B	B	A	B	B	B	B	A	A	B	B	B	A	B	B	A	A	B	B	B	B	B	B	B	A	B	B	A	A	A	A	A	0
B	B	B	A	A	B	B	A	B	B	A	A	A	B	B	A	B	B	B	B	A	A	B	B	A	A	B	B	A	A	B	B	B	B	B	B	B	A	B	B	A	A	A	A	A	0
B	B	B	A	A	B	B	A	B	B	A	A	A	B	B	A	B	B	B	B	A	A	B	B	A	A	B	B	A	A	B	B	B	B	B	B	B	A	B	B	A	A	A	A	A	0
B	B	B	A	A	B	B	A	B	B	A	A	A	B	B	A	B	B	B	B	A	A	B	B	A	A	B	B	A	A	B	B	B	B	B	B	B	A	B	B	A	A	A	A	A	0
B	B	B	A	A	B	B	A	B	B	A	A	A	B	B	A	B	B	B	B	A	A	B	B	A	A	A	B	A	A	B	B	B	B	B	B	B	A	B	B	A	A	A	A	A	0
B	B	B	A	A	B	B	A	B	B	A	A	A	B	B	A	B	B	B	B	A	A	B	B	A	A	A	B	A	A	B	B	B	B	B	B	B	A	B	B	A	A	A	A	A	0
A	B	B	A	A	B	B	A	B	B	A	A	A	B	B	A	B	B	B	B	A	A	B	B	A	B	A	B	A	B	B	B	B	B	B	B	B	A	B	B	A	A	A	A	A	0
A	B	B	A	A	B	B	A	B	B	A	A	A	B	B	A	B	B	B	B	A	A	B	B	A	B	A	B	A	B	B	B	B	B	B	A	B	A	B	B	A	A	A	A	A	0
A	B	B	A	A	B	B	A	B	B	A	A	A	B	B	A	B	B	B	B	A	A	B	B	A	B	A	B	A	B	B	B	B	B	B	A	B	A	B	B	A	A	A	A	A	0
A	B	B	A	A	B	B	A	B	B	A	A	A	B	B	A	B	B	B	B	A	A	B	B	A	B	A	B	A	B	B	B	B	B	B	A	B	A	B	B	A	A	A	A	A	0
A	B	B	A	A	B	B	A	B	B	A	A	A	B	B	A	B	B	B	B	A	A	B	B	A	B	A	B	A	B	B	B	B	B	B	A	B	A	B	B	A	A	A	A	A	0
A	B	B	A	A	B	B	A	B	B	A	A	A	B	B	A	B	B	B	B	A	A	B	B	A	B	A	B	A	B	B	B	B	B	B	A	B	A	B	B	A	A	A	A	A	0
A	B	B	A	A	B	B	A	B	B	A	A	A	B	B	A	B	B	B	B	A	A	B	B	A	B	A	B	A	B	B	B	B	B	B	A	B	A	B	B	A	A	A	A	A	0
A	B	B	A	A	B	B	A	B	B	A	A	A	B	B	A	B	B	B	B	A	A	B	B	A	B	A	B	A	B	B	B	B	B	B	A	B	A	B	B	A	A	A	A	A	0
A	B	B	A	A	B	B	A	B	B	A	A	A	B	B	A	B	B	B	B	A	A	B	B	A	B	A	B	A	B	B	B	B	B	B	A	B	B	B	B	A	A	A	A	A	0
A	B	B	A	A	B	B	A	B	B	A	A	A	B	B	A	B	B	B	B	A	A	B	B	A	B	A	B	A	B	B	B	B	B	B	A	B	B	B	B	A	A	A	A	A	0
A	B	A	B	A	A	A	B	B	A	B	B	B	A	B	B	A	A	B	B	B	B	A	A	A	A	0	A	A	A	A	B	B	A	A	B	A	B	B	B	B	A	B	A	B	B
A	B	A	B	A	A	A	B	B	A	B	B	B	B	B	B	A	A	B	B	B	B	A	A	A	A	0	A	A	A	A	B	B	A	A	B	A	B	B	B	B	A	B	A	B	B
A	B	A	B	A	A	A	B	B	A	B	B	B	B	B	B	A	A	B	B	B	B	A	A	A	A	B	A	A	A	A	B	B	A	A	B	A	B	B	B	B	A	B	A	B	B
A	B	A	B	A	A	A	B	B	A	B	B	B	B	B	B	A	A	B	B	B	B	A	A	A	A	B	A	A	A	A	B	B	A	A	B	A	B	B	B	B	A	B	A	B	B
A	B	A	B	A	A	A	B	B	A	B	B	B	B	B	B	A	A	B	B	B	B	A	A	A	A	B	A	A	A	A	B	B	A	A	B	A	B	B	B	B	A	B	A	B	B
A	B	A	B	A	A	A	B	B	A	B	B	B	B	B	B	A	A	B	B	B	B	B	A	A	A	B	A	A	A	A	B	B	A	A	B	A	B	B	B	B	A	B	A	B	B
A	B	A	B	A	A	A	B	B	A	B	B	B	B	B	B	A	A	B	B	B	B	B	A	A	A	B	A	A	A	A	B	B	A	A	B	A	B	B	B	B	A	B	A	B	B
A	B	A	B	A	A	A	B	B	A	B	B	B	B	A	B	A	A	B	B	B	B	B	A	A	A	B	A	A	A	A	B	B	A	A	B	A	B	B	B	B	A	B	A	B	B
A	B	A	B	A	A	A	B	B	A	B	B	B	B	A	B	A	A	B	B	B	B	B	A	A	A	B	A	A	A	A	B	B	A	A	B	A	B	B	B	B	A	B	A	B	B

A	B	A	B	A	A	A	B	B	A	B	B	B	B	A	B	A	A	B	B	B	B	B	A	A	A	B	A	A	A	A	B	B	A	A	B	A	B	B	B	B	A	B	A	B	B
A	B	A	B	A	A	A	B	B	A	B	B	B	B	A	B	A	A	B	B	B	B	B	A	A	A	B	A	A	A	A	B	B	A	A	B	A	B	B	B	B	A	B	A	B	B
A	B	A	B	A	A	B	B	B	A	B	B	B	B	A	B	A	A	B	B	B	B	B	A	A	A	B	A	A	A	A	B	B	A	A	B	A	B	B	B	B	A	B	A	B	B
A	B	A	B	A	A	B	B	B	A	B	B	B	B	A	B	A	A	B	B	B	B	B	A	A	A	B	A	A	A	A	B	B	A	A	B	A	B	B	B	B	A	B	A	B	B
A	B	A	B	A	A	B	B	B	A	B	B	B	B	A	B	A	A	B	B	B	B	B	A	A	A	B	A	A	A	A	B	B	A	A	A	A	B	B	B	B	A	B	A	B	B
A	B	A	B	A	B	B	B	B	A	B	B	B	B	A	B	A	A	B	B	B	B	B	A	A	A	B	A	A	A	A	B	B	A	A	A	A	B	B	B	B	A	B	A	B	B
A	B	A	B	B	B	B	B	B	A	B	B	B	B	A	B	A	A	B	B	B	B	B	A	A	A	B	A	A	A	A	B	B	A	A	A	A	B	B	B	B	A	B	A	B	B
A	B	A	B	B	B	B	B	B	A	B	B	B	B	A	B	A	A	B	B	B	B	B	A	A	A	B	A	A	A	A	B	B	A	A	A	A	B	B	B	B	A	B	A	B	B
A	B	A	B	B	B	B	B	B	A	B	B	B	B	A	B	A	A	B	B	B	B	B	A	A	A	B	A	A	A	A	B	B	A	A	A	A	B	B	B	B	A	B	A	B	B
A	B	B	B	B	B	B	B	B	B	B	B	B	B	A	B	A	A	B	B	B	B	B	A	A	A	B	A	A	A	A	B	B	A	A	A	A	B	B	B	B	A	B	A	B	B
A	B	B	B	B	B	B	B	B	B	B	B	B	B	A	B	A	A	B	B	B	B	B	A	A	A	B	A	A	A	A	B	B	A	A	A	A	B	B	B	B	A	B	A	B	B
A	B	B	B	B	B	B	B	B	B	B	B	B	B	A	B	A	A	B	B	B	B	B	A	A	A	B	A	A	A	A	B	B	A	A	A	A	B	B	B	B	A	B	A	B	B
A	B	B	B	B	B	B	B	B	B	B	B	B	B	A	B	A	A	B	B	B	B	B	A	A	A	B	A	A	A	A	B	B	A	A	A	A	B	B	B	B	A	B	A	B	B
A	B	B	B	B	B	B	B	B	B	B	B	B	B	A	B	A	A	B	B	B	B	B	A	A	A	B	A	A	A	A	B	B	A	A	A	A	B	B	B	B	A	B	A	B	B
A	B	B	B	B	B	B	B	B	B	B	B	B	B	A	B	A	A	B	B	B	B	B	A	A	A	B	A	A	B	A	B	B	A	A	A	A	B	B	B	B	A	B	A	B	B
A	B	B	B	B	B	B	B	B	B	B	B	B	B	A	B	A	A	B	B	B	B	B	A	A	A	B	A	A	B	A	B	B	A	A	A	A	A	B	B	B	A	B	A	B	B
A	B	B	B	B	B	B	B	B	B	B	B	B	B	A	B	A	A	B	B	B	B	B	A	A	A	B	A	A	B	A	B	B	A	A	A	A	A	B	B	B	A	B	A	B	B
A	B	B	B	B	B	B	B	B	B	B	B	B	B	A	B	A	A	B	B	B	B	B	A	A	A	B	A	A	B	A	B	B	A	A	A	A	A	B	B	B	A	B	A	B	B
A	B	B	B	B	B	B	B	B	B	B	B	B	B	A	B	A	A	B	B	B	B	B	A	A	A	B	A	A	B	A	B	B	A	A	A	A	A	B	B	B	A	B	A	B	B
A	B	B	A	B	B	B	B	B	B	B	B	B	B	A	B	A	A	B	B	B	B	B	A	A	A	B	A	A	B	A	B	B	A	A	A	A	A	B	B	B	A	B	A	B	B
A	B	B	A	B	B	B	B	B	B	B	B	B	B	A	B	A	A	B	B	B	B	B	A	A	A	B	A	A	B	A	B	B	B	A	A	A	A	B	B	B	A	B	A	B	B
A	B	B	A	B	B	B	B	B	B	B	B	B	B	A	B	A	A	B	B	B	B	B	A	A	B	B	A	A	B	A	B	B	B	A	A	A	A	B	B	B	A	B	A	B	B
A	B	B	A	B	B	B	B	B	B	B	B	B	B	A	B	A	A	B	B	B	B	B	A	A	B	B	A	B	B	A	B	B	B	A	A	A	A	B	B	B	A	B	A	B	B
A	B	B	A	B	B	B	B	B	B	B	B	B	B	A	B	A	A	B	B	B	B	B	A	A	B	B	A	B	B	A	B	B	B	A	A	A	A	B	B	B	A	B	A	B	B
A	B	B	A	B	B	B	B	B	B	B	B	B	B	A	B	A	A	B	B	B	B	A	A	A	B	B	A	B	B	A	B	B	B	A	A	A	A	B	B	B	A	B	A	B	B
A	B	B	A	B	B	B	B	B	B	B	B	B	B	A	B	A	A	B	B	B	B	A	A	A	B	B	A	B	B	A	B	B	B	A	A	A	A	B	B	B	A	B	A	B	B
A	B	B	A	B	B	B	B	B	B	B	B	B	B	A	B	A	A	B	B	B	B	A	A	A	B	B	A	B	B	A	B	B	B	A	A	A	A	B	B	B	A	B	A	B	B
A	B	B	A	B	B	B	B	B	B	B	B	B	B	A	B	A	A	B	B	B	B	A	A	A	B	B	A	B	B	A	B	B	B	A	A	A	A	B	B	B	A	B	A	B	B
A	B	B	A	B	B	B	B	B	B	B	B	B	B	A	B	A	A	B	B	B	B	A	A	A	B	B	A	B	B	A	B	B	B	A	A	A	A	B	B	B	A	B	A	B	B
A	B	B	A	B	B	B	B	B	B	B	B	B	B	A	B	A	A	B	B	B	B	A	A	A	B	B	A	B	B	A	B	B	B	A	A	A	A	B	B	B	A	B	A	B	B
A	B	B	A	B	B	B	B	B	B	B	B	B	B	A	B	A	A	B	B	B	B	A	A	B	B	B	A	B	B	A	B	B	B	A	A	A	A	B	B	B	A	B	A	B	B
A	B	B	A	B	A	B	B	B	B	B	B	B	B	A	B	A	A	B	B	B	B	A	A	B	B	B	A	B	B	A	A	B	B	A	A	A	A	B	B	B	A	B	A	B	B
A	B	B	A	B	A	B	B	B	B	B	B	B	B	A	B	A	A	B	B	B	B	A	A	B	B	B	A	B	B	A	A	B	B	A	A	A	A	B	B	B	A	B	A	B	B
A	B	B	A	B	A	B	B	B	B	B	B	B	B	A	B	A	A	B	B	B	B	A	A	B	B	B	A	B	B	A	A	B	B	A	A	A	A	B	B	B	A	B	A	B	B
A	B	B	A	B	A	B	B	B	B	B	B	B	B	A	B	B	B	B	B	B	B	A	B	B	B	B	A	B	B	B	A	B	B	B	A	A	A	B	B	B	A	B	A	B	B
A	B	B	A	B	A	B	B	B	B	B	B	B	B	A	B	B	B	B	B	B	B	A	B	B	B	B	A	B	B	B	A	B	B	B	A	A	B	B	B	B	A	B	A	B	B
A	B	B	A	B	A	B	B	B	B	B	B	B	B	B	B	B	B	B	B	B	B	A	B	B	B	B	A	B	B	B	A	B	B	B	A	A	B	B	B	B	A	B	A	B	B
A	B	B	A	B	A	B	B	B	B	B	B	B	B	B	B	B	B	B	B	B	B	A	B	B	B	B	A	B	B	B	A	B	B	B	A	A	B	B	B	B	A	B	A	B	B
A	B	B	A	B	A	B	B	B	B	B	B	B	B	B	B	B	B	A	B	B	B	A	B	B	B	B	A	B	B	B	A	A	B	B	A	A	B	B	B	B	A	B	A	B	B
A	B	B	A	B	A	B	B	B	B	B	B	B	B	B	B	B	B	A	B	B	B	A	B	B	B	B	A	B	B	B	A	A	B	B	A	A	B	B	B	B	A	B	A	B	B
A	B	B	A	B	A	B	B	B	B	A	B	B	B	B	B	B	B	A	B	B	B	A	B	B	B	B	A	B	B	B	A	A	B	B	A	A	B	B	B	A	A	B	A	B	B
A	B	B	A	B	A	B	B	B	B	A	B	B	B	B	B	B	B	A	B	B	B	A	B	B	B	B	A	B	B	B	A	A	B	B	A	A	B	B	B	A	A	B	A	B	B
A	B	B	A	B	A	B	B	B	B	A	B	B	B	B	B	B	B	A	B	B	B	A	B	B	B	B	A	B	B	B	A	A	B	B	A	A	B	B	B	A	A	B	A	B	B
A	B	B	A	B	A	B	B	B	B	A	B	B	B	B	B	B	B	A	B	B	B	A	B	B	B	B	A	B	B	B	A	A	B	B	A	A	B	B	B	A	A	B	A	B	B
A	B	B	A	B	A	B	B	B	B	A	B	B	B	B	B	B	B	A	B	B	A	A	B	B	B	B	A	B	B	B	A	A	B	B	A	A	B	B	B	A	A	B	A	B	B
A	B	B	A	B	A	B	B	B	B	A	B	B	B	B	B	B	B	A	B	B	A	A	B	B	B	B	A	B	B	B	A	A	B	B	A	A	B	B	B	A	A	B	A	B	B
A	B	B	A	B	A	B	B	B	B	A	B	B	B	B	B	B	B	A	B	A	A	A	B	B	B	B	A	B	B	B	A	A	B	B	A	A	B	B	B	A	A	B	A	B	B
A	B	B	A	B	A	B	B	B	B	A	B	B	B	B	B	B	B	A	B	A	A	A	B	B	B	B	A	B	B	B	A	A	B	B	A	A	B	B	B	A	A	B	A	B	B
A	B	B	A	B	A	B	B	B	B	A	B	B	B	B	B	B	B	A	B	A	A	A	B	B	B	B	A	B	B	B	A	A	B	B	A	A	B	B	B	A	A	B	A	B	B

A	B	B	A	B	A	B	B	B	B	A	B	B	B	B	B	B	B	A	B	A	A	A	B	B	B	B	A	B	B	B	A	A	B	B	A	A	B	B	B	A	A	B	A	B	B
A	B	B	A	B	A	B	B	B	B	A	B	B	B	B	B	B	B	A	B	A	A	A	B	B	B	B	A	B	B	B	A	A	B	B	A	A	B	B	B	A	A	B	A	B	B
A	B	B	A	B	A	B	B	B	B	A	B	B	B	B	B	B	B	A	B	A	A	A	B	B	B	B	A	B	B	B	A	A	B	B	A	A	B	B	A	A	A	B	A	B	B
A	B	B	B	B	A	B	B	B	B	A	B	B	B	B	B	B	B	A	B	A	A	A	B	B	B	B	A	B	B	B	A	A	B	B	A	A	B	B	A	A	A	B	A	B	B
A	B	A	B	B	A	B	B	B	A	A	B	B	B	B	B	B	B	A	B	A	A	A	B	B	B	B	A	B	B	B	A	A	B	B	A	A	B	B	A	A	A	B	A	B	B
A	B	A	B	B	A	B	B	B	A	A	A	B	B	B	B	B	B	A	B	A	A	A	B	B	B	B	A	B	B	B	A	A	B	B	A	A	B	B	A	A	A	B	A	B	B
A	B	A	B	B	A	B	B	B	A	A	A	B	B	B	B	B	B	A	B	A	A	A	B	B	B	B	A	B	B	B	A	A	A	B	A	A	B	B	A	A	A	B	A	B	B
A	B	A	B	B	A	B	B	B	A	A	A	B	B	B	B	B	B	A	B	A	A	A	B	B	B	B	A	B	B	B	B	A	A	B	A	A	B	B	A	A	A	B	A	B	B
A	B	A	B	B	A	B	B	B	A	A	A	B	B	B	B	B	B	A	B	A	A	A	B	B	B	B	B	B	B	B	B	A	A	B	A	A	B	B	A	A	A	B	A	B	B
A	B	A	B	B	A	B	B	B	A	A	A	B	B	B	B	B	B	A	B	A	A	A	B	B	B	B	B	B	B	B	B	A	A	B	B	A	B	B	A	A	A	B	A	B	B
A	B	A	B	B	A	B	B	B	A	A	A	B	B	B	B	B	B	A	B	A	A	A	B	B	B	B	B	B	B	B	B	A	A	B	B	A	B	B	A	A	A	B	B	B	B
A	B	A	B	B	A	B	B	B	A	A	A	B	B	B	B	B	B	A	B	A	A	A	B	B	B	B	B	B	A	B	B	A	A	B	B	A	B	B	A	A	A	B	B	B	B
A	B	A	B	B	A	B	B	B	A	A	A	B	B	B	B	B	B	A	B	A	A	A	B	B	B	B	B	B	A	B	B	A	A	B	B	A	B	B	A	A	A	B	B	B	B
A	B	A	B	B	A	B	B	B	A	A	A	B	B	B	B	B	B	A	B	A	A	A	B	A	B	B	B	B	A	B	B	A	A	B	B	A	B	B	A	A	A	B	B	B	B
A	B	A	B	B	A	B	B	B	A	A	A	B	B	B	B	B	B	A	B	A	A	A	B	A	B	B	B	B	A	B	B	A	A	B	B	A	B	B	A	A	A	B	B	B	B
A	B	A	B	B	A	B	B	B	A	A	A	B	A	B	B	B	B	A	B	A	A	A	B	A	B	B	B	B	A	B	B	A	A	B	B	A	B	B	A	A	A	B	B	B	B
A	B	A	B	B	A	B	B	B	A	A	A	B	A	B	B	B	B	A	B	A	A	A	B	A	B	B	A	B	A	B	B	A	A	B	B	A	B	B	A	A	A	B	B	B	B
A	B	A	B	B	A	B	B	B	A	A	A	B	A	B	B	B	B	A	B	A	A	A	B	A	B	B	A	B	A	B	B	A	A	B	B	A	B	B	A	A	A	B	B	B	B
A	B	A	B	B	A	B	B	B	A	A	A	B	A	B	B	B	B	A	B	A	A	A	B	A	B	B	A	B	A	B	B	A	A	B	B	A	B	B	A	A	A	B	B	B	B
A	B	A	B	B	A	B	B	B	A	A	A	B	A	B	B	B	B	A	B	A	A	A	B	A	B	B	A	B	A	B	B	A	A	B	B	A	B	B	A	A	A	B	B	B	B
A	B	A	B	B	A	A	B	B	A	A	A	B	A	B	B	B	B	A	B	A	A	A	B	A	B	B	A	B	A	B	B	A	A	B	B	A	B	B	A	A	A	B	B	B	B
A	B	A	B	B	A	A	B	B	A	A	A	B	A	B	B	B	B	A	B	A	A	A	B	A	B	B	A	B	A	B	B	A	A	B	B	A	B	B	A	A	A	B	B	B	B
A	B	A	B	B	A	A	B	B	A	A	A	B	A	B	B	B	B	A	B	A	A	A	B	A	B	A	A	B	A	B	B	A	A	B	B	A	B	B	A	A	A	B	B	B	B
A	B	A	B	B	A	A	B	B	A	A	A	B	A	B	B	B	B	A	B	A	A	A	B	A	B	A	A	B	A	B	B	A	A	B	B	A	B	B	A	A	A	B	B	B	B
A	B	A	B	B	A	A	B	B	A	A	A	B	A	B	B	B	B	A	B	A	A	A	B	A	B	A	A	B	A	B	B	A	A	B	B	A	B	B	A	A	A	B	B	B	B
A	B	A	B	B	A	A	B	B	A	A	A	B	A	B	B	B	B	A	B	A	A	A	B	A	B	A	A	B	A	B	B	A	A	B	B	A	B	B	A	A	A	B	B	B	B
A	B	A	B	B	A	A	B	B	A	A	A	B	A	B	B	B	B	A	B	A	A	A	B	A	B	A	A	B	A	B	B	A	A	B	B	A	B	B	A	A	A	B	B	B	B
A	B	A	B	B	A	A	B	B	A	A	A	B	A	B	B	B	B	A	B	A	A	A	B	A	B	A	A	B	A	B	B	A	A	B	B	A	B	B	A	A	A	B	B	B	B
A	B	A	B	B	A	A	B	B	A	A	A	B	A	B	B	B	B	A	B	A	A	A	B	A	B	A	A	B	A	B	B	A	A	B	B	A	B	B	A	A	A	B	B	B	B
A	B	A	B	B	A	A	B	B	A	A	A	B	A	B	B	B	B	A	B	A	A	A	B	A	B	A	A	B	A	B	B	A	A	B	B	A	B	B	A	A	A	B	B	B	B
A	B	A	B	B	A	A	B	B	A	A	A	B	A	B	B	A	A	A	B	A	A	A	A	A	B	A	A	B	A	A	B	A	A	A	B	A	B	B	A	A	A	B	B	B	B
A	B	A	B	B	A	A	B	B	A	A	A	B	A	B	B	A	A	A	B	A	A	A	A	A	B	A	A	B	A	A	B	A	A	A	B	A	B	B	A	A	A	B	B	B	B
A	B	A	B	B	A	A	B	B	A	A	A	B	A	B	B	A	A	A	B	A	A	A	A	A	B	A	A	B	A	A	B	A	A	A	B	A	B	B	A	A	A	B	B	B	B
A	B	A	B	B	A	A	B	B	A	A	A	B	A	B	B	A	A	A	B	A	A	A	A	A	B	A	A	B	A	A	B	A	A	A	B	A	B	B	A	A	A	B	B	B	B
A	B	A	B	B	A	A	B	B	A	A	A	B	A	B	B	A	A	A	B	A	A	A	A	A	B	A	A	B	A	A	B	A	A	A	B	A	B	B	A	A	A	B	B	B	B
A	B	A	B	B	A	A	B	B	A	A	A	B	A	B	B	A	A	A	B	A	A	A	A	A	B	A	A	B	A	A	B	A	A	A	B	A	B	B	A	A	A	B	B	B	B
B	A	B	A	A	B	A	A	A	B	B	B	A	B	A	A	B	B	B	A	B	A	A	B	B	B	A	B	A	B	B	B	B	A	B	A	B	A	B	B	B	B	B	A	A	A
B	A	B	A	A	B	A	A	A	B	B	B	A	B	A	A	B	B	B	A	B	A	A	B	B	B	A	B	A	B	B	B	B	A	B	A	B	A	B	B	B	B	B	A	A	A
B	A	B	A	A	B	B	A	A	B	B	B	A	B	A	A	B	B	B	A	B	A	A	B	B	B	A	B	A	B	B	B	B	A	B	A	B	A	B	B	B	B	B	A	A	A
B	A	B	A	A	B	B	A	A	B	B	B	A	B	A	A	B	B	B	A	B	A	A	B	B	B	A	B	A	B	B	B	B	A	B	A	B	A	B	B	B	B	B	A	A	A
B	A	B	A	A	B	B	A	A	B	B	B	A	B	A	A	B	B	B	A	B	A	A	B	B	B	A	B	A	B	B	B	B	A	B	A	B	A	B	B	B	B	B	A	A	A
B	A	B	A	A	B	B	A	A	B	B	B	A	B	A	A	B	B	B	A	B	A	A	B	B	B	A	B	A	B	B	B	B	A	B	A	B	A	B	B	B	B	B	A	A	A
B	A	B	A	A	B	B	A	A	B	B	B	A	B	A	A	B	B	B	A	B	A	A	B	B	B	A	B	A	B	B	B	B	A	B	A	B	A	B	B	B	B	B	A	A	A
B	A	B	A	A	B	B	A	A	B	B	B	A	B	A	A	B	B	B	A	B	A	A	B	B	B	A	B	A	B	B	A	B	A	B	A	B	A	B	B	B	B	B	A	A	A
B	A	B	A	A	B	B	A	A	B	B	B	A	B	A	A	B	B	B	A	B	A	A	B	B	B	A	B	A	B	B	A	B	A	B	A	B	A	B	B	B	B	B	A	A	A
B	A	B	A	A	B	B	A	A	B	B	B	A	B	A	A	B	B	B	A	B	A	A	B	B	B	A	B	A	B	B	A	B	A	B	A	B	A	B	B	B	B	B	A	A	A
B	A	B	A	A	B	B	A	A	B	B	B	A	A	A	A	B	B	B	A	B	A	A	B	B	B	A	B	A	B	B	A	B	A	B	A	B	A	B	B	B	B	B	A	A	A
B	A	B	A	A	B	B	A	A	B	B	B	A	A	A	A	B	B	B	A	B	A	A	B	B	B	A	B	A	B	B	A	B	A	B	A	B	A	B	B	B	B	B	A	A	A
B	A	B	A	A	B	B	A	A	B	B	B	A	A	A	A	B	B	B	A	B	A	A	B	B	B	A	B	A	B	B	A	B	A	B	A	B	A	B	B	B	B	B	A	A	A

B	A	B	A	A	B	B	A	A	B	B	A	A	A	A	A	B	B	B	A	B	A	A	B	B	B	A	B	A	B	B	A	B	A	B	A	B	A	B	B	B	B	B	A	A	A
B	A	B	A	A	B	B	A	A	B	B	A	A	A	A	A	B	B	B	A	B	A	A	B	B	B	A	B	A	B	B	A	B	A	B	A	B	A	B	A	B	B	B	A	A	A
B	A	B	A	A	B	B	A	A	B	B	A	A	A	A	A	B	B	B	A	B	A	A	B	B	B	A	B	A	B	B	A	B	A	B	A	B	A	B	A	B	B	B	A	A	A
B	A	B	A	A	B	B	A	A	B	B	A	A	A	A	A	B	B	B	A	B	A	A	B	B	B	A	B	A	B	B	A	B	A	B	A	B	A	B	A	B	B	B	A	A	A
B	A	B	A	A	B	B	A	A	B	B	A	A	A	A	A	B	B	B	A	B	A	A	B	B	B	A	B	A	B	B	A	B	A	B	A	B	A	B	A	B	B	B	A	A	A
B	A	B	A	A	B	B	A	A	B	B	A	A	A	A	A	B	B	B	A	B	A	A	B	B	B	A	B	A	B	B	A	B	A	B	A	B	A	B	A	B	B	B	A	A	A
B	A	B	A	A	B	B	A	A	B	B	A	A	A	A	A	B	B	B	A	B	A	A	B	B	B	A	B	A	B	B	A	B	A	B	A	B	A	B	A	B	B	B	A	A	A
B	A	B	A	A	B	B	A	A	B	B	A	A	A	A	A	B	B	B	A	B	A	A	B	B	B	A	B	A	B	B	A	B	A	B	A	B	A	A	A	B	B	B	A	A	A
B	A	B	A	A	B	B	A	A	B	B	A	A	A	A	A	B	B	B	A	B	A	A	B	B	B	A	B	A	B	B	A	B	A	B	A	B	A	A	A	B	B	B	A	A	A
B	A	B	A	A	B	B	A	A	B	B	A	A	A	A	A	B	B	B	A	B	A	A	B	B	B	A	B	A	B	B	A	B	A	B	A	B	A	A	A	B	B	B	A	A	A
B	A	B	A	A	B	B	A	A	B	B	A	A	A	A	A	B	B	B	A	B	A	A	B	B	B	A	B	A	B	B	A	B	A	B	A	B	A	A	A	B	B	B	A	A	A
B	A	B	A	A	B	B	A	A	B	B	A	A	A	A	A	B	B	B	A	B	A	A	B	B	B	A	B	B	B	B	A	B	A	B	A	B	A	A	A	B	B	B	A	A	A
B	A	B	A	A	B	B	A	A	B	B	A	A	A	A	A	B	B	B	A	B	A	A	B	B	B	A	B	B	B	B	A	B	A	B	A	B	A	A	A	B	B	B	A	B	A
B	A	B	A	A	B	B	A	A	B	B	A	A	A	A	A	B	B	B	A	B	A	A	B	B	B	A	B	B	B	B	A	B	A	B	A	B	B	A	A	B	B	B	A	B	A
B	A	B	A	A	B	B	A	A	B	B	A	A	A	A	A	B	B	B	A	B	A	A	B	B	B	A	B	B	B	B	A	B	A	B	A	B	B	A	A	B	B	B	A	B	A
B	A	B	A	A	B	B	A	A	B	B	A	A	A	A	A	B	B	B	A	B	A	A	B	B	B	A	B	B	B	B	A	B	A	B	A	B	B	A	A	B	B	B	A	B	A
B	A	B	A	A	B	B	A	A	B	B	A	A	A	A	A	B	B	B	A	B	A	A	B	B	B	A	B	B	B	B	A	B	A	B	A	B	B	A	A	B	B	B	A	B	A
B	A	B	A	A	B	B	A	A	B	B	A	A	A	A	A	B	B	B	A	B	A	A	B	B	B	A	B	B	B	B	A	B	A	B	A	B	B	A	A	B	B	B	A	B	A
B	A	B	A	A	B	B	A	A	B	B	A	A	A	A	A	B	B	B	A	B	A	A	B	B	B	A	B	B	B	B	A	B	A	B	A	B	B	A	A	B	B	B	A	B	A
B	B	B	A	A	B	B	A	B	B	B	A	A	A	A	A	B	B	B	A	B	A	A	B	B	B	A	B	B	B	B	A	B	A	B	A	B	B	A	A	B	B	B	A	B	A
B	B	B	A	A	B	B	A	B	B	B	A	A	A	A	A	B	B	B	A	B	A	A	B	B	B	A	B	B	B	B	A	B	A	B	A	B	B	A	A	B	B	B	B	B	A
B	B	B	A	A	B	B	A	B	B	B	A	A	A	A	A	B	B	B	A	B	A	A	B	B	B	A	B	B	B	B	A	B	A	B	A	B	B	A	A	B	B	B	B	B	A
B	B	B	A	A	B	B	A	B	B	B	A	A	A	A	A	B	B	B	A	B	A	A	B	B	B	A	B	B	B	B	A	B	A	B	A	B	B	A	A	B	A	B	B	B	A
B	B	B	A	A	B	B	A	B	B	B	A	A	A	A	A	B	B	B	A	B	A	A	B	B	B	A	B	B	B	B	A	B	A	B	A	B	B	A	A	B	A	B	B	B	A
B	B	B	A	A	B	B	A	B	B	B	A	A	A	A	A	B	B	B	A	B	A	A	B	B	B	A	B	B	B	B	A	B	A	B	A	B	B	A	A	B	A	B	B	B	A
B	B	B	A	A	B	B	A	B	B	B	A	A	A	A	A	B	B	B	A	B	A	A	B	B	B	A	B	B	B	B	A	B	A	B	A	B	B	A	A	B	A	B	B	B	A
B	B	B	A	A	B	B	A	B	B	B	A	A	B	A	A	B	B	B	A	B	A	A	B	B	B	A	B	B	B	B	A	B	A	B	A	B	B	A	A	B	A	B	B	B	A
B	B	B	A	A	B	B	A	B	B	B	A	A	B	A	A	B	B	B	A	B	A	A	B	B	B	A	B	B	B	B	A	B	A	B	A	B	B	A	A	0	A	B	B	B	A
B	B	B	A	A	B	B	A	B	B	B	A	A	B	A	A	B	B	B	A	B	A	A	B	B	A	A	B	B	B	B	A	B	A	B	A	B	B	A	A	0	A	B	B	B	A
B	B	B	A	A	B	B	A	B	B	B	A	A	B	A	A	B	B	B	A	B	A	A	B	B	A	A	B	B	B	B	A	B	A	B	A	B	B	A	A	0	A	B	B	B	A
B	B	B	A	A	B	B	A	B	B	B	A	A	B	A	A	B	B	B	A	B	A	A	B	B	A	A	B	B	B	B	A	B	A	B	A	B	B	A	A	0	A	B	B	B	A
B	B	B	A	A	B	B	A	B	B	B	A	A	B	A	A	B	B	B	A	B	A	A	B	B	A	A	B	B	B	B	A	B	A	B	A	B	B	A	A	0	A	B	B	B	A
B	B	B	A	A	B	B	A	B	B	B	A	A	B	A	A	B	B	B	A	B	A	A	B	B	A	A	B	B	B	B	A	B	A	B	A	B	B	A	A	0	A	B	B	B	A
B	B	B	A	A	B	B	A	B	B	B	A	A	B	A	A	B	B	B	A	B	A	A	B	B	A	A	A	B	B	B	A	B	A	B	A	B	B	A	A	0	A	B	B	B	A
B	B	B	A	A	B	B	A	B	B	B	A	A	B	A	A	B	B	B	B	B	A	A	B	B	A	A	A	B	B	B	A	B	A	B	A	B	B	A	A	0	A	B	B	B	A
B	B	B	A	A	B	B	A	B	B	B	A	A	B	A	A	B	B	B	B	B	A	A	B	B	A	A	A	B	B	B	A	B	A	B	A	B	B	A	A	0	A	B	B	B	A
B	B	B	A	A	B	B	A	B	B	B	A	A	B	A	A	B	B	B	B	B	A	A	B	B	A	A	A	B	B	B	A	B	A	B	A	A	B	A	A	0	A	B	B	B	A
B	A	B	A	A	B	B	B	A	B	A	A	B	B	A	A	B	B	A	B	B	A	B	B	A	A	A	A	A	B	B	A	A	B	B	B	A	A	A	A	A	B	B	B	A	A
B	A	B	A	A	B	B	B	A	B	A	A	B	B	A	A	B	B	A	B	B	A	B	B	A	A	A	A	A	B	B	A	A	B	B	B	A	A	A	A	A	B	B	B	A	A
B	A	B	A	A	B	B	B	A	B	A	A	B	B	A	A	B	B	A	B	B	A	B	B	A	A	A	A	A	B	B	A	A	B	B	B	A	A	A	A	A	B	B	B	B	A
B	A	B	A	A	B	B	B	A	B	A	A	B	B	A	A	B	B	A	B	B	A	B	B	A	A	A	A	A	B	B	A	A	B	B	B	A	A	A	A	A	B	B	B	B	A
B	A	B	A	A	B	B	B	A	B	A	A	B	B	A	A	B	B	A	B	B	A	B	B	A	A	A	A	A	B	B	A	A	B	B	B	A	A	A	A	A	B	B	B	B	A
B	A	B	A	A	B	B	B	A	B	A	A	B	B	A	A	B	B	A	B	B	A	B	B	A	A	A	A	A	B	B	A	A	B	B	A	A	A	B	A	A	B	B	B	B	A
B	A	B	A	A	B	B	B	A	B	A	A	B	B	A	A	B	B	A	B	B	A	B	B	A	A	A	A	A	B	B	A	A	B	B	A	A	A	B	A	A	B	B	B	B	A
B	A	B	A	A	B	B	B	A	B	A	A	B	B	A	A	B	B	A	B	B	A	B	B	A	A	A	A	A	B	B	A	A	B	B	A	A	A	B	A	A	B	B	B	B	A
B	A	B	A	A	B	B	B	A	B	A	A	A	B	A	A	B	B	A	B	B	A	B	B	A	A	A	A	A	B	B	A	A	B	B	A	B	A	B	A	A	B	B	B	B	A
B	A	B	A	A	B	B	B	A	B	A	A	A	B	A	A	B	B	A	B	B	A	B	B	A	A	A	A	A	B	B	A	A	B	B	A	B	A	B	A	A	B	B	B	B	A
B	A	B	A	A	B	B	B	A	B	A	A	A	B	A	A	B	B	A	B	B	A	B	B	A	A	A	A	A	B	B	A	A	B	B	A	B	A	B	A	A	B	B	B	B	A
B	A	B	A	A	B	B	B	A	B	A	A	A	B	A	A	B	B	A	B	B	A	A	B	A	A	A	A	A	B	B	A	A	B	B	A	B	A	B	A	A	B	B	B	B	A

B	A	B	A	A	B	A	B	A	B	A	A	A	B	A	A	B	B	A	B	B	A	A	B	A	A	A	A	A	B	B	A	A	B	B	A	B	A	B	A	A	B	B	B	B	A
B	A	B	A	A	B	A	B	A	B	A	A	A	B	A	A	B	B	A	B	B	A	A	B	A	A	A	A	A	B	B	A	A	B	B	A	B	A	B	A	A	B	B	B	B	A
B	A	B	A	A	B	A	B	A	B	A	A	A	B	A	A	B	B	A	B	B	A	A	B	A	A	A	A	A	B	B	B	A	B	B	A	B	A	B	A	A	A	B	B	B	A
B	A	B	A	A	B	A	B	A	B	A	A	A	B	A	A	B	B	B	B	B	A	A	B	A	A	A	A	A	B	B	B	B	B	B	A	B	A	B	A	A	A	B	B	B	A
B	A	B	A	A	B	A	B	A	B	A	A	A	B	A	A	B	B	B	B	B	A	A	B	A	A	A	A	A	B	B	B	B	B	B	A	B	A	B	A	A	A	B	B	B	A
B	A	B	A	A	B	A	B	A	B	A	A	A	B	A	A	B	B	B	B	B	A	A	B	A	A	A	A	A	B	B	B	B	B	B	A	B	A	B	A	A	A	B	B	B	A
B	A	B	A	B	A	A	B	A	B	A	A	A	B	A	A	B	B	B	B	B	A	A	B	A	B	A	A	A	B	B	B	B	B	B	A	B	A	B	A	A	A	B	B	B	A
B	A	B	A	B	A	A	B	A	B	A	A	A	B	A	A	B	B	B	B	B	A	A	B	A	B	A	A	A	B	B	B	B	B	B	A	B	A	B	A	A	A	B	B	B	A
B	A	A	A	B	A	A	B	A	A	A	A	A	B	A	A	B	B	B	B	B	A	A	B	A	B	A	A	A	B	B	B	B	B	B	A	B	A	B	A	A	A	B	B	B	A
B	A	A	A	B	A	A	B	A	A	A	A	A	B	A	A	B	B	B	B	A	A	A	B	A	B	A	A	A	B	B	B	B	B	B	A	B	A	B	A	A	A	B	B	B	A
B	A	A	A	B	A	A	B	A	A	A	A	A	B	A	A	B	B	B	B	A	A	A	B	A	B	A	A	B	B	B	B	B	B	B	A	B	A	B	A	A	A	B	B	B	A
B	A	A	A	B	A	A	B	A	A	A	A	A	B	A	A	B	B	B	B	A	A	A	B	A	B	A	A	B	B	B	B	B	B	B	A	B	A	B	A	A	A	B	B	B	A
B	A	A	A	B	A	A	B	A	A	A	A	A	B	A	A	B	B	B	B	A	A	A	B	A	B	A	A	B	B	B	B	B	B	B	A	B	A	B	A	A	A	B	B	B	A
B	A	A	A	B	A	A	B	A	A	A	A	A	B	A	A	B	B	B	B	A	A	A	B	A	B	A	A	B	B	B	B	B	B	B	A	B	A	B	A	A	A	B	B	B	A
B	A	A	A	B	A	A	B	A	A	A	A	A	B	A	A	B	B	B	A	A	A	A	B	A	B	A	A	B	B	B	B	B	B	B	A	B	A	B	A	A	A	B	B	B	A
B	A	A	A	B	A	A	B	A	A	A	A	A	B	A	A	B	B	B	A	A	A	A	B	A	B	A	A	B	B	B	B	B	B	B	A	B	B	B	A	A	A	B	B	B	A
B	A	A	A	B	A	A	B	A	A	A	A	A	B	A	A	B	B	B	A	A	A	A	B	A	B	A	A	B	B	B	B	B	B	B	A	B	B	B	A	A	A	B	B	B	A
B	A	A	A	B	A	A	B	A	A	A	A	A	B	A	A	B	B	B	A	A	A	A	B	A	B	A	A	B	B	B	B	B	B	B	A	B	B	B	A	A	A	B	B	B	A
B	A	A	A	B	A	A	B	A	A	A	A	A	B	A	A	B	B	B	A	A	A	A	B	A	B	A	A	B	B	B	B	B	B	B	A	B	B	B	A	A	A	B	B	B	A
B	A	A	A	B	A	A	B	A	A	A	A	A	B	A	A	B	B	B	A	A	A	A	B	A	B	A	A	B	B	B	B	B	B	B	A	B	B	B	A	A	A	B	B	B	A
B	A	A	A	B	A	A	B	A	A	A	A	A	B	A	A	B	B	B	A	A	A	A	B	A	B	A	A	B	B	B	B	B	B	B	A	B	B	B	A	A	A	B	B	B	A
B	A	A	A	B	A	A	B	A	A	A	A	A	B	A	A	B	B	B	A	A	A	A	B	A	B	A	A	B	B	B	B	B	B	B	A	B	B	B	B	A	A	B	B	B	A
B	A	A	B	B	A	A	B	A	A	A	A	A	B	A	A	B	B	B	A	A	A	A	B	A	B	A	A	B	B	B	B	B	B	B	A	B	B	B	B	A	A	B	B	B	A
B	A	A	B	B	A	A	B	A	A	A	A	A	B	A	A	B	B	B	A	A	A	A	B	A	B	A	A	B	B	B	B	B	B	B	A	B	B	B	B	A	A	B	B	B	A
B	A	A	B	B	A	A	B	A	A	A	A	A	B	A	A	B	B	B	A	A	A	A	B	A	B	A	A	B	B	B	B	B	B	B	A	B	B	B	B	A	A	B	B	B	A
B	A	A	B	B	A	A	B	A	A	A	A	A	B	B	A	B	B	B	A	A	A	A	B	A	B	A	A	B	B	B	B	B	B	B	A	B	B	B	B	A	A	B	B	B	A
B	A	A	B	B	A	A	B	A	A	A	A	A	B	B	A	B	B	B	A	A	A	A	B	A	B	A	A	B	B	B	B	B	B	B	A	B	B	B	B	A	A	B	B	B	A
B	A	A	B	B	A	A	B	A	A	A	A	A	B	B	A	B	B	B	A	A	A	A	B	A	B	A	A	B	B	B	B	B	B	B	A	B	B	B	B	A	A	B	B	B	A
B	A	A	B	B	A	A	B	A	A	A	A	A	B	B	A	B	B	B	A	A	A	A	B	A	B	A	A	B	B	B	B	B	B	B	A	B	B	B	B	A	A	B	B	B	A
B	A	A	B	B	A	A	B	A	A	A	A	A	B	A	A	B	B	B	A	A	A	A	B	A	B	A	A	B	A	B	B	B	B	B	A	B	B	B	B	A	A	B	B	B	A
B	A	A	B	B	A	A	B	A	A	A	A	A	B	A	A	B	B	B	A	A	A	A	B	A	B	A	A	B	A	B	B	B	B	B	A	B	B	B	B	A	A	B	B	B	A
B	A	A	B	B	A	A	B	A	A	A	A	A	B	A	A	B	B	B	A	A	A	A	B	A	B	A	A	B	A	B	B	B	B	B	A	B	B	B	B	A	A	B	B	B	A
B	A	A	B	B	A	B	B	A	A	A	A	A	B	A	A	B	B	B	A	A	A	A	B	A	B	A	A	B	A	B	B	B	B	B	A	B	B	B	B	A	A	B	B	B	A
B	A	A	B	B	A	B	B	A	A	A	A	A	B	A	A	B	B	B	A	A	A	A	B	A	B	A	A	B	A	B	B	B	B	B	A	B	B	B	B	A	A	B	B	B	A
B	A	A	B	B	A	B	B	A	A	A	A	A	B	A	B	B	B	B	A	A	A	A	B	A	B	A	A	B	A	B	B	B	B	B	A	B	B	B	B	A	A	B	B	B	A
B	B	A	B	B	A	B	B	B	A	A	A	A	B	A	B	B	B	B	A	A	A	A	B	A	B	A	A	B	A	B	B	B	B	B	A	B	B	B	B	A	A	B	B	B	A
B	B	A	B	B	A	B	B	B	A	A	A	A	B	A	B	B	B	B	A	A	A	A	B	A	B	A	A	B	A	B	B	B	B	B	A	B	B	B	B	A	B	B	B	B	A
B	B	A	B	B	A	B	B	B	A	A	A	A	B	A	B	B	B	B	A	A	A	A	B	A	B	A	A	B	A	B	B	B	B	B	A	B	B	B	B	A	B	B	B	B	A
B	B	A	B	B	A	B	B	B	A	A	A	A	B	A	B	B	B	B	A	A	A	A	B	A	B	A	A	B	A	B	B	B	B	B	A	B	B	B	B	A	B	B	B	B	A
B	B	A	B	B	A	B	B	B	A	A	A	A	B	A	B	B	B	B	A	A	A	A	B	A	B	A	A	B	A	B	B	B	B	B	A	B	B	B	B	A	B	B	B	B	B
B	B	A	B	B	A	B	B	B	A	A	A	A	B	A	B	B	B	B	A	A	A	A	B	A	B	A	A	B	A	B	B	B	B	B	A	B	B	B	B	A	B	A	B	B	B
B	B	A	B	B	A	B	B	B	A	A	A	B	B	A	B	B	B	B	A	A	A	A	B	A	B	A	A	B	A	B	B	B	B	B	A	B	B	B	B	A	B	A	B	B	B
B	B	A	B	B	A	B	B	B	A	B	A	B	B	A	B	B	B	B	A	A	A	A	B	A	B	A	A	B	A	B	A	B	B	B	A	B	B	B	B	B	B	A	B	B	B
B	B	A	B	B	A	B	B	B	A	B	A	B	A	A	B	B	B	B	A	A	A	A	B	A	B	A	A	B	A	B	A	B	B	B	A	B	B	B	B	B	B	A	B	B	B
B	B	A	A	B	A	B	B	B	A	B	A	B	A	A	B	B	B	B	A	A	A	A	B	A	B	A	A	B	A	B	A	B	B	B	A	B	B	B	B	B	B	A	B	B	B
B	B	A	A	B	A	B	B	B	A	B	A	B	A	A	B	A	A	B	A	A	A	A	A	A	B	A	A	B	A	A	A	B	B	A	A	B	B	B	B	B	B	A	B	B	B
B	B	A	A	B	A	B	B	B	A	B	A	B	A	A	B	A	A	B	A	A	A	A	A	A	B	A	A	B	A	A	A	B	B	A	A	B	B	B	B	B	B	A	B	B	B
B	B	A	A	B	A	B	B	B	A	B	A	B	A	A	B	A	A	B	A	A	A	A	A	A	B	A	A	B	A	A	A	B	B	A	A	B	A	B	B	B	B	A	B	B	B
B	B	A	A	B	A	B	B	B	A	B	A	B	A	A	B	A	A	B	A	A	A	A	A	A	B	A	A	B	A	A	A	B	B	A	A	B	A	B	B	B	B	0	B	B	B

B	B	A	A	B	A	B	A	B	A	B	A	B	A	A	B	A	A	B	A	A	A	A	A	B	B	A	A	B	A	A	A	B	B	A	A	B	A	B	B	B	B	0	B	B	B
B	B	A	A	B	A	B	A	B	A	B	A	B	A	A	B	A	A	B	A	A	A	A	A	B	B	A	A	B	A	A	A	B	B	A	A	B	A	B	B	B	B	0	B	B	B
B	B	A	A	B	A	B	A	B	A	B	A	B	A	A	B	A	A	B	A	A	A	A	A	B	B	A	A	B	A	A	A	B	B	A	A	A	A	B	B	B	B	0	B	B	B
B	B	A	A	B	A	B	A	B	A	B	A	B	A	A	B	A	A	B	A	A	A	A	A	B	B	A	A	B	A	A	A	B	B	A	A	A	A	B	B	B	B	0	B	B	B
B	B	A	A	B	A	B	A	B	A	B	A	B	A	A	B	A	A	B	A	A	A	A	A	B	B	A	A	B	A	A	A	B	B	A	A	A	A	B	B	B	B	0	B	B	B
B	B	A	A	B	A	B	A	B	A	B	A	B	A	A	B	A	A	B	A	A	A	A	A	B	B	A	A	B	A	A	A	B	B	A	A	A	A	B	B	B	B	0	B	B	B
B	B	A	A	B	A	B	A	B	A	B	A	B	A	A	B	A	A	B	A	A	A	A	A	B	B	A	A	B	A	A	A	B	B	A	A	A	A	B	B	B	B	0	B	B	B
B	B	A	A	A	A	B	A	B	A	B	A	B	A	A	B	A	A	B	A	A	A	A	A	B	B	A	A	B	A	A	A	B	B	A	A	A	A	B	B	B	B	0	B	B	B
B	B	A	A	A	A	B	A	B	A	B	A	B	A	A	B	A	A	B	A	A	A	A	A	B	B	A	A	B	A	A	A	B	B	A	A	A	A	B	B	B	B	0	B	B	B
B	B	A	A	A	A	B	A	B	A	B	A	B	A	A	B	A	A	B	A	A	A	A	A	B	B	A	B	B	A	A	A	B	B	A	A	A	A	B	B	B	B	0	B	B	B
B	B	A	A	A	A	B	A	B	A	B	A	B	A	A	B	A	A	B	A	A	A	A	A	B	B	A	B	B	A	A	A	B	B	A	A	A	A	B	B	B	B	0	B	B	B
B	B	A	A	A	A	B	A	B	A	B	A	B	A	A	B	A	A	B	A	A	A	A	A	B	B	A	B	B	A	A	A	B	B	A	A	A	A	B	B	B	B	0	B	B	B
B	B	A	A	A	A	B	A	B	A	B	A	B	A	A	B	A	A	B	A	A	A	A	A	B	B	A	B	B	A	A	A	B	B	A	A	A	A	B	B	B	B	0	B	B	B
B	B	A	A	A	A	B	A	B	A	B	A	B	A	A	B	A	A	B	A	A	A	A	A	B	B	A	B	0	A	A	A	B	A	A	A	A	A	B	B	B	B	0	A	B	B
B	B	A	A	A	A	B	A	B	A	B	A	B	A	A	B	A	A	B	A	A	A	A	A	B	B	B	B	0	A	A	A	B	A	A	A	A	A	B	B	B	B	0	A	B	B
B	B	A	A	A	A	B	A	B	A	B	A	B	A	A	B	A	A	B	A	A	A	A	A	B	B	B	B	0	A	A	A	B	A	A	A	A	A	B	B	B	B	0	A	B	B
B	B	A	A	A	A	B	A	B	A	B	A	B	A	A	B	A	A	B	A	A	A	A	A	B	B	B	B	0	A	A	A	B	A	A	A	A	A	B	B	B	B	0	A	B	B
A	A	A	A	B	B	B	B	A	A	A	B	B	B	A	A	B	B	B	B	A	B	B	B	A	A	B	B	B	A	B	B	B	A	B	B	B	A	B	A	A	A	B	B	B	B
A	A	A	A	B	B	B	B	A	A	A	B	B	B	A	A	B	B	B	B	A	B	B	B	A	A	B	B	B	A	B	B	B	A	B	B	B	A	B	A	A	A	B	B	B	B
A	A	A	A	B	B	B	B	A	A	A	B	B	B	A	A	B	B	B	B	A	B	B	B	B	A	B	B	B	A	B	B	B	A	B	B	B	A	B	A	A	A	B	B	B	B
A	A	A	A	B	B	B	B	A	A	A	B	B	B	A	A	B	B	B	B	A	B	B	B	B	A	B	B	B	A	B	B	B	A	B	B	B	A	B	A	A	A	B	B	B	B
A	A	A	A	B	B	B	B	A	A	A	B	B	B	A	A	B	B	B	B	A	B	B	B	B	A	B	B	B	A	B	B	B	A	B	B	B	A	B	A	A	A	B	B	B	B
A	A	A	A	B	A	B	B	A	A	A	B	B	B	A	A	B	B	B	B	A	B	B	B	B	A	B	B	B	A	B	B	B	A	B	B	B	A	B	A	A	A	B	B	B	B
A	A	A	A	B	A	B	B	A	A	A	B	B	B	A	A	B	B	B	B	A	B	B	B	B	A	B	B	B	A	B	B	B	A	B	B	B	A	B	A	A	A	B	B	B	B
A	A	A	A	B	A	B	B	A	A	A	B	B	B	A	A	B	B	B	B	A	B	B	B	B	A	B	B	B	A	B	B	B	A	B	B	B	A	B	A	A	A	B	B	A	B
A	A	A	A	B	A	B	B	A	A	A	B	B	B	A	A	B	B	B	B	A	B	B	B	B	A	B	B	B	A	B	B	B	A	B	B	A	A	B	A	A	A	B	B	A	B
A	A	A	A	B	A	B	B	A	A	A	B	B	B	A	B	B	B	B	B	A	B	B	B	B	A	B	B	B	A	B	B	B	A	B	B	A	A	B	A	A	A	B	B	A	B
A	A	A	A	B	A	B	B	A	A	A	B	B	B	A	B	A	A	B	B	A	B	B	A	B	A	B	B	B	A	A	B	B	A	A	B	A	A	B	A	A	A	B	B	A	B
A	A	A	A	B	A	B	B	A	A	A	B	B	B	A	B	A	A	A	B	A	B	B	A	B	A	B	B	B	A	A	B	A	A	A	B	A	A	B	A	A	A	B	B	A	B
A	A	A	A	B	A	B	B	A	A	A	B	B	B	A	B	A	A	A	B	A	B	B	A	B	A	B	B	B	A	A	B	A	A	A	B	A	A	B	A	A	A	B	B	A	B
A	A	A	A	B	A	B	B	A	A	A	B	B	B	A	B	A	A	A	B	A	B	B	A	B	A	B	B	B	A	A	B	A	A	A	B	A	A	B	A	A	A	B	B	A	B
A	A	A	A	B	A	B	B	A	A	A	B	B	B	A	B	A	A	A	B	A	B	B	A	B	A	B	B	B	A	A	B	A	A	A	B	A	A	A	A	A	A	B	B	A	B
A	A	A	A	B	A	B	B	A	A	A	B	B	B	A	B	A	A	A	B	A	B	B	A	B	A	B	B	B	A	A	B	A	A	A	B	A	A	A	A	A	A	B	B	A	B
A	A	A	A	B	A	B	B	A	A	A	B	B	B	A	B	A	A	A	A	A	B	B	A	B	A	B	B	B	A	A	B	A	A	A	B	A	A	A	B	A	A	B	B	A	B
A	A	A	A	B	A	B	B	A	A	A	B	B	B	A	B	A	A	A	A	A	A	B	A	B	A	B	B	B	A	A	B	A	A	A	B	A	A	A	B	A	A	B	B	A	B
A	A	A	A	B	A	B	B	A	A	A	B	B	B	A	B	A	A	A	A	A	A	B	A	B	A	A	B	B	A	A	B	A	A	A	B	A	A	A	B	A	A	B	B	A	B
A	A	A	A	B	A	B	B	A	A	A	B	B	B	A	B	A	A	A	A	A	A	B	A	B	A	A	B	B	A	A	B	A	A	A	B	A	A	A	B	A	A	B	B	A	B
A	A	A	A	B	A	B	B	A	A	A	B	B	A	A	B	A	A	A	A	A	A	B	A	B	A	A	B	B	A	A	B	A	A	A	B	A	A	A	B	A	A	B	B	A	B
A	A	A	A	B	A	B	B	A	A	A	B	B	A	A	B	A	A	A	A	A	A	B	A	B	A	A	B	B	A	A	B	A	A	A	B	A	A	A	B	A	A	B	B	A	B
A	A	A	A	B	A	B	B	A	A	A	B	B	A	A	B	A	A	A	A	A	A	B	A	B	A	A	B	A	A	A	B	A	A	A	B	A	A	A	B	A	A	B	B	A	B
A	A	A	A	B	A	B	B	A	A	A	B	B	A	A	B	A	A	A	A	A	A	B	A	B	A	A	B	A	A	A	B	A	A	A	B	A	A	A	B	A	A	B	B	A	B
A	A	A	A	B	A	B	B	A	A	A	B	B	A	A	B	A	A	A	A	A	A	B	A	B	A	A	A	A	A	A	B	A	A	A	B	A	A	A	B	A	A	B	B	A	B
A	A	A	A	B	A	B	B	A	A	A	B	B	A	A	B	A	A	A	A	A	A	B	A	B	A	A	A	A	A	A	B	A	A	A	B	A	A	A	B	A	A	B	B	A	B
A	A	A	A	A	A	B	B	A	A	A	B	B	A	A	B	A	A	A	A	A	A	B	A	B	A	A	A	A	A	A	B	A	A	A	B	A	A	A	B	A	A	B	B	A	B
A	A	A	A	A	A	B	B	A	A	A	B	B	A	A	B	A	A	A	A	A	A	B	A	B	A	A	A	A	A	A	B	A	A	A	B	A	A	A	B	A	A	B	B	A	B
A	A	A	A	A	A	B	B	A	A	A	B	B	A	A	B	A	A	A	A	A	A	B	A	B	A	A	A	A	A	A	B	A	A	A	B	A	A	A	B	A	A	B	B	A	B
A	A	A	A	A	A	B	B	A	A	A	B	B	A	A	B	A	A	A	A	A	A	B	A	B	A	A	A	A	A	A	B	A	A	A	B	A	A	A	B	A	A	B	B	A	B
A	A	A	A	A	A	B	B	A	A	A	B	B	A	A	B	A	A	A	A	A	A	B	A	B	A	A	A	A	A	A	B	A	A	A	B	A	A	A	B	A	A	B	B	A	B
A	A	A	A	A	A	B	B	A	A	A	B	B	A	A	B	A	A	A	A	A	A	B	A	B	A	A	A	A	A	A	B	A	A	A	B	A	A	A	B	A	A	B	B	A	B

A	A	A	A	A	A	B	B	A	A	A	B	B	A	A	B	A	A	A	A	A	A	B	A	B	A	A	A	A	A	A	B	A	A	A	B	A	A	A	B	A	A	B	B	A	B
A	A	A	A	A	A	B	B	A	A	A	B	B	A	A	B	A	A	A	A	A	A	B	A	B	A	A	A	A	A	A	B	A	A	A	B	A	A	A	B	A	A	A	B	A	B
A	A	A	A	A	A	B	B	A	A	A	B	B	A	A	B	A	A	A	A	A	A	B	A	B	B	A	A	A	A	A	B	A	A	A	B	A	A	A	B	A	A	A	B	A	B
A	A	A	A	A	A	B	B	A	A	A	B	B	A	A	B	A	A	A	A	A	A	B	A	B	B	A	A	A	A	A	B	A	A	A	B	A	A	A	B	A	A	A	B	A	B
A	A	A	A	A	A	B	B	A	A	A	B	B	A	A	B	A	A	A	A	A	A	B	A	B	B	A	A	A	A	A	B	A	A	A	B	A	A	A	B	A	A	A	B	A	B
A	A	A	A	A	A	B	B	A	A	A	B	B	A	A	B	A	A	A	A	A	A	B	A	B	B	A	A	A	A	A	B	A	A	A	B	A	A	A	B	A	A	A	B	A	B
A	A	A	A	A	A	B	B	A	A	A	B	B	A	A	B	A	A	A	A	A	A	B	A	B	B	A	A	A	A	A	B	A	A	A	B	A	A	A	B	A	A	A	B	A	B
A	A	A	A	A	A	B	B	A	A	A	B	B	A	A	B	A	A	A	A	A	A	B	A	B	B	A	A	A	A	A	B	A	A	A	B	A	A	A	B	A	A	A	B	A	B
A	A	A	A	A	A	B	B	A	A	A	B	B	A	A	B	A	A	A	A	A	A	B	A	B	B	A	A	A	A	A	B	A	A	A	B	A	A	A	B	A	A	A	B	A	B
A	A	A	A	A	A	B	B	A	A	A	B	B	A	A	B	A	A	A	A	A	A	B	A	B	B	A	A	A	A	A	B	A	B	A	B	A	A	A	B	A	A	A	B	A	B
A	A	A	A	A	A	B	B	A	A	A	B	B	A	A	B	A	A	A	A	A	A	B	A	B	B	A	A	A	A	A	B	A	B	A	B	A	A	A	B	A	A	A	B	A	B
A	A	A	A	A	A	B	B	A	A	A	B	B	A	A	B	A	A	A	A	A	A	B	A	B	B	A	A	A	A	A	B	A	B	A	B	A	A	A	B	A	A	A	B	A	B
A	A	A	A	A	A	B	A	A	A	A	B	B	A	A	B	A	A	A	A	A	A	B	A	B	B	A	A	A	A	A	B	A	B	A	B	A	A	A	B	A	A	A	B	A	B
A	A	A	A	A	A	B	A	A	A	A	B	B	A	A	B	A	A	A	A	A	A	B	A	B	B	A	A	A	A	A	B	A	B	A	B	A	B	A	B	A	A	A	B	A	B
A	A	A	A	A	A	B	A	A	A	A	B	B	A	A	B	A	A	A	A	A	A	B	A	B	B	A	A	A	A	A	B	A	B	A	B	A	B	A	B	A	A	A	A	A	B
A	A	A	A	A	A	B	A	A	A	A	B	B	A	A	B	A	A	A	A	A	A	B	A	B	B	A	A	A	A	A	B	A	B	A	B	A	B	A	B	A	A	A	A	A	B
A	A	A	A	A	A	B	A	A	A	A	B	B	A	A	B	A	A	A	A	A	A	B	A	B	B	A	A	A	A	A	B	A	B	A	B	A	B	A	B	A	A	A	A	A	B
A	A	A	A	A	A	B	A	A	A	A	B	B	A	A	B	A	A	A	A	A	A	B	A	B	B	A	A	A	A	A	B	A	B	A	B	A	B	A	B	A	A	A	A	A	B
A	A	A	A	A	A	B	A	A	A	A	B	B	A	A	B	A	A	A	A	A	A	B	A	B	B	A	A	A	A	A	B	A	B	A	B	A	B	A	B	A	A	A	A	A	B
A	A	A	A	A	A	B	A	A	A	A	B	B	A	A	B	A	A	A	A	A	A	B	A	B	B	A	A	A	A	A	B	A	B	A	B	A	B	A	B	A	A	A	A	A	B
A	A	A	A	A	A	B	A	A	A	A	B	B	A	A	B	A	A	A	A	A	A	B	A	B	B	A	A	A	A	A	B	A	B	A	B	A	B	A	B	A	A	A	A	A	B
A	A	A	A	A	A	B	A	A	A	A	B	B	A	A	B	A	A	A	A	A	A	B	A	B	B	A	A	A	A	A	B	A	B	A	B	A	B	A	B	A	A	A	A	A	B
A	A	A	A	A	A	B	A	A	A	A	B	B	A	A	B	A	A	A	A	A	A	B	A	B	B	A	A	A	A	A	B	A	B	A	B	A	B	A	B	A	A	A	A	A	B
A	A	A	A	A	A	B	A	A	A	A	B	B	A	A	B	A	A	A	A	A	A	B	A	B	B	A	A	A	A	A	B	A	B	A	B	A	B	A	B	A	A	A	A	A	B
A	A	A	A	A	A	B	A	A	A	A	B	B	A	A	B	A	A	A	A	A	A	B	A	B	B	A	A	A	A	A	B	A	B	A	B	A	B	A	B	A	A	A	A	A	B
A	A	A	A	A	A	B	A	A	A	A	B	B	A	A	B	A	A	A	A	A	A	B	A	B	B	A	A	A	A	A	B	A	B	A	B	A	B	A	B	A	A	A	A	A	B
A	A	A	A	A	A	B	A	A	A	A	B	B	A	A	B	A	A	A	A	A	A	B	A	B	B	A	B	A	A	A	B	A	B	A	B	A	B	A	B	A	A	A	A	A	B
A	A	A	A	A	A	B	A	A	A	A	B	B	A	A	B	A	A	A	A	A	A	B	A	B	B	A	B	A	A	A	B	A	B	A	B	A	B	A	B	A	A	A	A	A	B
A	A	A	A	A	A	B	A	A	A	A	B	B	A	A	B	A	A	A	A	A	A	B	A	B	B	A	B	A	A	A	B	A	B	A	B	A	B	A	B	A	A	A	A	A	B
A	A	A	A	A	A	B	A	A	A	A	B	B	A	A	B	A	A	A	A	A	A	B	A	B	B	A	B	A	A	A	B	A	B	A	B	A	B	A	B	A	A	A	A	A	B
A	A	A	A	A	A	B	A	A	A	A	B	B	A	A	B	A	A	A	A	A	A	B	A	B	B	A	B	A	A	A	B	A	B	A	B	A	B	A	B	A	A	A	A	A	B
A	A	A	A	A	A	B	A	A	A	A	B	B	A	A	B	A	A	A	A	A	A	B	A	B	B	A	B	A	A	A	B	A	B	A	B	A	B	A	B	A	A	A	A	A	B
B	A	A	A	A	A	B	A	A	A	A	B	B	A	A	B	A	A	A	A	A	A	B	A	B	B	A	B	A	A	A	B	A	B	A	B	A	B	A	B	A	A	A	A	A	B
B	A	A	A	A	A	B	A	A	A	A	B	B	A	A	B	A	A	A	A	A	A	B	A	B	B	A	B	A	A	A	B	A	B	A	B	A	B	A	B	A	A	A	A	A	B
B	A	A	A	A	A	B	A	A	A	A	B	B	A	A	B	A	A	A	A	A	A	B	A	B	B	A	B	A	A	A	B	A	B	A	B	A	B	A	B	A	A	A	A	A	B
B	A	A	A	A	A	B	A	A	A	A	B	B	A	A	B	A	A	A	A	A	A	B	A	B	B	A	B	A	A	A	B	A	B	A	B	A	B	A	B	A	A	A	A	A	B
B	A	A	A	A	A	B	A	A	A	A	B	B	A	A	A	A	A	A	A	A	A	B	A	B	B	A	B	A	A	A	B	A	B	A	B	A	B	A	B	A	A	A	A	A	B
B	B	A	A	A	A	B	A	B	A	A	B	B	A	A	A	A	A	A	A	A	A	B	A	B	B	A	B	A	A	A	B	A	B	A	B	A	B	A	B	A	A	A	A	A	B
B	B	A	A	A	B	B	A	B	A	A	B	B	A	A	A	A	A	A	A	A	A	B	A	B	B	A	B	A	A	A	B	A	B	A	B	A	B	A	B	A	A	A	A	A	B
B	B	A	A	A	B	B	A	B	A	A	B	B	A	A	A	A	A	A	A	A	A	B	A	B	B	A	B	A	A	A	B	A	B	A	B	A	B	A	B	A	A	A	A	A	B
B	B	A	A	A	B	B	A	B	A	A	B	B	A	A	A	A	A	A	A	A	A	B	A	B	B	A	B	A	A	A	B	A	B	A	B	A	B	A	B	A	A	B	A	A	B
B	B	A	A	A	B	B	A	B	A	A	B	B	A	A	A	A	A	A	A	A	A	B	A	B	B	A	B	B	A	A	B	A	B	A	B	A	B	A	B	A	A	B	A	A	B
B	B	A	A	A	B	B	A	B	A	A	B	B	A	A	A	A	A	A	A	A	A	B	A	B	B	A	B	B	A	A	B	A	B	A	B	A	B	A	B	A	A	B	A	A	A
B	B	A	A	A	B	B	A	B	A	A	B	B	A	A	A	A	A	A	A	A	A	B	A	B	B	A	B	B	A	A	B	A	B	A	B	A	B	A	B	A	A	B	A	A	A
B	B	A	A	A	B	B	A	B	A	A	B	B	A	A	A	A	A	A	A	A	A	B	A	B	B	A	B	B	A	A	B	A	B	A	B	A	B	A	B	A	A	B	A	A	A
B	B	A	A	A	B	B	A	B	A	A	B	B	A	A	A	A	A	A	A	A	A	B	A	B	B	A	B	B	A	A	B	A	B	A	B	A	B	A	B	A	A	B	A	A	A
B	B	A	A	A	B	B	A	B	A	A	B	B	A	A	A	A	A	A	A	A	A	B	A	B	B	A	A	B	A	A	B	A	B	A	B	A	B	A	B	A	A	B	A	A	A
B	B	A	A	A	B	B	A	B	A	A	B	B	A	A	A	A	A	A	A	A	A	B	A	B	B	A	A	B	A	A	B	A	B	A	B	A	B	A	B	A	A	B	A	A	A
B	B	A	A	A	B	B	A	B	A	A	B	B	A	A	A	A	A	A	B	A	A	B	A	B	B	A	A	B	A	A	B	A	B	A	B	A	B	A	B	A	A	B	A	A	A

B	B	A	A	A	B	B	A	B	A	A	B	B	A	A	A	A	A	A	B	A	A	B	A	B	B	A	A	B	A	A	B	A	B	A	B	A	B	A	B	A	A	B	A	A	A
B	B	A	A	A	B	B	A	B	A	A	B	B	A	A	A	A	A	A	B	A	A	B	A	B	B	A	A	B	A	A	B	A	B	A	B	A	B	A	B	A	A	B	A	A	A
B	B	A	A	A	B	B	A	B	A	A	B	B	A	A	A	A	A	B	B	A	A	B	A	B	B	A	A	B	A	A	B	B	B	A	B	A	B	A	B	A	A	B	A	A	A
B	B	A	A	A	B	B	A	B	A	A	B	B	A	A	A	A	A	B	B	A	A	B	A	A	B	A	A	B	A	A	B	B	B	A	B	A	B	A	B	A	A	B	A	A	A
B	B	A	A	A	B	B	A	B	A	A	B	B	A	A	A	A	A	B	B	A	A	B	A	A	B	A	A	B	A	A	B	B	B	A	B	A	B	A	B	A	A	B	A	A	A
B	B	A	A	A	B	B	A	B	A	A	B	B	A	A	A	A	A	B	B	A	A	B	A	A	B	A	A	B	A	A	B	B	B	A	B	A	B	A	A	A	A	B	A	A	A
B	B	A	A	A	B	B	B	B	A	A	B	B	A	A	A	A	A	B	B	A	A	B	A	A	B	A	A	B	A	A	B	B	B	A	B	A	B	A	A	A	A	B	A	A	A
B	B	A	A	A	B	B	B	B	A	A	B	B	A	A	A	A	A	B	B	A	A	B	A	A	B	A	A	B	A	A	B	B	B	A	B	A	B	A	A	A	A	B	A	A	A
B	B	B	B	A	B	B	A	B	B	A	A	B	B	A	0	B	B	B	A	0	B	B	B	B	B	B	B	A	B	B	B	B	B	B	B	B	B	B	B	A	B	B	B	A	B
B	B	B	B	A	B	B	A	B	B	A	A	B	B	A	0	B	B	B	A	0	B	B	B	B	B	B	B	A	B	B	B	B	B	B	B	B	B	B	B	A	B	B	B	A	B
B	B	B	B	A	B	B	A	B	B	A	A	B	B	A	0	B	B	B	A	0	B	B	B	B	B	B	B	A	B	B	B	B	B	B	B	B	B	B	B	A	B	B	B	A	B
B	B	B	B	A	B	B	A	B	B	A	A	B	B	A	0	B	B	B	A	0	B	B	B	B	B	B	B	A	B	B	B	B	B	B	B	B	B	B	B	A	B	B	B	A	B
B	B	B	B	A	B	B	A	B	B	A	A	B	B	A	0	B	B	B	A	0	B	B	B	B	B	B	B	A	B	B	B	B	B	B	B	B	B	B	B	A	B	B	B	A	B
B	B	B	B	A	B	B	A	B	B	A	A	B	B	A	0	B	B	B	A	0	B	B	B	B	B	B	B	A	B	B	B	B	B	B	B	B	B	B	B	A	B	B	B	A	B
B	B	B	B	A	B	B	A	B	B	A	A	B	B	A	0	B	B	B	A	0	B	B	B	B	B	B	B	A	B	B	B	B	B	B	B	B	B	B	B	A	B	B	B	A	B
B	B	B	B	A	B	B	A	B	B	A	A	B	B	A	0	B	B	B	A	0	B	B	B	B	B	B	B	A	B	B	B	B	B	B	B	B	B	B	B	A	B	B	B	A	B
B	B	B	B	A	B	B	A	B	B	A	A	B	B	A	0	B	B	B	A	0	B	B	B	B	B	B	B	A	B	B	B	B	B	B	B	B	B	B	B	A	B	B	B	A	B
B	B	B	B	A	B	B	A	B	B	A	A	B	B	A	0	B	B	B	A	0	B	B	B	B	B	B	B	A	B	B	B	B	B	B	B	B	B	B	B	A	B	B	B	A	B
B	B	B	B	A	B	B	A	B	B	A	A	B	B	A	0	B	B	B	A	0	B	B	B	B	B	B	B	A	B	B	B	B	B	B	B	B	B	B	B	A	B	B	B	A	B
B	B	B	B	A	B	B	A	B	B	A	A	B	B	A	0	B	B	B	A	0	B	B	B	B	B	B	B	A	B	B	B	B	B	B	B	B	B	B	B	A	B	B	B	A	B
B	B	B	B	A	B	B	A	B	B	A	A	B	B	A	0	B	B	B	A	0	B	B	B	B	B	A	B	A	B	B	B	B	B	B	B	B	B	B	B	A	B	B	B	A	B
B	B	B	B	A	B	B	A	B	B	A	A	B	B	A	0	B	B	B	A	0	B	B	B	B	B	A	B	A	B	B	B	B	B	B	B	B	B	B	B	A	B	B	B	A	B
B	B	B	B	A	B	B	A	B	B	A	A	B	B	A	0	B	B	B	A	0	B	B	B	B	B	A	B	A	B	B	B	B	B	B	B	B	B	B	B	A	B	B	B	A	B
B	B	B	B	A	B	B	A	B	B	A	A	B	B	A	0	B	B	B	B	0	B	B	B	B	B	A	B	A	B	B	B	B	B	B	B	B	B	B	B	A	B	0	B	A	B
B	B	B	B	A	B	B	A	B	B	A	A	B	B	A	0	B	B	B	B	0	B	B	B	B	B	A	B	A	B	B	B	B	B	B	B	B	A	B	B	A	B	0	B	A	B
B	B	B	B	A	B	B	A	B	B	A	A	B	B	A	0	B	B	B	B	0	B	B	B	B	B	A	B	B	B	B	B	B	B	B	B	B	A	B	B	A	B	0	B	A	B
B	B	B	B	A	B	B	A	B	B	A	A	B	B	A	0	B	B	B	B	0	B	B	B	B	B	A	B	B	B	B	B	B	B	B	B	B	A	B	B	A	B	0	B	A	B
B	B	B	B	A	B	B	A	B	B	A	A	B	B	A	0	B	B	B	B	0	B	B	B	B	B	A	B	B	B	B	B	B	B	B	B	B	A	B	B	A	B	0	B	A	B
B	B	B	B	A	B	B	A	B	B	A	A	B	B	A	0	B	B	B	B	0	B	B	B	B	B	A	B	B	B	B	B	B	B	B	B	B	A	B	B	A	B	0	B	A	B
B	B	B	B	A	B	B	A	B	B	A	A	B	B	A	0	B	B	B	B	0	B	B	B	B	B	A	B	B	B	B	B	B	B	B	B	B	A	B	B	A	B	0	B	A	B
B	B	B	B	A	B	B	A	B	B	A	A	B	B	A	0	B	B	B	B	0	B	B	B	B	B	A	B	B	B	B	B	B	B	B	B	B	A	B	B	A	B	0	B	A	B
B	B	B	B	A	B	B	B	B	B	A	A	B	B	A	0	B	B	B	B	0	B	B	B	B	B	A	B	B	B	B	B	B	B	B	B	B	A	B	B	A	B	0	B	A	B
B	B	B	B	A	B	B	B	B	B	A	A	B	B	A	0	B	B	B	B	0	B	B	B	B	B	A	B	B	B	B	B	B	B	B	B	B	A	B	B	A	B	0	B	A	B
B	B	B	B	A	B	B	B	B	B	0	A	B	B	A	0	B	B	B	B	0	B	B	B	B	B	A	B	B	B	B	B	B	B	B	B	B	A	B	B	0	B	0	B	A	B
B	B	B	B	A	B	B	B	B	B	0	A	B	B	A	0	B	B	B	B	0	B	B	B	B	B	A	B	B	B	B	B	B	B	B	B	B	A	B	B	0	B	0	B	A	B
B	B	B	B	A	A	B	B	B	B	0	A	B	B	A	0	B	B	B	B	0	B	B	B	B	B	A	B	B	B	B	B	B	B	B	B	B	A	B	B	0	B	0	B	A	B
B	B	B	B	A	A	B	B	B	B	0	A	B	B	A	0	B	B	B	B	0	B	B	B	B	B	A	B	B	B	B	B	B	B	B	B	B	A	B	B	0	B	0	B	A	B
B	B	B	B	A	A	B	B	B	B	0	A	B	B	A	0	B	B	B	B	0	B	B	B	B	B	A	B	B	B	B	B	B	B	B	B	B	A	B	B	0	B	0	B	A	B
B	B	B	B	A	A	B	B	B	B	0	A	B	B	A	0	B	B	B	B	0	B	B	B	B	B	A	B	B	B	B	B	B	B	B	B	B	A	B	B	0	B	0	B	A	B
B	B	B	B	A	A	B	B	B	B	0	A	B	B	A	0	B	B	B	B	0	B	B	B	B	B	A	B	B	B	B	B	B	B	B	A	B	A	B	B	0	B	0	B	A	B
0	B	B	B	A	A	B	B	B	B	0	A	B	B	A	0	B	B	B	B	0	B	B	B	B	B	A	B	B	B	B	B	B	B	B	A	0	A	B	B	0	B	0	B	A	B
0	B	B	B	A	A	B	B	B	B	0	A	B	B	A	0	B	B	B	B	0	B	B	B	B	B	A	B	B	B	B	B	B	B	B	0	0	A	B	B	0	B	0	B	A	B
0	B	B	B	A	A	B	B	B	B	0	A	B	B	A	0	B	B	B	B	0	B	B	B	B	B	A	B	A	B	B	B	B	B	B	0	0	A	B	B	0	B	0	B	A	B
0	B	B	B	A	A	B	B	B	B	0	A	B	B	A	0	B	B	B	B	0	B	B	B	B	B	B	B	A	B	B	B	B	B	B	0	0	A	B	B	0	B	0	B	A	B
0	B	B	B	A	A	B	B	B	B	0	A	B	B	A	0	B	B	B	B	0	B	B	B	B	B	B	B	A	B	B	B	B	B	B	0	0	A	B	B	0	B	0	B	A	B
0	B	B	B	A	A	B	B	B	B	0	A	B	B	A	A	B	B	B	B	0	B	B	B	B	B	B	B	A	B	B	B	B	B	B	0	0	A	B	B	0	B	0	B	A	B
B	B	B	0	A	A	B	B	B	B	0	A	B	B	A	A	B	B	B	B	0	B	0	B	B	B	B	0	A	B	B	B	B	B	B	0	0	A	B	B	0	B	A	B	A	B
B	B	B	0	A	A	B	B	B	B	0	A	B	B	A	A	B	B	B	B	0	B	0	B	B	B	B	0	A	B	B	B	B	B	B	0	0	A	B	B	0	B	A	B	A	B
B	B	B	0	A	A	B	B	B	B	0	A	B	B	B	A	B	B	B	B	0	B	0	B	B	B	B	0	A	B	B	B	B	B	B	0	0	A	B	B	0	B	A	B	A	B

B	B	B	0	A	A	A	B	B	B	0	A	B	B	B	A	A	A	B	B	0	B	0	A	B	B	B	0	A	B	A	B	B	B	A	0	0	A	B	B	0	B	A	B	A	B
B	A	B	0	A	A	A	B	A	B	0	A	B	B	B	A	A	A	B	B	0	B	0	A	B	B	B	0	A	A	A	B	B	B	A	0	0	A	B	B	0	B	A	B	A	B
B	A	B	0	A	A	A	B	A	B	0	A	B	B	B	A	A	A	B	B	0	B	0	A	B	B	B	0	A	A	A	B	B	B	A	0	0	A	B	B	0	B	A	B	A	B
B	A	B	0	A	A	A	B	A	B	0	A	B	B	B	A	A	A	B	B	0	B	0	A	B	B	B	0	A	A	A	B	B	B	A	0	0	A	B	B	0	B	A	B	A	B
0	A	B	0	A	A	A	B	A	B	0	A	B	B	B	A	A	A	B	B	B	B	0	A	B	B	B	0	A	A	A	B	B	0	A	0	0	A	B	B	0	B	A	B	A	B
B	A	A	A	A	B	A	A	A	A	A	A	A	B	B	A	B	B	B	B	A	B	A	B	A	A	A	A	A	A	B	A	B	A	B	B	B	B	A	B	A	B	A	A	A	B
B	A	A	A	A	B	A	A	A	A	A	A	A	B	B	A	B	B	B	B	A	B	A	B	A	A	A	A	A	A	B	A	B	A	B	B	B	B	A	B	A	B	A	A	A	B
B	A	A	A	A	B	A	A	A	A	A	A	A	B	B	A	B	B	B	B	A	B	A	B	A	A	A	A	A	A	B	A	B	A	B	B	B	B	A	B	A	B	A	A	A	B
B	A	A	A	A	B	B	A	A	A	A	A	A	B	B	A	B	B	B	B	A	B	A	B	A	A	A	A	A	A	B	A	B	A	B	B	B	B	A	B	A	B	A	A	A	B
B	A	A	A	A	B	B	A	A	A	A	A	A	B	B	A	B	B	B	B	A	B	A	B	A	A	A	A	A	A	B	A	B	A	B	B	B	B	A	B	A	B	A	A	A	B
B	A	A	A	A	B	B	A	A	A	A	A	A	B	B	A	B	B	B	B	A	B	A	B	A	A	A	A	A	A	B	A	B	A	B	B	B	B	A	B	A	B	A	A	A	B
B	A	A	A	A	B	B	A	A	A	A	A	A	B	B	A	B	B	B	B	A	A	A	B	A	A	A	A	A	A	B	A	B	A	B	B	B	B	A	B	A	B	A	A	A	B
B	A	A	A	A	B	B	A	A	A	A	A	A	A	B	A	B	B	B	B	A	A	A	B	A	A	A	A	A	A	B	A	B	A	B	B	B	B	A	B	A	B	A	A	A	B
B	A	A	A	A	B	B	A	A	A	A	A	A	A	B	A	B	B	B	B	A	A	A	B	A	A	A	B	A	B	B	A	B	A	B	B	B	B	A	B	A	B	A	A	A	B
B	A	A	B	A	B	B	A	A	A	A	A	A	A	B	A	B	B	B	B	A	A	A	B	A	A	A	B	A	B	B	A	B	A	B	B	B	B	A	B	A	B	A	A	A	B
B	A	A	B	A	B	A	A	A	A	A	A	A	A	B	A	B	B	B	B	A	A	A	B	A	A	A	B	A	B	B	A	B	A	B	B	B	B	A	B	A	B	A	A	A	B
B	A	A	B	A	B	A	A	A	A	A	A	A	A	B	A	B	B	B	B	A	A	A	B	A	A	A	B	A	B	B	A	B	A	B	B	B	B	A	B	A	B	A	A	A	B
B	A	A	B	A	B	A	A	A	A	A	A	A	A	B	A	B	B	B	B	A	A	A	B	A	A	A	B	A	B	B	A	B	A	B	B	B	A	A	B	A	B	A	A	A	B
B	A	A	B	A	B	A	A	A	A	A	A	A	A	B	A	B	B	B	B	B	A	A	B	A	A	A	B	A	B	B	A	B	A	B	B	B	A	A	B	A	B	A	A	A	B
B	A	A	B	A	B	A	A	A	A	A	B	A	A	B	A	B	B	B	B	B	A	A	B	A	A	A	B	A	B	B	A	B	A	B	B	B	A	A	B	A	B	A	A	A	B
B	A	A	B	A	B	A	A	A	A	A	B	A	A	B	A	B	B	B	B	B	A	A	B	A	A	A	B	A	B	B	A	B	A	B	B	B	A	A	B	A	B	B	A	A	B
B	A	A	B	A	B	A	A	A	A	A	B	A	A	B	A	B	B	B	B	B	A	A	B	A	B	A	B	A	B	B	A	B	A	B	B	B	A	A	B	A	B	B	A	A	B
B	A	A	B	A	B	A	A	A	A	A	B	A	A	B	A	B	B	B	B	B	A	A	B	A	B	A	B	A	B	B	A	B	A	B	B	B	A	A	B	A	B	B	A	A	B
B	A	A	B	A	B	A	A	A	A	A	B	A	A	B	A	B	B	B	B	B	A	A	B	A	B	A	B	A	B	B	A	B	A	B	B	B	A	A	B	A	B	B	A	A	B
B	A	A	B	A	B	A	A	A	A	A	B	A	A	B	A	B	B	B	B	B	A	A	B	A	B	A	B	A	B	B	A	B	A	B	B	B	A	A	B	A	B	B	A	A	B
B	A	A	B	A	B	A	A	A	A	A	B	A	A	B	A	B	B	B	B	B	A	A	B	A	B	A	B	A	B	B	A	B	A	B	B	B	A	A	B	A	B	B	A	A	B
B	A	A	B	A	B	A	A	A	A	A	B	A	A	B	A	B	B	B	B	B	A	A	B	A	B	A	B	A	B	B	A	B	A	B	B	B	A	A	B	A	B	B	A	A	B
B	A	A	B	B	B	A	A	A	A	A	B	A	A	B	A	B	B	B	B	B	A	A	B	A	B	A	B	A	B	B	A	B	A	B	B	B	A	A	B	A	B	B	A	A	B
B	A	A	B	B	B	A	A	A	A	A	B	A	A	B	A	B	B	B	B	B	A	A	B	A	B	A	B	A	B	B	A	B	A	B	B	B	A	A	B	A	B	B	A	A	B
B	A	A	B	B	B	A	A	A	A	A	B	A	A	B	A	B	B	B	B	B	A	A	B	A	B	B	B	A	B	B	A	B	A	B	B	B	A	A	B	A	B	B	A	A	B
B	A	A	B	B	B	A	A	A	A	A	B	A	A	B	A	B	B	B	B	B	A	A	B	A	B	B	B	A	B	B	A	B	A	B	B	A	A	A	B	A	B	B	A	A	B
B	A	A	B	B	B	A	A	A	A	A	B	A	A	B	A	B	B	B	B	B	A	A	B	A	B	B	B	A	B	B	A	B	A	B	B	A	A	A	B	A	B	B	A	A	B
B	A	A	B	B	B	A	A	A	A	A	B	A	A	B	A	B	B	B	B	B	A	A	B	B	B	B	B	A	B	B	A	B	A	B	B	A	A	A	B	A	B	B	A	A	B
B	A	A	B	B	B	A	A	A	A	A	A	A	A	B	A	B	B	B	B	B	A	A	B	B	B	B	B	A	B	B	A	B	A	B	B	A	A	A	B	A	B	B	B	A	B
A	A	A	B	B	B	A	A	A	A	A	A	A	A	B	A	B	B	B	B	B	A	A	B	B	B	B	B	A	B	B	A	B	A	B	B	A	A	A	B	A	B	B	B	A	B
A	A	A	B	B	B	A	A	A	A	A	A	A	A	B	A	B	B	B	B	B	A	A	B	B	B	B	B	A	B	B	A	B	A	B	B	A	A	A	B	A	B	A	B	A	B
A	A	A	B	B	B	A	A	A	A	A	A	A	A	B	A	B	B	A	B	B	A	A	B	B	B	B	B	A	B	B	A	A	A	B	B	A	A	A	B	A	B	A	B	A	B
A	A	A	B	B	B	A	A	A	A	A	A	A	A	B	A	A	A	A	B	B	A	A	A	B	B	B	B	A	B	A	A	A	A	A	B	A	A	A	B	A	B	A	B	A	B
A	A	A	B	B	B	A	A	A	A	A	A	A	A	B	A	A	A	A	B	B	A	A	A	B	B	B	B	A	B	A	A	A	A	A	B	A	A	A	B	A	B	A	B	A	B
A	B	A	B	B	B	A	A	B	A	A	A	A	A	B	A	A	A	A	B	B	A	A	A	B	B	B	B	A	B	A	A	A	A	A	B	A	A	A	B	A	B	A	B	A	B
A	B	A	B	B	B	A	A	B	A	A	A	A	A	B	A	A	A	A	B	B	A	A	A	B	B	B	B	A	B	A	A	A	A	A	B	A	A	A	B	A	B	A	B	A	B
A	B	A	B	B	B	A	A	B	A	A	A	A	A	B	A	A	A	A	B	B	A	A	A	B	B	B	B	A	B	A	A	A	A	A	A	A	A	A	B	A	B	A	B	A	B
A	B	A	B	B	B	A	A	B	A	B	A	A	A	B	A	A	A	A	B	B	A	A	A	B	B	B	B	A	B	A	A	A	A	A	A	A	B	A	B	B	B	A	B	A	B
A	B	A	B	B	B	A	B	B	A	B	A	A	A	B	A	A	A	A	B	B	A	A	A	B	B	B	B	A	B	A	A	A	A	A	A	A	B	A	B	B	B	A	B	A	B
A	B	A	B	B	B	A	B	B	A	B	A	A	A	B	A	A	A	A	B	B	A	A	A	B	B	B	B	A	B	A	A	A	A	A	A	A	B	A	B	B	B	A	B	A	B
A	B	A	B	B	B	A	B	B	A	B	A	A	A	B	A	A	A	A	B	B	A	A	A	B	B	B	B	A	B	A	A	A	A	A	A	A	B	A	B	B	B	A	B	A	B
A	B	A	B	B	A	A	B	B	A	B	A	A	A	B	A	A	A	A	B	B	A	A	A	B	B	B	B	A	B	A	A	A	A	A	A	A	B	A	B	B	B	A	B	A	B
A	B	A	B	B	A	A	B	B	A	B	A	A	A	B	A	A	A	A	B	B	A	A	A	B	B	B	B	A	B	A	A	A	A	A	A	A	B	A	B	B	B	A	B	A	B

A	B	A	B	B	A	A	B	B	A	B	A	A	A	B	A	A	A	A	B	B	A	A	A	B	B	B	B	A	B	A	A	A	A	A	A	A	B	A	B	B	B	A	B	A	B
A	B	A	B	B	A	A	B	B	A	B	A	A	A	B	A	A	A	A	B	B	A	A	A	B	B	B	B	A	B	A	A	A	A	A	A	A	B	A	B	B	B	A	B	A	B
A	B	A	B	B	A	A	B	B	A	B	A	A	A	B	A	A	A	A	B	B	A	A	A	B	B	B	B	A	B	A	A	A	A	A	A	A	B	A	B	B	B	A	B	A	B
A	B	A	B	A	A	A	B	B	A	B	A	A	A	B	A	A	A	A	B	B	A	A	A	B	B	B	B	A	B	A	B	A	A	A	A	A	B	A	B	A	B	A	B	A	B
A	B	A	B	B	A	A	B	B	A	B	A	B	A	B	A	A	A	A	B	B	A	A	A	B	B	B	B	A	B	A	B	A	A	A	A	A	B	B	B	B	B	A	B	A	B
A	B	A	B	B	A	A	B	B	A	B	A	B	A	B	A	A	A	A	B	B	A	A	A	B	B	B	B	A	B	A	B	A	A	A	A	A	B	B	B	B	B	A	B	A	B
A	B	A	B	B	A	B	B	B	A	B	A	B	A	B	A	A	A	A	B	B	A	A	A	B	B	B	B	A	B	A	B	A	A	A	A	A	B	B	B	B	B	A	B	A	B
A	B	A	B	B	A	B	B	B	A	B	A	B	A	B	B	A	A	A	B	B	A	A	A	B	B	B	B	A	B	A	B	A	A	A	A	A	B	B	B	B	B	A	B	A	B
A	B	A	B	B	A	B	B	B	A	B	A	B	A	B	B	A	A	A	B	B	A	A	A	B	B	B	B	A	B	A	B	A	A	A	A	A	B	B	B	B	A	A	B	A	B
A	B	A	B	B	A	B	B	B	A	B	A	B	A	B	B	A	A	A	B	B	A	A	A	B	B	B	B	A	B	A	B	A	A	A	A	A	A	B	B	B	A	A	B	A	B
A	B	A	B	B	A	B	B	B	A	B	A	B	A	B	B	A	A	A	B	B	A	A	A	B	A	B	B	A	B	A	B	A	A	A	A	A	A	B	B	B	A	A	B	B	B
A	B	A	B	B	A	B	B	B	A	B	A	B	B	B	B	A	A	A	B	B	A	A	A	B	A	B	B	A	B	A	B	A	A	A	A	A	A	B	B	B	A	A	B	B	B
B	B	A	A	B	0	B	B	B	A	A	B	A	A	B	A	B	B	B	B	A	B	B	B	A	B	0	A	A	A	B	A	B	A	B	B	B	A	B	B	A	A	0	A	A	A
A	B	A	A	B	0	B	B	B	A	A	B	A	A	B	A	B	B	B	B	A	B	B	B	A	B	0	A	A	A	B	A	B	A	B	B	B	A	B	B	A	A	0	A	A	A
A	B	A	B	B	0	B	B	B	A	A	B	A	A	B	A	B	B	B	B	A	B	B	B	A	B	0	A	A	A	B	A	B	A	B	B	B	A	B	B	A	A	0	A	A	A
A	B	A	B	B	0	B	B	B	A	A	B	A	A	B	A	B	B	B	B	A	B	B	B	A	B	0	A	A	A	B	A	B	A	B	B	B	A	B	B	A	A	0	A	A	A
A	B	A	B	B	0	B	B	B	A	A	B	A	A	B	A	B	B	B	B	A	B	B	B	A	B	0	A	A	A	B	A	B	A	B	B	B	A	B	B	A	A	0	A	A	A
A	B	A	B	B	0	B	B	B	A	A	B	A	A	B	A	B	B	B	B	A	B	B	B	A	B	0	A	A	A	B	A	B	A	B	B	B	A	B	B	A	A	0	A	A	A
A	A	A	B	B	0	B	B	A	A	A	B	A	A	B	A	B	B	B	B	A	B	B	B	A	B	0	A	A	A	B	A	B	A	B	B	B	A	B	A	A	A	0	A	A	A
A	A	A	B	A	0	B	B	A	A	A	B	A	A	B	A	B	B	B	B	A	B	B	B	A	B	0	A	A	A	B	A	B	A	B	B	B	A	B	A	A	A	0	A	A	A
A	A	A	B	A	A	B	B	A	A	A	B	A	A	B	A	B	B	B	B	A	A	B	B	A	B	B	A	A	A	B	A	B	A	B	B	B	A	B	A	A	A	A	A	A	A
A	A	A	B	A	A	B	B	A	A	A	A	A	A	B	A	B	B	B	B	A	A	B	B	A	B	B	A	A	A	B	A	B	A	B	B	B	A	B	A	A	A	A	A	A	A
A	A	A	B	A	A	B	B	A	A	A	A	A	A	B	A	B	B	B	B	A	A	B	B	A	B	B	A	A	A	B	A	B	A	B	B	B	A	B	A	A	A	A	A	A	A
A	A	A	B	A	A	B	B	A	A	A	A	A	A	B	A	B	B	B	B	A	A	B	B	A	B	B	A	A	A	B	A	B	A	B	B	B	A	B	A	A	A	A	A	A	A
A	A	A	B	A	A	B	B	A	A	A	A	A	A	A	A	B	B	B	A	A	A	B	B	A	B	B	A	A	A	B	A	B	B	B	A	B	A	B	A	A	A	A	A	A	A
A	A	A	B	A	A	B	B	A	A	A	A	A	A	A	A	B	B	B	A	A	A	B	B	A	B	B	A	A	A	B	A	B	B	B	A	B	A	B	A	A	A	A	A	A	A
A	A	A	B	A	A	B	B	A	A	A	A	A	A	A	A	B	B	B	A	A	A	B	B	A	B	B	A	A	A	B	A	B	B	B	A	A	A	B	A	A	A	A	A	A	A
A	A	A	B	A	A	B	B	A	A	A	A	A	A	A	A	B	B	B	A	A	A	B	B	A	B	B	A	A	A	B	A	B	B	B	A	A	A	B	A	A	A	A	A	A	A
A	A	A	B	A	A	B	B	A	A	A	A	A	A	A	A	B	B	B	A	A	A	B	B	A	B	B	A	A	A	B	A	B	B	B	A	A	A	B	A	A	A	A	A	A	A
A	A	A	B	A	A	B	B	A	A	A	A	A	A	A	A	B	B	B	A	A	A	B	B	A	B	B	A	A	A	B	A	B	B	B	A	A	A	B	A	A	A	A	A	A	A
A	A	A	B	A	A	B	B	A	A	A	A	A	A	A	A	B	B	B	A	B	A	B	B	A	B	B	A	A	A	B	A	B	B	B	A	A	A	B	A	A	A	A	A	A	A
A	A	A	B	A	A	B	B	A	A	A	A	A	A	A	A	B	B	B	B	B	A	B	B	A	B	B	A	A	A	B	A	B	B	B	A	A	A	B	A	A	A	A	A	A	A
A	A	A	B	A	A	B	B	A	A	A	A	A	A	A	A	B	B	B	B	B	A	B	B	A	B	B	A	A	A	B	A	B	B	B	A	A	A	B	A	A	A	B	A	A	A
A	A	A	B	A	A	A	B	A	A	A	A	A	A	A	A	B	B	B	B	B	A	B	B	A	B	B	A	A	A	B	A	B	B	B	A	A	A	B	A	A	A	B	A	A	A
A	A	A	B	A	A	A	B	A	A	A	A	A	A	A	A	B	B	B	B	B	A	B	B	A	B	B	A	B	A	B	A	B	B	B	A	A	A	B	A	A	A	B	A	A	A
A	A	A	B	A	A	A	B	A	A	A	A	A	A	A	A	B	B	B	B	B	A	B	B	A	B	B	A	B	A	B	A	B	B	B	A	A	A	B	A	A	A	B	A	A	A
A	A	A	B	A	A	A	B	A	A	A	A	A	A	A	A	B	B	B	B	B	A	B	B	A	B	B	A	B	A	B	A	B	B	B	A	A	A	B	A	A	A	B	A	A	A
A	A	A	B	A	A	A	B	A	A	A	A	A	A	A	A	B	B	B	B	B	A	B	B	A	B	B	A	B	A	B	A	B	B	B	A	A	A	B	A	A	A	B	A	A	A
A	A	A	A	A	A	A	B	A	A	A	A	A	A	A	A	B	B	B	B	B	A	B	B	A	B	B	A	B	A	B	A	B	B	B	A	A	A	B	A	A	A	B	A	A	A
A	A	A	A	A	A	A	B	A	A	A	A	A	A	A	A	B	B	B	B	A	A	B	B	A	B	B	A	B	A	B	A	B	B	B	A	A	A	B	A	A	A	B	A	A	A
A	A	A	A	A	A	A	B	A	A	A	A	A	A	A	A	B	B	B	B	A	A	B	B	A	B	B	A	B	A	B	A	B	B	B	A	A	A	B	A	A	A	B	A	A	A
A	A	A	A	A	A	A	B	A	A	A	A	A	A	A	A	B	B	B	B	A	A	B	B	A	B	B	A	B	A	B	A	B	B	B	A	A	A	B	A	A	A	B	A	A	A
A	A	A	A	A	A	A	B	A	A	A	A	A	A	A	A	B	B	B	B	A	A	B	B	A	B	B	A	B	A	B	A	B	B	B	A	A	A	B	A	A	A	B	A	A	A
A	A	A	A	A	A	A	B	A	A	A	A	A	A	A	A	B	B	B	B	A	A	B	B	A	B	B	A	B	A	B	A	B	B	B	A	A	A	B	A	A	A	B	A	A	A
A	A	A	A	A	A	A	B	A	A	A	A	A	A	A	A	B	B	B	B	A	A	B	B	A	B	B	A	B	A	B	A	B	B	B	A	A	A	B	A	A	A	B	A	A	A
A	A	A	A	A	A	A	B	A	A	A	A	A	A	A	A	B	B	B	B	A	A	B	B	A	B	B	A	B	A	B	A	B	B	B	A	A	A	B	A	A	A	B	A	A	A
A	A	A	A	A	A	A	B	A	A	A	A	A	A	A	A	B	B	B	B	A	A	B	B	A	B	B	A	B	A	B	A	B	B	B	A	A	A	B	A	A	A	B	A	A	A
A	A	A	A	A	A	A	B	A	A	A	A	A	A	A	A	B	B	B	B	A	A	B	B	A	B	B	A	B	A	B	A	B	B	B	A	A	A	B	A	A	A	B	A	A	A
A	A	A	A	A	A	A	B	A	A	A	A	A	A	A	A	B	B	B	B	A	A	B	B	A	B	B	A	B	A	B	A	B	B	B	A	A	A	B	A	A	A	B	A	A	A

B	A	A	A	A	A	A	B	A	A	A	A	A	A	A	A	B	B	B	B	A	A	B	B	A	B	B	A	B	A	B	A	B	B	B	A	A	A	B	A	A	A	B	A	A	A
B	A	A	A	A	A	A	B	A	A	A	A	A	A	A	A	B	B	B	B	A	A	B	B	A	B	B	A	B	A	B	A	B	B	B	A	A	A	B	A	A	A	B	A	A	A
B	A	A	A	A	A	A	B	A	A	A	A	A	A	A	A	B	B	B	B	A	A	B	B	A	B	B	A	B	A	B	A	B	B	B	A	A	A	B	A	A	A	B	A	A	A
B	A	A	A	A	A	A	B	A	A	A	A	A	A	A	A	B	B	B	B	A	B	B	B	A	B	B	A	B	A	B	A	B	B	B	A	A	A	B	A	A	A	B	A	A	A
B	A	A	A	A	A	A	B	A	A	A	A	A	A	A	A	B	B	B	B	A	B	B	B	A	B	B	A	B	A	B	A	B	B	B	A	A	A	B	A	A	A	B	A	A	A
B	A	A	A	A	A	B	B	A	A	A	A	A	A	A	A	B	B	B	B	A	B	B	B	A	B	B	A	B	A	B	A	B	B	B	A	A	A	B	A	A	A	B	A	A	A
B	A	A	A	A	A	B	B	A	A	A	A	A	A	A	A	B	B	B	B	A	B	B	B	A	B	B	A	B	A	B	A	B	B	B	A	A	A	B	A	A	A	B	A	A	A
B	A	A	A	A	A	B	B	A	A	A	A	A	A	A	A	B	B	B	B	A	B	B	B	A	B	B	A	B	A	B	A	B	B	B	A	A	A	B	A	A	A	B	A	A	A
B	A	A	A	A	A	B	B	A	A	A	A	A	A	A	A	B	B	B	B	A	B	B	B	A	B	B	A	B	A	B	A	B	B	B	A	A	A	B	A	A	A	B	A	A	A
B	A	A	A	A	A	B	B	A	A	A	A	A	A	A	A	B	B	B	B	A	B	B	B	A	B	B	A	B	A	B	A	B	B	B	A	A	A	B	A	A	A	B	A	A	A
B	A	A	A	A	A	B	B	A	A	A	B	A	A	A	A	B	B	B	B	A	B	B	B	A	B	B	A	B	A	B	A	B	B	B	A	A	A	B	A	A	A	B	A	A	A
B	A	A	A	A	A	B	B	A	A	A	B	A	A	A	A	B	B	B	B	B	B	B	B	A	B	B	A	B	A	B	A	B	B	B	A	A	A	B	A	A	A	B	A	A	A
B	A	A	A	A	A	B	B	A	A	A	B	A	A	A	A	B	B	B	B	B	B	B	B	A	B	B	A	B	A	B	A	B	B	B	A	A	A	B	A	A	A	B	A	A	A
B	A	A	A	A	A	B	B	A	A	A	B	A	A	A	A	B	B	B	B	B	B	B	B	A	B	B	A	B	A	B	A	B	B	B	A	A	A	B	A	A	A	B	A	A	A
B	A	A	A	A	A	B	B	A	A	A	B	A	A	A	A	B	B	B	B	B	B	B	B	A	B	B	A	B	A	B	A	B	B	B	A	A	A	B	A	A	A	B	A	A	A
B	A	A	A	A	A	B	B	A	A	A	B	A	B	A	A	B	B	B	B	B	B	B	B	A	B	B	A	B	A	B	A	B	B	B	A	A	A	B	A	A	A	B	A	A	A
B	A	A	A	A	A	B	B	A	A	A	B	A	B	A	A	B	B	B	B	B	B	B	B	A	B	B	B	B	A	B	A	B	B	B	A	A	A	B	A	A	A	B	A	A	A
B	A	A	A	A	A	B	B	A	A	A	B	A	B	A	A	B	B	B	B	B	B	B	B	A	B	B	B	B	A	B	A	B	B	B	A	A	A	B	A	A	A	B	A	A	A
B	A	A	A	A	A	A	B	A	A	A	B	A	B	A	A	B	B	B	B	B	B	B	B	A	B	B	B	B	A	B	A	B	B	B	A	A	A	B	A	A	A	B	A	A	A
B	A	A	A	A	A	A	B	A	A	A	B	A	B	A	A	B	B	B	B	B	B	B	B	A	B	B	B	B	A	B	A	B	B	B	A	A	A	A	A	A	A	B	A	A	A
B	A	A	A	A	A	A	B	A	A	A	B	A	B	A	A	B	B	B	B	B	B	B	B	A	B	B	B	B	A	B	A	B	B	B	A	A	A	A	A	A	A	B	A	A	A
B	A	A	A	A	A	A	B	A	A	A	B	A	B	A	A	B	B	B	B	B	B	B	B	A	B	B	B	B	A	B	A	B	B	B	A	A	A	A	A	A	A	B	A	B	A
B	A	A	A	A	A	A	B	A	A	A	B	A	B	A	A	B	B	B	B	B	B	B	B	A	B	B	B	B	A	B	A	B	B	B	A	A	A	A	A	A	A	A	A	B	A
B	A	A	A	A	A	A	B	A	A	A	B	A	B	A	A	B	B	B	B	B	B	B	B	A	A	B	B	B	A	B	A	B	B	B	A	A	A	A	A	A	A	A	A	B	A
B	A	A	A	A	A	A	B	A	A	A	B	A	B	A	A	B	B	B	B	B	B	B	B	A	A	B	B	B	A	B	A	B	B	B	A	A	A	A	A	A	A	A	A	B	A
B	A	A	A	A	A	A	B	A	A	A	B	A	B	A	A	B	B	B	B	B	B	B	B	A	A	B	B	B	A	B	A	B	B	B	A	A	A	A	A	A	A	A	B	B	A
B	A	A	A	A	A	A	A	A	A	A	B	A	B	A	A	B	B	B	A	B	B	B	B	A	A	B	B	B	A	B	A	B	B	B	A	A	A	A	A	A	A	A	B	B	A
B	A	A	A	A	A	A	A	A	A	A	B	A	B	A	A	B	B	B	A	B	B	B	B	A	A	B	B	B	A	B	A	B	B	B	A	A	A	A	A	A	A	A	B	B	A
B	A	A	A	A	A	A	A	A	A	A	B	A	B	A	A	B	B	B	A	B	B	B	B	A	A	B	B	B	A	B	A	B	B	B	A	A	A	A	A	A	A	A	B	B	A
B	A	A	A	A	A	A	A	A	A	A	B	A	B	A	A	B	B	B	A	B	B	B	B	A	A	B	B	B	A	B	A	B	B	B	A	A	A	A	A	A	A	A	B	B	A
B	A	A	A	A	A	A	A	A	A	A	B	A	B	A	A	B	B	B	A	B	B	B	B	A	A	B	B	B	A	B	A	B	B	B	A	A	A	A	A	A	A	A	B	B	B
B	A	A	A	A	B	A	A	A	A	A	B	A	B	A	A	B	B	A	A	B	B	B	B	A	A	B	B	B	A	B	A	A	B	B	A	A	A	A	A	A	A	A	B	B	B
B	A	A	A	A	B	A	A	A	A	A	B	A	B	A	A	B	B	A	A	B	B	B	B	A	A	B	B	B	A	B	A	A	B	B	A	A	A	A	A	A	A	A	B	B	B
B	A	A	A	A	B	A	A	A	A	A	B	A	A	A	A	B	B	A	A	B	B	B	B	A	A	B	B	B	A	B	A	A	B	B	A	A	A	A	A	A	A	A	B	B	B
B	A	A	A	A	B	A	A	A	A	A	B	A	A	A	A	B	B	A	A	A	B	B	B	A	A	B	B	B	A	B	A	A	B	B	A	A	A	A	A	A	A	A	B	B	B
B	A	A	A	A	B	A	A	A	A	A	B	A	A	A	A	B	B	A	A	A	B	B	B	A	A	B	B	B	A	B	A	A	B	B	A	A	A	A	A	A	A	A	B	B	B
B	A	A	A	A	B	A	A	A	A	A	B	A	A	A	A	B	B	A	A	A	B	B	B	A	A	B	B	B	A	B	A	A	B	B	A	A	A	A	A	A	A	A	B	B	B
B	A	A	A	A	B	A	A	A	A	A	B	A	A	A	A	B	B	A	A	A	B	B	B	A	A	B	B	B	A	B	A	A	B	B	B	A	A	A	A	A	A	A	B	B	B
B	A	A	A	A	B	A	B	A	A	A	B	A	A	A	A	B	B	A	A	A	B	B	B	A	A	B	B	B	A	B	A	A	B	B	B	A	A	A	A	A	A	A	B	B	B
B	A	A	A	A	B	A	B	A	A	A	B	A	A	A	B	B	B	A	A	A	B	B	B	A	A	B	B	B	A	B	A	A	B	B	B	A	A	A	A	A	A	A	B	B	B
B	A	A	B	A	B	A	B	A	A	A	B	A	A	A	B	B	B	A	A	A	B	B	B	A	A	B	B	B	A	B	A	A	B	B	B	A	A	A	A	A	A	A	B	B	B
B	A	A	B	A	B	A	B	A	A	A	B	A	A	A	B	B	B	A	A	A	B	B	B	A	A	B	B	B	A	B	B	A	B	B	B	A	A	A	A	A	A	A	B	B	B
B	A	A	B	A	B	A	B	A	A	A	B	B	A	A	B	B	B	A	A	A	B	B	B	A	A	B	B	B	A	B	B	A	B	B	B	A	A	A	A	A	A	A	B	B	B
B	A	A	B	A	B	A	B	A	A	A	B	B	A	A	B	B	B	A	A	A	B	B	B	A	A	B	B	B	A	B	B	A	B	B	B	A	A	A	A	A	A	A	B	B	B
B	A	A	B	A	B	A	B	A	A	A	B	B	A	A	B	B	B	A	A	A	B	B	B	A	A	B	B	B	A	B	B	A	B	B	B	A	A	A	A	A	A	A	B	B	B
B	A	A	B	A	B	A	B	A	A	A	B	B	A	A	B	B	B	A	A	A	B	B	B	A	A	B	B	B	A	B	B	A	B	B	B	A	A	A	A	A	A	A	B	B	B
B	A	A	B	B	B	A	B	A	A	A	B	B	A	A	B	B	B	A	A	A	B	B	B	A	A	B	B	B	A	B	B	A	B	B	B	A	A	A	A	A	A	A	B	B	B
B	A	A	B	B	B	A	B	A	A	A	B	B	A	A	B	B	B	A	A	A	B	B	B	A	A	B	B	B	A	B	B	A	B	B	B	A	A	A	A	A	A	A	B	B	B
B	A	A	B	B	B	A	B	A	A	A	B	B	A	A	B	B	B	A	A	A	B	B	B	A	A	B	B	B	A	B	B	A	B	B	B	A	A	A	A	A	A	A	B	B	B

B	A	A	B	B	B	A	B	A	A	A	B	B	A	A	B	B	B	A	A	A	B	B	B	A	A	B	B	B	A	B	B	A	B	B	B	A	A	A	A	A	A	A	B	B	B
B	A	A	B	B	B	A	B	A	A	A	B	B	A	A	B	B	B	A	A	A	B	B	B	A	A	B	B	B	A	B	B	A	B	B	B	A	A	A	A	A	A	A	B	B	B
B	A	A	B	B	B	A	B	A	A	A	B	B	A	A	B	B	B	A	A	A	B	B	B	A	A	B	B	B	A	B	B	A	B	B	B	B	A	A	A	A	A	A	B	B	B
A	A	A	A	B	A	A	A	A	A	A	A	B	B	A	A	A	A	A	A	B	B	A	A	B	A	B	B	A	A	A	B	A	A	A	A	A	B	A	A	A	A	A	A	A	A
A	A	A	A	B	A	A	A	A	A	A	A	B	B	A	A	A	A	A	A	B	B	A	A	B	A	B	B	A	A	A	B	A	A	A	A	A	B	A	A	A	A	A	A	A	A
A	A	A	A	B	A	A	A	A	A	A	A	B	B	A	A	A	A	A	A	B	B	A	A	B	A	B	B	A	A	A	B	A	A	A	A	A	B	A	A	A	A	A	A	A	A
A	A	A	A	B	A	A	A	A	A	A	A	B	B	A	A	A	A	A	A	B	B	A	A	B	A	B	B	A	A	A	B	A	A	A	A	A	B	A	A	A	A	A	A	A	A
A	A	A	A	B	A	A	A	A	A	A	A	B	B	A	A	A	A	A	A	B	B	A	A	B	A	B	B	A	A	A	B	A	A	A	A	A	B	A	A	A	A	A	B	A	A
A	A	A	A	B	A	A	A	A	A	A	A	B	B	A	A	A	A	A	A	B	B	A	A	B	A	B	B	A	A	A	B	A	A	A	A	A	B	A	A	A	A	A	B	A	A
A	A	A	A	B	A	A	A	A	A	A	A	B	B	A	A	A	A	A	A	B	B	A	A	B	A	B	B	A	A	A	B	A	A	A	A	A	B	A	A	A	A	A	B	A	A
A	A	A	A	B	A	A	A	A	A	A	A	B	B	A	A	A	A	A	A	B	B	A	A	B	A	B	B	A	A	A	B	A	A	A	A	A	B	A	A	A	A	A	B	A	A
A	A	A	A	B	A	A	A	A	A	A	A	B	B	A	A	A	A	A	A	B	B	A	A	B	A	B	B	A	A	A	B	A	A	A	A	A	B	A	A	A	A	A	B	A	A
A	A	A	A	B	A	A	A	A	A	A	A	B	B	A	A	A	A	A	A	B	B	A	A	B	A	B	B	A	A	A	B	A	A	A	A	A	B	A	A	A	A	A	B	A	A
A	A	A	A	B	A	A	A	A	A	A	A	B	B	A	A	A	A	A	A	B	B	A	A	B	A	B	B	A	A	A	B	A	A	A	A	A	B	A	A	A	A	A	B	A	A
A	A	A	A	B	A	A	A	A	A	A	A	B	B	A	A	A	A	A	A	B	B	A	A	B	A	B	B	A	A	A	B	A	A	A	A	A	B	A	A	A	A	A	B	A	A
A	A	A	A	B	A	A	A	A	A	A	A	B	B	A	A	A	A	A	A	B	B	A	A	B	A	B	B	A	A	A	B	A	A	A	A	A	B	A	A	A	A	A	B	A	A
A	A	A	A	B	A	A	A	A	A	A	A	B	B	A	A	A	A	A	A	B	B	A	A	B	A	B	B	A	A	A	B	A	A	0	A	A	B	A	A	A	A	A	B	A	A
A	A	A	A	B	A	A	A	A	A	A	A	B	B	A	A	A	A	A	A	B	B	A	A	B	A	B	B	A	A	A	B	A	A	0	A	A	B	A	A	A	A	A	B	A	A
A	A	A	A	B	A	A	A	A	A	A	A	B	B	A	A	A	A	A	A	B	B	A	A	B	A	B	B	A	A	A	B	A	A	0	A	A	B	A	A	A	A	A	B	A	A
A	A	A	A	B	A	A	A	A	A	A	A	B	B	B	A	A	A	A	A	B	B	A	A	B	A	B	B	A	A	A	B	A	A	0	A	A	B	A	A	A	A	A	B	A	A
A	A	A	A	B	A	A	A	A	A	A	A	B	B	B	A	A	A	A	A	B	B	A	A	B	A	B	B	A	A	A	B	A	A	0	A	A	B	A	A	A	A	A	B	A	A
A	A	A	A	B	A	A	A	A	A	A	A	B	B	B	A	A	A	A	A	B	B	A	A	B	A	B	B	A	A	A	B	A	A	0	A	A	B	A	A	A	A	A	B	A	A
A	A	A	A	B	A	A	A	A	A	A	A	B	B	B	A	A	A	A	A	B	B	A	A	B	A	B	B	A	A	A	B	A	A	0	A	A	B	A	A	A	A	A	B	A	A
A	A	A	A	B	A	A	A	A	A	A	A	B	B	B	A	A	A	A	A	B	B	A	A	B	A	B	B	B	A	A	B	A	A	0	A	A	B	A	A	A	A	A	B	A	A
A	A	A	A	B	A	A	A	A	A	A	A	B	B	B	A	A	A	A	A	B	B	A	A	B	A	B	B	B	A	A	B	A	A	A	B	A	B	A	A	A	A	A	B	A	A
A	A	A	A	B	A	A	A	A	A	A	A	B	B	B	A	A	A	A	A	B	B	A	A	B	A	B	B	B	A	A	B	A	A	A	B	A	B	A	A	A	A	A	B	A	A
A	A	A	A	B	A	A	A	A	A	A	A	B	B	B	A	A	A	A	A	B	B	A	A	B	A	B	B	B	A	A	B	A	A	A	B	A	B	A	A	A	A	A	B	A	A
A	A	A	A	B	A	A	A	A	A	A	A	B	B	B	A	A	A	A	A	B	B	A	A	B	A	B	B	B	A	A	B	A	A	A	B	A	B	A	A	A	A	A	B	A	A
A	A	A	A	B	A	A	A	A	A	A	B	B	B	B	A	A	A	A	A	B	B	A	A	B	A	B	B	B	A	A	B	A	A	A	B	A	B	A	A	A	A	A	B	A	A
A	A	A	A	B	A	A	A	A	A	A	B	B	B	B	A	A	A	A	A	B	B	A	A	B	A	B	B	B	A	A	B	A	A	A	B	A	B	A	A	A	A	A	B	A	A
A	A	A	A	B	A	A	A	A	A	A	B	B	B	B	A	A	A	A	A	B	B	A	A	B	A	B	B	B	A	A	B	A	A	A	B	A	B	A	A	A	A	A	B	A	A
A	A	A	A	B	A	A	A	A	A	A	B	B	B	B	A	A	A	A	A	B	B	A	A	B	A	B	B	B	A	A	B	A	A	A	B	A	B	A	A	A	A	A	B	A	A
A	A	A	A	B	A	A	A	A	A	A	B	B	B	B	A	A	A	A	A	B	B	A	A	B	A	B	B	B	A	A	B	A	A	A	B	A	B	A	A	A	A	A	B	A	B
A	A	A	A	B	A	A	A	A	A	A	B	B	B	B	A	A	A	B	A	B	B	A	A	B	A	B	B	B	A	A	B	B	A	A	B	A	B	A	A	A	A	A	B	A	B
A	A	A	A	B	A	A	A	A	A	A	B	B	B	B	A	A	A	B	A	B	B	A	A	B	A	B	B	B	A	A	B	B	A	A	B	A	B	A	A	A	A	A	B	A	B
A	A	A	A	B	A	A	A	A	A	A	B	B	B	B	B	A	A	B	A	B	B	A	A	B	A	B	B	B	A	A	B	B	A	A	B	A	B	A	A	A	A	A	B	A	B
A	A	A	A	B	A	A	A	A	A	A	B	B	B	B	B	A	A	B	A	B	B	A	A	B	A	B	B	B	A	A	B	B	A	A	B	A	B	A	A	A	A	A	B	A	B
A	A	A	A	B	A	A	A	A	A	A	B	B	B	B	B	A	A	B	A	B	A	A	A	B	A	B	B	B	A	A	B	B	A	A	B	A	B	A	A	A	A	A	B	A	B
A	A	A	A	B	A	A	A	A	A	A	B	B	B	B	B	A	A	B	A	B	A	A	A	B	A	B	B	B	A	A	B	B	A	A	B	A	B	A	A	A	A	A	B	A	B
A	A	A	A	B	A	A	A	A	A	A	B	B	B	B	B	A	A	B	A	B	A	A	A	B	A	B	B	B	A	A	A	B	A	A	B	A	B	A	A	A	A	A	B	A	B
A	A	A	A	B	A	A	A	A	A	A	B	B	B	B	B	A	A	B	A	B	A	A	A	B	A	B	B	B	A	A	A	B	A	A	B	A	B	A	A	A	A	A	B	A	B
A	A	A	A	B	A	A	A	A	A	A	B	A	B	B	B	A	A	B	A	B	A	A	A	B	A	B	B	B	A	A	A	B	A	A	B	A	B	A	A	A	A	A	B	A	B
A	A	A	A	B	A	A	A	A	A	A	B	A	B	B	B	A	A	B	A	B	A	A	A	B	A	B	B	B	A	A	A	B	A	A	B	A	B	A	A	A	A	A	B	A	B
A	A	A	A	B	A	A	A	A	A	A	B	A	B	B	B	A	A	B	A	B	A	A	A	B	A	B	B	B	A	A	A	B	A	A	B	A	B	A	A	A	A	A	B	A	B
A	A	A	A	B	A	A	A	A	A	A	B	A	B	B	B	A	A	B	A	B	A	A	A	B	A	B	B	B	A	A	A	B	A	A	B	A	B	A	A	A	A	A	B	A	B
A	A	A	A	B	A	A	A	A	A	A	B	A	B	B	B	A	A	B	A	B	A	A	A	A	A	B	B	B	A	A	A	B	A	A	B	A	B	A	A	A	A	A	B	A	B
A	A	A	A	B	A	A	A	A	A	A	B	A	B	B	B	A	A	B	A	B	A	A	A	A	A	B	B	B	A	A	A	B	A	A	B	A	B	A	A	A	A	A	B	A	B
A	A	A	A	0	A	A	A	A	A	A	B	A	A	B	B	A	A	B	A	B	A	A	A	A	A	B	B	B	A	A	A	B	A	A	B	A	B	A	A	A	A	A	B	A	B

A	A	A	A	0	A	A	A	A	A	A	B	A	A	B	B	A	A	B	A	B	A	A	A	A	A	B	B	B	A	A	A	B	A	A	B	A	B	A	A	A	A	A	B	A	B
A	A	A	A	0	A	B	A	A	A	A	B	A	A	B	B	A	A	B	A	B	A	A	A	A	A	A	B	B	A	A	A	B	A	A	B	A	B	B	A	A	A	A	B	A	B
A	A	A	A	0	A	B	A	A	A	A	B	A	A	B	B	A	A	B	A	B	A	A	A	A	A	A	B	B	A	A	A	B	A	A	B	A	B	B	A	A	A	A	B	A	B
A	A	A	A	0	A	B	A	A	A	A	B	A	A	B	B	A	A	B	A	B	A	A	A	A	A	A	A	B	A	A	A	B	A	A	B	A	B	B	A	A	A	A	B	A	B
A	A	A	A	0	A	B	A	A	A	A	B	A	A	B	B	A	A	B	A	B	A	A	A	A	A	A	A	B	A	A	A	B	A	A	B	A	B	B	A	A	A	A	A	A	B
A	A	A	A	0	A	B	A	A	A	A	B	A	A	B	B	A	A	B	A	B	A	A	A	A	A	A	A	B	A	A	A	B	A	A	B	A	B	B	A	A	A	A	A	A	B
A	A	A	A	0	A	B	A	A	A	A	B	A	A	B	B	A	A	B	A	B	A	A	A	A	A	A	A	B	A	A	A	B	A	A	B	A	B	B	A	A	A	A	A	A	B
A	A	A	A	0	A	B	A	A	A	A	B	A	A	B	B	A	A	B	A	B	A	A	A	A	A	A	A	B	A	A	A	B	A	A	B	A	B	B	A	A	A	A	A	A	B
A	A	A	A	0	A	B	A	A	A	A	B	A	A	B	B	A	A	B	A	B	A	A	A	A	A	A	A	B	A	A	A	B	A	A	B	A	B	B	A	A	A	A	A	A	B
A	A	A	A	0	A	B	A	A	A	A	B	A	A	B	B	A	A	B	A	B	A	A	A	A	A	A	A	B	A	A	A	B	A	A	B	A	B	B	A	A	A	A	A	A	B
A	A	A	A	0	A	B	A	A	A	A	B	A	A	B	B	A	A	B	A	B	A	A	A	A	A	A	A	B	A	A	A	B	A	A	B	A	B	B	A	A	A	A	A	A	B
A	A	A	A	0	A	B	A	A	A	A	B	A	A	B	B	A	A	B	A	B	A	A	A	A	A	A	A	B	A	A	A	B	A	A	B	A	B	B	A	A	A	A	A	A	B
A	A	A	A	0	A	B	A	A	A	A	B	A	A	B	B	A	A	B	A	B	A	A	A	A	A	A	A	B	A	A	A	B	A	A	B	A	B	B	A	A	A	A	A	A	B
A	A	A	A	0	A	B	A	A	A	A	B	A	A	B	B	A	A	B	A	B	A	A	A	A	A	A	A	B	A	A	A	B	A	A	B	A	B	B	A	A	A	A	A	A	B
A	A	A	A	B	A	B	A	A	A	A	B	A	A	B	B	A	A	B	A	B	A	A	A	A	A	A	A	B	A	A	A	B	A	A	B	A	B	B	A	A	A	A	A	A	B
A	A	B	A	B	A	B	A	A	B	B	B	A	B	B	B	A	A	B	A	B	A	B	A	A	A	A	B	B	A	A	A	B	A	A	B	A	B	B	A	A	A	A	A	A	B
A	A	B	A	B	A	B	A	A	B	B	A	A	B	B	B	A	A	B	B	B	A	B	A	A	A	A	B	B	A	A	A	B	A	A	B	A	B	B	A	A	A	A	A	A	B
A	A	A	B	B	B	A	A	A	0	B	B	A	A	A	B	A	0	A	B	B	B	B	0	B	B	B	B	B	A	A	A	A	0	A	A	B	A	B	B	B	B	B	A	B	A
A	A	A	B	B	B	A	A	A	0	B	B	A	A	A	B	A	0	A	B	B	B	B	0	B	B	B	B	B	A	A	A	A	0	A	A	B	A	B	B	B	B	B	A	B	A
A	A	A	B	B	B	B	A	A	0	B	B	A	A	A	B	A	0	A	A	B	B	B	0	B	B	B	B	B	A	A	A	A	0	A	A	B	A	B	B	B	B	B	A	B	A
A	A	A	B	B	B	B	A	A	A	B	B	A	A	A	B	A	A	A	A	A	B	B	0	B	B	A	B	B	A	A	A	A	0	A	A	B	A	B	B	B	B	B	A	B	A
A	A	A	B	A	B	B	A	A	A	B	B	A	A	A	B	A	A	B	A	A	B	B	0	B	B	A	B	B	A	A	A	B	0	A	A	B	A	B	B	B	B	B	A	B	A
A	B	A	B	A	B	B	A	B	A	B	B	A	A	A	B	A	A	B	A	A	B	B	0	B	B	A	B	B	A	A	A	B	0	A	A	B	A	B	B	B	B	B	A	B	A
A	B	A	B	A	B	B	A	B	A	B	B	A	A	A	B	A	A	B	A	A	B	B	0	B	B	A	B	B	A	A	A	B	0	A	A	B	A	B	B	B	B	B	A	B	A
A	B	B	B	A	B	B	A	B	B	B	B	A	A	A	B	A	A	B	A	A	B	B	0	B	B	A	B	B	A	A	A	B	0	A	A	B	A	B	B	B	B	B	A	B	A
A	B	B	B	A	B	B	A	B	B	B	B	A	A	A	B	A	A	B	A	A	B	B	A	B	B	A	B	B	A	A	A	B	0	A	A	B	A	B	A	B	B	0	A	B	A
A	B	B	B	A	B	B	A	B	B	B	B	A	A	A	B	A	A	B	A	A	B	B	A	B	B	A	B	B	A	A	A	B	0	A	A	B	A	0	A	B	B	0	A	B	A
A	B	B	B	A	B	B	A	B	B	B	B	A	B	A	B	A	A	B	A	A	B	B	A	B	B	A	B	B	A	A	A	B	0	A	A	B	A	0	A	B	B	0	A	B	A
A	B	B	B	A	B	B	A	B	B	B	B	A	B	A	A	A	A	B	A	A	B	B	A	B	B	A	B	B	A	A	A	B	0	A	A	B	A	0	A	B	B	0	A	B	A
A	B	B	B	A	B	B	A	B	B	B	B	A	B	A	A	A	A	B	A	A	B	A	A	B	B	A	B	B	A	A	A	B	0	A	A	B	A	0	A	B	B	0	A	B	A
A	B	B	B	A	B	B	A	B	B	B	B	A	B	A	A	A	A	B	A	A	B	A	A	B	B	A	B	B	A	A	A	B	0	A	A	B	A	0	A	B	B	0	A	B	A
A	B	B	B	A	B	B	A	B	B	B	B	A	B	A	A	A	A	B	A	A	B	A	A	B	B	A	A	B	A	A	A	B	0	A	A	B	A	0	A	B	B	0	A	B	A
A	B	B	B	A	B	B	A	B	B	B	B	A	B	A	A	A	A	B	A	A	B	A	A	B	B	A	A	B	A	A	A	B	0	A	A	B	A	0	A	B	B	0	A	B	A
A	B	B	B	A	B	B	A	B	B	B	B	A	B	A	A	A	A	B	A	A	B	A	A	B	B	A	A	B	A	A	A	B	0	A	A	B	A	0	A	B	B	0	A	B	A
A	B	B	B	A	B	B	A	B	B	B	A	A	B	A	A	A	A	B	A	A	B	A	A	B	B	A	A	B	A	A	A	B	0	A	A	B	A	0	A	B	B	0	A	B	A
A	B	B	B	A	B	B	A	B	B	A	A	A	B	A	A	A	A	B	A	A	B	A	A	B	B	A	A	B	A	A	A	B	0	A	A	B	A	0	A	A	B	0	A	B	A
A	B	B	B	A	B	B	A	B	B	A	A	A	B	A	A	A	A	B	A	A	B	A	A	B	B	A	A	B	A	A	A	B	0	A	A	B	A	0	A	A	B	0	A	B	A
A	B	B	B	A	B	B	A	B	B	A	A	A	B	A	A	A	A	B	A	A	B	A	A	B	B	A	A	B	A	A	A	B	0	A	A	B	A	0	A	A	B	0	A	B	A
A	B	B	B	A	B	B	A	B	B	A	A	A	B	A	A	A	A	B	A	A	B	A	A	B	B	A	A	B	A	A	A	B	0	A	A	B	A	0	A	A	B	0	A	B	A
A	B	B	B	A	B	B	A	B	B	A	A	A	B	A	A	A	A	B	A	A	B	A	A	B	A	A	A	0	A	A	A	B	0	A	A	A	A	0	A	A	B	0	A	B	A
A	B	B	A	A	B	B	A	B	B	A	A	A	B	A	A	A	A	B	A	A	B	A	A	B	A	A	A	0	A	A	A	B	0	A	A	A	A	0	A	A	B	0	A	B	A
A	B	B	A	A	B	B	A	B	B	A	A	A	B	A	A	A	A	B	A	A	B	A	A	A	A	A	A	0	A	A	A	B	0	A	A	A	A	0	A	A	B	0	A	B	A
A	B	B	A	A	B	B	A	B	B	A	A	A	B	A	A	A	A	B	A	A	B	A	A	A	A	A	A	0	A	A	A	B	0	A	A	A	A	0	A	A	A	0	A	B	A
A	B	B	A	A	B	B	A	B	B	A	B	A	B	A	A	A	A	B	A	A	B	A	A	A	A	A	A	0	A	A	A	B	0	A	A	A	A	0	A	A	A	0	A	B	A
A	B	B	A	A	B	B	A	B	B	A	B	A	B	A	A	A	A	B	A	A	B	A	A	A	A	A	A	0	A	A	A	B	0	A	A	A	A	0	A	A	A	0	A	B	A
A	B	B	A	A	B	B	A	B	B	A	B	A	B	A	A	A	A	B	A	A	B	A	A	A	A	A	A	B	A	A	A	B	0	A	A	A	A	0	A	A	A	0	A	B	A
A	B	B	A	A	B	B	A	B	B	A	B	A	B	A	A	A	A	B	A	A	B	A	A	A	A	A	A	B	A	A	A	B	0	A	A	A	A	0	A	A	A	0	A	B	A
A	B	B	A	A	B	B	A	B	B	A	B	A	B	A	A	A	A	B	A	A	B	A	A	A	A	A	A	B	A	A	A	B	0	A	A	A	A	0	A	A	A	0	A	B	A
A	B	B	A	A	B	B	A	B	B	A	B	A	B	A	A	A	A	B	A	A	B	A	A	A	A	A	A	B	A	A	A	B	0	A	A	A	A	0	A	A	A	0	A	B	A

A	B	B	A	A	B	B	A	B	B	A	B	A	B	A	A	A	A	B	A	A	B	A	A	A	A	B	A	B	A	A	A	B	0	A	A	A	A	0	A	A	A	0	A	B	A
A	B	B	A	A	B	B	A	B	B	A	B	A	B	A	A	A	A	B	A	A	B	A	A	A	A	B	A	B	A	A	A	B	0	A	A	A	A	0	A	A	A	0	B	B	A
A	A	B	A	A	B	B	A	A	B	A	B	A	B	A	A	A	A	B	A	A	B	A	A	A	A	B	A	B	A	A	A	B	0	A	A	A	A	0	A	A	A	0	B	B	A
A	A	B	A	A	B	B	A	A	B	A	B	A	B	A	A	A	A	B	A	A	B	A	A	A	A	B	A	B	A	A	A	B	0	A	A	A	A	0	A	A	A	A	B	B	A
A	A	B	A	A	B	B	A	A	B	A	B	A	B	A	A	A	A	B	A	A	B	A	A	A	A	B	A	B	A	A	A	B	0	A	A	A	A	0	A	A	A	A	B	B	A
A	A	B	A	A	B	B	A	A	B	A	B	A	B	A	A	A	A	B	A	A	B	A	A	A	A	B	A	B	A	A	A	B	0	A	A	A	A	0	B	A	A	0	B	B	A
A	A	B	B	A	B	B	A	A	B	A	B	A	B	A	A	A	A	B	A	A	B	A	A	A	A	B	A	B	A	A	A	B	A	A	A	A	A	0	B	A	A	0	B	B	A
A	A	B	B	A	B	B	A	A	B	A	B	A	A	A	A	A	A	B	A	A	B	A	A	A	A	B	A	B	A	A	A	B	A	A	A	A	A	0	B	A	A	0	B	B	A
A	A	B	B	A	B	B	A	A	B	A	B	A	A	A	A	A	A	B	A	A	B	A	A	A	A	B	A	B	A	A	A	B	A	A	A	A	A	0	B	A	A	A	B	B	A
A	A	B	B	A	B	B	A	A	B	A	B	A	A	A	A	A	A	B	A	A	B	A	A	A	A	B	A	B	A	A	A	B	A	A	A	A	A	0	B	A	A	A	B	B	A
A	A	B	B	A	B	B	A	A	B	A	B	A	A	A	A	A	A	B	A	A	B	A	A	A	A	B	A	B	A	A	A	B	A	A	A	A	A	0	B	A	A	A	B	B	A
A	A	B	B	A	B	B	A	A	B	A	B	A	A	A	A	A	A	B	A	A	B	A	A	A	A	B	A	B	A	A	A	B	A	A	A	A	A	0	B	A	A	A	B	B	A
A	A	B	B	A	B	B	A	A	B	A	B	A	A	A	A	A	A	B	A	A	B	A	A	A	A	B	A	B	A	A	A	B	A	A	A	A	A	0	B	A	A	A	A	B	A
A	A	B	B	A	B	B	A	A	B	A	B	A	A	A	A	A	A	B	A	A	B	A	A	A	A	B	A	B	A	A	A	B	A	A	A	A	A	0	B	A	A	A	A	B	A
A	A	A	A	A	B	A	B	A	A	A	A	B	A	A	A	A	A	A	A	A	A	A	A	B	A	A	A	B	A	A	A	A	B	A	A	A	A	A	A	A	A	A	A	A	B
A	A	A	A	A	B	A	B	A	A	A	A	A	A	A	A	A	A	A	A	A	A	A	A	B	A	A	A	B	A	B	A	A	B	A	A	A	A	A	A	A	A	A	A	A	B
A	A	A	A	A	B	A	B	A	A	A	A	A	A	A	A	A	A	A	A	A	A	A	A	B	B	A	A	B	A	0	A	A	B	A	A	A	A	A	A	A	A	A	A	A	B
A	A	A	A	A	B	A	B	A	A	A	A	A	A	A	A	A	A	A	A	A	A	A	A	B	B	A	A	A	A	0	A	A	B	A	A	A	A	A	A	A	A	A	A	A	B
A	A	A	A	A	B	A	B	A	A	A	A	A	A	A	A	A	A	A	A	A	A	A	A	B	B	A	A	A	A	0	A	A	B	A	A	A	A	A	A	A	A	A	A	A	A
A	A	A	A	A	B	A	B	A	A	A	A	A	A	A	A	A	A	A	A	A	A	A	A	A	B	A	A	A	A	0	A	A	B	A	A	A	A	A	A	A	A	A	A	A	A
A	A	A	A	A	B	A	B	0	A	A	A	A	A	A	A	A	A	A	A	A	A	A	A	A	B	A	A	A	A	0	A	A	B	A	A	A	A	A	A	A	A	A	A	A	A
A	A	A	A	A	B	A	B	0	A	A	A	A	A	A	A	A	A	A	A	A	A	A	A	A	B	A	A	A	A	0	A	A	A	A	A	A	A	A	A	A	A	A	A	A	A
A	A	A	A	A	B	A	B	0	A	A	A	A	A	A	A	A	A	A	A	A	A	A	A	A	B	A	A	A	A	0	A	A	A	A	A	A	A	A	A	A	A	A	A	A	A
A	A	A	A	A	B	A	B	0	A	A	A	A	A	A	A	A	A	A	A	A	A	A	A	A	B	A	A	A	A	0	A	A	A	A	A	A	A	A	A	A	A	A	A	A	A
A	A	A	A	A	B	A	B	0	A	A	A	A	A	A	A	A	A	A	A	A	A	A	A	A	B	A	A	A	A	0	A	A	A	A	A	A	A	A	A	A	A	A	A	A	A
A	A	A	A	A	B	A	B	0	A	A	A	A	A	A	A	A	A	B	A	A	A	A	A	A	B	A	A	A	A	0	A	B	A	A	A	A	A	A	A	A	A	A	A	A	A
A	A	A	A	A	B	A	B	0	A	A	A	A	A	A	A	A	A	B	A	A	A	A	A	A	B	A	A	A	A	0	A	B	A	A	A	A	A	A	A	A	A	A	A	A	A
A	A	A	A	A	B	A	B	0	A	A	A	A	A	A	A	A	A	B	A	A	A	A	A	A	B	A	A	A	A	0	A	B	A	A	A	A	A	A	A	A	A	A	A	A	A
A	A	A	A	A	B	A	B	0	A	A	A	A	A	A	A	A	A	B	A	A	A	A	A	A	B	B	A	A	A	0	A	B	A	A	A	A	A	A	A	A	A	A	A	A	A
A	A	A	A	A	B	A	B	0	A	A	A	A	A	A	A	A	A	B	A	A	A	A	A	A	B	B	A	A	A	0	A	B	A	A	A	A	A	A	A	A	A	A	A	A	A
A	A	A	A	A	B	A	B	0	A	A	A	A	A	A	A	A	A	B	A	A	B	A	A	A	B	B	A	A	A	0	A	B	A	A	A	A	A	A	A	A	A	A	A	A	A
A	A	A	A	A	B	A	B	0	A	A	A	A	A	A	A	A	A	B	A	A	B	A	A	A	B	B	A	A	A	0	A	B	A	A	A	A	A	A	A	A	A	A	A	A	A
A	A	A	A	A	B	A	B	0	A	A	A	A	A	A	A	A	A	B	A	A	B	A	A	A	B	B	A	A	A	0	A	B	A	A	A	A	A	A	A	A	A	A	A	A	A
A	A	A	A	A	B	A	B	0	A	A	A	A	A	A	A	A	A	A	A	A	B	A	A	A	B	B	A	A	A	0	A	A	A	A	A	A	A	A	A	A	A	A	A	A	A
A	A	A	A	A	B	A	B	0	A	A	B	A	A	A	A	A	A	A	A	A	B	A	A	A	B	B	A	A	A	0	A	A	A	A	A	A	A	A	A	A	A	A	A	A	A
A	A	A	A	A	B	A	B	0	A	A	B	A	A	A	A	A	A	A	A	A	B	A	A	A	B	B	A	A	A	0	A	A	A	A	A	A	A	A	A	A	A	A	A	A	A
A	A	A	A	A	B	A	A	0	A	A	B	A	A	A	A	A	A	A	A	A	B	A	A	A	B	B	A	A	A	0	A	A	A	A	A	A	A	A	A	A	A	A	A	A	A
A	A	B	A	A	B	A	A	A	B	A	B	A	A	A	A	A	A	A	A	A	B	A	A	A	B	B	A	A	A	0	A	A	A	A	A	A	A	A	A	A	A	A	A	A	A
A	A	B	A	A	B	A	A	A	B	A	B	A	A	A	A	A	A	A	A	A	B	A	A	A	B	B	A	A	A	0	A	A	A	A	A	A	A	A	A	A	A	A	A	A	A
A	A	B	A	A	B	A	A	A	B	A	B	A	A	A	A	A	A	A	A	A	B	A	A	A	B	B	A	A	A	0	A	A	A	A	A	A	A	A	A	A	A	A	A	A	A
A	A	B	A	A	B	A	A	A	B	A	B	A	A	A	A	A	A	A	A	A	B	A	A	A	B	B	A	A	A	0	A	A	A	A	A	A	A	A	A	A	B	A	A	A	A
A	A	B	A	A	B	A	A	A	B	A	B	A	A	A	A	A	A	A	A	A	B	A	A	A	B	B	A	A	A	0	A	A	A	A	A	A	A	A	A	A	B	A	A	A	A
A	A	B	A	A	B	A	A	A	B	A	B	A	A	A	A	A	A	A	A	A	B	A	A	A	B	B	A	A	B	0	A	A	A	A	A	A	A	A	A	A	B	A	A	A	A
A	A	B	A	B	B	A	A	A	B	A	B	A	A	A	A	A	A	A	A	A	B	A	A	A	B	B	A	A	B	0	A	A	A	A	A	A	A	A	A	A	B	A	A	A	A
A	A	B	A	B	B	A	A	A	B	A	B	A	A	A	A	A	A	A	A	A	B	A	A	A	B	B	A	A	B	0	A	A	A	A	A	A	A	A	B	A	B	A	A	A	A
A	A	B	A	B	B	A	A	A	B	A	B	A	A	A	A	A	A	A	A	A	B	A	A	A	B	B	A	A	B	0	B	A	A	A	A	A	A	A	B	A	B	A	A	A	A
A	A	B	A	B	B	A	A	A	B	A	B	A	A	A	B	A	A	A	A	A	B	A	A	A	B	B	A	A	B	0	B	A	A	A	A	A	A	A	B	A	B	A	B	A	A
A	A	B	A	B	B	A	A	A	B	A	B	A	A	A	B	B	B	A	A	A	B	A	B	A	B	B	A	A	B	0	B	A	A	B	A	A	A	A	B	A	B	B	B	A	A
A	A	B	A	B	B	A	A	A	B	A	B	A	A	A	B	B	B	A	A	A	B	A	B	A	B	B	A	A	B	0	B	A	A	B	A	A	A	A	B	A	B	B	B	A	A

A	A	B	A	B	B	A	A	A	B	A	B	A	A	A	B	B	B	A	A	A	B	A	B	A	B	B	A	A	B	0	B	A	A	B	A	A	A	A	B	A	B	B	B	A	A
A	A	B	A	B	B	A	A	A	B	A	B	A	A	A	B	B	B	A	A	A	B	A	B	A	B	B	A	A	B	0	B	A	A	B	A	A	A	A	B	A	B	B	B	A	A
A	A	B	A	B	B	A	A	A	B	A	B	A	A	A	B	B	B	A	A	A	B	A	B	A	B	B	A	A	B	0	B	A	A	B	A	A	A	A	B	A	B	B	B	A	A
A	A	B	A	B	B	A	A	A	B	A	B	A	A	A	B	B	B	A	A	A	B	A	B	A	A	B	A	A	B	0	B	A	A	B	A	A	A	A	B	A	B	B	B	A	B
A	A	B	A	B	B	A	A	A	B	A	B	A	A	A	B	B	B	A	A	A	B	A	B	A	A	B	A	A	B	0	B	A	A	B	B	A	A	A	B	A	B	B	B	B	B
A	A	B	A	B	B	A	A	A	B	A	B	A	A	A	B	B	B	A	A	A	B	A	B	A	A	B	A	A	B	0	B	A	A	B	B	A	A	A	B	A	B	B	B	B	B
A	A	B	A	B	B	A	A	A	B	A	B	B	A	A	B	B	B	A	A	A	B	A	B	A	A	B	A	A	B	B	B	A	A	B	B	A	A	A	B	A	B	B	B	B	B
A	A	B	A	B	B	A	A	A	B	A	B	B	A	A	B	B	B	A	A	A	B	A	B	A	A	B	A	A	B	B	B	A	A	B	B	A	A	A	B	A	B	B	B	B	B
A	A	B	A	B	B	A	A	A	B	A	B	B	A	A	B	B	B	A	A	B	B	A	B	A	A	B	A	A	B	B	B	A	A	B	B	A	A	A	B	A	B	B	B	B	B
A	A	B	A	B	B	A	A	A	B	A	B	B	A	A	B	B	B	A	A	B	B	A	B	A	A	B	A	A	B	B	B	A	A	B	B	A	A	A	B	A	B	B	B	B	B
A	A	B	A	B	B	A	A	A	B	A	B	B	A	A	B	B	B	A	A	B	B	A	B	A	A	B	A	A	B	B	B	A	A	B	B	A	A	A	B	A	B	B	B	B	B
A	B	B	A	B	B	A	A	B	B	A	B	B	A	A	B	B	B	A	A	B	B	A	B	A	A	B	A	A	B	B	B	A	A	B	B	A	A	A	B	A	B	B	B	B	B
A	A	B	A	A	A	A	B	A	B	B	B	A	B	B	B	A	A	B	B	B	B	B	A	B	B	B	B	A	A	A	B	B	A	A	B	A	B	B	B	B	B	B	A	B	B
A	A	A	A	A	A	A	B	A	A	B	B	B	B	B	B	A	A	B	B	B	B	B	A	B	B	B	B	A	A	A	B	B	A	A	B	A	B	B	B	B	B	B	A	B	B
A	A	A	A	A	A	A	B	A	A	B	B	B	B	B	B	A	A	B	B	B	B	B	A	B	B	B	B	A	A	A	B	B	A	A	B	A	B	B	B	B	B	B	A	B	B
A	A	A	A	A	A	A	B	B	A	B	B	B	B	B	B	A	A	B	B	B	B	B	A	B	B	B	B	A	A	A	B	B	A	A	B	A	B	B	B	B	B	B	A	B	B
A	A	A	A	A	A	A	B	B	A	B	B	B	B	B	B	A	A	B	B	B	B	B	A	B	B	B	B	A	A	A	B	B	A	A	B	A	B	B	B	B	B	B	A	B	B
A	A	A	A	A	A	A	B	B	A	B	B	B	B	B	B	A	A	B	B	B	B	B	A	B	B	B	B	A	A	A	B	B	A	A	B	A	B	B	B	B	B	B	A	B	A
B	A	A	A	A	A	A	B	B	A	B	B	B	B	B	B	A	A	B	B	B	B	B	A	A	B	B	B	A	A	A	B	B	A	A	B	A	B	B	B	B	B	B	B	B	A
B	A	A	A	A	A	A	B	A	A	B	B	B	B	B	B	A	A	B	B	B	A	B	A	A	B	B	B	A	A	A	B	B	A	A	B	A	B	B	B	B	B	A	B	B	A
B	A	A	A	A	A	A	B	A	A	B	B	B	B	B	B	A	A	B	B	B	A	B	A	A	B	B	B	A	A	A	B	B	A	A	B	B	B	B	B	B	B	A	B	B	A
B	A	A	A	A	A	A	B	A	A	B	B	B	B	B	B	A	A	A	B	B	A	B	A	A	B	B	B	A	A	A	B	A	A	A	B	B	B	B	B	B	B	A	B	B	A
B	A	A	A	A	A	A	B	A	A	B	B	B	B	B	B	A	A	A	B	B	A	B	A	A	B	B	B	A	A	A	B	A	A	A	B	B	B	B	B	B	B	A	B	B	A
B	A	A	A	A	A	A	B	A	A	B	B	B	B	B	B	A	A	A	B	B	A	B	A	A	B	B	B	A	A	A	B	A	A	A	B	B	B	B	B	B	B	A	B	B	A
B	A	A	A	A	A	A	B	A	A	B	B	B	B	B	B	A	A	A	B	B	A	B	A	A	B	B	B	B	A	A	B	A	A	A	B	B	B	B	B	B	B	A	B	B	A
B	A	A	A	A	A	A	B	A	A	B	B	B	B	B	B	A	A	A	B	B	A	B	A	A	B	B	B	B	A	A	B	A	A	A	B	B	B	B	B	B	B	A	B	B	A
B	A	A	A	A	A	A	B	A	A	B	B	B	B	B	B	A	A	A	B	B	A	B	A	A	B	B	B	B	A	A	B	A	B	A	B	B	B	B	B	B	B	A	B	B	A
B	A	A	A	A	A	A	B	A	A	B	B	B	B	B	B	A	A	A	B	B	A	B	A	A	B	B	B	B	A	A	B	A	B	A	B	B	B	B	B	B	B	A	B	B	A
B	A	A	A	A	A	A	B	A	A	B	B	B	B	B	B	A	A	A	B	B	A	B	A	A	B	B	B	B	A	A	B	A	B	A	B	B	B	B	B	B	B	A	B	B	A
B	A	A	A	A	A	A	B	A	A	B	B	B	B	B	B	A	A	A	B	B	A	B	A	A	B	B	B	B	A	A	B	A	B	A	B	B	B	B	B	B	B	A	B	B	A
B	A	A	A	A	A	A	B	A	A	B	B	B	B	B	B	A	A	A	B	B	A	B	A	A	B	B	B	B	A	A	B	A	B	A	B	B	B	B	B	B	B	A	B	B	A
B	A	A	A	A	A	A	B	A	A	B	B	B	B	B	B	A	A	A	B	B	A	B	A	A	B	B	B	B	A	A	B	A	B	A	B	B	B	B	B	B	B	A	B	B	A
B	A	A	A	A	A	B	B	A	A	B	B	B	B	B	B	A	A	A	B	B	A	B	A	A	B	B	B	B	A	A	B	A	B	A	B	B	B	B	B	B	B	A	B	B	A
B	A	A	A	A	A	B	B	A	A	B	B	B	B	B	B	A	A	A	B	B	A	B	A	A	B	B	B	B	A	A	B	A	B	A	B	B	B	B	B	B	B	A	B	B	A
B	A	A	A	A	A	B	B	A	A	B	B	B	B	B	B	A	A	A	B	B	A	B	A	A	B	B	B	B	A	A	B	A	B	A	B	B	B	B	B	B	B	A	B	B	A
B	A	A	A	A	A	B	B	A	A	B	B	B	B	B	B	A	A	A	B	B	A	B	A	A	B	B	B	B	A	A	B	A	B	A	B	B	B	B	B	B	B	A	B	B	A
B	A	A	A	A	A	B	B	A	A	B	B	B	B	B	B	A	A	A	B	B	A	B	A	A	B	B	B	B	A	A	B	A	B	A	B	B	B	B	B	B	B	A	B	B	A
B	A	A	A	A	A	B	B	A	A	B	B	B	B	B	B	A	A	A	B	B	A	B	A	A	B	B	A	B	A	A	B	A	B	A	B	B	B	B	B	B	B	A	B	B	A
B	A	A	A	A	A	B	B	A	A	B	B	B	B	B	B	A	A	A	B	B	A	B	A	A	B	B	A	0	A	A	B	A	B	A	B	B	B	B	B	B	B	A	B	B	A
B	A	A	A	A	A	B	B	A	A	B	B	B	B	B	B	A	A	A	B	B	A	B	A	A	B	B	A	0	A	A	B	A	B	A	B	B	B	B	B	B	B	A	B	B	A
B	A	A	A	A	A	B	B	A	A	B	B	B	B	B	B	A	A	A	B	B	A	B	A	A	B	B	A	0	A	A	B	A	B	A	B	B	B	B	B	B	B	A	B	B	A
B	A	A	A	A	A	B	B	A	A	B	B	B	B	B	B	A	A	A	B	B	A	B	A	A	B	B	A	0	A	A	B	A	B	A	B	B	B	B	B	B	B	A	B	B	A
B	A	A	A	A	A	B	B	A	A	B	B	B	B	B	B	B	B	A	B	B	A	B	B	A	B	B	A	0	A	B	B	A	B	B	B	B	B	B	B	B	B	A	B	B	A
B	A	A	A	A	A	B	B	A	A	B	B	B	B	B	B	B	B	A	B	B	A	B	B	A	B	B	A	0	A	B	B	A	B	B	B	B	B	B	B	B	B	A	B	B	A
A	A	A	A	A	A	B	B	A	A	A	B	B	B	B	B	B	B	A	B	B	A	B	B	A	B	B	A	0	A	B	B	A	B	B	B	B	B	B	B	A	B	A	B	B	A
A	A	A	A	A	A	B	B	A	A	A	B	B	B	B	B	B	B	A	B	B	A	B	B	A	B	B	A	0	A	B	B	A	B	B	B	B	B	B	B	A	B	A	B	B	A
A	A	A	B	A	A	B	B	A	A	A	B	B	B	B	B	B	B	A	B	B	A	B	B	A	B	B	A	0	A	B	B	A	B	B	B	B	B	B	B	A	B	A	B	B	B
A	A	A	B	A	A	B	B	A	A	A	B	B	B	B	B	B	B	A	B	B	A	B	B	A	B	B	A	A	A	B	B	A	B	B	B	B	B	B	B	A	B	A	B	B	B
A	A	A	B	A	B	B	B	A	A	A	B	B	B	B	B	B	B	A	B	B	A	B	B	A	B	B	A	A	A	B	B	A	B	B	B	B	B	B	B	A	B	A	B	B	B

A	A	A	B	A	B	B	B	A	A	A	B	B	B	B	B	B	B	A	B	B	A	B	B	A	B	B	A	A	A	B	B	A	B	B	B	B	B	B	B	A	B	A	B	B	B
A	A	A	B	A	B	B	B	A	A	A	B	B	B	B	B	B	B	A	B	B	A	B	B	A	B	B	A	A	A	B	B	A	B	B	A	B	B	B	B	A	B	A	B	B	B
A	A	A	B	A	B	B	B	A	A	A	B	B	B	B	B	B	B	A	B	B	B	B	B	A	B	B	A	A	A	B	B	A	B	B	A	B	B	B	B	A	B	A	B	B	B
A	A	A	B	A	B	B	A	A	A	A	B	B	B	B	B	B	B	A	B	B	B	B	B	A	B	B	A	A	A	B	B	A	B	B	A	B	B	B	B	A	B	A	B	B	B
A	A	A	B	A	B	B	A	A	A	A	B	B	B	B	B	B	B	A	B	B	B	B	B	A	B	B	A	A	A	B	B	A	B	B	A	B	B	B	B	A	B	A	B	B	B
A	A	A	B	A	B	B	A	A	A	A	B	B	B	B	B	B	B	A	B	B	B	B	B	A	B	B	A	A	A	B	B	A	B	B	A	B	B	B	B	A	B	A	B	B	B
A	A	A	B	A	B	B	A	A	A	A	B	B	B	B	B	B	B	A	B	B	B	B	B	A	B	B	A	A	A	B	B	A	B	B	A	A	B	B	B	A	B	A	B	B	B
A	A	A	B	A	B	B	A	A	A	A	B	B	B	B	B	B	B	A	B	B	B	B	B	A	B	B	A	A	B	B	B	A	B	B	A	A	B	B	A	A	B	A	B	B	B
A	B	A	B	A	B	B	A	B	A	A	B	B	B	B	B	B	B	A	B	B	B	B	B	A	B	B	A	A	B	B	B	A	B	B	A	A	B	B	A	A	B	A	B	B	B
A	B	A	B	A	B	B	A	B	A	A	B	B	B	B	B	B	B	A	B	B	B	B	B	A	B	B	A	A	B	B	B	A	B	B	A	A	B	B	A	A	B	A	B	B	B
A	B	A	B	A	B	B	A	B	A	A	B	B	B	B	B	B	B	A	B	B	B	B	B	A	B	B	A	A	B	B	B	A	B	B	A	A	B	B	A	A	B	A	B	B	B
A	B	A	A	A	B	B	A	B	A	A	B	B	B	B	B	B	B	A	B	B	B	B	B	A	B	B	A	A	B	B	B	A	B	B	A	A	B	B	A	A	B	A	B	B	B
A	B	A	A	A	B	B	A	B	A	A	B	B	B	B	B	B	B	A	B	B	B	B	B	A	B	B	A	B	B	B	B	A	B	B	A	A	B	B	A	A	B	A	B	B	B
A	B	A	A	A	B	B	A	B	A	A	B	B	B	B	B	B	B	A	B	B	B	B	B	A	B	B	A	B	B	B	B	A	B	B	A	A	B	B	A	A	B	A	B	B	B
A	B	A	A	A	B	B	A	B	A	A	B	B	B	B	B	B	B	A	B	B	B	B	B	A	B	B	A	B	B	B	B	A	B	B	A	A	B	B	A	A	B	A	B	B	B
A	B	A	A	A	B	B	A	B	A	B	B	B	B	B	B	B	B	A	B	B	B	B	B	A	B	B	A	B	B	B	B	A	B	B	A	A	B	B	A	B	B	A	B	B	B
A	B	A	A	A	B	B	A	B	A	B	B	B	B	B	B	B	B	A	B	B	B	B	B	A	B	B	A	B	B	B	B	A	B	B	A	A	B	B	A	B	B	A	B	B	B
A	B	A	A	A	B	B	A	B	A	B	B	B	B	B	B	B	B	A	B	B	B	B	B	B	B	B	A	B	B	B	B	A	B	B	A	A	B	B	A	B	B	A	B	B	B
A	B	A	A	A	B	B	A	B	A	B	B	B	B	B	B	B	B	A	B	B	B	B	B	B	B	B	A	B	B	B	B	A	B	B	A	A	B	B	A	B	B	A	B	B	B
A	B	A	A	A	B	B	A	B	A	B	B	B	B	B	B	B	B	A	B	B	B	B	B	B	B	B	A	B	B	B	B	A	A	B	A	A	B	B	A	B	B	A	B	B	B
A	B	A	A	A	B	B	A	B	A	B	B	B	B	B	B	B	B	A	B	B	B	B	B	B	B	B	A	A	B	B	B	A	A	B	A	A	B	B	A	B	B	A	B	B	B
A	B	A	A	A	B	B	A	B	A	B	B	B	B	B	B	B	B	A	B	B	B	B	B	B	B	B	A	A	B	B	B	A	A	B	A	A	B	B	A	B	B	A	B	B	B
A	B	A	A	A	B	B	B	B	A	B	B	B	B	B	B	B	B	A	B	B	B	B	B	B	B	B	A	A	B	B	B	A	A	B	A	A	B	B	A	B	B	A	B	B	B
A	B	A	A	A	B	B	B	B	A	B	B	B	B	B	B	B	B	A	B	B	B	B	B	B	B	B	A	A	B	B	B	A	A	B	A	A	B	B	A	B	B	A	B	B	B
A	B	A	A	A	B	B	B	B	A	B	B	B	B	B	B	B	B	A	B	B	B	B	B	B	B	B	A	A	B	B	B	A	A	B	A	A	B	B	A	B	B	A	B	B	B
A	B	A	A	A	B	B	B	B	A	B	B	B	B	A	B	B	B	A	B	B	B	B	B	B	B	B	A	A	B	B	B	A	A	B	A	A	B	B	A	B	B	A	B	B	B
A	B	A	A	A	B	A	B	B	A	B	B	B	B	A	B	B	B	A	B	B	B	B	B	B	B	B	A	A	B	B	B	A	A	B	A	A	B	B	A	B	B	A	B	B	B
A	B	A	A	A	B	A	B	B	A	B	B	B	A	A	B	B	B	A	B	B	B	B	B	B	B	B	A	A	B	B	B	A	A	B	A	A	B	B	A	B	B	A	B	B	B
A	B	A	A	A	B	A	B	B	A	B	B	B	A	A	B	B	B	A	B	B	B	B	B	B	B	B	A	A	B	B	B	A	A	B	A	A	B	B	A	B	B	A	B	B	B
A	B	A	A	A	B	A	B	B	A	B	B	B	A	A	B	B	B	A	A	B	B	B	B	B	B	B	A	A	B	B	B	A	A	B	A	A	B	B	A	B	B	A	B	B	B
A	B	A	A	A	B	A	B	B	A	B	B	B	A	A	B	B	B	A	A	B	B	B	B	B	B	B	A	A	B	B	B	A	A	B	A	A	B	B	A	B	B	A	B	B	B
A	B	A	A	A	B	A	B	B	A	B	B	B	A	A	B	B	B	A	A	B	B	B	B	B	B	B	A	A	B	B	B	A	A	B	A	A	B	B	A	B	B	A	B	B	B
A	B	A	A	B	B	A	B	B	A	B	B	B	A	A	B	B	B	A	A	B	B	B	B	B	B	B	A	A	B	B	B	A	A	B	A	A	B	B	A	B	B	A	B	B	B
A	B	A	A	B	B	A	B	B	A	B	B	B	A	A	B	B	B	A	A	B	A	B	B	B	B	B	A	A	B	B	B	A	A	B	A	A	B	B	A	B	B	A	B	B	B
A	B	A	A	B	B	A	B	B	A	B	B	B	A	A	B	B	B	B	A	B	A	B	B	B	B	B	A	A	B	B	B	B	A	B	A	A	B	B	A	B	B	A	B	B	B
A	B	A	A	B	B	A	B	B	A	B	B	B	A	A	B	B	B	B	A	B	A	B	B	B	B	B	A	A	B	B	B	B	A	B	A	A	B	B	A	B	B	A	B	B	B
A	B	A	A	B	B	A	B	B	A	B	B	B	A	A	B	B	B	B	A	B	A	B	B	B	B	B	A	A	B	B	A	B	A	B	A	A	B	B	A	B	B	A	B	B	B
A	B	A	A	B	B	A	B	B	A	B	B	B	A	A	B	B	B	B	A	B	A	B	B	B	B	B	A	A	B	B	A	B	A	B	A	A	B	B	A	B	B	A	B	B	B
A	B	A	A	B	B	A	B	B	A	B	B	B	A	A	B	B	B	B	A	B	A	B	B	B	B	B	A	A	B	B	A	B	A	B	A	A	B	B	A	B	B	A	B	B	B
A	B	A	A	B	B	A	B	B	A	B	B	B	A	A	B	B	B	B	A	B	A	B	B	B	B	B	B	A	B	B	A	B	A	B	A	A	B	B	A	B	B	A	B	B	B
A	B	A	A	B	B	A	B	B	A	B	B	B	A	A	B	B	B	B	A	B	A	B	B	B	B	B	B	A	B	B	A	B	A	B	A	A	B	B	A	B	B	A	B	B	B
A	B	A	A	B	B	A	B	B	A	B	B	B	A	A	B	B	B	B	A	B	A	B	B	B	B	B	B	A	B	B	A	B	A	B	A	A	B	B	A	B	B	A	B	B	B
A	B	A	A	B	B	A	B	B	A	B	B	B	A	A	B	B	B	B	A	B	A	B	B	B	B	B	B	A	B	B	A	B	A	B	A	A	B	B	A	B	B	A	B	B	B
A	B	A	A	B	B	A	B	B	A	B	B	B	A	A	B	B	B	B	A	B	A	B	B	B	B	B	B	A	B	B	A	B	A	B	A	A	B	B	A	B	B	A	B	B	B
A	B	A	A	B	B	A	B	B	A	B	B	B	A	A	B	B	B	B	A	B	A	B	B	B	B	B	B	A	B	B	A	B	A	B	A	A	B	B	A	B	A	A	B	B	B
A	B	A	A	B	B	A	B	B	A	B	B	B	A	A	B	B	B	B	A	B	A	B	B	B	B	B	B	A	B	B	A	B	A	B	A	A	B	B	A	B	A	A	B	B	B
B	B	A	A	B	B	A	B	B	A	B	B	B	A	A	B	B	B	B	A	B	A	B	B	B	B	B	B	A	B	B	A	B	A	B	A	A	B	B	A	B	A	A	B	B	B
B	B	A	A	B	B	A	B	B	A	B	B	B	B	A	B	B	B	B	A	B	A	B	B	B	B	B	B	A	B	B	A	B	A	B	A	A	B	B	A	B	A	A	B	B	B
B	B	A	B	B	B	A	B	B	A	B	B	B	B	A	B	B	B	B	A	B	A	B	B	B	B	B	B	A	B	B	A	B	A	B	A	A	B	B	A	B	A	A	B	B	B

B	B	A	B	B	B	A	B	B	A	A	B	B	B	A	B	B	B	B	A	B	A	B	B	B	B	B	B	A	B	B	A	B	A	B	A	A	B	B	A	A	A	A	B	B	B
B	B	A	B	B	B	A	B	B	A	A	B	B	B	A	B	B	B	B	A	B	A	B	B	B	B	B	B	A	B	B	A	B	A	B	A	A	B	B	A	A	A	A	B	B	B
B	B	A	B	B	B	A	B	B	A	A	B	B	B	A	B	B	B	B	A	A	A	B	B	B	B	B	B	A	B	B	A	B	A	B	A	A	B	B	A	A	A	A	B	B	B
B	B	A	B	B	B	A	B	B	A	A	B	B	B	A	A	B	B	B	A	A	A	B	B	B	A	B	B	A	B	B	A	B	A	B	B	A	B	B	A	A	A	A	B	B	B
B	B	A	B	B	A	A	B	B	A	A	B	B	B	A	A	B	B	B	A	A	A	A	B	B	A	B	B	A	B	B	A	B	A	B	B	A	B	B	A	A	A	A	B	B	B
B	B	A	B	B	A	A	B	B	A	A	B	B	B	A	A	B	B	B	A	A	A	A	B	B	A	B	B	A	B	B	A	B	A	B	B	A	B	B	A	A	A	A	B	B	B
B	B	A	B	B	A	B	B	B	A	A	B	B	B	A	A	B	B	B	A	A	A	A	B	B	A	B	B	A	B	B	A	B	A	B	B	A	B	B	A	A	A	A	B	B	B
B	B	A	B	B	A	B	B	B	A	A	B	B	B	A	A	B	B	B	A	A	A	A	B	B	A	B	B	A	B	B	A	B	A	B	B	A	B	B	A	A	A	A	B	B	B
B	B	A	B	B	A	B	B	B	A	A	B	B	B	A	A	B	B	B	A	A	A	A	B	B	A	B	B	A	B	B	A	B	A	B	B	A	B	B	A	A	A	A	B	B	B
B	B	A	B	B	A	B	B	B	A	A	B	B	B	A	A	B	B	B	A	A	A	A	B	B	A	B	B	A	B	B	A	B	A	B	B	A	B	B	A	A	A	B	B	B	B
B	B	A	B	B	A	B	B	B	A	A	B	B	B	A	A	B	B	B	A	A	A	A	B	B	A	B	B	A	B	B	A	B	A	B	B	A	B	B	A	A	A	B	B	A	B
B	B	A	B	B	A	B	B	B	A	A	B	B	B	A	A	B	B	B	A	A	A	A	B	B	A	B	B	A	B	B	A	B	A	B	B	A	B	A	A	A	A	B	B	A	B
B	B	A	B	B	A	B	B	B	A	A	B	B	B	A	A	B	B	B	A	A	A	A	B	B	A	B	B	A	B	B	A	B	A	B	B	B	B	A	A	A	A	B	B	A	B
0	B	A	B	B	A	B	B	B	A	B	B	A	B	A	A	B	B	B	B	B	B	B	B	B	B	A	B	A	B	B	A	B	B	B	B	B	0	B	B	B	B	B	A	A	B
0	B	B	B	B	A	B	B	B	B	B	B	A	B	A	A	B	B	B	B	B	B	B	B	B	B	A	B	A	B	B	A	B	B	B	B	B	0	B	B	B	B	B	A	A	B
0	B	B	B	B	A	B	B	B	B	B	B	A	A	A	A	B	B	B	B	B	B	B	B	B	B	A	B	A	B	B	A	B	B	B	B	B	0	B	B	B	B	B	A	A	B
0	B	B	B	B	B	B	B	B	B	B	B	A	A	A	A	B	B	B	B	B	B	B	B	B	B	A	B	A	B	B	A	B	0	B	B	B	0	B	B	B	B	B	A	A	B
0	B	B	B	B	B	B	B	B	B	B	B	A	A	A	A	B	B	B	B	B	B	B	B	B	B	A	B	A	B	B	A	B	0	B	B	B	0	B	B	B	B	B	A	A	B
0	B	B	B	B	B	B	B	B	B	B	B	A	A	A	A	B	B	B	B	B	B	B	B	B	B	A	B	A	B	B	A	B	0	B	B	B	0	B	B	B	B	B	A	A	B
0	B	B	B	B	B	B	B	B	B	B	B	B	A	A	A	B	B	B	B	B	B	B	B	B	B	A	B	A	B	B	A	B	B	B	B	B	B	B	B	B	B	B	A	A	B
0	B	B	B	B	B	B	B	B	B	B	B	B	A	B	A	B	B	B	B	B	B	B	B	B	B	A	B	A	B	B	A	B	B	B	B	B	B	B	B	B	B	B	A	A	B
0	B	B	B	B	B	B	B	B	B	B	B	B	A	B	A	B	B	B	B	B	B	B	B	B	B	A	B	B	B	B	A	B	B	B	B	B	B	B	B	B	B	B	A	B	B
0	B	B	B	B	B	B	B	B	B	B	B	B	A	B	A	B	B	B	B	B	B	A	B	B	B	A	B	B	B	B	A	B	B	B	B	B	B	B	B	B	B	B	A	B	B
0	B	B	B	B	B	A	B	B	B	B	B	B	A	B	A	B	B	A	B	B	B	A	B	B	B	A	B	B	B	B	A	A	B	B	B	B	B	B	B	B	B	B	A	B	B
0	B	B	B	B	B	A	B	B	B	B	B	B	A	B	A	B	B	A	B	B	B	A	B	B	B	A	B	B	B	B	A	A	A	B	B	B	B	B	B	B	B	B	A	B	B
0	B	B	B	B	B	A	B	B	B	B	B	B	A	B	A	B	B	A	B	B	B	A	B	A	B	A	B	B	B	B	A	A	A	B	B	B	B	B	B	B	B	B	A	B	B
0	B	B	B	B	B	A	B	B	B	B	B	B	A	B	A	B	B	A	B	B	B	A	B	A	B	A	B	B	B	B	A	A	A	B	B	B	B	B	B	B	B	B	A	B	B
0	B	B	B	B	B	A	B	B	B	B	B	B	A	B	A	B	B	A	B	B	B	A	B	A	B	A	B	B	B	B	A	A	A	B	B	B	B	B	B	B	B	B	A	B	B
0	B	B	B	B	B	A	B	B	B	B	B	B	A	B	A	B	B	A	B	B	B	A	B	A	B	A	B	B	B	B	A	A	A	B	B	B	B	B	B	B	B	B	A	B	B
0	B	B	B	B	B	A	B	B	B	B	B	B	A	B	A	B	B	A	B	B	B	A	B	A	B	A	B	B	B	B	A	A	A	B	B	B	B	B	B	B	B	B	A	B	B
B	B	B	B	B	B	A	B	B	B	B	B	B	A	B	A	B	B	A	B	B	B	A	B	A	B	A	B	B	B	B	A	A	A	B	B	B	B	B	B	B	B	B	A	B	B
B	B	B	B	B	B	A	B	B	B	B	B	B	A	B	A	B	B	A	B	B	B	A	B	A	B	A	B	B	B	B	A	A	A	B	B	B	B	B	B	B	B	B	A	B	B
B	B	B	B	B	B	A	B	B	B	B	B	B	A	B	A	B	B	A	B	B	B	A	B	A	B	A	B	B	B	B	A	A	A	B	B	B	B	B	B	B	B	B	A	B	B
B	B	B	B	B	B	A	B	B	B	B	B	B	A	B	A	B	B	A	B	B	B	A	B	A	B	A	B	B	A	B	A	A	A	B	B	B	B	B	B	B	B	B	A	B	B
B	B	B	B	B	B	A	B	B	B	B	B	B	A	B	A	B	B	A	B	B	B	A	B	A	B	A	B	B	A	B	A	A	A	B	B	B	B	B	B	B	B	B	A	B	B
B	B	B	B	B	B	A	B	B	B	B	B	B	A	B	A	B	B	A	B	B	B	A	B	A	B	A	B	B	A	B	A	A	A	B	B	B	B	B	B	B	B	B	A	B	B
B	B	B	B	B	B	A	B	B	B	B	B	B	A	B	A	B	B	A	B	B	B	A	B	A	B	A	B	B	A	B	A	A	A	B	B	B	B	B	B	B	B	B	A	B	B
B	B	B	B	B	B	A	B	B	B	B	B	B	A	B	A	B	B	A	B	B	B	A	B	A	B	A	B	B	A	B	A	A	A	B	B	B	B	B	B	B	B	B	A	B	B
B	B	B	B	B	B	A	B	B	B	B	B	B	A	B	A	B	B	A	B	B	B	A	B	A	B	A	B	B	A	B	A	A	A	B	B	B	B	B	B	B	B	B	A	B	B
B	B	B	B	B	B	A	B	B	B	B	B	B	A	B	A	B	B	A	B	B	B	A	B	A	B	A	B	B	A	B	A	A	A	B	B	B	B	B	B	B	A	B	A	B	B
B	B	B	B	B	B	B	B	B	B	B	B	B	A	B	A	B	B	A	B	B	B	A	B	A	B	A	B	B	A	B	A	A	A	B	B	A	B	B	B	B	A	B	A	B	B
B	B	B	B	B	B	B	B	B	B	B	B	B	A	B	A	B	B	A	B	B	B	A	B	A	B	A	B	B	A	B	A	A	A	B	B	A	B	B	B	B	A	B	A	B	B
B	B	B	B	B	B	B	B	B	B	B	B	B	A	B	A	B	B	A	B	B	B	A	B	A	B	A	A	B	A	B	A	A	A	B	B	A	B	B	B	B	A	B	A	B	B
B	B	B	B	B	B	B	B	B	B	B	B	B	A	B	A	B	B	A	B	B	B	A	B	A	B	A	A	B	A	B	A	A	A	B	B	A	B	B	B	B	A	B	A	B	B
B	B	B	B	B	B	B	A	B	B	B	B	B	A	B	A	B	B	A	B	B	B	A	B	A	B	A	A	B	A	B	A	A	A	B	B	A	B	B	B	B	A	B	A	B	B
B	B	B	B	B	B	B	A	B	B	B	B	B	A	B	A	B	B	A	B	B	B	A	B	A	B	A	A	B	A	B	A	A	A	B	B	A	B	B	B	B	A	B	A	B	B
B	B	B	B	B	B	B	A	B	B	B	B	B	A	B	A	B	B	A	B	B	B	A	B	A	B	A	A	B	A	B	A	A	A	B	B	A	B	B	B	B	A	B	A	B	B
B	B	B	A	B	B	B	A	B	B	B	B	B	A	B	A	B	B	A	B	B	B	A	B	A	B	A	A	B	A	B	A	A	A	B	B	A	B	B	B	B	A	B	A	B	B
B	B	B	A	B	B	B	A	B	B	B	B	B	A	B	A	B	B	A	B	B	B	A	B	A	B	A	A	B	A	B	A	A	A	B	B	A	B	B	B	B	A	B	A	B	B

B	B	B	A	B	B	B	A	B	B	B	B	B	A	B	A	B	B	A	B	B	B	A	B	A	B	A	A	B	A	B	A	A	A	B	B	A	B	B	B	B	A	B	A	B	B
B	B	B	A	B	B	B	A	B	B	B	B	B	A	B	A	B	B	A	B	A	B	A	B	A	B	A	A	B	A	B	A	A	A	B	B	A	B	B	B	B	A	B	A	B	B
B	B	B	A	B	B	B	A	B	B	B	B	B	A	B	A	A	A	A	B	A	B	A	A	A	B	A	A	B	A	A	A	A	A	A	B	A	B	B	B	B	A	B	A	B	B
B	B	B	A	B	B	B	A	B	B	B	B	B	A	B	A	A	A	A	B	A	B	A	A	A	B	A	A	B	A	A	A	A	A	A	B	A	B	B	B	B	A	B	A	B	B
B	B	B	A	B	B	B	A	B	B	B	B	B	A	B	A	A	A	A	B	A	B	A	A	A	B	A	A	B	A	A	A	A	A	A	B	A	B	B	B	B	A	B	A	B	B
B	B	B	A	B	B	B	A	B	B	B	B	B	A	B	A	A	A	A	B	A	B	A	A	A	B	A	A	B	A	A	A	A	A	A	B	A	B	B	B	B	A	B	A	B	B
B	B	B	A	B	B	B	A	B	B	B	B	B	A	B	A	A	A	A	B	A	B	A	A	A	B	A	A	B	A	A	A	A	A	A	B	A	B	B	B	B	A	B	A	A	B
B	B	B	A	B	B	B	A	B	B	B	B	B	A	B	A	A	A	A	B	A	B	A	A	A	B	A	A	B	A	A	A	A	A	A	B	A	B	B	B	B	A	B	A	A	B
B	B	B	A	B	B	B	A	B	B	B	B	B	A	B	A	A	A	A	B	A	B	A	A	A	B	A	A	B	A	A	A	A	A	A	B	A	B	B	B	B	B	B	A	A	B
B	B	B	A	B	B	B	A	B	B	B	B	B	A	B	A	A	A	A	B	A	B	A	A	A	B	A	A	B	A	A	A	A	A	A	B	A	B	B	B	B	B	B	A	A	B
B	B	B	A	B	B	B	A	B	B	B	B	B	A	B	A	A	A	A	B	A	B	A	A	A	B	A	A	B	A	A	A	A	A	A	B	A	B	B	B	B	B	B	A	A	B
B	B	B	A	B	B	B	A	B	B	B	B	B	A	B	A	A	A	A	B	A	B	A	A	A	B	A	A	A	A	A	A	A	A	A	B	A	B	B	B	B	B	B	A	A	B
B	B	B	A	B	B	B	A	B	B	B	B	B	A	B	A	A	A	A	B	A	B	A	A	A	B	A	A	A	A	A	A	A	A	A	B	A	B	B	B	B	B	B	A	A	B
B	B	B	A	B	B	B	A	B	B	B	B	B	A	B	A	A	A	A	B	A	B	A	A	A	B	A	A	A	A	A	A	A	A	A	A	A	B	B	B	B	B	B	A	A	B
B	B	A	A	B	B	B	A	B	A	B	B	B	A	B	A	A	A	A	B	A	B	A	A	A	B	A	A	A	A	A	A	A	A	A	A	A	B	B	B	B	B	B	A	A	B
B	B	A	A	B	B	B	A	B	A	B	B	B	A	A	A	A	A	A	B	A	B	A	A	A	B	A	A	A	A	A	A	A	A	A	A	A	B	B	B	B	B	B	A	A	B
A	B	A	A	B	B	B	A	B	A	B	B	B	A	A	A	A	A	A	B	A	B	A	A	A	B	A	A	A	A	A	A	A	A	A	A	A	B	B	B	B	B	B	A	A	B
A	B	A	A	B	B	B	A	B	A	B	B	B	A	A	A	A	A	A	B	A	B	A	A	A	B	A	A	A	A	A	A	A	A	A	A	A	B	B	B	B	B	0	A	A	B
A	B	A	A	B	B	B	A	B	A	B	B	B	A	A	A	A	A	A	B	A	B	A	A	A	B	A	A	A	A	A	A	A	A	A	A	A	B	B	B	B	B	0	A	A	B
A	B	A	A	B	B	B	A	B	A	B	B	B	A	A	A	A	A	A	B	A	B	A	A	A	B	A	A	A	A	A	A	A	A	A	A	A	B	B	B	B	B	0	A	A	B
A	B	A	A	B	B	B	A	B	A	B	B	B	A	A	A	A	A	A	B	A	B	A	A	A	B	A	A	A	A	A	A	A	A	A	A	A	A	B	B	B	B	0	A	A	B
A	B	A	A	B	B	B	A	B	A	B	B	B	A	A	A	A	A	A	B	A	B	A	A	A	B	A	A	A	A	A	A	A	A	A	A	A	A	B	B	B	B	B	A	A	B
A	B	A	A	B	B	B	A	B	A	B	B	B	A	A	A	A	A	A	B	A	B	A	A	A	B	A	A	A	A	A	A	A	A	A	A	A	A	B	B	B	B	B	A	A	B
A	B	A	B	B	B	B	A	B	A	B	B	B	A	A	A	A	A	A	B	A	B	A	A	A	B	A	A	B	A	A	A	A	A	A	A	A	A	B	B	B	B	B	A	A	B
A	B	A	B	B	B	B	A	B	A	B	B	B	A	A	A	A	A	A	B	A	B	A	A	A	B	A	A	B	A	A	A	A	A	A	A	A	A	B	B	B	B	B	A	A	B
A	B	A	B	B	B	B	A	B	A	B	B	B	A	A	A	A	A	A	B	A	B	A	A	A	B	A	A	B	A	A	A	A	A	A	A	A	A	B	B	B	B	B	A	A	B
A	B	A	B	B	B	B	A	B	A	B	B	B	A	A	A	A	A	A	B	A	B	A	A	A	B	A	A	B	A	A	A	A	A	A	A	A	A	B	B	B	B	B	A	A	B
A	B	A	B	B	B	B	A	B	A	B	B	B	A	A	A	A	A	A	B	A	B	A	A	A	B	A	A	B	A	A	A	A	A	A	A	A	A	B	B	B	B	B	A	A	B
A	B	A	B	B	B	B	A	B	A	B	B	B	A	A	B	A	A	A	B	A	B	A	A	A	B	A	A	B	A	A	A	A	A	A	A	A	A	B	B	B	B	B	A	A	B
A	B	A	B	B	B	B	A	B	A	B	B	B	A	A	B	A	A	A	B	A	A	A	A	A	B	A	A	B	A	A	A	A	A	A	A	A	A	B	B	B	B	B	A	A	B
A	B	A	B	B	B	A	A	B	A	B	B	B	B	A	B	A	A	A	B	A	A	A	A	A	A	A	A	B	A	A	A	A	A	A	A	A	A	B	B	B	B	A	A	A	B
A	B	A	B	B	B	A	A	B	A	B	B	B	B	A	B	A	A	A	B	A	A	A	A	A	A	A	A	B	A	A	A	A	A	A	A	A	A	B	B	B	B	A	A	A	B
A	B	A	B	B	B	A	A	B	A	B	B	B	B	A	B	A	A	A	B	A	A	A	A	A	A	A	A	B	A	A	A	A	A	A	A	A	A	B	B	B	B	A	A	A	B
A	B	A	B	B	B	A	A	B	A	B	B	B	B	A	B	A	A	A	B	A	A	A	A	A	A	A	A	B	A	A	A	A	A	A	A	A	A	B	B	B	B	A	A	A	B
A	B	A	B	B	B	A	A	B	A	B	B	B	B	A	B	A	A	A	B	A	A	A	A	A	A	A	A	B	A	A	A	A	A	A	A	A	A	B	B	B	B	A	A	A	B
A	B	A	B	B	B	A	A	B	A	B	B	B	B	A	B	A	A	A	B	A	A	A	A	A	A	A	B	B	A	A	A	A	A	A	A	A	A	B	B	B	B	A	A	A	B
A	B	A	B	B	B	A	A	B	A	B	B	B	B	A	B	A	A	B	B	A	A	A	A	A	A	A	B	B	A	A	A	B	A	A	A	A	A	B	B	B	B	A	A	A	B
A	B	A	B	B	B	A	A	B	A	B	A	B	B	A	B	B	B	B	B	A	A	A	B	A	A	B	B	A	A	B	A	B	A	B	A	A	A	B	B	B	B	A	A	A	B
A	B	A	B	B	B	A	A	B	A	B	A	B	B	A	B	B	B	B	B	A	A	A	B	A	A	B	B	A	A	B	A	B	A	B	A	A	A	B	B	B	B	A	A	B	B
A	B	A	B	B	B	A	A	B	A	B	A	B	B	A	B	B	B	B	B	A	A	A	B	A	A	B	B	A	A	B	A	B	A	B	A	A	A	B	B	B	B	A	A	B	B
A	B	A	B	B	B	A	A	B	A	B	A	B	B	A	B	B	B	B	B	A	A	A	B	A	A	B	B	A	A	B	A	B	A	B	A	B	A	B	B	B	B	A	A	B	B
A	B	A	B	B	B	A	A	B	A	B	A	B	B	A	B	B	B	B	B	A	A	A	B	A	A	B	B	A	A	B	A	B	A	B	A	B	A	B	B	B	B	A	A	B	B
B	A	A	A	A	B	B	B	A	A	B	A	B	B	B	A	B	B	B	A	B	A	B	B	A	B	B	B	B	B	B	B	B	A	B	A	B	A	A	B	B	A	B	B	B	B
B	A	A	A	A	B	B	B	A	A	B	A	B	B	B	A	B	B	B	A	B	A	B	B	A	B	B	B	B	B	B	B	B	A	B	A	B	A	A	B	B	A	B	B	B	B
B	A	A	A	A	B	B	B	A	A	B	A	B	B	B	A	B	B	B	A	B	A	B	B	A	B	B	B	B	B	B	B	B	A	B	A	B	A	A	B	B	A	B	B	B	B
B	A	A	A	A	B	B	B	A	A	B	A	B	B	B	A	B	B	B	A	B	A	B	B	A	B	B	B	B	B	B	B	B	A	B	A	B	A	A	B	B	A	B	B	B	B
B	A	A	A	A	B	B	B	A	A	B	A	B	B	B	A	B	B	B	A	B	A	B	B	A	B	B	B	B	B	B	B	B	A	B	A	B	A	A	B	B	A	B	B	B	B
B	A	A	A	A	B	A	B	A	A	B	A	B	B	B	A	B	B	B	A	B	A	B	B	A	B	B	B	A	B	B	B	B	A	B	A	B	A	A	B	B	A	0	B	B	B
B	A	A	A	A	A	A	B	A	A	B	A	B	B	B	A	A	A	B	A	B	A	A	A	A	B	B	B	A	B	A	B	B	A	A	A	B	A	A	B	B	A	A	B	B	B

B	A	A	A	A	A	A	B	A	A	B	A	B	B	B	A	A	A	B	A	B	A	A	A	A	B	B	B	A	B	A	B	B	A	A	A	A	A	A	B	B	A	A	B	B	B
B	A	A	A	A	A	A	B	A	A	B	A	B	B	B	A	A	A	B	A	B	A	A	A	A	B	B	B	A	B	A	B	B	A	A	A	A	A	A	B	B	A	A	B	B	B
B	A	A	A	A	A	A	A	A	A	B	A	B	B	B	A	A	A	B	A	B	A	A	A	A	B	B	B	A	B	A	B	B	A	A	A	A	A	A	B	B	A	A	B	B	B
B	A	A	A	A	A	A	A	A	A	B	A	B	B	B	A	A	A	B	A	B	A	A	A	A	B	B	B	A	B	A	B	B	A	A	A	A	A	A	B	B	A	A	B	B	B
B	A	A	A	A	A	A	A	A	A	B	A	B	B	B	A	A	A	B	A	B	A	A	A	A	B	B	A	A	B	A	B	B	A	A	A	A	A	A	B	B	A	A	B	B	B
B	A	A	A	A	A	A	A	A	A	B	A	B	B	B	A	A	A	B	A	B	A	A	A	A	B	B	A	A	B	A	B	B	A	A	A	A	A	A	B	B	A	A	B	B	B
B	A	A	A	A	A	A	A	A	A	B	A	A	B	B	A	A	A	B	A	B	A	A	A	A	B	B	A	A	B	A	B	B	A	A	A	A	A	A	B	B	A	A	B	B	B
B	A	A	A	A	A	A	A	A	A	B	A	A	B	B	A	A	A	B	A	A	A	A	A	A	B	B	A	A	B	A	B	B	A	A	A	A	A	A	A	B	A	A	B	B	B
B	A	A	A	A	A	A	A	A	A	B	A	A	B	B	A	A	A	B	A	A	A	A	A	A	B	B	A	A	B	A	B	B	A	A	A	A	A	A	A	B	A	A	B	B	B
A	A	A	A	A	A	A	A	A	A	B	A	A	B	B	A	A	A	B	A	A	A	A	A	A	B	B	A	A	B	A	B	B	A	A	A	A	A	A	A	B	A	A	B	B	B
A	A	A	A	A	A	A	A	A	A	B	A	A	B	B	A	A	A	B	A	A	A	A	A	A	B	B	A	A	B	A	A	B	A	A	A	A	A	A	A	B	A	A	B	B	B
A	A	A	A	A	A	A	A	A	A	B	A	A	B	A	A	A	A	B	A	A	A	A	A	A	B	B	A	A	B	A	A	B	A	A	A	A	A	A	A	B	A	A	B	B	B
A	A	A	A	A	A	A	A	A	A	B	A	A	B	A	A	A	A	B	A	A	A	A	A	A	B	B	A	A	B	A	A	B	A	A	A	A	A	A	A	B	A	A	A	A	B
A	A	A	A	A	A	A	A	A	A	B	A	A	B	A	A	A	A	B	A	A	A	A	A	A	A	B	A	A	B	A	A	B	A	A	A	A	A	A	A	B	A	A	A	A	B
A	A	A	A	A	A	A	A	A	A	B	A	A	B	A	A	A	A	B	A	A	A	A	A	A	A	B	A	A	B	A	A	B	A	A	A	A	A	A	A	B	A	A	A	A	B
A	A	A	A	A	A	A	A	A	A	B	A	A	B	A	A	A	A	B	A	A	A	A	A	A	A	B	A	A	B	A	A	B	A	A	A	A	A	A	A	B	A	A	A	A	B
A	A	A	A	A	A	A	A	A	A	B	A	A	B	A	A	A	A	B	A	A	A	A	A	A	A	A	A	A	B	A	A	B	A	A	A	A	A	A	A	B	A	A	A	A	B
A	A	A	A	A	A	A	A	A	A	B	A	A	B	A	A	A	A	B	A	A	A	A	A	A	A	A	A	A	B	A	A	B	A	A	A	A	A	A	A	B	A	A	A	A	A
A	A	A	A	A	A	A	A	A	A	B	A	A	B	A	A	A	A	B	A	A	A	A	A	A	A	A	A	A	B	A	A	B	A	A	A	A	A	A	A	B	A	A	A	A	A
A	A	A	A	A	A	A	A	A	A	B	A	A	B	A	A	A	A	B	A	A	A	A	A	A	A	A	A	A	B	A	A	B	A	A	A	A	A	A	A	B	A	A	A	A	A
A	A	A	A	A	A	A	A	A	A	B	A	A	B	A	A	A	A	B	A	A	A	A	A	A	A	A	A	A	B	A	A	B	A	A	A	A	A	A	A	B	A	A	A	A	A
A	A	A	A	A	A	A	A	A	A	B	A	A	A	A	A	A	A	B	A	A	A	A	A	A	A	A	A	A	B	A	A	B	A	A	A	A	A	A	A	B	A	A	A	A	A
A	A	A	A	A	A	A	A	A	A	B	A	A	A	A	A	A	A	B	A	A	A	A	A	A	A	A	A	A	B	A	A	B	A	A	A	A	A	A	A	B	A	A	A	A	A
A	A	A	A	A	A	A	A	A	A	B	A	A	A	A	A	A	A	B	A	A	A	A	A	A	A	A	A	A	B	A	A	B	A	A	A	A	A	A	A	B	A	A	A	A	A
A	A	A	A	A	A	A	A	A	A	B	A	A	A	A	A	A	A	B	A	A	A	A	A	A	A	A	A	A	B	A	A	B	A	A	A	A	A	A	A	B	A	A	A	A	A
A	A	A	A	A	A	A	A	A	A	B	A	A	A	A	A	A	A	B	A	A	A	A	A	A	A	A	A	A	B	A	A	B	A	A	A	A	A	A	A	B	A	A	A	A	A
A	A	A	A	A	A	A	A	A	A	B	A	A	A	A	A	A	A	B	A	A	A	A	A	A	A	A	A	A	B	A	A	B	A	A	A	A	A	A	A	B	A	A	A	A	A
A	A	A	A	A	A	A	A	A	A	B	A	A	A	A	A	A	A	B	A	A	A	A	A	A	A	A	A	A	B	A	A	B	A	A	A	A	A	A	A	B	A	A	A	A	A
A	A	A	A	A	A	A	A	A	A	B	A	A	A	A	A	A	A	B	A	A	A	A	A	A	A	A	A	A	B	A	A	B	A	A	A	A	B	A	A	B	A	A	A	A	A
A	A	A	A	A	A	A	A	A	A	B	A	A	A	A	A	A	A	B	A	A	A	A	A	A	A	A	A	A	B	A	A	B	A	A	A	A	B	A	A	B	A	A	A	A	A
B	A	A	A	A	A	A	A	A	A	B	A	A	A	A	A	A	A	B	A	A	A	A	A	A	A	A	A	A	B	A	A	B	A	A	A	A	B	A	A	B	A	A	A	A	A
B	A	A	A	A	A	A	A	A	A	B	A	A	A	B	A	A	A	B	A	A	A	A	A	A	A	A	A	A	B	A	A	B	A	A	A	A	B	A	A	B	A	A	A	A	A
B	A	A	A	A	A	A	A	A	A	B	A	A	A	B	A	A	A	B	A	A	A	A	A	A	A	A	A	A	B	A	A	B	A	A	A	A	B	A	A	B	A	A	A	A	A
B	A	A	A	A	A	A	A	A	A	B	A	A	A	B	A	A	A	B	A	A	A	A	A	A	A	A	A	A	B	A	A	B	A	A	A	A	B	A	A	B	A	A	A	A	A
B	A	A	A	A	A	A	A	A	A	B	A	A	A	B	A	A	A	B	A	A	A	A	A	A	A	A	A	A	B	A	A	B	A	A	A	A	B	A	A	B	A	A	A	A	A
B	A	A	A	A	A	A	A	A	A	B	A	A	A	B	A	A	A	B	A	A	A	A	A	A	A	A	A	A	B	A	A	B	A	A	A	A	B	A	A	B	A	A	A	A	A
B	A	A	A	A	A	A	A	A	A	B	A	A	A	B	A	A	A	B	A	A	A	A	A	A	A	A	A	A	B	A	A	B	A	A	A	A	B	A	A	B	A	A	A	A	A
B	A	A	A	A	A	A	A	A	A	B	A	A	A	B	A	A	A	B	A	A	A	A	A	A	A	A	A	A	B	A	B	B	A	A	A	A	B	A	A	B	A	A	A	A	A
B	A	A	A	A	A	A	A	A	A	B	A	A	A	B	A	A	A	B	A	A	A	A	A	A	A	A	A	A	B	A	B	B	A	A	A	A	B	A	A	B	A	A	A	A	A
B	A	A	A	A	A	A	A	A	A	B	A	A	A	B	A	A	A	B	A	A	A	A	A	A	A	A	A	A	B	A	B	B	A	A	A	A	B	A	A	B	A	A	A	A	A
B	A	A	A	A	A	A	A	A	A	B	A	A	A	B	A	A	A	B	A	A	A	B	A	A	A	A	A	A	B	A	B	B	A	A	A	A	B	A	A	B	A	A	A	A	A
B	A	A	A	A	A	A	A	A	A	B	A	A	A	B	A	A	A	B	B	A	A	B	A	A	A	A	A	A	B	A	B	B	A	A	A	A	B	A	A	B	A	A	A	A	B
B	A	B	A	A	A	A	A	A	B	A	A	A	A	B	A	A	A	B	B	A	A	B	A	A	A	A	A	A	B	A	B	B	A	A	A	A	B	A	A	A	A	A	A	A	B
B	B	B	A	A	A	A	A	B	B	A	A	A	A	B	A	A	A	B	B	A	A	B	A	A	A	A	A	A	B	A	B	B	A	A	A	A	B	A	A	A	A	A	A	A	B
B	B	B	A	A	A	A	A	B	B	A	A	A	A	B	A	A	A	B	B	A	A	B	A	A	A	A	A	A	B	A	B	B	A	A	A	A	B	A	A	A	A	A	A	A	B
B	B	B	A	A	A	A	A	B	B	A	A	A	A	B	A	A	A	B	B	A	A	B	A	A	A	A	A	A	B	A	B	B	B	A	A	A	B	A	A	A	A	A	A	A	B
A	B	B	A	A	A	A	A	B	B	A	A	A	A	B	A	A	A	B	B	A	A	B	A	A	A	A	A	A	B	A	B	B	B	A	A	A	B	A	A	A	A	A	A	A	B
A	B	B	A	A	A	A	A	B	B	A	A	A	A	B	A	A	A	B	B	A	A	B	A	A	A	A	A	A	B	A	B	B	B	A	A	A	B	A	A	A	A	A	B	A	B
A	B	B	A	A	A	A	A	B	B	A	A	A	B	B	A	A	A	B	B	A	A	B	A	A	A	A	A	A	B	A	B	B	B	A	A	A	B	A	A	A	A	A	B	A	B

A	B	B	A	A	A	A	A	B	B	A	A	A	B	B	A	A	A	B	B	B	A	B	A	A	B	A	A	A	B	A	B	B	B	A	A	A	B	A	A	A	A	A	B	A	B
A	B	B	A	A	A	A	A	B	B	A	A	A	B	A	A	A	A	B	B	B	A	B	A	A	B	A	A	A	B	A	B	B	B	A	A	A	B	A	A	A	A	A	B	A	B
A	B	B	A	A	B	A	A	B	B	A	A	A	B	A	A	A	A	B	B	B	A	B	A	A	B	A	A	A	B	A	B	B	B	A	A	A	B	A	A	A	A	A	B	A	B
A	B	B	A	A	B	A	A	B	B	A	A	A	B	A	A	A	A	B	B	B	A	B	A	A	B	A	A	A	B	A	B	B	B	A	A	A	A	A	A	A	A	A	B	A	B
A	B	B	A	A	B	A	A	B	B	A	A	A	B	A	A	A	A	B	B	B	A	B	A	B	B	A	A	A	B	A	B	B	B	A	A	A	A	A	A	A	A	A	B	A	B
A	B	B	A	A	B	A	A	B	B	A	A	A	B	A	A	A	A	B	B	B	A	B	A	B	B	A	B	A	B	A	B	B	B	A	A	A	A	A	A	A	A	A	B	A	B
A	B	B	A	A	B	A	A	B	B	A	A	A	B	A	A	A	A	B	B	B	A	B	A	B	B	A	B	A	B	A	B	B	B	A	A	A	A	A	A	A	A	A	B	A	B
A	B	B	A	A	B	A	B	B	B	A	A	A	B	A	A	A	A	B	B	B	A	B	A	B	B	A	B	A	B	A	B	B	B	A	A	A	A	A	A	A	A	A	B	A	B
A	B	B	A	B	B	A	B	B	B	A	A	A	B	A	A	A	A	B	B	B	A	B	A	B	B	A	B	A	B	A	B	B	B	A	A	A	A	A	A	A	A	A	B	A	B
0	A	A	0	B	B	A	B	A	A	0	A	A	0	A	A	A	A	A	A	A	A	0	A	B	B	B	0	A	A	A	A	A	B	A	A	0	A	A	A	0	A	A	A	B	A
0	A	A	0	B	B	A	B	A	A	0	A	A	0	A	A	A	A	A	A	A	A	0	A	B	B	B	0	A	A	A	A	A	B	A	A	0	A	A	A	0	A	A	A	B	A
0	A	A	0	B	B	A	B	A	A	0	A	A	0	A	A	A	A	A	A	A	A	0	A	B	A	B	0	A	A	A	A	A	B	A	A	0	A	A	A	0	A	B	A	B	A
0	A	A	0	B	B	A	B	A	A	0	A	A	0	A	A	A	A	A	A	A	A	0	A	B	A	B	0	A	A	A	A	A	B	A	A	0	A	A	A	0	A	B	A	B	A
0	A	A	0	B	B	A	B	A	A	0	A	A	0	A	A	A	A	A	A	A	A	0	A	B	A	B	0	A	A	A	A	A	B	A	A	0	A	A	A	0	A	B	A	B	A
0	A	A	0	B	B	A	A	A	A	0	A	A	0	A	A	A	A	A	A	A	A	0	A	B	A	B	0	A	A	A	A	A	B	A	A	0	A	A	A	0	A	B	A	B	A
0	A	A	0	B	B	A	A	A	A	0	A	A	0	A	A	A	A	A	A	A	A	0	A	B	A	B	0	A	A	A	A	A	B	A	A	0	A	A	A	0	A	B	A	B	A
0	A	A	0	B	B	A	A	A	A	0	A	A	0	A	A	A	A	A	A	A	A	0	A	B	A	B	0	A	A	A	A	A	B	A	A	0	A	A	A	0	A	B	A	B	A
0	A	A	0	B	A	A	A	A	A	0	A	A	0	A	A	A	A	A	A	A	A	0	A	B	A	B	0	A	A	A	A	A	B	A	A	0	A	A	A	0	A	B	A	B	A
0	A	A	0	B	A	A	A	A	A	0	A	A	0	A	A	A	A	A	A	A	A	0	A	B	A	B	0	A	A	A	A	A	B	A	A	0	A	A	A	0	A	B	A	A	A
0	A	A	0	B	A	A	A	A	A	0	A	A	0	A	A	A	A	A	A	A	A	0	A	B	A	B	0	A	A	A	A	A	B	A	A	0	A	A	A	0	A	B	A	A	A
0	A	A	A	B	A	A	A	A	A	0	A	A	A	A	0	A	A	A	A	A	A	A	A	B	A	B	A	A	A	A	A	A	B	A	A	0	A	A	A	0	A	0	A	A	A
0	A	A	A	B	A	A	A	A	A	0	A	A	A	A	0	A	A	A	A	A	A	A	A	B	A	B	A	A	A	A	A	A	B	A	A	0	A	A	A	0	A	0	A	A	A
0	A	A	A	B	A	A	A	A	A	0	A	A	A	A	0	A	A	A	A	A	A	A	A	B	A	B	A	A	A	A	A	A	B	A	A	0	A	A	A	0	A	0	A	A	A
0	A	A	A	B	A	A	A	A	A	0	A	A	A	A	0	A	A	A	A	A	A	A	A	B	A	B	A	A	A	A	A	A	B	A	A	0	A	A	A	0	A	0	A	A	A
0	A	A	A	B	A	A	A	A	A	0	A	A	A	A	0	A	A	A	A	A	A	A	A	B	A	A	A	A	A	A	A	A	B	A	A	0	A	A	A	0	A	0	A	A	A
A	A	A	A	B	A	A	A	A	A	0	A	A	A	A	0	A	A	A	A	A	A	A	A	B	A	A	A	A	A	A	A	A	B	A	A	0	A	A	A	0	A	0	A	A	A
A	A	A	A	B	A	A	A	A	A	0	A	A	A	A	0	A	A	A	A	A	A	A	A	B	A	A	A	A	A	A	A	A	B	A	A	0	A	A	A	0	A	0	A	A	A
A	A	A	A	B	A	A	A	A	A	0	A	A	A	A	0	A	A	A	A	A	A	A	A	B	A	A	A	A	A	A	A	A	B	A	A	A	A	A	A	0	A	0	A	A	A
A	A	A	A	B	A	A	A	A	A	0	A	A	A	A	0	A	A	A	A	A	A	A	A	B	A	A	A	A	A	A	A	A	B	A	A	A	A	A	A	0	A	0	A	A	A
A	A	A	A	B	A	A	A	A	A	0	A	A	A	A	0	A	A	A	A	A	A	A	A	B	A	A	A	A	A	A	A	A	B	A	A	A	A	A	A	0	A	0	A	A	A
A	A	A	A	B	A	A	A	A	A	0	A	A	A	A	0	A	A	A	A	A	A	A	A	B	A	A	A	A	A	A	A	A	B	A	A	A	A	A	A	0	A	0	A	A	A
A	A	A	A	B	A	A	A	A	A	0	A	A	A	A	0	A	A	A	A	A	A	A	A	B	A	A	A	A	A	A	A	A	B	A	A	A	A	A	A	0	A	0	A	A	A
A	A	A	A	B	A	A	A	A	A	A	A	A	A	A	0	A	A	A	A	A	A	A	A	B	A	A	A	A	A	A	A	A	B	A	A	A	A	A	A	A	A	0	A	A	A
A	A	A	A	B	A	A	A	A	A	A	A	A	A	A	0	A	A	A	A	A	A	A	A	B	A	A	A	A	A	A	A	A	B	A	A	A	A	A	A	A	A	0	A	A	A
A	A	A	A	B	A	A	A	A	A	A	A	A	A	A	0	A	A	A	A	A	A	A	A	A	A	A	A	A	A	A	A	A	B	A	A	A	A	A	A	A	A	0	A	A	A
A	A	A	A	B	A	A	A	A	A	A	A	A	A	A	0	A	A	A	A	A	A	A	A	A	A	A	A	A	A	A	A	A	B	A	A	A	A	A	A	A	A	0	A	A	A
A	A	A	A	B	A	A	A	A	A	A	A	A	A	A	0	A	A	A	A	A	A	A	A	A	A	A	A	A	A	A	A	A	B	A	A	A	A	A	A	A	A	0	A	A	B
A	A	A	A	B	A	A	A	A	A	A	A	A	A	A	0	A	A	A	A	A	A	A	A	A	A	A	A	A	A	A	A	A	B	A	A	A	A	A	A	A	A	0	A	A	B
A	A	A	A	B	A	A	A	A	A	A	A	A	A	A	0	A	A	A	A	A	A	A	A	A	A	A	A	A	A	A	A	A	B	A	A	A	A	A	A	A	A	0	A	A	B
A	A	A	A	B	A	A	A	A	A	A	A	A	A	A	0	A	A	A	A	A	A	A	A	A	A	A	A	A	A	A	A	A	B	A	A	A	A	A	A	A	A	0	A	A	B
A	A	A	A	B	A	A	A	A	A	A	A	A	A	A	0	A	A	A	A	A	A	A	A	A	A	A	A	A	A	A	A	A	B	A	A	A	A	A	A	A	A	0	A	A	B
A	A	A	A	B	A	A	A	A	A	A	A	A	A	A	0	A	A	A	A	A	A	A	A	A	A	A	A	A	A	A	A	A	B	A	A	A	A	A	A	A	A	0	A	A	B
A	A	A	A	B	A	A	A	A	A	A	A	A	A	A	0	A	A	A	A	A	A	A	A	A	A	A	A	A	A	A	A	A	B	A	A	A	A	A	A	A	A	0	A	A	B
A	A	A	A	B	A	A	A	A	A	A	A	A	A	A	0	A	A	A	A	A	A	A	A	A	A	A	A	A	A	A	A	A	B	A	A	A	A	A	A	A	A	0	A	A	B
A	A	A	A	B	A	A	A	A	A	A	A	A	A	A	0	A	A	A	A	A	A	A	A	A	A	A	A	A	A	A	A	A	B	A	A	A	A	A	A	A	A	0	A	A	B
A	A	A	A	B	A	A	A	A	A	A	A	A	A	A	0	A	A	A	A	A	A	A	A	A	A	A	A	A	A	A	A	A	B	A	A	A	A	A	A	A	A	0	A	A	B
A	A	A	A	B	A	A	A	A	A	A	A	A	A	A	0	A	A	A	A	A	A	A	A	A	A	A	A	A	A	A	A	A	B	A	A	A	A	A	A	A	A	0	A	A	B
A	A	A	A	B	A	A	A	A	A	A	A	A	A	A	0	A	A	A	A	A	A	A	A	A	A	A	A	A	A	A	A	A	B	A	A	A	A	A	A	A	A	0	A	A	B
A	A	A	A	B	A	A	A	A	A	A	A	A	A	A	0	A	A	A	A	A	A	A	A	A	A	A	A	A	A	A	A	A	B	A	A	A	A	A	A	A	A	0	A	A	B

B	A	A	A	A	A	A	B	A	A	B	B	B	B	A	B	B	B	B	B	A	A	B	B	B	A	B	B	A	A	B	A	B	A	B	B	B	A	B	B	B	A	A	A	A	B
B	A	A	B	A	A	A	B	A	A	B	B	B	B	A	B	B	B	B	B	A	A	B	B	B	A	B	B	A	A	B	A	B	A	B	B	B	A	B	B	B	A	A	A	A	B
B	A	A	B	A	A	A	B	A	A	B	B	B	B	A	B	B	B	B	B	A	A	B	B	B	A	B	B	A	A	B	A	B	A	B	B	B	A	B	B	B	A	A	A	A	B
B	A	A	B	A	A	A	B	A	A	B	B	B	B	A	B	B	B	B	B	A	A	B	B	B	A	B	B	A	A	B	A	B	A	B	B	B	A	B	B	B	A	A	A	A	B
B	A	A	B	A	A	A	B	A	A	B	B	B	B	A	B	B	B	B	B	A	A	B	B	B	A	B	B	A	A	B	A	B	A	B	A	B	A	B	B	B	A	A	A	A	B
B	A	A	B	A	A	A	B	A	A	B	B	B	B	A	B	B	B	B	B	A	A	B	B	B	A	B	B	A	A	B	A	B	A	B	A	B	A	B	B	B	A	A	A	A	B
B	A	A	B	A	A	A	B	A	A	B	B	B	B	A	B	B	B	B	B	A	A	B	B	B	A	B	B	A	A	B	A	B	A	B	A	B	A	B	B	B	A	A	A	A	B
B	A	A	B	A	A	A	B	A	A	B	B	B	B	A	B	B	B	B	B	A	A	B	B	B	A	B	B	A	A	B	A	B	A	B	A	B	A	B	B	B	A	A	A	A	B
B	A	A	B	A	A	A	B	A	A	B	B	B	B	A	B	B	B	B	B	A	A	B	B	B	A	B	B	A	A	B	A	B	A	B	A	B	A	B	B	B	A	A	A	A	B
B	A	A	B	A	A	A	B	A	A	B	B	B	B	A	B	B	B	B	B	A	A	B	B	B	A	B	B	A	A	B	A	B	A	B	A	B	A	B	B	B	A	A	A	A	B
B	A	A	B	A	A	A	B	A	A	B	B	B	B	A	B	B	B	B	B	A	A	B	B	B	A	B	B	A	A	B	A	B	A	B	A	B	B	B	B	B	A	A	A	A	B
B	A	A	B	A	A	A	B	A	A	B	B	B	B	A	B	B	B	B	B	A	A	B	B	B	A	B	B	A	A	B	A	B	A	B	A	B	B	B	B	B	A	A	A	A	B
B	A	A	B	A	A	A	B	A	A	B	B	B	B	A	B	B	B	B	B	A	A	B	B	B	A	B	B	A	A	B	A	B	A	B	A	B	B	B	B	B	A	A	A	A	B
B	A	A	B	A	A	A	B	A	A	B	B	B	B	A	B	B	B	B	B	A	A	B	B	B	A	B	B	A	A	B	A	B	A	B	A	B	B	B	B	B	A	A	A	A	B
B	A	A	B	A	A	A	B	A	A	B	B	B	B	A	B	B	B	B	B	A	A	B	B	B	A	B	B	A	A	B	A	B	A	B	A	B	B	B	B	B	A	A	A	A	B
B	A	A	B	A	A	A	B	A	A	B	B	B	B	A	B	B	B	B	B	A	A	B	B	B	A	B	B	A	A	B	A	B	A	B	A	B	B	B	B	B	A	A	A	A	B
B	A	A	B	A	A	A	B	A	A	B	B	B	B	A	B	B	B	B	B	A	A	B	B	A	A	A	B	A	A	B	A	B	A	B	A	B	B	B	B	B	A	A	A	A	B
B	A	A	B	A	A	A	B	A	A	B	B	B	B	A	B	B	B	B	B	A	A	B	B	A	A	A	B	A	A	B	A	B	A	B	A	B	B	B	B	B	A	A	A	A	B
B	A	A	B	A	A	A	B	A	A	B	B	B	B	A	B	B	B	B	B	A	B	B	B	A	A	A	B	B	A	B	A	B	A	B	A	B	B	B	B	B	A	B	A	A	B
B	A	A	B	A	A	A	B	A	A	B	B	B	B	A	B	B	B	B	B	A	B	B	B	A	A	A	B	B	A	B	A	B	A	B	A	B	B	B	B	B	A	B	A	A	B
B	A	A	B	A	A	A	B	A	A	B	B	B	B	A	B	B	B	B	B	A	B	A	B	A	A	A	B	B	A	B	A	B	A	B	A	B	B	B	B	B	A	B	A	A	B
B	A	A	B	A	A	A	B	A	A	B	B	B	B	A	B	B	B	B	B	A	B	A	B	A	A	A	B	B	A	B	A	B	A	B	A	B	B	B	B	B	A	B	A	A	B
B	A	A	B	A	B	A	B	A	A	B	B	B	B	A	B	B	B	B	B	A	B	A	B	A	A	A	B	B	A	B	A	B	A	B	A	B	B	B	B	B	A	B	A	A	B
B	A	A	B	A	B	A	A	A	A	B	B	B	B	A	B	B	B	B	B	A	B	A	B	A	A	A	B	B	A	B	A	B	A	B	A	B	B	B	B	B	A	B	A	A	B
B	A	A	B	A	B	A	A	A	A	B	B	B	B	A	B	B	B	B	B	A	B	A	B	A	A	A	B	B	A	B	B	B	A	B	A	B	B	B	B	B	A	B	A	A	B
B	A	A	B	A	B	B	A	A	A	B	B	B	B	A	B	B	B	B	B	A	B	A	B	A	A	A	B	B	A	B	B	B	A	B	A	B	B	B	B	B	A	B	A	A	B
B	A	A	B	A	B	B	A	A	A	B	B	B	B	A	B	B	B	B	B	A	B	A	B	A	A	A	B	B	A	B	B	B	A	B	A	B	B	B	B	B	A	B	A	A	B
B	A	A	B	A	B	B	A	A	A	B	B	B	B	A	B	B	B	B	B	A	B	A	B	A	A	A	B	B	A	B	B	B	A	B	A	B	B	B	B	B	A	B	A	A	B
B	A	A	B	A	B	B	A	A	A	B	B	B	B	A	B	B	B	B	B	A	B	A	B	A	A	A	B	B	A	B	B	B	A	B	A	B	B	B	B	B	A	B	A	A	B
B	A	A	B	B	B	B	A	A	A	B	B	B	B	A	B	B	B	B	B	A	B	A	B	A	A	A	B	B	A	B	B	B	A	B	A	B	B	B	B	B	A	B	A	A	B
B	A	A	B	B	B	B	A	A	A	B	B	B	B	A	B	B	B	B	B	A	B	B	B	A	A	A	B	B	A	B	B	B	A	B	A	B	B	B	B	B	A	B	A	A	B
B	A	A	B	B	B	B	A	A	A	B	B	B	B	A	B	B	B	B	B	A	B	B	B	A	A	A	B	B	A	B	B	B	A	B	A	B	A	B	B	B	A	B	A	A	B
B	A	A	B	B	B	B	A	A	A	B	B	B	B	A	B	B	B	B	B	A	B	B	B	A	A	A	B	B	A	B	B	B	A	B	A	B	A	A	B	B	A	B	A	A	B
B	A	A	B	B	B	B	A	A	A	B	B	B	B	A	B	B	B	B	B	A	B	B	B	A	A	A	B	B	A	B	B	B	A	B	A	B	A	A	B	B	A	B	A	A	B
B	A	A	B	B	B	B	A	A	A	B	B	B	B	A	B	B	B	A	B	A	B	B	B	A	A	A	B	B	A	B	B	A	A	B	A	B	A	A	B	B	A	B	A	A	B
B	A	A	B	B	B	B	A	A	A	B	B	B	B	A	B	B	B	A	B	A	B	B	B	A	A	A	B	B	A	B	B	A	A	B	A	B	A	A	A	B	A	B	A	A	B
B	A	A	B	B	B	B	A	A	A	B	B	B	B	A	B	B	B	A	B	A	B	B	B	A	A	A	B	B	A	B	B	A	A	B	A	B	A	A	A	B	A	B	A	A	B
B	A	A	B	B	B	B	A	A	A	B	B	B	B	A	B	B	B	A	B	A	B	B	B	A	A	A	B	B	A	B	B	A	A	B	A	B	A	A	A	B	A	B	A	A	B
B	A	A	B	B	B	B	A	A	A	B	B	B	B	A	B	B	B	A	B	A	B	B	B	A	A	A	B	B	A	B	B	A	A	B	A	B	A	A	A	B	A	B	A	A	B
B	A	A	B	B	B	B	A	A	A	B	B	B	B	A	B	B	B	A	B	A	B	B	B	A	A	A	B	B	A	B	B	A	A	B	A	B	A	A	A	B	A	B	A	A	B
B	A	A	B	B	B	B	A	A	A	B	B	B	B	A	B	B	B	A	B	A	B	B	B	A	A	A	B	B	B	B	B	A	A	B	A	B	A	A	A	B	A	B	A	A	B
B	A	A	B	B	B	B	A	A	A	B	B	B	B	A	B	B	B	A	B	A	B	B	B	A	A	A	B	B	B	B	B	A	A	B	A	B	A	A	A	B	A	B	A	A	B
B	A	A	B	B	B	B	A	A	A	B	B	B	B	A	B	B	B	A	B	A	B	B	B	A	A	A	B	B	B	B	B	A	A	B	A	B	A	A	A	B	A	B	A	A	B
B	A	A	B	B	B	B	A	A	A	B	B	B	B	A	B	B	B	A	B	A	B	B	B	A	A	A	B	B	B	B	B	A	A	B	A	B	A	A	A	B	A	B	A	A	B
B	A	A	B	B	B	B	A	A	A	B	B	B	B	A	B	B	B	A	B	A	B	B	B	A	A	A	B	B	B	B	B	A	A	B	A	B	A	A	A	B	A	B	A	B	B
B	A	A	B	B	B	B	A	A	A	B	B	B	B	B	B	B	B	A	B	A	B	B	B	A	A	A	B	B	B	B	B	A	A	B	A	B	A	A	A	B	A	B	A	B	B
B	A	A	B	B	B	B	A	A	A	B	B	B	B	B	B	B	B	A	B	A	B	B	B	A	A	A	B	B	B	B	B	A	A	B	A	B	A	A	A	B	A	B	A	B	B
B	A	A	B	B	B	B	A	A	A	B	B	B	B	B	B	B	B	A	B	A	B	B	B	A	A	A	B	B	B	B	B	A	A	B	A	B	A	A	A	B	A	B	A	B	B
B	A	A	B	B	B	B	A	A	A	B	B	B	B	B	B	B	B	A	B	A	B	B	B	A	A	A	B	B	B	B	B	A	A	B	A	B	A	A	A	B	A	B	A	B	B

B	A	A	B	B	B	B	A	A	A	B	B	B	B	B	B	B	B	A	B	A	B	B	B	A	A	A	B	B	B	B	B	A	A	B	A	B	A	A	A	B	A	B	A	B	B
B	A	A	A	B	B	B	A	A	A	B	B	B	B	B	B	B	B	A	B	A	B	B	B	A	A	A	B	B	B	B	B	A	A	B	A	B	A	A	A	B	A	B	A	B	B
B	A	A	A	B	B	B	A	A	A	B	B	B	B	B	B	B	B	A	B	A	B	B	B	A	A	A	B	B	B	B	B	A	A	B	A	B	A	A	A	B	A	B	A	B	B
A	A	A	A	B	B	B	A	A	A	B	B	B	B	B	B	B	B	A	B	A	B	B	B	A	A	A	B	B	B	B	B	A	A	B	A	B	A	A	A	B	A	B	A	B	B
A	A	A	A	B	B	B	A	A	A	B	B	B	B	B	B	B	B	A	B	A	B	B	B	A	A	A	B	B	B	B	B	A	A	B	A	B	A	A	A	B	A	B	A	B	B
A	A	A	A	B	B	B	A	A	A	B	B	B	B	B	B	B	B	A	B	A	B	B	B	A	A	A	B	B	B	B	B	A	A	B	A	B	A	A	A	B	A	B	A	B	B
A	A	A	A	B	B	B	A	A	A	B	B	B	B	B	B	B	B	A	B	A	B	B	B	A	A	A	B	B	B	B	B	A	A	B	A	B	A	A	A	B	A	B	A	B	B
A	A	A	A	B	B	B	A	A	A	A	B	B	B	B	B	B	B	A	B	A	B	B	B	A	A	A	B	B	B	B	B	A	A	B	A	B	A	A	A	A	A	B	A	B	B
A	A	A	A	B	B	B	A	A	A	A	B	B	B	B	B	B	B	A	B	A	B	B	B	A	A	A	B	B	B	B	B	A	A	B	A	B	A	B	A	A	A	B	A	B	B
A	A	A	A	B	B	B	A	A	A	A	B	B	B	B	B	B	B	A	B	A	B	B	B	A	A	A	B	B	B	B	B	A	A	B	A	B	A	B	A	A	A	B	A	B	B
A	A	A	A	B	B	B	A	A	A	A	B	B	B	B	B	B	B	A	B	A	B	B	B	A	A	A	B	B	B	B	B	A	A	B	A	B	A	B	A	A	A	B	A	B	B
A	A	A	A	B	B	B	A	A	A	A	B	B	B	B	B	B	B	A	B	A	B	B	B	A	A	A	B	B	B	B	B	A	A	B	A	B	A	B	A	A	B	B	A	B	B
A	A	A	A	B	B	B	A	A	A	A	B	B	B	B	B	B	B	B	B	A	B	B	B	A	A	A	B	B	B	B	B	B	A	B	A	B	A	B	A	A	B	B	A	B	B
A	A	A	A	B	B	B	A	A	A	A	B	B	B	B	B	B	B	B	B	A	B	B	B	A	A	A	B	B	B	B	B	B	A	B	A	B	A	B	A	A	B	B	A	B	B
A	A	A	A	B	B	B	A	A	A	A	B	B	B	B	B	B	B	B	B	A	B	B	B	A	A	A	B	B	B	B	B	B	A	B	A	B	A	B	A	A	B	B	A	B	B
A	A	B	A	B	B	B	A	A	B	A	B	B	B	B	B	B	B	B	B	A	B	B	B	A	A	A	B	B	B	B	B	B	A	B	A	B	B	B	A	A	B	B	A	B	B
A	A	B	A	B	B	B	A	A	B	A	B	B	B	B	B	B	B	B	B	A	B	B	B	A	A	A	B	B	B	B	B	B	A	B	A	B	B	B	A	A	B	B	A	B	B
A	A	B	A	B	B	B	A	A	B	A	B	B	B	B	B	B	B	B	B	A	B	B	B	A	A	A	B	B	B	B	B	B	A	B	A	B	B	B	A	A	B	B	A	B	B
A	A	B	A	B	B	B	A	A	B	A	B	B	B	B	B	B	B	B	B	A	B	B	B	A	A	A	B	B	B	B	B	B	A	B	A	B	B	B	A	A	B	B	A	A	B
A	A	B	A	B	B	B	A	A	B	A	B	B	B	B	B	B	B	B	B	A	B	B	B	A	A	A	B	B	B	B	B	B	A	B	A	B	B	B	A	A	B	B	A	A	B
A	A	B	A	B	B	B	A	A	B	A	B	B	B	B	B	B	B	B	B	A	B	B	B	B	A	A	B	B	B	B	B	B	A	B	A	B	B	B	A	A	B	B	A	A	B
A	A	B	A	B	B	B	B	A	B	A	B	B	B	B	B	B	B	B	B	B	B	B	B	B	A	A	B	B	B	B	B	B	A	B	A	B	B	B	A	A	B	B	A	A	B
A	A	B	A	B	B	B	B	A	B	A	B	B	B	B	B	B	B	B	B	B	B	B	B	B	A	A	B	B	B	B	B	B	A	B	A	B	B	B	A	A	B	B	A	A	B
A	A	B	A	B	A	B	B	A	B	A	B	B	B	B	B	B	B	B	B	B	B	B	B	B	A	A	B	B	B	B	B	B	A	B	A	B	B	B	A	A	B	B	A	A	B
A	A	B	A	B	A	B	B	A	B	A	B	B	B	B	B	B	B	B	B	B	B	B	B	B	A	A	B	B	B	B	B	B	A	B	A	B	B	B	A	A	B	B	A	A	B
A	A	B	A	B	A	B	B	A	B	A	B	B	B	B	B	B	B	B	B	B	B	B	B	B	A	A	B	B	B	B	B	B	A	B	A	B	B	B	A	A	B	B	A	A	B
A	A	B	A	B	A	B	B	A	B	A	A	B	B	B	B	B	B	B	B	B	B	B	B	B	A	A	B	B	B	B	B	B	A	B	A	B	B	B	A	A	B	B	A	A	B
A	A	B	A	B	A	B	B	A	B	A	A	B	B	B	B	B	B	B	B	B	B	B	B	B	A	A	B	B	B	B	B	B	A	B	A	B	B	B	A	A	B	B	B	A	B
A	A	B	A	B	A	B	B	A	B	A	A	B	B	B	B	B	B	B	B	B	B	B	B	B	A	A	B	B	B	B	B	B	A	B	A	B	B	B	A	A	B	B	B	A	B
A	A	B	A	B	A	B	B	A	B	A	A	B	B	B	B	B	B	B	B	B	B	B	B	B	A	A	B	B	B	B	B	B	A	B	A	B	B	B	A	A	B	B	B	A	B
A	A	B	A	B	A	B	B	A	B	A	A	B	B	B	B	B	B	B	B	B	B	B	B	B	A	A	B	B	B	B	B	B	A	B	A	B	B	B	A	A	B	B	B	A	B
A	A	B	A	B	A	B	B	A	B	A	A	B	B	B	B	B	B	B	B	B	B	B	B	B	A	A	B	A	B	B	B	B	A	B	A	B	B	B	A	A	B	B	B	A	B
A	A	B	A	B	A	B	B	A	B	A	A	B	B	B	B	B	B	B	B	B	B	B	B	B	A	A	B	A	B	B	B	B	A	B	A	B	B	B	A	A	B	B	B	A	B
B	A	A	A	B	A	A	A	A	A	B	B	A	B	B	A	B	B	B	A	B	A	A	B	B	B	A	A	A	A	B	A	B	A	B	B	A	B	A	A	B	A	B	B	B	A
B	A	A	A	B	A	A	A	A	A	B	B	A	B	B	A	B	B	B	A	B	A	A	B	B	B	A	A	A	A	B	A	B	A	B	B	A	B	A	A	B	A	A	B	B	A
B	A	A	A	B	A	A	A	A	A	B	B	A	B	B	A	B	B	B	A	B	A	A	B	B	B	A	A	A	A	B	A	B	A	B	B	A	B	A	A	B	A	A	B	B	A
B	A	A	A	B	A	A	A	A	A	B	B	A	B	B	A	B	B	B	A	B	A	A	B	B	B	A	A	A	A	B	A	B	A	B	B	A	B	A	A	B	A	A	B	B	A
B	A	A	A	B	A	A	A	A	A	B	B	A	B	B	A	B	B	B	A	B	A	A	B	B	B	A	A	A	A	B	A	B	A	B	B	A	B	A	A	B	A	A	B	B	A
B	A	A	A	B	A	A	A	A	A	B	B	A	B	B	A	B	B	B	A	B	A	A	B	B	B	A	A	A	A	B	A	B	A	B	B	A	B	A	A	B	A	A	B	B	A
B	A	A	A	B	A	A	A	A	A	B	B	A	B	B	A	B	B	B	A	B	A	A	B	B	B	A	A	A	A	B	A	B	A	B	B	A	B	A	A	B	A	A	B	B	A
B	A	A	A	B	A	A	A	A	A	B	B	A	B	B	A	B	B	B	A	B	A	A	B	B	B	A	A	A	A	B	A	B	A	B	B	A	B	A	A	B	A	A	B	B	A
B	A	A	B	B	A	A	A	A	A	B	B	A	B	B	A	B	B	B	A	B	A	A	B	B	B	A	A	A	A	B	A	B	A	B	B	A	B	A	A	B	A	A	B	B	A
B	A	A	B	B	A	A	A	A	A	B	B	A	B	B	A	B	B	B	A	B	A	A	B	B	B	A	A	A	A	B	A	B	A	B	B	A	B	A	A	B	A	A	B	B	A
B	A	A	B	B	A	A	A	A	A	B	B	A	B	B	A	B	B	A	A	B	A	A	B	B	B	A	A	A	A	B	A	A	A	B	B	A	B	A	A	B	A	A	B	B	A
B	B	A	B	B	A	A	A	B	A	B	B	A	B	B	A	B	B	A	A	B	A	A	B	B	B	A	A	A	A	B	A	A	A	B	B	A	B	A	A	B	A	A	A	B	A
B	B	A	B	B	A	A	A	B	A	B	B	A	B	B	A	B	B	A	A	A	A	A	B	B	B	A	A	A	A	B	A	A	A	B	B	A	B	A	A	B	A	A	A	B	A
B	B	A	B	B	A	A	A	B	A	B	B	A	B	B	A	B	B	A	A	A	A	A	B	B	B	A	A	A	A	B	A	A	A	B	B	A	B	A	A	B	A	A	A	B	B
B	B	A	B	B	A	A	B	B	A	B	B	A	B	B	A	B	B	A	A	A	A	A	B	B	B	A	A	A	A	B	A	A	A	B	B	A	B	A	A	B	A	A	A	B	B
B	B	A	B	B	A	A	B	B	A	B	B	A	B	B	A	B	B	A	B	A	B	A	B	B	B	A	A	A	A	B	A	A	A	B	B	A	B	A	B	B	A	A	A	B	B

B	B	A	B	B	A	A	B	B	A	B	B	A	B	B	A	A	A	A	B	A	B	A	A	B	B	A	A	A	A	A	A	A	A	A	B	A	B	A	B	B	A	A	A	B	B
B	B	A	B	B	A	A	B	B	A	B	B	A	B	A	A	A	A	A	B	A	B	A	A	B	B	A	A	A	A	A	A	A	A	A	B	A	B	A	B	B	A	A	A	B	B
B	B	A	B	B	A	A	B	B	A	B	B	A	B	A	A	A	A	A	B	A	B	A	A	A	B	A	A	A	A	A	A	A	A	A	B	A	B	A	B	B	A	A	A	B	B
B	B	A	B	B	A	A	B	B	A	B	B	A	B	A	A	A	A	A	B	A	B	A	A	A	B	A	A	A	A	A	A	A	A	A	B	A	B	A	B	B	A	A	A	B	B
B	B	A	B	B	A	A	B	B	A	B	B	A	B	A	A	A	A	A	B	A	B	A	A	A	B	A	A	A	A	A	A	A	A	A	B	A	B	A	B	B	A	A	A	B	B
B	B	B	B	B	A	A	B	B	B	B	A	A	B	A	A	A	A	A	B	A	B	A	A	A	B	A	B	A	A	A	A	A	A	A	B	A	B	A	B	B	A	A	A	B	B
B	B	B	B	B	A	A	B	B	B	A	A	A	B	A	A	A	A	A	B	A	B	A	A	A	B	B	B	A	A	A	A	A	A	A	B	A	B	A	B	A	A	A	A	B	B
B	B	B	B	B	A	A	B	B	B	A	A	A	B	A	A	A	A	B	B	A	B	A	A	A	B	B	B	A	A	A	A	B	A	A	B	A	B	A	B	A	A	A	A	B	B
B	B	B	B	B	A	A	B	B	B	A	A	A	B	A	A	A	A	B	B	A	B	A	A	A	B	B	B	A	A	A	A	B	A	A	B	A	B	A	B	A	A	A	A	B	B
B	B	B	B	B	A	B	B	B	B	A	A	A	B	A	A	A	A	B	B	A	B	A	A	A	B	B	B	A	A	A	A	B	A	A	B	A	B	A	B	A	A	A	A	B	B
B	B	B	B	B	A	B	B	B	B	A	A	A	B	A	A	A	A	B	B	A	B	A	A	A	B	B	B	A	A	A	A	B	A	A	B	A	B	A	B	A	A	A	A	B	B
B	B	B	B	B	A	B	B	B	B	A	A	A	B	A	A	A	A	B	B	A	B	A	A	A	B	B	B	A	A	A	A	B	A	A	B	A	B	A	B	A	A	A	A	B	B
B	B	B	B	B	A	B	B	B	B	A	A	A	B	A	A	A	A	B	B	A	B	A	A	A	B	B	B	A	A	A	A	B	A	A	B	A	B	A	B	A	A	A	A	B	B
B	B	B	B	B	A	B	B	B	B	A	A	A	B	A	A	A	A	B	B	A	B	A	A	A	A	B	B	A	A	A	A	B	A	A	B	A	B	A	B	A	A	A	A	B	B
B	B	B	B	B	A	B	B	B	B	B	A	A	B	A	A	A	A	B	B	A	B	A	A	A	A	B	B	A	A	A	A	B	A	A	B	A	B	A	B	B	A	A	A	B	B
B	B	B	B	B	A	B	B	B	B	B	A	A	B	A	A	A	A	B	B	A	B	A	A	A	A	B	B	A	A	A	A	B	A	A	B	A	B	A	B	B	A	A	A	B	B
A	B	B	B	B	A	B	B	B	B	B	A	A	B	A	A	A	A	B	B	A	B	A	A	A	A	B	B	A	A	A	A	B	A	A	B	A	B	A	B	B	A	A	A	B	B
A	B	B	B	B	A	B	B	B	B	B	A	A	B	A	A	A	A	B	B	A	B	A	A	A	A	B	B	A	A	A	A	B	A	A	B	A	B	A	B	B	A	A	A	B	B
A	B	B	B	B	A	B	B	B	B	B	A	A	B	A	A	A	A	B	B	A	B	A	A	A	A	B	B	A	A	A	A	B	A	A	B	A	B	A	B	B	A	A	A	A	B
A	B	B	B	B	A	B	B	B	B	B	A	A	B	A	A	A	A	B	B	A	B	A	A	A	A	B	B	A	A	A	A	B	A	A	B	A	B	A	B	B	A	A	A	A	B
A	B	B	A	B	A	B	B	B	B	B	A	A	B	A	A	A	A	B	B	A	B	A	A	A	A	B	B	A	A	A	A	B	A	A	B	A	B	A	B	B	A	A	A	A	B
A	B	B	A	B	A	B	B	B	B	B	A	A	B	A	A	A	A	B	B	A	B	A	A	A	A	B	B	A	A	A	A	B	A	A	B	A	B	A	B	B	A	A	A	A	B
A	B	B	A	B	A	B	B	B	B	B	A	A	B	A	A	A	A	B	B	A	B	A	A	A	A	B	B	A	A	A	A	B	A	A	B	A	B	A	B	B	A	A	A	A	B
A	B	B	A	B	A	B	B	B	B	B	A	A	B	A	A	A	A	B	B	A	B	A	A	A	A	B	B	A	A	A	A	B	A	A	B	A	A	A	B	B	A	A	A	A	B
A	B	B	A	B	A	B	B	B	B	B	A	A	B	A	A	A	A	B	B	A	B	A	A	A	A	B	B	A	A	A	A	B	A	A	B	A	A	A	B	B	A	A	A	A	B
A	B	B	A	B	A	B	B	B	B	B	A	A	B	A	A	A	A	B	B	A	B	A	A	A	A	B	B	A	A	A	A	B	A	A	B	A	A	A	B	B	A	A	A	A	B
A	B	B	A	B	A	B	B	B	B	B	A	A	B	A	A	A	A	B	B	A	B	A	A	A	A	B	B	A	A	A	A	B	A	A	B	A	A	A	B	B	A	A	A	A	B
A	B	B	A	B	A	B	B	B	B	B	A	A	B	A	A	A	A	B	B	A	B	A	A	A	A	B	B	A	A	A	A	B	A	A	B	A	A	A	B	B	A	A	A	A	0
A	B	B	A	B	A	B	B	B	B	B	A	A	B	A	A	A	A	B	B	A	B	A	A	A	A	B	B	A	A	A	A	B	A	A	B	A	A	A	B	B	A	A	A	A	0
A	B	B	A	B	B	B	B	B	B	B	A	A	B	A	A	A	A	B	B	A	B	A	A	A	A	B	B	A	A	A	B	B	A	A	B	A	A	A	B	B	A	A	A	A	A
A	B	B	A	B	B	B	B	B	B	B	A	A	B	A	B	A	A	B	B	B	B	A	A	A	A	B	B	A	A	A	B	B	A	A	B	A	0	A	B	B	A	A	A	A	A
A	B	B	A	B	B	B	B	B	B	B	A	A	B	A	B	B	B	B	B	B	B	A	B	A	A	B	B	A	A	B	B	B	A	B	B	A	0	A	B	B	A	A	A	A	A
A	B	B	A	B	B	B	B	B	B	B	A	A	B	A	B	B	B	B	B	B	B	A	B	A	A	B	B	A	A	B	B	B	A	B	B	A	0	A	B	B	A	A	A	A	A
A	B	B	A	B	B	B	B	B	B	B	A	A	B	A	B	B	B	B	B	B	B	A	B	A	A	B	B	A	A	B	B	B	A	B	B	A	0	A	B	B	B	A	A	A	A
A	B	B	A	B	B	B	B	B	B	B	A	A	B	A	B	B	B	B	B	B	B	A	B	A	A	B	B	A	A	B	B	B	A	B	B	A	0	A	B	B	B	A	B	A	A
A	B	B	A	B	B	B	B	B	B	B	A	A	B	B	B	B	B	B	B	B	B	A	B	B	0	B	B	A	A	B	B	B	A	B	B	A	0	A	B	B	B	A	B	A	A
A	B	B	A	B	B	B	B	B	B	B	A	A	A	B	B	B	B	B	B	B	B	A	B	B	0	B	B	A	A	B	B	B	A	B	A	A	0	A	B	B	B	A	B	A	A
A	B	B	A	B	B	B	B	B	B	B	A	A	A	B	B	B	B	B	B	B	B	A	B	B	0	B	B	A	A	B	B	B	A	B	A	A	0	A	B	B	B	A	B	A	A
A	B	B	A	B	0	B	A	B	B	B	B	B	A	B	B	A	A	A	B	B	B	B	A	B	B	B	A	A	B	A	A	A	B	A	B	A	B	A	A	B	A	A	A	B	A
A	B	B	B	B	0	B	A	B	B	B	B	B	A	B	B	A	A	A	B	B	B	B	A	B	B	B	A	A	B	A	A	A	B	A	B	A	B	A	A	B	A	A	A	B	A
A	B	B	B	B	0	B	A	B	B	B	B	B	A	B	B	A	A	A	B	B	B	B	A	B	B	B	A	0	B	A	A	A	B	A	B	A	B	A	A	B	A	A	A	B	A
A	B	B	B	B	0	B	A	B	B	B	A	B	A	B	B	A	A	A	B	B	B	B	A	B	B	B	A	0	B	A	A	A	B	A	B	A	B	B	A	B	A	A	A	B	A
B	B	B	B	B	0	B	A	B	B	B	A	B	A	B	B	A	A	A	B	B	B	B	A	B	B	B	A	A	B	A	A	A	B	A	B	A	B	B	A	B	A	A	A	B	A
B	B	B	B	A	B	B	A	B	B	B	A	B	A	B	B	A	A	A	B	B	B	B	A	B	B	B	A	A	B	A	A	A	B	A	B	A	B	B	A	B	A	A	A	B	A
B	B	B	B	A	B	B	A	B	B	B	A	B	A	B	B	A	A	B	B	B	B	B	A	B	B	B	A	A	B	A	B	A	B	A	B	A	B	B	B	B	A	A	A	B	A
B	B	B	B	A	B	B	A	B	B	B	A	B	A	B	B	A	A	B	B	B	B	B	A	B	B	B	A	A	B	A	B	B	B	A	B	A	B	B	B	B	A	A	A	B	A
B	B	B	B	A	B	B	A	B	B	B	A	B	A	B	B	A	A	B	B	B	B	B	A	B	B	B	A	A	B	A	B	B	B	A	A	A	B	B	B	B	A	A	A	B	A
B	B	B	B	A	B	B	A	B	B	B	A	B	A	B	B	A	A	B	B	B	B	B	A	B	B	B	A	A	B	A	B	B	B	A	A	A	B	B	B	B	A	A	A	B	A
B	B	B	B	A	B	B	A	B	B	B	A	B	A	B	B	A	A	B	B	B	B	B	A	B	B	B	A	A	B	A	B	B	B	A	A	A	B	B	B	B	A	A	A	B	A

B	B	B	B	A	B	B	A	B	B	B	A	B	A	B	B	A	A	B	B	B	B	B	A	B	B	B	A	A	B	A	B	B	B	A	A	A	B	B	B	B	A	A	A	B	A
B	B	B	B	A	B	B	A	B	B	B	A	B	A	B	B	A	A	B	B	B	B	B	A	B	B	B	A	A	B	A	B	B	B	A	A	A	B	B	B	B	A	A	A	B	A
B	B	B	B	A	B	B	A	B	B	B	A	B	A	B	B	A	A	B	B	B	B	B	A	B	B	B	A	A	B	A	B	B	B	A	A	A	B	B	B	B	A	A	A	B	A
B	B	B	B	A	B	B	A	B	B	B	A	B	A	B	A	A	A	B	B	B	B	B	A	B	B	B	A	A	B	A	B	B	A	A	A	A	B	B	B	B	A	A	A	B	A
B	B	B	B	A	B	B	A	B	B	B	A	B	A	B	A	B	B	B	B	B	B	B	B	B	B	B	A	A	B	B	B	B	A	B	A	A	B	B	B	B	A	A	A	B	A
B	B	B	B	A	B	B	A	B	B	B	A	B	A	B	A	B	B	B	B	B	B	B	B	B	B	B	A	B	B	B	B	B	A	B	A	A	B	B	B	B	A	A	A	B	A
B	B	B	B	A	B	B	A	B	B	B	A	B	A	B	A	B	B	B	B	B	B	B	B	B	B	B	A	B	B	B	B	B	A	B	A	A	B	B	B	B	A	A	A	B	A
B	B	B	B	A	B	B	A	B	B	B	A	B	A	B	A	B	B	B	B	A	B	B	B	B	B	B	A	B	B	B	B	B	A	B	A	A	B	B	B	B	A	A	A	B	A
B	B	B	B	A	B	B	A	B	B	B	A	B	A	B	A	B	B	B	B	A	B	B	B	B	B	B	A	B	B	B	B	B	A	B	A	A	B	B	B	B	A	A	A	B	A
B	B	B	B	A	B	B	A	B	B	B	A	A	A	B	A	B	B	B	B	A	B	B	B	B	B	B	A	B	B	B	B	B	A	B	A	A	B	B	B	B	A	A	A	B	A
B	B	B	B	A	B	B	A	B	B	B	A	A	A	B	A	B	B	B	B	A	B	B	B	B	B	B	A	B	B	B	B	B	A	B	A	A	B	B	B	B	A	A	A	B	B
B	B	B	B	A	B	B	A	B	B	B	A	A	A	B	A	B	B	B	B	A	B	B	B	B	B	B	A	B	B	B	B	B	A	B	A	A	B	B	B	B	A	A	A	B	B
B	B	B	B	A	B	B	A	B	B	B	A	A	A	B	A	B	B	B	B	A	B	B	B	B	B	B	A	B	B	B	B	B	A	B	A	A	B	B	B	B	A	A	A	B	B
B	B	B	B	A	B	B	A	B	B	B	A	A	A	B	A	B	B	B	A	A	B	B	B	B	B	B	A	B	B	B	B	B	B	B	A	A	B	B	B	B	A	A	A	B	B
B	B	B	B	A	B	B	A	B	B	B	A	A	A	B	A	B	B	B	A	A	B	B	B	B	B	B	A	B	B	B	B	B	B	B	A	A	B	B	B	B	A	A	A	B	B
B	B	B	B	A	B	B	A	B	B	B	A	A	A	B	A	B	B	B	A	A	B	B	B	B	B	B	A	B	B	B	B	B	B	B	A	A	B	B	B	B	A	A	A	B	B
B	B	B	B	A	B	B	A	B	B	B	A	A	A	B	A	B	B	B	A	A	B	B	B	B	B	B	A	B	B	B	B	B	B	B	A	A	B	B	B	B	A	A	A	B	B
B	B	B	B	A	B	B	A	B	B	B	A	A	A	B	A	B	B	A	A	A	B	B	B	B	B	B	A	B	B	B	B	A	B	B	A	A	B	B	B	B	A	A	A	B	B
B	B	B	B	A	B	B	A	B	B	B	A	A	A	B	A	B	B	A	A	A	B	B	B	B	B	B	A	B	B	B	B	A	B	B	A	A	B	B	B	B	A	A	A	B	B
B	B	B	B	A	B	B	A	B	B	B	A	A	A	B	A	B	B	A	A	A	B	B	B	B	B	B	A	B	B	B	B	A	B	B	A	A	B	B	B	B	A	A	A	B	B
B	B	B	B	A	B	B	A	B	B	B	A	A	A	B	A	B	B	A	A	A	B	B	B	B	B	B	A	B	B	B	B	A	B	B	A	A	A	B	B	B	A	A	A	B	B
B	B	B	B	A	B	B	A	B	B	B	A	A	A	B	A	B	B	A	A	A	B	B	B	B	B	B	A	B	B	B	B	A	B	B	A	A	A	B	B	B	A	A	A	B	B
B	A	A	B	A	B	B	A	A	A	B	A	A	A	B	A	B	B	A	A	A	B	B	B	B	B	B	A	B	B	B	B	A	B	B	A	A	A	B	B	B	A	A	A	B	B
B	A	A	B	A	B	B	A	A	A	B	A	A	A	B	A	B	B	A	A	A	B	B	B	B	B	B	A	B	B	B	B	A	B	B	A	A	A	B	A	B	A	A	A	B	B
B	A	A	B	A	B	B	A	A	A	B	A	A	A	B	A	B	B	A	A	A	B	B	B	B	B	B	A	A	B	B	B	A	B	B	A	A	A	B	A	B	A	A	A	B	B
B	A	A	B	A	B	B	A	A	A	B	A	A	A	B	A	B	B	A	A	A	B	B	B	B	B	B	A	A	B	B	B	A	B	B	A	A	A	B	A	B	A	A	A	B	B
B	A	A	B	A	B	B	A	A	A	B	A	A	A	B	A	B	B	A	A	A	B	B	B	B	B	B	A	A	B	B	B	A	B	B	A	A	A	B	A	B	A	A	A	B	B
B	A	A	B	A	B	B	A	A	A	B	A	A	A	B	A	B	B	A	A	A	B	B	B	B	B	B	A	A	B	B	B	A	B	B	A	A	A	B	A	B	A	A	A	B	B
B	A	A	B	A	B	B	A	A	A	B	A	A	A	B	A	B	B	A	A	A	B	B	B	B	A	B	A	A	B	B	B	A	B	B	A	A	A	B	A	B	A	A	B	B	B
B	A	A	B	A	B	B	A	A	A	B	A	A	A	B	A	A	A	A	A	A	B	B	A	B	A	B	A	A	B	A	B	A	B	A	A	A	A	B	A	B	A	A	B	B	B
B	A	A	B	A	B	B	A	A	A	B	A	A	A	B	A	A	A	A	A	A	B	B	A	B	A	B	A	A	B	A	B	A	B	A	A	A	A	B	A	B	A	A	B	B	B
B	A	A	B	A	B	B	A	A	A	B	A	A	A	B	B	A	A	A	A	A	B	B	A	B	A	B	A	A	B	A	B	A	B	A	A	A	A	B	A	B	A	A	B	B	B
A	A	A	B	A	B	B	A	A	A	B	A	A	A	B	B	A	A	A	A	A	B	B	A	B	A	B	A	A	B	A	B	A	B	A	A	A	A	B	A	B	A	A	B	B	A
A	A	A	B	A	B	B	A	A	A	B	A	A	A	B	B	A	A	A	A	A	B	B	A	B	A	B	A	A	B	A	B	A	B	A	A	A	A	B	A	B	A	A	B	B	A
A	A	A	B	A	B	B	A	A	A	B	A	A	A	B	B	A	A	A	A	A	B	B	A	B	A	B	A	A	B	A	B	A	B	A	A	A	A	B	A	B	A	A	B	B	A
A	A	A	B	A	B	B	A	A	A	B	A	A	A	B	B	A	A	A	A	A	B	B	A	B	A	B	A	A	B	A	B	A	B	A	A	A	A	B	A	B	A	A	B	B	A
A	A	A	B	A	B	B	A	A	A	B	A	A	A	B	B	A	A	A	A	A	B	B	A	B	A	B	A	A	B	A	B	A	B	A	A	A	A	B	A	B	A	A	B	B	A
A	A	A	B	0	B	B	A	A	A	B	A	A	A	B	B	A	A	A	A	A	B	B	A	B	A	B	A	A	B	A	B	A	0	A	A	A	A	B	A	B	A	A	A	A	A
A	A	A	B	0	B	B	A	A	A	B	A	A	A	B	B	A	A	A	A	A	B	B	A	B	A	B	A	A	B	A	B	A	0	A	A	A	A	B	A	B	A	A	A	A	A
A	A	A	B	0	B	B	A	A	A	B	A	A	A	A	B	A	A	A	A	A	B	B	A	B	A	B	A	A	B	A	B	A	0	A	A	A	A	B	A	B	A	A	A	A	A
A	A	A	B	0	B	B	A	A	A	B	A	A	A	A	B	A	A	A	A	A	B	B	A	B	A	B	A	A	B	A	B	A	0	A	A	A	A	B	A	B	A	A	A	A	A
A	A	A	B	0	B	B	A	A	A	B	A	A	A	A	B	A	A	A	A	A	A	B	A	B	A	B	A	A	B	A	B	A	0	A	A	A	A	B	A	B	A	A	A	A	A
A	A	A	B	0	B	B	A	A	A	B	A	A	B	A	B	A	A	A	A	A	A	B	A	B	A	B	A	A	B	A	B	A	0	A	A	A	A	B	A	B	A	A	A	A	A
A	A	A	B	0	B	B	A	A	A	B	A	A	B	A	B	A	A	A	A	A	A	B	A	B	A	B	A	A	B	A	B	A	0	A	A	A	A	B	A	B	A	A	A	A	A
A	A	A	B	0	B	B	A	A	A	B	B	A	B	A	B	A	A	A	A	A	A	B	A	B	A	B	A	A	B	A	B	A	0	A	A	A	A	B	A	B	A	A	A	A	A
A	A	A	B	0	B	B	A	A	A	B	B	A	B	A	B	A	A	A	A	A	A	B	A	B	A	B	A	A	A	A	B	A	0	A	B	A	A	B	A	B	A	A	A	A	A
A	A	B	A	0	A	B	A	A	B	B	B	A	B	A	B	A	A	A	A	A	A	B	A	B	A	B	B	A	A	A	B	A	0	A	B	A	A	B	A	B	A	A	A	A	A

SG_214	SG_22	SG_225	SG_249	SG_254	SG_270	SG_285	SG_30	SG_300	SG_309	SG_330	SG_333	SG_35	SG_351	SG_5	SG_75	SG_80	SG_85	SG_273	SG_276	SG_278	SG_281	SG_284	SG_294	SG_295	SG_303	SG_306	SG_317	SG_321	SG_46	SG_53	SG_69	SG_70	SG_87	SG_142	SG_289	SG_264	SG_25	SG_235	SG_315	SG_17	SG_230	SG_16	SG_58	SG_61	SG_79
A	A	0	A	B	B	A	0	A	A	B	B	B	A	0	A	A	B	A	0	0	B	B	B	A	A	A	A	B	A	A	0	0	A	B	B	B	B	A	A	B	B	0	B	B	B
A	A	0	A	B	B	A	0	A	A	B	A	B	A	0	A	A	B	A	0	0	B	B	B	A	A	A	A	B	A	A	0	0	A	B	B	B	B	A	A	B	B	0	B	B	B
A	A	0	A	B	B	A	0	A	A	B	A	B	A	0	A	A	B	A	0	0	B	B	B	A	A	A	A	B	0	0	0	0	A	B	B	B	B	A	A	B	B	0	B	B	B
A	A	0	A	B	B	A	0	A	A	B	A	B	A	0	A	A	B	A	0	0	B	A	B	A	A	A	A	B	0	0	0	0	A	B	B	B	B	A	A	B	B	0	B	B	B
A	A	0	A	B	B	A	0	A	A	B	A	B	A	0	A	B	B	A	0	0	B	A	B	A	A	A	A	B	0	0	0	0	A	B	B	B	B	A	A	B	B	0	B	B	B
A	A	0	A	B	B	A	0	A	A	B	A	B	A	0	A	B	B	A	0	0	B	A	B	A	A	A	A	B	0	0	0	0	A	B	B	B	B	A	A	B	B	0	B	B	B
A	A	0	A	B	B	A	A	A	A	B	A	B	B	0	A	B	B	A	0	0	B	A	B	A	A	A	A	B	0	0	0	0	A	B	B	B	B	A	A	B	B	0	B	B	B
A	A	0	A	B	B	A	A	A	A	B	A	B	B	0	A	B	B	A	0	0	B	A	B	B	A	A	A	B	0	0	0	0	A	B	B	B	B	A	A	B	B	0	B	B	B
A	A	0	A	B	B	A	A	A	A	B	A	B	B	0	A	B	B	A	0	0	B	A	B	B	A	A	A	B	0	0	0	0	A	B	B	B	B	A	A	B	B	0	B	B	B
0	A	0	A	B	B	A	A	A	A	B	A	B	B	0	A	B	B	A	0	0	B	A	B	B	A	A	A	B	0	0	0	0	A	B	B	B	B	A	A	B	A	0	B	B	B
0	A	0	A	B	B	A	A	A	A	B	A	B	B	0	A	B	B	A	B	B	B	A	B	B	A	A	A	B	0	0	0	0	A	B	B	B	B	A	A	B	A	0	B	B	0
0	A	0	A	B	B	A	A	A	A	B	A	B	B	0	A	B	B	A	B	B	B	A	B	B	A	A	A	A	0	0	0	0	A	B	B	B	B	A	A	B	A	0	B	B	0
0	A	0	A	B	B	A	A	A	A	B	A	B	B	0	A	B	B	B	B	B	B	A	B	B	A	A	A	A	0	0	0	0	A	B	B	B	B	A	A	B	A	0	B	B	0
0	A	0	A	B	B	A	A	A	A	B	A	B	B	0	A	B	B	B	B	B	B	A	B	B	A	A	B	A	0	0	0	0	A	B	B	B	B	A	A	B	A	0	B	B	0
0	A	0	A	B	B	A	A	A	A	B	A	B	B	0	A	B	B	B	B	B	B	A	B	B	A	A	B	A	0	0	0	0	A	B	B	B	B	A	A	B	A	0	B	B	0
0	A	0	A	B	B	A	A	A	A	B	A	B	B	0	A	B	B	B	B	B	B	A	B	B	A	A	B	A	0	0	0	0	A	B	B	B	B	A	A	B	A	0	B	B	0
0	A	0	A	B	B	A	A	A	A	B	A	B	B	0	A	B	B	B	B	B	B	A	B	B	A	A	B	A	B	B	0	0	A	B	B	B	B	A	A	B	A	0	B	B	0
0	A	0	A	B	B	A	A	A	A	B	A	B	B	0	A	B	B	B	B	B	B	A	B	B	A	A	B	A	B	B	0	0	A	B	B	B	B	A	A	B	A	0	B	B	0
0	A	0	A	B	B	A	A	A	A	B	A	B	B	0	A	B	0	B	B	B	B	A	B	B	B	A	B	A	B	B	0	0	A	B	B	B	B	A	A	B	A	0	B	B	0
0	A	0	A	B	B	A	A	A	A	B	A	B	B	0	A	B	A	B	B	B	B	A	B	B	B	A	B	A	B	B	A	A	A	B	B	B	B	A	A	B	A	A	B	B	0
A	A	0	A	B	B	A	A	A	A	B	A	B	B	0	A	B	A	B	B	B	B	A	B	B	B	A	B	A	B	B	A	A	A	B	B	B	B	A	A	B	A	A	B	B	0
A	A	0	A	B	B	A	A	A	A	B	A	B	B	0	A	B	A	B	B	B	B	A	B	B	B	A	B	A	B	B	A	A	A	B	B	B	B	A	A	B	A	A	B	B	0
0	A	0	A	B	B	A	A	A	A	B	A	B	B	0	A	B	A	B	B	B	B	A	B	B	B	A	B	A	B	B	A	A	A	B	B	B	B	A	A	B	A	A	B	B	0
0	A	0	A	B	B	A	A	A	A	A	A	B	B	0	A	B	A	B	B	B	B	A	B	B	B	A	B	A	B	B	A	A	A	B	B	B	B	A	A	B	A	A	B	B	0
0	A	0	A	B	B	A	A	A	A	A	A	B	B	0	A	B	A	B	B	B	B	A	B	B	B	A	B	A	B	B	A	A	A	B	B	B	B	A	A	B	A	A	B	B	0
0	A	0	A	A	B	A	A	A	A	A	A	B	B	0	A	B	A	B	B	B	B	A	B	B	B	A	B	A	B	B	A	A	A	B	B	A	B	A	A	B	A	A	B	B	0
0	A	0	A	A	B	A	A	A	A	A	A	B	B	0	A	B	A	B	B	B	B	A	B	B	B	A	B	A	B	B	A	A	A	B	B	A	B	A	A	A	A	A	B	B	0
0	A	0	A	A	B	A	A	A	B	A	A	B	B	0	A	B	A	B	B	B	B	A	B	B	B	A	B	A	B	B	A	A	A	B	B	A	B	A	A	A	A	0	B	B	0
0	A	0	A	A	B	A	A	A	B	A	A	B	B	0	A	B	A	B	B	B	B	A	B	B	B	A	B	A	B	B	A	A	A	B	B	A	B	A	A	A	A	0	B	B	0
0	A	0	A	A	B	A	A	A	B	A	A	B	B	0	A	B	A	B	B	B	B	A	B	B	B	A	B	A	B	B	A	A	A	B	B	A	B	A	A	A	A	0	B	B	0
0	A	0	A	A	B	A	A	A	B	A	A	B	B	0	A	B	A	B	B	B	B	A	B	B	B	A	B	A	B	B	A	A	A	B	B	A	B	A	A	A	A	0	B	B	0
0	A	0	A	A	B	A	A	A	B	A	A	B	B	0	A	B	A	B	B	B	B	A	B	B	B	A	B	A	B	B	A	A	B	B	B	A	B	A	A	A	A	0	B	B	0
0	A	0	A	A	B	A	A	A	B	A	A	B	B	0	A	B	A	B	B	B	B	A	B	B	B	A	B	A	B	B	A	A	B	B	B	A	B	A	A	A	A	0	B	B	0
0	A	0	A	A	B	A	A	A	B	A	A	A	B	0	A	B	A	B	B	B	B	A	B	B	B	A	B	A	B	B	A	A	B	B	B	A	B	A	A	A	A	0	B	B	0
0	A	0	A	A	B	A	A	A	B	A	A	A	B	0	A	B	A	B	B	B	B	A	B	B	B	A	B	A	B	B	A	A	B	B	B	A	B	A	A	A	A	0	B	B	0
0	A	0	A	A	B	A	A	A	B	A	A	A	B	0	A	B	A	B	B	B	B	A	B	B	B	A	B	A	B	B	A	A	B	B	B	A	B	A	A	A	A	0	B	B	0
0	A	0	A	A	B	A	A	A	B	A	A	A	B	0	A	B	A	B	B	B	B	A	B	B	B	A	B	A	B	B	A	A	B	B	B	A	B	A	A	A	B	0	B	B	0
0	A	0	A	A	B	A	A	A	B	A	A	A	B	0	A	B	A	B	B	B	B	A	B	B	B	A	B	B	B	B	A	A	B	B	B	A	B	A	A	A	B	0	B	B	0
0	A	0	A	A	B	A	B	A	B	A	A	A	B	0	A	B	A	B	B	B	B	A	B	B	B	A	B	B	B	B	A	A	B	B	B	A	B	A	A	A	B	0	B	B	0
0	A	0	A	A	B	A	B	A	B	A	B	A	B	0	A	B	A	B	B	B	B	A	B	B	B	A	B	B	B	B	A	A	B	B	B	A	B	A	A	A	B	0	B	B	0
0	A	0	A	A	B	A	B	A	B	A	B	A	B	0	A	B	A	B	B	B	B	A	B	B	B	B	B	B	B	B	A	A	B	B	B	A	B	A	A	A	B	0	B	B	0
0	A	0	A	A	B	A	B	A	B	A	B	A	B	0	A	B	A	B	B	B	B	A	B	B	B	B	B	B	B	B	A	A	B	B	B	A	B	A	A	A	B	0	B	B	0
0	A	0	A	A	B	A	B	A	B	A	B	A	B	0	A	A	A	B	B	B	B	A	B	B	B	B	B	B	B	B	A	A	B	B	B	A	B	A	A	A	B	0	B	B	0
0	A	0	A	A	B	A	B	A	B	A	B	A	B	0	A	A	A	B	B	B	B	A	B	B	B	B	B	B	B	B	A	A	A	B	B	A	B	A	A	A	B	0	B	B	0
0	A	A	A	A	B	A	B	A	B	A	B	A	B	0	A	A	A	B	B	B	B	A	B	B	B	B	B	B	B	B	A	A	A	B	B	0	B	A	A	A	B	0	B	B	0

0	A	A	A	A	B	A	B	A	B	A	B	A	B	0	A	A	A	B	B	B	B	A	B	B	B	B	B	B	B	B	A	A	A	B	B	0	B	A	A	A	B	0	B	B	0
0	A	A	A	A	B	A	B	A	B	A	B	A	B	0	A	A	A	B	B	B	B	A	A	B	B	B	B	B	B	B	A	A	A	B	B	0	B	A	A	A	B	0	B	B	0
0	A	A	A	A	B	A	B	A	B	A	B	A	B	0	A	A	A	B	B	B	B	A	A	B	B	B	B	B	B	B	A	A	A	B	B	0	B	A	A	A	B	0	B	B	0
0	A	A	A	A	B	A	B	A	B	A	B	A	B	0	A	A	A	B	B	B	B	A	A	B	B	B	B	B	B	B	A	A	A	B	B	0	B	A	A	A	B	0	B	B	0
0	A	A	A	A	B	A	B	A	B	A	B	A	B	0	A	A	A	B	B	B	B	A	A	B	B	B	B	B	B	B	A	A	A	B	B	0	B	A	A	A	B	0	B	B	0
0	A	A	A	A	B	A	B	A	B	A	B	A	B	0	A	A	A	B	B	B	B	B	A	B	B	B	B	B	B	B	A	A	A	B	B	0	B	A	A	A	A	0	B	B	0
A	A	A	A	A	B	A	B	A	B	A	B	A	B	0	A	A	A	B	B	B	B	B	A	B	B	B	B	B	B	B	B	B	A	B	B	0	B	A	A	A	A	0	B	B	0
A	A	A	A	A	B	A	B	A	A	A	B	A	B	0	A	A	A	B	B	B	B	B	A	B	B	B	B	B	B	B	B	B	A	B	B	0	B	A	A	A	A	0	B	B	0
A	A	A	A	A	B	A	B	A	A	A	B	A	B	0	A	A	A	B	B	B	B	B	A	B	B	B	B	B	B	B	B	B	A	B	B	0	B	A	A	A	A	0	B	B	0
A	A	A	A	A	B	A	B	A	A	A	B	A	B	0	A	A	A	B	A	A	B	B	A	B	B	B	B	B	B	B	B	B	A	B	B	0	B	B	A	A	A	0	B	B	0
A	A	A	A	A	B	A	B	A	A	A	B	A	B	0	A	A	A	B	A	A	B	B	A	B	B	B	B	B	B	B	B	B	A	B	B	0	B	B	A	A	A	0	B	B	0
A	A	A	A	A	B	A	B	A	A	A	B	A	B	0	A	A	A	B	A	A	B	B	A	B	B	B	B	B	B	B	B	B	A	B	B	0	B	B	A	A	A	0	B	B	0
A	A	A	A	A	B	A	B	A	A	A	B	A	B	0	A	A	A	B	A	A	B	B	A	B	B	B	B	B	B	B	B	B	A	B	B	0	A	B	A	A	A	0	B	B	0
A	A	A	A	A	B	A	B	A	A	A	B	A	A	0	A	A	A	B	A	A	B	B	A	B	B	B	B	B	B	B	B	B	A	B	B	0	A	B	A	A	A	0	B	B	0
A	A	A	B	A	B	A	B	A	A	A	B	A	A	0	A	A	A	B	A	A	B	B	A	B	B	B	B	B	B	B	B	B	A	B	B	0	A	B	A	A	A	0	B	B	0
B	A	B	B	B	A	B	B	B	B	B	B	A	A	A	A	B	B	A	B	B	B	A	B	A	B	B	B	B	B	B	B	B	A	A	B	A	B	A	B	A	A	B	B	B	B
B	A	B	B	B	A	0	B	B	0	B	B	A	A	A	A	B	B	A	B	B	B	A	B	A	B	B	B	B	B	B	B	B	A	A	B	A	B	A	B	A	A	B	B	B	B
B	A	B	B	B	A	0	B	B	0	B	B	A	A	A	A	B	B	A	B	B	B	A	A	A	B	B	B	B	B	B	B	B	A	A	B	A	B	A	B	A	A	B	A	A	B
B	A	B	B	B	A	0	B	B	0	B	B	A	A	A	A	B	B	A	B	B	B	A	A	A	B	B	B	B	B	B	B	B	A	A	B	A	B	A	B	A	A	B	A	A	B
B	A	B	B	B	A	0	B	B	0	B	B	A	A	A	A	B	B	A	B	B	B	A	A	A	B	B	B	B	B	B	B	B	A	A	B	A	B	A	B	A	A	B	A	A	B
B	A	B	B	B	A	0	B	B	0	B	B	A	A	A	A	B	B	A	B	B	B	A	A	A	B	B	B	A	B	B	B	B	A	A	B	A	B	A	B	A	A	B	A	A	B
B	A	B	B	B	A	0	B	B	A	B	B	A	B	A	A	B	B	A	B	B	B	A	A	A	B	B	B	A	B	B	B	B	A	A	B	A	B	A	B	A	A	B	A	A	B
B	A	B	B	B	A	0	B	B	A	B	B	A	B	A	A	B	B	A	B	B	B	A	A	A	B	B	B	A	B	B	B	B	A	A	B	A	B	A	B	A	A	B	A	A	B
B	A	B	B	B	A	0	B	B	A	B	B	A	B	A	A	B	B	A	B	B	B	A	A	A	B	B	B	A	B	B	B	B	A	A	B	A	B	A	B	A	A	B	A	A	B
B	A	B	B	B	A	0	B	B	A	B	B	A	B	A	A	B	B	A	B	B	B	A	A	A	B	B	B	A	B	B	B	B	A	A	B	A	B	A	B	A	A	B	A	A	B
B	B	B	B	B	A	0	B	B	A	B	B	A	B	A	A	B	B	A	B	B	B	A	A	A	B	B	B	A	B	B	B	B	A	A	B	A	B	A	B	A	A	B	A	A	B
B	B	B	B	B	A	0	B	B	A	B	B	A	B	A	A	B	B	A	B	B	B	A	A	A	B	B	B	A	B	B	B	B	A	A	B	A	B	A	B	A	A	B	A	A	B
B	B	B	B	B	A	0	B	B	A	B	B	A	B	A	A	B	B	A	B	B	B	A	A	A	B	B	B	A	B	B	B	B	A	B	B	A	B	A	B	A	A	B	A	A	B
B	B	B	B	B	A	0	B	B	A	B	B	A	B	A	A	B	B	A	B	B	B	A	A	A	B	B	B	A	B	B	B	B	A	B	B	A	B	A	B	A	A	B	A	A	B
B	B	B	B	B	A	0	B	B	B	B	B	A	B	A	A	B	B	A	B	B	B	A	A	A	B	B	B	A	B	B	B	B	A	B	B	A	B	A	B	A	A	B	A	A	B
B	B	B	B	B	A	0	B	B	B	B	B	A	B	A	A	B	B	A	B	B	B	A	A	A	B	B	B	A	B	B	B	B	A	B	B	A	B	A	B	A	A	B	A	A	B
B	B	B	B	B	A	0	B	B	B	B	B	A	B	A	A	B	B	A	B	B	B	A	A	A	B	B	B	A	B	B	B	B	A	B	B	A	B	A	B	A	A	B	A	A	B
B	B	B	B	B	A	0	B	B	B	B	B	A	B	A	A	B	B	A	B	B	B	A	A	A	B	B	B	A	B	B	B	B	A	B	B	A	B	B	B	A	A	B	A	A	B
B	B	B	B	B	A	0	B	B	B	B	B	A	B	A	A	B	B	A	B	B	B	A	A	A	B	B	B	A	B	B	B	B	A	B	B	A	B	B	B	A	A	B	A	A	B
B	B	B	B	B	A	0	B	B	B	B	B	A	B	A	A	B	B	A	B	B	B	A	A	A	B	B	B	A	B	B	B	B	A	B	B	A	B	B	B	A	A	B	A	A	B
B	B	B	B	B	A	0	B	B	B	B	B	A	B	A	A	B	B	A	B	B	B	A	A	A	B	B	B	A	B	B	B	B	A	B	A	A	B	B	B	A	A	B	A	A	B
B	B	B	B	B	A	B	B	B	B	B	B	B	B	A	A	B	B	A	B	B	B	A	A	A	A	B	B	A	B	B	B	B	A	B	A	A	B	B	B	A	A	B	A	A	B
B	B	B	B	B	A	B	B	B	B	B	A	B	B	A	A	B	B	A	B	B	B	A	A	A	A	B	B	A	B	B	B	B	A	B	A	B	B	B	B	A	A	B	A	A	B
B	B	A	B	B	A	B	B	B	B	B	A	B	B	A	A	B	B	A	B	B	B	A	A	A	A	B	B	A	B	B	B	B	A	B	A	B	B	B	B	A	A	B	A	A	B
B	B	A	B	B	A	B	B	B	B	B	A	B	B	A	A	B	B	A	B	B	B	A	A	A	A	B	B	A	B	B	B	B	A	B	A	B	B	B	B	A	A	B	A	A	B
B	B	A	B	B	A	B	B	B	B	B	A	B	B	A	A	B	B	A	B	B	A	A	A	A	A	B	B	A	B	B	B	B	A	B	A	B	B	B	B	A	A	B	A	A	B
B	B	A	B	B	A	B	B	B	B	B	A	B	B	A	A	B	B	A	B	B	A	A	A	A	A	B	B	A	B	B	B	B	A	B	A	B	B	B	B	B	A	B	A	A	B
B	B	A	B	B	A	B	B	B	B	B	A	B	B	A	A	B	B	A	B	B	A	A	A	A	A	B	B	A	B	B	B	B	A	B	A	B	B	B	B	B	A	B	A	A	B
B	B	A	B	B	A	B	B	B	B	B	A	B	B	A	A	B	B	A	B	B	A	A	A	A	A	B	B	B	B	B	B	B	A	B	A	B	B	B	B	B	A	B	A	A	B
B	B	A	B	B	A	B	B	B	B	B	A	B	B	B	A	B	B	A	B	B	A	A	A	A	A	B	B	B	B	B	B	B	A	B	A	B	B	B	B	B	A	B	A	A	B
B	B	A	B	B	A	B	B	B	B	B	A	B	B	B	A	B	B	A	B	B	A	A	A	A	A	B	B	B	B	B	B	B	A	B	A	B	B	B	B	B	A	B	A	A	B
B	B	A	B	B	A	B	B	B	B	B	A	B	B	B	A	B	B	A	B	B	A	A	A	A	A	B	B	B	B	B	B	B	A	B	A	B	B	B	B	B	A	B	A	A	A
B	B	A	B	B	A	B	B	B	B	B	A	B	B	B	A	B	B	A	B	B	A	A	A	A	A	B	B	B	B	B	B	B	A	B	A	B	B	B	B	B	A	B	A	A	A
B	B	A	B	B	A	B	B	B	B	B	A	B	B	B	A	B	B	A	B	B	A	A	A	A	A	B	B	B	B	B	B	B	A	B	A	B	B	B	B	B	A	B	A	A	A

B	B	A	B	B	A	B	B	B	B	B	A	B	B	B	A	B	B	A	B	B	A	A	A	A	A	B	B	B	B	B	B	B	A	B	A	B	A	B	B	B	A	B	A	A	A
B	B	A	B	B	A	B	B	B	B	B	A	B	B	B	A	B	B	A	B	B	A	A	A	A	A	B	B	B	B	B	B	B	A	B	A	B	A	B	B	B	A	B	A	A	A
B	B	A	B	B	A	B	B	B	B	B	A	B	B	B	A	B	B	A	B	B	A	A	A	A	A	B	B	B	B	B	B	B	A	B	A	B	A	B	B	B	A	B	A	A	A
B	B	A	B	B	A	B	B	B	B	B	A	B	B	B	A	B	B	A	B	B	A	A	A	A	A	B	B	B	B	B	B	B	A	B	A	B	A	B	B	B	A	B	A	A	A
B	B	A	B	B	A	B	B	B	B	B	A	B	B	B	A	B	B	A	B	B	A	A	A	A	A	B	B	B	B	B	B	B	A	B	A	B	A	B	B	B	A	B	A	A	A
B	B	A	B	B	A	B	B	B	B	B	A	B	B	B	A	B	B	A	B	B	A	A	A	A	A	B	B	B	B	B	B	B	A	B	A	B	A	B	B	B	A	B	A	A	A
B	B	A	B	B	A	B	B	B	B	B	A	B	B	B	A	B	B	A	B	B	A	A	A	A	A	B	B	B	B	B	B	B	A	B	A	B	A	B	B	B	A	A	A	A	A
B	B	A	B	B	A	B	B	B	B	B	A	B	B	B	A	B	B	A	B	B	A	A	A	A	A	B	B	B	B	B	B	B	A	B	B	B	A	B	B	B	A	A	A	A	A
B	B	A	B	B	A	B	B	B	B	B	A	A	B	B	A	B	B	A	B	B	A	A	A	A	A	B	B	B	B	B	B	B	A	B	B	B	A	B	B	B	A	A	A	A	A
B	B	A	B	B	A	B	B	B	B	B	A	A	B	B	A	B	B	A	B	B	A	A	A	A	A	B	B	B	B	B	B	B	A	B	B	B	A	B	B	B	B	A	A	A	A
B	B	A	B	B	A	B	B	B	B	B	A	A	B	B	A	B	B	A	B	B	A	A	A	A	A	B	B	B	B	B	B	B	B	B	B	B	A	B	B	B	B	A	A	A	A
B	B	A	B	B	A	B	B	B	B	B	A	A	B	B	A	B	B	A	B	B	A	A	A	A	A	B	B	B	B	B	B	B	B	B	B	B	A	B	B	B	B	A	A	A	A
B	B	A	B	B	A	B	B	B	B	B	A	A	B	B	A	B	B	A	B	B	A	A	A	A	A	B	B	B	B	B	B	B	B	B	B	B	A	B	B	B	B	A	A	A	B
B	A	A	B	B	A	B	B	B	B	B	A	A	B	B	A	B	B	A	B	B	A	A	A	A	A	B	B	B	B	B	B	B	B	B	B	B	A	B	B	B	B	A	A	A	B
B	A	A	B	B	A	B	B	B	B	B	A	A	B	B	A	B	B	A	B	B	A	A	A	A	A	B	B	B	B	B	B	B	B	B	B	B	A	B	B	B	B	A	A	A	B
B	A	A	B	B	A	A	B	B	B	B	A	A	B	B	A	B	B	A	B	B	A	A	A	A	A	B	B	B	B	B	B	B	B	B	B	B	A	B	B	B	B	A	A	A	B
B	A	A	B	B	A	A	B	B	B	B	A	A	B	B	A	B	B	A	B	B	A	A	A	A	A	B	B	B	B	B	B	B	B	B	B	B	A	B	B	B	B	A	A	A	B
B	A	A	B	B	A	A	B	B	B	B	A	A	B	B	A	B	B	A	B	B	A	A	A	A	A	B	A	B	B	B	B	B	B	B	B	B	A	B	B	B	B	A	A	A	B
B	A	A	B	B	A	A	B	B	B	B	A	A	B	B	A	B	B	A	B	B	A	A	A	A	A	B	A	B	B	B	B	B	B	B	B	B	A	B	B	B	B	A	A	A	B
B	A	A	B	B	A	A	B	B	B	B	A	A	B	B	A	B	B	A	B	B	A	A	A	A	A	B	A	B	B	B	B	B	B	B	B	B	A	B	B	B	B	A	A	A	B
B	A	A	B	B	A	A	B	B	B	B	A	A	B	B	A	B	B	A	B	B	A	A	A	A	A	B	A	B	B	B	B	B	B	B	B	B	B	B	B	B	B	A	A	A	B
B	A	A	B	B	A	A	B	B	B	B	A	A	B	B	B	B	B	A	B	B	A	A	A	A	A	B	A	B	B	B	B	B	B	B	B	B	B	B	B	B	B	A	A	A	B
B	A	A	B	B	A	A	B	B	B	B	A	A	B	B	B	B	B	A	B	B	A	A	A	A	A	B	A	B	B	B	B	B	B	B	B	B	B	B	B	B	B	A	A	A	B
B	A	A	B	B	A	A	B	B	B	B	B	A	B	B	B	B	B	A	B	B	A	A	A	A	A	B	A	B	B	B	B	B	B	B	B	B	B	B	B	B	B	A	A	A	B
B	A	A	B	B	A	A	B	B	B	B	B	A	B	A	B	B	B	A	B	B	A	A	A	A	A	B	A	B	B	B	B	B	B	B	B	B	B	B	B	B	B	A	A	A	B
B	A	A	B	B	A	A	B	B	B	B	B	A	B	A	B	B	B	A	B	B	A	A	A	A	A	B	A	B	B	B	B	B	B	B	B	B	B	B	B	B	B	A	A	A	B
B	A	A	B	B	A	A	B	B	B	B	B	A	B	A	B	B	B	A	B	B	A	A	A	A	A	B	A	B	B	B	A	A	B	B	B	B	B	B	B	B	B	A	A	A	B
B	A	A	B	B	B	A	B	B	B	B	B	A	B	A	B	B	B	A	B	B	A	A	A	A	A	B	A	B	B	B	A	A	B	B	B	B	B	B	B	B	B	A	A	A	B
B	A	A	B	B	B	A	B	B	B	B	B	A	B	A	B	B	B	A	B	B	A	A	A	A	A	B	A	B	B	B	A	A	B	B	B	B	B	B	B	B	B	A	A	A	B
B	A	A	B	B	B	A	B	B	B	B	B	A	B	A	B	B	B	A	B	B	A	A	A	A	A	B	A	B	B	B	A	A	B	B	B	B	B	B	B	B	B	A	A	A	B
B	A	A	B	B	B	A	B	B	B	B	B	A	B	A	B	B	B	A	B	B	A	A	A	A	A	B	A	B	B	B	A	A	B	B	B	B	B	B	B	B	B	A	A	A	B
B	A	A	B	B	B	A	B	B	B	B	B	A	B	A	B	B	B	A	B	B	A	A	A	A	A	B	A	B	B	B	A	A	B	B	B	B	B	B	B	B	B	A	A	A	B
B	A	A	B	A	B	A	B	B	B	B	B	A	B	A	B	B	B	A	B	B	A	A	A	A	A	B	A	B	B	B	A	A	B	B	B	B	B	B	B	B	B	A	A	A	B
B	A	A	B	A	B	A	B	B	B	B	B	A	B	A	B	B	B	A	B	B	A	A	A	A	A	B	A	B	B	B	A	A	B	B	B	B	B	B	B	B	B	A	A	A	B
B	A	A	B	A	B	A	B	B	B	B	B	A	B	A	B	B	B	A	A	A	A	A	A	A	A	B	A	B	B	B	A	A	B	B	B	B	B	B	B	B	B	A	A	A	B
B	A	A	B	A	B	A	A	B	B	B	B	A	B	A	B	B	B	A	A	A	A	A	A	A	A	B	A	B	B	B	A	A	B	B	B	B	B	B	B	B	B	B	A	A	B
B	A	A	B	A	B	A	A	B	B	B	B	A	B	A	B	B	B	A	A	A	A	A	B	A	A	B	A	B	B	B	A	A	B	B	B	B	B	B	B	B	B	B	A	A	B
B	A	A	B	A	B	A	A	B	B	B	B	A	B	A	B	B	B	A	A	A	A	A	B	A	A	B	A	A	B	B	A	A	B	B	B	B	B	B	B	B	B	B	A	A	B
B	B	A	B	A	B	A	0	B	B	B	B	A	A	A	B	B	B	A	A	A	A	A	B	A	A	B	A	A	B	B	A	A	B	B	B	B	B	B	B	B	B	B	A	A	B
B	B	A	B	A	B	A	0	B	B	B	B	A	A	A	B	B	B	A	A	A	A	A	B	A	A	B	A	A	B	B	A	A	B	B	B	B	B	B	B	B	B	B	A	A	B
B	B	B	B	A	B	B	B	A	A	A	B	B	B	B	A	B	A	A	B	B	B	0	B	B	B	A	A	A	A	A	B	B	B	B	A	A	A	B	A	B	A	B	A	A	A
B	B	B	B	A	B	B	B	A	A	A	B	B	B	B	A	B	A	A	B	B	B	0	B	B	B	A	A	A	A	A	B	B	B	B	A	A	A	B	A	B	A	B	A	A	A
B	B	B	B	A	B	B	B	A	A	A	B	B	B	B	A	B	A	A	B	B	B	0	B	B	B	A	A	A	A	A	B	B	B	B	A	A	A	B	A	B	A	B	A	A	A
B	B	B	B	A	B	B	B	A	A	A	B	B	B	B	A	B	A	A	B	B	B	0	B	B	B	A	A	A	A	A	B	B	B	B	A	A	A	B	A	B	A	B	B	B	A
B	B	A	B	A	B	B	B	0	A	A	B	B	B	B	A	B	A	A	B	B	B	0	B	B	B	A	A	A	A	A	B	B	B	B	A	A	A	B	A	B	A	B	B	B	A
B	B	A	B	A	B	B	B	0	A	A	B	B	B	B	A	B	A	A	B	B	B	A	B	B	B	A	A	A	A	A	B	B	B	B	A	A	A	B	A	B	A	B	B	B	A
B	B	A	B	A	B	B	B	0	A	A	B	B	B	B	A	B	A	A	B	B	B	A	B	B	B	A	A	A	A	A	B	B	B	B	A	A	A	B	A	B	A	B	B	B	A
B	B	A	B	A	B	B	B	0	A	A	B	B	B	B	A	B	A	A	B	B	B	A	B	B	B	A	A	A	A	A	B	B	B	B	A	A	A	B	A	B	A	B	B	B	A
B	B	A	B	B	B	B	B	0	A	A	B	B	B	B	A	B	A	A	B	B	B	A	B	B	B	A	A	A	A	A	B	B	B	B	A	A	A	B	A	B	A	B	B	B	A

B	B	A	B	B	B	B	B	0	A	A	B	B	B	B	A	B	A	A	B	B	B	A	B	B	B	A	A	A	A	A	B	B	B	B	A	A	A	B	A	B	A	B	B	B	A
B	B	A	B	B	B	B	B	0	A	A	B	B	B	B	A	B	A	A	B	B	B	A	B	B	B	A	A	A	A	A	B	B	B	B	A	A	A	B	A	B	A	B	B	B	A
B	B	A	B	B	B	B	B	0	A	A	B	B	B	B	A	B	A	A	B	B	B	A	B	B	B	A	A	A	B	B	B	B	B	B	A	A	A	B	A	B	A	B	B	B	A
B	B	A	B	B	B	A	B	0	A	A	B	B	B	B	A	B	A	A	B	B	B	A	B	B	B	A	A	A	B	B	B	B	B	B	A	A	A	B	A	B	A	B	B	B	A
B	B	A	B	B	B	A	B	0	A	A	B	B	B	B	A	B	A	A	B	B	B	A	B	B	B	A	A	A	B	B	B	B	B	B	A	A	A	B	A	B	A	B	B	B	A
B	B	A	B	B	B	A	B	0	A	A	B	B	B	B	A	B	A	A	B	B	A	A	B	B	B	A	A	A	B	B	B	B	B	B	A	A	A	B	A	B	A	B	B	B	A
B	B	A	B	B	B	A	B	0	A	A	B	B	B	B	A	B	A	A	B	B	A	A	B	B	B	A	A	A	B	B	B	B	B	B	A	A	A	B	A	B	A	B	B	B	A
B	B	A	B	B	B	A	B	0	A	A	B	B	B	B	B	B	A	A	B	B	A	A	B	B	B	A	A	A	B	B	B	B	B	B	A	A	A	B	A	B	A	B	B	B	A
B	B	A	B	B	B	A	B	0	A	A	B	B	B	B	B	B	A	A	B	B	B	A	B	B	B	A	A	A	B	B	B	B	B	B	A	A	A	B	A	B	A	B	B	B	A
B	B	A	B	B	B	A	B	0	A	A	B	B	B	B	B	B	A	A	B	B	B	A	B	B	B	A	A	A	B	B	B	B	B	B	A	A	A	B	A	B	A	B	B	B	A
B	B	A	B	B	B	A	B	0	A	A	B	B	B	B	B	B	A	A	B	B	B	A	B	B	B	A	A	A	B	B	B	B	B	B	A	A	A	B	A	B	A	B	B	B	A
B	B	A	B	B	B	A	B	0	A	A	B	B	B	A	B	B	A	A	B	B	B	A	B	B	B	A	A	A	B	B	B	B	B	B	A	A	A	B	A	B	A	B	B	B	A
B	B	A	B	B	B	A	B	0	A	A	B	B	B	A	B	B	A	A	B	B	B	A	B	B	B	A	A	A	B	B	B	B	B	B	A	A	A	B	A	B	A	B	B	B	A
B	B	A	B	B	B	A	B	0	B	A	B	B	B	A	B	B	A	A	B	B	B	A	B	B	B	A	A	A	B	B	B	B	B	B	A	A	A	B	A	B	A	B	B	B	A
B	B	A	B	B	B	A	B	0	B	A	B	B	B	A	B	B	A	A	B	B	B	A	B	B	B	A	A	A	B	B	B	B	B	B	A	A	A	B	A	B	B	B	B	B	A
B	A	A	B	B	B	A	B	0	B	A	B	B	B	A	B	B	A	A	B	B	B	A	B	B	B	A	A	A	B	B	B	B	B	B	A	A	A	B	A	B	B	B	B	B	A
B	A	A	B	B	A	A	B	0	B	A	B	B	B	A	B	B	A	A	B	B	B	A	B	B	B	A	A	A	B	B	B	B	B	B	A	A	A	B	A	B	B	B	B	B	A
B	A	A	B	B	A	A	B	0	B	A	B	B	B	A	B	B	A	A	B	B	B	A	B	B	B	A	A	A	B	B	B	B	B	B	A	A	A	0	A	B	B	B	B	B	B
B	A	A	B	B	A	B	B	0	B	A	B	B	B	A	B	B	A	A	B	B	B	A	B	B	B	A	A	B	B	B	B	B	B	B	A	A	A	0	A	B	B	B	B	B	B
B	A	A	B	B	A	B	B	0	B	A	B	B	B	A	B	B	A	A	B	B	B	A	B	B	B	A	A	B	B	B	B	B	B	B	A	A	A	0	A	B	B	B	B	B	B
B	A	A	B	B	A	B	B	0	B	A	B	B	B	A	B	B	A	A	B	B	B	A	B	B	B	A	A	B	B	B	B	B	B	B	A	A	A	0	A	B	B	B	B	B	B
B	A	A	B	B	A	B	B	0	B	A	B	B	B	A	B	B	A	A	B	B	B	A	B	A	B	A	A	B	B	B	B	B	B	B	A	A	A	0	A	B	B	B	B	B	B
B	A	A	B	B	A	B	B	0	B	A	B	B	B	A	B	B	A	A	B	B	B	A	B	A	B	A	B	B	B	B	B	B	B	B	A	A	A	0	A	B	B	B	B	B	B
B	A	A	B	B	A	B	B	A	B	A	B	B	B	A	B	B	A	A	B	B	B	A	B	A	B	A	B	B	B	B	B	B	B	B	A	A	A	0	A	B	B	B	B	B	B
B	A	A	B	B	A	B	B	A	B	A	B	B	B	A	B	B	A	A	B	B	B	A	B	A	B	A	B	B	B	B	B	B	B	B	A	A	A	0	A	B	B	B	B	B	B
B	A	A	B	B	A	B	B	A	B	A	B	B	B	A	B	B	A	A	B	B	B	A	B	A	B	A	B	B	B	B	B	B	B	B	A	A	B	0	A	B	B	B	B	B	B
B	A	A	B	B	A	B	B	A	B	A	B	B	B	A	B	B	A	A	B	B	B	A	B	A	B	A	B	B	B	B	B	B	B	B	A	A	B	0	A	B	B	B	B	B	B
B	A	A	B	B	A	A	B	A	B	A	B	B	B	A	B	B	A	A	B	B	B	A	B	A	B	A	B	B	B	B	B	B	B	B	A	A	B	0	A	B	B	B	B	B	B
B	A	A	B	B	A	A	B	A	B	A	B	B	B	A	B	B	A	A	B	B	B	A	B	A	B	A	B	B	B	B	B	B	B	A	A	A	B	A	A	B	B	B	B	B	B
B	A	A	A	B	A	A	B	A	B	A	B	B	B	A	B	B	A	A	B	B	B	A	B	A	B	A	B	B	B	B	B	B	B	A	A	A	B	A	A	B	B	B	B	B	B
B	A	A	A	B	A	A	B	A	B	A	B	B	B	A	B	B	A	A	B	B	B	A	B	A	B	A	B	B	B	B	B	B	B	A	A	A	B	A	A	B	B	B	B	B	B
B	A	A	A	B	A	A	B	A	B	A	B	B	B	A	B	B	A	A	B	B	B	A	B	A	B	A	B	B	B	B	B	B	B	A	A	A	B	A	A	B	B	B	B	B	B
B	A	A	A	B	A	A	B	A	A	A	B	B	B	A	B	B	A	A	B	B	B	A	B	A	B	A	B	B	B	B	B	B	B	A	A	A	B	A	A	B	B	B	B	B	B
B	A	A	A	B	A	A	B	A	A	A	B	B	B	A	B	B	A	A	B	B	B	A	B	A	B	A	B	B	B	B	B	B	B	A	A	A	B	A	A	B	B	B	B	B	B
B	A	A	A	B	A	A	B	A	A	A	B	B	B	A	B	B	A	A	B	B	B	A	B	A	B	A	B	B	B	B	B	B	B	A	A	A	B	A	A	B	B	B	B	B	B
B	B	A	A	B	A	A	B	A	A	A	B	B	B	A	B	B	A	A	B	B	B	A	B	A	B	A	B	B	B	B	B	B	B	A	A	A	B	A	A	B	B	B	B	B	B
B	B	A	A	B	A	A	B	A	A	A	B	B	B	A	B	B	A	A	B	B	B	A	B	A	B	A	B	B	B	B	B	B	B	A	A	A	B	A	A	B	B	B	B	B	B
B	B	A	A	B	A	A	B	A	A	A	B	B	B	A	B	B	A	B	B	B	B	A	B	A	B	A	B	B	B	B	B	B	B	A	A	A	B	A	A	B	B	B	B	B	B
B	B	A	A	B	A	A	B	A	A	A	B	B	B	A	B	B	A	B	B	B	B	A	B	A	B	A	B	B	B	B	B	B	B	A	A	A	B	A	A	B	B	B	B	B	B
B	B	A	A	B	A	A	B	A	A	A	B	B	B	A	A	B	A	B	B	B	B	A	B	A	B	A	B	B	B	B	B	B	B	A	A	A	B	A	A	B	B	B	B	B	B
B	B	A	A	B	A	A	B	A	A	A	B	B	B	A	A	B	A	B	B	B	B	A	B	A	B	A	B	B	B	B	B	B	B	A	A	A	B	A	A	B	B	B	B	B	B
B	B	A	A	B	A	A	B	A	A	A	B	B	B	A	A	B	A	B	B	B	B	A	B	A	B	A	B	B	B	B	B	B	B	A	A	A	B	A	A	B	B	B	B	B	B
B	B	A	A	B	A	A	B	A	A	A	B	B	B	A	A	B	A	B	B	B	B	A	B	A	B	A	B	B	B	B	B	B	B	A	A	A	B	A	A	B	B	B	B	B	B
B	B	A	A	B	A	B	B	A	A	A	B	B	B	A	A	B	A	B	B	B	B	A	B	A	B	A	B	B	B	B	B	B	B	A	A	A	B	A	A	B	B	B	B	B	B
B	B	A	A	B	A	B	B	A	A	A	B	B	B	A	A	B	A	B	B	B	B	A	B	A	B	A	B	B	B	B	B	B	B	A	A	A	B	A	A	B	B	B	B	B	B
B	B	A	A	B	B	B	B	A	A	A	B	B	B	A	A	B	A	B	B	B	B	A	B	A	B	A	B	B	B	B	B	B	B	A	A	A	B	A	A	B	B	B	B	B	B
B	B	A	A	B	B	B	B	A	A	A	B	B	B	A	A	B	A	B	B	B	B	A	B	A	B	A	B	B	B	B	B	B	B	A	A	A	B	A	A	B	B	B	B	B	B
B	B	A	A	B	B	B	B	A	A	A	B	B	B	A	A	B	A	B	B	B	B	A	B	A	B	A	B	B	B	B	B	B	B	A	A	A	B	A	A	B	B	B	B	B	B
B	B	A	A	B	B	B	B	A	A	A	B	B	B	B	A	B	A	B	B	B	B	A	B	A	B	A	B	B	B	B	B	B	B	A	A	A	B	A	A	B	B	B	B	B	B

B	B	A	A	B	B	B	B	A	A	A	B	B	B	B	A	B	A	B	B	B	B	A	B	A	B	A	B	B	B	B	B	B	B	B	A	A	B	A	A	B	B	B	B	B	B
B	B	A	A	B	B	B	B	A	A	A	B	B	B	B	A	B	A	B	B	B	B	A	B	A	B	A	B	B	B	B	B	B	B	B	A	A	B	A	A	B	B	B	B	B	B
B	B	A	A	B	B	B	B	A	A	A	B	B	B	B	A	B	A	B	B	B	B	A	B	A	B	A	B	B	B	B	B	B	B	B	A	A	B	A	A	B	B	B	B	B	B
B	B	A	A	B	B	B	B	A	A	A	B	B	B	B	A	B	A	B	B	B	B	A	B	A	B	A	B	B	B	B	B	B	B	B	A	A	B	A	A	B	B	B	B	B	B
B	B	A	A	B	B	B	B	A	A	A	B	B	B	B	A	B	A	B	B	B	B	A	B	A	B	A	B	B	B	B	B	B	B	B	A	A	B	A	A	B	B	B	B	B	B
B	B	A	A	B	B	B	B	A	A	A	B	B	B	B	A	B	A	B	B	B	B	A	B	A	B	A	B	B	B	B	B	B	B	B	A	A	B	A	A	B	B	B	B	B	B
B	B	A	A	B	B	B	B	A	A	A	B	B	B	B	A	B	A	B	B	B	B	A	B	A	B	A	B	B	B	B	B	B	B	B	A	A	B	A	A	B	B	B	B	B	B
B	B	A	A	B	B	B	B	A	A	A	B	B	B	B	A	B	A	B	B	B	B	A	B	A	B	A	B	B	B	B	B	B	B	B	A	A	B	A	A	B	B	B	B	B	B
B	B	A	A	B	B	B	B	A	A	A	B	B	B	B	A	B	A	B	B	B	B	A	B	A	B	A	B	B	B	B	B	B	B	B	A	A	B	A	A	B	B	B	B	B	B
B	B	A	A	B	B	B	B	A	A	A	B	B	B	B	A	B	A	B	B	B	B	A	B	A	B	A	B	B	B	B	B	B	B	B	A	A	B	A	A	B	B	B	B	B	B
B	B	A	A	B	B	B	B	A	A	A	B	B	B	B	A	B	A	B	B	B	B	A	B	A	B	A	B	B	B	B	B	B	B	B	A	A	B	A	A	B	B	B	B	B	B
B	B	A	A	B	B	B	B	A	A	A	B	B	B	B	A	B	A	B	B	B	B	A	B	A	B	A	B	B	B	B	B	B	B	B	A	A	B	A	A	B	B	B	B	B	B
B	B	A	A	B	B	B	B	A	A	A	B	B	B	B	A	B	A	B	B	B	B	A	B	B	B	A	B	B	B	B	B	B	B	B	A	A	B	A	A	B	B	B	B	B	B
B	B	A	A	B	B	B	B	A	A	A	B	B	B	B	A	B	A	B	B	B	B	A	B	B	B	A	B	B	B	B	B	B	B	B	A	A	B	A	A	B	B	B	B	B	B
B	B	A	A	B	B	B	B	A	A	A	B	A	B	B	A	B	A	B	B	B	B	A	B	B	B	A	B	B	B	B	B	B	B	B	A	A	B	A	A	B	B	B	B	B	B
B	B	A	A	B	B	B	B	A	A	A	B	A	B	B	A	B	A	B	B	B	B	A	B	B	B	A	B	B	B	B	B	B	B	B	A	A	B	A	A	B	B	B	B	B	B
B	B	A	A	B	B	B	B	A	A	A	B	A	B	B	A	B	A	B	B	B	B	A	B	B	A	A	B	B	B	B	B	B	B	B	A	A	B	A	A	B	B	B	B	B	B
B	B	A	A	B	B	B	B	A	A	A	B	A	B	B	A	B	A	B	B	B	B	A	B	B	A	A	B	B	B	B	B	B	B	B	A	A	B	A	A	B	B	B	B	B	B
B	B	A	A	B	B	A	B	A	A	A	B	A	B	B	A	B	A	B	B	B	B	A	B	B	A	A	B	B	B	B	B	B	B	B	A	A	B	A	A	B	B	B	B	B	B
B	B	B	A	B	B	A	B	A	A	A	B	A	B	B	A	B	A	B	B	B	B	A	B	B	A	A	B	B	B	B	B	B	B	B	A	A	B	A	A	B	B	B	B	B	B
B	B	B	A	B	B	A	B	A	A	A	B	A	B	B	A	B	A	B	B	B	B	A	B	B	A	A	B	B	A	A	B	B	B	B	A	A	B	A	A	B	B	B	B	B	B
B	B	B	A	B	B	A	B	A	A	A	B	A	B	A	A	B	A	B	B	B	B	A	B	B	A	A	B	B	A	A	B	B	B	B	A	A	B	A	A	B	B	B	B	B	B
B	B	B	A	B	B	A	B	A	A	A	B	A	B	A	A	B	A	B	B	B	B	A	B	B	A	A	B	B	A	A	B	B	B	B	A	A	B	A	A	B	B	B	B	B	B
B	B	B	A	B	B	A	B	A	B	A	B	A	B	A	A	B	A	B	B	B	B	A	B	B	A	A	B	B	A	A	B	B	B	B	A	A	B	A	A	B	B	B	B	B	B
B	B	B	A	B	B	A	B	A	B	A	B	A	B	A	A	B	A	B	B	B	B	A	B	B	A	A	B	A	A	A	B	B	B	B	A	A	B	A	A	B	B	B	B	B	B
B	B	B	A	B	B	A	B	A	B	A	B	A	A	A	A	B	A	B	B	B	B	A	B	B	A	A	B	A	A	A	B	B	B	B	A	A	B	A	A	B	B	B	B	B	B
B	B	B	A	B	B	A	B	A	B	A	B	A	A	A	A	B	A	B	B	B	B	A	B	B	A	A	B	A	A	A	B	B	B	B	A	A	B	A	A	B	A	B	B	B	B
B	B	B	A	B	B	A	B	A	B	A	B	A	A	A	A	B	B	B	B	B	B	A	B	B	A	A	B	A	A	A	B	B	B	B	A	A	B	A	A	B	A	B	B	B	B
B	B	B	A	B	B	A	B	A	B	A	B	A	A	A	A	B	B	B	B	B	B	A	B	B	A	A	B	A	A	A	B	B	B	B	A	A	B	A	A	B	A	A	B	B	B
B	B	B	A	B	B	A	B	A	B	A	B	A	A	A	A	B	B	B	B	B	B	A	B	B	A	A	B	A	A	A	B	B	B	B	A	A	B	A	A	B	A	A	B	B	B
B	B	B	A	B	B	A	B	A	B	A	B	A	A	A	A	B	B	B	B	B	B	A	B	B	A	A	B	A	A	A	B	B	B	B	A	A	B	A	A	B	A	A	B	B	B
B	B	B	B	B	B	A	B	A	B	A	B	A	A	A	A	B	B	B	B	B	B	A	B	B	A	A	B	A	A	A	B	B	B	B	A	A	B	A	A	B	A	A	B	B	B
B	B	B	B	B	B	A	B	B	B	A	B	A	A	A	A	B	B	B	B	B	B	A	B	B	A	A	B	A	A	A	B	B	B	B	A	A	B	A	A	B	A	A	B	B	B
B	B	B	B	B	B	A	B	B	B	A	B	A	A	A	A	B	B	B	B	B	B	A	B	B	A	A	B	A	A	A	B	B	B	B	A	A	B	A	A	B	A	A	B	B	B
B	B	B	B	B	B	A	B	B	B	A	B	A	A	A	A	B	B	B	B	B	B	B	B	B	A	A	B	A	A	A	B	B	B	B	A	A	B	A	A	B	A	A	B	B	B
B	B	B	B	B	B	A	B	B	B	A	B	A	A	A	A	B	B	B	B	B	B	B	B	B	B	0	B	A	A	A	B	B	B	B	A	A	B	A	A	B	A	A	B	B	B
A	A	A	A	B	B	A	B	B	A	A	A	A	A	B	A	A	B	B	B	B	A	A	B	A	B	A	A	B	A	A	A	A	A	B	B	A	B	A	B	A	A	A	B	B	A
A	A	A	A	B	B	A	B	B	A	A	B	A	A	B	A	A	B	B	B	B	A	A	B	A	B	A	A	B	A	A	A	A	A	B	B	A	B	A	B	A	A	A	B	B	A
A	A	A	A	B	B	A	B	B	A	A	B	A	A	B	A	A	B	B	B	B	A	A	B	A	B	A	A	B	B	B	A	A	A	B	B	A	B	A	B	A	A	A	B	B	A
A	A	A	A	B	B	A	B	B	A	A	B	A	A	B	B	A	B	B	B	B	A	A	B	A	B	A	A	B	B	B	A	A	A	B	B	A	B	A	B	A	A	A	B	B	A
A	A	A	A	B	B	A	B	B	A	A	B	A	A	B	B	A	B	B	B	B	A	A	B	A	B	A	A	B	B	B	A	A	A	B	B	A	B	A	B	A	A	B	B	B	A
A	A	A	A	B	B	A	B	B	A	A	B	A	A	B	B	A	B	B	B	B	A	A	B	A	B	A	A	B	B	B	A	A	A	B	B	A	B	A	B	A	A	B	B	B	A
A	A	B	A	B	B	A	B	B	A	A	B	A	A	B	B	A	B	B	B	B	A	A	B	A	B	A	A	B	B	B	A	A	A	B	B	A	B	A	B	A	A	B	B	B	A
A	A	B	A	B	B	A	B	B	A	A	B	A	A	B	B	A	B	B	B	B	A	A	B	A	B	A	A	B	B	B	A	A	A	B	B	A	B	A	B	A	A	B	B	B	A
A	A	B	A	B	B	A	B	B	A	A	B	B	A	B	B	A	B	B	B	B	A	A	B	A	B	A	A	B	B	B	A	A	A	B	B	A	B	A	B	A	A	B	B	B	A
A	A	B	A	B	B	A	B	B	A	A	B	B	A	B	B	A	A	B	B	B	A	A	B	A	B	A	A	B	B	B	A	A	A	B	B	A	B	A	B	A	A	B	B	B	A
A	A	B	A	B	B	A	B	B	A	A	B	B	A	B	B	A	A	B	B	B	A	A	B	A	B	A	A	B	B	B	A	A	A	B	B	A	B	A	B	A	A	B	B	B	A
A	A	B	A	B	B	A	B	B	A	A	B	B	A	B	B	A	A	B	B	B	A	A	B	A	B	A	A	B	B	B	A	A	A	B	B	A	B	A	B	A	A	B	B	B	A
A	A	B	A	B	B	A	B	B	A	A	B	B	A	B	B	A	A	B	B	B	A	A	B	A	B	A	A	B	B	B	B	B	A	B	B	A	B	A	B	A	A	B	B	B	A

A	A	B	A	B	B	A	B	B	A	A	B	B	A	B	B	A	A	B	B	B	A	A	B	A	B	A	A	B	B	B	B	B	A	B	B	A	B	A	B	A	A	B	B	B	A
A	A	B	A	B	A	A	B	B	A	A	B	B	A	B	B	A	A	B	B	B	A	A	B	A	B	A	A	B	B	B	B	B	A	B	B	A	B	A	B	A	A	B	B	B	A
A	A	B	A	B	A	A	B	B	A	A	B	B	A	B	B	A	A	B	B	B	A	A	B	A	B	A	A	B	B	B	B	B	A	B	B	A	B	A	B	A	A	B	B	B	A
A	A	B	A	B	A	A	B	B	A	A	B	B	A	A	B	A	A	B	B	B	A	A	B	A	B	A	A	B	B	B	B	B	A	B	B	A	B	A	B	A	A	B	B	B	A
A	A	B	A	B	A	A	B	B	A	A	B	B	A	A	B	A	A	B	B	B	A	A	B	A	B	A	A	B	B	B	B	B	A	B	B	A	A	A	B	A	A	B	B	B	A
A	A	B	A	B	A	A	B	B	A	A	B	B	A	A	B	A	A	B	B	B	A	A	B	A	B	A	A	B	B	B	B	B	A	B	B	A	A	A	B	A	A	B	B	B	A
A	A	B	A	B	A	A	B	B	A	A	B	B	A	A	B	A	A	B	B	B	A	A	B	A	B	A	A	B	B	B	B	B	A	B	B	A	A	A	B	A	A	B	B	B	A
A	A	B	A	B	A	A	B	B	A	A	B	B	A	A	B	A	A	B	B	B	A	A	B	A	B	A	A	B	B	B	B	B	A	B	B	A	A	A	B	A	A	B	B	B	A
A	A	B	A	B	A	A	B	B	A	A	B	B	A	A	B	A	A	B	B	B	A	A	B	A	B	A	A	B	B	B	B	B	A	B	B	A	A	A	B	A	A	B	B	B	B
A	A	B	A	B	A	A	B	B	A	A	B	B	A	A	B	A	A	B	B	B	A	A	B	A	B	A	A	B	B	B	B	B	A	B	B	A	A	A	B	A	A	B	B	B	B
A	A	B	A	B	A	A	B	B	A	A	B	B	A	A	B	A	B	B	B	B	A	A	B	A	B	A	A	B	B	B	B	B	A	B	B	A	A	A	B	A	A	B	B	B	B
A	A	B	A	B	A	A	B	B	A	A	B	B	A	A	B	A	B	B	B	B	A	A	B	A	B	A	B	B	B	B	B	B	A	B	B	A	A	A	B	A	A	B	B	B	B
A	A	A	A	B	A	A	B	B	A	A	B	B	A	A	B	A	B	B	B	B	A	A	B	A	B	A	B	B	B	B	B	B	A	B	B	A	A	A	B	A	A	B	B	B	B
A	B	A	A	B	A	A	B	B	A	A	B	B	A	A	B	A	B	B	B	B	A	A	B	A	B	A	B	B	B	B	B	B	A	B	B	A	A	A	B	A	A	B	B	B	B
A	B	A	A	B	A	A	B	B	A	A	B	B	A	A	B	A	B	B	B	B	A	A	B	A	B	A	B	B	B	B	B	B	A	B	B	A	A	A	B	A	A	B	B	B	B
A	B	A	A	B	A	A	B	B	A	A	B	B	A	A	B	A	B	B	B	B	A	A	B	A	B	A	B	B	B	B	B	B	A	B	B	A	A	A	B	A	A	B	B	B	B
A	B	A	A	B	A	A	A	B	A	A	B	B	A	A	B	A	B	B	B	B	A	A	B	A	B	A	B	B	B	B	B	B	A	B	B	A	A	A	B	A	A	B	B	B	B
A	B	A	A	B	A	A	A	B	A	A	B	B	A	A	B	A	B	B	B	B	A	A	B	B	B	A	B	B	B	B	B	B	A	B	B	A	A	A	B	A	A	B	B	B	B
A	B	A	B	B	A	A	A	B	A	A	B	B	A	A	B	A	B	B	B	B	A	A	B	B	B	A	B	B	B	B	B	B	A	B	B	A	A	A	B	A	A	B	B	B	B
A	B	A	B	B	A	A	A	B	A	A	B	B	A	A	B	A	B	B	B	B	A	A	B	B	B	A	B	B	B	B	B	B	A	B	B	A	A	A	B	A	A	B	B	B	B
A	B	A	B	B	A	A	A	B	A	A	B	B	A	A	B	A	B	B	B	B	A	A	B	B	B	A	B	B	B	B	B	B	A	B	B	A	A	A	B	A	A	B	B	B	B
A	B	A	B	B	A	0	A	B	A	A	B	B	A	A	B	A	B	B	B	B	A	A	B	B	B	A	B	B	B	B	B	B	A	B	B	A	A	A	B	A	A	B	B	B	B
A	B	A	B	B	A	0	A	B	A	A	B	B	A	A	B	A	B	B	B	B	A	A	B	B	B	A	B	B	B	B	B	B	A	B	B	A	A	A	B	A	A	B	B	B	B
A	B	A	B	B	A	0	A	B	A	A	B	B	A	A	B	A	B	B	B	B	A	B	B	B	B	A	B	B	B	B	B	B	A	B	B	A	A	A	B	A	A	B	B	B	B
A	B	A	B	B	A	B	A	B	A	A	B	B	A	A	B	A	B	B	B	B	A	B	B	B	B	A	B	B	B	B	B	B	A	B	B	A	A	A	B	A	A	B	B	B	B
A	B	A	B	B	A	B	A	B	A	A	B	B	A	A	B	A	B	B	B	B	A	B	B	B	B	A	B	A	B	B	B	B	A	B	B	A	A	A	B	A	A	B	B	B	B
A	B	A	B	B	A	B	A	B	A	A	B	B	A	A	B	A	B	B	B	B	A	B	B	B	B	A	B	A	B	B	B	B	A	B	B	A	A	A	B	A	A	B	B	B	B
A	B	A	B	B	A	B	A	B	A	A	B	B	B	A	B	A	B	B	B	B	A	B	B	B	B	A	B	A	B	B	B	B	A	B	B	A	A	A	B	A	A	B	B	B	B
A	B	A	B	B	A	B	A	B	A	A	B	B	B	A	B	A	B	B	B	B	A	B	B	B	B	A	B	A	B	B	B	B	A	B	B	A	A	A	B	A	A	B	B	B	B
A	B	A	B	B	A	B	A	A	A	A	B	B	B	A	B	A	B	B	B	B	A	B	B	B	B	A	B	A	B	B	B	B	A	B	B	A	A	A	B	A	A	B	B	B	B
A	B	A	B	B	A	B	A	A	A	A	B	B	B	A	B	A	B	B	B	B	A	B	B	B	B	A	B	A	B	B	B	B	A	A	B	A	A	A	B	A	A	B	B	B	B
A	B	A	B	B	A	B	A	A	A	A	B	B	B	A	B	A	B	B	B	B	A	B	B	B	B	A	B	A	B	B	B	B	B	A	B	A	A	A	B	A	A	B	B	B	B
A	B	A	B	B	A	B	A	A	A	A	B	B	B	A	B	A	B	B	B	B	A	B	B	B	B	A	B	A	B	B	B	B	B	A	B	A	A	A	B	A	A	B	B	B	B
A	B	A	B	B	A	B	A	A	A	B	B	B	B	A	B	A	B	B	B	B	A	B	B	B	B	A	B	A	B	B	B	B	B	A	B	A	A	A	B	A	A	B	B	B	B
A	B	A	B	B	A	B	A	A	A	B	B	B	B	A	B	A	B	B	B	B	A	B	B	B	B	A	B	A	B	B	B	B	B	A	B	A	A	A	B	A	A	B	B	B	B
A	B	A	B	B	A	B	A	A	A	B	B	B	B	A	B	A	B	B	B	B	A	B	B	B	B	A	B	A	B	B	B	B	B	A	B	A	B	A	B	A	A	B	B	B	B
A	B	A	B	B	A	B	A	A	A	B	B	B	B	A	B	A	B	B	B	B	A	B	B	B	B	A	B	A	B	B	B	B	B	A	B	A	B	A	B	A	A	B	B	B	B
A	A	B	A	A	0	A	B	A	B	B	A	B	A	B	A	B	A	B	A	A	B	B	A	A	A	B	A	A	B	B	B	B	A	0	A	B	B	B	B	B	A	B	A	A	B
A	A	B	A	A	0	A	B	A	B	B	A	B	A	B	A	B	A	B	A	A	B	B	A	A	A	B	A	A	B	B	B	B	A	0	A	B	B	B	B	B	A	B	A	A	A
A	A	B	A	A	0	A	B	A	B	B	A	B	A	B	A	B	A	B	A	A	B	B	A	A	A	B	A	A	B	B	B	B	A	0	0	B	B	B	B	B	A	B	A	A	A
A	A	B	A	A	0	A	B	A	B	B	A	B	A	B	A	B	A	B	A	A	B	B	A	A	A	B	A	A	B	B	B	B	A	B	0	B	B	B	B	B	A	B	A	A	A
A	A	B	A	A	0	A	B	A	B	B	A	B	A	B	A	B	A	B	A	A	B	B	A	A	A	B	A	A	B	B	B	B	A	B	B	B	B	B	B	B	A	B	A	A	A
A	A	B	A	A	A	A	B	A	B	B	A	B	A	B	A	B	A	B	A	A	B	B	A	A	A	B	A	A	B	B	B	B	A	B	B	B	B	B	B	B	A	B	A	A	A
A	A	B	A	A	A	A	B	A	B	B	A	B	A	B	A	B	A	B	A	A	B	B	A	A	A	B	A	A	B	B	B	B	A	B	B	B	B	B	B	B	A	B	B	B	A
A	A	B	A	A	A	A	B	A	B	B	A	B	A	B	A	B	A	A	A	A	B	B	A	A	A	B	A	A	B	B	B	B	A	B	B	B	B	B	B	B	A	B	B	B	A
A	A	B	A	A	A	A	B	A	B	B	A	B	A	B	A	B	A	A	A	A	B	B	A	A	A	B	A	A	B	B	B	B	A	B	B	B	B	B	B	B	A	A	B	B	A
A	A	B	A	A	A	A	B	A	B	B	A	B	A	B	A	B	A	A	A	A	B	B	A	A	A	B	A	A	B	B	B	B	A	B	B	B	B	B	B	B	A	A	B	B	A
A	A	B	A	A	A	A	B	A	A	B	A	B	A	B	A	B	A	A	A	A	B	B	A	A	A	B	A	A	B	B	B	B	A	B	B	B	B	B	B	B	A	A	B	B	A
B	A	B	A	A	A	A	B	A	A	B	A	B	A	B	A	B	A	A	A	A	B	B	A	A	A	B	A	A	B	B	B	B	A	B	B	B	B	B	B	B	A	A	B	B	A

B	A	B	A	A	A	A	B	A	A	B	A	B	A	B	A	B	A	A	A	A	B	B	A	A	A	B	A	A	A	A	B	B	A	B	B	B	B	B	B	B	A	A	B	B	A
B	A	B	A	A	A	A	B	A	A	B	A	B	A	B	A	B	A	A	A	A	B	B	A	A	A	B	A	A	A	A	B	B	A	B	B	B	B	B	A	B	A	A	B	B	A
B	A	B	A	A	A	A	B	A	A	B	A	B	A	B	A	B	A	A	A	A	B	B	A	A	A	B	A	A	A	A	B	B	A	B	B	B	B	B	A	B	A	A	B	B	A
B	A	B	A	A	A	A	B	A	A	B	A	B	A	B	A	B	A	A	A	A	B	B	A	A	A	B	A	A	A	A	B	B	A	B	B	B	B	B	A	B	A	A	B	B	A
B	A	B	A	A	A	A	B	A	A	B	A	B	B	B	A	B	A	A	A	A	B	B	A	A	A	B	A	A	A	A	B	B	A	B	B	B	B	B	A	B	A	A	B	B	A
B	A	B	A	A	A	A	B	A	A	B	A	B	B	B	A	B	A	A	A	A	B	B	A	A	A	A	A	A	A	A	B	B	A	B	B	B	B	B	A	B	A	A	B	B	A
B	A	B	A	A	A	A	B	A	A	B	A	B	B	B	A	B	A	A	A	A	B	B	A	A	A	A	A	A	A	A	B	B	A	B	B	B	B	B	A	B	A	A	B	B	A
B	A	B	A	A	A	A	B	A	A	B	A	B	B	B	A	B	A	A	A	A	B	B	A	A	A	A	A	A	A	A	B	B	A	B	B	B	B	B	A	B	A	A	B	B	A
B	A	B	A	A	A	A	B	A	A	B	A	B	B	B	A	B	A	A	A	A	B	B	A	A	A	A	A	A	A	A	B	B	A	B	B	B	B	B	A	B	A	A	B	B	A
B	A	B	A	A	A	A	B	A	A	B	A	B	B	B	A	B	A	A	A	A	B	B	A	A	A	A	A	A	A	A	B	B	A	B	B	B	B	B	A	B	A	A	B	B	A
B	A	B	A	A	A	A	B	A	A	B	A	B	B	B	A	B	A	A	A	A	B	B	A	A	A	A	B	A	A	A	B	B	A	B	B	B	B	B	A	B	A	A	B	B	A
B	A	B	A	A	A	A	B	B	A	B	A	B	B	B	A	B	A	A	A	A	B	B	A	A	A	A	B	A	A	A	B	B	A	B	B	B	B	B	A	B	A	A	B	B	A
B	A	B	A	A	A	A	B	B	A	B	A	B	B	B	A	B	A	A	A	A	B	B	A	A	A	A	B	A	A	A	B	B	A	B	B	B	B	B	A	B	A	A	B	B	A
B	A	B	A	A	A	A	B	B	A	B	A	B	B	B	A	B	A	A	A	A	B	B	A	A	A	A	B	A	A	A	B	B	A	B	B	B	B	B	A	B	A	A	B	B	A
B	A	B	A	A	A	A	B	B	A	B	A	B	B	B	A	B	A	A	A	A	B	B	A	A	A	A	B	A	A	A	B	B	A	B	B	B	B	B	A	B	A	A	B	B	A
B	B	B	A	A	A	A	B	B	A	B	A	B	B	B	A	B	A	A	A	A	B	A	A	A	A	A	B	A	A	A	B	B	A	B	B	B	B	B	A	B	A	A	B	B	A
B	B	B	A	A	A	A	B	B	A	B	A	B	B	B	A	B	A	A	A	A	B	A	A	A	A	A	B	A	A	A	B	B	A	B	B	B	B	B	A	B	A	A	B	B	A
B	B	B	A	A	A	A	B	B	A	B	A	B	B	B	A	B	A	A	A	A	B	A	A	A	A	A	B	A	A	A	B	B	A	B	B	B	B	B	A	B	A	A	B	B	A
B	B	B	A	A	A	A	B	B	A	B	A	B	B	B	A	B	A	A	A	A	A	A	A	A	A	A	B	A	A	A	B	B	A	B	B	B	B	B	A	B	A	A	B	B	A
B	B	B	A	A	A	A	B	B	A	B	A	B	B	B	A	B	A	A	A	A	A	A	A	A	A	A	B	A	A	A	B	B	A	B	B	B	B	B	A	B	A	A	B	B	A
B	B	B	B	A	A	A	B	B	A	B	A	B	B	B	A	B	A	A	A	A	A	A	A	A	A	A	B	A	A	A	B	B	A	B	B	B	B	B	A	B	A	A	B	B	A
B	B	B	B	A	A	A	B	B	A	B	A	B	B	B	A	B	A	A	A	A	A	A	A	A	A	A	B	A	A	A	B	B	A	B	B	B	B	B	A	B	A	A	B	B	A
B	B	B	B	A	A	A	B	B	A	B	A	B	B	B	A	B	A	A	A	A	A	A	A	A	A	A	B	A	A	A	B	B	A	B	B	B	B	B	A	B	A	A	B	B	A
B	B	B	B	A	A	A	B	B	A	B	A	B	B	B	A	B	A	A	A	A	A	A	A	A	A	A	B	A	A	A	B	B	A	B	B	A	B	B	A	A	A	A	B	B	A
B	B	B	B	A	A	A	B	B	A	B	A	A	B	B	A	B	A	A	A	A	A	A	A	A	A	A	B	A	A	A	B	B	A	B	B	A	B	B	A	A	A	A	B	B	A
B	B	B	B	A	A	A	B	B	A	B	A	A	B	B	A	B	A	A	A	A	A	A	A	A	A	A	B	A	A	A	B	B	A	B	B	A	B	B	A	A	A	A	B	B	A
B	B	B	B	A	A	A	B	B	A	B	A	A	B	B	A	B	A	A	A	A	A	A	A	A	A	A	B	A	A	A	B	B	A	B	B	A	B	B	A	A	A	B	B	B	A
B	B	B	B	A	A	A	B	B	A	B	A	A	B	B	A	B	A	A	A	A	B	A	A	A	A	A	B	A	A	A	B	B	A	B	B	A	B	B	A	A	A	B	B	B	A
B	B	B	B	A	A	A	B	B	A	B	A	A	B	B	A	B	A	A	A	A	B	A	A	A	A	A	B	A	A	A	B	B	B	B	B	A	B	B	A	A	A	B	B	B	A
B	B	B	B	A	A	A	B	B	A	B	A	A	B	B	A	B	A	A	A	A	B	A	A	A	A	A	B	A	A	A	B	B	B	B	B	A	B	B	A	A	A	B	B	B	A
B	B	B	B	A	A	A	A	B	A	B	A	A	B	B	A	B	A	A	A	A	B	A	A	A	A	A	B	A	A	A	B	B	B	B	B	A	B	B	A	A	A	B	B	B	A
B	B	B	B	A	A	A	A	B	A	B	A	A	B	B	A	B	A	A	A	A	B	A	A	A	A	A	B	A	A	A	B	B	B	B	B	B	B	B	A	A	A	B	B	B	A
B	B	B	B	A	A	A	A	B	A	B	A	A	B	B	A	B	A	A	A	A	B	A	A	A	A	A	B	A	B	B	B	B	B	B	B	B	B	B	A	A	A	A	B	B	A
B	B	B	B	A	A	A	A	B	A	B	A	A	B	B	A	B	B	A	A	A	B	A	A	A	A	A	B	A	B	B	B	B	B	B	B	B	B	B	A	A	A	A	B	B	A
B	B	B	B	A	A	A	A	B	A	B	A	A	B	B	A	B	B	A	A	A	B	A	A	A	A	A	B	A	B	B	B	B	B	B	B	B	B	B	A	A	A	A	B	B	A
B	B	B	B	A	A	A	A	B	A	B	A	A	B	B	A	B	B	A	A	A	B	A	A	A	A	A	B	A	B	B	B	B	B	B	B	B	B	B	A	A	A	A	B	B	A
B	B	B	B	B	A	A	A	B	A	B	A	A	B	B	A	B	B	A	A	A	B	A	A	A	A	A	B	A	B	B	B	B	B	B	B	B	B	B	A	A	A	A	B	B	A
B	B	B	B	B	A	A	B	B	A	B	A	A	B	B	A	B	B	A	A	A	B	A	A	A	A	A	B	A	B	B	B	B	B	B	B	B	B	B	A	A	A	A	B	B	A
B	B	B	B	B	A	A	B	B	A	B	A	A	B	B	A	B	B	A	A	A	B	A	A	A	B	A	B	A	B	B	B	B	B	B	B	B	B	B	A	A	A	A	B	B	A
B	B	B	B	B	A	A	B	B	A	B	A	A	B	B	A	B	B	A	A	A	B	A	A	A	B	A	B	A	B	B	B	B	B	B	B	B	B	B	A	A	A	A	B	B	A
A	B	B	B	B	A	A	B	B	A	B	A	A	B	B	A	B	B	A	A	A	B	A	A	A	B	A	B	A	B	B	B	B	B	B	B	B	B	B	A	A	A	A	B	B	A
A	B	B	B	B	A	A	B	B	A	B	A	A	B	B	A	B	B	A	A	A	B	A	A	A	B	A	B	A	B	B	B	B	B	B	B	B	B	B	A	A	A	A	B	B	A
A	B	B	B	B	A	A	B	B	A	B	A	A	B	B	A	B	B	A	A	A	B	A	A	A	B	A	B	A	B	B	B	B	B	B	B	B	B	B	A	A	A	A	B	B	A
A	B	B	B	B	A	A	B	B	A	B	A	A	B	B	A	B	B	A	A	A	B	A	A	A	B	A	B	A	B	B	B	B	B	B	B	B	B	B	A	A	A	A	B	B	A
A	B	B	B	B	A	A	B	B	A	B	A	A	B	B	A	B	B	A	A	A	B	A	A	A	B	A	B	A	B	B	B	B	B	B	B	B	B	B	A	A	A	A	B	B	A
A	B	B	B	B	A	A	B	B	A	B	A	A	B	B	A	B	B	A	A	A	B	A	A	A	B	A	B	A	B	B	B	B	B	B	B	B	B	B	A	A	A	A	B	B	A
A	B	B	B	B	A	A	B	B	A	A	A	A	B	B	A	B	B	A	A	A	B	A	A	A	B	A	B	A	B	B	B	B	B	B	B	B	B	B	A	A	A	A	B	B	A
A	A	B	B	B	A	A	B	B	A	A	A	A	B	B	A	B	B	A	A	A	B	A	A	A	B	A	B	A	B	B	B	B	B	B	B	B	B	B	A	A	A	A	B	B	A
A	A	B	B	B	A	A	B	B	A	A	A	A	B	B	A	B	B	A	A	A	B	A	A	A	B	A	B	A	B	B	B	B	B	B	B	B	0	B	A	A	A	A	B	B	A

A	A	B	B	B	A	A	B	B	A	A	A	A	B	B	A	B	B	A	A	A	B	A	A	A	B	A	B	A	B	B	B	B	B	B	B	B	0	B	A	A	A	A	B	B	A
A	A	B	B	B	A	B	B	B	A	A	A	A	B	B	A	B	B	A	A	A	B	A	A	A	B	A	B	A	B	B	B	B	B	B	B	B	0	B	A	A	A	A	B	B	A
A	A	B	B	B	A	B	B	B	A	A	A	A	B	B	A	B	B	A	A	A	B	A	A	A	B	A	B	A	B	B	B	B	B	B	B	B	0	B	A	A	A	A	B	B	A
A	A	B	B	B	A	B	B	B	A	A	A	A	B	B	A	B	B	A	A	A	B	A	B	A	B	A	B	A	B	B	B	B	B	B	B	B	0	B	A	A	A	A	B	B	A
A	A	B	B	B	A	B	B	B	A	A	A	A	B	B	A	B	B	A	A	A	B	A	B	A	B	A	B	A	B	B	B	B	B	B	B	B	0	B	A	A	A	A	B	B	A
A	A	B	B	B	A	B	B	B	A	A	A	A	B	B	A	B	B	A	A	A	B	A	B	A	B	A	B	A	B	B	B	B	B	A	B	B	0	B	A	A	A	A	B	B	A
A	A	B	B	B	A	B	B	B	A	A	A	A	B	B	A	A	B	A	A	A	B	A	B	A	B	A	B	A	B	B	B	B	B	A	B	B	0	B	A	A	A	A	B	B	A
A	A	B	B	B	A	B	B	B	A	A	A	A	B	B	A	A	B	A	A	A	B	A	B	A	B	A	B	A	B	B	B	B	B	A	B	B	0	B	A	A	A	A	B	B	A
A	A	B	B	B	A	B	B	B	A	A	A	A	B	B	A	A	B	A	A	A	B	A	B	B	B	A	B	A	B	B	B	B	B	A	B	B	0	B	A	A	A	A	B	B	A
A	A	B	B	B	A	B	B	B	A	A	A	A	B	B	A	A	B	A	A	A	B	A	B	B	B	A	B	A	B	B	B	B	B	A	B	B	0	B	A	A	A	A	B	B	A
A	A	B	B	B	A	B	B	B	A	A	B	A	B	B	A	A	B	A	A	A	B	A	B	B	B	A	B	A	B	B	B	B	B	A	B	B	0	B	A	A	A	A	B	B	A
A	A	B	B	B	A	B	B	B	A	A	B	A	B	B	A	A	B	A	A	A	B	A	B	B	B	A	B	A	B	B	B	B	B	A	B	B	0	B	A	A	A	A	B	B	A
A	A	A	B	B	A	B	B	B	A	A	B	A	B	B	A	A	B	A	A	A	B	A	B	B	B	A	B	A	B	B	B	B	B	A	B	B	0	B	A	A	A	A	B	B	A
A	A	A	B	B	A	B	B	B	A	A	B	A	B	B	A	A	B	A	A	A	B	A	B	B	B	A	B	A	B	B	B	B	B	A	B	B	0	B	A	A	A	A	B	B	A
A	A	A	B	B	A	B	B	B	A	A	B	A	A	B	A	A	B	A	A	A	B	A	B	B	B	A	B	A	B	B	B	B	B	A	B	B	0	B	A	A	A	A	B	B	A
A	A	A	B	B	A	B	B	B	A	A	B	A	A	B	B	A	B	A	A	A	B	A	B	B	B	A	B	A	B	B	B	B	B	A	B	B	0	B	A	A	A	A	B	B	A
A	A	A	B	B	A	B	B	B	A	A	B	A	A	B	B	A	B	A	A	A	B	A	B	B	B	A	B	A	B	B	B	B	B	A	B	B	0	B	A	A	A	A	B	B	A
B	A	A	B	B	A	A	B	B	A	A	A	A	A	B	B	A	A	A	A	A	A	A	A	B	B	B	B	B	B	B	B	B	A	A	A	B	B	B	A	B	A	A	A	A	A
B	A	A	B	B	A	A	B	B	A	A	A	A	B	B	B	A	A	A	A	A	A	A	A	B	B	B	B	B	B	B	B	B	A	A	A	B	B	B	A	B	A	A	A	A	A
B	A	A	B	B	A	A	B	B	A	A	A	A	B	B	B	A	A	A	A	A	A	A	A	B	B	B	B	B	B	B	B	B	A	A	A	B	B	B	A	B	A	A	A	A	A
B	A	A	B	B	A	A	B	B	A	A	A	A	B	B	B	A	A	A	A	A	A	A	A	B	B	B	B	B	B	B	B	B	A	A	A	B	B	B	A	B	A	A	A	A	A
B	A	A	B	B	A	A	B	B	A	A	A	A	B	B	B	A	A	A	A	A	A	A	A	B	B	B	B	B	B	B	B	B	A	A	A	B	B	B	A	B	A	A	A	A	A
B	A	A	B	B	A	A	B	B	A	A	A	A	B	B	B	A	A	A	A	A	A	A	A	B	B	B	B	B	B	B	B	B	A	A	A	B	B	B	A	B	A	A	A	A	A
B	A	A	B	B	A	A	B	B	A	A	A	A	B	B	B	A	A	A	A	A	A	A	A	B	B	B	B	B	B	B	B	B	A	A	A	B	B	B	A	B	A	A	A	A	A
B	A	A	B	B	A	A	B	B	A	A	A	A	B	B	B	A	A	A	A	A	A	A	A	B	B	B	B	B	B	B	B	B	A	A	A	B	B	B	A	B	A	A	A	A	A
B	A	A	B	B	A	A	B	B	A	A	A	A	B	B	B	A	A	A	A	A	A	A	A	B	B	B	B	B	B	B	B	B	A	A	A	B	B	B	A	B	A	A	A	A	A
B	A	A	B	B	A	A	B	B	A	A	A	A	B	B	B	A	A	A	A	A	A	A	A	B	B	B	B	B	B	B	B	B	A	A	A	B	B	B	A	B	A	A	A	A	A
B	A	A	B	B	A	A	B	B	A	A	A	A	B	B	B	A	A	A	A	A	A	A	A	B	B	B	B	B	B	B	B	B	A	A	A	B	B	B	A	B	A	A	A	A	A
B	A	A	B	A	A	A	B	B	A	A	A	A	B	B	B	A	A	A	A	A	A	A	A	B	B	B	B	B	B	B	B	B	A	A	A	B	B	B	A	B	A	A	A	A	A
B	A	A	B	A	A	A	B	B	A	A	A	A	B	B	B	A	A	A	A	A	A	A	A	B	B	B	B	B	B	B	A	A	A	A	A	B	B	B	A	B	A	A	A	A	A
B	A	A	B	A	A	A	B	B	A	A	A	A	B	B	B	A	A	A	A	A	A	A	B	B	B	B	B	B	B	B	A	A	A	A	A	B	B	B	A	B	A	A	A	A	A
B	A	A	B	A	A	A	B	B	A	A	A	A	B	B	B	A	A	A	A	A	A	A	B	B	B	B	B	B	B	B	A	A	A	A	A	B	B	B	A	B	A	A	A	A	A
B	A	A	B	A	A	A	B	B	B	A	A	A	B	B	B	A	A	A	A	A	A	A	B	B	B	B	B	B	B	B	A	A	A	A	A	B	B	B	A	B	A	A	A	A	A
B	A	A	B	A	A	A	B	B	B	A	A	A	B	B	B	A	B	A	A	A	A	A	B	B	B	B	B	B	B	B	A	A	A	A	A	B	B	B	A	B	A	A	A	A	A
B	A	A	B	A	A	A	B	B	B	A	A	A	B	B	B	A	B	A	A	A	A	A	B	B	B	B	B	B	B	B	A	A	A	A	A	B	B	B	A	B	A	A	A	A	A
B	A	A	B	A	A	A	B	B	B	A	A	A	B	B	B	A	B	A	A	A	A	A	B	B	B	B	B	B	B	B	A	A	A	A	A	B	B	B	A	B	A	A	A	A	A
B	A	A	B	A	A	A	B	B	B	A	A	A	B	B	A	A	B	A	A	A	A	A	B	A	B	B	B	B	B	B	A	A	A	A	A	B	B	B	A	B	A	A	A	A	A
B	A	A	B	A	A	A	B	B	B	A	A	A	B	B	A	A	B	A	A	A	A	A	B	A	B	B	B	B	B	B	A	A	A	A	A	B	B	B	A	B	A	A	A	A	A
B	A	A	B	A	A	A	B	B	B	A	A	A	B	B	A	A	B	A	A	A	A	A	B	A	B	B	B	B	B	B	A	A	A	A	A	B	B	A	A	B	A	A	A	A	A
B	A	A	B	A	A	A	B	B	B	A	A	A	B	B	A	A	B	A	A	A	A	A	B	A	B	B	A	B	B	B	A	A	A	A	A	B	B	A	A	B	A	A	A	A	A
B	A	A	B	A	A	A	B	B	B	A	A	A	B	B	A	A	B	A	A	A	A	A	B	A	B	B	A	B	B	B	A	A	A	A	A	B	B	A	A	B	B	A	A	A	A
B	A	A	B	A	A	A	B	B	B	A	A	A	B	B	A	A	B	A	A	A	A	A	B	A	B	B	A	B	B	B	A	A	A	A	A	B	B	A	A	B	B	A	A	A	A
B	A	A	B	A	A	A	B	B	B	A	A	A	B	B	A	A	B	A	A	A	A	A	B	A	B	B	A	B	B	B	A	A	A	B	A	B	B	A	A	B	B	A	A	A	A
B	A	A	B	A	A	A	B	B	B	A	A	A	B	B	A	A	B	A	A	A	A	A	B	A	B	B	A	B	B	B	A	A	A	B	A	B	B	A	A	B	B	A	A	A	A
B	A	B	B	A	A	A	B	B	B	A	A	A	B	B	A	A	B	A	A	A	A	A	B	A	B	B	A	B	B	B	A	A	A	B	A	B	B	A	A	B	B	A	A	A	A
B	A	B	B	A	A	A	B	B	B	A	A	A	B	B	A	A	B	A	A	A	B	A	B	A	B	B	A	B	B	B	A	A	A	B	A	B	B	A	A	B	B	A	A	A	A
B	A	B	B	A	A	A	B	B	B	A	A	A	B	B	A	A	B	A	A	A	B	A	B	A	B	B	A	B	B	B	A	A	A	B	A	B	B	A	A	B	B	A	A	A	A
B	A	B	B	A	A	A	B	B	B	A	A	A	B	B	A	A	B	A	A	A	B	A	B	A	B	B	A	B	B	B	A	A	A	B	A	B	B	A	A	A	B	A	A	A	A
B	A	B	B	A	A	A	B	B	B	A	A	A	B	B	A	A	B	A	A	A	B	A	B	A	B	B	A	B	B	B	A	A	A	B	A	A	A	A	A	A	B	A	A	A	A

B	A	B	B	A	A	A	B	B	B	A	A	A	B	B	A	A	B	A	A	A	B	A	B	A	B	B	A	B	B	B	A	A	A	B	A	A	A	A	A	A	B	A	A	A	A
B	A	B	B	A	A	A	B	B	B	A	A	A	B	B	A	A	B	A	A	A	B	A	B	A	B	B	A	B	B	B	A	A	A	B	A	A	A	A	A	A	B	A	A	A	A
B	A	B	B	A	A	A	B	B	B	A	A	A	B	B	A	A	B	A	A	A	B	A	B	A	B	B	A	B	B	B	A	A	A	B	A	A	A	A	A	A	B	A	A	A	A
B	A	B	B	A	A	A	B	B	B	A	A	A	B	B	A	A	B	A	A	A	B	A	B	A	B	B	A	B	B	B	A	A	A	B	A	A	A	A	A	A	B	A	A	A	A
B	A	B	B	A	A	A	B	B	B	A	A	A	B	B	A	A	B	A	A	A	B	A	B	A	B	A	A	B	B	B	A	A	A	B	A	A	A	A	A	A	B	A	A	A	A
B	A	B	B	A	A	B	B	B	B	A	A	A	B	B	A	A	B	A	A	A	B	A	B	A	B	A	A	B	B	B	A	A	A	B	A	A	A	A	A	A	B	A	A	A	A
B	A	B	B	A	A	B	B	B	B	A	A	A	B	B	A	B	B	A	A	A	B	A	B	A	B	A	A	B	B	B	A	A	A	B	A	A	A	A	A	A	B	A	A	A	A
B	A	B	B	A	A	B	B	B	B	A	A	A	B	B	A	B	B	A	A	A	B	A	B	A	B	A	A	B	B	B	A	A	A	B	A	A	A	A	A	A	B	A	A	A	A
B	A	B	B	A	A	B	B	B	B	A	A	A	B	B	A	B	B	A	A	A	B	A	B	A	B	A	A	B	B	B	A	A	A	B	A	A	A	A	A	A	B	A	A	A	A
B	A	B	B	A	A	B	B	B	B	A	A	A	B	B	A	B	B	A	A	A	B	A	B	A	B	A	A	B	B	B	A	A	A	B	A	A	A	A	A	A	B	A	A	A	A
B	A	B	B	A	A	B	B	B	B	A	A	A	B	B	A	B	B	A	A	A	B	A	B	A	B	A	A	B	B	B	A	A	A	B	A	A	A	A	A	A	B	A	A	A	A
B	A	B	B	A	A	B	B	B	B	A	A	A	B	B	A	B	B	A	A	A	B	A	B	A	B	A	A	B	B	B	A	A	A	B	A	A	A	A	A	A	B	A	B	B	A
B	A	B	B	A	A	B	B	B	B	A	A	A	B	B	A	B	B	A	A	A	B	A	B	A	B	A	A	B	B	B	A	A	A	B	A	A	A	A	A	A	B	A	B	B	A
B	B	B	B	A	A	B	B	B	B	A	A	A	B	B	A	B	B	A	A	A	B	A	B	A	B	A	A	B	B	B	A	A	A	B	A	A	A	A	A	A	B	A	B	B	A
B	B	B	B	A	A	B	B	B	B	A	A	B	B	B	A	B	B	A	A	A	B	A	B	A	B	A	A	B	B	B	A	A	A	B	A	A	A	A	A	A	B	A	B	B	A
B	B	B	B	A	A	A	B	B	B	A	A	B	B	B	A	B	B	A	A	A	B	A	B	A	B	A	A	B	B	B	A	A	A	B	A	A	A	A	A	A	B	A	B	B	A
B	B	B	A	A	A	A	B	B	B	A	A	B	B	B	A	B	B	A	A	A	B	A	B	A	B	A	A	B	B	B	A	A	A	B	A	A	A	A	A	A	B	A	B	B	A
B	B	B	A	A	A	A	B	B	B	A	A	B	B	B	A	B	B	A	A	A	B	A	B	A	B	A	A	B	B	B	A	A	A	B	A	A	A	A	A	A	A	A	B	B	A
B	B	B	A	A	A	A	B	B	B	A	A	B	B	B	A	B	B	A	A	A	B	A	B	A	B	A	A	B	B	B	A	A	A	B	A	A	A	A	A	A	A	A	B	B	A
B	B	B	A	A	A	A	B	B	A	A	A	B	B	B	A	B	B	A	A	A	B	A	B	A	B	A	A	B	B	B	A	A	A	B	A	A	A	A	A	A	A	A	B	B	A
B	B	B	A	A	A	A	B	B	A	A	A	B	B	B	A	B	B	A	A	A	B	A	B	A	B	A	A	B	B	B	A	A	A	B	A	A	A	B	A	A	A	A	B	B	A
B	B	B	A	A	A	A	B	B	A	A	A	B	B	B	A	B	B	A	A	A	B	A	B	A	B	A	A	B	B	B	A	A	A	B	A	A	A	B	A	A	A	A	B	B	A
B	B	B	A	A	A	A	B	B	A	A	A	B	B	B	A	B	B	A	A	A	B	A	B	A	B	A	A	A	B	B	A	A	A	B	A	A	A	B	A	A	A	A	B	B	A
B	B	B	A	A	A	A	B	B	A	B	A	B	B	B	A	B	B	A	A	A	B	A	B	A	B	A	A	A	B	B	A	A	A	B	A	A	A	B	A	A	A	A	B	B	A
B	B	B	A	A	A	A	B	B	A	B	A	B	B	B	A	B	B	A	B	B	B	A	B	A	B	A	A	A	B	B	A	A	A	B	A	A	A	B	A	A	A	A	B	B	A
B	B	B	A	A	A	A	B	B	A	B	A	B	B	B	A	B	B	A	B	B	B	A	B	A	B	A	A	A	B	B	A	A	A	B	A	A	A	B	A	A	A	A	B	B	A
B	B	B	A	A	A	A	B	B	A	B	A	B	B	B	A	B	B	A	B	B	B	A	B	A	B	A	A	A	B	B	A	A	A	B	A	A	A	B	A	A	A	A	B	B	A
B	B	A	A	A	A	A	B	B	A	B	A	B	B	B	A	B	B	A	B	B	B	A	B	A	B	A	A	A	B	B	A	A	A	B	A	A	A	B	A	A	A	A	B	B	A
B	B	A	A	A	A	A	B	B	A	B	A	B	B	B	A	B	A	A	B	B	B	A	B	A	B	A	A	A	B	B	A	A	A	B	A	A	A	B	A	A	A	A	B	B	A
B	B	A	A	A	A	A	B	B	A	B	A	B	B	B	A	B	A	A	B	B	B	A	B	A	B	A	A	A	B	B	A	A	A	B	A	A	A	B	A	A	A	A	B	B	A
B	B	A	A	A	A	A	B	B	A	B	A	B	B	B	A	B	A	A	B	B	B	A	A	A	B	A	A	A	B	B	A	A	A	B	A	A	A	B	A	A	A	A	B	B	A
B	B	A	A	A	A	A	B	B	A	B	A	B	B	B	A	B	A	A	B	B	B	A	A	A	B	A	A	A	B	B	A	A	A	B	A	A	A	B	A	A	A	A	B	B	A
B	B	A	A	A	A	A	B	B	A	B	A	B	B	B	A	B	A	A	B	B	B	A	A	A	B	A	A	A	B	B	A	A	A	B	A	A	A	B	A	A	A	A	B	B	A
B	B	A	A	A	A	A	B	B	A	B	A	B	B	B	A	B	A	A	B	B	B	A	A	A	B	A	A	A	B	B	A	A	A	B	A	A	A	B	A	A	A	A	B	B	A
B	B	A	A	A	B	A	B	B	A	B	A	A	B	B	A	B	A	A	B	B	B	A	A	A	B	A	A	A	B	B	A	A	A	B	A	A	A	B	A	A	A	A	B	B	A
B	B	A	A	A	B	A	B	B	A	B	A	A	B	B	A	B	A	A	B	B	B	A	A	B	B	A	A	A	B	B	A	A	A	B	A	A	A	B	A	A	A	A	B	B	A
B	B	A	A	A	B	A	B	B	A	B	A	A	B	B	A	B	A	A	B	B	B	A	A	B	B	A	A	A	B	B	A	A	A	B	A	A	A	B	A	A	A	A	B	B	A
B	B	A	A	A	B	A	B	B	A	B	A	A	B	B	A	B	A	A	B	B	B	A	A	B	B	A	A	A	B	B	A	A	A	B	A	A	A	B	A	A	A	A	B	B	A
B	B	A	A	A	B	A	B	B	A	B	A	A	B	B	A	B	A	A	B	B	B	A	A	B	B	A	A	A	B	B	A	A	A	B	A	A	A	B	A	A	A	A	B	B	A
B	B	A	A	A	B	A	A	B	A	B	A	A	B	B	A	A	A	A	B	B	B	A	A	B	B	A	A	A	B	B	A	A	A	B	A	A	A	B	A	A	A	A	B	B	A
B	B	A	A	A	B	A	A	B	A	B	A	A	B	B	A	A	A	A	B	B	B	A	A	B	B	A	A	A	B	B	A	A	A	B	A	A	A	B	A	A	A	A	B	B	A
B	B	A	A	A	B	A	A	B	A	B	A	A	B	B	A	A	A	A	B	B	B	A	A	B	B	A	B	A	B	B	A	A	A	B	A	A	A	B	A	A	A	A	B	B	A
B	B	A	A	A	B	A	A	B	A	B	A	A	B	B	A	A	A	A	B	B	B	A	A	B	B	A	B	A	B	B	A	A	A	B	A	A	A	B	A	A	A	A	B	B	A
A	B	A	A	A	B	A	A	B	A	B	A	A	B	B	A	A	A	A	B	B	B	A	A	B	B	B	B	A	B	B	A	A	A	B	A	A	A	B	A	A	A	A	B	B	A
A	B	A	A	B	B	A	A	B	A	B	A	A	B	B	A	A	A	A	B	B	B	A	A	B	B	B	B	A	B	B	A	A	A	B	A	A	A	B	A	A	A	A	B	B	A
A	B	A	A	B	B	A	A	B	A	B	A	A	B	B	A	A	A	A	B	B	A	A	A	B	B	B	B	A	B	B	A	A	A	B	A	A	A	B	A	A	A	A	B	B	A
A	B	A	A	B	B	A	A	B	A	B	A	A	B	B	A	A	A	A	B	B	A	A	A	B	B	B	B	A	B	B	A	A	A	B	A	A	A	B	A	A	A	A	B	B	A
A	B	A	A	B	B	A	A	B	A	B	A	A	B	B	A	A	A	A	B	B	A	A	A	B	B	B	B	A	B	B	A	A	A	B	A	A	A	B	A	A	A	A	B	B	A
A	B	A	A	B	B	A	A	B	A	B	A	A	B	B	A	A	A	A	B	B	A	A	A	B	B	B	B	A	B	B	A	A	A	B	A	A	A	B	A	A	A	A	B	B	A

A	B	A	A	B	B	A	A	B	A	B	A	A	B	B	A	A	A	A	B	B	A	A	A	B	B	B	B	A	B	B	A	A	A	B	A	A	A	B	A	A	A	A	B	B	A
A	B	A	A	B	B	A	A	B	A	B	A	A	B	B	A	A	A	A	B	B	A	A	A	B	B	B	B	A	B	B	A	A	A	B	A	A	A	B	A	A	A	A	B	B	A
A	B	A	A	B	B	A	A	B	A	B	A	A	B	B	B	A	A	A	B	B	A	A	A	B	B	B	B	A	B	B	A	A	A	B	A	A	A	B	A	A	A	A	B	B	A
A	B	A	A	B	B	A	A	B	A	B	A	A	B	B	B	A	A	A	B	B	A	A	A	B	B	B	B	A	B	B	A	A	A	B	A	A	A	B	A	A	A	A	B	B	A
A	B	A	A	B	B	A	A	B	A	B	A	A	B	B	B	A	A	A	B	B	A	A	A	B	B	B	B	A	B	B	A	A	A	B	A	A	B	B	A	A	A	A	B	B	A
A	B	A	A	B	B	A	A	B	A	B	A	A	B	B	B	A	A	A	B	B	A	A	A	B	B	B	B	A	B	B	A	A	A	B	A	A	B	B	A	A	A	A	B	B	A
A	B	A	A	B	B	A	A	B	A	B	A	A	B	B	B	A	A	A	B	B	A	A	A	B	B	B	B	A	B	B	A	A	A	B	A	A	B	B	A	A	A	A	B	B	A
A	B	A	A	B	B	A	A	B	A	B	A	A	B	A	B	A	A	A	B	B	A	A	A	B	B	B	B	A	B	B	A	A	A	B	A	A	B	B	A	A	A	A	B	B	A
B	B	A	B	B	B	B	B	A	A	B	B	B	A	B	A	B	B	A	B	B	B	A	A	B	A	B	A	B	B	B	0	0	B	B	B	B	B	B	A	B	B	B	B	B	B
B	B	A	B	B	B	B	B	A	A	B	B	B	A	B	A	B	B	A	B	B	B	A	A	B	A	B	A	B	B	B	0	0	B	A	B	B	B	B	A	B	B	B	B	B	B
B	B	A	B	B	B	B	B	A	A	B	B	B	A	B	A	B	B	A	B	B	B	A	A	B	A	B	A	B	B	B	0	0	B	A	B	B	B	B	A	A	B	B	B	B	B
B	B	A	B	B	B	B	B	A	A	B	B	B	A	B	A	B	B	A	B	B	B	A	A	B	A	B	A	B	B	B	0	0	B	A	B	B	B	B	A	A	B	B	B	B	B
B	B	A	B	B	B	B	B	A	A	B	B	B	A	B	A	B	B	A	B	B	B	A	A	B	A	B	A	B	B	B	0	0	B	A	B	B	B	B	A	B	B	B	B	B	B
B	B	A	B	B	B	B	B	A	A	B	B	B	A	B	A	B	B	A	B	B	B	A	A	B	A	B	A	B	B	B	0	0	B	A	B	B	B	B	A	B	B	B	B	B	B
B	B	A	B	B	B	B	B	A	A	B	B	B	A	B	A	B	B	A	B	B	B	A	A	B	A	B	A	B	B	B	0	0	B	A	B	B	B	B	A	A	B	0	B	B	B
B	B	A	B	B	B	B	B	A	A	B	B	B	A	B	A	B	B	A	B	B	A	A	A	B	A	B	A	B	B	B	A	A	B	A	B	B	B	B	A	A	B	0	B	B	B
A	B	B	B	B	B	B	B	A	A	B	B	B	A	B	A	B	B	A	B	B	A	A	A	B	A	B	A	B	B	B	A	A	B	A	B	B	B	B	A	A	B	0	B	B	B
A	B	B	B	B	B	B	B	A	A	B	B	B	A	B	A	B	B	A	B	B	A	A	A	B	A	B	A	B	B	B	A	A	B	A	B	B	B	B	A	A	B	0	B	B	B
A	B	B	B	B	B	B	B	A	A	B	B	B	A	B	A	B	B	A	B	B	A	A	B	B	A	B	A	B	B	B	A	A	B	A	B	B	B	B	A	A	B	0	B	B	B
A	B	B	B	B	B	A	B	A	A	B	B	B	A	B	A	B	B	A	B	B	A	A	B	B	A	B	A	B	B	B	A	A	B	A	B	B	B	B	A	A	B	0	B	B	B
A	B	B	B	B	B	A	B	A	A	B	B	B	A	B	A	B	B	A	B	B	A	A	B	B	A	B	A	B	B	B	A	A	B	A	B	B	B	B	A	A	B	0	B	B	B
A	B	B	B	B	B	A	B	A	A	B	B	B	A	B	A	B	B	A	B	B	A	A	B	B	B	B	A	B	B	B	A	A	B	A	B	B	B	B	A	A	B	0	B	B	B
A	B	B	B	B	B	A	B	A	A	B	B	B	A	B	A	B	B	A	B	B	A	A	B	B	B	B	A	B	B	B	A	A	B	A	B	B	B	B	A	A	B	0	B	B	B
A	B	B	B	B	B	A	B	A	A	B	B	B	A	B	A	B	B	A	B	B	A	A	B	B	B	B	A	B	B	B	A	A	B	A	B	B	B	B	A	A	B	0	B	B	B
A	A	B	B	B	B	A	B	A	A	B	B	B	A	B	A	B	B	A	B	B	A	A	B	B	B	B	A	B	B	B	A	A	B	A	B	B	B	B	A	A	B	0	B	B	B
A	A	B	B	B	B	A	B	A	A	B	B	B	A	B	A	B	B	A	B	B	A	A	B	B	B	B	B	B	B	B	A	A	B	A	B	B	B	B	A	A	B	0	B	B	B
A	A	B	B	B	B	A	B	A	A	B	B	B	A	B	A	B	B	A	B	B	A	A	B	B	B	B	B	B	B	B	A	A	B	A	B	B	B	B	A	A	B	0	B	B	B
A	A	B	B	B	B	A	B	B	A	B	B	B	A	B	A	B	B	A	B	B	A	A	B	B	B	B	B	B	B	B	A	A	B	A	B	B	B	B	A	A	B	0	B	B	B
B	A	B	B	B	B	A	B	B	A	B	B	B	A	B	A	B	B	A	B	B	A	A	B	B	B	B	B	B	B	B	A	A	B	A	B	B	B	B	A	A	B	0	B	B	B
B	A	B	B	B	B	A	B	B	A	B	B	B	A	B	A	B	B	A	B	B	A	A	B	B	B	B	B	B	B	B	A	A	B	A	B	A	B	B	A	A	B	0	B	B	B
B	A	B	B	B	B	A	B	B	A	B	B	B	A	B	A	B	B	A	B	B	A	A	B	B	B	B	B	B	B	B	A	A	B	A	B	A	B	B	A	0	B	0	B	B	B
B	A	B	B	B	B	A	B	B	A	B	B	B	A	B	A	B	B	A	B	B	A	A	B	B	B	B	B	B	B	B	A	A	B	A	B	A	B	B	A	0	B	0	B	B	B
B	A	B	B	B	B	A	B	B	A	B	B	B	A	B	A	B	B	A	B	B	A	A	B	B	B	B	B	B	B	B	A	A	B	A	B	A	B	B	A	0	B	0	B	B	B
B	A	B	B	B	B	A	B	B	A	B	B	B	A	B	A	B	B	A	B	B	A	A	B	B	B	B	B	B	B	B	A	A	B	A	B	A	B	B	A	0	B	0	B	B	B
B	A	B	B	B	B	A	B	B	A	B	B	B	A	B	A	B	B	B	B	B	A	A	B	B	B	B	B	B	B	B	A	A	B	A	B	A	B	B	A	0	B	0	B	B	B
B	A	B	B	B	B	A	B	B	A	B	B	B	A	B	A	B	B	B	B	B	A	A	B	B	B	B	B	B	B	B	A	A	B	A	B	A	B	B	A	0	B	0	B	B	B
B	A	B	B	B	B	A	B	B	A	B	B	B	A	B	A	B	B	B	B	B	A	A	B	B	B	B	B	B	B	B	A	A	B	A	B	A	B	B	A	0	B	0	B	B	B
B	A	B	B	B	B	A	B	B	A	B	B	B	A	B	A	B	B	B	B	B	A	A	B	B	B	B	B	B	B	B	A	A	B	A	B	A	B	B	A	0	B	0	B	B	B
B	A	B	B	B	B	B	B	B	A	B	B	B	A	B	A	B	B	B	B	B	A	A	B	B	B	B	B	B	B	B	A	A	B	A	B	A	B	B	A	0	B	0	B	B	B
B	A	B	A	B	B	B	B	B	A	B	B	B	A	B	A	B	B	B	B	B	A	A	B	B	B	B	B	B	B	B	A	A	B	A	B	A	B	B	A	0	B	0	B	B	B
B	A	B	A	B	B	B	B	B	A	B	B	B	A	B	A	B	B	B	B	B	A	A	B	B	B	B	B	B	B	B	A	A	B	B	B	A	B	B	A	0	B	0	B	B	B
B	A	B	A	B	B	B	B	B	A	B	B	B	A	B	A	B	B	B	B	B	A	A	B	B	B	B	B	B	B	B	A	A	B	B	B	A	B	B	A	0	B	0	B	B	B
B	A	B	A	B	B	B	B	B	A	B	B	B	A	A	A	B	B	B	B	B	A	A	B	B	B	B	A	B	B	B	A	A	B	B	B	A	B	B	A	0	B	0	B	B	B
B	A	B	A	B	B	B	B	B	A	B	B	B	A	A	A	B	B	B	B	B	A	A	B	B	B	B	A	B	B	B	A	A	B	B	B	A	B	B	A	0	B	0	B	B	B
B	A	B	A	B	B	B	B	B	A	B	B	B	A	A	A	B	B	B	B	B	B	A	B	B	B	B	A	B	B	B	A	A	B	B	B	A	B	B	A	A	B	0	B	B	B
B	A	B	A	B	B	B	B	B	A	B	B	B	A	A	A	B	B	B	B	B	B	A	B	B	B	B	A	B	B	B	A	A	B	B	B	A	B	B	A	A	B	0	A	A	B
B	A	A	A	B	B	B	B	B	A	B	B	0	A	A	A	B	B	B	B	B	B	A	B	B	B	B	A	B	B	B	A	A	B	B	B	A	B	B	A	A	B	0	A	A	B
B	A	A	A	B	B	B	B	B	A	B	B	0	A	A	A	B	B	B	B	B	B	A	B	B	B	B	A	B	B	B	A	A	A	B	B	A	B	B	A	A	B	0	A	A	B
B	A	A	A	B	B	B	B	B	A	B	B	0	A	A	A	B	B	B	B	B	B	A	B	B	B	B	A	B	B	B	A	A	A	B	B	A	B	B	A	A	B	0	A	A	B

B	A	A	A	B	B	B	B	B	A	B	B	0	A	A	A	B	B	B	B	B	B	A	B	B	B	B	A	B	A	A	A	A	A	B	B	A	B	B	A	A	B	0	A	A	B
B	A	A	A	B	B	B	0	B	A	B	B	0	A	A	A	B	B	B	B	B	B	A	B	B	B	B	A	B	A	A	A	A	A	B	B	A	B	B	A	A	B	0	A	A	B
B	A	A	A	B	B	B	0	B	A	B	B	0	A	A	A	B	B	B	B	B	B	A	B	B	B	B	A	B	A	A	0	0	A	B	B	A	B	B	A	0	B	0	A	A	B
B	A	A	A	B	B	B	0	B	A	B	B	0	A	A	B	B	B	B	B	B	B	A	B	B	B	B	A	B	A	A	0	0	A	B	B	A	B	B	A	0	B	0	A	A	B
B	A	A	A	B	B	B	0	B	A	B	B	0	A	A	B	B	B	B	B	B	B	A	B	B	B	A	A	B	A	A	0	0	A	B	B	A	B	B	A	0	B	0	A	A	B
B	B	A	B	A	A	A	B	A	A	A	B	B	A	B	A	B	B	A	B	B	A	B	A	A	A	A	A	A	A	A	A	A	B	A	B	B	B	B	A	B	A	A	B	B	A
B	B	A	B	A	A	A	B	A	A	A	B	B	A	B	A	B	B	A	B	B	A	B	A	A	A	A	A	A	A	A	A	A	B	A	B	B	B	B	A	B	A	A	B	B	A
B	B	A	B	A	A	A	B	A	A	A	B	B	A	B	A	B	B	A	B	B	A	B	A	A	A	A	A	A	A	A	A	A	B	A	B	B	B	B	A	B	A	A	B	B	A
B	B	A	B	A	A	A	B	A	A	A	B	B	A	B	A	B	B	A	B	B	A	B	A	A	A	A	A	A	B	B	A	A	B	A	B	B	B	B	A	B	A	A	B	B	A
B	B	A	B	A	A	A	B	A	A	A	B	B	A	B	A	B	B	A	B	B	A	B	A	A	A	A	A	B	B	B	A	A	B	A	B	B	B	B	A	B	A	A	B	B	A
B	B	A	B	A	A	A	B	A	A	A	B	B	A	B	A	B	B	A	B	B	A	B	A	A	A	A	A	B	B	B	A	A	B	A	B	B	B	B	A	B	A	A	B	B	A
B	B	A	B	A	A	A	B	A	A	A	B	B	A	B	A	B	B	A	B	B	A	B	A	A	A	A	A	B	B	B	A	A	B	A	B	B	B	B	A	B	A	A	B	B	A
B	B	A	B	A	A	A	B	A	A	A	B	B	A	B	A	B	B	A	B	B	A	B	A	A	A	A	A	B	B	B	A	A	B	A	B	B	B	B	A	B	A	A	B	B	A
B	B	A	B	A	A	A	A	A	A	A	B	B	A	B	A	B	B	A	B	B	A	B	A	A	A	A	A	B	B	B	A	A	B	A	B	B	B	B	A	B	A	A	B	B	A
B	B	A	B	A	A	A	A	A	A	A	B	B	A	B	A	B	B	A	B	B	A	B	A	A	A	A	A	B	B	B	A	A	B	A	B	B	B	B	A	B	A	A	B	B	A
B	B	A	B	A	A	A	A	A	A	A	B	B	A	B	A	B	B	A	B	B	A	B	A	A	A	A	A	B	A	A	A	A	B	A	B	B	B	B	A	B	A	A	B	B	A
B	B	A	B	A	A	A	A	A	A	A	B	B	A	B	A	B	B	A	B	B	A	B	A	A	A	A	A	B	A	A	A	A	B	A	B	B	B	B	A	B	A	A	B	B	B
B	A	A	B	A	A	A	A	A	A	A	B	B	A	B	A	B	B	A	B	B	A	B	A	A	A	A	A	B	A	A	A	A	B	A	B	B	B	B	A	B	A	A	B	B	B
B	A	A	B	A	A	A	A	A	A	A	B	B	A	B	A	B	B	A	B	B	A	B	A	A	A	A	A	B	A	A	A	A	B	A	B	B	B	B	A	B	A	A	B	B	B
B	A	A	B	A	A	A	A	A	A	A	B	B	A	B	A	B	B	A	B	B	A	B	A	A	A	A	A	B	A	A	A	A	B	A	B	B	B	B	A	B	A	A	B	B	B
B	A	A	B	A	A	A	A	A	A	A	B	B	A	B	A	B	B	A	B	B	A	B	A	A	A	A	A	B	A	A	A	A	B	A	B	B	B	B	A	B	A	A	B	B	B
B	A	A	B	A	A	A	A	A	A	A	B	B	A	B	A	B	B	A	B	B	A	B	A	A	A	A	A	B	A	A	A	A	B	A	B	B	B	B	A	B	A	A	B	B	B
B	A	A	B	A	A	A	A	A	A	A	B	B	A	B	A	B	B	A	B	B	A	B	A	A	A	A	A	B	A	A	A	A	A	A	B	B	B	B	A	B	A	A	B	B	B
B	A	A	B	A	A	A	A	A	A	A	B	B	A	B	A	B	B	A	B	B	A	B	A	A	B	A	A	B	A	A	A	A	A	A	B	B	B	B	A	B	A	A	B	B	B
B	A	A	A	A	A	A	A	A	A	A	B	B	A	B	A	B	B	A	B	B	A	B	A	A	B	A	A	B	A	A	A	A	A	A	B	B	B	B	A	B	A	A	B	B	B
B	A	A	A	A	A	A	A	A	A	A	B	B	A	B	A	B	B	A	B	B	A	B	A	A	B	B	A	B	A	A	A	A	A	A	B	B	B	B	A	B	A	A	B	B	B
B	A	A	A	A	A	A	A	A	A	A	B	B	A	B	A	B	B	A	B	B	A	B	A	A	B	B	A	B	A	A	A	A	A	A	B	B	B	B	A	B	A	A	B	B	B
B	A	A	A	A	A	A	A	A	A	A	B	B	A	B	A	B	B	A	B	B	A	B	A	A	B	B	A	B	A	A	A	A	A	A	B	B	B	B	A	B	A	A	B	B	B
B	A	A	A	A	A	A	A	A	A	A	B	B	A	B	A	B	B	A	B	B	A	B	A	A	B	B	A	B	A	A	A	A	A	A	B	B	B	B	A	B	A	A	B	B	B
B	A	A	A	A	A	A	A	A	A	A	B	B	A	B	A	B	B	A	B	B	A	B	A	A	B	B	A	B	A	A	A	A	A	A	B	B	B	B	A	B	A	A	B	B	B
B	A	A	A	A	A	A	A	A	A	A	B	B	A	B	A	B	B	A	B	B	A	B	A	A	B	B	A	B	A	A	A	A	A	A	B	B	B	B	A	B	A	A	B	B	B
B	A	A	A	A	A	A	A	A	A	A	B	B	A	B	A	B	B	A	B	B	A	B	A	A	B	B	A	B	A	A	A	A	A	A	B	B	B	B	A	B	A	A	B	B	B
B	A	A	A	A	A	A	A	A	A	A	B	B	A	B	A	B	B	A	B	B	A	B	A	A	B	B	A	B	A	A	A	A	A	A	B	B	B	B	A	B	A	A	B	B	B
B	A	A	A	A	A	A	A	A	A	A	B	B	A	A	A	B	B	A	B	B	A	B	A	A	B	B	A	B	A	A	A	A	A	A	B	B	B	B	A	B	A	A	B	B	B
B	A	A	A	A	A	A	A	A	A	A	B	B	A	A	A	B	B	A	B	B	A	B	A	A	B	B	A	B	A	A	A	A	A	A	B	B	B	B	A	B	A	A	B	B	B
B	A	A	A	A	A	A	A	A	A	A	B	B	A	A	A	B	B	A	B	B	A	B	A	A	B	B	A	B	A	A	A	A	A	A	B	B	B	B	A	B	A	A	B	B	B
B	A	A	A	A	A	A	A	A	A	A	B	B	A	A	A	B	B	A	B	B	A	B	A	A	B	B	A	B	A	A	A	A	A	A	B	B	B	B	A	B	A	A	B	B	B
B	A	A	A	A	A	A	A	A	A	A	B	B	A	A	A	B	B	A	B	B	A	B	A	A	B	B	A	B	A	A	A	A	A	A	B	B	B	B	A	B	A	A	B	B	B
B	A	A	A	A	A	A	A	A	A	A	B	A	A	A	A	B	B	A	B	B	A	B	A	A	B	B	A	B	A	A	A	A	A	A	B	B	B	B	A	B	A	A	B	B	B
B	A	A	A	A	A	A	A	A	A	A	B	A	A	A	A	B	B	A	B	B	A	B	A	A	B	B	A	B	A	A	A	A	A	A	B	B	B	B	A	B	A	A	B	B	B
B	A	A	A	A	A	A	A	A	A	A	B	A	A	A	A	B	B	A	B	B	A	B	A	A	B	B	A	A	A	A	A	A	A	A	B	B	B	B	A	B	A	A	B	B	B
B	A	A	A	A	A	A	A	A	A	A	B	A	A	A	A	B	B	A	B	B	A	B	A	A	B	B	A	A	A	A	A	A	A	A	B	B	B	B	A	B	A	A	B	B	B
B	B	A	A	A	A	A	A	A	A	A	B	A	A	A	A	B	A	A	B	B	A	B	A	A	B	B	A	A	A	A	A	A	A	A	B	B	B	B	A	B	A	A	B	B	B
B	B	A	A	A	A	A	A	A	A	A	B	A	B	A	A	B	A	A	B	B	A	B	A	A	B	B	A	A	A	A	A	A	A	A	B	B	B	B	A	B	A	A	B	B	B
B	B	A	A	A	A	A	A	A	A	A	B	A	B	A	A	B	A	A	B	B	A	B	A	A	B	B	A	A	A	A	A	A	A	A	B	B	B	B	A	B	A	A	B	B	B
B	B	A	A	A	A	A	A	A	A	A	B	A	B	A	A	B	A	A	B	B	A	B	A	A	B	B	A	A	A	A	A	A	A	A	B	B	B	B	A	B	A	A	B	B	B
B	B	A	A	A	A	A	A	A	A	A	B	A	B	A	A	B	A	A	B	B	A	B	A	A	B	B	A	A	A	A	A	A	A	A	B	B	B	B	A	B	A	A	A	A	B
B	B	A	A	A	A	A	A	A	A	B	B	A	B	A	A	B	A	B	B	B	B	B	A	A	B	B	A	A	A	A	A	A	A	A	B	B	B	B	A	B	A	A	A	A	B

B	B	A	A	A	A	A	A	B	A	B	B	A	B	A	A	B	A	B	B	B	B	B	A	A	B	B	A	A	A	A	A	A	A	A	B	B	B	B	B	B	A	A	A	A	B
B	B	A	A	A	A	A	A	B	A	B	B	A	B	A	A	B	A	B	A	A	B	B	A	A	B	B	A	A	A	A	A	A	A	A	B	B	B	B	B	B	A	A	A	A	B
B	B	A	A	A	B	A	A	B	A	B	B	A	B	A	A	B	A	B	A	A	B	B	A	A	B	B	A	A	A	A	A	A	A	A	B	B	B	B	B	B	A	A	A	A	B
B	B	A	A	A	B	A	A	B	A	B	B	A	B	A	A	B	A	B	A	A	B	B	A	A	B	B	A	A	A	A	A	A	A	A	B	B	B	B	B	B	A	A	A	A	B
B	B	A	A	A	B	A	A	B	A	B	B	A	B	A	A	B	A	B	A	A	B	B	A	A	B	B	A	A	A	A	A	A	A	A	B	B	B	B	B	B	A	A	A	A	B
B	B	A	A	A	B	A	A	B	A	B	B	A	B	A	A	B	A	B	A	A	B	B	A	A	B	B	A	A	A	A	A	A	A	A	B	B	B	B	B	B	A	A	A	A	B
B	B	A	A	A	B	A	A	B	A	B	B	A	B	A	A	B	A	B	A	A	B	B	A	A	B	B	A	A	B	B	A	A	A	A	B	B	B	B	B	B	A	A	A	A	B
B	B	A	A	A	B	A	A	B	A	B	B	A	B	A	A	B	A	B	A	A	B	B	A	A	B	B	A	A	B	B	A	A	A	A	B	B	B	B	B	B	A	A	A	A	B
B	B	A	A	A	B	A	A	B	A	B	B	A	B	A	A	B	A	B	A	A	B	B	A	A	B	B	A	A	B	B	A	A	A	A	B	B	B	B	B	B	A	A	A	A	B
B	A	A	B	A	B	A	A	B	A	B	B	A	B	A	A	B	A	B	A	A	B	B	A	A	B	B	A	A	B	B	A	A	A	A	B	B	B	B	B	B	A	A	A	A	B
B	A	A	B	A	B	A	B	B	A	B	B	A	B	A	A	B	A	B	A	A	B	B	A	A	B	B	A	A	B	B	A	A	A	A	B	B	B	B	B	B	A	A	A	A	B
B	A	A	B	A	B	A	B	B	A	B	B	A	B	A	A	B	A	B	A	A	B	B	A	A	B	B	A	A	B	B	A	A	A	A	B	B	B	B	B	B	A	A	A	A	B
A	A	B	A	A	A	A	B	A	A	B	B	A	A	A	B	A	B	B	B	B	B	A	A	A	A	B	A	A	B	B	B	B	A	A	A	B	A	A	B	A	A	A	A	A	B
A	A	B	A	A	A	A	B	A	A	B	B	A	A	A	B	A	B	B	B	B	B	A	A	A	A	B	A	A	B	B	B	B	A	A	A	B	A	A	B	A	A	A	A	A	B
A	A	B	A	A	A	A	B	A	A	B	B	A	A	A	B	A	B	B	B	B	B	A	A	A	A	B	A	0	B	B	B	B	A	A	A	B	A	A	0	A	A	A	A	A	B
A	A	B	A	A	A	A	B	A	A	B	B	A	A	A	A	A	B	B	B	B	B	A	A	A	A	B	A	0	B	B	B	B	A	A	A	B	A	A	A	A	A	A	A	A	B
A	A	B	A	A	A	A	B	A	A	B	B	A	A	A	A	A	B	B	B	B	B	A	A	A	A	B	A	0	B	B	B	B	A	A	A	B	A	A	A	A	A	A	A	A	B
A	A	B	A	A	A	A	B	A	A	B	B	A	A	A	A	A	B	A	B	B	B	A	A	A	A	B	A	0	B	B	B	B	A	A	A	B	A	A	A	A	A	A	A	A	B
A	A	B	A	A	A	A	B	A	A	B	B	A	A	A	A	A	B	A	B	B	B	A	A	A	A	B	A	0	B	B	B	B	A	A	A	B	A	A	A	A	A	A	A	A	B
A	A	B	A	A	A	A	B	A	A	B	B	A	A	A	A	A	B	A	B	B	B	A	A	A	A	B	A	0	B	B	B	B	A	A	A	B	A	A	A	A	A	A	A	A	B
A	A	B	A	A	A	A	B	A	A	B	B	A	A	A	A	A	B	A	B	B	B	A	A	A	A	B	A	0	B	B	B	B	A	A	A	B	A	A	A	A	A	A	A	A	B
A	A	B	A	A	A	A	B	A	A	B	B	A	A	A	A	A	B	A	B	B	B	A	A	A	A	B	A	0	B	B	B	B	A	A	A	B	A	A	A	A	A	A	A	A	B
A	A	B	A	A	A	A	B	A	A	B	B	A	A	A	A	A	B	A	A	A	B	A	A	A	A	B	A	B	B	B	B	B	A	A	A	B	A	A	A	A	A	A	A	A	B
A	A	B	A	A	A	A	B	A	A	B	B	A	A	A	A	A	B	A	A	A	B	A	A	A	A	B	A	B	B	B	B	B	A	A	A	B	A	A	A	A	A	A	A	A	B
A	A	B	A	A	A	B	B	A	A	B	B	B	A	A	A	A	B	A	A	A	B	A	A	A	A	A	A	B	B	B	A	A	A	A	A	B	A	B	A	A	A	B	A	A	A
A	A	B	A	A	A	B	B	A	A	B	B	B	A	A	A	A	B	A	A	A	B	A	A	A	A	A	A	B	B	B	A	A	A	A	A	B	A	B	A	A	A	B	A	A	A
A	A	B	A	A	A	B	B	A	A	B	B	B	A	A	A	A	B	A	A	A	B	A	A	A	A	A	A	B	B	B	A	A	A	A	A	B	A	B	A	A	A	B	A	A	A
A	A	B	A	A	A	B	B	A	A	B	B	B	A	A	A	A	B	A	A	A	B	A	B	A	A	A	A	B	B	B	A	A	A	A	A	B	A	B	A	A	A	B	A	A	A
A	A	B	A	A	A	B	B	A	A	B	A	B	A	A	A	A	B	A	A	A	B	A	B	A	A	A	A	B	B	B	A	A	A	A	A	B	B	B	A	A	A	B	A	A	A
A	A	B	A	A	A	B	B	A	A	B	A	B	A	A	A	A	B	A	A	A	B	A	B	A	A	A	A	B	B	B	A	A	A	A	A	B	B	B	A	A	A	B	A	A	A
A	A	B	A	A	A	B	B	A	A	B	A	B	A	A	A	A	B	A	A	A	B	A	B	A	A	A	A	B	B	B	A	A	A	A	A	B	B	B	A	A	A	B	A	A	A
A	A	B	A	A	A	B	B	A	A	B	A	B	A	A	A	A	B	A	A	A	B	A	B	A	A	A	A	B	B	B	A	A	A	A	A	B	B	B	A	A	A	B	A	A	A
A	A	B	A	A	A	B	B	A	A	B	A	B	A	A	A	A	A	A	A	A	B	A	B	A	A	A	A	B	B	B	A	A	A	A	A	B	B	B	A	A	A	B	A	A	A
A	A	B	A	A	A	B	B	A	A	B	A	B	A	A	A	A	A	A	A	A	B	A	B	A	A	A	A	B	A	A	A	A	A	A	A	B	B	B	A	A	A	B	A	A	A
A	A	B	A	A	A	B	B	A	A	B	A	B	A	A	A	A	A	A	A	A	B	A	B	A	A	A	B	B	A	A	A	A	A	A	A	B	B	B	A	A	A	B	A	A	A
A	A	B	A	A	A	B	B	A	A	B	A	B	A	A	A	A	A	A	A	A	B	A	B	A	A	A	B	B	A	A	A	A	A	A	A	B	B	B	A	A	A	B	A	A	A
A	A	B	A	A	A	B	B	A	A	B	A	B	A	A	A	A	A	A	A	A	A	A	B	A	A	A	B	B	A	A	A	A	A	A	A	B	B	B	A	A	A	B	A	A	A
A	A	B	A	A	A	B	B	A	A	B	A	B	A	A	A	A	A	A	A	A	A	A	B	A	A	A	B	B	A	A	A	A	A	A	A	B	B	B	A	A	A	B	A	A	A
A	A	B	A	A	A	B	B	A	A	B	A	B	A	A	A	A	A	A	A	A	A	A	B	A	A	A	B	B	A	A	A	A	A	A	A	B	B	B	A	A	A	B	A	A	A
A	A	B	A	A	A	B	B	A	B	B	A	B	A	A	A	A	A	A	A	A	A	A	B	A	A	A	B	B	A	A	A	A	A	A	A	B	B	B	A	A	A	B	A	A	A
A	A	B	A	A	A	B	B	A	B	B	A	B	A	A	A	A	A	A	A	A	A	A	B	A	A	A	B	B	A	A	A	A	A	A	A	B	B	B	A	A	A	B	A	A	A
A	A	B	A	A	A	B	B	A	B	B	A	B	A	A	A	A	A	A	A	A	A	A	B	A	A	A	B	B	A	A	A	A	A	A	A	B	B	B	A	A	A	B	A	A	A
A	A	B	A	A	A	B	B	A	B	B	A	B	A	A	A	A	A	A	A	A	A	A	B	A	A	A	B	B	A	A	A	A	A	A	A	B	B	B	A	A	A	B	A	A	A
A	A	B	A	A	A	B	B	A	B	B	A	B	A	B	A	A	A	A	A	A	A	A	B	A	A	A	B	B	A	A	A	A	A	A	A	B	B	B	A	A	A	B	A	A	A
A	A	B	A	A	A	B	B	A	B	B	A	B	A	B	A	A	A	A	A	A	A	A	B	A	A	A	B	B	A	A	A	A	A	A	A	B	B	B	A	A	A	B	B	B	A
A	A	B	A	A	A	B	B	A	B	B	A	B	A	B	A	A	A	A	A	A	A	A	B	A	A	A	B	B	A	A	A	A	A	A	A	B	B	B	A	A	A	B	B	B	A
A	A	B	A	A	A	B	B	A	B	B	A	B	A	B	A	A	A	A	A	A	A	A	B	A	A	A	B	B	A	A	A	A	A	A	A	B	B	B	A	A	A	A	B	B	A
A	A	B	A	A	A	B	B	A	B	B	A	B	A	B	A	A	A	A	A	A	A	A	B	A	A	A	B	B	A	A	A	A	A	A	A	B	B	B	A	A	A	A	B	B	B
A	A	B	A	A	A	B	B	A	B	B	A	B	A	B	A	A	A	A	A	A	A	A	B	A	A	A	B	B	A	A	A	A	A	A	A	B	B	B	A	A	A	A	B	B	B

A	A	B	A	A	A	B	B	A	B	B	A	B	A	B	A	A	A	A	A	A	A	A	B	A	A	A	B	B	A	A	A	A	A	A	A	B	B	B	A	A	A	A	B	B	B
A	A	B	A	A	A	B	B	A	B	B	A	B	A	B	A	A	A	A	A	A	A	A	B	A	A	A	B	B	A	A	A	A	A	A	A	B	B	B	A	A	A	A	B	B	B
A	A	B	A	A	A	B	B	A	B	B	A	B	A	B	A	A	A	A	A	A	A	A	B	A	A	A	B	B	A	A	A	A	A	A	A	B	B	B	B	A	A	A	B	B	B
A	A	B	A	A	A	B	B	A	B	B	A	B	A	B	A	A	A	A	A	A	A	A	B	A	A	A	B	B	A	A	A	A	A	A	A	B	B	B	B	A	A	A	B	B	B
A	A	B	A	A	A	B	B	A	B	B	A	B	A	B	A	A	A	A	A	A	A	A	B	A	A	A	B	B	A	A	B	B	A	A	A	B	B	B	B	A	A	A	B	B	B
A	A	B	A	A	A	B	B	A	B	B	A	B	A	B	A	A	A	A	A	A	A	A	B	A	A	A	B	B	B	B	B	B	B	A	A	B	B	B	B	A	A	A	B	B	B
A	A	B	A	A	A	B	B	A	B	B	A	B	A	B	A	A	A	A	A	A	A	A	B	A	A	A	B	B	B	B	B	B	B	B	A	B	B	B	B	A	A	A	B	B	B
A	A	B	A	A	A	B	B	A	B	B	A	B	A	B	A	A	A	A	A	A	A	B	B	A	A	A	B	B	B	B	B	B	B	B	B	B	B	B	B	A	A	A	B	B	B
A	A	B	A	A	A	B	B	A	B	B	A	B	A	B	A	A	A	A	A	A	A	B	B	A	A	A	B	B	B	B	B	B	B	B	B	A	B	B	B	A	A	A	B	B	B
A	A	B	A	A	A	B	B	A	A	B	A	B	A	B	A	A	A	A	A	A	A	B	B	A	A	A	B	B	B	B	B	B	B	B	B	A	B	B	B	A	A	A	B	B	B
A	A	B	A	A	A	B	B	A	A	B	A	B	A	B	A	A	A	A	A	A	A	B	B	A	A	A	B	B	B	B	B	B	B	B	B	A	B	B	B	A	A	A	B	B	B
A	A	B	A	A	A	B	B	A	A	B	A	B	A	B	A	A	A	A	A	A	A	B	B	A	A	A	B	B	B	B	B	B	B	B	B	A	B	B	B	A	A	A	B	B	B
A	A	B	A	A	A	B	B	A	A	B	A	B	A	B	A	A	A	A	A	A	A	B	B	A	A	A	B	B	B	B	B	B	B	B	B	A	B	B	B	B	A	A	B	B	B
A	A	B	A	A	A	B	B	A	A	B	A	B	A	B	A	A	A	A	A	A	A	B	B	A	A	A	B	B	B	B	B	B	B	B	B	A	B	B	B	B	A	A	B	B	B
A	A	B	A	A	A	B	B	A	A	B	A	B	A	B	A	A	A	A	A	A	A	B	B	A	A	A	B	B	B	B	B	B	B	B	B	A	B	B	B	B	A	A	B	B	B
A	A	B	A	A	A	B	B	A	A	B	A	B	A	B	A	A	A	A	A	A	A	B	B	A	A	A	B	B	B	B	B	B	B	B	B	A	B	B	B	B	A	A	B	B	B
A	A	B	A	A	A	B	B	A	A	B	A	B	A	B	A	A	A	A	A	A	A	B	B	A	A	A	B	B	B	B	B	B	B	B	B	A	B	B	B	B	A	A	B	B	B
A	A	B	A	A	A	B	B	A	A	B	A	B	A	B	A	A	A	A	A	A	A	B	B	A	A	A	B	B	B	B	B	B	B	B	B	A	B	B	B	B	A	A	B	B	B
A	A	B	A	A	A	B	B	A	A	B	A	B	A	B	A	A	A	A	A	A	A	B	B	A	A	A	B	B	A	A	B	B	B	B	B	A	B	B	B	B	A	A	B	B	B
A	A	B	A	A	A	B	B	A	A	B	A	B	A	B	A	A	A	A	A	A	A	B	B	A	A	A	B	B	A	A	B	B	B	B	B	A	B	B	B	B	A	A	B	B	B
A	A	B	A	A	A	B	B	A	A	B	A	B	A	B	A	A	A	A	B	B	A	B	B	A	A	A	B	B	A	A	B	B	B	B	B	A	B	B	B	B	A	A	B	B	B
A	A	B	A	A	A	B	B	A	A	B	A	B	A	B	A	A	A	A	B	B	A	B	B	A	A	A	B	B	A	A	B	B	B	B	B	A	B	B	B	B	A	A	B	B	B
A	A	B	A	A	A	B	B	A	A	B	A	B	A	B	A	A	A	A	B	B	A	B	B	A	A	A	B	B	A	A	B	B	B	B	B	A	B	B	B	B	A	A	B	B	B
A	A	B	A	A	A	B	B	A	A	B	A	B	A	B	A	A	A	A	B	B	A	B	B	A	A	A	B	B	A	A	B	B	B	B	B	A	B	B	B	B	A	A	B	B	B
A	A	B	A	A	A	B	B	A	A	B	A	B	A	B	A	A	A	A	B	B	A	B	B	A	A	A	B	B	A	A	B	B	B	B	B	A	B	B	B	B	A	A	B	B	B
A	A	B	A	A	A	B	B	A	A	B	A	B	A	B	A	A	A	A	B	B	A	B	B	A	A	A	B	B	A	A	B	B	B	B	B	A	B	B	B	B	A	A	B	B	B
A	A	B	A	A	A	B	B	A	A	B	A	B	A	B	A	A	A	A	B	B	A	B	B	A	A	A	B	B	A	A	B	B	B	B	B	A	B	B	B	B	A	A	B	B	B
A	A	B	A	A	A	B	B	A	A	B	A	B	A	B	A	A	A	A	B	B	A	B	B	A	A	A	B	B	A	A	B	B	B	B	B	A	B	B	B	B	A	A	B	B	B
A	A	B	A	A	A	B	B	A	A	B	A	A	A	B	A	A	A	A	B	B	A	B	B	A	A	A	B	B	A	A	B	B	B	0	B	A	B	B	B	B	A	A	B	B	B
A	A	B	A	A	B	B	B	A	A	B	A	A	A	B	A	A	A	A	B	B	A	B	B	A	A	A	B	B	A	A	B	B	B	0	B	A	B	B	B	B	A	A	B	B	B
A	A	B	A	A	B	B	B	A	A	B	A	A	A	B	A	A	A	A	B	B	A	B	B	A	A	A	B	B	A	A	B	B	B	0	B	A	B	B	B	B	A	A	B	B	B
A	A	B	A	A	B	B	B	A	A	B	A	A	A	B	A	A	A	A	B	B	A	B	B	A	A	A	B	B	A	A	B	B	B	0	B	A	B	B	B	B	A	A	B	B	B
A	A	B	A	A	B	B	B	A	A	B	A	A	A	B	A	A	A	A	B	B	A	B	B	A	A	A	B	B	A	A	B	B	B	0	B	A	B	A	B	B	A	A	B	B	B
A	A	B	A	A	B	B	B	A	A	B	A	A	A	B	A	A	A	A	B	B	A	B	B	A	A	A	B	B	A	A	B	B	B	0	B	A	B	A	B	B	A	A	B	B	B
A	A	B	A	A	B	B	A	A	A	B	A	A	A	B	A	A	A	A	B	B	A	B	B	A	A	A	B	B	A	A	B	B	B	0	B	A	B	A	B	B	A	A	B	B	B
A	A	B	A	A	B	B	A	A	A	A	A	A	A	B	A	A	A	A	B	B	A	B	B	A	A	A	B	B	A	A	B	B	B	0	B	A	B	A	B	B	A	A	B	B	B
A	A	B	A	A	B	B	A	A	A	A	A	A	B	B	A	A	A	A	B	B	A	B	B	A	A	A	B	B	A	A	B	B	B	0	B	A	B	A	B	B	A	A	B	B	B
A	A	B	A	A	B	B	A	A	A	A	A	A	B	B	A	A	A	A	B	B	A	B	B	A	A	A	B	B	A	A	B	B	B	0	B	A	B	A	B	B	A	A	B	B	B
A	A	B	A	A	B	B	A	A	A	A	A	A	B	A	A	A	A	A	B	B	A	B	B	A	A	A	B	B	A	A	B	B	B	0	B	A	B	A	B	B	A	A	B	B	B
A	A	B	A	A	B	B	A	A	A	A	A	A	B	A	A	A	A	A	B	B	A	B	B	A	A	A	B	B	A	A	B	B	B	0	B	B	B	A	B	B	A	A	B	B	B
A	A	B	A	A	B	B	A	A	A	A	A	A	B	A	A	A	A	0	B	B	A	B	B	A	A	A	B	B	A	A	B	B	B	0	B	B	B	A	B	B	A	A	B	B	B
A	A	B	A	A	B	B	A	A	A	A	A	A	B	A	A	A	A	0	B	B	A	B	B	A	A	A	B	B	A	A	B	B	B	0	B	B	B	A	B	B	A	A	B	B	B
A	A	B	A	A	B	B	A	A	A	A	A	A	B	A	A	A	A	0	B	B	A	B	B	A	A	A	B	B	A	A	B	B	B	0	B	B	B	A	B	B	A	A	B	B	B
A	A	B	A	A	B	B	A	A	A	A	A	A	B	A	A	A	A	0	B	B	A	B	B	A	A	A	B	B	A	A	B	B	B	0	B	B	B	A	B	B	A	A	B	B	B
A	A	B	A	B	B	B	A	A	A	A	A	A	B	A	A	A	A	0	B	B	A	B	B	A	A	A	B	B	A	A	B	B	B	0	B	B	B	A	B	B	A	A	B	B	B
A	A	B	A	B	B	B	A	A	A	A	B	A	B	A	A	A	A	0	B	B	A	B	B	A	A	A	B	B	A	A	B	B	B	0	B	B	B	A	B	B	A	A	B	B	B
A	A	B	A	B	B	B	A	A	A	A	B	A	B	A	A	A	A	0	B	B	A	B	B	B	A	A	B	B	A	A	B	B	B	0	B	B	B	A	B	B	A	A	B	B	B
A	A	B	A	B	B	B	A	A	A	A	B	A	B	A	A	A	A	0	B	B	A	B	B	B	A	A	B	B	A	A	B	B	B	0	B	B	B	A	B	B	A	A	B	B	B
A	A	B	A	B	B	B	A	A	A	A	B	A	B	A	A	A	A	0	B	B	A	B	B	B	A	A	B	B	A	A	B	B	B	0	0	B	B	A	B	B	A	A	B	B	B

B	A	B	A	B	B	B	A	A	A	A	B	A	B	A	A	A	A	0	B	B	A	B	B	B	A	A	B	B	A	A	B	B	B	0	0	B	B	A	B	B	A	A	B	B	B
B	A	B	A	B	B	B	A	A	A	A	B	A	B	A	A	A	A	0	B	B	B	B	B	B	A	A	B	B	A	A	B	B	A	0	0	B	B	A	A	A	A	A	A	A	A
B	A	B	A	B	B	B	B	B	A	A	B	A	B	A	A	A	A	0	B	B	B	B	B	B	A	A	B	B	A	A	B	B	A	0	0	B	B	A	A	A	A	A	A	A	A
A	B	B	A	A	A	A	A	A	A	A	A	A	A	0	A	B	B	A	A	A	A	A	B	A	B	A	A	A	A	A	A	A	A	A	A	B	A	A	B	A	A	B	A	A	A
A	B	B	A	A	A	A	A	A	A	A	A	A	A	0	A	B	B	A	A	A	A	A	A	A	B	A	A	A	A	A	A	A	A	A	A	B	A	A	B	A	A	B	A	A	A
A	B	B	A	A	A	A	A	A	A	A	A	A	A	A	A	B	B	A	A	A	A	A	A	A	B	A	A	A	A	A	A	A	A	A	A	B	A	A	B	A	A	B	A	A	A
A	B	B	A	A	A	A	A	A	A	A	A	A	A	A	A	B	B	A	A	A	A	A	A	A	A	A	A	A	A	A	A	A	A	A	A	B	A	A	B	A	A	B	A	A	A
A	B	B	A	A	A	A	A	A	A	A	A	A	A	A	A	B	B	A	A	A	A	A	A	A	A	A	A	A	A	A	A	A	A	A	A	B	A	A	B	A	A	B	A	A	A
A	B	B	A	A	A	A	A	A	A	A	A	A	A	A	A	B	B	A	A	A	A	A	A	A	A	A	A	A	A	A	A	A	A	A	A	B	A	A	B	A	A	B	A	A	A
A	B	B	A	A	A	A	A	A	A	A	A	A	A	A	A	B	B	A	A	A	A	A	B	A	A	A	A	A	A	A	A	A	A	A	A	B	A	A	B	A	A	B	A	A	A
A	B	B	A	A	A	A	A	A	A	A	A	A	A	A	A	B	B	A	A	A	A	A	B	A	A	A	A	A	A	A	A	A	A	A	A	B	A	A	A	A	A	B	A	A	A
A	B	B	A	A	A	A	A	A	A	A	A	A	A	A	A	B	B	A	A	A	A	A	B	A	A	A	A	A	A	A	A	A	A	A	A	B	A	A	A	A	A	B	A	A	A
A	B	B	A	A	A	A	A	A	A	A	A	A	A	A	A	B	B	A	A	A	A	A	B	A	A	A	A	A	A	A	A	A	A	A	A	B	A	A	A	A	A	B	A	A	A
A	B	B	A	A	A	A	A	A	A	A	A	A	A	A	A	B	B	A	A	A	A	A	A	A	A	A	A	A	A	A	A	A	A	A	A	B	A	A	A	A	A	B	A	A	A
A	B	A	A	A	A	A	A	A	A	A	A	A	A	A	A	B	B	A	A	A	A	A	A	A	A	A	A	A	A	A	A	A	A	A	A	B	A	A	A	A	A	B	A	A	A
A	B	A	A	A	A	A	A	A	A	A	A	A	A	A	A	B	B	A	A	A	A	A	A	A	A	A	A	A	A	A	A	A	A	A	A	B	A	A	A	A	A	B	A	A	A
A	B	A	A	A	A	A	A	A	A	A	A	A	A	A	A	B	B	A	A	A	A	A	A	A	A	A	A	A	A	A	A	A	A	A	A	B	A	A	A	A	A	B	A	A	A
A	B	A	A	A	A	A	A	A	A	A	A	A	A	A	A	B	B	A	A	A	A	A	A	A	A	A	A	A	A	A	A	A	A	A	A	B	A	A	A	A	A	B	A	A	A
A	B	A	A	A	A	A	A	A	A	A	A	B	A	A	A	B	B	A	A	A	A	A	A	A	A	A	A	A	A	A	A	A	A	A	A	B	A	A	A	A	A	B	A	A	A
A	B	A	A	A	A	A	A	A	A	A	A	B	A	A	A	B	B	A	A	A	A	A	A	A	A	A	A	A	A	A	A	A	A	A	A	B	A	A	A	A	B	B	A	A	A
A	B	A	A	A	A	A	A	A	A	A	A	B	A	A	A	B	B	A	A	A	A	A	A	A	A	A	A	A	A	A	A	A	A	A	A	B	A	A	A	A	B	B	A	A	A
A	B	A	A	A	A	A	A	A	A	A	A	B	A	A	A	B	B	A	A	A	A	A	A	A	A	B	A	A	A	A	A	A	A	A	A	B	A	A	A	A	B	B	A	A	A
B	B	A	A	A	A	A	A	A	A	A	A	B	A	A	A	B	B	A	A	A	A	A	A	A	A	B	A	A	A	A	A	A	A	A	A	B	A	A	A	A	B	B	A	A	A
B	B	A	A	A	A	A	A	A	A	A	A	B	A	A	A	B	B	A	A	A	A	A	A	A	A	B	A	A	A	A	A	A	A	A	A	B	A	A	A	B	B	B	A	A	A
B	B	A	A	A	A	A	A	A	A	A	A	B	A	A	A	B	B	A	A	A	A	A	A	A	B	B	A	A	A	A	A	A	A	A	A	B	A	A	A	B	B	B	A	A	A
B	B	A	A	A	A	A	A	A	A	A	A	B	A	A	A	B	B	A	A	A	A	A	A	A	B	B	A	A	A	A	A	A	A	A	A	B	A	A	A	B	B	B	A	A	A
B	B	A	A	A	A	A	A	A	A	A	A	B	A	A	A	B	0	A	A	A	A	A	A	A	B	B	A	A	A	A	A	A	A	A	A	A	A	A	A	B	B	B	A	A	A
B	B	A	A	A	A	A	A	A	A	A	A	B	A	A	A	B	0	A	A	A	A	A	A	A	B	B	A	A	B	B	A	A	A	A	A	A	A	A	A	B	B	B	A	A	A
B	B	A	A	A	A	A	A	A	A	A	A	B	A	A	A	B	0	A	A	A	A	A	A	A	B	B	A	A	B	B	A	A	A	A	A	A	A	A	A	B	B	B	A	A	A
B	B	A	A	A	A	A	A	A	A	A	A	B	A	A	A	B	0	A	A	A	A	A	A	A	B	B	A	A	B	B	A	A	A	A	A	A	A	A	A	B	B	B	A	A	A
B	B	A	A	A	A	A	A	A	A	A	A	B	A	A	A	B	0	A	A	A	A	A	A	A	B	B	A	A	B	B	A	A	A	A	A	A	A	A	A	B	B	B	A	A	A
B	B	A	A	A	A	A	A	A	A	A	A	B	A	A	A	B	0	A	A	A	A	A	A	A	B	B	A	A	B	B	A	A	A	A	A	A	A	A	A	B	B	B	A	A	A
B	B	A	A	A	A	A	A	A	A	A	A	B	A	A	A	B	0	A	A	A	A	A	A	A	B	B	A	A	B	B	A	A	A	A	A	A	A	A	A	B	B	B	B	B	A
B	B	A	A	A	A	A	A	A	A	A	A	B	A	A	A	B	0	A	A	A	A	A	A	A	B	B	A	A	B	B	A	A	A	A	A	A	A	A	A	B	B	B	B	B	A
B	B	A	A	A	A	A	A	A	A	A	A	B	A	A	A	B	0	A	A	A	A	A	A	A	B	B	A	A	B	B	A	A	A	A	A	A	A	A	A	B	B	B	B	B	A
B	B	A	A	A	A	A	A	A	A	A	A	B	A	A	A	B	0	A	A	A	A	A	B	A	A	A	B	B	A	A	B	B	A	A	A	A	A	A	A	B	B	B	B	B	A
B	B	A	A	A	A	A	A	A	A	A	A	B	A	A	A	B	0	A	A	A	B	A	B	A	A	A	B	B	A	A	B	B	A	A	A	A	A	A	A	B	B	B	B	B	A
B	B	A	A	A	A	A	A	A	A	A	A	B	A	A	A	B	0	A	A	A	B	A	B	A	A	A	B	B	A	A	B	B	B	A	A	A	A	A	A	B	B	B	B	B	A
B	B	A	A	A	A	A	A	A	A	A	A	B	A	A	A	B	0	A	A	A	B	A	B	A	A	A	B	B	A	A	B	B	B	A	A	A	A	A	A	B	B	B	B	B	A
B	B	A	A	A	A	A	A	A	A	A	A	B	A	A	A	B	0	A	A	A	B	A	B	A	A	A	B	B	A	A	B	B	B	A	A	A	B	A	A	B	B	B	B	B	A
B	B	A	A	A	A	A	A	A	A	A	A	B	A	A	A	B	0	A	A	A	B	A	B	A	A	A	B	B	A	A	B	B	B	A	A	A	B	A	A	B	B	B	B	B	A
B	B	A	A	A	A	A	A	A	A	A	A	B	A	A	A	B	0	A	A	A	B	A	B	A	A	A	B	B	A	A	B	B	B	A	B	A	B	A	A	B	B	B	B	B	A
B	B	A	A	A	A	A	A	A	A	A	A	B	A	A	A	B	0	A	A	A	B	A	B	A	A	A	B	B	A	A	B	B	B	A	B	A	B	A	A	B	B	B	B	B	A
B	B	A	A	A	B	A	A	A	A	A	A	B	A	A	A	B	0	A	A	A	B	A	B	A	A	A	B	B	A	A	B	B	B	A	B	A	B	A	A	B	B	B	B	B	A
B	B	A	A	A	B	A	A	A	A	A	A	B	A	A	A	B	0	A	B	B	B	A	B	A	A	A	B	B	A	A	B	B	B	A	B	A	B	A	A	B	B	B	B	B	A
B	B	A	A	A	B	A	A	A	A	A	A	B	A	A	A	B	0	A	B	B	B	A	B	A	A	A	B	B	A	A	B	B	B	A	B	A	B	A	A	B	B	B	B	B	A
B	B	A	A	A	B	A	A	A	A	A	A	B	A	A	A	B	0	A	B	B	B	A	B	A	A	A	B	B	A	A	B	B	B	A	B	A	B	A	A	B	B	B	B	B	A
B	B	A	A	B	B	A	A	A	A	A	A	B	A	A	A	B	0	A	B	B	B	A	B	A	A	A	B	B	A	A	B	B	B	A	B	A	B	A	A	B	B	B	B	B	A
B	B	A	A	B	B	A	A	A	A	A	A	B	A	A	A	B	0	A	B	B	B	A	B	0	A	A	B	B	A	A	B	B	B	A	B	A	B	A	A	B	B	B	B	B	A

B	B	A	A	B	B	A	A	A	A	A	A	B	A	A	A	B	0	A	B	B	B	B	B	0	A	A	B	B	A	A	B	B	B	A	B	A	B	A	A	B	B	B	B	B	A
B	B	A	A	B	B	A	A	A	A	A	A	B	A	A	A	B	0	A	B	B	B	B	B	0	A	A	B	B	B	B	B	B	B	A	B	A	B	A	A	B	B	B	B	B	A
B	B	A	A	B	B	A	A	A	A	A	A	B	A	A	A	B	0	A	B	B	B	B	B	0	A	A	B	B	B	B	B	B	B	A	B	A	B	A	A	B	B	B	B	B	A
B	B	A	A	B	B	A	A	A	A	A	A	B	A	A	A	B	0	A	B	B	B	B	B	0	A	A	B	B	B	B	B	B	B	A	B	A	B	A	A	B	B	B	B	B	A
B	B	A	A	B	B	A	A	A	A	A	A	B	A	A	A	B	0	A	B	B	B	B	B	0	A	A	B	B	B	B	B	B	B	A	B	A	B	A	A	B	B	B	B	B	A
B	B	A	A	B	B	A	A	A	B	A	A	B	A	A	A	B	0	A	B	B	B	B	B	0	A	A	B	B	B	B	B	B	B	A	B	A	B	A	A	B	B	B	B	B	A
B	B	A	A	B	B	A	A	A	B	A	A	B	A	A	A	B	0	A	B	B	B	B	B	0	A	A	B	B	B	B	B	B	B	A	B	A	B	A	A	B	B	B	B	B	A
B	B	A	A	B	B	A	A	A	B	A	A	B	A	A	A	B	0	A	B	B	B	B	B	0	A	A	B	B	B	B	B	B	B	A	B	A	B	A	A	B	B	B	B	B	A
A	B	A	A	B	B	A	A	A	B	A	A	B	A	A	A	B	0	A	B	B	B	B	B	0	A	A	B	B	B	B	B	B	B	A	B	A	B	A	A	B	B	B	B	B	A
A	B	A	A	B	B	A	A	A	B	B	A	B	A	A	A	B	0	A	B	B	B	B	B	0	A	A	B	B	B	B	B	B	B	A	B	A	B	A	A	B	B	B	B	B	A
A	B	A	A	B	B	A	A	A	B	B	A	B	A	A	A	B	0	A	B	B	B	B	B	0	A	A	B	B	B	B	B	B	B	A	B	A	B	A	A	B	B	B	B	B	A
A	B	A	A	B	B	A	A	A	B	B	B	B	A	A	A	B	0	A	B	B	B	B	B	0	A	A	B	B	B	B	B	B	B	A	B	A	B	A	A	B	B	B	B	B	A
A	B	A	A	B	B	A	A	A	B	B	B	A	A	A	A	B	0	A	B	B	B	B	B	0	A	A	B	B	B	B	B	B	B	A	B	A	B	A	A	B	B	B	B	B	A
A	B	A	A	B	B	A	A	A	B	B	B	A	A	A	A	B	0	A	B	B	B	B	B	B	A	A	B	B	B	B	B	B	B	B	B	A	B	A	A	B	B	B	B	B	A
A	B	A	A	B	B	A	B	A	B	B	B	A	A	A	A	B	0	A	B	B	B	B	B	B	A	A	B	B	B	B	B	B	B	B	B	A	B	A	A	B	B	B	B	B	A
A	B	A	A	B	B	B	B	A	B	B	B	B	A	B	A	B	0	A	B	B	B	B	B	B	A	A	B	B	B	B	B	B	B	B	B	A	B	A	A	B	B	B	B	B	B
A	B	A	A	B	B	B	B	A	B	B	B	B	A	B	A	B	0	A	B	B	B	B	B	B	A	A	B	B	B	B	B	B	B	B	B	A	B	A	A	B	B	B	B	B	B
B	A	0	A	A	B	B	B	A	B	B	A	A	A	B	A	A	B	A	B	B	B	B	B	A	A	B	B	B	A	A	B	B	A	B	B	A	B	A	A	B	B	B	0	A	B
B	A	0	A	A	B	B	B	A	B	B	A	A	A	B	A	A	B	A	B	B	B	B	B	A	A	B	B	B	A	A	B	B	A	B	B	A	B	A	B	B	B	B	0	A	B
B	A	0	A	A	B	B	B	A	B	B	A	A	A	B	A	A	B	A	B	B	B	B	B	A	A	B	B	B	B	B	B	B	A	B	B	A	B	A	B	B	B	B	0	A	B
B	A	0	A	A	B	B	B	A	B	B	A	A	A	B	A	A	B	A	B	B	B	B	B	A	A	B	B	B	B	B	B	B	A	B	B	A	B	A	B	B	B	B	0	A	B
B	A	0	A	A	B	B	A	A	B	B	A	A	A	B	A	0	B	A	B	B	B	B	B	A	A	B	B	B	B	B	B	B	A	B	B	A	B	A	B	B	B	B	0	A	B
B	A	0	A	A	B	B	A	A	B	B	A	A	A	A	A	B	B	A	A	A	B	B	B	A	A	B	B	B	B	B	B	B	A	B	B	A	B	A	B	B	B	B	0	A	B
B	A	0	A	A	B	B	A	A	B	B	A	A	B	A	A	B	B	A	A	A	B	B	B	A	A	B	B	B	B	B	B	B	A	B	B	A	B	A	B	B	B	B	0	A	B
B	A	0	A	A	B	B	A	B	B	B	A	A	B	A	A	B	B	A	A	A	B	B	B	A	A	B	B	B	B	B	B	B	A	B	A	A	B	A	B	B	B	B	0	A	B
0	A	0	A	A	B	B	A	B	B	B	A	A	B	0	A	0	B	A	A	A	B	B	B	A	A	B	B	B	B	B	B	B	A	B	A	A	B	A	B	B	B	B	A	A	0
0	A	0	A	A	B	B	A	B	B	B	A	A	B	0	A	0	B	A	A	A	B	B	B	A	A	B	B	B	B	B	B	B	A	B	A	A	B	A	B	B	B	B	A	A	0
0	A	0	A	A	B	B	A	B	B	B	A	A	B	0	A	0	B	A	A	A	B	B	B	A	A	B	B	B	B	B	B	B	A	B	A	A	B	A	B	B	B	B	A	A	0
0	A	0	A	A	B	B	A	B	B	B	A	A	B	0	A	0	B	A	A	A	B	B	B	A	A	B	B	B	B	B	B	B	A	B	A	A	B	A	B	B	B	B	A	A	0
0	A	0	A	A	B	B	A	B	B	B	A	A	B	0	A	0	B	A	A	A	B	B	B	A	A	B	B	B	B	B	B	B	A	B	A	0	B	A	B	B	B	B	A	A	0
0	A	0	A	A	B	B	A	B	B	B	A	A	B	0	B	0	B	A	A	A	B	B	B	A	A	B	B	B	B	B	B	B	A	B	A	B	B	A	B	B	B	B	A	A	0
0	A	0	A	A	B	B	A	B	B	B	A	A	B	0	B	0	B	A	A	A	B	B	B	A	A	B	B	B	B	B	B	B	A	B	A	B	B	A	B	B	B	B	A	A	0
0	A	0	A	A	B	B	A	B	B	B	A	A	B	0	B	0	B	A	A	A	B	B	B	A	A	B	B	B	B	B	B	B	A	B	A	B	B	A	B	B	A	0	A	A	0
0	A	0	A	A	B	B	A	B	B	B	A	A	B	0	B	0	A	A	A	A	B	B	B	A	A	B	B	B	B	B	B	B	A	B	A	B	B	A	B	B	A	0	A	A	0
0	A	0	A	A	B	B	A	B	B	B	A	A	B	0	B	0	A	A	A	A	B	B	B	A	A	B	B	B	B	B	B	B	A	B	A	B	B	A	B	B	A	0	A	A	0
0	A	0	A	A	B	B	A	B	B	B	A	A	B	0	B	0	A	A	A	A	B	B	B	A	A	B	B	B	B	B	B	B	A	B	A	B	B	A	B	B	A	0	A	A	0
0	A	0	A	A	B	B	A	B	B	B	A	B	B	0	B	0	A	A	A	A	B	B	B	A	A	B	B	B	B	B	B	B	A	B	A	B	B	A	B	B	A	0	A	A	0
0	A	0	A	A	B	B	A	B	B	B	A	B	B	0	B	0	A	A	A	A	B	B	B	A	A	B	B	B	B	B	B	B	B	B	A	B	B	A	B	B	A	0	A	A	0
0	A	0	A	A	B	B	A	B	B	B	A	B	B	0	B	0	A	A	A	A	B	B	B	A	A	B	B	B	B	B	B	B	B	B	B	B	B	A	B	B	A	0	A	A	0
0	A	0	A	A	B	B	A	B	B	A	A	B	B	0	B	0	A	A	A	A	B	B	B	A	A	B	B	B	B	B	B	B	B	B	B	B	B	A	B	B	A	0	A	A	0
0	A	0	A	A	B	B	A	B	B	A	A	B	B	0	B	0	A	A	A	A	B	B	B	A	A	B	B	B	B	B	B	B	B	B	B	B	B	A	B	B	A	0	A	A	0
0	A	0	A	A	B	B	A	B	B	A	A	B	B	B	B	0	A	A	A	A	B	B	B	A	A	B	B	B	B	B	B	B	B	B	B	B	B	A	B	B	A	0	A	A	0
0	A	0	A	A	B	B	A	B	B	A	A	B	B	B	B	0	A	A	A	A	B	B	B	A	A	B	B	B	B	B	B	B	B	B	B	B	B	A	B	B	A	0	A	A	0
0	A	0	A	A	B	B	A	B	B	A	A	B	B	B	B	0	A	A	A	A	B	B	B	A	A	A	B	B	B	B	B	B	B	B	B	B	B	A	B	B	A	0	A	A	0
0	A	0	A	A	B	B	A	B	B	A	A	B	B	B	B	0	A	A	A	A	B	B	B	A	A	A	B	B	B	B	B	B	B	B	B	B	B	A	B	B	A	0	B	B	0
0	A	0	A	A	B	B	A	B	B	A	A	B	B	B	B	0	A	A	A	A	B	B	B	A	A	A	B	B	B	B	B	B	B	B	B	B	B	A	B	B	A	0	B	B	0
0	A	0	A	A	B	B	A	B	B	A	A	B	B	B	B	0	A	A	A	A	B	B	B	A	A	A	B	B	B	B	B	B	B	B	B	B	B	A	B	B	A	0	B	B	0
0	A	0	A	A	B	B	A	B	B	A	A	B	B	B	B	0	A	A	A	A	B	B	B	A	A	A	B	B	B	B	B	B	B	B	B	B	B	A	B	B	A	0	B	B	0
0	A	0	A	A	B	B	0	B	B	A	A	B	A	0	B	A	A	A	A	A	B	B	B	A	A	A	B	B	B	B	B	B	B	B	B	B	B	A	B	B	A	0	B	B	0

0	A	0	A	A	B	B	0	B	B	A	A	B	A	0	B	A	A	A	A	A	B	B	B	A	A	A	B	B	B	B	B	B	B	B	B	B	B	A	B	B	A	0	B	B	0
0	A	0	A	A	B	B	0	B	B	A	A	B	A	0	B	A	A	A	A	A	B	B	B	A	A	A	B	B	B	B	B	B	B	B	B	B	B	A	B	B	A	0	B	B	0
0	A	0	A	A	B	B	0	B	B	A	A	B	A	0	B	A	A	A	A	A	B	B	B	A	A	A	B	B	B	B	B	B	B	B	B	B	B	A	B	B	A	0	B	B	0
0	A	0	A	A	B	B	0	B	B	A	A	B	A	0	B	A	A	A	A	A	B	B	B	A	A	A	B	B	B	B	B	B	B	B	B	B	B	A	B	B	A	0	B	B	B
0	A	0	A	A	B	B	0	B	B	A	A	B	A	0	B	A	A	A	A	A	B	B	B	A	A	A	B	B	B	B	B	B	B	B	B	0	B	A	B	B	A	0	B	B	B
0	A	0	A	A	B	B	0	B	B	A	A	B	A	0	B	A	A	A	A	A	B	B	B	A	A	A	B	B	B	B	B	B	B	B	B	0	B	A	B	B	A	0	B	B	0
0	A	B	A	A	B	B	0	B	B	A	A	B	A	0	B	A	A	A	A	A	B	B	B	A	A	A	B	B	B	B	B	B	B	B	B	0	B	A	B	B	A	0	B	B	0
0	A	B	A	A	B	B	0	B	B	A	A	B	A	0	B	A	A	A	A	A	B	B	B	A	A	A	B	B	B	B	B	B	B	B	B	0	B	A	B	B	A	0	B	B	0
A	A	B	A	A	B	B	0	B	A	A	A	B	A	0	B	A	A	A	A	A	B	B	B	A	A	A	B	B	B	B	B	B	B	B	B	0	B	A	B	B	A	0	B	B	0
A	A	B	A	A	B	B	0	B	A	A	A	B	A	0	B	A	A	A	A	A	B	B	B	A	A	A	B	B	B	B	A	A	B	B	B	0	B	A	B	B	A	0	B	B	0
A	A	B	A	A	B	B	0	B	A	A	A	B	A	0	B	A	A	A	A	A	B	B	B	A	A	A	B	B	B	B	A	A	B	B	B	0	B	A	B	B	A	0	B	B	0
A	A	B	A	A	B	B	0	B	A	A	A	B	A	0	A	A	A	A	A	A	B	B	B	A	A	A	B	B	B	B	A	A	B	B	B	0	B	A	B	B	A	0	B	B	0
A	A	B	A	A	B	B	0	B	A	A	A	B	A	0	A	A	A	A	A	A	B	B	B	A	A	A	B	B	B	B	A	A	B	B	B	0	B	A	B	B	A	0	B	B	0
A	A	B	A	A	B	B	0	B	A	A	A	B	A	0	A	A	A	A	A	A	B	B	B	A	A	A	B	B	B	B	A	A	B	B	B	0	B	A	B	B	A	0	B	B	0
A	A	B	A	A	A	A	A	B	B	B	0	A	A	A	A	A	A	A	A	A	B	B	A	A	A	B	B	B	A	A	A	A	0	A	A	B	A	0	B	A	B	A	A	A	A
A	A	B	A	A	A	A	A	B	B	B	0	A	A	A	A	A	A	A	A	A	B	B	A	A	A	B	B	B	A	A	A	A	0	A	A	B	A	0	B	A	B	A	A	A	A
A	A	B	A	A	A	A	A	B	B	B	0	A	A	A	A	A	A	A	A	A	B	B	A	A	A	B	B	B	A	A	A	A	0	A	A	B	A	0	B	A	B	A	A	A	A
A	A	B	A	A	A	A	A	B	B	B	0	A	A	A	A	A	A	A	A	A	B	B	A	A	A	A	A	B	A	A	A	A	0	A	A	B	A	0	B	A	B	A	A	A	A
A	A	B	A	A	A	A	A	B	B	B	0	A	A	A	A	A	A	A	A	A	B	B	A	A	A	A	A	B	A	A	A	A	0	A	A	B	A	0	B	A	B	A	A	A	A
A	A	B	A	A	A	A	A	B	B	B	0	A	A	A	A	A	A	A	A	A	0	0	A	A	A	A	A	B	A	A	A	A	0	A	0	B	A	0	B	A	B	A	A	A	A
A	A	B	A	A	A	A	A	B	B	B	0	A	A	A	A	A	A	A	A	A	A	A	B	A	A	A	A	B	A	A	A	A	0	A	B	B	A	0	B	A	B	A	A	A	A
A	A	B	A	A	A	A	A	B	B	B	0	A	A	A	A	A	A	A	A	A	A	A	B	A	A	A	A	B	A	A	A	A	0	A	B	B	A	0	B	A	B	A	A	A	A
A	A	B	A	A	A	A	A	B	B	B	0	A	A	A	A	A	A	A	A	A	A	A	B	A	A	A	A	B	A	A	A	A	0	A	B	B	A	0	B	A	B	A	A	A	A
A	A	B	A	A	A	B	A	B	B	B	0	A	A	0	A	A	A	A	A	A	A	A	B	A	A	A	A	B	A	A	A	A	0	A	B	B	A	0	B	A	B	A	A	A	A
A	A	B	A	A	A	B	A	B	A	B	0	A	A	0	A	A	A	A	A	A	A	A	B	A	A	A	A	B	A	A	A	A	0	A	B	B	A	0	B	A	B	A	A	A	A
A	A	B	A	A	A	B	A	B	A	B	0	A	A	0	A	A	A	A	A	A	A	A	B	B	A	A	A	B	A	A	A	A	0	A	B	B	A	0	B	A	B	A	A	A	A
A	A	B	A	A	A	B	A	B	A	B	0	A	A	0	A	A	A	A	A	A	A	A	B	B	A	A	A	B	A	A	A	A	0	A	B	B	A	0	B	A	B	A	A	A	A
A	A	B	A	A	A	A	A	B	A	A	0	A	A	0	A	A	A	A	A	A	A	A	B	B	A	A	A	B	A	A	A	A	0	A	B	B	A	0	B	A	B	A	A	A	A
A	A	A	A	A	A	A	A	B	A	A	0	A	A	0	A	A	A	A	A	A	A	A	B	B	A	A	A	B	A	A	A	A	0	A	B	B	A	0	B	A	B	A	A	A	A
A	A	A	A	A	A	A	A	B	A	A	0	A	A	0	A	A	A	A	A	A	A	A	B	B	A	A	A	B	A	A	A	A	0	A	B	B	A	0	B	A	B	A	A	A	A
A	A	A	A	A	A	A	A	B	A	A	0	A	A	0	A	A	A	A	A	A	A	A	B	B	A	A	A	B	A	A	A	A	0	A	B	B	A	0	B	A	B	A	A	A	A
A	A	A	A	A	A	A	A	B	A	A	0	A	A	0	A	A	A	A	A	A	A	A	B	B	A	A	A	B	A	A	A	A	0	A	B	B	A	0	B	A	A	A	A	A	A
A	A	A	A	A	A	A	A	B	A	A	0	A	A	0	A	A	A	A	A	A	A	A	B	B	A	A	A	B	A	A	A	A	0	A	A	B	A	0	B	A	A	A	A	A	A
A	A	A	A	A	A	A	A	A	A	B	0	A	A	A	A	A	A	A	A	A	A	A	B	B	A	A	A	B	A	A	A	A	0	A	A	B	A	0	B	A	A	A	A	A	A
A	A	A	A	A	A	A	A	A	A	B	0	A	A	A	A	A	A	A	A	A	A	A	B	B	A	A	A	B	A	A	A	A	0	A	A	B	A	0	B	A	A	A	A	A	A
A	A	A	A	A	A	A	A	A	A	B	0	A	A	A	A	A	A	A	A	A	A	A	B	B	A	A	A	B	A	A	A	A	A	A	A	B	A	0	B	A	A	A	A	A	A
A	A	A	A	A	A	A	A	A	A	B	0	A	A	A	A	A	A	A	A	A	A	A	B	B	A	A	A	B	A	A	A	A	A	A	A	B	A	0	B	A	A	A	A	A	A
A	A	A	A	A	A	A	A	A	A	B	0	A	A	A	A	A	A	A	A	A	A	A	B	B	A	A	A	B	A	A	A	A	A	A	A	B	A	0	B	A	A	A	A	A	A
A	A	A	A	A	A	A	A	A	A	B	0	A	A	A	A	A	A	A	A	A	A	A	B	B	B	A	A	B	A	A	A	A	A	A	A	B	A	0	B	A	A	A	A	A	A
A	A	A	A	A	A	A	A	A	A	B	0	A	A	A	A	A	A	A	A	A	A	A	B	B	B	A	A	B	A	A	B	B	A	A	A	B	A	0	B	A	A	A	A	A	A
A	A	A	A	A	A	A	A	A	A	B	0	A	A	A	A	A	A	A	A	A	A	A	B	B	B	A	A	B	A	A	B	B	A	A	A	B	A	0	B	A	A	A	A	A	A
A	A	A	A	A	A	A	A	A	A	B	0	A	A	A	A	A	A	A	A	A	A	A	B	B	B	A	A	B	A	A	B	B	A	A	A	B	A	0	B	A	A	A	A	A	A
A	A	A	A	A	A	A	A	A	A	B	0	A	A	A	A	A	A	A	A	A	A	A	B	B	B	A	A	B	A	A	B	B	A	A	A	B	A	0	B	A	A	A	A	A	A
A	A	A	A	A	A	A	A	A	A	B	0	A	A	A	A	A	A	A	A	A	A	A	B	B	B	A	A	B	A	A	B	B	A	A	A	B	A	B	B	A	A	A	A	A	A
A	A	A	A	A	A	A	A	A	A	B	0	A	A	A	A	A	A	A	A	A	A	A	B	B	B	A	A	B	A	A	B	B	A	B	A	B	A	B	B	A	A	A	A	A	A
A	A	A	A	A	A	A	A	A	A	B	0	A	A	0	A	A	A	A	A	A	A	A	B	B	B	A	A	B	A	A	B	B	A	B	A	B	A	B	B	A	A	A	A	A	A
A	A	A	A	A	A	A	A	A	A	B	0	A	A	0	A	A	A	A	A	A	A	A	B	B	B	A	A	B	A	A	B	B	A	B	A	B	A	B	B	A	B	A	A	A	A
A	A	A	A	A	A	A	A	A	A	B	0	A	A	0	A	A	A	A	A	A	A	A	B	B	B	A	A	B	A	A	B	B	A	B	A	B	A	B	B	A	B	A	A	A	A
A	A	A	A	A	A	A	A	A	A	B	0	A	A	0	A	A	A	A	A	A	A	A	B	B	B	A	A	B	A	A	B	B	A	B	A	B	A	B	B	A	B	A	A	A	A

A	A	A	A	A	A	A	A	A	A	B	0	A	A	0	A	A	A	A	A	A	A	A	B	B	B	A	A	B	A	A	B	B	A	B	A	B	A	B	B	A	B	A	A	A	A
A	A	A	A	A	A	A	A	A	A	B	0	A	A	0	A	A	A	A	A	A	A	A	B	B	B	A	A	B	A	A	B	B	A	B	A	B	B	B	B	A	B	A	A	A	A
A	A	A	A	A	A	A	A	A	A	B	0	A	A	0	A	A	A	A	A	A	A	A	B	B	B	A	A	B	A	A	B	B	A	B	A	B	B	B	B	A	B	A	A	A	A
A	A	A	A	A	A	A	A	A	A	B	0	A	A	0	A	A	A	A	A	A	A	A	B	B	B	A	A	B	A	A	B	B	A	B	A	B	B	B	B	A	B	B	A	A	A
A	A	A	A	A	A	A	A	A	A	B	0	A	A	0	A	A	A	A	A	A	A	A	B	B	B	A	A	B	A	A	B	B	A	B	A	B	B	B	B	B	B	B	A	A	A
A	A	A	A	A	A	A	A	A	A	B	0	A	A	0	A	A	A	A	A	A	A	A	B	B	B	A	A	B	A	A	B	B	A	B	A	B	B	B	A	B	B	B	A	A	A
A	A	A	A	A	A	A	A	A	A	B	0	A	A	0	A	A	A	A	A	A	A	A	B	B	B	A	A	B	A	A	B	B	A	B	A	B	B	B	A	B	B	B	A	A	A
A	A	A	A	A	A	A	A	A	A	B	0	A	A	0	A	A	A	A	A	A	A	A	B	B	B	A	A	B	A	A	B	B	A	B	A	B	B	B	A	B	B	B	A	A	A
A	A	A	A	A	A	A	A	A	A	B	0	A	A	0	A	A	A	A	A	A	A	A	B	B	B	A	A	B	A	A	B	B	A	B	A	B	B	B	A	B	B	B	A	A	A
A	A	A	A	A	A	A	A	A	A	B	0	A	A	0	A	A	A	A	A	A	A	A	B	B	B	A	A	B	A	A	B	B	A	B	A	B	B	B	A	B	B	B	A	A	A
A	A	A	A	A	A	A	A	A	A	B	0	A	A	0	A	A	A	A	A	A	A	A	A	B	B	A	A	B	A	A	B	B	A	B	A	B	B	B	A	B	B	B	A	A	A
A	A	A	A	A	A	A	A	A	A	B	0	A	A	0	A	A	A	A	A	A	A	A	A	B	B	A	A	B	A	A	B	B	A	B	A	B	B	B	A	B	B	B	A	A	A
B	B	A	B	B	B	B	A	B	B	B	B	A	A	A	B	A	B	B	B	B	B	A	B	B	A	A	A	B	A	A	B	B	A	A	A	B	A	A	A	B	A	B	B	B	B
B	B	A	B	B	B	B	A	B	B	B	B	A	A	A	B	A	B	B	B	B	B	A	B	B	A	A	A	B	A	A	B	B	A	A	A	B	A	A	A	B	A	B	A	A	B
B	B	A	A	B	B	B	A	B	B	B	B	A	A	A	B	A	B	B	B	B	B	A	B	B	A	A	A	B	A	A	B	B	A	A	A	B	A	A	A	B	A	B	A	A	B
B	B	A	A	B	B	B	0	B	B	B	B	A	A	0	A	A	B	B	B	B	B	A	B	B	A	A	A	B	A	A	B	B	A	A	A	B	A	A	A	B	0	B	A	A	B
B	B	A	A	B	B	B	0	B	B	B	B	A	A	0	A	A	B	B	B	B	A	A	B	B	A	A	A	B	A	A	B	B	A	A	A	B	A	A	A	B	0	B	A	A	B
B	B	A	A	B	B	B	A	B	B	B	B	A	A	0	A	A	B	B	B	B	A	A	B	B	A	A	A	B	A	A	B	B	A	A	A	B	A	A	A	B	0	B	A	A	B
B	B	A	A	B	B	B	A	B	B	B	B	A	A	0	A	A	B	B	B	B	A	A	B	B	A	A	A	B	A	A	B	B	A	A	A	B	A	B	A	B	0	B	A	A	B
B	B	A	A	B	B	B	A	B	B	B	B	A	A	0	A	A	B	B	B	B	A	A	B	A	A	A	A	B	A	A	B	B	A	A	A	B	A	B	A	B	0	B	A	A	B
B	B	A	A	B	B	B	A	B	B	B	B	A	A	0	A	A	B	B	B	B	A	A	B	A	A	A	A	B	A	A	B	B	A	A	A	B	A	B	A	B	0	B	A	A	B
B	B	A	A	B	B	B	A	B	B	B	B	A	A	0	A	A	B	B	B	B	A	A	B	A	A	A	A	A	A	A	B	B	A	A	A	B	A	B	A	B	A	B	A	A	B
B	B	A	A	B	B	B	A	B	B	B	B	A	A	0	A	A	A	B	B	B	A	A	B	A	A	A	A	A	A	A	B	B	A	A	A	B	A	B	A	B	A	B	A	A	B
B	B	A	A	B	B	B	A	B	B	B	B	A	A	0	A	A	A	B	B	B	A	A	B	A	A	A	A	A	A	A	B	B	A	A	B	B	A	B	A	B	A	B	A	A	B
B	B	A	A	B	B	B	A	B	B	B	B	A	A	0	A	A	A	B	B	B	A	A	B	A	A	A	A	A	A	A	B	B	B	A	B	B	A	B	A	B	A	B	A	A	B
B	B	A	A	B	B	B	A	B	B	B	B	A	A	0	A	A	A	B	B	B	A	A	B	A	A	A	A	A	A	A	B	B	B	A	B	A	A	B	A	B	A	B	A	A	B
B	B	A	A	B	B	B	A	B	B	B	B	A	A	0	A	A	A	B	B	B	A	A	B	A	A	A	A	A	A	A	B	B	B	A	B	A	A	B	A	B	A	B	A	A	B
B	B	A	A	B	B	B	A	B	B	B	B	A	A	0	A	A	A	B	B	B	A	A	B	A	A	A	A	A	A	A	B	B	B	A	B	A	A	B	A	B	A	B	A	A	B
B	B	A	A	B	B	B	A	B	B	B	A	A	A	B	A	A	A	B	B	B	A	A	B	A	A	A	A	A	A	A	B	B	B	A	B	A	A	B	A	B	A	B	A	A	B
B	B	A	A	B	B	B	A	B	B	B	A	A	A	B	A	A	A	B	B	B	A	A	B	A	A	B	A	A	A	A	B	B	B	A	B	A	A	B	A	B	A	B	A	A	B
B	B	B	A	B	B	B	A	B	B	B	A	A	A	B	A	A	A	B	B	B	A	A	B	A	A	B	A	A	A	A	B	B	B	A	B	A	A	B	A	B	A	B	A	A	B
B	B	B	A	B	B	B	A	B	B	B	A	A	A	B	A	A	A	B	B	B	A	A	B	A	A	B	A	A	A	A	B	B	B	A	B	A	A	B	A	B	A	B	A	A	B
B	B	B	A	B	B	B	A	B	B	B	A	A	A	B	A	A	A	B	B	B	A	A	B	A	A	B	A	A	B	B	B	B	B	A	B	A	A	B	A	B	A	B	A	A	B
B	B	B	A	B	B	B	A	B	B	B	A	A	A	B	A	A	A	B	A	A	A	A	B	A	A	B	A	A	B	B	B	B	B	A	B	A	A	B	A	B	A	B	A	A	B
B	B	B	A	B	B	B	A	B	B	B	B	A	A	B	A	A	A	B	A	A	A	A	B	A	A	B	A	A	B	B	B	B	B	A	B	A	A	B	A	B	A	B	A	A	B
B	B	B	A	B	B	B	A	B	B	B	B	A	A	B	A	A	A	B	A	A	A	A	B	A	A	B	A	A	B	B	B	B	B	A	B	A	A	B	A	B	A	B	A	A	B
B	B	B	A	B	B	B	A	B	B	B	B	A	A	B	A	A	A	B	A	A	A	A	B	A	A	B	A	A	B	B	B	B	B	A	B	A	A	B	A	B	A	B	A	A	B
B	B	B	A	B	B	B	A	B	B	B	B	A	A	B	A	A	A	B	A	A	A	A	B	A	A	B	A	A	B	B	B	B	B	A	B	A	A	A	A	B	A	B	A	A	B
B	B	B	A	B	B	B	A	B	B	B	B	A	A	B	A	A	A	B	A	A	A	A	B	A	A	B	A	A	B	B	B	B	B	A	B	A	A	B	A	B	A	B	A	A	B
B	B	B	A	B	A	B	A	B	B	B	B	A	A	B	A	A	A	B	A	A	B	A	B	A	A	B	A	A	B	B	B	B	B	A	B	A	A	B	A	B	A	B	A	A	B
B	B	B	A	B	A	B	A	B	B	B	B	A	A	B	A	A	A	B	A	A	B	A	B	A	A	B	A	A	B	B	B	B	B	A	B	A	B	B	A	B	A	B	A	A	B
B	B	B	A	B	A	B	A	B	B	B	B	A	A	B	A	A	A	B	A	A	B	A	B	A	A	B	A	A	B	B	B	B	B	A	B	A	B	B	A	B	A	B	A	A	B
B	B	B	A	B	A	B	A	B	B	B	B	A	A	B	A	A	A	B	A	A	B	A	B	A	A	B	A	A	B	B	B	B	B	A	B	B	B	B	A	B	A	B	A	A	B
B	B	B	A	B	A	B	B	B	B	B	B	A	A	B	A	A	A	B	A	A	B	A	B	A	A	B	A	A	B	B	B	B	B	A	B	B	B	B	A	B	A	B	A	A	B
B	B	B	A	B	A	B	B	B	B	B	B	A	A	B	A	A	A	B	A	A	B	A	B	A	A	B	A	A	B	B	B	B	B	A	B	B	B	B	A	B	A	B	A	A	B
B	B	A	A	B	A	B	B	B	B	B	B	A	A	B	A	A	A	B	A	A	B	A	B	A	A	B	A	A	B	B	B	B	B	A	B	B	B	B	A	B	A	B	A	A	B
B	B	A	A	B	A	B	B	B	B	B	B	A	A	B	A	A	A	B	A	A	B	A	B	A	A	B	A	A	B	B	B	B	B	A	B	B	B	B	A	B	A	B	A	A	B
B	B	A	A	B	A	B	B	B	B	B	B	A	A	B	A	B	A	B	A	A	B	A	B	A	A	B	A	A	B	B	B	B	B	A	B	B	B	B	A	B	A	B	A	A	B
B	B	A	A	B	A	B	B	B	B	B	B	A	A	B	A	B	A	B	A	A	B	A	B	A	A	B	A	A	B	B	A	A	B	A	B	B	B	B	A	B	A	B	B	B	B

B	B	A	A	B	A	B	B	B	B	B	B	B	A	B	A	B	A	B	A	A	B	A	B	A	A	B	A	A	B	B	A	A	B	A	B	B	B	B	A	B	A	B	B	B	B
B	B	A	A	B	A	B	B	B	B	B	B	B	A	B	A	B	A	B	A	A	B	A	B	A	A	B	A	A	B	B	A	A	B	A	B	B	B	B	A	B	A	B	B	B	B
B	B	A	A	B	A	B	B	B	B	B	B	B	A	B	A	B	A	B	A	A	B	A	B	A	A	B	A	B	B	B	A	A	B	A	B	B	B	B	A	B	A	B	B	B	B
B	B	A	A	B	A	B	B	B	B	B	B	B	A	B	A	B	A	B	A	A	B	A	B	A	A	B	A	B	B	B	A	A	B	A	B	B	B	B	A	B	A	B	B	B	B
B	B	A	A	B	A	B	B	B	B	B	B	B	A	B	A	B	A	B	A	A	B	A	B	A	A	B	A	B	B	B	A	A	B	A	B	B	B	B	A	B	A	B	B	B	B
B	B	A	A	B	A	B	B	B	B	B	B	B	A	A	A	B	A	B	A	A	B	A	B	A	A	B	A	B	B	B	A	A	B	A	B	B	B	B	A	B	A	B	B	B	B
B	B	A	A	B	A	B	B	B	B	B	B	B	A	A	A	B	A	B	A	A	B	A	B	A	A	B	A	B	B	B	A	A	B	A	B	B	B	B	A	B	A	B	B	B	B
B	B	A	A	B	A	B	B	B	B	B	B	B	A	A	A	B	A	B	A	A	B	A	B	A	A	B	A	B	B	B	A	A	B	A	B	B	B	B	A	B	A	B	B	B	B
B	B	A	A	B	A	B	B	B	B	B	B	B	A	A	A	B	A	B	A	A	B	A	B	A	A	B	A	B	B	B	A	A	B	A	B	B	B	B	A	B	A	B	B	B	B
B	B	A	A	B	A	B	B	B	B	B	B	B	A	A	A	B	A	B	A	A	B	A	B	B	A	B	A	B	B	B	A	A	B	A	B	B	B	B	A	B	A	B	B	B	B
B	B	A	A	B	A	B	B	B	B	B	B	B	A	A	A	B	A	B	A	A	B	A	B	B	A	B	A	B	B	B	A	A	B	A	B	B	B	B	B	B	A	B	B	B	B
B	B	A	A	B	A	B	B	B	B	B	B	B	A	A	A	B	A	B	A	A	B	A	B	B	A	B	A	B	B	B	A	A	B	A	B	B	B	B	B	B	A	B	B	B	B
B	B	A	A	B	A	B	B	B	B	B	B	B	A	A	A	B	A	B	A	A	B	A	B	B	A	B	B	B	B	B	A	A	B	A	B	B	B	B	B	B	A	B	B	B	B
B	B	A	A	B	A	B	B	B	B	B	B	B	A	A	B	B	A	B	A	A	B	A	B	B	A	B	B	B	B	B	A	A	B	A	B	B	B	B	B	B	A	B	B	B	B
B	B	A	A	B	A	B	B	B	B	B	B	B	A	A	B	B	A	B	A	A	B	A	B	B	A	B	B	B	B	B	A	A	B	A	B	B	B	B	B	B	A	B	B	B	B
B	B	A	A	B	A	B	B	B	B	B	B	B	A	A	B	B	A	B	A	A	B	A	B	B	A	B	B	B	B	B	A	A	B	A	B	B	B	B	B	B	A	B	B	B	B
B	B	A	A	B	A	B	B	B	B	B	B	B	A	A	B	B	A	B	A	A	B	A	B	B	A	B	B	B	B	B	A	A	B	A	B	B	B	B	B	B	A	B	B	B	B
B	B	A	A	B	A	B	B	B	B	B	B	B	A	A	B	B	A	B	A	A	B	A	B	B	A	B	B	B	B	B	A	A	B	A	B	B	B	B	B	B	A	B	B	B	B
B	B	A	A	B	A	B	B	B	B	B	B	B	A	A	B	B	A	B	A	A	B	A	B	B	A	B	B	B	B	B	A	A	B	A	B	B	B	B	B	B	A	B	B	B	B
B	B	A	A	B	A	B	B	B	B	B	B	B	A	A	B	B	A	B	A	A	B	A	B	B	A	B	B	A	B	B	A	A	B	A	B	B	A	B	B	B	A	B	B	B	B
A	B	A	A	B	A	B	B	B	B	B	B	B	A	A	B	B	A	B	A	A	B	A	B	B	A	B	A	A	B	B	A	A	B	A	B	B	A	B	B	B	A	B	B	B	B
A	B	A	A	B	A	B	B	B	B	B	B	B	A	A	B	B	A	B	A	A	B	A	B	B	A	B	A	A	B	B	A	A	B	A	B	B	A	B	B	B	A	B	B	B	B
A	B	A	A	B	A	B	B	B	B	B	B	B	A	A	B	B	A	B	A	A	B	A	B	B	A	B	A	A	B	B	A	A	B	A	B	A	A	B	B	B	A	B	B	B	B
A	B	A	A	B	A	B	B	B	B	B	B	B	A	A	B	B	A	B	A	A	B	A	B	B	A	B	A	A	B	B	A	A	B	A	B	A	A	B	B	B	A	B	B	B	B
A	B	A	A	B	A	B	B	B	B	B	B	B	A	A	B	B	A	B	A	A	B	A	B	B	A	B	A	A	B	B	A	A	A	A	B	A	A	B	B	B	A	B	B	B	B
A	B	A	A	B	A	B	B	B	B	B	B	B	A	A	B	B	A	B	A	A	B	A	B	B	A	B	A	A	B	B	A	A	A	A	B	A	A	B	B	B	A	B	B	B	B
A	B	A	A	B	A	B	B	B	B	B	B	B	A	B	B	B	A	B	A	A	B	A	A	B	A	B	A	A	A	A	A	A	A	A	B	A	A	B	B	B	A	B	B	B	B
A	B	A	A	B	A	B	B	B	B	B	B	B	A	B	B	B	A	B	A	A	B	A	A	B	A	B	A	A	A	A	A	A	A	A	B	A	A	B	B	B	A	B	B	B	B
A	B	A	A	B	A	B	B	A	B	B	B	B	A	B	B	B	A	B	A	A	B	A	A	B	A	B	A	A	A	A	A	A	A	A	B	A	A	B	B	B	A	B	B	B	B
A	B	A	A	B	A	B	B	A	B	B	B	B	A	B	B	B	A	B	A	A	B	A	A	B	A	B	A	A	A	A	A	A	A	A	B	A	A	B	B	B	A	B	B	B	B
A	B	A	A	B	A	B	A	A	B	B	B	B	A	B	B	B	A	B	A	A	B	A	A	B	A	B	A	A	A	A	A	A	A	A	B	A	A	B	B	B	A	B	B	B	B
A	B	A	A	B	A	B	A	A	B	B	B	B	A	B	B	B	A	B	A	A	B	A	A	B	A	B	A	A	A	A	A	A	A	B	B	A	A	B	B	B	A	B	B	B	B
A	B	A	A	B	A	B	A	A	B	B	B	B	A	B	B	B	A	A	A	A	B	A	A	B	A	B	A	A	A	A	A	A	A	B	B	A	A	B	B	B	A	B	B	B	B
A	B	A	A	B	A	B	A	A	B	B	B	B	A	B	B	B	A	A	A	A	B	A	A	B	A	B	A	A	A	A	A	A	A	B	B	A	A	B	B	B	A	B	B	B	B
A	B	A	A	B	A	B	A	A	B	B	B	B	A	B	B	B	A	A	A	A	B	A	A	B	A	B	A	A	A	A	A	A	A	B	B	A	A	B	B	B	A	B	B	B	B
A	B	A	A	B	A	B	A	A	B	B	B	B	A	B	B	B	A	A	A	A	B	A	A	B	A	B	A	A	A	A	A	A	A	B	B	A	A	B	B	B	A	B	B	B	B
A	B	A	A	B	A	B	A	A	B	B	B	B	A	B	B	B	A	A	A	A	B	A	A	B	A	B	A	A	A	A	A	A	A	B	B	A	A	B	B	B	A	B	B	B	B
A	B	A	A	B	A	B	A	A	B	B	B	B	A	B	B	B	A	A	A	A	B	A	A	B	A	B	A	A	A	A	A	A	A	B	B	A	A	B	B	B	A	B	B	B	B
A	B	A	A	B	A	B	A	A	B	B	B	B	A	B	B	B	A	A	A	A	B	A	A	B	A	B	A	A	A	A	A	A	A	B	B	A	A	B	B	B	A	B	B	B	B
A	B	A	A	B	A	B	A	A	B	B	B	B	A	B	B	B	A	A	A	A	B	A	A	B	A	B	A	A	A	A	A	A	A	B	B	A	A	B	B	B	A	B	B	B	B
A	B	A	A	B	A	B	A	A	B	B	B	B	A	B	B	B	A	A	A	A	B	A	A	B	A	B	A	A	A	A	A	A	A	B	B	A	A	B	B	B	A	B	B	B	B
A	B	A	A	B	A	B	A	A	B	B	A	B	A	B	B	B	A	A	A	A	B	A	A	B	A	B	A	A	A	A	A	A	A	B	B	A	A	B	B	B	A	B	B	B	B
A	B	A	A	A	A	B	A	A	B	B	A	B	A	B	B	B	A	A	A	A	B	A	A	B	A	B	A	A	A	A	A	A	A	B	B	A	A	B	B	B	A	B	B	B	B
A	B	A	A	A	A	B	A	A	B	A	A	B	A	B	B	B	A	A	A	A	B	A	A	B	A	B	A	A	A	A	A	A	A	B	B	A	A	B	B	B	A	B	B	B	B
A	B	A	A	A	A	B	A	A	B	A	A	B	A	B	B	B	A	A	A	A	B	A	A	B	A	B	A	A	A	A	A	A	A	B	B	A	A	B	B	B	A	B	B	B	B
A	B	A	A	A	A	B	A	A	B	A	A	B	A	B	B	B	A	A	A	A	B	A	A	B	A	B	A	A	A	A	A	A	A	B	B	A	A	B	B	B	A	B	B	B	B
A	B	B	A	A	A	B	A	A	B	A	A	B	A	B	B	B	A	A	A	A	B	A	A	B	A	B	A	A	A	A	A	A	A	B	B	A	A	B	B	B	A	B	B	B	B
A	B	B	A	A	A	B	A	A	B	A	A	B	A	B	B	B	A	A	A	A	B	A	A	B	A	B	A	A	A	A	A	A	A	B	B	A	A	B	B	B	A	B	B	B	B
A	B	B	A	A	A	B	A	A	B	A	A	B	A	B	B	B	A	A	B	B	B	A	A	B	A	B	A	A	A	A	A	A	A	B	B	A	A	A	B	B	A	B	B	B	B

A	B	B	A	A	A	B	A	A	B	A	A	B	A	B	B	B	A	A	B	B	B	A	A	B	A	B	A	A	A	A	A	A	A	B	B	A	A	A	B	B	A	B	B	B	B
A	B	B	A	A	A	B	A	A	B	A	A	A	A	B	B	B	A	A	B	B	B	A	A	B	A	B	A	A	A	A	A	A	A	B	B	A	A	A	B	B	A	B	B	B	B
A	B	B	A	A	A	B	A	A	B	A	A	A	A	B	B	B	A	A	B	B	B	A	A	B	A	B	A	A	A	A	A	A	A	B	B	A	A	A	B	B	A	B	B	B	B
A	B	B	A	A	A	B	A	A	B	A	A	A	A	B	B	B	A	A	B	B	B	B	A	B	A	B	A	A	A	A	A	A	A	B	B	A	A	A	B	B	A	B	B	B	B
A	B	B	A	A	A	B	A	A	B	A	A	A	A	B	B	B	A	A	B	B	B	B	A	B	A	B	A	A	A	A	A	A	A	B	B	A	A	A	B	B	A	B	B	B	B
A	B	B	A	A	A	B	A	A	B	A	A	A	A	B	B	B	A	A	B	B	B	B	A	B	A	B	A	A	A	A	A	A	A	B	B	A	B	A	B	B	A	B	B	B	B
A	B	B	A	A	A	B	A	A	B	A	A	A	A	B	B	B	A	A	B	B	B	B	A	B	A	B	A	A	B	B	A	A	A	B	B	A	B	A	B	B	A	B	B	B	B
A	B	B	A	A	A	B	A	A	B	A	A	A	A	B	B	B	A	A	B	B	B	B	A	A	A	B	A	A	B	B	A	A	A	B	B	A	B	A	B	B	A	B	B	B	B
A	B	B	A	A	A	B	A	A	B	A	A	A	A	B	B	B	A	A	B	B	B	B	A	A	A	B	A	A	B	B	A	A	A	B	B	A	B	A	B	B	A	B	B	B	B
A	B	B	A	A	A	B	A	A	B	A	A	A	A	B	B	B	A	A	B	B	B	B	A	A	A	B	A	A	B	B	A	A	A	B	B	A	B	A	B	B	A	B	B	B	B
A	B	B	A	A	A	B	A	A	B	A	A	A	A	B	B	B	A	A	B	B	B	B	A	A	A	B	A	A	B	B	A	A	A	B	B	A	B	A	B	B	B	B	B	B	B
A	B	B	A	A	A	B	A	A	B	A	A	A	A	B	B	B	A	A	B	B	B	B	A	A	A	B	A	A	B	B	A	A	A	B	B	A	B	A	B	B	B	B	B	B	B
A	B	B	A	A	A	B	A	A	B	A	A	A	A	B	B	B	A	A	B	B	B	B	A	A	A	B	A	A	B	B	A	A	A	B	B	A	B	A	B	B	B	B	B	B	B
B	B	A	B	B	0	B	A	B	B	B	A	A	B	A	A	B	B	B	A	A	B	A	A	B	A	B	A	A	B	B	B	B	A	0	A	A	A	B	B	B	A	B	B	B	B
B	B	A	B	B	0	B	A	B	B	B	A	A	B	A	A	B	B	B	A	A	B	A	A	B	A	B	A	A	B	B	B	B	A	0	A	A	A	B	B	B	A	B	B	B	B
B	B	A	B	B	0	B	A	B	B	B	A	A	B	A	A	B	B	B	A	A	B	A	A	B	A	B	A	A	B	B	B	B	A	0	A	A	A	B	B	B	A	B	B	B	B
B	B	A	B	B	0	B	A	B	B	B	A	A	B	A	A	B	B	B	A	A	B	A	A	B	A	B	A	A	B	B	B	B	A	0	A	A	A	B	B	B	A	B	B	B	B
B	B	A	B	B	0	B	A	B	B	B	A	A	B	A	A	B	B	B	A	A	B	A	A	B	A	B	A	A	B	B	B	B	A	0	A	A	A	B	B	B	A	B	B	B	B
B	B	A	B	B	0	B	A	B	B	B	A	A	B	A	A	B	B	B	B	B	B	A	A	B	A	B	A	A	B	B	B	B	A	0	0	A	A	B	B	B	A	B	B	B	B
B	B	A	B	B	0	B	A	B	B	B	A	A	B	A	A	B	B	B	B	B	B	A	A	B	A	B	A	A	B	B	B	B	A	A	0	A	A	B	B	B	A	B	B	B	B
B	B	A	B	B	B	B	A	B	B	B	A	A	B	A	A	B	B	B	B	B	B	A	A	B	A	B	A	A	B	B	B	B	A	A	B	A	A	B	B	B	A	B	B	B	B
B	B	A	B	B	B	B	A	B	B	B	A	A	B	A	A	B	B	B	B	B	B	A	A	B	A	B	B	A	B	B	B	B	A	A	B	A	A	B	B	B	A	B	B	B	B
B	B	A	B	B	B	B	A	B	B	B	A	A	B	A	A	B	B	B	B	B	B	B	A	B	A	B	B	A	B	B	B	B	A	A	B	A	A	B	B	B	A	B	B	B	B
B	B	A	B	B	B	B	A	B	A	B	A	A	B	A	A	B	B	B	B	B	B	B	A	B	A	B	B	A	A	A	B	B	A	A	B	A	A	B	B	B	A	B	B	B	B
B	B	A	B	B	B	B	A	B	A	B	A	A	B	A	A	B	B	B	B	B	B	B	A	B	A	B	B	A	A	A	B	B	A	A	B	A	A	B	B	B	A	B	B	B	B
B	B	A	B	B	B	B	A	B	A	B	A	A	B	A	A	B	B	B	B	B	B	B	A	B	A	B	B	A	A	A	B	B	A	A	B	A	A	B	B	B	A	B	B	B	B
B	B	A	B	B	B	B	A	B	A	B	A	A	A	A	A	B	B	B	B	B	B	B	A	B	A	B	B	A	A	A	B	B	A	A	B	A	A	B	B	B	A	B	B	B	B
B	B	A	B	B	B	B	A	B	A	B	A	A	A	A	A	B	A	B	B	B	B	B	A	B	A	B	B	A	A	A	B	B	A	A	B	A	A	B	B	B	A	B	B	B	B
B	B	A	B	B	B	B	A	B	A	B	B	A	A	A	A	B	A	B	B	B	B	B	A	B	A	B	B	A	A	A	B	B	A	A	B	A	A	B	B	B	A	B	B	B	B
B	B	A	B	B	B	B	A	B	A	B	B	A	A	A	A	B	A	B	B	B	B	B	A	B	A	B	B	A	A	A	B	B	A	A	B	A	A	B	B	B	A	B	B	B	B
B	B	A	B	B	B	B	A	B	A	B	B	A	A	B	A	B	A	B	B	B	B	B	A	B	A	B	B	A	A	A	B	B	A	A	B	A	A	B	B	B	A	B	B	B	B
B	B	A	B	B	B	B	A	B	A	B	B	A	A	B	A	B	A	B	B	B	B	B	A	B	A	B	B	A	A	A	B	B	A	A	B	A	A	B	B	B	A	B	B	B	A
B	B	A	B	B	B	B	A	B	A	B	B	A	A	B	A	B	A	B	B	B	B	B	A	B	A	B	B	A	A	A	B	B	A	A	B	A	A	B	B	B	A	B	B	B	A
B	B	A	B	B	B	B	A	B	A	B	B	A	A	B	A	B	A	B	B	B	B	B	A	B	A	B	B	A	A	A	B	B	A	A	B	A	A	B	B	B	A	B	B	B	A
B	B	A	B	B	B	B	A	B	A	B	B	A	A	B	A	B	A	B	B	B	A	B	A	B	A	B	B	A	A	A	B	B	A	A	B	A	A	B	B	B	A	B	B	B	A
B	B	A	B	B	B	B	A	B	A	B	B	A	A	B	A	B	A	B	B	B	A	B	A	B	A	B	B	A	A	A	B	B	A	A	B	A	A	B	B	B	A	B	B	B	A
B	B	A	B	B	B	B	A	B	A	B	B	A	A	B	A	B	A	B	B	B	A	B	A	B	A	B	B	A	A	A	B	B	A	A	B	A	A	B	B	B	A	B	B	B	A
B	B	B	B	B	B	B	A	B	A	B	B	A	A	B	A	B	A	B	B	B	A	B	A	B	A	B	B	A	A	A	B	B	A	A	B	A	A	B	B	B	A	B	B	B	A
B	B	B	B	B	B	B	A	B	A	B	B	A	A	B	A	A	A	B	B	B	A	B	A	B	A	B	B	A	A	A	B	B	A	A	B	A	A	B	B	B	A	B	B	B	A
B	B	B	B	B	B	B	A	B	A	B	B	A	A	B	A	A	A	B	B	B	A	B	A	B	A	B	B	A	A	A	B	B	A	A	B	A	A	B	B	B	A	B	B	B	A
B	B	B	B	B	B	B	A	B	A	B	B	A	A	B	A	A	A	B	B	B	A	B	A	B	A	B	B	A	B	B	B	B	A	A	B	A	A	B	B	B	A	B	B	B	A
B	B	B	B	B	B	B	A	B	A	B	B	A	A	B	A	A	A	B	B	B	A	B	A	B	A	B	B	A	B	B	B	B	A	A	B	A	A	B	A	B	A	B	B	B	A
B	B	B	B	B	B	B	A	B	A	B	B	A	A	B	A	A	A	B	B	B	A	B	A	B	A	B	B	A	B	B	B	B	A	A	B	A	A	B	A	B	A	B	B	B	A
B	B	B	B	B	B	B	A	B	A	B	B	A	A	B	A	A	A	B	B	B	A	B	A	B	A	B	B	A	B	B	B	B	A	A	B	B	A	B	A	B	A	B	B	B	A
B	B	B	B	B	B	B	A	B	A	B	B	A	A	B	A	A	A	B	B	B	A	B	A	A	A	B	B	A	B	B	B	B	A	A	B	B	A	B	A	B	A	B	B	B	A
B	B	B	B	B	B	B	A	B	A	B	B	A	A	B	A	A	A	B	B	B	A	B	A	A	A	B	B	A	B	B	B	B	A	A	B	B	A	B	A	B	A	B	B	B	A
B	B	B	B	B	B	B	A	B	A	B	B	A	A	B	A	A	A	B	B	B	A	B	A	A	A	B	B	A	B	B	A	A	A	A	B	B	A	B	A	B	A	B	B	B	A
B	B	B	B	B	B	B	A	B	A	B	B	A	A	B	A	A	A	B	B	B	A	B	A	A	A	B	B	A	B	B	A	A	A	A	B	B	A	B	A	B	A	B	B	B	A
B	B	B	B	B	B	B	A	B	A	B	B	A	A	B	A	A	A	B	B	B	A	B	A	A	A	B	B	A	B	B	A	A	A	A	B	B	A	B	A	B	A	B	A	A	A

B	B	B	B	B	B	B	A	B	A	B	B	A	A	B	A	A	A	B	B	B	A	B	A	A	A	B	B	A	B	B	A	A	A	A	B	B	A	B	A	B	A	B	A	A	A
B	B	B	B	B	B	B	A	B	A	B	B	A	A	B	A	A	A	B	B	B	A	B	A	A	A	B	B	A	B	B	A	A	A	A	B	B	A	B	A	B	A	B	A	A	A
B	B	B	B	B	B	B	A	B	B	B	B	A	A	B	A	A	A	B	B	B	A	B	A	A	A	B	B	A	B	B	A	A	A	A	B	B	A	B	A	B	A	B	A	A	A
B	B	B	B	B	B	B	A	B	B	B	B	A	A	B	A	A	A	B	B	B	A	B	A	A	A	B	B	A	B	B	A	A	A	A	B	B	A	B	A	B	A	B	A	A	A
B	B	B	B	B	B	B	A	B	B	B	B	B	A	B	A	A	A	B	B	B	A	B	A	A	A	B	B	B	B	B	A	A	A	A	B	B	A	B	A	B	A	B	A	A	A
B	B	B	B	B	B	B	A	B	B	B	B	B	A	B	A	A	A	B	B	B	A	B	A	A	A	B	B	B	B	B	B	B	A	A	B	B	A	B	A	B	A	B	A	A	A
B	B	B	B	B	B	B	A	B	B	B	B	B	A	B	A	A	A	B	B	B	A	B	A	A	A	B	B	B	B	B	B	B	A	A	B	B	A	B	A	B	A	B	A	A	A
B	B	B	B	B	A	B	A	B	B	B	B	B	A	B	A	A	A	B	B	B	A	B	A	A	A	B	B	B	B	B	B	B	A	A	B	B	A	B	A	B	A	B	A	A	A
B	B	B	B	B	A	B	A	B	B	B	B	B	A	B	A	A	A	B	B	B	A	B	A	A	A	A	B	B	B	B	B	B	A	A	B	B	A	B	A	B	A	B	A	A	A
B	B	B	B	B	A	B	A	B	B	B	B	B	A	B	A	A	A	B	B	B	A	B	A	A	A	A	B	B	B	B	B	B	A	A	B	B	A	B	A	B	A	B	A	A	A
B	B	B	B	B	A	B	A	B	B	B	B	B	A	B	A	A	A	B	B	B	A	B	A	A	A	A	B	B	B	B	B	B	A	A	B	B	A	B	A	B	A	B	A	A	A
B	B	B	B	B	A	B	A	B	B	B	B	B	A	B	A	A	A	B	B	B	A	B	A	A	A	A	A	B	B	B	B	B	A	A	B	B	A	B	A	B	A	B	A	A	A
B	B	B	B	B	A	B	A	B	B	B	B	B	A	B	A	A	A	B	B	B	A	B	A	A	A	A	A	B	B	B	B	B	A	A	B	B	A	B	A	B	A	B	A	A	A
B	B	B	B	A	A	B	A	B	B	B	B	B	A	B	A	A	A	B	B	B	A	B	A	A	A	A	A	B	B	B	B	B	A	A	B	B	A	B	A	B	A	B	A	A	A
B	B	B	A	A	A	B	A	B	B	B	B	B	A	B	A	A	A	B	B	B	A	B	A	A	A	A	A	B	B	B	B	B	A	A	B	B	A	B	A	B	A	B	A	A	A
B	B	B	A	A	A	B	A	B	B	B	B	B	A	B	A	A	A	B	B	B	A	B	A	A	A	A	A	B	B	B	B	B	A	A	B	B	A	B	A	B	A	B	A	A	A
B	B	B	A	A	A	B	A	B	B	B	B	B	A	B	A	A	A	B	B	B	A	B	A	A	A	A	A	B	B	B	B	B	A	A	B	B	A	B	A	B	A	B	A	A	A
B	B	B	A	A	A	B	A	B	B	B	B	B	A	B	A	A	A	B	B	B	A	B	A	A	A	A	A	B	B	B	B	B	A	A	B	B	A	B	A	B	A	B	A	A	A
B	B	B	A	A	A	B	B	B	B	B	B	B	A	B	A	A	A	B	B	B	A	B	A	A	A	A	A	B	B	B	B	B	B	A	B	B	A	B	A	B	A	B	A	A	A
B	B	B	A	A	A	B	B	B	B	B	B	B	A	B	A	A	A	B	B	B	A	B	A	A	A	A	A	B	B	B	B	B	B	A	B	B	A	B	A	B	A	B	A	A	A
B	A	B	A	A	A	B	B	B	B	B	B	B	A	B	A	A	A	B	B	B	A	B	A	A	A	A	A	B	B	B	B	B	B	A	B	B	A	B	A	B	A	B	A	A	A
B	A	B	A	A	A	B	B	B	B	B	B	B	A	B	A	A	A	B	B	B	A	B	A	A	A	B	A	B	B	B	B	B	B	A	B	B	A	B	A	B	A	B	A	A	A
B	A	B	A	A	A	B	B	B	B	B	B	B	A	B	A	A	A	B	B	B	A	B	A	A	A	B	A	B	B	B	A	A	B	A	B	B	A	B	A	B	A	B	A	A	A
B	A	B	A	A	A	B	B	B	B	B	B	B	A	B	A	A	A	B	B	B	A	B	A	A	A	B	A	B	B	B	A	A	B	A	B	B	A	B	A	B	A	B	A	A	B
B	A	B	A	A	A	B	B	B	B	B	B	B	A	B	A	A	A	B	B	B	A	B	A	A	A	B	A	B	B	B	A	A	B	A	B	B	A	B	A	B	A	B	A	A	B
B	A	B	A	A	A	B	B	B	B	B	B	B	A	B	A	A	A	B	B	B	B	B	A	A	A	B	A	B	B	B	A	A	B	A	B	B	A	B	A	B	A	B	A	A	B
B	A	B	A	A	A	B	B	A	B	B	B	B	A	B	A	A	A	B	B	B	B	B	A	A	A	B	A	B	B	B	A	A	B	A	B	B	A	B	A	B	A	B	A	A	B
B	A	B	A	A	A	B	B	A	B	B	B	B	A	B	A	A	A	B	B	B	B	B	A	A	A	B	A	B	B	B	A	A	B	A	B	B	B	B	A	B	A	B	A	A	B
B	A	B	A	A	A	B	B	A	B	B	B	B	A	B	A	A	A	B	B	B	B	B	A	A	A	B	A	B	B	B	A	A	B	A	B	B	B	B	A	B	A	B	A	A	B
B	A	B	A	A	A	B	B	A	B	B	B	B	A	B	A	A	A	B	B	B	B	B	A	A	A	B	A	B	B	B	A	A	B	A	B	B	B	B	A	B	A	B	A	A	B
B	A	B	A	A	A	B	B	A	B	B	B	B	A	B	A	A	A	B	B	B	B	B	A	A	A	B	A	B	A	A	A	A	B	A	B	B	B	B	A	B	A	B	A	A	B
B	A	B	A	A	A	B	B	A	B	B	B	B	A	B	A	A	A	B	B	B	B	A	A	A	A	B	A	A	A	A	A	A	B	A	B	B	B	B	A	B	A	B	A	A	B
B	A	B	A	A	A	B	B	A	B	B	A	B	A	B	A	A	A	A	B	B	B	A	A	A	A	B	A	A	A	A	A	A	B	A	B	B	B	B	B	B	A	B	B	B	B
B	A	B	A	A	A	A	B	A	B	B	A	B	A	B	A	A	A	A	B	B	B	A	A	A	A	B	A	A	A	A	A	A	B	A	B	B	B	A	B	B	A	B	B	B	B
B	A	B	A	A	A	A	B	A	B	B	A	B	A	B	A	A	A	A	B	B	B	A	A	A	A	B	A	A	A	A	A	A	B	A	B	B	B	A	B	B	A	B	B	B	B
B	A	B	A	A	A	A	B	A	B	B	A	B	A	B	A	A	A	A	B	B	B	A	A	A	A	B	A	A	A	A	A	A	B	A	B	B	B	A	B	B	A	B	B	B	B
B	A	B	A	A	A	A	B	A	B	B	A	B	A	B	A	A	A	A	B	B	B	A	A	A	A	B	A	A	A	A	A	A	B	A	B	B	B	A	B	B	A	B	B	B	B
B	A	B	A	A	A	A	B	A	B	B	A	B	A	B	A	A	A	A	B	B	B	A	A	A	A	B	A	A	A	A	A	A	B	A	B	B	B	A	B	B	A	B	A	A	B
B	A	B	A	A	A	A	B	A	B	B	A	B	A	B	A	A	A	A	B	B	B	A	A	A	A	B	A	A	A	A	A	A	B	A	B	B	B	A	B	B	A	B	A	A	B
B	A	B	A	A	A	A	B	A	B	B	A	B	A	B	A	A	A	A	B	B	B	A	A	A	A	B	A	A	A	A	A	A	B	A	B	B	B	A	B	B	A	B	A	A	B
B	A	B	A	B	A	A	B	A	B	B	A	B	A	B	A	A	A	A	B	B	B	A	A	A	A	B	A	A	A	A	A	A	B	A	B	B	B	A	B	B	A	B	A	A	B
B	A	B	A	B	A	A	B	A	B	B	A	B	A	A	A	A	A	A	B	B	B	A	A	A	A	B	A	A	A	A	A	A	B	A	B	B	B	A	B	B	A	B	A	A	B
B	A	A	B	B	A	B	A	B	A	B	A	A	B	B	B	A	B	B	B	B	B	B	B	A	A	A	B	A	B	B	B	B	B	B	A	B	B	A	A	B	A	B	B	B	B
B	A	A	B	B	A	B	A	B	A	B	A	A	B	B	B	A	B	B	B	B	B	B	B	A	A	A	B	A	B	B	B	B	B	B	A	B	B	A	A	B	A	A	B	B	B
B	A	A	B	B	A	B	A	B	A	B	A	A	B	B	B	A	B	A	B	B	B	B	B	A	A	A	B	A	B	B	B	B	B	B	A	B	B	A	A	B	A	A	B	B	B
B	A	A	B	B	A	B	A	B	A	B	A	A	B	B	B	A	B	A	B	B	B	B	A	A	A	A	B	A	B	B	B	B	B	B	A	B	B	A	A	B	A	A	B	B	B
B	A	A	B	B	A	B	A	B	A	B	A	A	B	B	B	A	B	A	B	B	B	B	A	A	A	A	B	A	B	B	B	B	A	A	B	A	A	A	B	A	A	B	B	B
B	A	A	B	B	A	B	A	B	A	B	A	A	B	B	B	A	B	A	B	B	A	B	A	A	A	A	A	A	A	A	B	B	B	A	A	B	A	A	A	B	A	A	B	B	B
B	A	A	B	B	A	B	A	B	A	B	A	A	B	B	B	A	B	A	B	B	A	B	A	A	A	A	A	A	A	A	B	B	B	A	A	B	A	A	A	B	A	A	B	B	B

B	A	A	B	B	A	B	A	B	A	B	A	A	B	B	B	A	B	A	B	B	A	B	A	A	A	A	A	A	A	A	B	B	B	A	A	B	A	A	A	B	A	A	B	B	B
B	A	A	B	B	A	B	A	B	A	B	A	A	B	B	B	B	A	A	B	B	A	B	A	A	A	A	A	A	A	A	B	B	B	A	A	B	A	A	A	B	A	A	B	B	B
B	A	A	B	B	A	B	A	B	A	B	A	A	B	B	B	B	A	A	B	B	A	B	A	A	A	A	A	A	A	A	B	B	B	A	A	B	A	A	A	B	A	A	B	B	B
B	A	A	B	B	A	B	A	B	A	B	A	A	B	B	A	B	A	A	B	B	A	B	A	A	A	A	A	A	A	A	B	B	B	A	A	B	A	A	A	B	A	A	B	B	B
B	A	A	B	B	A	B	A	B	A	B	A	A	B	B	A	B	A	A	B	B	A	B	A	A	A	A	A	A	A	A	B	B	B	A	A	B	A	A	A	B	A	A	B	B	B
B	A	A	B	A	A	B	A	B	A	B	A	A	B	B	A	B	A	A	B	B	A	B	A	A	A	A	A	A	A	A	B	B	B	A	A	B	A	A	A	B	A	A	B	B	B
B	A	A	B	A	A	B	A	B	A	B	A	A	B	B	A	B	A	A	A	A	A	B	A	A	A	A	A	A	A	A	B	B	B	A	A	A	A	A	B	B	A	A	B	B	B
B	A	A	B	A	A	A	A	B	A	B	A	A	B	A	A	B	A	A	A	A	A	B	A	A	A	A	A	A	A	A	B	B	B	A	A	A	A	A	B	B	A	A	B	B	B
B	A	A	B	A	A	A	A	B	A	B	A	A	B	A	A	B	A	A	A	A	A	B	A	A	A	A	A	A	A	A	B	B	B	A	A	A	A	A	B	B	A	A	B	B	B
B	A	A	B	A	A	A	A	B	A	B	A	A	B	A	A	B	A	A	A	A	A	B	A	A	A	A	A	A	A	A	B	B	B	A	A	A	A	A	B	B	A	A	B	B	B
B	A	A	B	A	A	A	A	B	A	B	A	A	A	A	A	B	A	A	A	A	A	B	A	A	A	A	A	A	A	A	B	B	B	A	A	A	A	A	B	B	A	A	B	B	B
B	A	A	B	A	A	A	A	B	A	B	A	A	A	A	A	B	A	A	A	A	A	B	A	A	A	A	A	A	A	A	B	B	B	A	A	A	A	A	B	B	A	A	B	B	B
B	A	A	B	A	A	A	A	B	A	A	A	A	A	A	A	B	A	A	A	A	A	B	A	A	A	A	A	A	A	A	B	B	B	A	A	A	A	A	B	B	A	A	A	A	B
B	A	A	B	A	A	A	A	B	A	A	A	A	A	A	A	B	A	A	A	A	A	B	A	B	A	A	A	A	A	A	B	B	B	A	A	A	A	A	B	B	A	A	A	A	A
B	A	A	B	A	A	A	A	B	A	A	A	A	A	A	A	B	A	A	A	A	A	B	A	B	A	A	A	A	A	A	B	B	B	A	A	A	A	A	B	B	A	A	A	A	A
B	A	A	B	A	A	A	A	B	A	A	A	A	A	A	A	B	A	A	A	A	A	B	A	B	A	A	A	A	A	A	B	B	B	A	A	A	A	A	B	B	A	A	A	A	A
B	A	A	B	A	A	A	A	B	A	A	A	A	A	A	A	B	A	A	A	A	A	B	A	B	A	A	A	A	A	A	B	B	B	A	A	A	A	A	B	B	A	A	A	A	A
B	A	A	B	A	A	A	A	A	A	A	A	A	A	A	A	B	A	A	A	A	A	B	A	B	A	A	A	A	A	A	A	A	B	A	A	A	A	A	B	B	A	A	A	A	A
B	A	A	B	A	A	A	A	A	A	A	A	A	A	A	A	B	A	A	A	A	A	B	A	B	A	A	A	A	A	A	A	A	B	A	A	A	A	A	B	B	A	A	A	A	A
B	A	A	B	A	A	A	A	A	A	A	A	A	A	A	A	B	A	A	A	A	A	B	A	B	A	A	A	A	A	A	A	A	B	A	A	A	A	A	B	B	A	A	A	A	A
B	A	A	B	A	A	B	A	A	A	A	A	A	A	A	A	B	A	A	A	A	A	B	A	B	A	A	A	A	A	A	A	A	B	A	A	A	A	A	B	B	A	A	A	A	A
B	A	A	B	A	A	B	A	A	A	A	A	A	A	A	A	B	A	A	A	A	A	B	A	B	A	A	A	A	A	A	A	A	B	A	A	A	A	A	B	B	A	A	A	A	A
B	A	A	B	A	A	B	A	A	A	A	A	A	A	A	A	B	A	A	A	A	A	B	A	B	A	A	A	A	A	A	A	A	B	A	A	A	A	A	B	B	A	A	A	A	A
B	A	A	B	A	B	B	A	A	A	A	A	A	A	A	A	B	A	A	A	A	A	B	A	B	A	A	A	A	A	A	A	A	B	A	A	A	A	A	B	B	A	A	A	A	A
B	A	A	B	A	B	B	A	A	A	A	A	A	A	A	A	B	A	A	A	A	A	B	A	B	A	A	A	A	A	A	A	A	B	A	B	A	A	A	B	A	A	A	A	A	A
B	A	A	B	A	B	B	A	A	A	A	A	A	A	A	A	B	A	A	B	B	A	B	A	B	A	A	A	A	A	A	A	A	B	B	B	A	A	A	B	A	A	A	A	A	A
B	A	A	B	A	B	A	A	A	A	A	A	A	A	A	A	B	A	A	B	B	A	B	A	B	A	A	A	A	A	A	A	A	B	B	B	A	A	A	B	A	A	A	A	A	A
B	A	A	B	A	B	A	A	A	A	A	A	A	A	A	A	B	A	A	B	B	A	B	A	B	A	A	A	A	A	A	A	A	B	B	B	A	A	A	B	A	A	A	A	A	A
B	B	A	B	A	B	A	A	A	A	A	A	A	A	A	A	B	A	A	B	B	A	B	A	B	A	A	A	A	A	A	A	A	B	B	B	A	A	A	B	A	A	A	A	A	A
B	B	A	B	A	B	A	A	A	A	A	A	A	A	A	A	B	A	A	B	B	A	B	A	B	A	A	A	A	A	A	A	A	B	B	B	A	A	A	B	A	A	A	A	A	A
B	B	A	B	A	B	A	A	A	A	A	A	A	A	A	A	B	A	A	B	B	A	B	A	B	A	A	A	A	A	A	A	A	B	B	B	A	A	A	B	A	A	A	A	A	A
B	B	A	B	A	B	A	A	A	A	A	A	A	A	A	A	B	A	A	B	B	A	B	A	B	A	A	A	A	A	A	A	A	B	B	B	A	A	A	B	A	A	A	A	A	A
B	B	A	B	A	B	A	A	A	A	A	A	A	A	A	A	B	A	A	B	B	A	B	A	B	A	A	A	A	A	A	A	A	B	B	B	A	A	A	B	A	A	A	A	A	A
B	B	A	B	A	B	A	A	A	A	A	A	A	A	A	A	B	A	A	B	B	A	B	A	B	A	A	A	A	A	A	A	A	B	B	B	A	A	A	B	A	A	A	B	A	A
B	B	A	B	A	B	A	A	A	A	A	A	A	A	A	A	B	A	A	B	B	A	B	A	B	A	A	A	A	A	A	A	A	B	B	B	A	A	A	B	A	A	A	B	B	A
B	B	A	B	A	B	A	A	A	A	A	A	A	A	0	A	B	A	A	B	B	A	B	A	B	A	A	A	A	A	A	A	A	B	B	B	A	A	A	B	A	A	A	B	B	A
B	B	A	B	A	B	A	A	B	B	A	A	A	A	0	A	B	A	A	B	B	A	B	A	B	A	A	A	A	A	A	A	A	B	B	B	A	A	A	B	A	A	A	B	B	A
B	B	A	B	A	B	A	A	B	B	A	A	A	A	0	A	B	A	A	B	B	A	B	A	B	A	A	A	A	A	A	A	A	B	B	B	A	A	A	B	A	A	A	B	B	A
B	B	B	B	A	B	A	A	B	B	A	A	A	A	0	A	B	A	A	B	B	A	B	A	B	A	A	A	A	A	A	A	A	B	B	B	A	A	A	B	A	A	A	B	B	A
B	B	B	B	A	B	A	A	B	B	A	A	A	A	0	A	B	A	A	B	B	A	B	A	B	A	A	A	A	A	A	A	A	B	B	B	A	A	A	B	A	A	A	B	B	A
B	B	B	B	A	B	A	A	B	B	B	A	A	A	0	A	B	B	A	B	B	A	B	A	B	A	A	A	A	A	A	A	A	B	B	B	A	A	A	B	A	A	A	B	B	A
B	B	B	B	A	B	A	A	B	B	B	A	A	A	0	A	B	B	A	B	B	A	B	A	B	A	A	A	A	A	A	A	A	B	B	B	A	A	A	B	A	A	A	B	B	A
B	B	B	B	A	B	A	A	B	A	B	A	A	A	0	A	B	B	A	B	B	A	B	A	B	A	A	A	A	A	A	A	A	B	B	B	A	A	A	B	A	A	A	B	B	A
B	B	B	B	A	B	A	A	B	A	B	A	A	A	0	A	B	B	A	B	B	A	B	A	B	A	A	A	A	A	A	A	A	B	B	B	A	A	A	B	A	A	A	B	B	A
B	B	B	B	A	B	A	A	B	A	B	A	A	A	0	A	B	B	A	B	B	A	B	A	B	A	A	A	A	A	A	A	A	B	B	B	A	0	A	B	A	A	A	A	A	A
B	B	B	B	A	B	A	A	B	A	B	A	A	A	0	A	B	B	A	B	B	A	B	A	A	A	A	A	A	A	A	A	A	B	B	B	A	0	A	B	A	A	A	A	A	A
B	B	B	B	A	B	A	A	B	A	B	A	A	A	0	A	B	B	A	B	B	A	B	A	A	A	A	A	A	A	A	A	A	B	B	B	A	0	A	B	A	A	A	A	A	A
B	B	B	B	A	B	A	A	B	A	B	A	A	A	0	A	B	B	A	B	B	A	B	A	A	A	A	A	A	A	A	A	A	B	B	B	A	0	A	B	A	A	A	A	A	A
B	B	B	B	A	A	A	A	B	A	B	A	A	A	0	A	B	B	A	B	B	A	B	A	A	A	A	A	A	A	A	A	A	B	B	B	A	0	A	B	A	A	A	A	A	A

B	B	B	B	A	A	A	A	B	A	B	A	A	A	0	B	B	B	A	B	B	A	B	A	A	A	A	A	A	A	A	A	A	B	B	B	A	0	A	B	A	A	A	A	A	A
B	B	B	B	A	A	A	A	B	A	B	A	A	A	0	B	B	B	A	B	B	A	B	A	A	A	A	A	A	A	A	B	B	B	B	B	A	0	A	B	A	A	A	A	A	A
B	B	B	B	A	A	A	A	B	A	B	A	A	A	0	B	B	B	A	B	B	A	B	A	A	A	A	A	A	A	A	B	B	B	B	B	A	0	A	B	A	A	A	B	B	A
B	A	B	B	A	A	A	A	B	A	B	A	A	A	0	B	B	B	A	B	B	A	B	A	A	A	A	A	A	A	A	B	B	B	B	B	A	0	A	B	A	A	A	B	B	A
B	A	B	B	A	A	A	A	B	A	B	A	A	A	0	B	B	B	A	B	B	A	A	A	A	A	A	A	A	A	A	B	B	B	B	B	A	0	B	B	A	A	A	B	B	A
B	A	B	B	A	A	A	A	B	A	B	A	A	A	0	B	B	B	A	B	B	A	A	A	A	A	A	A	A	A	A	B	B	B	B	B	A	0	B	B	A	A	A	B	B	A
B	A	B	B	A	A	A	A	B	A	B	A	A	A	0	B	B	B	A	B	B	A	A	B	A	A	A	A	A	A	A	B	B	B	B	B	A	0	B	A	A	A	A	B	B	A
B	A	B	B	A	A	A	A	B	A	B	A	A	A	0	B	B	B	A	B	B	A	A	B	A	A	A	A	A	A	A	B	B	B	B	B	A	0	B	A	A	A	A	B	B	A
B	A	B	B	A	A	A	A	B	A	B	A	A	A	0	B	B	B	A	B	B	A	A	B	A	A	A	A	A	A	A	B	B	B	B	B	A	0	B	A	A	A	A	B	B	A
A	A	B	B	B	A	0	0	B	B	A	A	0	0	A	B	B	A	A	B	B	B	B	A	A	B	A	A	B	A	A	0	0	B	B	A	B	A	B	A	A	A	A	B	B	A
A	A	B	B	B	A	0	0	B	B	A	A	0	0	A	B	B	A	A	B	B	B	B	A	A	B	A	A	B	A	A	0	0	B	B	A	B	A	B	B	A	A	A	B	B	A
A	A	B	B	B	A	A	0	B	B	A	A	0	0	A	B	B	A	A	B	B	B	B	A	A	B	A	A	B	A	A	0	0	B	B	A	B	A	B	B	A	A	A	B	B	A
A	A	B	B	B	A	A	0	B	B	A	A	0	0	A	B	B	A	A	B	B	B	B	A	A	B	A	A	B	A	A	0	0	B	B	A	B	A	B	B	A	A	A	B	B	A
A	A	B	B	B	A	A	A	B	B	A	A	0	0	A	A	B	A	A	B	B	B	B	A	A	B	A	A	B	A	A	0	0	B	B	A	B	A	B	B	A	A	A	B	B	A
A	A	B	B	B	A	A	A	B	B	A	A	0	0	A	A	B	A	A	B	B	B	B	A	A	B	A	A	B	A	A	0	0	B	B	A	B	A	B	B	A	A	A	B	B	A
A	A	B	B	B	A	A	A	B	B	A	A	0	0	A	A	B	A	A	B	B	B	B	A	A	B	A	A	B	A	A	0	0	B	B	A	B	A	B	B	A	A	A	A	A	A
A	A	B	B	B	A	A	A	B	B	A	A	0	0	A	A	B	A	A	B	B	B	B	A	A	B	B	A	B	A	A	0	0	B	B	A	B	A	B	B	A	A	A	A	A	A
A	A	B	B	B	A	A	A	B	B	A	A	0	0	A	A	B	A	A	B	B	B	B	A	A	B	B	A	B	A	A	0	0	B	B	A	B	A	B	B	A	A	A	A	A	A
A	A	B	B	B	A	A	A	B	B	A	A	0	0	A	A	B	A	A	B	B	B	B	A	A	B	B	A	B	A	A	0	0	B	B	A	B	A	B	B	A	A	A	A	A	A
A	A	B	B	B	A	A	A	B	B	A	A	0	0	A	A	B	A	A	B	B	B	B	A	A	B	B	A	B	A	A	0	0	B	B	A	B	A	B	B	A	A	A	A	A	A
A	A	B	B	B	A	A	A	B	A	A	A	A	0	A	A	0	A	A	B	B	B	B	A	A	B	B	A	B	A	A	0	0	0	0	A	B	A	B	B	A	A	A	A	A	A
A	A	B	B	B	A	A	A	B	A	A	A	A	0	A	A	A	A	A	B	B	B	B	A	A	B	B	A	B	A	A	0	0	A	A	A	B	A	B	B	A	B	A	A	A	A
A	A	B	B	B	A	A	A	A	A	A	A	A	0	A	A	A	A	A	B	B	B	B	A	A	B	B	A	B	A	A	0	0	A	A	A	B	A	B	B	A	B	A	A	A	A
A	A	B	B	B	A	A	A	A	A	A	A	A	0	A	A	A	A	A	B	B	B	A	A	A	B	B	A	B	A	A	0	0	A	A	A	B	A	B	B	A	B	A	A	A	A
A	A	B	B	B	A	A	A	A	A	A	A	A	0	A	A	A	A	A	B	B	B	A	A	A	B	B	A	B	A	A	0	0	A	A	A	B	A	B	B	A	B	A	A	A	A
A	A	B	B	B	A	A	A	A	A	A	A	A	0	A	A	A	A	A	B	B	B	A	A	A	B	B	A	B	A	A	0	0	A	A	A	B	A	B	B	A	B	A	A	A	A
A	A	B	B	B	A	A	A	A	A	A	A	A	A	A	A	A	A	A	B	B	B	A	A	A	B	B	A	B	A	A	0	0	A	A	A	B	A	B	B	A	B	A	A	A	A
A	A	B	B	B	A	A	A	A	A	A	A	A	A	A	A	A	A	A	B	B	B	A	A	A	B	B	A	B	A	A	0	0	A	A	A	B	A	B	B	A	B	A	A	A	A
A	A	B	B	B	A	A	A	A	A	A	A	A	A	A	A	A	A	A	A	A	B	A	A	A	B	B	A	B	A	A	0	0	A	A	A	B	A	B	B	A	B	A	A	A	A
A	A	B	B	B	A	A	A	A	A	A	A	A	A	A	A	A	A	A	A	A	B	A	A	A	B	B	A	B	A	A	0	0	A	A	A	B	A	B	B	A	B	A	A	A	A
A	A	B	B	B	A	A	A	A	A	A	A	A	A	A	A	A	A	A	A	A	B	A	A	A	B	B	A	B	A	A	0	0	A	A	A	A	A	B	B	A	B	A	A	A	A
A	A	B	B	B	A	B	A	A	A	A	A	A	A	A	A	A	A	A	A	A	B	A	A	A	B	B	A	B	A	A	0	0	A	A	A	A	A	B	B	A	B	A	A	A	A
A	A	B	B	B	A	B	A	A	A	A	A	A	A	A	A	A	A	A	A	A	B	A	A	A	B	B	A	B	A	A	0	0	A	A	A	A	A	B	B	A	B	A	A	A	A
A	A	B	B	B	A	B	A	A	A	A	A	A	A	A	A	A	A	A	A	A	B	A	A	A	B	B	A	B	A	A	0	0	A	A	A	A	A	B	B	A	B	A	A	A	A
A	A	B	B	B	A	B	A	A	A	A	A	A	A	A	A	A	A	A	A	A	B	A	A	A	B	B	A	B	A	A	0	0	A	A	A	A	A	B	B	A	B	A	A	A	A
A	A	B	B	B	A	B	A	A	A	A	A	A	A	A	A	A	A	A	A	A	A	A	A	A	B	B	A	B	A	A	0	0	A	A	A	A	A	B	B	A	B	A	A	A	A
A	A	B	B	B	A	B	A	A	A	A	A	A	A	A	A	A	A	A	A	A	A	A	A	A	B	B	A	B	A	A	0	0	A	A	A	A	A	B	B	A	B	A	A	A	A
A	A	B	B	B	A	B	A	A	A	A	A	A	A	A	A	A	A	A	A	A	A	A	A	A	B	B	A	B	A	A	0	0	A	A	A	B	A	B	B	A	B	A	A	A	A
A	A	B	B	B	A	B	A	A	A	A	A	A	A	A	A	A	A	A	A	A	A	A	A	A	B	B	A	B	A	A	0	0	A	A	A	B	A	B	B	A	B	A	A	A	A
A	A	B	B	B	B	B	A	A	A	A	A	A	A	A	A	A	A	A	A	A	A	A	A	A	B	B	A	B	A	A	0	0	A	A	A	B	A	B	B	A	B	A	A	A	A
A	A	B	B	A	B	B	A	A	A	A	A	A	A	A	A	A	A	A	A	A	A	A	A	A	B	B	A	B	A	A	0	0	A	A	A	B	A	B	B	A	B	A	A	A	A
A	A	A	B	A	B	B	A	A	A	A	A	A	A	A	A	A	A	A	A	A	A	A	A	A	B	B	A	B	A	A	0	0	A	A	A	B	A	B	B	A	B	A	A	A	A
A	A	A	A	A	B	B	A	A	A	A	A	A	A	A	A	A	A	A	A	A	A	A	A	A	B	B	A	B	A	A	0	0	A	A	A	B	A	B	B	A	B	A	A	A	A
A	A	A	A	A	B	B	A	A	A	A	A	A	A	A	A	A	A	A	A	A	A	A	A	A	B	B	A	B	A	A	0	0	A	A	A	B	A	B	B	A	B	A	A	A	A
A	A	A	A	A	B	B	A	A	A	A	A	A	A	A	A	A	A	A	A	A	A	A	A	A	B	B	A	B	A	A	0	0	A	A	A	B	A	B	B	A	A	A	A	A	A
A	A	A	A	A	B	B	A	A	A	A	A	A	A	A	A	A	A	A	B	B	A	A	A	A	B	B	A	B	A	A	0	0	A	A	A	B	A	B	B	A	A	A	A	A	A
B	A	A	A	A	B	B	A	A	A	A	A	A	A	A	A	A	A	A	B	B	A	A	A	A	B	B	A	B	A	A	0	0	A	A	A	B	A	B	B	A	A	A	A	A	A
B	A	A	A	A	B	B	A	A	A	A	A	A	A	A	A	A	A	A	B	B	A	A	A	A	B	A	A	B	A	A	0	0	A	A	A	B	A	B	B	A	A	A	A	A	A
B	A	A	A	A	B	B	A	A	A	A	A	A	A	A	A	A	A	A	B	B	A	A	A	B	B	A	A	B	A	A	0	0	A	A	A	B	A	B	B	A	A	A	A	A	A

B	A	A	A	A	B	B	B	B	B	A	A	B	A	B	B	B	A	A	A	A	B	B	A	B	B	B	A	B	A	A	B	B	A	B	B	A	A	A	A	A	B	B	A	A	A
B	A	A	A	A	B	B	B	B	B	A	A	B	A	B	B	B	A	A	A	A	B	B	A	B	B	B	A	B	A	A	B	B	A	B	B	A	A	A	A	A	B	B	A	A	A
B	A	A	A	A	B	B	B	B	B	A	A	B	A	B	B	B	A	A	B	B	B	B	A	B	B	B	A	B	A	A	B	B	A	B	B	A	A	A	A	A	B	B	A	A	A
B	A	A	A	A	B	B	A	B	B	A	A	B	A	B	B	B	A	A	B	B	B	B	A	B	B	B	A	B	A	A	B	B	A	B	B	A	A	A	A	A	B	B	A	A	A
B	A	A	A	A	B	B	A	B	B	A	A	B	A	B	B	B	A	A	B	B	B	B	A	B	B	B	A	B	A	A	B	B	A	B	B	A	A	A	A	A	B	B	A	A	A
B	A	A	A	A	B	B	A	B	B	A	A	B	A	B	B	B	A	A	B	B	B	B	A	B	B	B	A	B	A	A	B	B	A	B	B	A	A	A	A	A	B	B	A	A	A
B	A	A	B	A	B	B	A	B	B	A	A	B	A	B	B	B	A	A	B	B	B	B	A	B	B	B	A	B	A	A	B	B	A	B	B	A	A	A	A	A	B	B	A	A	A
B	A	A	B	A	B	B	A	B	B	A	A	B	A	B	B	B	A	A	B	B	B	B	A	B	B	B	A	B	A	A	B	B	A	B	B	A	A	A	A	A	B	B	A	A	A
B	A	A	B	A	B	B	A	B	B	A	A	B	A	B	B	B	A	A	B	B	B	B	A	B	B	B	A	B	A	A	B	B	A	B	B	A	A	A	A	A	A	B	A	A	A
B	A	A	B	A	B	B	A	B	B	A	A	B	A	B	B	B	A	A	B	B	B	B	A	B	B	B	A	B	A	A	B	B	A	B	B	A	A	A	A	B	A	B	A	A	A
B	B	A	B	A	B	B	A	B	B	A	A	B	A	B	B	B	A	A	B	B	B	B	A	B	B	B	A	B	A	A	B	B	A	B	B	A	A	A	A	B	A	B	A	A	A
B	B	A	B	A	B	B	A	B	B	A	A	B	A	B	B	B	A	A	B	B	B	B	A	B	B	A	A	B	A	A	B	B	A	B	B	A	A	A	A	B	A	B	A	A	A
B	B	A	B	A	B	B	A	B	B	A	A	B	A	B	B	B	A	A	B	B	B	B	A	B	B	A	A	B	A	A	B	B	A	B	B	A	B	A	A	B	A	B	A	A	A
B	B	A	B	A	B	B	A	B	B	A	B	B	A	B	B	B	A	A	B	B	B	B	A	B	B	A	A	B	A	A	B	B	A	B	B	A	B	A	A	B	A	B	A	A	A
B	B	A	B	A	B	B	A	B	B	A	B	B	A	B	B	B	A	A	B	B	B	B	A	B	B	A	A	B	A	A	B	B	A	B	B	B	B	A	A	B	A	B	A	A	A
B	B	A	B	A	B	B	A	B	B	A	B	B	A	B	B	B	A	A	B	B	B	B	A	B	B	A	A	B	A	A	B	B	A	B	B	B	B	A	A	B	A	B	A	A	A
B	B	A	B	A	B	B	A	B	B	A	B	B	A	B	B	B	A	A	B	B	A	B	A	B	B	A	A	B	A	A	B	B	A	B	B	B	B	A	A	B	A	B	A	A	A
B	B	A	B	A	B	B	A	B	B	A	B	B	A	B	B	B	A	A	B	B	A	B	A	B	B	A	A	B	A	A	B	B	A	B	B	B	B	A	A	B	A	B	A	A	A
B	B	A	B	A	B	B	A	B	B	A	B	B	A	B	B	B	A	A	B	B	A	B	A	B	B	A	B	B	A	A	B	B	A	B	B	B	B	A	A	B	A	B	A	A	A
B	B	A	B	A	B	B	A	B	B	A	B	B	A	B	B	B	A	A	B	B	A	B	A	B	B	A	B	B	A	A	B	B	A	B	B	B	B	A	A	B	A	A	A	A	A
B	B	A	B	A	B	B	A	B	B	A	B	B	A	B	B	B	A	A	B	B	A	B	A	B	B	A	B	B	A	A	B	B	A	B	B	B	B	A	A	B	A	A	A	A	A
B	B	A	B	B	B	B	A	B	B	A	B	B	A	B	B	B	A	A	B	B	A	B	A	B	B	A	B	B	A	A	B	B	A	A	B	B	B	A	A	B	A	A	A	A	A
B	B	A	B	B	B	B	A	B	B	A	B	B	A	B	B	B	A	A	B	B	A	B	A	B	B	A	B	B	A	A	B	B	A	A	B	B	B	A	A	B	A	A	A	A	A
B	B	A	B	B	B	B	A	B	B	A	B	B	A	B	B	B	A	A	B	B	A	B	A	B	B	A	B	B	A	A	B	B	A	A	B	B	B	A	A	B	A	A	A	A	A
B	B	A	B	B	B	B	A	B	B	A	B	B	A	B	B	B	A	A	B	B	A	B	A	B	B	A	B	B	A	A	B	B	A	A	B	B	B	A	A	B	A	A	A	A	A
B	B	A	B	B	B	B	A	B	B	A	B	B	A	B	B	B	A	A	B	B	A	B	A	B	B	A	B	B	B	B	B	B	A	A	B	B	B	A	A	B	A	A	A	A	A
B	B	A	B	B	B	B	A	B	B	A	B	B	B	B	B	B	A	A	B	B	A	B	A	B	B	A	B	B	B	B	B	B	A	A	B	B	B	A	A	B	A	A	A	A	A
B	B	A	B	B	B	B	A	A	B	A	B	B	B	B	B	B	A	A	B	B	A	B	A	B	B	A	B	B	B	B	B	B	A	A	B	B	B	A	A	B	A	A	A	A	A
B	B	A	B	B	B	B	A	A	B	A	B	B	B	B	B	B	A	A	B	B	A	B	A	B	B	A	B	B	B	B	B	B	A	A	B	B	B	B	A	B	A	A	A	A	A
B	B	A	B	B	B	B	A	A	B	A	B	B	B	B	B	B	A	A	B	B	A	B	A	B	B	A	B	B	B	B	B	B	A	A	B	B	B	B	A	B	A	A	A	A	A
B	B	A	B	B	B	B	A	A	B	A	B	B	B	B	B	B	A	A	B	B	A	B	A	B	B	A	B	B	B	B	B	B	A	A	B	B	B	B	A	B	A	A	A	A	A
B	A	A	B	B	B	B	A	A	B	A	B	B	B	B	B	B	A	A	B	B	A	B	A	B	B	A	B	B	B	B	B	B	A	A	B	B	B	B	A	B	A	A	A	A	A
B	A	A	B	B	B	B	A	A	B	A	B	B	B	B	B	B	A	A	B	B	A	B	A	B	B	A	B	B	B	B	B	B	A	A	B	B	B	B	A	B	A	A	A	A	A
B	A	A	B	B	B	B	B	A	B	A	B	B	B	B	B	B	A	A	B	B	A	B	A	B	B	A	B	B	B	B	B	B	A	A	B	B	B	B	A	B	A	A	A	A	A
B	A	A	B	B	B	B	B	A	B	A	B	B	B	B	B	B	A	A	B	B	A	B	A	B	B	A	B	B	B	B	B	B	A	A	B	B	B	B	A	B	A	A	A	A	A
B	A	A	B	B	B	B	B	A	B	A	B	B	B	B	B	B	A	A	B	B	A	B	A	B	B	A	B	B	B	B	B	B	A	A	B	B	B	B	A	B	A	A	A	A	A
B	A	A	B	B	B	B	B	A	B	B	B	B	B	B	B	B	A	A	B	B	A	B	A	B	B	A	B	B	B	B	B	B	A	A	B	B	B	B	A	B	A	A	A	A	A
B	A	A	B	B	B	B	B	A	B	B	B	B	B	B	B	B	A	A	B	B	A	B	A	B	B	A	B	B	B	B	B	B	A	A	B	B	B	B	A	B	A	A	A	A	A
B	A	A	B	B	B	B	B	A	B	B	B	B	B	B	B	B	A	A	B	B	A	B	A	B	B	A	B	B	B	B	B	B	A	A	B	B	B	B	A	B	A	A	A	A	A
B	A	A	B	B	B	B	B	A	B	B	B	B	B	B	B	B	A	A	B	B	A	B	A	B	B	A	B	B	B	B	B	B	A	A	B	B	B	B	A	B	A	A	A	A	A
B	A	A	B	B	B	B	B	A	B	B	B	B	B	B	B	B	A	A	B	B	A	B	A	B	B	A	B	B	B	B	B	B	A	A	B	B	B	B	A	B	A	A	A	A	A
B	A	A	B	B	B	B	B	A	B	B	B	B	B	B	B	B	A	A	B	B	A	B	A	B	B	A	B	B	B	B	B	B	A	A	B	B	B	B	B	B	A	A	A	A	A
B	A	A	B	B	B	B	B	A	B	B	B	B	B	B	B	B	A	A	B	B	A	B	A	B	B	A	B	B	B	B	B	B	A	A	B	B	B	B	B	B	A	A	A	A	A
B	A	A	B	B	B	B	B	A	B	B	B	B	B	B	B	B	A	A	B	B	A	B	A	B	B	A	B	B	B	B	B	B	A	A	A	B	B	B	B	B	A	A	A	A	A
B	A	A	B	B	B	A	B	A	B	B	B	B	B	B	B	B	A	A	B	B	A	B	A	B	B	A	B	B	B	B	B	B	A	A	A	B	B	B	B	B	A	A	A	A	A
B	A	A	B	B	B	A	B	A	B	B	B	A	B	B	B	B	A	A	B	B	A	B	A	B	B	A	B	B	B	B	B	B	A	A	A	B	B	B	B	B	A	A	A	A	A
B	A	A	A	B	B	A	B	A	B	B	B	A	B	B	B	B	A	A	B	B	B	B	A	B	B	A	B	B	B	B	B	B	A	A	A	B	B	B	B	B	A	A	A	A	A
B	A	A	A	B	B	A	B	A	B	B	B	A	B	B	B	B	A	A	B	B	B	B	A	B	B	A	B	B	B	B	B	B	A	A	A	B	B	B	B	A	A	A	A	A	A
B	A	A	A	B	B	A	B	A	B	B	B	A	B	B	B	B	A	A	B	B	B	B	A	B	A	A	B	B	B	B	B	B	A	A	A	B	B	B	B	A	A	A	A	A	A

B	A	A	A	B	B	A	B	A	B	B	B	A	B	B	A	B	A	A	B	B	B	B	A	B	A	A	B	B	B	B	B	B	A	A	A	B	B	B	B	A	A	A	A	A	A
B	A	A	A	B	B	A	B	A	B	B	B	A	B	B	A	B	A	A	B	B	B	B	A	B	A	A	B	B	B	B	B	B	A	A	A	B	B	B	B	A	A	A	A	A	A
B	A	A	A	B	B	A	B	A	B	B	B	A	B	B	A	B	A	A	B	B	B	A	A	B	A	A	B	B	B	B	B	B	A	A	A	B	B	B	B	A	A	A	A	A	A
B	A	A	A	B	B	A	B	A	B	B	B	A	B	B	A	B	A	A	B	B	B	A	A	B	A	A	B	B	B	B	B	B	A	A	A	B	B	B	B	A	A	A	A	A	A
B	A	A	A	B	B	A	B	A	B	B	B	A	B	B	A	B	A	A	B	B	B	A	A	B	A	A	B	B	B	B	B	B	A	A	A	B	B	B	B	A	A	B	B	B	A
B	A	A	A	A	B	A	B	A	B	B	B	A	B	B	A	B	A	A	B	B	B	A	A	B	A	A	B	B	B	B	B	B	A	A	A	B	B	B	B	A	A	B	B	B	A
B	A	A	A	A	B	A	B	A	B	B	B	A	B	B	A	B	A	A	B	B	B	A	A	B	A	A	B	B	B	B	B	B	A	A	A	B	B	B	B	B	A	B	B	B	A
B	A	A	A	A	B	A	B	A	B	B	B	A	B	B	A	B	A	A	B	B	B	A	A	B	A	A	B	B	B	B	B	B	A	A	A	B	B	B	B	B	A	B	B	B	A
B	A	A	A	A	B	A	B	A	B	B	B	A	B	B	A	B	A	A	B	B	B	A	A	B	A	A	B	B	B	B	B	B	A	A	A	B	B	B	B	B	A	B	B	B	A
B	A	A	A	A	B	A	B	A	B	B	B	A	B	B	A	B	A	A	B	B	B	A	A	B	A	A	B	B	B	B	B	B	A	A	A	B	B	B	B	B	A	B	B	B	A
B	A	A	A	A	B	A	B	A	B	B	B	A	B	B	A	B	A	A	B	B	B	A	A	B	A	A	B	B	B	B	B	B	A	A	A	B	B	B	B	B	A	B	B	B	A
B	A	A	A	A	B	A	B	A	B	B	B	A	B	B	A	B	A	A	B	B	B	A	A	B	A	A	B	B	B	B	B	B	A	A	A	B	B	B	B	B	A	B	B	B	A
B	A	A	A	A	B	A	B	A	B	B	B	A	B	B	A	B	B	A	B	B	B	A	A	B	A	A	B	B	B	B	B	B	A	A	A	B	B	B	B	B	A	B	B	B	A
B	A	A	A	A	B	A	B	A	B	B	B	A	B	B	A	B	B	A	B	B	B	A	A	B	A	A	B	B	B	B	B	B	A	A	A	A	B	B	B	B	A	B	B	B	A
B	A	A	A	A	B	A	B	A	B	B	B	A	A	B	A	B	B	A	B	B	B	A	A	B	A	A	B	B	B	B	B	B	A	A	A	A	B	B	B	B	A	B	B	B	A
B	B	A	A	A	B	A	B	A	B	B	B	A	A	B	A	B	B	A	B	B	B	A	A	B	A	A	B	B	B	B	B	B	A	A	A	A	B	B	B	B	A	B	B	B	A
B	B	A	A	A	B	A	B	A	B	B	B	A	A	B	A	B	B	A	B	B	B	A	A	B	A	A	B	B	B	B	A	A	A	A	A	A	B	B	B	B	A	B	B	B	A
B	B	A	A	A	B	A	B	A	B	B	B	A	A	B	A	B	B	A	B	B	B	A	A	B	A	A	B	B	B	B	A	A	A	A	A	A	B	B	B	B	A	B	B	B	A
B	B	A	A	A	B	A	B	A	B	B	B	A	A	B	A	B	B	A	B	B	B	A	A	B	A	A	B	B	B	B	A	A	A	A	A	A	B	B	B	B	A	B	B	B	A
B	B	A	A	A	B	A	B	A	B	A	B	A	A	B	A	B	B	A	B	B	B	A	A	B	A	A	B	B	B	B	A	A	A	A	A	A	B	B	B	B	A	B	B	B	A
B	B	A	A	A	B	A	B	A	B	A	B	A	A	B	A	B	B	A	B	B	B	A	A	B	A	A	B	B	B	B	A	A	A	A	A	A	B	B	B	B	A	B	B	B	A
B	B	A	A	A	B	A	B	A	A	A	B	A	A	B	A	B	B	A	B	B	B	A	A	B	A	A	B	B	B	B	A	A	A	B	A	A	B	B	B	B	A	B	B	B	A
B	B	A	A	A	B	A	B	A	A	A	B	A	A	B	A	B	B	A	B	B	B	A	A	B	A	B	B	B	B	B	A	A	A	B	A	A	B	B	B	B	A	B	B	B	A
B	B	A	A	A	B	A	B	A	A	A	B	A	A	B	A	B	B	A	B	B	B	A	A	B	A	B	B	B	B	B	A	A	A	B	A	A	B	B	B	B	A	B	B	B	A
B	B	A	A	A	B	A	0	A	A	A	B	A	A	B	A	B	B	A	B	B	B	A	A	B	A	B	B	B	B	B	A	A	A	B	A	A	B	B	B	B	A	B	B	B	A
B	B	A	A	A	B	A	A	A	A	A	B	A	A	B	A	B	B	A	B	B	B	A	A	B	A	B	B	B	B	B	A	A	A	B	A	A	B	B	B	B	A	B	B	B	A
B	B	A	A	A	B	A	A	A	A	A	B	A	A	B	A	B	B	A	B	B	B	A	A	B	A	B	B	B	B	B	A	A	A	B	A	A	B	B	B	B	A	B	B	B	A
B	B	A	A	A	B	A	A	A	A	A	B	A	A	B	A	B	B	A	B	B	B	A	A	B	A	B	B	B	B	B	A	A	A	B	A	A	B	B	B	B	A	B	B	B	A
B	B	A	A	A	B	A	A	A	A	A	B	A	A	B	A	B	B	A	B	B	B	A	A	B	A	B	B	B	B	B	A	A	A	B	A	A	B	B	B	B	A	B	B	B	A
B	B	A	A	A	B	A	A	A	A	A	B	A	A	B	A	B	B	A	B	B	B	A	A	B	A	B	B	B	B	B	A	A	A	B	A	A	B	B	B	B	A	B	B	B	A
B	B	A	A	A	B	A	A	A	A	A	B	A	A	B	A	B	B	A	B	B	B	A	A	B	A	B	B	B	B	B	A	A	A	B	A	A	B	B	B	B	A	B	B	B	A
B	B	A	A	A	B	A	A	A	A	A	A	A	A	B	A	B	B	A	B	B	B	A	A	B	A	B	A	B	B	B	A	A	A	B	A	A	B	B	B	B	A	B	B	B	A
B	B	A	A	A	B	A	A	A	A	A	A	A	A	B	A	B	B	A	B	B	B	A	A	B	A	B	A	B	B	B	A	A	A	B	A	A	B	B	B	B	A	B	B	B	A
B	B	B	B	A	A	A	A	A	A	A	A	B	B	B	A	A	A	A	A	A	A	B	B	B	B	A	A	B	A	A	A	A	B	A	B	A	A	A	A	A	A	A	A	A	A
B	B	B	B	A	A	A	A	A	A	A	A	B	B	B	A	A	A	A	A	A	A	B	B	B	B	A	A	B	A	A	A	A	B	A	B	A	A	A	A	A	A	B	A	A	A
B	B	B	B	A	A	A	A	A	A	A	A	B	B	B	A	A	A	A	A	A	A	B	B	B	B	A	A	B	A	A	A	A	B	A	B	A	A	A	A	A	A	B	A	A	A
B	B	B	B	A	A	A	A	A	A	A	A	B	B	B	A	A	A	A	A	A	A	B	B	B	B	A	A	B	A	A	A	A	B	A	B	A	A	A	A	A	A	A	A	A	A
B	B	B	B	A	A	A	A	A	A	A	A	B	B	B	A	A	A	A	A	A	A	B	B	B	B	A	A	B	A	A	A	A	B	A	B	A	A	A	A	A	A	B	A	A	A
B	B	B	B	A	A	A	A	A	A	A	A	B	B	B	A	A	A	A	A	A	A	B	B	B	B	A	A	B	A	A	A	A	B	A	A	A	A	A	A	A	A	B	A	A	A
B	B	B	B	A	A	A	A	A	A	A	A	B	B	B	A	A	A	A	A	A	A	B	B	B	B	A	A	B	A	A	A	A	B	A	A	A	A	A	A	A	A	B	A	A	B
B	B	B	B	A	A	A	A	A	A	A	A	B	B	B	A	A	A	A	A	A	A	B	B	B	B	A	A	B	A	A	A	A	B	A	A	A	A	A	A	A	A	B	A	A	B
B	B	B	B	A	A	A	A	A	A	A	A	B	B	B	A	A	A	A	A	A	A	B	B	B	B	A	A	B	A	A	A	A	B	A	A	A	A	A	A	A	A	B	A	A	B
B	B	B	B	A	A	A	A	A	A	A	A	B	B	B	A	A	A	A	A	A	A	B	B	B	B	A	A	B	A	A	A	A	B	A	A	A	A	A	A	A	A	B	A	A	B
B	B	B	B	A	A	A	A	A	A	A	A	B	B	B	A	A	A	A	A	A	A	B	B	B	B	A	A	B	A	A	A	A	B	A	A	A	A	A	A	A	A	B	A	A	B
B	B	B	B	B	A	A	A	A	A	A	A	B	B	B	A	A	A	A	A	A	A	B	B	B	B	A	A	B	A	A	A	A	A	A	A	A	A	A	A	A	A	B	A	A	B
B	B	B	B	B	A	A	A	A	A	A	A	B	B	B	A	A	A	A	A	A	A	B	B	B	B	A	A	B	A	A	A	A	A	A	A	A	A	A	A	A	A	B	A	A	B
B	B	B	B	B	A	A	A	A	A	A	A	B	B	B	A	A	A	A	A	A	A	B	B	B	B	A	A	B	A	A	A	A	A	A	A	A	A	A	A	A	A	B	A	A	B
B	B	B	B	B	A	A	A	A	A	A	A	B	B	B	A	A	A	A	B	B	A	B	B	B	B	A	A	B	A	A	A	A	A	A	A	A	A	A	A	A	A	B	A	A	B
B	B	B	B	B	A	A	A	A	A	A	A	B	B	B	A	B	A	A	B	B	A	B	B	B	B	A	A	B	A	A	A	A	A	A	A	A	A	A	A	A	A	B	A	A	B

B	B	B	B	B	A	A	A	A	A	A	A	B	B	B	A	B	A	A	B	B	A	B	B	B	B	A	A	B	A	A	A	A	A	A	A	A	A	A	A	A	A	B	A	A	B
B	B	B	B	B	A	A	A	A	A	A	A	B	B	B	A	B	A	A	B	B	A	B	B	B	B	A	A	B	A	A	A	A	A	A	A	A	A	A	A	A	A	B	A	A	B
A	B	B	B	B	A	A	A	A	A	A	A	B	B	B	A	B	A	A	B	B	A	B	B	B	B	A	A	B	A	A	A	A	A	A	A	A	A	A	A	A	A	B	A	A	B
A	B	B	B	B	A	A	A	A	A	A	A	B	B	B	A	B	A	A	B	B	A	B	B	B	B	A	A	B	A	A	A	A	A	A	A	A	A	A	A	A	A	B	A	A	B
A	B	B	B	B	A	A	A	A	A	A	A	B	A	B	A	B	A	A	B	B	A	B	B	B	B	A	A	B	A	A	A	A	A	A	A	A	A	A	A	A	A	B	A	A	B
A	B	B	B	B	A	A	A	A	A	A	A	B	A	B	A	B	A	A	B	B	A	B	B	B	B	A	A	B	A	A	A	A	A	A	A	A	A	A	A	A	A	B	A	A	B
A	B	B	B	B	A	A	A	A	A	A	A	B	A	B	A	B	A	A	B	B	A	B	B	B	B	A	A	B	A	A	A	A	A	A	A	A	A	A	A	A	A	B	A	A	B
A	B	B	B	B	A	A	A	A	A	A	A	B	A	B	A	B	A	A	B	B	A	B	B	B	B	A	A	B	A	A	A	A	A	A	A	A	A	B	A	A	A	B	A	A	B
A	B	B	B	B	A	A	A	A	A	A	A	B	A	B	A	B	A	A	B	B	A	B	B	B	B	A	A	B	A	A	A	A	A	A	A	A	B	B	A	A	A	B	A	A	B
A	B	B	B	B	A	A	A	A	A	A	A	B	A	B	A	B	A	A	B	B	A	B	B	B	B	A	A	B	B	B	A	A	A	A	A	A	B	B	A	A	A	B	A	A	B
A	B	B	B	B	A	A	A	A	A	A	A	B	A	B	A	B	A	A	B	B	A	B	B	B	B	A	A	B	B	B	A	A	A	A	A	A	B	B	A	A	A	B	A	A	B
A	B	B	B	B	A	A	A	A	A	A	A	B	A	B	A	B	A	A	B	B	A	B	B	B	B	A	A	B	B	B	A	A	B	A	A	A	B	B	A	A	A	B	A	A	B
A	B	B	B	B	A	A	A	A	A	A	A	B	A	B	A	B	A	A	B	B	A	B	B	B	B	A	A	B	B	B	A	A	B	A	A	A	B	B	A	A	A	B	A	A	B
A	B	B	B	B	A	A	A	A	A	A	A	B	A	B	A	B	A	A	B	B	A	B	B	B	B	A	A	B	B	B	A	A	B	A	A	A	B	B	A	A	A	B	A	A	B
A	B	B	B	B	A	A	A	A	A	A	A	B	A	B	A	B	A	A	B	B	A	B	B	B	B	A	A	B	B	B	A	A	B	A	A	A	B	B	A	A	A	B	A	A	B
A	B	B	B	B	A	A	A	A	A	A	A	B	A	B	A	B	A	A	B	B	A	B	B	B	B	A	A	A	B	B	A	A	B	A	A	A	B	B	A	A	A	B	A	A	B
A	B	B	B	B	A	A	A	A	A	A	A	B	A	B	A	B	A	A	B	B	A	B	B	B	B	A	A	A	B	B	A	A	B	A	A	A	B	B	A	A	A	B	A	A	B
A	B	A	B	B	A	A	A	A	A	A	A	B	A	B	A	B	A	A	B	B	A	A	B	B	B	A	A	A	B	B	A	A	B	A	A	A	B	B	A	A	A	B	A	A	B
A	B	A	B	B	A	A	A	A	A	A	A	B	A	B	A	B	A	A	B	B	A	A	B	B	B	A	A	A	B	B	A	A	B	A	A	A	B	B	A	A	A	B	A	A	B
A	B	A	B	B	A	A	A	A	A	A	A	B	A	B	A	B	A	A	B	B	A	A	B	B	B	B	A	A	B	B	A	A	B	A	A	A	B	B	A	A	A	B	A	A	B
A	B	A	B	B	A	A	A	A	A	A	A	B	A	B	A	B	A	A	B	B	A	A	B	B	B	B	A	A	B	B	A	A	B	A	A	A	B	B	A	A	A	B	A	A	B
A	B	A	B	B	A	A	A	A	A	A	A	B	A	B	A	B	A	A	B	B	A	A	B	A	B	B	A	A	B	B	A	A	B	A	A	A	B	B	A	A	A	B	A	A	B
B	B	A	B	B	A	A	A	A	A	A	A	B	A	B	A	B	A	A	B	B	A	A	B	A	B	B	A	A	B	B	A	A	B	A	A	A	B	B	A	A	A	B	A	A	B
B	A	A	B	B	A	A	A	A	A	A	A	B	A	B	A	B	A	A	B	B	A	A	B	A	B	B	A	A	B	B	A	A	B	A	A	A	B	B	A	A	A	B	A	A	B
B	A	A	B	B	A	A	A	A	A	A	A	B	A	B	A	B	A	A	B	B	A	A	B	A	B	B	A	A	B	B	A	A	B	A	A	A	B	B	A	A	A	A	A	A	B
B	A	A	B	B	A	A	A	A	A	A	A	B	A	B	A	B	A	A	B	B	A	A	B	A	B	B	A	A	B	B	A	A	B	A	A	A	B	B	A	A	A	A	A	A	B
B	A	A	B	B	A	A	A	A	A	A	A	B	A	B	A	B	A	A	B	B	A	A	B	A	B	B	A	A	B	B	A	A	B	A	A	A	B	B	A	A	A	A	A	A	B
B	A	A	B	B	A	A	A	A	A	B	A	B	A	B	A	B	A	A	B	B	A	A	B	A	A	B	A	A	B	B	A	A	B	A	A	A	B	B	A	A	A	A	A	A	B
B	A	A	B	B	A	A	A	A	A	B	A	A	A	B	A	B	A	A	B	B	A	A	B	A	A	B	A	A	B	B	A	A	B	A	A	A	B	B	A	A	A	A	A	A	B
B	A	A	B	B	A	A	A	A	A	B	A	A	A	B	A	B	A	A	B	B	A	A	B	A	A	B	A	A	B	B	A	A	B	A	A	A	B	B	A	A	A	A	A	A	B
B	A	A	B	B	A	A	A	0	A	B	A	A	A	B	A	B	A	A	B	B	A	A	B	A	A	B	A	B	B	B	A	A	B	A	A	A	B	B	A	A	A	A	A	A	B
B	A	A	B	B	A	A	A	0	A	B	A	A	A	B	A	B	A	A	B	B	A	A	B	A	A	B	A	B	B	B	A	A	B	A	A	A	B	B	A	A	A	A	A	A	B
B	A	A	B	B	A	A	A	0	A	B	A	A	A	B	A	B	A	A	B	B	A	A	B	A	A	B	A	B	B	B	A	A	B	A	A	A	B	B	A	A	A	A	B	B	B
B	A	A	B	B	A	A	A	0	A	B	A	A	A	B	A	B	A	A	B	B	A	A	B	A	A	B	A	B	B	B	A	A	B	A	A	A	B	B	A	A	A	A	B	B	B
B	A	A	B	B	A	A	A	0	A	B	A	A	A	B	A	B	A	A	B	B	A	A	B	A	A	B	A	B	B	B	A	A	B	A	A	A	B	B	A	A	A	A	B	B	B
B	A	A	B	B	A	A	A	0	A	B	A	A	A	B	A	B	A	A	B	B	A	A	B	A	A	B	A	B	B	B	A	A	B	A	A	A	B	B	A	A	A	A	B	B	B
B	B	A	B	B	A	A	A	0	A	B	B	A	A	B	A	B	A	A	B	B	A	A	B	A	A	B	A	B	B	B	A	A	B	A	B	A	B	A	A	A	A	A	B	B	B
B	B	A	B	A	A	A	A	0	A	B	B	A	A	B	A	B	A	A	B	B	A	A	B	A	A	B	A	B	B	B	A	A	B	A	B	A	B	A	A	A	A	A	B	B	B
B	B	B	B	B	B	A	A	B	A	B	A	B	B	B	B	B	B	B	A	A	B	A	A	B	A	B	A	0	B	B	B	B	A	A	A	A	B	B	A	B	A	A	B	B	A
B	B	B	B	B	B	B	A	B	A	B	A	B	B	A	B	B	B	B	A	A	B	A	A	B	A	B	A	0	B	B	B	B	A	A	B	A	B	B	A	B	A	A	B	B	A
B	B	B	B	B	B	B	A	B	B	B	A	B	B	A	B	B	B	B	A	A	B	A	A	B	A	B	A	0	B	B	B	B	A	A	B	A	B	B	B	B	A	A	B	B	A
B	B	B	B	B	B	B	A	B	B	B	A	B	B	A	A	B	B	B	A	A	B	A	A	B	A	B	A	0	B	B	B	B	A	A	B	A	B	B	B	B	B	A	B	B	A
B	B	B	B	B	B	B	A	B	B	B	B	B	B	A	A	B	B	B	A	A	B	A	A	B	A	B	A	0	B	B	B	B	A	A	B	A	B	B	B	B	B	A	B	B	A
B	B	B	B	B	B	B	A	B	B	B	B	B	B	A	A	B	B	B	A	A	B	A	A	B	A	B	A	0	B	B	B	B	A	A	B	A	B	B	B	B	B	A	B	B	A
B	B	B	B	B	B	B	B	B	B	B	B	B	B	A	A	B	B	A	B	B	B	A	A	B	B	B	A	A	B	B	B	B	A	A	B	A	A	B	B	B	B	B	B	B	A
B	B	B	B	B	B	B	B	B	B	B	B	B	B	A	A	B	B	A	B	B	B	A	A	B	B	B	A	A	B	B	B	B	A	A	B	A	A	B	B	B	B	B	B	B	A
B	B	B	B	B	B	B	B	B	B	B	B	B	B	A	A	B	B	A	B	B	B	A	A	B	B	B	A	A	B	B	B	B	A	A	B	A	A	B	B	B	B	B	B	B	A
B	B	B	B	B	B	B	B	B	B	B	B	B	B	A	A	B	B	A	B	B	B	A	A	B	B	B	A	A	B	B	B	B	A	A	B	A	A	B	B	B	B	B	B	B	A
B	B	B	B	B	B	B	B	B	B	A	B	B	B	A	A	B	B	A	B	B	B	A	A	B	B	B	A	A	B	B	B	B	A	A	B	A	A	B	B	B	B	B	B	B	A

B	B	B	B	B	B	B	B	B	B	A	B	B	B	A	A	B	B	A	B	B	B	A	A	B	B	B	A	A	B	B	B	B	A	A	B	A	A	B	B	B	B	B	B	B	A
B	B	B	B	A	B	B	B	B	B	A	B	B	B	A	A	B	B	A	B	B	B	A	A	B	B	B	A	A	B	B	B	B	A	A	B	A	A	B	B	B	B	B	B	B	A
B	B	B	B	A	B	B	B	B	B	A	B	B	B	A	A	B	B	A	B	B	B	A	A	A	B	B	A	A	B	B	B	B	A	A	B	A	A	B	B	B	B	B	B	B	A
B	B	B	B	A	B	B	B	B	B	A	B	B	B	A	A	B	B	A	B	B	B	A	A	A	B	B	A	A	B	B	B	B	A	A	B	A	A	B	B	B	B	B	B	B	A
B	B	B	B	A	B	B	B	B	B	A	B	B	B	A	A	B	B	A	B	B	B	A	A	A	B	B	A	A	B	B	B	B	A	A	B	A	A	B	B	B	B	B	B	B	A
B	B	B	B	A	B	B	B	B	B	A	B	B	B	A	A	B	B	A	B	B	B	A	A	A	B	B	B	A	B	B	B	B	A	A	B	A	A	B	B	B	B	B	B	B	A
B	B	B	B	A	B	B	B	B	B	A	B	B	B	A	A	B	B	A	B	B	B	A	A	A	B	B	B	A	B	B	B	B	A	A	B	A	A	B	B	B	B	B	B	B	A
B	B	B	B	A	B	B	B	B	B	A	B	B	B	A	A	B	B	A	B	B	B	A	A	A	B	B	B	A	B	B	B	B	A	A	B	A	A	B	B	B	B	B	B	B	A
B	B	B	B	A	B	B	B	B	B	A	B	B	B	A	A	B	B	A	B	B	B	A	A	A	B	B	B	A	B	B	B	B	A	A	B	A	A	B	B	B	B	B	B	B	A
B	B	B	B	A	B	B	B	B	B	A	B	B	B	A	A	B	B	A	B	B	B	A	A	A	B	B	B	A	B	B	B	B	A	A	B	A	A	B	B	B	A	B	B	B	A
B	B	B	B	A	B	B	B	B	B	A	B	B	B	A	A	B	B	A	B	B	B	A	A	A	B	B	B	A	B	B	B	B	A	A	B	A	A	B	B	B	A	B	B	B	A
B	B	B	B	A	B	B	A	B	B	A	B	B	B	A	A	B	B	A	B	B	B	A	A	A	B	B	B	A	B	B	B	B	A	A	B	A	A	B	B	B	A	B	B	B	A
B	B	B	B	A	B	B	A	B	B	A	B	B	B	A	A	B	B	A	B	B	B	A	A	A	B	B	B	A	B	B	B	B	A	A	B	A	A	B	B	B	A	B	B	B	A
B	B	B	B	A	B	B	A	B	B	A	B	B	B	A	A	B	B	A	B	B	B	A	A	A	B	B	B	A	B	B	A	A	A	A	B	A	A	B	B	B	A	B	B	B	A
B	B	B	B	A	B	B	A	B	B	A	B	B	B	A	A	B	B	A	B	B	B	A	A	A	B	B	B	A	B	B	A	A	A	A	B	A	A	B	B	B	A	B	B	B	A
B	B	B	B	A	B	B	A	B	B	A	B	B	B	B	A	B	B	A	B	B	B	A	A	A	B	B	B	A	B	B	A	A	A	A	B	A	A	B	B	B	A	A	B	B	A
B	B	B	B	A	B	B	A	B	B	A	B	B	A	B	A	B	B	A	B	B	B	A	A	A	B	B	B	A	B	B	A	A	A	A	B	A	A	B	B	B	A	A	B	B	A
B	B	B	B	A	B	B	A	B	B	A	B	B	A	B	A	B	B	A	B	B	B	A	A	A	B	B	B	A	B	B	A	A	A	A	B	A	A	B	B	B	A	A	B	B	A
B	B	B	B	A	B	B	A	B	B	A	B	B	A	B	A	A	B	A	B	B	B	A	A	A	B	B	B	A	B	B	A	A	A	A	B	A	A	B	B	B	A	A	B	B	A
B	B	B	B	A	A	B	A	B	B	A	B	B	A	B	A	A	B	A	B	B	B	A	A	A	B	B	B	A	B	B	A	A	A	A	B	A	A	B	B	B	A	A	B	B	A
B	A	B	B	A	A	B	A	B	B	A	B	B	A	B	A	A	B	A	B	B	B	A	A	A	B	B	B	A	B	B	A	A	A	A	B	A	A	B	B	B	A	A	B	B	A
B	A	B	B	A	A	B	A	B	B	A	B	B	A	B	A	A	B	A	B	B	B	A	A	A	B	B	B	A	B	B	A	A	A	A	B	A	A	B	B	B	A	A	B	B	A
B	A	B	B	A	A	B	A	B	B	A	B	B	A	B	A	A	B	A	B	B	B	A	A	A	B	B	B	A	B	B	A	A	A	A	B	A	A	B	A	B	A	A	B	B	A
B	A	B	B	A	A	B	A	B	B	A	B	B	A	B	A	A	B	A	B	B	B	A	A	A	B	B	B	A	B	B	A	A	A	A	B	A	A	B	A	B	A	A	B	B	A
B	A	B	B	A	A	B	A	B	B	A	B	B	A	B	A	A	B	A	B	B	B	A	A	A	B	B	A	A	B	B	A	A	A	A	B	A	A	B	A	B	A	A	B	B	A
B	A	B	B	A	A	A	A	B	B	A	B	B	A	B	A	A	B	A	B	B	B	A	A	A	B	B	A	A	B	B	A	A	A	A	B	A	A	B	A	B	A	A	B	B	A
B	A	B	B	A	A	A	A	B	B	A	B	B	A	B	A	A	B	A	B	B	B	A	A	A	A	B	A	A	B	B	A	A	A	A	B	A	A	B	A	B	A	A	B	B	A
B	A	B	B	A	A	A	A	B	B	A	B	B	A	B	A	A	B	A	B	B	A	A	A	A	A	B	A	A	B	B	A	A	A	A	B	A	A	B	A	B	A	A	B	B	A
B	A	B	B	A	A	A	A	B	B	A	B	B	A	B	A	A	B	A	A	A	A	A	A	A	A	B	A	A	B	B	A	A	A	A	B	A	A	B	A	B	A	A	B	B	A
B	A	B	B	A	A	A	A	B	B	A	B	B	A	B	A	A	B	A	A	A	A	A	A	A	A	B	A	A	B	B	A	A	A	A	B	A	A	B	A	B	A	A	B	B	A
A	A	B	B	A	A	A	A	B	B	A	B	B	A	B	A	A	B	A	A	A	A	A	A	A	A	B	A	A	B	B	A	A	A	A	B	A	A	B	A	B	A	A	B	B	A
A	A	B	B	A	A	A	A	B	B	A	B	B	A	B	A	A	B	A	A	A	A	A	A	A	A	B	A	A	B	B	A	A	A	A	B	A	A	B	A	B	A	A	B	B	A
A	A	B	B	A	A	A	A	B	B	A	B	B	A	B	A	A	B	A	A	A	A	A	A	A	A	B	A	A	B	B	A	A	A	A	A	A	A	B	A	B	A	A	B	B	A
A	A	B	B	A	A	A	A	B	B	A	B	B	A	B	A	A	B	A	A	A	A	A	A	A	A	B	A	A	B	B	A	A	A	A	A	A	A	B	A	B	A	A	B	B	A
A	A	B	B	A	A	A	A	B	B	A	B	B	A	B	A	A	B	A	A	A	A	A	A	A	A	B	A	A	B	B	A	A	A	A	A	A	A	B	A	B	A	A	B	B	A
A	A	B	B	A	A	A	A	B	B	A	B	B	A	B	A	A	B	A	A	A	A	A	A	A	A	B	A	A	B	B	A	A	A	A	A	A	A	A	A	B	A	A	B	B	A
A	A	B	B	A	A	A	A	B	B	A	B	B	A	B	A	A	B	A	A	A	A	A	A	A	A	B	A	A	B	B	A	A	A	A	A	A	A	A	A	B	A	A	B	B	A
A	A	B	B	A	A	A	A	B	B	A	B	B	A	B	A	A	B	A	A	A	A	A	A	0	A	B	A	A	B	B	A	A	A	A	A	A	A	A	A	B	A	A	B	B	A
A	A	B	B	A	A	A	A	B	B	A	B	B	A	B	A	A	B	A	A	A	A	A	A	0	A	B	A	A	B	B	A	A	A	A	A	A	A	A	A	B	A	A	B	B	A
A	A	B	B	A	A	A	A	B	B	A	B	B	A	B	A	A	B	A	A	A	A	A	A	0	A	B	A	A	B	B	A	A	A	A	A	A	A	A	A	B	A	A	B	B	A
A	A	B	B	A	A	A	A	B	B	A	B	B	A	B	A	A	B	A	A	A	A	A	A	0	A	B	A	A	B	B	A	A	A	A	A	A	A	A	A	B	A	A	B	B	A
A	A	B	B	A	A	A	A	B	B	A	B	B	A	B	A	A	B	A	A	A	A	A	A	0	A	B	A	A	B	B	A	A	A	A	A	A	A	A	A	B	A	A	B	B	A
A	A	B	B	A	A	A	A	B	B	A	B	B	A	B	A	A	B	A	A	A	A	A	A	0	A	B	A	A	B	B	A	A	A	A	A	A	A	A	A	B	A	A	B	B	A
B	A	B	B	A	A	A	A	B	B	A	B	B	A	B	A	A	B	A	A	A	A	A	A	0	A	B	A	A	B	B	A	A	A	A	A	A	A	A	A	B	A	A	B	B	A
B	A	B	B	A	A	A	A	B	B	A	B	B	A	B	A	A	B	A	A	A	A	A	A	0	A	B	A	A	B	B	A	A	A	A	A	A	A	A	A	B	A	A	B	B	A
B	A	B	B	A	A	A	A	B	B	A	B	B	A	B	A	A	B	A	A	A	A	A	A	0	A	B	A	A	B	B	A	A	A	A	A	A	A	A	A	B	A	A	B	B	A
B	A	B	B	A	A	A	A	B	0	A	B	0	A	B	A	A	0	B	A	A	A	A	A	0	A	B	A	A	B	B	A	A	A	A	A	A	A	A	A	B	A	A	B	B	B

SG_123	SG_126	SG_138	SG_139	SG_143	SG_153	SG_287	SG_72	SG_161	SG_228	SG_36
B	B	B	B	B	B	B	0	B	A	0
B	B	B	B	B	B	B	0	B	A	0
B	B	B	B	B	B	B	0	B	A	0
B	B	B	B	B	B	B	0	B	A	0
B	B	B	B	B	B	B	0	B	A	0
B	B	B	B	B	B	B	0	B	A	0
B	B	B	B	B	B	B	0	B	A	0
B	B	B	B	B	B	B	0	B	A	0
B	B	B	B	B	B	B	0	B	A	0
B	B	B	B	B	B	B	0	B	A	0
B	B	B	B	B	B	B	0	B	A	0
B	B	B	B	B	B	B	0	B	A	0
B	B	B	B	B	B	B	0	B	A	0
B	B	B	B	B	B	B	0	B	A	0
B	B	B	B	B	B	B	0	B	A	0
B	B	B	B	B	B	B	0	B	A	0
B	B	B	B	B	B	B	0	B	A	0
B	B	B	B	B	B	B	0	B	A	0
B	B	B	B	B	B	B	0	B	A	0
B	B	B	B	B	B	B	A	B	A	A
B	B	B	B	B	B	B	A	B	A	A
B	B	B	B	B	B	B	A	B	A	A
B	B	B	0	0	B	B	A	B	A	A
B	B	B	0	0	B	B	A	B	A	A
B	B	B	0	0	B	B	A	B	A	A
B	B	B	0	0	B	B	A	B	A	A
B	B	B	0	0	B	B	A	B	A	A
B	B	B	0	0	B	B	A	B	A	A
B	B	B	0	0	B	B	A	B	A	A
B	B	B	0	0	B	B	A	B	A	A
B	B	B	0	0	B	B	A	B	A	A
B	B	B	0	0	B	B	A	B	A	A
B	B	B	A	0	B	B	A	B	A	A
B	B	B	A	0	B	B	A	B	A	A
B	B	B	A	0	B	B	A	B	A	A
B	B	B	A	0	B	B	A	B	A	A
B	B	B	A	0	B	B	A	B	A	A
B	B	B	A	0	B	B	A	B	A	A
B	B	B	A	A	B	B	A	B	A	A
B	B	B	A	A	B	B	A	B	A	A
B	B	B	A	A	B	B	A	B	A	A
B	A	B	A	A	B	B	A	B	A	A
B	A	B	A	A	B	B	A	B	A	A
B	A	B	A	A	B	B	A	B	A	A
B	A	A	A	A	A	B	A	A	A	A

B	A	A	A	A	A	B	A	A	A	A
B	A	A	A	A	A	B	A	A	A	A
B	A	A	A	A	A	B	A	A	A	A
B	A	A	A	A	A	B	B	A	A	A
B	A	A	A	A	A	B	B	A	A	A
B	A	A	A	A	A	B	B	A	A	A
B	A	A	A	A	A	B	B	A	A	A
B	A	A	A	A	A	B	B	A	A	A
B	A	A	A	A	A	B	B	A	A	A
A	A	A	A	A	A	B	B	A	A	A
A	A	A	A	A	A	B	B	A	A	A
A	A	A	A	A	A	B	B	A	A	A
A	A	A	A	A	A	B	B	A	A	A
A	A	A	A	A	A	B	B	A	A	A
A	A	A	A	A	A	B	B	A	A	A
B	B	A	B	B	A	B	B	A	B	A
B	B	A	B	B	A	B	B	A	B	A
B	B	A	B	B	A	B	B	A	B	A
B	B	A	B	B	A	B	B	A	B	A
B	B	A	B	B	A	B	B	A	B	A
B	B	A	B	B	A	B	B	A	B	A
B	B	A	B	B	A	B	B	A	B	A
B	B	A	B	B	A	B	B	A	B	A
B	B	A	B	B	A	A	B	A	B	A
B	B	A	B	B	A	A	B	A	B	A
B	B	A	B	B	A	A	B	A	B	A
B	B	A	B	B	A	A	B	A	B	A
B	B	A	B	B	A	A	B	A	B	A
B	B	A	B	B	A	A	B	A	B	A
B	B	A	B	B	A	A	B	A	B	A
B	B	A	B	B	A	A	B	A	B	A
B	B	A	B	B	A	A	B	A	B	A
B	B	A	B	B	A	A	B	A	B	A
B	B	A	B	B	A	A	B	A	B	A
B	B	A	B	B	A	A	B	A	B	A
B	B	A	B	B	A	A	B	A	B	A
B	B	A	B	B	A	A	B	A	B	A
B	A	A	B	A	A	A	B	A	B	A
B	A	A	B	A	A	A	B	A	B	A
B	A	B	B	A	B	A	B	B	B	A
B	A	B	B	A	B	A	B	B	B	A
B	A	B	B	A	B	A	B	B	B	A
B	A	B	B	A	B	A	B	B	B	A
B	A	B	B	A	B	A	B	B	B	A
B	A	B	B	A	B	A	B	B	B	A
B	A	B	B	A	B	A	B	B	B	A
B	A	B	B	A	B	A	B	B	B	A
B	A	B	B	A	B	A	B	B	B	A
B	A	B	B	A	B	A	B	B	B	A

B	A	B	B	A	B	A	B	B	B	A
B	A	B	B	A	B	A	B	B	B	A
B	A	B	B	A	B	A	B	B	B	A
B	A	B	B	A	B	A	B	B	B	A
B	A	B	B	A	B	A	B	B	B	A
B	A	B	B	A	B	A	B	B	B	A
B	A	B	B	A	B	A	B	B	B	A
B	A	B	B	A	B	A	B	B	B	A
B	A	B	B	A	B	A	B	B	B	A
B	A	B	B	A	B	A	B	B	B	A
B	A	B	B	A	B	A	B	B	B	A
B	A	B	B	B	B	A	B	B	B	A
B	A	B	B	B	B	A	B	B	B	A
B	A	B	B	B	B	A	B	B	B	A
A	A	B	B	B	B	A	B	B	B	A
A	A	B	B	B	B	A	B	B	B	A
A	A	B	B	B	B	A	B	B	B	A
A	A	B	B	B	B	A	B	B	B	A
A	A	B	B	B	B	A	B	B	B	A
A	A	B	B	B	B	A	B	B	B	A
A	A	B	B	A	B	A	B	B	B	A
A	A	B	B	A	B	A	B	B	B	A
A	A	B	B	A	B	A	B	B	B	A
A	A	B	B	A	B	A	B	B	B	A
A	A	B	B	A	B	A	B	B	B	A
A	A	B	B	A	B	A	B	B	B	A
A	A	B	B	A	B	A	B	B	B	A
A	A	B	B	A	B	A	B	B	B	A
A	A	B	B	A	B	A	B	B	B	A
A	A	B	B	A	B	A	B	B	B	A
A	A	B	B	A	B	A	B	B	B	A
A	A	B	B	A	B	A	B	B	B	A
A	A	B	B	A	B	A	B	B	B	A
A	A	B	B	A	B	A	B	B	B	A
A	A	B	B	A	B	A	B	B	B	A
A	A	B	B	A	B	A	B	B	B	A
A	A	B	B	A	B	A	B	B	B	A
A	A	B	B	A	B	A	B	B	B	A
A	A	B	B	A	B	A	A	B	B	A
A	A	B	A	A	B	A	A	B	B	A
B	B	B	A	A	B	A	B	B	A	A
B	B	B	A	A	B	A	B	B	A	A
B	B	B	A	A	B	A	B	B	A	A
B	B	B	A	A	B	A	B	B	A	A
B	B	B	A	A	B	B	B	B	A	A
B	B	B	A	A	B	B	B	B	A	A
B	B	B	A	A	B	B	B	B	B	A
B	B	B	A	A	B	B	B	B	B	A
B	B	B	A	A	B	B	B	B	B	A

B	B	B	A	A	B	B	B	B	B	A
B	B	B	A	A	B	B	B	B	B	A
B	B	B	A	A	B	B	B	B	B	A
B	B	B	A	A	B	B	B	B	B	A
B	B	B	A	A	B	B	B	B	B	A
B	B	B	A	A	B	B	B	B	B	A
B	B	B	A	A	B	B	B	B	B	A
B	B	B	A	A	B	B	B	B	B	A
B	B	B	A	A	B	B	B	B	B	A
B	B	B	A	A	B	B	B	B	B	A
B	B	A	A	A	A	B	B	A	B	A
B	B	A	A	A	A	B	B	A	B	A
B	B	A	A	B	A	B	B	A	B	A
B	B	A	A	B	A	B	B	A	B	A
B	B	A	A	B	A	B	B	A	B	A
B	B	A	A	B	A	B	B	A	B	A
B	B	A	A	B	A	B	B	A	B	A
B	B	A	A	B	A	B	B	A	B	A
B	B	A	A	B	A	B	B	A	B	A
B	B	A	A	B	A	B	B	A	B	A
B	B	A	A	B	A	B	B	A	B	A
B	B	A	A	B	A	B	B	A	B	A
B	B	A	A	B	A	B	B	A	B	A
A	B	A	A	B	A	B	B	A	B	A
A	B	A	A	B	A	B	B	A	B	A
A	B	A	A	B	A	B	B	A	B	A
A	B	A	A	B	A	B	B	A	B	A
A	B	A	A	B	A	B	B	A	B	A
A	B	A	A	B	A	B	B	A	B	A
A	B	A	A	B	A	B	B	A	B	A
A	B	A	A	B	A	B	B	A	B	A
A	B	A	A	B	A	B	B	A	B	A
A	B	A	A	B	A	B	B	A	B	A
A	B	A	A	B	A	B	B	A	B	A
A	B	A	A	B	A	B	B	A	B	A
A	B	A	A	A	A	B	B	A	B	A
A	B	A	A	A	A	B	B	A	B	A
A	B	A	A	A	A	B	B	A	B	A
A	B	A	A	A	A	B	B	A	B	A
A	B	A	A	A	A	B	B	A	B	A
A	B	A	A	A	A	B	B	A	B	A
A	B	A	A	A	A	B	B	A	B	A
A	B	A	A	A	A	B	B	A	B	B
A	B	A	A	A	A	B	B	A	B	B
A	B	A	A	A	A	B	B	A	B	B
A	B	A	A	A	A	B	B	A	B	B
A	B	A	A	A	A	B	B	A	B	B
A	B	A	B	A	A	B	B	A	B	B
A	B	A	B	A	A	B	B	A	B	B

A	B	A	B	A	A	B	B	A	B	B
A	B	A	B	A	A	B	B	A	B	B
A	B	A	B	A	A	B	B	A	B	B
A	B	A	B	A	A	B	B	A	B	B
A	B	A	B	A	A	B	B	A	B	B
A	B	A	B	A	A	B	B	A	B	B
A	A	A	B	A	A	B	B	A	B	B
A	A	A	B	A	A	B	B	A	B	B
A	A	A	B	A	A	B	B	A	B	B
A	A	A	B	A	A	B	B	A	B	B
A	A	A	B	A	A	B	B	A	B	B
A	A	A	B	A	A	B	B	A	B	B
A	A	A	B	A	A	B	B	A	B	B
A	A	A	B	A	A	B	B	A	B	B
A	A	A	B	A	A	B	B	A	B	B
A	A	A	B	A	A	B	B	A	B	B
A	A	A	B	A	A	B	B	A	B	B
A	A	A	B	A	A	B	B	A	B	B
A	A	A	B	A	A	B	A	A	B	B
A	A	A	B	A	A	B	A	A	B	B
A	A	A	B	A	A	B	A	A	B	B
A	A	A	B	A	A	B	A	A	B	B
A	A	A	B	A	A	B	A	A	B	B
A	A	A	B	A	A	B	A	A	B	B
A	A	A	B	A	A	B	A	A	B	B
A	A	A	B	A	A	B	A	A	B	B
A	A	A	B	A	A	B	A	A	B	B
A	A	A	B	A	A	B	A	A	B	B
A	A	A	B	A	A	B	A	A	B	B
A	A	A	B	A	A	A	A	A	B	B
A	A	A	B	A	A	A	A	A	B	B
A	A	A	B	A	A	A	A	A	B	B
A	A	A	B	A	A	A	A	A	B	B
A	A	A	B	B	A	A	A	A	B	B
A	A	A	B	B	A	A	A	A	B	B
A	A	A	B	A	A	A	A	A	B	B
A	A	A	A	B	A	A	A	A	B	B
A	A	A	A	B	A	A	A	A	B	B
A	A	A	A	B	A	A	A	A	B	B
A	A	A	A	B	A	A	A	A	B	B
A	A	A	A	B	A	A	A	A	B	B
A	A	A	A	B	A	B	A	A	B	B
A	A	A	A	B	A	B	A	A	B	B
A	A	A	A	B	A	B	A	A	B	B
A	A	A	A	B	A	B	A	A	B	B
A	A	A	A	B	A	B	A	A	B	B
A	A	A	A	B	A	B	A	A	B	B
A	B	A	A	A	A	B	A	A	B	B
A	B	A	A	A	A	B	A	A	B	B

A	B	A	A	A	A	B	A	A	B	B
A	B	A	A	A	A	B	A	A	B	B
A	B	A	A	A	A	B	A	A	B	B
A	B	A	A	A	A	B	A	A	B	B
A	B	A	A	A	A	B	A	A	B	B
A	B	A	A	A	A	B	A	A	B	B
A	B	A	A	A	A	B	A	A	B	B
A	B	A	A	A	A	B	A	A	B	B
A	B	A	A	A	A	B	A	A	B	B
A	B	A	A	A	A	B	A	A	B	B
A	B	A	A	A	A	B	A	A	B	B
A	B	A	A	A	A	B	A	A	B	B
A	B	A	A	A	A	B	A	A	B	B
A	B	A	A	A	A	B	A	A	B	B
A	B	B	A	A	B	B	A	B	B	B
A	B	B	A	A	B	B	A	B	B	B
A	B	B	A	A	B	B	A	B	B	B
A	B	B	A	A	B	B	A	B	B	B
A	B	B	A	A	B	B	A	B	B	B
A	B	B	A	A	B	B	A	B	B	B
A	B	B	A	A	B	B	A	B	B	B
A	B	B	A	A	B	B	A	B	B	B
A	B	B	A	A	B	B	A	B	B	B
A	B	B	A	A	B	B	A	B	B	B
A	B	B	A	A	B	B	A	B	B	B
A	B	B	A	A	B	B	A	B	B	B
A	B	B	A	A	B	B	A	B	B	B
A	B	B	A	A	B	B	A	B	B	B
A	B	B	A	A	B	B	A	B	B	B
A	B	B	A	A	B	B	A	B	B	B
A	B	B	A	A	B	B	A	B	B	B
A	B	B	A	A	B	B	A	B	B	B
A	B	B	A	A	B	B	A	B	B	B
A	B	B	A	A	B	A	A	B	B	B
A	B	B	A	A	B	A	A	B	B	B
A	B	B	A	A	B	A	A	B	B	B
A	B	B	A	A	B	A	A	B	B	B
A	B	A	A	A	A	A	A	A	B	B
A	B	A	A	A	A	A	A	A	B	B
A	B	A	A	A	A	A	A	A	B	B
A	B	A	A	A	A	A	A	A	B	B
A	B	A	A	A	A	A	A	A	B	B
A	B	A	A	A	A	A	A	A	B	B
A	B	A	A	A	A	A	A	A	B	B
A	B	A	A	A	A	A	A	A	B	B
B	B	A	A	A	A	A	A	A	B	B
B	B	A	A	A	A	A	A	A	B	A
B	B	A	A	A	A	A	A	A	B	A
B	B	A	A	A	A	A	B	A	B	A

B	B	A	A	A	A	A	B	A	B	A
B	B	A	A	A	A	A	B	A	B	A
B	B	A	A	A	A	A	B	A	B	A
B	B	A	A	A	A	A	B	A	B	A
B	B	A	A	A	A	A	B	A	B	A
B	B	A	A	A	A	A	B	A	B	A
B	B	A	A	A	A	A	B	A	B	A
B	B	A	A	A	A	A	B	A	B	A
B	B	A	A	A	A	A	B	A	B	A
B	B	A	A	A	A	A	B	A	B	A
B	B	A	A	A	A	A	B	A	B	A
B	B	A	A	A	A	A	B	A	B	A
B	B	A	A	A	A	A	B	A	B	A
B	B	A	A	A	A	A	B	A	B	A
B	B	A	A	A	A	A	B	A	B	A
B	B	A	A	A	A	A	B	A	B	A
B	B	A	A	A	A	A	B	A	B	A
B	B	A	A	B	A	A	B	A	B	A
B	B	A	A	B	A	A	B	A	B	A
B	B	A	A	B	A	A	B	A	A	A
B	B	A	A	B	A	A	B	A	A	A
B	B	A	A	B	A	A	B	A	A	A
B	B	A	A	B	A	A	B	A	A	A
B	B	A	A	B	A	A	B	A	A	A
B	B	A	A	B	A	A	B	A	A	A
B	B	A	A	B	A	A	B	A	A	A
B	B	A	A	B	A	A	B	A	A	A
B	B	A	A	B	A	A	B	A	A	A
B	B	A	A	B	A	A	B	A	A	A
0	B	A	A	B	A	A	B	A	A	A
0	B	A	A	B	A	A	B	A	A	A
0	B	A	A	B	A	A	B	A	A	A
0	B	A	A	B	A	A	B	A	A	A
0	B	A	A	B	A	A	B	A	A	A
0	B	A	A	B	A	A	B	A	A	A
0	B	A	A	B	A	A	B	A	A	A
0	B	A	A	B	A	A	B	A	A	A
0	B	A	A	B	A	A	B	A	A	A
0	B	A	A	B	A	A	B	A	A	A
0	B	A	A	B	A	A	B	A	A	A
0	B	A	A	B	A	A	B	A	A	A
0	B	A	A	B	A	A	B	A	A	A
0	B	A	A	A	A	A	B	A	A	A
0	B	A	A	A	A	A	B	A	A	A
0	B	A	A	A	A	A	B	A	A	A
0	B	A	A	A	A	A	B	A	A	A
0	B	A	A	A	A	A	B	A	A	A
0	B	A	A	A	A	A	B	A	A	A
0	B	A	A	A	A	A	B	A	A	A

0	B	A	A	A	A	A	B	A	A	A
0	B	A	A	A	A	A	B	A	A	A
0	B	A	A	A	A	A	B	A	A	A
0	B	A	A	A	A	A	B	A	A	A
0	B	A	A	A	A	A	B	A	A	A
0	B	A	A	A	A	A	B	A	A	A
0	B	A	A	A	A	A	B	A	A	A
0	B	A	A	A	A	A	B	A	A	A
0	B	A	A	A	A	A	B	A	A	A
0	B	A	A	A	A	A	B	A	A	A
0	B	A	A	A	A	A	B	A	A	A
0	B	A	A	A	A	A	B	A	A	A
0	B	A	A	A	A	A	B	A	A	A
0	B	A	A	A	A	A	B	A	A	A
0	B	A	A	A	A	A	B	A	A	A
0	B	A	A	A	A	A	B	A	A	A
0	B	A	A	A	A	A	B	A	A	B
A	A	B	B	A	B	B	B	B	B	B
A	A	B	B	A	B	B	B	B	B	B
A	A	B	B	A	B	B	B	B	B	B
A	B	B	B	A	B	B	B	B	B	B
A	B	B	A	A	B	B	B	B	B	B
A	B	B	A	A	B	B	B	B	B	B
A	B	B	A	A	B	B	B	B	B	B
A	B	B	A	A	B	B	B	B	B	B
A	B	B	A	A	B	B	B	B	B	B
A	B	B	A	A	B	B	B	B	B	B
A	B	B	A	A	B	B	B	B	B	B
A	B	B	A	A	B	B	A	B	B	B
A	B	B	A	A	B	B	A	B	B	B
A	B	B	A	A	B	B	A	B	B	B
A	B	B	A	A	B	B	A	B	B	B
A	B	B	A	A	B	B	A	B	B	B
A	B	B	A	A	B	B	A	B	B	B
A	B	B	A	A	B	B	A	B	B	B
A	B	B	A	A	B	B	A	B	B	B
A	B	B	A	A	B	B	A	B	B	B
A	B	B	A	A	B	B	A	B	B	B
A	B	B	A	A	B	B	A	B	B	B
A	B	B	A	A	B	B	A	B	B	B
A	B	B	A	A	B	B	A	B	B	B
A	B	B	A	A	B	B	A	B	B	B
A	B	B	A	A	B	B	A	B	B	B
A	B	B	A	A	B	B	A	B	B	B
A	B	B	A	A	B	B	A	B	B	A
A	B	B	A	A	B	B	A	B	B	A
A	B	B	A	B	B	B	A	B	B	A
A	B	B	A	B	B	B	A	B	B	A
A	B	B	A	B	B	B	A	B	B	A

A	B	B	A	B	B	B	A	B	B	A
A	B	B	A	B	B	B	A	B	B	A
A	B	B	A	B	B	B	A	B	B	A
A	B	B	A	B	B	B	A	B	B	A
A	B	B	A	B	B	B	A	B	B	A
A	B	B	A	B	B	B	A	B	B	A
A	B	B	A	B	B	B	A	B	B	A
A	B	B	A	B	B	B	A	B	B	A
A	B	B	A	B	B	B	A	B	B	A
A	B	B	A	B	B	B	A	B	B	A
A	B	B	A	B	B	B	A	B	B	A
A	B	B	A	B	B	B	A	B	B	A
A	B	B	A	B	B	B	A	B	B	A
A	B	B	A	B	B	B	A	B	B	A
A	B	B	A	B	B	B	A	B	B	A
A	B	B	A	B	B	B	A	B	B	A
A	B	B	A	B	B	B	A	B	B	A
A	B	B	A	B	B	B	A	B	B	A
A	B	B	A	B	B	B	A	B	B	A
A	B	B	A	B	B	B	A	B	B	A
A	B	B	A	B	B	B	A	B	B	A
A	B	B	A	B	B	B	A	B	B	A
A	B	B	A	B	B	B	A	B	B	A
A	B	B	A	B	B	B	A	B	B	A
A	B	B	A	B	B	B	A	B	B	A
A	B	B	A	B	B	B	A	B	B	A
A	B	B	A	B	B	B	A	B	B	A
A	B	B	A	B	B	B	A	B	B	A
A	B	B	A	B	B	B	A	B	B	A
A	B	B	A	B	B	B	A	B	B	A
A	B	B	A	B	B	B	A	B	B	A
A	B	B	A	B	B	B	A	B	B	A
A	B	B	A	B	B	B	A	B	B	A
A	B	B	A	B	B	B	A	B	B	A
A	B	B	A	B	B	B	A	B	B	A
A	B	B	A	B	B	B	A	B	B	A
A	B	B	A	B	B	B	A	B	B	A
A	B	B	A	B	B	B	A	B	B	A
A	B	B	A	B	B	B	A	B	B	A
A	B	B	A	B	B	B	A	B	B	A
A	B	B	A	B	B	B	A	B	B	A
A	B	B	A	B	B	B	A	B	B	A
A	B	B	A	B	B	B	A	B	B	A
A	B	B	A	B	B	B	A	B	B	A
A	B	B	A	B	B	B	A	B	B	A
A	B	B	A	B	B	B	A	B	B	A
A	B	B	A	B	B	B	A	B	B	A
A	B	B	A	B	B	B	A	B	B	A
A	B	B	A	B	B	B	A	B	B	A

A	B	B	A	B	B	B	A	B	B	B
A	B	B	A	B	B	B	B	B	B	B
A	B	B	A	B	B	B	B	B	B	B
A	B	B	A	B	B	B	B	B	B	B
A	B	A	A	B	A	A	B	A	B	B
A	B	A	A	B	A	A	B	A	B	B
A	B	A	A	B	A	A	B	A	B	B
A	B	A	A	B	A	A	B	A	B	B
B	B	B	B	B	B	B	B	B	B	B
B	B	B	B	B	B	B	B	B	B	B
B	B	B	B	B	B	B	B	B	B	B
B	B	B	B	B	B	B	B	B	B	B
B	B	B	B	B	B	B	B	B	B	B
B	B	B	B	B	B	B	B	B	B	B
B	B	B	0	B	B	B	B	B	B	B
B	B	B	0	B	B	B	B	B	B	B
B	B	B	0	B	B	B	B	B	B	B
B	B	B	0	B	B	B	B	B	B	B
B	B	B	0	B	B	B	B	B	B	B
B	B	B	0	B	B	B	B	B	B	B
B	B	B	0	B	B	B	B	B	B	B
B	B	B	0	B	B	B	B	B	B	B
B	B	B	0	B	B	B	B	B	B	B
B	B	B	0	B	B	B	B	B	B	B
B	B	B	0	B	B	B	B	B	B	B
B	B	B	0	B	B	B	B	B	B	B
B	B	B	0	B	B	B	B	B	B	B
B	B	B	0	B	B	B	B	B	B	B
B	B	B	0	B	B	B	B	B	B	B
B	B	B	0	B	B	B	B	B	B	B
B	B	B	0	B	B	B	B	B	B	B
B	B	B	0	B	B	B	B	B	B	B
B	B	B	0	B	B	B	B	B	B	B
B	B	B	0	B	B	A	B	B	B	B
B	B	B	0	B	B	A	B	B	B	B
B	B	B	0	B	B	A	B	B	B	A
B	B	B	0	B	B	A	B	B	B	A
B	B	B	0	B	B	A	B	B	B	A
B	B	B	0	B	B	A	B	B	B	A
B	B	B	0	B	B	A	B	B	B	A
B	B	B	0	B	B	A	B	B	B	A
B	B	B	0	B	B	A	B	B	B	A
B	B	B	0	B	B	A	B	B	B	A
B	B	B	0	B	B	A	B	B	B	A
B	B	B	0	B	B	A	B	B	B	A
B	B	B	0	B	B	A	B	B	B	A
0	0	B	0	B	B	A	B	B	B	A
0	0	B	0	B	B	A	B	B	B	A
0	0	B	0	B	B	A	B	B	B	A

0	0	B	0	B	B	A	B	B	B	A
0	0	B	0	B	B	A	B	B	B	A
0	0	B	0	B	B	A	B	B	B	A
0	0	B	0	B	B	A	B	B	B	A
0	0	B	0	B	B	A	B	B	B	A
A	A	B	A	B	B	A	B	B	A	B
B	A	B	A	B	B	A	B	B	A	B
B	A	B	B	B	B	A	B	B	A	B
B	A	B	B	B	B	A	B	B	A	B
B	A	B	B	B	B	A	B	B	A	B
B	A	B	B	B	B	A	B	B	A	B
B	A	B	B	B	B	A	B	B	A	B
B	A	B	B	B	B	A	B	B	A	B
B	A	B	B	B	B	A	B	B	A	B
B	A	B	B	B	B	A	B	B	A	B
B	A	B	B	B	B	A	B	B	A	B
B	A	B	B	B	B	A	B	B	A	B
B	A	B	B	B	B	A	B	B	A	B
B	A	B	B	B	B	A	B	B	A	B
B	A	B	B	B	B	A	B	B	A	B
B	A	B	B	B	B	A	B	B	A	B
B	A	B	B	B	B	A	B	B	A	B
B	A	B	B	B	B	A	B	B	A	B
B	A	B	B	B	B	A	B	B	A	B
B	A	B	B	B	B	A	B	B	A	B
B	A	B	B	B	B	A	B	B	A	B
B	A	B	B	B	B	A	B	B	A	B
B	A	B	B	B	B	A	B	B	A	B
B	B	B	B	B	B	A	B	B	A	B
B	B	B	B	B	B	A	B	B	A	B
B	B	B	B	B	B	A	B	B	A	B
B	B	B	B	B	B	A	B	B	A	B
B	B	B	B	B	B	A	B	B	A	B
B	B	B	B	B	B	A	B	B	A	B
B	B	B	B	B	B	A	B	B	A	B
B	B	B	B	B	B	A	B	B	A	B
B	B	B	B	A	B	A	B	B	A	B
B	B	B	B	A	B	A	B	B	A	B
B	B	B	B	A	B	A	B	B	A	B
B	B	B	B	A	B	A	B	B	A	B
B	B	B	B	A	B	B	B	B	A	B
B	B	B	B	A	B	B	B	B	A	B
B	B	B	B	A	B	B	B	B	A	B
B	B	B	B	A	B	B	B	B	A	B
B	B	B	B	A	B	B	B	B	A	A
B	B	B	B	A	B	B	B	B	A	A
B	B	B	B	A	B	B	B	B	A	A
B	B	B	B	A	B	B	B	B	A	A

B	B	B	B	A	B	B	B	B	A	A
B	B	B	B	A	B	B	B	B	A	A
B	B	B	B	A	B	B	B	B	A	A
B	B	B	B	A	B	B	A	B	A	A
B	B	B	B	A	B	B	B	B	A	A
B	B	B	B	A	B	B	B	B	A	A
B	B	B	B	A	B	B	B	B	A	A
B	B	B	B	A	B	B	B	B	A	A
B	B	B	B	A	B	B	B	B	A	A
B	B	B	B	A	B	B	B	B	A	A
B	B	B	B	A	B	B	B	B	A	A
B	B	B	B	A	B	B	B	B	A	A
B	A	A	A	B	A	A	B	A	A	B
B	A	A	A	B	A	A	B	A	A	B
B	A	A	A	B	A	A	B	A	A	B
B	A	A	A	B	A	A	B	A	A	B
B	A	A	A	B	A	A	B	A	A	B
B	A	A	A	B	A	A	B	A	A	B
B	A	A	A	B	A	A	B	A	A	B
B	A	A	A	B	A	A	B	A	A	B
B	A	A	A	B	A	A	B	A	A	B
B	A	A	A	B	A	A	B	A	A	B
B	A	A	A	B	A	A	B	A	A	B
B	A	A	A	B	A	A	B	A	A	B
A	A	A	A	B	A	A	B	A	A	B
A	A	A	A	B	A	A	B	A	A	B
A	A	A	A	B	A	A	B	A	A	B
A	A	A	A	B	A	A	B	A	A	B
A	A	A	A	B	A	A	B	A	A	B
A	A	A	A	B	A	A	B	A	A	B
A	A	A	A	B	A	A	B	A	A	B
A	A	A	A	B	A	A	B	A	A	B
A	A	A	A	B	A	A	B	A	A	B
A	A	A	A	B	A	A	B	A	A	B
A	A	A	A	B	A	A	B	A	A	B
A	A	A	A	B	A	A	B	A	A	A
A	A	A	A	B	A	A	B	A	A	A
A	A	A	A	B	A	A	B	A	A	A
A	A	A	A	B	A	A	B	A	A	A
A	A	A	A	B	A	A	B	A	A	A
A	A	A	A	B	A	A	B	A	A	B
A	B	A	A	B	A	B	B	A	A	B
A	B	A	B	A	A	B	B	A	A	B
A	B	A	B	A	A	B	B	A	A	B
A	B	A	B	A	A	B	B	A	A	B
A	B	B	B	A	B	B	B	B	A	B
A	B	B	B	A	B	B	B	B	A	B
A	B	B	B	A	B	B	B	B	A	B
B	B	B	B	A	B	B	B	B	A	B

B	B	B	B	A	B	B	B	B	A	B
B	B	B	B	A	B	B	B	B	A	B
B	B	B	B	A	B	B	A	B	A	B
B	B	B	B	A	B	B	A	B	A	B
B	B	B	B	A	B	B	A	B	A	B
B	B	B	B	A	B	B	A	B	A	B
B	B	B	B	A	B	B	A	B	A	B
B	B	B	B	A	B	B	A	B	A	B
B	B	B	B	A	B	B	A	B	B	B
B	B	B	B	A	B	B	A	B	B	B
B	B	B	B	A	B	B	A	B	B	B
B	B	B	B	A	B	B	A	B	B	B
B	B	B	B	A	B	B	A	B	B	B
B	B	B	B	A	B	B	A	B	B	B
B	B	B	B	A	B	B	A	B	B	B
B	B	B	B	A	B	B	A	B	B	B
B	B	B	B	A	B	B	A	B	B	B
B	B	B	B	A	B	B	A	B	B	B
B	A	B	B	A	B	B	A	B	B	B
B	A	B	B	A	B	B	A	B	B	B
B	A	B	B	A	B	B	A	B	B	B
B	A	B	B	A	B	B	A	B	B	B
B	A	B	B	A	B	B	A	B	B	B
B	A	B	B	A	B	B	A	B	B	B
B	A	B	B	A	B	B	A	B	B	A
B	A	B	B	A	B	B	A	B	B	A
B	A	B	B	A	B	B	A	B	B	A
B	A	B	B	A	B	B	A	B	B	A
B	A	B	B	A	B	B	A	B	B	A
B	A	B	B	A	B	B	A	B	B	A
B	A	B	B	A	B	B	A	B	B	A
B	A	B	B	A	B	B	A	B	B	A
B	A	B	B	A	B	B	A	B	B	A
B	A	B	B	A	B	B	A	B	B	A
B	A	B	B	A	B	B	A	B	B	A
B	A	B	B	A	B	B	A	B	B	A
B	A	B	B	A	B	B	A	B	B	A
B	A	B	B	A	B	B	A	B	B	A
B	A	B	B	A	B	B	A	B	B	A
B	A	B	B	A	B	B	A	B	B	A
B	A	B	B	A	B	B	A	B	B	A
B	A	B	B	A	B	B	A	B	B	A
B	A	B	B	A	B	B	A	B	B	A
B	A	B	B	A	B	B	A	B	B	A
B	A	B	B	A	B	B	A	B	B	A
B	A	B	B	A	B	B	A	B	B	A
B	A	B	B	A	B	B	A	B	B	A
B	A	B	B	B	B	B	A	B	B	A
B	A	A	B	B	A	B	A	A	B	A

B	A	A	B	B	A	B	A	A	B	A
A	A	A	B	B	A	B	A	A	B	A
A	A	A	B	B	A	B	A	A	B	A
B	A	A	A	B	A	A	A	A	A	A
B	A	A	A	B	A	A	A	A	A	A
B	A	A	A	B	A	A	A	A	A	A
B	A	A	A	B	A	A	A	A	A	A
B	A	A	A	B	A	A	A	A	A	A
B	A	A	A	B	A	A	A	A	A	A
B	A	A	A	B	A	A	A	A	A	A
B	A	A	A	B	A	A	A	A	A	A
B	A	A	A	B	A	A	A	A	A	A
B	A	A	A	B	A	A	A	A	A	A
B	A	A	A	B	A	A	A	A	A	A
B	A	A	A	B	A	A	A	A	A	A
A	A	A	A	B	A	A	A	A	A	A
A	A	A	A	B	A	A	A	A	A	A
A	A	A	A	B	A	B	A	A	A	A
A	A	A	A	B	A	B	A	A	A	A
A	A	A	A	B	A	B	A	A	A	A
A	A	A	A	B	A	B	A	A	A	A
A	A	A	A	B	A	B	A	A	A	A
A	A	A	A	B	A	B	A	A	A	A
A	A	A	A	B	A	B	A	A	A	A
A	A	A	A	B	A	B	A	A	A	A
A	A	A	A	B	A	B	A	A	A	A
A	A	A	A	B	A	B	A	A	A	A
A	A	A	A	B	A	B	A	A	A	A
A	A	A	A	B	A	B	A	A	A	A
A	A	B	A	B	B	B	A	B	A	A
A	A	B	A	B	B	B	B	B	A	A
A	A	B	A	B	B	B	B	B	A	A
A	A	B	A	B	B	B	B	B	A	A
A	A	B	A	B	B	B	B	B	A	A
A	A	B	A	B	B	B	B	B	A	A
A	A	B	A	B	B	B	B	B	A	A
A	A	B	A	B	B	B	B	B	A	A
A	A	B	A	B	B	B	B	B	A	A
A	A	B	A	B	B	B	B	B	A	A
A	A	B	A	B	B	B	B	B	A	A
A	A	B	A	B	B	B	B	B	A	A
A	A	B	A	B	B	B	B	B	A	A
A	A	B	A	B	B	B	B	B	A	A
A	A	B	A	B	B	B	B	B	A	A
A	A	B	A	B	B	B	B	B	A	A
B	A	B	A	B	B	B	B	B	A	A
B	A	B	A	B	B	B	B	B	A	A
B	A	B	A	B	B	B	B	B	A	A
B	A	B	A	B	B	B	B	B	A	A

B	A	B	A	B	B	B	B	B	A	A
B	A	B	A	B	B	B	B	B	A	A
B	A	B	A	B	B	B	B	B	A	A
B	A	B	A	B	B	B	B	B	A	A
B	A	B	A	B	B	B	B	B	A	A
B	A	B	A	B	B	B	B	B	A	B
B	A	B	A	B	B	B	B	B	A	B
B	A	B	A	B	B	B	B	B	A	B
B	A	B	A	B	B	B	B	B	A	B
B	A	B	A	B	B	B	B	B	A	B
B	A	B	A	A	B	B	B	B	A	B
B	A	B	A	A	B	B	B	B	A	B
B	A	B	A	A	B	B	B	B	A	B
B	A	B	A	A	B	B	B	B	A	B
B	A	B	A	A	B	B	B	B	A	B
B	B	B	A	A	B	B	B	B	A	B
B	B	B	A	A	B	B	B	B	A	B
B	B	A	A	A	A	B	B	A	A	B
B	B	B	A	A	B	B	B	B	A	B
B	B	B	A	A	B	B	B	B	A	B
B	B	B	A	A	B	B	B	B	A	B
B	B	B	A	A	B	B	B	B	A	B
B	B	B	A	A	B	B	B	B	A	B
B	B	B	A	A	B	B	B	B	A	B
A	B	B	A	0	B	B	B	B	A	B
A	B	B	0	0	B	B	B	B	A	B
A	B	B	0	0	B	B	B	B	A	A
A	B	B	0	0	B	B	B	B	A	A
A	B	B	0	0	B	B	B	B	A	A
A	B	B	0	0	B	B	B	B	A	A
A	B	B	0	0	B	B	B	B	A	A
A	B	B	0	0	B	B	B	B	A	A
A	B	B	0	0	B	B	B	B	A	A
A	B	B	0	0	B	B	B	B	A	A
A	B	B	0	0	B	B	B	B	A	A
A	B	B	0	0	B	B	B	B	A	A
A	B	B	0	0	B	B	B	B	A	A
A	B	B	0	0	B	B	B	B	A	A
A	B	B	0	0	B	B	B	B	A	A
A	B	B	0	0	B	B	B	B	A	A
A	B	B	B	0	B	B	B	B	A	A
A	B	B	B	0	B	B	B	B	A	A
A	B	B	B	0	B	B	B	B	A	A
A	B	B	B	0	B	B	B	B	A	A
A	B	B	B	0	B	B	B	B	A	A
A	B	B	B	0	B	B	B	B	A	A
A	B	B	B	0	B	B	B	B	A	A
A	B	B	B	0	B	B	A	B	A	A
A	B	B	B	0	B	B	A	B	A	A

A	B	B	B	B	B	B	A	B	A	A
A	B	B	B	B	B	B	A	B	A	A
A	B	B	B	B	B	B	A	B	A	A
A	B	B	B	B	B	B	A	B	A	A
A	B	B	B	B	B	B	A	B	A	A
A	B	B	B	B	B	B	A	B	A	A
A	0	B	B	B	B	B	A	B	A	A
A	0	B	B	B	B	B	A	B	A	A
A	0	B	B	B	B	B	A	B	A	A
A	0	B	B	B	B	B	A	B	A	A
A	0	B	B	B	B	B	A	B	A	A
A	0	B	B	B	B	B	A	B	A	A
A	0	B	B	B	B	B	A	B	A	A
A	0	B	B	B	B	A	A	B	A	A
A	A	A	A	A	A	A	A	A	B	A
A	A	A	A	A	A	A	A	A	B	A
A	A	A	A	A	A	A	A	A	B	A
A	A	A	A	A	A	A	A	A	A	A
A	A	A	A	A	A	A	A	A	A	A
A	A	A	A	A	A	A	A	A	A	A
A	A	A	A	A	A	A	A	A	A	A
A	A	A	A	A	A	A	A	A	A	A
A	A	A	A	A	A	A	A	A	A	A
A	A	A	A	A	A	A	A	A	A	A
A	A	A	A	A	A	A	A	A	A	A
A	A	A	A	A	A	A	A	A	A	A
A	A	A	A	A	A	A	A	A	A	A
A	A	A	A	A	A	A	A	A	A	A
A	A	A	A	A	A	A	A	A	A	A
A	A	A	A	A	A	A	A	A	A	A
A	A	A	A	A	A	A	A	A	A	A
A	A	A	A	A	A	A	A	A	A	A
A	A	A	A	A	A	A	A	A	A	A
A	A	A	A	A	A	A	A	A	A	A
A	A	A	A	A	A	A	A	A	A	A
A	A	A	A	A	A	A	A	A	A	A
A	A	A	A	A	A	A	A	A	A	A
A	A	A	A	A	A	A	A	A	A	A
A	A	A	A	A	A	A	A	A	A	A
A	A	A	A	A	A	A	A	A	A	A
A	A	A	A	A	A	A	A	A	A	A
A	A	A	A	A	A	A	A	A	A	A
A	A	A	A	A	A	A	A	A	A	A
A	A	A	A	A	A	A	A	A	A	A
A	A	A	A	A	A	A	A	A	A	A
A	B	A	A	A	A	A	A	A	A	A
A	B	A	A	A	A	A	A	A	A	A
A	B	A	A	A	A	A	A	A	A	A
A	B	A	A	A	A	A	A	A	A	A

A	B	A	A	A	A	A	A	A	A	A
A	B	A	A	A	A	A	A	A	A	A
A	B	A	A	A	A	A	A	A	A	A
A	B	A	A	A	A	A	A	A	A	A
A	B	A	A	A	A	A	A	A	A	A
A	B	A	A	A	A	B	A	A	B	B
A	B	A	A	A	A	B	A	A	B	B
A	B	A	A	A	A	B	A	A	B	B
A	B	A	A	A	A	B	A	A	B	B
A	B	A	A	A	A	B	A	A	B	B
A	B	A	A	A	A	B	A	A	B	B
A	B	A	A	A	A	B	A	A	B	B
B	B	A	A	B	A	A	A	A	B	A
B	B	A	A	B	A	A	A	A	B	A
B	B	A	A	B	A	A	A	A	B	A
B	B	A	A	B	A	A	A	A	B	A
B	B	A	A	B	A	A	A	A	B	A
B	B	A	A	B	A	A	A	A	B	A
B	B	A	A	B	A	A	A	A	B	A
B	B	A	A	B	A	A	A	A	B	A
B	B	A	A	B	A	A	A	A	B	A
B	B	A	A	B	A	A	A	A	B	A
B	B	A	A	B	A	A	A	A	B	A
B	B	A	A	B	A	A	A	A	B	A
B	B	A	A	B	A	A	A	A	B	A
B	B	A	A	B	A	A	A	A	B	A
B	B	A	A	B	A	A	A	A	B	A
B	B	A	A	B	A	A	A	A	B	A
B	B	A	A	B	A	A	A	A	B	B
B	B	A	A	B	A	A	A	A	B	B
B	B	A	A	B	A	A	A	A	B	B
A	B	A	A	B	A	A	A	A	B	B
A	B	A	A	B	A	A	A	A	B	B
A	B	A	A	B	A	A	A	A	B	B
A	B	A	A	B	A	A	A	A	B	B
A	B	A	A	B	A	A	A	A	B	B
A	A	A	A	B	A	A	A	A	B	B
A	A	A	A	B	A	A	A	A	B	B
A	A	A	A	B	A	A	A	A	B	B
A	B	A	A	B	A	A	A	A	B	B
A	B	A	A	B	A	A	A	A	B	B
A	B	A	A	B	A	A	A	A	B	B
A	B	A	A	B	A	A	A	A	B	B
A	B	A	A	B	A	A	A	A	B	B
A	B	B	A	B	B	A	A	B	B	B
A	B	B	A	B	B	A	A	B	B	B
A	B	B	A	B	B	A	A	B	B	B
A	B	B	A	B	B	A	A	B	B	B
A	B	B	A	A	B	A	A	B	B	B

A	B	B	A	A	B	A	A	B	B	B
A	B	B	A	A	B	A	A	B	B	B
A	B	B	A	A	B	A	A	B	B	B
A	B	B	A	A	B	A	A	B	B	B
A	B	B	A	A	B	A	A	B	B	B
A	B	B	A	A	B	A	A	B	B	B
A	B	B	A	A	B	A	A	B	B	B
A	A	B	A	A	B	A	A	B	B	B
A	A	B	A	A	B	A	A	B	B	B
A	A	B	A	A	B	A	A	B	B	B
A	A	B	A	A	B	A	A	B	B	B
A	A	B	A	A	B	A	A	B	B	B
A	A	B	A	A	B	A	A	B	B	B
A	A	B	A	A	B	A	A	B	B	B
A	A	B	A	A	B	A	A	B	B	B
A	A	B	A	A	B	A	A	B	B	B
A	A	B	A	A	B	A	A	B	B	B
A	A	B	A	A	B	A	A	B	B	B
A	A	B	A	A	B	A	A	B	B	A
A	A	B	A	A	B	A	A	B	B	A
A	A	B	A	A	B	A	A	B	B	A
A	A	B	B	A	B	A	A	B	B	A
A	A	B	B	A	B	A	A	B	B	A
A	A	B	B	B	B	A	A	B	B	A
A	A	B	B	B	B	A	A	B	B	A
A	A	B	B	B	B	A	A	B	B	A
A	A	B	B	B	B	A	A	B	B	A
A	A	B	B	B	B	A	A	B	B	A
A	A	B	B	B	B	A	A	B	B	A
A	A	B	B	B	B	A	A	B	B	A
A	A	B	B	B	B	A	A	B	B	A
A	A	B	B	B	B	A	A	B	B	A
A	A	B	B	B	B	A	A	B	B	A
A	A	B	B	B	B	A	A	B	B	A
A	A	B	B	B	B	A	A	B	B	A
A	A	B	B	B	B	A	A	B	B	A
A	A	B	B	B	B	A	A	B	B	A
A	A	B	B	B	B	A	A	B	B	A
A	A	A	B	B	A	A	A	A	B	A
A	A	A	B	B	A	A	A	A	B	A
A	A	A	B	B	A	A	A	A	B	A
A	A	A	B	B	A	A	A	A	B	A
A	A	A	B	B	A	A	A	A	B	A
A	A	A	B	B	A	A	A	A	B	A
A	A	A	B	B	A	A	A	A	B	A
A	A	A	B	B	A	A	A	A	B	A
A	A	A	B	B	A	A	A	A	B	A
A	A	A	B	B	A	A	A	A	B	A
A	A	A	B	B	A	A	A	A	B	A

A	A	A	B	B	A	A	A	A	B	A
A	A	A	B	B	A	A	A	A	B	A
A	A	A	B	B	A	A	A	A	B	A
A	A	A	B	B	A	B	A	A	B	A
A	A	A	B	B	A	B	A	A	B	A
A	A	A	B	B	A	B	A	A	B	A
A	A	A	B	B	A	B	A	A	B	A
A	A	A	B	B	A	B	A	A	B	A
A	A	A	A	B	A	B	A	A	B	A
A	A	A	A	B	A	B	A	A	B	A
A	A	A	A	B	A	B	A	A	B	A
A	A	A	A	B	A	B	A	A	B	A
A	A	A	A	B	A	B	A	A	B	A
A	A	B	A	A	B	A	B	B	A	A
A	A	B	A	A	B	A	B	B	A	A
A	A	B	A	A	B	A	B	B	A	A
A	A	B	A	A	B	A	B	B	A	A
A	A	A	A	A	A	A	B	A	A	A
A	A	A	A	A	A	A	B	A	A	A
A	A	A	A	A	A	A	B	A	A	A
A	A	A	A	A	A	A	B	A	A	A
A	A	A	A	A	A	A	B	A	A	A
A	A	A	A	A	A	A	B	A	A	A
A	A	A	A	A	A	A	B	A	B	A
A	A	A	A	A	A	A	B	A	B	A
A	A	A	A	A	A	A	B	A	B	A
A	A	A	A	A	A	A	B	A	B	A
A	A	A	A	A	A	A	B	A	B	A
A	A	A	A	A	A	A	B	A	B	A
A	A	A	A	A	A	A	B	A	B	A
A	A	A	A	A	A	A	B	A	B	A
A	A	A	A	A	A	A	B	A	B	A
A	A	A	A	A	A	A	B	A	B	A
A	A	A	A	A	A	A	B	A	B	A
A	A	A	A	A	A	A	B	A	B	A
A	A	A	A	A	A	A	B	A	B	B
A	A	A	A	B	A	A	B	A	B	B
A	A	A	B	B	A	A	B	A	B	B
A	A	A	B	B	A	A	B	A	B	B
A	A	A	B	B	A	A	B	A	B	B
A	A	A	B	B	A	B	B	A	B	B
A	A	A	B	B	A	B	B	A	B	B
A	A	A	B	B	A	B	B	A	B	B
A	A	A	B	B	A	B	B	A	B	B
A	A	A	B	B	A	B	B	A	B	B
A	A	A	B	B	A	B	B	A	B	B
A	A	A	B	B	A	B	B	A	B	B
A	B	A	B	B	A	B	B	A	B	B
A	B	A	B	B	A	B	B	A	B	B

A	B	A	B	B	A	B	B	A	B	B
A	B	A	B	B	A	B	B	A	B	B
A	B	A	B	B	A	B	B	A	B	B
A	B	A	B	B	A	B	B	A	B	B
A	B	A	B	B	A	B	B	A	B	B
A	B	A	B	B	A	B	B	A	B	A
A	B	A	B	B	A	B	B	A	B	A
A	B	A	B	B	A	B	B	A	B	A
A	B	A	A	B	A	B	B	A	B	A
A	B	A	A	B	A	B	B	A	B	A
A	B	A	A	B	A	B	B	A	B	A
A	B	A	A	B	A	B	B	A	B	A
A	B	A	A	A	A	B	B	A	B	A
A	B	A	A	A	A	B	B	A	B	A
A	B	A	A	A	A	B	B	A	B	A
A	B	A	A	A	A	B	B	A	B	A
A	B	A	A	A	A	B	B	A	B	A
A	B	B	A	A	B	B	B	B	B	A
A	B	B	A	A	B	B	B	B	B	A
A	B	B	A	A	B	B	A	B	B	A
A	B	B	A	A	B	B	A	B	B	A
A	B	B	A	A	B	B	A	B	B	A
B	B	B	B	A	B	B	A	B	B	A
B	B	B	B	A	B	B	A	B	B	A
B	B	B	B	A	B	B	A	B	B	A
B	B	B	B	A	B	B	A	B	B	A
B	B	B	B	A	B	B	A	B	B	A
B	B	B	B	A	B	B	A	B	B	A
B	B	B	B	A	B	B	A	B	B	A
B	B	B	B	A	B	B	A	B	B	A
B	B	B	B	A	B	B	A	B	B	A
B	B	B	B	A	B	B	A	B	B	A
B	B	B	B	A	B	B	A	B	B	A
B	B	B	B	A	B	B	A	B	B	A
B	B	B	B	A	B	B	A	B	B	A
B	B	B	B	A	B	B	A	B	B	A
B	B	B	B	A	B	A	A	B	B	A
B	B	B	B	A	B	A	A	B	B	A
B	B	B	B	A	B	A	A	B	B	A
B	B	B	B	A	B	A	A	B	B	A
B	B	B	B	A	B	A	A	B	B	A
B	B	B	B	A	B	A	A	B	B	A
B	B	B	B	B	B	A	B	B	A	B
B	B	B	B	B	B	A	B	B	A	B
B	B	B	B	B	B	A	B	B	A	A
B	B	B	B	B	B	A	B	B	A	A
A	B	B	B	B	B	A	B	B	A	A
A	B	B	B	B	B	A	B	B	A	A
A	B	B	B	B	B	A	B	B	A	A

A	B	B	B	B	B	A	B	B	A	A
A	B	B	B	B	B	A	B	B	A	A
A	B	B	B	B	B	A	B	B	A	A
A	B	B	B	B	B	A	B	B	A	A
A	B	B	B	B	B	A	B	B	A	A
A	B	B	B	B	B	A	B	B	A	A
A	B	B	B	B	B	A	B	B	A	A
A	B	B	B	B	B	A	A	B	A	A
A	B	B	B	B	B	A	A	B	A	A
A	B	B	B	B	B	A	A	B	A	A
A	B	B	B	B	B	A	A	B	A	A
A	B	B	B	A	B	A	A	B	A	A
A	B	B	A	A	B	A	A	B	A	A
A	B	B	A	A	B	A	A	B	A	A
A	B	A	A	A	A	A	A	A	A	A
A	B	A	A	A	A	A	A	A	A	A
A	B	A	A	A	A	A	A	A	A	A
A	A	A	A	A	A	A	A	A	A	A
A	A	A	A	A	A	A	A	A	A	A
A	A	A	A	B	A	A	A	A	A	A
A	A	A	A	B	A	A	A	A	A	A
A	A	A	A	B	A	A	A	A	A	A
A	A	A	A	B	A	A	A	A	A	A
A	A	A	A	B	A	A	A	A	A	A
A	A	A	A	B	A	A	A	A	A	A
A	A	A	A	B	A	A	A	A	A	A
A	A	A	A	B	A	A	A	A	A	A
A	A	A	A	A	A	A	A	A	A	A
A	A	A	A	A	A	A	A	A	A	A
A	A	A	A	A	A	A	A	A	A	A
A	A	A	A	A	A	A	A	A	A	A
A	A	A	A	A	A	A	A	A	A	A
A	A	A	A	A	A	A	A	A	A	A
A	A	A	A	A	A	A	A	A	A	A
A	A	A	A	A	A	A	A	A	A	A
A	A	0	A	A	A	A	A	A	A	A
A	A	0	A	A	A	A	A	A	A	A
A	A	0	A	A	A	A	A	A	A	A
A	A	0	A	A	A	A	A	A	A	A
0	A	A	A	A	A	A	A	A	A	A
0	A	A	A	A	A	A	A	A	A	A
0	A	A	A	A	A	A	A	A	A	A
0	A	A	A	A	A	A	A	A	A	A
0	A	A	A	A	A	A	A	A	A	A
0	A	A	A	A	A	A	A	A	A	A
0	A	A	A	B	A	A	A	A	A	A
0	A	A	A	B	A	A	A	A	A	A
0	A	A	A	B	A	A	A	A	A	A
0	A	A	A	B	A	A	A	A	A	A

0	A	A	A	B	A	A	A	A	A	A
0	A	A	A	B	A	A	A	A	A	A
0	A	A	A	B	A	A	A	A	A	A
0	A	A	A	B	A	A	A	A	A	A
0	A	B	A	B	B	A	A	B	A	A
0	A	B	A	B	B	A	A	B	A	A
0	A	B	A	B	B	A	A	B	A	A
0	A	B	A	B	B	A	A	B	A	B
0	A	B	A	B	B	A	A	B	A	B
0	0	B	A	B	B	B	A	B	A	A
0	0	B	A	B	B	B	A	B	A	A
0	0	B	A	B	B	B	A	B	A	A
0	0	A	A	B	A	B	A	A	A	A
0	0	A	A	A	A	B	A	A	A	A
0	0	A	A	A	A	B	A	A	A	A
0	0	A	A	A	A	B	A	A	A	A
0	0	A	A	A	A	B	A	A	A	A
0	0	A	A	A	A	B	A	A	A	A
0	0	A	A	A	A	B	A	A	A	A
0	0	A	A	A	A	B	A	A	A	A
A	A	A	A	A	A	B	A	A	A	A
A	A	A	A	A	A	B	A	A	A	A
A	A	A	A	A	A	B	A	A	A	A
A	A	A	A	A	A	B	A	A	A	A
A	A	A	A	A	A	B	A	A	A	A
A	A	A	A	A	A	B	A	A	A	A
A	A	A	A	A	A	B	A	A	A	A
A	A	A	A	A	A	B	A	A	A	A
A	A	A	A	A	A	B	A	A	A	A
A	A	A	A	A	A	B	A	A	A	A
A	A	A	A	A	A	B	A	A	A	A
A	A	A	A	A	A	B	A	A	A	A
A	A	A	A	A	A	B	A	A	A	A
A	A	A	A	A	A	B	A	A	A	A
A	A	A	A	A	A	B	A	A	A	A
A	A	A	A	A	A	B	A	A	A	A
A	A	A	A	A	A	B	A	A	A	A
A	A	A	A	A	A	B	A	A	A	A
A	A	A	A	A	A	B	A	A	A	A
A	A	A	A	A	A	B	A	A	A	A
A	A	A	A	A	A	B	A	A	A	A
A	A	A	A	A	A	B	A	A	A	A
A	A	A	A	A	A	B	A	A	A	A
A	A	A	A	A	A	B	A	A	A	A
A	A	A	A	A	A	B	A	A	A	A
A	A	A	A	A	A	B	A	A	A	A
A	A	A	A	A	A	B	A	A	A	A
A	A	A	A	A	A	B	A	A	B	A
A	A	A	A	A	A	B	A	A	B	A

B	A	A	B	A	A	B	A	A	B	B
B	A	A	B	A	A	B	A	A	B	B
B	A	A	B	A	A	B	A	A	B	B
B	A	A	B	A	A	B	B	A	B	B
B	A	A	B	A	A	B	B	A	B	B
B	A	A	B	A	A	B	B	A	B	B
B	A	A	B	A	A	B	B	A	B	B
B	A	A	B	A	A	B	B	A	B	B
B	A	A	B	A	A	B	B	A	B	B
B	A	A	B	A	A	B	B	A	B	B
B	A	A	B	A	A	B	B	A	B	B
B	A	A	B	A	A	B	B	A	B	B
B	A	A	B	A	A	B	B	A	B	B
B	A	A	B	A	A	B	B	A	B	B
B	A	A	B	A	A	B	B	A	B	B
B	A	A	B	A	A	B	B	A	B	B
B	A	A	B	A	A	B	B	A	B	B
B	A	A	B	B	A	B	B	A	B	B
B	A	A	A	B	A	B	B	A	B	B
B	A	A	A	B	A	B	B	A	B	B
B	A	A	A	B	A	B	B	A	B	B
B	A	A	A	B	A	B	B	A	B	B
B	A	A	A	B	A	B	B	A	B	B
B	A	A	A	B	A	B	B	A	B	B
B	A	A	A	B	A	B	B	A	B	B
B	A	A	A	B	A	B	B	A	B	B
B	A	A	A	B	A	B	B	A	B	B
B	A	A	A	B	A	B	B	A	B	B
B	A	A	A	B	A	B	B	A	B	B
B	A	A	A	B	A	B	B	A	B	B
B	A	A	A	B	A	B	B	A	B	B
B	A	A	A	B	A	B	A	A	B	B
B	A	A	A	B	A	B	A	A	B	B
B	A	A	A	B	A	B	A	A	B	B
B	A	A	A	B	A	B	A	A	B	A
B	A	A	A	B	A	B	A	A	B	A
B	A	A	A	B	A	B	A	A	B	A
B	A	A	A	B	A	B	A	A	B	A
B	A	A	A	B	A	B	A	A	B	A
B	A	A	A	B	A	B	A	A	B	A
B	A	A	A	B	A	B	A	A	B	A
B	A	A	A	B	A	B	A	A	B	A
B	A	A	A	B	A	B	A	A	B	A
B	A	A	A	B	A	B	A	A	B	A
B	A	A	A	B	A	B	A	A	B	A
B	A	A	A	B	A	B	A	A	B	A
B	A	A	A	B	A	B	A	A	B	A
B	A	A	A	B	A	B	A	A	B	A
B	A	A	A	B	A	B	A	A	B	A

B	A	A	A	B	A	B	A	A	B	A
B	A	A	A	B	A	B	A	A	B	A
B	A	A	A	B	A	B	A	A	B	A
B	A	A	A	B	A	B	A	A	B	A
B	A	A	A	B	A	A	A	A	B	A
B	A	A	A	B	A	A	A	A	B	A
B	A	A	A	B	A	A	A	A	B	A
B	A	A	A	B	A	A	A	A	B	A
B	A	A	A	B	A	A	A	A	B	A
A	A	A	A	B	A	A	A	A	B	A
A	A	A	A	B	A	A	A	A	B	A
A	A	A	A	B	A	A	A	A	B	A
A	A	A	A	B	A	A	A	A	B	A
A	A	A	A	B	A	A	A	A	B	A
A	A	A	A	B	A	A	A	A	B	A
A	A	A	A	B	A	A	A	A	B	A
A	A	A	A	B	A	A	A	A	B	A
A	A	A	A	B	A	A	A	A	B	A
A	A	A	A	B	A	A	A	A	B	A
A	A	A	A	B	A	A	A	A	B	A
A	A	A	A	B	A	A	A	A	B	A
A	A	A	A	B	A	A	A	A	B	A
A	A	A	A	B	A	A	A	A	B	A
A	A	A	A	B	A	A	A	A	B	A
A	A	A	A	B	A	A	A	A	B	A
A	A	A	A	B	A	A	A	A	B	A
A	A	A	A	B	A	A	A	A	B	A
A	A	A	A	B	A	A	A	A	B	A
A	A	A	A	B	A	A	A	A	B	A
A	A	A	B	B	A	A	A	A	B	A
A	A	A	B	B	A	A	A	A	B	A
A	A	A	B	B	A	A	A	A	B	A
A	A	A	B	B	A	A	A	A	B	A
A	A	B	B	A	B	B	B	B	B	B
A	A	B	B	A	B	B	B	B	B	B
A	A	B	B	A	B	B	B	B	B	B
A	A	B	B	A	B	B	B	B	B	B
A	A	B	B	A	B	B	B	B	B	B
A	A	B	B	A	B	B	B	B	B	B
A	A	B	B	A	B	B	B	B	B	B
A	A	B	B	A	B	B	B	B	B	B
A	A	B	B	A	B	B	B	B	B	B
A	A	B	B	B	B	B	B	B	B	B
A	A	B	B	B	B	B	B	B	B	B
A	A	B	B	B	B	B	B	B	B	B
A	A	B	B	B	B	B	B	B	B	B
A	B	B	B	B	B	B	B	B	B	B
A	B	B	B	B	B	B	B	B	B	B
A	B	B	B	B	B	B	B	B	A	B
