# Supplementary material for: A high-density SNP genotyping array for Brassica napus and its ancestral diploid species based on optimised selection of single-locus markers in the allotetraploid genome
Source: Theor Appl Genet. 2016 Jun 30;129(10):1887–99. doi: 10.1007/s00122-016-2746-7 (PMC5025514; doi:10.1007/s00122-016-2746-7)
Supplement: Supplementary file 3 — Scoring metrics for SNP loci at TraitGenetics in B. napus, B. oleracea and B. rapa (PDF 9064 kb) [file 122_2016_2746_MOESM3_ESM.pdf]

Table 2: Scoring metrics for SNP loci at TraitGenetics in B. napus, B. oleracea and B. rapa

Index	Name	Scoring	napus		oleracea		rapa	
			Call Freq	GenTrain Scor	Call Freq	GenTrain Score	Call Freq	GenTrain Sco
1	Bn-A01-p10000230	A-genome specific (napus-rapa)	0.9904306	0.2665956	not scorable	not scorable	0.9917355	0.3447306
2	Bn-A01-p1000115	A-genome specific (napus-rapa)	0.9473684	0.669354	not scorable	not scorable	0.9752066	0.6498784
3	Bn-A01-p1001022	A-genome specific (napus-rapa)	1	0.4369346	not scorable	not scorable	0.9917355	0.6821402
4	Bn-A01-p10013185	A-genome specific (napus-rapa)	0.9880383	0.3689644	not scorable	not scorable	0.9917355	0.6091064
5	Bn-A01-p10026020	A-genome specific (napus-rapa)	0.9712918	0.3369454	not scorable	not scorable	0.9669421	0.7870976
6	Bn-A01-p10027328	A-genome specific (napus-rapa)	0.9617225	0.6239452	not scorable	not scorable	0.9504132	0.607905
7	Bn-A01-p10031169	A-genome specific (rapa only)	not scorable	not scorable	not scorable	not scorable	1	0.6865193
8	Bn-A01-p10036602	A-genome specific (napus-rapa)	0.9904306	0.7991401	not scorable	not scorable	1	0.6476043
9	Bn-A01-p100441	A-genome specific (napus-rapa)	0.9784689	0.8056656	not scorable	not scorable	0.9752066	0.7679359
10	Bn-A01-p10059043	genome unspecific (all in)	0.9641148	0.3950519	1	0.5893024	0.9586777	0.8467416
11	Bn-A01-p100693	A-genome specific (napus-rapa)	0.9760765	0.682655	not scorable	not scorable	0.9752066	0.7123271
12	Bn-A01-p10069493	A-genome specific (napus-rapa)	0.9880383	0.7955522	not scorable	not scorable	0.9917355	0.5615633
13	Bn-A01-p10071456	A-genome specific (napus-rapa)	0.9712918	0.6898434	not scorable	not scorable	0.7107438	0.848121
14	Bn-A01-p10071903	A-genome specific (napus-rapa)	0.9808612	0.6955411	not scorable	not scorable	1	0.8032902
15	Bn-A01-p101021	A-genome specific (napus-rapa)	0.9904306	0.7510921	not scorable	not scorable	1	0.6350701
16	Bn-A01-p10105499	A-genome specific (napus-rapa)	0.9880383	0.5330738	not scorable	not scorable	0.9669421	0.7831092
17	Bn-A01-p101094	A-genome specific (napus-rapa)	0.9880383	0.7635614	not scorable	not scorable	0.9917355	0.7450218
18	Bn-A01-p1012990	A-genome specific (napus-rapa)	0.9593301	0.3686254	not scorable	not scorable	0.9752066	0.6742238
19	Bn-A01-p101451	A-genome specific (napus-rapa)	0.9784689	0.7987067	not scorable	not scorable	0.9669421	0.7406716
20	Bn-A01-p10166739	A-genome specific (napus-rapa)	0.9665072	0.8488839	not scorable	not scorable	0.7355372	0.8519396
21	Bn-A01-p10181972	A-genome specific (napus-rapa)	0.9856459	0.7297298	not scorable	not scorable	0.9834711	0.7876438
22	Bn-A01-p101879	A-genome specific (napus-rapa)	0.9617225	0.7877749	not scorable	not scorable	0.9669421	0.7570555
23	Bn-A01-p101943	A-genome specific (napus-rapa)	0.9688995	0.7574532	not scorable	not scorable	0.9834711	0.7462464
24	Bn-A01-p102158	A-genome specific (napus-rapa)	0.9688995	0.6769506	not scorable	not scorable	0.9834711	0.7080495
25	Bn-A01-p102187	A-genome specific (napus-rapa)	0.9736842	0.763105	not scorable	not scorable	0.9752066	0.7424731
26	Bn-A01-p102249	A-genome specific (napus-rapa)	0.9832536	0.6805793	not scorable	not scorable	0.9917355	0.7638057
27	Bn-A01-p10231780	A-genome specific (napus-rapa)	0.9736842	0.7622875	not scorable	not scorable	1	0.7452666
28	Bn-A01-p10234366	A-genome specific (napus-rapa)	0.9043062	0.3499477	not scorable	not scorable	1	0.6643501
29	Bn-A01-p10238825	all not scorable	not scorable	not scorable	not scorable	not scorable	not scorable	not scorable
30	Bn-A01-p10242217	A-genome specific (napus-rapa)	0.9617225	0.793101	not scorable	not scorable	0.9917355	0.7951404

31	Bn-A01-p10242298	A-genome specific (napus-rapa)	0.8014354	0.7506034	not scorable	not scorable	0.9834711	0.7371384
32	Bn-A01-p10246312	A-genome specific (napus-rapa)	0.9928229	0.7311953	not scorable	not scorable	1	0.7193801
33	Bn-A01-p10249225	A-genome specific (napus-rapa)	0.9952153	0.3300748	not scorable	not scorable	1	0.9083468
34	Bn-A01-p10250255	A-genome specific (napus-rapa)	0.9880383	0.7561908	not scorable	not scorable	1	0.6893185
35	Bn-A01-p10251052	A-genome specific (napus-rapa)	0.9760765	0.7680904	not scorable	not scorable	1	0.7708634
36	Bn-A01-p10257919	A-genome specific (napus-rapa)	0.9665072	0.5213329	not scorable	not scorable	1	0.7956077
37	Bn-A01-p1026631	A-genome specific (napus-rapa)	0.9688995	0.4588441	not scorable	not scorable	1	0.7344612
38	Bn-A01-p10268662	A-genome specific (napus-rapa)	0.9760765	0.3181653	not scorable	not scorable	1	0.7622542
39	Bn-A01-p10278752	genome unspecific (all in)	0.9832536	0.4511455	1	0.7729614	1	0.8469009
40	Bn-A01-p10285074	A-genome specific (napus-rapa)	0.9784689	0.7776531	not scorable	not scorable	1	0.8155444
41	Bn-A01-p1029558	A-genome specific (napus-rapa)	0.9712918	0.7703233	not scorable	not scorable	0.9917355	0.7591832
42	Bn-A01-p1029668	A-genome specific (napus-rapa)	0.9856459	0.8019634	not scorable	not scorable	1	0.7948786
43	Bn-A01-p10297732	A-genome specific (napus-rapa)	0.9928229	0.4578273	not scorable	not scorable	1	0.8104565
44	Bn-A01-p10299187	A-genome specific (napus-rapa)	0.9688995	0.7587264	not scorable	not scorable	1	0.7222773
45	Bn-A01-p10340159	A-genome specific (napus-rapa)	0.9808612	0.6033359	not scorable	not scorable	0.9752066	0.7038876
46	Bn-A01-p10340283	A-genome specific (rapa only)	not scorable	not scorable	not scorable	not scorable	1	0.6538376
47	Bn-A01-p10340829	genome unspecific (all in)	0.9784689	0.4524876	1	0.6944774	1	0.8158014
48	Bn-A01-p10343401	A-genome specific (napus-rapa)	0.9665072	0.7711882	not scorable	not scorable	0.9917355	0.7655298
49	Bn-A01-p10344498	A-genome specific (napus-rapa)	0.9641148	0.7845517	not scorable	not scorable	0.9834711	0.8655694
50	Bn-A01-p10345430	A-genome specific (napus-rapa)	0.9928229	0.6684332	not scorable	not scorable	0.9917355	0.7227203
51	Bn-A01-p10346546	A-genome specific (napus-rapa)	0.9880383	0.4187292	not scorable	not scorable	0.9752066	0.5752468
52	Bn-A01-p10354645	A-genome specific (napus-rapa)	0.9880383	0.5061122	not scorable	not scorable	0.9834711	0.7026362
53	Bn-A01-p10392909	A-genome specific (rapa only)	not scorable	not scorable	not scorable	not scorable	1	0.7844879
54	Bn-A01-p10398937	A-genome specific (napus-rapa)	0.9569378	0.6135517	not scorable	not scorable	0.9917355	0.7251074
55	Bn-A01-p10399077	all not scorable	not scorable	not scorable	not scorable	not scorable	not scorable	not scorable
56	Bn-A01-p10406165	A-genome specific (napus-rapa)	0.9593301	0.2466998	not scorable	not scorable	0.9917355	0.7093098
57	Bn-A01-p10410665	A-genome specific (napus-rapa)	0.9808612	0.2381571	not scorable	not scorable	1	0.7641702
58	Bn-A01-p10415035	A-genome specific (rapa only)	not scorable	not scorable	not scorable	not scorable	1	0.6120533
59	Bn-A01-p1041947	A-genome specific (napus-rapa)	0.9784689	0.784063	not scorable	not scorable	1	0.7492238
60	Bn-A01-p10426647	A-genome specific (napus-rapa)	0.9784689	0.789389	not scorable	not scorable	0.9917355	0.7297331
61	Bn-A01-p10426714	A-genome specific (napus-rapa)	0.9736842	0.7676027	not scorable	not scorable	0.9917355	0.8170549
62	Bn-A01-p10427300	A-genome specific (rapa only)	not scorable	not scorable	not scorable	not scorable	1	0.7909042
63	Bn-A01-p10457944	A-genome specific (napus-rapa)	0.9904306	0.3923164	not scorable	not scorable	1	0.8196553

64	Bn-A01-p1045881	A-genome specific (napus-rapa)	0.9617225	0.777612	not scorable	not scorable	0.9834711	0.7430993
65	Bn-A01-p10493092	A-genome specific (rapa only)	not scorable	not scorable	not scorable	not scorable	1	0.8138223
66	Bn-A01-p10499882	genome unspecific (oleracea-rapa)	not scorable	not scorable	0.8992248	0.7567436	0.9834711	0.7394594
67	Bn-A01-p10513529	A-genome specific (napus-rapa)	0.9928229	0.7872128	not scorable	not scorable	0.9834711	0.7949384
68	Bn-A01-p10518110	A-genome specific (napus-rapa)	0.9952153	0.6645517	not scorable	not scorable	0.9834711	0.7841431
69	Bn-A01-p10523194	A-genome specific (napus-rapa)	0.9952153	0.8192077	not scorable	not scorable	0.9917355	0.7845644
70	Bn-A01-p10523264	A-genome specific (napus-rapa)	0.9976076	0.7840292	not scorable	not scorable	1	0.7216815
71	Bn-A01-p10523833	A-genome specific (napus-rapa)	0.9856459	0.3752156	not scorable	not scorable	0.9834711	0.7254374
72	Bn-A01-p10525172	A-genome specific (napus-rapa)	0.9880383	0.7530954	not scorable	not scorable	1	0.7573287
73	Bn-A01-p10525357	napus specific	0.9736842	0.3932988	not scorable	not scorable	not scorable	not scorable
74	Bn-A01-p10525805	napus specific	0.9138756	0.7695048	not scorable	not scorable	not scorable	not scorable
75	Bn-A01-p10526054	napus specific	0.9114832	0.7585191	not scorable	not scorable	not scorable	not scorable
76	Bn-A01-p10526561	A-genome specific (napus-rapa)	0.9976076	0.7347771	not scorable	not scorable	0.9917355	0.7274886
77	Bn-A01-p10543102	A-genome specific (napus-rapa)	0.9904306	0.6350611	not scorable	not scorable	0.9917355	0.7372921
78	Bn-A01-p10550486	A-genome specific (napus-rapa)	0.9952153	0.7756099	not scorable	not scorable	1	0.8130969
79	Bn-A01-p10559083	A-genome specific (napus-rapa)	0.9784689	0.2816452	not scorable	not scorable	0.9834711	0.7035483
80	Bn-A01-p1056386	A-genome specific (napus-rapa)	0.9712918	0.5567337	not scorable	not scorable	0.9834711	0.6834758
81	Bn-A01-p10590357	A-genome specific (napus-rapa)	0.9880383	0.7009175	not scorable	not scorable	0.7190083	0.750165
82	Bn-A01-p10611800	A-genome specific (napus-rapa)	0.9880383	0.7350952	not scorable	not scorable	1	0.7683663
83	Bn-A01-p1062520	A-genome specific (napus-rapa)	0.9808612	0.5263378	not scorable	not scorable	0.9917355	0.8171118
84	Bn-A01-p10636637	A-genome specific (napus-rapa)	0.9760765	0.6933311	not scorable	not scorable	0.9752066	0.7229741
85	Bn-A01-p10636994	A-genome specific (rapa only)	not scorable	not scorable	not scorable	not scorable	0.9669421	0.7712798
86	Bn-A01-p10638747	A-genome specific (rapa only)	not scorable	not scorable	not scorable	not scorable	1	0.7568499
87	Bn-A01-p10639421	A-genome specific (rapa only)	not scorable	not scorable	not scorable	not scorable	0.9421487	0.789074
88	Bn-A01-p10640093	A-genome specific (rapa only)	not scorable	not scorable	not scorable	not scorable	0.9421487	0.7718278
89	Bn-A01-p10640881	A-genome specific (napus-rapa)	0.9665072	0.3705567	not scorable	not scorable	1	0.7294249
90	Bn-A01-p10641656	A-genome specific (napus-rapa)	0.9162679	0.4478071	not scorable	not scorable	0.9834711	0.7104915
91	Bn-A01-p1067371	A-genome specific (napus-rapa)	0.9617225	0.7760444	not scorable	not scorable	0.9669421	0.8453609
92	Bn-A01-p10693617	A-genome specific (napus-rapa)	0.9521531	0.4207712	not scorable	not scorable	1	0.5723776
93	Bn-A01-p10693623	A-genome specific (napus-rapa)	0.9712918	0.4451212	not scorable	not scorable	1	0.8112289
94	Bn-A01-p10720611	A-genome specific (napus-rapa)	0.9354067	0.6733357	not scorable	not scorable	0.7190083	0.9279843
95	Bn-A01-p1073387	A-genome specific (napus-rapa)	0.9760765	0.3872616	not scorable	not scorable	0.9917355	0.7375655
96	Bn-A01-p10740084	A-genome specific (napus-rapa)	0.9569378	0.7093448	not scorable	not scorable	1	0.8074856

97	Bn-A01-p10786043	genome unspecific (oleracea-rapa)	not scorable	not scorable	0.9689922	0.3920207	1	0.9292135
98	Bn-A01-p108136	A-genome specific (napus-rapa)	0.9952153	0.5074401	not scorable	not scorable	0.9752066	0.7821275
99	Bn-A01-p108225	A-genome specific (napus-rapa)	0.9760765	0.773875	not scorable	not scorable	0.9917355	0.7596025
100	Bn-A01-p10835605	A-genome specific (napus-rapa)	0.9880383	0.7875881	not scorable	not scorable	0.9752066	0.7282169
101	Bn-A01-p10838493	A-genome specific (napus-rapa)	0.9904306	0.7826334	not scorable	not scorable	0.9917355	0.6839622
102	Bn-A01-p10853026	A-genome specific (napus-rapa)	1	0.9172003	not scorable	not scorable	0.9834711	0.9303316
103	Bn-A01-p10859527	A-genome specific (napus-rapa)	0.9808612	0.7705874	not scorable	not scorable	0.9917355	0.7856755
104	Bn-A01-p10876307	napus specific	1	0.2578293	not scorable	not scorable	not scorable	not scorable
105	Bn-A01-p10889788	A-genome specific (napus-rapa)	0.8827751	0.3836298	not scorable	not scorable	0.9834711	0.7057354
106	Bn-A01-p1089109	A-genome specific (napus-rapa)	1	0.7805272	not scorable	not scorable	1	0.8653851
107	Bn-A01-p10916044	A-genome specific (napus-rapa)	0.9904306	0.4869438	not scorable	not scorable	0.9917355	0.6763602
108	Bn-A01-p1091610	A-genome specific (napus-rapa)	0.9976076	0.4233577	not scorable	not scorable	0.9917355	0.7249529
109	Bn-A01-p10926129	A-genome specific (napus-rapa)	0.9976076	0.4364225	not scorable	not scorable	0.9917355	0.7858144
110	Bn-A01-p10933947	A-genome specific (napus-rapa)	0.9760765	0.3426332	not scorable	not scorable	0.9752066	0.6558676
111	Bn-A01-p10934377	A-genome specific (napus-rapa)	0.9808612	0.7126837	not scorable	not scorable	1	0.7573561
112	Bn-A01-p10938883	A-genome specific (napus-rapa)	0.9665072	0.7735888	not scorable	not scorable	1	0.7193962
113	Bn-A01-p10939144	A-genome specific (napus-rapa)	0.9832536	0.7809803	not scorable	not scorable	0.9917355	0.804975
114	Bn-A01-p10945930	A-genome specific (napus-rapa)	0.9928229	0.4515028	not scorable	not scorable	1	0.8009096
115	Bn-A01-p1095733	A-genome specific (napus-rapa)	0.9712918	0.7122016	not scorable	not scorable	0.9917355	0.7053235
116	Bn-A01-p10963536	A-genome specific (napus-rapa)	0.9880383	0.7486913	not scorable	not scorable	0.9834711	0.7680522
117	Bn-A01-p10991782	A-genome specific (napus-rapa)	0.9832536	0.7104169	not scorable	not scorable	0.9752066	0.7130669
118	Bn-A01-p11011196	A-genome specific (napus-rapa)	0.9832536	0.7513719	not scorable	not scorable	0.9834711	0.8446312
119	Bn-A01-p1104187	A-genome specific (napus-rapa)	0.9832536	0.7797337	not scorable	not scorable	0.9834711	0.7974988
120	Bn-A01-p11052248	A-genome specific (napus-rapa)	0.9856459	0.7830637	not scorable	not scorable	0.9752066	0.6894654
121	Bn-A01-p11054015	A-genome specific (napus-rapa)	0.9856459	0.7572004	not scorable	not scorable	0.9669421	0.6853675
122	Bn-A01-p11077627	A-genome specific (napus-rapa)	0.9784689	0.7391128	not scorable	not scorable	0.9834711	0.711687
123	Bn-A01-p11078406	A-genome specific (napus-rapa)	0.9617225	0.7677053	not scorable	not scorable	1	0.6199373
124	Bn-A01-p11091663	A-genome specific (napus-rapa)	0.9832536	0.4930466	not scorable	not scorable	1	0.8745553
125	Bn-A01-p11091816	A-genome specific (napus-rapa)	0.9736842	0.4552591	not scorable	not scorable	0.9917355	0.770445
126	Bn-A01-p11108443	A-genome specific (rapa only)	not scorable	not scorable	not scorable	not scorable	0.9752066	0.7657378
127	Bn-A01-p11119985	A-genome specific (rapa only)	not scorable	not scorable	not scorable	not scorable	0.9752066	0.7788876
128	Bn-A01-p11120400	A-genome specific (napus-rapa)	0.9928229	0.9126557	not scorable	not scorable	1	0.8144232
129	Bn-A01-p11120493	A-genome specific (napus-rapa)	0.9832536	0.3486197	not scorable	not scorable	0.9917355	0.7748761

130	Bn-A01-p11137395	A-genome specific (napus-rapa)	0.9832536	0.3058263	not scorable	not scorable	0.9917355	0.7863011
131	Bn-A01-p11142752	A-genome specific (napus-rapa)	0.9808612	0.7719147	not scorable	not scorable	1	0.7587181
132	Bn-A01-p11163785	A-genome specific (napus-rapa)	0.9545454	0.2719347	not scorable	not scorable	0.9917355	0.569109
133	Bn-A01-p11163980	A-genome specific (napus-rapa)	0.9688995	0.570121	not scorable	not scorable	0.9586777	0.6586577
134	Bn-A01-p11174997	A-genome specific (napus-rapa)	0.9545454	0.3162233	not scorable	not scorable	0.9586777	0.6028904
135	Bn-A01-p1119894	A-genome specific (napus-rapa)	0.9952153	0.2926795	not scorable	not scorable	1	0.7513643
136	Bn-A01-p11206467	A-genome specific (napus-rapa)	0.9665072	0.3842166	not scorable	not scorable	0.9917355	0.6783591
137	Bn-A01-p11233207	A-genome specific (napus-rapa)	0.9928229	0.8546239	not scorable	not scorable	0.9917355	0.7136764
138	Bn-A01-p11234159	A-genome specific (rapa only)	not scorable	not scorable	not scorable	not scorable	1	0.7829238
139	Bn-A01-p11238778	A-genome specific (napus-rapa)	0.9712918	0.2994429	not scorable	not scorable	0.9752066	0.3521244
140	Bn-A01-p11249993	A-genome specific (napus-rapa)	0.9593301	0.2973236	not scorable	not scorable	0.9917355	0.7709839
141	Bn-A01-p11253272	A-genome specific (rapa only)	not scorable	not scorable	not scorable	not scorable	0.9834711	0.6109979
142	Bn-A01-p1125565	A-genome specific (napus-rapa)	0.9688995	0.7639387	not scorable	not scorable	0.9917355	0.778431
143	Bn-A01-p1126874	A-genome specific (napus-rapa)	0.9641148	0.7268822	not scorable	not scorable	0.9917355	0.7429338
144	Bn-A01-p11273927	A-genome specific (rapa only)	not scorable	not scorable	not scorable	not scorable	0.9917355	0.6625919
145	Bn-A01-p11284636	A-genome specific (napus-rapa)	0.9928229	0.7981051	not scorable	not scorable	1	0.7081991
146	Bn-A01-p11302783	A-genome specific (rapa only)	not scorable	not scorable	not scorable	not scorable	1	0.8061329
147	Bn-A01-p11306490	A-genome specific (napus-rapa)	0.9617225	0.3862232	not scorable	not scorable	0.9917355	0.6777248
148	Bn-A01-p11309186	A-genome specific (rapa only)	not scorable	not scorable	not scorable	not scorable	1	0.7310278
149	Bn-A01-p11324370	A-genome specific (rapa only)	not scorable	not scorable	not scorable	not scorable	1	0.6410352
150	Bn-A01-p1132885	A-genome specific (napus-rapa)	0.9712918	0.7654447	not scorable	not scorable	0.9917355	0.6737197
151	Bn-A01-p11330661	A-genome specific (napus-rapa)	0.9736842	0.3595648	not scorable	not scorable	1	0.7052383
152	Bn-A01-p1133447	A-genome specific (napus-rapa)	0.9832536	0.7735092	not scorable	not scorable	0.9917355	0.8131012
153	Bn-A01-p11336370	A-genome specific (rapa only)	not scorable	not scorable	not scorable	not scorable	0.9917355	0.7437819
154	Bn-A01-p11347791	A-genome specific (napus-rapa)	1	0.8656827	not scorable	not scorable	1	0.863152
155	Bn-A01-p11355643	A-genome specific (rapa only)	not scorable	not scorable	not scorable	not scorable	1	0.9514361
156	Bn-A01-p11407167	A-genome specific (napus-rapa)	0.9904306	0.7098235	not scorable	not scorable	0.9752066	0.7002256
157	Bn-A01-p11434769	A-genome specific (rapa only)	not scorable	not scorable	not scorable	not scorable	0.9917355	0.8224482
158	Bn-A01-p11441851	A-genome specific (napus-rapa)	0.9736842	0.8038772	not scorable	not scorable	0.9917355	0.8008587
159	Bn-A01-p11444689	A-genome specific (rapa only)	not scorable	not scorable	not scorable	not scorable	1	0.6845859
160	Bn-A01-p1145674	A-genome specific (napus-rapa)	0.9784689	0.7559376	not scorable	not scorable	1	0.7285279
161	Bn-A01-p11459295	A-genome specific (rapa only)	not scorable	not scorable	not scorable	not scorable	1	0.7279535
162	Bn-A01-p11462315	A-genome specific (rapa only)	not scorable	not scorable	not scorable	not scorable	0.9752066	0.7117506

163	Bn-A01-p11469596	A-genome specific (rapa only)	not scorable	not scorable	not scorable	not scorable	0.9834711	0.6496505
164	Bn-A01-p11469992	A-genome specific (napus-rapa)	0.9497608	0.4587814	not scorable	not scorable	1	0.704862
165	Bn-A01-p11471489	genome unspecific (all in)	0.9569378	0.4844996	1	0.5508249	0.9917355	0.7345119
166	Bn-A01-p11479565	A-genome specific (napus-rapa)	0.9832536	0.4146444	not scorable	not scorable	0.5123967	0.6613363
167	Bn-A01-p11480814	A-genome specific (napus-rapa)	0.9688995	0.5216396	not scorable	not scorable	0.9586777	0.7981321
168	Bn-A01-p11483435	A-genome specific (napus-rapa)	1	0.7617218	not scorable	not scorable	1	0.8028744
169	Bn-A01-p1148760	A-genome specific (napus-rapa)	0.9856459	0.3684264	not scorable	not scorable	0.9834711	0.8941768
170	Bn-A01-p11492050	A-genome specific (napus-rapa)	0.9688995	0.335267	not scorable	not scorable	0.9917355	0.6780698
171	Bn-A01-p1149351	genome unspecific (all in)	0.923445	0.5852232	0.9534883	0.654623	1	0.8124242
172	Bn-A01-p1149698	genome unspecific (all in)	0.9617225	0.4704581	0.9379845	0.6799759	0.9752066	0.6659241
173	Bn-A01-p1150857	A-genome specific (napus-rapa)	0.8325359	0.7234866	not scorable	not scorable	0.9504132	0.6767076
174	Bn-A01-p1151961	A-genome specific (napus-rapa)	1	0.5312282	not scorable	not scorable	0.9917355	0.9019314
175	Bn-A01-p1152123	A-genome specific (napus-rapa)	0.9880383	0.7434763	not scorable	not scorable	0.9917355	0.720995
176	Bn-A01-p1152359	genome unspecific (all in)	0.9712918	0.7203477	0.9534883	0.7175227	0.9752066	0.5010673
177	Bn-A01-p1152369	A-genome specific (napus-rapa)	0.9521531	0.4297814	not scorable	not scorable	1	0.7564054
178	Bn-A01-p11543126	A-genome specific (napus-rapa)	0.9928229	0.8502678	not scorable	not scorable	0.9669421	0.8039905
179	Bn-A01-p11543191	A-genome specific (napus-rapa)	0.9928229	0.7317408	not scorable	not scorable	0.9917355	0.7064139
180	Bn-A01-p11559064	A-genome specific (rapa only)	not scorable	not scorable	not scorable	not scorable	0.9917355	0.7162943
181	Bn-A01-p11560385	A-genome specific (napus-rapa)	1	0.6953942	not scorable	not scorable	0.9917355	0.5080594
182	Bn-A01-p1156081	A-genome specific (napus-rapa)	0.9449761	0.2179753	not scorable	not scorable	0.9834711	0.6784428
183	Bn-A01-p1156979	A-genome specific (napus-rapa)	0.8492823	0.8454186	not scorable	not scorable	0.9752066	0.7879395
184	Bn-A01-p1158564	A-genome specific (napus-rapa)	0.9880383	0.7327312	not scorable	not scorable	0.9752066	0.722595
185	Bn-A01-p11613721	A-genome specific (napus-rapa)	1	0.4893315	not scorable	not scorable	1	0.4247669
186	Bn-A01-p11639506	A-genome specific (napus-rapa)	0.9952153	0.681012	not scorable	not scorable	0.9834711	0.8110971
187	Bn-A01-p11639917	A-genome specific (napus-rapa)	0.937799	0.6482093	not scorable	not scorable	1	0.7930498
188	Bn-A01-p11641669	all not scorable	not scorable	not scorable	not scorable	not scorable	not scorable	not scorable
189	Bn-A01-p11642664	A-genome specific (napus-rapa)	0.9976076	0.3638537	not scorable	not scorable	1	0.7609156
190	Bn-A01-p11642677	A-genome specific (napus-rapa)	1	0.9175813	not scorable	not scorable	1	0.8330763
191	Bn-A01-p11642772	A-genome specific (napus-rapa)	1	0.524255	not scorable	not scorable	1	0.8136061
192	Bn-A01-p11642789	napus specific	1	0.3111525	not scorable	not scorable	not scorable	not scorable
193	Bn-A01-p11652789	A-genome specific (napus-rapa)	0.9976076	0.3390766	not scorable	not scorable	0.9917355	0.7788732
194	Bn-A01-p11653461	A-genome specific (napus-rapa)	0.9976076	0.3562978	not scorable	not scorable	0.9834711	0.6805549
195	Bn-A01-p11702957	A-genome specific (napus-rapa)	0.9665072	0.7782543	not scorable	not scorable	1	0.7741857

196	Bn-A01-p1170567	A-genome specific (napus-rapa)	0.9784689	0.308269	not scorable	not scorable	0.9834711	0.6972505
197	Bn-A01-p11727190	A-genome specific (rapa only)	not scorable	not scorable	not scorable	not scorable	0.9917355	0.83433
198	Bn-A01-p11741112	A-genome specific (napus-rapa)	0.8827751	0.7705357	not scorable	not scorable	0.5371901	0.7974368
199	Bn-A01-p11744233	A-genome specific (napus-rapa)	0.9641148	0.2742572	not scorable	not scorable	0.9752066	0.7549212
200	Bn-A01-p1175428	A-genome specific (napus-rapa)	0.9856459	0.2533995	not scorable	not scorable	1	0.6986491
201	Bn-A01-p11807124	A-genome specific (napus-rapa)	0.9880383	0.849119	not scorable	not scorable	0.9834711	0.7056556
202	Bn-A01-p11808121	A-genome specific (napus-rapa)	0.9856459	0.8017023	not scorable	not scorable	0.9834711	0.7314562
203	Bn-A01-p11817698	A-genome specific (napus-rapa)	0.9880383	0.7665815	not scorable	not scorable	0.9917355	0.8112159
204	Bn-A01-p11818901	A-genome specific (napus-rapa)	0.9832536	0.782715	not scorable	not scorable	0.9752066	0.7018273
205	Bn-A01-p11825880	A-genome specific (napus-rapa)	0.9928229	0.8684071	not scorable	not scorable	0.9834711	0.6657122
206	Bn-A01-p11830957	A-genome specific (napus-rapa)	0.9784689	0.483146	not scorable	not scorable	0.9834711	0.740051
207	Bn-A01-p1187868	A-genome specific (napus-rapa)	0.9736842	0.6643248	not scorable	not scorable	0.9834711	0.7386498
208	Bn-A01-p1188004	A-genome specific (napus-rapa)	0.9784689	0.7499646	not scorable	not scorable	1	0.7097313
209	Bn-A01-p1188928	A-genome specific (napus-rapa)	0.9330143	0.6153653	not scorable	not scorable	1	0.7807196
210	Bn-A01-p1190844	A-genome specific (napus-rapa)	0.8588517	0.9205657	not scorable	not scorable	1	0.7594979
211	Bn-A01-p1192928	A-genome specific (napus-rapa)	0.9617225	0.4158099	not scorable	not scorable	1	0.7139555
212	Bn-A01-p11933	A-genome specific (napus-rapa)	0.9425837	0.6843772	not scorable	not scorable	0.9917355	0.6815898
213	Bn-A01-p11938379	A-genome specific (napus-rapa)	0.9904306	0.8265477	not scorable	not scorable	0.9834711	0.6481165
214	Bn-A01-p11939834	A-genome specific (rapa only)	not scorable	not scorable	not scorable	not scorable	0.9834711	0.9228295
215	Bn-A01-p1194009	A-genome specific (napus-rapa)	0.930622	0.5868458	not scorable	not scorable	0.9917355	0.7976093
216	Bn-A01-p11947255	A-genome specific (napus-rapa)	0.9928229	0.7053589	not scorable	not scorable	1	0.7524902
217	Bn-A01-p11951605	A-genome specific (napus-rapa)	0.9904306	0.8588542	not scorable	not scorable	0.9917355	0.8131865
218	Bn-A01-p11954334	A-genome specific (napus-rapa)	0.9928229	0.5757538	not scorable	not scorable	0.9338843	0.758755
219	Bn-A01-p11955645	A-genome specific (napus-rapa)	0.9928229	0.4916941	not scorable	not scorable	1	0.7414955
220	Bn-A01-p11965733	A-genome specific (rapa only)	not scorable	not scorable	not scorable	not scorable	0.9421487	0.7101336
221	Bn-A01-p11991521	A-genome specific (napus-rapa)	0.9856459	0.6375272	not scorable	not scorable	1	0.8737795
222	Bn-A01-p11992281	A-genome specific (napus-rapa)	0.9665072	0.3765419	not scorable	not scorable	0.9917355	0.7738066
223	Bn-A01-p11993589	A-genome specific (napus-rapa)	0.9952153	0.7460844	not scorable	not scorable	0.9834711	0.6876795
224	Bn-A01-p11996408	A-genome specific (napus-rapa)	0.7966507	0.7690449	not scorable	not scorable	0.6198347	0.8586335
225	Bn-A01-p11996672	A-genome specific (rapa only)	not scorable	not scorable	not scorable	not scorable	0.6280992	0.8808653
226	Bn-A01-p120225	A-genome specific (napus-rapa)	0.9904306	0.2652374	not scorable	not scorable	0.9917355	0.6753637
227	Bn-A01-p12066369	genome unspecific (all in)	0.9952153	0.3208931	0.9922481	0.5905417	1	0.8027052
228	Bn-A01-p12077110	A-genome specific (napus-rapa)	0.9904306	0.7698391	not scorable	not scorable	0.9917355	0.8636591

229	Bn-A01-p12081177	A-genome specific (napus-rapa)	0.9952153	0.4110411	not scorable	not scorable	1	0.7938477
230	Bn-A01-p1208515	A-genome specific (napus-rapa)	0.9976076	0.4442226	not scorable	not scorable	0.9917355	0.7727268
231	Bn-A01-p12098080	A-genome specific (napus-rapa)	0.9952153	0.420172	not scorable	not scorable	1	0.771117
232	Bn-A01-p12136794	A-genome specific (napus-rapa)	1	0.270754	not scorable	not scorable	1	0.5623716
233	Bn-A01-p12136908	A-genome specific (napus-rapa)	1	0.549558	not scorable	not scorable	1	0.4069836
234	Bn-A01-p12138089	A-genome specific (napus-rapa)	0.9928229	0.632485	not scorable	not scorable	1	0.5373095
235	Bn-A01-p12145148	A-genome specific (napus-rapa)	0.9952153	0.431631	not scorable	not scorable	1	0.8453875
236	Bn-A01-p12158810	A-genome specific (napus-rapa)	0.9952153	0.4761357	not scorable	not scorable	0.9917355	0.6880202
237	Bn-A01-p12178646	A-genome specific (napus-rapa)	0.9976076	0.4218055	not scorable	not scorable	1	0.7138398
238	Bn-A01-p12179784	A-genome specific (napus-rapa)	0.9904306	0.8303608	not scorable	not scorable	1	0.7847885
239	Bn-A01-p12192020	genome unspecific (all in)	0.9928229	0.6613205	0.3178295	0.6927192	0.9917355	0.7069837
240	Bn-A01-p12206449	napus specific	0.9856459	0.6708309	not scorable	not scorable	not scorable	not scorable
241	Bn-A01-p1220904	A-genome specific (napus-rapa)	1	0.6044071	not scorable	not scorable	1	0.7979612
242	Bn-A01-p12221698	A-genome specific (napus-rapa)	0.9904306	0.7802077	not scorable	not scorable	1	0.8099741
243	Bn-A01-p12222100	A-genome specific (napus-rapa)	0.9904306	0.7875617	not scorable	not scorable	0.9917355	0.770835
244	Bn-A01-p12222728	A-genome specific (napus-rapa)	0.9880383	0.7653283	not scorable	not scorable	0.9669421	0.7439612
245	Bn-A01-p12222806	A-genome specific (napus-rapa)	0.9880383	0.8772995	not scorable	not scorable	0.9917355	0.6783558
246	Bn-A01-p12224872	A-genome specific (napus-rapa)	0.9904306	0.8545919	not scorable	not scorable	0.9917355	0.7215734
247	Bn-A01-p12233209	A-genome specific (napus-rapa)	0.3062201	0.7394736	not scorable	not scorable	1	0.7454135
248	Bn-A01-p12237534	A-genome specific (napus-rapa)	1	0.5140246	not scorable	not scorable	1	0.7883869
249	Bn-A01-p12257699	A-genome specific (napus-rapa)	0.9976076	0.404594	not scorable	not scorable	0.9834711	0.8113015
250	Bn-A01-p12262504	A-genome specific (napus-rapa)	1	0.3449225	not scorable	not scorable	0.9752066	0.5642056
251	Bn-A01-p1226475	A-genome specific (napus-rapa)	0.9641148	0.4153487	not scorable	not scorable	1	0.8808117
252	Bn-A01-p1227000	A-genome specific (napus-rapa)	0.9904306	0.7547374	not scorable	not scorable	0.9917355	0.7880889
253	Bn-A01-p1227023	A-genome specific (napus-rapa)	0.9736842	0.7761165	not scorable	not scorable	1	0.8054933
254	Bn-A01-p12302708	A-genome specific (napus-rapa)	0.9928229	0.8611882	not scorable	not scorable	0.9917355	0.7236418
255	Bn-A01-p12302861	A-genome specific (napus-rapa)	0.923445	0.7147273	not scorable	not scorable	0.9752066	0.7385623
256	Bn-A01-p12305025	A-genome specific (napus-rapa)	0.9904306	0.8505798	not scorable	not scorable	1	0.816321
257	Bn-A01-p12305828	A-genome specific (napus-rapa)	0.9952153	0.3198976	not scorable	not scorable	1	0.4710016
258	Bn-A01-p12306195	A-genome specific (napus-rapa)	0.9880383	0.8063994	not scorable	not scorable	1	0.8220317
259	Bn-A01-p12308897	A-genome specific (napus-rapa)	0.9928229	0.7099838	not scorable	not scorable	1	0.6720178
260	Bn-A01-p12309767	A-genome specific (napus-rapa)	0.9904306	0.8443652	not scorable	not scorable	1	0.7676212
261	Bn-A01-p12314813	A-genome specific (napus-rapa)	0.8899522	0.8075957	not scorable	not scorable	0.9586777	0.6058481

262	Bn-A01-p1232064	A-genome specific (napus-rapa)	1	0.336769	not scorable	not scorable	0.9917355	0.7886637
263	Bn-A01-p12350104	A-genome specific (napus-rapa)	0.9880383	0.6635865	not scorable	not scorable	1	0.6303344
264	Bn-A01-p12352518	A-genome specific (napus-rapa)	0.9880383	0.6720262	not scorable	not scorable	1	0.6211244
265	Bn-A01-p1235334	A-genome specific (napus-rapa)	0.9665072	0.7371562	not scorable	not scorable	1	0.744643
266	Bn-A01-p1236927	A-genome specific (napus-rapa)	0.645933	0.9331403	not scorable	not scorable	0.9338843	0.8867427
267	Bn-A01-p12380936	A-genome specific (napus-rapa)	0.9880383	0.8092616	not scorable	not scorable	1	0.8592322
268	Bn-A01-p12387882	A-genome specific (napus-rapa)	0.9880383	0.8279986	not scorable	not scorable	0.9834711	0.8116725
269	Bn-A01-p12391243	A-genome specific (napus-rapa)	0.9904306	0.8344067	not scorable	not scorable	1	0.7828811
270	Bn-A01-p12399796	A-genome specific (napus-rapa)	0.9928229	0.8191944	not scorable	not scorable	1	0.8668937
271	Bn-A01-p12400477	A-genome specific (napus-rapa)	0.9880383	0.7945017	not scorable	not scorable	0.9917355	0.7392782
272	Bn-A01-p12427149	A-genome specific (napus-rapa)	0.9952153	0.5509108	not scorable	not scorable	1	0.71289
273	Bn-A01-p12454306	A-genome specific (napus-rapa)	0.9928229	0.6266865	not scorable	not scorable	1	0.6219013
274	Bn-A01-p12456089	A-genome specific (napus-rapa)	0.9928229	0.7882095	not scorable	not scorable	0.9917355	0.7803518
275	Bn-A01-p1247899	A-genome specific (napus-rapa)	0.9593301	0.7977593	not scorable	not scorable	1	0.8291255
276	Bn-A01-p1248141	A-genome specific (rapa only)	not scorable	not scorable	not scorable	not scorable	0.9834711	0.7837986
277	Bn-A01-p12484368	A-genome specific (napus-rapa)	0.9784689	0.583856	not scorable	not scorable	0.9834711	0.5942021
278	Bn-A01-p1249847	A-genome specific (napus-rapa)	0.9760765	0.7802738	not scorable	not scorable	0.9752066	0.8324441
279	Bn-A01-p1250595	A-genome specific (napus-rapa)	0.9019139	0.7718884	not scorable	not scorable	1	0.7470211
280	Bn-A01-p12510328	A-genome specific (napus-rapa)	0.9808612	0.7774609	not scorable	not scorable	0.9834711	0.7612363
281	Bn-A01-p12510566	A-genome specific (napus-rapa)	0.9976076	0.4475251	not scorable	not scorable	1	0.7615709
282	Bn-A01-p12516944	A-genome specific (napus-rapa)	0.9808612	0.6722834	not scorable	not scorable	0.9669421	0.6302709
283	Bn-A01-p12522369	A-genome specific (napus-rapa)	0.9808612	0.7599123	not scorable	not scorable	0.9834711	0.7753927
284	Bn-A01-p1252553	A-genome specific (rapa only)	not scorable	not scorable	not scorable	not scorable	1	0.6659275
285	Bn-A01-p12533991	A-genome specific (napus-rapa)	0.9904306	0.7667246	not scorable	not scorable	0.9586777	0.7200297
286	Bn-A01-p12535269	A-genome specific (napus-rapa)	0.9976076	0.4794829	not scorable	not scorable	0.9752066	0.7307885
287	Bn-A01-p12535612	A-genome specific (napus-rapa)	0.9904306	0.7829784	not scorable	not scorable	0.9834711	0.7756329
288	Bn-A01-p12539479	genome unspecific (all in)	1	0.5445863	0.9767442	0.678154	1	0.7936676
289	Bn-A01-p12539655	A-genome specific (napus-rapa)	0.9928229	0.7922541	not scorable	not scorable	0.9669421	0.7480221
290	Bn-A01-p12539953	napus specific	0.9880383	0.5494856	not scorable	not scorable	not scorable	not scorable
291	Bn-A01-p12543678	A-genome specific (napus-rapa)	1	0.47417	not scorable	not scorable	0.9834711	0.6800156
292	Bn-A01-p12544054	A-genome specific (napus-rapa)	0.9928229	0.7409139	not scorable	not scorable	1	0.7505876
293	Bn-A01-p12550092	A-genome specific (rapa only)	not scorable	not scorable	not scorable	not scorable	0.9752066	0.6806947
294	Bn-A01-p12560952	A-genome specific (napus-rapa)	0.9976076	0.4461923	not scorable	not scorable	1	0.6930565

295	Bn-A01-p12577268	A-genome specific (napus-rapa)	0.9928229	0.8848283	not scorable	not scorable	0.9752066	0.7341932
296	Bn-A01-p12580778	A-genome specific (napus-rapa)	0.9976076	0.5284097	not scorable	not scorable	0.9917355	0.6902963
297	Bn-A01-p12591168	A-genome specific (napus-rapa)	0.9832536	0.8065313	not scorable	not scorable	0.6859504	0.7888045
298	Bn-A01-p12593173	A-genome specific (napus-rapa)	0.9928229	0.7619047	not scorable	not scorable	1	0.6766812
299	Bn-A01-p12593802	A-genome specific (rapa only)	not scorable	not scorable	not scorable	not scorable	1	0.839635
300	Bn-A01-p1261017	A-genome specific (napus-rapa)	0.9904306	0.7708218	not scorable	not scorable	0.9917355	0.747498
301	Bn-A01-p12618357	A-genome specific (napus-rapa)	0.9976076	0.5196093	not scorable	not scorable	0.9834711	0.6811854
302	Bn-A01-p12622123	A-genome specific (napus-rapa)	0.9736842	0.8064575	not scorable	not scorable	1	0.7525831
303	Bn-A01-p12625892	A-genome specific (napus-rapa)	0.9928229	0.7502456	not scorable	not scorable	0.9834711	0.7789644
304	Bn-A01-p12653338	A-genome specific (napus-rapa)	0.9904306	0.4675172	not scorable	not scorable	0.9917355	0.8427517
305	Bn-A01-p12665045	A-genome specific (napus-rapa)	0.9928229	0.7249033	not scorable	not scorable	1	0.6900909
306	Bn-A01-p12665250	A-genome specific (napus-rapa)	0.9928229	0.7594367	not scorable	not scorable	0.9752066	0.7094528
307	Bn-A01-p12672899	A-genome specific (napus-rapa)	0.9976076	0.4629915	not scorable	not scorable	1	0.6967734
308	Bn-A01-p12734805	A-genome specific (napus-rapa)	0.9928229	0.7585934	not scorable	not scorable	1	0.8947011
309	Bn-A01-p1275284	A-genome specific (napus-rapa)	0.9688995	0.7700123	not scorable	not scorable	0.9917355	0.6936204
310	Bn-A01-p1276486	A-genome specific (napus-rapa)	0.9784689	0.7029064	not scorable	not scorable	1	0.7620607
311	Bn-A01-p1276504	A-genome specific (napus-rapa)	0.9712918	0.7991804	not scorable	not scorable	0.9917355	0.8100437
312	Bn-A01-p12774270	A-genome specific (napus-rapa)	0.9976076	0.5231249	not scorable	not scorable	0.9917355	0.8439682
313	Bn-A01-p12823550	A-genome specific (napus-rapa)	1	0.455857	not scorable	not scorable	0.9917355	0.8354748
314	Bn-A01-p12926196	A-genome specific (napus-rapa)	1	0.4634304	not scorable	not scorable	0.9917355	0.8035609
315	Bn-A01-p1296503	A-genome specific (rapa only)	not scorable	not scorable	not scorable	not scorable	0.9586777	0.3988096
316	Bn-A01-p1314194	A-genome specific (napus-rapa)	0.9880383	0.2333066	not scorable	not scorable	1	0.58727
317	Bn-A01-p1314440	A-genome specific (napus-rapa)	0.9760765	0.2707549	not scorable	not scorable	0.9752066	0.6625077
318	Bn-A01-p1320368	A-genome specific (napus-rapa)	0.8995215	0.797123	not scorable	not scorable	0.9834711	0.67584
319	Bn-A01-p13239976	A-genome specific (napus-rapa)	0.9832536	0.7094571	not scorable	not scorable	0.9917355	0.7518338
320	Bn-A01-p1324586	A-genome specific (napus-rapa)	0.9688995	0.7556233	not scorable	not scorable	0.9834711	0.7181178
321	Bn-A01-p13622238	A-genome specific (napus-rapa)	0.6315789	0.6731903	not scorable	not scorable	0.9421487	0.7406708
322	Bn-A01-p13709801	A-genome specific (napus-rapa)	0.9736842	0.7139389	not scorable	not scorable	1	0.5780196
323	Bn-A01-p1372430	A-genome specific (napus-rapa)	0.9401914	0.7634706	not scorable	not scorable	0.9752066	0.8155184
324	Bn-A01-p1373104	A-genome specific (napus-rapa)	0.9832536	0.7966446	not scorable	not scorable	0.9917355	0.8139654
325	Bn-A01-p1373239	A-genome specific (napus-rapa)	0.9784689	0.5574312	not scorable	not scorable	0.6280992	0.6954632
326	Bn-A01-p1373827	A-genome specific (napus-rapa)	0.9808612	0.7701248	not scorable	not scorable	0.9917355	0.7884952
327	Bn-A01-p1373838	A-genome specific (napus-rapa)	0.9784689	0.7812393	not scorable	not scorable	0.9917355	0.8319343

328	Bn-A01-p1373965	A-genome specific (napus-rapa)	0.9784689	0.71347	not scorable	not scorable	0.9669421	0.6786404
329	Bn-A01-p1376589	A-genome specific (napus-rapa)	0.9880383	0.7608371	not scorable	not scorable	0.9917355	0.7075
330	Bn-A01-p13775817	A-genome specific (napus-rapa)	0.9904306	0.7354486	not scorable	not scorable	0.9917355	0.7816562
331	Bn-A01-p137814	A-genome specific (napus-rapa)	0.9856459	0.6964834	not scorable	not scorable	0.9917355	0.6833126
332	Bn-A01-p137869	A-genome specific (napus-rapa)	0.9904306	0.6550323	not scorable	not scorable	1	0.6128799
333	Bn-A01-p13825941	A-genome specific (napus-rapa)	0.2464115	0.7578292	not scorable	not scorable	0.9586777	0.7199126
334	Bn-A01-p13827513	A-genome specific (napus-rapa)	0.9952153	0.6120517	not scorable	not scorable	0.9586777	0.7022258
335	Bn-A01-p13828017	A-genome specific (napus-rapa)	0.9808612	0.4143094	not scorable	not scorable	0.9752066	0.5753651
336	Bn-A01-p13828314	A-genome specific (napus-rapa)	0.9736842	0.5698635	not scorable	not scorable	0.9669421	0.8085203
337	Bn-A01-p1384609	A-genome specific (napus-rapa)	0.9880383	0.7425978	not scorable	not scorable	0.9917355	0.9091246
338	Bn-A01-p1385993	A-genome specific (napus-rapa)	0.9497608	0.5272615	not scorable	not scorable	1	0.9140136
339	Bn-A01-p1387174	A-genome specific (napus-rapa)	0.9784689	0.417858	not scorable	not scorable	1	0.7745267
340	Bn-A01-p138731	A-genome specific (napus-rapa)	0.9904306	0.6756431	not scorable	not scorable	0.9834711	0.7377116
341	Bn-A01-p1387769	A-genome specific (napus-rapa)	0.9784689	0.753564	not scorable	not scorable	0.9752066	0.8053254
342	Bn-A01-p13893020	A-genome specific (napus-rapa)	0.9880383	0.8205257	not scorable	not scorable	1	0.8404639
343	Bn-A01-p14001039	A-genome specific (napus-rapa)	1	0.4849428	not scorable	not scorable	1	0.7021827
344	Bn-A01-p14022855	A-genome specific (napus-rapa)	0.9928229	0.7837644	not scorable	not scorable	0.9834711	0.8055862
345	Bn-A01-p14046733	A-genome specific (napus-rapa)	0.9952153	0.4037965	not scorable	not scorable	0.9752066	0.558627
346	Bn-A01-p14057073	A-genome specific (rapa only)	not scorable	not scorable	not scorable	not scorable	0.9917355	0.6622183
347	Bn-A01-p14071179	A-genome specific (napus-rapa)	0.9904306	0.7523553	not scorable	not scorable	1	0.7740541
348	Bn-A01-p14071237	A-genome specific (napus-rapa)	0.9856459	0.83043	not scorable	not scorable	1	0.8182964
349	Bn-A01-p14074100	A-genome specific (napus-rapa)	0.9928229	0.7429424	not scorable	not scorable	1	0.7840061
350	Bn-A01-p14074268	A-genome specific (napus-rapa)	0.9928229	0.721787	not scorable	not scorable	1	0.7295138
351	Bn-A01-p14074391	A-genome specific (napus-rapa)	0.9856459	0.7404426	not scorable	not scorable	1	0.7592629
352	Bn-A01-p14076641	A-genome specific (napus-rapa)	0.9904306	0.8262197	not scorable	not scorable	1	0.8091902
353	Bn-A01-p14079006	A-genome specific (napus-rapa)	0.9880383	0.618178	not scorable	not scorable	1	0.7588036
354	Bn-A01-p14084579	A-genome specific (napus-rapa)	0.9808612	0.4481516	not scorable	not scorable	1	0.7093671
355	Bn-A01-p14090315	A-genome specific (napus-rapa)	0.9880383	0.3972804	not scorable	not scorable	0.9752066	0.5547916
356	Bn-A01-p1409723	A-genome specific (napus-rapa)	0.9928229	0.4407693	not scorable	not scorable	0.9834711	0.6924533
357	Bn-A01-p14102222	A-genome specific (napus-rapa)	0.9832536	0.8619328	not scorable	not scorable	1	0.8167796
358	Bn-A01-p14104219	genome unspecific (all in)	0.9952153	0.7884539	1	0.7146667	0.9834711	0.7699351
359	Bn-A01-p14104241	A-genome specific (napus-rapa)	1	0.4081207	not scorable	not scorable	0.9834711	0.4463008
360	Bn-A01-p14105205	A-genome specific (napus-rapa)	1	0.8373582	not scorable	not scorable	1	0.8525043

361	Bn-A01-p14106230	A-genome specific (napus-rapa)	1	0.4224357	not scorable	not scorable	0.9917355	0.4459139
362	Bn-A01-p14106856	A-genome specific (napus-rapa)	1	0.7212236	not scorable	not scorable	1	0.8079168
363	Bn-A01-p14128129	A-genome specific (napus-rapa)	0.9976076	0.4451966	not scorable	not scorable	0.9669421	0.7046128
364	Bn-A01-p14134232	A-genome specific (napus-rapa)	0.9928229	0.7758922	not scorable	not scorable	0.9917355	0.7049485
365	Bn-A01-p14138072	A-genome specific (napus-rapa)	0.9952153	0.5800401	not scorable	not scorable	0.9834711	0.7863265
366	Bn-A01-p1414695	A-genome specific (napus-rapa)	1	0.7173789	not scorable	not scorable	1	0.7565342
367	Bn-A01-p14148071	A-genome specific (napus-rapa)	0.9928229	0.7279299	not scorable	not scorable	0.9917355	0.6800501
368	Bn-A01-p14167866	A-genome specific (napus-rapa)	0.9880383	0.7617337	not scorable	not scorable	0.9752066	0.7794988
369	Bn-A01-p14168802	A-genome specific (napus-rapa)	0.9904306	0.6497484	not scorable	not scorable	0.9917355	0.7465103
370	Bn-A01-p14169412	A-genome specific (napus-rapa)	0.9784689	0.3887236	not scorable	not scorable	1	0.8047553
371	Bn-A01-p14181463	A-genome specific (napus-rapa)	0.9904306	0.7526248	not scorable	not scorable	0.9752066	0.6868008
372	Bn-A01-p14181651	A-genome specific (napus-rapa)	0.9928229	0.762764	not scorable	not scorable	0.9917355	0.6897457
373	Bn-A01-p14181829	A-genome specific (napus-rapa)	0.9928229	0.7675856	not scorable	not scorable	0.9917355	0.7165584
374	Bn-A01-p14191393	A-genome specific (napus-rapa)	0.9904306	0.5792308	not scorable	not scorable	0.9917355	0.6684284
375	Bn-A01-p14197545	A-genome specific (napus-rapa)	0.9976076	0.496622	not scorable	not scorable	0.9917355	0.777238
376	Bn-A01-p14200714	A-genome specific (napus-rapa)	0.9880383	0.7581865	not scorable	not scorable	0.9834711	0.733355
377	Bn-A01-p14200787	A-genome specific (napus-rapa)	0.9928229	0.7033648	not scorable	not scorable	0.9834711	0.6810399
378	Bn-A01-p14201581	A-genome specific (napus-rapa)	0.9856459	0.6761243	not scorable	not scorable	1	0.6566948
379	Bn-A01-p14210994	A-genome specific (napus-rapa)	0.9928229	0.6086816	not scorable	not scorable	0.9917355	0.679038
380	Bn-A01-p14223709	A-genome specific (napus-rapa)	0.9904306	0.605064	not scorable	not scorable	0.9917355	0.7369236
381	Bn-A01-p1422384	genome unspecific (all in)	0.9928229	0.3068601	1	0.6902146	1	0.8004208
382	Bn-A01-p1423163	A-genome specific (napus-rapa)	0.9784689	0.4976095	not scorable	not scorable	1	0.6994658
383	Bn-A01-p14232473	A-genome specific (napus-rapa)	0.9880383	0.8046031	not scorable	not scorable	0.9834711	0.7539325
384	Bn-A01-p14238948	A-genome specific (napus-rapa)	0.9784689	0.5621237	not scorable	not scorable	0.9917355	0.7249692
385	Bn-A01-p14241215	A-genome specific (napus-rapa)	0.9904306	0.7441103	not scorable	not scorable	0.9834711	0.7770978
386	Bn-A01-p14247718	A-genome specific (napus-rapa)	0.9617225	0.5910885	not scorable	not scorable	0.9834711	0.781979
387	Bn-A01-p14256104	A-genome specific (napus-rapa)	0.9952153	0.2883646	not scorable	not scorable	0.9917355	0.7211576
388	Bn-A01-p14328823	A-genome specific (napus-rapa)	1	0.5628255	not scorable	not scorable	0.9917355	0.615298
389	Bn-A01-p14339825	A-genome specific (napus-rapa)	0.9976076	0.2813169	not scorable	not scorable	0.9917355	0.8140109
390	Bn-A01-p1439621	A-genome specific (napus-rapa)	0.9760765	0.5267045	not scorable	not scorable	0.9834711	0.367469
391	Bn-A01-p1440009	A-genome specific (napus-rapa)	0.9808612	0.6311568	not scorable	not scorable	0.9834711	0.7109494
392	Bn-A01-p14402434	A-genome specific (napus-rapa)	0.937799	0.2134711	not scorable	not scorable	1	0.7746665
393	Bn-A01-p14415386	A-genome specific (napus-rapa)	1	0.3608276	not scorable	not scorable	0.9917355	0.677067

394	Bn-A01-p14419363	A-genome specific (napus-rapa)	0.9928229	0.835639	not scorable	not scorable	0.9917355	0.89465
395	Bn-A01-p1443389	A-genome specific (napus-rapa)	0.9617225	0.7277284	not scorable	not scorable	0.9917355	0.7347819
396	Bn-A01-p1448811	A-genome specific (rapa only)	not scorable	not scorable	not scorable	not scorable	1	0.7545834
397	Bn-A01-p1449865	A-genome specific (napus-rapa)	0.9665072	0.6526303	not scorable	not scorable	0.9917355	0.6696596
398	Bn-A01-p1453156	A-genome specific (napus-rapa)	0.9928229	0.820729	not scorable	not scorable	0.9917355	0.7191458
399	Bn-A01-p1453560	A-genome specific (napus-rapa)	0.8851675	0.8316536	not scorable	not scorable	0.9421487	0.7675593
400	Bn-A01-p14559770	A-genome specific (napus-rapa)	0.7894737	0.7637153	not scorable	not scorable	0.7190083	0.7365817
401	Bn-A01-p1456256	A-genome specific (napus-rapa)	0.9928229	0.8287286	not scorable	not scorable	0.9834711	0.6924742
402	Bn-A01-p145676	A-genome specific (napus-rapa)	0.9880383	0.7903903	not scorable	not scorable	0.9834711	0.7132263
403	Bn-A01-p14661055	A-genome specific (napus-rapa)	1	0.8004208	not scorable	not scorable	1	0.7739488
404	Bn-A01-p14661148	A-genome specific (napus-rapa)	1	0.8114662	not scorable	not scorable	1	0.8004208
405	Bn-A01-p14666643	A-genome specific (napus-rapa)	0.9904306	0.4931239	not scorable	not scorable	0.9834711	0.7937879
406	Bn-A01-p14687636	A-genome specific (napus-rapa)	0.9952153	0.5186229	not scorable	not scorable	1	0.820781
407	Bn-A01-p146963	A-genome specific (napus-rapa)	0.9688995	0.7917191	not scorable	not scorable	0.9917355	0.7564167
408	Bn-A01-p14704844	A-genome specific (napus-rapa)	0.9928229	0.4295946	not scorable	not scorable	1	0.8401869
409	Bn-A01-p14723306	A-genome specific (napus-rapa)	0.9952153	0.5226974	not scorable	not scorable	1	0.7832876
410	Bn-A01-p14727717	A-genome specific (napus-rapa)	0.9880383	0.7645015	not scorable	not scorable	0.9917355	0.7983885
411	Bn-A01-p1473188	A-genome specific (napus-rapa)	1	0.5945251	not scorable	not scorable	0.9917355	0.7232726
412	Bn-A01-p147360	A-genome specific (napus-rapa)	0.9760765	0.8084076	not scorable	not scorable	1	0.6635737
413	Bn-A01-p1476216	A-genome specific (napus-rapa)	0.9712918	0.4450222	not scorable	not scorable	1	0.8051416
414	Bn-A01-p147990	A-genome specific (napus-rapa)	0.9808612	0.8084252	not scorable	not scorable	1	0.7380052
415	Bn-A01-p14802709	A-genome specific (napus-rapa)	0.9856459	0.5062508	not scorable	not scorable	1	0.9175813
416	Bn-A01-p14859839	A-genome specific (napus-rapa)	0.9928229	0.7264214	not scorable	not scorable	1	0.9175813
417	Bn-A01-p14865565	A-genome specific (napus-rapa)	0.9928229	0.5397404	not scorable	not scorable	1	0.9068346
418	Bn-A01-p14901403	A-genome specific (napus-rapa)	0.9904306	0.7747275	not scorable	not scorable	0.9917355	0.7628844
419	Bn-A01-p14905298	A-genome specific (napus-rapa)	0.9808612	0.704729	not scorable	not scorable	0.9917355	0.6850774
420	Bn-A01-p14906403	A-genome specific (napus-rapa)	0.9928229	0.6796619	not scorable	not scorable	0.9752066	0.7699912
421	Bn-A01-p14906494	A-genome specific (napus-rapa)	0.9928229	0.7638305	not scorable	not scorable	1	0.8100366
422	Bn-A01-p14914475	A-genome specific (napus-rapa)	0.9928229	0.8088243	not scorable	not scorable	1	0.7370641
423	Bn-A01-p14917697	A-genome specific (napus-rapa)	0.9928229	0.810746	not scorable	not scorable	0.9917355	0.8215768
424	Bn-A01-p14934844	A-genome specific (napus-rapa)	0.9760765	0.8228506	not scorable	not scorable	1	0.8241322
425	Bn-A01-p14942681	A-genome specific (napus-rapa)	0.9928229	0.7083406	not scorable	not scorable	0.9834711	0.6553146
426	Bn-A01-p14943499	A-genome specific (napus-rapa)	0.9952153	0.5254089	not scorable	not scorable	0.9834711	0.6814213

427	Bn-A01-p14945302	A-genome specific (napus-rapa)	0.9952153	0.3859778	not scorable	not scorable	0.9917355	0.8088143
428	Bn-A01-p14947053	A-genome specific (napus-rapa)	0.9928229	0.7179595	not scorable	not scorable	0.9834711	0.7865661
429	Bn-A01-p14947418	A-genome specific (napus-rapa)	0.9952153	0.7861242	not scorable	not scorable	1	0.8765777
430	Bn-A01-p14948237	A-genome specific (napus-rapa)	0.9928229	0.8043585	not scorable	not scorable	0.9917355	0.7063561
431	Bn-A01-p14948379	A-genome specific (napus-rapa)	0.9928229	0.8044872	not scorable	not scorable	0.9917355	0.7598522
432	Bn-A01-p14948614	A-genome specific (napus-rapa)	0.9928229	0.7633395	not scorable	not scorable	0.9834711	0.6639513
433	Bn-A01-p14948748	A-genome specific (napus-rapa)	0.9928229	0.7529407	not scorable	not scorable	0.9917355	0.6355018
434	Bn-A01-p14949030	A-genome specific (napus-rapa)	0.9928229	0.6473867	not scorable	not scorable	0.9834711	0.6821839
435	Bn-A01-p14949121	A-genome specific (napus-rapa)	0.9904306	0.6645685	not scorable	not scorable	1	0.8004208
436	Bn-A01-p14949256	A-genome specific (napus-rapa)	0.9928229	0.7288114	not scorable	not scorable	0.9834711	0.6886961
437	Bn-A01-p14955594	A-genome specific (napus-rapa)	0.9928229	0.7838486	not scorable	not scorable	1	0.7819808
438	Bn-A01-p14959478	A-genome specific (napus-rapa)	0.9928229	0.7589554	not scorable	not scorable	0.9917355	0.7598228
439	Bn-A01-p14960233	A-genome specific (napus-rapa)	0.9952153	0.3823385	not scorable	not scorable	0.9917355	0.7391033
440	Bn-A01-p14965172	A-genome specific (napus-rapa)	0.9928229	0.3137937	not scorable	not scorable	0.9917355	0.6380635
441	Bn-A01-p14965596	A-genome specific (napus-rapa)	0.9976076	0.5253988	not scorable	not scorable	0.9917355	0.6897768
442	Bn-A01-p15000034	genome unspecific (all in)	0.9760765	0.6658307	0.9922481	0.6835211	0.677686	0.8119524
443	Bn-A01-p15000468	A-genome specific (napus-rapa)	0.9497608	0.7372293	not scorable	not scorable	0.9917355	0.7155222
444	Bn-A01-p150028	A-genome specific (napus-rapa)	0.9784689	0.7550246	not scorable	not scorable	1	0.6418651
445	Bn-A01-p15043967	genome unspecific (all in)	0.9832536	0.6693811	1	0.6971845	0.6942149	0.7494321
446	Bn-A01-p15086787	A-genome specific (napus-rapa)	0.9928229	0.7409608	not scorable	not scorable	0.9752066	0.7042561
447	Bn-A01-p15087045	A-genome specific (napus-rapa)	0.9928229	0.3108245	not scorable	not scorable	0.9421487	0.3693855
448	Bn-A01-p15087171	A-genome specific (napus-rapa)	0.9928229	0.7024454	not scorable	not scorable	0.9669421	0.6953952
449	Bn-A01-p15090383	A-genome specific (napus-rapa)	0.9976076	0.610445	not scorable	not scorable	0.9917355	0.7694124
450	Bn-A01-p15092778	A-genome specific (napus-rapa)	0.9976076	0.5632354	not scorable	not scorable	0.9917355	0.6819887
451	Bn-A01-p15095213	A-genome specific (napus-rapa)	0.9904306	0.7800793	not scorable	not scorable	1	0.6652268
452	Bn-A01-p15095314	A-genome specific (napus-rapa)	0.9904306	0.6482113	not scorable	not scorable	0.9834711	0.6881775
453	Bn-A01-p15095524	A-genome specific (napus-rapa)	0.9928229	0.783015	not scorable	not scorable	0.9917355	0.7791887
454	Bn-A01-p15114417	A-genome specific (napus-rapa)	0.9760765	0.4198054	not scorable	not scorable	0.9834711	0.5448056
455	Bn-A01-p15130843	A-genome specific (napus-rapa)	1	0.5823976	not scorable	not scorable	0.9917355	0.6272891
456	Bn-A01-p15179106	A-genome specific (napus-rapa)	0.9880383	0.7790681	not scorable	not scorable	1	0.7381263
457	Bn-A01-p15182302	A-genome specific (napus-rapa)	0.9808612	0.5141631	not scorable	not scorable	0.9752066	0.7187808
458	Bn-A01-p15193510	A-genome specific (napus-rapa)	0.9162679	0.7698487	not scorable	not scorable	0.9586777	0.8133104
459	Bn-A01-p15228282	A-genome specific (napus-rapa)	0.9928229	0.2350904	not scorable	not scorable	0.9504132	0.2707185

460	Bn-A01-p15233095	genome unspecific (all in)	0.9952153	0.4859448	1	0.7212361	1	0.7566166
461	Bn-A01-p15295874	genome unspecific (all in)	0.9856459	0.694932	1	0.7193761	0.9834711	0.7254611
462	Bn-A01-p1530160	A-genome specific (rapa only)	not scorable	not scorable	not scorable	not scorable	0.9917355	0.8077192
463	Bn-A01-p1531829	A-genome specific (napus-rapa)	0.9928229	0.3034803	not scorable	not scorable	0.9834711	0.521618
464	Bn-A01-p1538603	A-genome specific (rapa only)	not scorable	not scorable	not scorable	not scorable	0.9669421	0.7456784
465	Bn-A01-p15401259	A-genome specific (napus-rapa)	0.9928229	0.8076295	not scorable	not scorable	0.9917355	0.7794766
466	Bn-A01-p15402291	A-genome specific (napus-rapa)	0.9952153	0.4682174	not scorable	not scorable	1	0.9167765
467	Bn-A01-p15407905	A-genome specific (rapa only)	not scorable	not scorable	not scorable	not scorable	0.9752066	0.5063943
468	Bn-A01-p15408721	A-genome specific (napus-rapa)	0.9928229	0.6006129	not scorable	not scorable	0.9917355	0.7109582
469	Bn-A01-p15413589	A-genome specific (napus-rapa)	0.9928229	0.7687166	not scorable	not scorable	0.9917355	0.7175106
470	Bn-A01-p15432406	A-genome specific (napus-rapa)	0.9808612	0.4111577	not scorable	not scorable	1	0.7494321
471	Bn-A01-p15441407	A-genome specific (napus-rapa)	0.9880383	0.6540042	not scorable	not scorable	0.9834711	0.7575629
472	Bn-A01-p15446864	A-genome specific (napus-rapa)	1	0.4980409	not scorable	not scorable	0.9834711	0.778089
473	Bn-A01-p15468033	A-genome specific (napus-rapa)	1	0.5170386	not scorable	not scorable	0.9834711	0.7272325
474	Bn-A01-p1547232	A-genome specific (napus-rapa)	0.9904306	0.6331899	not scorable	not scorable	0.9669421	0.669001
475	Bn-A01-p15474986	A-genome specific (napus-rapa)	0.9976076	0.3677513	not scorable	not scorable	0.9669421	0.4768252
476	Bn-A01-p15475038	A-genome specific (napus-rapa)	0.9832536	0.7868354	not scorable	not scorable	0.6280992	0.8079355
477	Bn-A01-p15476640	A-genome specific (napus-rapa)	0.9928229	0.4840779	not scorable	not scorable	0.9586777	0.6968947
478	Bn-A01-p15481986	A-genome specific (napus-rapa)	0.9928229	0.7137043	not scorable	not scorable	0.9669421	0.7309846
479	Bn-A01-p1548460	A-genome specific (napus-rapa)	1	0.3601478	not scorable	not scorable	0.9917355	0.6790785
480	Bn-A01-p15487080	A-genome specific (napus-rapa)	0.9928229	0.7198843	not scorable	not scorable	1	0.7173789
481	Bn-A01-p15489266	A-genome specific (napus-rapa)	0.9928229	0.7487867	not scorable	not scorable	0.9669421	0.641044
482	Bn-A01-p15494260	A-genome specific (napus-rapa)	0.9904306	0.6933253	not scorable	not scorable	0.9917355	0.7529789
483	Bn-A01-p15494469	A-genome specific (napus-rapa)	0.9928229	0.5815339	not scorable	not scorable	0.9917355	0.7235553
484	Bn-A01-p15496639	A-genome specific (napus-rapa)	0.9880383	0.8594449	not scorable	not scorable	0.9917355	0.8350746
485	Bn-A01-p15496772	A-genome specific (napus-rapa)	0.9904306	0.7802292	not scorable	not scorable	0.9834711	0.8083651
486	Bn-A01-p15497190	A-genome specific (napus-rapa)	0.9880383	0.7668061	not scorable	not scorable	0.9917355	0.6968383
487	Bn-A01-p15502589	A-genome specific (napus-rapa)	0.9880383	0.7110991	not scorable	not scorable	0.677686	0.8436487
488	Bn-A01-p1551223	A-genome specific (napus-rapa)	0.9856459	0.734421	not scorable	not scorable	1	0.7074836
489	Bn-A01-p1551700	A-genome specific (napus-rapa)	0.9928229	0.7563044	not scorable	not scorable	0.9917355	0.7815472
490	Bn-A01-p15517260	genome unspecific (all in)	0.9976076	0.5997657	0.9922481	0.6040146	1	0.896279
491	Bn-A01-p15519090	A-genome specific (napus-rapa)	1	0.3679932	not scorable	not scorable	0.9917355	0.8227494
492	Bn-A01-p1551913	A-genome specific (napus-rapa)	0.9880383	0.6190057	not scorable	not scorable	0.9752066	0.7421802

493	Bn-A01-p1556145	A-genome specific (napus-rapa)	0.9952153	0.7384948	not scorable	not scorable	1	0.6906948
494	Bn-A01-p15568028	A-genome specific (napus-rapa)	0.9952153	0.6198791	not scorable	not scorable	1	0.7352715
495	Bn-A01-p15695152	A-genome specific (napus-rapa)	0.9904306	0.5570671	not scorable	not scorable	1	0.5452222
496	Bn-A01-p1570727	A-genome specific (rapa only)	not scorable	not scorable	not scorable	not scorable	0.9669421	0.8201513
497	Bn-A01-p1570761	A-genome specific (napus-rapa)	0.9928229	0.7646217	not scorable	not scorable	0.9917355	0.8193383
498	Bn-A01-p1578682	A-genome specific (napus-rapa)	0.9784689	0.3511277	not scorable	not scorable	1	0.7929845
499	Bn-A01-p1580970	A-genome specific (napus-rapa)	0.9952153	0.7842332	not scorable	not scorable	0.9917355	0.7591249
500	Bn-A01-p15865197	A-genome specific (napus-rapa)	0.9880383	0.683064	not scorable	not scorable	1	0.7001366
501	Bn-A01-p15957543	A-genome specific (napus-rapa)	0.9928229	0.7657695	not scorable	not scorable	1	0.7300923
502	Bn-A01-p159728	A-genome specific (rapa only)	not scorable	not scorable	not scorable	not scorable	0.7272727	0.8154013
503	Bn-A01-p16015382	A-genome specific (napus-rapa)	0.9976076	0.3258899	not scorable	not scorable	0.9669421	0.8871631
504	Bn-A01-p1606312	A-genome specific (napus-rapa)	0.9856459	0.3759683	not scorable	not scorable	0.9917355	0.6047406
505	Bn-A01-p16079153	A-genome specific (napus-rapa)	0.9928229	0.5610049	not scorable	not scorable	1	0.8062817
506	Bn-A01-p16079370	A-genome specific (napus-rapa)	0.9952153	0.2868307	not scorable	not scorable	0.9834711	0.5488765
507	Bn-A01-p16086381	A-genome specific (napus-rapa)	0.9928229	0.7185038	not scorable	not scorable	1	0.7241637
508	Bn-A01-p16088721	A-genome specific (napus-rapa)	0.9114832	0.7631961	not scorable	not scorable	1	0.736501
509	Bn-A01-p16093558	A-genome specific (napus-rapa)	0.9114832	0.7679684	not scorable	not scorable	0.9917355	0.8115
510	Bn-A01-p16093901	A-genome specific (napus-rapa)	0.9090909	0.8021525	not scorable	not scorable	0.9834711	0.7747257
511	Bn-A01-p16094037	A-genome specific (napus-rapa)	0.9114832	0.9077126	not scorable	not scorable	0.9917355	0.7579889
512	Bn-A01-p16103425	A-genome specific (napus-rapa)	0.9114832	0.820013	not scorable	not scorable	0.9917355	0.7282635
513	Bn-A01-p16106314	A-genome specific (napus-rapa)	0.9904306	0.7283542	not scorable	not scorable	0.9834711	0.7959835
514	Bn-A01-p16106372	A-genome specific (napus-rapa)	0.9952153	0.4838714	not scorable	not scorable	1	0.8413136
515	Bn-A01-p16111263	A-genome specific (napus-rapa)	0.9880383	0.6492066	not scorable	not scorable	0.9917355	0.7464206
516	Bn-A01-p16121664	A-genome specific (napus-rapa)	0.9856459	0.699172	not scorable	not scorable	1	0.6688023
517	Bn-A01-p16122923	A-genome specific (napus-rapa)	0.9856459	0.7803624	not scorable	not scorable	0.9834711	0.7789334
518	Bn-A01-p16123649	A-genome specific (napus-rapa)	0.9880383	0.6815299	not scorable	not scorable	1	0.6397638
519	Bn-A01-p161237	A-genome specific (napus-rapa)	0.9784689	0.4650359	not scorable	not scorable	0.9834711	0.8589982
520	Bn-A01-p16127871	A-genome specific (napus-rapa)	0.9784689	0.655113	not scorable	not scorable	0.9834711	0.6328713
521	Bn-A01-p16128165	A-genome specific (napus-rapa)	0.9880383	0.6388603	not scorable	not scorable	0.9834711	0.7871969
522	Bn-A01-p16128458	A-genome specific (napus-rapa)	0.9832536	0.786655	not scorable	not scorable	1	0.8214581
523	Bn-A01-p16131102	A-genome specific (rapa only)	not scorable	not scorable	not scorable	not scorable	1	0.5988446
524	Bn-A01-p16134540	A-genome specific (napus-rapa)	0.9808612	0.7730778	not scorable	not scorable	1	0.8002763
525	Bn-A01-p16134838	A-genome specific (napus-rapa)	0.9832536	0.6728048	not scorable	not scorable	0.9917355	0.636606

526	Bn-A01-p16135094	A-genome specific (napus-rapa)	0.9856459	0.8113371	not scorable	not scorable	0.9917355	0.8655304
527	Bn-A01-p16135537	A-genome specific (napus-rapa)	0.9904306	0.8074677	not scorable	not scorable	0.9834711	0.7440563
528	Bn-A01-p16138308	A-genome specific (napus-rapa)	0.9856459	0.7679947	not scorable	not scorable	1	0.6009578
529	Bn-A01-p16142196	A-genome specific (napus-rapa)	0.9880383	0.7657752	not scorable	not scorable	0.9917355	0.786679
530	Bn-A01-p16150568	A-genome specific (napus-rapa)	0.8971292	0.798889	not scorable	not scorable	0.9669421	0.6695432
531	Bn-A01-p16173770	A-genome specific (napus-rapa)	0.9880383	0.7032694	not scorable	not scorable	0.9834711	0.6449168
532	Bn-A01-p16174042	A-genome specific (napus-rapa)	0.9904306	0.7043581	not scorable	not scorable	0.9834711	0.6463727
533	Bn-A01-p16175909	A-genome specific (napus-rapa)	0.8971292	0.7245876	not scorable	not scorable	0.9834711	0.7453458
534	Bn-A01-p16181728	A-genome specific (napus-rapa)	0.9976076	0.6304978	not scorable	not scorable	0.9917355	0.7008156
535	Bn-A01-p16200217	A-genome specific (napus-rapa)	0.9904306	0.7541463	not scorable	not scorable	0.9834711	0.7937998
536	Bn-A01-p16217917	A-genome specific (napus-rapa)	0.9784689	0.7738739	not scorable	not scorable	0.9917355	0.8072308
537	Bn-A01-p16220364	A-genome specific (rapa only)	not scorable	not scorable	not scorable	not scorable	0.9586777	0.9140136
538	Bn-A01-p16221475	A-genome specific (napus-rapa)	0.8995215	0.7689804	not scorable	not scorable	0.9917355	0.7176483
539	Bn-A01-p16221732	A-genome specific (napus-rapa)	0.8995215	0.7762688	not scorable	not scorable	0.9917355	0.7419144
540	Bn-A01-p16222086	A-genome specific (napus-rapa)	0.9019139	0.7806044	not scorable	not scorable	0.9917355	0.763863
541	Bn-A01-p16222098	A-genome specific (napus-rapa)	0.8995215	0.7847332	not scorable	not scorable	0.9752066	0.7576745
542	Bn-A01-p16222167	A-genome specific (napus-rapa)	0.9019139	0.7986882	not scorable	not scorable	0.9917355	0.8212947
543	Bn-A01-p16222479	A-genome specific (napus-rapa)	0.8971292	0.7570435	not scorable	not scorable	0.9917355	0.8039848
544	Bn-A01-p16223356	A-genome specific (napus-rapa)	0.9449761	0.5053831	not scorable	not scorable	0.9834711	0.7781911
545	Bn-A01-p16228743	A-genome specific (rapa only)	not scorable	not scorable	not scorable	not scorable	1	0.7745675
546	Bn-A01-p16229096	A-genome specific (napus-rapa)	0.9856459	0.5102772	not scorable	not scorable	1	0.785867
547	Bn-A01-p16233062	A-genome specific (napus-rapa)	0.9904306	0.8196404	not scorable	not scorable	0.9917355	0.7845725
548	Bn-A01-p16253463	A-genome specific (napus-rapa)	0.9976076	0.4026324	not scorable	not scorable	0.9834711	0.7639982
549	Bn-A01-p16257043	A-genome specific (napus-rapa)	0.9856459	0.7180244	not scorable	not scorable	1	0.8146579
550	Bn-A01-p16260285	A-genome specific (napus-rapa)	0.2416268	0.7443817	not scorable	not scorable	0.9834711	0.6711024
551	Bn-A01-p16261297	A-genome specific (napus-rapa)	0.2416268	0.8141596	not scorable	not scorable	0.9834711	0.7326172
552	Bn-A01-p16262292	A-genome specific (napus-rapa)	0.2440191	0.3077965	not scorable	not scorable	0.9752066	0.4696523
553	Bn-A01-p16276961	A-genome specific (napus-rapa)	0.9904306	0.7830012	not scorable	not scorable	0.9917355	0.8340974
554	Bn-A01-p16278008	A-genome specific (napus-rapa)	0.9952153	0.417592	not scorable	not scorable	1	0.7161466
555	Bn-A01-p16303134	A-genome specific (napus-rapa)	0.9976076	0.4768853	not scorable	not scorable	0.9917355	0.7695389
556	Bn-A01-p16323375	A-genome specific (napus-rapa)	1	0.9136801	not scorable	not scorable	0.9917355	0.9063484
557	Bn-A01-p16323503	A-genome specific (napus-rapa)	1	0.8053868	not scorable	not scorable	1	0.8936522
558	Bn-A01-p16324010	A-genome specific (napus-rapa)	1	0.4965208	not scorable	not scorable	0.9917355	0.694369

559	Bn-A01-p1632686	A-genome specific (napus-rapa)	0.9952153	0.878007	not scorable	not scorable	1	0.8493479
560	Bn-A01-p16331424	A-genome specific (napus-rapa)	0.9928229	0.6178913	not scorable	not scorable	1	0.5856024
561	Bn-A01-p16343556	A-genome specific (napus-rapa)	0.9832536	0.7422501	not scorable	not scorable	0.9834711	0.7504656
562	Bn-A01-p16358989	A-genome specific (napus-rapa)	0.9856459	0.8287114	not scorable	not scorable	0.9834711	0.7684152
563	Bn-A01-p16366870	A-genome specific (rapa only)	not scorable	not scorable	not scorable	not scorable	0.9917355	0.7117968
564	Bn-A01-p1637234	A-genome specific (napus-rapa)	0.9976076	0.8748439	not scorable	not scorable	1	0.8785267
565	Bn-A01-p16374068	A-genome specific (napus-rapa)	0.9760765	0.7339548	not scorable	not scorable	0.9834711	0.6726437
566	Bn-A01-p16380496	A-genome specific (napus-rapa)	0.9880383	0.6769793	not scorable	not scorable	0.9917355	0.6108499
567	Bn-A01-p16389291	A-genome specific (napus-rapa)	0.9832536	0.780506	not scorable	not scorable	0.9834711	0.7797557
568	Bn-A01-p16393582	A-genome specific (napus-rapa)	1	0.9204846	not scorable	not scorable	1	0.7975187
569	Bn-A01-p16393856	genome unspecific (all in)	1	0.5319276	1	0.3029858	1	0.565518
570	Bn-A01-p16393939	A-genome specific (rapa only)	not scorable	not scorable	not scorable	not scorable	1	0.9129078
571	Bn-A01-p16394111	A-genome specific (rapa only)	not scorable	not scorable	not scorable	not scorable	1	0.6716551
572	Bn-A01-p16396926	A-genome specific (napus-rapa)	0.8995215	0.7681311	not scorable	not scorable	0.9586777	0.7467767
573	Bn-A01-p1639922	A-genome specific (napus-rapa)	0.9784689	0.9354553	not scorable	not scorable	1	0.7488236
574	Bn-A01-p16403023	A-genome specific (napus-rapa)	0.8995215	0.800343	not scorable	not scorable	0.9752066	0.7980971
575	Bn-A01-p16404320	A-genome specific (napus-rapa)	0.8588517	0.5856859	not scorable	not scorable	1	0.7935664
576	Bn-A01-p1641033	A-genome specific (napus-rapa)	0.9976076	0.3572757	not scorable	not scorable	1	0.6802424
577	Bn-A01-p16418499	A-genome specific (napus-rapa)	0.9952153	0.3521809	not scorable	not scorable	0.9917355	0.7357308
578	Bn-A01-p16432946	A-genome specific (napus-rapa)	0.9928229	0.4925531	not scorable	not scorable	0.9917355	0.7818975
579	Bn-A01-p16436191	A-genome specific (napus-rapa)	0.9904306	0.4915351	not scorable	not scorable	1	0.7461705
580	Bn-A01-p16454615	A-genome specific (napus-rapa)	0.9904306	0.8129848	not scorable	not scorable	1	0.6821043
581	Bn-A01-p16455409	A-genome specific (napus-rapa)	0.9928229	0.4026206	not scorable	not scorable	0.9917355	0.8313449
582	Bn-A01-p16461540	A-genome specific (napus-rapa)	0.9904306	0.7248526	not scorable	not scorable	1	0.86626
583	Bn-A01-p16461639	A-genome specific (napus-rapa)	0.9880383	0.7522252	not scorable	not scorable	1	0.7946908
584	Bn-A01-p16472273	A-genome specific (napus-rapa)	1	0.2862718	not scorable	not scorable	0.9834711	0.7589861
585	Bn-A01-p16474128	A-genome specific (napus-rapa)	0.2416268	0.7374499	not scorable	not scorable	1	0.716747
586	Bn-A01-p16476326	A-genome specific (napus-rapa)	1	0.3284398	not scorable	not scorable	0.9834711	0.6340655
587	Bn-A01-p1647693	all not scorable	not scorable	not scorable	not scorable	not scorable	not scorable	not scorable
588	Bn-A01-p16512818	A-genome specific (napus-rapa)	1	0.4885701	not scorable	not scorable	0.9917355	0.5887577
589	Bn-A01-p16529313	A-genome specific (napus-rapa)	0.9832536	0.7733735	not scorable	not scorable	1	0.7356086
590	Bn-A01-p16537769	A-genome specific (napus-rapa)	0.9928229	0.7152212	not scorable	not scorable	0.9917355	0.750037
591	Bn-A01-p16537792	A-genome specific (napus-rapa)	0.9880383	0.8508732	not scorable	not scorable	0.9834711	0.859604

592	Bn-A01-p16537866	A-genome specific (napus-rapa)	0.9904306	0.8043455	not scorable	not scorable	0.9834711	0.7957188
593	Bn-A01-p16538323	A-genome specific (napus-rapa)	0.9880383	0.7855483	not scorable	not scorable	0.9834711	0.7307933
594	Bn-A01-p16538587	A-genome specific (napus-rapa)	0.9928229	0.7088559	not scorable	not scorable	0.9917355	0.7447251
595	Bn-A01-p16539250	A-genome specific (napus-rapa)	0.9928229	0.7540243	not scorable	not scorable	0.7024794	0.8012853
596	Bn-A01-p16557636	A-genome specific (napus-rapa)	0.9928229	0.7834316	not scorable	not scorable	1	0.787746
597	Bn-A01-p16571536	all not scorable	not scorable	not scorable	not scorable	not scorable	not scorable	not scorable
598	Bn-A01-p16577721	A-genome specific (napus-rapa)	0.9832536	0.7134266	not scorable	not scorable	0.9917355	0.7008683
599	Bn-A01-p16580971	A-genome specific (napus-rapa)	1	0.3964088	not scorable	not scorable	0.9834711	0.6908677
600	Bn-A01-p16597123	napus specific	0.9880383	0.784229	not scorable	not scorable	not scorable	not scorable
601	Bn-A01-p16598652	A-genome specific (napus-rapa)	0.9928229	0.5979915	not scorable	not scorable	0.9669421	0.6860338
602	Bn-A01-p1661180	A-genome specific (napus-rapa)	0.9880383	0.8044863	not scorable	not scorable	0.9917355	0.7934247
603	Bn-A01-p16627043	A-genome specific (napus-rapa)	0.8995215	0.6405609	not scorable	not scorable	0.9917355	0.6457112
604	Bn-A01-p16630613	A-genome specific (napus-rapa)	0.9928229	0.3531196	not scorable	not scorable	0.9917355	0.7367932
605	Bn-A01-p16646241	A-genome specific (napus-rapa)	0.9760765	0.7370559	not scorable	not scorable	0.9752066	0.7813773
606	Bn-A01-p16656886	napus specific	0.9904306	0.3866979	not scorable	not scorable	not scorable	not scorable
607	Bn-A01-p16662544	A-genome specific (napus-rapa)	0.9880383	0.8032884	not scorable	not scorable	0.9752066	0.8668666
608	Bn-A01-p16665014	A-genome specific (napus-rapa)	0.9880383	0.5387177	not scorable	not scorable	0.9917355	0.6877334
609	Bn-A01-p16665036	A-genome specific (napus-rapa)	0.9856459	0.6043679	not scorable	not scorable	1	0.6807325
610	Bn-A01-p1666732	A-genome specific (napus-rapa)	0.9880383	0.7702525	not scorable	not scorable	0.661157	0.8177667
611	Bn-A01-p16669142	A-genome specific (napus-rapa)	1	0.5099291	not scorable	not scorable	0.9834711	0.7565833
612	Bn-A01-p16692819	A-genome specific (napus-rapa)	0.9928229	0.5488218	not scorable	not scorable	0.9834711	0.6984584
613	Bn-A01-p16692959	A-genome specific (napus-rapa)	0.9880383	0.567459	not scorable	not scorable	0.9917355	0.5289788
614	Bn-A01-p16694394	A-genome specific (napus-rapa)	0.9904306	0.6589631	not scorable	not scorable	0.9669421	0.7750105
615	Bn-A01-p16705936	A-genome specific (napus-rapa)	0.9976076	0.3731414	not scorable	not scorable	1	0.7746196
616	Bn-A01-p16711121	A-genome specific (napus-rapa)	0.9904306	0.8257817	not scorable	not scorable	0.9834711	0.7851875
617	Bn-A01-p16715637	A-genome specific (napus-rapa)	0.9904306	0.5700925	not scorable	not scorable	0.9834711	0.6775176
618	Bn-A01-p16717957	A-genome specific (napus-rapa)	1	0.425095	not scorable	not scorable	0.9917355	0.7935309
619	Bn-A01-p16720365	A-genome specific (napus-rapa)	0.9928229	0.4854914	not scorable	not scorable	1	0.8328984
620	Bn-A01-p16730334	A-genome specific (napus-rapa)	0.9736842	0.6023037	not scorable	not scorable	0.9834711	0.7142143
621	Bn-A01-p16749946	A-genome specific (napus-rapa)	0.9904306	0.5913548	not scorable	not scorable	1	0.8864269
622	Bn-A01-p16749951	A-genome specific (napus-rapa)	0.9904306	0.7618225	not scorable	not scorable	1	0.71694
623	Bn-A01-p16770716	A-genome specific (napus-rapa)	0.9976076	0.61774	not scorable	not scorable	0.9586777	0.7752942
624	Bn-A01-p16774157	A-genome specific (napus-rapa)	0.9904306	0.7051504	not scorable	not scorable	0.9917355	0.8027504

625	Bn-A01-p1678541	A-genome specific (napus-rapa)	0.9808612	0.817511	not scorable	not scorable	0.9917355	0.7182372
626	Bn-A01-p16829363	A-genome specific (napus-rapa)	0.9904306	0.6778446	not scorable	not scorable	1	0.7358214
627	Bn-A01-p1684485	A-genome specific (napus-rapa)	0.9928229	0.3096772	not scorable	not scorable	0.9917355	0.7501307
628	Bn-A01-p16847535	A-genome specific (napus-rapa)	0.9904306	0.7494892	not scorable	not scorable	0.9834711	0.6201832
629	Bn-A01-p16857183	A-genome specific (rapa only)	not scorable	not scorable	not scorable	not scorable	0.9834711	0.6362481
630	Bn-A01-p16857466	A-genome specific (napus-rapa)	0.9904306	0.812416	not scorable	not scorable	1	0.7273348
631	Bn-A01-p16869474	A-genome specific (napus-rapa)	0.7894737	0.7016385	not scorable	not scorable	0.9669421	0.6944501
632	Bn-A01-p16893578	A-genome specific (napus-rapa)	0.9976076	0.7652619	not scorable	not scorable	0.9917355	0.6816649
633	Bn-A01-p16911720	A-genome specific (napus-rapa)	0.9904306	0.7678972	not scorable	not scorable	0.9752066	0.8552126
634	Bn-A01-p16926592	A-genome specific (napus-rapa)	0.9808612	0.7393218	not scorable	not scorable	0.9917355	0.7746066
635	Bn-A01-p16935031	A-genome specific (napus-rapa)	0.9832536	0.3404019	not scorable	not scorable	0.9834711	0.7814434
636	Bn-A01-p16943370	A-genome specific (napus-rapa)	0.9880383	0.5710623	not scorable	not scorable	0.9917355	0.7750006
637	Bn-A01-p16951655	A-genome specific (napus-rapa)	0.9784689	0.7569799	not scorable	not scorable	0.9917355	0.8329853
638	Bn-A01-p16994459	A-genome specific (napus-rapa)	0.9880383	0.7282444	not scorable	not scorable	1	0.7173322
639	Bn-A01-p16997658	A-genome specific (napus-rapa)	0.9880383	0.5147389	not scorable	not scorable	0.9917355	0.7850258
640	Bn-A01-p17015383	A-genome specific (napus-rapa)	0.9952153	0.6912148	not scorable	not scorable	1	0.7097523
641	Bn-A01-p17036109	A-genome specific (napus-rapa)	0.9904306	0.3477275	not scorable	not scorable	0.9834711	0.7767258
642	Bn-A01-p17040457	A-genome specific (napus-rapa)	0.9952153	0.7442024	not scorable	not scorable	1	0.8760849
643	Bn-A01-p17049439	A-genome specific (napus-rapa)	0.9880383	0.8556809	not scorable	not scorable	1	0.7719007
644	Bn-A01-p17050461	A-genome specific (napus-rapa)	0.9856459	0.6832667	not scorable	not scorable	0.9917355	0.6741931
645	Bn-A01-p17050843	A-genome specific (rapa only)	not scorable	not scorable	not scorable	not scorable	1	0.8403057
646	Bn-A01-p17052809	A-genome specific (rapa only)	not scorable	not scorable	not scorable	not scorable	1	0.8387069
647	Bn-A01-p17081120	A-genome specific (napus-rapa)	0.9760765	0.7639223	not scorable	not scorable	0.9917355	0.8948524
648	Bn-A01-p17081703	A-genome specific (napus-rapa)	0.9593301	0.2894594	not scorable	not scorable	1	0.7197731
649	Bn-A01-p17097891	A-genome specific (napus-rapa)	0.9952153	0.6623596	not scorable	not scorable	1	0.6990979
650	Bn-A01-p17104282	A-genome specific (napus-rapa)	0.9688995	0.6718576	not scorable	not scorable	1	0.6820723
651	Bn-A01-p17105194	A-genome specific (napus-rapa)	0.9856459	0.7155052	not scorable	not scorable	1	0.6876032
652	Bn-A01-p17105372	A-genome specific (napus-rapa)	0.9784689	0.7416512	not scorable	not scorable	1	0.7899162
653	Bn-A01-p17105672	A-genome specific (napus-rapa)	0.9784689	0.7499294	not scorable	not scorable	1	0.8204867
654	Bn-A01-p17106985	A-genome specific (napus-rapa)	0.9665072	0.7396958	not scorable	not scorable	0.9917355	0.7168478
655	Bn-A01-p17110860	A-genome specific (rapa only)	not scorable	not scorable	not scorable	not scorable	0.9173554	0.7018179
656	Bn-A01-p17118647	A-genome specific (napus-rapa)	0.9736842	0.5511296	not scorable	not scorable	0.9834711	0.7015873
657	Bn-A01-p17118839	A-genome specific (napus-rapa)	0.9784689	0.7481247	not scorable	not scorable	1	0.7686127

658	Bn-A01-p17119024	A-genome specific (napus-rapa)	0.9641148	0.7682443	not scorable	not scorable	0.9834711	0.8368103
659	Bn-A01-p17119126	all not scorable	not scorable	not scorable	not scorable	not scorable	not scorable	not scorable
660	Bn-A01-p17121291	A-genome specific (napus-rapa)	0.9808612	0.7791226	not scorable	not scorable	0.9834711	0.7744188
661	Bn-A01-p17121775	A-genome specific (napus-rapa)	0.9665072	0.7750316	not scorable	not scorable	1	0.8301167
662	Bn-A01-p17121894	A-genome specific (napus-rapa)	0.9856459	0.7478482	not scorable	not scorable	0.9834711	0.8058835
663	Bn-A01-p17124558	A-genome specific (napus-rapa)	0.9784689	0.6160161	not scorable	not scorable	0.9917355	0.6343466
664	Bn-A01-p17124831	A-genome specific (napus-rapa)	0.9928229	0.7052152	not scorable	not scorable	0.9917355	0.7005992
665	Bn-A01-p17129906	A-genome specific (napus-rapa)	0.9760765	0.6670837	not scorable	not scorable	1	0.7983007
666	Bn-A01-p17136539	A-genome specific (napus-rapa)	0.8923445	0.6854421	not scorable	not scorable	0.9917355	0.7781309
667	Bn-A01-p17137863	A-genome specific (napus-rapa)	0.8875598	0.6679348	not scorable	not scorable	0.9834711	0.7010527
668	Bn-A01-p17150604	napus specific	0.9808612	0.7554874	not scorable	not scorable	not scorable	not scorable
669	Bn-A01-p17150875	A-genome specific (napus-rapa)	0.9904306	0.7777652	not scorable	not scorable	0.9834711	0.7707326
670	Bn-A01-p17156534	A-genome specific (napus-rapa)	0.9688995	0.7575676	not scorable	not scorable	0.9752066	0.704819
671	Bn-A01-p17157752	A-genome specific (napus-rapa)	0.9808612	0.7880577	not scorable	not scorable	0.9752066	0.6911551
672	Bn-A01-p17159109	A-genome specific (napus-rapa)	0.9760765	0.7516333	not scorable	not scorable	0.9917355	0.8127504
673	Bn-A01-p17160355	A-genome specific (napus-rapa)	0.9784689	0.7126247	not scorable	not scorable	0.9752066	0.6938723
674	Bn-A01-p17165052	A-genome specific (napus-rapa)	0.9593301	0.7211581	not scorable	not scorable	0.9752066	0.6923501
675	Bn-A01-p17165303	A-genome specific (napus-rapa)	0.9641148	0.7933876	not scorable	not scorable	0.9504132	0.7812968
676	Bn-A01-p17174111	A-genome specific (napus-rapa)	0.9090909	0.7931235	not scorable	not scorable	1	0.5289788
677	Bn-A01-p17187327	A-genome specific (napus-rapa)	0.9856459	0.7135414	not scorable	not scorable	0.9917355	0.8220242
678	Bn-A01-p1719658	A-genome specific (napus-rapa)	1	0.3501566	not scorable	not scorable	1	0.7568442
679	Bn-A01-p17216088	A-genome specific (napus-rapa)	0.9808612	0.6870565	not scorable	not scorable	1	0.633431
680	Bn-A01-p17216237	A-genome specific (napus-rapa)	0.9832536	0.8189083	not scorable	not scorable	0.9917355	0.7715991
681	Bn-A01-p17242218	A-genome specific (napus-rapa)	0.9976076	0.2425291	not scorable	not scorable	0.9917355	0.7018273
682	Bn-A01-p17258931	A-genome specific (napus-rapa)	0.9856459	0.644659	not scorable	not scorable	0.9917355	0.6937487
683	Bn-A01-p17271891	A-genome specific (napus-rapa)	0.9784689	0.7648455	not scorable	not scorable	0.9669421	0.7456536
684	Bn-A01-p17278340	A-genome specific (napus-rapa)	0.9904306	0.738239	not scorable	not scorable	1	0.7485788
685	Bn-A01-p17283208	A-genome specific (napus-rapa)	0.9928229	0.783596	not scorable	not scorable	0.9421487	0.8526646
686	Bn-A01-p17294955	A-genome specific (napus-rapa)	0.9904306	0.7997325	not scorable	not scorable	0.9834711	0.8220457
687	Bn-A01-p17295156	A-genome specific (napus-rapa)	0.9808612	0.7977918	not scorable	not scorable	1	0.8592536
688	Bn-A01-p17296147	A-genome specific (napus-rapa)	0.9904306	0.3554254	not scorable	not scorable	0.9834711	0.9116107
689	Bn-A01-p17306980	A-genome specific (napus-rapa)	0.9784689	0.4418235	not scorable	not scorable	0.9752066	0.9363571
690	Bn-A01-p17315519	A-genome specific (napus-rapa)	0.9880383	0.405986	not scorable	not scorable	1	0.8662788

691	Bn-A01-p17326645	A-genome specific (napus-rapa)	0.9904306	0.5211549	not scorable	not scorable	0.9917355	0.7663043
692	Bn-A01-p17377721	A-genome specific (napus-rapa)	0.9832536	0.6599445	not scorable	not scorable	1	0.7731497
693	Bn-A01-p17382168	A-genome specific (napus-rapa)	0.9976076	0.5330813	not scorable	not scorable	0.9834711	0.7722343
694	Bn-A01-p17408107	A-genome specific (napus-rapa)	0.9952153	0.6183806	not scorable	not scorable	0.9752066	0.7215518
695	Bn-A01-p17428120	A-genome specific (napus-rapa)	0.9928229	0.7673422	not scorable	not scorable	1	0.8026747
696	Bn-A01-p1742846	A-genome specific (napus-rapa)	0.9952153	0.7960058	not scorable	not scorable	0.9917355	0.879678
697	Bn-A01-p17432814	A-genome specific (napus-rapa)	0.9712918	0.6890628	not scorable	not scorable	1	0.7437273
698	Bn-A01-p17434748	A-genome specific (napus-rapa)	0.9928229	0.404459	not scorable	not scorable	1	0.6807756
699	Bn-A01-p17437603	A-genome specific (napus-rapa)	0.9880383	0.801483	not scorable	not scorable	1	0.8362373
700	Bn-A01-p17438645	A-genome specific (napus-rapa)	0.9880383	0.8329412	not scorable	not scorable	1	0.7834038
701	Bn-A01-p17445392	A-genome specific (napus-rapa)	0.9808612	0.7143654	not scorable	not scorable	0.9917355	0.6885723
702	Bn-A01-p1745099	A-genome specific (napus-rapa)	0.9186603	0.389883	not scorable	not scorable	1	0.7660935
703	Bn-A01-p1746226	A-genome specific (napus-rapa)	0.9784689	0.3277761	not scorable	not scorable	1	0.8191469
704	Bn-A01-p17476240	A-genome specific (napus-rapa)	0.9808612	0.5461645	not scorable	not scorable	0.9917355	0.8413854
705	Bn-A01-p17480096	A-genome specific (napus-rapa)	0.9856459	0.7888386	not scorable	not scorable	0.9669421	0.8617895
706	Bn-A01-p17481703	A-genome specific (napus-rapa)	0.9832536	0.7608973	not scorable	not scorable	0.9917355	0.7823467
707	Bn-A01-p17481804	A-genome specific (napus-rapa)	0.9928229	0.7337267	not scorable	not scorable	0.9669421	0.6711696
708	Bn-A01-p17482037	A-genome specific (napus-rapa)	0.9952153	0.6714442	not scorable	not scorable	0.9917355	0.7055385
709	Bn-A01-p17483006	A-genome specific (napus-rapa)	0.9880383	0.7622485	not scorable	not scorable	0.9834711	0.701647
710	Bn-A01-p17492348	genome unspecific (all in)	0.9736842	0.4429394	1	0.6910578	1	0.7721856
711	Bn-A01-p17494364	A-genome specific (napus-rapa)	0.9784689	0.7601151	not scorable	not scorable	0.7024794	0.8565337
712	Bn-A01-p17498057	A-genome specific (napus-rapa)	0.9976076	0.7821545	not scorable	not scorable	0.9586777	0.8342802
713	Bn-A01-p17506817	A-genome specific (napus-rapa)	0.9928229	0.7875162	not scorable	not scorable	1	0.7955745
714	Bn-A01-p17518104	A-genome specific (napus-rapa)	0.9928229	0.6490927	not scorable	not scorable	0.9834711	0.5204863
715	Bn-A01-p17518305	A-genome specific (napus-rapa)	0.9760765	0.7707673	not scorable	not scorable	1	0.7829878
716	Bn-A01-p17518468	A-genome specific (napus-rapa)	0.9880383	0.8095683	not scorable	not scorable	0.9752066	0.6595996
717	Bn-A01-p17527782	A-genome specific (napus-rapa)	0.9760765	0.7562634	not scorable	not scorable	1	0.7369925
718	Bn-A01-p17535899	genome unspecific (oleracea-rapa)	not scorable	not scorable	0.9689922	0.1840074	0.9834711	0.6616288
719	Bn-A01-p17536754	A-genome specific (napus-rapa)	0.9401914	0.2811816	not scorable	not scorable	0.9669421	0.6946872
720	Bn-A01-p17548021	A-genome specific (napus-rapa)	0.9760765	0.4789921	not scorable	not scorable	0.9586777	0.5041517
721	Bn-A01-p17548227	A-genome specific (napus-rapa)	0.9760765	0.654238	not scorable	not scorable	0.9752066	0.7411684
722	Bn-A01-p17549137	genome unspecific (all in)	1	0.4609133	1	0.7753507	1	0.9087456
723	Bn-A01-p17550125	genome unspecific (all in)	0.9976076	0.431604	0.9922481	0.6535215	0.7107438	0.8948976

724	Bn-A01-p17550588	A-genome specific (napus-rapa)	0.9952153	0.5061523	not scorable	not scorable	0.9834711	0.7631577
725	Bn-A01-p17552389	A-genome specific (napus-rapa)	0.9952153	0.5914578	not scorable	not scorable	1	0.7386726
726	Bn-A01-p17553462	A-genome specific (napus-rapa)	0.9856459	0.7288255	not scorable	not scorable	0.9834711	0.7968372
727	Bn-A01-p17555906	A-genome specific (napus-rapa)	0.9856459	0.7726346	not scorable	not scorable	0.9669421	0.7372245
728	Bn-A01-p17565834	A-genome specific (napus-rapa)	0.9904306	0.7580767	not scorable	not scorable	0.9834711	0.7330833
729	Bn-A01-p17574483	A-genome specific (napus-rapa)	0.9832536	0.8022539	not scorable	not scorable	0.9917355	0.7579209
730	Bn-A01-p17577426	A-genome specific (napus-rapa)	0.9928229	0.2949226	not scorable	not scorable	0.9834711	0.5145421
731	Bn-A01-p17577903	napus specific	0.9904306	0.4289759	not scorable	not scorable	not scorable	not scorable
732	Bn-A01-p17578169	A-genome specific (napus-rapa)	0.9808612	0.2543724	not scorable	not scorable	0.9834711	0.3464498
733	Bn-A01-p176052	A-genome specific (napus-rapa)	0.9784689	0.4352709	not scorable	not scorable	1	0.9171203
734	Bn-A01-p17625721	A-genome specific (napus-rapa)	0.9712918	0.6404186	not scorable	not scorable	0.9752066	0.7461996
735	Bn-A01-p17626052	A-genome specific (napus-rapa)	0.9688995	0.7650398	not scorable	not scorable	0.9834711	0.6583885
736	Bn-A01-p17629571	A-genome specific (napus-rapa)	0.9641148	0.4197555	not scorable	not scorable	0.9917355	0.8258931
737	Bn-A01-p17629628	A-genome specific (napus-rapa)	0.9856459	0.7722364	not scorable	not scorable	0.9669421	0.7829692
738	Bn-A01-p17636122	A-genome specific (rapa only)	not scorable	not scorable	not scorable	not scorable	0.9834711	0.8589982
739	Bn-A01-p17649002	A-genome specific (napus-rapa)	0.9976076	0.7422687	not scorable	not scorable	0.9917355	0.6844832
740	Bn-A01-p17677926	A-genome specific (napus-rapa)	0.9928229	0.306643	not scorable	not scorable	0.9752066	0.6886397
741	Bn-A01-p1768443	A-genome specific (napus-rapa)	0.9976076	0.5107654	not scorable	not scorable	1	0.7844037
742	Bn-A01-p17691799	A-genome specific (napus-rapa)	0.9808612	0.5229204	not scorable	not scorable	0.9834711	0.6625265
743	Bn-A01-p17693439	A-genome specific (napus-rapa)	0.9904306	0.823651	not scorable	not scorable	0.9834711	0.6941054
744	Bn-A01-p17696068	A-genome specific (napus-rapa)	1	0.7668517	not scorable	not scorable	1	0.8554994
745	Bn-A01-p17696358	A-genome specific (napus-rapa)	0.9880383	0.272892	not scorable	not scorable	1	0.4697423
746	Bn-A01-p1770291	A-genome specific (napus-rapa)	0.9952153	0.7055102	not scorable	not scorable	1	0.7542719
747	Bn-A01-p1770482	A-genome specific (napus-rapa)	0.9928229	0.7919643	not scorable	not scorable	0.9917355	0.7914497
748	Bn-A01-p17714021	A-genome specific (napus-rapa)	0.9856459	0.4330999	not scorable	not scorable	0.9834711	0.7611402
749	Bn-A01-p17716773	A-genome specific (napus-rapa)	0.9904306	0.731729	not scorable	not scorable	0.9834711	0.6709678
750	Bn-A01-p17717864	A-genome specific (napus-rapa)	0.9880383	0.752753	not scorable	not scorable	0.9752066	0.7137238
751	Bn-A01-p1772621	A-genome specific (napus-rapa)	0.9952153	0.7768759	not scorable	not scorable	0.9917355	0.724595
752	Bn-A01-p17728772	A-genome specific (napus-rapa)	0.9928229	0.8093067	not scorable	not scorable	0.9917355	0.6986303
753	Bn-A01-p17730468	A-genome specific (napus-rapa)	0.9928229	0.7961984	not scorable	not scorable	0.9752066	0.8658577
754	Bn-A01-p1773289	A-genome specific (napus-rapa)	0.9952153	0.7264529	not scorable	not scorable	0.9834711	0.7228026
755	Bn-A01-p17751647	A-genome specific (napus-rapa)	1	0.7246113	not scorable	not scorable	0.9917355	0.6953586
756	Bn-A01-p17755236	A-genome specific (napus-rapa)	0.9928229	0.5117599	not scorable	not scorable	1	0.7929777

757	Bn-A01-p17769742	A-genome specific (napus-rapa)	0.9952153	0.7658806	not scorable	not scorable	0.9917355	0.8323468
758	Bn-A01-p17771411	A-genome specific (napus-rapa)	0.9856459	0.7975176	not scorable	not scorable	0.9917355	0.7378754
759	Bn-A01-p17783808	A-genome specific (napus-rapa)	0.8923445	0.7792401	not scorable	not scorable	0.8264463	0.9501473
760	Bn-A01-p17783900	A-genome specific (napus-rapa)	0.8923445	0.7557725	not scorable	not scorable	0.8429752	0.8259932
761	Bn-A01-p17787446	A-genome specific (napus-rapa)	0.8899522	0.7335814	not scorable	not scorable	0.6694215	0.7373936
762	Bn-A01-p17806607	A-genome specific (napus-rapa)	0.9043062	0.7967382	not scorable	not scorable	0.677686	0.8546116
763	Bn-A01-p17820874	A-genome specific (napus-rapa)	0.9641148	0.6722628	not scorable	not scorable	0.9834711	0.5141634
764	Bn-A01-p17839637	A-genome specific (napus-rapa)	0.9928229	0.787922	not scorable	not scorable	1	0.7804823
765	Bn-A01-p17845660	A-genome specific (rapa only)	not scorable	not scorable	not scorable	not scorable	0.9586777	0.3844747
766	Bn-A01-p17849675	A-genome specific (napus-rapa)	0.9880383	0.7981772	not scorable	not scorable	0.9834711	0.6551153
767	Bn-A01-p178709	A-genome specific (napus-rapa)	0.9760765	0.6352564	not scorable	not scorable	0.9752066	0.649575
768	Bn-A01-p17881348	A-genome specific (napus-rapa)	0.9952153	0.73558	not scorable	not scorable	0.9917355	0.7549964
769	Bn-A01-p17885385	A-genome specific (napus-rapa)	0.9928229	0.7649127	not scorable	not scorable	0.9834711	0.7332052
770	Bn-A01-p17885812	A-genome specific (napus-rapa)	0.9784689	0.4682631	not scorable	not scorable	1	0.8203393
771	Bn-A01-p17886123	A-genome specific (napus-rapa)	0.9952153	0.7823653	not scorable	not scorable	0.9917355	0.8887201
772	Bn-A01-p17886595	A-genome specific (napus-rapa)	0.9904306	0.7056537	not scorable	not scorable	0.9834711	0.7402385
773	Bn-A01-p17887135	A-genome specific (napus-rapa)	0.9904306	0.807008	not scorable	not scorable	1	0.7553928
774	Bn-A01-p17887214	A-genome specific (napus-rapa)	0.9928229	0.7506796	not scorable	not scorable	1	0.7937827
775	Bn-A01-p17887935	A-genome specific (napus-rapa)	0.9880383	0.8086185	not scorable	not scorable	0.9917355	0.8238699
776	Bn-A01-p17888905	A-genome specific (napus-rapa)	0.9952153	0.8036841	not scorable	not scorable	1	0.7830911
777	Bn-A01-p17892299	A-genome specific (napus-rapa)	0.9808612	0.7417711	not scorable	not scorable	0.9834711	0.7635936
778	Bn-A01-p17899781	A-genome specific (napus-rapa)	0.9976076	0.6888735	not scorable	not scorable	0.9752066	0.811487
779	Bn-A01-p17905950	A-genome specific (napus-rapa)	0.9904306	0.7740884	not scorable	not scorable	0.9834711	0.8723322
780	Bn-A01-p17906322	A-genome specific (napus-rapa)	0.9760765	0.6116692	not scorable	not scorable	0.9834711	0.7694179
781	Bn-A01-p17906399	A-genome specific (napus-rapa)	0.9856459	0.7592572	not scorable	not scorable	0.9834711	0.7615721
782	Bn-A01-p17906765	A-genome specific (napus-rapa)	0.9904306	0.7874	not scorable	not scorable	1	0.827272
783	Bn-A01-p17907059	A-genome specific (napus-rapa)	0.9760765	0.67416	not scorable	not scorable	0.9834711	0.7752387
784	Bn-A01-p17907563	A-genome specific (napus-rapa)	0.9832536	0.7567583	not scorable	not scorable	0.9917355	0.812967
785	Bn-A01-p17910798	A-genome specific (napus-rapa)	0.9928229	0.6571036	not scorable	not scorable	0.9917355	0.8096161
786	Bn-A01-p17911199	A-genome specific (napus-rapa)	0.9904306	0.4846343	not scorable	not scorable	0.9917355	0.7983863
787	Bn-A01-p17911921	A-genome specific (napus-rapa)	0.9928229	0.3981737	not scorable	not scorable	0.9917355	0.7845946
788	Bn-A01-p17916004	A-genome specific (napus-rapa)	0.9928229	0.790007	not scorable	not scorable	1	0.7759329
789	Bn-A01-p17930058	A-genome specific (napus-rapa)	0.9808612	0.6466267	not scorable	not scorable	0.9834711	0.8730558

790	Bn-A01-p17931128	A-genome specific (napus-rapa)	0.9832536	0.6534733	not scorable	not scorable	1	0.7493571
791	Bn-A01-p17935219	A-genome specific (napus-rapa)	0.9880383	0.7043811	not scorable	not scorable	0.9917355	0.7096606
792	Bn-A01-p1793928	A-genome specific (napus-rapa)	0.4330144	0.7249883	not scorable	not scorable	1	0.8639467
793	Bn-A01-p17941441	napus specific	0.9162679	0.6844801	not scorable	not scorable	not scorable	not scorable
794	Bn-A01-p17997441	A-genome specific (napus-rapa)	0.9832536	0.7872486	not scorable	not scorable	1	0.7409858
795	Bn-A01-p17998573	A-genome specific (napus-rapa)	0.9784689	0.7373443	not scorable	not scorable	0.6942149	0.6990501
796	Bn-A01-p18006188	A-genome specific (napus-rapa)	0.9928229	0.8265212	not scorable	not scorable	1	0.811202
797	Bn-A01-p18008659	A-genome specific (napus-rapa)	0.9904306	0.3946294	not scorable	not scorable	0.9917355	0.7861206
798	Bn-A01-p18014946	A-genome specific (napus-rapa)	0.9928229	0.8300089	not scorable	not scorable	1	0.7501816
799	Bn-A01-p18015006	A-genome specific (napus-rapa)	0.9904306	0.6797619	not scorable	not scorable	1	0.7415134
800	Bn-A01-p18015162	A-genome specific (napus-rapa)	0.9880383	0.6277282	not scorable	not scorable	1	0.6875441
801	Bn-A01-p18015323	A-genome specific (napus-rapa)	0.284689	0.7125275	not scorable	not scorable	0.9834711	0.7640899
802	Bn-A01-p18019641	A-genome specific (napus-rapa)	0.9952153	0.6560127	not scorable	not scorable	1	0.6691702
803	Bn-A01-p18020258	A-genome specific (napus-rapa)	0.9952153	0.7839074	not scorable	not scorable	1	0.7293375
804	Bn-A01-p18020314	A-genome specific (napus-rapa)	0.9904306	0.7788019	not scorable	not scorable	1	0.719255
805	Bn-A01-p18020503	A-genome specific (napus-rapa)	0.9904306	0.7913567	not scorable	not scorable	1	0.6985102
806	Bn-A01-p18023959	A-genome specific (napus-rapa)	0.9832536	0.7964001	not scorable	not scorable	1	0.7600434
807	Bn-A01-p18025382	A-genome specific (rapa only)	not scorable	not scorable	not scorable	not scorable	1	0.8509145
808	Bn-A01-p18025988	A-genome specific (napus-rapa)	0.9808612	0.6371223	not scorable	not scorable	1	0.8545972
809	Bn-A01-p18027200	A-genome specific (napus-rapa)	0.9904306	0.811421	not scorable	not scorable	1	0.6716869
810	Bn-A01-p18027307	A-genome specific (napus-rapa)	0.9928229	0.7706072	not scorable	not scorable	1	0.7900006
811	Bn-A01-p18027398	A-genome specific (napus-rapa)	0.9904306	0.7966785	not scorable	not scorable	1	0.8003525
812	Bn-A01-p18028380	A-genome specific (napus-rapa)	0.9904306	0.8077947	not scorable	not scorable	0.9917355	0.7173789
813	Bn-A01-p18028483	A-genome specific (napus-rapa)	0.9904306	0.7106894	not scorable	not scorable	1	0.7746665
814	Bn-A01-p18030126	A-genome specific (napus-rapa)	0.9952153	0.7719436	not scorable	not scorable	1	0.8747157
815	Bn-A01-p18032386	A-genome specific (napus-rapa)	0.9856459	0.781758	not scorable	not scorable	0.9917355	0.8777807
816	Bn-A01-p18053077	A-genome specific (napus-rapa)	0.9928229	0.7944798	not scorable	not scorable	1	0.7239156
817	Bn-A01-p18063416	A-genome specific (napus-rapa)	0.9976076	0.3871094	not scorable	not scorable	0.9917355	0.8083651
818	Bn-A01-p18072231	A-genome specific (napus-rapa)	0.8157895	0.5525681	not scorable	not scorable	0.9752066	0.7569818
819	Bn-A01-p18072667	all not scorable	not scorable	not scorable	not scorable	not scorable	not scorable	not scorable
820	Bn-A01-p18073544	A-genome specific (napus-rapa)	0.9976076	0.8440844	not scorable	not scorable	1	0.7946706
821	Bn-A01-p18078718	A-genome specific (napus-rapa)	0.9832536	0.7190431	not scorable	not scorable	1	0.7069076
822	Bn-A01-p18084128	A-genome specific (napus-rapa)	0.8110048	0.7533848	not scorable	not scorable	0.9669421	0.8233715

823	Bn-A01-p18091225	A-genome specific (napus-rapa)	0.8253589	0.781181	not scorable	not scorable	0.9752066	0.7819396
824	Bn-A01-p18091559	A-genome specific (napus-rapa)	0.8277512	0.7534589	not scorable	not scorable	0.9752066	0.7929583
825	Bn-A01-p18091996	A-genome specific (napus-rapa)	0.8157895	0.7131996	not scorable	not scorable	0.9669421	0.7044402
826	Bn-A01-p18091997	A-genome specific (napus-rapa)	0.9880383	0.6160438	not scorable	not scorable	1	0.6266667
827	Bn-A01-p18096974	A-genome specific (napus-rapa)	0.9736842	0.7334434	not scorable	not scorable	0.9917355	0.8129012
828	Bn-A01-p18100900	A-genome specific (napus-rapa)	0.9832536	0.7256022	not scorable	not scorable	0.9834711	0.8351194
829	Bn-A01-p18102632	A-genome specific (napus-rapa)	0.9210526	0.6826652	not scorable	not scorable	0.7024794	0.8514571
830	Bn-A01-p18102711	A-genome specific (napus-rapa)	0.9162679	0.734462	not scorable	not scorable	0.7107438	0.7791697
831	Bn-A01-p18119989	A-genome specific (napus-rapa)	0.9832536	0.7077435	not scorable	not scorable	0.9917355	0.7442268
832	Bn-A01-p18121827	A-genome specific (napus-rapa)	0.9880383	0.7357628	not scorable	not scorable	0.9834711	0.8058633
833	Bn-A01-p18124795	A-genome specific (napus-rapa)	0.9641148	0.4797145	not scorable	not scorable	0.9834711	0.665405
834	Bn-A01-p18160311	A-genome specific (napus-rapa)	0.9090909	0.7251902	not scorable	not scorable	0.6859504	0.7432426
835	Bn-A01-p18178802	A-genome specific (napus-rapa)	0.9832536	0.7011608	not scorable	not scorable	0.9917355	0.6487134
836	Bn-A01-p18182766	A-genome specific (napus-rapa)	0.9904306	0.455505	not scorable	not scorable	1	0.7793339
837	Bn-A01-p18197352	A-genome specific (napus-rapa)	0.9760765	0.5295266	not scorable	not scorable	1	0.7870198
838	Bn-A01-p18200921	A-genome specific (napus-rapa)	0.9928229	0.8013641	not scorable	not scorable	0.9917355	0.6954973
839	Bn-A01-p18205455	A-genome specific (napus-rapa)	0.9832536	0.8144378	not scorable	not scorable	0.9917355	0.7042676
840	Bn-A01-p18218629	A-genome specific (napus-rapa)	0.8133971	0.8604687	not scorable	not scorable	0.892562	0.7958575
841	Bn-A01-p18230629	A-genome specific (napus-rapa)	0.9784689	0.409407	not scorable	not scorable	0.9834711	0.7828593
842	Bn-A01-p1823705	A-genome specific (napus-rapa)	0.9928229	0.6776361	not scorable	not scorable	0.9834711	0.6932496
843	Bn-A01-p18240444	genome unspecific (oleracea-rapa)	not scorable	not scorable	0.8992248	0.7777816	0.9421487	0.8071821
844	Bn-A01-p18260283	A-genome specific (napus-rapa)	0.9880383	0.6948167	not scorable	not scorable	0.9917355	0.7927071
845	Bn-A01-p18266537	A-genome specific (napus-rapa)	0.9569378	0.7025238	not scorable	not scorable	0.9752066	0.7452602
846	Bn-A01-p18285077	A-genome specific (napus-rapa)	0.9976076	0.6315592	not scorable	not scorable	1	0.8335571
847	Bn-A01-p18306617	A-genome specific (napus-rapa)	0.9760765	0.5190143	not scorable	not scorable	0.9917355	0.7201776
848	Bn-A01-p1831507	A-genome specific (napus-rapa)	0.8564593	0.6942256	not scorable	not scorable	0.9917355	0.725321
849	Bn-A01-p18327940	A-genome specific (napus-rapa)	0.9617225	0.3242286	not scorable	not scorable	1	0.7448562
850	Bn-A01-p18329609	A-genome specific (napus-rapa)	0.9521531	0.5385139	not scorable	not scorable	0.9834711	0.7768234
851	Bn-A01-p18329689	A-genome specific (napus-rapa)	0.9808612	0.794782	not scorable	not scorable	0.9917355	0.7565959
852	Bn-A01-p18329796	A-genome specific (napus-rapa)	0.9736842	0.7488375	not scorable	not scorable	0.9752066	0.6893721
853	Bn-A01-p18330598	A-genome specific (napus-rapa)	0.9856459	0.7238232	not scorable	not scorable	0.9917355	0.7127189
854	Bn-A01-p18367467	A-genome specific (napus-rapa)	0.9784689	0.2853943	not scorable	not scorable	0.9917355	0.7983629
855	Bn-A01-p18383994	A-genome specific (napus-rapa)	0.9784689	0.3066712	not scorable	not scorable	1	0.8416427

856	Bn-A01-p1838960	A-genome specific (napus-rapa)	0.9904306	0.4762939	not scorable	not scorable	0.9834711	0.7456536
857	Bn-A01-p18391341	A-genome specific (napus-rapa)	0.9760765	0.5622629	not scorable	not scorable	0.9090909	0.7507448
858	Bn-A01-p1842079	genome unspecific (all in)	0.9856459	0.585894	1	0.8213499	0.9834711	0.8713034
859	Bn-A01-p1842658	A-genome specific (napus-rapa)	0.9952153	0.2699037	not scorable	not scorable	0.9917355	0.7442436
860	Bn-A01-p18435876	A-genome specific (napus-rapa)	0.9760765	0.4218654	not scorable	not scorable	0.9917355	0.6071299
861	Bn-A01-p18438154	A-genome specific (rapa only)	not scorable	not scorable	not scorable	not scorable	1	0.7322186
862	Bn-A01-p1855903	A-genome specific (napus-rapa)	0.9928229	0.787994	not scorable	not scorable	0.9834711	0.7341285
863	Bn-A01-p1858279	A-genome specific (napus-rapa)	0.923445	0.6676201	not scorable	not scorable	1	0.9156699
864	Bn-A01-p1859204	genome unspecific (all in)	0.9186603	0.215144	0.9844961	0.4183614	0.9917355	0.8931121
865	Bn-A01-p18612031	A-genome specific (napus-rapa)	0.9880383	0.7805707	not scorable	not scorable	1	0.7260014
866	Bn-A01-p18635245	A-genome specific (napus-rapa)	0.9928229	0.656462	not scorable	not scorable	0.9917355	0.6712978
867	Bn-A01-p18635275	A-genome specific (napus-rapa)	0.9832536	0.7189415	not scorable	not scorable	1	0.6488666
868	Bn-A01-p18635430	A-genome specific (napus-rapa)	0.9976076	0.7476389	not scorable	not scorable	0.9917355	0.8074325
869	Bn-A01-p18635517	napus specific	0.9904306	0.5992392	not scorable	not scorable	not scorable	not scorable
870	Bn-A01-p18636804	A-genome specific (napus-rapa)	0.9880383	0.7262247	not scorable	not scorable	0.9917355	0.8057707
871	Bn-A01-p18639615	A-genome specific (napus-rapa)	0.9856459	0.7036929	not scorable	not scorable	0.9834711	0.7545809
872	Bn-A01-p1864705	A-genome specific (napus-rapa)	0.9832536	0.6450518	not scorable	not scorable	1	0.8141319
873	Bn-A01-p18662925	A-genome specific (napus-rapa)	0.9856459	0.7046435	not scorable	not scorable	1	0.8694528
874	Bn-A01-p18672984	A-genome specific (napus-rapa)	0.9904306	0.8026987	not scorable	not scorable	0.9752066	0.8181344
875	Bn-A01-p18673020	A-genome specific (napus-rapa)	0.9736842	0.5364887	not scorable	not scorable	0.9834711	0.7820559
876	Bn-A01-p18676639	A-genome specific (napus-rapa)	0.9952153	0.6584688	not scorable	not scorable	0.9752066	0.7819791
877	Bn-A01-p18691000	A-genome specific (napus-rapa)	0.9832536	0.7947907	not scorable	not scorable	1	0.8460613
878	Bn-A01-p18691142	A-genome specific (napus-rapa)	0.9904306	0.6689628	not scorable	not scorable	1	0.8731641
879	Bn-A01-p18692022	A-genome specific (napus-rapa)	0.9880383	0.687549	not scorable	not scorable	1	0.7885991
880	Bn-A01-p18692229	A-genome specific (napus-rapa)	0.9928229	0.7333393	not scorable	not scorable	0.9917355	0.7818957
881	Bn-A01-p18692686	A-genome specific (napus-rapa)	0.9928229	0.7528501	not scorable	not scorable	0.9752066	0.7499897
882	Bn-A01-p18694130	A-genome specific (napus-rapa)	0.9904306	0.5919966	not scorable	not scorable	1	0.9240721
883	Bn-A01-p18702163	A-genome specific (napus-rapa)	0.9928229	0.3885471	not scorable	not scorable	1	0.724835
884	Bn-A01-p18703183	A-genome specific (napus-rapa)	0.9928229	0.7347363	not scorable	not scorable	0.9752066	0.7189662
885	Bn-A01-p18703922	A-genome specific (napus-rapa)	0.9712918	0.7245755	not scorable	not scorable	0.9834711	0.8092477
886	Bn-A01-p18704205	A-genome specific (napus-rapa)	0.9760765	0.6896244	not scorable	not scorable	0.9669421	0.6764028
887	Bn-A01-p18704834	A-genome specific (napus-rapa)	0.9712918	0.6059431	not scorable	not scorable	0.9669421	0.578228
888	Bn-A01-p18705556	A-genome specific (napus-rapa)	1	0.6986961	not scorable	not scorable	1	0.7063369

889	Bn-A01-p18706337	A-genome specific (napus-rapa)	0.9904306	0.6432931	not scorable	not scorable	1	0.7174482
890	Bn-A01-p18711867	A-genome specific (napus-rapa)	0.9760765	0.3873529	not scorable	not scorable	0.9752066	0.7682264
891	Bn-A01-p18714050	A-genome specific (napus-rapa)	0.9641148	0.3656378	not scorable	not scorable	0.9669421	0.4078181
892	Bn-A01-p18714119	A-genome specific (napus-rapa)	0.9760765	0.3217244	not scorable	not scorable	0.9256198	0.4200652
893	Bn-A01-p18714182	A-genome specific (napus-rapa)	0.9760765	0.4828098	not scorable	not scorable	1	0.2942531
894	Bn-A01-p18715909	napus specific	0.9832536	0.7322287	not scorable	not scorable	not scorable	not scorable
895	Bn-A01-p18716258	A-genome specific (rapa only)	not scorable	not scorable	not scorable	not scorable	0.9834711	0.8782422
896	Bn-A01-p18727458	A-genome specific (napus-rapa)	0.9928229	0.5058958	not scorable	not scorable	0.9917355	0.7904691
897	Bn-A01-p18734383	A-genome specific (napus-rapa)	0.9832536	0.7749346	not scorable	not scorable	1	0.9458289
898	Bn-A01-p18739675	A-genome specific (napus-rapa)	0.9952153	0.7848468	not scorable	not scorable	0.9917355	0.6813199
899	Bn-A01-p1879401	A-genome specific (napus-rapa)	0.9712918	0.7648847	not scorable	not scorable	0.9834711	0.6770404
900	Bn-A01-p1880406	A-genome specific (napus-rapa)	0.9593301	0.8138466	not scorable	not scorable	0.9917355	0.7954207
901	Bn-A01-p18813615	A-genome specific (napus-rapa)	0.9880383	0.3614313	not scorable	not scorable	0.9917355	0.7286923
902	Bn-A01-p18813849	A-genome specific (napus-rapa)	0.9856459	0.2387568	not scorable	not scorable	0.9834711	0.6608431
903	Bn-A01-p18826838	A-genome specific (napus-rapa)	0.645933	0.8575372	not scorable	not scorable	0.9917355	0.826878
904	Bn-A01-p18826928	A-genome specific (rapa only)	not scorable	not scorable	not scorable	not scorable	0.9834711	0.8090869
905	Bn-A01-p18827383	A-genome specific (rapa only)	not scorable	not scorable	not scorable	not scorable	0.9917355	0.7033091
906	Bn-A01-p18828028	A-genome specific (rapa only)	not scorable	not scorable	not scorable	not scorable	0.9917355	0.7750772
907	Bn-A01-p18828081	A-genome specific (napus-rapa)	0.6578947	0.6999833	not scorable	not scorable	0.9917355	0.7311981
908	Bn-A01-p18828505	A-genome specific (napus-rapa)	0.6435407	0.8080457	not scorable	not scorable	0.9917355	0.6930169
909	Bn-A01-p18835025	A-genome specific (napus-rapa)	0.9928229	0.69782	not scorable	not scorable	0.9917355	0.6723409
910	Bn-A01-p18837660	A-genome specific (rapa only)	not scorable	not scorable	not scorable	not scorable	0.9917355	0.8218932
911	Bn-A01-p18850969	A-genome specific (napus-rapa)	0.9808612	0.4367225	not scorable	not scorable	0.9917355	0.6786802
912	Bn-A01-p18866149	A-genome specific (rapa only)	not scorable	not scorable	not scorable	not scorable	0.9752066	0.7313379
913	Bn-A01-p18870174	A-genome specific (napus-rapa)	0.9928229	0.6610835	not scorable	not scorable	0.9669421	0.7008046
914	Bn-A01-p18876940	A-genome specific (napus-rapa)	0.9784689	0.6623287	not scorable	not scorable	0.9834711	0.7647851
915	Bn-A01-p18881723	A-genome specific (napus-rapa)	0.9880383	0.4945321	not scorable	not scorable	0.9669421	0.7037584
916	Bn-A01-p18882626	A-genome specific (napus-rapa)	0.9401914	0.6798978	not scorable	not scorable	0.9669421	0.6893647
917	Bn-A01-p18890238	A-genome specific (napus-rapa)	0.9736842	0.6502059	not scorable	not scorable	0.9834711	0.7107322
918	Bn-A01-p18894627	A-genome specific (napus-rapa)	0.9904306	0.5921113	not scorable	not scorable	0.9752066	0.7936412
919	Bn-A01-p18896245	A-genome specific (napus-rapa)	0.9138756	0.7407206	not scorable	not scorable	0.9834711	0.7608529
920	Bn-A01-p1890102	A-genome specific (napus-rapa)	0.9784689	0.6894891	not scorable	not scorable	1	0.8045743
921	Bn-A01-p18902391	A-genome specific (napus-rapa)	0.9976076	0.773557	not scorable	not scorable	0.9752066	0.7193771

922	Bn-A01-p18914107	C-genome specific (napus-oleracea)	0.9856459	0.2657087	0.9302326	0.2311522	not scorable	not scorable
923	Bn-A01-p18914763	A-genome specific (napus-rapa)	1	0.9396719	not scorable	not scorable	1	0.8294522
924	Bn-A01-p18917698	A-genome specific (napus-rapa)	0.9928229	0.7933979	not scorable	not scorable	0.9834711	0.7172196
925	Bn-A01-p19097875	A-genome specific (napus-rapa)	0.9880383	0.5141646	not scorable	not scorable	0.9834711	0.7519721
926	Bn-A01-p19098195	A-genome specific (napus-rapa)	0.9976076	0.782424	not scorable	not scorable	0.9917355	0.7093671
927	Bn-A01-p19098776	A-genome specific (napus-rapa)	0.9880383	0.7726154	not scorable	not scorable	0.9834711	0.7545089
928	Bn-A01-p19107003	A-genome specific (napus-rapa)	0.9832536	0.8005287	not scorable	not scorable	0.9834711	0.733486
929	Bn-A01-p19107198	A-genome specific (napus-rapa)	0.9904306	0.7871521	not scorable	not scorable	0.9752066	0.7154981
930	Bn-A01-p19107773	A-genome specific (napus-rapa)	0.9832536	0.7488924	not scorable	not scorable	0.9504132	0.8055305
931	Bn-A01-p19108439	A-genome specific (napus-rapa)	0.9904306	0.7670153	not scorable	not scorable	0.9752066	0.6991875
932	Bn-A01-p19108743	A-genome specific (napus-rapa)	0.9952153	0.7104266	not scorable	not scorable	0.9834711	0.7913313
933	Bn-A01-p19108849	A-genome specific (napus-rapa)	0.9952153	0.8007604	not scorable	not scorable	0.9917355	0.7845291
934	Bn-A01-p19110384	A-genome specific (napus-rapa)	0.9880383	0.7701765	not scorable	not scorable	0.9834711	0.7314096
935	Bn-A01-p19110508	A-genome specific (napus-rapa)	0.9904306	0.7509905	not scorable	not scorable	0.9834711	0.7531759
936	Bn-A01-p19110596	A-genome specific (rapa only)	not scorable	not scorable	not scorable	not scorable	0.9834711	0.8929924
937	Bn-A01-p19111246	A-genome specific (napus-rapa)	0.9784689	0.7507916	not scorable	not scorable	0.9834711	0.7382591
938	Bn-A01-p19111305	A-genome specific (napus-rapa)	0.9880383	0.8098382	not scorable	not scorable	0.9834711	0.8127406
939	Bn-A01-p19114969	A-genome specific (napus-rapa)	0.9928229	0.8036723	not scorable	not scorable	0.9917355	0.810369
940	Bn-A01-p19115021	A-genome specific (napus-rapa)	0.9880383	0.8156418	not scorable	not scorable	0.9834711	0.8177392
941	Bn-A01-p19115242	A-genome specific (napus-rapa)	0.9952153	0.7833368	not scorable	not scorable	0.9586777	0.8200111
942	Bn-A01-p19115530	A-genome specific (napus-rapa)	0.9736842	0.764879	not scorable	not scorable	0.9834711	0.7498007
943	Bn-A01-p19115872	A-genome specific (napus-rapa)	0.9976076	0.8009316	not scorable	not scorable	0.9834711	0.8135641
944	Bn-A01-p19116006	A-genome specific (napus-rapa)	0.9952153	0.7864987	not scorable	not scorable	0.9834711	0.7867858
945	Bn-A01-p19116819	A-genome specific (napus-rapa)	0.9904306	0.7181495	not scorable	not scorable	0.9917355	0.7238413
946	Bn-A01-p19122048	A-genome specific (napus-rapa)	0.9760765	0.7708241	not scorable	not scorable	0.9586777	0.8079948
947	Bn-A01-p19125668	A-genome specific (napus-rapa)	0.9760765	0.7078291	not scorable	not scorable	0.9586777	0.6816537
948	Bn-A01-p19132144	A-genome specific (napus-rapa)	0.9880383	0.6337997	not scorable	not scorable	1	0.7944537
949	Bn-A01-p19139637	genome unspecific (all in)	0.9760765	0.6100909	1	0.5891986	0.9669421	0.7987592
950	Bn-A01-p19150923	A-genome specific (napus-rapa)	0.9880383	0.7907714	not scorable	not scorable	0.9917355	0.8049241
951	Bn-A01-p19153919	A-genome specific (napus-rapa)	0.9808612	0.7549415	not scorable	not scorable	0.9752066	0.7238413
952	Bn-A01-p19156177	A-genome specific (napus-rapa)	0.9736842	0.7111392	not scorable	not scorable	1	0.7988512
953	Bn-A01-p19158271	A-genome specific (napus-rapa)	0.9928229	0.7292264	not scorable	not scorable	1	0.7299778
954	Bn-A01-p19158362	A-genome specific (napus-rapa)	0.9952153	0.7194334	not scorable	not scorable	1	0.7325492

955	Bn-A01-p19162993	A-genome specific (napus-rapa)	0.9976076	0.479274	not scorable	not scorable	1	0.8552678
956	Bn-A01-p19163275	A-genome specific (napus-rapa)	0.9880383	0.5024969	not scorable	not scorable	0.9917355	0.7194584
957	Bn-A01-p19170760	A-genome specific (napus-rapa)	0.9856459	0.5509182	not scorable	not scorable	0.9917355	0.7876227
958	Bn-A01-p19174115	A-genome specific (napus-rapa)	0.9976076	0.7440745	not scorable	not scorable	1	0.7571859
959	Bn-A01-p1917414	A-genome specific (napus-rapa)	1	0.8339194	not scorable	not scorable	1	0.8020645
960	Bn-A01-p19185181	A-genome specific (napus-rapa)	0.9928229	0.7770296	not scorable	not scorable	1	0.8544614
961	Bn-A01-p19220693	A-genome specific (napus-rapa)	0.9736842	0.7194408	not scorable	not scorable	1	0.7635936
962	Bn-A01-p19283108	A-genome specific (napus-rapa)	0.9976076	0.7200807	not scorable	not scorable	1	0.768926
963	Bn-A01-p1929982	A-genome specific (napus-rapa)	0.9880383	0.5810862	not scorable	not scorable	1	0.6965786
964	Bn-A01-p1930111	A-genome specific (napus-rapa)	1	0.5229401	not scorable	not scorable	1	0.768311
965	Bn-A01-p19340658	A-genome specific (napus-rapa)	0.9952153	0.7972027	not scorable	not scorable	1	0.9414077
966	Bn-A01-p19355180	A-genome specific (napus-rapa)	0.9976076	0.3478079	not scorable	not scorable	1	0.6606453
967	Bn-A01-p19446599	A-genome specific (napus-rapa)	0.9760765	0.5129727	not scorable	not scorable	1	0.8975873
968	Bn-A01-p19447794	A-genome specific (napus-rapa)	0.9856459	0.2713615	not scorable	not scorable	1	0.8273389
969	Bn-A01-p19449612	A-genome specific (napus-rapa)	0.9952153	0.8004653	not scorable	not scorable	0.9917355	0.7587818
970	Bn-A01-p19498222	A-genome specific (napus-rapa)	0.9832536	0.3593376	not scorable	not scorable	0.9834711	0.7025349
971	Bn-A01-p19512499	A-genome specific (napus-rapa)	0.9928229	0.4705554	not scorable	not scorable	0.9917355	0.6777889
972	Bn-A01-p1951988	A-genome specific (napus-rapa)	0.9114832	0.5675307	not scorable	not scorable	0.9834711	0.5431461
973	Bn-A01-p195253	A-genome specific (napus-rapa)	0.9904306	0.7094556	not scorable	not scorable	1	0.7093971
974	Bn-A01-p19525594	A-genome specific (napus-rapa)	0.9832536	0.4937035	not scorable	not scorable	0.9917355	0.6975186
975	Bn-A01-p19527588	A-genome specific (napus-rapa)	0.9928229	0.526689	not scorable	not scorable	1	0.8165209
976	Bn-A01-p19528388	A-genome specific (napus-rapa)	0.9665072	0.681171	not scorable	not scorable	0.9917355	0.6935953
977	Bn-A01-p19533568	A-genome specific (napus-rapa)	0.9856459	0.4676928	not scorable	not scorable	0.9917355	0.6338909
978	Bn-A01-p19535456	A-genome specific (rapa only)	not scorable	not scorable	not scorable	not scorable	0.7107438	0.9041291
979	Bn-A01-p19540960	A-genome specific (napus-rapa)	0.9928229	0.5603268	not scorable	not scorable	1	0.8396539
980	Bn-A01-p19562202	A-genome specific (napus-rapa)	0.9928229	0.3951175	not scorable	not scorable	1	0.8226761
981	Bn-A01-p19564015	A-genome specific (napus-rapa)	0.9928229	0.6966766	not scorable	not scorable	1	0.7777089
982	Bn-A01-p19567680	A-genome specific (rapa only)	not scorable	not scorable	not scorable	not scorable	1	0.785926
983	Bn-A01-p19577901	A-genome specific (napus-rapa)	0.9976076	0.5612255	not scorable	not scorable	1	0.7961764
984	Bn-A01-p19579243	A-genome specific (napus-rapa)	0.9066986	0.749384	not scorable	not scorable	0.9834711	0.7030711
985	Bn-A01-p19579412	A-genome specific (napus-rapa)	0.9880383	0.6988412	not scorable	not scorable	1	0.716031
986	Bn-A01-p19581862	A-genome specific (napus-rapa)	0.9641148	0.7853197	not scorable	not scorable	0.9834711	0.7918388
987	Bn-A01-p19582940	A-genome specific (napus-rapa)	0.9832536	0.8202503	not scorable	not scorable	0.9917355	0.7567395

988	Bn-A01-p19583367	A-genome specific (napus-rapa)	0.9880383	0.8262744	not scorable	not scorable	0.9834711	0.747709
989	Bn-A01-p19583736	A-genome specific (napus-rapa)	0.9856459	0.5869185	not scorable	not scorable	1	0.8125831
990	Bn-A01-p19589837	A-genome specific (napus-rapa)	0.9928229	0.8125619	not scorable	not scorable	0.9834711	0.7501816
991	Bn-A01-p19589921	A-genome specific (napus-rapa)	0.9928229	0.8076128	not scorable	not scorable	1	0.7784025
992	Bn-A01-p19592804	A-genome specific (napus-rapa)	0.9784689	0.8402614	not scorable	not scorable	0.9834711	0.7235413
993	Bn-A01-p19593638	all not scorable	not scorable	not scorable	not scorable	not scorable	not scorable	not scorable
994	Bn-A01-p19593702	napus specific	0.9856459	0.6717374	not scorable	not scorable	not scorable	not scorable
995	Bn-A01-p19594423	A-genome specific (napus-rapa)	0.9832536	0.801175	not scorable	not scorable	0.9917355	0.7040766
996	Bn-A01-p19598162	napus specific	0.9784689	0.301473	not scorable	not scorable	not scorable	not scorable
997	Bn-A01-p19599270	A-genome specific (napus-rapa)	0.9832536	0.7222377	not scorable	not scorable	0.9586777	0.6799036
998	Bn-A01-p19600244	A-genome specific (napus-rapa)	0.9904306	0.7374802	not scorable	not scorable	0.9338843	0.7679144
999	Bn-A01-p19601137	A-genome specific (napus-rapa)	0.9688995	0.8285986	not scorable	not scorable	1	0.8059633
1000	Bn-A01-p19601542	A-genome specific (napus-rapa)	0.9736842	0.8026147	not scorable	not scorable	0.9752066	0.7471011
1001	Bn-A01-p19602112	A-genome specific (napus-rapa)	0.9856459	0.7406455	not scorable	not scorable	0.9917355	0.7089196
1002	Bn-A01-p19605587	napus specific	0.9808612	0.3241806	not scorable	not scorable	not scorable	not scorable
1003	Bn-A01-p19607256	A-genome specific (napus-rapa)	0.9784689	0.6934058	not scorable	not scorable	0.9917355	0.681553
1004	Bn-A01-p19617963	A-genome specific (napus-rapa)	0.9856459	0.757225	not scorable	not scorable	0.9917355	0.7512794
1005	Bn-A01-p19623880	A-genome specific (napus-rapa)	0.9904306	0.7152954	not scorable	not scorable	0.9752066	0.617974
1006	Bn-A01-p19623985	A-genome specific (napus-rapa)	0.9928229	0.7817439	not scorable	not scorable	0.9917355	0.7299535
1007	Bn-A01-p19624123	A-genome specific (napus-rapa)	0.9880383	0.6129854	not scorable	not scorable	0.9834711	0.5326691
1008	Bn-A01-p19624216	A-genome specific (napus-rapa)	0.9880383	0.6622064	not scorable	not scorable	0.9834711	0.6145394
1009	Bn-A01-p19633815	A-genome specific (napus-rapa)	0.9688995	0.7800902	not scorable	not scorable	0.9917355	0.8343857
1010	Bn-A01-p19650666	A-genome specific (napus-rapa)	0.9521531	0.5026596	not scorable	not scorable	0.9917355	0.8291974
1011	Bn-A01-p19661033	A-genome specific (napus-rapa)	0.9808612	0.4546458	not scorable	not scorable	0.9834711	0.8611981
1012	Bn-A01-p1966955	A-genome specific (napus-rapa)	0.9593301	0.4119262	not scorable	not scorable	0.9834711	0.7863845
1013	Bn-A01-p19700004	A-genome specific (napus-rapa)	0.9760765	0.6949056	not scorable	not scorable	1	0.6908952
1014	Bn-A01-p19712396	A-genome specific (napus-rapa)	1	0.9303331	not scorable	not scorable	0.9834711	0.8649814
1015	Bn-A01-p19722138	A-genome specific (napus-rapa)	0.9545454	0.6610944	not scorable	not scorable	0.9834711	0.752296
1016	Bn-A01-p19731678	A-genome specific (napus-rapa)	0.9760765	0.7362979	not scorable	not scorable	1	0.7518246
1017	Bn-A01-p1975231	A-genome specific (napus-rapa)	0.9952153	0.7850443	not scorable	not scorable	0.9917355	0.8627473
1018	Bn-A01-p19762477	A-genome specific (napus-rapa)	0.9856459	0.4133842	not scorable	not scorable	1	0.7227727
1019	Bn-A01-p19764240	A-genome specific (napus-rapa)	0.9617225	0.2927379	not scorable	not scorable	1	0.7933361
1020	Bn-A01-p1977270	A-genome specific (napus-rapa)	0.9904306	0.2892985	not scorable	not scorable	0.9834711	0.789915

1021	Bn-A01-p1980181	A-genome specific (napus-rapa)	0.9856459	0.7016599	not scorable	not scorable	1	0.7900167
1022	Bn-A01-p19803988	A-genome specific (napus-rapa)	0.9736842	0.7715852	not scorable	not scorable	0.9586777	0.7671582
1023	Bn-A01-p19813138	A-genome specific (napus-rapa)	0.9521531	0.3797322	not scorable	not scorable	0.9752066	0.7013641
1024	Bn-A01-p19840597	A-genome specific (napus-rapa)	0.9545454	0.6460978	not scorable	not scorable	0.9917355	0.8694528
1025	Bn-A01-p19855343	A-genome specific (napus-rapa)	0.4856459	0.6923867	not scorable	not scorable	1	0.7911531
1026	Bn-A01-p19858735	A-genome specific (napus-rapa)	0.7464115	0.7444174	not scorable	not scorable	0.9834711	0.6552684
1027	Bn-A01-p19879925	A-genome specific (napus-rapa)	0.9736842	0.7194312	not scorable	not scorable	1	0.9279277
1028	Bn-A01-p1990755	A-genome specific (napus-rapa)	0.9736842	0.5892439	not scorable	not scorable	0.9586777	0.5243856
1029	Bn-A01-p19929280	A-genome specific (rapa only)	not scorable	not scorable	not scorable	not scorable	0.9834711	0.7739415
1030	Bn-A01-p19931030	A-genome specific (napus-rapa)	0.9617225	0.7888451	not scorable	not scorable	0.9586777	0.8517572
1031	Bn-A01-p19931596	A-genome specific (napus-rapa)	0.9665072	0.7345183	not scorable	not scorable	0.9834711	0.8491015
1032	Bn-A01-p1993302	A-genome specific (napus-rapa)	0.9808612	0.7018445	not scorable	not scorable	0.9834711	0.7483851
1033	Bn-A01-p19946190	A-genome specific (napus-rapa)	0.9736842	0.7741898	not scorable	not scorable	1	0.7791921
1034	Bn-A01-p19955264	A-genome specific (napus-rapa)	0.9928229	0.7233712	not scorable	not scorable	1	0.7401661
1035	Bn-A01-p19961176	A-genome specific (napus-rapa)	0.9665072	0.3320428	not scorable	not scorable	0.9917355	0.7101301
1036	Bn-A01-p19983739	A-genome specific (napus-rapa)	0.9976076	0.6827177	not scorable	not scorable	1	0.7195216
1037	Bn-A01-p19993388	A-genome specific (napus-rapa)	0.9928229	0.7497161	not scorable	not scorable	0.9504132	0.7507055
1038	Bn-A01-p2004336	A-genome specific (napus-rapa)	0.9856459	0.7611501	not scorable	not scorable	1	0.7961465
1039	Bn-A01-p2009581	A-genome specific (rapa only)	not scorable	not scorable	not scorable	not scorable	1	0.8530514
1040	Bn-A01-p20133687	A-genome specific (napus-rapa)	0.9832536	0.3317388	not scorable	not scorable	0.9669421	0.478596
1041	Bn-A01-p20139530	A-genome specific (napus-rapa)	0.9665072	0.6784402	not scorable	not scorable	0.9917355	0.7801748
1042	Bn-A01-p20144346	A-genome specific (napus-rapa)	0.9593301	0.6712831	not scorable	not scorable	0.9834711	0.6230401
1043	Bn-A01-p20144596	A-genome specific (rapa only)	not scorable	not scorable	not scorable	not scorable	0.9586777	0.6346936
1044	Bn-A01-p20147405	A-genome specific (napus-rapa)	0.9784689	0.6703942	not scorable	not scorable	0.9834711	0.7626469
1045	Bn-A01-p20159960	A-genome specific (napus-rapa)	0.9641148	0.27909	not scorable	not scorable	1	0.6780497
1046	Bn-A01-p20166016	A-genome specific (napus-rapa)	0.9593301	0.6117621	not scorable	not scorable	1	0.7541876
1047	Bn-A01-p20167230	A-genome specific (napus-rapa)	0.9808612	0.7496573	not scorable	not scorable	0.9586777	0.7545766
1048	Bn-A01-p20181046	A-genome specific (napus-rapa)	0.9856459	0.7814912	not scorable	not scorable	1	0.8112941
1049	Bn-A01-p20181346	A-genome specific (napus-rapa)	0.9880383	0.4082313	not scorable	not scorable	0.9834711	0.7316437
1050	Bn-A01-p20196813	A-genome specific (napus-rapa)	0.9928229	0.4083154	not scorable	not scorable	1	0.731406
1051	Bn-A01-p20207440	A-genome specific (napus-rapa)	0.9904306	0.8127222	not scorable	not scorable	0.9917355	0.6975948
1052	Bn-A01-p20211329	A-genome specific (napus-rapa)	0.9760765	0.4697541	not scorable	not scorable	0.9917355	0.7364028
1053	Bn-A01-p20211387	A-genome specific (napus-rapa)	0.9688995	0.7742302	not scorable	not scorable	1	0.7985536

1054	Bn-A01-p20233113	A-genome specific (napus-rapa)	0.9712918	0.4945846	not scorable	not scorable	0.9504132	0.6739829
1055	Bn-A01-p20240168	all not scorable	not scorable	not scorable	not scorable	not scorable	not scorable	not scorable
1056	Bn-A01-p20240780	A-genome specific (napus-rapa)	0.9904306	0.4945932	not scorable	not scorable	1	0.8030509
1057	Bn-A01-p20246564	A-genome specific (napus-rapa)	0.9736842	0.6821868	not scorable	not scorable	0.9917355	0.7081411
1058	Bn-A01-p20246840	A-genome specific (napus-rapa)	0.9784689	0.7250506	not scorable	not scorable	0.9917355	0.697707
1059	Bn-A01-p20248379	A-genome specific (napus-rapa)	0.9712918	0.5714301	not scorable	not scorable	0.9834711	0.6713508
1060	Bn-A01-p2025400	A-genome specific (napus-rapa)	0.9856459	0.7888976	not scorable	not scorable	1	0.7452201
1061	Bn-A01-p20259868	A-genome specific (napus-rapa)	0.9808612	0.4723499	not scorable	not scorable	0.9917355	0.7848109
1062	Bn-A01-p20282705	A-genome specific (napus-rapa)	0.9473684	0.4994308	not scorable	not scorable	0.9752066	0.7833955
1063	Bn-A01-p20306930	A-genome specific (napus-rapa)	0.4090909	0.7258548	not scorable	not scorable	0.9752066	0.7300994
1064	Bn-A01-p20323059	A-genome specific (napus-rapa)	0.9736842	0.7895429	not scorable	not scorable	0.9834711	0.8649952
1065	Bn-A01-p20393111	A-genome specific (napus-rapa)	0.9401914	0.4292949	not scorable	not scorable	0.9834711	0.7033637
1066	Bn-A01-p20397807	A-genome specific (napus-rapa)	0.9832536	0.7810751	not scorable	not scorable	0.9917355	0.8071022
1067	Bn-A01-p20406782	A-genome specific (napus-rapa)	0.7583732	0.7681702	not scorable	not scorable	1	0.8911937
1068	Bn-A01-p2042209	A-genome specific (napus-rapa)	0.9330143	0.5054451	not scorable	not scorable	0.9090909	0.8088876
1069	Bn-A01-p20430407	A-genome specific (napus-rapa)	0.9736842	0.7353063	not scorable	not scorable	0.9917355	0.7894275
1070	Bn-A01-p20431228	A-genome specific (napus-rapa)	0.9856459	0.3802324	not scorable	not scorable	0.9917355	0.7413363
1071	Bn-A01-p20433111	A-genome specific (napus-rapa)	0.8157895	0.4689084	not scorable	not scorable	0.9917355	0.8403395
1072	Bn-A01-p20442457	A-genome specific (napus-rapa)	0.9736842	0.7752901	not scorable	not scorable	1	0.8271491
1073	Bn-A01-p2046061	A-genome specific (napus-rapa)	1	0.5705255	not scorable	not scorable	1	0.626141
1074	Bn-A01-p20471502	A-genome specific (napus-rapa)	0.9880383	0.7762998	not scorable	not scorable	1	0.790405
1075	Bn-A01-p20482459	A-genome specific (napus-rapa)	0.9880383	0.7130696	not scorable	not scorable	0.9917355	0.7138222
1076	Bn-A01-p20483938	A-genome specific (napus-rapa)	0.9880383	0.8052693	not scorable	not scorable	0.9917355	0.786477
1077	Bn-A01-p20497397	A-genome specific (rapa only)	not scorable	not scorable	not scorable	not scorable	1	0.7687935
1078	Bn-A01-p2053320	A-genome specific (napus-rapa)	0.9736842	0.4493733	not scorable	not scorable	1	0.7832971
1079	Bn-A01-p20564016	A-genome specific (napus-rapa)	0.9832536	0.4856167	not scorable	not scorable	0.9917355	0.8145845
1080	Bn-A01-p2065122	all not scorable	not scorable	not scorable	not scorable	not scorable	not scorable	not scorable
1081	Bn-A01-p206610	A-genome specific (napus-rapa)	0.9784689	0.7638199	not scorable	not scorable	1	0.735123
1082	Bn-A01-p2067006	A-genome specific (napus-rapa)	0.9976076	0.252827	not scorable	not scorable	1	0.7983102
1083	Bn-A01-p20679722	A-genome specific (napus-rapa)	0.9688995	0.2180236	not scorable	not scorable	0.9917355	0.7754571
1084	Bn-A01-p2069218	A-genome specific (napus-rapa)	0.9928229	0.786928	not scorable	not scorable	0.9834711	0.792945
1085	Bn-A01-p20692707	A-genome specific (napus-rapa)	0.8181818	0.6316313	not scorable	not scorable	1	0.7487122
1086	Bn-A01-p2069488	A-genome specific (napus-rapa)	0.9784689	0.784027	not scorable	not scorable	0.9917355	0.7749951

1087	Bn-A01-p20696971	A-genome specific (rapa only)	not scorable	not scorable	not scorable	not scorable	1	0.7800605
1088	Bn-A01-p207098	A-genome specific (napus-rapa)	0.9952153	0.656113	not scorable	not scorable	1	0.6902008
1089	Bn-A01-p207363	A-genome specific (napus-rapa)	0.9808612	0.7446876	not scorable	not scorable	0.9917355	0.888725
1090	Bn-A01-p20745330	A-genome specific (napus-rapa)	0.9808612	0.7583753	not scorable	not scorable	1	0.8036532
1091	Bn-A01-p20745820	A-genome specific (napus-rapa)	0.9641148	0.5903901	not scorable	not scorable	1	0.7613015
1092	Bn-A01-p20753932	A-genome specific (napus-rapa)	0.9808612	0.7379682	not scorable	not scorable	0.9669421	0.7893093
1093	Bn-A01-p20761933	A-genome specific (napus-rapa)	0.9736842	0.7071389	not scorable	not scorable	1	0.7244587
1094	Bn-A01-p20782963	A-genome specific (napus-rapa)	1	0.6287634	not scorable	not scorable	1	0.610312
1095	Bn-A01-p20783867	A-genome specific (rapa only)	not scorable	not scorable	not scorable	not scorable	0.9917355	0.4578595
1096	Bn-A01-p20784377	A-genome specific (rapa only)	not scorable	not scorable	not scorable	not scorable	1	0.7687175
1097	Bn-A01-p20789828	A-genome specific (napus-rapa)	0.9760765	0.6742715	not scorable	not scorable	0.9834711	0.7307041
1098	Bn-A01-p20805506	A-genome specific (napus-rapa)	0.9880383	0.773923	not scorable	not scorable	0.9917355	0.7421681
1099	Bn-A01-p20805613	A-genome specific (napus-rapa)	0.9760765	0.818741	not scorable	not scorable	1	0.6807423
1100	Bn-A01-p20814699	A-genome specific (napus-rapa)	0.9856459	0.7737158	not scorable	not scorable	1	0.714651
1101	Bn-A01-p20836664	A-genome specific (napus-rapa)	0.9832536	0.7845371	not scorable	not scorable	0.9917355	0.7840344
1102	Bn-A01-p20839901	A-genome specific (napus-rapa)	0.9928229	0.5653014	not scorable	not scorable	0.9917355	0.8402824
1103	Bn-A01-p20843887	A-genome specific (rapa only)	not scorable	not scorable	not scorable	not scorable	0.9586777	0.7429884
1104	Bn-A01-p20866022	A-genome specific (napus-rapa)	0.9856459	0.738369	not scorable	not scorable	0.9917355	0.7217659
1105	Bn-A01-p20866518	A-genome specific (napus-rapa)	0.9688995	0.7866235	not scorable	not scorable	0.9752066	0.7656797
1106	Bn-A01-p20873189	A-genome specific (rapa only)	not scorable	not scorable	not scorable	not scorable	0.9917355	0.7168004
1107	Bn-A01-p20873657	A-genome specific (napus-rapa)	1	0.3756197	not scorable	not scorable	0.9834711	0.7238929
1108	Bn-A01-p20879747	A-genome specific (rapa only)	not scorable	not scorable	not scorable	not scorable	0.9586777	0.7172893
1109	Bn-A01-p20890858	A-genome specific (napus-rapa)	0.9760765	0.6005332	not scorable	not scorable	0.9917355	0.5555217
1110	Bn-A01-p20892080	A-genome specific (napus-rapa)	0.9784689	0.6818768	not scorable	not scorable	0.9917355	0.7874163
1111	Bn-A01-p20892145	A-genome specific (napus-rapa)	0.9784689	0.7363312	not scorable	not scorable	0.9917355	0.7930954
1112	Bn-A01-p20899472	A-genome specific (napus-rapa)	0.9976076	0.5551814	not scorable	not scorable	1	0.7886671
1113	Bn-A01-p20909649	genome unspecific (all in)	0.9688995	0.3138359	1	0.7674054	1	0.8932936
1114	Bn-A01-p20909816	napus specific	0.9880383	0.2087191	not scorable	not scorable	not scorable	not scorable
1115	Bn-A01-p2091322	A-genome specific (napus-rapa)	0.9736842	0.7806415	not scorable	not scorable	0.9752066	0.8202122
1116	Bn-A01-p20924050	A-genome specific (napus-rapa)	0.9976076	0.4660888	not scorable	not scorable	0.9917355	0.7327726
1117	Bn-A01-p20933189	A-genome specific (napus-rapa)	0.9665072	0.7662805	not scorable	not scorable	0.6115702	0.778066
1118	Bn-A01-p2094793	A-genome specific (napus-rapa)	0.9712918	0.6656118	not scorable	not scorable	0.9669421	0.7207119
1119	Bn-A01-p20987149	A-genome specific (napus-rapa)	0.9976076	0.8346832	not scorable	not scorable	0.9917355	0.9000877

1120	Bn-A01-p20988977	A-genome specific (napus-rapa)	0.9928229	0.7715532	not scorable	not scorable	0.9669421	0.7195324
1121	Bn-A01-p20990218	A-genome specific (napus-rapa)	0.9712918	0.7728657	not scorable	not scorable	0.9834711	0.7967883
1122	Bn-A01-p2100996	A-genome specific (napus-rapa)	0.9712918	0.2475047	not scorable	not scorable	1	0.6512433
1123	Bn-A01-p21018746	A-genome specific (napus-rapa)	0.9736842	0.4308953	not scorable	not scorable	0.6942149	0.6457663
1124	Bn-A01-p21035670	A-genome specific (napus-rapa)	0.9832536	0.3988458	not scorable	not scorable	1	0.727766
1125	Bn-A01-p21038677	A-genome specific (napus-rapa)	0.9928229	0.5019218	not scorable	not scorable	1	0.7634613
1126	Bn-A01-p21075183	A-genome specific (napus-rapa)	0.6770335	0.7769992	not scorable	not scorable	0.6859504	0.709143
1127	Bn-A01-p21079129	A-genome specific (napus-rapa)	0.9880383	0.7778019	not scorable	not scorable	1	0.7988187
1128	Bn-A01-p21079348	A-genome specific (napus-rapa)	0.9617225	0.7981407	not scorable	not scorable	0.9917355	0.8157659
1129	Bn-A01-p21079774	A-genome specific (napus-rapa)	0.9856459	0.8170367	not scorable	not scorable	1	0.8276477
1130	Bn-A01-p21079909	A-genome specific (napus-rapa)	0.9784689	0.7816327	not scorable	not scorable	0.9917355	0.6867492
1131	Bn-A01-p21103014	A-genome specific (napus-rapa)	0.9641148	0.8139529	not scorable	not scorable	0.9752066	0.7972863
1132	Bn-A01-p21106413	A-genome specific (napus-rapa)	0.9521531	0.7441249	not scorable	not scorable	0.9917355	0.7250969
1133	Bn-A01-p21106687	A-genome specific (napus-rapa)	0.9760765	0.7919081	not scorable	not scorable	1	0.777253
1134	Bn-A01-p21107496	A-genome specific (napus-rapa)	0.9904306	0.7069155	not scorable	not scorable	1	0.7578943
1135	Bn-A01-p21108496	A-genome specific (napus-rapa)	0.9736842	0.6998052	not scorable	not scorable	1	0.7184181
1136	Bn-A01-p2111978	A-genome specific (napus-rapa)	0.9904306	0.8039233	not scorable	not scorable	0.9917355	0.6942841
1137	Bn-A01-p21135145	A-genome specific (napus-rapa)	0.9784689	0.7556403	not scorable	not scorable	1	0.7789075
1138	Bn-A01-p21136052	A-genome specific (napus-rapa)	0.9784689	0.7514102	not scorable	not scorable	0.9834711	0.6944429
1139	Bn-A01-p21141514	A-genome specific (napus-rapa)	0.9736842	0.7587781	not scorable	not scorable	0.9669421	0.7307457
1140	Bn-A01-p21145008	A-genome specific (napus-rapa)	0.9712918	0.7802681	not scorable	not scorable	1	0.8227912
1141	Bn-A01-p2114552	A-genome specific (napus-rapa)	0.9712918	0.69961	not scorable	not scorable	0.9752066	0.5983737
1142	Bn-A01-p2114803	A-genome specific (napus-rapa)	0.9784689	0.4147435	not scorable	not scorable	1	0.7219532
1143	Bn-A01-p2121224	A-genome specific (napus-rapa)	0.7057416	0.7774605	not scorable	not scorable	1	0.7649631
1144	Bn-A01-p21219526	A-genome specific (napus-rapa)	0.9401914	0.5173224	not scorable	not scorable	0.9752066	0.7057587
1145	Bn-A01-p21224647	A-genome specific (napus-rapa)	0.9617225	0.2291792	not scorable	not scorable	0.9917355	0.7580727
1146	Bn-A01-p21236322	A-genome specific (napus-rapa)	0.9736842	0.7753983	not scorable	not scorable	1	0.7901254
1147	Bn-A01-p21244027	A-genome specific (napus-rapa)	0.9688995	0.6903384	not scorable	not scorable	1	0.8680103
1148	Bn-A01-p21277841	genome unspecific (all in)	0.9473684	0.2243028	1	0.3785537	0.9834711	0.8054857
1149	Bn-A01-p21309566	A-genome specific (napus-rapa)	0.9832536	0.5416542	not scorable	not scorable	0.9834711	0.7373434
1150	Bn-A01-p21329900	A-genome specific (napus-rapa)	0.9832536	0.4604614	not scorable	not scorable	0.9917355	0.6681912
1151	Bn-A01-p21393260	A-genome specific (napus-rapa)	0.9928229	0.7849559	not scorable	not scorable	0.9669421	0.8439177
1152	Bn-A01-p21394483	A-genome specific (napus-rapa)	0.9808612	0.7956669	not scorable	not scorable	0.6694215	0.91594

1153	Bn-A01-p21430708	A-genome specific (napus-rapa)	0.9904306	0.4779043	not scorable	not scorable	0.9834711	0.6926004
1154	Bn-A01-p21444041	A-genome specific (napus-rapa)	0.9043062	0.8112793	not scorable	not scorable	0.9586777	0.7127911
1155	Bn-A01-p21473161	A-genome specific (napus-rapa)	0.9904306	0.4372155	not scorable	not scorable	1	0.6990392
1156	Bn-A01-p21479969	A-genome specific (napus-rapa)	0.9880383	0.7194398	not scorable	not scorable	1	0.8513337
1157	Bn-A01-p2148059	A-genome specific (napus-rapa)	0.9760765	0.2688045	not scorable	not scorable	0.9917355	0.7874246
1158	Bn-A01-p21484088	A-genome specific (napus-rapa)	0.9880383	0.7775188	not scorable	not scorable	1	0.7515343
1159	Bn-A01-p21484875	A-genome specific (napus-rapa)	0.9784689	0.8191733	not scorable	not scorable	1	0.8132238
1160	Bn-A01-p21499792	A-genome specific (napus-rapa)	0.9832536	0.7800277	not scorable	not scorable	1	0.8112231
1161	Bn-A01-p21500509	A-genome specific (napus-rapa)	0.9880383	0.7448571	not scorable	not scorable	1	0.7811202
1162	Bn-A01-p21500631	A-genome specific (napus-rapa)	0.9928229	0.7876289	not scorable	not scorable	1	0.8260823
1163	Bn-A01-p21512266	A-genome specific (napus-rapa)	0.9832536	0.7842101	not scorable	not scorable	0.9917355	0.8314618
1164	Bn-A01-p21512514	A-genome specific (napus-rapa)	0.9904306	0.7911364	not scorable	not scorable	1	0.8747709
1165	Bn-A01-p21512663	A-genome specific (napus-rapa)	0.9904306	0.7924657	not scorable	not scorable	0.9917355	0.7810264
1166	Bn-A01-p21513096	A-genome specific (napus-rapa)	0.9856459	0.7510304	not scorable	not scorable	1	0.7772545
1167	Bn-A01-p21517353	A-genome specific (napus-rapa)	0.9832536	0.7208664	not scorable	not scorable	0.9834711	0.7463361
1168	Bn-A01-p21519780	A-genome specific (napus-rapa)	0.9928229	0.4656622	not scorable	not scorable	0.9917355	0.7230756
1169	Bn-A01-p21524150	A-genome specific (napus-rapa)	0.9832536	0.7480121	not scorable	not scorable	0.9834711	0.7236288
1170	Bn-A01-p21527187	napus specific	0.9712918	0.7841974	not scorable	not scorable	not scorable	not scorable
1171	Bn-A01-p21532512	A-genome specific (napus-rapa)	0.9808612	0.8031058	not scorable	not scorable	0.9917355	0.7970411
1172	Bn-A01-p21556957	A-genome specific (napus-rapa)	0.9832536	0.7433251	not scorable	not scorable	1	0.8188446
1173	Bn-A01-p2158511	A-genome specific (napus-rapa)	0.9952153	0.4783701	not scorable	not scorable	0.9917355	0.6653698
1174	Bn-A01-p21605865	A-genome specific (napus-rapa)	0.9856459	0.7676363	not scorable	not scorable	0.9834711	0.7487302
1175	Bn-A01-p21633202	A-genome specific (napus-rapa)	0.9832536	0.2129229	not scorable	not scorable	0.7272727	0.726383
1176	Bn-A01-p21634024	A-genome specific (rapa only)	not scorable	not scorable	not scorable	not scorable	0.9256198	0.7160041
1177	Bn-A01-p21634235	A-genome specific (napus-rapa)	0.708134	0.6840537	not scorable	not scorable	0.9586777	0.7728974
1178	Bn-A01-p21634265	A-genome specific (napus-rapa)	0.6961722	0.7550347	not scorable	not scorable	0.8099173	0.8751275
1179	Bn-A01-p21634646	A-genome specific (napus-rapa)	0.9880383	0.5856106	not scorable	not scorable	0.9586777	0.8531295
1180	Bn-A01-p21635000	A-genome specific (napus-rapa)	0.6937799	0.6917459	not scorable	not scorable	0.9256198	0.7972612
1181	Bn-A01-p21635137	A-genome specific (napus-rapa)	0.6937799	0.7673885	not scorable	not scorable	0.7024794	0.8670852
1182	Bn-A01-p21635270	A-genome specific (napus-rapa)	0.7105263	0.7512083	not scorable	not scorable	0.6859504	0.8061119
1183	Bn-A01-p21636048	A-genome specific (napus-rapa)	0.6985646	0.7828644	not scorable	not scorable	0.8429752	0.8760504
1184	Bn-A01-p21638516	A-genome specific (napus-rapa)	0.6937799	0.7597344	not scorable	not scorable	0.6446281	0.7163467
1185	Bn-A01-p21643354	A-genome specific (napus-rapa)	0.9880383	0.7520018	not scorable	not scorable	1	0.8116826

1186	Bn-A01-p21644756	A-genome specific (napus-rapa)	0.9904306	0.7442039	not scorable	not scorable	0.9834711	0.7101955
1187	Bn-A01-p21651835	A-genome specific (napus-rapa)	0.9736842	0.4099847	not scorable	not scorable	1	0.8072308
1188	Bn-A01-p21663900	A-genome specific (napus-rapa)	0.9712918	0.3955137	not scorable	not scorable	1	0.8140981
1189	Bn-A01-p21708353	A-genome specific (napus-rapa)	0.9976076	0.2944632	not scorable	not scorable	0.9504132	0.6546811
1190	Bn-A01-p21709916	A-genome specific (napus-rapa)	0.9904306	0.7557947	not scorable	not scorable	0.9752066	0.7352013
1191	Bn-A01-p2171217	A-genome specific (napus-rapa)	0.9473684	0.7994196	not scorable	not scorable	0.9669421	0.8333422
1192	Bn-A01-p21715162	A-genome specific (napus-rapa)	0.9760765	0.797234	not scorable	not scorable	0.9834711	0.7415209
1193	Bn-A01-p21724511	A-genome specific (napus-rapa)	0.9976076	0.2613971	not scorable	not scorable	1	0.6889215
1194	Bn-A01-p21758046	A-genome specific (napus-rapa)	0.9808612	0.464204	not scorable	not scorable	0.9917355	0.7292978
1195	Bn-A01-p21776016	A-genome specific (napus-rapa)	0.9928229	0.6982969	not scorable	not scorable	0.9917355	0.6681895
1196	Bn-A01-p21776083	A-genome specific (napus-rapa)	0.9928229	0.729663	not scorable	not scorable	1	0.7921427
1197	Bn-A01-p21776155	A-genome specific (napus-rapa)	0.9688995	0.7190909	not scorable	not scorable	1	0.6886137
1198	Bn-A01-p21778610	A-genome specific (rapa only)	not scorable	not scorable	not scorable	not scorable	1	0.5856268
1199	Bn-A01-p2177864	A-genome specific (napus-rapa)	1	0.5031275	not scorable	not scorable	0.9917355	0.830166
1200	Bn-A01-p21791128	A-genome specific (napus-rapa)	0.9712918	0.7671278	not scorable	not scorable	0.9834711	0.8319019
1201	Bn-A01-p21797129	A-genome specific (napus-rapa)	0.9976076	0.7701522	not scorable	not scorable	1	0.8672051
1202	Bn-A01-p21812098	A-genome specific (rapa only)	not scorable	not scorable	not scorable	not scorable	0.9752066	0.7991732
1203	Bn-A01-p21846342	A-genome specific (napus-rapa)	0.9928229	0.2670345	not scorable	not scorable	0.9917355	0.7077151
1204	Bn-A01-p21855770	A-genome specific (napus-rapa)	0.9904306	0.807543	not scorable	not scorable	1	0.7378951
1205	Bn-A01-p21856899	A-genome specific (napus-rapa)	0.9976076	0.3288653	not scorable	not scorable	1	0.6415594
1206	Bn-A01-p21860162	A-genome specific (napus-rapa)	0.9593301	0.8079533	not scorable	not scorable	0.9834711	0.663301
1207	Bn-A01-p21869384	A-genome specific (napus-rapa)	0.9760765	0.4366409	not scorable	not scorable	1	0.8095888
1208	Bn-A01-p2187099	A-genome specific (napus-rapa)	0.9880383	0.8440625	not scorable	not scorable	0.9917355	0.8004754
1209	Bn-A01-p2188753	A-genome specific (napus-rapa)	0.9976076	0.5340092	not scorable	not scorable	0.9917355	0.7912415
1210	Bn-A01-p21902788	A-genome specific (napus-rapa)	1	0.5133105	not scorable	not scorable	1	0.8045593
1211	Bn-A01-p21923141	A-genome specific (napus-rapa)	0.9952153	0.2502425	not scorable	not scorable	0.9834711	0.7663959
1212	Bn-A01-p21926431	A-genome specific (napus-rapa)	0.9928229	0.7073368	not scorable	not scorable	0.9834711	0.6939745
1213	Bn-A01-p21928497	A-genome specific (napus-rapa)	0.9928229	0.8351394	not scorable	not scorable	0.9834711	0.7767712
1214	Bn-A01-p21928531	A-genome specific (napus-rapa)	0.9880383	0.7624127	not scorable	not scorable	0.9834711	0.7694218
1215	Bn-A01-p21928927	A-genome specific (napus-rapa)	0.9880383	0.6347137	not scorable	not scorable	0.9834711	0.7023777
1216	Bn-A01-p21930611	A-genome specific (napus-rapa)	0.9832536	0.7745256	not scorable	not scorable	0.9917355	0.7190839
1217	Bn-A01-p21930738	A-genome specific (napus-rapa)	0.9832536	0.8021903	not scorable	not scorable	0.9752066	0.7594448
1218	Bn-A01-p21930931	A-genome specific (napus-rapa)	0.9880383	0.7272469	not scorable	not scorable	0.9752066	0.7414829

1219	Bn-A01-p21931228	A-genome specific (napus-rapa)	0.9808612	0.8153423	not scorable	not scorable	1	0.7706913
1220	Bn-A01-p21931498	A-genome specific (napus-rapa)	0.9856459	0.7856403	not scorable	not scorable	1	0.7447198
1221	Bn-A01-p21931622	A-genome specific (napus-rapa)	0.9856459	0.7411048	not scorable	not scorable	0.9669421	0.7485299
1222	Bn-A01-p21931853	A-genome specific (napus-rapa)	0.9784689	0.7496225	not scorable	not scorable	0.9834711	0.787676
1223	Bn-A01-p21937985	A-genome specific (napus-rapa)	0.9784689	0.7510415	not scorable	not scorable	0.9917355	0.7683975
1224	Bn-A01-p21938751	A-genome specific (napus-rapa)	0.9928229	0.492867	not scorable	not scorable	0.9752066	0.751124
1225	Bn-A01-p21960901	A-genome specific (napus-rapa)	0.9832536	0.7993464	not scorable	not scorable	0.9669421	0.8676426
1226	Bn-A01-p21969405	A-genome specific (napus-rapa)	0.9832536	0.7057003	not scorable	not scorable	1	0.6501922
1227	Bn-A01-p2197707	A-genome specific (napus-rapa)	0.9976076	0.7362452	not scorable	not scorable	0.9834711	0.6864372
1228	Bn-A01-p2197976	A-genome specific (napus-rapa)	0.9832536	0.767311	not scorable	not scorable	0.9834711	0.7320065
1229	Bn-A01-p21980567	A-genome specific (napus-rapa)	0.9760765	0.413111	not scorable	not scorable	0.9834711	0.7200872
1230	Bn-A01-p21997686	A-genome specific (napus-rapa)	0.9712918	0.7510949	not scorable	not scorable	0.9504132	0.6874543
1231	Bn-A01-p21998517	all not scorable	not scorable	not scorable	not scorable	not scorable	not scorable	not scorable
1232	Bn-A01-p22014179	A-genome specific (napus-rapa)	0.9856459	0.3477323	not scorable	not scorable	0.9917355	0.5846771
1233	Bn-A01-p22014707	napus specific	0.9425837	0.2472964	not scorable	not scorable	not scorable	not scorable
1234	Bn-A01-p22016353	A-genome specific (napus-rapa)	0.9760765	0.3455385	not scorable	not scorable	0.9834711	0.6872441
1235	Bn-A01-p22018616	A-genome specific (napus-rapa)	0.9904306	0.290755	not scorable	not scorable	0.9917355	0.6475991
1236	Bn-A01-p22022023	A-genome specific (napus-rapa)	0.9904306	0.4735844	not scorable	not scorable	1	0.6638831
1237	Bn-A01-p2202853	A-genome specific (rapa only)	not scorable	not scorable	not scorable	not scorable	0.9669421	0.8262289
1238	Bn-A01-p22038749	A-genome specific (napus-rapa)	0.9880383	0.7787855	not scorable	not scorable	1	0.725246
1239	Bn-A01-p22038776	A-genome specific (napus-rapa)	0.9856459	0.7363254	not scorable	not scorable	0.9834711	0.6787181
1240	Bn-A01-p22051021	A-genome specific (napus-rapa)	1	0.7891633	not scorable	not scorable	1	0.840076
1241	Bn-A01-p22051502	A-genome specific (napus-rapa)	0.9928229	0.7686757	not scorable	not scorable	1	0.7942631
1242	Bn-A01-p22051510	A-genome specific (napus-rapa)	0.9928229	0.7875522	not scorable	not scorable	1	0.7692299
1243	Bn-A01-p220693	A-genome specific (napus-rapa)	0.9952153	0.2658252	not scorable	not scorable	0.9752066	0.5328778
1244	Bn-A01-p2207452	A-genome specific (napus-rapa)	0.9665072	0.7822148	not scorable	not scorable	0.9834711	0.823975
1245	Bn-A01-p22085117	A-genome specific (napus-rapa)	1	0.315121	not scorable	not scorable	1	0.7842602
1246	Bn-A01-p22086244	A-genome specific (napus-rapa)	0.9928229	0.8241183	not scorable	not scorable	1	0.7860596
1247	Bn-A01-p22087364	A-genome specific (napus-rapa)	0.9928229	0.7795103	not scorable	not scorable	1	0.8302346
1248	Bn-A01-p22087629	A-genome specific (napus-rapa)	0.9928229	0.7083049	not scorable	not scorable	0.9917355	0.7525083
1249	Bn-A01-p22156169	A-genome specific (rapa only)	not scorable	not scorable	not scorable	not scorable	0.9917355	0.5527267
1250	Bn-A01-p22182442	A-genome specific (napus-rapa)	0.9976076	0.4028099	not scorable	not scorable	1	0.6480865
1251	Bn-A01-p2221110	A-genome specific (rapa only)	not scorable	not scorable	not scorable	not scorable	0.9173554	0.8338553

1252	Bn-A01-p22257309	A-genome specific (napus-rapa)	0.9928229	0.819879	not scorable	not scorable	1	0.5891774
1253	Bn-A01-p22265175	A-genome specific (napus-rapa)	0.9976076	0.7633851	not scorable	not scorable	1	0.834482
1254	Bn-A01-p2230371	A-genome specific (napus-rapa)	1	0.5383748	not scorable	not scorable	1	0.844363
1255	Bn-A01-p22363809	A-genome specific (napus-rapa)	0.9904306	0.4252563	not scorable	not scorable	1	0.7043824
1256	Bn-A01-p2242890	A-genome specific (napus-rapa)	0.9688995	0.7682471	not scorable	not scorable	0.9917355	0.8421025
1257	Bn-A01-p2244327	A-genome specific (napus-rapa)	0.9808612	0.7545872	not scorable	not scorable	1	0.723166
1258	Bn-A01-p224469	A-genome specific (rapa only)	not scorable	not scorable	not scorable	not scorable	0.9669421	0.7711245
1259	Bn-A01-p2245780	A-genome specific (napus-rapa)	0.9880383	0.3480325	not scorable	not scorable	0.9917355	0.5946738
1260	Bn-A01-p2246317	A-genome specific (napus-rapa)	0.9808612	0.8361022	not scorable	not scorable	1	0.7960499
1261	Bn-A01-p2246373	A-genome specific (napus-rapa)	0.9856459	0.4741815	not scorable	not scorable	0.9917355	0.5605972
1262	Bn-A01-p2251106	A-genome specific (napus-rapa)	0.9928229	0.4362786	not scorable	not scorable	1	0.7343857
1263	Bn-A01-p2254004	A-genome specific (napus-rapa)	0.4473684	0.8042827	not scorable	not scorable	0.9752066	0.8380253
1264	Bn-A01-p2254072	A-genome specific (napus-rapa)	0.9736842	0.8085449	not scorable	not scorable	0.9917355	0.7631917
1265	Bn-A01-p2254235	A-genome specific (napus-rapa)	0.9688995	0.777378	not scorable	not scorable	0.9834711	0.7544801
1266	Bn-A01-p22602330	napus specific	0.9952153	0.8202555	not scorable	not scorable	not scorable	not scorable
1267	Bn-A01-p2260859	A-genome specific (napus-rapa)	0.9928229	0.3341701	not scorable	not scorable	0.9917355	0.8187154
1268	Bn-A01-p2261903	A-genome specific (napus-rapa)	0.9784689	0.7690903	not scorable	not scorable	0.9917355	0.7629737
1269	Bn-A01-p22681496	A-genome specific (napus-rapa)	0.9808612	0.7929315	not scorable	not scorable	0.9752066	0.8622975
1270	Bn-A01-p22684301	A-genome specific (napus-rapa)	0.9952153	0.4197113	not scorable	not scorable	1	0.7571332
1271	Bn-A01-p22685756	A-genome specific (napus-rapa)	0.9569378	0.8135445	not scorable	not scorable	1	0.811961
1272	Bn-A01-p22685858	A-genome specific (napus-rapa)	0.9641148	0.4163218	not scorable	not scorable	1	0.7369736
1273	Bn-A01-p22739887	A-genome specific (napus-rapa)	0.9665072	0.7374793	not scorable	not scorable	0.9504132	0.7456605
1274	Bn-A01-p22741943	A-genome specific (napus-rapa)	0.9497608	0.7704418	not scorable	not scorable	0.6942149	0.6868446
1275	Bn-A01-p22743403	A-genome specific (napus-rapa)	0.9760765	0.2887884	not scorable	not scorable	0.9669421	0.6822459
1276	Bn-A01-p22768523	A-genome specific (napus-rapa)	0.9976076	0.3621256	not scorable	not scorable	1	0.8224539
1277	Bn-A01-p2277290	A-genome specific (napus-rapa)	0.9976076	0.4743615	not scorable	not scorable	0.9917355	0.7758656
1278	Bn-A01-p22777288	genome unspecific (oleracea-rapa)	not scorable	not scorable	1	0.7666422	0.6446281	0.7944868
1279	Bn-A01-p22841111	A-genome specific (napus-rapa)	0.9856459	0.7810175	not scorable	not scorable	1	0.7337765
1280	Bn-A01-p22841314	A-genome specific (napus-rapa)	0.9712918	0.2228272	not scorable	not scorable	0.9752066	0.7119761
1281	Bn-A01-p2288902	A-genome specific (napus-rapa)	0.9545454	0.4458767	not scorable	not scorable	0.9834711	0.7043029
1282	Bn-A01-p2291940	A-genome specific (napus-rapa)	0.9832536	0.7156431	not scorable	not scorable	1	0.7420965
1283	Bn-A01-p2292174	A-genome specific (napus-rapa)	0.9449761	0.280975	not scorable	not scorable	1	0.717533
1284	Bn-A01-p22930245	A-genome specific (napus-rapa)	1	0.7612559	not scorable	not scorable	1	0.9205657

1285	Bn-A01-p22937843	A-genome specific (napus-rapa)	1	0.3052333	not scorable	not scorable	1	0.7650189
1286	Bn-A01-p22948925	genome unspecific (all in)	0.9712918	0.5550418	0.9844961	0.8127997	1	0.7510429
1287	Bn-A01-p22949106	A-genome specific (napus-rapa)	0.9784689	0.805405	not scorable	not scorable	0.9917355	0.7019351
1288	Bn-A01-p22982065	A-genome specific (rapa only)	not scorable	not scorable	not scorable	not scorable	0.9752066	0.5944711
1289	Bn-A01-p22983721	A-genome specific (napus-rapa)	0.9832536	0.4943672	not scorable	not scorable	1	0.8190554
1290	Bn-A01-p22999151	A-genome specific (napus-rapa)	0.9760765	0.7114803	not scorable	not scorable	1	0.8264934
1291	Bn-A01-p23020726	A-genome specific (napus-rapa)	0.9856459	0.7519872	not scorable	not scorable	1	0.8056981
1292	Bn-A01-p2303308	A-genome specific (napus-rapa)	0.9665072	0.7683836	not scorable	not scorable	1	0.8494412
1293	Bn-A01-p2306208	A-genome specific (napus-rapa)	0.8516746	0.4765505	not scorable	not scorable	1	0.7074097
1294	Bn-A01-p2306938	A-genome specific (napus-rapa)	0.9736842	0.7333896	not scorable	not scorable	0.9917355	0.6452415
1295	Bn-A01-p2307144	A-genome specific (napus-rapa)	0.9856459	0.4980933	not scorable	not scorable	1	0.6990011
1296	Bn-A01-p23078554	A-genome specific (napus-rapa)	0.9952153	0.4007292	not scorable	not scorable	0.9917355	0.7009389
1297	Bn-A01-p23080525	A-genome specific (napus-rapa)	0.9976076	0.4736131	not scorable	not scorable	1	0.7248024
1298	Bn-A01-p23085796	A-genome specific (napus-rapa)	0.9880383	0.802633	not scorable	not scorable	1	0.8084953
1299	Bn-A01-p23086253	A-genome specific (napus-rapa)	0.9688995	0.7590642	not scorable	not scorable	0.5454546	0.8287867
1300	Bn-A01-p23087044	A-genome specific (napus-rapa)	0.9617225	0.8082688	not scorable	not scorable	1	0.7904124
1301	Bn-A01-p23087707	A-genome specific (napus-rapa)	0.9688995	0.7960865	not scorable	not scorable	0.5041322	0.8489662
1302	Bn-A01-p2309179	A-genome specific (napus-rapa)	0.9904306	0.5221317	not scorable	not scorable	1	0.7740356
1303	Bn-A01-p23095567	A-genome specific (napus-rapa)	0.9880383	0.2759733	not scorable	not scorable	0.9917355	0.7131069
1304	Bn-A01-p23096147	A-genome specific (napus-rapa)	0.9904306	0.6500086	not scorable	not scorable	1	0.745021
1305	Bn-A01-p23097693	A-genome specific (napus-rapa)	0.9928229	0.7831156	not scorable	not scorable	0.9917355	0.7571821
1306	Bn-A01-p23103120	A-genome specific (napus-rapa)	0.9784689	0.8010248	not scorable	not scorable	0.9834711	0.7368048
1307	Bn-A01-p2311015	A-genome specific (napus-rapa)	0.9665072	0.7765666	not scorable	not scorable	0.9834711	0.7445812
1308	Bn-A01-p23111844	A-genome specific (napus-rapa)	0.9904306	0.4457023	not scorable	not scorable	1	0.7477413
1309	Bn-A01-p23120368	A-genome specific (napus-rapa)	0.9808612	0.5690736	not scorable	not scorable	0.7107438	0.8447563
1310	Bn-A01-p23122066	A-genome specific (napus-rapa)	1	0.6891087	not scorable	not scorable	1	0.731029
1311	Bn-A01-p23169607	A-genome specific (rapa only)	not scorable	not scorable	not scorable	not scorable	0.9917355	0.8546581
1312	Bn-A01-p23175111	A-genome specific (napus-rapa)	0.9952153	0.5584118	not scorable	not scorable	0.9917355	0.8065881
1313	Bn-A01-p23181293	A-genome specific (napus-rapa)	0.9784689	0.7416286	not scorable	not scorable	0.5041322	0.6868434
1314	Bn-A01-p2318972	A-genome specific (napus-rapa)	0.9688995	0.76538	not scorable	not scorable	0.9669421	0.7855306
1315	Bn-A01-p23191627	A-genome specific (napus-rapa)	1	0.7749949	not scorable	not scorable	0.9917355	0.8254227
1316	Bn-A01-p23195492	A-genome specific (napus-rapa)	0.9928229	0.7544676	not scorable	not scorable	1	0.7185012
1317	Bn-A01-p23196022	A-genome specific (napus-rapa)	0.9928229	0.5930946	not scorable	not scorable	0.9917355	0.7436028

1318	Bn-A01-p2323744	A-genome specific (napus-rapa)	0.9832536	0.3705159	not scorable	not scorable	0.9917355	0.7756329
1319	Bn-A01-p23253678	A-genome specific (napus-rapa)	0.9928229	0.7209864	not scorable	not scorable	0.9834711	0.7176437
1320	Bn-A01-p23259034	A-genome specific (napus-rapa)	0.9976076	0.4824677	not scorable	not scorable	1	0.6487527
1321	Bn-A01-p23259741	A-genome specific (napus-rapa)	0.9928229	0.5622385	not scorable	not scorable	0.9834711	0.5775352
1322	Bn-A01-p23279247	A-genome specific (napus-rapa)	0.9976076	0.6421199	not scorable	not scorable	0.9834711	0.653347
1323	Bn-A01-p23280094	A-genome specific (rapa only)	not scorable	not scorable	not scorable	not scorable	0.9834711	0.800547
1324	Bn-A01-p23281709	genome unspecific (oleracea-rapa)	not scorable	not scorable	0.8914729	0.571296	1	0.7627373
1325	Bn-A01-p23281878	A-genome specific (napus-rapa)	0.9976076	0.8442404	not scorable	not scorable	1	0.7397453
1326	Bn-A01-p23282001	A-genome specific (napus-rapa)	0.9976076	0.8044995	not scorable	not scorable	1	0.7788512
1327	Bn-A01-p23288238	A-genome specific (rapa only)	not scorable	not scorable	not scorable	not scorable	0.9834711	0.7931979
1328	Bn-A01-p2329932	A-genome specific (napus-rapa)	0.9712918	0.7792413	not scorable	not scorable	0.9586777	0.3619901
1329	Bn-A01-p23303284	A-genome specific (rapa only)	not scorable	not scorable	not scorable	not scorable	0.9917355	0.7199739
1330	Bn-A01-p23321507	A-genome specific (napus-rapa)	0.9928229	0.4771986	not scorable	not scorable	1	0.8599893
1331	Bn-A01-p23321771	A-genome specific (napus-rapa)	0.9832536	0.8004524	not scorable	not scorable	0.9917355	0.7372571
1332	Bn-A01-p23322377	A-genome specific (napus-rapa)	0.9856459	0.7945657	not scorable	not scorable	0.9669421	0.6650604
1333	Bn-A01-p23322438	A-genome specific (napus-rapa)	0.9928229	0.7724172	not scorable	not scorable	0.6859504	0.8044198
1334	Bn-A01-p23330944	napus specific	0.9641148	0.7854073	not scorable	not scorable	not scorable	not scorable
1335	Bn-A01-p23370802	A-genome specific (napus-rapa)	0.9090909	0.6774311	not scorable	not scorable	0.7851239	0.9175813
1336	Bn-A01-p2340048	A-genome specific (napus-rapa)	0.9736842	0.7568356	not scorable	not scorable	0.9917355	0.7776608
1337	Bn-A01-p2340359	A-genome specific (napus-rapa)	0.9688995	0.8000708	not scorable	not scorable	0.9917355	0.8062946
1338	Bn-A01-p2341408	A-genome specific (napus-rapa)	0.9688995	0.7963336	not scorable	not scorable	0.9917355	0.82846
1339	Bn-A01-p23448661	A-genome specific (napus-rapa)	1	0.8051615	not scorable	not scorable	1	0.7693356
1340	Bn-A01-p2345255	A-genome specific (napus-rapa)	0.9904306	0.3794476	not scorable	not scorable	1	0.7177778
1341	Bn-A01-p23472380	A-genome specific (napus-rapa)	0.9665072	0.7073492	not scorable	not scorable	0.7190083	0.8644511
1342	Bn-A01-p23472646	A-genome specific (napus-rapa)	0.9688995	0.780826	not scorable	not scorable	0.9752066	0.6808475
1343	Bn-A01-p23473514	A-genome specific (napus-rapa)	0.9856459	0.7871395	not scorable	not scorable	1	0.7663456
1344	Bn-A01-p23477194	A-genome specific (napus-rapa)	0.9784689	0.7894045	not scorable	not scorable	0.9917355	0.680047
1345	Bn-A01-p23478530	A-genome specific (napus-rapa)	0.9808612	0.7880544	not scorable	not scorable	0.5785124	0.8392923
1346	Bn-A01-p23479473	A-genome specific (napus-rapa)	0.9760765	0.4882902	not scorable	not scorable	1	0.9050438
1347	Bn-A01-p23486950	genome unspecific (oleracea-rapa)	not scorable	not scorable	0.379845	0.4147588	0.9586777	0.6875448
1348	Bn-A01-p234898	A-genome specific (napus-rapa)	0.9545454	0.5658053	not scorable	not scorable	1	0.7364199
1349	Bn-A01-p23493920	genome unspecific (all in)	0.9641148	0.354924	1	0.7483889	0.5867769	0.7977689
1350	Bn-A01-p235115	A-genome specific (napus-rapa)	0.9976076	0.3274618	not scorable	not scorable	0.9834711	0.8499259

1351	Bn-A01-p23536634	A-genome specific (napus-rapa)	0.7440192	0.9175813	not scorable	not scorable	1	0.8393767
1352	Bn-A01-p23538267	A-genome specific (napus-rapa)	0.9808612	0.3049752	not scorable	not scorable	0.9917355	0.719432
1353	Bn-A01-p23538856	A-genome specific (napus-rapa)	0.9832536	0.759492	not scorable	not scorable	0.9917355	0.8081059
1354	Bn-A01-p23539305	A-genome specific (napus-rapa)	0.9808612	0.7853466	not scorable	not scorable	0.9917355	0.7615933
1355	Bn-A01-p235484	A-genome specific (napus-rapa)	0.9808612	0.7788705	not scorable	not scorable	0.9504132	0.7387811
1356	Bn-A01-p23560747	A-genome specific (napus-rapa)	0.9880383	0.4264954	not scorable	not scorable	0.9917355	0.8612621
1357	Bn-A01-p23568963	A-genome specific (napus-rapa)	0.9569378	0.388804	not scorable	not scorable	1	0.7810875
1358	Bn-A01-p235781	A-genome specific (napus-rapa)	0.9617225	0.6839854	not scorable	not scorable	0.9752066	0.7545217
1359	Bn-A01-p23582571	A-genome specific (rapa only)	not scorable	not scorable	not scorable	not scorable	1	0.7810928
1360	Bn-A01-p23586266	A-genome specific (napus-rapa)	0.9880383	0.7936415	not scorable	not scorable	0.9917355	0.7195811
1361	Bn-A01-p23600621	A-genome specific (rapa only)	not scorable	not scorable	not scorable	not scorable	1	0.7362989
1362	Bn-A01-p23602361	A-genome specific (napus-rapa)	0.9258373	0.7903606	not scorable	not scorable	0.9917355	0.9209533
1363	Bn-A01-p2360909	A-genome specific (napus-rapa)	0.9282297	0.2840842	not scorable	not scorable	1	0.6060079
1364	Bn-A01-p23613283	A-genome specific (napus-rapa)	0.9641148	0.3217329	not scorable	not scorable	1	0.8210919
1365	Bn-A01-p23614494	A-genome specific (napus-rapa)	0.9784689	0.7881173	not scorable	not scorable	0.9917355	0.8503544
1366	Bn-A01-p23614887	A-genome specific (napus-rapa)	0.9856459	0.4750561	not scorable	not scorable	1	0.8401434
1367	Bn-A01-p23616839	A-genome specific (napus-rapa)	0.7200957	0.7503549	not scorable	not scorable	0.9834711	0.8459679
1368	Bn-A01-p23627526	A-genome specific (rapa only)	not scorable	not scorable	not scorable	not scorable	1	0.70992
1369	Bn-A01-p23634753	A-genome specific (napus-rapa)	0.3516746	0.6132114	not scorable	not scorable	0.9586777	0.677716
1370	Bn-A01-p23638070	A-genome specific (napus-rapa)	0.9736842	0.3647994	not scorable	not scorable	0.9917355	0.8165768
1371	Bn-A01-p23638107	A-genome specific (napus-rapa)	0.9832536	0.7521313	not scorable	not scorable	0.9834711	0.699226
1372	Bn-A01-p23640103	A-genome specific (napus-rapa)	0.9832536	0.3959498	not scorable	not scorable	0.9917355	0.75645
1373	Bn-A01-p23642494	genome unspecific (all in)	0.9952153	0.4736132	0.9922481	0.787617	1	0.8653798
1374	Bn-A01-p2365493	A-genome specific (napus-rapa)	0.9856459	0.7742472	not scorable	not scorable	0.9917355	0.8267537
1375	Bn-A01-p2365667	A-genome specific (napus-rapa)	0.9928229	0.8451989	not scorable	not scorable	0.9834711	0.7469729
1376	Bn-A01-p23658023	A-genome specific (napus-rapa)	0.9641148	0.4300889	not scorable	not scorable	1	0.7003947
1377	Bn-A01-p23672301	A-genome specific (napus-rapa)	0.9641148	0.3488769	not scorable	not scorable	1	0.5617584
1378	Bn-A01-p23685466	napus specific	0.9593301	0.6645694	not scorable	not scorable	not scorable	not scorable
1379	Bn-A01-p23685978	A-genome specific (napus-rapa)	1	0.5197503	not scorable	not scorable	0.9917355	0.6741084
1380	Bn-A01-p23694782	A-genome specific (napus-rapa)	0.9641148	0.7416436	not scorable	not scorable	0.5454546	0.7885255
1381	Bn-A01-p23718726	A-genome specific (napus-rapa)	0.9688995	0.5472277	not scorable	not scorable	0.5289256	0.7350129
1382	Bn-A01-p23719321	A-genome specific (napus-rapa)	0.9497608	0.6870394	not scorable	not scorable	0.9917355	0.6643477
1383	Bn-A01-p23728095	A-genome specific (napus-rapa)	0.9497608	0.3293611	not scorable	not scorable	0.6198347	0.6918504

1384	Bn-A01-p23730610	A-genome specific (napus-rapa)	0.9904306	0.4781381	not scorable	not scorable	0.9834711	0.7648607
1385	Bn-A01-p23736208	A-genome specific (napus-rapa)	0.9808612	0.5065973	not scorable	not scorable	1	0.7911221
1386	Bn-A01-p23739022	A-genome specific (napus-rapa)	0.9641148	0.7718753	not scorable	not scorable	1	0.795207
1387	Bn-A01-p23763782	A-genome specific (napus-rapa)	0.9952153	0.6070205	not scorable	not scorable	1	0.6270859
1388	Bn-A01-p23772904	A-genome specific (rapa only)	not scorable	not scorable	not scorable	not scorable	0.9834711	0.6901347
1389	Bn-A01-p23785561	A-genome specific (napus-rapa)	1	0.8298646	not scorable	not scorable	1	0.7831385
1390	Bn-A01-p23809071	A-genome specific (rapa only)	not scorable	not scorable	not scorable	not scorable	0.9834711	0.8070274
1391	Bn-A01-p23810423	A-genome specific (rapa only)	not scorable	not scorable	not scorable	not scorable	0.9834711	0.8393794
1392	Bn-A01-p23813837	A-genome specific (rapa only)	not scorable	not scorable	not scorable	not scorable	0.9834711	0.6367546
1393	Bn-A01-p23815268	A-genome specific (rapa only)	not scorable	not scorable	not scorable	not scorable	0.661157	0.7117022
1394	Bn-A01-p23819116	A-genome specific (rapa only)	not scorable	not scorable	not scorable	not scorable	0.6694215	0.7913548
1395	Bn-A01-p23842865	A-genome specific (napus-rapa)	0.9952153	0.7866972	not scorable	not scorable	0.9669421	0.753824
1396	Bn-A01-p23843030	napus specific	0.9258373	0.3085712	not scorable	not scorable	not scorable	not scorable
1397	Bn-A01-p23843222	A-genome specific (rapa only)	not scorable	not scorable	not scorable	not scorable	0.9917355	0.7847385
1398	Bn-A01-p23859890	all not scorable	not scorable	not scorable	not scorable	not scorable	not scorable	not scorable
1399	Bn-A01-p2387124	A-genome specific (napus-rapa)	0.9832536	0.8097206	not scorable	not scorable	0.9834711	0.7495213
1400	Bn-A01-p23873720	A-genome specific (napus-rapa)	0.930622	0.3679378	not scorable	not scorable	0.9834711	0.7237209
1401	Bn-A01-p23876801	A-genome specific (rapa only)	not scorable	not scorable	not scorable	not scorable	0.9917355	0.8050253
1402	Bn-A01-p23886020	A-genome specific (rapa only)	not scorable	not scorable	not scorable	not scorable	0.9917355	0.8253878
1403	Bn-A01-p23905191	A-genome specific (napus-rapa)	0.9928229	0.7011353	not scorable	not scorable	1	0.7309983
1404	Bn-A01-p23935076	A-genome specific (rapa only)	not scorable	not scorable	not scorable	not scorable	1	0.7061613
1405	Bn-A01-p2393817	A-genome specific (napus-rapa)	0.9832536	0.4068847	not scorable	not scorable	1	0.7451702
1406	Bn-A01-p23938571	A-genome specific (rapa only)	not scorable	not scorable	not scorable	not scorable	0.9586777	0.7761822
1407	Bn-A01-p23943508	A-genome specific (napus-rapa)	0.9928229	0.8453334	not scorable	not scorable	0.9917355	0.7493598
1408	Bn-A01-p23948008	A-genome specific (rapa only)	not scorable	not scorable	not scorable	not scorable	0.9917355	0.801908
1409	Bn-A01-p23950559	A-genome specific (napus-rapa)	0.9880383	0.8377019	not scorable	not scorable	0.6859504	0.7849017
1410	Bn-A01-p23961368	A-genome specific (rapa only)	not scorable	not scorable	not scorable	not scorable	1	0.784368
1411	Bn-A01-p23965220	A-genome specific (rapa only)	not scorable	not scorable	not scorable	not scorable	0.9834711	0.8097942
1412	Bn-A01-p23966537	A-genome specific (napus-rapa)	0.7392344	0.8097153	not scorable	not scorable	0.9338843	0.8991763
1413	Bn-A01-p23972100	A-genome specific (napus-rapa)	0.9976076	0.7458545	not scorable	not scorable	0.9834711	0.8803958
1414	Bn-A01-p24006789	A-genome specific (napus-rapa)	0.9904306	0.7994248	not scorable	not scorable	0.9834711	0.7649407
1415	Bn-A01-p24020373	A-genome specific (napus-rapa)	0.9904306	0.7989796	not scorable	not scorable	1	0.8170915
1416	Bn-A01-p24020451	A-genome specific (napus-rapa)	0.9808612	0.2440005	not scorable	not scorable	0.9669421	0.3683226

1417	Bn-A01-p2403471	A-genome specific (napus-rapa)	0.9808612	0.4790445	not scorable	not scorable	1	0.7357944
1418	Bn-A01-p24057724	A-genome specific (napus-rapa)	0.9856459	0.3349906	not scorable	not scorable	0.8842975	0.861779
1419	Bn-A01-p24070704	A-genome specific (napus-rapa)	0.9904306	0.7627197	not scorable	not scorable	0.9917355	0.8273427
1420	Bn-A01-p24107515	genome unspecific (all in)	0.9928229	0.3078613	1	0.656441	1	0.608694
1421	Bn-A01-p24145113	genome unspecific (all in)	1	0.5753938	0.9767442	0.1910557	1	0.6896119
1422	Bn-A01-p2421445	A-genome specific (napus-rapa)	0.9856459	0.5331212	not scorable	not scorable	0.9917355	0.767171
1423	Bn-A01-p24226107	A-genome specific (napus-rapa)	0.9473684	0.841207	not scorable	not scorable	0.7603306	0.7489003
1424	Bn-A01-p24229645	A-genome specific (napus-rapa)	0.9976076	0.5136648	not scorable	not scorable	1	0.7401398
1425	Bn-A01-p24308273	A-genome specific (rapa only)	not scorable	not scorable	not scorable	not scorable	1	0.8329957
1426	Bn-A01-p24308957	A-genome specific (rapa only)	not scorable	not scorable	not scorable	not scorable	0.9917355	0.6960069
1427	Bn-A01-p24309386	A-genome specific (rapa only)	not scorable	not scorable	not scorable	not scorable	0.9834711	0.6971646
1428	Bn-A01-p24310993	A-genome specific (napus-rapa)	0.9904306	0.6655776	not scorable	not scorable	0.9752066	0.6095613
1429	Bn-A01-p2431503	A-genome specific (napus-rapa)	0.9856459	0.5223921	not scorable	not scorable	0.9834711	0.6830099
1430	Bn-A01-p24319792	A-genome specific (napus-rapa)	0.923445	0.80387	not scorable	not scorable	0.9917355	0.7902024
1431	Bn-A01-p24320794	A-genome specific (napus-rapa)	0.923445	0.8141079	not scorable	not scorable	0.9917355	0.808334
1432	Bn-A01-p24346952	A-genome specific (napus-rapa)	0.9665072	0.6221851	not scorable	not scorable	1	0.8607013
1433	Bn-A01-p24360206	A-genome specific (napus-rapa)	0.9736842	0.4804409	not scorable	not scorable	0.9917355	0.7155857
1434	Bn-A01-p24369239	A-genome specific (napus-rapa)	0.9497608	0.6817468	not scorable	not scorable	0.9669421	0.8293341
1435	Bn-A01-p24369455	A-genome specific (napus-rapa)	0.9784689	0.7875039	not scorable	not scorable	0.9834711	0.7560163
1436	Bn-A01-p24372608	A-genome specific (napus-rapa)	0.9880383	0.5917053	not scorable	not scorable	1	0.7882738
1437	Bn-A01-p24390991	A-genome specific (napus-rapa)	0.9784689	0.2994004	not scorable	not scorable	1	0.5798491
1438	Bn-A01-p24394537	A-genome specific (napus-rapa)	0.9832536	0.3120457	not scorable	not scorable	0.9917355	0.695465
1439	Bn-A01-p24395406	A-genome specific (napus-rapa)	0.4976076	0.8100576	not scorable	not scorable	0.9834711	0.777389
1440	Bn-A01-p24396906	A-genome specific (napus-rapa)	0.9665072	0.7855649	not scorable	not scorable	1	0.7922278
1441	Bn-A01-p24408637	A-genome specific (napus-rapa)	0.9449761	0.6741723	not scorable	not scorable	0.9752066	0.7833724
1442	Bn-A01-p24416660	A-genome specific (napus-rapa)	0.9880383	0.5369902	not scorable	not scorable	0.9917355	0.83626
1443	Bn-A01-p24425084	A-genome specific (napus-rapa)	0.8373206	0.4140776	not scorable	not scorable	1	0.7186209
1444	Bn-A01-p24425211	A-genome specific (napus-rapa)	0.9784689	0.1855441	not scorable	not scorable	0.9834711	0.7880548
1445	Bn-A01-p24425829	A-genome specific (napus-rapa)	0.9736842	0.2893933	not scorable	not scorable	0.9669421	0.7113546
1446	Bn-A01-p24429653	genome unspecific (oleracea-rapa)	not scorable	not scorable	1	0.362717	0.9669421	0.3311764
1447	Bn-A01-p24431478	A-genome specific (napus-rapa)	1	0.3956327	not scorable	not scorable	1	0.7721409
1448	Bn-A01-p24435452	A-genome specific (napus-rapa)	1	0.8135551	not scorable	not scorable	1	0.7627373
1449	Bn-A01-p2445274	A-genome specific (rapa only)	not scorable	not scorable	not scorable	not scorable	0.6033058	0.6714416

1450	Bn-A01-p2445468	A-genome specific (napus-rapa)	0.9904306	0.8275579	not scorable	not scorable	1	0.7245526
1451	Bn-A01-p2445602	A-genome specific (napus-rapa)	0.9904306	0.675117	not scorable	not scorable	1	0.7778318
1452	Bn-A01-p2448162	A-genome specific (napus-rapa)	0.9832536	0.7567	not scorable	not scorable	0.9917355	0.7476665
1453	Bn-A01-p24487852	A-genome specific (napus-rapa)	0.9712918	0.7359673	not scorable	not scorable	0.9752066	0.7595801
1454	Bn-A01-p24504495	A-genome specific (napus-rapa)	0.9856459	0.8126721	not scorable	not scorable	1	0.8288649
1455	Bn-A01-p24504575	A-genome specific (napus-rapa)	0.9760765	0.7744135	not scorable	not scorable	1	0.8637648
1456	Bn-A01-p24507466	A-genome specific (napus-rapa)	0.9880383	0.301901	not scorable	not scorable	0.9834711	0.6863935
1457	Bn-A01-p24510656	A-genome specific (napus-rapa)	0.9880383	0.5812946	not scorable	not scorable	1	0.7785823
1458	Bn-A01-p24520244	A-genome specific (napus-rapa)	0.9952153	0.2084642	not scorable	not scorable	0.9834711	0.7292882
1459	Bn-A01-p24525303	genome unspecific (all in)	0.9832536	0.268355	0.9379845	0.2047486	0.9917355	0.4987241
1460	Bn-A01-p24526982	A-genome specific (napus-rapa)	0.9760765	0.4268059	not scorable	not scorable	0.9917355	0.7868028
1461	Bn-A01-p24529391	A-genome specific (rapa only)	not scorable	not scorable	not scorable	not scorable	1	0.8549314
1462	Bn-A01-p2453633	A-genome specific (napus-rapa)	0.9904306	0.7778233	not scorable	not scorable	1	0.8758393
1463	Bn-A01-p24536432	A-genome specific (rapa only)	not scorable	not scorable	not scorable	not scorable	0.6446281	0.797288
1464	Bn-A01-p2453758	A-genome specific (napus-rapa)	0.9736842	0.721588	not scorable	not scorable	1	0.710425
1465	Bn-A01-p24541095	A-genome specific (napus-rapa)	0.9521531	0.7622583	not scorable	not scorable	0.9669421	0.7658485
1466	Bn-A01-p2455219	A-genome specific (napus-rapa)	0.9976076	0.5782492	not scorable	not scorable	1	0.5620514
1467	Bn-A01-p24556230	A-genome specific (napus-rapa)	0.9856459	0.7371927	not scorable	not scorable	0.9917355	0.7270799
1468	Bn-A01-p24565084	A-genome specific (napus-rapa)	0.9784689	0.7746619	not scorable	not scorable	1	0.884536
1469	Bn-A01-p24565311	A-genome specific (napus-rapa)	0.9856459	0.7740189	not scorable	not scorable	1	0.8901196
1470	Bn-A01-p24570589	all not scorable	not scorable	not scorable	not scorable	not scorable	not scorable	not scorable
1471	Bn-A01-p24575577	A-genome specific (napus-rapa)	1	0.5044385	not scorable	not scorable	1	0.8423266
1472	Bn-A01-p24575679	A-genome specific (napus-rapa)	0.9808612	0.8052014	not scorable	not scorable	1	0.8594825
1473	Bn-A01-p2457750	A-genome specific (napus-rapa)	0.9569378	0.3759834	not scorable	not scorable	1	0.7167414
1474	Bn-A01-p24578644	A-genome specific (napus-rapa)	0.9784689	0.8072044	not scorable	not scorable	1	0.8659754
1475	Bn-A01-p2461470	A-genome specific (rapa only)	not scorable	not scorable	not scorable	not scorable	0.892562	0.7880238
1476	Bn-A01-p24634303	A-genome specific (napus-rapa)	0.9497608	0.808457	not scorable	not scorable	1	0.8315895
1477	Bn-A01-p24635378	A-genome specific (napus-rapa)	0.9569378	0.7524585	not scorable	not scorable	0.9834711	0.8615099
1478	Bn-A01-p24636537	A-genome specific (napus-rapa)	0.9521531	0.4982219	not scorable	not scorable	0.9752066	0.5981658
1479	Bn-A01-p24646334	A-genome specific (napus-rapa)	0.9569378	0.7587104	not scorable	not scorable	1	0.8688908
1480	Bn-A01-p24655110	A-genome specific (napus-rapa)	0.9712918	0.6192454	not scorable	not scorable	1	0.6744155
1481	Bn-A01-p24664331	A-genome specific (napus-rapa)	1	0.5751504	not scorable	not scorable	0.9834711	0.7478836
1482	Bn-A01-p24696367	A-genome specific (napus-rapa)	0.9856459	0.7572768	not scorable	not scorable	1	0.7326887

1483	Bn-A01-p24697185	A-genome specific (napus-rapa)	0.9593301	0.6373955	not scorable	not scorable	1	0.7636979
1484	Bn-A01-p24701885	A-genome specific (napus-rapa)	0.9593301	0.7391626	not scorable	not scorable	0.9834711	0.6563621
1485	Bn-A01-p24703150	A-genome specific (napus-rapa)	0.9665072	0.7775779	not scorable	not scorable	0.9669421	0.9010419
1486	Bn-A01-p24705790	A-genome specific (napus-rapa)	0.9066986	0.7817326	not scorable	not scorable	1	0.8373652
1487	Bn-A01-p24706378	A-genome specific (napus-rapa)	0.9808612	0.7276669	not scorable	not scorable	1	0.7582248
1488	Bn-A01-p24707425	A-genome specific (napus-rapa)	0.9665072	0.5010982	not scorable	not scorable	1	0.719188
1489	Bn-A01-p24707727	A-genome specific (napus-rapa)	0.9760765	0.7108399	not scorable	not scorable	1	0.7629914
1490	Bn-A01-p24720378	A-genome specific (rapa only)	not scorable	not scorable	not scorable	not scorable	0.9834711	0.7409135
1491	Bn-A01-p24737418	A-genome specific (rapa only)	not scorable	not scorable	not scorable	not scorable	0.7190083	0.7509561
1492	Bn-A01-p2475676	A-genome specific (napus-rapa)	0.9904306	0.8335802	not scorable	not scorable	0.9669421	0.7403755
1493	Bn-A01-p24756888	A-genome specific (rapa only)	not scorable	not scorable	not scorable	not scorable	0.9834711	0.7443166
1494	Bn-A01-p2478054	A-genome specific (napus-rapa)	0.9880383	0.8098974	not scorable	not scorable	0.9834711	0.7537001
1495	Bn-A01-p2478055	A-genome specific (napus-rapa)	0.9904306	0.7839454	not scorable	not scorable	0.9917355	0.8948976
1496	Bn-A01-p24811222	A-genome specific (napus-rapa)	0.9832536	0.7061973	not scorable	not scorable	0.9917355	0.7183754
1497	Bn-A01-p24812396	A-genome specific (rapa only)	not scorable	not scorable	not scorable	not scorable	1	0.7998822
1498	Bn-A01-p24813515	A-genome specific (napus-rapa)	0.9114832	0.7790854	not scorable	not scorable	1	0.7214473
1499	Bn-A01-p24828081	A-genome specific (napus-rapa)	0.8444976	0.6401235	not scorable	not scorable	0.9752066	0.8474625
1500	Bn-A01-p24829245	A-genome specific (napus-rapa)	0.9712918	0.7737567	not scorable	not scorable	0.9834711	0.6725538
1501	Bn-A01-p24829988	A-genome specific (napus-rapa)	0.9665072	0.7670236	not scorable	not scorable	0.9917355	0.7559603
1502	Bn-A01-p24830111	A-genome specific (napus-rapa)	0.9712918	0.7710227	not scorable	not scorable	1	0.7891905
1503	Bn-A01-p24830366	A-genome specific (napus-rapa)	0.9760765	0.7959251	not scorable	not scorable	0.9917355	0.7824134
1504	Bn-A01-p24835306	A-genome specific (napus-rapa)	0.9760765	0.7540577	not scorable	not scorable	0.9917355	0.7852557
1505	Bn-A01-p24835449	A-genome specific (napus-rapa)	0.9641148	0.76271	not scorable	not scorable	0.9917355	0.7823989
1506	Bn-A01-p24839451	A-genome specific (napus-rapa)	0.9712918	0.6969022	not scorable	not scorable	0.9669421	0.6999369
1507	Bn-A01-p24839905	all not scorable	not scorable	not scorable	not scorable	not scorable	not scorable	not scorable
1508	Bn-A01-p24840306	A-genome specific (napus-rapa)	0.9497608	0.7121454	not scorable	not scorable	0.9917355	0.7506587
1509	Bn-A01-p24848629	A-genome specific (napus-rapa)	0.9712918	0.4086661	not scorable	not scorable	0.9669421	0.6925956
1510	Bn-A01-p24849993	A-genome specific (napus-rapa)	0.9880383	0.928963	not scorable	not scorable	1	0.8751482
1511	Bn-A01-p24858036	A-genome specific (napus-rapa)	0.9712918	0.3744818	not scorable	not scorable	1	0.7467463
1512	Bn-A01-p24862401	A-genome specific (napus-rapa)	0.9904306	0.5692626	not scorable	not scorable	1	0.8646256
1513	Bn-A01-p24880356	A-genome specific (rapa only)	not scorable	not scorable	not scorable	not scorable	0.9752066	0.6948057
1514	Bn-A01-p24915384	A-genome specific (napus-rapa)	0.9928229	0.2656686	not scorable	not scorable	0.9917355	0.7081516
1515	Bn-A01-p2491976	genome unspecific (oleracea-rapa)	not scorable	not scorable	0.9689922	0.7763976	0.9669421	0.7203174

1516	Bn-A01-p24920662	A-genome specific (napus-rapa)	0.9784689	0.7795499	not scorable	not scorable	1	0.7853989
1517	Bn-A01-p24921162	A-genome specific (napus-rapa)	0.9880383	0.7734184	not scorable	not scorable	0.9669421	0.766748
1518	Bn-A01-p24921355	A-genome specific (napus-rapa)	0.9880383	0.7848532	not scorable	not scorable	1	0.7023785
1519	Bn-A01-p24950985	A-genome specific (napus-rapa)	0.9880383	0.6286371	not scorable	not scorable	0.9834711	0.8221104
1520	Bn-A01-p24974533	A-genome specific (rapa only)	not scorable	not scorable	not scorable	not scorable	1	0.7177046
1521	Bn-A01-p24976705	A-genome specific (rapa only)	not scorable	not scorable	not scorable	not scorable	1	0.7681012
1522	Bn-A01-p24981413	A-genome specific (rapa only)	not scorable	not scorable	not scorable	not scorable	1	0.8298145
1523	Bn-A01-p25007130	A-genome specific (rapa only)	not scorable	not scorable	not scorable	not scorable	1	0.7098919
1524	Bn-A01-p2501423	A-genome specific (napus-rapa)	0.937799	0.7924248	not scorable	not scorable	0.9917355	0.7531719
1525	Bn-A01-p25032772	A-genome specific (napus-rapa)	0.8325359	0.8315535	not scorable	not scorable	0.9669421	0.7183945
1526	Bn-A01-p25040577	A-genome specific (napus-rapa)	0.8181818	0.6237687	not scorable	not scorable	0.9917355	0.6249232
1527	Bn-A01-p2504370	A-genome specific (napus-rapa)	0.9665072	0.5298676	not scorable	not scorable	0.9669421	0.5865411
1528	Bn-A01-p25050109	napus specific	0.7942584	0.7649186	not scorable	not scorable	not scorable	not scorable
1529	Bn-A01-p25061137	A-genome specific (rapa only)	not scorable	not scorable	not scorable	not scorable	1	0.6876795
1530	Bn-A01-p25070443	A-genome specific (rapa only)	not scorable	not scorable	not scorable	not scorable	1	0.7276598
1531	Bn-A01-p25072800	A-genome specific (napus-rapa)	0.8732057	0.7136853	not scorable	not scorable	0.9834711	0.663849
1532	Bn-A01-p250759	A-genome specific (napus-rapa)	0.8516746	0.5970012	not scorable	not scorable	1	0.8675878
1533	Bn-A01-p25079412	A-genome specific (rapa only)	not scorable	not scorable	not scorable	not scorable	1	0.6824101
1534	Bn-A01-p25079505	genome unspecific (oleracea-rapa)	not scorable	not scorable	1	0.6769527	0.9917355	0.7303389
1535	Bn-A01-p2509163	A-genome specific (napus-rapa)	0.9904306	0.7483288	not scorable	not scorable	1	0.8552703
1536	Bn-A01-p25099980	A-genome specific (rapa only)	not scorable	not scorable	not scorable	not scorable	1	0.8293523
1537	Bn-A01-p25100490	A-genome specific (rapa only)	not scorable	not scorable	not scorable	not scorable	1	0.8545477
1538	Bn-A01-p25101023	all not scorable	not scorable	not scorable	not scorable	not scorable	not scorable	not scorable
1539	Bn-A01-p25117730	A-genome specific (rapa only)	not scorable	not scorable	not scorable	not scorable	0.9917355	0.6995957
1540	Bn-A01-p25143334	A-genome specific (rapa only)	not scorable	not scorable	not scorable	not scorable	0.9586777	0.7124214
1541	Bn-A01-p25171870	A-genome specific (napus-rapa)	0.9641148	0.673611	not scorable	not scorable	0.9669421	0.5965027
1542	Bn-A01-p25175675	A-genome specific (napus-rapa)	0.9904306	0.4201587	not scorable	not scorable	0.9834711	0.7826332
1543	Bn-A01-p2517658	A-genome specific (rapa only)	not scorable	not scorable	not scorable	not scorable	0.9669421	0.7787679
1544	Bn-A01-p25183707	A-genome specific (napus-rapa)	0.9760765	0.4232383	not scorable	not scorable	1	0.6547667
1545	Bn-A01-p25196502	A-genome specific (napus-rapa)	0.9880383	0.8362558	not scorable	not scorable	1	0.8185435
1546	Bn-A01-p25199395	A-genome specific (napus-rapa)	0.9760765	0.7669085	not scorable	not scorable	0.9917355	0.8103641
1547	Bn-A01-p25200386	A-genome specific (napus-rapa)	0.9880383	0.7959126	not scorable	not scorable	1	0.7050563
1548	Bn-A01-p25235998	A-genome specific (napus-rapa)	0.9880383	0.3322228	not scorable	not scorable	0.9669421	0.774264

1549	Bn-A01-p25255567	A-genome specific (rapa only)	not scorable	not scorable	not scorable	not scorable	1	0.6599271
1550	Bn-A01-p25258657	A-genome specific (rapa only)	not scorable	not scorable	not scorable	not scorable	0.9256198	0.7803772
1551	Bn-A01-p25259103	A-genome specific (rapa only)	not scorable	not scorable	not scorable	not scorable	0.9917355	0.7087636
1552	Bn-A01-p25259292	A-genome specific (rapa only)	not scorable	not scorable	not scorable	not scorable	0.9917355	0.7659664
1553	Bn-A01-p25260327	A-genome specific (rapa only)	not scorable	not scorable	not scorable	not scorable	1	0.8254527
1554	Bn-A01-p25260589	A-genome specific (rapa only)	not scorable	not scorable	not scorable	not scorable	1	0.7226265
1555	Bn-A01-p25260919	A-genome specific (rapa only)	not scorable	not scorable	not scorable	not scorable	0.9834711	0.7707136
1556	Bn-A01-p25267377	A-genome specific (napus-rapa)	0.9736842	0.6954525	not scorable	not scorable	1	0.7010509
1557	Bn-A01-p25279161	A-genome specific (napus-rapa)	0.6076555	0.809734	not scorable	not scorable	0.6198347	0.7973475
1558	Bn-A01-p25311527	A-genome specific (rapa only)	not scorable	not scorable	not scorable	not scorable	1	0.7161973
1559	Bn-A01-p25326722	A-genome specific (napus-rapa)	0.9856459	0.6084388	not scorable	not scorable	1	0.7342008
1560	Bn-A01-p25327241	A-genome specific (napus-rapa)	1	0.8293141	not scorable	not scorable	1	0.807766
1561	Bn-A01-p2533727	A-genome specific (napus-rapa)	0.9976076	0.4080127	not scorable	not scorable	0.9834711	0.6731198
1562	Bn-A01-p25349875	A-genome specific (napus-rapa)	0.9545454	0.2165197	not scorable	not scorable	0.9421487	0.7213356
1563	Bn-A01-p25350397	A-genome specific (napus-rapa)	0.8971292	0.5441456	not scorable	not scorable	0.9917355	0.8030928
1564	Bn-A01-p25351684	A-genome specific (napus-rapa)	0.9880383	0.784217	not scorable	not scorable	1	0.6944329
1565	Bn-A01-p25352753	A-genome specific (napus-rapa)	0.9808612	0.7879115	not scorable	not scorable	1	0.7608553
1566	Bn-A01-p25363566	A-genome specific (napus-rapa)	0.9784689	0.8070248	not scorable	not scorable	0.9752066	0.6411145
1567	Bn-A01-p25364218	A-genome specific (napus-rapa)	0.9808612	0.568985	not scorable	not scorable	1	0.6798471
1568	Bn-A01-p25377739	A-genome specific (napus-rapa)	0.9736842	0.6431644	not scorable	not scorable	1	0.521526
1569	Bn-A01-p2538180	A-genome specific (napus-rapa)	0.9760765	0.5830104	not scorable	not scorable	0.9669421	0.7880175
1570	Bn-A01-p25389497	all not scorable	not scorable	not scorable	not scorable	not scorable	not scorable	not scorable
1571	Bn-A01-p25391997	A-genome specific (napus-rapa)	0.9736842	0.7723963	not scorable	not scorable	0.9752066	0.8450002
1572	Bn-A01-p25397605	A-genome specific (napus-rapa)	0.9976076	0.7205103	not scorable	not scorable	0.9917355	0.7431399
1573	Bn-A01-p25405698	A-genome specific (napus-rapa)	0.9928229	0.289308	not scorable	not scorable	0.9834711	0.757373
1574	Bn-A01-p25428278	A-genome specific (napus-rapa)	0.9808612	0.7101657	not scorable	not scorable	0.9504132	0.7796092
1575	Bn-A01-p25429100	A-genome specific (napus-rapa)	0.9952153	0.8599785	not scorable	not scorable	0.7107438	0.8382545
1576	Bn-A01-p25444722	A-genome specific (rapa only)	not scorable	not scorable	not scorable	not scorable	0.9917355	0.7594981
1577	Bn-A01-p25453874	A-genome specific (napus-rapa)	0.9736842	0.4813457	not scorable	not scorable	1	0.6962444
1578	Bn-A01-p25462155	A-genome specific (rapa only)	not scorable	not scorable	not scorable	not scorable	1	0.8127098
1579	Bn-A01-p25470470	A-genome specific (napus-rapa)	0.9808612	0.7856573	not scorable	not scorable	0.9752066	0.7677757
1580	Bn-A01-p25480187	A-genome specific (rapa only)	not scorable	not scorable	not scorable	not scorable	0.9752066	0.7956141
1581	Bn-A01-p25480397	A-genome specific (rapa only)	not scorable	not scorable	not scorable	not scorable	0.9834711	0.6662053

1582	Bn-A01-p25509783	A-genome specific (rapa only)	not scorable	not scorable	not scorable	not scorable	1	0.7019389
1583	Bn-A01-p25512934	A-genome specific (napus-rapa)	0.9832536	0.8282065	not scorable	not scorable	0.6115702	0.7569256
1584	Bn-A01-p25513710	A-genome specific (napus-rapa)	0.9928229	0.7697304	not scorable	not scorable	1	0.7597374
1585	Bn-A01-p25517197	A-genome specific (napus-rapa)	0.9880383	0.5899979	not scorable	not scorable	0.5619835	0.6304076
1586	Bn-A01-p25517270	A-genome specific (rapa only)	not scorable	not scorable	not scorable	not scorable	0.9834711	0.8219146
1587	Bn-A01-p25518745	A-genome specific (rapa only)	not scorable	not scorable	not scorable	not scorable	0.9834711	0.8812889
1588	Bn-A01-p2553345	A-genome specific (napus-rapa)	0.9904306	0.8359692	not scorable	not scorable	0.9917355	0.7963902
1589	Bn-A01-p25555508	A-genome specific (napus-rapa)	0.8684211	0.77848	not scorable	not scorable	0.9834711	0.8703188
1590	Bn-A01-p25561204	A-genome specific (rapa only)	not scorable	not scorable	not scorable	not scorable	0.9834711	0.795249
1591	Bn-A01-p25562652	A-genome specific (rapa only)	not scorable	not scorable	not scorable	not scorable	0.9752066	0.7071002
1592	Bn-A01-p25570701	A-genome specific (rapa only)	not scorable	not scorable	not scorable	not scorable	0.9752066	0.775996
1593	Bn-A01-p25572254	A-genome specific (rapa only)	not scorable	not scorable	not scorable	not scorable	0.9834711	0.6962332
1594	Bn-A01-p25577348	A-genome specific (napus-rapa)	0.8660287	0.8042552	not scorable	not scorable	0.9669421	0.7670889
1595	Bn-A01-p25580651	A-genome specific (rapa only)	not scorable	not scorable	not scorable	not scorable	1	0.8513826
1596	Bn-A01-p25591508	A-genome specific (rapa only)	not scorable	not scorable	not scorable	not scorable	0.9834711	0.750864
1597	Bn-A01-p2569303	A-genome specific (napus-rapa)	0.9880383	0.7404099	not scorable	not scorable	0.9917355	0.7474413
1598	Bn-A01-p2574969	A-genome specific (napus-rapa)	0.9593301	0.4939444	not scorable	not scorable	0.9834711	0.6824396
1599	Bn-A01-p25750911	A-genome specific (napus-rapa)	0.9904306	0.7645283	not scorable	not scorable	1	0.7694198
1600	Bn-A01-p2575497	A-genome specific (napus-rapa)	0.9712918	0.6934321	not scorable	not scorable	1	0.8270477
1601	Bn-A01-p2587709	A-genome specific (napus-rapa)	0.9593301	0.6222298	not scorable	not scorable	1	0.7796671
1602	Bn-A01-p25898606	A-genome specific (napus-rapa)	1	0.5482896	not scorable	not scorable	1	0.5871008
1603	Bn-A01-p26010353	A-genome specific (napus-rapa)	0.9856459	0.615495	not scorable	not scorable	1	0.6975942
1604	Bn-A01-p26064479	A-genome specific (napus-rapa)	0.9856459	0.5602459	not scorable	not scorable	0.9917355	0.7934078
1605	Bn-A01-p2608660	A-genome specific (napus-rapa)	0.9904306	0.7366656	not scorable	not scorable	1	0.84435
1606	Bn-A01-p2624222	A-genome specific (napus-rapa)	0.9688995	0.3626452	not scorable	not scorable	1	0.8290663
1607	Bn-A01-p2626270	A-genome specific (napus-rapa)	0.9641148	0.7752636	not scorable	not scorable	1	0.6829811
1608	Bn-A01-p2634563	A-genome specific (napus-rapa)	0.9688995	0.2637914	not scorable	not scorable	0.9752066	0.6170012
1609	Bn-A01-p26366791	A-genome specific (napus-rapa)	0.9784689	0.6954595	not scorable	not scorable	0.9834711	0.6575026
1610	Bn-A01-p26369426	A-genome specific (napus-rapa)	0.6650718	0.9205657	not scorable	not scorable	0.7024794	0.8007924
1611	Bn-A01-p26369651	A-genome specific (napus-rapa)	1	0.8093156	not scorable	not scorable	0.9834711	0.6622275
1612	Bn-A01-p2637699	A-genome specific (napus-rapa)	0.6650718	0.8008664	not scorable	not scorable	1	0.8570511
1613	Bn-A01-p26380200	A-genome specific (rapa only)	not scorable	not scorable	not scorable	not scorable	0.9669421	0.740419
1614	Bn-A01-p26384777	A-genome specific (rapa only)	not scorable	not scorable	not scorable	not scorable	1	0.9077223

1615	Bn-A01-p26400154	A-genome specific (napus-rapa)	0.9688995	0.6682233	not scorable	not scorable	0.9917355	0.8313791
1616	Bn-A01-p26423587	A-genome specific (napus-rapa)	0.9688995	0.7842595	not scorable	not scorable	0.9834711	0.6842724
1617	Bn-A01-p26425810	A-genome specific (napus-rapa)	0.9665072	0.7088943	not scorable	not scorable	0.6942149	0.8734109
1618	Bn-A01-p26426004	A-genome specific (napus-rapa)	0.9760765	0.5383828	not scorable	not scorable	0.9752066	0.6775534
1619	Bn-A01-p26447438	A-genome specific (napus-rapa)	0.9880383	0.7560492	not scorable	not scorable	1	0.7910574
1620	Bn-A01-p26461873	A-genome specific (rapa only)	not scorable	not scorable	not scorable	not scorable	0.9834711	0.7643821
1621	Bn-A01-p2647221	A-genome specific (napus-rapa)	0.9976076	0.4508382	not scorable	not scorable	1	0.6124635
1622	Bn-A01-p26515072	A-genome specific (rapa only)	not scorable	not scorable	not scorable	not scorable	1	0.7033863
1623	Bn-A01-p26516918	A-genome specific (rapa only)	not scorable	not scorable	not scorable	not scorable	1	0.67151
1624	Bn-A01-p26518304	genome unspecific (all in)	0.9808612	0.3732749	0.9844961	0.6722978	0.9917355	0.702432
1625	Bn-A01-p26519047	A-genome specific (napus-rapa)	0.9688995	0.5022182	not scorable	not scorable	1	0.7389509
1626	Bn-A01-p26541761	A-genome specific (napus-rapa)	0.9832536	0.6988035	not scorable	not scorable	1	0.7355374
1627	Bn-A01-p26543758	A-genome specific (napus-rapa)	0.9760765	0.9241689	not scorable	not scorable	1	0.6946182
1628	Bn-A01-p26568738	A-genome specific (rapa only)	not scorable	not scorable	not scorable	not scorable	0.9834711	0.6525146
1629	Bn-A01-p26571596	A-genome specific (napus-rapa)	0.8444976	0.8652158	not scorable	not scorable	0.6528926	0.7360749
1630	Bn-A01-p26572331	A-genome specific (rapa only)	not scorable	not scorable	not scorable	not scorable	1	0.8116723
1631	Bn-A01-p26575652	genome unspecific (all in)	0.9569378	0.2610116	1	0.7531129	1	0.8193658
1632	Bn-A01-p26589959	A-genome specific (napus-rapa)	0.9952153	0.5221454	not scorable	not scorable	0.9917355	0.6602874
1633	Bn-A01-p26592021	genome unspecific (oleracea-rapa)	not scorable	not scorable	0.6046512	0.6249839	0.9917355	0.609157
1634	Bn-A01-p26594603	A-genome specific (napus-rapa)	0.854067	0.3610093	not scorable	not scorable	0.9917355	0.7388162
1635	Bn-A01-p26604864	A-genome specific (napus-rapa)	0.9473684	0.7415193	not scorable	not scorable	0.9669421	0.794245
1636	Bn-A01-p26614232	A-genome specific (napus-rapa)	0.937799	0.7755526	not scorable	not scorable	0.6859504	0.7463118
1637	Bn-A01-p26624315	A-genome specific (rapa only)	not scorable	not scorable	not scorable	not scorable	0.9917355	0.8170115
1638	Bn-A01-p26627091	A-genome specific (rapa only)	not scorable	not scorable	not scorable	not scorable	1	0.6677487
1639	Bn-A01-p26658327	A-genome specific (rapa only)	not scorable	not scorable	not scorable	not scorable	1	0.873747
1640	Bn-A01-p2667471	A-genome specific (napus-rapa)	0.9856459	0.723443	not scorable	not scorable	0.9752066	0.8243435
1641	Bn-A01-p2667691	A-genome specific (napus-rapa)	0.9808612	0.7911763	not scorable	not scorable	0.9834711	0.8258467
1642	Bn-A01-p2667767	genome unspecific (all in)	0.9736842	0.3710898	0.9302326	0.2758512	0.9752066	0.721679
1643	Bn-A01-p26685015	A-genome specific (rapa only)	not scorable	not scorable	not scorable	not scorable	0.9834711	0.7691421
1644	Bn-A01-p26685077	A-genome specific (rapa only)	not scorable	not scorable	not scorable	not scorable	0.9752066	0.81042
1645	Bn-A01-p26688333	A-genome specific (napus-rapa)	0.9808612	0.2916954	not scorable	not scorable	0.9834711	0.7743217
1646	Bn-A01-p2668951	A-genome specific (napus-rapa)	0.9808612	0.6611878	not scorable	not scorable	0.9917355	0.8018193
1647	Bn-A01-p26713360	A-genome specific (rapa only)	not scorable	not scorable	not scorable	not scorable	0.9917355	0.8115249

1648	Bn-A01-p26715665	A-genome specific (rapa only)	not scorable	not scorable	not scorable	not scorable	0.9669421	0.6890505
1649	Bn-A01-p26716060	A-genome specific (napus-rapa)	0.9712918	0.3123906	not scorable	not scorable	1	0.7099588
1650	Bn-A01-p26722319	A-genome specific (napus-rapa)	0.9808612	0.4783655	not scorable	not scorable	0.9917355	0.689772
1651	Bn-A01-p26759778	A-genome specific (napus-rapa)	0.9521531	0.7195595	not scorable	not scorable	0.9834711	0.6986067
1652	Bn-A01-p26760158	genome unspecific (oleracea-rapa)	not scorable	not scorable	1	0.7360497	0.9834711	0.6171024
1653	Bn-A01-p26767819	A-genome specific (rapa only)	not scorable	not scorable	not scorable	not scorable	0.9834711	0.779614
1654	Bn-A01-p26829665	all not scorable	not scorable	not scorable	not scorable	not scorable	not scorable	not scorable
1655	Bn-A01-p26837207	A-genome specific (napus-rapa)	0.9736842	0.8089336	not scorable	not scorable	0.9917355	0.7124652
1656	Bn-A01-p26853670	A-genome specific (rapa only)	not scorable	not scorable	not scorable	not scorable	0.9008265	0.7397743
1657	Bn-A01-p26866541	A-genome specific (rapa only)	not scorable	not scorable	not scorable	not scorable	0.9917355	0.7490847
1658	Bn-A01-p2688662	A-genome specific (napus-rapa)	0.9928229	0.5515639	not scorable	not scorable	0.6859504	0.7051241
1659	Bn-A01-p26910983	A-genome specific (rapa only)	not scorable	not scorable	not scorable	not scorable	1	0.7336277
1660	Bn-A01-p26914512	A-genome specific (napus-rapa)	0.9545454	0.6756006	not scorable	not scorable	0.9917355	0.5329408
1661	Bn-A01-p26914851	A-genome specific (napus-rapa)	0.9497608	0.2426034	not scorable	not scorable	0.9917355	0.6924549
1662	Bn-A01-p26921219	A-genome specific (napus-rapa)	0.9832536	0.3716201	not scorable	not scorable	0.9752066	0.5742437
1663	Bn-A01-p26921609	genome unspecific (all in)	0.9641148	0.4161336	1	0.6501446	1	0.6423814
1664	Bn-A01-p26940404	A-genome specific (napus-rapa)	0.9760765	0.6928773	not scorable	not scorable	1	0.6974153
1665	Bn-A01-p2694323	A-genome specific (napus-rapa)	0.9880383	0.4965835	not scorable	not scorable	0.9834711	0.7093155
1666	Bn-A01-p26945726	A-genome specific (rapa only)	not scorable	not scorable	not scorable	not scorable	0.9338843	0.7810817
1667	Bn-A01-p26946254	A-genome specific (rapa only)	not scorable	not scorable	not scorable	not scorable	0.9752066	0.7256585
1668	Bn-A01-p26956455	A-genome specific (napus-rapa)	0.9688995	0.7827863	not scorable	not scorable	1	0.8139744
1669	Bn-A01-p26965229	A-genome specific (rapa only)	not scorable	not scorable	not scorable	not scorable	0.9669421	0.7229632
1670	Bn-A01-p26966407	A-genome specific (napus-rapa)	0.9569378	0.8092843	not scorable	not scorable	0.9917355	0.8564767
1671	Bn-A01-p26968952	A-genome specific (napus-rapa)	0.9641148	0.7950211	not scorable	not scorable	0.8016529	0.9056543
1672	Bn-A01-p26969210	A-genome specific (napus-rapa)	0.9617225	0.3783558	not scorable	not scorable	1	0.7606742
1673	Bn-A01-p26973178	A-genome specific (napus-rapa)	0.9593301	0.4139059	not scorable	not scorable	1	0.7689937
1674	Bn-A01-p26985090	A-genome specific (napus-rapa)	0.9880383	0.2568854	not scorable	not scorable	0.9917355	0.8004847
1675	Bn-A01-p26990131	A-genome specific (napus-rapa)	0.9545454	0.7705088	not scorable	not scorable	0.9504132	0.7806082
1676	Bn-A01-p26996900	A-genome specific (rapa only)	not scorable	not scorable	not scorable	not scorable	1	0.8263984
1677	Bn-A01-p26998962	A-genome specific (napus-rapa)	0.9641148	0.7970244	not scorable	not scorable	0.9917355	0.7937827
1678	Bn-A01-p2699907	A-genome specific (napus-rapa)	0.9928229	0.5025808	not scorable	not scorable	1	0.6625305
1679	Bn-A01-p27004740	A-genome specific (napus-rapa)	0.9976076	0.4138593	not scorable	not scorable	0.9834711	0.6067383
1680	Bn-A01-p27012139	A-genome specific (rapa only)	not scorable	not scorable	not scorable	not scorable	0.9669421	0.6144933

1681	Bn-A01-p27017324	A-genome specific (napus-rapa)	0.9712918	0.7670439	not scorable	not scorable	0.9752066	0.8035139
1682	Bn-A01-p27017651	A-genome specific (napus-rapa)	0.7464115	0.9348852	not scorable	not scorable	0.7107438	0.8875333
1683	Bn-A01-p27019745	A-genome specific (napus-rapa)	0.9401914	0.2796735	not scorable	not scorable	1	0.6578092
1684	Bn-A01-p27029107	A-genome specific (napus-rapa)	0.9712918	0.7035935	not scorable	not scorable	0.9917355	0.6642047
1685	Bn-A01-p27029254	A-genome specific (napus-rapa)	0.9760765	0.7797901	not scorable	not scorable	0.9917355	0.7151884
1686	Bn-A01-p27037472	A-genome specific (napus-rapa)	0.9593301	0.7365149	not scorable	not scorable	0.9834711	0.777812
1687	Bn-A01-p27050083	A-genome specific (napus-rapa)	0.9808612	0.7951952	not scorable	not scorable	1	0.7197951
1688	Bn-A01-p27060364	A-genome specific (napus-rapa)	0.9473684	0.7583688	not scorable	not scorable	0.9834711	0.7008753
1689	Bn-A01-p27061075	A-genome specific (napus-rapa)	0.9617225	0.7225438	not scorable	not scorable	0.5702479	0.6977504
1690	Bn-A01-p27079797	A-genome specific (rapa only)	not scorable	not scorable	not scorable	not scorable	0.9834711	0.7477164
1691	Bn-A01-p27101289	A-genome specific (napus-rapa)	1	0.4302978	not scorable	not scorable	1	0.8129535
1692	Bn-A01-p27107081	A-genome specific (napus-rapa)	0.9856459	0.3985004	not scorable	not scorable	1	0.8831033
1693	Bn-A01-p27115455	A-genome specific (rapa only)	not scorable	not scorable	not scorable	not scorable	0.9917355	0.7271727
1694	Bn-A01-p27117954	A-genome specific (rapa only)	not scorable	not scorable	not scorable	not scorable	0.9917355	0.6898228
1695	Bn-A01-p27119250	genome unspecific (all in)	0.9856459	0.4498782	0.9922481	0.5605261	1	0.7542568
1696	Bn-A01-p27125649	A-genome specific (napus-rapa)	0.9880383	0.3834951	not scorable	not scorable	0.9917355	0.7938709
1697	Bn-A01-p2713439	A-genome specific (napus-rapa)	0.5598086	0.818283	not scorable	not scorable	0.9421487	0.7534826
1698	Bn-A01-p2713528	A-genome specific (napus-rapa)	0.9569378	0.8000318	not scorable	not scorable	0.9917355	0.7873488
1699	Bn-A01-p2713583	A-genome specific (napus-rapa)	0.9832536	0.7885799	not scorable	not scorable	0.9834711	0.7803199
1700	Bn-A01-p27147782	A-genome specific (napus-rapa)	0.8732057	0.3912995	not scorable	not scorable	0.9752066	0.7209196
1701	Bn-A01-p2715156	A-genome specific (rapa only)	not scorable	not scorable	not scorable	not scorable	0.8512397	0.8030821
1702	Bn-A01-p2718126	A-genome specific (napus-rapa)	0.9832536	0.8021598	not scorable	not scorable	0.9917355	0.820279
1703	Bn-A01-p27202461	A-genome specific (rapa only)	not scorable	not scorable	not scorable	not scorable	0.9917355	0.800547
1704	Bn-A01-p27220075	A-genome specific (rapa only)	not scorable	not scorable	not scorable	not scorable	1	0.7757145
1705	Bn-A01-p27225628	A-genome specific (napus-rapa)	0.9976076	0.4919578	not scorable	not scorable	1	0.7707056
1706	Bn-A01-p27263558	A-genome specific (rapa only)	not scorable	not scorable	not scorable	not scorable	0.9917355	0.7293021
1707	Bn-A01-p27272066	A-genome specific (napus-rapa)	0.9832536	0.2431741	not scorable	not scorable	0.9834711	0.7385784
1708	Bn-A01-p27280214	all not scorable	not scorable	not scorable	not scorable	not scorable	not scorable	not scorable
1709	Bn-A01-p27293125	A-genome specific (rapa only)	not scorable	not scorable	not scorable	not scorable	1	0.7710881
1710	Bn-A01-p27299339	A-genome specific (rapa only)	not scorable	not scorable	not scorable	not scorable	0.9752066	0.686775
1711	Bn-A01-p27301264	A-genome specific (napus-rapa)	0.9832536	0.4795091	not scorable	not scorable	0.9669421	0.7520791
1712	Bn-A01-p27301637	A-genome specific (rapa only)	not scorable	not scorable	not scorable	not scorable	0.9917355	0.7782217
1713	Bn-A01-p27301980	A-genome specific (rapa only)	not scorable	not scorable	not scorable	not scorable	0.9917355	0.7005582

1714	Bn-A01-p27302144	A-genome specific (rapa only)	not scorable	not scorable	not scorable	not scorable	0.9669421	0.7911367
1715	Bn-A01-p27318082	A-genome specific (rapa only)	not scorable	not scorable	not scorable	not scorable	0.9834711	0.638901
1716	Bn-A01-p27320515	A-genome specific (rapa only)	not scorable	not scorable	not scorable	not scorable	0.6942149	0.8224226
1717	Bn-A01-p27331467	A-genome specific (napus-rapa)	0.9641148	0.6307237	not scorable	not scorable	0.7024794	0.7799551
1718	Bn-A01-p27336799	A-genome specific (rapa only)	not scorable	not scorable	not scorable	not scorable	0.9917355	0.6770988
1719	Bn-A01-p27348128	A-genome specific (napus-rapa)	0.9856459	0.3866173	not scorable	not scorable	1	0.8445116
1720	Bn-A01-p27360503	A-genome specific (napus-rapa)	0.9688995	0.3792932	not scorable	not scorable	1	0.875935
1721	Bn-A01-p27361855	A-genome specific (napus-rapa)	0.9976076	0.555003	not scorable	not scorable	0.9834711	0.6815073
1722	Bn-A01-p27381111	A-genome specific (rapa only)	not scorable	not scorable	not scorable	not scorable	0.9917355	0.65742
1723	Bn-A01-p27389703	A-genome specific (rapa only)	not scorable	not scorable	not scorable	not scorable	1	0.7646528
1724	Bn-A01-p27392534	A-genome specific (napus-rapa)	0.9952153	0.4685433	not scorable	not scorable	0.9834711	0.7262161
1725	Bn-A01-p27417163	A-genome specific (napus-rapa)	1	0.4133775	not scorable	not scorable	0.9917355	0.5353597
1726	Bn-A01-p27463135	A-genome specific (napus-rapa)	0.9928229	0.8263522	not scorable	not scorable	1	0.7819124
1727	Bn-A01-p2746862	A-genome specific (rapa only)	not scorable	not scorable	not scorable	not scorable	0.9917355	0.7796991
1728	Bn-A01-p27522299	genome unspecific (oleracea-rapa)	not scorable	not scorable	1	0.2938209	0.9669421	0.7448333
1729	Bn-A01-p27570336	A-genome specific (rapa only)	not scorable	not scorable	not scorable	not scorable	0.9917355	0.7193253
1730	Bn-A01-p27570489	A-genome specific (rapa only)	not scorable	not scorable	not scorable	not scorable	1	0.8490494
1731	Bn-A01-p27573404	A-genome specific (rapa only)	not scorable	not scorable	not scorable	not scorable	1	0.7003347
1732	Bn-A01-p27580681	A-genome specific (napus-rapa)	0.4832536	0.6559853	not scorable	not scorable	0.6446281	0.7950267
1733	Bn-A01-p27581056	A-genome specific (napus-rapa)	0.492823	0.6759167	not scorable	not scorable	0.6694215	0.8353992
1734	Bn-A01-p27589456	A-genome specific (napus-rapa)	0.9976076	0.4253203	not scorable	not scorable	0.9917355	0.8792187
1735	Bn-A01-p27601050	A-genome specific (rapa only)	not scorable	not scorable	not scorable	not scorable	0.9669421	0.7694532
1736	Bn-A01-p27622120	A-genome specific (rapa only)	not scorable	not scorable	not scorable	not scorable	1	0.8334464
1737	Bn-A01-p27622168	A-genome specific (rapa only)	not scorable	not scorable	not scorable	not scorable	1	0.7784517
1738	Bn-A01-p27623686	A-genome specific (rapa only)	not scorable	not scorable	not scorable	not scorable	1	0.7635936
1739	Bn-A01-p27668070	all not scorable	not scorable	not scorable	not scorable	not scorable	not scorable	not scorable
1740	Bn-A01-p27673018	A-genome specific (rapa only)	not scorable	not scorable	not scorable	not scorable	0.9752066	0.7682135
1741	Bn-A01-p276866	A-genome specific (rapa only)	not scorable	not scorable	not scorable	not scorable	0.9917355	0.7236418
1742	Bn-A01-p27722989	A-genome specific (napus-rapa)	0.9497608	0.7698267	not scorable	not scorable	0.9834711	0.6762297
1743	Bn-A01-p2775635	A-genome specific (rapa only)	not scorable	not scorable	not scorable	not scorable	1	0.7546726
1744	Bn-A01-p27774124	A-genome specific (napus-rapa)	1	0.4061617	not scorable	not scorable	0.9752066	0.7862923
1745	Bn-A01-p27774386	A-genome specific (napus-rapa)	0.9688995	0.7730745	not scorable	not scorable	0.7024794	0.8366635
1746	Bn-A01-p27774666	genome unspecific (all in)	0.9760765	0.3004295	0.9922481	0.6603354	0.9752066	0.6336449

1747	Bn-A01-p27777763	A-genome specific (napus-rapa)	0.9665072	0.7987347	not scorable	not scorable	1	0.8344165
1748	Bn-A01-p27796877	A-genome specific (napus-rapa)	0.9880383	0.6402216	not scorable	not scorable	1	0.6431798
1749	Bn-A01-p27797076	A-genome specific (napus-rapa)	0.9808612	0.3111543	not scorable	not scorable	0.9917355	0.6791795
1750	Bn-A01-p27810962	A-genome specific (napus-rapa)	0.9976076	0.8167478	not scorable	not scorable	1	0.8240092
1751	Bn-A01-p27822000	A-genome specific (rapa only)	not scorable	not scorable	not scorable	not scorable	1	0.7553414
1752	Bn-A01-p27822790	A-genome specific (napus-rapa)	1	0.6021739	not scorable	not scorable	0.9834711	0.6872332
1753	Bn-A01-p27830372	A-genome specific (rapa only)	not scorable	not scorable	not scorable	not scorable	0.9752066	0.7691837
1754	Bn-A01-p27831569	A-genome specific (rapa only)	not scorable	not scorable	not scorable	not scorable	0.9917355	0.8169929
1755	Bn-A01-p27834392	A-genome specific (rapa only)	not scorable	not scorable	not scorable	not scorable	0.9586777	0.7613422
1756	Bn-A01-p27834452	A-genome specific (rapa only)	not scorable	not scorable	not scorable	not scorable	0.9421487	0.7178444
1757	Bn-A01-p2784074	A-genome specific (napus-rapa)	0.8755981	0.8158981	not scorable	not scorable	0.9669421	0.8508033
1758	Bn-A01-p27843242	A-genome specific (rapa only)	not scorable	not scorable	not scorable	not scorable	1	0.7580888
1759	Bn-A01-p27845673	A-genome specific (rapa only)	not scorable	not scorable	not scorable	not scorable	0.9917355	0.8069113
1760	Bn-A01-p27846002	A-genome specific (rapa only)	not scorable	not scorable	not scorable	not scorable	0.9834711	0.74037
1761	Bn-A01-p27846227	A-genome specific (rapa only)	not scorable	not scorable	not scorable	not scorable	0.9752066	0.7497923
1762	Bn-A01-p27846544	A-genome specific (rapa only)	not scorable	not scorable	not scorable	not scorable	0.9917355	0.8041285
1763	Bn-A01-p27854949	napus specific	0.9641148	0.2450865	not scorable	not scorable	not scorable	not scorable
1764	Bn-A01-p27866419	A-genome specific (napus-rapa)	0.8277512	0.860509	not scorable	not scorable	0.9338843	0.7838142
1765	Bn-A01-p27877194	A-genome specific (rapa only)	not scorable	not scorable	not scorable	not scorable	0.9669421	0.772181
1766	Bn-A01-p27893933	A-genome specific (rapa only)	not scorable	not scorable	not scorable	not scorable	0.9752066	0.6997312
1767	Bn-A01-p27905344	A-genome specific (napus-rapa)	0.9904306	0.856211	not scorable	not scorable	0.9834711	0.8231663
1768	Bn-A01-p27907325	A-genome specific (napus-rapa)	0.9641148	0.8043339	not scorable	not scorable	0.8595041	0.7993796
1769	Bn-A01-p27907422	A-genome specific (napus-rapa)	0.9904306	0.7374505	not scorable	not scorable	0.9834711	0.738986
1770	Bn-A01-p27926074	A-genome specific (rapa only)	not scorable	not scorable	not scorable	not scorable	0.9752066	0.6954615
1771	Bn-A01-p27926195	A-genome specific (rapa only)	not scorable	not scorable	not scorable	not scorable	0.9834711	0.8130386
1772	Bn-A01-p27926396	A-genome specific (rapa only)	not scorable	not scorable	not scorable	not scorable	0.6198347	0.7201397
1773	Bn-A01-p27926574	A-genome specific (napus-rapa)	0.9904306	0.903313	not scorable	not scorable	1	0.8407481
1774	Bn-A01-p27926866	A-genome specific (rapa only)	not scorable	not scorable	not scorable	not scorable	0.9752066	0.663447
1775	Bn-A01-p2792719	A-genome specific (rapa only)	not scorable	not scorable	not scorable	not scorable	1	0.8306577
1776	Bn-A01-p27928188	A-genome specific (rapa only)	not scorable	not scorable	not scorable	not scorable	0.9752066	0.7983577
1777	Bn-A01-p27928239	A-genome specific (rapa only)	not scorable	not scorable	not scorable	not scorable	0.9669421	0.614388
1778	Bn-A01-p27936360	A-genome specific (napus-rapa)	1	0.9205657	not scorable	not scorable	1	0.8921908
1779	Bn-A01-p27937532	A-genome specific (rapa only)	not scorable	not scorable	not scorable	not scorable	0.9834711	0.7537576

1780	Bn-A01-p27942610	A-genome specific (rapa only)	not scorable	not scorable	not scorable	not scorable	1	0.6849987
1781	Bn-A01-p27947381	A-genome specific (rapa only)	not scorable	not scorable	not scorable	not scorable	0.9834711	0.774467
1782	Bn-A01-p27948361	A-genome specific (rapa only)	not scorable	not scorable	not scorable	not scorable	0.9917355	0.8010319
1783	Bn-A01-p27953178	A-genome specific (rapa only)	not scorable	not scorable	not scorable	not scorable	0.9586777	0.6766823
1784	Bn-A01-p27953261	A-genome specific (rapa only)	not scorable	not scorable	not scorable	not scorable	0.9917355	0.8046464
1785	Bn-A01-p27954131	A-genome specific (rapa only)	not scorable	not scorable	not scorable	not scorable	0.9917355	0.7908595
1786	Bn-A01-p27954294	A-genome specific (napus-rapa)	0.9688995	0.3112217	not scorable	not scorable	0.9917355	0.7570941
1787	Bn-A01-p27956374	A-genome specific (rapa only)	not scorable	not scorable	not scorable	not scorable	0.6033058	0.6817184
1788	Bn-A01-p27965742	A-genome specific (rapa only)	not scorable	not scorable	not scorable	not scorable	0.9834711	0.6647778
1789	Bn-A01-p27968584	A-genome specific (rapa only)	not scorable	not scorable	not scorable	not scorable	0.553719	0.6842507
1790	Bn-A01-p27988223	A-genome specific (napus-rapa)	0.9880383	0.3950522	not scorable	not scorable	0.677686	0.7613192
1791	Bn-A01-p27989439	A-genome specific (rapa only)	not scorable	not scorable	not scorable	not scorable	0.9834711	0.7130436
1792	Bn-A01-p28047872	A-genome specific (napus-rapa)	0.9521531	0.6571617	not scorable	not scorable	0.9256198	0.7125588
1793	Bn-A01-p28049872	A-genome specific (napus-rapa)	1	0.6987723	not scorable	not scorable	0.9917355	0.7735245
1794	Bn-A01-p28089456	genome unspecific (all in)	0.9090909	0.6534238	0.9767442	0.7560699	1	0.7790902
1795	Bn-A01-p28102080	A-genome specific (napus-rapa)	0.9880383	0.7155705	not scorable	not scorable	0.9834711	0.7733118
1796	Bn-A01-p28110393	A-genome specific (rapa only)	not scorable	not scorable	not scorable	not scorable	1	0.7004535
1797	Bn-A01-p28147757	A-genome specific (rapa only)	not scorable	not scorable	not scorable	not scorable	0.9834711	0.7986389
1798	Bn-A01-p28161207	A-genome specific (napus-rapa)	0.9688995	0.4169863	not scorable	not scorable	0.9917355	0.7851829
1799	Bn-A01-p28186318	napus specific	0.9569378	0.5135412	not scorable	not scorable	not scorable	not scorable
1800	Bn-A01-p28193774	A-genome specific (rapa only)	not scorable	not scorable	not scorable	not scorable	0.9752066	0.6785234
1801	Bn-A01-p28205769	A-genome specific (rapa only)	not scorable	not scorable	not scorable	not scorable	0.9917355	0.7012425
1802	Bn-A01-p28207450	A-genome specific (napus-rapa)	0.9449761	0.7264074	not scorable	not scorable	1	0.7755895
1803	Bn-A01-p28217427	A-genome specific (napus-rapa)	0.9712918	0.7347751	not scorable	not scorable	0.9669421	0.6490854
1804	Bn-A01-p28220430	A-genome specific (napus-rapa)	0.9856459	0.8010601	not scorable	not scorable	0.7107438	0.7222805
1805	Bn-A01-p28222170	A-genome specific (napus-rapa)	0.9904306	0.7297508	not scorable	not scorable	0.6528926	0.7096924
1806	Bn-A01-p28245023	A-genome specific (rapa only)	not scorable	not scorable	not scorable	not scorable	1	0.8746086
1807	Bn-A01-p28249918	A-genome specific (rapa only)	not scorable	not scorable	not scorable	not scorable	0.9917355	0.8026363
1808	Bn-A01-p28250936	A-genome specific (rapa only)	not scorable	not scorable	not scorable	not scorable	0.9834711	0.8272757
1809	Bn-A01-p2825565	A-genome specific (napus-rapa)	0.9760765	0.632284	not scorable	not scorable	1	0.7229235
1810	Bn-A01-p28261145	A-genome specific (napus-rapa)	0.9880383	0.3644576	not scorable	not scorable	0.9834711	0.7709141
1811	Bn-A01-p28278192	A-genome specific (napus-rapa)	0.923445	0.7091743	not scorable	not scorable	0.9917355	0.9175813
1812	Bn-A01-p28285580	A-genome specific (rapa only)	not scorable	not scorable	not scorable	not scorable	0.9917355	0.7753977

1813	Bn-A01-p28326025	A-genome specific (rapa only)	not scorable	not scorable	not scorable	not scorable	0.9917355	0.6630344
1814	Bn-A01-p28333226	A-genome specific (rapa only)	not scorable	not scorable	not scorable	not scorable	1	0.7597438
1815	Bn-A01-p28336382	A-genome specific (rapa only)	not scorable	not scorable	not scorable	not scorable	0.9917355	0.818212
1816	Bn-A01-p28336754	A-genome specific (rapa only)	not scorable	not scorable	not scorable	not scorable	0.9917355	0.7392313
1817	Bn-A01-p28346931	A-genome specific (rapa only)	not scorable	not scorable	not scorable	not scorable	0.9752066	0.6803021
1818	Bn-A01-p28360889	A-genome specific (napus-rapa)	0.9736842	0.592301	not scorable	not scorable	0.9917355	0.7353469
1819	Bn-A01-p28364600	A-genome specific (rapa only)	not scorable	not scorable	not scorable	not scorable	1	0.7304878
1820	Bn-A01-p2836617	A-genome specific (napus-rapa)	0.9976076	0.6349795	not scorable	not scorable	1	0.840636
1821	Bn-A01-p28380414	A-genome specific (napus-rapa)	0.9473684	0.7675439	not scorable	not scorable	0.9917355	0.7852464
1822	Bn-A01-p28385501	napus specific	0.9330143	0.3633123	not scorable	not scorable	not scorable	not scorable
1823	Bn-A01-p28403438	all not scorable	not scorable	not scorable	not scorable	not scorable	not scorable	not scorable
1824	Bn-A01-p2841043	A-genome specific (napus-rapa)	0.9856459	0.7710929	not scorable	not scorable	0.9834711	0.8840709
1825	Bn-A01-p28426820	A-genome specific (napus-rapa)	0.8875598	0.6931239	not scorable	not scorable	0.9917355	0.7941848
1826	Bn-A01-p28447868	A-genome specific (rapa only)	not scorable	not scorable	not scorable	not scorable	1	0.8301668
1827	Bn-A01-p28462071	A-genome specific (napus-rapa)	0.9784689	0.7963622	not scorable	not scorable	0.9834711	0.7447215
1828	Bn-A01-p2846364	all not scorable	not scorable	not scorable	not scorable	not scorable	not scorable	not scorable
1829	Bn-A01-p2848253	all not scorable	not scorable	not scorable	not scorable	not scorable	not scorable	not scorable
1830	Bn-A01-p2849034	napus specific	0.6339713	0.6444719	not scorable	not scorable	not scorable	not scorable
1831	Bn-A01-p2849540	all not scorable	not scorable	not scorable	not scorable	not scorable	not scorable	not scorable
1832	Bn-A01-p28497362	A-genome specific (napus-rapa)	0.9641148	0.7879814	not scorable	not scorable	0.9752066	0.7086282
1833	Bn-A01-p28497900	A-genome specific (napus-rapa)	0.9928229	0.2773077	not scorable	not scorable	0.9917355	0.7122121
1834	Bn-A01-p28500378	A-genome specific (napus-rapa)	0.9354067	0.7364721	not scorable	not scorable	0.9917355	0.9449387
1835	Bn-A01-p28502173	A-genome specific (rapa only)	not scorable	not scorable	not scorable	not scorable	1	0.8346031
1836	Bn-A01-p285120	genome unspecific (oleracea-rapa)	not scorable	not scorable	1	0.1869946	1	0.6821967
1837	Bn-A01-p28534093	A-genome specific (rapa only)	not scorable	not scorable	not scorable	not scorable	0.9834711	0.7950118
1838	Bn-A01-p28551714	A-genome specific (rapa only)	not scorable	not scorable	not scorable	not scorable	1	0.715218
1839	Bn-A01-p28571117	A-genome specific (rapa only)	not scorable	not scorable	not scorable	not scorable	0.6115702	0.8330126
1840	Bn-A01-p28577026	A-genome specific (napus-rapa)	0.8684211	0.8513124	not scorable	not scorable	0.9834711	0.7906627
1841	Bn-A01-p28578023	A-genome specific (napus-rapa)	0.8397129	0.7480147	not scorable	not scorable	1	0.6930552
1842	Bn-A01-p28592050	A-genome specific (rapa only)	not scorable	not scorable	not scorable	not scorable	0.9917355	0.7818213
1843	Bn-A01-p2861502	A-genome specific (napus-rapa)	0.9952153	0.729397	not scorable	not scorable	0.9834711	0.8000473
1844	Bn-A01-p2864193	A-genome specific (napus-rapa)	0.9760765	0.5908046	not scorable	not scorable	1	0.7103306
1845	Bn-A01-p2867284	A-genome specific (napus-rapa)	0.9736842	0.7508223	not scorable	not scorable	0.9917355	0.6903467

1846	Bn-A01-p2873007	A-genome specific (napus-rapa)	0.9569378	0.4239145	not scorable	not scorable	0.9834711	0.7014819
1847	Bn-A01-p2874393	A-genome specific (napus-rapa)	0.8971292	0.7987571	not scorable	not scorable	0.9834711	0.734327
1848	Bn-A01-p2882270	A-genome specific (napus-rapa)	0.9688995	0.5760276	not scorable	not scorable	0.9586777	0.8383704
1849	Bn-A01-p2884934	A-genome specific (napus-rapa)	0.9521531	0.7886327	not scorable	not scorable	0.9917355	0.7683929
1850	Bn-A01-p2891863	A-genome specific (napus-rapa)	0.9760765	0.7732499	not scorable	not scorable	0.9669421	0.7369455
1851	Bn-A01-p289703	A-genome specific (napus-rapa)	0.9928229	0.7153968	not scorable	not scorable	0.9917355	0.7434375
1852	Bn-A01-p2897071	A-genome specific (napus-rapa)	0.9617225	0.3035728	not scorable	not scorable	0.9834711	0.7097909
1853	Bn-A01-p2910608	A-genome specific (napus-rapa)	0.9760765	0.7092775	not scorable	not scorable	0.9834711	0.6939689
1854	Bn-A01-p2910752	A-genome specific (napus-rapa)	0.9736842	0.8218167	not scorable	not scorable	1	0.7760538
1855	Bn-A01-p2915231	A-genome specific (napus-rapa)	0.9832536	0.4145631	not scorable	not scorable	1	0.8453924
1856	Bn-A01-p2915821	A-genome specific (napus-rapa)	0.9449761	0.6903124	not scorable	not scorable	0.9834711	0.8297606
1857	Bn-A01-p2928824	A-genome specific (napus-rapa)	0.8253589	0.7656881	not scorable	not scorable	0.9752066	0.7696398
1858	Bn-A01-p2934166	napus specific	0.9521531	0.4453172	not scorable	not scorable	not scorable	not scorable
1859	Bn-A01-p2956393	A-genome specific (napus-rapa)	0.9976076	0.6018646	not scorable	not scorable	1	0.7904034
1860	Bn-A01-p2957869	A-genome specific (napus-rapa)	0.9904306	0.7563173	not scorable	not scorable	0.9917355	0.7935365
1861	Bn-A01-p2961857	A-genome specific (napus-rapa)	0.9880383	0.7986737	not scorable	not scorable	1	0.739793
1862	Bn-A01-p2990104	A-genome specific (napus-rapa)	0.9665072	0.7831562	not scorable	not scorable	0.9917355	0.7596586
1863	Bn-A01-p3000989	A-genome specific (napus-rapa)	0.9856459	0.3878766	not scorable	not scorable	0.9917355	0.8493416
1864	Bn-A01-p3005310	A-genome specific (napus-rapa)	0.9186603	0.7661651	not scorable	not scorable	0.9586777	0.8256969
1865	Bn-A01-p3005793	A-genome specific (napus-rapa)	0.9976076	0.4922641	not scorable	not scorable	1	0.7715249
1866	Bn-A01-p3045214	A-genome specific (napus-rapa)	0.9904306	0.7866718	not scorable	not scorable	1	0.8005987
1867	Bn-A01-p3045984	A-genome specific (napus-rapa)	0.9904306	0.7010028	not scorable	not scorable	1	0.7621678
1868	Bn-A01-p3050613	A-genome specific (napus-rapa)	0.9808612	0.7057841	not scorable	not scorable	1	0.7628181
1869	Bn-A01-p3059596	A-genome specific (napus-rapa)	0.9904306	0.565281	not scorable	not scorable	0.9669421	0.7029747
1870	Bn-A01-p3061223	A-genome specific (napus-rapa)	0.9569378	0.7845698	not scorable	not scorable	0.9669421	0.7955427
1871	Bn-A01-p3069312	genome unspecific (oleracea-rapa)	not scorable	not scorable	1	0.7580108	0.9834711	0.7045255
1872	Bn-A01-p3070657	A-genome specific (napus-rapa)	0.9832536	0.6830578	not scorable	not scorable	0.9834711	0.649318
1873	Bn-A01-p3076531	A-genome specific (napus-rapa)	0.9712918	0.7489499	not scorable	not scorable	0.7272727	0.820873
1874	Bn-A01-p3077309	A-genome specific (napus-rapa)	0.9760765	0.2049619	not scorable	not scorable	0.9421487	0.5487303
1875	Bn-A01-p3078457	A-genome specific (napus-rapa)	0.9856459	0.5116394	not scorable	not scorable	0.9669421	0.7157542
1876	Bn-A01-p3085248	A-genome specific (rapa only)	not scorable	not scorable	not scorable	not scorable	0.6859504	0.8775727
1877	Bn-A01-p309524	A-genome specific (napus-rapa)	1	0.4592944	not scorable	not scorable	0.9917355	0.6722058
1878	Bn-A01-p3097105	A-genome specific (napus-rapa)	0.9976076	0.5499591	not scorable	not scorable	1	0.7545418

1879	Bn-A01-p3100346	A-genome specific (napus-rapa)	0.9712918	0.6972825	not scorable	not scorable	0.9834711	0.669937
1880	Bn-A01-p3105148	A-genome specific (rapa only)	not scorable	not scorable	not scorable	not scorable	0.9917355	0.7792274
1881	Bn-A01-p3105358	A-genome specific (rapa only)	not scorable	not scorable	not scorable	not scorable	0.9917355	0.6894561
1882	Bn-A01-p3111607	A-genome specific (napus-rapa)	0.9641148	0.8011817	not scorable	not scorable	0.9338843	0.8770106
1883	Bn-A01-p3123580	A-genome specific (rapa only)	not scorable	not scorable	not scorable	not scorable	0.9917355	0.7902868
1884	Bn-A01-p3134159	A-genome specific (napus-rapa)	0.9712918	0.7653059	not scorable	not scorable	0.9917355	0.8318024
1885	Bn-A01-p3136468	A-genome specific (napus-rapa)	0.9760765	0.7205639	not scorable	not scorable	0.9752066	0.7721062
1886	Bn-A01-p3137277	A-genome specific (rapa only)	not scorable	not scorable	not scorable	not scorable	0.9834711	0.7474329
1887	Bn-A01-p314983	A-genome specific (napus-rapa)	0.9904306	0.8063725	not scorable	not scorable	0.9586777	0.6512539
1888	Bn-A01-p3152636	A-genome specific (napus-rapa)	0.9952153	0.9109368	not scorable	not scorable	1	0.7812275
1889	Bn-A01-p3169855	A-genome specific (napus-rapa)	0.9928229	0.7696655	not scorable	not scorable	0.9917355	0.7947142
1890	Bn-A01-p3173607	A-genome specific (napus-rapa)	0.9354067	0.7420068	not scorable	not scorable	0.9834711	0.7570364
1891	Bn-A01-p3175614	A-genome specific (napus-rapa)	0.9593301	0.7831421	not scorable	not scorable	0.9834711	0.7247187
1892	Bn-A01-p3179210	genome unspecific (oleracea-rapa)	not scorable	not scorable	0.9922481	0.8127	1	0.8222544
1893	Bn-A01-p3179292	A-genome specific (napus-rapa)	0.9904306	0.7726401	not scorable	not scorable	0.7272727	0.9174899
1894	Bn-A01-p3183888	genome unspecific (all in)	0.9473684	0.5764778	1	0.7828524	0.9917355	0.7103003
1895	Bn-A01-p3195980	A-genome specific (napus-rapa)	1	0.4310189	not scorable	not scorable	0.9917355	0.6954912
1896	Bn-A01-p3223005	A-genome specific (rapa only)	not scorable	not scorable	not scorable	not scorable	0.9752066	0.7779692
1897	Bn-A01-p3226309	A-genome specific (napus-rapa)	0.9665072	0.6033128	not scorable	not scorable	1	0.8270521
1898	Bn-A01-p3232184	A-genome specific (napus-rapa)	0.9832536	0.8032655	not scorable	not scorable	0.9834711	0.7461516
1899	Bn-A01-p3232810	A-genome specific (rapa only)	not scorable	not scorable	not scorable	not scorable	1	0.7239926
1900	Bn-A01-p3232913	A-genome specific (rapa only)	not scorable	not scorable	not scorable	not scorable	0.9752066	0.7343474
1901	Bn-A01-p3233247	A-genome specific (napus-rapa)	0.9425837	0.4314358	not scorable	not scorable	1	0.6963357
1902	Bn-A01-p3233299	A-genome specific (napus-rapa)	0.9856459	0.7198768	not scorable	not scorable	0.9834711	0.7327123
1903	Bn-A01-p3240780	A-genome specific (napus-rapa)	0.9784689	0.6421887	not scorable	not scorable	0.9917355	0.7567704
1904	Bn-A01-p324562	A-genome specific (napus-rapa)	1	0.6530379	not scorable	not scorable	1	0.7470765
1905	Bn-A01-p3279678	A-genome specific (napus-rapa)	0.8181818	0.8100924	not scorable	not scorable	0.9752066	0.7968768
1906	Bn-A01-p3306526	A-genome specific (napus-rapa)	0.9641148	0.7555211	not scorable	not scorable	0.9834711	0.784013
1907	Bn-A01-p3307173	A-genome specific (napus-rapa)	0.9832536	0.4445606	not scorable	not scorable	0.9917355	0.6905535
1908	Bn-A01-p3323476	A-genome specific (napus-rapa)	0.9904306	0.8398063	not scorable	not scorable	0.9752066	0.6787765
1909	Bn-A01-p333856	A-genome specific (rapa only)	not scorable	not scorable	not scorable	not scorable	0.9752066	0.6435884
1910	Bn-A01-p3378332	genome unspecific (all in)	0.9880383	0.7875119	0.3643411	0.783221	1	0.6674224
1911	Bn-A01-p340954	A-genome specific (rapa only)	not scorable	not scorable	not scorable	not scorable	0.9752066	0.618829

1912	Bn-A01-p344884	A-genome specific (napus-rapa)	0.9976076	0.3728947	not scorable	not scorable	0.661157	0.7944186
1913	Bn-A01-p345182	A-genome specific (napus-rapa)	0.9952153	0.496635	not scorable	not scorable	1	0.6711772
1914	Bn-A01-p3487320	A-genome specific (napus-rapa)	0.9665072	0.6719519	not scorable	not scorable	0.9917355	0.7242321
1915	Bn-A01-p3495476	A-genome specific (napus-rapa)	0.9832536	0.6147087	not scorable	not scorable	1	0.7745915
1916	Bn-A01-p3567127	A-genome specific (napus-rapa)	0.9808612	0.8181761	not scorable	not scorable	1	0.7503445
1917	Bn-A01-p3573045	A-genome specific (napus-rapa)	0.9880383	0.4627736	not scorable	not scorable	1	0.9205657
1918	Bn-A01-p3576068	A-genome specific (napus-rapa)	0.9712918	0.8186768	not scorable	not scorable	0.9752066	0.7677296
1919	Bn-A01-p3579887	A-genome specific (napus-rapa)	0.9760765	0.7375665	not scorable	not scorable	1	0.7242132
1920	Bn-A01-p3586744	A-genome specific (napus-rapa)	0.9736842	0.7886152	not scorable	not scorable	0.9917355	0.8249918
1921	Bn-A01-p3597929	A-genome specific (napus-rapa)	0.9856459	0.790275	not scorable	not scorable	0.9917355	0.8355395
1922	Bn-A01-p3598097	A-genome specific (napus-rapa)	0.9880383	0.7476067	not scorable	not scorable	0.9917355	0.6963976
1923	Bn-A01-p3598515	A-genome specific (napus-rapa)	0.9856459	0.7742741	not scorable	not scorable	1	0.7201275
1924	Bn-A01-p3598533	A-genome specific (napus-rapa)	0.9880383	0.7938583	not scorable	not scorable	1	0.7693703
1925	Bn-A01-p3599097	A-genome specific (napus-rapa)	0.9976076	0.4714738	not scorable	not scorable	0.9917355	0.6789401
1926	Bn-A01-p3610530	A-genome specific (napus-rapa)	0.9952153	0.4284107	not scorable	not scorable	1	0.7069653
1927	Bn-A01-p3619975	A-genome specific (napus-rapa)	0.9808612	0.8189453	not scorable	not scorable	0.9834711	0.7821474
1928	Bn-A01-p3626821	A-genome specific (napus-rapa)	0.9904306	0.5621526	not scorable	not scorable	0.9917355	0.5647066
1929	Bn-A01-p3631990	A-genome specific (napus-rapa)	0.9976076	0.4501518	not scorable	not scorable	0.9917355	0.7579525
1930	Bn-A01-p3640191	A-genome specific (rapa only)	not scorable	not scorable	not scorable	not scorable	0.9917355	0.6538121
1931	Bn-A01-p3643352	A-genome specific (napus-rapa)	0.9808612	0.3401394	not scorable	not scorable	1	0.5272361
1932	Bn-A01-p3644770	A-genome specific (napus-rapa)	0.9904306	0.3707033	not scorable	not scorable	0.9917355	0.7095698
1933	Bn-A01-p3664698	A-genome specific (napus-rapa)	0.9976076	0.5467758	not scorable	not scorable	1	0.8300927
1934	Bn-A01-p3666241	A-genome specific (napus-rapa)	0.9976076	0.4607628	not scorable	not scorable	0.9917355	0.646854
1935	Bn-A01-p3703676	A-genome specific (napus-rapa)	1	0.8579717	not scorable	not scorable	1	0.8272811
1936	Bn-A01-p3707366	A-genome specific (napus-rapa)	0.9880383	0.3699408	not scorable	not scorable	1	0.8083752
1937	Bn-A01-p3713266	A-genome specific (napus-rapa)	0.9808612	0.3131576	not scorable	not scorable	0.9917355	0.6393247
1938	Bn-A01-p3737522	A-genome specific (napus-rapa)	0.9712918	0.6801396	not scorable	not scorable	0.9917355	0.7063222
1939	Bn-A01-p3744726	A-genome specific (napus-rapa)	0.9880383	0.2810631	not scorable	not scorable	0.9669421	0.6993529
1940	Bn-A01-p3755849	A-genome specific (napus-rapa)	0.9760765	0.7949107	not scorable	not scorable	0.9834711	0.7691048
1941	Bn-A01-p3758784	A-genome specific (napus-rapa)	0.9760765	0.8023077	not scorable	not scorable	0.9834711	0.6898049
1942	Bn-A01-p3782356	A-genome specific (napus-rapa)	1	0.4761784	not scorable	not scorable	0.9917355	0.734802
1943	Bn-A01-p3789302	A-genome specific (napus-rapa)	0.9856459	0.7144525	not scorable	not scorable	1	0.7264047
1944	Bn-A01-p3791028	A-genome specific (napus-rapa)	0.9928229	0.6827036	not scorable	not scorable	1	0.8175309

1945	Bn-A01-p3829226	A-genome specific (rapa only)	not scorable	not scorable	not scorable	not scorable	0.9752066	0.7902855
1946	Bn-A01-p3829785	A-genome specific (napus-rapa)	0.9569378	0.2908964	not scorable	not scorable	1	0.6784846
1947	Bn-A01-p3858902	A-genome specific (napus-rapa)	0.9904306	0.3400923	not scorable	not scorable	1	0.7006515
1948	Bn-A01-p3859106	A-genome specific (napus-rapa)	0.9904306	0.6617844	not scorable	not scorable	0.9917355	0.6067287
1949	Bn-A01-p3859610	A-genome specific (napus-rapa)	0.9904306	0.7908252	not scorable	not scorable	0.9834711	0.830327
1950	Bn-A01-p3889035	A-genome specific (napus-rapa)	0.9976076	0.6098653	not scorable	not scorable	0.9917355	0.6420069
1951	Bn-A01-p3892184	A-genome specific (napus-rapa)	0.9952153	0.5233259	not scorable	not scorable	0.9917355	0.7596947
1952	Bn-A01-p3904495	A-genome specific (napus-rapa)	0.9880383	0.8255144	not scorable	not scorable	0.9834711	0.7869604
1953	Bn-A01-p3906965	A-genome specific (napus-rapa)	0.9928229	0.7953025	not scorable	not scorable	0.9834711	0.7001463
1954	Bn-A01-p3935187	A-genome specific (napus-rapa)	0.0430622	0.7860854	not scorable	not scorable	0.6280992	0.8128915
1955	Bn-A01-p3938241	A-genome specific (napus-rapa)	0.9952153	0.7635738	not scorable	not scorable	0.7190083	0.8052166
1956	Bn-A01-p3939508	A-genome specific (napus-rapa)	0.9952153	0.6447311	not scorable	not scorable	0.9917355	0.7087289
1957	Bn-A01-p3948389	A-genome specific (napus-rapa)	0.9976076	0.3568731	not scorable	not scorable	1	0.8207335
1958	Bn-A01-p3984534	A-genome specific (napus-rapa)	0.9784689	0.6183667	not scorable	not scorable	0.9586777	0.8106123
1959	Bn-A01-p3993078	A-genome specific (napus-rapa)	0.9976076	0.3399651	not scorable	not scorable	1	0.7353838
1960	Bn-A01-p4003991	A-genome specific (napus-rapa)	0.9617225	0.8126613	not scorable	not scorable	0.9669421	0.6949151
1961	Bn-A01-p4023655	A-genome specific (napus-rapa)	0.9928229	0.7605813	not scorable	not scorable	1	0.7286872
1962	Bn-A01-p4027402	A-genome specific (napus-rapa)	0.9928229	0.7933981	not scorable	not scorable	0.9834711	0.7935458
1963	Bn-A01-p4041055	A-genome specific (napus-rapa)	0.9665072	0.7576659	not scorable	not scorable	0.9917355	0.6273046
1964	Bn-A01-p4070059	A-genome specific (napus-rapa)	0.9688995	0.733361	not scorable	not scorable	0.9752066	0.7774941
1965	Bn-A01-p4077105	A-genome specific (napus-rapa)	0.7416268	0.7789842	not scorable	not scorable	0.9752066	0.6770623
1966	Bn-A01-p4084366	A-genome specific (napus-rapa)	0.9760765	0.686331	not scorable	not scorable	0.9917355	0.6950665
1967	Bn-A01-p4084739	A-genome specific (napus-rapa)	0.9832536	0.2971842	not scorable	not scorable	0.9917355	0.6656501
1968	Bn-A01-p4087533	A-genome specific (napus-rapa)	0.9688995	0.793394	not scorable	not scorable	1	0.777238
1969	Bn-A01-p4109157	A-genome specific (napus-rapa)	0.9976076	0.4794883	not scorable	not scorable	1	0.6742547
1970	Bn-A01-p4113202	A-genome specific (napus-rapa)	0.8923445	0.7826226	not scorable	not scorable	0.9917355	0.7657975
1971	Bn-A01-p4116110	A-genome specific (napus-rapa)	0.9904306	0.2814051	not scorable	not scorable	0.9917355	0.654273
1972	Bn-A01-p4117153	A-genome specific (rapa only)	not scorable	not scorable	not scorable	not scorable	0.6363636	0.8286211
1973	Bn-A01-p4164843	A-genome specific (napus-rapa)	0.9928229	0.8209696	not scorable	not scorable	1	0.7588842
1974	Bn-A01-p4167795	A-genome specific (rapa only)	not scorable	not scorable	not scorable	not scorable	1	0.9272014
1975	Bn-A01-p4173668	A-genome specific (napus-rapa)	0.9784689	0.7497423	not scorable	not scorable	0.9834711	0.6916872
1976	Bn-A01-p4177941	A-genome specific (napus-rapa)	0.9928229	0.724332	not scorable	not scorable	1	0.7427279
1977	Bn-A01-p4188629	A-genome specific (napus-rapa)	0.9808612	0.5971491	not scorable	not scorable	1	0.7580498

1978	Bn-A01-p4220987	A-genome specific (napus-rapa)	0.6889952	0.4224357	not scorable	not scorable	1	0.9115393
1979	Bn-A01-p4227703	A-genome specific (napus-rapa)	0.9832536	0.5570098	not scorable	not scorable	0.9752066	0.5284312
1980	Bn-A01-p4228985	A-genome specific (napus-rapa)	0.9952153	0.7630062	not scorable	not scorable	1	0.6986982
1981	Bn-A01-p4230829	A-genome specific (napus-rapa)	0.9880383	0.5348946	not scorable	not scorable	0.9752066	0.6185827
1982	Bn-A01-p4233306	A-genome specific (napus-rapa)	0.9641148	0.7929199	not scorable	not scorable	1	0.6154903
1983	Bn-A01-p4235273	A-genome specific (napus-rapa)	0.9904306	0.4592314	not scorable	not scorable	1	0.73063
1984	Bn-A01-p4242269	A-genome specific (napus-rapa)	0.9736842	0.5961131	not scorable	not scorable	1	0.7361456
1985	Bn-A01-p4246586	A-genome specific (napus-rapa)	0.9760765	0.4423441	not scorable	not scorable	0.9917355	0.7927324
1986	Bn-A01-p4258195	A-genome specific (napus-rapa)	0.9569378	0.3858768	not scorable	not scorable	0.9834711	0.7729093
1987	Bn-A01-p4284958	A-genome specific (napus-rapa)	0.9760765	0.6106357	not scorable	not scorable	1	0.6961098
1988	Bn-A01-p4288869	A-genome specific (rapa only)	not scorable	not scorable	not scorable	not scorable	0.9752066	0.8584366
1989	Bn-A01-p4288938	A-genome specific (rapa only)	not scorable	not scorable	not scorable	not scorable	0.9752066	0.6310735
1990	Bn-A01-p4289535	A-genome specific (rapa only)	not scorable	not scorable	not scorable	not scorable	0.7024794	0.7914145
1991	Bn-A01-p4293847	A-genome specific (napus-rapa)	0.9952153	0.5026233	not scorable	not scorable	1	0.7940474
1992	Bn-A01-p4293878	A-genome specific (napus-rapa)	0.9688995	0.7835191	not scorable	not scorable	1	0.7495431
1993	Bn-A01-p4303527	A-genome specific (napus-rapa)	0.9641148	0.3598139	not scorable	not scorable	0.9752066	0.6726535
1994	Bn-A01-p4310302	A-genome specific (napus-rapa)	0.9976076	0.6587597	not scorable	not scorable	0.9834711	0.7331177
1995	Bn-A01-p4311134	A-genome specific (napus-rapa)	0.9497608	0.7890669	not scorable	not scorable	0.9834711	0.8010122
1996	Bn-A01-p4322340	A-genome specific (napus-rapa)	0.8301435	0.8004053	not scorable	not scorable	0.7272727	0.8122094
1997	Bn-A01-p4325162	genome unspecific (oleracea-rapa)	not scorable	not scorable	0.9767442	0.3321356	1	0.7602208
1998	Bn-A01-p4335144	A-genome specific (rapa only)	not scorable	not scorable	not scorable	not scorable	1	0.7234482
1999	Bn-A01-p4341541	A-genome specific (napus-rapa)	0.9976076	0.5103784	not scorable	not scorable	0.9834711	0.6772383
2000	Bn-A01-p4353657	A-genome specific (napus-rapa)	0.9928229	0.7368858	not scorable	not scorable	0.9917355	0.7499076
2001	Bn-A01-p4354753	A-genome specific (napus-rapa)	0.9832536	0.7596107	not scorable	not scorable	0.9917355	0.8092792
2002	Bn-A01-p436141	A-genome specific (napus-rapa)	0.9808612	0.6967064	not scorable	not scorable	0.9834711	0.7605804
2003	Bn-A01-p4377760	A-genome specific (napus-rapa)	0.9808612	0.6977333	not scorable	not scorable	1	0.7723418
2004	Bn-A01-p4378321	A-genome specific (napus-rapa)	0.9808612	0.6627697	not scorable	not scorable	0.9917355	0.876231
2005	Bn-A01-p4390611	A-genome specific (napus-rapa)	1	0.4343343	not scorable	not scorable	1	0.7546347
2006	Bn-A01-p4394660	A-genome specific (napus-rapa)	1	0.3066774	not scorable	not scorable	1	0.7326872
2007	Bn-A01-p4419443	A-genome specific (napus-rapa)	0.9162679	0.670675	not scorable	not scorable	0.9834711	0.6981179
2008	Bn-A01-p442492	A-genome specific (napus-rapa)	0.9904306	0.3415146	not scorable	not scorable	1	0.7647003
2009	Bn-A01-p4452779	A-genome specific (napus-rapa)	0.9641148	0.668288	not scorable	not scorable	0.9504132	0.5985798
2010	Bn-A01-p4499485	A-genome specific (napus-rapa)	0.9808612	0.741653	not scorable	not scorable	0.9917355	0.7932042

2011	Bn-A01-p4506540	A-genome specific (napus-rapa)	0.9808612	0.3264243	not scorable	not scorable	1	0.8013151
2012	Bn-A01-p4518460	A-genome specific (napus-rapa)	0.9688995	0.4225649	not scorable	not scorable	0.9917355	0.7643929
2013	Bn-A01-p4519762	A-genome specific (napus-rapa)	0.9856459	0.4314763	not scorable	not scorable	0.9917355	0.6781538
2014	Bn-A01-p4549564	A-genome specific (napus-rapa)	0.9808612	0.7590743	not scorable	not scorable	0.9834711	0.7560032
2015	Bn-A01-p4580719	A-genome specific (napus-rapa)	0.8564593	0.6595703	not scorable	not scorable	1	0.8018597
2016	Bn-A01-p460072	A-genome specific (napus-rapa)	0.9712918	0.76019	not scorable	not scorable	0.6115702	0.691606
2017	Bn-A01-p4600975	A-genome specific (napus-rapa)	0.9880383	0.45643	not scorable	not scorable	1	0.8114882
2018	Bn-A01-p4612881	A-genome specific (napus-rapa)	0.9808612	0.7932433	not scorable	not scorable	1	0.7341067
2019	Bn-A01-p4635210	A-genome specific (rapa only)	not scorable	not scorable	not scorable	not scorable	0.9917355	0.6508822
2020	Bn-A01-p4641747	A-genome specific (napus-rapa)	0.9856459	0.7474229	not scorable	not scorable	0.9917355	0.8024733
2021	Bn-A01-p4641802	A-genome specific (napus-rapa)	0.9856459	0.7496878	not scorable	not scorable	0.9917355	0.6713664
2022	Bn-A01-p4651746	A-genome specific (napus-rapa)	0.9760765	0.737113	not scorable	not scorable	1	0.6510744
2023	Bn-A01-p465783	A-genome specific (rapa only)	not scorable	not scorable	not scorable	not scorable	0.9917355	0.7984232
2024	Bn-A01-p4659972	A-genome specific (napus-rapa)	0.9928229	0.6505904	not scorable	not scorable	0.9752066	0.6963493
2025	Bn-A01-p467816	A-genome specific (napus-rapa)	0.9832536	0.7639633	not scorable	not scorable	0.9917355	0.7160303
2026	Bn-A01-p4734188	A-genome specific (napus-rapa)	0.9976076	0.5797902	not scorable	not scorable	0.9917355	0.721589
2027	Bn-A01-p4736924	A-genome specific (napus-rapa)	0.9449761	0.9006857	not scorable	not scorable	0.9834711	0.8178453
2028	Bn-A01-p4747874	A-genome specific (napus-rapa)	0.9784689	0.7041543	not scorable	not scorable	0.9752066	0.7544323
2029	Bn-A01-p4750734	A-genome specific (napus-rapa)	0.9736842	0.80375	not scorable	not scorable	0.9917355	0.8354823
2030	Bn-A01-p4759559	A-genome specific (rapa only)	not scorable	not scorable	not scorable	not scorable	1	0.8309966
2031	Bn-A01-p4773772	A-genome specific (napus-rapa)	0.9880383	0.7483223	not scorable	not scorable	0.9917355	0.8155614
2032	Bn-A01-p4774065	A-genome specific (napus-rapa)	0.9904306	0.8091096	not scorable	not scorable	0.9917355	0.7436564
2033	Bn-A01-p4785261	A-genome specific (napus-rapa)	0.9856459	0.7720748	not scorable	not scorable	0.9834711	0.7886267
2034	Bn-A01-p4786653	A-genome specific (napus-rapa)	0.9976076	0.6571602	not scorable	not scorable	1	0.7455136
2035	Bn-A01-p4786754	A-genome specific (napus-rapa)	0.9976076	0.7961323	not scorable	not scorable	0.9834711	0.7601196
2036	Bn-A01-p4786787	A-genome specific (napus-rapa)	0.9880383	0.7768333	not scorable	not scorable	0.9834711	0.6978854
2037	Bn-A01-p4787515	A-genome specific (napus-rapa)	0.9928229	0.483084	not scorable	not scorable	1	0.6188304
2038	Bn-A01-p4799050	A-genome specific (napus-rapa)	0.9904306	0.7891912	not scorable	not scorable	0.9834711	0.7973623
2039	Bn-A01-p4800440	A-genome specific (napus-rapa)	0.9904306	0.2792964	not scorable	not scorable	0.9917355	0.730725
2040	Bn-A01-p4803773	A-genome specific (napus-rapa)	0.9832536	0.4494584	not scorable	not scorable	1	0.802505
2041	Bn-A01-p4808404	A-genome specific (napus-rapa)	0.9736842	0.7407727	not scorable	not scorable	0.9917355	0.7580041
2042	Bn-A01-p4812269	A-genome specific (napus-rapa)	0.9760765	0.5209119	not scorable	not scorable	0.9834711	0.7646228
2043	Bn-A01-p4820187	A-genome specific (napus-rapa)	0.9952153	0.4907919	not scorable	not scorable	0.9917355	0.8330544

2044	Bn-A01-p4826685	A-genome specific (rapa only)	not scorable	not scorable	not scorable	not scorable	0.8595041	0.832737
2045	Bn-A01-p4832074	A-genome specific (napus-rapa)	0.9928229	0.7329871	not scorable	not scorable	1	0.8088252
2046	Bn-A01-p4833020	A-genome specific (napus-rapa)	0.9856459	0.8222839	not scorable	not scorable	0.9917355	0.7895895
2047	Bn-A01-p483495	A-genome specific (napus-rapa)	1	0.3150097	not scorable	not scorable	0.9834711	0.7635671
2048	Bn-A01-p4836338	A-genome specific (napus-rapa)	0.9880383	0.7789082	not scorable	not scorable	0.9752066	0.7507509
2049	Bn-A01-p4840973	A-genome specific (napus-rapa)	1	0.4826368	not scorable	not scorable	1	0.7042567
2050	Bn-A01-p4869879	A-genome specific (napus-rapa)	0.9593301	0.6718714	not scorable	not scorable	0.9752066	0.8061771
2051	Bn-A01-p4874972	A-genome specific (napus-rapa)	0.9449761	0.4059836	not scorable	not scorable	0.9834711	0.7601165
2052	Bn-A01-p4877343	A-genome specific (napus-rapa)	0.9736842	0.3981194	not scorable	not scorable	0.9669421	0.7427683
2053	Bn-A01-p4883188	A-genome specific (napus-rapa)	0.9880383	0.7749712	not scorable	not scorable	0.9834711	0.7397466
2054	Bn-A01-p4908924	A-genome specific (napus-rapa)	0.9784689	0.7889029	not scorable	not scorable	0.9834711	0.7143246
2055	Bn-A01-p4909065	A-genome specific (napus-rapa)	0.9856459	0.7772519	not scorable	not scorable	1	0.7478473
2056	Bn-A01-p4920622	A-genome specific (napus-rapa)	0.9904306	0.3423496	not scorable	not scorable	0.9917355	0.7863657
2057	Bn-A01-p4922557	A-genome specific (napus-rapa)	0.9952153	0.4569855	not scorable	not scorable	1	0.7854537
2058	Bn-A01-p4925004	A-genome specific (napus-rapa)	0.9880383	0.3037469	not scorable	not scorable	0.9917355	0.7730688
2059	Bn-A01-p4940549	A-genome specific (napus-rapa)	0.9928229	0.7686237	not scorable	not scorable	1	0.7127941
2060	Bn-A01-p4947577	A-genome specific (napus-rapa)	0.9880383	0.4240515	not scorable	not scorable	0.9917355	0.6772852
2061	Bn-A01-p4948316	genome unspecific (oleracea-rapa)	not scorable	not scorable	1	0.7319669	1	0.7374497
2062	Bn-A01-p4963297	A-genome specific (napus-rapa)	0.9832536	0.6070957	not scorable	not scorable	0.9917355	0.8610334
2063	Bn-A01-p4985329	A-genome specific (napus-rapa)	0.9784689	0.8136621	not scorable	not scorable	0.9669421	0.8289551
2064	Bn-A01-p4998346	A-genome specific (rapa only)	not scorable	not scorable	not scorable	not scorable	1	0.7691226
2065	Bn-A01-p5017444	A-genome specific (napus-rapa)	1	0.4861147	not scorable	not scorable	1	0.8230196
2066	Bn-A01-p5017446	A-genome specific (napus-rapa)	1	0.4244876	not scorable	not scorable	0.9917355	0.775295
2067	Bn-A01-p5020890	A-genome specific (napus-rapa)	1	0.4880226	not scorable	not scorable	0.9669421	0.8105909
2068	Bn-A01-p5057753	A-genome specific (napus-rapa)	0.9904306	0.460929	not scorable	not scorable	1	0.74534
2069	Bn-A01-p5069371	A-genome specific (napus-rapa)	0.9904306	0.3857467	not scorable	not scorable	0.9669421	0.5834985
2070	Bn-A01-p5070565	A-genome specific (napus-rapa)	0.9880383	0.7840532	not scorable	not scorable	0.9752066	0.7417479
2071	Bn-A01-p5070836	A-genome specific (napus-rapa)	0.9904306	0.7360534	not scorable	not scorable	0.9752066	0.6108016
2072	Bn-A01-p5071350	A-genome specific (napus-rapa)	0.9928229	0.7461768	not scorable	not scorable	0.9834711	0.7423385
2073	Bn-A01-p5071658	A-genome specific (rapa only)	not scorable	not scorable	not scorable	not scorable	0.9917355	0.6843054
2074	Bn-A01-p5075984	A-genome specific (napus-rapa)	0.9880383	0.8043486	not scorable	not scorable	0.9752066	0.7140822
2075	Bn-A01-p5089377	A-genome specific (napus-rapa)	0.9976076	0.4774405	not scorable	not scorable	1	0.7809237
2076	Bn-A01-p5101078	A-genome specific (napus-rapa)	0.9928229	0.8002905	not scorable	not scorable	0.9917355	0.7929338

2077	Bn-A01-p5106042	A-genome specific (napus-rapa)	0.9856459	0.4505196	not scorable	not scorable	0.9917355	0.6782633
2078	Bn-A01-p511682	A-genome specific (napus-rapa)	1	0.2659173	not scorable	not scorable	0.9834711	0.7973625
2079	Bn-A01-p515600	A-genome specific (napus-rapa)	0.9688995	0.4822006	not scorable	not scorable	1	0.7068847
2080	Bn-A01-p5193639	A-genome specific (napus-rapa)	0.9976076	0.4310189	not scorable	not scorable	1	0.6963694
2081	Bn-A01-p5212238	A-genome specific (rapa only)	not scorable	not scorable	not scorable	not scorable	0.9834711	0.7183639
2082	Bn-A01-p5215209	A-genome specific (napus-rapa)	1	0.3623964	not scorable	not scorable	1	0.7779456
2083	Bn-A01-p5238564	A-genome specific (napus-rapa)	0.9928229	0.8818117	not scorable	not scorable	1	0.7597861
2084	Bn-A01-p5239959	A-genome specific (napus-rapa)	0.9976076	0.7925668	not scorable	not scorable	1	0.7448562
2085	Bn-A01-p5242967	A-genome specific (napus-rapa)	0.9880383	0.5644107	not scorable	not scorable	1	0.671823
2086	Bn-A01-p5243029	A-genome specific (napus-rapa)	0.9880383	0.8224536	not scorable	not scorable	0.9917355	0.8157201
2087	Bn-A01-p5243036	A-genome specific (napus-rapa)	0.9832536	0.6195478	not scorable	not scorable	0.9752066	0.6610458
2088	Bn-A01-p5243181	A-genome specific (napus-rapa)	0.9880383	0.7904221	not scorable	not scorable	1	0.755037
2089	Bn-A01-p5245778	A-genome specific (napus-rapa)	0.9976076	0.6725068	not scorable	not scorable	0.9834711	0.6601241
2090	Bn-A01-p5246582	A-genome specific (napus-rapa)	0.9976076	0.473164	not scorable	not scorable	1	0.6867018
2091	Bn-A01-p5259856	A-genome specific (napus-rapa)	0.9976076	0.4592904	not scorable	not scorable	1	0.7813702
2092	Bn-A01-p5265598	A-genome specific (napus-rapa)	0.9832536	0.778321	not scorable	not scorable	0.9917355	0.7906627
2093	Bn-A01-p5268871	A-genome specific (napus-rapa)	0.9976076	0.6607196	not scorable	not scorable	0.9834711	0.7921939
2094	Bn-A01-p5269368	A-genome specific (napus-rapa)	0.9688995	0.421522	not scorable	not scorable	1	0.6962355
2095	Bn-A01-p5284702	A-genome specific (napus-rapa)	0.9928229	0.7771178	not scorable	not scorable	0.9834711	0.7523236
2096	Bn-A01-p5296564	A-genome specific (napus-rapa)	0.9832536	0.7891576	not scorable	not scorable	0.9917355	0.8578618
2097	Bn-A01-p5298283	A-genome specific (napus-rapa)	0.9856459	0.3430477	not scorable	not scorable	0.9917355	0.7904419
2098	Bn-A01-p5318059	A-genome specific (napus-rapa)	0.9736842	0.7707634	not scorable	not scorable	0.9834711	0.7396865
2099	Bn-A01-p5323726	A-genome specific (napus-rapa)	0.9856459	0.5614401	not scorable	not scorable	0.9834711	0.5872219
2100	Bn-A01-p5324399	A-genome specific (napus-rapa)	0.9856459	0.7514811	not scorable	not scorable	0.9917355	0.6677213
2101	Bn-A01-p5324472	A-genome specific (napus-rapa)	0.9880383	0.7730073	not scorable	not scorable	0.9586777	0.7589369
2102	Bn-A01-p5328220	A-genome specific (napus-rapa)	0.9856459	0.823927	not scorable	not scorable	0.9917355	0.8094317
2103	Bn-A01-p5328679	A-genome specific (napus-rapa)	0.9856459	0.8220761	not scorable	not scorable	1	0.8985579
2104	Bn-A01-p5330192	A-genome specific (napus-rapa)	0.9808612	0.8155247	not scorable	not scorable	1	0.8294564
2105	Bn-A01-p5332773	A-genome specific (napus-rapa)	0.9856459	0.8290957	not scorable	not scorable	0.9917355	0.7827479
2106	Bn-A01-p5334474	A-genome specific (napus-rapa)	0.9856459	0.769523	not scorable	not scorable	1	0.7415857
2107	Bn-A01-p5335218	A-genome specific (napus-rapa)	0.9832536	0.7847264	not scorable	not scorable	1	0.7409155
2108	Bn-A01-p5335323	A-genome specific (napus-rapa)	0.9880383	0.8104	not scorable	not scorable	0.9504132	0.7273586
2109	Bn-A01-p5335372	A-genome specific (napus-rapa)	0.9928229	0.4337811	not scorable	not scorable	1	0.8146218

2110	Bn-A01-p5340953	A-genome specific (napus-rapa)	0.9904306	0.5399374	not scorable	not scorable	0.9917355	0.8212364
2111	Bn-A01-p5357589	A-genome specific (napus-rapa)	0.9952153	0.5376253	not scorable	not scorable	1	0.7292859
2112	Bn-A01-p5374990	A-genome specific (napus-rapa)	1	0.4482889	not scorable	not scorable	1	0.4893315
2113	Bn-A01-p5378430	A-genome specific (napus-rapa)	0.9904306	0.7723417	not scorable	not scorable	1	0.7518216
2114	Bn-A01-p5385928	A-genome specific (napus-rapa)	1	0.3575064	not scorable	not scorable	1	0.8192832
2115	Bn-A01-p5391551	A-genome specific (napus-rapa)	0.9904306	0.7306954	not scorable	not scorable	0.9917355	0.745652
2116	Bn-A01-p5418396	A-genome specific (napus-rapa)	0.9904306	0.7510388	not scorable	not scorable	0.9917355	0.6882154
2117	Bn-A01-p5469727	A-genome specific (napus-rapa)	0.9952153	0.7539974	not scorable	not scorable	0.9834711	0.7471559
2118	Bn-A01-p5480514	A-genome specific (napus-rapa)	0.9760765	0.8059107	not scorable	not scorable	0.9917355	0.7204049
2119	Bn-A01-p5480517	A-genome specific (napus-rapa)	0.9736842	0.7777247	not scorable	not scorable	1	0.6935188
2120	Bn-A01-p5481004	A-genome specific (napus-rapa)	0.9880383	0.8053382	not scorable	not scorable	0.9834711	0.7625436
2121	Bn-A01-p5485980	A-genome specific (napus-rapa)	0.9856459	0.6330682	not scorable	not scorable	0.9752066	0.656383
2122	Bn-A01-p5486104	A-genome specific (napus-rapa)	0.9880383	0.6369889	not scorable	not scorable	0.9752066	0.6756431
2123	Bn-A01-p5499353	A-genome specific (napus-rapa)	0.9976076	0.3134699	not scorable	not scorable	1	0.7913718
2124	Bn-A01-p5506421	genome unspecific (oleracea-rapa)	not scorable	not scorable	0.9147287	0.7307861	1	0.8190892
2125	Bn-A01-p5508840	A-genome specific (napus-rapa)	0.9808612	0.3190888	not scorable	not scorable	0.9752066	0.7559415
2126	Bn-A01-p5508998	A-genome specific (napus-rapa)	0.9760765	0.4693629	not scorable	not scorable	0.9421487	0.8740647
2127	Bn-A01-p5512	A-genome specific (napus-rapa)	0.9497608	0.7785608	not scorable	not scorable	0.9752066	0.759106
2128	Bn-A01-p5522403	A-genome specific (napus-rapa)	0.9569378	0.4154296	not scorable	not scorable	0.9752066	0.7721131
2129	Bn-A01-p5522870	A-genome specific (napus-rapa)	0.9665072	0.7970471	not scorable	not scorable	1	0.8100895
2130	Bn-A01-p5535282	A-genome specific (rapa only)	not scorable	not scorable	not scorable	not scorable	1	0.951578
2131	Bn-A01-p5536695	A-genome specific (napus-rapa)	0.9641148	0.5886007	not scorable	not scorable	1	0.7606366
2132	Bn-A01-p5550226	A-genome specific (napus-rapa)	0.9736842	0.7764096	not scorable	not scorable	0.9834711	0.7165308
2133	Bn-A01-p5553918	A-genome specific (napus-rapa)	0.9976076	0.2813197	not scorable	not scorable	0.9917355	0.7434332
2134	Bn-A01-p5567077	A-genome specific (napus-rapa)	0.9808612	0.7492032	not scorable	not scorable	0.9504132	0.8040844
2135	Bn-A01-p5567840	A-genome specific (napus-rapa)	0.5645933	0.7692157	not scorable	not scorable	1	0.7827143
2136	Bn-A01-p5570110	A-genome specific (napus-rapa)	0.9784689	0.6138938	not scorable	not scorable	0.9917355	0.6514556
2137	Bn-A01-p5570221	A-genome specific (napus-rapa)	0.9258373	0.7212295	not scorable	not scorable	0.9917355	0.8147714
2138	Bn-A01-p5579695	A-genome specific (napus-rapa)	1	0.8207097	not scorable	not scorable	1	0.8207246
2139	Bn-A01-p5585071	A-genome specific (napus-rapa)	0.9736842	0.4355158	not scorable	not scorable	0.9917355	0.788171
2140	Bn-A01-p5585933	A-genome specific (napus-rapa)	0.6961722	0.7830566	not scorable	not scorable	0.9504132	0.8255201
2141	Bn-A01-p5586618	A-genome specific (napus-rapa)	0.9617225	0.7954295	not scorable	not scorable	0.9752066	0.7845778
2142	Bn-A01-p5586671	A-genome specific (napus-rapa)	0.9545454	0.7930225	not scorable	not scorable	0.9834711	0.7634333

2143	Bn-A01-p5586854	A-genome specific (napus-rapa)	0.9808612	0.7582862	not scorable	not scorable	0.9917355	0.6768746
2144	Bn-A01-p5587840	A-genome specific (napus-rapa)	0.9784689	0.7797007	not scorable	not scorable	0.9917355	0.7020786
2145	Bn-A01-p5590998	A-genome specific (napus-rapa)	0.9665072	0.7757873	not scorable	not scorable	0.9917355	0.8386104
2146	Bn-A01-p5591950	A-genome specific (rapa only)	not scorable	not scorable	not scorable	not scorable	0.9917355	0.6990972
2147	Bn-A01-p5593317	A-genome specific (napus-rapa)	0.9186603	0.1788501	not scorable	not scorable	0.9669421	0.8678293
2148	Bn-A01-p5593369	A-genome specific (rapa only)	not scorable	not scorable	not scorable	not scorable	0.9917355	0.7192603
2149	Bn-A01-p5595319	A-genome specific (napus-rapa)	0.9641148	0.6990168	not scorable	not scorable	1	0.8317366
2150	Bn-A01-p5599369	A-genome specific (napus-rapa)	0.9808612	0.4604861	not scorable	not scorable	0.9917355	0.74865
2151	Bn-A01-p5601232	A-genome specific (napus-rapa)	0.9712918	0.7683066	not scorable	not scorable	0.9834711	0.7597882
2152	Bn-A01-p5621392	A-genome specific (napus-rapa)	1	0.5693927	not scorable	not scorable	0.9669421	0.5432907
2153	Bn-A01-p5635471	A-genome specific (napus-rapa)	0.9904306	0.3406782	not scorable	not scorable	0.9917355	0.7091282
2154	Bn-A01-p5647795	A-genome specific (napus-rapa)	0.9736842	0.7877428	not scorable	not scorable	0.9834711	0.7709503
2155	Bn-A01-p5648120	A-genome specific (napus-rapa)	0.9736842	0.3239561	not scorable	not scorable	0.9917355	0.7437051
2156	Bn-A01-p5658004	A-genome specific (napus-rapa)	0.9043062	0.342047	not scorable	not scorable	0.9586777	0.4271367
2157	Bn-A01-p5659083	A-genome specific (napus-rapa)	0.9593301	0.255041	not scorable	not scorable	0.9917355	0.7796705
2158	Bn-A01-p5660238	A-genome specific (napus-rapa)	0.9976076	0.265656	not scorable	not scorable	0.9752066	0.751285
2159	Bn-A01-p5661294	A-genome specific (napus-rapa)	0.9880383	0.7955012	not scorable	not scorable	1	0.802054
2160	Bn-A01-p5663319	A-genome specific (napus-rapa)	0.9832536	0.6906137	not scorable	not scorable	1	0.7714279
2161	Bn-A01-p5665774	A-genome specific (napus-rapa)	0.9856459	0.7763845	not scorable	not scorable	1	0.8286185
2162	Bn-A01-p5678678	A-genome specific (napus-rapa)	0.9832536	0.7891049	not scorable	not scorable	1	0.7650721
2163	Bn-A01-p5679389	A-genome specific (napus-rapa)	0.9832536	0.7918617	not scorable	not scorable	1	0.7799536
2164	Bn-A01-p5680581	A-genome specific (napus-rapa)	0.9856459	0.5880549	not scorable	not scorable	1	0.7164133
2165	Bn-A01-p5680730	A-genome specific (napus-rapa)	0.9832536	0.6427014	not scorable	not scorable	1	0.7245708
2166	Bn-A01-p5688168	A-genome specific (napus-rapa)	0.9832536	0.8187027	not scorable	not scorable	0.9752066	0.8422864
2167	Bn-A01-p5697529	genome unspecific (oleracea-rapa)	not scorable	not scorable	1	0.8311134	1	0.7713938
2168	Bn-A01-p5697706	A-genome specific (napus-rapa)	0.9569378	0.4799112	not scorable	not scorable	0.9834711	0.7375431
2169	Bn-A01-p5699110	A-genome specific (napus-rapa)	0.9832536	0.7823582	not scorable	not scorable	0.9917355	0.8342558
2170	Bn-A01-p5711920	A-genome specific (napus-rapa)	0.9952153	0.3571143	not scorable	not scorable	1	0.7794638
2171	Bn-A01-p5715141	A-genome specific (napus-rapa)	0.9904306	0.7464477	not scorable	not scorable	1	0.8172321
2172	Bn-A01-p5718049	A-genome specific (napus-rapa)	0.9808612	0.7441888	not scorable	not scorable	1	0.8043071
2173	Bn-A01-p5730640	A-genome specific (napus-rapa)	0.9808612	0.3155487	not scorable	not scorable	0.9752066	0.5122095
2174	Bn-A01-p5731429	A-genome specific (napus-rapa)	0.9401914	0.7437899	not scorable	not scorable	0.9917355	0.7120453
2175	Bn-A01-p5731932	A-genome specific (napus-rapa)	0.9832536	0.6795269	not scorable	not scorable	1	0.6095803

2176	Bn-A01-p5732226	A-genome specific (napus-rapa)	0.9593301	0.7638625	not scorable	not scorable	0.9834711	0.8456624
2177	Bn-A01-p5737901	A-genome specific (napus-rapa)	0.9856459	0.7434707	not scorable	not scorable	0.9752066	0.7544049
2178	Bn-A01-p5752342	A-genome specific (napus-rapa)	0.9832536	0.7855351	not scorable	not scorable	1	0.7941739
2179	Bn-A01-p5762190	A-genome specific (napus-rapa)	0.9425837	0.7217531	not scorable	not scorable	0.9834711	0.7455346
2180	Bn-A01-p5775331	A-genome specific (napus-rapa)	0.9880383	0.8044859	not scorable	not scorable	0.9917355	0.7391033
2181	Bn-A01-p5777117	A-genome specific (napus-rapa)	0.9904306	0.7685633	not scorable	not scorable	1	0.7447929
2182	Bn-A01-p5781261	A-genome specific (napus-rapa)	0.9545454	0.6577423	not scorable	not scorable	0.9834711	0.7491552
2183	Bn-A01-p5787591	A-genome specific (napus-rapa)	0.9904306	0.7816634	not scorable	not scorable	1	0.7606892
2184	Bn-A01-p5795016	A-genome specific (napus-rapa)	0.9904306	0.7579244	not scorable	not scorable	1	0.7593027
2185	Bn-A01-p5796314	all not scorable	not scorable	not scorable	not scorable	not scorable	not scorable	not scorable
2186	Bn-A01-p5796425	A-genome specific (napus-rapa)	0.9425837	0.6601626	not scorable	not scorable	0.9917355	0.6673819
2187	Bn-A01-p5797296	napus specific	0.9880383	0.3113802	not scorable	not scorable	not scorable	not scorable
2188	Bn-A01-p5797810	genome unspecific (oleracea-rapa)	not scorable	not scorable	0.9069768	0.7020756	0.9669421	0.8012508
2189	Bn-A01-p5805154	A-genome specific (napus-rapa)	0.9880383	0.767651	not scorable	not scorable	1	0.6725802
2190	Bn-A01-p5806635	A-genome specific (napus-rapa)	0.9952153	0.5076189	not scorable	not scorable	0.9917355	0.8203074
2191	Bn-A01-p5808036	A-genome specific (napus-rapa)	0.9665072	0.7336619	not scorable	not scorable	0.9421487	0.8017119
2192	Bn-A01-p5821651	A-genome specific (napus-rapa)	0.9617225	0.4636785	not scorable	not scorable	0.9917355	0.8140017
2193	Bn-A01-p5822015	A-genome specific (napus-rapa)	0.9593301	0.7860306	not scorable	not scorable	0.9669421	0.8255906
2194	Bn-A01-p5835558	A-genome specific (napus-rapa)	0.9856459	0.7031496	not scorable	not scorable	0.9917355	0.7530049
2195	Bn-A01-p5862558	A-genome specific (napus-rapa)	0.9808612	0.8018116	not scorable	not scorable	0.9752066	0.8050262
2196	Bn-A01-p5868598	A-genome specific (napus-rapa)	0.9712918	0.7711254	not scorable	not scorable	0.9586777	0.69955
2197	Bn-A01-p5873504	A-genome specific (napus-rapa)	0.9904306	0.7734785	not scorable	not scorable	0.9917355	0.8293523
2198	Bn-A01-p5878485	A-genome specific (napus-rapa)	0.9784689	0.7783518	not scorable	not scorable	0.9834711	0.7171305
2199	Bn-A01-p5886182	A-genome specific (napus-rapa)	1	0.4112878	not scorable	not scorable	1	0.7999297
2200	Bn-A01-p5899092	A-genome specific (napus-rapa)	0.9138756	0.6433278	not scorable	not scorable	1	0.7680961
2201	Bn-A01-p5903527	A-genome specific (napus-rapa)	0.9928229	0.3913113	not scorable	not scorable	0.9834711	0.6958624
2202	Bn-A01-p5917688	A-genome specific (napus-rapa)	0.9832536	0.6234019	not scorable	not scorable	0.9669421	0.6150268
2203	Bn-A01-p5918340	A-genome specific (napus-rapa)	0.9976076	0.7736933	not scorable	not scorable	0.9752066	0.832279
2204	Bn-A01-p5921497	A-genome specific (napus-rapa)	0.9832536	0.6767806	not scorable	not scorable	0.9752066	0.7048401
2205	Bn-A01-p5930704	A-genome specific (napus-rapa)	1	0.5172032	not scorable	not scorable	0.9752066	0.7344397
2206	Bn-A01-p5943392	A-genome specific (napus-rapa)	0.9952153	0.4961517	not scorable	not scorable	0.9669421	0.6773928
2207	Bn-A01-p5944398	A-genome specific (napus-rapa)	0.9808612	0.826543	not scorable	not scorable	0.9917355	0.7882035
2208	Bn-A01-p5949498	A-genome specific (napus-rapa)	0.9952153	0.4866338	not scorable	not scorable	0.9917355	0.8151706

2209	Bn-A01-p5949582	A-genome specific (napus-rapa)	0.9856459	0.4707538	not scorable	not scorable	0.9917355	0.7562752
2210	Bn-A01-p5949665	A-genome specific (napus-rapa)	0.9856459	0.4663477	not scorable	not scorable	1	0.6962676
2211	Bn-A01-p595492	A-genome specific (rapa only)	not scorable	not scorable	not scorable	not scorable	0.9256198	0.7305183
2212	Bn-A01-p595651	A-genome specific (napus-rapa)	0.9760765	0.7674024	not scorable	not scorable	0.9917355	0.7977939
2213	Bn-A01-p5959700	A-genome specific (napus-rapa)	0.9976076	0.4378778	not scorable	not scorable	1	0.7427151
2214	Bn-A01-p597363	A-genome specific (napus-rapa)	1	0.3204771	not scorable	not scorable	1	0.7883846
2215	Bn-A01-p5973643	A-genome specific (rapa only)	not scorable	not scorable	not scorable	not scorable	0.9752066	0.8495506
2216	Bn-A01-p5977595	A-genome specific (rapa only)	not scorable	not scorable	not scorable	not scorable	0.9834711	0.9175813
2217	Bn-A01-p5977820	A-genome specific (napus-rapa)	0.9856459	0.7262964	not scorable	not scorable	0.9752066	0.6786442
2218	Bn-A01-p598242	A-genome specific (napus-rapa)	0.9712918	0.5376529	not scorable	not scorable	0.9917355	0.7963605
2219	Bn-A01-p5990203	A-genome specific (napus-rapa)	0.9928229	0.4151998	not scorable	not scorable	1	0.6994829
2220	Bn-A01-p5994840	A-genome specific (napus-rapa)	0.9641148	0.7607844	not scorable	not scorable	0.9917355	0.7378923
2221	Bn-A01-p5994980	A-genome specific (napus-rapa)	0.9688995	0.7006634	not scorable	not scorable	0.9917355	0.6849162
2222	Bn-A01-p599910	A-genome specific (napus-rapa)	0.9712918	0.731526	not scorable	not scorable	0.9752066	0.5852711
2223	Bn-A01-p6007093	A-genome specific (napus-rapa)	0.9832536	0.7782392	not scorable	not scorable	0.9917355	0.8103148
2224	Bn-A01-p6035491	A-genome specific (napus-rapa)	0.9856459	0.758619	not scorable	not scorable	0.9834711	0.7686905
2225	Bn-A01-p6038249	A-genome specific (napus-rapa)	0.9832536	0.7453348	not scorable	not scorable	0.9752066	0.801205
2226	Bn-A01-p6040667	A-genome specific (napus-rapa)	0.9784689	0.6878166	not scorable	not scorable	0.9669421	0.8186894
2227	Bn-A01-p6044475	A-genome specific (napus-rapa)	0.9904306	0.7295846	not scorable	not scorable	0.9834711	0.7857201
2228	Bn-A01-p6053633	A-genome specific (napus-rapa)	0.9952153	0.7652755	not scorable	not scorable	1	0.7901177
2229	Bn-A01-p6066325	A-genome specific (napus-rapa)	1	0.5403682	not scorable	not scorable	0.9917355	0.6978582
2230	Bn-A01-p6081978	A-genome specific (napus-rapa)	0.9808612	0.7582098	not scorable	not scorable	0.9917355	0.7664619
2231	Bn-A01-p6083015	A-genome specific (napus-rapa)	1	0.1826068	not scorable	not scorable	0.9834711	0.81692
2232	Bn-A01-p6093123	A-genome specific (napus-rapa)	0.9832536	0.4043821	not scorable	not scorable	0.9752066	0.6965731
2233	Bn-A01-p609857	A-genome specific (napus-rapa)	0.9688995	0.7655809	not scorable	not scorable	1	0.7170258
2234	Bn-A01-p6111016	A-genome specific (napus-rapa)	0.9928229	0.5186349	not scorable	not scorable	1	0.7932262
2235	Bn-A01-p611977	A-genome specific (napus-rapa)	0.9736842	0.7615775	not scorable	not scorable	1	0.8450921
2236	Bn-A01-p6158889	A-genome specific (napus-rapa)	0.9904306	0.7524049	not scorable	not scorable	0.9752066	0.6986917
2237	Bn-A01-p6168163	A-genome specific (napus-rapa)	0.9784689	0.7616544	not scorable	not scorable	0.9917355	0.7859291
2238	Bn-A01-p6168191	all not scorable	not scorable	not scorable	not scorable	not scorable	not scorable	not scorable
2239	Bn-A01-p6180257	A-genome specific (napus-rapa)	0.9688995	0.7374685	not scorable	not scorable	0.9917355	0.8134049
2240	Bn-A01-p6182710	A-genome specific (napus-rapa)	0.9928229	0.7673516	not scorable	not scorable	0.9917355	0.7216988
2241	Bn-A01-p6188347	A-genome specific (napus-rapa)	0.9784689	0.7286863	not scorable	not scorable	1	0.6928239

2242	Bn-A01-p6199422	A-genome specific (rapa only)	not scorable	not scorable	not scorable	not scorable	0.9834711	0.9205657
2243	Bn-A01-p6215196	A-genome specific (napus-rapa)	1	0.3676657	not scorable	not scorable	1	0.7867026
2244	Bn-A01-p6216424	A-genome specific (napus-rapa)	1	0.304164	not scorable	not scorable	0.9917355	0.8134012
2245	Bn-A01-p6217011	A-genome specific (napus-rapa)	0.9904306	0.2954578	not scorable	not scorable	0.9917355	0.7512081
2246	Bn-A01-p6220667	A-genome specific (napus-rapa)	0.9976076	0.5097005	not scorable	not scorable	0.9917355	0.8104565
2247	Bn-A01-p622550	A-genome specific (napus-rapa)	0.9904306	0.7812953	not scorable	not scorable	0.9834711	0.8074109
2248	Bn-A01-p6232061	A-genome specific (napus-rapa)	0.9976076	0.472675	not scorable	not scorable	0.9669421	0.6426827
2249	Bn-A01-p623568	A-genome specific (napus-rapa)	1	0.8177386	not scorable	not scorable	1	0.8135551
2250	Bn-A01-p6239	A-genome specific (napus-rapa)	0.9401914	0.8139242	not scorable	not scorable	0.9834711	0.7995675
2251	Bn-A01-p6270054	A-genome specific (napus-rapa)	0.9928229	0.5981111	not scorable	not scorable	0.9917355	0.6299887
2252	Bn-A01-p6271245	A-genome specific (napus-rapa)	1	0.5554703	not scorable	not scorable	0.9834711	0.6639184
2253	Bn-A01-p6272642	A-genome specific (napus-rapa)	0.9904306	0.7962251	not scorable	not scorable	0.9834711	0.8045162
2254	Bn-A01-p6274135	A-genome specific (napus-rapa)	0.9976076	0.7401276	not scorable	not scorable	0.9752066	0.7640173
2255	Bn-A01-p6275784	A-genome specific (napus-rapa)	0.9928229	0.7751971	not scorable	not scorable	0.9917355	0.8285038
2256	Bn-A01-p6290508	A-genome specific (rapa only)	not scorable	not scorable	not scorable	not scorable	0.9834711	0.3114929
2257	Bn-A01-p6333810	A-genome specific (napus-rapa)	0.9904306	0.7003289	not scorable	not scorable	1	0.7423966
2258	Bn-A01-p6333910	A-genome specific (napus-rapa)	0.9832536	0.7911803	not scorable	not scorable	1	0.707109
2259	Bn-A01-p634701	A-genome specific (napus-rapa)	1	0.6687379	not scorable	not scorable	1	0.7590938
2260	Bn-A01-p6349933	A-genome specific (napus-rapa)	0.9688995	0.787617	not scorable	not scorable	0.9752066	0.8284212
2261	Bn-A01-p6369633	A-genome specific (rapa only)	not scorable	not scorable	not scorable	not scorable	0.9421487	0.7599422
2262	Bn-A01-p637586	A-genome specific (napus-rapa)	0.9569378	0.3589388	not scorable	not scorable	0.9834711	0.7750542
2263	Bn-A01-p6376105	A-genome specific (napus-rapa)	1	0.7951425	not scorable	not scorable	1	0.7650967
2264	Bn-A01-p6380934	A-genome specific (napus-rapa)	0.9665072	0.4092464	not scorable	not scorable	0.9752066	0.7127312
2265	Bn-A01-p6388431	A-genome specific (napus-rapa)	0.9832536	0.5766192	not scorable	not scorable	0.7272727	0.7358895
2266	Bn-A01-p6390236	A-genome specific (napus-rapa)	0.9976076	0.5965861	not scorable	not scorable	0.7272727	0.6775459
2267	Bn-A01-p6439605	A-genome specific (napus-rapa)	0.9880383	0.2116308	not scorable	not scorable	1	0.7611951
2268	Bn-A01-p644410	A-genome specific (napus-rapa)	0.9736842	0.7594993	not scorable	not scorable	1	0.7881269
2269	Bn-A01-p645951	A-genome specific (napus-rapa)	0.6770335	0.7597431	not scorable	not scorable	0.661157	0.7736719
2270	Bn-A01-p6478922	A-genome specific (napus-rapa)	0.9928229	0.2762364	not scorable	not scorable	0.9669421	0.7037422
2271	Bn-A01-p6479735	A-genome specific (napus-rapa)	0.9880383	0.8213089	not scorable	not scorable	0.9669421	0.7185031
2272	Bn-A01-p648196	A-genome specific (napus-rapa)	0.9832536	0.782642	not scorable	not scorable	1	0.7671788
2273	Bn-A01-p6482543	A-genome specific (napus-rapa)	0.9832536	0.7830471	not scorable	not scorable	0.9752066	0.769574
2274	Bn-A01-p6487075	A-genome specific (napus-rapa)	0.9952153	0.475727	not scorable	not scorable	1	0.8067995

2275	Bn-A01-p649805	A-genome specific (napus-rapa)	0.9497608	0.358359	not scorable	not scorable	0.9917355	0.7158309
2276	Bn-A01-p6505754	A-genome specific (rapa only)	not scorable	not scorable	not scorable	not scorable	1	0.9158837
2277	Bn-A01-p6507432	A-genome specific (napus-rapa)	0.9928229	0.6174532	not scorable	not scorable	0.9917355	0.796374
2278	Bn-A01-p6512377	C-genome specific (napus-oleracea)	0.9928229	0.7809948	0.9844961	0.8054167	not scorable	not scorable
2279	Bn-A01-p6530903	A-genome specific (napus-rapa)	0.9928229	0.8076142	not scorable	not scorable	0.9917355	0.8047049
2280	Bn-A01-p6538362	A-genome specific (napus-rapa)	1	0.4858949	not scorable	not scorable	1	0.8601731
2281	Bn-A01-p6546245	A-genome specific (napus-rapa)	0.9832536	0.6770661	not scorable	not scorable	0.9834711	0.7246336
2282	Bn-A01-p6547052	A-genome specific (napus-rapa)	0.9904306	0.7185974	not scorable	not scorable	0.9917355	0.7078228
2283	Bn-A01-p6552596	A-genome specific (napus-rapa)	1	0.4380204	not scorable	not scorable	0.9917355	0.5749632
2284	Bn-A01-p6558843	A-genome specific (napus-rapa)	1	0.3575321	not scorable	not scorable	0.9917355	0.8506843
2285	Bn-A01-p6560270	A-genome specific (napus-rapa)	0.9904306	0.7903701	not scorable	not scorable	0.9917355	0.7395895
2286	Bn-A01-p657852	A-genome specific (napus-rapa)	0.9904306	0.7476627	not scorable	not scorable	1	0.8160439
2287	Bn-A01-p6604419	A-genome specific (napus-rapa)	0.9880383	0.4487656	not scorable	not scorable	0.9917355	0.6779995
2288	Bn-A01-p6625349	A-genome specific (napus-rapa)	0.9928229	0.6404023	not scorable	not scorable	0.9834711	0.6447065
2289	Bn-A01-p6628035	A-genome specific (napus-rapa)	0.9712918	0.7749171	not scorable	not scorable	0.9752066	0.690061
2290	Bn-A01-p663088	A-genome specific (napus-rapa)	0.9760765	0.5883609	not scorable	not scorable	1	0.6916187
2291	Bn-A01-p6643749	A-genome specific (napus-rapa)	0.9641148	0.6942798	not scorable	not scorable	1	0.835351
2292	Bn-A01-p6664608	A-genome specific (napus-rapa)	0.9904306	0.4035521	not scorable	not scorable	1	0.7573897
2293	Bn-A01-p6678914	A-genome specific (napus-rapa)	0.9856459	0.5675757	not scorable	not scorable	1	0.7343488
2294	Bn-A01-p6679394	A-genome specific (napus-rapa)	0.9641148	0.7160898	not scorable	not scorable	0.9834711	0.7293248
2295	Bn-A01-p6679669	genome unspecific (all in)	0.9856459	0.3020632	1	0.658442	1	0.7157381
2296	Bn-A01-p6687707	A-genome specific (napus-rapa)	0.9928229	0.7410733	not scorable	not scorable	0.9834711	0.7221861
2297	Bn-A01-p6687839	A-genome specific (napus-rapa)	0.9736842	0.8030968	not scorable	not scorable	0.9917355	0.7910724
2298	Bn-A01-p6699539	A-genome specific (napus-rapa)	0.9712918	0.4604871	not scorable	not scorable	1	0.8626049
2299	Bn-A01-p6702060	A-genome specific (rapa only)	not scorable	not scorable	not scorable	not scorable	0.7520661	0.8046659
2300	Bn-A01-p6708539	A-genome specific (napus-rapa)	0.9928229	0.778225	not scorable	not scorable	0.9752066	0.7503335
2301	Bn-A01-p6713213	A-genome specific (napus-rapa)	0.9760765	0.7126238	not scorable	not scorable	1	0.6824932
2302	Bn-A01-p6714740	A-genome specific (napus-rapa)	0.9354067	0.7851816	not scorable	not scorable	0.9834711	0.6891667
2303	Bn-A01-p6715317	A-genome specific (napus-rapa)	0.9808612	0.6300939	not scorable	not scorable	0.9834711	0.6613318
2304	Bn-A01-p6747706	A-genome specific (napus-rapa)	0.9904306	0.770321	not scorable	not scorable	0.9917355	0.7062156
2305	Bn-A01-p6773750	A-genome specific (napus-rapa)	0.9784689	0.7805338	not scorable	not scorable	0.9586777	0.7638553
2306	Bn-A01-p6773949	A-genome specific (napus-rapa)	0.9760765	0.7639586	not scorable	not scorable	0.9752066	0.866415
2307	Bn-A01-p6774097	A-genome specific (napus-rapa)	1	0.4762239	not scorable	not scorable	1	0.7718182

2308	Bn-A01-p6774837	A-genome specific (napus-rapa)	0.9784689	0.7782727	not scorable	not scorable	0.9669421	0.7567326
2309	Bn-A01-p6776634	A-genome specific (napus-rapa)	0.9449761	0.6310245	not scorable	not scorable	0.9834711	0.7802128
2310	Bn-A01-p6776950	A-genome specific (napus-rapa)	1	0.4195857	not scorable	not scorable	1	0.6981117
2311	Bn-A01-p678018	A-genome specific (rapa only)	not scorable	not scorable	not scorable	not scorable	1	0.8108955
2312	Bn-A01-p6787691	A-genome specific (napus-rapa)	0.9688995	0.4664785	not scorable	not scorable	1	0.7965355
2313	Bn-A01-p6787930	A-genome specific (napus-rapa)	0.9688995	0.6154464	not scorable	not scorable	1	0.7818822
2314	Bn-A01-p6788044	A-genome specific (rapa only)	not scorable	not scorable	not scorable	not scorable	1	0.6968842
2315	Bn-A01-p6801449	A-genome specific (napus-rapa)	0.9401914	0.4269576	not scorable	not scorable	0.9917355	0.8644511
2316	Bn-A01-p6825673	A-genome specific (napus-rapa)	0.9665072	0.8130674	not scorable	not scorable	0.9834711	0.7297969
2317	Bn-A01-p6826327	A-genome specific (napus-rapa)	0.9784689	0.6887788	not scorable	not scorable	0.9586777	0.7197458
2318	Bn-A01-p6838738	A-genome specific (napus-rapa)	0.9832536	0.7929561	not scorable	not scorable	0.7107438	0.7452965
2319	Bn-A01-p6841235	A-genome specific (napus-rapa)	1	0.5621869	not scorable	not scorable	0.9917355	0.7724892
2320	Bn-A01-p6854917	A-genome specific (napus-rapa)	0.9856459	0.5191624	not scorable	not scorable	0.9917355	0.7998236
2321	Bn-A01-p6863835	A-genome specific (napus-rapa)	0.9760765	0.494791	not scorable	not scorable	0.9752066	0.725516
2322	Bn-A01-p6877281	A-genome specific (napus-rapa)	0.9952153	0.4794303	not scorable	not scorable	1	0.7268496
2323	Bn-A01-p688355	A-genome specific (napus-rapa)	0.9688995	0.6550958	not scorable	not scorable	0.9834711	0.658986
2324	Bn-A01-p6898291	A-genome specific (napus-rapa)	0.9880383	0.7674453	not scorable	not scorable	0.9917355	0.849797
2325	Bn-A01-p6898690	A-genome specific (napus-rapa)	0.9736842	0.7089965	not scorable	not scorable	0.9586777	0.740084
2326	Bn-A01-p6899980	A-genome specific (rapa only)	not scorable	not scorable	not scorable	not scorable	0.7024794	0.829312
2327	Bn-A01-p6900616	A-genome specific (napus-rapa)	0.923445	0.7537299	not scorable	not scorable	0.7024794	0.7531074
2328	Bn-A01-p6907522	A-genome specific (napus-rapa)	0.9210526	0.7828278	not scorable	not scorable	0.7520661	0.8056048
2329	Bn-A01-p6920456	A-genome specific (napus-rapa)	0.9904306	0.4195472	not scorable	not scorable	0.5289256	0.7684046
2330	Bn-A01-p6925167	A-genome specific (napus-rapa)	0.9928229	0.5100092	not scorable	not scorable	0.9752066	0.7864066
2331	Bn-A01-p6925436	A-genome specific (napus-rapa)	0.9928229	0.2755999	not scorable	not scorable	1	0.8039197
2332	Bn-A01-p6950868	A-genome specific (napus-rapa)	0.9952153	0.3965079	not scorable	not scorable	0.9834711	0.7228364
2333	Bn-A01-p6958863	A-genome specific (napus-rapa)	0.9904306	0.7921842	not scorable	not scorable	1	0.7584904
2334	Bn-A01-p6959248	A-genome specific (napus-rapa)	0.9832536	0.6995357	not scorable	not scorable	0.9834711	0.6788211
2335	Bn-A01-p6962542	A-genome specific (napus-rapa)	0.9928229	0.3783413	not scorable	not scorable	0.9917355	0.7766539
2336	Bn-A01-p6965221	A-genome specific (napus-rapa)	0.9856459	0.7610687	not scorable	not scorable	0.9917355	0.7300522
2337	Bn-A01-p6968751	A-genome specific (napus-rapa)	0.9856459	0.6658638	not scorable	not scorable	1	0.6847009
2338	Bn-A01-p6969197	A-genome specific (napus-rapa)	0.9904306	0.7772461	not scorable	not scorable	0.9917355	0.7385896
2339	Bn-A01-p6969284	A-genome specific (napus-rapa)	0.9736842	0.7234355	not scorable	not scorable	0.9917355	0.6965255
2340	Bn-A01-p6969358	A-genome specific (napus-rapa)	0.9808612	0.739107	not scorable	not scorable	0.9834711	0.7076883

2341	Bn-A01-p6972779	A-genome specific (napus-rapa)	0.9832536	0.8286869	not scorable	not scorable	0.9834711	0.7356253
2342	Bn-A01-p6976938	genome unspecific (all in)	0.9832536	0.4903604	1	0.6895438	1	0.7092124
2343	Bn-A01-p6986405	A-genome specific (napus-rapa)	1	0.3554479	not scorable	not scorable	1	0.5891774
2344	Bn-A01-p7026162	A-genome specific (napus-rapa)	0.9952153	0.3324807	not scorable	not scorable	0.9917355	0.7743288
2345	Bn-A01-p704525	A-genome specific (napus-rapa)	0.9688995	0.342135	not scorable	not scorable	0.9917355	0.7644219
2346	Bn-A01-p7068752	A-genome specific (napus-rapa)	0.9880383	0.7759447	not scorable	not scorable	1	0.8433055
2347	Bn-A01-p7069263	A-genome specific (napus-rapa)	0.9904306	0.6863875	not scorable	not scorable	0.9834711	0.7667808
2348	Bn-A01-p7086543	A-genome specific (napus-rapa)	0.9856459	0.8168805	not scorable	not scorable	0.9917355	0.7059886
2349	Bn-A01-p7087000	A-genome specific (napus-rapa)	1	0.6823635	not scorable	not scorable	1	0.7235581
2350	Bn-A01-p7089762	A-genome specific (napus-rapa)	0.3516746	0.8145286	not scorable	not scorable	0.9917355	0.7944399
2351	Bn-A01-p7089826	A-genome specific (rapa only)	not scorable	not scorable	not scorable	not scorable	0.9917355	0.6463398
2352	Bn-A01-p710239	all not scorable	not scorable	not scorable	not scorable	not scorable	not scorable	not scorable
2353	Bn-A01-p71129	A-genome specific (napus-rapa)	0.9880383	0.3098471	not scorable	not scorable	1	0.6927842
2354	Bn-A01-p7121832	genome unspecific (all in)	0.9784689	0.1980119	0.9147287	0.3581319	0.9669421	0.752364
2355	Bn-A01-p7122036	A-genome specific (napus-rapa)	1	0.3673542	not scorable	not scorable	1	0.7641606
2356	Bn-A01-p7132729	A-genome specific (napus-rapa)	0.9976076	0.7540873	not scorable	not scorable	0.9917355	0.7186126
2357	Bn-A01-p713562	A-genome specific (napus-rapa)	0.9736842	0.4626265	not scorable	not scorable	0.9917355	0.7072161
2358	Bn-A01-p7137897	A-genome specific (napus-rapa)	0.9784689	0.4893922	not scorable	not scorable	0.9917355	0.7713531
2359	Bn-A01-p7150748	A-genome specific (napus-rapa)	0.9665072	0.7311332	not scorable	not scorable	1	0.9398885
2360	Bn-A01-p7151096	A-genome specific (napus-rapa)	1	0.7625467	not scorable	not scorable	0.9917355	0.7858406
2361	Bn-A01-p7160702	A-genome specific (napus-rapa)	0.9617225	0.6795166	not scorable	not scorable	0.9752066	0.6693636
2362	Bn-A01-p7205595	A-genome specific (napus-rapa)	0.9760765	0.7124651	not scorable	not scorable	1	0.8169149
2363	Bn-A01-p733483	A-genome specific (rapa only)	not scorable	not scorable	not scorable	not scorable	0.892562	0.8194628
2364	Bn-A01-p7363480	A-genome specific (napus-rapa)	0.9641148	0.7205732	not scorable	not scorable	1	0.6812111
2365	Bn-A01-p7367598	A-genome specific (napus-rapa)	1	0.4494559	not scorable	not scorable	0.9834711	0.8006749
2366	Bn-A01-p7369125	A-genome specific (napus-rapa)	0.9928229	0.7529274	not scorable	not scorable	0.9834711	0.718548
2367	Bn-A01-p7372570	A-genome specific (napus-rapa)	0.6866029	0.7958463	not scorable	not scorable	0.7272727	0.8594449
2368	Bn-A01-p7372653	A-genome specific (napus-rapa)	1	0.7662389	not scorable	not scorable	1	0.8422474
2369	Bn-A01-p7373427	A-genome specific (napus-rapa)	0.9832536	0.7566932	not scorable	not scorable	0.9917355	0.6678384
2370	Bn-A01-p7374290	A-genome specific (napus-rapa)	0.9976076	0.3018439	not scorable	not scorable	0.9752066	0.5473303
2371	Bn-A01-p7374589	A-genome specific (napus-rapa)	0.9760765	0.8052503	not scorable	not scorable	0.9834711	0.7312777
2372	Bn-A01-p737560	A-genome specific (napus-rapa)	0.9736842	0.8045892	not scorable	not scorable	0.9752066	0.7409469
2373	Bn-A01-p7383179	A-genome specific (napus-rapa)	0.6698565	0.8109727	not scorable	not scorable	0.7438017	0.8231242

2374	Bn-A01-p7385409	A-genome specific (napus-rapa)	0.6961722	0.8022107	not scorable	not scorable	0.7190083	0.8580421
2375	Bn-A01-p7385725	A-genome specific (napus-rapa)	0.9497608	0.3903539	not scorable	not scorable	0.7438017	0.71036
2376	Bn-A01-p7385797	A-genome specific (napus-rapa)	0.9952153	0.6736407	not scorable	not scorable	0.7272727	0.8570039
2377	Bn-A01-p7386111	A-genome specific (rapa only)	not scorable	not scorable	not scorable	not scorable	0.7355372	0.9192614
2378	Bn-A01-p739098	A-genome specific (napus-rapa)	0.9856459	0.7232225	not scorable	not scorable	1	0.7234554
2379	Bn-A01-p7399827	A-genome specific (napus-rapa)	1	0.6071301	not scorable	not scorable	0.8016529	0.7444668
2380	Bn-A01-p7405147	A-genome specific (napus-rapa)	0.9808612	0.7481248	not scorable	not scorable	0.9834711	0.7427847
2381	Bn-A01-p7405753	A-genome specific (napus-rapa)	0.6961722	0.7977476	not scorable	not scorable	0.7355372	0.7548996
2382	Bn-A01-p7405882	A-genome specific (napus-rapa)	0.9066986	0.6878061	not scorable	not scorable	0.9504132	0.6386759
2383	Bn-A01-p7406041	A-genome specific (napus-rapa)	0.6866029	0.7968957	not scorable	not scorable	0.768595	0.8521869
2384	Bn-A01-p7430242	A-genome specific (napus-rapa)	0.9712918	0.7152056	not scorable	not scorable	1	0.7297956
2385	Bn-A01-p7430311	A-genome specific (napus-rapa)	0.9784689	0.7761593	not scorable	not scorable	0.9917355	0.7492446
2386	Bn-A01-p7440935	A-genome specific (rapa only)	not scorable	not scorable	not scorable	not scorable	0.9834711	0.7212331
2387	Bn-A01-p7452338	A-genome specific (napus-rapa)	0.9808612	0.5051401	not scorable	not scorable	0.9669421	0.8339436
2388	Bn-A01-p7455231	A-genome specific (napus-rapa)	0.9880383	0.5607159	not scorable	not scorable	0.9834711	0.8695884
2389	Bn-A01-p7455852	A-genome specific (napus-rapa)	0.9880383	0.8828124	not scorable	not scorable	1	0.7701377
2390	Bn-A01-p7473265	A-genome specific (napus-rapa)	1	0.2407155	not scorable	not scorable	1	0.8040091
2391	Bn-A01-p7479483	A-genome specific (napus-rapa)	0.9952153	0.627915	not scorable	not scorable	0.9917355	0.6491689
2392	Bn-A01-p7487861	A-genome specific (napus-rapa)	0.9880383	0.7675837	not scorable	not scorable	0.9917355	0.7947931
2393	Bn-A01-p7493373	A-genome specific (napus-rapa)	0.9760765	0.302868	not scorable	not scorable	0.9834711	0.6171378
2394	Bn-A01-p7512557	A-genome specific (napus-rapa)	1	0.5472876	not scorable	not scorable	0.9917355	0.8259965
2395	Bn-A01-p751573	A-genome specific (napus-rapa)	0.9784689	0.7365752	not scorable	not scorable	0.677686	0.7394518
2396	Bn-A01-p7530968	A-genome specific (napus-rapa)	0.9928229	0.6045443	not scorable	not scorable	1	0.7420533
2397	Bn-A01-p7533971	A-genome specific (napus-rapa)	0.9880383	0.458242	not scorable	not scorable	0.9917355	0.6422251
2398	Bn-A01-p7548806	A-genome specific (rapa only)	not scorable	not scorable	not scorable	not scorable	0.9917355	0.6982405
2399	Bn-A01-p7551163	A-genome specific (napus-rapa)	0.9880383	0.6721168	not scorable	not scorable	0.9752066	0.6448169
2400	Bn-A01-p7554673	A-genome specific (napus-rapa)	0.9928229	0.7458624	not scorable	not scorable	1	0.6728373
2401	Bn-A01-p7557422	A-genome specific (napus-rapa)	0.6842105	0.7584272	not scorable	not scorable	1	0.7497242
2402	Bn-A01-p7558012	A-genome specific (napus-rapa)	0.9928229	0.774186	not scorable	not scorable	1	0.7174791
2403	Bn-A01-p7558345	A-genome specific (napus-rapa)	0.9856459	0.8008437	not scorable	not scorable	1	0.8039128
2404	Bn-A01-p7559946	A-genome specific (napus-rapa)	0.9832536	0.7810608	not scorable	not scorable	0.9917355	0.8125475
2405	Bn-A01-p7560785	A-genome specific (napus-rapa)	0.9641148	0.6054804	not scorable	not scorable	0.9917355	0.717272
2406	Bn-A01-p7573871	A-genome specific (napus-rapa)	0.9114832	0.2995738	not scorable	not scorable	1	0.7884533

2407	Bn-A01-p7574355	A-genome specific (napus-rapa)	0.9688995	0.7985705	not scorable	not scorable	0.9917355	0.8095371
2408	Bn-A01-p7582017	A-genome specific (napus-rapa)	0.9545454	0.7742874	not scorable	not scorable	1	0.812126
2409	Bn-A01-p7585849	A-genome specific (napus-rapa)	0.6196172	0.6646404	not scorable	not scorable	0.9917355	0.6248508
2410	Bn-A01-p7586821	A-genome specific (napus-rapa)	0.6172249	0.8140727	not scorable	not scorable	0.9917355	0.7355657
2411	Bn-A01-p7587840	A-genome specific (napus-rapa)	0.6291866	0.6795503	not scorable	not scorable	0.9917355	0.7298859
2412	Bn-A01-p7588413	A-genome specific (napus-rapa)	0.6172249	0.8374454	not scorable	not scorable	0.9917355	0.7436024
2413	Bn-A01-p7588937	A-genome specific (napus-rapa)	0.6196172	0.665211	not scorable	not scorable	0.9669421	0.6965741
2414	Bn-A01-p7588958	A-genome specific (napus-rapa)	0.6196172	0.550025	not scorable	not scorable	0.9917355	0.7090922
2415	Bn-A01-p7593180	A-genome specific (napus-rapa)	0.9593301	0.7883755	not scorable	not scorable	0.9917355	0.697901
2416	Bn-A01-p7594170	A-genome specific (napus-rapa)	0.9760765	0.3142905	not scorable	not scorable	1	0.6846697
2417	Bn-A01-p7603030	A-genome specific (napus-rapa)	0.9712918	0.657733	not scorable	not scorable	1	0.7022686
2418	Bn-A01-p7615707	A-genome specific (napus-rapa)	0.9928229	0.7525604	not scorable	not scorable	1	0.8236455
2419	Bn-A01-p7619726	A-genome specific (napus-rapa)	0.9904306	0.699182	not scorable	not scorable	1	0.7219252
2420	Bn-A01-p7643514	A-genome specific (napus-rapa)	0.9952153	0.459986	not scorable	not scorable	0.9669421	0.6585762
2421	Bn-A01-p7643571	A-genome specific (napus-rapa)	0.9952153	0.5340405	not scorable	not scorable	0.9917355	0.6922877
2422	Bn-A01-p7646601	A-genome specific (napus-rapa)	0.8899522	0.7886694	not scorable	not scorable	0.8347107	0.7315326
2423	Bn-A01-p7648929	A-genome specific (napus-rapa)	0.9928229	0.7824821	not scorable	not scorable	0.9752066	0.6878925
2424	Bn-A01-p7656521	A-genome specific (napus-rapa)	0.9904306	0.6184712	not scorable	not scorable	1	0.7726986
2425	Bn-A01-p7667896	A-genome specific (napus-rapa)	0.9928229	0.7623014	not scorable	not scorable	1	0.8065068
2426	Bn-A01-p7698558	A-genome specific (napus-rapa)	0.9976076	0.3634282	not scorable	not scorable	1	0.8851715
2427	Bn-A01-p7699159	A-genome specific (napus-rapa)	0.9928229	0.6679955	not scorable	not scorable	1	0.6790781
2428	Bn-A01-p7702471	A-genome specific (napus-rapa)	0.9928229	0.786551	not scorable	not scorable	0.9834711	0.6382802
2429	Bn-A01-p7714470	A-genome specific (napus-rapa)	0.9904306	0.7398877	not scorable	not scorable	0.9917355	0.7031711
2430	Bn-A01-p7717618	genome unspecific (oleracea-rapa)	not scorable	not scorable	0.9302326	0.7176385	1	0.7546726
2431	Bn-A01-p7721497	A-genome specific (napus-rapa)	0.9880383	0.5481173	not scorable	not scorable	0.9504132	0.7639257
2432	Bn-A01-p7724859	genome unspecific (all in)	0.9952153	0.4273613	1	0.8923284	0.9917355	0.8226846
2433	Bn-A01-p7745080	napus specific	0.9641148	0.7598489	not scorable	not scorable	not scorable	not scorable
2434	Bn-A01-p7768351	A-genome specific (napus-rapa)	0.9904306	0.7137059	not scorable	not scorable	0.9834711	0.7438502
2435	Bn-A01-p777123	A-genome specific (napus-rapa)	0.9856459	0.4237095	not scorable	not scorable	1	0.8014378
2436	Bn-A01-p7771617	A-genome specific (napus-rapa)	0.9904306	0.5054722	not scorable	not scorable	0.9669421	0.7344456
2437	Bn-A01-p7772345	A-genome specific (napus-rapa)	0.9976076	0.5243252	not scorable	not scorable	0.9917355	0.7194836
2438	Bn-A01-p778048	A-genome specific (napus-rapa)	0.9760765	0.5793232	not scorable	not scorable	1	0.8928617
2439	Bn-A01-p7787153	A-genome specific (napus-rapa)	0.9808612	0.3531474	not scorable	not scorable	1	0.766865

2440	Bn-A01-p7800839	A-genome specific (napus-rapa)	0.9760765	0.7722787	not scorable	not scorable	0.9917355	0.7331263
2441	Bn-A01-p7825044	A-genome specific (napus-rapa)	0.9736842	0.7719089	not scorable	not scorable	0.9917355	0.7183975
2442	Bn-A01-p7827260	A-genome specific (napus-rapa)	0.9712918	0.2763461	not scorable	not scorable	1	0.663735
2443	Bn-A01-p7831289	A-genome specific (napus-rapa)	0.9832536	0.2693953	not scorable	not scorable	1	0.7397969
2444	Bn-A01-p7834871	A-genome specific (napus-rapa)	0.9545454	0.304858	not scorable	not scorable	1	0.731966
2445	Bn-A01-p7850092	A-genome specific (napus-rapa)	1	0.4901769	not scorable	not scorable	1	0.7899298
2446	Bn-A01-p7852440	A-genome specific (napus-rapa)	1	0.3275238	not scorable	not scorable	0.9917355	0.731602
2447	Bn-A01-p7861283	A-genome specific (napus-rapa)	0.9976076	0.7270315	not scorable	not scorable	1	0.5793777
2448	Bn-A01-p7875358	A-genome specific (napus-rapa)	1	0.3032759	not scorable	not scorable	0.9752066	0.6764334
2449	Bn-A01-p7889385	A-genome specific (napus-rapa)	0.9928229	0.5966011	not scorable	not scorable	1	0.6941039
2450	Bn-A01-p7890174	A-genome specific (napus-rapa)	0.9952153	0.543226	not scorable	not scorable	0.9834711	0.7313213
2451	Bn-A01-p7897804	A-genome specific (napus-rapa)	0.9952153	0.2542506	not scorable	not scorable	0.9752066	0.6869955
2452	Bn-A01-p7897805	A-genome specific (rapa only)	not scorable	not scorable	not scorable	not scorable	0.9669421	0.7058876
2453	Bn-A01-p7926287	A-genome specific (napus-rapa)	0.9904306	0.4442226	not scorable	not scorable	1	0.6871758
2454	Bn-A01-p7942548	A-genome specific (napus-rapa)	0.9952153	0.6533158	not scorable	not scorable	1	0.7888203
2455	Bn-A01-p7953001	A-genome specific (napus-rapa)	1	0.5438352	not scorable	not scorable	0.9917355	0.7806042
2456	Bn-A01-p796004	A-genome specific (napus-rapa)	1	0.5622409	not scorable	not scorable	1	0.9176469
2457	Bn-A01-p7961620	A-genome specific (napus-rapa)	0.9856459	0.6350563	not scorable	not scorable	0.9504132	0.5597297
2458	Bn-A01-p7973418	genome unspecific (all in)	0.9808612	0.7144896	0	0.808931	0.9834711	0.7087855
2459	Bn-A01-p7974551	A-genome specific (napus-rapa)	0.9641148	0.6452731	not scorable	not scorable	0.9834711	0.7545086
2460	Bn-A01-p797684	A-genome specific (rapa only)	not scorable	not scorable	not scorable	not scorable	1	0.7742329
2461	Bn-A01-p7979458	genome unspecific (all in)	0.9928229	0.564781	1	0.3299505	0.9917355	0.7008063
2462	Bn-A01-p7982220	A-genome specific (napus-rapa)	0.9928229	0.7455831	not scorable	not scorable	0.9917355	0.6441367
2463	Bn-A01-p7983241	A-genome specific (napus-rapa)	0.9928229	0.4124842	not scorable	not scorable	1	0.8243906
2464	Bn-A01-p7987687	A-genome specific (napus-rapa)	0.9856459	0.8060251	not scorable	not scorable	0.9752066	0.6797213
2465	Bn-A01-p8014995	A-genome specific (napus-rapa)	0.9952153	0.7170584	not scorable	not scorable	0.9752066	0.7845222
2466	Bn-A01-p8016433	A-genome specific (napus-rapa)	1	0.232866	not scorable	not scorable	0.9834711	0.7348057
2467	Bn-A01-p8017380	A-genome specific (napus-rapa)	0.9952153	0.7561173	not scorable	not scorable	0.9917355	0.692532
2468	Bn-A01-p80385	A-genome specific (napus-rapa)	0.9617225	0.351738	not scorable	not scorable	0.9917355	0.7612265
2469	Bn-A01-p8043776	A-genome specific (napus-rapa)	0.9856459	0.3712352	not scorable	not scorable	0.9834711	0.6108911
2470	Bn-A01-p8045760	A-genome specific (napus-rapa)	0.9760765	0.7887551	not scorable	not scorable	0.9834711	0.7928751
2471	Bn-A01-p8046142	A-genome specific (napus-rapa)	0.9808612	0.6899778	not scorable	not scorable	0.9917355	0.6347992
2472	Bn-A01-p8051029	A-genome specific (napus-rapa)	0.9138756	0.9405676	not scorable	not scorable	0.9586777	0.8292131

2473	Bn-A01-p8051804	A-genome specific (napus-rapa)	0.9114832	0.7925414	not scorable	not scorable	0.9752066	0.7722662
2474	Bn-A01-p8058255	A-genome specific (napus-rapa)	0.9784689	0.7729293	not scorable	not scorable	1	0.745807
2475	Bn-A01-p8058475	A-genome specific (napus-rapa)	0.9665072	0.7575583	not scorable	not scorable	1	0.7943444
2476	Bn-A01-p8063652	A-genome specific (napus-rapa)	0.9808612	0.8112089	not scorable	not scorable	0.9834711	0.7694861
2477	Bn-A01-p8076080	A-genome specific (napus-rapa)	0.9880383	0.3597575	not scorable	not scorable	0.9669421	0.4141327
2478	Bn-A01-p8080531	A-genome specific (napus-rapa)	1	0.5336986	not scorable	not scorable	0.9917355	0.7904381
2479	Bn-A01-p8108178	A-genome specific (napus-rapa)	0.9880383	0.6630409	not scorable	not scorable	1	0.6783065
2480	Bn-A01-p8111196	A-genome specific (napus-rapa)	0.9880383	0.8067618	not scorable	not scorable	0.9834711	0.721589
2481	Bn-A01-p8115849	A-genome specific (napus-rapa)	0.9880383	0.7497345	not scorable	not scorable	0.9834711	0.7582858
2482	Bn-A01-p8116314	A-genome specific (napus-rapa)	0.9856459	0.4136742	not scorable	not scorable	0.9752066	0.6267422
2483	Bn-A01-p8121877	A-genome specific (napus-rapa)	1	0.474205	not scorable	not scorable	1	0.9171228
2484	Bn-A01-p8122779	A-genome specific (napus-rapa)	0.9928229	0.8090187	not scorable	not scorable	1	0.7367046
2485	Bn-A01-p8129876	A-genome specific (napus-rapa)	0.9928229	0.6896059	not scorable	not scorable	1	0.7358541
2486	Bn-A01-p8150111	A-genome specific (napus-rapa)	0.9784689	0.1952544	not scorable	not scorable	1	0.8676265
2487	Bn-A01-p8154524	genome unspecific (all in)	0.9928229	0.3190248	1	0.7664712	1	0.8948976
2488	Bn-A01-p816488	A-genome specific (napus-rapa)	1	0.8060362	not scorable	not scorable	1	0.8806193
2489	Bn-A01-p820317	A-genome specific (napus-rapa)	0.9688995	0.6036797	not scorable	not scorable	1	0.662836
2490	Bn-A01-p8203740	A-genome specific (rapa only)	not scorable	not scorable	not scorable	not scorable	0.9669421	0.7472444
2491	Bn-A01-p8204552	A-genome specific (rapa only)	not scorable	not scorable	not scorable	not scorable	0.9752066	0.7003862
2492	Bn-A01-p8205728	A-genome specific (rapa only)	not scorable	not scorable	not scorable	not scorable	0.9752066	0.6931832
2493	Bn-A01-p8206136	A-genome specific (rapa only)	not scorable	not scorable	not scorable	not scorable	0.9752066	0.6621579
2494	Bn-A01-p8206374	A-genome specific (rapa only)	not scorable	not scorable	not scorable	not scorable	0.9752066	0.7004539
2495	Bn-A01-p8207083	A-genome specific (rapa only)	not scorable	not scorable	not scorable	not scorable	0.9669421	0.6886162
2496	Bn-A01-p821328	A-genome specific (napus-rapa)	0.9688995	0.5216793	not scorable	not scorable	0.9917355	0.7409398
2497	Bn-A01-p8221245	A-genome specific (napus-rapa)	0.9904306	0.8066382	not scorable	not scorable	0.9917355	0.806761
2498	Bn-A01-p8240363	A-genome specific (napus-rapa)	0.9928229	0.7980362	not scorable	not scorable	1	0.8143303
2499	Bn-A01-p8242690	A-genome specific (napus-rapa)	1	0.7224955	not scorable	not scorable	1	0.7035602
2500	Bn-A01-p8246641	A-genome specific (napus-rapa)	0.9928229	0.7678831	not scorable	not scorable	1	0.8675844
2501	Bn-A01-p8263964	A-genome specific (napus-rapa)	0.9928229	0.7404135	not scorable	not scorable	0.9917355	0.8341498
2502	Bn-A01-p8276793	A-genome specific (napus-rapa)	1	0.5136053	not scorable	not scorable	1	0.8451606
2503	Bn-A01-p8276922	A-genome specific (napus-rapa)	1	0.447688	not scorable	not scorable	0.9917355	0.7292544
2504	Bn-A01-p8278925	A-genome specific (napus-rapa)	1	0.6328043	not scorable	not scorable	0.9917355	0.7798906
2505	Bn-A01-p830221	A-genome specific (napus-rapa)	0.9856459	0.7956669	not scorable	not scorable	1	0.745196

2506	Bn-A01-p8335308	A-genome specific (napus-rapa)	0.9976076	0.4142104	not scorable	not scorable	0.9917355	0.7550115
2507	Bn-A01-p8353426	A-genome specific (napus-rapa)	1	0.5826371	not scorable	not scorable	0.9917355	0.8050923
2508	Bn-A01-p8354196	A-genome specific (napus-rapa)	1	0.476792	not scorable	not scorable	1	0.730772
2509	Bn-A01-p8378967	A-genome specific (napus-rapa)	0.9928229	0.7276884	not scorable	not scorable	1	0.8415009
2510	Bn-A01-p8402568	A-genome specific (napus-rapa)	0.9928229	0.8039323	not scorable	not scorable	0.9917355	0.6903467
2511	Bn-A01-p8404588	A-genome specific (napus-rapa)	0.9928229	0.7046103	not scorable	not scorable	0.9752066	0.6969791
2512	Bn-A01-p8417520	A-genome specific (napus-rapa)	0.9928229	0.759161	not scorable	not scorable	0.9834711	0.7092545
2513	Bn-A01-p8418102	A-genome specific (napus-rapa)	0.9928229	0.7589638	not scorable	not scorable	1	0.74563
2514	Bn-A01-p8439363	A-genome specific (napus-rapa)	0.9976076	0.5437354	not scorable	not scorable	0.9669421	0.7212059
2515	Bn-A01-p8450346	A-genome specific (napus-rapa)	0.9736842	0.2757879	not scorable	not scorable	0.9834711	0.8572526
2516	Bn-A01-p8451192	A-genome specific (rapa only)	not scorable	not scorable	not scorable	not scorable	0.892562	0.9031098
2517	Bn-A01-p8452366	A-genome specific (napus-rapa)	0.9928229	0.6835634	not scorable	not scorable	0.9917355	0.8382507
2518	Bn-A01-p8452493	A-genome specific (rapa only)	not scorable	not scorable	not scorable	not scorable	0.9752066	0.6934153
2519	Bn-A01-p8452981	A-genome specific (napus-rapa)	0.9808612	0.7122888	not scorable	not scorable	1	0.6697664
2520	Bn-A01-p8453146	A-genome specific (napus-rapa)	0.2009569	0.8126745	not scorable	not scorable	0.9008265	0.7006959
2521	Bn-A01-p8470314	A-genome specific (napus-rapa)	0.9856459	0.399159	not scorable	not scorable	0.9917355	0.7456675
2522	Bn-A01-p849125	A-genome specific (napus-rapa)	0.9712918	0.7621524	not scorable	not scorable	1	0.7442658
2523	Bn-A01-p8501753	A-genome specific (napus-rapa)	0.9928229	0.744251	not scorable	not scorable	1	0.7448832
2524	Bn-A01-p8504134	A-genome specific (napus-rapa)	0.9976076	0.3563309	not scorable	not scorable	1	0.6914352
2525	Bn-A01-p8515129	A-genome specific (napus-rapa)	0.9832536	0.646772	not scorable	not scorable	0.9834711	0.705299
2526	Bn-A01-p8543512	A-genome specific (napus-rapa)	0.9928229	0.5613573	not scorable	not scorable	0.9586777	0.600333
2527	Bn-A01-p8564149	A-genome specific (napus-rapa)	1	0.5112184	not scorable	not scorable	1	0.6868309
2528	Bn-A01-p857771	A-genome specific (rapa only)	not scorable	not scorable	not scorable	not scorable	0.9917355	0.8379771
2529	Bn-A01-p8593291	A-genome specific (rapa only)	not scorable	not scorable	not scorable	not scorable	0.9834711	0.7600462
2530	Bn-A01-p8594736	A-genome specific (rapa only)	not scorable	not scorable	not scorable	not scorable	0.9669421	0.7750521
2531	Bn-A01-p8594955	A-genome specific (napus-rapa)	0.9258373	0.6900374	not scorable	not scorable	0.9586777	0.7211653
2532	Bn-A01-p8595450	A-genome specific (napus-rapa)	0.923445	0.754145	not scorable	not scorable	0.9586777	0.7254611
2533	Bn-A01-p8595895	A-genome specific (napus-rapa)	0.923445	0.8595558	not scorable	not scorable	0.9256198	0.8174812
2534	Bn-A01-p8596463	A-genome specific (napus-rapa)	0.9282297	0.6795592	not scorable	not scorable	0.9338843	0.6791383
2535	Bn-A01-p8596650	A-genome specific (napus-rapa)	0.9138756	0.634635	not scorable	not scorable	0.9504132	0.7755718
2536	Bn-A01-p8647625	A-genome specific (napus-rapa)	0.9928229	0.2701297	not scorable	not scorable	0.9752066	0.6576771
2537	Bn-A01-p866260	A-genome specific (napus-rapa)	0.9641148	0.7459672	not scorable	not scorable	0.9917355	0.6692497
2538	Bn-A01-p8675601	A-genome specific (napus-rapa)	0.9904306	0.8351841	not scorable	not scorable	0.9917355	0.7229037

2539	Bn-A01-p8675930	A-genome specific (napus-rapa)	0.9832536	0.4794636	not scorable	not scorable	0.9834711	0.7567218
2540	Bn-A01-p8676024	A-genome specific (napus-rapa)	0.9832536	0.8228162	not scorable	not scorable	0.9917355	0.7094581
2541	Bn-A01-p8676144	A-genome specific (napus-rapa)	0.9784689	0.759104	not scorable	not scorable	0.9834711	0.7854255
2542	Bn-A01-p8681309	A-genome specific (napus-rapa)	0.9880383	0.7955467	not scorable	not scorable	0.9834711	0.7120237
2543	Bn-A01-p8681556	A-genome specific (napus-rapa)	0.9904306	0.7907762	not scorable	not scorable	1	0.7470711
2544	Bn-A01-p8681757	A-genome specific (napus-rapa)	0.9928229	0.7421923	not scorable	not scorable	0.9917355	0.6885744
2545	Bn-A01-p8683487	A-genome specific (napus-rapa)	1	0.3350065	not scorable	not scorable	0.9917355	0.7198656
2546	Bn-A01-p872436	A-genome specific (napus-rapa)	0.9808612	0.4215149	not scorable	not scorable	1	0.7339357
2547	Bn-A01-p877322	A-genome specific (napus-rapa)	0.9808612	0.7545708	not scorable	not scorable	0.9834711	0.6719007
2548	Bn-A01-p878256	A-genome specific (napus-rapa)	0.9904306	0.2900791	not scorable	not scorable	1	0.8042273
2549	Bn-A01-p8782649	A-genome specific (napus-rapa)	0.9952153	0.4333103	not scorable	not scorable	1	0.7693055
2550	Bn-A01-p8788567	A-genome specific (napus-rapa)	0.9976076	0.6562223	not scorable	not scorable	0.9834711	0.6253553
2551	Bn-A01-p8795719	A-genome specific (napus-rapa)	0.9976076	0.511342	not scorable	not scorable	1	0.7645436
2552	Bn-A01-p879755	A-genome specific (napus-rapa)	0.9593301	0.7835568	not scorable	not scorable	0.677686	0.7467541
2553	Bn-A01-p8805177	A-genome specific (napus-rapa)	0.9569378	0.7094924	not scorable	not scorable	0.9586777	0.7014148
2554	Bn-A01-p8806524	A-genome specific (napus-rapa)	0.9617225	0.8632586	not scorable	not scorable	0.9669421	0.8109026
2555	Bn-A01-p8821722	A-genome specific (napus-rapa)	0.9641148	0.7543002	not scorable	not scorable	0.9504132	0.6673809
2556	Bn-A01-p8836783	A-genome specific (napus-rapa)	0.9880383	0.6576318	not scorable	not scorable	0.9834711	0.7938799
2557	Bn-A01-p8839183	A-genome specific (napus-rapa)	0.9593301	0.7752286	not scorable	not scorable	0.9586777	0.9217552
2558	Bn-A01-p8859217	A-genome specific (napus-rapa)	0.9521531	0.8547055	not scorable	not scorable	0.9504132	0.7056898
2559	Bn-A01-p88608	A-genome specific (napus-rapa)	0.9712918	0.7518556	not scorable	not scorable	0.9834711	0.561146
2560	Bn-A01-p8874473	A-genome specific (napus-rapa)	0.9569378	0.7515191	not scorable	not scorable	0.9586777	0.6925956
2561	Bn-A01-p887815	A-genome specific (napus-rapa)	0.9736842	0.6064315	not scorable	not scorable	1	0.8186453
2562	Bn-A01-p8901327	A-genome specific (napus-rapa)	0.9856459	0.7420153	not scorable	not scorable	0.9752066	0.7356703
2563	Bn-A01-p8901490	A-genome specific (napus-rapa)	0.9928229	0.5579286	not scorable	not scorable	0.9917355	0.7018243
2564	Bn-A01-p8914737	A-genome specific (napus-rapa)	0.9641148	0.7574978	not scorable	not scorable	0.9504132	0.7086438
2565	Bn-A01-p8960719	A-genome specific (napus-rapa)	1	0.9204677	not scorable	not scorable	1	0.8850337
2566	Bn-A01-p8977767	A-genome specific (napus-rapa)	0.8803828	0.7757977	not scorable	not scorable	1	0.8694186
2567	Bn-A01-p8982996	A-genome specific (napus-rapa)	0.9952153	0.3588107	not scorable	not scorable	1	0.7053689
2568	Bn-A01-p89956	A-genome specific (napus-rapa)	0.9976076	0.5809868	not scorable	not scorable	0.9917355	0.7549776
2569	Bn-A01-p9004629	A-genome specific (napus-rapa)	0.9832536	0.7539826	not scorable	not scorable	0.9834711	0.7929008
2570	Bn-A01-p9030262	genome unspecific (all in)	0.9904306	0.3257509	0.9922481	0.7548456	1	0.9072468
2571	Bn-A01-p9065564	A-genome specific (napus-rapa)	0.9976076	0.8793069	not scorable	not scorable	1	0.8687926

2572	Bn-A01-p9075803	A-genome specific (napus-rapa)	0.9641148	0.7687171	not scorable	not scorable	0.9834711	0.7346492
2573	Bn-A01-p9075931	A-genome specific (napus-rapa)	0.9641148	0.7872018	not scorable	not scorable	0.9752066	0.8098927
2574	Bn-A01-p907721	genome unspecific (all in)	0.9880383	0.4234551	0.9844961	0.79218	1	0.8237056
2575	Bn-A01-p9080359	A-genome specific (napus-rapa)	0.9904306	0.5151495	not scorable	not scorable	0.9917355	0.6973079
2576	Bn-A01-p9080573	A-genome specific (napus-rapa)	0.9832536	0.408157	not scorable	not scorable	0.9752066	0.7570655
2577	Bn-A01-p912298	A-genome specific (napus-rapa)	0.9665072	0.7901939	not scorable	not scorable	0.9752066	0.802668
2578	Bn-A01-p9125819	A-genome specific (napus-rapa)	0.9952153	0.6438332	not scorable	not scorable	0.9917355	0.702389
2579	Bn-A01-p9129693	A-genome specific (napus-rapa)	0.9688995	0.7014509	not scorable	not scorable	1	0.8009096
2580	Bn-A01-p9130391	A-genome specific (napus-rapa)	0.9736842	0.7490004	not scorable	not scorable	0.9917355	0.7654656
2581	Bn-A01-p9138209	A-genome specific (rapa only)	not scorable	not scorable	not scorable	not scorable	0.9421487	0.8454501
2582	Bn-A01-p9145211	A-genome specific (napus-rapa)	0.9641148	0.5722004	not scorable	not scorable	1	0.8004572
2583	Bn-A01-p9145630	A-genome specific (napus-rapa)	0.9569378	0.7104997	not scorable	not scorable	0.9834711	0.6699126
2584	Bn-A01-p9146490	A-genome specific (napus-rapa)	0.9808612	0.7422884	not scorable	not scorable	0.9917355	0.6891719
2585	Bn-A01-p9147096	A-genome specific (rapa only)	not scorable	not scorable	not scorable	not scorable	0.9834711	0.6999326
2586	Bn-A01-p9149601	A-genome specific (napus-rapa)	0.9808612	0.7714487	not scorable	not scorable	0.9917355	0.708837
2587	Bn-A01-p9150695	A-genome specific (napus-rapa)	0.9736842	0.7677061	not scorable	not scorable	0.9834711	0.810416
2588	Bn-A01-p9155327	A-genome specific (rapa only)	not scorable	not scorable	not scorable	not scorable	0.9834711	0.7497222
2589	Bn-A01-p916774	A-genome specific (napus-rapa)	0.7799043	0.8346233	not scorable	not scorable	0.9586777	0.7504229
2590	Bn-A01-p916852	A-genome specific (napus-rapa)	0.9569378	0.4246368	not scorable	not scorable	1	0.753805
2591	Bn-A01-p9170858	A-genome specific (napus-rapa)	0.9928229	0.4531369	not scorable	not scorable	0.9917355	0.6248583
2592	Bn-A01-p9176104	A-genome specific (napus-rapa)	0.9904306	0.3004888	not scorable	not scorable	0.9752066	0.6629816
2593	Bn-A01-p9180340	A-genome specific (napus-rapa)	0.9976076	0.5139866	not scorable	not scorable	0.9917355	0.7039695
2594	Bn-A01-p9180828	A-genome specific (napus-rapa)	0.9904306	0.7768031	not scorable	not scorable	0.9917355	0.7104266
2595	Bn-A01-p9203096	A-genome specific (napus-rapa)	0.9976076	0.5895977	not scorable	not scorable	0.9669421	0.7215685
2596	Bn-A01-p9220977	A-genome specific (napus-rapa)	0.9928229	0.7969592	not scorable	not scorable	0.9669421	0.7793942
2597	Bn-A01-p9223420	A-genome specific (napus-rapa)	0.9880383	0.7529009	not scorable	not scorable	0.9834711	0.830092
2598	Bn-A01-p9227229	A-genome specific (napus-rapa)	0.9808612	0.7488626	not scorable	not scorable	0.9752066	0.7170734
2599	Bn-A01-p923356	A-genome specific (napus-rapa)	0.9736842	0.4025852	not scorable	not scorable	1	0.7469699
2600	Bn-A01-p9254925	A-genome specific (napus-rapa)	0.9880383	0.808206	not scorable	not scorable	0.9834711	0.7040766
2601	Bn-A01-p9256011	A-genome specific (napus-rapa)	0.9928229	0.474391	not scorable	not scorable	1	0.8234416
2602	Bn-A01-p9258144	A-genome specific (napus-rapa)	0.9904306	0.5806828	not scorable	not scorable	1	0.7516875
2603	Bn-A01-p9259611	A-genome specific (napus-rapa)	0.9760765	0.6485757	not scorable	not scorable	0.9752066	0.6364136
2604	Bn-A01-p9260073	A-genome specific (napus-rapa)	0.9856459	0.7871568	not scorable	not scorable	0.9917355	0.7391517

2605	Bn-A01-p9264081	A-genome specific (napus-rapa)	0.9928229	0.7438051	not scorable	not scorable	1	0.7140734
2606	Bn-A01-p9269627	A-genome specific (napus-rapa)	0.9928229	0.8345587	not scorable	not scorable	1	0.7156405
2607	Bn-A01-p9303710	A-genome specific (napus-rapa)	0.9952153	0.404116	not scorable	not scorable	0.9917355	0.7147431
2608	Bn-A01-p9325719	A-genome specific (napus-rapa)	0.9976076	0.6660621	not scorable	not scorable	0.9834711	0.8911073
2609	Bn-A01-p9326695	A-genome specific (napus-rapa)	0.9856459	0.8196874	not scorable	not scorable	0.9917355	0.6952415
2610	Bn-A01-p9331463	A-genome specific (napus-rapa)	0.9665072	0.5058377	not scorable	not scorable	1	0.7508058
2611	Bn-A01-p9333956	A-genome specific (napus-rapa)	0.715311	0.8552695	not scorable	not scorable	0.9917355	0.789065
2612	Bn-A01-p9337252	A-genome specific (napus-rapa)	0.9688995	0.7778311	not scorable	not scorable	1	0.7055126
2613	Bn-A01-p933795	genome unspecific (all in)	0.9665072	0.2856917	1	0.7653105	0.9834711	0.7510444
2614	Bn-A01-p9360043	A-genome specific (napus-rapa)	0.9880383	0.5856451	not scorable	not scorable	0.9917355	0.6853399
2615	Bn-A01-p936014	genome unspecific (oleracea-rapa)	not scorable	not scorable	0.875969	0.7430966	0.9917355	0.8470932
2616	Bn-A01-p9360971	A-genome specific (napus-rapa)	0.9928229	0.9043303	not scorable	not scorable	1	0.8273721
2617	Bn-A01-p9369257	A-genome specific (napus-rapa)	0.9641148	0.8053153	not scorable	not scorable	1	0.8127
2618	Bn-A01-p9371414	A-genome specific (rapa only)	not scorable	not scorable	not scorable	not scorable	1	0.8173693
2619	Bn-A01-p938887	A-genome specific (napus-rapa)	0.9880383	0.4487089	not scorable	not scorable	0.9917355	0.6863895
2620	Bn-A01-p939202	A-genome specific (napus-rapa)	0.9880383	0.7098374	not scorable	not scorable	0.9752066	0.6949719
2621	Bn-A01-p9408018	A-genome specific (napus-rapa)	1	0.6137596	not scorable	not scorable	0.9917355	0.6506451
2622	Bn-A01-p941792	A-genome specific (napus-rapa)	0.9736842	0.3842787	not scorable	not scorable	1	0.8856928
2623	Bn-A01-p9436886	A-genome specific (rapa only)	not scorable	not scorable	not scorable	not scorable	0.6198347	0.8336314
2624	Bn-A01-p9442471	A-genome specific (napus-rapa)	0.9880383	0.8291274	not scorable	not scorable	0.9834711	0.7246636
2625	Bn-A01-p9443863	A-genome specific (rapa only)	not scorable	not scorable	not scorable	not scorable	0.9917355	0.7512324
2626	Bn-A01-p9445754	A-genome specific (napus-rapa)	0.9928229	0.8633167	not scorable	not scorable	0.9834711	0.7547623
2627	Bn-A01-p9469933	A-genome specific (napus-rapa)	0.9976076	0.4200247	not scorable	not scorable	0.9917355	0.7909877
2628	Bn-A01-p9476520	napus specific	0.9449761	0.1835868	not scorable	not scorable	not scorable	not scorable
2629	Bn-A01-p9499100	A-genome specific (napus-rapa)	0.9497608	0.4286865	not scorable	not scorable	1	0.8834755
2630	Bn-A01-p951063	A-genome specific (napus-rapa)	0.9688995	0.4920422	not scorable	not scorable	1	0.8791605
2631	Bn-A01-p951341	A-genome specific (napus-rapa)	0.9401914	0.2838576	not scorable	not scorable	1	0.6675612
2632	Bn-A01-p9519478	A-genome specific (napus-rapa)	0.9641148	0.6942161	not scorable	not scorable	0.9917355	0.6849335
2633	Bn-A01-p9519660	A-genome specific (napus-rapa)	0.9641148	0.7019097	not scorable	not scorable	0.9917355	0.8588858
2634	Bn-A01-p9520409	genome unspecific (all in)	0.9784689	0.3140631	1	0.8037983	1	0.7157279
2635	Bn-A01-p9523572	A-genome specific (napus-rapa)	0.9928229	0.6623319	not scorable	not scorable	1	0.6594497
2636	Bn-A01-p9551341	A-genome specific (napus-rapa)	0.9808612	0.7527544	not scorable	not scorable	0.9173554	0.70767
2637	Bn-A01-p9565360	A-genome specific (napus-rapa)	0.9928229	0.8328172	not scorable	not scorable	0.9834711	0.8324143

2638	Bn-A01-p9565804	A-genome specific (napus-rapa)	1	0.4570931	not scorable	not scorable	1	0.7501816
2639	Bn-A01-p9568888	A-genome specific (napus-rapa)	0.9784689	0.8387133	not scorable	not scorable	1	0.76025
2640	Bn-A01-p95718	A-genome specific (napus-rapa)	0.9784689	0.8050982	not scorable	not scorable	0.9834711	0.8011191
2641	Bn-A01-p9599644	A-genome specific (rapa only)	not scorable	not scorable	not scorable	not scorable	0.9669421	0.9064599
2642	Bn-A01-p9614730	A-genome specific (napus-rapa)	0.9760765	0.7618071	not scorable	not scorable	1	0.7269471
2643	Bn-A01-p9619932	A-genome specific (napus-rapa)	1	0.8786843	not scorable	not scorable	1	0.7780618
2644	Bn-A01-p9621623	A-genome specific (napus-rapa)	0.9784689	0.7825742	not scorable	not scorable	1	0.8332121
2645	Bn-A01-p968378	A-genome specific (rapa only)	not scorable	not scorable	not scorable	not scorable	0.9917355	0.7188525
2646	Bn-A01-p968465	A-genome specific (napus-rapa)	0.9760765	0.8218916	not scorable	not scorable	0.9834711	0.7817704
2647	Bn-A01-p9699152	A-genome specific (napus-rapa)	0.9784689	0.8165813	not scorable	not scorable	0.9917355	0.8720146
2648	Bn-A01-p970103	A-genome specific (napus-rapa)	0.9736842	0.7788212	not scorable	not scorable	1	0.7826774
2649	Bn-A01-p9711809	A-genome specific (napus-rapa)	0.9665072	0.7503656	not scorable	not scorable	0.9917355	0.7686307
2650	Bn-A01-p9712591	A-genome specific (napus-rapa)	0.8684211	0.7848732	not scorable	not scorable	1	0.8381248
2651	Bn-A01-p974934	A-genome specific (napus-rapa)	0.9808612	0.7761122	not scorable	not scorable	0.9669421	0.7349759
2652	Bn-A01-p975213	A-genome specific (napus-rapa)	0.7129186	0.7942976	not scorable	not scorable	0.9917355	0.804921
2653	Bn-A01-p975640	A-genome specific (rapa only)	not scorable	not scorable	not scorable	not scorable	0.9917355	0.7672531
2654	Bn-A01-p97663	A-genome specific (napus-rapa)	0.9617225	0.7998995	not scorable	not scorable	0.9669421	0.7627535
2655	Bn-A01-p97751	A-genome specific (rapa only)	not scorable	not scorable	not scorable	not scorable	0.9834711	0.778963
2656	Bn-A01-p977861	A-genome specific (rapa only)	not scorable	not scorable	not scorable	not scorable	0.9752066	0.8843359
2657	Bn-A01-p978860	A-genome specific (napus-rapa)	0.9784689	0.7921835	not scorable	not scorable	1	0.7054566
2658	Bn-A01-p9810552	genome unspecific (oleracea-rapa)	not scorable	not scorable	0.9922481	0.7761868	1	0.7562456
2659	Bn-A01-p9853365	A-genome specific (napus-rapa)	0.9904306	0.5016139	not scorable	not scorable	0.9917355	0.5414737
2660	Bn-A01-p9863114	A-genome specific (napus-rapa)	0.9880383	0.3176528	not scorable	not scorable	0.9834711	0.8269134
2661	Bn-A01-p9866468	A-genome specific (napus-rapa)	0.9832536	0.7630193	not scorable	not scorable	0.9834711	0.7889405
2662	Bn-A01-p9884449	A-genome specific (napus-rapa)	0.8947368	0.8031442	not scorable	not scorable	1	0.9175813
2663	Bn-A01-p9922741	A-genome specific (napus-rapa)	0.9880383	0.8095062	not scorable	not scorable	1	0.7075329
2664	Bn-A01-p994722	A-genome specific (napus-rapa)	0.9880383	0.649506	not scorable	not scorable	0.9917355	0.6135442
2665	Bn-A01-p9951633	A-genome specific (napus-rapa)	0.9880383	0.3974268	not scorable	not scorable	0.9669421	0.7194839
2666	Bn-A01-p9961425	A-genome specific (napus-rapa)	0.9976076	0.4783892	not scorable	not scorable	1	0.6939689
2667	Bn-A01-p9961553	A-genome specific (napus-rapa)	0.9952153	0.5215012	not scorable	not scorable	1	0.7492537
2668	Bn-A01-p9982115	A-genome specific (napus-rapa)	0.9856459	0.353414	not scorable	not scorable	0.9917355	0.8076326
2669	Bn-A01-p9989240	A-genome specific (napus-rapa)	0.9784689	0.8365031	not scorable	not scorable	0.9834711	0.7052579
2670	Bn-A01-p9996240	A-genome specific (napus-rapa)	0.9856459	0.7447909	not scorable	not scorable	0.9834711	0.676405

2671	Bn-A01-p9996458	A-genome specific (napus-rapa)	0.9904306	0.8417633	not scorable	not scorable	0.9834711	0.6754791
2672	Bn-A01-p9999485	A-genome specific (napus-rapa)	0.930622	0.5098737	not scorable	not scorable	1	0.7076181
2673	Bn-A02-p10019636	A-genome specific (napus-rapa)	0.9832536	0.6768479	not scorable	not scorable	1	0.5130539
2674	Bn-A02-p10020231	A-genome specific (napus-rapa)	0.9712918	0.7959	not scorable	not scorable	0.9917355	0.7730865
2675	Bn-A02-p10067386	A-genome specific (napus-rapa)	0.9760765	0.5348075	not scorable	not scorable	0.9834711	0.7040934
2676	Bn-A02-p10078171	A-genome specific (napus-rapa)	0.9736842	0.2962688	not scorable	not scorable	0.9917355	0.8180001
2677	Bn-A02-p10092191	A-genome specific (napus-rapa)	0.9545454	0.3095171	not scorable	not scorable	0.9834711	0.7040308
2678	Bn-A02-p10096185	A-genome specific (napus-rapa)	0.9784689	0.8123909	not scorable	not scorable	0.9752066	0.718857
2679	Bn-A02-p10105701	A-genome specific (napus-rapa)	0.9712918	0.49967	not scorable	not scorable	0.9752066	0.6521387
2680	Bn-A02-p10107504	A-genome specific (napus-rapa)	0.9808612	0.7367874	not scorable	not scorable	1	0.7105102
2681	Bn-A02-p10110769	A-genome specific (napus-rapa)	0.9736842	0.4548682	not scorable	not scorable	1	0.7229961
2682	Bn-A02-p10111305	A-genome specific (napus-rapa)	0.9880383	0.7430297	not scorable	not scorable	0.9669421	0.7044492
2683	Bn-A02-p10126530	A-genome specific (napus-rapa)	0.9808612	0.4145238	not scorable	not scorable	0.9917355	0.7274572
2684	Bn-A02-p10129605	A-genome specific (napus-rapa)	0.9856459	0.7700725	not scorable	not scorable	0.9834711	0.7212927
2685	Bn-A02-p10133353	A-genome specific (napus-rapa)	0.9976076	0.7499518	not scorable	not scorable	1	0.781088
2686	Bn-A02-p10138982	A-genome specific (napus-rapa)	0.9808612	0.3945711	not scorable	not scorable	0.9834711	0.7971616
2687	Bn-A02-p10140065	A-genome specific (rapa only)	not scorable	not scorable	not scorable	not scorable	1	0.7252899
2688	Bn-A02-p10153359	genome unspecific (all in)	0.9976076	0.2801223	0.9767442	0.2563767	1	0.9101796
2689	Bn-A02-p10156025	A-genome specific (napus-rapa)	0.9808612	0.8138556	not scorable	not scorable	1	0.7896795
2690	Bn-A02-p10176579	A-genome specific (napus-rapa)	0.9688995	0.8134037	not scorable	not scorable	0.9834711	0.773312
2691	Bn-A02-p10176749	A-genome specific (napus-rapa)	0.9760765	0.7891621	not scorable	not scorable	0.9834711	0.787383
2692	Bn-A02-p10181448	A-genome specific (napus-rapa)	0.9712918	0.380966	not scorable	not scorable	0.9752066	0.7876717
2693	Bn-A02-p10184110	A-genome specific (napus-rapa)	0.9952153	0.2823426	not scorable	not scorable	0.9752066	0.7566882
2694	Bn-A02-p10186244	A-genome specific (napus-rapa)	0.9641148	0.7965204	not scorable	not scorable	0.9752066	0.7650018
2695	Bn-A02-p10186594	A-genome specific (napus-rapa)	0.9593301	0.7766776	not scorable	not scorable	0.9917355	0.7914191
2696	Bn-A02-p10193709	A-genome specific (napus-rapa)	0.9904306	0.5009422	not scorable	not scorable	1	0.7423257
2697	Bn-A02-p1020806	A-genome specific (napus-rapa)	0.9856459	0.8437583	not scorable	not scorable	0.5041322	0.8503447
2698	Bn-A02-p10218812	A-genome specific (napus-rapa)	0.9928229	0.7201415	not scorable	not scorable	1	0.9007735
2699	Bn-A02-p10227986	A-genome specific (napus-rapa)	0.9904306	0.7557117	not scorable	not scorable	0.9917355	0.753494
2700	Bn-A02-p10228189	A-genome specific (napus-rapa)	1	0.4886748	not scorable	not scorable	0.9917355	0.7458565
2701	Bn-A02-p10231298	A-genome specific (napus-rapa)	0.9641148	0.4686856	not scorable	not scorable	0.9917355	0.7815917
2702	Bn-A02-p10247756	A-genome specific (napus-rapa)	0.9808612	0.2493556	not scorable	not scorable	0.9834711	0.7064714
2703	Bn-A02-p1025518	A-genome specific (rapa only)	not scorable	not scorable	not scorable	not scorable	0.8595041	0.8203158

2704	Bn-A02-p10255955	A-genome specific (napus-rapa)	0.9808612	0.2803211	not scorable	not scorable	0.9917355	0.6078122
2705	Bn-A02-p10259746	A-genome specific (napus-rapa)	0.9736842	0.3071546	not scorable	not scorable	0.9834711	0.70717
2706	Bn-A02-p1027570	A-genome specific (napus-rapa)	1	0.4916743	not scorable	not scorable	0.9669421	0.7017869
2707	Bn-A02-p1028349	A-genome specific (napus-rapa)	1	0.5580248	not scorable	not scorable	0.9586777	0.5250807
2708	Bn-A02-p10289331	A-genome specific (napus-rapa)	0.9593301	0.7756767	not scorable	not scorable	1	0.7583615
2709	Bn-A02-p10323998	A-genome specific (napus-rapa)	0.9880383	0.6987922	not scorable	not scorable	0.9834711	0.7097909
2710	Bn-A02-p10325260	genome unspecific (all in)	0.9880383	0.3900229	1	0.6717914	0.9752066	0.6823048
2711	Bn-A02-p10332863	genome unspecific (all in)	0.9880383	0.3265109	0.6124031	0.7499385	1	0.7759184
2712	Bn-A02-p10338350	A-genome specific (rapa only)	not scorable	not scorable	not scorable	not scorable	0.9752066	0.6816012
2713	Bn-A02-p10377179	A-genome specific (napus-rapa)	0.9952153	0.3298168	not scorable	not scorable	1	0.6632591
2714	Bn-A02-p10395108	A-genome specific (napus-rapa)	0.9832536	0.7160507	not scorable	not scorable	0.5206612	0.6552821
2715	Bn-A02-p10396907	A-genome specific (napus-rapa)	0.9904306	0.7796504	not scorable	not scorable	0.9917355	0.7234836
2716	Bn-A02-p10404084	A-genome specific (napus-rapa)	0.9904306	0.6718618	not scorable	not scorable	0.9834711	0.6729136
2717	Bn-A02-p10409103	A-genome specific (napus-rapa)	0.9904306	0.4713656	not scorable	not scorable	0.9752066	0.8531474
2718	Bn-A02-p10418131	A-genome specific (napus-rapa)	0.9880383	0.2555766	not scorable	not scorable	0.9917355	0.7020359
2719	Bn-A02-p10444138	A-genome specific (napus-rapa)	0.9832536	0.764904	not scorable	not scorable	0.9917355	0.7129886
2720	Bn-A02-p10452854	A-genome specific (rapa only)	not scorable	not scorable	not scorable	not scorable	0.5041322	0.8365592
2721	Bn-A02-p10477549	A-genome specific (napus-rapa)	0.9665072	0.7591661	not scorable	not scorable	0.9752066	0.7014977
2722	Bn-A02-p10485644	A-genome specific (napus-rapa)	0.9904306	0.7794305	not scorable	not scorable	0.9917355	0.7535936
2723	Bn-A02-p10493685	genome unspecific (all in)	0.9665072	0.6924852	0.9844961	0.7629939	0.9834711	0.7207382
2724	Bn-A02-p10507801	A-genome specific (napus-rapa)	0.9736842	0.7457719	not scorable	not scorable	0.9752066	0.7184493
2725	Bn-A02-p10507864	A-genome specific (napus-rapa)	0.9736842	0.7900165	not scorable	not scorable	0.9752066	0.7258007
2726	Bn-A02-p10508075	A-genome specific (napus-rapa)	0.9593301	0.7859077	not scorable	not scorable	0.9917355	0.8068569
2727	Bn-A02-p10510996	A-genome specific (napus-rapa)	0.9904306	0.7696472	not scorable	not scorable	0.9917355	0.7112234
2728	Bn-A02-p10515029	A-genome specific (napus-rapa)	0.9665072	0.6581645	not scorable	not scorable	0.9586777	0.6531467
2729	Bn-A02-p10521287	A-genome specific (rapa only)	not scorable	not scorable	not scorable	not scorable	0.9752066	0.832016
2730	Bn-A02-p10524842	A-genome specific (napus-rapa)	0.9808612	0.7277724	not scorable	not scorable	0.9834711	0.6959049
2731	Bn-A02-p10527075	A-genome specific (napus-rapa)	0.9712918	0.7875571	not scorable	not scorable	0.9834711	0.7003807
2732	Bn-A02-p10528586	A-genome specific (napus-rapa)	0.9641148	0.7890535	not scorable	not scorable	0.9917355	0.75708
2733	Bn-A02-p10530978	A-genome specific (napus-rapa)	0.9712918	0.7926415	not scorable	not scorable	0.9752066	0.7487559
2734	Bn-A02-p10533300	A-genome specific (napus-rapa)	0.9688995	0.7206933	not scorable	not scorable	0.9917355	0.7208403
2735	Bn-A02-p10540052	A-genome specific (napus-rapa)	0.9784689	0.7893047	not scorable	not scorable	0.9917355	0.7183622
2736	Bn-A02-p10540148	A-genome specific (napus-rapa)	0.9832536	0.7607111	not scorable	not scorable	0.9752066	0.8009692

2737	Bn-A02-p10556758	A-genome specific (napus-rapa)	0.9545454	0.6535953	not scorable	not scorable	0.9752066	0.6365817
2738	Bn-A02-p10568947	A-genome specific (napus-rapa)	0.9880383	0.3953613	not scorable	not scorable	1	0.7785489
2739	Bn-A02-p10574526	A-genome specific (napus-rapa)	0.9641148	0.7928859	not scorable	not scorable	0.9421487	0.6956645
2740	Bn-A02-p10587170	A-genome specific (napus-rapa)	0.9736842	0.8007827	not scorable	not scorable	0.9917355	0.7218029
2741	Bn-A02-p10587232	A-genome specific (napus-rapa)	0.9808612	0.8027452	not scorable	not scorable	0.9917355	0.707949
2742	Bn-A02-p10587536	genome unspecific (oleracea-rapa)	not scorable	not scorable	1	0.7922735	0.9586777	0.6254619
2743	Bn-A02-p10591779	A-genome specific (napus-rapa)	0.9976076	0.4737935	not scorable	not scorable	1	0.7207334
2744	Bn-A02-p10614602	A-genome specific (napus-rapa)	0.9593301	0.7765902	not scorable	not scorable	0.9917355	0.8198096
2745	Bn-A02-p10616871	A-genome specific (napus-rapa)	0.9593301	0.6324186	not scorable	not scorable	0.9834711	0.6896921
2746	Bn-A02-p10621504	A-genome specific (napus-rapa)	0.9712918	0.8007813	not scorable	not scorable	0.9917355	0.7963939
2747	Bn-A02-p10622338	A-genome specific (napus-rapa)	0.9043062	0.7439107	not scorable	not scorable	0.9421487	0.6466249
2748	Bn-A02-p10623838	A-genome specific (napus-rapa)	0.9784689	0.8121132	not scorable	not scorable	0.9917355	0.7430676
2749	Bn-A02-p10624373	A-genome specific (napus-rapa)	0.9760765	0.6364824	not scorable	not scorable	0.9917355	0.7832156
2750	Bn-A02-p10625720	A-genome specific (napus-rapa)	0.9617225	0.6920608	not scorable	not scorable	0.9917355	0.7600092
2751	Bn-A02-p10626782	A-genome specific (napus-rapa)	0.9880383	0.6974671	not scorable	not scorable	0.9669421	0.6698877
2752	Bn-A02-p10629886	A-genome specific (napus-rapa)	0.9856459	0.7639012	not scorable	not scorable	0.9917355	0.7615372
2753	Bn-A02-p10637202	A-genome specific (napus-rapa)	0.9736842	0.8084061	not scorable	not scorable	0.9669421	0.696144
2754	Bn-A02-p10638989	A-genome specific (napus-rapa)	0.9521531	0.8145033	not scorable	not scorable	0.9834711	0.7489321
2755	Bn-A02-p10641032	A-genome specific (rapa only)	not scorable	not scorable	not scorable	not scorable	0.8099173	0.7194871
2756	Bn-A02-p10652585	A-genome specific (napus-rapa)	0.9593301	0.4640046	not scorable	not scorable	0.9834711	0.6824505
2757	Bn-A02-p10668400	A-genome specific (napus-rapa)	0.9545454	0.6338026	not scorable	not scorable	1	0.7554175
2758	Bn-A02-p10681015	A-genome specific (napus-rapa)	0.9736842	0.5088671	not scorable	not scorable	0.9669421	0.7497477
2759	Bn-A02-p1068542	A-genome specific (napus-rapa)	0.9952153	0.6929082	not scorable	not scorable	0.9586777	0.7232896
2760	Bn-A02-p10690394	A-genome specific (napus-rapa)	0.9760765	0.8052262	not scorable	not scorable	0.9752066	0.7920424
2761	Bn-A02-p10690477	A-genome specific (napus-rapa)	0.9282297	0.8134491	not scorable	not scorable	0.9586777	0.7888144
2762	Bn-A02-p10699604	A-genome specific (napus-rapa)	0.6889952	0.7059219	not scorable	not scorable	0.9586777	0.6969976
2763	Bn-A02-p10700544	A-genome specific (napus-rapa)	0.9832536	0.7736111	not scorable	not scorable	0.9917355	0.7982529
2764	Bn-A02-p10711353	A-genome specific (napus-rapa)	0.9832536	0.7857351	not scorable	not scorable	0.9917355	0.786674
2765	Bn-A02-p10711442	A-genome specific (napus-rapa)	0.9904306	0.7956988	not scorable	not scorable	0.9834711	0.7153298
2766	Bn-A02-p10711565	A-genome specific (napus-rapa)	0.9760765	0.7996149	not scorable	not scorable	1	0.8851421
2767	Bn-A02-p10719296	A-genome specific (napus-rapa)	0.9545454	0.6446798	not scorable	not scorable	0.9834711	0.6717822
2768	Bn-A02-p10742809	A-genome specific (napus-rapa)	0.9593301	0.516248	not scorable	not scorable	0.9421487	0.5033373
2769	Bn-A02-p10745711	A-genome specific (napus-rapa)	0.9688995	0.4523543	not scorable	not scorable	1	0.8420836

2770	Bn-A02-p10746556	A-genome specific (napus-rapa)	0.7464115	0.8351284	not scorable	not scorable	0.9917355	0.8498756
2771	Bn-A02-p10753552	A-genome specific (napus-rapa)	0.9832536	0.3150043	not scorable	not scorable	0.9917355	0.6645859
2772	Bn-A02-p1075920	A-genome specific (rapa only)	not scorable	not scorable	not scorable	not scorable	0.9917355	0.6832442
2773	Bn-A02-p10763116	A-genome specific (napus-rapa)	0.9617225	0.5694524	not scorable	not scorable	0.9917355	0.8066405
2774	Bn-A02-p10775283	genome unspecific (all in)	0.9832536	0.4724845	1	0.8460527	1	0.8948976
2775	Bn-A02-p10780709	genome unspecific (oleracea-rapa)	not scorable	not scorable	0.9844961	0.8925639	0.9834711	0.7848444
2776	Bn-A02-p10781906	A-genome specific (napus-rapa)	0.9665072	0.7488509	not scorable	not scorable	0.9834711	0.7508231
2777	Bn-A02-p10786230	A-genome specific (napus-rapa)	0.9688995	0.6598713	not scorable	not scorable	0.9834711	0.7079984
2778	Bn-A02-p10787104	A-genome specific (napus-rapa)	0.9688995	0.8031533	not scorable	not scorable	0.9752066	0.7152818
2779	Bn-A02-p10787380	A-genome specific (napus-rapa)	0.9880383	0.814478	not scorable	not scorable	0.9917355	0.7956197
2780	Bn-A02-p10788285	A-genome specific (napus-rapa)	0.9736842	0.3972595	not scorable	not scorable	1	0.7906294
2781	Bn-A02-p10790263	A-genome specific (napus-rapa)	0.9736842	0.7650067	not scorable	not scorable	0.9834711	0.7881014
2782	Bn-A02-p10793326	A-genome specific (napus-rapa)	0.9688995	0.7895914	not scorable	not scorable	0.9917355	0.6720709
2783	Bn-A02-p10796317	napus specific	0.9641148	0.5793405	not scorable	not scorable	not scorable	not scorable
2784	Bn-A02-p10796462	A-genome specific (napus-rapa)	0.9832536	0.7570568	not scorable	not scorable	0.9917355	0.7536912
2785	Bn-A02-p10806477	A-genome specific (napus-rapa)	0.9736842	0.5485599	not scorable	not scorable	1	0.7544218
2786	Bn-A02-p10811918	A-genome specific (napus-rapa)	0.9665072	0.590257	not scorable	not scorable	0.9917355	0.7296093
2787	Bn-A02-p10815211	A-genome specific (napus-rapa)	0.9880383	0.8048449	not scorable	not scorable	0.9917355	0.7565199
2788	Bn-A02-p10815571	A-genome specific (napus-rapa)	0.9928229	0.5668868	not scorable	not scorable	0.9752066	0.7146045
2789	Bn-A02-p10815969	A-genome specific (napus-rapa)	0.9832536	0.6772779	not scorable	not scorable	1	0.8796692
2790	Bn-A02-p10818801	A-genome specific (napus-rapa)	0.9928229	0.4794307	not scorable	not scorable	1	0.9175798
2791	Bn-A02-p10822102	A-genome specific (napus-rapa)	0.9497608	0.5050519	not scorable	not scorable	0.9917355	0.9175813
2792	Bn-A02-p10839131	A-genome specific (napus-rapa)	0.9617225	0.7706677	not scorable	not scorable	1	0.8052031
2793	Bn-A02-p10850012	A-genome specific (napus-rapa)	0.9904306	0.5145055	not scorable	not scorable	1	0.6923397
2794	Bn-A02-p10850323	A-genome specific (napus-rapa)	0.9952153	0.3639618	not scorable	not scorable	1	0.7544973
2795	Bn-A02-p1085455	A-genome specific (napus-rapa)	0.9808612	0.8290815	not scorable	not scorable	1	0.7229961
2796	Bn-A02-p10857857	A-genome specific (napus-rapa)	0.9808612	0.7506896	not scorable	not scorable	0.9752066	0.6707141
2797	Bn-A02-p10858522	A-genome specific (rapa only)	not scorable	not scorable	not scorable	not scorable	0.9669421	0.4402857
2798	Bn-A02-p10861090	A-genome specific (napus-rapa)	0.9952153	0.7483519	not scorable	not scorable	1	0.6497253
2799	Bn-A02-p10866642	A-genome specific (napus-rapa)	0.9880383	0.7501666	not scorable	not scorable	0.9834711	0.740935
2800	Bn-A02-p10869120	A-genome specific (napus-rapa)	0.9880383	0.5414088	not scorable	not scorable	0.9917355	0.7046836
2801	Bn-A02-p10879119	A-genome specific (napus-rapa)	0.9162679	0.734138	not scorable	not scorable	0.9669421	0.6380289
2802	Bn-A02-p108936	A-genome specific (napus-rapa)	1	0.7189614	not scorable	not scorable	0.9834711	0.6850874

2803	Bn-A02-p10895698	A-genome specific (napus-rapa)	0.9856459	0.6615065	not scorable	not scorable	1	0.9175813
2804	Bn-A02-p10906851	A-genome specific (napus-rapa)	0.9928229	0.3369223	not scorable	not scorable	1	0.7347764
2805	Bn-A02-p1091779	A-genome specific (rapa only)	not scorable	not scorable	not scorable	not scorable	0.9834711	0.6601198
2806	Bn-A02-p1092146	A-genome specific (napus-rapa)	0.9928229	0.7744465	not scorable	not scorable	1	0.6967734
2807	Bn-A02-p10930249	A-genome specific (napus-rapa)	0.9114832	0.8129231	not scorable	not scorable	0.5289256	0.7493338
2808	Bn-A02-p10949322	A-genome specific (napus-rapa)	0.9880383	0.4706188	not scorable	not scorable	0.9586777	0.6240984
2809	Bn-A02-p10956599	A-genome specific (napus-rapa)	0.9497608	0.7206351	not scorable	not scorable	0.5371901	0.811419
2810	Bn-A02-p10959141	A-genome specific (napus-rapa)	0.9952153	0.4101261	not scorable	not scorable	1	0.795513
2811	Bn-A02-p1096082	A-genome specific (napus-rapa)	0.9928229	0.7764494	not scorable	not scorable	0.9834711	0.7484605
2812	Bn-A02-p10983922	A-genome specific (napus-rapa)	0.9904306	0.2231961	not scorable	not scorable	0.9669421	0.3928776
2813	Bn-A02-p10992266	A-genome specific (napus-rapa)	0.8349282	0.7761034	not scorable	not scorable	0.4876033	0.7822044
2814	Bn-A02-p10992866	napus specific	0.9330143	0.8007172	not scorable	not scorable	not scorable	not scorable
2815	Bn-A02-p10996151	A-genome specific (napus-rapa)	0.9808612	0.2712472	not scorable	not scorable	0.9834711	0.6900607
2816	Bn-A02-p11014885	A-genome specific (napus-rapa)	0.9545454	0.5390819	not scorable	not scorable	0.9834711	0.7383878
2817	Bn-A02-p11038401	A-genome specific (napus-rapa)	0.9760765	0.8103774	not scorable	not scorable	0.9752066	0.7814685
2818	Bn-A02-p11039119	A-genome specific (napus-rapa)	0.9880383	0.5246774	not scorable	not scorable	0.9090909	0.5560069
2819	Bn-A02-p11041638	A-genome specific (napus-rapa)	0.9736842	0.439959	not scorable	not scorable	0.9917355	0.6725169
2820	Bn-A02-p11041726	A-genome specific (napus-rapa)	0.9928229	0.4879316	not scorable	not scorable	0.9917355	0.7675335
2821	Bn-A02-p11053817	A-genome specific (napus-rapa)	0.9210526	0.7571442	not scorable	not scorable	1	0.8138818
2822	Bn-A02-p11060975	A-genome specific (napus-rapa)	0.9928229	0.81266	not scorable	not scorable	0.9752066	0.5140325
2823	Bn-A02-p11062457	A-genome specific (napus-rapa)	0.9617225	0.7984988	not scorable	not scorable	1	0.7689113
2824	Bn-A02-p11068431	A-genome specific (napus-rapa)	0.9952153	0.5549823	not scorable	not scorable	1	0.6978682
2825	Bn-A02-p11070906	A-genome specific (napus-rapa)	0.9880383	0.7532225	not scorable	not scorable	0.9917355	0.7022554
2826	Bn-A02-p11073162	A-genome specific (napus-rapa)	0.9856459	0.7307814	not scorable	not scorable	0.9586777	0.6609219
2827	Bn-A02-p11073326	A-genome specific (napus-rapa)	0.9832536	0.3749127	not scorable	not scorable	0.9917355	0.6800783
2828	Bn-A02-p11080706	A-genome specific (napus-rapa)	0.9856459	0.7338021	not scorable	not scorable	0.9752066	0.7186599
2829	Bn-A02-p11086855	A-genome specific (napus-rapa)	0.9976076	0.5488379	not scorable	not scorable	0.9917355	0.822943
2830	Bn-A02-p11087046	A-genome specific (napus-rapa)	0.9952153	0.8911709	not scorable	not scorable	0.9917355	0.9435292
2831	Bn-A02-p11087388	A-genome specific (napus-rapa)	1	0.5896162	not scorable	not scorable	0.9834711	0.7022027
2832	Bn-A02-p11096221	A-genome specific (napus-rapa)	0.9880383	0.7519594	not scorable	not scorable	0.9752066	0.8098366
2833	Bn-A02-p11153317	A-genome specific (napus-rapa)	0.9114832	0.3976288	not scorable	not scorable	0.9586777	0.3803256
2834	Bn-A02-p11155686	A-genome specific (napus-rapa)	0.9976076	0.7610021	not scorable	not scorable	0.9752066	0.6341022
2835	Bn-A02-p11156474	A-genome specific (napus-rapa)	0.9928229	0.7569256	not scorable	not scorable	0.9917355	0.7017822

2836	Bn-A02-p11159353	A-genome specific (napus-rapa)	0.9952153	0.7625158	not scorable	not scorable	1	0.7760359
2837	Bn-A02-p11160726	A-genome specific (napus-rapa)	0.9904306	0.7619434	not scorable	not scorable	0.9504132	0.7258481
2838	Bn-A02-p11161716	A-genome specific (napus-rapa)	0.9976076	0.6558157	not scorable	not scorable	0.9917355	0.7105656
2839	Bn-A02-p11170230	A-genome specific (napus-rapa)	0.9928229	0.4909083	not scorable	not scorable	1	0.6815073
2840	Bn-A02-p11182703	A-genome specific (napus-rapa)	0.7535885	0.7147064	not scorable	not scorable	0.9669421	0.6667518
2841	Bn-A02-p11187572	A-genome specific (napus-rapa)	0.9976076	0.814931	not scorable	not scorable	1	0.8978928
2842	Bn-A02-p11192451	A-genome specific (napus-rapa)	0.708134	0.8059431	not scorable	not scorable	0.9256198	0.7171036
2843	Bn-A02-p11195341	A-genome specific (napus-rapa)	0.923445	0.7832264	not scorable	not scorable	0.9669421	0.748989
2844	Bn-A02-p11200305	A-genome specific (napus-rapa)	0.9808612	0.7618113	not scorable	not scorable	0.9917355	0.7497262
2845	Bn-A02-p11209194	A-genome specific (napus-rapa)	0.9784689	0.497162	not scorable	not scorable	0.9917355	0.5300997
2846	Bn-A02-p11223862	A-genome specific (napus-rapa)	0.9808612	0.4996364	not scorable	not scorable	0.9917355	0.7754756
2847	Bn-A02-p11227845	A-genome specific (napus-rapa)	0.9617225	0.5126876	not scorable	not scorable	0.9917355	0.7600925
2848	Bn-A02-p11235969	A-genome specific (napus-rapa)	0.7727273	0.7533811	not scorable	not scorable	0.9669421	0.6997408
2849	Bn-A02-p11237912	A-genome specific (napus-rapa)	0.9641148	0.6966767	not scorable	not scorable	0.9834711	0.6931527
2850	Bn-A02-p11238010	A-genome specific (napus-rapa)	0.9856459	0.66574	not scorable	not scorable	0.9752066	0.732523
2851	Bn-A02-p11238896	A-genome specific (napus-rapa)	0.9880383	0.7811423	not scorable	not scorable	0.9917355	0.6957834
2852	Bn-A02-p11248280	A-genome specific (napus-rapa)	0.9808612	0.7456627	not scorable	not scorable	0.9669421	0.744024
2853	Bn-A02-p11248642	A-genome specific (napus-rapa)	0.9952153	0.845449	not scorable	not scorable	1	0.704862
2854	Bn-A02-p11248742	A-genome specific (napus-rapa)	0.9904306	0.7550974	not scorable	not scorable	0.9834711	0.7947085
2855	Bn-A02-p11248828	A-genome specific (napus-rapa)	0.9832536	0.7696138	not scorable	not scorable	0.9752066	0.7062414
2856	Bn-A02-p11259297	A-genome specific (napus-rapa)	0.9880383	0.7764443	not scorable	not scorable	0.9752066	0.684204
2857	Bn-A02-p11262178	A-genome specific (napus-rapa)	0.9641148	0.6542574	not scorable	not scorable	1	0.5850273
2858	Bn-A02-p11265573	A-genome specific (rapa only)	not scorable	not scorable	not scorable	not scorable	0.9752066	0.7400513
2859	Bn-A02-p11278529	A-genome specific (napus-rapa)	0.9808612	0.3572665	not scorable	not scorable	0.9917355	0.7376901
2860	Bn-A02-p11284285	A-genome specific (napus-rapa)	0.9808612	0.8224531	not scorable	not scorable	1	0.7647761
2861	Bn-A02-p11284503	A-genome specific (napus-rapa)	0.9736842	0.7608749	not scorable	not scorable	0.9917355	0.6876206
2862	Bn-A02-p11291606	A-genome specific (napus-rapa)	0.9904306	0.6919408	not scorable	not scorable	0.9917355	0.7130901
2863	Bn-A02-p11293986	A-genome specific (napus-rapa)	0.9904306	0.3272583	not scorable	not scorable	1	0.6816372
2864	Bn-A02-p11320789	A-genome specific (napus-rapa)	0.9665072	0.6465892	not scorable	not scorable	0.9752066	0.6608645
2865	Bn-A02-p11328718	A-genome specific (napus-rapa)	0.9832536	0.8043924	not scorable	not scorable	0.9834711	0.7811155
2866	Bn-A02-p11342711	A-genome specific (napus-rapa)	0.8014354	0.6517679	not scorable	not scorable	0.5454546	0.6414672
2867	Bn-A02-p11345557	A-genome specific (napus-rapa)	0.9043062	0.72053	not scorable	not scorable	0.9917355	0.697055
2868	Bn-A02-p11346389	A-genome specific (napus-rapa)	0.9473684	0.6544337	not scorable	not scorable	1	0.6833037

2869	Bn-A02-p1135078	A-genome specific (napus-rapa)	0.9928229	0.7934042	not scorable	not scorable	0.9834711	0.7985696
2870	Bn-A02-p11355276	A-genome specific (napus-rapa)	0.9784689	0.2296644	not scorable	not scorable	0.9917355	0.2892561
2871	Bn-A02-p11356944	A-genome specific (napus-rapa)	0.8516746	0.797935	not scorable	not scorable	0.9504132	0.7663594
2872	Bn-A02-p11365167	A-genome specific (napus-rapa)	0.9880383	0.863392	not scorable	not scorable	0.9917355	0.7964792
2873	Bn-A02-p11415015	A-genome specific (napus-rapa)	0.9904306	0.5148746	not scorable	not scorable	0.9752066	0.7052752
2874	Bn-A02-p11439590	A-genome specific (napus-rapa)	0.9856459	0.8014436	not scorable	not scorable	1	0.8020115
2875	Bn-A02-p11440895	A-genome specific (napus-rapa)	0.9712918	0.7340946	not scorable	not scorable	0.9917355	0.667549
2876	Bn-A02-p11441711	A-genome specific (napus-rapa)	0.9736842	0.807888	not scorable	not scorable	0.9834711	0.7124315
2877	Bn-A02-p11449348	A-genome specific (napus-rapa)	0.9952153	0.7607928	not scorable	not scorable	1	0.7302788
2878	Bn-A02-p11449488	A-genome specific (napus-rapa)	0.9808612	0.7433488	not scorable	not scorable	0.9669421	0.6871973
2879	Bn-A02-p11450032	A-genome specific (napus-rapa)	0.9856459	0.7887045	not scorable	not scorable	0.9834711	0.7806471
2880	Bn-A02-p11450463	A-genome specific (napus-rapa)	0.9832536	0.7445564	not scorable	not scorable	0.9752066	0.7299915
2881	Bn-A02-p11451035	A-genome specific (napus-rapa)	0.9784689	0.6973041	not scorable	not scorable	0.9752066	0.6546887
2882	Bn-A02-p11451261	A-genome specific (napus-rapa)	0.9904306	0.7776282	not scorable	not scorable	0.9421487	0.7071161
2883	Bn-A02-p11454010	A-genome specific (napus-rapa)	0.9784689	0.49856	not scorable	not scorable	0.9917355	0.7399143
2884	Bn-A02-p11454573	A-genome specific (napus-rapa)	0.9736842	0.8019562	not scorable	not scorable	0.9834711	0.7659231
2885	Bn-A02-p11454658	A-genome specific (napus-rapa)	0.9665072	0.4799769	not scorable	not scorable	0.9834711	0.6375329
2886	Bn-A02-p11454746	A-genome specific (napus-rapa)	0.9976076	0.5088444	not scorable	not scorable	0.9917355	0.7639434
2887	Bn-A02-p11455068	A-genome specific (napus-rapa)	0.9856459	0.8754658	not scorable	not scorable	1	0.726869
2888	Bn-A02-p11455310	A-genome specific (napus-rapa)	0.9760765	0.7452747	not scorable	not scorable	0.9834711	0.6808891
2889	Bn-A02-p11455369	A-genome specific (napus-rapa)	0.9976076	0.6914675	not scorable	not scorable	1	0.7416446
2890	Bn-A02-p11455659	A-genome specific (napus-rapa)	0.9760765	0.7828279	not scorable	not scorable	0.9917355	0.7620965
2891	Bn-A02-p11457727	A-genome specific (napus-rapa)	0.9880383	0.7685125	not scorable	not scorable	0.9834711	0.8020721
2892	Bn-A02-p11463288	A-genome specific (napus-rapa)	0.9593301	0.5707067	not scorable	not scorable	1	0.7846172
2893	Bn-A02-p11479169	A-genome specific (napus-rapa)	0.9808612	0.2665071	not scorable	not scorable	1	0.728054
2894	Bn-A02-p11479678	A-genome specific (napus-rapa)	0.9808612	0.481438	not scorable	not scorable	0.9752066	0.6666704
2895	Bn-A02-p11492320	A-genome specific (napus-rapa)	0.9808612	0.8083671	not scorable	not scorable	0.9752066	0.7859519
2896	Bn-A02-p11513420	A-genome specific (napus-rapa)	0.9665072	0.6642956	not scorable	not scorable	0.9917355	0.7619538
2897	Bn-A02-p11517785	A-genome specific (napus-rapa)	0.9808612	0.4592679	not scorable	not scorable	0.9834711	0.7182298
2898	Bn-A02-p11521800	A-genome specific (napus-rapa)	0.9665072	0.7816321	not scorable	not scorable	0.9752066	0.7947698
2899	Bn-A02-p11535570	A-genome specific (napus-rapa)	0.9928229	0.264915	not scorable	not scorable	0.9834711	0.7512991
2900	Bn-A02-p11550877	A-genome specific (rapa only)	not scorable	not scorable	not scorable	not scorable	1	0.7329642
2901	Bn-A02-p1155656	A-genome specific (rapa only)	not scorable	not scorable	not scorable	not scorable	0.9917355	0.9057445

2902	Bn-A02-p1157373	genome unspecific (all in)	0.923445	0.3294582	0.9689922	0.4151027	1	0.8004208
2903	Bn-A02-p11585751	A-genome specific (napus-rapa)	0.9162679	0.771548	not scorable	not scorable	0.9669421	0.8051392
2904	Bn-A02-p11616676	A-genome specific (napus-rapa)	0.9832536	0.5424441	not scorable	not scorable	0.9834711	0.7579786
2905	Bn-A02-p11646535	A-genome specific (napus-rapa)	0.9880383	0.7761496	not scorable	not scorable	1	0.8266833
2906	Bn-A02-p11648522	A-genome specific (napus-rapa)	0.9904306	0.7490399	not scorable	not scorable	1	0.7088333
2907	Bn-A02-p11660867	A-genome specific (napus-rapa)	0.9856459	0.2779405	not scorable	not scorable	1	0.7242132
2908	Bn-A02-p11666688	A-genome specific (napus-rapa)	0.9904306	0.4843172	not scorable	not scorable	1	0.8815182
2909	Bn-A02-p11673455	napus specific	0.8755981	0.8049057	not scorable	not scorable	not scorable	not scorable
2910	Bn-A02-p11682974	A-genome specific (napus-rapa)	0.9880383	0.7270359	not scorable	not scorable	1	0.8275138
2911	Bn-A02-p11683024	A-genome specific (napus-rapa)	0.9928229	0.8446832	not scorable	not scorable	1	0.8287739
2912	Bn-A02-p11683481	A-genome specific (napus-rapa)	0.9952153	0.7875241	not scorable	not scorable	0.9917355	0.7862288
2913	Bn-A02-p11703005	A-genome specific (napus-rapa)	0.9856459	0.7646531	not scorable	not scorable	1	0.7421271
2914	Bn-A02-p11713324	A-genome specific (napus-rapa)	0.9928229	0.6708755	not scorable	not scorable	0.9752066	0.6934128
2915	Bn-A02-p11713499	A-genome specific (napus-rapa)	0.9928229	0.3315977	not scorable	not scorable	0.9834711	0.7698158
2916	Bn-A02-p11723698	A-genome specific (napus-rapa)	0.9976076	0.3505815	not scorable	not scorable	0.9834711	0.7173629
2917	Bn-A02-p1173704	A-genome specific (napus-rapa)	0.9904306	0.7684156	not scorable	not scorable	0.9669421	0.6529559
2918	Bn-A02-p11737945	A-genome specific (napus-rapa)	0.9593301	0.7974638	not scorable	not scorable	0.9917355	0.7521831
2919	Bn-A02-p11738896	A-genome specific (napus-rapa)	0.9665072	0.6754242	not scorable	not scorable	0.9834711	0.7129886
2920	Bn-A02-p11746891	A-genome specific (napus-rapa)	0.9880383	0.5269552	not scorable	not scorable	0.9834711	0.7232494
2921	Bn-A02-p11748602	A-genome specific (napus-rapa)	0.9904306	0.7632337	not scorable	not scorable	1	0.7375398
2922	Bn-A02-p11750740	A-genome specific (napus-rapa)	0.9952153	0.4899747	not scorable	not scorable	1	0.7425883
2923	Bn-A02-p11759281	A-genome specific (napus-rapa)	1	0.3314945	not scorable	not scorable	1	0.7138557
2924	Bn-A02-p11765114	A-genome specific (napus-rapa)	0.9928229	0.7497788	not scorable	not scorable	0.9834711	0.6990585
2925	Bn-A02-p11772983	genome unspecific (all in)	0.9736842	0.7786677	0.9302326	0.7067049	0.9752066	0.7286063
2926	Bn-A02-p11786253	A-genome specific (napus-rapa)	0.8516746	0.7011642	not scorable	not scorable	0.5206612	0.7193962
2927	Bn-A02-p11786572	A-genome specific (napus-rapa)	0.8349282	0.8233702	not scorable	not scorable	0.5206612	0.7136915
2928	Bn-A02-p11789023	A-genome specific (napus-rapa)	0.9856459	0.7425846	not scorable	not scorable	0.9834711	0.7670333
2929	Bn-A02-p11793499	A-genome specific (napus-rapa)	0.9856459	0.4941938	not scorable	not scorable	1	0.7909877
2930	Bn-A02-p11816790	A-genome specific (napus-rapa)	0.9880383	0.7559376	not scorable	not scorable	0.9752066	0.7964983
2931	Bn-A02-p11817742	A-genome specific (napus-rapa)	0.9665072	0.7764336	not scorable	not scorable	0.9834711	0.7596439
2932	Bn-A02-p11829280	A-genome specific (napus-rapa)	0.9904306	0.7819287	not scorable	not scorable	0.9834711	0.7220608
2933	Bn-A02-p11842725	A-genome specific (napus-rapa)	0.9928229	0.5599157	not scorable	not scorable	0.9752066	0.705859
2934	Bn-A02-p11847986	A-genome specific (napus-rapa)	0.9736842	0.3778673	not scorable	not scorable	0.9669421	0.6833251

2935	Bn-A02-p11849409	A-genome specific (napus-rapa)	0.9928229	0.7760916	not scorable	not scorable	1	0.6869434
2936	Bn-A02-p11849571	A-genome specific (napus-rapa)	0.9928229	0.7626822	not scorable	not scorable	0.9917355	0.6886213
2937	Bn-A02-p11859430	A-genome specific (napus-rapa)	0.9856459	0.4207686	not scorable	not scorable	1	0.7350504
2938	Bn-A02-p11863349	A-genome specific (napus-rapa)	0.9688995	0.7503841	not scorable	not scorable	0.9834711	0.7157601
2939	Bn-A02-p11863681	A-genome specific (napus-rapa)	0.9808612	0.7406486	not scorable	not scorable	0.9504132	0.6547279
2940	Bn-A02-p11870177	A-genome specific (napus-rapa)	0.9880383	0.4452708	not scorable	not scorable	0.9752066	0.7755873
2941	Bn-A02-p1188292	A-genome specific (rapa only)	not scorable	not scorable	not scorable	not scorable	1	0.7992046
2942	Bn-A02-p11896294	A-genome specific (napus-rapa)	0.9808612	0.7787958	not scorable	not scorable	0.9917355	0.6990761
2943	Bn-A02-p11896538	A-genome specific (napus-rapa)	0.9760765	0.4258346	not scorable	not scorable	0.9917355	0.8072101
2944	Bn-A02-p11901091	A-genome specific (rapa only)	not scorable	not scorable	not scorable	not scorable	0.9834711	0.8028865
2945	Bn-A02-p11906704	A-genome specific (napus-rapa)	0.9665072	0.762642	not scorable	not scorable	0.9917355	0.7633223
2946	Bn-A02-p11909870	A-genome specific (napus-rapa)	0.9904306	0.4768819	not scorable	not scorable	1	0.7397082
2947	Bn-A02-p11918483	A-genome specific (napus-rapa)	1	0.4609109	not scorable	not scorable	1	0.8705826
2948	Bn-A02-p11929538	A-genome specific (napus-rapa)	0.9617225	0.3704279	not scorable	not scorable	0.9504132	0.6249714
2949	Bn-A02-p11935002	napus specific	0.6220096	0.6733309	not scorable	not scorable	not scorable	not scorable
2950	Bn-A02-p11936177	A-genome specific (napus-rapa)	0.9856459	0.3989817	not scorable	not scorable	0.9504132	0.6615893
2951	Bn-A02-p11937221	A-genome specific (napus-rapa)	0.9641148	0.6867681	not scorable	not scorable	0.9752066	0.6587664
2952	Bn-A02-p11939715	A-genome specific (napus-rapa)	0.9760765	0.7611288	not scorable	not scorable	0.9504132	0.719249
2953	Bn-A02-p11939983	A-genome specific (napus-rapa)	0.9473684	0.773665	not scorable	not scorable	0.9834711	0.8140697
2954	Bn-A02-p11960741	A-genome specific (napus-rapa)	0.9928229	0.6541438	not scorable	not scorable	0.9917355	0.8216027
2955	Bn-A02-p11961581	A-genome specific (napus-rapa)	0.9808612	0.4033592	not scorable	not scorable	0.9917355	0.8282794
2956	Bn-A02-p1198	A-genome specific (napus-rapa)	1	0.5418917	not scorable	not scorable	1	0.7599372
2957	Bn-A02-p11986866	genome unspecific (oleracea-rapa)	not scorable	not scorable	1	0.8301461	1	0.8432821
2958	Bn-A02-p11994999	A-genome specific (napus-rapa)	0.9593301	0.4591976	not scorable	not scorable	1	0.7292421
2959	Bn-A02-p11995673	A-genome specific (napus-rapa)	0.9856459	0.389854	not scorable	not scorable	1	0.8379921
2960	Bn-A02-p11996587	A-genome specific (napus-rapa)	1	0.9300349	not scorable	not scorable	1	0.9248517
2961	Bn-A02-p11998345	A-genome specific (napus-rapa)	0.9736842	0.8021038	not scorable	not scorable	0.9917355	0.7332684
2962	Bn-A02-p11998540	A-genome specific (napus-rapa)	0.9808612	0.7896866	not scorable	not scorable	0.9917355	0.7973562
2963	Bn-A02-p11998921	A-genome specific (napus-rapa)	0.9688995	0.760342	not scorable	not scorable	0.9752066	0.6896471
2964	Bn-A02-p12003125	A-genome specific (napus-rapa)	0.9832536	0.7981007	not scorable	not scorable	0.9834711	0.7544396
2965	Bn-A02-p12003217	A-genome specific (napus-rapa)	0.4019139	0.9498829	not scorable	not scorable	0.9586777	0.7825954
2966	Bn-A02-p12028821	A-genome specific (napus-rapa)	0.8038278	0.7886043	not scorable	not scorable	1	0.8230875
2967	Bn-A02-p12044265	A-genome specific (napus-rapa)	0.9928229	0.556847	not scorable	not scorable	0.9917355	0.5414523

2968	Bn-A02-p12061350	A-genome specific (napus-rapa)	0.9641148	0.7026444	not scorable	not scorable	0.9917355	0.7122235
2969	Bn-A02-p12081581	A-genome specific (napus-rapa)	0.9665072	0.79785	not scorable	not scorable	1	0.7521147
2970	Bn-A02-p12105374	A-genome specific (napus-rapa)	0.9784689	0.4860516	not scorable	not scorable	1	0.8135551
2971	Bn-A02-p12106636	A-genome specific (napus-rapa)	0.9904306	0.2968828	not scorable	not scorable	0.9834711	0.7397626
2972	Bn-A02-p12107746	A-genome specific (napus-rapa)	0.9856459	0.7380853	not scorable	not scorable	0.9917355	0.8289013
2973	Bn-A02-p12107868	A-genome specific (napus-rapa)	0.9808612	0.5027643	not scorable	not scorable	0.9917355	0.8566363
2974	Bn-A02-p12145607	A-genome specific (napus-rapa)	0.6770335	0.7081192	not scorable	not scorable	0.9752066	0.8176157
2975	Bn-A02-p12149042	A-genome specific (napus-rapa)	0.9976076	0.4936244	not scorable	not scorable	1	0.7785498
2976	Bn-A02-p12150733	A-genome specific (napus-rapa)	0.8971292	0.7192385	not scorable	not scorable	0.9834711	0.7370347
2977	Bn-A02-p12157799	A-genome specific (napus-rapa)	0.9856459	0.6507718	not scorable	not scorable	1	0.7474164
2978	Bn-A02-p12164268	A-genome specific (napus-rapa)	0.9425837	0.720846	not scorable	not scorable	0.9834711	0.7778099
2979	Bn-A02-p12169696	A-genome specific (napus-rapa)	0.9832536	0.5474809	not scorable	not scorable	0.9834711	0.7844557
2980	Bn-A02-p12169776	A-genome specific (rapa only)	not scorable	not scorable	not scorable	not scorable	0.9917355	0.7397237
2981	Bn-A02-p12179107	A-genome specific (napus-rapa)	0.9952153	0.7621909	not scorable	not scorable	1	0.7417945
2982	Bn-A02-p12179579	A-genome specific (napus-rapa)	0.9856459	0.8037829	not scorable	not scorable	0.9917355	0.8098742
2983	Bn-A02-p12192921	A-genome specific (napus-rapa)	0.9976076	0.748126	not scorable	not scorable	1	0.7654577
2984	Bn-A02-p12196899	A-genome specific (rapa only)	not scorable	not scorable	not scorable	not scorable	1	0.829446
2985	Bn-A02-p12198897	A-genome specific (napus-rapa)	0.9928229	0.4897215	not scorable	not scorable	0.9917355	0.743686
2986	Bn-A02-p1220275	A-genome specific (napus-rapa)	0.9880383	0.4864532	not scorable	not scorable	0.9752066	0.7591179
2987	Bn-A02-p12219666	A-genome specific (napus-rapa)	1	0.751255	not scorable	not scorable	1	0.7271354
2988	Bn-A02-p12220436	A-genome specific (napus-rapa)	0.9880383	0.7704843	not scorable	not scorable	0.9834711	0.7068547
2989	Bn-A02-p12241488	A-genome specific (rapa only)	not scorable	not scorable	not scorable	not scorable	0.9917355	0.8034954
2990	Bn-A02-p12269431	A-genome specific (napus-rapa)	0.9856459	0.7404609	not scorable	not scorable	0.9752066	0.6860862
2991	Bn-A02-p12278577	A-genome specific (napus-rapa)	0.9976076	0.458028	not scorable	not scorable	0.9917355	0.6696428
2992	Bn-A02-p12284078	A-genome specific (napus-rapa)	1	0.4342639	not scorable	not scorable	0.9669421	0.6744222
2993	Bn-A02-p12284671	A-genome specific (napus-rapa)	0.9880383	0.5247986	not scorable	not scorable	1	0.6188555
2994	Bn-A02-p12284882	napus specific	0.9904306	0.2994366	not scorable	not scorable	not scorable	not scorable
2995	Bn-A02-p12287615	A-genome specific (napus-rapa)	0.9760765	0.393047	not scorable	not scorable	0.9834711	0.6791325
2996	Bn-A02-p12292026	A-genome specific (napus-rapa)	0.9904306	0.8245637	not scorable	not scorable	0.9917355	0.7018297
2997	Bn-A02-p12292768	A-genome specific (napus-rapa)	0.9880383	0.8497826	not scorable	not scorable	0.9917355	0.7110556
2998	Bn-A02-p12293388	A-genome specific (napus-rapa)	1	0.7727818	not scorable	not scorable	0.9834711	0.7991754
2999	Bn-A02-p12295281	A-genome specific (napus-rapa)	0.9784689	0.8003728	not scorable	not scorable	0.9917355	0.8062111
3000	Bn-A02-p12295682	A-genome specific (napus-rapa)	0.9904306	0.7848144	not scorable	not scorable	1	0.8008788

3001	Bn-A02-p12297473	A-genome specific (napus-rapa)	0.9904306	0.7904986	not scorable	not scorable	0.9917355	0.8090288
3002	Bn-A02-p12314542	A-genome specific (napus-rapa)	0.9808612	0.7941186	not scorable	not scorable	0.9752066	0.7202161
3003	Bn-A02-p1232964	A-genome specific (napus-rapa)	0.9904306	0.765703	not scorable	not scorable	0.9917355	0.7993867
3004	Bn-A02-p12336037	A-genome specific (napus-rapa)	0.9593301	0.5993348	not scorable	not scorable	0.9669421	0.6836075
3005	Bn-A02-p12342256	A-genome specific (napus-rapa)	0.9808612	0.8060136	not scorable	not scorable	0.5206612	0.7377832
3006	Bn-A02-p1235480	A-genome specific (napus-rapa)	1	0.7858552	not scorable	not scorable	0.9669421	0.7332122
3007	Bn-A02-p12363140	A-genome specific (napus-rapa)	0.9928229	0.7272876	not scorable	not scorable	0.9834711	0.6330875
3008	Bn-A02-p12393535	A-genome specific (napus-rapa)	0.9904306	0.7753468	not scorable	not scorable	0.9917355	0.717861
3009	Bn-A02-p12393617	A-genome specific (napus-rapa)	0.9736842	0.6194615	not scorable	not scorable	0.9834711	0.7509158
3010	Bn-A02-p12397326	A-genome specific (napus-rapa)	0.9784689	0.4361291	not scorable	not scorable	0.9256198	0.8802001
3011	Bn-A02-p12397585	A-genome specific (napus-rapa)	0.9641148	0.4943984	not scorable	not scorable	0.9669421	0.8064753
3012	Bn-A02-p12415095	A-genome specific (napus-rapa)	0.9736842	0.559009	not scorable	not scorable	0.9917355	0.7808155
3013	Bn-A02-p12443767	A-genome specific (napus-rapa)	0.9808612	0.7532993	not scorable	not scorable	1	0.8028949
3014	Bn-A02-p12449263	A-genome specific (napus-rapa)	0.9856459	0.2742781	not scorable	not scorable	0.9917355	0.6262633
3015	Bn-A02-p12452799	A-genome specific (napus-rapa)	0.9928229	0.7202903	not scorable	not scorable	1	0.8318203
3016	Bn-A02-p12460628	A-genome specific (napus-rapa)	0.9976076	0.6967202	not scorable	not scorable	0.9752066	0.856594
3017	Bn-A02-p1250673	all not scorable	not scorable	not scorable	not scorable	not scorable	not scorable	not scorable
3018	Bn-A02-p12508852	A-genome specific (napus-rapa)	0.9665072	0.8058401	not scorable	not scorable	1	0.8185257
3019	Bn-A02-p12512160	A-genome specific (napus-rapa)	0.9880383	0.749584	not scorable	not scorable	0.9917355	0.7018969
3020	Bn-A02-p12512328	A-genome specific (napus-rapa)	0.9688995	0.7579747	not scorable	not scorable	1	0.8930789
3021	Bn-A02-p12512533	A-genome specific (napus-rapa)	0.9928229	0.5883021	not scorable	not scorable	1	0.81844
3022	Bn-A02-p12514179	A-genome specific (napus-rapa)	0.9545454	0.7207578	not scorable	not scorable	1	0.7512512
3023	Bn-A02-p12516859	A-genome specific (napus-rapa)	0.9784689	0.6744137	not scorable	not scorable	1	0.7575754
3024	Bn-A02-p12520861	A-genome specific (napus-rapa)	1	0.493536	not scorable	not scorable	1	0.738891
3025	Bn-A02-p12521545	A-genome specific (napus-rapa)	0.9904306	0.3644065	not scorable	not scorable	1	0.6803178
3026	Bn-A02-p12524852	genome unspecific (all in)	0.9760765	0.348583	1	0.882567	1	0.9272014
3027	Bn-A02-p12534375	napus specific	0.9784689	0.4074126	not scorable	not scorable	not scorable	not scorable
3028	Bn-A02-p12550795	A-genome specific (napus-rapa)	0.9760765	0.7516208	not scorable	not scorable	0.9752066	0.6732684
3029	Bn-A02-p12552323	A-genome specific (napus-rapa)	0.9760765	0.7907307	not scorable	not scorable	0.9586777	0.7391577
3030	Bn-A02-p12554865	A-genome specific (napus-rapa)	0.9856459	0.6909371	not scorable	not scorable	0.9752066	0.6912593
3031	Bn-A02-p12559982	A-genome specific (napus-rapa)	0.6818182	0.804282	not scorable	not scorable	0.8677686	0.9761626
3032	Bn-A02-p12581342	A-genome specific (rapa only)	not scorable	not scorable	not scorable	not scorable	0.9752066	0.8258571
3033	Bn-A02-p12585867	A-genome specific (napus-rapa)	1	0.9373254	not scorable	not scorable	0.9917355	0.8933609

3034	Bn-A02-p12585930	A-genome specific (napus-rapa)	1	0.6154503	not scorable	not scorable	0.9752066	0.6100948
3035	Bn-A02-p12589304	A-genome specific (napus-rapa)	0.9688995	0.2623686	not scorable	not scorable	0.9834711	0.7913849
3036	Bn-A02-p12595278	napus specific	0.9928229	0.4228345	not scorable	not scorable	not scorable	not scorable
3037	Bn-A02-p12597733	A-genome specific (napus-rapa)	0.9832536	0.773325	not scorable	not scorable	0.9586777	0.7685441
3038	Bn-A02-p1259929	A-genome specific (napus-rapa)	0.9784689	0.6306293	not scorable	not scorable	0.9834711	0.7018437
3039	Bn-A02-p126006	A-genome specific (napus-rapa)	0.9784689	0.3625441	not scorable	not scorable	0.9917355	0.8044072
3040	Bn-A02-p12608089	A-genome specific (napus-rapa)	0.9832536	0.5572177	not scorable	not scorable	0.9669421	0.7795289
3041	Bn-A02-p12627047	A-genome specific (napus-rapa)	0.9976076	0.7910479	not scorable	not scorable	0.9834711	0.7728977
3042	Bn-A02-p12628141	A-genome specific (napus-rapa)	0.9808612	0.6876252	not scorable	not scorable	1	0.7473743
3043	Bn-A02-p12632166	A-genome specific (napus-rapa)	0.9593301	0.7653671	not scorable	not scorable	0.9917355	0.8112254
3044	Bn-A02-p12637974	A-genome specific (napus-rapa)	0.9808612	0.8006718	not scorable	not scorable	1	0.8108238
3045	Bn-A02-p12642708	A-genome specific (napus-rapa)	0.9593301	0.6746796	not scorable	not scorable	0.9834711	0.7056495
3046	Bn-A02-p1264906	A-genome specific (napus-rapa)	0.9856459	0.2903114	not scorable	not scorable	0.9834711	0.7050172
3047	Bn-A02-p1265949	A-genome specific (napus-rapa)	0.9736842	0.6890687	not scorable	not scorable	0.9834711	0.7542964
3048	Bn-A02-p12666196	A-genome specific (napus-rapa)	0.9641148	0.6424054	not scorable	not scorable	1	0.8061697
3049	Bn-A02-p12678353	A-genome specific (napus-rapa)	1	0.4934493	not scorable	not scorable	1	0.9248387
3050	Bn-A02-p12680940	A-genome specific (napus-rapa)	0.9712918	0.7547821	not scorable	not scorable	1	0.7326435
3051	Bn-A02-p12681201	A-genome specific (napus-rapa)	0.9688995	0.7319032	not scorable	not scorable	1	0.5587328
3052	Bn-A02-p12681247	A-genome specific (napus-rapa)	0.9808612	0.3738289	not scorable	not scorable	0.5371901	0.8077816
3053	Bn-A02-p12686417	A-genome specific (napus-rapa)	0.9904306	0.4061546	not scorable	not scorable	0.9917355	0.8540307
3054	Bn-A02-p12686781	A-genome specific (napus-rapa)	0.9904306	0.752461	not scorable	not scorable	1	0.916114
3055	Bn-A02-p12688495	A-genome specific (napus-rapa)	0.9928229	0.5041894	not scorable	not scorable	0.9834711	0.8124056
3056	Bn-A02-p12688906	A-genome specific (napus-rapa)	0.9976076	0.3862539	not scorable	not scorable	0.9834711	0.7362109
3057	Bn-A02-p12693516	A-genome specific (napus-rapa)	1	0.3463797	not scorable	not scorable	1	0.6576501
3058	Bn-A02-p12739678	A-genome specific (napus-rapa)	0.9832536	0.374083	not scorable	not scorable	0.9834711	0.7574135
3059	Bn-A02-p12752915	A-genome specific (napus-rapa)	1	0.4721927	not scorable	not scorable	1	0.629589
3060	Bn-A02-p12756887	A-genome specific (napus-rapa)	0.9976076	0.4264193	not scorable	not scorable	0.9834711	0.7309587
3061	Bn-A02-p1276717	napus specific	0.3014354	0.798218	not scorable	not scorable	not scorable	not scorable
3062	Bn-A02-p1276995	napus specific	0.9904306	0.8092516	not scorable	not scorable	not scorable	not scorable
3063	Bn-A02-p12801838	A-genome specific (napus-rapa)	0.9928229	0.3043488	not scorable	not scorable	1	0.6181029
3064	Bn-A02-p12809029	all not scorable	not scorable	not scorable	not scorable	not scorable	not scorable	not scorable
3065	Bn-A02-p12810041	A-genome specific (rapa only)	not scorable	not scorable	not scorable	not scorable	0.9834711	0.7567164
3066	Bn-A02-p12815817	A-genome specific (napus-rapa)	0.9736842	0.4311776	not scorable	not scorable	0.9917355	0.6685718

3067	Bn-A02-p12819772	A-genome specific (napus-rapa)	0.9760765	0.3336272	not scorable	not scorable	0.9917355	0.7716679
3068	Bn-A02-p12821038	A-genome specific (napus-rapa)	1	0.3549289	not scorable	not scorable	0.9917355	0.7908466
3069	Bn-A02-p12822311	A-genome specific (napus-rapa)	0.9688995	0.2569763	not scorable	not scorable	1	0.7157181
3070	Bn-A02-p12822315	A-genome specific (rapa only)	not scorable	not scorable	not scorable	not scorable	0.9752066	0.8205012
3071	Bn-A02-p12848961	A-genome specific (napus-rapa)	0.9736842	0.6456749	not scorable	not scorable	0.9752066	0.7791468
3072	Bn-A02-p12851818	A-genome specific (napus-rapa)	0.9904306	0.7944171	not scorable	not scorable	0.9917355	0.7243189
3073	Bn-A02-p1290847	A-genome specific (napus-rapa)	0.9425837	0.3386586	not scorable	not scorable	1	0.5631257
3074	Bn-A02-p1292023	A-genome specific (rapa only)	not scorable	not scorable	not scorable	not scorable	1	0.7729655
3075	Bn-A02-p12939509	napus specific	0.9665072	0.4091599	not scorable	not scorable	not scorable	not scorable
3076	Bn-A02-p12943042	A-genome specific (napus-rapa)	0.9665072	0.7594435	not scorable	not scorable	0.9669421	0.7312071
3077	Bn-A02-p12945561	A-genome specific (napus-rapa)	0.930622	0.4862561	not scorable	not scorable	0.9917355	0.7703245
3078	Bn-A02-p12962845	A-genome specific (napus-rapa)	0.8277512	0.6209021	not scorable	not scorable	0.9917355	0.7740173
3079	Bn-A02-p12971726	A-genome specific (napus-rapa)	0.9856459	0.7625436	not scorable	not scorable	0.9669421	0.8265869
3080	Bn-A02-p12972954	A-genome specific (napus-rapa)	0.9808612	0.7495691	not scorable	not scorable	0.9917355	0.8686861
3081	Bn-A02-p12976547	A-genome specific (napus-rapa)	0.9090909	0.5285606	not scorable	not scorable	1	0.7832621
3082	Bn-A02-p12986045	A-genome specific (napus-rapa)	0.9617225	0.3835713	not scorable	not scorable	0.9917355	0.7294025
3083	Bn-A02-p12996998	A-genome specific (napus-rapa)	0.9880383	0.7194604	not scorable	not scorable	0.9669421	0.7697719
3084	Bn-A02-p12997693	A-genome specific (napus-rapa)	0.9856459	0.729233	not scorable	not scorable	1	0.7322882
3085	Bn-A02-p12999856	A-genome specific (napus-rapa)	0.9928229	0.8175188	not scorable	not scorable	1	0.7091442
3086	Bn-A02-p13000494	A-genome specific (napus-rapa)	0.9952153	0.7763442	not scorable	not scorable	1	0.7785882
3087	Bn-A02-p13016695	A-genome specific (napus-rapa)	0.9736842	0.302451	not scorable	not scorable	0.9421487	0.8466056
3088	Bn-A02-p13019236	A-genome specific (napus-rapa)	0.9856459	0.4044824	not scorable	not scorable	0.9834711	0.6865157
3089	Bn-A02-p13067449	A-genome specific (napus-rapa)	0.9784689	0.7781981	not scorable	not scorable	1	0.7503625
3090	Bn-A02-p13068355	A-genome specific (napus-rapa)	0.9736842	0.7938458	not scorable	not scorable	0.9669421	0.7307636
3091	Bn-A02-p13091073	A-genome specific (rapa only)	not scorable	not scorable	not scorable	not scorable	1	0.7828613
3092	Bn-A02-p13118529	A-genome specific (napus-rapa)	0.9569378	0.2390958	not scorable	not scorable	1	0.7353324
3093	Bn-A02-p13121161	A-genome specific (napus-rapa)	0.9497608	0.273148	not scorable	not scorable	0.9834711	0.7903211
3094	Bn-A02-p13123802	A-genome specific (napus-rapa)	0.9712918	0.7986241	not scorable	not scorable	0.9834711	0.776059
3095	Bn-A02-p13124058	A-genome specific (napus-rapa)	0.937799	0.5927464	not scorable	not scorable	0.9834711	0.7872227
3096	Bn-A02-p13130726	napus specific	0.9330143	0.8038674	not scorable	not scorable	not scorable	not scorable
3097	Bn-A02-p13153436	A-genome specific (napus-rapa)	1	0.4686905	not scorable	not scorable	1	0.7272668
3098	Bn-A02-p13167989	A-genome specific (napus-rapa)	0.9712918	0.3887221	not scorable	not scorable	1	0.8348
3099	Bn-A02-p1319039	A-genome specific (napus-rapa)	1	0.6933597	not scorable	not scorable	0.9917355	0.7070914

3100	Bn-A02-p13195248	A-genome specific (napus-rapa)	1	0.8101473	not scorable	not scorable	1	0.7743075
3101	Bn-A02-p13197154	A-genome specific (napus-rapa)	0.9138756	0.7388572	not scorable	not scorable	0.9421487	0.8354583
3102	Bn-A02-p13212386	A-genome specific (napus-rapa)	0.9521531	0.6497005	not scorable	not scorable	0.9917355	0.8486647
3103	Bn-A02-p1321904	A-genome specific (napus-rapa)	0.9976076	0.7331814	not scorable	not scorable	0.9917355	0.6991895
3104	Bn-A02-p1324368	A-genome specific (rapa only)	not scorable	not scorable	not scorable	not scorable	1	0.8299943
3105	Bn-A02-p13252027	A-genome specific (napus-rapa)	0.9880383	0.8114662	not scorable	not scorable	0.9669421	0.8135551
3106	Bn-A02-p1325213	A-genome specific (napus-rapa)	0.9808612	0.7558952	not scorable	not scorable	1	0.8009962
3107	Bn-A02-p13252137	A-genome specific (napus-rapa)	0.9952153	0.5226454	not scorable	not scorable	0.9338843	0.7734648
3108	Bn-A02-p13252354	all not scorable	not scorable	not scorable	not scorable	not scorable	not scorable	not scorable
3109	Bn-A02-p13252652	all not scorable	not scorable	not scorable	not scorable	not scorable	not scorable	not scorable
3110	Bn-A02-p1326013	A-genome specific (napus-rapa)	0.9617225	0.7765158	not scorable	not scorable	0.9917355	0.7835029
3111	Bn-A02-p1327787	A-genome specific (napus-rapa)	0.9760765	0.6300305	not scorable	not scorable	1	0.6114821
3112	Bn-A02-p1335862	A-genome specific (napus-rapa)	0.9736842	0.3646343	not scorable	not scorable	1	0.8194499
3113	Bn-A02-p1337706	A-genome specific (napus-rapa)	0.9976076	0.781209	not scorable	not scorable	0.9834711	0.70269
3114	Bn-A02-p13382591	A-genome specific (napus-rapa)	1	0.7914145	not scorable	not scorable	1	0.7751204
3115	Bn-A02-p1341101	A-genome specific (napus-rapa)	0.861244	0.7869154	not scorable	not scorable	0.9173554	0.85241
3116	Bn-A02-p13411303	A-genome specific (napus-rapa)	0.9976076	0.7329584	not scorable	not scorable	1	0.7269387
3117	Bn-A02-p1344870	genome unspecific (all in)	0.9880383	0.7564654	0.9922481	0.8008927	0.9752066	0.7187182
3118	Bn-A02-p13498319	A-genome specific (napus-rapa)	0.9641148	0.5731714	not scorable	not scorable	0.9834711	0.7220136
3119	Bn-A02-p13556392	genome unspecific (all in)	0.9952153	0.4762699	0.9922481	0.8615783	1	0.7766667
3120	Bn-A02-p13556497	A-genome specific (napus-rapa)	0.9928229	0.5689869	not scorable	not scorable	0.9834711	0.7158391
3121	Bn-A02-p13570742	A-genome specific (napus-rapa)	0.9760765	0.7889354	not scorable	not scorable	1	0.8047741
3122	Bn-A02-p13572191	A-genome specific (napus-rapa)	1	0.5138949	not scorable	not scorable	1	0.7666658
3123	Bn-A02-p13593944	A-genome specific (napus-rapa)	0.9880383	0.8059001	not scorable	not scorable	0.9917355	0.7374982
3124	Bn-A02-p13598238	A-genome specific (napus-rapa)	0.9952153	0.7114246	not scorable	not scorable	0.9669421	0.6806829
3125	Bn-A02-p13625461	A-genome specific (napus-rapa)	0.9904306	0.7997314	not scorable	not scorable	0.9917355	0.7884254
3126	Bn-A02-p13626431	A-genome specific (napus-rapa)	0.9904306	0.800919	not scorable	not scorable	1	0.8815737
3127	Bn-A02-p13627045	A-genome specific (napus-rapa)	0.9976076	0.3082423	not scorable	not scorable	0.9834711	0.6562842
3128	Bn-A02-p13637436	A-genome specific (napus-rapa)	0.9928229	0.9285347	not scorable	not scorable	0.9917355	0.9140795
3129	Bn-A02-p13662225	A-genome specific (napus-rapa)	0.9928229	0.4142511	not scorable	not scorable	0.9834711	0.7867023
3130	Bn-A02-p1370581	genome unspecific (all in)	0.9617225	0.3378389	1	0.8841014	1	0.7788354
3131	Bn-A02-p1372727	A-genome specific (napus-rapa)	0.9688995	0.6618596	not scorable	not scorable	0.9917355	0.810988
3132	Bn-A02-p13751737	A-genome specific (napus-rapa)	0.9952153	0.7630872	not scorable	not scorable	0.9917355	0.7146024

3133	Bn-A02-p13782884	A-genome specific (napus-rapa)	0.9928229	0.678608	not scorable	not scorable	1	0.7051241
3134	Bn-A02-p13794668	A-genome specific (napus-rapa)	0.9976076	0.4710896	not scorable	not scorable	0.9752066	0.7030707
3135	Bn-A02-p13798655	A-genome specific (napus-rapa)	0.9928229	0.7804482	not scorable	not scorable	0.9834711	0.8088208
3136	Bn-A02-p13803383	A-genome specific (napus-rapa)	0.9928229	0.8160556	not scorable	not scorable	0.9917355	0.7613291
3137	Bn-A02-p13803435	A-genome specific (napus-rapa)	0.9904306	0.8010759	not scorable	not scorable	0.9586777	0.6625916
3138	Bn-A02-p13822105	A-genome specific (napus-rapa)	0.9976076	0.589919	not scorable	not scorable	0.9834711	0.7539659
3139	Bn-A02-p13874627	A-genome specific (napus-rapa)	1	0.6508506	not scorable	not scorable	0.9834711	0.7062038
3140	Bn-A02-p13875459	A-genome specific (napus-rapa)	0.9880383	0.3205053	not scorable	not scorable	1	0.7531955
3141	Bn-A02-p13951646	A-genome specific (napus-rapa)	1	0.8778391	not scorable	not scorable	1	0.905901
3142	Bn-A02-p13958436	A-genome specific (rapa only)	not scorable	not scorable	not scorable	not scorable	0.9917355	0.7924424
3143	Bn-A02-p1396892	A-genome specific (napus-rapa)	1	0.6305942	not scorable	not scorable	1	0.7350931
3144	Bn-A02-p13972998	A-genome specific (napus-rapa)	0.9976076	0.2268504	not scorable	not scorable	0.9586777	0.7717934
3145	Bn-A02-p13999424	A-genome specific (napus-rapa)	1	0.4013976	not scorable	not scorable	1	0.8388037
3146	Bn-A02-p14024462	A-genome specific (rapa only)	not scorable	not scorable	not scorable	not scorable	1	0.6423919
3147	Bn-A02-p14025825	A-genome specific (napus-rapa)	0.9952153	0.8215231	not scorable	not scorable	0.9917355	0.8860154
3148	Bn-A02-p14026570	A-genome specific (napus-rapa)	0.9952153	0.7107612	not scorable	not scorable	1	0.8211767
3149	Bn-A02-p1402685	A-genome specific (napus-rapa)	0.9784689	0.7155414	not scorable	not scorable	0.9917355	0.7257178
3150	Bn-A02-p14028000	A-genome specific (napus-rapa)	0.9928229	0.8161988	not scorable	not scorable	1	0.8306969
3151	Bn-A02-p14028770	A-genome specific (napus-rapa)	0.9904306	0.8026987	not scorable	not scorable	0.9917355	0.7695999
3152	Bn-A02-p14040220	napus specific	0.9952153	0.7734257	not scorable	not scorable	not scorable	not scorable
3153	Bn-A02-p14053180	A-genome specific (rapa only)	not scorable	not scorable	not scorable	not scorable	0.9752066	0.8068985
3154	Bn-A02-p14078497	A-genome specific (napus-rapa)	0.9952153	0.753142	not scorable	not scorable	0.9834711	0.7835267
3155	Bn-A02-p14103759	A-genome specific (napus-rapa)	0.9928229	0.7090805	not scorable	not scorable	0.9586777	0.7887905
3156	Bn-A02-p14104250	A-genome specific (napus-rapa)	0.9832536	0.715404	not scorable	not scorable	0.9917355	0.7885925
3157	Bn-A02-p1411508	A-genome specific (napus-rapa)	0.9832536	0.7886575	not scorable	not scorable	0.9834711	0.7385584
3158	Bn-A02-p14127042	A-genome specific (napus-rapa)	0.9904306	0.602768	not scorable	not scorable	0.9917355	0.7735261
3159	Bn-A02-p14130758	A-genome specific (napus-rapa)	1	0.7834822	not scorable	not scorable	0.9834711	0.7546473
3160	Bn-A02-p14134708	A-genome specific (rapa only)	not scorable	not scorable	not scorable	not scorable	0.9917355	0.7866042
3161	Bn-A02-p14145586	A-genome specific (napus-rapa)	0.9976076	0.5545668	not scorable	not scorable	1	0.78807
3162	Bn-A02-p14148076	A-genome specific (napus-rapa)	0.9976076	0.485128	not scorable	not scorable	0.9917355	0.7263379
3163	Bn-A02-p14151274	A-genome specific (napus-rapa)	0.9952153	0.7993426	not scorable	not scorable	0.9917355	0.7700318
3164	Bn-A02-p14162326	A-genome specific (rapa only)	not scorable	not scorable	not scorable	not scorable	0.9834711	0.7902763
3165	Bn-A02-p14167623	napus specific	0.9162679	0.708674	not scorable	not scorable	not scorable	not scorable

3166	Bn-A02-p14170476	A-genome specific (napus-rapa)	1	0.5086023	not scorable	not scorable	1	0.7456457
3167	Bn-A02-p14174686	A-genome specific (rapa only)	not scorable	not scorable	not scorable	not scorable	0.9917355	0.9146796
3168	Bn-A02-p14175730	A-genome specific (napus-rapa)	0.9952153	0.7994079	not scorable	not scorable	0.9834711	0.7641769
3169	Bn-A02-p14177785	all not scorable	not scorable	not scorable	not scorable	not scorable	not scorable	not scorable
3170	Bn-A02-p14178166	A-genome specific (napus-rapa)	0.9952153	0.7640718	not scorable	not scorable	0.9917355	0.7337866
3171	Bn-A02-p14208801	A-genome specific (napus-rapa)	0.9976076	0.6125628	not scorable	not scorable	0.9752066	0.671567
3172	Bn-A02-p14215327	A-genome specific (napus-rapa)	0.9952153	0.7308566	not scorable	not scorable	0.9917355	0.7086867
3173	Bn-A02-p14227678	A-genome specific (napus-rapa)	1	0.4773371	not scorable	not scorable	0.9834711	0.7871575
3174	Bn-A02-p14229894	napus specific	0.9928229	0.4736191	not scorable	not scorable	not scorable	not scorable
3175	Bn-A02-p14232495	A-genome specific (rapa only)	not scorable	not scorable	not scorable	not scorable	0.9834711	0.716597
3176	Bn-A02-p14287347	A-genome specific (napus-rapa)	0.9928229	0.5181399	not scorable	not scorable	1	0.7908058
3177	Bn-A02-p14287428	A-genome specific (napus-rapa)	1	0.3091168	not scorable	not scorable	0.9752066	0.6256217
3178	Bn-A02-p14293315	A-genome specific (napus-rapa)	0.9952153	0.8029899	not scorable	not scorable	0.9917355	0.8364806
3179	Bn-A02-p14328410	A-genome specific (napus-rapa)	0.9856459	0.2954222	not scorable	not scorable	1	0.8746864
3180	Bn-A02-p14500945	all not scorable	not scorable	not scorable	not scorable	not scorable	not scorable	not scorable
3181	Bn-A02-p14526876	A-genome specific (napus-rapa)	0.9976076	0.7718452	not scorable	not scorable	0.9504132	0.8171834
3182	Bn-A02-p14526990	A-genome specific (napus-rapa)	0.9976076	0.6941428	not scorable	not scorable	0.9504132	0.8804815
3183	Bn-A02-p14527781	A-genome specific (napus-rapa)	0.9976076	0.739652	not scorable	not scorable	0.9504132	0.682395
3184	Bn-A02-p14529778	A-genome specific (napus-rapa)	0.9808612	0.3523288	not scorable	not scorable	0.9504132	0.6567213
3185	Bn-A02-p14530693	A-genome specific (napus-rapa)	0.9952153	0.4602996	not scorable	not scorable	0.9586777	0.7497717
3186	Bn-A02-p14540346	A-genome specific (napus-rapa)	0.9976076	0.5967311	not scorable	not scorable	0.9917355	0.8113362
3187	Bn-A02-p14564880	A-genome specific (napus-rapa)	0.9736842	0.7544039	not scorable	not scorable	0.9917355	0.7916464
3188	Bn-A02-p14575017	A-genome specific (rapa only)	not scorable	not scorable	not scorable	not scorable	1	0.7892429
3189	Bn-A02-p14576479	A-genome specific (napus-rapa)	0.9617225	0.6179405	not scorable	not scorable	0.9834711	0.6857461
3190	Bn-A02-p14579685	A-genome specific (napus-rapa)	0.9712918	0.7896586	not scorable	not scorable	1	0.6303774
3191	Bn-A02-p14592602	napus specific	0.9976076	0.4610621	not scorable	not scorable	not scorable	not scorable
3192	Bn-A02-p14605944	A-genome specific (napus-rapa)	0.9976076	0.7861257	not scorable	not scorable	0.9834711	0.7907084
3193	Bn-A02-p14608076	A-genome specific (napus-rapa)	0.9617225	0.4318213	not scorable	not scorable	1	0.7371429
3194	Bn-A02-p14612382	A-genome specific (rapa only)	not scorable	not scorable	not scorable	not scorable	1	0.675705
3195	Bn-A02-p14615703	A-genome specific (napus-rapa)	0.9976076	0.4392627	not scorable	not scorable	1	0.8022124
3196	Bn-A02-p14650284	A-genome specific (napus-rapa)	0.9593301	0.4010577	not scorable	not scorable	1	0.8922935
3197	Bn-A02-p14653729	A-genome specific (napus-rapa)	0.9928229	0.4022931	not scorable	not scorable	0.9834711	0.7064978
3198	Bn-A02-p14695760	A-genome specific (napus-rapa)	0.9952153	0.7374635	not scorable	not scorable	1	0.7495615

3199	Bn-A02-p14707749	napus specific	0.9976076	0.6386841	not scorable	not scorable	not scorable	not scorable
3200	Bn-A02-p14708741	A-genome specific (rapa only)	not scorable	not scorable	not scorable	not scorable	0.9752066	0.6924533
3201	Bn-A02-p14736669	A-genome specific (napus-rapa)	0.9952153	0.2526194	not scorable	not scorable	0.9752066	0.7054088
3202	Bn-A02-p14775699	A-genome specific (napus-rapa)	0.9928229	0.6674984	not scorable	not scorable	0.9752066	0.6047301
3203	Bn-A02-p14781371	A-genome specific (napus-rapa)	0.9976076	0.776486	not scorable	not scorable	0.9917355	0.7931407
3204	Bn-A02-p1485915	A-genome specific (napus-rapa)	0.9904306	0.8188727	not scorable	not scorable	1	0.7730481
3205	Bn-A02-p14886507	A-genome specific (napus-rapa)	0.9952153	0.7729254	not scorable	not scorable	0.9917355	0.7292245
3206	Bn-A02-p14896274	A-genome specific (napus-rapa)	0.9976076	0.3076343	not scorable	not scorable	1	0.8496791
3207	Bn-A02-p14898010	A-genome specific (napus-rapa)	0.9952153	0.8113019	not scorable	not scorable	1	0.773995
3208	Bn-A02-p14902806	A-genome specific (napus-rapa)	0.9976076	0.8330638	not scorable	not scorable	0.9752066	0.6937109
3209	Bn-A02-p14903483	napus specific	0.9186603	0.7637809	not scorable	not scorable	not scorable	not scorable
3210	Bn-A02-p14903643	A-genome specific (napus-rapa)	1	0.4668023	not scorable	not scorable	0.9917355	0.7659745
3211	Bn-A02-p14905419	A-genome specific (napus-rapa)	0.9162679	0.7156558	not scorable	not scorable	0.9669421	0.729185
3212	Bn-A02-p14925274	A-genome specific (napus-rapa)	0.9976076	0.5879623	not scorable	not scorable	0.9917355	0.7749733
3213	Bn-A02-p14926973	A-genome specific (napus-rapa)	1	0.4753223	not scorable	not scorable	1	0.7666692
3214	Bn-A02-p14938768	A-genome specific (napus-rapa)	0.7679426	0.6964341	not scorable	not scorable	0.9752066	0.7950392
3215	Bn-A02-p14938941	A-genome specific (napus-rapa)	0.7727273	0.7503761	not scorable	not scorable	0.9834711	0.8349305
3216	Bn-A02-p14939071	A-genome specific (napus-rapa)	0.7679426	0.7303702	not scorable	not scorable	0.9834711	0.694302
3217	Bn-A02-p14939204	A-genome specific (napus-rapa)	0.7679426	0.7392656	not scorable	not scorable	0.9834711	0.6875225
3218	Bn-A02-p14939483	A-genome specific (napus-rapa)	0.7679426	0.7080011	not scorable	not scorable	0.9752066	0.7187466
3219	Bn-A02-p14940026	A-genome specific (napus-rapa)	0.7655503	0.712921	not scorable	not scorable	0.9917355	0.7143264
3220	Bn-A02-p14940029	A-genome specific (napus-rapa)	0.7703349	0.7341982	not scorable	not scorable	0.9917355	0.7132887
3221	Bn-A02-p14940149	A-genome specific (napus-rapa)	0.7679426	0.7375392	not scorable	not scorable	0.9834711	0.7064788
3222	Bn-A02-p14940218	A-genome specific (napus-rapa)	0.7703349	0.7889026	not scorable	not scorable	0.9917355	0.7719629
3223	Bn-A02-p14941541	A-genome specific (napus-rapa)	0.7655503	0.7290855	not scorable	not scorable	0.9917355	0.7591025
3224	Bn-A02-p14941595	genome unspecific (all in)	0.784689	0.556541	0.9689922	0.2911764	0.9752066	0.7178582
3225	Bn-A02-p14941620	A-genome specific (napus-rapa)	0.7679426	0.742449	not scorable	not scorable	0.9834711	0.7869908
3226	Bn-A02-p14942048	A-genome specific (napus-rapa)	0.7679426	0.6525595	not scorable	not scorable	0.9834711	0.6345238
3227	Bn-A02-p14944551	A-genome specific (napus-rapa)	0.9976076	0.7801242	not scorable	not scorable	0.9917355	0.7728239
3228	Bn-A02-p14946150	A-genome specific (napus-rapa)	0.9952153	0.4909858	not scorable	not scorable	0.9917355	0.7757232
3229	Bn-A02-p14956864	A-genome specific (napus-rapa)	0.9976076	0.7656525	not scorable	not scorable	0.9917355	0.8042552
3230	Bn-A02-p14992706	A-genome specific (napus-rapa)	0.9952153	0.7701067	not scorable	not scorable	0.9917355	0.754338
3231	Bn-A02-p14992781	A-genome specific (napus-rapa)	0.9928229	0.6437628	not scorable	not scorable	0.9834711	0.6896743

3232	Bn-A02-p14992802	A-genome specific (rapa only)	not scorable	not scorable	not scorable	not scorable	0.9834711	0.7107058
3233	Bn-A02-p14996107	A-genome specific (napus-rapa)	0.9832536	0.4112699	not scorable	not scorable	0.9834711	0.7167431
3234	Bn-A02-p1506579	A-genome specific (rapa only)	not scorable	not scorable	not scorable	not scorable	1	0.7349294
3235	Bn-A02-p1517113	genome unspecific (oleracea-rapa)	not scorable	not scorable	1	0.4316438	0.9917355	0.8447355
3236	Bn-A02-p15184172	A-genome specific (napus-rapa)	0.9808612	0.5725501	not scorable	not scorable	1	0.7195811
3237	Bn-A02-p1528341	A-genome specific (rapa only)	not scorable	not scorable	not scorable	not scorable	0.9917355	0.8606058
3238	Bn-A02-p1530996	A-genome specific (napus-rapa)	0.9976076	0.7850553	not scorable	not scorable	1	0.8521883
3239	Bn-A02-p15322404	A-genome specific (napus-rapa)	0.9976076	0.8518915	not scorable	not scorable	1	0.869809
3240	Bn-A02-p15323930	A-genome specific (napus-rapa)	0.9856459	0.8466671	not scorable	not scorable	0.9834711	0.7481999
3241	Bn-A02-p15323936	A-genome specific (napus-rapa)	0.9976076	0.9260141	not scorable	not scorable	1	0.922092
3242	Bn-A02-p15457681	A-genome specific (napus-rapa)	0.9688995	0.4769859	not scorable	not scorable	0.9586777	0.8188943
3243	Bn-A02-p1552305	A-genome specific (napus-rapa)	0.9856459	0.7942645	not scorable	not scorable	1	0.8949617
3244	Bn-A02-p15569271	A-genome specific (napus-rapa)	0.9856459	0.5730578	not scorable	not scorable	0.9752066	0.6869631
3245	Bn-A02-p15628202	napus specific	0.5598086	0.8948976	not scorable	not scorable	not scorable	not scorable
3246	Bn-A02-p15637932	A-genome specific (napus-rapa)	0.9928229	0.4018365	not scorable	not scorable	0.9917355	0.6668006
3247	Bn-A02-p15693192	A-genome specific (napus-rapa)	0.9952153	0.7194458	not scorable	not scorable	0.9917355	0.7284528
3248	Bn-A02-p15717143	A-genome specific (napus-rapa)	0.9976076	0.3885508	not scorable	not scorable	0.9917355	0.8207138
3249	Bn-A02-p15745628	A-genome specific (napus-rapa)	0.9832536	0.5656829	not scorable	not scorable	1	0.7645996
3250	Bn-A02-p15750257	A-genome specific (napus-rapa)	0.9880383	0.4720289	not scorable	not scorable	0.9917355	0.7631801
3251	Bn-A02-p15756475	A-genome specific (napus-rapa)	0.9784689	0.6451641	not scorable	not scorable	0.9669421	0.6379052
3252	Bn-A02-p15758928	A-genome specific (napus-rapa)	0.930622	0.3192373	not scorable	not scorable	0.9669421	0.768541
3253	Bn-A02-p15760465	A-genome specific (napus-rapa)	0.9449761	0.5432864	not scorable	not scorable	0.9834711	0.7620175
3254	Bn-A02-p1576350	A-genome specific (napus-rapa)	0.9832536	0.710338	not scorable	not scorable	0.9917355	0.7532918
3255	Bn-A02-p15764198	A-genome specific (napus-rapa)	0.9808612	0.8299569	not scorable	not scorable	0.9917355	0.8014634
3256	Bn-A02-p15766649	A-genome specific (napus-rapa)	0.9904306	0.4476127	not scorable	not scorable	0.9752066	0.7028346
3257	Bn-A02-p15789088	A-genome specific (napus-rapa)	0.9880383	0.369144	not scorable	not scorable	0.9917355	0.8578107
3258	Bn-A02-p15806083	A-genome specific (rapa only)	not scorable	not scorable	not scorable	not scorable	0.5041322	0.8272053
3259	Bn-A02-p15820017	A-genome specific (napus-rapa)	0.9736842	0.2763368	not scorable	not scorable	1	0.7772811
3260	Bn-A02-p15832342	A-genome specific (napus-rapa)	1	0.7871383	not scorable	not scorable	1	0.759752
3261	Bn-A02-p15870327	A-genome specific (napus-rapa)	0.9880383	0.4586517	not scorable	not scorable	0.9834711	0.7576363
3262	Bn-A02-p15884717	A-genome specific (napus-rapa)	0.9856459	0.7434567	not scorable	not scorable	0.9834711	0.7521971
3263	Bn-A02-p15898839	A-genome specific (napus-rapa)	0.9928229	0.3479863	not scorable	not scorable	1	0.7975821
3264	Bn-A02-p15912199	A-genome specific (napus-rapa)	0.9880383	0.4438224	not scorable	not scorable	0.9834711	0.8111171

3265	Bn-A02-p15928585	A-genome specific (napus-rapa)	0.8421053	0.7538716	not scorable	not scorable	0.7190083	0.7534271
3266	Bn-A02-p159653	A-genome specific (napus-rapa)	0.9712918	0.3828684	not scorable	not scorable	1	0.7571688
3267	Bn-A02-p16058769	A-genome specific (napus-rapa)	1	0.7785135	not scorable	not scorable	1	0.7494321
3268	Bn-A02-p16083197	A-genome specific (napus-rapa)	0.9904306	0.3283503	not scorable	not scorable	0.9917355	0.7055491
3269	Bn-A02-p16209439	A-genome specific (napus-rapa)	0.9904306	0.6745288	not scorable	not scorable	1	0.6475382
3270	Bn-A02-p16220163	A-genome specific (napus-rapa)	0.9952153	0.7883631	not scorable	not scorable	0.9917355	0.7088114
3271	Bn-A02-p16223740	A-genome specific (napus-rapa)	0.8205742	0.6892622	not scorable	not scorable	0.9917355	0.6945941
3272	Bn-A02-p16259084	A-genome specific (napus-rapa)	0.9952153	0.7750432	not scorable	not scorable	0.9752066	0.7747369
3273	Bn-A02-p16259298	A-genome specific (napus-rapa)	0.9928229	0.2517718	not scorable	not scorable	0.9586777	0.6845247
3274	Bn-A02-p1629448	A-genome specific (napus-rapa)	1	0.8139551	not scorable	not scorable	1	0.8534235
3275	Bn-A02-p16311535	A-genome specific (napus-rapa)	0.9904306	0.8190353	not scorable	not scorable	1	0.9175564
3276	Bn-A02-p16339698	genome unspecific (all in)	0.9976076	0.7515985	1	0.60638	0.9752066	0.6622245
3277	Bn-A02-p16428766	napus specific	0.9736842	0.9258286	not scorable	not scorable	not scorable	not scorable
3278	Bn-A02-p16486966	A-genome specific (napus-rapa)	1	0.7914145	not scorable	not scorable	1	0.7927939
3279	Bn-A02-p16520874	A-genome specific (napus-rapa)	0.9641148	0.6786538	not scorable	not scorable	0.5206612	0.9088613
3280	Bn-A02-p16521871	A-genome specific (napus-rapa)	0.9688995	0.5906556	not scorable	not scorable	0.5206612	0.8557174
3281	Bn-A02-p16526928	A-genome specific (napus-rapa)	0.9976076	0.4644936	not scorable	not scorable	0.9752066	0.5394788
3282	Bn-A02-p16528486	A-genome specific (napus-rapa)	0.9425837	0.5674553	not scorable	not scorable	0.553719	0.8826532
3283	Bn-A02-p16529092	A-genome specific (napus-rapa)	0.9760765	0.8183839	not scorable	not scorable	0.9504132	0.8272623
3284	Bn-A02-p16571709	A-genome specific (napus-rapa)	0.9952153	0.8356315	not scorable	not scorable	0.9752066	0.7506766
3285	Bn-A02-p16572757	A-genome specific (napus-rapa)	0.9904306	0.7791448	not scorable	not scorable	1	0.759035
3286	Bn-A02-p16666866	A-genome specific (napus-rapa)	0.9808612	0.6835147	not scorable	not scorable	0.9917355	0.7209759
3287	Bn-A02-p1671689	A-genome specific (napus-rapa)	0.9593301	0.7804402	not scorable	not scorable	1	0.7844547
3288	Bn-A02-p1684625	A-genome specific (rapa only)	not scorable	not scorable	not scorable	not scorable	0.9834711	0.7595637
3289	Bn-A02-p1687398	A-genome specific (napus-rapa)	0.9665072	0.7374522	not scorable	not scorable	0.9917355	0.758269
3290	Bn-A02-p1698015	A-genome specific (napus-rapa)	0.9569378	0.6934527	not scorable	not scorable	0.9917355	0.7447706
3291	Bn-A02-p17012938	all not scorable	not scorable	not scorable	not scorable	not scorable	not scorable	not scorable
3292	Bn-A02-p1705187	A-genome specific (napus-rapa)	0.9928229	0.4218378	not scorable	not scorable	1	0.9062111
3293	Bn-A02-p1707420	A-genome specific (napus-rapa)	0.9832536	0.7512879	not scorable	not scorable	0.9421487	0.8276614
3294	Bn-A02-p17112647	A-genome specific (napus-rapa)	0.9952153	0.8849835	not scorable	not scorable	1	0.8621171
3295	Bn-A02-p17156661	A-genome specific (napus-rapa)	0.9904306	0.2969875	not scorable	not scorable	0.9669421	0.7041948
3296	Bn-A02-p17189095	A-genome specific (rapa only)	not scorable	not scorable	not scorable	not scorable	0.553719	0.8566031
3297	Bn-A02-p17189164	A-genome specific (rapa only)	not scorable	not scorable	not scorable	not scorable	0.677686	0.8487107

3298	Bn-A02-p17224721	genome unspecific (oleracea-rapa)	not scorable	not scorable	0.9457364	0.303142	1	0.7320092
3299	Bn-A02-p17229398	genome unspecific (all in)	0.9976076	0.6051318	1	0.8194432	0.9917355	0.8813978
3300	Bn-A02-p17333549	genome unspecific (all in)	1	0.5355057	0.8837209	0.6940374	1	0.9171556
3301	Bn-A02-p17381818	A-genome specific (napus-rapa)	0.9856459	0.7751049	not scorable	not scorable	1	0.9175813
3302	Bn-A02-p174249	A-genome specific (napus-rapa)	0.9856459	0.3675432	not scorable	not scorable	1	0.6804243
3303	Bn-A02-p17532943	A-genome specific (napus-rapa)	0.9330143	0.2241565	not scorable	not scorable	1	0.614313
3304	Bn-A02-p175605	A-genome specific (napus-rapa)	0.9856459	0.7376103	not scorable	not scorable	0.9917355	0.727749
3305	Bn-A02-p1761218	A-genome specific (rapa only)	not scorable	not scorable	not scorable	not scorable	1	0.7419717
3306	Bn-A02-p1788566	A-genome specific (napus-rapa)	0.9808612	0.7657143	not scorable	not scorable	1	0.8607456
3307	Bn-A02-p17910994	napus specific	0.9784689	0.6188984	not scorable	not scorable	not scorable	not scorable
3308	Bn-A02-p17938666	A-genome specific (napus-rapa)	0.9736842	0.4440767	not scorable	not scorable	0.5289256	0.7542993
3309	Bn-A02-p179557	A-genome specific (napus-rapa)	0.9736842	0.7552561	not scorable	not scorable	0.9917355	0.7326435
3310	Bn-A02-p17996122	A-genome specific (napus-rapa)	0.9952153	0.8217659	not scorable	not scorable	0.8760331	0.8410667
3311	Bn-A02-p18076499	A-genome specific (napus-rapa)	0.9952153	0.8135217	not scorable	not scorable	1	0.8000314
3312	Bn-A02-p18101171	A-genome specific (napus-rapa)	0.9808612	0.6833199	not scorable	not scorable	1	0.7609816
3313	Bn-A02-p182447	A-genome specific (napus-rapa)	1	0.78006	not scorable	not scorable	1	0.7387633
3314	Bn-A02-p182618	A-genome specific (napus-rapa)	0.9952153	0.7005345	not scorable	not scorable	0.9917355	0.7436823
3315	Bn-A02-p18310675	A-genome specific (napus-rapa)	1	0.4247035	not scorable	not scorable	0.9917355	0.7122792
3316	Bn-A02-p183438	A-genome specific (napus-rapa)	0.9856459	0.8136101	not scorable	not scorable	1	0.7503253
3317	Bn-A02-p183586	A-genome specific (napus-rapa)	0.9784689	0.5067708	not scorable	not scorable	0.9917355	0.7391412
3318	Bn-A02-p183840	A-genome specific (napus-rapa)	1	0.811821	not scorable	not scorable	0.9917355	0.6969276
3319	Bn-A02-p18438691	A-genome specific (napus-rapa)	0.9880383	0.6433415	not scorable	not scorable	1	0.7591171
3320	Bn-A02-p18571872	napus specific	0.569378	0.7873629	not scorable	not scorable	not scorable	not scorable
3321	Bn-A02-p185917	A-genome specific (napus-rapa)	0.8277512	0.787751	not scorable	not scorable	0.9834711	0.8356722
3322	Bn-A02-p18599005	A-genome specific (napus-rapa)	1	0.3046076	not scorable	not scorable	0.9917355	0.7935658
3323	Bn-A02-p187256	A-genome specific (napus-rapa)	0.8349282	0.7739975	not scorable	not scorable	0.9338843	0.8139877
3324	Bn-A02-p188268	A-genome specific (napus-rapa)	0.8397129	0.8014279	not scorable	not scorable	0.9752066	0.82846
3325	Bn-A02-p188454	A-genome specific (napus-rapa)	0.8397129	0.7736885	not scorable	not scorable	0.9834711	0.8050065
3326	Bn-A02-p1890913	A-genome specific (rapa only)	not scorable	not scorable	not scorable	not scorable	1	0.9270482
3327	Bn-A02-p18917772	A-genome specific (napus-rapa)	0.9880383	0.5712082	not scorable	not scorable	0.9834711	0.754685
3328	Bn-A02-p1894727	A-genome specific (napus-rapa)	0.9976076	0.9007395	not scorable	not scorable	0.6280992	0.9082332
3329	Bn-A02-p18949958	A-genome specific (napus-rapa)	0.9880383	0.6829301	not scorable	not scorable	1	0.8575348
3330	Bn-A02-p18966918	genome unspecific (all in)	0.9880383	0.6974407	0.1317829	0.7355374	1	0.7381126

3331	Bn-A02-p18974322	A-genome specific (napus-rapa)	0.9856459	0.7299376	not scorable	not scorable	0.9669421	0.7591202
3332	Bn-A02-p18979982	A-genome specific (napus-rapa)	0.4330144	0.7270218	not scorable	not scorable	0.5289256	0.723753
3333	Bn-A02-p18984484	A-genome specific (rapa only)	not scorable	not scorable	not scorable	not scorable	0.9752066	0.7668897
3334	Bn-A02-p18985754	A-genome specific (napus-rapa)	1	0.6766622	not scorable	not scorable	0.9834711	0.7739437
3335	Bn-A02-p18989840	A-genome specific (napus-rapa)	0.9976076	0.3219452	not scorable	not scorable	1	0.7293474
3336	Bn-A02-p18994242	A-genome specific (rapa only)	not scorable	not scorable	not scorable	not scorable	0.9008265	0.8783097
3337	Bn-A02-p18994312	A-genome specific (napus-rapa)	0.9665072	0.7423475	not scorable	not scorable	1	0.792895
3338	Bn-A02-p18994472	A-genome specific (napus-rapa)	0.9880383	0.7758804	not scorable	not scorable	1	0.8369021
3339	Bn-A02-p18998627	A-genome specific (napus-rapa)	0.9904306	0.7807118	not scorable	not scorable	0.9669421	0.7903531
3340	Bn-A02-p19003944	A-genome specific (napus-rapa)	0.9808612	0.4376461	not scorable	not scorable	0.9917355	0.7898582
3341	Bn-A02-p19045730	A-genome specific (napus-rapa)	0.423445	0.7574914	not scorable	not scorable	0.9669421	0.7010869
3342	Bn-A02-p19049506	A-genome specific (napus-rapa)	0.9712918	0.7938126	not scorable	not scorable	1	0.7669932
3343	Bn-A02-p19049989	A-genome specific (napus-rapa)	0.9952153	0.338118	not scorable	not scorable	1	0.7443598
3344	Bn-A02-p19052873	A-genome specific (napus-rapa)	0.9832536	0.675292	not scorable	not scorable	0.9752066	0.8368086
3345	Bn-A02-p19053770	all not scorable	not scorable	not scorable	not scorable	not scorable	not scorable	not scorable
3346	Bn-A02-p19058136	A-genome specific (rapa only)	not scorable	not scorable	not scorable	not scorable	0.9917355	0.5807959
3347	Bn-A02-p19070958	A-genome specific (napus-rapa)	0.9928229	0.4264335	not scorable	not scorable	0.9834711	0.8310072
3348	Bn-A02-p19123194	A-genome specific (rapa only)	not scorable	not scorable	not scorable	not scorable	1	0.662511
3349	Bn-A02-p19125023	A-genome specific (napus-rapa)	0.9928229	0.6135465	not scorable	not scorable	1	0.7106043
3350	Bn-A02-p19160671	A-genome specific (rapa only)	not scorable	not scorable	not scorable	not scorable	1	0.7419274
3351	Bn-A02-p19170689	A-genome specific (napus-rapa)	0.9784689	0.7197862	not scorable	not scorable	0.9917355	0.754686
3352	Bn-A02-p19174964	A-genome specific (napus-rapa)	0.9952153	0.6125658	not scorable	not scorable	0.9752066	0.7376505
3353	Bn-A02-p19175402	A-genome specific (napus-rapa)	0.9832536	0.335347	not scorable	not scorable	0.9917355	0.6871848
3354	Bn-A02-p19190669	A-genome specific (napus-rapa)	0.708134	0.7142869	not scorable	not scorable	0.9834711	0.7804465
3355	Bn-A02-p19201685	A-genome specific (napus-rapa)	0.9736842	0.6717947	not scorable	not scorable	0.9586777	0.7534206
3356	Bn-A02-p19207431	A-genome specific (napus-rapa)	0.9641148	0.5406559	not scorable	not scorable	1	0.674446
3357	Bn-A02-p19209964	A-genome specific (napus-rapa)	0.9952153	0.2666814	not scorable	not scorable	1	0.8197358
3358	Bn-A02-p19212766	A-genome specific (napus-rapa)	0.9784689	0.6571057	not scorable	not scorable	1	0.766425
3359	Bn-A02-p19218108	A-genome specific (napus-rapa)	0.9976076	0.3613857	not scorable	not scorable	1	0.7301149
3360	Bn-A02-p19244695	A-genome specific (napus-rapa)	0.9904306	0.8008955	not scorable	not scorable	1	0.7503625
3361	Bn-A02-p19260818	A-genome specific (napus-rapa)	0.9928229	0.7832009	not scorable	not scorable	0.9917355	0.7601098
3362	Bn-A02-p19261853	A-genome specific (napus-rapa)	0.9976076	0.607985	not scorable	not scorable	0.9752066	0.7628533
3363	Bn-A02-p19285211	A-genome specific (napus-rapa)	0.9928229	0.4477962	not scorable	not scorable	1	0.7857104

3364	Bn-A02-p19294461	A-genome specific (napus-rapa)	0.9904306	0.7697594	not scorable	not scorable	1	0.69863
3365	Bn-A02-p19299222	A-genome specific (napus-rapa)	0.9784689	0.8348282	not scorable	not scorable	0.9586777	0.8073711
3366	Bn-A02-p19299455	A-genome specific (napus-rapa)	0.9856459	0.8115287	not scorable	not scorable	0.9917355	0.7871199
3367	Bn-A02-p19316657	A-genome specific (napus-rapa)	0.9928229	0.6827853	not scorable	not scorable	1	0.8138443
3368	Bn-A02-p19317643	A-genome specific (napus-rapa)	0.9928229	0.4772309	not scorable	not scorable	0.9834711	0.7266084
3369	Bn-A02-p19343485	A-genome specific (napus-rapa)	0.9952153	0.827432	not scorable	not scorable	1	0.8773471
3370	Bn-A02-p19348348	A-genome specific (napus-rapa)	0.9880383	0.7427712	not scorable	not scorable	0.9917355	0.74309
3371	Bn-A02-p19373757	A-genome specific (napus-rapa)	0.9928229	0.7929814	not scorable	not scorable	1	0.7975748
3372	Bn-A02-p19373825	A-genome specific (napus-rapa)	0.9832536	0.7524077	not scorable	not scorable	0.9917355	0.8111154
3373	Bn-A02-p19374782	A-genome specific (napus-rapa)	0.9856459	0.6161359	not scorable	not scorable	0.9917355	0.7184696
3374	Bn-A02-p19375860	A-genome specific (napus-rapa)	0.9880383	0.7419744	not scorable	not scorable	0.9834711	0.6856686
3375	Bn-A02-p19385896	genome unspecific (all in)	0.9880383	0.5695209	0.9379845	0.6722535	0.9256198	0.644646
3376	Bn-A02-p19385986	A-genome specific (napus-rapa)	0.9952153	0.6765476	not scorable	not scorable	0.9752066	0.7185709
3377	Bn-A02-p19395795	A-genome specific (napus-rapa)	0.9904306	0.7975338	not scorable	not scorable	0.9752066	0.759536
3378	Bn-A02-p19401844	A-genome specific (napus-rapa)	0.9904306	0.7386193	not scorable	not scorable	0.9834711	0.8475552
3379	Bn-A02-p19402089	C-genome specific (napus-oleracea)	0.9952153	0.6208922	0.9922481	0.7522202	not scorable	not scorable
3380	Bn-A02-p19408740	A-genome specific (rapa only)	not scorable	not scorable	not scorable	not scorable	0.9834711	0.7160276
3381	Bn-A02-p19410459	A-genome specific (napus-rapa)	0.9904306	0.6650201	not scorable	not scorable	0.9917355	0.5514108
3382	Bn-A02-p19450017	A-genome specific (napus-rapa)	0.9928229	0.556492	not scorable	not scorable	0.9834711	0.644587
3383	Bn-A02-p19450418	A-genome specific (napus-rapa)	1	0.3706591	not scorable	not scorable	0.9504132	0.6996231
3384	Bn-A02-p19451380	A-genome specific (napus-rapa)	0.9904306	0.7379296	not scorable	not scorable	0.9421487	0.7155941
3385	Bn-A02-p19460299	napus specific	0.9928229	0.5402043	not scorable	not scorable	not scorable	not scorable
3386	Bn-A02-p19521910	genome unspecific (all in)	0.9928229	0.4065026	0.5581396	0.5237762	1	0.730982
3387	Bn-A02-p19534217	A-genome specific (napus-rapa)	0.9856459	0.743274	not scorable	not scorable	0.9586777	0.4080552
3388	Bn-A02-p19549587	A-genome specific (napus-rapa)	0.9952153	0.7742425	not scorable	not scorable	1	0.8410764
3389	Bn-A02-p19566951	A-genome specific (napus-rapa)	0.9784689	0.8074201	not scorable	not scorable	0.9669421	0.707109
3390	Bn-A02-p19573867	A-genome specific (napus-rapa)	0.9952153	0.7664746	not scorable	not scorable	0.9917355	0.757426
3391	Bn-A02-p19575587	A-genome specific (napus-rapa)	0.9952153	0.2564941	not scorable	not scorable	0.9917355	0.7480944
3392	Bn-A02-p19587474	A-genome specific (napus-rapa)	0.9928229	0.7116289	not scorable	not scorable	0.9917355	0.6727926
3393	Bn-A02-p19587812	A-genome specific (napus-rapa)	0.9856459	0.7730435	not scorable	not scorable	1	0.6846573
3394	Bn-A02-p19589605	A-genome specific (napus-rapa)	0.9354067	0.6928881	not scorable	not scorable	0.9752066	0.6963306
3395	Bn-A02-p19602796	A-genome specific (napus-rapa)	0.930622	0.6446189	not scorable	not scorable	0.9586777	0.7259034
3396	Bn-A02-p19619823	A-genome specific (napus-rapa)	0.9880383	0.439704	not scorable	not scorable	0.9917355	0.7504635

3397	Bn-A02-p19630208	A-genome specific (napus-rapa)	0.9976076	0.3444678	not scorable	not scorable	1	0.7159357
3398	Bn-A02-p19645773	A-genome specific (napus-rapa)	0.9976076	0.408344	not scorable	not scorable	0.9834711	0.7211741
3399	Bn-A02-p19662482	A-genome specific (napus-rapa)	0.9952153	0.8010619	not scorable	not scorable	1	0.793086
3400	Bn-A02-p19674831	A-genome specific (napus-rapa)	0.9928229	0.7267082	not scorable	not scorable	0.9917355	0.6194032
3401	Bn-A02-p19682273	A-genome specific (napus-rapa)	0.9952153	0.7087796	not scorable	not scorable	0.9917355	0.8172332
3402	Bn-A02-p19682984	A-genome specific (napus-rapa)	0.9880383	0.8256827	not scorable	not scorable	0.9917355	0.7260362
3403	Bn-A02-p19683093	A-genome specific (napus-rapa)	0.9856459	0.8360622	not scorable	not scorable	0.9752066	0.6650287
3404	Bn-A02-p19702955	A-genome specific (napus-rapa)	0.9880383	0.5580875	not scorable	not scorable	0.9917355	0.7339699
3405	Bn-A02-p19704677	A-genome specific (napus-rapa)	0.9904306	0.8179314	not scorable	not scorable	1	0.6896189
3406	Bn-A02-p19709973	genome unspecific (all in)	0.9665072	0.3614577	0.5658914	0.760924	1	0.9175813
3407	Bn-A02-p19717843	genome unspecific (all in)	0.9832536	0.2837937	0.5968992	0.8157162	1	0.9205657
3408	Bn-A02-p19731316	A-genome specific (rapa only)	not scorable	not scorable	not scorable	not scorable	0.9917355	0.7562061
3409	Bn-A02-p19756499	A-genome specific (napus-rapa)	0.9904306	0.3150766	not scorable	not scorable	1	0.8169969
3410	Bn-A02-p19872956	A-genome specific (napus-rapa)	0.9736842	0.7928926	not scorable	not scorable	0.9917355	0.8035119
3411	Bn-A02-p19874478	A-genome specific (napus-rapa)	0.9712918	0.7010235	not scorable	not scorable	0.9834711	0.7755477
3412	Bn-A02-p19875345	A-genome specific (napus-rapa)	0.9641148	0.7393757	not scorable	not scorable	0.9669421	0.4932909
3413	Bn-A02-p19886585	A-genome specific (napus-rapa)	0.9832536	0.8011352	not scorable	not scorable	0.9752066	0.7887095
3414	Bn-A02-p19889192	A-genome specific (napus-rapa)	0.9641148	0.7445493	not scorable	not scorable	0.9917355	0.8343771
3415	Bn-A02-p19892433	A-genome specific (napus-rapa)	0.9712918	0.7249332	not scorable	not scorable	0.9586777	0.7938417
3416	Bn-A02-p19892632	A-genome specific (napus-rapa)	0.9545454	0.7664846	not scorable	not scorable	0.9752066	0.8041441
3417	Bn-A02-p19892839	A-genome specific (napus-rapa)	0.9665072	0.7512187	not scorable	not scorable	0.9834711	0.7997946
3418	Bn-A02-p19927952	A-genome specific (napus-rapa)	0.9904306	0.5425022	not scorable	not scorable	0.9834711	0.8147336
3419	Bn-A02-p1997812	A-genome specific (napus-rapa)	0.9952153	0.755885	not scorable	not scorable	1	0.7570357
3420	Bn-A02-p20055831	A-genome specific (napus-rapa)	0.6842105	0.7686288	not scorable	not scorable	0.7024794	0.8239623
3421	Bn-A02-p20060311	A-genome specific (rapa only)	not scorable	not scorable	not scorable	not scorable	1	0.869534
3422	Bn-A02-p20062461	A-genome specific (napus-rapa)	1	0.5037426	not scorable	not scorable	1	0.7533351
3423	Bn-A02-p20062658	genome unspecific (all in)	0.9952153	0.3305332	0.8914729	0.7284315	1	0.9123241
3424	Bn-A02-p20062733	genome unspecific (all in)	0.8851675	0.3023143	0.875969	0.7023808	1	0.8004208
3425	Bn-A02-p20063202	A-genome specific (napus-rapa)	1	0.7514131	not scorable	not scorable	1	0.774545
3426	Bn-A02-p20157985	A-genome specific (napus-rapa)	0.9760765	0.7897193	not scorable	not scorable	0.9752066	0.7636895
3427	Bn-A02-p20158101	A-genome specific (napus-rapa)	0.9808612	0.7741902	not scorable	not scorable	0.9917355	0.7398732
3428	Bn-A02-p20159139	A-genome specific (rapa only)	not scorable	not scorable	not scorable	not scorable	0.9752066	0.7484978
3429	Bn-A02-p20166067	A-genome specific (napus-rapa)	0.9712918	0.4491982	not scorable	not scorable	0.9917355	0.782876

3430	Bn-A02-p20196208	A-genome specific (napus-rapa)	0.9688995	0.7952898	not scorable	not scorable	0.9752066	0.7849531
3431	Bn-A02-p201968	A-genome specific (napus-rapa)	0.9712918	0.5378693	not scorable	not scorable	0.9834711	0.8129182
3432	Bn-A02-p20200723	A-genome specific (napus-rapa)	0.9856459	0.7912079	not scorable	not scorable	0.9834711	0.7303801
3433	Bn-A02-p20201241	A-genome specific (napus-rapa)	0.9665072	0.8028215	not scorable	not scorable	0.9421487	0.7625163
3434	Bn-A02-p20203160	A-genome specific (napus-rapa)	0.9760765	0.7381895	not scorable	not scorable	0.9834711	0.6785634
3435	Bn-A02-p20207870	A-genome specific (napus-rapa)	0.9856459	0.5584565	not scorable	not scorable	0.9917355	0.6824547
3436	Bn-A02-p202316	A-genome specific (napus-rapa)	0.9688995	0.5232906	not scorable	not scorable	1	0.7914523
3437	Bn-A02-p20242753	A-genome specific (napus-rapa)	0.9976076	0.4398999	not scorable	not scorable	1	0.5503383
3438	Bn-A02-p202493	A-genome specific (napus-rapa)	0.9856459	0.4822587	not scorable	not scorable	1	0.7453754
3439	Bn-A02-p202819	A-genome specific (napus-rapa)	0.9665072	0.7669547	not scorable	not scorable	0.9834711	0.7628406
3440	Bn-A02-p20363776	A-genome specific (napus-rapa)	0.9760765	0.7788219	not scorable	not scorable	0.9917355	0.8338223
3441	Bn-A02-p20371730	A-genome specific (napus-rapa)	0.9904306	0.7706293	not scorable	not scorable	0.9917355	0.7780712
3442	Bn-A02-p20371774	A-genome specific (napus-rapa)	1	0.4714387	not scorable	not scorable	0.9752066	0.7148634
3443	Bn-A02-p20396880	A-genome specific (napus-rapa)	0.9928229	0.4084851	not scorable	not scorable	1	0.7438364
3444	Bn-A02-p20398	A-genome specific (napus-rapa)	0.9832536	0.5807173	not scorable	not scorable	0.9917355	0.7939435
3445	Bn-A02-p20461477	A-genome specific (napus-rapa)	0.9952153	0.7283329	not scorable	not scorable	1	0.8100535
3446	Bn-A02-p20462487	A-genome specific (napus-rapa)	0.9928229	0.682543	not scorable	not scorable	1	0.7235581
3447	Bn-A02-p20526431	A-genome specific (napus-rapa)	0.9952153	0.4436027	not scorable	not scorable	1	0.8543575
3448	Bn-A02-p20536885	A-genome specific (napus-rapa)	0.9976076	0.8315644	not scorable	not scorable	0.9917355	0.8129056
3449	Bn-A02-p20561132	A-genome specific (rapa only)	not scorable	not scorable	not scorable	not scorable	0.9586777	0.5810268
3450	Bn-A02-p20568585	A-genome specific (napus-rapa)	0.9976076	0.7771861	not scorable	not scorable	1	0.7742361
3451	Bn-A02-p20571280	A-genome specific (napus-rapa)	0.9976076	0.4347409	not scorable	not scorable	0.9834711	0.7250179
3452	Bn-A02-p20580541	all not scorable	not scorable	not scorable	not scorable	not scorable	not scorable	not scorable
3453	Bn-A02-p20634433	A-genome specific (napus-rapa)	0.9856459	0.7399609	not scorable	not scorable	0.9752066	0.8000385
3454	Bn-A02-p20678898	A-genome specific (napus-rapa)	0.9856459	0.808339	not scorable	not scorable	0.9834711	0.813789
3455	Bn-A02-p20700463	A-genome specific (napus-rapa)	0.9880383	0.7585077	not scorable	not scorable	0.9917355	0.8314418
3456	Bn-A02-p20718571	A-genome specific (napus-rapa)	0.9952153	0.533386	not scorable	not scorable	0.9669421	0.7482151
3457	Bn-A02-p20718651	A-genome specific (napus-rapa)	0.9952153	0.8433724	not scorable	not scorable	0.9834711	0.7140613
3458	Bn-A02-p20735027	A-genome specific (napus-rapa)	1	0.6916012	not scorable	not scorable	1	0.6733703
3459	Bn-A02-p20746287	A-genome specific (rapa only)	not scorable	not scorable	not scorable	not scorable	0.9752066	0.7791169
3460	Bn-A02-p2075762	A-genome specific (rapa only)	not scorable	not scorable	not scorable	not scorable	1	0.7238167
3461	Bn-A02-p20762322	A-genome specific (napus-rapa)	0.9976076	0.4611103	not scorable	not scorable	0.9917355	0.7794813
3462	Bn-A02-p20776266	A-genome specific (napus-rapa)	0.9856459	0.6891719	not scorable	not scorable	1	0.6668433

3463	Bn-A02-p20832528	A-genome specific (napus-rapa)	0.9904306	0.4638848	not scorable	not scorable	1	0.7473689
3464	Bn-A02-p20845905	A-genome specific (napus-rapa)	0.9856459	0.7550212	not scorable	not scorable	0.9917355	0.6871008
3465	Bn-A02-p20846941	A-genome specific (rapa only)	not scorable	not scorable	not scorable	not scorable	0.9917355	0.7095971
3466	Bn-A02-p20882001	A-genome specific (napus-rapa)	0.9928229	0.731735	not scorable	not scorable	0.9834711	0.6641613
3467	Bn-A02-p20882153	A-genome specific (napus-rapa)	0.9928229	0.7754462	not scorable	not scorable	0.9917355	0.7567177
3468	Bn-A02-p209474	napus specific	0.9330143	0.7922223	not scorable	not scorable	not scorable	not scorable
3469	Bn-A02-p21009288	A-genome specific (napus-rapa)	1	0.3163469	not scorable	not scorable	0.9917355	0.7067649
3470	Bn-A02-p21023727	A-genome specific (napus-rapa)	0.9952153	0.3315013	not scorable	not scorable	0.9834711	0.6480788
3471	Bn-A02-p21032956	A-genome specific (napus-rapa)	0.9736842	0.7808708	not scorable	not scorable	0.5123967	0.7518522
3472	Bn-A02-p2105937	A-genome specific (napus-rapa)	0.9593301	0.358872	not scorable	not scorable	0.8677686	0.8040534
3473	Bn-A02-p21061002	A-genome specific (napus-rapa)	0.9832536	0.7263495	not scorable	not scorable	0.5454546	0.7184159
3474	Bn-A02-p21062567	A-genome specific (napus-rapa)	0.9952153	0.8275415	not scorable	not scorable	1	0.7770717
3475	Bn-A02-p21062857	genome unspecific (all in)	0.9258373	0.399449	1	0.4648754	0.9917355	0.6189544
3476	Bn-A02-p21073755	A-genome specific (napus-rapa)	1	0.7067085	not scorable	not scorable	1	0.7420408
3477	Bn-A02-p21207148	A-genome specific (napus-rapa)	0.9928229	0.7890028	not scorable	not scorable	0.9834711	0.7668741
3478	Bn-A02-p21211198	A-genome specific (napus-rapa)	0.9928229	0.6645795	not scorable	not scorable	0.9752066	0.6894016
3479	Bn-A02-p2121360	A-genome specific (napus-rapa)	0.9330143	0.6232838	not scorable	not scorable	0.9752066	0.6727062
3480	Bn-A02-p2122414	A-genome specific (napus-rapa)	0.9545454	0.7675133	not scorable	not scorable	1	0.7018533
3481	Bn-A02-p2122850	A-genome specific (napus-rapa)	0.84689	0.685052	not scorable	not scorable	0.9586777	0.7266377
3482	Bn-A02-p2124245	A-genome specific (napus-rapa)	0.9784689	0.7692166	not scorable	not scorable	1	0.7127941
3483	Bn-A02-p2124328	A-genome specific (napus-rapa)	0.9784689	0.7605591	not scorable	not scorable	0.9834711	0.7091021
3484	Bn-A02-p2124421	A-genome specific (napus-rapa)	0.9832536	0.7772292	not scorable	not scorable	0.9752066	0.7871816
3485	Bn-A02-p21256748	A-genome specific (napus-rapa)	1	0.3078872	not scorable	not scorable	0.9834711	0.3533215
3486	Bn-A02-p21263855	A-genome specific (napus-rapa)	0.9832536	0.5715723	not scorable	not scorable	0.9834711	0.8376518
3487	Bn-A02-p2128775	A-genome specific (napus-rapa)	0.9784689	0.7890088	not scorable	not scorable	1	0.7925525
3488	Bn-A02-p212959	genome unspecific (all in)	0.9928229	0.3280488	0.9922481	0.7566465	1	0.7546111
3489	Bn-A02-p2130611	A-genome specific (rapa only)	not scorable	not scorable	not scorable	not scorable	1	0.7432612
3490	Bn-A02-p2130925	A-genome specific (rapa only)	not scorable	not scorable	not scorable	not scorable	1	0.7803956
3491	Bn-A02-p21322148	napus specific	0.9665072	0.6898457	not scorable	not scorable	not scorable	not scorable
3492	Bn-A02-p21324539	A-genome specific (napus-rapa)	0.9712918	0.6929569	not scorable	not scorable	0.9834711	0.7816266
3493	Bn-A02-p21325110	A-genome specific (napus-rapa)	0.9928229	0.5332254	not scorable	not scorable	1	0.6490307
3494	Bn-A02-p2134112	A-genome specific (napus-rapa)	0.9784689	0.7852402	not scorable	not scorable	1	0.8140467
3495	Bn-A02-p2135053	A-genome specific (napus-rapa)	0.7894737	0.7417116	not scorable	not scorable	0.9256198	0.895869

3496	Bn-A02-p2136039	A-genome specific (napus-rapa)	0.9712918	0.8001956	not scorable	not scorable	0.9834711	0.8629477
3497	Bn-A02-p21378450	A-genome specific (napus-rapa)	0.9665072	0.7622777	not scorable	not scorable	0.9917355	0.8349451
3498	Bn-A02-p21379810	A-genome specific (napus-rapa)	0.9856459	0.7353065	not scorable	not scorable	1	0.8885454
3499	Bn-A02-p2138408	A-genome specific (napus-rapa)	0.9712918	0.7956358	not scorable	not scorable	1	0.8249322
3500	Bn-A02-p21409149	A-genome specific (napus-rapa)	0.8827751	0.3138179	not scorable	not scorable	1	0.7023624
3501	Bn-A02-p21409508	A-genome specific (napus-rapa)	0.9210526	0.2678368	not scorable	not scorable	0.9917355	0.8170711
3502	Bn-A02-p21410424	A-genome specific (napus-rapa)	0.9473684	0.3920943	not scorable	not scorable	0.9752066	0.6448124
3503	Bn-A02-p2146210	A-genome specific (napus-rapa)	0.9952153	0.7934194	not scorable	not scorable	1	0.8060201
3504	Bn-A02-p21467101	A-genome specific (rapa only)	not scorable	not scorable	not scorable	not scorable	1	0.7052949
3505	Bn-A02-p21468	A-genome specific (napus-rapa)	0.9832536	0.6331475	not scorable	not scorable	1	0.6980684
3506	Bn-A02-p21489620	A-genome specific (napus-rapa)	0.9641148	0.7534441	not scorable	not scorable	0.9917355	0.8208557
3507	Bn-A02-p21490663	A-genome specific (napus-rapa)	0.9856459	0.7722486	not scorable	not scorable	0.9917355	0.7376631
3508	Bn-A02-p21491139	A-genome specific (napus-rapa)	0.9880383	0.4959746	not scorable	not scorable	1	0.6642843
3509	Bn-A02-p21510968	A-genome specific (napus-rapa)	1	0.5654504	not scorable	not scorable	1	0.7322224
3510	Bn-A02-p21544735	A-genome specific (napus-rapa)	0.9784689	0.8002014	not scorable	not scorable	0.6942149	0.8549622
3511	Bn-A02-p2154644	A-genome specific (rapa only)	not scorable	not scorable	not scorable	not scorable	1	0.7229298
3512	Bn-A02-p21563911	A-genome specific (napus-rapa)	0.9952153	0.8029575	not scorable	not scorable	1	0.9205657
3513	Bn-A02-p21570784	A-genome specific (rapa only)	not scorable	not scorable	not scorable	not scorable	1	0.6758623
3514	Bn-A02-p21595382	A-genome specific (napus-rapa)	1	0.511056	not scorable	not scorable	1	0.6431578
3515	Bn-A02-p21598341	A-genome specific (napus-rapa)	0.9784689	0.9095823	not scorable	not scorable	1	0.7492611
3516	Bn-A02-p21616494	A-genome specific (rapa only)	not scorable	not scorable	not scorable	not scorable	0.9834711	0.7769882
3517	Bn-A02-p21625125	A-genome specific (napus-rapa)	0.7655503	0.5280212	not scorable	not scorable	0.9917355	0.6256972
3518	Bn-A02-p21639869	A-genome specific (napus-rapa)	0.9019139	0.6518254	not scorable	not scorable	0.9669421	0.8198121
3519	Bn-A02-p21648504	A-genome specific (napus-rapa)	1	0.379426	not scorable	not scorable	1	0.6690158
3520	Bn-A02-p2165239	A-genome specific (napus-rapa)	0.9760765	0.7507719	not scorable	not scorable	0.9834711	0.6987672
3521	Bn-A02-p21701259	genome unspecific (oleracea-rapa)	not scorable	not scorable	0.8837209	0.615404	1	0.8118467
3522	Bn-A02-p21713756	genome unspecific (all in)	0.9952153	0.4614603	0.9689922	0.9031397	1	0.9036642
3523	Bn-A02-p21713818	genome unspecific (all in)	0.9928229	0.3574382	0.9922481	0.8090666	1	0.8644261
3524	Bn-A02-p21736387	A-genome specific (napus-rapa)	1	0.522947	not scorable	not scorable	0.9834711	0.8085203
3525	Bn-A02-p21737275	A-genome specific (napus-rapa)	1	0.7206468	not scorable	not scorable	0.9752066	0.7038418
3526	Bn-A02-p2182742	A-genome specific (napus-rapa)	0.9880383	0.7673079	not scorable	not scorable	1	0.7020361
3527	Bn-A02-p21891973	A-genome specific (napus-rapa)	1	0.9022347	not scorable	not scorable	1	0.8549153
3528	Bn-A02-p21930156	A-genome specific (napus-rapa)	0.9928229	0.7939026	not scorable	not scorable	0.9917355	0.7642924

3529	Bn-A02-p22296426	A-genome specific (napus-rapa)	0.9880383	0.4016187	not scorable	not scorable	0.9917355	0.8134547
3530	Bn-A02-p22332961	A-genome specific (napus-rapa)	0.9688995	0.7772213	not scorable	not scorable	0.9834711	0.8097115
3531	Bn-A02-p22351644	A-genome specific (napus-rapa)	1	0.521263	not scorable	not scorable	0.9834711	0.9244554
3532	Bn-A02-p22352518	A-genome specific (napus-rapa)	0.9904306	0.6195386	not scorable	not scorable	1	0.7112254
3533	Bn-A02-p22357265	A-genome specific (napus-rapa)	0.9952153	0.7974623	not scorable	not scorable	1	0.7683881
3534	Bn-A02-p22365337	A-genome specific (napus-rapa)	0.9808612	0.7768333	not scorable	not scorable	0.9586777	0.7517447
3535	Bn-A02-p22371956	A-genome specific (napus-rapa)	0.9952153	0.6454414	not scorable	not scorable	0.9752066	0.7074341
3536	Bn-A02-p22375400	A-genome specific (napus-rapa)	0.9760765	0.8156456	not scorable	not scorable	1	0.9175813
3537	Bn-A02-p22375887	A-genome specific (napus-rapa)	0.9832536	0.8675371	not scorable	not scorable	0.9917355	0.7958832
3538	Bn-A02-p22376180	all not scorable	not scorable	not scorable	not scorable	not scorable	not scorable	not scorable
3539	Bn-A02-p22383771	A-genome specific (napus-rapa)	0.9712918	0.781617	not scorable	not scorable	0.9752066	0.6862211
3540	Bn-A02-p22386317	A-genome specific (napus-rapa)	0.9880383	0.2899284	not scorable	not scorable	0.9752066	0.7603972
3541	Bn-A02-p22388092	A-genome specific (napus-rapa)	0.9904306	0.7147967	not scorable	not scorable	1	0.7279547
3542	Bn-A02-p22394284	A-genome specific (napus-rapa)	0.9736842	0.7772864	not scorable	not scorable	0.9917355	0.7686312
3543	Bn-A02-p22394632	A-genome specific (napus-rapa)	0.9760765	0.8140411	not scorable	not scorable	0.9917355	0.7002051
3544	Bn-A02-p22395060	A-genome specific (napus-rapa)	0.9760765	0.7007563	not scorable	not scorable	0.9834711	0.678377
3545	Bn-A02-p22398620	A-genome specific (napus-rapa)	0.9832536	0.319207	not scorable	not scorable	1	0.7202674
3546	Bn-A02-p22409779	A-genome specific (napus-rapa)	0.9784689	0.7726023	not scorable	not scorable	0.9834711	0.7979118
3547	Bn-A02-p22426466	genome unspecific (oleracea-rapa)	not scorable	not scorable	0.9689922	0.7172679	0.9669421	0.7040766
3548	Bn-A02-p22427387	A-genome specific (rapa only)	not scorable	not scorable	not scorable	not scorable	0.9669421	0.6758683
3549	Bn-A02-p22429308	A-genome specific (napus-rapa)	0.9760765	0.6585421	not scorable	not scorable	0.9834711	0.6453311
3550	Bn-A02-p22429563	A-genome specific (napus-rapa)	0.9880383	0.6526489	not scorable	not scorable	0.9917355	0.7820048
3551	Bn-A02-p22429871	A-genome specific (napus-rapa)	0.9856459	0.7876871	not scorable	not scorable	1	0.800707
3552	Bn-A02-p22433753	A-genome specific (napus-rapa)	0.9545454	0.8027224	not scorable	not scorable	0.6280992	0.7676806
3553	Bn-A02-p22443535	A-genome specific (napus-rapa)	0.9736842	0.6848145	not scorable	not scorable	1	0.6422119
3554	Bn-A02-p22460758	A-genome specific (napus-rapa)	0.9928229	0.7566096	not scorable	not scorable	0.9504132	0.7140741
3555	Bn-A02-p22478377	A-genome specific (napus-rapa)	0.9976076	0.8351101	not scorable	not scorable	0.9669421	0.7273648
3556	Bn-A02-p22479272	A-genome specific (napus-rapa)	0.9976076	0.7845125	not scorable	not scorable	0.9917355	0.7804822
3557	Bn-A02-p22485435	A-genome specific (napus-rapa)	0.9928229	0.4354958	not scorable	not scorable	0.9669421	0.7040677
3558	Bn-A02-p22485606	A-genome specific (napus-rapa)	0.9856459	0.7628941	not scorable	not scorable	1	0.7635186
3559	Bn-A02-p22487030	A-genome specific (napus-rapa)	0.9880383	0.6580428	not scorable	not scorable	0.9834711	0.8791919
3560	Bn-A02-p22492507	A-genome specific (napus-rapa)	1	0.8593741	not scorable	not scorable	0.9917355	0.7778147
3561	Bn-A02-p22493338	A-genome specific (napus-rapa)	0.9880383	0.8755674	not scorable	not scorable	0.9834711	0.7810593

3562	Bn-A02-p22501359	A-genome specific (napus-rapa)	1	0.7809012	not scorable	not scorable	0.9834711	0.7490339
3563	Bn-A02-p22507142	A-genome specific (napus-rapa)	0.9976076	0.4334134	not scorable	not scorable	1	0.7098995
3564	Bn-A02-p22508460	A-genome specific (napus-rapa)	0.9952153	0.6864519	not scorable	not scorable	0.9752066	0.7185382
3565	Bn-A02-p22508870	A-genome specific (napus-rapa)	1	0.8053889	not scorable	not scorable	0.9917355	0.7334594
3566	Bn-A02-p22509521	A-genome specific (rapa only)	not scorable	not scorable	not scorable	not scorable	0.5371901	0.9242454
3567	Bn-A02-p22515745	A-genome specific (napus-rapa)	0.9976076	0.4849932	not scorable	not scorable	0.9834711	0.7041288
3568	Bn-A02-p22518385	A-genome specific (napus-rapa)	1	0.8135514	not scorable	not scorable	0.9834711	0.7203573
3569	Bn-A02-p22519689	all not scorable	not scorable	not scorable	not scorable	not scorable	not scorable	not scorable
3570	Bn-A02-p22520110	A-genome specific (napus-rapa)	1	0.7712844	not scorable	not scorable	0.9917355	0.7420133
3571	Bn-A02-p22520724	A-genome specific (napus-rapa)	0.9952153	0.6150697	not scorable	not scorable	0.9669421	0.8071707
3572	Bn-A02-p22521362	A-genome specific (napus-rapa)	1	0.615611	not scorable	not scorable	0.9752066	0.7252113
3573	Bn-A02-p22521802	A-genome specific (napus-rapa)	0.9976076	0.6711379	not scorable	not scorable	0.9917355	0.7826753
3574	Bn-A02-p22528701	A-genome specific (napus-rapa)	1	0.7649238	not scorable	not scorable	0.9834711	0.7305132
3575	Bn-A02-p22534152	A-genome specific (napus-rapa)	0.9976076	0.7978523	not scorable	not scorable	0.9586777	0.6992367
3576	Bn-A02-p22534735	A-genome specific (napus-rapa)	0.9928229	0.7065177	not scorable	not scorable	0.9586777	0.7675989
3577	Bn-A02-p22543683	A-genome specific (napus-rapa)	1	0.7523718	not scorable	not scorable	0.9752066	0.7157784
3578	Bn-A02-p22550542	A-genome specific (napus-rapa)	1	0.763524	not scorable	not scorable	0.9917355	0.6447003
3579	Bn-A02-p22552916	A-genome specific (napus-rapa)	1	0.5895082	not scorable	not scorable	0.9834711	0.7469853
3580	Bn-A02-p22557981	A-genome specific (napus-rapa)	0.9928229	0.6069931	not scorable	not scorable	0.9834711	0.7471687
3581	Bn-A02-p22558530	A-genome specific (napus-rapa)	1	0.8180031	not scorable	not scorable	0.9917355	0.7753686
3582	Bn-A02-p22560577	A-genome specific (napus-rapa)	0.9976076	0.7836257	not scorable	not scorable	0.9752066	0.765547
3583	Bn-A02-p22563469	A-genome specific (napus-rapa)	1	0.8127581	not scorable	not scorable	0.9752066	0.6963291
3584	Bn-A02-p22564964	A-genome specific (napus-rapa)	1	0.7732013	not scorable	not scorable	0.9586777	0.6477479
3585	Bn-A02-p22565755	A-genome specific (napus-rapa)	0.9976076	0.7792986	not scorable	not scorable	0.9917355	0.7740891
3586	Bn-A02-p22570273	napus specific	1	0.5955835	not scorable	not scorable	not scorable	not scorable
3587	Bn-A02-p22571187	A-genome specific (napus-rapa)	1	0.7799062	not scorable	not scorable	1	0.6635286
3588	Bn-A02-p22571726	A-genome specific (napus-rapa)	1	0.8218698	not scorable	not scorable	0.9834711	0.7351703
3589	Bn-A02-p22576112	A-genome specific (napus-rapa)	1	0.7431802	not scorable	not scorable	0.9669421	0.731402
3590	Bn-A02-p22576511	A-genome specific (napus-rapa)	1	0.7854228	not scorable	not scorable	0.9834711	0.773126
3591	Bn-A02-p22577127	A-genome specific (napus-rapa)	0.9976076	0.8376964	not scorable	not scorable	0.9917355	0.7398707
3592	Bn-A02-p22581557	A-genome specific (napus-rapa)	0.9976076	0.7541991	not scorable	not scorable	0.9917355	0.7806268
3593	Bn-A02-p2258714	A-genome specific (napus-rapa)	0.9904306	0.8655387	not scorable	not scorable	1	0.7099429
3594	Bn-A02-p22590101	napus specific	1	0.6253963	not scorable	not scorable	not scorable	not scorable

3595	Bn-A02-p22596234	A-genome specific (napus-rapa)	0.9856459	0.77826	not scorable	not scorable	0.9917355	0.67696
3596	Bn-A02-p22596523	A-genome specific (napus-rapa)	0.9976076	0.6691222	not scorable	not scorable	0.9834711	0.6366681
3597	Bn-A02-p22597014	A-genome specific (napus-rapa)	0.9952153	0.7698966	not scorable	not scorable	0.9752066	0.688451
3598	Bn-A02-p22599833	A-genome specific (napus-rapa)	0.9928229	0.8114292	not scorable	not scorable	0.9752066	0.7526598
3599	Bn-A02-p22601555	A-genome specific (napus-rapa)	0.9760765	0.7174469	not scorable	not scorable	1	0.7961111
3600	Bn-A02-p22602317	A-genome specific (napus-rapa)	1	0.6976327	not scorable	not scorable	0.9834711	0.7263689
3601	Bn-A02-p22637438	A-genome specific (napus-rapa)	0.9880383	0.7778311	not scorable	not scorable	0.9834711	0.6971074
3602	Bn-A02-p22637504	A-genome specific (napus-rapa)	1	0.6926453	not scorable	not scorable	0.9752066	0.7289565
3603	Bn-A02-p22638512	A-genome specific (napus-rapa)	1	0.7764199	not scorable	not scorable	0.9752066	0.7406246
3604	Bn-A02-p22641483	A-genome specific (napus-rapa)	1	0.7388332	not scorable	not scorable	0.9752066	0.700188
3605	Bn-A02-p22642101	A-genome specific (napus-rapa)	0.9976076	0.7936939	not scorable	not scorable	0.9834711	0.7386996
3606	Bn-A02-p22650896	A-genome specific (napus-rapa)	0.9904306	0.7715876	not scorable	not scorable	0.9669421	0.7585784
3607	Bn-A02-p22651355	A-genome specific (napus-rapa)	1	0.6935971	not scorable	not scorable	0.9834711	0.7686504
3608	Bn-A02-p22654465	A-genome specific (napus-rapa)	1	0.7784702	not scorable	not scorable	0.9834711	0.7760611
3609	Bn-A02-p22670299	A-genome specific (napus-rapa)	1	0.7654476	not scorable	not scorable	1	0.6273827
3610	Bn-A02-p22682931	genome unspecific (all in)	1	0.2682992	1	0.3285714	0.9834711	0.6714574
3611	Bn-A02-p22693351	A-genome specific (napus-rapa)	0.9832536	0.2681309	not scorable	not scorable	1	0.8646921
3612	Bn-A02-p22697509	A-genome specific (napus-rapa)	0.9880383	0.6055601	not scorable	not scorable	1	0.8815182
3613	Bn-A02-p22702484	A-genome specific (napus-rapa)	1	0.8077227	not scorable	not scorable	0.9752066	0.7370024
3614	Bn-A02-p22741797	A-genome specific (napus-rapa)	0.9976076	0.6988018	not scorable	not scorable	1	0.8180268
3615	Bn-A02-p2274901	genome unspecific (oleracea-rapa)	not scorable	not scorable	1	0.7594399	1	0.774431
3616	Bn-A02-p22757902	A-genome specific (napus-rapa)	0.9880383	0.7844929	not scorable	not scorable	0.9752066	0.6552019
3617	Bn-A02-p2277616	A-genome specific (napus-rapa)	0.9784689	0.4426889	not scorable	not scorable	0.9917355	0.8382434
3618	Bn-A02-p22787085	A-genome specific (napus-rapa)	0.9904306	0.5408834	not scorable	not scorable	0.9752066	0.7192839
3619	Bn-A02-p22798107	A-genome specific (napus-rapa)	0.9856459	0.4326584	not scorable	not scorable	0.9669421	0.7222261
3620	Bn-A02-p22811512	A-genome specific (napus-rapa)	0.9928229	0.4735287	not scorable	not scorable	0.9917355	0.7642992
3621	Bn-A02-p22843446	A-genome specific (napus-rapa)	0.9880383	0.7982895	not scorable	not scorable	0.9917355	0.8337346
3622	Bn-A02-p22847040	A-genome specific (napus-rapa)	0.9856459	0.8084412	not scorable	not scorable	0.9669421	0.7761505
3623	Bn-A02-p22847472	A-genome specific (napus-rapa)	0.9952153	0.5778109	not scorable	not scorable	0.9752066	0.7539784
3624	Bn-A02-p22863292	A-genome specific (rapa only)	not scorable	not scorable	not scorable	not scorable	0.5289256	0.7726357
3625	Bn-A02-p22863757	napus specific	0.5885168	0.6622717	not scorable	not scorable	not scorable	not scorable
3626	Bn-A02-p22863981	A-genome specific (rapa only)	not scorable	not scorable	not scorable	not scorable	0.5371901	0.7949208
3627	Bn-A02-p22872607	A-genome specific (napus-rapa)	0.9736842	0.7882505	not scorable	not scorable	0.9752066	0.8781655

3628	Bn-A02-p22875408	A-genome specific (napus-rapa)	0.9665072	0.7596585	not scorable	not scorable	0.9834711	0.8201935
3629	Bn-A02-p2287712	A-genome specific (napus-rapa)	0.8875598	0.5745402	not scorable	not scorable	1	0.7285279
3630	Bn-A02-p2288207	A-genome specific (napus-rapa)	0.9688995	0.7309657	not scorable	not scorable	1	0.7236208
3631	Bn-A02-p2288216	A-genome specific (napus-rapa)	0.9784689	0.5747801	not scorable	not scorable	0.9917355	0.5979134
3632	Bn-A02-p22899292	A-genome specific (napus-rapa)	0.9665072	0.7846684	not scorable	not scorable	0.9917355	0.8520135
3633	Bn-A02-p22899854	A-genome specific (napus-rapa)	0.930622	0.7088029	not scorable	not scorable	0.9917355	0.7071448
3634	Bn-A02-p22906452	A-genome specific (napus-rapa)	0.9688995	0.7716946	not scorable	not scorable	0.9917355	0.6991569
3635	Bn-A02-p22906797	A-genome specific (napus-rapa)	0.9688995	0.7731929	not scorable	not scorable	0.9917355	0.6942787
3636	Bn-A02-p2292871	A-genome specific (napus-rapa)	0.9808612	0.7769397	not scorable	not scorable	1	0.8756821
3637	Bn-A02-p23092476	A-genome specific (napus-rapa)	0.9712918	0.2117586	not scorable	not scorable	0.9669421	0.6954831
3638	Bn-A02-p23095572	A-genome specific (napus-rapa)	0.9808612	0.4715748	not scorable	not scorable	1	0.8203941
3639	Bn-A02-p23098985	A-genome specific (rapa only)	not scorable	not scorable	not scorable	not scorable	0.6280992	0.7293175
3640	Bn-A02-p23099281	A-genome specific (napus-rapa)	0.9354067	0.7661719	not scorable	not scorable	0.9834711	0.801636
3641	Bn-A02-p23101889	A-genome specific (napus-rapa)	0.9354067	0.733131	not scorable	not scorable	0.9917355	0.7304488
3642	Bn-A02-p23108517	A-genome specific (napus-rapa)	0.9617225	0.2822814	not scorable	not scorable	0.9917355	0.7038341
3643	Bn-A02-p23110903	A-genome specific (napus-rapa)	0.9712918	0.491399	not scorable	not scorable	1	0.8131012
3644	Bn-A02-p23112746	A-genome specific (napus-rapa)	0.9736842	0.3258263	not scorable	not scorable	0.9917355	0.7377492
3645	Bn-A02-p23128571	A-genome specific (napus-rapa)	0.9712918	0.3058508	not scorable	not scorable	1	0.73063
3646	Bn-A02-p23135500	A-genome specific (napus-rapa)	0.9449761	0.7044726	not scorable	not scorable	1	0.9470829
3647	Bn-A02-p23161914	A-genome specific (napus-rapa)	0.9090909	0.6420701	not scorable	not scorable	0.9834711	0.7223239
3648	Bn-A02-p23163100	A-genome specific (napus-rapa)	0.9330143	0.6656118	not scorable	not scorable	1	0.7713397
3649	Bn-A02-p23168213	A-genome specific (napus-rapa)	0.9401914	0.8384131	not scorable	not scorable	1	0.8259038
3650	Bn-A02-p23237139	A-genome specific (napus-rapa)	0.9856459	0.7869931	not scorable	not scorable	0.9917355	0.7693927
3651	Bn-A02-p23251431	A-genome specific (napus-rapa)	0.9760765	0.6727031	not scorable	not scorable	0.9834711	0.7975906
3652	Bn-A02-p23257505	A-genome specific (rapa only)	not scorable	not scorable	not scorable	not scorable	1	0.7584332
3653	Bn-A02-p23277919	A-genome specific (rapa only)	not scorable	not scorable	not scorable	not scorable	1	0.8554686
3654	Bn-A02-p23279287	A-genome specific (napus-rapa)	0.9832536	0.462583	not scorable	not scorable	1	0.6909724
3655	Bn-A02-p23280472	A-genome specific (napus-rapa)	0.9880383	0.7590026	not scorable	not scorable	0.9917355	0.7927341
3656	Bn-A02-p23280665	A-genome specific (rapa only)	not scorable	not scorable	not scorable	not scorable	1	0.7560042
3657	Bn-A02-p23280869	A-genome specific (napus-rapa)	1	0.2597172	not scorable	not scorable	0.9834711	0.8783981
3658	Bn-A02-p23295254	A-genome specific (napus-rapa)	0.9497608	0.8023953	not scorable	not scorable	0.677686	0.7945569
3659	Bn-A02-p23297690	A-genome specific (napus-rapa)	0.8086125	0.7823358	not scorable	not scorable	0.6198347	0.819187
3660	Bn-A02-p23297789	A-genome specific (napus-rapa)	0.9665072	0.250282	not scorable	not scorable	1	0.7194965

3661	Bn-A02-p23309610	A-genome specific (napus-rapa)	0.9904306	0.7803795	not scorable	not scorable	1	0.8100291
3662	Bn-A02-p23362842	A-genome specific (napus-rapa)	1	0.5559785	not scorable	not scorable	1	0.8388335
3663	Bn-A02-p23408870	A-genome specific (napus-rapa)	0.9928229	0.7027501	not scorable	not scorable	1	0.7159663
3664	Bn-A02-p23411852	A-genome specific (napus-rapa)	0.9928229	0.8472601	not scorable	not scorable	0.9834711	0.8058628
3665	Bn-A02-p23414384	A-genome specific (rapa only)	not scorable	not scorable	not scorable	not scorable	0.9917355	0.812347
3666	Bn-A02-p23414610	A-genome specific (rapa only)	not scorable	not scorable	not scorable	not scorable	0.9917355	0.9208769
3667	Bn-A02-p23414614	A-genome specific (napus-rapa)	0.9928229	0.8138618	not scorable	not scorable	1	0.6801172
3668	Bn-A02-p2341934	A-genome specific (rapa only)	not scorable	not scorable	not scorable	not scorable	0.9834711	0.7390162
3669	Bn-A02-p23423377	A-genome specific (napus-rapa)	0.9928229	0.7672445	not scorable	not scorable	0.9917355	0.7537945
3670	Bn-A02-p23425517	A-genome specific (napus-rapa)	0.9928229	0.7828693	not scorable	not scorable	0.9917355	0.8001636
3671	Bn-A02-p23428461	A-genome specific (napus-rapa)	0.9952153	0.4188091	not scorable	not scorable	1	0.7133143
3672	Bn-A02-p23432823	A-genome specific (napus-rapa)	0.9880383	0.7859029	not scorable	not scorable	0.9504132	0.7570602
3673	Bn-A02-p23436829	A-genome specific (napus-rapa)	0.9904306	0.7535886	not scorable	not scorable	0.9669421	0.7323167
3674	Bn-A02-p23448271	A-genome specific (napus-rapa)	0.9928229	0.4397616	not scorable	not scorable	0.9834711	0.7345325
3675	Bn-A02-p23454935	C-genome specific (oleracea only)	not scorable	not scorable	0.9767442	0.4313002	not scorable	not scorable
3676	Bn-A02-p23456103	A-genome specific (napus-rapa)	0.9928229	0.781303	not scorable	not scorable	0.9917355	0.7226713
3677	Bn-A02-p23472944	A-genome specific (napus-rapa)	0.9976076	0.3908518	not scorable	not scorable	1	0.7799541
3678	Bn-A02-p23473807	A-genome specific (napus-rapa)	0.9904306	0.8068834	not scorable	not scorable	0.9917355	0.7606095
3679	Bn-A02-p23474309	A-genome specific (napus-rapa)	0.9928229	0.8063899	not scorable	not scorable	1	0.7272524
3680	Bn-A02-p23477774	A-genome specific (napus-rapa)	0.9976076	0.335554	not scorable	not scorable	0.9752066	0.77592
3681	Bn-A02-p23477968	genome unspecific (all in)	0.9928229	0.6834925	0.9767442	0.6711627	0.9752066	0.7387694
3682	Bn-A02-p23479432	napus specific	0.9976076	0.3585258	not scorable	not scorable	not scorable	not scorable
3683	Bn-A02-p23482499	A-genome specific (napus-rapa)	0.9904306	0.7096521	not scorable	not scorable	0.5454546	0.8180267
3684	Bn-A02-p23486694	A-genome specific (napus-rapa)	0.9904306	0.7726752	not scorable	not scorable	0.9669421	0.7529763
3685	Bn-A02-p23488731	A-genome specific (napus-rapa)	1	0.4068048	not scorable	not scorable	1	0.7977145
3686	Bn-A02-p23491463	A-genome specific (napus-rapa)	0.9401914	0.7705586	not scorable	not scorable	0.6446281	0.7256376
3687	Bn-A02-p23494185	A-genome specific (napus-rapa)	0.9880383	0.7259326	not scorable	not scorable	0.9834711	0.7514516
3688	Bn-A02-p23495061	A-genome specific (napus-rapa)	0.9090909	0.8014886	not scorable	not scorable	0.9256198	0.7067049
3689	Bn-A02-p23497111	A-genome specific (napus-rapa)	0.9928229	0.453399	not scorable	not scorable	0.9917355	0.7909767
3690	Bn-A02-p23509687	A-genome specific (napus-rapa)	0.9928229	0.7584389	not scorable	not scorable	0.9917355	0.8594449
3691	Bn-A02-p23509986	A-genome specific (napus-rapa)	0.9904306	0.8138064	not scorable	not scorable	1	0.7759688
3692	Bn-A02-p23510178	A-genome specific (napus-rapa)	0.9928229	0.7674699	not scorable	not scorable	1	0.768249
3693	Bn-A02-p23523709	A-genome specific (napus-rapa)	0.9928229	0.4664733	not scorable	not scorable	0.9834711	0.7735984

3694	Bn-A02-p2364069	A-genome specific (napus-rapa)	0.9952153	0.8401033	not scorable	not scorable	0.9917355	0.7232671
3695	Bn-A02-p2364479	A-genome specific (rapa only)	not scorable	not scorable	not scorable	not scorable	0.9834711	0.7082456
3696	Bn-A02-p23668607	A-genome specific (napus-rapa)	0.9784689	0.7050626	not scorable	not scorable	0.5867769	0.7432435
3697	Bn-A02-p23669898	A-genome specific (napus-rapa)	0.9928229	0.7446899	not scorable	not scorable	0.9917355	0.8124055
3698	Bn-A02-p23685036	A-genome specific (napus-rapa)	0.9545454	0.5623235	not scorable	not scorable	0.9669421	0.7203811
3699	Bn-A02-p23686560	A-genome specific (napus-rapa)	0.9449761	0.7810077	not scorable	not scorable	0.9834711	0.824479
3700	Bn-A02-p23700858	A-genome specific (napus-rapa)	0.9928229	0.7699556	not scorable	not scorable	1	0.7058686
3701	Bn-A02-p23708117	A-genome specific (napus-rapa)	0.9066986	0.7721456	not scorable	not scorable	0.9834711	0.850803
3702	Bn-A02-p23708299	A-genome specific (napus-rapa)	0.8971292	0.8265429	not scorable	not scorable	1	0.7654849
3703	Bn-A02-p23708516	A-genome specific (napus-rapa)	0.9138756	0.7960063	not scorable	not scorable	1	0.7929248
3704	Bn-A02-p23708678	A-genome specific (napus-rapa)	0.9019139	0.7473363	not scorable	not scorable	0.9752066	0.8091279
3705	Bn-A02-p23726200	A-genome specific (napus-rapa)	0.8923445	0.7673007	not scorable	not scorable	0.9917355	0.7483892
3706	Bn-A02-p23728173	C-genome specific (napus-oleracea)	0.9976076	0.2992877	1	0.2808201	not scorable	not scorable
3707	Bn-A02-p23729046	A-genome specific (napus-rapa)	0.9162679	0.7776386	not scorable	not scorable	0.9834711	0.7980347
3708	Bn-A02-p23730904	A-genome specific (napus-rapa)	0.9688995	0.5461779	not scorable	not scorable	0.9669421	0.7071556
3709	Bn-A02-p23743267	A-genome specific (rapa only)	not scorable	not scorable	not scorable	not scorable	1	0.7484516
3710	Bn-A02-p23757876	A-genome specific (napus-rapa)	0.9904306	0.2645536	not scorable	not scorable	0.9917355	0.7627375
3711	Bn-A02-p23788301	A-genome specific (napus-rapa)	0.9258373	0.7790092	not scorable	not scorable	0.5206612	0.7645022
3712	Bn-A02-p23792703	A-genome specific (napus-rapa)	0.9808612	0.7995489	not scorable	not scorable	0.9917355	0.7536179
3713	Bn-A02-p23819701	A-genome specific (napus-rapa)	0.9665072	0.3651035	not scorable	not scorable	0.9834711	0.7610574
3714	Bn-A02-p23819797	A-genome specific (napus-rapa)	0.9688995	0.8039054	not scorable	not scorable	0.9917355	0.7417265
3715	Bn-A02-p23824343	A-genome specific (napus-rapa)	0.9473684	0.8027064	not scorable	not scorable	0.9834711	0.7375073
3716	Bn-A02-p23826324	A-genome specific (napus-rapa)	0.9688995	0.8016993	not scorable	not scorable	0.9917355	0.7797013
3717	Bn-A02-p23831173	A-genome specific (napus-rapa)	0.9665072	0.800363	not scorable	not scorable	0.9917355	0.7404003
3718	Bn-A02-p23832547	A-genome specific (napus-rapa)	0.9784689	0.2573805	not scorable	not scorable	0.9752066	0.7778892
3719	Bn-A02-p23835683	A-genome specific (napus-rapa)	0.9688995	0.8042824	not scorable	not scorable	1	0.776125
3720	Bn-A02-p23836232	A-genome specific (napus-rapa)	0.9856459	0.7916022	not scorable	not scorable	1	0.6906022
3721	Bn-A02-p23842103	A-genome specific (napus-rapa)	0.9688995	0.5502855	not scorable	not scorable	0.9586777	0.7913043
3722	Bn-A02-p23842763	A-genome specific (rapa only)	not scorable	not scorable	not scorable	not scorable	0.9917355	0.7079534
3723	Bn-A02-p23856500	A-genome specific (napus-rapa)	0.9736842	0.8020059	not scorable	not scorable	1	0.8033827
3724	Bn-A02-p23857384	A-genome specific (napus-rapa)	0.9904306	0.7934136	not scorable	not scorable	1	0.7751837
3725	Bn-A02-p23875682	A-genome specific (napus-rapa)	0.9569378	0.4061098	not scorable	not scorable	0.9917355	0.7782946
3726	Bn-A02-p23889188	A-genome specific (napus-rapa)	0.9736842	0.3557959	not scorable	not scorable	1	0.6828266

3727	Bn-A02-p2389418	A-genome specific (rapa only)	not scorable	not scorable	not scorable	not scorable	0.9834711	0.7429992
3728	Bn-A02-p23897014	A-genome specific (napus-rapa)	0.9856459	0.7154508	not scorable	not scorable	1	0.7913748
3729	Bn-A02-p23910066	A-genome specific (napus-rapa)	0.7942584	0.7545238	not scorable	not scorable	0.9421487	0.819399
3730	Bn-A02-p23920155	A-genome specific (napus-rapa)	0.9928229	0.392696	not scorable	not scorable	0.9917355	0.7806912
3731	Bn-A02-p23925334	A-genome specific (napus-rapa)	0.9880383	0.3813853	not scorable	not scorable	0.9917355	0.6662903
3732	Bn-A02-p24015556	A-genome specific (napus-rapa)	0.937799	0.5419008	not scorable	not scorable	0.5702479	0.9061049
3733	Bn-A02-p24178094	A-genome specific (napus-rapa)	0.9928229	0.7320278	not scorable	not scorable	0.9752066	0.7523103
3734	Bn-A02-p24216905	A-genome specific (napus-rapa)	1	0.6643068	not scorable	not scorable	1	0.7376376
3735	Bn-A02-p2421888	A-genome specific (rapa only)	not scorable	not scorable	not scorable	not scorable	1	0.7537644
3736	Bn-A02-p24237359	A-genome specific (napus-rapa)	1	0.5665131	not scorable	not scorable	1	0.7232355
3737	Bn-A02-p24268496	A-genome specific (napus-rapa)	0.9976076	0.5206932	not scorable	not scorable	0.9917355	0.716031
3738	Bn-A02-p24269435	A-genome specific (napus-rapa)	0.9712918	0.7855024	not scorable	not scorable	0.9752066	0.7267952
3739	Bn-A02-p24316498	A-genome specific (napus-rapa)	0.9904306	0.3959908	not scorable	not scorable	0.9917355	0.6321682
3740	Bn-A02-p24317870	A-genome specific (napus-rapa)	0.9712918	0.7354863	not scorable	not scorable	1	0.6083272
3741	Bn-A02-p2434462	A-genome specific (rapa only)	not scorable	not scorable	not scorable	not scorable	0.9917355	0.7366205
3742	Bn-A02-p24345550	A-genome specific (napus-rapa)	0.9856459	0.3550808	not scorable	not scorable	0.9917355	0.8072528
3743	Bn-A02-p24345838	A-genome specific (napus-rapa)	0.9641148	0.6958523	not scorable	not scorable	0.9917355	0.8327926
3744	Bn-A02-p24351316	A-genome specific (napus-rapa)	0.9688995	0.7990696	not scorable	not scorable	0.9752066	0.749428
3745	Bn-A02-p24353955	A-genome specific (napus-rapa)	0.9736842	0.7045437	not scorable	not scorable	1	0.7591577
3746	Bn-A02-p24353997	A-genome specific (napus-rapa)	0.9665072	0.7995799	not scorable	not scorable	1	0.7117163
3747	Bn-A02-p24354876	A-genome specific (napus-rapa)	0.9688995	0.6773205	not scorable	not scorable	0.9752066	0.6877344
3748	Bn-A02-p24359370	A-genome specific (napus-rapa)	0.9497608	0.7032784	not scorable	not scorable	0.9917355	0.7386569
3749	Bn-A02-p24366221	A-genome specific (napus-rapa)	0.9928229	0.7302223	not scorable	not scorable	1	0.7658132
3750	Bn-A02-p24378297	A-genome specific (napus-rapa)	0.9856459	0.5205678	not scorable	not scorable	1	0.8302463
3751	Bn-A02-p24386848	A-genome specific (napus-rapa)	0.9617225	0.7679321	not scorable	not scorable	0.9917355	0.8334917
3752	Bn-A02-p24399308	A-genome specific (napus-rapa)	0.9617225	0.7601669	not scorable	not scorable	1	0.825245
3753	Bn-A02-p24401267	A-genome specific (napus-rapa)	0.9497608	0.7585703	not scorable	not scorable	0.5702479	0.7939715
3754	Bn-A02-p24402133	A-genome specific (napus-rapa)	0.9808612	0.7526482	not scorable	not scorable	1	0.7709031
3755	Bn-A02-p24415454	A-genome specific (napus-rapa)	0.9808612	0.789212	not scorable	not scorable	0.9504132	0.7628745
3756	Bn-A02-p24417020	A-genome specific (napus-rapa)	0.9952153	0.3483326	not scorable	not scorable	1	0.7442658
3757	Bn-A02-p2443019	A-genome specific (napus-rapa)	0.9760765	0.7584864	not scorable	not scorable	0.9917355	0.8422563
3758	Bn-A02-p2443275	A-genome specific (napus-rapa)	0.9449761	0.7744979	not scorable	not scorable	0.9752066	0.7906436
3759	Bn-A02-p24438115	A-genome specific (napus-rapa)	1	0.6174138	not scorable	not scorable	0.9752066	0.7790318

3760	Bn-A02-p24449690	A-genome specific (napus-rapa)	0.9473684	0.4058479	not scorable	not scorable	0.9669421	0.58711
3761	Bn-A02-p24462762	A-genome specific (napus-rapa)	0.9760765	0.5152972	not scorable	not scorable	0.9752066	0.5898604
3762	Bn-A02-p24477743	all not scorable	not scorable	not scorable	not scorable	not scorable	not scorable	not scorable
3763	Bn-A02-p24505252	A-genome specific (napus-rapa)	0.9904306	0.7258546	not scorable	not scorable	0.9917355	0.7190342
3764	Bn-A02-p24508488	A-genome specific (napus-rapa)	0.9904306	0.7945114	not scorable	not scorable	0.9917355	0.7995239
3765	Bn-A02-p2451470	A-genome specific (napus-rapa)	0.9736842	0.7273471	not scorable	not scorable	0.9834711	0.6920629
3766	Bn-A02-p24522983	A-genome specific (napus-rapa)	0.9952153	0.3993677	not scorable	not scorable	1	0.7361935
3767	Bn-A02-p24532892	A-genome specific (napus-rapa)	0.9688995	0.8634405	not scorable	not scorable	0.6528926	0.8086928
3768	Bn-A02-p24533751	A-genome specific (napus-rapa)	0.9066986	0.8011951	not scorable	not scorable	0.9173554	0.8075153
3769	Bn-A02-p24543172	A-genome specific (napus-rapa)	0.9880383	0.8754367	not scorable	not scorable	0.9917355	0.7396324
3770	Bn-A02-p24548194	A-genome specific (rapa only)	not scorable	not scorable	not scorable	not scorable	0.9917355	0.7788241
3771	Bn-A02-p24552203	A-genome specific (napus-rapa)	0.9760765	0.7576782	not scorable	not scorable	1	0.8251155
3772	Bn-A02-p24552476	A-genome specific (napus-rapa)	0.9928229	0.7689822	not scorable	not scorable	1	0.7931144
3773	Bn-A02-p24552542	A-genome specific (napus-rapa)	0.9832536	0.7658389	not scorable	not scorable	1	0.7597877
3774	Bn-A02-p24552639	A-genome specific (napus-rapa)	0.9856459	0.80213	not scorable	not scorable	1	0.7398953
3775	Bn-A02-p24552721	napus specific	0.9019139	0.9047894	not scorable	not scorable	not scorable	not scorable
3776	Bn-A02-p24552840	A-genome specific (napus-rapa)	0.9832536	0.8093157	not scorable	not scorable	1	0.781226
3777	Bn-A02-p24552843	A-genome specific (napus-rapa)	0.9880383	0.7193589	not scorable	not scorable	1	0.7668512
3778	Bn-A02-p24559792	A-genome specific (napus-rapa)	0.9928229	0.7475585	not scorable	not scorable	1	0.7790264
3779	Bn-A02-p24575998	A-genome specific (napus-rapa)	0.9856459	0.7743512	not scorable	not scorable	1	0.7161565
3780	Bn-A02-p24577932	A-genome specific (napus-rapa)	0.9904306	0.7846226	not scorable	not scorable	1	0.6941665
3781	Bn-A02-p24584058	A-genome specific (napus-rapa)	1	0.5375031	not scorable	not scorable	1	0.8226336
3782	Bn-A02-p2466355	A-genome specific (rapa only)	not scorable	not scorable	not scorable	not scorable	0.9834711	0.7707361
3783	Bn-A02-p24675182	A-genome specific (napus-rapa)	0.9952153	0.7475734	not scorable	not scorable	0.677686	0.6845919
3784	Bn-A02-p24687309	A-genome specific (napus-rapa)	0.9832536	0.7240523	not scorable	not scorable	0.9917355	0.8019288
3785	Bn-A02-p24688870	A-genome specific (napus-rapa)	0.9976076	0.9227535	not scorable	not scorable	1	0.9172798
3786	Bn-A02-p24700081	A-genome specific (napus-rapa)	0.9617225	0.3645316	not scorable	not scorable	0.9752066	0.7151247
3787	Bn-A02-p24700485	A-genome specific (rapa only)	not scorable	not scorable	not scorable	not scorable	0.9752066	0.7033099
3788	Bn-A02-p24700670	A-genome specific (napus-rapa)	0.9688995	0.4008781	not scorable	not scorable	0.9421487	0.7673577
3789	Bn-A02-p24700750	A-genome specific (rapa only)	not scorable	not scorable	not scorable	not scorable	0.9338843	0.7215623
3790	Bn-A02-p24702518	C-genome specific (oleracea only)	not scorable	not scorable	0.9922481	0.2352519	not scorable	not scorable
3791	Bn-A02-p24702986	A-genome specific (napus-rapa)	0.9784689	0.4731687	not scorable	not scorable	0.9669421	0.7972026
3792	Bn-A02-p24703050	A-genome specific (rapa only)	not scorable	not scorable	not scorable	not scorable	0.9586777	0.7641606

3793	Bn-A02-p24706646	A-genome specific (napus-rapa)	0.9808612	0.4346285	not scorable	not scorable	0.9834711	0.6902314
3794	Bn-A02-p24708197	A-genome specific (napus-rapa)	0.9832536	0.3769797	not scorable	not scorable	0.9917355	0.6938098
3795	Bn-A02-p24718110	A-genome specific (napus-rapa)	0.9856459	0.5957897	not scorable	not scorable	0.9669421	0.654136
3796	Bn-A02-p24722684	A-genome specific (napus-rapa)	0.9808612	0.8465989	not scorable	not scorable	1	0.8125221
3797	Bn-A02-p24731379	genome unspecific (all in)	0.9545454	0.3791173	1	0.3393075	0.9834711	0.7734646
3798	Bn-A02-p2475208	A-genome specific (napus-rapa)	0.9832536	0.7964051	not scorable	not scorable	1	0.808856
3799	Bn-A02-p2480400	A-genome specific (rapa only)	not scorable	not scorable	not scorable	not scorable	0.9917355	0.7158222
3800	Bn-A02-p24809526	A-genome specific (napus-rapa)	0.9880383	0.735046	not scorable	not scorable	0.9917355	0.6636193
3801	Bn-A02-p24810609	A-genome specific (napus-rapa)	0.9904306	0.7623301	not scorable	not scorable	0.9917355	0.7824732
3802	Bn-A02-p24827362	A-genome specific (napus-rapa)	0.9856459	0.4755225	not scorable	not scorable	1	0.7868106
3803	Bn-A02-p24844291	A-genome specific (napus-rapa)	0.9832536	0.5374362	not scorable	not scorable	1	0.7736591
3804	Bn-A02-p24851624	A-genome specific (napus-rapa)	0.9976076	0.4736698	not scorable	not scorable	1	0.7015013
3805	Bn-A02-p24878657	A-genome specific (napus-rapa)	0.9952153	0.224447	not scorable	not scorable	0.9669421	0.5929585
3806	Bn-A02-p2489530	A-genome specific (rapa only)	not scorable	not scorable	not scorable	not scorable	1	0.7618606
3807	Bn-A02-p2490293	A-genome specific (napus-rapa)	0.9736842	0.5408491	not scorable	not scorable	1	0.7359998
3808	Bn-A02-p24909771	A-genome specific (napus-rapa)	0.9928229	0.8024475	not scorable	not scorable	0.9834711	0.7870135
3809	Bn-A02-p24911505	A-genome specific (napus-rapa)	0.9904306	0.7679711	not scorable	not scorable	0.9504132	0.7485819
3810	Bn-A02-p24912792	A-genome specific (napus-rapa)	0.9928229	0.7205454	not scorable	not scorable	1	0.7901271
3811	Bn-A02-p24914083	A-genome specific (napus-rapa)	1	0.7059814	not scorable	not scorable	1	0.8004208
3812	Bn-A02-p24919366	A-genome specific (rapa only)	not scorable	not scorable	not scorable	not scorable	0.6446281	0.9446526
3813	Bn-A02-p24928201	A-genome specific (rapa only)	not scorable	not scorable	not scorable	not scorable	0.9917355	0.7351401
3814	Bn-A02-p24928311	A-genome specific (napus-rapa)	0.8708134	0.7632811	not scorable	not scorable	0.8760331	0.7590061
3815	Bn-A02-p24945785	A-genome specific (napus-rapa)	0.9784689	0.422927	not scorable	not scorable	0.9834711	0.6968016
3816	Bn-A02-p24948938	A-genome specific (napus-rapa)	0.9593301	0.820463	not scorable	not scorable	1	0.7932958
3817	Bn-A02-p24967958	A-genome specific (napus-rapa)	1	0.4569711	not scorable	not scorable	0.9834711	0.8208371
3818	Bn-A02-p24971607	A-genome specific (napus-rapa)	0.9808612	0.5161635	not scorable	not scorable	0.9917355	0.6083936
3819	Bn-A02-p25022333	all not scorable	not scorable	not scorable	not scorable	not scorable	not scorable	not scorable
3820	Bn-A02-p25022921	genome unspecific (oleracea-rapa)	not scorable	not scorable	0.9767442	0.5230137	0.9834711	0.525265
3821	Bn-A02-p25027576	genome unspecific (all in)	0.9832536	0.3075857	1	0.8095853	1	0.8009473
3822	Bn-A02-p2502950	A-genome specific (napus-rapa)	0.937799	0.5792095	not scorable	not scorable	1	0.7601622
3823	Bn-A02-p25079431	A-genome specific (napus-rapa)	0.9976076	0.704473	not scorable	not scorable	0.9917355	0.7331099
3824	Bn-A02-p25081911	A-genome specific (rapa only)	not scorable	not scorable	not scorable	not scorable	1	0.827769
3825	Bn-A02-p25081916	A-genome specific (napus-rapa)	1	0.3411662	not scorable	not scorable	0.9834711	0.7254934

3826	Bn-A02-p2509802	A-genome specific (napus-rapa)	0.9449761	0.7629402	not scorable	not scorable	0.9834711	0.6931663
3827	Bn-A02-p25100451	A-genome specific (napus-rapa)	0.9904306	0.7879258	not scorable	not scorable	1	0.7935399
3828	Bn-A02-p25105527	A-genome specific (napus-rapa)	1	0.4670247	not scorable	not scorable	1	0.7273133
3829	Bn-A02-p25109769	A-genome specific (napus-rapa)	0.9928229	0.9235229	not scorable	not scorable	1	0.7847053
3830	Bn-A02-p25118816	A-genome specific (napus-rapa)	0.9904306	0.4378158	not scorable	not scorable	0.9834711	0.4827474
3831	Bn-A02-p251558	A-genome specific (napus-rapa)	0.9928229	0.5861614	not scorable	not scorable	0.9752066	0.677668
3832	Bn-A02-p25174583	genome unspecific (all in)	0.9641148	0.3216029	0.9534883	0.7254546	0.9917355	0.5180019
3833	Bn-A02-p25180996	A-genome specific (napus-rapa)	0.9545454	0.7543437	not scorable	not scorable	0.9917355	0.7621441
3834	Bn-A02-p25181474	A-genome specific (napus-rapa)	0.9808612	0.444824	not scorable	not scorable	1	0.7543489
3835	Bn-A02-p25181726	genome unspecific (all in)	0.9856459	0.3598177	1	0.6156915	1	0.875934
3836	Bn-A02-p25188041	A-genome specific (napus-rapa)	0.9545454	0.7840285	not scorable	not scorable	0.9752066	0.7019362
3837	Bn-A02-p25190854	A-genome specific (napus-rapa)	0.9808612	0.7632776	not scorable	not scorable	0.9834711	0.7260795
3838	Bn-A02-p25199281	genome unspecific (oleracea-rapa)	not scorable	not scorable	1	0.751542	0.9752066	0.657665
3839	Bn-A02-p25201372	A-genome specific (napus-rapa)	0.9928229	0.6143605	not scorable	not scorable	0.9669421	0.6023389
3840	Bn-A02-p2520421	A-genome specific (napus-rapa)	0.9688995	0.6893967	not scorable	not scorable	0.9834711	0.8861109
3841	Bn-A02-p25205468	A-genome specific (napus-rapa)	0.9808612	0.3969807	not scorable	not scorable	0.9834711	0.6045682
3842	Bn-A02-p25206495	A-genome specific (napus-rapa)	0.9808612	0.4619537	not scorable	not scorable	1	0.7418953
3843	Bn-A02-p25206605	A-genome specific (napus-rapa)	0.9760765	0.6348951	not scorable	not scorable	0.9834711	0.7883735
3844	Bn-A02-p25216530	A-genome specific (napus-rapa)	0.9808612	0.2618947	not scorable	not scorable	0.9834711	0.8351889
3845	Bn-A02-p25218817	A-genome specific (napus-rapa)	0.9282297	0.5755724	not scorable	not scorable	0.9752066	0.6840392
3846	Bn-A02-p25260066	A-genome specific (napus-rapa)	0.9904306	0.3791234	not scorable	not scorable	1	0.7450714
3847	Bn-A02-p2527082	A-genome specific (napus-rapa)	0.9784689	0.7182524	not scorable	not scorable	1	0.6417865
3848	Bn-A02-p2527735	A-genome specific (napus-rapa)	0.9688995	0.7504268	not scorable	not scorable	1	0.8490733
3849	Bn-A02-p25285941	A-genome specific (rapa only)	not scorable	not scorable	not scorable	not scorable	1	0.7436799
3850	Bn-A02-p25286323	A-genome specific (rapa only)	not scorable	not scorable	not scorable	not scorable	0.5206612	0.8930295
3851	Bn-A02-p25286373	A-genome specific (napus-rapa)	0.8947368	0.3031206	not scorable	not scorable	0.5206612	0.692642
3852	Bn-A02-p25332661	A-genome specific (rapa only)	not scorable	not scorable	not scorable	not scorable	1	0.8309836
3853	Bn-A02-p25334100	genome unspecific (all in)	0.9856459	0.2618202	0.9922481	0.2440073	0.9752066	0.466137
3854	Bn-A02-p25334266	A-genome specific (napus-rapa)	0.9688995	0.7898447	not scorable	not scorable	0.9752066	0.7802529
3855	Bn-A02-p25334271	A-genome specific (napus-rapa)	0.9784689	0.8132872	not scorable	not scorable	0.9669421	0.7840019
3856	Bn-A02-p25350701	A-genome specific (napus-rapa)	0.7583732	0.6717579	not scorable	not scorable	0.9917355	0.6839571
3857	Bn-A02-p2535232	A-genome specific (rapa only)	not scorable	not scorable	not scorable	not scorable	1	0.7257899
3858	Bn-A02-p2536779	A-genome specific (napus-rapa)	0.9736842	0.7274444	not scorable	not scorable	0.9752066	0.73816

3859	Bn-A02-p25367886	A-genome specific (napus-rapa)	0.9760765	0.6727452	not scorable	not scorable	1	0.6955542
3860	Bn-A02-p25375825	A-genome specific (napus-rapa)	0.9760765	0.651063	not scorable	not scorable	1	0.7161332
3861	Bn-A02-p25389902	A-genome specific (napus-rapa)	0.9760765	0.7423301	not scorable	not scorable	1	0.812272
3862	Bn-A02-p25400515	A-genome specific (napus-rapa)	0.9497608	0.7730032	not scorable	not scorable	0.9834711	0.7955602
3863	Bn-A02-p25400591	A-genome specific (napus-rapa)	0.9425837	0.7311572	not scorable	not scorable	0.9917355	0.7134288
3864	Bn-A02-p25401060	A-genome specific (rapa only)	not scorable	not scorable	not scorable	not scorable	0.9834711	0.6469969
3865	Bn-A02-p25402286	A-genome specific (napus-rapa)	0.9593301	0.8104648	not scorable	not scorable	0.9917355	0.7402028
3866	Bn-A02-p25402971	A-genome specific (napus-rapa)	0.9712918	0.7307721	not scorable	not scorable	0.9834711	0.8236844
3867	Bn-A02-p25413153	all not scorable	not scorable	not scorable	not scorable	not scorable	not scorable	not scorable
3868	Bn-A02-p25419536	A-genome specific (napus-rapa)	0.9808612	0.3150016	not scorable	not scorable	1	0.8684912
3869	Bn-A02-p2542065	A-genome specific (napus-rapa)	0.9665072	0.7713618	not scorable	not scorable	0.9669421	0.7538679
3870	Bn-A02-p25429624	A-genome specific (napus-rapa)	0.9832536	0.4774918	not scorable	not scorable	0.9917355	0.7816078
3871	Bn-A02-p25429837	A-genome specific (napus-rapa)	0.9569378	0.2545027	not scorable	not scorable	0.9917355	0.6324435
3872	Bn-A02-p25436510	A-genome specific (napus-rapa)	0.9832536	0.3485574	not scorable	not scorable	1	0.7474809
3873	Bn-A02-p25438684	A-genome specific (napus-rapa)	0.9712918	0.7670354	not scorable	not scorable	0.9917355	0.7036653
3874	Bn-A02-p2544070	A-genome specific (napus-rapa)	0.9808612	0.7641829	not scorable	not scorable	0.9834711	0.6910309
3875	Bn-A02-p25445157	A-genome specific (napus-rapa)	0.9688995	0.2448494	not scorable	not scorable	0.9917355	0.673512
3876	Bn-A02-p25446521	A-genome specific (napus-rapa)	0.9760765	0.7983402	not scorable	not scorable	0.9917355	0.7629623
3877	Bn-A02-p25448092	A-genome specific (napus-rapa)	0.9832536	0.7914116	not scorable	not scorable	1	0.8009782
3878	Bn-A02-p25453070	A-genome specific (napus-rapa)	0.9832536	0.7821937	not scorable	not scorable	0.9917355	0.7001942
3879	Bn-A02-p25453447	A-genome specific (napus-rapa)	0.9688995	0.7808092	not scorable	not scorable	0.9752066	0.717015
3880	Bn-A02-p25454242	A-genome specific (napus-rapa)	0.9712918	0.7595798	not scorable	not scorable	0.9917355	0.7700318
3881	Bn-A02-p25454499	A-genome specific (rapa only)	not scorable	not scorable	not scorable	not scorable	0.9917355	0.7417291
3882	Bn-A02-p25454882	A-genome specific (napus-rapa)	0.9712918	0.7419256	not scorable	not scorable	0.9834711	0.7072452
3883	Bn-A02-p25456985	A-genome specific (napus-rapa)	0.9832536	0.649024	not scorable	not scorable	0.9834711	0.6549544
3884	Bn-A02-p25457045	A-genome specific (napus-rapa)	0.9736842	0.8153956	not scorable	not scorable	1	0.7757701
3885	Bn-A02-p25457843	A-genome specific (napus-rapa)	0.9832536	0.7692493	not scorable	not scorable	0.9834711	0.8290427
3886	Bn-A02-p25463582	A-genome specific (napus-rapa)	0.9736842	0.7814487	not scorable	not scorable	0.9834711	0.7188965
3887	Bn-A02-p25465508	A-genome specific (napus-rapa)	0.9593301	0.8011951	not scorable	not scorable	0.9917355	0.8009939
3888	Bn-A02-p25465589	A-genome specific (napus-rapa)	0.9641148	0.6783643	not scorable	not scorable	0.9752066	0.835183
3889	Bn-A02-p25477417	A-genome specific (rapa only)	not scorable	not scorable	not scorable	not scorable	1	0.8107145
3890	Bn-A02-p25479893	A-genome specific (napus-rapa)	0.9665072	0.7336133	not scorable	not scorable	1	0.8385277
3891	Bn-A02-p25484876	A-genome specific (napus-rapa)	0.9736842	0.4614016	not scorable	not scorable	1	0.7779287

3892	Bn-A02-p25485627	A-genome specific (napus-rapa)	0.9760765	0.6849786	not scorable	not scorable	0.9917355	0.7877229
3893	Bn-A02-p25488435	A-genome specific (napus-rapa)	0.9880383	0.4579249	not scorable	not scorable	1	0.7004995
3894	Bn-A02-p2549141	A-genome specific (napus-rapa)	0.9760765	0.7501812	not scorable	not scorable	0.9917355	0.7150395
3895	Bn-A02-p25497743	A-genome specific (napus-rapa)	0.9665072	0.4827575	not scorable	not scorable	0.9752066	0.7531053
3896	Bn-A02-p2551178	A-genome specific (napus-rapa)	1	0.6997946	not scorable	not scorable	0.9917355	0.7211741
3897	Bn-A02-p25530111	A-genome specific (napus-rapa)	0.9688995	0.7659855	not scorable	not scorable	0.9917355	0.8344188
3898	Bn-A02-p25530994	genome unspecific (all in)	0.9688995	0.3538687	0.9767442	0.5843842	1	0.6534097
3899	Bn-A02-p25554878	A-genome specific (napus-rapa)	0.9784689	0.6235612	not scorable	not scorable	0.9256198	0.7623515
3900	Bn-A02-p25564792	A-genome specific (napus-rapa)	0.9043062	0.5261033	not scorable	not scorable	0.9834711	0.6805814
3901	Bn-A02-p25572877	A-genome specific (napus-rapa)	0.9856459	0.2909664	not scorable	not scorable	0.9917355	0.7619383
3902	Bn-A02-p2557799	genome unspecific (oleracea-rapa)	not scorable	not scorable	0.9922481	0.7083627	1	0.7010074
3903	Bn-A02-p25612808	A-genome specific (napus-rapa)	0.9832536	0.3084299	not scorable	not scorable	1	0.7717501
3904	Bn-A02-p25615285	A-genome specific (napus-rapa)	0.9617225	0.7701499	not scorable	not scorable	1	0.8086112
3905	Bn-A02-p25625124	A-genome specific (napus-rapa)	0.5263158	0.7663057	not scorable	not scorable	0.6280992	0.8006331
3906	Bn-A02-p25625846	A-genome specific (napus-rapa)	0.9928229	0.5837622	not scorable	not scorable	0.6033058	0.8064268
3907	Bn-A02-p25626500	A-genome specific (napus-rapa)	0.569378	0.8204875	not scorable	not scorable	0.6033058	0.8046752
3908	Bn-A02-p25630745	A-genome specific (napus-rapa)	0.5741627	0.7672402	not scorable	not scorable	0.5950413	0.8365013
3909	Bn-A02-p25631431	A-genome specific (napus-rapa)	0.5717703	0.7990752	not scorable	not scorable	0.6198347	0.7510429
3910	Bn-A02-p25632681	A-genome specific (napus-rapa)	0.5717703	0.6927576	not scorable	not scorable	0.6528926	0.8233868
3911	Bn-A02-p25636038	A-genome specific (napus-rapa)	0.5358852	0.7991782	not scorable	not scorable	0.5702479	0.725287
3912	Bn-A02-p25636136	A-genome specific (napus-rapa)	0.5550239	0.7480863	not scorable	not scorable	0.6198347	0.750002
3913	Bn-A02-p25644999	all not scorable	not scorable	not scorable	not scorable	not scorable	not scorable	not scorable
3914	Bn-A02-p25652516	A-genome specific (napus-rapa)	0.9665072	0.5213373	not scorable	not scorable	0.9669421	0.6927272
3915	Bn-A02-p25655528	A-genome specific (napus-rapa)	0.923445	0.4746001	not scorable	not scorable	1	0.7063446
3916	Bn-A02-p25670193	A-genome specific (napus-rapa)	0.9688995	0.4428025	not scorable	not scorable	0.9834711	0.7674047
3917	Bn-A02-p25678882	A-genome specific (napus-rapa)	0.9712918	0.4364111	not scorable	not scorable	1	0.712101
3918	Bn-A02-p25681480	A-genome specific (napus-rapa)	0.9784689	0.7493784	not scorable	not scorable	0.9421487	0.7899156
3919	Bn-A02-p25687470	A-genome specific (napus-rapa)	0.9617225	0.1909869	not scorable	not scorable	0.9752066	0.7376403
3920	Bn-A02-p2568989	A-genome specific (rapa only)	not scorable	not scorable	not scorable	not scorable	0.9834711	0.5355994
3921	Bn-A02-p2572194	A-genome specific (napus-rapa)	0.9976076	0.8292307	not scorable	not scorable	1	0.8640971
3922	Bn-A02-p2572625	A-genome specific (napus-rapa)	0.9976076	0.7241075	not scorable	not scorable	0.9917355	0.7682964
3923	Bn-A02-p25734367	A-genome specific (napus-rapa)	0.9712918	0.4049708	not scorable	not scorable	0.9917355	0.716334
3924	Bn-A02-p25735700	A-genome specific (napus-rapa)	0.9282297	0.7727267	not scorable	not scorable	0.6528926	0.8785214

3925	Bn-A02-p25736307	A-genome specific (napus-rapa)	0.5669857	0.810605	not scorable	not scorable	1	0.7757788
3926	Bn-A02-p25739024	A-genome specific (napus-rapa)	0.9784689	0.3976241	not scorable	not scorable	0.9834711	0.7549633
3927	Bn-A02-p25741878	A-genome specific (napus-rapa)	0.9688995	0.3933358	not scorable	not scorable	0.9917355	0.7895689
3928	Bn-A02-p25746504	A-genome specific (napus-rapa)	1	0.5243186	not scorable	not scorable	1	0.7490153
3929	Bn-A02-p25747937	A-genome specific (rapa only)	not scorable	not scorable	not scorable	not scorable	1	0.883854
3930	Bn-A02-p25762081	A-genome specific (napus-rapa)	0.9952153	0.2743968	not scorable	not scorable	0.9917355	0.759901
3931	Bn-A02-p25795588	A-genome specific (napus-rapa)	0.9641148	0.3011917	not scorable	not scorable	1	0.6392568
3932	Bn-A02-p25814719	A-genome specific (napus-rapa)	0.9712918	0.4716518	not scorable	not scorable	0.9752066	0.763435
3933	Bn-A02-p25820450	A-genome specific (napus-rapa)	0.9760765	0.7957777	not scorable	not scorable	1	0.8083437
3934	Bn-A02-p25821750	A-genome specific (napus-rapa)	0.9880383	0.8817617	not scorable	not scorable	1	0.792043
3935	Bn-A02-p25826690	A-genome specific (napus-rapa)	0.9760765	0.7687581	not scorable	not scorable	0.9834711	0.7344646
3936	Bn-A02-p25827208	A-genome specific (napus-rapa)	0.9904306	0.7642173	not scorable	not scorable	0.9834711	0.7254342
3937	Bn-A02-p2582999	A-genome specific (napus-rapa)	0.9665072	0.6571609	not scorable	not scorable	1	0.7168393
3938	Bn-A02-p25841130	A-genome specific (napus-rapa)	0.930622	0.6638005	not scorable	not scorable	1	0.6396332
3939	Bn-A02-p25841252	A-genome specific (napus-rapa)	0.930622	0.716061	not scorable	not scorable	0.5206612	0.7955049
3940	Bn-A02-p25841382	A-genome specific (napus-rapa)	0.9497608	0.7266648	not scorable	not scorable	0.5454546	0.6909549
3941	Bn-A02-p25841722	A-genome specific (napus-rapa)	0.9449761	0.6642765	not scorable	not scorable	0.5619835	0.6384211
3942	Bn-A02-p25842875	A-genome specific (napus-rapa)	0.9808612	0.7622928	not scorable	not scorable	0.9917355	0.7260741
3943	Bn-A02-p25844300	A-genome specific (napus-rapa)	0.9665072	0.785914	not scorable	not scorable	1	0.7415183
3944	Bn-A02-p25845339	A-genome specific (napus-rapa)	0.9760765	0.7935207	not scorable	not scorable	0.9752066	0.7594121
3945	Bn-A02-p25857471	A-genome specific (napus-rapa)	0.9066986	0.4629106	not scorable	not scorable	0.5123967	0.699336
3946	Bn-A02-p25864051	A-genome specific (napus-rapa)	0.9641148	0.6404436	not scorable	not scorable	0.9752066	0.8045942
3947	Bn-A02-p25864853	A-genome specific (napus-rapa)	0.854067	0.7860294	not scorable	not scorable	0.6198347	0.7113413
3948	Bn-A02-p25865360	A-genome specific (napus-rapa)	0.9760765	0.5010147	not scorable	not scorable	1	0.7286872
3949	Bn-A02-p25876712	A-genome specific (napus-rapa)	1	0.5214758	not scorable	not scorable	1	0.8333946
3950	Bn-A02-p25881031	A-genome specific (napus-rapa)	0.9928229	0.7448925	not scorable	not scorable	1	0.6961026
3951	Bn-A02-p25881408	A-genome specific (napus-rapa)	0.9736842	0.4496895	not scorable	not scorable	0.9917355	0.7178582
3952	Bn-A02-p25883567	A-genome specific (napus-rapa)	0.9784689	0.8083759	not scorable	not scorable	0.9917355	0.7647892
3953	Bn-A02-p25966536	genome unspecific (oleracea-rapa)	not scorable	not scorable	1	0.5178636	1	0.3993078
3954	Bn-A02-p26013503	A-genome specific (napus-rapa)	0.84689	0.7475929	not scorable	not scorable	0.9669421	0.7360693
3955	Bn-A02-p2608125	A-genome specific (napus-rapa)	0.9976076	0.7863283	not scorable	not scorable	0.9917355	0.7411901
3956	Bn-A02-p26154897	A-genome specific (napus-rapa)	0.9712918	0.6476855	not scorable	not scorable	0.9917355	0.7736945
3957	Bn-A02-p26154951	A-genome specific (napus-rapa)	0.9904306	0.7867417	not scorable	not scorable	0.9917355	0.8218488

3958	Bn-A02-p2615923	A-genome specific (rapa only)	not scorable	not scorable	not scorable	not scorable	1	0.801878
3959	Bn-A02-p2617701	A-genome specific (napus-rapa)	0.9808612	0.7945674	not scorable	not scorable	0.9669421	0.8006936
3960	Bn-A02-p2617781	A-genome specific (napus-rapa)	0.9784689	0.7875533	not scorable	not scorable	0.9752066	0.7834601
3961	Bn-A02-p26193572	A-genome specific (napus-rapa)	0.8875598	0.6917698	not scorable	not scorable	0.9752066	0.8332381
3962	Bn-A02-p2620397	A-genome specific (rapa only)	not scorable	not scorable	not scorable	not scorable	1	0.5637079
3963	Bn-A02-p26214379	A-genome specific (rapa only)	not scorable	not scorable	not scorable	not scorable	1	0.6990784
3964	Bn-A02-p2624375	A-genome specific (napus-rapa)	0.9473684	0.8466316	not scorable	not scorable	0.6528926	0.6872752
3965	Bn-A02-p26304354	A-genome specific (napus-rapa)	0.9808612	0.399991	not scorable	not scorable	0.9090909	0.7015182
3966	Bn-A02-p26307761	all not scorable	not scorable	not scorable	not scorable	not scorable	not scorable	not scorable
3967	Bn-A02-p26322916	A-genome specific (napus-rapa)	0.9832536	0.7588039	not scorable	not scorable	0.9834711	0.7526912
3968	Bn-A02-p26335941	A-genome specific (rapa only)	not scorable	not scorable	not scorable	not scorable	0.9917355	0.7744511
3969	Bn-A02-p26345558	A-genome specific (napus-rapa)	0.9641148	0.4395147	not scorable	not scorable	1	0.9277734
3970	Bn-A02-p26373377	A-genome specific (rapa only)	not scorable	not scorable	not scorable	not scorable	0.9586777	0.7892213
3971	Bn-A02-p2638031	A-genome specific (napus-rapa)	0.9497608	0.7690462	not scorable	not scorable	0.661157	0.7666137
3972	Bn-A02-p26380384	A-genome specific (napus-rapa)	0.9760765	0.30195	not scorable	not scorable	0.9917355	0.7596891
3973	Bn-A02-p26398002	A-genome specific (napus-rapa)	0.9832536	0.792685	not scorable	not scorable	0.9669421	0.8037483
3974	Bn-A02-p26411585	A-genome specific (napus-rapa)	0.9736842	0.2365642	not scorable	not scorable	0.9504132	0.4493165
3975	Bn-A02-p26412693	A-genome specific (napus-rapa)	0.9641148	0.2491794	not scorable	not scorable	0.9917355	0.6738153
3976	Bn-A02-p26414199	genome unspecific (oleracea-rapa)	not scorable	not scorable	1	0.8132226	1	0.9205657
3977	Bn-A02-p26419529	A-genome specific (rapa only)	not scorable	not scorable	not scorable	not scorable	0.9917355	0.7722063
3978	Bn-A02-p26421790	A-genome specific (rapa only)	not scorable	not scorable	not scorable	not scorable	1	0.7378635
3979	Bn-A02-p26425347	A-genome specific (rapa only)	not scorable	not scorable	not scorable	not scorable	1	0.8065163
3980	Bn-A02-p2642904	A-genome specific (napus-rapa)	0.9665072	0.7193154	not scorable	not scorable	1	0.7948084
3981	Bn-A02-p2643078	A-genome specific (napus-rapa)	0.9569378	0.8174909	not scorable	not scorable	0.9834711	0.8277231
3982	Bn-A02-p26431587	A-genome specific (rapa only)	not scorable	not scorable	not scorable	not scorable	0.9917355	0.7846536
3983	Bn-A02-p26435743	A-genome specific (rapa only)	not scorable	not scorable	not scorable	not scorable	0.9917355	0.7133168
3984	Bn-A02-p26446178	A-genome specific (rapa only)	not scorable	not scorable	not scorable	not scorable	0.9752066	0.7688381
3985	Bn-A02-p2646929	genome unspecific (oleracea-rapa)	not scorable	not scorable	0.9844961	0.827836	0.9917355	0.680208
3986	Bn-A02-p26512828	A-genome specific (rapa only)	not scorable	not scorable	not scorable	not scorable	1	0.757892
3987	Bn-A02-p26512923	A-genome specific (rapa only)	not scorable	not scorable	not scorable	not scorable	0.9834711	0.7818587
3988	Bn-A02-p26514827	A-genome specific (rapa only)	not scorable	not scorable	not scorable	not scorable	0.553719	0.9267779
3989	Bn-A02-p26535192	A-genome specific (rapa only)	not scorable	not scorable	not scorable	not scorable	0.677686	0.8058625
3990	Bn-A02-p26539305	A-genome specific (rapa only)	not scorable	not scorable	not scorable	not scorable	1	0.8042592

3991	Bn-A02-p26547428	A-genome specific (rapa only)	not scorable	not scorable	not scorable	not scorable	1	0.7725431
3992	Bn-A02-p2654991	A-genome specific (napus-rapa)	0.9545454	0.6550132	not scorable	not scorable	0.9834711	0.7215709
3993	Bn-A02-p26554539	genome unspecific (oleracea-rapa)	not scorable	not scorable	1	0.6706535	0.9752066	0.6763287
3994	Bn-A02-p26564337	A-genome specific (rapa only)	not scorable	not scorable	not scorable	not scorable	0.9917355	0.7519459
3995	Bn-A02-p26567426	A-genome specific (rapa only)	not scorable	not scorable	not scorable	not scorable	0.9917355	0.816599
3996	Bn-A02-p26571107	A-genome specific (rapa only)	not scorable	not scorable	not scorable	not scorable	0.9917355	0.7906119
3997	Bn-A02-p266070	A-genome specific (napus-rapa)	0.9976076	0.6167102	not scorable	not scorable	0.9421487	0.6662418
3998	Bn-A02-p266121	A-genome specific (napus-rapa)	0.9904306	0.6227802	not scorable	not scorable	0.9752066	0.6053193
3999	Bn-A02-p26620662	A-genome specific (napus-rapa)	0.9832536	0.7806922	not scorable	not scorable	1	0.7642689
4000	Bn-A02-p26621160	A-genome specific (napus-rapa)	0.9808612	0.7907835	not scorable	not scorable	0.9917355	0.8348098
4001	Bn-A02-p26625153	A-genome specific (napus-rapa)	0.9928229	0.5479173	not scorable	not scorable	0.9917355	0.8063736
4002	Bn-A02-p26625696	A-genome specific (napus-rapa)	0.9952153	0.4624704	not scorable	not scorable	0.9917355	0.7242765
4003	Bn-A02-p26630032	A-genome specific (rapa only)	not scorable	not scorable	not scorable	not scorable	0.9917355	0.7779773
4004	Bn-A02-p26640222	A-genome specific (napus-rapa)	0.9688995	0.5788972	not scorable	not scorable	0.9917355	0.619308
4005	Bn-A02-p26641996	A-genome specific (napus-rapa)	0.9712918	0.7847545	not scorable	not scorable	1	0.8028569
4006	Bn-A02-p26644297	genome unspecific (oleracea-rapa)	not scorable	not scorable	1	0.7580749	0.9834711	0.7418215
4007	Bn-A02-p26645956	A-genome specific (napus-rapa)	0.9521531	0.7631143	not scorable	not scorable	1	0.5694045
4008	Bn-A02-p26646040	napus specific	0.8803828	0.746186	not scorable	not scorable	not scorable	not scorable
4009	Bn-A02-p26646436	A-genome specific (napus-rapa)	0.9784689	0.7430586	not scorable	not scorable	1	0.8842213
4010	Bn-A02-p26651278	A-genome specific (napus-rapa)	0.9688995	0.7659454	not scorable	not scorable	1	0.8722966
4011	Bn-A02-p26653626	A-genome specific (napus-rapa)	0.9593301	0.7489499	not scorable	not scorable	1	0.897248
4012	Bn-A02-p26654006	A-genome specific (napus-rapa)	0.9569378	0.6947718	not scorable	not scorable	1	0.8088461
4013	Bn-A02-p26656067	A-genome specific (napus-rapa)	0.9665072	0.7541266	not scorable	not scorable	0.9834711	0.8084803
4014	Bn-A02-p26656981	A-genome specific (napus-rapa)	0.9880383	0.7365009	not scorable	not scorable	0.9917355	0.6713122
4015	Bn-A02-p26658350	A-genome specific (napus-rapa)	0.9904306	0.2795922	not scorable	not scorable	0.9917355	0.7690216
4016	Bn-A02-p26665282	A-genome specific (napus-rapa)	0.9784689	0.834662	not scorable	not scorable	0.9917355	0.8041646
4017	Bn-A02-p26668289	A-genome specific (napus-rapa)	0.9736842	0.4320352	not scorable	not scorable	0.9917355	0.740729
4018	Bn-A02-p26669888	A-genome specific (napus-rapa)	0.9569378	0.770789	not scorable	not scorable	1	0.861191
4019	Bn-A02-p26686239	A-genome specific (napus-rapa)	0.9641148	0.5426888	not scorable	not scorable	1	0.6891931
4020	Bn-A02-p26689319	napus specific	0.9473684	0.7657803	not scorable	not scorable	not scorable	not scorable
4021	Bn-A02-p26710300	A-genome specific (napus-rapa)	0.9856459	0.4745325	not scorable	not scorable	1	0.7859391
4022	Bn-A02-p26713316	A-genome specific (napus-rapa)	0.9808612	0.5081707	not scorable	not scorable	0.9917355	0.8199698
4023	Bn-A02-p26733511	A-genome specific (napus-rapa)	0.3014354	0.8632697	not scorable	not scorable	0.6033058	0.7495869

4024	Bn-A02-p2673795	A-genome specific (rapa only)	not scorable	not scorable	not scorable	not scorable	0.9917355	0.8189792
4025	Bn-A02-p26792851	A-genome specific (napus-rapa)	0.9880383	0.3599333	not scorable	not scorable	1	0.7368796
4026	Bn-A02-p26803902	A-genome specific (napus-rapa)	0.9880383	0.4413253	not scorable	not scorable	0.9834711	0.7269258
4027	Bn-A02-p26804091	napus specific	0.9641148	0.753601	not scorable	not scorable	not scorable	not scorable
4028	Bn-A02-p26816101	A-genome specific (napus-rapa)	0.9856459	0.620794	not scorable	not scorable	1	0.8381849
4029	Bn-A02-p26816506	A-genome specific (napus-rapa)	0.9712918	0.7593117	not scorable	not scorable	0.9752066	0.7046372
4030	Bn-A02-p26819130	A-genome specific (napus-rapa)	0.9641148	0.6796762	not scorable	not scorable	0.9669421	0.6601005
4031	Bn-A02-p26819608	A-genome specific (napus-rapa)	0.9736842	0.6938714	not scorable	not scorable	1	0.7155285
4032	Bn-A02-p26824239	A-genome specific (napus-rapa)	0.9545454	0.6782708	not scorable	not scorable	0.9752066	0.7695445
4033	Bn-A02-p26824300	A-genome specific (napus-rapa)	0.9760765	0.6812587	not scorable	not scorable	0.9834711	0.7296497
4034	Bn-A02-p26824823	A-genome specific (napus-rapa)	0.9880383	0.5716254	not scorable	not scorable	1	0.8088076
4035	Bn-A02-p26832454	A-genome specific (napus-rapa)	0.9665072	0.7540839	not scorable	not scorable	0.9834711	0.6486282
4036	Bn-A02-p26845132	A-genome specific (napus-rapa)	0.9784689	0.3136747	not scorable	not scorable	1	0.9175813
4037	Bn-A02-p26853555	A-genome specific (napus-rapa)	0.9784689	0.4481732	not scorable	not scorable	0.9917355	0.7805899
4038	Bn-A02-p26854880	A-genome specific (napus-rapa)	0.9736842	0.7967595	not scorable	not scorable	0.9752066	0.7930169
4039	Bn-A02-p26854933	A-genome specific (napus-rapa)	0.9736842	0.8090507	not scorable	not scorable	1	0.8546475
4040	Bn-A02-p26857230	A-genome specific (napus-rapa)	0.9760765	0.5307622	not scorable	not scorable	0.9752066	0.6929754
4041	Bn-A02-p26883269	A-genome specific (napus-rapa)	0.9832536	0.7466351	not scorable	not scorable	1	0.7359638
4042	Bn-A02-p26887073	A-genome specific (rapa only)	not scorable	not scorable	not scorable	not scorable	1	0.7684813
4043	Bn-A02-p26890561	genome unspecific (all in)	0.9808612	0.4807646	0.9379845	0.657937	1	0.7935476
4044	Bn-A02-p26892575	A-genome specific (napus-rapa)	0.9928229	0.7423341	not scorable	not scorable	0.9917355	0.7790258
4045	Bn-A02-p26900937	A-genome specific (napus-rapa)	0.9928229	0.7546605	not scorable	not scorable	0.9669421	0.6675804
4046	Bn-A02-p26901243	A-genome specific (napus-rapa)	0.9736842	0.851343	not scorable	not scorable	0.9834711	0.7954875
4047	Bn-A02-p2692116	A-genome specific (napus-rapa)	0.9808612	0.7156996	not scorable	not scorable	1	0.6999377
4048	Bn-A02-p26921384	A-genome specific (napus-rapa)	0.9808612	0.7524504	not scorable	not scorable	1	0.9040579
4049	Bn-A02-p26946005	A-genome specific (napus-rapa)	0.9760765	0.3013268	not scorable	not scorable	0.9834711	0.5502778
4050	Bn-A02-p26947254	A-genome specific (napus-rapa)	0.9952153	0.7655926	not scorable	not scorable	0.9917355	0.6948284
4051	Bn-A02-p2695870	A-genome specific (napus-rapa)	0.9808612	0.8047065	not scorable	not scorable	0.9752066	0.742887
4052	Bn-A02-p26975795	A-genome specific (napus-rapa)	0.9976076	0.9081466	not scorable	not scorable	1	0.9072468
4053	Bn-A02-p2698217	A-genome specific (napus-rapa)	0.9952153	0.7598017	not scorable	not scorable	1	0.8049851
4054	Bn-A02-p26982864	A-genome specific (napus-rapa)	0.9736842	0.7551115	not scorable	not scorable	1	0.7686984
4055	Bn-A02-p26986319	A-genome specific (rapa only)	not scorable	not scorable	not scorable	not scorable	0.9917355	0.7323602
4056	Bn-A02-p26987864	A-genome specific (napus-rapa)	0.9904306	0.4711652	not scorable	not scorable	1	0.811287

4057	Bn-A02-p27022909	A-genome specific (napus-rapa)	0.9712918	0.7675508	not scorable	not scorable	1	0.8020658
4058	Bn-A02-p27024443	A-genome specific (rapa only)	not scorable	not scorable	not scorable	not scorable	0.9917355	0.7196941
4059	Bn-A02-p27030941	A-genome specific (rapa only)	not scorable	not scorable	not scorable	not scorable	1	0.7651234
4060	Bn-A02-p27035366	A-genome specific (rapa only)	not scorable	not scorable	not scorable	not scorable	0.9917355	0.6993365
4061	Bn-A02-p27035425	A-genome specific (rapa only)	not scorable	not scorable	not scorable	not scorable	0.9586777	0.693325
4062	Bn-A02-p27042108	A-genome specific (rapa only)	not scorable	not scorable	not scorable	not scorable	0.9917355	0.6703722
4063	Bn-A02-p27043712	A-genome specific (rapa only)	not scorable	not scorable	not scorable	not scorable	0.9669421	0.7058506
4064	Bn-A02-p27051969	A-genome specific (rapa only)	not scorable	not scorable	not scorable	not scorable	0.5454546	0.9033917
4065	Bn-A02-p27052532	A-genome specific (rapa only)	not scorable	not scorable	not scorable	not scorable	0.9917355	0.7801372
4066	Bn-A02-p27052742	A-genome specific (napus-rapa)	0.937799	0.7954038	not scorable	not scorable	0.9917355	0.7201011
4067	Bn-A02-p27055844	A-genome specific (napus-rapa)	0.9976076	0.7761796	not scorable	not scorable	0.9834711	0.7095855
4068	Bn-A02-p27055872	A-genome specific (napus-rapa)	0.9976076	0.6695601	not scorable	not scorable	0.9834711	0.6932496
4069	Bn-A02-p2707979	A-genome specific (napus-rapa)	0.8755981	0.7478034	not scorable	not scorable	0.9669421	0.7306691
4070	Bn-A02-p2708161	A-genome specific (napus-rapa)	0.8851675	0.7656389	not scorable	not scorable	0.9834711	0.6962705
4071	Bn-A02-p27109424	A-genome specific (napus-rapa)	0.9976076	0.7050758	not scorable	not scorable	1	0.8116624
4072	Bn-A02-p27109713	A-genome specific (napus-rapa)	0.9880383	0.7930319	not scorable	not scorable	1	0.7193862
4073	Bn-A02-p27109858	A-genome specific (napus-rapa)	0.9808612	0.786954	not scorable	not scorable	0.9752066	0.667107
4074	Bn-A02-p27110006	A-genome specific (napus-rapa)	0.9976076	0.7360944	not scorable	not scorable	0.9917355	0.8136193
4075	Bn-A02-p27110104	A-genome specific (rapa only)	not scorable	not scorable	not scorable	not scorable	1	0.8036969
4076	Bn-A02-p27111886	A-genome specific (napus-rapa)	0.9904306	0.705619	not scorable	not scorable	1	0.6223736
4077	Bn-A02-p27111963	A-genome specific (napus-rapa)	0.9856459	0.800321	not scorable	not scorable	0.9834711	0.6771937
4078	Bn-A02-p27121126	A-genome specific (rapa only)	not scorable	not scorable	not scorable	not scorable	0.9917355	0.758953
4079	Bn-A02-p27122935	A-genome specific (napus-rapa)	0.9354067	0.7579655	not scorable	not scorable	0.9586777	0.8869727
4080	Bn-A02-p27124090	A-genome specific (napus-rapa)	0.9928229	0.7467044	not scorable	not scorable	0.9917355	0.7432273
4081	Bn-A02-p27125319	A-genome specific (napus-rapa)	0.9952153	0.7443404	not scorable	not scorable	0.9752066	0.7277436
4082	Bn-A02-p27134251	A-genome specific (rapa only)	not scorable	not scorable	not scorable	not scorable	0.9917355	0.8322234
4083	Bn-A02-p27144	A-genome specific (napus-rapa)	1	0.9454014	not scorable	not scorable	0.9834711	0.9129306
4084	Bn-A02-p27149778	A-genome specific (napus-rapa)	0.9736842	0.6724017	not scorable	not scorable	1	0.7319797
4085	Bn-A02-p27149888	A-genome specific (napus-rapa)	0.9832536	0.760445	not scorable	not scorable	1	0.6918393
4086	Bn-A02-p27158239	A-genome specific (napus-rapa)	1	0.4580872	not scorable	not scorable	0.9917355	0.7487189
4087	Bn-A02-p27158781	A-genome specific (rapa only)	not scorable	not scorable	not scorable	not scorable	0.9752066	0.7163467
4088	Bn-A02-p27171199	A-genome specific (rapa only)	not scorable	not scorable	not scorable	not scorable	0.9834711	0.8014399
4089	Bn-A02-p27172338	A-genome specific (napus-rapa)	0.9330143	0.7801768	not scorable	not scorable	0.9586777	0.7515734

4090	Bn-A02-p27179350	A-genome specific (napus-rapa)	0.9688995	0.8147829	not scorable	not scorable	0.9834711	0.6780698
4091	Bn-A02-p27180049	A-genome specific (napus-rapa)	0.9760765	0.7316093	not scorable	not scorable	0.9752066	0.688991
4092	Bn-A02-p2718786	A-genome specific (rapa only)	not scorable	not scorable	not scorable	not scorable	0.9917355	0.7767615
4093	Bn-A02-p27187948	A-genome specific (rapa only)	not scorable	not scorable	not scorable	not scorable	0.9834711	0.7809755
4094	Bn-A02-p27203061	A-genome specific (napus-rapa)	0.9712918	0.8052812	not scorable	not scorable	0.9752066	0.7218333
4095	Bn-A02-p27215745	A-genome specific (napus-rapa)	0.9856459	0.8049582	not scorable	not scorable	1	0.9024585
4096	Bn-A02-p27220702	A-genome specific (rapa only)	not scorable	not scorable	not scorable	not scorable	0.9752066	0.715555
4097	Bn-A02-p27226579	A-genome specific (napus-rapa)	0.9976076	0.8044042	not scorable	not scorable	0.9917355	0.7104958
4098	Bn-A02-p27245861	A-genome specific (napus-rapa)	0.9808612	0.7580105	not scorable	not scorable	0.9917355	0.7575112
4099	Bn-A02-p27246186	A-genome specific (rapa only)	not scorable	not scorable	not scorable	not scorable	0.9834711	0.9168805
4100	Bn-A02-p27255197	A-genome specific (napus-rapa)	1	0.7989994	not scorable	not scorable	1	0.9175813
4101	Bn-A02-p27262588	A-genome specific (napus-rapa)	0.9784689	0.6537481	not scorable	not scorable	0.9834711	0.6727278
4102	Bn-A02-p2727560	A-genome specific (napus-rapa)	0.9760765	0.6094089	not scorable	not scorable	1	0.6658591
4103	Bn-A02-p27286923	A-genome specific (napus-rapa)	0.9952153	0.6910018	not scorable	not scorable	0.9752066	0.7611907
4104	Bn-A02-p27288011	A-genome specific (napus-rapa)	0.9976076	0.7435452	not scorable	not scorable	0.9834711	0.7656165
4105	Bn-A02-p27288044	A-genome specific (napus-rapa)	0.9976076	0.790016	not scorable	not scorable	0.9917355	0.8122747
4106	Bn-A02-p27288121	A-genome specific (napus-rapa)	0.9976076	0.7893304	not scorable	not scorable	0.9834711	0.7964598
4107	Bn-A02-p27303569	A-genome specific (rapa only)	not scorable	not scorable	not scorable	not scorable	0.5041322	0.8875917
4108	Bn-A02-p27304105	A-genome specific (napus-rapa)	0.9952153	0.7667795	not scorable	not scorable	0.9917355	0.8034995
4109	Bn-A02-p27321599	A-genome specific (napus-rapa)	0.930622	0.3027512	not scorable	not scorable	1	0.7252899
4110	Bn-A02-p27327804	A-genome specific (napus-rapa)	0.9593301	0.4393972	not scorable	not scorable	0.9917355	0.7394917
4111	Bn-A02-p27336483	A-genome specific (napus-rapa)	0.9473684	0.4737795	not scorable	not scorable	0.9752066	0.7756771
4112	Bn-A02-p27358734	A-genome specific (napus-rapa)	0.8708134	0.7463854	not scorable	not scorable	0.9834711	0.7799899
4113	Bn-A02-p27360365	A-genome specific (rapa only)	not scorable	not scorable	not scorable	not scorable	0.9917355	0.7288406
4114	Bn-A02-p2737068	A-genome specific (rapa only)	not scorable	not scorable	not scorable	not scorable	0.5371901	0.7728091
4115	Bn-A02-p2738923	A-genome specific (rapa only)	not scorable	not scorable	not scorable	not scorable	1	0.8204298
4116	Bn-A02-p2739888	A-genome specific (napus-rapa)	1	0.7562031	not scorable	not scorable	0.9917355	0.8115303
4117	Bn-A02-p27399448	A-genome specific (napus-rapa)	0.9808612	0.780218	not scorable	not scorable	0.9917355	0.7127644
4118	Bn-A02-p27403121	A-genome specific (napus-rapa)	0.9976076	0.7450931	not scorable	not scorable	0.9752066	0.7051202
4119	Bn-A02-p27405658	A-genome specific (rapa only)	not scorable	not scorable	not scorable	not scorable	1	0.7221172
4120	Bn-A02-p27408435	A-genome specific (rapa only)	not scorable	not scorable	not scorable	not scorable	0.9917355	0.7757141
4121	Bn-A02-p27414529	A-genome specific (rapa only)	not scorable	not scorable	not scorable	not scorable	0.9917355	0.7387944
4122	Bn-A02-p27414794	A-genome specific (rapa only)	not scorable	not scorable	not scorable	not scorable	0.9917355	0.7883318

4123	Bn-A02-p27420743	A-genome specific (rapa only)	not scorable	not scorable	not scorable	not scorable	0.9669421	0.7712587
4124	Bn-A02-p27420799	A-genome specific (rapa only)	not scorable	not scorable	not scorable	not scorable	0.9834711	0.698632
4125	Bn-A02-p27427939	A-genome specific (rapa only)	not scorable	not scorable	not scorable	not scorable	1	0.9039531
4126	Bn-A02-p27433275	A-genome specific (rapa only)	not scorable	not scorable	not scorable	not scorable	1	0.9175813
4127	Bn-A02-p27450764	A-genome specific (napus-rapa)	1	0.3825184	not scorable	not scorable	0.9834711	0.5923205
4128	Bn-A02-p27490396	A-genome specific (napus-rapa)	0.9952153	0.3973916	not scorable	not scorable	0.9834711	0.8189586
4129	Bn-A02-p27503846	A-genome specific (napus-rapa)	0.9976076	0.6860216	not scorable	not scorable	1	0.8237322
4130	Bn-A02-p27504018	A-genome specific (napus-rapa)	1	0.3064729	not scorable	not scorable	0.9917355	0.7062199
4131	Bn-A02-p27514432	all not scorable	not scorable	not scorable	not scorable	not scorable	not scorable	not scorable
4132	Bn-A02-p27514988	A-genome specific (napus-rapa)	0.9952153	0.7789278	not scorable	not scorable	1	0.8208517
4133	Bn-A02-p27515530	A-genome specific (napus-rapa)	0.9856459	0.5207881	not scorable	not scorable	1	0.7389831
4134	Bn-A02-p27525531	A-genome specific (napus-rapa)	0.9952153	0.7512988	not scorable	not scorable	1	0.759341
4135	Bn-A02-p2753403	A-genome specific (napus-rapa)	0.9186603	0.5924847	not scorable	not scorable	0.9917355	0.709433
4136	Bn-A02-p2753972	A-genome specific (napus-rapa)	0.9832536	0.7203191	not scorable	not scorable	0.9917355	0.8249404
4137	Bn-A02-p27565132	A-genome specific (napus-rapa)	0.9904306	0.6114936	not scorable	not scorable	0.9917355	0.7840218
4138	Bn-A02-p27567204	A-genome specific (napus-rapa)	0.9928229	0.7207499	not scorable	not scorable	0.9917355	0.7182376
4139	Bn-A02-p27567264	A-genome specific (napus-rapa)	1	0.4454421	not scorable	not scorable	1	0.8673873
4140	Bn-A02-p27569069	A-genome specific (napus-rapa)	0.9928229	0.7483461	not scorable	not scorable	0.9834711	0.8154327
4141	Bn-A02-p27613710	A-genome specific (napus-rapa)	0.9952153	0.4459811	not scorable	not scorable	0.9834711	0.6928372
4142	Bn-A02-p27623430	A-genome specific (rapa only)	not scorable	not scorable	not scorable	not scorable	0.9917355	0.7893071
4143	Bn-A02-p27625682	A-genome specific (rapa only)	not scorable	not scorable	not scorable	not scorable	0.9917355	0.7201275
4144	Bn-A02-p27633795	A-genome specific (rapa only)	not scorable	not scorable	not scorable	not scorable	0.5206612	0.8931723
4145	Bn-A02-p27645672	A-genome specific (napus-rapa)	0.9736842	0.3794462	not scorable	not scorable	0.9917355	0.7384122
4146	Bn-A02-p27651489	A-genome specific (rapa only)	not scorable	not scorable	not scorable	not scorable	1	0.7450505
4147	Bn-A02-p27653095	A-genome specific (rapa only)	not scorable	not scorable	not scorable	not scorable	0.9917355	0.730344
4148	Bn-A02-p27653419	A-genome specific (rapa only)	not scorable	not scorable	not scorable	not scorable	0.9834711	0.77314
4149	Bn-A02-p276572	A-genome specific (napus-rapa)	1	0.4936258	not scorable	not scorable	0.9834711	0.6660089
4150	Bn-A02-p27679891	A-genome specific (rapa only)	not scorable	not scorable	not scorable	not scorable	0.9504132	0.7439188
4151	Bn-A02-p27679897	A-genome specific (rapa only)	not scorable	not scorable	not scorable	not scorable	1	0.8615742
4152	Bn-A02-p27680472	A-genome specific (napus-rapa)	0.9904306	0.8541971	not scorable	not scorable	1	0.8004208
4153	Bn-A02-p27682762	A-genome specific (napus-rapa)	0.9186603	0.3652399	not scorable	not scorable	1	0.8744652
4154	Bn-A02-p27702997	A-genome specific (rapa only)	not scorable	not scorable	not scorable	not scorable	1	0.8418922
4155	Bn-A02-p27714758	A-genome specific (rapa only)	not scorable	not scorable	not scorable	not scorable	1	0.7429466

4156	Bn-A02-p27743910	A-genome specific (napus-rapa)	0.9449761	0.3921739	not scorable	not scorable	1	0.6640704
4157	Bn-A02-p27749719	A-genome specific (rapa only)	not scorable	not scorable	not scorable	not scorable	1	0.7236234
4158	Bn-A02-p27755843	A-genome specific (napus-rapa)	0.9880383	0.6966766	not scorable	not scorable	0.9338843	0.8427827
4159	Bn-A02-p2776432	A-genome specific (napus-rapa)	0.9808612	0.6893966	not scorable	not scorable	0.9752066	0.7962028
4160	Bn-A02-p2776634	genome unspecific (all in)	0.9832536	0.4275755	0.9844961	0.775808	1	0.8376198
4161	Bn-A02-p27781100	A-genome specific (rapa only)	not scorable	not scorable	not scorable	not scorable	1	0.7988073
4162	Bn-A02-p27786739	A-genome specific (napus-rapa)	0.9784689	0.8302444	not scorable	not scorable	1	0.8284417
4163	Bn-A02-p27792444	A-genome specific (napus-rapa)	0.9856459	0.7039253	not scorable	not scorable	0.9917355	0.7092206
4164	Bn-A02-p27799727	A-genome specific (rapa only)	not scorable	not scorable	not scorable	not scorable	0.9834711	0.812578
4165	Bn-A02-p2782176	A-genome specific (rapa only)	not scorable	not scorable	not scorable	not scorable	0.9752066	0.7574002
4166	Bn-A02-p27831174	A-genome specific (napus-rapa)	0.9856459	0.8409862	not scorable	not scorable	1	0.7349294
4167	Bn-A02-p27833071	A-genome specific (rapa only)	not scorable	not scorable	not scorable	not scorable	1	0.7982418
4168	Bn-A02-p27834072	A-genome specific (rapa only)	not scorable	not scorable	not scorable	not scorable	1	0.7453524
4169	Bn-A02-p2784335	A-genome specific (napus-rapa)	0.9784689	0.6609194	not scorable	not scorable	0.9752066	0.5288004
4170	Bn-A02-p27845279	A-genome specific (rapa only)	not scorable	not scorable	not scorable	not scorable	1	0.765373
4171	Bn-A02-p2785185	napus specific	0.9593301	0.7411431	not scorable	not scorable	not scorable	not scorable
4172	Bn-A02-p2788144	A-genome specific (rapa only)	not scorable	not scorable	not scorable	not scorable	0.9917355	0.7647673
4173	Bn-A02-p2799146	A-genome specific (napus-rapa)	0.9688995	0.7581848	not scorable	not scorable	0.9917355	0.9226131
4174	Bn-A02-p2800227	A-genome specific (napus-rapa)	0.9952153	0.72118	not scorable	not scorable	0.9669421	0.6331613
4175	Bn-A02-p2800579	A-genome specific (napus-rapa)	0.8181818	0.7795267	not scorable	not scorable	0.6033058	0.8494496
4176	Bn-A02-p2800698	all not scorable	not scorable	not scorable	not scorable	not scorable	not scorable	not scorable
4177	Bn-A02-p2817281	A-genome specific (napus-rapa)	0.9784689	0.6710376	not scorable	not scorable	0.9834711	0.7629179
4178	Bn-A02-p2817294	A-genome specific (napus-rapa)	0.9856459	0.7422963	not scorable	not scorable	1	0.7883999
4179	Bn-A02-p2818741	A-genome specific (napus-rapa)	0.9736842	0.750652	not scorable	not scorable	0.9834711	0.6630738
4180	Bn-A02-p284968	A-genome specific (napus-rapa)	0.9952153	0.7675233	not scorable	not scorable	0.9917355	0.6820813
4181	Bn-A02-p2851231	A-genome specific (napus-rapa)	1	0.9235846	not scorable	not scorable	1	0.8193799
4182	Bn-A02-p2867545	A-genome specific (rapa only)	not scorable	not scorable	not scorable	not scorable	0.9917355	0.7483432
4183	Bn-A02-p2877222	A-genome specific (napus-rapa)	0.9760765	0.763966	not scorable	not scorable	0.553719	0.8948171
4184	Bn-A02-p2923889	A-genome specific (napus-rapa)	0.9880383	0.7452393	not scorable	not scorable	0.9917355	0.7043781
4185	Bn-A02-p2927357	A-genome specific (napus-rapa)	0.4760765	0.6279205	not scorable	not scorable	0.9917355	0.6815047
4186	Bn-A02-p2931725	A-genome specific (napus-rapa)	0.9904306	0.7252479	not scorable	not scorable	0.9917355	0.8230082
4187	Bn-A02-p293860	genome unspecific (oleracea-rapa)	not scorable	not scorable	0.9922481	0.6929381	0.9917355	0.736891
4188	Bn-A02-p294984	A-genome specific (napus-rapa)	0.9904306	0.3565961	not scorable	not scorable	0.9917355	0.6810721

4189	Bn-A02-p2962298	A-genome specific (rapa only)	not scorable	not scorable	not scorable	not scorable	0.9917355	0.8164601
4190	Bn-A02-p298761	A-genome specific (rapa only)	not scorable	not scorable	not scorable	not scorable	1	0.809144
4191	Bn-A02-p300727	A-genome specific (napus-rapa)	0.9712918	0.7969199	not scorable	not scorable	0.9834711	0.8792441
4192	Bn-A02-p3011103	A-genome specific (napus-rapa)	0.9904306	0.7613142	not scorable	not scorable	1	0.7414308
4193	Bn-A02-p302582	A-genome specific (napus-rapa)	0.9904306	0.7871293	not scorable	not scorable	0.9917355	0.8073703
4194	Bn-A02-p303085	A-genome specific (napus-rapa)	0.9808612	0.7691188	not scorable	not scorable	1	0.7979436
4195	Bn-A02-p303243	A-genome specific (napus-rapa)	0.9904306	0.7603363	not scorable	not scorable	1	0.7996552
4196	Bn-A02-p303330	A-genome specific (napus-rapa)	0.9832536	0.8243567	not scorable	not scorable	1	0.7496144
4197	Bn-A02-p3033620	A-genome specific (napus-rapa)	0.9760765	0.3926562	not scorable	not scorable	0.9834711	0.6992005
4198	Bn-A02-p303375	A-genome specific (napus-rapa)	0.9880383	0.8044316	not scorable	not scorable	1	0.7909131
4199	Bn-A02-p303515	A-genome specific (napus-rapa)	0.9904306	0.7783655	not scorable	not scorable	0.9917355	0.770577
4200	Bn-A02-p3035603	A-genome specific (napus-rapa)	0.7200957	0.7854255	not scorable	not scorable	1	0.924587
4201	Bn-A02-p304056	A-genome specific (napus-rapa)	0.9952153	0.4231332	not scorable	not scorable	1	0.8353633
4202	Bn-A02-p305007	A-genome specific (napus-rapa)	0.9736842	0.6939866	not scorable	not scorable	0.9917355	0.7499908
4203	Bn-A02-p3050150	A-genome specific (rapa only)	not scorable	not scorable	not scorable	not scorable	1	0.7978084
4204	Bn-A02-p3072415	A-genome specific (rapa only)	not scorable	not scorable	not scorable	not scorable	0.9917355	0.7901576
4205	Bn-A02-p307342	A-genome specific (napus-rapa)	1	0.6154287	not scorable	not scorable	1	0.7980505
4206	Bn-A02-p3074016	A-genome specific (napus-rapa)	0.9808612	0.7776757	not scorable	not scorable	1	0.7513423
4207	Bn-A02-p307657	A-genome specific (napus-rapa)	0.9880383	0.8106052	not scorable	not scorable	0.9917355	0.7432435
4208	Bn-A02-p3078017	A-genome specific (napus-rapa)	1	0.9272014	not scorable	not scorable	1	0.9144938
4209	Bn-A02-p3080150	A-genome specific (napus-rapa)	0.9856459	0.450667	not scorable	not scorable	1	0.7641606
4210	Bn-A02-p308370	A-genome specific (napus-rapa)	0.9856459	0.8209551	not scorable	not scorable	1	0.9156529
4211	Bn-A02-p3085246	A-genome specific (rapa only)	not scorable	not scorable	not scorable	not scorable	0.9834711	0.7977921
4212	Bn-A02-p3092363	all not scorable	not scorable	not scorable	not scorable	not scorable	not scorable	not scorable
4213	Bn-A02-p3092949	all not scorable	not scorable	not scorable	not scorable	not scorable	not scorable	not scorable
4214	Bn-A02-p3096890	A-genome specific (rapa only)	not scorable	not scorable	not scorable	not scorable	0.9338843	0.7577526
4215	Bn-A02-p3121742	A-genome specific (napus-rapa)	0.9880383	0.7164178	not scorable	not scorable	0.9917355	0.7737699
4216	Bn-A02-p3138043	A-genome specific (napus-rapa)	0.9904306	0.7673922	not scorable	not scorable	0.9917355	0.7984076
4217	Bn-A02-p3142416	A-genome specific (rapa only)	not scorable	not scorable	not scorable	not scorable	1	0.7428162
4218	Bn-A02-p3145923	A-genome specific (napus-rapa)	0.9976076	0.8273849	not scorable	not scorable	1	0.8248339
4219	Bn-A02-p3148346	A-genome specific (rapa only)	not scorable	not scorable	not scorable	not scorable	0.9338843	0.7786475
4220	Bn-A02-p3150860	napus specific	0.854067	0.6689566	not scorable	not scorable	not scorable	not scorable
4221	Bn-A02-p3160461	A-genome specific (napus-rapa)	0.9665072	0.6257343	not scorable	not scorable	0.9917355	0.8386027

4222	Bn-A02-p3167622	A-genome specific (napus-rapa)	0.9736842	0.855147	not scorable	not scorable	0.9834711	0.6926764
4223	Bn-A02-p3167673	A-genome specific (rapa only)	not scorable	not scorable	not scorable	not scorable	0.9752066	0.7908163
4224	Bn-A02-p3174	A-genome specific (napus-rapa)	0.8755981	0.7005323	not scorable	not scorable	0.9752066	0.7232825
4225	Bn-A02-p3175610	A-genome specific (napus-rapa)	0.9976076	0.8603156	not scorable	not scorable	1	0.7788567
4226	Bn-A02-p3180162	A-genome specific (napus-rapa)	0.9880383	0.7390934	not scorable	not scorable	0.9752066	0.7269111
4227	Bn-A02-p3181443	A-genome specific (napus-rapa)	0.9784689	0.7715141	not scorable	not scorable	1	0.7705671
4228	Bn-A02-p3183179	A-genome specific (rapa only)	not scorable	not scorable	not scorable	not scorable	0.9586777	0.7391528
4229	Bn-A02-p3190650	A-genome specific (napus-rapa)	0.9736842	0.468041	not scorable	not scorable	0.9834711	0.6957653
4230	Bn-A02-p319267	A-genome specific (napus-rapa)	0.9856459	0.7585956	not scorable	not scorable	1	0.8240879
4231	Bn-A02-p3207085	A-genome specific (napus-rapa)	1	0.8127784	not scorable	not scorable	1	0.8219877
4232	Bn-A02-p3208275	A-genome specific (napus-rapa)	0.9928229	0.8324371	not scorable	not scorable	1	0.776773
4233	Bn-A02-p3214095	A-genome specific (napus-rapa)	0.9641148	0.7986619	not scorable	not scorable	1	0.8148509
4234	Bn-A02-p3214903	A-genome specific (napus-rapa)	0.9880383	0.4980189	not scorable	not scorable	0.9917355	0.8277064
4235	Bn-A02-p3221060	A-genome specific (rapa only)	not scorable	not scorable	not scorable	not scorable	0.9917355	0.7456936
4236	Bn-A02-p3221634	A-genome specific (napus-rapa)	0.9928229	0.7656881	not scorable	not scorable	1	0.6442078
4237	Bn-A02-p3233816	A-genome specific (napus-rapa)	0.9832536	0.7968277	not scorable	not scorable	0.9834711	0.7868679
4238	Bn-A02-p325065	A-genome specific (napus-rapa)	0.9928229	0.8065077	not scorable	not scorable	1	0.8488413
4239	Bn-A02-p325788	A-genome specific (napus-rapa)	0.9904306	0.7758175	not scorable	not scorable	1	0.8708174
4240	Bn-A02-p3260536	A-genome specific (napus-rapa)	0.9880383	0.5524712	not scorable	not scorable	1	0.8296832
4241	Bn-A02-p3281338	A-genome specific (napus-rapa)	0.9808612	0.5225887	not scorable	not scorable	0.9752066	0.776704
4242	Bn-A02-p3281452	A-genome specific (napus-rapa)	0.9904306	0.7788058	not scorable	not scorable	0.9834711	0.7505839
4243	Bn-A02-p3294682	A-genome specific (napus-rapa)	0.2272727	0.8130986	not scorable	not scorable	0.9586777	0.896002
4244	Bn-A02-p3295898	A-genome specific (napus-rapa)	0.9784689	0.8221808	not scorable	not scorable	0.9752066	0.6195815
4245	Bn-A02-p3297592	A-genome specific (napus-rapa)	0.9760765	0.5186864	not scorable	not scorable	0.9917355	0.7758402
4246	Bn-A02-p3299206	A-genome specific (napus-rapa)	0.9904306	0.8332821	not scorable	not scorable	1	0.8194165
4247	Bn-A02-p3300731	A-genome specific (napus-rapa)	0.9928229	0.6305459	not scorable	not scorable	0.9669421	0.7460604
4248	Bn-A02-p3302725	A-genome specific (napus-rapa)	0.9952153	0.8101256	not scorable	not scorable	1	0.9272014
4249	Bn-A02-p3306076	A-genome specific (napus-rapa)	0.9808612	0.7137041	not scorable	not scorable	1	0.7075891
4250	Bn-A02-p3344176	A-genome specific (napus-rapa)	0.9952153	0.7584064	not scorable	not scorable	0.9834711	0.7916382
4251	Bn-A02-p3349026	A-genome specific (napus-rapa)	0.9880383	0.3954459	not scorable	not scorable	1	0.7681049
4252	Bn-A02-p335197	A-genome specific (napus-rapa)	0.9976076	0.7851082	not scorable	not scorable	1	0.8405633
4253	Bn-A02-p3352863	genome unspecific (all in)	0.9928229	0.640462	0.9922481	0.5913485	1	0.7755569
4254	Bn-A02-p3354301	A-genome specific (napus-rapa)	1	0.4468471	not scorable	not scorable	1	0.7238976

4255	Bn-A02-p3361391	A-genome specific (napus-rapa)	1	0.754914	not scorable	not scorable	1	0.7748008
4256	Bn-A02-p337110	A-genome specific (napus-rapa)	0.9856459	0.7886564	not scorable	not scorable	1	0.8232
4257	Bn-A02-p337457	A-genome specific (rapa only)	not scorable	not scorable	not scorable	not scorable	1	0.7973372
4258	Bn-A02-p3374678	A-genome specific (napus-rapa)	0.9880383	0.7693029	not scorable	not scorable	1	0.7105102
4259	Bn-A02-p3380510	A-genome specific (napus-rapa)	0.9904306	0.516042	not scorable	not scorable	0.9834711	0.7625436
4260	Bn-A02-p3398506	A-genome specific (napus-rapa)	0.9976076	0.7494109	not scorable	not scorable	0.9834711	0.7326435
4261	Bn-A02-p3398710	A-genome specific (napus-rapa)	0.9976076	0.5974189	not scorable	not scorable	0.9917355	0.7036746
4262	Bn-A02-p3422005	A-genome specific (rapa only)	not scorable	not scorable	not scorable	not scorable	1	0.7102833
4263	Bn-A02-p3423823	A-genome specific (napus-rapa)	1	0.8228431	not scorable	not scorable	1	0.7282277
4264	Bn-A02-p3427640	A-genome specific (napus-rapa)	0.9952153	0.8528981	not scorable	not scorable	1	0.899518
4265	Bn-A02-p3430069	A-genome specific (napus-rapa)	1	0.7241183	not scorable	not scorable	1	0.7172196
4266	Bn-A02-p3446017	A-genome specific (napus-rapa)	0.9976076	0.6881834	not scorable	not scorable	0.9834711	0.7549419
4267	Bn-A02-p3458391	napus specific	0.9808612	0.2580661	not scorable	not scorable	not scorable	not scorable
4268	Bn-A02-p3478450	A-genome specific (napus-rapa)	0.9976076	0.7733645	not scorable	not scorable	0.5454546	0.8910362
4269	Bn-A02-p3488996	A-genome specific (napus-rapa)	1	0.8129396	not scorable	not scorable	1	0.7840965
4270	Bn-A02-p3489974	A-genome specific (napus-rapa)	0.9832536	0.7442302	not scorable	not scorable	0.6942149	0.6591148
4271	Bn-A02-p3493190	A-genome specific (napus-rapa)	0.9808612	0.2336812	not scorable	not scorable	0.9834711	0.8945882
4272	Bn-A02-p3503647	genome unspecific (oleracea-rapa)	not scorable	not scorable	0.9689922	0.8445874	0.9917355	0.8168836
4273	Bn-A02-p3507057	genome unspecific (all in)	0.9043062	0.2368324	0.9844961	0.3313471	1	0.7367962
4274	Bn-A02-p3530703	A-genome specific (napus-rapa)	0.9401914	0.6041773	not scorable	not scorable	0.9669421	0.7302511
4275	Bn-A02-p3534963	A-genome specific (napus-rapa)	0.9928229	0.7801194	not scorable	not scorable	1	0.804667
4276	Bn-A02-p3535142	A-genome specific (rapa only)	not scorable	not scorable	not scorable	not scorable	1	0.8456472
4277	Bn-A02-p3539297	A-genome specific (napus-rapa)	0.9952153	0.6513321	not scorable	not scorable	1	0.772202
4278	Bn-A02-p3539377	A-genome specific (napus-rapa)	1	0.7857051	not scorable	not scorable	1	0.8352838
4279	Bn-A02-p3542024	A-genome specific (napus-rapa)	0.9976076	0.825833	not scorable	not scorable	0.9834711	0.7103617
4280	Bn-A02-p3543810	A-genome specific (napus-rapa)	0.9928229	0.7002599	not scorable	not scorable	0.9834711	0.786476
4281	Bn-A02-p3558502	A-genome specific (napus-rapa)	0.9904306	0.521555	not scorable	not scorable	1	0.9175813
4282	Bn-A02-p3571230	A-genome specific (napus-rapa)	0.9880383	0.7158828	not scorable	not scorable	0.9917355	0.6966808
4283	Bn-A02-p3597721	A-genome specific (napus-rapa)	0.9330143	0.7997871	not scorable	not scorable	0.9669421	0.6885561
4284	Bn-A02-p3606635	A-genome specific (napus-rapa)	1	0.4585992	not scorable	not scorable	0.9752066	0.7800079
4285	Bn-A02-p3607564	A-genome specific (napus-rapa)	0.9976076	0.7771602	not scorable	not scorable	1	0.7457581
4286	Bn-A02-p3614673	A-genome specific (napus-rapa)	0.9928229	0.6678221	not scorable	not scorable	0.9752066	0.7443328
4287	Bn-A02-p3627856	A-genome specific (rapa only)	not scorable	not scorable	not scorable	not scorable	1	0.8051801

4288	Bn-A02-p3633790	A-genome specific (napus-rapa)	0.9904306	0.7093559	not scorable	not scorable	0.9917355	0.6521871
4289	Bn-A02-p3635114	A-genome specific (napus-rapa)	0.9904306	0.4526619	not scorable	not scorable	1	0.7277146
4290	Bn-A02-p3640490	A-genome specific (napus-rapa)	1	0.7649073	not scorable	not scorable	0.9917355	0.4554196
4291	Bn-A02-p3656969	A-genome specific (napus-rapa)	1	0.7190414	not scorable	not scorable	1	0.7217764
4292	Bn-A02-p3658139	A-genome specific (napus-rapa)	1	0.7561354	not scorable	not scorable	0.9834711	0.6945941
4293	Bn-A02-p3679028	A-genome specific (rapa only)	not scorable	not scorable	not scorable	not scorable	1	0.7210601
4294	Bn-A02-p3708263	A-genome specific (napus-rapa)	0.9952153	0.8437808	not scorable	not scorable	1	0.8158038
4295	Bn-A02-p3711684	A-genome specific (napus-rapa)	0.9976076	0.7873632	not scorable	not scorable	0.9752066	0.6790326
4296	Bn-A02-p3716898	A-genome specific (napus-rapa)	0.9976076	0.7628529	not scorable	not scorable	0.9752066	0.7966264
4297	Bn-A02-p3717426	A-genome specific (napus-rapa)	0.9760765	0.7119119	not scorable	not scorable	1	0.8830408
4298	Bn-A02-p3718340	A-genome specific (napus-rapa)	0.9928229	0.7781079	not scorable	not scorable	0.9917355	0.7873951
4299	Bn-A02-p3728882	A-genome specific (napus-rapa)	0.9976076	0.9352732	not scorable	not scorable	0.9752066	0.6932694
4300	Bn-A02-p3730794	A-genome specific (napus-rapa)	0.9976076	0.7651848	not scorable	not scorable	0.9752066	0.5937151
4301	Bn-A02-p3735101	A-genome specific (napus-rapa)	0.9928229	0.5343648	not scorable	not scorable	0.9834711	0.6856036
4302	Bn-A02-p3742509	A-genome specific (napus-rapa)	1	0.7804589	not scorable	not scorable	0.9917355	0.8533995
4303	Bn-A02-p3759462	A-genome specific (napus-rapa)	0.9976076	0.8623892	not scorable	not scorable	1	0.8057495
4304	Bn-A02-p3760177	A-genome specific (napus-rapa)	0.9952153	0.7505469	not scorable	not scorable	0.9917355	0.7195768
4305	Bn-A02-p3760247	A-genome specific (napus-rapa)	1	0.7479872	not scorable	not scorable	0.9917355	0.6816152
4306	Bn-A02-p3774623	A-genome specific (napus-rapa)	1	0.5972399	not scorable	not scorable	1	0.5904108
4307	Bn-A02-p3781878	A-genome specific (napus-rapa)	0.9952153	0.5107217	not scorable	not scorable	1	0.7023802
4308	Bn-A02-p3792136	A-genome specific (rapa only)	not scorable	not scorable	not scorable	not scorable	1	0.7644401
4309	Bn-A02-p3796695	napus specific	0.9976076	0.8089832	not scorable	not scorable	not scorable	not scorable
4310	Bn-A02-p379918	A-genome specific (napus-rapa)	0.9880383	0.6593289	not scorable	not scorable	1	0.7607631
4311	Bn-A02-p3801428	genome unspecific (oleracea-rapa)	not scorable	not scorable	1	0.7420871	1	0.7255807
4312	Bn-A02-p3803198	A-genome specific (napus-rapa)	0.9904306	0.7752225	not scorable	not scorable	0.9834711	0.8004208
4313	Bn-A02-p3803477	A-genome specific (napus-rapa)	0.9976076	0.7706614	not scorable	not scorable	0.9917355	0.9175813
4314	Bn-A02-p3807577	A-genome specific (rapa only)	not scorable	not scorable	not scorable	not scorable	0.9752066	0.7975124
4315	Bn-A02-p3809970	A-genome specific (rapa only)	not scorable	not scorable	not scorable	not scorable	1	0.6843715
4316	Bn-A02-p3810028	A-genome specific (rapa only)	not scorable	not scorable	not scorable	not scorable	1	0.8380908
4317	Bn-A02-p3810078	genome unspecific (oleracea-rapa)	not scorable	not scorable	0.9844961	0.7952663	1	0.8158219
4318	Bn-A02-p3821814	A-genome specific (rapa only)	not scorable	not scorable	not scorable	not scorable	0.9669421	0.6861063
4319	Bn-A02-p3821944	A-genome specific (napus-rapa)	0.9904306	0.6041161	not scorable	not scorable	0.9669421	0.6444383
4320	Bn-A02-p3822985	A-genome specific (napus-rapa)	0.9258373	0.7677257	not scorable	not scorable	0.9752066	0.6591173

4321	Bn-A02-p3859399	A-genome specific (napus-rapa)	0.9497608	0.6619011	not scorable	not scorable	1	0.8032478
4322	Bn-A02-p3860483	A-genome specific (napus-rapa)	0.9904306	0.7303491	not scorable	not scorable	0.9834711	0.7451994
4323	Bn-A02-p38907	A-genome specific (napus-rapa)	0.8301435	0.622017	not scorable	not scorable	0.9917355	0.7852495
4324	Bn-A02-p3897213	A-genome specific (napus-rapa)	0.9976076	0.7561886	not scorable	not scorable	0.9917355	0.7629454
4325	Bn-A02-p3897546	A-genome specific (napus-rapa)	0.9976076	0.5592635	not scorable	not scorable	0.9917355	0.6194165
4326	Bn-A02-p3898766	A-genome specific (rapa only)	not scorable	not scorable	not scorable	not scorable	1	0.7308679
4327	Bn-A02-p3914269	A-genome specific (napus-rapa)	1	0.6252947	not scorable	not scorable	1	0.7368252
4328	Bn-A02-p3934729	A-genome specific (napus-rapa)	0.9952153	0.8670811	not scorable	not scorable	1	0.6852039
4329	Bn-A02-p393636	A-genome specific (napus-rapa)	0.9952153	0.4601662	not scorable	not scorable	0.9917355	0.6525966
4330	Bn-A02-p3950581	A-genome specific (napus-rapa)	0.777512	0.7868301	not scorable	not scorable	0.9917355	0.7088592
4331	Bn-A02-p3965286	A-genome specific (rapa only)	not scorable	not scorable	not scorable	not scorable	0.9669421	0.3925121
4332	Bn-A02-p39778	A-genome specific (napus-rapa)	0.8516746	0.8284452	not scorable	not scorable	0.9917355	0.6835809
4333	Bn-A02-p3978921	A-genome specific (rapa only)	not scorable	not scorable	not scorable	not scorable	0.9834711	0.563055
4334	Bn-A02-p39833	A-genome specific (napus-rapa)	0.84689	0.8048167	not scorable	not scorable	1	0.7941261
4335	Bn-A02-p4035808	A-genome specific (napus-rapa)	0.9904306	0.790561	not scorable	not scorable	1	0.777971
4336	Bn-A02-p4037028	A-genome specific (napus-rapa)	0.9497608	0.7303379	not scorable	not scorable	0.9504132	0.5240539
4337	Bn-A02-p4038224	A-genome specific (napus-rapa)	0.9760765	0.768791	not scorable	not scorable	0.9917355	0.5708975
4338	Bn-A02-p4044933	A-genome specific (napus-rapa)	0.9784689	0.4838615	not scorable	not scorable	1	0.7535345
4339	Bn-A02-p4045217	A-genome specific (rapa only)	not scorable	not scorable	not scorable	not scorable	1	0.7389865
4340	Bn-A02-p4046982	all not scorable	not scorable	not scorable	not scorable	not scorable	not scorable	not scorable
4341	Bn-A02-p4065755	genome unspecific (oleracea-rapa)	not scorable	not scorable	0.9922481	0.2403945	0.9834711	0.7167103
4342	Bn-A02-p4102727	A-genome specific (napus-rapa)	0.9952153	0.3080755	not scorable	not scorable	0.9421487	0.4265235
4343	Bn-A02-p4103096	A-genome specific (rapa only)	not scorable	not scorable	not scorable	not scorable	0.9834711	0.628665
4344	Bn-A02-p4103158	A-genome specific (rapa only)	not scorable	not scorable	not scorable	not scorable	0.9917355	0.7288734
4345	Bn-A02-p4103543	genome unspecific (all in)	0.9832536	0.3944136	0.8604651	0.8573309	0.9917355	0.7623614
4346	Bn-A02-p4104054	genome unspecific (all in)	0.9856459	0.4846509	1	0.7248943	1	0.775696
4347	Bn-A02-p4110181	A-genome specific (napus-rapa)	0.9880383	0.7696335	not scorable	not scorable	1	0.8103651
4348	Bn-A02-p4110757	A-genome specific (napus-rapa)	0.9952153	0.8184463	not scorable	not scorable	0.9586777	0.8297745
4349	Bn-A02-p4118946	A-genome specific (napus-rapa)	0.8492823	0.7822152	not scorable	not scorable	0.9338843	0.744895
4350	Bn-A02-p4151292	A-genome specific (napus-rapa)	1	0.8317466	not scorable	not scorable	1	0.7899064
4351	Bn-A02-p416231	A-genome specific (napus-rapa)	0.9808612	0.4206856	not scorable	not scorable	1	0.7746207
4352	Bn-A02-p4174594	A-genome specific (rapa only)	not scorable	not scorable	not scorable	not scorable	1	0.8251065
4353	Bn-A02-p4183553	A-genome specific (napus-rapa)	0.9665072	0.7514824	not scorable	not scorable	1	0.701698

4354	Bn-A02-p4199792	A-genome specific (napus-rapa)	0.9928229	0.3374906	not scorable	not scorable	0.9752066	0.7997442
4355	Bn-A02-p4201122	A-genome specific (napus-rapa)	0.9856459	0.7741914	not scorable	not scorable	0.9834711	0.7821755
4356	Bn-A02-p4210188	A-genome specific (rapa only)	not scorable	not scorable	not scorable	not scorable	0.9752066	0.7926081
4357	Bn-A02-p4210810	A-genome specific (napus-rapa)	0.9880383	0.4968298	not scorable	not scorable	0.9917355	0.7129104
4358	Bn-A02-p4220181	A-genome specific (napus-rapa)	0.9808612	0.5292759	not scorable	not scorable	0.9752066	0.6516504
4359	Bn-A02-p4232093	napus specific	0.9976076	0.5522467	not scorable	not scorable	not scorable	not scorable
4360	Bn-A02-p4237047	genome unspecific (all in)	0.9904306	0.2906427	0.9922481	0.3304989	0.9834711	0.6496409
4361	Bn-A02-p4241100	A-genome specific (napus-rapa)	0.9904306	0.6594497	not scorable	not scorable	0.9917355	0.6687542
4362	Bn-A02-p4243717	A-genome specific (napus-rapa)	0.9952153	0.7974913	not scorable	not scorable	0.9834711	0.6770139
4363	Bn-A02-p425861	A-genome specific (napus-rapa)	0.9856459	0.5498161	not scorable	not scorable	0.9834711	0.7188483
4364	Bn-A02-p4259381	A-genome specific (napus-rapa)	0.9856459	0.781203	not scorable	not scorable	0.9917355	0.7498073
4365	Bn-A02-p4262796	A-genome specific (napus-rapa)	0.9784689	0.698232	not scorable	not scorable	1	0.7622395
4366	Bn-A02-p4262907	A-genome specific (napus-rapa)	0.9904306	0.7871943	not scorable	not scorable	1	0.7046912
4367	Bn-A02-p4277649	A-genome specific (napus-rapa)	0.9880383	0.6826527	not scorable	not scorable	0.9917355	0.6763458
4368	Bn-A02-p4290375	A-genome specific (napus-rapa)	0.9832536	0.7800839	not scorable	not scorable	0.9917355	0.8130093
4369	Bn-A02-p4291278	A-genome specific (rapa only)	not scorable	not scorable	not scorable	not scorable	0.9669421	0.6735589
4370	Bn-A02-p4295967	A-genome specific (napus-rapa)	0.9976076	0.8604002	not scorable	not scorable	1	0.8306279
4371	Bn-A02-p4298976	A-genome specific (napus-rapa)	0.9138756	0.8424721	not scorable	not scorable	0.9917355	0.7464924
4372	Bn-A02-p4299085	A-genome specific (napus-rapa)	0.9138756	0.8138258	not scorable	not scorable	0.9917355	0.7331789
4373	Bn-A02-p4299411	A-genome specific (napus-rapa)	0.923445	0.8410183	not scorable	not scorable	1	0.7903213
4374	Bn-A02-p4306196	A-genome specific (napus-rapa)	0.9904306	0.6218811	not scorable	not scorable	0.9917355	0.6430467
4375	Bn-A02-p4308819	A-genome specific (rapa only)	not scorable	not scorable	not scorable	not scorable	0.9752066	0.9273664
4376	Bn-A02-p4343365	A-genome specific (napus-rapa)	1	0.4463344	not scorable	not scorable	0.9917355	0.7953724
4377	Bn-A02-p4345046	A-genome specific (napus-rapa)	0.9736842	0.1926384	not scorable	not scorable	0.9917355	0.6237074
4378	Bn-A02-p4345098	A-genome specific (napus-rapa)	0.9928229	0.3173415	not scorable	not scorable	0.9834711	0.6631392
4379	Bn-A02-p4351893	genome unspecific (all in)	0.9760765	0.5212235	0.9922481	0.7509216	1	0.8744286
4380	Bn-A02-p4352396	A-genome specific (napus-rapa)	0.9952153	0.778622	not scorable	not scorable	1	0.7919223
4381	Bn-A02-p4374253	genome unspecific (all in)	0.9976076	0.3012469	0.8992248	0.7545024	1	0.7554235
4382	Bn-A02-p441751	A-genome specific (napus-rapa)	0.9880383	0.2781225	not scorable	not scorable	0.9917355	0.8054933
4383	Bn-A02-p4488937	A-genome specific (napus-rapa)	0.9736842	0.3653258	not scorable	not scorable	0.9834711	0.8055197
4384	Bn-A02-p4567454	A-genome specific (rapa only)	not scorable	not scorable	not scorable	not scorable	0.9338843	0.6885831
4385	Bn-A02-p4568023	A-genome specific (napus-rapa)	0.9880383	0.8814222	not scorable	not scorable	0.9917355	0.852074
4386	Bn-A02-p459981	A-genome specific (napus-rapa)	0.9952153	0.3350696	not scorable	not scorable	0.9834711	0.7767122

4387	Bn-A02-p4611221	genome unspecific (all in)	0.9880383	0.2152308	1	0.6156929	1	0.7914145
4388	Bn-A02-p463348	A-genome specific (napus-rapa)	0.9952153	0.7979576	not scorable	not scorable	0.9917355	0.7448519
4389	Bn-A02-p4681731	A-genome specific (rapa only)	not scorable	not scorable	not scorable	not scorable	0.9834711	0.788332
4390	Bn-A02-p4698141	A-genome specific (napus-rapa)	0.9976076	0.7886842	not scorable	not scorable	0.9752066	0.8378487
4391	Bn-A02-p4704233	A-genome specific (rapa only)	not scorable	not scorable	not scorable	not scorable	1	0.7932316
4392	Bn-A02-p4712683	A-genome specific (napus-rapa)	0.9904306	0.750589	not scorable	not scorable	0.9917355	0.7666252
4393	Bn-A02-p4716855	A-genome specific (rapa only)	not scorable	not scorable	not scorable	not scorable	0.9917355	0.6470522
4394	Bn-A02-p4721726	A-genome specific (napus-rapa)	0.5191388	0.8085547	not scorable	not scorable	0.9669421	0.7927424
4395	Bn-A02-p4729892	A-genome specific (rapa only)	not scorable	not scorable	not scorable	not scorable	1	0.8375739
4396	Bn-A02-p4744168	A-genome specific (rapa only)	not scorable	not scorable	not scorable	not scorable	0.9917355	0.7469485
4397	Bn-A02-p474724	A-genome specific (napus-rapa)	0.9688995	0.4096408	not scorable	not scorable	0.9421487	0.6735286
4398	Bn-A02-p4747805	A-genome specific (rapa only)	not scorable	not scorable	not scorable	not scorable	1	0.8569115
4399	Bn-A02-p476011	A-genome specific (napus-rapa)	0.9928229	0.4853908	not scorable	not scorable	1	0.7022258
4400	Bn-A02-p4761483	A-genome specific (napus-rapa)	0.9641148	0.6097913	not scorable	not scorable	1	0.6943445
4401	Bn-A02-p4773307	A-genome specific (napus-rapa)	0.9952153	0.9294605	not scorable	not scorable	0.9834711	0.9270522
4402	Bn-A02-p4774095	genome unspecific (all in)	0.9282297	0.4472634	0.9767442	0.71777	0.9834711	0.6755278
4403	Bn-A02-p4774099	genome unspecific (all in)	0.9282297	0.4093938	0.9844961	0.4128196	0.9338843	0.4871291
4404	Bn-A02-p4777053	A-genome specific (napus-rapa)	0.8229665	0.7404048	not scorable	not scorable	0.8760331	0.7252899
4405	Bn-A02-p4779271	A-genome specific (rapa only)	not scorable	not scorable	not scorable	not scorable	1	0.7209949
4406	Bn-A02-p4783596	A-genome specific (napus-rapa)	0.9976076	0.820315	not scorable	not scorable	1	0.7955751
4407	Bn-A02-p4881323	A-genome specific (napus-rapa)	1	0.7337472	not scorable	not scorable	0.9834711	0.7914415
4408	Bn-A02-p488160	A-genome specific (napus-rapa)	0.9904306	0.3373286	not scorable	not scorable	1	0.735561
4409	Bn-A02-p490065	A-genome specific (napus-rapa)	0.9665072	0.3130467	not scorable	not scorable	0.9834711	0.6885369
4410	Bn-A02-p4913845	genome unspecific (oleracea-rapa)	not scorable	not scorable	0.9922481	0.2782879	0.9752066	0.7756873
4411	Bn-A02-p4928982	A-genome specific (napus-rapa)	1	0.6091886	not scorable	not scorable	1	0.6921083
4412	Bn-A02-p500418	A-genome specific (napus-rapa)	0.9880383	0.7193102	not scorable	not scorable	0.9669421	0.6337368
4413	Bn-A02-p501869	A-genome specific (napus-rapa)	0.9880383	0.3425539	not scorable	not scorable	0.9669421	0.7903363
4414	Bn-A02-p5040691	A-genome specific (napus-rapa)	0.9593301	0.3963282	not scorable	not scorable	1	0.7677222
4415	Bn-A02-p5063790	A-genome specific (napus-rapa)	0.923445	0.4887169	not scorable	not scorable	1	0.5706043
4416	Bn-A02-p510727	A-genome specific (napus-rapa)	0.9976076	0.7427027	not scorable	not scorable	0.9586777	0.6744213
4417	Bn-A02-p5144074	A-genome specific (napus-rapa)	1	0.811515	not scorable	not scorable	1	0.894883
4418	Bn-A02-p5166772	A-genome specific (rapa only)	not scorable	not scorable	not scorable	not scorable	1	0.693492
4419	Bn-A02-p518958	A-genome specific (napus-rapa)	0.9976076	0.7830816	not scorable	not scorable	0.9669421	0.7571258

4420	Bn-A02-p5201568	A-genome specific (napus-rapa)	0.9473684	0.3404023	not scorable	not scorable	0.9752066	0.7629492
4421	Bn-A02-p5242706	A-genome specific (napus-rapa)	0.9521531	0.2572013	not scorable	not scorable	0.9917355	0.6547562
4422	Bn-A02-p5271340	genome unspecific (oleracea-rapa)	not scorable	not scorable	0.9922481	0.4320933	0.9834711	0.6876038
4423	Bn-A02-p5304300	A-genome specific (rapa only)	not scorable	not scorable	not scorable	not scorable	1	0.8477979
4424	Bn-A02-p5308168	A-genome specific (rapa only)	not scorable	not scorable	not scorable	not scorable	1	0.7675935
4425	Bn-A02-p5308657	A-genome specific (rapa only)	not scorable	not scorable	not scorable	not scorable	0.9752066	0.8052266
4426	Bn-A02-p5325556	A-genome specific (napus-rapa)	0.9976076	0.896828	not scorable	not scorable	1	0.9093074
4427	Bn-A02-p5326021	A-genome specific (napus-rapa)	0.9784689	0.9216133	not scorable	not scorable	0.8760331	0.9083843
4428	Bn-A02-p5326222	A-genome specific (napus-rapa)	0.9904306	0.6871462	not scorable	not scorable	1	0.6674343
4429	Bn-A02-p5327209	napus specific	0.9808612	0.6934028	not scorable	not scorable	not scorable	not scorable
4430	Bn-A02-p5360273	A-genome specific (rapa only)	not scorable	not scorable	not scorable	not scorable	0.9752066	0.6917183
4431	Bn-A02-p5394958	A-genome specific (napus-rapa)	0.9832536	0.2611039	not scorable	not scorable	1	0.7401002
4432	Bn-A02-p5429003	A-genome specific (napus-rapa)	0.9928229	0.619529	not scorable	not scorable	0.9917355	0.6574569
4433	Bn-A02-p5436943	A-genome specific (napus-rapa)	0.9904306	0.9396824	not scorable	not scorable	1	0.9205657
4434	Bn-A02-p5439746	A-genome specific (rapa only)	not scorable	not scorable	not scorable	not scorable	0.9917355	0.8759143
4435	Bn-A02-p5442618	A-genome specific (rapa only)	not scorable	not scorable	not scorable	not scorable	1	0.6362951
4436	Bn-A02-p545783	A-genome specific (napus-rapa)	0.9976076	0.7253315	not scorable	not scorable	0.9917355	0.7748725
4437	Bn-A02-p548469	A-genome specific (napus-rapa)	1	0.6908416	not scorable	not scorable	0.9917355	0.7442268
4438	Bn-A02-p5485978	A-genome specific (napus-rapa)	0.9784689	0.3173281	not scorable	not scorable	1	0.854561
4439	Bn-A02-p5500107	A-genome specific (napus-rapa)	0.9401914	0.5539218	not scorable	not scorable	1	0.8589143
4440	Bn-A02-p5501733	A-genome specific (napus-rapa)	0.9904306	0.8083949	not scorable	not scorable	1	0.8550768
4441	Bn-A02-p5507193	A-genome specific (napus-rapa)	0.9904306	0.7308789	not scorable	not scorable	1	0.7412943
4442	Bn-A02-p5507530	A-genome specific (napus-rapa)	0.9928229	0.7874572	not scorable	not scorable	1	0.7433375
4443	Bn-A02-p551481	A-genome specific (napus-rapa)	1	0.8026631	not scorable	not scorable	1	0.7169277
4444	Bn-A02-p5516551	A-genome specific (napus-rapa)	0.9904306	0.4253047	not scorable	not scorable	0.9834711	0.8260332
4445	Bn-A02-p5517878	A-genome specific (napus-rapa)	0.6076555	0.7627512	not scorable	not scorable	0.892562	0.788415
4446	Bn-A02-p5519388	A-genome specific (napus-rapa)	0.9760765	0.7563108	not scorable	not scorable	0.9834711	0.7393679
4447	Bn-A02-p5519417	A-genome specific (napus-rapa)	0.9808612	0.7604249	not scorable	not scorable	0.9917355	0.8382361
4448	Bn-A02-p552098	A-genome specific (rapa only)	not scorable	not scorable	not scorable	not scorable	0.9834711	0.7012425
4449	Bn-A02-p5541204	A-genome specific (napus-rapa)	0.9904306	0.7818286	not scorable	not scorable	0.9917355	0.7302324
4450	Bn-A02-p5551332	A-genome specific (napus-rapa)	0.9736842	0.8041474	not scorable	not scorable	0.9917355	0.8230613
4451	Bn-A02-p5553077	A-genome specific (napus-rapa)	0.9904306	0.8060101	not scorable	not scorable	1	0.7995954
4452	Bn-A02-p5553663	genome unspecific (oleracea-rapa)	not scorable	not scorable	0.9767442	0.7221775	0.9917355	0.7603016

4453	Bn-A02-p5561369	A-genome specific (rapa only)	not scorable	not scorable	not scorable	not scorable	0.9752066	0.8170031
4454	Bn-A02-p5570144	A-genome specific (napus-rapa)	0.9808612	0.5246951	not scorable	not scorable	1	0.7487973
4455	Bn-A02-p5571981	A-genome specific (napus-rapa)	0.9760765	0.7667593	not scorable	not scorable	0.9752066	0.7836084
4456	Bn-A02-p5574727	A-genome specific (napus-rapa)	0.9784689	0.7808352	not scorable	not scorable	0.9834711	0.6910924
4457	Bn-A02-p557523	napus specific	0.9066986	0.8211706	not scorable	not scorable	not scorable	not scorable
4458	Bn-A02-p5582861	A-genome specific (napus-rapa)	0.9856459	0.7693852	not scorable	not scorable	0.9917355	0.7902237
4459	Bn-A02-p5592678	A-genome specific (rapa only)	not scorable	not scorable	not scorable	not scorable	0.9752066	0.7212086
4460	Bn-A02-p5601991	A-genome specific (napus-rapa)	0.9928229	0.7301419	not scorable	not scorable	0.9917355	0.7443858
4461	Bn-A02-p5602271	A-genome specific (napus-rapa)	0.9760765	0.3972452	not scorable	not scorable	1	0.7543278
4462	Bn-A02-p5604926	all not scorable	not scorable	not scorable	not scorable	not scorable	not scorable	not scorable
4463	Bn-A02-p5612147	all not scorable	not scorable	not scorable	not scorable	not scorable	not scorable	not scorable
4464	Bn-A02-p561309	A-genome specific (napus-rapa)	0.9928229	0.8494334	not scorable	not scorable	0.9834711	0.7867317
4465	Bn-A02-p5625172	A-genome specific (rapa only)	not scorable	not scorable	not scorable	not scorable	1	0.7344813
4466	Bn-A02-p562671	A-genome specific (napus-rapa)	0.9928229	0.685403	not scorable	not scorable	0.9834711	0.7060453
4467	Bn-A02-p5628094	genome unspecific (all in)	0.9808612	0.3281028	0.9302326	0.7890494	1	0.7906294
4468	Bn-A02-p5628473	A-genome specific (napus-rapa)	0.9569378	0.4606788	not scorable	not scorable	0.9917355	0.7612749
4469	Bn-A02-p5628535	A-genome specific (rapa only)	not scorable	not scorable	not scorable	not scorable	1	0.7258791
4470	Bn-A02-p5640408	A-genome specific (napus-rapa)	0.9856459	0.6717374	not scorable	not scorable	0.7851239	0.8105487
4471	Bn-A02-p5644858	A-genome specific (rapa only)	not scorable	not scorable	not scorable	not scorable	0.9421487	0.7434226
4472	Bn-A02-p5651799	A-genome specific (rapa only)	not scorable	not scorable	not scorable	not scorable	1	0.7480727
4473	Bn-A02-p5660188	A-genome specific (napus-rapa)	0.9952153	0.7504759	not scorable	not scorable	1	0.7823016
4474	Bn-A02-p5661524	A-genome specific (rapa only)	not scorable	not scorable	not scorable	not scorable	1	0.7491434
4475	Bn-A02-p5664842	A-genome specific (rapa only)	not scorable	not scorable	not scorable	not scorable	0.9834711	0.7210259
4476	Bn-A02-p5666475	A-genome specific (rapa only)	not scorable	not scorable	not scorable	not scorable	0.9256198	0.7581453
4477	Bn-A02-p5694092	A-genome specific (rapa only)	not scorable	not scorable	not scorable	not scorable	0.9917355	0.6366179
4478	Bn-A02-p5705619	A-genome specific (rapa only)	not scorable	not scorable	not scorable	not scorable	1	0.8282704
4479	Bn-A02-p5716304	A-genome specific (napus-rapa)	0.8971292	0.6756388	not scorable	not scorable	0.9586777	0.6974347
4480	Bn-A02-p5722550	A-genome specific (rapa only)	not scorable	not scorable	not scorable	not scorable	0.9008265	0.8617846
4481	Bn-A02-p5731499	genome unspecific (all in)	0.9880383	0.3241851	0.9767442	0.7650697	0.9917355	0.69218
4482	Bn-A02-p5743951	A-genome specific (napus-rapa)	0.9976076	0.7768667	not scorable	not scorable	1	0.7128059
4483	Bn-A02-p576204	A-genome specific (napus-rapa)	0.9856459	0.5391067	not scorable	not scorable	0.9917355	0.7723142
4484	Bn-A02-p5765840	A-genome specific (napus-rapa)	0.9976076	0.8604025	not scorable	not scorable	1	0.8159983
4485	Bn-A02-p5772588	A-genome specific (rapa only)	not scorable	not scorable	not scorable	not scorable	1	0.8007826

4486	Bn-A02-p5772793	A-genome specific (napus-rapa)	0.9976076	0.8481009	not scorable	not scorable	1	0.8455975
4487	Bn-A02-p5773812	A-genome specific (napus-rapa)	0.9760765	0.3380639	not scorable	not scorable	0.9834711	0.6019617
4488	Bn-A02-p5774569	A-genome specific (napus-rapa)	0.9736842	0.272102	not scorable	not scorable	1	0.7661864
4489	Bn-A02-p5797724	A-genome specific (rapa only)	not scorable	not scorable	not scorable	not scorable	0.9834711	0.7996386
4490	Bn-A02-p5799113	A-genome specific (napus-rapa)	0.9497608	0.7670333	not scorable	not scorable	1	0.8455574
4491	Bn-A02-p5802612	A-genome specific (napus-rapa)	0.9976076	0.8196452	not scorable	not scorable	0.9669421	0.7364082
4492	Bn-A02-p5802772	A-genome specific (napus-rapa)	0.9545454	0.4164293	not scorable	not scorable	0.9752066	0.6933086
4493	Bn-A02-p5803164	A-genome specific (napus-rapa)	0.9736842	0.7416502	not scorable	not scorable	0.9917355	0.7104843
4494	Bn-A02-p5803432	A-genome specific (rapa only)	not scorable	not scorable	not scorable	not scorable	0.6694215	0.8562906
4495	Bn-A02-p5807792	genome unspecific (oleracea-rapa)	not scorable	not scorable	0.9844961	0.4755484	0.9752066	0.8486922
4496	Bn-A02-p5812506	A-genome specific (napus-rapa)	0.9952153	0.464436	not scorable	not scorable	0.9917355	0.6618773
4497	Bn-A02-p5814583	A-genome specific (napus-rapa)	0.9976076	0.9205657	not scorable	not scorable	1	0.9175813
4498	Bn-A02-p5826306	A-genome specific (rapa only)	not scorable	not scorable	not scorable	not scorable	1	0.5362331
4499	Bn-A02-p5826505	A-genome specific (napus-rapa)	0.9976076	0.2186389	not scorable	not scorable	0.9834711	0.7754879
4500	Bn-A02-p5826541	A-genome specific (napus-rapa)	1	0.4347724	not scorable	not scorable	1	0.4843079
4501	Bn-A02-p5832870	A-genome specific (napus-rapa)	0.9904306	0.5430917	not scorable	not scorable	0.9917355	0.5658643
4502	Bn-A02-p584200	A-genome specific (napus-rapa)	0.9976076	0.7156122	not scorable	not scorable	1	0.7221863
4503	Bn-A02-p5859447	A-genome specific (napus-rapa)	0.9856459	0.5417457	not scorable	not scorable	0.9834711	0.7634062
4504	Bn-A02-p5866325	A-genome specific (rapa only)	not scorable	not scorable	not scorable	not scorable	0.9917355	0.5073043
4505	Bn-A02-p5867250	A-genome specific (napus-rapa)	0.8732057	0.8548781	not scorable	not scorable	0.8181818	0.7813128
4506	Bn-A02-p588093	A-genome specific (napus-rapa)	0.9976076	0.5081543	not scorable	not scorable	0.9917355	0.7910755
4507	Bn-A02-p5882799	A-genome specific (rapa only)	not scorable	not scorable	not scorable	not scorable	1	0.7054497
4508	Bn-A02-p5907701	A-genome specific (napus-rapa)	0.9952153	0.8019977	not scorable	not scorable	1	0.7940277
4509	Bn-A02-p5917045	A-genome specific (napus-rapa)	0.9928229	0.3213164	not scorable	not scorable	1	0.7545345
4510	Bn-A02-p5923794	A-genome specific (rapa only)	not scorable	not scorable	not scorable	not scorable	0.9834711	0.7477874
4511	Bn-A02-p5923878	A-genome specific (napus-rapa)	0.9952153	0.7679284	not scorable	not scorable	0.9917355	0.701698
4512	Bn-A02-p5924483	A-genome specific (rapa only)	not scorable	not scorable	not scorable	not scorable	0.9752066	0.7249451
4513	Bn-A02-p5925648	A-genome specific (napus-rapa)	0.9928229	0.7785779	not scorable	not scorable	0.9917355	0.7698848
4514	Bn-A02-p5925715	A-genome specific (rapa only)	not scorable	not scorable	not scorable	not scorable	0.9834711	0.7883452
4515	Bn-A02-p5925766	A-genome specific (napus-rapa)	0.9976076	0.8441619	not scorable	not scorable	1	0.8305959
4516	Bn-A02-p5933593	A-genome specific (napus-rapa)	0.9354067	0.547087	not scorable	not scorable	0.9834711	0.7794496
4517	Bn-A02-p5938385	A-genome specific (napus-rapa)	0.9976076	0.8689145	not scorable	not scorable	1	0.8207097
4518	Bn-A02-p594109	A-genome specific (napus-rapa)	0.9952153	0.8526723	not scorable	not scorable	1	0.8321144

4519	Bn-A02-p594700	A-genome specific (napus-rapa)	0.9856459	0.6705382	not scorable	not scorable	0.9917355	0.7121257
4520	Bn-A02-p5958923	A-genome specific (rapa only)	not scorable	not scorable	not scorable	not scorable	1	0.8313413
4521	Bn-A02-p5985314	A-genome specific (napus-rapa)	0.9880383	0.7800914	not scorable	not scorable	0.9834711	0.7874767
4522	Bn-A02-p5985692	A-genome specific (napus-rapa)	0.9928229	0.7644014	not scorable	not scorable	0.9834711	0.8584728
4523	Bn-A02-p5991465	A-genome specific (napus-rapa)	0.9688995	0.7176875	not scorable	not scorable	1	0.7293733
4524	Bn-A02-p6039769	A-genome specific (napus-rapa)	0.9497608	0.6976555	not scorable	not scorable	0.9669421	0.802623
4525	Bn-A02-p6084757	A-genome specific (napus-rapa)	0.9856459	0.7431587	not scorable	not scorable	0.9834711	0.6957084
4526	Bn-A02-p6097441	A-genome specific (napus-rapa)	0.9928229	0.6320357	not scorable	not scorable	0.9917355	0.6976976
4527	Bn-A02-p6099790	A-genome specific (napus-rapa)	0.9784689	0.2118287	not scorable	not scorable	1	0.8377007
4528	Bn-A02-p6100412	genome unspecific (all in)	0.9258373	0.2497963	0.9922481	0.6364007	1	0.8653501
4529	Bn-A02-p6105931	A-genome specific (napus-rapa)	1	0.7914145	not scorable	not scorable	1	0.8793069
4530	Bn-A02-p6147730	A-genome specific (napus-rapa)	0.9808612	0.7141695	not scorable	not scorable	0.9917355	0.6520399
4531	Bn-A02-p6147823	A-genome specific (napus-rapa)	0.9784689	0.6429716	not scorable	not scorable	0.9834711	0.5949283
4532	Bn-A02-p6149358	A-genome specific (rapa only)	not scorable	not scorable	not scorable	not scorable	0.9586777	0.6377702
4533	Bn-A02-p615312	A-genome specific (napus-rapa)	0.9952153	0.3213072	not scorable	not scorable	0.9917355	0.7689164
4534	Bn-A02-p6156818	A-genome specific (napus-rapa)	0.9808612	0.5805482	not scorable	not scorable	0.9917355	0.7837216
4535	Bn-A02-p616247	A-genome specific (napus-rapa)	0.9880383	0.4667118	not scorable	not scorable	1	0.794138
4536	Bn-A02-p6164030	A-genome specific (napus-rapa)	0.9665072	0.619513	not scorable	not scorable	1	0.7757052
4537	Bn-A02-p6166043	A-genome specific (napus-rapa)	0.9952153	0.7389088	not scorable	not scorable	0.9752066	0.6468085
4538	Bn-A02-p6166584	A-genome specific (napus-rapa)	0.9114832	0.807421	not scorable	not scorable	0.9834711	0.7459374
4539	Bn-A02-p6167491	A-genome specific (napus-rapa)	0.9688995	0.5337244	not scorable	not scorable	0.9752066	0.615742
4540	Bn-A02-p6171263	A-genome specific (napus-rapa)	0.9832536	0.3229727	not scorable	not scorable	0.9834711	0.8367396
4541	Bn-A02-p6181965	A-genome specific (napus-rapa)	0.9569378	0.7621058	not scorable	not scorable	0.9834711	0.7694786
4542	Bn-A02-p6182038	A-genome specific (napus-rapa)	0.9641148	0.5949909	not scorable	not scorable	0.9834711	0.7015014
4543	Bn-A02-p6182486	A-genome specific (rapa only)	not scorable	not scorable	not scorable	not scorable	1	0.8397807
4544	Bn-A02-p61866	A-genome specific (napus-rapa)	0.9856459	0.7465323	not scorable	not scorable	0.9917355	0.7017055
4545	Bn-A02-p6202881	A-genome specific (napus-rapa)	0.9880383	0.8191638	not scorable	not scorable	0.9917355	0.7973462
4546	Bn-A02-p6203059	A-genome specific (napus-rapa)	0.9952153	0.7212951	not scorable	not scorable	1	0.7105698
4547	Bn-A02-p6206533	A-genome specific (napus-rapa)	0.9545454	0.7936383	not scorable	not scorable	0.9669421	0.7062147
4548	Bn-A02-p6206785	A-genome specific (napus-rapa)	0.9736842	0.7782666	not scorable	not scorable	0.9752066	0.663608
4549	Bn-A02-p6209563	A-genome specific (napus-rapa)	0.9856459	0.5697475	not scorable	not scorable	0.9752066	0.6660384
4550	Bn-A02-p6233	A-genome specific (napus-rapa)	1	0.5769446	not scorable	not scorable	1	0.8098003
4551	Bn-A02-p6237474	A-genome specific (napus-rapa)	0.9856459	0.8074326	not scorable	not scorable	0.9917355	0.7682201

4552	Bn-A02-p6244013	A-genome specific (napus-rapa)	0.923445	0.6209527	not scorable	not scorable	0.9834711	0.6713664
4553	Bn-A02-p624595	genome unspecific (oleracea-rapa)	not scorable	not scorable	0.9767442	0.2261281	1	0.9095823
4554	Bn-A02-p6252196	A-genome specific (napus-rapa)	0.9928229	0.299654	not scorable	not scorable	1	0.6684769
4555	Bn-A02-p6254332	A-genome specific (rapa only)	not scorable	not scorable	not scorable	not scorable	0.9917355	0.6902054
4556	Bn-A02-p6256499	A-genome specific (napus-rapa)	0.9736842	0.7784863	not scorable	not scorable	0.9834711	0.6970574
4557	Bn-A02-p6265274	A-genome specific (napus-rapa)	0.9880383	0.7995563	not scorable	not scorable	0.9752066	0.755883
4558	Bn-A02-p6267188	A-genome specific (napus-rapa)	0.9617225	0.8089084	not scorable	not scorable	0.7355372	0.7669171
4559	Bn-A02-p6267300	A-genome specific (napus-rapa)	0.9928229	0.7435931	not scorable	not scorable	1	0.7785914
4560	Bn-A02-p6274305	A-genome specific (napus-rapa)	0.9760765	0.7441811	not scorable	not scorable	0.9834711	0.7594367
4561	Bn-A02-p6277812	A-genome specific (napus-rapa)	0.9952153	0.8073602	not scorable	not scorable	0.9917355	0.6898733
4562	Bn-A02-p6280517	napus specific	0.9952153	0.6966712	not scorable	not scorable	not scorable	not scorable
4563	Bn-A02-p6285260	A-genome specific (rapa only)	not scorable	not scorable	not scorable	not scorable	0.5371901	0.7326007
4564	Bn-A02-p6300284	A-genome specific (napus-rapa)	0.9784689	0.7454118	not scorable	not scorable	0.9834711	0.8496284
4565	Bn-A02-p6310054	A-genome specific (napus-rapa)	0.9665072	0.5060724	not scorable	not scorable	0.9834711	0.7148749
4566	Bn-A02-p631297	A-genome specific (napus-rapa)	0.9952153	0.749391	not scorable	not scorable	0.9669421	0.8361734
4567	Bn-A02-p6315194	A-genome specific (napus-rapa)	0.9808612	0.5279381	not scorable	not scorable	1	0.7138557
4568	Bn-A02-p6315978	A-genome specific (napus-rapa)	0.9928229	0.7804682	not scorable	not scorable	1	0.7631087
4569	Bn-A02-p634256	napus specific	0.9736842	0.763501	not scorable	not scorable	not scorable	not scorable
4570	Bn-A02-p6360867	A-genome specific (napus-rapa)	0.9688995	0.3896244	not scorable	not scorable	1	0.7404224
4571	Bn-A02-p636428	A-genome specific (napus-rapa)	0.9928229	0.371872	not scorable	not scorable	1	0.5858629
4572	Bn-A02-p6366262	A-genome specific (napus-rapa)	0.9736842	0.3002524	not scorable	not scorable	0.9834711	0.6362446
4573	Bn-A02-p6373647	A-genome specific (rapa only)	not scorable	not scorable	not scorable	not scorable	0.9752066	0.6891651
4574	Bn-A02-p6375486	A-genome specific (rapa only)	not scorable	not scorable	not scorable	not scorable	0.9917355	0.7289358
4575	Bn-A02-p6384663	A-genome specific (napus-rapa)	0.9569378	0.6048188	not scorable	not scorable	0.9834711	0.6799752
4576	Bn-A02-p6390043	A-genome specific (napus-rapa)	0.7894737	0.6738291	not scorable	not scorable	0.7107438	0.8229404
4577	Bn-A02-p6391751	A-genome specific (napus-rapa)	0.9808612	0.7732618	not scorable	not scorable	0.9834711	0.8046186
4578	Bn-A02-p6392450	A-genome specific (napus-rapa)	0.9880383	0.7118236	not scorable	not scorable	1	0.7200636
4579	Bn-A02-p6399820	A-genome specific (napus-rapa)	0.9784689	0.3577301	not scorable	not scorable	0.9917355	0.819568
4580	Bn-A02-p6439295	A-genome specific (napus-rapa)	0.8732057	0.7733414	not scorable	not scorable	0.6859504	0.7693124
4581	Bn-A02-p6439449	A-genome specific (napus-rapa)	0.8779904	0.8220137	not scorable	not scorable	0.7603306	0.744128
4582	Bn-A02-p6440070	A-genome specific (napus-rapa)	0.8755981	0.8131681	not scorable	not scorable	0.7107438	0.7444963
4583	Bn-A02-p6441318	A-genome specific (napus-rapa)	0.8755981	0.8652211	not scorable	not scorable	0.7603306	0.8172553
4584	Bn-A02-p6461773	A-genome specific (napus-rapa)	0.9976076	0.6988143	not scorable	not scorable	1	0.8354879

4585	Bn-A02-p647024	A-genome specific (napus-rapa)	0.9282297	0.7713887	not scorable	not scorable	0.9586777	0.7350385
4586	Bn-A02-p6473898	A-genome specific (napus-rapa)	0.9976076	0.7752768	not scorable	not scorable	1	0.8413565
4587	Bn-A02-p6474102	A-genome specific (napus-rapa)	0.9904306	0.5423152	not scorable	not scorable	0.9917355	0.7640417
4588	Bn-A02-p6476748	A-genome specific (napus-rapa)	0.9736842	0.358921	not scorable	not scorable	0.9834711	0.6330484
4589	Bn-A02-p647706	A-genome specific (napus-rapa)	0.9904306	0.4872692	not scorable	not scorable	0.9504132	0.6511233
4590	Bn-A02-p6478309	A-genome specific (napus-rapa)	0.9928229	0.6101801	not scorable	not scorable	0.9586777	0.6377709
4591	Bn-A02-p6483805	A-genome specific (napus-rapa)	0.9425837	0.5696764	not scorable	not scorable	0.9917355	0.7122976
4592	Bn-A02-p6500009	A-genome specific (napus-rapa)	0.9354067	0.2619227	not scorable	not scorable	0.9752066	0.6501539
4593	Bn-A02-p65078	A-genome specific (napus-rapa)	0.9952153	0.6668917	not scorable	not scorable	0.9834711	0.8454812
4594	Bn-A02-p6523411	A-genome specific (napus-rapa)	0.9784689	0.2798371	not scorable	not scorable	0.9917355	0.7663219
4595	Bn-A02-p6523609	genome unspecific (all in)	0.9808612	0.5341781	1	0.6987798	0.9917355	0.63166
4596	Bn-A02-p652623	A-genome specific (napus-rapa)	0.9784689	0.3485759	not scorable	not scorable	1	0.7472227
4597	Bn-A02-p6555729	A-genome specific (napus-rapa)	0.9856459	0.3686316	not scorable	not scorable	1	0.8027746
4598	Bn-A02-p655765	A-genome specific (napus-rapa)	0.9880383	0.5550263	not scorable	not scorable	0.9917355	0.7321919
4599	Bn-A02-p6564856	A-genome specific (napus-rapa)	0.9952153	0.6878751	not scorable	not scorable	1	0.7124805
4600	Bn-A02-p6564861	A-genome specific (napus-rapa)	0.9952153	0.8035287	not scorable	not scorable	1	0.7157857
4601	Bn-A02-p6570881	A-genome specific (napus-rapa)	0.9928229	0.7494606	not scorable	not scorable	1	0.6830564
4602	Bn-A02-p6576389	A-genome specific (napus-rapa)	0.9928229	0.4598082	not scorable	not scorable	0.5206612	0.7139429
4603	Bn-A02-p6576872	A-genome specific (napus-rapa)	0.9784689	0.2617305	not scorable	not scorable	0.5123967	0.7275748
4604	Bn-A02-p6577065	A-genome specific (napus-rapa)	0.8947368	0.8137456	not scorable	not scorable	0.5041322	0.8098675
4605	Bn-A02-p6578877	A-genome specific (napus-rapa)	0.9976076	0.8984276	not scorable	not scorable	0.9752066	0.7803598
4606	Bn-A02-p6584506	A-genome specific (napus-rapa)	0.9952153	0.5712456	not scorable	not scorable	1	0.6940579
4607	Bn-A02-p6587201	A-genome specific (napus-rapa)	0.9928229	0.8060136	not scorable	not scorable	0.9752066	0.7849017
4608	Bn-A02-p6615821	A-genome specific (napus-rapa)	0.9976076	0.721914	not scorable	not scorable	0.9917355	0.7995842
4609	Bn-A02-p6627382	A-genome specific (napus-rapa)	0.9976076	0.6875388	not scorable	not scorable	1	0.7181782
4610	Bn-A02-p6633	A-genome specific (napus-rapa)	0.9856459	0.4457311	not scorable	not scorable	1	0.8392644
4611	Bn-A02-p6653554	A-genome specific (napus-rapa)	0.9856459	0.6781244	not scorable	not scorable	1	0.774173
4612	Bn-A02-p6658392	A-genome specific (napus-rapa)	0.9784689	0.7642959	not scorable	not scorable	0.9917355	0.8067613
4613	Bn-A02-p6689427	A-genome specific (napus-rapa)	0.9784689	0.4580777	not scorable	not scorable	1	0.7765206
4614	Bn-A02-p6692347	A-genome specific (napus-rapa)	0.9904306	0.7958043	not scorable	not scorable	0.9917355	0.7130685
4615	Bn-A02-p6697045	A-genome specific (napus-rapa)	0.9760765	0.3930667	not scorable	not scorable	1	0.7876993
4616	Bn-A02-p6711579	A-genome specific (napus-rapa)	1	0.7776538	not scorable	not scorable	1	0.9272014
4617	Bn-A02-p6711900	A-genome specific (napus-rapa)	0.9880383	0.3324243	not scorable	not scorable	0.9834711	0.777401

4618	Bn-A02-p6751482	A-genome specific (napus-rapa)	0.9952153	0.6591077	not scorable	not scorable	0.9752066	0.6666946
4619	Bn-A02-p6751605	A-genome specific (napus-rapa)	0.9976076	0.7521054	not scorable	not scorable	0.9834711	0.7640524
4620	Bn-A02-p6764001	A-genome specific (napus-rapa)	0.9976076	0.4450783	not scorable	not scorable	0.9917355	0.7753879
4621	Bn-A02-p6766615	A-genome specific (napus-rapa)	0.9952153	0.7420125	not scorable	not scorable	0.9917355	0.68961
4622	Bn-A02-p676719	A-genome specific (napus-rapa)	0.9904306	0.7880341	not scorable	not scorable	1	0.7855743
4623	Bn-A02-p6770000	A-genome specific (rapa only)	not scorable	not scorable	not scorable	not scorable	0.9834711	0.7649528
4624	Bn-A02-p677501	A-genome specific (napus-rapa)	0.9784689	0.4593813	not scorable	not scorable	1	0.7973509
4625	Bn-A02-p6794013	A-genome specific (napus-rapa)	0.9952153	0.3576344	not scorable	not scorable	0.9834711	0.7456623
4626	Bn-A02-p6813	A-genome specific (napus-rapa)	1	0.8794669	not scorable	not scorable	1	0.8527914
4627	Bn-A02-p6816192	A-genome specific (napus-rapa)	0.9952153	0.5480705	not scorable	not scorable	0.9834711	0.793625
4628	Bn-A02-p6816262	A-genome specific (napus-rapa)	0.9976076	0.8376912	not scorable	not scorable	0.9669421	0.7804608
4629	Bn-A02-p6820464	A-genome specific (rapa only)	not scorable	not scorable	not scorable	not scorable	0.9834711	0.693504
4630	Bn-A02-p6839427	A-genome specific (napus-rapa)	0.9856459	0.7948368	not scorable	not scorable	1	0.7325586
4631	Bn-A02-p6843407	A-genome specific (napus-rapa)	0.9641148	0.7362293	not scorable	not scorable	0.9917355	0.7070044
4632	Bn-A02-p6844873	A-genome specific (napus-rapa)	0.9832536	0.7439936	not scorable	not scorable	0.9917355	0.6598505
4633	Bn-A02-p6844903	A-genome specific (napus-rapa)	0.9736842	0.7262095	not scorable	not scorable	0.9752066	0.7188483
4634	Bn-A02-p6845049	A-genome specific (napus-rapa)	0.9760765	0.741873	not scorable	not scorable	0.9752066	0.6872027
4635	Bn-A02-p6845122	A-genome specific (napus-rapa)	0.9808612	0.6641968	not scorable	not scorable	0.9586777	0.6685803
4636	Bn-A02-p6845953	A-genome specific (napus-rapa)	0.9856459	0.8436761	not scorable	not scorable	0.9834711	0.8762286
4637	Bn-A02-p6845958	A-genome specific (napus-rapa)	0.9808612	0.7665581	not scorable	not scorable	1	0.7521828
4638	Bn-A02-p6852756	A-genome specific (napus-rapa)	0.9808612	0.7790608	not scorable	not scorable	0.9752066	0.8439336
4639	Bn-A02-p6859259	genome unspecific (all in)	0.9904306	0.5174167	1	0.7194259	0.9669421	0.7409862
4640	Bn-A02-p6862619	napus specific	0.9712918	0.1894621	not scorable	not scorable	not scorable	not scorable
4641	Bn-A02-p6868939	A-genome specific (rapa only)	not scorable	not scorable	not scorable	not scorable	0.9917355	0.7443218
4642	Bn-A02-p6871477	A-genome specific (rapa only)	not scorable	not scorable	not scorable	not scorable	1	0.7646332
4643	Bn-A02-p6898425	A-genome specific (rapa only)	not scorable	not scorable	not scorable	not scorable	0.9917355	0.7576258
4644	Bn-A02-p6901814	napus specific	0.9545454	0.7065215	not scorable	not scorable	not scorable	not scorable
4645	Bn-A02-p6917044	A-genome specific (napus-rapa)	0.9856459	0.7451756	not scorable	not scorable	0.9834711	0.7902066
4646	Bn-A02-p6930285	A-genome specific (rapa only)	not scorable	not scorable	not scorable	not scorable	1	0.7362921
4647	Bn-A02-p6995796	A-genome specific (napus-rapa)	0.9928229	0.6266125	not scorable	not scorable	0.9834711	0.7795001
4648	Bn-A02-p7004091	A-genome specific (napus-rapa)	0.9856459	0.4681264	not scorable	not scorable	0.5123967	0.7975696
4649	Bn-A02-p7012860	A-genome specific (napus-rapa)	0.9856459	0.7295506	not scorable	not scorable	0.9834711	0.6994348
4650	Bn-A02-p7014257	genome unspecific (all in)	0.9784689	0.2926778	0.9922481	0.7100427	0.9834711	0.7134168

4651	Bn-A02-p7029950	A-genome specific (rapa only)	not scorable	not scorable	not scorable	not scorable	0.9586777	0.7991558
4652	Bn-A02-p7041297	A-genome specific (napus-rapa)	0.9880383	0.3653447	not scorable	not scorable	1	0.7388051
4653	Bn-A02-p7043311	A-genome specific (napus-rapa)	0.9784689	0.7472472	not scorable	not scorable	0.9834711	0.7724172
4654	Bn-A02-p7043901	A-genome specific (napus-rapa)	0.9928229	0.5941372	not scorable	not scorable	0.9834711	0.6892305
4655	Bn-A02-p7056194	A-genome specific (napus-rapa)	0.9856459	0.7829198	not scorable	not scorable	0.9917355	0.7168388
4656	Bn-A02-p7057571	A-genome specific (napus-rapa)	0.9928229	0.813883	not scorable	not scorable	0.9834711	0.7023647
4657	Bn-A02-p7067463	A-genome specific (napus-rapa)	0.9832536	0.6481613	not scorable	not scorable	1	0.8131424
4658	Bn-A02-p709952	A-genome specific (napus-rapa)	0.9976076	0.4975208	not scorable	not scorable	1	0.8378811
4659	Bn-A02-p7104549	A-genome specific (rapa only)	not scorable	not scorable	not scorable	not scorable	1	0.8418266
4660	Bn-A02-p7105435	A-genome specific (napus-rapa)	0.9760765	0.2741919	not scorable	not scorable	0.9917355	0.7189451
4661	Bn-A02-p7138093	A-genome specific (rapa only)	not scorable	not scorable	not scorable	not scorable	0.9752066	0.8211529
4662	Bn-A02-p7138191	A-genome specific (rapa only)	not scorable	not scorable	not scorable	not scorable	0.9917355	0.797233
4663	Bn-A02-p7139447	A-genome specific (napus-rapa)	0.9952153	0.7956669	not scorable	not scorable	0.9917355	0.7349489
4664	Bn-A02-p7147282	genome unspecific (oleracea-rapa)	not scorable	not scorable	1	0.5896284	0.9669421	0.3954763
4665	Bn-A02-p714791	A-genome specific (napus-rapa)	1	0.5002283	not scorable	not scorable	0.9917355	0.7496533
4666	Bn-A02-p7183200	A-genome specific (napus-rapa)	0.9976076	0.4379927	not scorable	not scorable	1	0.8339192
4667	Bn-A02-p7207945	A-genome specific (rapa only)	not scorable	not scorable	not scorable	not scorable	0.9917355	0.7125625
4668	Bn-A02-p7236427	A-genome specific (napus-rapa)	0.4808612	0.8332496	not scorable	not scorable	0.9834711	0.7765396
4669	Bn-A02-p7242957	A-genome specific (rapa only)	not scorable	not scorable	not scorable	not scorable	0.9917355	0.7567341
4670	Bn-A02-p7243236	A-genome specific (napus-rapa)	0.9928229	0.7470311	not scorable	not scorable	0.9752066	0.8084701
4671	Bn-A02-p7245587	A-genome specific (napus-rapa)	1	0.3631712	not scorable	not scorable	0.9917355	0.5879409
4672	Bn-A02-p7245763	A-genome specific (napus-rapa)	0.9880383	0.7696891	not scorable	not scorable	1	0.6945117
4673	Bn-A02-p7250607	A-genome specific (napus-rapa)	0.9114832	0.6063761	not scorable	not scorable	1	0.8160156
4674	Bn-A02-p725112	A-genome specific (napus-rapa)	0.9904306	0.8017733	not scorable	not scorable	0.9917355	0.8002675
4675	Bn-A02-p7260577	A-genome specific (napus-rapa)	0.9784689	0.8253279	not scorable	not scorable	0.7438017	0.9062533
4676	Bn-A02-p7260946	A-genome specific (napus-rapa)	0.9952153	0.7700814	not scorable	not scorable	0.9834711	0.6947733
4677	Bn-A02-p7264855	A-genome specific (napus-rapa)	0.9712918	0.3267059	not scorable	not scorable	1	0.8261173
4678	Bn-A02-p727584	A-genome specific (rapa only)	not scorable	not scorable	not scorable	not scorable	0.9917355	0.6587366
4679	Bn-A02-p7278213	A-genome specific (napus-rapa)	0.9832536	0.3320951	not scorable	not scorable	1	0.7145144
4680	Bn-A02-p7278330	A-genome specific (rapa only)	not scorable	not scorable	not scorable	not scorable	0.9917355	0.8152286
4681	Bn-A02-p7279011	A-genome specific (rapa only)	not scorable	not scorable	not scorable	not scorable	0.661157	0.876044
4682	Bn-A02-p7281627	A-genome specific (napus-rapa)	0.9736842	0.5013642	not scorable	not scorable	1	0.8649814
4683	Bn-A02-p7282115	A-genome specific (napus-rapa)	0.9928229	0.7853137	not scorable	not scorable	1	0.8737969

4684	Bn-A02-p7297184	A-genome specific (napus-rapa)	0.9736842	0.7731378	not scorable	not scorable	0.9008265	0.8804122
4685	Bn-A02-p7306278	A-genome specific (rapa only)	not scorable	not scorable	not scorable	not scorable	1	0.6945083
4686	Bn-A02-p7319800	A-genome specific (napus-rapa)	0.9808612	0.7977852	not scorable	not scorable	0.9917355	0.7964792
4687	Bn-A02-p7324958	A-genome specific (napus-rapa)	0.9976076	0.3081795	not scorable	not scorable	0.9669421	0.7791222
4688	Bn-A02-p7349063	A-genome specific (napus-rapa)	0.9832536	0.6922182	not scorable	not scorable	1	0.7902352
4689	Bn-A02-p7350064	A-genome specific (napus-rapa)	0.9832536	0.8015566	not scorable	not scorable	0.9834711	0.7667254
4690	Bn-A02-p7350199	A-genome specific (napus-rapa)	0.9808612	0.7851732	not scorable	not scorable	0.9917355	0.7564395
4691	Bn-A02-p7351109	A-genome specific (napus-rapa)	0.9354067	0.6073132	not scorable	not scorable	1	0.7789128
4692	Bn-A02-p7351619	A-genome specific (napus-rapa)	0.9928229	0.7625743	not scorable	not scorable	0.9834711	0.7774591
4693	Bn-A02-p7351746	A-genome specific (napus-rapa)	0.9832536	0.7448601	not scorable	not scorable	0.9917355	0.7728441
4694	Bn-A02-p7361744	A-genome specific (napus-rapa)	0.9784689	0.726295	not scorable	not scorable	1	0.8533447
4695	Bn-A02-p7363071	A-genome specific (napus-rapa)	0.9856459	0.7735092	not scorable	not scorable	1	0.8100545
4696	Bn-A02-p7368167	A-genome specific (rapa only)	not scorable	not scorable	not scorable	not scorable	0.9917355	0.721895
4697	Bn-A02-p7371108	A-genome specific (napus-rapa)	0.937799	0.6195772	not scorable	not scorable	0.9917355	0.7371387
4698	Bn-A02-p7396082	A-genome specific (napus-rapa)	0.9688995	0.753009	not scorable	not scorable	1	0.6973409
4699	Bn-A02-p7396400	A-genome specific (rapa only)	not scorable	not scorable	not scorable	not scorable	0.9752066	0.699058
4700	Bn-A02-p7409699	A-genome specific (rapa only)	not scorable	not scorable	not scorable	not scorable	0.9834711	0.4547226
4701	Bn-A02-p7439205	napus specific	0.9186603	0.5750291	not scorable	not scorable	not scorable	not scorable
4702	Bn-A02-p7453258	A-genome specific (napus-rapa)	0.9832536	0.398471	not scorable	not scorable	0.9917355	0.793022
4703	Bn-A02-p745468	A-genome specific (napus-rapa)	1	0.7634817	not scorable	not scorable	1	0.7167994
4704	Bn-A02-p7463965	A-genome specific (napus-rapa)	0.9210526	0.7899924	not scorable	not scorable	0.9586777	0.8533713
4705	Bn-A02-p7464840	A-genome specific (napus-rapa)	0.9521531	0.6051677	not scorable	not scorable	0.9669421	0.7679975
4706	Bn-A02-p7464997	A-genome specific (napus-rapa)	0.9066986	0.6930822	not scorable	not scorable	0.9669421	0.8337642
4707	Bn-A02-p7475028	A-genome specific (napus-rapa)	0.5071771	0.6843799	not scorable	not scorable	0.9669421	0.6100981
4708	Bn-A02-p7476709	A-genome specific (napus-rapa)	0.9880383	0.5378415	not scorable	not scorable	0.9421487	0.7577009
4709	Bn-A02-p7482915	A-genome specific (napus-rapa)	0.9736842	0.8048622	not scorable	not scorable	0.9834711	0.6957147
4710	Bn-A02-p7483107	A-genome specific (napus-rapa)	0.9688995	0.7878407	not scorable	not scorable	1	0.7962533
4711	Bn-A02-p7483376	A-genome specific (rapa only)	not scorable	not scorable	not scorable	not scorable	0.9917355	0.8259604
4712	Bn-A02-p7498192	A-genome specific (napus-rapa)	0.9832536	0.3554854	not scorable	not scorable	0.9917355	0.7371905
4713	Bn-A02-p7510882	A-genome specific (napus-rapa)	0.9856459	0.6959207	not scorable	not scorable	0.9834711	0.7712981
4714	Bn-A02-p7514338	A-genome specific (rapa only)	not scorable	not scorable	not scorable	not scorable	0.9834711	0.7690032
4715	Bn-A02-p7516226	A-genome specific (napus-rapa)	0.9330143	0.6878141	not scorable	not scorable	0.9834711	0.7514516
4716	Bn-A02-p7516732	A-genome specific (napus-rapa)	0.9641148	0.2267042	not scorable	not scorable	0.9917355	0.6486743

4717	Bn-A02-p7520783	A-genome specific (napus-rapa)	0.9784689	0.2833979	not scorable	not scorable	0.9917355	0.64094
4718	Bn-A02-p7525472	A-genome specific (rapa only)	not scorable	not scorable	not scorable	not scorable	0.9586777	0.8314947
4719	Bn-A02-p7531907	A-genome specific (napus-rapa)	0.9976076	0.5109164	not scorable	not scorable	0.9917355	0.84812
4720	Bn-A02-p7534591	A-genome specific (rapa only)	not scorable	not scorable	not scorable	not scorable	0.9917355	0.8015221
4721	Bn-A02-p7575736	A-genome specific (napus-rapa)	0.9760765	0.7653556	not scorable	not scorable	1	0.7466948
4722	Bn-A02-p7589454	A-genome specific (rapa only)	not scorable	not scorable	not scorable	not scorable	0.9669421	0.8385628
4723	Bn-A02-p759031	A-genome specific (napus-rapa)	0.9162679	0.7249252	not scorable	not scorable	0.9421487	0.8133634
4724	Bn-A02-p7595911	A-genome specific (napus-rapa)	0.9784689	0.7827367	not scorable	not scorable	1	0.7086748
4725	Bn-A02-p7597314	A-genome specific (napus-rapa)	0.9880383	0.8045736	not scorable	not scorable	1	0.784313
4726	Bn-A02-p761524	A-genome specific (napus-rapa)	0.9904306	0.8139089	not scorable	not scorable	0.9834711	0.7433274
4727	Bn-A02-p7647776	A-genome specific (napus-rapa)	1	0.520664	not scorable	not scorable	1	0.8241445
4728	Bn-A02-p7675384	A-genome specific (napus-rapa)	0.9904306	0.8122706	not scorable	not scorable	0.9834711	0.7235599
4729	Bn-A02-p7675505	A-genome specific (napus-rapa)	0.9928229	0.5661506	not scorable	not scorable	0.9917355	0.8729188
4730	Bn-A02-p770821	A-genome specific (rapa only)	not scorable	not scorable	not scorable	not scorable	0.9834711	0.6544235
4731	Bn-A02-p771019	A-genome specific (rapa only)	not scorable	not scorable	not scorable	not scorable	0.9586777	0.5612607
4732	Bn-A02-p7712926	A-genome specific (napus-rapa)	0.9401914	0.7799253	not scorable	not scorable	0.9669421	0.7193478
4733	Bn-A02-p771313	A-genome specific (napus-rapa)	0.9760765	0.5494034	not scorable	not scorable	0.9752066	0.6921548
4734	Bn-A02-p771951	A-genome specific (napus-rapa)	0.9976076	0.7461146	not scorable	not scorable	1	0.6980916
4735	Bn-A02-p7736604	A-genome specific (napus-rapa)	0.9832536	0.6744211	not scorable	not scorable	0.9917355	0.7271112
4736	Bn-A02-p7738248	A-genome specific (napus-rapa)	0.9760765	0.7665671	not scorable	not scorable	1	0.8824477
4737	Bn-A02-p7750368	A-genome specific (napus-rapa)	0.9904306	0.5737354	not scorable	not scorable	1	0.8440034
4738	Bn-A02-p7753579	A-genome specific (napus-rapa)	0.9832536	0.8317832	not scorable	not scorable	1	0.6907074
4739	Bn-A02-p7756308	A-genome specific (napus-rapa)	0.9425837	0.7772068	not scorable	not scorable	0.9752066	0.9074243
4740	Bn-A02-p7775332	A-genome specific (napus-rapa)	0.9760765	0.7645133	not scorable	not scorable	0.9834711	0.7020374
4741	Bn-A02-p7784914	A-genome specific (napus-rapa)	0.9952153	0.6933859	not scorable	not scorable	1	0.8234416
4742	Bn-A02-p7786183	A-genome specific (napus-rapa)	0.9808612	0.7772229	not scorable	not scorable	0.9834711	0.7531403
4743	Bn-A02-p7788379	A-genome specific (napus-rapa)	0.9832536	0.3166976	not scorable	not scorable	0.9834711	0.7241945
4744	Bn-A02-p7788840	A-genome specific (napus-rapa)	0.9856459	0.7429016	not scorable	not scorable	1	0.8407756
4745	Bn-A02-p7801111	A-genome specific (napus-rapa)	0.9497608	0.7901652	not scorable	not scorable	1	0.8085507
4746	Bn-A02-p7804291	A-genome specific (napus-rapa)	0.9401914	0.7688072	not scorable	not scorable	0.9752066	0.7591489
4747	Bn-A02-p7812681	A-genome specific (rapa only)	not scorable	not scorable	not scorable	not scorable	1	0.7149075
4748	Bn-A02-p78265	A-genome specific (rapa only)	not scorable	not scorable	not scorable	not scorable	0.9834711	0.8095888
4749	Bn-A02-p7831145	C-genome specific (oleracea only)	not scorable	not scorable	0.9689922	0.4413385	not scorable	not scorable

4750	Bn-A02-p7840077	A-genome specific (rapa only)	not scorable	not scorable	not scorable	not scorable	0.9834711	0.7420868
4751	Bn-A02-p7848143	A-genome specific (napus-rapa)	0.9904306	0.7275792	not scorable	not scorable	0.9834711	0.7327777
4752	Bn-A02-p7853194	A-genome specific (napus-rapa)	0.9928229	0.7486134	not scorable	not scorable	1	0.772261
4753	Bn-A02-p785930	all not scorable	not scorable	not scorable	not scorable	not scorable	not scorable	not scorable
4754	Bn-A02-p7862412	A-genome specific (napus-rapa)	0.9712918	0.3328695	not scorable	not scorable	0.9834711	0.7011391
4755	Bn-A02-p7878229	A-genome specific (rapa only)	not scorable	not scorable	not scorable	not scorable	1	0.8070189
4756	Bn-A02-p7882027	A-genome specific (napus-rapa)	0.6100478	0.8126702	not scorable	not scorable	0.7272727	0.6993932
4757	Bn-A02-p7885731	A-genome specific (napus-rapa)	0.9521531	0.7212026	not scorable	not scorable	0.9917355	0.7851168
4758	Bn-A02-p7886882	napus specific	0.9904306	0.7931737	not scorable	not scorable	not scorable	not scorable
4759	Bn-A02-p7892272	A-genome specific (napus-rapa)	0.9904306	0.8000088	not scorable	not scorable	0.9917355	0.8076818
4760	Bn-A02-p7892561	A-genome specific (napus-rapa)	0.9880383	0.7351797	not scorable	not scorable	0.9504132	0.7372491
4761	Bn-A02-p7893025	A-genome specific (rapa only)	not scorable	not scorable	not scorable	not scorable	0.9917355	0.716031
4762	Bn-A02-p7893461	A-genome specific (rapa only)	not scorable	not scorable	not scorable	not scorable	1	0.7461784
4763	Bn-A02-p7893901	A-genome specific (rapa only)	not scorable	not scorable	not scorable	not scorable	1	0.7789708
4764	Bn-A02-p7894216	A-genome specific (rapa only)	not scorable	not scorable	not scorable	not scorable	0.9917355	0.7923428
4765	Bn-A02-p7894339	A-genome specific (rapa only)	not scorable	not scorable	not scorable	not scorable	0.9917355	0.7945102
4766	Bn-A02-p7896923	A-genome specific (napus-rapa)	0.9784689	0.5332489	not scorable	not scorable	0.9917355	0.7669969
4767	Bn-A02-p7904444	A-genome specific (napus-rapa)	0.9832536	0.793214	not scorable	not scorable	0.9834711	0.7326415
4768	Bn-A02-p7926823	A-genome specific (napus-rapa)	0.9760765	0.743062	not scorable	not scorable	0.9917355	0.7631068
4769	Bn-A02-p7935221	A-genome specific (napus-rapa)	0.9976076	0.3749342	not scorable	not scorable	0.9752066	0.7526984
4770	Bn-A02-p7947982	A-genome specific (napus-rapa)	0.9784689	0.7641205	not scorable	not scorable	0.9752066	0.6839395
4771	Bn-A02-p7952281	A-genome specific (napus-rapa)	0.9928229	0.7124779	not scorable	not scorable	1	0.7197591
4772	Bn-A02-p7956983	A-genome specific (rapa only)	not scorable	not scorable	not scorable	not scorable	0.9752066	0.7352474
4773	Bn-A02-p7958421	A-genome specific (napus-rapa)	0.9904306	0.4171607	not scorable	not scorable	1	0.814585
4774	Bn-A02-p7970283	A-genome specific (napus-rapa)	0.6291866	0.7940232	not scorable	not scorable	0.7355372	0.8040105
4775	Bn-A02-p7975165	A-genome specific (napus-rapa)	0.9952153	0.7289852	not scorable	not scorable	0.9834711	0.7655351
4776	Bn-A02-p7976278	A-genome specific (napus-rapa)	0.9856459	0.5153814	not scorable	not scorable	0.9917355	0.664094
4777	Bn-A02-p7978230	A-genome specific (napus-rapa)	0.9976076	0.4148431	not scorable	not scorable	0.9917355	0.6981266
4778	Bn-A02-p799930	A-genome specific (napus-rapa)	0.9904306	0.6834955	not scorable	not scorable	0.9917355	0.8329262
4779	Bn-A02-p8011131	A-genome specific (napus-rapa)	0.9856459	0.3153768	not scorable	not scorable	1	0.6632591
4780	Bn-A02-p801257	napus specific	0.8516746	0.7581462	not scorable	not scorable	not scorable	not scorable
4781	Bn-A02-p8015745	A-genome specific (rapa only)	not scorable	not scorable	not scorable	not scorable	0.9586777	0.718304
4782	Bn-A02-p8024157	A-genome specific (napus-rapa)	0.9617225	0.7603827	not scorable	not scorable	0.9752066	0.7269179

4783	Bn-A02-p8024784	A-genome specific (napus-rapa)	0.9976076	0.9150075	not scorable	not scorable	1	0.9267293
4784	Bn-A02-p8026465	A-genome specific (napus-rapa)	0.9952153	0.9272014	not scorable	not scorable	1	0.9205657
4785	Bn-A02-p8057234	A-genome specific (napus-rapa)	0.9976076	0.4670551	not scorable	not scorable	0.9834711	0.7954453
4786	Bn-A02-p806025	A-genome specific (napus-rapa)	0.9593301	0.8267122	not scorable	not scorable	0.9338843	0.8624802
4787	Bn-A02-p806949	A-genome specific (napus-rapa)	0.9976076	0.746634	not scorable	not scorable	0.9917355	0.7883527
4788	Bn-A02-p8084362	A-genome specific (napus-rapa)	0.9880383	0.5006534	not scorable	not scorable	1	0.77835
4789	Bn-A02-p8084605	A-genome specific (napus-rapa)	1	0.4267537	not scorable	not scorable	1	0.8605337
4790	Bn-A02-p808711	A-genome specific (napus-rapa)	0.9952153	0.7540577	not scorable	not scorable	1	0.7609124
4791	Bn-A02-p8090196	A-genome specific (napus-rapa)	0.9832536	0.7745731	not scorable	not scorable	0.9752066	0.7508547
4792	Bn-A02-p809627	A-genome specific (napus-rapa)	0.9928229	0.7338062	not scorable	not scorable	0.9917355	0.7338463
4793	Bn-A02-p8111245	A-genome specific (napus-rapa)	0.9497608	0.5355018	not scorable	not scorable	0.9834711	0.6968412
4794	Bn-A02-p8140997	A-genome specific (napus-rapa)	0.9808612	0.7376928	not scorable	not scorable	0.9917355	0.814316
4795	Bn-A02-p8141909	A-genome specific (napus-rapa)	0.9880383	0.6842383	not scorable	not scorable	0.9834711	0.7324694
4796	Bn-A02-p8145676	A-genome specific (napus-rapa)	0.9808612	0.7428272	not scorable	not scorable	1	0.7580712
4797	Bn-A02-p8160522	A-genome specific (napus-rapa)	0.9736842	0.3464864	not scorable	not scorable	1	0.8466807
4798	Bn-A02-p8169424	A-genome specific (napus-rapa)	0.9880383	0.691925	not scorable	not scorable	0.9669421	0.7177985
4799	Bn-A02-p8169665	A-genome specific (napus-rapa)	0.9736842	0.4415961	not scorable	not scorable	0.9834711	0.8673841
4800	Bn-A02-p8176181	A-genome specific (napus-rapa)	0.9736842	0.5366587	not scorable	not scorable	1	0.7941383
4801	Bn-A02-p8177809	A-genome specific (napus-rapa)	0.9976076	0.4444549	not scorable	not scorable	0.9917355	0.7065331
4802	Bn-A02-p8179492	all not scorable	not scorable	not scorable	not scorable	not scorable	not scorable	not scorable
4803	Bn-A02-p8190375	A-genome specific (napus-rapa)	0.9473684	0.7682696	not scorable	not scorable	0.9752066	0.7602542
4804	Bn-A02-p8191099	A-genome specific (napus-rapa)	0.9736842	0.7887865	not scorable	not scorable	0.9586777	0.779614
4805	Bn-A02-p8199891	A-genome specific (napus-rapa)	0.9880383	0.8242561	not scorable	not scorable	0.9917355	0.8053056
4806	Bn-A02-p8200381	A-genome specific (napus-rapa)	0.9856459	0.7659872	not scorable	not scorable	0.9421487	0.7305777
4807	Bn-A02-p8204490	A-genome specific (napus-rapa)	0.9880383	0.5524054	not scorable	not scorable	0.9917355	0.7635288
4808	Bn-A02-p8208117	A-genome specific (napus-rapa)	1	0.8191374	not scorable	not scorable	1	0.7971621
4809	Bn-A02-p8215285	A-genome specific (napus-rapa)	0.9760765	0.8012247	not scorable	not scorable	0.9917355	0.7342598
4810	Bn-A02-p8234333	A-genome specific (napus-rapa)	0.9641148	0.7151495	not scorable	not scorable	0.9669421	0.7437899
4811	Bn-A02-p8234485	A-genome specific (napus-rapa)	0.9449761	0.761075	not scorable	not scorable	0.9752066	0.7008019
4812	Bn-A02-p8234978	A-genome specific (napus-rapa)	0.9736842	0.793328	not scorable	not scorable	0.9917355	0.6924239
4813	Bn-A02-p8235110	A-genome specific (rapa only)	not scorable	not scorable	not scorable	not scorable	0.9834711	0.6876985
4814	Bn-A02-p823586	napus specific	0.9258373	0.7739003	not scorable	not scorable	not scorable	not scorable
4815	Bn-A02-p8243789	A-genome specific (napus-rapa)	0.9593301	0.5693166	not scorable	not scorable	0.9917355	0.7230725

4816	Bn-A02-p8249838	A-genome specific (rapa only)	not scorable	not scorable	not scorable	not scorable	1	0.7638761
4817	Bn-A02-p8250856	A-genome specific (napus-rapa)	0.9928229	0.7187208	not scorable	not scorable	1	0.7286923
4818	Bn-A02-p8251258	A-genome specific (napus-rapa)	0.9952153	0.4134906	not scorable	not scorable	0.9917355	0.7947058
4819	Bn-A02-p8264334	all not scorable	not scorable	not scorable	not scorable	not scorable	not scorable	not scorable
4820	Bn-A02-p8273655	A-genome specific (rapa only)	not scorable	not scorable	not scorable	not scorable	1	0.698265
4821	Bn-A02-p8277939	A-genome specific (napus-rapa)	0.9904306	0.4458942	not scorable	not scorable	1	0.7953373
4822	Bn-A02-p8284992	A-genome specific (napus-rapa)	0.9784689	0.7553542	not scorable	not scorable	0.9917355	0.7771038
4823	Bn-A02-p8285151	A-genome specific (napus-rapa)	0.9808612	0.7415641	not scorable	not scorable	1	0.7416434
4824	Bn-A02-p8289331	A-genome specific (napus-rapa)	0.9952153	0.3183582	not scorable	not scorable	0.9752066	0.7616993
4825	Bn-A02-p8294934	A-genome specific (napus-rapa)	0.9808612	0.7157307	not scorable	not scorable	0.5371901	0.8269897
4826	Bn-A02-p8297807	A-genome specific (napus-rapa)	0.9665072	0.7890869	not scorable	not scorable	1	0.7888252
4827	Bn-A02-p8313782	A-genome specific (napus-rapa)	0.9760765	0.4816065	not scorable	not scorable	1	0.814971
4828	Bn-A02-p8323616	A-genome specific (napus-rapa)	0.9808612	0.8115861	not scorable	not scorable	1	0.7183553
4829	Bn-A02-p8324359	A-genome specific (napus-rapa)	0.9760765	0.7803825	not scorable	not scorable	0.9917355	0.6995066
4830	Bn-A02-p8335312	A-genome specific (napus-rapa)	0.9688995	0.7704459	not scorable	not scorable	0.9917355	0.7934188
4831	Bn-A02-p8336294	A-genome specific (napus-rapa)	0.9641148	0.7805146	not scorable	not scorable	0.9752066	0.7969964
4832	Bn-A02-p8338189	A-genome specific (napus-rapa)	0.9760765	0.6283789	not scorable	not scorable	1	0.7533931
4833	Bn-A02-p8344540	A-genome specific (napus-rapa)	0.9258373	0.7418282	not scorable	not scorable	0.9917355	0.6777365
4834	Bn-A02-p8391383	A-genome specific (napus-rapa)	0.9832536	0.773262	not scorable	not scorable	1	0.6896096
4835	Bn-A02-p8391574	A-genome specific (napus-rapa)	0.9808612	0.7027655	not scorable	not scorable	0.9752066	0.7185681
4836	Bn-A02-p839171	A-genome specific (napus-rapa)	0.9976076	0.3595916	not scorable	not scorable	0.9752066	0.6978591
4837	Bn-A02-p8396864	A-genome specific (napus-rapa)	0.2105263	0.778595	not scorable	not scorable	0.9834711	0.7934849
4838	Bn-A02-p8404663	A-genome specific (rapa only)	not scorable	not scorable	not scorable	not scorable	0.9504132	0.7742965
4839	Bn-A02-p8412911	A-genome specific (napus-rapa)	0.9952153	0.6589787	not scorable	not scorable	0.9917355	0.6743745
4840	Bn-A02-p8413531	A-genome specific (rapa only)	not scorable	not scorable	not scorable	not scorable	1	0.7965445
4841	Bn-A02-p8440451	napus specific	0.8971292	0.7837322	not scorable	not scorable	not scorable	not scorable
4842	Bn-A02-p8443355	A-genome specific (rapa only)	not scorable	not scorable	not scorable	not scorable	1	0.7207681
4843	Bn-A02-p8444558	A-genome specific (rapa only)	not scorable	not scorable	not scorable	not scorable	0.9917355	0.7097507
4844	Bn-A02-p8453414	A-genome specific (napus-rapa)	0.9784689	0.7611449	not scorable	not scorable	1	0.7239645
4845	Bn-A02-p8453695	A-genome specific (napus-rapa)	0.8947368	0.4902489	not scorable	not scorable	0.9504132	0.3262676
4846	Bn-A02-p8466214	A-genome specific (napus-rapa)	0.9736842	0.797366	not scorable	not scorable	0.9834711	0.7368887
4847	Bn-A02-p8471639	all not scorable	not scorable	not scorable	not scorable	not scorable	not scorable	not scorable
4848	Bn-A02-p8472166	A-genome specific (napus-rapa)	0.9736842	0.7792907	not scorable	not scorable	0.9917355	0.7467239

4849	Bn-A02-p8473119	A-genome specific (napus-rapa)	0.9808612	0.720535	not scorable	not scorable	0.9917355	0.7277477
4850	Bn-A02-p847638	A-genome specific (napus-rapa)	1	0.5046297	not scorable	not scorable	0.9917355	0.6686085
4851	Bn-A02-p8481759	A-genome specific (napus-rapa)	0.9760765	0.7173734	not scorable	not scorable	1	0.7015526
4852	Bn-A02-p848731	A-genome specific (napus-rapa)	0.9976076	0.7477041	not scorable	not scorable	0.9917355	0.7712649
4853	Bn-A02-p849524	A-genome specific (napus-rapa)	0.9904306	0.6546049	not scorable	not scorable	0.9917355	0.7267894
4854	Bn-A02-p849709	A-genome specific (rapa only)	not scorable	not scorable	not scorable	not scorable	0.9917355	0.8305345
4855	Bn-A02-p8501267	A-genome specific (napus-rapa)	0.9760765	0.7358354	not scorable	not scorable	1	0.9237478
4856	Bn-A02-p8509407	A-genome specific (napus-rapa)	0.9665072	0.6734825	not scorable	not scorable	1	0.7550055
4857	Bn-A02-p8509694	A-genome specific (rapa only)	not scorable	not scorable	not scorable	not scorable	1	0.7099557
4858	Bn-A02-p8513186	A-genome specific (napus-rapa)	0.9712918	0.7282321	not scorable	not scorable	0.9834711	0.752672
4859	Bn-A02-p8517135	A-genome specific (napus-rapa)	0.8827751	0.7714842	not scorable	not scorable	0.8842975	0.7534347
4860	Bn-A02-p852096	all not scorable	not scorable	not scorable	not scorable	not scorable	not scorable	not scorable
4861	Bn-A02-p8527757	A-genome specific (napus-rapa)	0.9952153	0.5384931	not scorable	not scorable	0.9834711	0.6437333
4862	Bn-A02-p8530142	A-genome specific (napus-rapa)	0.8086125	0.7919436	not scorable	not scorable	0.9834711	0.8904615
4863	Bn-A02-p8532901	A-genome specific (napus-rapa)	0.9330143	0.5372787	not scorable	not scorable	0.9752066	0.7926612
4864	Bn-A02-p8538437	A-genome specific (napus-rapa)	0.9856459	0.6686826	not scorable	not scorable	0.9752066	0.7670333
4865	Bn-A02-p8541841	A-genome specific (napus-rapa)	0.9976076	0.3236063	not scorable	not scorable	0.9917355	0.7523773
4866	Bn-A02-p8549373	A-genome specific (napus-rapa)	0.9545454	0.7367265	not scorable	not scorable	1	0.8335581
4867	Bn-A02-p8549867	A-genome specific (napus-rapa)	0.9760765	0.7863663	not scorable	not scorable	0.9752066	0.8164532
4868	Bn-A02-p8554870	A-genome specific (napus-rapa)	0.9473684	0.2962263	not scorable	not scorable	0.9834711	0.7239497
4869	Bn-A02-p8559435	A-genome specific (napus-rapa)	0.9043062	0.7104662	not scorable	not scorable	0.9752066	0.8039837
4870	Bn-A02-p8560119	A-genome specific (napus-rapa)	0.9617225	0.8069634	not scorable	not scorable	0.9917355	0.7794464
4871	Bn-A02-p8561027	A-genome specific (napus-rapa)	0.9665072	0.8011537	not scorable	not scorable	0.9752066	0.7057483
4872	Bn-A02-p8561181	A-genome specific (napus-rapa)	0.5789474	0.7616862	not scorable	not scorable	0.9834711	0.824221
4873	Bn-A02-p8567245	A-genome specific (rapa only)	not scorable	not scorable	not scorable	not scorable	0.9917355	0.6489592
4874	Bn-A02-p8570729	A-genome specific (napus-rapa)	0.9569378	0.7714831	not scorable	not scorable	0.9173554	0.6793488
4875	Bn-A02-p8571485	A-genome specific (napus-rapa)	0.9593301	0.6382663	not scorable	not scorable	0.9917355	0.6964638
4876	Bn-A02-p8571577	A-genome specific (napus-rapa)	0.9617225	0.783915	not scorable	not scorable	0.9586777	0.7203005
4877	Bn-A02-p85725	A-genome specific (napus-rapa)	0.9808612	0.3422756	not scorable	not scorable	0.9834711	0.7246202
4878	Bn-A02-p8575325	A-genome specific (napus-rapa)	0.9832536	0.7588373	not scorable	not scorable	0.9669421	0.6796128
4879	Bn-A02-p859079	napus specific	1	0.478964	not scorable	not scorable	not scorable	not scorable
4880	Bn-A02-p8621264	A-genome specific (rapa only)	not scorable	not scorable	not scorable	not scorable	0.9752066	0.7035534
4881	Bn-A02-p8624200	A-genome specific (napus-rapa)	1	0.8475392	not scorable	not scorable	1	0.8095871

4882	Bn-A02-p8625851	A-genome specific (napus-rapa)	0.9928229	0.718559	not scorable	not scorable	0.9917355	0.7996876
4883	Bn-A02-p8633119	A-genome specific (napus-rapa)	0.9665072	0.698976	not scorable	not scorable	0.9834711	0.8351535
4884	Bn-A02-p8634642	A-genome specific (napus-rapa)	0.9904306	0.7561342	not scorable	not scorable	0.9834711	0.7780765
4885	Bn-A02-p8637915	A-genome specific (napus-rapa)	0.9856459	0.7927399	not scorable	not scorable	0.9834711	0.77153
4886	Bn-A02-p8660234	A-genome specific (napus-rapa)	0.9569378	0.6187571	not scorable	not scorable	0.9586777	0.5864879
4887	Bn-A02-p8660285	A-genome specific (rapa only)	not scorable	not scorable	not scorable	not scorable	0.9586777	0.6975725
4888	Bn-A02-p8660632	A-genome specific (napus-rapa)	0.9832536	0.8076782	not scorable	not scorable	0.9917355	0.7670717
4889	Bn-A02-p8663297	A-genome specific (napus-rapa)	0.9952153	0.6865365	not scorable	not scorable	0.9917355	0.7680466
4890	Bn-A02-p8667676	A-genome specific (napus-rapa)	0.9952153	0.9033464	not scorable	not scorable	1	0.8149705
4891	Bn-A02-p8676211	A-genome specific (rapa only)	not scorable	not scorable	not scorable	not scorable	0.9834711	0.2653292
4892	Bn-A02-p8678600	A-genome specific (napus-rapa)	0.9617225	0.7642558	not scorable	not scorable	0.9752066	0.817524
4893	Bn-A02-p8682290	A-genome specific (napus-rapa)	0.9712918	0.7554324	not scorable	not scorable	1	0.7368765
4894	Bn-A02-p8688298	A-genome specific (rapa only)	not scorable	not scorable	not scorable	not scorable	1	0.7040934
4895	Bn-A02-p8702373	A-genome specific (napus-rapa)	0.9688995	0.7898713	not scorable	not scorable	1	0.730248
4896	Bn-A02-p8703446	A-genome specific (napus-rapa)	0.9760765	0.5815477	not scorable	not scorable	0.9917355	0.8475636
4897	Bn-A02-p870494	napus specific	0.9760765	0.6542935	not scorable	not scorable	not scorable	not scorable
4898	Bn-A02-p8711609	A-genome specific (napus-rapa)	0.9856459	0.7840903	not scorable	not scorable	1	0.738374
4899	Bn-A02-p8720739	A-genome specific (rapa only)	not scorable	not scorable	not scorable	not scorable	0.9669421	0.7304225
4900	Bn-A02-p8724443	A-genome specific (napus-rapa)	0.9569378	0.4934731	not scorable	not scorable	0.9752066	0.8381849
4901	Bn-A02-p8726802	A-genome specific (napus-rapa)	0.9688995	0.7439119	not scorable	not scorable	0.9917355	0.7210738
4902	Bn-A02-p8732961	A-genome specific (napus-rapa)	0.861244	0.8682185	not scorable	not scorable	0.9834711	0.7684597
4903	Bn-A02-p8735305	A-genome specific (napus-rapa)	0.9712918	0.3013057	not scorable	not scorable	0.9834711	0.6596815
4904	Bn-A02-p873656	A-genome specific (napus-rapa)	0.9760765	0.8196452	not scorable	not scorable	1	0.734683
4905	Bn-A02-p873770	A-genome specific (napus-rapa)	0.9904306	0.8123478	not scorable	not scorable	0.9917355	0.8047553
4906	Bn-A02-p8747010	A-genome specific (napus-rapa)	0.9904306	0.5924079	not scorable	not scorable	0.9586777	0.7683663
4907	Bn-A02-p8754935	A-genome specific (napus-rapa)	0.9832536	0.4749767	not scorable	not scorable	0.9752066	0.7340579
4908	Bn-A02-p8767145	A-genome specific (napus-rapa)	0.9760765	0.7727231	not scorable	not scorable	0.9917355	0.7515374
4909	Bn-A02-p8777846	A-genome specific (napus-rapa)	0.9760765	0.7966446	not scorable	not scorable	0.9834711	0.742353
4910	Bn-A02-p8799151	A-genome specific (napus-rapa)	0.9736842	0.7784495	not scorable	not scorable	1	0.876192
4911	Bn-A02-p8802671	A-genome specific (napus-rapa)	0.9688995	0.7899616	not scorable	not scorable	1	0.8378905
4912	Bn-A02-p8804225	A-genome specific (napus-rapa)	0.9856459	0.3960864	not scorable	not scorable	1	0.842582
4913	Bn-A02-p8827347	A-genome specific (napus-rapa)	0.9832536	0.382514	not scorable	not scorable	0.9917355	0.7884539
4914	Bn-A02-p8833760	A-genome specific (napus-rapa)	0.9760765	0.6850253	not scorable	not scorable	0.9917355	0.8032025

4915	Bn-A02-p8834915	A-genome specific (napus-rapa)	0.9904306	0.7288857	not scorable	not scorable	1	0.7250962
4916	Bn-A02-p8836098	A-genome specific (napus-rapa)	0.9856459	0.8020059	not scorable	not scorable	1	0.7586786
4917	Bn-A02-p8849037	A-genome specific (napus-rapa)	0.9665072	0.2983027	not scorable	not scorable	1	0.6409478
4918	Bn-A02-p8865764	A-genome specific (napus-rapa)	0.9856459	0.7690871	not scorable	not scorable	1	0.787617
4919	Bn-A02-p8865968	A-genome specific (rapa only)	not scorable	not scorable	not scorable	not scorable	0.9917355	0.7017485
4920	Bn-A02-p8867093	A-genome specific (rapa only)	not scorable	not scorable	not scorable	not scorable	0.9917355	0.7152845
4921	Bn-A02-p8867507	A-genome specific (napus-rapa)	0.9712918	0.7863376	not scorable	not scorable	0.9752066	0.6603634
4922	Bn-A02-p8867662	A-genome specific (napus-rapa)	0.9593301	0.7845464	not scorable	not scorable	1	0.7205973
4923	Bn-A02-p8872271	A-genome specific (napus-rapa)	0.9856459	0.8036887	not scorable	not scorable	1	0.7485667
4924	Bn-A02-p8873630	A-genome specific (napus-rapa)	0.9760765	0.6572811	not scorable	not scorable	1	0.720804
4925	Bn-A02-p8873864	A-genome specific (napus-rapa)	0.9712918	0.7957613	not scorable	not scorable	1	0.7436157
4926	Bn-A02-p8873993	A-genome specific (napus-rapa)	0.9736842	0.7118471	not scorable	not scorable	0.9834711	0.6862456
4927	Bn-A02-p8874016	A-genome specific (napus-rapa)	0.9808612	0.7950137	not scorable	not scorable	0.9917355	0.6646068
4928	Bn-A02-p8874086	A-genome specific (napus-rapa)	0.9736842	0.7226784	not scorable	not scorable	1	0.6919687
4929	Bn-A02-p8874299	A-genome specific (napus-rapa)	0.9736842	0.79494	not scorable	not scorable	0.9917355	0.8521953
4930	Bn-A02-p8874380	A-genome specific (napus-rapa)	0.9712918	0.7818184	not scorable	not scorable	0.9917355	0.7235523
4931	Bn-A02-p8876332	A-genome specific (rapa only)	not scorable	not scorable	not scorable	not scorable	1	0.6843128
4932	Bn-A02-p8877059	genome unspecific (all in)	0.9712918	0.706939	0	0.8113948	0.9504132	0.8172697
4933	Bn-A02-p8877587	A-genome specific (napus-rapa)	0.9593301	0.8076585	not scorable	not scorable	0.9917355	0.7701694
4934	Bn-A02-p8877788	A-genome specific (napus-rapa)	0.9688995	0.7278332	not scorable	not scorable	1	0.7987592
4935	Bn-A02-p8878326	A-genome specific (napus-rapa)	0.8899522	0.7969353	not scorable	not scorable	0.9834711	0.6872807
4936	Bn-A02-p8880230	A-genome specific (napus-rapa)	0.9736842	0.7921325	not scorable	not scorable	0.9752066	0.6945366
4937	Bn-A02-p8880464	A-genome specific (napus-rapa)	0.9617225	0.7861856	not scorable	not scorable	0.9752066	0.72905
4938	Bn-A02-p8881070	A-genome specific (napus-rapa)	0.9330143	0.764646	not scorable	not scorable	0.9917355	0.6877344
4939	Bn-A02-p8881251	A-genome specific (napus-rapa)	0.9665072	0.7859368	not scorable	not scorable	0.9669421	0.6697457
4940	Bn-A02-p8883792	A-genome specific (napus-rapa)	0.9665072	0.8145517	not scorable	not scorable	1	0.7010351
4941	Bn-A02-p8884242	A-genome specific (napus-rapa)	0.9545454	0.8238764	not scorable	not scorable	0.9917355	0.7827922
4942	Bn-A02-p8884712	A-genome specific (napus-rapa)	0.9784689	0.7232582	not scorable	not scorable	0.9917355	0.6940895
4943	Bn-A02-p8885004	A-genome specific (napus-rapa)	0.9497608	0.3307886	not scorable	not scorable	0.9917355	0.7487202
4944	Bn-A02-p8885082	A-genome specific (rapa only)	not scorable	not scorable	not scorable	not scorable	0.9834711	0.7810735
4945	Bn-A02-p8885209	A-genome specific (napus-rapa)	0.9593301	0.6352014	not scorable	not scorable	0.9917355	0.781507
4946	Bn-A02-p8885908	A-genome specific (napus-rapa)	0.9665072	0.7939116	not scorable	not scorable	0.9834711	0.6617929
4947	Bn-A02-p8886238	A-genome specific (napus-rapa)	0.9736842	0.7833209	not scorable	not scorable	0.9917355	0.6785312

4948	Bn-A02-p8891703	A-genome specific (napus-rapa)	0.9736842	0.6968184	not scorable	not scorable	0.9669421	0.6883987
4949	Bn-A02-p8893045	all not scorable	not scorable	not scorable	not scorable	not scorable	not scorable	not scorable
4950	Bn-A02-p8893096	A-genome specific (rapa only)	not scorable	not scorable	not scorable	not scorable	0.9669421	0.6837302
4951	Bn-A02-p8895388	A-genome specific (napus-rapa)	0.9760765	0.8127044	not scorable	not scorable	0.9917355	0.7527145
4952	Bn-A02-p8899305	A-genome specific (napus-rapa)	0.9330143	0.4404597	not scorable	not scorable	0.9917355	0.647458
4953	Bn-A02-p8912747	A-genome specific (napus-rapa)	0.9665072	0.7831338	not scorable	not scorable	0.9917355	0.7793103
4954	Bn-A02-p8915005	A-genome specific (napus-rapa)	0.9736842	0.59136	not scorable	not scorable	1	0.8237346
4955	Bn-A02-p8924447	A-genome specific (napus-rapa)	0.9688995	0.7810876	not scorable	not scorable	0.9917355	0.7595123
4956	Bn-A02-p8925092	A-genome specific (napus-rapa)	0.9617225	0.3177333	not scorable	not scorable	1	0.8572899
4957	Bn-A02-p892640	A-genome specific (napus-rapa)	1	0.6572978	not scorable	not scorable	1	0.8333569
4958	Bn-A02-p8934314	A-genome specific (napus-rapa)	0.9760765	0.2788229	not scorable	not scorable	0.9917355	0.7001159
4959	Bn-A02-p8934537	A-genome specific (napus-rapa)	0.9712918	0.6562421	not scorable	not scorable	1	0.7762496
4960	Bn-A02-p8937580	A-genome specific (napus-rapa)	0.9976076	0.8694758	not scorable	not scorable	0.9752066	0.7626801
4961	Bn-A02-p8949465	A-genome specific (napus-rapa)	0.9736842	0.4009407	not scorable	not scorable	0.9834711	0.7783774
4962	Bn-A02-p8950348	A-genome specific (napus-rapa)	0.9928229	0.6693106	not scorable	not scorable	0.9834711	0.6793928
4963	Bn-A02-p8954851	A-genome specific (napus-rapa)	0.9688995	0.777397	not scorable	not scorable	0.9917355	0.7377263
4964	Bn-A02-p8955992	A-genome specific (napus-rapa)	0.9593301	0.7447118	not scorable	not scorable	0.9917355	0.7058651
4965	Bn-A02-p8967830	C-genome specific (napus-oleracea)	0.9210526	0.7796193	0.6589147	0.8494952	not scorable	not scorable
4966	Bn-A02-p8999771	A-genome specific (napus-rapa)	0.9856459	0.7328517	not scorable	not scorable	0.9917355	0.6987185
4967	Bn-A02-p9000565	A-genome specific (napus-rapa)	0.9832536	0.7067785	not scorable	not scorable	0.9669421	0.6719358
4968	Bn-A02-p9000921	A-genome specific (napus-rapa)	0.9928229	0.6272995	not scorable	not scorable	0.9752066	0.6600125
4969	Bn-A02-p9000998	A-genome specific (napus-rapa)	0.9952153	0.7395847	not scorable	not scorable	1	0.7134259
4970	Bn-A02-p9006677	A-genome specific (napus-rapa)	0.9712918	0.3774639	not scorable	not scorable	1	0.9205657
4971	Bn-A02-p9022920	A-genome specific (napus-rapa)	0.9736842	0.4857047	not scorable	not scorable	0.9834711	0.9272014
4972	Bn-A02-p9051321	A-genome specific (napus-rapa)	1	0.9175768	not scorable	not scorable	1	0.8840809
4973	Bn-A02-p9064726	A-genome specific (napus-rapa)	1	0.7090018	not scorable	not scorable	0.9917355	0.8523772
4974	Bn-A02-p9071076	A-genome specific (rapa only)	not scorable	not scorable	not scorable	not scorable	0.9834711	0.6841179
4975	Bn-A02-p9080423	A-genome specific (napus-rapa)	0.9928229	0.695892	not scorable	not scorable	1	0.8126447
4976	Bn-A02-p9108719	napus specific	0.9688995	0.3636605	not scorable	not scorable	not scorable	not scorable
4977	Bn-A02-p9135710	A-genome specific (napus-rapa)	0.9521531	0.7869809	not scorable	not scorable	0.9586777	0.7641803
4978	Bn-A02-p9140734	A-genome specific (napus-rapa)	0.9784689	0.3136055	not scorable	not scorable	0.9586777	0.6647474
4979	Bn-A02-p9143216	A-genome specific (rapa only)	not scorable	not scorable	not scorable	not scorable	0.9917355	0.5247383
4980	Bn-A02-p9148801	A-genome specific (napus-rapa)	0.9880383	0.4756634	not scorable	not scorable	0.9586777	0.6771753

4981	Bn-A02-p9160227	A-genome specific (napus-rapa)	0.9808612	0.2608811	not scorable	not scorable	0.9752066	0.7255831
4982	Bn-A02-p9161498	A-genome specific (napus-rapa)	0.8660287	0.7773438	not scorable	not scorable	0.9834711	0.7711986
4983	Bn-A02-p918384	A-genome specific (napus-rapa)	0.9976076	0.7655144	not scorable	not scorable	0.9752066	0.7845314
4984	Bn-A02-p9183872	A-genome specific (napus-rapa)	0.9760765	0.4758004	not scorable	not scorable	0.9669421	0.8517894
4985	Bn-A02-p9187768	A-genome specific (napus-rapa)	0.9928229	0.5838235	not scorable	not scorable	1	0.8343691
4986	Bn-A02-p9195997	genome unspecific (all in)	0.9712918	0.2334578	0.9689922	0.6652421	0.9917355	0.5713479
4987	Bn-A02-p9206062	genome unspecific (oleracea-rapa)	not scorable	not scorable	0.8139535	0.6963674	1	0.7494321
4988	Bn-A02-p9206254	A-genome specific (napus-rapa)	0.9976076	0.5156251	not scorable	not scorable	1	0.786476
4989	Bn-A02-p9206548	A-genome specific (napus-rapa)	0.9928229	0.5255143	not scorable	not scorable	1	0.7397041
4990	Bn-A02-p9223722	A-genome specific (napus-rapa)	0.9784689	0.7596304	not scorable	not scorable	0.9917355	0.7886162
4991	Bn-A02-p9224016	A-genome specific (napus-rapa)	0.9832536	0.7509986	not scorable	not scorable	0.9917355	0.7002389
4992	Bn-A02-p9225798	A-genome specific (napus-rapa)	0.9808612	0.2610306	not scorable	not scorable	0.9917355	0.5986961
4993	Bn-A02-p9229113	A-genome specific (napus-rapa)	0.9688995	0.7302253	not scorable	not scorable	1	0.7854271
4994	Bn-A02-p9231115	A-genome specific (napus-rapa)	0.9712918	0.5493301	not scorable	not scorable	0.9917355	0.7077852
4995	Bn-A02-p9239879	A-genome specific (napus-rapa)	0.9593301	0.7846327	not scorable	not scorable	0.9752066	0.693035
4996	Bn-A02-p9240182	A-genome specific (napus-rapa)	0.9736842	0.7751217	not scorable	not scorable	0.9834711	0.9338203
4997	Bn-A02-p9242308	A-genome specific (napus-rapa)	0.9856459	0.7768582	not scorable	not scorable	0.9834711	0.7938426
4998	Bn-A02-p9246552	A-genome specific (napus-rapa)	0.9736842	0.5621161	not scorable	not scorable	0.9586777	0.6450936
4999	Bn-A02-p925207	napus specific	0.9330143	0.8118936	not scorable	not scorable	not scorable	not scorable
5000	Bn-A02-p9254995	A-genome specific (napus-rapa)	0.9665072	0.2997098	not scorable	not scorable	0.9917355	0.7039331
5001	Bn-A02-p9255686	A-genome specific (napus-rapa)	0.9712918	0.737632	not scorable	not scorable	0.9917355	0.8072408
5002	Bn-A02-p9256040	A-genome specific (napus-rapa)	0.9760765	0.7672445	not scorable	not scorable	1	0.7542315
5003	Bn-A02-p9258117	A-genome specific (napus-rapa)	0.9712918	0.7800077	not scorable	not scorable	0.9834711	0.7725549
5004	Bn-A02-p9259325	A-genome specific (napus-rapa)	0.9712918	0.7439548	not scorable	not scorable	0.9504132	0.6833032
5005	Bn-A02-p9265512	A-genome specific (napus-rapa)	0.9066986	0.5795457	not scorable	not scorable	0.9917355	0.7580718
5006	Bn-A02-p9274719	genome unspecific (all in)	0.9976076	0.542537	1	0.8225538	1	0.7224851
5007	Bn-A02-p9281731	napus specific	0.9066986	0.6560249	not scorable	not scorable	not scorable	not scorable
5008	Bn-A02-p9308233	A-genome specific (napus-rapa)	0.6315789	0.7479566	not scorable	not scorable	0.9586777	0.6988206
5009	Bn-A02-p9319210	A-genome specific (rapa only)	not scorable	not scorable	not scorable	not scorable	0.9504132	0.5468885
5010	Bn-A02-p9322468	A-genome specific (rapa only)	not scorable	not scorable	not scorable	not scorable	0.9421487	0.9106142
5011	Bn-A02-p9339288	napus specific	0.9043062	0.7675657	not scorable	not scorable	not scorable	not scorable
5012	Bn-A02-p9339514	A-genome specific (napus-rapa)	0.9832536	0.6492009	not scorable	not scorable	0.9917355	0.6525173
5013	Bn-A02-p9340002	A-genome specific (napus-rapa)	0.9928229	0.7730449	not scorable	not scorable	0.9752066	0.8154225

5014	Bn-A02-p9341487	A-genome specific (napus-rapa)	0.9952153	0.8653359	not scorable	not scorable	1	0.8242752
5015	Bn-A02-p9346140	A-genome specific (napus-rapa)	0.9856459	0.5455258	not scorable	not scorable	0.9917355	0.7884895
5016	Bn-A02-p9351376	A-genome specific (rapa only)	not scorable	not scorable	not scorable	not scorable	1	0.7450076
5017	Bn-A02-p9353942	A-genome specific (napus-rapa)	0.9880383	0.2247259	not scorable	not scorable	0.9669421	0.6955034
5018	Bn-A02-p9361967	A-genome specific (napus-rapa)	0.923445	0.744975	not scorable	not scorable	0.9834711	0.7014447
5019	Bn-A02-p9371363	A-genome specific (napus-rapa)	0.9856459	0.7793347	not scorable	not scorable	0.9917355	0.7990386
5020	Bn-A02-p9371633	A-genome specific (napus-rapa)	0.9880383	0.7021627	not scorable	not scorable	0.9917355	0.6871121
5021	Bn-A02-p9371841	A-genome specific (napus-rapa)	0.9904306	0.7379723	not scorable	not scorable	0.9834711	0.7526949
5022	Bn-A02-p9371930	A-genome specific (napus-rapa)	0.9617225	0.7309755	not scorable	not scorable	1	0.7721822
5023	Bn-A02-p9371948	A-genome specific (napus-rapa)	0.9593301	0.6600273	not scorable	not scorable	0.9834711	0.7073705
5024	Bn-A02-p9396655	napus specific	0.7129186	0.7936415	not scorable	not scorable	not scorable	not scorable
5025	Bn-A02-p9408651	A-genome specific (napus-rapa)	0.9832536	0.3293611	not scorable	not scorable	1	0.6871157
5026	Bn-A02-p9409799	A-genome specific (napus-rapa)	0.9712918	0.7992056	not scorable	not scorable	0.9917355	0.6805574
5027	Bn-A02-p9415380	A-genome specific (napus-rapa)	0.9928229	0.2596824	not scorable	not scorable	1	0.6770098
5028	Bn-A02-p9415636	A-genome specific (napus-rapa)	0.9736842	0.3919061	not scorable	not scorable	0.9752066	0.6684452
5029	Bn-A02-p9415901	A-genome specific (napus-rapa)	0.9808612	0.3232072	not scorable	not scorable	0.9834711	0.7325208
5030	Bn-A02-p9416095	A-genome specific (napus-rapa)	0.9832536	0.7488766	not scorable	not scorable	1	0.7041416
5031	Bn-A02-p9420837	A-genome specific (napus-rapa)	0.9976076	0.7760484	not scorable	not scorable	1	0.801774
5032	Bn-A02-p9422112	A-genome specific (napus-rapa)	0.9760765	0.3308937	not scorable	not scorable	1	0.6877309
5033	Bn-A02-p9425125	A-genome specific (napus-rapa)	0.9832536	0.7117971	not scorable	not scorable	0.9917355	0.6986142
5034	Bn-A02-p9434869	A-genome specific (napus-rapa)	0.9808612	0.5108179	not scorable	not scorable	0.9917355	0.7067189
5035	Bn-A02-p9464293	A-genome specific (napus-rapa)	0.6794258	0.945904	not scorable	not scorable	0.9917355	0.8876017
5036	Bn-A02-p9465668	A-genome specific (napus-rapa)	0.9784689	0.7924582	not scorable	not scorable	1	0.8008413
5037	Bn-A02-p9480465	A-genome specific (napus-rapa)	1	0.4593177	not scorable	not scorable	0.9834711	0.6881039
5038	Bn-A02-p9497598	A-genome specific (napus-rapa)	0.8779904	0.7278972	not scorable	not scorable	0.9421487	0.6449527
5039	Bn-A02-p9505646	A-genome specific (napus-rapa)	0.9736842	0.7756432	not scorable	not scorable	0.9834711	0.82829
5040	Bn-A02-p9506103	A-genome specific (napus-rapa)	0.9784689	0.7672995	not scorable	not scorable	0.9834711	0.8257592
5041	Bn-A02-p9513250	A-genome specific (napus-rapa)	0.9641148	0.681105	not scorable	not scorable	0.9917355	0.7670318
5042	Bn-A02-p9519366	A-genome specific (rapa only)	not scorable	not scorable	not scorable	not scorable	0.9917355	0.7981747
5043	Bn-A02-p9526960	A-genome specific (napus-rapa)	0.9976076	0.6011081	not scorable	not scorable	0.9834711	0.7315794
5044	Bn-A02-p9558338	A-genome specific (napus-rapa)	0.9856459	0.733752	not scorable	not scorable	0.9752066	0.7167961
5045	Bn-A02-p9558523	A-genome specific (napus-rapa)	0.5430622	0.7401857	not scorable	not scorable	0.9834711	0.8094644
5046	Bn-A02-p9559304	A-genome specific (napus-rapa)	0.9904306	0.6920804	not scorable	not scorable	1	0.8064953

5047	Bn-A02-p9560736	A-genome specific (napus-rapa)	0.9856459	0.5930505	not scorable	not scorable	0.9834711	0.8103212
5048	Bn-A02-p9566560	A-genome specific (napus-rapa)	0.9880383	0.3657166	not scorable	not scorable	0.9917355	0.8537922
5049	Bn-A02-p9599263	A-genome specific (napus-rapa)	0.9162679	0.6811639	not scorable	not scorable	0.9256198	0.7497954
5050	Bn-A02-p9607043	A-genome specific (napus-rapa)	0.9688995	0.7822963	not scorable	not scorable	0.9752066	0.7917066
5051	Bn-A02-p9607756	A-genome specific (napus-rapa)	0.9282297	0.2962231	not scorable	not scorable	0.9917355	0.8077894
5052	Bn-A02-p9608319	A-genome specific (napus-rapa)	0.9665072	0.7714535	not scorable	not scorable	1	0.6758727
5053	Bn-A02-p9608730	napus specific	0.9401914	0.6664383	not scorable	not scorable	not scorable	not scorable
5054	Bn-A02-p9610453	A-genome specific (napus-rapa)	0.9641148	0.8031533	not scorable	not scorable	0.9917355	0.7072586
5055	Bn-A02-p9622161	A-genome specific (napus-rapa)	0.9617225	0.7823756	not scorable	not scorable	0.9917355	0.7343901
5056	Bn-A02-p9625502	A-genome specific (napus-rapa)	0.9665072	0.4461489	not scorable	not scorable	1	0.7937247
5057	Bn-A02-p9635795	A-genome specific (napus-rapa)	0.8205742	0.6419194	not scorable	not scorable	0.9669421	0.7361779
5058	Bn-A02-p9635953	A-genome specific (napus-rapa)	0.8181818	0.7770723	not scorable	not scorable	0.9752066	0.7846726
5059	Bn-A02-p9636219	A-genome specific (napus-rapa)	0.9808612	0.7702147	not scorable	not scorable	0.9917355	0.8343857
5060	Bn-A02-p9640434	A-genome specific (napus-rapa)	0.9712918	0.3644251	not scorable	not scorable	0.9669421	0.7664369
5061	Bn-A02-p9643734	A-genome specific (napus-rapa)	0.9856459	0.2134844	not scorable	not scorable	0.9834711	0.8139505
5062	Bn-A02-p9646068	A-genome specific (napus-rapa)	0.9928229	0.5001884	not scorable	not scorable	0.9917355	0.7465184
5063	Bn-A02-p9648484	A-genome specific (napus-rapa)	0.9880383	0.456204	not scorable	not scorable	0.9917355	0.7378272
5064	Bn-A02-p9664226	A-genome specific (napus-rapa)	0.9736842	0.3049676	not scorable	not scorable	0.9834711	0.7570844
5065	Bn-A02-p9684927	A-genome specific (napus-rapa)	0.9497608	0.5149683	not scorable	not scorable	0.9669421	0.6981347
5066	Bn-A02-p9703128	A-genome specific (napus-rapa)	0.9569378	0.6929321	not scorable	not scorable	0.9834711	0.6924984
5067	Bn-A02-p9706613	A-genome specific (napus-rapa)	0.9593301	0.2768548	not scorable	not scorable	1	0.6496807
5068	Bn-A02-p9707309	A-genome specific (napus-rapa)	0.9497608	0.5908216	not scorable	not scorable	0.9917355	0.604704
5069	Bn-A02-p9719196	A-genome specific (napus-rapa)	0.930622	0.2214538	not scorable	not scorable	0.9834711	0.7352247
5070	Bn-A02-p9722276	A-genome specific (napus-rapa)	0.9760765	0.3235612	not scorable	not scorable	1	0.8764072
5071	Bn-A02-p9726742	A-genome specific (napus-rapa)	0.9832536	0.7788599	not scorable	not scorable	1	0.8189065
5072	Bn-A02-p972896	A-genome specific (napus-rapa)	0.9114832	0.7506474	not scorable	not scorable	0.9752066	0.7043232
5073	Bn-A02-p9740120	genome unspecific (all in)	0.9976076	0.471016	0.9689922	0.814975	1	0.8815182
5074	Bn-A02-p975209	A-genome specific (napus-rapa)	0.9688995	0.351389	not scorable	not scorable	0.9752066	0.6246641
5075	Bn-A02-p9778318	A-genome specific (napus-rapa)	0.9760765	0.7124499	not scorable	not scorable	1	0.9272014
5076	Bn-A02-p978741	A-genome specific (napus-rapa)	0.9976076	0.7207088	not scorable	not scorable	0.9917355	0.6466544
5077	Bn-A02-p980390	A-genome specific (napus-rapa)	0.9928229	0.7120554	not scorable	not scorable	0.9917355	0.7619979
5078	Bn-A02-p9829719	A-genome specific (napus-rapa)	0.930622	0.6622922	not scorable	not scorable	1	0.7909383
5079	Bn-A02-p9846691	A-genome specific (napus-rapa)	0.9856459	0.3124525	not scorable	not scorable	0.9917355	0.6956154

5080	Bn-A02-p9846899	A-genome specific (napus-rapa)	0.9976076	0.5643549	not scorable	not scorable	0.9752066	0.7678604
5081	Bn-A02-p985366	A-genome specific (napus-rapa)	0.9928229	0.5698414	not scorable	not scorable	0.9917355	0.6743953
5082	Bn-A02-p9885351	A-genome specific (napus-rapa)	0.9665072	0.7918265	not scorable	not scorable	0.9834711	0.7267699
5083	Bn-A02-p9886564	A-genome specific (napus-rapa)	0.9832536	0.2930706	not scorable	not scorable	0.9752066	0.7100459
5084	Bn-A02-p9888208	A-genome specific (napus-rapa)	0.9856459	0.2426255	not scorable	not scorable	1	0.7156282
5085	Bn-A02-p9891733	A-genome specific (napus-rapa)	0.9066986	0.7524282	not scorable	not scorable	0.9669421	0.7861187
5086	Bn-A02-p9927808	A-genome specific (napus-rapa)	0.930622	0.7163373	not scorable	not scorable	0.9669421	0.8277952
5087	Bn-A02-p9959992	A-genome specific (napus-rapa)	0.9928229	0.7713007	not scorable	not scorable	0.9834711	0.7899249
5088	Bn-A02-p9961743	napus specific	0.9832536	0.7417537	not scorable	not scorable	not scorable	not scorable
5089	Bn-A02-p9970625	A-genome specific (napus-rapa)	0.9880383	0.367826	not scorable	not scorable	0.9917355	0.7483729
5090	Bn-A02-p9970848	A-genome specific (napus-rapa)	0.9808612	0.2768814	not scorable	not scorable	0.9917355	0.7713221
5091	Bn-A02-p9973775	A-genome specific (rapa only)	not scorable	not scorable	not scorable	not scorable	0.9834711	0.8004208
5092	Bn-A02-p9973927	A-genome specific (napus-rapa)	0.9282297	0.2411183	not scorable	not scorable	0.9834711	0.7349294
5093	Bn-A02-p99883	A-genome specific (napus-rapa)	1	0.6802951	not scorable	not scorable	1	0.6503867
5094	Bn-A02-p9990613	A-genome specific (napus-rapa)	0.9760765	0.2269715	not scorable	not scorable	1	0.7566578
5095	Bn-A02-p9993945	A-genome specific (napus-rapa)	0.9641148	0.2989485	not scorable	not scorable	1	0.7528283
5096	Bn-A02-p9996703	A-genome specific (napus-rapa)	0.9760765	0.7730789	not scorable	not scorable	0.9752066	0.7927519
5097	Bn-A02-p9997237	napus specific	0.9856459	0.2436382	not scorable	not scorable	not scorable	not scorable
5098	Bn-A03-p10002791	A-genome specific (napus-rapa)	0.9904306	0.4369407	not scorable	not scorable	1	0.7494321
5099	Bn-A03-p10008905	A-genome specific (napus-rapa)	0.9688995	0.4513174	not scorable	not scorable	0.9834711	0.7129212
5100	Bn-A03-p10010569	A-genome specific (napus-rapa)	0.9952153	0.7856001	not scorable	not scorable	0.9834711	0.8028401
5101	Bn-A03-p10011955	A-genome specific (napus-rapa)	0.9880383	0.6975461	not scorable	not scorable	0.9917355	0.8015306
5102	Bn-A03-p10023639	A-genome specific (napus-rapa)	0.9760765	0.709915	not scorable	not scorable	1	0.7380836
5103	Bn-A03-p10027020	A-genome specific (napus-rapa)	0.9760765	0.532495	not scorable	not scorable	0.9752066	0.6972185
5104	Bn-A03-p10045527	genome unspecific (all in)	0.9904306	0.305382	0.9844961	0.5803345	1	0.9316995
5105	Bn-A03-p10047160	A-genome specific (napus-rapa)	0.9928229	0.8468072	not scorable	not scorable	1	0.7791812
5106	Bn-A03-p10047998	A-genome specific (napus-rapa)	0.9952153	0.5821193	not scorable	not scorable	1	0.7071238
5107	Bn-A03-p1004918	A-genome specific (rapa only)	not scorable	not scorable	not scorable	not scorable	0.9752066	0.6627242
5108	Bn-A03-p10062838	A-genome specific (napus-rapa)	0.9952153	0.2569478	not scorable	not scorable	0.9752066	0.4449755
5109	Bn-A03-p10076616	napus specific	1	0.2863811	not scorable	not scorable	not scorable	not scorable
5110	Bn-A03-p10081359	genome unspecific (oleracea-rapa)	not scorable	not scorable	0.9922481	0.8276236	0.9752066	0.7019902
5111	Bn-A03-p1008445	A-genome specific (napus-rapa)	0.937799	0.8034447	not scorable	not scorable	0.9752066	0.7608739
5112	Bn-A03-p10084501	genome unspecific (all in)	0.9928229	0.5920501	0.9922481	0.7134535	1	0.6859793

5113	Bn-A03-p10086496	A-genome specific (napus-rapa)	0.9928229	0.8305932	not scorable	not scorable	1	0.7515859
5114	Bn-A03-p10087723	A-genome specific (rapa only)	not scorable	not scorable	not scorable	not scorable	1	0.6799615
5115	Bn-A03-p10092390	A-genome specific (napus-rapa)	0.9880383	0.3959139	not scorable	not scorable	1	0.851064
5116	Bn-A03-p10092885	genome unspecific (all in)	0.9952153	0.5089732	1	0.5726631	1	0.7075
5117	Bn-A03-p10110586	A-genome specific (napus-rapa)	0.9976076	0.7987271	not scorable	not scorable	0.9917355	0.7786188
5118	Bn-A03-p10113984	A-genome specific (napus-rapa)	0.3564593	0.7933518	not scorable	not scorable	0.9256198	0.8246439
5119	Bn-A03-p10117543	A-genome specific (napus-rapa)	0.9617225	0.8963926	not scorable	not scorable	0.9834711	0.6658646
5120	Bn-A03-p10129836	A-genome specific (napus-rapa)	0.9425837	0.4671274	not scorable	not scorable	0.9917355	0.6048279
5121	Bn-A03-p10130816	A-genome specific (napus-rapa)	1	0.5209166	not scorable	not scorable	0.9917355	0.7870192
5122	Bn-A03-p1013445	A-genome specific (rapa only)	not scorable	not scorable	not scorable	not scorable	1	0.8134261
5123	Bn-A03-p10218040	genome unspecific (all in)	0.9904306	0.3423601	0.9689922	0.4026267	1	0.8692989
5124	Bn-A03-p10248162	A-genome specific (napus-rapa)	0.9665072	0.5798329	not scorable	not scorable	0.9834711	0.7586334
5125	Bn-A03-p10273355	A-genome specific (napus-rapa)	0.9712918	0.5473375	not scorable	not scorable	1	0.9175813
5126	Bn-A03-p10285809	A-genome specific (napus-rapa)	0.9856459	0.7585521	not scorable	not scorable	0.9917355	0.7438421
5127	Bn-A03-p10288202	A-genome specific (napus-rapa)	0.9712918	0.5421591	not scorable	not scorable	1	0.7639437
5128	Bn-A03-p10293071	A-genome specific (napus-rapa)	0.9760765	0.796537	not scorable	not scorable	1	0.7257573
5129	Bn-A03-p10296969	A-genome specific (napus-rapa)	0.9712918	0.3834068	not scorable	not scorable	0.9917355	0.7526135
5130	Bn-A03-p10313368	A-genome specific (napus-rapa)	0.9976076	0.7105801	not scorable	not scorable	1	0.8095524
5131	Bn-A03-p10321263	A-genome specific (napus-rapa)	0.9952153	0.3379588	not scorable	not scorable	1	0.7663543
5132	Bn-A03-p10321416	A-genome specific (napus-rapa)	0.9976076	0.6965511	not scorable	not scorable	0.9834711	0.6862957
5133	Bn-A03-p10342088	A-genome specific (napus-rapa)	0.9473684	0.4222451	not scorable	not scorable	0.8429752	0.7829915
5134	Bn-A03-p10350207	A-genome specific (rapa only)	not scorable	not scorable	not scorable	not scorable	1	0.7801202
5135	Bn-A03-p10355167	A-genome specific (napus-rapa)	0.9856459	0.6615505	not scorable	not scorable	1	0.7108301
5136	Bn-A03-p10376862	A-genome specific (napus-rapa)	0.9712918	0.3064386	not scorable	not scorable	0.9669421	0.650941
5137	Bn-A03-p10388827	A-genome specific (napus-rapa)	0.9952153	0.4445018	not scorable	not scorable	0.9669421	0.6687172
5138	Bn-A03-p10393461	A-genome specific (napus-rapa)	0.9952153	0.5799794	not scorable	not scorable	0.9834711	0.6805213
5139	Bn-A03-p10400341	A-genome specific (napus-rapa)	0.9952153	0.6966792	not scorable	not scorable	0.9917355	0.4865592
5140	Bn-A03-p10402639	A-genome specific (napus-rapa)	0.9976076	0.7570226	not scorable	not scorable	0.9917355	0.6226847
5141	Bn-A03-p10414176	A-genome specific (napus-rapa)	0.8660287	0.5933778	not scorable	not scorable	1	0.9475539
5142	Bn-A03-p10445051	A-genome specific (napus-rapa)	0.9976076	0.4600951	not scorable	not scorable	1	0.7455676
5143	Bn-A03-p10448318	A-genome specific (napus-rapa)	0.9952153	0.7364935	not scorable	not scorable	0.9917355	0.7078092
5144	Bn-A03-p10449972	A-genome specific (napus-rapa)	0.9928229	0.5898635	not scorable	not scorable	0.9421487	0.571029
5145	Bn-A03-p10458923	A-genome specific (napus-rapa)	0.9856459	0.7496867	not scorable	not scorable	0.9834711	0.6795592

5146	Bn-A03-p10464551	A-genome specific (napus-rapa)	0.9952153	0.4340621	not scorable	not scorable	0.9917355	0.7509199
5147	Bn-A03-p10466633	A-genome specific (napus-rapa)	0.9952153	0.6763363	not scorable	not scorable	1	0.6536661
5148	Bn-A03-p1046864	A-genome specific (napus-rapa)	0.9569378	0.6579955	not scorable	not scorable	1	0.8833086
5149	Bn-A03-p10482765	A-genome specific (napus-rapa)	0.9952153	0.7696385	not scorable	not scorable	0.9917355	0.77306
5150	Bn-A03-p10487754	A-genome specific (napus-rapa)	0.9856459	0.5900955	not scorable	not scorable	1	0.7101598
5151	Bn-A03-p1050437	A-genome specific (napus-rapa)	0.9114832	0.6664365	not scorable	not scorable	0.9834711	0.6996428
5152	Bn-A03-p1050893	A-genome specific (napus-rapa)	0.9043062	0.7562289	not scorable	not scorable	1	0.7581046
5153	Bn-A03-p10517056	A-genome specific (napus-rapa)	0.9712918	0.8157431	not scorable	not scorable	0.9834711	0.7420409
5154	Bn-A03-p10517505	A-genome specific (rapa only)	not scorable	not scorable	not scorable	not scorable	0.9834711	0.7477279
5155	Bn-A03-p1051774	A-genome specific (rapa only)	not scorable	not scorable	not scorable	not scorable	1	0.7390981
5156	Bn-A03-p10533775	A-genome specific (napus-rapa)	0.9856459	0.775816	not scorable	not scorable	0.9917355	0.6888669
5157	Bn-A03-p10547280	A-genome specific (napus-rapa)	0.9688995	0.4582045	not scorable	not scorable	0.9669421	0.530384
5158	Bn-A03-p10562274	A-genome specific (napus-rapa)	0.9282297	0.3036279	not scorable	not scorable	1	0.636762
5159	Bn-A03-p10565738	A-genome specific (napus-rapa)	0.9856459	0.6583429	not scorable	not scorable	1	0.7071266
5160	Bn-A03-p10566503	A-genome specific (napus-rapa)	0.9760765	0.680471	not scorable	not scorable	1	0.7485616
5161	Bn-A03-p10573912	A-genome specific (napus-rapa)	0.9928229	0.3987852	not scorable	not scorable	1	0.8336737
5162	Bn-A03-p10583411	A-genome specific (napus-rapa)	0.8732057	0.7733875	not scorable	not scorable	0.9834711	0.9057256
5163	Bn-A03-p10584028	A-genome specific (napus-rapa)	0.9258373	0.7291861	not scorable	not scorable	1	0.8424219
5164	Bn-A03-p10587547	A-genome specific (napus-rapa)	0.9521531	0.7806543	not scorable	not scorable	1	0.6817682
5165	Bn-A03-p10600103	A-genome specific (napus-rapa)	0.9641148	0.2724372	not scorable	not scorable	1	0.7498477
5166	Bn-A03-p10600567	napus specific	0.9210526	0.6899465	not scorable	not scorable	not scorable	not scorable
5167	Bn-A03-p10607288	A-genome specific (napus-rapa)	0.9593301	0.6844414	not scorable	not scorable	1	0.7240503
5168	Bn-A03-p10608519	A-genome specific (napus-rapa)	0.7296651	0.7067349	not scorable	not scorable	1	0.7428197
5169	Bn-A03-p10617866	A-genome specific (napus-rapa)	0.9832536	0.7344964	not scorable	not scorable	1	0.7591079
5170	Bn-A03-p10623167	A-genome specific (napus-rapa)	0.5598086	0.7900027	not scorable	not scorable	1	0.7642848
5171	Bn-A03-p10640254	A-genome specific (napus-rapa)	0.9904306	0.7523452	not scorable	not scorable	0.9917355	0.7294865
5172	Bn-A03-p10640643	A-genome specific (napus-rapa)	0.9665072	0.7525132	not scorable	not scorable	0.9834711	0.7146232
5173	Bn-A03-p10641561	napus specific	0.5454546	0.9183553	not scorable	not scorable	not scorable	not scorable
5174	Bn-A03-p10641836	A-genome specific (napus-rapa)	0.9712918	0.7117614	not scorable	not scorable	1	0.7591307
5175	Bn-A03-p10649410	A-genome specific (napus-rapa)	0.9976076	0.4964367	not scorable	not scorable	0.9669421	0.7111429
5176	Bn-A03-p1065055	A-genome specific (napus-rapa)	0.9832536	0.4295581	not scorable	not scorable	1	0.8234133
5177	Bn-A03-p10651420	A-genome specific (napus-rapa)	0.9569378	0.726617	not scorable	not scorable	1	0.8007333
5178	Bn-A03-p10664726	A-genome specific (napus-rapa)	0.9258373	0.4499105	not scorable	not scorable	0.9834711	0.8144349

5179	Bn-A03-p1067313	A-genome specific (napus-rapa)	0.9282297	0.7697099	not scorable	not scorable	0.9917355	0.8741567
5180	Bn-A03-p10676505	A-genome specific (napus-rapa)	0.9760765	0.5602989	not scorable	not scorable	0.9917355	0.8051141
5181	Bn-A03-p10680539	A-genome specific (napus-rapa)	0.9808612	0.7273422	not scorable	not scorable	1	0.8264885
5182	Bn-A03-p1068165	A-genome specific (napus-rapa)	0.9186603	0.7748325	not scorable	not scorable	1	0.8023399
5183	Bn-A03-p10682159	A-genome specific (napus-rapa)	0.9808612	0.7240335	not scorable	not scorable	0.9917355	0.7898132
5184	Bn-A03-p10683528	napus specific	0.5287082	0.8634806	not scorable	not scorable	not scorable	not scorable
5185	Bn-A03-p10692514	A-genome specific (napus-rapa)	0.9736842	0.6444696	not scorable	not scorable	1	0.7060363
5186	Bn-A03-p10703085	A-genome specific (napus-rapa)	0.9928229	0.772752	not scorable	not scorable	0.9917355	0.7545361
5187	Bn-A03-p1070680	A-genome specific (napus-rapa)	0.9808612	0.5434826	not scorable	not scorable	1	0.7004576
5188	Bn-A03-p10714724	napus specific	0.937799	0.7147045	not scorable	not scorable	not scorable	not scorable
5189	Bn-A03-p10723392	A-genome specific (napus-rapa)	0.7822967	0.7702524	not scorable	not scorable	0.9752066	0.8523399
5190	Bn-A03-p10723484	A-genome specific (napus-rapa)	0.7751196	0.7760659	not scorable	not scorable	0.9917355	0.8407545
5191	Bn-A03-p10732934	A-genome specific (napus-rapa)	0.9354067	0.7264069	not scorable	not scorable	0.9834711	0.6598726
5192	Bn-A03-p10749391	A-genome specific (napus-rapa)	0.9688995	0.3528845	not scorable	not scorable	0.9834711	0.6596674
5193	Bn-A03-p10757525	A-genome specific (napus-rapa)	0.9760765	0.7875881	not scorable	not scorable	1	0.7383026
5194	Bn-A03-p1075837	A-genome specific (napus-rapa)	0.9521531	0.5350001	not scorable	not scorable	1	0.51849
5195	Bn-A03-p10758970	A-genome specific (napus-rapa)	0.9617225	0.808253	not scorable	not scorable	1	0.8542958
5196	Bn-A03-p10761386	A-genome specific (napus-rapa)	0.9952153	0.5001145	not scorable	not scorable	1	0.694041
5197	Bn-A03-p10761460	A-genome specific (napus-rapa)	0.9904306	0.5271561	not scorable	not scorable	0.9504132	0.5644894
5198	Bn-A03-p10773321	A-genome specific (napus-rapa)	0.9904306	0.450622	not scorable	not scorable	1	0.6759783
5199	Bn-A03-p10773339	A-genome specific (napus-rapa)	0.9976076	0.7701179	not scorable	not scorable	0.9917355	0.7748146
5200	Bn-A03-p10794513	napus specific	0.9425837	0.4827262	not scorable	not scorable	not scorable	not scorable
5201	Bn-A03-p10794985	A-genome specific (napus-rapa)	0.9808612	0.7518843	not scorable	not scorable	0.9834711	0.7504583
5202	Bn-A03-p10797405	A-genome specific (napus-rapa)	0.9784689	0.5351871	not scorable	not scorable	0.9917355	0.5099606
5203	Bn-A03-p10802728	A-genome specific (napus-rapa)	0.9976076	0.466033	not scorable	not scorable	0.9834711	0.7786132
5204	Bn-A03-p10803927	A-genome specific (napus-rapa)	0.9856459	0.399814	not scorable	not scorable	0.9917355	0.7481225
5205	Bn-A03-p10814591	A-genome specific (napus-rapa)	0.9928229	0.7702935	not scorable	not scorable	1	0.8013009
5206	Bn-A03-p10819432	A-genome specific (napus-rapa)	0.9928229	0.742265	not scorable	not scorable	1	0.7286986
5207	Bn-A03-p10823720	A-genome specific (napus-rapa)	0.9856459	0.5328443	not scorable	not scorable	1	0.7636456
5208	Bn-A03-p10830360	A-genome specific (napus-rapa)	0.9641148	0.7510456	not scorable	not scorable	0.9917355	0.7301316
5209	Bn-A03-p10833240	A-genome specific (napus-rapa)	0.9928229	0.8954517	not scorable	not scorable	0.9917355	0.7212858
5210	Bn-A03-p1084819	A-genome specific (napus-rapa)	0.9210526	0.7678473	not scorable	not scorable	1	0.8743911
5211	Bn-A03-p10853616	A-genome specific (napus-rapa)	0.9856459	0.7324257	not scorable	not scorable	0.9834711	0.6658531

5212	Bn-A03-p10855422	A-genome specific (napus-rapa)	1	0.7208948	not scorable	not scorable	1	0.6957343
5213	Bn-A03-p10862251	A-genome specific (napus-rapa)	0.9928229	0.8510907	not scorable	not scorable	1	0.6960684
5214	Bn-A03-p10873434	A-genome specific (napus-rapa)	0.9808612	0.7538314	not scorable	not scorable	0.8264463	0.7195574
5215	Bn-A03-p10883930	genome unspecific (all in)	0.9976076	0.361945	0.9767442	0.3745004	1	0.7578155
5216	Bn-A03-p10889554	A-genome specific (napus-rapa)	0.2583732	0.8736487	not scorable	not scorable	0.9917355	0.8269143
5217	Bn-A03-p10897572	A-genome specific (napus-rapa)	0.9952153	0.7888409	not scorable	not scorable	1	0.7595835
5218	Bn-A03-p10898289	A-genome specific (napus-rapa)	0.9976076	0.8274082	not scorable	not scorable	0.9834711	0.7513597
5219	Bn-A03-p1089875	A-genome specific (napus-rapa)	0.9282297	0.7706058	not scorable	not scorable	1	0.8045731
5220	Bn-A03-p10900301	A-genome specific (napus-rapa)	0.9976076	0.5491616	not scorable	not scorable	0.9752066	0.7895783
5221	Bn-A03-p10900813	A-genome specific (napus-rapa)	0.9928229	0.7789853	not scorable	not scorable	0.9834711	0.8842712
5222	Bn-A03-p10907793	A-genome specific (napus-rapa)	0.9928229	0.8101681	not scorable	not scorable	0.9752066	0.7146232
5223	Bn-A03-p10912453	A-genome specific (napus-rapa)	0.9904306	0.7070758	not scorable	not scorable	1	0.7987826
5224	Bn-A03-p10912815	A-genome specific (napus-rapa)	0.9976076	0.4802548	not scorable	not scorable	1	0.7937169
5225	Bn-A03-p10928669	A-genome specific (napus-rapa)	0.9904306	0.5314842	not scorable	not scorable	0.9917355	0.8049473
5226	Bn-A03-p10928990	A-genome specific (napus-rapa)	0.9162679	0.8207017	not scorable	not scorable	1	0.7711915
5227	Bn-A03-p10930541	A-genome specific (napus-rapa)	0.9928229	0.5416912	not scorable	not scorable	0.9834711	0.72797
5228	Bn-A03-p10947710	genome unspecific (oleracea-rapa)	not scorable	not scorable	0.8372093	0.84755	0.9834711	0.8124357
5229	Bn-A03-p10952575	A-genome specific (napus-rapa)	0.9952153	0.4075186	not scorable	not scorable	1	0.8410593
5230	Bn-A03-p10958194	A-genome specific (napus-rapa)	1	0.727371	not scorable	not scorable	1	0.7362441
5231	Bn-A03-p10959387	A-genome specific (napus-rapa)	0.9976076	0.7027575	not scorable	not scorable	1	0.8062757
5232	Bn-A03-p10959439	A-genome specific (napus-rapa)	0.9928229	0.7249324	not scorable	not scorable	1	0.7989482
5233	Bn-A03-p10962307	A-genome specific (napus-rapa)	0.9760765	0.7153134	not scorable	not scorable	0.8264463	0.8269348
5234	Bn-A03-p10962645	A-genome specific (napus-rapa)	0.9712918	0.8104526	not scorable	not scorable	0.8264463	0.8379447
5235	Bn-A03-p10962969	A-genome specific (napus-rapa)	0.9641148	0.7838488	not scorable	not scorable	0.8099173	0.750002
5236	Bn-A03-p10963046	A-genome specific (rapa only)	not scorable	not scorable	not scorable	not scorable	0.8347107	0.7599619
5237	Bn-A03-p10964394	A-genome specific (napus-rapa)	0.9712918	0.8041307	not scorable	not scorable	0.8016529	0.7759728
5238	Bn-A03-p10964511	A-genome specific (napus-rapa)	0.9832536	0.8158737	not scorable	not scorable	0.9586777	0.752816
5239	Bn-A03-p10964682	A-genome specific (napus-rapa)	0.9688995	0.7365783	not scorable	not scorable	0.8512397	0.7401044
5240	Bn-A03-p10967268	A-genome specific (napus-rapa)	0.9904306	0.3666797	not scorable	not scorable	0.8181818	0.7268057
5241	Bn-A03-p10969109	A-genome specific (napus-rapa)	0.9952153	0.5169186	not scorable	not scorable	1	0.7779718
5242	Bn-A03-p1098531	A-genome specific (napus-rapa)	1	0.858917	not scorable	not scorable	1	0.7516536
5243	Bn-A03-p10993904	A-genome specific (napus-rapa)	0.9856459	0.7525268	not scorable	not scorable	1	0.9078432
5244	Bn-A03-p11027825	A-genome specific (napus-rapa)	1	0.7040766	not scorable	not scorable	1	0.8796402

5245	Bn-A03-p11051970	napus specific	0.7416268	0.7625728	not scorable	not scorable	not scorable	not scorable
5246	Bn-A03-p11053758	A-genome specific (napus-rapa)	0.9784689	0.7210378	not scorable	not scorable	0.9917355	0.7042054
5247	Bn-A03-p11053857	A-genome specific (napus-rapa)	0.9712918	0.7358117	not scorable	not scorable	1	0.6926724
5248	Bn-A03-p11065674	A-genome specific (napus-rapa)	0.9928229	0.5306202	not scorable	not scorable	1	0.7296067
5249	Bn-A03-p11070576	A-genome specific (napus-rapa)	0.9904306	0.6397136	not scorable	not scorable	0.9834711	0.6687723
5250	Bn-A03-p11070958	A-genome specific (napus-rapa)	1	0.7929812	not scorable	not scorable	1	0.7708455
5251	Bn-A03-p11071010	A-genome specific (napus-rapa)	0.9952153	0.8150311	not scorable	not scorable	1	0.8585363
5252	Bn-A03-p11071320	A-genome specific (napus-rapa)	0.9952153	0.8373995	not scorable	not scorable	1	0.7776622
5253	Bn-A03-p11071640	A-genome specific (napus-rapa)	1	0.8568761	not scorable	not scorable	1	0.758513
5254	Bn-A03-p11086751	A-genome specific (napus-rapa)	0.9832536	0.6624835	not scorable	not scorable	0.9586777	0.5446155
5255	Bn-A03-p11087692	A-genome specific (napus-rapa)	0.9904306	0.7798703	not scorable	not scorable	1	0.794051
5256	Bn-A03-p11122273	C-genome specific (napus-oleracea)	0.9186603	0.3395446	0.9379845	0.3942117	not scorable	not scorable
5257	Bn-A03-p11133743	A-genome specific (napus-rapa)	0.9976076	0.7565389	not scorable	not scorable	0.9834711	0.6928803
5258	Bn-A03-p11138137	A-genome specific (napus-rapa)	0.9808612	0.3407292	not scorable	not scorable	0.9917355	0.7248131
5259	Bn-A03-p11143696	A-genome specific (napus-rapa)	0.2942584	0.8642073	not scorable	not scorable	0.9917355	0.7833296
5260	Bn-A03-p11156293	A-genome specific (napus-rapa)	0.9904306	0.5983151	not scorable	not scorable	0.9917355	0.7647294
5261	Bn-A03-p11171067	A-genome specific (napus-rapa)	0.9904306	0.7249979	not scorable	not scorable	0.9917355	0.690599
5262	Bn-A03-p11177892	A-genome specific (napus-rapa)	0.9952153	0.4820233	not scorable	not scorable	1	0.7097556
5263	Bn-A03-p11188500	A-genome specific (napus-rapa)	0.8181818	0.856598	not scorable	not scorable	0.8842975	0.9022073
5264	Bn-A03-p11191805	A-genome specific (napus-rapa)	0.9856459	0.590327	not scorable	not scorable	0.9586777	0.7615929
5265	Bn-A03-p11202719	A-genome specific (napus-rapa)	1	0.2742017	not scorable	not scorable	0.9834711	0.7079362
5266	Bn-A03-p11217606	A-genome specific (rapa only)	not scorable	not scorable	not scorable	not scorable	1	0.7558123
5267	Bn-A03-p11218468	A-genome specific (napus-rapa)	0.9952153	0.3259405	not scorable	not scorable	0.9917355	0.2867653
5268	Bn-A03-p11227755	A-genome specific (napus-rapa)	0.9952153	0.7815886	not scorable	not scorable	1	0.7548996
5269	Bn-A03-p11232586	A-genome specific (napus-rapa)	0.9904306	0.635788	not scorable	not scorable	0.9917355	0.7681777
5270	Bn-A03-p11247615	A-genome specific (napus-rapa)	0.9904306	0.8046045	not scorable	not scorable	0.9917355	0.7090014
5271	Bn-A03-p11256138	A-genome specific (napus-rapa)	0.9976076	0.8029923	not scorable	not scorable	0.9917355	0.8379771
5272	Bn-A03-p11256392	A-genome specific (napus-rapa)	0.9928229	0.8011888	not scorable	not scorable	0.9917355	0.8272159
5273	Bn-A03-p11256664	A-genome specific (napus-rapa)	0.9976076	0.787027	not scorable	not scorable	0.9917355	0.7674003
5274	Bn-A03-p11257708	A-genome specific (napus-rapa)	0.9856459	0.7697231	not scorable	not scorable	0.9917355	0.7650257
5275	Bn-A03-p11294370	A-genome specific (napus-rapa)	0.9952153	0.5161114	not scorable	not scorable	0.9917355	0.7534155
5276	Bn-A03-p11294858	A-genome specific (napus-rapa)	0.9928229	0.473326	not scorable	not scorable	1	0.7826302
5277	Bn-A03-p11319722	A-genome specific (napus-rapa)	0.9976076	0.7894596	not scorable	not scorable	1	0.8113519

5278	Bn-A03-p11319901	A-genome specific (napus-rapa)	0.9856459	0.8079349	not scorable	not scorable	1	0.7369935
5279	Bn-A03-p11325897	A-genome specific (napus-rapa)	0.9162679	0.778622	not scorable	not scorable	1	0.8317298
5280	Bn-A03-p11345845	A-genome specific (napus-rapa)	0.9952153	0.7709465	not scorable	not scorable	0.9008265	0.8522056
5281	Bn-A03-p11347564	A-genome specific (rapa only)	not scorable	not scorable	not scorable	not scorable	0.9338843	0.8261573
5282	Bn-A03-p11356180	A-genome specific (napus-rapa)	0.9784689	0.7586975	not scorable	not scorable	1	0.8818015
5283	Bn-A03-p11364656	napus specific	0.9856459	0.8942379	not scorable	not scorable	not scorable	not scorable
5284	Bn-A03-p11381125	A-genome specific (napus-rapa)	0.9880383	0.7882114	not scorable	not scorable	0.9834711	0.6976214
5285	Bn-A03-p11383138	A-genome specific (napus-rapa)	0.9617225	0.452123	not scorable	not scorable	0.9917355	0.7168742
5286	Bn-A03-p11391930	A-genome specific (napus-rapa)	0.9784689	0.7674896	not scorable	not scorable	0.9917355	0.7167431
5287	Bn-A03-p11392385	A-genome specific (rapa only)	not scorable	not scorable	not scorable	not scorable	0.9917355	0.7067898
5288	Bn-A03-p11399915	A-genome specific (napus-rapa)	0.9952153	0.7555442	not scorable	not scorable	0.9834711	0.7980237
5289	Bn-A03-p11402714	A-genome specific (napus-rapa)	0.9976076	0.5265287	not scorable	not scorable	1	0.6362857
5290	Bn-A03-p11418849	A-genome specific (napus-rapa)	0.9952153	0.342601	not scorable	not scorable	1	0.7736256
5291	Bn-A03-p11421511	A-genome specific (napus-rapa)	0.9976076	0.8522483	not scorable	not scorable	0.9669421	0.7761122
5292	Bn-A03-p11423310	A-genome specific (napus-rapa)	0.9880383	0.6718942	not scorable	not scorable	0.9834711	0.7916752
5293	Bn-A03-p11429520	A-genome specific (napus-rapa)	1	0.5276634	not scorable	not scorable	0.9752066	0.717873
5294	Bn-A03-p11444081	A-genome specific (napus-rapa)	1	0.7654977	not scorable	not scorable	1	0.7557611
5295	Bn-A03-p11444389	A-genome specific (napus-rapa)	1	0.3525551	not scorable	not scorable	0.9917355	0.7121141
5296	Bn-A03-p11456115	A-genome specific (napus-rapa)	0.9856459	0.7553195	not scorable	not scorable	1	0.7741286
5297	Bn-A03-p11468741	A-genome specific (napus-rapa)	0.9760765	0.3434114	not scorable	not scorable	0.9917355	0.7976286
5298	Bn-A03-p11499523	A-genome specific (napus-rapa)	0.9904306	0.510186	not scorable	not scorable	1	0.9493449
5299	Bn-A03-p11505658	A-genome specific (napus-rapa)	0.9952153	0.5080623	not scorable	not scorable	0.9917355	0.7239868
5300	Bn-A03-p11515810	A-genome specific (napus-rapa)	0.9904306	0.769222	not scorable	not scorable	1	0.8403501
5301	Bn-A03-p11517597	A-genome specific (napus-rapa)	0.9832536	0.7974146	not scorable	not scorable	0.9917355	0.7821614
5302	Bn-A03-p11519012	A-genome specific (napus-rapa)	0.9928229	0.8161196	not scorable	not scorable	1	0.6907297
5303	Bn-A03-p11519264	A-genome specific (napus-rapa)	0.9976076	0.7696153	not scorable	not scorable	0.9917355	0.7411231
5304	Bn-A03-p11520156	A-genome specific (napus-rapa)	0.9904306	0.5554656	not scorable	not scorable	1	0.7920699
5305	Bn-A03-p11534030	A-genome specific (napus-rapa)	0.9736842	0.5493516	not scorable	not scorable	0.9586777	0.8408164
5306	Bn-A03-p11544253	A-genome specific (napus-rapa)	0.9856459	0.5519262	not scorable	not scorable	1	0.7959908
5307	Bn-A03-p11546520	A-genome specific (napus-rapa)	0.9832536	0.7670555	not scorable	not scorable	0.9834711	0.7487155
5308	Bn-A03-p11549018	A-genome specific (napus-rapa)	0.9904306	0.7890614	not scorable	not scorable	0.9834711	0.7610225
5309	Bn-A03-p11549288	A-genome specific (napus-rapa)	0.9760765	0.758216	not scorable	not scorable	0.9752066	0.7888584
5310	Bn-A03-p11572347	A-genome specific (napus-rapa)	0.9760765	0.7478341	not scorable	not scorable	1	0.7103665

5311	Bn-A03-p11578563	A-genome specific (napus-rapa)	0.9952153	0.4625314	not scorable	not scorable	0.9752066	0.5359061
5312	Bn-A03-p11583086	A-genome specific (napus-rapa)	0.9856459	0.6954622	not scorable	not scorable	0.9917355	0.756093
5313	Bn-A03-p11590315	A-genome specific (napus-rapa)	0.8995215	0.7078218	not scorable	not scorable	0.8595041	0.7018247
5314	Bn-A03-p11605644	A-genome specific (napus-rapa)	0.9856459	0.2481291	not scorable	not scorable	0.9834711	0.8187895
5315	Bn-A03-p11613167	A-genome specific (napus-rapa)	0.9928229	0.3266001	not scorable	not scorable	1	0.7098928
5316	Bn-A03-p11613865	A-genome specific (napus-rapa)	0.8755981	0.7328466	not scorable	not scorable	1	0.7868593
5317	Bn-A03-p11617534	A-genome specific (napus-rapa)	0.9952153	0.7821622	not scorable	not scorable	1	0.7471316
5318	Bn-A03-p11618241	A-genome specific (napus-rapa)	1	0.73057	not scorable	not scorable	1	0.8249638
5319	Bn-A03-p11621375	A-genome specific (napus-rapa)	0.9808612	0.3059969	not scorable	not scorable	1	0.6820162
5320	Bn-A03-p11623083	A-genome specific (napus-rapa)	0.9928229	0.4268504	not scorable	not scorable	0.892562	0.7944839
5321	Bn-A03-p11624578	A-genome specific (napus-rapa)	0.9090909	0.803053	not scorable	not scorable	0.892562	0.9165112
5322	Bn-A03-p11626015	A-genome specific (napus-rapa)	0.9952153	0.7538181	not scorable	not scorable	1	0.7991292
5323	Bn-A03-p11626279	A-genome specific (napus-rapa)	0.9904306	0.5367107	not scorable	not scorable	0.9917355	0.7539644
5324	Bn-A03-p11633959	napus specific	0.9210526	0.7694378	not scorable	not scorable	not scorable	not scorable
5325	Bn-A03-p11640168	genome unspecific (all in)	0.9832536	0.6016328	1	0.7989718	0.9917355	0.7286076
5326	Bn-A03-p11646564	all not scorable	not scorable	not scorable	not scorable	not scorable	not scorable	not scorable
5327	Bn-A03-p11655161	A-genome specific (napus-rapa)	0.9545454	0.564651	not scorable	not scorable	1	0.6705018
5328	Bn-A03-p11655433	A-genome specific (napus-rapa)	0.9832536	0.7781782	not scorable	not scorable	0.9834711	0.8498148
5329	Bn-A03-p11655749	A-genome specific (napus-rapa)	0.9952153	0.7113739	not scorable	not scorable	1	0.7526731
5330	Bn-A03-p11656333	A-genome specific (napus-rapa)	0.9401914	0.8599369	not scorable	not scorable	0.9752066	0.8130879
5331	Bn-A03-p11683045	A-genome specific (napus-rapa)	0.9880383	0.3834499	not scorable	not scorable	1	0.7995946
5332	Bn-A03-p11684352	A-genome specific (napus-rapa)	0.923445	0.8009381	not scorable	not scorable	0.9834711	0.7981747
5333	Bn-A03-p11687251	A-genome specific (napus-rapa)	0.9186603	0.7300445	not scorable	not scorable	1	0.7006202
5334	Bn-A03-p11704125	A-genome specific (napus-rapa)	0.9976076	0.7270659	not scorable	not scorable	0.9834711	0.661199
5335	Bn-A03-p11704711	A-genome specific (napus-rapa)	0.9784689	0.3660067	not scorable	not scorable	0.9669421	0.6936245
5336	Bn-A03-p11708366	A-genome specific (rapa only)	not scorable	not scorable	not scorable	not scorable	0.9752066	0.652494
5337	Bn-A03-p11739768	A-genome specific (napus-rapa)	0.9904306	0.506255	not scorable	not scorable	1	0.8180857
5338	Bn-A03-p11744082	A-genome specific (napus-rapa)	0.9928229	0.6664226	not scorable	not scorable	0.9834711	0.7653599
5339	Bn-A03-p11751694	A-genome specific (napus-rapa)	0.9856459	0.518812	not scorable	not scorable	0.9834711	0.7396557
5340	Bn-A03-p11765203	all not scorable	not scorable	not scorable	not scorable	not scorable	not scorable	not scorable
5341	Bn-A03-p11771139	A-genome specific (napus-rapa)	0.9976076	0.3892892	not scorable	not scorable	0.9669421	0.9483778
5342	Bn-A03-p11772387	genome unspecific (all in)	0.9904306	0.2941328	0.9767442	0.5585905	1	0.7641606
5343	Bn-A03-p11785159	A-genome specific (napus-rapa)	0.9928229	0.6886181	not scorable	not scorable	1	0.6813985

5344	Bn-A03-p11792328	A-genome specific (napus-rapa)	1	0.3266852	not scorable	not scorable	0.9586777	0.7307112
5345	Bn-A03-p11798309	A-genome specific (napus-rapa)	0.9832536	0.4521256	not scorable	not scorable	0.9752066	0.7513744
5346	Bn-A03-p11822847	all not scorable	not scorable	not scorable	not scorable	not scorable	not scorable	not scorable
5347	Bn-A03-p11831815	A-genome specific (napus-rapa)	0.9856459	0.2458735	not scorable	not scorable	1	0.6995863
5348	Bn-A03-p11883572	A-genome specific (napus-rapa)	1	0.8484479	not scorable	not scorable	0.9917355	0.7096455
5349	Bn-A03-p1194072	A-genome specific (rapa only)	not scorable	not scorable	not scorable	not scorable	0.9917355	0.707278
5350	Bn-A03-p1195712	genome unspecific (oleracea-rapa)	not scorable	not scorable	0.8062016	0.846328	0.9917355	0.798512
5351	Bn-A03-p12008511	A-genome specific (napus-rapa)	0.9904306	0.4370525	not scorable	not scorable	0.9752066	0.7146838
5352	Bn-A03-p12026	A-genome specific (napus-rapa)	0.8421053	0.7745283	not scorable	not scorable	0.9834711	0.6785688
5353	Bn-A03-p12065947	A-genome specific (napus-rapa)	0.9808612	0.7540565	not scorable	not scorable	0.9834711	0.7485709
5354	Bn-A03-p12076661	A-genome specific (napus-rapa)	0.9952153	0.3229261	not scorable	not scorable	0.9917355	0.8114336
5355	Bn-A03-p1218291	A-genome specific (napus-rapa)	0.9282297	0.7873467	not scorable	not scorable	0.9834711	0.8236184
5356	Bn-A03-p12186958	A-genome specific (napus-rapa)	0.9952153	0.7441103	not scorable	not scorable	1	0.8246635
5357	Bn-A03-p12201775	A-genome specific (napus-rapa)	0.9856459	0.5317661	not scorable	not scorable	0.9752066	0.6073731
5358	Bn-A03-p12213267	A-genome specific (napus-rapa)	0.9880383	0.764293	not scorable	not scorable	0.9834711	0.7754347
5359	Bn-A03-p12219523	A-genome specific (napus-rapa)	0.9952153	0.3804826	not scorable	not scorable	1	0.7836584
5360	Bn-A03-p12250401	A-genome specific (napus-rapa)	0.9928229	0.7676533	not scorable	not scorable	0.9917355	0.8905174
5361	Bn-A03-p12279241	A-genome specific (napus-rapa)	1	0.7835542	not scorable	not scorable	1	0.8904615
5362	Bn-A03-p12279706	A-genome specific (napus-rapa)	0.9880383	0.7328691	not scorable	not scorable	1	0.8294268
5363	Bn-A03-p12286718	A-genome specific (napus-rapa)	0.9976076	0.7198862	not scorable	not scorable	0.9917355	0.6772168
5364	Bn-A03-p12287907	A-genome specific (napus-rapa)	0.9976076	0.806498	not scorable	not scorable	1	0.8131604
5365	Bn-A03-p12287911	A-genome specific (napus-rapa)	0.9880383	0.7397896	not scorable	not scorable	1	0.6906343
5366	Bn-A03-p12296442	A-genome specific (napus-rapa)	1	0.614265	not scorable	not scorable	0.9834711	0.5578845
5367	Bn-A03-p12296651	A-genome specific (napus-rapa)	0.9904306	0.24645	not scorable	not scorable	1	0.7601145
5368	Bn-A03-p12302996	A-genome specific (napus-rapa)	0.9808612	0.7689352	not scorable	not scorable	0.9917355	0.7222427
5369	Bn-A03-p12308046	A-genome specific (napus-rapa)	0.9880383	0.6991484	not scorable	not scorable	0.9917355	0.7072919
5370	Bn-A03-p12308158	A-genome specific (napus-rapa)	0.9952153	0.8054594	not scorable	not scorable	0.9834711	0.8439984
5371	Bn-A03-p12308276	A-genome specific (napus-rapa)	0.9904306	0.8167464	not scorable	not scorable	0.9917355	0.6840516
5372	Bn-A03-p12308684	A-genome specific (napus-rapa)	0.9976076	0.7417129	not scorable	not scorable	1	0.7710169
5373	Bn-A03-p12308737	A-genome specific (napus-rapa)	0.9952153	0.6595648	not scorable	not scorable	1	0.6380289
5374	Bn-A03-p12309478	A-genome specific (napus-rapa)	0.9043062	0.7343063	not scorable	not scorable	1	0.7661504
5375	Bn-A03-p12311830	A-genome specific (napus-rapa)	0.9665072	0.7749124	not scorable	not scorable	1	0.7145388
5376	Bn-A03-p12313036	A-genome specific (napus-rapa)	1	0.7020041	not scorable	not scorable	0.9917355	0.7266544

5377	Bn-A03-p12313330	A-genome specific (napus-rapa)	1	0.7153094	not scorable	not scorable	1	0.7312977
5378	Bn-A03-p12319647	A-genome specific (napus-rapa)	0.9880383	0.5280263	not scorable	not scorable	1	0.7356917
5379	Bn-A03-p12322277	A-genome specific (napus-rapa)	0.9880383	0.8434859	not scorable	not scorable	0.9917355	0.7931738
5380	Bn-A03-p12331321	A-genome specific (napus-rapa)	0.9521531	0.6258197	not scorable	not scorable	0.9173554	0.630042
5381	Bn-A03-p12331460	A-genome specific (napus-rapa)	0.9521531	0.697512	not scorable	not scorable	0.9752066	0.7733748
5382	Bn-A03-p12331575	A-genome specific (napus-rapa)	0.9090909	0.7676178	not scorable	not scorable	0.9834711	0.7290828
5383	Bn-A03-p12332552	A-genome specific (napus-rapa)	0.9019139	0.7542918	not scorable	not scorable	0.9917355	0.6788916
5384	Bn-A03-p12342812	A-genome specific (napus-rapa)	0.9784689	0.7981699	not scorable	not scorable	0.9669421	0.7868872
5385	Bn-A03-p12343742	A-genome specific (napus-rapa)	0.9952153	0.3964042	not scorable	not scorable	0.9669421	0.7839566
5386	Bn-A03-p12353218	A-genome specific (napus-rapa)	0.9904306	0.6915604	not scorable	not scorable	0.9917355	0.6715325
5387	Bn-A03-p12353370	A-genome specific (napus-rapa)	0.9832536	0.4654876	not scorable	not scorable	0.9917355	0.8285162
5388	Bn-A03-p12359936	A-genome specific (napus-rapa)	0.9904306	0.6844398	not scorable	not scorable	1	0.6976528
5389	Bn-A03-p1238329	A-genome specific (napus-rapa)	0.9210526	0.6514074	not scorable	not scorable	0.9917355	0.660017
5390	Bn-A03-p12393626	A-genome specific (napus-rapa)	0.9808612	0.3891913	not scorable	not scorable	1	0.8050153
5391	Bn-A03-p12393711	A-genome specific (napus-rapa)	0.9832536	0.7893108	not scorable	not scorable	0.9917355	0.7345523
5392	Bn-A03-p12402349	genome unspecific (oleracea-rapa)	not scorable	not scorable	0.9922481	0.6164997	0.9917355	0.7526404
5393	Bn-A03-p12411362	A-genome specific (napus-rapa)	0.9832536	0.3419674	not scorable	not scorable	0.9834711	0.7833
5394	Bn-A03-p12426461	A-genome specific (napus-rapa)	0.9760765	0.4430735	not scorable	not scorable	0.9834711	0.8299392
5395	Bn-A03-p12429205	A-genome specific (napus-rapa)	0.9928229	0.7123672	not scorable	not scorable	1	0.7436125
5396	Bn-A03-p12431376	A-genome specific (napus-rapa)	0.9928229	0.5014616	not scorable	not scorable	1	0.9175813
5397	Bn-A03-p12439824	A-genome specific (napus-rapa)	1	0.5507981	not scorable	not scorable	0.9917355	0.5755402
5398	Bn-A03-p12444739	A-genome specific (napus-rapa)	0.9904306	0.7940341	not scorable	not scorable	1	0.9175813
5399	Bn-A03-p12453877	A-genome specific (napus-rapa)	0.9760765	0.4141209	not scorable	not scorable	1	0.8250605
5400	Bn-A03-p12457385	A-genome specific (napus-rapa)	0.9904306	0.8196128	not scorable	not scorable	0.9834711	0.7022214
5401	Bn-A03-p12457751	A-genome specific (napus-rapa)	0.9976076	0.6652936	not scorable	not scorable	0.9917355	0.7541751
5402	Bn-A03-p12471513	A-genome specific (napus-rapa)	0.9808612	0.7662895	not scorable	not scorable	0.9504132	0.8564795
5403	Bn-A03-p12477334	A-genome specific (napus-rapa)	0.9736842	0.6007537	not scorable	not scorable	1	0.8140023
5404	Bn-A03-p12482349	A-genome specific (napus-rapa)	0.9808612	0.530714	not scorable	not scorable	0.9669421	0.7761641
5405	Bn-A03-p12511746	A-genome specific (napus-rapa)	0.9856459	0.4961472	not scorable	not scorable	0.9917355	0.709359
5406	Bn-A03-p12512134	A-genome specific (napus-rapa)	0.9760765	0.6887558	not scorable	not scorable	0.9752066	0.6475088
5407	Bn-A03-p12518314	napus specific	0.9880383	0.7215762	not scorable	not scorable	not scorable	not scorable
5408	Bn-A03-p12532686	A-genome specific (napus-rapa)	0.9856459	0.2383776	not scorable	not scorable	0.9917355	0.7848483
5409	Bn-A03-p12541037	A-genome specific (napus-rapa)	0.9712918	0.2832885	not scorable	not scorable	0.9752066	0.7017043

5410	Bn-A03-p12541207	A-genome specific (napus-rapa)	0.9832536	0.5727046	not scorable	not scorable	0.9917355	0.7235581
5411	Bn-A03-p12541337	A-genome specific (napus-rapa)	0.9904306	0.493327	not scorable	not scorable	1	0.7702418
5412	Bn-A03-p1254871	A-genome specific (rapa only)	not scorable	not scorable	not scorable	not scorable	1	0.7720746
5413	Bn-A03-p12622050	A-genome specific (napus-rapa)	0.9856459	0.7873049	not scorable	not scorable	0.9586777	0.8310348
5414	Bn-A03-p12623286	A-genome specific (napus-rapa)	0.9736842	0.7667772	not scorable	not scorable	0.9669421	0.7985176
5415	Bn-A03-p12628781	A-genome specific (napus-rapa)	0.9808612	0.6317586	not scorable	not scorable	0.9917355	0.7436985
5416	Bn-A03-p12631124	genome unspecific (oleracea-rapa)	not scorable	not scorable	0.9844961	0.7691123	0.9917355	0.7360191
5417	Bn-A03-p12638944	A-genome specific (napus-rapa)	0.9784689	0.6388492	not scorable	not scorable	0.9917355	0.8265692
5418	Bn-A03-p12639200	A-genome specific (napus-rapa)	0.9856459	0.7831169	not scorable	not scorable	1	0.6787928
5419	Bn-A03-p12639319	A-genome specific (napus-rapa)	0.9904306	0.7773882	not scorable	not scorable	1	0.8138083
5420	Bn-A03-p12737336	genome unspecific (all in)	0.9760765	0.6574421	0.744186	0.7042676	1	0.7723419
5421	Bn-A03-p12783093	A-genome specific (napus-rapa)	0.9976076	0.7700087	not scorable	not scorable	1	0.7941356
5422	Bn-A03-p12784925	A-genome specific (napus-rapa)	0.9976076	0.7800601	not scorable	not scorable	1	0.7423106
5423	Bn-A03-p12820905	A-genome specific (napus-rapa)	0.9760765	0.7878451	not scorable	not scorable	0.9669421	0.7505035
5424	Bn-A03-p12826949	A-genome specific (napus-rapa)	0.9760765	0.4618206	not scorable	not scorable	0.9752066	0.8013898
5425	Bn-A03-p12859516	A-genome specific (napus-rapa)	0.9760765	0.2996366	not scorable	not scorable	0.9834711	0.8416808
5426	Bn-A03-p12863264	A-genome specific (napus-rapa)	1	0.8406045	not scorable	not scorable	0.9752066	0.7046431
5427	Bn-A03-p12863642	A-genome specific (napus-rapa)	0.9976076	0.7384824	not scorable	not scorable	1	0.703868
5428	Bn-A03-p12863684	A-genome specific (napus-rapa)	0.9976076	0.6936817	not scorable	not scorable	0.9834711	0.6851025
5429	Bn-A03-p12869911	A-genome specific (napus-rapa)	0.9952153	0.796129	not scorable	not scorable	0.9917355	0.7869104
5430	Bn-A03-p12903591	A-genome specific (napus-rapa)	0.9904306	0.7986871	not scorable	not scorable	0.9834711	0.7465758
5431	Bn-A03-p12932399	A-genome specific (napus-rapa)	0.9928229	0.7815844	not scorable	not scorable	1	0.7678177
5432	Bn-A03-p12932760	A-genome specific (rapa only)	not scorable	not scorable	not scorable	not scorable	1	0.8147472
5433	Bn-A03-p12933099	A-genome specific (napus-rapa)	0.9976076	0.8017709	not scorable	not scorable	1	0.7613931
5434	Bn-A03-p12935447	A-genome specific (napus-rapa)	0.9880383	0.7791921	not scorable	not scorable	0.9917355	0.7463617
5435	Bn-A03-p12941404	A-genome specific (napus-rapa)	0.9832536	0.7646145	not scorable	not scorable	1	0.7931048
5436	Bn-A03-p12945091	A-genome specific (napus-rapa)	1	0.7832142	not scorable	not scorable	1	0.8687926
5437	Bn-A03-p12973462	A-genome specific (napus-rapa)	0.9976076	0.8429547	not scorable	not scorable	0.9669421	0.6962659
5438	Bn-A03-p1297873	A-genome specific (rapa only)	not scorable	not scorable	not scorable	not scorable	0.9834711	0.8588021
5439	Bn-A03-p12981348	A-genome specific (napus-rapa)	0.9712918	0.5782443	not scorable	not scorable	0.9917355	0.9387571
5440	Bn-A03-p12990610	A-genome specific (napus-rapa)	0.9976076	0.7942174	not scorable	not scorable	1	0.7194485
5441	Bn-A03-p12993600	A-genome specific (napus-rapa)	0.9808612	0.7555156	not scorable	not scorable	0.9917355	0.751028
5442	Bn-A03-p12994885	A-genome specific (napus-rapa)	0.9641148	0.5852197	not scorable	not scorable	1	0.6510744

5443	Bn-A03-p13016324	A-genome specific (napus-rapa)	0.6339713	0.8027261	not scorable	not scorable	0.8595041	0.7470858
5444	Bn-A03-p13017512	A-genome specific (napus-rapa)	0.9617225	0.4284885	not scorable	not scorable	0.9917355	0.6560733
5445	Bn-A03-p13017704	A-genome specific (napus-rapa)	0.9712918	0.7600284	not scorable	not scorable	0.9917355	0.7455516
5446	Bn-A03-p13018172	genome unspecific (oleracea-rapa)	not scorable	not scorable	0.9689922	0.2966666	0.9834711	0.6869229
5447	Bn-A03-p13032037	A-genome specific (napus-rapa)	0.9832536	0.7658719	not scorable	not scorable	0.9917355	0.7422404
5448	Bn-A03-p13032092	A-genome specific (napus-rapa)	0.9904306	0.6968184	not scorable	not scorable	0.9917355	0.7993341
5449	Bn-A03-p13037975	A-genome specific (napus-rapa)	0.9760765	0.7079893	not scorable	not scorable	1	0.8891756
5450	Bn-A03-p13039000	A-genome specific (napus-rapa)	0.9880383	0.7549368	not scorable	not scorable	0.9917355	0.7707877
5451	Bn-A03-p13039285	A-genome specific (napus-rapa)	0.9952153	0.8191442	not scorable	not scorable	0.9917355	0.8052118
5452	Bn-A03-p13043164	A-genome specific (napus-rapa)	0.9401914	0.5375264	not scorable	not scorable	0.9834711	0.626734
5453	Bn-A03-p13044950	A-genome specific (napus-rapa)	0.777512	0.8106512	not scorable	not scorable	1	0.9269007
5454	Bn-A03-p13050570	A-genome specific (rapa only)	not scorable	not scorable	not scorable	not scorable	0.9834711	0.7451766
5455	Bn-A03-p13058729	A-genome specific (napus-rapa)	0.9617225	0.4325492	not scorable	not scorable	0.8595041	0.7930796
5456	Bn-A03-p13061709	A-genome specific (napus-rapa)	0.9784689	0.5482695	not scorable	not scorable	0.9834711	0.6541692
5457	Bn-A03-p1308820	A-genome specific (napus-rapa)	0.9138756	0.737713	not scorable	not scorable	0.9917355	0.7170929
5458	Bn-A03-p13094043	A-genome specific (napus-rapa)	0.9138756	0.7610633	not scorable	not scorable	0.8842975	0.8087493
5459	Bn-A03-p13099476	A-genome specific (napus-rapa)	0.9952153	0.8050456	not scorable	not scorable	1	0.8257377
5460	Bn-A03-p13104283	A-genome specific (napus-rapa)	0.9928229	0.7234445	not scorable	not scorable	1	0.6676938
5461	Bn-A03-p13107268	A-genome specific (napus-rapa)	0.9976076	0.6178387	not scorable	not scorable	0.9834711	0.8067495
5462	Bn-A03-p13109267	A-genome specific (napus-rapa)	0.9976076	0.7923573	not scorable	not scorable	0.9834711	0.7238204
5463	Bn-A03-p13131453	A-genome specific (napus-rapa)	0.9665072	0.7766892	not scorable	not scorable	1	0.7681646
5464	Bn-A03-p13145923	A-genome specific (napus-rapa)	0.9665072	0.320095	not scorable	not scorable	0.9421487	0.543936
5465	Bn-A03-p13160528	A-genome specific (napus-rapa)	0.9880383	0.5374056	not scorable	not scorable	0.9917355	0.7803829
5466	Bn-A03-p13169138	A-genome specific (napus-rapa)	0.9712918	0.7496251	not scorable	not scorable	1	0.798901
5467	Bn-A03-p13169346	A-genome specific (napus-rapa)	0.9904306	0.7261339	not scorable	not scorable	0.9834711	0.7545496
5468	Bn-A03-p13177232	A-genome specific (napus-rapa)	0.8205742	0.797794	not scorable	not scorable	0.9669421	0.6495573
5469	Bn-A03-p13189171	A-genome specific (napus-rapa)	0.9641148	0.3557096	not scorable	not scorable	1	0.7248943
5470	Bn-A03-p13216785	genome unspecific (oleracea-rapa)	not scorable	not scorable	1	0.5129972	1	0.7146787
5471	Bn-A03-p13218451	A-genome specific (napus-rapa)	0.8397129	0.8176482	not scorable	not scorable	0.9586777	0.7561886
5472	Bn-A03-p13219815	A-genome specific (napus-rapa)	0.8492823	0.7979313	not scorable	not scorable	0.9669421	0.7862451
5473	Bn-A03-p13221584	A-genome specific (napus-rapa)	0.8373206	0.7272601	not scorable	not scorable	0.9834711	0.6307871
5474	Bn-A03-p13223204	A-genome specific (napus-rapa)	0.8325359	0.8085008	not scorable	not scorable	0.9669421	0.7589105
5475	Bn-A03-p13236840	A-genome specific (napus-rapa)	0.9880383	0.7197053	not scorable	not scorable	0.9917355	0.6955878

5476	Bn-A03-p13253155	A-genome specific (napus-rapa)	0.9736842	0.4488608	not scorable	not scorable	1	0.7229308
5477	Bn-A03-p13254564	A-genome specific (napus-rapa)	0.9712918	0.4297775	not scorable	not scorable	0.9752066	0.8038705
5478	Bn-A03-p13266036	A-genome specific (napus-rapa)	0.9688995	0.7279103	not scorable	not scorable	0.9834711	0.9090296
5479	Bn-A03-p13286574	A-genome specific (napus-rapa)	0.9976076	0.8166432	not scorable	not scorable	0.9917355	0.7436472
5480	Bn-A03-p13287670	A-genome specific (napus-rapa)	0.9856459	0.7370334	not scorable	not scorable	0.9834711	0.7315952
5481	Bn-A03-p13288429	A-genome specific (napus-rapa)	0.9880383	0.7271395	not scorable	not scorable	1	0.7617276
5482	Bn-A03-p13291935	A-genome specific (napus-rapa)	0.9473684	0.7384024	not scorable	not scorable	0.8595041	0.7880812
5483	Bn-A03-p13298734	A-genome specific (napus-rapa)	0.9856459	0.6595703	not scorable	not scorable	0.9917355	0.7043232
5484	Bn-A03-p13302773	A-genome specific (rapa only)	not scorable	not scorable	not scorable	not scorable	1	0.7432435
5485	Bn-A03-p13302882	genome unspecific (all in)	0.576555	0.8793352	0	0.7813661	0.9504132	0.8552643
5486	Bn-A03-p13306819	A-genome specific (napus-rapa)	0.9880383	0.3131319	not scorable	not scorable	0.9834711	0.7816145
5487	Bn-A03-p13324647	A-genome specific (napus-rapa)	0.7655503	0.8495582	not scorable	not scorable	1	0.8019731
5488	Bn-A03-p13325522	A-genome specific (napus-rapa)	0.7631579	0.8007662	not scorable	not scorable	1	0.8154832
5489	Bn-A03-p13335275	A-genome specific (napus-rapa)	0.9832536	0.5067183	not scorable	not scorable	0.9752066	0.7409006
5490	Bn-A03-p13335946	A-genome specific (napus-rapa)	0.9928229	0.4231012	not scorable	not scorable	0.9917355	0.6920217
5491	Bn-A03-p13365712	A-genome specific (napus-rapa)	0.9521531	0.4719522	not scorable	not scorable	0.9834711	0.6980245
5492	Bn-A03-p13421039	A-genome specific (napus-rapa)	0.9736842	0.7898866	not scorable	not scorable	0.9586777	0.8185682
5493	Bn-A03-p13440035	A-genome specific (napus-rapa)	0.9569378	0.5555617	not scorable	not scorable	0.9752066	0.7261897
5494	Bn-A03-p13451851	A-genome specific (napus-rapa)	0.9856459	0.7665362	not scorable	not scorable	0.9917355	0.720566
5495	Bn-A03-p13465062	A-genome specific (napus-rapa)	0.9856459	0.6989835	not scorable	not scorable	0.9504132	0.5669611
5496	Bn-A03-p13469770	A-genome specific (rapa only)	not scorable	not scorable	not scorable	not scorable	1	0.7871423
5497	Bn-A03-p13492986	genome unspecific (oleracea-rapa)	not scorable	not scorable	0.9224806	0.2585334	0.9917355	0.8324326
5498	Bn-A03-p13502336	A-genome specific (napus-rapa)	0.9784689	0.5150532	not scorable	not scorable	1	0.7712471
5499	Bn-A03-p13502830	A-genome specific (napus-rapa)	0.9832536	0.3917311	not scorable	not scorable	1	0.7432263
5500	Bn-A03-p135145	genome unspecific (oleracea-rapa)	not scorable	not scorable	0.9612403	0.2493363	0.9752066	0.633635
5501	Bn-A03-p13514947	A-genome specific (napus-rapa)	0.9688995	0.2851632	not scorable	not scorable	0.9917355	0.6936299
5502	Bn-A03-p13523343	A-genome specific (napus-rapa)	0.9928229	0.2722597	not scorable	not scorable	0.9834711	0.6532474
5503	Bn-A03-p1352996	A-genome specific (napus-rapa)	0.9258373	0.7539104	not scorable	not scorable	1	0.779401
5504	Bn-A03-p1353309	A-genome specific (napus-rapa)	0.9760765	0.6323574	not scorable	not scorable	1	0.7502307
5505	Bn-A03-p13533904	A-genome specific (napus-rapa)	0.9976076	0.8043466	not scorable	not scorable	0.9917355	0.7397541
5506	Bn-A03-p1354206	A-genome specific (napus-rapa)	0.8205742	0.7939495	not scorable	not scorable	0.9752066	0.8259369
5507	Bn-A03-p1354375	A-genome specific (napus-rapa)	0.9258373	0.7201173	not scorable	not scorable	1	0.7776101
5508	Bn-A03-p13545223	A-genome specific (napus-rapa)	0.9856459	0.7475673	not scorable	not scorable	1	0.7341583

5509	Bn-A03-p13546174	A-genome specific (rapa only)	not scorable	not scorable	not scorable	not scorable	0.9256198	0.7897152
5510	Bn-A03-p13567559	A-genome specific (napus-rapa)	0.9521531	0.7850012	not scorable	not scorable	0.9834711	0.6556188
5511	Bn-A03-p13572495	A-genome specific (napus-rapa)	0.9904306	0.7550953	not scorable	not scorable	0.9834711	0.7901838
5512	Bn-A03-p13585261	A-genome specific (napus-rapa)	0.9904306	0.6324359	not scorable	not scorable	1	0.6591645
5513	Bn-A03-p13605344	A-genome specific (napus-rapa)	0.8205742	0.7957346	not scorable	not scorable	0.9669421	0.8044536
5514	Bn-A03-p13610858	A-genome specific (napus-rapa)	0.9856459	0.3683416	not scorable	not scorable	0.9917355	0.7316198
5515	Bn-A03-p13616280	A-genome specific (napus-rapa)	0.9712918	0.3851596	not scorable	not scorable	0.9834711	0.6910375
5516	Bn-A03-p13624310	A-genome specific (napus-rapa)	0.9880383	0.7689049	not scorable	not scorable	1	0.7942903
5517	Bn-A03-p13630048	A-genome specific (napus-rapa)	0.708134	0.8208213	not scorable	not scorable	1	0.6942151
5518	Bn-A03-p13640859	A-genome specific (napus-rapa)	0.9521531	0.3585845	not scorable	not scorable	0.9917355	0.7248549
5519	Bn-A03-p13653603	A-genome specific (napus-rapa)	0.6961722	0.6386769	not scorable	not scorable	0.9917355	0.7124016
5520	Bn-A03-p13658460	A-genome specific (napus-rapa)	0.9760765	0.3058629	not scorable	not scorable	0.9834711	0.7036489
5521	Bn-A03-p13664500	A-genome specific (napus-rapa)	0.9904306	0.7809391	not scorable	not scorable	1	0.8004379
5522	Bn-A03-p13664657	A-genome specific (napus-rapa)	0.9856459	0.7614344	not scorable	not scorable	1	0.8096587
5523	Bn-A03-p13678735	A-genome specific (napus-rapa)	0.9904306	0.2356496	not scorable	not scorable	0.9669421	0.7648913
5524	Bn-A03-p13679854	A-genome specific (napus-rapa)	0.9808612	0.3194575	not scorable	not scorable	0.9752066	0.7138126
5525	Bn-A03-p1373885	A-genome specific (rapa only)	not scorable	not scorable	not scorable	not scorable	0.9917355	0.8151305
5526	Bn-A03-p13746713	A-genome specific (napus-rapa)	0.9904306	0.7644034	not scorable	not scorable	0.9834711	0.7075557
5527	Bn-A03-p13747074	A-genome specific (napus-rapa)	0.9569378	0.6721485	not scorable	not scorable	0.9669421	0.6966495
5528	Bn-A03-p13755726	A-genome specific (napus-rapa)	0.9904306	0.8009173	not scorable	not scorable	0.9256198	0.6906074
5529	Bn-A03-p13758392	A-genome specific (napus-rapa)	0.9904306	0.8019578	not scorable	not scorable	0.9421487	0.6907854
5530	Bn-A03-p13773583	A-genome specific (napus-rapa)	0.9904306	0.7904152	not scorable	not scorable	0.8595041	0.7094761
5531	Bn-A03-p13777203	genome unspecific (all in)	0.9880383	0.4100632	0.9224806	0.5359281	0.9834711	0.7040216
5532	Bn-A03-p13778786	A-genome specific (napus-rapa)	0.9880383	0.4727136	not scorable	not scorable	0.9917355	0.7680721
5533	Bn-A03-p13783263	A-genome specific (napus-rapa)	0.9808612	0.4066428	not scorable	not scorable	0.9917355	0.7238167
5534	Bn-A03-p13802162	A-genome specific (napus-rapa)	0.9808612	0.7617787	not scorable	not scorable	0.9752066	0.6973376
5535	Bn-A03-p13810458	A-genome specific (napus-rapa)	1	0.7586714	not scorable	not scorable	0.8347107	0.890564
5536	Bn-A03-p13814543	A-genome specific (napus-rapa)	0.9832536	0.3215439	not scorable	not scorable	0.8264463	0.9209731
5537	Bn-A03-p13815665	A-genome specific (napus-rapa)	1	0.2961185	not scorable	not scorable	0.9917355	0.5903504
5538	Bn-A03-p13853558	A-genome specific (napus-rapa)	0.9880383	0.7477842	not scorable	not scorable	0.9669421	0.7439271
5539	Bn-A03-p13859927	genome unspecific (oleracea-rapa)	not scorable	not scorable	0.9922481	0.7938771	0.9752066	0.8544804
5540	Bn-A03-p13861679	A-genome specific (napus-rapa)	0.5813397	0.6621116	not scorable	not scorable	0.9917355	0.767139
5541	Bn-A03-p13861749	A-genome specific (napus-rapa)	1	0.8086978	not scorable	not scorable	1	0.7721077

5542	Bn-A03-p13863828	A-genome specific (napus-rapa)	0.930622	0.2622168	not scorable	not scorable	0.9917355	0.7585171
5543	Bn-A03-p13883754	A-genome specific (napus-rapa)	0.9808612	0.7763981	not scorable	not scorable	0.9752066	0.7595723
5544	Bn-A03-p13906492	A-genome specific (napus-rapa)	0.9880383	0.4191443	not scorable	not scorable	0.9917355	0.7659603
5545	Bn-A03-p13908787	A-genome specific (napus-rapa)	0.9928229	0.4923468	not scorable	not scorable	0.9917355	0.7575203
5546	Bn-A03-p13912476	A-genome specific (napus-rapa)	0.9641148	0.7107902	not scorable	not scorable	1	0.74534
5547	Bn-A03-p13922223	A-genome specific (napus-rapa)	0.9641148	0.623268	not scorable	not scorable	1	0.7375211
5548	Bn-A03-p13922405	A-genome specific (napus-rapa)	0.9880383	0.7825214	not scorable	not scorable	0.9834711	0.7619595
5549	Bn-A03-p13925731	A-genome specific (napus-rapa)	0.9976076	0.6870852	not scorable	not scorable	1	0.6231178
5550	Bn-A03-p13943992	A-genome specific (napus-rapa)	0.9880383	0.7358934	not scorable	not scorable	1	0.7277293
5551	Bn-A03-p13968921	A-genome specific (napus-rapa)	0.9880383	0.3160003	not scorable	not scorable	0.9917355	0.7945459
5552	Bn-A03-p13970114	A-genome specific (napus-rapa)	0.9880383	0.8090085	not scorable	not scorable	1	0.7802013
5553	Bn-A03-p13970168	A-genome specific (napus-rapa)	0.9952153	0.8578107	not scorable	not scorable	1	0.9037206
5554	Bn-A03-p13974725	A-genome specific (napus-rapa)	0.9736842	0.7978377	not scorable	not scorable	0.9917355	0.8080547
5555	Bn-A03-p13975945	A-genome specific (napus-rapa)	0.9952153	0.6803098	not scorable	not scorable	1	0.7234979
5556	Bn-A03-p13977740	A-genome specific (napus-rapa)	0.9760765	0.6493017	not scorable	not scorable	0.8842975	0.7062998
5557	Bn-A03-p13987679	A-genome specific (napus-rapa)	0.9880383	0.6738646	not scorable	not scorable	0.9917355	0.8088477
5558	Bn-A03-p13996014	A-genome specific (napus-rapa)	0.9976076	0.6778447	not scorable	not scorable	0.9669421	0.6658475
5559	Bn-A03-p14003701	A-genome specific (napus-rapa)	1	0.8339772	not scorable	not scorable	1	0.8444647
5560	Bn-A03-p14009871	A-genome specific (napus-rapa)	0.9880383	0.6976369	not scorable	not scorable	0.9917355	0.7022238
5561	Bn-A03-p14015779	A-genome specific (napus-rapa)	0.9832536	0.4871463	not scorable	not scorable	0.9834711	0.7179611
5562	Bn-A03-p14023392	A-genome specific (napus-rapa)	0.9928229	0.7555622	not scorable	not scorable	0.9917355	0.7395499
5563	Bn-A03-p14024778	A-genome specific (napus-rapa)	0.569378	0.6711851	not scorable	not scorable	0.9752066	0.7475929
5564	Bn-A03-p14026999	A-genome specific (napus-rapa)	0.9114832	0.7844557	not scorable	not scorable	0.9834711	0.8433641
5565	Bn-A03-p14031468	A-genome specific (napus-rapa)	0.9066986	0.7507783	not scorable	not scorable	0.9834711	0.826178
5566	Bn-A03-p14034228	A-genome specific (napus-rapa)	0.9736842	0.5849665	not scorable	not scorable	0.9917355	0.7298668
5567	Bn-A03-p14037892	A-genome specific (napus-rapa)	0.9856459	0.6491271	not scorable	not scorable	0.9669421	0.7144052
5568	Bn-A03-p14041361	A-genome specific (napus-rapa)	0.9545454	0.8000417	not scorable	not scorable	1	0.8258333
5569	Bn-A03-p14041931	A-genome specific (napus-rapa)	0.9545454	0.410037	not scorable	not scorable	0.9917355	0.6684541
5570	Bn-A03-p14092692	A-genome specific (napus-rapa)	0.9688995	0.4871914	not scorable	not scorable	0.9917355	0.8051483
5571	Bn-A03-p14095948	A-genome specific (napus-rapa)	0.9880383	0.724889	not scorable	not scorable	0.9834711	0.7857534
5572	Bn-A03-p14112910	A-genome specific (napus-rapa)	0.9497608	0.7906131	not scorable	not scorable	0.9917355	0.6849222
5573	Bn-A03-p14113489	A-genome specific (napus-rapa)	0.9688995	0.8285137	not scorable	not scorable	0.9917355	0.7255349
5574	Bn-A03-p14121492	A-genome specific (napus-rapa)	0.9928229	0.5724918	not scorable	not scorable	1	0.7507635

5575	Bn-A03-p14127649	A-genome specific (napus-rapa)	0.9952153	0.622758	not scorable	not scorable	0.9752066	0.5975209
5576	Bn-A03-p14128333	A-genome specific (napus-rapa)	0.9880383	0.3287923	not scorable	not scorable	1	0.7134259
5577	Bn-A03-p14153283	A-genome specific (napus-rapa)	0.9976076	0.8687427	not scorable	not scorable	1	0.9517783
5578	Bn-A03-p14153306	A-genome specific (napus-rapa)	0.9976076	0.8035696	not scorable	not scorable	0.9834711	0.6600227
5579	Bn-A03-p14153376	A-genome specific (napus-rapa)	0.8373206	0.7136337	not scorable	not scorable	0.9752066	0.7867898
5580	Bn-A03-p14154555	A-genome specific (napus-rapa)	0.9952153	0.7690284	not scorable	not scorable	0.9752066	0.7331636
5581	Bn-A03-p14154912	A-genome specific (napus-rapa)	0.9976076	0.8985654	not scorable	not scorable	0.9834711	0.7598843
5582	Bn-A03-p14155274	A-genome specific (napus-rapa)	1	0.8123643	not scorable	not scorable	0.9917355	0.7361144
5583	Bn-A03-p141681	A-genome specific (napus-rapa)	0.9880383	0.5229543	not scorable	not scorable	0.9669421	0.6846697
5584	Bn-A03-p14175904	A-genome specific (napus-rapa)	0.9760765	0.7793086	not scorable	not scorable	1	0.8220586
5585	Bn-A03-p14176469	A-genome specific (napus-rapa)	0.9952153	0.749972	not scorable	not scorable	0.9669421	0.7744361
5586	Bn-A03-p14196859	A-genome specific (napus-rapa)	0.9880383	0.7472756	not scorable	not scorable	0.9834711	0.6724088
5587	Bn-A03-p14197151	A-genome specific (napus-rapa)	0.9928229	0.7728011	not scorable	not scorable	1	0.738019
5588	Bn-A03-p14201146	A-genome specific (napus-rapa)	0.9114832	0.8100545	not scorable	not scorable	1	0.895025
5589	Bn-A03-p14209971	A-genome specific (napus-rapa)	0.9665072	0.7987092	not scorable	not scorable	0.9834711	0.8942698
5590	Bn-A03-p14212196	A-genome specific (rapa only)	not scorable	not scorable	not scorable	not scorable	1	0.8220702
5591	Bn-A03-p14251137	A-genome specific (napus-rapa)	0.9952153	0.7820045	not scorable	not scorable	1	0.7419274
5592	Bn-A03-p14261914	A-genome specific (napus-rapa)	0.9784689	0.7012179	not scorable	not scorable	1	0.6996191
5593	Bn-A03-p14262103	A-genome specific (napus-rapa)	0.9856459	0.3898825	not scorable	not scorable	1	0.707387
5594	Bn-A03-p14273821	A-genome specific (napus-rapa)	0.9665072	0.69795	not scorable	not scorable	1	0.7108782
5595	Bn-A03-p14276328	A-genome specific (napus-rapa)	0.9784689	0.4558971	not scorable	not scorable	1	0.7252982
5596	Bn-A03-p14282460	A-genome specific (napus-rapa)	0.9880383	0.7106097	not scorable	not scorable	0.9834711	0.6710642
5597	Bn-A03-p14286873	A-genome specific (napus-rapa)	0.9569378	0.310583	not scorable	not scorable	0.9834711	0.7155547
5598	Bn-A03-p14290741	A-genome specific (napus-rapa)	0.9760765	0.7024059	not scorable	not scorable	0.8842975	0.722693
5599	Bn-A03-p14291952	genome unspecific (all in)	0.9688995	0.3534776	0.9767442	0.7120925	0.9834711	0.7110288
5600	Bn-A03-p14295089	A-genome specific (napus-rapa)	0.9952153	0.3625909	not scorable	not scorable	1	0.8107109
5601	Bn-A03-p14295396	A-genome specific (napus-rapa)	0.9856459	0.6979052	not scorable	not scorable	0.9917355	0.7312261
5602	Bn-A03-p14306440	A-genome specific (napus-rapa)	0.9712918	0.5816492	not scorable	not scorable	0.9917355	0.6859793
5603	Bn-A03-p14309803	A-genome specific (napus-rapa)	0.9880383	0.7647578	not scorable	not scorable	0.9752066	0.7296809
5604	Bn-A03-p14310386	A-genome specific (napus-rapa)	0.9952153	0.7946287	not scorable	not scorable	0.9917355	0.7160908
5605	Bn-A03-p14310805	A-genome specific (napus-rapa)	0.8779904	0.8153975	not scorable	not scorable	0.9834711	0.6939947
5606	Bn-A03-p14330609	A-genome specific (napus-rapa)	0.9928229	0.3079938	not scorable	not scorable	0.9834711	0.6302835
5607	Bn-A03-p14353080	napus specific	0.8851675	0.6714671	not scorable	not scorable	not scorable	not scorable

5608	Bn-A03-p14355646	A-genome specific (napus-rapa)	0.9904306	0.7248414	not scorable	not scorable	0.9752066	0.6567682
5609	Bn-A03-p1435886	A-genome specific (napus-rapa)	0.9162679	0.6739193	not scorable	not scorable	1	0.676922
5610	Bn-A03-p14363525	all not scorable	not scorable	not scorable	not scorable	not scorable	not scorable	not scorable
5611	Bn-A03-p14369287	A-genome specific (napus-rapa)	0.9928229	0.717856	not scorable	not scorable	0.9752066	0.8358058
5612	Bn-A03-p14385006	A-genome specific (napus-rapa)	0.9976076	0.7217564	not scorable	not scorable	1	0.6676899
5613	Bn-A03-p14387177	A-genome specific (napus-rapa)	0.9784689	0.4337255	not scorable	not scorable	1	0.8219444
5614	Bn-A03-p14398744	A-genome specific (napus-rapa)	0.9832536	0.3954021	not scorable	not scorable	1	0.7322186
5615	Bn-A03-p14399972	A-genome specific (napus-rapa)	0.9952153	0.6774447	not scorable	not scorable	0.9917355	0.6786544
5616	Bn-A03-p14400020	A-genome specific (napus-rapa)	0.9832536	0.8121197	not scorable	not scorable	0.9834711	0.725991
5617	Bn-A03-p14402509	A-genome specific (napus-rapa)	0.9832536	0.3938435	not scorable	not scorable	1	0.6781039
5618	Bn-A03-p14413451	genome unspecific (all in)	0.8684211	0.582573	0.9844961	0.280252	1	0.7966102
5619	Bn-A03-p14418556	A-genome specific (napus-rapa)	0.9569378	0.3492815	not scorable	not scorable	1	0.7088731
5620	Bn-A03-p14418650	A-genome specific (napus-rapa)	0.9952153	0.4613407	not scorable	not scorable	1	0.8180235
5621	Bn-A03-p14423477	A-genome specific (napus-rapa)	0.9784689	0.7276827	not scorable	not scorable	0.9917355	0.8317313
5622	Bn-A03-p14434665	A-genome specific (napus-rapa)	0.9952153	0.8050964	not scorable	not scorable	0.9834711	0.8200701
5623	Bn-A03-p14447238	A-genome specific (napus-rapa)	0.9880383	0.7488167	not scorable	not scorable	1	0.8136883
5624	Bn-A03-p14458139	A-genome specific (napus-rapa)	0.9856459	0.622539	not scorable	not scorable	1	0.8436139
5625	Bn-A03-p14461008	A-genome specific (napus-rapa)	0.9952153	0.574797	not scorable	not scorable	0.9834711	0.7148626
5626	Bn-A03-p14462148	A-genome specific (napus-rapa)	0.9952153	0.8337399	not scorable	not scorable	1	0.8752148
5627	Bn-A03-p14462570	A-genome specific (napus-rapa)	1	0.6659186	not scorable	not scorable	1	0.6420932
5628	Bn-A03-p14472406	A-genome specific (napus-rapa)	1	0.7481881	not scorable	not scorable	0.8760331	0.7151247
5629	Bn-A03-p14473184	napus specific	1	0.5623639	not scorable	not scorable	not scorable	not scorable
5630	Bn-A03-p14473296	A-genome specific (napus-rapa)	0.9760765	0.6871446	not scorable	not scorable	0.9917355	0.6352119
5631	Bn-A03-p14474478	A-genome specific (napus-rapa)	0.9617225	0.7667105	not scorable	not scorable	1	0.8335571
5632	Bn-A03-p14511431	A-genome specific (napus-rapa)	0.9904306	0.774935	not scorable	not scorable	1	0.72925
5633	Bn-A03-p14552086	A-genome specific (napus-rapa)	0.9928229	0.5263312	not scorable	not scorable	1	0.7709211
5634	Bn-A03-p14557663	A-genome specific (napus-rapa)	0.9808612	0.5876682	not scorable	not scorable	0.9917355	0.7431487
5635	Bn-A03-p14577690	A-genome specific (napus-rapa)	0.9688995	0.3514619	not scorable	not scorable	0.9834711	0.6644379
5636	Bn-A03-p14583041	A-genome specific (napus-rapa)	0.9880383	0.8107138	not scorable	not scorable	1	0.8385125
5637	Bn-A03-p14585799	A-genome specific (napus-rapa)	0.9928229	0.7772273	not scorable	not scorable	0.9917355	0.796831
5638	Bn-A03-p14586392	A-genome specific (napus-rapa)	0.9832536	0.4746354	not scorable	not scorable	0.9917355	0.8356195
5639	Bn-A03-p14598476	A-genome specific (napus-rapa)	0.9617225	0.2552498	not scorable	not scorable	1	0.7032276
5640	Bn-A03-p14611641	A-genome specific (napus-rapa)	0.9904306	0.7164478	not scorable	not scorable	1	0.7243329

5641	Bn-A03-p1461633	A-genome specific (napus-rapa)	0.9354067	0.7939495	not scorable	not scorable	1	0.7344691
5642	Bn-A03-p1466010	A-genome specific (rapa only)	not scorable	not scorable	not scorable	not scorable	0.9834711	0.5258038
5643	Bn-A03-p14684924	A-genome specific (rapa only)	not scorable	not scorable	not scorable	not scorable	0.9669421	0.7001394
5644	Bn-A03-p14685500	A-genome specific (napus-rapa)	0.8779904	0.7309419	not scorable	not scorable	0.7768595	0.7855022
5645	Bn-A03-p14692579	A-genome specific (napus-rapa)	0.8923445	0.9450443	not scorable	not scorable	0.9834711	0.9309483
5646	Bn-A03-p14710981	A-genome specific (napus-rapa)	0.9952153	0.7886893	not scorable	not scorable	1	0.8135551
5647	Bn-A03-p14735831	A-genome specific (napus-rapa)	0.9904306	0.7048963	not scorable	not scorable	0.9917355	0.6952731
5648	Bn-A03-p14736161	A-genome specific (napus-rapa)	0.9784689	0.424578	not scorable	not scorable	0.9752066	0.8142088
5649	Bn-A03-p14742926	A-genome specific (napus-rapa)	0.7559808	0.7870395	not scorable	not scorable	0.9752066	0.7637817
5650	Bn-A03-p14747263	A-genome specific (napus-rapa)	0.9904306	0.7483796	not scorable	not scorable	1	0.8131497
5651	Bn-A03-p1475495	A-genome specific (rapa only)	not scorable	not scorable	not scorable	not scorable	0.9834711	0.7726238
5652	Bn-A03-p14758285	genome unspecific (all in)	0.9784689	0.2935005	1	0.3217177	0.9752066	0.6095991
5653	Bn-A03-p14759764	A-genome specific (napus-rapa)	0.9928229	0.7803418	not scorable	not scorable	1	0.6893014
5654	Bn-A03-p14760518	A-genome specific (rapa only)	not scorable	not scorable	not scorable	not scorable	0.8347107	0.9272014
5655	Bn-A03-p14784764	A-genome specific (napus-rapa)	0.9856459	0.3099771	not scorable	not scorable	0.9669421	0.8127
5656	Bn-A03-p14792548	A-genome specific (rapa only)	not scorable	not scorable	not scorable	not scorable	1	0.7491972
5657	Bn-A03-p14803521	A-genome specific (rapa only)	not scorable	not scorable	not scorable	not scorable	0.8016529	0.8910999
5658	Bn-A03-p14811204	A-genome specific (napus-rapa)	0.9401914	0.2399881	not scorable	not scorable	1	0.7603807
5659	Bn-A03-p14811653	A-genome specific (napus-rapa)	0.9593301	0.5079381	not scorable	not scorable	1	0.7259326
5660	Bn-A03-p14816541	genome unspecific (oleracea-rapa)	not scorable	not scorable	1	0.837801	0.9917355	0.7517515
5661	Bn-A03-p14839497	A-genome specific (napus-rapa)	0.9808612	0.4461769	not scorable	not scorable	0.9917355	0.6580603
5662	Bn-A03-p14839583	A-genome specific (napus-rapa)	0.9928229	0.8175452	not scorable	not scorable	0.9917355	0.718304
5663	Bn-A03-p14848117	genome unspecific (all in)	0.9784689	0.3038665	0.9922481	0.7093671	0.9834711	0.7567326
5664	Bn-A03-p14852785	A-genome specific (napus-rapa)	0.9904306	0.6757932	not scorable	not scorable	0.9917355	0.6839479
5665	Bn-A03-p14870270	A-genome specific (napus-rapa)	0.9760765	0.649468	not scorable	not scorable	1	0.662836
5666	Bn-A03-p14870763	A-genome specific (rapa only)	not scorable	not scorable	not scorable	not scorable	0.9834711	0.8213806
5667	Bn-A03-p14871005	napus specific	0.8205742	0.8221628	not scorable	not scorable	not scorable	not scorable
5668	Bn-A03-p1488306	A-genome specific (napus-rapa)	0.9210526	0.4899762	not scorable	not scorable	0.9917355	0.8117021
5669	Bn-A03-p14885241	A-genome specific (napus-rapa)	0.9952153	0.7662086	not scorable	not scorable	0.9917355	0.7178061
5670	Bn-A03-p14888403	A-genome specific (napus-rapa)	0.9880383	0.2803631	not scorable	not scorable	0.9834711	0.7696
5671	Bn-A03-p14903814	A-genome specific (napus-rapa)	0.9952153	0.3717571	not scorable	not scorable	0.9917355	0.7831675
5672	Bn-A03-p14927037	A-genome specific (napus-rapa)	0.9880383	0.4381097	not scorable	not scorable	1	0.7634497
5673	Bn-A03-p14949812	A-genome specific (napus-rapa)	1	0.7996386	not scorable	not scorable	1	0.8187039

5674	Bn-A03-p14962288	A-genome specific (napus-rapa)	0.9473684	0.7845681	not scorable	not scorable	0.8016529	0.6963878
5675	Bn-A03-p14968153	A-genome specific (napus-rapa)	0.9712918	0.5191349	not scorable	not scorable	0.9752066	0.6832536
5676	Bn-A03-p15002477	A-genome specific (napus-rapa)	0.9712918	0.6804684	not scorable	not scorable	0.9917355	0.7264439
5677	Bn-A03-p15004059	A-genome specific (napus-rapa)	0.9760765	0.7517055	not scorable	not scorable	0.9917355	0.7765962
5678	Bn-A03-p15015706	A-genome specific (napus-rapa)	0.9976076	0.8209934	not scorable	not scorable	0.9752066	0.7204394
5679	Bn-A03-p1503462	genome unspecific (all in)	0.9928229	0.4794303	1	0.7639066	0.9669421	0.7233297
5680	Bn-A03-p15038774	A-genome specific (napus-rapa)	0.9712918	0.7477467	not scorable	not scorable	0.9917355	0.7957059
5681	Bn-A03-p15044158	A-genome specific (napus-rapa)	0.9880383	0.4475853	not scorable	not scorable	0.9917355	0.5921194
5682	Bn-A03-p1506511	A-genome specific (napus-rapa)	0.854067	0.9129604	not scorable	not scorable	0.9834711	0.9275912
5683	Bn-A03-p15070818	A-genome specific (napus-rapa)	0.2703349	0.8790218	not scorable	not scorable	0.8181818	0.8604698
5684	Bn-A03-p15077006	A-genome specific (napus-rapa)	0.9904306	0.7561331	not scorable	not scorable	1	0.7214014
5685	Bn-A03-p1507769	A-genome specific (napus-rapa)	0.9258373	0.7666782	not scorable	not scorable	0.9752066	0.745841
5686	Bn-A03-p15102212	A-genome specific (napus-rapa)	0.9665072	0.3867507	not scorable	not scorable	1	0.7221172
5687	Bn-A03-p15103575	A-genome specific (napus-rapa)	0.9880383	0.2589812	not scorable	not scorable	0.9752066	0.7408481
5688	Bn-A03-p15107284	all not scorable	not scorable	not scorable	not scorable	not scorable	not scorable	not scorable
5689	Bn-A03-p15120410	A-genome specific (napus-rapa)	0.9641148	0.8001947	not scorable	not scorable	0.8595041	0.6801624
5690	Bn-A03-p15139190	A-genome specific (napus-rapa)	0.777512	0.7662652	not scorable	not scorable	0.9586777	0.7215816
5691	Bn-A03-p15139484	A-genome specific (napus-rapa)	1	0.5433649	not scorable	not scorable	0.9917355	0.8404831
5692	Bn-A03-p15141821	A-genome specific (napus-rapa)	0.9712918	0.7536452	not scorable	not scorable	0.8429752	0.6504474
5693	Bn-A03-p1514927	A-genome specific (napus-rapa)	0.9282297	0.5494355	not scorable	not scorable	0.9917355	0.8025808
5694	Bn-A03-p15149454	A-genome specific (napus-rapa)	0.9736842	0.5544051	not scorable	not scorable	1	0.8154362
5695	Bn-A03-p15156393	A-genome specific (napus-rapa)	0.8995215	0.7620121	not scorable	not scorable	0.9917355	0.6760501
5696	Bn-A03-p15174986	A-genome specific (napus-rapa)	0.777512	0.7827256	not scorable	not scorable	1	0.8398426
5697	Bn-A03-p15184379	A-genome specific (napus-rapa)	0.9760765	0.5470327	not scorable	not scorable	0.9834711	0.7353612
5698	Bn-A03-p15200901	A-genome specific (napus-rapa)	0.9832536	0.6751266	not scorable	not scorable	1	0.7009098
5699	Bn-A03-p15201279	A-genome specific (napus-rapa)	0.9856459	0.3372405	not scorable	not scorable	0.9917355	0.807839
5700	Bn-A03-p15207594	A-genome specific (napus-rapa)	0.9856459	0.328628	not scorable	not scorable	0.9834711	0.572859
5701	Bn-A03-p15229988	A-genome specific (napus-rapa)	0.9641148	0.6857461	not scorable	not scorable	0.9669421	0.7181081
5702	Bn-A03-p15233860	A-genome specific (napus-rapa)	0.9090909	0.736141	not scorable	not scorable	0.7933884	0.723796
5703	Bn-A03-p15237693	A-genome specific (napus-rapa)	0.9904306	0.7515081	not scorable	not scorable	0.9917355	0.7944423
5704	Bn-A03-p15240794	A-genome specific (napus-rapa)	0.9856459	0.6728234	not scorable	not scorable	1	0.7037767
5705	Bn-A03-p15240992	A-genome specific (napus-rapa)	1	0.4536684	not scorable	not scorable	0.9834711	0.6094438
5706	Bn-A03-p15245600	A-genome specific (napus-rapa)	0.9282297	0.7623614	not scorable	not scorable	0.9669421	0.8710286

5707	Bn-A03-p15265791	A-genome specific (napus-rapa)	0.9593301	0.5790476	not scorable	not scorable	1	0.8182366
5708	Bn-A03-p15266403	A-genome specific (napus-rapa)	0.9976076	0.8013346	not scorable	not scorable	0.9917355	0.730126
5709	Bn-A03-p15300653	A-genome specific (napus-rapa)	0.9952153	0.7078519	not scorable	not scorable	1	0.7750097
5710	Bn-A03-p1532114	A-genome specific (napus-rapa)	0.9928229	0.5590882	not scorable	not scorable	0.9834711	0.642509
5711	Bn-A03-p15326805	A-genome specific (napus-rapa)	1	0.5069947	not scorable	not scorable	0.9834711	0.6907421
5712	Bn-A03-p15327817	A-genome specific (napus-rapa)	0.9976076	0.6554469	not scorable	not scorable	0.9917355	0.7374666
5713	Bn-A03-p15327921	A-genome specific (napus-rapa)	0.9976076	0.7899165	not scorable	not scorable	1	0.7012504
5714	Bn-A03-p15337088	A-genome specific (napus-rapa)	0.9760765	0.2826414	not scorable	not scorable	0.9752066	0.7571762
5715	Bn-A03-p15346153	A-genome specific (napus-rapa)	0.9952153	0.7068107	not scorable	not scorable	0.9917355	0.8496006
5716	Bn-A03-p15347967	A-genome specific (napus-rapa)	0.9976076	0.7004927	not scorable	not scorable	0.9917355	0.6930565
5717	Bn-A03-p15349925	genome unspecific (all in)	0.9880383	0.5159891	0.4573644	0.7350179	0.9917355	0.740214
5718	Bn-A03-p15351982	A-genome specific (napus-rapa)	0.9832536	0.8041285	not scorable	not scorable	1	0.7890749
5719	Bn-A03-p15359867	A-genome specific (napus-rapa)	0.9904306	0.6512235	not scorable	not scorable	1	0.7450662
5720	Bn-A03-p15363919	A-genome specific (napus-rapa)	0.9808612	0.4389783	not scorable	not scorable	1	0.7643426
5721	Bn-A03-p15365259	A-genome specific (napus-rapa)	0.9952153	0.7956763	not scorable	not scorable	0.9834711	0.6946486
5722	Bn-A03-p15368328	A-genome specific (napus-rapa)	0.9952153	0.5564671	not scorable	not scorable	0.9834711	0.5495553
5723	Bn-A03-p1538555	A-genome specific (napus-rapa)	0.9114832	0.6784859	not scorable	not scorable	0.9917355	0.7462659
5724	Bn-A03-p15397187	A-genome specific (napus-rapa)	0.9832536	0.6791142	not scorable	not scorable	0.9834711	0.8034778
5725	Bn-A03-p15404736	A-genome specific (napus-rapa)	0.9904306	0.7703875	not scorable	not scorable	0.9834711	0.7902734
5726	Bn-A03-p15405498	A-genome specific (napus-rapa)	0.9473684	0.7249722	not scorable	not scorable	0.9752066	0.736288
5727	Bn-A03-p15406047	A-genome specific (napus-rapa)	0.9928229	0.6817471	not scorable	not scorable	1	0.8276423
5728	Bn-A03-p15416728	A-genome specific (napus-rapa)	0.9880383	0.6631147	not scorable	not scorable	1	0.698265
5729	Bn-A03-p15417676	genome unspecific (all in)	0.9976076	0.4700363	1	0.238809	1	0.7320092
5730	Bn-A03-p15424536	A-genome specific (napus-rapa)	0.937799	0.8125674	not scorable	not scorable	0.9834711	0.7829943
5731	Bn-A03-p15435114	A-genome specific (napus-rapa)	0.9928229	0.339014	not scorable	not scorable	0.9752066	0.6457492
5732	Bn-A03-p15435174	A-genome specific (napus-rapa)	0.9904306	0.4871615	not scorable	not scorable	0.9834711	0.6360325
5733	Bn-A03-p15435442	A-genome specific (napus-rapa)	1	0.7472403	not scorable	not scorable	0.9917355	0.656527
5734	Bn-A03-p15481319	A-genome specific (napus-rapa)	0.9665072	0.4600129	not scorable	not scorable	0.9917355	0.7272258
5735	Bn-A03-p15481682	A-genome specific (napus-rapa)	0.9976076	0.7355552	not scorable	not scorable	1	0.7620569
5736	Bn-A03-p15496608	A-genome specific (napus-rapa)	0.9880383	0.5596464	not scorable	not scorable	1	0.7371384
5737	Bn-A03-p15496962	A-genome specific (napus-rapa)	0.9952153	0.7085512	not scorable	not scorable	1	0.7451537
5738	Bn-A03-p15499288	A-genome specific (napus-rapa)	0.9952153	0.7748116	not scorable	not scorable	0.9917355	0.809986
5739	Bn-A03-p15546590	A-genome specific (napus-rapa)	1	0.8077878	not scorable	not scorable	0.9917355	0.823669

5740	Bn-A03-p15548506	A-genome specific (napus-rapa)	0.9928229	0.7761807	not scorable	not scorable	0.9917355	0.7633445
5741	Bn-A03-p15548953	A-genome specific (napus-rapa)	0.9976076	0.5442027	not scorable	not scorable	1	0.7333017
5742	Bn-A03-p15559809	A-genome specific (napus-rapa)	0.9784689	0.7781263	not scorable	not scorable	0.9834711	0.6999873
5743	Bn-A03-p15576074	A-genome specific (napus-rapa)	0.923445	0.6568506	not scorable	not scorable	0.9917355	0.7013826
5744	Bn-A03-p15577849	A-genome specific (napus-rapa)	0.9976076	0.7831069	not scorable	not scorable	0.9752066	0.7673208
5745	Bn-A03-p15593483	A-genome specific (napus-rapa)	1	0.5617753	not scorable	not scorable	0.9917355	0.7782555
5746	Bn-A03-p1559742	A-genome specific (napus-rapa)	0.9186603	0.7672096	not scorable	not scorable	0.7024794	0.9079534
5747	Bn-A03-p15605355	A-genome specific (napus-rapa)	1	0.5572206	not scorable	not scorable	1	0.7730535
5748	Bn-A03-p15615278	A-genome specific (napus-rapa)	0.9808612	0.4956777	not scorable	not scorable	1	0.7427022
5749	Bn-A03-p1562119	A-genome specific (napus-rapa)	0.9162679	0.777845	not scorable	not scorable	0.6280992	0.8443323
5750	Bn-A03-p15622267	A-genome specific (napus-rapa)	0.9808612	0.8511497	not scorable	not scorable	1	0.8241596
5751	Bn-A03-p15624030	A-genome specific (napus-rapa)	0.9880383	0.707849	not scorable	not scorable	1	0.7393419
5752	Bn-A03-p15624379	A-genome specific (napus-rapa)	0.9952153	0.7125828	not scorable	not scorable	0.9917355	0.6714318
5753	Bn-A03-p15627698	A-genome specific (napus-rapa)	0.9736842	0.7754917	not scorable	not scorable	1	0.7903121
5754	Bn-A03-p15634432	A-genome specific (napus-rapa)	0.9569378	0.3715838	not scorable	not scorable	0.9917355	0.7342305
5755	Bn-A03-p1563613	A-genome specific (napus-rapa)	0.9186603	0.8060953	not scorable	not scorable	0.6859504	0.8912069
5756	Bn-A03-p15675871	A-genome specific (napus-rapa)	0.9880383	0.5198693	not scorable	not scorable	0.9834711	0.653852
5757	Bn-A03-p15689225	A-genome specific (napus-rapa)	1	0.5081469	not scorable	not scorable	1	0.6305527
5758	Bn-A03-p15702943	A-genome specific (napus-rapa)	0.9904306	0.6146862	not scorable	not scorable	0.9917355	0.9281533
5759	Bn-A03-p15704793	A-genome specific (napus-rapa)	0.9760765	0.3832133	not scorable	not scorable	0.9917355	0.6943207
5760	Bn-A03-p15704830	A-genome specific (napus-rapa)	0.9641148	0.5219854	not scorable	not scorable	1	0.7103293
5761	Bn-A03-p15705021	C-genome specific (oleracea only)	not scorable	not scorable	0.9922481	0.3368704	not scorable	not scorable
5762	Bn-A03-p15708192	A-genome specific (napus-rapa)	0.9928229	0.7072939	not scorable	not scorable	1	0.6718319
5763	Bn-A03-p15708289	A-genome specific (napus-rapa)	1	0.849976	not scorable	not scorable	0.9834711	0.7447924
5764	Bn-A03-p15725925	A-genome specific (napus-rapa)	0.9784689	0.7446344	not scorable	not scorable	0.9917355	0.7825384
5765	Bn-A03-p15732	A-genome specific (napus-rapa)	0.8229665	0.8143427	not scorable	not scorable	0.9834711	0.8273963
5766	Bn-A03-p15739646	A-genome specific (napus-rapa)	0.9880383	0.7851327	not scorable	not scorable	0.9669421	0.7509246
5767	Bn-A03-p15744983	A-genome specific (napus-rapa)	0.9856459	0.7238212	not scorable	not scorable	0.9917355	0.6929718
5768	Bn-A03-p15748819	A-genome specific (napus-rapa)	0.9976076	0.8017119	not scorable	not scorable	0.9917355	0.7475286
5769	Bn-A03-p15749083	A-genome specific (napus-rapa)	0.9904306	0.7736924	not scorable	not scorable	1	0.7984229
5770	Bn-A03-p15766386	A-genome specific (napus-rapa)	1	0.5856662	not scorable	not scorable	0.9917355	0.7227655
5771	Bn-A03-p15768536	A-genome specific (napus-rapa)	0.9832536	0.256184	not scorable	not scorable	0.9669421	0.5986605
5772	Bn-A03-p1577561	A-genome specific (rapa only)	not scorable	not scorable	not scorable	not scorable	0.9917355	0.6334302

5773	Bn-A03-p15776521	A-genome specific (napus-rapa)	0.9976076	0.551823	not scorable	not scorable	0.9504132	0.53808
5774	Bn-A03-p15776756	A-genome specific (napus-rapa)	0.9760765	0.6135227	not scorable	not scorable	0.9917355	0.7062946
5775	Bn-A03-p15801208	A-genome specific (napus-rapa)	0.9330143	0.6865979	not scorable	not scorable	0.8264463	0.7518073
5776	Bn-A03-p15811901	A-genome specific (napus-rapa)	0.9976076	0.4115214	not scorable	not scorable	1	0.6955462
5777	Bn-A03-p15815965	A-genome specific (napus-rapa)	0.9665072	0.3551319	not scorable	not scorable	0.9586777	0.7047315
5778	Bn-A03-p15818896	A-genome specific (napus-rapa)	0.9928229	0.4340068	not scorable	not scorable	1	0.7021772
5779	Bn-A03-p15824796	A-genome specific (napus-rapa)	0.9808612	0.7744122	not scorable	not scorable	1	0.7825404
5780	Bn-A03-p15827113	A-genome specific (napus-rapa)	0.9832536	0.7078003	not scorable	not scorable	0.9917355	0.8336643
5781	Bn-A03-p15828397	A-genome specific (napus-rapa)	0.9832536	0.7639653	not scorable	not scorable	0.9917355	0.743686
5782	Bn-A03-p15852439	A-genome specific (napus-rapa)	0.9808612	0.2874022	not scorable	not scorable	0.9752066	0.7401809
5783	Bn-A03-p15853765	A-genome specific (napus-rapa)	0.9784689	0.4476235	not scorable	not scorable	0.9917355	0.8188219
5784	Bn-A03-p15871284	A-genome specific (napus-rapa)	0.9665072	0.4337922	not scorable	not scorable	1	0.7136886
5785	Bn-A03-p15875404	A-genome specific (napus-rapa)	0.9688995	0.8308064	not scorable	not scorable	0.9834711	0.7567569
5786	Bn-A03-p1588735	A-genome specific (rapa only)	not scorable	not scorable	not scorable	not scorable	0.9834711	0.685509
5787	Bn-A03-p15892296	A-genome specific (napus-rapa)	0.84689	0.8783022	not scorable	not scorable	0.9586777	0.8064034
5788	Bn-A03-p15893952	A-genome specific (napus-rapa)	0.9976076	0.7574086	not scorable	not scorable	1	0.6928743
5789	Bn-A03-p15894013	A-genome specific (napus-rapa)	0.9856459	0.7843164	not scorable	not scorable	0.9917355	0.756395
5790	Bn-A03-p15900189	A-genome specific (napus-rapa)	0.9952153	0.7789081	not scorable	not scorable	0.9917355	0.7818949
5791	Bn-A03-p15906810	A-genome specific (napus-rapa)	0.9952153	0.7712682	not scorable	not scorable	0.9917355	0.6829743
5792	Bn-A03-p15916054	A-genome specific (napus-rapa)	0.9784689	0.7678409	not scorable	not scorable	0.9917355	0.8149601
5793	Bn-A03-p15916258	A-genome specific (napus-rapa)	0.9784689	0.7608996	not scorable	not scorable	0.9917355	0.7556771
5794	Bn-A03-p15923626	A-genome specific (napus-rapa)	0.9856459	0.3586591	not scorable	not scorable	1	0.8740078
5795	Bn-A03-p15936354	A-genome specific (napus-rapa)	0.8732057	0.8808252	not scorable	not scorable	0.9834711	0.7776138
5796	Bn-A03-p15954297	A-genome specific (napus-rapa)	0.9832536	0.4021505	not scorable	not scorable	0.9917355	0.7595155
5797	Bn-A03-p15957657	A-genome specific (napus-rapa)	0.9928229	0.7754202	not scorable	not scorable	1	0.7142359
5798	Bn-A03-p15958035	A-genome specific (napus-rapa)	0.9856459	0.4773967	not scorable	not scorable	1	0.7742409
5799	Bn-A03-p15960001	A-genome specific (napus-rapa)	0.9904306	0.789126	not scorable	not scorable	1	0.7167431
5800	Bn-A03-p15960376	A-genome specific (napus-rapa)	0.9760765	0.6953256	not scorable	not scorable	0.9752066	0.7994384
5801	Bn-A03-p1596091	A-genome specific (napus-rapa)	0.923445	0.7765236	not scorable	not scorable	0.9917355	0.7429354
5802	Bn-A03-p15968189	A-genome specific (napus-rapa)	0.8851675	0.7968677	not scorable	not scorable	0.9256198	0.7547897
5803	Bn-A03-p15973005	A-genome specific (napus-rapa)	0.9736842	0.6498584	not scorable	not scorable	0.9752066	0.8266627
5804	Bn-A03-p15974991	A-genome specific (napus-rapa)	0.9784689	0.8270382	not scorable	not scorable	0.9917355	0.5909037
5805	Bn-A03-p15979252	A-genome specific (napus-rapa)	0.8444976	0.6860555	not scorable	not scorable	0.9752066	0.6890675

5806	Bn-A03-p15983019	A-genome specific (napus-rapa)	0.9952153	0.7497607	not scorable	not scorable	0.9752066	0.7629418
5807	Bn-A03-p16000194	genome unspecific (all in)	1	0.3238211	0.9844961	0.3306791	1	0.7888938
5808	Bn-A03-p16017186	A-genome specific (napus-rapa)	0.9904306	0.8434092	not scorable	not scorable	1	0.8069542
5809	Bn-A03-p16038553	A-genome specific (napus-rapa)	0.9569378	0.8636244	not scorable	not scorable	1	0.8210682
5810	Bn-A03-p16051661	A-genome specific (napus-rapa)	1	0.6308175	not scorable	not scorable	0.9917355	0.7086748
5811	Bn-A03-p16055930	all not scorable	not scorable	not scorable	not scorable	not scorable	not scorable	not scorable
5812	Bn-A03-p16056039	napus specific	0.9856459	0.5439663	not scorable	not scorable	not scorable	not scorable
5813	Bn-A03-p16056249	A-genome specific (napus-rapa)	0.9665072	0.3264222	not scorable	not scorable	0.9917355	0.6957013
5814	Bn-A03-p16056460	A-genome specific (napus-rapa)	0.9880383	0.3676674	not scorable	not scorable	0.9917355	0.7115262
5815	Bn-A03-p16056621	A-genome specific (napus-rapa)	0.9904306	0.4629203	not scorable	not scorable	1	0.6119907
5816	Bn-A03-p16070543	A-genome specific (napus-rapa)	0.9928229	0.6828198	not scorable	not scorable	0.9917355	0.6958005
5817	Bn-A03-p16079386	A-genome specific (napus-rapa)	0.9904306	0.7370976	not scorable	not scorable	0.9917355	0.7973625
5818	Bn-A03-p16081434	A-genome specific (napus-rapa)	0.9760765	0.7037004	not scorable	not scorable	0.9834711	0.8008798
5819	Bn-A03-p16084302	A-genome specific (napus-rapa)	0.9976076	0.8506913	not scorable	not scorable	0.9917355	0.8015116
5820	Bn-A03-p16098207	A-genome specific (napus-rapa)	0.9712918	0.7022911	not scorable	not scorable	1	0.8795602
5821	Bn-A03-p16103891	A-genome specific (napus-rapa)	0.9832536	0.7711818	not scorable	not scorable	1	0.7465637
5822	Bn-A03-p16123758	A-genome specific (napus-rapa)	0.9904306	0.6619513	not scorable	not scorable	1	0.7235709
5823	Bn-A03-p16126013	genome unspecific (all in)	0.9712918	0.4219336	1	0.7823842	0.9834711	0.7285361
5824	Bn-A03-p16134415	A-genome specific (napus-rapa)	0.9832536	0.4449373	not scorable	not scorable	0.9834711	0.7294535
5825	Bn-A03-p16158248	A-genome specific (napus-rapa)	0.9688995	0.5906062	not scorable	not scorable	0.8429752	0.5966659
5826	Bn-A03-p16162908	A-genome specific (napus-rapa)	1	0.736278	not scorable	not scorable	1	0.9084849
5827	Bn-A03-p16167780	all not scorable	not scorable	not scorable	not scorable	not scorable	not scorable	not scorable
5828	Bn-A03-p16170389	A-genome specific (rapa only)	not scorable	not scorable	not scorable	not scorable	0.7355372	0.5411618
5829	Bn-A03-p16185537	A-genome specific (napus-rapa)	0.9832536	0.7412998	not scorable	not scorable	1	0.7161438
5830	Bn-A03-p16190643	A-genome specific (napus-rapa)	0.9928229	0.7603837	not scorable	not scorable	1	0.8494194
5831	Bn-A03-p16211692	A-genome specific (napus-rapa)	0.9880383	0.8083097	not scorable	not scorable	1	0.7690955
5832	Bn-A03-p162225	A-genome specific (napus-rapa)	0.9473684	0.3193581	not scorable	not scorable	0.9917355	0.6868628
5833	Bn-A03-p16229294	A-genome specific (napus-rapa)	0.9545454	0.3971421	not scorable	not scorable	0.9917355	0.6895267
5834	Bn-A03-p16262184	A-genome specific (napus-rapa)	0.9976076	0.387283	not scorable	not scorable	0.9917355	0.7795519
5835	Bn-A03-p16269745	napus specific	0.9760765	0.7556658	not scorable	not scorable	not scorable	not scorable
5836	Bn-A03-p16275650	A-genome specific (napus-rapa)	0.9928229	0.668286	not scorable	not scorable	1	0.6983143
5837	Bn-A03-p16278251	A-genome specific (napus-rapa)	0.9904306	0.7760227	not scorable	not scorable	0.9917355	0.782773
5838	Bn-A03-p16284595	A-genome specific (napus-rapa)	0.9856459	0.7047896	not scorable	not scorable	0.9917355	0.7207536

5839	Bn-A03-p16289300	all not scorable	not scorable	not scorable	not scorable	not scorable	not scorable	not scorable
5840	Bn-A03-p16301988	A-genome specific (napus-rapa)	0.9593301	0.3784592	not scorable	not scorable	0.9917355	0.7722343
5841	Bn-A03-p16303181	A-genome specific (napus-rapa)	0.9856459	0.3492493	not scorable	not scorable	1	0.7279542
5842	Bn-A03-p16303780	A-genome specific (napus-rapa)	0.9808612	0.7920244	not scorable	not scorable	0.9834711	0.8327581
5843	Bn-A03-p16309	A-genome specific (rapa only)	not scorable	not scorable	not scorable	not scorable	0.9504132	0.6329616
5844	Bn-A03-p16342771	A-genome specific (napus-rapa)	0.9904306	0.5533428	not scorable	not scorable	0.7851239	0.6732634
5845	Bn-A03-p16355867	A-genome specific (napus-rapa)	0.9856459	0.6793572	not scorable	not scorable	0.9917355	0.6867253
5846	Bn-A03-p16371467	A-genome specific (napus-rapa)	0.9976076	0.8243888	not scorable	not scorable	0.9752066	0.7924223
5847	Bn-A03-p16378233	A-genome specific (napus-rapa)	0.9832536	0.4709962	not scorable	not scorable	1	0.7808928
5848	Bn-A03-p16379045	A-genome specific (napus-rapa)	0.9760765	0.7439343	not scorable	not scorable	0.9421487	0.6569786
5849	Bn-A03-p16380304	A-genome specific (napus-rapa)	0.9832536	0.6913049	not scorable	not scorable	0.9917355	0.5697977
5850	Bn-A03-p1638326	A-genome specific (rapa only)	not scorable	not scorable	not scorable	not scorable	1	0.6537102
5851	Bn-A03-p1639446	genome unspecific (oleracea-rapa)	not scorable	not scorable	0.9844961	0.6593229	0.9834711	0.6956896
5852	Bn-A03-p16404390	A-genome specific (napus-rapa)	0.9976076	0.7140145	not scorable	not scorable	1	0.681006
5853	Bn-A03-p16404538	A-genome specific (napus-rapa)	0.9832536	0.5336586	not scorable	not scorable	0.9752066	0.7792552
5854	Bn-A03-p16404871	all not scorable	not scorable	not scorable	not scorable	not scorable	not scorable	not scorable
5855	Bn-A03-p16405269	A-genome specific (napus-rapa)	0.9976076	0.8308399	not scorable	not scorable	1	0.7016065
5856	Bn-A03-p16409353	A-genome specific (napus-rapa)	0.9904306	0.7417583	not scorable	not scorable	0.9917355	0.7726752
5857	Bn-A03-p16416948	A-genome specific (napus-rapa)	0.9976076	0.7819876	not scorable	not scorable	1	0.788431
5858	Bn-A03-p16422604	A-genome specific (napus-rapa)	0.9784689	0.4000248	not scorable	not scorable	0.9834711	0.6955087
5859	Bn-A03-p16423239	A-genome specific (napus-rapa)	0.9832536	0.7007966	not scorable	not scorable	0.8016529	0.7622204
5860	Bn-A03-p16431100	A-genome specific (napus-rapa)	0.9736842	0.7618128	not scorable	not scorable	0.9917355	0.7124017
5861	Bn-A03-p16437979	A-genome specific (napus-rapa)	0.9641148	0.7509831	not scorable	not scorable	0.7851239	0.8866258
5862	Bn-A03-p16444510	napus specific	0.9976076	0.7917167	not scorable	not scorable	not scorable	not scorable
5863	Bn-A03-p16454331	A-genome specific (napus-rapa)	1	0.7989338	not scorable	not scorable	0.9834711	0.7336798
5864	Bn-A03-p16481995	A-genome specific (napus-rapa)	1	0.6652509	not scorable	not scorable	0.9917355	0.7063071
5865	Bn-A03-p16482731	A-genome specific (napus-rapa)	1	0.5187945	not scorable	not scorable	0.9917355	0.7232355
5866	Bn-A03-p16501946	A-genome specific (napus-rapa)	0.9760765	0.7124595	not scorable	not scorable	0.9752066	0.8068278
5867	Bn-A03-p1652490	A-genome specific (rapa only)	not scorable	not scorable	not scorable	not scorable	0.9834711	0.6770979
5868	Bn-A03-p16530135	A-genome specific (napus-rapa)	1	0.553915	not scorable	not scorable	1	0.6927592
5869	Bn-A03-p16565487	A-genome specific (napus-rapa)	0.9808612	0.281171	not scorable	not scorable	0.9752066	0.6554057
5870	Bn-A03-p16607714	A-genome specific (napus-rapa)	0.9784689	0.4582933	not scorable	not scorable	0.9917355	0.7042211
5871	Bn-A03-p16610235	A-genome specific (rapa only)	not scorable	not scorable	not scorable	not scorable	0.9834711	0.6933311

5872	Bn-A03-p16611349	A-genome specific (napus-rapa)	0.9904306	0.7420169	not scorable	not scorable	0.9917355	0.7453818
5873	Bn-A03-p16613382	A-genome specific (napus-rapa)	0.9976076	0.4803152	not scorable	not scorable	1	0.4691428
5874	Bn-A03-p166292	A-genome specific (napus-rapa)	0.8373206	0.8067325	not scorable	not scorable	1	0.7243997
5875	Bn-A03-p166402	A-genome specific (napus-rapa)	0.8397129	0.8196601	not scorable	not scorable	0.9834711	0.7853113
5876	Bn-A03-p16742011	A-genome specific (napus-rapa)	0.9928229	0.755663	not scorable	not scorable	1	0.793067
5877	Bn-A03-p16758311	A-genome specific (napus-rapa)	0.9856459	0.3771826	not scorable	not scorable	1	0.6275153
5878	Bn-A03-p16759411	napus specific	0.9688995	0.5400857	not scorable	not scorable	not scorable	not scorable
5879	Bn-A03-p16769908	A-genome specific (rapa only)	not scorable	not scorable	not scorable	not scorable	0.9834711	0.6836593
5880	Bn-A03-p16800145	A-genome specific (napus-rapa)	0.9282297	0.6720465	not scorable	not scorable	0.9669421	0.6081598
5881	Bn-A03-p16811562	A-genome specific (napus-rapa)	0.9928229	0.6886096	not scorable	not scorable	0.9917355	0.7236227
5882	Bn-A03-p16812953	A-genome specific (napus-rapa)	0.9808612	0.4658723	not scorable	not scorable	0.9917355	0.6645512
5883	Bn-A03-p16852209	A-genome specific (napus-rapa)	0.9928229	0.6901168	not scorable	not scorable	0.9917355	0.676823
5884	Bn-A03-p1687166	A-genome specific (rapa only)	not scorable	not scorable	not scorable	not scorable	0.9917355	0.7303081
5885	Bn-A03-p16901041	A-genome specific (napus-rapa)	0.9856459	0.7783247	not scorable	not scorable	1	0.7819281
5886	Bn-A03-p16915803	A-genome specific (rapa only)	not scorable	not scorable	not scorable	not scorable	0.9917355	0.8455941
5887	Bn-A03-p16915877	A-genome specific (napus-rapa)	0.9545454	0.8684124	not scorable	not scorable	1	0.729519
5888	Bn-A03-p16955732	A-genome specific (napus-rapa)	0.9688995	0.3632278	not scorable	not scorable	0.9834711	0.7008772
5889	Bn-A03-p16969084	A-genome specific (napus-rapa)	0.9784689	0.5458037	not scorable	not scorable	1	0.7719373
5890	Bn-A03-p16976391	A-genome specific (rapa only)	not scorable	not scorable	not scorable	not scorable	1	0.8383706
5891	Bn-A03-p1698016	A-genome specific (napus-rapa)	0.9162679	0.6094505	not scorable	not scorable	0.9834711	0.5717454
5892	Bn-A03-p16981919	A-genome specific (napus-rapa)	1	0.7637413	not scorable	not scorable	1	0.7864357
5893	Bn-A03-p16989353	A-genome specific (napus-rapa)	0.9808612	0.7027869	not scorable	not scorable	1	0.6433784
5894	Bn-A03-p16990947	genome unspecific (all in)	0.9928229	0.3104821	1	0.8112317	1	0.8296907
5895	Bn-A03-p17019251	A-genome specific (napus-rapa)	0.9545454	0.5636312	not scorable	not scorable	1	0.8124371
5896	Bn-A03-p17038064	A-genome specific (napus-rapa)	0.9904306	0.3187398	not scorable	not scorable	0.9917355	0.8496033
5897	Bn-A03-p17038887	A-genome specific (napus-rapa)	0.9760765	0.7407515	not scorable	not scorable	0.9173554	0.6858595
5898	Bn-A03-p17038965	A-genome specific (napus-rapa)	0.9856459	0.7904156	not scorable	not scorable	0.9173554	0.8394261
5899	Bn-A03-p17056395	A-genome specific (napus-rapa)	0.9736842	0.7162153	not scorable	not scorable	1	0.7794396
5900	Bn-A03-p1711856	A-genome specific (napus-rapa)	0.9330143	0.6051639	not scorable	not scorable	1	0.6803021
5901	Bn-A03-p17125715	all not scorable	not scorable	not scorable	not scorable	not scorable	not scorable	not scorable
5902	Bn-A03-p17139931	A-genome specific (napus-rapa)	0.9904306	0.5969656	not scorable	not scorable	0.9917355	0.6325836
5903	Bn-A03-p1716217	A-genome specific (napus-rapa)	0.923445	0.7468333	not scorable	not scorable	0.9752066	0.7976837
5904	Bn-A03-p17176782	all not scorable	not scorable	not scorable	not scorable	not scorable	not scorable	not scorable

5905	Bn-A03-p17182494	A-genome specific (napus-rapa)	0.9976076	0.7876688	not scorable	not scorable	0.9752066	0.6970707
5906	Bn-A03-p17183616	A-genome specific (napus-rapa)	1	0.7053196	not scorable	not scorable	1	0.8135551
5907	Bn-A03-p17184441	A-genome specific (napus-rapa)	0.9976076	0.7322975	not scorable	not scorable	0.9917355	0.6272221
5908	Bn-A03-p17190733	A-genome specific (rapa only)	not scorable	not scorable	not scorable	not scorable	0.9917355	0.8646078
5909	Bn-A03-p17194139	A-genome specific (napus-rapa)	1	0.4959221	not scorable	not scorable	1	0.808534
5910	Bn-A03-p1719791	A-genome specific (napus-rapa)	0.2583732	0.8442286	not scorable	not scorable	0.9669421	0.8843412
5911	Bn-A03-p17207547	napus specific	0.715311	0.7953328	not scorable	not scorable	not scorable	not scorable
5912	Bn-A03-p17213825	A-genome specific (napus-rapa)	0.9760765	0.4408043	not scorable	not scorable	0.9917355	0.6703242
5913	Bn-A03-p17217528	A-genome specific (napus-rapa)	0.9760765	0.3073706	not scorable	not scorable	0.9917355	0.6981302
5914	Bn-A03-p17281617	A-genome specific (napus-rapa)	0.9880383	0.7505705	not scorable	not scorable	1	0.7419678
5915	Bn-A03-p17334423	napus specific	0.9162679	0.33171	not scorable	not scorable	not scorable	not scorable
5916	Bn-A03-p17336041	A-genome specific (napus-rapa)	1	0.3784633	not scorable	not scorable	0.9421487	0.7608163
5917	Bn-A03-p1741556	A-genome specific (rapa only)	not scorable	not scorable	not scorable	not scorable	0.7272727	0.7320971
5918	Bn-A03-p17532322	A-genome specific (napus-rapa)	0.9880383	0.7174388	not scorable	not scorable	1	0.8136467
5919	Bn-A03-p1755651	A-genome specific (napus-rapa)	0.9521531	0.4247928	not scorable	not scorable	0.9834711	0.7561532
5920	Bn-A03-p17588	genome unspecific (oleracea-rapa)	not scorable	not scorable	0.8062016	0.7828159	0.9917355	0.8317524
5921	Bn-A03-p1762980	A-genome specific (napus-rapa)	0.9593301	0.5796103	not scorable	not scorable	0.9917355	0.5846988
5922	Bn-A03-p1777701	A-genome specific (napus-rapa)	0.9976076	0.8637566	not scorable	not scorable	0.9834711	0.8122839
5923	Bn-A03-p17782292	A-genome specific (napus-rapa)	0.9880383	0.3478327	not scorable	not scorable	0.9834711	0.7519507
5924	Bn-A03-p17790084	A-genome specific (napus-rapa)	0.9928229	0.4302098	not scorable	not scorable	0.9669421	0.6913705
5925	Bn-A03-p17921239	A-genome specific (napus-rapa)	1	0.7980775	not scorable	not scorable	0.9917355	0.81765
5926	Bn-A03-p17926123	A-genome specific (napus-rapa)	0.9952153	0.7382676	not scorable	not scorable	1	0.6610279
5927	Bn-A03-p17985410	A-genome specific (napus-rapa)	0.9976076	0.7107227	not scorable	not scorable	0.9752066	0.6485186
5928	Bn-A03-p17986611	A-genome specific (napus-rapa)	0.9928229	0.8617132	not scorable	not scorable	1	0.7781072
5929	Bn-A03-p18014273	A-genome specific (napus-rapa)	0.9760765	0.3950177	not scorable	not scorable	0.9504132	0.5878692
5930	Bn-A03-p18019822	A-genome specific (napus-rapa)	0.9808612	0.5332072	not scorable	not scorable	1	0.552564
5931	Bn-A03-p18025565	A-genome specific (napus-rapa)	0.9904306	0.8491425	not scorable	not scorable	0.9917355	0.6834776
5932	Bn-A03-p18025753	A-genome specific (napus-rapa)	0.9425837	0.4191322	not scorable	not scorable	0.9669421	0.7563342
5933	Bn-A03-p1805828	A-genome specific (rapa only)	not scorable	not scorable	not scorable	not scorable	0.9834711	0.7012051
5934	Bn-A03-p18120938	A-genome specific (napus-rapa)	0.9976076	0.3656736	not scorable	not scorable	0.9917355	0.8146527
5935	Bn-A03-p1813564	A-genome specific (napus-rapa)	0.9593301	0.2842583	not scorable	not scorable	1	0.6849828
5936	Bn-A03-p1817556	A-genome specific (napus-rapa)	0.9354067	0.3027873	not scorable	not scorable	0.9917355	0.4166303
5937	Bn-A03-p18183550	A-genome specific (napus-rapa)	1	0.5612964	not scorable	not scorable	1	0.7235482

5938	Bn-A03-p18198495	A-genome specific (napus-rapa)	1	0.3341349	not scorable	not scorable	0.9917355	0.9078106
5939	Bn-A03-p18208425	A-genome specific (napus-rapa)	0.9976076	0.3448997	not scorable	not scorable	1	0.792897
5940	Bn-A03-p1831525	A-genome specific (rapa only)	not scorable	not scorable	not scorable	not scorable	0.9834711	0.7714021
5941	Bn-A03-p18376493	A-genome specific (napus-rapa)	0.9904306	0.3937381	not scorable	not scorable	0.9752066	0.6526463
5942	Bn-A03-p18383780	A-genome specific (rapa only)	not scorable	not scorable	not scorable	not scorable	0.9338843	0.3575154
5943	Bn-A03-p1839932	A-genome specific (rapa only)	not scorable	not scorable	not scorable	not scorable	0.9917355	0.8015463
5944	Bn-A03-p18405869	A-genome specific (napus-rapa)	0.9808612	0.8277916	not scorable	not scorable	0.8264463	0.821041
5945	Bn-A03-p18414643	A-genome specific (napus-rapa)	0.9904306	0.3270931	not scorable	not scorable	0.9834711	0.6325362
5946	Bn-A03-p18451316	A-genome specific (napus-rapa)	0.9952153	0.6760358	not scorable	not scorable	1	0.7252899
5947	Bn-A03-p18455020	A-genome specific (napus-rapa)	0.9952153	0.7941719	not scorable	not scorable	0.9917355	0.7985208
5948	Bn-A03-p18455932	A-genome specific (napus-rapa)	0.9952153	0.7440118	not scorable	not scorable	0.9834711	0.7045221
5949	Bn-A03-p18461597	A-genome specific (napus-rapa)	0.9856459	0.7949721	not scorable	not scorable	0.8264463	0.7094161
5950	Bn-A03-p18461778	A-genome specific (napus-rapa)	0.9880383	0.7892097	not scorable	not scorable	0.8429752	0.7491046
5951	Bn-A03-p18462261	A-genome specific (napus-rapa)	0.9856459	0.6079261	not scorable	not scorable	0.8429752	0.7533877
5952	Bn-A03-p18468633	A-genome specific (napus-rapa)	0.9832536	0.5413554	not scorable	not scorable	0.8347107	0.7911589
5953	Bn-A03-p18471820	A-genome specific (napus-rapa)	0.9880383	0.7169248	not scorable	not scorable	1	0.6558676
5954	Bn-A03-p18570046	A-genome specific (napus-rapa)	0.9976076	0.6385927	not scorable	not scorable	0.9752066	0.8265762
5955	Bn-A03-p18570852	A-genome specific (napus-rapa)	0.9976076	0.3695284	not scorable	not scorable	0.9834711	0.7976577
5956	Bn-A03-p18571065	genome unspecific (all in)	0.9976076	0.3102164	1	0.2856168	1	0.7679
5957	Bn-A03-p18579721	A-genome specific (napus-rapa)	0.9952153	0.3568533	not scorable	not scorable	1	0.7355271
5958	Bn-A03-p18592597	A-genome specific (napus-rapa)	0.9904306	0.4536687	not scorable	not scorable	1	0.7497537
5959	Bn-A03-p18593371	A-genome specific (napus-rapa)	0.9904306	0.7123775	not scorable	not scorable	0.9752066	0.6515277
5960	Bn-A03-p18593905	A-genome specific (napus-rapa)	0.9976076	0.3994464	not scorable	not scorable	1	0.687777
5961	Bn-A03-p18594273	A-genome specific (napus-rapa)	0.9617225	0.3510044	not scorable	not scorable	0.9834711	0.7642049
5962	Bn-A03-p18595122	napus specific	0.9808612	0.2614416	not scorable	not scorable	not scorable	not scorable
5963	Bn-A03-p18602597	A-genome specific (napus-rapa)	0.9880383	0.5841707	not scorable	not scorable	0.9917355	0.57105
5964	Bn-A03-p18606297	A-genome specific (napus-rapa)	1	0.6599659	not scorable	not scorable	0.9752066	0.6776835
5965	Bn-A03-p18618117	A-genome specific (napus-rapa)	0.9832536	0.7178324	not scorable	not scorable	0.9669421	0.6909164
5966	Bn-A03-p18618990	A-genome specific (napus-rapa)	0.9856459	0.7230592	not scorable	not scorable	0.9752066	0.6512918
5967	Bn-A03-p18622329	A-genome specific (napus-rapa)	0.9736842	0.5718836	not scorable	not scorable	0.9256198	0.8697541
5968	Bn-A03-p18638146	A-genome specific (napus-rapa)	0.9904306	0.8092182	not scorable	not scorable	1	0.8199876
5969	Bn-A03-p18668408	A-genome specific (napus-rapa)	1	0.7975907	not scorable	not scorable	1	0.8815182
5970	Bn-A03-p18682521	A-genome specific (napus-rapa)	0.9976076	0.7871521	not scorable	not scorable	0.9834711	0.7701083

5971	Bn-A03-p1869248	genome unspecific (all in)	0.930622	0.5747726	1	0.7672392	0.9752066	0.7502021
5972	Bn-A03-p18692928	A-genome specific (napus-rapa)	0.9832536	0.5211822	not scorable	not scorable	1	0.8331708
5973	Bn-A03-p187017	A-genome specific (napus-rapa)	0.9928229	0.5452315	not scorable	not scorable	1	0.7820035
5974	Bn-A03-p18706654	A-genome specific (napus-rapa)	0.9736842	0.2380497	not scorable	not scorable	0.9752066	0.713761
5975	Bn-A03-p1871012	A-genome specific (rapa only)	not scorable	not scorable	not scorable	not scorable	0.9834711	0.7617037
5976	Bn-A03-p18737967	A-genome specific (napus-rapa)	0.9784689	0.543836	not scorable	not scorable	0.9917355	0.7147654
5977	Bn-A03-p18744023	napus specific	0.9832536	0.7971055	not scorable	not scorable	not scorable	not scorable
5978	Bn-A03-p18744159	A-genome specific (napus-rapa)	0.9832536	0.7175259	not scorable	not scorable	0.9752066	0.6860651
5979	Bn-A03-p18745119	A-genome specific (napus-rapa)	1	0.8296717	not scorable	not scorable	0.9917355	0.7232494
5980	Bn-A03-p18746523	A-genome specific (napus-rapa)	0.9808612	0.6861332	not scorable	not scorable	0.9834711	0.8949589
5981	Bn-A03-p18746612	A-genome specific (napus-rapa)	0.9976076	0.7717363	not scorable	not scorable	0.9669421	0.8834158
5982	Bn-A03-p18746881	A-genome specific (napus-rapa)	0.9904306	0.8288935	not scorable	not scorable	0.9752066	0.7490746
5983	Bn-A03-p18747847	all not scorable	not scorable	not scorable	not scorable	not scorable	not scorable	not scorable
5984	Bn-A03-p18749943	A-genome specific (napus-rapa)	0.9856459	0.8402233	not scorable	not scorable	0.9834711	0.6724489
5985	Bn-A03-p18749952	A-genome specific (napus-rapa)	0.9904306	0.8196344	not scorable	not scorable	0.9917355	0.6710151
5986	Bn-A03-p18750371	A-genome specific (napus-rapa)	0.9928229	0.7971272	not scorable	not scorable	0.9917355	0.6904303
5987	Bn-A03-p18752710	A-genome specific (napus-rapa)	0.9880383	0.7547866	not scorable	not scorable	0.9917355	0.7020488
5988	Bn-A03-p18753036	napus specific	0.9808612	0.830726	not scorable	not scorable	not scorable	not scorable
5989	Bn-A03-p18762440	A-genome specific (napus-rapa)	0.9784689	0.5197375	not scorable	not scorable	0.9834711	0.6920379
5990	Bn-A03-p18762453	A-genome specific (napus-rapa)	0.9856459	0.7083723	not scorable	not scorable	0.9834711	0.7400401
5991	Bn-A03-p18762984	A-genome specific (napus-rapa)	0.9976076	0.7644388	not scorable	not scorable	0.9917355	0.6809168
5992	Bn-A03-p18764384	A-genome specific (napus-rapa)	0.8660287	0.7793524	not scorable	not scorable	1	0.9302652
5993	Bn-A03-p18765971	A-genome specific (napus-rapa)	0.9688995	0.6821235	not scorable	not scorable	0.9504132	0.6991098
5994	Bn-A03-p18766028	A-genome specific (napus-rapa)	0.9138756	0.6455745	not scorable	not scorable	0.9917355	0.6800793
5995	Bn-A03-p18768321	A-genome specific (napus-rapa)	0.9880383	0.7971544	not scorable	not scorable	0.9834711	0.777375
5996	Bn-A03-p18770617	A-genome specific (napus-rapa)	0.9904306	0.7659637	not scorable	not scorable	0.9917355	0.7930048
5997	Bn-A03-p18770982	A-genome specific (napus-rapa)	0.9952153	0.7832958	not scorable	not scorable	0.9917355	0.7998117
5998	Bn-A03-p18775919	A-genome specific (napus-rapa)	0.9880383	0.7789081	not scorable	not scorable	1	0.7112501
5999	Bn-A03-p18776071	A-genome specific (napus-rapa)	0.9952153	0.6397488	not scorable	not scorable	0.9834711	0.6499786
6000	Bn-A03-p18782352	A-genome specific (napus-rapa)	0.9880383	0.7566057	not scorable	not scorable	0.9917355	0.786381
6001	Bn-A03-p18782416	A-genome specific (napus-rapa)	0.9856459	0.7578342	not scorable	not scorable	1	0.7136897
6002	Bn-A03-p18788929	napus specific	0.5430622	0.7588344	not scorable	not scorable	not scorable	not scorable
6003	Bn-A03-p187972	A-genome specific (rapa only)	not scorable	not scorable	not scorable	not scorable	1	0.6663824

6004	Bn-A03-p18803630	all not scorable	not scorable	not scorable	not scorable	not scorable	not scorable	not scorable
6005	Bn-A03-p18807150	A-genome specific (napus-rapa)	0.9856459	0.5484413	not scorable	not scorable	0.9917355	0.7697235
6006	Bn-A03-p18810251	A-genome specific (napus-rapa)	0.9976076	0.7507116	not scorable	not scorable	0.9917355	0.6961094
6007	Bn-A03-p18811487	A-genome specific (napus-rapa)	0.9832536	0.7511508	not scorable	not scorable	1	0.9180558
6008	Bn-A03-p18816507	A-genome specific (napus-rapa)	0.9976076	0.4373566	not scorable	not scorable	0.9917355	0.8063063
6009	Bn-A03-p18818925	A-genome specific (napus-rapa)	1	0.8117172	not scorable	not scorable	1	0.8175144
6010	Bn-A03-p18819844	A-genome specific (napus-rapa)	0.9952153	0.8220329	not scorable	not scorable	0.9834711	0.7070054
6011	Bn-A03-p18820592	A-genome specific (napus-rapa)	0.9904306	0.7495844	not scorable	not scorable	0.9917355	0.7989676
6012	Bn-A03-p18824580	A-genome specific (napus-rapa)	0.3708134	0.9408662	not scorable	not scorable	0.9338843	0.8345996
6013	Bn-A03-p18826478	A-genome specific (napus-rapa)	0.9593301	0.3471732	not scorable	not scorable	0.9917355	0.7415839
6014	Bn-A03-p18828645	A-genome specific (napus-rapa)	0.9856459	0.793972	not scorable	not scorable	0.9917355	0.7868921
6015	Bn-A03-p18830716	A-genome specific (napus-rapa)	0.8923445	0.7831449	not scorable	not scorable	0.9917355	0.8752664
6016	Bn-A03-p18834464	A-genome specific (napus-rapa)	0.9904306	0.7841095	not scorable	not scorable	0.9917355	0.8675472
6017	Bn-A03-p18836509	A-genome specific (napus-rapa)	0.9784689	0.3861595	not scorable	not scorable	1	0.7495229
6018	Bn-A03-p18846268	A-genome specific (napus-rapa)	0.9760765	0.7756373	not scorable	not scorable	1	0.8475112
6019	Bn-A03-p18848732	A-genome specific (napus-rapa)	0.9641148	0.5980548	not scorable	not scorable	0.9917355	0.6926912
6020	Bn-A03-p18855689	A-genome specific (napus-rapa)	0.9832536	0.7642395	not scorable	not scorable	0.9834711	0.8278041
6021	Bn-A03-p18859928	A-genome specific (napus-rapa)	0.9904306	0.8042495	not scorable	not scorable	1	0.7779072
6022	Bn-A03-p18873704	A-genome specific (napus-rapa)	0.9784689	0.4155857	not scorable	not scorable	0.9834711	0.7445134
6023	Bn-A03-p18883097	napus specific	0.9688995	0.4081837	not scorable	not scorable	not scorable	not scorable
6024	Bn-A03-p18883151	C-genome specific (napus-oleracea)	0.9114832	0.2868923	0.255814	0.6635755	not scorable	not scorable
6025	Bn-A03-p18886205	napus specific	0.937799	0.7247446	not scorable	not scorable	not scorable	not scorable
6026	Bn-A03-p18899251	napus specific	0.9186603	0.7587651	not scorable	not scorable	not scorable	not scorable
6027	Bn-A03-p18899284	napus specific	0.9114832	0.7655693	not scorable	not scorable	not scorable	not scorable
6028	Bn-A03-p1894119	A-genome specific (napus-rapa)	0.9688995	0.5487162	not scorable	not scorable	1	0.7267181
6029	Bn-A03-p1899032	A-genome specific (napus-rapa)	0.9090909	0.7472325	not scorable	not scorable	0.9586777	0.8200139
6030	Bn-A03-p19045801	A-genome specific (napus-rapa)	0.9856459	0.7685573	not scorable	not scorable	1	0.8039527
6031	Bn-A03-p19046345	all not scorable	not scorable	not scorable	not scorable	not scorable	not scorable	not scorable
6032	Bn-A03-p19047537	A-genome specific (napus-rapa)	0.9880383	0.7155481	not scorable	not scorable	0.9834711	0.8207924
6033	Bn-A03-p1904916	A-genome specific (napus-rapa)	0.937799	0.6480187	not scorable	not scorable	0.9917355	0.7586453
6034	Bn-A03-p19065774	A-genome specific (napus-rapa)	0.9880383	0.7673887	not scorable	not scorable	1	0.7415125
6035	Bn-A03-p19066579	A-genome specific (napus-rapa)	0.9808612	0.7505156	not scorable	not scorable	1	0.6989763
6036	Bn-A03-p19068030	A-genome specific (napus-rapa)	0.9808612	0.3741009	not scorable	not scorable	0.9917355	0.7393765

6037	Bn-A03-p19071304	A-genome specific (napus-rapa)	0.9712918	0.7512957	not scorable	not scorable	0.9834711	0.8303624
6038	Bn-A03-p19071572	A-genome specific (napus-rapa)	0.9880383	0.5213324	not scorable	not scorable	0.9917355	0.8137031
6039	Bn-A03-p19078721	A-genome specific (napus-rapa)	0.7966507	0.8299983	not scorable	not scorable	0.9669421	0.706614
6040	Bn-A03-p19091667	A-genome specific (napus-rapa)	0.9712918	0.2540083	not scorable	not scorable	1	0.7089314
6041	Bn-A03-p19094554	A-genome specific (napus-rapa)	1	0.8618054	not scorable	not scorable	0.9669421	0.7783759
6042	Bn-A03-p19129006	A-genome specific (napus-rapa)	0.9856459	0.3986091	not scorable	not scorable	0.9917355	0.8830506
6043	Bn-A03-p19132431	A-genome specific (napus-rapa)	0.9856459	0.7832031	not scorable	not scorable	1	0.7867216
6044	Bn-A03-p19147644	A-genome specific (napus-rapa)	0.9928229	0.4278602	not scorable	not scorable	1	0.7789389
6045	Bn-A03-p19160298	A-genome specific (napus-rapa)	0.9665072	0.5445761	not scorable	not scorable	0.9834711	0.7719007
6046	Bn-A03-p19166062	A-genome specific (napus-rapa)	0.9712918	0.2878649	not scorable	not scorable	0.9834711	0.6715563
6047	Bn-A03-p1918536	A-genome specific (rapa only)	not scorable	not scorable	not scorable	not scorable	0.9834711	0.7190452
6048	Bn-A03-p19189483	A-genome specific (napus-rapa)	0.9880383	0.7662436	not scorable	not scorable	0.9834711	0.7904102
6049	Bn-A03-p19191616	A-genome specific (rapa only)	not scorable	not scorable	not scorable	not scorable	0.9752066	0.6877309
6050	Bn-A03-p19198939	A-genome specific (napus-rapa)	0.9688995	0.773173	not scorable	not scorable	1	0.7875286
6051	Bn-A03-p19202899	A-genome specific (napus-rapa)	0.9545454	0.7675167	not scorable	not scorable	0.9669421	0.7168917
6052	Bn-A03-p19203494	A-genome specific (napus-rapa)	0.9688995	0.7651234	not scorable	not scorable	0.9834711	0.786392
6053	Bn-A03-p19204483	A-genome specific (napus-rapa)	0.9593301	0.6393643	not scorable	not scorable	0.9752066	0.6758332
6054	Bn-A03-p19210113	A-genome specific (napus-rapa)	0.9856459	0.721864	not scorable	not scorable	1	0.7183708
6055	Bn-A03-p1922712	A-genome specific (napus-rapa)	0.9712918	0.7695417	not scorable	not scorable	0.9917355	0.8058044
6056	Bn-A03-p1923025	A-genome specific (napus-rapa)	0.9617225	0.7801909	not scorable	not scorable	0.9504132	0.8158876
6057	Bn-A03-p19243266	A-genome specific (napus-rapa)	0.9808612	0.7757382	not scorable	not scorable	0.9917355	0.7791938
6058	Bn-A03-p19252196	A-genome specific (napus-rapa)	0.9880383	0.7599619	not scorable	not scorable	1	0.7002952
6059	Bn-A03-p19254792	A-genome specific (napus-rapa)	0.9569378	0.6242054	not scorable	not scorable	0.9917355	0.7129078
6060	Bn-A03-p1926809	A-genome specific (napus-rapa)	0.9688995	0.7979753	not scorable	not scorable	0.9669421	0.7753451
6061	Bn-A03-p19274997	A-genome specific (napus-rapa)	0.9688995	0.7692891	not scorable	not scorable	1	0.8848283
6062	Bn-A03-p19323656	A-genome specific (napus-rapa)	0.9808612	0.4017629	not scorable	not scorable	0.9752066	0.7390499
6063	Bn-A03-p19333794	A-genome specific (napus-rapa)	0.9688995	0.5613789	not scorable	not scorable	0.9917355	0.7972284
6064	Bn-A03-p19344007	A-genome specific (napus-rapa)	0.9712918	0.5003403	not scorable	not scorable	0.9917355	0.8099015
6065	Bn-A03-p19359327	A-genome specific (napus-rapa)	0.8732057	0.9311934	not scorable	not scorable	0.7272727	0.7467983
6066	Bn-A03-p19366010	A-genome specific (napus-rapa)	0.9952153	0.8100545	not scorable	not scorable	1	0.7133861
6067	Bn-A03-p19375820	A-genome specific (napus-rapa)	0.9186603	0.7617968	not scorable	not scorable	0.9752066	0.7376153
6068	Bn-A03-p19380485	A-genome specific (napus-rapa)	1	0.661095	not scorable	not scorable	0.9669421	0.6145211
6069	Bn-A03-p19380801	A-genome specific (napus-rapa)	0.9976076	0.7438509	not scorable	not scorable	0.9752066	0.8119538

6070	Bn-A03-p19389076	A-genome specific (napus-rapa)	0.9808612	0.7064304	not scorable	not scorable	0.9834711	0.7078112
6071	Bn-A03-p19408999	A-genome specific (napus-rapa)	0.9760765	0.4452616	not scorable	not scorable	1	0.6651956
6072	Bn-A03-p19438796	A-genome specific (napus-rapa)	0.9904306	0.7384716	not scorable	not scorable	0.9917355	0.7086143
6073	Bn-A03-p19441544	A-genome specific (napus-rapa)	0.9832536	0.6752714	not scorable	not scorable	1	0.6582553
6074	Bn-A03-p19441620	A-genome specific (napus-rapa)	0.9808612	0.7604288	not scorable	not scorable	0.9834711	0.7663515
6075	Bn-A03-p19442945	A-genome specific (napus-rapa)	0.9952153	0.357811	not scorable	not scorable	1	0.7830779
6076	Bn-A03-p19462036	A-genome specific (napus-rapa)	0.9569378	0.7339234	not scorable	not scorable	0.9917355	0.699551
6077	Bn-A03-p19475069	A-genome specific (napus-rapa)	0.9688995	0.458533	not scorable	not scorable	1	0.8934759
6078	Bn-A03-p19501529	A-genome specific (napus-rapa)	0.3133971	0.84958	not scorable	not scorable	0.7272727	0.8663617
6079	Bn-A03-p19510486	A-genome specific (napus-rapa)	0.9952153	0.8361156	not scorable	not scorable	0.9917355	0.7753705
6080	Bn-A03-p19512402	A-genome specific (napus-rapa)	0.8947368	0.5866775	not scorable	not scorable	0.9917355	0.6844546
6081	Bn-A03-p1951453	A-genome specific (rapa only)	not scorable	not scorable	not scorable	not scorable	0.9669421	0.6780698
6082	Bn-A03-p19519674	A-genome specific (napus-rapa)	0.9880383	0.7584445	not scorable	not scorable	1	0.700622
6083	Bn-A03-p19526788	A-genome specific (napus-rapa)	0.9784689	0.720786	not scorable	not scorable	0.9834711	0.6623063
6084	Bn-A03-p19527199	A-genome specific (napus-rapa)	0.9952153	0.7564561	not scorable	not scorable	1	0.6980743
6085	Bn-A03-p19536775	A-genome specific (napus-rapa)	0.9808612	0.8098843	not scorable	not scorable	0.9917355	0.7459868
6086	Bn-A03-p19542946	A-genome specific (napus-rapa)	1	0.5076848	not scorable	not scorable	0.9834711	0.7060285
6087	Bn-A03-p1954639	A-genome specific (napus-rapa)	0.9521531	0.5947318	not scorable	not scorable	1	0.7578901
6088	Bn-A03-p19559007	A-genome specific (napus-rapa)	0.9904306	0.7568452	not scorable	not scorable	0.9917355	0.6700526
6089	Bn-A03-p19560497	A-genome specific (napus-rapa)	0.9880383	0.7574773	not scorable	not scorable	0.9834711	0.735888
6090	Bn-A03-p19560700	A-genome specific (napus-rapa)	0.9760765	0.7615058	not scorable	not scorable	0.9752066	0.7110013
6091	Bn-A03-p19564317	A-genome specific (napus-rapa)	0.9712918	0.7082992	not scorable	not scorable	0.9752066	0.6261423
6092	Bn-A03-p19564333	A-genome specific (napus-rapa)	0.9162679	0.8328186	not scorable	not scorable	0.9834711	0.7613197
6093	Bn-A03-p19573902	A-genome specific (napus-rapa)	0.9808612	0.3522361	not scorable	not scorable	1	0.7227835
6094	Bn-A03-p19582066	A-genome specific (napus-rapa)	0.9808612	0.3615849	not scorable	not scorable	1	0.8213951
6095	Bn-A03-p1959964	A-genome specific (napus-rapa)	0.9665072	0.6872754	not scorable	not scorable	0.9834711	0.6392025
6096	Bn-A03-p1960313	A-genome specific (napus-rapa)	0.9593301	0.6660919	not scorable	not scorable	0.9834711	0.773732
6097	Bn-A03-p1960694	A-genome specific (napus-rapa)	0.9712918	0.6596957	not scorable	not scorable	0.9917355	0.688472
6098	Bn-A03-p196636	A-genome specific (napus-rapa)	0.6818182	0.7556645	not scorable	not scorable	0.9834711	0.707594
6099	Bn-A03-p19727558	A-genome specific (napus-rapa)	0.9976076	0.9304118	not scorable	not scorable	0.9834711	0.7890996
6100	Bn-A03-p19740654	A-genome specific (rapa only)	not scorable	not scorable	not scorable	not scorable	0.9917355	0.6764538
6101	Bn-A03-p19751864	A-genome specific (napus-rapa)	0.9162679	0.8084127	not scorable	not scorable	0.9504132	0.7249707
6102	Bn-A03-p19752316	A-genome specific (napus-rapa)	0.9401914	0.8548384	not scorable	not scorable	0.9090909	0.9171779

6103	Bn-A03-p19753364	A-genome specific (napus-rapa)	1	0.7808523	not scorable	not scorable	0.9917355	0.8134644
6104	Bn-A03-p19753436	A-genome specific (napus-rapa)	1	0.6794679	not scorable	not scorable	1	0.7252899
6105	Bn-A03-p19753470	A-genome specific (napus-rapa)	1	0.4541752	not scorable	not scorable	0.9669421	0.5258642
6106	Bn-A03-p19766214	napus specific	0.9928229	0.3283196	not scorable	not scorable	not scorable	not scorable
6107	Bn-A03-p19768447	A-genome specific (napus-rapa)	1	0.5056018	not scorable	not scorable	0.9669421	0.793625
6108	Bn-A03-p19769998	A-genome specific (napus-rapa)	1	0.7992011	not scorable	not scorable	1	0.8048838
6109	Bn-A03-p19772056	A-genome specific (napus-rapa)	0.9928229	0.6309823	not scorable	not scorable	0.9917355	0.7635841
6110	Bn-A03-p19776651	A-genome specific (napus-rapa)	0.9976076	0.9655425	not scorable	not scorable	1	0.7930965
6111	Bn-A03-p19793770	A-genome specific (napus-rapa)	0.930622	0.8751893	not scorable	not scorable	0.9917355	0.7627573
6112	Bn-A03-p19796541	A-genome specific (napus-rapa)	0.9258373	0.8203386	not scorable	not scorable	0.9834711	0.7526286
6113	Bn-A03-p19798208	A-genome specific (napus-rapa)	0.9976076	0.5190509	not scorable	not scorable	0.9669421	0.6662316
6114	Bn-A03-p19805933	A-genome specific (napus-rapa)	1	0.309222	not scorable	not scorable	0.9834711	0.7181162
6115	Bn-A03-p19806018	A-genome specific (napus-rapa)	1	0.3695414	not scorable	not scorable	0.9834711	0.7312811
6116	Bn-A03-p19819975	A-genome specific (napus-rapa)	1	0.5634728	not scorable	not scorable	1	0.6937931
6117	Bn-A03-p19832980	A-genome specific (napus-rapa)	1	0.7917327	not scorable	not scorable	0.9917355	0.8020663
6118	Bn-A03-p19844661	A-genome specific (napus-rapa)	0.9330143	0.8104562	not scorable	not scorable	0.7933884	0.7802427
6119	Bn-A03-p19850198	A-genome specific (napus-rapa)	0.9976076	0.4634023	not scorable	not scorable	1	0.7905928
6120	Bn-A03-p19851089	A-genome specific (napus-rapa)	0.9354067	0.7529355	not scorable	not scorable	0.8016529	0.7763917
6121	Bn-A03-p19868485	A-genome specific (napus-rapa)	0.9952153	0.4001562	not scorable	not scorable	0.9834711	0.7286804
6122	Bn-A03-p19874058	A-genome specific (napus-rapa)	0.9928229	0.7663468	not scorable	not scorable	0.9917355	0.691284
6123	Bn-A03-p19880918	A-genome specific (napus-rapa)	1	0.5895585	not scorable	not scorable	0.9834711	0.6455349
6124	Bn-A03-p198822	A-genome specific (napus-rapa)	0.6818182	0.7771927	not scorable	not scorable	0.9917355	0.7162221
6125	Bn-A03-p19896843	napus specific	0.7464115	0.7341067	not scorable	not scorable	not scorable	not scorable
6126	Bn-A03-p19897251	A-genome specific (napus-rapa)	1	0.2993476	not scorable	not scorable	0.9834711	0.7047676
6127	Bn-A03-p19902796	A-genome specific (napus-rapa)	0.9832536	0.3626459	not scorable	not scorable	0.9917355	0.6950014
6128	Bn-A03-p1990974	genome unspecific (all in)	0.9832536	0.3297311	1	0.7970625	0.9917355	0.7411686
6129	Bn-A03-p19916449	A-genome specific (napus-rapa)	0.9976076	0.307027	not scorable	not scorable	0.9752066	0.5567098
6130	Bn-A03-p19925593	A-genome specific (napus-rapa)	0.9856459	0.7799436	not scorable	not scorable	0.9917355	0.7674381
6131	Bn-A03-p19928269	A-genome specific (napus-rapa)	0.9928229	0.7676277	not scorable	not scorable	1	0.8436524
6132	Bn-A03-p19935973	A-genome specific (napus-rapa)	0.9808612	0.7727245	not scorable	not scorable	1	0.7786573
6133	Bn-A03-p19942172	A-genome specific (napus-rapa)	0.9904306	0.7741914	not scorable	not scorable	0.9752066	0.7838417
6134	Bn-A03-p19951011	A-genome specific (napus-rapa)	0.9258373	0.2531542	not scorable	not scorable	1	0.7607262
6135	Bn-A03-p19953514	A-genome specific (napus-rapa)	0.7416268	0.7429729	not scorable	not scorable	0.9752066	0.7107991

6136	Bn-A03-p19956358	napus specific	1	0.3323511	not scorable	not scorable	not scorable	not scorable
6137	Bn-A03-p19973423	A-genome specific (napus-rapa)	0.9856459	0.4428411	not scorable	not scorable	0.9752066	0.6967553
6138	Bn-A03-p19974784	A-genome specific (napus-rapa)	0.9928229	0.7574182	not scorable	not scorable	0.9917355	0.7372297
6139	Bn-A03-p19975539	A-genome specific (napus-rapa)	0.9736842	0.2830442	not scorable	not scorable	0.9917355	0.6927454
6140	Bn-A03-p20000930	A-genome specific (napus-rapa)	0.9593301	0.6181672	not scorable	not scorable	0.9669421	0.6513372
6141	Bn-A03-p20005216	A-genome specific (napus-rapa)	0.9712918	0.5260543	not scorable	not scorable	0.9917355	0.7219275
6142	Bn-A03-p20007146	A-genome specific (napus-rapa)	0.9688995	0.3568729	not scorable	not scorable	1	0.5964255
6143	Bn-A03-p20008868	A-genome specific (napus-rapa)	1	0.5485577	not scorable	not scorable	0.9752066	0.7209976
6144	Bn-A03-p20025367	A-genome specific (napus-rapa)	1	0.7649662	not scorable	not scorable	1	0.8493798
6145	Bn-A03-p20033408	A-genome specific (napus-rapa)	1	0.8576522	not scorable	not scorable	1	0.8211405
6146	Bn-A03-p2003715	A-genome specific (napus-rapa)	0.9808612	0.8376459	not scorable	not scorable	0.9917355	0.779722
6147	Bn-A03-p20044499	A-genome specific (rapa only)	not scorable	not scorable	not scorable	not scorable	0.9917355	0.7282605
6148	Bn-A03-p2004585	A-genome specific (napus-rapa)	0.9976076	0.3985095	not scorable	not scorable	0.9917355	0.7541243
6149	Bn-A03-p2005352	A-genome specific (rapa only)	not scorable	not scorable	not scorable	not scorable	0.9917355	0.7801163
6150	Bn-A03-p20079160	A-genome specific (rapa only)	not scorable	not scorable	not scorable	not scorable	0.9338843	0.7564192
6151	Bn-A03-p20079452	A-genome specific (napus-rapa)	0.9952153	0.6825824	not scorable	not scorable	0.9669421	0.7240042
6152	Bn-A03-p20080800	A-genome specific (napus-rapa)	0.9976076	0.740008	not scorable	not scorable	0.9917355	0.6594836
6153	Bn-A03-p20086767	A-genome specific (napus-rapa)	0.9497608	0.2990162	not scorable	not scorable	0.9834711	0.6448386
6154	Bn-A03-p20100490	A-genome specific (napus-rapa)	0.9760765	0.4651143	not scorable	not scorable	1	0.6391391
6155	Bn-A03-p201146	A-genome specific (rapa only)	not scorable	not scorable	not scorable	not scorable	1	0.9272014
6156	Bn-A03-p20114910	A-genome specific (napus-rapa)	0.9736842	0.4287883	not scorable	not scorable	0.9834711	0.7848179
6157	Bn-A03-p20122616	A-genome specific (napus-rapa)	0.9832536	0.3489036	not scorable	not scorable	0.9834711	0.7483006
6158	Bn-A03-p20125361	napus specific	0.8397129	0.8807803	not scorable	not scorable	not scorable	not scorable
6159	Bn-A03-p20145024	A-genome specific (napus-rapa)	0.9808612	0.3058938	not scorable	not scorable	0.9834711	0.7309557
6160	Bn-A03-p20150479	A-genome specific (napus-rapa)	0.9641148	0.7557421	not scorable	not scorable	0.9504132	0.7576761
6161	Bn-A03-p20151244	A-genome specific (napus-rapa)	0.9712918	0.7182727	not scorable	not scorable	1	0.727949
6162	Bn-A03-p20158806	A-genome specific (napus-rapa)	0.9808612	0.3911626	not scorable	not scorable	1	0.7656837
6163	Bn-A03-p20164751	A-genome specific (napus-rapa)	0.9928229	0.4320413	not scorable	not scorable	1	0.7047713
6164	Bn-A03-p20180463	A-genome specific (napus-rapa)	0.9760765	0.2332338	not scorable	not scorable	1	0.7379075
6165	Bn-A03-p2018577	A-genome specific (rapa only)	not scorable	not scorable	not scorable	not scorable	1	0.7163702
6166	Bn-A03-p20186336	A-genome specific (rapa only)	not scorable	not scorable	not scorable	not scorable	1	0.7977625
6167	Bn-A03-p20213689	A-genome specific (napus-rapa)	0.9712918	0.5965237	not scorable	not scorable	0.9834711	0.6457031
6168	Bn-A03-p20216550	A-genome specific (napus-rapa)	0.9617225	0.4678882	not scorable	not scorable	1	0.7347732

6169	Bn-A03-p20221017	napus specific	0.9282297	0.7110363	not scorable	not scorable	not scorable	not scorable
6170	Bn-A03-p20225335	A-genome specific (napus-rapa)	0.9952153	0.3243728	not scorable	not scorable	1	0.6370981
6171	Bn-A03-p20237681	A-genome specific (napus-rapa)	0.9617225	0.2422656	not scorable	not scorable	0.9834711	0.9131089
6172	Bn-A03-p20262635	A-genome specific (rapa only)	not scorable	not scorable	not scorable	not scorable	0.7933884	0.7073662
6173	Bn-A03-p20269025	A-genome specific (napus-rapa)	1	0.3923702	not scorable	not scorable	1	0.7286923
6174	Bn-A03-p20286113	A-genome specific (napus-rapa)	0.9952153	0.8009252	not scorable	not scorable	0.9834711	0.7892681
6175	Bn-A03-p203000	A-genome specific (napus-rapa)	0.9258373	0.7603936	not scorable	not scorable	1	0.7193484
6176	Bn-A03-p2030676	A-genome specific (napus-rapa)	0.9976076	0.5457158	not scorable	not scorable	1	0.6476653
6177	Bn-A03-p20318892	A-genome specific (napus-rapa)	0.9760765	0.7576782	not scorable	not scorable	0.9421487	0.7993273
6178	Bn-A03-p20319347	A-genome specific (napus-rapa)	0.9784689	0.7931784	not scorable	not scorable	0.9752066	0.7586836
6179	Bn-A03-p20324951	A-genome specific (napus-rapa)	1	0.8534212	not scorable	not scorable	1	0.7934078
6180	Bn-A03-p20350690	A-genome specific (napus-rapa)	0.9952153	0.8337978	not scorable	not scorable	1	0.7898603
6181	Bn-A03-p20358050	A-genome specific (napus-rapa)	0.9688995	0.5710906	not scorable	not scorable	0.9669421	0.8567631
6182	Bn-A03-p20361721	A-genome specific (napus-rapa)	0.9066986	0.7137193	not scorable	not scorable	0.9752066	0.6831111
6183	Bn-A03-p20369417	A-genome specific (napus-rapa)	0.9904306	0.4587319	not scorable	not scorable	1	0.8337883
6184	Bn-A03-p20369521	A-genome specific (napus-rapa)	0.9832536	0.7970738	not scorable	not scorable	0.9917355	0.6586018
6185	Bn-A03-p20375108	genome unspecific (all in)	0.9665072	0.4841399	0.9767442	0.8174405	1	0.9095823
6186	Bn-A03-p20375514	A-genome specific (napus-rapa)	0.9473684	0.5790371	not scorable	not scorable	1	0.8815182
6187	Bn-A03-p20383979	A-genome specific (napus-rapa)	0.9952153	0.8178393	not scorable	not scorable	0.9917355	0.806816
6188	Bn-A03-p20395531	A-genome specific (napus-rapa)	1	0.7107133	not scorable	not scorable	0.9917355	0.6751812
6189	Bn-A03-p20395584	A-genome specific (napus-rapa)	0.9976076	0.6955988	not scorable	not scorable	1	0.7698651
6190	Bn-A03-p20398340	A-genome specific (napus-rapa)	0.9688995	0.7482507	not scorable	not scorable	0.9752066	0.6013519
6191	Bn-A03-p20398711	A-genome specific (napus-rapa)	0.9760765	0.7532679	not scorable	not scorable	0.9752066	0.7983918
6192	Bn-A03-p20409233	A-genome specific (napus-rapa)	0.9736842	0.3795091	not scorable	not scorable	1	0.8593605
6193	Bn-A03-p20413103	A-genome specific (napus-rapa)	0.9952153	0.7079429	not scorable	not scorable	1	0.7304603
6194	Bn-A03-p20417630	A-genome specific (napus-rapa)	0.9928229	0.5051054	not scorable	not scorable	0.8181818	0.7801041
6195	Bn-A03-p20424880	A-genome specific (napus-rapa)	0.9928229	0.7996594	not scorable	not scorable	0.9834711	0.692294
6196	Bn-A03-p20427554	A-genome specific (napus-rapa)	0.9904306	0.8604092	not scorable	not scorable	0.9669421	0.7713128
6197	Bn-A03-p20429794	A-genome specific (napus-rapa)	0.9952153	0.713192	not scorable	not scorable	0.9917355	0.6902151
6198	Bn-A03-p20442807	A-genome specific (rapa only)	not scorable	not scorable	not scorable	not scorable	0.9752066	0.6444087
6199	Bn-A03-p20477726	A-genome specific (napus-rapa)	1	0.3432764	not scorable	not scorable	0.9834711	0.8211704
6200	Bn-A03-p20495253	A-genome specific (napus-rapa)	0.9976076	0.864391	not scorable	not scorable	0.9752066	0.7017789
6201	Bn-A03-p20495700	A-genome specific (napus-rapa)	0.9928229	0.7726158	not scorable	not scorable	0.9917355	0.745065

6202	Bn-A03-p20514460	napus specific	0.9593301	0.3554783	not scorable	not scorable	not scorable	not scorable
6203	Bn-A03-p20518761	A-genome specific (napus-rapa)	0.9497608	0.5317938	not scorable	not scorable	0.9586777	0.7241183
6204	Bn-A03-p20523474	A-genome specific (rapa only)	not scorable	not scorable	not scorable	not scorable	0.9586777	0.5277287
6205	Bn-A03-p20523585	A-genome specific (napus-rapa)	0.9880383	0.7465385	not scorable	not scorable	0.9834711	0.6238458
6206	Bn-A03-p20524035	genome unspecific (all in)	0.9760765	0.2529562	0.9767442	0.3153403	1	0.8114529
6207	Bn-A03-p20525474	A-genome specific (napus-rapa)	0.9617225	0.7032795	not scorable	not scorable	0.9834711	0.6479452
6208	Bn-A03-p20525591	A-genome specific (napus-rapa)	0.9856459	0.7816616	not scorable	not scorable	0.9834711	0.823826
6209	Bn-A03-p2054412	A-genome specific (napus-rapa)	0.9712918	0.7530861	not scorable	not scorable	0.9752066	0.7312261
6210	Bn-A03-p20550791	A-genome specific (rapa only)	not scorable	not scorable	not scorable	not scorable	0.9752066	0.7471964
6211	Bn-A03-p20551216	A-genome specific (napus-rapa)	0.9976076	0.8236186	not scorable	not scorable	0.9752066	0.7600628
6212	Bn-A03-p2055179	A-genome specific (rapa only)	not scorable	not scorable	not scorable	not scorable	0.9834711	0.7889516
6213	Bn-A03-p20560569	A-genome specific (napus-rapa)	0.9736842	0.463465	not scorable	not scorable	0.9586777	0.6049393
6214	Bn-A03-p20568764	A-genome specific (napus-rapa)	0.9784689	0.733285	not scorable	not scorable	1	0.7304791
6215	Bn-A03-p20584351	genome unspecific (all in)	0.9712918	0.4877732	1	0.7966806	0.9917355	0.7941987
6216	Bn-A03-p20604949	A-genome specific (napus-rapa)	0.9712918	0.4202984	not scorable	not scorable	0.9917355	0.7782079
6217	Bn-A03-p20609910	A-genome specific (napus-rapa)	0.9736842	0.6487846	not scorable	not scorable	1	0.7701765
6218	Bn-A03-p20627333	A-genome specific (napus-rapa)	0.9952153	0.6966182	not scorable	not scorable	0.9752066	0.6630704
6219	Bn-A03-p20656717	A-genome specific (napus-rapa)	0.9593301	0.4956236	not scorable	not scorable	1	0.7757955
6220	Bn-A03-p20662048	A-genome specific (napus-rapa)	0.9784689	0.7987103	not scorable	not scorable	0.9834711	0.7269177
6221	Bn-A03-p20664670	A-genome specific (napus-rapa)	0.9784689	0.4998975	not scorable	not scorable	0.9834711	0.7746316
6222	Bn-A03-p20670561	A-genome specific (napus-rapa)	0.9856459	0.6916338	not scorable	not scorable	0.9752066	0.6891034
6223	Bn-A03-p20673486	A-genome specific (napus-rapa)	0.9808612	0.315338	not scorable	not scorable	0.9586777	0.7195854
6224	Bn-A03-p20678223	A-genome specific (napus-rapa)	0.9808612	0.7631621	not scorable	not scorable	0.9917355	0.8921801
6225	Bn-A03-p20678571	A-genome specific (napus-rapa)	0.9784689	0.7527214	not scorable	not scorable	0.9834711	0.8366571
6226	Bn-A03-p20691359	A-genome specific (napus-rapa)	0.9641148	0.5129788	not scorable	not scorable	1	0.7880695
6227	Bn-A03-p20693752	A-genome specific (napus-rapa)	0.9760765	0.4608092	not scorable	not scorable	1	0.8342676
6228	Bn-A03-p20701991	A-genome specific (napus-rapa)	0.9928229	0.7775438	not scorable	not scorable	0.9917355	0.7711118
6229	Bn-A03-p20702469	A-genome specific (napus-rapa)	1	0.692532	not scorable	not scorable	1	0.7396948
6230	Bn-A03-p2071494	A-genome specific (napus-rapa)	1	0.4960324	not scorable	not scorable	0.9917355	0.729495
6231	Bn-A03-p20727133	A-genome specific (napus-rapa)	0.9928229	0.3749401	not scorable	not scorable	0.9752066	0.7807903
6232	Bn-A03-p20730830	A-genome specific (napus-rapa)	0.9856459	0.7553341	not scorable	not scorable	1	0.9175813
6233	Bn-A03-p2073180	A-genome specific (napus-rapa)	1	0.4905245	not scorable	not scorable	1	0.7664936
6234	Bn-A03-p20736965	A-genome specific (napus-rapa)	0.9736842	0.5409086	not scorable	not scorable	1	0.7523996

6235	Bn-A03-p2074890	A-genome specific (napus-rapa)	0.9688995	0.7589542	not scorable	not scorable	0.9834711	0.7769175
6236	Bn-A03-p20749815	A-genome specific (napus-rapa)	0.9808612	0.3823527	not scorable	not scorable	1	0.8296082
6237	Bn-A03-p20762376	A-genome specific (napus-rapa)	0.9784689	0.463612	not scorable	not scorable	1	0.9205657
6238	Bn-A03-p207748	genome unspecific (oleracea-rapa)	not scorable	not scorable	0.8914729	0.7466597	1	0.7914145
6239	Bn-A03-p20786060	A-genome specific (napus-rapa)	0.9425837	0.7526995	not scorable	not scorable	0.9338843	0.7819484
6240	Bn-A03-p20848887	all not scorable	not scorable	not scorable	not scorable	not scorable	not scorable	not scorable
6241	Bn-A03-p20909567	A-genome specific (napus-rapa)	0.9545454	0.3245638	not scorable	not scorable	0.9834711	0.8221763
6242	Bn-A03-p20937268	A-genome specific (napus-rapa)	0.9928229	0.2755845	not scorable	not scorable	0.9669421	0.7514905
6243	Bn-A03-p20983297	A-genome specific (napus-rapa)	0.9928229	0.7758434	not scorable	not scorable	1	0.8474829
6244	Bn-A03-p20983398	A-genome specific (napus-rapa)	0.9952153	0.3939326	not scorable	not scorable	1	0.682759
6245	Bn-A03-p2100226	A-genome specific (rapa only)	not scorable	not scorable	not scorable	not scorable	0.9834711	0.8539659
6246	Bn-A03-p21075664	A-genome specific (napus-rapa)	0.9880383	0.4918177	not scorable	not scorable	0.9917355	0.776694
6247	Bn-A03-p21076805	A-genome specific (napus-rapa)	1	0.4126867	not scorable	not scorable	0.9834711	0.5815508
6248	Bn-A03-p21155851	A-genome specific (napus-rapa)	1	0.7876927	not scorable	not scorable	1	0.9175813
6249	Bn-A03-p21199018	genome unspecific (oleracea-rapa)	not scorable	not scorable	1	0.7179766	1	0.9422481
6250	Bn-A03-p21205471	A-genome specific (napus-rapa)	0.9952153	0.4264135	not scorable	not scorable	0.9917355	0.7357489
6251	Bn-A03-p21210189	A-genome specific (napus-rapa)	1	0.3241173	not scorable	not scorable	1	0.7559249
6252	Bn-A03-p21225846	A-genome specific (napus-rapa)	0.9043062	0.6870103	not scorable	not scorable	0.9669421	0.7285719
6253	Bn-A03-p21226758	A-genome specific (rapa only)	not scorable	not scorable	not scorable	not scorable	0.9917355	0.7117158
6254	Bn-A03-p21234281	A-genome specific (napus-rapa)	0.9832536	0.7910416	not scorable	not scorable	1	0.7515329
6255	Bn-A03-p21237671	A-genome specific (napus-rapa)	0.9856459	0.6612093	not scorable	not scorable	1	0.7770717
6256	Bn-A03-p21238466	A-genome specific (napus-rapa)	0.9904306	0.6936077	not scorable	not scorable	1	0.7235095
6257	Bn-A03-p21241827	A-genome specific (napus-rapa)	1	0.7662671	not scorable	not scorable	1	0.8519569
6258	Bn-A03-p21252366	A-genome specific (napus-rapa)	0.9928229	0.554938	not scorable	not scorable	1	0.761601
6259	Bn-A03-p21259337	A-genome specific (napus-rapa)	1	0.7414662	not scorable	not scorable	1	0.9089059
6260	Bn-A03-p21271941	A-genome specific (napus-rapa)	0.9784689	0.488311	not scorable	not scorable	1	0.7644803
6261	Bn-A03-p21283570	A-genome specific (napus-rapa)	0.9569378	0.5407825	not scorable	not scorable	0.9917355	0.7183611
6262	Bn-A03-p21284312	A-genome specific (napus-rapa)	0.9712918	0.8437614	not scorable	not scorable	0.9669421	0.8666806
6263	Bn-A03-p21292773	A-genome specific (napus-rapa)	0.9880383	0.6843174	not scorable	not scorable	0.9917355	0.6850431
6264	Bn-A03-p21294836	A-genome specific (napus-rapa)	1	0.5138816	not scorable	not scorable	1	0.5861297
6265	Bn-A03-p21298611	A-genome specific (napus-rapa)	0.9832536	0.8315659	not scorable	not scorable	0.9917355	0.8625424
6266	Bn-A03-p2130281	A-genome specific (napus-rapa)	0.9593301	0.7817718	not scorable	not scorable	0.9752066	0.7514432
6267	Bn-A03-p21329715	A-genome specific (napus-rapa)	0.9808612	0.7225955	not scorable	not scorable	0.9834711	0.7309238

6268	Bn-A03-p21344477	A-genome specific (napus-rapa)	0.9593301	0.440294	not scorable	not scorable	0.9834711	0.6951214
6269	Bn-A03-p21353686	A-genome specific (napus-rapa)	0.9593301	0.3525266	not scorable	not scorable	1	0.6925776
6270	Bn-A03-p21357849	A-genome specific (napus-rapa)	0.9617225	0.550795	not scorable	not scorable	0.9917355	0.8347137
6271	Bn-A03-p21375969	A-genome specific (napus-rapa)	0.9952153	0.3572702	not scorable	not scorable	1	0.7455035
6272	Bn-A03-p21377430	A-genome specific (napus-rapa)	0.9641148	0.4677371	not scorable	not scorable	0.9917355	0.700554
6273	Bn-A03-p2140542	A-genome specific (napus-rapa)	1	0.5125045	not scorable	not scorable	0.9917355	0.7812234
6274	Bn-A03-p21414316	A-genome specific (napus-rapa)	0.9162679	0.3614666	not scorable	not scorable	0.9586777	0.598104
6275	Bn-A03-p21422696	A-genome specific (napus-rapa)	0.9856459	0.7600757	not scorable	not scorable	0.9752066	0.7248582
6276	Bn-A03-p21428952	A-genome specific (napus-rapa)	0.9928229	0.81681	not scorable	not scorable	0.9917355	0.9498444
6277	Bn-A03-p21436299	A-genome specific (napus-rapa)	0.9928229	0.7816932	not scorable	not scorable	0.9834711	0.8611281
6278	Bn-A03-p21478889	A-genome specific (napus-rapa)	0.9784689	0.8303384	not scorable	not scorable	0.9917355	0.85388
6279	Bn-A03-p21487106	A-genome specific (napus-rapa)	0.9832536	0.453779	not scorable	not scorable	0.9173554	0.7763742
6280	Bn-A03-p21503102	napus specific	0.7296651	0.7117328	not scorable	not scorable	not scorable	not scorable
6281	Bn-A03-p21504871	A-genome specific (napus-rapa)	0.9808612	0.5327719	not scorable	not scorable	1	0.8582115
6282	Bn-A03-p21505363	A-genome specific (napus-rapa)	0.9880383	0.7802736	not scorable	not scorable	1	0.8357486
6283	Bn-A03-p21505431	A-genome specific (napus-rapa)	0.9880383	0.7864668	not scorable	not scorable	1	0.7983146
6284	Bn-A03-p21505440	A-genome specific (napus-rapa)	0.9880383	0.7941719	not scorable	not scorable	1	0.8338675
6285	Bn-A03-p21505530	A-genome specific (napus-rapa)	0.9665072	0.7511067	not scorable	not scorable	0.9504132	0.8098857
6286	Bn-A03-p21507834	A-genome specific (napus-rapa)	0.9593301	0.5762652	not scorable	not scorable	0.9917355	0.5834825
6287	Bn-A03-p21507915	A-genome specific (napus-rapa)	0.9665072	0.2600136	not scorable	not scorable	0.9834711	0.6805131
6288	Bn-A03-p21508594	A-genome specific (napus-rapa)	0.9736842	0.3599712	not scorable	not scorable	1	0.7124087
6289	Bn-A03-p21536745	A-genome specific (napus-rapa)	0.9928229	0.7666442	not scorable	not scorable	0.9504132	0.687184
6290	Bn-A03-p21540310	A-genome specific (napus-rapa)	0.9928229	0.7675065	not scorable	not scorable	0.9917355	0.766727
6291	Bn-A03-p21570061	A-genome specific (napus-rapa)	0.9712918	0.7844723	not scorable	not scorable	1	0.7872455
6292	Bn-A03-p2159699	A-genome specific (rapa only)	not scorable	not scorable	not scorable	not scorable	0.9669421	0.7171257
6293	Bn-A03-p21614714	A-genome specific (napus-rapa)	0.9736842	0.5486966	not scorable	not scorable	0.9917355	0.7602798
6294	Bn-A03-p21615990	A-genome specific (napus-rapa)	0.9904306	0.7830507	not scorable	not scorable	0.9834711	0.8167623
6295	Bn-A03-p21619999	A-genome specific (napus-rapa)	0.9880383	0.7981629	not scorable	not scorable	0.9917355	0.7762188
6296	Bn-A03-p21632587	A-genome specific (napus-rapa)	0.9832536	0.6657807	not scorable	not scorable	0.9917355	0.6694291
6297	Bn-A03-p21660490	A-genome specific (napus-rapa)	0.9904306	0.746069	not scorable	not scorable	0.9917355	0.7537252
6298	Bn-A03-p21666750	A-genome specific (napus-rapa)	0.9784689	0.278403	not scorable	not scorable	0.9834711	0.7569873
6299	Bn-A03-p21666983	A-genome specific (napus-rapa)	0.9952153	0.7979863	not scorable	not scorable	1	0.7778046
6300	Bn-A03-p21669774	A-genome specific (napus-rapa)	0.9856459	0.8075654	not scorable	not scorable	1	0.8208194

6301	Bn-A03-p21675680	A-genome specific (rapa only)	not scorable	not scorable	not scorable	not scorable	0.9256198	0.8312004
6302	Bn-A03-p21676845	A-genome specific (napus-rapa)	0.9928229	0.5182927	not scorable	not scorable	0.9917355	0.6493702
6303	Bn-A03-p21685480	A-genome specific (napus-rapa)	0.9880383	0.3029352	not scorable	not scorable	1	0.6600704
6304	Bn-A03-p2171944	A-genome specific (napus-rapa)	0.9784689	0.77803	not scorable	not scorable	0.9834711	0.746554
6305	Bn-A03-p21744993	A-genome specific (napus-rapa)	0.9832536	0.4591534	not scorable	not scorable	0.9917355	0.7415015
6306	Bn-A03-p21750559	A-genome specific (napus-rapa)	0.9976076	0.8096412	not scorable	not scorable	0.9834711	0.7321907
6307	Bn-A03-p21750922	A-genome specific (napus-rapa)	0.9210526	0.7551218	not scorable	not scorable	0.9752066	0.7293375
6308	Bn-A03-p21752115	napus specific	0.9808612	0.9014213	not scorable	not scorable	not scorable	not scorable
6309	Bn-A03-p21752426	A-genome specific (napus-rapa)	0.9976076	0.8703268	not scorable	not scorable	0.9834711	0.704466
6310	Bn-A03-p21770898	A-genome specific (rapa only)	not scorable	not scorable	not scorable	not scorable	0.9338843	0.6870328
6311	Bn-A03-p21771317	A-genome specific (napus-rapa)	0.9904306	0.798027	not scorable	not scorable	1	0.7296703
6312	Bn-A03-p21782249	napus specific	0.9784689	0.7019429	not scorable	not scorable	not scorable	not scorable
6313	Bn-A03-p21783015	A-genome specific (napus-rapa)	0.9880383	0.5116287	not scorable	not scorable	1	0.7630137
6314	Bn-A03-p21786943	A-genome specific (napus-rapa)	0.9330143	0.6289027	not scorable	not scorable	0.9586777	0.6245322
6315	Bn-A03-p21787407	A-genome specific (napus-rapa)	0.9354067	0.7796378	not scorable	not scorable	1	0.7356615
6316	Bn-A03-p21787813	napus specific	0.7296651	0.7939495	not scorable	not scorable	not scorable	not scorable
6317	Bn-A03-p21798303	A-genome specific (napus-rapa)	1	0.8077108	not scorable	not scorable	1	0.7887701
6318	Bn-A03-p21813117	A-genome specific (napus-rapa)	0.9952153	0.7582909	not scorable	not scorable	0.9586777	0.6755568
6319	Bn-A03-p21820847	A-genome specific (napus-rapa)	0.9928229	0.7874824	not scorable	not scorable	0.9917355	0.8086653
6320	Bn-A03-p21830330	A-genome specific (napus-rapa)	0.9186603	0.7358611	not scorable	not scorable	0.9834711	0.7947387
6321	Bn-A03-p21832912	A-genome specific (napus-rapa)	0.9808612	0.3596093	not scorable	not scorable	0.9917355	0.8580785
6322	Bn-A03-p21839200	A-genome specific (napus-rapa)	0.9569378	0.8154865	not scorable	not scorable	0.9504132	0.8594111
6323	Bn-A03-p21841965	A-genome specific (napus-rapa)	0.9641148	0.5846424	not scorable	not scorable	0.9917355	0.719255
6324	Bn-A03-p21846413	A-genome specific (napus-rapa)	0.9808612	0.6768082	not scorable	not scorable	0.9586777	0.8098153
6325	Bn-A03-p21860400	A-genome specific (napus-rapa)	0.9688995	0.4328285	not scorable	not scorable	1	0.766824
6326	Bn-A03-p21862988	A-genome specific (napus-rapa)	0.9904306	0.8426129	not scorable	not scorable	1	0.8855926
6327	Bn-A03-p21864294	A-genome specific (napus-rapa)	0.9976076	0.2862117	not scorable	not scorable	0.9834711	0.5994023
6328	Bn-A03-p21904842	napus specific	0.9641148	0.4594987	not scorable	not scorable	not scorable	not scorable
6329	Bn-A03-p2190638	A-genome specific (napus-rapa)	0.9712918	0.7831275	not scorable	not scorable	0.9917355	0.6898537
6330	Bn-A03-p21907151	A-genome specific (napus-rapa)	0.9976076	0.8289795	not scorable	not scorable	0.9917355	0.7300616
6331	Bn-A03-p21909917	A-genome specific (napus-rapa)	0.9928229	0.6287314	not scorable	not scorable	1	0.6946223
6332	Bn-A03-p21921653	A-genome specific (napus-rapa)	0.9856459	0.312009	not scorable	not scorable	1	0.696214
6333	Bn-A03-p21927724	A-genome specific (napus-rapa)	0.9712918	0.6462619	not scorable	not scorable	0.9917355	0.7466308

6334	Bn-A03-p21937030	A-genome specific (napus-rapa)	0.9856459	0.7508786	not scorable	not scorable	0.8181818	0.7356452
6335	Bn-A03-p21941473	A-genome specific (napus-rapa)	0.9832536	0.3764886	not scorable	not scorable	0.9917355	0.7344292
6336	Bn-A03-p21942870	A-genome specific (napus-rapa)	0.9784689	0.2997	not scorable	not scorable	0.9834711	0.79971
6337	Bn-A03-p21945987	A-genome specific (napus-rapa)	0.9736842	0.6612054	not scorable	not scorable	0.9669421	0.6070964
6338	Bn-A03-p21964085	A-genome specific (napus-rapa)	0.5861244	0.9261189	not scorable	not scorable	0.9834711	0.7562438
6339	Bn-A03-p21967946	A-genome specific (napus-rapa)	0.9832536	0.4185989	not scorable	not scorable	0.9834711	0.7193655
6340	Bn-A03-p21979477	C-genome specific (napus-oleracea)	0.9856459	0.2599811	1	0.7659699	not scorable	not scorable
6341	Bn-A03-p21980336	A-genome specific (napus-rapa)	1	0.3883741	not scorable	not scorable	0.9834711	0.5898842
6342	Bn-A03-p22002284	A-genome specific (rapa only)	not scorable	not scorable	not scorable	not scorable	0.9586777	0.6683499
6343	Bn-A03-p22003165	A-genome specific (napus-rapa)	0.9952153	0.8550834	not scorable	not scorable	0.9917355	0.7354049
6344	Bn-A03-p22012070	A-genome specific (napus-rapa)	0.9976076	0.8217029	not scorable	not scorable	0.9917355	0.7982837
6345	Bn-A03-p22014622	A-genome specific (napus-rapa)	0.9545454	0.3774506	not scorable	not scorable	1	0.8461323
6346	Bn-A03-p22028778	A-genome specific (napus-rapa)	0.9736842	0.3741097	not scorable	not scorable	1	0.6725635
6347	Bn-A03-p2203942	A-genome specific (napus-rapa)	0.9665072	0.7790984	not scorable	not scorable	0.9752066	0.7439688
6348	Bn-A03-p2206256	genome unspecific (all in)	0.8923445	0.2756155	0.9922481	0.823165	1	0.7204181
6349	Bn-A03-p22065637	A-genome specific (napus-rapa)	0.9904306	0.3656313	not scorable	not scorable	1	0.7087905
6350	Bn-A03-p22072036	A-genome specific (napus-rapa)	0.9641148	0.3077792	not scorable	not scorable	0.9917355	0.6161923
6351	Bn-A03-p22088201	A-genome specific (napus-rapa)	0.9856459	0.6445189	not scorable	not scorable	0.9917355	0.6962284
6352	Bn-A03-p22090414	A-genome specific (napus-rapa)	0.9856459	0.5518949	not scorable	not scorable	0.9752066	0.8125991
6353	Bn-A03-p22098429	A-genome specific (napus-rapa)	0.9832536	0.5766641	not scorable	not scorable	0.9917355	0.6877048
6354	Bn-A03-p22102124	A-genome specific (napus-rapa)	0.9665072	0.2893229	not scorable	not scorable	0.9917355	0.6015636
6355	Bn-A03-p22112518	genome unspecific (oleracea-rapa)	not scorable	not scorable	0.9922481	0.7189572	1	0.7703759
6356	Bn-A03-p221207	A-genome specific (napus-rapa)	0.8732057	0.7716154	not scorable	not scorable	0.9752066	0.8568738
6357	Bn-A03-p22121980	A-genome specific (napus-rapa)	0.9928229	0.3504651	not scorable	not scorable	0.9917355	0.8087388
6358	Bn-A03-p22125583	napus specific	0.8660287	0.6723101	not scorable	not scorable	not scorable	not scorable
6359	Bn-A03-p22132164	A-genome specific (napus-rapa)	0.9808612	0.5093468	not scorable	not scorable	0.9834711	0.7250662
6360	Bn-A03-p22132555	A-genome specific (napus-rapa)	0.9832536	0.7400139	not scorable	not scorable	0.9669421	0.6942552
6361	Bn-A03-p22132662	A-genome specific (napus-rapa)	0.9952153	0.7128378	not scorable	not scorable	0.9752066	0.770838
6362	Bn-A03-p22138059	A-genome specific (napus-rapa)	0.9880383	0.7652116	not scorable	not scorable	1	0.890921
6363	Bn-A03-p22145945	A-genome specific (napus-rapa)	0.9688995	0.7254025	not scorable	not scorable	0.9917355	0.7200636
6364	Bn-A03-p22149000	A-genome specific (napus-rapa)	0.9928229	0.7903253	not scorable	not scorable	0.9917355	0.7348881
6365	Bn-A03-p22164764	A-genome specific (napus-rapa)	0.9760765	0.7393739	not scorable	not scorable	1	0.8570282
6366	Bn-A03-p22183905	A-genome specific (napus-rapa)	0.9880383	0.4522366	not scorable	not scorable	1	0.8679707

6367	Bn-A03-p22227279	A-genome specific (napus-rapa)	0.9258373	0.6984127	not scorable	not scorable	0.7768595	0.8460121
6368	Bn-A03-p22237846	A-genome specific (napus-rapa)	0.9856459	0.7959632	not scorable	not scorable	0.9917355	0.8647583
6369	Bn-A03-p22238801	A-genome specific (napus-rapa)	0.9712918	0.5692192	not scorable	not scorable	0.9917355	0.5410329
6370	Bn-A03-p22272850	A-genome specific (napus-rapa)	0.9401914	0.7211033	not scorable	not scorable	1	0.7770748
6371	Bn-A03-p22285105	A-genome specific (napus-rapa)	0.9784689	0.2984741	not scorable	not scorable	1	0.71514
6372	Bn-A03-p2228813	A-genome specific (napus-rapa)	0.9976076	0.4629818	not scorable	not scorable	0.9752066	0.7875568
6373	Bn-A03-p22299476	A-genome specific (napus-rapa)	0.9856459	0.7830136	not scorable	not scorable	0.9752066	0.7665268
6374	Bn-A03-p22300372	A-genome specific (napus-rapa)	0.9712918	0.3406335	not scorable	not scorable	0.9421487	0.4362547
6375	Bn-A03-p2230320	A-genome specific (napus-rapa)	0.9712918	0.7822981	not scorable	not scorable	1	0.7291278
6376	Bn-A03-p22304555	A-genome specific (napus-rapa)	0.9569378	0.2915057	not scorable	not scorable	0.9834711	0.6862457
6377	Bn-A03-p22306212	A-genome specific (napus-rapa)	0.9688995	0.7806745	not scorable	not scorable	0.9834711	0.8223407
6378	Bn-A03-p22306743	A-genome specific (napus-rapa)	0.9545454	0.3764042	not scorable	not scorable	0.8181818	0.7849016
6379	Bn-A03-p22313737	A-genome specific (napus-rapa)	0.9760765	0.3153366	not scorable	not scorable	0.9669421	0.664616
6380	Bn-A03-p22323976	A-genome specific (napus-rapa)	0.9497608	0.5754092	not scorable	not scorable	1	0.6722468
6381	Bn-A03-p22339476	A-genome specific (napus-rapa)	0.9880383	0.8506054	not scorable	not scorable	1	0.6805297
6382	Bn-A03-p22339742	A-genome specific (rapa only)	not scorable	not scorable	not scorable	not scorable	0.9917355	0.9417582
6383	Bn-A03-p22349705	A-genome specific (napus-rapa)	0.9832536	0.7702117	not scorable	not scorable	0.9752066	0.6805447
6384	Bn-A03-p22369823	A-genome specific (napus-rapa)	0.9976076	0.5268403	not scorable	not scorable	0.9917355	0.6614335
6385	Bn-A03-p22378361	A-genome specific (napus-rapa)	0.9952153	0.7479324	not scorable	not scorable	0.9834711	0.7447708
6386	Bn-A03-p22382259	A-genome specific (napus-rapa)	1	0.312561	not scorable	not scorable	1	0.7297021
6387	Bn-A03-p22382828	A-genome specific (napus-rapa)	0.9808612	0.5064759	not scorable	not scorable	0.9917355	0.7372329
6388	Bn-A03-p22385937	A-genome specific (napus-rapa)	1	0.7724019	not scorable	not scorable	1	0.8146399
6389	Bn-A03-p22398141	A-genome specific (napus-rapa)	0.9832536	0.3562434	not scorable	not scorable	0.9917355	0.6048486
6390	Bn-A03-p22465067	A-genome specific (napus-rapa)	0.9952153	0.8265594	not scorable	not scorable	0.9834711	0.8111263
6391	Bn-A03-p22465690	A-genome specific (napus-rapa)	0.9976076	0.7660604	not scorable	not scorable	0.9834711	0.7135148
6392	Bn-A03-p22465764	A-genome specific (napus-rapa)	0.9976076	0.8998451	not scorable	not scorable	0.9917355	0.7577426
6393	Bn-A03-p22506637	A-genome specific (napus-rapa)	1	0.5249521	not scorable	not scorable	1	0.7714251
6394	Bn-A03-p22556722	A-genome specific (napus-rapa)	0.9928229	0.4870737	not scorable	not scorable	0.9586777	0.7637552
6395	Bn-A03-p22574983	A-genome specific (napus-rapa)	0.9832536	0.7662134	not scorable	not scorable	1	0.9272014
6396	Bn-A03-p22582234	A-genome specific (napus-rapa)	0.8971292	0.7180863	not scorable	not scorable	0.9504132	0.7508155
6397	Bn-A03-p22582673	A-genome specific (napus-rapa)	0.9114832	0.6783901	not scorable	not scorable	0.9504132	0.6794808
6398	Bn-A03-p22582862	A-genome specific (napus-rapa)	0.9066986	0.6511111	not scorable	not scorable	0.9256198	0.6436167
6399	Bn-A03-p22588041	A-genome specific (napus-rapa)	1	0.5016941	not scorable	not scorable	1	0.8085111

6400	Bn-A03-p22590442	A-genome specific (napus-rapa)	0.9808612	0.7705176	not scorable	not scorable	0.9917355	0.7310675
6401	Bn-A03-p22595213	A-genome specific (napus-rapa)	0.9760765	0.484121	not scorable	not scorable	0.9917355	0.8936757
6402	Bn-A03-p22610222	A-genome specific (napus-rapa)	0.9760765	0.4829943	not scorable	not scorable	1	0.7629493
6403	Bn-A03-p22614031	genome unspecific (all in)	0.9904306	0.1914603	0.9379845	0.4726573	0.9752066	0.8008627
6404	Bn-A03-p22619997	A-genome specific (napus-rapa)	0.9832536	0.7418314	not scorable	not scorable	0.9917355	0.7648365
6405	Bn-A03-p2262214	A-genome specific (rapa only)	not scorable	not scorable	not scorable	not scorable	0.9917355	0.8358978
6406	Bn-A03-p22626240	A-genome specific (napus-rapa)	0.9784689	0.526287	not scorable	not scorable	0.9917355	0.7633111
6407	Bn-A03-p22627731	A-genome specific (napus-rapa)	0.9784689	0.7889062	not scorable	not scorable	0.9669421	0.7729505
6408	Bn-A03-p22628435	A-genome specific (rapa only)	not scorable	not scorable	not scorable	not scorable	0.9917355	0.7364721
6409	Bn-A03-p22630782	A-genome specific (napus-rapa)	1	0.7750576	not scorable	not scorable	0.9917355	0.691558
6410	Bn-A03-p22633445	A-genome specific (napus-rapa)	0.9880383	0.8077011	not scorable	not scorable	1	0.8203164
6411	Bn-A03-p22635426	A-genome specific (napus-rapa)	0.9832536	0.7417774	not scorable	not scorable	1	0.730357
6412	Bn-A03-p22643433	A-genome specific (napus-rapa)	0.9808612	0.6783279	not scorable	not scorable	0.9917355	0.7479311
6413	Bn-A03-p22651625	A-genome specific (napus-rapa)	0.9856459	0.7490229	not scorable	not scorable	1	0.8018248
6414	Bn-A03-p22655575	A-genome specific (napus-rapa)	0.9856459	0.4820206	not scorable	not scorable	1	0.7932408
6415	Bn-A03-p22679507	A-genome specific (napus-rapa)	0.9856459	0.7042384	not scorable	not scorable	0.9917355	0.7677697
6416	Bn-A03-p22708175	A-genome specific (napus-rapa)	1	0.7671781	not scorable	not scorable	1	0.8653011
6417	Bn-A03-p2271873	A-genome specific (napus-rapa)	0.9521531	0.7987809	not scorable	not scorable	0.9752066	0.8401836
6418	Bn-A03-p22729	A-genome specific (rapa only)	not scorable	not scorable	not scorable	not scorable	0.9834711	0.775111
6419	Bn-A03-p22730208	A-genome specific (rapa only)	not scorable	not scorable	not scorable	not scorable	0.9752066	0.6834903
6420	Bn-A03-p22737711	A-genome specific (rapa only)	not scorable	not scorable	not scorable	not scorable	0.7438017	0.7618164
6421	Bn-A03-p22739253	A-genome specific (napus-rapa)	0.9760765	0.4007961	not scorable	not scorable	1	0.776496
6422	Bn-A03-p22739529	A-genome specific (napus-rapa)	0.9569378	0.7661553	not scorable	not scorable	1	0.8345648
6423	Bn-A03-p22744766	A-genome specific (napus-rapa)	0.9760765	0.6737286	not scorable	not scorable	1	0.7686638
6424	Bn-A03-p22750972	A-genome specific (napus-rapa)	0.9521531	0.7643581	not scorable	not scorable	0.7603306	0.8103977
6425	Bn-A03-p22752812	A-genome specific (napus-rapa)	0.9449761	0.7050996	not scorable	not scorable	0.7603306	0.7855605
6426	Bn-A03-p22752871	A-genome specific (napus-rapa)	0.9497608	0.7584514	not scorable	not scorable	0.7438017	0.8229498
6427	Bn-A03-p22776355	A-genome specific (rapa only)	not scorable	not scorable	not scorable	not scorable	0.9669421	0.7323347
6428	Bn-A03-p22779259	A-genome specific (napus-rapa)	0.5478469	0.8765852	not scorable	not scorable	0.6859504	0.9724753
6429	Bn-A03-p22816593	napus specific	0.6746411	0.802617	not scorable	not scorable	not scorable	not scorable
6430	Bn-A03-p22816713	napus specific	0.6722488	0.8211461	not scorable	not scorable	not scorable	not scorable
6431	Bn-A03-p22816724	napus specific	0.6889952	0.8005238	not scorable	not scorable	not scorable	not scorable
6432	Bn-A03-p22816854	napus specific	0.6698565	0.8395578	not scorable	not scorable	not scorable	not scorable

6433	Bn-A03-p22818464	A-genome specific (napus-rapa)	0.9856459	0.3015084	not scorable	not scorable	0.9917355	0.6741338
6434	Bn-A03-p22820800	napus specific	0.8899522	0.853515	not scorable	not scorable	not scorable	not scorable
6435	Bn-A03-p22820867	napus specific	0.6674641	0.7510757	not scorable	not scorable	not scorable	not scorable
6436	Bn-A03-p22820943	A-genome specific (napus-rapa)	0.9856459	0.7614925	not scorable	not scorable	1	0.7721574
6437	Bn-A03-p22820996	A-genome specific (napus-rapa)	0.9617225	0.7828537	not scorable	not scorable	1	0.7771863
6438	Bn-A03-p22821346	A-genome specific (napus-rapa)	0.9976076	0.8176157	not scorable	not scorable	1	0.7497553
6439	Bn-A03-p22838495	A-genome specific (napus-rapa)	0.9617225	0.7881553	not scorable	not scorable	0.9752066	0.6544216
6440	Bn-A03-p22841929	A-genome specific (napus-rapa)	0.9880383	0.3395004	not scorable	not scorable	0.9834711	0.7111582
6441	Bn-A03-p22858823	A-genome specific (napus-rapa)	0.9712918	0.7052743	not scorable	not scorable	0.9917355	0.8040303
6442	Bn-A03-p22882585	A-genome specific (napus-rapa)	0.9928229	0.2344454	not scorable	not scorable	0.9834711	0.7321667
6443	Bn-A03-p22886478	all not scorable	not scorable	not scorable	not scorable	not scorable	not scorable	not scorable
6444	Bn-A03-p2297079	A-genome specific (rapa only)	not scorable	not scorable	not scorable	not scorable	0.9834711	0.7613336
6445	Bn-A03-p2297145	A-genome specific (napus-rapa)	0.9641148	0.9209452	not scorable	not scorable	0.9917355	0.8415254
6446	Bn-A03-p22974798	A-genome specific (napus-rapa)	1	0.320936	not scorable	not scorable	1	0.8078685
6447	Bn-A03-p22975377	A-genome specific (napus-rapa)	0.9976076	0.5475405	not scorable	not scorable	0.9917355	0.7485106
6448	Bn-A03-p22976259	A-genome specific (napus-rapa)	0.9976076	0.3943763	not scorable	not scorable	1	0.669937
6449	Bn-A03-p22976648	A-genome specific (napus-rapa)	0.9952153	0.5405343	not scorable	not scorable	0.9669421	0.6221597
6450	Bn-A03-p23017354	A-genome specific (napus-rapa)	0.9880383	0.5785322	not scorable	not scorable	0.9917355	0.7817844
6451	Bn-A03-p23020431	A-genome specific (napus-rapa)	0.9832536	0.724619	not scorable	not scorable	0.9669421	0.6784481
6452	Bn-A03-p23023607	A-genome specific (napus-rapa)	0.9736842	0.7666983	not scorable	not scorable	0.9834711	0.7978314
6453	Bn-A03-p23023930	A-genome specific (napus-rapa)	0.9712918	0.7535623	not scorable	not scorable	0.9669421	0.7688043
6454	Bn-A03-p23025381	A-genome specific (napus-rapa)	0.9880383	0.7719436	not scorable	not scorable	0.9917355	0.8847975
6455	Bn-A03-p23026734	A-genome specific (napus-rapa)	1	0.800547	not scorable	not scorable	1	0.8473544
6456	Bn-A03-p23027465	A-genome specific (napus-rapa)	0.9760765	0.6976238	not scorable	not scorable	1	0.7935653
6457	Bn-A03-p23036027	A-genome specific (napus-rapa)	0.9401914	0.7485551	not scorable	not scorable	0.9338843	0.8121871
6458	Bn-A03-p23039421	A-genome specific (napus-rapa)	1	0.3329871	not scorable	not scorable	0.9834711	0.7977852
6459	Bn-A03-p23041622	A-genome specific (napus-rapa)	0.9784689	0.7204294	not scorable	not scorable	1	0.7425357
6460	Bn-A03-p23041853	A-genome specific (napus-rapa)	0.9952153	0.8764942	not scorable	not scorable	1	0.7588842
6461	Bn-A03-p23043608	A-genome specific (napus-rapa)	0.9904306	0.7924223	not scorable	not scorable	0.9586777	0.6823445
6462	Bn-A03-p23046592	A-genome specific (napus-rapa)	0.9832536	0.7762009	not scorable	not scorable	0.9917355	0.8176394
6463	Bn-A03-p23047749	A-genome specific (napus-rapa)	0.8971292	0.7130809	not scorable	not scorable	0.768595	0.7108089
6464	Bn-A03-p23061800	all not scorable	not scorable	not scorable	not scorable	not scorable	not scorable	not scorable
6465	Bn-A03-p23065216	A-genome specific (napus-rapa)	0.9808612	0.7353194	not scorable	not scorable	0.9917355	0.7850741

6466	Bn-A03-p23065321	A-genome specific (napus-rapa)	0.9712918	0.617568	not scorable	not scorable	1	0.7352853
6467	Bn-A03-p23077236	A-genome specific (napus-rapa)	0.9210526	0.7277477	not scorable	not scorable	0.9752066	0.7167404
6468	Bn-A03-p23080487	A-genome specific (napus-rapa)	1	0.3167184	not scorable	not scorable	0.9752066	0.5542271
6469	Bn-A03-p23086442	A-genome specific (napus-rapa)	0.9138756	0.9256723	not scorable	not scorable	0.9586777	0.8661759
6470	Bn-A03-p23088716	A-genome specific (napus-rapa)	0.9880383	0.44187	not scorable	not scorable	0.9669421	0.7827646
6471	Bn-A03-p2311607	A-genome specific (napus-rapa)	0.9688995	0.7115034	not scorable	not scorable	0.9834711	0.7372549
6472	Bn-A03-p23123050	A-genome specific (napus-rapa)	0.9976076	0.8142844	not scorable	not scorable	0.9917355	0.789754
6473	Bn-A03-p23123128	A-genome specific (napus-rapa)	1	0.6563773	not scorable	not scorable	0.9504132	0.7063643
6474	Bn-A03-p23127658	A-genome specific (napus-rapa)	1	0.5585528	not scorable	not scorable	0.9669421	0.7749835
6475	Bn-A03-p23129458	A-genome specific (napus-rapa)	0.9952153	0.3596164	not scorable	not scorable	0.9917355	0.7739381
6476	Bn-A03-p23130971	A-genome specific (napus-rapa)	1	0.7101598	not scorable	not scorable	1	0.7175964
6477	Bn-A03-p2313208	A-genome specific (napus-rapa)	0.9808612	0.7570496	not scorable	not scorable	0.9917355	0.8090098
6478	Bn-A03-p23143134	A-genome specific (napus-rapa)	0.9928229	0.3708405	not scorable	not scorable	0.9917355	0.7936793
6479	Bn-A03-p23156409	A-genome specific (napus-rapa)	0.6411483	0.8340276	not scorable	not scorable	0.9834711	0.7055126
6480	Bn-A03-p23156512	A-genome specific (napus-rapa)	0.4210526	0.7452894	not scorable	not scorable	1	0.8429463
6481	Bn-A03-p23157775	A-genome specific (napus-rapa)	0.708134	0.8274667	not scorable	not scorable	0.9917355	0.8321756
6482	Bn-A03-p23159350	A-genome specific (napus-rapa)	0.638756	0.8650714	not scorable	not scorable	1	0.729888
6483	Bn-A03-p23160671	A-genome specific (napus-rapa)	0.5956938	0.807777	not scorable	not scorable	0.9834711	0.7017589
6484	Bn-A03-p23170401	A-genome specific (napus-rapa)	0.9808612	0.5152809	not scorable	not scorable	0.9834711	0.6950313
6485	Bn-A03-p23181984	A-genome specific (napus-rapa)	0.9138756	0.4811173	not scorable	not scorable	0.9834711	0.7956775
6486	Bn-A03-p23185327	A-genome specific (napus-rapa)	0.9832536	0.49613	not scorable	not scorable	0.9752066	0.7635037
6487	Bn-A03-p23193342	A-genome specific (napus-rapa)	0.9784689	0.4584962	not scorable	not scorable	0.9752066	0.7820773
6488	Bn-A03-p23205791	A-genome specific (napus-rapa)	0.9688995	0.3860783	not scorable	not scorable	0.9834711	0.7063694
6489	Bn-A03-p23218296	A-genome specific (napus-rapa)	0.9688995	0.7719693	not scorable	not scorable	0.9917355	0.8484495
6490	Bn-A03-p23222657	A-genome specific (napus-rapa)	0.8923445	0.1901414	not scorable	not scorable	1	0.4904395
6491	Bn-A03-p23240923	A-genome specific (napus-rapa)	0.9712918	0.7868927	not scorable	not scorable	0.9834711	0.7699184
6492	Bn-A03-p23243420	A-genome specific (napus-rapa)	0.9760765	0.2870198	not scorable	not scorable	0.9834711	0.7446321
6493	Bn-A03-p23253636	A-genome specific (napus-rapa)	0.9904306	0.7741188	not scorable	not scorable	0.9917355	0.7942212
6494	Bn-A03-p23286977	A-genome specific (napus-rapa)	0.9760765	0.2697973	not scorable	not scorable	0.9752066	0.6906274
6495	Bn-A03-p23303062	A-genome specific (napus-rapa)	0.9832536	0.4553734	not scorable	not scorable	1	0.7970527
6496	Bn-A03-p23318910	A-genome specific (napus-rapa)	0.9784689	0.5078291	not scorable	not scorable	0.9917355	0.7793401
6497	Bn-A03-p23319135	A-genome specific (napus-rapa)	0.9856459	0.5727573	not scorable	not scorable	0.9669421	0.8178099
6498	Bn-A03-p23396260	A-genome specific (napus-rapa)	0.9952153	0.8014634	not scorable	not scorable	0.9917355	0.8243392

6499	Bn-A03-p23414886	A-genome specific (napus-rapa)	0.8014354	0.6830094	not scorable	not scorable	0.9834711	0.6186244
6500	Bn-A03-p23426911	A-genome specific (napus-rapa)	0.9952153	0.7079076	not scorable	not scorable	0.9917355	0.861829
6501	Bn-A03-p2342965	A-genome specific (rapa only)	not scorable	not scorable	not scorable	not scorable	1	0.7916567
6502	Bn-A03-p23432067	A-genome specific (napus-rapa)	1	0.4448519	not scorable	not scorable	0.9917355	0.8136197
6503	Bn-A03-p2344066	A-genome specific (napus-rapa)	0.9880383	0.5083225	not scorable	not scorable	0.9669421	0.5845135
6504	Bn-A03-p23454434	all not scorable	not scorable	not scorable	not scorable	not scorable	not scorable	not scorable
6505	Bn-A03-p23485055	A-genome specific (napus-rapa)	0.9976076	0.7848318	not scorable	not scorable	0.9752066	0.7951664
6506	Bn-A03-p23485435	A-genome specific (napus-rapa)	0.9880383	0.7667989	not scorable	not scorable	0.9834711	0.7025059
6507	Bn-A03-p23503118	A-genome specific (napus-rapa)	0.9712918	0.687502	not scorable	not scorable	1	0.7912294
6508	Bn-A03-p235039	A-genome specific (napus-rapa)	0.9090909	0.6865657	not scorable	not scorable	0.9834711	0.698902
6509	Bn-A03-p23522509	A-genome specific (napus-rapa)	0.9784689	0.7817625	not scorable	not scorable	0.9834711	0.6656432
6510	Bn-A03-p23526338	A-genome specific (napus-rapa)	0.9952153	0.7768538	not scorable	not scorable	0.9917355	0.7952038
6511	Bn-A03-p23526756	A-genome specific (napus-rapa)	0.9784689	0.8050382	not scorable	not scorable	0.9834711	0.8056173
6512	Bn-A03-p23526967	A-genome specific (napus-rapa)	0.9832536	0.8074094	not scorable	not scorable	1	0.83028
6513	Bn-A03-p23528989	A-genome specific (rapa only)	not scorable	not scorable	not scorable	not scorable	0.9834711	0.8103784
6514	Bn-A03-p23529225	A-genome specific (napus-rapa)	0.9808612	0.7732179	not scorable	not scorable	0.9917355	0.7836375
6515	Bn-A03-p23550322	genome unspecific (all in)	0.9856459	0.5230834	0.8139535	0.8171942	0.9917355	0.7851355
6516	Bn-A03-p23552464	A-genome specific (napus-rapa)	0.9976076	0.7062023	not scorable	not scorable	0.9834711	0.7684326
6517	Bn-A03-p23552601	A-genome specific (napus-rapa)	0.9928229	0.7582803	not scorable	not scorable	0.9917355	0.6838505
6518	Bn-A03-p23562008	A-genome specific (napus-rapa)	0.8875598	0.4303952	not scorable	not scorable	0.9669421	0.6852541
6519	Bn-A03-p23596892	A-genome specific (napus-rapa)	0.9904306	0.7674566	not scorable	not scorable	0.9752066	0.7786945
6520	Bn-A03-p23599149	A-genome specific (napus-rapa)	0.9976076	0.2558829	not scorable	not scorable	0.9752066	0.7329832
6521	Bn-A03-p23607037	A-genome specific (napus-rapa)	0.9976076	0.6927492	not scorable	not scorable	0.9834711	0.6976467
6522	Bn-A03-p23608564	A-genome specific (napus-rapa)	0.9928229	0.2944959	not scorable	not scorable	0.9834711	0.7143667
6523	Bn-A03-p23609377	A-genome specific (napus-rapa)	1	0.686775	not scorable	not scorable	0.9834711	0.6767924
6524	Bn-A03-p23609934	A-genome specific (napus-rapa)	0.9928229	0.7692418	not scorable	not scorable	0.9752066	0.7848398
6525	Bn-A03-p23621455	A-genome specific (napus-rapa)	0.9856459	0.3991474	not scorable	not scorable	1	0.9205657
6526	Bn-A03-p23634723	A-genome specific (rapa only)	not scorable	not scorable	not scorable	not scorable	0.9421487	0.8028761
6527	Bn-A03-p23635020	A-genome specific (rapa only)	not scorable	not scorable	not scorable	not scorable	0.6280992	0.7289358
6528	Bn-A03-p23663605	A-genome specific (napus-rapa)	0.9808612	0.7938348	not scorable	not scorable	0.9917355	0.7267323
6529	Bn-A03-p2366510	A-genome specific (napus-rapa)	0.9712918	0.8248184	not scorable	not scorable	0.9834711	0.771007
6530	Bn-A03-p23666221	A-genome specific (rapa only)	not scorable	not scorable	not scorable	not scorable	0.9917355	0.7269418
6531	Bn-A03-p23672735	A-genome specific (napus-rapa)	0.9665072	0.7687989	not scorable	not scorable	0.9834711	0.7417922

6532	Bn-A03-p23674889	A-genome specific (napus-rapa)	0.9952153	0.7836125	not scorable	not scorable	0.9917355	0.7136915
6533	Bn-A03-p23685798	A-genome specific (napus-rapa)	0.9354067	0.3446471	not scorable	not scorable	1	0.8241557
6534	Bn-A03-p23696737	A-genome specific (napus-rapa)	0.9784689	0.3153011	not scorable	not scorable	0.9917355	0.7806168
6535	Bn-A03-p23704255	A-genome specific (napus-rapa)	0.9760765	0.510873	not scorable	not scorable	0.892562	0.7778919
6536	Bn-A03-p23708487	A-genome specific (napus-rapa)	0.9521531	0.7509792	not scorable	not scorable	0.9752066	0.7140445
6537	Bn-A03-p23711362	A-genome specific (napus-rapa)	0.9928229	0.7698783	not scorable	not scorable	0.9669421	0.7892889
6538	Bn-A03-p23715660	A-genome specific (napus-rapa)	0.9904306	0.7702344	not scorable	not scorable	0.9917355	0.8182154
6539	Bn-A03-p23719771	A-genome specific (napus-rapa)	0.9617225	0.6481889	not scorable	not scorable	0.9917355	0.6578873
6540	Bn-A03-p23735715	A-genome specific (napus-rapa)	0.9808612	0.4543206	not scorable	not scorable	0.9917355	0.7749013
6541	Bn-A03-p23736096	A-genome specific (napus-rapa)	0.9976076	0.2980876	not scorable	not scorable	0.9752066	0.6123064
6542	Bn-A03-p23736392	A-genome specific (rapa only)	not scorable	not scorable	not scorable	not scorable	0.9834711	0.7204025
6543	Bn-A03-p23760564	A-genome specific (napus-rapa)	0.9976076	0.7670866	not scorable	not scorable	0.9917355	0.6642581
6544	Bn-A03-p23798235	A-genome specific (napus-rapa)	0.9904306	0.7717593	not scorable	not scorable	1	0.8047553
6545	Bn-A03-p23799968	A-genome specific (napus-rapa)	0.9976076	0.7730271	not scorable	not scorable	0.9752066	0.6944774
6546	Bn-A03-p23801324	napus specific	0.9162679	0.7821022	not scorable	not scorable	not scorable	not scorable
6547	Bn-A03-p23809317	A-genome specific (napus-rapa)	0.9808612	0.7160119	not scorable	not scorable	0.9752066	0.8231553
6548	Bn-A03-p23809595	A-genome specific (napus-rapa)	0.9928229	0.7088347	not scorable	not scorable	0.9834711	0.7513597
6549	Bn-A03-p23810014	A-genome specific (napus-rapa)	0.9904306	0.7962388	not scorable	not scorable	0.9586777	0.7829553
6550	Bn-A03-p23822093	A-genome specific (napus-rapa)	0.9736842	0.7625387	not scorable	not scorable	0.9421487	0.758621
6551	Bn-A03-p23853396	A-genome specific (rapa only)	not scorable	not scorable	not scorable	not scorable	0.9669421	0.8302457
6552	Bn-A03-p23860825	A-genome specific (napus-rapa)	0.9976076	0.7748017	not scorable	not scorable	1	0.6352153
6553	Bn-A03-p23861481	A-genome specific (napus-rapa)	0.9856459	0.7044101	not scorable	not scorable	0.9834711	0.6949393
6554	Bn-A03-p23862018	A-genome specific (napus-rapa)	0.9952153	0.8029972	not scorable	not scorable	0.9834711	0.7045221
6555	Bn-A03-p23862683	A-genome specific (napus-rapa)	0.9976076	0.774298	not scorable	not scorable	0.9834711	0.7822146
6556	Bn-A03-p23868030	A-genome specific (napus-rapa)	1	0.7935309	not scorable	not scorable	0.9752066	0.7918096
6557	Bn-A03-p23887373	A-genome specific (napus-rapa)	0.9736842	0.7892883	not scorable	not scorable	0.9917355	0.6838019
6558	Bn-A03-p23890040	A-genome specific (napus-rapa)	0.9928229	0.7708631	not scorable	not scorable	0.9752066	0.6660798
6559	Bn-A03-p23899	A-genome specific (napus-rapa)	0.9258373	0.7694713	not scorable	not scorable	1	0.7778862
6560	Bn-A03-p23917701	A-genome specific (napus-rapa)	0.9832536	0.3891894	not scorable	not scorable	1	0.7697716
6561	Bn-A03-p23921932	A-genome specific (napus-rapa)	0.9856459	0.7860337	not scorable	not scorable	0.9834711	0.6995802
6562	Bn-A03-p23973475	napus specific	0.9258373	0.8941005	not scorable	not scorable	not scorable	not scorable
6563	Bn-A03-p23980725	A-genome specific (napus-rapa)	0.9760765	0.3097561	not scorable	not scorable	1	0.7412145
6564	Bn-A03-p23980912	A-genome specific (napus-rapa)	0.9784689	0.7603488	not scorable	not scorable	0.9917355	0.9142475

6565	Bn-A03-p23984129	A-genome specific (napus-rapa)	0.7703349	0.7418365	not scorable	not scorable	0.9834711	0.8413941
6566	Bn-A03-p23992075	A-genome specific (napus-rapa)	0.9880383	0.7651425	not scorable	not scorable	1	0.7070044
6567	Bn-A03-p23996628	A-genome specific (napus-rapa)	0.930622	0.8504254	not scorable	not scorable	0.7768595	0.8496944
6568	Bn-A03-p24002443	A-genome specific (napus-rapa)	0.9473684	0.7575043	not scorable	not scorable	0.9917355	0.9112235
6569	Bn-A03-p24016551	A-genome specific (napus-rapa)	0.9665072	0.6842436	not scorable	not scorable	1	0.7372954
6570	Bn-A03-p24017311	A-genome specific (napus-rapa)	0.854067	0.3563369	not scorable	not scorable	0.9834711	0.7376646
6571	Bn-A03-p24017859	A-genome specific (napus-rapa)	0.9976076	0.5676636	not scorable	not scorable	1	0.7955081
6572	Bn-A03-p24017924	A-genome specific (napus-rapa)	1	0.7091545	not scorable	not scorable	1	0.8235429
6573	Bn-A03-p24022648	A-genome specific (napus-rapa)	0.9928229	0.5055766	not scorable	not scorable	0.9834711	0.7996072
6574	Bn-A03-p2402265	A-genome specific (rapa only)	not scorable	not scorable	not scorable	not scorable	0.9834711	0.7335652
6575	Bn-A03-p24032564	A-genome specific (napus-rapa)	0.9784689	0.7691477	not scorable	not scorable	1	0.7067118
6576	Bn-A03-p24046319	A-genome specific (rapa only)	not scorable	not scorable	not scorable	not scorable	1	0.8054308
6577	Bn-A03-p24046452	A-genome specific (napus-rapa)	0.9808612	0.7913827	not scorable	not scorable	1	0.7256375
6578	Bn-A03-p24048132	A-genome specific (napus-rapa)	0.9904306	0.7527011	not scorable	not scorable	1	0.7570085
6579	Bn-A03-p2405726	A-genome specific (rapa only)	not scorable	not scorable	not scorable	not scorable	0.9917355	0.7453861
6580	Bn-A03-p24059470	A-genome specific (rapa only)	not scorable	not scorable	not scorable	not scorable	1	0.7277196
6581	Bn-A03-p24090020	A-genome specific (napus-rapa)	0.9832536	0.3460981	not scorable	not scorable	1	0.7371384
6582	Bn-A03-p24092986	A-genome specific (napus-rapa)	0.9832536	0.6357081	not scorable	not scorable	0.9917355	0.7225231
6583	Bn-A03-p24104930	A-genome specific (napus-rapa)	1	0.7418646	not scorable	not scorable	1	0.7510702
6584	Bn-A03-p24117554	A-genome specific (napus-rapa)	0.9952153	0.4200491	not scorable	not scorable	0.9917355	0.7217508
6585	Bn-A03-p2412263	A-genome specific (napus-rapa)	0.9569378	0.5755259	not scorable	not scorable	1	0.775237
6586	Bn-A03-p24124318	napus specific	0.8971292	0.7795846	not scorable	not scorable	not scorable	not scorable
6587	Bn-A03-p24134454	napus specific	0.9952153	0.317534	not scorable	not scorable	not scorable	not scorable
6588	Bn-A03-p24154359	A-genome specific (napus-rapa)	0.9066986	0.7960536	not scorable	not scorable	0.9669421	0.7218816
6589	Bn-A03-p24169327	napus specific	0.9258373	0.5291593	not scorable	not scorable	not scorable	not scorable
6590	Bn-A03-p24175395	A-genome specific (rapa only)	not scorable	not scorable	not scorable	not scorable	1	0.6969588
6591	Bn-A03-p24176332	A-genome specific (napus-rapa)	0.9736842	0.7735883	not scorable	not scorable	0.9917355	0.7736937
6592	Bn-A03-p24179750	A-genome specific (napus-rapa)	0.9952153	0.7563667	not scorable	not scorable	0.9917355	0.7743954
6593	Bn-A03-p24181488	A-genome specific (napus-rapa)	0.9928229	0.7708838	not scorable	not scorable	0.9834711	0.798441
6594	Bn-A03-p24181569	A-genome specific (napus-rapa)	0.9976076	0.7749998	not scorable	not scorable	1	0.7880737
6595	Bn-A03-p24218520	A-genome specific (rapa only)	not scorable	not scorable	not scorable	not scorable	0.9752066	0.7232825
6596	Bn-A03-p2423548	A-genome specific (rapa only)	not scorable	not scorable	not scorable	not scorable	0.9586777	0.6759076
6597	Bn-A03-p24249333	A-genome specific (napus-rapa)	0.8995215	0.7744742	not scorable	not scorable	0.9834711	0.7492133

6598	Bn-A03-p24255868	A-genome specific (napus-rapa)	0.9880383	0.7224682	not scorable	not scorable	1	0.7622278
6599	Bn-A03-p2427713	A-genome specific (napus-rapa)	0.708134	0.6424924	not scorable	not scorable	0.9586777	0.6638596
6600	Bn-A03-p24294571	A-genome specific (napus-rapa)	0.9976076	0.5479292	not scorable	not scorable	1	0.7316726
6601	Bn-A03-p24298999	A-genome specific (napus-rapa)	0.923445	0.6835436	not scorable	not scorable	0.9834711	0.7401499
6602	Bn-A03-p2431404	A-genome specific (napus-rapa)	0.9736842	0.2814147	not scorable	not scorable	1	0.7296267
6603	Bn-A03-p24345237	A-genome specific (napus-rapa)	0.9545454	0.706633	not scorable	not scorable	0.8099173	0.7231381
6604	Bn-A03-p24349898	A-genome specific (napus-rapa)	0.9808612	0.4174549	not scorable	not scorable	1	0.787483
6605	Bn-A03-p24355827	A-genome specific (napus-rapa)	0.9569378	0.5907879	not scorable	not scorable	0.9834711	0.7473179
6606	Bn-A03-p24355878	A-genome specific (napus-rapa)	0.9736842	0.5505385	not scorable	not scorable	0.9752066	0.6328842
6607	Bn-A03-p24363260	A-genome specific (napus-rapa)	0.9952153	0.7580212	not scorable	not scorable	0.9752066	0.7676342
6608	Bn-A03-p24363811	A-genome specific (napus-rapa)	0.9808612	0.7302264	not scorable	not scorable	0.9834711	0.762
6609	Bn-A03-p24365383	A-genome specific (napus-rapa)	0.9665072	0.5213048	not scorable	not scorable	0.9917355	0.524058
6610	Bn-A03-p24392068	A-genome specific (napus-rapa)	0.9976076	0.7611596	not scorable	not scorable	0.9834711	0.7716715
6611	Bn-A03-p24394796	A-genome specific (napus-rapa)	0.9808612	0.7212237	not scorable	not scorable	0.9834711	0.7574471
6612	Bn-A03-p24395668	A-genome specific (napus-rapa)	0.9928229	0.7437569	not scorable	not scorable	0.9834711	0.6757309
6613	Bn-A03-p24396004	A-genome specific (napus-rapa)	0.9880383	0.7388577	not scorable	not scorable	0.9834711	0.6558907
6614	Bn-A03-p24397912	A-genome specific (napus-rapa)	0.9880383	0.7995637	not scorable	not scorable	0.9586777	0.7421635
6615	Bn-A03-p24397916	A-genome specific (napus-rapa)	0.9952153	0.7781911	not scorable	not scorable	0.9917355	0.777849
6616	Bn-A03-p24398419	A-genome specific (napus-rapa)	0.9880383	0.7853137	not scorable	not scorable	0.9669421	0.7497286
6617	Bn-A03-p24400338	A-genome specific (napus-rapa)	0.9928229	0.7855397	not scorable	not scorable	0.9917355	0.7177429
6618	Bn-A03-p24405388	A-genome specific (rapa only)	not scorable	not scorable	not scorable	not scorable	0.9834711	0.8320355
6619	Bn-A03-p24410521	A-genome specific (napus-rapa)	0.9856459	0.7664156	not scorable	not scorable	0.9917355	0.7606129
6620	Bn-A03-p24411222	A-genome specific (napus-rapa)	0.9832536	0.8042824	not scorable	not scorable	0.9834711	0.7851459
6621	Bn-A03-p24415246	A-genome specific (napus-rapa)	0.9952153	0.7788144	not scorable	not scorable	0.9917355	0.6986021
6622	Bn-A03-p24417118	A-genome specific (napus-rapa)	0.930622	0.7650862	not scorable	not scorable	0.9917355	0.6678255
6623	Bn-A03-p24419229	A-genome specific (napus-rapa)	0.9880383	0.7954323	not scorable	not scorable	0.9752066	0.7792856
6624	Bn-A03-p24420093	A-genome specific (napus-rapa)	0.9928229	0.7789081	not scorable	not scorable	0.9917355	0.8614453
6625	Bn-A03-p24420842	A-genome specific (napus-rapa)	0.9808612	0.7540386	not scorable	not scorable	0.9917355	0.7871201
6626	Bn-A03-p24422801	A-genome specific (napus-rapa)	0.9856459	0.7401875	not scorable	not scorable	1	0.7411011
6627	Bn-A03-p24423536	A-genome specific (napus-rapa)	0.9904306	0.8110406	not scorable	not scorable	0.9917355	0.7948937
6628	Bn-A03-p24423762	A-genome specific (napus-rapa)	0.9952153	0.6865418	not scorable	not scorable	0.9917355	0.6487774
6629	Bn-A03-p24423797	A-genome specific (napus-rapa)	0.9904306	0.7778602	not scorable	not scorable	0.9834711	0.6776635
6630	Bn-A03-p24424174	A-genome specific (napus-rapa)	0.9928229	0.8183743	not scorable	not scorable	0.9834711	0.7110603

6631	Bn-A03-p24424231	A-genome specific (napus-rapa)	0.9784689	0.779777	not scorable	not scorable	0.9752066	0.7162258
6632	Bn-A03-p24424286	A-genome specific (napus-rapa)	0.9880383	0.7768571	not scorable	not scorable	0.9917355	0.73553
6633	Bn-A03-p24424807	A-genome specific (napus-rapa)	0.9784689	0.7244657	not scorable	not scorable	0.9917355	0.7418869
6634	Bn-A03-p24426088	A-genome specific (napus-rapa)	0.9904306	0.7729529	not scorable	not scorable	1	0.7531262
6635	Bn-A03-p24445966	napus specific	0.3995215	0.8148175	not scorable	not scorable	not scorable	not scorable
6636	Bn-A03-p24482649	A-genome specific (napus-rapa)	0.9952153	0.7767802	not scorable	not scorable	1	0.7414125
6637	Bn-A03-p24486881	A-genome specific (napus-rapa)	0.9976076	0.7680234	not scorable	not scorable	0.9834711	0.7595137
6638	Bn-A03-p2449290	A-genome specific (napus-rapa)	1	0.4754882	not scorable	not scorable	1	0.7494448
6639	Bn-A03-p24494026	A-genome specific (napus-rapa)	0.8444976	0.8110638	not scorable	not scorable	0.9752066	0.787383
6640	Bn-A03-p24494224	A-genome specific (napus-rapa)	0.9282297	0.8322148	not scorable	not scorable	0.9669421	0.7381436
6641	Bn-A03-p24497262	A-genome specific (napus-rapa)	0.9952153	0.744936	not scorable	not scorable	0.9421487	0.7786604
6642	Bn-A03-p24512946	A-genome specific (napus-rapa)	0.9019139	0.8957767	not scorable	not scorable	1	0.8114341
6643	Bn-A03-p24526574	A-genome specific (napus-rapa)	0.9952153	0.5153062	not scorable	not scorable	0.9752066	0.7604197
6644	Bn-A03-p24527313	A-genome specific (napus-rapa)	1	0.6452897	not scorable	not scorable	0.9917355	0.5981495
6645	Bn-A03-p24537588	A-genome specific (napus-rapa)	0.9330143	0.7836428	not scorable	not scorable	0.7851239	0.7209138
6646	Bn-A03-p2453901	A-genome specific (rapa only)	not scorable	not scorable	not scorable	not scorable	1	0.8448456
6647	Bn-A03-p24544179	A-genome specific (napus-rapa)	0.9736842	0.327416	not scorable	not scorable	1	0.7278167
6648	Bn-A03-p24565421	A-genome specific (napus-rapa)	1	0.3343455	not scorable	not scorable	1	0.7956936
6649	Bn-A03-p24576670	A-genome specific (napus-rapa)	0.9473684	0.6447119	not scorable	not scorable	0.9834711	0.7154917
6650	Bn-A03-p2457869	A-genome specific (napus-rapa)	0.9665072	0.7285771	not scorable	not scorable	0.9834711	0.6776835
6651	Bn-A03-p2457934	A-genome specific (napus-rapa)	0.9665072	0.8408797	not scorable	not scorable	0.9917355	0.7355909
6652	Bn-A03-p24581655	A-genome specific (napus-rapa)	0.9593301	0.6387448	not scorable	not scorable	0.9917355	0.7010389
6653	Bn-A03-p24588576	A-genome specific (rapa only)	not scorable	not scorable	not scorable	not scorable	0.9917355	0.7109206
6654	Bn-A03-p24596982	A-genome specific (rapa only)	not scorable	not scorable	not scorable	not scorable	0.9752066	0.799085
6655	Bn-A03-p2460475	A-genome specific (napus-rapa)	0.9641148	0.7700267	not scorable	not scorable	1	0.7969586
6656	Bn-A03-p24606883	A-genome specific (napus-rapa)	0.8684211	0.9407511	not scorable	not scorable	0.9669421	0.7466294
6657	Bn-A03-p24612593	A-genome specific (napus-rapa)	1	0.924385	not scorable	not scorable	1	0.8794277
6658	Bn-A03-p24613172	A-genome specific (napus-rapa)	0.9904306	0.7253217	not scorable	not scorable	1	0.7584611
6659	Bn-A03-p24626484	A-genome specific (napus-rapa)	0.8708134	0.9285722	not scorable	not scorable	0.9752066	0.7708727
6660	Bn-A03-p24627047	A-genome specific (napus-rapa)	0.9760765	0.5440142	not scorable	not scorable	1	0.9264424
6661	Bn-A03-p24650359	A-genome specific (napus-rapa)	0.9569378	0.4088935	not scorable	not scorable	0.9917355	0.8189151
6662	Bn-A03-p24669270	A-genome specific (napus-rapa)	0.8755981	0.9019492	not scorable	not scorable	0.9752066	0.8634915
6663	Bn-A03-p24688180	A-genome specific (napus-rapa)	0.9832536	0.8504308	not scorable	not scorable	0.9834711	0.8867637

6664	Bn-A03-p2468983	genome unspecific (oleracea-rapa)	not scorable	not scorable	0.8914729	0.1443294	0.9752066	0.5309913
6665	Bn-A03-p24690789	A-genome specific (napus-rapa)	0.5669857	0.9238845	not scorable	not scorable	1	0.8151029
6666	Bn-A03-p2469223	A-genome specific (napus-rapa)	0.9928229	0.4302405	not scorable	not scorable	0.9834711	0.8057071
6667	Bn-A03-p24704126	A-genome specific (napus-rapa)	0.8205742	0.8293523	not scorable	not scorable	1	0.7634205
6668	Bn-A03-p24710055	A-genome specific (napus-rapa)	0.9976076	0.7177405	not scorable	not scorable	1	0.7012724
6669	Bn-A03-p24739525	A-genome specific (napus-rapa)	0.9928229	0.7478398	not scorable	not scorable	0.9917355	0.7245228
6670	Bn-A03-p24745951	A-genome specific (napus-rapa)	0.9928229	0.7689065	not scorable	not scorable	0.9917355	0.8573797
6671	Bn-A03-p24746007	A-genome specific (napus-rapa)	0.9904306	0.7518393	not scorable	not scorable	0.9917355	0.8664003
6672	Bn-A03-p24748004	A-genome specific (napus-rapa)	0.9712918	0.2494912	not scorable	not scorable	0.9917355	0.6805801
6673	Bn-A03-p24753667	A-genome specific (napus-rapa)	0.9760765	0.8025423	not scorable	not scorable	0.9917355	0.7638793
6674	Bn-A03-p24753829	A-genome specific (napus-rapa)	0.9760765	0.7258437	not scorable	not scorable	0.9917355	0.7317423
6675	Bn-A03-p24763656	A-genome specific (rapa only)	not scorable	not scorable	not scorable	not scorable	0.9586777	0.8156029
6676	Bn-A03-p24773584	A-genome specific (napus-rapa)	0.9976076	0.5180653	not scorable	not scorable	0.9917355	0.7889594
6677	Bn-A03-p24780067	napus specific	0.9904306	0.7459771	not scorable	not scorable	not scorable	not scorable
6678	Bn-A03-p2479213	napus specific	0.9449761	0.7499059	not scorable	not scorable	not scorable	not scorable
6679	Bn-A03-p24800655	A-genome specific (napus-rapa)	1	0.5467187	not scorable	not scorable	0.9917355	0.7207055
6680	Bn-A03-p24805476	A-genome specific (napus-rapa)	0.9856459	0.5785417	not scorable	not scorable	0.9917355	0.6551772
6681	Bn-A03-p24809290	A-genome specific (napus-rapa)	0.9856459	0.7662671	not scorable	not scorable	0.9917355	0.7481571
6682	Bn-A03-p24811187	A-genome specific (napus-rapa)	0.9976076	0.855454	not scorable	not scorable	0.9752066	0.7945815
6683	Bn-A03-p24823015	A-genome specific (napus-rapa)	0.9952153	0.7882114	not scorable	not scorable	0.9917355	0.7172629
6684	Bn-A03-p24824357	A-genome specific (napus-rapa)	0.9641148	0.801886	not scorable	not scorable	0.9008265	0.8868099
6685	Bn-A03-p24827646	genome unspecific (all in)	0.9904306	0.5232289	0.9689922	0.8129192	1	0.7073078
6686	Bn-A03-p24829281	A-genome specific (napus-rapa)	0.9688995	0.3696975	not scorable	not scorable	0.9834711	0.709146
6687	Bn-A03-p2483644	A-genome specific (napus-rapa)	0.9784689	0.5844431	not scorable	not scorable	0.9669421	0.6319522
6688	Bn-A03-p24842021	A-genome specific (napus-rapa)	1	0.7692734	not scorable	not scorable	1	0.7140905
6689	Bn-A03-p24864494	A-genome specific (napus-rapa)	0.3110048	0.7871903	not scorable	not scorable	0.9008265	0.7816744
6690	Bn-A03-p24896477	A-genome specific (napus-rapa)	0.9138756	0.8411083	not scorable	not scorable	0.9752066	0.8180065
6691	Bn-A03-p24896531	A-genome specific (napus-rapa)	0.9162679	0.7905918	not scorable	not scorable	0.9834711	0.7903524
6692	Bn-A03-p24896769	A-genome specific (napus-rapa)	0.9090909	0.7748513	not scorable	not scorable	0.9669421	0.7399241
6693	Bn-A03-p24896884	A-genome specific (napus-rapa)	0.9162679	0.8371346	not scorable	not scorable	0.9669421	0.8107114
6694	Bn-A03-p24897111	A-genome specific (napus-rapa)	0.9162679	0.7743694	not scorable	not scorable	0.9669421	0.7331177
6695	Bn-A03-p24897118	A-genome specific (napus-rapa)	0.9138756	0.7929528	not scorable	not scorable	0.9669421	0.7012326
6696	Bn-A03-p24897286	A-genome specific (napus-rapa)	0.9162679	0.8032023	not scorable	not scorable	0.9834711	0.8049498

6697	Bn-A03-p24900647	A-genome specific (napus-rapa)	0.9162679	0.7930267	not scorable	not scorable	0.9752066	0.7520571
6698	Bn-A03-p24902152	A-genome specific (napus-rapa)	0.9162679	0.8104087	not scorable	not scorable	0.9752066	0.7386913
6699	Bn-A03-p24902631	A-genome specific (napus-rapa)	0.9162679	0.7193407	not scorable	not scorable	0.9834711	0.7033099
6700	Bn-A03-p24906226	A-genome specific (napus-rapa)	0.9928229	0.4535144	not scorable	not scorable	1	0.789242
6701	Bn-A03-p2491346	A-genome specific (rapa only)	not scorable	not scorable	not scorable	not scorable	0.9917355	0.6706721
6702	Bn-A03-p24920415	A-genome specific (napus-rapa)	1	0.419773	not scorable	not scorable	0.9917355	0.7479537
6703	Bn-A03-p24928989	A-genome specific (napus-rapa)	0.9952153	0.6587282	not scorable	not scorable	0.9917355	0.7071024
6704	Bn-A03-p2493541	A-genome specific (napus-rapa)	0.9688995	0.6543214	not scorable	not scorable	1	0.7432543
6705	Bn-A03-p24965131	A-genome specific (napus-rapa)	1	0.5820072	not scorable	not scorable	0.9917355	0.6805493
6706	Bn-A03-p24971161	A-genome specific (napus-rapa)	1	0.7123237	not scorable	not scorable	0.9917355	0.7226265
6707	Bn-A03-p24971360	A-genome specific (napus-rapa)	0.9760765	0.7650027	not scorable	not scorable	0.9917355	0.8602692
6708	Bn-A03-p24985700	A-genome specific (napus-rapa)	0.9952153	0.7498276	not scorable	not scorable	0.9834711	0.7640035
6709	Bn-A03-p24986472	A-genome specific (napus-rapa)	0.9976076	0.8994301	not scorable	not scorable	0.9917355	0.775649
6710	Bn-A03-p24990059	A-genome specific (napus-rapa)	0.9736842	0.3895088	not scorable	not scorable	0.9834711	0.6775794
6711	Bn-A03-p24998976	A-genome specific (napus-rapa)	0.9808612	0.6447199	not scorable	not scorable	0.9917355	0.690023
6712	Bn-A03-p24999716	A-genome specific (napus-rapa)	0.4808612	0.779134	not scorable	not scorable	0.7851239	0.7942743
6713	Bn-A03-p25008908	A-genome specific (napus-rapa)	0.9497608	0.714157	not scorable	not scorable	0.9917355	0.6735455
6714	Bn-A03-p25009736	A-genome specific (napus-rapa)	0.9976076	0.8183227	not scorable	not scorable	0.9917355	0.7711441
6715	Bn-A03-p25009919	A-genome specific (napus-rapa)	0.9976076	0.7632269	not scorable	not scorable	0.9752066	0.7353338
6716	Bn-A03-p25009925	A-genome specific (napus-rapa)	0.9904306	0.7889529	not scorable	not scorable	0.9917355	0.7823338
6717	Bn-A03-p25024647	A-genome specific (napus-rapa)	0.9880383	0.7658893	not scorable	not scorable	0.9917355	0.7651227
6718	Bn-A03-p25024749	A-genome specific (napus-rapa)	0.9760765	0.6174672	not scorable	not scorable	0.9917355	0.7014967
6719	Bn-A03-p25024928	A-genome specific (napus-rapa)	0.9928229	0.7730804	not scorable	not scorable	0.9917355	0.7447109
6720	Bn-A03-p25025360	A-genome specific (napus-rapa)	0.9904306	0.7985497	not scorable	not scorable	0.9834711	0.78022
6721	Bn-A03-p25032014	A-genome specific (napus-rapa)	0.4760765	0.7160954	not scorable	not scorable	0.8181818	0.6777143
6722	Bn-A03-p25033468	A-genome specific (napus-rapa)	0.4688995	0.8519772	not scorable	not scorable	0.8677686	0.7776775
6723	Bn-A03-p25037423	A-genome specific (napus-rapa)	0.9186603	0.6794637	not scorable	not scorable	0.9834711	0.8246735
6724	Bn-A03-p25043466	A-genome specific (napus-rapa)	0.9545454	0.5195987	not scorable	not scorable	0.9917355	0.706578
6725	Bn-A03-p25046120	A-genome specific (napus-rapa)	0.9330143	0.5248874	not scorable	not scorable	0.9917355	0.8076674
6726	Bn-A03-p25047750	A-genome specific (napus-rapa)	0.9832536	0.3872924	not scorable	not scorable	0.9917355	0.7050795
6727	Bn-A03-p25055647	A-genome specific (napus-rapa)	0.9856459	0.3799444	not scorable	not scorable	1	0.7277759
6728	Bn-A03-p25071259	A-genome specific (napus-rapa)	0.9952153	0.8408425	not scorable	not scorable	0.9834711	0.7655048
6729	Bn-A03-p25073363	A-genome specific (napus-rapa)	1	0.7562859	not scorable	not scorable	0.9917355	0.7041311

6730	Bn-A03-p25082044	A-genome specific (napus-rapa)	0.9976076	0.5740218	not scorable	not scorable	0.9917355	0.7705
6731	Bn-A03-p25111028	A-genome specific (napus-rapa)	0.9928229	0.4311489	not scorable	not scorable	1	0.7081072
6732	Bn-A03-p25111171	A-genome specific (napus-rapa)	1	0.7293203	not scorable	not scorable	0.9834711	0.8019294
6733	Bn-A03-p2511868	A-genome specific (napus-rapa)	1	0.7071137	not scorable	not scorable	1	0.7193207
6734	Bn-A03-p25119771	A-genome specific (napus-rapa)	0.9976076	0.408127	not scorable	not scorable	1	0.7791945
6735	Bn-A03-p2520008	A-genome specific (napus-rapa)	0.9641148	0.5266188	not scorable	not scorable	0.9917355	0.7052752
6736	Bn-A03-p2521128	A-genome specific (napus-rapa)	0.9545454	0.7649608	not scorable	not scorable	1	0.7406269
6737	Bn-A03-p25265364	A-genome specific (napus-rapa)	0.9473684	0.3411439	not scorable	not scorable	0.9669421	0.489955
6738	Bn-A03-p25296332	A-genome specific (napus-rapa)	0.9976076	0.4726601	not scorable	not scorable	1	0.8176416
6739	Bn-A03-p25314490	A-genome specific (napus-rapa)	0.9688995	0.7207966	not scorable	not scorable	0.9504132	0.619914
6740	Bn-A03-p25314570	A-genome specific (napus-rapa)	0.9497608	0.6824952	not scorable	not scorable	0.9834711	0.812075
6741	Bn-A03-p2532699	A-genome specific (napus-rapa)	0.9928229	0.4587076	not scorable	not scorable	1	0.6748204
6742	Bn-A03-p25330901	A-genome specific (napus-rapa)	0.9952153	0.7343946	not scorable	not scorable	0.9834711	0.8567975
6743	Bn-A03-p25337594	A-genome specific (napus-rapa)	0.9976076	0.7948563	not scorable	not scorable	1	0.7743765
6744	Bn-A03-p25342507	A-genome specific (napus-rapa)	0.9904306	0.3955503	not scorable	not scorable	0.9834711	0.7077503
6745	Bn-A03-p25343812	A-genome specific (napus-rapa)	0.9952153	0.8380256	not scorable	not scorable	0.9834711	0.8076818
6746	Bn-A03-p25361950	A-genome specific (napus-rapa)	0.9880383	0.5241575	not scorable	not scorable	1	0.8598664
6747	Bn-A03-p25362364	A-genome specific (napus-rapa)	0.9952153	0.5350069	not scorable	not scorable	0.9834711	0.7898923
6748	Bn-A03-p25365003	A-genome specific (napus-rapa)	0.9952153	0.8402349	not scorable	not scorable	1	0.752243
6749	Bn-A03-p25393494	A-genome specific (napus-rapa)	0.9736842	0.7469046	not scorable	not scorable	0.9669421	0.860481
6750	Bn-A03-p25402301	A-genome specific (napus-rapa)	0.9784689	0.7053856	not scorable	not scorable	1	0.747857
6751	Bn-A03-p25402717	A-genome specific (napus-rapa)	0.9856459	0.7672871	not scorable	not scorable	0.9752066	0.7252939
6752	Bn-A03-p25403786	A-genome specific (napus-rapa)	0.9521531	0.2843831	not scorable	not scorable	0.9669421	0.7203365
6753	Bn-A03-p25408779	A-genome specific (napus-rapa)	0.9712918	0.6047825	not scorable	not scorable	1	0.769795
6754	Bn-A03-p25423612	A-genome specific (napus-rapa)	0.9928229	0.7919471	not scorable	not scorable	1	0.9298731
6755	Bn-A03-p25437069	A-genome specific (napus-rapa)	0.9976076	0.8890684	not scorable	not scorable	1	0.9454979
6756	Bn-A03-p25471848	A-genome specific (napus-rapa)	1	0.3907432	not scorable	not scorable	0.9917355	0.8217604
6757	Bn-A03-p25473832	A-genome specific (napus-rapa)	1	0.451246	not scorable	not scorable	1	0.8447355
6758	Bn-A03-p25474850	A-genome specific (napus-rapa)	0.9832536	0.6371432	not scorable	not scorable	0.9917355	0.6303114
6759	Bn-A03-p2548187	A-genome specific (napus-rapa)	0.9665072	0.539252	not scorable	not scorable	1	0.7037888
6760	Bn-A03-p25489551	A-genome specific (napus-rapa)	0.9880383	0.8479936	not scorable	not scorable	0.9917355	0.7167284
6761	Bn-A03-p25494539	A-genome specific (napus-rapa)	0.9784689	0.7259338	not scorable	not scorable	0.9834711	0.7865135
6762	Bn-A03-p25511978	A-genome specific (napus-rapa)	0.9952153	0.8457839	not scorable	not scorable	0.9834711	0.7027249

6763	Bn-A03-p25521989	A-genome specific (napus-rapa)	0.9808612	0.574041	not scorable	not scorable	0.9834711	0.6542021
6764	Bn-A03-p25525612	A-genome specific (napus-rapa)	0.9928229	0.4244581	not scorable	not scorable	0.9834711	0.5805591
6765	Bn-A03-p25555423	A-genome specific (napus-rapa)	0.9928229	0.8022624	not scorable	not scorable	0.9834711	0.8207097
6766	Bn-A03-p2557607	A-genome specific (rapa only)	not scorable	not scorable	not scorable	not scorable	0.9834711	0.7793936
6767	Bn-A03-p25582691	A-genome specific (napus-rapa)	0.9928229	0.3984939	not scorable	not scorable	1	0.825563
6768	Bn-A03-p25584526	A-genome specific (napus-rapa)	0.9904306	0.7711689	not scorable	not scorable	1	0.840514
6769	Bn-A03-p25591818	A-genome specific (napus-rapa)	0.9952153	0.7376582	not scorable	not scorable	0.9917355	0.7359099
6770	Bn-A03-p25599340	A-genome specific (napus-rapa)	0.9928229	0.881541	not scorable	not scorable	0.9834711	0.7774724
6771	Bn-A03-p25604179	A-genome specific (rapa only)	not scorable	not scorable	not scorable	not scorable	0.9834711	0.7855322
6772	Bn-A03-p25606900	A-genome specific (napus-rapa)	0.9976076	0.3176388	not scorable	not scorable	0.9917355	0.6795604
6773	Bn-A03-p25608893	A-genome specific (napus-rapa)	0.9976076	0.8006701	not scorable	not scorable	1	0.828581
6774	Bn-A03-p25614406	A-genome specific (napus-rapa)	0.9449761	0.8415254	not scorable	not scorable	0.9504132	0.868379
6775	Bn-A03-p25625732	A-genome specific (rapa only)	not scorable	not scorable	not scorable	not scorable	0.9917355	0.7766615
6776	Bn-A03-p25629067	A-genome specific (napus-rapa)	0.9976076	0.680786	not scorable	not scorable	0.9834711	0.7774729
6777	Bn-A03-p25631316	A-genome specific (napus-rapa)	0.9880383	0.7775806	not scorable	not scorable	0.9917355	0.7925289
6778	Bn-A03-p25636300	A-genome specific (napus-rapa)	0.9904306	0.601392	not scorable	not scorable	0.9669421	0.6347953
6779	Bn-A03-p25637279	A-genome specific (napus-rapa)	0.9784689	0.7510444	not scorable	not scorable	1	0.7365086
6780	Bn-A03-p25640844	A-genome specific (napus-rapa)	0.9904306	0.7188381	not scorable	not scorable	0.9834711	0.7224126
6781	Bn-A03-p25668597	A-genome specific (napus-rapa)	1	0.5108613	not scorable	not scorable	0.9834711	0.6673809
6782	Bn-A03-p25671744	A-genome specific (napus-rapa)	0.9832536	0.3228954	not scorable	not scorable	0.9669421	0.5717062
6783	Bn-A03-p25679775	A-genome specific (napus-rapa)	0.9641148	0.7193376	not scorable	not scorable	0.7603306	0.8694186
6784	Bn-A03-p25681343	A-genome specific (napus-rapa)	1	0.3304496	not scorable	not scorable	1	0.8078775
6785	Bn-A03-p25683964	A-genome specific (rapa only)	not scorable	not scorable	not scorable	not scorable	0.8760331	0.7884539
6786	Bn-A03-p25695855	A-genome specific (napus-rapa)	0.9856459	0.394556	not scorable	not scorable	1	0.7804914
6787	Bn-A03-p25704793	A-genome specific (napus-rapa)	0.9904306	0.3877018	not scorable	not scorable	0.9834711	0.583651
6788	Bn-A03-p25708944	A-genome specific (napus-rapa)	0.9665072	0.820559	not scorable	not scorable	1	0.8369554
6789	Bn-A03-p25715049	A-genome specific (napus-rapa)	0.9210526	0.8697508	not scorable	not scorable	0.9917355	0.8575163
6790	Bn-A03-p25718518	A-genome specific (napus-rapa)	0.9904306	0.3670449	not scorable	not scorable	1	0.7320683
6791	Bn-A03-p25718716	A-genome specific (napus-rapa)	0.9976076	0.7384736	not scorable	not scorable	1	0.8295
6792	Bn-A03-p25729227	A-genome specific (napus-rapa)	0.9976076	0.8185547	not scorable	not scorable	1	0.7339805
6793	Bn-A03-p25729305	A-genome specific (napus-rapa)	0.9976076	0.8402116	not scorable	not scorable	0.9917355	0.7584571
6794	Bn-A03-p25734907	A-genome specific (napus-rapa)	0.9976076	0.76619	not scorable	not scorable	1	0.8157343
6795	Bn-A03-p25751759	A-genome specific (napus-rapa)	0.9952153	0.7675278	not scorable	not scorable	0.9834711	0.7505261

6796	Bn-A03-p25756310	A-genome specific (napus-rapa)	0.9928229	0.743876	not scorable	not scorable	0.9834711	0.8010354
6797	Bn-A03-p25765687	A-genome specific (rapa only)	not scorable	not scorable	not scorable	not scorable	0.9752066	0.7443224
6798	Bn-A03-p25774773	A-genome specific (napus-rapa)	0.9856459	0.5365525	not scorable	not scorable	0.9834711	0.6948738
6799	Bn-A03-p25774827	C-genome specific (oleracea only)	not scorable	not scorable	0.9844961	0.7793023	not scorable	not scorable
6800	Bn-A03-p25779411	genome unspecific (oleracea-rapa)	not scorable	not scorable	0.7209302	0.6888492	0.9834711	0.6966167
6801	Bn-A03-p25785875	A-genome specific (napus-rapa)	0.9952153	0.4903269	not scorable	not scorable	0.9752066	0.6751118
6802	Bn-A03-p2579866	napus specific	0.9521531	0.7848895	not scorable	not scorable	not scorable	not scorable
6803	Bn-A03-p25801783	A-genome specific (napus-rapa)	0.9665072	0.6965187	not scorable	not scorable	0.9917355	0.6834372
6804	Bn-A03-p25805632	A-genome specific (napus-rapa)	0.9425837	0.8701583	not scorable	not scorable	0.9834711	0.7873433
6805	Bn-A03-p25810585	A-genome specific (napus-rapa)	1	0.7812222	not scorable	not scorable	0.9917355	0.7063597
6806	Bn-A03-p25819955	A-genome specific (napus-rapa)	0.9928229	0.3887921	not scorable	not scorable	0.9834711	0.818069
6807	Bn-A03-p25825392	A-genome specific (napus-rapa)	0.9904306	0.7841604	not scorable	not scorable	1	0.8017315
6808	Bn-A03-p25826578	A-genome specific (napus-rapa)	0.9880383	0.7935843	not scorable	not scorable	0.9752066	0.6838543
6809	Bn-A03-p25827210	A-genome specific (napus-rapa)	0.9880383	0.7471937	not scorable	not scorable	0.9752066	0.6832657
6810	Bn-A03-p25827833	A-genome specific (napus-rapa)	1	0.7345186	not scorable	not scorable	1	0.6406283
6811	Bn-A03-p25828479	napus specific	0.708134	0.8215497	not scorable	not scorable	not scorable	not scorable
6812	Bn-A03-p25839360	napus specific	0.9593301	0.428109	not scorable	not scorable	not scorable	not scorable
6813	Bn-A03-p25841013	A-genome specific (rapa only)	not scorable	not scorable	not scorable	not scorable	0.9917355	0.3333955
6814	Bn-A03-p25841615	A-genome specific (napus-rapa)	0.9904306	0.3985109	not scorable	not scorable	0.9834711	0.8428062
6815	Bn-A03-p25843937	A-genome specific (napus-rapa)	0.9904306	0.7541863	not scorable	not scorable	0.9917355	0.776098
6816	Bn-A03-p25849429	A-genome specific (napus-rapa)	0.9856459	0.5017938	not scorable	not scorable	0.9917355	0.580787
6817	Bn-A03-p25849463	A-genome specific (napus-rapa)	0.9808612	0.8131489	not scorable	not scorable	0.9834711	0.7338386
6818	Bn-A03-p25850146	genome unspecific (all in)	0.9856459	0.4132021	0.9612403	0.5904712	0.9834711	0.7476213
6819	Bn-A03-p25851789	A-genome specific (napus-rapa)	0.9521531	0.6022698	not scorable	not scorable	0.9917355	0.7232682
6820	Bn-A03-p25858075	A-genome specific (rapa only)	not scorable	not scorable	not scorable	not scorable	0.9834711	0.4864223
6821	Bn-A03-p25868537	A-genome specific (napus-rapa)	0.9904306	0.7868593	not scorable	not scorable	1	0.6773683
6822	Bn-A03-p25879876	A-genome specific (napus-rapa)	0.9808612	0.7370031	not scorable	not scorable	0.9834711	0.7203544
6823	Bn-A03-p25885330	napus specific	0.9856459	0.8587439	not scorable	not scorable	not scorable	not scorable
6824	Bn-A03-p2589077	A-genome specific (rapa only)	not scorable	not scorable	not scorable	not scorable	0.9917355	0.7975518
6825	Bn-A03-p25949532	napus specific	0.9665072	0.5365107	not scorable	not scorable	not scorable	not scorable
6826	Bn-A03-p25949676	A-genome specific (napus-rapa)	0.9880383	0.5965263	not scorable	not scorable	0.9834711	0.6803482
6827	Bn-A03-p25951191	A-genome specific (napus-rapa)	0.9784689	0.699569	not scorable	not scorable	0.9338843	0.6602532
6828	Bn-A03-p25952725	A-genome specific (napus-rapa)	0.9952153	0.7196659	not scorable	not scorable	1	0.731602

6829	Bn-A03-p25957465	A-genome specific (napus-rapa)	0.9904306	0.3994641	not scorable	not scorable	1	0.8100411
6830	Bn-A03-p2596410	A-genome specific (napus-rapa)	0.9043062	0.8245699	not scorable	not scorable	0.9917355	0.7741908
6831	Bn-A03-p25993601	A-genome specific (napus-rapa)	0.9736842	0.7725473	not scorable	not scorable	0.9669421	0.7286393
6832	Bn-A03-p26001258	A-genome specific (napus-rapa)	0.9736842	0.7355054	not scorable	not scorable	0.9586777	0.7007593
6833	Bn-A03-p26004171	A-genome specific (napus-rapa)	0.9904306	0.6480551	not scorable	not scorable	0.9917355	0.7915948
6834	Bn-A03-p26013651	A-genome specific (napus-rapa)	0.9856459	0.3118527	not scorable	not scorable	1	0.7047822
6835	Bn-A03-p26014745	A-genome specific (napus-rapa)	0.9952153	0.7933949	not scorable	not scorable	1	0.7126471
6836	Bn-A03-p26021585	A-genome specific (napus-rapa)	0.9545454	0.5247356	not scorable	not scorable	0.9834711	0.6472479
6837	Bn-A03-p26055806	A-genome specific (napus-rapa)	0.9712918	0.741665	not scorable	not scorable	1	0.793758
6838	Bn-A03-p2606192	A-genome specific (napus-rapa)	0.4162679	0.729663	not scorable	not scorable	0.7851239	0.7589241
6839	Bn-A03-p26066930	A-genome specific (napus-rapa)	0.9904306	0.7704231	not scorable	not scorable	1	0.8491004
6840	Bn-A03-p26071059	A-genome specific (napus-rapa)	0.9928229	0.7766228	not scorable	not scorable	0.9834711	0.7586592
6841	Bn-A03-p26071126	A-genome specific (napus-rapa)	0.9952153	0.8086145	not scorable	not scorable	0.9917355	0.7759608
6842	Bn-A03-p26075478	A-genome specific (napus-rapa)	0.9736842	0.670863	not scorable	not scorable	0.9917355	0.7247779
6843	Bn-A03-p26075535	A-genome specific (napus-rapa)	0.9665072	0.4842155	not scorable	not scorable	0.9834711	0.7629414
6844	Bn-A03-p26079114	A-genome specific (napus-rapa)	0.9832536	0.5846496	not scorable	not scorable	1	0.7045545
6845	Bn-A03-p26081548	A-genome specific (napus-rapa)	0.9880383	0.5393499	not scorable	not scorable	1	0.7757495
6846	Bn-A03-p26090388	A-genome specific (napus-rapa)	0.9952153	0.4719716	not scorable	not scorable	1	0.837967
6847	Bn-A03-p26100548	A-genome specific (rapa only)	not scorable	not scorable	not scorable	not scorable	1	0.8522928
6848	Bn-A03-p26100814	A-genome specific (napus-rapa)	0.9808612	0.6988091	not scorable	not scorable	0.9834711	0.7615478
6849	Bn-A03-p26116027	A-genome specific (napus-rapa)	0.9904306	0.3484017	not scorable	not scorable	0.9834711	0.7468299
6850	Bn-A03-p26117920	A-genome specific (napus-rapa)	0.9976076	0.8454746	not scorable	not scorable	0.9917355	0.7616268
6851	Bn-A03-p26119193	A-genome specific (napus-rapa)	0.9784689	0.7946625	not scorable	not scorable	0.9917355	0.7900776
6852	Bn-A03-p26121502	A-genome specific (napus-rapa)	0.9904306	0.734126	not scorable	not scorable	1	0.8095435
6853	Bn-A03-p26131473	A-genome specific (napus-rapa)	1	0.7161857	not scorable	not scorable	1	0.6707532
6854	Bn-A03-p26132481	A-genome specific (napus-rapa)	0.9928229	0.8183736	not scorable	not scorable	1	0.7053532
6855	Bn-A03-p26133142	A-genome specific (napus-rapa)	0.9952153	0.6921923	not scorable	not scorable	0.9917355	0.6241058
6856	Bn-A03-p26133346	A-genome specific (napus-rapa)	0.9928229	0.3146702	not scorable	not scorable	0.9586777	0.8039298
6857	Bn-A03-p26148876	all not scorable	not scorable	not scorable	not scorable	not scorable	not scorable	not scorable
6858	Bn-A03-p2616068	genome unspecific (oleracea-rapa)	not scorable	not scorable	1	0.2758499	1	0.6459131
6859	Bn-A03-p26167847	genome unspecific (all in)	0.9712918	0.2595774	0.9844961	0.2700552	0.9917355	0.7070636
6860	Bn-A03-p26167992	A-genome specific (napus-rapa)	1	0.4293925	not scorable	not scorable	0.9504132	0.5407359
6861	Bn-A03-p26168881	A-genome specific (napus-rapa)	0.9952153	0.4548941	not scorable	not scorable	0.9669421	0.5443752

6862	Bn-A03-p26195875	A-genome specific (napus-rapa)	0.9856459	0.8025534	not scorable	not scorable	0.9752066	0.6937616
6863	Bn-A03-p26197815	A-genome specific (napus-rapa)	0.9928229	0.6836517	not scorable	not scorable	0.9669421	0.6768839
6864	Bn-A03-p26202244	A-genome specific (napus-rapa)	0.9808612	0.7560939	not scorable	not scorable	0.9834711	0.7826273
6865	Bn-A03-p2621677	A-genome specific (napus-rapa)	0.9258373	0.4502969	not scorable	not scorable	0.9834711	0.7815526
6866	Bn-A03-p26216950	A-genome specific (napus-rapa)	0.7799043	0.9031515	not scorable	not scorable	0.9917355	0.7515881
6867	Bn-A03-p26232681	A-genome specific (napus-rapa)	1	0.4373125	not scorable	not scorable	1	0.5383495
6868	Bn-A03-p26248671	A-genome specific (napus-rapa)	0.9545454	0.4741703	not scorable	not scorable	0.9752066	0.6797703
6869	Bn-A03-p26264242	A-genome specific (napus-rapa)	0.9880383	0.5044744	not scorable	not scorable	0.9752066	0.7670268
6870	Bn-A03-p26272560	napus specific	0.9497608	0.203819	not scorable	not scorable	not scorable	not scorable
6871	Bn-A03-p26275444	A-genome specific (napus-rapa)	0.7009569	0.8216316	not scorable	not scorable	0.7355372	0.793963
6872	Bn-A03-p2627552	A-genome specific (napus-rapa)	0.9665072	0.803582	not scorable	not scorable	1	0.9175813
6873	Bn-A03-p26275615	A-genome specific (napus-rapa)	0.6937799	0.7685632	not scorable	not scorable	0.7520661	0.7452087
6874	Bn-A03-p26276349	A-genome specific (napus-rapa)	0.9425837	0.4405088	not scorable	not scorable	0.9917355	0.6655075
6875	Bn-A03-p262793	A-genome specific (rapa only)	not scorable	not scorable	not scorable	not scorable	0.9917355	0.7908501
6876	Bn-A03-p26284129	A-genome specific (napus-rapa)	0.9928229	0.7244878	not scorable	not scorable	0.9669421	0.6572834
6877	Bn-A03-p26284449	A-genome specific (napus-rapa)	1	0.6214259	not scorable	not scorable	0.9917355	0.7626569
6878	Bn-A03-p26284882	A-genome specific (napus-rapa)	0.9928229	0.7061277	not scorable	not scorable	1	0.7426893
6879	Bn-A03-p26286301	A-genome specific (napus-rapa)	0.9856459	0.7270816	not scorable	not scorable	0.9834711	0.6942053
6880	Bn-A03-p26288769	A-genome specific (napus-rapa)	0.9665072	0.7068239	not scorable	not scorable	0.9917355	0.7297269
6881	Bn-A03-p26309336	A-genome specific (napus-rapa)	0.9569378	0.4783708	not scorable	not scorable	0.9917355	0.7556286
6882	Bn-A03-p26309550	A-genome specific (napus-rapa)	0.9856459	0.7664756	not scorable	not scorable	0.9834711	0.7348804
6883	Bn-A03-p26310351	A-genome specific (napus-rapa)	0.9665072	0.6987505	not scorable	not scorable	0.9917355	0.8069491
6884	Bn-A03-p26311193	A-genome specific (napus-rapa)	0.9904306	0.7821362	not scorable	not scorable	1	0.8052432
6885	Bn-A03-p26319168	napus specific	0.8205742	0.7010623	not scorable	not scorable	not scorable	not scorable
6886	Bn-A03-p26359851	A-genome specific (napus-rapa)	0.9928229	0.4600107	not scorable	not scorable	0.9917355	0.8041645
6887	Bn-A03-p2638510	A-genome specific (rapa only)	not scorable	not scorable	not scorable	not scorable	0.9917355	0.6927592
6888	Bn-A03-p26405367	napus specific	0.9162679	0.7248186	not scorable	not scorable	not scorable	not scorable
6889	Bn-A03-p26406372	A-genome specific (napus-rapa)	0.9641148	0.6426784	not scorable	not scorable	0.9917355	0.6273827
6890	Bn-A03-p2643134	A-genome specific (napus-rapa)	0.9425837	0.362263	not scorable	not scorable	0.9752066	0.6994933
6891	Bn-A03-p26433672	A-genome specific (napus-rapa)	0.7105263	0.7559118	not scorable	not scorable	0.9752066	0.7808021
6892	Bn-A03-p26433849	A-genome specific (napus-rapa)	0.9952153	0.7687855	not scorable	not scorable	1	0.8133682
6893	Bn-A03-p26433912	A-genome specific (napus-rapa)	0.9928229	0.6766505	not scorable	not scorable	1	0.6741807
6894	Bn-A03-p26433948	A-genome specific (napus-rapa)	0.9880383	0.7566537	not scorable	not scorable	1	0.813203

6895	Bn-A03-p26437565	A-genome specific (napus-rapa)	0.9880383	0.758953	not scorable	not scorable	0.9834711	0.8212726
6896	Bn-A03-p26437680	A-genome specific (napus-rapa)	0.9952153	0.7290215	not scorable	not scorable	0.9917355	0.7624756
6897	Bn-A03-p26437883	A-genome specific (napus-rapa)	1	0.777526	not scorable	not scorable	0.9669421	0.775204
6898	Bn-A03-p26439962	genome unspecific (oleracea-rapa)	not scorable	not scorable	1	0.726634	0.9917355	0.6544634
6899	Bn-A03-p26444464	A-genome specific (napus-rapa)	0.9401914	0.6385937	not scorable	not scorable	1	0.6798831
6900	Bn-A03-p26444945	A-genome specific (napus-rapa)	0.9928229	0.7162951	not scorable	not scorable	0.5041322	0.7600735
6901	Bn-A03-p26445028	A-genome specific (napus-rapa)	0.9856459	0.7649498	not scorable	not scorable	1	0.7528773
6902	Bn-A03-p2645391	A-genome specific (napus-rapa)	0.9545454	0.7080087	not scorable	not scorable	0.9834711	0.6686319
6903	Bn-A03-p26477533	A-genome specific (napus-rapa)	0.9617225	0.7520068	not scorable	not scorable	0.9669421	0.784013
6904	Bn-A03-p26478842	A-genome specific (napus-rapa)	0.9784689	0.6849964	not scorable	not scorable	0.9586777	0.7096214
6905	Bn-A03-p26506472	genome unspecific (all in)	0.9808612	0.4113053	1	0.806362	1	0.8687758
6906	Bn-A03-p26510093	A-genome specific (napus-rapa)	1	0.803435	not scorable	not scorable	1	0.729994
6907	Bn-A03-p26528958	A-genome specific (napus-rapa)	0.9808612	0.732834	not scorable	not scorable	0.9834711	0.7480645
6908	Bn-A03-p26546744	genome unspecific (all in)	0.9641148	0.3827632	1	0.6900312	1	0.7700782
6909	Bn-A03-p26560194	A-genome specific (napus-rapa)	0.9856459	0.7960782	not scorable	not scorable	1	0.7778792
6910	Bn-A03-p26567111	A-genome specific (napus-rapa)	1	0.3512424	not scorable	not scorable	1	0.6504184
6911	Bn-A03-p26567993	A-genome specific (napus-rapa)	0.9641148	0.2559953	not scorable	not scorable	0.9917355	0.6866909
6912	Bn-A03-p26571083	A-genome specific (napus-rapa)	0.9832536	0.6627277	not scorable	not scorable	0.9586777	0.632782
6913	Bn-A03-p26572622	A-genome specific (napus-rapa)	0.9784689	0.7811325	not scorable	not scorable	0.9917355	0.8067438
6914	Bn-A03-p26577952	genome unspecific (oleracea-rapa)	not scorable	not scorable	0.9922481	0.8580986	0.9917355	0.7089232
6915	Bn-A03-p26603077	A-genome specific (napus-rapa)	0.9928229	0.6825198	not scorable	not scorable	0.9917355	0.6211556
6916	Bn-A03-p26604097	A-genome specific (napus-rapa)	0.9976076	0.7801243	not scorable	not scorable	1	0.800683
6917	Bn-A03-p26604578	A-genome specific (napus-rapa)	0.9880383	0.3092309	not scorable	not scorable	0.9504132	0.5986605
6918	Bn-A03-p26607921	A-genome specific (napus-rapa)	0.9976076	0.7745147	not scorable	not scorable	0.9917355	0.6981117
6919	Bn-A03-p2662675	A-genome specific (napus-rapa)	0.9904306	0.7263095	not scorable	not scorable	0.9917355	0.7857051
6920	Bn-A03-p2665731	A-genome specific (napus-rapa)	0.8947368	0.6912753	not scorable	not scorable	0.9834711	0.6499636
6921	Bn-A03-p2666271	A-genome specific (napus-rapa)	0.9425837	0.70749	not scorable	not scorable	1	0.7315536
6922	Bn-A03-p2667726	A-genome specific (napus-rapa)	0.8923445	0.8550065	not scorable	not scorable	1	0.7467236
6923	Bn-A03-p2668128	A-genome specific (napus-rapa)	0.9617225	0.7612469	not scorable	not scorable	1	0.8079165
6924	Bn-A03-p26740723	A-genome specific (napus-rapa)	0.9904306	0.7091329	not scorable	not scorable	1	0.8927264
6925	Bn-A03-p26742006	A-genome specific (napus-rapa)	0.9976076	0.8672755	not scorable	not scorable	1	0.8087255
6926	Bn-A03-p26756701	A-genome specific (rapa only)	not scorable	not scorable	not scorable	not scorable	0.5206612	0.8783935
6927	Bn-A03-p26763577	A-genome specific (napus-rapa)	0.9784689	0.7593756	not scorable	not scorable	0.9752066	0.7473724

6928	Bn-A03-p26764606	A-genome specific (napus-rapa)	0.9856459	0.5562426	not scorable	not scorable	0.9917355	0.5541473
6929	Bn-A03-p26771482	A-genome specific (napus-rapa)	0.9832536	0.8376549	not scorable	not scorable	0.9834711	0.8001143
6930	Bn-A03-p26775994	A-genome specific (napus-rapa)	0.9832536	0.8415766	not scorable	not scorable	0.9504132	0.7123927
6931	Bn-A03-p26786549	A-genome specific (napus-rapa)	0.9832536	0.7385593	not scorable	not scorable	0.9834711	0.7538136
6932	Bn-A03-p26788875	A-genome specific (napus-rapa)	0.9808612	0.8791263	not scorable	not scorable	0.9752066	0.7437811
6933	Bn-A03-p26789755	A-genome specific (napus-rapa)	0.9808612	0.8416991	not scorable	not scorable	0.9752066	0.8084202
6934	Bn-A03-p26798652	A-genome specific (napus-rapa)	0.9856459	0.6861849	not scorable	not scorable	0.9834711	0.7165272
6935	Bn-A03-p26800384	A-genome specific (napus-rapa)	0.9784689	0.7653916	not scorable	not scorable	0.9834711	0.8016018
6936	Bn-A03-p26803262	A-genome specific (napus-rapa)	0.9976076	0.482747	not scorable	not scorable	1	0.5601621
6937	Bn-A03-p26809706	A-genome specific (napus-rapa)	0.9688995	0.4604504	not scorable	not scorable	0.9834711	0.7731143
6938	Bn-A03-p26810924	A-genome specific (napus-rapa)	1	0.855454	not scorable	not scorable	1	0.8131094
6939	Bn-A03-p26811025	A-genome specific (napus-rapa)	0.9593301	0.8007044	not scorable	not scorable	0.9421487	0.7196259
6940	Bn-A03-p26811385	A-genome specific (rapa only)	not scorable	not scorable	not scorable	not scorable	0.9669421	0.7715239
6941	Bn-A03-p26815896	A-genome specific (napus-rapa)	0.9856459	0.6578658	not scorable	not scorable	0.9669421	0.6235002
6942	Bn-A03-p26822285	A-genome specific (napus-rapa)	0.9832536	0.7411423	not scorable	not scorable	0.9669421	0.7708023
6943	Bn-A03-p26822384	A-genome specific (napus-rapa)	0.9928229	0.7723818	not scorable	not scorable	0.9669421	0.7589203
6944	Bn-A03-p26824343	A-genome specific (napus-rapa)	0.9832536	0.8012585	not scorable	not scorable	0.9669421	0.7365518
6945	Bn-A03-p26833555	A-genome specific (napus-rapa)	0.9880383	0.7408515	not scorable	not scorable	1	0.7145385
6946	Bn-A03-p26833841	A-genome specific (napus-rapa)	0.9808612	0.5010561	not scorable	not scorable	1	0.7752328
6947	Bn-A03-p2683534	A-genome specific (napus-rapa)	0.9282297	0.6759418	not scorable	not scorable	0.9917355	0.8159829
6948	Bn-A03-p26839758	A-genome specific (napus-rapa)	0.9952153	0.6641985	not scorable	not scorable	1	0.7106516
6949	Bn-A03-p26840269	A-genome specific (napus-rapa)	0.9952153	0.5319017	not scorable	not scorable	1	0.7183553
6950	Bn-A03-p26848326	A-genome specific (napus-rapa)	1	0.4762031	not scorable	not scorable	0.9917355	0.4571813
6951	Bn-A03-p26852795	A-genome specific (napus-rapa)	1	0.5265277	not scorable	not scorable	0.9917355	0.6783493
6952	Bn-A03-p2691383	A-genome specific (napus-rapa)	0.9521531	0.3539826	not scorable	not scorable	1	0.598024
6953	Bn-A03-p26931199	A-genome specific (napus-rapa)	0.9784689	0.5114753	not scorable	not scorable	1	0.7746196
6954	Bn-A03-p2695311	A-genome specific (napus-rapa)	0.9425837	0.761053	not scorable	not scorable	1	0.7035822
6955	Bn-A03-p27002187	A-genome specific (napus-rapa)	0.9019139	0.7281234	not scorable	not scorable	0.9917355	0.7836207
6956	Bn-A03-p27006222	A-genome specific (napus-rapa)	0.9712918	0.8117684	not scorable	not scorable	1	0.7509883
6957	Bn-A03-p27008420	A-genome specific (napus-rapa)	0.9880383	0.7626038	not scorable	not scorable	0.9917355	0.7926454
6958	Bn-A03-p27028300	A-genome specific (rapa only)	not scorable	not scorable	not scorable	not scorable	1	0.8147027
6959	Bn-A03-p27033682	A-genome specific (rapa only)	not scorable	not scorable	not scorable	not scorable	0.9834711	0.8754691
6960	Bn-A03-p2704795	A-genome specific (napus-rapa)	0.9688995	0.4099247	not scorable	not scorable	1	0.6896008

6961	Bn-A03-p27066000	A-genome specific (napus-rapa)	0.9712918	0.3901446	not scorable	not scorable	1	0.7529513
6962	Bn-A03-p27070872	A-genome specific (napus-rapa)	0.9736842	0.300966	not scorable	not scorable	1	0.7216338
6963	Bn-A03-p27101177	A-genome specific (napus-rapa)	0.9665072	0.7722501	not scorable	not scorable	0.9586777	0.7041734
6964	Bn-A03-p27104733	A-genome specific (napus-rapa)	0.9808612	0.3841944	not scorable	not scorable	0.9834711	0.703747
6965	Bn-A03-p27111541	A-genome specific (napus-rapa)	0.9760765	0.7797049	not scorable	not scorable	0.9504132	0.7288821
6966	Bn-A03-p27112789	A-genome specific (napus-rapa)	0.9856459	0.783897	not scorable	not scorable	0.9504132	0.6896096
6967	Bn-A03-p2714251	A-genome specific (napus-rapa)	0.9641148	0.5954776	not scorable	not scorable	0.9917355	0.7058203
6968	Bn-A03-p27146568	A-genome specific (napus-rapa)	1	0.6099746	not scorable	not scorable	0.9834711	0.77184
6969	Bn-A03-p27156983	A-genome specific (napus-rapa)	1	0.3399675	not scorable	not scorable	1	0.6766819
6970	Bn-A03-p27164178	A-genome specific (napus-rapa)	0.9856459	0.8822398	not scorable	not scorable	0.9752066	0.7472021
6971	Bn-A03-p27168311	A-genome specific (napus-rapa)	0.9904306	0.8216213	not scorable	not scorable	0.9917355	0.7742347
6972	Bn-A03-p27171824	A-genome specific (napus-rapa)	0.9976076	0.8129576	not scorable	not scorable	1	0.7395731
6973	Bn-A03-p27172113	A-genome specific (napus-rapa)	0.9976076	0.8040757	not scorable	not scorable	0.9834711	0.8489268
6974	Bn-A03-p27172168	A-genome specific (napus-rapa)	0.9976076	0.8094168	not scorable	not scorable	0.9752066	0.84435
6975	Bn-A03-p27179255	A-genome specific (napus-rapa)	0.9904306	0.7480031	not scorable	not scorable	1	0.7746897
6976	Bn-A03-p27179357	A-genome specific (napus-rapa)	1	0.6772203	not scorable	not scorable	1	0.8044871
6977	Bn-A03-p27181751	A-genome specific (napus-rapa)	0.9928229	0.7431011	not scorable	not scorable	0.9752066	0.696816
6978	Bn-A03-p27182289	A-genome specific (napus-rapa)	0.9976076	0.8771244	not scorable	not scorable	0.9917355	0.7292225
6979	Bn-A03-p27192830	A-genome specific (napus-rapa)	0.9976076	0.7931052	not scorable	not scorable	0.9834711	0.8129033
6980	Bn-A03-p27193587	A-genome specific (napus-rapa)	1	0.6785228	not scorable	not scorable	1	0.7089376
6981	Bn-A03-p2719384	A-genome specific (napus-rapa)	0.8803828	0.8367904	not scorable	not scorable	0.9338843	0.8638873
6982	Bn-A03-p27218655	A-genome specific (napus-rapa)	0.9952153	0.8216213	not scorable	not scorable	0.9752066	0.7980337
6983	Bn-A03-p27253035	A-genome specific (napus-rapa)	0.9928229	0.4479863	not scorable	not scorable	1	0.6967041
6984	Bn-A03-p27253678	A-genome specific (napus-rapa)	1	0.8352608	not scorable	not scorable	1	0.864952
6985	Bn-A03-p27255790	A-genome specific (napus-rapa)	0.9976076	0.8169866	not scorable	not scorable	1	0.8463818
6986	Bn-A03-p27256355	A-genome specific (napus-rapa)	0.9904306	0.8182196	not scorable	not scorable	0.9917355	0.8277613
6987	Bn-A03-p27257933	A-genome specific (napus-rapa)	0.9880383	0.773239	not scorable	not scorable	0.9834711	0.8280368
6988	Bn-A03-p27269401	A-genome specific (napus-rapa)	0.9952153	0.8198647	not scorable	not scorable	0.9917355	0.7685498
6989	Bn-A03-p2727877	A-genome specific (napus-rapa)	0.9449761	0.6629925	not scorable	not scorable	0.9834711	0.772486
6990	Bn-A03-p27288521	A-genome specific (napus-rapa)	0.9880383	0.7762017	not scorable	not scorable	0.9834711	0.860003
6991	Bn-A03-p27289437	A-genome specific (napus-rapa)	1	0.7533017	not scorable	not scorable	0.9917355	0.7171793
6992	Bn-A03-p27298810	A-genome specific (rapa only)	not scorable	not scorable	not scorable	not scorable	0.9917355	0.7906627
6993	Bn-A03-p27304692	A-genome specific (napus-rapa)	0.9880383	0.3042431	not scorable	not scorable	0.9917355	0.7136481

6994	Bn-A03-p2731426	A-genome specific (rapa only)	not scorable	not scorable	not scorable	not scorable	0.9752066	0.5488179
6995	Bn-A03-p27337536	A-genome specific (napus-rapa)	1	0.5538231	not scorable	not scorable	1	0.7181345
6996	Bn-A03-p27339890	A-genome specific (napus-rapa)	0.9952153	0.5209069	not scorable	not scorable	1	0.8084198
6997	Bn-A03-p27354494	A-genome specific (napus-rapa)	0.9904306	0.5840589	not scorable	not scorable	1	0.6669952
6998	Bn-A03-p27356417	A-genome specific (napus-rapa)	0.9928229	0.7347983	not scorable	not scorable	0.9917355	0.7829773
6999	Bn-A03-p27356726	A-genome specific (napus-rapa)	0.9880383	0.7173883	not scorable	not scorable	0.9917355	0.8169715
7000	Bn-A03-p27357082	A-genome specific (napus-rapa)	0.9808612	0.7527719	not scorable	not scorable	1	0.7427746
7001	Bn-A03-p27358390	A-genome specific (napus-rapa)	0.9856459	0.7578005	not scorable	not scorable	1	0.8538238
7002	Bn-A03-p27360321	A-genome specific (napus-rapa)	0.9760765	0.807938	not scorable	not scorable	1	0.703074
7003	Bn-A03-p27361855	A-genome specific (napus-rapa)	0.9808612	0.7501829	not scorable	not scorable	1	0.8169141
7004	Bn-A03-p27365971	A-genome specific (napus-rapa)	1	0.7593862	not scorable	not scorable	0.9917355	0.7831937
7005	Bn-A03-p2736960	A-genome specific (rapa only)	not scorable	not scorable	not scorable	not scorable	1	0.6230366
7006	Bn-A03-p27369889	A-genome specific (napus-rapa)	0.9880383	0.6661528	not scorable	not scorable	0.9834711	0.7149803
7007	Bn-A03-p2737116	A-genome specific (napus-rapa)	1	0.6097013	not scorable	not scorable	1	0.8254912
7008	Bn-A03-p2737190	genome unspecific (all in)	0.9401914	0.5622235	0.9379845	0.5291947	1	0.882794
7009	Bn-A03-p27372281	A-genome specific (napus-rapa)	0.9952153	0.8094139	not scorable	not scorable	0.9834711	0.7765123
7010	Bn-A03-p2737338	A-genome specific (napus-rapa)	0.9688995	0.801573	not scorable	not scorable	0.9917355	0.7151299
7011	Bn-A03-p27376850	A-genome specific (napus-rapa)	0.9832536	0.7825384	not scorable	not scorable	0.7272727	0.8277064
7012	Bn-A03-p27377729	A-genome specific (napus-rapa)	0.9712918	0.5838571	not scorable	not scorable	0.7190083	0.5786133
7013	Bn-A03-p27377839	A-genome specific (napus-rapa)	0.9808612	0.7654952	not scorable	not scorable	0.8429752	0.8042278
7014	Bn-A03-p27377892	A-genome specific (napus-rapa)	0.9784689	0.6663824	not scorable	not scorable	0.7107438	0.6681738
7015	Bn-A03-p27378026	A-genome specific (napus-rapa)	0.9832536	0.7002315	not scorable	not scorable	0.7272727	0.6999639
7016	Bn-A03-p27378198	A-genome specific (napus-rapa)	0.9736842	0.5799665	not scorable	not scorable	0.7272727	0.563747
7017	Bn-A03-p27378353	A-genome specific (napus-rapa)	0.9808612	0.7535151	not scorable	not scorable	0.7272727	0.7807822
7018	Bn-A03-p27379193	A-genome specific (napus-rapa)	0.9784689	0.7311361	not scorable	not scorable	0.7355372	0.7388902
7019	Bn-A03-p27379303	A-genome specific (napus-rapa)	0.930622	0.7811382	not scorable	not scorable	0.7190083	0.8943027
7020	Bn-A03-p27379570	A-genome specific (napus-rapa)	0.9665072	0.7570744	not scorable	not scorable	0.7272727	0.7782164
7021	Bn-A03-p27380908	A-genome specific (napus-rapa)	0.9784689	0.7825954	not scorable	not scorable	0.8016529	0.814934
7022	Bn-A03-p27381126	A-genome specific (napus-rapa)	0.9808612	0.7898132	not scorable	not scorable	0.7107438	0.8435183
7023	Bn-A03-p27381378	A-genome specific (napus-rapa)	0.9760765	0.7259022	not scorable	not scorable	0.7024794	0.7408311
7024	Bn-A03-p27385155	genome unspecific (oleracea-rapa)	not scorable	not scorable	1	0.7500497	0.9834711	0.7796244
7025	Bn-A03-p27387912	A-genome specific (napus-rapa)	0.9712918	0.5390388	not scorable	not scorable	0.9834711	0.561349
7026	Bn-A03-p27397690	all not scorable	not scorable	not scorable	not scorable	not scorable	not scorable	not scorable

7027	Bn-A03-p27398341	all not scorable	not scorable	not scorable	not scorable	not scorable	not scorable	not scorable
7028	Bn-A03-p27398352	A-genome specific (napus-rapa)	0.9784689	0.8037688	not scorable	not scorable	0.9752066	0.6944774
7029	Bn-A03-p27398935	A-genome specific (rapa only)	not scorable	not scorable	not scorable	not scorable	0.7438017	0.7740446
7030	Bn-A03-p27399719	A-genome specific (napus-rapa)	0.9784689	0.8117824	not scorable	not scorable	0.9834711	0.7524701
7031	Bn-A03-p27406806	A-genome specific (rapa only)	not scorable	not scorable	not scorable	not scorable	0.9917355	0.7665129
7032	Bn-A03-p27416815	A-genome specific (napus-rapa)	0.9952153	0.7858635	not scorable	not scorable	0.9834711	0.6959602
7033	Bn-A03-p27419226	napus specific	0.9856459	0.879243	not scorable	not scorable	not scorable	not scorable
7034	Bn-A03-p27419285	A-genome specific (napus-rapa)	0.9904306	0.8373511	not scorable	not scorable	0.9917355	0.7180871
7035	Bn-A03-p27420794	A-genome specific (napus-rapa)	0.9904306	0.8535629	not scorable	not scorable	0.9752066	0.762654
7036	Bn-A03-p27423379	A-genome specific (napus-rapa)	0.9832536	0.456253	not scorable	not scorable	0.9917355	0.6995439
7037	Bn-A03-p27438081	A-genome specific (napus-rapa)	0.9832536	0.7741535	not scorable	not scorable	0.9834711	0.7906648
7038	Bn-A03-p27445819	A-genome specific (napus-rapa)	0.9258373	0.1661237	not scorable	not scorable	1	0.6724718
7039	Bn-A03-p27454930	A-genome specific (napus-rapa)	0.9808612	0.578975	not scorable	not scorable	0.9917355	0.7476752
7040	Bn-A03-p27455156	A-genome specific (napus-rapa)	0.9928229	0.6616126	not scorable	not scorable	1	0.6643892
7041	Bn-A03-p27484868	genome unspecific (all in)	0.9736842	0.6293373	1	0.7732232	0.9834711	0.7902876
7042	Bn-A03-p2748923	A-genome specific (rapa only)	not scorable	not scorable	not scorable	not scorable	1	0.7392583
7043	Bn-A03-p27489686	A-genome specific (napus-rapa)	0.9808612	0.4531082	not scorable	not scorable	0.9917355	0.8039277
7044	Bn-A03-p27516492	A-genome specific (napus-rapa)	1	0.7875707	not scorable	not scorable	1	0.9271749
7045	Bn-A03-p27541115	A-genome specific (napus-rapa)	0.9976076	0.7814572	not scorable	not scorable	1	0.726869
7046	Bn-A03-p27546971	A-genome specific (napus-rapa)	0.9880383	0.714899	not scorable	not scorable	1	0.7799388
7047	Bn-A03-p27547270	A-genome specific (napus-rapa)	0.9952153	0.7309559	not scorable	not scorable	1	0.9173692
7048	Bn-A03-p27564692	A-genome specific (napus-rapa)	0.9760765	0.7877326	not scorable	not scorable	1	0.7145227
7049	Bn-A03-p27568225	A-genome specific (napus-rapa)	0.9449761	0.5741157	not scorable	not scorable	0.9917355	0.7939925
7050	Bn-A03-p27568580	A-genome specific (napus-rapa)	0.9832536	0.628589	not scorable	not scorable	1	0.8144061
7051	Bn-A03-p27581773	A-genome specific (napus-rapa)	0.9569378	0.4641013	not scorable	not scorable	0.9669421	0.7949653
7052	Bn-A03-p27582705	A-genome specific (napus-rapa)	0.9832536	0.7572169	not scorable	not scorable	0.9917355	0.6910838
7053	Bn-A03-p27596068	A-genome specific (napus-rapa)	0.9880383	0.30397	not scorable	not scorable	1	0.789576
7054	Bn-A03-p27600547	A-genome specific (napus-rapa)	0.9904306	0.6697934	not scorable	not scorable	0.9669421	0.5540885
7055	Bn-A03-p27607709	A-genome specific (napus-rapa)	0.9688995	0.6382255	not scorable	not scorable	0.9917355	0.8056468
7056	Bn-A03-p27612791	A-genome specific (napus-rapa)	0.9712918	0.6236287	not scorable	not scorable	1	0.8460862
7057	Bn-A03-p27616297	A-genome specific (napus-rapa)	0.9688995	0.7382997	not scorable	not scorable	0.7272727	0.7197168
7058	Bn-A03-p27629415	A-genome specific (napus-rapa)	0.9808612	0.4580355	not scorable	not scorable	0.9917355	0.7441819
7059	Bn-A03-p27650553	A-genome specific (napus-rapa)	0.9688995	0.4572642	not scorable	not scorable	1	0.8387879

7060	Bn-A03-p27662317	A-genome specific (napus-rapa)	0.9856459	0.7706242	not scorable	not scorable	1	0.8382767
7061	Bn-A03-p27663888	A-genome specific (napus-rapa)	0.9880383	0.7389907	not scorable	not scorable	0.9917355	0.7358662
7062	Bn-A03-p27665971	genome unspecific (oleracea-rapa)	not scorable	not scorable	1	0.7703836	0.9917355	0.7536673
7063	Bn-A03-p27683723	A-genome specific (napus-rapa)	0.9928229	0.7747034	not scorable	not scorable	0.9834711	0.7669605
7064	Bn-A03-p27683903	A-genome specific (napus-rapa)	0.9832536	0.702018	not scorable	not scorable	0.9834711	0.790482
7065	Bn-A03-p27685078	A-genome specific (napus-rapa)	0.9712918	0.3405987	not scorable	not scorable	0.9834711	0.7834053
7066	Bn-A03-p27686257	A-genome specific (napus-rapa)	0.9904306	0.7805742	not scorable	not scorable	0.9917355	0.7190969
7067	Bn-A03-p27686487	A-genome specific (napus-rapa)	0.9808612	0.8038026	not scorable	not scorable	0.9752066	0.8142092
7068	Bn-A03-p27686879	A-genome specific (napus-rapa)	0.7703349	0.8045428	not scorable	not scorable	0.5371901	0.7640303
7069	Bn-A03-p27712521	A-genome specific (napus-rapa)	0.9712918	0.7771339	not scorable	not scorable	0.7272727	0.7481439
7070	Bn-A03-p27713307	A-genome specific (napus-rapa)	0.9641148	0.7741648	not scorable	not scorable	0.7190083	0.8094397
7071	Bn-A03-p27713975	A-genome specific (napus-rapa)	0.9449761	0.7795983	not scorable	not scorable	0.7107438	0.8662751
7072	Bn-A03-p27715593	A-genome specific (napus-rapa)	0.937799	0.7570992	not scorable	not scorable	0.7107438	0.7473218
7073	Bn-A03-p27719757	A-genome specific (napus-rapa)	0.9497608	0.4115874	not scorable	not scorable	1	0.7045447
7074	Bn-A03-p27720045	A-genome specific (napus-rapa)	0.9354067	0.4395901	not scorable	not scorable	1	0.7248667
7075	Bn-A03-p27741110	A-genome specific (napus-rapa)	0.9784689	0.7681945	not scorable	not scorable	0.9917355	0.7829702
7076	Bn-A03-p27742950	A-genome specific (napus-rapa)	0.9832536	0.4788291	not scorable	not scorable	0.9669421	0.6681253
7077	Bn-A03-p27746955	genome unspecific (all in)	0.9976076	0.264052	1	0.7269822	1	0.5029543
7078	Bn-A03-p27747178	A-genome specific (napus-rapa)	0.9665072	0.2357372	not scorable	not scorable	0.9917355	0.705092
7079	Bn-A03-p27747229	A-genome specific (rapa only)	not scorable	not scorable	not scorable	not scorable	0.9917355	0.6978273
7080	Bn-A03-p27752076	A-genome specific (napus-rapa)	0.9736842	0.7674094	not scorable	not scorable	1	0.8192819
7081	Bn-A03-p27752857	A-genome specific (napus-rapa)	0.9880383	0.7731963	not scorable	not scorable	0.9834711	0.7553476
7082	Bn-A03-p27791357	A-genome specific (napus-rapa)	0.9952153	0.8201092	not scorable	not scorable	0.9834711	0.7225638
7083	Bn-A03-p27791437	A-genome specific (napus-rapa)	0.9832536	0.7800751	not scorable	not scorable	0.9917355	0.785115
7084	Bn-A03-p27793125	A-genome specific (napus-rapa)	0.9736842	0.7266456	not scorable	not scorable	0.9917355	0.6913665
7085	Bn-A03-p27794041	A-genome specific (napus-rapa)	0.9736842	0.6427104	not scorable	not scorable	0.9834711	0.6237078
7086	Bn-A03-p27794263	A-genome specific (napus-rapa)	0.9760765	0.7191436	not scorable	not scorable	0.9917355	0.6929486
7087	Bn-A03-p27794657	A-genome specific (napus-rapa)	0.9760765	0.6543214	not scorable	not scorable	0.9752066	0.6233765
7088	Bn-A03-p27794772	A-genome specific (napus-rapa)	0.9856459	0.7770101	not scorable	not scorable	0.9834711	0.807207
7089	Bn-A03-p27794775	A-genome specific (napus-rapa)	0.9880383	0.7636347	not scorable	not scorable	0.9834711	0.7354633
7090	Bn-A03-p27794848	A-genome specific (napus-rapa)	0.9808612	0.798434	not scorable	not scorable	0.9917355	0.8053529
7091	Bn-A03-p27796940	A-genome specific (napus-rapa)	0.9904306	0.8143449	not scorable	not scorable	0.9669421	0.7759143
7092	Bn-A03-p27805759	A-genome specific (napus-rapa)	0.9856459	0.775235	not scorable	not scorable	1	0.79044

7093	Bn-A03-p27824257	A-genome specific (napus-rapa)	0.9928229	0.8047447	not scorable	not scorable	0.9917355	0.8195639
7094	Bn-A03-p27825739	A-genome specific (napus-rapa)	0.9904306	0.7155367	not scorable	not scorable	0.9917355	0.6461827
7095	Bn-A03-p27828889	A-genome specific (napus-rapa)	0.9832536	0.7180378	not scorable	not scorable	0.9834711	0.787192
7096	Bn-A03-p27838177	A-genome specific (napus-rapa)	0.9808612	0.3786797	not scorable	not scorable	1	0.9205657
7097	Bn-A03-p27840169	A-genome specific (napus-rapa)	0.9880383	0.7728361	not scorable	not scorable	1	0.7521731
7098	Bn-A03-p27841728	A-genome specific (napus-rapa)	0.9904306	0.7306871	not scorable	not scorable	0.9834711	0.7014851
7099	Bn-A03-p27853312	A-genome specific (napus-rapa)	0.9736842	0.4202016	not scorable	not scorable	0.9917355	0.6752047
7100	Bn-A03-p27866782	A-genome specific (napus-rapa)	0.9904306	0.8099139	not scorable	not scorable	0.9917355	0.7336286
7101	Bn-A03-p2789113	A-genome specific (rapa only)	not scorable	not scorable	not scorable	not scorable	1	0.7624819
7102	Bn-A03-p27906760	A-genome specific (napus-rapa)	0.9545454	0.2278894	not scorable	not scorable	0.9834711	0.5567296
7103	Bn-A03-p27917455	A-genome specific (napus-rapa)	0.9760765	0.3924345	not scorable	not scorable	1	0.824479
7104	Bn-A03-p27927967	A-genome specific (napus-rapa)	0.9760765	0.7458301	not scorable	not scorable	0.9917355	0.7811404
7105	Bn-A03-p27928232	A-genome specific (napus-rapa)	0.6842105	0.920714	not scorable	not scorable	0.9834711	0.9154006
7106	Bn-A03-p2793288	A-genome specific (napus-rapa)	0.9976076	0.5323982	not scorable	not scorable	0.9421487	0.7830532
7107	Bn-A03-p27951283	A-genome specific (napus-rapa)	0.9473684	0.4916507	not scorable	not scorable	1	0.6493279
7108	Bn-A03-p27951979	A-genome specific (napus-rapa)	0.9856459	0.7780089	not scorable	not scorable	1	0.7522417
7109	Bn-A03-p27972408	genome unspecific (oleracea-rapa)	not scorable	not scorable	1	0.7275194	0.9917355	0.7361933
7110	Bn-A03-p27972738	A-genome specific (napus-rapa)	0.9856459	0.729419	not scorable	not scorable	0.9917355	0.6577612
7111	Bn-A03-p28036367	A-genome specific (napus-rapa)	0.9928229	0.7737016	not scorable	not scorable	1	0.8143556
7112	Bn-A03-p28039749	A-genome specific (napus-rapa)	0.9856459	0.7531274	not scorable	not scorable	1	0.8229404
7113	Bn-A03-p28043216	A-genome specific (napus-rapa)	0.9856459	0.7254896	not scorable	not scorable	0.9586777	0.6057239
7114	Bn-A03-p28046101	A-genome specific (napus-rapa)	0.9736842	0.4523977	not scorable	not scorable	0.9917355	0.7401661
7115	Bn-A03-p28091909	A-genome specific (napus-rapa)	0.9665072	0.7601923	not scorable	not scorable	0.9834711	0.799817
7116	Bn-A03-p28097052	A-genome specific (napus-rapa)	0.9712918	0.4672724	not scorable	not scorable	1	0.7211741
7117	Bn-A03-p28109103	A-genome specific (napus-rapa)	0.9712918	0.7290346	not scorable	not scorable	0.9917355	0.6604959
7118	Bn-A03-p28117836	A-genome specific (napus-rapa)	0.9880383	0.5744061	not scorable	not scorable	1	0.7413988
7119	Bn-A03-p28126287	napus specific	0.9832536	0.7181222	not scorable	not scorable	not scorable	not scorable
7120	Bn-A03-p28127216	A-genome specific (napus-rapa)	0.9736842	0.6039369	not scorable	not scorable	1	0.4958567
7121	Bn-A03-p28153736	A-genome specific (napus-rapa)	0.9808612	0.4470077	not scorable	not scorable	0.9834711	0.6936432
7122	Bn-A03-p28153797	A-genome specific (napus-rapa)	0.9712918	0.6438315	not scorable	not scorable	1	0.6877341
7123	Bn-A03-p28154053	A-genome specific (napus-rapa)	0.9736842	0.3183069	not scorable	not scorable	0.9752066	0.5865586
7124	Bn-A03-p28154830	A-genome specific (napus-rapa)	0.9952153	0.7955617	not scorable	not scorable	0.9917355	0.7168419
7125	Bn-A03-p28154970	A-genome specific (napus-rapa)	0.3875598	0.8578239	not scorable	not scorable	0.5289256	0.8158457

7126	Bn-A03-p28155412	A-genome specific (napus-rapa)	0.9928229	0.7692649	not scorable	not scorable	1	0.8310312
7127	Bn-A03-p28184972	A-genome specific (napus-rapa)	0.9832536	0.3112537	not scorable	not scorable	1	0.6751164
7128	Bn-A03-p28202050	A-genome specific (napus-rapa)	0.9545454	0.6056274	not scorable	not scorable	1	0.7861024
7129	Bn-A03-p28207594	A-genome specific (napus-rapa)	0.9688995	0.2742949	not scorable	not scorable	0.9504132	0.7785954
7130	Bn-A03-p28221433	A-genome specific (napus-rapa)	0.9976076	0.5621893	not scorable	not scorable	1	0.7208881
7131	Bn-A03-p28222096	A-genome specific (napus-rapa)	1	0.675622	not scorable	not scorable	0.9834711	0.7502331
7132	Bn-A03-p28227026	A-genome specific (napus-rapa)	0.9784689	0.6979976	not scorable	not scorable	1	0.6960055
7133	Bn-A03-p28229360	A-genome specific (napus-rapa)	0.9688995	0.7755161	not scorable	not scorable	0.9504132	0.7281106
7134	Bn-A03-p28232863	A-genome specific (napus-rapa)	0.9952153	0.7112629	not scorable	not scorable	0.9917355	0.7435111
7135	Bn-A03-p28233201	A-genome specific (napus-rapa)	0.9760765	0.7051514	not scorable	not scorable	1	0.7223274
7136	Bn-A03-p28233701	A-genome specific (napus-rapa)	0.9856459	0.7429647	not scorable	not scorable	1	0.7946225
7137	Bn-A03-p28234166	A-genome specific (napus-rapa)	0.9808612	0.7539203	not scorable	not scorable	1	0.7873787
7138	Bn-A03-p28234586	A-genome specific (napus-rapa)	0.9952153	0.8321174	not scorable	not scorable	0.9917355	0.7326576
7139	Bn-A03-p28242905	A-genome specific (napus-rapa)	1	0.8031629	not scorable	not scorable	1	0.7498396
7140	Bn-A03-p28243306	A-genome specific (napus-rapa)	0.9856459	0.8576143	not scorable	not scorable	1	0.807159
7141	Bn-A03-p28256169	A-genome specific (napus-rapa)	0.9712918	0.6829509	not scorable	not scorable	0.9917355	0.795688
7142	Bn-A03-p28265602	genome unspecific (all in)	0.9521531	0.1937395	0.9379845	0.3726288	1	0.7591531
7143	Bn-A03-p28276873	all not scorable	not scorable	not scorable	not scorable	not scorable	not scorable	not scorable
7144	Bn-A03-p28279796	A-genome specific (napus-rapa)	0.9904306	0.7203869	not scorable	not scorable	1	0.7195519
7145	Bn-A03-p28297407	A-genome specific (napus-rapa)	0.9976076	0.4653637	not scorable	not scorable	1	0.7974627
7146	Bn-A03-p28302949	A-genome specific (napus-rapa)	0.9976076	0.6006212	not scorable	not scorable	0.9917355	0.7615933
7147	Bn-A03-p2834684	A-genome specific (napus-rapa)	0.9856459	0.5045836	not scorable	not scorable	1	0.7593281
7148	Bn-A03-p28360281	A-genome specific (napus-rapa)	1	0.7635936	not scorable	not scorable	1	0.8135551
7149	Bn-A03-p28389539	A-genome specific (napus-rapa)	0.9904306	0.3998596	not scorable	not scorable	1	0.753969
7150	Bn-A03-p2843108	A-genome specific (napus-rapa)	0.9545454	0.7818289	not scorable	not scorable	0.9917355	0.811019
7151	Bn-A03-p28464995	A-genome specific (napus-rapa)	0.9760765	0.7325476	not scorable	not scorable	0.9917355	0.7201521
7152	Bn-A03-p28468317	A-genome specific (napus-rapa)	1	0.7063881	not scorable	not scorable	1	0.7802441
7153	Bn-A03-p28469194	A-genome specific (napus-rapa)	0.9449761	0.3289601	not scorable	not scorable	0.9834711	0.6889063
7154	Bn-A03-p28491294	A-genome specific (napus-rapa)	0.9401914	0.796029	not scorable	not scorable	0.9669421	0.786539
7155	Bn-A03-p28491779	A-genome specific (rapa only)	not scorable	not scorable	not scorable	not scorable	1	0.77314
7156	Bn-A03-p28498617	A-genome specific (napus-rapa)	0.9856459	0.7755794	not scorable	not scorable	1	0.9205657
7157	Bn-A03-p28498755	A-genome specific (napus-rapa)	0.9856459	0.7543649	not scorable	not scorable	0.9917355	0.6885571
7158	Bn-A03-p28499049	A-genome specific (napus-rapa)	0.9665072	0.4431145	not scorable	not scorable	1	0.6770748

7159	Bn-A03-p28499141	A-genome specific (napus-rapa)	0.9856459	0.7413908	not scorable	not scorable	0.9834711	0.8041093
7160	Bn-A03-p28535638	A-genome specific (napus-rapa)	0.9593301	0.6671326	not scorable	not scorable	0.9834711	0.7626065
7161	Bn-A03-p28541411	A-genome specific (napus-rapa)	0.9712918	0.5021151	not scorable	not scorable	0.9917355	0.7114221
7162	Bn-A03-p28551517	A-genome specific (rapa only)	not scorable	not scorable	not scorable	not scorable	0.9917355	0.7847934
7163	Bn-A03-p28551626	A-genome specific (napus-rapa)	0.9976076	0.7840836	not scorable	not scorable	0.9834711	0.7838938
7164	Bn-A03-p28551775	A-genome specific (napus-rapa)	0.9617225	0.7759422	not scorable	not scorable	0.9917355	0.8073231
7165	Bn-A03-p28560125	A-genome specific (napus-rapa)	0.9736842	0.6304501	not scorable	not scorable	0.9917355	0.731862
7166	Bn-A03-p28560659	A-genome specific (napus-rapa)	0.9784689	0.2696207	not scorable	not scorable	0.9917355	0.6887593
7167	Bn-A03-p2856933	A-genome specific (rapa only)	not scorable	not scorable	not scorable	not scorable	0.9834711	0.7855918
7168	Bn-A03-p28570053	A-genome specific (napus-rapa)	0.8803828	0.8177989	not scorable	not scorable	0.7024794	0.7618618
7169	Bn-A03-p28583587	A-genome specific (napus-rapa)	0.9880383	0.5598829	not scorable	not scorable	0.9752066	0.5884658
7170	Bn-A03-p28583685	A-genome specific (napus-rapa)	0.9904306	0.881644	not scorable	not scorable	0.9669421	0.7512952
7171	Bn-A03-p28588701	A-genome specific (napus-rapa)	0.9832536	0.5333548	not scorable	not scorable	0.9752066	0.7008672
7172	Bn-A03-p2858882	A-genome specific (napus-rapa)	0.9641148	0.6424435	not scorable	not scorable	0.9669421	0.622668
7173	Bn-A03-p2858972	A-genome specific (rapa only)	not scorable	not scorable	not scorable	not scorable	0.9834711	0.7391571
7174	Bn-A03-p28590688	genome unspecific (all in)	0.9665072	0.4761234	1	0.798078	0.9752066	0.7135509
7175	Bn-A03-p28590746	A-genome specific (napus-rapa)	0.9880383	0.6407571	not scorable	not scorable	0.9917355	0.6884
7176	Bn-A03-p28594732	A-genome specific (napus-rapa)	0.9808612	0.7382373	not scorable	not scorable	0.9917355	0.8298221
7177	Bn-A03-p2860489	A-genome specific (napus-rapa)	0.9617225	0.7056313	not scorable	not scorable	0.9917355	0.760975
7178	Bn-A03-p28616355	A-genome specific (napus-rapa)	0.9976076	0.4192386	not scorable	not scorable	0.9917355	0.8256459
7179	Bn-A03-p2862687	A-genome specific (napus-rapa)	0.9641148	0.7049245	not scorable	not scorable	0.9834711	0.7526577
7180	Bn-A03-p28698732	napus specific	0.9832536	0.5525395	not scorable	not scorable	not scorable	not scorable
7181	Bn-A03-p2872671	A-genome specific (napus-rapa)	0.9593301	0.5435914	not scorable	not scorable	0.9834711	0.7172602
7182	Bn-A03-p2879773	A-genome specific (napus-rapa)	0.9928229	0.5626425	not scorable	not scorable	0.9917355	0.6738516
7183	Bn-A03-p28808705	A-genome specific (napus-rapa)	0.9928229	0.7681407	not scorable	not scorable	0.9917355	0.8123032
7184	Bn-A03-p28840414	A-genome specific (napus-rapa)	0.9449761	0.4473711	not scorable	not scorable	1	0.7677687
7185	Bn-A03-p28843588	A-genome specific (napus-rapa)	0.9688995	0.7426613	not scorable	not scorable	1	0.7543925
7186	Bn-A03-p28847903	A-genome specific (napus-rapa)	0.9856459	0.7596955	not scorable	not scorable	1	0.7531066
7187	Bn-A03-p28851691	A-genome specific (napus-rapa)	0.9019139	0.938517	not scorable	not scorable	0.9917355	0.8441256
7188	Bn-A03-p288544	A-genome specific (rapa only)	not scorable	not scorable	not scorable	not scorable	1	0.9205657
7189	Bn-A03-p28873781	all not scorable	not scorable	not scorable	not scorable	not scorable	not scorable	not scorable
7190	Bn-A03-p28877626	A-genome specific (napus-rapa)	0.9904306	0.7554089	not scorable	not scorable	1	0.7023286
7191	Bn-A03-p28877719	A-genome specific (napus-rapa)	0.9880383	0.7517628	not scorable	not scorable	0.9917355	0.7848888

7192	Bn-A03-p28878501	A-genome specific (napus-rapa)	0.9712918	0.5849917	not scorable	not scorable	0.9917355	0.7145746
7193	Bn-A03-p28878698	A-genome specific (napus-rapa)	0.9904306	0.6747506	not scorable	not scorable	1	0.7523732
7194	Bn-A03-p28878703	A-genome specific (napus-rapa)	0.9784689	0.7422957	not scorable	not scorable	0.9917355	0.7390368
7195	Bn-A03-p28879610	A-genome specific (napus-rapa)	0.9784689	0.6709555	not scorable	not scorable	0.9917355	0.7249307
7196	Bn-A03-p28882585	A-genome specific (napus-rapa)	0.9760765	0.7693222	not scorable	not scorable	0.9752066	0.7361351
7197	Bn-A03-p28883986	A-genome specific (napus-rapa)	0.9712918	0.5098153	not scorable	not scorable	0.9752066	0.6310138
7198	Bn-A03-p28910384	A-genome specific (napus-rapa)	0.3253588	0.7872123	not scorable	not scorable	0.9834711	0.8387734
7199	Bn-A03-p28910430	A-genome specific (napus-rapa)	0.9688995	0.7772505	not scorable	not scorable	1	0.7144622
7200	Bn-A03-p2891056	A-genome specific (napus-rapa)	0.9641148	0.4711859	not scorable	not scorable	0.9917355	0.7393948
7201	Bn-A03-p2891934	A-genome specific (napus-rapa)	0.9545454	0.6779337	not scorable	not scorable	1	0.7698004
7202	Bn-A03-p28924123	A-genome specific (napus-rapa)	0.9856459	0.6041095	not scorable	not scorable	1	0.7401604
7203	Bn-A03-p28926445	A-genome specific (napus-rapa)	0.9521531	0.4186138	not scorable	not scorable	1	0.7233305
7204	Bn-A03-p28928570	A-genome specific (napus-rapa)	0.9952153	0.702576	not scorable	not scorable	0.9834711	0.7183622
7205	Bn-A03-p28948799	A-genome specific (napus-rapa)	0.9952153	0.7663476	not scorable	not scorable	0.9917355	0.7386804
7206	Bn-A03-p28963072	A-genome specific (napus-rapa)	0.9832536	0.4001698	not scorable	not scorable	0.9834711	0.5891729
7207	Bn-A03-p2897610	A-genome specific (napus-rapa)	0.9688995	0.7648936	not scorable	not scorable	0.9586777	0.7667674
7208	Bn-A03-p28977058	A-genome specific (napus-rapa)	0.9569378	0.456908	not scorable	not scorable	1	0.7709086
7209	Bn-A03-p2898043	A-genome specific (napus-rapa)	1	0.5426577	not scorable	not scorable	1	0.7591149
7210	Bn-A03-p28981296	A-genome specific (napus-rapa)	0.9904306	0.7907872	not scorable	not scorable	0.9752066	0.793385
7211	Bn-A03-p28982546	A-genome specific (napus-rapa)	0.9952153	0.7591814	not scorable	not scorable	0.9917355	0.7118359
7212	Bn-A03-p28995612	genome unspecific (all in)	0.9928229	0.349898	0.9922481	0.5993966	1	0.5918231
7213	Bn-A03-p28996082	A-genome specific (rapa only)	not scorable	not scorable	not scorable	not scorable	0.9917355	0.5829232
7214	Bn-A03-p29002618	A-genome specific (napus-rapa)	0.9808612	0.6950197	not scorable	not scorable	0.9917355	0.7956129
7215	Bn-A03-p29005979	A-genome specific (napus-rapa)	0.9856459	0.7662292	not scorable	not scorable	0.9917355	0.7204551
7216	Bn-A03-p29007606	A-genome specific (rapa only)	not scorable	not scorable	not scorable	not scorable	1	0.7286919
7217	Bn-A03-p29009401	A-genome specific (napus-rapa)	0.9808612	0.740739	not scorable	not scorable	1	0.7667289
7218	Bn-A03-p29046284	A-genome specific (napus-rapa)	0.9904306	0.7638041	not scorable	not scorable	1	0.7869431
7219	Bn-A03-p29050002	A-genome specific (napus-rapa)	0.9043062	0.5878133	not scorable	not scorable	1	0.7753394
7220	Bn-A03-p29053385	A-genome specific (napus-rapa)	0.9880383	0.7665234	not scorable	not scorable	0.9917355	0.7308035
7221	Bn-A03-p29060027	A-genome specific (napus-rapa)	0.9904306	0.7771861	not scorable	not scorable	0.9917355	0.9081098
7222	Bn-A03-p29060253	A-genome specific (napus-rapa)	0.9952153	0.784619	not scorable	not scorable	1	0.8428769
7223	Bn-A03-p29065314	A-genome specific (napus-rapa)	0.85588517	0.8049759	not scorable	not scorable	0.9917355	0.7866986
7224	Bn-A03-p29066510	A-genome specific (napus-rapa)	0.9688995	0.7361217	not scorable	not scorable	0.9834711	0.7800515

7225	Bn-A03-p29067657	A-genome specific (napus-rapa)	0.8588517	0.7468092	not scorable	not scorable	0.9752066	0.7072048
7226	Bn-A03-p29068059	A-genome specific (napus-rapa)	0.8373206	0.6029469	not scorable	not scorable	0.9917355	0.7781275
7227	Bn-A03-p29068198	A-genome specific (napus-rapa)	0.8636364	0.8974518	not scorable	not scorable	0.9917355	0.7600738
7228	Bn-A03-p29075333	A-genome specific (napus-rapa)	0.861244	0.7693104	not scorable	not scorable	0.9917355	0.694396
7229	Bn-A03-p29081614	A-genome specific (napus-rapa)	0.9904306	0.7786232	not scorable	not scorable	1	0.7739069
7230	Bn-A03-p29097772	A-genome specific (napus-rapa)	0.861244	0.8097457	not scorable	not scorable	0.9917355	0.8154153
7231	Bn-A03-p29102865	A-genome specific (napus-rapa)	0.8301435	0.7673451	not scorable	not scorable	0.9090909	0.7372139
7232	Bn-A03-p29118954	A-genome specific (napus-rapa)	0.9904306	0.7539196	not scorable	not scorable	0.9917355	0.8142101
7233	Bn-A03-p2914046	A-genome specific (napus-rapa)	0.9832536	0.5664155	not scorable	not scorable	0.8677686	0.7586442
7234	Bn-A03-p2917477	A-genome specific (napus-rapa)	0.9928229	0.7969561	not scorable	not scorable	0.8264463	0.7934078
7235	Bn-A03-p29198982	A-genome specific (napus-rapa)	0.9688995	0.7120817	not scorable	not scorable	1	0.7580948
7236	Bn-A03-p29212835	A-genome specific (napus-rapa)	0.9593301	0.6416954	not scorable	not scorable	0.9669421	0.7852247
7237	Bn-A03-p29216506	A-genome specific (napus-rapa)	0.9784689	0.4670393	not scorable	not scorable	1	0.7512187
7238	Bn-A03-p29216612	A-genome specific (napus-rapa)	0.9784689	0.7371357	not scorable	not scorable	0.9669421	0.8144518
7239	Bn-A03-p29234677	A-genome specific (napus-rapa)	0.9904306	0.528591	not scorable	not scorable	0.9504132	0.8324642
7240	Bn-A03-p29264946	A-genome specific (napus-rapa)	0.9593301	0.6186846	not scorable	not scorable	1	0.7170045
7241	Bn-A03-p29269385	A-genome specific (napus-rapa)	0.9449761	0.4979934	not scorable	not scorable	1	0.8365017
7242	Bn-A03-p29271424	A-genome specific (napus-rapa)	0.9880383	0.7818581	not scorable	not scorable	0.9917355	0.7457815
7243	Bn-A03-p29271737	A-genome specific (napus-rapa)	0.9617225	0.7689042	not scorable	not scorable	1	0.9272014
7244	Bn-A03-p29274532	A-genome specific (napus-rapa)	0.9808612	0.7666342	not scorable	not scorable	1	0.8287388
7245	Bn-A03-p29279909	A-genome specific (napus-rapa)	0.9736842	0.7692649	not scorable	not scorable	1	0.9095823
7246	Bn-A03-p2929711	A-genome specific (napus-rapa)	0.2631579	0.7658883	not scorable	not scorable	0.768595	0.8123304
7247	Bn-A03-p29326525	A-genome specific (napus-rapa)	0.9880383	0.8137729	not scorable	not scorable	0.9917355	0.895059
7248	Bn-A03-p29335920	A-genome specific (napus-rapa)	0.9808612	0.4391937	not scorable	not scorable	1	0.8766605
7249	Bn-A03-p29338454	A-genome specific (napus-rapa)	0.9784689	0.7294619	not scorable	not scorable	1	0.7597827
7250	Bn-A03-p29342929	A-genome specific (napus-rapa)	0.9665072	0.3763268	not scorable	not scorable	1	0.6945017
7251	Bn-A03-p29346125	A-genome specific (napus-rapa)	0.9856459	0.5306969	not scorable	not scorable	0.9917355	0.8363402
7252	Bn-A03-p29347368	A-genome specific (napus-rapa)	0.9569378	0.6044278	not scorable	not scorable	0.9917355	0.7525958
7253	Bn-A03-p29364596	A-genome specific (napus-rapa)	0.9712918	0.5721333	not scorable	not scorable	0.9752066	0.6476204
7254	Bn-A03-p29367826	napus specific	0.9760765	0.7366489	not scorable	not scorable	not scorable	not scorable
7255	Bn-A03-p29390022	genome unspecific (all in)	0.9808612	0.7658425	0	0.6805282	1	0.7795187
7256	Bn-A03-p29391849	A-genome specific (napus-rapa)	0.9736842	0.7217233	not scorable	not scorable	0.6942149	0.8036858
7257	Bn-A03-p2946358	A-genome specific (napus-rapa)	1	0.6211592	not scorable	not scorable	1	0.7760504

7258	Bn-A03-p2948394	A-genome specific (napus-rapa)	0.9354067	0.6339281	not scorable	not scorable	0.8181818	0.7120833
7259	Bn-A03-p2948695	A-genome specific (napus-rapa)	0.9497608	0.6818768	not scorable	not scorable	0.8347107	0.8127915
7260	Bn-A03-p29564970	A-genome specific (napus-rapa)	0.9808612	0.6863119	not scorable	not scorable	0.9917355	0.7128249
7261	Bn-A03-p29565087	A-genome specific (napus-rapa)	0.9856459	0.7531183	not scorable	not scorable	0.9917355	0.7522025
7262	Bn-A03-p29566168	A-genome specific (napus-rapa)	0.9880383	0.560161	not scorable	not scorable	1	0.6432272
7263	Bn-A03-p29571938	napus specific	0.9880383	0.8135673	not scorable	not scorable	not scorable	not scorable
7264	Bn-A03-p29573204	A-genome specific (napus-rapa)	0.9904306	0.8737755	not scorable	not scorable	1	0.6888322
7265	Bn-A03-p29583640	A-genome specific (napus-rapa)	0.9952153	0.8547808	not scorable	not scorable	0.9917355	0.8151352
7266	Bn-A03-p29587388	A-genome specific (napus-rapa)	0.9688995	0.6370021	not scorable	not scorable	0.9834711	0.6661862
7267	Bn-A03-p29587945	A-genome specific (napus-rapa)	0.9593301	0.7906181	not scorable	not scorable	0.5206612	0.7181526
7268	Bn-A03-p29590349	A-genome specific (napus-rapa)	0.9904306	0.764203	not scorable	not scorable	0.9917355	0.7196062
7269	Bn-A03-p29593434	A-genome specific (napus-rapa)	0.9880383	0.6907109	not scorable	not scorable	0.9917355	0.656527
7270	Bn-A03-p29595276	A-genome specific (napus-rapa)	0.9856459	0.7840836	not scorable	not scorable	0.9834711	0.793843
7271	Bn-A03-p29598801	A-genome specific (napus-rapa)	0.9665072	0.5468537	not scorable	not scorable	0.9834711	0.6829081
7272	Bn-A03-p29606245	A-genome specific (napus-rapa)	0.9641148	0.8147309	not scorable	not scorable	0.9834711	0.7197455
7273	Bn-A03-p29614729	A-genome specific (napus-rapa)	0.9808612	0.7880369	not scorable	not scorable	1	0.6931245
7274	Bn-A03-p29629982	A-genome specific (napus-rapa)	0.9928229	0.7660485	not scorable	not scorable	0.9834711	0.7627978
7275	Bn-A03-p29632526	A-genome specific (napus-rapa)	0.9593301	0.4500183	not scorable	not scorable	1	0.7244065
7276	Bn-A03-p29656801	A-genome specific (napus-rapa)	0.8803828	0.7446745	not scorable	not scorable	0.9917355	0.787953
7277	Bn-A03-p29661431	A-genome specific (napus-rapa)	0.9928229	0.8393649	not scorable	not scorable	1	0.9029373
7278	Bn-A03-p2971192	A-genome specific (napus-rapa)	1	0.5213755	not scorable	not scorable	0.9917355	0.8864086
7279	Bn-A03-p29764052	A-genome specific (napus-rapa)	0.9928229	0.806209	not scorable	not scorable	1	0.8287753
7280	Bn-A03-p2976496	A-genome specific (rapa only)	not scorable	not scorable	not scorable	not scorable	1	0.6941791
7281	Bn-A03-p29776900	A-genome specific (napus-rapa)	0.937799	0.819433	not scorable	not scorable	1	0.8694528
7282	Bn-A03-p29780293	A-genome specific (napus-rapa)	0.9354067	0.714115	not scorable	not scorable	0.9752066	0.7938456
7283	Bn-A03-p29781065	A-genome specific (napus-rapa)	0.9162679	0.7127428	not scorable	not scorable	0.9752066	0.7694935
7284	Bn-A03-p29784941	A-genome specific (napus-rapa)	0.9712918	0.7793172	not scorable	not scorable	0.9752066	0.9070715
7285	Bn-A03-p2978588	A-genome specific (napus-rapa)	0.9784689	0.5245405	not scorable	not scorable	0.9917355	0.697923
7286	Bn-A03-p29790183	A-genome specific (napus-rapa)	0.9617225	0.405933	not scorable	not scorable	0.9586777	0.8412624
7287	Bn-A03-p29802913	A-genome specific (napus-rapa)	0.9497608	0.7634909	not scorable	not scorable	0.9917355	0.6825903
7288	Bn-A03-p29803241	A-genome specific (napus-rapa)	0.9282297	0.6450936	not scorable	not scorable	0.9917355	0.7445741
7289	Bn-A03-p29803351	A-genome specific (napus-rapa)	0.9497608	0.7874149	not scorable	not scorable	0.9917355	0.7890589
7290	Bn-A03-p29805598	A-genome specific (napus-rapa)	0.9425837	0.7708021	not scorable	not scorable	0.9917355	0.762583

7291	Bn-A03-p29806105	A-genome specific (napus-rapa)	0.9449761	0.7796823	not scorable	not scorable	0.9917355	0.8487194
7292	Bn-A03-p29816785	A-genome specific (napus-rapa)	0.9354067	0.7750766	not scorable	not scorable	0.9586777	0.8437102
7293	Bn-A03-p29823747	A-genome specific (napus-rapa)	0.9473684	0.7535161	not scorable	not scorable	0.9752066	0.9039531
7294	Bn-A03-p298309	A-genome specific (napus-rapa)	0.9952153	0.5088559	not scorable	not scorable	1	0.7739488
7295	Bn-A03-p29834625	A-genome specific (napus-rapa)	0.9952153	0.7632703	not scorable	not scorable	0.9917355	0.7582729
7296	Bn-A03-p29840518	A-genome specific (napus-rapa)	0.9904306	0.3875247	not scorable	not scorable	0.9917355	0.7152185
7297	Bn-A03-p29849117	A-genome specific (napus-rapa)	0.9976076	0.7762936	not scorable	not scorable	1	0.7775881
7298	Bn-A03-p29849191	A-genome specific (napus-rapa)	0.9952153	0.6915416	not scorable	not scorable	0.9917355	0.6492524
7299	Bn-A03-p29855378	A-genome specific (napus-rapa)	0.9784689	0.3719263	not scorable	not scorable	1	0.8920393
7300	Bn-A03-p29866453	genome unspecific (all in)	0.9952153	0.5086149	0.6744186	0.7118279	1	0.7200772
7301	Bn-A03-p29868254	A-genome specific (napus-rapa)	0.9497608	0.7709776	not scorable	not scorable	0.9752066	0.7413903
7302	Bn-A03-p29873919	A-genome specific (napus-rapa)	0.9808612	0.6785958	not scorable	not scorable	1	0.6963812
7303	Bn-A03-p29874052	A-genome specific (napus-rapa)	0.9952153	0.6786919	not scorable	not scorable	1	0.6943514
7304	Bn-A03-p29883989	A-genome specific (napus-rapa)	0.930622	0.7600673	not scorable	not scorable	0.9586777	0.8555742
7305	Bn-A03-p29896540	A-genome specific (napus-rapa)	0.9928229	0.3182597	not scorable	not scorable	0.9917355	0.8210978
7306	Bn-A03-p29899721	A-genome specific (napus-rapa)	0.9330143	0.7211718	not scorable	not scorable	0.9752066	0.8053306
7307	Bn-A03-p29900824	A-genome specific (rapa only)	not scorable	not scorable	not scorable	not scorable	1	0.9175813
7308	Bn-A03-p29926117	A-genome specific (napus-rapa)	0.937799	0.7676384	not scorable	not scorable	0.9917355	0.8501012
7309	Bn-A03-p29938	A-genome specific (rapa only)	not scorable	not scorable	not scorable	not scorable	0.9669421	0.7304058
7310	Bn-A03-p29956906	A-genome specific (napus-rapa)	0.930622	0.718242	not scorable	not scorable	0.9752066	0.7918587
7311	Bn-A03-p29964330	A-genome specific (rapa only)	not scorable	not scorable	not scorable	not scorable	1	0.8807709
7312	Bn-A03-p2997270	A-genome specific (napus-rapa)	0.9904306	0.4739395	not scorable	not scorable	0.9834711	0.7568986
7313	Bn-A03-p29998247	A-genome specific (napus-rapa)	1	0.9241819	not scorable	not scorable	1	0.8492144
7314	Bn-A03-p29999738	A-genome specific (napus-rapa)	0.9976076	0.8537236	not scorable	not scorable	1	0.7801048
7315	Bn-A03-p3001467	napus specific	0.9521531	0.9018	not scorable	not scorable	not scorable	not scorable
7316	Bn-A03-p30029704	A-genome specific (napus-rapa)	0.9976076	0.7954653	not scorable	not scorable	0.9917355	0.7469117
7317	Bn-A03-p30052524	A-genome specific (napus-rapa)	0.9976076	0.3725545	not scorable	not scorable	1	0.6586422
7318	Bn-A03-p30057461	genome unspecific (all in)	0.9832536	0.4776487	0.9844961	0.1838472	1	0.6222353
7319	Bn-A03-p30170124	A-genome specific (napus-rapa)	0.9856459	0.7641972	not scorable	not scorable	1	0.7595674
7320	Bn-A03-p30183760	A-genome specific (napus-rapa)	0.9665072	0.3902398	not scorable	not scorable	0.9669421	0.4814632
7321	Bn-A03-p30183937	A-genome specific (napus-rapa)	0.9712918	0.43295	not scorable	not scorable	0.9917355	0.8316599
7322	Bn-A03-p30243799	A-genome specific (napus-rapa)	0.9904306	0.2951837	not scorable	not scorable	0.9834711	0.7766962
7323	Bn-A03-p30291719	A-genome specific (napus-rapa)	1	0.7966691	not scorable	not scorable	0.9917355	0.711896

7324	Bn-A03-p30351345	A-genome specific (napus-rapa)	1	0.566712	not scorable	not scorable	1	0.8532568
7325	Bn-A03-p30358487	genome unspecific (all in)	0.9449761	0.3163812	0.8604651	0.7119734	0.9917355	0.8004208
7326	Bn-A03-p3036102	A-genome specific (napus-rapa)	0.9497608	0.3268545	not scorable	not scorable	0.9586777	0.7098031
7327	Bn-A03-p3036446	A-genome specific (rapa only)	not scorable	not scorable	not scorable	not scorable	0.9752066	0.6340719
7328	Bn-A03-p3052882	A-genome specific (napus-rapa)	1	0.5801196	not scorable	not scorable	0.9917355	0.627337
7329	Bn-A03-p3053702	A-genome specific (napus-rapa)	1	0.6781774	not scorable	not scorable	1	0.6593966
7330	Bn-A03-p30564852	A-genome specific (napus-rapa)	0.9928229	0.7367018	not scorable	not scorable	1	0.7524662
7331	Bn-A03-p30658785	A-genome specific (napus-rapa)	0.9976076	0.7743611	not scorable	not scorable	0.9917355	0.8109788
7332	Bn-A03-p30663365	A-genome specific (napus-rapa)	0.9928229	0.8114098	not scorable	not scorable	0.9917355	0.7867317
7333	Bn-A03-p30667694	A-genome specific (napus-rapa)	0.9880383	0.6799687	not scorable	not scorable	0.9752066	0.7446882
7334	Bn-A03-p306814	A-genome specific (rapa only)	not scorable	not scorable	not scorable	not scorable	1	0.9171866
7335	Bn-A03-p30693412	A-genome specific (napus-rapa)	0.9904306	0.3624463	not scorable	not scorable	1	0.785064
7336	Bn-A03-p3073797	napus specific	0.9976076	0.771359	not scorable	not scorable	not scorable	not scorable
7337	Bn-A03-p3074927	napus specific	0.9952153	0.9073427	not scorable	not scorable	not scorable	not scorable
7338	Bn-A03-p3075427	A-genome specific (napus-rapa)	0.9976076	0.9041845	not scorable	not scorable	0.9917355	0.7842876
7339	Bn-A03-p30831188	A-genome specific (napus-rapa)	0.9856459	0.7755584	not scorable	not scorable	0.9090909	0.7897342
7340	Bn-A03-p3084593	napus specific	0.9808612	0.8051857	not scorable	not scorable	not scorable	not scorable
7341	Bn-A03-p3084866	napus specific	0.9952153	0.8515416	not scorable	not scorable	not scorable	not scorable
7342	Bn-A03-p30852922	A-genome specific (napus-rapa)	0.9976076	0.7506766	not scorable	not scorable	0.9338843	0.8085203
7343	Bn-A03-p30873818	A-genome specific (rapa only)	not scorable	not scorable	not scorable	not scorable	1	0.9110658
7344	Bn-A03-p30873956	genome unspecific (oleracea-rapa)	not scorable	not scorable	0.9767442	0.7185478	1	0.8933567
7345	Bn-A03-p30874086	A-genome specific (napus-rapa)	0.9760765	0.7056215	not scorable	not scorable	0.9834711	0.697804
7346	Bn-A03-p30876909	A-genome specific (napus-rapa)	0.9928229	0.7840836	not scorable	not scorable	0.9917355	0.812594
7347	Bn-A03-p30879004	A-genome specific (napus-rapa)	0.9641148	0.7896242	not scorable	not scorable	0.9586777	0.6705306
7348	Bn-A03-p30879164	A-genome specific (napus-rapa)	0.9832536	0.4556359	not scorable	not scorable	0.9752066	0.705884
7349	Bn-A03-p30888263	A-genome specific (napus-rapa)	0.9928229	0.698755	not scorable	not scorable	0.9834711	0.6865193
7350	Bn-A03-p30897153	A-genome specific (napus-rapa)	0.9808612	0.3836831	not scorable	not scorable	0.9752066	0.6731411
7351	Bn-A03-p30905429	A-genome specific (napus-rapa)	0.9832536	0.7667378	not scorable	not scorable	0.9917355	0.7327078
7352	Bn-A03-p30906755	A-genome specific (napus-rapa)	0.9784689	0.7487896	not scorable	not scorable	0.9917355	0.781985
7353	Bn-A03-p3091011	napus specific	0.9928229	0.6404029	not scorable	not scorable	not scorable	not scorable
7354	Bn-A03-p30910234	A-genome specific (napus-rapa)	0.9712918	0.7465215	not scorable	not scorable	0.9917355	0.7014378
7355	Bn-A03-p30920789	A-genome specific (napus-rapa)	0.9880383	0.3679796	not scorable	not scorable	0.9917355	0.7866022
7356	Bn-A03-p30925102	A-genome specific (napus-rapa)	0.9976076	0.7399983	not scorable	not scorable	0.9917355	0.8017028

7357	Bn-A03-p30951542	A-genome specific (napus-rapa)	0.9760765	0.7741535	not scorable	not scorable	1	0.8129897
7358	Bn-A03-p30974143	A-genome specific (napus-rapa)	1	0.7006917	not scorable	not scorable	1	0.6676938
7359	Bn-A03-p30974251	A-genome specific (napus-rapa)	1	0.8894587	not scorable	not scorable	1	0.7419274
7360	Bn-A03-p3097703	napus specific	0.9952153	0.7768877	not scorable	not scorable	not scorable	not scorable
7361	Bn-A03-p3098147	A-genome specific (napus-rapa)	1	0.4308606	not scorable	not scorable	0.9504132	0.5643108
7362	Bn-A03-p3098813	napus specific	0.9952153	0.8062941	not scorable	not scorable	not scorable	not scorable
7363	Bn-A03-p3099901	napus specific	0.9880383	0.6711724	not scorable	not scorable	not scorable	not scorable
7364	Bn-A03-p31030338	A-genome specific (napus-rapa)	0.9641148	0.3674453	not scorable	not scorable	0.9834711	0.7863241
7365	Bn-A03-p31049094	A-genome specific (napus-rapa)	0.9736842	0.2855645	not scorable	not scorable	1	0.7216815
7366	Bn-A03-p31076742	napus specific	0.9856459	0.4421882	not scorable	not scorable	not scorable	not scorable
7367	Bn-A03-p31076904	A-genome specific (napus-rapa)	0.9808612	0.7619962	not scorable	not scorable	0.9834711	0.8556731
7368	Bn-A03-p31077034	A-genome specific (napus-rapa)	0.9760765	0.7832189	not scorable	not scorable	0.9917355	0.7354562
7369	Bn-A03-p31078490	A-genome specific (napus-rapa)	1	0.7827932	not scorable	not scorable	1	0.8105487
7370	Bn-A03-p31088060	A-genome specific (napus-rapa)	0.9784689	0.7735092	not scorable	not scorable	1	0.8232071
7371	Bn-A03-p31088264	A-genome specific (napus-rapa)	0.9928229	0.7695096	not scorable	not scorable	0.9917355	0.7313921
7372	Bn-A03-p31091148	A-genome specific (napus-rapa)	0.8564593	0.8360246	not scorable	not scorable	1	0.7165963
7373	Bn-A03-p31093169	A-genome specific (napus-rapa)	1	0.3026855	not scorable	not scorable	1	0.7884539
7374	Bn-A03-p31095221	A-genome specific (napus-rapa)	0.9832536	0.7706004	not scorable	not scorable	1	0.7888339
7375	Bn-A03-p31113968	A-genome specific (napus-rapa)	0.9904306	0.8733141	not scorable	not scorable	1	0.6243924
7376	Bn-A03-p31147953	A-genome specific (napus-rapa)	0.9688995	0.7692309	not scorable	not scorable	0.8099173	0.8662917
7377	Bn-A03-p3115732	all not scorable	not scorable	not scorable	not scorable	not scorable	not scorable	not scorable
7378	Bn-A03-p31160337	A-genome specific (napus-rapa)	0.9617225	0.4683604	not scorable	not scorable	0.7603306	0.7156561
7379	Bn-A03-p3116829	napus specific	0.9593301	0.7793429	not scorable	not scorable	not scorable	not scorable
7380	Bn-A03-p31179815	A-genome specific (napus-rapa)	0.9760765	0.3598176	not scorable	not scorable	0.9917355	0.7653556
7381	Bn-A03-p31212393	A-genome specific (napus-rapa)	0.9928229	0.6963856	not scorable	not scorable	0.9917355	0.6557474
7382	Bn-A03-p31242297	A-genome specific (napus-rapa)	0.9904306	0.8045593	not scorable	not scorable	0.6859504	0.8378905
7383	Bn-A03-p31242854	A-genome specific (rapa only)	not scorable	not scorable	not scorable	not scorable	0.6446281	0.8210481
7384	Bn-A03-p31246266	A-genome specific (rapa only)	not scorable	not scorable	not scorable	not scorable	0.6528926	0.6682963
7385	Bn-A03-p312762	A-genome specific (napus-rapa)	0.9354067	0.7956669	not scorable	not scorable	0.9917355	0.8317735
7386	Bn-A03-p3131450	napus specific	0.9712918	0.4333505	not scorable	not scorable	not scorable	not scorable
7387	Bn-A03-p31327366	A-genome specific (napus-rapa)	0.9425837	0.5657234	not scorable	not scorable	1	0.8640254
7388	Bn-A03-p31535298	A-genome specific (napus-rapa)	0.9617225	0.3001902	not scorable	not scorable	1	0.8422567
7389	Bn-A03-p31540985	A-genome specific (napus-rapa)	0.9976076	0.7813376	not scorable	not scorable	0.9834711	0.7424419

7390	Bn-A03-p31546901	A-genome specific (napus-rapa)	1	0.8642864	not scorable	not scorable	1	0.8965881
7391	Bn-A03-p3156872	A-genome specific (napus-rapa)	0.9952153	0.3808227	not scorable	not scorable	0.9917355	0.6865885
7392	Bn-A03-p31579593	A-genome specific (napus-rapa)	1	0.8496538	not scorable	not scorable	1	0.7964512
7393	Bn-A03-p31651142	A-genome specific (rapa only)	not scorable	not scorable	not scorable	not scorable	0.9834711	0.5385464
7394	Bn-A03-p31654131	napus specific	0.9665072	0.2757368	not scorable	not scorable	not scorable	not scorable
7395	Bn-A03-p3189093	A-genome specific (napus-rapa)	0.9832536	0.7849496	not scorable	not scorable	1	0.7705146
7396	Bn-A03-p3189333	A-genome specific (napus-rapa)	0.9952153	0.7471203	not scorable	not scorable	1	0.7294715
7397	Bn-A03-p319304	A-genome specific (rapa only)	not scorable	not scorable	not scorable	not scorable	0.9917355	0.5783283
7398	Bn-A03-p3219484	A-genome specific (napus-rapa)	0.937799	0.4705165	not scorable	not scorable	0.9834711	0.6797649
7399	Bn-A03-p3272346	A-genome specific (napus-rapa)	0.9736842	0.8213861	not scorable	not scorable	0.9834711	0.8431111
7400	Bn-A03-p3273629	A-genome specific (rapa only)	not scorable	not scorable	not scorable	not scorable	1	0.7030702
7401	Bn-A03-p3319357	A-genome specific (napus-rapa)	0.9976076	0.8361261	not scorable	not scorable	0.9917355	0.8397495
7402	Bn-A03-p334010	A-genome specific (rapa only)	not scorable	not scorable	not scorable	not scorable	0.9917355	0.7829539
7403	Bn-A03-p3381122	A-genome specific (napus-rapa)	0.9856459	0.3129415	not scorable	not scorable	1	0.9084126
7404	Bn-A03-p3392103	A-genome specific (napus-rapa)	0.9928229	0.8190565	not scorable	not scorable	0.9752066	0.8681164
7405	Bn-A03-p3476060	A-genome specific (rapa only)	not scorable	not scorable	not scorable	not scorable	0.8760331	0.7789106
7406	Bn-A03-p3476410	A-genome specific (napus-rapa)	0.9952153	0.8346981	not scorable	not scorable	0.8429752	0.7211741
7407	Bn-A03-p3489437	A-genome specific (rapa only)	not scorable	not scorable	not scorable	not scorable	0.9917355	0.6864532
7408	Bn-A03-p3492107	A-genome specific (rapa only)	not scorable	not scorable	not scorable	not scorable	1	0.7386974
7409	Bn-A03-p3507222	A-genome specific (rapa only)	not scorable	not scorable	not scorable	not scorable	1	0.6878806
7410	Bn-A03-p3517306	A-genome specific (napus-rapa)	1	0.8207132	not scorable	not scorable	0.9834711	0.8173782
7411	Bn-A03-p3544165	A-genome specific (rapa only)	not scorable	not scorable	not scorable	not scorable	1	0.7022559
7412	Bn-A03-p3544245	A-genome specific (napus-rapa)	0.9976076	0.790868	not scorable	not scorable	0.9752066	0.7849029
7413	Bn-A03-p3561270	A-genome specific (napus-rapa)	1	0.9261386	not scorable	not scorable	1	0.9174846
7414	Bn-A03-p3571622	genome unspecific (all in)	0.8229665	0.7497075	0.4031008	0.8061886	0.9917355	0.9159064
7415	Bn-A03-p3571859	A-genome specific (rapa only)	not scorable	not scorable	not scorable	not scorable	0.9752066	0.5783415
7416	Bn-A03-p360880	A-genome specific (napus-rapa)	0.923445	0.8077155	not scorable	not scorable	0.9917355	0.8367031
7417	Bn-A03-p3674265	A-genome specific (napus-rapa)	0.9976076	0.8055902	not scorable	not scorable	1	0.8065564
7418	Bn-A03-p3692048	A-genome specific (napus-rapa)	0.9593301	0.6570483	not scorable	not scorable	0.9669421	0.6710246
7419	Bn-A03-p3698620	A-genome specific (napus-rapa)	0.9832536	0.7668395	not scorable	not scorable	0.9917355	0.7277821
7420	Bn-A03-p373134	A-genome specific (napus-rapa)	0.9043062	0.7963603	not scorable	not scorable	0.9586777	0.7559701
7421	Bn-A03-p373153	A-genome specific (napus-rapa)	0.8397129	0.8162653	not scorable	not scorable	0.9586777	0.8350886
7422	Bn-A03-p3795299	A-genome specific (napus-rapa)	0.9832536	0.7647683	not scorable	not scorable	0.9752066	0.7420568

7423	Bn-A03-p3795523	A-genome specific (napus-rapa)	0.9928229	0.8317405	not scorable	not scorable	0.9917355	0.798208
7424	Bn-A03-p3796394	all not scorable	not scorable	not scorable	not scorable	not scorable	not scorable	not scorable
7425	Bn-A03-p3796557	A-genome specific (napus-rapa)	0.9928229	0.8178231	not scorable	not scorable	1	0.8090347
7426	Bn-A03-p3796624	A-genome specific (napus-rapa)	0.9952153	0.7192219	not scorable	not scorable	0.9834711	0.7131919
7427	Bn-A03-p3799804	A-genome specific (napus-rapa)	0.9928229	0.7384247	not scorable	not scorable	1	0.8085203
7428	Bn-A03-p3801626	napus specific	0.8875598	0.7811927	not scorable	not scorable	not scorable	not scorable
7429	Bn-A03-p3808441	A-genome specific (napus-rapa)	0.9928229	0.793898	not scorable	not scorable	0.9917355	0.8062909
7430	Bn-A03-p3819549	A-genome specific (napus-rapa)	0.9976076	0.4191545	not scorable	not scorable	1	0.7973999
7431	Bn-A03-p3823071	A-genome specific (napus-rapa)	0.9497608	0.4489898	not scorable	not scorable	0.9917355	0.8061205
7432	Bn-A03-p3839084	A-genome specific (rapa only)	not scorable	not scorable	not scorable	not scorable	0.8099173	0.9174042
7433	Bn-A03-p3851195	A-genome specific (napus-rapa)	0.9952153	0.825492	not scorable	not scorable	1	0.7423896
7434	Bn-A03-p3857752	A-genome specific (napus-rapa)	0.9928229	0.4850564	not scorable	not scorable	0.9917355	0.699442
7435	Bn-A03-p3861991	A-genome specific (napus-rapa)	0.9928229	0.8351377	not scorable	not scorable	1	0.8656023
7436	Bn-A03-p3862302	A-genome specific (napus-rapa)	0.9976076	0.86612	not scorable	not scorable	1	0.9185661
7437	Bn-A03-p3862406	A-genome specific (rapa only)	not scorable	not scorable	not scorable	not scorable	0.9917355	0.72885
7438	Bn-A03-p3862928	A-genome specific (napus-rapa)	0.9760765	0.7193097	not scorable	not scorable	0.8264463	0.6743186
7439	Bn-A03-p386542	napus specific	0.9976076	0.309222	not scorable	not scorable	not scorable	not scorable
7440	Bn-A03-p3877500	A-genome specific (napus-rapa)	0.9665072	0.7332357	not scorable	not scorable	0.9586777	0.7405977
7441	Bn-A03-p3877572	A-genome specific (rapa only)	not scorable	not scorable	not scorable	not scorable	1	0.7663021
7442	Bn-A03-p3951623	A-genome specific (napus-rapa)	0.9904306	0.7584762	not scorable	not scorable	0.9917355	0.7614423
7443	Bn-A03-p3951816	A-genome specific (napus-rapa)	0.9904306	0.5718336	not scorable	not scorable	0.9917355	0.7045814
7444	Bn-A03-p3969037	A-genome specific (napus-rapa)	0.9976076	0.9178608	not scorable	not scorable	0.9917355	0.7826211
7445	Bn-A03-p3969278	A-genome specific (napus-rapa)	0.9976076	0.4478361	not scorable	not scorable	0.9752066	0.7365075
7446	Bn-A03-p3984795	all not scorable	not scorable	not scorable	not scorable	not scorable	not scorable	not scorable
7447	Bn-A03-p3994857	A-genome specific (napus-rapa)	1	0.833072	not scorable	not scorable	1	0.8848283
7448	Bn-A03-p4008078	napus specific	0.9473684	0.6965044	not scorable	not scorable	not scorable	not scorable
7449	Bn-A03-p403559	A-genome specific (napus-rapa)	0.9330143	0.7870512	not scorable	not scorable	1	0.8571517
7450	Bn-A03-p4036118	A-genome specific (rapa only)	not scorable	not scorable	not scorable	not scorable	0.9586777	0.8122351
7451	Bn-A03-p4036196	A-genome specific (napus-rapa)	0.9832536	0.7309472	not scorable	not scorable	0.9917355	0.7294192
7452	Bn-A03-p4050929	napus specific	0.6650718	0.6892128	not scorable	not scorable	not scorable	not scorable
7453	Bn-A03-p4092312	A-genome specific (rapa only)	not scorable	not scorable	not scorable	not scorable	1	0.7807286
7454	Bn-A03-p4096019	A-genome specific (napus-rapa)	0.9712918	0.7570184	not scorable	not scorable	0.9752066	0.779534
7455	Bn-A03-p4100027	A-genome specific (rapa only)	not scorable	not scorable	not scorable	not scorable	0.9669421	0.6978019

7456	Bn-A03-p4116886	A-genome specific (napus-rapa)	0.9880383	0.7458222	not scorable	not scorable	0.9752066	0.7983682
7457	Bn-A03-p4118881	A-genome specific (rapa only)	not scorable	not scorable	not scorable	not scorable	0.9752066	0.7186745
7458	Bn-A03-p4123164	A-genome specific (napus-rapa)	0.9354067	0.7384563	not scorable	not scorable	0.9917355	0.7338673
7459	Bn-A03-p4123209	A-genome specific (napus-rapa)	0.9856459	0.7800994	not scorable	not scorable	0.9752066	0.7938052
7460	Bn-A03-p4130585	A-genome specific (rapa only)	not scorable	not scorable	not scorable	not scorable	0.9917355	0.8010893
7461	Bn-A03-p4138735	A-genome specific (napus-rapa)	0.9832536	0.5220073	not scorable	not scorable	0.9834711	0.751825
7462	Bn-A03-p4153697	A-genome specific (rapa only)	not scorable	not scorable	not scorable	not scorable	0.9752066	0.8081412
7463	Bn-A03-p4167644	A-genome specific (napus-rapa)	0.9593301	0.7695863	not scorable	not scorable	0.9834711	0.7611853
7464	Bn-A03-p4177118	A-genome specific (napus-rapa)	0.9784689	0.3540428	not scorable	not scorable	0.9917355	0.4965771
7465	Bn-A03-p4180932	genome unspecific (oleracea-rapa)	not scorable	not scorable	1	0.8619709	1	0.8527403
7466	Bn-A03-p4183056	A-genome specific (rapa only)	not scorable	not scorable	not scorable	not scorable	1	0.7018657
7467	Bn-A03-p419250	A-genome specific (napus-rapa)	0.9952153	0.4492301	not scorable	not scorable	1	0.8602356
7468	Bn-A03-p4193022	napus specific	0.9593301	0.7873563	not scorable	not scorable	not scorable	not scorable
7469	Bn-A03-p4196989	A-genome specific (napus-rapa)	0.9784689	0.7868638	not scorable	not scorable	0.9917355	0.8168967
7470	Bn-A03-p4202242	A-genome specific (rapa only)	not scorable	not scorable	not scorable	not scorable	0.9752066	0.5849507
7471	Bn-A03-p4205159	A-genome specific (napus-rapa)	0.9856459	0.7409237	not scorable	not scorable	0.9752066	0.7354854
7472	Bn-A03-p420642	A-genome specific (napus-rapa)	0.9928229	0.4941792	not scorable	not scorable	1	0.8597022
7473	Bn-A03-p4211072	A-genome specific (napus-rapa)	0.9760765	0.7550015	not scorable	not scorable	0.9834711	0.6907074
7474	Bn-A03-p4215720	A-genome specific (rapa only)	not scorable	not scorable	not scorable	not scorable	0.9504132	0.6121618
7475	Bn-A03-p4216444	A-genome specific (napus-rapa)	0.9425837	0.5429848	not scorable	not scorable	0.9917355	0.7278751
7476	Bn-A03-p4232493	A-genome specific (napus-rapa)	0.8684211	0.7604216	not scorable	not scorable	0.9752066	0.723519
7477	Bn-A03-p4235658	A-genome specific (napus-rapa)	0.9090909	0.4272245	not scorable	not scorable	0.9834711	0.6468084
7478	Bn-A03-p4244641	A-genome specific (napus-rapa)	1	0.7506877	not scorable	not scorable	0.9917355	0.7521315
7479	Bn-A03-p4256940	genome unspecific (oleracea-rapa)	not scorable	not scorable	0.9922481	0.7301472	1	0.7355853
7480	Bn-A03-p4279693	A-genome specific (napus-rapa)	0.9736842	0.7255251	not scorable	not scorable	0.9834711	0.6686319
7481	Bn-A03-p4279836	A-genome specific (rapa only)	not scorable	not scorable	not scorable	not scorable	0.9917355	0.6698927
7482	Bn-A03-p4292459	A-genome specific (napus-rapa)	0.9976076	0.7845509	not scorable	not scorable	1	0.6975146
7483	Bn-A03-p4295145	A-genome specific (rapa only)	not scorable	not scorable	not scorable	not scorable	0.8181818	0.7584581
7484	Bn-A03-p4309562	A-genome specific (napus-rapa)	0.9976076	0.8131651	not scorable	not scorable	0.9834711	0.7924426
7485	Bn-A03-p4335536	A-genome specific (napus-rapa)	0.9186603	0.5150786	not scorable	not scorable	0.9834711	0.7622114
7486	Bn-A03-p4339301	A-genome specific (napus-rapa)	0.9952153	0.3713767	not scorable	not scorable	0.9586777	0.5536273
7487	Bn-A03-p4342338	A-genome specific (napus-rapa)	0.9856459	0.7673541	not scorable	not scorable	0.9917355	0.7554075
7488	Bn-A03-p4348997	napus specific	0.9665072	0.7706622	not scorable	not scorable	not scorable	not scorable

7489	Bn-A03-p4352837	A-genome specific (napus-rapa)	0.9736842	0.782464	not scorable	not scorable	0.9917355	0.7207875
7490	Bn-A03-p4378124	A-genome specific (rapa only)	not scorable	not scorable	not scorable	not scorable	0.9917355	0.7696424
7491	Bn-A03-p4382093	A-genome specific (napus-rapa)	0.9736842	0.8152851	not scorable	not scorable	1	0.7655828
7492	Bn-A03-p4387230	A-genome specific (napus-rapa)	0.9976076	0.7720975	not scorable	not scorable	0.9834711	0.7136315
7493	Bn-A03-p4399285	A-genome specific (napus-rapa)	0.9617225	0.8134522	not scorable	not scorable	0.8347107	0.7443467
7494	Bn-A03-p4407987	A-genome specific (napus-rapa)	0.9784689	0.7005998	not scorable	not scorable	0.9917355	0.8963528
7495	Bn-A03-p4408006	A-genome specific (napus-rapa)	0.9712918	0.6952382	not scorable	not scorable	0.9834711	0.722072
7496	Bn-A03-p4408895	A-genome specific (napus-rapa)	0.9880383	0.7228676	not scorable	not scorable	1	0.7608284
7497	Bn-A03-p4409606	A-genome specific (napus-rapa)	0.9952153	0.7896671	not scorable	not scorable	1	0.7605999
7498	Bn-A03-p4409970	A-genome specific (rapa only)	not scorable	not scorable	not scorable	not scorable	0.9917355	0.6062437
7499	Bn-A03-p4416817	A-genome specific (napus-rapa)	0.9497608	0.3722148	not scorable	not scorable	1	0.7361338
7500	Bn-A03-p4423212	A-genome specific (napus-rapa)	0.9808612	0.7590605	not scorable	not scorable	0.9917355	0.8290427
7501	Bn-A03-p4423604	A-genome specific (napus-rapa)	0.9952153	0.7677105	not scorable	not scorable	0.9834711	0.8054933
7502	Bn-A03-p4425566	A-genome specific (napus-rapa)	0.9904306	0.6061164	not scorable	not scorable	0.9834711	0.7758104
7503	Bn-A03-p4425819	A-genome specific (napus-rapa)	0.9952153	0.7762482	not scorable	not scorable	0.9917355	0.7679095
7504	Bn-A03-p4437907	A-genome specific (napus-rapa)	0.9928229	0.612386	not scorable	not scorable	0.9917355	0.7161686
7505	Bn-A03-p4473205	A-genome specific (napus-rapa)	0.9904306	0.4387455	not scorable	not scorable	0.9834711	0.733959
7506	Bn-A03-p4488639	A-genome specific (napus-rapa)	0.9497608	0.7110998	not scorable	not scorable	1	0.8264596
7507	Bn-A03-p4498766	A-genome specific (napus-rapa)	0.8660287	0.7371564	not scorable	not scorable	0.8677686	0.7121256
7508	Bn-A03-p4507718	A-genome specific (napus-rapa)	0.9760765	0.7723679	not scorable	not scorable	0.9834711	0.6824206
7509	Bn-A03-p4509102	A-genome specific (napus-rapa)	0.9856459	0.7400692	not scorable	not scorable	1	0.8190148
7510	Bn-A03-p4548924	A-genome specific (napus-rapa)	0.9880383	0.72348	not scorable	not scorable	0.9917355	0.7426291
7511	Bn-A03-p4549344	A-genome specific (rapa only)	not scorable	not scorable	not scorable	not scorable	1	0.7140883
7512	Bn-A03-p4550270	A-genome specific (rapa only)	not scorable	not scorable	not scorable	not scorable	0.9917355	0.7593805
7513	Bn-A03-p4560576	A-genome specific (rapa only)	not scorable	not scorable	not scorable	not scorable	1	0.6621513
7514	Bn-A03-p4567578	A-genome specific (napus-rapa)	0.9043062	0.4334243	not scorable	not scorable	0.9917355	0.6896756
7515	Bn-A03-p4572110	A-genome specific (napus-rapa)	0.9808612	0.4066466	not scorable	not scorable	0.9917355	0.716942
7516	Bn-A03-p4575317	napus specific	0.8349282	0.6528487	not scorable	not scorable	not scorable	not scorable
7517	Bn-A03-p4590174	A-genome specific (napus-rapa)	0.9712918	0.7019057	not scorable	not scorable	1	0.7443007
7518	Bn-A03-p4593298	A-genome specific (rapa only)	not scorable	not scorable	not scorable	not scorable	0.9917355	0.7635609
7519	Bn-A03-p4593331	A-genome specific (rapa only)	not scorable	not scorable	not scorable	not scorable	0.9834711	0.7097092
7520	Bn-A03-p4594763	A-genome specific (napus-rapa)	0.9952153	0.4865949	not scorable	not scorable	0.9834711	0.630109
7521	Bn-A03-p4605838	A-genome specific (rapa only)	not scorable	not scorable	not scorable	not scorable	0.9834711	0.7916752

7522	Bn-A03-p4606213	A-genome specific (rapa only)	not scorable	not scorable	not scorable	not scorable	0.9669421	0.795204
7523	Bn-A03-p4607120	A-genome specific (napus-rapa)	0.9760765	0.6457719	not scorable	not scorable	0.9586777	0.6408432
7524	Bn-A03-p4607947	A-genome specific (napus-rapa)	0.8181818	0.915972	not scorable	not scorable	0.9752066	0.9098665
7525	Bn-A03-p4620459	A-genome specific (napus-rapa)	0.9832536	0.7515733	not scorable	not scorable	1	0.7921442
7526	Bn-A03-p4638893	A-genome specific (rapa only)	not scorable	not scorable	not scorable	not scorable	0.9917355	0.7505503
7527	Bn-A03-p4659068	A-genome specific (napus-rapa)	0.9497608	0.6456177	not scorable	not scorable	0.9917355	0.7774001
7528	Bn-A03-p4673503	A-genome specific (napus-rapa)	0.9449761	0.5026833	not scorable	not scorable	0.9834711	0.5808347
7529	Bn-A03-p4673508	A-genome specific (napus-rapa)	0.9952153	0.8007945	not scorable	not scorable	0.9834711	0.6946002
7530	Bn-A03-p4682207	A-genome specific (napus-rapa)	0.9832536	0.2984687	not scorable	not scorable	0.9834711	0.5423722
7531	Bn-A03-p471570	A-genome specific (napus-rapa)	0.9569378	0.5187739	not scorable	not scorable	1	0.7045401
7532	Bn-A03-p4753001	A-genome specific (rapa only)	not scorable	not scorable	not scorable	not scorable	1	0.7775756
7533	Bn-A03-p4769653	A-genome specific (napus-rapa)	0.9928229	0.8140981	not scorable	not scorable	0.9752066	0.7942212
7534	Bn-A03-p4783485	A-genome specific (napus-rapa)	0.9904306	0.7794448	not scorable	not scorable	1	0.6291124
7535	Bn-A03-p478702	A-genome specific (napus-rapa)	0.930622	0.7558073	not scorable	not scorable	0.9917355	0.7653976
7536	Bn-A03-p4798485	A-genome specific (rapa only)	not scorable	not scorable	not scorable	not scorable	1	0.699198
7537	Bn-A03-p4799142	A-genome specific (napus-rapa)	0.9952153	0.5554939	not scorable	not scorable	0.9917355	0.7076653
7538	Bn-A03-p4809473	A-genome specific (rapa only)	not scorable	not scorable	not scorable	not scorable	0.9917355	0.8551057
7539	Bn-A03-p4827846	A-genome specific (napus-rapa)	0.9760765	0.7097555	not scorable	not scorable	1	0.8002217
7540	Bn-A03-p4829139	A-genome specific (rapa only)	not scorable	not scorable	not scorable	not scorable	1	0.8395246
7541	Bn-A03-p4861136	A-genome specific (napus-rapa)	0.9760765	0.3054993	not scorable	not scorable	0.9752066	0.7061461
7542	Bn-A03-p4871103	A-genome specific (napus-rapa)	0.9952153	0.7390259	not scorable	not scorable	1	0.7673323
7543	Bn-A03-p487666	A-genome specific (rapa only)	not scorable	not scorable	not scorable	not scorable	0.9834711	0.8213049
7544	Bn-A03-p4879812	A-genome specific (napus-rapa)	0.9641148	0.7393047	not scorable	not scorable	0.9669421	0.6856222
7545	Bn-A03-p4881229	A-genome specific (rapa only)	not scorable	not scorable	not scorable	not scorable	0.9834711	0.7830136
7546	Bn-A03-p49020	A-genome specific (rapa only)	not scorable	not scorable	not scorable	not scorable	0.9834711	0.7723494
7547	Bn-A03-p4909233	A-genome specific (napus-rapa)	0.9832536	0.75986	not scorable	not scorable	0.9752066	0.7603261
7548	Bn-A03-p4909313	A-genome specific (rapa only)	not scorable	not scorable	not scorable	not scorable	0.9834711	0.7297021
7549	Bn-A03-p4909522	A-genome specific (rapa only)	not scorable	not scorable	not scorable	not scorable	0.9586777	0.7460187
7550	Bn-A03-p4916224	A-genome specific (napus-rapa)	0.6124402	0.8795152	not scorable	not scorable	0.9752066	0.7748017
7551	Bn-A03-p4934887	A-genome specific (rapa only)	not scorable	not scorable	not scorable	not scorable	0.9752066	0.8006725
7552	Bn-A03-p493766	A-genome specific (napus-rapa)	0.9210526	0.4904248	not scorable	not scorable	1	0.8513054
7553	Bn-A03-p494427	genome unspecific (oleracea-rapa)	not scorable	not scorable	0.9844961	0.40434	1	0.7401911
7554	Bn-A03-p4944913	A-genome specific (napus-rapa)	0.9952153	0.7145084	not scorable	not scorable	0.9752066	0.7206949

7555	Bn-A03-p498320	A-genome specific (napus-rapa)	0.8636364	0.7401044	not scorable	not scorable	1	0.8134705
7556	Bn-A03-p4989769	A-genome specific (napus-rapa)	0.7344497	0.8284623	not scorable	not scorable	0.9834711	0.8940818
7557	Bn-A03-p4992039	A-genome specific (rapa only)	not scorable	not scorable	not scorable	not scorable	0.9752066	0.7506456
7558	Bn-A03-p4997351	A-genome specific (rapa only)	not scorable	not scorable	not scorable	not scorable	0.9669421	0.6049495
7559	Bn-A03-p4999662	A-genome specific (rapa only)	not scorable	not scorable	not scorable	not scorable	0.9917355	0.6903507
7560	Bn-A03-p5006544	A-genome specific (rapa only)	not scorable	not scorable	not scorable	not scorable	1	0.7349294
7561	Bn-A03-p5023169	napus specific	0.7631579	0.7465882	not scorable	not scorable	not scorable	not scorable
7562	Bn-A03-p5025156	A-genome specific (napus-rapa)	0.9688995	0.5639485	not scorable	not scorable	0.9834711	0.6179368
7563	Bn-A03-p5038403	A-genome specific (napus-rapa)	0.6339713	0.9158995	not scorable	not scorable	0.8595041	0.8201163
7564	Bn-A03-p5039235	A-genome specific (napus-rapa)	0.9784689	0.4879007	not scorable	not scorable	1	0.9110646
7565	Bn-A03-p5039586	A-genome specific (napus-rapa)	0.9641148	0.7705398	not scorable	not scorable	1	0.7923465
7566	Bn-A03-p5042719	A-genome specific (napus-rapa)	0.9449761	0.4365304	not scorable	not scorable	1	0.7129645
7567	Bn-A03-p5045095	A-genome specific (napus-rapa)	0.9330143	0.3949938	not scorable	not scorable	0.9834711	0.6846312
7568	Bn-A03-p5052289	A-genome specific (napus-rapa)	0.7535885	0.8195979	not scorable	not scorable	0.9669421	0.8135254
7569	Bn-A03-p5067556	A-genome specific (napus-rapa)	0.923445	0.4162782	not scorable	not scorable	0.9917355	0.6957626
7570	Bn-A03-p5072729	A-genome specific (napus-rapa)	1	0.4789127	not scorable	not scorable	0.9917355	0.6915346
7571	Bn-A03-p5078310	A-genome specific (napus-rapa)	0.9856459	0.8392569	not scorable	not scorable	1	0.7351667
7572	Bn-A03-p5083198	A-genome specific (napus-rapa)	0.9832536	0.8282107	not scorable	not scorable	0.8760331	0.8126996
7573	Bn-A03-p5083386	A-genome specific (napus-rapa)	0.9832536	0.7605443	not scorable	not scorable	0.8760331	0.8306364
7574	Bn-A03-p5083430	A-genome specific (napus-rapa)	0.9832536	0.745198	not scorable	not scorable	0.892562	0.7880469
7575	Bn-A03-p5111446	A-genome specific (napus-rapa)	1	0.3496064	not scorable	not scorable	0.9752066	0.6835081
7576	Bn-A03-p5115321	A-genome specific (napus-rapa)	0.9665072	0.4286527	not scorable	not scorable	0.9834711	0.8201146
7577	Bn-A03-p5131282	A-genome specific (napus-rapa)	0.9832536	0.7473509	not scorable	not scorable	0.9752066	0.6861427
7578	Bn-A03-p5131371	A-genome specific (napus-rapa)	0.9784689	0.6954108	not scorable	not scorable	0.9917355	0.7205139
7579	Bn-A03-p5133977	A-genome specific (napus-rapa)	0.9066986	0.5278134	not scorable	not scorable	0.9834711	0.8154917
7580	Bn-A03-p5135902	A-genome specific (napus-rapa)	0.9593301	0.2716399	not scorable	not scorable	0.9917355	0.7132878
7581	Bn-A03-p5140330	A-genome specific (rapa only)	not scorable	not scorable	not scorable	not scorable	0.9834711	0.8352724
7582	Bn-A03-p5140947	A-genome specific (napus-rapa)	0.9832536	0.7468907	not scorable	not scorable	0.9917355	0.8015907
7583	Bn-A03-p5144171	A-genome specific (napus-rapa)	0.9856459	0.8331536	not scorable	not scorable	1	0.7692884
7584	Bn-A03-p5153735	A-genome specific (napus-rapa)	0.9808612	0.3676153	not scorable	not scorable	0.9752066	0.6608645
7585	Bn-A03-p5159930	A-genome specific (napus-rapa)	0.9832536	0.3291049	not scorable	not scorable	0.9421487	0.4762597
7586	Bn-A03-p5160088	A-genome specific (napus-rapa)	0.9856459	0.8008282	not scorable	not scorable	0.9917355	0.7239057
7587	Bn-A03-p5163056	A-genome specific (napus-rapa)	0.9760765	0.4545608	not scorable	not scorable	0.9917355	0.7009826

7588	Bn-A03-p5170706	napus specific	0.9832536	0.7802194	not scorable	not scorable	not scorable	not scorable
7589	Bn-A03-p5177830	A-genome specific (napus-rapa)	0.9904306	0.4417276	not scorable	not scorable	0.9834711	0.787963
7590	Bn-A03-p5180655	A-genome specific (napus-rapa)	0.9832536	0.6906459	not scorable	not scorable	0.9834711	0.7566171
7591	Bn-A03-p5182850	genome unspecific (all in)	1	0.2989883	0.9922481	0.7417857	0.9834711	0.677817
7592	Bn-A03-p5222291	A-genome specific (napus-rapa)	0.9904306	0.4853771	not scorable	not scorable	1	0.6948672
7593	Bn-A03-p5227700	A-genome specific (napus-rapa)	0.9425837	0.7589675	not scorable	not scorable	0.9917355	0.8446013
7594	Bn-A03-p5230726	A-genome specific (napus-rapa)	0.9545454	0.5746073	not scorable	not scorable	1	0.6942185
7595	Bn-A03-p5263828	A-genome specific (napus-rapa)	0.9880383	0.3956468	not scorable	not scorable	1	0.7234806
7596	Bn-A03-p5268272	A-genome specific (napus-rapa)	0.9976076	0.5160456	not scorable	not scorable	0.9834711	0.8202378
7597	Bn-A03-p5273046	A-genome specific (rapa only)	not scorable	not scorable	not scorable	not scorable	0.9752066	0.797078
7598	Bn-A03-p528276	A-genome specific (napus-rapa)	0.9162679	0.7452217	not scorable	not scorable	0.9917355	0.8402592
7599	Bn-A03-p532166	A-genome specific (napus-rapa)	0.7272727	0.7899777	not scorable	not scorable	0.9917355	0.7971246
7600	Bn-A03-p5333699	A-genome specific (napus-rapa)	0.9952153	0.8347282	not scorable	not scorable	0.9834711	0.7905619
7601	Bn-A03-p5341139	A-genome specific (napus-rapa)	0.9928229	0.7916658	not scorable	not scorable	0.9669421	0.7027308
7602	Bn-A03-p5357737	genome unspecific (all in)	0.6124402	0.7744598	1	0.8297678	0.9917355	0.655319
7603	Bn-A03-p5360744	A-genome specific (rapa only)	not scorable	not scorable	not scorable	not scorable	0.9752066	0.7681804
7604	Bn-A03-p5374417	A-genome specific (napus-rapa)	0.9976076	0.814248	not scorable	not scorable	1	0.6559938
7605	Bn-A03-p537905	A-genome specific (napus-rapa)	0.8971292	0.7208124	not scorable	not scorable	1	0.778098
7606	Bn-A03-p5380857	A-genome specific (napus-rapa)	0.9928229	0.8735576	not scorable	not scorable	0.9917355	0.8310575
7607	Bn-A03-p5408678	A-genome specific (napus-rapa)	0.9904306	0.3527291	not scorable	not scorable	1	0.7370149
7608	Bn-A03-p5414946	A-genome specific (napus-rapa)	0.9880383	0.785073	not scorable	not scorable	1	0.7032702
7609	Bn-A03-p5423318	A-genome specific (rapa only)	not scorable	not scorable	not scorable	not scorable	1	0.6382402
7610	Bn-A03-p5434265	napus specific	0.9617225	0.9395323	not scorable	not scorable	not scorable	not scorable
7611	Bn-A03-p5439400	A-genome specific (napus-rapa)	1	0.8231412	not scorable	not scorable	0.9917355	0.7209238
7612	Bn-A03-p5454100	napus specific	0.9904306	0.4696753	not scorable	not scorable	not scorable	not scorable
7613	Bn-A03-p5455069	A-genome specific (napus-rapa)	0.9952153	0.7235022	not scorable	not scorable	1	0.6482639
7614	Bn-A03-p5462418	A-genome specific (napus-rapa)	0.9784689	0.6060026	not scorable	not scorable	1	0.7626569
7615	Bn-A03-p5467712	A-genome specific (napus-rapa)	0.9928229	0.7519439	not scorable	not scorable	0.9752066	0.8078017
7616	Bn-A03-p5468447	A-genome specific (napus-rapa)	0.9928229	0.6672873	not scorable	not scorable	1	0.6238344
7617	Bn-A03-p5471743	genome unspecific (all in)	0.9760765	0.6652225	0.9457364	0.7108734	0.9752066	0.8212636
7618	Bn-A03-p5502308	A-genome specific (napus-rapa)	0.9952153	0.7460867	not scorable	not scorable	1	0.7245347
7619	Bn-A03-p5503857	A-genome specific (napus-rapa)	0.9832536	0.7147524	not scorable	not scorable	0.8512397	0.7197788
7620	Bn-A03-p5523239	A-genome specific (napus-rapa)	0.9952153	0.7797544	not scorable	not scorable	0.9586777	0.6684033

7621	Bn-A03-p5523813	A-genome specific (napus-rapa)	0.9952153	0.6902739	not scorable	not scorable	1	0.6626507
7622	Bn-A03-p5523943	A-genome specific (napus-rapa)	0.9952153	0.7863435	not scorable	not scorable	0.9752066	0.6979521
7623	Bn-A03-p5532043	A-genome specific (napus-rapa)	0.9976076	0.6598164	not scorable	not scorable	0.9669421	0.6211019
7624	Bn-A03-p5548413	A-genome specific (rapa only)	not scorable	not scorable	not scorable	not scorable	1	0.7026088
7625	Bn-A03-p5551018	A-genome specific (napus-rapa)	0.9712918	0.7843596	not scorable	not scorable	0.9752066	0.7562535
7626	Bn-A03-p5563280	A-genome specific (rapa only)	not scorable	not scorable	not scorable	not scorable	1	0.7262031
7627	Bn-A03-p5564849	A-genome specific (napus-rapa)	0.9880383	0.7540259	not scorable	not scorable	0.9834711	0.790215
7628	Bn-A03-p5578777	A-genome specific (napus-rapa)	0.9928229	0.4088874	not scorable	not scorable	1	0.651049
7629	Bn-A03-p5597551	A-genome specific (napus-rapa)	0.9856459	0.5018368	not scorable	not scorable	0.9917355	0.6488491
7630	Bn-A03-p560769	A-genome specific (napus-rapa)	0.9138756	0.7024938	not scorable	not scorable	1	0.784973
7631	Bn-A03-p5608927	napus specific	0.9808612	0.7422436	not scorable	not scorable	not scorable	not scorable
7632	Bn-A03-p560941	A-genome specific (napus-rapa)	0.9330143	0.7251384	not scorable	not scorable	1	0.7420298
7633	Bn-A03-p5611940	A-genome specific (napus-rapa)	0.9808612	0.3990846	not scorable	not scorable	0.9504132	0.7212554
7634	Bn-A03-p5612769	napus specific	0.9928229	0.7015483	not scorable	not scorable	not scorable	not scorable
7635	Bn-A03-p5613665	napus specific	0.9808612	0.7945741	not scorable	not scorable	not scorable	not scorable
7636	Bn-A03-p5614097	all not scorable	not scorable	not scorable	not scorable	not scorable	not scorable	not scorable
7637	Bn-A03-p5626575	A-genome specific (napus-rapa)	0.9784689	0.6800024	not scorable	not scorable	0.9917355	0.7092251
7638	Bn-A03-p5626694	A-genome specific (napus-rapa)	1	0.5089189	not scorable	not scorable	1	0.753419
7639	Bn-A03-p5627074	A-genome specific (napus-rapa)	0.9904306	0.7714169	not scorable	not scorable	0.9917355	0.7448741
7640	Bn-A03-p5627585	A-genome specific (napus-rapa)	1	0.7101301	not scorable	not scorable	1	0.7770717
7641	Bn-A03-p563787	A-genome specific (napus-rapa)	0.9641148	0.4873606	not scorable	not scorable	0.9669421	0.7325926
7642	Bn-A03-p5643775	A-genome specific (napus-rapa)	0.9832536	0.7604436	not scorable	not scorable	0.9917355	0.7450931
7643	Bn-A03-p5649178	A-genome specific (napus-rapa)	0.9952153	0.2716594	not scorable	not scorable	0.9834711	0.7157406
7644	Bn-A03-p565187	A-genome specific (napus-rapa)	0.9545454	0.4646529	not scorable	not scorable	0.9752066	0.7442171
7645	Bn-A03-p565363	genome unspecific (oleracea-rapa)	not scorable	not scorable	1	0.7804757	1	0.6987483
7646	Bn-A03-p5654220	A-genome specific (rapa only)	not scorable	not scorable	not scorable	not scorable	0.9752066	0.6857201
7647	Bn-A03-p565637	A-genome specific (rapa only)	not scorable	not scorable	not scorable	not scorable	0.9917355	0.6213671
7648	Bn-A03-p5669661	napus specific	0.9641148	0.8873985	not scorable	not scorable	not scorable	not scorable
7649	Bn-A03-p5682911	A-genome specific (napus-rapa)	1	0.4009607	not scorable	not scorable	1	0.7407764
7650	Bn-A03-p5694401	A-genome specific (napus-rapa)	0.9712918	0.6057469	not scorable	not scorable	1	0.7001542
7651	Bn-A03-p5696470	A-genome specific (rapa only)	not scorable	not scorable	not scorable	not scorable	1	0.8180838
7652	Bn-A03-p5711554	A-genome specific (napus-rapa)	0.9928229	0.7332344	not scorable	not scorable	1	0.7167833
7653	Bn-A03-p5711923	A-genome specific (napus-rapa)	0.9712918	0.7394128	not scorable	not scorable	0.9834711	0.7323701

7654	Bn-A03-p5713023	A-genome specific (napus-rapa)	0.9688995	0.7675729	not scorable	not scorable	0.9752066	0.7785958
7655	Bn-A03-p5721171	A-genome specific (napus-rapa)	0.9952153	0.7383321	not scorable	not scorable	0.9834711	0.6850379
7656	Bn-A03-p5721231	A-genome specific (napus-rapa)	0.9928229	0.7257864	not scorable	not scorable	0.9834711	0.7240035
7657	Bn-A03-p5721443	A-genome specific (napus-rapa)	0.9928229	0.6802424	not scorable	not scorable	0.9752066	0.657653
7658	Bn-A03-p5721617	A-genome specific (napus-rapa)	0.9928229	0.7017218	not scorable	not scorable	0.9917355	0.6438657
7659	Bn-A03-p5722009	A-genome specific (napus-rapa)	0.9808612	0.8182721	not scorable	not scorable	0.9173554	0.8394191
7660	Bn-A03-p5722579	A-genome specific (napus-rapa)	0.6578947	0.8087062	not scorable	not scorable	0.9173554	0.7569847
7661	Bn-A03-p5741340	A-genome specific (napus-rapa)	0.9808612	0.6766212	not scorable	not scorable	0.9917355	0.6753191
7662	Bn-A03-p5741875	A-genome specific (napus-rapa)	0.9593301	0.6407257	not scorable	not scorable	1	0.7332143
7663	Bn-A03-p5742880	A-genome specific (napus-rapa)	0.9808612	0.7775691	not scorable	not scorable	0.9834711	0.7497889
7664	Bn-A03-p5742916	A-genome specific (napus-rapa)	0.9736842	0.8094077	not scorable	not scorable	0.9834711	0.773612
7665	Bn-A03-p5743020	A-genome specific (napus-rapa)	0.6291866	0.8918571	not scorable	not scorable	0.9504132	0.8022243
7666	Bn-A03-p5744242	A-genome specific (napus-rapa)	0.9808612	0.7668223	not scorable	not scorable	0.9586777	0.7821004
7667	Bn-A03-p5744750	A-genome specific (napus-rapa)	0.9832536	0.6882675	not scorable	not scorable	1	0.7082319
7668	Bn-A03-p5745392	A-genome specific (napus-rapa)	0.9808612	0.7589923	not scorable	not scorable	1	0.8049096
7669	Bn-A03-p5745449	A-genome specific (napus-rapa)	0.9808612	0.7711915	not scorable	not scorable	1	0.8199109
7670	Bn-A03-p5746114	A-genome specific (napus-rapa)	0.937799	0.712346	not scorable	not scorable	0.9917355	0.6518111
7671	Bn-A03-p5748366	A-genome specific (napus-rapa)	0.9593301	0.7932428	not scorable	not scorable	1	0.8184522
7672	Bn-A03-p5748775	A-genome specific (napus-rapa)	0.9688995	0.7906005	not scorable	not scorable	1	0.8387375
7673	Bn-A03-p5748884	A-genome specific (napus-rapa)	0.9425837	0.7400977	not scorable	not scorable	0.9669421	0.7659114
7674	Bn-A03-p5749121	A-genome specific (napus-rapa)	0.937799	0.6597643	not scorable	not scorable	1	0.8096516
7675	Bn-A03-p5749213	A-genome specific (napus-rapa)	0.9593301	0.7252596	not scorable	not scorable	0.9834711	0.7200157
7676	Bn-A03-p5749663	A-genome specific (napus-rapa)	0.9808612	0.7740886	not scorable	not scorable	1	0.7736467
7677	Bn-A03-p5749832	A-genome specific (napus-rapa)	0.9258373	0.8235879	not scorable	not scorable	0.9834711	0.8677401
7678	Bn-A03-p5750321	A-genome specific (napus-rapa)	0.9760765	0.7092806	not scorable	not scorable	1	0.7177447
7679	Bn-A03-p5751160	A-genome specific (napus-rapa)	0.9401914	0.722494	not scorable	not scorable	0.9752066	0.789126
7680	Bn-A03-p5751992	A-genome specific (napus-rapa)	0.9641148	0.7591591	not scorable	not scorable	0.9834711	0.707387
7681	Bn-A03-p5752052	genome unspecific (all in)	0.9449761	0.7822777	0	0.6957793	0.9834711	0.7532101
7682	Bn-A03-p5752371	A-genome specific (rapa only)	not scorable	not scorable	not scorable	not scorable	0.9669421	0.754569
7683	Bn-A03-p5762915	A-genome specific (rapa only)	not scorable	not scorable	not scorable	not scorable	0.9834711	0.7183402
7684	Bn-A03-p5770833	A-genome specific (napus-rapa)	0.9449761	0.4158233	not scorable	not scorable	0.9669421	0.7087587
7685	Bn-A03-p5784532	A-genome specific (napus-rapa)	0.9641148	0.7948966	not scorable	not scorable	0.9586777	0.751454
7686	Bn-A03-p5796033	A-genome specific (napus-rapa)	0.9641148	0.4620031	not scorable	not scorable	1	0.7253703

7687	Bn-A03-p5800566	genome unspecific (all in)	0.9880383	0.477366	1	0.4860544	1	0.7252899
7688	Bn-A03-p5809786	A-genome specific (napus-rapa)	0.9688995	0.5077896	not scorable	not scorable	0.9834711	0.7426553
7689	Bn-A03-p5809860	A-genome specific (napus-rapa)	0.9808612	0.7963656	not scorable	not scorable	0.9752066	0.7490408
7690	Bn-A03-p5814447	A-genome specific (napus-rapa)	0.9880383	0.756735	not scorable	not scorable	0.9917355	0.7081367
7691	Bn-A03-p5866896	genome unspecific (oleracea-rapa)	not scorable	not scorable	1	0.6823338	1	0.6421195
7692	Bn-A03-p5892557	A-genome specific (rapa only)	not scorable	not scorable	not scorable	not scorable	1	0.7009104
7693	Bn-A03-p5893002	A-genome specific (napus-rapa)	0.9736842	0.9006504	not scorable	not scorable	0.9917355	0.7643259
7694	Bn-A03-p5903435	A-genome specific (napus-rapa)	0.9928229	0.5589588	not scorable	not scorable	1	0.6952415
7695	Bn-A03-p5912430	A-genome specific (rapa only)	not scorable	not scorable	not scorable	not scorable	0.9752066	0.792782
7696	Bn-A03-p5952923	A-genome specific (rapa only)	not scorable	not scorable	not scorable	not scorable	0.9752066	0.6297615
7697	Bn-A03-p5956851	A-genome specific (napus-rapa)	0.9928229	0.7068547	not scorable	not scorable	0.9752066	0.7471516
7698	Bn-A03-p5956934	A-genome specific (napus-rapa)	0.9832536	0.7215312	not scorable	not scorable	1	0.7494771
7699	Bn-A03-p5984123	A-genome specific (napus-rapa)	0.923445	0.5989958	not scorable	not scorable	1	0.7783204
7700	Bn-A03-p6036521	A-genome specific (napus-rapa)	0.9712918	0.8171275	not scorable	not scorable	0.9752066	0.7714523
7701	Bn-A03-p6039029	A-genome specific (napus-rapa)	0.9952153	0.7918741	not scorable	not scorable	1	0.7293366
7702	Bn-A03-p6057369	A-genome specific (napus-rapa)	1	0.6707018	not scorable	not scorable	0.9917355	0.8286775
7703	Bn-A03-p6126012	A-genome specific (napus-rapa)	1	0.3284041	not scorable	not scorable	1	0.5708936
7704	Bn-A03-p6132626	A-genome specific (rapa only)	not scorable	not scorable	not scorable	not scorable	1	0.87866
7705	Bn-A03-p6136484	A-genome specific (napus-rapa)	0.9928229	0.5015988	not scorable	not scorable	1	0.709865
7706	Bn-A03-p6138644	A-genome specific (rapa only)	not scorable	not scorable	not scorable	not scorable	0.9834711	0.7308469
7707	Bn-A03-p6149485	A-genome specific (napus-rapa)	0.9784689	0.729452	not scorable	not scorable	0.9917355	0.749133
7708	Bn-A03-p6157864	A-genome specific (napus-rapa)	0.9736842	0.6212168	not scorable	not scorable	1	0.7885433
7709	Bn-A03-p6158211	A-genome specific (napus-rapa)	0.9784689	0.3321478	not scorable	not scorable	1	0.7712401
7710	Bn-A03-p6182289	A-genome specific (rapa only)	not scorable	not scorable	not scorable	not scorable	1	0.7442658
7711	Bn-A03-p6204498	napus specific	1	0.5770498	not scorable	not scorable	not scorable	not scorable
7712	Bn-A03-p6224340	A-genome specific (rapa only)	not scorable	not scorable	not scorable	not scorable	0.9752066	0.8199376
7713	Bn-A03-p6228570	A-genome specific (napus-rapa)	0.9928229	0.8592411	not scorable	not scorable	0.9586777	0.7800742
7714	Bn-A03-p623390	A-genome specific (napus-rapa)	0.9808612	0.2333404	not scorable	not scorable	1	0.7466799
7715	Bn-A03-p6265064	A-genome specific (napus-rapa)	1	0.5446512	not scorable	not scorable	0.9917355	0.5692675
7716	Bn-A03-p6274828	A-genome specific (napus-rapa)	0.9832536	0.7945466	not scorable	not scorable	0.9173554	0.7593935
7717	Bn-A03-p6276385	A-genome specific (napus-rapa)	0.9952153	0.784013	not scorable	not scorable	1	0.8848283
7718	Bn-A03-p6286363	A-genome specific (napus-rapa)	0.9856459	0.7597181	not scorable	not scorable	1	0.7036739
7719	Bn-A03-p6288173	A-genome specific (napus-rapa)	0.9712918	0.7191334	not scorable	not scorable	0.9917355	0.7273776

7720	Bn-A03-p6299523	A-genome specific (napus-rapa)	0.9904306	0.5775436	not scorable	not scorable	1	0.7618905
7721	Bn-A03-p630979	A-genome specific (napus-rapa)	0.930622	0.7795375	not scorable	not scorable	0.9834711	0.7428126
7722	Bn-A03-p6317002	A-genome specific (napus-rapa)	0.9617225	0.3640856	not scorable	not scorable	0.9917355	0.7899418
7723	Bn-A03-p6319963	A-genome specific (napus-rapa)	0.9928229	0.7754805	not scorable	not scorable	1	0.6523157
7724	Bn-A03-p6324760	A-genome specific (napus-rapa)	1	0.6786327	not scorable	not scorable	1	0.7920848
7725	Bn-A03-p6332797	A-genome specific (napus-rapa)	0.937799	0.6997382	not scorable	not scorable	1	0.6406797
7726	Bn-A03-p6335597	A-genome specific (napus-rapa)	0.9976076	0.7587337	not scorable	not scorable	0.9917355	0.7591255
7727	Bn-A03-p6338496	A-genome specific (napus-rapa)	0.9569378	0.6025947	not scorable	not scorable	1	0.7654959
7728	Bn-A03-p6354338	A-genome specific (napus-rapa)	0.9569378	0.3636395	not scorable	not scorable	1	0.7516617
7729	Bn-A03-p6358993	A-genome specific (rapa only)	not scorable	not scorable	not scorable	not scorable	1	0.8786294
7730	Bn-A03-p6384649	A-genome specific (rapa only)	not scorable	not scorable	not scorable	not scorable	0.9834711	0.5907344
7731	Bn-A03-p6385958	A-genome specific (napus-rapa)	0.9736842	0.7536376	not scorable	not scorable	0.9917355	0.6859278
7732	Bn-A03-p6387774	A-genome specific (rapa only)	not scorable	not scorable	not scorable	not scorable	1	0.7683919
7733	Bn-A03-p6388604	A-genome specific (napus-rapa)	0.9952153	0.8128043	not scorable	not scorable	1	0.7224071
7734	Bn-A03-p6404659	genome unspecific (oleracea-rapa)	not scorable	not scorable	0.9302326	0.5090902	0.9917355	0.6582912
7735	Bn-A03-p6404903	A-genome specific (napus-rapa)	0.9808612	0.7040495	not scorable	not scorable	0.9917355	0.7700132
7736	Bn-A03-p6408156	A-genome specific (rapa only)	not scorable	not scorable	not scorable	not scorable	0.8264463	0.8384824
7737	Bn-A03-p641927	A-genome specific (rapa only)	not scorable	not scorable	not scorable	not scorable	0.9834711	0.7197356
7738	Bn-A03-p6420355	A-genome specific (napus-rapa)	0.9784689	0.8237109	not scorable	not scorable	1	0.7403933
7739	Bn-A03-p6423049	A-genome specific (napus-rapa)	0.9856459	0.8040795	not scorable	not scorable	0.9917355	0.7844661
7740	Bn-A03-p6423198	A-genome specific (napus-rapa)	0.9832536	0.6089762	not scorable	not scorable	0.9752066	0.6009976
7741	Bn-A03-p6423795	A-genome specific (napus-rapa)	0.9832536	0.8189678	not scorable	not scorable	0.9586777	0.7880545
7742	Bn-A03-p6424980	napus specific	0.9712918	0.4553261	not scorable	not scorable	not scorable	not scorable
7743	Bn-A03-p6425315	A-genome specific (napus-rapa)	0.9952153	0.8751906	not scorable	not scorable	1	0.8272818
7744	Bn-A03-p6425858	A-genome specific (napus-rapa)	0.9688995	0.7051423	not scorable	not scorable	0.9752066	0.7280242
7745	Bn-A03-p6427905	A-genome specific (rapa only)	not scorable	not scorable	not scorable	not scorable	1	0.6877584
7746	Bn-A03-p6428220	A-genome specific (napus-rapa)	0.9928229	0.7895075	not scorable	not scorable	1	0.785244
7747	Bn-A03-p6439432	A-genome specific (napus-rapa)	0.9880383	0.4580778	not scorable	not scorable	0.9586777	0.697583
7748	Bn-A03-p6456584	A-genome specific (napus-rapa)	0.9760765	0.3310408	not scorable	not scorable	0.9752066	0.6821544
7749	Bn-A03-p6484137	A-genome specific (napus-rapa)	0.9736842	0.3664588	not scorable	not scorable	0.9752066	0.6856814
7750	Bn-A03-p6498305	A-genome specific (rapa only)	not scorable	not scorable	not scorable	not scorable	1	0.6585189
7751	Bn-A03-p6502463	A-genome specific (napus-rapa)	0.9808612	0.6337031	not scorable	not scorable	1	0.7765067
7752	Bn-A03-p6514858	A-genome specific (rapa only)	not scorable	not scorable	not scorable	not scorable	0.9834711	0.7720999

7753	Bn-A03-p6522454	A-genome specific (napus-rapa)	0.9808612	0.6806252	not scorable	not scorable	0.9834711	0.6385329
7754	Bn-A03-p6528914	A-genome specific (napus-rapa)	1	0.3859816	not scorable	not scorable	0.9917355	0.7362677
7755	Bn-A03-p654363	A-genome specific (rapa only)	not scorable	not scorable	not scorable	not scorable	0.9917355	0.6888669
7756	Bn-A03-p6556647	A-genome specific (napus-rapa)	0.9521531	0.280574	not scorable	not scorable	1	0.7634516
7757	Bn-A03-p657593	A-genome specific (napus-rapa)	0.8660287	0.8035964	not scorable	not scorable	0.6859504	0.7414052
7758	Bn-A03-p6576575	A-genome specific (napus-rapa)	0.9784689	0.7915924	not scorable	not scorable	0.9917355	0.8152432
7759	Bn-A03-p6577213	A-genome specific (napus-rapa)	0.9832536	0.8422651	not scorable	not scorable	1	0.7615284
7760	Bn-A03-p6578752	A-genome specific (napus-rapa)	0.9952153	0.8335571	not scorable	not scorable	1	0.8946469
7761	Bn-A03-p6583208	A-genome specific (napus-rapa)	0.9928229	0.8190148	not scorable	not scorable	1	0.8687926
7762	Bn-A03-p6585645	A-genome specific (napus-rapa)	0.9784689	0.8056495	not scorable	not scorable	1	0.7335952
7763	Bn-A03-p6586436	A-genome specific (napus-rapa)	0.9760765	0.7923179	not scorable	not scorable	1	0.8529477
7764	Bn-A03-p6590051	A-genome specific (napus-rapa)	0.9760765	0.8023167	not scorable	not scorable	1	0.7660844
7765	Bn-A03-p6591745	genome unspecific (all in)	0.9808612	0.7587294	1	0.7190649	1	0.6741855
7766	Bn-A03-p6602787	A-genome specific (rapa only)	not scorable	not scorable	not scorable	not scorable	1	0.6745787
7767	Bn-A03-p6602851	A-genome specific (napus-rapa)	0.9832536	0.5261881	not scorable	not scorable	0.9917355	0.7614741
7768	Bn-A03-p6636780	A-genome specific (napus-rapa)	0.9832536	0.8496484	not scorable	not scorable	0.9917355	0.7395672
7769	Bn-A03-p6646094	A-genome specific (napus-rapa)	0.9976076	0.532305	not scorable	not scorable	1	0.7602962
7770	Bn-A03-p6668218	napus specific	0.9904306	0.4715981	not scorable	not scorable	not scorable	not scorable
7771	Bn-A03-p6668259	A-genome specific (napus-rapa)	0.9784689	0.2491457	not scorable	not scorable	0.9917355	0.7262536
7772	Bn-A03-p6672024	A-genome specific (napus-rapa)	0.9928229	0.7527573	not scorable	not scorable	1	0.7811348
7773	Bn-A03-p6677819	A-genome specific (napus-rapa)	0.9832536	0.7128766	not scorable	not scorable	0.9917355	0.6868538
7774	Bn-A03-p6681953	A-genome specific (napus-rapa)	0.9784689	0.7798386	not scorable	not scorable	0.9834711	0.6523717
7775	Bn-A03-p6706586	A-genome specific (rapa only)	not scorable	not scorable	not scorable	not scorable	1	0.9188636
7776	Bn-A03-p6708217	A-genome specific (napus-rapa)	0.9760765	0.5172084	not scorable	not scorable	0.9917355	0.6682555
7777	Bn-A03-p6708475	A-genome specific (rapa only)	not scorable	not scorable	not scorable	not scorable	0.9917355	0.7638845
7778	Bn-A03-p6710409	A-genome specific (napus-rapa)	0.9808612	0.7290791	not scorable	not scorable	0.9834711	0.704776
7779	Bn-A03-p6710651	A-genome specific (napus-rapa)	0.9760765	0.8184522	not scorable	not scorable	0.9834711	0.7412955
7780	Bn-A03-p671279	A-genome specific (napus-rapa)	0.9880383	0.4935156	not scorable	not scorable	0.9917355	0.7924836
7781	Bn-A03-p6717546	A-genome specific (rapa only)	not scorable	not scorable	not scorable	not scorable	0.9917355	0.6960269
7782	Bn-A03-p6717785	A-genome specific (napus-rapa)	0.9952153	0.66571	not scorable	not scorable	0.9917355	0.7103029
7783	Bn-A03-p6744274	A-genome specific (napus-rapa)	0.9856459	0.8007197	not scorable	not scorable	1	0.8153307
7784	Bn-A03-p6744344	A-genome specific (napus-rapa)	0.9784689	0.8188414	not scorable	not scorable	1	0.8334719
7785	Bn-A03-p6760051	A-genome specific (napus-rapa)	0.9928229	0.74318	not scorable	not scorable	0.9752066	0.7032975

7786	Bn-A03-p6765024	A-genome specific (napus-rapa)	0.9904306	0.6783697	not scorable	not scorable	0.9917355	0.6882072
7787	Bn-A03-p6771876	A-genome specific (napus-rapa)	0.9928229	0.6878965	not scorable	not scorable	0.9834711	0.6901347
7788	Bn-A03-p6772326	A-genome specific (napus-rapa)	0.9808612	0.7773609	not scorable	not scorable	1	0.8035665
7789	Bn-A03-p6781156	A-genome specific (napus-rapa)	0.9832536	0.7223697	not scorable	not scorable	1	0.7799522
7790	Bn-A03-p6782910	A-genome specific (rapa only)	not scorable	not scorable	not scorable	not scorable	0.9752066	0.5520744
7791	Bn-A03-p6782961	A-genome specific (rapa only)	not scorable	not scorable	not scorable	not scorable	0.9917355	0.8328607
7792	Bn-A03-p6783685	A-genome specific (napus-rapa)	0.9521531	0.5803976	not scorable	not scorable	0.9752066	0.7419853
7793	Bn-A03-p6789784	A-genome specific (napus-rapa)	0.9952153	0.2798916	not scorable	not scorable	1	0.6751118
7794	Bn-A03-p6791851	A-genome specific (napus-rapa)	0.9808612	0.7624815	not scorable	not scorable	0.9834711	0.7057595
7795	Bn-A03-p6804536	A-genome specific (napus-rapa)	0.9808612	0.794342	not scorable	not scorable	0.9917355	0.8217803
7796	Bn-A03-p6804857	A-genome specific (napus-rapa)	0.9641148	0.8086624	not scorable	not scorable	1	0.7912036
7797	Bn-A03-p6807075	A-genome specific (napus-rapa)	0.9856459	0.715036	not scorable	not scorable	0.9256198	0.8330972
7798	Bn-A03-p6834343	A-genome specific (napus-rapa)	1	0.3140541	not scorable	not scorable	1	0.742772
7799	Bn-A03-p6845546	A-genome specific (rapa only)	not scorable	not scorable	not scorable	not scorable	0.9504132	0.6257551
7800	Bn-A03-p6849607	A-genome specific (napus-rapa)	0.9617225	0.4570783	not scorable	not scorable	0.9917355	0.8129342
7801	Bn-A03-p6888670	napus specific	0.9066986	0.8362017	not scorable	not scorable	not scorable	not scorable
7802	Bn-A03-p6895946	all not scorable	not scorable	not scorable	not scorable	not scorable	not scorable	not scorable
7803	Bn-A03-p6897034	A-genome specific (rapa only)	not scorable	not scorable	not scorable	not scorable	0.9834711	0.7672023
7804	Bn-A03-p6898220	A-genome specific (napus-rapa)	0.9928229	0.8671735	not scorable	not scorable	1	0.8630779
7805	Bn-A03-p6907743	A-genome specific (napus-rapa)	0.9880383	0.6319141	not scorable	not scorable	0.9917355	0.7497693
7806	Bn-A03-p6909237	A-genome specific (napus-rapa)	0.9593301	0.6266375	not scorable	not scorable	0.9834711	0.8052031
7807	Bn-A03-p6910492	A-genome specific (rapa only)	not scorable	not scorable	not scorable	not scorable	1	0.7955713
7808	Bn-A03-p6919500	A-genome specific (napus-rapa)	1	0.3282791	not scorable	not scorable	1	0.8660193
7809	Bn-A03-p6934438	A-genome specific (napus-rapa)	0.9856459	0.6963912	not scorable	not scorable	0.9917355	0.7082966
7810	Bn-A03-p6934751	A-genome specific (rapa only)	not scorable	not scorable	not scorable	not scorable	1	0.7009504
7811	Bn-A03-p6937615	A-genome specific (rapa only)	not scorable	not scorable	not scorable	not scorable	0.9752066	0.7622582
7812	Bn-A03-p6967307	A-genome specific (napus-rapa)	1	0.3870788	not scorable	not scorable	0.9834711	0.9253247
7813	Bn-A03-p6984742	A-genome specific (napus-rapa)	0.9808612	0.2919358	not scorable	not scorable	0.9834711	0.7307633
7814	Bn-A03-p6994294	A-genome specific (napus-rapa)	0.9904306	0.343385	not scorable	not scorable	0.9834711	0.6234235
7815	Bn-A03-p7007376	A-genome specific (napus-rapa)	0.9808612	0.8265488	not scorable	not scorable	0.9256198	0.8753491
7816	Bn-A03-p7007377	A-genome specific (napus-rapa)	0.9808612	0.8358084	not scorable	not scorable	0.9090909	0.8002372
7817	Bn-A03-p7011698	A-genome specific (rapa only)	not scorable	not scorable	not scorable	not scorable	0.9917355	0.692975
7818	Bn-A03-p7011746	A-genome specific (napus-rapa)	1	0.555096	not scorable	not scorable	1	0.5013781

7819	Bn-A03-p7012232	C-genome specific (oleracea only)	not scorable	not scorable	0.9767442	0.2309433	not scorable	not scorable
7820	Bn-A03-p7015536	A-genome specific (napus-rapa)	0.9928229	0.3272834	not scorable	not scorable	1	0.8231977
7821	Bn-A03-p7049396	A-genome specific (napus-rapa)	0.1650718	0.7937773	not scorable	not scorable	1	0.861973
7822	Bn-A03-p7054553	A-genome specific (napus-rapa)	0.9952153	0.414262	not scorable	not scorable	1	0.7273511
7823	Bn-A03-p7058001	A-genome specific (napus-rapa)	0.9952153	0.7145224	not scorable	not scorable	1	0.6893721
7824	Bn-A03-p7067563	A-genome specific (napus-rapa)	0.9952153	0.345826	not scorable	not scorable	0.9834711	0.6995296
7825	Bn-A03-p7074078	A-genome specific (rapa only)	not scorable	not scorable	not scorable	not scorable	0.9917355	0.7504783
7826	Bn-A03-p7075595	A-genome specific (napus-rapa)	0.9976076	0.5182511	not scorable	not scorable	1	0.6876625
7827	Bn-A03-p7079712	genome unspecific (all in)	0.9856459	0.5330578	0.9922481	0.6257297	0.9752066	0.632522
7828	Bn-A03-p7085943	A-genome specific (napus-rapa)	0.9928229	0.7964124	not scorable	not scorable	1	0.7073128
7829	Bn-A03-p708787	A-genome specific (napus-rapa)	0.9066986	0.826536	not scorable	not scorable	1	0.741662
7830	Bn-A03-p7088375	A-genome specific (napus-rapa)	0.9473684	0.6002557	not scorable	not scorable	1	0.8142083
7831	Bn-A03-p709005	A-genome specific (napus-rapa)	0.9186603	0.7810993	not scorable	not scorable	0.9338843	0.8616534
7832	Bn-A03-p7090745	A-genome specific (rapa only)	not scorable	not scorable	not scorable	not scorable	0.9917355	0.7459779
7833	Bn-A03-p709332	A-genome specific (rapa only)	not scorable	not scorable	not scorable	not scorable	0.9834711	0.7633051
7834	Bn-A03-p7094698	A-genome specific (napus-rapa)	0.9928229	0.8692414	not scorable	not scorable	1	0.7700555
7835	Bn-A03-p7110332	A-genome specific (napus-rapa)	0.9856459	0.8862039	not scorable	not scorable	0.9834711	0.8053902
7836	Bn-A03-p711408	A-genome specific (rapa only)	not scorable	not scorable	not scorable	not scorable	1	0.6884168
7837	Bn-A03-p712308	A-genome specific (napus-rapa)	1	0.8479601	not scorable	not scorable	1	0.7142797
7838	Bn-A03-p7157714	A-genome specific (napus-rapa)	0.9904306	0.474685	not scorable	not scorable	0.9834711	0.8012645
7839	Bn-A03-p7157782	A-genome specific (napus-rapa)	0.9928229	0.4641683	not scorable	not scorable	1	0.6855757
7840	Bn-A03-p7159916	A-genome specific (napus-rapa)	0.923445	0.4012836	not scorable	not scorable	1	0.7281422
7841	Bn-A03-p7167442	A-genome specific (napus-rapa)	0.9688995	0.7769015	not scorable	not scorable	1	0.7721235
7842	Bn-A03-p7177161	A-genome specific (rapa only)	not scorable	not scorable	not scorable	not scorable	0.9752066	0.7400542
7843	Bn-A03-p7177504	A-genome specific (napus-rapa)	0.9904306	0.7107428	not scorable	not scorable	0.9834711	0.7164374
7844	Bn-A03-p7177776	A-genome specific (napus-rapa)	0.9928229	0.7667168	not scorable	not scorable	1	0.8241898
7845	Bn-A03-p7178551	A-genome specific (napus-rapa)	0.9736842	0.4197768	not scorable	not scorable	0.9834711	0.5970958
7846	Bn-A03-p7178650	A-genome specific (napus-rapa)	0.9880383	0.7814372	not scorable	not scorable	0.9917355	0.7935368
7847	Bn-A03-p7178917	A-genome specific (napus-rapa)	0.9952153	0.6905993	not scorable	not scorable	0.9917355	0.6450099
7848	Bn-A03-p7180288	A-genome specific (napus-rapa)	0.9354067	0.6637082	not scorable	not scorable	0.9586777	0.6264626
7849	Bn-A03-p7186958	A-genome specific (napus-rapa)	0.9258373	0.8471591	not scorable	not scorable	0.9752066	0.7062156
7850	Bn-A03-p7187473	napus specific	0.930622	0.4184518	not scorable	not scorable	not scorable	not scorable
7851	Bn-A03-p7218582	A-genome specific (napus-rapa)	0.9210526	0.5867325	not scorable	not scorable	1	0.8618941

7852	Bn-A03-p7224854	A-genome specific (napus-rapa)	0.9449761	0.309217	not scorable	not scorable	0.9256198	0.5548381
7853	Bn-A03-p7228211	A-genome specific (napus-rapa)	0.9760765	0.7348103	not scorable	not scorable	0.9917355	0.8001279
7854	Bn-A03-p7228783	A-genome specific (napus-rapa)	0.9928229	0.8081543	not scorable	not scorable	1	0.6297082
7855	Bn-A03-p7234862	A-genome specific (napus-rapa)	0.9904306	0.7955063	not scorable	not scorable	0.9834711	0.8025838
7856	Bn-A03-p7234988	A-genome specific (napus-rapa)	0.9928229	0.2749851	not scorable	not scorable	0.9834711	0.6965818
7857	Bn-A03-p7235826	A-genome specific (napus-rapa)	0.9952153	0.7408643	not scorable	not scorable	1	0.7441986
7858	Bn-A03-p7236103	A-genome specific (napus-rapa)	0.9736842	0.7096326	not scorable	not scorable	0.9917355	0.7369606
7859	Bn-A03-p7239205	A-genome specific (napus-rapa)	0.9904306	0.8235979	not scorable	not scorable	0.9917355	0.7279946
7860	Bn-A03-p7239260	A-genome specific (napus-rapa)	0.9856459	0.8112721	not scorable	not scorable	0.9917355	0.719924
7861	Bn-A03-p7248970	A-genome specific (napus-rapa)	0.9904306	0.7920889	not scorable	not scorable	0.9586777	0.7223118
7862	Bn-A03-p7253298	A-genome specific (napus-rapa)	0.9904306	0.3306782	not scorable	not scorable	0.9834711	0.7065586
7863	Bn-A03-p726812	A-genome specific (napus-rapa)	0.9880383	0.3461441	not scorable	not scorable	1	0.5755651
7864	Bn-A03-p7281096	A-genome specific (napus-rapa)	0.9473684	0.4519103	not scorable	not scorable	0.9917355	0.8257833
7865	Bn-A03-p7284090	A-genome specific (napus-rapa)	0.9904306	0.7283169	not scorable	not scorable	1	0.7275584
7866	Bn-A03-p72933	A-genome specific (rapa only)	not scorable	not scorable	not scorable	not scorable	0.9834711	0.6942123
7867	Bn-A03-p7295912	A-genome specific (rapa only)	not scorable	not scorable	not scorable	not scorable	0.9917355	0.6802698
7868	Bn-A03-p731558	A-genome specific (napus-rapa)	0.8660287	0.3825243	not scorable	not scorable	0.9917355	0.7076031
7869	Bn-A03-p7319235	A-genome specific (napus-rapa)	0.9210526	0.5451888	not scorable	not scorable	0.9917355	0.7155814
7870	Bn-A03-p7326202	A-genome specific (napus-rapa)	0.9952153	0.6600214	not scorable	not scorable	0.9834711	0.6684946
7871	Bn-A03-p7331109	A-genome specific (napus-rapa)	0.9880383	0.7623122	not scorable	not scorable	0.9917355	0.7248695
7872	Bn-A03-p7331517	A-genome specific (napus-rapa)	0.9665072	0.5396728	not scorable	not scorable	0.9917355	0.7341067
7873	Bn-A03-p7356882	A-genome specific (napus-rapa)	0.9712918	0.9313904	not scorable	not scorable	0.9917355	0.7929051
7874	Bn-A03-p7357257	A-genome specific (napus-rapa)	0.638756	0.9248657	not scorable	not scorable	0.9669421	0.7596024
7875	Bn-A03-p7366616	A-genome specific (napus-rapa)	0.9976076	0.3706979	not scorable	not scorable	1	0.7443042
7876	Bn-A03-p7366783	A-genome specific (napus-rapa)	0.9712918	0.7896255	not scorable	not scorable	0.9834711	0.7296342
7877	Bn-A03-p7378514	A-genome specific (napus-rapa)	0.9593301	0.4882496	not scorable	not scorable	0.9834711	0.5874348
7878	Bn-A03-p7382887	A-genome specific (napus-rapa)	0.9521531	0.7130471	not scorable	not scorable	0.9834711	0.7210281
7879	Bn-A03-p7411648	A-genome specific (napus-rapa)	0.861244	0.7363209	not scorable	not scorable	0.9421487	0.7981498
7880	Bn-A03-p7420835	A-genome specific (napus-rapa)	0.6913875	0.7361311	not scorable	not scorable	0.9917355	0.6960069
7881	Bn-A03-p7421178	A-genome specific (napus-rapa)	0.6913875	0.8177536	not scorable	not scorable	1	0.7663583
7882	Bn-A03-p7428803	genome unspecific (all in)	0.9904306	0.3207513	0.8992248	0.2607104	1	0.7111256
7883	Bn-A03-p7434488	A-genome specific (napus-rapa)	0.9736842	0.7590962	not scorable	not scorable	0.9917355	0.7470349
7884	Bn-A03-p7434950	A-genome specific (napus-rapa)	0.9593301	0.7741464	not scorable	not scorable	0.9752066	0.770776

7885	Bn-A03-p7442744	A-genome specific (rapa only)	not scorable	not scorable	not scorable	not scorable	0.9917355	0.8087432
7886	Bn-A03-p7445206	A-genome specific (napus-rapa)	0.9688995	0.538993	not scorable	not scorable	1	0.7421709
7887	Bn-A03-p7458972	A-genome specific (napus-rapa)	0.9808612	0.2663221	not scorable	not scorable	0.9834711	0.8142844
7888	Bn-A03-p7460244	napus specific	0.8971292	0.7798976	not scorable	not scorable	not scorable	not scorable
7889	Bn-A03-p7467515	A-genome specific (napus-rapa)	0.9593301	0.4718303	not scorable	not scorable	1	0.7751601
7890	Bn-A03-p7478438	napus specific	0.9688995	0.8177654	not scorable	not scorable	not scorable	not scorable
7891	Bn-A03-p7480105	napus specific	0.9617225	0.7940962	not scorable	not scorable	not scorable	not scorable
7892	Bn-A03-p7482812	A-genome specific (napus-rapa)	0.9569378	0.3202595	not scorable	not scorable	0.9917355	0.6474978
7893	Bn-A03-p7485450	A-genome specific (rapa only)	not scorable	not scorable	not scorable	not scorable	1	0.6459444
7894	Bn-A03-p7491211	napus specific	0.9880383	0.7397998	not scorable	not scorable	not scorable	not scorable
7895	Bn-A03-p7493664	A-genome specific (napus-rapa)	0.9688995	0.7613888	not scorable	not scorable	0.9669421	0.7379045
7896	Bn-A03-p7500107	A-genome specific (napus-rapa)	0.9569378	0.4783834	not scorable	not scorable	0.9917355	0.7180824
7897	Bn-A03-p7501352	A-genome specific (napus-rapa)	0.9880383	0.8642383	not scorable	not scorable	0.9917355	0.7869141
7898	Bn-A03-p7505194	A-genome specific (napus-rapa)	0.4736842	0.7601249	not scorable	not scorable	0.9669421	0.7676057
7899	Bn-A03-p7506855	A-genome specific (napus-rapa)	0.9904306	0.5208967	not scorable	not scorable	0.9752066	0.809961
7900	Bn-A03-p7514571	A-genome specific (napus-rapa)	0.9497608	0.7155401	not scorable	not scorable	0.9586777	0.7044402
7901	Bn-A03-p7514678	A-genome specific (napus-rapa)	0.9904306	0.796658	not scorable	not scorable	1	0.8149011
7902	Bn-A03-p7514862	A-genome specific (napus-rapa)	0.9856459	0.6718059	not scorable	not scorable	1	0.8182911
7903	Bn-A03-p7521958	A-genome specific (napus-rapa)	0.9066986	0.6398383	not scorable	not scorable	0.9008265	0.6616946
7904	Bn-A03-p7528981	A-genome specific (napus-rapa)	0.9976076	0.5059961	not scorable	not scorable	1	0.6929526
7905	Bn-A03-p7532415	A-genome specific (napus-rapa)	0.9976076	0.448343	not scorable	not scorable	0.9752066	0.6053073
7906	Bn-A03-p7543362	genome unspecific (all in)	0.9808612	0.341412	1	0.6602734	0.9834711	0.6805757
7907	Bn-A03-p755100	A-genome specific (rapa only)	not scorable	not scorable	not scorable	not scorable	1	0.9002686
7908	Bn-A03-p7551518	A-genome specific (napus-rapa)	0.9784689	0.3172227	not scorable	not scorable	1	0.6937545
7909	Bn-A03-p7555264	A-genome specific (napus-rapa)	0.9856459	0.8141341	not scorable	not scorable	0.9917355	0.7917782
7910	Bn-A03-p7571364	A-genome specific (napus-rapa)	0.9832536	0.7520587	not scorable	not scorable	1	0.8258477
7911	Bn-A03-p7572109	A-genome specific (napus-rapa)	0.9880383	0.7813368	not scorable	not scorable	0.9752066	0.7855919
7912	Bn-A03-p7572128	A-genome specific (napus-rapa)	0.9521531	0.7166634	not scorable	not scorable	0.9256198	0.6872754
7913	Bn-A03-p7575620	A-genome specific (napus-rapa)	0.9952153	0.3622191	not scorable	not scorable	0.9834711	0.686775
7914	Bn-A03-p757592	A-genome specific (napus-rapa)	0.6196172	0.7273234	not scorable	not scorable	0.9586777	0.7081448
7915	Bn-A03-p7581383	A-genome specific (napus-rapa)	0.9258373	0.7431417	not scorable	not scorable	0.892562	0.7974854
7916	Bn-A03-p7582235	A-genome specific (napus-rapa)	0.9712918	0.7577997	not scorable	not scorable	0.9917355	0.7223673
7917	Bn-A03-p7583703	A-genome specific (napus-rapa)	0.9784689	0.331875	not scorable	not scorable	1	0.7438364

7918	Bn-A03-p7588123	A-genome specific (napus-rapa)	0.9688995	0.7998334	not scorable	not scorable	0.9586777	0.7161086
7919	Bn-A03-p7595626	A-genome specific (napus-rapa)	0.9880383	0.3507471	not scorable	not scorable	0.9752066	0.7036184
7920	Bn-A03-p7597598	A-genome specific (napus-rapa)	0.9473684	0.774967	not scorable	not scorable	0.9834711	0.8072164
7921	Bn-A03-p7613494	A-genome specific (napus-rapa)	0.7870814	0.7882515	not scorable	not scorable	0.8842975	0.8468915
7922	Bn-A03-p7614596	A-genome specific (napus-rapa)	0.9832536	0.7469115	not scorable	not scorable	1	0.7945759
7923	Bn-A03-p7618555	A-genome specific (napus-rapa)	0.9856459	0.7852318	not scorable	not scorable	0.9752066	0.6915144
7924	Bn-A03-p7627590	A-genome specific (napus-rapa)	0.9880383	0.4902492	not scorable	not scorable	1	0.7462053
7925	Bn-A03-p7628749	A-genome specific (napus-rapa)	0.9808612	0.3179613	not scorable	not scorable	1	0.7790431
7926	Bn-A03-p7628905	A-genome specific (napus-rapa)	0.9545454	0.6884062	not scorable	not scorable	1	0.7683609
7927	Bn-A03-p7629219	A-genome specific (napus-rapa)	0.9066986	0.6685717	not scorable	not scorable	0.9669421	0.7053821
7928	Bn-A03-p7630376	A-genome specific (napus-rapa)	0.930622	0.5820872	not scorable	not scorable	0.9834711	0.7606472
7929	Bn-A03-p7630628	A-genome specific (napus-rapa)	0.9784689	0.4727942	not scorable	not scorable	0.9917355	0.6558676
7930	Bn-A03-p7631083	A-genome specific (napus-rapa)	0.9736842	0.7604119	not scorable	not scorable	0.8760331	0.7134827
7931	Bn-A03-p7631177	A-genome specific (napus-rapa)	0.9952153	0.5393506	not scorable	not scorable	1	0.8293539
7932	Bn-A03-p7641673	A-genome specific (napus-rapa)	0.9712918	0.7624017	not scorable	not scorable	0.9917355	0.8140582
7933	Bn-A03-p7642519	A-genome specific (napus-rapa)	0.9641148	0.7805383	not scorable	not scorable	1	0.7829676
7934	Bn-A03-p7642692	A-genome specific (napus-rapa)	0.9688995	0.7818435	not scorable	not scorable	0.9752066	0.7955495
7935	Bn-A03-p764274	genome unspecific (oleracea-rapa)	not scorable	not scorable	0.8217054	0.3564879	1	0.7281514
7936	Bn-A03-p7643795	genome unspecific (all in)	0.9641148	0.554243	1	0.5639431	1	0.730028
7937	Bn-A03-p765077	A-genome specific (napus-rapa)	0.9162679	0.8465989	not scorable	not scorable	1	0.8448888
7938	Bn-A03-p7652326	A-genome specific (napus-rapa)	0.9808612	0.8162246	not scorable	not scorable	0.9917355	0.746737
7939	Bn-A03-p766322	A-genome specific (napus-rapa)	0.8947368	0.7363375	not scorable	not scorable	0.9834711	0.6976467
7940	Bn-A03-p7672403	A-genome specific (napus-rapa)	0.9784689	0.7421128	not scorable	not scorable	0.9752066	0.8550293
7941	Bn-A03-p7675340	genome unspecific (all in)	0.9688995	0.2801807	1	0.333893	0.9917355	0.6773373
7942	Bn-A03-p7678159	A-genome specific (napus-rapa)	0.9617225	0.3958095	not scorable	not scorable	0.9256198	0.7303997
7943	Bn-A03-p7685279	A-genome specific (napus-rapa)	0.9928229	0.2687158	not scorable	not scorable	0.9917355	0.6681796
7944	Bn-A03-p7688578	A-genome specific (napus-rapa)	0.9090909	0.4068252	not scorable	not scorable	0.9917355	0.7214007
7945	Bn-A03-p7692467	all not scorable	not scorable	not scorable	not scorable	not scorable	not scorable	not scorable
7946	Bn-A03-p77099	genome unspecific (oleracea-rapa)	not scorable	not scorable	1	0.7718685	0.9917355	0.7827933
7947	Bn-A03-p7738300	A-genome specific (napus-rapa)	0.9784689	0.7533256	not scorable	not scorable	0.9834711	0.6728689
7948	Bn-A03-p7757095	A-genome specific (rapa only)	not scorable	not scorable	not scorable	not scorable	0.9752066	0.6170102
7949	Bn-A03-p7760102	A-genome specific (napus-rapa)	0.9832536	0.8584554	not scorable	not scorable	0.9669421	0.8288376
7950	Bn-A03-p7761061	A-genome specific (rapa only)	not scorable	not scorable	not scorable	not scorable	0.9752066	0.6404971

7951	Bn-A03-p7766116	A-genome specific (napus-rapa)	0.9569378	0.2146632	not scorable	not scorable	1	0.6995631
7952	Bn-A03-p7773812	A-genome specific (napus-rapa)	0.9688995	0.7613217	not scorable	not scorable	0.9586777	0.8175377
7953	Bn-A03-p7788800	A-genome specific (napus-rapa)	0.9688995	0.7726347	not scorable	not scorable	0.9917355	0.7859753
7954	Bn-A03-p7789818	A-genome specific (napus-rapa)	0.9665072	0.6634905	not scorable	not scorable	0.9917355	0.8148888
7955	Bn-A03-p7793076	genome unspecific (all in)	0.9952153	0.2924091	1	0.5418264	0.9752066	0.6456934
7956	Bn-A03-p7799483	A-genome specific (napus-rapa)	0.9952153	0.3850054	not scorable	not scorable	0.9834711	0.9005287
7957	Bn-A03-p7806058	genome unspecific (all in)	0.9665072	0.3719357	1	0.8925079	0.9917355	0.8772941
7958	Bn-A03-p7814324	A-genome specific (napus-rapa)	0.84689	0.7953589	not scorable	not scorable	1	0.7412948
7959	Bn-A03-p7818081	A-genome specific (napus-rapa)	0.8708134	0.6082478	not scorable	not scorable	1	0.6643581
7960	Bn-A03-p7818503	A-genome specific (napus-rapa)	0.9904306	0.7104915	not scorable	not scorable	0.9834711	0.718382
7961	Bn-A03-p7823490	A-genome specific (napus-rapa)	0.9712918	0.3136446	not scorable	not scorable	0.9917355	0.7073662
7962	Bn-A03-p7835836	A-genome specific (napus-rapa)	0.9832536	0.643238	not scorable	not scorable	1	0.7027575
7963	Bn-A03-p7838070	A-genome specific (napus-rapa)	0.9928229	0.3629685	not scorable	not scorable	0.9669421	0.8379777
7964	Bn-A03-p7840758	A-genome specific (napus-rapa)	0.9904306	0.7863585	not scorable	not scorable	0.9834711	0.7848645
7965	Bn-A03-p7851577	A-genome specific (napus-rapa)	0.9880383	0.5297433	not scorable	not scorable	0.9834711	0.6410352
7966	Bn-A03-p78559	A-genome specific (rapa only)	not scorable	not scorable	not scorable	not scorable	0.9834711	0.7763156
7967	Bn-A03-p7857447	A-genome specific (napus-rapa)	0.9928229	0.3716141	not scorable	not scorable	0.9586777	0.8426623
7968	Bn-A03-p7870307	A-genome specific (napus-rapa)	0.9880383	0.7293568	not scorable	not scorable	1	0.7486603
7969	Bn-A03-p7870541	A-genome specific (napus-rapa)	0.9832536	0.7231628	not scorable	not scorable	0.9834711	0.7117054
7970	Bn-A03-p7873426	A-genome specific (napus-rapa)	0.9976076	0.5273734	not scorable	not scorable	1	0.7895192
7971	Bn-A03-p7878888	A-genome specific (napus-rapa)	0.9928229	0.7995591	not scorable	not scorable	0.9752066	0.7207244
7972	Bn-A03-p7883846	A-genome specific (napus-rapa)	0.9497608	0.5426535	not scorable	not scorable	0.9917355	0.7397
7973	Bn-A03-p7887578	A-genome specific (napus-rapa)	0.8923445	0.717517	not scorable	not scorable	0.9834711	0.7958792
7974	Bn-A03-p7897822	A-genome specific (napus-rapa)	0.9784689	0.4297853	not scorable	not scorable	0.9586777	0.662388
7975	Bn-A03-p7902457	A-genome specific (napus-rapa)	0.9928229	0.8593473	not scorable	not scorable	1	0.7110505
7976	Bn-A03-p7919195	A-genome specific (napus-rapa)	0.9880383	0.7775519	not scorable	not scorable	0.9586777	0.7437963
7977	Bn-A03-p7919322	A-genome specific (napus-rapa)	0.9856459	0.7459603	not scorable	not scorable	0.9917355	0.7563326
7978	Bn-A03-p7920650	A-genome specific (napus-rapa)	0.9976076	0.3032302	not scorable	not scorable	0.9834711	0.7916691
7979	Bn-A03-p7939679	A-genome specific (napus-rapa)	0.9832536	0.8767146	not scorable	not scorable	1	0.7861291
7980	Bn-A03-p7940082	A-genome specific (napus-rapa)	0.9856459	0.8793382	not scorable	not scorable	0.9917355	0.7218574
7981	Bn-A03-p7943450	A-genome specific (napus-rapa)	0.9856459	0.8901269	not scorable	not scorable	1	0.8421362
7982	Bn-A03-p7946505	A-genome specific (napus-rapa)	0.9904306	0.9012498	not scorable	not scorable	0.9504132	0.7703752
7983	Bn-A03-p7949241	all not scorable	not scorable	not scorable	not scorable	not scorable	not scorable	not scorable

7984	Bn-A03-p7961581	A-genome specific (napus-rapa)	0.9976076	0.423016	not scorable	not scorable	1	0.746624
7985	Bn-A03-p7963101	A-genome specific (rapa only)	not scorable	not scorable	not scorable	not scorable	0.9586777	0.6969824
7986	Bn-A03-p7964361	A-genome specific (napus-rapa)	0.9856459	0.8757187	not scorable	not scorable	0.9834711	0.7637922
7987	Bn-A03-p7964519	A-genome specific (napus-rapa)	0.9928229	0.9042242	not scorable	not scorable	0.9917355	0.7245597
7988	Bn-A03-p7964789	A-genome specific (napus-rapa)	0.9904306	0.8629914	not scorable	not scorable	0.9917355	0.7236944
7989	Bn-A03-p7965237	A-genome specific (napus-rapa)	0.9880383	0.6358898	not scorable	not scorable	0.9752066	0.7363251
7990	Bn-A03-p7966805	A-genome specific (napus-rapa)	0.9904306	0.727407	not scorable	not scorable	0.9917355	0.7719597
7991	Bn-A03-p7969685	A-genome specific (napus-rapa)	0.9928229	0.7756539	not scorable	not scorable	0.9834711	0.7815326
7992	Bn-A03-p7974602	A-genome specific (napus-rapa)	0.9473684	0.5788326	not scorable	not scorable	0.9917355	0.7771342
7993	Bn-A03-p7983071	A-genome specific (rapa only)	not scorable	not scorable	not scorable	not scorable	1	0.8145293
7994	Bn-A03-p7985158	A-genome specific (rapa only)	not scorable	not scorable	not scorable	not scorable	1	0.6209045
7995	Bn-A03-p7988522	A-genome specific (napus-rapa)	0.9617225	0.812738	not scorable	not scorable	0.9586777	0.7288146
7996	Bn-A03-p7988667	A-genome specific (napus-rapa)	0.9449761	0.8378785	not scorable	not scorable	0.9752066	0.7220598
7997	Bn-A03-p7988820	A-genome specific (napus-rapa)	0.9593301	0.8512008	not scorable	not scorable	0.9834711	0.6926226
7998	Bn-A03-p8008867	A-genome specific (napus-rapa)	0.9928229	0.8100467	not scorable	not scorable	1	0.722063
7999	Bn-A03-p8022406	A-genome specific (napus-rapa)	0.9928229	0.8666855	not scorable	not scorable	1	0.7135404
8000	Bn-A03-p8032849	A-genome specific (napus-rapa)	0.9688995	0.7248591	not scorable	not scorable	0.9752066	0.7370734
8001	Bn-A03-p8033095	A-genome specific (napus-rapa)	0.9521531	0.7423527	not scorable	not scorable	0.9917355	0.8050555
8002	Bn-A03-p8038504	A-genome specific (rapa only)	not scorable	not scorable	not scorable	not scorable	0.9752066	0.7011166
8003	Bn-A03-p8039401	A-genome specific (napus-rapa)	0.9880383	0.4662207	not scorable	not scorable	1	0.6702687
8004	Bn-A03-p8043620	A-genome specific (napus-rapa)	0.9928229	0.6037242	not scorable	not scorable	1	0.7454115
8005	Bn-A03-p8043983	A-genome specific (napus-rapa)	0.9545454	0.5438173	not scorable	not scorable	0.9917355	0.7818972
8006	Bn-A03-p8059035	A-genome specific (napus-rapa)	0.9832536	0.6072763	not scorable	not scorable	1	0.8532906
8007	Bn-A03-p8070883	napus specific	0.9976076	0.3785277	not scorable	not scorable	not scorable	not scorable
8008	Bn-A03-p8075228	A-genome specific (rapa only)	not scorable	not scorable	not scorable	not scorable	0.9752066	0.7116282
8009	Bn-A03-p8080680	A-genome specific (napus-rapa)	1	0.8165222	not scorable	not scorable	1	0.9272014
8010	Bn-A03-p8083083	A-genome specific (rapa only)	not scorable	not scorable	not scorable	not scorable	0.9173554	0.8953075
8011	Bn-A03-p8084586	A-genome specific (rapa only)	not scorable	not scorable	not scorable	not scorable	0.9504132	0.7922245
8012	Bn-A03-p8095796	A-genome specific (napus-rapa)	0.8923445	0.630339	not scorable	not scorable	0.9917355	0.6721433
8013	Bn-A03-p8103457	A-genome specific (napus-rapa)	0.9688995	0.860061	not scorable	not scorable	0.9917355	0.6951423
8014	Bn-A03-p8109045	A-genome specific (napus-rapa)	0.9976076	0.5465681	not scorable	not scorable	1	0.7277114
8015	Bn-A03-p8112016	napus specific	0.9066986	0.7465459	not scorable	not scorable	not scorable	not scorable
8016	Bn-A03-p8122222	A-genome specific (napus-rapa)	0.9904306	0.6508276	not scorable	not scorable	0.9752066	0.6908095

8017	Bn-A03-p8133096	A-genome specific (napus-rapa)	0.9593301	0.7272726	not scorable	not scorable	1	0.7253703
8018	Bn-A03-p8148926	A-genome specific (rapa only)	not scorable	not scorable	not scorable	not scorable	1	0.6952136
8019	Bn-A03-p8152842	A-genome specific (napus-rapa)	0.9808612	0.562276	not scorable	not scorable	0.9834711	0.7199671
8020	Bn-A03-p8158265	genome unspecific (all in)	0.9808612	0.3333014	0.9844961	0.628476	1	0.6688465
8021	Bn-A03-p8169179	A-genome specific (napus-rapa)	0.9736842	0.4531946	not scorable	not scorable	1	0.7482402
8022	Bn-A03-p8170734	A-genome specific (napus-rapa)	0.9832536	0.8113705	not scorable	not scorable	0.9917355	0.781993
8023	Bn-A03-p8171211	A-genome specific (napus-rapa)	0.9832536	0.6838223	not scorable	not scorable	0.9917355	0.6747683
8024	Bn-A03-p8171248	A-genome specific (napus-rapa)	0.930622	0.6925251	not scorable	not scorable	1	0.7072228
8025	Bn-A03-p8177644	A-genome specific (napus-rapa)	0.9665072	0.6665681	not scorable	not scorable	0.9834711	0.7905504
8026	Bn-A03-p8177695	A-genome specific (rapa only)	not scorable	not scorable	not scorable	not scorable	1	0.839259
8027	Bn-A03-p8181125	A-genome specific (napus-rapa)	0.9880383	0.8252659	not scorable	not scorable	1	0.8376619
8028	Bn-A03-p8181187	A-genome specific (napus-rapa)	0.9880383	0.8416769	not scorable	not scorable	1	0.7709971
8029	Bn-A03-p8181778	A-genome specific (napus-rapa)	0.9880383	0.9206545	not scorable	not scorable	1	0.8655627
8030	Bn-A03-p8190847	A-genome specific (napus-rapa)	0.9593301	0.5388932	not scorable	not scorable	1	0.781415
8031	Bn-A03-p8204479	napus specific	0.9665072	0.3874211	not scorable	not scorable	not scorable	not scorable
8032	Bn-A03-p8206961	A-genome specific (napus-rapa)	0.9832536	0.7390412	not scorable	not scorable	0.9834711	0.7284867
8033	Bn-A03-p8217276	A-genome specific (napus-rapa)	0.9497608	0.8225138	not scorable	not scorable	0.9338843	0.7087269
8034	Bn-A03-p8217358	A-genome specific (napus-rapa)	0.9593301	0.7060575	not scorable	not scorable	0.9421487	0.7454649
8035	Bn-A03-p8218946	A-genome specific (napus-rapa)	0.9808612	0.4847307	not scorable	not scorable	0.9834711	0.6894066
8036	Bn-A03-p8260551	genome unspecific (all in)	0.9904306	0.5266603	0.8139535	0.8230354	0.9917355	0.7760841
8037	Bn-A03-p8279849	A-genome specific (napus-rapa)	0.9928229	0.2759137	not scorable	not scorable	0.9669421	0.5983175
8038	Bn-A03-p8282545	A-genome specific (napus-rapa)	0.9760765	0.6716564	not scorable	not scorable	0.9669421	0.7433973
8039	Bn-A03-p8285385	A-genome specific (napus-rapa)	0.9736842	0.3082609	not scorable	not scorable	0.9917355	0.6946752
8040	Bn-A03-p8288895	A-genome specific (napus-rapa)	0.9760765	0.3961723	not scorable	not scorable	0.9752066	0.7154225
8041	Bn-A03-p8298067	A-genome specific (napus-rapa)	0.9904306	0.5942143	not scorable	not scorable	0.9917355	0.819873
8042	Bn-A03-p8298336	A-genome specific (napus-rapa)	0.9856459	0.8289244	not scorable	not scorable	0.9834711	0.7844557
8043	Bn-A03-p830116	A-genome specific (napus-rapa)	0.9114832	0.7808228	not scorable	not scorable	0.9752066	0.8012742
8044	Bn-A03-p830487	A-genome specific (napus-rapa)	0.9665072	0.7296516	not scorable	not scorable	0.9917355	0.8125748
8045	Bn-A03-p8308938	A-genome specific (napus-rapa)	0.9880383	0.8061008	not scorable	not scorable	1	0.8952507
8046	Bn-A03-p8314005	A-genome specific (napus-rapa)	0.9928229	0.7073323	not scorable	not scorable	1	0.8135551
8047	Bn-A03-p831433	A-genome specific (napus-rapa)	0.9736842	0.3172887	not scorable	not scorable	1	0.7266697
8048	Bn-A03-p832473	A-genome specific (napus-rapa)	0.930622	0.7404763	not scorable	not scorable	0.9834711	0.7968811
8049	Bn-A03-p8329291	A-genome specific (napus-rapa)	1	0.288366	not scorable	not scorable	1	0.7538685

8050	Bn-A03-p8333536	A-genome specific (napus-rapa)	0.8851675	0.8173244	not scorable	not scorable	0.9917355	0.8176495
8051	Bn-A03-p8337135	A-genome specific (napus-rapa)	0.9569378	0.7496994	not scorable	not scorable	0.9917355	0.7231776
8052	Bn-A03-p8337378	A-genome specific (napus-rapa)	1	0.2480136	not scorable	not scorable	0.9917355	0.8207901
8053	Bn-A03-p834379	A-genome specific (napus-rapa)	0.8947368	0.7469566	not scorable	not scorable	1	0.8073053
8054	Bn-A03-p8347370	A-genome specific (napus-rapa)	1	0.6323521	not scorable	not scorable	0.9834711	0.8290746
8055	Bn-A03-p835162	A-genome specific (napus-rapa)	0.9090909	0.8144715	not scorable	not scorable	0.9834711	0.7926462
8056	Bn-A03-p8359092	genome unspecific (all in)	0.9832536	0.4480899	0.9922481	0.7010086	1	0.6915832
8057	Bn-A03-p835987	A-genome specific (rapa only)	not scorable	not scorable	not scorable	not scorable	0.9917355	0.7512562
8058	Bn-A03-p8360894	A-genome specific (napus-rapa)	0.7703349	0.7254874	not scorable	not scorable	1	0.6452205
8059	Bn-A03-p836873	A-genome specific (napus-rapa)	0.9330143	0.7468809	not scorable	not scorable	1	0.750535
8060	Bn-A03-p8372559	A-genome specific (rapa only)	not scorable	not scorable	not scorable	not scorable	1	0.6499091
8061	Bn-A03-p8380550	A-genome specific (napus-rapa)	0.9210526	0.7673404	not scorable	not scorable	0.9586777	0.634596
8062	Bn-A03-p8381012	A-genome specific (napus-rapa)	0.9569378	0.8029826	not scorable	not scorable	1	0.8129317
8063	Bn-A03-p8394184	A-genome specific (napus-rapa)	0.9880383	0.8539055	not scorable	not scorable	0.9917355	0.8112677
8064	Bn-A03-p8395621	A-genome specific (napus-rapa)	0.9760765	0.7593513	not scorable	not scorable	1	0.7997159
8065	Bn-A03-p8396525	A-genome specific (napus-rapa)	0.9425837	0.503436	not scorable	not scorable	0.9834711	0.6872858
8066	Bn-A03-p8398790	A-genome specific (napus-rapa)	0.9880383	0.8186218	not scorable	not scorable	1	0.7011481
8067	Bn-A03-p8403705	A-genome specific (rapa only)	not scorable	not scorable	not scorable	not scorable	0.9669421	0.7536424
8068	Bn-A03-p8406930	A-genome specific (napus-rapa)	0.9282297	0.8363864	not scorable	not scorable	0.9834711	0.7729632
8069	Bn-A03-p8407999	A-genome specific (napus-rapa)	0.9856459	0.7204257	not scorable	not scorable	0.9752066	0.7485912
8070	Bn-A03-p8408815	A-genome specific (napus-rapa)	0.9760765	0.4676016	not scorable	not scorable	0.9586777	0.5687731
8071	Bn-A03-p8420805	A-genome specific (napus-rapa)	0.7177033	0.7934078	not scorable	not scorable	0.9917355	0.9205657
8072	Bn-A03-p8439865	A-genome specific (napus-rapa)	0.9952153	0.862923	not scorable	not scorable	0.9917355	0.7611737
8073	Bn-A03-p8454293	A-genome specific (rapa only)	not scorable	not scorable	not scorable	not scorable	0.9917355	0.7845222
8074	Bn-A03-p8467104	A-genome specific (napus-rapa)	0.9904306	0.6917644	not scorable	not scorable	0.9917355	0.6659946
8075	Bn-A03-p8475614	genome unspecific (all in)	0.9545454	0.3609864	0.9844961	0.8061702	1	0.8762643
8076	Bn-A03-p8479803	genome unspecific (all in)	0.9928229	0.3155419	1	0.762161	1	0.9095522
8077	Bn-A03-p8480669	A-genome specific (napus-rapa)	0.923445	0.4297373	not scorable	not scorable	0.9834711	0.6956323
8078	Bn-A03-p8483706	A-genome specific (napus-rapa)	0.9880383	0.8093553	not scorable	not scorable	0.9834711	0.7568502
8079	Bn-A03-p8497543	A-genome specific (napus-rapa)	0.9856459	0.7652116	not scorable	not scorable	0.9917355	0.6539943
8080	Bn-A03-p8554739	A-genome specific (napus-rapa)	0.9736842	0.7544846	not scorable	not scorable	0.9917355	0.7149889
8081	Bn-A03-p8559617	A-genome specific (napus-rapa)	0.9473684	0.7055877	not scorable	not scorable	0.9917355	0.6857461
8082	Bn-A03-p8559794	A-genome specific (napus-rapa)	0.9808612	0.4732807	not scorable	not scorable	1	0.6763545

8083	Bn-A03-p8585196	A-genome specific (rapa only)	not scorable	not scorable	not scorable	not scorable	0.9834711	0.765916
8084	Bn-A03-p858910	A-genome specific (napus-rapa)	0.9976076	0.8514487	not scorable	not scorable	1	0.8497714
8085	Bn-A03-p8620584	A-genome specific (napus-rapa)	0.9832536	0.4373923	not scorable	not scorable	1	0.9005054
8086	Bn-A03-p8629992	A-genome specific (rapa only)	not scorable	not scorable	not scorable	not scorable	1	0.8289879
8087	Bn-A03-p8631833	A-genome specific (napus-rapa)	0.9880383	0.8025541	not scorable	not scorable	0.9917355	0.6806947
8088	Bn-A03-p8632710	A-genome specific (rapa only)	not scorable	not scorable	not scorable	not scorable	0.9338843	0.9205657
8089	Bn-A03-p8637914	A-genome specific (napus-rapa)	0.9665072	0.7387698	not scorable	not scorable	0.9752066	0.7786468
8090	Bn-A03-p8638217	A-genome specific (napus-rapa)	0.9736842	0.7155128	not scorable	not scorable	0.9917355	0.7819756
8091	Bn-A03-p8642052	A-genome specific (rapa only)	not scorable	not scorable	not scorable	not scorable	0.9752066	0.7835432
8092	Bn-A03-p8645654	A-genome specific (napus-rapa)	0.9545454	0.6775714	not scorable	not scorable	0.9834711	0.7561993
8093	Bn-A03-p8654614	A-genome specific (napus-rapa)	0.9736842	0.5815858	not scorable	not scorable	0.9752066	0.761075
8094	Bn-A03-p8663803	A-genome specific (napus-rapa)	0.9880383	0.8783022	not scorable	not scorable	0.9917355	0.8349034
8095	Bn-A03-p8665794	A-genome specific (napus-rapa)	0.9808612	0.8101007	not scorable	not scorable	0.9917355	0.6446292
8096	Bn-A03-p8671468	A-genome specific (napus-rapa)	0.9976076	0.4231066	not scorable	not scorable	0.9752066	0.7455958
8097	Bn-A03-p8671697	A-genome specific (napus-rapa)	0.9808612	0.5885524	not scorable	not scorable	0.9834711	0.7932267
8098	Bn-A03-p8681690	A-genome specific (napus-rapa)	0.9760765	0.369589	not scorable	not scorable	0.9917355	0.7109153
8099	Bn-A03-p8733974	genome unspecific (all in)	0.9712918	0.2463539	0.9922481	0.6779475	0.9917355	0.6373128
8100	Bn-A03-p8735039	A-genome specific (napus-rapa)	0.9880383	0.4804429	not scorable	not scorable	0.9586777	0.6910145
8101	Bn-A03-p8741978	A-genome specific (napus-rapa)	0.9832536	0.7843004	not scorable	not scorable	0.9834711	0.7230071
8102	Bn-A03-p8741996	A-genome specific (napus-rapa)	0.9952153	0.5569785	not scorable	not scorable	0.9834711	0.7475901
8103	Bn-A03-p8745839	genome unspecific (all in)	0.9425837	0.4220534	0.9612403	0.7897397	0.9090909	0.8268548
8104	Bn-A03-p8756590	A-genome specific (napus-rapa)	0.9880383	0.911702	not scorable	not scorable	0.9917355	0.7954699
8105	Bn-A03-p8764251	A-genome specific (napus-rapa)	0.9856459	0.781794	not scorable	not scorable	0.9834711	0.8124008
8106	Bn-A03-p8764481	A-genome specific (napus-rapa)	0.9880383	0.8000137	not scorable	not scorable	0.9752066	0.7511868
8107	Bn-A03-p8769576	A-genome specific (napus-rapa)	0.9808612	0.7925531	not scorable	not scorable	0.9752066	0.7328518
8108	Bn-A03-p8802360	A-genome specific (napus-rapa)	0.9880383	0.830029	not scorable	not scorable	0.9834711	0.7302321
8109	Bn-A03-p8805441	A-genome specific (napus-rapa)	0.9928229	0.7682233	not scorable	not scorable	0.9917355	0.7076876
8110	Bn-A03-p8812807	A-genome specific (napus-rapa)	0.9928229	0.7059764	not scorable	not scorable	0.9669421	0.6588802
8111	Bn-A03-p8822734	A-genome specific (napus-rapa)	0.9545454	0.5315421	not scorable	not scorable	1	0.802894
8112	Bn-A03-p8823042	all not scorable	not scorable	not scorable	not scorable	not scorable	not scorable	not scorable
8113	Bn-A03-p8833016	A-genome specific (napus-rapa)	0.9856459	0.7759858	not scorable	not scorable	0.9834711	0.6910642
8114	Bn-A03-p8836751	A-genome specific (napus-rapa)	0.937799	0.3300055	not scorable	not scorable	0.9752066	0.7521774
8115	Bn-A03-p8842546	A-genome specific (napus-rapa)	0.9976076	0.4330713	not scorable	not scorable	1	0.794439

8116	Bn-A03-p8850004	A-genome specific (napus-rapa)	0.930622	0.6505194	not scorable	not scorable	1	0.8976545
8117	Bn-A03-p8850377	A-genome specific (napus-rapa)	0.9904306	0.8256539	not scorable	not scorable	1	0.8529564
8118	Bn-A03-p8851043	A-genome specific (napus-rapa)	0.9258373	0.5602669	not scorable	not scorable	0.9504132	0.7088173
8119	Bn-A03-p8851142	A-genome specific (napus-rapa)	0.9856459	0.7756077	not scorable	not scorable	0.9917355	0.7463047
8120	Bn-A03-p8860638	A-genome specific (napus-rapa)	0.9712918	0.7110343	not scorable	not scorable	0.9834711	0.7140822
8121	Bn-A03-p8861495	A-genome specific (rapa only)	not scorable	not scorable	not scorable	not scorable	0.9834711	0.8360152
8122	Bn-A03-p8881377	A-genome specific (napus-rapa)	0.9808612	0.6276909	not scorable	not scorable	0.9669421	0.7762519
8123	Bn-A03-p8881585	A-genome specific (napus-rapa)	0.9856459	0.7473072	not scorable	not scorable	0.9834711	0.7268212
8124	Bn-A03-p8883782	A-genome specific (napus-rapa)	0.9928229	0.8087612	not scorable	not scorable	0.9917355	0.839403
8125	Bn-A03-p8883841	A-genome specific (rapa only)	not scorable	not scorable	not scorable	not scorable	0.9752066	0.7288934
8126	Bn-A03-p8897362	A-genome specific (napus-rapa)	0.9736842	0.725117	not scorable	not scorable	0.9917355	0.7080775
8127	Bn-A03-p8906751	A-genome specific (napus-rapa)	0.9880383	0.7912863	not scorable	not scorable	0.9917355	0.7019222
8128	Bn-A03-p8915240	A-genome specific (napus-rapa)	0.9904306	0.7710129	not scorable	not scorable	0.9917355	0.761231
8129	Bn-A03-p8924852	A-genome specific (napus-rapa)	0.9712918	0.5209178	not scorable	not scorable	0.9669421	0.7831932
8130	Bn-A03-p8952053	A-genome specific (napus-rapa)	0.9760765	0.3549931	not scorable	not scorable	0.9752066	0.7886239
8131	Bn-A03-p8956355	A-genome specific (napus-rapa)	0.9832536	0.8501896	not scorable	not scorable	0.9669421	0.7751827
8132	Bn-A03-p8957734	A-genome specific (napus-rapa)	0.9497608	0.7021428	not scorable	not scorable	1	0.756869
8133	Bn-A03-p8960332	A-genome specific (napus-rapa)	0.9856459	0.5009062	not scorable	not scorable	0.9917355	0.753154
8134	Bn-A03-p8973137	A-genome specific (napus-rapa)	0.9928229	0.4283671	not scorable	not scorable	1	0.6861063
8135	Bn-A03-p8978761	napus specific	0.9545454	0.9373336	not scorable	not scorable	not scorable	not scorable
8136	Bn-A03-p898067	A-genome specific (napus-rapa)	0.9952153	0.5487018	not scorable	not scorable	0.9669421	0.7880925
8137	Bn-A03-p8983947	A-genome specific (napus-rapa)	1	0.6971599	not scorable	not scorable	1	0.6006638
8138	Bn-A03-p8987098	A-genome specific (rapa only)	not scorable	not scorable	not scorable	not scorable	0.9834711	0.7425229
8139	Bn-A03-p8987269	A-genome specific (napus-rapa)	0.7942584	0.9177062	not scorable	not scorable	0.9256198	0.8233967
8140	Bn-A03-p8987820	A-genome specific (rapa only)	not scorable	not scorable	not scorable	not scorable	0.9338843	0.9172689
8141	Bn-A03-p9011801	A-genome specific (rapa only)	not scorable	not scorable	not scorable	not scorable	0.9917355	0.7833462
8142	Bn-A03-p9011966	A-genome specific (napus-rapa)	0.9473684	0.8213773	not scorable	not scorable	0.9917355	0.7067118
8143	Bn-A03-p9020167	A-genome specific (napus-rapa)	0.9665072	0.8092616	not scorable	not scorable	0.9917355	0.7681311
8144	Bn-A03-p9020291	A-genome specific (napus-rapa)	0.9736842	0.7745702	not scorable	not scorable	1	0.7303197
8145	Bn-A03-p90436	A-genome specific (napus-rapa)	0.923445	0.7357419	not scorable	not scorable	0.9917355	0.7506878
8146	Bn-A03-p9047380	A-genome specific (napus-rapa)	0.9545454	0.8764279	not scorable	not scorable	0.9256198	0.7893785
8147	Bn-A03-p9093699	A-genome specific (napus-rapa)	0.9928229	0.7951738	not scorable	not scorable	0.9752066	0.695935
8148	Bn-A03-p9098576	A-genome specific (napus-rapa)	0.6818182	0.8076818	not scorable	not scorable	0.9504132	0.9272014

8149	Bn-A03-p9098773	A-genome specific (napus-rapa)	0.9784689	0.5690199	not scorable	not scorable	0.9917355	0.7165427
8150	Bn-A03-p9100699	A-genome specific (rapa only)	not scorable	not scorable	not scorable	not scorable	0.9917355	0.7610134
8151	Bn-A03-p9115020	A-genome specific (napus-rapa)	0.9760765	0.7626681	not scorable	not scorable	1	0.8720064
8152	Bn-A03-p912314	A-genome specific (napus-rapa)	0.9354067	0.7941422	not scorable	not scorable	0.9834711	0.8197823
8153	Bn-A03-p9123542	A-genome specific (napus-rapa)	0.9617225	0.6339982	not scorable	not scorable	0.9834711	0.7958812
8154	Bn-A03-p9124799	A-genome specific (napus-rapa)	0.9880383	0.7759107	not scorable	not scorable	0.9834711	0.8263959
8155	Bn-A03-p9129107	A-genome specific (napus-rapa)	0.9641148	0.4291966	not scorable	not scorable	0.9586777	0.6976671
8156	Bn-A03-p9143356	A-genome specific (napus-rapa)	0.9832536	0.8076818	not scorable	not scorable	0.9917355	0.7954005
8157	Bn-A03-p9148822	A-genome specific (napus-rapa)	0.9593301	0.4197842	not scorable	not scorable	0.9917355	0.7096302
8158	Bn-A03-p9170934	A-genome specific (napus-rapa)	1	0.4784003	not scorable	not scorable	0.9669421	0.7862108
8159	Bn-A03-p9179284	napus specific	0.9952153	0.6522878	not scorable	not scorable	not scorable	not scorable
8160	Bn-A03-p9179896	A-genome specific (napus-rapa)	0.9808612	0.3712496	not scorable	not scorable	1	0.7416984
8161	Bn-A03-p9186605	A-genome specific (napus-rapa)	0.9880383	0.8187077	not scorable	not scorable	1	0.8566714
8162	Bn-A03-p9191787	A-genome specific (napus-rapa)	0.9904306	0.3934736	not scorable	not scorable	0.9834711	0.6987166
8163	Bn-A03-p9203389	A-genome specific (napus-rapa)	0.5023924	0.9074499	not scorable	not scorable	0.9752066	0.809663
8164	Bn-A03-p9203536	A-genome specific (napus-rapa)	0.5	0.8673111	not scorable	not scorable	0.9917355	0.8327085
8165	Bn-A03-p9203834	A-genome specific (napus-rapa)	0.5023924	0.8927962	not scorable	not scorable	0.9917355	0.7881667
8166	Bn-A03-p9203992	A-genome specific (napus-rapa)	0.5	0.7895767	not scorable	not scorable	0.9586777	0.726112
8167	Bn-A03-p9203999	A-genome specific (napus-rapa)	0.5023924	0.8285478	not scorable	not scorable	0.9834711	0.7294878
8168	Bn-A03-p9208735	A-genome specific (napus-rapa)	0.9832536	0.7881516	not scorable	not scorable	0.9917355	0.8189138
8169	Bn-A03-p9233645	A-genome specific (napus-rapa)	0.8181818	0.7980588	not scorable	not scorable	0.8512397	0.9045029
8170	Bn-A03-p9243701	A-genome specific (napus-rapa)	0.9736842	0.5357692	not scorable	not scorable	0.9752066	0.8135148
8171	Bn-A03-p9244195	A-genome specific (napus-rapa)	0.9545454	0.4738123	not scorable	not scorable	0.9917355	0.7388391
8172	Bn-A03-p9245937	A-genome specific (napus-rapa)	0.9928229	0.5050273	not scorable	not scorable	1	0.7377167
8173	Bn-A03-p9246222	A-genome specific (napus-rapa)	0.9784689	0.8299648	not scorable	not scorable	1	0.8685864
8174	Bn-A03-p924676	A-genome specific (napus-rapa)	0.937799	0.82362	not scorable	not scorable	1	0.7849017
8175	Bn-A03-p9247666	A-genome specific (napus-rapa)	0.9808612	0.8251132	not scorable	not scorable	1	0.8045345
8176	Bn-A03-p9247798	A-genome specific (napus-rapa)	0.9760765	0.8211127	not scorable	not scorable	0.9834711	0.7969552
8177	Bn-A03-p924780	A-genome specific (napus-rapa)	0.9258373	0.7988252	not scorable	not scorable	0.9917355	0.7193992
8178	Bn-A03-p9250832	genome unspecific (all in)	0.9641148	0.6307454	0.9767442	0.2193727	0.9669421	0.6678938
8179	Bn-A03-p9255139	A-genome specific (napus-rapa)	0.9880383	0.7556924	not scorable	not scorable	1	0.7673302
8180	Bn-A03-p9265554	A-genome specific (napus-rapa)	0.9904306	0.5388063	not scorable	not scorable	1	0.8319378
8181	Bn-A03-p9266328	A-genome specific (napus-rapa)	0.9856459	0.8347671	not scorable	not scorable	0.9917355	0.7165243

8182	Bn-A03-p927517	A-genome specific (rapa only)	not scorable	not scorable	not scorable	not scorable	1	0.5875754
8183	Bn-A03-p928009	A-genome specific (napus-rapa)	0.9210526	0.7507027	not scorable	not scorable	0.9834711	0.7298775
8184	Bn-A03-p928274	A-genome specific (napus-rapa)	0.9186603	0.7185768	not scorable	not scorable	0.9917355	0.8020242
8185	Bn-A03-p9300575	A-genome specific (napus-rapa)	0.9808612	0.4227254	not scorable	not scorable	0.9917355	0.724448
8186	Bn-A03-p9301541	A-genome specific (napus-rapa)	0.9617225	0.651466	not scorable	not scorable	1	0.7045599
8187	Bn-A03-p9303850	A-genome specific (napus-rapa)	0.4162679	0.9453188	not scorable	not scorable	0.9586777	0.7642528
8188	Bn-A03-p9304563	A-genome specific (napus-rapa)	0.9784689	0.771987	not scorable	not scorable	0.9834711	0.7881145
8189	Bn-A03-p9304885	genome unspecific (all in)	0.9784689	0.219511	0.9922481	0.6636861	1	0.768774
8190	Bn-A03-p9324653	A-genome specific (napus-rapa)	1	0.4331245	not scorable	not scorable	1	0.7229989
8191	Bn-A03-p9328470	A-genome specific (napus-rapa)	0.9976076	0.2860115	not scorable	not scorable	0.9917355	0.6501398
8192	Bn-A03-p9338698	A-genome specific (napus-rapa)	0.9904306	0.8710861	not scorable	not scorable	1	0.7626578
8193	Bn-A03-p9343295	A-genome specific (napus-rapa)	0.9904306	0.7682336	not scorable	not scorable	0.9834711	0.6707665
8194	Bn-A03-p9343716	A-genome specific (napus-rapa)	0.9976076	0.4753319	not scorable	not scorable	0.9917355	0.7652662
8195	Bn-A03-p9357075	A-genome specific (napus-rapa)	0.9976076	0.5371368	not scorable	not scorable	1	0.7124085
8196	Bn-A03-p9361280	A-genome specific (napus-rapa)	0.9880383	0.8418941	not scorable	not scorable	0.9586777	0.7349766
8197	Bn-A03-p9382281	A-genome specific (napus-rapa)	0.9928229	0.8051531	not scorable	not scorable	0.9917355	0.9476676
8198	Bn-A03-p9383602	genome unspecific (all in)	1	0.4999238	0.9922481	0.3170122	1	0.4357498
8199	Bn-A03-p9429437	A-genome specific (napus-rapa)	0.9928229	0.8178275	not scorable	not scorable	0.9834711	0.7696753
8200	Bn-A03-p943531	A-genome specific (napus-rapa)	0.3325359	0.8559471	not scorable	not scorable	0.9834711	0.7129991
8201	Bn-A03-p9439354	A-genome specific (napus-rapa)	0.9928229	0.5440177	not scorable	not scorable	1	0.7325223
8202	Bn-A03-p9439893	A-genome specific (napus-rapa)	0.9880383	0.6743492	not scorable	not scorable	0.9752066	0.6523845
8203	Bn-A03-p9448206	A-genome specific (napus-rapa)	0.9928229	0.6158956	not scorable	not scorable	0.9917355	0.5663684
8204	Bn-A03-p9449316	A-genome specific (napus-rapa)	0.9904306	0.4329319	not scorable	not scorable	0.9917355	0.7178582
8205	Bn-A03-p9468025	A-genome specific (napus-rapa)	0.9808612	0.8243108	not scorable	not scorable	0.9834711	0.8135014
8206	Bn-A03-p9476053	A-genome specific (napus-rapa)	1	0.4381896	not scorable	not scorable	0.9752066	0.785815
8207	Bn-A03-p9480203	A-genome specific (napus-rapa)	0.9808612	0.7954422	not scorable	not scorable	1	0.7580955
8208	Bn-A03-p9485365	A-genome specific (napus-rapa)	0.9856459	0.504979	not scorable	not scorable	0.9917355	0.719432
8209	Bn-A03-p9488435	A-genome specific (napus-rapa)	0.9712918	0.7200912	not scorable	not scorable	0.9917355	0.7169514
8210	Bn-A03-p9497697	A-genome specific (napus-rapa)	0.9928229	0.8077534	not scorable	not scorable	1	0.7906294
8211	Bn-A03-p9497821	A-genome specific (napus-rapa)	0.9808612	0.7028803	not scorable	not scorable	0.9917355	0.7636057
8212	Bn-A03-p950999	A-genome specific (napus-rapa)	0.937799	0.6629262	not scorable	not scorable	1	0.7252899
8213	Bn-A03-p951365	A-genome specific (napus-rapa)	0.9258373	0.7399897	not scorable	not scorable	1	0.694619
8214	Bn-A03-p952091	A-genome specific (napus-rapa)	0.9282297	0.7751124	not scorable	not scorable	1	0.7791197

8215	Bn-A03-p9522072	A-genome specific (napus-rapa)	0.9976076	0.5446739	not scorable	not scorable	0.9917355	0.787383
8216	Bn-A03-p9525820	A-genome specific (napus-rapa)	0.9784689	0.5567996	not scorable	not scorable	1	0.8379921
8217	Bn-A03-p9537155	A-genome specific (napus-rapa)	0.9665072	0.7811695	not scorable	not scorable	0.9669421	0.7156999
8218	Bn-A03-p9537618	A-genome specific (napus-rapa)	0.9904306	0.8132609	not scorable	not scorable	0.9917355	0.73758
8219	Bn-A03-p955176	A-genome specific (napus-rapa)	0.923445	0.8029299	not scorable	not scorable	0.9917355	0.7592824
8220	Bn-A03-p9564515	genome unspecific (all in)	0.8851675	0.6878916	0.875969	0.6828715	0.9917355	0.8827738
8221	Bn-A03-p9567767	A-genome specific (napus-rapa)	0.9832536	0.4896149	not scorable	not scorable	0.9669421	0.5896956
8222	Bn-A03-p9601581	A-genome specific (napus-rapa)	0.9760765	0.8438662	not scorable	not scorable	0.9752066	0.7880756
8223	Bn-A03-p9602074	A-genome specific (napus-rapa)	0.9736842	0.85719	not scorable	not scorable	0.9338843	0.7268355
8224	Bn-A03-p9605319	A-genome specific (napus-rapa)	0.9880383	0.7125748	not scorable	not scorable	0.9917355	0.6493331
8225	Bn-A03-p9613933	A-genome specific (napus-rapa)	1	0.5445434	not scorable	not scorable	0.9669421	0.5570701
8226	Bn-A03-p9615126	A-genome specific (napus-rapa)	0.9593301	0.207033	not scorable	not scorable	0.9834711	0.7785882
8227	Bn-A03-p9618630	A-genome specific (napus-rapa)	0.9904306	0.7337993	not scorable	not scorable	1	0.7019977
8228	Bn-A03-p9626063	A-genome specific (napus-rapa)	0.9330143	0.4540745	not scorable	not scorable	1	0.716634
8229	Bn-A03-p9627365	A-genome specific (napus-rapa)	0.9880383	0.8284228	not scorable	not scorable	0.9834711	0.8236209
8230	Bn-A03-p9637487	A-genome specific (napus-rapa)	0.9856459	0.773469	not scorable	not scorable	0.9752066	0.7566584
8231	Bn-A03-p9638708	A-genome specific (napus-rapa)	0.9784689	0.7591976	not scorable	not scorable	1	0.7067255
8232	Bn-A03-p9638726	A-genome specific (napus-rapa)	0.9784689	0.6730143	not scorable	not scorable	1	0.6701939
8233	Bn-A03-p9641671	A-genome specific (napus-rapa)	0.9665072	0.7145602	not scorable	not scorable	0.9834711	0.8776289
8234	Bn-A03-p9651526	A-genome specific (napus-rapa)	0.9617225	0.5007778	not scorable	not scorable	1	0.7901848
8235	Bn-A03-p9657864	A-genome specific (napus-rapa)	0.9880383	0.3346985	not scorable	not scorable	1	0.8214939
8236	Bn-A03-p9660869	A-genome specific (napus-rapa)	0.9904306	0.822892	not scorable	not scorable	0.9917355	0.7876235
8237	Bn-A03-p9677969	A-genome specific (napus-rapa)	0.9832536	0.7930467	not scorable	not scorable	0.9917355	0.7781708
8238	Bn-A03-p9678981	A-genome specific (napus-rapa)	0.9904306	0.5264527	not scorable	not scorable	0.9752066	0.856342
8239	Bn-A03-p9680882	A-genome specific (napus-rapa)	0.9832536	0.7821287	not scorable	not scorable	0.9669421	0.8207156
8240	Bn-A03-p9705481	A-genome specific (napus-rapa)	0.9856459	0.3125615	not scorable	not scorable	0.9917355	0.7238167
8241	Bn-A03-p9706640	A-genome specific (napus-rapa)	0.9976076	0.5269832	not scorable	not scorable	1	0.6799201
8242	Bn-A03-p9734588	A-genome specific (napus-rapa)	0.9880383	0.75969	not scorable	not scorable	0.9834711	0.7822676
8243	Bn-A03-p9747710	A-genome specific (napus-rapa)	0.9904306	0.8018699	not scorable	not scorable	0.9917355	0.7112521
8244	Bn-A03-p9747824	A-genome specific (napus-rapa)	0.9856459	0.7656601	not scorable	not scorable	1	0.7134107
8245	Bn-A03-p974894	A-genome specific (napus-rapa)	0.9019139	0.5746238	not scorable	not scorable	0.9917355	0.6071299
8246	Bn-A03-p9751621	A-genome specific (napus-rapa)	0.9904306	0.7924676	not scorable	not scorable	0.9917355	0.7797557
8247	Bn-A03-p9758514	A-genome specific (napus-rapa)	0.9832536	0.813787	not scorable	not scorable	0.9752066	0.7772134

8248	Bn-A03-p9764680	A-genome specific (napus-rapa)	0.9904306	0.3219271	not scorable	not scorable	1	0.6783624
8249	Bn-A03-p9765420	A-genome specific (napus-rapa)	0.9928229	0.8496934	not scorable	not scorable	0.9917355	0.824485
8250	Bn-A03-p9765623	A-genome specific (napus-rapa)	0.9928229	0.7278595	not scorable	not scorable	0.9752066	0.858018
8251	Bn-A03-p9818699	A-genome specific (napus-rapa)	0.9928229	0.7417248	not scorable	not scorable	0.9752066	0.708764
8252	Bn-A03-p9819365	A-genome specific (napus-rapa)	0.9832536	0.8683464	not scorable	not scorable	0.9917355	0.7144147
8253	Bn-A03-p9819389	A-genome specific (napus-rapa)	0.9952153	0.7879774	not scorable	not scorable	0.9834711	0.7665128
8254	Bn-A03-p9823618	A-genome specific (napus-rapa)	0.9976076	0.422529	not scorable	not scorable	0.9834711	0.7146232
8255	Bn-A03-p9825032	A-genome specific (napus-rapa)	1	0.3567328	not scorable	not scorable	1	0.7154919
8256	Bn-A03-p9833648	A-genome specific (napus-rapa)	0.9808612	0.7212242	not scorable	not scorable	0.9834711	0.8197204
8257	Bn-A03-p9836757	A-genome specific (napus-rapa)	0.9856459	0.7402512	not scorable	not scorable	0.9834711	0.703786
8258	Bn-A03-p9839212	A-genome specific (napus-rapa)	0.9976076	0.4739356	not scorable	not scorable	1	0.8268818
8259	Bn-A03-p9847680	A-genome specific (napus-rapa)	0.9904306	0.4366435	not scorable	not scorable	0.9917355	0.8222803
8260	Bn-A03-p9856465	A-genome specific (napus-rapa)	0.9760765	0.8058246	not scorable	not scorable	0.9917355	0.8192184
8261	Bn-A03-p9856923	A-genome specific (napus-rapa)	0.9952153	0.7556314	not scorable	not scorable	1	0.7578101
8262	Bn-A03-p9863138	A-genome specific (napus-rapa)	0.9712918	0.7400446	not scorable	not scorable	0.9917355	0.6763602
8263	Bn-A03-p9863553	A-genome specific (napus-rapa)	0.9808612	0.8178373	not scorable	not scorable	0.9834711	0.819433
8264	Bn-A03-p9863600	A-genome specific (napus-rapa)	0.9784689	0.7930661	not scorable	not scorable	0.9917355	0.8327429
8265	Bn-A03-p9869156	A-genome specific (napus-rapa)	0.9832536	0.7287478	not scorable	not scorable	0.9917355	0.7020041
8266	Bn-A03-p9869182	genome unspecific (oleracea-rapa)	not scorable	not scorable	1	0.74485	0.9917355	0.6656052
8267	Bn-A03-p9873327	A-genome specific (napus-rapa)	0.9760765	0.7071537	not scorable	not scorable	0.9917355	0.6880409
8268	Bn-A03-p9873384	A-genome specific (napus-rapa)	0.9497608	0.7196521	not scorable	not scorable	1	0.7514223
8269	Bn-A03-p9876378	napus specific	0.9641148	0.3372435	not scorable	not scorable	not scorable	not scorable
8270	Bn-A03-p9883544	A-genome specific (napus-rapa)	1	0.5696082	not scorable	not scorable	0.9917355	0.8124406
8271	Bn-A03-p9899417	A-genome specific (napus-rapa)	0.9904306	0.5360056	not scorable	not scorable	0.9917355	0.6932496
8272	Bn-A03-p991949	A-genome specific (napus-rapa)	0.937799	0.7882087	not scorable	not scorable	0.9917355	0.6646572
8273	Bn-A03-p9920847	A-genome specific (napus-rapa)	0.9856459	0.7390603	not scorable	not scorable	0.9917355	0.7579179
8274	Bn-A03-p9921379	A-genome specific (napus-rapa)	0.9736842	0.7923144	not scorable	not scorable	1	0.72553
8275	Bn-A03-p9921928	A-genome specific (napus-rapa)	0.9808612	0.7132422	not scorable	not scorable	0.9669421	0.8813163
8276	Bn-A03-p9924047	A-genome specific (napus-rapa)	0.9665072	0.8160322	not scorable	not scorable	1	0.834636
8277	Bn-A03-p9925289	A-genome specific (napus-rapa)	0.9784689	0.7936907	not scorable	not scorable	0.8429752	0.8380996
8278	Bn-A03-p9925550	A-genome specific (napus-rapa)	0.9784689	0.7453971	not scorable	not scorable	0.9917355	0.7933823
8279	Bn-A03-p9926783	A-genome specific (napus-rapa)	0.9928229	0.7663506	not scorable	not scorable	0.9917355	0.841835
8280	Bn-A03-p9939018	A-genome specific (napus-rapa)	0.9736842	0.7517345	not scorable	not scorable	0.9669421	0.759119

8281	Bn-A03-p9966625	A-genome specific (napus-rapa)	1	0.4490254	not scorable	not scorable	1	0.8289795
8282	Bn-A03-p9969989	A-genome specific (napus-rapa)	0.9952153	0.6578659	not scorable	not scorable	1	0.7479122
8283	Bn-A04-p1001237	A-genome specific (napus-rapa)	0.9952153	0.8207692	not scorable	not scorable	1	0.7500455
8284	Bn-A04-p10016854	A-genome specific (napus-rapa)	0.9976076	0.8386101	not scorable	not scorable	1	0.813361
8285	Bn-A04-p10043859	A-genome specific (napus-rapa)	0.9952153	0.7882869	not scorable	not scorable	0.9917355	0.7368809
8286	Bn-A04-p10049865	A-genome specific (napus-rapa)	0.9928229	0.843116	not scorable	not scorable	1	0.8045247
8287	Bn-A04-p10049966	A-genome specific (napus-rapa)	0.9952153	0.8420658	not scorable	not scorable	0.9752066	0.7369736
8288	Bn-A04-p10054968	A-genome specific (napus-rapa)	0.9976076	0.843972	not scorable	not scorable	1	0.7769243
8289	Bn-A04-p10056980	A-genome specific (napus-rapa)	0.9976076	0.8318204	not scorable	not scorable	1	0.8339064
8290	Bn-A04-p10058358	A-genome specific (napus-rapa)	0.9976076	0.8516029	not scorable	not scorable	1	0.724456
8291	Bn-A04-p10059165	A-genome specific (napus-rapa)	0.9976076	0.8318616	not scorable	not scorable	0.9917355	0.7225745
8292	Bn-A04-p10069116	A-genome specific (napus-rapa)	0.9952153	0.7280481	not scorable	not scorable	0.9752066	0.7994025
8293	Bn-A04-p10071085	A-genome specific (napus-rapa)	0.9952153	0.8072441	not scorable	not scorable	0.9917355	0.7467128
8294	Bn-A04-p1007534	A-genome specific (napus-rapa)	1	0.8366807	not scorable	not scorable	0.9669421	0.7097182
8295	Bn-A04-p10085135	A-genome specific (napus-rapa)	0.9952153	0.8560814	not scorable	not scorable	0.9917355	0.8062136
8296	Bn-A04-p10085844	A-genome specific (napus-rapa)	0.9928229	0.6696359	not scorable	not scorable	0.9834711	0.7779803
8297	Bn-A04-p10087754	A-genome specific (napus-rapa)	0.9976076	0.8179201	not scorable	not scorable	0.9917355	0.7036551
8298	Bn-A04-p10088142	A-genome specific (napus-rapa)	0.9976076	0.7769604	not scorable	not scorable	1	0.7135277
8299	Bn-A04-p10088385	A-genome specific (napus-rapa)	0.9952153	0.7859275	not scorable	not scorable	0.9834711	0.8122377
8300	Bn-A04-p10088445	A-genome specific (napus-rapa)	0.9976076	0.7772551	not scorable	not scorable	0.9917355	0.7788066
8301	Bn-A04-p10088570	A-genome specific (napus-rapa)	0.9976076	0.6487384	not scorable	not scorable	0.9834711	0.6547102
8302	Bn-A04-p10089027	A-genome specific (napus-rapa)	0.9928229	0.8231288	not scorable	not scorable	0.9834711	0.6673149
8303	Bn-A04-p10089238	A-genome specific (napus-rapa)	0.9976076	0.8389751	not scorable	not scorable	0.9917355	0.7967852
8304	Bn-A04-p10089301	A-genome specific (napus-rapa)	0.9904306	0.7732738	not scorable	not scorable	0.9917355	0.7759476
8305	Bn-A04-p10089384	A-genome specific (napus-rapa)	0.9976076	0.6814407	not scorable	not scorable	0.9917355	0.7043613
8306	Bn-A04-p10089680	A-genome specific (napus-rapa)	0.9976076	0.8483146	not scorable	not scorable	0.9917355	0.8021637
8307	Bn-A04-p10089715	A-genome specific (napus-rapa)	0.9976076	0.568394	not scorable	not scorable	1	0.7419648
8308	Bn-A04-p10090470	A-genome specific (napus-rapa)	0.9976076	0.7574509	not scorable	not scorable	1	0.5943417
8309	Bn-A04-p10093618	A-genome specific (napus-rapa)	0.9952153	0.8437599	not scorable	not scorable	0.9917355	0.6536983
8310	Bn-A04-p10094831	A-genome specific (napus-rapa)	0.9976076	0.7979577	not scorable	not scorable	1	0.7247049
8311	Bn-A04-p10099096	A-genome specific (napus-rapa)	0.9976076	0.4986041	not scorable	not scorable	0.9917355	0.7146772
8312	Bn-A04-p1009976	A-genome specific (napus-rapa)	0.9976076	0.8549963	not scorable	not scorable	1	0.8335614
8313	Bn-A04-p10101293	A-genome specific (napus-rapa)	0.9976076	0.8632371	not scorable	not scorable	0.9917355	0.7997406

8314	Bn-A04-p10103888	A-genome specific (napus-rapa)	0.9952153	0.8219081	not scorable	not scorable	0.9834711	0.7100585
8315	Bn-A04-p10104666	A-genome specific (napus-rapa)	0.9976076	0.8416846	not scorable	not scorable	0.9917355	0.7556812
8316	Bn-A04-p10108610	A-genome specific (napus-rapa)	0.9976076	0.7929857	not scorable	not scorable	0.9917355	0.8036009
8317	Bn-A04-p10113831	A-genome specific (napus-rapa)	0.9976076	0.7879074	not scorable	not scorable	0.9917355	0.7410045
8318	Bn-A04-p10113969	A-genome specific (napus-rapa)	0.9976076	0.7823327	not scorable	not scorable	0.9917355	0.765747
8319	Bn-A04-p10129538	A-genome specific (napus-rapa)	0.9808612	0.8399643	not scorable	not scorable	0.6528926	0.7571167
8320	Bn-A04-p10154400	A-genome specific (napus-rapa)	1	0.5451833	not scorable	not scorable	1	0.6887994
8321	Bn-A04-p10155907	A-genome specific (napus-rapa)	0.9976076	0.7024054	not scorable	not scorable	1	0.6694872
8322	Bn-A04-p10176387	A-genome specific (napus-rapa)	0.9928229	0.8513263	not scorable	not scorable	0.9834711	0.9176717
8323	Bn-A04-p10182453	A-genome specific (napus-rapa)	0.9976076	0.8641753	not scorable	not scorable	0.9834711	0.7448161
8324	Bn-A04-p1018807	A-genome specific (napus-rapa)	0.9952153	0.8483796	not scorable	not scorable	0.9834711	0.7734122
8325	Bn-A04-p10188827	A-genome specific (napus-rapa)	0.9976076	0.8437311	not scorable	not scorable	1	0.6903697
8326	Bn-A04-p10196289	A-genome specific (napus-rapa)	0.9952153	0.3453643	not scorable	not scorable	1	0.6625491
8327	Bn-A04-p1021879	A-genome specific (napus-rapa)	0.9832536	0.6289667	not scorable	not scorable	0.9669421	0.7084547
8328	Bn-A04-p10250633	A-genome specific (napus-rapa)	0.9976076	0.8435445	not scorable	not scorable	0.9917355	0.6931905
8329	Bn-A04-p10261874	A-genome specific (napus-rapa)	1	0.4218935	not scorable	not scorable	0.9752066	0.679301
8330	Bn-A04-p10286035	A-genome specific (rapa only)	not scorable	not scorable	not scorable	not scorable	0.9173554	0.6801589
8331	Bn-A04-p10288476	napus specific	0.8779904	0.5105038	not scorable	not scorable	not scorable	not scorable
8332	Bn-A04-p1032336	A-genome specific (napus-rapa)	0.9976076	0.8285049	not scorable	not scorable	1	0.6066822
8333	Bn-A04-p1036308	A-genome specific (napus-rapa)	0.9904306	0.316713	not scorable	not scorable	0.9917355	0.8012068
8334	Bn-A04-p10372116	A-genome specific (napus-rapa)	0.9928229	0.8525689	not scorable	not scorable	0.9917355	0.7980971
8335	Bn-A04-p10383434	A-genome specific (napus-rapa)	0.9641148	0.3800404	not scorable	not scorable	0.9917355	0.7010307
8336	Bn-A04-p10387804	A-genome specific (napus-rapa)	0.9736842	0.85525	not scorable	not scorable	1	0.6937472
8337	Bn-A04-p10393460	A-genome specific (napus-rapa)	0.9665072	0.8327757	not scorable	not scorable	0.9752066	0.7197168
8338	Bn-A04-p1040916	genome unspecific (oleracea-rapa)	not scorable	not scorable	0.9844961	0.2614505	0.9917355	0.6735559
8339	Bn-A04-p1042273	A-genome specific (napus-rapa)	0.9832536	0.7321724	not scorable	not scorable	0.9669421	0.6387057
8340	Bn-A04-p1043111	A-genome specific (napus-rapa)	0.9856459	0.7037452	not scorable	not scorable	1	0.786476
8341	Bn-A04-p10434805	A-genome specific (napus-rapa)	1	0.466299	not scorable	not scorable	0.9752066	0.7657341
8342	Bn-A04-p10458946	A-genome specific (napus-rapa)	0.9665072	0.7514317	not scorable	not scorable	0.9586777	0.7845129
8343	Bn-A04-p10464942	napus specific	0.9976076	0.7759518	not scorable	not scorable	not scorable	not scorable
8344	Bn-A04-p1048288	A-genome specific (napus-rapa)	1	0.8376964	not scorable	not scorable	0.9752066	0.7805875
8345	Bn-A04-p10490223	A-genome specific (napus-rapa)	0.9928229	0.7575678	not scorable	not scorable	0.9752066	0.7975682
8346	Bn-A04-p10490380	A-genome specific (napus-rapa)	0.9473684	0.7800913	not scorable	not scorable	1	0.8081351

8347	Bn-A04-p10494765	A-genome specific (napus-rapa)	0.9521531	0.7182139	not scorable	not scorable	0.9917355	0.724129
8348	Bn-A04-p10502015	A-genome specific (napus-rapa)	0.9784689	0.7156128	not scorable	not scorable	1	0.6882113
8349	Bn-A04-p10503082	A-genome specific (napus-rapa)	0.9904306	0.7761877	not scorable	not scorable	0.9504132	0.679795
8350	Bn-A04-p10515374	A-genome specific (napus-rapa)	0.9736842	0.7316802	not scorable	not scorable	1	0.7532828
8351	Bn-A04-p10541268	A-genome specific (napus-rapa)	0.9784689	0.6421807	not scorable	not scorable	0.9917355	0.7863185
8352	Bn-A04-p10555408	A-genome specific (napus-rapa)	0.9545454	0.4772646	not scorable	not scorable	0.9669421	0.6877676
8353	Bn-A04-p10559481	A-genome specific (napus-rapa)	0.9880383	0.7976924	not scorable	not scorable	0.9669421	0.6385577
8354	Bn-A04-p10560896	A-genome specific (napus-rapa)	0.9904306	0.7484185	not scorable	not scorable	0.9752066	0.6769026
8355	Bn-A04-p10566018	A-genome specific (napus-rapa)	0.9928229	0.5253142	not scorable	not scorable	1	0.7069696
8356	Bn-A04-p10592914	all not scorable	not scorable	not scorable	not scorable	not scorable	not scorable	not scorable
8357	Bn-A04-p10600455	A-genome specific (napus-rapa)	0.9569378	0.5473564	not scorable	not scorable	0.9917355	0.8063386
8358	Bn-A04-p10602815	genome unspecific (oleracea-rapa)	not scorable	not scorable	0.9844961	0.6821816	0.9752066	0.6242504
8359	Bn-A04-p10608772	A-genome specific (napus-rapa)	0.9617225	0.7889425	not scorable	not scorable	1	0.64634
8360	Bn-A04-p1062119	A-genome specific (napus-rapa)	1	0.8512694	not scorable	not scorable	0.9917355	0.7979999
8361	Bn-A04-p10628643	A-genome specific (napus-rapa)	0.7966507	0.8336589	not scorable	not scorable	0.9834711	0.6700917
8362	Bn-A04-p10628977	A-genome specific (napus-rapa)	0.9808612	0.7951728	not scorable	not scorable	1	0.7051874
8363	Bn-A04-p10630934	A-genome specific (napus-rapa)	0.9880383	0.762882	not scorable	not scorable	0.9834711	0.7000063
8364	Bn-A04-p10649540	A-genome specific (napus-rapa)	0.9641148	0.7857676	not scorable	not scorable	0.9917355	0.7255757
8365	Bn-A04-p1065480	A-genome specific (napus-rapa)	1	0.359257	not scorable	not scorable	1	0.7893053
8366	Bn-A04-p10661890	A-genome specific (napus-rapa)	0.9593301	0.6469404	not scorable	not scorable	1	0.7895319
8367	Bn-A04-p10661931	A-genome specific (napus-rapa)	0.9904306	0.7546182	not scorable	not scorable	0.9917355	0.7384813
8368	Bn-A04-p10662404	A-genome specific (napus-rapa)	0.9665072	0.7383147	not scorable	not scorable	0.9834711	0.6824803
8369	Bn-A04-p10671164	A-genome specific (napus-rapa)	0.9880383	0.688552	not scorable	not scorable	0.9752066	0.6951733
8370	Bn-A04-p10671721	A-genome specific (napus-rapa)	0.9688995	0.8310868	not scorable	not scorable	1	0.708733
8371	Bn-A04-p10680523	A-genome specific (napus-rapa)	0.9545454	0.8276861	not scorable	not scorable	0.9752066	0.81874
8372	Bn-A04-p10688504	A-genome specific (napus-rapa)	0.9688995	0.424396	not scorable	not scorable	0.9752066	0.7267343
8373	Bn-A04-p1070815	A-genome specific (napus-rapa)	0.9760765	0.8646542	not scorable	not scorable	0.9752066	0.781979
8374	Bn-A04-p10718521	A-genome specific (napus-rapa)	0.9952153	0.7794726	not scorable	not scorable	1	0.6799201
8375	Bn-A04-p10719274	A-genome specific (napus-rapa)	0.9880383	0.7935993	not scorable	not scorable	0.9834711	0.7245079
8376	Bn-A04-p10719331	A-genome specific (napus-rapa)	0.9952153	0.8056173	not scorable	not scorable	0.9917355	0.666648
8377	Bn-A04-p10744003	A-genome specific (napus-rapa)	0.9928229	0.838342	not scorable	not scorable	0.9669421	0.7194893
8378	Bn-A04-p10748559	A-genome specific (napus-rapa)	0.9808612	0.8149921	not scorable	not scorable	0.9834711	0.6947346
8379	Bn-A04-p10759605	A-genome specific (napus-rapa)	0.9808612	0.8049639	not scorable	not scorable	0.9834711	0.7862172

8380	Bn-A04-p10759783	A-genome specific (napus-rapa)	0.9904306	0.7786547	not scorable	not scorable	0.9834711	0.7103407
8381	Bn-A04-p10760138	A-genome specific (napus-rapa)	0.9712918	0.858559	not scorable	not scorable	0.9917355	0.7498785
8382	Bn-A04-p10760352	A-genome specific (napus-rapa)	0.9760765	0.7394434	not scorable	not scorable	0.9834711	0.7699208
8383	Bn-A04-p10760790	A-genome specific (napus-rapa)	0.9808612	0.2792164	not scorable	not scorable	0.9917355	0.8339955
8384	Bn-A04-p10769811	A-genome specific (napus-rapa)	0.9856459	0.8510734	not scorable	not scorable	0.5371901	0.8237607
8385	Bn-A04-p10774641	A-genome specific (napus-rapa)	0.9880383	0.6498886	not scorable	not scorable	0.9917355	0.7958391
8386	Bn-A04-p1077522	A-genome specific (napus-rapa)	0.9928229	0.9211312	not scorable	not scorable	0.9917355	0.799288
8387	Bn-A04-p10786704	A-genome specific (napus-rapa)	0.9976076	0.8363218	not scorable	not scorable	0.9917355	0.7370206
8388	Bn-A04-p10792201	A-genome specific (napus-rapa)	0.9928229	0.756969	not scorable	not scorable	0.9917355	0.7524305
8389	Bn-A04-p10808508	A-genome specific (napus-rapa)	0.9952153	0.9055482	not scorable	not scorable	1	0.7676948
8390	Bn-A04-p10808805	A-genome specific (napus-rapa)	0.9952153	0.3410371	not scorable	not scorable	1	0.8109607
8391	Bn-A04-p10809637	A-genome specific (napus-rapa)	0.9904306	0.4565796	not scorable	not scorable	0.9917355	0.6924493
8392	Bn-A04-p10833713	A-genome specific (napus-rapa)	0.9952153	0.7133706	not scorable	not scorable	1	0.7409858
8393	Bn-A04-p10841936	A-genome specific (napus-rapa)	0.9593301	0.3679833	not scorable	not scorable	1	0.7481895
8394	Bn-A04-p10852032	A-genome specific (napus-rapa)	0.9712918	0.4671388	not scorable	not scorable	1	0.7719324
8395	Bn-A04-p10861826	A-genome specific (napus-rapa)	0.9784689	0.8145068	not scorable	not scorable	0.9834711	0.7358518
8396	Bn-A04-p10867512	genome unspecific (all in)	0.9736842	0.352234	1	0.7783606	0.9917355	0.8028898
8397	Bn-A04-p10870734	A-genome specific (napus-rapa)	0.9665072	0.7660088	not scorable	not scorable	1	0.8021563
8398	Bn-A04-p10871341	A-genome specific (napus-rapa)	0.9904306	0.5109445	not scorable	not scorable	1	0.3467439
8399	Bn-A04-p10871494	A-genome specific (napus-rapa)	0.9808612	0.8157527	not scorable	not scorable	0.9917355	0.8313978
8400	Bn-A04-p10872405	A-genome specific (napus-rapa)	0.9928229	0.7619505	not scorable	not scorable	0.9917355	0.6816216
8401	Bn-A04-p10881502	A-genome specific (napus-rapa)	0.9784689	0.8564889	not scorable	not scorable	1	0.7427967
8402	Bn-A04-p10902724	A-genome specific (napus-rapa)	0.9688995	0.4296813	not scorable	not scorable	1	0.7094886
8403	Bn-A04-p10903353	genome unspecific (oleracea-rapa)	not scorable	not scorable	0.9767442	0.7446627	1	0.8085706
8404	Bn-A04-p10912425	A-genome specific (napus-rapa)	0.5311005	0.7969366	not scorable	not scorable	0.9752066	0.7349294
8405	Bn-A04-p10914108	A-genome specific (napus-rapa)	0.9784689	0.8111229	not scorable	not scorable	0.9917355	0.8102296
8406	Bn-A04-p1091727	A-genome specific (napus-rapa)	0.9617225	0.7933137	not scorable	not scorable	0.9917355	0.8004746
8407	Bn-A04-p10975832	A-genome specific (napus-rapa)	0.9976076	0.5447663	not scorable	not scorable	0.9917355	0.7871124
8408	Bn-A04-p10976023	A-genome specific (rapa only)	not scorable	not scorable	not scorable	not scorable	0.9917355	0.6558319
8409	Bn-A04-p10980342	A-genome specific (napus-rapa)	0.9952153	0.8843005	not scorable	not scorable	0.9917355	0.8661726
8410	Bn-A04-p10983805	A-genome specific (napus-rapa)	0.9976076	0.8190384	not scorable	not scorable	0.9917355	0.831573
8411	Bn-A04-p11015443	A-genome specific (napus-rapa)	0.9928229	0.3293518	not scorable	not scorable	1	0.7668723
8412	Bn-A04-p11017537	A-genome specific (napus-rapa)	0.9784689	0.7119262	not scorable	not scorable	1	0.8115246

8413	Bn-A04-p11018682	A-genome specific (napus-rapa)	0.9760765	0.7434618	not scorable	not scorable	0.9917355	0.6633283
8414	Bn-A04-p11020444	A-genome specific (napus-rapa)	0.9832536	0.6799475	not scorable	not scorable	0.9917355	0.6218448
8415	Bn-A04-p11026664	A-genome specific (napus-rapa)	0.9976076	0.6752296	not scorable	not scorable	0.9834711	0.7336089
8416	Bn-A04-p1104385	A-genome specific (napus-rapa)	0.9952153	0.8376332	not scorable	not scorable	0.9752066	0.7301727
8417	Bn-A04-p11058870	A-genome specific (napus-rapa)	0.3014354	0.826343	not scorable	not scorable	0.6528926	0.7627692
8418	Bn-A04-p11119216	A-genome specific (napus-rapa)	0.9593301	0.8023747	not scorable	not scorable	0.9669421	0.5952334
8419	Bn-A04-p11119582	A-genome specific (napus-rapa)	0.9665072	0.3541713	not scorable	not scorable	0.9917355	0.6930982
8420	Bn-A04-p11164883	A-genome specific (napus-rapa)	0.8516746	0.798871	not scorable	not scorable	0.9917355	0.7897629
8421	Bn-A04-p11191754	A-genome specific (napus-rapa)	0.9688995	0.7349456	not scorable	not scorable	1	0.7518212
8422	Bn-A04-p11192862	A-genome specific (napus-rapa)	0.9856459	0.7489851	not scorable	not scorable	0.9917355	0.6202541
8423	Bn-A04-p11197346	A-genome specific (napus-rapa)	0.9760765	0.7901875	not scorable	not scorable	0.9834711	0.7315583
8424	Bn-A04-p11197707	A-genome specific (napus-rapa)	0.9856459	0.7327107	not scorable	not scorable	0.9834711	0.6689033
8425	Bn-A04-p11203305	A-genome specific (napus-rapa)	0.9904306	0.4841329	not scorable	not scorable	0.9917355	0.7806171
8426	Bn-A04-p11209663	A-genome specific (napus-rapa)	0.9832536	0.4417794	not scorable	not scorable	0.9917355	0.846046
8427	Bn-A04-p11209773	A-genome specific (napus-rapa)	0.9952153	0.403861	not scorable	not scorable	1	0.8113095
8428	Bn-A04-p11209827	A-genome specific (napus-rapa)	0.9832536	0.348819	not scorable	not scorable	1	0.7906191
8429	Bn-A04-p11213108	A-genome specific (napus-rapa)	0.9784689	0.7754679	not scorable	not scorable	1	0.8296761
8430	Bn-A04-p11243983	A-genome specific (napus-rapa)	0.9976076	0.6729593	not scorable	not scorable	0.9669421	0.6608245
8431	Bn-A04-p11243994	A-genome specific (napus-rapa)	0.9976076	0.7634824	not scorable	not scorable	1	0.780166
8432	Bn-A04-p11244643	A-genome specific (napus-rapa)	1	0.6319475	not scorable	not scorable	0.9917355	0.7229488
8433	Bn-A04-p11244673	A-genome specific (napus-rapa)	0.9952153	0.4235312	not scorable	not scorable	0.9834711	0.6734545
8434	Bn-A04-p11245906	A-genome specific (napus-rapa)	1	0.6183447	not scorable	not scorable	1	0.6291033
8435	Bn-A04-p11247206	A-genome specific (napus-rapa)	0.9593301	0.8027782	not scorable	not scorable	0.5702479	0.8055053
8436	Bn-A04-p11250035	A-genome specific (napus-rapa)	0.9665072	0.7782564	not scorable	not scorable	0.6033058	0.7751748
8437	Bn-A04-p11250141	A-genome specific (napus-rapa)	0.9665072	0.7988107	not scorable	not scorable	0.5702479	0.8357344
8438	Bn-A04-p11256083	all not scorable	not scorable	not scorable	not scorable	not scorable	not scorable	not scorable
8439	Bn-A04-p11274255	A-genome specific (napus-rapa)	0.9856459	0.6918575	not scorable	not scorable	0.9834711	0.7057195
8440	Bn-A04-p11291596	A-genome specific (napus-rapa)	0.9545454	0.5077589	not scorable	not scorable	0.6033058	0.7397362
8441	Bn-A04-p11293892	A-genome specific (napus-rapa)	0.9665072	0.4283896	not scorable	not scorable	1	0.7810965
8442	Bn-A04-p11319703	A-genome specific (napus-rapa)	0.3779904	0.8715475	not scorable	not scorable	0.9669421	0.837379
8443	Bn-A04-p11320045	A-genome specific (napus-rapa)	0.361244	0.8987191	not scorable	not scorable	0.9669421	0.8408843
8444	Bn-A04-p11325008	A-genome specific (napus-rapa)	0.9760765	0.2713946	not scorable	not scorable	1	0.7620342
8445	Bn-A04-p11329756	A-genome specific (napus-rapa)	0.9712918	0.6339489	not scorable	not scorable	0.9917355	0.5242216

8446	Bn-A04-p11352544	A-genome specific (napus-rapa)	0.9330143	0.4189594	not scorable	not scorable	0.9917355	0.7152346
8447	Bn-A04-p11359006	A-genome specific (napus-rapa)	0.9976076	0.8961288	not scorable	not scorable	0.9917355	0.7383954
8448	Bn-A04-p113641	A-genome specific (napus-rapa)	0.9880383	0.8019829	not scorable	not scorable	0.9917355	0.764704
8449	Bn-A04-p11364790	A-genome specific (napus-rapa)	0.9856459	0.799143	not scorable	not scorable	1	0.8154098
8450	Bn-A04-p11376488	A-genome specific (napus-rapa)	0.9904306	0.5416701	not scorable	not scorable	0.9834711	0.4465471
8451	Bn-A04-p11377702	A-genome specific (napus-rapa)	0.9976076	0.8929359	not scorable	not scorable	0.9586777	0.7357397
8452	Bn-A04-p11377775	A-genome specific (napus-rapa)	0.9976076	0.7869737	not scorable	not scorable	1	0.7448992
8453	Bn-A04-p11386737	A-genome specific (napus-rapa)	0.9976076	0.7497301	not scorable	not scorable	1	0.7224936
8454	Bn-A04-p11402649	A-genome specific (napus-rapa)	0.9976076	0.8229404	not scorable	not scorable	0.9834711	0.8387681
8455	Bn-A04-p11413704	A-genome specific (napus-rapa)	0.9976076	0.6421454	not scorable	not scorable	0.9917355	0.7937956
8456	Bn-A04-p11420466	A-genome specific (napus-rapa)	0.9808612	0.7545582	not scorable	not scorable	0.9834711	0.8153421
8457	Bn-A04-p11425304	A-genome specific (napus-rapa)	0.9832536	0.7470962	not scorable	not scorable	0.9834711	0.7572152
8458	Bn-A04-p11428085	A-genome specific (napus-rapa)	0.9712918	0.3755125	not scorable	not scorable	1	0.7598017
8459	Bn-A04-p11437131	A-genome specific (rapa only)	not scorable	not scorable	not scorable	not scorable	0.7190083	0.6616288
8460	Bn-A04-p11449464	A-genome specific (napus-rapa)	0.9880383	0.5621551	not scorable	not scorable	1	0.7828462
8461	Bn-A04-p1145007	A-genome specific (napus-rapa)	0.9880383	0.7787584	not scorable	not scorable	0.9917355	0.7894613
8462	Bn-A04-p11450339	A-genome specific (napus-rapa)	0.9856459	0.4106717	not scorable	not scorable	1	0.6537138
8463	Bn-A04-p11452549	A-genome specific (napus-rapa)	0.9832536	0.8007146	not scorable	not scorable	0.6280992	0.8014024
8464	Bn-A04-p11453319	A-genome specific (napus-rapa)	0.9760765	0.7636005	not scorable	not scorable	0.9752066	0.7483381
8465	Bn-A04-p11463316	A-genome specific (napus-rapa)	0.9832536	0.8077514	not scorable	not scorable	0.9917355	0.7443219
8466	Bn-A04-p11465588	A-genome specific (napus-rapa)	0.9760765	0.8033053	not scorable	not scorable	0.9834711	0.7806956
8467	Bn-A04-p11503303	A-genome specific (napus-rapa)	0.9569378	0.4752349	not scorable	not scorable	0.9917355	0.6312414
8468	Bn-A04-p11509775	A-genome specific (napus-rapa)	0.9928229	0.7518989	not scorable	not scorable	1	0.8014507
8469	Bn-A04-p11510797	A-genome specific (napus-rapa)	0.9928229	0.8274636	not scorable	not scorable	0.9917355	0.7951212
8470	Bn-A04-p11512379	A-genome specific (napus-rapa)	0.9808612	0.3933196	not scorable	not scorable	1	0.7762159
8471	Bn-A04-p11513352	A-genome specific (napus-rapa)	0.9976076	0.5019376	not scorable	not scorable	1	0.7811896
8472	Bn-A04-p1156390	A-genome specific (napus-rapa)	0.9976076	0.8349912	not scorable	not scorable	1	0.9175813
8473	Bn-A04-p11585127	A-genome specific (napus-rapa)	0.8971292	0.359635	not scorable	not scorable	0.9834711	0.6958169
8474	Bn-A04-p11586565	A-genome specific (rapa only)	not scorable	not scorable	not scorable	not scorable	1	0.8225167
8475	Bn-A04-p11588227	A-genome specific (napus-rapa)	1	0.3847252	not scorable	not scorable	1	0.8885585
8476	Bn-A04-p11595428	A-genome specific (napus-rapa)	0.9880383	0.3101252	not scorable	not scorable	0.9834711	0.6206599
8477	Bn-A04-p1159662	A-genome specific (napus-rapa)	0.9976076	0.4716674	not scorable	not scorable	1	0.7302722
8478	Bn-A04-p1159924	A-genome specific (napus-rapa)	1	0.8780655	not scorable	not scorable	0.9834711	0.716031

8479	Bn-A04-p1161563	A-genome specific (napus-rapa)	1	0.5672154	not scorable	not scorable	0.9834711	0.7119878
8480	Bn-A04-p11633209	A-genome specific (napus-rapa)	0.9976076	0.8097162	not scorable	not scorable	0.9834711	0.7852401
8481	Bn-A04-p11633439	napus specific	0.9856459	0.8110777	not scorable	not scorable	not scorable	not scorable
8482	Bn-A04-p11693521	A-genome specific (napus-rapa)	0.9545454	0.545512	not scorable	not scorable	0.9917355	0.6889182
8483	Bn-A04-p11734372	A-genome specific (napus-rapa)	0.9976076	0.8854356	not scorable	not scorable	0.9752066	0.7678203
8484	Bn-A04-p1176453	A-genome specific (napus-rapa)	0.9952153	0.842691	not scorable	not scorable	0.9834711	0.6823388
8485	Bn-A04-p11765380	A-genome specific (napus-rapa)	0.9880383	0.3325762	not scorable	not scorable	0.9917355	0.7874572
8486	Bn-A04-p11791366	A-genome specific (napus-rapa)	0.9593301	0.7645989	not scorable	not scorable	0.9917355	0.8616549
8487	Bn-A04-p11794986	A-genome specific (napus-rapa)	0.9856459	0.3557006	not scorable	not scorable	1	0.7020434
8488	Bn-A04-p11795779	A-genome specific (napus-rapa)	0.9952153	0.8616337	not scorable	not scorable	0.9917355	0.7747407
8489	Bn-A04-p11802638	A-genome specific (napus-rapa)	0.9521531	0.5070018	not scorable	not scorable	1	0.7100585
8490	Bn-A04-p11810574	A-genome specific (napus-rapa)	0.9976076	0.6263047	not scorable	not scorable	0.9917355	0.6756958
8491	Bn-A04-p11818064	A-genome specific (napus-rapa)	0.9880383	0.8015811	not scorable	not scorable	0.9917355	0.7505407
8492	Bn-A04-p11818085	A-genome specific (napus-rapa)	0.9880383	0.8031575	not scorable	not scorable	0.9669421	0.8119537
8493	Bn-A04-p11865854	A-genome specific (napus-rapa)	0.9976076	0.6386148	not scorable	not scorable	0.9917355	0.7938323
8494	Bn-A04-p1187598	genome unspecific (oleracea-rapa)	not scorable	not scorable	0.9689922	0.31451	0.9752066	0.820682
8495	Bn-A04-p11888044	A-genome specific (napus-rapa)	0.9952153	0.8239522	not scorable	not scorable	1	0.8415254
8496	Bn-A04-p11890416	A-genome specific (napus-rapa)	0.9952153	0.7846714	not scorable	not scorable	0.9586777	0.6934159
8497	Bn-A04-p11896581	A-genome specific (napus-rapa)	0.9641148	0.7131069	not scorable	not scorable	0.9834711	0.6972656
8498	Bn-A04-p11896696	A-genome specific (napus-rapa)	0.9521531	0.5982027	not scorable	not scorable	0.9834711	0.666065
8499	Bn-A04-p11897079	A-genome specific (napus-rapa)	0.9928229	0.836853	not scorable	not scorable	1	0.8194156
8500	Bn-A04-p11900571	A-genome specific (napus-rapa)	0.7894737	0.7905681	not scorable	not scorable	0.9752066	0.7072074
8501	Bn-A04-p11902113	A-genome specific (napus-rapa)	0.784689	0.8736613	not scorable	not scorable	0.9917355	0.749165
8502	Bn-A04-p11907565	A-genome specific (napus-rapa)	0.9712918	0.8030925	not scorable	not scorable	0.9917355	0.7898548
8503	Bn-A04-p11922910	A-genome specific (napus-rapa)	0.9856459	0.6438789	not scorable	not scorable	0.9504132	0.8122483
8504	Bn-A04-p11923310	A-genome specific (napus-rapa)	0.9497608	0.2436112	not scorable	not scorable	0.9917355	0.5414732
8505	Bn-A04-p11923511	genome unspecific (all in)	0.9736842	0.6563888	0.6899225	0.1654596	0.9917355	0.6818378
8506	Bn-A04-p11924219	A-genome specific (napus-rapa)	0.9665072	0.5526964	not scorable	not scorable	0.9752066	0.6458265
8507	Bn-A04-p11957518	A-genome specific (napus-rapa)	0.9808612	0.7100198	not scorable	not scorable	0.9917355	0.7365537
8508	Bn-A04-p11958047	A-genome specific (napus-rapa)	0.9856459	0.7228459	not scorable	not scorable	0.9752066	0.7192405
8509	Bn-A04-p11961175	A-genome specific (napus-rapa)	0.9760765	0.6170105	not scorable	not scorable	1	0.6869503
8510	Bn-A04-p11988057	A-genome specific (napus-rapa)	0.9641148	0.8002757	not scorable	not scorable	0.9917355	0.8090409
8511	Bn-A04-p12010066	A-genome specific (napus-rapa)	0.9856459	0.7861364	not scorable	not scorable	0.9917355	0.7332153

8512	Bn-A04-p12011281	A-genome specific (napus-rapa)	0.9688995	0.2105072	not scorable	not scorable	0.9669421	0.8100608
8513	Bn-A04-p12037515	A-genome specific (napus-rapa)	0.6435407	0.7702129	not scorable	not scorable	0.9669421	0.7403468
8514	Bn-A04-p12051277	A-genome specific (napus-rapa)	0.9784689	0.5457255	not scorable	not scorable	0.9917355	0.7975391
8515	Bn-A04-p12084855	A-genome specific (napus-rapa)	0.9665072	0.6273301	not scorable	not scorable	1	0.8591565
8516	Bn-A04-p12087644	A-genome specific (napus-rapa)	0.9712918	0.4148239	not scorable	not scorable	1	0.8135551
8517	Bn-A04-p12112148	A-genome specific (napus-rapa)	0.9856459	0.7860578	not scorable	not scorable	0.9917355	0.7683342
8518	Bn-A04-p12112787	A-genome specific (napus-rapa)	0.9760765	0.8157622	not scorable	not scorable	1	0.6999747
8519	Bn-A04-p12119805	A-genome specific (napus-rapa)	0.9784689	0.8352177	not scorable	not scorable	1	0.9616575
8520	Bn-A04-p1212589	A-genome specific (napus-rapa)	1	0.8316116	not scorable	not scorable	0.9917355	0.7770296
8521	Bn-A04-p1213196	A-genome specific (napus-rapa)	1	0.7703892	not scorable	not scorable	1	0.652612
8522	Bn-A04-p12151317	A-genome specific (napus-rapa)	0.9736842	0.7835966	not scorable	not scorable	0.9669421	0.7586418
8523	Bn-A04-p1215863	A-genome specific (rapa only)	not scorable	not scorable	not scorable	not scorable	1	0.6379946
8524	Bn-A04-p12159795	A-genome specific (napus-rapa)	0.6076555	0.7812938	not scorable	not scorable	0.9752066	0.851019
8525	Bn-A04-p12163578	A-genome specific (rapa only)	not scorable	not scorable	not scorable	not scorable	0.9834711	0.7744899
8526	Bn-A04-p12163967	genome unspecific (all in)	0.9521531	0.4200505	0.9922481	0.7514443	1	0.6995161
8527	Bn-A04-p12170206	A-genome specific (rapa only)	not scorable	not scorable	not scorable	not scorable	0.9752066	0.686855
8528	Bn-A04-p12204775	A-genome specific (napus-rapa)	0.9856459	0.7040867	not scorable	not scorable	0.9752066	0.6316977
8529	Bn-A04-p12205378	A-genome specific (napus-rapa)	0.9832536	0.5396996	not scorable	not scorable	1	0.7597284
8530	Bn-A04-p12210199	A-genome specific (napus-rapa)	0.9617225	0.6493184	not scorable	not scorable	1	0.7635127
8531	Bn-A04-p12211993	A-genome specific (napus-rapa)	0.9736842	0.4197427	not scorable	not scorable	0.9834711	0.8088477
8532	Bn-A04-p122280	A-genome specific (napus-rapa)	0.9808612	0.8018345	not scorable	not scorable	1	0.8522458
8533	Bn-A04-p12236240	A-genome specific (napus-rapa)	0.9808612	0.6702024	not scorable	not scorable	0.9752066	0.6860204
8534	Bn-A04-p12243311	A-genome specific (napus-rapa)	1	0.7572199	not scorable	not scorable	0.9834711	0.7137662
8535	Bn-A04-p12251256	A-genome specific (napus-rapa)	0.9808612	0.7875952	not scorable	not scorable	0.9586777	0.732173
8536	Bn-A04-p12256481	A-genome specific (napus-rapa)	0.9760765	0.7809999	not scorable	not scorable	0.9834711	0.6754586
8537	Bn-A04-p12256574	A-genome specific (napus-rapa)	0.9856459	0.7477446	not scorable	not scorable	0.9917355	0.6901799
8538	Bn-A04-p12256636	A-genome specific (napus-rapa)	0.9808612	0.7094451	not scorable	not scorable	0.9917355	0.7629602
8539	Bn-A04-p12256681	A-genome specific (napus-rapa)	0.9808612	0.8087395	not scorable	not scorable	0.9586777	0.6695005
8540	Bn-A04-p12259499	A-genome specific (napus-rapa)	0.9976076	0.6996875	not scorable	not scorable	0.9586777	0.6530643
8541	Bn-A04-p12263920	A-genome specific (rapa only)	not scorable	not scorable	not scorable	not scorable	0.9669421	0.5374677
8542	Bn-A04-p12283561	A-genome specific (napus-rapa)	0.9880383	0.7673404	not scorable	not scorable	1	0.8345054
8543	Bn-A04-p12286484	A-genome specific (napus-rapa)	1	0.8517051	not scorable	not scorable	0.9917355	0.7330995
8544	Bn-A04-p12288635	napus specific	0.9736842	0.7843238	not scorable	not scorable	not scorable	not scorable

8545	Bn-A04-p1231047	A-genome specific (napus-rapa)	0.9928229	0.8660761	not scorable	not scorable	1	0.8238707
8546	Bn-A04-p1231127	A-genome specific (napus-rapa)	1	0.576185	not scorable	not scorable	0.9917355	0.7684141
8547	Bn-A04-p12314901	A-genome specific (napus-rapa)	0.9760765	0.7342036	not scorable	not scorable	1	0.757929
8548	Bn-A04-p12314909	A-genome specific (napus-rapa)	0.9880383	0.7784563	not scorable	not scorable	0.9917355	0.7529894
8549	Bn-A04-p12328218	A-genome specific (napus-rapa)	0.9760765	0.4669247	not scorable	not scorable	0.9917355	0.7670028
8550	Bn-A04-p12333381	genome unspecific (all in)	0.9688995	0.5162895	0.9767442	0.6989626	0.9752066	0.7991898
8551	Bn-A04-p12341429	A-genome specific (napus-rapa)	0.9976076	0.5802559	not scorable	not scorable	1	0.7467578
8552	Bn-A04-p12344157	A-genome specific (napus-rapa)	0.9856459	0.7042174	not scorable	not scorable	0.9752066	0.6783651
8553	Bn-A04-p12344342	A-genome specific (napus-rapa)	0.9712918	0.7812368	not scorable	not scorable	0.9669421	0.7731624
8554	Bn-A04-p12344614	A-genome specific (napus-rapa)	0.9976076	0.6740161	not scorable	not scorable	1	0.702597
8555	Bn-A04-p12348826	A-genome specific (napus-rapa)	0.9880383	0.7575199	not scorable	not scorable	0.9834711	0.7098398
8556	Bn-A04-p12350578	A-genome specific (napus-rapa)	0.9928229	0.7566296	not scorable	not scorable	1	0.7356002
8557	Bn-A04-p12360869	A-genome specific (napus-rapa)	0.9832536	0.5093851	not scorable	not scorable	0.9834711	0.6558722
8558	Bn-A04-p12365264	A-genome specific (napus-rapa)	0.9976076	0.856564	not scorable	not scorable	1	0.8741148
8559	Bn-A04-p12372810	A-genome specific (napus-rapa)	0.9784689	0.778657	not scorable	not scorable	0.9917355	0.7854293
8560	Bn-A04-p1239789	A-genome specific (napus-rapa)	0.9952153	0.7754754	not scorable	not scorable	1	0.679424
8561	Bn-A04-p1240196	A-genome specific (napus-rapa)	0.9976076	0.894206	not scorable	not scorable	0.9752066	0.6878929
8562	Bn-A04-p12411454	A-genome specific (napus-rapa)	1	0.5601169	not scorable	not scorable	1	0.5782492
8563	Bn-A04-p1244890	A-genome specific (napus-rapa)	1	0.8515416	not scorable	not scorable	1	0.8843345
8564	Bn-A04-p12452535	A-genome specific (napus-rapa)	0.9904306	0.7998144	not scorable	not scorable	1	0.7260639
8565	Bn-A04-p12452755	A-genome specific (napus-rapa)	0.8851675	0.7591785	not scorable	not scorable	0.6198347	0.7910275
8566	Bn-A04-p12509027	A-genome specific (napus-rapa)	0.9928229	0.7594662	not scorable	not scorable	0.9834711	0.6766143
8567	Bn-A04-p12526741	A-genome specific (napus-rapa)	0.9880383	0.789998	not scorable	not scorable	0.9834711	0.6664869
8568	Bn-A04-p12527154	A-genome specific (napus-rapa)	0.9976076	0.8056944	not scorable	not scorable	1	0.7598791
8569	Bn-A04-p12540627	A-genome specific (napus-rapa)	0.9952153	0.8501765	not scorable	not scorable	0.9834711	0.6892497
8570	Bn-A04-p12541660	A-genome specific (napus-rapa)	0.9952153	0.4851846	not scorable	not scorable	0.9917355	0.7704691
8571	Bn-A04-p12542184	A-genome specific (napus-rapa)	1	0.8581874	not scorable	not scorable	1	0.8291336
8572	Bn-A04-p12547910	A-genome specific (napus-rapa)	1	0.7870187	not scorable	not scorable	0.9917355	0.6672594
8573	Bn-A04-p12550756	A-genome specific (napus-rapa)	0.9976076	0.5321653	not scorable	not scorable	0.9834711	0.662327
8574	Bn-A04-p12551118	A-genome specific (napus-rapa)	0.9976076	0.819128	not scorable	not scorable	0.9752066	0.7076954
8575	Bn-A04-p12555320	A-genome specific (napus-rapa)	0.923445	0.3905267	not scorable	not scorable	0.9669421	0.7253155
8576	Bn-A04-p12572164	A-genome specific (napus-rapa)	1	0.7876078	not scorable	not scorable	1	0.7825954
8577	Bn-A04-p12572310	all not scorable	not scorable	not scorable	not scorable	not scorable	not scorable	not scorable

8578	Bn-A04-p12572376	A-genome specific (napus-rapa)	0.9976076	0.8813428	not scorable	not scorable	0.9917355	0.7133514
8579	Bn-A04-p12572577	A-genome specific (napus-rapa)	0.9904306	0.7700216	not scorable	not scorable	0.9586777	0.7588212
8580	Bn-A04-p12575050	A-genome specific (napus-rapa)	1	0.84174	not scorable	not scorable	0.9586777	0.6798798
8581	Bn-A04-p12575115	A-genome specific (napus-rapa)	0.9904306	0.4145241	not scorable	not scorable	1	0.7552096
8582	Bn-A04-p12578822	A-genome specific (napus-rapa)	0.9928229	0.348518	not scorable	not scorable	0.9834711	0.8048924
8583	Bn-A04-p12580768	A-genome specific (napus-rapa)	1	0.887202	not scorable	not scorable	0.9917355	0.8017709
8584	Bn-A04-p12584051	A-genome specific (rapa only)	not scorable	not scorable	not scorable	not scorable	0.9586777	0.7690871
8585	Bn-A04-p12586651	A-genome specific (napus-rapa)	0.9138756	0.782023	not scorable	not scorable	0.5371901	0.8636483
8586	Bn-A04-p12599627	napus specific	0.9593301	0.201881	not scorable	not scorable	not scorable	not scorable
8587	Bn-A04-p12599843	A-genome specific (napus-rapa)	0.9808612	0.4784869	not scorable	not scorable	0.9669421	0.6997253
8588	Bn-A04-p12600635	A-genome specific (napus-rapa)	0.9880383	0.3595544	not scorable	not scorable	1	0.6998697
8589	Bn-A04-p12631523	A-genome specific (napus-rapa)	0.9712918	0.4057275	not scorable	not scorable	0.9917355	0.2845516
8590	Bn-A04-p12634330	A-genome specific (napus-rapa)	0.9904306	0.7383524	not scorable	not scorable	0.9834711	0.684243
8591	Bn-A04-p12634856	A-genome specific (napus-rapa)	0.9928229	0.7878991	not scorable	not scorable	0.9752066	0.7330651
8592	Bn-A04-p12635106	A-genome specific (napus-rapa)	0.9952153	0.7771924	not scorable	not scorable	0.9917355	0.6822492
8593	Bn-A04-p12635986	A-genome specific (napus-rapa)	0.9976076	0.7705176	not scorable	not scorable	1	0.7445042
8594	Bn-A04-p12639105	A-genome specific (napus-rapa)	0.9808612	0.6479716	not scorable	not scorable	0.9917355	0.7485714
8595	Bn-A04-p12651819	A-genome specific (napus-rapa)	0.9617225	0.4140143	not scorable	not scorable	1	0.6902404
8596	Bn-A04-p12655669	A-genome specific (napus-rapa)	1	0.5282421	not scorable	not scorable	0.9917355	0.7970411
8597	Bn-A04-p12659254	A-genome specific (napus-rapa)	0.9856459	0.7964299	not scorable	not scorable	0.9834711	0.8356597
8598	Bn-A04-p12659402	A-genome specific (napus-rapa)	0.9784689	0.7081259	not scorable	not scorable	0.9752066	0.7742743
8599	Bn-A04-p12670129	A-genome specific (napus-rapa)	1	0.5453542	not scorable	not scorable	0.9834711	0.7792304
8600	Bn-A04-p12671369	genome unspecific (all in)	0.9712918	0.3244569	0.9767442	0.7909235	0.9917355	0.8045309
8601	Bn-A04-p12675088	A-genome specific (napus-rapa)	0.9976076	0.7527285	not scorable	not scorable	0.9917355	0.7881291
8602	Bn-A04-p12677961	A-genome specific (napus-rapa)	0.5885168	0.6016579	not scorable	not scorable	0.9090909	0.4309601
8603	Bn-A04-p12680156	A-genome specific (napus-rapa)	0.9904306	0.7572992	not scorable	not scorable	1	0.7216191
8604	Bn-A04-p12687510	A-genome specific (napus-rapa)	0.9880383	0.3896342	not scorable	not scorable	1	0.7158871
8605	Bn-A04-p12690759	A-genome specific (napus-rapa)	0.9856459	0.8504075	not scorable	not scorable	1	0.6923243
8606	Bn-A04-p12691245	A-genome specific (napus-rapa)	0.9736842	0.870918	not scorable	not scorable	0.9834711	0.6862206
8607	Bn-A04-p12712772	A-genome specific (napus-rapa)	0.9928229	0.7840798	not scorable	not scorable	0.9917355	0.7836695
8608	Bn-A04-p12715859	A-genome specific (napus-rapa)	0.9904306	0.661178	not scorable	not scorable	0.9917355	0.7749835
8609	Bn-A04-p12717815	all not scorable	not scorable	not scorable	not scorable	not scorable	not scorable	not scorable
8610	Bn-A04-p12719682	A-genome specific (napus-rapa)	0.9928229	0.7902774	not scorable	not scorable	0.9917355	0.7896356

8611	Bn-A04-p12724637	A-genome specific (napus-rapa)	0.9880383	0.8367066	not scorable	not scorable	1	0.8039333
8612	Bn-A04-p12725596	A-genome specific (napus-rapa)	0.9880383	0.5598685	not scorable	not scorable	0.9834711	0.7019476
8613	Bn-A04-p12726067	A-genome specific (napus-rapa)	0.9976076	0.7111446	not scorable	not scorable	0.9834711	0.6448683
8614	Bn-A04-p12726676	A-genome specific (napus-rapa)	1	0.5776842	not scorable	not scorable	1	0.7357392
8615	Bn-A04-p12728362	A-genome specific (napus-rapa)	0.9904306	0.8155922	not scorable	not scorable	0.9917355	0.8109586
8616	Bn-A04-p12734346	A-genome specific (napus-rapa)	0.9904306	0.8220102	not scorable	not scorable	0.9917355	0.8147585
8617	Bn-A04-p12735594	A-genome specific (napus-rapa)	1	0.534942	not scorable	not scorable	0.9834711	0.7131827
8618	Bn-A04-p1274596	A-genome specific (napus-rapa)	0.9832536	0.8256963	not scorable	not scorable	0.5785124	0.8367513
8619	Bn-A04-p12749707	A-genome specific (napus-rapa)	1	0.4388056	not scorable	not scorable	0.9834711	0.6762528
8620	Bn-A04-p12751774	genome unspecific (all in)	0.9904306	0.4347689	0.9922481	0.7704939	0.6363636	0.7284935
8621	Bn-A04-p12756666	A-genome specific (napus-rapa)	0.9880383	0.7085101	not scorable	not scorable	0.9917355	0.7306235
8622	Bn-A04-p12759219	napus specific	0.9665072	0.9007835	not scorable	not scorable	not scorable	not scorable
8623	Bn-A04-p12759499	genome unspecific (oleracea-rapa)	not scorable	not scorable	0.9457364	0.749599	0.9917355	0.7714852
8624	Bn-A04-p1276308	A-genome specific (napus-rapa)	0.9904306	0.7677081	not scorable	not scorable	0.9917355	0.7819209
8625	Bn-A04-p12764046	A-genome specific (napus-rapa)	0.9808612	0.7533298	not scorable	not scorable	0.9256198	0.8407952
8626	Bn-A04-p12769407	A-genome specific (napus-rapa)	0.9880383	0.8697634	not scorable	not scorable	0.9586777	0.7432804
8627	Bn-A04-p12772506	A-genome specific (napus-rapa)	0.05263158	0.8013936	not scorable	not scorable	0.9917355	0.7865393
8628	Bn-A04-p12782652	A-genome specific (rapa only)	not scorable	not scorable	not scorable	not scorable	0.9917355	0.7775161
8629	Bn-A04-p12793333	A-genome specific (napus-rapa)	0.9928229	0.7679189	not scorable	not scorable	0.9917355	0.8519257
8630	Bn-A04-p12793456	A-genome specific (napus-rapa)	0.9904306	0.3446214	not scorable	not scorable	0.9752066	0.6652234
8631	Bn-A04-p12793487	A-genome specific (napus-rapa)	0.9880383	0.6725469	not scorable	not scorable	1	0.7793756
8632	Bn-A04-p1279752	A-genome specific (rapa only)	not scorable	not scorable	not scorable	not scorable	0.9917355	0.7881186
8633	Bn-A04-p12798976	A-genome specific (napus-rapa)	0.9904306	0.8269345	not scorable	not scorable	1	0.665654
8634	Bn-A04-p12802914	A-genome specific (napus-rapa)	0.9784689	0.3349117	not scorable	not scorable	0.9752066	0.7696701
8635	Bn-A04-p12818353	A-genome specific (napus-rapa)	0.9736842	0.7500497	not scorable	not scorable	1	0.7554216
8636	Bn-A04-p12818639	A-genome specific (napus-rapa)	0.9832536	0.7678173	not scorable	not scorable	0.9917355	0.6911854
8637	Bn-A04-p12819355	A-genome specific (napus-rapa)	0.9832536	0.8111658	not scorable	not scorable	0.9917355	0.7915535
8638	Bn-A04-p12820145	napus specific	0.8875598	0.6984441	not scorable	not scorable	not scorable	not scorable
8639	Bn-A04-p12822929	A-genome specific (napus-rapa)	0.9760765	0.8296344	not scorable	not scorable	0.9834711	0.6924461
8640	Bn-A04-p12824172	A-genome specific (napus-rapa)	0.9688995	0.746143	not scorable	not scorable	0.9917355	0.7352476
8641	Bn-A04-p1282528	A-genome specific (napus-rapa)	1	0.3911941	not scorable	not scorable	0.9752066	0.7070133
8642	Bn-A04-p12825295	A-genome specific (napus-rapa)	0.9736842	0.7659827	not scorable	not scorable	1	0.7064683
8643	Bn-A04-p12826986	A-genome specific (napus-rapa)	0.9808612	0.8069044	not scorable	not scorable	0.9834711	0.7321525

8644	Bn-A04-p12827056	A-genome specific (napus-rapa)	0.9688995	0.7772141	not scorable	not scorable	1	0.7813382
8645	Bn-A04-p12827460	A-genome specific (napus-rapa)	0.9712918	0.7868294	not scorable	not scorable	1	0.6534175
8646	Bn-A04-p12829159	A-genome specific (napus-rapa)	0.9760765	0.7121423	not scorable	not scorable	0.9917355	0.6745057
8647	Bn-A04-p12829470	A-genome specific (napus-rapa)	0.9808612	0.7660738	not scorable	not scorable	0.9917355	0.8163778
8648	Bn-A04-p12840183	genome unspecific (all in)	0.9880383	0.5810961	0.9767442	0.7127693	0.9752066	0.6296021
8649	Bn-A04-p12857210	A-genome specific (rapa only)	not scorable	not scorable	not scorable	not scorable	0.9917355	0.7645889
8650	Bn-A04-p12857974	A-genome specific (napus-rapa)	0.9904306	0.5482713	not scorable	not scorable	0.9917355	0.685105
8651	Bn-A04-p12868057	A-genome specific (napus-rapa)	0.9808612	0.4549513	not scorable	not scorable	0.9752066	0.7079678
8652	Bn-A04-p12877134	A-genome specific (napus-rapa)	0.9641148	0.3385034	not scorable	not scorable	1	0.6998524
8653	Bn-A04-p12888388	genome unspecific (all in)	0.9330143	0.2337195	1	0.1970024	1	0.6242925
8654	Bn-A04-p12891881	A-genome specific (napus-rapa)	0.854067	0.8536795	not scorable	not scorable	0.5785124	0.8305143
8655	Bn-A04-p12893331	A-genome specific (napus-rapa)	0.9880383	0.7850089	not scorable	not scorable	0.9834711	0.6968538
8656	Bn-A04-p12893630	A-genome specific (napus-rapa)	0.9497608	0.8350449	not scorable	not scorable	0.9834711	0.7015526
8657	Bn-A04-p12893753	A-genome specific (napus-rapa)	0.9736842	0.8238004	not scorable	not scorable	0.9834711	0.7710534
8658	Bn-A04-p12894555	A-genome specific (napus-rapa)	1	0.8443248	not scorable	not scorable	0.9834711	0.7292119
8659	Bn-A04-p12897769	A-genome specific (napus-rapa)	0.3516746	0.8721944	not scorable	not scorable	1	0.7732308
8660	Bn-A04-p12899754	A-genome specific (napus-rapa)	0.9832536	0.7109562	not scorable	not scorable	1	0.6983694
8661	Bn-A04-p12899869	A-genome specific (napus-rapa)	0.9808612	0.4843763	not scorable	not scorable	1	0.7689446
8662	Bn-A04-p12901227	A-genome specific (napus-rapa)	0.9617225	0.7143648	not scorable	not scorable	0.9917355	0.7945212
8663	Bn-A04-p1291618	A-genome specific (napus-rapa)	0.9952153	0.635577	not scorable	not scorable	0.9917355	0.6505196
8664	Bn-A04-p12917949	genome unspecific (all in)	0.9928229	0.6301141	0.9844961	0.7352682	1	0.5818597
8665	Bn-A04-p12942400	A-genome specific (napus-rapa)	1	0.4237649	not scorable	not scorable	1	0.6743978
8666	Bn-A04-p12953418	A-genome specific (napus-rapa)	1	0.7397565	not scorable	not scorable	1	0.826506
8667	Bn-A04-p12959668	A-genome specific (napus-rapa)	0.9688995	0.7584568	not scorable	not scorable	0.9917355	0.8393419
8668	Bn-A04-p12985030	all not scorable	not scorable	not scorable	not scorable	not scorable	not scorable	not scorable
8669	Bn-A04-p12987331	A-genome specific (napus-rapa)	0.9712918	0.356707	not scorable	not scorable	0.9256198	0.7178574
8670	Bn-A04-p12994676	A-genome specific (rapa only)	not scorable	not scorable	not scorable	not scorable	1	0.689185
8671	Bn-A04-p13009893	A-genome specific (napus-rapa)	0.9665072	0.7515081	not scorable	not scorable	0.9917355	0.6543237
8672	Bn-A04-p13050240	A-genome specific (napus-rapa)	0.9784689	0.5026264	not scorable	not scorable	0.7438017	0.7940515
8673	Bn-A04-p13052013	A-genome specific (napus-rapa)	0.9521531	0.3152596	not scorable	not scorable	0.9917355	0.7780165
8674	Bn-A04-p13056296	A-genome specific (napus-rapa)	0.9808612	0.788011	not scorable	not scorable	0.9834711	0.7397541
8675	Bn-A04-p13056382	A-genome specific (napus-rapa)	0.9688995	0.7818384	not scorable	not scorable	0.9752066	0.7636859
8676	Bn-A04-p13056438	all not scorable	not scorable	not scorable	not scorable	not scorable	not scorable	not scorable

8677	Bn-A04-p13059166	A-genome specific (napus-rapa)	0.9497608	0.740001	not scorable	not scorable	1	0.8356096
8678	Bn-A04-p13059485	A-genome specific (napus-rapa)	0.9928229	0.7723844	not scorable	not scorable	0.9917355	0.8338223
8679	Bn-A04-p13061139	A-genome specific (napus-rapa)	0.9952153	0.7673975	not scorable	not scorable	1	0.7832446
8680	Bn-A04-p13062081	A-genome specific (napus-rapa)	0.9617225	0.6712302	not scorable	not scorable	0.9586777	0.7570057
8681	Bn-A04-p13063145	A-genome specific (napus-rapa)	0.9880383	0.7554143	not scorable	not scorable	0.9917355	0.6814371
8682	Bn-A04-p13068545	A-genome specific (napus-rapa)	0.9569378	0.6437039	not scorable	not scorable	0.9834711	0.8295368
8683	Bn-A04-p13069741	A-genome specific (napus-rapa)	0.9760765	0.4607904	not scorable	not scorable	1	0.7015526
8684	Bn-A04-p13078078	A-genome specific (napus-rapa)	0.9425837	0.5950263	not scorable	not scorable	1	0.8085203
8685	Bn-A04-p13091624	A-genome specific (napus-rapa)	0.9760765	0.6470073	not scorable	not scorable	0.9917355	0.5802294
8686	Bn-A04-p13096878	A-genome specific (napus-rapa)	0.9928229	0.7962025	not scorable	not scorable	0.9834711	0.7447971
8687	Bn-A04-p13107522	A-genome specific (napus-rapa)	0.9928229	0.5788236	not scorable	not scorable	0.9834711	0.7612342
8688	Bn-A04-p13112913	A-genome specific (napus-rapa)	0.9928229	0.6347869	not scorable	not scorable	0.9917355	0.7952991
8689	Bn-A04-p1311487	A-genome specific (napus-rapa)	0.9952153	0.6997204	not scorable	not scorable	0.9917355	0.6630068
8690	Bn-A04-p1311567	A-genome specific (napus-rapa)	0.9928229	0.7247425	not scorable	not scorable	1	0.6756914
8691	Bn-A04-p13116453	genome unspecific (all in)	0.8732057	0.5862358	1	0.8160813	0.9917355	0.6668433
8692	Bn-A04-p13118144	A-genome specific (napus-rapa)	0.9976076	0.7129986	not scorable	not scorable	1	0.6874943
8693	Bn-A04-p13119516	genome unspecific (all in)	0.9952153	0.5772075	0.8372093	0.8750586	0.9917355	0.7052052
8694	Bn-A04-p13122589	A-genome specific (napus-rapa)	0.9784689	0.7765417	not scorable	not scorable	0.9917355	0.824479
8695	Bn-A04-p13125820	A-genome specific (napus-rapa)	0.9832536	0.7866411	not scorable	not scorable	1	0.8492312
8696	Bn-A04-p13128840	A-genome specific (napus-rapa)	0.9473684	0.6920022	not scorable	not scorable	0.9917355	0.6851635
8697	Bn-A04-p13129093	A-genome specific (napus-rapa)	0.9760765	0.4086539	not scorable	not scorable	0.9917355	0.7748213
8698	Bn-A04-p13129306	A-genome specific (napus-rapa)	0.9808612	0.7822734	not scorable	not scorable	0.9834711	0.7596551
8699	Bn-A04-p13129601	A-genome specific (napus-rapa)	0.9880383	0.7583378	not scorable	not scorable	0.9917355	0.8260867
8700	Bn-A04-p13129741	A-genome specific (napus-rapa)	0.9832536	0.8099283	not scorable	not scorable	0.9917355	0.7835218
8701	Bn-A04-p13140266	A-genome specific (napus-rapa)	0.9425837	0.5901919	not scorable	not scorable	1	0.7931106
8702	Bn-A04-p13145539	A-genome specific (napus-rapa)	0.9688995	0.5638658	not scorable	not scorable	0.9834711	0.715457
8703	Bn-A04-p13173919	A-genome specific (napus-rapa)	0.9832536	0.8129992	not scorable	not scorable	0.9917355	0.7114246
8704	Bn-A04-p1317446	genome unspecific (all in)	0.9808612	0.4493911	0.9612403	0.484121	0.9834711	0.7016078
8705	Bn-A04-p1318613	A-genome specific (napus-rapa)	0.9952153	0.7837514	not scorable	not scorable	0.9834711	0.7764203
8706	Bn-A04-p13196638	A-genome specific (rapa only)	not scorable	not scorable	not scorable	not scorable	0.9752066	0.6712735
8707	Bn-A04-p13206432	A-genome specific (napus-rapa)	0.9569378	0.5369815	not scorable	not scorable	0.9834711	0.707193
8708	Bn-A04-p13207715	A-genome specific (napus-rapa)	0.9928229	0.3652149	not scorable	not scorable	1	0.55558
8709	Bn-A04-p13208442	A-genome specific (napus-rapa)	0.9210526	0.3328519	not scorable	not scorable	0.9421487	0.6106737

8710	Bn-A04-p13215929	A-genome specific (napus-rapa)	0.9856459	0.7888114	not scorable	not scorable	0.9917355	0.7608863
8711	Bn-A04-p13216114	A-genome specific (napus-rapa)	0.9832536	0.7922563	not scorable	not scorable	0.9917355	0.7896386
8712	Bn-A04-p1322758	A-genome specific (napus-rapa)	0.9976076	0.7649282	not scorable	not scorable	0.9586777	0.6355415
8713	Bn-A04-p13235708	A-genome specific (napus-rapa)	0.9856459	0.8409498	not scorable	not scorable	0.9669421	0.782364
8714	Bn-A04-p1323611	A-genome specific (napus-rapa)	0.9784689	0.933138	not scorable	not scorable	0.9917355	0.6883734
8715	Bn-A04-p13241959	A-genome specific (napus-rapa)	0.9425837	0.2512828	not scorable	not scorable	0.9917355	0.6633003
8716	Bn-A04-p1324549	A-genome specific (napus-rapa)	0.9497608	0.8019532	not scorable	not scorable	0.9586777	0.6592495
8717	Bn-A04-p13245778	A-genome specific (napus-rapa)	0.9473684	0.5452483	not scorable	not scorable	0.9669421	0.6492565
8718	Bn-A04-p13246454	A-genome specific (napus-rapa)	0.9760765	0.5265793	not scorable	not scorable	0.9917355	0.7909213
8719	Bn-A04-p13247108	A-genome specific (napus-rapa)	0.9210526	0.8356755	not scorable	not scorable	0.9834711	0.817649
8720	Bn-A04-p13249011	A-genome specific (napus-rapa)	0.9784689	0.7507092	not scorable	not scorable	1	0.7756329
8721	Bn-A04-p13269135	A-genome specific (napus-rapa)	0.9808612	0.7297376	not scorable	not scorable	1	0.4341539
8722	Bn-A04-p13286494	A-genome specific (napus-rapa)	0.9952153	0.7799457	not scorable	not scorable	0.9752066	0.8097162
8723	Bn-A04-p1329352	A-genome specific (napus-rapa)	0.9976076	0.8132388	not scorable	not scorable	0.6033058	0.6661707
8724	Bn-A04-p13293545	A-genome specific (napus-rapa)	0.9569378	0.577386	not scorable	not scorable	1	0.7725486
8725	Bn-A04-p13293640	A-genome specific (napus-rapa)	0.9688995	0.6529618	not scorable	not scorable	1	0.7933806
8726	Bn-A04-p13294322	A-genome specific (napus-rapa)	0.9760765	0.7063718	not scorable	not scorable	0.6280992	0.6914117
8727	Bn-A04-p1329438	A-genome specific (napus-rapa)	0.9976076	0.5550905	not scorable	not scorable	0.6198347	0.6314523
8728	Bn-A04-p13302511	A-genome specific (napus-rapa)	0.9928229	0.78348	not scorable	not scorable	1	0.7660799
8729	Bn-A04-p1330321	A-genome specific (napus-rapa)	0.9832536	0.6773221	not scorable	not scorable	0.9834711	0.7964904
8730	Bn-A04-p13306369	A-genome specific (napus-rapa)	0.9688995	0.7725218	not scorable	not scorable	0.9917355	0.7251441
8731	Bn-A04-p13330392	A-genome specific (napus-rapa)	0.9138756	0.534896	not scorable	not scorable	0.6446281	0.8413619
8732	Bn-A04-p13336924	A-genome specific (napus-rapa)	0.9760765	0.7862427	not scorable	not scorable	1	0.8537028
8733	Bn-A04-p13368998	A-genome specific (napus-rapa)	0.9952153	0.7176596	not scorable	not scorable	0.9834711	0.7003902
8734	Bn-A04-p13370433	A-genome specific (napus-rapa)	0.9784689	0.7979206	not scorable	not scorable	1	0.7933572
8735	Bn-A04-p13372886	A-genome specific (napus-rapa)	0.9832536	0.7647925	not scorable	not scorable	1	0.7714852
8736	Bn-A04-p13384606	A-genome specific (napus-rapa)	0.9760765	0.740822	not scorable	not scorable	1	0.7381898
8737	Bn-A04-p13389366	A-genome specific (napus-rapa)	0.9832536	0.7011939	not scorable	not scorable	0.9752066	0.6765223
8738	Bn-A04-p13389622	A-genome specific (napus-rapa)	0.9880383	0.7084573	not scorable	not scorable	1	0.7139006
8739	Bn-A04-p13389743	A-genome specific (napus-rapa)	0.9856459	0.7794688	not scorable	not scorable	1	0.791757
8740	Bn-A04-p13389784	A-genome specific (napus-rapa)	0.9928229	0.7883298	not scorable	not scorable	0.9917355	0.8145373
8741	Bn-A04-p13390838	A-genome specific (napus-rapa)	0.9665072	0.7692496	not scorable	not scorable	1	0.8222939
8742	Bn-A04-p13394479	A-genome specific (napus-rapa)	0.9880383	0.6972967	not scorable	not scorable	0.9917355	0.7416757

8743	Bn-A04-p13398568	A-genome specific (napus-rapa)	0.9808612	0.7532398	not scorable	not scorable	0.9917355	0.7918411
8744	Bn-A04-p13418563	A-genome specific (napus-rapa)	0.9904306	0.773893	not scorable	not scorable	0.9917355	0.7629912
8745	Bn-A04-p13428137	A-genome specific (napus-rapa)	0.9736842	0.7791185	not scorable	not scorable	1	0.823853
8746	Bn-A04-p13428243	A-genome specific (napus-rapa)	0.9880383	0.7864855	not scorable	not scorable	1	0.7442375
8747	Bn-A04-p13428324	A-genome specific (napus-rapa)	0.9808612	0.7360375	not scorable	not scorable	1	0.7960946
8748	Bn-A04-p13429557	A-genome specific (napus-rapa)	0.9545454	0.3825476	not scorable	not scorable	1	0.6558536
8749	Bn-A04-p13430155	A-genome specific (napus-rapa)	0.9856459	0.7677682	not scorable	not scorable	1	0.7701179
8750	Bn-A04-p13430376	A-genome specific (napus-rapa)	0.9832536	0.7854255	not scorable	not scorable	0.9917355	0.7924891
8751	Bn-A04-p13430818	A-genome specific (napus-rapa)	0.9928229	0.7541033	not scorable	not scorable	0.9917355	0.808763
8752	Bn-A04-p13431005	A-genome specific (napus-rapa)	0.9784689	0.763966	not scorable	not scorable	1	0.7095973
8753	Bn-A04-p13461501	A-genome specific (napus-rapa)	0.9880383	0.4052474	not scorable	not scorable	0.9834711	0.7485737
8754	Bn-A04-p1346738	A-genome specific (napus-rapa)	0.9904306	0.8585643	not scorable	not scorable	1	0.8165873
8755	Bn-A04-p13490747	A-genome specific (napus-rapa)	0.9736842	0.3502563	not scorable	not scorable	0.9752066	0.7025554
8756	Bn-A04-p1349255	A-genome specific (napus-rapa)	0.9928229	0.333895	not scorable	not scorable	0.9752066	0.6834141
8757	Bn-A04-p13493883	A-genome specific (rapa only)	not scorable	not scorable	not scorable	not scorable	1	0.7171257
8758	Bn-A04-p13497608	A-genome specific (napus-rapa)	0.9856459	0.7837802	not scorable	not scorable	0.9917355	0.8219877
8759	Bn-A04-p13501943	A-genome specific (napus-rapa)	0.9976076	0.5632286	not scorable	not scorable	1	0.8370017
8760	Bn-A04-p13504670	A-genome specific (napus-rapa)	1	0.771015	not scorable	not scorable	0.9834711	0.8017088
8761	Bn-A04-p13505017	A-genome specific (napus-rapa)	0.9928229	0.5897554	not scorable	not scorable	0.9834711	0.7983969
8762	Bn-A04-p13510301	A-genome specific (napus-rapa)	0.9976076	0.4915183	not scorable	not scorable	0.9834711	0.8000414
8763	Bn-A04-p1351337	A-genome specific (napus-rapa)	1	0.7278644	not scorable	not scorable	1	0.7056748
8764	Bn-A04-p13516223	A-genome specific (napus-rapa)	0.9952153	0.5690843	not scorable	not scorable	0.9504132	0.4879051
8765	Bn-A04-p13516274	A-genome specific (napus-rapa)	0.9354067	0.6560977	not scorable	not scorable	1	0.5571658
8766	Bn-A04-p13516285	A-genome specific (napus-rapa)	0.9976076	0.8222326	not scorable	not scorable	0.9586777	0.72562
8767	Bn-A04-p13516411	A-genome specific (napus-rapa)	1	0.8198941	not scorable	not scorable	0.9834711	0.7302921
8768	Bn-A04-p13517158	A-genome specific (napus-rapa)	0.9976076	0.8421408	not scorable	not scorable	0.9586777	0.7205924
8769	Bn-A04-p13520736	A-genome specific (napus-rapa)	0.9952153	0.890413	not scorable	not scorable	1	0.7099233
8770	Bn-A04-p13521376	A-genome specific (napus-rapa)	0.9976076	0.8757938	not scorable	not scorable	0.9752066	0.7975124
8771	Bn-A04-p13521405	A-genome specific (napus-rapa)	0.9928229	0.7897244	not scorable	not scorable	0.9834711	0.7771454
8772	Bn-A04-p1353175	A-genome specific (rapa only)	not scorable	not scorable	not scorable	not scorable	0.9834711	0.7040766
8773	Bn-A04-p1353238	A-genome specific (napus-rapa)	0.9952153	0.8877921	not scorable	not scorable	1	0.8387109
8774	Bn-A04-p13532829	A-genome specific (napus-rapa)	1	0.3716237	not scorable	not scorable	1	0.7949167
8775	Bn-A04-p13534631	all not scorable	not scorable	not scorable	not scorable	not scorable	not scorable	not scorable

8776	Bn-A04-p13541465	A-genome specific (napus-rapa)	1	0.7668424	not scorable	not scorable	0.9834711	0.7095779
8777	Bn-A04-p13552122	A-genome specific (napus-rapa)	1	0.4140143	not scorable	not scorable	0.9917355	0.6581989
8778	Bn-A04-p1355502	A-genome specific (napus-rapa)	1	0.7587216	not scorable	not scorable	0.9752066	0.6418401
8779	Bn-A04-p1356122	A-genome specific (napus-rapa)	0.9760765	0.6029772	not scorable	not scorable	0.9669421	0.6607398
8780	Bn-A04-p13562332	A-genome specific (napus-rapa)	1	0.3485843	not scorable	not scorable	0.9752066	0.7939828
8781	Bn-A04-p13572341	A-genome specific (napus-rapa)	0.9760765	0.7850365	not scorable	not scorable	0.7272727	0.828771
8782	Bn-A04-p13578411	A-genome specific (napus-rapa)	0.9976076	0.4900584	not scorable	not scorable	0.9917355	0.8312033
8783	Bn-A04-p13583325	A-genome specific (napus-rapa)	0.9784689	0.6803538	not scorable	not scorable	0.9834711	0.7169108
8784	Bn-A04-p13589498	A-genome specific (napus-rapa)	0.9976076	0.3747656	not scorable	not scorable	0.9834711	0.6746309
8785	Bn-A04-p13600415	A-genome specific (napus-rapa)	1	0.320516	not scorable	not scorable	0.9917355	0.5273464
8786	Bn-A04-p13615482	A-genome specific (napus-rapa)	1	0.6960961	not scorable	not scorable	1	0.812517
8787	Bn-A04-p13645293	A-genome specific (napus-rapa)	0.9641148	0.411984	not scorable	not scorable	0.9917355	0.3943763
8788	Bn-A04-p13655958	A-genome specific (napus-rapa)	0.9952153	0.787613	not scorable	not scorable	1	0.7034411
8789	Bn-A04-p13659690	A-genome specific (napus-rapa)	0.9952153	0.8111996	not scorable	not scorable	1	0.7787471
8790	Bn-A04-p13665489	A-genome specific (napus-rapa)	0.9665072	0.4916672	not scorable	not scorable	0.9917355	0.7540017
8791	Bn-A04-p13670045	A-genome specific (napus-rapa)	0.9952153	0.4756983	not scorable	not scorable	0.9917355	0.7915851
8792	Bn-A04-p13685561	A-genome specific (napus-rapa)	0.9808612	0.7610233	not scorable	not scorable	1	0.8138438
8793	Bn-A04-p13689816	A-genome specific (napus-rapa)	0.9760765	0.8012473	not scorable	not scorable	0.9834711	0.9360203
8794	Bn-A04-p13705410	A-genome specific (napus-rapa)	0.9784689	0.450924	not scorable	not scorable	1	0.7932116
8795	Bn-A04-p13705548	A-genome specific (napus-rapa)	0.9784689	0.6924239	not scorable	not scorable	0.9834711	0.5871264
8796	Bn-A04-p13705636	A-genome specific (napus-rapa)	0.9952153	0.7597603	not scorable	not scorable	1	0.7812651
8797	Bn-A04-p13705760	A-genome specific (napus-rapa)	0.9976076	0.7714643	not scorable	not scorable	0.9834711	0.7710491
8798	Bn-A04-p13711282	A-genome specific (napus-rapa)	0.9832536	0.7933822	not scorable	not scorable	0.9917355	0.8299816
8799	Bn-A04-p13726056	genome unspecific (all in)	0.937799	0.4401062	0.9767442	0.7996681	0.9917355	0.7209969
8800	Bn-A04-p13738279	A-genome specific (napus-rapa)	0.9880383	0.8017275	not scorable	not scorable	0.9834711	0.8184816
8801	Bn-A04-p13761897	A-genome specific (napus-rapa)	0.8229665	0.762412	not scorable	not scorable	0.9834711	0.808535
8802	Bn-A04-p13767868	A-genome specific (napus-rapa)	0.8277512	0.8096746	not scorable	not scorable	0.9834711	0.8228822
8803	Bn-A04-p1377226	A-genome specific (napus-rapa)	0.9976076	0.5630221	not scorable	not scorable	0.9917355	0.7894242
8804	Bn-A04-p13856134	A-genome specific (napus-rapa)	1	0.5565655	not scorable	not scorable	0.9752066	0.5433732
8805	Bn-A04-p13859935	A-genome specific (napus-rapa)	1	0.7825954	not scorable	not scorable	1	0.7584673
8806	Bn-A04-p13866861	A-genome specific (napus-rapa)	0.9210526	0.8000322	not scorable	not scorable	0.9917355	0.914403
8807	Bn-A04-p13899576	A-genome specific (napus-rapa)	0.9976076	0.4361346	not scorable	not scorable	1	0.8263542
8808	Bn-A04-p13900963	A-genome specific (napus-rapa)	0.9976076	0.8466083	not scorable	not scorable	0.9752066	0.6504048

8809	Bn-A04-p13901008	A-genome specific (napus-rapa)	0.9976076	0.8965474	not scorable	not scorable	0.9752066	0.7032007
8810	Bn-A04-p1390449	A-genome specific (napus-rapa)	0.9952153	0.5500242	not scorable	not scorable	1	0.6708648
8811	Bn-A04-p13910168	A-genome specific (napus-rapa)	1	0.8595471	not scorable	not scorable	1	0.6798391
8812	Bn-A04-p13910822	A-genome specific (rapa only)	not scorable	not scorable	not scorable	not scorable	0.9752066	0.741864
8813	Bn-A04-p13927295	A-genome specific (rapa only)	not scorable	not scorable	not scorable	not scorable	0.9669421	0.8095517
8814	Bn-A04-p13930713	A-genome specific (rapa only)	not scorable	not scorable	not scorable	not scorable	0.9917355	0.6521096
8815	Bn-A04-p13992889	A-genome specific (napus-rapa)	0.9952153	0.3358991	not scorable	not scorable	0.9917355	0.7116842
8816	Bn-A04-p13993666	A-genome specific (napus-rapa)	1	0.8533637	not scorable	not scorable	0.9917355	0.7772585
8817	Bn-A04-p13993683	A-genome specific (napus-rapa)	1	0.8535424	not scorable	not scorable	0.9917355	0.7222244
8818	Bn-A04-p13995098	napus specific	1	0.757318	not scorable	not scorable	not scorable	not scorable
8819	Bn-A04-p14008077	A-genome specific (napus-rapa)	1	0.7271194	not scorable	not scorable	1	0.8213393
8820	Bn-A04-p14018867	A-genome specific (napus-rapa)	1	0.9373063	not scorable	not scorable	1	0.7610074
8821	Bn-A04-p14020738	A-genome specific (napus-rapa)	1	0.8047376	not scorable	not scorable	1	0.6965014
8822	Bn-A04-p14020855	napus specific	0.9880383	0.9122071	not scorable	not scorable	not scorable	not scorable
8823	Bn-A04-p14023419	A-genome specific (napus-rapa)	0.9976076	0.8546386	not scorable	not scorable	0.661157	0.7589057
8824	Bn-A04-p14026143	A-genome specific (napus-rapa)	1	0.6673465	not scorable	not scorable	0.9834711	0.662568
8825	Bn-A04-p14060096	A-genome specific (napus-rapa)	1	0.7592424	not scorable	not scorable	1	0.7705707
8826	Bn-A04-p14092385	genome unspecific (all in)	0.9928229	0.3395887	0.9922481	0.83543	1	0.8948976
8827	Bn-A04-p14156506	A-genome specific (napus-rapa)	0.9808612	0.7853199	not scorable	not scorable	0.9669421	0.8099291
8828	Bn-A04-p14159293	A-genome specific (napus-rapa)	0.9832536	0.5484189	not scorable	not scorable	1	0.7437668
8829	Bn-A04-p14159494	A-genome specific (rapa only)	not scorable	not scorable	not scorable	not scorable	0.9752066	0.7406795
8830	Bn-A04-p14159558	A-genome specific (napus-rapa)	0.9736842	0.5130865	not scorable	not scorable	0.9834711	0.6786793
8831	Bn-A04-p1416287	A-genome specific (napus-rapa)	0.9856459	0.6835722	not scorable	not scorable	1	0.7125415
8832	Bn-A04-p1417481	A-genome specific (napus-rapa)	0.9952153	0.4594841	not scorable	not scorable	1	0.7728057
8833	Bn-A04-p14226310	A-genome specific (napus-rapa)	1	0.7012544	not scorable	not scorable	0.9834711	0.7769274
8834	Bn-A04-p14255771	A-genome specific (napus-rapa)	1	0.6149848	not scorable	not scorable	0.9669421	0.4718842
8835	Bn-A04-p14271109	A-genome specific (rapa only)	not scorable	not scorable	not scorable	not scorable	0.6528926	0.6085756
8836	Bn-A04-p14271110	A-genome specific (napus-rapa)	1	0.7752897	not scorable	not scorable	0.9917355	0.7002603
8837	Bn-A04-p14271161	A-genome specific (napus-rapa)	0.9928229	0.7585056	not scorable	not scorable	0.9752066	0.7125888
8838	Bn-A04-p14289058	A-genome specific (napus-rapa)	1	0.8327709	not scorable	not scorable	1	0.6784759
8839	Bn-A04-p1429850	A-genome specific (napus-rapa)	0.9976076	0.7378104	not scorable	not scorable	0.6033058	0.8033731
8840	Bn-A04-p14312019	A-genome specific (napus-rapa)	1	0.84258	not scorable	not scorable	1	0.7755565
8841	Bn-A04-p14315336	A-genome specific (napus-rapa)	0.9856459	0.4180059	not scorable	not scorable	0.9917355	0.7846863

8842	Bn-A04-p14332759	A-genome specific (napus-rapa)	0.9521531	0.4363637	not scorable	not scorable	0.9752066	0.6968851
8843	Bn-A04-p14333001	A-genome specific (napus-rapa)	0.9784689	0.341587	not scorable	not scorable	1	0.7111393
8844	Bn-A04-p14353463	A-genome specific (napus-rapa)	1	0.3971906	not scorable	not scorable	0.9917355	0.6136271
8845	Bn-A04-p14357191	A-genome specific (napus-rapa)	0.9904306	0.7903373	not scorable	not scorable	0.9834711	0.752178
8846	Bn-A04-p14357445	A-genome specific (napus-rapa)	0.9880383	0.3883097	not scorable	not scorable	0.9917355	0.6916431
8847	Bn-A04-p14360385	A-genome specific (napus-rapa)	0.9808612	0.776	not scorable	not scorable	0.9834711	0.7325151
8848	Bn-A04-p14360799	A-genome specific (napus-rapa)	0.9617225	0.4874062	not scorable	not scorable	1	0.8318096
8849	Bn-A04-p14365868	A-genome specific (napus-rapa)	0.9880383	0.8163774	not scorable	not scorable	0.9752066	0.7795016
8850	Bn-A04-p14380140	A-genome specific (napus-rapa)	0.9784689	0.7819045	not scorable	not scorable	1	0.8394032
8851	Bn-A04-p14397957	A-genome specific (napus-rapa)	0.9736842	0.6998108	not scorable	not scorable	0.9669421	0.6785831
8852	Bn-A04-p14398335	A-genome specific (napus-rapa)	0.9856459	0.8058596	not scorable	not scorable	0.9752066	0.8375925
8853	Bn-A04-p14410667	A-genome specific (napus-rapa)	0.9832536	0.7819387	not scorable	not scorable	1	0.7518701
8854	Bn-A04-p14478925	A-genome specific (napus-rapa)	0.9832536	0.7692937	not scorable	not scorable	0.9917355	0.6623275
8855	Bn-A04-p14479867	A-genome specific (napus-rapa)	0.9976076	0.46312	not scorable	not scorable	0.9834711	0.6397657
8856	Bn-A04-p14483727	A-genome specific (napus-rapa)	0.9952153	0.8228639	not scorable	not scorable	0.9834711	0.7672791
8857	Bn-A04-p1451594	A-genome specific (napus-rapa)	0.9880383	0.8188927	not scorable	not scorable	0.6694215	0.7177484
8858	Bn-A04-p14528871	A-genome specific (napus-rapa)	0.9665072	0.5166218	not scorable	not scorable	0.9917355	0.849865
8859	Bn-A04-p14532353	A-genome specific (napus-rapa)	0.9449761	0.66468	not scorable	not scorable	1	0.8815182
8860	Bn-A04-p1454434	A-genome specific (napus-rapa)	1	0.8628727	not scorable	not scorable	1	0.7907986
8861	Bn-A04-p14559897	A-genome specific (napus-rapa)	0.9952153	0.3454482	not scorable	not scorable	0.9834711	0.7780501
8862	Bn-A04-p14563007	A-genome specific (napus-rapa)	0.5526316	0.921045	not scorable	not scorable	0.661157	0.9240721
8863	Bn-A04-p14565138	A-genome specific (napus-rapa)	0.9928229	0.7123283	not scorable	not scorable	0.9504132	0.6689837
8864	Bn-A04-p14565883	A-genome specific (napus-rapa)	0.9856459	0.8046634	not scorable	not scorable	0.9834711	0.8255138
8865	Bn-A04-p14572391	A-genome specific (napus-rapa)	1	0.5506166	not scorable	not scorable	0.9834711	0.822262
8866	Bn-A04-p14582431	genome unspecific (oleracea-rapa)	not scorable	not scorable	1	0.6185355	1	0.7172861
8867	Bn-A04-p14582584	A-genome specific (napus-rapa)	0.9832536	0.5569968	not scorable	not scorable	0.9669421	0.6119093
8868	Bn-A04-p14586235	A-genome specific (napus-rapa)	0.9473684	0.5395532	not scorable	not scorable	1	0.6168636
8869	Bn-A04-p14588183	A-genome specific (napus-rapa)	0.9832536	0.2461415	not scorable	not scorable	1	0.4406723
8870	Bn-A04-p14622218	napus specific	0.9545454	0.7289078	not scorable	not scorable	not scorable	not scorable
8871	Bn-A04-p14630533	A-genome specific (napus-rapa)	0.9210526	0.2241433	not scorable	not scorable	0.9669421	0.6798057
8872	Bn-A04-p14630759	genome unspecific (oleracea-rapa)	not scorable	not scorable	0.620155	0.6463355	0.9586777	0.8118961
8873	Bn-A04-p14631343	A-genome specific (napus-rapa)	1	0.7741535	not scorable	not scorable	0.9834711	0.9175813
8874	Bn-A04-p14631586	A-genome specific (napus-rapa)	0.937799	0.7899167	not scorable	not scorable	0.9669421	0.8393922

8875	Bn-A04-p14640007	napus specific	0.9736842	0.2430935	not scorable	not scorable	not scorable	not scorable
8876	Bn-A04-p14642152	A-genome specific (napus-rapa)	0.9928229	0.2688541	not scorable	not scorable	1	0.6886604
8877	Bn-A04-p1465207	A-genome specific (napus-rapa)	0.9617225	0.7372109	not scorable	not scorable	0.5619835	0.6746701
8878	Bn-A04-p14652223	A-genome specific (napus-rapa)	0.9856459	0.7517949	not scorable	not scorable	1	0.7839529
8879	Bn-A04-p14661285	A-genome specific (napus-rapa)	0.9880383	0.442535	not scorable	not scorable	1	0.7219486
8880	Bn-A04-p14662924	A-genome specific (napus-rapa)	0.9880383	0.5894179	not scorable	not scorable	0.9834711	0.9287174
8881	Bn-A04-p14670237	A-genome specific (napus-rapa)	0.9856459	0.8007197	not scorable	not scorable	0.9834711	0.7178417
8882	Bn-A04-p14670992	A-genome specific (napus-rapa)	0.9665072	0.5149094	not scorable	not scorable	0.9752066	0.6248713
8883	Bn-A04-p14673783	A-genome specific (napus-rapa)	0.9952153	0.7928311	not scorable	not scorable	0.9752066	0.793625
8884	Bn-A04-p14687930	A-genome specific (napus-rapa)	0.9952153	0.8053312	not scorable	not scorable	0.9586777	0.7706864
8885	Bn-A04-p1470844	A-genome specific (napus-rapa)	0.9952153	0.8176733	not scorable	not scorable	0.9669421	0.7707459
8886	Bn-A04-p1471607	A-genome specific (napus-rapa)	1	0.6979212	not scorable	not scorable	0.9917355	0.7699408
8887	Bn-A04-p1471982	A-genome specific (napus-rapa)	0.9952153	0.79048	not scorable	not scorable	1	0.684007
8888	Bn-A04-p1472416	A-genome specific (napus-rapa)	0.9928229	0.674539	not scorable	not scorable	0.9586777	0.5636415
8889	Bn-A04-p1472739	A-genome specific (napus-rapa)	0.9665072	0.3088323	not scorable	not scorable	0.9834711	0.6878969
8890	Bn-A04-p14742779	A-genome specific (napus-rapa)	0.9784689	0.7279728	not scorable	not scorable	0.9917355	0.7575227
8891	Bn-A04-p14756001	A-genome specific (napus-rapa)	0.9760765	0.7832031	not scorable	not scorable	0.9752066	0.808744
8892	Bn-A04-p14756394	A-genome specific (napus-rapa)	0.9832536	0.7193728	not scorable	not scorable	0.9752066	0.7475697
8893	Bn-A04-p14777980	A-genome specific (napus-rapa)	0.9976076	0.8765668	not scorable	not scorable	0.9834711	0.6999747
8894	Bn-A04-p14778146	A-genome specific (napus-rapa)	0.9976076	0.8469702	not scorable	not scorable	0.9917355	0.7347717
8895	Bn-A04-p1479491	A-genome specific (rapa only)	not scorable	not scorable	not scorable	not scorable	0.9752066	0.7138944
8896	Bn-A04-p1480226	A-genome specific (napus-rapa)	1	0.6448796	not scorable	not scorable	1	0.8164276
8897	Bn-A04-p14807002	A-genome specific (napus-rapa)	0.9928229	0.5445462	not scorable	not scorable	0.9421487	0.7312317
8898	Bn-A04-p14807150	A-genome specific (napus-rapa)	0.9856459	0.5047338	not scorable	not scorable	0.9586777	0.8268245
8899	Bn-A04-p14807561	A-genome specific (napus-rapa)	0.9976076	0.3733341	not scorable	not scorable	1	0.7529748
8900	Bn-A04-p14808819	A-genome specific (napus-rapa)	0.9784689	0.5201086	not scorable	not scorable	1	0.8602008
8901	Bn-A04-p1481110	A-genome specific (napus-rapa)	1	0.8596519	not scorable	not scorable	0.9834711	0.7064376
8902	Bn-A04-p14815251	A-genome specific (napus-rapa)	0.9976076	0.4453147	not scorable	not scorable	0.9917355	0.7308435
8903	Bn-A04-p14822521	A-genome specific (napus-rapa)	0.9928229	0.5396577	not scorable	not scorable	0.9752066	0.6183447
8904	Bn-A04-p14826595	A-genome specific (napus-rapa)	0.9258373	0.7816034	not scorable	not scorable	0.9752066	0.7277337
8905	Bn-A04-p14829686	A-genome specific (rapa only)	not scorable	not scorable	not scorable	not scorable	1	0.6342785
8906	Bn-A04-p14867427	A-genome specific (napus-rapa)	0.8181818	0.8924959	not scorable	not scorable	1	0.9205657
8907	Bn-A04-p14877869	A-genome specific (napus-rapa)	0.9784689	0.4724688	not scorable	not scorable	0.9834711	0.7466301

8908	Bn-A04-p14893870	A-genome specific (napus-rapa)	0.9928229	0.8062928	not scorable	not scorable	0.9917355	0.8303288
8909	Bn-A04-p1490485	A-genome specific (napus-rapa)	0.9736842	0.357735	not scorable	not scorable	1	0.7070534
8910	Bn-A04-p14908542	A-genome specific (napus-rapa)	0.9928229	0.8123332	not scorable	not scorable	0.9669421	0.7541844
8911	Bn-A04-p14917114	A-genome specific (napus-rapa)	0.9736842	0.7611674	not scorable	not scorable	0.9917355	0.8119168
8912	Bn-A04-p14922562	A-genome specific (napus-rapa)	0.9736842	0.2890656	not scorable	not scorable	0.9917355	0.5810626
8913	Bn-A04-p14923168	A-genome specific (napus-rapa)	0.9641148	0.4672006	not scorable	not scorable	0.9917355	0.7964969
8914	Bn-A04-p14944336	A-genome specific (napus-rapa)	0.9928229	0.7148775	not scorable	not scorable	0.9834711	0.6753877
8915	Bn-A04-p14944421	A-genome specific (napus-rapa)	0.9952153	0.7682916	not scorable	not scorable	0.9917355	0.7734752
8916	Bn-A04-p14954537	A-genome specific (napus-rapa)	0.9904306	0.7682233	not scorable	not scorable	0.9669421	0.7555351
8917	Bn-A04-p14961814	A-genome specific (napus-rapa)	0.9832536	0.7764475	not scorable	not scorable	0.9917355	0.8490673
8918	Bn-A04-p14965926	A-genome specific (napus-rapa)	0.9856459	0.7438592	not scorable	not scorable	1	0.700806
8919	Bn-A04-p14972825	A-genome specific (napus-rapa)	0.9928229	0.8437278	not scorable	not scorable	0.9752066	0.7766623
8920	Bn-A04-p149787	genome unspecific (all in)	0.9784689	0.303927	0.9922481	0.6747913	1	0.7914145
8921	Bn-A04-p14986920	A-genome specific (napus-rapa)	0.9760765	0.4248636	not scorable	not scorable	0.9917355	0.7250165
8922	Bn-A04-p14987934	A-genome specific (napus-rapa)	0.9832536	0.6600332	not scorable	not scorable	0.9917355	0.6890939
8923	Bn-A04-p14988304	A-genome specific (napus-rapa)	0.9736842	0.3083933	not scorable	not scorable	1	0.7751698
8924	Bn-A04-p14999714	A-genome specific (napus-rapa)	0.9880383	0.7560533	not scorable	not scorable	0.9917355	0.7688572
8925	Bn-A04-p14999768	all not scorable	not scorable	not scorable	not scorable	not scorable	not scorable	not scorable
8926	Bn-A04-p15001710	A-genome specific (napus-rapa)	0.9641148	0.6927934	not scorable	not scorable	1	0.6857525
8927	Bn-A04-p15007146	A-genome specific (napus-rapa)	0.937799	0.7691654	not scorable	not scorable	0.9834711	0.6955795
8928	Bn-A04-p15009639	A-genome specific (napus-rapa)	0.9641148	0.6388344	not scorable	not scorable	0.9834711	0.746902
8929	Bn-A04-p15011193	A-genome specific (napus-rapa)	0.9856459	0.4417515	not scorable	not scorable	1	0.8323125
8930	Bn-A04-p15030355	A-genome specific (napus-rapa)	0.9688995	0.5768256	not scorable	not scorable	1	0.7208149
8931	Bn-A04-p15030899	A-genome specific (rapa only)	not scorable	not scorable	not scorable	not scorable	0.9917355	0.7297885
8932	Bn-A04-p15030963	A-genome specific (napus-rapa)	0.9617225	0.3941147	not scorable	not scorable	0.9917355	0.7351688
8933	Bn-A04-p15041769	A-genome specific (napus-rapa)	0.9688995	0.7473382	not scorable	not scorable	0.9834711	0.6657705
8934	Bn-A04-p15048204	A-genome specific (napus-rapa)	0.9593301	0.4951431	not scorable	not scorable	0.9834711	0.813601
8935	Bn-A04-p15066563	A-genome specific (rapa only)	not scorable	not scorable	not scorable	not scorable	0.9669421	0.635949
8936	Bn-A04-p15070651	A-genome specific (rapa only)	not scorable	not scorable	not scorable	not scorable	0.9917355	0.7690042
8937	Bn-A04-p15108913	A-genome specific (napus-rapa)	0.9425837	0.3975478	not scorable	not scorable	0.9669421	0.6982431
8938	Bn-A04-p15122412	all not scorable	not scorable	not scorable	not scorable	not scorable	not scorable	not scorable
8939	Bn-A04-p15134578	all not scorable	not scorable	not scorable	not scorable	not scorable	not scorable	not scorable
8940	Bn-A04-p15150564	A-genome specific (napus-rapa)	0.9832536	0.2521837	not scorable	not scorable	0.9917355	0.6601775

8941	Bn-A04-p15152016	A-genome specific (napus-rapa)	0.8636364	0.3612198	not scorable	not scorable	0.9834711	0.6596007
8942	Bn-A04-p15164920	A-genome specific (rapa only)	not scorable	not scorable	not scorable	not scorable	0.9669421	0.8009343
8943	Bn-A04-p15168889	A-genome specific (napus-rapa)	0.9976076	0.4718766	not scorable	not scorable	1	0.8063467
8944	Bn-A04-p15184744	A-genome specific (rapa only)	not scorable	not scorable	not scorable	not scorable	0.9834711	0.7594317
8945	Bn-A04-p15190648	A-genome specific (rapa only)	not scorable	not scorable	not scorable	not scorable	0.9917355	0.7941428
8946	Bn-A04-p15195920	A-genome specific (rapa only)	not scorable	not scorable	not scorable	not scorable	0.9917355	0.8040481
8947	Bn-A04-p15207252	A-genome specific (rapa only)	not scorable	not scorable	not scorable	not scorable	0.9917355	0.7280351
8948	Bn-A04-p15244369	A-genome specific (napus-rapa)	0.9976076	0.3748531	not scorable	not scorable	0.9917355	0.7797454
8949	Bn-A04-p15287200	A-genome specific (napus-rapa)	0.9976076	0.8265352	not scorable	not scorable	0.9917355	0.7858993
8950	Bn-A04-p15296217	A-genome specific (napus-rapa)	0.9808612	0.6057025	not scorable	not scorable	1	0.9050301
8951	Bn-A04-p15298740	A-genome specific (napus-rapa)	0.9976076	0.8805987	not scorable	not scorable	0.9752066	0.7331897
8952	Bn-A04-p15310116	A-genome specific (napus-rapa)	1	0.579002	not scorable	not scorable	0.9917355	0.864643
8953	Bn-A04-p15312437	A-genome specific (napus-rapa)	0.361244	0.8274619	not scorable	not scorable	0.8760331	0.7963597
8954	Bn-A04-p15315594	A-genome specific (napus-rapa)	0.9880383	0.4213975	not scorable	not scorable	1	0.7005973
8955	Bn-A04-p15341169	A-genome specific (rapa only)	not scorable	not scorable	not scorable	not scorable	1	0.8318462
8956	Bn-A04-p15341244	A-genome specific (rapa only)	not scorable	not scorable	not scorable	not scorable	0.9752066	0.7745593
8957	Bn-A04-p15367046	A-genome specific (napus-rapa)	0.9593301	0.4754821	not scorable	not scorable	1	0.828523
8958	Bn-A04-p15377693	A-genome specific (rapa only)	not scorable	not scorable	not scorable	not scorable	1	0.7806923
8959	Bn-A04-p15378781	A-genome specific (rapa only)	not scorable	not scorable	not scorable	not scorable	1	0.7553833
8960	Bn-A04-p15399118	genome unspecific (all in)	0.9688995	0.4317366	0.9922481	0.2693038	0.9917355	0.6900888
8961	Bn-A04-p15404568	A-genome specific (napus-rapa)	0.9736842	0.7705973	not scorable	not scorable	0.9917355	0.7175075
8962	Bn-A04-p15409836	A-genome specific (napus-rapa)	0.9952153	0.8255778	not scorable	not scorable	1	0.8592454
8963	Bn-A04-p15411699	A-genome specific (napus-rapa)	0.9880383	0.7481518	not scorable	not scorable	0.9752066	0.7824817
8964	Bn-A04-p15413213	A-genome specific (napus-rapa)	0.9545454	0.6505222	not scorable	not scorable	1	0.8042552
8965	Bn-A04-p15414343	A-genome specific (napus-rapa)	0.9617225	0.7986905	not scorable	not scorable	0.9917355	0.7818269
8966	Bn-A04-p15420126	A-genome specific (napus-rapa)	0.9617225	0.2420422	not scorable	not scorable	0.9421487	0.4464254
8967	Bn-A04-p15429891	A-genome specific (napus-rapa)	0.9808612	0.3387024	not scorable	not scorable	1	0.7824845
8968	Bn-A04-p15436139	A-genome specific (napus-rapa)	0.9928229	0.4957927	not scorable	not scorable	0.9917355	0.8073221
8969	Bn-A04-p15441336	A-genome specific (napus-rapa)	0.9928229	0.8675869	not scorable	not scorable	0.9752066	0.7738849
8970	Bn-A04-p15456188	A-genome specific (napus-rapa)	0.9928229	0.3531757	not scorable	not scorable	0.9834711	0.7432657
8971	Bn-A04-p15466343	A-genome specific (napus-rapa)	0.9928229	0.3888402	not scorable	not scorable	0.9752066	0.7849017
8972	Bn-A04-p15471964	A-genome specific (napus-rapa)	0.9904306	0.553331	not scorable	not scorable	0.9917355	0.7183611
8973	Bn-A04-p15475759	A-genome specific (napus-rapa)	0.9928229	0.8503884	not scorable	not scorable	1	0.6720875

8974	Bn-A04-p15485629	A-genome specific (napus-rapa)	0.9832536	0.4889628	not scorable	not scorable	0.9834711	0.7197552
8975	Bn-A04-p15492182	A-genome specific (napus-rapa)	0.9569378	0.8238503	not scorable	not scorable	0.9917355	0.8284803
8976	Bn-A04-p15498393	A-genome specific (napus-rapa)	0.9832536	0.4516468	not scorable	not scorable	0.9917355	0.7465746
8977	Bn-A04-p15500804	A-genome specific (napus-rapa)	0.9808612	0.8274619	not scorable	not scorable	0.6115702	0.8291611
8978	Bn-A04-p15514869	A-genome specific (napus-rapa)	0.9665072	0.7837225	not scorable	not scorable	0.9586777	0.8555552
8979	Bn-A04-p15519877	A-genome specific (napus-rapa)	0.9760765	0.5630146	not scorable	not scorable	0.9834711	0.7425946
8980	Bn-A04-p15530059	A-genome specific (napus-rapa)	0.9712918	0.4097189	not scorable	not scorable	0.9669421	0.7251468
8981	Bn-A04-p15530609	A-genome specific (napus-rapa)	0.9282297	0.6327026	not scorable	not scorable	0.9834711	0.7189613
8982	Bn-A04-p15530625	A-genome specific (napus-rapa)	0.9712918	0.7573868	not scorable	not scorable	1	0.7488809
8983	Bn-A04-p15534626	A-genome specific (napus-rapa)	0.9952153	0.8694858	not scorable	not scorable	0.9834711	0.7954171
8984	Bn-A04-p15534921	A-genome specific (rapa only)	not scorable	not scorable	not scorable	not scorable	0.9669421	0.9249945
8985	Bn-A04-p15535011	A-genome specific (napus-rapa)	0.9952153	0.8011312	not scorable	not scorable	0.9917355	0.6266003
8986	Bn-A04-p15555443	A-genome specific (napus-rapa)	0.9832536	0.4954794	not scorable	not scorable	0.9834711	0.7202361
8987	Bn-A04-p15556246	A-genome specific (napus-rapa)	0.9904306	0.4349702	not scorable	not scorable	0.9834711	0.7843896
8988	Bn-A04-p15559138	genome unspecific (oleracea-rapa)	not scorable	not scorable	0.9302326	0.7215804	0.9586777	0.7506922
8989	Bn-A04-p15597988	A-genome specific (napus-rapa)	0.9784689	0.7855307	not scorable	not scorable	0.9917355	0.7710512
8990	Bn-A04-p15600337	A-genome specific (napus-rapa)	0.9688995	0.7857604	not scorable	not scorable	0.9917355	0.7370362
8991	Bn-A04-p15617379	A-genome specific (napus-rapa)	0.9928229	0.4393173	not scorable	not scorable	0.9834711	0.7063597
8992	Bn-A04-p15619897	A-genome specific (napus-rapa)	0.9856459	0.8061854	not scorable	not scorable	0.9834711	0.7880082
8993	Bn-A04-p15630537	A-genome specific (napus-rapa)	1	0.7776763	not scorable	not scorable	1	0.7739488
8994	Bn-A04-p15631388	A-genome specific (napus-rapa)	0.9856459	0.7892735	not scorable	not scorable	0.9504132	0.7711592
8995	Bn-A04-p15643031	A-genome specific (napus-rapa)	0.9856459	0.3994646	not scorable	not scorable	0.9586777	0.7832878
8996	Bn-A04-p15655213	A-genome specific (napus-rapa)	0.9784689	0.7783051	not scorable	not scorable	0.9917355	0.6700102
8997	Bn-A04-p15658143	A-genome specific (rapa only)	not scorable	not scorable	not scorable	not scorable	0.9917355	0.7228674
8998	Bn-A04-p15661972	A-genome specific (napus-rapa)	0.9952153	0.8268484	not scorable	not scorable	0.9917355	0.7969698
8999	Bn-A04-p15662133	A-genome specific (rapa only)	not scorable	not scorable	not scorable	not scorable	1	0.7560207
9000	Bn-A04-p15670183	A-genome specific (napus-rapa)	0.9928229	0.8028214	not scorable	not scorable	1	0.8575141
9001	Bn-A04-p15670197	genome unspecific (all in)	0.9904306	0.598814	0.8372093	0.772759	0.9834711	0.836568
9002	Bn-A04-p15701924	A-genome specific (rapa only)	not scorable	not scorable	not scorable	not scorable	0.6694215	0.7527181
9003	Bn-A04-p15707649	A-genome specific (napus-rapa)	0.9952153	0.8562291	not scorable	not scorable	1	0.7934078
9004	Bn-A04-p15710186	A-genome specific (napus-rapa)	0.9952153	0.7781971	not scorable	not scorable	0.9834711	0.752719
9005	Bn-A04-p15713429	A-genome specific (napus-rapa)	0.9856459	0.780229	not scorable	not scorable	0.9917355	0.7755706
9006	Bn-A04-p15727549	A-genome specific (napus-rapa)	0.9856459	0.3169387	not scorable	not scorable	0.9834711	0.6191358

9007	Bn-A04-p15731996	A-genome specific (rapa only)	not scorable	not scorable	not scorable	not scorable	0.9917355	0.7219242
9008	Bn-A04-p15742373	A-genome specific (napus-rapa)	0.9856459	0.3684909	not scorable	not scorable	0.9917355	0.7239241
9009	Bn-A04-p15745275	A-genome specific (napus-rapa)	0.9880383	0.4952703	not scorable	not scorable	0.9834711	0.8191153
9010	Bn-A04-p1576564	A-genome specific (napus-rapa)	0.9976076	0.4568068	not scorable	not scorable	1	0.6691948
9011	Bn-A04-p15835310	napus specific	0.9665072	0.7248076	not scorable	not scorable	not scorable	not scorable
9012	Bn-A04-p15840589	A-genome specific (rapa only)	not scorable	not scorable	not scorable	not scorable	0.9752066	0.7052125
9013	Bn-A04-p1585447	A-genome specific (napus-rapa)	0.9880383	0.8136523	not scorable	not scorable	1	0.7555221
9014	Bn-A04-p15870508	A-genome specific (napus-rapa)	0.9808612	0.5506707	not scorable	not scorable	1	0.8276071
9015	Bn-A04-p15877051	A-genome specific (napus-rapa)	0.4186603	0.8247074	not scorable	not scorable	0.9834711	0.8079519
9016	Bn-A04-p15885427	A-genome specific (napus-rapa)	0.9617225	0.3203213	not scorable	not scorable	0.9917355	0.7625296
9017	Bn-A04-p1589006	A-genome specific (napus-rapa)	0.9952153	0.7933808	not scorable	not scorable	1	0.7830779
9018	Bn-A04-p15893499	A-genome specific (napus-rapa)	0.937799	0.2057027	not scorable	not scorable	1	0.7680414
9019	Bn-A04-p15895339	A-genome specific (napus-rapa)	0.7105263	0.8232837	not scorable	not scorable	0.9752066	0.7590252
9020	Bn-A04-p15895510	A-genome specific (napus-rapa)	0.9856459	0.4960383	not scorable	not scorable	0.9917355	0.7418856
9021	Bn-A04-p15895735	A-genome specific (napus-rapa)	0.6818182	0.6693903	not scorable	not scorable	0.9586777	0.6479997
9022	Bn-A04-p1590169	A-genome specific (napus-rapa)	0.9952153	0.8418182	not scorable	not scorable	0.9834711	0.7519378
9023	Bn-A04-p1590669	A-genome specific (napus-rapa)	0.9784689	0.6616009	not scorable	not scorable	0.6446281	0.7480513
9024	Bn-A04-p15906747	A-genome specific (napus-rapa)	0.9832536	0.3805396	not scorable	not scorable	0.9834711	0.7332237
9025	Bn-A04-p1590931	A-genome specific (napus-rapa)	0.8157895	0.8177654	not scorable	not scorable	0.6033058	0.734874
9026	Bn-A04-p15933265	A-genome specific (napus-rapa)	1	0.7226316	not scorable	not scorable	1	0.8209013
9027	Bn-A04-p15939956	A-genome specific (napus-rapa)	0.9880383	0.4430558	not scorable	not scorable	0.9917355	0.8343889
9028	Bn-A04-p15942668	A-genome specific (rapa only)	not scorable	not scorable	not scorable	not scorable	0.9834711	0.797233
9029	Bn-A04-p15951138	A-genome specific (napus-rapa)	0.9976076	0.2739176	not scorable	not scorable	0.9669421	0.631776
9030	Bn-A04-p15953263	A-genome specific (rapa only)	not scorable	not scorable	not scorable	not scorable	0.9504132	0.6552808
9031	Bn-A04-p15970355	A-genome specific (napus-rapa)	0.9330143	0.2541671	not scorable	not scorable	0.9917355	0.6497782
9032	Bn-A04-p16000929	A-genome specific (napus-rapa)	0.9880383	0.5212955	not scorable	not scorable	0.9834711	0.798773
9033	Bn-A04-p16014198	A-genome specific (napus-rapa)	1	0.7110427	not scorable	not scorable	1	0.7394592
9034	Bn-A04-p16022528	A-genome specific (rapa only)	not scorable	not scorable	not scorable	not scorable	0.9752066	0.751667
9035	Bn-A04-p16025591	A-genome specific (napus-rapa)	0.9952153	0.5243186	not scorable	not scorable	1	0.6766161
9036	Bn-A04-p16056186	A-genome specific (napus-rapa)	0.9976076	0.3251873	not scorable	not scorable	0.9586777	0.6609219
9037	Bn-A04-p16074808	A-genome specific (rapa only)	not scorable	not scorable	not scorable	not scorable	0.9917355	0.7956432
9038	Bn-A04-p16090875	A-genome specific (napus-rapa)	0.9784689	0.4039671	not scorable	not scorable	0.9834711	0.6929324
9039	Bn-A04-p16104213	A-genome specific (napus-rapa)	0.9784689	0.7652541	not scorable	not scorable	0.9834711	0.8186907

9040	Bn-A04-p16104616	A-genome specific (napus-rapa)	0.9760765	0.7912861	not scorable	not scorable	0.9752066	0.7970411
9041	Bn-A04-p16107844	A-genome specific (rapa only)	not scorable	not scorable	not scorable	not scorable	1	0.8207097
9042	Bn-A04-p16109558	genome unspecific (all in)	0.9904306	0.3151409	0.9689922	0.4156069	1	0.7622204
9043	Bn-A04-p16119082	genome unspecific (oleracea-rapa)	not scorable	not scorable	0.9922481	0.7060951	1	0.9180714
9044	Bn-A04-p16120092	genome unspecific (oleracea-rapa)	not scorable	not scorable	0.9922481	0.6392727	0.9917355	0.7252899
9045	Bn-A04-p16123695	A-genome specific (napus-rapa)	0.9808612	0.4287671	not scorable	not scorable	1	0.8092792
9046	Bn-A04-p16131108	A-genome specific (napus-rapa)	0.9330143	0.7396515	not scorable	not scorable	0.9752066	0.6020277
9047	Bn-A04-p16137916	A-genome specific (napus-rapa)	0.9808612	0.297788	not scorable	not scorable	1	0.7251201
9048	Bn-A04-p16143324	A-genome specific (napus-rapa)	1	0.8105522	not scorable	not scorable	1	0.8867181
9049	Bn-A04-p16155604	A-genome specific (napus-rapa)	0.9928229	0.452481	not scorable	not scorable	0.9917355	0.7555377
9050	Bn-A04-p16156157	A-genome specific (napus-rapa)	0.9688995	0.7905537	not scorable	not scorable	0.6363636	0.7196677
9051	Bn-A04-p16157904	A-genome specific (napus-rapa)	0.9904306	0.3286742	not scorable	not scorable	0.9752066	0.7865062
9052	Bn-A04-p16166839	A-genome specific (napus-rapa)	0.9545454	0.5754302	not scorable	not scorable	0.9338843	0.8087256
9053	Bn-A04-p16224941	A-genome specific (napus-rapa)	0.9928229	0.5272621	not scorable	not scorable	1	0.7376546
9054	Bn-A04-p1623151	A-genome specific (napus-rapa)	0.9904306	0.5945843	not scorable	not scorable	1	0.7526131
9055	Bn-A04-p16233024	A-genome specific (napus-rapa)	0.9784689	0.7560669	not scorable	not scorable	0.9752066	0.7822965
9056	Bn-A04-p16233377	A-genome specific (napus-rapa)	0.9832536	0.7673827	not scorable	not scorable	0.9834711	0.810416
9057	Bn-A04-p16237245	A-genome specific (napus-rapa)	1	0.4094593	not scorable	not scorable	0.9421487	0.7714387
9058	Bn-A04-p16259439	A-genome specific (napus-rapa)	0.9904306	0.7317196	not scorable	not scorable	1	0.7738628
9059	Bn-A04-p16264494	A-genome specific (napus-rapa)	0.9449761	0.8168051	not scorable	not scorable	0.9752066	0.6923664
9060	Bn-A04-p16268792	A-genome specific (napus-rapa)	0.9928229	0.7988904	not scorable	not scorable	0.9834711	0.8011487
9061	Bn-A04-p16275472	A-genome specific (napus-rapa)	0.9952153	0.7683555	not scorable	not scorable	0.9752066	0.7473217
9062	Bn-A04-p16275979	A-genome specific (napus-rapa)	0.9760765	0.6940623	not scorable	not scorable	0.9752066	0.7150274
9063	Bn-A04-p16276156	A-genome specific (napus-rapa)	0.9665072	0.6689013	not scorable	not scorable	0.9834711	0.6570787
9064	Bn-A04-p16276464	A-genome specific (napus-rapa)	0.9928229	0.6911103	not scorable	not scorable	0.9917355	0.8652223
9065	Bn-A04-p16277340	A-genome specific (napus-rapa)	0.9760765	0.7334817	not scorable	not scorable	0.9752066	0.7988884
9066	Bn-A04-p16280279	A-genome specific (napus-rapa)	0.9641148	0.7654166	not scorable	not scorable	0.9917355	0.8184033
9067	Bn-A04-p16282039	A-genome specific (napus-rapa)	0.9736842	0.8036469	not scorable	not scorable	0.9834711	0.8424072
9068	Bn-A04-p16283972	A-genome specific (napus-rapa)	0.9545454	0.4788777	not scorable	not scorable	0.9752066	0.6095938
9069	Bn-A04-p16287850	A-genome specific (napus-rapa)	0.9712918	0.5572191	not scorable	not scorable	1	0.7736467
9070	Bn-A04-p16288681	A-genome specific (napus-rapa)	0.9688995	0.8059821	not scorable	not scorable	0.9834711	0.797058
9071	Bn-A04-p16303678	A-genome specific (napus-rapa)	0.9784689	0.7592261	not scorable	not scorable	0.9834711	0.7900144
9072	Bn-A04-p16303728	A-genome specific (napus-rapa)	0.9712918	0.4969774	not scorable	not scorable	0.9834711	0.6983337

9073	Bn-A04-p16313477	A-genome specific (napus-rapa)	0.9904306	0.4821763	not scorable	not scorable	1	0.803342
9074	Bn-A04-p16320671	A-genome specific (napus-rapa)	0.9808612	0.3562808	not scorable	not scorable	0.9917355	0.6095246
9075	Bn-A04-p1633159	A-genome specific (napus-rapa)	1	0.7986	not scorable	not scorable	1	0.9105069
9076	Bn-A04-p16336301	A-genome specific (napus-rapa)	0.9808612	0.7883286	not scorable	not scorable	1	0.7205747
9077	Bn-A04-p16337105	all not scorable	not scorable	not scorable	not scorable	not scorable	not scorable	not scorable
9078	Bn-A04-p16365226	A-genome specific (napus-rapa)	0.9641148	0.7618787	not scorable	not scorable	0.9917355	0.7544466
9079	Bn-A04-p16368407	A-genome specific (napus-rapa)	0.9617225	0.5301584	not scorable	not scorable	0.9834711	0.8044382
9080	Bn-A04-p16368852	A-genome specific (napus-rapa)	0.9736842	0.5225792	not scorable	not scorable	0.9834711	0.7175061
9081	Bn-A04-p16382491	A-genome specific (rapa only)	not scorable	not scorable	not scorable	not scorable	0.9834711	0.7189265
9082	Bn-A04-p16394755	A-genome specific (napus-rapa)	0.9760765	0.5281989	not scorable	not scorable	0.9752066	0.7523744
9083	Bn-A04-p16403131	A-genome specific (napus-rapa)	0.9808612	0.4637301	not scorable	not scorable	0.9752066	0.6675128
9084	Bn-A04-p1640389	A-genome specific (napus-rapa)	0.791866	0.8334967	not scorable	not scorable	0.6280992	0.7151299
9085	Bn-A04-p16405335	A-genome specific (napus-rapa)	0.9497608	0.3173717	not scorable	not scorable	0.9917355	0.6665735
9086	Bn-A04-p16416104	A-genome specific (napus-rapa)	0.9736842	0.6532468	not scorable	not scorable	0.9834711	0.778954
9087	Bn-A04-p16427350	A-genome specific (napus-rapa)	0.9736842	0.5670363	not scorable	not scorable	0.9917355	0.7875672
9088	Bn-A04-p16431550	all not scorable	not scorable	not scorable	not scorable	not scorable	not scorable	not scorable
9089	Bn-A04-p16445225	A-genome specific (napus-rapa)	0.9928229	0.9192502	not scorable	not scorable	0.9917355	0.8060234
9090	Bn-A04-p16445602	A-genome specific (napus-rapa)	0.9904306	0.7662759	not scorable	not scorable	0.9752066	0.7135587
9091	Bn-A04-p16445675	A-genome specific (napus-rapa)	0.9904306	0.7884324	not scorable	not scorable	0.9917355	0.8376818
9092	Bn-A04-p16446976	A-genome specific (napus-rapa)	0.7320574	0.8136343	not scorable	not scorable	0.5867769	0.89616
9093	Bn-A04-p16447217	A-genome specific (napus-rapa)	0.9952153	0.8872474	not scorable	not scorable	0.9834711	0.8513421
9094	Bn-A04-p16451992	A-genome specific (napus-rapa)	0.9880383	0.716279	not scorable	not scorable	1	0.6619216
9095	Bn-A04-p16452098	A-genome specific (napus-rapa)	0.9880383	0.8007171	not scorable	not scorable	0.9917355	0.7386625
9096	Bn-A04-p16459969	A-genome specific (napus-rapa)	0.9712918	0.7880909	not scorable	not scorable	0.9917355	0.7237646
9097	Bn-A04-p16477853	A-genome specific (napus-rapa)	0.9617225	0.5674626	not scorable	not scorable	0.9917355	0.7901692
9098	Bn-A04-p16479236	A-genome specific (napus-rapa)	0.9856459	0.2797354	not scorable	not scorable	1	0.6886033
9099	Bn-A04-p16486340	A-genome specific (napus-rapa)	0.8325359	0.7807469	not scorable	not scorable	1	0.9175813
9100	Bn-A04-p16503326	A-genome specific (napus-rapa)	0.9688995	0.5961856	not scorable	not scorable	0.9917355	0.7653733
9101	Bn-A04-p16506148	A-genome specific (napus-rapa)	0.9976076	0.4075226	not scorable	not scorable	1	0.8048211
9102	Bn-A04-p16508914	A-genome specific (napus-rapa)	1	0.413096	not scorable	not scorable	0.9917355	0.6873556
9103	Bn-A04-p16514488	A-genome specific (napus-rapa)	0.9952153	0.8754031	not scorable	not scorable	0.9834711	0.7123968
9104	Bn-A04-p16517396	A-genome specific (napus-rapa)	0.9976076	0.449349	not scorable	not scorable	0.9917355	0.711579
9105	Bn-A04-p16523010	A-genome specific (napus-rapa)	0.9952153	0.7148441	not scorable	not scorable	1	0.7342599

9106	Bn-A04-p16523116	A-genome specific (napus-rapa)	0.9617225	0.7593277	not scorable	not scorable	1	0.8359115
9107	Bn-A04-p16528010	A-genome specific (napus-rapa)	0.9808612	0.8005027	not scorable	not scorable	1	0.7201062
9108	Bn-A04-p16528107	A-genome specific (napus-rapa)	0.9904306	0.7767451	not scorable	not scorable	0.9917355	0.8306848
9109	Bn-A04-p16540340	A-genome specific (napus-rapa)	0.9736842	0.4869212	not scorable	not scorable	1	0.7682258
9110	Bn-A04-p16545584	A-genome specific (napus-rapa)	0.9928229	0.538789	not scorable	not scorable	1	0.7712092
9111	Bn-A04-p16552002	A-genome specific (napus-rapa)	0.9712918	0.8806614	not scorable	not scorable	1	0.8550907
9112	Bn-A04-p16553214	A-genome specific (napus-rapa)	0.9832536	0.7728311	not scorable	not scorable	0.9834711	0.7551324
9113	Bn-A04-p16553276	A-genome specific (napus-rapa)	0.9880383	0.7522739	not scorable	not scorable	0.9917355	0.7815723
9114	Bn-A04-p1655416	A-genome specific (napus-rapa)	1	0.7894776	not scorable	not scorable	0.9669421	0.6749547
9115	Bn-A04-p16555982	A-genome specific (napus-rapa)	0.9760765	0.8012777	not scorable	not scorable	0.9917355	0.8385908
9116	Bn-A04-p16556868	A-genome specific (napus-rapa)	0.9904306	0.8083851	not scorable	not scorable	0.9917355	0.7289358
9117	Bn-A04-p16558888	A-genome specific (napus-rapa)	0.9473684	0.5247774	not scorable	not scorable	0.9834711	0.6983034
9118	Bn-A04-p16563543	A-genome specific (napus-rapa)	0.9641148	0.7335649	not scorable	not scorable	1	0.7911353
9119	Bn-A04-p16565696	A-genome specific (napus-rapa)	0.9904306	0.8416278	not scorable	not scorable	0.9834711	0.7948382
9120	Bn-A04-p16579551	A-genome specific (napus-rapa)	0.9569378	0.7153713	not scorable	not scorable	0.9421487	0.7440684
9121	Bn-A04-p16584084	A-genome specific (napus-rapa)	0.9688995	0.3080729	not scorable	not scorable	0.9917355	0.6665162
9122	Bn-A04-p16595064	A-genome specific (napus-rapa)	0.9856459	0.7171603	not scorable	not scorable	1	0.8497944
9123	Bn-A04-p16598514	A-genome specific (napus-rapa)	0.9808612	0.587745	not scorable	not scorable	0.9834711	0.7915269
9124	Bn-A04-p16605277	A-genome specific (napus-rapa)	0.9688995	0.4714673	not scorable	not scorable	1	0.7218091
9125	Bn-A04-p16607433	A-genome specific (napus-rapa)	0.9832536	0.2925589	not scorable	not scorable	0.9917355	0.7480614
9126	Bn-A04-p16608584	A-genome specific (napus-rapa)	0.9856459	0.7705176	not scorable	not scorable	0.9917355	0.8201232
9127	Bn-A04-p16621448	A-genome specific (napus-rapa)	1	0.5528759	not scorable	not scorable	1	0.621411
9128	Bn-A04-p16627363	genome unspecific (all in)	0.9832536	0.4278835	1	0.3121269	0.9917355	0.749171
9129	Bn-A04-p16651306	A-genome specific (napus-rapa)	0.9928229	0.8096758	not scorable	not scorable	0.9834711	0.7765445
9130	Bn-A04-p16663552	A-genome specific (napus-rapa)	0.9832536	0.8161658	not scorable	not scorable	1	0.7831389
9131	Bn-A04-p16677612	A-genome specific (napus-rapa)	0.9976076	0.7330693	not scorable	not scorable	0.9834711	0.8070533
9132	Bn-A04-p16678843	A-genome specific (napus-rapa)	0.9976076	0.7804426	not scorable	not scorable	0.9917355	0.850601
9133	Bn-A04-p16684288	A-genome specific (napus-rapa)	0.9976076	0.2560492	not scorable	not scorable	0.9669421	0.6476616
9134	Bn-A04-p16711900	A-genome specific (napus-rapa)	0.9976076	0.713241	not scorable	not scorable	0.9917355	0.6702768
9135	Bn-A04-p16719721	A-genome specific (napus-rapa)	0.9976076	0.3065865	not scorable	not scorable	0.9752066	0.5069157
9136	Bn-A04-p16719955	A-genome specific (napus-rapa)	1	0.5058821	not scorable	not scorable	0.9917355	0.6931867
9137	Bn-A04-p16743976	A-genome specific (napus-rapa)	0.9712918	0.8717489	not scorable	not scorable	1	0.7639438
9138	Bn-A04-p16744035	A-genome specific (napus-rapa)	0.9665072	0.8291883	not scorable	not scorable	1	0.6761023

9139	Bn-A04-p16745356	A-genome specific (napus-rapa)	0.9712918	0.8297076	not scorable	not scorable	0.9917355	0.8291255
9140	Bn-A04-p1674763	A-genome specific (napus-rapa)	0.9832536	0.4698481	not scorable	not scorable	1	0.7439995
9141	Bn-A04-p16748162	A-genome specific (napus-rapa)	0.9712918	0.7756525	not scorable	not scorable	1	0.8368417
9142	Bn-A04-p16751677	A-genome specific (napus-rapa)	0.9904306	0.8125824	not scorable	not scorable	0.9752066	0.6576722
9143	Bn-A04-p16791082	A-genome specific (napus-rapa)	0.9952153	0.8318138	not scorable	not scorable	0.9917355	0.8784877
9144	Bn-A04-p16798852	A-genome specific (napus-rapa)	0.9952153	0.7555044	not scorable	not scorable	0.9917355	0.5861406
9145	Bn-A04-p1680506	A-genome specific (napus-rapa)	1	0.7418743	not scorable	not scorable	1	0.6688542
9146	Bn-A04-p1680518	A-genome specific (rapa only)	not scorable	not scorable	not scorable	not scorable	0.9917355	0.8215771
9147	Bn-A04-p16805391	A-genome specific (napus-rapa)	0.9904306	0.383598	not scorable	not scorable	1	0.7349452
9148	Bn-A04-p1680673	A-genome specific (napus-rapa)	0.9449761	0.7928798	not scorable	not scorable	1	0.739696
9149	Bn-A04-p16819445	A-genome specific (napus-rapa)	0.9880383	0.7820782	not scorable	not scorable	0.9917355	0.8184493
9150	Bn-A04-p16821442	A-genome specific (napus-rapa)	0.9952153	0.7034137	not scorable	not scorable	0.9752066	0.6568198
9151	Bn-A04-p16830389	genome unspecific (all in)	0.9880383	0.4994667	1	0.7293031	0.9834711	0.6510171
9152	Bn-A04-p1683090	A-genome specific (napus-rapa)	1	0.826373	not scorable	not scorable	1	0.8841101
9153	Bn-A04-p16832545	genome unspecific (all in)	1	0.5132703	0.9379845	0.6009685	1	0.738565
9154	Bn-A04-p16841462	A-genome specific (napus-rapa)	0.9952153	0.5057462	not scorable	not scorable	0.9834711	0.7964279
9155	Bn-A04-p1684695	A-genome specific (napus-rapa)	1	0.7648603	not scorable	not scorable	0.9752066	0.6854421
9156	Bn-A04-p16892915	A-genome specific (napus-rapa)	0.9425837	0.8199479	not scorable	not scorable	0.9669421	0.8552856
9157	Bn-A04-p16919648	A-genome specific (napus-rapa)	0.9928229	0.5252975	not scorable	not scorable	0.6528926	0.8841216
9158	Bn-A04-p16919672	genome unspecific (all in)	1	0.7410883	1	0.7624856	0.9834711	0.6756677
9159	Bn-A04-p16946306	A-genome specific (napus-rapa)	1	0.8148901	not scorable	not scorable	0.9834711	0.7867321
9160	Bn-A04-p16980928	A-genome specific (napus-rapa)	0.9952153	0.3370576	not scorable	not scorable	0.9752066	0.6836994
9161	Bn-A04-p16998111	A-genome specific (napus-rapa)	0.9976076	0.8370125	not scorable	not scorable	1	0.6827254
9162	Bn-A04-p17003395	A-genome specific (napus-rapa)	0.9952153	0.5619785	not scorable	not scorable	0.9834711	0.7775808
9163	Bn-A04-p17019529	A-genome specific (napus-rapa)	1	0.323826	not scorable	not scorable	1	0.8194088
9164	Bn-A04-p17044228	A-genome specific (napus-rapa)	0.9952153	0.7619458	not scorable	not scorable	1	0.7170051
9165	Bn-A04-p17045348	A-genome specific (napus-rapa)	0.9952153	0.7594439	not scorable	not scorable	0.9834711	0.7740433
9166	Bn-A04-p17046250	A-genome specific (napus-rapa)	0.9976076	0.8112252	not scorable	not scorable	0.9752066	0.6964207
9167	Bn-A04-p17047140	A-genome specific (rapa only)	not scorable	not scorable	not scorable	not scorable	0.9917355	0.8332147
9168	Bn-A04-p17052071	A-genome specific (napus-rapa)	0.9976076	0.8790981	not scorable	not scorable	0.9917355	0.8068503
9169	Bn-A04-p17058367	A-genome specific (napus-rapa)	0.9976076	0.3719747	not scorable	not scorable	1	0.8425243
9170	Bn-A04-p17105440	A-genome specific (napus-rapa)	1	0.427574	not scorable	not scorable	0.9752066	0.7082671
9171	Bn-A04-p17106064	A-genome specific (napus-rapa)	0.9976076	0.6892042	not scorable	not scorable	0.9752066	0.7431537

9172	Bn-A04-p17107045	A-genome specific (napus-rapa)	0.9952153	0.3327017	not scorable	not scorable	1	0.8207603
9173	Bn-A04-p17125513	A-genome specific (napus-rapa)	1	0.5922579	not scorable	not scorable	1	0.7522939
9174	Bn-A04-p17142570	A-genome specific (napus-rapa)	0.9976076	0.7493378	not scorable	not scorable	1	0.6986762
9175	Bn-A04-p17155901	A-genome specific (napus-rapa)	0.9976076	0.8105419	not scorable	not scorable	1	0.7950195
9176	Bn-A04-p17157304	A-genome specific (napus-rapa)	0.9952153	0.8371145	not scorable	not scorable	1	0.7828301
9177	Bn-A04-p17170974	A-genome specific (napus-rapa)	0.9473684	0.4424879	not scorable	not scorable	0.9752066	0.6896485
9178	Bn-A04-p17171349	A-genome specific (napus-rapa)	0.9952153	0.871802	not scorable	not scorable	0.9917355	0.7684325
9179	Bn-A04-p17172246	A-genome specific (napus-rapa)	1	0.8263673	not scorable	not scorable	0.9834711	0.769586
9180	Bn-A04-p17174846	A-genome specific (napus-rapa)	0.9928229	0.9002177	not scorable	not scorable	0.9752066	0.8416573
9181	Bn-A04-p17179870	A-genome specific (napus-rapa)	0.9976076	0.3356032	not scorable	not scorable	1	0.732608
9182	Bn-A04-p1718231	A-genome specific (napus-rapa)	1	0.7037888	not scorable	not scorable	0.9917355	0.6583381
9183	Bn-A04-p1718248	A-genome specific (napus-rapa)	0.9952153	0.7812059	not scorable	not scorable	0.9834711	0.6742517
9184	Bn-A04-p17212731	A-genome specific (napus-rapa)	0.9952153	0.6740559	not scorable	not scorable	0.9752066	0.6938457
9185	Bn-A04-p17217840	all not scorable	not scorable	not scorable	not scorable	not scorable	not scorable	not scorable
9186	Bn-A04-p17219361	napus specific	0.9808612	0.771708	not scorable	not scorable	not scorable	not scorable
9187	Bn-A04-p17219743	all not scorable	not scorable	not scorable	not scorable	not scorable	not scorable	not scorable
9188	Bn-A04-p17220068	napus specific	0.9880383	0.7727132	not scorable	not scorable	not scorable	not scorable
9189	Bn-A04-p17221332	napus specific	0.9856459	0.7123217	not scorable	not scorable	not scorable	not scorable
9190	Bn-A04-p1722346	A-genome specific (napus-rapa)	0.9880383	0.8090347	not scorable	not scorable	0.7520661	0.8127477
9191	Bn-A04-p17223885	A-genome specific (napus-rapa)	0.9808612	0.7372922	not scorable	not scorable	0.9586777	0.7661338
9192	Bn-A04-p17231009	A-genome specific (napus-rapa)	0.9880383	0.7914444	not scorable	not scorable	0.9752066	0.763038
9193	Bn-A04-p17238605	A-genome specific (napus-rapa)	0.9856459	0.71657	not scorable	not scorable	0.9834711	0.7793567
9194	Bn-A04-p17239071	A-genome specific (napus-rapa)	0.9856459	0.7552015	not scorable	not scorable	1	0.80383
9195	Bn-A04-p17239509	A-genome specific (napus-rapa)	0.9736842	0.787952	not scorable	not scorable	0.9752066	0.7964415
9196	Bn-A04-p17273076	genome unspecific (oleracea-rapa)	not scorable	not scorable	0.9922481	0.4757029	0.9917355	0.5461337
9197	Bn-A04-p17277010	genome unspecific (all in)	0.9784689	0.2859913	1	0.7475697	0.9917355	0.8004208
9198	Bn-A04-p17278229	genome unspecific (all in)	0.9784689	0.5420578	0.9767442	0.7440732	0.9917355	0.6701708
9199	Bn-A04-p17281577	A-genome specific (napus-rapa)	0.9736842	0.8332546	not scorable	not scorable	1	0.8114095
9200	Bn-A04-p17295665	genome unspecific (all in)	0.9569378	0.6091447	0.9767442	0.726351	0.9917355	0.6534202
9201	Bn-A04-p17300068	A-genome specific (napus-rapa)	0.9760765	0.3884032	not scorable	not scorable	0.9917355	0.6596727
9202	Bn-A04-p17312041	A-genome specific (napus-rapa)	1	0.5309883	not scorable	not scorable	0.9917355	0.7332304
9203	Bn-A04-p17320026	A-genome specific (napus-rapa)	0.9976076	0.4501354	not scorable	not scorable	1	0.7955703
9204	Bn-A04-p17322241	A-genome specific (napus-rapa)	0.9976076	0.7763656	not scorable	not scorable	0.9834711	0.6931029

9205	Bn-A04-p17331915	A-genome specific (napus-rapa)	0.9617225	0.4042864	not scorable	not scorable	0.9834711	0.6394106
9206	Bn-A04-p17358519	A-genome specific (napus-rapa)	0.9712918	0.4889482	not scorable	not scorable	1	0.8001173
9207	Bn-A04-p17372718	genome unspecific (oleracea-rapa)	not scorable	not scorable	1	0.7715026	0.9834711	0.7500796
9208	Bn-A04-p1738118	A-genome specific (napus-rapa)	0.9760765	0.8141175	not scorable	not scorable	0.9752066	0.7339138
9209	Bn-A04-p17383739	A-genome specific (napus-rapa)	0.9665072	0.3048913	not scorable	not scorable	1	0.7664859
9210	Bn-A04-p1738861	A-genome specific (rapa only)	not scorable	not scorable	not scorable	not scorable	0.9834711	0.6850112
9211	Bn-A04-p17391232	A-genome specific (napus-rapa)	0.9880383	0.4808972	not scorable	not scorable	0.9917355	0.782317
9212	Bn-A04-p17391802	napus specific	0.9808612	0.4710767	not scorable	not scorable	not scorable	not scorable
9213	Bn-A04-p17394552	genome unspecific (all in)	0.9354067	0.3282011	0.9612403	0.5709252	0.9834711	0.6888669
9214	Bn-A04-p17394736	A-genome specific (napus-rapa)	0.9665072	0.3871326	not scorable	not scorable	1	0.7610028
9215	Bn-A04-p17401632	A-genome specific (napus-rapa)	0.9808612	0.766139	not scorable	not scorable	0.9917355	0.7552312
9216	Bn-A04-p17412823	A-genome specific (napus-rapa)	0.9617225	0.3924695	not scorable	not scorable	1	0.6986724
9217	Bn-A04-p17416243	A-genome specific (napus-rapa)	0.9856459	0.7190037	not scorable	not scorable	1	0.7188333
9218	Bn-A04-p17417142	A-genome specific (napus-rapa)	0.9808612	0.3384117	not scorable	not scorable	1	0.835825
9219	Bn-A04-p17422800	A-genome specific (napus-rapa)	0.9856459	0.7849482	not scorable	not scorable	0.9752066	0.7973136
9220	Bn-A04-p17425190	A-genome specific (napus-rapa)	0.9784689	0.6943787	not scorable	not scorable	0.9834711	0.7054963
9221	Bn-A04-p17439839	A-genome specific (napus-rapa)	0.9928229	0.7619459	not scorable	not scorable	1	0.8105936
9222	Bn-A04-p1745832	A-genome specific (napus-rapa)	0.9449761	0.7983341	not scorable	not scorable	0.9752066	0.7761122
9223	Bn-A04-p17475728	genome unspecific (all in)	0.9928229	0.3846953	0.9922481	0.8508529	1	0.8056206
9224	Bn-A04-p17487757	A-genome specific (napus-rapa)	0.9856459	0.4817092	not scorable	not scorable	0.9669421	0.7563528
9225	Bn-A04-p17489063	all not scorable	not scorable	not scorable	not scorable	not scorable	not scorable	not scorable
9226	Bn-A04-p17490425	A-genome specific (napus-rapa)	0.9880383	0.7604005	not scorable	not scorable	0.9917355	0.814182
9227	Bn-A04-p17496899	A-genome specific (napus-rapa)	0.9976076	0.8837978	not scorable	not scorable	1	0.8458923
9228	Bn-A04-p17530621	genome unspecific (all in)	0.9736842	0.568487	1	0.614495	1	0.7030984
9229	Bn-A04-p17535104	A-genome specific (napus-rapa)	0.9904306	0.7880712	not scorable	not scorable	0.6528926	0.7216272
9230	Bn-A04-p17541500	genome unspecific (all in)	0.9808612	0.2776755	0.9922481	0.6945408	0.9669421	0.6465729
9231	Bn-A04-p17541623	A-genome specific (napus-rapa)	0.9784689	0.5757022	not scorable	not scorable	0.9834711	0.7612407
9232	Bn-A04-p17550209	A-genome specific (napus-rapa)	0.9736842	0.4063496	not scorable	not scorable	0.9834711	0.80571
9233	Bn-A04-p1756591	napus specific	0.9354067	0.3709655	not scorable	not scorable	not scorable	not scorable
9234	Bn-A04-p17574162	A-genome specific (napus-rapa)	0.9784689	0.7623603	not scorable	not scorable	0.9917355	0.8143303
9235	Bn-A04-p17576444	A-genome specific (napus-rapa)	0.9880383	0.7487143	not scorable	not scorable	1	0.7794484
9236	Bn-A04-p17577700	A-genome specific (napus-rapa)	0.9760765	0.3619131	not scorable	not scorable	1	0.6498604
9237	Bn-A04-p17578444	A-genome specific (napus-rapa)	0.9425837	0.7892169	not scorable	not scorable	0.9834711	0.6851476

9238	Bn-A04-p17580804	A-genome specific (napus-rapa)	0.9808612	0.6691713	not scorable	not scorable	0.9917355	0.6957108
9239	Bn-A04-p1758570	A-genome specific (napus-rapa)	0.9617225	0.5611995	not scorable	not scorable	0.9917355	0.8268179
9240	Bn-A04-p1759257	A-genome specific (napus-rapa)	0.9784689	0.7690881	not scorable	not scorable	0.9917355	0.7682269
9241	Bn-A04-p17613869	A-genome specific (napus-rapa)	0.9736842	0.7083262	not scorable	not scorable	0.9917355	0.824894
9242	Bn-A04-p17614504	A-genome specific (napus-rapa)	0.930622	0.7508956	not scorable	not scorable	1	0.7457941
9243	Bn-A04-p17615088	A-genome specific (napus-rapa)	0.9210526	0.7526013	not scorable	not scorable	1	0.8698636
9244	Bn-A04-p17623914	A-genome specific (napus-rapa)	0.9473684	0.4055259	not scorable	not scorable	0.9834711	0.6934316
9245	Bn-A04-p17624469	genome unspecific (oleracea-rapa)	not scorable	not scorable	0.9379845	0.3292428	0.9917355	0.3654249
9246	Bn-A04-p17624526	A-genome specific (rapa only)	not scorable	not scorable	not scorable	not scorable	0.9917355	0.7047639
9247	Bn-A04-p17632009	A-genome specific (napus-rapa)	0.9856459	0.7922968	not scorable	not scorable	0.9917355	0.8095888
9248	Bn-A04-p17634469	A-genome specific (napus-rapa)	0.9593301	0.4022683	not scorable	not scorable	0.9917355	0.7894939
9249	Bn-A04-p1764916	A-genome specific (napus-rapa)	0.9808612	0.7904852	not scorable	not scorable	0.9834711	0.7043457
9250	Bn-A04-p1765163	A-genome specific (napus-rapa)	0.9808612	0.7940629	not scorable	not scorable	0.9834711	0.6877309
9251	Bn-A04-p17658206	A-genome specific (napus-rapa)	0.7679426	0.7688709	not scorable	not scorable	0.9586777	0.8058131
9252	Bn-A04-p17685210	A-genome specific (rapa only)	not scorable	not scorable	not scorable	not scorable	1	0.7533008
9253	Bn-A04-p17686228	A-genome specific (napus-rapa)	0.9808612	0.8109291	not scorable	not scorable	0.9752066	0.7732124
9254	Bn-A04-p17689002	A-genome specific (napus-rapa)	0.9856459	0.7096539	not scorable	not scorable	0.9834711	0.7649984
9255	Bn-A04-p17689324	A-genome specific (napus-rapa)	1	0.7371257	not scorable	not scorable	1	0.8234757
9256	Bn-A04-p17692138	A-genome specific (napus-rapa)	0.9784689	0.6827103	not scorable	not scorable	1	0.6984116
9257	Bn-A04-p17698780	A-genome specific (napus-rapa)	0.9928229	0.809095	not scorable	not scorable	0.9917355	0.7481785
9258	Bn-A04-p17704458	A-genome specific (napus-rapa)	0.9712918	0.5686407	not scorable	not scorable	1	0.8023478
9259	Bn-A04-p17706333	A-genome specific (napus-rapa)	0.937799	0.7629701	not scorable	not scorable	0.9669421	0.7973309
9260	Bn-A04-p17712184	A-genome specific (napus-rapa)	0.9832536	0.7548684	not scorable	not scorable	0.9917355	0.8186453
9261	Bn-A04-p1773333	A-genome specific (napus-rapa)	0.9928229	0.7347106	not scorable	not scorable	1	0.713299
9262	Bn-A04-p17736387	A-genome specific (napus-rapa)	0.9641148	0.3719573	not scorable	not scorable	0.9752066	0.5446155
9263	Bn-A04-p17736770	A-genome specific (napus-rapa)	0.9880383	0.7763885	not scorable	not scorable	0.9834711	0.6605806
9264	Bn-A04-p17738097	A-genome specific (napus-rapa)	0.9976076	0.7940474	not scorable	not scorable	0.9917355	0.7818289
9265	Bn-A04-p1774031	A-genome specific (napus-rapa)	0.9784689	0.7893559	not scorable	not scorable	0.9834711	0.7624306
9266	Bn-A04-p17740663	genome unspecific (all in)	0.9425837	0.4804407	0.9767442	0.6810263	0.9504132	0.6841583
9267	Bn-A04-p17742799	A-genome specific (napus-rapa)	0.9473684	0.4634572	not scorable	not scorable	0.9586777	0.676002
9268	Bn-A04-p1774926	A-genome specific (napus-rapa)	0.9904306	0.7742343	not scorable	not scorable	1	0.7556958
9269	Bn-A04-p1775155	A-genome specific (napus-rapa)	0.9856459	0.5195107	not scorable	not scorable	0.9917355	0.8368692
9270	Bn-A04-p17756059	A-genome specific (napus-rapa)	0.9856459	0.7263153	not scorable	not scorable	0.9917355	0.6229188

9271	Bn-A04-p17760280	napus specific	0.9593301	0.4725342	not scorable	not scorable	not scorable	not scorable
9272	Bn-A04-p17760869	A-genome specific (napus-rapa)	0.9808612	0.3724486	not scorable	not scorable	0.9917355	0.7871463
9273	Bn-A04-p17766173	genome unspecific (all in)	0.9856459	0.7261904	1	0.574428	0.9586777	0.8483949
9274	Bn-A04-p17768404	A-genome specific (napus-rapa)	0.9712918	0.5458004	not scorable	not scorable	1	0.8344214
9275	Bn-A04-p17770281	A-genome specific (napus-rapa)	0.9976076	0.8183022	not scorable	not scorable	1	0.8543575
9276	Bn-A04-p1777948	A-genome specific (napus-rapa)	1	0.8730361	not scorable	not scorable	1	0.9199983
9277	Bn-A04-p17790228	A-genome specific (napus-rapa)	0.9880383	0.3945232	not scorable	not scorable	0.9917355	0.7689141
9278	Bn-A04-p1780149	A-genome specific (napus-rapa)	0.9593301	0.3534143	not scorable	not scorable	0.9834711	0.7713329
9279	Bn-A04-p17811740	A-genome specific (napus-rapa)	0.9473684	0.6990018	not scorable	not scorable	1	0.7195191
9280	Bn-A04-p17814849	A-genome specific (napus-rapa)	0.791866	0.8416464	not scorable	not scorable	0.9917355	0.7414146
9281	Bn-A04-p17816796	A-genome specific (napus-rapa)	0.9665072	0.6559324	not scorable	not scorable	0.9917355	0.5829142
9282	Bn-A04-p17816969	genome unspecific (all in)	0.9665072	0.5660895	0.9844961	0.8552722	0.9752066	0.7807314
9283	Bn-A04-p17820683	A-genome specific (napus-rapa)	1	0.6150594	not scorable	not scorable	1	0.8569579
9284	Bn-A04-p17832455	genome unspecific (oleracea-rapa)	not scorable	not scorable	1	0.8178433	0.9917355	0.7492447
9285	Bn-A04-p1783485	A-genome specific (napus-rapa)	0.9832536	0.3124115	not scorable	not scorable	0.9834711	0.7559544
9286	Bn-A04-p17845366	A-genome specific (napus-rapa)	1	0.5164079	not scorable	not scorable	1	0.7068847
9287	Bn-A04-p17858149	A-genome specific (napus-rapa)	0.9665072	0.7801339	not scorable	not scorable	0.9834711	0.7179871
9288	Bn-A04-p17864572	A-genome specific (napus-rapa)	0.9784689	0.7750976	not scorable	not scorable	0.9917355	0.7724181
9289	Bn-A04-p17864879	A-genome specific (napus-rapa)	0.9760765	0.8020527	not scorable	not scorable	0.9752066	0.6438812
9290	Bn-A04-p17870056	A-genome specific (napus-rapa)	0.8899522	0.8125927	not scorable	not scorable	0.661157	0.8444123
9291	Bn-A04-p17871505	A-genome specific (napus-rapa)	0.9760765	0.7973577	not scorable	not scorable	0.9917355	0.8243164
9292	Bn-A04-p17885659	A-genome specific (rapa only)	not scorable	not scorable	not scorable	not scorable	0.9917355	0.7211996
9293	Bn-A04-p17896333	genome unspecific (all in)	0.9808612	0.3373762	0.9922481	0.7062766	1	0.7586259
9294	Bn-A04-p17910578	A-genome specific (napus-rapa)	1	0.6171024	not scorable	not scorable	1	0.6066825
9295	Bn-A04-p17934648	A-genome specific (napus-rapa)	0.9856459	0.7921286	not scorable	not scorable	0.9917355	0.7168507
9296	Bn-A04-p17940697	A-genome specific (napus-rapa)	0.9808612	0.782281	not scorable	not scorable	0.9917355	0.796643
9297	Bn-A04-p17940767	A-genome specific (napus-rapa)	0.9712918	0.551406	not scorable	not scorable	0.9917355	0.7232706
9298	Bn-A04-p17968847	A-genome specific (napus-rapa)	0.9904306	0.73083	not scorable	not scorable	0.9834711	0.6117924
9299	Bn-A04-p17969838	A-genome specific (napus-rapa)	0.9712918	0.6820135	not scorable	not scorable	0.9917355	0.6840917
9300	Bn-A04-p17976746	A-genome specific (napus-rapa)	0.9688995	0.591197	not scorable	not scorable	0.9834711	0.6313891
9301	Bn-A04-p18003170	A-genome specific (napus-rapa)	0.9808612	0.763015	not scorable	not scorable	1	0.6622107
9302	Bn-A04-p18003813	A-genome specific (napus-rapa)	0.9569378	0.8846033	not scorable	not scorable	0.553719	0.7853353
9303	Bn-A04-p18007716	napus specific	0.7344497	0.7232249	not scorable	not scorable	not scorable	not scorable

9304	Bn-A04-p18009131	A-genome specific (napus-rapa)	0.9641148	0.7968261	not scorable	not scorable	0.5950413	0.8320854
9305	Bn-A04-p18012102	A-genome specific (napus-rapa)	0.9784689	0.6004086	not scorable	not scorable	0.9917355	0.7839343
9306	Bn-A04-p18013923	A-genome specific (napus-rapa)	0.9784689	0.7722824	not scorable	not scorable	0.9917355	0.7659952
9307	Bn-A04-p18015189	A-genome specific (napus-rapa)	0.9736842	0.7822166	not scorable	not scorable	1	0.8471489
9308	Bn-A04-p18027570	A-genome specific (napus-rapa)	0.9760765	0.7871521	not scorable	not scorable	1	0.7737237
9309	Bn-A04-p18031439	A-genome specific (napus-rapa)	1	0.5329401	not scorable	not scorable	0.9834711	0.663646
9310	Bn-A04-p18038306	A-genome specific (napus-rapa)	0.9736842	0.6891751	not scorable	not scorable	0.9752066	0.7156014
9311	Bn-A04-p18047327	A-genome specific (napus-rapa)	0.9784689	0.2376818	not scorable	not scorable	1	0.749177
9312	Bn-A04-p18047606	A-genome specific (napus-rapa)	0.9210526	0.3096215	not scorable	not scorable	0.9917355	0.7592556
9313	Bn-A04-p18055770	genome unspecific (all in)	0.9186603	0.4081582	0.9922481	0.7631772	0.9586777	0.7923121
9314	Bn-A04-p18062365	A-genome specific (napus-rapa)	0.9832536	0.8513764	not scorable	not scorable	0.9421487	0.6639891
9315	Bn-A04-p18065518	A-genome specific (rapa only)	not scorable	not scorable	not scorable	not scorable	1	0.7697366
9316	Bn-A04-p18065896	A-genome specific (napus-rapa)	0.9688995	0.7668924	not scorable	not scorable	0.9917355	0.6893607
9317	Bn-A04-p18071582	A-genome specific (rapa only)	not scorable	not scorable	not scorable	not scorable	0.5950413	0.9034117
9318	Bn-A04-p18071791	A-genome specific (napus-rapa)	0.9784689	0.7740461	not scorable	not scorable	0.5867769	0.8148199
9319	Bn-A04-p18075680	genome unspecific (all in)	0.9736842	0.8108997	0.6589147	0.7548283	1	0.8223149
9320	Bn-A04-p18081163	A-genome specific (napus-rapa)	0.9760765	0.7579353	not scorable	not scorable	0.9834711	0.7779294
9321	Bn-A04-p18083568	A-genome specific (napus-rapa)	0.9976076	0.3565235	not scorable	not scorable	0.9752066	0.6521329
9322	Bn-A04-p18085734	A-genome specific (napus-rapa)	0.9593301	0.8723353	not scorable	not scorable	0.9669421	0.7333806
9323	Bn-A04-p18086224	A-genome specific (napus-rapa)	0.9736842	0.7558789	not scorable	not scorable	1	0.7853137
9324	Bn-A04-p18087342	A-genome specific (napus-rapa)	0.9186603	0.7205167	not scorable	not scorable	0.9586777	0.6936534
9325	Bn-A04-p18087738	A-genome specific (napus-rapa)	0.9904306	0.7436739	not scorable	not scorable	0.9917355	0.7851833
9326	Bn-A04-p18089854	A-genome specific (napus-rapa)	0.9784689	0.7827268	not scorable	not scorable	0.9752066	0.8230858
9327	Bn-A04-p18090397	A-genome specific (napus-rapa)	0.9856459	0.7235605	not scorable	not scorable	0.9752066	0.6923385
9328	Bn-A04-p18097103	genome unspecific (oleracea-rapa)	not scorable	not scorable	0.9069768	0.3917983	0.9834711	0.7128335
9329	Bn-A04-p18097421	A-genome specific (napus-rapa)	1	0.4144393	not scorable	not scorable	1	0.7452957
9330	Bn-A04-p18112711	A-genome specific (napus-rapa)	0.9832536	0.784529	not scorable	not scorable	0.9917355	0.7907945
9331	Bn-A04-p18124053	A-genome specific (napus-rapa)	0.9712918	0.4249277	not scorable	not scorable	0.9917355	0.8154249
9332	Bn-A04-p18125528	A-genome specific (napus-rapa)	0.9617225	0.7216362	not scorable	not scorable	1	0.7539133
9333	Bn-A04-p18125679	A-genome specific (napus-rapa)	0.9784689	0.7003452	not scorable	not scorable	0.9752066	0.6569011
9334	Bn-A04-p18129552	A-genome specific (napus-rapa)	0.9856459	0.6119404	not scorable	not scorable	0.9834711	0.8353892
9335	Bn-A04-p18136301	A-genome specific (napus-rapa)	0.9665072	0.5278142	not scorable	not scorable	1	0.8233715
9336	Bn-A04-p18152854	A-genome specific (napus-rapa)	0.9928229	0.7483749	not scorable	not scorable	0.9917355	0.8131679

9337	Bn-A04-p1815404	A-genome specific (napus-rapa)	0.9521531	0.4739307	not scorable	not scorable	0.9917355	0.7547195
9338	Bn-A04-p18156458	A-genome specific (napus-rapa)	0.9712918	0.8481968	not scorable	not scorable	0.9752066	0.7234749
9339	Bn-A04-p18167535	A-genome specific (napus-rapa)	0.9952153	0.3223836	not scorable	not scorable	0.9917355	0.6869836
9340	Bn-A04-p18167614	A-genome specific (napus-rapa)	0.9784689	0.5104159	not scorable	not scorable	0.9917355	0.7796663
9341	Bn-A04-p18168733	A-genome specific (napus-rapa)	0.8851675	0.7785262	not scorable	not scorable	1	0.8611665
9342	Bn-A04-p18178609	A-genome specific (napus-rapa)	0.9736842	0.8045176	not scorable	not scorable	1	0.9002636
9343	Bn-A04-p18180777	A-genome specific (napus-rapa)	0.9641148	0.5545123	not scorable	not scorable	0.661157	0.6913545
9344	Bn-A04-p1824456	A-genome specific (napus-rapa)	0.9928229	0.7039209	not scorable	not scorable	0.9917355	0.7353681
9345	Bn-A04-p18252317	A-genome specific (rapa only)	not scorable	not scorable	not scorable	not scorable	1	0.7553875
9346	Bn-A04-p18256793	A-genome specific (napus-rapa)	0.8397129	0.6416445	not scorable	not scorable	0.9586777	0.6417928
9347	Bn-A04-p1827230	A-genome specific (napus-rapa)	0.9976076	0.7858793	not scorable	not scorable	0.9917355	0.8146861
9348	Bn-A04-p18273048	A-genome specific (napus-rapa)	0.8971292	0.5324244	not scorable	not scorable	1	0.853812
9349	Bn-A04-p1829219	A-genome specific (rapa only)	not scorable	not scorable	not scorable	not scorable	0.9834711	0.7268962
9350	Bn-A04-p18354325	A-genome specific (napus-rapa)	0.9832536	0.7207896	not scorable	not scorable	0.9917355	0.7356425
9351	Bn-A04-p18358463	A-genome specific (napus-rapa)	1	0.5419363	not scorable	not scorable	1	0.6022679
9352	Bn-A04-p1837535	napus specific	0.5143541	0.7901528	not scorable	not scorable	not scorable	not scorable
9353	Bn-A04-p18377757	A-genome specific (napus-rapa)	0.9760765	0.376796	not scorable	not scorable	0.9917355	0.7922279
9354	Bn-A04-p18390795	A-genome specific (napus-rapa)	0.9808612	0.8326987	not scorable	not scorable	0.9917355	0.7538387
9355	Bn-A04-p18409410	A-genome specific (napus-rapa)	0.9808612	0.6210484	not scorable	not scorable	0.9917355	0.7567343
9356	Bn-A04-p18417786	A-genome specific (napus-rapa)	0.9760765	0.7386652	not scorable	not scorable	1	0.7134618
9357	Bn-A04-p18429573	A-genome specific (napus-rapa)	0.9784689	0.7627969	not scorable	not scorable	0.9752066	0.7619901
9358	Bn-A04-p18455291	A-genome specific (napus-rapa)	0.9976076	0.7742743	not scorable	not scorable	1	0.8543575
9359	Bn-A04-p18457550	A-genome specific (napus-rapa)	0.9736842	0.7270214	not scorable	not scorable	1	0.7860953
9360	Bn-A04-p18458146	A-genome specific (napus-rapa)	0.9856459	0.62256	not scorable	not scorable	0.9752066	0.7660415
9361	Bn-A04-p18467235	A-genome specific (napus-rapa)	0.9784689	0.8484657	not scorable	not scorable	1	0.8019052
9362	Bn-A04-p18471424	A-genome specific (napus-rapa)	0.9832536	0.5735394	not scorable	not scorable	1	0.7524127
9363	Bn-A04-p18472857	A-genome specific (napus-rapa)	0.7511961	0.8125546	not scorable	not scorable	0.9421487	0.840508
9364	Bn-A04-p18487715	A-genome specific (napus-rapa)	0.9856459	0.6005326	not scorable	not scorable	0.9917355	0.7544709
9365	Bn-A04-p18499666	A-genome specific (napus-rapa)	0.9665072	0.7675864	not scorable	not scorable	0.9834711	0.8289795
9366	Bn-A04-p18502854	A-genome specific (napus-rapa)	0.9712918	0.7453173	not scorable	not scorable	0.9834711	0.7962135
9367	Bn-A04-p18515323	A-genome specific (rapa only)	not scorable	not scorable	not scorable	not scorable	1	0.8304235
9368	Bn-A04-p18522691	A-genome specific (napus-rapa)	0.9665072	0.5274078	not scorable	not scorable	0.9752066	0.7389593
9369	Bn-A04-p18530009	A-genome specific (napus-rapa)	0.9808612	0.7832899	not scorable	not scorable	1	0.7686359

9370	Bn-A04-p1854718	A-genome specific (napus-rapa)	0.9688995	0.6981506	not scorable	not scorable	0.9669421	0.6981179
9371	Bn-A04-p18552075	A-genome specific (rapa only)	not scorable	not scorable	not scorable	not scorable	0.9917355	0.8478519
9372	Bn-A04-p18562244	A-genome specific (napus-rapa)	0.9736842	0.6083112	not scorable	not scorable	0.9752066	0.6437557
9373	Bn-A04-p18572841	A-genome specific (napus-rapa)	0.9856459	0.3579233	not scorable	not scorable	0.9669421	0.5806885
9374	Bn-A04-p18573606	A-genome specific (napus-rapa)	0.9617225	0.7783461	not scorable	not scorable	0.9834711	0.7747121
9375	Bn-A04-p18577466	A-genome specific (napus-rapa)	0.9712918	0.4843069	not scorable	not scorable	1	0.8039973
9376	Bn-A04-p1858140	A-genome specific (napus-rapa)	0.9473684	0.8093418	not scorable	not scorable	0.6115702	0.8021932
9377	Bn-A04-p18585603	A-genome specific (napus-rapa)	0.9808612	0.7196856	not scorable	not scorable	0.9834711	0.6702854
9378	Bn-A04-p18595858	A-genome specific (napus-rapa)	0.9784689	0.2949174	not scorable	not scorable	0.9834711	0.787617
9379	Bn-A04-p18606660	A-genome specific (napus-rapa)	0.9880383	0.5421918	not scorable	not scorable	1	0.8031628
9380	Bn-A04-p18613344	A-genome specific (napus-rapa)	0.9856459	0.3524694	not scorable	not scorable	1	0.7698085
9381	Bn-A04-p18617343	A-genome specific (napus-rapa)	0.9928229	0.791268	not scorable	not scorable	0.9917355	0.8367441
9382	Bn-A04-p18620226	A-genome specific (napus-rapa)	0.9880383	0.3630414	not scorable	not scorable	0.9917355	0.7168025
9383	Bn-A04-p18621492	genome unspecific (all in)	0.9617225	0.5757912	0.9844961	0.6875192	0.9917355	0.827274
9384	Bn-A04-p18639840	A-genome specific (napus-rapa)	1	0.370384	not scorable	not scorable	0.9834711	0.6649366
9385	Bn-A04-p18639922	A-genome specific (rapa only)	not scorable	not scorable	not scorable	not scorable	1	0.7288427
9386	Bn-A04-p1865434	A-genome specific (napus-rapa)	0.9976076	0.5109836	not scorable	not scorable	0.9752066	0.8020285
9387	Bn-A04-p18673015	A-genome specific (napus-rapa)	1	0.4650257	not scorable	not scorable	1	0.9172017
9388	Bn-A04-p1867423	A-genome specific (napus-rapa)	0.9952153	0.9065347	not scorable	not scorable	0.9917355	0.7515473
9389	Bn-A04-p18693211	A-genome specific (napus-rapa)	0.9976076	0.637215	not scorable	not scorable	0.9917355	0.7354065
9390	Bn-A04-p18694989	A-genome specific (napus-rapa)	0.9976076	0.7163467	not scorable	not scorable	1	0.7299266
9391	Bn-A04-p1869508	A-genome specific (napus-rapa)	0.9928229	0.5185612	not scorable	not scorable	0.9752066	0.6888708
9392	Bn-A04-p18702920	A-genome specific (rapa only)	not scorable	not scorable	not scorable	not scorable	1	0.7448068
9393	Bn-A04-p18731541	A-genome specific (napus-rapa)	1	0.5030417	not scorable	not scorable	0.9917355	0.6422077
9394	Bn-A04-p18763435	A-genome specific (napus-rapa)	0.9928229	0.5907952	not scorable	not scorable	0.9834711	0.7735179
9395	Bn-A04-p18789871	A-genome specific (rapa only)	not scorable	not scorable	not scorable	not scorable	1	0.7948923
9396	Bn-A04-p1880654	A-genome specific (napus-rapa)	0.9976076	0.740625	not scorable	not scorable	0.9834711	0.7037346
9397	Bn-A04-p18806834	A-genome specific (napus-rapa)	1	0.6974877	not scorable	not scorable	0.9752066	0.6947444
9398	Bn-A04-p1882240	A-genome specific (napus-rapa)	0.9880383	0.7585191	not scorable	not scorable	0.9669421	0.8018925
9399	Bn-A04-p18867216	A-genome specific (napus-rapa)	0.9952153	0.7146183	not scorable	not scorable	1	0.7739488
9400	Bn-A04-p18913495	A-genome specific (napus-rapa)	0.9832536	0.6570467	not scorable	not scorable	0.9834711	0.589
9401	Bn-A04-p18918997	genome unspecific (all in)	0.8971292	0.3304026	0.9922481	0.5211179	1	0.5440472
9402	Bn-A04-p1893968	A-genome specific (napus-rapa)	0.7535885	0.8571028	not scorable	not scorable	0.9752066	0.7513897

9403	Bn-A04-p18943386	A-genome specific (napus-rapa)	0.9976076	0.7341207	not scorable	not scorable	1	0.7034611
9404	Bn-A04-p18946513	A-genome specific (napus-rapa)	1	0.7550989	not scorable	not scorable	1	0.7739488
9405	Bn-A04-p1895018	A-genome specific (napus-rapa)	0.7416268	0.8385868	not scorable	not scorable	0.9917355	0.7028005
9406	Bn-A04-p18960154	A-genome specific (napus-rapa)	0.9952153	0.5318099	not scorable	not scorable	1	0.6826699
9407	Bn-A04-p18961456	A-genome specific (napus-rapa)	0.9856459	0.6222124	not scorable	not scorable	0.9917355	0.7530153
9408	Bn-A04-p18961945	genome unspecific (all in)	0.9282297	0.472239	0.9922481	0.791706	1	0.9175813
9409	Bn-A04-p18961947	A-genome specific (napus-rapa)	0.9545454	0.4880107	not scorable	not scorable	1	0.8751518
9410	Bn-A04-p18962305	A-genome specific (napus-rapa)	0.8875598	0.4742662	not scorable	not scorable	1	0.9045871
9411	Bn-A04-p18962378	A-genome specific (napus-rapa)	0.9712918	0.5074472	not scorable	not scorable	1	0.9174577
9412	Bn-A04-p18962442	A-genome specific (napus-rapa)	0.9736842	0.4072584	not scorable	not scorable	1	0.9062158
9413	Bn-A04-p1897541	A-genome specific (napus-rapa)	0.7440192	0.7304007	not scorable	not scorable	1	0.7156159
9414	Bn-A04-p1900422	A-genome specific (napus-rapa)	0.3492823	0.7240215	not scorable	not scorable	0.9834711	0.7506039
9415	Bn-A04-p1913537	A-genome specific (napus-rapa)	0.6770335	0.6039565	not scorable	not scorable	0.9917355	0.6560127
9416	Bn-A04-p1927138	A-genome specific (napus-rapa)	0.7464115	0.8825182	not scorable	not scorable	0.9834711	0.6488328
9417	Bn-A04-p1927832	A-genome specific (napus-rapa)	0.7655503	0.8910024	not scorable	not scorable	0.9917355	0.9169714
9418	Bn-A04-p1928290	A-genome specific (napus-rapa)	0.7488039	0.6184193	not scorable	not scorable	0.9834711	0.7381606
9419	Bn-A04-p1931502	A-genome specific (napus-rapa)	0.9808612	0.7575074	not scorable	not scorable	0.9669421	0.7980855
9420	Bn-A04-p1932300	A-genome specific (napus-rapa)	0.7559808	0.7339693	not scorable	not scorable	0.9834711	0.7159997
9421	Bn-A04-p1932352	A-genome specific (napus-rapa)	0.7535885	0.7055543	not scorable	not scorable	0.9917355	0.7359238
9422	Bn-A04-p1935147	A-genome specific (napus-rapa)	0.7511961	0.7771561	not scorable	not scorable	0.9917355	0.7374033
9423	Bn-A04-p1938332	A-genome specific (napus-rapa)	0.9856459	0.5309194	not scorable	not scorable	0.9917355	0.7033768
9424	Bn-A04-p1941188	A-genome specific (napus-rapa)	0.7535885	0.8031276	not scorable	not scorable	0.9917355	0.6655442
9425	Bn-A04-p1943749	A-genome specific (napus-rapa)	0.7583732	0.648775	not scorable	not scorable	0.9917355	0.8508542
9426	Bn-A04-p1948034	A-genome specific (napus-rapa)	0.7511961	0.77904	not scorable	not scorable	0.9917355	0.8282349
9427	Bn-A04-p1950544	A-genome specific (napus-rapa)	0.9976076	0.8626078	not scorable	not scorable	1	0.8090609
9428	Bn-A04-p1962888	A-genome specific (napus-rapa)	0.9952153	0.284406	not scorable	not scorable	0.9669421	0.7412119
9429	Bn-A04-p1971842	A-genome specific (napus-rapa)	0.9760765	0.7932827	not scorable	not scorable	0.9752066	0.8103147
9430	Bn-A04-p1976999	napus specific	0.7416268	0.8063838	not scorable	not scorable	not scorable	not scorable
9431	Bn-A04-p1978703	A-genome specific (napus-rapa)	0.9736842	0.3083358	not scorable	not scorable	1	0.8046467
9432	Bn-A04-p1979834	A-genome specific (napus-rapa)	0.9760765	0.7673513	not scorable	not scorable	0.9834711	0.7859978
9433	Bn-A04-p1983164	A-genome specific (napus-rapa)	1	0.7278885	not scorable	not scorable	0.9917355	0.6793128
9434	Bn-A04-p1991843	A-genome specific (napus-rapa)	0.9569378	0.2886991	not scorable	not scorable	0.9834711	0.7372728
9435	Bn-A04-p1993598	A-genome specific (napus-rapa)	0.9976076	0.7717809	not scorable	not scorable	0.9834711	0.7365086

9436	Bn-A04-p2000268	A-genome specific (napus-rapa)	0.9593301	0.535801	not scorable	not scorable	1	0.68033
9437	Bn-A04-p2009476	A-genome specific (napus-rapa)	0.9928229	0.7619805	not scorable	not scorable	0.9586777	0.7405311
9438	Bn-A04-p2010971	A-genome specific (napus-rapa)	0.9401914	0.6525037	not scorable	not scorable	1	0.811681
9439	Bn-A04-p2029030	napus specific	0.5717703	0.9137297	not scorable	not scorable	not scorable	not scorable
9440	Bn-A04-p2037077	A-genome specific (napus-rapa)	1	0.8327038	not scorable	not scorable	0.9917355	0.7358753
9441	Bn-A04-p2048587	A-genome specific (napus-rapa)	0.9354067	0.326625	not scorable	not scorable	0.9917355	0.6839201
9442	Bn-A04-p2094469	A-genome specific (napus-rapa)	0.9425837	0.4467099	not scorable	not scorable	0.9917355	0.7089894
9443	Bn-A04-p2094818	A-genome specific (napus-rapa)	0.9856459	0.7519183	not scorable	not scorable	0.9834711	0.7932144
9444	Bn-A04-p2104072	A-genome specific (napus-rapa)	0.9617225	0.7662662	not scorable	not scorable	1	0.7771659
9445	Bn-A04-p2111647	A-genome specific (napus-rapa)	0.9904306	0.7343224	not scorable	not scorable	1	0.7697691
9446	Bn-A04-p2122324	A-genome specific (napus-rapa)	1	0.3789478	not scorable	not scorable	0.9752066	0.5582925
9447	Bn-A04-p2124879	napus specific	0.2057416	0.8477142	not scorable	not scorable	not scorable	not scorable
9448	Bn-A04-p2126232	A-genome specific (napus-rapa)	0.9976076	0.776185	not scorable	not scorable	0.9834711	0.8142525
9449	Bn-A04-p2126294	A-genome specific (napus-rapa)	0.9688995	0.4974627	not scorable	not scorable	0.6198347	0.7825954
9450	Bn-A04-p2135204	A-genome specific (napus-rapa)	0.9976076	0.4804099	not scorable	not scorable	1	0.772817
9451	Bn-A04-p2137317	A-genome specific (napus-rapa)	1	0.8344694	not scorable	not scorable	0.9917355	0.7394388
9452	Bn-A04-p2146459	A-genome specific (napus-rapa)	0.9952153	0.7319627	not scorable	not scorable	0.9834711	0.6985237
9453	Bn-A04-p2149134	A-genome specific (napus-rapa)	0.9545454	0.4646099	not scorable	not scorable	1	0.9157093
9454	Bn-A04-p2149214	A-genome specific (napus-rapa)	0.9904306	0.3017809	not scorable	not scorable	0.9834711	0.676232
9455	Bn-A04-p2151567	A-genome specific (napus-rapa)	0.9952153	0.8203091	not scorable	not scorable	1	0.7278452
9456	Bn-A04-p2152171	A-genome specific (napus-rapa)	0.9593301	0.7641389	not scorable	not scorable	0.9917355	0.6832229
9457	Bn-A04-p2152528	A-genome specific (napus-rapa)	0.9928229	0.7917723	not scorable	not scorable	0.9917355	0.8159432
9458	Bn-A04-p2178146	napus specific	0.9114832	0.7122878	not scorable	not scorable	not scorable	not scorable
9459	Bn-A04-p2191612	A-genome specific (napus-rapa)	0.9904306	0.6390353	not scorable	not scorable	0.9834711	0.5627925
9460	Bn-A04-p2192192	A-genome specific (napus-rapa)	0.9904306	0.674539	not scorable	not scorable	0.6528926	0.7080037
9461	Bn-A04-p2200043	A-genome specific (napus-rapa)	0.9856459	0.7563377	not scorable	not scorable	1	0.7087176
9462	Bn-A04-p2213805	A-genome specific (napus-rapa)	0.9904306	0.4540154	not scorable	not scorable	0.9834711	0.817902
9463	Bn-A04-p2215376	A-genome specific (napus-rapa)	1	0.4623215	not scorable	not scorable	0.9669421	0.3027811
9464	Bn-A04-p2215559	A-genome specific (napus-rapa)	0.9449761	0.2626651	not scorable	not scorable	0.9669421	0.619914
9465	Bn-A04-p2218115	A-genome specific (napus-rapa)	1	0.8164203	not scorable	not scorable	0.9917355	0.7112229
9466	Bn-A04-p2222852	A-genome specific (napus-rapa)	0.9976076	0.4066476	not scorable	not scorable	0.9917355	0.8223843
9467	Bn-A04-p223001	A-genome specific (napus-rapa)	1	0.8904615	not scorable	not scorable	0.9917355	0.753701
9468	Bn-A04-p225323	A-genome specific (napus-rapa)	1	0.5888935	not scorable	not scorable	0.9917355	0.8241305

9469	Bn-A04-p2270213	A-genome specific (napus-rapa)	0.9952153	0.7807232	not scorable	not scorable	0.9917355	0.9039531
9470	Bn-A04-p2282199	A-genome specific (napus-rapa)	0.9928229	0.4734727	not scorable	not scorable	0.9834711	0.7976328
9471	Bn-A04-p2288114	A-genome specific (rapa only)	not scorable	not scorable	not scorable	not scorable	0.9917355	0.7814125
9472	Bn-A04-p2309625	A-genome specific (napus-rapa)	0.9617225	0.1878528	not scorable	not scorable	0.9504132	0.272849
9473	Bn-A04-p2325988	A-genome specific (napus-rapa)	0.9952153	0.7790074	not scorable	not scorable	0.9834711	0.8140697
9474	Bn-A04-p2330084	A-genome specific (napus-rapa)	0.937799	0.6990896	not scorable	not scorable	0.9834711	0.6775374
9475	Bn-A04-p2334920	A-genome specific (rapa only)	not scorable	not scorable	not scorable	not scorable	0.9834711	0.7767055
9476	Bn-A04-p233588	A-genome specific (napus-rapa)	0.9904306	0.6691502	not scorable	not scorable	0.9917355	0.6715612
9477	Bn-A04-p2342131	A-genome specific (napus-rapa)	0.9880383	0.6735913	not scorable	not scorable	0.9834711	0.6240643
9478	Bn-A04-p234664	A-genome specific (napus-rapa)	0.9952153	0.6436669	not scorable	not scorable	1	0.8587458
9479	Bn-A04-p2353879	A-genome specific (napus-rapa)	0.9521531	0.412494	not scorable	not scorable	0.9752066	0.6599309
9480	Bn-A04-p2367374	A-genome specific (napus-rapa)	0.9736842	0.7731407	not scorable	not scorable	0.6859504	0.8719174
9481	Bn-A04-p2401413	A-genome specific (napus-rapa)	0.9545454	0.635132	not scorable	not scorable	0.9669421	0.7034123
9482	Bn-A04-p2411039	A-genome specific (napus-rapa)	0.937799	0.8439403	not scorable	not scorable	0.9586777	0.9173898
9483	Bn-A04-p2415169	A-genome specific (rapa only)	not scorable	not scorable	not scorable	not scorable	1	0.7306921
9484	Bn-A04-p2429853	A-genome specific (napus-rapa)	1	0.6686773	not scorable	not scorable	0.9752066	0.7752491
9485	Bn-A04-p2434674	A-genome specific (napus-rapa)	1	0.8997231	not scorable	not scorable	0.9834711	0.7657718
9486	Bn-A04-p24368	A-genome specific (napus-rapa)	1	0.8297507	not scorable	not scorable	0.9917355	0.7447408
9487	Bn-A04-p2439163	A-genome specific (napus-rapa)	0.9976076	0.826654	not scorable	not scorable	0.9917355	0.7295072
9488	Bn-A04-p24411	A-genome specific (napus-rapa)	0.9976076	0.6727062	not scorable	not scorable	0.9917355	0.5694439
9489	Bn-A04-p2448580	A-genome specific (napus-rapa)	1	0.8427519	not scorable	not scorable	1	0.7790077
9490	Bn-A04-p2456200	A-genome specific (rapa only)	not scorable	not scorable	not scorable	not scorable	0.9917355	0.7168925
9491	Bn-A04-p2475793	A-genome specific (napus-rapa)	1	0.7676928	not scorable	not scorable	1	0.8343173
9492	Bn-A04-p248884	A-genome specific (napus-rapa)	0.9952153	0.4766282	not scorable	not scorable	1	0.7383217
9493	Bn-A04-p2498254	A-genome specific (napus-rapa)	0.9880383	0.7251781	not scorable	not scorable	0.9834711	0.6546672
9494	Bn-A04-p251383	A-genome specific (napus-rapa)	0.9928229	0.8272741	not scorable	not scorable	1	0.8727394
9495	Bn-A04-p2537756	A-genome specific (napus-rapa)	0.9856459	0.5380934	not scorable	not scorable	0.9669421	0.7627054
9496	Bn-A04-p2542000	A-genome specific (napus-rapa)	1	0.8663135	not scorable	not scorable	0.9917355	0.7016385
9497	Bn-A04-p2552293	A-genome specific (napus-rapa)	1	0.7317032	not scorable	not scorable	1	0.7771295
9498	Bn-A04-p2562005	A-genome specific (napus-rapa)	1	0.8335621	not scorable	not scorable	1	0.7860581
9499	Bn-A04-p2566546	A-genome specific (napus-rapa)	0.9952153	0.3018834	not scorable	not scorable	0.9752066	0.8288633
9500	Bn-A04-p2568394	A-genome specific (napus-rapa)	0.9976076	0.4785771	not scorable	not scorable	0.9834711	0.7898469
9501	Bn-A04-p2592215	A-genome specific (napus-rapa)	1	0.8281221	not scorable	not scorable	1	0.8552262

9502	Bn-A04-p2593658	genome unspecific (all in)	0.9880383	0.7125414	0.6899225	0.7805941	0.9421487	0.6749262
9503	Bn-A04-p2603671	A-genome specific (napus-rapa)	1	0.8167262	not scorable	not scorable	1	0.7819862
9504	Bn-A04-p2611718	A-genome specific (napus-rapa)	1	0.8350718	not scorable	not scorable	0.9752066	0.695935
9505	Bn-A04-p261340	A-genome specific (napus-rapa)	0.9617225	0.8275406	not scorable	not scorable	0.9669421	0.7846559
9506	Bn-A04-p2616582	A-genome specific (napus-rapa)	1	0.8485806	not scorable	not scorable	0.6363636	0.8613519
9507	Bn-A04-p2623116	A-genome specific (rapa only)	not scorable	not scorable	not scorable	not scorable	0.9917355	0.7823381
9508	Bn-A04-p262404	A-genome specific (napus-rapa)	0.9904306	0.5352684	not scorable	not scorable	0.9917355	0.7330095
9509	Bn-A04-p2648823	A-genome specific (napus-rapa)	1	0.8381849	not scorable	not scorable	0.9752066	0.7106797
9510	Bn-A04-p2650427	C-genome specific (napus-oleracea)	0.9880383	0.2994321	0.9922481	0.2138192	not scorable	not scorable
9511	Bn-A04-p2651171	A-genome specific (napus-rapa)	1	0.7638833	not scorable	not scorable	0.9917355	0.7585063
9512	Bn-A04-p2657695	A-genome specific (napus-rapa)	0.9952153	0.8520935	not scorable	not scorable	0.9834711	0.7134477
9513	Bn-A04-p2658023	A-genome specific (napus-rapa)	0.9856459	0.8402416	not scorable	not scorable	0.9752066	0.7553384
9514	Bn-A04-p2660465	A-genome specific (napus-rapa)	0.8755981	0.615896	not scorable	not scorable	0.9669421	0.7305559
9515	Bn-A04-p2662208	A-genome specific (napus-rapa)	0.9928229	0.7814817	not scorable	not scorable	1	0.7206949
9516	Bn-A04-p2664124	A-genome specific (napus-rapa)	0.9904306	0.8719767	not scorable	not scorable	0.9752066	0.7276515
9517	Bn-A04-p2665421	A-genome specific (napus-rapa)	0.9521531	0.3646606	not scorable	not scorable	0.9256198	0.6543438
9518	Bn-A04-p2666754	A-genome specific (napus-rapa)	0.9832536	0.9051176	not scorable	not scorable	0.9834711	0.7071137
9519	Bn-A04-p2668053	A-genome specific (napus-rapa)	0.9952153	0.3278828	not scorable	not scorable	0.9752066	0.7391689
9520	Bn-A04-p2669971	A-genome specific (napus-rapa)	0.9928229	0.7138222	not scorable	not scorable	0.9917355	0.7119705
9521	Bn-A04-p2670078	A-genome specific (napus-rapa)	0.9808612	0.5696267	not scorable	not scorable	0.9917355	0.5836712
9522	Bn-A04-p2670318	A-genome specific (napus-rapa)	0.8779904	0.4526289	not scorable	not scorable	0.9834711	0.6826093
9523	Bn-A04-p2671273	A-genome specific (napus-rapa)	0.9832536	0.8560692	not scorable	not scorable	0.9834711	0.7603245
9524	Bn-A04-p267294	A-genome specific (napus-rapa)	0.9928229	0.7613875	not scorable	not scorable	1	0.8187364
9525	Bn-A04-p2680732	A-genome specific (napus-rapa)	0.9952153	0.6863958	not scorable	not scorable	1	0.764473
9526	Bn-A04-p2689124	A-genome specific (napus-rapa)	0.9952153	0.7902796	not scorable	not scorable	0.9834711	0.7597603
9527	Bn-A04-p2692131	A-genome specific (napus-rapa)	0.9904306	0.8663232	not scorable	not scorable	1	0.8325943
9528	Bn-A04-p2713517	A-genome specific (napus-rapa)	0.9952153	0.7981277	not scorable	not scorable	0.9669421	0.8200515
9529	Bn-A04-p2713564	A-genome specific (napus-rapa)	0.9880383	0.4474392	not scorable	not scorable	0.9752066	0.7984909
9530	Bn-A04-p2723787	A-genome specific (napus-rapa)	1	0.7664241	not scorable	not scorable	0.9917355	0.8182597
9531	Bn-A04-p2730690	A-genome specific (napus-rapa)	0.8636364	0.6293261	not scorable	not scorable	0.9752066	0.5933949
9532	Bn-A04-p2748876	A-genome specific (rapa only)	not scorable	not scorable	not scorable	not scorable	0.9090909	0.7965723
9533	Bn-A04-p2750401	A-genome specific (napus-rapa)	0.9880383	0.7679557	not scorable	not scorable	0.9669421	0.7664316
9534	Bn-A04-p2751097	A-genome specific (napus-rapa)	0.9952153	0.816592	not scorable	not scorable	0.9834711	0.7876455

9535	Bn-A04-p2753796	A-genome specific (napus-rapa)	0.9880383	0.7799343	not scorable	not scorable	0.9834711	0.6818096
9536	Bn-A04-p2753824	A-genome specific (napus-rapa)	0.9928229	0.7277877	not scorable	not scorable	0.9834711	0.7291099
9537	Bn-A04-p2760477	A-genome specific (napus-rapa)	0.5717703	0.8434469	not scorable	not scorable	1	0.7900175
9538	Bn-A04-p2761067	A-genome specific (napus-rapa)	0.5717703	0.8090534	not scorable	not scorable	0.9752066	0.7447679
9539	Bn-A04-p2762891	A-genome specific (napus-rapa)	0.5717703	0.8499875	not scorable	not scorable	0.9917355	0.7929817
9540	Bn-A04-p2763423	A-genome specific (napus-rapa)	0.569378	0.7674242	not scorable	not scorable	0.9834711	0.7189919
9541	Bn-A04-p2765547	A-genome specific (napus-rapa)	1	0.7539171	not scorable	not scorable	0.9917355	0.7561262
9542	Bn-A04-p2780974	A-genome specific (napus-rapa)	0.9928229	0.8703667	not scorable	not scorable	0.9834711	0.7900752
9543	Bn-A04-p2781853	napus specific	0.9928229	0.8312163	not scorable	not scorable	not scorable	not scorable
9544	Bn-A04-p2782258	A-genome specific (napus-rapa)	0.9784689	0.859454	not scorable	not scorable	0.9917355	0.6891034
9545	Bn-A04-p2783207	A-genome specific (napus-rapa)	0.9952153	0.8717191	not scorable	not scorable	1	0.6725802
9546	Bn-A04-p2789250	A-genome specific (napus-rapa)	1	0.8908999	not scorable	not scorable	1	0.6723114
9547	Bn-A04-p2793807	A-genome specific (napus-rapa)	0.9712918	0.6131735	not scorable	not scorable	0.9917355	0.6984304
9548	Bn-A04-p2802411	A-genome specific (napus-rapa)	0.9856459	0.5570931	not scorable	not scorable	0.9669421	0.741774
9549	Bn-A04-p2807465	A-genome specific (napus-rapa)	1	0.4821323	not scorable	not scorable	0.9669421	0.6388381
9550	Bn-A04-p2817722	A-genome specific (napus-rapa)	0.9808612	0.8714933	not scorable	not scorable	0.9834711	0.7397835
9551	Bn-A04-p2818095	A-genome specific (napus-rapa)	0.9784689	0.3937797	not scorable	not scorable	1	0.6995043
9552	Bn-A04-p2823217	A-genome specific (rapa only)	not scorable	not scorable	not scorable	not scorable	0.9917355	0.6949153
9553	Bn-A04-p2825294	A-genome specific (napus-rapa)	0.9928229	0.4378979	not scorable	not scorable	0.9917355	0.8275031
9554	Bn-A04-p2825362	A-genome specific (napus-rapa)	0.9976076	0.8656128	not scorable	not scorable	0.9917355	0.7592086
9555	Bn-A04-p2833736	A-genome specific (napus-rapa)	0.9784689	0.4374253	not scorable	not scorable	0.9834711	0.6926391
9556	Bn-A04-p2841305	A-genome specific (napus-rapa)	0.9545454	0.5106031	not scorable	not scorable	0.9917355	0.7826812
9557	Bn-A04-p2841433	A-genome specific (rapa only)	not scorable	not scorable	not scorable	not scorable	0.9752066	0.7049065
9558	Bn-A04-p2843802	A-genome specific (napus-rapa)	0.9904306	0.7318231	not scorable	not scorable	0.9752066	0.7436404
9559	Bn-A04-p2845855	A-genome specific (napus-rapa)	0.9952153	0.7758907	not scorable	not scorable	0.9834711	0.7620555
9560	Bn-A04-p2847914	A-genome specific (napus-rapa)	0.9832536	0.8247396	not scorable	not scorable	0.9834711	0.7043232
9561	Bn-A04-p2881354	A-genome specific (napus-rapa)	0.9808612	0.7598998	not scorable	not scorable	0.9917355	0.811658
9562	Bn-A04-p288906	A-genome specific (napus-rapa)	0.9976076	0.8664871	not scorable	not scorable	0.9917355	0.790215
9563	Bn-A04-p2889239	A-genome specific (napus-rapa)	0.9880383	0.7880844	not scorable	not scorable	0.9917355	0.7702582
9564	Bn-A04-p2892557	A-genome specific (napus-rapa)	0.9904306	0.7414456	not scorable	not scorable	0.9752066	0.6576321
9565	Bn-A04-p2892722	A-genome specific (napus-rapa)	0.9928229	0.7429333	not scorable	not scorable	0.9917355	0.7875235
9566	Bn-A04-p2892985	A-genome specific (napus-rapa)	0.9976076	0.8004358	not scorable	not scorable	1	0.5972179
9567	Bn-A04-p2893053	A-genome specific (napus-rapa)	1	0.7760101	not scorable	not scorable	0.9834711	0.7528777

9568	Bn-A04-p289974	A-genome specific (napus-rapa)	0.9114832	0.8632445	not scorable	not scorable	0.9752066	0.8131607
9569	Bn-A04-p2903530	A-genome specific (napus-rapa)	0.9688995	0.6445517	not scorable	not scorable	0.9834711	0.6959602
9570	Bn-A04-p2906681	A-genome specific (napus-rapa)	0.9880383	0.5479018	not scorable	not scorable	0.6859504	0.6176571
9571	Bn-A04-p2909242	A-genome specific (napus-rapa)	0.9904306	0.5744396	not scorable	not scorable	0.6198347	0.7985611
9572	Bn-A04-p2913939	A-genome specific (napus-rapa)	0.9832536	0.7473009	not scorable	not scorable	0.9917355	0.7100779
9573	Bn-A04-p2914409	A-genome specific (napus-rapa)	0.9976076	0.7739198	not scorable	not scorable	0.9917355	0.7785292
9574	Bn-A04-p2921313	A-genome specific (napus-rapa)	0.9976076	0.7598693	not scorable	not scorable	0.9834711	0.7208499
9575	Bn-A04-p2921682	A-genome specific (napus-rapa)	0.9808612	0.7717234	not scorable	not scorable	1	0.7523933
9576	Bn-A04-p2921832	A-genome specific (napus-rapa)	0.9808612	0.7628785	not scorable	not scorable	1	0.7415655
9577	Bn-A04-p2923396	A-genome specific (napus-rapa)	0.9928229	0.773458	not scorable	not scorable	0.9834711	0.7745193
9578	Bn-A04-p2925273	A-genome specific (napus-rapa)	0.9904306	0.7640589	not scorable	not scorable	1	0.7040766
9579	Bn-A04-p2932086	A-genome specific (napus-rapa)	0.9784689	0.7483853	not scorable	not scorable	0.9834711	0.7067118
9580	Bn-A04-p2933140	A-genome specific (napus-rapa)	0.9904306	0.7628951	not scorable	not scorable	0.9834711	0.7882631
9581	Bn-A04-p2941201	genome unspecific (all in)	0.9952153	0.5264153	1	0.7475585	1	0.6308588
9582	Bn-A04-p2944723	A-genome specific (napus-rapa)	0.9952153	0.7635173	not scorable	not scorable	1	0.8256688
9583	Bn-A04-p2947993	A-genome specific (napus-rapa)	0.9904306	0.8388332	not scorable	not scorable	0.9917355	0.7684254
9584	Bn-A04-p2948936	A-genome specific (napus-rapa)	0.9928229	0.8439397	not scorable	not scorable	0.9752066	0.7488836
9585	Bn-A04-p2949084	A-genome specific (napus-rapa)	0.9784689	0.7794812	not scorable	not scorable	0.9917355	0.6824558
9586	Bn-A04-p2949339	A-genome specific (napus-rapa)	0.9832536	0.771849	not scorable	not scorable	0.9834711	0.7362629
9587	Bn-A04-p2954791	A-genome specific (napus-rapa)	0.9760765	0.32821	not scorable	not scorable	0.9834711	0.7735373
9588	Bn-A04-p2954824	A-genome specific (napus-rapa)	0.9617225	0.5287805	not scorable	not scorable	0.9669421	0.7611408
9589	Bn-A04-p2957928	napus specific	1	0.7919362	not scorable	not scorable	not scorable	not scorable
9590	Bn-A04-p2957984	A-genome specific (napus-rapa)	0.9952153	0.7972959	not scorable	not scorable	0.9834711	0.4753979
9591	Bn-A04-p2960723	A-genome specific (napus-rapa)	0.9880383	0.6027597	not scorable	not scorable	0.9752066	0.6625611
9592	Bn-A04-p2963387	A-genome specific (napus-rapa)	0.9569378	0.3881554	not scorable	not scorable	0.9834711	0.7738208
9593	Bn-A04-p2969081	A-genome specific (napus-rapa)	0.9904306	0.5233365	not scorable	not scorable	0.9669421	0.4605447
9594	Bn-A04-p2983187	napus specific	0.9521531	0.4215306	not scorable	not scorable	not scorable	not scorable
9595	Bn-A04-p2999394	A-genome specific (napus-rapa)	0.9736842	0.7565537	not scorable	not scorable	1	0.9397733
9596	Bn-A04-p3002670	A-genome specific (napus-rapa)	0.9808612	0.3134426	not scorable	not scorable	0.9752066	0.595712
9597	Bn-A04-p3004068	A-genome specific (napus-rapa)	0.9808612	0.3576762	not scorable	not scorable	1	0.777019
9598	Bn-A04-p3016121	A-genome specific (napus-rapa)	0.9665072	0.4919949	not scorable	not scorable	1	0.7907543
9599	Bn-A04-p3021710	A-genome specific (napus-rapa)	0.9952153	0.8231564	not scorable	not scorable	0.9834711	0.7821741
9600	Bn-A04-p3022681	A-genome specific (napus-rapa)	0.9904306	0.5689077	not scorable	not scorable	1	0.6983941

9601	Bn-A04-p3095652	A-genome specific (napus-rapa)	0.9808612	0.7372097	not scorable	not scorable	0.9917355	0.7457149
9602	Bn-A04-p3116993	A-genome specific (rapa only)	not scorable	not scorable	not scorable	not scorable	0.9917355	0.8159167
9603	Bn-A04-p3118075	A-genome specific (napus-rapa)	1	0.7953196	not scorable	not scorable	1	0.8409084
9604	Bn-A04-p312324	A-genome specific (napus-rapa)	0.9952153	0.842741	not scorable	not scorable	0.9917355	0.8422567
9605	Bn-A04-p3126855	A-genome specific (napus-rapa)	0.9712918	0.4353352	not scorable	not scorable	0.9834711	0.8293211
9606	Bn-A04-p3128038	A-genome specific (napus-rapa)	0.9569378	0.6284894	not scorable	not scorable	0.9834711	0.6509814
9607	Bn-A04-p313091	A-genome specific (napus-rapa)	0.9952153	0.810208	not scorable	not scorable	0.9917355	0.7386276
9608	Bn-A04-p313410	A-genome specific (napus-rapa)	0.9976076	0.7754975	not scorable	not scorable	0.9917355	0.8053572
9609	Bn-A04-p3135512	A-genome specific (napus-rapa)	0.9641148	0.5115613	not scorable	not scorable	0.9917355	0.8104939
9610	Bn-A04-p3135978	A-genome specific (napus-rapa)	0.9784689	0.6924659	not scorable	not scorable	0.9834711	0.6947585
9611	Bn-A04-p3138111	A-genome specific (napus-rapa)	0.9688995	0.6079832	not scorable	not scorable	0.9917355	0.8030513
9612	Bn-A04-p3138462	A-genome specific (napus-rapa)	0.9856459	0.7549686	not scorable	not scorable	0.9917355	0.7850422
9613	Bn-A04-p313935	A-genome specific (napus-rapa)	1	0.7925267	not scorable	not scorable	1	0.6683086
9614	Bn-A04-p3140726	A-genome specific (napus-rapa)	0.9856459	0.7561897	not scorable	not scorable	1	0.7110297
9615	Bn-A04-p314440	A-genome specific (napus-rapa)	1	0.767509	not scorable	not scorable	0.9669421	0.7318206
9616	Bn-A04-p3144839	A-genome specific (napus-rapa)	0.9497608	0.3521485	not scorable	not scorable	0.9917355	0.7202103
9617	Bn-A04-p314536	A-genome specific (napus-rapa)	0.9928229	0.6678345	not scorable	not scorable	1	0.5823967
9618	Bn-A04-p314872	A-genome specific (napus-rapa)	0.9952153	0.6921808	not scorable	not scorable	0.9669421	0.4962315
9619	Bn-A04-p3152494	A-genome specific (napus-rapa)	0.9712918	0.4383987	not scorable	not scorable	0.9917355	0.7212923
9620	Bn-A04-p3152554	A-genome specific (napus-rapa)	0.9617225	0.6366324	not scorable	not scorable	0.9917355	0.7431666
9621	Bn-A04-p3172854	A-genome specific (napus-rapa)	0.9976076	0.7145781	not scorable	not scorable	1	0.7717832
9622	Bn-A04-p3181336	A-genome specific (napus-rapa)	0.9976076	0.3238432	not scorable	not scorable	1	0.7481608
9623	Bn-A04-p3182956	A-genome specific (napus-rapa)	0.9090909	0.7343262	not scorable	not scorable	0.9752066	0.7482621
9624	Bn-A04-p3190463	A-genome specific (napus-rapa)	0.8349282	0.7240528	not scorable	not scorable	0.9834711	0.6891936
9625	Bn-A04-p320972	A-genome specific (napus-rapa)	0.9976076	0.3709218	not scorable	not scorable	1	0.7819138
9626	Bn-A04-p3219148	A-genome specific (napus-rapa)	0.9497608	0.8237522	not scorable	not scorable	0.9834711	0.8183623
9627	Bn-A04-p3220424	napus specific	1	0.2904207	not scorable	not scorable	not scorable	not scorable
9628	Bn-A04-p3220819	A-genome specific (napus-rapa)	1	0.7237918	not scorable	not scorable	1	0.7890415
9629	Bn-A04-p3225119	A-genome specific (napus-rapa)	0.9665072	0.3322402	not scorable	not scorable	0.9834711	0.5679683
9630	Bn-A04-p322820	A-genome specific (napus-rapa)	0.9617225	0.3106201	not scorable	not scorable	0.9834711	0.7809242
9631	Bn-A04-p3229572	A-genome specific (napus-rapa)	0.9928229	0.5491607	not scorable	not scorable	0.8429752	0.6602194
9632	Bn-A04-p3235336	A-genome specific (napus-rapa)	0.9569378	0.4326471	not scorable	not scorable	0.9917355	0.6624446
9633	Bn-A04-p324144	A-genome specific (napus-rapa)	1	0.8535089	not scorable	not scorable	0.9917355	0.8142882

9634	Bn-A04-p325019	A-genome specific (napus-rapa)	0.9856459	0.8690729	not scorable	not scorable	1	0.689499
9635	Bn-A04-p3254177	A-genome specific (napus-rapa)	0.9856459	0.2469944	not scorable	not scorable	0.553719	0.5592213
9636	Bn-A04-p3255140	A-genome specific (napus-rapa)	0.8779904	0.7130182	not scorable	not scorable	0.5619835	0.762321
9637	Bn-A04-p3255172	A-genome specific (napus-rapa)	0.8373206	0.4981945	not scorable	not scorable	0.5371901	0.6907253
9638	Bn-A04-p326007	A-genome specific (napus-rapa)	0.9952153	0.8205206	not scorable	not scorable	0.9917355	0.792846
9639	Bn-A04-p3262432	A-genome specific (napus-rapa)	0.9808612	0.7822923	not scorable	not scorable	0.9917355	0.7973625
9640	Bn-A04-p3263085	A-genome specific (napus-rapa)	0.9808612	0.5163279	not scorable	not scorable	1	0.6415797
9641	Bn-A04-p3267803	A-genome specific (napus-rapa)	0.9760765	0.8148065	not scorable	not scorable	1	0.8561852
9642	Bn-A04-p3272740	genome unspecific (all in)	0.9880383	0.7129759	1	0.6543147	0.9834711	0.7067428
9643	Bn-A04-p3273187	A-genome specific (napus-rapa)	1	0.7283937	not scorable	not scorable	0.9917355	0.746606
9644	Bn-A04-p3274507	A-genome specific (napus-rapa)	1	0.5940585	not scorable	not scorable	0.9917355	0.7608721
9645	Bn-A04-p3283282	A-genome specific (napus-rapa)	0.9880383	0.2793228	not scorable	not scorable	0.9917355	0.7658658
9646	Bn-A04-p3294004	A-genome specific (napus-rapa)	1	0.6732786	not scorable	not scorable	0.9752066	0.7480511
9647	Bn-A04-p3297115	A-genome specific (napus-rapa)	0.9952153	0.4511201	not scorable	not scorable	1	0.7978051
9648	Bn-A04-p3298487	A-genome specific (napus-rapa)	0.9952153	0.5420992	not scorable	not scorable	1	0.7656361
9649	Bn-A04-p330341	A-genome specific (napus-rapa)	0.9880383	0.266967	not scorable	not scorable	1	0.7114117
9650	Bn-A04-p3306253	genome unspecific (oleracea-rapa)	not scorable	not scorable	0.9379845	0.4681461	0.9421487	0.7813604
9651	Bn-A04-p3308299	A-genome specific (rapa only)	not scorable	not scorable	not scorable	not scorable	0.5867769	0.8340232
9652	Bn-A04-p3331930	A-genome specific (napus-rapa)	0.9880383	0.7167162	not scorable	not scorable	1	0.6032355
9653	Bn-A04-p3331969	A-genome specific (napus-rapa)	0.9904306	0.4958424	not scorable	not scorable	0.9834711	0.8074395
9654	Bn-A04-p3332039	A-genome specific (napus-rapa)	0.9736842	0.7028045	not scorable	not scorable	0.9669421	0.698546
9655	Bn-A04-p3332042	A-genome specific (napus-rapa)	0.9832536	0.8346835	not scorable	not scorable	0.9917355	0.8272724
9656	Bn-A04-p3345530	A-genome specific (napus-rapa)	0.9665072	0.2968284	not scorable	not scorable	1	0.6649233
9657	Bn-A04-p3351067	A-genome specific (napus-rapa)	0.9880383	0.8082699	not scorable	not scorable	0.9834711	0.7952991
9658	Bn-A04-p3351150	A-genome specific (napus-rapa)	0.9736842	0.7455055	not scorable	not scorable	0.9752066	0.7599207
9659	Bn-A04-p3357098	A-genome specific (napus-rapa)	0.9688995	0.4591659	not scorable	not scorable	0.9917355	0.7944338
9660	Bn-A04-p3357519	A-genome specific (napus-rapa)	1	0.9166866	not scorable	not scorable	1	0.8343857
9661	Bn-A04-p3357700	A-genome specific (napus-rapa)	0.9760765	0.8923251	not scorable	not scorable	1	0.7752552
9662	Bn-A04-p3358441	A-genome specific (rapa only)	not scorable	not scorable	not scorable	not scorable	0.5123967	0.8018306
9663	Bn-A04-p3363190	A-genome specific (napus-rapa)	1	0.8345768	not scorable	not scorable	0.9917355	0.6878995
9664	Bn-A04-p3379410	A-genome specific (napus-rapa)	0.9760765	0.8111658	not scorable	not scorable	0.9752066	0.8474989
9665	Bn-A04-p3390331	A-genome specific (napus-rapa)	0.9497608	0.4796315	not scorable	not scorable	0.9834711	0.7371351
9666	Bn-A04-p3395103	A-genome specific (napus-rapa)	0.9784689	0.7286358	not scorable	not scorable	0.9752066	0.6794516

9667	Bn-A04-p3413548	A-genome specific (napus-rapa)	0.9808612	0.8150966	not scorable	not scorable	0.9834711	0.700367
9668	Bn-A04-p3414140	A-genome specific (napus-rapa)	0.9641148	0.7400357	not scorable	not scorable	1	0.6520115
9669	Bn-A04-p3414232	A-genome specific (napus-rapa)	0.9952153	0.7394629	not scorable	not scorable	0.9917355	0.6892272
9670	Bn-A04-p3414449	A-genome specific (napus-rapa)	0.9784689	0.8362392	not scorable	not scorable	0.9917355	0.7336606
9671	Bn-A04-p3420532	A-genome specific (napus-rapa)	0.9832536	0.7832367	not scorable	not scorable	1	0.7412711
9672	Bn-A04-p3421117	A-genome specific (napus-rapa)	0.9760765	0.7056122	not scorable	not scorable	1	0.6796372
9673	Bn-A04-p3448682	A-genome specific (napus-rapa)	1	0.843249	not scorable	not scorable	1	0.7359045
9674	Bn-A04-p3449764	A-genome specific (napus-rapa)	0.9976076	0.9182991	not scorable	not scorable	1	0.8533314
9675	Bn-A04-p3457753	A-genome specific (napus-rapa)	0.9401914	0.7852687	not scorable	not scorable	0.9917355	0.7562483
9676	Bn-A04-p3462758	A-genome specific (napus-rapa)	0.9832536	0.5873974	not scorable	not scorable	0.9917355	0.7719791
9677	Bn-A04-p3463785	A-genome specific (napus-rapa)	0.9928229	0.5595241	not scorable	not scorable	0.9917355	0.599916
9678	Bn-A04-p3471699	A-genome specific (napus-rapa)	0.9856459	0.791226	not scorable	not scorable	0.9586777	0.6553779
9679	Bn-A04-p3473990	A-genome specific (napus-rapa)	0.9856459	0.7897729	not scorable	not scorable	0.9752066	0.7045884
9680	Bn-A04-p3483694	A-genome specific (napus-rapa)	1	0.8070485	not scorable	not scorable	0.9834711	0.8291102
9681	Bn-A04-p3483780	A-genome specific (napus-rapa)	0.9832536	0.6768233	not scorable	not scorable	0.9834711	0.7027584
9682	Bn-A04-p3485611	A-genome specific (napus-rapa)	0.9976076	0.821795	not scorable	not scorable	0.9834711	0.7286923
9683	Bn-A04-p3509049	napus specific	0.8397129	0.6622617	not scorable	not scorable	not scorable	not scorable
9684	Bn-A04-p3528213	A-genome specific (napus-rapa)	0.9569378	0.3505399	not scorable	not scorable	0.9752066	0.688382
9685	Bn-A04-p3540163	A-genome specific (napus-rapa)	0.9976076	0.7931589	not scorable	not scorable	0.5785124	0.870795
9686	Bn-A04-p354688	A-genome specific (napus-rapa)	0.9808612	0.8486626	not scorable	not scorable	0.6198347	0.8127691
9687	Bn-A04-p3551969	A-genome specific (napus-rapa)	0.9880383	0.7521334	not scorable	not scorable	1	0.7590011
9688	Bn-A04-p3563096	A-genome specific (napus-rapa)	0.9904306	0.7159402	not scorable	not scorable	0.9917355	0.708402
9689	Bn-A04-p356570	A-genome specific (napus-rapa)	0.9784689	0.9011632	not scorable	not scorable	0.9752066	0.7546584
9690	Bn-A04-p3571259	A-genome specific (napus-rapa)	0.9449761	0.6791484	not scorable	not scorable	0.9669421	0.6931794
9691	Bn-A04-p3572717	A-genome specific (napus-rapa)	0.9497608	0.7308953	not scorable	not scorable	0.9752066	0.6905558
9692	Bn-A04-p357272	A-genome specific (napus-rapa)	0.9952153	0.7421556	not scorable	not scorable	0.9586777	0.7707107
9693	Bn-A04-p3572777	A-genome specific (napus-rapa)	0.9808612	0.7235262	not scorable	not scorable	0.9917355	0.7708753
9694	Bn-A04-p3573078	A-genome specific (rapa only)	not scorable	not scorable	not scorable	not scorable	0.9834711	0.6871217
9695	Bn-A04-p3583216	A-genome specific (napus-rapa)	0.9354067	0.6651208	not scorable	not scorable	0.9917355	0.7590817
9696	Bn-A04-p3589494	A-genome specific (napus-rapa)	0.9976076	0.3944502	not scorable	not scorable	1	0.690158
9697	Bn-A04-p3602299	A-genome specific (napus-rapa)	0.9665072	0.7454916	not scorable	not scorable	0.9834711	0.7091731
9698	Bn-A04-p3615799	napus specific	1	0.5498412	not scorable	not scorable	not scorable	not scorable
9699	Bn-A04-p3618090	napus specific	0.9952153	0.5200757	not scorable	not scorable	not scorable	not scorable

9700	Bn-A04-p3628682	A-genome specific (napus-rapa)	0.9593301	0.6987593	not scorable	not scorable	1	0.8096865
9701	Bn-A04-p3630793	A-genome specific (napus-rapa)	0.9928229	0.5142915	not scorable	not scorable	1	0.7006238
9702	Bn-A04-p3631563	A-genome specific (napus-rapa)	0.9736842	0.7348714	not scorable	not scorable	0.9917355	0.6737842
9703	Bn-A04-p3632226	A-genome specific (napus-rapa)	0.9880383	0.7441453	not scorable	not scorable	0.9917355	0.6383561
9704	Bn-A04-p3632611	A-genome specific (napus-rapa)	0.9952153	0.7638794	not scorable	not scorable	1	0.7177677
9705	Bn-A04-p3651589	A-genome specific (napus-rapa)	0.9521531	0.3363951	not scorable	not scorable	0.9834711	0.7252181
9706	Bn-A04-p3668627	A-genome specific (napus-rapa)	0.9784689	0.8279315	not scorable	not scorable	1	0.8445701
9707	Bn-A04-p3690435	A-genome specific (napus-rapa)	0.9832536	0.5158319	not scorable	not scorable	0.9917355	0.7831922
9708	Bn-A04-p3695547	A-genome specific (napus-rapa)	0.4688995	0.8840098	not scorable	not scorable	0.9256198	0.79131
9709	Bn-A04-p3702357	A-genome specific (napus-rapa)	0.9760765	0.6328984	not scorable	not scorable	0.9917355	0.8569925
9710	Bn-A04-p3702896	A-genome specific (napus-rapa)	0.9760765	0.2963188	not scorable	not scorable	0.9669421	0.6159005
9711	Bn-A04-p370297	A-genome specific (napus-rapa)	0.9928229	0.7338368	not scorable	not scorable	0.9586777	0.6154082
9712	Bn-A04-p3719381	A-genome specific (napus-rapa)	0.9928229	0.7935023	not scorable	not scorable	1	0.8458138
9713	Bn-A04-p3721587	A-genome specific (napus-rapa)	1	0.8098124	not scorable	not scorable	0.9834711	0.7324129
9714	Bn-A04-p3724573	A-genome specific (napus-rapa)	1	0.4763272	not scorable	not scorable	1	0.8012655
9715	Bn-A04-p3743549	A-genome specific (napus-rapa)	0.9928229	0.7134449	not scorable	not scorable	0.9752066	0.6592058
9716	Bn-A04-p3750975	A-genome specific (napus-rapa)	0.9808612	0.5248904	not scorable	not scorable	1	0.8206075
9717	Bn-A04-p375224	A-genome specific (napus-rapa)	0.9880383	0.8160862	not scorable	not scorable	0.9586777	0.7933186
9718	Bn-A04-p375518	A-genome specific (napus-rapa)	0.9952153	0.8331373	not scorable	not scorable	0.9834711	0.8238301
9719	Bn-A04-p3757486	A-genome specific (napus-rapa)	0.9928229	0.6882309	not scorable	not scorable	0.9834711	0.7053689
9720	Bn-A04-p3761523	A-genome specific (napus-rapa)	0.9952153	0.8013068	not scorable	not scorable	0.9834711	0.8510177
9721	Bn-A04-p3820381	A-genome specific (napus-rapa)	0.9856459	0.73953	not scorable	not scorable	0.9917355	0.7098398
9722	Bn-A04-p3851969	A-genome specific (napus-rapa)	0.7703349	0.8607996	not scorable	not scorable	0.9917355	0.7917648
9723	Bn-A04-p3852124	napus specific	0.7631579	0.9124498	not scorable	not scorable	not scorable	not scorable
9724	Bn-A04-p3853621	napus specific	0.7679426	0.7272235	not scorable	not scorable	not scorable	not scorable
9725	Bn-A04-p3875034	A-genome specific (napus-rapa)	0.9808612	0.7815894	not scorable	not scorable	0.9917355	0.6289822
9726	Bn-A04-p3915978	napus specific	1	0.5584974	not scorable	not scorable	not scorable	not scorable
9727	Bn-A04-p3923982	A-genome specific (rapa only)	not scorable	not scorable	not scorable	not scorable	0.5950413	0.7240272
9728	Bn-A04-p3933714	A-genome specific (napus-rapa)	0.9904306	0.7633109	not scorable	not scorable	1	0.6769987
9729	Bn-A04-p3934671	A-genome specific (napus-rapa)	0.9808612	0.7172512	not scorable	not scorable	0.9917355	0.6734592
9730	Bn-A04-p397523	A-genome specific (napus-rapa)	0.9952153	0.6883276	not scorable	not scorable	0.9917355	0.6598009
9731	Bn-A04-p398807	A-genome specific (napus-rapa)	0.9976076	0.5090746	not scorable	not scorable	1	0.707249
9732	Bn-A04-p3990611	A-genome specific (napus-rapa)	0.9856459	0.7086554	not scorable	not scorable	1	0.7245188

9733	Bn-A04-p3990828	A-genome specific (napus-rapa)	0.9856459	0.7751051	not scorable	not scorable	1	0.7197421
9734	Bn-A04-p3991261	A-genome specific (napus-rapa)	0.9880383	0.7758925	not scorable	not scorable	1	0.8293419
9735	Bn-A04-p4002585	A-genome specific (napus-rapa)	0.9688995	0.7339039	not scorable	not scorable	1	0.5660856
9736	Bn-A04-p4034426	A-genome specific (napus-rapa)	0.9928229	0.8028546	not scorable	not scorable	0.9834711	0.8058633
9737	Bn-A04-p4035141	A-genome specific (rapa only)	not scorable	not scorable	not scorable	not scorable	0.9917355	0.7770618
9738	Bn-A04-p404042	all not scorable	not scorable	not scorable	not scorable	not scorable	not scorable	not scorable
9739	Bn-A04-p405060	A-genome specific (napus-rapa)	0.9880383	0.6296661	not scorable	not scorable	0.9752066	0.578559
9740	Bn-A04-p4056517	A-genome specific (napus-rapa)	1	0.8227402	not scorable	not scorable	0.9834711	0.7267053
9741	Bn-A04-p4060806	A-genome specific (napus-rapa)	0.9880383	0.7782921	not scorable	not scorable	0.9752066	0.764575
9742	Bn-A04-p4074166	A-genome specific (napus-rapa)	0.9976076	0.223732	not scorable	not scorable	1	0.528818
9743	Bn-A04-p4074703	A-genome specific (napus-rapa)	0.9617225	0.5453034	not scorable	not scorable	1	0.8668658
9744	Bn-A04-p4082416	A-genome specific (rapa only)	not scorable	not scorable	not scorable	not scorable	0.9834711	0.6448815
9745	Bn-A04-p4087475	A-genome specific (rapa only)	not scorable	not scorable	not scorable	not scorable	0.9090909	0.4266796
9746	Bn-A04-p4090389	A-genome specific (napus-rapa)	0.9976076	0.447558	not scorable	not scorable	0.9834711	0.7945956
9747	Bn-A04-p4097272	A-genome specific (napus-rapa)	0.9808612	0.7983378	not scorable	not scorable	0.9586777	0.7204002
9748	Bn-A04-p4099958	A-genome specific (napus-rapa)	0.9880383	0.7956931	not scorable	not scorable	1	0.7757955
9749	Bn-A04-p4101266	A-genome specific (napus-rapa)	0.9665072	0.7125781	not scorable	not scorable	0.9752066	0.6319184
9750	Bn-A04-p4101473	A-genome specific (napus-rapa)	0.9784689	0.3777509	not scorable	not scorable	0.9834711	0.7278553
9751	Bn-A04-p4102094	A-genome specific (napus-rapa)	0.9784689	0.7552872	not scorable	not scorable	0.9752066	0.6973829
9752	Bn-A04-p4107072	A-genome specific (napus-rapa)	0.9808612	0.7831545	not scorable	not scorable	0.9834711	0.7410726
9753	Bn-A04-p4112460	A-genome specific (napus-rapa)	0.9856459	0.7972761	not scorable	not scorable	1	0.7807541
9754	Bn-A04-p4117100	A-genome specific (napus-rapa)	0.9808612	0.7453884	not scorable	not scorable	1	0.6795201
9755	Bn-A04-p4121578	A-genome specific (napus-rapa)	0.9976076	0.7499337	not scorable	not scorable	1	0.6930039
9756	Bn-A04-p4124082	A-genome specific (napus-rapa)	0.9736842	0.6360599	not scorable	not scorable	0.9669421	0.5565655
9757	Bn-A04-p4124240	napus specific	0.9641148	0.5240147	not scorable	not scorable	not scorable	not scorable
9758	Bn-A04-p4131451	A-genome specific (napus-rapa)	0.9760765	0.772599	not scorable	not scorable	0.9917355	0.7774039
9759	Bn-A04-p4150340	napus specific	0.9688995	0.2382677	not scorable	not scorable	not scorable	not scorable
9760	Bn-A04-p4152511	A-genome specific (napus-rapa)	0.9497608	0.4941343	not scorable	not scorable	0.9917355	0.7016693
9761	Bn-A04-p4169131	A-genome specific (napus-rapa)	0.9593301	0.4229332	not scorable	not scorable	0.9834711	0.7743751
9762	Bn-A04-p4179724	genome unspecific (oleracea-rapa)	not scorable	not scorable	0.9689922	0.1459306	0.9586777	0.7096977
9763	Bn-A04-p4179915	A-genome specific (napus-rapa)	0.9545454	0.512719	not scorable	not scorable	0.9917355	0.6160439
9764	Bn-A04-p4200354	A-genome specific (napus-rapa)	0.9976076	0.7976544	not scorable	not scorable	0.9834711	0.6958786
9765	Bn-A04-p4203564	A-genome specific (napus-rapa)	0.9808612	0.7739424	not scorable	not scorable	0.9752066	0.7022622

9766	Bn-A04-p4204119	A-genome specific (napus-rapa)	0.9808612	0.7932532	not scorable	not scorable	0.9834711	0.7639515
9767	Bn-A04-p4204775	A-genome specific (napus-rapa)	0.9258373	0.554356	not scorable	not scorable	0.9752066	0.6448771
9768	Bn-A04-p4207198	A-genome specific (napus-rapa)	0.9904306	0.7523361	not scorable	not scorable	0.9834711	0.7191718
9769	Bn-A04-p4210431	A-genome specific (napus-rapa)	1	0.759294	not scorable	not scorable	0.9917355	0.730921
9770	Bn-A04-p4217227	A-genome specific (napus-rapa)	0.9928229	0.319452	not scorable	not scorable	1	0.6219663
9771	Bn-A04-p4221603	A-genome specific (napus-rapa)	0.9784689	0.7481617	not scorable	not scorable	1	0.742234
9772	Bn-A04-p4222491	A-genome specific (napus-rapa)	1	0.4819123	not scorable	not scorable	1	0.7065566
9773	Bn-A04-p4223859	A-genome specific (napus-rapa)	0.9736842	0.4806241	not scorable	not scorable	0.9917355	0.7880316
9774	Bn-A04-p4224646	A-genome specific (napus-rapa)	0.562201	0.8135828	not scorable	not scorable	0.9752066	0.7918426
9775	Bn-A04-p4224705	A-genome specific (napus-rapa)	0.562201	0.8414686	not scorable	not scorable	0.9917355	0.7345473
9776	Bn-A04-p4244824	A-genome specific (napus-rapa)	1	0.4458955	not scorable	not scorable	0.9834711	0.4173105
9777	Bn-A04-p4249132	A-genome specific (napus-rapa)	1	0.6055367	not scorable	not scorable	0.9834711	0.6871157
9778	Bn-A04-p4254366	A-genome specific (napus-rapa)	1	0.5333191	not scorable	not scorable	0.9669421	0.7209556
9779	Bn-A04-p4265054	A-genome specific (napus-rapa)	1	0.3207493	not scorable	not scorable	1	0.7530859
9780	Bn-A04-p4265606	A-genome specific (napus-rapa)	0.9928229	0.3418558	not scorable	not scorable	0.9752066	0.7594211
9781	Bn-A04-p4276334	A-genome specific (napus-rapa)	0.9593301	0.3814976	not scorable	not scorable	0.9917355	0.7816851
9782	Bn-A04-p4286343	A-genome specific (napus-rapa)	0.9976076	0.797215	not scorable	not scorable	0.9917355	0.7879608
9783	Bn-A04-p4294569	A-genome specific (napus-rapa)	1	0.8437496	not scorable	not scorable	1	0.9039531
9784	Bn-A04-p4307559	A-genome specific (napus-rapa)	0.9856459	0.6352119	not scorable	not scorable	0.9917355	0.5085835
9785	Bn-A04-p4319736	genome unspecific (oleracea-rapa)	not scorable	not scorable	0.9922481	0.3165402	0.9586777	0.5628939
9786	Bn-A04-p4325390	A-genome specific (napus-rapa)	0.9593301	0.5915003	not scorable	not scorable	1	0.7061199
9787	Bn-A04-p4330419	A-genome specific (napus-rapa)	0.9952153	0.4140308	not scorable	not scorable	1	0.6995957
9788	Bn-A04-p4332836	A-genome specific (napus-rapa)	1	0.8024035	not scorable	not scorable	1	0.7674087
9789	Bn-A04-p4346492	A-genome specific (napus-rapa)	1	0.8925295	not scorable	not scorable	0.9834711	0.8139626
9790	Bn-A04-p4349715	A-genome specific (napus-rapa)	0.9976076	0.7705802	not scorable	not scorable	1	0.7417137
9791	Bn-A04-p4350372	A-genome specific (napus-rapa)	1	0.846877	not scorable	not scorable	0.9669421	0.772532
9792	Bn-A04-p4350555	A-genome specific (napus-rapa)	0.9976076	0.8569781	not scorable	not scorable	0.9834711	0.70071
9793	Bn-A04-p4382202	A-genome specific (napus-rapa)	1	0.8587587	not scorable	not scorable	0.9834711	0.809307
9794	Bn-A04-p4392113	A-genome specific (napus-rapa)	0.9880383	0.8623146	not scorable	not scorable	0.9917355	0.7862728
9795	Bn-A04-p4409348	A-genome specific (napus-rapa)	0.9952153	0.5518695	not scorable	not scorable	1	0.6903692
9796	Bn-A04-p4409463	A-genome specific (napus-rapa)	1	0.8665085	not scorable	not scorable	1	0.7267236
9797	Bn-A04-p4409752	A-genome specific (napus-rapa)	1	0.8512385	not scorable	not scorable	0.9834711	0.7056103
9798	Bn-A04-p4410113	A-genome specific (napus-rapa)	1	0.8684896	not scorable	not scorable	0.9834711	0.7185551

9799	Bn-A04-p4410144	A-genome specific (napus-rapa)	0.9976076	0.7749025	not scorable	not scorable	0.9752066	0.7003623
9800	Bn-A04-p4410196	A-genome specific (napus-rapa)	0.9856459	0.8900737	not scorable	not scorable	0.9917355	0.6597216
9801	Bn-A04-p4418670	A-genome specific (napus-rapa)	1	0.7807335	not scorable	not scorable	1	0.6909857
9802	Bn-A04-p4418715	A-genome specific (napus-rapa)	0.9976076	0.4672951	not scorable	not scorable	0.9917355	0.6015807
9803	Bn-A04-p4529360	A-genome specific (napus-rapa)	0.9976076	0.7739609	not scorable	not scorable	0.9504132	0.618674
9804	Bn-A04-p4531566	napus specific	0.6578947	0.7762067	not scorable	not scorable	not scorable	not scorable
9805	Bn-A04-p4532802	all not scorable	not scorable	not scorable	not scorable	not scorable	not scorable	not scorable
9806	Bn-A04-p4544600	A-genome specific (napus-rapa)	0.8325359	0.7813753	not scorable	not scorable	1	0.7518713
9807	Bn-A04-p455869	A-genome specific (napus-rapa)	1	0.7916754	not scorable	not scorable	0.9752066	0.7002707
9808	Bn-A04-p459395	A-genome specific (rapa only)	not scorable	not scorable	not scorable	not scorable	0.7438017	0.7764386
9809	Bn-A04-p4598738	A-genome specific (napus-rapa)	0.9354067	0.4549383	not scorable	not scorable	0.9834711	0.8527662
9810	Bn-A04-p4598943	A-genome specific (napus-rapa)	0.9760765	0.4621509	not scorable	not scorable	0.9834711	0.7865753
9811	Bn-A04-p4599093	A-genome specific (napus-rapa)	0.9162679	0.3712881	not scorable	not scorable	0.9834711	0.7127799
9812	Bn-A04-p4599664	A-genome specific (napus-rapa)	0.9425837	0.3275432	not scorable	not scorable	0.9834711	0.7140323
9813	Bn-A04-p4599814	A-genome specific (napus-rapa)	0.9401914	0.3193439	not scorable	not scorable	0.9752066	0.7096587
9814	Bn-A04-p4599916	A-genome specific (rapa only)	not scorable	not scorable	not scorable	not scorable	0.9917355	0.6891796
9815	Bn-A04-p4599988	A-genome specific (napus-rapa)	0.9354067	0.3619776	not scorable	not scorable	0.9586777	0.7845204
9816	Bn-A04-p4600223	A-genome specific (napus-rapa)	0.937799	0.737289	not scorable	not scorable	0.9752066	0.7395847
9817	Bn-A04-p4601980	A-genome specific (napus-rapa)	0.923445	0.5134644	not scorable	not scorable	0.9917355	0.7442976
9818	Bn-A04-p4602694	A-genome specific (napus-rapa)	0.9186603	0.5689141	not scorable	not scorable	0.9586777	0.7948715
9819	Bn-A04-p461679	napus specific	0.9186603	0.4716844	not scorable	not scorable	not scorable	not scorable
9820	Bn-A04-p462219	A-genome specific (rapa only)	not scorable	not scorable	not scorable	not scorable	0.768595	0.9197958
9821	Bn-A04-p462966	A-genome specific (napus-rapa)	0.9545454	0.2508653	not scorable	not scorable	0.6859504	0.7453435
9822	Bn-A04-p463864	A-genome specific (napus-rapa)	1	0.9018453	not scorable	not scorable	1	0.707625
9823	Bn-A04-p4693254	A-genome specific (napus-rapa)	0.9952153	0.9151071	not scorable	not scorable	0.9917355	0.7107084
9824	Bn-A04-p4717909	A-genome specific (napus-rapa)	1	0.7645525	not scorable	not scorable	1	0.8839908
9825	Bn-A04-p4730302	A-genome specific (napus-rapa)	0.9569378	0.9091653	not scorable	not scorable	0.9752066	0.7941489
9826	Bn-A04-p4736023	A-genome specific (napus-rapa)	0.9976076	0.8694049	not scorable	not scorable	0.9834711	0.7844154
9827	Bn-A04-p4742377	A-genome specific (napus-rapa)	0.9808612	0.8521061	not scorable	not scorable	0.9586777	0.738763
9828	Bn-A04-p4742432	A-genome specific (napus-rapa)	0.9808612	0.7986385	not scorable	not scorable	0.9752066	0.828782
9829	Bn-A04-p4823556	A-genome specific (napus-rapa)	0.9952153	0.753897	not scorable	not scorable	0.9917355	0.7912108
9830	Bn-A04-p4825669	A-genome specific (napus-rapa)	0.9760765	0.8084666	not scorable	not scorable	0.9752066	0.7542337
9831	Bn-A04-p4834943	A-genome specific (napus-rapa)	0.9880383	0.7192727	not scorable	not scorable	0.9752066	0.7370261

9832	Bn-A04-p4835096	A-genome specific (napus-rapa)	0.9856459	0.7839423	not scorable	not scorable	0.9917355	0.6749162
9833	Bn-A04-p4835244	A-genome specific (napus-rapa)	0.9880383	0.7847956	not scorable	not scorable	0.9586777	0.7749012
9834	Bn-A04-p4835883	A-genome specific (napus-rapa)	0.9904306	0.7957003	not scorable	not scorable	0.9834711	0.6796759
9835	Bn-A04-p4836499	A-genome specific (napus-rapa)	0.9952153	0.8154332	not scorable	not scorable	0.9917355	0.8035693
9836	Bn-A04-p4848743	A-genome specific (napus-rapa)	0.9952153	0.8003404	not scorable	not scorable	0.9586777	0.8317357
9837	Bn-A04-p4864900	A-genome specific (napus-rapa)	0.9976076	0.8397645	not scorable	not scorable	0.9917355	0.751178
9838	Bn-A04-p4866786	A-genome specific (napus-rapa)	0.9976076	0.8346374	not scorable	not scorable	0.9917355	0.7236252
9839	Bn-A04-p4872836	A-genome specific (napus-rapa)	0.9832536	0.7690726	not scorable	not scorable	0.9421487	0.8739601
9840	Bn-A04-p4874261	A-genome specific (napus-rapa)	1	0.8284246	not scorable	not scorable	0.9917355	0.8004699
9841	Bn-A04-p4874840	A-genome specific (napus-rapa)	0.9832536	0.7904475	not scorable	not scorable	0.9504132	0.7446173
9842	Bn-A04-p487513	A-genome specific (napus-rapa)	1	0.891062	not scorable	not scorable	0.9752066	0.7600093
9843	Bn-A04-p4879262	A-genome specific (napus-rapa)	0.9952153	0.8054369	not scorable	not scorable	0.9917355	0.7226719
9844	Bn-A04-p4880759	A-genome specific (napus-rapa)	0.9904306	0.5864121	not scorable	not scorable	1	0.7323372
9845	Bn-A04-p4881168	A-genome specific (napus-rapa)	0.9952153	0.860281	not scorable	not scorable	1	0.8010378
9846	Bn-A04-p4881807	A-genome specific (napus-rapa)	0.9832536	0.6726725	not scorable	not scorable	0.9917355	0.76532
9847	Bn-A04-p4891169	A-genome specific (napus-rapa)	0.8660287	0.5657546	not scorable	not scorable	0.6280992	0.7008231
9848	Bn-A04-p4897489	A-genome specific (napus-rapa)	0.9784689	0.7685058	not scorable	not scorable	0.6694215	0.6766605
9849	Bn-A04-p4909402	A-genome specific (napus-rapa)	0.9880383	0.7551913	not scorable	not scorable	0.6363636	0.6933745
9850	Bn-A04-p490967	A-genome specific (rapa only)	not scorable	not scorable	not scorable	not scorable	0.9834711	0.7521201
9851	Bn-A04-p4910033	A-genome specific (napus-rapa)	0.9928229	0.7786417	not scorable	not scorable	0.6363636	0.6378961
9852	Bn-A04-p4932716	A-genome specific (napus-rapa)	0.9688995	0.5220121	not scorable	not scorable	0.9669421	0.5437787
9853	Bn-A04-p4976932	A-genome specific (napus-rapa)	0.9856459	0.6974087	not scorable	not scorable	0.9752066	0.6940176
9854	Bn-A04-p4977488	A-genome specific (napus-rapa)	1	0.9048023	not scorable	not scorable	1	0.6956778
9855	Bn-A04-p4978478	A-genome specific (napus-rapa)	0.9976076	0.9353137	not scorable	not scorable	0.9834711	0.7379379
9856	Bn-A04-p4978492	A-genome specific (napus-rapa)	0.9976076	0.7940474	not scorable	not scorable	0.9917355	0.7178526
9857	Bn-A04-p4978780	A-genome specific (napus-rapa)	0.9976076	0.7619479	not scorable	not scorable	0.9917355	0.7284321
9858	Bn-A04-p5133653	A-genome specific (napus-rapa)	0.9952153	0.7939724	not scorable	not scorable	0.9834711	0.736361
9859	Bn-A04-p5137321	genome unspecific (all in)	0.9832536	0.4653326	1	0.834457	0.9917355	0.7587286
9860	Bn-A04-p5183306	A-genome specific (napus-rapa)	0.9784689	0.7701285	not scorable	not scorable	0.9752066	0.7219929
9861	Bn-A04-p5223849	C-genome specific (oleracea only)	not scorable	not scorable	0.8527132	0.5250189	not scorable	not scorable
9862	Bn-A04-p5270881	A-genome specific (napus-rapa)	0.9593301	0.4592729	not scorable	not scorable	0.9834711	0.691402
9863	Bn-A04-p5273199	A-genome specific (napus-rapa)	0.9784689	0.3468392	not scorable	not scorable	0.9917355	0.7274802
9864	Bn-A04-p534430	A-genome specific (napus-rapa)	0.9904306	0.805127	not scorable	not scorable	0.6115702	0.7714831

9865	Bn-A04-p534845	A-genome specific (napus-rapa)	1	0.8235574	not scorable	not scorable	1	0.7033026
9866	Bn-A04-p5378408	A-genome specific (napus-rapa)	0.9952153	0.574847	not scorable	not scorable	1	0.8342283
9867	Bn-A04-p538802	A-genome specific (rapa only)	not scorable	not scorable	not scorable	not scorable	0.9669421	0.7396516
9868	Bn-A04-p5398352	A-genome specific (napus-rapa)	0.9880383	0.6685718	not scorable	not scorable	0.9834711	0.6575862
9869	Bn-A04-p541336	A-genome specific (napus-rapa)	0.9928229	0.8598717	not scorable	not scorable	0.8181818	0.7086295
9870	Bn-A04-p5501342	all not scorable	not scorable	not scorable	not scorable	not scorable	not scorable	not scorable
9871	Bn-A04-p5501870	A-genome specific (rapa only)	not scorable	not scorable	not scorable	not scorable	1	0.5171568
9872	Bn-A04-p5553976	A-genome specific (rapa only)	not scorable	not scorable	not scorable	not scorable	1	0.6753637
9873	Bn-A04-p5554444	A-genome specific (napus-rapa)	0.9928229	0.5301726	not scorable	not scorable	1	0.678672
9874	Bn-A04-p556991	A-genome specific (napus-rapa)	1	0.8675472	not scorable	not scorable	0.9752066	0.773154
9875	Bn-A04-p557097	A-genome specific (napus-rapa)	0.9976076	0.7265286	not scorable	not scorable	0.9917355	0.7062421
9876	Bn-A04-p5576673	A-genome specific (napus-rapa)	0.9880383	0.5809089	not scorable	not scorable	1	0.8722882
9877	Bn-A04-p560622	A-genome specific (napus-rapa)	0.8875598	0.7777887	not scorable	not scorable	0.5867769	0.7101301
9878	Bn-A04-p5646833	A-genome specific (napus-rapa)	1	0.5817558	not scorable	not scorable	1	0.7970801
9879	Bn-A04-p570250	napus specific	0.937799	0.7836981	not scorable	not scorable	not scorable	not scorable
9880	Bn-A04-p5715780	A-genome specific (napus-rapa)	0.9904306	0.8154697	not scorable	not scorable	0.9917355	0.7531332
9881	Bn-A04-p5788908	A-genome specific (napus-rapa)	0.9976076	0.3163436	not scorable	not scorable	0.9917355	0.6137172
9882	Bn-A04-p5809264	A-genome specific (napus-rapa)	0.9952153	0.8477324	not scorable	not scorable	1	0.8330486
9883	Bn-A04-p581704	A-genome specific (napus-rapa)	0.9760765	0.7221511	not scorable	not scorable	0.9917355	0.7125766
9884	Bn-A04-p582626	A-genome specific (napus-rapa)	0.8779904	0.8360122	not scorable	not scorable	0.9917355	0.7359703
9885	Bn-A04-p5827901	A-genome specific (napus-rapa)	0.9976076	0.5218312	not scorable	not scorable	1	0.6871443
9886	Bn-A04-p582979	A-genome specific (napus-rapa)	0.8875598	0.809747	not scorable	not scorable	0.9752066	0.7321706
9887	Bn-A04-p583036	A-genome specific (napus-rapa)	0.8851675	0.8938024	not scorable	not scorable	0.9917355	0.7962971
9888	Bn-A04-p583242	A-genome specific (napus-rapa)	0.8827751	0.85905	not scorable	not scorable	1	0.812957
9889	Bn-A04-p583254	A-genome specific (napus-rapa)	0.861244	0.7743843	not scorable	not scorable	0.5950413	0.7277901
9890	Bn-A04-p583798	A-genome specific (rapa only)	not scorable	not scorable	not scorable	not scorable	0.5702479	0.7915887
9891	Bn-A04-p5843077	A-genome specific (rapa only)	not scorable	not scorable	not scorable	not scorable	0.9917355	0.8474121
9892	Bn-A04-p5852413	A-genome specific (napus-rapa)	0.9952153	0.8030596	not scorable	not scorable	1	0.7313014
9893	Bn-A04-p5853514	A-genome specific (napus-rapa)	0.9976076	0.7908846	not scorable	not scorable	1	0.720183
9894	Bn-A04-p5872564	A-genome specific (napus-rapa)	0.9880383	0.7862628	not scorable	not scorable	0.9834711	0.728904
9895	Bn-A04-p5873190	A-genome specific (napus-rapa)	0.9760765	0.8440688	not scorable	not scorable	0.9752066	0.7359368
9896	Bn-A04-p5880992	A-genome specific (napus-rapa)	0.9736842	0.9294937	not scorable	not scorable	1	0.7593716
9897	Bn-A04-p5885917	A-genome specific (napus-rapa)	1	0.8018859	not scorable	not scorable	0.5454546	0.9061333

9898	Bn-A04-p5919648	A-genome specific (napus-rapa)	0.9712918	0.8805773	not scorable	not scorable	0.9421487	0.7700459
9899	Bn-A04-p5938125	A-genome specific (napus-rapa)	0.9952153	0.4340712	not scorable	not scorable	1	0.9039531
9900	Bn-A04-p5938245	A-genome specific (napus-rapa)	0.9856459	0.6724878	not scorable	not scorable	0.9917355	0.8404178
9901	Bn-A04-p5959753	A-genome specific (napus-rapa)	0.9832536	0.7346559	not scorable	not scorable	0.9917355	0.8154227
9902	Bn-A04-p5964175	A-genome specific (napus-rapa)	1	0.9181998	not scorable	not scorable	1	0.7282632
9903	Bn-A04-p5965590	A-genome specific (napus-rapa)	0.9808612	0.6542808	not scorable	not scorable	1	0.6985441
9904	Bn-A04-p5967828	A-genome specific (napus-rapa)	0.9856459	0.6762845	not scorable	not scorable	1	0.66468
9905	Bn-A04-p5971620	A-genome specific (napus-rapa)	0.9952153	0.5202371	not scorable	not scorable	0.9917355	0.8227199
9906	Bn-A04-p597296	A-genome specific (napus-rapa)	0.9976076	0.841257	not scorable	not scorable	0.9834711	0.7501634
9907	Bn-A04-p5973348	A-genome specific (napus-rapa)	0.9928229	0.7223004	not scorable	not scorable	0.9834711	0.8211411
9908	Bn-A04-p5973919	A-genome specific (napus-rapa)	1	0.9148182	not scorable	not scorable	0.9834711	0.7691472
9909	Bn-A04-p5974195	A-genome specific (napus-rapa)	0.9880383	0.6380497	not scorable	not scorable	1	0.8177654
9910	Bn-A04-p5979118	A-genome specific (napus-rapa)	0.9928229	0.8629093	not scorable	not scorable	0.9917355	0.8601335
9911	Bn-A04-p5982123	A-genome specific (napus-rapa)	0.9736842	0.725991	not scorable	not scorable	0.9917355	0.6329935
9912	Bn-A04-p5983577	A-genome specific (napus-rapa)	1	0.8800282	not scorable	not scorable	1	0.7221214
9913	Bn-A04-p5985513	A-genome specific (napus-rapa)	1	0.4845143	not scorable	not scorable	1	0.7050616
9914	Bn-A04-p5987463	A-genome specific (napus-rapa)	0.9473684	0.9255054	not scorable	not scorable	0.9752066	0.718918
9915	Bn-A04-p599845	A-genome specific (napus-rapa)	0.9976076	0.8630112	not scorable	not scorable	0.9834711	0.8095143
9916	Bn-A04-p5999405	A-genome specific (napus-rapa)	0.9641148	0.527492	not scorable	not scorable	0.9834711	0.7505626
9917	Bn-A04-p600223	A-genome specific (napus-rapa)	1	0.8772717	not scorable	not scorable	0.9917355	0.7897659
9918	Bn-A04-p6004690	A-genome specific (napus-rapa)	0.9354067	0.7739547	not scorable	not scorable	0.9917355	0.6727043
9919	Bn-A04-p6008358	A-genome specific (rapa only)	not scorable	not scorable	not scorable	not scorable	0.9669421	0.7934078
9920	Bn-A04-p6008778	A-genome specific (napus-rapa)	0.930622	0.8857938	not scorable	not scorable	0.9256198	0.8506994
9921	Bn-A04-p6017218	A-genome specific (napus-rapa)	0.937799	0.5871725	not scorable	not scorable	0.9834711	0.5966064
9922	Bn-A04-p6017599	A-genome specific (napus-rapa)	0.9617225	0.3882974	not scorable	not scorable	1	0.5586416
9923	Bn-A04-p6023914	A-genome specific (napus-rapa)	0.9976076	0.7645338	not scorable	not scorable	0.9917355	0.7552441
9924	Bn-A04-p6027878	A-genome specific (napus-rapa)	0.9641148	0.6985116	not scorable	not scorable	0.9752066	0.7447017
9925	Bn-A04-p6028097	A-genome specific (napus-rapa)	0.9904306	0.7908722	not scorable	not scorable	0.9752066	0.7105474
9926	Bn-A04-p6028334	A-genome specific (napus-rapa)	0.9928229	0.7789082	not scorable	not scorable	0.9752066	0.797245
9927	Bn-A04-p6029797	A-genome specific (napus-rapa)	0.9425837	0.7922821	not scorable	not scorable	0.9586777	0.6776279
9928	Bn-A04-p6030108	A-genome specific (rapa only)	not scorable	not scorable	not scorable	not scorable	0.5950413	0.9354743
9929	Bn-A04-p6042013	A-genome specific (rapa only)	not scorable	not scorable	not scorable	not scorable	0.9917355	0.7639738
9930	Bn-A04-p6047600	A-genome specific (napus-rapa)	0.9784689	0.7461309	not scorable	not scorable	0.9917355	0.7409972

9931	Bn-A04-p6050616	A-genome specific (napus-rapa)	0.9617225	0.4251138	not scorable	not scorable	0.9256198	0.7061945
9932	Bn-A04-p6052986	A-genome specific (rapa only)	not scorable	not scorable	not scorable	not scorable	0.9586777	0.6597604
9933	Bn-A04-p6053997	A-genome specific (napus-rapa)	0.9832536	0.7716251	not scorable	not scorable	0.9917355	0.783999
9934	Bn-A04-p6063409	A-genome specific (napus-rapa)	0.930622	0.279572	not scorable	not scorable	0.6198347	0.8845037
9935	Bn-A04-p6100884	A-genome specific (napus-rapa)	0.9665072	0.8093098	not scorable	not scorable	0.9917355	0.8799968
9936	Bn-A04-p6165743	A-genome specific (napus-rapa)	0.9928229	0.8573222	not scorable	not scorable	1	0.7556916
9937	Bn-A04-p619001	A-genome specific (napus-rapa)	1	0.8582115	not scorable	not scorable	0.9669421	0.6748194
9938	Bn-A04-p6228211	A-genome specific (napus-rapa)	0.9641148	0.3793482	not scorable	not scorable	1	0.7545423
9939	Bn-A04-p6240855	A-genome specific (napus-rapa)	0.9641148	0.2621892	not scorable	not scorable	0.9834711	0.7025011
9940	Bn-A04-p6255997	A-genome specific (napus-rapa)	0.9784689	0.8076194	not scorable	not scorable	1	0.7960291
9941	Bn-A04-p6263702	A-genome specific (napus-rapa)	0.5119617	0.908464	not scorable	not scorable	0.9917355	0.6863167
9942	Bn-A04-p6264043	A-genome specific (napus-rapa)	0.9856459	0.6856831	not scorable	not scorable	1	0.9001486
9943	Bn-A04-p6277634	A-genome specific (napus-rapa)	0.9760765	0.4505779	not scorable	not scorable	1	0.7981288
9944	Bn-A04-p6284377	A-genome specific (rapa only)	not scorable	not scorable	not scorable	not scorable	1	0.7773446
9945	Bn-A04-p6296526	A-genome specific (napus-rapa)	0.9712918	0.8283322	not scorable	not scorable	0.9917355	0.7898396
9946	Bn-A04-p6319430	A-genome specific (napus-rapa)	0.9976076	0.6159064	not scorable	not scorable	0.9917355	0.7505349
9947	Bn-A04-p6340854	A-genome specific (napus-rapa)	0.9736842	0.7706004	not scorable	not scorable	0.9834711	0.6992329
9948	Bn-A04-p6341516	A-genome specific (napus-rapa)	0.9617225	0.3808186	not scorable	not scorable	1	0.7891354
9949	Bn-A04-p6376472	A-genome specific (napus-rapa)	0.9545454	0.8802879	not scorable	not scorable	0.9752066	0.7973285
9950	Bn-A04-p637683	A-genome specific (napus-rapa)	0.9856459	0.8774217	not scorable	not scorable	0.9752066	0.7078871
9951	Bn-A04-p6389136	A-genome specific (napus-rapa)	0.9641148	0.5246497	not scorable	not scorable	0.9834711	0.7341309
9952	Bn-A04-p6395765	A-genome specific (napus-rapa)	0.5669857	0.8266092	not scorable	not scorable	0.9586777	0.7618849
9953	Bn-A04-p6402808	A-genome specific (napus-rapa)	0.5669857	0.7382709	not scorable	not scorable	0.9834711	0.7158242
9954	Bn-A04-p641998	A-genome specific (napus-rapa)	0.9976076	0.8628868	not scorable	not scorable	1	0.7774823
9955	Bn-A04-p6422450	A-genome specific (napus-rapa)	0.9784689	0.9128547	not scorable	not scorable	0.9917355	0.765166
9956	Bn-A04-p6428686	A-genome specific (napus-rapa)	0.9832536	0.4368686	not scorable	not scorable	0.9669421	0.7796715
9957	Bn-A04-p643876	A-genome specific (napus-rapa)	1	0.7253414	not scorable	not scorable	0.9834711	0.6911283
9958	Bn-A04-p6445083	A-genome specific (rapa only)	not scorable	not scorable	not scorable	not scorable	1	0.7547234
9959	Bn-A04-p6448172	A-genome specific (napus-rapa)	0.9736842	0.9136612	not scorable	not scorable	0.9669421	0.7958726
9960	Bn-A04-p6453979	A-genome specific (napus-rapa)	0.9832536	0.8232718	not scorable	not scorable	0.9752066	0.6871821
9961	Bn-A04-p6454976	A-genome specific (napus-rapa)	0.9449761	0.8999277	not scorable	not scorable	0.9752066	0.7872966
9962	Bn-A04-p6455109	A-genome specific (napus-rapa)	0.9784689	0.8771845	not scorable	not scorable	0.9917355	0.7903211
9963	Bn-A04-p6456456	A-genome specific (napus-rapa)	0.9784689	0.8515025	not scorable	not scorable	0.9917355	0.6986197

9964	Bn-A04-p645702	A-genome specific (napus-rapa)	1	0.8248156	not scorable	not scorable	0.9834711	0.7886414
9965	Bn-A04-p646064	A-genome specific (napus-rapa)	1	0.7401044	not scorable	not scorable	1	0.8004208
9966	Bn-A04-p646105	all not scorable	not scorable	not scorable	not scorable	not scorable	not scorable	not scorable
9967	Bn-A04-p6463878	A-genome specific (napus-rapa)	0.9952153	0.7676194	not scorable	not scorable	0.9917355	0.7106016
9968	Bn-A04-p6465442	A-genome specific (napus-rapa)	0.9832536	0.7657553	not scorable	not scorable	0.9834711	0.8112298
9969	Bn-A04-p648152	A-genome specific (napus-rapa)	1	0.7870384	not scorable	not scorable	1	0.7712533
9970	Bn-A04-p6486540	napus specific	0.5598086	0.9152238	not scorable	not scorable	not scorable	not scorable
9971	Bn-A04-p6487804	A-genome specific (napus-rapa)	0.7607656	0.7515029	not scorable	not scorable	0.9917355	0.7588108
9972	Bn-A04-p652225	A-genome specific (rapa only)	not scorable	not scorable	not scorable	not scorable	0.9752066	0.8545262
9973	Bn-A04-p6533593	A-genome specific (napus-rapa)	0.9928229	0.762158	not scorable	not scorable	1	0.7287537
9974	Bn-A04-p6550369	A-genome specific (napus-rapa)	0.9784689	0.7639387	not scorable	not scorable	1	0.7247278
9975	Bn-A04-p6550757	A-genome specific (napus-rapa)	0.861244	0.8748199	not scorable	not scorable	0.9917355	0.7452475
9976	Bn-A04-p6555913	A-genome specific (napus-rapa)	0.9832536	0.7496696	not scorable	not scorable	0.9834711	0.799786
9977	Bn-A04-p6556405	A-genome specific (napus-rapa)	0.9593301	0.5875356	not scorable	not scorable	0.9834711	0.8334903
9978	Bn-A04-p6563107	A-genome specific (rapa only)	not scorable	not scorable	not scorable	not scorable	1	0.7279266
9979	Bn-A04-p6563200	A-genome specific (napus-rapa)	0.9904306	0.3387341	not scorable	not scorable	0.9834711	0.829729
9980	Bn-A04-p6588935	A-genome specific (napus-rapa)	0.9904306	0.7315736	not scorable	not scorable	0.9834711	0.7602701
9981	Bn-A04-p6632126	A-genome specific (napus-rapa)	0.9856459	0.5697042	not scorable	not scorable	1	0.8135915
9982	Bn-A04-p6637458	A-genome specific (napus-rapa)	0.9760765	0.7329367	not scorable	not scorable	1	0.7349604
9983	Bn-A04-p6640990	A-genome specific (napus-rapa)	0.9688995	0.4465581	not scorable	not scorable	1	0.8411725
9984	Bn-A04-p6646210	all not scorable	not scorable	not scorable	not scorable	not scorable	not scorable	not scorable
9985	Bn-A04-p664994	A-genome specific (napus-rapa)	0.9760765	0.3959174	not scorable	not scorable	0.9586777	0.7460113
9986	Bn-A04-p6653508	A-genome specific (napus-rapa)	0.9712918	0.794568	not scorable	not scorable	0.9834711	0.7155414
9987	Bn-A04-p6657342	A-genome specific (napus-rapa)	0.9832536	0.3898592	not scorable	not scorable	0.9752066	0.7938295
9988	Bn-A04-p665754	genome unspecific (all in)	0.9880383	0.4300866	0.9922481	0.4884036	0.9834711	0.5358047
9989	Bn-A04-p665932	A-genome specific (napus-rapa)	0.9952153	0.3966294	not scorable	not scorable	0.9586777	0.467429
9990	Bn-A04-p665967	A-genome specific (rapa only)	not scorable	not scorable	not scorable	not scorable	0.9752066	0.4856958
9991	Bn-A04-p6673120	A-genome specific (napus-rapa)	0.6028708	0.5820294	not scorable	not scorable	0.9917355	0.7252836
9992	Bn-A04-p6678797	A-genome specific (napus-rapa)	0.8588517	0.395766	not scorable	not scorable	0.9917355	0.7362986
9993	Bn-A04-p6679263	A-genome specific (rapa only)	not scorable	not scorable	not scorable	not scorable	0.9504132	0.478887
9994	Bn-A04-p6679568	A-genome specific (napus-rapa)	0.9784689	0.290753	not scorable	not scorable	1	0.6113716
9995	Bn-A04-p6682658	A-genome specific (napus-rapa)	0.9425837	0.8142837	not scorable	not scorable	0.9752066	0.803691
9996	Bn-A04-p6682728	A-genome specific (napus-rapa)	0.9497608	0.8800325	not scorable	not scorable	1	0.8622939

9997	Bn-A04-p6683009	A-genome specific (napus-rapa)	0.9760765	0.9023381	not scorable	not scorable	0.9917355	0.8341916
9998	Bn-A04-p668378	A-genome specific (napus-rapa)	1	0.4709941	not scorable	not scorable	0.9834711	0.7793339
9999	Bn-A04-p6684054	A-genome specific (napus-rapa)	0.9521531	0.9124076	not scorable	not scorable	1	0.749783
####	Bn-A04-p6685027	A-genome specific (napus-rapa)	0.9665072	0.9114733	not scorable	not scorable	1	0.8524694
####	Bn-A04-p6685062	A-genome specific (napus-rapa)	0.9832536	0.8274801	not scorable	not scorable	1	0.8213645
####	Bn-A04-p6685294	A-genome specific (napus-rapa)	0.9784689	0.7606586	not scorable	not scorable	0.9917355	0.6078041
####	Bn-A04-p6685371	A-genome specific (napus-rapa)	0.9784689	0.8236449	not scorable	not scorable	1	0.753652
####	Bn-A04-p669182	A-genome specific (napus-rapa)	0.9880383	0.5731716	not scorable	not scorable	0.9834711	0.6663988
####	Bn-A04-p6697580	A-genome specific (napus-rapa)	0.9856459	0.8477979	not scorable	not scorable	1	0.765373
####	Bn-A04-p6718464	A-genome specific (napus-rapa)	0.7942584	0.8897412	not scorable	not scorable	0.9834711	0.8290797
####	Bn-A04-p6719949	A-genome specific (napus-rapa)	0.9976076	0.8471035	not scorable	not scorable	0.9669421	0.7914112
####	Bn-A04-p6747807	A-genome specific (napus-rapa)	0.9760765	0.8199897	not scorable	not scorable	0.9669421	0.9464173
####	Bn-A04-p6748822	A-genome specific (napus-rapa)	0.9760765	0.4440337	not scorable	not scorable	0.9752066	0.7378531
####	Bn-A04-p6749073	A-genome specific (napus-rapa)	0.9832536	0.8735242	not scorable	not scorable	0.9669421	0.8702711
####	Bn-A04-p6749114	A-genome specific (napus-rapa)	0.9856459	0.8695291	not scorable	not scorable	0.9669421	0.9212252
####	Bn-A04-p6749322	A-genome specific (napus-rapa)	0.9712918	0.9001594	not scorable	not scorable	0.9504132	0.7788189
####	Bn-A04-p6752299	A-genome specific (napus-rapa)	0.9808612	0.748781	not scorable	not scorable	0.9421487	0.7760171
####	Bn-A04-p6752979	A-genome specific (napus-rapa)	0.9641148	0.7401796	not scorable	not scorable	0.9669421	0.8045593
####	Bn-A04-p6760294	A-genome specific (napus-rapa)	0.9832536	0.8392224	not scorable	not scorable	0.9669421	0.868216
####	Bn-A04-p6764233	A-genome specific (napus-rapa)	0.9808612	0.7111743	not scorable	not scorable	0.9752066	0.8058044
####	Bn-A04-p6765045	genome unspecific (all in)	0.9784689	0.672936	0.9147287	0.5974009	0.9834711	0.6931816
####	Bn-A04-p6771264	napus specific	0.9736842	0.735633	not scorable	not scorable	not scorable	not scorable
####	Bn-A04-p6773984	A-genome specific (napus-rapa)	0.9856459	0.6987329	not scorable	not scorable	0.9669421	0.8133746
####	Bn-A04-p6775008	A-genome specific (napus-rapa)	0.9688995	0.6521707	not scorable	not scorable	0.9586777	0.7985248
####	Bn-A04-p6776597	A-genome specific (napus-rapa)	0.9784689	0.7792198	not scorable	not scorable	0.9586777	0.7552744
####	Bn-A04-p6776786	A-genome specific (napus-rapa)	0.9928229	0.7324047	not scorable	not scorable	0.9752066	0.8397954
####	Bn-A04-p6778593	A-genome specific (napus-rapa)	0.8947368	0.8122345	not scorable	not scorable	0.9669421	0.8290346
####	Bn-A04-p6780163	A-genome specific (napus-rapa)	0.9904306	0.4469636	not scorable	not scorable	0.9752066	0.9033059
####	Bn-A04-p6783717	A-genome specific (napus-rapa)	0.9880383	0.8150943	not scorable	not scorable	0.9834711	0.8595492
####	Bn-A04-p6790373	A-genome specific (napus-rapa)	0.9952153	0.5695329	not scorable	not scorable	0.9917355	0.6775374
####	Bn-A04-p6929056	A-genome specific (napus-rapa)	0.9976076	0.7278077	not scorable	not scorable	0.9752066	0.6922396
####	Bn-A04-p6953674	A-genome specific (napus-rapa)	1	0.7804081	not scorable	not scorable	1	0.7641612
####	Bn-A04-p6958157	A-genome specific (rapa only)	not scorable	not scorable	not scorable	not scorable	0.5950413	0.9175813

####	Bn-A04-p6966154	A-genome specific (napus-rapa)	0.9976076	0.8965958	not scorable	not scorable	0.9917355	0.7112095
####	Bn-A04-p6966782	A-genome specific (napus-rapa)	1	0.856232	not scorable	not scorable	1	0.9227971
####	Bn-A04-p6968899	A-genome specific (napus-rapa)	1	0.9118298	not scorable	not scorable	1	0.9175813
####	Bn-A04-p6973605	A-genome specific (napus-rapa)	0.9928229	0.7844111	not scorable	not scorable	1	0.9398479
####	Bn-A04-p6973712	A-genome specific (napus-rapa)	0.9976076	0.9009352	not scorable	not scorable	1	0.9468859
####	Bn-A04-p6974206	A-genome specific (napus-rapa)	1	0.8796032	not scorable	not scorable	1	0.9142227
####	Bn-A04-p6975646	A-genome specific (napus-rapa)	0.9952153	0.8429987	not scorable	not scorable	1	0.9175813
####	Bn-A04-p6978108	A-genome specific (napus-rapa)	0.9976076	0.7583425	not scorable	not scorable	1	0.9360299
####	Bn-A04-p6978163	A-genome specific (napus-rapa)	1	0.8218337	not scorable	not scorable	1	0.9178212
####	Bn-A04-p6979129	A-genome specific (napus-rapa)	1	0.90432	not scorable	not scorable	1	0.9419204
####	Bn-A04-p6980459	A-genome specific (napus-rapa)	0.9952153	0.8312419	not scorable	not scorable	1	0.9076689
####	Bn-A04-p7003715	A-genome specific (napus-rapa)	1	0.483662	not scorable	not scorable	0.9917355	0.6387082
####	Bn-A04-p7026999	A-genome specific (rapa only)	not scorable	not scorable	not scorable	not scorable	1	0.8815182
####	Bn-A04-p7027225	A-genome specific (rapa only)	not scorable	not scorable	not scorable	not scorable	0.6280992	0.7904024
####	Bn-A04-p7027307	all not scorable	not scorable	not scorable	not scorable	not scorable	not scorable	not scorable
####	Bn-A04-p7044446	A-genome specific (napus-rapa)	0.9808612	0.4780554	not scorable	not scorable	0.9834711	0.7182308
####	Bn-A04-p7066181	A-genome specific (napus-rapa)	0.9736842	0.6650302	not scorable	not scorable	0.9834711	0.7178236
####	Bn-A04-p7066595	A-genome specific (napus-rapa)	0.9736842	0.5823061	not scorable	not scorable	1	0.7966691
####	Bn-A04-p7068079	A-genome specific (napus-rapa)	0.9832536	0.5404111	not scorable	not scorable	0.9834711	0.6997826
####	Bn-A04-p7077586	A-genome specific (napus-rapa)	0.9976076	0.4038846	not scorable	not scorable	1	0.9125418
####	Bn-A04-p7088336	A-genome specific (napus-rapa)	0.9784689	0.4733645	not scorable	not scorable	0.9834711	0.8500586
####	Bn-A04-p7090997	A-genome specific (napus-rapa)	0.9760765	0.7503874	not scorable	not scorable	0.9917355	0.7580069
####	Bn-A04-p7093491	A-genome specific (napus-rapa)	0.9976076	0.3170142	not scorable	not scorable	0.9834711	0.5131946
####	Bn-A04-p7107858	A-genome specific (napus-rapa)	0.9832536	0.7716262	not scorable	not scorable	0.9834711	0.73132
####	Bn-A04-p7109346	A-genome specific (napus-rapa)	0.9904306	0.7769073	not scorable	not scorable	0.9834711	0.6910355
####	Bn-A04-p7109625	A-genome specific (napus-rapa)	0.9736842	0.7926259	not scorable	not scorable	0.9917355	0.7642915
####	Bn-A04-p7112803	A-genome specific (napus-rapa)	0.9952153	0.7362097	not scorable	not scorable	0.9669421	0.703155
####	Bn-A04-p7113045	A-genome specific (napus-rapa)	0.9712918	0.7333022	not scorable	not scorable	0.9504132	0.5492328
####	Bn-A04-p7113156	A-genome specific (napus-rapa)	0.9928229	0.7662893	not scorable	not scorable	1	0.6766212
####	Bn-A04-p7113281	genome unspecific (all in)	0.9952153	0.8078924	0	0.6140522	1	0.6776279
####	Bn-A04-p7113498	A-genome specific (napus-rapa)	0.9880383	0.7760915	not scorable	not scorable	0.9834711	0.8062617
####	Bn-A04-p7113584	genome unspecific (all in)	0.9928229	0.913235	0	0.7204491	0.9834711	0.6879769
####	Bn-A04-p7133952	A-genome specific (napus-rapa)	0.9784689	0.7311476	not scorable	not scorable	0.9834711	0.7128029

####	Bn-A04-p7134022	A-genome specific (napus-rapa)	0.9928229	0.7762072	not scorable	not scorable	0.9917355	0.6683936
####	Bn-A04-p7139140	A-genome specific (napus-rapa)	0.9904306	0.7271534	not scorable	not scorable	0.9834711	0.7426725
####	Bn-A04-p7141171	A-genome specific (napus-rapa)	0.9736842	0.6285266	not scorable	not scorable	0.9917355	0.7130669
####	Bn-A04-p7142597	A-genome specific (napus-rapa)	0.9928229	0.77657	not scorable	not scorable	0.9834711	0.7744859
####	Bn-A04-p7143891	A-genome specific (napus-rapa)	0.9928229	0.7668345	not scorable	not scorable	0.9834711	0.8073388
####	Bn-A04-p7149396	A-genome specific (napus-rapa)	0.9593301	0.5597114	not scorable	not scorable	0.9834711	0.7088029
####	Bn-A04-p7150064	A-genome specific (napus-rapa)	0.9808612	0.7186714	not scorable	not scorable	0.9917355	0.7096214
####	Bn-A04-p7150104	A-genome specific (napus-rapa)	0.9856459	0.7084139	not scorable	not scorable	0.9917355	0.697928
####	Bn-A04-p7150329	A-genome specific (napus-rapa)	0.9641148	0.6301583	not scorable	not scorable	0.9752066	0.6678376
####	Bn-A04-p7157859	A-genome specific (napus-rapa)	0.9856459	0.7396079	not scorable	not scorable	0.9834711	0.6882576
####	Bn-A04-p7161326	A-genome specific (napus-rapa)	1	0.7861754	not scorable	not scorable	1	0.8002234
####	Bn-A04-p7162690	A-genome specific (napus-rapa)	0.9880383	0.7415367	not scorable	not scorable	1	0.8204935
####	Bn-A04-p716889	A-genome specific (napus-rapa)	0.9976076	0.4554918	not scorable	not scorable	0.9834711	0.8365848
####	Bn-A04-p7212436	A-genome specific (napus-rapa)	0.9952153	0.3994882	not scorable	not scorable	0.6694215	0.6970574
####	Bn-A04-p7233978	A-genome specific (napus-rapa)	0.9952153	0.8007117	not scorable	not scorable	0.9917355	0.8081447
####	Bn-A04-p7251871	A-genome specific (napus-rapa)	0.9976076	0.8749103	not scorable	not scorable	0.9834711	0.7945439
####	Bn-A04-p726356	A-genome specific (napus-rapa)	0.9952153	0.3590641	not scorable	not scorable	1	0.9175813
####	Bn-A04-p7275318	genome unspecific (all in)	1	0.615882	0.9379845	0.2732761	0.9917355	0.6927312
####	Bn-A04-p7297317	A-genome specific (napus-rapa)	1	0.6054923	not scorable	not scorable	1	0.7485933
####	Bn-A04-p7322000	A-genome specific (napus-rapa)	0.9976076	0.3754301	not scorable	not scorable	0.9917355	0.6986197
####	Bn-A04-p7322497	A-genome specific (napus-rapa)	1	0.5437943	not scorable	not scorable	1	0.7594932
####	Bn-A04-p7323275	A-genome specific (napus-rapa)	0.9976076	0.4262182	not scorable	not scorable	1	0.8431817
####	Bn-A04-p7349115	A-genome specific (napus-rapa)	0.9019139	0.7640037	not scorable	not scorable	1	0.7499316
####	Bn-A04-p7349794	A-genome specific (napus-rapa)	0.9258373	0.7609718	not scorable	not scorable	1	0.8468006
####	Bn-A04-p7350870	A-genome specific (napus-rapa)	0.9282297	0.8474121	not scorable	not scorable	0.9917355	0.7377093
####	Bn-A04-p7373248	A-genome specific (napus-rapa)	0.9808612	0.5564902	not scorable	not scorable	1	0.6803967
####	Bn-A04-p7389149	A-genome specific (napus-rapa)	1	0.5958052	not scorable	not scorable	1	0.7106146
####	Bn-A04-p7407657	A-genome specific (napus-rapa)	0.9880383	0.6392852	not scorable	not scorable	1	0.8143092
####	Bn-A04-p7410699	A-genome specific (napus-rapa)	0.9976076	0.9321206	not scorable	not scorable	0.9834711	0.7415906
####	Bn-A04-p741528	A-genome specific (napus-rapa)	0.9832536	0.5304719	not scorable	not scorable	0.9917355	0.770471
####	Bn-A04-p7422354	A-genome specific (napus-rapa)	0.9832536	0.8444116	not scorable	not scorable	0.9834711	0.7772185
####	Bn-A04-p7425311	A-genome specific (rapa only)	not scorable	not scorable	not scorable	not scorable	0.9669421	0.8804176
####	Bn-A04-p7425359	A-genome specific (napus-rapa)	0.9832536	0.825327	not scorable	not scorable	0.9834711	0.7959595

####	Bn-A04-p7425697	A-genome specific (napus-rapa)	0.9760765	0.8060524	not scorable	not scorable	0.9917355	0.8162266
####	Bn-A04-p7426527	napus specific	0.9976076	0.8104141	not scorable	not scorable	not scorable	not scorable
####	Bn-A04-p7431245	A-genome specific (napus-rapa)	0.9952153	0.9080831	not scorable	not scorable	0.9834711	0.7900194
####	Bn-A04-p7431816	A-genome specific (napus-rapa)	0.9928229	0.8423564	not scorable	not scorable	0.9834711	0.7790921
####	Bn-A04-p7441018	A-genome specific (napus-rapa)	0.9952153	0.7113469	not scorable	not scorable	1	0.7389982
####	Bn-A04-p7441659	A-genome specific (napus-rapa)	0.9904306	0.7866982	not scorable	not scorable	0.9917355	0.5471747
####	Bn-A04-p7442353	A-genome specific (napus-rapa)	0.9952153	0.7438589	not scorable	not scorable	0.9917355	0.673363
####	Bn-A04-p7442886	A-genome specific (napus-rapa)	1	0.8512269	not scorable	not scorable	0.9917355	0.756297
####	Bn-A04-p7443395	A-genome specific (napus-rapa)	0.9928229	0.8648512	not scorable	not scorable	0.9669421	0.7866274
####	Bn-A04-p7446849	napus specific	1	0.4349402	not scorable	not scorable	not scorable	not scorable
####	Bn-A04-p745024	A-genome specific (napus-rapa)	0.9497608	0.2553417	not scorable	not scorable	0.9917355	0.6930565
####	Bn-A04-p7450678	genome unspecific (all in)	0.8923445	0.6293741	0.9534883	0.6267408	0.9834711	0.6918287
####	Bn-A04-p7471870	A-genome specific (napus-rapa)	0.9952153	0.8356263	not scorable	not scorable	1	0.7661656
####	Bn-A04-p7474589	A-genome specific (napus-rapa)	0.9760765	0.4599922	not scorable	not scorable	1	0.7508737
####	Bn-A04-p7482491	A-genome specific (napus-rapa)	0.9784689	0.7952471	not scorable	not scorable	0.6198347	0.8367791
####	Bn-A04-p7488675	A-genome specific (napus-rapa)	0.9952153	0.8127908	not scorable	not scorable	0.9917355	0.769349
####	Bn-A04-p7490390	A-genome specific (napus-rapa)	1	0.8391998	not scorable	not scorable	0.5123967	0.8836357
####	Bn-A04-p7493966	A-genome specific (napus-rapa)	1	0.8408002	not scorable	not scorable	1	0.7511113
####	Bn-A04-p7500685	napus specific	0.9019139	0.8028878	not scorable	not scorable	not scorable	not scorable
####	Bn-A04-p7503042	A-genome specific (napus-rapa)	1	0.8872287	not scorable	not scorable	0.9917355	0.7947276
####	Bn-A04-p7519541	A-genome specific (napus-rapa)	0.9617225	0.6296679	not scorable	not scorable	0.9338843	0.7685955
####	Bn-A04-p7520330	A-genome specific (napus-rapa)	1	0.7809185	not scorable	not scorable	0.9834711	0.6799062
####	Bn-A04-p7540834	napus specific	0.9688995	0.5406189	not scorable	not scorable	not scorable	not scorable
####	Bn-A04-p7541325	A-genome specific (napus-rapa)	0.9712918	0.772532	not scorable	not scorable	0.6198347	0.7827822
####	Bn-A04-p7541762	A-genome specific (napus-rapa)	0.9952153	0.8833086	not scorable	not scorable	0.6446281	0.7848005
####	Bn-A04-p7542594	A-genome specific (napus-rapa)	0.9928229	0.8664813	not scorable	not scorable	0.677686	0.7717489
####	Bn-A04-p7556327	all not scorable	not scorable	not scorable	not scorable	not scorable	not scorable	not scorable
####	Bn-A04-p757668	A-genome specific (napus-rapa)	0.9952153	0.8052458	not scorable	not scorable	0.9586777	0.7402171
####	Bn-A04-p7582986	A-genome specific (napus-rapa)	1	0.8571876	not scorable	not scorable	0.9586777	0.7517944
####	Bn-A04-p7584788	napus specific	0.2368421	0.8033082	not scorable	not scorable	not scorable	not scorable
####	Bn-A04-p7586476	A-genome specific (rapa only)	not scorable	not scorable	not scorable	not scorable	0.9834711	0.6431442
####	Bn-A04-p7588820	all not scorable	not scorable	not scorable	not scorable	not scorable	not scorable	not scorable
####	Bn-A04-p7596556	A-genome specific (napus-rapa)	0.9784689	0.8916296	not scorable	not scorable	1	0.8252487

####	Bn-A04-p7598934	A-genome specific (napus-rapa)	0.9904306	0.8089281	not scorable	not scorable	0.9917355	0.7541262
####	Bn-A04-p7601638	A-genome specific (napus-rapa)	0.9928229	0.7956967	not scorable	not scorable	0.9834711	0.7693331
####	Bn-A04-p7601731	A-genome specific (napus-rapa)	0.9928229	0.8182912	not scorable	not scorable	0.9917355	0.8088477
####	Bn-A04-p761383	A-genome specific (napus-rapa)	0.9928229	0.5236396	not scorable	not scorable	0.9586777	0.756209
####	Bn-A04-p7616574	A-genome specific (napus-rapa)	0.9808612	0.8833086	not scorable	not scorable	0.9834711	0.7585436
####	Bn-A04-p7617794	A-genome specific (napus-rapa)	0.9808612	0.8166683	not scorable	not scorable	1	0.6721445
####	Bn-A04-p7625503	A-genome specific (napus-rapa)	0.9952153	0.7114244	not scorable	not scorable	1	0.6936299
####	Bn-A04-p7629926	A-genome specific (rapa only)	not scorable	not scorable	not scorable	not scorable	0.7024794	0.9029506
####	Bn-A04-p7635112	genome unspecific (all in)	0.9617225	0.3983181	1	0.7484283	0.9669421	0.6149928
####	Bn-A04-p7643584	all not scorable	not scorable	not scorable	not scorable	not scorable	not scorable	not scorable
####	Bn-A04-p7656041	A-genome specific (napus-rapa)	1	0.7630587	not scorable	not scorable	1	0.6186112
####	Bn-A04-p7666812	A-genome specific (napus-rapa)	0.9904306	0.8209378	not scorable	not scorable	0.9917355	0.7360542
####	Bn-A04-p7666981	A-genome specific (napus-rapa)	0.9928229	0.8113848	not scorable	not scorable	0.9917355	0.7937239
####	Bn-A04-p7670170	A-genome specific (napus-rapa)	0.9736842	0.9388269	not scorable	not scorable	0.9834711	0.7650318
####	Bn-A04-p7670852	A-genome specific (napus-rapa)	1	0.8646756	not scorable	not scorable	0.9917355	0.6895545
####	Bn-A04-p7671731	A-genome specific (napus-rapa)	0.9856459	0.816581	not scorable	not scorable	0.9917355	0.7079362
####	Bn-A04-p7672240	A-genome specific (napus-rapa)	0.9952153	0.8096704	not scorable	not scorable	1	0.7383842
####	Bn-A04-p7680446	A-genome specific (napus-rapa)	0.9976076	0.798659	not scorable	not scorable	0.9752066	0.8343484
####	Bn-A04-p7681107	A-genome specific (napus-rapa)	0.9832536	0.8143232	not scorable	not scorable	0.9917355	0.7496772
####	Bn-A04-p7681563	A-genome specific (napus-rapa)	1	0.6009593	not scorable	not scorable	1	0.6637822
####	Bn-A04-p7681694	A-genome specific (napus-rapa)	0.9880383	0.8084921	not scorable	not scorable	1	0.8562489
####	Bn-A04-p7683677	A-genome specific (napus-rapa)	0.9880383	0.8064625	not scorable	not scorable	0.9917355	0.7640833
####	Bn-A04-p7684111	A-genome specific (napus-rapa)	1	0.7867724	not scorable	not scorable	1	0.8476073
####	Bn-A04-p7685115	A-genome specific (napus-rapa)	0.9952153	0.8163102	not scorable	not scorable	1	0.7567353
####	Bn-A04-p7694288	A-genome specific (napus-rapa)	0.9665072	0.3307825	not scorable	not scorable	1	0.7779458
####	Bn-A04-p7760488	A-genome specific (napus-rapa)	0.9832536	0.7927777	not scorable	not scorable	0.9917355	0.7451807
####	Bn-A04-p7760541	A-genome specific (napus-rapa)	0.9856459	0.7810854	not scorable	not scorable	0.9752066	0.7268405
####	Bn-A04-p7769990	A-genome specific (napus-rapa)	0.9904306	0.4741548	not scorable	not scorable	0.9834711	0.764693
####	Bn-A04-p7770904	A-genome specific (napus-rapa)	0.9952153	0.8132417	not scorable	not scorable	0.9834711	0.734351
####	Bn-A04-p7776319	A-genome specific (napus-rapa)	0.9856459	0.3826382	not scorable	not scorable	0.9752066	0.856419
####	Bn-A04-p7790919	A-genome specific (rapa only)	not scorable	not scorable	not scorable	not scorable	0.9586777	0.9175813
####	Bn-A04-p7791214	A-genome specific (napus-rapa)	0.9904306	0.4645264	not scorable	not scorable	0.9834711	0.7904528
####	Bn-A04-p7797300	A-genome specific (napus-rapa)	0.9832536	0.5335478	not scorable	not scorable	0.9669421	0.8519897

####	Bn-A04-p7808804	A-genome specific (napus-rapa)	0.9545454	0.8310137	not scorable	not scorable	0.9917355	0.690971
####	Bn-A04-p7813672	A-genome specific (napus-rapa)	0.9904306	0.827877	not scorable	not scorable	0.9917355	0.8060883
####	Bn-A04-p7818700	A-genome specific (rapa only)	not scorable	not scorable	not scorable	not scorable	0.9917355	0.8526634
####	Bn-A04-p7819391	A-genome specific (rapa only)	not scorable	not scorable	not scorable	not scorable	0.9669421	0.8297783
####	Bn-A04-p7819525	A-genome specific (napus-rapa)	0.9952153	0.8313876	not scorable	not scorable	0.9917355	0.7712851
####	Bn-A04-p7822519	A-genome specific (napus-rapa)	0.9976076	0.8454219	not scorable	not scorable	0.9917355	0.7753746
####	Bn-A04-p7822585	A-genome specific (napus-rapa)	0.9928229	0.8603637	not scorable	not scorable	1	0.8282386
####	Bn-A04-p783339	A-genome specific (napus-rapa)	1	0.5803968	not scorable	not scorable	0.9752066	0.6993493
####	Bn-A04-p784708	A-genome specific (napus-rapa)	1	0.8884304	not scorable	not scorable	0.9834711	0.8181403
####	Bn-A04-p784894	all not scorable	not scorable	not scorable	not scorable	not scorable	not scorable	not scorable
####	Bn-A04-p785628	A-genome specific (napus-rapa)	0.9976076	0.6728179	not scorable	not scorable	0.9834711	0.7597919
####	Bn-A04-p7857679	A-genome specific (napus-rapa)	0.9760765	0.4480949	not scorable	not scorable	0.9834711	0.7050518
####	Bn-A04-p7857722	genome unspecific (all in)	0.9880383	0.5405815	0.9767442	0.69373	0.6363636	0.8505408
####	Bn-A04-p7858224	A-genome specific (napus-rapa)	1	0.5880607	not scorable	not scorable	0.5950413	0.7930255
####	Bn-A04-p786588	A-genome specific (napus-rapa)	0.9114832	0.7920969	not scorable	not scorable	1	0.5647836
####	Bn-A04-p7870001	A-genome specific (napus-rapa)	0.9856459	0.4580181	not scorable	not scorable	0.9917355	0.7963138
####	Bn-A04-p7883120	A-genome specific (napus-rapa)	0.9760765	0.3672342	not scorable	not scorable	0.9917355	0.7438364
####	Bn-A04-p7886817	A-genome specific (napus-rapa)	0.9856459	0.7639872	not scorable	not scorable	1	0.8101128
####	Bn-A04-p7887561	A-genome specific (napus-rapa)	0.9856459	0.8009068	not scorable	not scorable	1	0.7210601
####	Bn-A04-p7888986	A-genome specific (napus-rapa)	0.9904306	0.7392183	not scorable	not scorable	1	0.767114
####	Bn-A04-p7889763	A-genome specific (napus-rapa)	0.9784689	0.4434755	not scorable	not scorable	1	0.8539606
####	Bn-A04-p7897465	A-genome specific (napus-rapa)	0.9808612	0.8277351	not scorable	not scorable	1	0.7973625
####	Bn-A04-p7914869	A-genome specific (napus-rapa)	0.9976076	0.5364723	not scorable	not scorable	1	0.7501513
####	Bn-A04-p792399	A-genome specific (napus-rapa)	0.9976076	0.8968745	not scorable	not scorable	1	0.8145378
####	Bn-A04-p7930087	A-genome specific (napus-rapa)	0.9928229	0.7955263	not scorable	not scorable	0.9917355	0.7151299
####	Bn-A04-p7931854	A-genome specific (napus-rapa)	0.9904306	0.8425085	not scorable	not scorable	0.9752066	0.7417024
####	Bn-A04-p7933628	A-genome specific (napus-rapa)	0.9976076	0.4957593	not scorable	not scorable	0.9917355	0.7868304
####	Bn-A04-p7955059	A-genome specific (napus-rapa)	0.9593301	0.7531537	not scorable	not scorable	0.5785124	0.789211
####	Bn-A04-p7965197	A-genome specific (napus-rapa)	0.9928229	0.6470641	not scorable	not scorable	0.9917355	0.8068677
####	Bn-A04-p7984004	A-genome specific (napus-rapa)	0.9856459	0.5271731	not scorable	not scorable	0.9917355	0.7830393
####	Bn-A04-p7990037	A-genome specific (rapa only)	not scorable	not scorable	not scorable	not scorable	0.9338843	0.929083
####	Bn-A04-p7990273	A-genome specific (napus-rapa)	0.9976076	0.8231098	not scorable	not scorable	0.9834711	0.6911854
####	Bn-A04-p8004085	A-genome specific (napus-rapa)	0.9976076	0.5162324	not scorable	not scorable	1	0.7170125

####	Bn-A04-p8009543	A-genome specific (napus-rapa)	0.9976076	0.8133294	not scorable	not scorable	0.9834711	0.81576
####	Bn-A04-p8017096	A-genome specific (napus-rapa)	0.9712918	0.9213765	not scorable	not scorable	0.6363636	0.8572638
####	Bn-A04-p803315	A-genome specific (napus-rapa)	1	0.7587425	not scorable	not scorable	0.9752066	0.6693327
####	Bn-A04-p808824	A-genome specific (napus-rapa)	0.9736842	0.7504764	not scorable	not scorable	0.9752066	0.519604
####	Bn-A04-p808834	A-genome specific (napus-rapa)	0.9832536	0.8006344	not scorable	not scorable	0.9917355	0.7503625
####	Bn-A04-p809984	A-genome specific (napus-rapa)	0.9880383	0.8694727	not scorable	not scorable	0.9834711	0.7809902
####	Bn-A04-p810039	A-genome specific (napus-rapa)	0.9354067	0.8546536	not scorable	not scorable	0.9917355	0.675675
####	Bn-A04-p810166	A-genome specific (napus-rapa)	0.9832536	0.8693039	not scorable	not scorable	0.9917355	0.8493357
####	Bn-A04-p810935	A-genome specific (napus-rapa)	0.9976076	0.8362382	not scorable	not scorable	1	0.8187394
####	Bn-A04-p8114074	A-genome specific (napus-rapa)	0.9808612	0.2343308	not scorable	not scorable	0.6528926	0.7713266
####	Bn-A04-p8114079	A-genome specific (napus-rapa)	0.9401914	0.4925568	not scorable	not scorable	0.6033058	0.8028675
####	Bn-A04-p8116942	A-genome specific (napus-rapa)	0.9904306	0.6797017	not scorable	not scorable	0.9917355	0.7038804
####	Bn-A04-p8117244	A-genome specific (napus-rapa)	0.9617225	0.7128887	not scorable	not scorable	0.9834711	0.8165557
####	Bn-A04-p813173	A-genome specific (napus-rapa)	0.9976076	0.7707787	not scorable	not scorable	1	0.6409599
####	Bn-A04-p814033	A-genome specific (napus-rapa)	1	0.8171469	not scorable	not scorable	0.6446281	0.7564514
####	Bn-A04-p8178053	genome unspecific (all in)	0.9976076	0.574803	1	0.8519285	1	0.8996406
####	Bn-A04-p8178997	A-genome specific (napus-rapa)	1	0.5061432	not scorable	not scorable	1	0.8107701
####	Bn-A04-p8186888	A-genome specific (napus-rapa)	0.9784689	0.7789908	not scorable	not scorable	0.9752066	0.7129104
####	Bn-A04-p8208799	A-genome specific (napus-rapa)	0.9952153	0.8867826	not scorable	not scorable	1	0.8063325
####	Bn-A04-p8285381	A-genome specific (rapa only)	not scorable	not scorable	not scorable	not scorable	0.9256198	0.8678751
####	Bn-A04-p8287776	A-genome specific (napus-rapa)	0.9952153	0.8853577	not scorable	not scorable	0.9917355	0.8115923
####	Bn-A04-p8287906	A-genome specific (napus-rapa)	0.9928229	0.8595376	not scorable	not scorable	1	0.6956896
####	Bn-A04-p8292499	A-genome specific (napus-rapa)	0.9688995	0.7806461	not scorable	not scorable	0.9669421	0.7773674
####	Bn-A04-p8299391	A-genome specific (napus-rapa)	0.9880383	0.3366669	not scorable	not scorable	0.9917355	0.7313297
####	Bn-A04-p8310242	A-genome specific (napus-rapa)	0.9904306	0.4868661	not scorable	not scorable	1	0.7559428
####	Bn-A04-p8310328	A-genome specific (napus-rapa)	0.9976076	0.7898337	not scorable	not scorable	0.9669421	0.6809998
####	Bn-A04-p831099	napus specific	1	0.4989774	not scorable	not scorable	not scorable	not scorable
####	Bn-A04-p8312347	A-genome specific (napus-rapa)	0.9832536	0.5547321	not scorable	not scorable	0.9917355	0.7683265
####	Bn-A04-p8328216	A-genome specific (napus-rapa)	0.9928229	0.5286749	not scorable	not scorable	0.9834711	0.7913663
####	Bn-A04-p8329549	A-genome specific (napus-rapa)	0.9856459	0.5460216	not scorable	not scorable	0.9669421	0.69318
####	Bn-A04-p8344866	A-genome specific (napus-rapa)	0.9904306	0.8069627	not scorable	not scorable	0.9752066	0.680862
####	Bn-A04-p8345666	A-genome specific (napus-rapa)	0.9976076	0.905706	not scorable	not scorable	0.9917355	0.7739204
####	Bn-A04-p8348145	A-genome specific (napus-rapa)	1	0.8899168	not scorable	not scorable	0.9834711	0.7438365

####	Bn-A04-p8348750	A-genome specific (napus-rapa)	0.9832536	0.2045122	not scorable	not scorable	1	0.7878866
####	Bn-A04-p8348806	A-genome specific (napus-rapa)	1	0.536495	not scorable	not scorable	0.9834711	0.6691662
####	Bn-A04-p8403728	napus specific	0.9808612	0.5115303	not scorable	not scorable	not scorable	not scorable
####	Bn-A04-p8407945	A-genome specific (napus-rapa)	0.9760765	0.3598927	not scorable	not scorable	0.9834711	0.7133861
####	Bn-A04-p8441932	A-genome specific (napus-rapa)	0.9760765	0.6885573	not scorable	not scorable	1	0.7590942
####	Bn-A04-p8473768	A-genome specific (napus-rapa)	0.9808612	0.4162657	not scorable	not scorable	1	0.7688319
####	Bn-A04-p8481222	A-genome specific (napus-rapa)	0.9784689	0.8817683	not scorable	not scorable	0.9834711	0.7993042
####	Bn-A04-p8555881	all not scorable	not scorable	not scorable	not scorable	not scorable	not scorable	not scorable
####	Bn-A04-p8559580	A-genome specific (napus-rapa)	0.9928229	0.3533401	not scorable	not scorable	0.9586777	0.6995912
####	Bn-A04-p8564072	A-genome specific (napus-rapa)	0.9976076	0.5773626	not scorable	not scorable	0.9917355	0.7942156
####	Bn-A04-p8575908	A-genome specific (napus-rapa)	1	0.7663167	not scorable	not scorable	0.9752066	0.696036
####	Bn-A04-p8575962	A-genome specific (napus-rapa)	1	0.7807058	not scorable	not scorable	0.9834711	0.6868559
####	Bn-A04-p8587019	A-genome specific (napus-rapa)	0.9641148	0.831047	not scorable	not scorable	0.9917355	0.7955283
####	Bn-A04-p8595035	A-genome specific (napus-rapa)	1	0.2690754	not scorable	not scorable	0.9917355	0.6896948
####	Bn-A04-p860212	A-genome specific (napus-rapa)	0.9976076	0.555096	not scorable	not scorable	0.9917355	0.6489385
####	Bn-A04-p8609799	A-genome specific (napus-rapa)	0.9784689	0.5611215	not scorable	not scorable	1	0.7035398
####	Bn-A04-p8612311	A-genome specific (napus-rapa)	0.2559808	0.9407187	not scorable	not scorable	0.9586777	0.7994237
####	Bn-A04-p8615093	A-genome specific (rapa only)	not scorable	not scorable	not scorable	not scorable	0.892562	0.9095823
####	Bn-A04-p865058	A-genome specific (napus-rapa)	1	0.8923261	not scorable	not scorable	0.9669421	0.7993027
####	Bn-A04-p8686116	A-genome specific (napus-rapa)	1	0.9146931	not scorable	not scorable	0.9669421	0.7992495
####	Bn-A04-p8686934	genome unspecific (all in)	0.9904306	0.395905	0.9922481	0.8177934	0.9834711	0.8256018
####	Bn-A04-p869491	A-genome specific (napus-rapa)	1	0.7871816	not scorable	not scorable	0.9834711	0.7781474
####	Bn-A04-p8700128	A-genome specific (napus-rapa)	0.9952153	0.7936482	not scorable	not scorable	0.9504132	0.8224537
####	Bn-A04-p870365	A-genome specific (napus-rapa)	0.9976076	0.7287188	not scorable	not scorable	0.9917355	0.6644652
####	Bn-A04-p8705840	A-genome specific (napus-rapa)	0.9976076	0.7673804	not scorable	not scorable	0.9917355	0.7613204
####	Bn-A04-p8706392	A-genome specific (napus-rapa)	0.9880383	0.7701946	not scorable	not scorable	0.9917355	0.663013
####	Bn-A04-p8709391	A-genome specific (napus-rapa)	0.9880383	0.7593687	not scorable	not scorable	1	0.7322744
####	Bn-A04-p8713733	A-genome specific (napus-rapa)	0.9880383	0.7646733	not scorable	not scorable	1	0.6876985
####	Bn-A04-p8718115	A-genome specific (napus-rapa)	0.9760765	0.3915617	not scorable	not scorable	1	0.6986263
####	Bn-A04-p8729788	A-genome specific (napus-rapa)	0.9641148	0.5679501	not scorable	not scorable	0.9834711	0.7908595
####	Bn-A04-p874008	A-genome specific (napus-rapa)	0.9952153	0.6224654	not scorable	not scorable	0.9834711	0.6537809
####	Bn-A04-p8761898	A-genome specific (napus-rapa)	0.9473684	0.3352092	not scorable	not scorable	0.9917355	0.7340437
####	Bn-A04-p8773241	A-genome specific (napus-rapa)	0.9928229	0.8324577	not scorable	not scorable	0.9917355	0.74115

####	Bn-A04-p8773286	A-genome specific (napus-rapa)	0.9904306	0.7273647	not scorable	not scorable	0.9834711	0.7478666
####	Bn-A04-p8782104	A-genome specific (napus-rapa)	0.9712918	0.4582036	not scorable	not scorable	0.9917355	0.8176341
####	Bn-A04-p8786765	A-genome specific (napus-rapa)	1	0.8400073	not scorable	not scorable	0.9834711	0.9195408
####	Bn-A04-p8788445	A-genome specific (napus-rapa)	0.9760765	0.3741866	not scorable	not scorable	0.9834711	0.8543459
####	Bn-A04-p8790105	A-genome specific (napus-rapa)	0.9952153	0.7656885	not scorable	not scorable	1	0.6902189
####	Bn-A04-p8790433	A-genome specific (napus-rapa)	0.9808612	0.5290859	not scorable	not scorable	1	0.8198267
####	Bn-A04-p8796688	A-genome specific (napus-rapa)	0.9736842	0.720816	not scorable	not scorable	1	0.6164368
####	Bn-A04-p8796706	A-genome specific (napus-rapa)	0.9904306	0.7732147	not scorable	not scorable	1	0.7306563
####	Bn-A04-p8803630	A-genome specific (napus-rapa)	0.9976076	0.778626	not scorable	not scorable	0.9917355	0.7335028
####	Bn-A04-p8807708	A-genome specific (napus-rapa)	0.9784689	0.7814562	not scorable	not scorable	1	0.7346032
####	Bn-A04-p8811740	A-genome specific (napus-rapa)	0.9712918	0.3259647	not scorable	not scorable	1	0.8570947
####	Bn-A04-p8813038	A-genome specific (napus-rapa)	0.9928229	0.7203408	not scorable	not scorable	0.9752066	0.6865944
####	Bn-A04-p8817223	A-genome specific (rapa only)	not scorable	not scorable	not scorable	not scorable	0.9834711	0.9117482
####	Bn-A04-p8817780	A-genome specific (napus-rapa)	0.9880383	0.4556274	not scorable	not scorable	1	0.83672
####	Bn-A04-p8824695	A-genome specific (napus-rapa)	0.9952153	0.8014634	not scorable	not scorable	0.9917355	0.7514749
####	Bn-A04-p8828395	A-genome specific (napus-rapa)	0.9832536	0.2826765	not scorable	not scorable	1	0.7246916
####	Bn-A04-p8828462	A-genome specific (napus-rapa)	0.9688995	0.6805991	not scorable	not scorable	1	0.7441957
####	Bn-A04-p8830454	A-genome specific (napus-rapa)	1	0.8825166	not scorable	not scorable	0.9917355	0.7235581
####	Bn-A04-p8834077	A-genome specific (napus-rapa)	1	0.8000051	not scorable	not scorable	0.9917355	0.793542
####	Bn-A04-p8834259	A-genome specific (napus-rapa)	0.9784689	0.7352648	not scorable	not scorable	0.9917355	0.758696
####	Bn-A04-p8840146	A-genome specific (napus-rapa)	0.9688995	0.5638545	not scorable	not scorable	1	0.7625474
####	Bn-A04-p8847485	A-genome specific (napus-rapa)	0.9665072	0.7959204	not scorable	not scorable	1	0.8684258
####	Bn-A04-p8850036	A-genome specific (napus-rapa)	0.9784689	0.4603537	not scorable	not scorable	0.9917355	0.699442
####	Bn-A04-p8865894	A-genome specific (napus-rapa)	1	0.8604447	not scorable	not scorable	0.9917355	0.7062998
####	Bn-A04-p887081	A-genome specific (napus-rapa)	0.9880383	0.5241237	not scorable	not scorable	0.9834711	0.768783
####	Bn-A04-p8888969	A-genome specific (rapa only)	not scorable	not scorable	not scorable	not scorable	0.9752066	0.723342
####	Bn-A04-p8889253	A-genome specific (napus-rapa)	1	0.6881747	not scorable	not scorable	0.9917355	0.6993302
####	Bn-A04-p8892416	A-genome specific (napus-rapa)	0.9952153	0.4842038	not scorable	not scorable	0.9834711	0.665067
####	Bn-A04-p8906594	A-genome specific (napus-rapa)	1	0.8323046	not scorable	not scorable	1	0.7239684
####	Bn-A04-p8974361	A-genome specific (rapa only)	not scorable	not scorable	not scorable	not scorable	1	0.917867
####	Bn-A04-p9024009	A-genome specific (napus-rapa)	1	0.7517868	not scorable	not scorable	0.9917355	0.7625978
####	Bn-A04-p9086923	A-genome specific (napus-rapa)	1	0.6299996	not scorable	not scorable	1	0.7966477
####	Bn-A04-p9088043	A-genome specific (napus-rapa)	0.9976076	0.887599	not scorable	not scorable	1	0.7919511

####	Bn-A04-p9088760	A-genome specific (napus-rapa)	0.9976076	0.785426	not scorable	not scorable	1	0.7002039
####	Bn-A04-p9097344	A-genome specific (napus-rapa)	1	0.3353213	not scorable	not scorable	0.9917355	0.6841851
####	Bn-A04-p9098709	A-genome specific (napus-rapa)	1	0.7168217	not scorable	not scorable	1	0.7854597
####	Bn-A04-p9099209	A-genome specific (napus-rapa)	1	0.6073417	not scorable	not scorable	1	0.6622245
####	Bn-A04-p9107147	A-genome specific (napus-rapa)	0.9521531	0.3930911	not scorable	not scorable	0.9752066	0.8016405
####	Bn-A04-p9114826	A-genome specific (napus-rapa)	0.9617225	0.2688745	not scorable	not scorable	0.9917355	0.771207
####	Bn-A04-p9119079	A-genome specific (napus-rapa)	0.9521531	0.9521978	not scorable	not scorable	0.9917355	0.7039872
####	Bn-A04-p9119142	A-genome specific (napus-rapa)	0.9856459	0.6387358	not scorable	not scorable	0.9834711	0.628647
####	Bn-A04-p9119675	A-genome specific (napus-rapa)	1	0.8142598	not scorable	not scorable	1	0.7210986
####	Bn-A04-p9119867	A-genome specific (napus-rapa)	0.9856459	0.7847597	not scorable	not scorable	0.9917355	0.8202969
####	Bn-A04-p9174273	A-genome specific (napus-rapa)	0.9880383	0.6059897	not scorable	not scorable	0.9917355	0.653261
####	Bn-A04-p922828	A-genome specific (napus-rapa)	1	0.7974275	not scorable	not scorable	0.9917355	0.7160897
####	Bn-A04-p9231842	A-genome specific (rapa only)	not scorable	not scorable	not scorable	not scorable	0.9917355	0.6512775
####	Bn-A04-p9232024	A-genome specific (napus-rapa)	0.9832536	0.3077331	not scorable	not scorable	0.9917355	0.7868775
####	Bn-A04-p9232365	A-genome specific (rapa only)	not scorable	not scorable	not scorable	not scorable	1	0.4968174
####	Bn-A04-p9232828	A-genome specific (napus-rapa)	1	0.6882874	not scorable	not scorable	1	0.8207097
####	Bn-A04-p9245980	A-genome specific (napus-rapa)	1	0.5795714	not scorable	not scorable	1	0.5987751
####	Bn-A04-p926916	A-genome specific (napus-rapa)	0.9904306	0.3209788	not scorable	not scorable	1	0.8268133
####	Bn-A04-p928350	A-genome specific (napus-rapa)	0.9904306	0.7980775	not scorable	not scorable	0.9834711	0.7754667
####	Bn-A04-p9296314	A-genome specific (napus-rapa)	0.9904306	0.8298323	not scorable	not scorable	0.9917355	0.8050555
####	Bn-A04-p9296465	A-genome specific (napus-rapa)	0.9736842	0.2799123	not scorable	not scorable	0.9834711	0.793625
####	Bn-A04-p9309213	A-genome specific (napus-rapa)	0.9521531	0.905031	not scorable	not scorable	1	0.9183635
####	Bn-A04-p9317798	A-genome specific (napus-rapa)	0.9952153	0.694189	not scorable	not scorable	0.9917355	0.7417953
####	Bn-A04-p9322160	A-genome specific (napus-rapa)	0.9856459	0.7892161	not scorable	not scorable	0.9752066	0.8019537
####	Bn-A04-p932865	A-genome specific (napus-rapa)	0.9952153	0.5507236	not scorable	not scorable	1	0.7952886
####	Bn-A04-p9331682	A-genome specific (napus-rapa)	0.9138756	0.7715955	not scorable	not scorable	0.8512397	0.8207097
####	Bn-A04-p9337637	A-genome specific (rapa only)	not scorable	not scorable	not scorable	not scorable	0.9421487	0.6834289
####	Bn-A04-p9339599	A-genome specific (napus-rapa)	0.8636364	0.8010102	not scorable	not scorable	0.8677686	0.7371542
####	Bn-A04-p9340809	all not scorable	not scorable	not scorable	not scorable	not scorable	not scorable	not scorable
####	Bn-A04-p934150	A-genome specific (napus-rapa)	0.9952153	0.5777289	not scorable	not scorable	0.9917355	0.6809824
####	Bn-A04-p9345530	A-genome specific (napus-rapa)	0.9952153	0.8150251	not scorable	not scorable	0.9917355	0.6937414
####	Bn-A04-p9355248	A-genome specific (napus-rapa)	1	0.841675	not scorable	not scorable	0.9917355	0.8758779
####	Bn-A04-p9363550	A-genome specific (napus-rapa)	0.9904306	0.3691185	not scorable	not scorable	0.9917355	0.7145512

####	Bn-A04-p9379937	A-genome specific (napus-rapa)	0.9138756	0.3482456	not scorable	not scorable	1	0.7705146
####	Bn-A04-p9392577	A-genome specific (napus-rapa)	0.9641148	0.53807	not scorable	not scorable	0.9917355	0.7897233
####	Bn-A04-p9400393	A-genome specific (napus-rapa)	0.9665072	0.5128107	not scorable	not scorable	0.9917355	0.7880455
####	Bn-A04-p9422027	A-genome specific (napus-rapa)	0.9808612	0.7664756	not scorable	not scorable	0.9834711	0.7977133
####	Bn-A04-p9426523	napus specific	0.9808612	0.7615884	not scorable	not scorable	not scorable	not scorable
####	Bn-A04-p9450472	all not scorable	not scorable	not scorable	not scorable	not scorable	not scorable	not scorable
####	Bn-A04-p9484747	A-genome specific (napus-rapa)	0.9904306	0.5516437	not scorable	not scorable	0.9752066	0.6492524
####	Bn-A04-p9505012	A-genome specific (rapa only)	not scorable	not scorable	not scorable	not scorable	0.9752066	0.6284665
####	Bn-A04-p9508676	A-genome specific (napus-rapa)	0.9976076	0.6537102	not scorable	not scorable	0.9917355	0.5897086
####	Bn-A04-p9509486	A-genome specific (napus-rapa)	0.9880383	0.7921632	not scorable	not scorable	1	0.7992977
####	Bn-A04-p9510223	A-genome specific (napus-rapa)	0.9832536	0.7733799	not scorable	not scorable	0.9834711	0.6951471
####	Bn-A04-p9510750	A-genome specific (napus-rapa)	0.9904306	0.7236553	not scorable	not scorable	0.9669421	0.7728162
####	Bn-A04-p9510764	A-genome specific (napus-rapa)	0.9928229	0.692579	not scorable	not scorable	0.9752066	0.6869572
####	Bn-A04-p9511386	A-genome specific (napus-rapa)	0.9856459	0.7634513	not scorable	not scorable	0.9834711	0.7638118
####	Bn-A04-p9522781	A-genome specific (rapa only)	not scorable	not scorable	not scorable	not scorable	0.9586777	0.6194744
####	Bn-A04-p9524800	A-genome specific (napus-rapa)	0.6602871	0.8614707	not scorable	not scorable	0.9917355	0.8302645
####	Bn-A04-p9532027	A-genome specific (napus-rapa)	0.9976076	0.4450374	not scorable	not scorable	0.9834711	0.8185435
####	Bn-A04-p9532255	A-genome specific (napus-rapa)	0.9976076	0.8423527	not scorable	not scorable	1	0.7097299
####	Bn-A04-p9543062	A-genome specific (napus-rapa)	0.9952153	0.7187647	not scorable	not scorable	1	0.7462972
####	Bn-A04-p9543969	A-genome specific (napus-rapa)	1	0.6057992	not scorable	not scorable	1	0.8353348
####	Bn-A04-p9566708	A-genome specific (napus-rapa)	0.9952153	0.6662956	not scorable	not scorable	1	0.8047553
####	Bn-A04-p9582109	A-genome specific (napus-rapa)	1	0.8624684	not scorable	not scorable	1	0.8448273
####	Bn-A04-p9606190	A-genome specific (napus-rapa)	0.9976076	0.6862993	not scorable	not scorable	1	0.6469069
####	Bn-A04-p9623505	A-genome specific (napus-rapa)	0.9928229	0.7551577	not scorable	not scorable	0.9834711	0.6819919
####	Bn-A04-p9623913	A-genome specific (napus-rapa)	0.9760765	0.6052546	not scorable	not scorable	1	0.63592
####	Bn-A04-p9632786	A-genome specific (napus-rapa)	0.9497608	0.7873529	not scorable	not scorable	0.6115702	0.8656352
####	Bn-A04-p9639648	A-genome specific (napus-rapa)	1	0.61053	not scorable	not scorable	1	0.7871554
####	Bn-A04-p9658559	A-genome specific (napus-rapa)	0.9784689	0.658468	not scorable	not scorable	0.9917355	0.6753122
####	Bn-A04-p9670525	A-genome specific (napus-rapa)	0.9760765	0.7996546	not scorable	not scorable	0.9834711	0.8391967
####	Bn-A04-p9673337	A-genome specific (napus-rapa)	0.6626794	0.8388265	not scorable	not scorable	0.9338843	0.811949
####	Bn-A04-p9675790	A-genome specific (napus-rapa)	0.9593301	0.4168999	not scorable	not scorable	0.9834711	0.7084561
####	Bn-A04-p9716870	A-genome specific (napus-rapa)	1	0.5442087	not scorable	not scorable	0.9917355	0.7000874
####	Bn-A04-p9746407	A-genome specific (napus-rapa)	1	0.5834023	not scorable	not scorable	0.9834711	0.6432823

####	Bn-A04-p9802131	A-genome specific (napus-rapa)	0.9952153	0.537937	not scorable	not scorable	1	0.7337655
####	Bn-A04-p9808875	A-genome specific (napus-rapa)	0.9545454	0.4453346	not scorable	not scorable	0.9917355	0.721589
####	Bn-A04-p980952	A-genome specific (napus-rapa)	0.9928229	0.8485228	not scorable	not scorable	0.9917355	0.7222196
####	Bn-A04-p9811506	A-genome specific (napus-rapa)	0.930622	0.3106559	not scorable	not scorable	0.9834711	0.8163984
####	Bn-A04-p9814236	A-genome specific (napus-rapa)	0.9593301	0.3300521	not scorable	not scorable	0.9917355	0.7012927
####	Bn-A04-p9814284	A-genome specific (napus-rapa)	0.9760765	0.5995137	not scorable	not scorable	1	0.6529687
####	Bn-A04-p9815052	A-genome specific (napus-rapa)	0.9665072	0.3830069	not scorable	not scorable	0.9917355	0.6686319
####	Bn-A04-p9819724	A-genome specific (napus-rapa)	1	0.3170858	not scorable	not scorable	0.9834711	0.7652212
####	Bn-A04-p9819885	A-genome specific (napus-rapa)	0.9856459	0.7640828	not scorable	not scorable	1	0.8040874
####	Bn-A04-p982068	A-genome specific (napus-rapa)	0.9952153	0.8071095	not scorable	not scorable	0.9669421	0.726182
####	Bn-A04-p9830766	A-genome specific (rapa only)	not scorable	not scorable	not scorable	not scorable	0.9917355	0.7269744
####	Bn-A04-p9841094	A-genome specific (napus-rapa)	0.9736842	0.6381893	not scorable	not scorable	1	0.6701869
####	Bn-A04-p9844020	A-genome specific (napus-rapa)	0.9952153	0.4953309	not scorable	not scorable	0.9586777	0.7701138
####	Bn-A04-p9847875	A-genome specific (napus-rapa)	0.9904306	0.5638334	not scorable	not scorable	0.9669421	0.5768443
####	Bn-A04-p9848849	A-genome specific (napus-rapa)	1	0.7803863	not scorable	not scorable	0.9834711	0.7040766
####	Bn-A04-p9861037	A-genome specific (napus-rapa)	0.9928229	0.7915911	not scorable	not scorable	1	0.6864107
####	Bn-A04-p9863329	A-genome specific (napus-rapa)	0.9904306	0.8071716	not scorable	not scorable	0.9917355	0.663902
####	Bn-A04-p9864811	A-genome specific (napus-rapa)	0.9952153	0.8489355	not scorable	not scorable	0.9917355	0.6606812
####	Bn-A04-p9864983	A-genome specific (napus-rapa)	0.9904306	0.8392501	not scorable	not scorable	0.9834711	0.7136915
####	Bn-A04-p9865629	A-genome specific (napus-rapa)	1	0.7732459	not scorable	not scorable	0.9834711	0.6956896
####	Bn-A04-p9865712	A-genome specific (napus-rapa)	0.9976076	0.8476278	not scorable	not scorable	0.9917355	0.6753443
####	Bn-A04-p9866201	A-genome specific (napus-rapa)	0.9976076	0.8249698	not scorable	not scorable	1	0.7983621
####	Bn-A04-p9868258	A-genome specific (napus-rapa)	0.9952153	0.7446294	not scorable	not scorable	0.9586777	0.7967765
####	Bn-A04-p9870167	A-genome specific (napus-rapa)	0.9952153	0.7990296	not scorable	not scorable	0.9917355	0.6813985
####	Bn-A04-p9870879	A-genome specific (napus-rapa)	0.9688995	0.7680029	not scorable	not scorable	1	0.8077747
####	Bn-A04-p9878192	A-genome specific (napus-rapa)	0.9497608	0.8386382	not scorable	not scorable	0.9669421	0.6833032
####	Bn-A04-p9878444	A-genome specific (napus-rapa)	0.9976076	0.8070768	not scorable	not scorable	0.9917355	0.8059325
####	Bn-A04-p9879436	A-genome specific (napus-rapa)	1	0.5113595	not scorable	not scorable	1	0.7163417
####	Bn-A04-p9920827	A-genome specific (napus-rapa)	0.9952153	0.8515794	not scorable	not scorable	0.9752066	0.7506722
####	Bn-A04-p9920873	A-genome specific (napus-rapa)	0.9976076	0.9483694	not scorable	not scorable	0.9917355	0.7679619
####	Bn-A04-p9921876	A-genome specific (napus-rapa)	1	0.505382	not scorable	not scorable	1	0.681392
####	Bn-A04-p9930371	A-genome specific (napus-rapa)	0.9976076	0.4993044	not scorable	not scorable	1	0.8109526
####	Bn-A04-p9932606	A-genome specific (napus-rapa)	0.9952153	0.8101181	not scorable	not scorable	1	0.8076404

####	Bn-A04-p9983165	A-genome specific (napus-rapa)	0.9976076	0.8907107	not scorable	not scorable	1	0.8255098
####	Bn-A04-p9991606	A-genome specific (napus-rapa)	0.9952153	0.8370666	not scorable	not scorable	0.9917355	0.8026443
####	Bn-A05-p10009197	A-genome specific (napus-rapa)	0.9784689	0.8228517	not scorable	not scorable	0.6528926	0.7877723
####	Bn-A05-p10009485	A-genome specific (napus-rapa)	0.9880383	0.8407717	not scorable	not scorable	0.9917355	0.7830843
####	Bn-A05-p1006754	A-genome specific (napus-rapa)	0.9808612	0.3251211	not scorable	not scorable	0.9834711	0.6717662
####	Bn-A05-p10101040	A-genome specific (napus-rapa)	0.9952153	0.8525339	not scorable	not scorable	0.9752066	0.7983692
####	Bn-A05-p10103295	A-genome specific (napus-rapa)	0.9928229	0.7692301	not scorable	not scorable	1	0.732154
####	Bn-A05-p10171248	A-genome specific (napus-rapa)	0.9856459	0.780487	not scorable	not scorable	0.9834711	0.6768451
####	Bn-A05-p10179640	A-genome specific (napus-rapa)	0.9904306	0.7738579	not scorable	not scorable	0.9917355	0.7902774
####	Bn-A05-p10181096	A-genome specific (napus-rapa)	0.9928229	0.7908362	not scorable	not scorable	0.9752066	0.7849041
####	Bn-A05-p10214000	A-genome specific (napus-rapa)	0.9832536	0.8311532	not scorable	not scorable	1	0.8541407
####	Bn-A05-p10214398	A-genome specific (napus-rapa)	0.9976076	0.7280865	not scorable	not scorable	0.9917355	0.7061196
####	Bn-A05-p10241814	A-genome specific (rapa only)	not scorable	not scorable	not scorable	not scorable	0.9834711	0.8350291
####	Bn-A05-p10264427	A-genome specific (napus-rapa)	0.9952153	0.7572432	not scorable	not scorable	0.9834711	0.8491581
####	Bn-A05-p10264494	A-genome specific (napus-rapa)	0.9880383	0.7961781	not scorable	not scorable	0.9917355	0.7816985
####	Bn-A05-p10267278	A-genome specific (napus-rapa)	0.7009569	0.6446386	not scorable	not scorable	0.9917355	0.8514802
####	Bn-A05-p10269046	A-genome specific (napus-rapa)	0.9880383	0.7053419	not scorable	not scorable	1	0.7045303
####	Bn-A05-p10278245	A-genome specific (napus-rapa)	0.9904306	0.817386	not scorable	not scorable	1	0.7319714
####	Bn-A05-p10281337	A-genome specific (napus-rapa)	0.9880383	0.8695367	not scorable	not scorable	0.9834711	0.6860555
####	Bn-A05-p10292778	napus specific	0.9114832	0.5879735	not scorable	not scorable	not scorable	not scorable
####	Bn-A05-p10296509	A-genome specific (rapa only)	not scorable	not scorable	not scorable	not scorable	0.9917355	0.7259103
####	Bn-A05-p1030058	A-genome specific (napus-rapa)	0.9760765	0.4672996	not scorable	not scorable	0.9917355	0.719144
####	Bn-A05-p10305352	A-genome specific (napus-rapa)	1	0.3156064	not scorable	not scorable	0.9917355	0.7129645
####	Bn-A05-p10320143	A-genome specific (napus-rapa)	0.9928229	0.4388309	not scorable	not scorable	0.9917355	0.7289489
####	Bn-A05-p10320501	A-genome specific (napus-rapa)	0.9808612	0.479975	not scorable	not scorable	0.9752066	0.7753587
####	Bn-A05-p10321304	A-genome specific (napus-rapa)	0.9976076	0.9311632	not scorable	not scorable	1	0.9531466
####	Bn-A05-p10321580	A-genome specific (napus-rapa)	0.9736842	0.8081185	not scorable	not scorable	0.9586777	0.7983829
####	Bn-A05-p10331539	A-genome specific (rapa only)	not scorable	not scorable	not scorable	not scorable	0.9917355	0.5493351
####	Bn-A05-p10349551	A-genome specific (napus-rapa)	0.9832536	0.8400269	not scorable	not scorable	1	0.7355271
####	Bn-A05-p1035167	A-genome specific (napus-rapa)	0.9904306	0.3684653	not scorable	not scorable	0.9917355	0.8001444
####	Bn-A05-p10357316	A-genome specific (napus-rapa)	0.9904306	0.8200872	not scorable	not scorable	0.9917355	0.8041285
####	Bn-A05-p10364013	A-genome specific (napus-rapa)	0.9904306	0.7999232	not scorable	not scorable	1	0.8028173
####	Bn-A05-p10365857	A-genome specific (napus-rapa)	0.9976076	0.6964929	not scorable	not scorable	1	0.7362399

####	Bn-A05-p10367279	A-genome specific (napus-rapa)	0.9856459	0.6697623	not scorable	not scorable	0.9917355	0.7017483
####	Bn-A05-p10368686	A-genome specific (napus-rapa)	0.9904306	0.6932496	not scorable	not scorable	0.9834711	0.6866765
####	Bn-A05-p103817	A-genome specific (napus-rapa)	0.9617225	0.5990162	not scorable	not scorable	0.9917355	0.6673574
####	Bn-A05-p10403917	napus specific	0.9784689	0.6967798	not scorable	not scorable	not scorable	not scorable
####	Bn-A05-p10413113	A-genome specific (napus-rapa)	0.9880383	0.7812539	not scorable	not scorable	0.9917355	0.7152185
####	Bn-A05-p10413574	A-genome specific (napus-rapa)	0.9736842	0.7236876	not scorable	not scorable	0.9834711	0.7653875
####	Bn-A05-p10423564	A-genome specific (napus-rapa)	0.9856459	0.4268348	not scorable	not scorable	0.9917355	0.5865664
####	Bn-A05-p10425198	A-genome specific (napus-rapa)	1	0.3380069	not scorable	not scorable	1	0.9205657
####	Bn-A05-p10426347	A-genome specific (napus-rapa)	0.9736842	0.8450391	not scorable	not scorable	0.7107438	0.8306848
####	Bn-A05-p10426618	A-genome specific (napus-rapa)	1	0.5947348	not scorable	not scorable	1	0.653881
####	Bn-A05-p10437937	A-genome specific (napus-rapa)	0.9760765	0.8395281	not scorable	not scorable	0.9256198	0.7150277
####	Bn-A05-p10452570	A-genome specific (napus-rapa)	0.9904306	0.3781323	not scorable	not scorable	1	0.7421775
####	Bn-A05-p10622526	A-genome specific (napus-rapa)	0.9832536	0.6384492	not scorable	not scorable	1	0.6074274
####	Bn-A05-p10624916	A-genome specific (napus-rapa)	0.9808612	0.7680653	not scorable	not scorable	0.9917355	0.8149812
####	Bn-A05-p10625034	A-genome specific (napus-rapa)	0.6746411	0.9512877	not scorable	not scorable	0.9917355	0.7820156
####	Bn-A05-p10625215	A-genome specific (rapa only)	not scorable	not scorable	not scorable	not scorable	1	0.8542037
####	Bn-A05-p10625236	A-genome specific (napus-rapa)	0.9952153	0.7870179	not scorable	not scorable	1	0.9269587
####	Bn-A05-p10665044	A-genome specific (napus-rapa)	0.9880383	0.5658607	not scorable	not scorable	0.9834711	0.7108423
####	Bn-A05-p10694572	A-genome specific (napus-rapa)	1	0.4907801	not scorable	not scorable	1	0.7849252
####	Bn-A05-p10697862	A-genome specific (napus-rapa)	0.9856459	0.399174	not scorable	not scorable	0.9669421	0.6212759
####	Bn-A05-p1071371	A-genome specific (napus-rapa)	0.9688995	0.6816048	not scorable	not scorable	0.9917355	0.7867506
####	Bn-A05-p10719967	A-genome specific (napus-rapa)	1	0.6594534	not scorable	not scorable	1	0.7279152
####	Bn-A05-p10734565	A-genome specific (napus-rapa)	0.9808612	0.4804975	not scorable	not scorable	0.9752066	0.4132078
####	Bn-A05-p1074864	A-genome specific (napus-rapa)	0.9665072	0.6936206	not scorable	not scorable	0.9917355	0.732167
####	Bn-A05-p10752346	A-genome specific (napus-rapa)	0.9665072	0.3609956	not scorable	not scorable	0.9917355	0.7215093
####	Bn-A05-p10760570	A-genome specific (napus-rapa)	0.9976076	0.4728328	not scorable	not scorable	0.9917355	0.7976328
####	Bn-A05-p10778859	A-genome specific (napus-rapa)	0.9880383	0.4745528	not scorable	not scorable	0.9834711	0.7800552
####	Bn-A05-p10780480	A-genome specific (napus-rapa)	0.9928229	0.8679422	not scorable	not scorable	0.9834711	0.6146014
####	Bn-A05-p10783057	A-genome specific (napus-rapa)	0.9904306	0.7144561	not scorable	not scorable	0.9586777	0.6455203
####	Bn-A05-p10798961	A-genome specific (napus-rapa)	0.9784689	0.8021863	not scorable	not scorable	0.9917355	0.8384817
####	Bn-A05-p10804278	A-genome specific (napus-rapa)	0.9736842	0.7732871	not scorable	not scorable	0.9917355	0.7815014
####	Bn-A05-p10820194	A-genome specific (napus-rapa)	0.9928229	0.5564591	not scorable	not scorable	1	0.891554
####	Bn-A05-p10840480	napus specific	0.9425837	0.2722276	not scorable	not scorable	not scorable	not scorable

####	Bn-A05-p1084980	A-genome specific (napus-rapa)	0.9545454	0.5764614	not scorable	not scorable	0.9752066	0.7083077
####	Bn-A05-p108510	A-genome specific (napus-rapa)	0.9784689	0.3971686	not scorable	not scorable	0.9834711	0.7645464
####	Bn-A05-p10868044	A-genome specific (napus-rapa)	0.9928229	0.311801	not scorable	not scorable	1	0.8200477
####	Bn-A05-p10922835	A-genome specific (napus-rapa)	0.9832536	0.3018378	not scorable	not scorable	1	0.8350435
####	Bn-A05-p10924105	A-genome specific (napus-rapa)	0.9856459	0.6882429	not scorable	not scorable	1	0.6341765
####	Bn-A05-p10930228	A-genome specific (napus-rapa)	1	0.7345608	not scorable	not scorable	1	0.7991373
####	Bn-A05-p10939686	A-genome specific (napus-rapa)	0.9928229	0.6961426	not scorable	not scorable	0.9917355	0.606396
####	Bn-A05-p10939740	A-genome specific (napus-rapa)	0.9832536	0.4492921	not scorable	not scorable	1	0.6784759
####	Bn-A05-p10940401	A-genome specific (napus-rapa)	0.9904306	0.802958	not scorable	not scorable	1	0.8546536
####	Bn-A05-p10945933	A-genome specific (napus-rapa)	1	0.9503593	not scorable	not scorable	1	0.9475388
####	Bn-A05-p10946327	A-genome specific (napus-rapa)	1	0.9309	not scorable	not scorable	1	0.9035182
####	Bn-A05-p10947783	A-genome specific (napus-rapa)	0.9760765	0.4956893	not scorable	not scorable	1	0.8736501
####	Bn-A05-p10948934	A-genome specific (napus-rapa)	0.9736842	0.6442204	not scorable	not scorable	0.9917355	0.750405
####	Bn-A05-p10951155	A-genome specific (rapa only)	not scorable	not scorable	not scorable	not scorable	0.9917355	0.7947016
####	Bn-A05-p10951975	A-genome specific (napus-rapa)	1	0.7698122	not scorable	not scorable	1	0.8252758
####	Bn-A05-p10964994	A-genome specific (napus-rapa)	0.9808612	0.7640045	not scorable	not scorable	0.9917355	0.7376109
####	Bn-A05-p10966950	A-genome specific (napus-rapa)	0.9808612	0.6866301	not scorable	not scorable	1	0.753532
####	Bn-A05-p1096935	A-genome specific (napus-rapa)	0.9904306	0.7216619	not scorable	not scorable	0.9834711	0.8318064
####	Bn-A05-p1097088	A-genome specific (napus-rapa)	0.9736842	0.3094587	not scorable	not scorable	0.9917355	0.7943903
####	Bn-A05-p10981427	A-genome specific (napus-rapa)	0.9856459	0.7471141	not scorable	not scorable	1	0.7975445
####	Bn-A05-p109998	A-genome specific (napus-rapa)	0.9712918	0.6913311	not scorable	not scorable	0.9834711	0.7008945
####	Bn-A05-p10999977	A-genome specific (napus-rapa)	0.9928229	0.7575833	not scorable	not scorable	0.9917355	0.7174992
####	Bn-A05-p11000036	A-genome specific (napus-rapa)	0.9784689	0.8012666	not scorable	not scorable	1	0.8584123
####	Bn-A05-p11000261	A-genome specific (napus-rapa)	0.9688995	0.3418545	not scorable	not scorable	1	0.6735432
####	Bn-A05-p11012409	A-genome specific (napus-rapa)	0.9952153	0.8030952	not scorable	not scorable	0.9834711	0.772213
####	Bn-A05-p11013933	A-genome specific (napus-rapa)	0.9808612	0.6185138	not scorable	not scorable	1	0.7846502
####	Bn-A05-p11013981	A-genome specific (napus-rapa)	1	0.3953399	not scorable	not scorable	0.9917355	0.7565321
####	Bn-A05-p11044537	A-genome specific (napus-rapa)	0.9952153	0.4340661	not scorable	not scorable	1	0.7935139
####	Bn-A05-p11048761	A-genome specific (napus-rapa)	1	0.3613172	not scorable	not scorable	0.9917355	0.7520851
####	Bn-A05-p11056573	A-genome specific (napus-rapa)	0.9976076	0.5238574	not scorable	not scorable	1	0.6719183
####	Bn-A05-p11057820	A-genome specific (rapa only)	not scorable	not scorable	not scorable	not scorable	0.9834711	0.781804
####	Bn-A05-p11068866	A-genome specific (napus-rapa)	0.9928229	0.6895443	not scorable	not scorable	0.9917355	0.872438
####	Bn-A05-p1107888	A-genome specific (napus-rapa)	0.9760765	0.8050262	not scorable	not scorable	0.6033058	0.8641129

####	Bn-A05-p11084469	A-genome specific (napus-rapa)	0.9880383	0.4779801	not scorable	not scorable	0.9834711	0.8632979
####	Bn-A05-p11088814	A-genome specific (napus-rapa)	0.9832536	0.3985411	not scorable	not scorable	1	0.8302616
####	Bn-A05-p11120195	A-genome specific (napus-rapa)	0.9712918	0.7769009	not scorable	not scorable	0.9338843	0.7469814
####	Bn-A05-p11120274	A-genome specific (napus-rapa)	0.9784689	0.3511234	not scorable	not scorable	0.9834711	0.7205312
####	Bn-A05-p11143078	A-genome specific (napus-rapa)	0.9808612	0.7403288	not scorable	not scorable	0.7933884	0.6244924
####	Bn-A05-p11146315	A-genome specific (napus-rapa)	0.9832536	0.317552	not scorable	not scorable	0.9917355	0.8853623
####	Bn-A05-p11146571	A-genome specific (napus-rapa)	0.9808612	0.6606387	not scorable	not scorable	1	0.7135057
####	Bn-A05-p11151246	A-genome specific (napus-rapa)	0.9904306	0.7100585	not scorable	not scorable	1	0.7573233
####	Bn-A05-p11164954	A-genome specific (napus-rapa)	1	0.5560554	not scorable	not scorable	0.9834711	0.7011353
####	Bn-A05-p11168272	A-genome specific (napus-rapa)	0.8660287	0.4882196	not scorable	not scorable	0.9917355	0.723166
####	Bn-A05-p11168423	A-genome specific (napus-rapa)	0.8875598	0.4474941	not scorable	not scorable	0.9338843	0.8526633
####	Bn-A05-p11225095	A-genome specific (napus-rapa)	1	0.9247398	not scorable	not scorable	1	0.7898901
####	Bn-A05-p11226462	A-genome specific (napus-rapa)	0.9904306	0.7625824	not scorable	not scorable	1	0.8448205
####	Bn-A05-p11254494	A-genome specific (napus-rapa)	0.9976076	0.7568642	not scorable	not scorable	0.9752066	0.8331724
####	Bn-A05-p11255103	A-genome specific (napus-rapa)	0.9976076	0.803587	not scorable	not scorable	0.9586777	0.6572919
####	Bn-A05-p11279325	A-genome specific (napus-rapa)	0.9736842	0.8526029	not scorable	not scorable	1	0.8344012
####	Bn-A05-p11279669	A-genome specific (napus-rapa)	0.9856459	0.4569578	not scorable	not scorable	1	0.711734
####	Bn-A05-p11283530	A-genome specific (napus-rapa)	0.9928229	0.6571167	not scorable	not scorable	1	0.8664528
####	Bn-A05-p11297100	A-genome specific (napus-rapa)	0.9832536	0.4468037	not scorable	not scorable	0.9917355	0.6539873
####	Bn-A05-p1134104	A-genome specific (napus-rapa)	0.9832536	0.660798	not scorable	not scorable	0.9586777	0.8077363
####	Bn-A05-p11372809	A-genome specific (napus-rapa)	0.9784689	0.7797241	not scorable	not scorable	0.9504132	0.7548708
####	Bn-A05-p11377111	A-genome specific (napus-rapa)	1	0.7323557	not scorable	not scorable	0.9834711	0.8058489
####	Bn-A05-p11384651	A-genome specific (napus-rapa)	1	0.5799218	not scorable	not scorable	0.9834711	0.640321
####	Bn-A05-p11426194	A-genome specific (napus-rapa)	0.9808612	0.7528992	not scorable	not scorable	1	0.8240975
####	Bn-A05-p11429310	A-genome specific (napus-rapa)	1	0.7844516	not scorable	not scorable	1	0.8275546
####	Bn-A05-p114598	A-genome specific (napus-rapa)	0.9138756	0.630439	not scorable	not scorable	0.9752066	0.6763995
####	Bn-A05-p11514604	A-genome specific (napus-rapa)	1	0.5801526	not scorable	not scorable	1	0.8532773
####	Bn-A05-p11562034	A-genome specific (napus-rapa)	1	0.7536629	not scorable	not scorable	0.9917355	0.7237091
####	Bn-A05-p11593933	A-genome specific (napus-rapa)	0.9976076	0.3307559	not scorable	not scorable	1	0.6350003
####	Bn-A05-p11606554	A-genome specific (napus-rapa)	0.9952153	0.7309262	not scorable	not scorable	1	0.7408684
####	Bn-A05-p11607344	A-genome specific (napus-rapa)	0.9808612	0.6591588	not scorable	not scorable	0.9669421	0.7114879
####	Bn-A05-p11626174	A-genome specific (napus-rapa)	0.784689	0.6752737	not scorable	not scorable	0.9834711	0.8358579
####	Bn-A05-p11686214	A-genome specific (napus-rapa)	1	0.8200702	not scorable	not scorable	0.9917355	0.7604609

####	Bn-A05-p11687380	A-genome specific (napus-rapa)	0.9808612	0.2602273	not scorable	not scorable	1	0.7562124
####	Bn-A05-p11689852	A-genome specific (napus-rapa)	0.9976076	0.512587	not scorable	not scorable	1	0.7315711
####	Bn-A05-p11690317	A-genome specific (napus-rapa)	1	0.4908344	not scorable	not scorable	0.9917355	0.7906306
####	Bn-A05-p11702049	A-genome specific (napus-rapa)	0.9210526	0.4428999	not scorable	not scorable	1	0.7025673
####	Bn-A05-p11735505	A-genome specific (napus-rapa)	0.9952153	0.2897251	not scorable	not scorable	0.9917355	0.6549749
####	Bn-A05-p11736698	A-genome specific (napus-rapa)	0.9832536	0.3752197	not scorable	not scorable	1	0.7154679
####	Bn-A05-p11737404	A-genome specific (napus-rapa)	0.9712918	0.6717739	not scorable	not scorable	0.9917355	0.8337364
####	Bn-A05-p11738337	A-genome specific (napus-rapa)	0.9258373	0.4318279	not scorable	not scorable	0.9834711	0.6354367
####	Bn-A05-p11740536	A-genome specific (napus-rapa)	0.9449761	0.2184676	not scorable	not scorable	1	0.6928614
####	Bn-A05-p11751401	A-genome specific (napus-rapa)	0.9832536	0.279249	not scorable	not scorable	1	0.8018968
####	Bn-A05-p11767399	A-genome specific (napus-rapa)	1	0.6436768	not scorable	not scorable	1	0.5904204
####	Bn-A05-p11776888	A-genome specific (napus-rapa)	0.3636364	0.7117825	not scorable	not scorable	0.9504132	0.7958187
####	Bn-A05-p11781784	all not scorable	not scorable	not scorable	not scorable	not scorable	not scorable	not scorable
####	Bn-A05-p11785796	A-genome specific (napus-rapa)	0.9832536	0.5086489	not scorable	not scorable	0.9917355	0.7321043
####	Bn-A05-p11786537	A-genome specific (napus-rapa)	0.9712918	0.6408622	not scorable	not scorable	0.9834711	0.6683499
####	Bn-A05-p11812178	A-genome specific (napus-rapa)	0.9904306	0.3612525	not scorable	not scorable	0.9917355	0.6519752
####	Bn-A05-p11812265	A-genome specific (napus-rapa)	0.9880383	0.5173039	not scorable	not scorable	0.9834711	0.8749894
####	Bn-A05-p11818095	A-genome specific (napus-rapa)	0.9880383	0.3236778	not scorable	not scorable	1	0.643315
####	Bn-A05-p11840240	A-genome specific (napus-rapa)	0.9856459	0.3569155	not scorable	not scorable	1	0.604719
####	Bn-A05-p11856345	A-genome specific (napus-rapa)	0.9784689	0.2876118	not scorable	not scorable	1	0.7033995
####	Bn-A05-p11910270	A-genome specific (napus-rapa)	0.5717703	0.6970498	not scorable	not scorable	0.9917355	0.782876
####	Bn-A05-p11926097	A-genome specific (napus-rapa)	0.9832536	0.3781281	not scorable	not scorable	1	0.8733965
####	Bn-A05-p1192706	A-genome specific (napus-rapa)	0.8803828	0.6934756	not scorable	not scorable	0.9917355	0.8071598
####	Bn-A05-p11927100	A-genome specific (napus-rapa)	0.9665072	0.7542313	not scorable	not scorable	0.9917355	0.668591
####	Bn-A05-p11927159	A-genome specific (napus-rapa)	0.9904306	0.7146924	not scorable	not scorable	1	0.7375373
####	Bn-A05-p11927165	A-genome specific (napus-rapa)	0.9952153	0.6632242	not scorable	not scorable	1	0.5584884
####	Bn-A05-p11942356	A-genome specific (napus-rapa)	0.9736842	0.4197618	not scorable	not scorable	0.9917355	0.6936432
####	Bn-A05-p11961924	A-genome specific (napus-rapa)	0.8014354	0.6956896	not scorable	not scorable	0.9917355	0.8062737
####	Bn-A05-p11966222	A-genome specific (napus-rapa)	1	0.4630724	not scorable	not scorable	1	0.8092235
####	Bn-A05-p1196947	A-genome specific (napus-rapa)	0.9904306	0.4780737	not scorable	not scorable	1	0.7318985
####	Bn-A05-p11975840	A-genome specific (napus-rapa)	0.9808612	0.2445872	not scorable	not scorable	0.9917355	0.7094512
####	Bn-A05-p11990369	all not scorable	not scorable	not scorable	not scorable	not scorable	not scorable	not scorable
####	Bn-A05-p12002033	A-genome specific (napus-rapa)	0.9904306	0.79955	not scorable	not scorable	1	0.7749903

####	Bn-A05-p12018989	A-genome specific (napus-rapa)	0.930622	0.7136176	not scorable	not scorable	0.9669421	0.6731555
####	Bn-A05-p12019087	A-genome specific (napus-rapa)	0.9928229	0.7387426	not scorable	not scorable	1	0.7598882
####	Bn-A05-p12019708	A-genome specific (napus-rapa)	0.9282297	0.6132887	not scorable	not scorable	0.9917355	0.6137882
####	Bn-A05-p12021730	A-genome specific (napus-rapa)	0.9712918	0.5945939	not scorable	not scorable	1	0.783363
####	Bn-A05-p12021976	A-genome specific (napus-rapa)	0.9330143	0.727056	not scorable	not scorable	0.9917355	0.6938709
####	Bn-A05-p12022144	A-genome specific (rapa only)	not scorable	not scorable	not scorable	not scorable	1	0.6642559
####	Bn-A05-p12022540	A-genome specific (napus-rapa)	0.9832536	0.5735331	not scorable	not scorable	1	0.7856574
####	Bn-A05-p12024011	A-genome specific (rapa only)	not scorable	not scorable	not scorable	not scorable	0.9752066	0.6680171
####	Bn-A05-p12026117	A-genome specific (napus-rapa)	0.9856459	0.7323353	not scorable	not scorable	0.9834711	0.7741836
####	Bn-A05-p12032938	A-genome specific (napus-rapa)	0.9808612	0.4830529	not scorable	not scorable	0.9752066	0.5800495
####	Bn-A05-p12046650	A-genome specific (napus-rapa)	0.9880383	0.7948073	not scorable	not scorable	0.9834711	0.8256641
####	Bn-A05-p1204987	A-genome specific (napus-rapa)	0.9521531	0.5445216	not scorable	not scorable	0.9917355	0.8132794
####	Bn-A05-p12052571	A-genome specific (napus-rapa)	0.9760765	0.647494	not scorable	not scorable	1	0.7699569
####	Bn-A05-p12056452	A-genome specific (napus-rapa)	0.9832536	0.3264702	not scorable	not scorable	1	0.7706463
####	Bn-A05-p12059250	A-genome specific (napus-rapa)	0.9880383	0.4038003	not scorable	not scorable	0.9504132	0.3437998
####	Bn-A05-p12067093	A-genome specific (napus-rapa)	0.9784689	0.6427388	not scorable	not scorable	1	0.6878925
####	Bn-A05-p12069010	A-genome specific (napus-rapa)	0.9880383	0.3897271	not scorable	not scorable	0.9917355	0.7048109
####	Bn-A05-p12102885	A-genome specific (napus-rapa)	0.9808612	0.6965085	not scorable	not scorable	0.9917355	0.8101091
####	Bn-A05-p12103432	A-genome specific (napus-rapa)	0.9856459	0.7847863	not scorable	not scorable	1	0.7180848
####	Bn-A05-p12104940	A-genome specific (napus-rapa)	0.9928229	0.7226744	not scorable	not scorable	1	0.7067276
####	Bn-A05-p12105780	A-genome specific (napus-rapa)	0.9880383	0.7468796	not scorable	not scorable	1	0.8738732
####	Bn-A05-p12115583	A-genome specific (napus-rapa)	0.9928229	0.7238235	not scorable	not scorable	1	0.7776101
####	Bn-A05-p12119364	A-genome specific (napus-rapa)	0.9641148	0.7349157	not scorable	not scorable	0.9917355	0.7893811
####	Bn-A05-p12120855	A-genome specific (napus-rapa)	0.9904306	0.4658964	not scorable	not scorable	1	0.6680119
####	Bn-A05-p12131090	genome unspecific (all in)	0.9976076	0.5546744	1	0.8611138	1	0.8567975
####	Bn-A05-p1214053	A-genome specific (napus-rapa)	0.9665072	0.5312838	not scorable	not scorable	0.9834711	0.7992573
####	Bn-A05-p12141401	A-genome specific (napus-rapa)	0.7583732	0.6458821	not scorable	not scorable	0.9917355	0.9205657
####	Bn-A05-p12158169	A-genome specific (napus-rapa)	0.9832536	0.3755104	not scorable	not scorable	0.9834711	0.6338977
####	Bn-A05-p1216828	A-genome specific (napus-rapa)	0.9545454	0.3080547	not scorable	not scorable	0.9834711	0.6533034
####	Bn-A05-p12170375	A-genome specific (napus-rapa)	0.9952153	0.8476576	not scorable	not scorable	1	0.7412776
####	Bn-A05-p12193915	A-genome specific (napus-rapa)	0.9904306	0.6844695	not scorable	not scorable	0.9834711	0.7229283
####	Bn-A05-p12199879	A-genome specific (napus-rapa)	0.9808612	0.7940825	not scorable	not scorable	0.9917355	0.7894837
####	Bn-A05-p1220485	A-genome specific (napus-rapa)	0.9952153	0.7324972	not scorable	not scorable	0.9917355	0.7131635

####	Bn-A05-p12205591	A-genome specific (napus-rapa)	0.9928229	0.8675802	not scorable	not scorable	0.9917355	0.7907953
####	Bn-A05-p12207497	A-genome specific (napus-rapa)	0.9904306	0.7710915	not scorable	not scorable	0.9917355	0.7886205
####	Bn-A05-p12208604	A-genome specific (rapa only)	not scorable	not scorable	not scorable	not scorable	0.9917355	0.6806947
####	Bn-A05-p12208728	A-genome specific (napus-rapa)	0.9808612	0.6977511	not scorable	not scorable	0.9917355	0.6158109
####	Bn-A05-p12208840	A-genome specific (napus-rapa)	0.9880383	0.8236844	not scorable	not scorable	0.9917355	0.7944437
####	Bn-A05-p12209129	A-genome specific (napus-rapa)	0.9976076	0.7228634	not scorable	not scorable	0.9917355	0.7724233
####	Bn-A05-p12209466	A-genome specific (napus-rapa)	0.9880383	0.7562965	not scorable	not scorable	0.9917355	0.6949515
####	Bn-A05-p12216620	A-genome specific (napus-rapa)	0.9808612	0.4155895	not scorable	not scorable	0.9752066	0.4500768
####	Bn-A05-p12246566	A-genome specific (napus-rapa)	0.9928229	0.2806034	not scorable	not scorable	0.9834711	0.7626072
####	Bn-A05-p12248987	A-genome specific (napus-rapa)	0.9593301	0.6909347	not scorable	not scorable	0.9834711	0.7086985
####	Bn-A05-p12298408	A-genome specific (napus-rapa)	0.937799	0.8502172	not scorable	not scorable	0.9752066	0.8394201
####	Bn-A05-p12321766	A-genome specific (napus-rapa)	0.9904306	0.2832311	not scorable	not scorable	1	0.8411826
####	Bn-A05-p12362411	A-genome specific (napus-rapa)	0.9928229	0.4449223	not scorable	not scorable	1	0.7785457
####	Bn-A05-p1237296	A-genome specific (napus-rapa)	0.9736842	0.4250365	not scorable	not scorable	0.9917355	0.625229
####	Bn-A05-p12378382	A-genome specific (napus-rapa)	0.9808612	0.7729486	not scorable	not scorable	0.9586777	0.7323682
####	Bn-A05-p12395933	A-genome specific (napus-rapa)	0.9928229	0.4513147	not scorable	not scorable	0.9917355	0.8177226
####	Bn-A05-p12408504	A-genome specific (napus-rapa)	0.9976076	0.3931613	not scorable	not scorable	1	0.7534895
####	Bn-A05-p12418607	A-genome specific (napus-rapa)	0.9617225	0.458407	not scorable	not scorable	0.9752066	0.8636117
####	Bn-A05-p12418761	A-genome specific (napus-rapa)	0.9880383	0.8026497	not scorable	not scorable	0.9917355	0.7104266
####	Bn-A05-p12418828	A-genome specific (napus-rapa)	0.9880383	0.9092134	not scorable	not scorable	0.9834711	0.7526832
####	Bn-A05-p12436060	A-genome specific (napus-rapa)	0.9832536	0.6399797	not scorable	not scorable	1	0.7494859
####	Bn-A05-p12447042	A-genome specific (napus-rapa)	0.9952153	0.4593855	not scorable	not scorable	1	0.6949418
####	Bn-A05-p12447170	A-genome specific (napus-rapa)	0.9904306	0.3445522	not scorable	not scorable	1	0.7308116
####	Bn-A05-p12450816	A-genome specific (napus-rapa)	0.9976076	0.8048355	not scorable	not scorable	1	0.7709633
####	Bn-A05-p12453362	A-genome specific (napus-rapa)	0.9856459	0.7879313	not scorable	not scorable	0.9669421	0.6849987
####	Bn-A05-p12457036	A-genome specific (napus-rapa)	0.9856459	0.3241183	not scorable	not scorable	1	0.7911202
####	Bn-A05-p12457173	A-genome specific (napus-rapa)	0.9880383	0.3713906	not scorable	not scorable	1	0.702443
####	Bn-A05-p12457670	A-genome specific (napus-rapa)	0.9880383	0.2918586	not scorable	not scorable	0.9834711	0.6968593
####	Bn-A05-p12489885	A-genome specific (rapa only)	not scorable	not scorable	not scorable	not scorable	0.9752066	0.7083436
####	Bn-A05-p12493072	A-genome specific (napus-rapa)	0.9736842	0.8115861	not scorable	not scorable	0.9917355	0.7029465
####	Bn-A05-p12497800	A-genome specific (napus-rapa)	0.8660287	0.6470056	not scorable	not scorable	0.9834711	0.6358404
####	Bn-A05-p12507206	A-genome specific (napus-rapa)	0.9928229	0.3180775	not scorable	not scorable	0.9917355	0.7851636
####	Bn-A05-p12511706	A-genome specific (napus-rapa)	0.9880383	0.3850041	not scorable	not scorable	0.9917355	0.6697007

####	Bn-A05-p12519886	A-genome specific (rapa only)	not scorable	not scorable	not scorable	not scorable	0.9917355	0.7288145
####	Bn-A05-p12529959	A-genome specific (napus-rapa)	1	0.7611011	not scorable	not scorable	1	0.9158497
####	Bn-A05-p1254185	A-genome specific (napus-rapa)	0.9928229	0.7687845	not scorable	not scorable	0.9834711	0.7160311
####	Bn-A05-p12546760	A-genome specific (napus-rapa)	0.9665072	0.7981407	not scorable	not scorable	1	0.6761603
####	Bn-A05-p12553495	A-genome specific (napus-rapa)	0.9832536	0.6272883	not scorable	not scorable	1	0.7849402
####	Bn-A05-p12553851	A-genome specific (napus-rapa)	0.9904306	0.7633795	not scorable	not scorable	0.9834711	0.6744097
####	Bn-A05-p12554299	A-genome specific (napus-rapa)	0.9808612	0.7774656	not scorable	not scorable	1	0.7397594
####	Bn-A05-p12573416	A-genome specific (napus-rapa)	0.9880383	0.3838162	not scorable	not scorable	0.9834711	0.7412516
####	Bn-A05-p12592066	A-genome specific (napus-rapa)	0.9976076	0.5060132	not scorable	not scorable	1	0.811729
####	Bn-A05-p12597194	A-genome specific (napus-rapa)	0.9880383	0.5311761	not scorable	not scorable	0.9752066	0.7964972
####	Bn-A05-p12597426	A-genome specific (napus-rapa)	0.9736842	0.8158063	not scorable	not scorable	0.9834711	0.7834632
####	Bn-A05-p1259869	A-genome specific (napus-rapa)	0.9808612	0.7864878	not scorable	not scorable	1	0.8198699
####	Bn-A05-p1260228	A-genome specific (napus-rapa)	0.9856459	0.799269	not scorable	not scorable	1	0.8647583
####	Bn-A05-p1260305	A-genome specific (napus-rapa)	0.9880383	0.6872016	not scorable	not scorable	0.9917355	0.7127189
####	Bn-A05-p1260565	A-genome specific (napus-rapa)	0.9617225	0.7873439	not scorable	not scorable	0.9752066	0.7040371
####	Bn-A05-p12608926	A-genome specific (napus-rapa)	0.9928229	0.4431527	not scorable	not scorable	1	0.7110881
####	Bn-A05-p12643056	A-genome specific (napus-rapa)	0.9880383	0.4527169	not scorable	not scorable	1	0.7007595
####	Bn-A05-p1267683	A-genome specific (napus-rapa)	0.9808612	0.4915771	not scorable	not scorable	1	0.8508493
####	Bn-A05-p12684736	A-genome specific (napus-rapa)	0.9928229	0.3137266	not scorable	not scorable	0.9917355	0.8041816
####	Bn-A05-p12734993	A-genome specific (napus-rapa)	0.9856459	0.5767543	not scorable	not scorable	0.9917355	0.8002324
####	Bn-A05-p1279304	A-genome specific (napus-rapa)	0.9712918	0.2987345	not scorable	not scorable	0.9917355	0.7284475
####	Bn-A05-p1281138	A-genome specific (napus-rapa)	0.6602871	0.9126741	not scorable	not scorable	0.9090909	0.9032104
####	Bn-A05-p12821101	A-genome specific (napus-rapa)	0.9904306	0.5412003	not scorable	not scorable	0.9917355	0.6170105
####	Bn-A05-p12821172	A-genome specific (napus-rapa)	0.9808612	0.680163	not scorable	not scorable	0.9834711	0.6844396
####	Bn-A05-p12838668	A-genome specific (rapa only)	not scorable	not scorable	not scorable	not scorable	1	0.6241857
####	Bn-A05-p12845300	A-genome specific (napus-rapa)	0.9904306	0.4443542	not scorable	not scorable	1	0.7444597
####	Bn-A05-p12867516	A-genome specific (napus-rapa)	0.9904306	0.6560721	not scorable	not scorable	0.9834711	0.6922773
####	Bn-A05-p12868006	A-genome specific (napus-rapa)	0.9808612	0.8216829	not scorable	not scorable	1	0.6916436
####	Bn-A05-p12878356	A-genome specific (napus-rapa)	0.9832536	0.6306806	not scorable	not scorable	0.9834711	0.8142368
####	Bn-A05-p12888273	A-genome specific (napus-rapa)	0.9832536	0.7663379	not scorable	not scorable	1	0.7354251
####	Bn-A05-p12888422	A-genome specific (napus-rapa)	0.9736842	0.7228274	not scorable	not scorable	1	0.7129829
####	Bn-A05-p12889434	A-genome specific (napus-rapa)	0.9952153	0.457323	not scorable	not scorable	0.9917355	0.6840535
####	Bn-A05-p12890298	A-genome specific (napus-rapa)	0.9880383	0.7477623	not scorable	not scorable	1	0.7751675

####	Bn-A05-p12890362	A-genome specific (napus-rapa)	0.9808612	0.6319359	not scorable	not scorable	0.9669421	0.690979
####	Bn-A05-p12891251	A-genome specific (napus-rapa)	1	0.5344025	not scorable	not scorable	0.9834711	0.8115477
####	Bn-A05-p12891987	A-genome specific (napus-rapa)	0.9880383	0.8566455	not scorable	not scorable	1	0.9080831
####	Bn-A05-p12895483	A-genome specific (napus-rapa)	0.9904306	0.669691	not scorable	not scorable	0.9917355	0.7172675
####	Bn-A05-p12911399	A-genome specific (napus-rapa)	0.9736842	0.6437307	not scorable	not scorable	0.9834711	0.6914029
####	Bn-A05-p12930251	A-genome specific (napus-rapa)	0.354067	0.7725613	not scorable	not scorable	0.9834711	0.9175813
####	Bn-A05-p12952248	A-genome specific (napus-rapa)	0.9952153	0.6756845	not scorable	not scorable	1	0.7287537
####	Bn-A05-p1299570	A-genome specific (rapa only)	not scorable	not scorable	not scorable	not scorable	0.9834711	0.7113431
####	Bn-A05-p13000538	A-genome specific (napus-rapa)	0.3851674	0.7832317	not scorable	not scorable	0.9752066	0.7999963
####	Bn-A05-p13013349	A-genome specific (napus-rapa)	0.3803828	0.7069109	not scorable	not scorable	0.9669421	0.7808555
####	Bn-A05-p1308471	A-genome specific (napus-rapa)	0.9808612	0.685629	not scorable	not scorable	0.9752066	0.6307886
####	Bn-A05-p13094470	A-genome specific (rapa only)	not scorable	not scorable	not scorable	not scorable	0.9917355	0.675553
####	Bn-A05-p13098758	A-genome specific (napus-rapa)	0.9808612	0.794416	not scorable	not scorable	1	0.7727491
####	Bn-A05-p1310329	A-genome specific (napus-rapa)	0.9186603	0.7898545	not scorable	not scorable	0.9917355	0.6103619
####	Bn-A05-p1311652	A-genome specific (napus-rapa)	0.9904306	0.4460801	not scorable	not scorable	1	0.8329018
####	Bn-A05-p13119450	A-genome specific (napus-rapa)	0.9712918	0.7932056	not scorable	not scorable	0.9917355	0.7230601
####	Bn-A05-p13126937	A-genome specific (napus-rapa)	0.9832536	0.3106436	not scorable	not scorable	0.9917355	0.7105414
####	Bn-A05-p13165406	napus specific	0.9665072	0.5090244	not scorable	not scorable	not scorable	not scorable
####	Bn-A05-p13168139	A-genome specific (napus-rapa)	0.9880383	0.7873908	not scorable	not scorable	1	0.8366613
####	Bn-A05-p13175224	A-genome specific (napus-rapa)	0.9760765	0.6961217	not scorable	not scorable	1	0.76025
####	Bn-A05-p13178934	A-genome specific (napus-rapa)	0.9808612	0.8046561	not scorable	not scorable	1	0.8252965
####	Bn-A05-p13178955	A-genome specific (napus-rapa)	0.9856459	0.7747124	not scorable	not scorable	0.9917355	0.8701311
####	Bn-A05-p13179258	A-genome specific (napus-rapa)	0.9736842	0.7925007	not scorable	not scorable	0.9917355	0.7546523
####	Bn-A05-p13184792	A-genome specific (napus-rapa)	0.9952153	0.4030424	not scorable	not scorable	1	0.8898563
####	Bn-A05-p13187401	A-genome specific (napus-rapa)	0.9904306	0.7997032	not scorable	not scorable	1	0.8224833
####	Bn-A05-p13190945	A-genome specific (napus-rapa)	0.9856459	0.2190625	not scorable	not scorable	0.9917355	0.7122391
####	Bn-A05-p13191000	A-genome specific (napus-rapa)	0.9641148	0.7673834	not scorable	not scorable	0.9917355	0.830798
####	Bn-A05-p13224690	A-genome specific (napus-rapa)	0.9928229	0.5053673	not scorable	not scorable	1	0.8457354
####	Bn-A05-p13227178	A-genome specific (napus-rapa)	0.9952153	0.501025	not scorable	not scorable	0.9834711	0.837082
####	Bn-A05-p13282990	A-genome specific (napus-rapa)	0.9952153	0.7747018	not scorable	not scorable	1	0.7926157
####	Bn-A05-p13301511	A-genome specific (napus-rapa)	0.3971292	0.8172142	not scorable	not scorable	1	0.7864866
####	Bn-A05-p1335771	A-genome specific (napus-rapa)	0.9880383	0.3658217	not scorable	not scorable	1	0.8123307
####	Bn-A05-p13372155	A-genome specific (napus-rapa)	0.3995215	0.7192481	not scorable	not scorable	0.9834711	0.776039

####	Bn-A05-p13453538	A-genome specific (napus-rapa)	0.9904306	0.496338	not scorable	not scorable	0.9834711	0.6998889
####	Bn-A05-p13460617	A-genome specific (napus-rapa)	0.9976076	0.490584	not scorable	not scorable	0.9834711	0.895432
####	Bn-A05-p1347246	A-genome specific (napus-rapa)	0.9617225	0.7890999	not scorable	not scorable	0.9586777	0.8063037
####	Bn-A05-p13482596	A-genome specific (napus-rapa)	0.9904306	0.7835218	not scorable	not scorable	1	0.8207101
####	Bn-A05-p13483874	A-genome specific (napus-rapa)	0.9832536	0.7677003	not scorable	not scorable	0.9669421	0.7230237
####	Bn-A05-p13483931	A-genome specific (napus-rapa)	0.9856459	0.8002555	not scorable	not scorable	0.9752066	0.7280045
####	Bn-A05-p13488514	A-genome specific (napus-rapa)	0.9784689	0.8188689	not scorable	not scorable	0.9917355	0.8433141
####	Bn-A05-p13488725	A-genome specific (napus-rapa)	0.9904306	0.8027456	not scorable	not scorable	0.9917355	0.7335148
####	Bn-A05-p13489002	A-genome specific (napus-rapa)	0.9880383	0.8091476	not scorable	not scorable	0.9917355	0.7338857
####	Bn-A05-p13493260	A-genome specific (napus-rapa)	0.9856459	0.8161368	not scorable	not scorable	1	0.7481321
####	Bn-A05-p13493392	A-genome specific (napus-rapa)	0.9832536	0.7362406	not scorable	not scorable	1	0.8094467
####	Bn-A05-p13493704	A-genome specific (napus-rapa)	0.9832536	0.7749788	not scorable	not scorable	0.9834711	0.8763453
####	Bn-A05-p13493781	A-genome specific (napus-rapa)	0.9832536	0.7189589	not scorable	not scorable	1	0.8160059
####	Bn-A05-p13493836	A-genome specific (napus-rapa)	0.9856459	0.7921391	not scorable	not scorable	1	0.891156
####	Bn-A05-p13494389	A-genome specific (napus-rapa)	0.9928229	0.6618043	not scorable	not scorable	0.9834711	0.731228
####	Bn-A05-p13494414	A-genome specific (napus-rapa)	0.9904306	0.7100684	not scorable	not scorable	0.9917355	0.7601068
####	Bn-A05-p1349520	A-genome specific (napus-rapa)	0.9760765	0.7388194	not scorable	not scorable	0.9752066	0.8102825
####	Bn-A05-p13504387	A-genome specific (napus-rapa)	0.9928229	0.4699766	not scorable	not scorable	1	0.8465989
####	Bn-A05-p1350976	A-genome specific (napus-rapa)	0.9712918	0.7458147	not scorable	not scorable	1	0.8200578
####	Bn-A05-p13514214	A-genome specific (napus-rapa)	0.9952153	0.4063192	not scorable	not scorable	0.9834711	0.7681223
####	Bn-A05-p13526183	A-genome specific (napus-rapa)	0.9832536	0.7413812	not scorable	not scorable	0.9834711	0.7767414
####	Bn-A05-p13528032	A-genome specific (napus-rapa)	0.861244	0.7728866	not scorable	not scorable	0.9834711	0.7235278
####	Bn-A05-p1353104	C-genome specific (oleracea only)	not scorable	not scorable	1	0.7331931	not scorable	not scorable
####	Bn-A05-p13532974	A-genome specific (napus-rapa)	0.9856459	0.7951331	not scorable	not scorable	0.9917355	0.683278
####	Bn-A05-p13533202	A-genome specific (napus-rapa)	0.9617225	0.7897811	not scorable	not scorable	0.9834711	0.7711689
####	Bn-A05-p13533449	A-genome specific (napus-rapa)	0.9880383	0.7867874	not scorable	not scorable	0.9752066	0.7913948
####	Bn-A05-p13541711	A-genome specific (napus-rapa)	0.9880383	0.6913	not scorable	not scorable	1	0.7446905
####	Bn-A05-p13552586	A-genome specific (napus-rapa)	0.9904306	0.8032731	not scorable	not scorable	0.9834711	0.8620332
####	Bn-A05-p13553481	A-genome specific (napus-rapa)	0.9425837	0.7166058	not scorable	not scorable	1	0.7578908
####	Bn-A05-p13553743	A-genome specific (napus-rapa)	0.9976076	0.7855683	not scorable	not scorable	1	0.7515587
####	Bn-A05-p13554854	A-genome specific (napus-rapa)	0.9760765	0.6102501	not scorable	not scorable	0.9834711	0.74719
####	Bn-A05-p1356378	A-genome specific (napus-rapa)	0.9545454	0.4927146	not scorable	not scorable	1	0.7227259
####	Bn-A05-p13565564	A-genome specific (napus-rapa)	0.9928229	0.8003382	not scorable	not scorable	1	0.7951249

####	Bn-A05-p1368115	A-genome specific (napus-rapa)	0.9617225	0.7514594	not scorable	not scorable	1	0.8613519
####	Bn-A05-p13687507	A-genome specific (napus-rapa)	0.9904306	0.4794829	not scorable	not scorable	0.9917355	0.7135841
####	Bn-A05-p13688426	A-genome specific (napus-rapa)	0.9904306	0.7326518	not scorable	not scorable	0.9586777	0.662137
####	Bn-A05-p13688982	A-genome specific (napus-rapa)	0.9904306	0.3676944	not scorable	not scorable	0.9917355	0.7063069
####	Bn-A05-p13690413	A-genome specific (napus-rapa)	0.9928229	0.272142	not scorable	not scorable	0.9917355	0.699124
####	Bn-A05-p1372057	A-genome specific (napus-rapa)	0.9425837	0.4685318	not scorable	not scorable	1	0.6533017
####	Bn-A05-p137314	A-genome specific (napus-rapa)	0.6937799	0.7736022	not scorable	not scorable	0.9917355	0.7314861
####	Bn-A05-p13751450	A-genome specific (napus-rapa)	0.9856459	0.8160366	not scorable	not scorable	1	0.8079789
####	Bn-A05-p13760191	A-genome specific (napus-rapa)	0.9617225	0.41025	not scorable	not scorable	0.9917355	0.7282501
####	Bn-A05-p13760281	A-genome specific (napus-rapa)	0.8564593	0.8104867	not scorable	not scorable	0.9917355	0.8700463
####	Bn-A05-p13766174	A-genome specific (napus-rapa)	0.9832536	0.8136856	not scorable	not scorable	0.9834711	0.7176499
####	Bn-A05-p13774122	A-genome specific (napus-rapa)	0.9808612	0.5089132	not scorable	not scorable	1	0.1978836
####	Bn-A05-p13774324	A-genome specific (napus-rapa)	0.7870814	0.839577	not scorable	not scorable	0.9917355	0.7914145
####	Bn-A05-p13778123	A-genome specific (napus-rapa)	0.8708134	0.678169	not scorable	not scorable	0.9834711	0.8529011
####	Bn-A05-p13778371	A-genome specific (napus-rapa)	0.8755981	0.7763469	not scorable	not scorable	1	0.8425163
####	Bn-A05-p13778538	A-genome specific (napus-rapa)	0.8803828	0.6764037	not scorable	not scorable	0.9752066	0.8451946
####	Bn-A05-p13809267	A-genome specific (napus-rapa)	0.9760765	0.5502014	not scorable	not scorable	0.9834711	0.6976266
####	Bn-A05-p13811322	A-genome specific (napus-rapa)	0.9880383	0.6552807	not scorable	not scorable	0.9834711	0.7940351
####	Bn-A05-p13811525	A-genome specific (napus-rapa)	0.9712918	0.7712513	not scorable	not scorable	0.9834711	0.8404107
####	Bn-A05-p13813146	A-genome specific (napus-rapa)	0.9952153	0.3812588	not scorable	not scorable	1	0.7364012
####	Bn-A05-p13816756	A-genome specific (napus-rapa)	0.9784689	0.7378824	not scorable	not scorable	0.9917355	0.6621579
####	Bn-A05-p13816826	A-genome specific (napus-rapa)	0.9832536	0.7385879	not scorable	not scorable	0.9834711	0.7130393
####	Bn-A05-p13816842	A-genome specific (napus-rapa)	0.9832536	0.6734825	not scorable	not scorable	1	0.6872042
####	Bn-A05-p13817153	A-genome specific (napus-rapa)	0.9808612	0.7846883	not scorable	not scorable	1	0.7473196
####	Bn-A05-p13817451	A-genome specific (napus-rapa)	0.9928229	0.7258217	not scorable	not scorable	0.9834711	0.6874065
####	Bn-A05-p13822414	A-genome specific (napus-rapa)	0.9832536	0.6532624	not scorable	not scorable	1	0.7766234
####	Bn-A05-p13822616	A-genome specific (napus-rapa)	0.9904306	0.8201162	not scorable	not scorable	0.9917355	0.8231684
####	Bn-A05-p13823408	A-genome specific (napus-rapa)	0.9976076	0.7245713	not scorable	not scorable	0.9752066	0.6923596
####	Bn-A05-p13825402	A-genome specific (napus-rapa)	0.9784689	0.8118443	not scorable	not scorable	0.9917355	0.653144
####	Bn-A05-p13826013	A-genome specific (napus-rapa)	0.9832536	0.5139144	not scorable	not scorable	1	0.7233354
####	Bn-A05-p13834175	A-genome specific (napus-rapa)	1	0.5192636	not scorable	not scorable	0.9834711	0.7326576
####	Bn-A05-p13834240	A-genome specific (rapa only)	not scorable	not scorable	not scorable	not scorable	0.9917355	0.8351378
####	Bn-A05-p1384000	A-genome specific (napus-rapa)	0.9688995	0.6978064	not scorable	not scorable	0.9834711	0.7716873

####	Bn-A05-p13844605	A-genome specific (napus-rapa)	1	0.5999731	not scorable	not scorable	1	0.6028796
####	Bn-A05-p13845255	A-genome specific (napus-rapa)	0.9928229	0.6469375	not scorable	not scorable	0.9752066	0.7577213
####	Bn-A05-p1384677	A-genome specific (napus-rapa)	0.9808612	0.7675519	not scorable	not scorable	0.9917355	0.7773376
####	Bn-A05-p1385188	A-genome specific (napus-rapa)	0.9808612	0.7623653	not scorable	not scorable	0.9834711	0.715282
####	Bn-A05-p13852930	A-genome specific (napus-rapa)	0.9856459	0.7852725	not scorable	not scorable	1	0.7941309
####	Bn-A05-p13853329	A-genome specific (napus-rapa)	0.8732057	0.8379179	not scorable	not scorable	0.9917355	0.7798659
####	Bn-A05-p13856683	A-genome specific (napus-rapa)	0.9760765	0.3584197	not scorable	not scorable	0.9752066	0.493851
####	Bn-A05-p13863624	A-genome specific (napus-rapa)	0.9880383	0.7436077	not scorable	not scorable	1	0.7346509
####	Bn-A05-p13863673	A-genome specific (napus-rapa)	0.9952153	0.6098399	not scorable	not scorable	0.9917355	0.7088531
####	Bn-A05-p13863975	A-genome specific (napus-rapa)	0.9928229	0.4700585	not scorable	not scorable	1	0.6507345
####	Bn-A05-p13864064	A-genome specific (napus-rapa)	0.9736842	0.6167814	not scorable	not scorable	1	0.8678269
####	Bn-A05-p13864133	A-genome specific (napus-rapa)	0.9808612	0.6631585	not scorable	not scorable	0.9917355	0.6643708
####	Bn-A05-p13864467	A-genome specific (napus-rapa)	0.9856459	0.768726	not scorable	not scorable	0.9917355	0.8722557
####	Bn-A05-p13869168	A-genome specific (napus-rapa)	0.9952153	0.6563772	not scorable	not scorable	1	0.799539
####	Bn-A05-p13876782	A-genome specific (napus-rapa)	0.9904306	0.6782483	not scorable	not scorable	1	0.78584
####	Bn-A05-p13880377	A-genome specific (napus-rapa)	0.9952153	0.7413821	not scorable	not scorable	1	0.8029795
####	Bn-A05-p13882697	A-genome specific (napus-rapa)	0.9880383	0.3930521	not scorable	not scorable	1	0.8673864
####	Bn-A05-p13883109	A-genome specific (napus-rapa)	0.9425837	0.356817	not scorable	not scorable	1	0.7221759
####	Bn-A05-p13886960	A-genome specific (napus-rapa)	0.9808612	0.8075964	not scorable	not scorable	0.9834711	0.74742
####	Bn-A05-p13891848	A-genome specific (napus-rapa)	0.9880383	0.7937678	not scorable	not scorable	0.9917355	0.7512119
####	Bn-A05-p13895100	A-genome specific (napus-rapa)	0.9928229	0.6979634	not scorable	not scorable	1	0.7765287
####	Bn-A05-p13895153	A-genome specific (napus-rapa)	0.9832536	0.8050931	not scorable	not scorable	0.9834711	0.8698773
####	Bn-A05-p13896924	A-genome specific (napus-rapa)	0.9928229	0.7534214	not scorable	not scorable	1	0.8116738
####	Bn-A05-p13897888	A-genome specific (napus-rapa)	0.9976076	0.7385688	not scorable	not scorable	0.9917355	0.7780793
####	Bn-A05-p13900766	A-genome specific (napus-rapa)	0.9928229	0.7996481	not scorable	not scorable	1	0.7500615
####	Bn-A05-p13907424	A-genome specific (napus-rapa)	0.9832536	0.1752356	not scorable	not scorable	0.9669421	0.7884697
####	Bn-A05-p13908499	A-genome specific (napus-rapa)	0.9952153	0.3651779	not scorable	not scorable	1	0.5023226
####	Bn-A05-p13931810	A-genome specific (napus-rapa)	0.9928229	0.4967313	not scorable	not scorable	0.9834711	0.7134245
####	Bn-A05-p13948854	A-genome specific (napus-rapa)	0.9952153	0.7995563	not scorable	not scorable	0.9917355	0.7551011
####	Bn-A05-p1396162	A-genome specific (napus-rapa)	0.9043062	0.7500815	not scorable	not scorable	0.5867769	0.9166433
####	Bn-A05-p13962356	A-genome specific (napus-rapa)	0.9688995	0.8108222	not scorable	not scorable	1	0.7492601
####	Bn-A05-p13963172	A-genome specific (napus-rapa)	0.6650718	0.8151597	not scorable	not scorable	1	0.8207138
####	Bn-A05-p13965323	A-genome specific (napus-rapa)	0.9952153	0.771007	not scorable	not scorable	1	0.9271286

####	Bn-A05-p1396567	A-genome specific (napus-rapa)	0.8971292	0.7661606	not scorable	not scorable	0.5454546	0.7890839
####	Bn-A05-p13968337	A-genome specific (napus-rapa)	0.9856459	0.7526824	not scorable	not scorable	1	0.8129922
####	Bn-A05-p13969222	A-genome specific (napus-rapa)	0.9569378	0.7754179	not scorable	not scorable	1	0.8217288
####	Bn-A05-p1396959	all not scorable	not scorable	not scorable	not scorable	not scorable	not scorable	not scorable
####	Bn-A05-p13973628	A-genome specific (napus-rapa)	0.9928229	0.516964	not scorable	not scorable	0.9917355	0.7507907
####	Bn-A05-p13975657	A-genome specific (napus-rapa)	0.9784689	0.2027846	not scorable	not scorable	0.9834711	0.7254837
####	Bn-A05-p14021272	A-genome specific (napus-rapa)	0.9880383	0.7540577	not scorable	not scorable	0.9917355	0.815152
####	Bn-A05-p14029097	A-genome specific (napus-rapa)	0.9784689	0.7808251	not scorable	not scorable	0.9917355	0.7537515
####	Bn-A05-p14029214	all not scorable	not scorable	not scorable	not scorable	not scorable	not scorable	not scorable
####	Bn-A05-p14040257	A-genome specific (napus-rapa)	0.9736842	0.4005092	not scorable	not scorable	0.9669421	0.4212311
####	Bn-A05-p14040907	A-genome specific (napus-rapa)	0.9808612	0.4296155	not scorable	not scorable	0.9752066	0.6714398
####	Bn-A05-p14042687	A-genome specific (napus-rapa)	0.9712918	0.5099954	not scorable	not scorable	0.9834711	0.7495871
####	Bn-A05-p140798	A-genome specific (napus-rapa)	0.9258373	0.6342245	not scorable	not scorable	0.9917355	0.6893881
####	Bn-A05-p14104827	A-genome specific (napus-rapa)	1	0.8021232	not scorable	not scorable	1	0.8361113
####	Bn-A05-p14106384	A-genome specific (napus-rapa)	1	0.4595138	not scorable	not scorable	1	0.8090124
####	Bn-A05-p14121401	A-genome specific (napus-rapa)	0.7822967	0.7564514	not scorable	not scorable	0.9917355	0.5940767
####	Bn-A05-p14131155	A-genome specific (napus-rapa)	0.9832536	0.7562683	not scorable	not scorable	1	0.7514383
####	Bn-A05-p14144406	A-genome specific (napus-rapa)	0.7894737	0.7740884	not scorable	not scorable	1	0.8120064
####	Bn-A05-p14146305	A-genome specific (napus-rapa)	0.9904306	0.7616547	not scorable	not scorable	1	0.7247769
####	Bn-A05-p14175152	napus specific	0.9808612	0.3778795	not scorable	not scorable	not scorable	not scorable
####	Bn-A05-p14198626	A-genome specific (napus-rapa)	1	0.8170009	not scorable	not scorable	0.9834711	0.8392849
####	Bn-A05-p14202849	A-genome specific (napus-rapa)	1	0.3898809	not scorable	not scorable	1	0.717967
####	Bn-A05-p14205845	A-genome specific (napus-rapa)	0.9832536	0.715423	not scorable	not scorable	0.9917355	0.7272967
####	Bn-A05-p14206065	A-genome specific (napus-rapa)	0.9832536	0.7855651	not scorable	not scorable	0.9834711	0.7542132
####	Bn-A05-p14206169	A-genome specific (napus-rapa)	0.9976076	0.9034107	not scorable	not scorable	1	0.8346071
####	Bn-A05-p14206731	A-genome specific (napus-rapa)	0.9976076	0.7868271	not scorable	not scorable	1	0.7494321
####	Bn-A05-p14207371	A-genome specific (napus-rapa)	0.9976076	0.8384841	not scorable	not scorable	1	0.8369067
####	Bn-A05-p14207788	A-genome specific (napus-rapa)	0.9976076	0.9237373	not scorable	not scorable	1	0.8667399
####	Bn-A05-p14208091	A-genome specific (napus-rapa)	0.9976076	0.7819678	not scorable	not scorable	1	0.7622204
####	Bn-A05-p14208624	A-genome specific (napus-rapa)	0.9976076	0.8506218	not scorable	not scorable	1	0.8117813
####	Bn-A05-p14208906	A-genome specific (napus-rapa)	0.9856459	0.7174041	not scorable	not scorable	0.9917355	0.6997458
####	Bn-A05-p14213049	A-genome specific (napus-rapa)	0.9856459	0.4249578	not scorable	not scorable	0.9917355	0.6465485
####	Bn-A05-p14253804	napus specific	0.9856459	0.919146	not scorable	not scorable	not scorable	not scorable

####	Bn-A05-p14255879	napus specific	0.9928229	0.8932253	not scorable	not scorable	not scorable	not scorable
####	Bn-A05-p14256951	A-genome specific (napus-rapa)	0.9832536	0.6772866	not scorable	not scorable	1	0.6926029
####	Bn-A05-p14285804	A-genome specific (rapa only)	not scorable	not scorable	not scorable	not scorable	0.9752066	0.8013329
####	Bn-A05-p14285873	A-genome specific (rapa only)	not scorable	not scorable	not scorable	not scorable	0.9834711	0.8469042
####	Bn-A05-p14313107	A-genome specific (napus-rapa)	0.9928229	0.4286734	not scorable	not scorable	1	0.6025474
####	Bn-A05-p14325043	A-genome specific (napus-rapa)	1	0.60592	not scorable	not scorable	0.9917355	0.735331
####	Bn-A05-p14332800	A-genome specific (napus-rapa)	1	0.506248	not scorable	not scorable	0.9917355	0.7971937
####	Bn-A05-p14336877	A-genome specific (napus-rapa)	0.9856459	0.7438463	not scorable	not scorable	0.9917355	0.7396191
####	Bn-A05-p14338060	A-genome specific (napus-rapa)	0.9928229	0.7387361	not scorable	not scorable	0.9834711	0.7587919
####	Bn-A05-p14347292	A-genome specific (napus-rapa)	0.9808612	0.414051	not scorable	not scorable	0.9834711	0.7776639
####	Bn-A05-p14347603	A-genome specific (napus-rapa)	0.9808612	0.8123105	not scorable	not scorable	0.9917355	0.8151368
####	Bn-A05-p14347674	A-genome specific (napus-rapa)	0.9904306	0.731306	not scorable	not scorable	0.9834711	0.7675073
####	Bn-A05-p1434959	A-genome specific (napus-rapa)	0.9904306	0.4842895	not scorable	not scorable	0.9834711	0.7519047
####	Bn-A05-p14355487	A-genome specific (rapa only)	not scorable	not scorable	not scorable	not scorable	1	0.7443701
####	Bn-A05-p143572	A-genome specific (napus-rapa)	0.9688995	0.784629	not scorable	not scorable	0.9752066	0.7121841
####	Bn-A05-p1436011	A-genome specific (napus-rapa)	0.9904306	0.5177342	not scorable	not scorable	0.9834711	0.7729131
####	Bn-A05-p14364345	A-genome specific (napus-rapa)	0.9784689	0.3349369	not scorable	not scorable	0.9917355	0.7784424
####	Bn-A05-p14364568	all not scorable	not scorable	not scorable	not scorable	not scorable	not scorable	not scorable
####	Bn-A05-p14372759	A-genome specific (napus-rapa)	0.9784689	0.3221039	not scorable	not scorable	1	0.7074004
####	Bn-A05-p14374199	A-genome specific (napus-rapa)	0.9808612	0.814245	not scorable	not scorable	1	0.6876312
####	Bn-A05-p14374632	A-genome specific (napus-rapa)	0.9808612	0.7934174	not scorable	not scorable	1	0.7716556
####	Bn-A05-p14380049	A-genome specific (napus-rapa)	0.9880383	0.3719637	not scorable	not scorable	1	0.7265942
####	Bn-A05-p14394157	A-genome specific (napus-rapa)	0.9808612	0.6792425	not scorable	not scorable	0.9752066	0.5497229
####	Bn-A05-p14394895	A-genome specific (napus-rapa)	0.9928229	0.7681825	not scorable	not scorable	0.9834711	0.5876268
####	Bn-A05-p14402155	A-genome specific (napus-rapa)	0.9736842	0.8202082	not scorable	not scorable	1	0.7421642
####	Bn-A05-p14426042	A-genome specific (napus-rapa)	0.9952153	0.4348619	not scorable	not scorable	0.9669421	0.5778209
####	Bn-A05-p14426121	A-genome specific (napus-rapa)	0.9856459	0.784063	not scorable	not scorable	1	0.7790133
####	Bn-A05-p14435461	A-genome specific (napus-rapa)	0.9856459	0.8269274	not scorable	not scorable	0.9917355	0.7587654
####	Bn-A05-p14443915	A-genome specific (napus-rapa)	0.9904306	0.3103996	not scorable	not scorable	0.9586777	0.6236752
####	Bn-A05-p14444179	A-genome specific (napus-rapa)	0.9904306	0.2955016	not scorable	not scorable	0.9586777	0.6518297
####	Bn-A05-p14447476	A-genome specific (napus-rapa)	0.9928229	0.5022085	not scorable	not scorable	0.9834711	0.6915604
####	Bn-A05-p14464244	A-genome specific (napus-rapa)	0.9928229	0.4584039	not scorable	not scorable	0.9917355	0.7704409
####	Bn-A05-p14464476	A-genome specific (napus-rapa)	0.9928229	0.3744709	not scorable	not scorable	1	0.7681217

####	Bn-A05-p14477462	A-genome specific (napus-rapa)	0.9832536	0.4693806	not scorable	not scorable	1	0.6758462
####	Bn-A05-p14480752	A-genome specific (napus-rapa)	0.9425837	0.7793456	not scorable	not scorable	0.9586777	0.8289343
####	Bn-A05-p14482654	A-genome specific (napus-rapa)	0.9832536	0.5779714	not scorable	not scorable	0.9917355	0.4764111
####	Bn-A05-p14482858	A-genome specific (napus-rapa)	0.9880383	0.7399379	not scorable	not scorable	0.9917355	0.863204
####	Bn-A05-p14483099	A-genome specific (napus-rapa)	0.8349282	0.7658073	not scorable	not scorable	0.9586777	0.8031629
####	Bn-A05-p14483962	A-genome specific (napus-rapa)	0.9736842	0.7380367	not scorable	not scorable	0.9752066	0.6580356
####	Bn-A05-p14496771	A-genome specific (napus-rapa)	0.8373206	0.7288017	not scorable	not scorable	0.9752066	0.7277868
####	Bn-A05-p14505946	A-genome specific (napus-rapa)	0.9976076	0.7708385	not scorable	not scorable	0.9834711	0.7193513
####	Bn-A05-p14516105	A-genome specific (napus-rapa)	0.9784689	0.7091001	not scorable	not scorable	0.9917355	0.750927
####	Bn-A05-p1451750	A-genome specific (napus-rapa)	1	0.5406895	not scorable	not scorable	1	0.7658601
####	Bn-A05-p14521315	A-genome specific (napus-rapa)	0.9832536	0.318531	not scorable	not scorable	1	0.6997939
####	Bn-A05-p14525918	A-genome specific (napus-rapa)	0.8421053	0.8169802	not scorable	not scorable	0.9834711	0.7190273
####	Bn-A05-p14529716	A-genome specific (napus-rapa)	0.9904306	0.542789	not scorable	not scorable	0.9917355	0.7279447
####	Bn-A05-p14532593	A-genome specific (rapa only)	not scorable	not scorable	not scorable	not scorable	0.9669421	0.8086067
####	Bn-A05-p14533489	A-genome specific (napus-rapa)	0.8373206	0.758665	not scorable	not scorable	0.9669421	0.7592772
####	Bn-A05-p14537104	A-genome specific (napus-rapa)	0.9976076	0.6153359	not scorable	not scorable	0.9834711	0.689976
####	Bn-A05-p14546189	A-genome specific (napus-rapa)	0.9617225	0.2713633	not scorable	not scorable	0.9834711	0.7709047
####	Bn-A05-p14547107	A-genome specific (napus-rapa)	0.8373206	0.7661392	not scorable	not scorable	0.9752066	0.6386008
####	Bn-A05-p14555358	A-genome specific (napus-rapa)	0.8397129	0.8024165	not scorable	not scorable	0.9752066	0.7788349
####	Bn-A05-p14557992	A-genome specific (napus-rapa)	0.8397129	0.8255709	not scorable	not scorable	0.9752066	0.7840218
####	Bn-A05-p14558091	A-genome specific (napus-rapa)	0.8373206	0.5990408	not scorable	not scorable	0.9834711	0.5514354
####	Bn-A05-p14558121	A-genome specific (rapa only)	not scorable	not scorable	not scorable	not scorable	0.9752066	0.7268059
####	Bn-A05-p1457058	A-genome specific (napus-rapa)	0.9832536	0.7419155	not scorable	not scorable	0.9917355	0.854089
####	Bn-A05-p14579391	A-genome specific (napus-rapa)	0.9880383	0.7644255	not scorable	not scorable	1	0.8538787
####	Bn-A05-p14580151	A-genome specific (napus-rapa)	0.9904306	0.3185287	not scorable	not scorable	1	0.8449438
####	Bn-A05-p14588431	A-genome specific (napus-rapa)	0.9928229	0.8693936	not scorable	not scorable	0.9090909	0.706449
####	Bn-A05-p14591985	A-genome specific (napus-rapa)	0.8564593	0.7890928	not scorable	not scorable	0.6198347	0.6992605
####	Bn-A05-p14592407	A-genome specific (napus-rapa)	0.9928229	0.7781494	not scorable	not scorable	1	0.7518246
####	Bn-A05-p14599830	A-genome specific (napus-rapa)	0.9904306	0.7608089	not scorable	not scorable	0.9752066	0.7815613
####	Bn-A05-p14600386	A-genome specific (napus-rapa)	0.9952153	0.5137135	not scorable	not scorable	1	0.8320046
####	Bn-A05-p14602709	A-genome specific (napus-rapa)	0.9856459	0.7673253	not scorable	not scorable	0.9834711	0.787383
####	Bn-A05-p14602869	A-genome specific (napus-rapa)	0.9880383	0.7483007	not scorable	not scorable	0.9917355	0.7345109
####	Bn-A05-p14603280	A-genome specific (napus-rapa)	0.9976076	0.8350514	not scorable	not scorable	1	0.7561697

####	Bn-A05-p14612446	A-genome specific (napus-rapa)	0.9880383	0.5248339	not scorable	not scorable	0.9917355	0.7501578
####	Bn-A05-p14620205	A-genome specific (rapa only)	not scorable	not scorable	not scorable	not scorable	0.9752066	0.9095823
####	Bn-A05-p14624727	A-genome specific (napus-rapa)	0.9688995	0.6074086	not scorable	not scorable	0.9669421	0.5866516
####	Bn-A05-p14624784	A-genome specific (napus-rapa)	0.9856459	0.7651913	not scorable	not scorable	0.6115702	0.7332695
####	Bn-A05-p1462945	A-genome specific (napus-rapa)	0.9760765	0.4114179	not scorable	not scorable	0.9917355	0.7029889
####	Bn-A05-p14636717	A-genome specific (napus-rapa)	0.9497608	0.6557966	not scorable	not scorable	0.9669421	0.7349476
####	Bn-A05-p14662656	A-genome specific (napus-rapa)	0.9928229	0.3799554	not scorable	not scorable	0.9917355	0.6579516
####	Bn-A05-p14663154	A-genome specific (napus-rapa)	0.9976076	0.7225737	not scorable	not scorable	0.7107438	0.7009459
####	Bn-A05-p14673407	A-genome specific (napus-rapa)	0.9784689	0.4298753	not scorable	not scorable	0.9917355	0.8042552
####	Bn-A05-p14709014	A-genome specific (napus-rapa)	0.9688995	0.8146971	not scorable	not scorable	1	0.8318565
####	Bn-A05-p14709370	A-genome specific (napus-rapa)	0.9880383	0.720078	not scorable	not scorable	0.9834711	0.7056816
####	Bn-A05-p14712935	A-genome specific (napus-rapa)	0.9784689	0.8067452	not scorable	not scorable	1	0.8397262
####	Bn-A05-p14715097	A-genome specific (napus-rapa)	0.9712918	0.6511468	not scorable	not scorable	1	0.8587967
####	Bn-A05-p14723523	A-genome specific (napus-rapa)	0.9712918	0.5362268	not scorable	not scorable	0.9917355	0.6893369
####	Bn-A05-p14726545	A-genome specific (napus-rapa)	0.9880383	0.347479	not scorable	not scorable	0.9917355	0.7839159
####	Bn-A05-p14726690	A-genome specific (napus-rapa)	0.9760765	0.7617753	not scorable	not scorable	0.9917355	0.7839109
####	Bn-A05-p1473472	A-genome specific (rapa only)	not scorable	not scorable	not scorable	not scorable	1	0.7366139
####	Bn-A05-p14734937	A-genome specific (napus-rapa)	0.9784689	0.7810981	not scorable	not scorable	0.9917355	0.8014634
####	Bn-A05-p14739008	A-genome specific (napus-rapa)	0.9138756	0.6958975	not scorable	not scorable	0.9834711	0.711966
####	Bn-A05-p14739080	A-genome specific (napus-rapa)	0.9832536	0.6573217	not scorable	not scorable	1	0.5689861
####	Bn-A05-p14740992	A-genome specific (napus-rapa)	0.9665072	0.8022601	not scorable	not scorable	1	0.8177823
####	Bn-A05-p14742715	A-genome specific (napus-rapa)	0.9904306	0.6834508	not scorable	not scorable	0.9917355	0.8206556
####	Bn-A05-p14743027	A-genome specific (napus-rapa)	0.9856459	0.794562	not scorable	not scorable	0.9917355	0.7314629
####	Bn-A05-p14747774	A-genome specific (napus-rapa)	0.9784689	0.7959195	not scorable	not scorable	1	0.7810712
####	Bn-A05-p1475369	A-genome specific (napus-rapa)	0.9545454	0.2426655	not scorable	not scorable	1	0.6989659
####	Bn-A05-p14755242	A-genome specific (napus-rapa)	0.9808612	0.4162999	not scorable	not scorable	0.9834711	0.7997415
####	Bn-A05-p14764932	A-genome specific (napus-rapa)	0.7200957	0.8674166	not scorable	not scorable	0.5785124	0.7819356
####	Bn-A05-p14786195	A-genome specific (napus-rapa)	0.9736842	0.5182476	not scorable	not scorable	1	0.7645805
####	Bn-A05-p14787361	A-genome specific (napus-rapa)	0.9808612	0.795471	not scorable	not scorable	0.9917355	0.7428662
####	Bn-A05-p1479325	A-genome specific (napus-rapa)	0.9784689	0.3422905	not scorable	not scorable	0.9917355	0.7766304
####	Bn-A05-p14801451	A-genome specific (napus-rapa)	0.9784689	0.7164723	not scorable	not scorable	0.6446281	0.7567889
####	Bn-A05-p14813049	A-genome specific (napus-rapa)	0.9569378	0.7689926	not scorable	not scorable	0.9752066	0.5455063
####	Bn-A05-p14813106	A-genome specific (napus-rapa)	0.9593301	0.8010921	not scorable	not scorable	0.9834711	0.662568

####	Bn-A05-p14821280	A-genome specific (napus-rapa)	0.9784689	0.7403466	not scorable	not scorable	1	0.6082642
####	Bn-A05-p14821998	A-genome specific (napus-rapa)	0.9593301	0.7614154	not scorable	not scorable	0.9917355	0.695935
####	Bn-A05-p14830384	A-genome specific (napus-rapa)	0.9736842	0.682444	not scorable	not scorable	0.9917355	0.6359735
####	Bn-A05-p14840113	A-genome specific (napus-rapa)	0.9760765	0.4991404	not scorable	not scorable	0.9917355	0.7922906
####	Bn-A05-p14844746	A-genome specific (napus-rapa)	0.9808612	0.4812004	not scorable	not scorable	0.9917355	0.7631871
####	Bn-A05-p14882151	A-genome specific (napus-rapa)	0.9856459	0.7831926	not scorable	not scorable	0.9917355	0.7953966
####	Bn-A05-p14890882	A-genome specific (napus-rapa)	0.6052632	0.910757	not scorable	not scorable	0.9917355	0.8575654
####	Bn-A05-p14899711	A-genome specific (napus-rapa)	1	0.5515773	not scorable	not scorable	0.9834711	0.6927553
####	Bn-A05-p14906734	A-genome specific (napus-rapa)	0.9904306	0.7836866	not scorable	not scorable	1	0.7636451
####	Bn-A05-p1492327	napus specific	1	0.2782149	not scorable	not scorable	not scorable	not scorable
####	Bn-A05-p14951722	A-genome specific (napus-rapa)	0.9784689	0.5346705	not scorable	not scorable	1	0.7943577
####	Bn-A05-p14959506	A-genome specific (napus-rapa)	0.854067	0.7845822	not scorable	not scorable	1	0.8514328
####	Bn-A05-p14960328	A-genome specific (napus-rapa)	0.8732057	0.7913759	not scorable	not scorable	0.9917355	0.8220202
####	Bn-A05-p1496037	A-genome specific (napus-rapa)	0.9784689	0.4256454	not scorable	not scorable	0.9917355	0.6649013
####	Bn-A05-p14991084	A-genome specific (rapa only)	not scorable	not scorable	not scorable	not scorable	1	0.7439071
####	Bn-A05-p1499192	A-genome specific (napus-rapa)	0.9856459	0.3200792	not scorable	not scorable	1	0.6739376
####	Bn-A05-p15004033	A-genome specific (napus-rapa)	0.9880383	0.5505275	not scorable	not scorable	1	0.6769819
####	Bn-A05-p15013585	A-genome specific (napus-rapa)	0.9976076	0.4027723	not scorable	not scorable	0.9917355	0.8552632
####	Bn-A05-p15015962	A-genome specific (napus-rapa)	0.9808612	0.621562	not scorable	not scorable	1	0.7016079
####	Bn-A05-p15016066	A-genome specific (napus-rapa)	0.9688995	0.5459198	not scorable	not scorable	0.9917355	0.6292179
####	Bn-A05-p15035058	A-genome specific (napus-rapa)	0.9593301	0.7695797	not scorable	not scorable	0.5867769	0.8307579
####	Bn-A05-p15082396	A-genome specific (napus-rapa)	0.9880383	0.7367738	not scorable	not scorable	1	0.6410413
####	Bn-A05-p15086331	A-genome specific (napus-rapa)	0.9760765	0.6199493	not scorable	not scorable	1	0.7077931
####	Bn-A05-p15099108	A-genome specific (napus-rapa)	0.9928229	0.7848312	not scorable	not scorable	1	0.6463428
####	Bn-A05-p15103134	A-genome specific (napus-rapa)	0.9736842	0.7672597	not scorable	not scorable	0.9917355	0.7165173
####	Bn-A05-p15107406	A-genome specific (napus-rapa)	0.9688995	0.7677789	not scorable	not scorable	0.9752066	0.8237666
####	Bn-A05-p15108699	all not scorable	not scorable	not scorable	not scorable	not scorable	not scorable	not scorable
####	Bn-A05-p15113044	A-genome specific (napus-rapa)	0.9832536	0.7744453	not scorable	not scorable	0.9917355	0.7128341
####	Bn-A05-p15113541	A-genome specific (napus-rapa)	0.9736842	0.7806722	not scorable	not scorable	1	0.6716575
####	Bn-A05-p15113570	A-genome specific (napus-rapa)	0.9760765	0.7931147	not scorable	not scorable	1	0.7076277
####	Bn-A05-p15113653	A-genome specific (napus-rapa)	0.9736842	0.6775535	not scorable	not scorable	0.9586777	0.70492
####	Bn-A05-p15115420	A-genome specific (napus-rapa)	0.9569378	0.7764308	not scorable	not scorable	0.9917355	0.7767562
####	Bn-A05-p15116155	A-genome specific (napus-rapa)	0.9712918	0.7779978	not scorable	not scorable	0.9917355	0.7329232

####	Bn-A05-p15116220	A-genome specific (napus-rapa)	0.9904306	0.4398892	not scorable	not scorable	0.9834711	0.7153529
####	Bn-A05-p15126127	A-genome specific (napus-rapa)	0.9808612	0.5139937	not scorable	not scorable	0.9586777	0.6475566
####	Bn-A05-p15136372	napus specific	1	0.9205657	not scorable	not scorable	not scorable	not scorable
####	Bn-A05-p15157665	A-genome specific (napus-rapa)	0.9808612	0.7897446	not scorable	not scorable	0.9917355	0.7877035
####	Bn-A05-p15160819	napus specific	0.3468899	0.7877973	not scorable	not scorable	not scorable	not scorable
####	Bn-A05-p15169260	A-genome specific (rapa only)	not scorable	not scorable	not scorable	not scorable	1	0.797058
####	Bn-A05-p15169361	A-genome specific (napus-rapa)	0.9665072	0.7839491	not scorable	not scorable	0.9917355	0.7369743
####	Bn-A05-p15187671	A-genome specific (napus-rapa)	0.9856459	0.5736871	not scorable	not scorable	0.9917355	0.7453722
####	Bn-A05-p15218436	A-genome specific (napus-rapa)	0.9641148	0.787951	not scorable	not scorable	1	0.8012621
####	Bn-A05-p15224458	A-genome specific (napus-rapa)	1	0.633243	not scorable	not scorable	1	0.646846
####	Bn-A05-p15227777	A-genome specific (napus-rapa)	0.9784689	0.6610716	not scorable	not scorable	0.9917355	0.7694553
####	Bn-A05-p15232488	A-genome specific (napus-rapa)	0.8779904	0.7285617	not scorable	not scorable	0.9834711	0.7112391
####	Bn-A05-p15236669	napus specific	0.9641148	0.6989807	not scorable	not scorable	not scorable	not scorable
####	Bn-A05-p15250834	A-genome specific (napus-rapa)	0.9665072	0.3429927	not scorable	not scorable	0.9917355	0.7360603
####	Bn-A05-p15281306	A-genome specific (napus-rapa)	0.9784689	0.5877738	not scorable	not scorable	0.9834711	0.7516944
####	Bn-A05-p15281766	A-genome specific (napus-rapa)	0.9904306	0.7733926	not scorable	not scorable	0.9834711	0.8325318
####	Bn-A05-p15282156	genome unspecific (all in)	0.9952153	0.6063809	0.4341085	0.7980374	0.9917355	0.6948407
####	Bn-A05-p15290524	A-genome specific (napus-rapa)	0.9808612	0.6668944	not scorable	not scorable	0.9917355	0.7845024
####	Bn-A05-p15297460	A-genome specific (napus-rapa)	0.9832536	0.7291475	not scorable	not scorable	1	0.754338
####	Bn-A05-p15320768	A-genome specific (napus-rapa)	0.9832536	0.3868776	not scorable	not scorable	0.9917355	0.919155
####	Bn-A05-p15322236	A-genome specific (napus-rapa)	0.9545454	0.7000575	not scorable	not scorable	0.6033058	0.8050629
####	Bn-A05-p15343768	A-genome specific (napus-rapa)	0.9688995	0.7579447	not scorable	not scorable	0.9917355	0.8942854
####	Bn-A05-p15351341	A-genome specific (napus-rapa)	0.9736842	0.780077	not scorable	not scorable	0.9917355	0.8272399
####	Bn-A05-p15369031	A-genome specific (napus-rapa)	0.9760765	0.4481802	not scorable	not scorable	0.9834711	0.746865
####	Bn-A05-p15374255	A-genome specific (napus-rapa)	0.9688995	0.7440934	not scorable	not scorable	1	0.7804538
####	Bn-A05-p15375132	A-genome specific (napus-rapa)	0.9784689	0.6917923	not scorable	not scorable	0.9752066	0.7001899
####	Bn-A05-p15383133	A-genome specific (napus-rapa)	1	0.5476851	not scorable	not scorable	1	0.6591572
####	Bn-A05-p15383563	A-genome specific (napus-rapa)	0.9880383	0.7710983	not scorable	not scorable	1	0.7433065
####	Bn-A05-p15385848	A-genome specific (napus-rapa)	1	0.7314773	not scorable	not scorable	1	0.8115661
####	Bn-A05-p15387386	A-genome specific (napus-rapa)	0.9904306	0.7090982	not scorable	not scorable	1	0.7925208
####	Bn-A05-p15397425	A-genome specific (napus-rapa)	0.8110048	0.7347764	not scorable	not scorable	0.9752066	0.6457372
####	Bn-A05-p15426487	A-genome specific (napus-rapa)	0.9521531	0.796814	not scorable	not scorable	0.9256198	0.8752542
####	Bn-A05-p15443959	genome unspecific (all in)	0.9952153	0.4008607	1	0.8458923	0.9917355	0.8191499

####	Bn-A05-p15445826	A-genome specific (napus-rapa)	0.9784689	0.7543095	not scorable	not scorable	1	0.7583339
####	Bn-A05-p15446452	A-genome specific (napus-rapa)	0.9928229	0.6822941	not scorable	not scorable	0.9917355	0.5668756
####	Bn-A05-p15446585	A-genome specific (napus-rapa)	0.9832536	0.6377189	not scorable	not scorable	0.9834711	0.6136621
####	Bn-A05-p15446739	A-genome specific (napus-rapa)	0.9976076	0.7183458	not scorable	not scorable	0.9917355	0.6591359
####	Bn-A05-p15447107	A-genome specific (napus-rapa)	0.9928229	0.7100793	not scorable	not scorable	0.9834711	0.6509095
####	Bn-A05-p15447772	A-genome specific (napus-rapa)	0.9880383	0.7853509	not scorable	not scorable	0.9917355	0.7651446
####	Bn-A05-p15459231	A-genome specific (napus-rapa)	0.9808612	0.6417672	not scorable	not scorable	1	0.6665895
####	Bn-A05-p15459604	A-genome specific (napus-rapa)	0.9928229	0.758574	not scorable	not scorable	0.9917355	0.8425643
####	Bn-A05-p15459993	A-genome specific (napus-rapa)	0.9880383	0.7832555	not scorable	not scorable	1	0.8146937
####	Bn-A05-p15460116	A-genome specific (napus-rapa)	0.9928229	0.7840836	not scorable	not scorable	0.9917355	0.8362582
####	Bn-A05-p15460178	A-genome specific (napus-rapa)	0.9880383	0.7007706	not scorable	not scorable	0.9834711	0.6738854
####	Bn-A05-p15460626	A-genome specific (napus-rapa)	0.9856459	0.4954044	not scorable	not scorable	0.9504132	0.3861498
####	Bn-A05-p15460677	A-genome specific (napus-rapa)	0.9760765	0.5765041	not scorable	not scorable	0.9752066	0.4807346
####	Bn-A05-p15461137	A-genome specific (napus-rapa)	0.9808612	0.7967417	not scorable	not scorable	0.9917355	0.7943989
####	Bn-A05-p15463933	A-genome specific (napus-rapa)	0.9928229	0.8284883	not scorable	not scorable	1	0.8665701
####	Bn-A05-p15464720	A-genome specific (napus-rapa)	0.9856459	0.7598712	not scorable	not scorable	0.9834711	0.7615276
####	Bn-A05-p15465648	napus specific	0.8181818	0.7671464	not scorable	not scorable	not scorable	not scorable
####	Bn-A05-p15466228	A-genome specific (napus-rapa)	0.9808612	0.5147618	not scorable	not scorable	0.9917355	0.8581793
####	Bn-A05-p15472992	A-genome specific (napus-rapa)	0.9832536	0.5021293	not scorable	not scorable	1	0.8222046
####	Bn-A05-p15475096	A-genome specific (napus-rapa)	1	0.6625175	not scorable	not scorable	0.9752066	0.7573017
####	Bn-A05-p15478674	A-genome specific (napus-rapa)	0.9832536	0.7978693	not scorable	not scorable	0.9834711	0.7508439
####	Bn-A05-p15478769	A-genome specific (napus-rapa)	0.9760765	0.4896141	not scorable	not scorable	0.9917355	0.7891018
####	Bn-A05-p15491528	A-genome specific (napus-rapa)	0.9880383	0.5245419	not scorable	not scorable	1	0.7526832
####	Bn-A05-p15510479	A-genome specific (napus-rapa)	0.9736842	0.7057055	not scorable	not scorable	1	0.815388
####	Bn-A05-p15511775	A-genome specific (napus-rapa)	0.9928229	0.8198833	not scorable	not scorable	1	0.844681
####	Bn-A05-p15521397	A-genome specific (napus-rapa)	0.9760765	0.8144602	not scorable	not scorable	1	0.9095823
####	Bn-A05-p15532294	A-genome specific (napus-rapa)	0.9952153	0.7700274	not scorable	not scorable	1	0.7461237
####	Bn-A05-p1554943	A-genome specific (napus-rapa)	0.9832536	0.7725419	not scorable	not scorable	0.9917355	0.7532787
####	Bn-A05-p15551715	A-genome specific (napus-rapa)	0.9760765	0.2803045	not scorable	not scorable	0.9834711	0.750066
####	Bn-A05-p1561518	A-genome specific (rapa only)	not scorable	not scorable	not scorable	not scorable	1	0.8855544
####	Bn-A05-p1566005	A-genome specific (rapa only)	not scorable	not scorable	not scorable	not scorable	1	0.8566455
####	Bn-A05-p15663987	A-genome specific (napus-rapa)	1	0.8448036	not scorable	not scorable	1	0.7673309
####	Bn-A05-p15670035	A-genome specific (napus-rapa)	0.9952153	0.7650651	not scorable	not scorable	0.9917355	0.8241062

####	Bn-A05-p15676718	A-genome specific (napus-rapa)	1	0.616855	not scorable	not scorable	0.9834711	0.8207097
####	Bn-A05-p15679327	A-genome specific (napus-rapa)	0.9904306	0.7569318	not scorable	not scorable	0.9917355	0.8894345
####	Bn-A05-p15702744	A-genome specific (napus-rapa)	1	0.9138703	not scorable	not scorable	1	0.7590037
####	Bn-A05-p1578074	A-genome specific (napus-rapa)	0.9880383	0.8222545	not scorable	not scorable	0.9917355	0.7236798
####	Bn-A05-p15780983	A-genome specific (napus-rapa)	0.9976076	0.5722775	not scorable	not scorable	0.9752066	0.6745558
####	Bn-A05-p15789974	A-genome specific (napus-rapa)	1	0.7099044	not scorable	not scorable	0.9834711	0.7584304
####	Bn-A05-p1579155	A-genome specific (napus-rapa)	0.9880383	0.7970954	not scorable	not scorable	1	0.827274
####	Bn-A05-p1579208	A-genome specific (napus-rapa)	0.9904306	0.7943621	not scorable	not scorable	1	0.7182841
####	Bn-A05-p15797839	A-genome specific (napus-rapa)	1	0.7414995	not scorable	not scorable	1	0.7989914
####	Bn-A05-p15802321	A-genome specific (napus-rapa)	1	0.7275501	not scorable	not scorable	0.9917355	0.7508512
####	Bn-A05-p1580246	A-genome specific (napus-rapa)	0.9856459	0.4330649	not scorable	not scorable	1	0.567142
####	Bn-A05-p15805806	A-genome specific (napus-rapa)	1	0.7254026	not scorable	not scorable	1	0.7254937
####	Bn-A05-p15805908	A-genome specific (napus-rapa)	0.9976076	0.7906715	not scorable	not scorable	0.9752066	0.7895731
####	Bn-A05-p15808942	A-genome specific (napus-rapa)	0.9952153	0.7964587	not scorable	not scorable	0.9917355	0.7472867
####	Bn-A05-p15812933	A-genome specific (napus-rapa)	1	0.5405614	not scorable	not scorable	0.9834711	0.736235
####	Bn-A05-p15859690	A-genome specific (napus-rapa)	1	0.5161004	not scorable	not scorable	0.9834711	0.1651634
####	Bn-A05-p15867343	A-genome specific (napus-rapa)	0.9760765	0.4371895	not scorable	not scorable	0.9917355	0.755999
####	Bn-A05-p15868478	A-genome specific (napus-rapa)	0.9641148	0.5863327	not scorable	not scorable	0.9752066	0.7075087
####	Bn-A05-p15894915	A-genome specific (napus-rapa)	0.9808612	0.4722785	not scorable	not scorable	1	0.8414277
####	Bn-A05-p15905187	A-genome specific (napus-rapa)	0.9856459	0.7799413	not scorable	not scorable	0.9834711	0.7659622
####	Bn-A05-p15906659	A-genome specific (napus-rapa)	0.9832536	0.3729982	not scorable	not scorable	0.9752066	0.5912985
####	Bn-A05-p15938967	A-genome specific (rapa only)	not scorable	not scorable	not scorable	not scorable	0.9752066	0.7252899
####	Bn-A05-p15940232	A-genome specific (napus-rapa)	0.9617225	0.7653453	not scorable	not scorable	0.9917355	0.841803
####	Bn-A05-p15948690	A-genome specific (napus-rapa)	0.9641148	0.2776361	not scorable	not scorable	1	0.6763525
####	Bn-A05-p16026452	A-genome specific (napus-rapa)	0.9856459	0.5221619	not scorable	not scorable	0.9917355	0.7448257
####	Bn-A05-p16030815	A-genome specific (napus-rapa)	0.9832536	0.6568515	not scorable	not scorable	0.9834711	0.6936035
####	Bn-A05-p16039471	A-genome specific (napus-rapa)	0.9856459	0.4687572	not scorable	not scorable	1	0.5967325
####	Bn-A05-p16051992	A-genome specific (napus-rapa)	0.9952153	0.4489051	not scorable	not scorable	0.5702479	0.7993333
####	Bn-A05-p16054367	A-genome specific (napus-rapa)	0.9832536	0.2861169	not scorable	not scorable	0.9917355	0.7242475
####	Bn-A05-p16092993	A-genome specific (napus-rapa)	0.9832536	0.7552835	not scorable	not scorable	0.9752066	0.7811423
####	Bn-A05-p16098564	A-genome specific (napus-rapa)	0.9712918	0.4569332	not scorable	not scorable	0.9834711	0.6892272
####	Bn-A05-p16099943	A-genome specific (napus-rapa)	0.9784689	0.5994108	not scorable	not scorable	0.9752066	0.5960335
####	Bn-A05-p16099945	A-genome specific (napus-rapa)	0.9880383	0.6694568	not scorable	not scorable	0.9504132	0.6247362

####	Bn-A05-p16106702	A-genome specific (napus-rapa)	0.9808612	0.7536811	not scorable	not scorable	0.9752066	0.7725515
####	Bn-A05-p16106977	A-genome specific (napus-rapa)	0.9856459	0.7788095	not scorable	not scorable	0.9834711	0.7843596
####	Bn-A05-p16109920	A-genome specific (napus-rapa)	0.9760765	0.7084873	not scorable	not scorable	0.9586777	0.7022573
####	Bn-A05-p16167725	A-genome specific (napus-rapa)	0.9665072	0.4833287	not scorable	not scorable	0.9421487	0.5372674
####	Bn-A05-p16183886	A-genome specific (napus-rapa)	0.9712918	0.1909826	not scorable	not scorable	0.9834711	0.7687289
####	Bn-A05-p16186321	A-genome specific (napus-rapa)	0.9712918	0.7193025	not scorable	not scorable	0.9917355	0.752258
####	Bn-A05-p16195531	A-genome specific (napus-rapa)	0.9712918	0.3349337	not scorable	not scorable	0.9917355	0.7427092
####	Bn-A05-p16196113	A-genome specific (napus-rapa)	0.9760765	0.3908678	not scorable	not scorable	0.9834711	0.7953966
####	Bn-A05-p16209763	A-genome specific (napus-rapa)	0.9808612	0.7459595	not scorable	not scorable	1	0.8141569
####	Bn-A05-p16224115	A-genome specific (napus-rapa)	0.9832536	0.7737777	not scorable	not scorable	1	0.7656431
####	Bn-A05-p16224273	A-genome specific (napus-rapa)	0.9784689	0.6120229	not scorable	not scorable	0.9669421	0.682959
####	Bn-A05-p16225090	A-genome specific (napus-rapa)	0.9832536	0.7134193	not scorable	not scorable	0.9669421	0.6872365
####	Bn-A05-p16230123	A-genome specific (napus-rapa)	0.9880383	0.6459444	not scorable	not scorable	0.9917355	0.6649489
####	Bn-A05-p16234543	A-genome specific (napus-rapa)	0.9856459	0.6698401	not scorable	not scorable	0.9917355	0.8235812
####	Bn-A05-p16240970	A-genome specific (napus-rapa)	0.9760765	0.7849498	not scorable	not scorable	0.9834711	0.7071137
####	Bn-A05-p16244846	A-genome specific (napus-rapa)	0.9784689	0.4673198	not scorable	not scorable	0.9752066	0.5983363
####	Bn-A05-p16245038	A-genome specific (napus-rapa)	0.9808612	0.7848592	not scorable	not scorable	0.9834711	0.7905645
####	Bn-A05-p16260356	A-genome specific (napus-rapa)	0.9593301	0.4803911	not scorable	not scorable	1	0.7976328
####	Bn-A05-p16260894	A-genome specific (napus-rapa)	0.9760765	0.773262	not scorable	not scorable	0.9917355	0.7936016
####	Bn-A05-p16272686	A-genome specific (napus-rapa)	0.9976076	0.4462278	not scorable	not scorable	0.9917355	0.7161742
####	Bn-A05-p16274076	A-genome specific (napus-rapa)	1	0.8394041	not scorable	not scorable	0.9834711	0.7511635
####	Bn-A05-p1630204	A-genome specific (napus-rapa)	1	0.3513969	not scorable	not scorable	1	0.7705783
####	Bn-A05-p16305594	A-genome specific (napus-rapa)	1	0.5788181	not scorable	not scorable	1	0.5959508
####	Bn-A05-p16338133	A-genome specific (napus-rapa)	1	0.8122911	not scorable	not scorable	0.9338843	0.7600561
####	Bn-A05-p16359545	A-genome specific (napus-rapa)	1	0.4620944	not scorable	not scorable	0.9834711	0.7584956
####	Bn-A05-p16392699	A-genome specific (napus-rapa)	0.9904306	0.504046	not scorable	not scorable	0.9834711	0.8498172
####	Bn-A05-p16406388	A-genome specific (napus-rapa)	1	0.7764187	not scorable	not scorable	0.9834711	0.8940311
####	Bn-A05-p16422457	A-genome specific (napus-rapa)	0.9976076	0.5096543	not scorable	not scorable	1	0.6684838
####	Bn-A05-p16460167	A-genome specific (napus-rapa)	0.9976076	0.7073627	not scorable	not scorable	0.9752066	0.7077425
####	Bn-A05-p16468152	A-genome specific (rapa only)	not scorable	not scorable	not scorable	not scorable	0.6363636	0.8308049
####	Bn-A05-p16471616	A-genome specific (napus-rapa)	0.9952153	0.5745972	not scorable	not scorable	0.9834711	0.7855946
####	Bn-A05-p16477848	A-genome specific (napus-rapa)	0.9952153	0.8168548	not scorable	not scorable	0.6363636	0.7369214
####	Bn-A05-p16489462	A-genome specific (napus-rapa)	0.9665072	0.6599779	not scorable	not scorable	0.9917355	0.88727

####	Bn-A05-p16490679	A-genome specific (napus-rapa)	0.9856459	0.7090759	not scorable	not scorable	1	0.7238823
####	Bn-A05-p16506888	A-genome specific (napus-rapa)	0.9880383	0.5703408	not scorable	not scorable	0.9752066	0.6959504
####	Bn-A05-p16507044	A-genome specific (napus-rapa)	0.9904306	0.7790682	not scorable	not scorable	1	0.6710737
####	Bn-A05-p16507306	A-genome specific (napus-rapa)	0.9952153	0.7963493	not scorable	not scorable	0.9669421	0.7681305
####	Bn-A05-p16509283	A-genome specific (napus-rapa)	0.9952153	0.5517134	not scorable	not scorable	1	0.7711137
####	Bn-A05-p16538605	A-genome specific (napus-rapa)	0.9880383	0.3249863	not scorable	not scorable	0.9669421	0.4571435
####	Bn-A05-p16544454	A-genome specific (napus-rapa)	1	0.6414527	not scorable	not scorable	0.9834711	0.7508258
####	Bn-A05-p16545436	A-genome specific (napus-rapa)	0.9952153	0.8058719	not scorable	not scorable	0.9669421	0.7720716
####	Bn-A05-p16546416	A-genome specific (napus-rapa)	0.9976076	0.7037584	not scorable	not scorable	0.9917355	0.7293484
####	Bn-A05-p16553141	A-genome specific (napus-rapa)	0.9928229	0.7601622	not scorable	not scorable	0.9917355	0.7748176
####	Bn-A05-p16561237	A-genome specific (napus-rapa)	0.9880383	0.3928767	not scorable	not scorable	0.9917355	0.7400256
####	Bn-A05-p16581289	A-genome specific (napus-rapa)	0.9928229	0.6671382	not scorable	not scorable	0.9752066	0.7299423
####	Bn-A05-p16588569	A-genome specific (napus-rapa)	0.9832536	0.793039	not scorable	not scorable	0.9834711	0.7318531
####	Bn-A05-p16590105	A-genome specific (napus-rapa)	0.9976076	0.5835805	not scorable	not scorable	1	0.830209
####	Bn-A05-p16625783	A-genome specific (napus-rapa)	0.9976076	0.5292512	not scorable	not scorable	0.9917355	0.6822277
####	Bn-A05-p16637476	A-genome specific (napus-rapa)	0.9976076	0.4658909	not scorable	not scorable	0.9834711	0.863726
####	Bn-A05-p16648406	A-genome specific (rapa only)	not scorable	not scorable	not scorable	not scorable	0.9834711	0.7949702
####	Bn-A05-p16651858	A-genome specific (napus-rapa)	0.9904306	0.6212329	not scorable	not scorable	0.9504132	0.7778232
####	Bn-A05-p16652401	A-genome specific (napus-rapa)	0.9952153	0.7843031	not scorable	not scorable	0.9752066	0.5959725
####	Bn-A05-p16664411	A-genome specific (rapa only)	not scorable	not scorable	not scorable	not scorable	0.9752066	0.5841213
####	Bn-A05-p16671522	A-genome specific (napus-rapa)	0.9904306	0.8205975	not scorable	not scorable	0.9917355	0.8478391
####	Bn-A05-p1671823	A-genome specific (napus-rapa)	1	0.8179615	not scorable	not scorable	1	0.7789278
####	Bn-A05-p16735186	A-genome specific (napus-rapa)	0.9976076	0.7844908	not scorable	not scorable	0.9256198	0.8222046
####	Bn-A05-p16738292	A-genome specific (napus-rapa)	0.9856459	0.5365632	not scorable	not scorable	1	0.7554443
####	Bn-A05-p16738871	A-genome specific (napus-rapa)	0.9856459	0.7097429	not scorable	not scorable	1	0.7977925
####	Bn-A05-p16747703	A-genome specific (napus-rapa)	0.9736842	0.2536981	not scorable	not scorable	1	0.7399379
####	Bn-A05-p16747797	A-genome specific (napus-rapa)	0.9784689	0.7949107	not scorable	not scorable	0.9917355	0.7079124
####	Bn-A05-p16754780	A-genome specific (napus-rapa)	0.9760765	0.7943084	not scorable	not scorable	0.9917355	0.7343701
####	Bn-A05-p16755625	A-genome specific (napus-rapa)	0.9712918	0.5163674	not scorable	not scorable	0.9421487	0.7700818
####	Bn-A05-p1676024	A-genome specific (napus-rapa)	0.9880383	0.8057048	not scorable	not scorable	0.6694215	0.85427
####	Bn-A05-p16775295	all not scorable	not scorable	not scorable	not scorable	not scorable	not scorable	not scorable
####	Bn-A05-p16789633	A-genome specific (napus-rapa)	0.9808612	0.688322	not scorable	not scorable	0.9917355	0.694905
####	Bn-A05-p16789808	A-genome specific (napus-rapa)	0.9784689	0.7539029	not scorable	not scorable	0.9834711	0.7533532

####	Bn-A05-p1679589	A-genome specific (napus-rapa)	0.9976076	0.5277396	not scorable	not scorable	0.9752066	0.8038052
####	Bn-A05-p16801582	genome unspecific (oleracea-rapa)	not scorable	not scorable	0.7829458	0.7604458	1	0.9244093
####	Bn-A05-p16811156	A-genome specific (napus-rapa)	0.9688995	0.4077357	not scorable	not scorable	1	0.8722049
####	Bn-A05-p16833526	A-genome specific (napus-rapa)	0.9856459	0.7660205	not scorable	not scorable	1	0.9157199
####	Bn-A05-p16834136	A-genome specific (napus-rapa)	0.9617225	0.3424505	not scorable	not scorable	1	0.8064858
####	Bn-A05-p16835570	A-genome specific (napus-rapa)	0.9784689	0.7846854	not scorable	not scorable	0.9917355	0.8325015
####	Bn-A05-p16836518	A-genome specific (napus-rapa)	0.9808612	0.7881526	not scorable	not scorable	1	0.6742994
####	Bn-A05-p16842656	A-genome specific (napus-rapa)	0.9712918	0.5759544	not scorable	not scorable	0.9917355	0.7929825
####	Bn-A05-p16844203	A-genome specific (napus-rapa)	0.9401914	0.3952431	not scorable	not scorable	0.9917355	0.6075105
####	Bn-A05-p16844504	A-genome specific (napus-rapa)	0.9784689	0.7800228	not scorable	not scorable	0.9917355	0.7452869
####	Bn-A05-p16846880	A-genome specific (napus-rapa)	0.9856459	0.7552933	not scorable	not scorable	0.9752066	0.8392952
####	Bn-A05-p16848755	A-genome specific (napus-rapa)	0.9808612	0.6118512	not scorable	not scorable	1	0.801263
####	Bn-A05-p16862109	A-genome specific (napus-rapa)	0.9760765	0.5934374	not scorable	not scorable	1	0.9175813
####	Bn-A05-p16863502	A-genome specific (napus-rapa)	0.576555	0.8212944	not scorable	not scorable	0.6280992	0.7867437
####	Bn-A05-p16867347	A-genome specific (napus-rapa)	0.9688995	0.7496771	not scorable	not scorable	1	0.7554558
####	Bn-A05-p16892759	A-genome specific (napus-rapa)	0.9904306	0.7620708	not scorable	not scorable	0.9834711	0.6522584
####	Bn-A05-p1689277	A-genome specific (napus-rapa)	0.9497608	0.7717922	not scorable	not scorable	0.9752066	0.7805854
####	Bn-A05-p16897600	A-genome specific (napus-rapa)	0.937799	0.7428461	not scorable	not scorable	0.5702479	0.6744905
####	Bn-A05-p16898305	A-genome specific (napus-rapa)	1	0.7450127	not scorable	not scorable	0.9752066	0.8274895
####	Bn-A05-p16900864	A-genome specific (napus-rapa)	0.9760765	0.7494133	not scorable	not scorable	1	0.7868506
####	Bn-A05-p1690598	A-genome specific (napus-rapa)	0.9976076	0.6642578	not scorable	not scorable	0.9917355	0.6813819
####	Bn-A05-p16909798	A-genome specific (napus-rapa)	0.9784689	0.6453311	not scorable	not scorable	0.9504132	0.5804911
####	Bn-A05-p16909956	genome unspecific (all in)	0.9952153	0.2493585	0.9457364	0.2621864	1	0.76249
####	Bn-A05-p1692254	A-genome specific (napus-rapa)	0.9736842	0.7878061	not scorable	not scorable	0.9834711	0.7818343
####	Bn-A05-p1692692	A-genome specific (napus-rapa)	0.9856459	0.7880577	not scorable	not scorable	0.9752066	0.7233731
####	Bn-A05-p1693000	A-genome specific (napus-rapa)	0.9760765	0.8224052	not scorable	not scorable	1	0.794052
####	Bn-A05-p1693196	A-genome specific (napus-rapa)	0.9808612	0.6897439	not scorable	not scorable	0.9752066	0.7935562
####	Bn-A05-p16935999	A-genome specific (napus-rapa)	0.9928229	0.7149521	not scorable	not scorable	0.9834711	0.7774039
####	Bn-A05-p16938263	A-genome specific (napus-rapa)	0.9904306	0.5446244	not scorable	not scorable	1	0.9055923
####	Bn-A05-p16942305	A-genome specific (rapa only)	not scorable	not scorable	not scorable	not scorable	0.9917355	0.895419
####	Bn-A05-p16953641	A-genome specific (napus-rapa)	0.5358852	0.7500644	not scorable	not scorable	0.9917355	0.7161742
####	Bn-A05-p1695394	A-genome specific (napus-rapa)	0.9784689	0.7629402	not scorable	not scorable	0.9917355	0.750471
####	Bn-A05-p1695552	A-genome specific (napus-rapa)	0.9880383	0.7842772	not scorable	not scorable	1	0.7796711

####	Bn-A05-p1696330	A-genome specific (napus-rapa)	0.9832536	0.7748728	not scorable	not scorable	1	0.7703344
####	Bn-A05-p1696411	A-genome specific (napus-rapa)	0.9736842	0.7862639	not scorable	not scorable	1	0.7787101
####	Bn-A05-p1696613	A-genome specific (napus-rapa)	0.9760765	0.7705297	not scorable	not scorable	1	0.7559938
####	Bn-A05-p1697018	A-genome specific (napus-rapa)	0.9880383	0.7715172	not scorable	not scorable	0.9917355	0.7758816
####	Bn-A05-p1697401	A-genome specific (napus-rapa)	1	0.5611373	not scorable	not scorable	0.9917355	0.7371728
####	Bn-A05-p1701030	A-genome specific (napus-rapa)	0.8349282	0.7862386	not scorable	not scorable	0.6280992	0.7525893
####	Bn-A05-p17093469	A-genome specific (napus-rapa)	1	0.6037034	not scorable	not scorable	1	0.7603049
####	Bn-A05-p17108506	genome unspecific (all in)	0.9976076	0.4627327	0.9922481	0.7803835	0.9917355	0.6951741
####	Bn-A05-p17202622	A-genome specific (napus-rapa)	0.9952153	0.7829521	not scorable	not scorable	1	0.9205657
####	Bn-A05-p17203695	A-genome specific (napus-rapa)	1	0.5051728	not scorable	not scorable	1	0.7245421
####	Bn-A05-p17215153	A-genome specific (napus-rapa)	1	0.6774176	not scorable	not scorable	0.9917355	0.7125033
####	Bn-A05-p17241091	A-genome specific (napus-rapa)	0.9856459	0.5002236	not scorable	not scorable	1	0.7279716
####	Bn-A05-p17243214	A-genome specific (napus-rapa)	0.9928229	0.8194101	not scorable	not scorable	1	0.7231084
####	Bn-A05-p17262653	A-genome specific (napus-rapa)	0.9880383	0.326676	not scorable	not scorable	1	0.7319344
####	Bn-A05-p17292224	A-genome specific (napus-rapa)	0.9832536	0.5085568	not scorable	not scorable	0.9917355	0.5967978
####	Bn-A05-p17330707	napus specific	0.9617225	0.6767837	not scorable	not scorable	not scorable	not scorable
####	Bn-A05-p17366544	A-genome specific (napus-rapa)	0.2990431	0.4987206	not scorable	not scorable	0.9256198	0.8912995
####	Bn-A05-p17368155	A-genome specific (napus-rapa)	0.9928229	0.7637491	not scorable	not scorable	0.9917355	0.7006459
####	Bn-A05-p17392998	A-genome specific (napus-rapa)	0.9904306	0.8037758	not scorable	not scorable	0.9917355	0.8049123
####	Bn-A05-p17399423	A-genome specific (napus-rapa)	0.9712918	0.6582587	not scorable	not scorable	1	0.8056542
####	Bn-A05-p17402697	A-genome specific (napus-rapa)	0.9736842	0.4541373	not scorable	not scorable	1	0.7795539
####	Bn-A05-p17417422	A-genome specific (napus-rapa)	0.9736842	0.3474081	not scorable	not scorable	1	0.8360887
####	Bn-A05-p17419591	A-genome specific (napus-rapa)	0.8397129	0.7464461	not scorable	not scorable	0.9752066	0.715128
####	Bn-A05-p17420484	A-genome specific (napus-rapa)	0.9880383	0.5239101	not scorable	not scorable	0.9834711	0.7253301
####	Bn-A05-p17420613	A-genome specific (napus-rapa)	0.9832536	0.73572	not scorable	not scorable	1	0.7278512
####	Bn-A05-p17442162	A-genome specific (napus-rapa)	0.9952153	0.792631	not scorable	not scorable	0.8099173	0.9174066
####	Bn-A05-p1744534	A-genome specific (napus-rapa)	0.9617225	0.7774826	not scorable	not scorable	0.9669421	0.7252018
####	Bn-A05-p17445937	A-genome specific (napus-rapa)	1	0.5558235	not scorable	not scorable	0.9752066	0.8257785
####	Bn-A05-p17482579	A-genome specific (napus-rapa)	1	0.4511294	not scorable	not scorable	1	0.7933664
####	Bn-A05-p17511337	A-genome specific (napus-rapa)	0.9880383	0.8150228	not scorable	not scorable	1	0.8239708
####	Bn-A05-p17514353	A-genome specific (napus-rapa)	0.9976076	0.8090102	not scorable	not scorable	0.9834711	0.7426201
####	Bn-A05-p17542638	A-genome specific (napus-rapa)	1	0.4473538	not scorable	not scorable	1	0.7482538
####	Bn-A05-p17544675	A-genome specific (napus-rapa)	1	0.6870948	not scorable	not scorable	0.9834711	0.6459777

####	Bn-A05-p17564038	A-genome specific (napus-rapa)	0.9904306	0.6127589	not scorable	not scorable	0.9752066	0.7137067
####	Bn-A05-p17566846	A-genome specific (napus-rapa)	0.9952153	0.3123011	not scorable	not scorable	0.9917355	0.6951149
####	Bn-A05-p17567179	A-genome specific (napus-rapa)	0.9808612	0.5152463	not scorable	not scorable	0.9917355	0.8127797
####	Bn-A05-p17568606	A-genome specific (napus-rapa)	0.9928229	0.7597451	not scorable	not scorable	1	0.7856432
####	Bn-A05-p17587730	genome unspecific (oleracea-rapa)	not scorable	not scorable	0.9844961	0.7984183	1	0.6624619
####	Bn-A05-p1761473	A-genome specific (napus-rapa)	0.9712918	0.7745822	not scorable	not scorable	0.9917355	0.7014567
####	Bn-A05-p17614751	A-genome specific (napus-rapa)	0.9952153	0.6897087	not scorable	not scorable	1	0.6641342
####	Bn-A05-p17615064	A-genome specific (napus-rapa)	0.9976076	0.5212131	not scorable	not scorable	1	0.8379771
####	Bn-A05-p17616044	A-genome specific (napus-rapa)	0.9976076	0.8161885	not scorable	not scorable	1	0.9222978
####	Bn-A05-p17616132	A-genome specific (napus-rapa)	0.9904306	0.7743224	not scorable	not scorable	0.9917355	0.740004
####	Bn-A05-p17617527	A-genome specific (napus-rapa)	1	0.4264731	not scorable	not scorable	1	0.8815182
####	Bn-A05-p17617724	A-genome specific (napus-rapa)	1	0.7299884	not scorable	not scorable	0.9917355	0.766555
####	Bn-A05-p1762229	A-genome specific (napus-rapa)	0.9688995	0.6995797	not scorable	not scorable	0.6528926	0.7378951
####	Bn-A05-p17624348	A-genome specific (napus-rapa)	0.9952153	0.8596416	not scorable	not scorable	1	0.9235974
####	Bn-A05-p17625934	A-genome specific (napus-rapa)	0.9952153	0.8139869	not scorable	not scorable	1	0.902242
####	Bn-A05-p17625943	A-genome specific (napus-rapa)	0.9976076	0.8286158	not scorable	not scorable	0.9917355	0.9022775
####	Bn-A05-p1764530	A-genome specific (napus-rapa)	0.9856459	0.4993658	not scorable	not scorable	0.9917355	0.4631481
####	Bn-A05-p1765880	A-genome specific (napus-rapa)	0.9665072	0.664854	not scorable	not scorable	0.9834711	0.642917
####	Bn-A05-p1766223	A-genome specific (napus-rapa)	0.9425837	0.7807313	not scorable	not scorable	0.9669421	0.7673622
####	Bn-A05-p17675829	A-genome specific (napus-rapa)	1	0.4401192	not scorable	not scorable	0.9917355	0.7295379
####	Bn-A05-p17708970	A-genome specific (napus-rapa)	1	0.3174834	not scorable	not scorable	0.9669421	0.7841673
####	Bn-A05-p17739455	A-genome specific (napus-rapa)	1	0.7352515	not scorable	not scorable	0.9917355	0.7067034
####	Bn-A05-p17750022	A-genome specific (napus-rapa)	0.9976076	0.7796417	not scorable	not scorable	0.9834711	0.8053109
####	Bn-A05-p1775540	A-genome specific (napus-rapa)	0.9856459	0.5699703	not scorable	not scorable	0.9834711	0.7337215
####	Bn-A05-p17758084	A-genome specific (napus-rapa)	1	0.26791	not scorable	not scorable	0.9834711	0.531085
####	Bn-A05-p17761769	A-genome specific (napus-rapa)	0.9976076	0.7003805	not scorable	not scorable	0.9917355	0.8154237
####	Bn-A05-p17770256	A-genome specific (napus-rapa)	0.9928229	0.7709633	not scorable	not scorable	0.9834711	0.6791053
####	Bn-A05-p17774694	A-genome specific (napus-rapa)	0.9880383	0.5366027	not scorable	not scorable	0.9917355	0.6648225
####	Bn-A05-p17799506	A-genome specific (napus-rapa)	1	0.4596072	not scorable	not scorable	0.9917355	0.7995193
####	Bn-A05-p17822322	A-genome specific (napus-rapa)	0.9904306	0.2578293	not scorable	not scorable	1	0.4994062
####	Bn-A05-p17830597	A-genome specific (napus-rapa)	0.9952153	0.7972015	not scorable	not scorable	0.9917355	0.7214636
####	Bn-A05-p17835705	napus specific	0.9712918	0.5217221	not scorable	not scorable	not scorable	not scorable
####	Bn-A05-p17880874	A-genome specific (napus-rapa)	0.9952153	0.7459006	not scorable	not scorable	0.9834711	0.6697192

####	Bn-A05-p17894045	A-genome specific (napus-rapa)	1	0.2957292	not scorable	not scorable	1	0.7984077
####	Bn-A05-p17895197	A-genome specific (napus-rapa)	0.9928229	0.7835103	not scorable	not scorable	1	0.8408566
####	Bn-A05-p17913760	A-genome specific (napus-rapa)	0.9976076	0.4657085	not scorable	not scorable	1	0.7769049
####	Bn-A05-p17928363	A-genome specific (rapa only)	not scorable	not scorable	not scorable	not scorable	0.9669421	0.6306389
####	Bn-A05-p17931505	A-genome specific (napus-rapa)	0.9617225	0.7360166	not scorable	not scorable	0.9586777	0.6121697
####	Bn-A05-p1806574	A-genome specific (napus-rapa)	0.9760765	0.784379	not scorable	not scorable	0.9834711	0.6773961
####	Bn-A05-p18066693	A-genome specific (napus-rapa)	1	0.8848283	not scorable	not scorable	1	0.8687926
####	Bn-A05-p18088421	A-genome specific (napus-rapa)	0.9976076	0.334849	not scorable	not scorable	0.9917355	0.7883866
####	Bn-A05-p18101769	A-genome specific (napus-rapa)	0.9688995	0.7995363	not scorable	not scorable	1	0.7945122
####	Bn-A05-p18109527	A-genome specific (napus-rapa)	0.9832536	0.7878743	not scorable	not scorable	1	0.8055421
####	Bn-A05-p18109536	A-genome specific (napus-rapa)	0.9880383	0.7339863	not scorable	not scorable	0.9917355	0.758664
####	Bn-A05-p18112139	A-genome specific (napus-rapa)	0.9880383	0.7971956	not scorable	not scorable	0.9917355	0.7398812
####	Bn-A05-p18118640	A-genome specific (napus-rapa)	0.9401914	0.4276387	not scorable	not scorable	0.9917355	0.7683113
####	Bn-A05-p18125663	A-genome specific (napus-rapa)	0.9712918	0.7962593	not scorable	not scorable	0.9752066	0.8088316
####	Bn-A05-p18135244	A-genome specific (napus-rapa)	0.9043062	0.7670919	not scorable	not scorable	0.5785124	0.6815456
####	Bn-A05-p18137410	A-genome specific (napus-rapa)	0.9736842	0.837493	not scorable	not scorable	0.9669421	0.7091826
####	Bn-A05-p18139526	A-genome specific (napus-rapa)	0.9569378	0.704061	not scorable	not scorable	0.6694215	0.6691558
####	Bn-A05-p18147040	A-genome specific (napus-rapa)	0.9928229	0.6531543	not scorable	not scorable	0.6528926	0.7110949
####	Bn-A05-p18161591	A-genome specific (napus-rapa)	0.9449761	0.7081791	not scorable	not scorable	0.9834711	0.7166088
####	Bn-A05-p1818846	A-genome specific (napus-rapa)	0.9880383	0.524544	not scorable	not scorable	0.9917355	0.7604629
####	Bn-A05-p18191062	A-genome specific (napus-rapa)	0.9736842	0.5829559	not scorable	not scorable	1	0.8098131
####	Bn-A05-p18196942	A-genome specific (napus-rapa)	0.9856459	0.2930158	not scorable	not scorable	0.9917355	0.6575781
####	Bn-A05-p18214148	A-genome specific (napus-rapa)	0.9880383	0.7865417	not scorable	not scorable	0.9834711	0.6974153
####	Bn-A05-p18223406	A-genome specific (napus-rapa)	0.9688995	0.4363306	not scorable	not scorable	0.9917355	0.6892233
####	Bn-A05-p18229790	A-genome specific (napus-rapa)	0.9904306	0.6918726	not scorable	not scorable	0.9669421	0.7156951
####	Bn-A05-p182664	A-genome specific (napus-rapa)	0.9258373	0.3784495	not scorable	not scorable	0.9917355	0.6883869
####	Bn-A05-p18306160	A-genome specific (napus-rapa)	0.9904306	0.5774844	not scorable	not scorable	0.9917355	0.7680172
####	Bn-A05-p18309576	A-genome specific (napus-rapa)	0.9569378	0.5543652	not scorable	not scorable	0.9917355	0.7454807
####	Bn-A05-p18310658	A-genome specific (napus-rapa)	0.9736842	0.4239044	not scorable	not scorable	0.9834711	0.7794924
####	Bn-A05-p18310838	A-genome specific (napus-rapa)	0.9641148	0.7562679	not scorable	not scorable	1	0.7997552
####	Bn-A05-p18349076	genome unspecific (oleracea-rapa)	not scorable	not scorable	0.9534883	0.2591399	0.7272727	0.5172655
####	Bn-A05-p18376815	genome unspecific (oleracea-rapa)	not scorable	not scorable	1	0.8176717	1	0.9280821
####	Bn-A05-p18391243	A-genome specific (napus-rapa)	0.9832536	0.3704997	not scorable	not scorable	1	0.6827843

####	Bn-A05-p18391581	A-genome specific (napus-rapa)	0.9688995	0.8121782	not scorable	not scorable	1	0.8106832
####	Bn-A05-p18452462	A-genome specific (napus-rapa)	0.9904306	0.5025557	not scorable	not scorable	1	0.9057909
####	Bn-A05-p18452563	A-genome specific (napus-rapa)	0.9808612	0.2969941	not scorable	not scorable	1	0.7571141
####	Bn-A05-p18453140	A-genome specific (napus-rapa)	0.9760765	0.3244177	not scorable	not scorable	0.9834711	0.6703537
####	Bn-A05-p18458181	A-genome specific (napus-rapa)	0.9760765	0.28783	not scorable	not scorable	0.9917355	0.6964967
####	Bn-A05-p18475836	A-genome specific (napus-rapa)	0.9856459	0.4475583	not scorable	not scorable	0.9834711	0.8390219
####	Bn-A05-p18480152	A-genome specific (napus-rapa)	0.9712918	0.2669426	not scorable	not scorable	0.9834711	0.7198011
####	Bn-A05-p18490530	A-genome specific (napus-rapa)	0.9688995	0.7209903	not scorable	not scorable	0.9834711	0.8227723
####	Bn-A05-p18496803	A-genome specific (napus-rapa)	0.9856459	0.3156546	not scorable	not scorable	0.9917355	0.7618081
####	Bn-A05-p18505935	A-genome specific (napus-rapa)	0.9760765	0.7335835	not scorable	not scorable	1	0.711411
####	Bn-A05-p18528112	A-genome specific (napus-rapa)	0.9856459	0.3836942	not scorable	not scorable	1	0.7009844
####	Bn-A05-p18543237	A-genome specific (napus-rapa)	0.9736842	0.6104291	not scorable	not scorable	0.9917355	0.5937957
####	Bn-A05-p18543933	A-genome specific (napus-rapa)	0.9784689	0.7968438	not scorable	not scorable	0.9669421	0.751047
####	Bn-A05-p1854510	A-genome specific (napus-rapa)	0.9665072	0.8455062	not scorable	not scorable	1	0.7174947
####	Bn-A05-p18547040	A-genome specific (napus-rapa)	0.9952153	0.4680369	not scorable	not scorable	1	0.7574129
####	Bn-A05-p1857946	A-genome specific (napus-rapa)	0.9952153	0.7790803	not scorable	not scorable	0.9834711	0.7758102
####	Bn-A05-p18590326	A-genome specific (napus-rapa)	0.9832536	0.5050199	not scorable	not scorable	0.9917355	0.7871014
####	Bn-A05-p18598001	A-genome specific (napus-rapa)	0.9665072	0.2669114	not scorable	not scorable	0.9917355	0.7021827
####	Bn-A05-p18615644	A-genome specific (napus-rapa)	0.9832536	0.7493223	not scorable	not scorable	1	0.6383966
####	Bn-A05-p18616260	A-genome specific (napus-rapa)	0.9760765	0.7105904	not scorable	not scorable	1	0.9036612
####	Bn-A05-p18627784	A-genome specific (napus-rapa)	0.9976076	0.237393	not scorable	not scorable	0.9834711	0.2942947
####	Bn-A05-p18637087	A-genome specific (napus-rapa)	0.9928229	0.6785161	not scorable	not scorable	1	0.6441295
####	Bn-A05-p18642842	A-genome specific (napus-rapa)	0.8684211	0.8694867	not scorable	not scorable	0.9586777	0.8305073
####	Bn-A05-p18643459	A-genome specific (rapa only)	not scorable	not scorable	not scorable	not scorable	0.9752066	0.7495101
####	Bn-A05-p18682729	A-genome specific (napus-rapa)	0.9736842	0.7901246	not scorable	not scorable	0.6363636	0.7793339
####	Bn-A05-p18683201	A-genome specific (rapa only)	not scorable	not scorable	not scorable	not scorable	0.661157	0.8288758
####	Bn-A05-p18687031	A-genome specific (napus-rapa)	0.9641148	0.8402984	not scorable	not scorable	0.9917355	0.8087777
####	Bn-A05-p18689306	A-genome specific (napus-rapa)	0.9880383	0.3364558	not scorable	not scorable	0.9917355	0.8159032
####	Bn-A05-p18704009	A-genome specific (napus-rapa)	0.9952153	0.7843069	not scorable	not scorable	1	0.7341428
####	Bn-A05-p18704064	A-genome specific (napus-rapa)	0.9832536	0.6833819	not scorable	not scorable	0.9917355	0.6273827
####	Bn-A05-p18704349	A-genome specific (napus-rapa)	0.9904306	0.7359627	not scorable	not scorable	1	0.7461109
####	Bn-A05-p18730286	A-genome specific (napus-rapa)	0.9928229	0.4749652	not scorable	not scorable	1	0.8024037
####	Bn-A05-p18744312	A-genome specific (napus-rapa)	0.9928229	0.7930564	not scorable	not scorable	0.9917355	0.6972361

#### Bn-A05-p18744372	A-genome specific (napus-rapa)	0.9976076	0.8387947	not scorable	not scorable	0.9834711	0.7821184
#### Bn-A05-p18753257	A-genome specific (napus-rapa)	0.9856459	0.7386575	not scorable	not scorable	0.9834711	0.6705433
#### Bn-A05-p18759144	A-genome specific (napus-rapa)	0.9880383	0.5555689	not scorable	not scorable	1	0.8431962
#### Bn-A05-p18761878	A-genome specific (napus-rapa)	0.9784689	0.712483	not scorable	not scorable	0.9917355	0.6847045
#### Bn-A05-p18768801	A-genome specific (napus-rapa)	0.9880383	0.7929792	not scorable	not scorable	0.9917355	0.7793105
#### Bn-A05-p18770393	A-genome specific (rapa only)	not scorable	not scorable	not scorable	not scorable	0.9669421	0.4998976
#### Bn-A05-p18773930	A-genome specific (rapa only)	not scorable	not scorable	not scorable	not scorable	0.9834711	0.7098026
#### Bn-A05-p18793322	A-genome specific (napus-rapa)	0.9856459	0.4054185	not scorable	not scorable	1	0.823873
#### Bn-A05-p18807426	A-genome specific (napus-rapa)	0.9760765	0.8084629	not scorable	not scorable	0.9917355	0.8016225
#### Bn-A05-p18819337	A-genome specific (napus-rapa)	0.9784689	0.7621922	not scorable	not scorable	1	0.8214605
#### Bn-A05-p18819519	A-genome specific (napus-rapa)	0.9712918	0.6016858	not scorable	not scorable	0.9917355	0.6027501
#### Bn-A05-p18819673	A-genome specific (napus-rapa)	0.9736842	0.6826335	not scorable	not scorable	0.9752066	0.653946
#### Bn-A05-p18820057	A-genome specific (napus-rapa)	0.9808612	0.6931797	not scorable	not scorable	1	0.7088397
#### Bn-A05-p18820492	A-genome specific (napus-rapa)	0.9808612	0.7802718	not scorable	not scorable	1	0.7440118
#### Bn-A05-p18828578	A-genome specific (napus-rapa)	0.937799	0.6682511	not scorable	not scorable	0.9669421	0.683764
#### Bn-A05-p18828589	A-genome specific (napus-rapa)	0.9665072	0.5048072	not scorable	not scorable	0.9834711	0.5205449
#### Bn-A05-p18905070	A-genome specific (napus-rapa)	0.9880383	0.5100516	not scorable	not scorable	1	0.7456774
#### Bn-A05-p18922154	A-genome specific (napus-rapa)	0.1650718	0.8128185	not scorable	not scorable	0.9917355	0.6545202
#### Bn-A05-p1893613	A-genome specific (rapa only)	not scorable	not scorable	not scorable	not scorable	0.9917355	0.775069
#### Bn-A05-p18958059	A-genome specific (napus-rapa)	0.9856459	0.4368172	not scorable	not scorable	0.9834711	0.7022696
#### Bn-A05-p18958083	A-genome specific (napus-rapa)	0.9880383	0.6894801	not scorable	not scorable	0.9917355	0.6084838
#### Bn-A05-p1898273	A-genome specific (napus-rapa)	0.9617225	0.816856	not scorable	not scorable	1	0.839677
#### Bn-A05-p19001293	A-genome specific (napus-rapa)	0.9856459	0.6972432	not scorable	not scorable	1	0.8030552
#### Bn-A05-p19017469	A-genome specific (rapa only)	not scorable	not scorable	not scorable	not scorable	0.8842975	0.5867121
#### Bn-A05-p19029505	A-genome specific (napus-rapa)	0.9880383	0.8400073	not scorable	not scorable	0.9752066	0.781377
#### Bn-A05-p19031269	A-genome specific (napus-rapa)	0.9665072	0.7906581	not scorable	not scorable	0.6115702	0.7822942
#### Bn-A05-p19032247	A-genome specific (napus-rapa)	0.9904306	0.4876139	not scorable	not scorable	1	0.6950285
#### Bn-A05-p19041695	A-genome specific (napus-rapa)	0.9928229	0.3772976	not scorable	not scorable	0.9834711	0.7942212
#### Bn-A05-p19048941	A-genome specific (napus-rapa)	0.9736842	0.4222291	not scorable	not scorable	0.9917355	0.7461041
#### Bn-A05-p19064234	A-genome specific (napus-rapa)	0.9832536	0.3355565	not scorable	not scorable	1	0.8202433
#### Bn-A05-p19067421	A-genome specific (napus-rapa)	1	0.8146383	not scorable	not scorable	1	0.839835
#### Bn-A05-p19070214	A-genome specific (napus-rapa)	0.9880383	0.8027963	not scorable	not scorable	0.9917355	0.8378819
#### Bn-A05-p19070284	A-genome specific (napus-rapa)	0.9856459	0.7942677	not scorable	not scorable	0.9834711	0.8151299

####	Bn-A05-p19071118	A-genome specific (rapa only)	not scorable	not scorable	not scorable	not scorable	1	0.8089101
####	Bn-A05-p19096242	A-genome specific (napus-rapa)	0.9904306	0.7253518	not scorable	not scorable	0.9834711	0.7074528
####	Bn-A05-p19105275	A-genome specific (napus-rapa)	0.9904306	0.7743271	not scorable	not scorable	0.9917355	0.8093156
####	Bn-A05-p19105857	A-genome specific (napus-rapa)	0.9904306	0.7165952	not scorable	not scorable	0.9917355	0.6813741
####	Bn-A05-p19116891	genome unspecific (all in)	0.9952153	0.7018828	0.5271318	0.8990383	0.9917355	0.8093607
####	Bn-A05-p19128595	A-genome specific (napus-rapa)	0.9784689	0.7674341	not scorable	not scorable	0.9917355	0.7785748
####	Bn-A05-p19130774	A-genome specific (napus-rapa)	0.9665072	0.8104416	not scorable	not scorable	0.9752066	0.731097
####	Bn-A05-p19178139	A-genome specific (napus-rapa)	0.9928229	0.7858185	not scorable	not scorable	1	0.7645862
####	Bn-A05-p19186596	A-genome specific (napus-rapa)	0.9880383	0.3609344	not scorable	not scorable	1	0.7827481
####	Bn-A05-p19187149	A-genome specific (napus-rapa)	1	0.9137889	not scorable	not scorable	1	0.8031005
####	Bn-A05-p1919060	A-genome specific (napus-rapa)	0.9952153	0.8255541	not scorable	not scorable	0.9917355	0.7356588
####	Bn-A05-p19195181	A-genome specific (napus-rapa)	0.9832536	0.7276098	not scorable	not scorable	0.9834711	0.746573
####	Bn-A05-p19207735	A-genome specific (napus-rapa)	0.9856459	0.7369217	not scorable	not scorable	1	0.8877785
####	Bn-A05-p19209866	A-genome specific (napus-rapa)	0.9808612	0.2709426	not scorable	not scorable	0.9669421	0.7014334
####	Bn-A05-p19224833	A-genome specific (napus-rapa)	0.9880383	0.3494681	not scorable	not scorable	0.9834711	0.54172
####	Bn-A05-p19238078	A-genome specific (napus-rapa)	0.9976076	0.7904353	not scorable	not scorable	1	0.7989678
####	Bn-A05-p19238134	A-genome specific (napus-rapa)	0.9952153	0.7720228	not scorable	not scorable	0.9834711	0.7047588
####	Bn-A05-p19238534	A-genome specific (napus-rapa)	0.9736842	0.4631932	not scorable	not scorable	0.9917355	0.8773267
####	Bn-A05-p19242100	A-genome specific (napus-rapa)	0.9880383	0.8190317	not scorable	not scorable	0.9917355	0.751514
####	Bn-A05-p19242397	A-genome specific (napus-rapa)	0.9856459	0.7980725	not scorable	not scorable	0.9752066	0.7860961
####	Bn-A05-p19246371	A-genome specific (napus-rapa)	0.9832536	0.6741466	not scorable	not scorable	0.9421487	0.6237388
####	Bn-A05-p19250569	A-genome specific (napus-rapa)	0.9880383	0.7601599	not scorable	not scorable	0.9669421	0.7229794
####	Bn-A05-p19251131	A-genome specific (napus-rapa)	0.9904306	0.8021458	not scorable	not scorable	0.9586777	0.8041587
####	Bn-A05-p19267100	A-genome specific (napus-rapa)	0.9832536	0.7817973	not scorable	not scorable	0.9752066	0.7792676
####	Bn-A05-p19267153	A-genome specific (napus-rapa)	0.9880383	0.7503397	not scorable	not scorable	0.9917355	0.7667828
####	Bn-A05-p19267475	A-genome specific (napus-rapa)	0.9904306	0.4392189	not scorable	not scorable	1	0.788645
####	Bn-A05-p19268141	A-genome specific (napus-rapa)	0.9736842	0.7103897	not scorable	not scorable	1	0.6443118
####	Bn-A05-p19278249	A-genome specific (napus-rapa)	0.9736842	0.5218727	not scorable	not scorable	1	0.7544576
####	Bn-A05-p19278438	A-genome specific (napus-rapa)	0.9760765	0.7480281	not scorable	not scorable	0.9834711	0.7629552
####	Bn-A05-p19359433	A-genome specific (napus-rapa)	0.9832536	0.7811325	not scorable	not scorable	0.9834711	0.8282984
####	Bn-A05-p19359443	A-genome specific (napus-rapa)	0.9880383	0.7932043	not scorable	not scorable	0.9834711	0.7902157
####	Bn-A05-p19362820	A-genome specific (napus-rapa)	0.9904306	0.3887517	not scorable	not scorable	0.9917355	0.7937153
####	Bn-A05-p1942242	A-genome specific (napus-rapa)	0.9928229	0.7065078	not scorable	not scorable	0.9917355	0.8143404

#### Bn-A05-p19425901	A-genome specific (napus-rapa)	0.9952153	0.7571525	not scorable	not scorable	1	0.7581795
#### Bn-A05-p1943093	A-genome specific (napus-rapa)	0.9928229	0.468921	not scorable	not scorable	0.9917355	0.8022016
#### Bn-A05-p19436126	A-genome specific (napus-rapa)	0.9832536	0.8082582	not scorable	not scorable	0.9917355	0.8727548
#### Bn-A05-p19445367	A-genome specific (napus-rapa)	0.9760765	0.7043135	not scorable	not scorable	0.9669421	0.7168652
#### Bn-A05-p19448529	A-genome specific (napus-rapa)	0.9808612	0.8131216	not scorable	not scorable	0.9834711	0.7119912
#### Bn-A05-p19473882	genome unspecific (oleracea-rapa)	not scorable	not scorable	1	0.6678175	1	0.6811868
#### Bn-A05-p19514459	A-genome specific (napus-rapa)	0.9712918	0.603533	not scorable	not scorable	0.9834711	0.8118221
#### Bn-A05-p19530568	A-genome specific (napus-rapa)	0.9952153	0.7137901	not scorable	not scorable	0.9917355	0.8636814
#### Bn-A05-p19532660	A-genome specific (napus-rapa)	0.9952153	0.7368143	not scorable	not scorable	1	0.713484
#### Bn-A05-p19539167	A-genome specific (napus-rapa)	0.9880383	0.7475441	not scorable	not scorable	0.9834711	0.6041471
#### Bn-A05-p19539235	A-genome specific (napus-rapa)	0.9904306	0.7932793	not scorable	not scorable	0.9752066	0.7947152
#### Bn-A05-p19539677	A-genome specific (rapa only)	not scorable	not scorable	not scorable	not scorable	0.9338843	0.9086833
#### Bn-A05-p19547378	A-genome specific (napus-rapa)	0.9856459	0.3961389	not scorable	not scorable	0.6363636	0.7372702
#### Bn-A05-p19550645	A-genome specific (napus-rapa)	0.9641148	0.5505758	not scorable	not scorable	1	0.6817572
#### Bn-A05-p19554281	A-genome specific (napus-rapa)	0.9928229	0.8087645	not scorable	not scorable	0.9917355	0.8203865
#### Bn-A05-p19571798	A-genome specific (napus-rapa)	0.9928229	0.7838237	not scorable	not scorable	0.9917355	0.8076818
#### Bn-A05-p19575123	A-genome specific (napus-rapa)	0.9808612	0.408687	not scorable	not scorable	0.9917355	0.7882144
#### Bn-A05-p19583714	A-genome specific (napus-rapa)	0.9904306	0.7711592	not scorable	not scorable	0.9834711	0.7552525
#### Bn-A05-p19599390	A-genome specific (napus-rapa)	0.9641148	0.6753418	not scorable	not scorable	1	0.6834372
#### Bn-A05-p19599809	A-genome specific (napus-rapa)	0.9904306	0.7909828	not scorable	not scorable	0.9917355	0.8582645
#### Bn-A05-p19612275	A-genome specific (napus-rapa)	0.9880383	0.294282	not scorable	not scorable	1	0.736665
#### Bn-A05-p19622826	A-genome specific (napus-rapa)	0.9736842	0.4491403	not scorable	not scorable	1	0.7850553
#### Bn-A05-p19623018	A-genome specific (napus-rapa)	0.9856459	0.775817	not scorable	not scorable	0.9752066	0.7384774
#### Bn-A05-p19650213	A-genome specific (napus-rapa)	1	0.7801627	not scorable	not scorable	0.9834711	0.8294867
#### Bn-A05-p19650278	A-genome specific (napus-rapa)	0.9904306	0.7563848	not scorable	not scorable	0.9752066	0.7030701
#### Bn-A05-p19650965	A-genome specific (napus-rapa)	0.9976076	0.8002308	not scorable	not scorable	0.9752066	0.7565655
#### Bn-A05-p19653084	A-genome specific (napus-rapa)	0.9904306	0.7296695	not scorable	not scorable	0.9917355	0.6920334
#### Bn-A05-p19660820	A-genome specific (napus-rapa)	0.9880383	0.7889743	not scorable	not scorable	0.9834711	0.6860847
#### Bn-A05-p19666434	A-genome specific (napus-rapa)	0.9880383	0.7207434	not scorable	not scorable	0.9917355	0.8140488
#### Bn-A05-p19668317	A-genome specific (napus-rapa)	0.9904306	0.7663769	not scorable	not scorable	0.9917355	0.7738497
#### Bn-A05-p19668632	A-genome specific (napus-rapa)	0.9880383	0.8202624	not scorable	not scorable	1	0.798358
#### Bn-A05-p19728614	A-genome specific (napus-rapa)	0.9688995	0.7175491	not scorable	not scorable	0.9917355	0.5924731
#### Bn-A05-p19730291	genome unspecific (oleracea-rapa)	not scorable	not scorable	1	0.5098581	0.9917355	0.7524529

####	Bn-A05-p1974178	A-genome specific (napus-rapa)	0.9856459	0.49788	not scorable	not scorable	0.6446281	0.8392317
####	Bn-A05-p19745674	napus specific	0.9162679	0.770963	not scorable	not scorable	not scorable	not scorable
####	Bn-A05-p1975785	A-genome specific (napus-rapa)	0.9976076	0.7140692	not scorable	not scorable	0.9834711	0.7319134
####	Bn-A05-p19777231	A-genome specific (napus-rapa)	0.9808612	0.6733628	not scorable	not scorable	1	0.7389225
####	Bn-A05-p19777547	A-genome specific (napus-rapa)	0.9856459	0.6516067	not scorable	not scorable	1	0.8216771
####	Bn-A05-p1978957	A-genome specific (napus-rapa)	0.9928229	0.8288244	not scorable	not scorable	0.9917355	0.7230591
####	Bn-A05-p19802577	A-genome specific (napus-rapa)	0.9904306	0.6090744	not scorable	not scorable	0.9917355	0.7235928
####	Bn-A05-p19806333	A-genome specific (napus-rapa)	0.9832536	0.692484	not scorable	not scorable	0.9917355	0.6951473
####	Bn-A05-p1981647	A-genome specific (napus-rapa)	0.9665072	0.6750557	not scorable	not scorable	0.9421487	0.7101196
####	Bn-A05-p19846575	A-genome specific (rapa only)	not scorable	not scorable	not scorable	not scorable	1	0.7876082
####	Bn-A05-p19857351	A-genome specific (napus-rapa)	0.9545454	0.4825408	not scorable	not scorable	0.9834711	0.5126354
####	Bn-A05-p19857532	A-genome specific (napus-rapa)	1	0.9293141	not scorable	not scorable	1	0.9272014
####	Bn-A05-p19873242	A-genome specific (napus-rapa)	0.9904306	0.5724945	not scorable	not scorable	1	0.7133751
####	Bn-A05-p19886554	A-genome specific (napus-rapa)	0.9808612	0.7535625	not scorable	not scorable	0.9752066	0.7823407
####	Bn-A05-p19887308	A-genome specific (napus-rapa)	0.9952153	0.5443494	not scorable	not scorable	1	0.7948728
####	Bn-A05-p19897379	A-genome specific (napus-rapa)	0.9880383	0.7325768	not scorable	not scorable	0.9917355	0.7469649
####	Bn-A05-p19909249	A-genome specific (napus-rapa)	0.9808612	0.4889305	not scorable	not scorable	1	0.7802272
####	Bn-A05-p19919111	A-genome specific (napus-rapa)	0.9736842	0.7657433	not scorable	not scorable	0.9834711	0.7334405
####	Bn-A05-p19919179	A-genome specific (napus-rapa)	0.9760765	0.8020984	not scorable	not scorable	0.9752066	0.7395979
####	Bn-A05-p19930910	A-genome specific (napus-rapa)	0.9976076	0.7693241	not scorable	not scorable	0.9752066	0.7839488
####	Bn-A05-p19943528	genome unspecific (all in)	0.9712918	0.318673	0.9844961	0.693344	1	0.8547045
####	Bn-A05-p19946413	A-genome specific (napus-rapa)	0.9712918	0.4089649	not scorable	not scorable	1	0.7831712
####	Bn-A05-p19967151	A-genome specific (napus-rapa)	0.9856459	0.2613275	not scorable	not scorable	0.9917355	0.8088612
####	Bn-A05-p19973413	A-genome specific (napus-rapa)	0.9593301	0.2435013	not scorable	not scorable	0.9917355	0.7411395
####	Bn-A05-p19978706	A-genome specific (napus-rapa)	0.9521531	0.7928636	not scorable	not scorable	1	0.7841166
####	Bn-A05-p19990020	A-genome specific (napus-rapa)	0.9760765	0.496685	not scorable	not scorable	0.9834711	0.7898074
####	Bn-A05-p19990560	A-genome specific (rapa only)	not scorable	not scorable	not scorable	not scorable	1	0.790464
####	Bn-A05-p20009925	A-genome specific (napus-rapa)	0.9928229	0.7848729	not scorable	not scorable	0.9752066	0.7938465
####	Bn-A05-p20019130	A-genome specific (napus-rapa)	0.9712918	0.7967959	not scorable	not scorable	0.9917355	0.8150891
####	Bn-A05-p20020286	A-genome specific (napus-rapa)	0.9952153	0.6312669	not scorable	not scorable	1	0.8691807
####	Bn-A05-p200254	A-genome specific (napus-rapa)	0.9928229	0.7902161	not scorable	not scorable	0.9917355	0.7328228
####	Bn-A05-p20074144	A-genome specific (napus-rapa)	1	0.8135734	not scorable	not scorable	1	0.7493539
####	Bn-A05-p20075053	A-genome specific (napus-rapa)	0.9784689	0.7830873	not scorable	not scorable	1	0.7151078

####	Bn-A05-p20100234	A-genome specific (napus-rapa)	0.9665072	0.3752645	not scorable	not scorable	0.9917355	0.73515
####	Bn-A05-p20105944	A-genome specific (napus-rapa)	0.9904306	0.8153329	not scorable	not scorable	1	0.7803828
####	Bn-A05-p20109948	A-genome specific (napus-rapa)	0.9856459	0.6754115	not scorable	not scorable	0.9752066	0.7042676
####	Bn-A05-p20113630	A-genome specific (napus-rapa)	0.9784689	0.5466524	not scorable	not scorable	1	0.9174437
####	Bn-A05-p20124425	A-genome specific (napus-rapa)	0.9952153	0.7914414	not scorable	not scorable	1	0.819771
####	Bn-A05-p20125478	A-genome specific (napus-rapa)	0.9832536	0.7983338	not scorable	not scorable	1	0.9272014
####	Bn-A05-p20125982	A-genome specific (napus-rapa)	0.9688995	0.7763443	not scorable	not scorable	0.9669421	0.7509598
####	Bn-A05-p20126071	A-genome specific (napus-rapa)	0.9736842	0.7990362	not scorable	not scorable	1	0.8321881
####	Bn-A05-p20127293	A-genome specific (napus-rapa)	0.8444976	0.8520336	not scorable	not scorable	0.9917355	0.8467577
####	Bn-A05-p20127547	A-genome specific (napus-rapa)	0.9880383	0.7509729	not scorable	not scorable	1	0.8546867
####	Bn-A05-p20128796	A-genome specific (napus-rapa)	0.9904306	0.7946774	not scorable	not scorable	1	0.7485278
####	Bn-A05-p20129266	A-genome specific (napus-rapa)	0.9760765	0.7836324	not scorable	not scorable	0.9834711	0.7888456
####	Bn-A05-p20138451	A-genome specific (napus-rapa)	0.9904306	0.8086479	not scorable	not scorable	1	0.7256749
####	Bn-A05-p20139872	A-genome specific (napus-rapa)	0.9880383	0.7532134	not scorable	not scorable	0.9752066	0.6368245
####	Bn-A05-p20141883	A-genome specific (napus-rapa)	0.9928229	0.5119429	not scorable	not scorable	1	0.4670898
####	Bn-A05-p20142804	A-genome specific (napus-rapa)	0.9760765	0.3626936	not scorable	not scorable	0.9917355	0.665702
####	Bn-A05-p20161210	genome unspecific (all in)	0.9545454	0.435711	0.9612403	0.2465464	0.9669421	0.7331205
####	Bn-A05-p20163118	A-genome specific (napus-rapa)	0.9712918	0.7182986	not scorable	not scorable	0.9752066	0.6363951
####	Bn-A05-p20167176	A-genome specific (napus-rapa)	0.9880383	0.8047754	not scorable	not scorable	0.9752066	0.7708302
####	Bn-A05-p20168806	A-genome specific (napus-rapa)	0.9856459	0.4766909	not scorable	not scorable	1	0.6893203
####	Bn-A05-p20168909	A-genome specific (napus-rapa)	0.9593301	0.4621453	not scorable	not scorable	0.9917355	0.5466226
####	Bn-A05-p20174313	genome unspecific (oleracea-rapa)	not scorable	not scorable	1	0.6476261	0.9834711	0.6396965
####	Bn-A05-p20178007	A-genome specific (napus-rapa)	0.9736842	0.7182854	not scorable	not scorable	0.9752066	0.7030895
####	Bn-A05-p20179255	A-genome specific (rapa only)	not scorable	not scorable	not scorable	not scorable	0.9834711	0.6770346
####	Bn-A05-p20232989	A-genome specific (napus-rapa)	0.9808612	0.6763161	not scorable	not scorable	1	0.6912112
####	Bn-A05-p2024975	A-genome specific (napus-rapa)	0.9880383	0.6235477	not scorable	not scorable	0.9669421	0.7517704
####	Bn-A05-p20254809	A-genome specific (napus-rapa)	0.9880383	0.7832338	not scorable	not scorable	0.9917355	0.7335973
####	Bn-A05-p20283717	A-genome specific (napus-rapa)	0.9688995	0.7979661	not scorable	not scorable	0.6033058	0.8163134
####	Bn-A05-p20283817	A-genome specific (napus-rapa)	0.9688995	0.7293816	not scorable	not scorable	0.9504132	0.5072837
####	Bn-A05-p20290091	A-genome specific (napus-rapa)	0.9593301	0.5055331	not scorable	not scorable	1	0.8800009
####	Bn-A05-p20292751	genome unspecific (oleracea-rapa)	not scorable	not scorable	0.9689922	0.1657277	0.9917355	0.267318
####	Bn-A05-p20296253	A-genome specific (napus-rapa)	0.9904306	0.5022858	not scorable	not scorable	1	0.8149554
####	Bn-A05-p20300638	A-genome specific (napus-rapa)	0.930622	0.8085203	not scorable	not scorable	1	0.8135064

####	Bn-A05-p20302128	A-genome specific (napus-rapa)	0.9760765	0.6715361	not scorable	not scorable	1	0.648831
####	Bn-A05-p2030789	A-genome specific (napus-rapa)	0.9784689	0.3704401	not scorable	not scorable	0.9752066	0.7370691
####	Bn-A05-p20314128	A-genome specific (napus-rapa)	0.9952153	0.8022643	not scorable	not scorable	1	0.7174681
####	Bn-A05-p20316923	A-genome specific (napus-rapa)	0.9832536	0.3256988	not scorable	not scorable	1	0.7699323
####	Bn-A05-p20319622	A-genome specific (napus-rapa)	0.9665072	0.7280776	not scorable	not scorable	0.9752066	0.7875085
####	Bn-A05-p20328249	A-genome specific (napus-rapa)	0.9665072	0.4088381	not scorable	not scorable	0.9752066	0.6655978
####	Bn-A05-p20328443	A-genome specific (napus-rapa)	0.9856459	0.4476026	not scorable	not scorable	0.9917355	0.6830243
####	Bn-A05-p20337405	A-genome specific (napus-rapa)	0.9976076	0.5321876	not scorable	not scorable	1	0.7530559
####	Bn-A05-p20354606	A-genome specific (napus-rapa)	0.9928229	0.4471853	not scorable	not scorable	0.9752066	0.7229759
####	Bn-A05-p20359381	A-genome specific (napus-rapa)	0.9760765	0.819029	not scorable	not scorable	1	0.8759238
####	Bn-A05-p20359553	A-genome specific (napus-rapa)	0.9880383	0.8108431	not scorable	not scorable	1	0.7006989
####	Bn-A05-p20365953	A-genome specific (napus-rapa)	0.9856459	0.4544694	not scorable	not scorable	0.9752066	0.6775002
####	Bn-A05-p20372600	A-genome specific (napus-rapa)	1	0.5826588	not scorable	not scorable	1	0.6202648
####	Bn-A05-p20375812	A-genome specific (napus-rapa)	0.9832536	0.4208603	not scorable	not scorable	1	0.8089778
####	Bn-A05-p20377770	A-genome specific (napus-rapa)	0.9880383	0.7193465	not scorable	not scorable	1	0.7157628
####	Bn-A05-p20379970	A-genome specific (napus-rapa)	0.8827751	0.6723114	not scorable	not scorable	0.6033058	0.8003798
####	Bn-A05-p20380629	A-genome specific (napus-rapa)	0.8708134	0.797788	not scorable	not scorable	0.6033058	0.8167256
####	Bn-A05-p20386700	A-genome specific (napus-rapa)	0.9952153	0.3097196	not scorable	not scorable	0.9917355	0.7365292
####	Bn-A05-p20390129	A-genome specific (napus-rapa)	0.9928229	0.7852919	not scorable	not scorable	0.9752066	0.7857522
####	Bn-A05-p20394162	genome unspecific (all in)	0.9665072	0.4083105	0.9844961	0.2497406	0.9752066	0.7805901
####	Bn-A05-p2039690	A-genome specific (napus-rapa)	0.9904306	0.8505738	not scorable	not scorable	0.9834711	0.8360972
####	Bn-A05-p20399452	A-genome specific (napus-rapa)	0.9593301	0.5287408	not scorable	not scorable	0.9752066	0.6864699
####	Bn-A05-p20404099	A-genome specific (napus-rapa)	0.9928229	0.6677064	not scorable	not scorable	1	0.7645778
####	Bn-A05-p20422081	A-genome specific (napus-rapa)	0.9952153	0.4992225	not scorable	not scorable	1	0.6608376
####	Bn-A05-p20425452	A-genome specific (napus-rapa)	0.9880383	0.3434804	not scorable	not scorable	0.9834711	0.7118058
####	Bn-A05-p20425648	A-genome specific (napus-rapa)	0.9760765	0.3273316	not scorable	not scorable	1	0.6400863
####	Bn-A05-p20429403	A-genome specific (napus-rapa)	0.9952153	0.8572267	not scorable	not scorable	1	0.8349755
####	Bn-A05-p20443393	A-genome specific (napus-rapa)	1	0.431834	not scorable	not scorable	1	0.6674994
####	Bn-A05-p20449058	A-genome specific (napus-rapa)	0.9784689	0.4197202	not scorable	not scorable	1	0.671567
####	Bn-A05-p20458590	A-genome specific (napus-rapa)	0.9904306	0.7864174	not scorable	not scorable	1	0.7807422
####	Bn-A05-p20461416	A-genome specific (napus-rapa)	0.9760765	0.6551969	not scorable	not scorable	0.9752066	0.8027548
####	Bn-A05-p20461727	A-genome specific (napus-rapa)	0.9712918	0.7965148	not scorable	not scorable	0.9917355	0.7794971
####	Bn-A05-p20467809	A-genome specific (napus-rapa)	0.9665072	0.7856001	not scorable	not scorable	1	0.7120411

####	Bn-A05-p20478486	A-genome specific (rapa only)	not scorable	not scorable	not scorable	not scorable	1	0.7882326
####	Bn-A05-p20479068	A-genome specific (rapa only)	not scorable	not scorable	not scorable	not scorable	1	0.7473264
####	Bn-A05-p20485998	A-genome specific (napus-rapa)	0.9904306	0.7871493	not scorable	not scorable	0.9752066	0.7532809
####	Bn-A05-p20487983	A-genome specific (napus-rapa)	0.9832536	0.814613	not scorable	not scorable	0.9834711	0.6874883
####	Bn-A05-p20492525	A-genome specific (napus-rapa)	0.9976076	0.5457357	not scorable	not scorable	1	0.6541435
####	Bn-A05-p20503767	A-genome specific (napus-rapa)	1	0.7752416	not scorable	not scorable	1	0.7422752
####	Bn-A05-p20534431	A-genome specific (napus-rapa)	0.9856459	0.7770748	not scorable	not scorable	0.9834711	0.8340049
####	Bn-A05-p20541690	A-genome specific (napus-rapa)	1	0.9205657	not scorable	not scorable	1	0.9175813
####	Bn-A05-p20541987	A-genome specific (napus-rapa)	0.9904306	0.7663167	not scorable	not scorable	1	0.8494952
####	Bn-A05-p20549826	A-genome specific (napus-rapa)	0.4282297	0.6536983	not scorable	not scorable	0.9917355	0.6212941
####	Bn-A05-p20550694	A-genome specific (napus-rapa)	0.9952153	0.72338	not scorable	not scorable	1	0.6951995
####	Bn-A05-p20554567	genome unspecific (all in)	0.9952153	0.7852837	0	0.8007098	0.9752066	0.7708607
####	Bn-A05-p20555996	A-genome specific (napus-rapa)	0.9952153	0.7231936	not scorable	not scorable	0.9917355	0.6714237
####	Bn-A05-p20563653	A-genome specific (napus-rapa)	0.9952153	0.4542452	not scorable	not scorable	0.9917355	0.7747972
####	Bn-A05-p20564729	A-genome specific (napus-rapa)	0.9665072	0.3319836	not scorable	not scorable	0.9917355	0.7465069
####	Bn-A05-p20591895	A-genome specific (napus-rapa)	0.9760765	0.5500098	not scorable	not scorable	1	0.7904748
####	Bn-A05-p20591964	A-genome specific (napus-rapa)	0.9617225	0.7880247	not scorable	not scorable	0.6033058	0.7417902
####	Bn-A05-p20594196	A-genome specific (napus-rapa)	0.9784689	0.5079296	not scorable	not scorable	1	0.7770717
####	Bn-A05-p20597839	A-genome specific (napus-rapa)	0.9928229	0.4501688	not scorable	not scorable	0.9752066	0.7544127
####	Bn-A05-p20601139	A-genome specific (napus-rapa)	0.9856459	0.775849	not scorable	not scorable	1	0.8259001
####	Bn-A05-p20623573	A-genome specific (napus-rapa)	0.9952153	0.7981007	not scorable	not scorable	1	0.7360603
####	Bn-A05-p20626818	A-genome specific (napus-rapa)	1	0.7727537	not scorable	not scorable	1	0.7619172
####	Bn-A05-p2062917	A-genome specific (napus-rapa)	0.9808612	0.7832018	not scorable	not scorable	0.9917355	0.709864
####	Bn-A05-p20632153	A-genome specific (napus-rapa)	1	0.7782727	not scorable	not scorable	1	0.7137816
####	Bn-A05-p20673293	A-genome specific (napus-rapa)	1	0.7602968	not scorable	not scorable	1	0.7257791
####	Bn-A05-p20676409	napus specific	0.9665072	0.8126962	not scorable	not scorable	not scorable	not scorable
####	Bn-A05-p2067722	A-genome specific (napus-rapa)	0.9928229	0.7870454	not scorable	not scorable	0.9834711	0.7444382
####	Bn-A05-p20681871	A-genome specific (napus-rapa)	1	0.7554034	not scorable	not scorable	1	0.8508033
####	Bn-A05-p20690058	A-genome specific (napus-rapa)	1	0.7746114	not scorable	not scorable	0.9917355	0.7405595
####	Bn-A05-p20692714	A-genome specific (napus-rapa)	0.9976076	0.654015	not scorable	not scorable	1	0.7819035
####	Bn-A05-p20697145	A-genome specific (napus-rapa)	0.9952153	0.7117223	not scorable	not scorable	1	0.7247998
####	Bn-A05-p20697410	A-genome specific (napus-rapa)	1	0.7353347	not scorable	not scorable	0.9917355	0.8288288
####	Bn-A05-p20697473	A-genome specific (napus-rapa)	0.9976076	0.7759578	not scorable	not scorable	1	0.7882201

####	Bn-A05-p20697824	A-genome specific (napus-rapa)	1	0.7232289	not scorable	not scorable	0.9917355	0.8047732
####	Bn-A05-p20697908	A-genome specific (napus-rapa)	0.9928229	0.7377468	not scorable	not scorable	1	0.8051713
####	Bn-A05-p20698928	A-genome specific (rapa only)	not scorable	not scorable	not scorable	not scorable	1	0.6957591
####	Bn-A05-p20699671	A-genome specific (napus-rapa)	0.9976076	0.7901343	not scorable	not scorable	1	0.7452181
####	Bn-A05-p20705675	A-genome specific (rapa only)	not scorable	not scorable	not scorable	not scorable	1	0.7860979
####	Bn-A05-p20712388	A-genome specific (napus-rapa)	0.9808612	0.5235363	not scorable	not scorable	1	0.7863717
####	Bn-A05-p20714207	A-genome specific (napus-rapa)	1	0.5625229	not scorable	not scorable	0.9917355	0.7708303
####	Bn-A05-p20714302	A-genome specific (napus-rapa)	0.9976076	0.3776084	not scorable	not scorable	0.9917355	0.7072175
####	Bn-A05-p20714581	A-genome specific (napus-rapa)	1	0.8025625	not scorable	not scorable	0.9834711	0.7581809
####	Bn-A05-p20716500	A-genome specific (napus-rapa)	0.9976076	0.6833518	not scorable	not scorable	1	0.8132641
####	Bn-A05-p20718776	A-genome specific (napus-rapa)	0.9976076	0.7629884	not scorable	not scorable	1	0.7851137
####	Bn-A05-p20719343	A-genome specific (napus-rapa)	0.9808612	0.7337445	not scorable	not scorable	0.9338843	0.909426
####	Bn-A05-p20728232	A-genome specific (napus-rapa)	0.9473684	0.6045958	not scorable	not scorable	1	0.7402759
####	Bn-A05-p20728284	A-genome specific (rapa only)	not scorable	not scorable	not scorable	not scorable	0.9669421	0.8469902
####	Bn-A05-p20734549	A-genome specific (rapa only)	not scorable	not scorable	not scorable	not scorable	0.9834711	0.7496812
####	Bn-A05-p20747913	A-genome specific (napus-rapa)	0.9856459	0.7515344	not scorable	not scorable	0.6033058	0.7955049
####	Bn-A05-p20790867	A-genome specific (napus-rapa)	0.9928229	0.7206525	not scorable	not scorable	1	0.7059751
####	Bn-A05-p20791306	A-genome specific (napus-rapa)	0.9976076	0.7794048	not scorable	not scorable	1	0.6834136
####	Bn-A05-p20794306	A-genome specific (napus-rapa)	1	0.8687871	not scorable	not scorable	1	0.8653011
####	Bn-A05-p20808964	A-genome specific (rapa only)	not scorable	not scorable	not scorable	not scorable	0.9834711	0.7332327
####	Bn-A05-p20809217	A-genome specific (rapa only)	not scorable	not scorable	not scorable	not scorable	0.9834711	0.7161915
####	Bn-A05-p20809327	A-genome specific (rapa only)	not scorable	not scorable	not scorable	not scorable	0.9834711	0.8123746
####	Bn-A05-p20811510	A-genome specific (rapa only)	not scorable	not scorable	not scorable	not scorable	0.9917355	0.7600672
####	Bn-A05-p20818194	A-genome specific (rapa only)	not scorable	not scorable	not scorable	not scorable	0.9586777	0.7629373
####	Bn-A05-p20818989	A-genome specific (napus-rapa)	1	0.7705563	not scorable	not scorable	1	0.9022536
####	Bn-A05-p20824936	A-genome specific (napus-rapa)	0.9856459	0.7600218	not scorable	not scorable	0.9752066	0.6711195
####	Bn-A05-p20825280	A-genome specific (napus-rapa)	0.9521531	0.7395855	not scorable	not scorable	0.9752066	0.6695426
####	Bn-A05-p20825449	A-genome specific (rapa only)	not scorable	not scorable	not scorable	not scorable	0.9834711	0.7079834
####	Bn-A05-p20828693	A-genome specific (napus-rapa)	1	0.7702438	not scorable	not scorable	0.9834711	0.6761604
####	Bn-A05-p20839044	A-genome specific (napus-rapa)	0.9928229	0.7961762	not scorable	not scorable	0.9752066	0.7195259
####	Bn-A05-p20843453	A-genome specific (rapa only)	not scorable	not scorable	not scorable	not scorable	1	0.7066184
####	Bn-A05-p20844496	A-genome specific (napus-rapa)	1	0.7192586	not scorable	not scorable	0.9917355	0.7224618
####	Bn-A05-p20844938	A-genome specific (rapa only)	not scorable	not scorable	not scorable	not scorable	0.9917355	0.7266377

####	Bn-A05-p20852240	A-genome specific (napus-rapa)	0.9449761	0.6600185	not scorable	not scorable	0.9669421	0.7666233
####	Bn-A05-p20888695	A-genome specific (rapa only)	not scorable	not scorable	not scorable	not scorable	1	0.8130783
####	Bn-A05-p20894465	A-genome specific (rapa only)	not scorable	not scorable	not scorable	not scorable	0.8595041	0.8137174
####	Bn-A05-p20903214	A-genome specific (rapa only)	not scorable	not scorable	not scorable	not scorable	1	0.836808
####	Bn-A05-p20903608	A-genome specific (napus-rapa)	0.9952153	0.3238211	not scorable	not scorable	1	0.7622204
####	Bn-A05-p2091308	A-genome specific (napus-rapa)	0.9976076	0.7697294	not scorable	not scorable	0.9834711	0.8054408
####	Bn-A05-p20940971	A-genome specific (napus-rapa)	0.9856459	0.4615396	not scorable	not scorable	0.6115702	0.719925
####	Bn-A05-p20955184	A-genome specific (napus-rapa)	0.9808612	0.5152993	not scorable	not scorable	0.9834711	0.8527468
####	Bn-A05-p20972649	A-genome specific (napus-rapa)	0.9856459	0.8033336	not scorable	not scorable	1	0.7487295
####	Bn-A05-p20974054	A-genome specific (napus-rapa)	0.9808612	0.664434	not scorable	not scorable	0.9917355	0.6520352
####	Bn-A05-p20998848	A-genome specific (rapa only)	not scorable	not scorable	not scorable	not scorable	1	0.8364183
####	Bn-A05-p21000008	A-genome specific (napus-rapa)	0.9904306	0.7671291	not scorable	not scorable	0.9669421	0.7257819
####	Bn-A05-p21000614	A-genome specific (napus-rapa)	0.9880383	0.7912342	not scorable	not scorable	1	0.8335571
####	Bn-A05-p21006621	A-genome specific (rapa only)	not scorable	not scorable	not scorable	not scorable	0.9834711	0.7047226
####	Bn-A05-p2102634	A-genome specific (napus-rapa)	0.9952153	0.3961241	not scorable	not scorable	1	0.6062281
####	Bn-A05-p21028051	A-genome specific (napus-rapa)	0.9904306	0.795072	not scorable	not scorable	0.9917355	0.8064644
####	Bn-A05-p21029850	A-genome specific (napus-rapa)	0.9952153	0.8328664	not scorable	not scorable	1	0.8227471
####	Bn-A05-p21029910	A-genome specific (napus-rapa)	0.9952153	0.808516	not scorable	not scorable	0.6033058	0.752282
####	Bn-A05-p21036828	A-genome specific (napus-rapa)	0.9832536	0.5192206	not scorable	not scorable	0.9834711	0.7137486
####	Bn-A05-p21037865	A-genome specific (napus-rapa)	0.9736842	0.8352256	not scorable	not scorable	0.9834711	0.7685616
####	Bn-A05-p21047802	A-genome specific (napus-rapa)	0.9952153	0.7495071	not scorable	not scorable	0.9834711	0.7348757
####	Bn-A05-p21052552	A-genome specific (napus-rapa)	1	0.5293095	not scorable	not scorable	0.9917355	0.7350201
####	Bn-A05-p21054101	A-genome specific (napus-rapa)	0.9832536	0.7928503	not scorable	not scorable	1	0.8416532
####	Bn-A05-p21056938	A-genome specific (napus-rapa)	0.9976076	0.8414966	not scorable	not scorable	1	0.8032891
####	Bn-A05-p21057936	A-genome specific (napus-rapa)	0.9976076	0.410116	not scorable	not scorable	0.9752066	0.621749
####	Bn-A05-p21058156	A-genome specific (napus-rapa)	0.9928229	0.7922674	not scorable	not scorable	0.9917355	0.8185
####	Bn-A05-p21058435	A-genome specific (napus-rapa)	0.9928229	0.8365861	not scorable	not scorable	0.9834711	0.8371058
####	Bn-A05-p21064254	A-genome specific (rapa only)	not scorable	not scorable	not scorable	not scorable	1	0.6823571
####	Bn-A05-p21065087	A-genome specific (napus-rapa)	0.9019139	0.7869024	not scorable	not scorable	0.9752066	0.7942155
####	Bn-A05-p2106680	A-genome specific (napus-rapa)	0.9976076	0.7434528	not scorable	not scorable	0.9917355	0.7792991
####	Bn-A05-p21070302	A-genome specific (napus-rapa)	0.9976076	0.702597	not scorable	not scorable	0.9752066	0.6549986
####	Bn-A05-p21070991	A-genome specific (napus-rapa)	1	0.6763301	not scorable	not scorable	1	0.7727653
####	Bn-A05-p2107808	A-genome specific (napus-rapa)	0.9976076	0.7726344	not scorable	not scorable	0.9834711	0.7135004

####	Bn-A05-p21081145	A-genome specific (napus-rapa)	0.9186603	0.8262522	not scorable	not scorable	0.9752066	0.8215418
####	Bn-A05-p2108713	A-genome specific (napus-rapa)	0.9904306	0.669964	not scorable	not scorable	0.9834711	0.6704814
####	Bn-A05-p21093954	A-genome specific (napus-rapa)	0.9832536	0.4445618	not scorable	not scorable	0.9834711	0.6530712
####	Bn-A05-p2109546	A-genome specific (napus-rapa)	0.9976076	0.7664476	not scorable	not scorable	0.9917355	0.7320964
####	Bn-A05-p2110255	A-genome specific (napus-rapa)	0.9976076	0.6807659	not scorable	not scorable	1	0.7216478
####	Bn-A05-p2110344	A-genome specific (napus-rapa)	0.9976076	0.6357534	not scorable	not scorable	0.9917355	0.6743599
####	Bn-A05-p21106566	A-genome specific (napus-rapa)	0.9784689	0.5225418	not scorable	not scorable	0.9917355	0.8646462
####	Bn-A05-p21107181	A-genome specific (napus-rapa)	0.9665072	0.2894852	not scorable	not scorable	1	0.7045832
####	Bn-A05-p2111165	A-genome specific (napus-rapa)	0.9880383	0.5099034	not scorable	not scorable	1	0.7208876
####	Bn-A05-p21163226	A-genome specific (napus-rapa)	1	0.7776649	not scorable	not scorable	1	0.7909042
####	Bn-A05-p21176831	A-genome specific (napus-rapa)	0.9784689	0.7205074	not scorable	not scorable	1	0.716603
####	Bn-A05-p21182276	A-genome specific (napus-rapa)	0.9760765	0.4191424	not scorable	not scorable	0.9917355	0.7322522
####	Bn-A05-p21188442	A-genome specific (napus-rapa)	0.9880383	0.4976288	not scorable	not scorable	0.9917355	0.7365086
####	Bn-A05-p21193491	A-genome specific (napus-rapa)	0.9928229	0.7595256	not scorable	not scorable	1	0.8420879
####	Bn-A05-p21201908	A-genome specific (napus-rapa)	0.9904306	0.6795592	not scorable	not scorable	0.9917355	0.6544748
####	Bn-A05-p21201956	A-genome specific (napus-rapa)	0.9928229	0.706045	not scorable	not scorable	1	0.6532649
####	Bn-A05-p21208394	A-genome specific (napus-rapa)	1	0.7773431	not scorable	not scorable	0.9752066	0.7551941
####	Bn-A05-p21210078	A-genome specific (napus-rapa)	0.9880383	0.332107	not scorable	not scorable	0.9752066	0.6795902
####	Bn-A05-p21221296	A-genome specific (napus-rapa)	0.9856459	0.7859538	not scorable	not scorable	0.9834711	0.7522865
####	Bn-A05-p21233666	A-genome specific (napus-rapa)	0.9976076	0.3983047	not scorable	not scorable	0.9834711	0.5923949
####	Bn-A05-p21240761	A-genome specific (napus-rapa)	1	0.8096263	not scorable	not scorable	0.9834711	0.7924066
####	Bn-A05-p21243931	A-genome specific (napus-rapa)	0.9928229	0.7775173	not scorable	not scorable	0.9834711	0.7910011
####	Bn-A05-p21248382	A-genome specific (napus-rapa)	1	0.7730108	not scorable	not scorable	0.9834711	0.856598
####	Bn-A05-p21248720	A-genome specific (napus-rapa)	0.9832536	0.7494116	not scorable	not scorable	0.9834711	0.8046125
####	Bn-A05-p21267598	A-genome specific (napus-rapa)	0.9832536	0.3948153	not scorable	not scorable	0.9834711	0.7206949
####	Bn-A05-p21299910	A-genome specific (napus-rapa)	0.9928229	0.7539886	not scorable	not scorable	0.9752066	0.7752903
####	Bn-A05-p21313618	A-genome specific (napus-rapa)	1	0.7451401	not scorable	not scorable	0.9586777	0.7349232
####	Bn-A05-p21319243	A-genome specific (napus-rapa)	0.9952153	0.5112509	not scorable	not scorable	0.9917355	0.6711031
####	Bn-A05-p21325400	A-genome specific (napus-rapa)	0.9928229	0.7900273	not scorable	not scorable	1	0.9175813
####	Bn-A05-p21326091	A-genome specific (napus-rapa)	1	0.6878671	not scorable	not scorable	0.9917355	0.7766346
####	Bn-A05-p21326775	A-genome specific (napus-rapa)	0.9928229	0.5187322	not scorable	not scorable	1	0.8521883
####	Bn-A05-p21345579	A-genome specific (rapa only)	not scorable	not scorable	not scorable	not scorable	0.9917355	0.8713833
####	Bn-A05-p21347821	A-genome specific (napus-rapa)	0.9856459	0.7239614	not scorable	not scorable	0.9834711	0.7814583

####	Bn-A05-p21370435	A-genome specific (napus-rapa)	0.9688995	0.3178908	not scorable	not scorable	0.9917355	0.7465438
####	Bn-A05-p21370618	A-genome specific (napus-rapa)	0.9832536	0.7726155	not scorable	not scorable	0.9752066	0.6337259
####	Bn-A05-p21375513	napus specific	0.9138756	0.2569491	not scorable	not scorable	not scorable	not scorable
####	Bn-A05-p21375514	napus specific	0.9330143	0.3076274	not scorable	not scorable	not scorable	not scorable
####	Bn-A05-p21377682	genome unspecific (all in)	0.9832536	0.4470023	1	0.8345801	0.9917355	0.6790586
####	Bn-A05-p21381782	A-genome specific (napus-rapa)	0.9282297	0.7677541	not scorable	not scorable	0.9917355	0.7657691
####	Bn-A05-p2138342	A-genome specific (napus-rapa)	1	0.7135787	not scorable	not scorable	1	0.7726238
####	Bn-A05-p21384657	A-genome specific (napus-rapa)	0.9760765	0.2509238	not scorable	not scorable	0.9752066	0.798973
####	Bn-A05-p21385524	A-genome specific (napus-rapa)	0.9856459	0.5043669	not scorable	not scorable	1	0.8116566
####	Bn-A05-p2139529	A-genome specific (rapa only)	not scorable	not scorable	not scorable	not scorable	0.9917355	0.5694224
####	Bn-A05-p2142102	A-genome specific (napus-rapa)	0.9856459	0.7580984	not scorable	not scorable	0.9752066	0.8223481
####	Bn-A05-p2143241	A-genome specific (napus-rapa)	0.437799	0.6308175	not scorable	not scorable	0.9421487	0.5064331
####	Bn-A05-p2143292	A-genome specific (rapa only)	not scorable	not scorable	not scorable	not scorable	0.9421487	0.7769517
####	Bn-A05-p21463902	genome unspecific (oleracea-rapa)	not scorable	not scorable	1	0.6000344	1	0.5187793
####	Bn-A05-p21470614	A-genome specific (napus-rapa)	0.9976076	0.742601	not scorable	not scorable	0.9917355	0.7722381
####	Bn-A05-p21480994	A-genome specific (napus-rapa)	0.9928229	0.4171317	not scorable	not scorable	0.9917355	0.7164915
####	Bn-A05-p21484113	A-genome specific (napus-rapa)	0.9976076	0.7598017	not scorable	not scorable	1	0.836735
####	Bn-A05-p21491115	A-genome specific (napus-rapa)	0.9904306	0.5458049	not scorable	not scorable	1	0.7316357
####	Bn-A05-p21493851	A-genome specific (napus-rapa)	0.9808612	0.7372672	not scorable	not scorable	0.5619835	0.7653882
####	Bn-A05-p2151202	A-genome specific (napus-rapa)	0.9976076	0.7449302	not scorable	not scorable	1	0.7936785
####	Bn-A05-p21532839	A-genome specific (napus-rapa)	0.9856459	0.5268254	not scorable	not scorable	0.9917355	0.7915397
####	Bn-A05-p21538700	A-genome specific (napus-rapa)	0.9952153	0.7670899	not scorable	not scorable	0.9834711	0.7445341
####	Bn-A05-p2155466	A-genome specific (napus-rapa)	0.9976076	0.4434382	not scorable	not scorable	1	0.8148221
####	Bn-A05-p21579642	A-genome specific (napus-rapa)	0.9976076	0.4027491	not scorable	not scorable	1	0.7701805
####	Bn-A05-p21587317	A-genome specific (napus-rapa)	0.9976076	0.7936804	not scorable	not scorable	0.9669421	0.7507553
####	Bn-A05-p21600657	A-genome specific (napus-rapa)	1	0.6807379	not scorable	not scorable	0.9752066	0.7791275
####	Bn-A05-p21600954	A-genome specific (napus-rapa)	0.9976076	0.4951656	not scorable	not scorable	0.9917355	0.8177617
####	Bn-A05-p21631586	A-genome specific (napus-rapa)	1	0.6203411	not scorable	not scorable	0.9917355	0.7126814
####	Bn-A05-p21633055	A-genome specific (napus-rapa)	0.8803828	0.5077754	not scorable	not scorable	0.9834711	0.7425181
####	Bn-A05-p21638427	A-genome specific (napus-rapa)	0.9928229	0.7092291	not scorable	not scorable	0.9834711	0.8488882
####	Bn-A05-p21641604	A-genome specific (napus-rapa)	0.9928229	0.774497	not scorable	not scorable	1	0.7822919
####	Bn-A05-p21641616	A-genome specific (napus-rapa)	0.9976076	0.519976	not scorable	not scorable	0.9917355	0.6418307
####	Bn-A05-p21646684	A-genome specific (napus-rapa)	0.9162679	0.7904294	not scorable	not scorable	0.9752066	0.7793182

####	Bn-A05-p21658509	A-genome specific (rapa only)	not scorable	not scorable	not scorable	not scorable	0.9421487	0.7769966
####	Bn-A05-p21660030	A-genome specific (napus-rapa)	0.9760765	0.8042824	not scorable	not scorable	0.9586777	0.6996052
####	Bn-A05-p21660088	A-genome specific (napus-rapa)	0.9880383	0.7108365	not scorable	not scorable	0.9917355	0.7411529
####	Bn-A05-p21661113	A-genome specific (napus-rapa)	0.9928229	0.7525829	not scorable	not scorable	0.9917355	0.6838576
####	Bn-A05-p2166681	A-genome specific (rapa only)	not scorable	not scorable	not scorable	not scorable	0.9917355	0.7819025
####	Bn-A05-p21668872	A-genome specific (napus-rapa)	1	0.4041414	not scorable	not scorable	0.9834711	0.7819542
####	Bn-A05-p2167188	A-genome specific (napus-rapa)	0.9976076	0.7683138	not scorable	not scorable	0.9917355	0.7997399
####	Bn-A05-p2167269	A-genome specific (napus-rapa)	0.930622	0.8306848	not scorable	not scorable	0.9752066	0.846468
####	Bn-A05-p2167540	A-genome specific (napus-rapa)	0.9976076	0.7877693	not scorable	not scorable	0.9834711	0.8136072
####	Bn-A05-p21698558	A-genome specific (napus-rapa)	1	0.49882	not scorable	not scorable	0.9834711	0.7208527
####	Bn-A05-p21699314	A-genome specific (napus-rapa)	0.9904306	0.7705401	not scorable	not scorable	0.9917355	0.7775611
####	Bn-A05-p2172320	A-genome specific (napus-rapa)	1	0.5257344	not scorable	not scorable	0.9752066	0.7061011
####	Bn-A05-p21806633	A-genome specific (napus-rapa)	0.9952153	0.69458	not scorable	not scorable	0.9834711	0.734471
####	Bn-A05-p21806801	A-genome specific (napus-rapa)	0.9952153	0.5898287	not scorable	not scorable	1	0.7937827
####	Bn-A05-p21809718	A-genome specific (napus-rapa)	0.9976076	0.8536648	not scorable	not scorable	0.9834711	0.7489479
####	Bn-A05-p21814557	A-genome specific (napus-rapa)	1	0.5700043	not scorable	not scorable	1	0.7504022
####	Bn-A05-p21848003	A-genome specific (rapa only)	not scorable	not scorable	not scorable	not scorable	0.9752066	0.7820434
####	Bn-A05-p21852014	A-genome specific (napus-rapa)	0.9976076	0.7914261	not scorable	not scorable	1	0.7369435
####	Bn-A05-p21852563	A-genome specific (napus-rapa)	0.9928229	0.8486146	not scorable	not scorable	0.9834711	0.6912694
####	Bn-A05-p21853739	napus specific	0.9688995	0.7908984	not scorable	not scorable	not scorable	not scorable
####	Bn-A05-p21865451	A-genome specific (napus-rapa)	1	0.4477971	not scorable	not scorable	0.9917355	0.7345971
####	Bn-A05-p21886422	A-genome specific (napus-rapa)	0.8947368	0.537281	not scorable	not scorable	0.9917355	0.7030191
####	Bn-A05-p21887311	A-genome specific (napus-rapa)	0.9904306	0.4551728	not scorable	not scorable	0.9834711	0.7781072
####	Bn-A05-p21889289	A-genome specific (napus-rapa)	0.9808612	0.6830326	not scorable	not scorable	0.9834711	0.7096455
####	Bn-A05-p21915455	genome unspecific (oleracea-rapa)	not scorable	not scorable	0.9922481	0.3139133	0.9421487	0.6590759
####	Bn-A05-p21930978	A-genome specific (napus-rapa)	1	0.620148	not scorable	not scorable	0.9586777	0.7267897
####	Bn-A05-p21973951	A-genome specific (napus-rapa)	0.8995215	0.7815474	not scorable	not scorable	0.7768595	0.7941189
####	Bn-A05-p22015412	A-genome specific (napus-rapa)	0.9976076	0.1640322	not scorable	not scorable	0.9834711	0.6303926
####	Bn-A05-p22021177	A-genome specific (napus-rapa)	1	0.6568402	not scorable	not scorable	0.9752066	0.7416866
####	Bn-A05-p22027389	A-genome specific (napus-rapa)	0.9976076	0.4874499	not scorable	not scorable	0.9669421	0.6166462
####	Bn-A05-p22028401	A-genome specific (napus-rapa)	1	0.8104924	not scorable	not scorable	0.6033058	0.8254998
####	Bn-A05-p22060825	A-genome specific (napus-rapa)	1	0.7892071	not scorable	not scorable	1	0.8137309
####	Bn-A05-p2208960	A-genome specific (napus-rapa)	0.9952153	0.873731	not scorable	not scorable	0.9504132	0.7929228

####	Bn-A05-p22089650	A-genome specific (napus-rapa)	0.9665072	0.7888787	not scorable	not scorable	0.9834711	0.7721342
####	Bn-A05-p22101592	A-genome specific (napus-rapa)	0.8971292	0.7844431	not scorable	not scorable	0.9752066	0.7825384
####	Bn-A05-p22111286	A-genome specific (napus-rapa)	0.9784689	0.234571	not scorable	not scorable	0.9752066	0.5864163
####	Bn-A05-p22111345	A-genome specific (rapa only)	not scorable	not scorable	not scorable	not scorable	0.9586777	0.5490675
####	Bn-A05-p22114137	A-genome specific (rapa only)	not scorable	not scorable	not scorable	not scorable	0.9586777	0.7816538
####	Bn-A05-p22117809	A-genome specific (rapa only)	not scorable	not scorable	not scorable	not scorable	0.9586777	0.6645556
####	Bn-A05-p221189	A-genome specific (napus-rapa)	0.5454546	0.862361	not scorable	not scorable	0.9669421	0.7192402
####	Bn-A05-p22132297	A-genome specific (napus-rapa)	0.9712918	0.4790277	not scorable	not scorable	0.9834711	0.8276204
####	Bn-A05-p22135265	genome unspecific (all in)	0.9784689	0.8138227	0	0.4607987	0.9917355	0.8274808
####	Bn-A05-p22141046	A-genome specific (napus-rapa)	0.9808612	0.5483452	not scorable	not scorable	0.9917355	0.6880242
####	Bn-A05-p22142609	A-genome specific (rapa only)	not scorable	not scorable	not scorable	not scorable	0.9917355	0.6666172
####	Bn-A05-p22145624	A-genome specific (rapa only)	not scorable	not scorable	not scorable	not scorable	0.9834711	0.7035027
####	Bn-A05-p22152482	A-genome specific (napus-rapa)	0.9904306	0.3749974	not scorable	not scorable	0.9752066	0.8042552
####	Bn-A05-p22153383	A-genome specific (rapa only)	not scorable	not scorable	not scorable	not scorable	0.9669421	0.8040302
####	Bn-A05-p22159613	A-genome specific (napus-rapa)	0.9880383	0.7251481	not scorable	not scorable	0.9917355	0.7521194
####	Bn-A05-p22169560	A-genome specific (rapa only)	not scorable	not scorable	not scorable	not scorable	0.9917355	0.621645
####	Bn-A05-p22182628	A-genome specific (napus-rapa)	0.9856459	0.7051139	not scorable	not scorable	0.9917355	0.5906218
####	Bn-A05-p22218776	A-genome specific (napus-rapa)	0.9928229	0.7933466	not scorable	not scorable	1	0.7415362
####	Bn-A05-p22221563	A-genome specific (napus-rapa)	0.9760765	0.7929196	not scorable	not scorable	1	0.7795539
####	Bn-A05-p22224811	A-genome specific (napus-rapa)	1	0.7413105	not scorable	not scorable	1	0.8088477
####	Bn-A05-p22249401	A-genome specific (napus-rapa)	0.9784689	0.5096595	not scorable	not scorable	1	0.7789563
####	Bn-A05-p22265537	A-genome specific (napus-rapa)	0.9808612	0.7759255	not scorable	not scorable	0.9834711	0.7848645
####	Bn-A05-p22266340	A-genome specific (napus-rapa)	0.9617225	0.6425937	not scorable	not scorable	0.9917355	0.6752724
####	Bn-A05-p22266933	A-genome specific (napus-rapa)	0.9497608	0.7471942	not scorable	not scorable	0.9338843	0.7040766
####	Bn-A05-p22269511	A-genome specific (napus-rapa)	0.9258373	0.8017946	not scorable	not scorable	1	0.7862009
####	Bn-A05-p2227024	A-genome specific (napus-rapa)	0.9904306	0.7797565	not scorable	not scorable	0.9917355	0.8057628
####	Bn-A05-p22276626	A-genome specific (napus-rapa)	0.9808612	0.2848408	not scorable	not scorable	1	0.710373
####	Bn-A05-p22282750	A-genome specific (napus-rapa)	0.9904306	0.8126651	not scorable	not scorable	0.9917355	0.8914528
####	Bn-A05-p22282814	A-genome specific (napus-rapa)	0.9019139	0.7565764	not scorable	not scorable	0.9917355	0.7396141
####	Bn-A05-p22284501	A-genome specific (napus-rapa)	0.9880383	0.7566727	not scorable	not scorable	0.9917355	0.7012435
####	Bn-A05-p22298146	A-genome specific (napus-rapa)	0.9545454	0.1958308	not scorable	not scorable	1	0.5715545
####	Bn-A05-p22299077	A-genome specific (rapa only)	not scorable	not scorable	not scorable	not scorable	0.9752066	0.4721518
####	Bn-A05-p22299214	A-genome specific (rapa only)	not scorable	not scorable	not scorable	not scorable	0.9834711	0.6176679

####	Bn-A05-p22310050	A-genome specific (rapa only)	not scorable	not scorable	not scorable	not scorable	0.9917355	0.7187123
####	Bn-A05-p22318660	A-genome specific (napus-rapa)	0.9880383	0.8013927	not scorable	not scorable	0.6033058	0.8004208
####	Bn-A05-p22320477	all not scorable	not scorable	not scorable	not scorable	not scorable	not scorable	not scorable
####	Bn-A05-p22324761	A-genome specific (napus-rapa)	0.9736842	0.3963413	not scorable	not scorable	0.9752066	0.6690314
####	Bn-A05-p2233637	A-genome specific (napus-rapa)	0.9928229	0.8317678	not scorable	not scorable	0.9917355	0.7916046
####	Bn-A05-p2233700	A-genome specific (napus-rapa)	0.9880383	0.7833233	not scorable	not scorable	0.9834711	0.7703477
####	Bn-A05-p2234082	A-genome specific (napus-rapa)	0.9928229	0.7385961	not scorable	not scorable	0.9917355	0.6878072
####	Bn-A05-p22350368	A-genome specific (rapa only)	not scorable	not scorable	not scorable	not scorable	0.8842975	0.83925
####	Bn-A05-p22351135	A-genome specific (napus-rapa)	0.9808612	0.8755383	not scorable	not scorable	0.9504132	0.7221171
####	Bn-A05-p22392750	A-genome specific (rapa only)	not scorable	not scorable	not scorable	not scorable	0.6115702	0.7724408
####	Bn-A05-p22406985	A-genome specific (rapa only)	not scorable	not scorable	not scorable	not scorable	1	0.7708783
####	Bn-A05-p22427412	A-genome specific (napus-rapa)	0.9952153	0.3635646	not scorable	not scorable	0.9917355	0.6710666
####	Bn-A05-p22452120	all not scorable	not scorable	not scorable	not scorable	not scorable	not scorable	not scorable
####	Bn-A05-p22454211	A-genome specific (napus-rapa)	0.9569378	0.7320339	not scorable	not scorable	0.9586777	0.7344668
####	Bn-A05-p22454557	A-genome specific (napus-rapa)	0.9832536	0.7811071	not scorable	not scorable	0.9752066	0.7615733
####	Bn-A05-p22459438	A-genome specific (napus-rapa)	0.9784689	0.8313856	not scorable	not scorable	0.9586777	0.7304482
####	Bn-A05-p2246038	A-genome specific (napus-rapa)	0.9928229	0.7849702	not scorable	not scorable	0.9917355	0.7576417
####	Bn-A05-p22463256	A-genome specific (napus-rapa)	0.9425837	0.5035987	not scorable	not scorable	0.9834711	0.5577688
####	Bn-A05-p22476414	A-genome specific (napus-rapa)	0.9928229	0.5296592	not scorable	not scorable	0.9752066	0.7995027
####	Bn-A05-p22478538	A-genome specific (napus-rapa)	0.9952153	0.4276024	not scorable	not scorable	0.9752066	0.6946626
####	Bn-A05-p22480535	A-genome specific (napus-rapa)	0.9976076	0.4092898	not scorable	not scorable	0.9669421	0.77586
####	Bn-A05-p22488454	A-genome specific (napus-rapa)	0.9952153	0.5340592	not scorable	not scorable	1	0.7580737
####	Bn-A05-p22498838	A-genome specific (napus-rapa)	1	0.6679631	not scorable	not scorable	0.9752066	0.6324893
####	Bn-A05-p22506006	A-genome specific (napus-rapa)	1	0.5484684	not scorable	not scorable	0.6363636	0.7906653
####	Bn-A05-p22511813	A-genome specific (napus-rapa)	0.9952153	0.7485923	not scorable	not scorable	0.6280992	0.7735133
####	Bn-A05-p22513075	A-genome specific (napus-rapa)	0.9952153	0.6842329	not scorable	not scorable	0.6033058	0.7607438
####	Bn-A05-p22519751	A-genome specific (napus-rapa)	0.9952153	0.2751799	not scorable	not scorable	0.9917355	0.8236753
####	Bn-A05-p22519836	A-genome specific (napus-rapa)	0.9832536	0.7055925	not scorable	not scorable	1	0.772859
####	Bn-A05-p2254100	A-genome specific (napus-rapa)	0.9976076	0.7945387	not scorable	not scorable	1	0.7825052
####	Bn-A05-p22553631	napus specific	0.9976076	0.4702678	not scorable	not scorable	not scorable	not scorable
####	Bn-A05-p22563719	A-genome specific (napus-rapa)	0.9856459	0.6494826	not scorable	not scorable	0.9421487	0.7428306
####	Bn-A05-p22585118	A-genome specific (napus-rapa)	0.9976076	0.814613	not scorable	not scorable	0.9917355	0.7457741
####	Bn-A05-p22589874	A-genome specific (napus-rapa)	0.9617225	0.6215775	not scorable	not scorable	0.9669421	0.6610469

####	Bn-A05-p22591072	A-genome specific (napus-rapa)	0.9904306	0.8847374	not scorable	not scorable	0.9834711	0.8422496
####	Bn-A05-p22591626	A-genome specific (napus-rapa)	1	0.7675446	not scorable	not scorable	1	0.7635067
####	Bn-A05-p22599241	A-genome specific (napus-rapa)	0.9952153	0.2845806	not scorable	not scorable	0.9917355	0.774188
####	Bn-A05-p22613926	A-genome specific (napus-rapa)	0.9880383	0.76738	not scorable	not scorable	0.9917355	0.8168433
####	Bn-A05-p2261738	A-genome specific (napus-rapa)	0.9976076	0.7402956	not scorable	not scorable	1	0.8306921
####	Bn-A05-p22622143	A-genome specific (rapa only)	not scorable	not scorable	not scorable	not scorable	1	0.6048745
####	Bn-A05-p2262914	A-genome specific (napus-rapa)	0.9976076	0.3671518	not scorable	not scorable	0.9917355	0.7397051
####	Bn-A05-p22636722	A-genome specific (napus-rapa)	0.9976076	0.430973	not scorable	not scorable	0.9917355	0.8232467
####	Bn-A05-p22650582	A-genome specific (napus-rapa)	0.9928229	0.8681046	not scorable	not scorable	0.9834711	0.802765
####	Bn-A05-p22651145	A-genome specific (napus-rapa)	0.9904306	0.2977657	not scorable	not scorable	0.9504132	0.6113445
####	Bn-A05-p22653151	A-genome specific (napus-rapa)	0.9952153	0.7313808	not scorable	not scorable	0.9752066	0.7022651
####	Bn-A05-p22657528	A-genome specific (napus-rapa)	0.9449761	0.7330033	not scorable	not scorable	0.9421487	0.6958111
####	Bn-A05-p22658512	A-genome specific (napus-rapa)	0.9928229	0.7995687	not scorable	not scorable	0.9917355	0.8458923
####	Bn-A05-p22660561	all not scorable	not scorable	not scorable	not scorable	not scorable	not scorable	not scorable
####	Bn-A05-p22669409	napus specific	0.9904306	0.5690113	not scorable	not scorable	not scorable	not scorable
####	Bn-A05-p22677701	A-genome specific (napus-rapa)	0.9928229	0.661962	not scorable	not scorable	0.9752066	0.8399903
####	Bn-A05-p22677829	A-genome specific (napus-rapa)	0.9952153	0.8165326	not scorable	not scorable	0.9917355	0.7954273
####	Bn-A05-p22677937	A-genome specific (napus-rapa)	0.9976076	0.8343228	not scorable	not scorable	0.9752066	0.7478139
####	Bn-A05-p22682587	A-genome specific (napus-rapa)	0.9976076	0.6520808	not scorable	not scorable	0.9917355	0.6212825
####	Bn-A05-p22682639	A-genome specific (napus-rapa)	0.9952153	0.5789832	not scorable	not scorable	1	0.7407741
####	Bn-A05-p22689848	A-genome specific (napus-rapa)	0.9952153	0.6445189	not scorable	not scorable	0.9834711	0.7312164
####	Bn-A05-p22691469	A-genome specific (napus-rapa)	0.9928229	0.7812486	not scorable	not scorable	0.9917355	0.79443
####	Bn-A05-p22706491	A-genome specific (napus-rapa)	0.9545454	0.6963454	not scorable	not scorable	0.9917355	0.7546661
####	Bn-A05-p22712783	A-genome specific (napus-rapa)	0.9904306	0.2508829	not scorable	not scorable	1	0.7040576
####	Bn-A05-p2272518	A-genome specific (napus-rapa)	0.9976076	0.7209546	not scorable	not scorable	1	0.7030711
####	Bn-A05-p22727640	A-genome specific (napus-rapa)	0.9976076	0.7258548	not scorable	not scorable	1	0.7015526
####	Bn-A05-p2273196	A-genome specific (napus-rapa)	0.9928229	0.8041633	not scorable	not scorable	0.9834711	0.8163401
####	Bn-A05-p2274301	A-genome specific (napus-rapa)	1	0.3344539	not scorable	not scorable	0.9834711	0.7692136
####	Bn-A05-p22766713	A-genome specific (napus-rapa)	0.9904306	0.7295586	not scorable	not scorable	1	0.6895437
####	Bn-A05-p22766965	A-genome specific (napus-rapa)	0.9904306	0.8455086	not scorable	not scorable	0.9752066	0.7396585
####	Bn-A05-p22767027	A-genome specific (napus-rapa)	0.9856459	0.8483468	not scorable	not scorable	0.9834711	0.8161229
####	Bn-A05-p22767106	A-genome specific (napus-rapa)	0.9952153	0.7881978	not scorable	not scorable	0.9917355	0.828397
####	Bn-A05-p22771419	A-genome specific (napus-rapa)	0.9904306	0.5750239	not scorable	not scorable	0.9669421	0.7233328

####	Bn-A05-p22780601	A-genome specific (napus-rapa)	0.9258373	0.7603143	not scorable	not scorable	0.661157	0.8731608
####	Bn-A05-p22780670	A-genome specific (napus-rapa)	0.9665072	0.7261226	not scorable	not scorable	0.661157	0.7764697
####	Bn-A05-p22786339	A-genome specific (napus-rapa)	0.9976076	0.8509513	not scorable	not scorable	0.9669421	0.6952189
####	Bn-A05-p22789344	A-genome specific (napus-rapa)	0.5550239	0.7057846	not scorable	not scorable	0.9421487	0.6796387
####	Bn-A05-p22789711	A-genome specific (napus-rapa)	0.9760765	0.8125226	not scorable	not scorable	0.9752066	0.8158158
####	Bn-A05-p22789974	A-genome specific (napus-rapa)	0.9784689	0.7555637	not scorable	not scorable	0.9669421	0.6943771
####	Bn-A05-p22797664	A-genome specific (napus-rapa)	0.9856459	0.7540249	not scorable	not scorable	0.9917355	0.7634553
####	Bn-A05-p22804927	A-genome specific (napus-rapa)	0.9880383	0.5109917	not scorable	not scorable	1	0.814081
####	Bn-A05-p2280549	A-genome specific (napus-rapa)	0.9976076	0.6281154	not scorable	not scorable	0.9752066	0.7679021
####	Bn-A05-p22812957	A-genome specific (napus-rapa)	1	0.6609219	not scorable	not scorable	1	0.7739488
####	Bn-A05-p22813120	A-genome specific (napus-rapa)	0.9952153	0.9438366	not scorable	not scorable	1	0.8604133
####	Bn-A05-p22813805	genome unspecific (all in)	0.9856459	0.4101639	1	0.7905083	0.9752066	0.6975757
####	Bn-A05-p22826924	A-genome specific (napus-rapa)	0.9928229	0.7387625	not scorable	not scorable	0.9752066	0.6997511
####	Bn-A05-p22853415	A-genome specific (napus-rapa)	0.9473684	0.3411839	not scorable	not scorable	0.9752066	0.6384286
####	Bn-A05-p228555	A-genome specific (napus-rapa)	0.9808612	0.815669	not scorable	not scorable	0.9834711	0.7364951
####	Bn-A05-p2285903	genome unspecific (all in)	0.9952153	0.3339771	1	0.8260807	1	0.8848283
####	Bn-A05-p22863641	A-genome specific (napus-rapa)	0.9904306	0.395654	not scorable	not scorable	0.9917355	0.6621847
####	Bn-A05-p22868682	A-genome specific (napus-rapa)	0.9904306	0.7768592	not scorable	not scorable	0.9834711	0.7630409
####	Bn-A05-p22868951	A-genome specific (napus-rapa)	0.9856459	0.7994657	not scorable	not scorable	0.9834711	0.8311573
####	Bn-A05-p22874045	genome unspecific (all in)	1	0.7428617	0.8449612	0.6617243	0.9834711	0.6947252
####	Bn-A05-p2287815	A-genome specific (napus-rapa)	0.9976076	0.8395217	not scorable	not scorable	0.9834711	0.6707829
####	Bn-A05-p22889788	A-genome specific (napus-rapa)	0.9569378	0.4011386	not scorable	not scorable	0.9917355	0.7368926
####	Bn-A05-p22899595	A-genome specific (napus-rapa)	0.9856459	0.7586249	not scorable	not scorable	0.9917355	0.8500487
####	Bn-A05-p22911906	A-genome specific (napus-rapa)	0.9521531	0.7017894	not scorable	not scorable	0.9917355	0.7193606
####	Bn-A05-p22912544	A-genome specific (napus-rapa)	0.9641148	0.3944719	not scorable	not scorable	1	0.6956896
####	Bn-A05-p22913147	A-genome specific (napus-rapa)	0.9976076	0.8058243	not scorable	not scorable	1	0.702931
####	Bn-A05-p22913571	A-genome specific (napus-rapa)	1	0.7675736	not scorable	not scorable	1	0.9048258
####	Bn-A05-p22913874	A-genome specific (napus-rapa)	0.9952153	0.7786604	not scorable	not scorable	1	0.7453237
####	Bn-A05-p22915357	A-genome specific (napus-rapa)	0.9928229	0.3714773	not scorable	not scorable	1	0.8114662
####	Bn-A05-p22917436	A-genome specific (napus-rapa)	0.9952153	0.8394711	not scorable	not scorable	1	0.8325576
####	Bn-A05-p22920228	A-genome specific (napus-rapa)	1	0.7847597	not scorable	not scorable	1	0.7760417
####	Bn-A05-p22934224	A-genome specific (napus-rapa)	0.9760765	0.3045922	not scorable	not scorable	1	0.7180079
####	Bn-A05-p2293577	A-genome specific (napus-rapa)	0.4425837	0.7886499	not scorable	not scorable	1	0.7649547

####	Bn-A05-p22938228	A-genome specific (napus-rapa)	0.9617225	0.5224178	not scorable	not scorable	0.9917355	0.6127103
####	Bn-A05-p22943215	A-genome specific (napus-rapa)	0.9880383	0.7238548	not scorable	not scorable	0.9834711	0.7074269
####	Bn-A05-p22946715	napus specific	0.5263158	0.8659872	not scorable	not scorable	not scorable	not scorable
####	Bn-A05-p22946858	A-genome specific (napus-rapa)	0.9760765	0.6395214	not scorable	not scorable	0.9834711	0.7772235
####	Bn-A05-p22948751	A-genome specific (napus-rapa)	0.9354067	0.2743224	not scorable	not scorable	0.9834711	0.7820827
####	Bn-A05-p2296307	A-genome specific (napus-rapa)	0.9928229	0.7033357	not scorable	not scorable	0.9917355	0.6460242
####	Bn-A05-p22969236	A-genome specific (napus-rapa)	1	0.6644443	not scorable	not scorable	1	0.6461107
####	Bn-A05-p22969550	A-genome specific (napus-rapa)	0.9688995	0.7875746	not scorable	not scorable	1	0.7857482
####	Bn-A05-p22973324	A-genome specific (napus-rapa)	0.9712918	0.7805417	not scorable	not scorable	0.9917355	0.7270243
####	Bn-A05-p2297395	A-genome specific (napus-rapa)	0.9928229	0.6505721	not scorable	not scorable	1	0.6231968
####	Bn-A05-p22976431	A-genome specific (napus-rapa)	0.9952153	0.7606364	not scorable	not scorable	1	0.7793296
####	Bn-A05-p22977061	A-genome specific (napus-rapa)	1	0.4247281	not scorable	not scorable	1	0.8217116
####	Bn-A05-p22981266	A-genome specific (napus-rapa)	0.9688995	0.4603234	not scorable	not scorable	1	0.7869714
####	Bn-A05-p2299167	A-genome specific (napus-rapa)	0.9904306	0.7875724	not scorable	not scorable	1	0.705898
####	Bn-A05-p2299442	A-genome specific (napus-rapa)	0.9736842	0.8443511	not scorable	not scorable	0.9008265	0.8126721
####	Bn-A05-p2300076	A-genome specific (napus-rapa)	0.9952153	0.7731925	not scorable	not scorable	0.9917355	0.7896207
####	Bn-A05-p2301167	A-genome specific (napus-rapa)	1	0.6628417	not scorable	not scorable	1	0.6214259
####	Bn-A05-p23019620	A-genome specific (napus-rapa)	0.9880383	0.7247303	not scorable	not scorable	0.9752066	0.6808112
####	Bn-A05-p23020463	A-genome specific (rapa only)	not scorable	not scorable	not scorable	not scorable	0.5041322	0.8869507
####	Bn-A05-p23020834	A-genome specific (napus-rapa)	0.9952153	0.7800123	not scorable	not scorable	0.9834711	0.7115383
####	Bn-A05-p23027881	A-genome specific (napus-rapa)	0.9521531	0.3005885	not scorable	not scorable	1	0.6528514
####	Bn-A05-p23030143	A-genome specific (napus-rapa)	0.9952153	0.4945874	not scorable	not scorable	1	0.8193437
####	Bn-A05-p2303112	A-genome specific (napus-rapa)	0.9928229	0.6062984	not scorable	not scorable	1	0.6444352
####	Bn-A05-p23032803	A-genome specific (napus-rapa)	0.9904306	0.7808572	not scorable	not scorable	0.9917355	0.8220969
####	Bn-A05-p23044521	A-genome specific (napus-rapa)	0.9952153	0.8038412	not scorable	not scorable	0.9834711	0.7540687
####	Bn-A05-p23079485	A-genome specific (napus-rapa)	1	0.4628164	not scorable	not scorable	0.9752066	0.7464112
####	Bn-A05-p23081875	A-genome specific (napus-rapa)	0.9832536	0.3126375	not scorable	not scorable	0.9917355	0.7382128
####	Bn-A05-p23102053	genome unspecific (all in)	1	0.3415891	0.9224806	0.4624231	1	0.930966
####	Bn-A05-p23126108	A-genome specific (napus-rapa)	0.9856459	0.6845897	not scorable	not scorable	0.6280992	0.7347293
####	Bn-A05-p23140205	A-genome specific (napus-rapa)	0.9976076	0.6502038	not scorable	not scorable	0.9917355	0.6519747
####	Bn-A05-p23141080	A-genome specific (napus-rapa)	0.9832536	0.6419073	not scorable	not scorable	0.9752066	0.6830118
####	Bn-A05-p23143046	A-genome specific (napus-rapa)	0.9904306	0.8207465	not scorable	not scorable	0.9752066	0.742853
####	Bn-A05-p23145678	A-genome specific (rapa only)	not scorable	not scorable	not scorable	not scorable	1	0.8073645

####	Bn-A05-p23146882	A-genome specific (napus-rapa)	0.9976076	0.5902624	not scorable	not scorable	0.9834711	0.5176431
####	Bn-A05-p23154172	A-genome specific (napus-rapa)	0.9928229	0.5456713	not scorable	not scorable	1	0.6392193
####	Bn-A05-p23159292	genome unspecific (all in)	0.9688995	0.4220355	1	0.7186732	0.9917355	0.8205839
####	Bn-A05-p23165568	A-genome specific (napus-rapa)	0.9832536	0.7834677	not scorable	not scorable	1	0.828151
####	Bn-A05-p23166612	A-genome specific (napus-rapa)	0.9928229	0.7672309	not scorable	not scorable	0.9917355	0.6131639
####	Bn-A05-p23166708	A-genome specific (napus-rapa)	0.9928229	0.7877322	not scorable	not scorable	1	0.7255321
####	Bn-A05-p23167201	A-genome specific (napus-rapa)	0.8803828	0.8186991	not scorable	not scorable	0.8512397	0.7911698
####	Bn-A05-p23172786	A-genome specific (napus-rapa)	0.9736842	0.2467696	not scorable	not scorable	0.9669421	0.6358763
####	Bn-A05-p23179980	A-genome specific (napus-rapa)	0.9665072	0.7030742	not scorable	not scorable	0.9752066	0.6195934
####	Bn-A05-p23188259	A-genome specific (napus-rapa)	0.9976076	0.7992148	not scorable	not scorable	0.9917355	0.771064
####	Bn-A05-p23188406	A-genome specific (napus-rapa)	0.9952153	0.5092274	not scorable	not scorable	1	0.5618805
####	Bn-A05-p23196656	A-genome specific (napus-rapa)	0.9928229	0.7156864	not scorable	not scorable	1	0.6975278
####	Bn-A05-p23207708	A-genome specific (rapa only)	not scorable	not scorable	not scorable	not scorable	1	0.803066
####	Bn-A05-p23253826	A-genome specific (napus-rapa)	0.9354067	0.744895	not scorable	not scorable	0.9669421	0.7433818
####	Bn-A05-p23276053	A-genome specific (napus-rapa)	0.9330143	0.7757209	not scorable	not scorable	0.9917355	0.7499852
####	Bn-A05-p23285926	A-genome specific (napus-rapa)	0.9856459	0.3318587	not scorable	not scorable	1	0.6409353
####	Bn-A05-p23288913	napus specific	0.784689	0.6076639	not scorable	not scorable	not scorable	not scorable
####	Bn-A05-p23290863	A-genome specific (napus-rapa)	0.9784689	0.7835447	not scorable	not scorable	0.9752066	0.785302
####	Bn-A05-p2329965	genome unspecific (oleracea-rapa)	not scorable	not scorable	0.9922481	0.3731116	0.8760331	0.9412735
####	Bn-A05-p23314738	A-genome specific (napus-rapa)	0.9712918	0.2453709	not scorable	not scorable	1	0.684404
####	Bn-A05-p23318038	A-genome specific (napus-rapa)	1	0.7833564	not scorable	not scorable	1	0.9196517
####	Bn-A05-p23340419	A-genome specific (napus-rapa)	0.9832536	0.7927031	not scorable	not scorable	1	0.8421641
####	Bn-A05-p23358283	A-genome specific (napus-rapa)	0.9688995	0.5917117	not scorable	not scorable	0.5950413	0.5466749
####	Bn-A05-p23372051	A-genome specific (napus-rapa)	0.9952153	0.7640694	not scorable	not scorable	1	0.7845569
####	Bn-A05-p2337684	A-genome specific (napus-rapa)	0.9904306	0.8493359	not scorable	not scorable	0.9917355	0.7586541
####	Bn-A05-p23377419	A-genome specific (napus-rapa)	0.9952153	0.3796244	not scorable	not scorable	0.9917355	0.8441574
####	Bn-A05-p23397606	A-genome specific (napus-rapa)	0.9904306	0.8007011	not scorable	not scorable	0.9834711	0.766562
####	Bn-A05-p23405117	A-genome specific (napus-rapa)	0.9856459	0.7413768	not scorable	not scorable	0.9834711	0.6588489
####	Bn-A05-p2340565	A-genome specific (napus-rapa)	0.423445	0.8328705	not scorable	not scorable	0.9752066	0.7977849
####	Bn-A05-p23415646	A-genome specific (napus-rapa)	0.9976076	0.336408	not scorable	not scorable	0.9917355	0.7945306
####	Bn-A05-p23424482	A-genome specific (rapa only)	not scorable	not scorable	not scorable	not scorable	1	0.7444375
####	Bn-A05-p23433947	A-genome specific (napus-rapa)	0.9904306	0.3968226	not scorable	not scorable	1	0.8180188
####	Bn-A05-p23445454	A-genome specific (napus-rapa)	0.9952153	0.372411	not scorable	not scorable	1	0.7431428

####	Bn-A05-p2345233	A-genome specific (napus-rapa)	0.6722488	0.6780389	not scorable	not scorable	0.9917355	0.7961171
####	Bn-A05-p2345849	A-genome specific (napus-rapa)	0.9976076	0.3395363	not scorable	not scorable	0.9917355	0.6822758
####	Bn-A05-p23465599	A-genome specific (rapa only)	not scorable	not scorable	not scorable	not scorable	0.9917355	0.831443
####	Bn-A05-p23466337	A-genome specific (rapa only)	not scorable	not scorable	not scorable	not scorable	1	0.2763056
####	Bn-A05-p23478913	A-genome specific (napus-rapa)	0.9760765	0.7508786	not scorable	not scorable	1	0.7716454
####	Bn-A05-p23488683	A-genome specific (napus-rapa)	0.9617225	0.8047315	not scorable	not scorable	0.9917355	0.6813025
####	Bn-A05-p23498540	A-genome specific (napus-rapa)	0.9880383	0.7994444	not scorable	not scorable	1	0.8852836
####	Bn-A05-p2350131	A-genome specific (napus-rapa)	0.9712918	0.7374497	not scorable	not scorable	0.9917355	0.7220762
####	Bn-A05-p23502993	A-genome specific (napus-rapa)	0.9856459	0.7766336	not scorable	not scorable	1	0.8236775
####	Bn-A05-p23512396	A-genome specific (napus-rapa)	0.9736842	0.7557615	not scorable	not scorable	0.6033058	0.7420741
####	Bn-A05-p23512931	napus specific	0.9856459	0.7598492	not scorable	not scorable	not scorable	not scorable
####	Bn-A05-p2351545	A-genome specific (napus-rapa)	0.9976076	0.2861604	not scorable	not scorable	1	0.7926196
####	Bn-A05-p23529753	A-genome specific (napus-rapa)	0.9736842	0.7686282	not scorable	not scorable	1	0.7840353
####	Bn-A05-p23529824	A-genome specific (napus-rapa)	0.9784689	0.6313235	not scorable	not scorable	0.9752066	0.7448338
####	Bn-A05-p23529883	A-genome specific (napus-rapa)	0.9808612	0.788775	not scorable	not scorable	1	0.6647314
####	Bn-A05-p23530018	A-genome specific (napus-rapa)	0.9880383	0.7887045	not scorable	not scorable	0.9752066	0.7810897
####	Bn-A05-p23530095	A-genome specific (napus-rapa)	0.9808612	0.7996424	not scorable	not scorable	1	0.7034416
####	Bn-A05-p23531991	A-genome specific (napus-rapa)	0.9784689	0.8067618	not scorable	not scorable	0.9917355	0.7172812
####	Bn-A05-p23532126	A-genome specific (napus-rapa)	0.9808612	0.7725722	not scorable	not scorable	0.9917355	0.7330098
####	Bn-A05-p23532489	A-genome specific (napus-rapa)	0.9928229	0.7669217	not scorable	not scorable	1	0.8007853
####	Bn-A05-p23533696	all not scorable	not scorable	not scorable	not scorable	not scorable	not scorable	not scorable
####	Bn-A05-p2353656	A-genome specific (napus-rapa)	0.9928229	0.8010672	not scorable	not scorable	0.9917355	0.6917989
####	Bn-A05-p23543751	A-genome specific (napus-rapa)	0.9832536	0.7781438	not scorable	not scorable	0.9834711	0.7185529
####	Bn-A05-p23547791	A-genome specific (napus-rapa)	0.9928229	0.3867505	not scorable	not scorable	0.9834711	0.7957595
####	Bn-A05-p23553029	A-genome specific (napus-rapa)	0.9856459	0.7925563	not scorable	not scorable	0.9917355	0.7326556
####	Bn-A05-p23562940	A-genome specific (napus-rapa)	0.9928229	0.7135322	not scorable	not scorable	1	0.9063296
####	Bn-A05-p23587018	A-genome specific (napus-rapa)	0.9617225	0.6405453	not scorable	not scorable	1	0.7185994
####	Bn-A05-p23587547	A-genome specific (napus-rapa)	0.9736842	0.7510253	not scorable	not scorable	0.9917355	0.7714632
####	Bn-A05-p23588992	A-genome specific (napus-rapa)	0.9665072	0.6657295	not scorable	not scorable	0.9917355	0.6366118
####	Bn-A05-p23593836	A-genome specific (napus-rapa)	0.9832536	0.7880369	not scorable	not scorable	1	0.8499617
####	Bn-A05-p23598510	A-genome specific (napus-rapa)	0.3923445	0.6619669	not scorable	not scorable	0.6033058	0.6373359
####	Bn-A05-p23607588	A-genome specific (napus-rapa)	0.9880383	0.5126613	not scorable	not scorable	1	0.7859963
####	Bn-A05-p2367876	A-genome specific (napus-rapa)	0.9688995	0.5550761	not scorable	not scorable	0.9834711	0.6929861

####	Bn-A05-p23680925	A-genome specific (napus-rapa)	0.8803828	0.7859167	not scorable	not scorable	0.8512397	0.822635
####	Bn-A05-p23691939	A-genome specific (napus-rapa)	0.9832536	0.3169344	not scorable	not scorable	0.9917355	0.7562806
####	Bn-A05-p23692249	A-genome specific (napus-rapa)	0.9665072	0.7746539	not scorable	not scorable	0.9834711	0.8272757
####	Bn-A05-p23693572	A-genome specific (napus-rapa)	0.9736842	0.4139259	not scorable	not scorable	0.9917355	0.7164802
####	Bn-A05-p23699894	A-genome specific (napus-rapa)	0.9641148	0.6927411	not scorable	not scorable	1	0.8170996
####	Bn-A05-p23701898	A-genome specific (rapa only)	not scorable	not scorable	not scorable	not scorable	0.9917355	0.545823
####	Bn-A05-p23702049	A-genome specific (napus-rapa)	0.9617225	0.7894464	not scorable	not scorable	1	0.8014603
####	Bn-A05-p23702275	A-genome specific (rapa only)	not scorable	not scorable	not scorable	not scorable	1	0.7854686
####	Bn-A05-p23703740	A-genome specific (napus-rapa)	0.9641148	0.803811	not scorable	not scorable	1	0.817339
####	Bn-A05-p23704743	A-genome specific (napus-rapa)	0.9808612	0.9268792	not scorable	not scorable	0.5950413	0.9458012
####	Bn-A05-p23717457	A-genome specific (rapa only)	not scorable	not scorable	not scorable	not scorable	0.9834711	0.6571057
####	Bn-A05-p23718363	A-genome specific (napus-rapa)	0.9808612	0.784701	not scorable	not scorable	0.9917355	0.7905719
####	Bn-A05-p23726137	A-genome specific (rapa only)	not scorable	not scorable	not scorable	not scorable	0.9834711	0.8130754
####	Bn-A05-p23745136	A-genome specific (rapa only)	not scorable	not scorable	not scorable	not scorable	0.9834711	0.7343995
####	Bn-A05-p2376801	A-genome specific (napus-rapa)	0.9904306	0.815001	not scorable	not scorable	0.9752066	0.7108423
####	Bn-A05-p23776343	A-genome specific (napus-rapa)	0.9736842	0.7018662	not scorable	not scorable	1	0.7682801
####	Bn-A05-p23776869	A-genome specific (napus-rapa)	0.9784689	0.738869	not scorable	not scorable	0.9917355	0.8472357
####	Bn-A05-p23782123	A-genome specific (napus-rapa)	0.9712918	0.8232322	not scorable	not scorable	0.5785124	0.8589618
####	Bn-A05-p23782304	A-genome specific (napus-rapa)	0.9760765	0.7808185	not scorable	not scorable	0.9752066	0.7006547
####	Bn-A05-p23790987	A-genome specific (rapa only)	not scorable	not scorable	not scorable	not scorable	1	0.9029719
####	Bn-A05-p23791467	A-genome specific (napus-rapa)	0.9784689	0.7675696	not scorable	not scorable	1	0.7962339
####	Bn-A05-p23802326	A-genome specific (napus-rapa)	0.9736842	0.4184909	not scorable	not scorable	0.9752066	0.5859402
####	Bn-A05-p23838032	A-genome specific (napus-rapa)	0.9736842	0.3907262	not scorable	not scorable	1	0.8177654
####	Bn-A05-p23844016	A-genome specific (napus-rapa)	0.9210526	0.7035195	not scorable	not scorable	1	0.6986886
####	Bn-A05-p23844225	A-genome specific (napus-rapa)	0.8971292	0.5920736	not scorable	not scorable	0.9834711	0.7840218
####	Bn-A05-p23848037	A-genome specific (napus-rapa)	0.9665072	0.3917488	not scorable	not scorable	0.9586777	0.696164
####	Bn-A05-p23855991	A-genome specific (napus-rapa)	0.9593301	0.7654794	not scorable	not scorable	0.9669421	0.7048714
####	Bn-A05-p23873413	A-genome specific (napus-rapa)	0.9473684	0.7606381	not scorable	not scorable	1	0.8063037
####	Bn-A05-p23876386	A-genome specific (napus-rapa)	0.9545454	0.7733989	not scorable	not scorable	0.9752066	0.769637
####	Bn-A05-p23893594	napus specific	0.9401914	0.7760084	not scorable	not scorable	not scorable	not scorable
####	Bn-A05-p23899052	A-genome specific (rapa only)	not scorable	not scorable	not scorable	not scorable	1	0.7556387
####	Bn-A05-p23899654	A-genome specific (rapa only)	not scorable	not scorable	not scorable	not scorable	1	0.8132989
####	Bn-A05-p23906880	A-genome specific (rapa only)	not scorable	not scorable	not scorable	not scorable	1	0.9095823

####	Bn-A05-p23909209	A-genome specific (rapa only)	not scorable	not scorable	not scorable	not scorable	0.9917355	0.712705
####	Bn-A05-p23928643	A-genome specific (napus-rapa)	0.9641148	0.7736719	not scorable	not scorable	0.9834711	0.7780471
####	Bn-A05-p2393098	A-genome specific (napus-rapa)	0.9904306	0.7314749	not scorable	not scorable	1	0.8280163
####	Bn-A05-p2394919	A-genome specific (napus-rapa)	0.9401914	0.7789187	not scorable	not scorable	0.9834711	0.6658624
####	Bn-A05-p2416806	A-genome specific (napus-rapa)	0.9736842	0.3280345	not scorable	not scorable	1	0.7681864
####	Bn-A05-p2422387	A-genome specific (napus-rapa)	0.9952153	0.5480961	not scorable	not scorable	1	0.7003313
####	Bn-A05-p2423005	A-genome specific (napus-rapa)	0.9904306	0.7817843	not scorable	not scorable	0.9834711	0.7939165
####	Bn-A05-p2431338	A-genome specific (napus-rapa)	0.9952153	0.7717138	not scorable	not scorable	1	0.7717351
####	Bn-A05-p2434176	napus specific	0.9808612	0.5088442	not scorable	not scorable	not scorable	not scorable
####	Bn-A05-p244051	A-genome specific (napus-rapa)	0.9928229	0.3492232	not scorable	not scorable	0.9917355	0.7717753
####	Bn-A05-p2440512	A-genome specific (napus-rapa)	0.9856459	0.4270251	not scorable	not scorable	0.9917355	0.7947931
####	Bn-A05-p2460244	A-genome specific (napus-rapa)	0.9736842	0.565502	not scorable	not scorable	0.9917355	0.8196729
####	Bn-A05-p2469338	A-genome specific (napus-rapa)	0.9473684	0.5384806	not scorable	not scorable	1	0.6877302
####	Bn-A05-p2472050	A-genome specific (napus-rapa)	0.9760765	0.6752376	not scorable	not scorable	1	0.7236546
####	Bn-A05-p2472584	genome unspecific (all in)	1	0.8257477	0.6046512	0.7736955	0.9752066	0.7309579
####	Bn-A05-p2488952	A-genome specific (napus-rapa)	0.9712918	0.52394	not scorable	not scorable	1	0.8483881
####	Bn-A05-p2489101	A-genome specific (napus-rapa)	0.9808612	0.44293	not scorable	not scorable	0.9752066	0.7494167
####	Bn-A05-p2492451	A-genome specific (napus-rapa)	0.9760765	0.4935141	not scorable	not scorable	0.9834711	0.8013238
####	Bn-A05-p249420	A-genome specific (napus-rapa)	0.9712918	0.7924633	not scorable	not scorable	0.6859504	0.750376
####	Bn-A05-p2494204	A-genome specific (napus-rapa)	1	0.4434827	not scorable	not scorable	0.9669421	0.2788869
####	Bn-A05-p2495066	A-genome specific (napus-rapa)	0.9784689	0.6106819	not scorable	not scorable	1	0.8035387
####	Bn-A05-p2495419	A-genome specific (napus-rapa)	0.8708134	0.3498313	not scorable	not scorable	1	0.7010313
####	Bn-A05-p2495697	A-genome specific (napus-rapa)	0.9928229	0.56278	not scorable	not scorable	1	0.7709854
####	Bn-A05-p2497466	A-genome specific (napus-rapa)	0.9976076	0.4663571	not scorable	not scorable	0.9917355	0.6722499
####	Bn-A05-p2501988	A-genome specific (napus-rapa)	0.9641148	0.8103759	not scorable	not scorable	0.9834711	0.7328052
####	Bn-A05-p2523070	A-genome specific (napus-rapa)	0.9976076	0.7947698	not scorable	not scorable	1	0.7488022
####	Bn-A05-p2523179	A-genome specific (napus-rapa)	0.4473684	0.7680776	not scorable	not scorable	0.9586777	0.8233419
####	Bn-A05-p2525969	A-genome specific (rapa only)	not scorable	not scorable	not scorable	not scorable	0.9917355	0.7322124
####	Bn-A05-p2534219	A-genome specific (napus-rapa)	1	0.4512076	not scorable	not scorable	0.9834711	0.7872394
####	Bn-A05-p253584	A-genome specific (napus-rapa)	0.9760765	0.4557344	not scorable	not scorable	0.9917355	0.7305357
####	Bn-A05-p25416	A-genome specific (napus-rapa)	0.9904306	0.8229336	not scorable	not scorable	0.7190083	0.8270081
####	Bn-A05-p25420	A-genome specific (napus-rapa)	0.9928229	0.8146019	not scorable	not scorable	0.9917355	0.7793977
####	Bn-A05-p2555885	A-genome specific (rapa only)	not scorable	not scorable	not scorable	not scorable	1	0.6865501

####	Bn-A05-p2563320	A-genome specific (napus-rapa)	0.9856459	0.7190225	not scorable	not scorable	1	0.7169521
####	Bn-A05-p2565185	A-genome specific (napus-rapa)	0.9904306	0.5145594	not scorable	not scorable	1	0.7155109
####	Bn-A05-p2565586	A-genome specific (napus-rapa)	0.9856459	0.8437333	not scorable	not scorable	0.9834711	0.763512
####	Bn-A05-p2565706	A-genome specific (napus-rapa)	0.9880383	0.6537374	not scorable	not scorable	0.9752066	0.663453
####	Bn-A05-p2565761	A-genome specific (napus-rapa)	0.9928229	0.7516063	not scorable	not scorable	0.9917355	0.7809256
####	Bn-A05-p2582423	A-genome specific (napus-rapa)	0.9856459	0.565093	not scorable	not scorable	0.9917355	0.5022524
####	Bn-A05-p2592847	genome unspecific (all in)	0.9880383	0.6977961	1	0.7624332	1	0.685295
####	Bn-A05-p2600118	A-genome specific (napus-rapa)	0.9712918	0.7719892	not scorable	not scorable	0.9752066	0.7950472
####	Bn-A05-p2604531	genome unspecific (all in)	0.9712918	0.5858039	1	0.7002443	0.9752066	0.7457366
####	Bn-A05-p2604957	A-genome specific (napus-rapa)	0.3444976	0.7906983	not scorable	not scorable	0.9752066	0.7057136
####	Bn-A05-p2606660	A-genome specific (napus-rapa)	0.9736842	0.478859	not scorable	not scorable	0.9752066	0.7283066
####	Bn-A05-p2610006	A-genome specific (napus-rapa)	0.9425837	0.8004273	not scorable	not scorable	0.9669421	0.7442045
####	Bn-A05-p262934	C-genome specific (oleracea only)	not scorable	not scorable	0.9457364	0.3201617	not scorable	not scorable
####	Bn-A05-p2641800	A-genome specific (napus-rapa)	0.9976076	0.7511835	not scorable	not scorable	1	0.9080027
####	Bn-A05-p2645307	A-genome specific (napus-rapa)	0.9808612	0.4063636	not scorable	not scorable	1	0.7661255
####	Bn-A05-p2645365	A-genome specific (napus-rapa)	0.8923445	0.7715023	not scorable	not scorable	1	0.7687295
####	Bn-A05-p2648679	genome unspecific (all in)	0.9904306	0.5575504	1	0.744995	0.9669421	0.5783864
####	Bn-A05-p2653139	A-genome specific (napus-rapa)	0.9856459	0.4438694	not scorable	not scorable	0.9917355	0.6693183
####	Bn-A05-p2656042	A-genome specific (napus-rapa)	0.9760765	0.4989264	not scorable	not scorable	1	0.7007509
####	Bn-A05-p2671921	A-genome specific (napus-rapa)	0.9976076	0.519507	not scorable	not scorable	0.6528926	0.723908
####	Bn-A05-p267915	A-genome specific (napus-rapa)	0.9904306	0.4218368	not scorable	not scorable	1	0.7966787
####	Bn-A05-p2688101	A-genome specific (napus-rapa)	0.9952153	0.3774758	not scorable	not scorable	0.5950413	0.7855997
####	Bn-A05-p2711007	A-genome specific (napus-rapa)	0.9952153	0.7755628	not scorable	not scorable	0.9917355	0.7754411
####	Bn-A05-p271108	A-genome specific (napus-rapa)	0.9808612	0.2950563	not scorable	not scorable	1	0.9272014
####	Bn-A05-p2711208	A-genome specific (napus-rapa)	0.9928229	0.3813375	not scorable	not scorable	0.9917355	0.6469216
####	Bn-A05-p2718015	A-genome specific (napus-rapa)	0.9904306	0.5096283	not scorable	not scorable	0.9834711	0.7924049
####	Bn-A05-p2726301	A-genome specific (napus-rapa)	0.9904306	0.7670671	not scorable	not scorable	0.9834711	0.7541008
####	Bn-A05-p2732409	A-genome specific (napus-rapa)	0.9856459	0.2966138	not scorable	not scorable	0.9586777	0.8117022
####	Bn-A05-p2735134	A-genome specific (napus-rapa)	0.9832536	0.8026188	not scorable	not scorable	0.9586777	0.8083189
####	Bn-A05-p2746921	A-genome specific (napus-rapa)	0.9808612	0.7475392	not scorable	not scorable	1	0.6875693
####	Bn-A05-p2751864	A-genome specific (napus-rapa)	1	0.4297657	not scorable	not scorable	1	0.8919886
####	Bn-A05-p2765988	A-genome specific (napus-rapa)	0.9952153	0.483954	not scorable	not scorable	1	0.7542082
####	Bn-A05-p2773820	A-genome specific (napus-rapa)	0.9952153	0.3757491	not scorable	not scorable	1	0.7467764

####	Bn-A05-p2777110	A-genome specific (napus-rapa)	0.9114832	0.7869422	not scorable	not scorable	0.9917355	0.707423
####	Bn-A05-p2777824	A-genome specific (napus-rapa)	0.9808612	0.7813799	not scorable	not scorable	1	0.8672201
####	Bn-A05-p2777912	A-genome specific (napus-rapa)	0.9784689	0.7961776	not scorable	not scorable	1	0.7482163
####	Bn-A05-p2778040	A-genome specific (napus-rapa)	0.9784689	0.7809176	not scorable	not scorable	0.9917355	0.7817007
####	Bn-A05-p2778230	A-genome specific (napus-rapa)	0.9712918	0.7976192	not scorable	not scorable	0.9917355	0.8100824
####	Bn-A05-p2778577	A-genome specific (napus-rapa)	0.9641148	0.7918816	not scorable	not scorable	0.9917355	0.7955325
####	Bn-A05-p2778679	A-genome specific (napus-rapa)	0.9880383	0.7723442	not scorable	not scorable	1	0.7768531
####	Bn-A05-p2778737	A-genome specific (napus-rapa)	0.9880383	0.7880577	not scorable	not scorable	1	0.836551
####	Bn-A05-p2779142	A-genome specific (napus-rapa)	0.9808612	0.3813139	not scorable	not scorable	1	0.766578
####	Bn-A05-p2791353	A-genome specific (napus-rapa)	0.5909091	0.773262	not scorable	not scorable	0.9917355	0.7376583
####	Bn-A05-p2794364	A-genome specific (napus-rapa)	0.9904306	0.5126869	not scorable	not scorable	1	0.7613098
####	Bn-A05-p2804703	A-genome specific (napus-rapa)	0.9976076	0.7785249	not scorable	not scorable	0.9917355	0.7353749
####	Bn-A05-p280800	A-genome specific (napus-rapa)	0.9856459	0.6939957	not scorable	not scorable	0.9752066	0.7133701
####	Bn-A05-p2830103	A-genome specific (napus-rapa)	0.9617225	0.30877	not scorable	not scorable	1	0.6827766
####	Bn-A05-p2839985	A-genome specific (napus-rapa)	0.6698565	0.6712462	not scorable	not scorable	0.9917355	0.8961731
####	Bn-A05-p2840537	A-genome specific (napus-rapa)	0.9952153	0.7569368	not scorable	not scorable	0.9917355	0.7831008
####	Bn-A05-p2840734	A-genome specific (napus-rapa)	0.9976076	0.7781259	not scorable	not scorable	1	0.7880946
####	Bn-A05-p2841024	A-genome specific (napus-rapa)	0.9976076	0.6610551	not scorable	not scorable	0.9917355	0.7817025
####	Bn-A05-p2843347	A-genome specific (napus-rapa)	0.9904306	0.7686352	not scorable	not scorable	1	0.86847
####	Bn-A05-p2844059	A-genome specific (napus-rapa)	0.9808612	0.7885288	not scorable	not scorable	0.9917355	0.7894838
####	Bn-A05-p2849284	A-genome specific (napus-rapa)	1	0.5752714	not scorable	not scorable	1	0.8260863
####	Bn-A05-p2852544	A-genome specific (napus-rapa)	0.9832536	0.2833515	not scorable	not scorable	0.9834711	0.6638611
####	Bn-A05-p2860757	A-genome specific (napus-rapa)	0.9952153	0.7616597	not scorable	not scorable	0.9917355	0.8343795
####	Bn-A05-p2863301	A-genome specific (napus-rapa)	0.9952153	0.7006729	not scorable	not scorable	0.9752066	0.6844829
####	Bn-A05-p2863674	A-genome specific (napus-rapa)	0.9952153	0.6066533	not scorable	not scorable	0.9834711	0.702307
####	Bn-A05-p2864686	A-genome specific (napus-rapa)	0.9952153	0.7876695	not scorable	not scorable	0.9917355	0.7941719
####	Bn-A05-p2865380	A-genome specific (napus-rapa)	0.9952153	0.7966254	not scorable	not scorable	0.9917355	0.8502555
####	Bn-A05-p2865634	A-genome specific (napus-rapa)	0.9952153	0.8073664	not scorable	not scorable	0.9669421	0.7225314
####	Bn-A05-p2865716	A-genome specific (napus-rapa)	0.9952153	0.801136	not scorable	not scorable	0.9752066	0.6728813
####	Bn-A05-p2868976	A-genome specific (napus-rapa)	0.9952153	0.6688196	not scorable	not scorable	1	0.7449527
####	Bn-A05-p2898061	genome unspecific (oleracea-rapa)	not scorable	not scorable	1	0.6624733	0.9917355	0.6877553
####	Bn-A05-p2924872	A-genome specific (napus-rapa)	0.6722488	0.789874	not scorable	not scorable	0.9917355	0.7774907
####	Bn-A05-p2925195	A-genome specific (napus-rapa)	0.9736842	0.784622	not scorable	not scorable	0.9752066	0.7869414

####	Bn-A05-p2929698	A-genome specific (napus-rapa)	0.9856459	0.4213647	not scorable	not scorable	1	0.7545244
####	Bn-A05-p2937826	A-genome specific (napus-rapa)	0.9904306	0.5692319	not scorable	not scorable	0.9917355	0.7883516
####	Bn-A05-p2937829	A-genome specific (rapa only)	not scorable	not scorable	not scorable	not scorable	0.9834711	0.5733733
####	Bn-A05-p2940969	A-genome specific (rapa only)	not scorable	not scorable	not scorable	not scorable	1	0.8106322
####	Bn-A05-p2984656	all not scorable	not scorable	not scorable	not scorable	not scorable	not scorable	not scorable
####	Bn-A05-p3002824	A-genome specific (napus-rapa)	0.9808612	0.348067	not scorable	not scorable	0.6363636	0.7793971
####	Bn-A05-p3005109	A-genome specific (napus-rapa)	0.9545454	0.3150868	not scorable	not scorable	0.9917355	0.7581408
####	Bn-A05-p3019980	A-genome specific (napus-rapa)	0.9832536	0.7760325	not scorable	not scorable	0.9917355	0.7758887
####	Bn-A05-p3021668	A-genome specific (napus-rapa)	0.9688995	0.6707211	not scorable	not scorable	0.9752066	0.6478857
####	Bn-A05-p3042172	A-genome specific (napus-rapa)	0.9952153	0.8578289	not scorable	not scorable	1	0.8437781
####	Bn-A05-p3042173	A-genome specific (napus-rapa)	0.9880383	0.8248671	not scorable	not scorable	0.9834711	0.7889662
####	Bn-A05-p3043365	A-genome specific (napus-rapa)	0.9880383	0.4755261	not scorable	not scorable	1	0.7684873
####	Bn-A05-p3045689	A-genome specific (napus-rapa)	0.9114832	0.7995032	not scorable	not scorable	0.9752066	0.8235878
####	Bn-A05-p3053025	A-genome specific (napus-rapa)	0.9976076	0.7745945	not scorable	not scorable	1	0.7242321
####	Bn-A05-p3053859	A-genome specific (napus-rapa)	0.9976076	0.7594656	not scorable	not scorable	1	0.8076131
####	Bn-A05-p3061052	A-genome specific (napus-rapa)	0.9976076	0.6755899	not scorable	not scorable	0.9834711	0.6472185
####	Bn-A05-p3068418	A-genome specific (napus-rapa)	0.3277512	0.8248016	not scorable	not scorable	0.9504132	0.8525023
####	Bn-A05-p3071868	A-genome specific (napus-rapa)	0.9832536	0.3722265	not scorable	not scorable	1	0.8076818
####	Bn-A05-p3099878	A-genome specific (napus-rapa)	0.9832536	0.5896843	not scorable	not scorable	0.9917355	0.634932
####	Bn-A05-p3112401	A-genome specific (napus-rapa)	0.9976076	0.781545	not scorable	not scorable	0.9917355	0.7799048
####	Bn-A05-p3117520	A-genome specific (napus-rapa)	0.9952153	0.3388641	not scorable	not scorable	1	0.8052797
####	Bn-A05-p3129492	A-genome specific (napus-rapa)	0.9856459	0.8004558	not scorable	not scorable	0.9669421	0.687094
####	Bn-A05-p3130062	A-genome specific (napus-rapa)	0.9712918	0.6795881	not scorable	not scorable	0.9834711	0.7245228
####	Bn-A05-p3135130	A-genome specific (napus-rapa)	0.9760765	0.3800686	not scorable	not scorable	1	0.677421
####	Bn-A05-p3135213	A-genome specific (napus-rapa)	0.9832536	0.4945431	not scorable	not scorable	0.9917355	0.7302743
####	Bn-A05-p3163568	A-genome specific (napus-rapa)	0.9808612	0.3816141	not scorable	not scorable	0.9917355	0.6674753
####	Bn-A05-p3166433	genome unspecific (oleracea-rapa)	not scorable	not scorable	1	0.799049	1	0.7562295
####	Bn-A05-p3188172	A-genome specific (napus-rapa)	0.9712918	0.6477227	not scorable	not scorable	0.9917355	0.650798
####	Bn-A05-p3188714	A-genome specific (napus-rapa)	1	0.3946687	not scorable	not scorable	0.9917355	0.7742113
####	Bn-A05-p3191390	A-genome specific (napus-rapa)	0.9928229	0.7226068	not scorable	not scorable	0.9917355	0.6829407
####	Bn-A05-p3198955	genome unspecific (all in)	0.9928229	0.4597722	0.9922481	0.7310675	1	0.6704807
####	Bn-A05-p3242299	A-genome specific (napus-rapa)	0.9976076	0.8227099	not scorable	not scorable	0.9917355	0.6832201
####	Bn-A05-p3246311	A-genome specific (napus-rapa)	0.9832536	0.2901366	not scorable	not scorable	0.9834711	0.7514731

####	Bn-A05-p3248878	A-genome specific (napus-rapa)	0.9688995	0.6249004	not scorable	not scorable	0.5950413	0.7615864
####	Bn-A05-p3251117	A-genome specific (napus-rapa)	0.7535885	0.9007937	not scorable	not scorable	0.9752066	0.818193
####	Bn-A05-p3258566	A-genome specific (napus-rapa)	0.9928229	0.312549	not scorable	not scorable	0.9917355	0.8290188
####	Bn-A05-p3264129	A-genome specific (napus-rapa)	0.9593301	0.3437693	not scorable	not scorable	0.9669421	0.7990826
####	Bn-A05-p3275289	A-genome specific (napus-rapa)	0.9880383	0.4555812	not scorable	not scorable	1	0.8571696
####	Bn-A05-p3278023	A-genome specific (napus-rapa)	0.9928229	0.8041607	not scorable	not scorable	0.9917355	0.7828347
####	Bn-A05-p3278514	A-genome specific (napus-rapa)	0.9856459	0.8233534	not scorable	not scorable	1	0.8343857
####	Bn-A05-p3280592	A-genome specific (napus-rapa)	0.9688995	0.7906918	not scorable	not scorable	0.6115702	0.7991788
####	Bn-A05-p3282089	A-genome specific (napus-rapa)	0.9976076	0.4091158	not scorable	not scorable	1	0.8008999
####	Bn-A05-p3300112	A-genome specific (napus-rapa)	0.9808612	0.7861837	not scorable	not scorable	0.9834711	0.7737466
####	Bn-A05-p3307736	A-genome specific (napus-rapa)	0.9641148	0.8097274	not scorable	not scorable	0.6528926	0.7116662
####	Bn-A05-p3311786	A-genome specific (napus-rapa)	0.9880383	0.4359278	not scorable	not scorable	0.9752066	0.6907184
####	Bn-A05-p3311869	A-genome specific (napus-rapa)	0.9856459	0.8272244	not scorable	not scorable	0.9834711	0.7842609
####	Bn-A05-p3312679	A-genome specific (napus-rapa)	1	0.4742499	not scorable	not scorable	1	0.7168373
####	Bn-A05-p3312786	A-genome specific (napus-rapa)	1	0.7202613	not scorable	not scorable	1	0.7010086
####	Bn-A05-p3320783	A-genome specific (napus-rapa)	0.9904306	0.5543631	not scorable	not scorable	0.9917355	0.7051241
####	Bn-A05-p3322798	A-genome specific (rapa only)	not scorable	not scorable	not scorable	not scorable	0.8347107	0.9316226
####	Bn-A05-p3323878	A-genome specific (rapa only)	not scorable	not scorable	not scorable	not scorable	0.9917355	0.8438382
####	Bn-A05-p3330640	A-genome specific (napus-rapa)	0.9593301	0.6288496	not scorable	not scorable	1	0.7682366
####	Bn-A05-p3361741	A-genome specific (napus-rapa)	0.9832536	0.4568853	not scorable	not scorable	0.6198347	0.8152189
####	Bn-A05-p3369681	A-genome specific (rapa only)	not scorable	not scorable	not scorable	not scorable	0.9586777	0.7008532
####	Bn-A05-p3394311	A-genome specific (napus-rapa)	0.9712918	0.7691963	not scorable	not scorable	0.9669421	0.7264202
####	Bn-A05-p3401393	A-genome specific (napus-rapa)	0.9976076	0.8011637	not scorable	not scorable	0.9834711	0.7955781
####	Bn-A05-p3406573	A-genome specific (napus-rapa)	0.9952153	0.6795959	not scorable	not scorable	0.9834711	0.7937259
####	Bn-A05-p3414540	A-genome specific (napus-rapa)	0.9952153	0.7213245	not scorable	not scorable	0.9834711	0.8229404
####	Bn-A05-p3421628	A-genome specific (napus-rapa)	0.5956938	0.6998192	not scorable	not scorable	0.9917355	0.6752687
####	Bn-A05-p342596	A-genome specific (napus-rapa)	0.9832536	0.3299021	not scorable	not scorable	0.9834711	0.8031581
####	Bn-A05-p3427883	A-genome specific (napus-rapa)	0.9928229	0.7586371	not scorable	not scorable	1	0.7024336
####	Bn-A05-p3427993	A-genome specific (napus-rapa)	0.9425837	0.791457	not scorable	not scorable	0.6528926	0.7577688
####	Bn-A05-p3428986	A-genome specific (rapa only)	not scorable	not scorable	not scorable	not scorable	0.9917355	0.6743433
####	Bn-A05-p3429612	A-genome specific (napus-rapa)	0.9641148	0.2792437	not scorable	not scorable	1	0.7342988
####	Bn-A05-p3450929	A-genome specific (rapa only)	not scorable	not scorable	not scorable	not scorable	0.9669421	0.7679964
####	Bn-A05-p3454754	A-genome specific (napus-rapa)	0.9688995	0.7995518	not scorable	not scorable	0.9917355	0.7878326

####	Bn-A05-p3456024	A-genome specific (napus-rapa)	0.9808612	0.8060433	not scorable	not scorable	1	0.7407264
####	Bn-A05-p3456661	A-genome specific (napus-rapa)	0.9856459	0.783475	not scorable	not scorable	0.9834711	0.774552
####	Bn-A05-p3467036	A-genome specific (napus-rapa)	0.9808612	0.593524	not scorable	not scorable	0.9834711	0.7202281
####	Bn-A05-p3467060	A-genome specific (napus-rapa)	0.9712918	0.8095763	not scorable	not scorable	0.9752066	0.8306469
####	Bn-A05-p3467688	A-genome specific (napus-rapa)	0.9090909	0.7945712	not scorable	not scorable	0.9338843	0.6951165
####	Bn-A05-p3468129	A-genome specific (napus-rapa)	0.9138756	0.7987754	not scorable	not scorable	0.9752066	0.7906311
####	Bn-A05-p3468204	A-genome specific (napus-rapa)	0.9066986	0.8287258	not scorable	not scorable	0.9669421	0.8233879
####	Bn-A05-p346822	A-genome specific (napus-rapa)	0.9665072	0.4170791	not scorable	not scorable	0.9917355	0.7077696
####	Bn-A05-p3469996	A-genome specific (napus-rapa)	0.8995215	0.8013204	not scorable	not scorable	0.9586777	0.8112935
####	Bn-A05-p347486	A-genome specific (rapa only)	not scorable	not scorable	not scorable	not scorable	0.9917355	0.829697
####	Bn-A05-p3481481	A-genome specific (napus-rapa)	0.9808612	0.3751002	not scorable	not scorable	1	0.7393478
####	Bn-A05-p3481611	A-genome specific (napus-rapa)	0.9928229	0.8035924	not scorable	not scorable	1	0.7939289
####	Bn-A05-p3482813	A-genome specific (napus-rapa)	0.9808612	0.3690387	not scorable	not scorable	1	0.7700332
####	Bn-A05-p3494729	A-genome specific (rapa only)	not scorable	not scorable	not scorable	not scorable	0.9834711	0.7099763
####	Bn-A05-p3507831	A-genome specific (napus-rapa)	0.9928229	0.8087324	not scorable	not scorable	1	0.7054136
####	Bn-A05-p3516602	A-genome specific (napus-rapa)	0.9880383	0.8096855	not scorable	not scorable	0.9834711	0.7566053
####	Bn-A05-p3518253	A-genome specific (napus-rapa)	0.9736842	0.5480176	not scorable	not scorable	0.9917355	0.4206098
####	Bn-A05-p3548736	A-genome specific (napus-rapa)	0.9856459	0.4976004	not scorable	not scorable	0.9752066	0.5202888
####	Bn-A05-p3550203	A-genome specific (napus-rapa)	0.9952153	0.3889967	not scorable	not scorable	1	0.8449665
####	Bn-A05-p355200	A-genome specific (napus-rapa)	0.9832536	0.3992393	not scorable	not scorable	0.9752066	0.6934315
####	Bn-A05-p3557797	A-genome specific (napus-rapa)	0.9784689	0.8101119	not scorable	not scorable	0.9834711	0.760955
####	Bn-A05-p3598362	A-genome specific (napus-rapa)	0.9952153	0.7524389	not scorable	not scorable	0.9834711	0.7724001
####	Bn-A05-p3598758	A-genome specific (napus-rapa)	0.9976076	0.7286766	not scorable	not scorable	1	0.7217363
####	Bn-A05-p3599315	A-genome specific (napus-rapa)	0.9952153	0.8402058	not scorable	not scorable	1	0.6955948
####	Bn-A05-p3605605	A-genome specific (napus-rapa)	0.9976076	0.8023859	not scorable	not scorable	0.9834711	0.7601281
####	Bn-A05-p3610111	A-genome specific (napus-rapa)	0.9976076	0.8049498	not scorable	not scorable	0.9752066	0.7664462
####	Bn-A05-p3611878	A-genome specific (napus-rapa)	0.9952153	0.5530657	not scorable	not scorable	0.9917355	0.7026774
####	Bn-A05-p3617320	A-genome specific (napus-rapa)	0.9976076	0.4559218	not scorable	not scorable	0.9917355	0.728605
####	Bn-A05-p3618356	A-genome specific (napus-rapa)	0.7320574	0.9072468	not scorable	not scorable	0.6446281	0.8190282
####	Bn-A05-p3629424	A-genome specific (rapa only)	not scorable	not scorable	not scorable	not scorable	0.9504132	0.7536385
####	Bn-A05-p363138	napus specific	0.9497608	0.5625359	not scorable	not scorable	not scorable	not scorable
####	Bn-A05-p3649985	A-genome specific (napus-rapa)	0.9928229	0.8952301	not scorable	not scorable	1	0.7891211
####	Bn-A05-p3650066	A-genome specific (napus-rapa)	0.9904306	0.8428977	not scorable	not scorable	0.9669421	0.7653947

####	Bn-A05-p3659292	A-genome specific (napus-rapa)	0.9952153	0.6293502	not scorable	not scorable	0.9586777	0.7737158
####	Bn-A05-p3659796	A-genome specific (napus-rapa)	0.9976076	0.267253	not scorable	not scorable	0.9669421	0.6955644
####	Bn-A05-p3670158	A-genome specific (napus-rapa)	0.9928229	0.4979096	not scorable	not scorable	1	0.8379771
####	Bn-A05-p3681637	A-genome specific (napus-rapa)	0.8373206	0.3705647	not scorable	not scorable	0.9752066	0.7852494
####	Bn-A05-p3684668	A-genome specific (napus-rapa)	0.9952153	0.7469894	not scorable	not scorable	1	0.865162
####	Bn-A05-p3703643	A-genome specific (napus-rapa)	0.9808612	0.4320617	not scorable	not scorable	1	0.8021294
####	Bn-A05-p3719423	A-genome specific (rapa only)	not scorable	not scorable	not scorable	not scorable	0.9917355	0.7630112
####	Bn-A05-p3754543	A-genome specific (napus-rapa)	0.930622	0.6769214	not scorable	not scorable	0.7024794	0.7991043
####	Bn-A05-p3759320	A-genome specific (napus-rapa)	0.9617225	0.7117105	not scorable	not scorable	0.9834711	0.6990387
####	Bn-A05-p377542	A-genome specific (napus-rapa)	0.9760765	0.5942039	not scorable	not scorable	0.9586777	0.5984696
####	Bn-A05-p3781544	A-genome specific (napus-rapa)	0.9976076	0.486738	not scorable	not scorable	0.9917355	0.7536452
####	Bn-A05-p3796206	genome unspecific (all in)	0.9904306	0.5314343	0.5348837	0.7262002	0.9256198	0.7739488
####	Bn-A05-p3808836	A-genome specific (napus-rapa)	0.9976076	0.9127215	not scorable	not scorable	1	0.8167268
####	Bn-A05-p3812776	A-genome specific (napus-rapa)	0.9976076	0.9175813	not scorable	not scorable	0.9834711	0.8135673
####	Bn-A05-p3833073	A-genome specific (rapa only)	not scorable	not scorable	not scorable	not scorable	0.9752066	0.7199836
####	Bn-A05-p3833308	genome unspecific (all in)	0.9019139	0.3787898	0.9767442	0.7471843	1	0.821539
####	Bn-A05-p3834445	A-genome specific (napus-rapa)	0.9880383	0.8052018	not scorable	not scorable	0.9917355	0.7686269
####	Bn-A05-p3834639	A-genome specific (napus-rapa)	0.3110048	0.7667319	not scorable	not scorable	0.9834711	0.7949042
####	Bn-A05-p3846005	A-genome specific (napus-rapa)	0.9904306	0.7634487	not scorable	not scorable	1	0.7718545
####	Bn-A05-p3847996	A-genome specific (rapa only)	not scorable	not scorable	not scorable	not scorable	0.9917355	0.6813985
####	Bn-A05-p3871998	A-genome specific (napus-rapa)	1	0.5071949	not scorable	not scorable	0.9752066	0.8024748
####	Bn-A05-p3882826	A-genome specific (rapa only)	not scorable	not scorable	not scorable	not scorable	0.9917355	0.7749658
####	Bn-A05-p389230	A-genome specific (napus-rapa)	0.9665072	0.5343968	not scorable	not scorable	0.9504132	0.5389776
####	Bn-A05-p3901565	A-genome specific (napus-rapa)	0.9928229	0.3769887	not scorable	not scorable	1	0.7594963
####	Bn-A05-p3919804	all not scorable	not scorable	not scorable	not scorable	not scorable	not scorable	not scorable
####	Bn-A05-p3920198	all not scorable	not scorable	not scorable	not scorable	not scorable	not scorable	not scorable
####	Bn-A05-p392053	A-genome specific (napus-rapa)	0.9880383	0.7695165	not scorable	not scorable	0.9917355	0.7808229
####	Bn-A05-p3923692	A-genome specific (napus-rapa)	0.9617225	0.4427902	not scorable	not scorable	0.9834711	0.5364295
####	Bn-A05-p3923814	A-genome specific (napus-rapa)	0.9736842	0.8666215	not scorable	not scorable	1	0.8828484
####	Bn-A05-p3939102	A-genome specific (napus-rapa)	0.9880383	0.8403008	not scorable	not scorable	1	0.7807861
####	Bn-A05-p3953066	A-genome specific (napus-rapa)	0.8708134	0.779858	not scorable	not scorable	0.9752066	0.8354011
####	Bn-A05-p3963790	A-genome specific (napus-rapa)	0.6674641	0.8312193	not scorable	not scorable	0.9504132	0.7220723
####	Bn-A05-p3966848	A-genome specific (napus-rapa)	0.9856459	0.5215058	not scorable	not scorable	0.9834711	0.6832324

####	Bn-A05-p3988218	A-genome specific (napus-rapa)	0.9904306	0.7421105	not scorable	not scorable	1	0.6914105
####	Bn-A05-p3988279	A-genome specific (napus-rapa)	0.9880383	0.8032272	not scorable	not scorable	0.9917355	0.7999281
####	Bn-A05-p3988958	A-genome specific (napus-rapa)	0.9880383	0.3325582	not scorable	not scorable	0.9917355	0.8205577
####	Bn-A05-p3989555	A-genome specific (napus-rapa)	0.9904306	0.7155205	not scorable	not scorable	1	0.6075637
####	Bn-A05-p4006770	A-genome specific (napus-rapa)	1	0.5134318	not scorable	not scorable	1	0.8006559
####	Bn-A05-p4016172	A-genome specific (rapa only)	not scorable	not scorable	not scorable	not scorable	0.9917355	0.6800712
####	Bn-A05-p4017270	A-genome specific (napus-rapa)	0.9976076	0.7677189	not scorable	not scorable	0.6363636	0.6948223
####	Bn-A05-p4022440	A-genome specific (rapa only)	not scorable	not scorable	not scorable	not scorable	0.9834711	0.6967734
####	Bn-A05-p4023464	A-genome specific (napus-rapa)	0.7009569	0.6209862	not scorable	not scorable	0.9917355	0.606728
####	Bn-A05-p4036806	A-genome specific (napus-rapa)	0.9952153	0.7323389	not scorable	not scorable	1	0.6970947
####	Bn-A05-p4043474	A-genome specific (napus-rapa)	0.9712918	0.5272076	not scorable	not scorable	1	0.6288794
####	Bn-A05-p4046605	A-genome specific (napus-rapa)	0.9904306	0.7003254	not scorable	not scorable	1	0.7105125
####	Bn-A05-p4053502	A-genome specific (napus-rapa)	0.9784689	0.7397651	not scorable	not scorable	0.9917355	0.7471753
####	Bn-A05-p4054823	A-genome specific (napus-rapa)	0.9880383	0.4770927	not scorable	not scorable	0.9917355	0.7372529
####	Bn-A05-p4055656	A-genome specific (napus-rapa)	0.9904306	0.2386352	not scorable	not scorable	0.9669421	0.6087569
####	Bn-A05-p4055839	A-genome specific (napus-rapa)	0.9856459	0.5861222	not scorable	not scorable	1	0.7399741
####	Bn-A05-p4059534	A-genome specific (napus-rapa)	0.9808612	0.792668	not scorable	not scorable	1	0.804073
####	Bn-A05-p4061730	A-genome specific (napus-rapa)	0.9880383	0.7330705	not scorable	not scorable	0.9834711	0.8051432
####	Bn-A05-p4061978	A-genome specific (napus-rapa)	0.9880383	0.8112355	not scorable	not scorable	0.9917355	0.8439929
####	Bn-A05-p4062134	A-genome specific (napus-rapa)	0.9976076	0.7699234	not scorable	not scorable	1	0.9153172
####	Bn-A05-p4068277	A-genome specific (napus-rapa)	0.9880383	0.7676619	not scorable	not scorable	0.9917355	0.6670371
####	Bn-A05-p4069460	A-genome specific (napus-rapa)	0.9736842	0.3033395	not scorable	not scorable	0.9917355	0.8202339
####	Bn-A05-p4073230	A-genome specific (napus-rapa)	0.9952153	0.3732508	not scorable	not scorable	1	0.7611668
####	Bn-A05-p4073997	A-genome specific (napus-rapa)	0.9904306	0.7797557	not scorable	not scorable	1	0.709715
####	Bn-A05-p4078496	A-genome specific (napus-rapa)	0.9976076	0.437979	not scorable	not scorable	1	0.8624078
####	Bn-A05-p4080798	A-genome specific (napus-rapa)	0.9952153	0.7415245	not scorable	not scorable	1	0.6769364
####	Bn-A05-p4085121	A-genome specific (napus-rapa)	0.937799	0.7691876	not scorable	not scorable	0.6115702	0.778749
####	Bn-A05-p4086144	A-genome specific (napus-rapa)	0.9880383	0.7337471	not scorable	not scorable	0.9752066	0.836688
####	Bn-A05-p4093306	A-genome specific (napus-rapa)	0.9904306	0.4645639	not scorable	not scorable	0.9917355	0.7702807
####	Bn-A05-p4102906	A-genome specific (napus-rapa)	0.9952153	0.7668298	not scorable	not scorable	1	0.6828028
####	Bn-A05-p4104070	A-genome specific (napus-rapa)	0.9928229	0.2909306	not scorable	not scorable	0.9669421	0.5938171
####	Bn-A05-p4108818	A-genome specific (napus-rapa)	0.9832536	0.6139143	not scorable	not scorable	0.9917355	0.7667087
####	Bn-A05-p4110015	A-genome specific (napus-rapa)	1	0.3718556	not scorable	not scorable	0.9917355	0.7370849

####	Bn-A05-p4121158	A-genome specific (napus-rapa)	0.930622	0.6926226	not scorable	not scorable	0.9834711	0.6969336
####	Bn-A05-p4176921	genome unspecific (all in)	0.9856459	0.4738692	0.9922481	0.5829095	1	0.7935928
####	Bn-A05-p4196214	A-genome specific (napus-rapa)	0.9976076	0.8038533	not scorable	not scorable	0.677686	0.7847714
####	Bn-A05-p4197697	A-genome specific (napus-rapa)	0.9784689	0.8213857	not scorable	not scorable	1	0.8833862
####	Bn-A05-p4212213	A-genome specific (napus-rapa)	0.9856459	0.3297706	not scorable	not scorable	1	0.7602192
####	Bn-A05-p4230422	A-genome specific (napus-rapa)	1	0.437949	not scorable	not scorable	1	0.8893603
####	Bn-A05-p4290068	A-genome specific (napus-rapa)	0.5095694	0.8442473	not scorable	not scorable	0.9090909	0.9208351
####	Bn-A05-p4304172	genome unspecific (all in)	0.9928229	0.7313564	0.9689922	0.6964487	0.9421487	0.8085063
####	Bn-A05-p4307316	genome unspecific (oleracea-rapa)	not scorable	not scorable	0.9069768	0.761631	1	0.9207501
####	Bn-A05-p4316642	A-genome specific (napus-rapa)	0.9904306	0.485852	not scorable	not scorable	0.9752066	0.7756222
####	Bn-A05-p4316943	A-genome specific (napus-rapa)	0.9521531	0.8025826	not scorable	not scorable	0.9917355	0.8208314
####	Bn-A05-p4333409	A-genome specific (napus-rapa)	0.9928229	0.8091696	not scorable	not scorable	1	0.7468417
####	Bn-A05-p4336667	A-genome specific (napus-rapa)	0.9856459	0.7581415	not scorable	not scorable	1	0.7645065
####	Bn-A05-p4353454	A-genome specific (napus-rapa)	0.9856459	0.7578623	not scorable	not scorable	1	0.7559701
####	Bn-A05-p4354442	A-genome specific (napus-rapa)	0.9832536	0.5834324	not scorable	not scorable	0.9917355	0.6909901
####	Bn-A05-p4355047	A-genome specific (napus-rapa)	0.9832536	0.5654079	not scorable	not scorable	1	0.7252899
####	Bn-A05-p4373694	A-genome specific (napus-rapa)	0.9928229	0.8360434	not scorable	not scorable	0.9917355	0.7197514
####	Bn-A05-p4376029	A-genome specific (napus-rapa)	0.9019139	0.7736164	not scorable	not scorable	0.9917355	0.677412
####	Bn-A05-p4398134	A-genome specific (napus-rapa)	0.9952153	0.8033118	not scorable	not scorable	1	0.811842
####	Bn-A05-p4399949	A-genome specific (napus-rapa)	0.9928229	0.3202833	not scorable	not scorable	1	0.7686591
####	Bn-A05-p4418703	A-genome specific (napus-rapa)	1	0.3292082	not scorable	not scorable	1	0.7318229
####	Bn-A05-p4422786	A-genome specific (napus-rapa)	1	0.6984933	not scorable	not scorable	0.9917355	0.6806584
####	Bn-A05-p44690	A-genome specific (napus-rapa)	1	0.9208159	not scorable	not scorable	1	0.9175813
####	Bn-A05-p449086	A-genome specific (napus-rapa)	0.9904306	0.5557899	not scorable	not scorable	1	0.7975834
####	Bn-A05-p4498290	A-genome specific (napus-rapa)	0.9808612	0.2800896	not scorable	not scorable	1	0.8185809
####	Bn-A05-p4498398	A-genome specific (napus-rapa)	0.9210526	0.8246281	not scorable	not scorable	0.5950413	0.8055689
####	Bn-A05-p4516526	A-genome specific (napus-rapa)	0.9928229	0.5057297	not scorable	not scorable	1	0.751707
####	Bn-A05-p4516748	A-genome specific (rapa only)	not scorable	not scorable	not scorable	not scorable	0.5950413	0.8095464
####	Bn-A05-p4520844	A-genome specific (napus-rapa)	0.9880383	0.2398178	not scorable	not scorable	0.9917355	0.6121123
####	Bn-A05-p4524286	A-genome specific (napus-rapa)	0.9808612	0.5798689	not scorable	not scorable	1	0.7902774
####	Bn-A05-p4525705	A-genome specific (napus-rapa)	0.9665072	0.8010295	not scorable	not scorable	0.9338843	0.7183189
####	Bn-A05-p452939	genome unspecific (oleracea-rapa)	not scorable	not scorable	0.9534883	0.745786	1	0.7650468
####	Bn-A05-p4542257	A-genome specific (napus-rapa)	0.9976076	0.4687764	not scorable	not scorable	0.9917355	0.8074523

####	Bn-A05-p4549959	A-genome specific (napus-rapa)	1	0.7016702	not scorable	not scorable	0.9834711	0.7275646
####	Bn-A05-p4550465	A-genome specific (napus-rapa)	0.9808612	0.7742141	not scorable	not scorable	0.9752066	0.6939689
####	Bn-A05-p4569670	A-genome specific (napus-rapa)	0.9808612	0.7412094	not scorable	not scorable	0.9834711	0.6824313
####	Bn-A05-p458357	C-genome specific (napus-oleracea)	0.9258373	0.7462918	0.9922481	0.8526646	not scorable	not scorable
####	Bn-A05-p4590055	A-genome specific (napus-rapa)	0.9904306	0.8067362	not scorable	not scorable	0.9917355	0.8186828
####	Bn-A05-p4590326	A-genome specific (napus-rapa)	0.9880383	0.6497977	not scorable	not scorable	0.9752066	0.7595804
####	Bn-A05-p4593001	A-genome specific (napus-rapa)	0.9856459	0.4793262	not scorable	not scorable	0.9834711	0.5802915
####	Bn-A05-p4612911	A-genome specific (napus-rapa)	0.9880383	0.4905775	not scorable	not scorable	1	0.7434353
####	Bn-A05-p4616151	A-genome specific (napus-rapa)	0.9904306	0.588962	not scorable	not scorable	0.9917355	0.8148105
####	Bn-A05-p461633	A-genome specific (napus-rapa)	0.9665072	0.4848723	not scorable	not scorable	1	0.8906373
####	Bn-A05-p4618158	A-genome specific (napus-rapa)	1	0.7632905	not scorable	not scorable	0.9834711	0.7271518
####	Bn-A05-p4619303	A-genome specific (napus-rapa)	0.9976076	0.52302	not scorable	not scorable	0.9834711	0.8037351
####	Bn-A05-p4621087	A-genome specific (napus-rapa)	1	0.7420156	not scorable	not scorable	0.9917355	0.7805594
####	Bn-A05-p4621949	A-genome specific (napus-rapa)	0.9928229	0.7732162	not scorable	not scorable	1	0.712615
####	Bn-A05-p4625019	A-genome specific (napus-rapa)	0.9784689	0.5050447	not scorable	not scorable	1	0.6948495
####	Bn-A05-p4632407	A-genome specific (napus-rapa)	0.9760765	0.7745882	not scorable	not scorable	0.9421487	0.8486249
####	Bn-A05-p4644984	A-genome specific (napus-rapa)	0.9952153	0.3719256	not scorable	not scorable	1	0.5629493
####	Bn-A05-p4653714	A-genome specific (rapa only)	not scorable	not scorable	not scorable	not scorable	0.9752066	0.5860605
####	Bn-A05-p4655845	A-genome specific (napus-rapa)	1	0.6904525	not scorable	not scorable	1	0.8528472
####	Bn-A05-p4665099	A-genome specific (napus-rapa)	1	0.7936761	not scorable	not scorable	1	0.7228909
####	Bn-A05-p4679663	A-genome specific (napus-rapa)	0.9976076	0.5178118	not scorable	not scorable	1	0.7543517
####	Bn-A05-p468551	A-genome specific (napus-rapa)	0.9617225	0.4698649	not scorable	not scorable	0.9917355	0.7805789
####	Bn-A05-p4696360	A-genome specific (napus-rapa)	0.9928229	0.7912977	not scorable	not scorable	0.6528926	0.8410159
####	Bn-A05-p4715599	genome unspecific (all in)	0.9976076	0.3098338	0.9922481	0.8547986	0.9917355	0.7894143
####	Bn-A05-p4717725	A-genome specific (napus-rapa)	0.9976076	0.8287444	not scorable	not scorable	0.9917355	0.7754467
####	Bn-A05-p4717815	A-genome specific (napus-rapa)	0.9904306	0.7311299	not scorable	not scorable	0.9752066	0.7903441
####	Bn-A05-p472271	genome unspecific (all in)	0.9760765	0.7046981	0.4806201	0.8073233	1	0.6445189
####	Bn-A05-p4728882	A-genome specific (napus-rapa)	1	0.7759413	not scorable	not scorable	0.9834711	0.7240552
####	Bn-A05-p4729566	A-genome specific (napus-rapa)	0.9832536	0.6323283	not scorable	not scorable	0.9834711	0.7224679
####	Bn-A05-p4732914	A-genome specific (napus-rapa)	1	0.7892722	not scorable	not scorable	0.9917355	0.7886006
####	Bn-A05-p4736340	A-genome specific (napus-rapa)	0.9952153	0.8242797	not scorable	not scorable	0.9917355	0.743686
####	Bn-A05-p4737634	A-genome specific (rapa only)	not scorable	not scorable	not scorable	not scorable	0.9752066	0.7674559
####	Bn-A05-p474257	A-genome specific (napus-rapa)	0.9832536	0.6842052	not scorable	not scorable	0.9917355	0.8155786

####	Bn-A05-p4749718	A-genome specific (napus-rapa)	0.9904306	0.5278285	not scorable	not scorable	0.9917355	0.7486592
####	Bn-A05-p4762044	A-genome specific (napus-rapa)	0.9976076	0.5199819	not scorable	not scorable	0.9834711	0.818361
####	Bn-A05-p4772468	A-genome specific (napus-rapa)	1	0.7239965	not scorable	not scorable	0.9834711	0.8150748
####	Bn-A05-p4792988	A-genome specific (napus-rapa)	0.9976076	0.6683721	not scorable	not scorable	1	0.7639329
####	Bn-A05-p4793033	A-genome specific (napus-rapa)	1	0.7405659	not scorable	not scorable	0.9834711	0.7751213
####	Bn-A05-p4794141	A-genome specific (napus-rapa)	0.9904306	0.3112699	not scorable	not scorable	0.9917355	0.8205397
####	Bn-A05-p4820365	A-genome specific (napus-rapa)	1	0.7049519	not scorable	not scorable	1	0.6951041
####	Bn-A05-p482473	A-genome specific (napus-rapa)	0.9952153	0.5469098	not scorable	not scorable	0.9917355	0.8302022
####	Bn-A05-p484591	A-genome specific (napus-rapa)	0.9784689	0.3902336	not scorable	not scorable	1	0.6366912
####	Bn-A05-p4871527	A-genome specific (napus-rapa)	0.9736842	0.8832948	not scorable	not scorable	1	0.7727718
####	Bn-A05-p4904405	A-genome specific (napus-rapa)	0.9832536	0.9174066	not scorable	not scorable	0.9834711	0.7671781
####	Bn-A05-p4915516	A-genome specific (napus-rapa)	0.9904306	0.8395197	not scorable	not scorable	0.6280992	0.8327757
####	Bn-A05-p4919899	A-genome specific (napus-rapa)	0.9545454	0.783209	not scorable	not scorable	0.9917355	0.8098593
####	Bn-A05-p4924856	A-genome specific (napus-rapa)	1	0.7765347	not scorable	not scorable	1	0.7007138
####	Bn-A05-p4937055	A-genome specific (napus-rapa)	0.9904306	0.7788116	not scorable	not scorable	0.9834711	0.7730682
####	Bn-A05-p4940676	A-genome specific (napus-rapa)	0.9928229	0.7967482	not scorable	not scorable	1	0.8110461
####	Bn-A05-p4940713	A-genome specific (napus-rapa)	0.9928229	0.8025123	not scorable	not scorable	0.9917355	0.830782
####	Bn-A05-p4940779	A-genome specific (napus-rapa)	0.9976076	0.7690636	not scorable	not scorable	1	0.8061883
####	Bn-A05-p4941234	A-genome specific (napus-rapa)	0.9952153	0.7803927	not scorable	not scorable	1	0.8291568
####	Bn-A05-p4959282	A-genome specific (napus-rapa)	1	0.7265302	not scorable	not scorable	0.9752066	0.8177654
####	Bn-A05-p4961402	A-genome specific (napus-rapa)	0.9928229	0.2369486	not scorable	not scorable	0.9752066	0.6553057
####	Bn-A05-p4973912	A-genome specific (napus-rapa)	0.9784689	0.7456518	not scorable	not scorable	0.6363636	0.6720281
####	Bn-A05-p4984837	A-genome specific (napus-rapa)	0.9904306	0.3096625	not scorable	not scorable	0.9834711	0.7409236
####	Bn-A05-p5001194	A-genome specific (rapa only)	not scorable	not scorable	not scorable	not scorable	0.6694215	0.5974919
####	Bn-A05-p5002988	A-genome specific (napus-rapa)	0.9904306	0.838789	not scorable	not scorable	1	0.787343
####	Bn-A05-p5004187	A-genome specific (napus-rapa)	0.9688995	0.5026452	not scorable	not scorable	1	0.7988282
####	Bn-A05-p5020058	A-genome specific (napus-rapa)	0.9856459	0.5424193	not scorable	not scorable	1	0.7243367
####	Bn-A05-p5026139	A-genome specific (napus-rapa)	0.9904306	0.2649752	not scorable	not scorable	1	0.6340655
####	Bn-A05-p5037839	A-genome specific (napus-rapa)	0.9976076	0.7643007	not scorable	not scorable	0.9752066	0.7481176
####	Bn-A05-p5038148	A-genome specific (napus-rapa)	0.9880383	0.8075311	not scorable	not scorable	0.9917355	0.730388
####	Bn-A05-p5041159	A-genome specific (napus-rapa)	0.9904306	0.825842	not scorable	not scorable	0.9917355	0.677913
####	Bn-A05-p5043499	genome unspecific (oleracea-rapa)	not scorable	not scorable	1	0.7170734	1	0.7574295
####	Bn-A05-p5057096	A-genome specific (napus-rapa)	0.9952153	0.7060942	not scorable	not scorable	0.9834711	0.7596684

####	Bn-A05-p5057141	A-genome specific (napus-rapa)	0.9712918	0.468114	not scorable	not scorable	0.9917355	0.7499781
####	Bn-A05-p5058143	A-genome specific (napus-rapa)	0.9928229	0.5408046	not scorable	not scorable	0.9752066	0.5831441
####	Bn-A05-p5093548	A-genome specific (napus-rapa)	0.9641148	0.7200636	not scorable	not scorable	0.6115702	0.8191477
####	Bn-A05-p5100352	A-genome specific (napus-rapa)	0.9712918	0.238547	not scorable	not scorable	0.9752066	0.7892913
####	Bn-A05-p5102120	A-genome specific (napus-rapa)	0.9976076	0.6686502	not scorable	not scorable	1	0.8821031
####	Bn-A05-p5102147	A-genome specific (napus-rapa)	0.9928229	0.3779608	not scorable	not scorable	1	0.6987064
####	Bn-A05-p5104821	A-genome specific (napus-rapa)	0.9880383	0.6548197	not scorable	not scorable	0.9917355	0.703967
####	Bn-A05-p5133417	A-genome specific (napus-rapa)	0.9952153	0.4179525	not scorable	not scorable	1	0.8437105
####	Bn-A05-p5135426	A-genome specific (napus-rapa)	0.9880383	0.5508231	not scorable	not scorable	0.9917355	0.7604856
####	Bn-A05-p518923	A-genome specific (rapa only)	not scorable	not scorable	not scorable	not scorable	0.9834711	0.7748172
####	Bn-A05-p5251613	A-genome specific (napus-rapa)	0.9760765	0.2796916	not scorable	not scorable	0.9834711	0.7649689
####	Bn-A05-p5252542	A-genome specific (napus-rapa)	0.9856459	0.8303989	not scorable	not scorable	0.9834711	0.8090895
####	Bn-A05-p5264904	A-genome specific (napus-rapa)	0.9928229	0.7877973	not scorable	not scorable	1	0.8613048
####	Bn-A05-p5279229	A-genome specific (napus-rapa)	0.9856459	0.5830094	not scorable	not scorable	1	0.8385333
####	Bn-A05-p5280016	A-genome specific (napus-rapa)	0.9736842	0.5574831	not scorable	not scorable	0.9669421	0.675553
####	Bn-A05-p5281478	A-genome specific (napus-rapa)	0.9880383	0.6975561	not scorable	not scorable	0.9917355	0.723166
####	Bn-A05-p5283304	A-genome specific (napus-rapa)	0.9880383	0.4820598	not scorable	not scorable	0.9586777	0.6301776
####	Bn-A05-p529716	A-genome specific (napus-rapa)	0.9760765	0.8003569	not scorable	not scorable	1	0.8788541
####	Bn-A05-p5328959	A-genome specific (napus-rapa)	0.9856459	0.4628373	not scorable	not scorable	1	0.7696513
####	Bn-A05-p5337620	A-genome specific (napus-rapa)	0.9976076	0.2854094	not scorable	not scorable	0.9752066	0.6233408
####	Bn-A05-p5344859	A-genome specific (napus-rapa)	0.9976076	0.8063635	not scorable	not scorable	0.9834711	0.7331893
####	Bn-A05-p5349944	A-genome specific (napus-rapa)	0.9928229	0.7114276	not scorable	not scorable	0.9917355	0.8096758
####	Bn-A05-p5350567	A-genome specific (napus-rapa)	0.9880383	0.8110018	not scorable	not scorable	0.9917355	0.7957904
####	Bn-A05-p5350892	A-genome specific (napus-rapa)	0.9736842	0.7889077	not scorable	not scorable	0.9834711	0.8015345
####	Bn-A05-p5372015	A-genome specific (napus-rapa)	1	0.7798709	not scorable	not scorable	0.9504132	0.7789019
####	Bn-A05-p5374000	A-genome specific (napus-rapa)	0.9952153	0.7103273	not scorable	not scorable	0.9917355	0.6717374
####	Bn-A05-p5391937	A-genome specific (napus-rapa)	1	0.8083164	not scorable	not scorable	0.9834711	0.7355098
####	Bn-A05-p541565	A-genome specific (napus-rapa)	0.9784689	0.5517754	not scorable	not scorable	0.9669421	0.6874883
####	Bn-A05-p5437257	A-genome specific (napus-rapa)	0.9832536	0.3715005	not scorable	not scorable	0.9834711	0.7342843
####	Bn-A05-p5445252	A-genome specific (napus-rapa)	0.9904306	0.7646507	not scorable	not scorable	1	0.708764
####	Bn-A05-p5446122	A-genome specific (napus-rapa)	0.9976076	0.7831202	not scorable	not scorable	1	0.706614
####	Bn-A05-p5457877	A-genome specific (napus-rapa)	0.9976076	0.8569642	not scorable	not scorable	0.9917355	0.7591975
####	Bn-A05-p547582	A-genome specific (napus-rapa)	0.9688995	0.6681643	not scorable	not scorable	0.9834711	0.7019902

####	Bn-A05-p5475992	A-genome specific (napus-rapa)	0.9952153	0.8615543	not scorable	not scorable	0.9917355	0.728601
####	Bn-A05-p5483612	A-genome specific (napus-rapa)	0.9952153	0.5158451	not scorable	not scorable	1	0.6144376
####	Bn-A05-p5492491	A-genome specific (napus-rapa)	1	0.8458923	not scorable	not scorable	0.9917355	0.8011296
####	Bn-A05-p550807	genome unspecific (all in)	0.9545454	0.771167	0.8914729	0.7189392	0.9669421	0.7022595
####	Bn-A05-p5519729	A-genome specific (napus-rapa)	0.9928229	0.7635285	not scorable	not scorable	0.9917355	0.717526
####	Bn-A05-p5521242	A-genome specific (napus-rapa)	0.9952153	0.4846175	not scorable	not scorable	0.9834711	0.6666117
####	Bn-A05-p5522210	genome unspecific (all in)	0.9976076	0.5576342	0.9767442	0.7742203	0.9834711	0.702941
####	Bn-A05-p5533987	A-genome specific (napus-rapa)	0.9976076	0.7485694	not scorable	not scorable	1	0.8855474
####	Bn-A05-p5535142	A-genome specific (napus-rapa)	0.9760765	0.8099664	not scorable	not scorable	0.9669421	0.6966965
####	Bn-A05-p5537385	A-genome specific (napus-rapa)	0.9784689	0.7407898	not scorable	not scorable	0.9421487	0.7778892
####	Bn-A05-p5537609	A-genome specific (napus-rapa)	0.9928229	0.822769	not scorable	not scorable	1	0.7726629
####	Bn-A05-p5537751	A-genome specific (napus-rapa)	1	0.6489761	not scorable	not scorable	0.9917355	0.633616
####	Bn-A05-p5541232	A-genome specific (napus-rapa)	0.9904306	0.7009401	not scorable	not scorable	0.9834711	0.6236969
####	Bn-A05-p5542434	A-genome specific (napus-rapa)	0.9928229	0.4665029	not scorable	not scorable	0.9917355	0.7219897
####	Bn-A05-p5552246	A-genome specific (napus-rapa)	0.9928229	0.4445888	not scorable	not scorable	0.9917355	0.6707439
####	Bn-A05-p556585	A-genome specific (rapa only)	not scorable	not scorable	not scorable	not scorable	0.9834711	0.6689662
####	Bn-A05-p5565945	A-genome specific (napus-rapa)	0.9928229	0.2367571	not scorable	not scorable	0.9917355	0.6732364
####	Bn-A05-p5566193	A-genome specific (napus-rapa)	0.9784689	0.7650709	not scorable	not scorable	0.9669421	0.7854449
####	Bn-A05-p5575695	A-genome specific (napus-rapa)	0.9904306	0.7988507	not scorable	not scorable	1	0.7444711
####	Bn-A05-p5586839	A-genome specific (napus-rapa)	0.9904306	0.7729803	not scorable	not scorable	0.9752066	0.797488
####	Bn-A05-p5588271	A-genome specific (napus-rapa)	0.9784689	0.8021218	not scorable	not scorable	0.9752066	0.7177495
####	Bn-A05-p5595403	A-genome specific (napus-rapa)	0.9856459	0.7961553	not scorable	not scorable	0.9586777	0.7053341
####	Bn-A05-p5595578	A-genome specific (napus-rapa)	0.9928229	0.8138397	not scorable	not scorable	0.9834711	0.7627122
####	Bn-A05-p559597	A-genome specific (napus-rapa)	0.9832536	0.7928916	not scorable	not scorable	0.9752066	0.6760932
####	Bn-A05-p5614651	A-genome specific (napus-rapa)	0.9856459	0.6714244	not scorable	not scorable	1	0.6574759
####	Bn-A05-p5615444	A-genome specific (napus-rapa)	1	0.9306033	not scorable	not scorable	1	0.9090905
####	Bn-A05-p5627413	A-genome specific (napus-rapa)	0.9856459	0.7817304	not scorable	not scorable	1	0.8684785
####	Bn-A05-p5644210	A-genome specific (napus-rapa)	1	0.7643037	not scorable	not scorable	0.9752066	0.738128
####	Bn-A05-p5645000	A-genome specific (napus-rapa)	1	0.7764401	not scorable	not scorable	0.9752066	0.7834842
####	Bn-A05-p5647262	A-genome specific (rapa only)	not scorable	not scorable	not scorable	not scorable	0.9917355	0.5493351
####	Bn-A05-p565719	A-genome specific (napus-rapa)	0.9641148	0.7366168	not scorable	not scorable	0.9586777	0.6894202
####	Bn-A05-p5657708	A-genome specific (napus-rapa)	0.9952153	0.7353714	not scorable	not scorable	1	0.7009387
####	Bn-A05-p5661136	A-genome specific (napus-rapa)	0.9904306	0.3982007	not scorable	not scorable	1	0.8160369

####	Bn-A05-p5662601	A-genome specific (napus-rapa)	0.9880383	0.7063412	not scorable	not scorable	0.9917355	0.8761218
####	Bn-A05-p5664658	A-genome specific (napus-rapa)	0.9928229	0.7242243	not scorable	not scorable	1	0.7129104
####	Bn-A05-p5670181	A-genome specific (napus-rapa)	0.9976076	0.816709	not scorable	not scorable	0.9917355	0.7355717
####	Bn-A05-p5684860	A-genome specific (napus-rapa)	0.9928229	0.6914271	not scorable	not scorable	1	0.7698848
####	Bn-A05-p5687833	A-genome specific (napus-rapa)	0.9665072	0.7874331	not scorable	not scorable	0.9256198	0.6338614
####	Bn-A05-p5689705	A-genome specific (napus-rapa)	0.9856459	0.7425504	not scorable	not scorable	1	0.6509471
####	Bn-A05-p5691374	A-genome specific (napus-rapa)	0.9856459	0.7868741	not scorable	not scorable	0.9834711	0.7357318
####	Bn-A05-p5693743	A-genome specific (napus-rapa)	0.9880383	0.8607521	not scorable	not scorable	0.9669421	0.6499743
####	Bn-A05-p5700216	A-genome specific (napus-rapa)	0.9928229	0.8958366	not scorable	not scorable	0.9834711	0.7554693
####	Bn-A05-p5700891	A-genome specific (napus-rapa)	0.9880383	0.778435	not scorable	not scorable	0.9917355	0.8445532
####	Bn-A05-p5714702	A-genome specific (napus-rapa)	0.9736842	0.651947	not scorable	not scorable	1	0.7468688
####	Bn-A05-p5714756	A-genome specific (napus-rapa)	0.9784689	0.7674046	not scorable	not scorable	0.9917355	0.8524482
####	Bn-A05-p5714836	A-genome specific (napus-rapa)	0.9760765	0.8463846	not scorable	not scorable	0.9834711	0.7745014
####	Bn-A05-p5715403	A-genome specific (napus-rapa)	0.9856459	0.2988797	not scorable	not scorable	0.9669421	0.6477179
####	Bn-A05-p575873	A-genome specific (napus-rapa)	0.9736842	0.3295884	not scorable	not scorable	0.9834711	0.7255786
####	Bn-A05-p5764156	A-genome specific (rapa only)	not scorable	not scorable	not scorable	not scorable	0.9917355	0.7034169
####	Bn-A05-p5770114	A-genome specific (napus-rapa)	0.9976076	0.810151	not scorable	not scorable	0.9917355	0.8233715
####	Bn-A05-p5778158	A-genome specific (napus-rapa)	0.9976076	0.4741229	not scorable	not scorable	0.9752066	0.6184489
####	Bn-A05-p5790313	napus specific	0.9665072	0.3541712	not scorable	not scorable	not scorable	not scorable
####	Bn-A05-p5796678	napus specific	0.9784689	0.8015411	not scorable	not scorable	not scorable	not scorable
####	Bn-A05-p579882	A-genome specific (napus-rapa)	0.9808612	0.6367021	not scorable	not scorable	0.9917355	0.7132058
####	Bn-A05-p5805445	A-genome specific (napus-rapa)	0.9856459	0.4234855	not scorable	not scorable	1	0.8073739
####	Bn-A05-p5808654	A-genome specific (napus-rapa)	0.9832536	0.725927	not scorable	not scorable	1	0.8137786
####	Bn-A05-p5808895	A-genome specific (napus-rapa)	0.9856459	0.4120608	not scorable	not scorable	0.9917355	0.7860762
####	Bn-A05-p5809043	A-genome specific (rapa only)	not scorable	not scorable	not scorable	not scorable	1	0.8237792
####	Bn-A05-p5812537	A-genome specific (napus-rapa)	0.9928229	0.7550341	not scorable	not scorable	1	0.7392774
####	Bn-A05-p5817231	A-genome specific (napus-rapa)	0.9641148	0.6717362	not scorable	not scorable	0.9586777	0.5416139
####	Bn-A05-p5831673	A-genome specific (napus-rapa)	0.9952153	0.4942217	not scorable	not scorable	0.9917355	0.7745402
####	Bn-A05-p5838524	A-genome specific (napus-rapa)	0.9976076	0.7330959	not scorable	not scorable	1	0.8100908
####	Bn-A05-p5839222	A-genome specific (napus-rapa)	0.9928229	0.7168773	not scorable	not scorable	0.9917355	0.8095312
####	Bn-A05-p5839438	A-genome specific (napus-rapa)	1	0.7484082	not scorable	not scorable	0.9917355	0.7910023
####	Bn-A05-p583972	A-genome specific (napus-rapa)	0.9712918	0.3655559	not scorable	not scorable	0.9752066	0.6930552
####	Bn-A05-p5842980	A-genome specific (napus-rapa)	0.9904306	0.4735969	not scorable	not scorable	0.9917355	0.7523423

####	Bn-A05-p5865143	A-genome specific (napus-rapa)	0.9976076	0.658154	not scorable	not scorable	0.9917355	0.7902774
####	Bn-A05-p5878329	A-genome specific (napus-rapa)	0.9880383	0.4424873	not scorable	not scorable	0.9834711	0.8033706
####	Bn-A05-p589658	A-genome specific (napus-rapa)	0.9736842	0.6748786	not scorable	not scorable	1	0.7139092
####	Bn-A05-p5897513	A-genome specific (napus-rapa)	0.9952153	0.7781377	not scorable	not scorable	1	0.6956896
####	Bn-A05-p58992	A-genome specific (napus-rapa)	0.9904306	0.818263	not scorable	not scorable	1	0.7877918
####	Bn-A05-p59055	A-genome specific (napus-rapa)	0.9880383	0.7297782	not scorable	not scorable	1	0.6853239
####	Bn-A05-p59178	A-genome specific (napus-rapa)	0.9736842	0.3222489	not scorable	not scorable	1	0.719448
####	Bn-A05-p5924319	A-genome specific (napus-rapa)	1	0.7488141	not scorable	not scorable	1	0.6902477
####	Bn-A05-p5926631	genome unspecific (all in)	0.9784689	0.3078931	0.7286822	0.7272372	1	0.7402923
####	Bn-A05-p5936565	A-genome specific (rapa only)	not scorable	not scorable	not scorable	not scorable	1	0.7669629
####	Bn-A05-p5970916	A-genome specific (napus-rapa)	0.854067	0.7392447	not scorable	not scorable	0.6280992	0.7339198
####	Bn-A05-p5973528	A-genome specific (napus-rapa)	0.9904306	0.5513769	not scorable	not scorable	0.9834711	0.736039
####	Bn-A05-p5973876	A-genome specific (napus-rapa)	0.9904306	0.7882492	not scorable	not scorable	1	0.7899277
####	Bn-A05-p5992612	A-genome specific (napus-rapa)	0.9760765	0.51521	not scorable	not scorable	0.9917355	0.6953899
####	Bn-A05-p600196	A-genome specific (napus-rapa)	0.9808612	0.3326063	not scorable	not scorable	1	0.7902759
####	Bn-A05-p6004356	A-genome specific (napus-rapa)	0.9665072	0.341258	not scorable	not scorable	0.9917355	0.7070044
####	Bn-A05-p6020205	A-genome specific (napus-rapa)	0.9880383	0.7503774	not scorable	not scorable	0.9917355	0.6761893
####	Bn-A05-p6021593	A-genome specific (napus-rapa)	0.9976076	0.3164911	not scorable	not scorable	1	0.7774823
####	Bn-A05-p6055523	A-genome specific (napus-rapa)	0.9928229	0.70705	not scorable	not scorable	0.9834711	0.6101249
####	Bn-A05-p6061277	napus specific	0.9856459	0.483722	not scorable	not scorable	not scorable	not scorable
####	Bn-A05-p6063715	A-genome specific (napus-rapa)	0.9976076	0.5391663	not scorable	not scorable	0.9834711	0.8120726
####	Bn-A05-p6074427	A-genome specific (rapa only)	not scorable	not scorable	not scorable	not scorable	1	0.7314882
####	Bn-A05-p61233	A-genome specific (napus-rapa)	0.9521531	0.7106193	not scorable	not scorable	0.9504132	0.7574944
####	Bn-A05-p6154871	A-genome specific (napus-rapa)	0.9928229	0.4898474	not scorable	not scorable	0.9917355	0.5449288
####	Bn-A05-p6155799	A-genome specific (napus-rapa)	0.9856459	0.6689135	not scorable	not scorable	1	0.7917845
####	Bn-A05-p6155901	A-genome specific (napus-rapa)	0.9808612	0.6382338	not scorable	not scorable	0.9834711	0.7955763
####	Bn-A05-p6176943	A-genome specific (napus-rapa)	0.9928229	0.7721756	not scorable	not scorable	1	0.6539372
####	Bn-A05-p619336	A-genome specific (napus-rapa)	0.9665072	0.3372836	not scorable	not scorable	0.9917355	0.6179622
####	Bn-A05-p621136	A-genome specific (napus-rapa)	0.9904306	0.7074847	not scorable	not scorable	1	0.6573179
####	Bn-A05-p6236807	A-genome specific (napus-rapa)	0.9928229	0.4197509	not scorable	not scorable	0.9834711	0.9175813
####	Bn-A05-p6261796	A-genome specific (napus-rapa)	0.9976076	0.8113485	not scorable	not scorable	0.9752066	0.7763813
####	Bn-A05-p6269991	A-genome specific (napus-rapa)	0.9832536	0.6596448	not scorable	not scorable	1	0.6761098
####	Bn-A05-p6271443	A-genome specific (napus-rapa)	0.9952153	0.7837559	not scorable	not scorable	0.9834711	0.797317

####	Bn-A05-p6284593	A-genome specific (napus-rapa)	0.9928229	0.7729984	not scorable	not scorable	0.9834711	0.7511476
####	Bn-A05-p6288330	A-genome specific (napus-rapa)	0.9928229	0.362331	not scorable	not scorable	1	0.7744332
####	Bn-A05-p6308471	A-genome specific (napus-rapa)	0.9880383	0.8012438	not scorable	not scorable	0.9917355	0.759675
####	Bn-A05-p6308543	A-genome specific (napus-rapa)	0.9832536	0.6832628	not scorable	not scorable	1	0.5699365
####	Bn-A05-p6335652	A-genome specific (napus-rapa)	0.9784689	0.5997311	not scorable	not scorable	1	0.8701721
####	Bn-A05-p6337412	A-genome specific (napus-rapa)	0.9904306	0.354295	not scorable	not scorable	1	0.7327957
####	Bn-A05-p6402777	A-genome specific (napus-rapa)	1	0.8042387	not scorable	not scorable	0.9917355	0.7725101
####	Bn-A05-p6404424	A-genome specific (napus-rapa)	0.9880383	0.7127104	not scorable	not scorable	0.9917355	0.7763684
####	Bn-A05-p6404595	A-genome specific (napus-rapa)	0.9832536	0.7065465	not scorable	not scorable	0.9834711	0.7517272
####	Bn-A05-p6404775	C-genome specific (oleracea only)	not scorable	not scorable	0.9844961	0.1861193	not scorable	not scorable
####	Bn-A05-p6405021	A-genome specific (napus-rapa)	0.9712918	0.5235622	not scorable	not scorable	0.9834711	0.7377089
####	Bn-A05-p6405095	genome unspecific (all in)	0.9784689	0.4445553	1	0.6951364	0.9917355	0.7945669
####	Bn-A05-p6405336	A-genome specific (napus-rapa)	0.9736842	0.4605604	not scorable	not scorable	0.9834711	0.7322435
####	Bn-A05-p6405415	A-genome specific (napus-rapa)	0.9928229	0.8201808	not scorable	not scorable	0.9834711	0.8042438
####	Bn-A05-p6412530	A-genome specific (napus-rapa)	0.9880383	0.7917836	not scorable	not scorable	0.9834711	0.7608896
####	Bn-A05-p6415983	A-genome specific (napus-rapa)	0.9832536	0.8092616	not scorable	not scorable	0.9752066	0.8296482
####	Bn-A05-p6416604	A-genome specific (napus-rapa)	0.9760765	0.8030288	not scorable	not scorable	1	0.735843
####	Bn-A05-p6423355	A-genome specific (napus-rapa)	0.9760765	0.6865757	not scorable	not scorable	1	0.8159418
####	Bn-A05-p6423710	A-genome specific (napus-rapa)	0.9880383	0.401017	not scorable	not scorable	0.9586777	0.830142
####	Bn-A05-p6438367	A-genome specific (rapa only)	not scorable	not scorable	not scorable	not scorable	0.9586777	0.7089773
####	Bn-A05-p6441813	A-genome specific (napus-rapa)	0.9856459	0.5071548	not scorable	not scorable	0.9834711	0.6701323
####	Bn-A05-p6457465	genome unspecific (all in)	0.9784689	0.2835187	1	0.76615	0.9917355	0.793777
####	Bn-A05-p6473296	A-genome specific (napus-rapa)	0.9904306	0.4176446	not scorable	not scorable	0.9917355	0.5762315
####	Bn-A05-p6479603	A-genome specific (napus-rapa)	1	0.9205657	not scorable	not scorable	1	0.9173338
####	Bn-A05-p6480168	A-genome specific (napus-rapa)	0.9880383	0.4652334	not scorable	not scorable	0.9834711	0.7362641
####	Bn-A05-p6480907	A-genome specific (napus-rapa)	0.9904306	0.4886409	not scorable	not scorable	0.9917355	0.7112398
####	Bn-A05-p6480981	A-genome specific (napus-rapa)	0.9904306	0.7865103	not scorable	not scorable	0.9752066	0.7438797
####	Bn-A05-p6494313	A-genome specific (napus-rapa)	0.9880383	0.6675031	not scorable	not scorable	1	0.7673253
####	Bn-A05-p652475	genome unspecific (all in)	0.9928229	0.3437648	1	0.7685573	0.9917355	0.81151
####	Bn-A05-p6534544	A-genome specific (napus-rapa)	0.9856459	0.3679169	not scorable	not scorable	1	0.7788004
####	Bn-A05-p6535233	genome unspecific (oleracea-rapa)	not scorable	not scorable	1	0.7331263	0.9917355	0.6652145
####	Bn-A05-p653606	A-genome specific (napus-rapa)	0.7272727	0.7907175	not scorable	not scorable	0.9752066	0.7811267
####	Bn-A05-p6539730	A-genome specific (napus-rapa)	0.9952153	0.7789644	not scorable	not scorable	1	0.7342378

####	Bn-A05-p6541429	A-genome specific (napus-rapa)	0.9952153	0.76833	not scorable	not scorable	0.9917355	0.7223668
####	Bn-A05-p6542040	A-genome specific (napus-rapa)	0.9808612	0.4455372	not scorable	not scorable	0.9917355	0.7590788
####	Bn-A05-p6558171	A-genome specific (napus-rapa)	0.9832536	0.748695	not scorable	not scorable	0.9669421	0.7808446
####	Bn-A05-p6562415	A-genome specific (napus-rapa)	0.9880383	0.5455779	not scorable	not scorable	0.9917355	0.7167006
####	Bn-A05-p6571327	A-genome specific (napus-rapa)	0.9856459	0.6603037	not scorable	not scorable	0.9834711	0.6700168
####	Bn-A05-p6571330	A-genome specific (napus-rapa)	0.9904306	0.5890545	not scorable	not scorable	0.9917355	0.7995171
####	Bn-A05-p6654285	A-genome specific (napus-rapa)	0.9856459	0.5031602	not scorable	not scorable	0.9752066	0.6066801
####	Bn-A05-p6654419	A-genome specific (napus-rapa)	0.9952153	0.7783493	not scorable	not scorable	0.9834711	0.7856833
####	Bn-A05-p6672638	A-genome specific (napus-rapa)	0.923445	0.7494321	not scorable	not scorable	0.9421487	0.7349294
####	Bn-A05-p6674758	A-genome specific (napus-rapa)	0.9880383	0.3938569	not scorable	not scorable	0.9917355	0.689648
####	Bn-A05-p6682796	A-genome specific (napus-rapa)	0.9904306	0.3358619	not scorable	not scorable	0.9917355	0.7964735
####	Bn-A05-p6688695	A-genome specific (napus-rapa)	0.9904306	0.798718	not scorable	not scorable	1	0.8744026
####	Bn-A05-p671910	A-genome specific (napus-rapa)	0.9832536	0.7492642	not scorable	not scorable	1	0.6425663
####	Bn-A05-p673050	A-genome specific (napus-rapa)	0.9593301	0.3352894	not scorable	not scorable	1	0.6982101
####	Bn-A05-p6739093	A-genome specific (napus-rapa)	0.9712918	0.7812341	not scorable	not scorable	0.9917355	0.6749376
####	Bn-A05-p6739341	A-genome specific (napus-rapa)	0.9760765	0.7811968	not scorable	not scorable	1	0.7540129
####	Bn-A05-p6748150	A-genome specific (napus-rapa)	0.9928229	0.7119003	not scorable	not scorable	0.9834711	0.7113937
####	Bn-A05-p6750239	A-genome specific (napus-rapa)	0.9856459	0.7743136	not scorable	not scorable	0.9834711	0.7660106
####	Bn-A05-p6750458	A-genome specific (napus-rapa)	0.9952153	0.7878842	not scorable	not scorable	1	0.8332047
####	Bn-A05-p6751337	A-genome specific (napus-rapa)	0.9832536	0.7228219	not scorable	not scorable	1	0.8525218
####	Bn-A05-p6751841	A-genome specific (napus-rapa)	0.9952153	0.7654794	not scorable	not scorable	0.9917355	0.8147027
####	Bn-A05-p6757087	A-genome specific (napus-rapa)	1	0.8115413	not scorable	not scorable	0.9917355	0.7245347
####	Bn-A05-p6774249	A-genome specific (rapa only)	not scorable	not scorable	not scorable	not scorable	0.9917355	0.7874564
####	Bn-A05-p6775737	A-genome specific (napus-rapa)	0.9760765	0.4190556	not scorable	not scorable	1	0.8003169
####	Bn-A05-p677619	A-genome specific (napus-rapa)	0.9425837	0.2504087	not scorable	not scorable	0.9834711	0.5762169
####	Bn-A05-p6776954	A-genome specific (napus-rapa)	0.9832536	0.7631521	not scorable	not scorable	1	0.8597855
####	Bn-A05-p6786862	A-genome specific (napus-rapa)	0.9952153	0.7771927	not scorable	not scorable	1	0.8543575
####	Bn-A05-p6787164	A-genome specific (napus-rapa)	0.9928229	0.7936219	not scorable	not scorable	0.9917355	0.8396341
####	Bn-A05-p679803	A-genome specific (napus-rapa)	0.9665072	0.7858111	not scorable	not scorable	0.9752066	0.7689065
####	Bn-A05-p6822861	A-genome specific (rapa only)	not scorable	not scorable	not scorable	not scorable	0.9669421	0.6809804
####	Bn-A05-p6845189	A-genome specific (napus-rapa)	0.9928229	0.8092186	not scorable	not scorable	0.9752066	0.6964218
####	Bn-A05-p6867531	A-genome specific (napus-rapa)	0.9952153	0.4438299	not scorable	not scorable	1	0.7098165
####	Bn-A05-p686912	A-genome specific (napus-rapa)	0.9808612	0.7913456	not scorable	not scorable	0.9834711	0.7977321

####	Bn-A05-p6877058	A-genome specific (napus-rapa)	0.9904306	0.7863536	not scorable	not scorable	0.9917355	0.7464744
####	Bn-A05-p687790	A-genome specific (napus-rapa)	0.9808612	0.7371425	not scorable	not scorable	0.9834711	0.6832592
####	Bn-A05-p6878538	A-genome specific (napus-rapa)	1	0.5065711	not scorable	not scorable	0.9834711	0.6652026
####	Bn-A05-p6882852	A-genome specific (napus-rapa)	0.9952153	0.6977416	not scorable	not scorable	1	0.889635
####	Bn-A05-p6897871	A-genome specific (napus-rapa)	0.9952153	0.860123	not scorable	not scorable	0.9834711	0.774188
####	Bn-A05-p6898696	A-genome specific (napus-rapa)	0.9641148	0.4964765	not scorable	not scorable	0.9586777	0.6817704
####	Bn-A05-p6910402	A-genome specific (napus-rapa)	0.9880383	0.2418688	not scorable	not scorable	0.6694215	0.7165339
####	Bn-A05-p6918694	A-genome specific (napus-rapa)	0.923445	0.3251061	not scorable	not scorable	0.9917355	0.7513396
####	Bn-A05-p6951707	A-genome specific (napus-rapa)	1	0.7001775	not scorable	not scorable	1	0.7677348
####	Bn-A05-p6955996	A-genome specific (napus-rapa)	1	0.8125369	not scorable	not scorable	0.9669421	0.8028789
####	Bn-A05-p6957948	A-genome specific (napus-rapa)	0.9712918	0.7864401	not scorable	not scorable	0.5206612	0.8739935
####	Bn-A05-p6975353	A-genome specific (napus-rapa)	1	0.7178167	not scorable	not scorable	1	0.8937193
####	Bn-A05-p6984362	A-genome specific (napus-rapa)	0.9569378	0.7622342	not scorable	not scorable	0.9504132	0.6845619
####	Bn-A05-p6995896	A-genome specific (napus-rapa)	0.9928229	0.7861632	not scorable	not scorable	0.9917355	0.8054572
####	Bn-A05-p7011916	A-genome specific (napus-rapa)	0.9688995	0.7046986	not scorable	not scorable	0.5785124	0.8553948
####	Bn-A05-p701389	A-genome specific (napus-rapa)	0.9856459	0.7813323	not scorable	not scorable	0.9917355	0.8049595
####	Bn-A05-p7031053	A-genome specific (napus-rapa)	0.9688995	0.4107797	not scorable	not scorable	1	0.8007129
####	Bn-A05-p7032286	A-genome specific (napus-rapa)	0.9904306	0.7985684	not scorable	not scorable	0.9752066	0.8097327
####	Bn-A05-p7038463	A-genome specific (napus-rapa)	0.9808612	0.5140685	not scorable	not scorable	0.9917355	0.7096664
####	Bn-A05-p7048173	A-genome specific (napus-rapa)	0.9856459	0.3608137	not scorable	not scorable	0.9834711	0.6892272
####	Bn-A05-p7052463	A-genome specific (napus-rapa)	0.9976076	0.7554284	not scorable	not scorable	0.9834711	0.8345281
####	Bn-A05-p7059671	A-genome specific (napus-rapa)	0.9904306	0.7112923	not scorable	not scorable	0.9834711	0.6944501
####	Bn-A05-p7062575	A-genome specific (napus-rapa)	0.9784689	0.4274725	not scorable	not scorable	1	0.6696357
####	Bn-A05-p7062784	A-genome specific (napus-rapa)	0.9665072	0.4845349	not scorable	not scorable	1	0.8218998
####	Bn-A05-p7076442	A-genome specific (napus-rapa)	0.6315789	0.7961786	not scorable	not scorable	0.9090909	0.6639194
####	Bn-A05-p7098949	A-genome specific (napus-rapa)	0.9904306	0.7432833	not scorable	not scorable	0.9834711	0.6282696
####	Bn-A05-p7105221	A-genome specific (napus-rapa)	0.9736842	0.3574639	not scorable	not scorable	0.9917355	0.7593442
####	Bn-A05-p7119719	A-genome specific (napus-rapa)	0.9952153	0.818427	not scorable	not scorable	1	0.8623074
####	Bn-A05-p7125379	A-genome specific (napus-rapa)	0.5574163	0.7426743	not scorable	not scorable	0.9008265	0.7273611
####	Bn-A05-p7137614	A-genome specific (napus-rapa)	0.9617225	0.6670837	not scorable	not scorable	0.9338843	0.6717063
####	Bn-A05-p7140511	A-genome specific (napus-rapa)	0.9880383	0.7890329	not scorable	not scorable	0.9834711	0.8410932
####	Bn-A05-p7141123	A-genome specific (napus-rapa)	0.9976076	0.7797557	not scorable	not scorable	0.9917355	0.7808607
####	Bn-A05-p7154689	A-genome specific (napus-rapa)	0.9832536	0.4025866	not scorable	not scorable	0.9917355	0.6866934

####	Bn-A05-p717416	A-genome specific (rapa only)	not scorable	not scorable	not scorable	not scorable	0.6859504	0.9097302
####	Bn-A05-p7187425	A-genome specific (napus-rapa)	0.9617225	0.6996811	not scorable	not scorable	1	0.691284
####	Bn-A05-p7222795	A-genome specific (napus-rapa)	0.9928229	0.6545577	not scorable	not scorable	1	0.8059039
####	Bn-A05-p7224750	A-genome specific (napus-rapa)	0.9928229	0.7752569	not scorable	not scorable	0.9752066	0.7360302
####	Bn-A05-p7227877	A-genome specific (napus-rapa)	0.9665072	0.7973682	not scorable	not scorable	0.9504132	0.7823923
####	Bn-A05-p7231369	A-genome specific (napus-rapa)	0.9856459	0.8006775	not scorable	not scorable	1	0.7777886
####	Bn-A05-p7246705	A-genome specific (napus-rapa)	0.9976076	0.5808491	not scorable	not scorable	1	0.7277791
####	Bn-A05-p7255746	A-genome specific (napus-rapa)	0.9808612	0.7642801	not scorable	not scorable	0.9834711	0.7437173
####	Bn-A05-p726481	A-genome specific (napus-rapa)	0.9090909	0.7348965	not scorable	not scorable	0.9917355	0.7584748
####	Bn-A05-p7346478	A-genome specific (napus-rapa)	0.9952153	0.8106422	not scorable	not scorable	0.9917355	0.6970574
####	Bn-A05-p7352554	A-genome specific (rapa only)	not scorable	not scorable	not scorable	not scorable	0.9752066	0.7456172
####	Bn-A05-p7370869	A-genome specific (napus-rapa)	0.9832536	0.6645064	not scorable	not scorable	0.6033058	0.8857612
####	Bn-A05-p7434500	genome unspecific (all in)	0.5550239	0.8215581	0.8217054	0.6141299	0.9008265	0.6870269
####	Bn-A05-p7472918	A-genome specific (rapa only)	not scorable	not scorable	not scorable	not scorable	0.9586777	0.8517691
####	Bn-A05-p7491426	A-genome specific (rapa only)	not scorable	not scorable	not scorable	not scorable	0.9917355	0.7581525
####	Bn-A05-p7496554	A-genome specific (napus-rapa)	0.9832536	0.5635771	not scorable	not scorable	1	0.6221592
####	Bn-A05-p7552614	A-genome specific (napus-rapa)	0.9736842	0.5914077	not scorable	not scorable	1	0.8004208
####	Bn-A05-p7558647	A-genome specific (napus-rapa)	0.9904306	0.4010132	not scorable	not scorable	1	0.7788401
####	Bn-A05-p757624	A-genome specific (napus-rapa)	0.9760765	0.3231009	not scorable	not scorable	0.9834711	0.8269936
####	Bn-A05-p7589125	A-genome specific (napus-rapa)	0.9569378	0.3449278	not scorable	not scorable	0.9834711	0.4481883
####	Bn-A05-p7589257	A-genome specific (napus-rapa)	0.9784689	0.2155385	not scorable	not scorable	0.9917355	0.3538447
####	Bn-A05-p7591501	A-genome specific (napus-rapa)	0.9641148	0.6202886	not scorable	not scorable	0.661157	0.9102081
####	Bn-A05-p761283	A-genome specific (napus-rapa)	0.9521531	0.536536	not scorable	not scorable	0.9917355	0.7889036
####	Bn-A05-p7613069	A-genome specific (napus-rapa)	0.9976076	0.6039445	not scorable	not scorable	1	0.9151567
####	Bn-A05-p761376	A-genome specific (napus-rapa)	0.9952153	0.555096	not scorable	not scorable	1	0.5966557
####	Bn-A05-p7618158	all not scorable	not scorable	not scorable	not scorable	not scorable	not scorable	not scorable
####	Bn-A05-p7632429	A-genome specific (napus-rapa)	1	0.9071735	not scorable	not scorable	1	0.8693638
####	Bn-A05-p76428	A-genome specific (napus-rapa)	0.9760765	0.3828594	not scorable	not scorable	1	0.8137967
####	Bn-A05-p7651411	A-genome specific (napus-rapa)	1	0.7639561	not scorable	not scorable	1	0.9205657
####	Bn-A05-p7662456	napus specific	0.9665072	0.8282643	not scorable	not scorable	not scorable	not scorable
####	Bn-A05-p7705565	A-genome specific (napus-rapa)	0.9904306	0.8111199	not scorable	not scorable	0.9917355	0.7608135
####	Bn-A05-p771943	A-genome specific (napus-rapa)	0.9952153	0.8498164	not scorable	not scorable	0.9917355	0.8465595
####	Bn-A05-p772777	A-genome specific (rapa only)	not scorable	not scorable	not scorable	not scorable	0.9752066	0.7847687

####	Bn-A05-p7729367	A-genome specific (napus-rapa)	0.9832536	0.825826	not scorable	not scorable	0.9917355	0.7442175
####	Bn-A05-p7741244	A-genome specific (napus-rapa)	0.9688995	0.4069836	not scorable	not scorable	0.9917355	0.6509188
####	Bn-A05-p7778186	A-genome specific (napus-rapa)	0.9904306	0.319869	not scorable	not scorable	0.9917355	0.8269624
####	Bn-A05-p7781811	A-genome specific (napus-rapa)	0.9832536	0.3384133	not scorable	not scorable	0.9834711	0.5950561
####	Bn-A05-p7783330	A-genome specific (napus-rapa)	0.9928229	0.8666626	not scorable	not scorable	1	0.7518041
####	Bn-A05-p7791550	A-genome specific (napus-rapa)	0.9712918	0.7620973	not scorable	not scorable	0.5785124	0.7717942
####	Bn-A05-p779415	A-genome specific (napus-rapa)	0.9904306	0.8512001	not scorable	not scorable	1	0.6652964
####	Bn-A05-p780844	A-genome specific (napus-rapa)	0.9760765	0.5280765	not scorable	not scorable	1	0.8463393
####	Bn-A05-p7809870	A-genome specific (napus-rapa)	0.9569378	0.3162602	not scorable	not scorable	0.9917355	0.7057541
####	Bn-A05-p7811409	A-genome specific (napus-rapa)	0.9712918	0.2466919	not scorable	not scorable	1	0.7010397
####	Bn-A05-p7826230	A-genome specific (napus-rapa)	0.9832536	0.7678627	not scorable	not scorable	1	0.7553875
####	Bn-A05-p7840300	A-genome specific (napus-rapa)	0.9904306	0.7061687	not scorable	not scorable	0.9752066	0.7135404
####	Bn-A05-p7841073	A-genome specific (napus-rapa)	1	0.926601	not scorable	not scorable	1	0.9173834
####	Bn-A05-p784331	A-genome specific (napus-rapa)	0.9808612	0.3813459	not scorable	not scorable	0.9917355	0.7277915
####	Bn-A05-p7844876	A-genome specific (napus-rapa)	0.9856459	0.7311944	not scorable	not scorable	0.9752066	0.7440302
####	Bn-A05-p7855766	A-genome specific (napus-rapa)	0.9832536	0.7026118	not scorable	not scorable	0.9917355	0.7245739
####	Bn-A05-p7873080	A-genome specific (napus-rapa)	0.9904306	0.662837	not scorable	not scorable	0.9834711	0.752169
####	Bn-A05-p7874994	A-genome specific (napus-rapa)	0.9736842	0.7936383	not scorable	not scorable	0.9834711	0.725942
####	Bn-A05-p7901866	A-genome specific (napus-rapa)	0.9760765	0.2627688	not scorable	not scorable	1	0.7021827
####	Bn-A05-p7903619	A-genome specific (napus-rapa)	0.9952153	0.7732146	not scorable	not scorable	0.9917355	0.7774354
####	Bn-A05-p7911054	A-genome specific (napus-rapa)	0.9808612	0.5386556	not scorable	not scorable	0.9917355	0.7679034
####	Bn-A05-p7926510	A-genome specific (napus-rapa)	0.9904306	0.3013154	not scorable	not scorable	0.9834711	0.7210625
####	Bn-A05-p794760	A-genome specific (napus-rapa)	0.9282297	0.4796498	not scorable	not scorable	0.9834711	0.7398528
####	Bn-A05-p800515	A-genome specific (napus-rapa)	0.9688995	0.7052349	not scorable	not scorable	1	0.7156722
####	Bn-A05-p800549	A-genome specific (napus-rapa)	0.9425837	0.7872672	not scorable	not scorable	0.9586777	0.8605999
####	Bn-A05-p802111	A-genome specific (napus-rapa)	0.9832536	0.659824	not scorable	not scorable	1	0.7136589
####	Bn-A05-p8120795	A-genome specific (napus-rapa)	0.9712918	0.2666841	not scorable	not scorable	0.9834711	0.800547
####	Bn-A05-p8120997	A-genome specific (napus-rapa)	0.9856459	0.8104685	not scorable	not scorable	1	0.8360305
####	Bn-A05-p8135828	A-genome specific (napus-rapa)	1	0.7504071	not scorable	not scorable	0.9917355	0.7691384
####	Bn-A05-p81414	A-genome specific (rapa only)	not scorable	not scorable	not scorable	not scorable	1	0.7895902
####	Bn-A05-p815322	A-genome specific (napus-rapa)	0.9832536	0.734825	not scorable	not scorable	0.9917355	0.7046005
####	Bn-A05-p8165715	A-genome specific (rapa only)	not scorable	not scorable	not scorable	not scorable	0.9504132	0.3731641
####	Bn-A05-p816963	A-genome specific (napus-rapa)	0.9880383	0.8028721	not scorable	not scorable	1	0.8057212

####	Bn-A05-p817036	A-genome specific (napus-rapa)	0.9832536	0.7344827	not scorable	not scorable	0.9834711	0.7613885
####	Bn-A05-p8202536	A-genome specific (napus-rapa)	0.9904306	0.7349455	not scorable	not scorable	1	0.8134261
####	Bn-A05-p8206349	A-genome specific (napus-rapa)	1	0.6923679	not scorable	not scorable	1	0.7855819
####	Bn-A05-p8210067	A-genome specific (napus-rapa)	0.9952153	0.3349418	not scorable	not scorable	0.9752066	0.6781526
####	Bn-A05-p822087	genome unspecific (all in)	0.9473684	0.2233801	1	0.7563062	1	0.9137406
####	Bn-A05-p8222879	A-genome specific (napus-rapa)	1	0.6085679	not scorable	not scorable	0.9917355	0.7677385
####	Bn-A05-p822597	A-genome specific (napus-rapa)	0.9641148	0.4175113	not scorable	not scorable	0.9917355	0.7879268
####	Bn-A05-p8245454	A-genome specific (napus-rapa)	1	0.3775623	not scorable	not scorable	1	0.6073804
####	Bn-A05-p825088	A-genome specific (rapa only)	not scorable	not scorable	not scorable	not scorable	1	0.924392
####	Bn-A05-p825915	A-genome specific (napus-rapa)	0.9952153	0.8042147	not scorable	not scorable	1	0.9061772
####	Bn-A05-p8261872	A-genome specific (napus-rapa)	0.9976076	0.7794029	not scorable	not scorable	0.9917355	0.884536
####	Bn-A05-p8290002	A-genome specific (napus-rapa)	1	0.3553061	not scorable	not scorable	1	0.7439139
####	Bn-A05-p8297352	A-genome specific (napus-rapa)	0.9976076	0.4869464	not scorable	not scorable	1	0.7212299
####	Bn-A05-p8297563	genome unspecific (all in)	1	0.6663287	1	0.6209557	1	0.6675953
####	Bn-A05-p8318433	A-genome specific (napus-rapa)	1	0.58222	not scorable	not scorable	1	0.8300765
####	Bn-A05-p8320167	A-genome specific (napus-rapa)	1	0.943249	not scorable	not scorable	1	0.9012891
####	Bn-A05-p8322435	A-genome specific (napus-rapa)	0.9784689	0.5673671	not scorable	not scorable	0.9834711	0.735953
####	Bn-A05-p8327216	genome unspecific (all in)	0.9952153	0.4449646	0.9922481	0.6970797	0.9917355	0.8098354
####	Bn-A05-p8329506	A-genome specific (napus-rapa)	1	0.770074	not scorable	not scorable	1	0.8127157
####	Bn-A05-p837013	A-genome specific (napus-rapa)	0.9976076	0.3746752	not scorable	not scorable	1	0.852886
####	Bn-A05-p8376865	A-genome specific (napus-rapa)	0.9952153	0.491284	not scorable	not scorable	0.9917355	0.9180959
####	Bn-A05-p849712	A-genome specific (napus-rapa)	0.9952153	0.5020889	not scorable	not scorable	0.9917355	0.5856405
####	Bn-A05-p8511229	A-genome specific (napus-rapa)	1	0.7343102	not scorable	not scorable	1	0.8530596
####	Bn-A05-p8522584	A-genome specific (napus-rapa)	1	0.7594639	not scorable	not scorable	0.9834711	0.8858985
####	Bn-A05-p853364	A-genome specific (napus-rapa)	0.9952153	0.6690196	not scorable	not scorable	1	0.8918399
####	Bn-A05-p8542569	genome unspecific (all in)	0.9904306	0.4445888	0.9844961	0.8429019	1	0.8822209
####	Bn-A05-p8786891	A-genome specific (napus-rapa)	0.8564593	0.9051473	not scorable	not scorable	0.8842975	0.8142846
####	Bn-A05-p8798628	genome unspecific (all in)	0.9114832	0.3214868	1	0.5915157	1	0.7647092
####	Bn-A05-p8844881	A-genome specific (rapa only)	not scorable	not scorable	not scorable	not scorable	1	0.8510558
####	Bn-A05-p8851115	A-genome specific (napus-rapa)	0.9928229	0.6987026	not scorable	not scorable	1	0.8521883
####	Bn-A05-p8855134	A-genome specific (napus-rapa)	0.9976076	0.6784146	not scorable	not scorable	0.9090909	0.786476
####	Bn-A05-p8865637	all not scorable	not scorable	not scorable	not scorable	not scorable	not scorable	not scorable
####	Bn-A05-p8892295	A-genome specific (napus-rapa)	0.9952153	0.8944422	not scorable	not scorable	0.9917355	0.7449288

####	Bn-A05-p889343	A-genome specific (napus-rapa)	0.9665072	0.7180633	not scorable	not scorable	0.9917355	0.7194705
####	Bn-A05-p892520	A-genome specific (napus-rapa)	0.9736842	0.7607111	not scorable	not scorable	0.7520661	0.8040103
####	Bn-A05-p8943009	A-genome specific (napus-rapa)	0.9593301	0.4380862	not scorable	not scorable	0.9917355	0.9078884
####	Bn-A05-p894768	genome unspecific (all in)	0.9832536	0.3488854	0.9844961	0.6930037	0.9834711	0.7518154
####	Bn-A05-p895276	napus specific	0.9665072	0.5753163	not scorable	not scorable	not scorable	not scorable
####	Bn-A05-p895522	A-genome specific (napus-rapa)	0.9880383	0.4437819	not scorable	not scorable	1	0.6759462
####	Bn-A05-p904751	A-genome specific (napus-rapa)	0.9090909	0.84183	not scorable	not scorable	0.6280992	0.9260694
####	Bn-A05-p9051665	A-genome specific (rapa only)	not scorable	not scorable	not scorable	not scorable	0.9834711	0.6895393
####	Bn-A05-p9058906	A-genome specific (napus-rapa)	0.9880383	0.3685065	not scorable	not scorable	1	0.7652725
####	Bn-A05-p9063726	A-genome specific (napus-rapa)	0.9808612	0.3022112	not scorable	not scorable	0.9917355	0.7277582
####	Bn-A05-p907258	A-genome specific (napus-rapa)	0.9880383	0.3561211	not scorable	not scorable	0.9834711	0.7950127
####	Bn-A05-p9076153	A-genome specific (napus-rapa)	0.9928229	0.6749092	not scorable	not scorable	1	0.6324637
####	Bn-A05-p9076704	A-genome specific (napus-rapa)	0.861244	0.7651475	not scorable	not scorable	0.8760331	0.8531401
####	Bn-A05-p9077059	A-genome specific (napus-rapa)	0.8660287	0.845473	not scorable	not scorable	0.8677686	0.7930093
####	Bn-A05-p9078281	A-genome specific (napus-rapa)	0.9928229	0.6563681	not scorable	not scorable	0.9834711	0.691478
####	Bn-A05-p9078313	A-genome specific (napus-rapa)	0.9952153	0.7267309	not scorable	not scorable	1	0.6681523
####	Bn-A05-p9078783	A-genome specific (napus-rapa)	0.8684211	0.8788785	not scorable	not scorable	0.8677686	0.8447933
####	Bn-A05-p9079126	A-genome specific (napus-rapa)	0.9952153	0.7281143	not scorable	not scorable	1	0.8092738
####	Bn-A05-p9079762	A-genome specific (napus-rapa)	0.9832536	0.6731461	not scorable	not scorable	0.9752066	0.7046655
####	Bn-A05-p9080340	A-genome specific (napus-rapa)	0.9928229	0.3786621	not scorable	not scorable	0.9917355	0.7528778
####	Bn-A05-p910039	A-genome specific (napus-rapa)	0.9832536	0.3318798	not scorable	not scorable	1	0.7732701
####	Bn-A05-p9116236	A-genome specific (rapa only)	not scorable	not scorable	not scorable	not scorable	1	0.7733118
####	Bn-A05-p9118240	A-genome specific (napus-rapa)	0.9880383	0.6610653	not scorable	not scorable	0.9917355	0.7230952
####	Bn-A05-p917769	A-genome specific (napus-rapa)	0.9880383	0.3356301	not scorable	not scorable	0.9752066	0.6796146
####	Bn-A05-p9209378	genome unspecific (oleracea-rapa)	not scorable	not scorable	1	0.7820644	1	0.8271996
####	Bn-A05-p9224719	A-genome specific (napus-rapa)	0.9856459	0.4088669	not scorable	not scorable	1	0.7848381
####	Bn-A05-p9225459	A-genome specific (napus-rapa)	0.9808612	0.7433777	not scorable	not scorable	1	0.8014938
####	Bn-A05-p9226284	A-genome specific (napus-rapa)	0.9832536	0.6438946	not scorable	not scorable	0.9421487	0.6271072
####	Bn-A05-p922813	A-genome specific (napus-rapa)	0.9928229	0.790132	not scorable	not scorable	0.9917355	0.7960008
####	Bn-A05-p9231059	A-genome specific (rapa only)	not scorable	not scorable	not scorable	not scorable	0.9090909	0.886083
####	Bn-A05-p9247748	A-genome specific (napus-rapa)	0.9904306	0.4679045	not scorable	not scorable	0.9917355	0.7498806
####	Bn-A05-p9250358	A-genome specific (napus-rapa)	0.9880383	0.7666081	not scorable	not scorable	0.9834711	0.7687178
####	Bn-A05-p9253661	all not scorable	not scorable	not scorable	not scorable	not scorable	not scorable	not scorable

####	Bn-A05-p926361	A-genome specific (napus-rapa)	0.9808612	0.2743495	not scorable	not scorable	0.9752066	0.8189422
####	Bn-A05-p9266289	A-genome specific (rapa only)	not scorable	not scorable	not scorable	not scorable	0.9669421	0.6853701
####	Bn-A05-p9277011	A-genome specific (napus-rapa)	0.3755981	0.6125389	not scorable	not scorable	0.9917355	0.6557037
####	Bn-A05-p9286929	A-genome specific (napus-rapa)	0.9354067	0.4749032	not scorable	not scorable	0.9669421	0.9022794
####	Bn-A05-p9319851	A-genome specific (napus-rapa)	0.9952153	0.4492071	not scorable	not scorable	1	0.6073198
####	Bn-A05-p933536	A-genome specific (napus-rapa)	0.9904306	0.5653206	not scorable	not scorable	1	0.7613786
####	Bn-A05-p934600	A-genome specific (rapa only)	not scorable	not scorable	not scorable	not scorable	1	0.7629318
####	Bn-A05-p9347113	A-genome specific (napus-rapa)	0.9880383	0.4592353	not scorable	not scorable	1	0.6379285
####	Bn-A05-p9364170	A-genome specific (napus-rapa)	0.9904306	0.7530767	not scorable	not scorable	0.9917355	0.6750506
####	Bn-A05-p9370169	A-genome specific (rapa only)	not scorable	not scorable	not scorable	not scorable	1	0.6003873
####	Bn-A05-p9376879	A-genome specific (napus-rapa)	0.9952153	0.4925472	not scorable	not scorable	0.9834711	0.7426457
####	Bn-A05-p9384270	A-genome specific (napus-rapa)	0.9928229	0.7864153	not scorable	not scorable	0.6528926	0.8190794
####	Bn-A05-p9385617	A-genome specific (napus-rapa)	0.9952153	0.7065486	not scorable	not scorable	0.6694215	0.7205435
####	Bn-A05-p9389251	A-genome specific (napus-rapa)	0.9952153	0.6154464	not scorable	not scorable	0.9834711	0.7714587
####	Bn-A05-p9408634	A-genome specific (napus-rapa)	1	0.4476099	not scorable	not scorable	1	0.556193
####	Bn-A05-p9409136	A-genome specific (napus-rapa)	0.9976076	0.3470266	not scorable	not scorable	0.9834711	0.7718474
####	Bn-A05-p9431593	A-genome specific (napus-rapa)	0.9904306	0.7957033	not scorable	not scorable	0.9752066	0.7723736
####	Bn-A05-p9451391	A-genome specific (napus-rapa)	0.9976076	0.5411686	not scorable	not scorable	1	0.7594934
####	Bn-A05-p9455372	A-genome specific (napus-rapa)	0.9976076	0.7777707	not scorable	not scorable	1	0.5720658
####	Bn-A05-p9472646	A-genome specific (napus-rapa)	1	0.8632373	not scorable	not scorable	0.9834711	0.7086816
####	Bn-A05-p9472820	A-genome specific (napus-rapa)	1	0.6876233	not scorable	not scorable	1	0.7733137
####	Bn-A05-p9495579	A-genome specific (napus-rapa)	0.9952153	0.3299994	not scorable	not scorable	1	0.6966471
####	Bn-A05-p9502471	napus specific	0.9976076	0.5445434	not scorable	not scorable	not scorable	not scorable
####	Bn-A05-p9502893	A-genome specific (napus-rapa)	1	0.4083284	not scorable	not scorable	1	0.8368209
####	Bn-A05-p95346	A-genome specific (napus-rapa)	0.9856459	0.7401052	not scorable	not scorable	0.9752066	0.7575313
####	Bn-A05-p9555189	A-genome specific (napus-rapa)	0.9976076	0.560594	not scorable	not scorable	0.6942149	0.8049531
####	Bn-A05-p956298	A-genome specific (napus-rapa)	0.9952153	0.8398163	not scorable	not scorable	0.9917355	0.8670748
####	Bn-A05-p958740	A-genome specific (napus-rapa)	0.9904306	0.8351308	not scorable	not scorable	0.9917355	0.7492925
####	Bn-A05-p9602710	A-genome specific (napus-rapa)	0.7727273	0.7511754	not scorable	not scorable	0.9090909	0.8086715
####	Bn-A05-p9602897	A-genome specific (napus-rapa)	0.7870814	0.8015583	not scorable	not scorable	0.9421487	0.7753088
####	Bn-A05-p9623131	A-genome specific (napus-rapa)	0.9904306	0.3430033	not scorable	not scorable	0.9586777	0.6792218
####	Bn-A05-p9638089	A-genome specific (napus-rapa)	0.9952153	0.5364432	not scorable	not scorable	0.9917355	0.7654104
####	Bn-A05-p9638802	A-genome specific (napus-rapa)	0.9904306	0.810706	not scorable	not scorable	0.9834711	0.8138697

####	Bn-A05-p9646716	A-genome specific (napus-rapa)	0.9569378	0.2214656	not scorable	not scorable	0.9917355	0.8886898
####	Bn-A05-p9660152	A-genome specific (napus-rapa)	0.9880383	0.797058	not scorable	not scorable	1	0.8008999
####	Bn-A05-p966483	A-genome specific (napus-rapa)	0.9928229	0.4168855	not scorable	not scorable	0.9917355	0.7482154
####	Bn-A05-p9668222	genome unspecific (all in)	0.9928229	0.3821594	1	0.7867702	0.9834711	0.7542949
####	Bn-A05-p967131	A-genome specific (napus-rapa)	0.9449761	0.7762658	not scorable	not scorable	0.9421487	0.8261644
####	Bn-A05-p967575	A-genome specific (napus-rapa)	0.9114832	0.5136297	not scorable	not scorable	0.9338843	0.7119064
####	Bn-A05-p970816	A-genome specific (napus-rapa)	0.9162679	0.7677682	not scorable	not scorable	0.5950413	0.7938615
####	Bn-A05-p9758517	A-genome specific (napus-rapa)	0.9928229	0.7618899	not scorable	not scorable	0.9834711	0.8077748
####	Bn-A05-p9765872	A-genome specific (napus-rapa)	0.9928229	0.6955811	not scorable	not scorable	0.9917355	0.8765541
####	Bn-A05-p9766332	A-genome specific (napus-rapa)	0.9856459	0.7742776	not scorable	not scorable	1	0.6941759
####	Bn-A05-p9766628	A-genome specific (napus-rapa)	0.9904306	0.8496368	not scorable	not scorable	0.9917355	0.6659892
####	Bn-A05-p9773183	A-genome specific (napus-rapa)	0.9832536	0.6154975	not scorable	not scorable	1	0.7981734
####	Bn-A05-p9777066	napus specific	0.9688995	0.6134226	not scorable	not scorable	not scorable	not scorable
####	Bn-A05-p9777250	A-genome specific (napus-rapa)	0.9736842	0.2522809	not scorable	not scorable	1	0.7657952
####	Bn-A05-p983618	A-genome specific (rapa only)	not scorable	not scorable	not scorable	not scorable	1	0.8203638
####	Bn-A05-p9849049	A-genome specific (napus-rapa)	0.9856459	0.4875813	not scorable	not scorable	0.9834711	0.8220464
####	Bn-A05-p9849822	A-genome specific (napus-rapa)	0.9904306	0.3899673	not scorable	not scorable	1	0.7915296
####	Bn-A05-p9849941	A-genome specific (napus-rapa)	0.9832536	0.3376244	not scorable	not scorable	1	0.70673
####	Bn-A05-p9860991	A-genome specific (napus-rapa)	1	0.4797985	not scorable	not scorable	1	0.4693085
####	Bn-A05-p9877872	A-genome specific (napus-rapa)	0.9976076	0.8531508	not scorable	not scorable	1	0.6580876
####	Bn-A05-p9890807	A-genome specific (napus-rapa)	0.9904306	0.7820143	not scorable	not scorable	0.9917355	0.8573067
####	Bn-A05-p9900987	A-genome specific (napus-rapa)	0.9928229	0.810416	not scorable	not scorable	0.9917355	0.8289263
####	Bn-A05-p9937099	A-genome specific (napus-rapa)	1	0.4370553	not scorable	not scorable	1	0.8004208
####	Bn-A05-p996188	A-genome specific (rapa only)	not scorable	not scorable	not scorable	not scorable	0.9917355	0.7383497
####	Bn-A06-p10005161	A-genome specific (rapa only)	not scorable	not scorable	not scorable	not scorable	0.9917355	0.757585
####	Bn-A06-p10018992	A-genome specific (napus-rapa)	0.9928229	0.6779389	not scorable	not scorable	0.5867769	0.7382953
####	Bn-A06-p10019580	A-genome specific (napus-rapa)	0.9904306	0.7286653	not scorable	not scorable	0.5206612	0.7047048
####	Bn-A06-p10021272	A-genome specific (napus-rapa)	0.9904306	0.7362314	not scorable	not scorable	0.5123967	0.7969393
####	Bn-A06-p10021339	A-genome specific (napus-rapa)	0.9856459	0.819232	not scorable	not scorable	0.5785124	0.7559571
####	Bn-A06-p10021393	A-genome specific (napus-rapa)	0.9832536	0.8574103	not scorable	not scorable	0.5123967	0.8414418
####	Bn-A06-p10022213	A-genome specific (napus-rapa)	0.9808612	0.8076153	not scorable	not scorable	0.5206612	0.8362778
####	Bn-A06-p10022347	A-genome specific (napus-rapa)	0.9688995	0.750725	not scorable	not scorable	0.5123967	0.7304225
####	Bn-A06-p10022751	A-genome specific (napus-rapa)	0.9688995	0.7433733	not scorable	not scorable	0.5123967	0.7365086

####	Bn-A06-p10023556	A-genome specific (napus-rapa)	0.9952153	0.8005991	not scorable	not scorable	0.6033058	0.8322143
####	Bn-A06-p10031447	A-genome specific (napus-rapa)	0.9928229	0.7324781	not scorable	not scorable	0.5123967	0.8258424
####	Bn-A06-p10031660	A-genome specific (napus-rapa)	0.9880383	0.7948594	not scorable	not scorable	0.6115702	0.7985566
####	Bn-A06-p10032901	A-genome specific (napus-rapa)	0.9976076	0.8525339	not scorable	not scorable	0.8760331	0.9109087
####	Bn-A06-p10033398	A-genome specific (napus-rapa)	0.9928229	0.7041888	not scorable	not scorable	0.5206612	0.8174314
####	Bn-A06-p10033718	A-genome specific (napus-rapa)	0.9976076	0.7230359	not scorable	not scorable	0.5950413	0.7559296
####	Bn-A06-p10033903	A-genome specific (napus-rapa)	0.9880383	0.7998355	not scorable	not scorable	0.5206612	0.7571789
####	Bn-A06-p10040506	A-genome specific (napus-rapa)	0.9641148	0.7815504	not scorable	not scorable	0.9834711	0.6905599
####	Bn-A06-p10046575	A-genome specific (napus-rapa)	0.9928229	0.4476666	not scorable	not scorable	0.9834711	0.6486661
####	Bn-A06-p10046691	A-genome specific (napus-rapa)	0.9880383	0.4370077	not scorable	not scorable	0.9752066	0.5682449
####	Bn-A06-p10053057	A-genome specific (napus-rapa)	0.9784689	0.7132966	not scorable	not scorable	0.9917355	0.720109
####	Bn-A06-p10053801	A-genome specific (napus-rapa)	0.9832536	0.5524017	not scorable	not scorable	0.9917355	0.7330312
####	Bn-A06-p10064955	A-genome specific (napus-rapa)	0.9856459	0.7514418	not scorable	not scorable	1	0.7635981
####	Bn-A06-p10068703	A-genome specific (napus-rapa)	0.9904306	0.7648885	not scorable	not scorable	1	0.707705
####	Bn-A06-p10069150	A-genome specific (napus-rapa)	0.9904306	0.7644212	not scorable	not scorable	1	0.7453828
####	Bn-A06-p10085385	A-genome specific (napus-rapa)	0.9904306	0.724895	not scorable	not scorable	1	0.7030152
####	Bn-A06-p10085689	A-genome specific (napus-rapa)	0.9880383	0.8199857	not scorable	not scorable	0.9669421	0.8285653
####	Bn-A06-p10086667	A-genome specific (napus-rapa)	0.9904306	0.6935779	not scorable	not scorable	0.9504132	0.6436834
####	Bn-A06-p10124135	A-genome specific (napus-rapa)	0.9712918	0.4147213	not scorable	not scorable	0.9834711	0.6309807
####	Bn-A06-p10129250	A-genome specific (napus-rapa)	0.9976076	0.7594367	not scorable	not scorable	0.9917355	0.8421437
####	Bn-A06-p10135140	A-genome specific (napus-rapa)	0.9928229	0.4291705	not scorable	not scorable	0.9917355	0.6676522
####	Bn-A06-p10146008	A-genome specific (napus-rapa)	0.9904306	0.7528565	not scorable	not scorable	1	0.7192583
####	Bn-A06-p1015217	A-genome specific (napus-rapa)	0.9784689	0.7015271	not scorable	not scorable	0.9752066	0.678677
####	Bn-A06-p1016417	A-genome specific (napus-rapa)	0.9928229	0.7824732	not scorable	not scorable	1	0.8485919
####	Bn-A06-p10169231	A-genome specific (napus-rapa)	1	0.6946233	not scorable	not scorable	0.9917355	0.9083077
####	Bn-A06-p10182944	A-genome specific (napus-rapa)	0.9832536	0.3672982	not scorable	not scorable	0.9834711	0.6685633
####	Bn-A06-p10200920	A-genome specific (napus-rapa)	0.9928229	0.7788835	not scorable	not scorable	1	0.782432
####	Bn-A06-p10202557	A-genome specific (napus-rapa)	0.9880383	0.8108371	not scorable	not scorable	0.9917355	0.8001542
####	Bn-A06-p10210368	A-genome specific (napus-rapa)	0.9952153	0.8249696	not scorable	not scorable	1	0.9293141
####	Bn-A06-p10238741	A-genome specific (napus-rapa)	0.9928229	0.8214073	not scorable	not scorable	0.9586777	0.6514975
####	Bn-A06-p10238985	A-genome specific (napus-rapa)	0.9880383	0.5791875	not scorable	not scorable	0.9752066	0.7777372
####	Bn-A06-p10240329	A-genome specific (napus-rapa)	0.9976076	0.676163	not scorable	not scorable	0.9669421	0.6577469
####	Bn-A06-p10251899	A-genome specific (napus-rapa)	0.9856459	0.5322762	not scorable	not scorable	1	0.7553007

####	Bn-A06-p10296909	A-genome specific (napus-rapa)	0.9928229	0.7292485	not scorable	not scorable	0.9834711	0.7573762
####	Bn-A06-p10297153	A-genome specific (napus-rapa)	0.9880383	0.7499089	not scorable	not scorable	0.9917355	0.7056975
####	Bn-A06-p10300664	A-genome specific (napus-rapa)	0.9832536	0.6908392	not scorable	not scorable	0.9917355	0.7566491
####	Bn-A06-p10308910	napus specific	0.9641148	0.3299563	not scorable	not scorable	not scorable	not scorable
####	Bn-A06-p10320803	A-genome specific (rapa only)	not scorable	not scorable	not scorable	not scorable	0.9834711	0.7655988
####	Bn-A06-p10327909	A-genome specific (napus-rapa)	0.9808612	0.5509072	not scorable	not scorable	0.9752066	0.6856446
####	Bn-A06-p10330066	A-genome specific (napus-rapa)	0.9880383	0.7426164	not scorable	not scorable	0.9834711	0.8008162
####	Bn-A06-p10349427	A-genome specific (napus-rapa)	0.9832536	0.7123818	not scorable	not scorable	1	0.7020658
####	Bn-A06-p10351604	A-genome specific (napus-rapa)	0.9712918	0.7811277	not scorable	not scorable	0.9834711	0.6856064
####	Bn-A06-p10377145	all not scorable	not scorable	not scorable	not scorable	not scorable	not scorable	not scorable
####	Bn-A06-p10391346	A-genome specific (napus-rapa)	0.9784689	0.3586276	not scorable	not scorable	0.9834711	0.8245668
####	Bn-A06-p10400171	A-genome specific (napus-rapa)	1	0.7325938	not scorable	not scorable	1	0.7134259
####	Bn-A06-p10401376	A-genome specific (rapa only)	not scorable	not scorable	not scorable	not scorable	1	0.9095776
####	Bn-A06-p10403901	A-genome specific (napus-rapa)	0.9473684	0.648475	not scorable	not scorable	0.9834711	0.6145394
####	Bn-A06-p10416354	A-genome specific (napus-rapa)	1	0.7480313	not scorable	not scorable	1	0.8307312
####	Bn-A06-p10442596	A-genome specific (napus-rapa)	0.9928229	0.7814094	not scorable	not scorable	0.9917355	0.7386752
####	Bn-A06-p10443399	A-genome specific (napus-rapa)	0.9832536	0.7932527	not scorable	not scorable	0.9917355	0.7828946
####	Bn-A06-p10452758	A-genome specific (rapa only)	not scorable	not scorable	not scorable	not scorable	1	0.6569073
####	Bn-A06-p10453182	A-genome specific (napus-rapa)	0.9952153	0.6733881	not scorable	not scorable	0.9917355	0.7825242
####	Bn-A06-p10457871	napus specific	0.9856459	0.3273372	not scorable	not scorable	not scorable	not scorable
####	Bn-A06-p10470779	A-genome specific (rapa only)	not scorable	not scorable	not scorable	not scorable	0.9917355	0.9058921
####	Bn-A06-p10480404	A-genome specific (napus-rapa)	0.9856459	0.5408666	not scorable	not scorable	0.5206612	0.7030397
####	Bn-A06-p10499003	A-genome specific (napus-rapa)	0.9976076	0.4347561	not scorable	not scorable	0.9917355	0.5654813
####	Bn-A06-p1054104	A-genome specific (napus-rapa)	0.9976076	0.4959051	not scorable	not scorable	0.9834711	0.7066976
####	Bn-A06-p10574739	A-genome specific (napus-rapa)	0.9808612	0.7851234	not scorable	not scorable	0.9586777	0.7553866
####	Bn-A06-p10577973	A-genome specific (napus-rapa)	0.9904306	0.809202	not scorable	not scorable	1	0.8948976
####	Bn-A06-p10578969	A-genome specific (napus-rapa)	0.9856459	0.7670246	not scorable	not scorable	0.9834711	0.822782
####	Bn-A06-p10579615	A-genome specific (napus-rapa)	0.9712918	0.4500825	not scorable	not scorable	0.9834711	0.7369534
####	Bn-A06-p10588412	A-genome specific (rapa only)	not scorable	not scorable	not scorable	not scorable	0.9834711	0.8020135
####	Bn-A06-p10594170	A-genome specific (napus-rapa)	0.9712918	0.3262079	not scorable	not scorable	1	0.7672472
####	Bn-A06-p10606807	A-genome specific (napus-rapa)	0.9928229	0.7777972	not scorable	not scorable	1	0.8270501
####	Bn-A06-p10609574	A-genome specific (napus-rapa)	0.9928229	0.7547899	not scorable	not scorable	0.9917355	0.7159379
####	Bn-A06-p10609641	genome unspecific (all in)	0.9976076	0.3285409	0.9844961	0.8113595	1	0.8697782

####	Bn-A06-p10642761	A-genome specific (napus-rapa)	0.9617225	0.3745417	not scorable	not scorable	0.9917355	0.7121279
####	Bn-A06-p1071392	A-genome specific (napus-rapa)	0.9928229	0.3871962	not scorable	not scorable	1	0.7996386
####	Bn-A06-p10781335	A-genome specific (napus-rapa)	0.9784689	0.503312	not scorable	not scorable	1	0.8028117
####	Bn-A06-p10795156	A-genome specific (napus-rapa)	0.9904306	0.4693213	not scorable	not scorable	1	0.9115109
####	Bn-A06-p10818634	A-genome specific (napus-rapa)	0.8277512	0.5849885	not scorable	not scorable	0.9917355	0.6691458
####	Bn-A06-p1082496	A-genome specific (napus-rapa)	1	0.7985817	not scorable	not scorable	0.9917355	0.7981288
####	Bn-A06-p1082655	A-genome specific (napus-rapa)	0.9904306	0.7600288	not scorable	not scorable	0.9669421	0.7481756
####	Bn-A06-p10832056	A-genome specific (napus-rapa)	0.8899522	0.7787101	not scorable	not scorable	1	0.7529556
####	Bn-A06-p10833219	A-genome specific (napus-rapa)	0.9976076	0.4945572	not scorable	not scorable	1	0.7979036
####	Bn-A06-p10836353	A-genome specific (napus-rapa)	0.9904306	0.3777954	not scorable	not scorable	0.9917355	0.7131884
####	Bn-A06-p1083892	A-genome specific (napus-rapa)	1	0.786446	not scorable	not scorable	0.9834711	0.7670099
####	Bn-A06-p10854312	A-genome specific (napus-rapa)	0.9904306	0.5327448	not scorable	not scorable	0.9917355	0.7038196
####	Bn-A06-p10859915	A-genome specific (napus-rapa)	0.9760765	0.7411894	not scorable	not scorable	1	0.7187554
####	Bn-A06-p10863673	A-genome specific (napus-rapa)	0.9856459	0.8190123	not scorable	not scorable	0.9917355	0.7454397
####	Bn-A06-p10873048	A-genome specific (napus-rapa)	0.9880383	0.605126	not scorable	not scorable	0.9669421	0.6793934
####	Bn-A06-p1094608	A-genome specific (rapa only)	not scorable	not scorable	not scorable	not scorable	0.9669421	0.7048389
####	Bn-A06-p1099469	A-genome specific (napus-rapa)	1	0.6853533	not scorable	not scorable	1	0.6726277
####	Bn-A06-p1099939	A-genome specific (napus-rapa)	0.9976076	0.6649001	not scorable	not scorable	0.9917355	0.7140136
####	Bn-A06-p11021085	A-genome specific (napus-rapa)	0.9856459	0.636482	not scorable	not scorable	1	0.7642207
####	Bn-A06-p11024576	A-genome specific (napus-rapa)	0.9066986	0.4887941	not scorable	not scorable	1	0.6886236
####	Bn-A06-p11025660	A-genome specific (napus-rapa)	0.9354067	0.3770125	not scorable	not scorable	0.9834711	0.7245347
####	Bn-A06-p1106605	A-genome specific (napus-rapa)	0.9976076	0.6071167	not scorable	not scorable	0.9834711	0.710424
####	Bn-A06-p1115978	A-genome specific (napus-rapa)	1	0.9158275	not scorable	not scorable	1	0.9181228
####	Bn-A06-p11165324	A-genome specific (napus-rapa)	0.9760765	0.685284	not scorable	not scorable	0.9917355	0.702776
####	Bn-A06-p11165375	A-genome specific (napus-rapa)	0.9784689	0.7657825	not scorable	not scorable	1	0.7510055
####	Bn-A06-p1116784	A-genome specific (napus-rapa)	0.9952153	0.7222624	not scorable	not scorable	0.9917355	0.7158061
####	Bn-A06-p1116867	A-genome specific (napus-rapa)	1	0.8828264	not scorable	not scorable	1	0.8378905
####	Bn-A06-p1116930	A-genome specific (napus-rapa)	1	0.755897	not scorable	not scorable	1	0.8307881
####	Bn-A06-p1119681	A-genome specific (napus-rapa)	0.9976076	0.7638442	not scorable	not scorable	0.9834711	0.8110157
####	Bn-A06-p1119751	A-genome specific (napus-rapa)	1	0.4877147	not scorable	not scorable	0.9917355	0.7989585
####	Bn-A06-p1120102	A-genome specific (napus-rapa)	1	0.7950849	not scorable	not scorable	0.9917355	0.715754
####	Bn-A06-p1120107	A-genome specific (napus-rapa)	1	0.7730531	not scorable	not scorable	0.9917355	0.6995957
####	Bn-A06-p11231528	A-genome specific (napus-rapa)	0.9736842	0.4788904	not scorable	not scorable	1	0.7419868

####	Bn-A06-p112339	A-genome specific (napus-rapa)	0.9952153	0.564059	not scorable	not scorable	1	0.6142499
####	Bn-A06-p11239689	A-genome specific (napus-rapa)	0.9736842	0.228742	not scorable	not scorable	0.9917355	0.8115126
####	Bn-A06-p11291712	A-genome specific (napus-rapa)	0.9784689	0.7900804	not scorable	not scorable	0.7603306	0.8059192
####	Bn-A06-p11297663	A-genome specific (napus-rapa)	0.9904306	0.6288998	not scorable	not scorable	0.7603306	0.7540566
####	Bn-A06-p11301532	A-genome specific (napus-rapa)	0.9569378	0.7663103	not scorable	not scorable	1	0.8850875
####	Bn-A06-p11343875	A-genome specific (napus-rapa)	0.9665072	0.7698197	not scorable	not scorable	1	0.7954777
####	Bn-A06-p11344945	A-genome specific (napus-rapa)	0.9736842	0.6059076	not scorable	not scorable	1	0.8593845
####	Bn-A06-p11356047	A-genome specific (napus-rapa)	0.9832536	0.6646058	not scorable	not scorable	1	0.640595
####	Bn-A06-p11356414	A-genome specific (napus-rapa)	0.9832536	0.7946937	not scorable	not scorable	1	0.7201476
####	Bn-A06-p11356524	A-genome specific (napus-rapa)	0.9976076	0.8008898	not scorable	not scorable	0.9917355	0.8333257
####	Bn-A06-p1135789	A-genome specific (napus-rapa)	0.9928229	0.6717916	not scorable	not scorable	0.9834711	0.6808389
####	Bn-A06-p11362290	A-genome specific (napus-rapa)	0.9617225	0.3690567	not scorable	not scorable	1	0.7087374
####	Bn-A06-p11366920	A-genome specific (napus-rapa)	1	0.7647003	not scorable	not scorable	1	0.9175813
####	Bn-A06-p11368090	A-genome specific (napus-rapa)	0.8516746	0.8155918	not scorable	not scorable	0.6694215	0.7241411
####	Bn-A06-p11380334	A-genome specific (rapa only)	not scorable	not scorable	not scorable	not scorable	0.9752066	0.576641
####	Bn-A06-p11380775	A-genome specific (rapa only)	not scorable	not scorable	not scorable	not scorable	0.7520661	0.5657402
####	Bn-A06-p11381026	A-genome specific (napus-rapa)	1	0.7043434	not scorable	not scorable	1	0.7367962
####	Bn-A06-p113900	A-genome specific (napus-rapa)	0.9880383	0.2549745	not scorable	not scorable	0.9752066	0.6413375
####	Bn-A06-p11415584	A-genome specific (napus-rapa)	0.9784689	0.723091	not scorable	not scorable	1	0.797233
####	Bn-A06-p1142557	A-genome specific (napus-rapa)	0.9066986	0.7789338	not scorable	not scorable	0.9917355	0.7524039
####	Bn-A06-p11432316	A-genome specific (napus-rapa)	0.9784689	0.4630884	not scorable	not scorable	0.9504132	0.5443395
####	Bn-A06-p11455914	A-genome specific (napus-rapa)	1	0.7155162	not scorable	not scorable	0.9834711	0.8049601
####	Bn-A06-p114634	A-genome specific (napus-rapa)	0.9976076	0.4366549	not scorable	not scorable	0.6942149	0.8367572
####	Bn-A06-p1149034	A-genome specific (napus-rapa)	1	0.7789344	not scorable	not scorable	0.6942149	0.8005329
####	Bn-A06-p11500765	A-genome specific (napus-rapa)	0.9856459	0.5563209	not scorable	not scorable	1	0.7320092
####	Bn-A06-p1154840	A-genome specific (napus-rapa)	0.9952153	0.5568834	not scorable	not scorable	0.9917355	0.7325417
####	Bn-A06-p115737	A-genome specific (rapa only)	not scorable	not scorable	not scorable	not scorable	0.6694215	0.8624666
####	Bn-A06-p11588495	A-genome specific (napus-rapa)	1	0.3320274	not scorable	not scorable	0.9834711	0.6929848
####	Bn-A06-p115948	A-genome specific (napus-rapa)	0.9880383	0.7896861	not scorable	not scorable	1	0.7521878
####	Bn-A06-p1160984	A-genome specific (napus-rapa)	1	0.471817	not scorable	not scorable	0.9917355	0.7668743
####	Bn-A06-p1168447	napus specific	1	0.4249248	not scorable	not scorable	not scorable	not scorable
####	Bn-A06-p11704401	A-genome specific (napus-rapa)	0.9856459	0.7748103	not scorable	not scorable	0.9834711	0.8326044
####	Bn-A06-p11708819	napus specific	0.9808612	0.8019677	not scorable	not scorable	not scorable	not scorable

####	Bn-A06-p1173126	A-genome specific (napus-rapa)	0.9928229	0.8591606	not scorable	not scorable	0.9752066	0.7483622
####	Bn-A06-p1176062	A-genome specific (napus-rapa)	0.9952153	0.3308119	not scorable	not scorable	0.9917355	0.6588989
####	Bn-A06-p11776560	A-genome specific (napus-rapa)	1	0.635902	not scorable	not scorable	1	0.7739488
####	Bn-A06-p11808857	A-genome specific (rapa only)	not scorable	not scorable	not scorable	not scorable	0.9834711	0.6502585
####	Bn-A06-p11819912	A-genome specific (napus-rapa)	0.9784689	0.4961949	not scorable	not scorable	0.9834711	0.8148219
####	Bn-A06-p11819954	A-genome specific (napus-rapa)	0.9832536	0.3038135	not scorable	not scorable	0.9917355	0.7737194
####	Bn-A06-p11840908	A-genome specific (napus-rapa)	0.9976076	0.7613404	not scorable	not scorable	0.9917355	0.720181
####	Bn-A06-p1184282	A-genome specific (napus-rapa)	0.9976076	0.6958141	not scorable	not scorable	0.9917355	0.8346409
####	Bn-A06-p11848553	A-genome specific (napus-rapa)	0.9976076	0.7671664	not scorable	not scorable	1	0.737825
####	Bn-A06-p11848767	A-genome specific (napus-rapa)	0.9736842	0.6856481	not scorable	not scorable	0.9917355	0.8164111
####	Bn-A06-p11848770	A-genome specific (napus-rapa)	0.9784689	0.6121548	not scorable	not scorable	0.9917355	0.7745563
####	Bn-A06-p1185371	A-genome specific (napus-rapa)	0.9976076	0.4150164	not scorable	not scorable	0.9834711	0.7433071
####	Bn-A06-p1187915	C-genome specific (oleracea only)	not scorable	not scorable	0.9922481	0.6929926	not scorable	not scorable
####	Bn-A06-p11919396	A-genome specific (napus-rapa)	0.9952153	0.3445336	not scorable	not scorable	0.9752066	0.8395817
####	Bn-A06-p11995250	A-genome specific (napus-rapa)	1	0.5789745	not scorable	not scorable	1	0.5976474
####	Bn-A06-p12030020	A-genome specific (napus-rapa)	1	0.7614368	not scorable	not scorable	1	0.7591512
####	Bn-A06-p12078648	A-genome specific (napus-rapa)	0.8660287	0.7197168	not scorable	not scorable	0.7520661	0.7286038
####	Bn-A06-p12093192	A-genome specific (napus-rapa)	0.9880383	0.7244185	not scorable	not scorable	0.9917355	0.8740839
####	Bn-A06-p12135104	A-genome specific (napus-rapa)	0.9976076	0.6648868	not scorable	not scorable	0.9834711	0.6603483
####	Bn-A06-p12142438	A-genome specific (rapa only)	not scorable	not scorable	not scorable	not scorable	1	0.7533373
####	Bn-A06-p121547	A-genome specific (napus-rapa)	0.9928229	0.7853238	not scorable	not scorable	0.9917355	0.7681255
####	Bn-A06-p1217543	A-genome specific (napus-rapa)	0.9593301	0.7509734	not scorable	not scorable	0.661157	0.8204749
####	Bn-A06-p12202203	A-genome specific (napus-rapa)	0.9952153	0.3539796	not scorable	not scorable	0.9834711	0.7232896
####	Bn-A06-p12203381	A-genome specific (napus-rapa)	0.9880383	0.721136	not scorable	not scorable	0.9834711	0.7471445
####	Bn-A06-p12205865	A-genome specific (napus-rapa)	0.9976076	0.7670361	not scorable	not scorable	0.9917355	0.8164662
####	Bn-A06-p12213032	A-genome specific (napus-rapa)	0.9760765	0.768383	not scorable	not scorable	0.9917355	0.7115262
####	Bn-A06-p1225010	genome unspecific (oleracea-rapa)	not scorable	not scorable	0.9689922	0.7457433	0.7603306	0.8906642
####	Bn-A06-p1232267	A-genome specific (napus-rapa)	0.9976076	0.7996382	not scorable	not scorable	1	0.8012844
####	Bn-A06-p12337022	A-genome specific (napus-rapa)	0.9880383	0.7888277	not scorable	not scorable	0.9834711	0.7812226
####	Bn-A06-p12337596	A-genome specific (napus-rapa)	0.9952153	0.6485423	not scorable	not scorable	0.9834711	0.6113846
####	Bn-A06-p12337598	A-genome specific (napus-rapa)	0.9904306	0.5710512	not scorable	not scorable	0.9834711	0.6976759
####	Bn-A06-p12346760	A-genome specific (napus-rapa)	0.9760765	0.6087637	not scorable	not scorable	0.9669421	0.6330386
####	Bn-A06-p12348915	A-genome specific (napus-rapa)	0.9928229	0.7845942	not scorable	not scorable	0.9917355	0.8134061

####	Bn-A06-p12354309	A-genome specific (napus-rapa)	0.9832536	0.7853431	not scorable	not scorable	0.9752066	0.8267855
####	Bn-A06-p12354524	napus specific	0.9880383	0.6749924	not scorable	not scorable	not scorable	not scorable
####	Bn-A06-p12354582	A-genome specific (napus-rapa)	0.9952153	0.6736845	not scorable	not scorable	0.9669421	0.6859751
####	Bn-A06-p12355438	A-genome specific (napus-rapa)	0.9928229	0.7872537	not scorable	not scorable	0.9752066	0.7600623
####	Bn-A06-p12355762	A-genome specific (napus-rapa)	0.9928229	0.7610742	not scorable	not scorable	0.9752066	0.7340579
####	Bn-A06-p12356277	A-genome specific (napus-rapa)	0.9952153	0.8009423	not scorable	not scorable	0.9752066	0.8157302
####	Bn-A06-p12356525	A-genome specific (napus-rapa)	0.9832536	0.7396562	not scorable	not scorable	0.9752066	0.5668947
####	Bn-A06-p12356708	A-genome specific (rapa only)	not scorable	not scorable	not scorable	not scorable	0.9669421	0.7616207
####	Bn-A06-p12357497	A-genome specific (napus-rapa)	0.9904306	0.4722738	not scorable	not scorable	0.9834711	0.5847653
####	Bn-A06-p12358748	A-genome specific (napus-rapa)	0.9832536	0.7894202	not scorable	not scorable	0.9834711	0.8285961
####	Bn-A06-p12362853	A-genome specific (napus-rapa)	0.9856459	0.7481425	not scorable	not scorable	0.9834711	0.7500029
####	Bn-A06-p12363034	A-genome specific (napus-rapa)	0.9928229	0.7937927	not scorable	not scorable	0.9669421	0.7936831
####	Bn-A06-p12364597	A-genome specific (napus-rapa)	0.9928229	0.7800971	not scorable	not scorable	0.5289256	0.7043585
####	Bn-A06-p12364605	A-genome specific (rapa only)	not scorable	not scorable	not scorable	not scorable	0.892562	0.8862464
####	Bn-A06-p12376674	A-genome specific (napus-rapa)	0.9784689	0.6522495	not scorable	not scorable	0.9834711	0.6686524
####	Bn-A06-p12402009	A-genome specific (napus-rapa)	0.9928229	0.6910045	not scorable	not scorable	0.9752066	0.6983269
####	Bn-A06-p12404563	A-genome specific (napus-rapa)	0.9904306	0.7921799	not scorable	not scorable	0.9917355	0.7654617
####	Bn-A06-p12404570	A-genome specific (napus-rapa)	0.9952153	0.7330528	not scorable	not scorable	1	0.7640449
####	Bn-A06-p12404848	A-genome specific (napus-rapa)	0.9928229	0.6942918	not scorable	not scorable	1	0.8085203
####	Bn-A06-p12436092	A-genome specific (napus-rapa)	0.9904306	0.7813781	not scorable	not scorable	0.9917355	0.7952865
####	Bn-A06-p12438791	A-genome specific (napus-rapa)	0.9880383	0.7947542	not scorable	not scorable	1	0.917563
####	Bn-A06-p12441450	A-genome specific (napus-rapa)	0.9928229	0.7917563	not scorable	not scorable	0.9834711	0.7991471
####	Bn-A06-p12451649	A-genome specific (napus-rapa)	0.9832536	0.8325339	not scorable	not scorable	1	0.8167799
####	Bn-A06-p12464905	A-genome specific (napus-rapa)	0.9832536	0.4510816	not scorable	not scorable	1	0.7309964
####	Bn-A06-p12517420	A-genome specific (napus-rapa)	0.9928229	0.4458174	not scorable	not scorable	0.9669421	0.6896096
####	Bn-A06-p12534357	A-genome specific (napus-rapa)	0.9976076	0.4955723	not scorable	not scorable	0.9917355	0.784013
####	Bn-A06-p12609046	A-genome specific (napus-rapa)	0.9928229	0.393588	not scorable	not scorable	0.9917355	0.8045535
####	Bn-A06-p12627660	A-genome specific (napus-rapa)	0.9904306	0.3191718	not scorable	not scorable	1	0.7560848
####	Bn-A06-p12632207	A-genome specific (napus-rapa)	0.9808612	0.7236586	not scorable	not scorable	1	0.8049245
####	Bn-A06-p12634012	A-genome specific (napus-rapa)	0.9976076	0.7308255	not scorable	not scorable	1	0.7152911
####	Bn-A06-p12634097	A-genome specific (napus-rapa)	0.9952153	0.6698314	not scorable	not scorable	1	0.6212335
####	Bn-A06-p12641317	A-genome specific (napus-rapa)	0.9880383	0.8220268	not scorable	not scorable	0.9752066	0.7623531
####	Bn-A06-p12641905	A-genome specific (napus-rapa)	0.9952153	0.6881461	not scorable	not scorable	0.9917355	0.7351651

####	Bn-A06-p12645785	A-genome specific (napus-rapa)	0.9976076	0.6919374	not scorable	not scorable	0.9917355	0.6662588
####	Bn-A06-p12658161	A-genome specific (napus-rapa)	0.9808612	0.7381235	not scorable	not scorable	0.9752066	0.6265146
####	Bn-A06-p12696157	A-genome specific (napus-rapa)	0.9928229	0.6007544	not scorable	not scorable	0.9917355	0.7030661
####	Bn-A06-p12718659	A-genome specific (napus-rapa)	0.9976076	0.4325381	not scorable	not scorable	0.9917355	0.8123731
####	Bn-A06-p12732487	A-genome specific (napus-rapa)	0.9904306	0.4290624	not scorable	not scorable	0.9917355	0.8056887
####	Bn-A06-p12734894	A-genome specific (napus-rapa)	1	0.7226511	not scorable	not scorable	0.9669421	0.7358505
####	Bn-A06-p12748821	A-genome specific (napus-rapa)	0.9952153	0.7680824	not scorable	not scorable	0.9834711	0.7971296
####	Bn-A06-p12768136	A-genome specific (napus-rapa)	0.9832536	0.8058218	not scorable	not scorable	0.9834711	0.773975
####	Bn-A06-p12775306	A-genome specific (napus-rapa)	0.9952153	0.7606934	not scorable	not scorable	0.9834711	0.7206949
####	Bn-A06-p12775616	A-genome specific (napus-rapa)	0.9904306	0.8324084	not scorable	not scorable	0.9752066	0.7026841
####	Bn-A06-p12775665	A-genome specific (napus-rapa)	0.9856459	0.7219978	not scorable	not scorable	0.9669421	0.6444806
####	Bn-A06-p12775802	A-genome specific (napus-rapa)	0.9904306	0.8007179	not scorable	not scorable	1	0.8327143
####	Bn-A06-p1278419	A-genome specific (napus-rapa)	0.9952153	0.7274017	not scorable	not scorable	1	0.747323
####	Bn-A06-p12824031	A-genome specific (napus-rapa)	1	0.7356847	not scorable	not scorable	1	0.5940111
####	Bn-A06-p12843916	A-genome specific (napus-rapa)	0.9880383	0.8008307	not scorable	not scorable	0.9834711	0.7688105
####	Bn-A06-p12846574	A-genome specific (napus-rapa)	0.9856459	0.6324841	not scorable	not scorable	0.9834711	0.7516447
####	Bn-A06-p12868864	A-genome specific (napus-rapa)	0.9952153	0.789197	not scorable	not scorable	0.9917355	0.7647055
####	Bn-A06-p12876501	A-genome specific (napus-rapa)	0.9928229	0.7802979	not scorable	not scorable	0.9917355	0.7614141
####	Bn-A06-p12880298	A-genome specific (napus-rapa)	0.9808612	0.719653	not scorable	not scorable	0.9834711	0.6878283
####	Bn-A06-p12884492	A-genome specific (napus-rapa)	0.9808612	0.6015235	not scorable	not scorable	0.9834711	0.6671287
####	Bn-A06-p12884843	A-genome specific (napus-rapa)	1	0.9095823	not scorable	not scorable	1	0.9175813
####	Bn-A06-p12886443	A-genome specific (napus-rapa)	0.9952153	0.5282201	not scorable	not scorable	1	0.7472173
####	Bn-A06-p12886553	A-genome specific (napus-rapa)	0.9952153	0.5751531	not scorable	not scorable	0.9834711	0.7613427
####	Bn-A06-p12896905	A-genome specific (napus-rapa)	0.9904306	0.7935735	not scorable	not scorable	0.9669421	0.7731049
####	Bn-A06-p12911577	A-genome specific (napus-rapa)	0.9832536	0.4476945	not scorable	not scorable	0.9834711	0.7709775
####	Bn-A06-p1291667	A-genome specific (napus-rapa)	0.9976076	0.762328	not scorable	not scorable	1	0.8223467
####	Bn-A06-p1292241	A-genome specific (napus-rapa)	0.9904306	0.7520941	not scorable	not scorable	1	0.7025625
####	Bn-A06-p12980841	A-genome specific (rapa only)	not scorable	not scorable	not scorable	not scorable	0.8429752	0.7915418
####	Bn-A06-p12986192	A-genome specific (napus-rapa)	0.9904306	0.5063318	not scorable	not scorable	0.9917355	0.7220628
####	Bn-A06-p12995133	A-genome specific (napus-rapa)	0.9928229	0.6492539	not scorable	not scorable	0.9752066	0.6909733
####	Bn-A06-p13007607	A-genome specific (napus-rapa)	0.9928229	0.6879587	not scorable	not scorable	0.9917355	0.7241918
####	Bn-A06-p13008545	A-genome specific (napus-rapa)	0.9952153	0.7714634	not scorable	not scorable	0.9917355	0.7231992
####	Bn-A06-p13009409	A-genome specific (napus-rapa)	0.9880383	0.8101414	not scorable	not scorable	0.9917355	0.714877

####	Bn-A06-p13021244	A-genome specific (napus-rapa)	0.9736842	0.7813579	not scorable	not scorable	0.9669421	0.7623345
####	Bn-A06-p13105867	A-genome specific (napus-rapa)	0.9736842	0.7197819	not scorable	not scorable	0.7768595	0.7024059
####	Bn-A06-p13112819	A-genome specific (napus-rapa)	0.9880383	0.3857996	not scorable	not scorable	0.7520661	0.666582
####	Bn-A06-p1312619	A-genome specific (napus-rapa)	1	0.5414092	not scorable	not scorable	0.9834711	0.7589697
####	Bn-A06-p13126839	A-genome specific (napus-rapa)	0.9808612	0.8130695	not scorable	not scorable	0.7438017	0.6740149
####	Bn-A06-p13144922	A-genome specific (napus-rapa)	0.9545454	0.7878996	not scorable	not scorable	0.768595	0.8879762
####	Bn-A06-p13147955	A-genome specific (napus-rapa)	0.9688995	0.6904839	not scorable	not scorable	0.7438017	0.687968
####	Bn-A06-p13159551	A-genome specific (napus-rapa)	0.9952153	0.8062796	not scorable	not scorable	0.9917355	0.7947181
####	Bn-A06-p13162770	A-genome specific (napus-rapa)	0.9928229	0.5188691	not scorable	not scorable	0.9917355	0.7664203
####	Bn-A06-p13190352	A-genome specific (napus-rapa)	0.9904306	0.4019636	not scorable	not scorable	1	0.8484885
####	Bn-A06-p13194586	A-genome specific (napus-rapa)	0.9832536	0.7319274	not scorable	not scorable	0.9917355	0.6923604
####	Bn-A06-p13204772	A-genome specific (napus-rapa)	0.9976076	0.8050264	not scorable	not scorable	1	0.8013928
####	Bn-A06-p1322724	A-genome specific (napus-rapa)	0.9784689	0.4109783	not scorable	not scorable	0.6694215	0.7427732
####	Bn-A06-p13230875	A-genome specific (napus-rapa)	0.9904306	0.5164688	not scorable	not scorable	1	0.7067118
####	Bn-A06-p13230989	A-genome specific (napus-rapa)	0.9928229	0.4350449	not scorable	not scorable	0.9834711	0.7657516
####	Bn-A06-p1330567	A-genome specific (napus-rapa)	0.9808612	0.407299	not scorable	not scorable	0.9834711	0.7576386
####	Bn-A06-p1345642	A-genome specific (napus-rapa)	0.9808612	0.4551361	not scorable	not scorable	0.7272727	0.8089982
####	Bn-A06-p1348404	A-genome specific (napus-rapa)	0.9904306	0.7759456	not scorable	not scorable	0.9834711	0.7209508
####	Bn-A06-p1350786	A-genome specific (napus-rapa)	0.9928229	0.7708716	not scorable	not scorable	1	0.8219877
####	Bn-A06-p13583056	all not scorable	not scorable	not scorable	not scorable	not scorable	not scorable	not scorable
####	Bn-A06-p1358955	A-genome specific (napus-rapa)	0.9952153	0.795873	not scorable	not scorable	0.9752066	0.7155437
####	Bn-A06-p13610210	A-genome specific (napus-rapa)	0.9856459	0.3489999	not scorable	not scorable	1	0.8168827
####	Bn-A06-p1366713	A-genome specific (napus-rapa)	0.9808612	0.3811374	not scorable	not scorable	0.9917355	0.7749607
####	Bn-A06-p13726921	A-genome specific (napus-rapa)	0.9712918	0.5642921	not scorable	not scorable	1	0.8330827
####	Bn-A06-p13728321	A-genome specific (napus-rapa)	0.9904306	0.262479	not scorable	not scorable	0.9752066	0.7024423
####	Bn-A06-p1373016	A-genome specific (rapa only)	not scorable	not scorable	not scorable	not scorable	0.9917355	0.694411
####	Bn-A06-p13732668	A-genome specific (napus-rapa)	0.9784689	0.7261752	not scorable	not scorable	0.9917355	0.6924533
####	Bn-A06-p137614	A-genome specific (napus-rapa)	0.9952153	0.803583	not scorable	not scorable	0.9917355	0.721624
####	Bn-A06-p13787740	A-genome specific (napus-rapa)	1	0.554594	not scorable	not scorable	0.9917355	0.8443128
####	Bn-A06-p1378913	A-genome specific (napus-rapa)	0.9976076	0.7010535	not scorable	not scorable	1	0.7808867
####	Bn-A06-p13825200	A-genome specific (napus-rapa)	0.9928229	0.6931776	not scorable	not scorable	1	0.6199228
####	Bn-A06-p1383275	A-genome specific (napus-rapa)	0.9784689	0.789272	not scorable	not scorable	1	0.8033431
####	Bn-A06-p13850939	napus specific	0.937799	0.267318	not scorable	not scorable	not scorable	not scorable

####	Bn-A06-p13859891	A-genome specific (napus-rapa)	0.9641148	0.8077102	not scorable	not scorable	1	0.760376
####	Bn-A06-p1386474	A-genome specific (rapa only)	not scorable	not scorable	not scorable	not scorable	0.677686	0.9175554
####	Bn-A06-p13870364	A-genome specific (napus-rapa)	0.9832536	0.4489942	not scorable	not scorable	0.9834711	0.6769026
####	Bn-A06-p13872191	A-genome specific (napus-rapa)	0.9880383	0.6999618	not scorable	not scorable	0.9917355	0.7003243
####	Bn-A06-p139033	A-genome specific (napus-rapa)	1	0.6192982	not scorable	not scorable	0.9917355	0.8436432
####	Bn-A06-p13910548	A-genome specific (napus-rapa)	0.9904306	0.6967127	not scorable	not scorable	0.9834711	0.5734083
####	Bn-A06-p13913895	A-genome specific (napus-rapa)	0.9736842	0.8101604	not scorable	not scorable	1	0.7693269
####	Bn-A06-p13958812	A-genome specific (napus-rapa)	0.9258373	0.6859965	not scorable	not scorable	0.9338843	0.6680776
####	Bn-A06-p1403969	A-genome specific (napus-rapa)	1	0.374563	not scorable	not scorable	0.9834711	0.7782195
####	Bn-A06-p1404859	A-genome specific (napus-rapa)	0.9712918	0.7188787	not scorable	not scorable	0.5619835	0.7851781
####	Bn-A06-p1408273	A-genome specific (napus-rapa)	0.9784689	0.4574404	not scorable	not scorable	1	0.6308318
####	Bn-A06-p14121814	A-genome specific (napus-rapa)	0.9641148	0.7722188	not scorable	not scorable	0.9917355	0.7432481
####	Bn-A06-p14163715	A-genome specific (napus-rapa)	0.9808612	0.1966075	not scorable	not scorable	1	0.9515939
####	Bn-A06-p14179943	A-genome specific (napus-rapa)	0.9736842	0.794936	not scorable	not scorable	1	0.927162
####	Bn-A06-p14189236	A-genome specific (napus-rapa)	0.9856459	0.523357	not scorable	not scorable	0.9834711	0.7366242
####	Bn-A06-p14192915	A-genome specific (napus-rapa)	0.9904306	0.2542224	not scorable	not scorable	0.9917355	0.8117295
####	Bn-A06-p14193381	A-genome specific (napus-rapa)	0.8875598	0.7948895	not scorable	not scorable	0.9834711	0.753765
####	Bn-A06-p14198240	A-genome specific (napus-rapa)	0.9856459	0.4615354	not scorable	not scorable	1	0.7118057
####	Bn-A06-p14207547	A-genome specific (napus-rapa)	0.9688995	0.8198035	not scorable	not scorable	0.9834711	0.7526106
####	Bn-A06-p14212186	A-genome specific (napus-rapa)	0.9569378	0.6843207	not scorable	not scorable	0.9752066	0.7321373
####	Bn-A06-p14214930	A-genome specific (napus-rapa)	0.9545454	0.8288864	not scorable	not scorable	0.9669421	0.8011191
####	Bn-A06-p14232702	A-genome specific (napus-rapa)	0.5334928	0.6206563	not scorable	not scorable	0.9669421	0.6445115
####	Bn-A06-p14232703	A-genome specific (napus-rapa)	0.5550239	0.7066329	not scorable	not scorable	0.9586777	0.8436641
####	Bn-A06-p14241294	A-genome specific (napus-rapa)	0.9760765	0.775151	not scorable	not scorable	0.9752066	0.7459099
####	Bn-A06-p14266102	A-genome specific (napus-rapa)	1	0.4791672	not scorable	not scorable	0.9669421	0.7612398
####	Bn-A06-p14269763	A-genome specific (napus-rapa)	0.9832536	0.3308119	not scorable	not scorable	0.9917355	0.7608328
####	Bn-A06-p1427206	A-genome specific (napus-rapa)	0.9736842	0.7954577	not scorable	not scorable	0.9834711	0.8214294
####	Bn-A06-p14281185	A-genome specific (napus-rapa)	0.9832536	0.6103412	not scorable	not scorable	1	0.7816716
####	Bn-A06-p14285203	A-genome specific (napus-rapa)	0.9497608	0.7474042	not scorable	not scorable	0.9504132	0.6851076
####	Bn-A06-p14300543	A-genome specific (napus-rapa)	0.9952153	0.5159616	not scorable	not scorable	0.9917355	0.7302115
####	Bn-A06-p14300642	A-genome specific (napus-rapa)	0.9784689	0.4495222	not scorable	not scorable	1	0.8116663
####	Bn-A06-p14300919	A-genome specific (napus-rapa)	0.9545454	0.8661194	not scorable	not scorable	1	0.7707667
####	Bn-A06-p14316307	A-genome specific (napus-rapa)	0.9880383	0.7206984	not scorable	not scorable	0.9421487	0.707125

####	Bn-A06-p1432446	A-genome specific (napus-rapa)	0.9736842	0.8108827	not scorable	not scorable	0.9917355	0.8008413
####	Bn-A06-p14327501	A-genome specific (napus-rapa)	0.9976076	0.528057	not scorable	not scorable	1	0.8363531
####	Bn-A06-p1433021	A-genome specific (napus-rapa)	0.9880383	0.7491498	not scorable	not scorable	1	0.6983862
####	Bn-A06-p14369166	A-genome specific (napus-rapa)	0.9880383	0.4778179	not scorable	not scorable	0.9917355	0.8464755
####	Bn-A06-p14373935	A-genome specific (napus-rapa)	0.9880383	0.565223	not scorable	not scorable	0.9917355	0.7490245
####	Bn-A06-p14375425	A-genome specific (napus-rapa)	0.9904306	0.5628347	not scorable	not scorable	0.9834711	0.7631255
####	Bn-A06-p14375498	A-genome specific (napus-rapa)	0.9832536	0.7791786	not scorable	not scorable	0.9917355	0.8019738
####	Bn-A06-p14389679	A-genome specific (napus-rapa)	0.9928229	0.5136651	not scorable	not scorable	0.9917355	0.8639689
####	Bn-A06-p14391905	A-genome specific (napus-rapa)	0.9760765	0.6822259	not scorable	not scorable	0.9752066	0.7820169
####	Bn-A06-p14399524	A-genome specific (napus-rapa)	0.9354067	0.7612922	not scorable	not scorable	0.9586777	0.796928
####	Bn-A06-p14399853	all not scorable	not scorable	not scorable	not scorable	not scorable	not scorable	not scorable
####	Bn-A06-p14402043	napus specific	0.9712918	0.793607	not scorable	not scorable	not scorable	not scorable
####	Bn-A06-p14417437	A-genome specific (napus-rapa)	0.9856459	0.5853306	not scorable	not scorable	0.9917355	0.6968291
####	Bn-A06-p14428186	A-genome specific (napus-rapa)	0.9856459	0.4585965	not scorable	not scorable	0.9917355	0.8191147
####	Bn-A06-p14428293	A-genome specific (napus-rapa)	0.9928229	0.4666623	not scorable	not scorable	0.9917355	0.6929011
####	Bn-A06-p1443684	A-genome specific (napus-rapa)	0.9784689	0.2595223	not scorable	not scorable	0.9834711	0.7009473
####	Bn-A06-p14439178	A-genome specific (napus-rapa)	0.9880383	0.4974527	not scorable	not scorable	0.9917355	0.7384785
####	Bn-A06-p14439400	A-genome specific (napus-rapa)	1	0.5125557	not scorable	not scorable	0.9752066	0.6440314
####	Bn-A06-p14441081	A-genome specific (napus-rapa)	1	0.4584518	not scorable	not scorable	0.9752066	0.6872303
####	Bn-A06-p14457099	A-genome specific (napus-rapa)	0.9712918	0.4667186	not scorable	not scorable	1	0.7087782
####	Bn-A06-p14469354	A-genome specific (napus-rapa)	0.9904306	0.7506744	not scorable	not scorable	0.7603306	0.6354762
####	Bn-A06-p14474461	A-genome specific (napus-rapa)	0.9880383	0.6214383	not scorable	not scorable	0.9752066	0.7686826
####	Bn-A06-p14495889	A-genome specific (napus-rapa)	0.9976076	0.7886099	not scorable	not scorable	0.9834711	0.6886729
####	Bn-A06-p1450741	A-genome specific (napus-rapa)	0.9688995	0.6489722	not scorable	not scorable	0.9586777	0.6716062
####	Bn-A06-p14510328	A-genome specific (napus-rapa)	0.9952153	0.7417997	not scorable	not scorable	0.9917355	0.7113537
####	Bn-A06-p14527757	A-genome specific (napus-rapa)	1	0.4847424	not scorable	not scorable	0.9669421	0.761632
####	Bn-A06-p14546946	A-genome specific (napus-rapa)	0.9976076	0.750188	not scorable	not scorable	0.9834711	0.7955766
####	Bn-A06-p1455751	A-genome specific (napus-rapa)	0.9880383	0.7682316	not scorable	not scorable	0.6942149	0.7208323
####	Bn-A06-p1455781	A-genome specific (napus-rapa)	0.9928229	0.7793891	not scorable	not scorable	0.7190083	0.8176259
####	Bn-A06-p1456521	A-genome specific (napus-rapa)	0.9665072	0.5936332	not scorable	not scorable	0.7024794	0.7474849
####	Bn-A06-p1457087	A-genome specific (napus-rapa)	1	0.829748	not scorable	not scorable	0.9917355	0.748491
####	Bn-A06-p1457383	A-genome specific (napus-rapa)	0.9976076	0.7574754	not scorable	not scorable	0.9917355	0.6861063
####	Bn-A06-p14576278	genome unspecific (all in)	1	0.4846798	1	0.7319725	0.9917355	0.7285104

####	Bn-A06-p14578177	A-genome specific (napus-rapa)	1	0.3348734	not scorable	not scorable	0.9917355	0.4843373
####	Bn-A06-p14578303	A-genome specific (napus-rapa)	1	0.615422	not scorable	not scorable	0.9834711	0.8031524
####	Bn-A06-p14588321	A-genome specific (napus-rapa)	0.9976076	0.7487138	not scorable	not scorable	1	0.8079238
####	Bn-A06-p14594965	A-genome specific (napus-rapa)	1	0.6171259	not scorable	not scorable	0.9752066	0.5931935
####	Bn-A06-p14595126	A-genome specific (napus-rapa)	1	0.49661	not scorable	not scorable	1	0.7254171
####	Bn-A06-p1460039	A-genome specific (napus-rapa)	0.9904306	0.7678543	not scorable	not scorable	0.9421487	0.6831894
####	Bn-A06-p14616522	A-genome specific (napus-rapa)	0.9832536	0.781324	not scorable	not scorable	1	0.9272779
####	Bn-A06-p14621640	A-genome specific (napus-rapa)	0.937799	0.4416581	not scorable	not scorable	0.7024794	0.7744794
####	Bn-A06-p14664213	A-genome specific (napus-rapa)	0.9880383	0.4156978	not scorable	not scorable	1	0.7884539
####	Bn-A06-p14676716	A-genome specific (napus-rapa)	1	0.3219499	not scorable	not scorable	0.9917355	0.7753065
####	Bn-A06-p14690714	A-genome specific (rapa only)	not scorable	not scorable	not scorable	not scorable	1	0.3583842
####	Bn-A06-p14737621	A-genome specific (rapa only)	not scorable	not scorable	not scorable	not scorable	1	0.8684879
####	Bn-A06-p14773179	A-genome specific (napus-rapa)	1	0.5459703	not scorable	not scorable	1	0.609028
####	Bn-A06-p14809503	A-genome specific (napus-rapa)	1	0.76289	not scorable	not scorable	0.9917355	0.8565431
####	Bn-A06-p1481362	A-genome specific (napus-rapa)	0.9497608	0.7063059	not scorable	not scorable	0.9917355	0.7575313
####	Bn-A06-p14813811	A-genome specific (napus-rapa)	0.9904306	0.7976006	not scorable	not scorable	0.9917355	0.8136404
####	Bn-A06-p14816143	A-genome specific (napus-rapa)	0.9784689	0.4046156	not scorable	not scorable	1	0.7656946
####	Bn-A06-p14819465	A-genome specific (napus-rapa)	1	0.5601844	not scorable	not scorable	0.9917355	0.8521241
####	Bn-A06-p14829925	A-genome specific (napus-rapa)	0.9712918	0.7619435	not scorable	not scorable	1	0.670136
####	Bn-A06-p14834424	A-genome specific (napus-rapa)	0.9880383	0.3575363	not scorable	not scorable	1	0.807014
####	Bn-A06-p14836066	A-genome specific (napus-rapa)	0.9832536	0.8210949	not scorable	not scorable	0.9834711	0.6963905
####	Bn-A06-p14836486	A-genome specific (napus-rapa)	0.9904306	0.7869103	not scorable	not scorable	1	0.8004529
####	Bn-A06-p14843109	A-genome specific (napus-rapa)	0.9952153	0.2688068	not scorable	not scorable	0.9421487	0.2317262
####	Bn-A06-p14848662	A-genome specific (napus-rapa)	0.9856459	0.6390084	not scorable	not scorable	0.9917355	0.7926773
####	Bn-A06-p14850300	A-genome specific (rapa only)	not scorable	not scorable	not scorable	not scorable	0.9834711	0.5709409
####	Bn-A06-p14852091	A-genome specific (napus-rapa)	0.9904306	0.7832155	not scorable	not scorable	0.9834711	0.7396818
####	Bn-A06-p14852157	A-genome specific (napus-rapa)	0.9904306	0.8196893	not scorable	not scorable	0.9752066	0.7534373
####	Bn-A06-p1485525	A-genome specific (napus-rapa)	0.9976076	0.7547109	not scorable	not scorable	0.9834711	0.6994703
####	Bn-A06-p14864165	A-genome specific (napus-rapa)	0.8899522	0.7619857	not scorable	not scorable	0.9917355	0.8059069
####	Bn-A06-p14878721	A-genome specific (napus-rapa)	0.9736842	0.5197877	not scorable	not scorable	1	0.7458668
####	Bn-A06-p14880259	napus specific	0.9952153	0.7151111	not scorable	not scorable	not scorable	not scorable
####	Bn-A06-p14880848	A-genome specific (napus-rapa)	0.9880383	0.3200532	not scorable	not scorable	0.9917355	0.7114244
####	Bn-A06-p1490969	A-genome specific (napus-rapa)	0.9688995	0.358557	not scorable	not scorable	0.9917355	0.7364417

####	Bn-A06-p149145	A-genome specific (napus-rapa)	0.9928229	0.7958696	not scorable	not scorable	0.9917355	0.8283616
####	Bn-A06-p15006796	A-genome specific (napus-rapa)	0.9856459	0.6006454	not scorable	not scorable	1	0.7940072
####	Bn-A06-p15013369	genome unspecific (all in)	0.9880383	0.2372204	0.9922481	0.7339854	1	0.6831905
####	Bn-A06-p15037349	A-genome specific (napus-rapa)	0.9569378	0.7693295	not scorable	not scorable	0.9752066	0.6333217
####	Bn-A06-p15048	A-genome specific (napus-rapa)	0.9856459	0.7220254	not scorable	not scorable	1	0.7382159
####	Bn-A06-p15059166	A-genome specific (napus-rapa)	0.9880383	0.8142627	not scorable	not scorable	0.9834711	0.7445339
####	Bn-A06-p15065078	A-genome specific (napus-rapa)	0.9856459	0.3943763	not scorable	not scorable	0.9917355	0.8096535
####	Bn-A06-p1507197	A-genome specific (napus-rapa)	1	0.5790953	not scorable	not scorable	0.9834711	0.836962
####	Bn-A06-p15101156	A-genome specific (napus-rapa)	0.9688995	0.7929416	not scorable	not scorable	0.9917355	0.7893556
####	Bn-A06-p15112901	A-genome specific (rapa only)	not scorable	not scorable	not scorable	not scorable	0.9338843	0.7468821
####	Bn-A06-p1517263	A-genome specific (rapa only)	not scorable	not scorable	not scorable	not scorable	0.9834711	0.7371452
####	Bn-A06-p15210596	A-genome specific (napus-rapa)	0.937799	0.6820722	not scorable	not scorable	0.9586777	0.7456779
####	Bn-A06-p15258692	A-genome specific (napus-rapa)	0.9736842	0.2958046	not scorable	not scorable	1	0.7357686
####	Bn-A06-p15262271	A-genome specific (napus-rapa)	0.9952153	0.5158182	not scorable	not scorable	1	0.8354266
####	Bn-A06-p15285770	A-genome specific (napus-rapa)	0.9880383	0.6718265	not scorable	not scorable	0.9669421	0.5730987
####	Bn-A06-p15294160	A-genome specific (napus-rapa)	0.9760765	0.80081	not scorable	not scorable	0.9917355	0.714762
####	Bn-A06-p1530126	A-genome specific (napus-rapa)	1	0.4529341	not scorable	not scorable	0.9586777	0.6754574
####	Bn-A06-p15339222	A-genome specific (napus-rapa)	0.9808612	0.8271491	not scorable	not scorable	1	0.7333737
####	Bn-A06-p15358902	A-genome specific (napus-rapa)	0.9808612	0.429766	not scorable	not scorable	0.9917355	0.7305586
####	Bn-A06-p15360859	A-genome specific (napus-rapa)	0.9784689	0.7622387	not scorable	not scorable	0.9917355	0.7271897
####	Bn-A06-p15375170	A-genome specific (napus-rapa)	0.9736842	0.2998629	not scorable	not scorable	0.9917355	0.6485454
####	Bn-A06-p15375395	A-genome specific (napus-rapa)	0.9736842	0.4435343	not scorable	not scorable	0.9834711	0.7720932
####	Bn-A06-p15406244	A-genome specific (rapa only)	not scorable	not scorable	not scorable	not scorable	0.8595041	0.9053316
####	Bn-A06-p15415078	A-genome specific (napus-rapa)	0.9808612	0.3517488	not scorable	not scorable	0.9917355	0.7408474
####	Bn-A06-p15420581	A-genome specific (napus-rapa)	0.9832536	0.3873282	not scorable	not scorable	0.9917355	0.8055603
####	Bn-A06-p15421020	A-genome specific (napus-rapa)	0.9856459	0.7718892	not scorable	not scorable	0.9669421	0.6940722
####	Bn-A06-p15432171	A-genome specific (napus-rapa)	0.9258373	0.593458	not scorable	not scorable	1	0.8702592
####	Bn-A06-p15434445	A-genome specific (napus-rapa)	0.9569378	0.7686326	not scorable	not scorable	1	0.8094236
####	Bn-A06-p15470965	A-genome specific (napus-rapa)	0.9928229	0.4960161	not scorable	not scorable	0.9752066	0.772032
####	Bn-A06-p15489475	A-genome specific (napus-rapa)	0.9856459	0.7997606	not scorable	not scorable	0.9917355	0.7937827
####	Bn-A06-p15513288	A-genome specific (napus-rapa)	1	0.5107986	not scorable	not scorable	0.9917355	0.7956564
####	Bn-A06-p15515345	A-genome specific (napus-rapa)	0.9904306	0.717018	not scorable	not scorable	0.9834711	0.7648371
####	Bn-A06-p15581849	A-genome specific (napus-rapa)	0.9832536	0.7060107	not scorable	not scorable	0.9917355	0.7491596

####	Bn-A06-p15582577	A-genome specific (napus-rapa)	0.9976076	0.5034127	not scorable	not scorable	0.9834711	0.816063
####	Bn-A06-p15582617	A-genome specific (napus-rapa)	0.9712918	0.3404703	not scorable	not scorable	0.9917355	0.6603951
####	Bn-A06-p15584456	A-genome specific (napus-rapa)	0.9760765	0.7817284	not scorable	not scorable	0.9834711	0.773729
####	Bn-A06-p15632434	A-genome specific (napus-rapa)	0.9904306	0.7903177	not scorable	not scorable	0.9917355	0.8145425
####	Bn-A06-p15649917	A-genome specific (napus-rapa)	0.9784689	0.3170709	not scorable	not scorable	0.9669421	0.703772
####	Bn-A06-p15661345	A-genome specific (napus-rapa)	0.9952153	0.7876096	not scorable	not scorable	0.9917355	0.8083969
####	Bn-A06-p15662331	A-genome specific (napus-rapa)	0.9736842	0.7936228	not scorable	not scorable	0.661157	0.8287914
####	Bn-A06-p15691701	A-genome specific (napus-rapa)	0.9976076	0.5604097	not scorable	not scorable	1	0.8177654
####	Bn-A06-p15700525	A-genome specific (napus-rapa)	0.9952153	0.6883085	not scorable	not scorable	1	0.8086844
####	Bn-A06-p15701094	A-genome specific (napus-rapa)	0.9856459	0.7505224	not scorable	not scorable	0.9917355	0.7508853
####	Bn-A06-p15703535	A-genome specific (napus-rapa)	0.9832536	0.6276017	not scorable	not scorable	0.661157	0.8214749
####	Bn-A06-p15709579	genome unspecific (all in)	0.9976076	0.4452079	1	0.8207103	1	0.8145207
####	Bn-A06-p15712062	A-genome specific (napus-rapa)	0.9904306	0.7748302	not scorable	not scorable	0.9752066	0.8260106
####	Bn-A06-p15743629	A-genome specific (napus-rapa)	0.9521531	0.7693118	not scorable	not scorable	0.9669421	0.6781133
####	Bn-A06-p15748291	A-genome specific (napus-rapa)	0.9569378	0.6590814	not scorable	not scorable	0.9752066	0.7451802
####	Bn-A06-p15749428	A-genome specific (napus-rapa)	1	0.8336033	not scorable	not scorable	0.9917355	0.7204518
####	Bn-A06-p15760165	A-genome specific (napus-rapa)	0.9976076	0.3684029	not scorable	not scorable	0.9834711	0.7030522
####	Bn-A06-p15775625	A-genome specific (napus-rapa)	0.9976076	0.7858106	not scorable	not scorable	1	0.7236875
####	Bn-A06-p15778397	A-genome specific (napus-rapa)	0.9832536	0.3476014	not scorable	not scorable	0.9586777	0.8333383
####	Bn-A06-p15791244	A-genome specific (napus-rapa)	0.9760765	0.6102226	not scorable	not scorable	0.9752066	0.7762588
####	Bn-A06-p15801373	A-genome specific (napus-rapa)	0.9976076	0.7673511	not scorable	not scorable	0.9917355	0.7884806
####	Bn-A06-p15893014	A-genome specific (napus-rapa)	0.9904306	0.6443722	not scorable	not scorable	0.9834711	0.5724069
####	Bn-A06-p15893221	A-genome specific (napus-rapa)	0.9784689	0.2740359	not scorable	not scorable	0.9752066	0.5878999
####	Bn-A06-p15894458	A-genome specific (rapa only)	not scorable	not scorable	not scorable	not scorable	0.9917355	0.7480733
####	Bn-A06-p15894910	A-genome specific (napus-rapa)	0.9760765	0.3472844	not scorable	not scorable	1	0.7538385
####	Bn-A06-p15913910	A-genome specific (rapa only)	not scorable	not scorable	not scorable	not scorable	0.9834711	0.7553031
####	Bn-A06-p15921988	A-genome specific (napus-rapa)	0.9880383	0.4079188	not scorable	not scorable	0.9669421	0.5445014
####	Bn-A06-p15922683	A-genome specific (napus-rapa)	0.9808612	0.3923483	not scorable	not scorable	0.9752066	0.7946898
####	Bn-A06-p15966650	A-genome specific (napus-rapa)	0.9976076	0.6893014	not scorable	not scorable	0.9834711	0.8566131
####	Bn-A06-p15967529	A-genome specific (napus-rapa)	1	0.7865977	not scorable	not scorable	0.9669421	0.760443
####	Bn-A06-p15967682	A-genome specific (napus-rapa)	1	0.7933219	not scorable	not scorable	0.9834711	0.7036652
####	Bn-A06-p15988623	A-genome specific (napus-rapa)	1	0.5609792	not scorable	not scorable	0.9917355	0.7907662
####	Bn-A06-p15990313	A-genome specific (napus-rapa)	1	0.4885826	not scorable	not scorable	0.9917355	0.8057448

####	Bn-A06-p16000523	A-genome specific (rapa only)	not scorable	not scorable	not scorable	not scorable	0.9917355	0.7772956
####	Bn-A06-p16009894	A-genome specific (napus-rapa)	0.9976076	0.7800343	not scorable	not scorable	0.9669421	0.6932905
####	Bn-A06-p16013571	A-genome specific (napus-rapa)	0.9617225	0.6288615	not scorable	not scorable	0.9669421	0.6809539
####	Bn-A06-p16013796	A-genome specific (napus-rapa)	0.9665072	0.5355712	not scorable	not scorable	0.9917355	0.6742523
####	Bn-A06-p16014870	A-genome specific (napus-rapa)	0.9880383	0.6577537	not scorable	not scorable	0.9752066	0.6265906
####	Bn-A06-p16014941	A-genome specific (napus-rapa)	0.9904306	0.6792614	not scorable	not scorable	0.9669421	0.67022
####	Bn-A06-p16015026	A-genome specific (napus-rapa)	0.9952153	0.7109914	not scorable	not scorable	0.9752066	0.7356135
####	Bn-A06-p16015727	A-genome specific (napus-rapa)	0.9952153	0.677512	not scorable	not scorable	0.9917355	0.6759912
####	Bn-A06-p16015795	A-genome specific (napus-rapa)	0.6052632	0.8337684	not scorable	not scorable	0.7024794	0.7821928
####	Bn-A06-p16016634	A-genome specific (napus-rapa)	0.9928229	0.7771403	not scorable	not scorable	0.9917355	0.7905771
####	Bn-A06-p16025514	A-genome specific (napus-rapa)	0.9880383	0.6876359	not scorable	not scorable	0.9669421	0.6951582
####	Bn-A06-p16030994	A-genome specific (napus-rapa)	0.9784689	0.676307	not scorable	not scorable	1	0.6805803
####	Bn-A06-p16032028	A-genome specific (napus-rapa)	0.9760765	0.7824032	not scorable	not scorable	0.9669421	0.7094271
####	Bn-A06-p16042593	napus specific	0.9641148	0.9371856	not scorable	not scorable	not scorable	not scorable
####	Bn-A06-p16064239	A-genome specific (napus-rapa)	0.9497608	0.3711144	not scorable	not scorable	0.9917355	0.5681765
####	Bn-A06-p16071214	A-genome specific (napus-rapa)	0.9928229	0.704638	not scorable	not scorable	0.9669421	0.6239372
####	Bn-A06-p16102726	A-genome specific (napus-rapa)	0.9497608	0.6016836	not scorable	not scorable	0.9917355	0.7688866
####	Bn-A06-p16106230	A-genome specific (napus-rapa)	1	0.6587121	not scorable	not scorable	1	0.6720757
####	Bn-A06-p1612900	A-genome specific (rapa only)	not scorable	not scorable	not scorable	not scorable	0.9669421	0.9243378
####	Bn-A06-p16131586	A-genome specific (napus-rapa)	1	0.8006784	not scorable	not scorable	0.9917355	0.7430053
####	Bn-A06-p16133328	napus specific	0.9593301	0.6090599	not scorable	not scorable	not scorable	not scorable
####	Bn-A06-p16134293	A-genome specific (rapa only)	not scorable	not scorable	not scorable	not scorable	0.9752066	0.6779389
####	Bn-A06-p16148736	A-genome specific (napus-rapa)	0.9904306	0.8042262	not scorable	not scorable	0.9834711	0.7899241
####	Bn-A06-p16149093	A-genome specific (napus-rapa)	1	0.8135551	not scorable	not scorable	1	0.8135551
####	Bn-A06-p16150365	A-genome specific (napus-rapa)	0.9976076	0.7549687	not scorable	not scorable	0.9834711	0.7075345
####	Bn-A06-p16150919	A-genome specific (napus-rapa)	1	0.5551252	not scorable	not scorable	1	0.8004208
####	Bn-A06-p1615726	A-genome specific (napus-rapa)	0.9641148	0.5137805	not scorable	not scorable	1	0.6597274
####	Bn-A06-p16166965	A-genome specific (napus-rapa)	0.9976076	0.5130699	not scorable	not scorable	1	0.7427495
####	Bn-A06-p16168931	A-genome specific (napus-rapa)	1	0.7884359	not scorable	not scorable	1	0.7696534
####	Bn-A06-p16169045	A-genome specific (napus-rapa)	0.9976076	0.8268663	not scorable	not scorable	0.9917355	0.763027
####	Bn-A06-p16169367	A-genome specific (napus-rapa)	0.9880383	0.8011565	not scorable	not scorable	1	0.7046239
####	Bn-A06-p16169669	A-genome specific (napus-rapa)	0.9952153	0.7653556	not scorable	not scorable	0.9752066	0.7169248
####	Bn-A06-p16170755	A-genome specific (napus-rapa)	1	0.814613	not scorable	not scorable	0.9917355	0.7633869

####	Bn-A06-p16170813	A-genome specific (napus-rapa)	0.9976076	0.753828	not scorable	not scorable	0.9917355	0.7560859
####	Bn-A06-p16173797	A-genome specific (napus-rapa)	0.9904306	0.704466	not scorable	not scorable	0.9834711	0.7245347
####	Bn-A06-p16181242	A-genome specific (napus-rapa)	0.9832536	0.8068777	not scorable	not scorable	0.9669421	0.7793317
####	Bn-A06-p16188512	A-genome specific (napus-rapa)	0.9976076	0.7323664	not scorable	not scorable	1	0.765983
####	Bn-A06-p16237929	A-genome specific (napus-rapa)	0.9569378	0.7659086	not scorable	not scorable	0.9917355	0.7412319
####	Bn-A06-p16256906	A-genome specific (napus-rapa)	0.9976076	0.330162	not scorable	not scorable	0.9917355	0.6969356
####	Bn-A06-p1626020	A-genome specific (napus-rapa)	1	0.55515	not scorable	not scorable	1	0.7680972
####	Bn-A06-p16262957	A-genome specific (napus-rapa)	0.8636364	0.7787807	not scorable	not scorable	0.6363636	0.8215211
####	Bn-A06-p16283909	A-genome specific (napus-rapa)	0.9952153	0.7987685	not scorable	not scorable	0.5785124	0.8146011
####	Bn-A06-p16284324	A-genome specific (napus-rapa)	0.9976076	0.747008	not scorable	not scorable	1	0.6671334
####	Bn-A06-p16284610	A-genome specific (napus-rapa)	0.9880383	0.517243	not scorable	not scorable	1	0.7889764
####	Bn-A06-p16303805	A-genome specific (napus-rapa)	0.9952153	0.6358169	not scorable	not scorable	0.9504132	0.6003107
####	Bn-A06-p16303860	A-genome specific (napus-rapa)	0.9976076	0.7820808	not scorable	not scorable	0.9917355	0.7216529
####	Bn-A06-p16308544	A-genome specific (napus-rapa)	0.9976076	0.7764606	not scorable	not scorable	0.9669421	0.7460765
####	Bn-A06-p16314064	genome unspecific (all in)	0.9665072	0.3968066	1	0.4700956	0.9917355	0.8301404
####	Bn-A06-p16325882	A-genome specific (rapa only)	not scorable	not scorable	not scorable	not scorable	0.9917355	0.7901847
####	Bn-A06-p16325934	A-genome specific (napus-rapa)	0.9856459	0.7585959	not scorable	not scorable	1	0.6648738
####	Bn-A06-p16330377	A-genome specific (napus-rapa)	0.9832536	0.5988952	not scorable	not scorable	0.9917355	0.8228956
####	Bn-A06-p163388	A-genome specific (napus-rapa)	0.9760765	0.5409738	not scorable	not scorable	0.9504132	0.7393414
####	Bn-A06-p16342269	A-genome specific (rapa only)	not scorable	not scorable	not scorable	not scorable	0.9917355	0.8254753
####	Bn-A06-p16362555	A-genome specific (napus-rapa)	0.9880383	0.7954009	not scorable	not scorable	0.9834711	0.773917
####	Bn-A06-p1636449	A-genome specific (napus-rapa)	0.9928229	0.31699	not scorable	not scorable	0.9917355	0.7357985
####	Bn-A06-p16381299	A-genome specific (napus-rapa)	0.9928229	0.6663723	not scorable	not scorable	1	0.6890678
####	Bn-A06-p16386782	A-genome specific (napus-rapa)	0.9760765	0.7775438	not scorable	not scorable	0.9917355	0.816465
####	Bn-A06-p16386992	A-genome specific (napus-rapa)	0.9712918	0.7840853	not scorable	not scorable	0.9834711	0.8200318
####	Bn-A06-p16406113	A-genome specific (napus-rapa)	0.9904306	0.4294029	not scorable	not scorable	0.9669421	0.6740993
####	Bn-A06-p16449642	A-genome specific (napus-rapa)	0.9808612	0.7830929	not scorable	not scorable	0.9752066	0.777311
####	Bn-A06-p16460575	A-genome specific (napus-rapa)	0.9760765	0.7564679	not scorable	not scorable	0.9504132	0.6583517
####	Bn-A06-p16461885	A-genome specific (napus-rapa)	0.9808612	0.7222949	not scorable	not scorable	0.9752066	0.6781062
####	Bn-A06-p1650453	A-genome specific (napus-rapa)	0.9856459	0.7559413	not scorable	not scorable	0.9917355	0.6762386
####	Bn-A06-p1650457	A-genome specific (napus-rapa)	0.9856459	0.7668051	not scorable	not scorable	0.9834711	0.7953139
####	Bn-A06-p1650529	A-genome specific (napus-rapa)	0.9880383	0.9154827	not scorable	not scorable	0.9834711	0.7774786
####	Bn-A06-p1650786	A-genome specific (napus-rapa)	0.9952153	0.5077711	not scorable	not scorable	0.9752066	0.7738416

####	Bn-A06-p1652088	A-genome specific (napus-rapa)	0.9976076	0.7861372	not scorable	not scorable	0.9834711	0.7285472
####	Bn-A06-p16553412	A-genome specific (napus-rapa)	0.9808612	0.4776695	not scorable	not scorable	0.9834711	0.6487134
####	Bn-A06-p16553640	A-genome specific (napus-rapa)	0.9832536	0.5152501	not scorable	not scorable	0.9917355	0.6998188
####	Bn-A06-p16554297	A-genome specific (napus-rapa)	0.9904306	0.7926969	not scorable	not scorable	0.9917355	0.8641534
####	Bn-A06-p1658095	napus specific	0.9043062	0.54857	not scorable	not scorable	not scorable	not scorable
####	Bn-A06-p16588120	A-genome specific (napus-rapa)	0.9808612	0.6799524	not scorable	not scorable	0.9917355	0.7794802
####	Bn-A06-p16588154	napus specific	0.9641148	0.5498086	not scorable	not scorable	not scorable	not scorable
####	Bn-A06-p16613538	A-genome specific (napus-rapa)	0.9736842	0.6770887	not scorable	not scorable	0.9752066	0.6712872
####	Bn-A06-p1661477	A-genome specific (napus-rapa)	0.9090909	0.7082462	not scorable	not scorable	0.9669421	0.6202248
####	Bn-A06-p16620858	A-genome specific (napus-rapa)	0.9641148	0.2463419	not scorable	not scorable	0.9834711	0.7017635
####	Bn-A06-p16620984	A-genome specific (napus-rapa)	0.9856459	0.3237579	not scorable	not scorable	0.9917355	0.8110601
####	Bn-A06-p16621639	A-genome specific (napus-rapa)	0.9832536	0.7819065	not scorable	not scorable	0.9917355	0.8509741
####	Bn-A06-p16624574	A-genome specific (napus-rapa)	0.9856459	0.6861864	not scorable	not scorable	0.9917355	0.6345529
####	Bn-A06-p16629547	A-genome specific (napus-rapa)	0.9641148	0.3549107	not scorable	not scorable	0.9834711	0.7820685
####	Bn-A06-p16645035	A-genome specific (napus-rapa)	0.9928229	0.7582259	not scorable	not scorable	0.9917355	0.8235909
####	Bn-A06-p16645140	A-genome specific (napus-rapa)	0.9904306	0.5151128	not scorable	not scorable	0.9917355	0.8375668
####	Bn-A06-p16656745	genome unspecific (all in)	0.9856459	0.5116102	1	0.7760428	0.9917355	0.7242395
####	Bn-A06-p16657894	A-genome specific (napus-rapa)	0.9904306	0.7989181	not scorable	not scorable	1	0.7299228
####	Bn-A06-p16660065	A-genome specific (napus-rapa)	0.9736842	0.3040999	not scorable	not scorable	0.9917355	0.7493256
####	Bn-A06-p16678748	A-genome specific (napus-rapa)	0.9808612	0.7866086	not scorable	not scorable	1	0.7994226
####	Bn-A06-p16682224	A-genome specific (napus-rapa)	0.9928229	0.5212793	not scorable	not scorable	1	0.6942437
####	Bn-A06-p16689717	A-genome specific (napus-rapa)	0.9832536	0.5150467	not scorable	not scorable	0.9917355	0.7581329
####	Bn-A06-p16701773	A-genome specific (napus-rapa)	1	0.7024977	not scorable	not scorable	0.9834711	0.6945397
####	Bn-A06-p16702471	A-genome specific (napus-rapa)	0.9832536	0.5317633	not scorable	not scorable	1	0.6962484
####	Bn-A06-p16703929	A-genome specific (napus-rapa)	0.9904306	0.3986781	not scorable	not scorable	0.9917355	0.6901155
####	Bn-A06-p16718752	genome unspecific (all in)	0.9928229	0.5125078	1	0.7557063	0.6859504	0.7031403
####	Bn-A06-p16728885	A-genome specific (napus-rapa)	0.9976076	0.3571929	not scorable	not scorable	0.9917355	0.7053624
####	Bn-A06-p167382	A-genome specific (napus-rapa)	0.9904306	0.4215459	not scorable	not scorable	1	0.7788153
####	Bn-A06-p16829292	A-genome specific (napus-rapa)	0.9832536	0.446236	not scorable	not scorable	1	0.8893794
####	Bn-A06-p16840167	A-genome specific (napus-rapa)	0.9952153	0.792918	not scorable	not scorable	0.9917355	0.7687623
####	Bn-A06-p16847178	A-genome specific (napus-rapa)	1	0.7320092	not scorable	not scorable	1	0.7462659
####	Bn-A06-p16847229	A-genome specific (napus-rapa)	1	0.49915	not scorable	not scorable	0.9752066	0.6682746
####	Bn-A06-p1685018	A-genome specific (napus-rapa)	0.9856459	0.3285702	not scorable	not scorable	0.9586777	0.825318

####	Bn-A06-p1685192	A-genome specific (napus-rapa)	0.9808612	0.5958149	not scorable	not scorable	0.9586777	0.646979
####	Bn-A06-p16875263	A-genome specific (rapa only)	not scorable	not scorable	not scorable	not scorable	0.9917355	0.7264067
####	Bn-A06-p16933880	A-genome specific (napus-rapa)	0.9952153	0.5219405	not scorable	not scorable	0.9586777	0.7769821
####	Bn-A06-p16985788	A-genome specific (napus-rapa)	0.9928229	0.529933	not scorable	not scorable	1	0.716581
####	Bn-A06-p16992951	A-genome specific (napus-rapa)	0.9952153	0.7767684	not scorable	not scorable	0.9917355	0.6991709
####	Bn-A06-p16993923	A-genome specific (napus-rapa)	0.9904306	0.7562174	not scorable	not scorable	0.9917355	0.7593259
####	Bn-A06-p16994210	A-genome specific (napus-rapa)	0.9904306	0.3625469	not scorable	not scorable	0.9752066	0.798762
####	Bn-A06-p17003685	A-genome specific (napus-rapa)	0.9832536	0.460525	not scorable	not scorable	0.9917355	0.8311084
####	Bn-A06-p17006799	A-genome specific (napus-rapa)	0.9856459	0.7520738	not scorable	not scorable	1	0.7738914
####	Bn-A06-p17007775	A-genome specific (napus-rapa)	0.9760765	0.7357221	not scorable	not scorable	0.9669421	0.752656
####	Bn-A06-p17010360	A-genome specific (napus-rapa)	0.9784689	0.3767497	not scorable	not scorable	0.9917355	0.6951944
####	Bn-A06-p17011382	genome unspecific (all in)	0.9808612	0.4401713	1	0.7603709	1	0.6763377
####	Bn-A06-p17021956	A-genome specific (napus-rapa)	0.9952153	0.7969892	not scorable	not scorable	1	0.780094
####	Bn-A06-p17023559	A-genome specific (rapa only)	not scorable	not scorable	not scorable	not scorable	0.7024794	0.7414954
####	Bn-A06-p17024179	A-genome specific (napus-rapa)	0.9832536	0.6867579	not scorable	not scorable	0.9669421	0.6915157
####	Bn-A06-p17026294	A-genome specific (napus-rapa)	0.9784689	0.7406293	not scorable	not scorable	0.9752066	0.7910517
####	Bn-A06-p17026843	A-genome specific (rapa only)	not scorable	not scorable	not scorable	not scorable	0.9752066	0.6244431
####	Bn-A06-p17032583	A-genome specific (napus-rapa)	0.9808612	0.5624855	not scorable	not scorable	0.9917355	0.7642815
####	Bn-A06-p17033313	A-genome specific (napus-rapa)	0.9712918	0.6890773	not scorable	not scorable	0.9752066	0.6871214
####	Bn-A06-p17037739	genome unspecific (all in)	0.9856459	0.4811082	1	0.7871097	1	0.7534428
####	Bn-A06-p17037912	A-genome specific (napus-rapa)	0.9928229	0.4022129	not scorable	not scorable	0.9338843	0.6658676
####	Bn-A06-p17042021	A-genome specific (napus-rapa)	0.9856459	0.8023536	not scorable	not scorable	0.9834711	0.7903157
####	Bn-A06-p17042110	A-genome specific (napus-rapa)	0.9736842	0.6325307	not scorable	not scorable	0.9752066	0.7849693
####	Bn-A06-p17049401	A-genome specific (napus-rapa)	0.9856459	0.8054748	not scorable	not scorable	1	0.8035294
####	Bn-A06-p17058018	A-genome specific (napus-rapa)	0.9880383	0.7957971	not scorable	not scorable	0.5123967	0.7096606
####	Bn-A06-p17058537	A-genome specific (napus-rapa)	0.9736842	0.7213836	not scorable	not scorable	0.5206612	0.721753
####	Bn-A06-p17059130	A-genome specific (napus-rapa)	0.9952153	0.7962995	not scorable	not scorable	0.7107438	0.7779452
####	Bn-A06-p17059461	A-genome specific (napus-rapa)	0.9856459	0.690636	not scorable	not scorable	0.5041322	0.6995872
####	Bn-A06-p17065808	A-genome specific (napus-rapa)	0.9880383	0.8042824	not scorable	not scorable	0.6033058	0.8586174
####	Bn-A06-p17093738	A-genome specific (napus-rapa)	0.9928229	0.3475238	not scorable	not scorable	0.9669421	0.786568
####	Bn-A06-p17097734	A-genome specific (napus-rapa)	0.9808612	0.7112521	not scorable	not scorable	1	0.6937376
####	Bn-A06-p17099451	A-genome specific (napus-rapa)	0.9832536	0.5752718	not scorable	not scorable	0.9586777	0.6450936
####	Bn-A06-p17109583	A-genome specific (napus-rapa)	0.9952153	0.8038881	not scorable	not scorable	0.9752066	0.8189644

####	Bn-A06-p17112322	A-genome specific (napus-rapa)	1	0.8022394	not scorable	not scorable	0.9917355	0.7528518
####	Bn-A06-p17114458	A-genome specific (napus-rapa)	0.9976076	0.798973	not scorable	not scorable	0.553719	0.8408964
####	Bn-A06-p17143376	A-genome specific (napus-rapa)	0.2607656	0.8343296	not scorable	not scorable	1	0.8485305
####	Bn-A06-p17156091	A-genome specific (rapa only)	not scorable	not scorable	not scorable	not scorable	1	0.5747117
####	Bn-A06-p17173552	A-genome specific (napus-rapa)	0.9856459	0.5047378	not scorable	not scorable	1	0.8323764
####	Bn-A06-p17175388	A-genome specific (napus-rapa)	0.9904306	0.2982488	not scorable	not scorable	0.9834711	0.6719483
####	Bn-A06-p17176086	A-genome specific (napus-rapa)	1	0.6347812	not scorable	not scorable	0.9917355	0.662836
####	Bn-A06-p17190074	all not scorable	not scorable	not scorable	not scorable	not scorable	not scorable	not scorable
####	Bn-A06-p17208027	A-genome specific (napus-rapa)	0.9880383	0.7099763	not scorable	not scorable	0.5454546	0.6944501
####	Bn-A06-p172171	A-genome specific (napus-rapa)	0.9665072	0.7879169	not scorable	not scorable	0.9586777	0.6744213
####	Bn-A06-p17220153	A-genome specific (napus-rapa)	0.9856459	0.5504892	not scorable	not scorable	0.5702479	0.7181352
####	Bn-A06-p1722627	A-genome specific (napus-rapa)	0.9736842	0.8077073	not scorable	not scorable	0.9090909	0.7627784
####	Bn-A06-p17226648	A-genome specific (napus-rapa)	0.9952153	0.7989584	not scorable	not scorable	1	0.7504681
####	Bn-A06-p17242102	A-genome specific (napus-rapa)	0.8971292	0.5605977	not scorable	not scorable	0.8842975	0.7884649
####	Bn-A06-p17242631	all not scorable	not scorable	not scorable	not scorable	not scorable	not scorable	not scorable
####	Bn-A06-p17260160	A-genome specific (napus-rapa)	0.9904306	0.503556	not scorable	not scorable	1	0.7922361
####	Bn-A06-p17260573	A-genome specific (napus-rapa)	0.9808612	0.67841	not scorable	not scorable	0.9917355	0.8996682
####	Bn-A06-p17262265	A-genome specific (napus-rapa)	0.9736842	0.8249155	not scorable	not scorable	0.9917355	0.9212999
####	Bn-A06-p17267787	A-genome specific (napus-rapa)	0.9952153	0.7721497	not scorable	not scorable	0.9752066	0.7419204
####	Bn-A06-p172731	A-genome specific (napus-rapa)	0.9712918	0.7757637	not scorable	not scorable	1	0.6995161
####	Bn-A06-p17289947	A-genome specific (napus-rapa)	0.9928229	0.3172973	not scorable	not scorable	1	0.8516589
####	Bn-A06-p17291868	A-genome specific (napus-rapa)	0.9976076	0.84392	not scorable	not scorable	1	0.7142364
####	Bn-A06-p17301456	A-genome specific (napus-rapa)	0.9856459	0.3124814	not scorable	not scorable	1	0.7600912
####	Bn-A06-p17313345	A-genome specific (napus-rapa)	0.9928229	0.3456605	not scorable	not scorable	0.9917355	0.7812183
####	Bn-A06-p17321987	A-genome specific (napus-rapa)	0.9880383	0.768483	not scorable	not scorable	0.9586777	0.6961991
####	Bn-A06-p17331074	A-genome specific (napus-rapa)	0.9904306	0.4482886	not scorable	not scorable	1	0.7863377
####	Bn-A06-p17332795	A-genome specific (napus-rapa)	0.9928229	0.5705034	not scorable	not scorable	0.9669421	0.6275762
####	Bn-A06-p17337842	A-genome specific (napus-rapa)	0.9880383	0.3627049	not scorable	not scorable	0.9917355	0.7805368
####	Bn-A06-p17346654	A-genome specific (napus-rapa)	0.9736842	0.7343631	not scorable	not scorable	1	0.8137556
####	Bn-A06-p17375407	A-genome specific (napus-rapa)	0.9832536	0.3481195	not scorable	not scorable	1	0.7624283
####	Bn-A06-p17375882	A-genome specific (napus-rapa)	0.9856459	0.7378044	not scorable	not scorable	1	0.6036078
####	Bn-A06-p17411706	A-genome specific (napus-rapa)	0.9856459	0.7880903	not scorable	not scorable	0.9834711	0.6892179
####	Bn-A06-p17415359	A-genome specific (napus-rapa)	0.9856459	0.361008	not scorable	not scorable	0.9917355	0.7954502

####	Bn-A06-p17430491	genome unspecific (all in)	0.9425837	0.563417	0.9457364	0.3960749	0.9917355	0.8026219
####	Bn-A06-p1743446	A-genome specific (napus-rapa)	0.9784689	0.7319359	not scorable	not scorable	0.9834711	0.6432585
####	Bn-A06-p17452087	A-genome specific (napus-rapa)	0.9832536	0.512971	not scorable	not scorable	1	0.7739488
####	Bn-A06-p17455962	A-genome specific (napus-rapa)	0.9784689	0.4775787	not scorable	not scorable	1	0.8461192
####	Bn-A06-p17548258	genome unspecific (all in)	0.9760765	0.5652488	1	0.7617062	0.9917355	0.6502439
####	Bn-A06-p17548894	A-genome specific (napus-rapa)	0.9856459	0.4155801	not scorable	not scorable	0.9917355	0.740381
####	Bn-A06-p17549347	A-genome specific (napus-rapa)	0.9665072	0.4055918	not scorable	not scorable	0.9917355	0.7726756
####	Bn-A06-p17549603	A-genome specific (napus-rapa)	0.9880383	0.6795291	not scorable	not scorable	0.9752066	0.5866033
####	Bn-A06-p17566259	A-genome specific (napus-rapa)	0.9904306	0.5719148	not scorable	not scorable	1	0.6909539
####	Bn-A06-p17659127	A-genome specific (napus-rapa)	0.9880383	0.532942	not scorable	not scorable	0.9834711	0.8177654
####	Bn-A06-p17671185	A-genome specific (napus-rapa)	0.9856459	0.4119997	not scorable	not scorable	0.9917355	0.7792268
####	Bn-A06-p17681092	A-genome specific (napus-rapa)	0.9712918	0.7553742	not scorable	not scorable	0.9917355	0.7386914
####	Bn-A06-p1768254	A-genome specific (napus-rapa)	0.9880383	0.7857155	not scorable	not scorable	0.9752066	0.7275296
####	Bn-A06-p17684443	A-genome specific (napus-rapa)	0.9760765	0.3713014	not scorable	not scorable	0.9752066	0.4561963
####	Bn-A06-p17689034	A-genome specific (napus-rapa)	0.6842105	0.8082798	not scorable	not scorable	0.9834711	0.773594
####	Bn-A06-p1768918	A-genome specific (napus-rapa)	0.9856459	0.7711685	not scorable	not scorable	0.9752066	0.6552749
####	Bn-A06-p17689415	A-genome specific (napus-rapa)	0.9688995	0.6225581	not scorable	not scorable	0.9917355	0.6219917
####	Bn-A06-p17697154	A-genome specific (napus-rapa)	0.9808612	0.6385843	not scorable	not scorable	0.9834711	0.7781585
####	Bn-A06-p17699971	A-genome specific (napus-rapa)	0.9569378	0.6913501	not scorable	not scorable	0.9834711	0.707481
####	Bn-A06-p17710201	A-genome specific (rapa only)	not scorable	not scorable	not scorable	not scorable	0.9834711	0.7905051
####	Bn-A06-p17721678	A-genome specific (rapa only)	not scorable	not scorable	not scorable	not scorable	0.9917355	0.7419018
####	Bn-A06-p17729064	A-genome specific (napus-rapa)	0.9928229	0.3015568	not scorable	not scorable	0.9917355	0.7997445
####	Bn-A06-p17734402	A-genome specific (napus-rapa)	0.9832536	0.4417477	not scorable	not scorable	0.9917355	0.7591076
####	Bn-A06-p17734904	A-genome specific (napus-rapa)	0.9880383	0.802748	not scorable	not scorable	0.9917355	0.7012135
####	Bn-A06-p17737199	A-genome specific (rapa only)	not scorable	not scorable	not scorable	not scorable	1	0.6701952
####	Bn-A06-p17741868	A-genome specific (napus-rapa)	0.9880383	0.439115	not scorable	not scorable	0.9669421	0.6402496
####	Bn-A06-p17741952	A-genome specific (rapa only)	not scorable	not scorable	not scorable	not scorable	1	0.7051603
####	Bn-A06-p17744170	A-genome specific (napus-rapa)	0.9832536	0.3935951	not scorable	not scorable	0.6363636	0.7317601
####	Bn-A06-p17755205	A-genome specific (napus-rapa)	0.9928229	0.7709687	not scorable	not scorable	0.9834711	0.7878355
####	Bn-A06-p1776668	A-genome specific (napus-rapa)	1	0.7696667	not scorable	not scorable	1	0.6435617
####	Bn-A06-p1777127	A-genome specific (napus-rapa)	0.9736842	0.3885179	not scorable	not scorable	1	0.8521883
####	Bn-A06-p17778079	A-genome specific (napus-rapa)	0.9904306	0.2647348	not scorable	not scorable	0.9669421	0.6308004
####	Bn-A06-p17778210	A-genome specific (napus-rapa)	0.9832536	0.4448838	not scorable	not scorable	0.9917355	0.6289672

####	Bn-A06-p17785331	A-genome specific (napus-rapa)	0.9736842	0.322933	not scorable	not scorable	1	0.7231945
####	Bn-A06-p17790290	A-genome specific (napus-rapa)	0.9688995	0.7703403	not scorable	not scorable	0.9586777	0.7634347
####	Bn-A06-p17791496	A-genome specific (napus-rapa)	0.9832536	0.6984524	not scorable	not scorable	1	0.6247691
####	Bn-A06-p17791746	A-genome specific (napus-rapa)	0.9952153	0.7437948	not scorable	not scorable	0.9669421	0.701064
####	Bn-A06-p17823115	A-genome specific (napus-rapa)	0.9808612	0.4195192	not scorable	not scorable	1	0.7265286
####	Bn-A06-p17831661	A-genome specific (napus-rapa)	0.9784689	0.4501364	not scorable	not scorable	1	0.7012355
####	Bn-A06-p17863217	all not scorable	not scorable	not scorable	not scorable	not scorable	not scorable	not scorable
####	Bn-A06-p17887060	A-genome specific (napus-rapa)	0.9808612	0.4103791	not scorable	not scorable	1	0.6673359
####	Bn-A06-p17888567	A-genome specific (rapa only)	not scorable	not scorable	not scorable	not scorable	0.9504132	0.5677139
####	Bn-A06-p17889311	A-genome specific (napus-rapa)	0.9832536	0.6970931	not scorable	not scorable	1	0.5998946
####	Bn-A06-p17908957	A-genome specific (napus-rapa)	0.9760765	0.8095837	not scorable	not scorable	0.9338843	0.6954914
####	Bn-A06-p17920968	A-genome specific (napus-rapa)	0.9928229	0.7936275	not scorable	not scorable	0.9834711	0.798132
####	Bn-A06-p17929532	A-genome specific (napus-rapa)	0.9808612	0.590741	not scorable	not scorable	1	0.7815756
####	Bn-A06-p17932959	A-genome specific (napus-rapa)	0.9904306	0.2223562	not scorable	not scorable	0.9917355	0.686147
####	Bn-A06-p1793586	A-genome specific (napus-rapa)	0.9832536	0.7515153	not scorable	not scorable	0.9917355	0.7395804
####	Bn-A06-p17938497	A-genome specific (napus-rapa)	0.9952153	0.4317956	not scorable	not scorable	0.9834711	0.5992818
####	Bn-A06-p17939632	A-genome specific (napus-rapa)	0.9904306	0.8265953	not scorable	not scorable	1	0.7846893
####	Bn-A06-p17956497	A-genome specific (rapa only)	not scorable	not scorable	not scorable	not scorable	0.553719	0.6768451
####	Bn-A06-p17959919	A-genome specific (napus-rapa)	0.9952153	0.8089006	not scorable	not scorable	1	0.7364206
####	Bn-A06-p17967505	A-genome specific (napus-rapa)	0.9880383	0.6937433	not scorable	not scorable	0.9504132	0.6940042
####	Bn-A06-p18000403	A-genome specific (napus-rapa)	0.9928229	0.6857706	not scorable	not scorable	0.9917355	0.7401475
####	Bn-A06-p18000461	A-genome specific (napus-rapa)	0.9904306	0.6971313	not scorable	not scorable	0.9917355	0.6829788
####	Bn-A06-p1800467	A-genome specific (napus-rapa)	0.7583732	0.8040736	not scorable	not scorable	0.9173554	0.9136915
####	Bn-A06-p18006686	A-genome specific (napus-rapa)	0.9928229	0.7132073	not scorable	not scorable	1	0.8655702
####	Bn-A06-p18009980	A-genome specific (napus-rapa)	1	0.5291753	not scorable	not scorable	0.5867769	0.8085375
####	Bn-A06-p1802123	A-genome specific (napus-rapa)	0.9784689	0.2939378	not scorable	not scorable	1	0.8249864
####	Bn-A06-p18022188	A-genome specific (napus-rapa)	1	0.6656172	not scorable	not scorable	0.9834711	0.8335571
####	Bn-A06-p18025751	A-genome specific (napus-rapa)	0.9952153	0.4189116	not scorable	not scorable	0.9917355	0.710943
####	Bn-A06-p18028791	A-genome specific (napus-rapa)	1	0.8005349	not scorable	not scorable	0.9917355	0.767074
####	Bn-A06-p18028879	A-genome specific (napus-rapa)	0.8947368	0.7823271	not scorable	not scorable	0.9917355	0.7979024
####	Bn-A06-p18028921	A-genome specific (napus-rapa)	0.9928229	0.7547078	not scorable	not scorable	0.9834711	0.6995164
####	Bn-A06-p18029021	A-genome specific (napus-rapa)	0.9952153	0.7767658	not scorable	not scorable	0.9834711	0.7417904
####	Bn-A06-p18029621	A-genome specific (napus-rapa)	1	0.735266	not scorable	not scorable	1	0.7454732

####	Bn-A06-p18033006	A-genome specific (napus-rapa)	1	0.7479001	not scorable	not scorable	0.9669421	0.6729136
####	Bn-A06-p18040363	A-genome specific (napus-rapa)	0.9760765	0.7976784	not scorable	not scorable	0.9917355	0.7086518
####	Bn-A06-p18040423	A-genome specific (napus-rapa)	0.9736842	0.5453208	not scorable	not scorable	0.9834711	0.6132494
####	Bn-A06-p18058671	A-genome specific (napus-rapa)	0.9856459	0.7411953	not scorable	not scorable	1	0.7832034
####	Bn-A06-p18059278	A-genome specific (napus-rapa)	1	0.5448344	not scorable	not scorable	1	0.9091015
####	Bn-A06-p18059565	A-genome specific (napus-rapa)	0.9784689	0.7013718	not scorable	not scorable	0.9834711	0.6290873
####	Bn-A06-p18059678	A-genome specific (napus-rapa)	0.9904306	0.7027781	not scorable	not scorable	0.9917355	0.7504464
####	Bn-A06-p18065846	A-genome specific (napus-rapa)	0.9952153	0.5112348	not scorable	not scorable	0.9917355	0.7126986
####	Bn-A06-p18089377	A-genome specific (napus-rapa)	0.9593301	0.2185774	not scorable	not scorable	0.9834711	0.2757821
####	Bn-A06-p18102164	A-genome specific (napus-rapa)	0.9641148	0.7922965	not scorable	not scorable	0.9917355	0.7876217
####	Bn-A06-p18113924	A-genome specific (napus-rapa)	0.9832536	0.4810372	not scorable	not scorable	0.9752066	0.7857338
####	Bn-A06-p18115817	A-genome specific (napus-rapa)	0.9593301	0.2858504	not scorable	not scorable	0.9917355	0.7444384
####	Bn-A06-p18120189	A-genome specific (rapa only)	not scorable	not scorable	not scorable	not scorable	0.9917355	0.7702867
####	Bn-A06-p18120195	all not scorable	not scorable	not scorable	not scorable	not scorable	not scorable	not scorable
####	Bn-A06-p18127830	A-genome specific (napus-rapa)	0.9784689	0.8067102	not scorable	not scorable	0.9834711	0.704466
####	Bn-A06-p18129979	A-genome specific (napus-rapa)	0.9832536	0.7194813	not scorable	not scorable	0.9917355	0.7175804
####	Bn-A06-p18131525	A-genome specific (napus-rapa)	0.9712918	0.3714509	not scorable	not scorable	0.9752066	0.7763614
####	Bn-A06-p18136235	A-genome specific (napus-rapa)	0.9952153	0.7511073	not scorable	not scorable	0.9752066	0.7548258
####	Bn-A06-p18141167	A-genome specific (napus-rapa)	0.9904306	0.5778937	not scorable	not scorable	0.9834711	0.8293068
####	Bn-A06-p18144127	A-genome specific (napus-rapa)	0.9928229	0.6971865	not scorable	not scorable	0.9917355	0.6716666
####	Bn-A06-p18152497	A-genome specific (napus-rapa)	0.9808612	0.7709536	not scorable	not scorable	0.9917355	0.7789357
####	Bn-A06-p18182395	A-genome specific (napus-rapa)	0.9784689	0.6777947	not scorable	not scorable	0.9917355	0.6914294
####	Bn-A06-p18188412	A-genome specific (napus-rapa)	0.9904306	0.7851401	not scorable	not scorable	0.9917355	0.7144638
####	Bn-A06-p18188496	A-genome specific (napus-rapa)	0.9808612	0.4653105	not scorable	not scorable	0.9917355	0.7367048
####	Bn-A06-p18207917	A-genome specific (napus-rapa)	0.9569378	0.6837332	not scorable	not scorable	1	0.6655804
####	Bn-A06-p18219362	A-genome specific (napus-rapa)	0.9928229	0.8538665	not scorable	not scorable	1	0.7775072
####	Bn-A06-p18220327	A-genome specific (napus-rapa)	0.9186603	0.7954096	not scorable	not scorable	0.9917355	0.7642493
####	Bn-A06-p18232202	A-genome specific (rapa only)	not scorable	not scorable	not scorable	not scorable	0.9917355	0.7481443
####	Bn-A06-p18235310	A-genome specific (napus-rapa)	0.9952153	0.8165818	not scorable	not scorable	1	0.7548383
####	Bn-A06-p18237512	A-genome specific (napus-rapa)	1	0.6636746	not scorable	not scorable	0.9917355	0.7586554
####	Bn-A06-p18246821	A-genome specific (napus-rapa)	0.9665072	0.7635297	not scorable	not scorable	0.5950413	0.7949292
####	Bn-A06-p18247490	A-genome specific (napus-rapa)	0.8301435	0.7802194	not scorable	not scorable	0.5950413	0.7618685
####	Bn-A06-p18248917	A-genome specific (napus-rapa)	0.8588517	0.8104475	not scorable	not scorable	1	0.7480721

####	Bn-A06-p18249054	A-genome specific (napus-rapa)	0.854067	0.8036721	not scorable	not scorable	1	0.7709448
####	Bn-A06-p18252711	A-genome specific (napus-rapa)	0.9832536	0.6904163	not scorable	not scorable	1	0.6919689
####	Bn-A06-p18254877	A-genome specific (napus-rapa)	0.9688995	0.3515235	not scorable	not scorable	0.9834711	0.6896096
####	Bn-A06-p18260387	A-genome specific (napus-rapa)	0.9760765	0.4884132	not scorable	not scorable	1	0.7605261
####	Bn-A06-p18277724	A-genome specific (napus-rapa)	0.9928229	0.5829328	not scorable	not scorable	0.9752066	0.7854813
####	Bn-A06-p18277802	A-genome specific (napus-rapa)	1	0.402841	not scorable	not scorable	0.9752066	0.7809825
####	Bn-A06-p1834863	A-genome specific (napus-rapa)	1	0.8042074	not scorable	not scorable	1	0.9030796
####	Bn-A06-p18364834	A-genome specific (napus-rapa)	0.9880383	0.7417517	not scorable	not scorable	0.9834711	0.9059584
####	Bn-A06-p18369013	A-genome specific (napus-rapa)	0.9880383	0.7482964	not scorable	not scorable	0.9834711	0.8088477
####	Bn-A06-p18380196	A-genome specific (napus-rapa)	0.9808612	0.3050797	not scorable	not scorable	0.9586777	0.6481637
####	Bn-A06-p183811	A-genome specific (napus-rapa)	0.9736842	0.6255818	not scorable	not scorable	0.9917355	0.7556905
####	Bn-A06-p18388041	A-genome specific (napus-rapa)	0.9808612	0.8331169	not scorable	not scorable	0.9834711	0.7431734
####	Bn-A06-p18388778	genome unspecific (all in)	0.9856459	0.4047633	1	0.7316474	0.9834711	0.7585323
####	Bn-A06-p18390889	A-genome specific (napus-rapa)	0.9928229	0.7790163	not scorable	not scorable	0.9917355	0.7113248
####	Bn-A06-p18392463	A-genome specific (napus-rapa)	0.9497608	0.5927677	not scorable	not scorable	0.9834711	0.7830848
####	Bn-A06-p18394892	A-genome specific (napus-rapa)	0.9880383	0.6755649	not scorable	not scorable	1	0.6707243
####	Bn-A06-p18397592	A-genome specific (napus-rapa)	0.9880383	0.7038523	not scorable	not scorable	1	0.7522502
####	Bn-A06-p18402713	A-genome specific (napus-rapa)	0.9976076	0.7136945	not scorable	not scorable	0.9834711	0.6747848
####	Bn-A06-p18403451	A-genome specific (rapa only)	not scorable	not scorable	not scorable	not scorable	1	0.7463304
####	Bn-A06-p18408257	A-genome specific (napus-rapa)	0.9688995	0.3018214	not scorable	not scorable	1	0.8030666
####	Bn-A06-p18430792	A-genome specific (napus-rapa)	0.9832536	0.451726	not scorable	not scorable	1	0.7669737
####	Bn-A06-p18432812	A-genome specific (napus-rapa)	0.9976076	0.7306182	not scorable	not scorable	1	0.7612836
####	Bn-A06-p18438509	A-genome specific (napus-rapa)	0.9928229	0.7553815	not scorable	not scorable	0.9834711	0.7977165
####	Bn-A06-p18445250	A-genome specific (napus-rapa)	0.9880383	0.5409557	not scorable	not scorable	0.9917355	0.7779592
####	Bn-A06-p18454864	A-genome specific (rapa only)	not scorable	not scorable	not scorable	not scorable	0.9421487	0.7863449
####	Bn-A06-p18455125	A-genome specific (napus-rapa)	0.9688995	0.4016844	not scorable	not scorable	0.9752066	0.7910416
####	Bn-A06-p18500429	genome unspecific (oleracea-rapa)	not scorable	not scorable	0.9689922	0.7061687	0.9834711	0.6676789
####	Bn-A06-p18500596	A-genome specific (napus-rapa)	0.9641148	0.7976274	not scorable	not scorable	1	0.7395962
####	Bn-A06-p18500625	A-genome specific (napus-rapa)	0.9665072	0.3479977	not scorable	not scorable	0.9917355	0.8005264
####	Bn-A06-p18502274	A-genome specific (napus-rapa)	0.9880383	0.5381089	not scorable	not scorable	1	0.7865542
####	Bn-A06-p18502399	A-genome specific (napus-rapa)	0.9641148	0.2976414	not scorable	not scorable	1	0.7135133
####	Bn-A06-p18563686	A-genome specific (rapa only)	not scorable	not scorable	not scorable	not scorable	0.9917355	0.791281
####	Bn-A06-p18573338	A-genome specific (napus-rapa)	0.937799	0.2840751	not scorable	not scorable	0.9586777	0.6813247

####	Bn-A06-p18600460	A-genome specific (napus-rapa)	0.9641148	0.7609589	not scorable	not scorable	0.9917355	0.8145428
####	Bn-A06-p18614110	A-genome specific (napus-rapa)	0.9784689	0.2891992	not scorable	not scorable	0.661157	0.7808442
####	Bn-A06-p18624741	A-genome specific (napus-rapa)	0.9641148	0.5369933	not scorable	not scorable	0.9834711	0.6638477
####	Bn-A06-p18634396	A-genome specific (napus-rapa)	0.9688995	0.6871526	not scorable	not scorable	0.9917355	0.7785769
####	Bn-A06-p18642085	A-genome specific (napus-rapa)	0.9545454	0.3282853	not scorable	not scorable	1	0.6650838
####	Bn-A06-p18659358	A-genome specific (napus-rapa)	0.9832536	0.6857125	not scorable	not scorable	0.9917355	0.7968631
####	Bn-A06-p18692755	A-genome specific (napus-rapa)	0.9832536	0.827432	not scorable	not scorable	0.9834711	0.7762491
####	Bn-A06-p18706102	A-genome specific (napus-rapa)	0.9593301	0.5417364	not scorable	not scorable	0.9917355	0.8378816
####	Bn-A06-p18709596	napus specific	0.9330143	0.6786534	not scorable	not scorable	not scorable	not scorable
####	Bn-A06-p18717323	A-genome specific (napus-rapa)	0.9688995	0.3430781	not scorable	not scorable	0.9669421	0.5963017
####	Bn-A06-p18718829	A-genome specific (napus-rapa)	0.9904306	0.7736471	not scorable	not scorable	1	0.7951118
####	Bn-A06-p1872484	A-genome specific (napus-rapa)	0.9976076	0.6745178	not scorable	not scorable	0.9917355	0.7322186
####	Bn-A06-p1872597	A-genome specific (napus-rapa)	0.9880383	0.7718801	not scorable	not scorable	0.9752066	0.7277194
####	Bn-A06-p18729660	A-genome specific (napus-rapa)	0.9904306	0.7808825	not scorable	not scorable	1	0.7803028
####	Bn-A06-p18740524	A-genome specific (napus-rapa)	0.9760765	0.7806745	not scorable	not scorable	0.9752066	0.7940474
####	Bn-A06-p1874196	A-genome specific (napus-rapa)	0.9712918	0.7013798	not scorable	not scorable	0.9752066	0.9095823
####	Bn-A06-p18742389	A-genome specific (napus-rapa)	0.9641148	0.4342854	not scorable	not scorable	0.9917355	0.7907822
####	Bn-A06-p18754657	A-genome specific (napus-rapa)	0.9736842	0.5027965	not scorable	not scorable	0.9917355	0.7390832
####	Bn-A06-p18755269	A-genome specific (napus-rapa)	0.7511961	0.9464626	not scorable	not scorable	0.9586777	0.7572669
####	Bn-A06-p18755859	A-genome specific (napus-rapa)	0.9569378	0.2758512	not scorable	not scorable	1	0.6995457
####	Bn-A06-p18757339	A-genome specific (napus-rapa)	0.8995215	0.7634357	not scorable	not scorable	0.9834711	0.7145087
####	Bn-A06-p1875896	A-genome specific (napus-rapa)	0.9545454	0.6697966	not scorable	not scorable	0.9504132	0.6776387
####	Bn-A06-p18776948	genome unspecific (all in)	0.9665072	0.4297526	1	0.7470369	1	0.720088
####	Bn-A06-p18777001	A-genome specific (napus-rapa)	0.9808612	0.5051112	not scorable	not scorable	1	0.6640143
####	Bn-A06-p18783	A-genome specific (napus-rapa)	0.9832536	0.7305045	not scorable	not scorable	0.9917355	0.8166034
####	Bn-A06-p18785908	A-genome specific (napus-rapa)	0.9688995	0.6844227	not scorable	not scorable	0.9917355	0.7598116
####	Bn-A06-p18794208	A-genome specific (napus-rapa)	0.9712918	0.4739237	not scorable	not scorable	1	0.706701
####	Bn-A06-p18807305	A-genome specific (napus-rapa)	0.9976076	0.7208838	not scorable	not scorable	1	0.7802867
####	Bn-A06-p18813274	A-genome specific (napus-rapa)	0.9904306	0.597259	not scorable	not scorable	0.9834711	0.7830454
####	Bn-A06-p18867395	A-genome specific (napus-rapa)	0.9904306	0.2604821	not scorable	not scorable	1	0.8331373
####	Bn-A06-p18872147	A-genome specific (napus-rapa)	0.9856459	0.5564669	not scorable	not scorable	1	0.8458794
####	Bn-A06-p18880950	napus specific	0.9665072	0.6845963	not scorable	not scorable	not scorable	not scorable
####	Bn-A06-p18882138	A-genome specific (napus-rapa)	0.9665072	0.6305875	not scorable	not scorable	0.8677686	0.7244219

####	Bn-A06-p18885366	A-genome specific (napus-rapa)	0.9976076	0.7373943	not scorable	not scorable	1	0.6552948
####	Bn-A06-p18898902	A-genome specific (napus-rapa)	0.9832536	0.7536705	not scorable	not scorable	0.5123967	0.7731714
####	Bn-A06-p1892397	A-genome specific (napus-rapa)	0.9856459	0.3637568	not scorable	not scorable	1	0.7276234
####	Bn-A06-p1892464	A-genome specific (napus-rapa)	0.9760765	0.6482869	not scorable	not scorable	0.9917355	0.8592248
####	Bn-A06-p18998806	A-genome specific (napus-rapa)	0.9712918	0.6988216	not scorable	not scorable	1	0.8206883
####	Bn-A06-p19021973	A-genome specific (napus-rapa)	0.9808612	0.7568182	not scorable	not scorable	1	0.7855273
####	Bn-A06-p1903170	A-genome specific (napus-rapa)	1	0.7419457	not scorable	not scorable	1	0.9263889
####	Bn-A06-p1903532	A-genome specific (napus-rapa)	1	0.7786627	not scorable	not scorable	0.9752066	0.6854145
####	Bn-A06-p1908583	A-genome specific (rapa only)	not scorable	not scorable	not scorable	not scorable	1	0.910212
####	Bn-A06-p1908707	A-genome specific (napus-rapa)	0.9880383	0.6865292	not scorable	not scorable	0.9752066	0.7061555
####	Bn-A06-p19089918	A-genome specific (rapa only)	not scorable	not scorable	not scorable	not scorable	0.9338843	0.8505956
####	Bn-A06-p1909050	A-genome specific (napus-rapa)	0.9904306	0.6917045	not scorable	not scorable	1	0.7598165
####	Bn-A06-p19102612	A-genome specific (napus-rapa)	0.9808612	0.5070884	not scorable	not scorable	0.9256198	0.833535
####	Bn-A06-p19102725	A-genome specific (napus-rapa)	0.9641148	0.3065965	not scorable	not scorable	0.9256198	0.7462659
####	Bn-A06-p19130048	A-genome specific (napus-rapa)	0.9760765	0.7634797	not scorable	not scorable	0.892562	0.8042249
####	Bn-A06-p19151996	A-genome specific (napus-rapa)	0.9784689	0.7629507	not scorable	not scorable	0.9752066	0.880185
####	Bn-A06-p19153385	A-genome specific (napus-rapa)	0.9904306	0.7486759	not scorable	not scorable	0.9834711	0.7997234
####	Bn-A06-p19153719	A-genome specific (napus-rapa)	1	0.8666431	not scorable	not scorable	1	0.8159295
####	Bn-A06-p19163919	A-genome specific (napus-rapa)	0.9545454	0.4285963	not scorable	not scorable	0.9917355	0.6850684
####	Bn-A06-p19184868	A-genome specific (napus-rapa)	0.7799043	0.9095823	not scorable	not scorable	0.8181818	0.8635932
####	Bn-A06-p19185585	A-genome specific (napus-rapa)	0.9665072	0.5660708	not scorable	not scorable	0.9917355	0.6770072
####	Bn-A06-p19197269	A-genome specific (napus-rapa)	0.9952153	0.6872579	not scorable	not scorable	0.9917355	0.686269
####	Bn-A06-p19226794	A-genome specific (napus-rapa)	0.9736842	0.6155817	not scorable	not scorable	1	0.739498
####	Bn-A06-p19245025	A-genome specific (napus-rapa)	0.9952153	0.3904046	not scorable	not scorable	0.9752066	0.6919105
####	Bn-A06-p19256933	A-genome specific (napus-rapa)	0.9928229	0.2712645	not scorable	not scorable	0.9669421	0.7908595
####	Bn-A06-p1925858	A-genome specific (napus-rapa)	0.9952153	0.7962403	not scorable	not scorable	1	0.7966691
####	Bn-A06-p19263581	A-genome specific (napus-rapa)	0.9928229	0.3951331	not scorable	not scorable	0.9834711	0.8033799
####	Bn-A06-p19265815	A-genome specific (napus-rapa)	0.9832536	0.7166491	not scorable	not scorable	0.9917355	0.7570407
####	Bn-A06-p19291105	all not scorable	not scorable	not scorable	not scorable	not scorable	not scorable	not scorable
####	Bn-A06-p19292104	A-genome specific (napus-rapa)	0.9665072	0.2111718	not scorable	not scorable	0.9917355	0.8716812
####	Bn-A06-p19296157	A-genome specific (napus-rapa)	0.8636364	0.661546	not scorable	not scorable	0.9752066	0.8646256
####	Bn-A06-p19329736	A-genome specific (napus-rapa)	0.9784689	0.3716876	not scorable	not scorable	0.9834711	0.7747613
####	Bn-A06-p19333055	A-genome specific (napus-rapa)	0.9832536	0.5692393	not scorable	not scorable	0.9917355	0.6990505

####	Bn-A06-p19333117	A-genome specific (napus-rapa)	0.9952153	0.7677808	not scorable	not scorable	0.9917355	0.7531571
####	Bn-A06-p19333349	A-genome specific (napus-rapa)	0.9928229	0.7283338	not scorable	not scorable	0.9917355	0.6896072
####	Bn-A06-p19392651	A-genome specific (napus-rapa)	0.9880383	0.2369612	not scorable	not scorable	0.9834711	0.7475029
####	Bn-A06-p19395565	A-genome specific (napus-rapa)	0.9784689	0.7348083	not scorable	not scorable	0.9834711	0.8011216
####	Bn-A06-p19395890	A-genome specific (napus-rapa)	0.9832536	0.6002275	not scorable	not scorable	1	0.7635154
####	Bn-A06-p19396032	A-genome specific (napus-rapa)	0.9880383	0.3507533	not scorable	not scorable	1	0.777316
####	Bn-A06-p19404012	A-genome specific (napus-rapa)	0.9928229	0.7857079	not scorable	not scorable	0.9917355	0.7871282
####	Bn-A06-p19405765	A-genome specific (napus-rapa)	0.9976076	0.7626438	not scorable	not scorable	0.9917355	0.788398
####	Bn-A06-p19411124	A-genome specific (napus-rapa)	0.9641148	0.6208773	not scorable	not scorable	1	0.6846505
####	Bn-A06-p19416292	A-genome specific (napus-rapa)	0.9904306	0.5356665	not scorable	not scorable	0.9834711	0.7291237
####	Bn-A06-p19418423	A-genome specific (napus-rapa)	0.9784689	0.6066359	not scorable	not scorable	0.9917355	0.7041054
####	Bn-A06-p19419215	A-genome specific (napus-rapa)	0.5143541	0.7938844	not scorable	not scorable	0.9917355	0.7992279
####	Bn-A06-p19434905	A-genome specific (napus-rapa)	0.9856459	0.7373469	not scorable	not scorable	1	0.8686684
####	Bn-A06-p19437625	napus specific	0.791866	0.7642791	not scorable	not scorable	not scorable	not scorable
####	Bn-A06-p1947247	A-genome specific (napus-rapa)	1	0.4923992	not scorable	not scorable	0.9752066	0.6928769
####	Bn-A06-p19485623	A-genome specific (napus-rapa)	0.9952153	0.8226718	not scorable	not scorable	0.9669421	0.7041288
####	Bn-A06-p19489870	A-genome specific (rapa only)	not scorable	not scorable	not scorable	not scorable	0.9834711	0.5871974
####	Bn-A06-p19501594	A-genome specific (napus-rapa)	0.9688995	0.3466427	not scorable	not scorable	0.9752066	0.7825384
####	Bn-A06-p19502324	A-genome specific (napus-rapa)	0.9808612	0.38028	not scorable	not scorable	1	0.7106495
####	Bn-A06-p19508949	A-genome specific (napus-rapa)	0.5526316	0.7532428	not scorable	not scorable	0.9504132	0.7439496
####	Bn-A06-p19533298	A-genome specific (napus-rapa)	0.9473684	0.5469618	not scorable	not scorable	0.768595	0.7816594
####	Bn-A06-p195449	A-genome specific (napus-rapa)	0.9545454	0.6520118	not scorable	not scorable	0.9917355	0.6448559
####	Bn-A06-p19549093	A-genome specific (napus-rapa)	0.9760765	0.4753434	not scorable	not scorable	0.9917355	0.7659851
####	Bn-A06-p19549571	A-genome specific (napus-rapa)	0.9832536	0.6123109	not scorable	not scorable	0.9917355	0.6501432
####	Bn-A06-p19555116	A-genome specific (napus-rapa)	0.9904306	0.7935762	not scorable	not scorable	0.9669421	0.8104015
####	Bn-A06-p19562187	A-genome specific (napus-rapa)	0.930622	0.542289	not scorable	not scorable	0.9752066	0.6067291
####	Bn-A06-p19574863	A-genome specific (napus-rapa)	0.9976076	0.3686921	not scorable	not scorable	0.9917355	0.8230137
####	Bn-A06-p19579765	napus specific	0.9760765	0.5616598	not scorable	not scorable	not scorable	not scorable
####	Bn-A06-p19593266	A-genome specific (napus-rapa)	0.9760765	0.799538	not scorable	not scorable	1	0.7148065
####	Bn-A06-p19598035	A-genome specific (napus-rapa)	0.5861244	0.7935341	not scorable	not scorable	0.9669421	0.6908799
####	Bn-A06-p19602022	A-genome specific (napus-rapa)	0.5813397	0.6475175	not scorable	not scorable	1	0.7443811
####	Bn-A06-p1961013	A-genome specific (napus-rapa)	1	0.2919384	not scorable	not scorable	1	0.7532086
####	Bn-A06-p19623637	A-genome specific (rapa only)	not scorable	not scorable	not scorable	not scorable	1	0.9114538

####	Bn-A06-p19635380	A-genome specific (napus-rapa)	0.9952153	0.8093771	not scorable	not scorable	1	0.7707854
####	Bn-A06-p19646183	A-genome specific (napus-rapa)	0.9665072	0.2970377	not scorable	not scorable	0.9917355	0.7255021
####	Bn-A06-p19647126	A-genome specific (napus-rapa)	0.9856459	0.8109831	not scorable	not scorable	0.9917355	0.5964394
####	Bn-A06-p19647330	A-genome specific (napus-rapa)	0.7057416	0.8754691	not scorable	not scorable	0.9669421	0.8135107
####	Bn-A06-p19647388	A-genome specific (napus-rapa)	0.9808612	0.7452978	not scorable	not scorable	0.9834711	0.7078381
####	Bn-A06-p19647543	A-genome specific (napus-rapa)	0.9880383	0.7867172	not scorable	not scorable	0.9917355	0.7397462
####	Bn-A06-p19648186	A-genome specific (napus-rapa)	0.9282297	0.7251095	not scorable	not scorable	0.9586777	0.7819775
####	Bn-A06-p19648433	A-genome specific (napus-rapa)	0.9760765	0.3899417	not scorable	not scorable	0.9834711	0.7818428
####	Bn-A06-p19656363	A-genome specific (napus-rapa)	0.9904306	0.7738494	not scorable	not scorable	0.9917355	0.8034204
####	Bn-A06-p19658177	A-genome specific (napus-rapa)	0.9928229	0.7246714	not scorable	not scorable	1	0.7091008
####	Bn-A06-p19680250	A-genome specific (napus-rapa)	0.9928229	0.5603641	not scorable	not scorable	1	0.8166372
####	Bn-A06-p19715332	A-genome specific (napus-rapa)	0.9976076	0.3093178	not scorable	not scorable	1	0.7042676
####	Bn-A06-p1973105	A-genome specific (napus-rapa)	1	0.7099991	not scorable	not scorable	1	0.7999869
####	Bn-A06-p19739501	A-genome specific (napus-rapa)	0.9784689	0.267318	not scorable	not scorable	1	0.7444403
####	Bn-A06-p19747056	A-genome specific (napus-rapa)	0.9617225	0.8198293	not scorable	not scorable	0.9669421	0.8216213
####	Bn-A06-p19757422	A-genome specific (napus-rapa)	0.9952153	0.5804777	not scorable	not scorable	1	0.6221481
####	Bn-A06-p19759161	A-genome specific (napus-rapa)	0.9952153	0.7744001	not scorable	not scorable	0.9917355	0.791353
####	Bn-A06-p197604	A-genome specific (napus-rapa)	0.9617225	0.7286828	not scorable	not scorable	0.9669421	0.652746
####	Bn-A06-p19788556	A-genome specific (napus-rapa)	0.9928229	0.2895405	not scorable	not scorable	1	0.6805297
####	Bn-A06-p19792160	A-genome specific (napus-rapa)	1	0.7944196	not scorable	not scorable	1	0.9046174
####	Bn-A06-p19794728	A-genome specific (napus-rapa)	1	0.5867994	not scorable	not scorable	1	0.6163771
####	Bn-A06-p19804872	A-genome specific (napus-rapa)	0.9497608	0.6324359	not scorable	not scorable	0.9917355	0.6952382
####	Bn-A06-p19833393	A-genome specific (napus-rapa)	0.9665072	0.8179824	not scorable	not scorable	0.9669421	0.8325562
####	Bn-A06-p19838015	A-genome specific (napus-rapa)	1	0.8456079	not scorable	not scorable	1	0.889194
####	Bn-A06-p19885379	A-genome specific (napus-rapa)	0.9880383	0.2160652	not scorable	not scorable	1	0.9094265
####	Bn-A06-p19943194	A-genome specific (napus-rapa)	0.9880383	0.7726677	not scorable	not scorable	0.9834711	0.7007644
####	Bn-A06-p19968113	genome unspecific (all in)	1	0.214835	0.8914729	0.4755139	1	0.7227655
####	Bn-A06-p19971717	genome unspecific (all in)	0.9928229	0.2560492	0.8837209	0.2400374	0.9834711	0.6386963
####	Bn-A06-p19972403	A-genome specific (napus-rapa)	1	0.7532282	not scorable	not scorable	1	0.7703719
####	Bn-A06-p19976368	A-genome specific (napus-rapa)	1	0.6320699	not scorable	not scorable	1	0.6328461
####	Bn-A06-p20177226	A-genome specific (napus-rapa)	0.9976076	0.6426485	not scorable	not scorable	1	0.6510744
####	Bn-A06-p20177791	A-genome specific (rapa only)	not scorable	not scorable	not scorable	not scorable	1	0.4874006
####	Bn-A06-p20211283	A-genome specific (napus-rapa)	0.9904306	0.8044565	not scorable	not scorable	0.9834711	0.8354682

####	Bn-A06-p20320209	A-genome specific (napus-rapa)	0.9114832	0.4749121	not scorable	not scorable	0.9752066	0.6775332
####	Bn-A06-p20320258	A-genome specific (napus-rapa)	0.9880383	0.838596	not scorable	not scorable	0.9917355	0.7657278
####	Bn-A06-p20476713	A-genome specific (napus-rapa)	0.9832536	0.7395071	not scorable	not scorable	0.9917355	0.8139145
####	Bn-A06-p20494935	A-genome specific (napus-rapa)	0.9880383	0.2550202	not scorable	not scorable	1	0.6864058
####	Bn-A06-p20495127	A-genome specific (napus-rapa)	0.7655503	0.820134	not scorable	not scorable	0.9173554	0.9492106
####	Bn-A06-p205050	A-genome specific (napus-rapa)	0.9832536	0.463542	not scorable	not scorable	0.9669421	0.5689569
####	Bn-A06-p2060583	A-genome specific (napus-rapa)	1	0.426027	not scorable	not scorable	1	0.8063016
####	Bn-A06-p20633479	A-genome specific (rapa only)	not scorable	not scorable	not scorable	not scorable	0.9917355	0.7450555
####	Bn-A06-p2065936	A-genome specific (napus-rapa)	0.9952153	0.823295	not scorable	not scorable	1	0.8528919
####	Bn-A06-p20696441	A-genome specific (napus-rapa)	0.9976076	0.3493758	not scorable	not scorable	1	0.6870549
####	Bn-A06-p20696691	all not scorable	not scorable	not scorable	not scorable	not scorable	not scorable	not scorable
####	Bn-A06-p2077171	A-genome specific (napus-rapa)	0.9976076	0.3118794	not scorable	not scorable	0.9917355	0.7576765
####	Bn-A06-p20877321	genome unspecific (all in)	0.9688995	0.3527241	0.9844961	0.6829675	1	0.7376376
####	Bn-A06-p20877670	A-genome specific (napus-rapa)	0.854067	0.3210518	not scorable	not scorable	1	0.7295224
####	Bn-A06-p20998201	A-genome specific (napus-rapa)	0.9952153	0.7805338	not scorable	not scorable	0.8512397	0.7625126
####	Bn-A06-p21031038	A-genome specific (napus-rapa)	1	0.6018038	not scorable	not scorable	1	0.6547505
####	Bn-A06-p21041632	A-genome specific (napus-rapa)	0.9880383	0.7722193	not scorable	not scorable	0.9669421	0.7685857
####	Bn-A06-p21045315	A-genome specific (napus-rapa)	0.9617225	0.3913358	not scorable	not scorable	0.9752066	0.8068424
####	Bn-A06-p21060490	A-genome specific (napus-rapa)	0.9904306	0.7800975	not scorable	not scorable	0.9834711	0.6915432
####	Bn-A06-p21062265	A-genome specific (napus-rapa)	0.9138756	0.7305983	not scorable	not scorable	0.677686	0.8075364
####	Bn-A06-p21062492	A-genome specific (napus-rapa)	0.9162679	0.7780567	not scorable	not scorable	0.8099173	0.7831137
####	Bn-A06-p21064129	A-genome specific (napus-rapa)	0.9976076	0.792733	not scorable	not scorable	0.9834711	0.8247338
####	Bn-A06-p21064168	A-genome specific (napus-rapa)	0.9736842	0.3436185	not scorable	not scorable	0.9752066	0.8276739
####	Bn-A06-p21064290	A-genome specific (napus-rapa)	0.9832536	0.3008423	not scorable	not scorable	0.9834711	0.5878294
####	Bn-A06-p21064341	A-genome specific (napus-rapa)	0.9521531	0.3781022	not scorable	not scorable	0.9752066	0.5444675
####	Bn-A06-p21065019	A-genome specific (napus-rapa)	0.9856459	0.6881166	not scorable	not scorable	0.9752066	0.7473243
####	Bn-A06-p21069394	A-genome specific (napus-rapa)	0.9856459	0.7876945	not scorable	not scorable	0.9834711	0.8385129
####	Bn-A06-p2107176	A-genome specific (rapa only)	not scorable	not scorable	not scorable	not scorable	0.9917355	0.7065499
####	Bn-A06-p21072392	A-genome specific (napus-rapa)	0.9952153	0.741163	not scorable	not scorable	0.9917355	0.7936143
####	Bn-A06-p2107610	A-genome specific (napus-rapa)	0.9665072	0.7955374	not scorable	not scorable	0.9834711	0.9113625
####	Bn-A06-p21080778	A-genome specific (napus-rapa)	0.9832536	0.7810466	not scorable	not scorable	0.9834711	0.8216213
####	Bn-A06-p21080915	A-genome specific (napus-rapa)	0.9712918	0.7817146	not scorable	not scorable	0.9917355	0.7558359
####	Bn-A06-p21083249	napus specific	0.9784689	0.8006104	not scorable	not scorable	not scorable	not scorable

####	Bn-A06-p21083748	napus specific	0.9665072	0.7507926	not scorable	not scorable	not scorable	not scorable
####	Bn-A06-p21085532	A-genome specific (napus-rapa)	0.9832536	0.7947452	not scorable	not scorable	1	0.8486626
####	Bn-A06-p21086685	A-genome specific (napus-rapa)	0.9808612	0.7707045	not scorable	not scorable	1	0.6794615
####	Bn-A06-p21098677	genome unspecific (all in)	0.9928229	0.4802193	1	0.8082134	0.9917355	0.7124265
####	Bn-A06-p21101957	A-genome specific (napus-rapa)	0.9904306	0.4021807	not scorable	not scorable	0.9834711	0.5794188
####	Bn-A06-p21105437	A-genome specific (napus-rapa)	0.9641148	0.5281621	not scorable	not scorable	0.9917355	0.7740345
####	Bn-A06-p21116438	A-genome specific (napus-rapa)	0.9856459	0.8132126	not scorable	not scorable	0.9669421	0.7040084
####	Bn-A06-p21126329	A-genome specific (napus-rapa)	0.9808612	0.7639666	not scorable	not scorable	0.9834711	0.733636
####	Bn-A06-p21127138	A-genome specific (napus-rapa)	0.9760765	0.7619255	not scorable	not scorable	0.9917355	0.8098002
####	Bn-A06-p21127779	A-genome specific (napus-rapa)	0.9760765	0.4622948	not scorable	not scorable	0.9834711	0.6284818
####	Bn-A06-p21127805	A-genome specific (napus-rapa)	0.9952153	0.7430481	not scorable	not scorable	0.9917355	0.7973977
####	Bn-A06-p21127973	A-genome specific (napus-rapa)	0.9712918	0.7516951	not scorable	not scorable	0.9834711	0.8013431
####	Bn-A06-p21132765	A-genome specific (napus-rapa)	0.9760765	0.7968733	not scorable	not scorable	1	0.7179279
####	Bn-A06-p21134315	A-genome specific (napus-rapa)	0.9928229	0.7663901	not scorable	not scorable	1	0.8023173
####	Bn-A06-p21136673	A-genome specific (napus-rapa)	0.9880383	0.7977899	not scorable	not scorable	1	0.8099538
####	Bn-A06-p21150459	A-genome specific (napus-rapa)	0.9712918	0.3790761	not scorable	not scorable	1	0.7156364
####	Bn-A06-p21150622	A-genome specific (napus-rapa)	0.9904306	0.7484589	not scorable	not scorable	0.9752066	0.7023996
####	Bn-A06-p21155152	all not scorable	not scorable	not scorable	not scorable	not scorable	not scorable	not scorable
####	Bn-A06-p21162770	A-genome specific (napus-rapa)	0.9904306	0.7533696	not scorable	not scorable	1	0.6932312
####	Bn-A06-p21163037	A-genome specific (napus-rapa)	0.9808612	0.3869669	not scorable	not scorable	1	0.6315923
####	Bn-A06-p21163950	A-genome specific (napus-rapa)	0.9880383	0.4834919	not scorable	not scorable	1	0.7583529
####	Bn-A06-p21163993	A-genome specific (napus-rapa)	0.9928229	0.4787954	not scorable	not scorable	1	0.7304303
####	Bn-A06-p21164043	A-genome specific (rapa only)	not scorable	not scorable	not scorable	not scorable	0.9917355	0.6820268
####	Bn-A06-p21164284	A-genome specific (napus-rapa)	0.9545454	0.696143	not scorable	not scorable	1	0.7007295
####	Bn-A06-p21164289	A-genome specific (napus-rapa)	0.9976076	0.5394581	not scorable	not scorable	1	0.8105727
####	Bn-A06-p21183207	genome unspecific (all in)	0.9904306	0.2590947	0.9457364	0.6975202	0.9834711	0.6526677
####	Bn-A06-p21187999	A-genome specific (napus-rapa)	0.9808612	0.7304664	not scorable	not scorable	0.9338843	0.6561165
####	Bn-A06-p21191463	A-genome specific (napus-rapa)	0.9784689	0.5909143	not scorable	not scorable	1	0.6099425
####	Bn-A06-p21194827	A-genome specific (napus-rapa)	1	0.4028627	not scorable	not scorable	0.9917355	0.6728882
####	Bn-A06-p21195115	A-genome specific (napus-rapa)	0.9760765	0.3960669	not scorable	not scorable	0.9586777	0.7251166
####	Bn-A06-p21196739	A-genome specific (napus-rapa)	0.9688995	0.579423	not scorable	not scorable	0.9917355	0.6800721
####	Bn-A06-p21221869	napus specific	0.9880383	0.8122085	not scorable	not scorable	not scorable	not scorable
####	Bn-A06-p21224575	A-genome specific (napus-rapa)	0.9832536	0.418537	not scorable	not scorable	1	0.8168764

####	Bn-A06-p21238199	A-genome specific (rapa only)	not scorable	not scorable	not scorable	not scorable	1	0.8864327
####	Bn-A06-p21247593	A-genome specific (napus-rapa)	0.9784689	0.4038316	not scorable	not scorable	1	0.8135532
####	Bn-A06-p21252883	A-genome specific (napus-rapa)	0.9856459	0.7312089	not scorable	not scorable	0.9834711	0.6703395
####	Bn-A06-p21260739	A-genome specific (napus-rapa)	0.9904306	0.4597152	not scorable	not scorable	0.9917355	0.7289231
####	Bn-A06-p21262030	A-genome specific (napus-rapa)	0.9688995	0.6277261	not scorable	not scorable	1	0.7770801
####	Bn-A06-p21268699	A-genome specific (rapa only)	not scorable	not scorable	not scorable	not scorable	1	0.909694
####	Bn-A06-p21268967	A-genome specific (napus-rapa)	0.9928229	0.5820765	not scorable	not scorable	1	0.5676258
####	Bn-A06-p21269830	A-genome specific (napus-rapa)	0.9832536	0.6500201	not scorable	not scorable	0.9586777	0.5729207
####	Bn-A06-p21270132	A-genome specific (napus-rapa)	0.9760765	0.7749662	not scorable	not scorable	1	0.687643
####	Bn-A06-p21273211	A-genome specific (napus-rapa)	0.9760765	0.5881068	not scorable	not scorable	1	0.6182877
####	Bn-A06-p21277332	A-genome specific (napus-rapa)	0.9641148	0.2695112	not scorable	not scorable	0.9917355	0.8023721
####	Bn-A06-p21278243	A-genome specific (napus-rapa)	0.9856459	0.8019257	not scorable	not scorable	0.9834711	0.7363553
####	Bn-A06-p21280471	A-genome specific (napus-rapa)	0.9928229	0.4752955	not scorable	not scorable	0.9917355	0.7998558
####	Bn-A06-p21280942	A-genome specific (napus-rapa)	0.9856459	0.7534977	not scorable	not scorable	1	0.6936527
####	Bn-A06-p21281098	napus specific	0.937799	0.8020887	not scorable	not scorable	not scorable	not scorable
####	Bn-A06-p21293821	A-genome specific (napus-rapa)	0.9712918	0.4404388	not scorable	not scorable	0.9917355	0.6881834
####	Bn-A06-p21301745	A-genome specific (napus-rapa)	0.9904306	0.5374666	not scorable	not scorable	0.9917355	0.7843279
####	Bn-A06-p21302070	A-genome specific (rapa only)	not scorable	not scorable	not scorable	not scorable	0.9586777	0.4320623
####	Bn-A06-p21308348	A-genome specific (napus-rapa)	0.9928229	0.7287587	not scorable	not scorable	1	0.6985735
####	Bn-A06-p21310030	A-genome specific (napus-rapa)	0.9976076	0.5123106	not scorable	not scorable	0.9917355	0.7769424
####	Bn-A06-p21311925	A-genome specific (napus-rapa)	0.9952153	0.6906182	not scorable	not scorable	0.9586777	0.6699104
####	Bn-A06-p21315016	A-genome specific (napus-rapa)	0.9019139	0.7067087	not scorable	not scorable	0.9504132	0.7169248
####	Bn-A06-p21318925	A-genome specific (napus-rapa)	0.9593301	0.2656312	not scorable	not scorable	0.9752066	0.68916
####	Bn-A06-p21321797	A-genome specific (napus-rapa)	0.9090909	0.7376631	not scorable	not scorable	0.9834711	0.7627113
####	Bn-A06-p21324859	A-genome specific (napus-rapa)	0.9593301	0.7680596	not scorable	not scorable	0.6033058	0.7181293
####	Bn-A06-p21349507	A-genome specific (napus-rapa)	0.9856459	0.7901061	not scorable	not scorable	0.9752066	0.7320295
####	Bn-A06-p21415201	A-genome specific (napus-rapa)	0.9928229	0.6966767	not scorable	not scorable	0.9834711	0.6733619
####	Bn-A06-p21422671	A-genome specific (napus-rapa)	0.9952153	0.6725802	not scorable	not scorable	0.9917355	0.6740808
####	Bn-A06-p21426650	A-genome specific (napus-rapa)	0.9952153	0.792155	not scorable	not scorable	0.9834711	0.7730765
####	Bn-A06-p21438687	genome unspecific (all in)	0.9832536	0.4680392	0.9147287	0.6182296	0.9917355	0.7874941
####	Bn-A06-p21445218	A-genome specific (napus-rapa)	0.9856459	0.7633827	not scorable	not scorable	0.9917355	0.7934758
####	Bn-A06-p21445439	A-genome specific (napus-rapa)	0.9856459	0.4987118	not scorable	not scorable	1	0.7729406
####	Bn-A06-p21462006	A-genome specific (napus-rapa)	0.9688995	0.324174	not scorable	not scorable	0.9917355	0.4536872

####	Bn-A06-p21463076	napus specific	0.5980861	0.8178911	not scorable	not scorable	not scorable	not scorable
####	Bn-A06-p21471436	A-genome specific (napus-rapa)	0.9473684	0.4310223	not scorable	not scorable	1	0.8084679
####	Bn-A06-p21472562	A-genome specific (napus-rapa)	1	0.8393996	not scorable	not scorable	0.9917355	0.7509659
####	Bn-A06-p21475556	A-genome specific (napus-rapa)	0.9617225	0.3431004	not scorable	not scorable	1	0.7255074
####	Bn-A06-p21478202	A-genome specific (rapa only)	not scorable	not scorable	not scorable	not scorable	1	0.7644343
####	Bn-A06-p21490506	A-genome specific (napus-rapa)	0.9784689	0.3950372	not scorable	not scorable	0.9917355	0.7418157
####	Bn-A06-p21491454	A-genome specific (napus-rapa)	0.9712918	0.2717429	not scorable	not scorable	0.6280992	0.7318531
####	Bn-A06-p21501350	A-genome specific (napus-rapa)	0.9952153	0.4694072	not scorable	not scorable	1	0.884657
####	Bn-A06-p21509934	A-genome specific (napus-rapa)	0.9593301	0.5669113	not scorable	not scorable	0.9834711	0.6662092
####	Bn-A06-p21525576	A-genome specific (napus-rapa)	0.9952153	0.4283035	not scorable	not scorable	0.9917355	0.7857097
####	Bn-A06-p21550710	A-genome specific (napus-rapa)	0.9928229	0.3748721	not scorable	not scorable	0.9834711	0.6868999
####	Bn-A06-p21572310	A-genome specific (rapa only)	not scorable	not scorable	not scorable	not scorable	0.9917355	0.622836
####	Bn-A06-p21573640	A-genome specific (rapa only)	not scorable	not scorable	not scorable	not scorable	0.9834711	0.8135204
####	Bn-A06-p21574157	A-genome specific (napus-rapa)	0.9952153	0.7383929	not scorable	not scorable	0.9917355	0.7830412
####	Bn-A06-p21589512	A-genome specific (rapa only)	not scorable	not scorable	not scorable	not scorable	0.9917355	0.8008413
####	Bn-A06-p21591920	A-genome specific (napus-rapa)	0.9736842	0.7603014	not scorable	not scorable	1	0.8674148
####	Bn-A06-p21597170	A-genome specific (napus-rapa)	0.9784689	0.6898045	not scorable	not scorable	0.9669421	0.8284779
####	Bn-A06-p21600894	A-genome specific (napus-rapa)	0.9832536	0.7313558	not scorable	not scorable	0.9834711	0.6441015
####	Bn-A06-p21602381	A-genome specific (napus-rapa)	0.9832536	0.3559659	not scorable	not scorable	1	0.8798255
####	Bn-A06-p21613020	A-genome specific (napus-rapa)	0.9688995	0.3778238	not scorable	not scorable	0.9834711	0.8436733
####	Bn-A06-p21650617	A-genome specific (napus-rapa)	0.9856459	0.5190089	not scorable	not scorable	0.9917355	0.7120868
####	Bn-A06-p2165351	A-genome specific (napus-rapa)	1	0.3942173	not scorable	not scorable	0.9917355	0.6840373
####	Bn-A06-p21653611	A-genome specific (napus-rapa)	0.9641148	0.478348	not scorable	not scorable	0.9917355	0.8211638
####	Bn-A06-p21663690	A-genome specific (napus-rapa)	0.9976076	0.398362	not scorable	not scorable	0.9917355	0.7274289
####	Bn-A06-p21677028	A-genome specific (napus-rapa)	1	0.4955086	not scorable	not scorable	1	0.7612059
####	Bn-A06-p2168634	A-genome specific (rapa only)	not scorable	not scorable	not scorable	not scorable	1	0.7134245
####	Bn-A06-p21796008	A-genome specific (napus-rapa)	0.9904306	0.8114603	not scorable	not scorable	1	0.7301449
####	Bn-A06-p21797963	A-genome specific (napus-rapa)	0.9904306	0.7853025	not scorable	not scorable	1	0.8101285
####	Bn-A06-p21800812	A-genome specific (napus-rapa)	0.9856459	0.7225661	not scorable	not scorable	1	0.6692769
####	Bn-A06-p21800927	A-genome specific (napus-rapa)	0.9928229	0.7095326	not scorable	not scorable	0.9752066	0.6678817
####	Bn-A06-p21801638	A-genome specific (napus-rapa)	1	0.7741914	not scorable	not scorable	0.9834711	0.8351377
####	Bn-A06-p21804318	A-genome specific (napus-rapa)	0.9880383	0.7569664	not scorable	not scorable	0.9917355	0.7463443
####	Bn-A06-p21822204	A-genome specific (napus-rapa)	1	0.273265	not scorable	not scorable	1	0.6851047

####	Bn-A06-p21825957	A-genome specific (napus-rapa)	0.9617225	0.8006789	not scorable	not scorable	0.6033058	0.7864977
####	Bn-A06-p21826106	A-genome specific (rapa only)	not scorable	not scorable	not scorable	not scorable	0.5867769	0.8710383
####	Bn-A06-p21826609	A-genome specific (napus-rapa)	0.9760765	0.8092616	not scorable	not scorable	0.9917355	0.6939957
####	Bn-A06-p21826671	A-genome specific (napus-rapa)	0.9880383	0.7019902	not scorable	not scorable	1	0.7799422
####	Bn-A06-p21826830	A-genome specific (napus-rapa)	0.9593301	0.5281987	not scorable	not scorable	0.9752066	0.7150626
####	Bn-A06-p21826979	A-genome specific (napus-rapa)	0.9928229	0.7980903	not scorable	not scorable	0.9917355	0.796352
####	Bn-A06-p21827359	A-genome specific (napus-rapa)	0.9832536	0.7992296	not scorable	not scorable	0.9752066	0.7880515
####	Bn-A06-p21829647	A-genome specific (napus-rapa)	0.9784689	0.811658	not scorable	not scorable	1	0.7956669
####	Bn-A06-p21846491	A-genome specific (napus-rapa)	0.6818182	0.7068456	not scorable	not scorable	0.6115702	0.6572533
####	Bn-A06-p21848747	A-genome specific (napus-rapa)	0.9760765	0.6256702	not scorable	not scorable	0.9834711	0.7188126
####	Bn-A06-p2185099	A-genome specific (napus-rapa)	0.9688995	0.8690061	not scorable	not scorable	0.9834711	0.8737018
####	Bn-A06-p21852261	A-genome specific (napus-rapa)	0.9521531	0.5807966	not scorable	not scorable	0.9917355	0.8016173
####	Bn-A06-p21884761	A-genome specific (napus-rapa)	0.9880383	0.7742633	not scorable	not scorable	0.6528926	0.7730228
####	Bn-A06-p21888532	A-genome specific (napus-rapa)	0.9688995	0.4897531	not scorable	not scorable	1	0.8784661
####	Bn-A06-p21896359	A-genome specific (napus-rapa)	0.9760765	0.7328776	not scorable	not scorable	0.9834711	0.7238802
####	Bn-A06-p21896871	A-genome specific (napus-rapa)	0.9808612	0.7584976	not scorable	not scorable	0.9917355	0.8238913
####	Bn-A06-p21899421	A-genome specific (napus-rapa)	0.9952153	0.8050625	not scorable	not scorable	1	0.9052851
####	Bn-A06-p21902421	A-genome specific (napus-rapa)	0.9832536	0.7868257	not scorable	not scorable	1	0.6549867
####	Bn-A06-p21902773	A-genome specific (napus-rapa)	0.9784689	0.8052423	not scorable	not scorable	0.9834711	0.8192739
####	Bn-A06-p21903683	A-genome specific (napus-rapa)	0.9736842	0.7849514	not scorable	not scorable	1	0.7729177
####	Bn-A06-p21904019	A-genome specific (napus-rapa)	0.9880383	0.8071038	not scorable	not scorable	1	0.9146456
####	Bn-A06-p21904162	A-genome specific (napus-rapa)	0.9784689	0.6918477	not scorable	not scorable	0.9917355	0.7465235
####	Bn-A06-p21917612	A-genome specific (napus-rapa)	0.9736842	0.5197405	not scorable	not scorable	0.5206612	0.8392041
####	Bn-A06-p21926982	A-genome specific (napus-rapa)	0.9856459	0.6646758	not scorable	not scorable	0.9586777	0.6346143
####	Bn-A06-p21930942	A-genome specific (napus-rapa)	0.9880383	0.3698871	not scorable	not scorable	1	0.6919689
####	Bn-A06-p21933122	A-genome specific (napus-rapa)	1	0.5750912	not scorable	not scorable	0.9834711	0.8046679
####	Bn-A06-p21933776	A-genome specific (napus-rapa)	0.9952153	0.4777787	not scorable	not scorable	0.9834711	0.7492527
####	Bn-A06-p21933808	A-genome specific (napus-rapa)	1	0.7969946	not scorable	not scorable	0.9834711	0.7749467
####	Bn-A06-p21933870	A-genome specific (napus-rapa)	1	0.7083493	not scorable	not scorable	1	0.7488707
####	Bn-A06-p21953510	A-genome specific (napus-rapa)	1	0.8114662	not scorable	not scorable	1	0.7474564
####	Bn-A06-p21954315	A-genome specific (napus-rapa)	1	0.6515502	not scorable	not scorable	0.9917355	0.7105888
####	Bn-A06-p21955208	A-genome specific (napus-rapa)	1	0.6990795	not scorable	not scorable	0.9917355	0.6994056
####	Bn-A06-p21980483	A-genome specific (rapa only)	not scorable	not scorable	not scorable	not scorable	1	0.6824719

####	Bn-A06-p21980699	A-genome specific (napus-rapa)	0.9976076	0.3897233	not scorable	not scorable	1	0.7500832
####	Bn-A06-p21983885	A-genome specific (napus-rapa)	0.9904306	0.4978341	not scorable	not scorable	0.9834711	0.8298522
####	Bn-A06-p21986550	A-genome specific (napus-rapa)	0.9736842	0.7752701	not scorable	not scorable	0.9917355	0.6833972
####	Bn-A06-p21992536	A-genome specific (napus-rapa)	1	0.790081	not scorable	not scorable	0.9834711	0.7885493
####	Bn-A06-p21993050	A-genome specific (napus-rapa)	0.930622	0.7128178	not scorable	not scorable	1	0.7453101
####	Bn-A06-p22001193	A-genome specific (napus-rapa)	0.9760765	0.3092453	not scorable	not scorable	0.9917355	0.7098652
####	Bn-A06-p22051862	A-genome specific (napus-rapa)	0.9760765	0.4227062	not scorable	not scorable	0.9917355	0.7087814
####	Bn-A06-p22054532	A-genome specific (napus-rapa)	0.6363636	0.8905491	not scorable	not scorable	0.9917355	0.8583596
####	Bn-A06-p22056809	genome unspecific (all in)	0.9880383	0.2565279	1	0.2193336	1	0.6940234
####	Bn-A06-p22063657	A-genome specific (rapa only)	not scorable	not scorable	not scorable	not scorable	1	0.7440118
####	Bn-A06-p22074841	genome unspecific (all in)	0.9330143	0.2777138	0.9844961	0.6106088	0.9752066	0.5598418
####	Bn-A06-p22077748	A-genome specific (napus-rapa)	0.9880383	0.7323995	not scorable	not scorable	0.9669421	0.839055
####	Bn-A06-p22088550	A-genome specific (napus-rapa)	0.9880383	0.5401381	not scorable	not scorable	0.9834711	0.7096933
####	Bn-A06-p22096615	A-genome specific (napus-rapa)	0.9904306	0.3694408	not scorable	not scorable	0.9917355	0.6150306
####	Bn-A06-p22105035	napus specific	0.9880383	0.2993841	not scorable	not scorable	not scorable	not scorable
####	Bn-A06-p22121666	A-genome specific (napus-rapa)	0.9976076	0.2598789	not scorable	not scorable	1	0.7094886
####	Bn-A06-p22160452	A-genome specific (napus-rapa)	0.9880383	0.6269611	not scorable	not scorable	0.9917355	0.8496951
####	Bn-A06-p22163702	A-genome specific (napus-rapa)	0.9832536	0.2684243	not scorable	not scorable	0.9917355	0.7886636
####	Bn-A06-p22173832	A-genome specific (napus-rapa)	0.9976076	0.3968476	not scorable	not scorable	1	0.826859
####	Bn-A06-p2217578	A-genome specific (napus-rapa)	0.9928229	0.4004328	not scorable	not scorable	1	0.7175488
####	Bn-A06-p2218956	A-genome specific (napus-rapa)	0.9832536	0.584325	not scorable	not scorable	1	0.6789079
####	Bn-A06-p22209347	A-genome specific (napus-rapa)	0.9593301	0.5568881	not scorable	not scorable	0.9917355	0.7910571
####	Bn-A06-p2222872	A-genome specific (rapa only)	not scorable	not scorable	not scorable	not scorable	1	0.7884539
####	Bn-A06-p22231737	A-genome specific (napus-rapa)	0.9784689	0.7005237	not scorable	not scorable	0.9669421	0.8223557
####	Bn-A06-p22262511	A-genome specific (napus-rapa)	0.9784689	0.7894836	not scorable	not scorable	0.9834711	0.7995051
####	Bn-A06-p22267850	A-genome specific (napus-rapa)	1	0.9272014	not scorable	not scorable	0.9917355	0.9272014
####	Bn-A06-p22268128	A-genome specific (napus-rapa)	0.9856459	0.3041212	not scorable	not scorable	0.9752066	0.7819726
####	Bn-A06-p22271048	A-genome specific (napus-rapa)	0.9760765	0.2987354	not scorable	not scorable	0.9917355	0.6917776
####	Bn-A06-p22278059	A-genome specific (napus-rapa)	0.9880383	0.6133742	not scorable	not scorable	0.9917355	0.7196835
####	Bn-A06-p22278468	A-genome specific (napus-rapa)	0.9880383	0.5345018	not scorable	not scorable	0.9752066	0.7094762
####	Bn-A06-p22279513	A-genome specific (napus-rapa)	0.9976076	0.6832656	not scorable	not scorable	0.9917355	0.6990988
####	Bn-A06-p22324968	A-genome specific (napus-rapa)	0.9712918	0.758703	not scorable	not scorable	0.9669421	0.7949031
####	Bn-A06-p22326059	A-genome specific (napus-rapa)	0.9832536	0.7157491	not scorable	not scorable	0.9917355	0.7217886

####	Bn-A06-p22329026	A-genome specific (napus-rapa)	0.9928229	0.502389	not scorable	not scorable	0.9834711	0.8122377
####	Bn-A06-p22329819	A-genome specific (napus-rapa)	0.9904306	0.5097431	not scorable	not scorable	1	0.7515808
####	Bn-A06-p22330861	A-genome specific (napus-rapa)	1	0.8279113	not scorable	not scorable	1	0.8049911
####	Bn-A06-p22331680	A-genome specific (napus-rapa)	0.9976076	0.4326962	not scorable	not scorable	1	0.7173514
####	Bn-A06-p2234696	A-genome specific (napus-rapa)	0.8851675	0.7869537	not scorable	not scorable	0.9256198	0.7732797
####	Bn-A06-p2234718	A-genome specific (napus-rapa)	0.9569378	0.825357	not scorable	not scorable	0.9256198	0.7523549
####	Bn-A06-p22383556	A-genome specific (napus-rapa)	0.9832536	0.5125602	not scorable	not scorable	1	0.7850402
####	Bn-A06-p22383722	A-genome specific (napus-rapa)	0.9330143	0.5844208	not scorable	not scorable	0.9752066	0.6731301
####	Bn-A06-p22391357	genome unspecific (all in)	0.9808612	0.3056332	1	0.656002	1	0.8376571
####	Bn-A06-p22397061	A-genome specific (napus-rapa)	1	0.6626829	not scorable	not scorable	0.9917355	0.6592323
####	Bn-A06-p22503987	A-genome specific (napus-rapa)	0.9784689	0.551122	not scorable	not scorable	0.9917355	0.7753481
####	Bn-A06-p22505664	A-genome specific (napus-rapa)	0.9712918	0.523587	not scorable	not scorable	0.9917355	0.7711571
####	Bn-A06-p22505810	genome unspecific (all in)	0.9904306	0.4963154	1	0.8481144	0.9834711	0.8218268
####	Bn-A06-p2251910	A-genome specific (napus-rapa)	0.9665072	0.599645	not scorable	not scorable	0.661157	0.6186296
####	Bn-A06-p2252815	A-genome specific (napus-rapa)	0.9952153	0.3311453	not scorable	not scorable	0.9834711	0.7325816
####	Bn-A06-p22554741	A-genome specific (napus-rapa)	1	0.8304602	not scorable	not scorable	1	0.9175813
####	Bn-A06-p22555168	A-genome specific (napus-rapa)	0.9712918	0.5948875	not scorable	not scorable	1	0.6999635
####	Bn-A06-p22557239	A-genome specific (napus-rapa)	0.9736842	0.3231167	not scorable	not scorable	0.9834711	0.7087324
####	Bn-A06-p22559895	A-genome specific (napus-rapa)	0.9904306	0.6226589	not scorable	not scorable	0.9917355	0.6322215
####	Bn-A06-p22577587	A-genome specific (napus-rapa)	0.9880383	0.7462591	not scorable	not scorable	0.9917355	0.7569162
####	Bn-A06-p22582397	A-genome specific (napus-rapa)	0.9473684	0.5405324	not scorable	not scorable	0.9834711	0.6675481
####	Bn-A06-p22588868	A-genome specific (napus-rapa)	0.9856459	0.7789547	not scorable	not scorable	0.9917355	0.7228335
####	Bn-A06-p22589709	A-genome specific (napus-rapa)	0.9856459	0.7664944	not scorable	not scorable	0.9834711	0.7443291
####	Bn-A06-p22594488	A-genome specific (napus-rapa)	0.9545454	0.5016845	not scorable	not scorable	0.9752066	0.7725552
####	Bn-A06-p22595032	A-genome specific (napus-rapa)	0.7679426	0.7808175	not scorable	not scorable	0.6033058	0.6472887
####	Bn-A06-p22597668	A-genome specific (napus-rapa)	0.9688995	0.5357071	not scorable	not scorable	0.9917355	0.8633536
####	Bn-A06-p22601511	A-genome specific (napus-rapa)	0.9760765	0.4287783	not scorable	not scorable	0.9834711	0.7651327
####	Bn-A06-p22601589	A-genome specific (napus-rapa)	0.9904306	0.7768082	not scorable	not scorable	0.9669421	0.7140841
####	Bn-A06-p22605217	A-genome specific (napus-rapa)	0.9665072	0.6326937	not scorable	not scorable	0.9917355	0.6842645
####	Bn-A06-p22608192	A-genome specific (napus-rapa)	0.9856459	0.6730262	not scorable	not scorable	0.9834711	0.6658371
####	Bn-A06-p22644580	A-genome specific (napus-rapa)	0.9545454	0.8165238	not scorable	not scorable	0.9669421	0.8731697
####	Bn-A06-p22647955	A-genome specific (napus-rapa)	0.9904306	0.2856245	not scorable	not scorable	1	0.8211561
####	Bn-A06-p2268393	A-genome specific (napus-rapa)	0.9617225	0.7060627	not scorable	not scorable	0.9917355	0.7069958

####	Bn-A06-p22691250	A-genome specific (napus-rapa)	1	0.752691	not scorable	not scorable	1	0.9205657
####	Bn-A06-p22709184	genome unspecific (all in)	0.9784689	0.7797378	0.8682171	0.7570032	1	0.8409123
####	Bn-A06-p22709872	A-genome specific (napus-rapa)	1	0.3960792	not scorable	not scorable	0.9669421	0.7446026
####	Bn-A06-p2274318	A-genome specific (napus-rapa)	0.9904306	0.3607583	not scorable	not scorable	0.6942149	0.8568431
####	Bn-A06-p2275754	napus specific	0.5095694	0.777229	not scorable	not scorable	not scorable	not scorable
####	Bn-A06-p22773574	A-genome specific (napus-rapa)	0.9976076	0.4123129	not scorable	not scorable	0.9752066	0.7645047
####	Bn-A06-p22773576	A-genome specific (napus-rapa)	0.9976076	0.5770319	not scorable	not scorable	0.9834711	0.6940374
####	Bn-A06-p22781886	A-genome specific (napus-rapa)	0.9066986	0.5583483	not scorable	not scorable	0.6198347	0.6921057
####	Bn-A06-p22782335	A-genome specific (napus-rapa)	0.9641148	0.8132621	not scorable	not scorable	0.6115702	0.7008032
####	Bn-A06-p22784485	A-genome specific (napus-rapa)	0.6746411	0.714874	not scorable	not scorable	0.892562	0.6823767
####	Bn-A06-p22786948	A-genome specific (napus-rapa)	0.9760765	0.7822806	not scorable	not scorable	0.6198347	0.7517534
####	Bn-A06-p22788890	A-genome specific (napus-rapa)	0.9425837	0.7118186	not scorable	not scorable	0.9834711	0.6942552
####	Bn-A06-p22796718	genome unspecific (all in)	0.9760765	0.4126065	1	0.7355843	0.9834711	0.6716518
####	Bn-A06-p22806976	A-genome specific (napus-rapa)	0.9976076	0.7120022	not scorable	not scorable	1	0.6683499
####	Bn-A06-p22808095	A-genome specific (napus-rapa)	0.9760765	0.3261864	not scorable	not scorable	1	0.4626161
####	Bn-A06-p22809449	A-genome specific (napus-rapa)	0.9545454	0.6369309	not scorable	not scorable	0.9834711	0.77964
####	Bn-A06-p22816078	A-genome specific (napus-rapa)	0.9497608	0.5518503	not scorable	not scorable	0.9917355	0.8373659
####	Bn-A06-p22835281	A-genome specific (rapa only)	not scorable	not scorable	not scorable	not scorable	0.9917355	0.7165428
####	Bn-A06-p22847296	A-genome specific (napus-rapa)	0.9712918	0.374049	not scorable	not scorable	0.9834711	0.6694064
####	Bn-A06-p22882207	A-genome specific (napus-rapa)	0.9904306	0.7592138	not scorable	not scorable	0.9917355	0.6023511
####	Bn-A06-p22894392	A-genome specific (rapa only)	not scorable	not scorable	not scorable	not scorable	0.9752066	0.6975516
####	Bn-A06-p22910339	A-genome specific (napus-rapa)	0.9808612	0.4182937	not scorable	not scorable	0.9917355	0.6240761
####	Bn-A06-p22913862	A-genome specific (napus-rapa)	0.9784689	0.7338065	not scorable	not scorable	0.9834711	0.6828651
####	Bn-A06-p22914032	A-genome specific (napus-rapa)	0.9832536	0.7012511	not scorable	not scorable	0.9834711	0.6990761
####	Bn-A06-p22914117	A-genome specific (napus-rapa)	0.9856459	0.6923657	not scorable	not scorable	0.9586777	0.7093671
####	Bn-A06-p22917369	A-genome specific (napus-rapa)	0.9641148	0.7616261	not scorable	not scorable	0.9834711	0.7901378
####	Bn-A06-p22921908	A-genome specific (napus-rapa)	0.9832536	0.6794688	not scorable	not scorable	0.9917355	0.8013307
####	Bn-A06-p22922325	A-genome specific (napus-rapa)	0.9880383	0.7759107	not scorable	not scorable	1	0.9240721
####	Bn-A06-p2292578	A-genome specific (napus-rapa)	0.9401914	0.3481184	not scorable	not scorable	1	0.6500306
####	Bn-A06-p2293069	A-genome specific (napus-rapa)	0.9760765	0.8195679	not scorable	not scorable	1	0.8012809
####	Bn-A06-p22938934	A-genome specific (napus-rapa)	0.9688995	0.2358641	not scorable	not scorable	0.9752066	0.569261
####	Bn-A06-p22947499	A-genome specific (napus-rapa)	0.9617225	0.6171902	not scorable	not scorable	0.9917355	0.8018759
####	Bn-A06-p2300342	A-genome specific (napus-rapa)	0.9497608	0.7454369	not scorable	not scorable	0.9917355	0.8177654

####	Bn-A06-p2303914	A-genome specific (napus-rapa)	0.9545454	0.7636623	not scorable	not scorable	0.9586777	0.7777188
####	Bn-A06-p23041811	A-genome specific (napus-rapa)	0.9976076	0.7219982	not scorable	not scorable	0.9917355	0.6904709
####	Bn-A06-p23042397	A-genome specific (napus-rapa)	1	0.6746852	not scorable	not scorable	0.9917355	0.7225248
####	Bn-A06-p23042849	A-genome specific (napus-rapa)	0.9976076	0.801504	not scorable	not scorable	0.9917355	0.8148065
####	Bn-A06-p23043135	A-genome specific (napus-rapa)	0.9976076	0.8109214	not scorable	not scorable	0.9834711	0.7837855
####	Bn-A06-p2304435	A-genome specific (napus-rapa)	0.9976076	0.2655419	not scorable	not scorable	0.9834711	0.7964625
####	Bn-A06-p2305844	A-genome specific (napus-rapa)	0.9784689	0.3823975	not scorable	not scorable	0.9586777	0.7348355
####	Bn-A06-p2306499	A-genome specific (napus-rapa)	1	0.4666708	not scorable	not scorable	0.9834711	0.8250861
####	Bn-A06-p2307291	A-genome specific (napus-rapa)	0.9497608	0.8188066	not scorable	not scorable	0.9834711	0.8577474
####	Bn-A06-p2316252	A-genome specific (napus-rapa)	0.9473684	0.3444099	not scorable	not scorable	1	0.7808376
####	Bn-A06-p23216260	A-genome specific (napus-rapa)	0.9808612	0.8264181	not scorable	not scorable	0.9917355	0.7162943
####	Bn-A06-p23218510	A-genome specific (napus-rapa)	0.9617225	0.7825961	not scorable	not scorable	1	0.7543278
####	Bn-A06-p23219303	A-genome specific (napus-rapa)	0.9784689	0.8080567	not scorable	not scorable	1	0.8103186
####	Bn-A06-p23219336	A-genome specific (napus-rapa)	0.9593301	0.4510284	not scorable	not scorable	1	0.7410041
####	Bn-A06-p23219420	A-genome specific (napus-rapa)	0.9904306	0.8240921	not scorable	not scorable	1	0.7606184
####	Bn-A06-p23226757	A-genome specific (napus-rapa)	0.9952153	0.7345465	not scorable	not scorable	1	0.7063227
####	Bn-A06-p23226981	A-genome specific (napus-rapa)	0.9617225	0.7228265	not scorable	not scorable	1	0.8525698
####	Bn-A06-p23227091	A-genome specific (napus-rapa)	0.9784689	0.769268	not scorable	not scorable	1	0.7736149
####	Bn-A06-p23229650	A-genome specific (napus-rapa)	0.9665072	0.4458218	not scorable	not scorable	0.9834711	0.794191
####	Bn-A06-p23250542	A-genome specific (napus-rapa)	0.9952153	0.8103112	not scorable	not scorable	1	0.6782417
####	Bn-A06-p23258948	A-genome specific (rapa only)	not scorable	not scorable	not scorable	not scorable	1	0.7307554
####	Bn-A06-p23259546	A-genome specific (napus-rapa)	0.9880383	0.7547672	not scorable	not scorable	0.9917355	0.7317971
####	Bn-A06-p23268021	A-genome specific (napus-rapa)	0.9688995	0.6622695	not scorable	not scorable	0.9834711	0.8006958
####	Bn-A06-p23295405	A-genome specific (napus-rapa)	0.9784689	0.7934908	not scorable	not scorable	0.9917355	0.8321213
####	Bn-A06-p23341715	A-genome specific (napus-rapa)	0.9641148	0.7969531	not scorable	not scorable	0.9586777	0.7736661
####	Bn-A06-p23347767	A-genome specific (napus-rapa)	0.9904306	0.6970072	not scorable	not scorable	0.9917355	0.6929789
####	Bn-A06-p23350246	A-genome specific (napus-rapa)	0.9928229	0.8128534	not scorable	not scorable	0.9917355	0.8135626
####	Bn-A06-p23406582	A-genome specific (napus-rapa)	1	0.9205657	not scorable	not scorable	1	0.916608
####	Bn-A06-p2341154	A-genome specific (napus-rapa)	0.9832536	0.8126062	not scorable	not scorable	0.9752066	0.6947213
####	Bn-A06-p23442751	A-genome specific (napus-rapa)	0.9952153	0.4427902	not scorable	not scorable	1	0.8085203
####	Bn-A06-p2345966	A-genome specific (napus-rapa)	0.9832536	0.7527182	not scorable	not scorable	1	0.8422567
####	Bn-A06-p23468803	A-genome specific (napus-rapa)	0.9952153	0.7937553	not scorable	not scorable	1	0.7396594
####	Bn-A06-p2347754	A-genome specific (napus-rapa)	0.9688995	0.7010462	not scorable	not scorable	1	0.6793889

####	Bn-A06-p2348428	A-genome specific (napus-rapa)	0.9784689	0.4037814	not scorable	not scorable	0.9917355	0.7903694
####	Bn-A06-p23542209	A-genome specific (napus-rapa)	0.9976076	0.8102468	not scorable	not scorable	0.9834711	0.8047117
####	Bn-A06-p2357647	A-genome specific (napus-rapa)	0.9688995	0.7521178	not scorable	not scorable	1	0.8024052
####	Bn-A06-p23585772	A-genome specific (napus-rapa)	0.9760765	0.3811812	not scorable	not scorable	0.9752066	0.7761122
####	Bn-A06-p23586019	A-genome specific (napus-rapa)	1	0.5353746	not scorable	not scorable	0.9834711	0.5762067
####	Bn-A06-p23590868	A-genome specific (napus-rapa)	0.9904306	0.347268	not scorable	not scorable	1	0.7052888
####	Bn-A06-p23591616	A-genome specific (napus-rapa)	0.9760765	0.7132003	not scorable	not scorable	0.9917355	0.6967156
####	Bn-A06-p23595655	A-genome specific (napus-rapa)	0.9904306	0.802133	not scorable	not scorable	1	0.8153849
####	Bn-A06-p23612730	A-genome specific (napus-rapa)	0.9808612	0.7639281	not scorable	not scorable	1	0.8521708
####	Bn-A06-p23621651	A-genome specific (napus-rapa)	0.9712918	0.670655	not scorable	not scorable	0.9917355	0.7763514
####	Bn-A06-p23625426	A-genome specific (napus-rapa)	0.9330143	0.8034906	not scorable	not scorable	0.6115702	0.803229
####	Bn-A06-p23657129	A-genome specific (napus-rapa)	0.9784689	0.3808871	not scorable	not scorable	1	0.7354022
####	Bn-A06-p2365869	A-genome specific (napus-rapa)	0.9952153	0.7711585	not scorable	not scorable	0.9917355	0.8517414
####	Bn-A06-p23660656	A-genome specific (napus-rapa)	0.9712918	0.7373261	not scorable	not scorable	1	0.8592515
####	Bn-A06-p23661679	genome unspecific (oleracea-rapa)	not scorable	not scorable	1	0.699091	0.9917355	0.7998371
####	Bn-A06-p23665384	A-genome specific (napus-rapa)	0.9832536	0.3532295	not scorable	not scorable	0.6363636	0.8310879
####	Bn-A06-p23665564	A-genome specific (napus-rapa)	0.6483254	0.7533282	not scorable	not scorable	0.6694215	0.6998192
####	Bn-A06-p23666378	A-genome specific (napus-rapa)	0.6507177	0.8610106	not scorable	not scorable	0.6694215	0.8162478
####	Bn-A06-p23674542	A-genome specific (napus-rapa)	0.9856459	0.7621419	not scorable	not scorable	1	0.7255844
####	Bn-A06-p23677178	A-genome specific (napus-rapa)	0.9904306	0.7481931	not scorable	not scorable	1	0.6611145
####	Bn-A06-p23677200	A-genome specific (napus-rapa)	0.9928229	0.7532716	not scorable	not scorable	1	0.7818686
####	Bn-A06-p23681386	A-genome specific (napus-rapa)	0.9880383	0.7157691	not scorable	not scorable	1	0.6885931
####	Bn-A06-p23684217	A-genome specific (napus-rapa)	0.9784689	0.7621069	not scorable	not scorable	1	0.8291757
####	Bn-A06-p23684770	A-genome specific (napus-rapa)	0.9928229	0.7599296	not scorable	not scorable	1	0.8186069
####	Bn-A06-p23687609	A-genome specific (napus-rapa)	0.9952153	0.7531577	not scorable	not scorable	1	0.7956324
####	Bn-A06-p23688372	A-genome specific (napus-rapa)	0.9856459	0.7620777	not scorable	not scorable	1	0.7502258
####	Bn-A06-p23695500	A-genome specific (napus-rapa)	0.9760765	0.6462757	not scorable	not scorable	1	0.7836249
####	Bn-A06-p23695665	A-genome specific (napus-rapa)	0.9856459	0.7559047	not scorable	not scorable	1	0.8235264
####	Bn-A06-p23697182	A-genome specific (napus-rapa)	0.9760765	0.7507638	not scorable	not scorable	1	0.7582187
####	Bn-A06-p23697605	A-genome specific (napus-rapa)	0.9760765	0.7593462	not scorable	not scorable	1	0.6761311
####	Bn-A06-p23699003	A-genome specific (napus-rapa)	0.9928229	0.7846684	not scorable	not scorable	0.9834711	0.7774679
####	Bn-A06-p23699420	A-genome specific (napus-rapa)	0.9736842	0.6880916	not scorable	not scorable	0.9917355	0.7672326
####	Bn-A06-p23706979	A-genome specific (rapa only)	not scorable	not scorable	not scorable	not scorable	0.9834711	0.7644212

####	Bn-A06-p23710466	A-genome specific (napus-rapa)	0.9473684	0.6795021	not scorable	not scorable	0.9917355	0.7520569
####	Bn-A06-p23710506	A-genome specific (napus-rapa)	0.9952153	0.7464174	not scorable	not scorable	1	0.8086776
####	Bn-A06-p23741737	A-genome specific (napus-rapa)	0.9569378	0.6973283	not scorable	not scorable	0.9917355	0.659147
####	Bn-A06-p23744370	A-genome specific (napus-rapa)	0.03827751	0.7145514	not scorable	not scorable	0.9834711	0.6968536
####	Bn-A06-p23746948	A-genome specific (napus-rapa)	0.9952153	0.7534229	not scorable	not scorable	1	0.9240721
####	Bn-A06-p23748804	A-genome specific (napus-rapa)	0.9712918	0.5657801	not scorable	not scorable	0.9669421	0.7882775
####	Bn-A06-p23750653	A-genome specific (napus-rapa)	0.9521531	0.7523856	not scorable	not scorable	1	0.7608893
####	Bn-A06-p23759352	A-genome specific (napus-rapa)	0.9593301	0.6766212	not scorable	not scorable	0.9834711	0.9507774
####	Bn-A06-p23768610	A-genome specific (napus-rapa)	0.9832536	0.3197401	not scorable	not scorable	1	0.7320846
####	Bn-A06-p23776151	A-genome specific (napus-rapa)	0.9880383	0.6614456	not scorable	not scorable	0.9917355	0.8288725
####	Bn-A06-p23795599	A-genome specific (napus-rapa)	1	0.7491307	not scorable	not scorable	0.9752066	0.8230965
####	Bn-A06-p23795763	A-genome specific (napus-rapa)	0.9904306	0.8271506	not scorable	not scorable	0.9917355	0.8023478
####	Bn-A06-p23796020	A-genome specific (napus-rapa)	0.9904306	0.8661372	not scorable	not scorable	0.9917355	0.7957217
####	Bn-A06-p23806343	A-genome specific (napus-rapa)	0.9808612	0.4950114	not scorable	not scorable	0.9752066	0.5297749
####	Bn-A06-p23852270	A-genome specific (napus-rapa)	0.6842105	0.7750737	not scorable	not scorable	0.9504132	0.8161226
####	Bn-A06-p23857345	A-genome specific (napus-rapa)	0.9665072	0.4038264	not scorable	not scorable	0.9917355	0.7704021
####	Bn-A06-p23865356	A-genome specific (napus-rapa)	0.9928229	0.6824156	not scorable	not scorable	0.9834711	0.7200165
####	Bn-A06-p23873505	A-genome specific (napus-rapa)	0.6842105	0.8214992	not scorable	not scorable	0.9586777	0.8233764
####	Bn-A06-p23892315	A-genome specific (napus-rapa)	0.9904306	0.7632224	not scorable	not scorable	1	0.7323081
####	Bn-A06-p23892404	A-genome specific (napus-rapa)	0.9856459	0.7643707	not scorable	not scorable	0.9834711	0.7304637
####	Bn-A06-p2390609	A-genome specific (napus-rapa)	0.9928229	0.7813344	not scorable	not scorable	0.9917355	0.8547498
####	Bn-A06-p23944667	A-genome specific (napus-rapa)	0.9952153	0.7527133	not scorable	not scorable	1	0.7457933
####	Bn-A06-p23968879	A-genome specific (napus-rapa)	0.9880383	0.7399921	not scorable	not scorable	0.9834711	0.6907133
####	Bn-A06-p23972440	A-genome specific (napus-rapa)	0.9569378	0.4123479	not scorable	not scorable	0.9917355	0.7838849
####	Bn-A06-p23983648	A-genome specific (napus-rapa)	0.9784689	0.4675966	not scorable	not scorable	1	0.7320513
####	Bn-A06-p23988702	A-genome specific (napus-rapa)	0.9904306	0.4737935	not scorable	not scorable	1	0.8008183
####	Bn-A06-p24000840	A-genome specific (napus-rapa)	0.4952153	0.7729893	not scorable	not scorable	0.8842975	0.6698728
####	Bn-A06-p24005581	A-genome specific (napus-rapa)	0.9952153	0.510698	not scorable	not scorable	0.9752066	0.7795855
####	Bn-A06-p24006085	A-genome specific (rapa only)	not scorable	not scorable	not scorable	not scorable	0.9917355	0.6944862
####	Bn-A06-p24017545	all not scorable	not scorable	not scorable	not scorable	not scorable	not scorable	not scorable
####	Bn-A06-p24032427	A-genome specific (napus-rapa)	0.9712918	0.6964451	not scorable	not scorable	0.9834711	0.7926037
####	Bn-A06-p24040939	A-genome specific (napus-rapa)	0.9712918	0.7136787	not scorable	not scorable	0.9752066	0.7167431
####	Bn-A06-p24041870	genome unspecific (all in)	0.9952153	0.7806701	0	0.8419402	1	0.8067898

####	Bn-A06-p24043170	A-genome specific (napus-rapa)	0.9760765	0.4213769	not scorable	not scorable	0.9917355	0.7879115
####	Bn-A06-p24045825	A-genome specific (napus-rapa)	0.9832536	0.7527015	not scorable	not scorable	1	0.8020123
####	Bn-A06-p24046488	A-genome specific (napus-rapa)	0.9784689	0.3761618	not scorable	not scorable	0.9917355	0.4908206
####	Bn-A06-p2405022	C-genome specific (napus-oleracea)	0.9760765	0.7020916	0.9534883	0.7829805	not scorable	not scorable
####	Bn-A06-p24068524	genome unspecific (all in)	0.9784689	0.3539623	0.9844961	0.6033046	0.9834711	0.7407122
####	Bn-A06-p24070714	A-genome specific (napus-rapa)	0.9856459	0.7956859	not scorable	not scorable	0.9834711	0.7117031
####	Bn-A06-p2407080	A-genome specific (napus-rapa)	0.9832536	0.7327422	not scorable	not scorable	0.9917355	0.7528263
####	Bn-A06-p24075375	A-genome specific (napus-rapa)	0.854067	0.7805812	not scorable	not scorable	0.9834711	0.847411
####	Bn-A06-p24076443	A-genome specific (napus-rapa)	0.9736842	0.7658553	not scorable	not scorable	0.9834711	0.7631356
####	Bn-A06-p24099288	A-genome specific (napus-rapa)	0.9736842	0.6623705	not scorable	not scorable	0.9834711	0.7282557
####	Bn-A06-p2412998	A-genome specific (napus-rapa)	0.9904306	0.4997921	not scorable	not scorable	0.9917355	0.793184
####	Bn-A06-p24132842	A-genome specific (napus-rapa)	0.9952153	0.6984201	not scorable	not scorable	0.9917355	0.6863167
####	Bn-A06-p24132956	A-genome specific (napus-rapa)	0.9521531	0.4897759	not scorable	not scorable	0.9917355	0.7314074
####	Bn-A06-p2413971	A-genome specific (napus-rapa)	0.9880383	0.8155088	not scorable	not scorable	0.9834711	0.7966136
####	Bn-A06-p24142937	A-genome specific (napus-rapa)	0.9880383	0.7705176	not scorable	not scorable	0.9917355	0.7434394
####	Bn-A06-p24145208	A-genome specific (napus-rapa)	0.9832536	0.7220418	not scorable	not scorable	1	0.7846798
####	Bn-A06-p24147820	A-genome specific (napus-rapa)	0.9880383	0.7578976	not scorable	not scorable	0.9917355	0.7214114
####	Bn-A06-p24152122	A-genome specific (napus-rapa)	0.9736842	0.3817016	not scorable	not scorable	1	0.7841223
####	Bn-A06-p24156940	A-genome specific (napus-rapa)	0.9880383	0.7725638	not scorable	not scorable	1	0.7387109
####	Bn-A06-p24156988	A-genome specific (napus-rapa)	0.9904306	0.7732701	not scorable	not scorable	0.9917355	0.8162115
####	Bn-A06-p24167592	genome unspecific (all in)	0.9832536	0.5228553	0.9922481	0.7142245	0.9504132	0.6530532
####	Bn-A06-p24168470	A-genome specific (napus-rapa)	0.9569378	0.2581909	not scorable	not scorable	1	0.6989014
####	Bn-A06-p24170361	A-genome specific (napus-rapa)	0.9856459	0.8041015	not scorable	not scorable	0.9917355	0.7425246
####	Bn-A06-p24178982	A-genome specific (napus-rapa)	0.9784689	0.7979935	not scorable	not scorable	0.9752066	0.7627319
####	Bn-A06-p24204030	A-genome specific (napus-rapa)	0.9712918	0.7567242	not scorable	not scorable	1	0.7827548
####	Bn-A06-p24206019	A-genome specific (napus-rapa)	0.9760765	0.6906354	not scorable	not scorable	1	0.6784909
####	Bn-A06-p24206556	A-genome specific (napus-rapa)	0.9904306	0.7811074	not scorable	not scorable	1	0.7806654
####	Bn-A06-p24207308	genome unspecific (all in)	0.9808612	0.7700121	1	0.7066216	1	0.7630315
####	Bn-A06-p24210014	A-genome specific (napus-rapa)	0.9760765	0.7853403	not scorable	not scorable	0.9752066	0.8409306
####	Bn-A06-p24218304	A-genome specific (rapa only)	not scorable	not scorable	not scorable	not scorable	1	0.68419
####	Bn-A06-p24220121	A-genome specific (napus-rapa)	0.9712918	0.7681912	not scorable	not scorable	0.9917355	0.8310581
####	Bn-A06-p24222064	A-genome specific (napus-rapa)	0.9736842	0.4229615	not scorable	not scorable	0.9917355	0.7052028
####	Bn-A06-p24236385	A-genome specific (napus-rapa)	0.9928229	0.7824283	not scorable	not scorable	1	0.7816136

####	Bn-A06-p24237899	A-genome specific (napus-rapa)	0.9856459	0.5273543	not scorable	not scorable	0.9917355	0.7447951
####	Bn-A06-p24240603	A-genome specific (napus-rapa)	0.9688995	0.3621333	not scorable	not scorable	0.9917355	0.6660392
####	Bn-A06-p24245615	napus specific	0.9186603	0.6723401	not scorable	not scorable	not scorable	not scorable
####	Bn-A06-p24257262	A-genome specific (napus-rapa)	0.9808612	0.4894132	not scorable	not scorable	0.9917355	0.7019793
####	Bn-A06-p24267492	A-genome specific (napus-rapa)	0.9856459	0.4328229	not scorable	not scorable	1	0.7916889
####	Bn-A06-p24267499	A-genome specific (napus-rapa)	0.9832536	0.7580826	not scorable	not scorable	0.9834711	0.7597477
####	Bn-A06-p24267745	A-genome specific (napus-rapa)	0.9784689	0.7363952	not scorable	not scorable	0.9917355	0.7601219
####	Bn-A06-p24270718	A-genome specific (napus-rapa)	0.9736842	0.5481899	not scorable	not scorable	0.9834711	0.6788129
####	Bn-A06-p24274213	A-genome specific (napus-rapa)	0.9904306	0.8064559	not scorable	not scorable	0.9917355	0.8053553
####	Bn-A06-p24278510	A-genome specific (napus-rapa)	0.9952153	0.7204055	not scorable	not scorable	0.9752066	0.7096606
####	Bn-A06-p24284014	A-genome specific (napus-rapa)	0.9784689	0.4552818	not scorable	not scorable	0.9917355	0.7352065
####	Bn-A06-p24286025	A-genome specific (napus-rapa)	0.9808612	0.7074205	not scorable	not scorable	1	0.8006257
####	Bn-A06-p24297216	A-genome specific (napus-rapa)	0.9904306	0.8008186	not scorable	not scorable	1	0.6877922
####	Bn-A06-p24307250	A-genome specific (napus-rapa)	0.9880383	0.4293169	not scorable	not scorable	0.9834711	0.7258022
####	Bn-A06-p24319329	A-genome specific (napus-rapa)	0.84689	0.5147618	not scorable	not scorable	0.9917355	0.5765904
####	Bn-A06-p24327317	A-genome specific (napus-rapa)	0.9928229	0.4083067	not scorable	not scorable	0.9752066	0.6493217
####	Bn-A06-p24334015	A-genome specific (napus-rapa)	0.9832536	0.4737988	not scorable	not scorable	0.9917355	0.75764
####	Bn-A06-p24340685	A-genome specific (napus-rapa)	0.9784689	0.4739423	not scorable	not scorable	0.6446281	0.8013015
####	Bn-A06-p24340830	A-genome specific (napus-rapa)	0.9760765	0.4280463	not scorable	not scorable	0.9834711	0.760085
####	Bn-A06-p2434155	genome unspecific (all in)	1	0.3416681	0.9922481	0.8132935	1	0.8687926
####	Bn-A06-p24370009	A-genome specific (napus-rapa)	0.9736842	0.7653058	not scorable	not scorable	0.6694215	0.8104508
####	Bn-A06-p24371980	A-genome specific (napus-rapa)	0.9784689	0.5481317	not scorable	not scorable	0.9917355	0.6101058
####	Bn-A06-p2437284	A-genome specific (napus-rapa)	0.9976076	0.8089186	not scorable	not scorable	1	0.7078187
####	Bn-A06-p24386467	A-genome specific (napus-rapa)	0.9665072	0.5693668	not scorable	not scorable	0.9917355	0.5415506
####	Bn-A06-p24395608	A-genome specific (napus-rapa)	0.9856459	0.7508473	not scorable	not scorable	1	0.7220084
####	Bn-A06-p24401543	A-genome specific (napus-rapa)	0.9808612	0.7789615	not scorable	not scorable	0.9834711	0.7596446
####	Bn-A06-p24404374	A-genome specific (napus-rapa)	0.9330143	0.764757	not scorable	not scorable	0.9669421	0.7354829
####	Bn-A06-p24404996	A-genome specific (napus-rapa)	0.9641148	0.7880833	not scorable	not scorable	0.9834711	0.8022919
####	Bn-A06-p24405065	A-genome specific (napus-rapa)	0.9736842	0.7199325	not scorable	not scorable	0.9917355	0.7341916
####	Bn-A06-p24407165	A-genome specific (napus-rapa)	0.9641148	0.7386729	not scorable	not scorable	0.9917355	0.750797
####	Bn-A06-p24407535	A-genome specific (napus-rapa)	0.9665072	0.7890869	not scorable	not scorable	0.9917355	0.812771
####	Bn-A06-p24407609	A-genome specific (napus-rapa)	0.9665072	0.7799134	not scorable	not scorable	0.9917355	0.780642
####	Bn-A06-p24410007	A-genome specific (napus-rapa)	0.9880383	0.8015509	not scorable	not scorable	1	0.7470555

####	Bn-A06-p24428073	A-genome specific (napus-rapa)	0.9760765	0.3326877	not scorable	not scorable	0.9834711	0.7390581
####	Bn-A06-p24430448	A-genome specific (napus-rapa)	0.9784689	0.79985	not scorable	not scorable	0.9917355	0.8186901
####	Bn-A06-p24430453	A-genome specific (napus-rapa)	0.9880383	0.7830415	not scorable	not scorable	1	0.7520554
####	Bn-A06-p24431577	A-genome specific (napus-rapa)	0.9736842	0.7798895	not scorable	not scorable	0.9834711	0.7840755
####	Bn-A06-p24432451	A-genome specific (napus-rapa)	0.9593301	0.7778897	not scorable	not scorable	0.9917355	0.7769164
####	Bn-A06-p24432909	A-genome specific (napus-rapa)	0.9712918	0.7921971	not scorable	not scorable	0.9917355	0.6570231
####	Bn-A06-p24433131	A-genome specific (napus-rapa)	0.9712918	0.8167499	not scorable	not scorable	0.9834711	0.787383
####	Bn-A06-p24441882	A-genome specific (napus-rapa)	0.9832536	0.3056305	not scorable	not scorable	0.9917355	0.6923874
####	Bn-A06-p24453423	A-genome specific (napus-rapa)	0.9784689	0.2533611	not scorable	not scorable	0.9504132	0.7467681
####	Bn-A06-p24465494	A-genome specific (napus-rapa)	0.9521531	0.3466715	not scorable	not scorable	1	0.6000783
####	Bn-A06-p24466136	A-genome specific (napus-rapa)	0.9880383	0.7866471	not scorable	not scorable	0.9834711	0.7743333
####	Bn-A06-p24467652	A-genome specific (napus-rapa)	0.9928229	0.7882712	not scorable	not scorable	1	0.7133151
####	Bn-A06-p24496051	A-genome specific (napus-rapa)	0.9712918	0.5640677	not scorable	not scorable	1	0.8402684
####	Bn-A06-p24501315	A-genome specific (napus-rapa)	0.9617225	0.7895656	not scorable	not scorable	0.9917355	0.7548246
####	Bn-A06-p24501540	A-genome specific (rapa only)	not scorable	not scorable	not scorable	not scorable	1	0.8342927
####	Bn-A06-p24503033	A-genome specific (napus-rapa)	0.9688995	0.6983396	not scorable	not scorable	0.9834711	0.6839688
####	Bn-A06-p24508464	A-genome specific (napus-rapa)	0.9521531	0.7558073	not scorable	not scorable	1	0.8318305
####	Bn-A06-p24536499	A-genome specific (napus-rapa)	0.9928229	0.7754807	not scorable	not scorable	1	0.7281514
####	Bn-A06-p24544753	A-genome specific (napus-rapa)	0.9593301	0.7455648	not scorable	not scorable	1	0.787078
####	Bn-A06-p24551529	A-genome specific (napus-rapa)	0.9712918	0.7455115	not scorable	not scorable	0.9917355	0.7871619
####	Bn-A06-p24557108	A-genome specific (napus-rapa)	0.9354067	0.6098844	not scorable	not scorable	0.9834711	0.7855092
####	Bn-A06-p24557193	A-genome specific (napus-rapa)	0.9784689	0.5869064	not scorable	not scorable	0.9834711	0.7452355
####	Bn-A06-p24567939	A-genome specific (napus-rapa)	0.9880383	0.7400342	not scorable	not scorable	0.9917355	0.8879275
####	Bn-A06-p24570179	A-genome specific (napus-rapa)	0.8062201	0.8208902	not scorable	not scorable	0.9669421	0.7985221
####	Bn-A06-p24579442	A-genome specific (napus-rapa)	0.9880383	0.8181059	not scorable	not scorable	0.9834711	0.6504189
####	Bn-A06-p24580393	A-genome specific (napus-rapa)	0.9784689	0.7815068	not scorable	not scorable	0.9834711	0.7639838
####	Bn-A06-p24588298	A-genome specific (napus-rapa)	0.9904306	0.4381678	not scorable	not scorable	0.9917355	0.8442788
####	Bn-A06-p24599972	A-genome specific (napus-rapa)	0.9712918	0.6896733	not scorable	not scorable	0.9917355	0.7287537
####	Bn-A06-p24600019	A-genome specific (napus-rapa)	0.9688995	0.3473459	not scorable	not scorable	0.9752066	0.7272967
####	Bn-A06-p24600140	A-genome specific (napus-rapa)	0.9832536	0.7006138	not scorable	not scorable	0.9917355	0.7544801
####	Bn-A06-p24602106	A-genome specific (napus-rapa)	0.9856459	0.7926656	not scorable	not scorable	1	0.6689139
####	Bn-A06-p24602719	A-genome specific (napus-rapa)	0.9736842	0.6859457	not scorable	not scorable	1	0.6887476
####	Bn-A06-p24607556	A-genome specific (napus-rapa)	0.9832536	0.7538398	not scorable	not scorable	0.9586777	0.7552096

####	Bn-A06-p24618096	genome unspecific (all in)	0.9760765	0.2591633	1	0.7966581	0.9917355	0.7994234
####	Bn-A06-p24678077	A-genome specific (napus-rapa)	0.9976076	0.4567027	not scorable	not scorable	0.9917355	0.7807196
####	Bn-A06-p24695860	A-genome specific (napus-rapa)	0.9736842	0.5209771	not scorable	not scorable	0.9834711	0.7853186
####	Bn-A06-p24705718	A-genome specific (napus-rapa)	0.9138756	0.6859515	not scorable	not scorable	0.6528926	0.7639838
####	Bn-A06-p24709822	A-genome specific (napus-rapa)	0.9832536	0.7480705	not scorable	not scorable	0.9917355	0.8594416
####	Bn-A06-p24713872	A-genome specific (napus-rapa)	0.9832536	0.7750278	not scorable	not scorable	0.9834711	0.7489637
####	Bn-A06-p24717767	A-genome specific (napus-rapa)	0.9617225	0.6845276	not scorable	not scorable	0.9917355	0.7134931
####	Bn-A06-p24726203	genome unspecific (all in)	0.9665072	0.3857822	1	0.7529412	1	0.8348742
####	Bn-A06-p24730935	A-genome specific (napus-rapa)	1	0.2653577	not scorable	not scorable	1	0.7743655
####	Bn-A06-p24744523	A-genome specific (napus-rapa)	0.9617225	0.6452928	not scorable	not scorable	1	0.864459
####	Bn-A06-p24746974	A-genome specific (napus-rapa)	0.9712918	0.7664129	not scorable	not scorable	0.9834711	0.7446961
####	Bn-A06-p24814906	A-genome specific (napus-rapa)	0.9856459	0.7167431	not scorable	not scorable	0.9917355	0.6860588
####	Bn-A06-p24822573	A-genome specific (rapa only)	not scorable	not scorable	not scorable	not scorable	0.9917355	0.7269214
####	Bn-A06-p24825884	A-genome specific (napus-rapa)	0.9688995	0.6836185	not scorable	not scorable	0.9834711	0.7297958
####	Bn-A06-p24827051	A-genome specific (napus-rapa)	0.9952153	0.4814776	not scorable	not scorable	1	0.8845848
####	Bn-A06-p24827783	A-genome specific (napus-rapa)	0.9688995	0.7907486	not scorable	not scorable	0.9834711	0.7466914
####	Bn-A06-p24831071	A-genome specific (napus-rapa)	0.9784689	0.7108649	not scorable	not scorable	0.9917355	0.7639838
####	Bn-A06-p24834963	A-genome specific (napus-rapa)	0.9497608	0.3862358	not scorable	not scorable	0.9917355	0.7012126
####	Bn-A06-p24836495	A-genome specific (napus-rapa)	0.9880383	0.2657792	not scorable	not scorable	0.9917355	0.711806
####	Bn-A06-p24849468	A-genome specific (napus-rapa)	0.9641148	0.4645701	not scorable	not scorable	0.9586777	0.7832031
####	Bn-A06-p24886436	A-genome specific (napus-rapa)	0.9665072	0.3117276	not scorable	not scorable	0.9917355	0.6566592
####	Bn-A06-p24890908	A-genome specific (napus-rapa)	0.9856459	0.2800292	not scorable	not scorable	1	0.7078133
####	Bn-A06-p24913140	A-genome specific (napus-rapa)	0.9880383	0.306381	not scorable	not scorable	0.9917355	0.7672659
####	Bn-A06-p24922072	A-genome specific (napus-rapa)	0.9784689	0.7435666	not scorable	not scorable	0.9917355	0.7087088
####	Bn-A06-p24929938	A-genome specific (napus-rapa)	1	0.7454508	not scorable	not scorable	1	0.7914145
####	Bn-A06-p24934604	A-genome specific (napus-rapa)	0.9928229	0.8169877	not scorable	not scorable	1	0.7292976
####	Bn-A06-p24939059	A-genome specific (napus-rapa)	0.9784689	0.2558593	not scorable	not scorable	1	0.6663646
####	Bn-A06-p24973824	A-genome specific (napus-rapa)	0.9904306	0.7362843	not scorable	not scorable	0.9752066	0.7337518
####	Bn-A06-p24976434	A-genome specific (napus-rapa)	0.854067	0.4508709	not scorable	not scorable	0.9917355	0.6025972
####	Bn-A06-p2514723	A-genome specific (napus-rapa)	1	0.4976095	not scorable	not scorable	0.9834711	0.8001024
####	Bn-A06-p2521454	A-genome specific (napus-rapa)	0.9904306	0.8022602	not scorable	not scorable	0.9917355	0.7088029
####	Bn-A06-p2521668	A-genome specific (napus-rapa)	0.9760765	0.5481434	not scorable	not scorable	0.9917355	0.6438779
####	Bn-A06-p2525037	A-genome specific (napus-rapa)	0.9784689	0.6386495	not scorable	not scorable	1	0.8550336

####	Bn-A06-p2526552	A-genome specific (napus-rapa)	0.9904306	0.7495234	not scorable	not scorable	1	0.687061
####	Bn-A06-p25329425	A-genome specific (napus-rapa)	0.9688995	0.6205255	not scorable	not scorable	1	0.6815433
####	Bn-A06-p25343454	A-genome specific (napus-rapa)	0.9880383	0.510487	not scorable	not scorable	0.9917355	0.5930505
####	Bn-A06-p25345586	A-genome specific (napus-rapa)	0.9904306	0.7550738	not scorable	not scorable	0.9834711	0.7642198
####	Bn-A06-p25349874	A-genome specific (napus-rapa)	0.9832536	0.5222439	not scorable	not scorable	1	0.7933608
####	Bn-A06-p25349932	A-genome specific (napus-rapa)	0.9904306	0.7673638	not scorable	not scorable	1	0.7240891
####	Bn-A06-p25350033	A-genome specific (napus-rapa)	0.9904306	0.7208919	not scorable	not scorable	1	0.744217
####	Bn-A06-p25351980	A-genome specific (napus-rapa)	0.9904306	0.380904	not scorable	not scorable	0.9917355	0.7104467
####	Bn-A06-p25369364	napus specific	0.9282297	0.7210104	not scorable	not scorable	not scorable	not scorable
####	Bn-A06-p25369668	A-genome specific (napus-rapa)	0.9162679	0.6763486	not scorable	not scorable	1	0.7601068
####	Bn-A06-p25369719	genome unspecific (oleracea-rapa)	not scorable	not scorable	0.9534883	0.5544293	1	0.6704551
####	Bn-A06-p25377321	A-genome specific (napus-rapa)	0.9880383	0.6151441	not scorable	not scorable	1	0.7501194
####	Bn-A06-p25409852	A-genome specific (napus-rapa)	0.9928229	0.8395917	not scorable	not scorable	1	0.8310137
####	Bn-A06-p2542722	A-genome specific (napus-rapa)	0.9425837	0.4989112	not scorable	not scorable	0.9752066	0.7490253
####	Bn-A06-p25471463	A-genome specific (napus-rapa)	0.9665072	0.6680034	not scorable	not scorable	1	0.9440146
####	Bn-A06-p2563025	A-genome specific (napus-rapa)	1	0.3093325	not scorable	not scorable	0.9752066	0.5248225
####	Bn-A06-p2570378	A-genome specific (napus-rapa)	1	0.8544095	not scorable	not scorable	0.9917355	0.8176654
####	Bn-A06-p25786733	A-genome specific (napus-rapa)	0.9928229	0.65985	not scorable	not scorable	0.9834711	0.8000291
####	Bn-A06-p2582613	A-genome specific (napus-rapa)	0.9880383	0.7736222	not scorable	not scorable	0.9917355	0.7283458
####	Bn-A06-p25840740	A-genome specific (napus-rapa)	0.9928229	0.6616498	not scorable	not scorable	0.9752066	0.6269112
####	Bn-A06-p2586825	A-genome specific (napus-rapa)	1	0.7256602	not scorable	not scorable	1	0.8493412
####	Bn-A06-p25878721	A-genome specific (napus-rapa)	0.9736842	0.7482065	not scorable	not scorable	1	0.7666197
####	Bn-A06-p2599060	A-genome specific (napus-rapa)	1	0.7305337	not scorable	not scorable	0.9917355	0.8108302
####	Bn-A06-p2599150	A-genome specific (napus-rapa)	0.9928229	0.3280707	not scorable	not scorable	0.9834711	0.7538703
####	Bn-A06-p26011034	A-genome specific (napus-rapa)	0.9928229	0.7848144	not scorable	not scorable	1	0.9205657
####	Bn-A06-p26023391	A-genome specific (napus-rapa)	0.9952153	0.3582511	not scorable	not scorable	1	0.8164408
####	Bn-A06-p26039465	A-genome specific (rapa only)	not scorable	not scorable	not scorable	not scorable	0.9917355	0.8211779
####	Bn-A06-p26043562	A-genome specific (rapa only)	not scorable	not scorable	not scorable	not scorable	0.9504132	0.9148563
####	Bn-A06-p26045429	A-genome specific (napus-rapa)	0.9186603	0.6999741	not scorable	not scorable	0.9669421	0.7690442
####	Bn-A06-p26058372	A-genome specific (napus-rapa)	0.9928229	0.7098094	not scorable	not scorable	0.9917355	0.8199377
####	Bn-A06-p26058643	A-genome specific (napus-rapa)	1	0.3594028	not scorable	not scorable	0.9834711	0.7087224
####	Bn-A06-p26066533	A-genome specific (napus-rapa)	0.9904306	0.8260399	not scorable	not scorable	1	0.7085158
####	Bn-A06-p26080915	A-genome specific (napus-rapa)	0.9976076	0.3068143	not scorable	not scorable	1	0.7655848

####	Bn-A06-p26081617	A-genome specific (napus-rapa)	0.9066986	0.5803629	not scorable	not scorable	0.9917355	0.7391866
####	Bn-A06-p26083498	A-genome specific (napus-rapa)	0.9736842	0.7286274	not scorable	not scorable	0.9669421	0.7217246
####	Bn-A06-p26096667	A-genome specific (napus-rapa)	0.9952153	0.4549983	not scorable	not scorable	1	0.793473
####	Bn-A06-p26096963	A-genome specific (napus-rapa)	0.9210526	0.5800685	not scorable	not scorable	1	0.8004208
####	Bn-A06-p26097037	A-genome specific (rapa only)	not scorable	not scorable	not scorable	not scorable	1	0.718655
####	Bn-A06-p26100486	A-genome specific (napus-rapa)	0.9832536	0.7337466	not scorable	not scorable	1	0.7263482
####	Bn-A06-p26100652	A-genome specific (napus-rapa)	0.9760765	0.7908077	not scorable	not scorable	0.9752066	0.6948265
####	Bn-A06-p26100738	A-genome specific (napus-rapa)	0.9784689	0.7998211	not scorable	not scorable	1	0.7971106
####	Bn-A06-p26100812	A-genome specific (napus-rapa)	0.9808612	0.7648637	not scorable	not scorable	0.9669421	0.7623633
####	Bn-A06-p26101019	A-genome specific (napus-rapa)	0.9665072	0.8398883	not scorable	not scorable	0.9834711	0.7398025
####	Bn-A06-p26101080	A-genome specific (napus-rapa)	0.9832536	0.7959415	not scorable	not scorable	1	0.755209
####	Bn-A06-p26101272	A-genome specific (napus-rapa)	0.9928229	0.7701377	not scorable	not scorable	1	0.8317302
####	Bn-A06-p26108669	A-genome specific (napus-rapa)	0.9880383	0.4125436	not scorable	not scorable	0.9834711	0.7857311
####	Bn-A06-p26113448	A-genome specific (napus-rapa)	0.9808612	0.4965005	not scorable	not scorable	0.9834711	0.726025
####	Bn-A06-p26125430	all not scorable	not scorable	not scorable	not scorable	not scorable	not scorable	not scorable
####	Bn-A06-p26135022	A-genome specific (napus-rapa)	0.9904306	0.7597941	not scorable	not scorable	0.9917355	0.765302
####	Bn-A06-p26135038	A-genome specific (napus-rapa)	0.9976076	0.6387275	not scorable	not scorable	1	0.7229707
####	Bn-A06-p26135283	A-genome specific (napus-rapa)	0.9976076	0.5679134	not scorable	not scorable	0.9917355	0.7516038
####	Bn-A06-p26135324	A-genome specific (napus-rapa)	0.9904306	0.7888578	not scorable	not scorable	0.9917355	0.7930112
####	Bn-A06-p26139717	A-genome specific (napus-rapa)	0.9928229	0.8989305	not scorable	not scorable	1	0.7387427
####	Bn-A06-p26144134	A-genome specific (napus-rapa)	0.9904306	0.2579812	not scorable	not scorable	1	0.8019215
####	Bn-A06-p26144417	A-genome specific (napus-rapa)	1	0.4028936	not scorable	not scorable	0.9917355	0.7406995
####	Bn-A06-p26144731	A-genome specific (napus-rapa)	1	0.5251905	not scorable	not scorable	1	0.7412815
####	Bn-A06-p26152237	A-genome specific (napus-rapa)	0.9976076	0.5874063	not scorable	not scorable	0.9834711	0.80871
####	Bn-A06-p26154413	A-genome specific (napus-rapa)	0.9928229	0.4960452	not scorable	not scorable	0.9917355	0.7505927
####	Bn-A06-p26155068	A-genome specific (napus-rapa)	1	0.3792681	not scorable	not scorable	0.9834711	0.8087606
####	Bn-A06-p26159702	A-genome specific (napus-rapa)	0.9928229	0.7461846	not scorable	not scorable	0.9917355	0.7253973
####	Bn-A06-p2617079	A-genome specific (napus-rapa)	0.9832536	0.4598214	not scorable	not scorable	1	0.7614174
####	Bn-A06-p26173206	A-genome specific (napus-rapa)	0.9928229	0.6977573	not scorable	not scorable	0.9917355	0.7015341
####	Bn-A06-p26186229	A-genome specific (napus-rapa)	1	0.4045323	not scorable	not scorable	0.9834711	0.6657921
####	Bn-A06-p2619089	A-genome specific (napus-rapa)	0.9808612	0.7997314	not scorable	not scorable	0.9917355	0.778774
####	Bn-A06-p26212942	A-genome specific (napus-rapa)	1	0.3093872	not scorable	not scorable	1	0.7720919
####	Bn-A06-p26213014	A-genome specific (napus-rapa)	0.9952153	0.6634726	not scorable	not scorable	0.9917355	0.8367406

####	Bn-A06-p26213205	A-genome specific (rapa only)	not scorable	not scorable	not scorable	not scorable	1	0.8854492
####	Bn-A06-p26213281	A-genome specific (napus-rapa)	1	0.8546153	not scorable	not scorable	1	0.8591195
####	Bn-A06-p26219274	A-genome specific (napus-rapa)	1	0.5504327	not scorable	not scorable	0.9669421	0.7221036
####	Bn-A06-p26219617	A-genome specific (napus-rapa)	1	0.2113188	not scorable	not scorable	0.9752066	0.6817759
####	Bn-A06-p26222718	A-genome specific (napus-rapa)	0.9928229	0.6971142	not scorable	not scorable	1	0.8416879
####	Bn-A06-p26248182	A-genome specific (napus-rapa)	0.9904306	0.3770535	not scorable	not scorable	1	0.6527258
####	Bn-A06-p2630468	A-genome specific (napus-rapa)	0.9569378	0.4105567	not scorable	not scorable	0.9752066	0.5139383
####	Bn-A06-p2633224	A-genome specific (napus-rapa)	0.9856459	0.6777666	not scorable	not scorable	0.9917355	0.6745058
####	Bn-A06-p2633764	A-genome specific (napus-rapa)	1	0.8219877	not scorable	not scorable	0.9917355	0.7344885
####	Bn-A06-p2643305	A-genome specific (napus-rapa)	0.9976076	0.7391319	not scorable	not scorable	0.9834711	0.8159941
####	Bn-A06-p2643564	A-genome specific (napus-rapa)	0.9976076	0.7878247	not scorable	not scorable	0.9917355	0.8604648
####	Bn-A06-p2646572	genome unspecific (all in)	0.9760765	0.4263291	0.9922481	0.7997813	0.9752066	0.7435926
####	Bn-A06-p2650442	A-genome specific (napus-rapa)	0.9952153	0.2991098	not scorable	not scorable	1	0.80042
####	Bn-A06-p2651708	A-genome specific (napus-rapa)	1	0.4664699	not scorable	not scorable	1	0.7686933
####	Bn-A06-p2676314	A-genome specific (napus-rapa)	0.9976076	0.7671963	not scorable	not scorable	1	0.8017633
####	Bn-A06-p2676798	A-genome specific (napus-rapa)	0.9880383	0.7244194	not scorable	not scorable	1	0.8423881
####	Bn-A06-p267765	A-genome specific (napus-rapa)	0.9736842	0.7295835	not scorable	not scorable	0.9586777	0.6883175
####	Bn-A06-p2682929	A-genome specific (napus-rapa)	0.9784689	0.8340902	not scorable	not scorable	0.9917355	0.7977284
####	Bn-A06-p2698067	A-genome specific (napus-rapa)	0.9904306	0.3971766	not scorable	not scorable	0.9917355	0.7439876
####	Bn-A06-p2709177	A-genome specific (napus-rapa)	0.9880383	0.8196452	not scorable	not scorable	0.9917355	0.7301987
####	Bn-A06-p2714610	A-genome specific (napus-rapa)	0.9952153	0.4159195	not scorable	not scorable	0.9752066	0.6235566
####	Bn-A06-p2717967	A-genome specific (napus-rapa)	0.9952153	0.7396959	not scorable	not scorable	0.9752066	0.6811808
####	Bn-A06-p2718852	napus specific	0.9928229	0.7476975	not scorable	not scorable	not scorable	not scorable
####	Bn-A06-p2729577	A-genome specific (napus-rapa)	0.9832536	0.7801434	not scorable	not scorable	1	0.9240721
####	Bn-A06-p2732731	napus specific	0.8708134	0.4792121	not scorable	not scorable	not scorable	not scorable
####	Bn-A06-p2743076	A-genome specific (napus-rapa)	1	0.7513835	not scorable	not scorable	1	0.7904987
####	Bn-A06-p2748311	A-genome specific (napus-rapa)	0.9856459	0.4707588	not scorable	not scorable	1	0.8538328
####	Bn-A06-p2756247	A-genome specific (napus-rapa)	0.9856459	0.8038998	not scorable	not scorable	1	0.7843317
####	Bn-A06-p2756792	A-genome specific (napus-rapa)	0.9952153	0.709574	not scorable	not scorable	1	0.824177
####	Bn-A06-p2758495	A-genome specific (napus-rapa)	0.9928229	0.7379979	not scorable	not scorable	0.9834711	0.7079761
####	Bn-A06-p275949	A-genome specific (napus-rapa)	0.9952153	0.7801319	not scorable	not scorable	0.9752066	0.795041
####	Bn-A06-p2778072	A-genome specific (napus-rapa)	1	0.7493146	not scorable	not scorable	0.9834711	0.7316881
####	Bn-A06-p2778829	A-genome specific (napus-rapa)	1	0.7201904	not scorable	not scorable	0.9917355	0.7147341

####	Bn-A06-p2778837	A-genome specific (napus-rapa)	1	0.7323093	not scorable	not scorable	0.9834711	0.6661494
####	Bn-A06-p2779243	A-genome specific (napus-rapa)	0.9401914	0.6891719	not scorable	not scorable	1	0.7272689
####	Bn-A06-p2788585	A-genome specific (napus-rapa)	0.9976076	0.4946675	not scorable	not scorable	0.9586777	0.5837711
####	Bn-A06-p279045	A-genome specific (napus-rapa)	0.9928229	0.768324	not scorable	not scorable	0.9752066	0.8154362
####	Bn-A06-p2793899	A-genome specific (rapa only)	not scorable	not scorable	not scorable	not scorable	0.9834711	0.7969111
####	Bn-A06-p2801792	A-genome specific (napus-rapa)	1	0.7973794	not scorable	not scorable	0.9834711	0.7773519
####	Bn-A06-p280351	A-genome specific (napus-rapa)	0.9712918	0.7863003	not scorable	not scorable	0.9669421	0.6761603
####	Bn-A06-p2807759	A-genome specific (napus-rapa)	0.9880383	0.3079911	not scorable	not scorable	0.9917355	0.7513038
####	Bn-A06-p281559	napus specific	0.9784689	0.7342743	not scorable	not scorable	not scorable	not scorable
####	Bn-A06-p281874	napus specific	0.9928229	0.7197951	not scorable	not scorable	not scorable	not scorable
####	Bn-A06-p2823066	A-genome specific (napus-rapa)	0.9832536	0.8049476	not scorable	not scorable	0.9752066	0.8157909
####	Bn-A06-p2824500	A-genome specific (napus-rapa)	0.9952153	0.3446837	not scorable	not scorable	0.9752066	0.6788507
####	Bn-A06-p2826315	A-genome specific (napus-rapa)	0.9832536	0.3900352	not scorable	not scorable	0.9917355	0.6984608
####	Bn-A06-p2826630	A-genome specific (napus-rapa)	0.9760765	0.7750993	not scorable	not scorable	0.9834711	0.8224351
####	Bn-A06-p282853	A-genome specific (napus-rapa)	0.9784689	0.8276066	not scorable	not scorable	0.9752066	0.8023524
####	Bn-A06-p283425	A-genome specific (napus-rapa)	0.9736842	0.7740304	not scorable	not scorable	0.9752066	0.7923967
####	Bn-A06-p283640	A-genome specific (napus-rapa)	0.9808612	0.8207017	not scorable	not scorable	0.9834711	0.798973
####	Bn-A06-p2842757	A-genome specific (napus-rapa)	1	0.906397	not scorable	not scorable	1	0.7478409
####	Bn-A06-p284408	A-genome specific (napus-rapa)	0.9808612	0.7189634	not scorable	not scorable	0.9669421	0.6664119
####	Bn-A06-p2851834	A-genome specific (napus-rapa)	0.9712918	0.3397568	not scorable	not scorable	1	0.7406615
####	Bn-A06-p285515	A-genome specific (napus-rapa)	0.9784689	0.5718048	not scorable	not scorable	0.9586777	0.5392188
####	Bn-A06-p2870413	A-genome specific (napus-rapa)	0.9976076	0.7675655	not scorable	not scorable	0.9834711	0.7513582
####	Bn-A06-p287053	A-genome specific (napus-rapa)	0.9976076	0.656612	not scorable	not scorable	0.9917355	0.6803098
####	Bn-A06-p2874216	A-genome specific (napus-rapa)	0.9952153	0.4939228	not scorable	not scorable	0.9752066	0.6945729
####	Bn-A06-p2883231	A-genome specific (napus-rapa)	1	0.5738116	not scorable	not scorable	1	0.7783666
####	Bn-A06-p2883297	A-genome specific (napus-rapa)	0.9952153	0.5738513	not scorable	not scorable	1	0.8072396
####	Bn-A06-p288377	A-genome specific (napus-rapa)	0.9976076	0.7877859	not scorable	not scorable	0.9834711	0.8098057
####	Bn-A06-p2890703	A-genome specific (napus-rapa)	0.9569378	0.7758219	not scorable	not scorable	1	0.7549031
####	Bn-A06-p2891175	A-genome specific (napus-rapa)	0.930622	0.3316183	not scorable	not scorable	0.9752066	0.7637617
####	Bn-A06-p2891290	A-genome specific (napus-rapa)	0.9760765	0.7907544	not scorable	not scorable	0.9752066	0.7541506
####	Bn-A06-p289876	A-genome specific (napus-rapa)	0.9928229	0.7316931	not scorable	not scorable	0.9834711	0.7330132
####	Bn-A06-p2901305	A-genome specific (napus-rapa)	0.9976076	0.8035473	not scorable	not scorable	1	0.8148115
####	Bn-A06-p2903017	A-genome specific (napus-rapa)	0.9808612	0.342283	not scorable	not scorable	0.9834711	0.736963

####	Bn-A06-p290355	A-genome specific (napus-rapa)	0.9904306	0.7652098	not scorable	not scorable	1	0.9000877
####	Bn-A06-p2917807	A-genome specific (napus-rapa)	1	0.7558554	not scorable	not scorable	0.9917355	0.7723379
####	Bn-A06-p291943	A-genome specific (rapa only)	not scorable	not scorable	not scorable	not scorable	0.9752066	0.9240721
####	Bn-A06-p2929852	A-genome specific (napus-rapa)	0.9928229	0.484681	not scorable	not scorable	0.9917355	0.8510869
####	Bn-A06-p2930156	A-genome specific (napus-rapa)	1	0.7862228	not scorable	not scorable	0.9917355	0.6847303
####	Bn-A06-p2936438	A-genome specific (napus-rapa)	0.9976076	0.8259058	not scorable	not scorable	1	0.7069986
####	Bn-A06-p2938450	A-genome specific (napus-rapa)	0.9952153	0.5574459	not scorable	not scorable	1	0.665437
####	Bn-A06-p2938890	A-genome specific (napus-rapa)	1	0.4474623	not scorable	not scorable	0.9917355	0.5986149
####	Bn-A06-p2940743	A-genome specific (napus-rapa)	0.9928229	0.8692706	not scorable	not scorable	0.9669421	0.8513851
####	Bn-A06-p2949562	A-genome specific (napus-rapa)	1	0.7113797	not scorable	not scorable	0.9834711	0.778075
####	Bn-A06-p2949781	A-genome specific (napus-rapa)	0.9904306	0.7333906	not scorable	not scorable	0.9917355	0.7172619
####	Bn-A06-p2949858	A-genome specific (napus-rapa)	0.9904306	0.7633107	not scorable	not scorable	0.9834711	0.7848645
####	Bn-A06-p2954752	A-genome specific (napus-rapa)	1	0.7452237	not scorable	not scorable	0.9917355	0.8338599
####	Bn-A06-p2956891	A-genome specific (napus-rapa)	0.9760765	0.7527828	not scorable	not scorable	1	0.790238
####	Bn-A06-p2958095	A-genome specific (napus-rapa)	0.9856459	0.6854145	not scorable	not scorable	0.9917355	0.7545387
####	Bn-A06-p2966556	A-genome specific (napus-rapa)	0.9976076	0.7583834	not scorable	not scorable	0.9917355	0.8008767
####	Bn-A06-p2973074	A-genome specific (napus-rapa)	1	0.7459583	not scorable	not scorable	0.9834711	0.794817
####	Bn-A06-p2981395	A-genome specific (napus-rapa)	0.9808612	0.3988191	not scorable	not scorable	1	0.831035
####	Bn-A06-p2984184	C-genome specific (napus-oleracea)	0.9354067	0.7395762	0.6434109	0.7932946	not scorable	not scorable
####	Bn-A06-p2987202	A-genome specific (napus-rapa)	0.9760765	0.3288996	not scorable	not scorable	0.9917355	0.8349713
####	Bn-A06-p2993800	A-genome specific (napus-rapa)	0.9952153	0.4602275	not scorable	not scorable	1	0.769322
####	Bn-A06-p2995114	A-genome specific (napus-rapa)	0.9904306	0.7910628	not scorable	not scorable	0.9834711	0.7829219
####	Bn-A06-p3005047	A-genome specific (napus-rapa)	0.9880383	0.7917076	not scorable	not scorable	1	0.7100134
####	Bn-A06-p3005981	A-genome specific (napus-rapa)	0.9856459	0.8065036	not scorable	not scorable	0.9752066	0.6874833
####	Bn-A06-p3009854	A-genome specific (napus-rapa)	0.9928229	0.8143823	not scorable	not scorable	0.9834711	0.703272
####	Bn-A06-p3010221	A-genome specific (napus-rapa)	1	0.7319198	not scorable	not scorable	1	0.8135551
####	Bn-A06-p3012876	A-genome specific (napus-rapa)	0.9712918	0.7590306	not scorable	not scorable	0.9834711	0.6615953
####	Bn-A06-p3013547	A-genome specific (napus-rapa)	0.9952153	0.8121059	not scorable	not scorable	0.9752066	0.7888305
####	Bn-A06-p301366	A-genome specific (napus-rapa)	0.8803828	0.782281	not scorable	not scorable	0.9504132	0.8149335
####	Bn-A06-p3016186	A-genome specific (napus-rapa)	0.9928229	0.4099914	not scorable	not scorable	0.9917355	0.5507392
####	Bn-A06-p3016356	A-genome specific (napus-rapa)	0.9952153	0.7692254	not scorable	not scorable	1	0.8018759
####	Bn-A06-p3031327	A-genome specific (napus-rapa)	0.9976076	0.3836949	not scorable	not scorable	1	0.6577333
####	Bn-A06-p3049884	A-genome specific (napus-rapa)	1	0.3315896	not scorable	not scorable	0.9586777	0.6171182

####	Bn-A06-p3050205	A-genome specific (napus-rapa)	0.9880383	0.4294781	not scorable	not scorable	0.9834711	0.6586065
####	Bn-A06-p3050747	A-genome specific (napus-rapa)	0.9856459	0.7795768	not scorable	not scorable	0.9752066	0.7536905
####	Bn-A06-p3051257	A-genome specific (napus-rapa)	0.9976076	0.7466363	not scorable	not scorable	0.9917355	0.6989515
####	Bn-A06-p3051799	A-genome specific (napus-rapa)	1	0.7037053	not scorable	not scorable	1	0.5768476
####	Bn-A06-p3051928	A-genome specific (napus-rapa)	0.9952153	0.6313193	not scorable	not scorable	0.9752066	0.6814733
####	Bn-A06-p3065773	all not scorable	not scorable	not scorable	not scorable	not scorable	not scorable	not scorable
####	Bn-A06-p3066038	A-genome specific (napus-rapa)	0.9952153	0.7079026	not scorable	not scorable	0.9834711	0.6623591
####	Bn-A06-p3066372	A-genome specific (napus-rapa)	1	0.652509	not scorable	not scorable	0.9834711	0.8452606
####	Bn-A06-p3072724	A-genome specific (napus-rapa)	0.9880383	0.4416096	not scorable	not scorable	0.9917355	0.7365789
####	Bn-A06-p307436	A-genome specific (napus-rapa)	0.9856459	0.7164564	not scorable	not scorable	0.9917355	0.7097896
####	Bn-A06-p3088460	A-genome specific (napus-rapa)	0.9976076	0.2651271	not scorable	not scorable	0.9917355	0.7920059
####	Bn-A06-p3090078	A-genome specific (napus-rapa)	0.9401914	0.4144637	not scorable	not scorable	0.9752066	0.7156762
####	Bn-A06-p3093741	A-genome specific (napus-rapa)	0.9808612	0.818186	not scorable	not scorable	0.9752066	0.8298243
####	Bn-A06-p3096813	A-genome specific (napus-rapa)	0.9952153	0.7457037	not scorable	not scorable	0.9834711	0.8010721
####	Bn-A06-p3105589	A-genome specific (napus-rapa)	1	0.8185498	not scorable	not scorable	0.9834711	0.7883303
####	Bn-A06-p3114818	A-genome specific (napus-rapa)	0.9952153	0.7581507	not scorable	not scorable	1	0.9167013
####	Bn-A06-p3115038	A-genome specific (napus-rapa)	1	0.7262398	not scorable	not scorable	1	0.7135793
####	Bn-A06-p3118627	A-genome specific (napus-rapa)	0.9904306	0.7378588	not scorable	not scorable	0.9917355	0.8054758
####	Bn-A06-p3125473	A-genome specific (napus-rapa)	0.9808612	0.2748263	not scorable	not scorable	0.9834711	0.6985419
####	Bn-A06-p3132058	A-genome specific (napus-rapa)	0.9928229	0.8254164	not scorable	not scorable	1	0.6545114
####	Bn-A06-p3132860	A-genome specific (napus-rapa)	1	0.7885466	not scorable	not scorable	0.9917355	0.6686315
####	Bn-A06-p313319	A-genome specific (napus-rapa)	0.9880383	0.8273999	not scorable	not scorable	1	0.8079896
####	Bn-A06-p313835	A-genome specific (napus-rapa)	0.9760765	0.5284073	not scorable	not scorable	0.9917355	0.6830483
####	Bn-A06-p3146367	A-genome specific (napus-rapa)	0.9976076	0.754553	not scorable	not scorable	0.9834711	0.687094
####	Bn-A06-p3146720	A-genome specific (napus-rapa)	0.9832536	0.7451017	not scorable	not scorable	1	0.6875853
####	Bn-A06-p3167411	A-genome specific (napus-rapa)	0.9641148	0.7144848	not scorable	not scorable	0.9586777	0.7266851
####	Bn-A06-p3177392	A-genome specific (rapa only)	not scorable	not scorable	not scorable	not scorable	0.9421487	0.8840718
####	Bn-A06-p3177472	A-genome specific (napus-rapa)	0.9832536	0.7909437	not scorable	not scorable	1	0.7848222
####	Bn-A06-p3177570	A-genome specific (napus-rapa)	1	0.7857878	not scorable	not scorable	1	0.6843576
####	Bn-A06-p318331	A-genome specific (napus-rapa)	0.9569378	0.7647647	not scorable	not scorable	0.9834711	0.7724217
####	Bn-A06-p3184395	A-genome specific (napus-rapa)	0.9928229	0.8162605	not scorable	not scorable	0.6694215	0.8242667
####	Bn-A06-p3188819	A-genome specific (napus-rapa)	0.9856459	0.7951933	not scorable	not scorable	0.9834711	0.7849894
####	Bn-A06-p3189490	A-genome specific (napus-rapa)	0.9808612	0.362575	not scorable	not scorable	0.9752066	0.6472974

####	Bn-A06-p3202098	A-genome specific (napus-rapa)	0.9856459	0.7787027	not scorable	not scorable	0.9917355	0.8130777
####	Bn-A06-p3202615	A-genome specific (napus-rapa)	0.9665072	0.8001187	not scorable	not scorable	0.9834711	0.7483226
####	Bn-A06-p3206017	A-genome specific (napus-rapa)	1	0.4045249	not scorable	not scorable	0.9669421	0.7563871
####	Bn-A06-p3207623	A-genome specific (napus-rapa)	0.9904306	0.7489994	not scorable	not scorable	1	0.7921959
####	Bn-A06-p3210791	A-genome specific (napus-rapa)	0.9832536	0.5105319	not scorable	not scorable	0.9834711	0.7414125
####	Bn-A06-p3226693	A-genome specific (napus-rapa)	0.9808612	0.3313046	not scorable	not scorable	0.9917355	0.569905
####	Bn-A06-p3227752	A-genome specific (napus-rapa)	0.9784689	0.7716161	not scorable	not scorable	0.9834711	0.8028246
####	Bn-A06-p3230963	A-genome specific (rapa only)	not scorable	not scorable	not scorable	not scorable	0.6280992	0.750198
####	Bn-A06-p3239087	A-genome specific (napus-rapa)	0.9688995	0.7803575	not scorable	not scorable	0.5454546	0.7683636
####	Bn-A06-p3255819	A-genome specific (napus-rapa)	1	0.7917964	not scorable	not scorable	0.9917355	0.7324688
####	Bn-A06-p3256782	A-genome specific (napus-rapa)	0.9976076	0.6814371	not scorable	not scorable	0.9917355	0.7604615
####	Bn-A06-p3257163	A-genome specific (napus-rapa)	0.9952153	0.6921464	not scorable	not scorable	0.9669421	0.6786869
####	Bn-A06-p3269320	A-genome specific (napus-rapa)	1	0.767672	not scorable	not scorable	0.9669421	0.738236
####	Bn-A06-p3278682	A-genome specific (napus-rapa)	0.9976076	0.236427	not scorable	not scorable	0.9669421	0.6958703
####	Bn-A06-p3287018	A-genome specific (napus-rapa)	0.2799043	0.7809828	not scorable	not scorable	0.5867769	0.8664527
####	Bn-A06-p3291936	A-genome specific (napus-rapa)	0.9952153	0.8012258	not scorable	not scorable	0.9917355	0.8321635
####	Bn-A06-p329217	A-genome specific (napus-rapa)	0.854067	0.8471783	not scorable	not scorable	0.7768595	0.804925
####	Bn-A06-p3295431	A-genome specific (napus-rapa)	0.8253589	0.8056777	not scorable	not scorable	0.6280992	0.7766134
####	Bn-A06-p3302657	napus specific	0.6483254	0.6622101	not scorable	not scorable	not scorable	not scorable
####	Bn-A06-p330826	A-genome specific (rapa only)	not scorable	not scorable	not scorable	not scorable	0.9917355	0.6509212
####	Bn-A06-p3308398	A-genome specific (napus-rapa)	0.9784689	0.7618369	not scorable	not scorable	0.9834711	0.8192011
####	Bn-A06-p3316040	A-genome specific (napus-rapa)	0.6435407	0.7964104	not scorable	not scorable	0.9586777	0.8642471
####	Bn-A06-p3316207	A-genome specific (napus-rapa)	0.6172249	0.8259059	not scorable	not scorable	0.9504132	0.8083546
####	Bn-A06-p3318611	A-genome specific (napus-rapa)	0.9569378	0.3075124	not scorable	not scorable	0.9834711	0.5873021
####	Bn-A06-p3325502	A-genome specific (napus-rapa)	0.9808612	0.4534445	not scorable	not scorable	0.9917355	0.8392493
####	Bn-A06-p3325566	A-genome specific (napus-rapa)	0.9784689	0.365392	not scorable	not scorable	1	0.8142724
####	Bn-A06-p3329211	genome unspecific (all in)	1	0.4123001	0.9922481	0.8130574	0.9917355	0.7504174
####	Bn-A06-p3339435	A-genome specific (napus-rapa)	0.7894737	0.7842003	not scorable	not scorable	0.5867769	0.7863934
####	Bn-A06-p3353405	A-genome specific (napus-rapa)	0.9880383	0.5777375	not scorable	not scorable	0.9669421	0.6478892
####	Bn-A06-p3354754	A-genome specific (napus-rapa)	1	0.5356272	not scorable	not scorable	0.9917355	0.6374834
####	Bn-A06-p3361377	A-genome specific (napus-rapa)	1	0.7199516	not scorable	not scorable	0.9917355	0.7898132
####	Bn-A06-p3363188	A-genome specific (napus-rapa)	0.9976076	0.7909075	not scorable	not scorable	0.9917355	0.8109992
####	Bn-A06-p3370349	napus specific	1	0.7875881	not scorable	not scorable	not scorable	not scorable

####	Bn-A06-p337583	A-genome specific (napus-rapa)	0.9928229	0.5137511	not scorable	not scorable	0.9834711	0.8084265
####	Bn-A06-p3380495	A-genome specific (napus-rapa)	1	0.4767966	not scorable	not scorable	1	0.7926856
####	Bn-A06-p3399869	A-genome specific (napus-rapa)	1	0.7901269	not scorable	not scorable	1	0.8305473
####	Bn-A06-p3399973	A-genome specific (napus-rapa)	1	0.4330519	not scorable	not scorable	1	0.7271869
####	Bn-A06-p3405779	A-genome specific (napus-rapa)	0.9904306	0.7569005	not scorable	not scorable	0.9917355	0.7494027
####	Bn-A06-p3406041	napus specific	0.9114832	0.7724089	not scorable	not scorable	not scorable	not scorable
####	Bn-A06-p3414128	A-genome specific (napus-rapa)	0.9760765	0.786456	not scorable	not scorable	1	0.8815182
####	Bn-A06-p3416803	A-genome specific (napus-rapa)	0.9760765	0.7997735	not scorable	not scorable	1	0.9175546
####	Bn-A06-p3428214	A-genome specific (napus-rapa)	0.9497608	0.6506487	not scorable	not scorable	1	0.9166579
####	Bn-A06-p3434498	A-genome specific (napus-rapa)	1	0.7593729	not scorable	not scorable	0.9834711	0.7928383
####	Bn-A06-p3435985	A-genome specific (napus-rapa)	1	0.7950717	not scorable	not scorable	0.9917355	0.8089182
####	Bn-A06-p3437409	A-genome specific (napus-rapa)	1	0.7910791	not scorable	not scorable	1	0.7959436
####	Bn-A06-p3442742	A-genome specific (napus-rapa)	0.9976076	0.6638182	not scorable	not scorable	0.9917355	0.7746989
####	Bn-A06-p3444868	A-genome specific (napus-rapa)	1	0.7430891	not scorable	not scorable	1	0.7752895
####	Bn-A06-p3446956	A-genome specific (napus-rapa)	0.9976076	0.8196024	not scorable	not scorable	0.9834711	0.8043666
####	Bn-A06-p3447648	A-genome specific (napus-rapa)	1	0.3446561	not scorable	not scorable	0.9752066	0.7043564
####	Bn-A06-p3452904	A-genome specific (napus-rapa)	0.9952153	0.5932927	not scorable	not scorable	1	0.8554157
####	Bn-A06-p3468374	A-genome specific (napus-rapa)	0.9976076	0.330204	not scorable	not scorable	0.9917355	0.7680559
####	Bn-A06-p3483249	A-genome specific (napus-rapa)	0.9904306	0.4560266	not scorable	not scorable	1	0.6645095
####	Bn-A06-p3483382	A-genome specific (napus-rapa)	0.9952153	0.6318588	not scorable	not scorable	1	0.7433437
####	Bn-A06-p3491241	A-genome specific (napus-rapa)	1	0.5570792	not scorable	not scorable	0.9917355	0.7972407
####	Bn-A06-p3492295	A-genome specific (napus-rapa)	0.9976076	0.8249155	not scorable	not scorable	1	0.7901596
####	Bn-A06-p3496759	A-genome specific (napus-rapa)	0.9832536	0.4046704	not scorable	not scorable	0.9752066	0.8110504
####	Bn-A06-p3528559	A-genome specific (napus-rapa)	0.9928229	0.805824	not scorable	not scorable	0.9669421	0.7906119
####	Bn-A06-p3528628	A-genome specific (napus-rapa)	0.9928229	0.8011081	not scorable	not scorable	0.9917355	0.8001914
####	Bn-A06-p3532353	A-genome specific (napus-rapa)	0.9976076	0.612304	not scorable	not scorable	0.9834711	0.7486777
####	Bn-A06-p3561531	A-genome specific (napus-rapa)	0.9736842	0.3531477	not scorable	not scorable	0.9917355	0.8107828
####	Bn-A06-p3567180	A-genome specific (napus-rapa)	0.9736842	0.3782267	not scorable	not scorable	1	0.8402112
####	Bn-A06-p3584489	A-genome specific (napus-rapa)	0.9928229	0.7946991	not scorable	not scorable	0.9917355	0.7869681
####	Bn-A06-p3586728	A-genome specific (napus-rapa)	0.9952153	0.4734154	not scorable	not scorable	0.9917355	0.8257994
####	Bn-A06-p3587032	A-genome specific (napus-rapa)	0.9688995	0.5210065	not scorable	not scorable	0.9917355	0.8151843
####	Bn-A06-p3611806	A-genome specific (napus-rapa)	0.9688995	0.3628463	not scorable	not scorable	0.9917355	0.7920029
####	Bn-A06-p3612397	A-genome specific (napus-rapa)	0.9952153	0.5554092	not scorable	not scorable	1	0.7446254

####	Bn-A06-p362402	A-genome specific (napus-rapa)	0.8995215	0.7225267	not scorable	not scorable	0.9256198	0.6647897
####	Bn-A06-p3625328	A-genome specific (napus-rapa)	1	0.5151601	not scorable	not scorable	0.9917355	0.6486297
####	Bn-A06-p3628159	A-genome specific (napus-rapa)	0.9856459	0.3633574	not scorable	not scorable	0.9834711	0.7781549
####	Bn-A06-p364061	A-genome specific (napus-rapa)	0.9808612	0.3734849	not scorable	not scorable	0.9752066	0.6935074
####	Bn-A06-p3651074	A-genome specific (napus-rapa)	0.9856459	0.208482	not scorable	not scorable	1	0.8759552
####	Bn-A06-p3651473	A-genome specific (napus-rapa)	1	0.4211016	not scorable	not scorable	0.9752066	0.644226
####	Bn-A06-p3660997	all not scorable	not scorable	not scorable	not scorable	not scorable	not scorable	not scorable
####	Bn-A06-p3661134	A-genome specific (rapa only)	not scorable	not scorable	not scorable	not scorable	0.9834711	0.7224717
####	Bn-A06-p3661342	A-genome specific (napus-rapa)	0.9904306	0.5007264	not scorable	not scorable	1	0.7139118
####	Bn-A06-p3667300	A-genome specific (napus-rapa)	0.9186603	0.7192096	not scorable	not scorable	0.9834711	0.8566722
####	Bn-A06-p3677526	all not scorable	not scorable	not scorable	not scorable	not scorable	not scorable	not scorable
####	Bn-A06-p3691343	A-genome specific (napus-rapa)	0.9760765	0.5634008	not scorable	not scorable	0.9834711	0.6454048
####	Bn-A06-p369746	A-genome specific (napus-rapa)	0.9808612	0.7961401	not scorable	not scorable	0.9834711	0.6452215
####	Bn-A06-p3703307	A-genome specific (napus-rapa)	0.9928229	0.8039243	not scorable	not scorable	1	0.7846264
####	Bn-A06-p370376	A-genome specific (napus-rapa)	0.9880383	0.8162041	not scorable	not scorable	0.9752066	0.7103748
####	Bn-A06-p3708256	napus specific	0.9760765	0.3182925	not scorable	not scorable	not scorable	not scorable
####	Bn-A06-p3712646	A-genome specific (napus-rapa)	0.9928229	0.7763527	not scorable	not scorable	0.9834711	0.8438915
####	Bn-A06-p3723340	A-genome specific (napus-rapa)	0.9856459	0.4624	not scorable	not scorable	0.9834711	0.7717051
####	Bn-A06-p3731147	A-genome specific (napus-rapa)	1	0.4929586	not scorable	not scorable	0.9752066	0.6718804
####	Bn-A06-p3759020	A-genome specific (rapa only)	not scorable	not scorable	not scorable	not scorable	0.9834711	0.7475555
####	Bn-A06-p3805018	A-genome specific (napus-rapa)	0.9760765	0.6074849	not scorable	not scorable	0.9834711	0.6971861
####	Bn-A06-p3809199	A-genome specific (napus-rapa)	0.9928229	0.3584149	not scorable	not scorable	0.9669421	0.7658163
####	Bn-A06-p3818421	A-genome specific (napus-rapa)	0.9688995	0.5978594	not scorable	not scorable	0.9834711	0.7911736
####	Bn-A06-p3818484	A-genome specific (napus-rapa)	0.9856459	0.7941719	not scorable	not scorable	0.9752066	0.7634313
####	Bn-A06-p3825492	A-genome specific (napus-rapa)	0.9880383	0.3300152	not scorable	not scorable	0.9834711	0.7109948
####	Bn-A06-p3834324	A-genome specific (napus-rapa)	0.9928229	0.7175583	not scorable	not scorable	0.9586777	0.6053187
####	Bn-A06-p3837553	A-genome specific (napus-rapa)	0.9952153	0.7421918	not scorable	not scorable	0.9917355	0.7627959
####	Bn-A06-p3839293	A-genome specific (napus-rapa)	1	0.7392813	not scorable	not scorable	1	0.7817926
####	Bn-A06-p3867353	A-genome specific (napus-rapa)	0.9952153	0.8319956	not scorable	not scorable	0.9917355	0.7829617
####	Bn-A06-p3871984	A-genome specific (napus-rapa)	1	0.2822185	not scorable	not scorable	0.9917355	0.6697611
####	Bn-A06-p3900404	A-genome specific (napus-rapa)	0.9665072	0.7540386	not scorable	not scorable	1	0.689375
####	Bn-A06-p3913280	A-genome specific (napus-rapa)	1	0.4300812	not scorable	not scorable	0.9752066	0.7790034
####	Bn-A06-p3928714	napus specific	1	0.6880783	not scorable	not scorable	not scorable	not scorable

####	Bn-A06-p393328	A-genome specific (napus-rapa)	0.9952153	0.4704306	not scorable	not scorable	0.9669421	0.739553
####	Bn-A06-p3934890	A-genome specific (napus-rapa)	1	0.364909	not scorable	not scorable	0.9917355	0.7999551
####	Bn-A06-p3953661	A-genome specific (napus-rapa)	0.9784689	0.453999	not scorable	not scorable	0.9752066	0.6063034
####	Bn-A06-p3970972	A-genome specific (napus-rapa)	0.9760765	0.7247944	not scorable	not scorable	0.9752066	0.7942811
####	Bn-A06-p3976759	A-genome specific (napus-rapa)	0.9760765	0.3466649	not scorable	not scorable	0.9917355	0.619302
####	Bn-A06-p3986320	A-genome specific (napus-rapa)	0.9712918	0.4777187	not scorable	not scorable	1	0.8550683
####	Bn-A06-p3987067	A-genome specific (napus-rapa)	0.9832536	0.7481972	not scorable	not scorable	1	0.8682142
####	Bn-A06-p3988352	A-genome specific (napus-rapa)	1	0.7234792	not scorable	not scorable	0.9917355	0.6059493
####	Bn-A06-p3992289	A-genome specific (napus-rapa)	0.9401914	0.7020786	not scorable	not scorable	0.6528926	0.9175813
####	Bn-A06-p3997307	A-genome specific (napus-rapa)	0.9976076	0.7959026	not scorable	not scorable	0.9834711	0.7512732
####	Bn-A06-p3998593	A-genome specific (napus-rapa)	0.9880383	0.7518017	not scorable	not scorable	0.9834711	0.8202201
####	Bn-A06-p3999986	A-genome specific (napus-rapa)	0.9593301	0.525153	not scorable	not scorable	1	0.6080307
####	Bn-A06-p4001378	A-genome specific (napus-rapa)	0.9952153	0.4284897	not scorable	not scorable	1	0.6729115
####	Bn-A06-p4013010	A-genome specific (napus-rapa)	0.9904306	0.7668226	not scorable	not scorable	0.9917355	0.7997132
####	Bn-A06-p4016422	A-genome specific (napus-rapa)	0.9952153	0.7554193	not scorable	not scorable	0.9917355	0.7938406
####	Bn-A06-p4017460	A-genome specific (napus-rapa)	0.9641148	0.3343937	not scorable	not scorable	0.9834711	0.7864506
####	Bn-A06-p4019827	A-genome specific (napus-rapa)	0.9712918	0.320877	not scorable	not scorable	0.9917355	0.8135881
####	Bn-A06-p4021930	A-genome specific (napus-rapa)	0.9856459	0.8020997	not scorable	not scorable	0.9917355	0.7156382
####	Bn-A06-p4025613	A-genome specific (napus-rapa)	0.9856459	0.7910369	not scorable	not scorable	0.9834711	0.7602957
####	Bn-A06-p4026453	A-genome specific (napus-rapa)	0.9760765	0.7606373	not scorable	not scorable	0.9917355	0.7810404
####	Bn-A06-p4027051	A-genome specific (napus-rapa)	0.9593301	0.6036029	not scorable	not scorable	0.9669421	0.6795421
####	Bn-A06-p4039206	A-genome specific (napus-rapa)	0.9665072	0.3037911	not scorable	not scorable	0.9752066	0.6381266
####	Bn-A06-p4040976	A-genome specific (napus-rapa)	0.9784689	0.2679523	not scorable	not scorable	1	0.7535797
####	Bn-A06-p4056446	A-genome specific (napus-rapa)	0.9832536	0.778047	not scorable	not scorable	0.9917355	0.8372037
####	Bn-A06-p4060202	A-genome specific (napus-rapa)	0.9760765	0.4180308	not scorable	not scorable	0.9834711	0.6559128
####	Bn-A06-p4060310	A-genome specific (napus-rapa)	0.9784689	0.6316126	not scorable	not scorable	0.9834711	0.782944
####	Bn-A06-p4072818	A-genome specific (napus-rapa)	0.9880383	0.7808565	not scorable	not scorable	0.9669421	0.6943995
####	Bn-A06-p4098532	A-genome specific (napus-rapa)	0.9760765	0.6398569	not scorable	not scorable	1	0.7612875
####	Bn-A06-p4098800	A-genome specific (napus-rapa)	0.9904306	0.6194757	not scorable	not scorable	1	0.7282501
####	Bn-A06-p410058	A-genome specific (napus-rapa)	0.9904306	0.263812	not scorable	not scorable	0.5950413	0.7616834
####	Bn-A06-p4179318	A-genome specific (napus-rapa)	0.9880383	0.7737371	not scorable	not scorable	0.9917355	0.7894714
####	Bn-A06-p4195181	A-genome specific (napus-rapa)	1	0.8993629	not scorable	not scorable	0.9917355	0.9094582
####	Bn-A06-p4196008	A-genome specific (napus-rapa)	0.9808612	0.7532066	not scorable	not scorable	1	0.8918202

####	Bn-A06-p4211266	A-genome specific (napus-rapa)	0.9688995	0.678075	not scorable	not scorable	1	0.7003915
####	Bn-A06-p4218801	A-genome specific (napus-rapa)	0.8397129	0.7446243	not scorable	not scorable	0.9917355	0.7552251
####	Bn-A06-p4229919	A-genome specific (napus-rapa)	0.9904306	0.4308419	not scorable	not scorable	1	0.7749302
####	Bn-A06-p428400	A-genome specific (napus-rapa)	0.9736842	0.7619545	not scorable	not scorable	1	0.8912938
####	Bn-A06-p4315934	napus specific	0.6602871	0.6819515	not scorable	not scorable	not scorable	not scorable
####	Bn-A06-p4316905	A-genome specific (napus-rapa)	0.9856459	0.8143935	not scorable	not scorable	1	0.7507112
####	Bn-A06-p4317300	A-genome specific (napus-rapa)	1	0.7278983	not scorable	not scorable	0.9752066	0.7086738
####	Bn-A06-p4331202	A-genome specific (napus-rapa)	0.9856459	0.7995563	not scorable	not scorable	0.9917355	0.7252168
####	Bn-A06-p4332149	A-genome specific (napus-rapa)	0.9760765	0.4390059	not scorable	not scorable	0.9834711	0.8016818
####	Bn-A06-p4332223	A-genome specific (napus-rapa)	0.9856459	0.7832235	not scorable	not scorable	1	0.6778251
####	Bn-A06-p4333047	A-genome specific (napus-rapa)	0.8995215	0.7921216	not scorable	not scorable	0.5702479	0.6769026
####	Bn-A06-p4333144	A-genome specific (napus-rapa)	0.784689	0.7612076	not scorable	not scorable	0.9752066	0.840328
####	Bn-A06-p4333227	A-genome specific (napus-rapa)	0.9856459	0.7591707	not scorable	not scorable	0.9834711	0.7567682
####	Bn-A06-p4337931	A-genome specific (napus-rapa)	0.9784689	0.7216286	not scorable	not scorable	0.9917355	0.7515649
####	Bn-A06-p4340434	A-genome specific (napus-rapa)	0.9043062	0.7452542	not scorable	not scorable	0.553719	0.6765379
####	Bn-A06-p4340819	A-genome specific (napus-rapa)	0.9019139	0.7606668	not scorable	not scorable	0.6198347	0.6843472
####	Bn-A06-p4361865	A-genome specific (napus-rapa)	0.9880383	0.7789082	not scorable	not scorable	0.9834711	0.8416675
####	Bn-A06-p4362245	A-genome specific (napus-rapa)	0.9976076	0.3097715	not scorable	not scorable	1	0.6568727
####	Bn-A06-p4362386	A-genome specific (napus-rapa)	1	0.2916716	not scorable	not scorable	0.9834711	0.5582227
####	Bn-A06-p4362700	A-genome specific (napus-rapa)	0.9928229	0.7061266	not scorable	not scorable	1	0.734123
####	Bn-A06-p4365836	A-genome specific (napus-rapa)	0.9210526	0.3047065	not scorable	not scorable	0.9917355	0.712837
####	Bn-A06-p4365949	A-genome specific (napus-rapa)	0.9760765	0.2506799	not scorable	not scorable	0.9586777	0.5808154
####	Bn-A06-p4371627	A-genome specific (napus-rapa)	0.9808612	0.6922006	not scorable	not scorable	0.9834711	0.7800862
####	Bn-A06-p4409355	A-genome specific (napus-rapa)	0.9880383	0.4178815	not scorable	not scorable	1	0.7985421
####	Bn-A06-p4410177	A-genome specific (napus-rapa)	0.9736842	0.5992252	not scorable	not scorable	0.9917355	0.6104478
####	Bn-A06-p4418231	A-genome specific (napus-rapa)	0.9880383	0.707617	not scorable	not scorable	0.9917355	0.6525763
####	Bn-A06-p4419571	A-genome specific (napus-rapa)	0.9784689	0.5592513	not scorable	not scorable	0.9586777	0.759639
####	Bn-A06-p4420263	A-genome specific (napus-rapa)	0.9928229	0.7793623	not scorable	not scorable	1	0.6406884
####	Bn-A06-p4421214	A-genome specific (napus-rapa)	0.9832536	0.4924951	not scorable	not scorable	0.9752066	0.7904896
####	Bn-A06-p4428703	A-genome specific (napus-rapa)	0.9712918	0.7682348	not scorable	not scorable	0.9917355	0.704819
####	Bn-A06-p4435926	A-genome specific (napus-rapa)	0.9641148	0.3334932	not scorable	not scorable	0.9834711	0.7094157
####	Bn-A06-p4436168	A-genome specific (napus-rapa)	0.9952153	0.7524182	not scorable	not scorable	1	0.7004576
####	Bn-A06-p4437120	A-genome specific (napus-rapa)	0.9904306	0.7750767	not scorable	not scorable	1	0.7569137

####	Bn-A06-p4452778	A-genome specific (napus-rapa)	0.9760765	0.4778323	not scorable	not scorable	0.9834711	0.5756618
####	Bn-A06-p4461241	A-genome specific (napus-rapa)	0.9808612	0.7889364	not scorable	not scorable	1	0.8423122
####	Bn-A06-p4466057	all not scorable	not scorable	not scorable	not scorable	not scorable	not scorable	not scorable
####	Bn-A06-p4508774	A-genome specific (napus-rapa)	0.9856459	0.4707777	not scorable	not scorable	1	0.9154155
####	Bn-A06-p4509926	A-genome specific (rapa only)	not scorable	not scorable	not scorable	not scorable	0.9752066	0.9062178
####	Bn-A06-p4517457	A-genome specific (napus-rapa)	0.9665072	0.7768134	not scorable	not scorable	0.9917355	0.745486
####	Bn-A06-p4558209	A-genome specific (napus-rapa)	0.9928229	0.4368514	not scorable	not scorable	0.9669421	0.7709749
####	Bn-A06-p4597746	A-genome specific (napus-rapa)	0.9928229	0.7794246	not scorable	not scorable	1	0.84488
####	Bn-A06-p4619013	A-genome specific (napus-rapa)	0.9760765	0.4090758	not scorable	not scorable	0.9917355	0.6874133
####	Bn-A06-p4623401	A-genome specific (napus-rapa)	0.9880383	0.7607247	not scorable	not scorable	0.9834711	0.7822542
####	Bn-A06-p4703366	A-genome specific (napus-rapa)	0.9736842	0.6889068	not scorable	not scorable	0.9917355	0.6578664
####	Bn-A06-p4729500	A-genome specific (napus-rapa)	0.9569378	0.5523856	not scorable	not scorable	0.9917355	0.8349156
####	Bn-A06-p4731996	A-genome specific (napus-rapa)	0.7870814	0.7575244	not scorable	not scorable	0.9834711	0.7636389
####	Bn-A06-p4837796	A-genome specific (rapa only)	not scorable	not scorable	not scorable	not scorable	0.9917355	0.7333669
####	Bn-A06-p4838544	A-genome specific (napus-rapa)	0.9880383	0.7420257	not scorable	not scorable	0.9752066	0.7592574
####	Bn-A06-p4844167	A-genome specific (napus-rapa)	0.9736842	0.8158969	not scorable	not scorable	1	0.8208792
####	Bn-A06-p4874556	A-genome specific (napus-rapa)	0.9856459	0.7972259	not scorable	not scorable	0.9752066	0.782245
####	Bn-A06-p4881785	A-genome specific (napus-rapa)	1	0.6423515	not scorable	not scorable	1	0.6912151
####	Bn-A06-p4896084	A-genome specific (napus-rapa)	0.9521531	0.8121677	not scorable	not scorable	0.6033058	0.8943655
####	Bn-A06-p4901091	A-genome specific (napus-rapa)	0.9186603	0.775032	not scorable	not scorable	0.9752066	0.7186431
####	Bn-A06-p4901457	A-genome specific (napus-rapa)	0.8947368	0.8157951	not scorable	not scorable	0.9504132	0.6494765
####	Bn-A06-p4927344	A-genome specific (napus-rapa)	0.9760765	0.3249969	not scorable	not scorable	0.9834711	0.7254934
####	Bn-A06-p49287	napus specific	0.9904306	0.7124816	not scorable	not scorable	not scorable	not scorable
####	Bn-A06-p4929018	A-genome specific (napus-rapa)	0.9401914	0.4423695	not scorable	not scorable	0.9752066	0.7905645
####	Bn-A06-p4933932	A-genome specific (napus-rapa)	0.9880383	0.7564929	not scorable	not scorable	1	0.8662829
####	Bn-A06-p49438	napus specific	0.9736842	0.5542111	not scorable	not scorable	not scorable	not scorable
####	Bn-A06-p4976377	A-genome specific (napus-rapa)	0.8779904	0.6549259	not scorable	not scorable	0.9917355	0.6751723
####	Bn-A06-p5020498	A-genome specific (napus-rapa)	0.9952153	0.7895814	not scorable	not scorable	1	0.8145315
####	Bn-A06-p5020605	A-genome specific (napus-rapa)	0.9856459	0.311483	not scorable	not scorable	0.9669421	0.7203286
####	Bn-A06-p5020675	A-genome specific (napus-rapa)	0.9880383	0.7445997	not scorable	not scorable	1	0.8481613
####	Bn-A06-p50386	napus specific	0.9832536	0.7701079	not scorable	not scorable	not scorable	not scorable
####	Bn-A06-p5040441	A-genome specific (napus-rapa)	0.9784689	0.7895907	not scorable	not scorable	1	0.648029
####	Bn-A06-p5047871	A-genome specific (napus-rapa)	0.9928229	0.3980387	not scorable	not scorable	1	0.7209747

####	Bn-A06-p5053748	A-genome specific (napus-rapa)	0.9928229	0.7813157	not scorable	not scorable	0.9917355	0.7890258
####	Bn-A06-p506366	A-genome specific (napus-rapa)	0.8062201	0.8817921	not scorable	not scorable	1	0.7788738
####	Bn-A06-p5077968	A-genome specific (rapa only)	not scorable	not scorable	not scorable	not scorable	0.9504132	0.6804008
####	Bn-A06-p5091252	A-genome specific (napus-rapa)	0.9641148	0.3566382	not scorable	not scorable	1	0.765574
####	Bn-A06-p5094790	A-genome specific (napus-rapa)	0.9904306	0.7779871	not scorable	not scorable	0.9834711	0.8385081
####	Bn-A06-p5103824	A-genome specific (napus-rapa)	0.9688995	0.7114758	not scorable	not scorable	1	0.6910558
####	Bn-A06-p5114238	A-genome specific (napus-rapa)	0.9784689	0.3608175	not scorable	not scorable	0.9752066	0.5602247
####	Bn-A06-p5114770	A-genome specific (napus-rapa)	0.9043062	0.6766579	not scorable	not scorable	0.9752066	0.7133502
####	Bn-A06-p5130670	A-genome specific (napus-rapa)	0.9569378	0.5069199	not scorable	not scorable	0.9917355	0.7651305
####	Bn-A06-p5147575	A-genome specific (napus-rapa)	0.9832536	0.4830559	not scorable	not scorable	0.9917355	0.7809055
####	Bn-A06-p5151254	A-genome specific (napus-rapa)	0.9928229	0.7783281	not scorable	not scorable	1	0.779168
####	Bn-A06-p5151337	A-genome specific (napus-rapa)	0.9856459	0.5545317	not scorable	not scorable	0.9917355	0.7159153
####	Bn-A06-p5179267	A-genome specific (napus-rapa)	0.9832536	0.3432046	not scorable	not scorable	0.9917355	0.7846431
####	Bn-A06-p5182164	A-genome specific (napus-rapa)	0.9760765	0.7623614	not scorable	not scorable	0.9917355	0.6214764
####	Bn-A06-p5182253	A-genome specific (napus-rapa)	0.9784689	0.7504745	not scorable	not scorable	1	0.7374942
####	Bn-A06-p5182315	A-genome specific (napus-rapa)	0.9736842	0.7928563	not scorable	not scorable	0.9917355	0.7174947
####	Bn-A06-p5183425	A-genome specific (napus-rapa)	0.9928229	0.747763	not scorable	not scorable	0.9917355	0.8007044
####	Bn-A06-p5184830	A-genome specific (napus-rapa)	0.9952153	0.7561083	not scorable	not scorable	0.9834711	0.7445864
####	Bn-A06-p5187939	A-genome specific (napus-rapa)	0.9856459	0.7719108	not scorable	not scorable	0.9917355	0.8113057
####	Bn-A06-p5188207	A-genome specific (napus-rapa)	0.9856459	0.787071	not scorable	not scorable	0.9917355	0.7889054
####	Bn-A06-p5188627	A-genome specific (napus-rapa)	0.9665072	0.574296	not scorable	not scorable	0.9834711	0.5456266
####	Bn-A06-p5190285	A-genome specific (rapa only)	not scorable	not scorable	not scorable	not scorable	1	0.6298696
####	Bn-A06-p5190418	A-genome specific (rapa only)	not scorable	not scorable	not scorable	not scorable	0.9752066	0.6886467
####	Bn-A06-p5190514	A-genome specific (rapa only)	not scorable	not scorable	not scorable	not scorable	0.9917355	0.7054226
####	Bn-A06-p5192434	A-genome specific (napus-rapa)	0.9832536	0.7936383	not scorable	not scorable	0.9834711	0.774188
####	Bn-A06-p5197572	A-genome specific (napus-rapa)	0.9784689	0.7627431	not scorable	not scorable	0.9917355	0.7869879
####	Bn-A06-p5197898	A-genome specific (napus-rapa)	0.9760765	0.7780508	not scorable	not scorable	1	0.7453712
####	Bn-A06-p5201102	A-genome specific (napus-rapa)	0.9928229	0.3471432	not scorable	not scorable	0.9917355	0.769951
####	Bn-A06-p5217195	A-genome specific (napus-rapa)	0.9712918	0.5308471	not scorable	not scorable	1	0.794974
####	Bn-A06-p5226816	A-genome specific (napus-rapa)	0.9688995	0.6275808	not scorable	not scorable	0.9917355	0.6855088
####	Bn-A06-p5227178	A-genome specific (napus-rapa)	0.9784689	0.7358917	not scorable	not scorable	0.9834711	0.7170555
####	Bn-A06-p5227365	A-genome specific (napus-rapa)	0.9856459	0.7240264	not scorable	not scorable	0.9917355	0.7200636
####	Bn-A06-p5232462	A-genome specific (napus-rapa)	0.9808612	0.6758243	not scorable	not scorable	0.9917355	0.7172196

####	Bn-A06-p5232488	A-genome specific (napus-rapa)	0.9856459	0.6570908	not scorable	not scorable	0.9834711	0.7058906
####	Bn-A06-p5232937	A-genome specific (napus-rapa)	0.9832536	0.6746269	not scorable	not scorable	0.9834711	0.7310367
####	Bn-A06-p5233115	A-genome specific (napus-rapa)	0.9856459	0.7300066	not scorable	not scorable	0.9669421	0.7219447
####	Bn-A06-p5233226	A-genome specific (napus-rapa)	0.9928229	0.7496697	not scorable	not scorable	0.9917355	0.7555073
####	Bn-A06-p5235813	A-genome specific (napus-rapa)	0.9856459	0.7586971	not scorable	not scorable	0.9834711	0.7884734
####	Bn-A06-p5235889	A-genome specific (napus-rapa)	0.9856459	0.7773178	not scorable	not scorable	0.9834711	0.7868083
####	Bn-A06-p5236016	A-genome specific (napus-rapa)	0.9808612	0.7860653	not scorable	not scorable	1	0.878257
####	Bn-A06-p5236102	A-genome specific (napus-rapa)	0.9904306	0.7437516	not scorable	not scorable	0.9834711	0.7160677
####	Bn-A06-p5236283	A-genome specific (napus-rapa)	0.9880383	0.7541274	not scorable	not scorable	0.9917355	0.7534823
####	Bn-A06-p5236645	A-genome specific (napus-rapa)	0.9808612	0.5422934	not scorable	not scorable	0.9917355	0.8282238
####	Bn-A06-p5246637	A-genome specific (rapa only)	not scorable	not scorable	not scorable	not scorable	0.9917355	0.8192415
####	Bn-A06-p5260549	A-genome specific (napus-rapa)	0.9808612	0.3537495	not scorable	not scorable	1	0.7878213
####	Bn-A06-p5274160	A-genome specific (napus-rapa)	0.9832536	0.6610661	not scorable	not scorable	1	0.5574672
####	Bn-A06-p5275416	A-genome specific (rapa only)	not scorable	not scorable	not scorable	not scorable	0.553719	0.8046426
####	Bn-A06-p5275803	A-genome specific (napus-rapa)	0.9760765	0.7638571	not scorable	not scorable	0.9917355	0.8168549
####	Bn-A06-p5276150	A-genome specific (napus-rapa)	0.9808612	0.7408746	not scorable	not scorable	0.9834711	0.7891298
####	Bn-A06-p5276251	A-genome specific (napus-rapa)	0.9760765	0.7040401	not scorable	not scorable	0.9917355	0.7083914
####	Bn-A06-p5276641	A-genome specific (napus-rapa)	0.9856459	0.7339445	not scorable	not scorable	0.9917355	0.6986303
####	Bn-A06-p5276713	A-genome specific (napus-rapa)	0.9808612	0.626475	not scorable	not scorable	0.9669421	0.5579931
####	Bn-A06-p5276751	A-genome specific (napus-rapa)	0.9904306	0.7108434	not scorable	not scorable	0.9834711	0.7229997
####	Bn-A06-p5277548	A-genome specific (napus-rapa)	0.9904306	0.7823472	not scorable	not scorable	1	0.7481564
####	Bn-A06-p5278752	A-genome specific (napus-rapa)	0.9880383	0.7793317	not scorable	not scorable	0.9834711	0.7266651
####	Bn-A06-p5279490	A-genome specific (napus-rapa)	0.9856459	0.7156757	not scorable	not scorable	0.9917355	0.7201172
####	Bn-A06-p5280041	A-genome specific (napus-rapa)	0.9712918	0.7185276	not scorable	not scorable	0.9834711	0.6893438
####	Bn-A06-p5280225	A-genome specific (napus-rapa)	0.9712918	0.6073774	not scorable	not scorable	0.9917355	0.5690406
####	Bn-A06-p5284260	A-genome specific (napus-rapa)	0.9832536	0.6713755	not scorable	not scorable	0.9834711	0.8318305
####	Bn-A06-p5285976	A-genome specific (napus-rapa)	0.9760765	0.6840172	not scorable	not scorable	0.9917355	0.7153134
####	Bn-A06-p5299890	A-genome specific (napus-rapa)	0.9688995	0.2442499	not scorable	not scorable	0.6033058	0.7246003
####	Bn-A06-p5300943	A-genome specific (rapa only)	not scorable	not scorable	not scorable	not scorable	0.9917355	0.7549197
####	Bn-A06-p5301714	A-genome specific (napus-rapa)	0.9760765	0.7852103	not scorable	not scorable	0.9586777	0.7835553
####	Bn-A06-p5305727	A-genome specific (napus-rapa)	0.9760765	0.782874	not scorable	not scorable	1	0.8101183
####	Bn-A06-p5305781	A-genome specific (napus-rapa)	0.9928229	0.8006167	not scorable	not scorable	1	0.7903211
####	Bn-A06-p5306445	A-genome specific (napus-rapa)	0.9784689	0.677368	not scorable	not scorable	0.9834711	0.7116496

####	Bn-A06-p5310499	A-genome specific (napus-rapa)	0.9928229	0.8139647	not scorable	not scorable	0.9669421	0.7933717
####	Bn-A06-p5310521	A-genome specific (napus-rapa)	0.9856459	0.788574	not scorable	not scorable	0.9586777	0.7577538
####	Bn-A06-p5310579	A-genome specific (napus-rapa)	0.9832536	0.7929791	not scorable	not scorable	0.9669421	0.7238618
####	Bn-A06-p5311852	A-genome specific (napus-rapa)	0.9688995	0.6744235	not scorable	not scorable	0.9669421	0.7328676
####	Bn-A06-p5314603	A-genome specific (napus-rapa)	0.9760765	0.7834277	not scorable	not scorable	1	0.7330021
####	Bn-A06-p5315085	A-genome specific (napus-rapa)	0.9856459	0.7773328	not scorable	not scorable	1	0.7633339
####	Bn-A06-p5315140	A-genome specific (napus-rapa)	0.9760765	0.665778	not scorable	not scorable	0.9669421	0.6804513
####	Bn-A06-p5320572	A-genome specific (napus-rapa)	0.9832536	0.6714153	not scorable	not scorable	0.9917355	0.6533853
####	Bn-A06-p5321651	A-genome specific (napus-rapa)	0.9952153	0.3630171	not scorable	not scorable	0.9917355	0.6815509
####	Bn-A06-p5325547	A-genome specific (napus-rapa)	0.9712918	0.7630296	not scorable	not scorable	0.9834711	0.7480104
####	Bn-A06-p5331686	all not scorable	not scorable	not scorable	not scorable	not scorable	not scorable	not scorable
####	Bn-A06-p5333246	A-genome specific (napus-rapa)	0.9856459	0.6714153	not scorable	not scorable	0.9834711	0.7045974
####	Bn-A06-p5333348	A-genome specific (napus-rapa)	0.9760765	0.5519631	not scorable	not scorable	0.9752066	0.6561409
####	Bn-A06-p5335605	A-genome specific (napus-rapa)	0.9665072	0.6698004	not scorable	not scorable	0.9752066	0.7513966
####	Bn-A06-p5339433	A-genome specific (napus-rapa)	0.9832536	0.708905	not scorable	not scorable	1	0.6875546
####	Bn-A06-p5340584	A-genome specific (napus-rapa)	0.9808612	0.3071604	not scorable	not scorable	0.9834711	0.7097926
####	Bn-A06-p5344950	A-genome specific (napus-rapa)	0.9952153	0.5155717	not scorable	not scorable	0.9917355	0.6320549
####	Bn-A06-p5345401	A-genome specific (napus-rapa)	0.9904306	0.7396421	not scorable	not scorable	1	0.7677622
####	Bn-A06-p5346654	A-genome specific (napus-rapa)	0.9760765	0.4618848	not scorable	not scorable	0.9834711	0.7989571
####	Bn-A06-p5346849	A-genome specific (napus-rapa)	0.9736842	0.7323399	not scorable	not scorable	0.9917355	0.8092807
####	Bn-A06-p53517	napus specific	0.9856459	0.7369973	not scorable	not scorable	not scorable	not scorable
####	Bn-A06-p5372653	A-genome specific (napus-rapa)	0.9665072	0.8078989	not scorable	not scorable	0.9504132	0.7801714
####	Bn-A06-p5384725	A-genome specific (napus-rapa)	0.9808612	0.6426238	not scorable	not scorable	0.9917355	0.8063737
####	Bn-A06-p5386579	A-genome specific (napus-rapa)	0.9784689	0.8138018	not scorable	not scorable	0.9669421	0.7601424
####	Bn-A06-p5389758	A-genome specific (napus-rapa)	0.9641148	0.3694029	not scorable	not scorable	0.9917355	0.804377
####	Bn-A06-p5390277	A-genome specific (napus-rapa)	0.9808612	0.7250764	not scorable	not scorable	0.9504132	0.7124087
####	Bn-A06-p5393231	A-genome specific (napus-rapa)	0.9665072	0.565684	not scorable	not scorable	0.9752066	0.7812384
####	Bn-A06-p5427889	A-genome specific (napus-rapa)	0.9736842	0.7983542	not scorable	not scorable	0.9752066	0.7577651
####	Bn-A06-p543411	A-genome specific (napus-rapa)	0.9186603	0.7609383	not scorable	not scorable	0.9421487	0.6502959
####	Bn-A06-p5436800	A-genome specific (napus-rapa)	0.9784689	0.5815776	not scorable	not scorable	0.9834711	0.7561668
####	Bn-A06-p5438131	A-genome specific (napus-rapa)	0.9928229	0.7044265	not scorable	not scorable	1	0.7671019
####	Bn-A06-p5441991	A-genome specific (napus-rapa)	0.9856459	0.3541784	not scorable	not scorable	1	0.8748588
####	Bn-A06-p54687	napus specific	0.9712918	0.7010231	not scorable	not scorable	not scorable	not scorable

####	Bn-A06-p55225	napus specific	0.9736842	0.7352313	not scorable	not scorable	not scorable	not scorable
####	Bn-A06-p5535537	A-genome specific (napus-rapa)	0.9928229	0.7865396	not scorable	not scorable	1	0.7668408
####	Bn-A06-p5540327	A-genome specific (napus-rapa)	0.9880383	0.7661971	not scorable	not scorable	0.9917355	0.7044402
####	Bn-A06-p5546411	A-genome specific (napus-rapa)	0.291866	0.7376167	not scorable	not scorable	0.9504132	0.7007855
####	Bn-A06-p556859	A-genome specific (napus-rapa)	0.9186603	0.7177663	not scorable	not scorable	0.9834711	0.7856927
####	Bn-A06-p557358	A-genome specific (napus-rapa)	1	0.4402171	not scorable	not scorable	0.9834711	0.7043457
####	Bn-A06-p557754	A-genome specific (napus-rapa)	1	0.6982906	not scorable	not scorable	0.9834711	0.7111467
####	Bn-A06-p5577934	A-genome specific (napus-rapa)	0.9688995	0.4146676	not scorable	not scorable	1	0.704372
####	Bn-A06-p558792	A-genome specific (napus-rapa)	1	0.7892064	not scorable	not scorable	0.9834711	0.7860712
####	Bn-A06-p558853	A-genome specific (napus-rapa)	1	0.7934239	not scorable	not scorable	0.9917355	0.7884638
####	Bn-A06-p559060	A-genome specific (napus-rapa)	1	0.7321733	not scorable	not scorable	0.9752066	0.7922378
####	Bn-A06-p5593050	A-genome specific (napus-rapa)	1	0.7761238	not scorable	not scorable	0.9917355	0.7441496
####	Bn-A06-p559417	A-genome specific (napus-rapa)	0.9976076	0.6988571	not scorable	not scorable	0.9917355	0.768494
####	Bn-A06-p5600636	A-genome specific (napus-rapa)	0.9952153	0.7576929	not scorable	not scorable	0.9752066	0.7096564
####	Bn-A06-p5604858	A-genome specific (napus-rapa)	0.9952153	0.7562709	not scorable	not scorable	0.9752066	0.6840566
####	Bn-A06-p5616164	A-genome specific (napus-rapa)	0.9952153	0.7712396	not scorable	not scorable	0.9669421	0.7376125
####	Bn-A06-p5618302	A-genome specific (napus-rapa)	0.9808612	0.762643	not scorable	not scorable	0.9917355	0.6929061
####	Bn-A06-p5641863	A-genome specific (napus-rapa)	0.9976076	0.8732565	not scorable	not scorable	1	0.7851261
####	Bn-A06-p5669960	A-genome specific (napus-rapa)	0.9952153	0.7930985	not scorable	not scorable	0.9834711	0.6860991
####	Bn-A06-p568101	A-genome specific (napus-rapa)	1	0.7925012	not scorable	not scorable	1	0.7669629
####	Bn-A06-p5690066	A-genome specific (napus-rapa)	0.9712918	0.4606819	not scorable	not scorable	0.9917355	0.592959
####	Bn-A06-p5693735	A-genome specific (napus-rapa)	0.9856459	0.4815084	not scorable	not scorable	0.9917355	0.5109566
####	Bn-A06-p569582	A-genome specific (napus-rapa)	0.9976076	0.4660817	not scorable	not scorable	0.9256198	0.723671
####	Bn-A06-p5739682	A-genome specific (napus-rapa)	0.9928229	0.7576851	not scorable	not scorable	0.9834711	0.8192005
####	Bn-A06-p5741557	A-genome specific (napus-rapa)	0.9736842	0.7559947	not scorable	not scorable	1	0.7336461
####	Bn-A06-p5747694	A-genome specific (napus-rapa)	0.9904306	0.3743708	not scorable	not scorable	0.9834711	0.5009422
####	Bn-A06-p5758576	A-genome specific (napus-rapa)	0.9736842	0.5737105	not scorable	not scorable	0.9917355	0.822475
####	Bn-A06-p5761019	A-genome specific (rapa only)	not scorable	not scorable	not scorable	not scorable	0.9917355	0.6177276
####	Bn-A06-p5761097	A-genome specific (rapa only)	not scorable	not scorable	not scorable	not scorable	0.9917355	0.7502389
####	Bn-A06-p5762475	A-genome specific (rapa only)	not scorable	not scorable	not scorable	not scorable	0.9834711	0.8860479
####	Bn-A06-p5762802	A-genome specific (rapa only)	not scorable	not scorable	not scorable	not scorable	0.9834711	0.7025824
####	Bn-A06-p5767097	A-genome specific (napus-rapa)	0.9736842	0.5174576	not scorable	not scorable	0.6446281	0.8004208
####	Bn-A06-p576740	A-genome specific (napus-rapa)	0.9952153	0.5699016	not scorable	not scorable	0.9834711	0.6324359

####	Bn-A06-p5780982	A-genome specific (napus-rapa)	0.9617225	0.406638	not scorable	not scorable	1	0.7210048
####	Bn-A06-p5785209	genome unspecific (all in)	0.9952153	0.4223553	0.9844961	0.7470106	1	0.9296917
####	Bn-A06-p5824546	genome unspecific (all in)	0.9856459	0.4737935	0.9767442	0.7955338	1	0.744592
[truncated: 525,236 more chars]
