# Supplementary material for: A high-density SNP genotyping array for Brassica napus and its ancestral diploid species based on optimised selection of single-locus markers in the allotetraploid genome
Source: Theor Appl Genet. 2016 Jun 30;129(10):1887–99. doi: 10.1007/s00122-016-2746-7 (PMC5025514; doi:10.1007/s00122-016-2746-7)
Supplement: Supplementary file 4 — Physical position of each SNP locus in the Brassica napus genome based on BLAT analyses. SNP ID in B. napus genome is based on chromosome (or scaffold) name followed by base-pair position in the associated pseudo-molecule or scaffold. (PDF 6437 kb) [file 122_2016_2746_MOESM4_ESM.pdf]

Table 3: Physical Position of each SNP locus in the Brassica napus genome based on BLAT analyses. SNP ID in B. napus genome is based on chromosome (or scaffold) name followed by base pair position in the assoicated pseudomolecule or scaffold.

Illumina SNP_ID	DH12075v4 Sequence Identity	Darmor Sequence Identity	DH12075v4_ID	Darmor_ID
Bn-A01-p5512	0.750830565	0.757475083	Bn-Scaffold01043-p1	Bn-chrAnn_random-p188581
Bn-A01-p6239	0.990033223	0.720930233	Bn-Scaffold01043-p1	Bn-chrAnn_random-p188588
Bn-A01-p11933	0.975124378	0.945273632	Bn-Scaffold01043-p3	Bn-chrAnn_random-p188650
Bn-A01-p71129	0.996677741	0.986710963	Bn-N1-p68429	Bn-chrA01-p2439241
Bn-A01-p80385	0.991735537	0.983471074	Bn-N1-p77986	Bn-chrA01-p2449284
Bn-A01-p88608	0.909090909	0.768595041	Bn-N1-p87954	Bn-chrA05-p3397211
Bn-A01-p89956	0.991735537	0.933884298	Bn-N1-p88996	Bn-chrA05-p3398205
Bn-A01-p95718	0.983471074	0.991735537	Bn-N1-p101308	Bn-chrA01-p2465573
Bn-A01-p97663	1	0.94214876	Bn-N1-p103253	Bn-chrA01-p2467261
Bn-A01-p97751	0.975206612	0.983471074	Bn-N1-p103341	Bn-chrA01-p2467349
Bn-A01-p100441	1	0.991735537	Bn-N1-p106019	Bn-chrA01-p2468965
Bn-A01-p100693	0.975206612	0.966942149	Bn-N1-p106271	Bn-chrA01-p2469216
Bn-A01-p101021	0.980099502	0.970149254	Bn-N1-p106599	Bn-chrA01-p2469547
Bn-A01-p101094	0.983471074	0.991735537	Bn-N1-p106672	Bn-chrA01-p2469620
Bn-A01-p101451	0.983471074	0.991735537	Bn-N1-p107029	Bn-chrA01-p2469977
Bn-A01-p101879	0.991735537	0.983471074	Bn-N1-p107457	Bn-chrA01-p2470407
Bn-A01-p101943	0.975206612	0.983471074	Bn-N1-p107521	Bn-chrA01-p2470471
Bn-A01-p102158	0.991735537	0.983471074	Bn-N1-p107736	Bn-chrA01-p2470694
Bn-A01-p102187	0.983471074	0.975206612	Bn-N1-p107765	Bn-chrA01-p2470723
Bn-A01-p102249	0.983471074	0.991735537	Bn-N1-p107827	Bn-chrA01-p2470785
Bn-A01-p108136	0.986710963	0.973421927	Bn-N1-p118146	Bn-chrA01-p2489554
Bn-A01-p108225	0.983471074	0.975206612	Bn-N1-p118235	Bn-chrA01-p2489643
Bn-A01-p120225	0.990033223	0.990033223	Bn-N1-p131604	Bn-chrA01-p2501673
Bn-A01-p137814	0.958677686	0.966942149	Bn-N1-p148713	Bn-chrA01-p2518687
Bn-A01-p137869	0.966942149	0.710743802	Bn-N1-p148768	Bn-chrA01-p2518742
Bn-A01-p138731	0.990033223	0.986710963	Bn-N1-p149646	Bn-chrA01-p2519586
Bn-A01-p145676	0.975206612	0.950413223	Bn-N1-p156399	Bn-chrA01-p2527606
Bn-A01-p146963	0.983471074	0.991735537	Bn-N11-p1381796	Bn-chrA01-p2528886
Bn-A01-p147360	0.966942149	0.975206612	Bn-N1-p158083	Bn-chrA01-p2529282
Bn-A01-p147990	0.983471074	0.991735537	Bn-N1-p158713	Bn-chrA01-p2529914
Bn-A01-p150028	0.980066445	0.993355482	Bn-N1-p160751	Bn-chrA01-p2535566
Bn-A01-p159728	0.950413223	0.669421488	Bn-N1-p168480	Bn-chrA01-p2543129
Bn-A01-p161237	0.958677686	0.966942149	Bn-N1-p169827	Bn-chrA01-p2544476
Bn-A01-p176052	0.983471074	0.975206612	Bn-N1-p187213	Bn-chrA01-p2560144
Bn-A01-p178709	0.993355482	0.993355482	Bn-N1-p189666	Bn-chrA01-p2562774
Bn-A01-p195253	0.990033223	0.990033223	Bn-N1-p208162	Bn-chrA01-p2579347
Bn-A01-p206610	0.991735537	1	Bn-N1-p219257	Bn-chrA01-p2590705
Bn-A01-p207098	0.996677741	0.996677741	Bn-N1-p219745	Bn-chrA01-p2591193
Bn-A01-p207363	0.996677741	1	Bn-N1-p220010	Bn-chrA01-p2591458

Bn-A01-p220693	0.991735537	0.991735537	Bn-N1-p233449	Bn-chrA01-p2604767
Bn-A01-p224469	1	1	Bn-N1-p235934	Bn-chrA01-p2607252
Bn-A01-p234898	1	1	Bn-N1-p240997	Bn-chrC02-p8029946
Bn-A01-p235115	0.958677686	0.958677686	Bn-N1-p241218	Bn-chrC02-p8030167
Bn-A01-p235484	0.958677686	0.958677686	Bn-N1-p241562	Bn-chrA01-p2619078
Bn-A01-p235781	0.975206612	0.975206612	Bn-N1-p241855	Bn-chrA01-p2619371
Bn-A01-p250759	0.991735537	0.991735537	Bn-N1-p251681	Bn-chrA01-p2628362
Bn-A01-p276866	0.991735537	0.991735537	Bn-N1-p276123	Bn-chrA01-p2652468
Bn-A01-p285120	0.993355482	0.993355482	Bn-N1-p284373	Bn-chrA01-p2660716
Bn-A01-p289703	0.993355482	0.993355482	Bn-N1-p288958	Bn-chrA01-p2665301
Bn-A01-p309524	0.991735537	0.991735537	Bn-N1-p308842	Bn-chrA01-p2683477
Bn-A01-p314983	0.991735537	0.991735537	Bn-N1-p313165	Bn-chrA01-p2687800
Bn-A01-p324562	0.94214876	0.94214876	Bn-N1-p325704	Bn-chrA01-p2700421
Bn-A01-p333856	0.990033223	0.990033223	Bn-N1-p335464	Bn-chrA01_random-p83941
Bn-A01-p340954	1	1	Bn-N1-p338592	Bn-chrA01_random-p80813
Bn-A01-p344884	0.990033223	0.983388704	Bn-N1-p342591	Bn-chrC01-p300071
Bn-A01-p345182	0.991735537	0.991735537	Bn-N1-p342887	Bn-chrA01_random-p79093
Bn-A01-p436141	0.975124378	0.975124378	Bn-N1-p435708	Bn-chrA01-p29929
Bn-A01-p442492	0.991735537	0.991735537	Bn-N1-p442085	Bn-chrA01-p36516
Bn-A01-p460072	0.966942149	0.966942149	Bn-N1-p455627	Bn-chrA01-p50056
Bn-A01-p465783	0.94214876	0.94214876	Bn-N1-p461295	Bn-chrA01-p55724
Bn-A01-p467816	1	1	Bn-N1-p463330	Bn-chrA01-p57759
Bn-A01-p483495	1	1	Bn-N1-p478984	Bn-chrA01-p73410
Bn-A01-p511682	0.989583333	0.989583333	Bn-N1-p499736	Bn-chrA01-p93900
Bn-A01-p515600	0.985074627	0.985074627	Bn-N1-p504157	Bn-chrA01-p98316
Bn-A01-p595492	0.818181818	0.917355372	Bn-N1-p595343	Bn-chrA01-p175727
Bn-A01-p595651	1	1	Bn-N1-p595502	Bn-chrA01-p175886
Bn-A01-p597363	0.990033223	0.990033223	Bn-N1-p601625	Bn-chrA01-p180114
Bn-A01-p598242	0.950248756	0.950248756	Bn-N1-p602504	Bn-chrA01-p180993
Bn-A01-p599910	0.991735537	0.991735537	Bn-N1-p604190	Bn-chrA01-p182679
Bn-A01-p609857	0.975206612	0.975206612	Bn-N1-p613419	Bn-chrA01-p198823
Bn-A01-p611977	0.983471074	0.983471074	Bn-N1-p615539	Bn-chrA01-p200943
Bn-A01-p622550	0.975206612	0.975206612	Bn-N1-p627350	Bn-chrA01-p212754
Bn-A01-p623568	0.950166113	0.950166113	Bn-N1-p628366	Bn-chrA01-p213770
Bn-A01-p634701	0.993355482	0.993355482	Bn-N1-p639188	Bn-chrA01-p225446
Bn-A01-p637586	0.995024876	1	Bn-N1-p642087	Bn-chrA01-p228344
Bn-A01-p644410	0.983471074	0.983471074	Bn-N1-p648675	Bn-chrA01-p235192
Bn-A01-p645951	0.975206612	0.975206612	Bn-N1-p650216	Bn-chrA01-p236733
Bn-A01-p648196	0.970099668	0.970099668	Bn-N1-p652446	Bn-chrA01-p238963
Bn-A01-p649805	0.96345515	0.96345515	Bn-N1-p654018	Bn-chrA01-p240535
Bn-A01-p657852	0.975206612	0.975206612	Bn-N1-p657755	Bn-chrA01-p244272
Bn-A01-p663088	0.958677686	0.958677686	Bn-N1-p663269	Bn-chrA01-p250051
Bn-A01-p678018	0.946843854	0.956810631	Bn-N1-p678856	Bn-chrA01-p265644
Bn-A01-p688355	1	0.991735537	Bn-N1-p687818	Bn-chrA01-p282228
Bn-A01-p704525	0.966942149	0.950413223	Bn-N1-p704843	Bn-chrA01-p304646
Bn-A01-p710239	0.950413223	0.991735537	Bn-N11-p2083109	Bn-chrA01-p310387

Bn-A01-p713562	1	0.995024876	Bn-N1-p728364	Bn-chrA01-p326977
Bn-A01-p733483	0.743801653	0.743801653	Bn-N11-p2139723	Bn-chrC01-p802763
Bn-A01-p737560	0.966942149	0.975206612	Bn-N1-p752694	Bn-chrA01-p358468
Bn-A01-p739098	0.955223881	0.960199005	Bn-N1-p754255	Bn-chrA01-p360009
Bn-A01-p751573	0.993355482	0.993355482	Bn-N1-p765606	Bn-chrA01-p370162
Bn-A01-p777123	0.983471074	0.983471074	Bn-N1-p791511	Bn-chrA01-p397553
Bn-A01-p778048	0.991735537	0.991735537	Bn-N1-p792427	Bn-chrA01-p398469
Bn-A01-p796004	0.980099502	0.980099502	Bn-N1-p807924	Bn-chrA01_random-p92804
Bn-A01-p797684	0.975206612	0.975206612	Bn-N11-p2210789	Bn-chrC01-p875606
Bn-A01-p816488	0.993355482	0.993355482	Bn-N1-p836147	Bn-chrA01-p445113
Bn-A01-p820317	0.983388704	0.990033223	Bn-N1-p839949	Bn-chrA01-p449129
Bn-A01-p821328	0.993355482	0.993355482	Bn-N1-p840960	Bn-chrA01-p450140
Bn-A01-p830221	0.935323383	0.945273632	Bn-N1-p849820	Bn-chrAnn_random-p41074[illegible]
Bn-A01-p849125	0.991735537	0.991735537	Bn-N1-p874317	Bn-chrA01-p478991
Bn-A01-p857771	0.760330579	0.966942149	Bn-N1-p882928	Bn-chrA01-p489134
Bn-A01-p866260	0.983388704	0.993355482	Bn-N1-p891435	Bn-chrA01-p497515
Bn-A01-p872436	0.92039801	0.92039801	Bn-N11-p2309864	Bn-chrC01-p974076
Bn-A01-p877322	0.995024876	0.995024876	Bn-N1-p899365	Bn-chrA01-p504546
Bn-A01-p878256	0.985074627	0.985074627	Bn-N1-p900297	Bn-chrA01-p505478
Bn-A01-p879755	0.966942149	0.966942149	Bn-N1-p902872	Bn-chrA01_random-p97621
Bn-A01-p887815	0.995024876	0.995024876	Bn-N1-p910193	Bn-chrA01-p513142
Bn-A01-p907721	0.980066445	0.976744186	Bn-N11-p2335230	Bn-chrC01-p1000551
Bn-A01-p912298	0.975206612	0.975206612	Bn-N1-p932870	Bn-chrA01-p530592
Bn-A01-p916774	0.975206612	0.983471074	Bn-N1-p936377	Bn-chrA01-p534099
Bn-A01-p916852	0.986710963	0.993355482	Bn-N1-p936455	Bn-chrA01-p534177
Bn-A01-p923356	0.966942149	0.975206612	Bn-N1-p942965	Bn-chrA01-p540994
Bn-A01-p933795	0.976744186	0.797342193	Bn-N11-p2369057	Bn-chrC01-p1024757
Bn-A01-p936014	0.94214876	0.900826446	Bn-N1-p955576	Bn-chrA01-p551702
Bn-A01-p938887	0.990049751	0.985074627	Bn-N1-p958851	Bn-chrA01-p554681
Bn-A01-p939202	0.975206612	0.859504132	Bn-N1-p959173	Bn-chrA01-p554994
Bn-A01-p941792	0.611940299	0.611940299	Bn-N1-p966110	Bn-chrA01-p557582
Bn-A01-p951063	0.973421927	0.973421927	Bn-N1-p975265	Bn-chrA01-p566757
Bn-A01-p951341	0.992982456	0.992982456	Bn-N1-p975544	Bn-chrA01-p567036
Bn-A01-p968378	0.980066445	0.980066445	Bn-N1-p996064	Bn-chrA01-p586074
Bn-A01-p968465	0.983471074	0.983471074	Bn-N1-p996151	Bn-chrA01-p586161
Bn-A01-p970103	0.983471074	0.983471074	Bn-N1-p998054	Bn-chrA01-p588064
Bn-A01-p974934	0.916943522	0.916943522	Bn-N1-p1001647	Bn-chrA01-p591657
Bn-A01-p975213	0.813953488	0.903654485	Bn-N11-p2427698	Bn-chrC01-p1084272
Bn-A01-p975640	0.976744186	0.976744186	Bn-N1-p1002287	Bn-chrA01-p592297
Bn-A01-p977861	0.892561983	0.892561983	Bn-N11-p2437391	Bn-chrC01-p1097523
Bn-A01-p978860	0.528239203	0.993355482	Bn-N1-p1005541	Bn-chrA01-p595540
Bn-A01-p994722	0.993355482	0.993355482	Bn-N1-p1025240	Bn-chrA01-p611457
Bn-A01-p1000115	0.975124378	0.995024876	Bn-N1-p1030519	Bn-chrA01-p617758
Bn-A01-p1001022	0.956810631	0.96013289	Bn-N1-p1031427	Bn-chrA01-p618662
Bn-A01-p1012990	1	1	Bn-N1-p1038774	Bn-chrA01-p631968
Bn-A01-p1026631	0.970099668	0.976744186	Bn-N1-p1053515	Bn-chrA01_random-p24011

Bn-A01-p1029558	0.955223881	0.955223881	Bn-N1-p1056422	Bn-chrA01_random-p21095
Bn-A01-p1029668	0.973421927	0.986710963	Bn-N1-p1056532	Bn-chrA01_random-p20985
Bn-A01-p1041947	1	0.983471074	Bn-N1-p1069090	Bn-chrA01_random-p6482
Bn-A01-p1045881	0.966942149	0.884297521	Bn-N1-p1073882	Bn-chrA01_random-p2304
Bn-A01-p1056386	0.975206612	0.958677686	Bn-N1-p1084599	Bn-chrA01-p640502
Bn-A01-p1062520	0.950413223	0.966942149	Bn-N1-p1094128	Bn-chrA01-p647648
Bn-A01-p1067371	0.991735537	0.975206612	Bn-N1-p1099188	Bn-chrA01-p652869
Bn-A01-p1073387	1	0.995024876	Bn-N1-p1104160	Bn-chrA01-p658833
Bn-A01-p1089109	1	1	Bn-N1-p1124822	Bn-chrA01-p687338
Bn-A01-p1091610	0.986710963	0.986710963	Bn-N1-p1127323	Bn-chrC01-p1202103
Bn-A01-p1095733	0.991735537	0.983471074	Bn-N1-p1131446	Bn-chrA01-p695488
Bn-A01-p1104187	0.990033223	0.990033223	Bn-N1-p1140701	Bn-chrA01-p700541
Bn-A01-p1119894	0.980066445	0.983388704	Bn-N1-p1156939	Bn-chrA01-p717053
Bn-A01-p1125565	0.983471074	0.991735537	Bn-N1-p1162568	Bn-chrA01-p723009
Bn-A01-p1126874	0.975206612	0.966942149	Bn-N1-p1163645	Bn-chrA01-p724070
Bn-A01-p1132885	0.975206612	0.966942149	Bn-N1-p1169679	Bn-chrA01-p730062
Bn-A01-p1133447	1	0.991735537	Bn-N1-p1170241	Bn-chrA01-p730624
Bn-A01-p1145674	0.958677686	0.966942149	Bn-N1-p1193266	Bn-chrA01-p749100
Bn-A01-p1148760	1	0.995024876	Bn-N1-p1196607	Bn-chrA01-p752441
Bn-A01-p1149351	0.955223881	0.960199005	Bn-N1-p1197198	Bn-chrA01-p753032
Bn-A01-p1149698	0.975124378	0.970149254	Bn-N1-p1197545	Bn-chrA01-p753379
Bn-A01-p1150857	0.958677686	0.966942149	Bn-N1-p1198704	Bn-chrA01-p754538
Bn-A01-p1151961	0.895522388	0.900497512	Bn-N1-p1199814	Bn-chrA01-p755645
Bn-A01-p1152123	0.800664452	0.800664452	Bn-N1-p1199976	Bn-chrA01-p755807
Bn-A01-p1152359	0.995024876	0.990049751	Bn-N1-p1200214	Bn-chrA01-p756045
Bn-A01-p1152369	0.990049751	0.995024876	Bn-N1-p1200224	Bn-chrA01-p756055
Bn-A01-p1156081	0.966777409	0.980066445	Bn-N1-p1203925	Bn-chrA01-p759759
Bn-A01-p1156979	0.975206612	0.983471074	Bn-N1-p1204822	Bn-chrA01-p760656
Bn-A01-p1158564	0.975206612	0.966942149	Bn-N1-p1206415	Bn-chrA01-p762276
Bn-A01-p1170567	0.975206612	0.983471074	Bn-N1-p1219909	Bn-chrC01-p1294139
Bn-A01-p1175428	0.990033223	0.986710963	Bn-N1-p1224767	Bn-chrA01-p774559
Bn-A01-p1187868	0.990033223	0.990033223	Bn-N1-p1237997	Bn-chrA01-p786675
Bn-A01-p1188004	0.983388704	0.990033223	Bn-N1-p1238133	Bn-chrA01-p786811
Bn-A01-p1188928	0.950166113	0.950166113	Bn-N11-p2713205	Bn-chrC01-p1319057
Bn-A01-p1190844	0.973421927	0.980066445	Bn-N1-p1240680	Bn-chrA01-p789340
Bn-A01-p1192928	0.976744186	0.96345515	Bn-N1-p1242752	Bn-chrA01-p791408
Bn-A01-p1194009	0.996677741	0.993355482	Bn-N1-p1243827	Bn-chrA01-p792483
Bn-A01-p1208515	0.945273632	0.940298507	Bn-N1-p1255249	Bn-chrA01-p805970
Bn-A01-p1220904	0.976744186	0.976744186	Bn-Scaffold01553-p1	Bn-chrA01-p818672
Bn-A01-p1226475	0.966942149	0.958677686	Bn-N1-p1271278	Bn-chrA01-p824240
Bn-A01-p1227000	0.990033223	0.996677741	Bn-N1-p1271805	Bn-chrA01-p824767
Bn-A01-p1227023	0.983471074	0.991735537	Bn-N1-p1271828	Bn-chrA01-p824790
Bn-A01-p1232064	1	1	Bn-N1-p1276870	Bn-chrA01-p830190
Bn-A01-p1235334	0.980099502	0.980099502	Bn-N1-p1280151	Bn-chrA01-p833504
Bn-A01-p1236927	0.975206612	0.991735537	Bn-N1-p1281775	Bn-chrA01-p839406
Bn-A01-p1247899	0.975206612	0.958677686	Bn-N1-p1290014	Bn-chrA01-p848430

Bn-A01-p1248141	0.830564784	0.840531561	Bn-N1-p1290257	Bn-chrA01-p848672
Bn-A01-p1249847	0.966942149	0.834710744	Bn-N1-p1291963	Bn-chrA01-p850387
Bn-A01-p1250595	0.983471074	0.975206612	Bn-N1-p1292716	Bn-chrA01-p851175
Bn-A01-p1252553	0.940298507	1	Bn-N11-p2776803	Bn-chrA01-p853144
Bn-A01-p1261017	0.96345515	0.96345515	Bn-N1-p1304643	Bn-chrA01-p861463
Bn-A01-p1275284	0.991735537	1	Bn-N1-p1318149	Bn-chrA01-p873225
Bn-A01-p1276486	0.975206612	0.983471074	Bn-N1-p1319351	Bn-chrA01-p874438
Bn-A01-p1276504	0.991735537	0.983471074	Bn-N1-p1319369	Bn-chrA01-p874457
Bn-A01-p1296503	0.976744186	0.990033223	Bn-N11-p2881419	Bn-chrA01-p899412
Bn-A01-p1314194	0.953488372	0.953488372	Bn-N1-p1355312	Bn-chrA01-p915451
Bn-A01-p1314440	0.993355482	0.993355482	Bn-N1-p1355558	Bn-chrA01-p915697
Bn-A01-p1320368	0.867768595	0.867768595	Bn-N11-p2907936	Bn-chrC01-p1521321
Bn-A01-p1324586	0.906976744	0.910299003	Bn-N1-p1368115	Bn-chrA01-p925769
Bn-A01-p1372430	0.975206612	0.975206612	Bn-N1-p1418752	Bn-chrA01-p973535
Bn-A01-p1373104	0.983471074	0.983471074	Bn-N1-p1419426	Bn-chrA01-p974209
Bn-A01-p1373239	0.983471074	0.983471074	Bn-N1-p1419561	Bn-chrA01-p974344
Bn-A01-p1373827	0.991735537	0.991735537	Bn-N1-p1420152	Bn-chrA01-p974935
Bn-A01-p1373838	0.983471074	0.983471074	Bn-N1-p1420163	Bn-chrA01-p974946
Bn-A01-p1373965	0.958677686	0.958677686	Bn-N1-p1420294	Bn-chrA01-p975077
Bn-A01-p1376589	0.991735537	0.983471074	Bn-N1-p1423222	Bn-chrA01-p977752
Bn-A01-p1384609	0.983471074	0.975206612	Bn-N1-p1430257	Bn-chrA01-p985267
Bn-A01-p1385993	0.966942149	0.958677686	Bn-N1-p1431704	Bn-chrA01-p986712
Bn-A01-p1387174	0.990049751	0.985074627	Bn-N1-p1432886	Bn-chrA01-p987889
Bn-A01-p1387769	0.966942149	0.867768595	Bn-N1-p1433483	Bn-chrA01-p988482
Bn-A01-p1409723	0.991735537	0.991735537	Bn-N1-p1464686	Bn-chrA01-p1006969
Bn-A01-p1414695	0.930232558	0.990033223	Bn-N1-p1469639	Bn-chrA01-p1013353
Bn-A01-p1422384	0.99122807	0.99122807	Bn-N11-p3033609	Bn-chrC01-p1639343
Bn-A01-p1423163	0.983471074	0.975206612	Bn-N1-p1477507	Bn-chrA01-p1021287
Bn-A01-p1439621	0.990049751	0.985074627	Bn-N1-p1495430	Bn-chrA01-p1035645
Bn-A01-p1440009	0.960199005	0.955223881	Bn-N1-p1495817	Bn-chrA01-p1036032
Bn-A01-p1443389	0.986710963	0.993355482	Bn-N1-p1499226	Bn-chrCnn_random-p472875
Bn-A01-p1448811	0.993355482	0.993355482	Bn-N1-p1504639	Bn-chrA01-p1044861
Bn-A01-p1449865	0.980066445	0.970099668	Bn-N1-p1505698	Bn-chrA01-p1045916
Bn-A01-p1453156	0.966942149	0.975206612	Bn-N1-p1509145	Bn-chrA01-p1048424
Bn-A01-p1453560	0.966942149	0.958677686	Bn-N1-p1509590	Bn-chrA01-p1048828
Bn-A01-p1456256	0.950248756	0.965174129	Bn-N1-p1512420	Bn-chrA01-p1051673
Bn-A01-p1473188	0.980066445	0.986710963	Bn-N1-p1530325	Bn-chrA01-p1067702
Bn-A01-p1476216	0.990049751	0.990049751	Bn-N1-p1533136	Bn-chrA01-p1070537
Bn-A01-p1530160	0.909090909	0.909090909	Bn-N1-p1571664	Bn-chrAnn_random-p12544[illegible]
Bn-A01-p1531829	0.943521595	0.943521595	Bn-N1-p1573337	Bn-chrAnn_random-p12546[illegible]
Bn-A01-p1538603	0.966942149	0.884297521	Bn-N1-p1581192	Bn-chrAnn_random-p12555[illegible]
Bn-A01-p1547232	0.94214876	0.958677686	Bn-N1-p1589760	Bn-chrAnn_random-p12564[illegible]
Bn-A01-p1548460	0.826446281	0.991735537	Bn-N1-p1590677	Bn-chrAnn_random-p12566[illegible]
Bn-A01-p1551223	1	0.991735537	Bn-N1-p1593438	Bn-chrAnn_random-p12568[illegible]
Bn-A01-p1551700	0.991735537	1	Bn-N1-p1593859	Bn-chrAnn_random-p12569[illegible]
Bn-A01-p1551913	0.983471074	0.991735537	Bn-N1-p1594074	Bn-chrAnn_random-p12569[illegible]

Bn-A01-p1556145	0.958677686	0.966942149	Bn-N1-p1596831	Bn-chrAnn_random-p125717[illegible]
Bn-A01-p1570727	0.983471074	0.983471074	Bn-N1-p1618072	Bn-chrAnn_random-p125911[illegible]
Bn-A01-p1570761	0.983471074	0.983471074	Bn-N1-p1618106	Bn-chrAnn_random-p125911[illegible]
Bn-A01-p1578682	0.983471074	0.983471074	Bn-N1-p1627035	Bn-chrAnn_random-p12599[illegible]
Bn-A01-p1580970	0.986710963	0.990033223	Bn-N1-p1629324	Bn-chrAnn_random-p126013[illegible]
Bn-A01-p1606312	0.966777409	0.96345515	Bn-N1-p1648516	Bn-chrA01-p1145157
Bn-A01-p1632686	0.956810631	0.956810631	Bn-N1-p1674784	Bn-chrA09_random-p237927[illegible]
Bn-A01-p1637234	0.554817276	0.857142857	Bn-N1-p1679347	Bn-chrA01-p1175444
Bn-A01-p1639922	0.943521595	0.976744186	Bn-N11-p3255994	Bn-chrA01-p1178182
Bn-A01-p1641033	0.955223881	0.955223881	Bn-N1-p1683157	Bn-chrA01-p1179285
Bn-A01-p1647693	0.991735537	0.983471074	Bn-N1-p1690247	Bn-chrA01-p1195643
Bn-A01-p1661180	0.950413223	0.950413223	Bn-N1-p1703757	Bn-chrA01-p1208671
Bn-A01-p1666732	0.94214876	0.94214876	Bn-N1-p1709347	Bn-chrA01-p1214276
Bn-A01-p1678541	0.595041322	0.595041322	Bn-N11-p3326058	Bn-chrCnn_random-p717654[illegible]
Bn-A01-p1684485	0.975206612	0.975206612	Bn-N11-p3334929	Bn-chrC01-p1898637
Bn-A01-p1719658	0.990033223	0.990033223	Bn-N1-p1781685	Bn-chrA01-p1261804
Bn-A01-p1742846	0.561983471	0.561983471	Bn-N1-p1806273	Bn-chrA01-p1289063
Bn-A01-p1745099	0.983471074	0.983471074	Bn-N1-p1807914	Bn-chrA01-p1290704
Bn-A01-p1746226	0.976744186	0.973421927	Bn-N1-p1812677	Bn-chrA01-p1291831
Bn-A01-p1768443	0.996268657	0.996268657	Bn-N1-p1838760	Bn-chrA01-p1308411
Bn-A01-p1770291	0.983471074	0.983471074	Bn-N1-p1840607	Bn-chrA01-p1310265
Bn-A01-p1770482	0.983471074	0.983471074	Bn-N1-p1840799	Bn-chrA01-p1310457
Bn-A01-p1772621	0.966942149	0.966942149	Bn-N1-p1841352	Bn-chrA01-p1311010
Bn-A01-p1773289	0.958677686	0.958677686	Bn-N1-p1842065	Bn-chrA01-p1311722
Bn-A01-p1793928	0.965174129	0.950248756	Bn-N1-p1864630	Bn-chrA01-p1331404
Bn-A01-p1823705	0.958677686	0.950413223	Bn-N1-p1897288	Bn-chrA01-p1358952
Bn-A01-p1831507	0.983471074	0.983471074	Bn-N1-p1905152	Bn-chrA01-p1366450
Bn-A01-p1838960	0.859504132	0.859504132	Bn-N1-p1911681	Bn-chrA01-p1372669
Bn-A01-p1842079	0.996677741	0.996677741	Bn-N1-p1914802	Bn-chrA01-p1375790
Bn-A01-p1842658	0.990033223	0.990033223	Bn-N11-p3576771	Bn-chrA01-p1376367
Bn-A01-p1855903	0.834710744	0.826446281	Bn-N1-p1931677	Bn-chrA01-p1394072
Bn-A01-p1858279	0.991735537	0.958677686	Bn-N1-p1934009	Bn-chrA01-p1396447
Bn-A01-p1859204	0.986710963	0.986710963	Bn-N11-p4472911	Bn-chrC01-p3041931
Bn-A01-p1864705	0.867768595	0.975206612	Bn-N11-p3606007	Bn-chrA01-p1400752
Bn-A01-p1879401	0.943521595	0.943521595	Bn-N1-p1955958	Bn-chrA01-p1413459
Bn-A01-p1880406	0.991735537	1	Bn-N1-p1960659	Bn-chrA01-p1414503
Bn-A01-p1890102	0.991735537	1	Bn-N1-p1969454	Bn-chrA01-p1424620
Bn-A01-p1917414	0.976744186	0.990033223	Bn-N11-p3657049	Bn-chrA01-p1441928
Bn-A01-p1929982	0.990049751	0.995024876	Bn-N1-p1996291	Bn-chrA01-p1450528
Bn-A01-p1930111	0.990049751	0.990049751	Bn-N1-p1996420	Bn-chrA01-p1450657
Bn-A01-p1951988	0.990049751	0.995024876	Bn-N1-p2007372	Bn-chrA01-p1461636
Bn-A01-p1966955	0.955223881	0.950248756	Bn-N1-p2016917	Bn-chrA01-p1472135
Bn-A01-p1975231	0.986710963	0.986710963	Bn-N1-p2025297	Bn-chrA01-p1480280
Bn-A01-p1977270	0.996677741	0.996677741	Bn-N1-p2027327	Bn-chrA01-p1482310
Bn-A01-p1980181	0.996677741	0.996677741	Bn-N1-p2030241	Bn-chrA01-p1485222
Bn-A01-p1990755	0.966942149	0.966942149	Bn-N1-p2040832	Bn-chrAnn_random-p30455[illegible]

Bn-A01-p1993302	0.983471074	0.983471074	Bn-N1-p2044259	Bn-chrA01_random-p103884
Bn-A01-p2004336	0.983471074	0.975206612	Bn-N1-p2055534	Bn-chrA01-p1502176
Bn-A01-p2009581	0.933884298	0.933884298	Bn-N1-p2062288	Bn-chrA01-p1508709
Bn-A01-p2025400	0.950413223	0.950413223	Bn-N1-p2079512	Bn-chrA01-p1525039
Bn-A01-p2042209	0.975206612	0.975206612	Bn-N1-p2093225	Bn-chrA01-p1539200
Bn-A01-p2046061	0.983471074	0.983471074	Bn-N1-p2097038	Bn-chrA01-p1543004
Bn-A01-p2053320	0.983471074	0.983471074	Bn-N1-p2104938	Bn-chrA01-p1550903
Bn-A01-p2065122	0.661157025	0.462809917	Bn-N1-p2113151	Bn-chrA01-p1557612
Bn-A01-p2067006	0.950413223	0.958677686	Bn-N1-p2114732	Bn-chrA01-p1559279
Bn-A01-p2069218	0.983471074	0.983471074	Bn-N1-p2120534	Bn-chrA01-p1561518
Bn-A01-p2069488	0.983471074	0.991735537	Bn-N1-p2120803	Bn-chrA01-p1561788
Bn-A01-p2091322	0.975206612	0.975206612	Bn-N1-p2141306	Bn-chrA01-p1582609
Bn-A01-p2094793	0.983471074	0.991735537	Bn-N1-p2145056	Bn-chrA01-p1586633
Bn-A01-p2100996	0.983471074	0.975206612	Bn-N1-p2151261	Bn-chrA01-p1592840
Bn-A01-p2111978	0.644628099	0.710743802	Bn-N1-p2158333	Bn-chrA01-p1599912
Bn-A01-p2114552	0.996677741	0.996677741	Bn-N1-p2160898	Bn-chrA01-p1602491
Bn-A01-p2114803	0.990033223	0.990033223	Bn-N1-p2161149	Bn-chrA01-p1602742
Bn-A01-p2121224	0.743801653	0.909090909	Bn-N11-p3917104	Bn-chrA01-p1609506
Bn-A01-p2148059	0.973421927	0.973421927	Bn-N1-p2186745	Bn-chrA01-p1630377
Bn-A01-p2158511	0.980066445	0.980066445	Bn-N1-p2198901	Bn-chrA01-p1643742
Bn-A01-p2171217	0.983471074	0.983471074	Bn-N1-p2211868	Bn-chrA01-p1656717
Bn-A01-p2177864	0.983471074	0.983471074	Bn-N11-p3967814	Bn-chrC01-p2505476
Bn-A01-p2187099	0.892561983	0.892561983	Bn-N1-p2227901	Bn-chrA01-p1672657
Bn-A01-p2188753	0.990049751	0.990049751	Bn-N1-p2229534	Bn-chrA01-p1674290
Bn-A01-p2197707	0.875621891	0.875621891	Bn-N1-p2235975	Bn-chrA01-p1680441
Bn-A01-p2197976	0.958677686	0.950413223	Bn-N1-p2236253	Bn-chrA01-p1680730
Bn-A01-p2202853	0.983471074	0.983471074	Bn-N1-p2242175	Bn-chrA01-p1685778
Bn-A01-p2207452	0.966942149	0.909090909	Bn-N1-p2246891	Bn-chrC01-p2545298
Bn-A01-p2221110	0.94214876	0.958677686	Bn-N1-p2261367	Bn-chrA01-p1704124
Bn-A01-p2230371	1	1	Bn-N1-p2270625	Bn-chrA01-p1712925
Bn-A01-p2242890	0.985074627	0.990049751	Bn-N1-p2284563	Bn-chrA01-p1727139
Bn-A01-p2244327	0.950413223	0.917355372	Bn-N1-p2290291	Bn-chrA01-p1728533
Bn-A01-p2245780	0.980099502	0.985074627	Bn-N1-p2291747	Bn-chrA01-p1729966
Bn-A01-p2246317	0.970149254	0.970149254	Bn-N1-p2292283	Bn-chrA01-p1730502
Bn-A01-p2246373	0.970149254	0.970149254	Bn-N1-p2292339	Bn-chrA01-p1730558
Bn-A01-p2251106	0.983388704	0.983388704	Bn-N1-p2297029	Bn-chrA01-p1735248
Bn-A01-p2254004	0.933884298	0.933884298	Bn-N1-p2299641	Bn-chrA01-p1737860
Bn-A01-p2254072	0.950413223	0.950413223	Bn-N1-p2299709	Bn-chrA01-p1737928
Bn-A01-p2254235	0.983471074	0.983471074	Bn-N1-p2299872	Bn-chrA01-p1738091
Bn-A01-p2260859	0.975206612	0.975206612	Bn-N1-p2308181	Bn-chrA01-p1744726
Bn-A01-p2261903	0.958677686	0.958677686	Bn-N1-p2309230	Bn-chrA01-p1745775
Bn-A01-p2277290	0.976744186	0.976744186	Bn-N1-p2319617	Bn-chrA01-p1756160
Bn-A01-p2288902	0.990033223	0.986710963	Bn-N1-p2331102	Bn-chrA01-p1765752
Bn-A01-p2291940	0.94214876	0.94214876	Bn-N1-p2334384	Bn-chrA01-p1768759
Bn-A01-p2292174	0.995024876	0.930348259	Bn-N1-p2334618	Bn-chrC01-p2644797
Bn-A01-p2303308	0.966942149	0.966942149	Bn-N1-p2341606	Bn-chrA01-p1775948

Bn-A01-p2306208	0.965174129	0.885572139	Bn-N1-p2344506	Bn-chrC01-p2654110
Bn-A01-p2306938	0.966942149	0.975206612	Bn-N1-p2345236	Bn-chrA01-p1779641
Bn-A01-p2307144	0.983471074	0.991735537	Bn-N1-p2345442	Bn-chrA01-p1779848
Bn-A01-p2309179	0.925619835	0.925619835	Bn-N11-p4126437	Bn-chrC01-p2657014
Bn-A01-p2311015	0.991735537	1	Bn-N1-p2349273	Bn-chrA01-p1783685
Bn-A01-p2318972	0.973421927	0.990033223	Bn-N1-p2361058	Bn-chrA01-p1793043
Bn-A01-p2323744	0.990033223	0.986710963	Bn-N1-p2365999	Bn-chrA01-p1797827
Bn-A01-p2329932	0.975206612	0.983471074	Bn-N1-p2369712	Bn-chrA01-p1804477
Bn-A01-p2340048	0.991735537	1	Bn-N1-p2375360	Bn-chrA01-p1819172
Bn-A01-p2340359	0.917355372	0.975206612	Bn-N1-p2375614	Bn-chrA01-p1827218
Bn-A01-p2341408	0.991735537	0.983471074	Bn-N1-p2376710	Bn-chrA01-p1827987
Bn-A01-p2345255	0.840531561	0.976744186	Bn-N11-p4198517	Bn-chrA01-p1831598
Bn-A01-p2360909	0.958677686	0.958677686	Bn-N1-p2401000	Bn-chrA01-p1854227
Bn-A01-p2365493	0.909090909	0.909090909	Bn-N1-p2405268	Bn-chrA01-p1858493
Bn-A01-p2365667	0.991735537	0.991735537	Bn-N1-p2405441	Bn-chrA01-p1858666
Bn-A01-p2387124	0.993355482	0.993355482	Bn-N1-p2424897	Bn-chrA01-p1876775
Bn-A01-p2393817	0.996677741	0.996677741	Bn-N1-p2427793	Bn-chrA01-p1879671
Bn-A01-p2403471	0.983471074	0.983471074	Bn-N1-p2439812	Bn-chrA01-p1894127
Bn-A01-p2421445	0.958677686	0.958677686	Bn-N1-p2457821	Bn-chrA01-p1912045
Bn-A01-p2431503	0.970149254	0.970149254	Bn-N1-p2465850	Bn-chrA01-p1921210
Bn-A01-p2445274	0.996677741	0.993355482	Bn-N1-p2603063	Bn-chrA01-p1936143
Bn-A01-p2445468	0.990033223	0.990033223	Bn-N1-p2602869	Bn-chrA01-p1936337
Bn-A01-p2445602	0.983471074	0.983471074	Bn-N1-p2602735	Bn-chrA01-p1936471
Bn-A01-p2448162	1	1	Bn-N1-p2600258	Bn-chrA01-p1939000
Bn-A01-p2453633	0.993355482	0.993355482	Bn-N1-p2599772	Bn-chrA01-p1939486
Bn-A01-p2453758	0.993355482	0.990033223	Bn-N1-p2599647	Bn-chrA01-p1939611
Bn-A01-p2455219	0.970149254	0.970149254	Bn-N1-p2598186	Bn-chrA01-p1941072
Bn-A01-p2457750	0.986710963	0.983388704	Bn-N1-p2595584	Bn-chrA01-p1943675
Bn-A01-p2461470	0.664451827	0.777408638	Bn-N13-p52694603	Bn-chrAnn_random-p366721
Bn-A01-p2475676	0.945273632	0.945273632	Bn-N1-p2579612	Bn-chrA01_random-p108704
Bn-A01-p2478054	0.983471074	0.983471074	Bn-N1-p2577241	Bn-chrA01-p1965984
Bn-A01-p2478055	0.990033223	0.990033223	Bn-N1-p2577240	Bn-chrA01-p1965985
Bn-A01-p2491976	0.975206612	0.975206612	Bn-N1-p2562581	Bn-chrA01-p1979998
Bn-A01-p2501423	0.975206612	0.975206612	Bn-N1-p2552952	Bn-chrA01-p1989624
Bn-A01-p2504370	0.983471074	0.983471074	Bn-N1-p2550026	Bn-chrA01-p1992550
Bn-A01-p2509163	0.986710963	0.986710963	Bn-N1-p2543483	Bn-chrA01-p1998742
Bn-A01-p2517658	0.975206612	0.983471074	Bn-N1-p2535164	Bn-chrA01-p2012482
Bn-A01-p2533727	0.925373134	0.925373134	Bn-N1-p2516058	Bn-chrA01-p2028654
Bn-A01-p2538180	0.975206612	0.983471074	Bn-N1-p2509374	Bn-chrA01-p2035080
Bn-A01-p2553345	1	1	Bn-N1-p2490275	Bn-chrA01-p2054660
Bn-A01-p2569303	0.933884298	0.933884298	Bn-N1-p2609944	Bn-chrA01-p2070693
Bn-A01-p2574969	0.991735537	1	Bn-N1-p2616127	Bn-chrA01-p2076602
Bn-A01-p2575497	0.884297521	0.966942149	Bn-N1-p2616644	Bn-chrA01-p2077123
Bn-A01-p2587709	0.996677741	0.993355482	Bn-N1-p2637711	Bn-chrA01-p2094564
Bn-A01-p2608660	0.983471074	0.983471074	Bn-N1-p2657233	Bn-chrA01-p2115096
Bn-A01-p2624222	0.983471074	0.975206612	Bn-N1-p2671811	Bn-chrA01-p2128844

Bn-A01-p2626270	0.986710963	0.986710963	Bn-N1-p2675493	Bn-chrA01-p2132864
Bn-A01-p2634563	0.990033223	0.966777409	Bn-N1-p2683740	Bn-chrA01-p2141160
Bn-A01-p2637699	0.950413223	0.958677686	Bn-N1-p2686959	Bn-chrA01-p2144328
Bn-A01-p2647221	0.976744186	0.976744186	Bn-N1-p2697173	Bn-chrA01-p2154578
Bn-A01-p2667471	0.958677686	0.958677686	Bn-N1-p2720419	Bn-chrA01_random-p110915
Bn-A01-p2667691	0.996677741	0.996677741	Bn-N1-p2720635	Bn-chrA01_random-p111131
Bn-A01-p2667767	0.983471074	0.983471074	Bn-N1-p2720711	Bn-chrA01_random-p111207
Bn-A01-p2668951	0.983471074	0.983471074	Bn-N1-p2721895	Bn-chrA01_random-p112391
Bn-A01-p2688662	0.991735537	0.991735537	Bn-N1-p2742283	Bn-chrA01-p2194592
Bn-A01-p2694323	0.993355482	0.993355482	Bn-N1-p2747936	Bn-chrA01-p2200245
Bn-A01-p2699907	0.986710963	0.986710963	Bn-N1-p2753537	Bn-chrA01-p2205846
Bn-A01-p2713439	0.752066116	0.446280992	Bn-N8-p12837754	Bn-chrC01-p3314876
Bn-A01-p2713528	0.79338843	0.79338843	Bn-N1-p2770797	Bn-chrA01-p2220656
Bn-A01-p2713583	0.94214876	0.94214876	Bn-N1-p2770852	Bn-chrA01-p2220711
Bn-A01-p2715156	0.950248756	0.950248756	Bn-N1-p2772407	Bn-chrA01-p2222266
Bn-A01-p2718126	0.950413223	0.950413223	Bn-N1-p2775946	Bn-chrA01-p2225805
Bn-A01-p2746862	0.966942149	0.966942149	Bn-N1-p2808336	Bn-chrA01-p2253604
Bn-A01-p2775635	0.857142857	0.857142857	Bn-N1-p2829094	Bn-chrA01-p2273781
Bn-A01-p2784074	0.983471074	0.983471074	Bn-N1-p2841666	Bn-chrA01-p2282288
Bn-A01-p2792719	0.996677741	1	Bn-N1-p2850328	Bn-chrA01-p2290958
Bn-A01-p2825565	0.966942149	0.966942149	Bn-N1-p2879286	Bn-chrA01-p2327568
Bn-A01-p2836617	0.983471074	0.958677686	Bn-N1-p2893515	Bn-chrC01-p3438241
Bn-A01-p2841043	0.975206612	0.975206612	Bn-N1-p2900995	Bn-chrA01-p2350166
Bn-A01-p2846364	0.933884298	0.983471074	Bn-N14-p31103366	Bn-chrA03-p3693958
Bn-A01-p2848253	0.859504132	0.991735537	Bn-N14-p31101559	Bn-chrA03-p3695847
Bn-A01-p2849034	0.900826446	0.991735537	Bn-N14-p31100778	Bn-chrA03-p3696628
Bn-A01-p2849540	0.79338843	0.975206612	Bn-N14-p31100270	Bn-chrA03-p3697134
Bn-A01-p2861502	0.993355482	0.993355482	Bn-N1-p2916077	Bn-chrA01-p2364238
Bn-A01-p2864193	0.980066445	0.980066445	Bn-N1-p2918757	Bn-chrA01-p2366918
Bn-A01-p2867284	0.991735537	0.991735537	Bn-N1-p2921846	Bn-chrA01-p2370007
Bn-A01-p2873007	1	1	Bn-N1-p2927409	Bn-chrA01-p2375378
Bn-A01-p2874393	0.996677741	0.996677741	Bn-N1-p2928793	Bn-chrA01-p2376761
Bn-A01-p2882270	0.983471074	0.983471074	Bn-N1-p2938627	Bn-chrA01-p2386261
Bn-A01-p2884934	0.996677741	0.996677741	Bn-N1-p2941286	Bn-chrA01-p2388920
Bn-A01-p2891863	0.975206612	0.975206612	Bn-N1-p2948111	Bn-chrA01-p2395849
Bn-A01-p2897071	0.975206612	0.975206612	Bn-N1-p2953448	Bn-chrA01-p2401390
Bn-A01-p2910608	0.975206612	0.975206612	Bn-N1-p2976096	Bn-chrA01_random-p117420
Bn-A01-p2910752	0.956810631	0.890365449	Bn-N1-p2976240	Bn-chrA01_random-p117276
Bn-A01-p2915231	1	1	Bn-N1-p2981735	Bn-chrA01_random-p162800
Bn-A01-p2915821	0.991735537	0.991735537	Bn-N1-p2982319	Bn-chrA01_random-p162216
Bn-A01-p2928824	0.991735537	0.991735537	Bn-N1-p2997150	Bn-chrA01_random-p148871
Bn-A01-p2934166	0.976744186	0.976744186	Bn-N1-p3002527	Bn-chrA01_random-p143492
Bn-A01-p2956393	0.976744186	0.976744186	Bn-N1-p3018205	Bn-chrA01_random-p127199
Bn-A01-p2957869	0.983471074	0.983471074	Bn-N1-p3019681	Bn-chrA01_random-p125723
Bn-A01-p2961857	0.996677741	0.996677741	Bn-N1-p3027194	Bn-chrA01_random-p165382
Bn-A01-p2990104	0.892561983	0.892561983	Bn-N1-p3051730	Bn-chrA01-p2702388

Bn-A01-p3000989	0.903654485	0.903654485	Bn-N1-p3061785	Bn-chrA01-p2712442
Bn-A01-p3005310	0.950413223	0.950413223	Bn-N1-p3065971	Bn-chrA01-p2716627
Bn-A01-p3005793	0.950413223	0.950413223	Bn-N1-p3066454	Bn-chrA01-p2717110
Bn-A01-p3045214	0.966942149	0.966942149	Bn-N1-p3104967	Bn-chrA01-p2751675
Bn-A01-p3045984	0.975206612	0.975206612	Bn-N1-p3105729	Bn-chrA01-p2752437
Bn-A01-p3050613	0.993355482	0.993355482	Bn-N1-p3110102	Bn-chrA01-p2756809
Bn-A01-p3059596	1	1	Bn-N1-p3118136	Bn-chrA01-p2764840
Bn-A01-p3061223	0.990033223	0.990033223	Bn-N1-p3119720	Bn-chrA01-p2766424
Bn-A01-p3069312	0.983388704	0.983388704	Bn-N1-p3127809	Bn-chrA01-p2774513
Bn-A01-p3070657	0.991735537	0.991735537	Bn-N1-p3129154	Bn-chrA01-p2775858
Bn-A01-p3076531	0.975206612	0.975206612	Bn-N1-p3134899	Bn-chrC01-p3769821
Bn-A01-p3077309	0.996677741	0.996677741	Bn-N1-p3135680	Bn-chrC01-p3770602
Bn-A01-p3078457	0.990033223	0.990033223	Bn-N1-p3136822	Bn-chrA01-p2783526
Bn-A01-p3085248	0.966942149	0.966942149	Bn-N1-p3143561	Bn-chrA01-p2790265
Bn-A01-p3097105	0.996677741	0.996677741	Bn-N1-p3154795	Bn-chrA01-p2801303
Bn-A01-p3100346	1	0.979166667	Bn-N1-p3159422	Bn-chrA01-p2804505
Bn-A01-p3105148	0.975124378	0.970149254	Bn-N9-p30697279	Bn-chrAnn_random-p28611(
Bn-A01-p3105358	0.975124378	0.970149254	Bn-N9-p30697069	Bn-chrA01-p2808155
Bn-A01-p3111607	0.991735537	0.983471074	Bn-N1-p3174757	Bn-chrA01-p2814404
Bn-A01-p3123580	0.990033223	0.993355482	Bn-N1-p3186759	Bn-chrA01-p2826512
Bn-A01-p3134159	0.980099502	0.995024876	Bn-N1-p3197028	Bn-chrA01-p2837204
Bn-A01-p3136468	0.851239669	0.958677686	Bn-N1-p3199609	Bn-chrA01-p2839481
Bn-A01-p3137277	0.983471074	0.983471074	Bn-N1-p3200398	Bn-chrA01-p2840290
Bn-A01-p3152636	0.926910299	0.926910299	Bn-Scaffold23228-p2	Bn-chrA01-p2864980
Bn-A01-p3169855	0.983388704	0.990033223	Bn-N1-p3238299	Bn-chrA01-p2876625
Bn-A01-p3173607	0.995024876	1	Bn-Scaffold03534-p1	Bn-chrA01-p2883501
Bn-A01-p3175614	0.983471074	0.975206612	Bn-Scaffold03534-p2	Bn-chrA01-p2885919
Bn-A01-p3179210	0.940298507	0.925373134	Bn-Scaffold08692-p2	Bn-chrC01-p3934925
Bn-A01-p3179292	0.970149254	0.955223881	Bn-Scaffold08692-p1	Bn-chrA01-p2889585
Bn-A01-p3183888	0.983471074	0.975206612	Bn-N1-p3247018	Bn-chrA01-p2894177
Bn-A01-p3195980	0.995024876	0.995024876	Bn-N1-p3267173	Bn-chrA01-p2902094
Bn-A01-p3223005	0.983471074	0.966942149	Bn-N1-p3281849	Bn-chrC01-p3966286
Bn-A01-p3226309	0.966942149	0.966942149	Bn-N1-p3286889	Bn-chrA01-p2920700
Bn-A01-p3232184	0.975206612	0.975206612	Bn-N1-p3287088	Bn-chrA01-p2920899
Bn-A01-p3232810	0.991735537	0.991735537	Bn-N1-p3287712	Bn-chrA01-p2921523
Bn-A01-p3232913	0.966942149	0.966942149	Bn-N1-p3287815	Bn-chrA01-p2921626
Bn-A01-p3233247	0.958677686	0.958677686	Bn-N1-p3288149	Bn-chrA01-p2921960
Bn-A01-p3233299	0.975206612	0.975206612	Bn-N1-p3288201	Bn-chrA01-p2922012
Bn-A01-p3240780	0.991735537	0.991735537	Bn-N1-p3288610	Bn-chrA01-p2922421
Bn-A01-p3279678	1	0.991735537	Bn-N1-p3328131	Bn-chrAnn_random-p370601
Bn-A01-p3306526	0.68159204	0.990049751	Bn-N1-p3356185	Bn-chrA01-p2992971
Bn-A01-p3307173	0.94214876	0.94214876	Bn-N1-p3356763	Bn-chrA01-p2993618
Bn-A01-p3323476	0.983471074	0.983471074	Bn-N1-p3363215	Bn-chrA01-p3000070
Bn-A01-p3378332	0.975206612	0.975206612	Bn-N1-p3408988	Bn-chrA01-p3044599
Bn-A01-p3487320	0.996677741	1	Bn-N1-p3522836	Bn-chrA01-p3153918
Bn-A01-p3495476	0.991735537	0.983471074	Bn-N1-p3534473	Bn-chrA01-p3164781

Bn-A01-p3567127	0.975206612	0.975206612	Bn-N1-p3612730	Bn-chrA01-p3239160
Bn-A01-p3573045	0.975206612	0.975206612	Bn-N1-p3618633	Bn-chrA01-p3245063
Bn-A01-p3576068	0.983471074	0.983471074	Bn-N1-p3621665	Bn-chrAnn_random-p384643
Bn-A01-p3579887	0.958677686	0.958677686	Bn-N1-p3626398	Bn-chrAnn_random-p349933
Bn-A01-p3586744	0.983471074	0.983471074	Bn-N1-p3643891	Bn-chrA01-p3253994
Bn-A01-p3597929	0.966942149	0.966942149	Bn-N1-p3655047	Bn-chrA01-p3265150
Bn-A01-p3598097	0.975206612	0.975206612	Bn-N1-p3655215	Bn-chrA01-p3265318
Bn-A01-p3598515	0.983471074	0.983471074	Bn-N1-p3655632	Bn-chrA01-p3265735
Bn-A01-p3598533	0.983471074	0.983471074	Bn-N1-p3655650	Bn-chrA01-p3265753
Bn-A01-p3599097	0.975206612	0.975206612	Bn-N1-p3656214	Bn-chrA01-p3266317
Bn-A01-p3610530	0.975206612	0.975206612	Bn-N1-p3665703	Bn-chrA01-p3275805
Bn-A01-p3619975	0.991735537	0.983471074	Bn-N1-p3672616	Bn-chrA01-p3282715
Bn-A01-p3626821	0.983471074	0.991735537	Bn-N1-p3679483	Bn-chrA01-p3289581
Bn-A01-p3631990	0.950413223	0.958677686	Bn-N1-p3690151	Bn-chrA01-p3295907
Bn-A01-p3640191	0.734219269	1	Bn-N1-p3696984	Bn-chrA01-p3308163
Bn-A01-p3643352	0.909090909	0.909090909	Bn-N1-p3700085	Bn-chrA01-p3311268
Bn-A01-p3644770	0.983471074	0.983471074	Bn-N1-p3701452	Bn-chrA01-p3312635
Bn-A01-p3664698	0.983471074	0.983471074	Bn-N1-p3718064	Bn-chrAnn_random-p301126
Bn-A01-p3666241	0.966942149	0.966942149	Bn-N1-p3719607	Bn-chrAnn_random-p301111
Bn-A01-p3703676	0.980099502	0.980099502	Bn-N1-p3749245	Bn-chrA01-p3349308
Bn-A01-p3707366	0.975124378	0.975124378	Bn-N1-p3752956	Bn-chrA01-p3353019
Bn-A01-p3713266	0.950248756	0.950248756	Bn-N1-p3759089	Bn-chrA01-p3359152
Bn-A01-p3737522	0.975206612	0.983471074	Bn-N1-p3782540	Bn-chrA01-p3391005
Bn-A01-p3744726	0.970099668	0.976744186	Bn-N1-p3789910	Bn-chrA01-p3397751
Bn-A01-p3755849	0.917355372	0.917355372	Bn-N11-p6336373	Bn-chrC01-p4968134
Bn-A01-p3758784	0.983471074	0.975206612	Bn-N1-p3803774	Bn-chrA01-p3412484
Bn-A01-p3782356	0.975206612	0.983471074	Bn-N1-p3824864	Bn-chrA01-p3432062
Bn-A01-p3789302	0.975206612	0.983471074	Bn-N1-p3833917	Bn-chrA01-p3436321
Bn-A01-p3791028	0.975206612	0.859504132	Bn-N1-p3835643	Bn-chrA01-p3438048
Bn-A01-p3829226	1	1	Bn-N11-p6484251	Bn-chrC01-p5129208
Bn-A01-p3829785	0.975206612	0.983471074	Bn-N1-p3869223	Bn-chrA01-p3456113
Bn-A01-p3858902	0.870431894	0.870431894	Bn-N1-p3896021	Bn-chrA01-p3481753
Bn-A01-p3859106	0.966942149	0.966942149	Bn-N11-p6524804	Bn-chrA01-p3481957
Bn-A01-p3859610	0.983471074	0.983471074	Bn-N1-p3896729	Bn-chrA01-p3482461
Bn-A01-p3889035	0.983471074	0.983471074	Bn-N1-p3932629	Bn-chrA01-p3509966
Bn-A01-p3892184	0.983471074	0.983471074	Bn-N1-p3935131	Bn-chrA01-p3512468
Bn-A01-p3904495	0.975206612	0.975206612	Bn-N1-p3952570	Bn-chrA01-p3530446
Bn-A01-p3906965	1	1	Bn-N1-p3955040	Bn-chrA01-p3533140
Bn-A01-p3935187	1	1	Bn-N1-p3978250	Bn-chrA01-p3556690
Bn-A01-p3938241	0.960199005	0.960199005	Bn-N1-p3986378	Bn-chrA01-p3563774
Bn-A01-p3939508	0.983388704	0.983388704	Bn-N1-p3987645	Bn-chrA01-p3565041
Bn-A01-p3948389	0.983471074	0.983471074	Bn-N1-p3989780	Bn-chrA01-p3567176
Bn-A01-p3984534	0.990033223	0.990033223	Bn-N1-p4036442	Bn-chrA01-p3618764
Bn-A01-p3993078	1	1	Bn-N1-p4046378	Bn-chrA01-p3627722
Bn-A01-p4003991	0.990033223	0.990033223	Bn-N1-p4057880	Bn-chrA01-p3638550
Bn-A01-p4023655	0.990033223	0.990033223	Bn-N1-p4080706	Bn-chrA01_random-p184645

Bn-A01-p4027402	0.993355482	0.993355482	Bn-N1-p4084437	Bn-chrA01_random-p180914
Bn-A01-p4041055	0.983471074	0.983471074	Bn-N1-p4097706	Bn-chrA01_random-p204742
Bn-A01-p4070059	0.965174129	0.965174129	Bn-N1-p4129839	Bn-chrA01-p3734014
Bn-A01-p4077105	0.975206612	0.975206612	Bn-N1-p4136147	Bn-chrA01-p3740479
Bn-A01-p4084366	0.966942149	0.966942149	Bn-N1-p4143665	Bn-chrA01-p3747996
Bn-A01-p4084739	0.958677686	0.958677686	Bn-N1-p4144038	Bn-chrA01-p3748369
Bn-A01-p4087533	0.966942149	0.966942149	Bn-N1-p4147074	Bn-chrA01-p3751404
Bn-A01-p4109157	0.993355482	0.993355482	Bn-N1-p4164079	Bn-chrA01-p3772826
Bn-A01-p4113202	0.986710963	0.973421927	Bn-N1-p4168098	Bn-chrA01-p3788485
Bn-A01-p4116110	0.966777409	0.953488372	Bn-N1-p4171006	Bn-chrA01-p3794217
Bn-A01-p4117153	0.900332226	0.900332226	Bn-N1-p4172049	Bn-chrA01-p3795275
Bn-A01-p4164843	0.965174129	0.965174129	Bn-N1-p4215555	Bn-chrA01-p3844731
Bn-A01-p4167795	0.993355482	0.993355482	Bn-N1-p4219923	Bn-chrA01-p3847688
Bn-A01-p4173668	0.966942149	0.966942149	Bn-N1-p4226689	Bn-chrA01-p3853180
Bn-A01-p4177941	0.983471074	0.991735537	Bn-N1-p4230993	Bn-chrA01-p3857501
Bn-A01-p4188629	0.996677741	0.996677741	Bn-N1-p4241830	Bn-chrA01-p3870162
Bn-A01-p4220987	1	1	Bn-N11-p7086451	Bn-chrCnn_random-p470777
Bn-A01-p4227703	0.975206612	0.975206612	Bn-N1-p4274490	Bn-chrA01-p3904559
Bn-A01-p4228985	0.970099668	0.970099668	Bn-N1-p4275774	Bn-chrA01-p3905840
Bn-A01-p4230829	0.993355482	0.970099668	Bn-N1-p4277619	Bn-chrA01-p3907683
Bn-A01-p4233306	0.975206612	0.966942149	Bn-N1-p4285505	Bn-chrA01-p3911409
Bn-A01-p4235273	0.983471074	0.983471074	Bn-N1-p4287583	Bn-chrA01-p3914964
Bn-A01-p4242269	0.983471074	0.991735537	Bn-N1-p4293684	Bn-chrA01-p3921895
Bn-A01-p4246586	0.993355482	0.993355482	Bn-N1-p4297042	Bn-chrA01-p3927770
Bn-A01-p4258195	0.966777409	0.976744186	Bn-N1-p4304559	Bn-chrA01-p3935696
Bn-A01-p4284958	0.958677686	0.958677686	Bn-N1-p4325179	Bn-chrA01-p3956260
Bn-A01-p4288869	0.975124378	0.975124378	Bn-N1-p4328973	Bn-chrA01-p3960053
Bn-A01-p4288938	0.975124378	0.975124378	Bn-N1-p4329042	Bn-chrA01-p3960122
Bn-A01-p4289535	0.975124378	0.975124378	Bn-N17-p30092309	Bn-chrC07-p27349272
Bn-A01-p4293847	0.975124378	0.975124378	Bn-N1-p4333914	Bn-chrA01-p3966898
Bn-A01-p4293878	0.960199005	0.965174129	Bn-N1-p4333945	Bn-chrA01-p3966929
Bn-A01-p4303527	0.993355482	0.996677741	Bn-N1-p4345361	Bn-chrA01-p3984266
Bn-A01-p4310302	0.993355482	0.993355482	Bn-N1-p4353578	Bn-chrA01-p3996734
Bn-A01-p4311134	0.991735537	0.983471074	Bn-N1-p4354411	Bn-chrA01-p3997738
Bn-A01-p4322340	0.983471074	0.727272727	Bn-N1-p4367773	Bn-chrA01-p4009607
Bn-A01-p4325162	0.966942149	0.966942149	Bn-N1-p4372440	Bn-chrA01-p4012430
Bn-A01-p4335144	0.993355482	0.996677741	Bn-N1-p4380902	Bn-chrA01-p4020809
Bn-A01-p4341541	0.933884298	0.933884298	Bn-N11-p7236992	Bn-chrC01-p5889293
Bn-A01-p4353657	0.966942149	0.966942149	Bn-N1-p4399471	Bn-chrA01-p4039381
Bn-A01-p4354753	0.950248756	0.955223881	Bn-N1-p4400568	Bn-chrA01-p4040460
Bn-A01-p4377760	0.986710963	0.986710963	Bn-N1-p4421854	Bn-chrA01_random-p218676
Bn-A01-p4378321	0.993355482	0.993355482	Bn-N1-p4422415	Bn-chrA01_random-p218115
Bn-A01-p4390611	0.996677741	0.996677741	Bn-N1-p4436209	Bn-chrAnn_random-p235910
Bn-A01-p4394660	0.877076412	0.893687708	Bn-N11-p7302695	Bn-chrAnn_random-p235869
Bn-A01-p4419443	0.996677741	0.996677741	Bn-N1-p4463816	Bn-chrA01-p4049901
Bn-A01-p4452779	0.935323383	0.935323383	Bn-N1-p4488299	Bn-chrA01-p4076835

Bn-A01-p4499485	0.958677686	0.925619835	Bn-N1-p4529031	Bn-chrUnn_random-p819575
Bn-A01-p4506540	0.975206612	0.966942149	Bn-N1-p4536082	Bn-chrA01-p4125332
Bn-A01-p4518460	0.975124378	0.985074627	Bn-N1-p4548194	Bn-chrA01-p4148204
Bn-A01-p4519762	0.990033223	0.993355482	Bn-N1-p4549496	Bn-chrA01-p4149495
Bn-A01-p4549564	0.892561983	0.884297521	Bn-N1-p4588787	Bn-chrA01-p4179812
Bn-A01-p4580719	0.991735537	0.991735537	Bn-N1-p4614510	Bn-chrA01-p4200395
Bn-A01-p4600975	0.986710963	0.986710963	Bn-N1-p4642962	Bn-chrA01-p4227188
Bn-A01-p4612881	0.913621262	0.913621262	Bn-N1-p4651489	Bn-chrA01-p4235714
Bn-A01-p4635210	0.986710963	0.986710963	Bn-N1-p4673232	Bn-chrA01-p4254871
Bn-A01-p4641747	0.966942149	0.966942149	Bn-N1-p4679669	Bn-chrA01-p4261277
Bn-A01-p4641802	0.983471074	0.983471074	Bn-N1-p4679724	Bn-chrA01-p4261332
Bn-A01-p4651746	1	0.996677741	Bn-N1-p4690531	Bn-chrA01-p4273529
Bn-A01-p4659972	0.991735537	0.991735537	Bn-N1-p4696982	Bn-chrA01-p4279894
Bn-A01-p4734188	0.990033223	0.943521595	Bn-N1-p4744328	Bn-chrA01-p4337616
Bn-A01-p4736924	0.96345515	0.96345515	Bn-N1-p4747053	Bn-chrA01-p4340342
Bn-A01-p4747874	0.958677686	0.950413223	Bn-N1-p4757019	Bn-chrA01-p4350529
Bn-A01-p4750734	0.966942149	0.975206612	Bn-N1-p4759874	Bn-chrA01-p4353390
Bn-A01-p4759559	0.96013289	0.986710963	Bn-N11-p7885354	Bn-chrA01-p4363332
Bn-A01-p4773772	0.986710963	0.966777409	Bn-N1-p4785853	Bn-chrA01-p4379934
Bn-A01-p4774065	0.975206612	0.975206612	Bn-N1-p4786146	Bn-chrA01-p4380227
Bn-A01-p4785261	0.958677686	0.950413223	Bn-N1-p4801260	Bn-chrA01-p4397190
Bn-A01-p4786653	0.980066445	0.980066445	Bn-N1-p4802652	Bn-chrA01-p4398591
Bn-A01-p4786754	0.976744186	0.976744186	Bn-N1-p4802753	Bn-chrA01-p4398692
Bn-A01-p4786787	0.966942149	0.966942149	Bn-N1-p4802786	Bn-chrA01-p4398725
Bn-A01-p4787515	0.980066445	0.980066445	Bn-N1-p4803559	Bn-chrA01-p4399498
Bn-A01-p4799050	0.958677686	0.958677686	Bn-N1-p4814469	Bn-chrA01-p4410586
Bn-A01-p4800440	0.983471074	0.983471074	Bn-N11-p8026971	Bn-chrC01-p6459230
Bn-A01-p4803773	0.983388704	0.983388704	Bn-N1-p4819255	Bn-chrA01-p4413051
Bn-A01-p4808404	0.991735537	0.991735537	Bn-N1-p4824255	Bn-chrA01-p4417669
Bn-A01-p4812269	0.975124378	0.975124378	Bn-N1-p4828077	Bn-chrA01-p4421491
Bn-A01-p4820187	0.983471074	0.983471074	Bn-N1-p4830975	Bn-chrA01-p4424388
Bn-A01-p4826685	0.975206612	0.975206612	Bn-N1-p4838310	Bn-chrA01-p4433164
Bn-A01-p4832074	0.983471074	0.983471074	Bn-N1-p4839663	Bn-chrA01-p4434517
Bn-A01-p4833020	0.958677686	0.958677686	Bn-N1-p4840575	Bn-chrA01-p4435429
Bn-A01-p4836338	0.975206612	0.975206612	Bn-N1-p4844234	Bn-chrA01-p4439204
Bn-A01-p4840973	1	1	Bn-N1-p4847934	Bn-chrA01-p4442904
Bn-A01-p4869879	0.991735537	1	Bn-N1-p4876219	Bn-chrA01-p4468683
Bn-A01-p4874972	0.983471074	0.975206612	Bn-N1-p4881303	Bn-chrA01-p4474056
Bn-A01-p4877343	0.933884298	0.975206612	Bn-N1-p4888241	Bn-chrA01-p4476429
Bn-A01-p4883188	0.933884298	0.958677686	Bn-N1-p4892640	Bn-chrA01-p4482273
Bn-A01-p4908924	0.983471074	0.991735537	Bn-N1-p4918604	Bn-chrA01-p4512396
Bn-A01-p4909065	0.975206612	0.966942149	Bn-N1-p4918744	Bn-chrA01-p4512538
Bn-A01-p4920622	0.991735537	1	Bn-N1-p4922079	Bn-chrA01-p4515874
Bn-A01-p4922557	0.975206612	0.975206612	Bn-N1-p4924007	Bn-chrA01-p4517704
Bn-A01-p4925004	0.985074627	0.975124378	Bn-N1-p4926465	Bn-chrA01-p4520168
Bn-A01-p4940549	0.996677741	0.996677741	Bn-N1-p4944359	Bn-chrA01-p4541876

Bn-A01-p4947577	0.975124378	0.980099502	Bn-N1-p4952041	Bn-chrA01-p4549207
Bn-A01-p4948316	0.955223881	0.955223881	Bn-N1-p4952735	Bn-chrA01-p4549946
Bn-A01-p4963297	0.966942149	0.975206612	Bn-N1-p4969732	Bn-chrA01-p4566478
Bn-A01-p4985329	0.983471074	0.975206612	Bn-N1-p4983719	Bn-chrA01_random-p265519
Bn-A01-p4998346	0.996677741	0.990033223	Bn-N1-p4990305	Bn-chrAnn_random-p307601
Bn-A01-p5017444	0.955223881	0.925373134	Bn-N1-p5004859	Bn-chrC01-p6793387
Bn-A01-p5017446	0.955223881	0.935323383	Bn-N1-p5004861	Bn-chrA01-p4611547
Bn-A01-p5020890	0.991735537	0.991735537	Bn-N1-p5008304	Bn-chrA01-p4615003
Bn-A01-p5057753	1	1	Bn-N1-p5034368	Bn-chrA01-p4643123
Bn-A01-p5069371	0.985074627	0.985074627	Bn-N1-p5045938	Bn-chrA01-p4654693
Bn-A01-p5070565	0.991735537	0.991735537	Bn-N1-p5047130	Bn-chrA01-p4655885
Bn-A01-p5070836	1	1	Bn-N1-p5047400	Bn-chrA01-p4656155
Bn-A01-p5071350	0.983471074	0.983471074	Bn-N1-p5047914	Bn-chrA01-p4656669
Bn-A01-p5071658	0.991735537	0.991735537	Bn-N1-p5048223	Bn-chrA01-p4656978
Bn-A01-p5075984	1	0.859504132	Bn-N1-p5056324	Bn-chrC01-p6903574
Bn-A01-p5089377	0.983471074	0.702479339	Bn-N1-p5061670	Bn-chrC01-p6916435
Bn-A01-p5101078	1	1	Bn-N1-p5073789	Bn-chrA01-p4678834
Bn-A01-p5106042	0.965174129	0.965174129	Bn-N1-p5078759	Bn-chrA01-p4683804
Bn-A01-p5193639	1	1	Bn-N1-p5157847	Bn-chrA01-p4781765
Bn-A01-p5212238	0.993355482	0.993355482	Bn-N1-p5178801	Bn-chrA01-p4799202
Bn-A01-p5215209	0.870431894	0.870431894	Bn-N1-p5181745	Bn-chrC09-p22027228
Bn-A01-p5238564	0.996677741	0.996677741	Bn-N1-p5202234	Bn-chrA01-p4821807
Bn-A01-p5239959	0.990033223	0.990033223	Bn-N1-p5203630	Bn-chrA01-p4823203
Bn-A01-p5242967	0.990033223	0.990033223	Bn-N1-p5206638	Bn-chrA01-p4826211
Bn-A01-p5243029	0.990033223	0.990033223	Bn-N1-p5206700	Bn-chrA01-p4826273
Bn-A01-p5243036	0.983471074	0.983471074	Bn-N1-p5206707	Bn-chrA01-p4826280
Bn-A01-p5243181	0.983388704	0.983388704	Bn-N1-p5206852	Bn-chrA01-p4826425
Bn-A01-p5245778	0.953488372	0.953488372	Bn-N11-p8580681	Bn-chrC01-p7148597
Bn-A01-p5246582	0.980066445	0.980066445	Bn-N11-p8581506	Bn-chrC01-p7149422
Bn-A01-p5259856	0.966777409	0.966777409	Bn-N1-p5226110	Bn-chrA01-p4843199
Bn-A01-p5265598	0.975206612	0.975206612	Bn-N1-p5236162	Bn-chrA01-p4856648
Bn-A01-p5268871	0.970149254	0.970149254	Bn-N1-p5239470	Bn-chrA01-p4859956
Bn-A01-p5269368	0.986710963	0.986710963	Bn-N1-p5239967	Bn-chrA01-p4860453
Bn-A01-p5284702	0.980066445	0.980066445	Bn-N1-p5260212	Bn-chrA01-p4880514
Bn-A01-p5296564	0.966942149	0.975206612	Bn-N1-p5270686	Bn-chrA01-p4890976
Bn-A01-p5298283	0.958677686	0.958677686	Bn-N1-p5272405	Bn-chrA01-p4892688
Bn-A01-p5318059	0.958677686	0.958677686	Bn-N1-p5289158	Bn-chrA01-p4907007
Bn-A01-p5323726	1	1	Bn-N1-p5294832	Bn-chrA01-p4912681
Bn-A01-p5324399	1	1	Bn-N1-p5295505	Bn-chrA01-p4913354
Bn-A01-p5324472	0.993355482	0.993355482	Bn-N1-p5295578	Bn-chrA01-p4913427
Bn-A01-p5328220	0.966942149	0.966942149	Bn-N1-p5300332	Bn-chrA01_random-p247077
Bn-A01-p5328679	0.996677741	0.996677741	Bn-N1-p5300791	Bn-chrA01_random-p247536
Bn-A01-p5330192	0.933884298	0.933884298	Bn-N1-p5302294	Bn-chrC09-p34301636
Bn-A01-p5332773	1	1	Bn-N1-p5305947	Bn-chrA01_random-p252691
Bn-A01-p5334474	0.991735537	0.991735537	Bn-N1-p5307651	Bn-chrA01_random-p254395
Bn-A01-p5335218	0.991735537	0.991735537	Bn-N1-p5309927	Bn-chrA01_random-p256671

Bn-A01-p5335323	0.991735537	0.991735537	Bn-N1-p5310032	Bn-chrA01_random-p256776
Bn-A01-p5335372	0.983471074	0.983471074	Bn-N1-p5310082	Bn-chrA01_random-p256826
Bn-A01-p5340953	0.983471074	0.983471074	Bn-N1-p5315656	Bn-chrAnn_random-p311950
Bn-A01-p5357589	0.990033223	0.990033223	Bn-N1-p5336362	Bn-chrAnn_random-p403987
Bn-A01-p5374990	0.813953488	0.780730897	Bn-N1-p22910453	Bn-chrA01-p19372713
Bn-A01-p5378430	0.996677741	0.993355482	Bn-N1-p5359089	Bn-chrC01-p7332882
Bn-A01-p5385928	0.966777409	0.966777409	Bn-N11-p8814470	Bn-chrC01-p7348832
Bn-A01-p5391551	0.996677741	0.996677741	Bn-N1-p5370787	Bn-chrA01-p4963244
Bn-A01-p5418396	0.993355482	0.993355482	Bn-N1-p5397830	Bn-chrA01-p4990465
Bn-A01-p5469727	0.983471074	0.983471074	Bn-N1-p5442893	Bn-chrA01-p5034468
Bn-A01-p5480514	0.983471074	0.975206612	Bn-N1-p5451629	Bn-chrA01-p5042606
Bn-A01-p5480517	0.983471074	0.975206612	Bn-N1-p5451632	Bn-chrA01-p5042609
Bn-A01-p5481004	1	1	Bn-N1-p5452118	Bn-chrA01-p5043095
Bn-A01-p5485980	1	1	Bn-N1-p5456726	Bn-chrA01-p5047391
Bn-A01-p5486104	0.983471074	0.983471074	Bn-N1-p5456850	Bn-chrA01-p5047515
Bn-A01-p5499353	0.985074627	0.990049751	Bn-N19-p39038734	Bn-chrC01-p29419935
Bn-A01-p5506421	0.970149254	0.970149254	Bn-N1-p5479028	Bn-chrA01-p5068532
Bn-A01-p5508840	0.980099502	0.985074627	Bn-N1-p5481447	Bn-chrA01-p5070951
Bn-A01-p5508998	0.980099502	0.980099502	Bn-N1-p5481605	Bn-chrA01-p5071109
Bn-A01-p5522403	0.975206612	0.966942149	Bn-N1-p5499937	Bn-chrA01-p5082080
Bn-A01-p5522870	0.636363636	0.644628099	Bn-N1-p5500406	Bn-chrA01-p5082549
Bn-A01-p5535282	0.991735537	0.983471074	Bn-N1-p5509421	Bn-chrAnn_random-p370056
Bn-A01-p5536695	0.960199005	0.985074627	Bn-N1-p5510839	Bn-chrA01_random-p346634
Bn-A01-p5550226	0.950413223	0.933884298	Bn-N1-p5539534	Bn-chrA01_random-p335899
Bn-A01-p5553918	0.880398671	0.840531561	Bn-N9-p13057674	Bn-chrC08-p17347966
Bn-A01-p5567077	0.958677686	0.966942149	Bn-N1-p5556653	Bn-chrA01_random-p322699
Bn-A01-p5567840	0.851239669	0.876033058	Bn-N1-p5557170	Bn-chrA01_random-p321933
Bn-A01-p5570110	1	0.991735537	Bn-N1-p5562764	Bn-chrA01_random-p319411
Bn-A01-p5570221	0.975206612	0.975206612	Bn-N1-p5562878	Bn-chrA01_random-p319300
Bn-A01-p5579695	0.990033223	0.990033223	Bn-N1-p5576501	Bn-chrA01_random-p309426
Bn-A01-p5585071	0.995024876	0.980099502	Bn-N1-p5582231	Bn-chrA01_random-p304686
Bn-A01-p5585933	0.945273632	0.621890547	Bn-N1-p5583095	Bn-chrA01_random-p303822
Bn-A01-p5586618	0.958677686	0.950413223	Bn-N1-p5584066	Bn-chrA01_random-p302665
Bn-A01-p5586671	0.975206612	0.975206612	Bn-N1-p5584119	Bn-chrA01_random-p302612
Bn-A01-p5586854	0.966942149	0.975206612	Bn-N1-p5584302	Bn-chrA01_random-p302443
Bn-A01-p5587840	0.975206612	0.975206612	Bn-N1-p5585331	Bn-chrA01_random-p301224
Bn-A01-p5590998	0.975124378	0.965174129	Bn-N1-p5588331	Bn-chrA01_random-p300980
Bn-A01-p5591950	0.975206612	0.983471074	Bn-N1-p5589288	Bn-chrA01_random-p299000
Bn-A01-p5593317	0.984615385	0.984615385	Bn-N7-p13261996	Bn-chrC05-p6351853
Bn-A01-p5593369	0.989010989	0.989010989	Bn-N7-p13262048	Bn-chrAnn_random-p439721
Bn-A01-p5595319	1	0.991735537	Bn-N1-p5592645	Bn-chrA01_random-p296156
Bn-A01-p5599369	0.990033223	0.986710963	Bn-N1-p5594557	Bn-chrA01_random-p294062
Bn-A01-p5601232	0.990033223	0.986710963	Bn-N1-p5596422	Bn-chrA01-p5121858
Bn-A01-p5621392	0.986710963	0.990033223	Bn-N1-p5613276	Bn-chrA01-p5140924
Bn-A01-p5635471	0.975206612	0.983471074	Bn-Scaffold02141-p9	Bn-chrA01-p5145075
Bn-A01-p5647795	0.990033223	0.993355482	Bn-N1-p5631458	Bn-chrA01-p5154600

Bn-A01-p5648120	0.983388704	1	Bn-N1-p5631783	Bn-chrA01-p5154925
Bn-A01-p5658004	0.810945274	0.766169154	Bn-N1-p5642894	Bn-chrA01-p5165863
Bn-A01-p5659083	0.980066445	0.980066445	Bn-N1-p5644004	Bn-chrA01-p5166963
Bn-A01-p5660238	0.970099668	0.970099668	Bn-N1-p5645159	Bn-chrA01-p5168118
Bn-A01-p5661294	0.983388704	0.983388704	Bn-N1-p5646214	Bn-chrA01-p5169173
Bn-A01-p5663319	0.995024876	0.995024876	Bn-N1-p5648239	Bn-chrA01-p5171198
Bn-A01-p5665774	0.877076412	0.877076412	Bn-N1-p5650694	Bn-chrA01-p5173650
Bn-A01-p5678678	0.991735537	0.991735537	Bn-N1-p5668444	Bn-chrA01-p5185883
Bn-A01-p5679389	1	1	Bn-N1-p5669155	Bn-chrA01-p5186594
Bn-A01-p5680581	0.983471074	0.983471074	Bn-N1-p5670681	Bn-chrA01-p5187694
Bn-A01-p5680730	0.991735537	0.991735537	Bn-N1-p5670830	Bn-chrA01-p5187843
Bn-A01-p5688168	0.980099502	0.980099502	Bn-N1-p5679756	Bn-chrA01-p5193844
Bn-A01-p5697529	0.975206612	0.975206612	Bn-N1-p5687445	Bn-chrA01-p5201532
Bn-A01-p5697706	0.993355482	0.993355482	Bn-N1-p5687622	Bn-chrA01-p5201709
Bn-A01-p5699110	0.991735537	0.991735537	Bn-N1-p5689025	Bn-chrA01-p5203112
Bn-A01-p5711920	0.990033223	0.990033223	Bn-N1-p5700098	Bn-chrA01-p5214185
Bn-A01-p5715141	0.975206612	0.975206612	Bn-N1-p5703321	Bn-chrA01-p5217408
Bn-A01-p5718049	0.867768595	0.867768595	Bn-N11-p9349312	Bn-chrCnn_random-p172671
Bn-A01-p5730640	0.983471074	0.983471074	Bn-N1-p5719044	Bn-chrA01_random-p352044
Bn-A01-p5731429	1	1	Bn-N1-p5719833	Bn-chrA01_random-p351255
Bn-A01-p5731932	0.990033223	0.990033223	Bn-N1-p5720336	Bn-chrA01_random-p350752
Bn-A01-p5732226	0.991735537	0.991735537	Bn-N1-p5720630	Bn-chrA01_random-p350458
Bn-A01-p5737901	0.975206612	0.975206612	Bn-N1-p5728610	Bn-chrA01_random-p370504
Bn-A01-p5752342	0.966942149	0.966942149	Bn-N1-p5743225	Bn-chrA01_random-p356172
Bn-A01-p5762190	1	1	Bn-N1-p5759503	Bn-chrA01-p5236859
Bn-A01-p5775331	0.991735537	0.991735537	Bn-N1-p5766660	Bn-chrA01-p5244017
Bn-A01-p5777117	1	1	Bn-N1-p5768493	Bn-chrA01-p5245850
Bn-A01-p5781261	0.975206612	0.975206612	Bn-N1-p5772854	Bn-chrA01-p5250211
Bn-A01-p5787591	0.993355482	0.993355482	Bn-N1-p5787701	Bn-chrA01-p5266871
Bn-A01-p5795016	0.905472637	0.905472637	Bn-N1-p5795297	Bn-chrA01-p5274467
Bn-A01-p5796314	0.84079602	0.84079602	Bn-N1-p5796499	Bn-chrA01-p5275669
Bn-A01-p5796425	0.950413223	0.950413223	Bn-N1-p5796610	Bn-chrA01-p5275780
Bn-A01-p5797296	0.980099502	0.980099502	Bn-N1-p5797482	Bn-chrA01-p5276652
Bn-A01-p5797810	0.985074627	0.985074627	Bn-N1-p5797996	Bn-chrA01-p5277166
Bn-A01-p5805154	0.991735537	0.983471074	Bn-N1-p5805475	Bn-chrA01-p5284429
Bn-A01-p5806635	0.991735537	0.991735537	Bn-N1-p5806986	Bn-chrA01-p5285941
Bn-A01-p5808036	0.958677686	0.966942149	Bn-N1-p5808395	Bn-chrA01-p5287347
Bn-A01-p5821651	0.940298507	0.945273632	Bn-N1-p5822192	Bn-chrA01-p5301143
Bn-A01-p5822015	0.975206612	0.983471074	Bn-N1-p5822556	Bn-chrA01-p5301507
Bn-A01-p5835558	0.966942149	0.966942149	Bn-N1-p5839444	Bn-chrA01-p5321516
Bn-A01-p5862558	0.966942149	0.966942149	Bn-N1-p5873767	Bn-chrA01-p5346699
Bn-A01-p5868598	0.983471074	0.983471074	Bn-N1-p5878881	Bn-chrA01-p5351811
Bn-A01-p5873504	0.991735537	0.991735537	Bn-N1-p5883788	Bn-chrA01-p5356717
Bn-A01-p5878485	1	1	Bn-N1-p5888445	Bn-chrA01-p5361374
Bn-A01-p5886182	0.990033223	0.990033223	Bn-N1-p5896146	Bn-chrA01-p5369075
Bn-A01-p5899092	0.983471074	0.983471074	Bn-N1-p5914797	Bn-chrA01-p5394095

Bn-A01-p5903527	0.966942149	0.966942149	Bn-N1-p5919004	Bn-chrA01-p5398133
Bn-A01-p5917688	0.975206612	0.983471074	Bn-N1-p5927079	Bn-chrA01-p5405648
Bn-A01-p5918340	0.950413223	0.983471074	Bn-N11-p9648251	Bn-chrA01-p5406300
Bn-A01-p5921497	0.980066445	0.980066445	Bn-N1-p5930921	Bn-chrA01-p5409483
Bn-A01-p5930704	0.986710963	0.986710963	Bn-N1-p5940336	Bn-chrA01-p5418777
Bn-A01-p5943392	0.983471074	0.991735537	Bn-N1-p5953742	Bn-chrA01-p5433317
Bn-A01-p5944398	0.993355482	0.990033223	Bn-N1-p5954774	Bn-chrA01-p5434377
Bn-A01-p5949498	0.985074627	0.985074627	Bn-N1-p5959596	Bn-chrA01-p5439190
Bn-A01-p5949582	0.970149254	0.970149254	Bn-N1-p5959680	Bn-chrA01-p5439274
Bn-A01-p5949665	0.965174129	0.965174129	Bn-N1-p5959763	Bn-chrA01-p5439357
Bn-A01-p5959700	0.996677741	0.996677741	Bn-N1-p5963407	Bn-chrA01-p5443834
Bn-A01-p5973643	0.966942149	0.966942149	Bn-N1-p5979115	Bn-chrA01-p5461301
Bn-A01-p5977595	0.933884298	0.628099174	Bn-N1-p5991791	Bn-chrC01-p7971065
Bn-A01-p5977820	0.94214876	0.900826446	Bn-N1-p5992032	Bn-chrA01-p5463912
Bn-A01-p5990203	0.950413223	0.958677686	Bn-N1-p6006584	Bn-chrA01-p5473153
Bn-A01-p5994840	1	1	Bn-N1-p6011242	Bn-chrA01-p5477860
Bn-A01-p5994980	0.958677686	0.958677686	Bn-N1-p6011382	Bn-chrA01-p5478000
Bn-A01-p6007093	0.925619835	0.950413223	Bn-N1-p6023692	Bn-chrA01-p5491039
Bn-A01-p6035491	0.975206612	0.975206612	Bn-N1-p6057148	Bn-chrA01-p5519429
Bn-A01-p6038249	0.976744186	0.976744186	Bn-N1-p6059919	Bn-chrA01-p5522200
Bn-A01-p6040667	0.991735537	0.991735537	Bn-N1-p6062283	Bn-chrA01-p5524564
Bn-A01-p6044475	0.975206612	0.975206612	Bn-N1-p6066094	Bn-chrA01-p5528375
Bn-A01-p6053633	0.996677741	0.996677741	Bn-N1-p6078399	Bn-chrA01-p5539348
Bn-A01-p6066325	0.985074627	0.985074627	Bn-N1-p6089799	Bn-chrA01-p5549255
Bn-A01-p6081978	0.973421927	0.973421927	Bn-N1-p6101127	Bn-chrA01-p5560856
Bn-A01-p6083015	0.983471074	0.983471074	Bn-N1-p6102164	Bn-chrA01-p5561893
Bn-A01-p6093123	0.993355482	0.993355482	Bn-N1-p6114454	Bn-chrA01-p5574157
Bn-A01-p6111016	0.983471074	0.983471074	Bn-N1-p6134186	Bn-chrA01-p5594599
Bn-A01-p6158889	0.975206612	0.975206612	Bn-N1-p6185603	Bn-chrA01-p5644856
Bn-A01-p6168163	0.955223881	0.955223881	Bn-N1-p6192158	Bn-chrA01-p5651413
Bn-A01-p6168191	0.975206612	0.975206612	Bn-N1-p6192186	Bn-chrA01-p5651441
Bn-A01-p6180257	0.876033058	0.876033058	Bn-N1-p6204502	Bn-chrA01-p5663657
Bn-A01-p6182710	0.983388704	0.983388704	Bn-N1-p6206809	Bn-chrA01-p5665964
Bn-A01-p6188347	0.910299003	0.906976744	Bn-N1-p6212733	Bn-chrA01-p5671593
Bn-A01-p6199422	0.925619835	0.925619835	Bn-N1-p6225257	Bn-chrA01-p5684126
Bn-A01-p6215196	1	1	Bn-N1-p6237449	Bn-chrA01-p5696810
Bn-A01-p6216424	0.995024876	0.995024876	Bn-N11-p10168763	Bn-chrA01-p5698097
Bn-A01-p6217011	0.880597015	0.880597015	Bn-N1-p6240730	Bn-chrA01-p5698697
Bn-A01-p6220667	0.983471074	0.983471074	Bn-N1-p6244388	Bn-chrA01-p5702355
Bn-A01-p6232061	0.995024876	0.995024876	Bn-N1-p6255985	Bn-chrA01-p5714482
Bn-A01-p6270054	0.983471074	0.983471074	Bn-N1-p6282220	Bn-chrA01-p5738635
Bn-A01-p6271245	0.990033223	0.990033223	Bn-N1-p6283411	Bn-chrA01-p5739826
Bn-A01-p6272642	0.966942149	0.966942149	Bn-N1-p6284809	Bn-chrA01-p5741224
Bn-A01-p6274135	0.993355482	0.993355482	Bn-N1-p6286302	Bn-chrA01-p5742717
Bn-A01-p6275784	0.983471074	0.983471074	Bn-N1-p6287952	Bn-chrA01-p5744367
Bn-A01-p6290508	0.985074627	0.985074627	Bn-N1-p6303043	Bn-chrA01-p5759670

Bn-A01-p6333810	0.975206612	0.975206612	Bn-N1-p6331851	Bn-chrA01-p5789821
Bn-A01-p6333910	0.975206612	0.975206612	Bn-N1-p6331950	Bn-chrA01-p5789920
Bn-A01-p6349933	0.983471074	0.983471074	Bn-N1-p6348505	Bn-chrCnn_random-p611287
Bn-A01-p6369633	0.966942149	0.966942149	Bn-N1-p6371209	Bn-chrA01-p5828346
Bn-A01-p6376105	0.950248756	0.950248756	Bn-N1-p6377618	Bn-chrA01-p5834749
Bn-A01-p6380934	0.983471074	0.975206612	Bn-N1-p6382383	Bn-chrA01-p5841882
Bn-A01-p6388431	0.717607973	0.990033223	Bn-N11-p10420355	Bn-chrA01-p5849311
Bn-A01-p6390236	0.925619835	0.983471074	Bn-N11-p10420987	Bn-chrA01-p5851109
Bn-A01-p6439605	0.950413223	0.958677686	Bn-Scaffold01598-p2	Bn-chrA01-p5927786
Bn-A01-p6478922	1	0.991735537	Bn-N1-p6479723	Bn-chrA01-p5956270
Bn-A01-p6479735	0.975206612	0.966942149	Bn-N1-p6480559	Bn-chrA01-p5957083
Bn-A01-p6482543	0.983471074	0.975206612	Bn-N1-p6482538	Bn-chrA01-p5959061
Bn-A01-p6487075	0.991735537	0.983471074	Bn-N1-p6489048	Bn-chrA01-p5964206
Bn-A01-p6505754	0.966942149	0.966942149	Bn-N1-p6512936	Bn-chrA01-p5976139
Bn-A01-p6507432	0.958677686	0.958677686	Bn-N11-p10670241	Bn-chrC01-p8881969
Bn-A01-p6512377	0.995024876	0.995024876	Bn-N16-p6880042	Bn-chrC06-p6547683
Bn-A01-p6530903	0.991735537	0.991735537	Bn-N1-p6528242	Bn-chrA01-p5995366
Bn-A01-p6538362	0.983471074	0.983471074	Bn-N1-p6535423	Bn-chrA01-p6002546
Bn-A01-p6546245	0.940298507	0.940298507	Bn-N1-p6542526	Bn-chrA01-p6010206
Bn-A01-p6547052	0.975206612	0.966942149	Bn-N1-p6543334	Bn-chrA01-p6011013
Bn-A01-p6552596	0.966942149	0.966942149	Bn-N1-p6548875	Bn-chrA01-p6016894
Bn-A01-p6558843	0.991735537	0.991735537	Bn-N1-p6555124	Bn-chrA01-p6023144
Bn-A01-p6560270	0.983471074	0.983471074	Bn-N1-p6556551	Bn-chrA01-p6024571
Bn-A01-p6604419	0.975206612	0.983471074	Bn-N1-p6589736	Bn-chrA01-p6051921
Bn-A01-p6625349	0.925619835	0.801652893	Bn-N1-p6605923	Bn-chrA01-p6073160
Bn-A01-p6628035	0.966942149	0.975206612	Bn-N1-p6608609	Bn-chrA01-p6080336
Bn-A01-p6643749	0.970149254	1	Bn-N1-p6624569	Bn-chrA01-p6096242
Bn-A01-p6664608	0.991735537	0.983471074	Bn-N1-p6648803	Bn-chrA01-p6115949
Bn-A01-p6678914	0.958677686	0.950413223	Bn-N1-p6660632	Bn-chrA01-p6119402
Bn-A01-p6679394	0.990049751	1	Bn-N1-p6661113	Bn-chrA01-p6119883
Bn-A01-p6679669	0.980099502	0.950248756	Bn-N1-p6661389	Bn-chrA01-p6120161
Bn-A01-p6687707	0.926910299	0.976744186	Bn-N1-p6671771	Bn-chrA01-p6129231
Bn-A01-p6687839	0.993355482	0.990033223	Bn-N1-p6671903	Bn-chrA01-p6129363
Bn-A01-p6699539	0.983471074	0.983471074	Bn-N1-p6682012	Bn-chrA01-p6139862
Bn-A01-p6702060	0.84079602	0.582089552	Bn-N1-p6684545	Bn-chrC01-p9193465
Bn-A01-p6708539	0.965174129	0.970149254	Bn-N1-p6691596	Bn-chrA01-p6149644
Bn-A01-p6713213	0.991735537	0.991735537	Bn-N1-p6700220	Bn-chrA01-p6154320
Bn-A01-p6714740	0.983471074	0.983471074	Bn-N1-p6701747	Bn-chrA01-p6155847
Bn-A01-p6715317	0.983471074	0.983471074	Bn-N1-p6702324	Bn-chrA01-p6156424
Bn-A01-p6747706	0.990049751	0.900497512	Bn-N1-p6723020	Bn-chrA01-p6173561
Bn-A01-p6773750	0.950413223	0.917355372	Bn-N1-p6753096	Bn-chrA01-p6193165
Bn-A01-p6773949	0.966942149	0.975206612	Bn-N1-p6753295	Bn-chrA01-p6193364
Bn-A01-p6774097	0.958677686	0.966942149	Bn-N1-p6753441	Bn-chrA01-p6193512
Bn-A01-p6774837	0.975206612	0.958677686	Bn-N1-p6754182	Bn-chrA01-p6194250
Bn-A01-p6776634	0.980099502	0.960199005	Bn-N1-p6755980	Bn-chrA01-p6196049
Bn-A01-p6776950	0.980066445	0.973421927	Bn-N1-p6756296	Bn-chrA01-p6196365

Bn-A01-p6787691	0.960199005	0.925373134	Bn-N1-p6778779	Bn-chrC01-p9330432
Bn-A01-p6787930	0.985074627	0.965174129	Bn-N1-p6779018	Bn-chrC01-p9330669
Bn-A01-p6788044	0.985074627	0.970149254	Bn-N1-p6779131	Bn-chrC01-p9330782
Bn-A01-p6801449	0.980099502	0.950248756	Bn-N1-p6771861	Bn-chrC01-p9330657
Bn-A01-p6825673	0.983471074	0.975206612	Bn-N1-p6810101	Bn-chrA01-p6237991
Bn-A01-p6826327	0.983471074	0.991735537	Bn-N1-p6810755	Bn-chrA01-p6238642
Bn-A01-p6838738	0.900497512	0.900497512	Bn-N1-p6828366	Bn-chrA01-p6251013
Bn-A01-p6841235	0.996677741	0.996677741	Bn-N1-p6830489	Bn-chrA01-p6253475
Bn-A01-p6854917	1	0.743801653	Bn-N1-p6844587	Bn-chrA01-p6267309
Bn-A01-p6863835	1	0.991735537	Bn-N1-p6857101	Bn-chrA01-p6282214
Bn-A01-p6877281	0.92039801	0.980099502	Bn-N11-p11291882	Bn-chrA01-p6295794
Bn-A01-p6898291	0.986710963	0.970099668	Bn-N1-p6894209	Bn-chrA01-p6314129
Bn-A01-p6898690	0.975206612	0.983471074	Bn-N1-p6894608	Bn-chrA01-p6314539
Bn-A01-p6899980	0.621262458	0.777408638	Bn-N11-p11341765	Bn-chrC01_random-p546805
Bn-A01-p6900616	0.67768595	0.900826446	Bn-N3-p25195348	Bn-chrA01-p6317757
Bn-A01-p6907522	0.909090909	0.983471074	Bn-N1-p6896133	Bn-chrA01-p6325663
Bn-A01-p6920456	0.993355482	0.936877076	Bn-N1-p6908046	Bn-chrC01-p9662214
Bn-A01-p6925167	0.980099502	0.980099502	Bn-N1-p6911249	Bn-chrA01-p6341213
Bn-A01-p6925436	0.966942149	0.975206612	Bn-N1-p6911513	Bn-chrA01-p6341482
Bn-A01-p6950868	0.966777409	0.993355482	Bn-N11-p11409994	Bn-chrA01-p6363827
Bn-A01-p6958863	0.966942149	0.966942149	Bn-N1-p6946745	Bn-chrA01-p6371822
Bn-A01-p6959248	0.884297521	0.966942149	Bn-N1-p6947123	Bn-chrA01-p6372207
Bn-A01-p6962542	0.975206612	0.983471074	Bn-N1-p6949268	Bn-chrA01-p6375502
Bn-A01-p6965221	0.58677686	0.983471074	Bn-N1-p6952513	Bn-chrA01-p6378181
Bn-A01-p6968751	0.975206612	0.966942149	Bn-N1-p6955983	Bn-chrA01-p6381711
Bn-A01-p6969197	0.975206612	0.966942149	Bn-N1-p6956429	Bn-chrA01-p6382157
Bn-A01-p6969284	0.975206612	0.966942149	Bn-N1-p6956516	Bn-chrA01-p6382244
Bn-A01-p6969358	0.983471074	0.991735537	Bn-N1-p6956590	Bn-chrA01-p6382318
Bn-A01-p6972779	0.956810631	0.993355482	Bn-N1-p6961785	Bn-chrA01-p6393067
Bn-A01-p6976938	0.96013289	0.956810631	Bn-N1-p6971482	Bn-chrA01-p6397251
Bn-A01-p6986405	0.989583333	0.989583333	Bn-N1-p6981704	Bn-chrA01-p6406968
Bn-A01-p7026162	0.983471074	0.991735537	Bn-N1-p7018820	Bn-chrA01-p6432028
Bn-A01-p7068752	0.975206612	0.966942149	Bn-N1-p7060343	Bn-chrA01-p6472468
Bn-A01-p7069263	0.983471074	0.983471074	Bn-N1-p7060854	Bn-chrA01-p6472970
Bn-A01-p7086543	0.975206612	0.983471074	Bn-N1-p7076616	Bn-chrA01-p6487162
Bn-A01-p7087000	0.966942149	0.958677686	Bn-N1-p7077073	Bn-chrA01-p6487619
Bn-A01-p7089762	1	0.520661157	Bn-N1-p7079968	Bn-chrA07-p7625545
Bn-A01-p7089826	0.991735537	0.347107438	Bn-N1-p7080032	Unknown
Bn-A01-p7121832	0.990049751	0.990049751	Bn-N1-p7130180	Bn-chrC01-p10077455
Bn-A01-p7122036	0.995024876	0.995024876	Bn-N1-p7130384	Bn-chrC01-p10077659
Bn-A01-p7132729	0.983388704	0.983388704	Bn-N1-p7146539	Bn-chrA01-p6512785
Bn-A01-p7137897	0.975206612	0.966942149	Bn-N1-p7151712	Bn-chrA01-p6517958
Bn-A01-p7150748	0.945273632	0.995024876	Bn-N1-p7159741	Bn-chrC01-p10154461
Bn-A01-p7151096	0.815920398	1	Bn-N1-p7160087	Bn-chrC01-p10154802
Bn-A01-p7160702	0.966942149	0.975206612	Bn-N1-p7166601	Bn-chrA01-p6535701
Bn-A01-p7205595	0.990049751	0.990049751	Bn-N1-p7219932	Bn-chrA01-p6574483

Bn-A01-p7363480	0.955223881	0.955223881	Bn-N1-p7373856	Bn-chrA01-p6730564
Bn-A01-p7367598	0.970149254	0.970149254	Bn-N1-p7377974	Bn-chrA01-p6734682
Bn-A01-p7369125	0.940298507	0.940298507	Bn-N1-p7379533	Bn-chrA01-p6736241
Bn-A01-p7372570	0.975206612	0.975206612	Bn-N1-p7383038	Bn-chrA01-p6739746
Bn-A01-p7372653	0.905472637	0.905472637	Bn-N1-p7383121	Bn-chrA01-p6739829
Bn-A01-p7373427	0.983471074	0.983471074	Bn-N1-p7383894	Bn-chrA01-p6740602
Bn-A01-p7374290	0.970099668	0.970099668	Bn-N1-p7384740	Bn-chrA01-p6741448
Bn-A01-p7374589	0.966777409	0.966777409	Bn-N1-p7385039	Bn-chrA01-p6741747
Bn-A01-p7383179	0.983471074	0.983471074	Bn-N1-p7391780	Bn-chrA01-p6747608
Bn-A01-p7385409	0.983471074	0.983471074	Bn-N1-p7397323	Bn-chrA01-p6751739
Bn-A01-p7385725	0.925619835	0.925619835	Bn-N1-p7397662	Bn-chrA01-p6752078
Bn-A01-p7385797	0.925619835	0.925619835	Bn-N1-p7397728	Bn-chrA01-p6752144
Bn-A01-p7386111	0.809917355	0.314049587	Bn-N1-p7407499	Unknown
Bn-A01-p7399827	0.996677741	0.996677741	Bn-N1-p7417558	Bn-chrAnn_random-p310118
Bn-A01-p7405147	0.991735537	0.991735537	Bn-N1-p7423799	Bn-chrAnn_random-p388461
Bn-A01-p7405753	1	1	Bn-N1-p7430979	Bn-chrA01-p6753657
Bn-A01-p7405882	0.991735537	0.991735537	Bn-N1-p7431108	Bn-chrA01-p6753786
Bn-A01-p7406041	0.966942149	0.966942149	Bn-N1-p7431267	Bn-chrA01-p6753945
Bn-A01-p7430242	0.950413223	0.950413223	Bn-N1-p7471095	Bn-chrA01-p6794634
Bn-A01-p7430311	0.94214876	0.94214876	Bn-N1-p7471164	Bn-chrA01-p6794703
Bn-A01-p7440935	0.890365449	0.870431894	Bn-N1-p7476634	Bn-chrA01-p6800178
Bn-A01-p7452338	0.909090909	0.925619835	Bn-N1-p7483807	Bn-chrC01-p10669118
Bn-A01-p7455231	1	1	Bn-N1-p7488564	Bn-chrA01-p6812297
Bn-A01-p7455852	0.966942149	0.966942149	Bn-N1-p7489185	Bn-chrA01-p6812918
Bn-A01-p7473265	0.96345515	0.96345515	Bn-N1-p7507779	Bn-chrA01-p6831342
Bn-A01-p7479483	0.956810631	0.956810631	Bn-N1-p7515195	Bn-chrA01-p6837560
Bn-A01-p7487861	0.950413223	0.950413223	Bn-N1-p7523127	Bn-chrA01-p6845492
Bn-A01-p7493373	0.870431894	0.870431894	Bn-N1-p7527425	Bn-chrA01-p6849790
Bn-A01-p7512557	0.980099502	0.980099502	Bn-N1-p7539289	Bn-chrA01-p6861651
Bn-A01-p7530968	0.993355482	0.993355482	Bn-N1-p7557701	Bn-chrA01-p6880062
Bn-A01-p7533971	0.970149254	1	Bn-N1-p7562581	Bn-chrA01-p6884170
Bn-A01-p7548806	0.983388704	0.970099668	Bn-N1-p7577062	Bn-chrA01-p6889363
Bn-A01-p7551163	0.993355482	0.993355482	Bn-N1-p7579411	Bn-chrA01-p6891789
Bn-A01-p7554673	0.993355482	0.993355482	Bn-N1-p7582871	Bn-chrA01-p6895227
Bn-A01-p7557422	0.991735537	0.991735537	Bn-N1-p7585620	Bn-chrA01-p6898043
Bn-A01-p7558012	0.996677741	0.996677741	Bn-N1-p7586211	Bn-chrA01-p6898631
Bn-A01-p7558345	0.991735537	1	Bn-N1-p7586544	Bn-chrA01-p6898964
Bn-A01-p7559946	0.990033223	0.986710963	Bn-N1-p7588145	Bn-chrA01-p6900567
Bn-A01-p7560785	1	0.991735537	Bn-N1-p7588984	Bn-chrA01-p6901133
Bn-A01-p7573871	0.983388704	0.983388704	Bn-N1-p7606082	Bn-chrA01-p6916160
Bn-A01-p7574355	0.752066116	0.752066116	Bn-N1-p7606566	Bn-chrA01-p6916644
Bn-A01-p7582017	0.986710963	0.986710963	Bn-N1-p7616371	Bn-chrAnn_random-p40769[illegible]
Bn-A01-p7585849	0.893687708	0.651162791	Bn-N1-p23158511	Bn-chrC09-p13196064
Bn-A01-p7586821	0.764119601	0.764119601	Bn-N19-p17075652	Bn-chrC09-p13195093
Bn-A01-p7587840	0.746268657	0.746268657	Bn-N19-p17074632	Bn-chrC09-p13194073
Bn-A01-p7588413	0.777408638	0.328903654	Bn-N8-p8302634	Bn-chrC03-p56837490

Bn-A01-p7588937	0.637873754	0.156146179	Bn-N8-p8302107	Unknown
Bn-A01-p7588958	0.574750831	0.079734219	Bn-N8-p8302086	Unknown
Bn-A01-p7593180	0.890365449	0.96345515	Bn-N1-p7624917	Bn-chrA01_random-p395780
Bn-A01-p7594170	1	1	Bn-N1-p7625913	Bn-chrA01_random-p394784
Bn-A01-p7603030	0.993355482	0.993355482	Bn-N1-p7632937	Bn-chrA01_random-p387762
Bn-A01-p7615707	0.976744186	0.980066445	Bn-N1-p7645721	Bn-chrAnn_random-p464995
Bn-A01-p7619726	0.986710963	0.986710963	Bn-N1-p7649889	Bn-chrA08-p9978124
Bn-A01-p7643514	0.990049751	0.990049751	Bn-N1-p7681242	Bn-chrA08-p9946266
Bn-A01-p7643571	0.983471074	0.983471074	Bn-N1-p7681299	Bn-chrA08-p9946209
Bn-A01-p7646601	0.506578947	0.973684211	Unknown	Bn-chrA08-p9943123
Bn-A01-p7648929	0.995024876	1	Bn-N1-p7686806	Bn-chrA08-p9940730
Bn-A01-p7656521	1	1	Bn-N1-p7696624	Bn-chrA08-p9933069
Bn-A01-p7667896	0.990049751	0.990049751	Bn-N1-p7708636	Bn-chrA01-p6923086
Bn-A01-p7698558	0.983471074	0.991735537	Bn-N11-p12789926	Bn-chrC01-p11040845
Bn-A01-p7699159	0.991735537	1	Bn-N1-p7741280	Bn-chrC01-p11038059
Bn-A01-p7702471	0.975124378	0.980099502	Bn-N1-p7744599	Bn-chrA01-p6951719
Bn-A01-p7714470	0.993355482	0.993355482	Bn-N1-p7759078	Bn-chrA01-p6966540
Bn-A01-p7717618	0.820598007	0.823920266	Bn-N11-p12871562	Bn-chrA01-p6971242
Bn-A01-p7721497	0.990033223	0.990033223	Bn-N1-p7767388	Bn-chrA01-p6975452
Bn-A01-p7724859	0.958677686	0.966942149	Bn-N1-p7770754	Bn-chrA01-p6978807
Bn-A01-p7745080	1	0.995024876	Bn-N1-p7816543	Bn-chrA01-p7014266
Bn-A01-p7768351	0.985074627	0.980099502	Bn-N1-p7844467	Bn-chrA01-p7036995
Bn-A01-p7771617	0.995024876	1	Bn-N1-p7849593	Bn-chrA01-p7044017
Bn-A01-p7772345	0.993355482	0.946843854	Bn-N1-p7850321	Bn-chrA01-p7044748
Bn-A01-p7787153	0.990033223	0.990033223	Bn-N1-p7857203	Bn-chrA01-p7049040
Bn-A01-p7800839	0.983471074	0.983471074	Bn-N1-p7867517	Bn-chrAnn_random-p397960
Bn-A01-p7825044	0.983471074	0.983471074	Bn-N1-p7886156	Bn-chrA01_random-p418895
Bn-A01-p7827260	0.983388704	0.983388704	Bn-N1-p7888408	Bn-chrA01_random-p421146
Bn-A01-p7831289	0.990049751	0.990049751	Bn-N1-p7892564	Bn-chrA01_random-p425302
Bn-A01-p7834871	0.980099502	0.980099502	Bn-N1-p7896286	Bn-chrA01_random-p429024
Bn-A01-p7850092	0.930232558	0.936877076	Bn-N11-p13083414	Bn-chrC01_random-p669458
Bn-A01-p7852440	0.986710963	0.986710963	Bn-N1-p7911511	Bn-chrA01_random-p443776
Bn-A01-p7861283	0.990033223	0.990033223	Bn-N1-p7920296	Bn-chrA01-p7054916
Bn-A01-p7875358	0.983388704	0.983388704	Bn-N1-p7934380	Bn-chrA01-p7069135
Bn-A01-p7889385	0.990049751	0.990049751	Bn-N1-p7947519	Bn-chrA01-p7082077
Bn-A01-p7890174	0.990049751	0.990049751	Bn-N1-p7948308	Bn-chrA01-p7082866
Bn-A01-p7897804	0.980099502	0.980099502	Bn-N1-p7953013	Bn-chrA01-p7087572
Bn-A01-p7897805	0.975124378	0.975124378	Bn-N1-p7953014	Bn-chrA01-p7087573
Bn-A01-p7926287	0.975206612	0.975206612	Bn-N1-p7973148	Bn-chrA01-p7102643
Bn-A01-p7942548	0.980099502	0.980099502	Bn-N1-p7988862	Bn-chrA01-p7118271
Bn-A01-p7953001	0.975206612	0.975206612	Bn-N1-p7998583	Bn-chrA01-p7128068
Bn-A01-p7961620	0.976744186	0.976744186	Bn-N1-p8008195	Bn-chrA01-p7136643
Bn-A01-p7973418	0.996677741	0.996677741	Bn-N1-p8025818	Bn-chrA01-p7155781
Bn-A01-p7974551	1	1	Bn-N1-p8027772	Bn-chrA01-p7156915
Bn-A01-p7979458	0.818181818	0.818181818	Bn-N1-p8033912	Bn-chrA01-p7163055
Bn-A01-p7982220	0.975206612	0.975206612	Bn-N11-p13267055	Bn-chrA01-p7165840

Bn-A01-p7983241	0.983471074	0.983471074	Bn-N1-p8037687	Bn-chrA01-p7166830
Bn-A01-p7987687	0.925619835	0.925619835	Bn-N1-p8044316	Bn-chrA01-p7172128
Bn-A01-p8014995	0.986710963	0.986710963	Bn-N1-p8069849	Bn-chrA01-p7196146
Bn-A01-p8016433	0.996677741	0.996677741	Bn-N1-p8071288	Bn-chrA01-p7197578
Bn-A01-p8017380	0.996677741	0.996677741	Bn-N1-p8072235	Bn-chrA01-p7198525
Bn-A01-p8043776	0.837209302	0.837209302	Bn-N1-p8093914	Bn-chrA01-p7219664
Bn-A01-p8045760	0.975206612	0.975206612	Bn-N1-p8095898	Bn-chrA01-p7221648
Bn-A01-p8046142	0.784053156	0.784053156	Bn-N1-p8096280	Bn-chrA01-p7222030
Bn-A01-p8051029	1	1	Bn-N1-p8105843	Bn-chrC07-p41058503
Bn-A01-p8051804	0.989583333	0.989583333	Bn-N1-p8106618	Bn-chrC07-p41059278
Bn-A01-p8058255	0.892561983	0.892561983	Bn-N1-p8113120	Bn-chrA01-p7238499
Bn-A01-p8058475	0.975206612	0.975206612	Bn-N1-p8113339	Bn-chrA01-p7238718
Bn-A01-p8063652	0.966942149	0.966942149	Bn-N1-p8117459	Bn-chrA01-p7242838
Bn-A01-p8076080	1	1	Bn-N1-p8129897	Bn-chrA01-p7255275
Bn-A01-p8080531	0.993355482	0.993355482	Bn-N1-p8134219	Bn-chrA01-p7259596
Bn-A01-p8108178	0.966942149	0.966942149	Bn-N1-p8150073	Bn-chrA01-p7283813
Bn-A01-p8111196	0.652892562	0.652892562	Bn-N1-p8153093	Bn-chrA01-p7286833
Bn-A01-p8115849	0.991735537	0.991735537	Bn-N1-p8154622	Bn-chrA01-p7288362
Bn-A01-p8116314	0.980066445	0.980066445	Bn-N1-p8155064	Bn-chrA01-p7288804
Bn-A01-p8121877	0.996677741	0.996677741	Bn-N1-p8160621	Bn-chrA01-p7294361
Bn-A01-p8122779	0.993355482	0.993355482	Bn-N1-p8161525	Bn-chrA01-p7295265
Bn-A01-p8129876	0.990033223	0.990033223	Bn-N1-p8169670	Bn-chrA01-p7303410
Bn-A01-p8150111	0.966942149	0.975206612	Bn-N11-p13554024	Bn-chrA01-p7333522
Bn-A01-p8154524	0.990033223	0.990033223	Bn-N1-p8199131	Bn-chrA01-p7338018
Bn-A01-p8203740	0.983471074	0.983471074	Bn-N5-p19091302	Bn-chrA05-p16400128
Bn-A01-p8204552	0.990033223	0.990033223	Bn-N5-p19090490	Bn-chrA05-p16399316
Bn-A01-p8205728	0.980099502	0.980099502	Bn-N5-p19089314	Bn-chrA05-p16398140
Bn-A01-p8206136	0.970149254	0.970149254	Bn-N5-p19088906	Bn-chrA05-p16397732
Bn-A01-p8206374	0.970149254	0.970149254	Bn-N5-p19088668	Bn-chrA05-p16397494
Bn-A01-p8207083	0.96013289	0.986710963	Bn-N5-p19087961	Bn-chrA09-p9508538
Bn-A01-p8221245	0.985074627	0.985074627	Bn-N1-p8246043	Bn-chrA01-p7386200
Bn-A01-p8240363	0.986710963	0.986710963	Bn-N1-p8256146	Bn-chrA01-p7397322
Bn-A01-p8242690	0.973421927	0.973421927	Bn-Scaffold01941-p1	Bn-chrA01-p7406700
Bn-A01-p8246641	0.983388704	0.983388704	Bn-N1-p8273622	Bn-chrA01-p7420319
Bn-A01-p8263964	0.990033223	0.990033223	Bn-N1-p8302293	Bn-chrA01_random-p505876
Bn-A01-p8276793	0.970149254	0.970149254	Bn-N1-p8323848	Bn-chrA01_random-p487925
Bn-A01-p8276922	0.986710963	0.986710963	Bn-N1-p8323977	Bn-chrA01_random-p487796
Bn-A01-p8278925	0.983388704	0.983388704	Bn-N1-p8326546	Bn-chrA01_random-p481977
Bn-A01-p8335308	0.980066445	0.980066445	Bn-N1-p8372080	Bn-chrA01-p7450568
Bn-A01-p8353426	0.983388704	0.983388704	Bn-N1-p8396150	Bn-chrA01-p7470783
Bn-A01-p8354196	0.965174129	0.985074627	Bn-N11-p13880397	Bn-chrA01-p7471553
Bn-A01-p8378967	0.966777409	0.966777409	Bn-N1-p8419568	Bn-chrA01-p7493349
Bn-A01-p8402568	0.905472637	0.905472637	Bn-N1-p8446285	Bn-chrA01-p7516478
Bn-A01-p8404588	0.903654485	0.903654485	Bn-N1-p8448286	Bn-chrA01-p7518486
Bn-A01-p8417520	0.863787375	0.863787375	Bn-N1-p8460647	Bn-chrA01-p7530186
Bn-A01-p8418102	0.956810631	0.956810631	Bn-N1-p8461229	Bn-chrA01-p7530768

Bn-A01-p8439363	0.976744186	0.973421927	Bn-N1-p8481766	Bn-chrAnn_random-p300993
Bn-A01-p8450346	0.983471074	0.958677686	Bn-Scaffold36634-p1	Bn-chrC01-p12152927
Bn-A01-p8451192	0.933884298	0.933884298	Bn-N11-p14033294	Bn-chrC01-p12153772
Bn-A01-p8452366	0.966942149	0.966942149	Bn-N1-p8497468	Bn-chrA01-p7614126
Bn-A01-p8452493	0.809917355	0.809917355	Bn-N1-p8497595	Bn-chrA01-p7614253
Bn-A01-p8452981	0.958677686	0.958677686	Bn-N1-p8499567	Bn-chrA01-p7615693
Bn-A01-p8453146	0.817275748	0.817275748	Bn-N1-p8499692	Bn-chrA01-p7615818
Bn-A01-p8470314	0.970149254	0.970149254	Bn-N1-p8518161	Bn-chrA01-p7634112
Bn-A01-p8501753	0.973421927	0.973421927	Bn-N1-p8547388	Bn-chrA01-p7667287
Bn-A01-p8504134	0.996677741	0.996677741	Bn-N1-p8549776	Bn-chrA01-p7669675
Bn-A01-p8515129	0.993355482	0.993355482	Bn-N1-p8562814	Bn-chrA01-p7689564
Bn-A01-p8543512	0.750830565	0.750830565	Bn-N1-p8581598	Bn-chrA01-p7708346
Bn-A01-p8564149	0.966777409	0.966777409	Bn-N1-p8600760	Bn-chrA01-p7727524
Bn-A01-p8593291	0.966942149	0.966942149	Bn-N4-p2606739	Bn-chrA04-p2398257
Bn-A01-p8594736	0.966942149	0.966942149	Bn-N4-p2608184	Bn-chrA04-p2399702
Bn-A01-p8594955	1	1	Bn-Scaffold05191-p1	Bn-chrA04-p2399921
Bn-A01-p8595450	0.991735537	0.991735537	Bn-N4-p2608898	Bn-chrA04-p2400416
Bn-A01-p8595895	0.991735537	0.983471074	Bn-Scaffold05191-p5	Bn-chrA04-p2400861
Bn-A01-p8596463	0.768595041	1	Bn-N18-p10605319	Bn-chrA04-p2401429
Bn-A01-p8596650	1	0.991735537	Bn-Scaffold51445-p1	Bn-chrA04-p2401616
Bn-A01-p8647625	0.986710963	0.990033223	Bn-N1-p8687484	Bn-chrA01_random-p529366
Bn-A01-p8675601	0.991735537	0.727272727	Bn-N1-p8721483	Bn-chrA01-p7835169
Bn-A01-p8675930	0.975206612	0.983471074	Bn-N1-p8721812	Bn-chrA01-p7835721
Bn-A01-p8676024	0.983471074	0.991735537	Bn-N1-p8721906	Bn-chrA01-p7835815
Bn-A01-p8676144	0.983471074	0.975206612	Bn-N1-p8722026	Bn-chrA01-p7835935
Bn-A01-p8681309	0.983471074	0.991735537	Bn-N1-p8725314	Bn-chrA01-p7837781
Bn-A01-p8681556	1	0.991735537	Bn-N1-p8725561	Bn-chrA01-p7838028
Bn-A01-p8681757	0.955223881	0.960199005	Bn-N1-p8725762	Bn-chrA01-p7838230
Bn-A01-p8683487	0.996677741	0.996677741	Bn-N1-p8727492	Bn-chrA01-p7839954
Bn-A01-p8782649	0.991735537	0.983471074	Bn-N10-p4468256	Bn-chrA09_random-p186763
Bn-A01-p8788567	0.983471074	0.975206612	Bn-N9-p18588951	Bn-chrA09_random-p187355
Bn-A01-p8795719	0.990033223	0.990033223	Bn-N9-p18596033	Bn-chrA09_random-p188084
Bn-A01-p8805177	1	0.991735537	Bn-N9-p18605492	Bn-chrA09_random-p189029
Bn-A01-p8806524	1	0.991735537	Bn-N9-p18606839	Bn-chrA09_random-p189164
Bn-A01-p8821722	1	0.991735537	Bn-N9-p18625383	Bn-chrA09_random-p177019
Bn-A01-p8836783	0.991735537	0.983471074	Bn-N9-p18640527	Bn-chrA09_random-p175376
Bn-A01-p8839183	1	0.991735537	Bn-N9-p18642933	Bn-chrA09_random-p175136
Bn-A01-p8859217	1	0.991735537	Bn-N9-p18661213	Bn-chrCnn_random-p445910
Bn-A01-p8874473	0.991735537	1	Bn-N9-p18681639	Bn-chrCnn_random-p446093
Bn-A01-p8901327	0.975206612	0.966942149	Bn-N9-p18728866	Bn-chrA09_random-p168724
Bn-A01-p8901490	0.983471074	0.975206612	Bn-N9-p18729029	Bn-chrA09_random-p168707
Bn-A01-p8914737	0.991735537	1	Bn-N9-p18745608	Bn-chrA09_random-p167461
Bn-A01-p8960719	0.975206612	0.975206612	Bn-N9-p3108263	Bn-chrA09-p2625559
Bn-A01-p8977767	0.983471074	0.983471074	Bn-N9-p3090809	Bn-chrC09-p2832282
Bn-A01-p8982996	0.991735537	0.991735537	Bn-N9-p3088749	Bn-chrC09-p2830160
Bn-A01-p9004629	1	1	Bn-N9-p3062150	Bn-chrA09-p2580835

Bn-A01-p9030262	0.928143713	0.928143713	Bn-N9-p3044810	Bn-chrA09-p2563497
Bn-A01-p9065564	0.995024876	0.995024876	Bn-Scaffold06532-p1	Bn-chrA09-p2519782
Bn-A01-p9075803	0.991735537	0.983471074	Bn-N9-p2991521	Bn-chrA09-p2511838
Bn-A01-p9075931	0.975206612	0.966942149	Bn-N9-p2991393	Bn-chrA09-p2511710
Bn-A01-p9080359	1	0.933884298	Bn-N9-p2986898	Bn-chrA09-p2507320
Bn-A01-p9080573	0.900332226	0.950166113	Bn-N9-p2986684	Bn-chrA09-p2507104
Bn-A01-p9125819	0.94214876	0.94214876	Bn-N9-p2930756	Bn-chrA09-p2472808
Bn-A01-p9129693	0.980066445	0.983388704	Bn-N9-p2927594	Bn-chrA09-p2469614
Bn-A01-p9130391	1	0.983471074	Bn-N9-p2926885	Bn-chrA09-p2468909
Bn-A01-p9138209	0.909090909	0.950413223	Bn-N9-p2919064	Bn-chrA09-p2461096
Bn-A01-p9145211	1	0.995024876	Bn-N9-p2913161	Bn-chrA09-p2455247
Bn-A01-p9145630	0.970149254	0.930348259	Bn-N9-p2912742	Bn-chrA09-p2454827
Bn-A01-p9146490	0.975206612	0.983471074	Bn-N9-p2911878	Bn-chrA09-p2453962
Bn-A01-p9147096	0.975206612	0.966942149	Bn-N9-p2911272	Bn-chrA09-p2453356
Bn-A01-p9149601	0.958677686	0.966942149	Bn-N9-p2908776	Bn-chrA09-p2450831
Bn-A01-p9150695	0.991735537	0.983471074	Bn-N9-p2907683	Bn-chrA09-p2449731
Bn-A01-p9155327	0.975206612	0.975206612	Bn-N9-p2893302	Bn-chrA09-p2435088
Bn-A01-p9170858	1	0.996677741	Bn-N1-p8737520	Bn-chrA01-p7849996
Bn-A01-p9176104	0.966942149	0.975206612	Bn-N1-p8743891	Bn-chrA01-p7859833
Bn-A01-p9180340	0.945273632	0.940298507	Bn-N1-p8751591	Bn-chrA01_random-p522244
Bn-A01-p9180828	0.990049751	0.985074627	Bn-N1-p8752082	Bn-chrA01_random-p522735
Bn-A01-p9203096	0.996677741	0.996677741	Bn-N1-p8773790	Bn-chrA01-p7873510
Bn-A01-p9220977	0.991735537	1	Bn-N1-p8783356	Bn-chrA01-p7888809
Bn-A01-p9223420	0.96345515	0.970099668	Bn-N1-p8785906	Bn-chrA01-p7891359
Bn-A01-p9227229	0.950413223	0.983471074	Bn-N1-p8789674	Bn-chrA01-p7895148
Bn-A01-p9254925	0.985074627	0.990049751	Bn-N1-p8807849	Bn-chrA01-p7912026
Bn-A01-p9256011	0.975206612	0.983471074	Bn-N1-p8808969	Bn-chrA01-p7913112
Bn-A01-p9258144	0.991735537	0.983471074	Bn-N1-p8811102	Bn-chrA01-p7921856
Bn-A01-p9259611	0.991735537	0.983471074	Bn-N1-p8812569	Bn-chrA01-p7923304
Bn-A01-p9260073	0.958677686	0.950413223	Bn-N1-p8813031	Bn-chrA01-p7923766
Bn-A01-p9264081	0.876033058	0.975206612	Bn-N1-p8822872	Bn-chrA01-p7927613
Bn-A01-p9269627	0.96345515	0.96345515	Bn-N1-p8841697	Bn-chrA01-p7944235
Bn-A01-p9303710	0.993355482	0.993355482	Bn-N1-p8867189	Bn-chrA01-p7967727
Bn-A01-p9325719	0.986710963	0.990033223	Bn-N1-p8878832	Bn-chrA01-p7983028
Bn-A01-p9326695	0.966777409	0.966777409	Bn-N1-p8879780	Bn-chrA01-p7983984
Bn-A01-p9331463	0.980066445	0.983388704	Bn-N1-p8884533	Bn-chrA01-p7988751
Bn-A01-p9333956	0.958677686	0.917355372	Bn-N1-p8886114	Bn-chrA01-p7989766
Bn-A01-p9337252	0.975206612	0.983471074	Bn-N1-p8887264	Bn-chrAnn_random-p354603
Bn-A01-p9360043	0.943521595	0.943521595	Bn-N1-p8904052	Bn-chrA01_random-p526022
Bn-A01-p9360971	0.983388704	0.983388704	Bn-N1-p8904980	Bn-chrA01_random-p526950
Bn-A01-p9369257	0.925619835	0.917355372	Bn-N1-p8913447	Bn-chrA03-p26575287
Bn-A01-p9371414	0.985074627	0.985074627	Bn-N11-p14792142	Bn-chrC01-p12964792
Bn-A01-p9408018	0.966942149	0.966942149	Bn-N1-p8952049	Bn-chrA01-p8053704
Bn-A01-p9436886	0.669421488	0.669421488	Bn-N1-p8990931	Bn-chrA01-p8087944
Bn-A01-p9442471	0.975206612	0.975206612	Bn-N1-p8995481	Bn-chrA01-p8092397
Bn-A01-p9443863	0.983471074	0.983471074	Bn-N1-p8995668	Bn-chrA01-p8092584

Bn-A01-p9445754	1	1	Bn-N1-p8997612	Bn-chrA01-p8094528
Bn-A01-p9469933	0.956810631	0.956810631	Bn-N1-p9007626	Bn-chrA01-p8103593
Bn-A01-p9476520	0.990049751	0.990049751	Bn-N1-p9013686	Bn-chrA01-p8110280
Bn-A01-p9499100	0.958677686	0.950413223	Bn-N1-p9029564	Bn-chrA01-p8132236
Bn-A01-p9519478	0.983471074	0.983471074	Bn-N1-p9047680	Bn-chrA01-p8155867
Bn-A01-p9519660	0.94214876	0.94214876	Bn-N1-p9047862	Bn-chrA01-p8156049
Bn-A01-p9520409	0.991735537	0.991735537	Bn-N1-p9048617	Bn-chrA01-p8156987
Bn-A01-p9523572	0.880597015	0.880597015	Bn-N1-p9051751	Bn-chrA01-p8160121
Bn-A01-p9551341	0.966942149	0.975206612	Bn-N1-p9076656	Bn-chrA01-p8186347
Bn-A01-p9565360	0.966942149	0.975206612	Bn-N1-p9080805	Bn-chrA01-p8189148
Bn-A01-p9565804	0.991735537	0.983471074	Bn-N1-p9081251	Bn-chrA01-p8189592
Bn-A01-p9568888	0.983471074	0.975206612	Bn-N1-p9084007	Bn-chrA01-p8192676
Bn-A01-p9599644	0.933884298	0.933884298	Bn-N1-p9110966	Bn-chrA01-p8214774
Bn-A01-p9614730	0.815920398	0.810945274	Bn-N1-p9123417	Bn-chrA01-p8227276
Bn-A01-p9619932	0.993355482	0.993355482	Bn-N1-p9128645	Bn-chrA01-p8232640
Bn-A01-p9621623	1	0.989583333	Bn-N1-p9130259	Bn-chrA01-p8234254
Bn-A01-p9699152	0.983471074	0.991735537	Bn-N1-p9213589	Bn-chrA01-p8316136
Bn-A01-p9711809	0.94214876	0.933884298	Bn-N1-p9221164	Bn-chrA01-p8334397
Bn-A01-p9712591	0.975206612	0.983471074	Bn-N1-p9221963	Bn-chrA01-p8335140
Bn-A01-p9810552	0.983471074	0.983471074	Bn-N1-p9320614	Bn-chrA01-p8432722
Bn-A01-p9853365	0.975206612	0.975206612	Bn-N1-p9366090	Bn-chrA01-p8480162
Bn-A01-p9863114	0.933884298	0.933884298	Bn-N1-p9375414	Bn-chrA01-p8488625
Bn-A01-p9866468	0.925619835	0.925619835	Bn-N1-p9378768	Bn-chrA01-p8491979
Bn-A01-p9884449	0.958677686	0.958677686	Bn-N1-p9398357	Bn-chrA01-p8511262
Bn-A01-p9922741	0.940298507	0.940298507	Bn-N1-p9438573	Bn-chrA01-p8553421
Bn-A01-p9951633	0.657807309	0.524916944	Bn-N1-p9477503	Bn-chrA01-p8591700
Bn-A01-p9961425	0.990033223	0.990033223	Bn-N1-p9496705	Bn-chrAnn_random-p374683
Bn-A01-p9961553	0.970149254	0.970149254	Bn-N1-p9496833	Bn-chrAnn_random-p374684
Bn-A01-p9982115	0.958677686	0.958677686	Bn-N1-p9522795	Bn-chrAnn_random-p12866[illegible]
Bn-A01-p9989240	0.991735537	0.991735537	Bn-N1-p9529910	Bn-chrAnn_random-p128731
Bn-A01-p9996240	0.94214876	0.94214876	Bn-N1-p9540400	Bn-chrAnn_random-p128845
Bn-A01-p9996458	0.986710963	0.986710963	Bn-N1-p9540618	Bn-chrAnn_random-p128847
Bn-A01-p9999485	0.966942149	0.966942149	Bn-N1-p9549138	Bn-chrAnn_random-p128986
Bn-A01-p10000230	0.993355482	0.993355482	Bn-N1-p9549887	Bn-chrAnn_random-p128993
Bn-A01-p10013185	0.993355482	0.996677741	Bn-N1-p9563049	Bn-chrAnn_random-p12912[illegible]
Bn-A01-p10026020	0.991735537	0.991735537	Bn-N1-p9579604	Bn-chrAnn_random-p146746
Bn-A01-p10027328	0.975206612	0.975206612	Bn-N1-p9580912	Bn-chrAnn_random-p14675[illegible]
Bn-A01-p10031169	0.983471074	0.983471074	Bn-N1-p9585317	Bn-chrAnn_random-p146801
Bn-A01-p10036602	0.985074627	0.985074627	Bn-N1-p9590741	Bn-chrAnn_random-p146864
Bn-A01-p10059043	0.990049751	0.995024876	Bn-N18-p12616173	Bn-chrCnn_random-p839952
Bn-A01-p10069493	0.933884298	0.933884298	Bn-N1-p9620973	Bn-chrAnn_random-p147162
Bn-A01-p10071456	0.983471074	0.983471074	Bn-N1-p9622923	Bn-chrAnn_random-p147182
Bn-A01-p10071903	0.966942149	0.966942149	Bn-N1-p9623369	Bn-chrAnn_random-p147186
Bn-A01-p10105499	0.983471074	0.983471074	Bn-N1-p9656392	Bn-chrA01-p8689511
Bn-A01-p10166739	0.900497512	0.915422886	Bn-N11-p44256383	Bn-chrA01-p21625177
Bn-A01-p10181972	0.996677741	0.996677741	Bn-N1-p9743446	Bn-chrA01-p8782944

Bn-A01-p10231780	0.933884298	0.983471074	Bn-N1-p9782678	Bn-chrA01-p8828045
Bn-A01-p10234366	0.983388704	0.916943522	Bn-N1-p9784407	Bn-chrC01-p14449718
Bn-A01-p10238825	0.991735537	0.834710744	Bn-N1-p9788866	Bn-chrA01-p8835739
Bn-A01-p10242217	0.991735537	0.983471074	Bn-N1-p9793020	Bn-chrA01-p8840200
Bn-A01-p10242298	0.991735537	0.785123967	Bn-N1-p9793104	Bn-chrA01-p8840284
Bn-A01-p10246312	0.925619835	0.884297521	Bn-N1-p9794780	Bn-chrA01-p8848850
Bn-A01-p10249225	0.958677686	0.975206612	Bn-N1-p9797752	Bn-chrA01-p8852208
Bn-A01-p10250255	1	0.991735537	Bn-Scaffold18942-p4	Bn-chrA01-p8853237
Bn-A01-p10251052	0.983471074	0.991735537	Bn-N1-p9799309	Bn-chrA01-p8854034
Bn-A01-p10257919	0.975124378	0.980099502	Bn-N1-p9805260	Bn-chrA01-p8861357
Bn-A01-p10268662	0.991735537	0.966942149	Bn-N1-p9819446	Bn-chrC01-p14512664
Bn-A01-p10278752	0.985074627	0.990049751	Bn-N1-p9830448	Bn-chrA01-p8904034
Bn-A01-p10285074	0.983471074	0.991735537	Bn-N1-p9839493	Bn-chrA01-p8910719
Bn-A01-p10297732	0.842975207	0.809917355	Bn-N1-p9847473	Bn-chrC01-p14542530
Bn-A01-p10299187	0.966942149	0.966942149	Bn-N1-p9848920	Bn-chrA01-p8930734
Bn-A01-p10340159	0.975124378	0.980099502	Bn-N1-p9907693	Bn-chrC09-p43424846
Bn-A01-p10340283	0.990049751	0.990049751	Bn-N1-p9907816	Bn-chrC09-p43424969
Bn-A01-p10340829	0.990033223	0.990033223	Bn-N11-p16168231	Bn-chrCnn_random-p798659
Bn-A01-p10343401	0.975206612	0.966942149	Bn-N1-p9915747	Bn-chrC09-p43428088
Bn-A01-p10344498	0.983471074	0.991735537	Bn-N1-p9916844	Bn-chrC09-p43429185
Bn-A01-p10345430	0.995024876	0.995024876	Bn-N1-p9917777	Bn-chrC09-p43430117
Bn-A01-p10346546	0.986710963	0.897009967	Bn-N1-p9918900	Bn-chrAnn_random-p267797
Bn-A01-p10354645	0.980099502	0.985074627	Bn-N1-p9922762	Bn-chrAnn_random-p267782
Bn-A01-p10392909	0.958677686	0.966942149	Bn-N1-p9952292	Bn-chrAnn_random-p16588C
Bn-A01-p10398937	1	0.979020979	Bn-N1-p9958899	Bn-chrAnn_random-p165832
Bn-A01-p10399077	1	0.991735537	Bn-N1-p9959053	Bn-chrAnn_random-p165831
Bn-A01-p10406165	0.990049751	0.985074627	Bn-N1-p9965092	Bn-chrAnn_random-p165752
Bn-A01-p10410665	0.970149254	0.965174129	Bn-N1-p9970782	Bn-chrAnn_random-p165708
Bn-A01-p10415035	0.940298507	0.950248756	Bn-Scaffold22665-p1	Bn-chrCnn_random-p360074
Bn-A01-p10426647	0.983471074	0.991735537	Bn-N1-p9982395	Bn-chrA01_random-p549050
Bn-A01-p10426714	0.975206612	0.950413223	Bn-N1-p9982461	Bn-chrA01_random-p549117
Bn-A01-p10427300	0.867768595	0.975206612	Bn-N1-p9983049	Bn-chrA01_random-p549697
Bn-A01-p10457944	0.96345515	0.96345515	Bn-N1-p10049950	Bn-chrA01-p9436653
Bn-A01-p10493092	0.995024876	0.995024876	Bn-N1-p10087131	Bn-chrA01-p9471507
Bn-A01-p10499882	0.983388704	0.983388704	Bn-N1-p10100505	Bn-chrA01-p9484805
Bn-A01-p10513529	0.958677686	0.958677686	Bn-N7-p7136845	Bn-chrA07-p7462126
Bn-A01-p10518110	0.943521595	0.943521595	Bn-N7-p7142992	Bn-chrA07_random-p430212
Bn-A01-p10523194	0.983471074	0.983471074	Bn-N7-p7148322	Bn-chrA07-p7475388
Bn-A01-p10523264	0.950413223	0.950413223	Bn-N7-p7148392	Bn-chrA07-p7475458
Bn-A01-p10523833	0.983471074	0.983471074	Bn-N7-p7148961	Bn-chrA07-p7476027
Bn-A01-p10525172	0.487603306	0.487603306	Unknown	Unknown
Bn-A01-p10525357	0.991735537	0.892561983	Bn-N7-p7153279	Bn-chrC07-p13917541
Bn-A01-p10525805	0.991735537	0.991735537	Bn-N7-p7153728	Bn-chrA07-p7480794
Bn-A01-p10526054	1	1	Bn-N7-p7153977	Bn-chrA07-p7481043
Bn-A01-p10526561	0.986710963	0.986710963	Bn-N7-p7154482	Bn-chrA07-p7481548
Bn-A01-p10543102	0.983471074	0.983471074	Bn-N7-p7166365	Bn-chrA07-p7493428

Bn-A01-p10550486	0.976744186	0.976744186	Bn-N7-p7173907	Bn-chrA07-p7505801
Bn-A01-p10559083	0.966942149	0.966942149	Bn-N7-p7192886	Bn-chrA07-p7526838
Bn-A01-p10590357	0.916943522	0.916943522	Bn-N1-p10129316	Bn-chrA01-p9511106
Bn-A01-p10611800	0.960199005	0.960199005	Bn-N1-p10147556	Bn-chrA01-p9529405
Bn-A01-p10636637	0.983471074	0.991735537	Bn-N1-p10175252	Bn-chrA01-p9549776
Bn-A01-p10636994	0.975206612	0.94214876	Bn-N1-p10175609	Bn-chrA01-p9550132
Bn-A01-p10638747	0.995024876	0.990049751	Bn-N1-p10177362	Bn-chrA01-p9551872
Bn-A01-p10639421	1	0.991735537	Bn-N1-p10178036	Bn-chrA01-p9552543
Bn-A01-p10640093	0.975124378	0.905472637	Bn-N1-p10178708	Bn-chrA01-p9553216
Bn-A01-p10640881	0.975124378	0.970149254	Bn-N1-p10179496	Bn-chrC01-p14903309
Bn-A01-p10641656	0.983471074	0.991735537	Bn-N1-p10189977	Bn-chrA01-p9554954
Bn-A01-p10693617	0.991735537	0.983471074	Bn-N1-p10245371	Bn-chrA01-p9609388
Bn-A01-p10693623	0.983471074	0.991735537	Bn-N1-p10245377	Bn-chrA01-p9609394
Bn-A01-p10720611	0.991735537	1	Bn-N1-p10270324	Bn-chrA01-p9633959
Bn-A01-p10740084	1	0.991735537	Bn-N1-p10291363	Bn-chrA01-p9654097
Bn-A01-p10786043	0.990049751	0.990049751	Bn-Scaffold11235-p2	Bn-chrC08-p30904377
Bn-A01-p10835605	0.983471074	0.94214876	Bn-N1-p10379201	Bn-chrAnn_random-p143496
Bn-A01-p10838493	0.995024876	1	Bn-N1-p10382337	Bn-chrAnn_random-p436517
Bn-A01-p10853026	0.980099502	0.980099502	Bn-N1-p10395332	Bn-chrA01-p9020617
Bn-A01-p10859527	0.983471074	0.983471074	Bn-N1-p10401833	Bn-chrA01-p9008972
Bn-A01-p10876307	0.970149254	0.970149254	Bn-N1-p10414490	Bn-chrA01-p8992501
Bn-A01-p10889788	0.991735537	1	Bn-N1-p10427892	Bn-chrA01-p8978767
Bn-A01-p10916044	0.950413223	0.950413223	Bn-N1-p10473619	Bn-chrA01_random-p593852
Bn-A01-p10926129	0.983471074	0.983471074	Bn-N1-p10483194	Bn-chrA01-p9032376
Bn-A01-p10933947	0.895522388	1	Bn-N1-p10489280	Bn-chrA01-p9038471
Bn-A01-p10934377	0.94214876	0.975206612	Bn-N1-p10489679	Bn-chrA01-p9038901
Bn-A01-p10938883	0.925619835	0.933884298	Bn-N1-p10503300	Bn-chrA01-p9043403
Bn-A01-p10939144	0.958677686	0.966942149	Bn-N1-p10503591	Bn-chrA01-p9043664
Bn-A01-p10945930	0.975206612	0.983471074	Bn-N1-p10507360	Bn-chrA01-p9046595
Bn-A01-p10963536	0.958677686	0.966942149	Bn-N1-p10519075	Bn-chrA01-p9073519
Bn-A01-p10991782	0.996677741	0.996677741	Bn-N1-p10544311	Bn-chrA01-p9094081
Bn-A01-p11011196	0.983471074	0.991735537	Bn-N1-p10564116	Bn-chrA01-p9134447
Bn-A01-p11052248	0.851239669	0.851239669	Bn-Scaffold09710-p7	Bn-chrA01-p9167261
Bn-A01-p11054015	0.993355482	0.993355482	Bn-N1-p10597648	Bn-chrA01-p9173272
Bn-A01-p11077627	0.966942149	0.966942149	Bn-N1-p10604872	Bn-chrA01-p9180495
Bn-A01-p11078406	0.975206612	0.975206612	Bn-N1-p10605349	Bn-chrA01-p9180972
Bn-A01-p11091663	0.996677741	0.996677741	Bn-N1-p10608511	Bn-chrA01-p9184134
Bn-A01-p11091816	0.966942149	0.966942149	Bn-N1-p10608664	Bn-chrA01-p9184287
Bn-A01-p11108443	0.94214876	0.950413223	Bn-N1-p10623142	Bn-chrA01-p9200904
Bn-A01-p11119985	0.975124378	0.970149254	Bn-N1-p10628286	Bn-chrA01-p9206110
Bn-A01-p11120400	0.985074627	0.985074627	Bn-N1-p10628701	Bn-chrA01-p9206529
Bn-A01-p11120493	0.991525424	0.983050847	Bn-N1-p10628794	Bn-chrA01-p9206622
Bn-A01-p11137395	0.996677741	0.983388704	Bn-N1-p10642349	Bn-chrA01-p9216766
Bn-A01-p11142752	0.958677686	0.966942149	Bn-N1-p10656374	Bn-chrA01-p9218480
Bn-A01-p11163785	0.980099502	0.985074627	Bn-N1-p10671550	Bn-chrA01-p9233299
Bn-A01-p11163980	0.975124378	0.980099502	Bn-Scaffold43302-p2	Bn-chrA01-p9233494

Bn-A01-p11174997	0.851239669	0.991735537	Bn-N11-p17540927	Bn-chrA01_random-p569144
Bn-A01-p11206467	0.983471074	0.991735537	Bn-N11-p17565747	Bn-chrA01-p9274307
Bn-A01-p11233207	1	1	Bn-N1-p10742834	Bn-chrA01-p9297623
Bn-A01-p11234159	0.900497512	0.905472637	Bn-N1-p10743860	Bn-chrA01-p9298649
Bn-A01-p11238778	0.980066445	0.953488372	Bn-N5-p15435464	Bn-chrA01-p9303233
Bn-A01-p11249993	1	1	Bn-N1-p10758006	Bn-chrA01-p9312527
Bn-A01-p11253272	0.993355482	0.996677741	Bn-N1-p10762480	Bn-chrA01-p9317196
Bn-A01-p11273927	1	0.991735537	Bn-N1-p10787223	Bn-chrA01-p9340817
Bn-A01-p11284636	0.985074627	0.985074627	Bn-N1-p10810019	Bn-chrA01-p9362728
Bn-A01-p11302783	0.996677741	0.993355482	Bn-N1-p10838458	Bn-chrA01-p9389222
Bn-A01-p11306490	1	0.995024876	Bn-N1-p10842165	Bn-chrA01-p9392926
Bn-A01-p11309186	0.983471074	0.991735537	Bn-N1-p10844861	Bn-chrA01-p9395622
Bn-A01-p11324370	1	0.933884298	Bn-N1-p10854162	Bn-chrA01-p9405925
Bn-A01-p11330661	0.991735537	0.801652893	Bn-N1-p10860453	Bn-chrCnn_random-p657582
Bn-A01-p11336370	0.996677741	0.993355482	Bn-N1-p10866235	Bn-chrAnn_random-p358255
Bn-A01-p11347791	0.936877076	0.936877076	Bn-N1-p10873198	Bn-chrA01-p9681230
Bn-A01-p11355643	0.966942149	0.280991736	Bn-N1-p10882064	Bn-chrA06-p4854987
Bn-A01-p11407167	0.996677741	0.996677741	Bn-N1-p10936827	Bn-chrA01-p9751502
Bn-A01-p11434769	0.975206612	0.983471074	Bn-N1-p10964947	Bn-chrA01-p9788080
Bn-A01-p11441851	0.991735537	0.975206612	Bn-N1-p10971971	Bn-chrA01-p9794256
Bn-A01-p11444689	0.970149254	0.965174129	Bn-N1-p10974768	Bn-chrA01-p9797051
Bn-A01-p11459295	1	0.983471074	Bn-N1-p10989868	Bn-chrA01-p9806983
Bn-A01-p11462315	0.990049751	0.995024876	Bn-N1-p10993030	Bn-chrA01-p9810141
Bn-A01-p11469596	0.975206612	0.983471074	Bn-N1-p11000328	Bn-chrA01-p9817422
Bn-A01-p11469992	0.975206612	0.991735537	Bn-N1-p11000718	Bn-chrA01-p9817818
Bn-A01-p11471489	1	0.995024876	Bn-N1-p11002226	Bn-chrA01-p9819529
Bn-A01-p11479565	0.976744186	0.976744186	Bn-N1-p11009759	Bn-chrA01-p9827000
Bn-A01-p11480814	0.975124378	0.975124378	Bn-N1-p11014535	Bn-chrA01-p9831935
Bn-A01-p11483435	0.963302752	0.963302752	Bn-N1-p11017255	Bn-chrA01-p9834655
Bn-A01-p11492050	0.834710744	0.834710744	Bn-Scaffold20063-p5	Bn-chrC01-p15716756
Bn-A01-p11543126	1	1	Bn-N1-p11101231	Bn-chrA01_random-p652607
Bn-A01-p11543191	0.989583333	0.989583333	Bn-N1-p11101339	Bn-chrA01_random-p652715
Bn-A01-p11559064	1	1	Bn-N1-p11118034	Bn-chrAnn_random-p382882
Bn-A01-p11560385	0.993355482	0.993355482	Bn-N1-p11119355	Bn-chrAnn_random-p382869
Bn-A01-p11613721	0.987603306	0.987603306	Bn-N7-p4981618	Bn-chrAnn_random-p406274
Bn-A01-p11639506	0.975206612	0.983471074	Bn-N4-p4905255	Bn-chrCnn_random-p161486
Bn-A01-p11639917	0.991735537	0.925619835	Bn-N4-p4904844	Bn-chrCnn_random-p161490
Bn-A01-p11641669	0.991735537	1	Bn-N4-p4903092	Bn-chrCnn_random-p161507
Bn-A01-p11642664	0.990049751	0.990049751	Bn-N4-p4902101	Bn-chrCnn_random-p161517
Bn-A01-p11642677	0.980099502	0.980099502	Bn-N4-p4902089	Bn-chrCnn_random-p161517
Bn-A01-p11642772	0.985074627	0.985074627	Bn-N4-p4901993	Bn-chrCnn_random-p161518
Bn-A01-p11642789	0.995024876	0.995024876	Bn-N4-p4901976	Bn-chrCnn_random-p161518
Bn-A01-p11652789	0.976744186	0.976744186	Bn-N4-p4891849	Bn-chrCnn_random-p161611
Bn-A01-p11653461	0.990049751	0.990049751	Bn-N4-p4891177	Bn-chrCnn_random-p161618
Bn-A01-p11702957	0.990049751	0.990049751	Bn-N1-p11187449	Bn-chrA01-p9984798
Bn-A01-p11727190	0.958677686	0.94214876	Bn-N1-p11211254	Bn-chrCnn_random-p263832

Bn-A01-p11741112	0.995024876	0.995024876	Bn-N8-p556921	Bn-chrA08-p332364
Bn-A01-p11744233	0.917355372	0.900826446	Bn-N1-p11228267	Bn-chrC08-p14821098
Bn-A01-p11807124	0.983471074	0.983471074	Bn-N1-p11294556	Bn-chrA01-p10121073
Bn-A01-p11808121	0.983471074	0.818181818	Bn-N1-p11295584	Bn-chrA01-p10122176
Bn-A01-p11817698	0.966942149	0.958677686	Bn-N1-p11305925	Bn-chrA01-p10131743
Bn-A01-p11818901	0.983471074	0.975206612	Bn-N1-p11307133	Bn-chrA01-p10132945
Bn-A01-p11825880	0.983471074	0.991735537	Bn-N1-p11321978	Bn-chrA01-p10137457
Bn-A01-p11830957	0.955223881	0.995024876	Bn-N1-p11327102	Bn-chrA01-p10142601
Bn-A01-p11938379	0.985074627	0.985074627	Bn-N1-p11446368	Bn-chrA01-p10271080
Bn-A01-p11939834	0.876033058	0.876033058	Bn-N1-p11447825	Bn-chrA01-p10272536
Bn-A01-p11947255	0.983471074	0.991735537	Bn-N1-p11456056	Bn-chrA01-p10281087
Bn-A01-p11951605	0.990033223	0.996677741	Bn-N1-p11460402	Bn-chrA01-p10290337
Bn-A01-p11954334	0.983388704	0.990033223	Bn-N1-p11463122	Bn-chrC01-p16050939
Bn-A01-p11955645	0.975206612	0.983471074	Bn-N1-p11464433	Bn-chrA01-p10294376
Bn-A01-p11965733	0.958677686	0.950413223	Bn-N1-p11473220	Bn-chrA01-p10307722
Bn-A01-p11991521	0.975206612	0.975206612	Bn-N5-p9087685	Bn-chrA05_random-p784171
Bn-A01-p11992281	0.966942149	0.966942149	Bn-N5-p9086994	Bn-chrA05_random-p784862
Bn-A01-p11993589	0.986710963	0.986710963	Bn-N5-p9085684	Bn-chrA05_random-p786172
Bn-A01-p11996408	0.966942149	0.966942149	Bn-N5-p9084379	Bn-chrA05_random-p787477
Bn-A01-p11996672	0.983471074	0.983471074	Bn-N5-p9084114	Bn-chrA05_random-p787741
Bn-A01-p12066369	0.986710963	0.976744186	Bn-N1-p11497027	Bn-chrC01-p16116960
Bn-A01-p12077110	0.991735537	0.975206612	Bn-N1-p11508229	Bn-chrAnn_random-p112962
Bn-A01-p12081177	0.991735537	1	Bn-N1-p11512467	Bn-chrAnn_random-p112753
Bn-A01-p12098080	1	0.991735537	Bn-N1-p11530143	Bn-chrAnn_random-p112591
Bn-A01-p12136794	0.965174129	0.975124378	Bn-Scaffold16730-p2	Bn-chrAnn_random-p433388
Bn-A01-p12136908	0.950248756	0.950248756	Bn-N1-p12038742	Bn-chrAnn_random-p433387
Bn-A01-p12138089	0.986710963	0.986710963	Bn-N1-p11561468	Bn-chrA01-p10335500
Bn-A01-p12145148	0.990033223	0.993355482	Bn-N1-p11567265	Bn-chrA01-p10351942
Bn-A01-p12158810	0.986710963	0.986710963	Bn-N1-p11580929	Bn-chrAnn_random-p302213
Bn-A01-p12178646	0.980066445	0.980066445	Bn-N1-p11606752	Bn-chrA01-p10388628
Bn-A01-p12179784	0.983471074	0.975206612	Bn-N1-p11607855	Bn-chrA01-p10389764
Bn-A01-p12192020	0.991735537	0.983471074	Bn-N1-p11617868	Bn-chrA01-p10399308
Bn-A01-p12206449	0.983471074	0.983471074	Bn-N1-p11622924	Bn-chrA01-p10404352
Bn-A01-p12221698	0.991735537	0.991735537	Bn-N1-p11625035	Bn-chrA01-p10406845
Bn-A01-p12222100	0.966942149	0.966942149	Bn-N1-p11625435	Bn-chrA01-p10407242
Bn-A01-p12222728	0.966942149	0.966942149	Bn-N1-p11626103	Bn-chrA01-p10407913
Bn-A01-p12222806	0.975206612	0.975206612	Bn-N1-p11626179	Bn-chrA01-p10407989
Bn-A01-p12224872	0.830882353	0.819852941	Bn-N1-p11627344	Bn-chrUnn_random-p700611
Bn-A01-p12233209	0.986710963	0.936877076	Bn-N1-p11635701	Bn-chrA01-p10417484
Bn-A01-p12237534	0.94214876	0.966942149	Bn-N1-p11640047	Bn-chrA01-p10421790
Bn-A01-p12257699	0.991735537	0.975206612	Bn-N1-p11661093	Bn-chrA01-p10446561
Bn-A01-p12262504	0.991735537	0.958677686	Bn-N1-p11665618	Bn-chrC01_random-p127437
Bn-A01-p12302708	0.975206612	0.966942149	Bn-N1-p11698424	Bn-chrA01-p10487998
Bn-A01-p12302861	0.966942149	0.917355372	Bn-N1-p11702332	Bn-chrA01-p10488142
Bn-A01-p12305025	0.975206612	0.966942149	Bn-N1-p11703847	Bn-chrA01-p10489606
Bn-A01-p12305828	0.975206612	0.966942149	Bn-N1-p11704650	Bn-chrA01-p10490408

Bn-A01-p12306195	0.983471074	0.975206612	Bn-N1-p11705015	Bn-chrA01-p10490777
Bn-A01-p12308897	0.983471074	0.991735537	Bn-N1-p11707740	Bn-chrA01-p10492492
Bn-A01-p12309767	0.975206612	0.975206612	Bn-N1-p11708590	Bn-chrA01-p10493351
Bn-A01-p12314813	0.983471074	0.991735537	Bn-N1-p11714916	Bn-chrA01-p10499025
Bn-A01-p12350104	0.975206612	0.966942149	Bn-N1-p11741704	Bn-chrA01-p10537555
Bn-A01-p12352518	0.958677686	0.950413223	Bn-N1-p11754287	Bn-chrA01-p10539966
Bn-A01-p12380936	1	0.991735537	Bn-N1-p11775221	Bn-chrA01-p10569854
Bn-A01-p12387882	1	0.991735537	Bn-N1-p11791223	Bn-chrA01-p10584171
Bn-A01-p12391243	0.975206612	0.983471074	Bn-N1-p11795651	Bn-chrA01-p10587334
Bn-A01-p12399796	0.966942149	0.958677686	Bn-N1-p11805754	Bn-chrA01-p10605609
Bn-A01-p12400477	0.995024876	0.995024876	Bn-N1-p11807912	Bn-chrA01-p10606289
Bn-A01-p12427149	1	1	Bn-Scaffold36590-p2	Bn-chrA01-p10633916
Bn-A01-p12454306	0.955223881	0.955223881	Bn-N1-p11862661	Bn-chrA01-p10662642
Bn-A01-p12456089	0.966942149	0.966942149	Bn-N1-p11866943	Bn-chrA01-p10666542
Bn-A01-p12484368	1	1	Bn-N1-p11892376	Bn-chrA01-p10692073
Bn-A01-p12510328	0.991735537	0.991735537	Bn-N1-p11922690	Bn-chrA01-p10725263
Bn-A01-p12510566	0.966942149	0.966942149	Bn-N1-p11922928	Bn-chrA01-p10725501
Bn-A01-p12516944	0.975206612	0.975206612	Bn-N1-p11891352	Bn-chrA01-p10691053
Bn-A01-p12522369	0.950413223	0.950413223	Bn-N1-p11939222	Bn-chrA01-p10741070
Bn-A01-p12533991	0.958677686	0.958677686	Bn-N1-p11942661	Bn-chrA01-p10744498
Bn-A01-p12535269	0.993355482	0.993355482	Bn-N1-p11944135	Bn-chrA01-p10745972
Bn-A01-p12535612	0.983471074	0.983471074	Bn-N1-p11944482	Bn-chrA01-p10746319
Bn-A01-p12539479	0.909090909	0.909090909	Bn-N1-p11949008	Bn-chrA01-p10751083
Bn-A01-p12539655	0.975206612	0.975206612	Bn-N1-p11949187	Bn-chrA01-p10751262
Bn-A01-p12539953	0.975206612	0.975206612	Bn-N1-p11949787	Bn-chrA01-p10751560
Bn-A01-p12543678	0.950413223	0.950413223	Bn-N1-p11953827	Bn-chrA01-p10755625
Bn-A01-p12544054	0.94214876	0.94214876	Bn-N1-p11954216	Bn-chrA01-p10756014
Bn-A01-p12550092	0.954545455	0.948051948	Bn-Scaffold86145-p1	Bn-chrA02_random-p471511
Bn-A01-p12560952	0.925619835	0.925619835	Bn-N1-p11966937	Bn-chrA01-p10769520
Bn-A01-p12577268	0.993355482	0.993355482	Bn-N1-p11976112	Bn-chrA01-p10778597
Bn-A01-p12580778	0.990033223	0.990033223	Bn-N1-p11989462	Bn-chrA01-p10789584
Bn-A01-p12591168	0.909090909	0.909090909	Bn-N1-p12013538	Bn-chrA01-p10813660
Bn-A01-p12593173	0.990049751	0.990049751	Bn-N1-p12015522	Bn-chrA01-p10815644
Bn-A01-p12593802	0.958677686	0.966942149	Bn-N11-p19616656	Bn-chrC01-p16875949
Bn-A01-p12618357	0.983471074	0.983471074	Bn-N1-p12095406	Bn-chrA01-p10868515
Bn-A01-p12622123	0.958677686	0.958677686	Bn-N1-p12099404	Bn-chrA01-p10872512
Bn-A01-p12625892	0.985074627	0.985074627	Bn-N1-p12103289	Bn-chrA01-p10876397
Bn-A01-p12653338	0.986710963	0.986710963	Bn-N1-p12130885	Bn-chrA01-p10904989
Bn-A01-p12665045	0.980099502	0.980099502	Bn-N1-p12143466	Bn-chrA01-p10918499
Bn-A01-p12665250	0.991735537	0.991735537	Bn-N1-p12143671	Bn-chrA01-p10918704
Bn-A01-p12672899	0.993355482	0.993355482	Bn-N1-p12154035	Bn-chrA01-p10929347
Bn-A01-p12734805	0.975206612	0.975206612	Bn-N1-p12210563	Bn-chrA01-p10993535
Bn-A01-p12774270	0.995024876	0.975124378	Bn-N1-p12261697	Bn-chrA01-p11042463
Bn-A01-p12823550	0.910299003	0.910299003	Bn-N1-p12290126	Bn-chrA01-p11069418
Bn-A01-p12926196	0.996677741	0.996677741	Bn-N1-p12416534	Bn-chrA01-p11192051
Bn-A01-p13239976	0.996677741	1	Bn-N1-p12813137	Bn-chrAnn_random-p345508

Bn-A01-p13622238	0.991735537	1	Bn-N2-p181357	Bn-chrA07-p407234
Bn-A01-p13709801	0.940199336	0.936877076	Bn-N1-p13197936	Bn-chrA01-p11278841
Bn-A01-p13775817	0.986710963	0.990033223	Bn-N1-p13254584	Bn-chrA01-p11334902
Bn-A01-p13825941	0.810631229	0.810631229	Bn-N19-p51507834	Bn-chrC09_random-p428918
Bn-A01-p13827513	0.926910299	0.926910299	Bn-N19-p51509406	Bn-chrC09_random-p428760
Bn-A01-p13828017	0.956810631	0.956810631	Bn-N19-p51509910	Bn-chrC09_random-p428710
Bn-A01-p13828314	0.956810631	0.956810631	Bn-N19-p51510207	Bn-chrC09_random-p428680
Bn-A01-p13893020	0.719367589	0.71541502	Bn-N1-p13399572	Bn-chrA01-p11432787
Bn-A01-p14001039	0.980066445	0.943521595	Bn-N1-p13554075	Bn-chrA01-p11571915
Bn-A01-p14022855	0.996677741	0.980066445	Bn-N1-p13583032	Bn-chrA01-p11618842
Bn-A01-p14046733	0.986710963	0.956810631	Bn-Scaffold45718-p1	Bn-chrA01-p11644479
Bn-A01-p14057073	0.993355482	0.990033223	Bn-N1-p13638203	Bn-chrA01-p11661082
Bn-A01-p14071179	0.980066445	0.983388704	Bn-N1-p13650295	Bn-chrA01-p11667406
Bn-A01-p14071237	0.990033223	0.986710963	Bn-N1-p13650353	Bn-chrA01-p11667464
Bn-A01-p14074100	0.989583333	1	Bn-N1-p13653216	Bn-chrA01-p11670319
Bn-A01-p14074268	0.986710963	0.976744186	Bn-N1-p13653384	Bn-chrA01-p11670489
Bn-A01-p14074391	0.986710963	0.976744186	Bn-N1-p13653507	Bn-chrA01-p11670612
Bn-A01-p14076641	0.993355482	0.993355482	Bn-N1-p13657556	Bn-chrA01-p11672864
Bn-A01-p14079006	0.993355482	0.966777409	Bn-N1-p13659975	Bn-chrA01-p11675274
Bn-A01-p14084579	0.986710963	0.973421927	Bn-N1-p13665565	Bn-chrA01-p11681706
Bn-A01-p14090315	0.993355482	0.976744186	Bn-N1-p13671304	Bn-chrAnn_random-p269812
Bn-A01-p14102222	1	1	Bn-N1-p13683365	Bn-chrAnn_random-p268811
Bn-A01-p14104219	0.975124378	0.965174129	Bn-N1-p13685362	Bn-chrAnn_random-p268792
Bn-A01-p14104241	0.975124378	0.960199005	Bn-N1-p13685384	Bn-chrAnn_random-p268792
Bn-A01-p14105205	0.970149254	0.970149254	Bn-N1-p13686348	Bn-chrAnn_random-p268782
Bn-A01-p14106230	0.970149254	0.980099502	Bn-Scaffold01114-p3	Bn-chrAnn_random-p400988
Bn-A01-p14106856	0.970149254	0.970149254	Bn-N1-p13687999	Bn-chrC04-p6583826
Bn-A01-p14128129	0.986710963	0.986710963	Bn-N1-p13709307	Bn-chrAnn_random-p429347
Bn-A01-p14134232	0.96013289	0.986710963	Bn-N1-p13715421	Bn-chrA01-p11717043
Bn-A01-p14138072	0.96013289	0.980066445	Bn-N1-p13717849	Bn-chrA01-p11719402
Bn-A01-p14148071	0.953488372	0.986710963	Bn-N1-p13727990	Bn-chrA01-p11729856
Bn-A01-p14167866	0.800664452	0.993355482	Bn-N1-p13746841	Bn-chrA01-p11742567
Bn-A01-p14168802	0.976744186	0.986710963	Bn-N1-p13747777	Bn-chrA01-p11745409
Bn-A01-p14169412	0.950248756	0.950248756	Bn-N1-p13748387	Bn-chrA01-p11746017
Bn-A01-p14181463	0.950166113	0.970099668	Bn-N14-p49614979	Bn-chrA01-p11750241
Bn-A01-p14181651	0.966777409	0.990033223	Bn-N14-p49615167	Bn-chrA01-p11750429
Bn-A01-p14181829	0.956810631	0.993355482	Bn-N14-p49615345	Bn-chrA01-p11750610
Bn-A01-p14191393	0.980066445	0.986710963	Bn-N1-p13759660	Bn-chrA04-p7244923
Bn-A01-p14197545	0.990033223	0.993355482	Bn-N1-p13766078	Bn-chrA01-p11782873
Bn-A01-p14200714	0.983388704	0.986710963	Bn-N1-p13769421	Bn-chrA01-p11786918
Bn-A01-p14200787	0.983388704	0.983388704	Bn-N1-p13769494	Bn-chrA01-p11786991
Bn-A01-p14201581	0.976744186	0.990033223	Bn-N1-p13770288	Bn-chrA01-p11787785
Bn-A01-p14210994	0.970099668	0.996677741	Bn-N1-p13779649	Bn-chrAnn_random-p276661
Bn-A01-p14223709	0.946843854	0.986710963	Bn-N1-p13797566	Bn-chrA01-p11808100
Bn-A01-p14232473	0.860696517	0.985074627	Bn-N1-p13806329	Bn-chrA01-p11812928
Bn-A01-p14238948	0.993355482	0.943521595	Bn-N1-p13839796	Bn-chrA01-p11856037

Bn-A01-p14241215	0.980066445	1	Bn-N1-p13842063	Bn-chrA01-p11863020
Bn-A01-p14247718	0.96345515	0.956810631	Bn-N1-p13848567	Bn-chrA01-p11868535
Bn-A01-p14256104	0.990033223	0.993355482	Bn-N1-p13857145	Bn-chrA01-p11889252
Bn-A01-p14328823	0.990033223	0.996677741	Bn-N1-p13929322	Bn-chrA01-p12203164
Bn-A01-p14339825	0.976744186	0.973421927	Bn-N1-p13940499	Bn-chrA01-p12192990
Bn-A01-p14402434	0.975124378	0.975124378	Bn-N17-p12307568	Bn-chrC05-p32768653
Bn-A01-p14415386	1	0.996677741	Bn-N1-p13989587	Bn-chrAnn_random-p160285
Bn-A01-p14419363	0.985074627	0.950248756	Bn-N1-p13993480	Bn-chrAnn_random-p160217
Bn-A01-p14559770	0.950413223	0.958677686	Bn-N1-p1722929	Bn-chrA04-p13825972
Bn-A01-p14661055	0.990049751	0.990049751	Bn-N1-p14178768	Bn-chrAnn_random-p133186
Bn-A01-p14661148	0.985074627	0.985074627	Bn-N1-p14178861	Bn-chrAnn_random-p133185
Bn-A01-p14666643	0.995024876	0.990049751	Bn-N1-p14184521	Bn-chrAnn_random-p133134
Bn-A01-p14687636	0.983388704	0.983388704	Bn-N1-p14206347	Bn-chrAnn_random-p178503
Bn-A01-p14704844	0.996677741	0.986710963	Bn-N1-p14221219	Bn-chrA01-p12210254
Bn-A01-p14723306	0.983388704	0.986710963	Bn-N1-p14239993	Bn-chrA01-p12235606
Bn-A01-p14727717	0.986710963	0.976744186	Bn-N1-p14244403	Bn-chrA01-p12239999
Bn-A01-p14802709	0.991735537	0.983471074	Bn-N1-p14312647	Bn-chrA01-p12315278
Bn-A01-p14859839	0.958677686	0.950413223	Bn-N1-p14366238	Bn-chrA01-p12342920
Bn-A01-p14865565	0.991735537	0.983471074	Bn-N1-p14371963	Bn-chrA01-p12349702
Bn-A01-p14901403	0.983471074	0.975206612	Bn-N1-p14398108	Bn-chrA01-p12386121
Bn-A01-p14905298	0.966942149	0.975206612	Bn-N1-p14402784	Bn-chrA01-p12390905
Bn-A01-p14906403	0.993355482	0.92358804	Bn-N1-p14404126	Bn-chrA01-p12392297
Bn-A01-p14906494	0.925619835	0.933884298	Bn-N1-p14404217	Bn-chrA01-p12392388
Bn-A01-p14914475	0.980066445	0.946843854	Bn-N1-p14412214	Bn-chrA01-p12402099
Bn-A01-p14917697	0.955223881	0.960199005	Bn-N1-p14415406	Bn-chrA01-p12406288
Bn-A01-p14934844	0.975206612	0.966942149	Bn-N1-p14433010	Bn-chrAnn_random-p291410
Bn-A01-p14942681	0.966942149	0.958677686	Bn-N1-p14441031	Bn-chrA01-p12447746
Bn-A01-p14943499	0.983471074	0.991735537	Bn-N1-p14441849	Bn-chrA01-p12448568
Bn-A01-p14945302	1	0.991735537	Bn-N1-p14443652	Bn-chrA01-p12450364
Bn-A01-p14947053	0.990033223	0.990033223	Bn-N1-p14445402	Bn-chrA01-p12452115
Bn-A01-p14947418	1	0.996677741	Bn-N1-p14445767	Bn-chrA01-p12452480
Bn-A01-p14948237	0.983471074	0.991735537	Bn-N1-p14446586	Bn-chrA01-p12453302
Bn-A01-p14948379	1	0.991735537	Bn-N1-p14446728	Bn-chrA01-p12453444
Bn-A01-p14948614	0.991735537	1	Bn-N1-p14446963	Bn-chrA01-p12453679
Bn-A01-p14948748	0.983471074	0.975206612	Bn-N1-p14447097	Bn-chrA01-p12453813
Bn-A01-p14949030	0.983471074	0.975206612	Bn-N1-p14447379	Bn-chrA01-p12454095
Bn-A01-p14949121	0.975206612	0.975206612	Bn-N1-p14447470	Bn-chrA01-p12454186
Bn-A01-p14949256	0.991735537	0.983471074	Bn-N1-p14447605	Bn-chrA01-p12454321
Bn-A01-p14955594	0.966942149	0.975206612	Bn-N1-p14453956	Bn-chrA01-p12458348
Bn-A01-p14959478	0.958677686	0.966942149	Bn-N1-p14458294	Bn-chrA01-p12462566
Bn-A01-p14960233	0.996677741	0.990033223	Bn-N1-p14459360	Bn-chrA01-p12463323
Bn-A01-p14965172	0.990033223	0.980066445	Bn-N1-p14464498	Bn-chrA01-p12473353
Bn-A01-p14965596	0.983388704	0.973421927	Bn-N1-p14464922	Bn-chrC01_random-p152309
Bn-A01-p15000034	0.983388704	0.983388704	Bn-N11-p25098816	Bn-chrC01_random-p150608
Bn-A01-p15000468	0.975206612	0.950413223	Bn-N1-p14499762	Bn-chrA01-p12491512
Bn-A01-p15043967	0.980066445	0.980066445	Bn-N11-p25172656	Bn-chrC01-p22253866

Bn-A01-p15086787	0.980099502	0.980099502	Bn-N1-p14616211	Bn-chrA01-p12597407
Bn-A01-p15087045	0.980099502	0.980099502	Bn-N1-p14616469	Bn-chrA01-p12597665
Bn-A01-p15087171	0.973421927	0.973421927	Bn-N1-p14616595	Bn-chrA01-p12597791
Bn-A01-p15090383	0.980099502	0.980099502	Bn-N1-p14619802	Bn-chrA01-p12600998
Bn-A01-p15092778	0.993355482	0.993355482	Bn-N1-p14622199	Bn-chrA01-p12603395
Bn-A01-p15095213	0.976744186	0.976744186	Bn-N1-p14624637	Bn-chrA01-p12605833
Bn-A01-p15095314	0.877076412	0.877076412	Bn-N1-p14624738	Bn-chrA01-p12605934
Bn-A01-p15095524	0.933884298	0.933884298	Bn-N1-p14624914	Bn-chrA01-p12606110
Bn-A01-p15114417	0.985074627	0.990049751	Bn-N1-p14655740	Bn-chrA01-p12625253
Bn-A01-p15130843	0.965174129	0.985074627	Bn-N1-p14694618	Bn-chrC01-p22465827
Bn-A01-p15179106	0.863787375	0.860465116	Bn-N1-p14737237	Bn-chrA01-p12691742
Bn-A01-p15182302	1	0.989583333	Bn-N1-p14742625	Bn-chrA01-p12694938
Bn-A01-p15193510	0.719008264	0.983471074	Bn-N11-p25506553	Bn-chrA01-p12715760
Bn-A01-p15228282	0.976744186	0.986710963	Bn-N16-p1335530	Bn-chrA09-p11944215
Bn-A01-p15233095	0.986710963	0.980066445	Bn-N1-p14770954	Bn-chrC01-p22691873
Bn-A01-p15295874	0.791044776	0.791044776	Bn-N11-p25656722	Bn-chrC01_random-p176791
Bn-A01-p15401259	0.995024876	0.995024876	Bn-N1-p14920683	Bn-chrA01-p12837841
Bn-A01-p15402291	0.991735537	0.991735537	Bn-N1-p14921746	Bn-chrA01-p12838904
Bn-A01-p15407905	0.990033223	0.990033223	Bn-N1-p14927398	Bn-chrA01-p12844556
Bn-A01-p15408721	0.970099668	0.970099668	Bn-N1-p14928214	Bn-chrA01-p12845372
Bn-A01-p15413589	0.975206612	0.975206612	Bn-N1-p14933346	Bn-chrA01-p12850280
Bn-A01-p15432406	0.991735537	0.991735537	Bn-N1-p14962911	Bn-chrA01-p12878995
Bn-A01-p15441407	0.92358804	0.88372093	Bn-N1-p14971773	Bn-chrA01-p12887486
Bn-A01-p15446864	0.983471074	0.983471074	Bn-N1-p14978764	Bn-chrA01-p12894492
Bn-A01-p15468033	0.990033223	0.990033223	Bn-N1-p15006427	Bn-chrA01-p12916723
Bn-A01-p15474986	0.983471074	0.983471074	Bn-N1-p15012196	Bn-chrC01-p23201417
Bn-A01-p15475038	0.950413223	0.950413223	Bn-N1-p15012249	Bn-chrC01-p23201470
Bn-A01-p15476640	0.980066445	0.980066445	Bn-N1-p15013837	Bn-chrCnn_random-p723579
Bn-A01-p15481986	0.975206612	0.975206612	Bn-N1-p15020118	Bn-chrAnn_random-p330082
Bn-A01-p15487080	0.917355372	0.644628099	Bn-N1-p15024042	Bn-chrC01-p23217537
Bn-A01-p15489266	0.983471074	0.983471074	Bn-N1-p15030987	Bn-chrAnn_random-p391757
Bn-A01-p15494260	1	1	Bn-N1-p15038338	Bn-chrA01-p12938831
Bn-A01-p15494469	0.975206612	0.975206612	Bn-N1-p15038547	Bn-chrA01-p12939040
Bn-A01-p15496639	0.983471074	0.983471074	Bn-N1-p15040901	Bn-chrA01-p12941394
Bn-A01-p15496772	0.958677686	0.958677686	Bn-N1-p15041035	Bn-chrA01-p12941528
Bn-A01-p15497190	0.966942149	0.966942149	Bn-N1-p15041452	Bn-chrA01-p12941945
Bn-A01-p15502589	0.991735537	0.991735537	Bn-N1-p15042884	Bn-chrA01-p12943376
Bn-A01-p15517260	0.991735537	0.991735537	Bn-N1-p15057423	Bn-chrA01-p12957915
Bn-A01-p15519090	0.983471074	0.983471074	Bn-N1-p15059254	Bn-chrA01-p12959746
Bn-A01-p15568028	1	1	Bn-N1-p15097995	Bn-chrA01-p12993848
Bn-A01-p15695152	0.983471074	0.983471074	Bn-N1-p15206806	Bn-chrA01-p13102994
Bn-A01-p15865197	0.980099502	0.980099502	Bn-N1-p12541186	Bn-chrA01_random-p791100
Bn-A01-p15957543	0.990049751	0.995024876	Bn-N1-p16512681	Bn-chrA01-p13863734
Bn-A01-p16015382	0.986710963	0.970099668	Bn-N1-p16585172	Bn-chrAnn_random-p243209
Bn-A01-p16079153	0.983471074	0.272727273	Bn-N11-p28050120	Bn-chrA03_random-p581272
Bn-A01-p16079370	0.993355482	0.375415282	Bn-N11-p28050344	Bn-chrA09-p8626680

Bn-A01-p16086381	0.983471074	0.975206612	Bn-N1-p16669166	Bn-chrA01-p14038654
Bn-A01-p16088721	0.94214876	0.991735537	Bn-Scaffold05442-p4	Bn-chrA01-p14040998
Bn-A01-p16093558	0.198347107	0.975206612	Bn-N13-p39160420	Bn-chrA01-p14045453
Bn-A01-p16093901	0.280991736	0.818181818	Bn-N14-p31433813	Bn-chrA01-p14045796
Bn-A01-p16094037	0.446280992	0.983471074	Unknown	Bn-chrA01-p14045897
Bn-A01-p16103425	0.454545455	0.991735537	Bn-N11-p28075665	Bn-chrA01-p14051452
Bn-A01-p16106314	0.935323383	0.990049751	Bn-N1-p16697490	Bn-chrA01-p14052515
Bn-A01-p16106372	0.983388704	0.930232558	Bn-N1-p16697548	Bn-chrA01-p14052573
Bn-A01-p16111263	0.702479339	0.661157025	Bn-N1-p16698816	Bn-chrA01-p14053830
Bn-A01-p16121664	0.983471074	0.991735537	Bn-N1-p16721258	Bn-chrA01-p14059621
Bn-A01-p16122923	1	0.991735537	Bn-N1-p16722517	Bn-chrA01-p14060881
Bn-A01-p16123649	0.990033223	0.993355482	Bn-N1-p16723243	Bn-chrA01-p14061607
Bn-A01-p16127871	0.993355482	0.986710963	Bn-N1-p16727698	Bn-chrA01_random-p131901
Bn-A01-p16128165	0.975206612	0.983471074	Bn-N1-p16727992	Bn-chrA01_random-p131871
Bn-A01-p16128458	0.983471074	0.991735537	Bn-N1-p16728285	Bn-chrA01_random-p131842
Bn-A01-p16131102	0.970099668	0.973421927	Bn-N1-p16729165	Bn-chrA01_random-p131754
Bn-A01-p16134540	0.983471074	0.975206612	Bn-N1-p16735807	Bn-chrA01_random-p134584
Bn-A01-p16134838	0.991735537	0.983471074	Bn-N1-p16736097	Bn-chrA01_random-p134555
Bn-A01-p16135094	0.991735537	0.983471074	Bn-N1-p16736353	Bn-chrA01_random-p134529
Bn-A01-p16135537	0.991735537	0.983471074	Bn-N1-p16736796	Bn-chrA01_random-p134485
Bn-A01-p16138308	0.94214876	0.950413223	Bn-N1-p16739565	Bn-chrA01_random-p134071
Bn-A01-p16142196	0.983471074	0.983471074	Bn-N1-p16744182	Bn-chrA01_random-p133679
Bn-A01-p16150568	0.867768595	0.966942149	Bn-N1-p16749259	Bn-chrA01_random-p128916
Bn-A01-p16173770	0.851239669	0.975206612	Bn-N1-p16764051	Bn-chrA01-p14074814
Bn-A01-p16174042	0.966942149	0.983471074	Bn-N1-p16764321	Bn-chrA01-p14075086
Bn-A01-p16175909	0.917355372	0.991735537	Bn-N1-p16766349	Bn-chrA01-p14076950
Bn-A01-p16181728	0.933884298	0.966942149	Bn-N11-p27835335	Bn-chrA01-p14083969
Bn-A01-p16200217	0.842975207	0.966942149	Bn-N1-p16785995	Bn-chrA01-p14114223
Bn-A01-p16217917	0.94214876	0.983471074	Bn-N1-p16804153	Bn-chrA01-p14131049
Bn-A01-p16220364	0.67768595	0.694214876	Bn-N11-p27076827	Bn-chrC01-p24057869
Bn-A01-p16221475	0.371900826	0.991735537	Bn-N17-p45120061	Bn-chrA01-p14133581
Bn-A01-p16221732	0.148760331	0.933884298	Unknown	Bn-chrA01-p14133840
Bn-A01-p16222086	0.199004975	0.980099502	Bn-N1-p16805316	Bn-chrA01-p14134195
Bn-A01-p16222098	0.198347107	0.975206612	Unknown	Bn-chrA01-p14134207
Bn-A01-p16222167	0.338842975	0.94214876	Bn-N1-p16805397	Bn-chrA01-p14134275
Bn-A01-p16222479	0.437810945	0.955223881	Bn-N13-p67705972	Bn-chrA01-p14134590
Bn-A01-p16223356	0.910447761	0.92039801	Bn-N11-p27777155	Bn-chrC01-p25827037
Bn-A01-p16228743	0.860465116	0.897009967	Bn-N11-p29056823	Bn-chrA01-p14142011
Bn-A01-p16229096	0.991735537	0.983471074	Bn-N1-p16815822	Bn-chrC01-p25835131
Bn-A01-p16233062	0.970099668	0.973421927	Bn-N1-p16819825	Bn-chrA01-p14146374
Bn-A01-p16253463	0.940199336	0.936877076	Bn-Scaffold04479-p1	Bn-chrC01-p25846938
Bn-A01-p16257043	0.975206612	0.909090909	Bn-N1-p16830009	Bn-chrC01-p25850494
Bn-A01-p16260285	0.973421927	0.089700997	Bn-Scaffold07305-p5	Unknown
Bn-A01-p16261297	0.970099668	0.760797342	Bn-Scaffold04366-p3	Bn-chrC05_random-p159447
Bn-A01-p16262292	0.983388704	0.255813953	Bn-Scaffold04366-p1	Bn-chrA09-p17126371
Bn-A01-p16276961	0.983471074	0.975206612	Bn-N1-p16849435	Bn-chrA01_random-p128224

Bn-A01-p16278008	0.975206612	0.983471074	Bn-N1-p16850441	Bn-chrA01-p14169142
Bn-A01-p16303134	0.993355482	0.936877076	Bn-N1-p16877802	Bn-chrC01-p25895195
Bn-A01-p16323375	0.975206612	0.983471074	Bn-N1-p16897385	Bn-chrA01-p14233511
Bn-A01-p16323503	0.964646465	0.964646465	Bn-N1-p16897513	Bn-chrA01-p14233639
Bn-A01-p16324010	0.995024876	0.995024876	Bn-N1-p16898020	Bn-chrC04-p18413200
Bn-A01-p16331424	0.975206612	0.966942149	Bn-N1-p16906784	Bn-chrA01-p14240261
Bn-A01-p16343556	0.975206612	0.966942149	Bn-N1-p16918936	Bn-chrA01-p14250417
Bn-A01-p16358989	0.991735537	0.909090909	Bn-N1-p16941496	Bn-chrA01-p14264234
Bn-A01-p16366870	0.983388704	0.990033223	Bn-N1-p16950425	Bn-chrA01-p14280310
Bn-A01-p16374068	1	0.991735537	Bn-N1-p16958282	Bn-chrA01-p14293677
Bn-A01-p16380496	0.983471074	0.975206612	Bn-N1-p16964705	Bn-chrA01_random-p131333
Bn-A01-p16389291	0.966942149	0.909090909	Bn-N1-p16976972	Bn-chrC01-p26017794
Bn-A01-p16393582	1	1	Bn-N1-p16981289	Bn-chrA01_random-p130026
Bn-A01-p16393856	0.985074627	0.985074627	Bn-N1-p16981543	Bn-chrA01_random-p129999
Bn-A01-p16393939	0.980099502	0.980099502	Bn-N1-p16981626	Bn-chrA01_random-p129991
Bn-A01-p16394111	0.965174129	0.970149254	Bn-N1-p16981800	Bn-chrA01_random-p129973
Bn-A01-p16396926	0.884297521	0.983471074	Bn-N1-p16984666	Bn-chrA01_random-p129688
Bn-A01-p16403023	0.404958678	0.685950413	Bn-N4-p3587238	Bn-chrA01-p14298608
Bn-A01-p16404320	0.94214876	0.983471074	Bn-N11-p29288024	Bn-chrA01-p14299899
Bn-A01-p16418499	1	0.991735537	Bn-N1-p17016754	Bn-chrAnn_random-p40870C
Bn-A01-p16432946	0.900332226	0.900332226	Bn-N1-p17035841	Bn-chrA01-p14327208
Bn-A01-p16436191	0.950413223	0.975206612	Bn-N1-p17039064	Bn-chrCnn_random-p477538
Bn-A01-p16454615	0.980099502	0.990049751	Bn-N1-p17055050	Bn-chrA01-p14358520
Bn-A01-p16455409	0.990033223	0.990033223	Bn-N1-p17055849	Bn-chrA01-p14359315
Bn-A01-p16461540	0.975206612	0.983471074	Bn-N1-p17061967	Bn-chrA01-p14365463
Bn-A01-p16461639	0.991735537	0.859504132	Bn-N1-p17062066	Bn-chrA01-p14365562
Bn-A01-p16472273	0.990033223	0.887043189	Bn-N1-p17072572	Bn-chrA02_random-p798782
Bn-A01-p16474128	0.993355482	0.860465116	Bn-N1-p17074427	Bn-chrA01-p14367737
Bn-A01-p16476326	0.960199005	0.925373134	Bn-N1-p17076799	Bn-chrA01-p14374248
Bn-A01-p16512818	0.990033223	0.96345515	Bn-N1-p17110858	Bn-chrC01_random-p268350
Bn-A01-p16529313	0.925619835	0.991735537	Bn-N1-p17119831	Bn-chrA01-p14410166
Bn-A01-p16537769	0.996677741	0.993355482	Bn-N1-p17135543	Bn-chrA01-p14420041
Bn-A01-p16537792	0.983471074	0.975206612	Bn-N1-p17135568	Bn-chrA01-p14420066
Bn-A01-p16537866	0.986710963	0.980066445	Bn-N1-p17135642	Bn-chrA01-p14420140
Bn-A01-p16538323	0.990033223	0.993355482	Bn-N1-p17136099	Bn-chrA01-p14420597
Bn-A01-p16538587	0.990033223	0.986710963	Bn-N1-p17136363	Bn-chrA01-p14420861
Bn-A01-p16539250	0.990033223	0.993355482	Bn-N1-p17137026	Bn-chrA01-p14421524
Bn-A01-p16557636	0.996677741	0.993355482	Bn-N1-p17154159	Bn-chrA01-p14436519
Bn-A01-p16571536	0.986710963	0.983388704	Bn-N1-p17168783	Bn-chrA01-p14452383
Bn-A01-p16577721	0.993355482	0.212624585	Bn-N1-p17174924	Bn-chrA01-p14454929
Bn-A01-p16580971	0.976744186	0.980066445	Bn-N1-p17178201	Bn-chrA01-p14462524
Bn-A01-p16597123	0.983388704	0.993355482	Bn-N1-p17194917	Bn-chrA01-p14476069
Bn-A01-p16598652	0.976744186	0.983388704	Bn-N1-p17196443	Bn-chrA01-p14478823
Bn-A01-p16627043	0.917355372	0.983471074	Bn-N2-p10453749	Bn-chrA02-p7791887
Bn-A01-p16630613	0.975206612	0.975206612	Bn-N2-p10444355	Bn-chrA02-p7788321
Bn-A01-p16646241	0.983471074	0.975206612	Bn-N1-p17207434	Bn-chrA01-p14492109

Bn-A01-p16656886	0.983388704	0.993355482	Bn-N1-p17224184	Bn-chrA01-p14502829
Bn-A01-p16662544	0.983388704	0.986710963	Bn-N1-p17229848	Bn-chrA01-p14508831
Bn-A01-p16665014	0.993355482	0.990033223	Bn-N1-p17232442	Bn-chrA01-p14510754
Bn-A01-p16665036	0.990033223	0.993355482	Bn-N1-p17232464	Bn-chrA01-p14510776
Bn-A01-p16669142	0.983471074	0.975206612	Bn-N1-p17236570	Bn-chrA01-p14514883
Bn-A01-p16692819	0.990033223	0.996677741	Bn-N1-p17275453	Bn-chrA01-p14542903
Bn-A01-p16692959	0.986710963	0.990033223	Bn-N1-p17275593	Bn-chrA01-p14543043
Bn-A01-p16694394	0.986710963	0.983388704	Bn-N1-p17277060	Bn-chrA01-p14544510
Bn-A01-p16705936	0.991735537	1	Bn-N1-p17291479	Bn-chrA01-p14557803
Bn-A01-p16711121	0.991735537	0.983471074	Bn-N1-p17296764	Bn-chrA01-p14563922
Bn-A01-p16715637	0.991735537	0.983471074	Bn-N1-p17302028	Bn-chrA01-p14568433
Bn-A01-p16717957	0.930348259	0.975124378	Bn-N11-p29731164	Bn-chrA01-p14570752
Bn-A01-p16720365	0.966942149	0.958677686	Bn-N1-p17305907	Bn-chrA01-p14573453
Bn-A01-p16730334	0.985074627	0.970149254	Bn-N1-p17333950	Bn-chrA01-p14587549
Bn-A01-p16749946	0.966942149	0.975206612	Bn-N1-p17349538	Bn-chrA01-p14602802
Bn-A01-p16749951	0.966942149	0.958677686	Bn-N1-p17349543	Bn-chrA01-p14602807
Bn-A01-p16770716	0.956810631	0.993355482	Bn-N1-p17385502	Bn-chrA01-p14637449
Bn-A01-p16774157	0.980066445	0.983388704	Bn-N1-p17393353	Bn-chrA01-p14640791
Bn-A01-p16829363	0.983471074	0.983471074	Bn-N1-p17412271	Bn-chrA01-p14653681
Bn-A01-p16847535	0.970149254	0.970149254	Bn-N1-p17428973	Bn-chrA01-p14671327
Bn-A01-p16857183	0.990049751	0.985074627	Bn-N1-p17446015	Bn-chrA01-p14680871
Bn-A01-p16857466	0.970238095	0.964285714	Bn-N1-p17446297	Bn-chrA01-p14681171
Bn-A01-p16869474	0.975206612	0.925619835	Bn-N1-p17459802	Bn-chrC01-p26464545
Bn-A01-p16893578	0.996677741	0.996677741	Bn-N1-p17482527	Bn-chrA01-p14714622
Bn-A01-p16911720	0.966942149	0.504132231	Bn-N1-p17512257	Bn-chrC03-p55090928
Bn-A01-p16926592	0.867768595	0.966942149	Bn-N1-p17532356	Bn-chrA01-p14776966
Bn-A01-p16935031	0.991735537	0.983471074	Bn-N1-p17543406	Bn-chrA01-p14786202
Bn-A01-p16943370	0.991735537	0.983471074	Bn-N1-p17551434	Bn-chrA01-p14794535
Bn-A01-p16951655	0.983471074	0.975206612	Bn-N1-p17559830	Bn-chrA01-p14802949
Bn-A01-p16994459	0.661157025	0.925619835	Bn-N1-p17592603	Bn-chrA01-p14844467
Bn-A01-p16997658	0.993355482	0.990033223	Bn-N1-p17595887	Bn-chrA01-p14848522
Bn-A01-p17015383	0.995024876	0.995024876	Bn-N1-p17625591	Bn-chrA01-p14879747
Bn-A01-p17036109	0.980066445	0.976744186	Bn-N1-p17653794	Bn-chrAnn_random-p448288
Bn-A01-p17040457	0.96013289	0.990033223	Bn-N1-p17668847	Bn-chrA01_random-p879286
Bn-A01-p17049439	0.96013289	0.986710963	Bn-N1-p17677345	Bn-chrA01_random-p863810
Bn-A01-p17050461	0.970099668	0.986710963	Bn-N1-p17678314	Bn-chrA01_random-p862818
Bn-A01-p17050843	0.983471074	0.966942149	Bn-N1-p17678696	Bn-chrA01_random-p862437
Bn-A01-p17052809	1	0.986710963	Bn-N7-p14454790	Bn-chrA07_random-p134999
Bn-A01-p17081120	0.965174129	0.970149254	Bn-N1-p17714097	Bn-chrA01-p15453898
Bn-A01-p17081703	0.993355482	0.996677741	Bn-N1-p17714680	Bn-chrA01-p15454481
Bn-A01-p17097891	0.996677741	0.996677741	Bn-N1-p17727346	Bn-chrA01-p15479317
Bn-A01-p17104282	0.975206612	0.983471074	Bn-N1-p17733859	Bn-chrA01-p15485831
Bn-A01-p17105194	0.983471074	0.975206612	Bn-N1-p17734691	Bn-chrA01-p15486663
Bn-A01-p17105372	0.991735537	1	Bn-N1-p17734869	Bn-chrA01-p15486841
Bn-A01-p17105672	0.545454545	0.925619835	Bn-N1-p17735169	Bn-chrA01-p15487141
Bn-A01-p17106985	0.958677686	0.958677686	Bn-N1-p17737844	Bn-chrA01-p15495106

Bn-A01-p17110860	0.950413223	0.983471074	Bn-N1-p17742019	Bn-chrA01-p15498984
Bn-A01-p17118647	0.975206612	0.983471074	Bn-N1-p17745092	Bn-chrA01-p15506771
Bn-A01-p17118839	0.966942149	0.975206612	Bn-N1-p17745284	Bn-chrA01-p15506963
Bn-A01-p17119024	0.950413223	0.950413223	Bn-N1-p17745469	Bn-chrA01-p15507148
Bn-A01-p17119126	0.991735537	0.983471074	Bn-N1-p17745570	Bn-chrA01-p15507250
Bn-A01-p17121291	0.975206612	0.983471074	Bn-N1-p17746769	Bn-chrA01-p15509434
Bn-A01-p17121775	0.946843854	0.993355482	Bn-N1-p17747255	Bn-chrA01-p15509918
Bn-A01-p17121894	0.94214876	0.966942149	Bn-N1-p17747372	Bn-chrA01-p15510037
Bn-A01-p17124558	0.930232558	0.986710963	Bn-N1-p17750057	Bn-chrA01-p15512701
Bn-A01-p17124831	0.950166113	0.993355482	Bn-N1-p17750328	Bn-chrA01-p15512974
Bn-A01-p17129906	0.975206612	0.983471074	Bn-N1-p17753257	Bn-chrA01-p15518053
Bn-A01-p17136539	0.47107438	0.991735537	Bn-N13-p26682708	Bn-chrA01-p15526208
Bn-A01-p17137863	0.925619835	0.991735537	Bn-N11-p30524343	Bn-chrA01-p15527532
Bn-A01-p17150604	0.966942149	0.966942149	Bn-N1-p17764703	Bn-chrA01-p15538587
Bn-A01-p17150875	0.986710963	0.986710963	Bn-N1-p17764974	Bn-chrA01-p15538858
Bn-A01-p17156534	0.958677686	0.958677686	Bn-N1-p17772446	Bn-chrA01-p15544408
Bn-A01-p17157752	0.975206612	0.983471074	Bn-N1-p17773666	Bn-chrA01-p15545628
Bn-A01-p17159109	0.975206612	0.966942149	Bn-N1-p17775041	Bn-chrA01-p15547003
Bn-A01-p17160355	0.983471074	0.983471074	Bn-N1-p17776308	Bn-chrA01-p15551512
Bn-A01-p17165052	0.950413223	0.975206612	Bn-N1-p17781589	Bn-chrA01-p15556122
Bn-A01-p17165303	0.991735537	0.991735537	Bn-N1-p17781871	Bn-chrA01-p15556380
Bn-A01-p17174111	0.958677686	0.900826446	Bn-N1-p17794376	Bn-chrA01-p15564525
Bn-A01-p17187327	0.933554817	0.993355482	Bn-N1-p17820486	Bn-chrA01-p15577786
Bn-A01-p17216088	0.975206612	0.975206612	Bn-N1-p17856909	Bn-chrAnn_random-p190359
Bn-A01-p17216237	0.983471074	0.983471074	Bn-N1-p17857059	Bn-chrAnn_random-p190361
Bn-A01-p17242218	0.975206612	0.975206612	Bn-N1-p17887632	Bn-chrA01_random-p151251
Bn-A01-p17258931	0.958677686	0.958677686	Bn-N1-p17910900	Bn-chrA01_random-p153019
Bn-A01-p17271891	0.975206612	0.975206612	Bn-N11-p30805076	Bn-chrC01-p27172980
Bn-A01-p17278340	0.950413223	0.958677686	Bn-N1-p17931198	Bn-chrA01_random-p156481
Bn-A01-p17283208	0.976744186	0.993355482	Bn-N1-p17936190	Bn-chrA01_random-p157042
Bn-A01-p17294955	0.960199005	0.955223881	Bn-N1-p17949350	Bn-chrA01_random-p158249
Bn-A01-p17295156	0.966942149	0.958677686	Bn-N1-p17949551	Bn-chrA01_random-p158269
Bn-A01-p17296147	0.980066445	0.970099668	Bn-N1-p17954850	Bn-chrA01_random-p158348
Bn-A01-p17306980	0.975206612	0.966942149	Bn-Scaffold14773-p7	Bn-chrA01_random-p150604
Bn-A01-p17315519	0.990033223	0.990033223	Bn-N1-p17973483	Bn-chrA01_random-p150143
Bn-A01-p17326645	0.975206612	0.975206612	Bn-N1-p17985376	Bn-chrA01_random-p148928
Bn-A01-p17377721	0.983471074	0.991735537	Bn-N1-p18040148	Bn-chrA01_random-p143394
Bn-A01-p17382168	0.983471074	0.958677686	Bn-N1-p18044720	Bn-chrA01_random-p142934
Bn-A01-p17408107	0.950413223	0.958677686	Bn-N1-p18069353	Bn-chrA01_random-p140209
Bn-A01-p17428120	0.975206612	0.975206612	Bn-N1-p18081079	Bn-chrA01_random-p138987
Bn-A01-p17432814	0.966942149	0.966942149	Bn-N1-p18085755	Bn-chrA01_random-p138481
Bn-A01-p17434748	0.975206612	0.975206612	Bn-N1-p18087431	Bn-chrA01_random-p138313
Bn-A01-p17437603	0.975206612	0.975206612	Bn-N1-p18090288	Bn-chrA01_random-p138027
Bn-A01-p17438645	1	1	Bn-N1-p18091335	Bn-chrA01_random-p137923
Bn-A01-p17445392	0.990049751	0.985074627	Bn-N1-p18098323	Bn-chrA01_random-p150837
Bn-A01-p17476240	0.991735537	0.991735537	Bn-N1-p18134364	Bn-chrA01-p14922772

Bn-A01-p17480096	0.983471074	0.983471074	Bn-N1-p18138087	Bn-chrA01-p14927917
Bn-A01-p17481703	0.991735537	0.991735537	Bn-N1-p18140244	Bn-chrA01-p14930068
Bn-A01-p17481804	1	1	Bn-N1-p18140345	Bn-chrA01-p14930169
Bn-A01-p17482037	0.975206612	0.975206612	Bn-N1-p18140578	Bn-chrA01-p14930402
Bn-A01-p17483006	0.966942149	0.966942149	Bn-N1-p18141526	Bn-chrA01-p14931350
Bn-A01-p17492348	0.991735537	0.983471074	Bn-N1-p18150937	Bn-chrA01-p14940761
Bn-A01-p17494364	0.975206612	0.983471074	Bn-N1-p18153341	Bn-chrA01-p14943779
Bn-A01-p17498057	0.996677741	0.996677741	Bn-N1-p18157155	Bn-chrA01-p14947415
Bn-A01-p17506817	0.983471074	0.958677686	Bn-N1-p18163081	Bn-chrA01-p14951790
Bn-A01-p17518104	0.96345515	0.966777409	Bn-N1-p18166634	Bn-chrA01-p14955341
Bn-A01-p17518305	0.991735537	0.991735537	Bn-N1-p18166843	Bn-chrA01-p14955550
Bn-A01-p17518468	1	1	Bn-N1-p18167006	Bn-chrA01-p14955713
Bn-A01-p17527782	0.985074627	0.950248756	Bn-N1-p18176351	Bn-chrA01-p14959412
Bn-A01-p17535899	0.970149254	0.965174129	Bn-N1-p18183385	Bn-chrA01-p14962895
Bn-A01-p17536754	0.986710963	0.986710963	Bn-N1-p18184240	Bn-chrA01-p14963752
Bn-A01-p17548021	0.965174129	0.965174129	Bn-N1-p18188383	Bn-chrA01-p14967907
Bn-A01-p17548227	1	1	Bn-N1-p18188590	Bn-chrA01-p14968114
Bn-A01-p17549137	0.985074627	0.616915423	Bn-N11-p31369543	Bn-chrA03_random-p469254
Bn-A01-p17550125	0.985074627	0.985074627	Bn-N1-p18190500	Bn-chrA01-p14969836
Bn-A01-p17550588	0.970149254	0.975124378	Bn-N1-p18190963	Bn-chrA01-p14970299
Bn-A01-p17552389	0.990049751	0.985074627	Bn-N1-p18192762	Bn-chrA01-p14972098
Bn-A01-p17553462	0.975206612	0.975206612	Bn-N1-p18193852	Bn-chrA01-p14973171
Bn-A01-p17555906	0.975206612	0.966942149	Bn-N1-p18196281	Bn-chrA01-p14975603
Bn-A01-p17565834	1	1	Bn-N1-p18220168	Bn-chrA01-p15009588
Bn-A01-p17574483	0.983471074	0.983471074	Bn-N1-p18222096	Bn-chrA01-p15011514
Bn-A01-p17577426	0.965174129	0.980099502	Bn-N1-p18225040	Bn-chrA01-p15014120
Bn-A01-p17577903	0.985074627	0.985074627	Bn-N1-p18225517	Bn-chrA01-p15014597
Bn-A01-p17578169	0.981707317	0.969512195	Bn-N1-p18225783	Bn-chrA01-p15014864
Bn-A01-p17625721	0.983471074	0.975206612	Bn-N1-p18303544	Bn-chrA01-p15122568
Bn-A01-p17626052	0.958677686	0.94214876	Bn-N1-p18303875	Bn-chrA01-p15122899
Bn-A01-p17629571	0.993355482	0.990033223	Bn-N1-p18307392	Bn-chrA01-p15126416
Bn-A01-p17629628	0.991735537	0.991735537	Bn-N1-p18307449	Bn-chrA01-p15126473
Bn-A01-p17636122	0.809917355	0.809917355	Bn-N1-p18314487	Bn-chrA01-p15134606
Bn-A01-p17649002	0.975206612	0.975206612	Bn-N1-p18323834	Bn-chrA01-p15142705
Bn-A01-p17677926	0.991735537	0.991735537	Bn-N1-p18356262	Bn-chrA01-p15172597
Bn-A01-p17691799	0.991735537	0.991735537	Bn-N1-p18375745	Bn-chrA01-p15188778
Bn-A01-p17693439	0.950413223	0.950413223	Bn-N1-p18377386	Bn-chrA01-p15190419
Bn-A01-p17696068	0.985074627	0.985074627	Bn-N1-p18378510	Bn-chrA01-p15191542
Bn-A01-p17696358	0.975124378	0.975124378	Bn-N1-p18378797	Bn-chrA01-p15191829
Bn-A01-p17714021	1	1	Bn-N1-p18396319	Bn-chrAnn_random-p447918
Bn-A01-p17716773	1	1	Bn-N1-p18402890	Bn-chrA01_random-p159439
Bn-A01-p17717864	0.983471074	0.975206612	Bn-N1-p18403981	Bn-chrA01_random-p159548
Bn-A01-p17728772	0.991735537	0.991735537	Bn-N1-p18414830	Bn-chrA01_random-p160634
Bn-A01-p17730468	0.966942149	0.975206612	Bn-N1-p18416576	Bn-chrA01_random-p160809
Bn-A01-p17751647	0.990033223	0.986710963	Bn-N1-p18434525	Bn-chrA01_random-p162690
Bn-A01-p17755236	0.975206612	0.983471074	Bn-N1-p18438320	Bn-chrA01_random-p163049

Bn-A01-p17769742	0.917355372	0.925619835	Bn-N1-p18441884	Bn-chrA01_random-p163545
Bn-A01-p17771411	0.975206612	0.966942149	Bn-N1-p18443557	Bn-chrA01_random-p163712
Bn-A01-p17783808	0.148760331	0.991735537	Unknown	Bn-chrA01_random-p165374
Bn-A01-p17783900	0.140495868	0.975206612	Unknown	Bn-chrA01_random-p165384
Bn-A01-p17787446	0.314049587	0.975206612	Bn-N12-p42320001	Bn-chrA01_random-p165739
Bn-A01-p17806607	0.20661157	0.966942149	Bn-N9-p12835994	Bn-chrA01_random-p166661
Bn-A01-p17820874	0.933884298	0.933884298	Bn-N1-p18473106	Bn-chrA01_random-p200312
Bn-A01-p17839637	0.986710963	0.986710963	Bn-Scaffold07653-p6	Bn-chrA01_random-p201779
Bn-A01-p17845660	0.986710963	1	Bn-N1-p18498879	Bn-chrA01_random-p202178
Bn-A01-p17849675	0.966942149	0.966942149	Bn-N1-p18503139	Bn-chrA01_random-p173772
Bn-A01-p17881348	0.94214876	0.94214876	Bn-N1-p18531589	Bn-chrA01_random-p169984
Bn-A01-p17885385	0.950413223	0.958677686	Bn-N1-p18535795	Bn-chrA01_random-p169543
Bn-A01-p17885812	0.986710963	0.980066445	Bn-N1-p18536222	Bn-chrA01_random-p169500
Bn-A01-p17886123	0.990033223	0.990033223	Bn-N1-p18536533	Bn-chrA01_random-p169469
Bn-A01-p17886595	0.983388704	0.980066445	Bn-N1-p18537005	Bn-chrA01_random-p169422
Bn-A01-p17887135	0.958677686	0.966942149	Bn-N1-p18537545	Bn-chrA01_random-p169368
Bn-A01-p17887214	0.983471074	0.975206612	Bn-N1-p18537624	Bn-chrA01_random-p169360
Bn-A01-p17887935	0.958677686	0.966942149	Bn-N1-p18538345	Bn-chrA01_random-p169288
Bn-A01-p17888905	0.983471074	0.991735537	Bn-N1-p18539315	Bn-chrA01_random-p169191
Bn-A01-p17892299	1	0.991735537	Bn-N1-p18542985	Bn-chrA01_random-p168860
Bn-A01-p17899781	0.995024876	0.990049751	Bn-N1-p18550465	Bn-chrA01_random-p168079
Bn-A01-p17905950	0.975206612	0.966942149	Bn-N1-p18559022	Bn-chrA01-p15206005
Bn-A01-p17906322	0.991735537	0.975206612	Bn-N1-p18559394	Bn-chrA01-p15206635
Bn-A01-p17906399	0.991735537	0.983471074	Bn-N1-p18559471	Bn-chrA01-p15206712
Bn-A01-p17906765	0.975124378	0.980099502	Bn-N1-p18559837	Bn-chrA01-p15207080
Bn-A01-p17907059	0.958677686	0.950413223	Bn-N1-p18560131	Bn-chrA01-p15207374
Bn-A01-p17907563	0.958677686	0.950413223	Bn-N1-p18560635	Bn-chrA01-p15207878
Bn-A01-p17910798	0.966942149	0.958677686	Bn-N1-p18563874	Bn-chrA01-p15220514
Bn-A01-p17911199	0.993355482	0.990033223	Bn-N1-p18564275	Bn-chrA01-p15220912
Bn-A01-p17911921	0.986710963	0.983388704	Bn-N1-p18564997	Bn-chrA01-p15221605
Bn-A01-p17916004	0.983471074	0.991735537	Bn-N1-p18569082	Bn-chrA01-p15232902
Bn-A01-p17930058	0.950413223	0.958677686	Bn-N1-p18577929	Bn-chrA01-p15236220
Bn-A01-p17931128	0.983471074	0.752066116	Bn-N1-p18578999	Bn-chrA01-p15237290
Bn-A01-p17935219	0.975206612	0.909090909	Bn-N1-p18583111	Bn-chrC01-p28367512
Bn-A01-p17941441	0.960199005	0.955223881	Bn-N1-p18593240	Bn-chrA01-p15249586
Bn-A01-p17997441	0.970149254	0.975124378	Bn-N1-p18656229	Bn-chrA01-p15326784
Bn-A01-p17998573	0.975206612	0.983471074	Bn-N1-p18657361	Bn-chrA01-p15327908
Bn-A01-p18006188	0.966942149	0.958677686	Bn-N1-p18659664	Bn-chrA01-p15330214
Bn-A01-p18008659	0.980066445	0.96013289	Bn-N1-p18662134	Bn-chrA01-p15332688
Bn-A01-p18014946	0.983471074	0.975206612	Bn-N1-p18670345	Bn-chrA01-p15335748
Bn-A01-p18015006	0.991735537	0.983471074	Bn-N1-p18670405	Bn-chrA01-p15335808
Bn-A01-p18015162	0.975206612	0.892561983	Bn-N1-p18670561	Bn-chrA01-p15335961
Bn-A01-p18015323	0.950413223	0.933884298	Bn-N1-p18670722	Bn-chrA01-p15336359
Bn-A01-p18019641	0.966942149	0.958677686	Bn-N1-p18675095	Bn-chrA01-p15340082
Bn-A01-p18020258	0.925619835	0.933884298	Bn-N1-p18675712	Bn-chrA01-p15340700
Bn-A01-p18020314	0.933884298	0.917355372	Bn-N1-p18675768	Bn-chrA01-p15340756

Bn-A01-p18020503	0.983471074	0.909090909	Bn-N1-p18675961	Bn-chrA01-p15340939
Bn-A01-p18023959	0.958677686	0.958677686	Bn-N1-p18680459	Bn-chrA01-p15343709
Bn-A01-p18025382	0.975206612	0.628099174	Bn-N1-p18681882	Bn-chrA01-p15345125
Bn-A01-p18025988	0.991735537	0.983471074	Bn-N1-p18682493	Bn-chrA01-p15345441
Bn-A01-p18027200	0.966942149	0.958677686	Bn-N1-p18683704	Bn-chrA01-p15346627
Bn-A01-p18027307	0.975206612	0.958677686	Bn-N1-p18683811	Bn-chrA01-p15346736
Bn-A01-p18027398	0.966942149	0.975206612	Bn-N1-p18683902	Bn-chrA01-p15346827
Bn-A01-p18028380	0.966942149	0.975206612	Bn-N1-p18684884	Bn-chrA01-p15347828
Bn-A01-p18028483	0.991735537	0.983471074	Bn-N1-p18684987	Bn-chrA01-p15347931
Bn-A01-p18030126	0.975206612	0.966942149	Bn-N1-p18686121	Bn-chrA01-p15349092
Bn-A01-p18032386	0.983471074	0.975206612	Bn-N1-p18688384	Bn-chrA01-p15351972
Bn-A01-p18053077	0.966942149	0.966942149	Bn-N1-p18725795	Bn-chrA01-p15361513
Bn-A01-p18063416	0.983471074	0.983471074	Bn-N1-p18742084	Bn-chrA01-p15371856
Bn-A01-p18072231	0.975206612	0.975206612	Bn-N1-p18747408	Bn-chrA01-p15375755
Bn-A01-p18072667	0.995024876	0.995024876	Bn-N5-p23334896	Bn-chrA03-p1566974
Bn-A01-p18073544	0.985074627	0.990049751	Bn-Scaffold09539-p9	Bn-chrA01-p15377068
Bn-A01-p18078718	0.975206612	0.975206612	Bn-N1-p18750823	Bn-chrA01-p15382241
Bn-A01-p18084128	0.603305785	0.975206612	Bn-Scaffold00992-p1	Bn-chrAnn_random-p18526(
Bn-A01-p18091225	0.32231405	0.991735537	Bn-N17-p35940274	Bn-chrA01-p15583310
Bn-A01-p18091559	0.454545455	0.958677686	Bn-N13-p65584888	Bn-chrA01-p15583644
Bn-A01-p18091996	0.79338843	0.983471074	Bn-N1-p18759950	Bn-chrA01-p15584081
Bn-A01-p18091997	0.801652893	0.991735537	Bn-N1-p18759951	Bn-chrA01-p15584082
Bn-A01-p18096974	0.973421927	0.976744186	Bn-N1-p18763566	Bn-chrA01-p15587712
Bn-A01-p18100900	0.933884298	0.966942149	Bn-N1-p18772147	Bn-chrA01-p15596549
Bn-A01-p18102632	0.991735537	0.983471074	Bn-N1-p18776620	Bn-chrA01-p15598032
Bn-A01-p18102711	0.857142857	0.910299003	Bn-N1-p18776699	Bn-chrA01-p15598111
Bn-A01-p18119989	0.94214876	0.925619835	Bn-N1-p18779446	Bn-chrA01-p15605728
Bn-A01-p18121827	0.980066445	0.996677741	Bn-N11-p32273256	Bn-chrA01-p15607592
Bn-A01-p18124795	0.991735537	0.94214876	Bn-N1-p18784280	Bn-chrC01-p28653712
Bn-A01-p18160311	0.512437811	0.985074627	Bn-N1-p2018972	Bn-chrA01-p15631200
Bn-A01-p18178802	0.975206612	0.983471074	Bn-N1-p18826458	Bn-chrA01-p15665184
Bn-A01-p18182766	0.983471074	0.991735537	Bn-N1-p18840496	Bn-chrA01-p15669812
Bn-A01-p18197352	0.975206612	0.983471074	Bn-N1-p18850715	Bn-chrC01-p28759812
Bn-A01-p18200921	0.965174129	0.980099502	Bn-N1-p18852932	Bn-chrA01-p15685473
Bn-A01-p18205455	0.983471074	0.975206612	Bn-N1-p18860025	Bn-chrA01-p15691812
Bn-A01-p18218629	0.134328358	1	Bn-N4-p6007661	Bn-chrA01-p15706974
Bn-A01-p18230629	0.917355372	0.958677686	Bn-N11-p32395415	Bn-chrC01-p28819007
Bn-A01-p18240444	0.966777409	0.976744186	Bn-N4-p19005396	Bn-chrA01-p15744443
Bn-A01-p18260283	0.884297521	0.975206612	Bn-N11-p32466843	Bn-chrA01-p15763518
Bn-A01-p18266537	0.975206612	0.966942149	Bn-N1-p18907894	Bn-chrA01-p15769768
Bn-A01-p18285077	0.990033223	0.990033223	Bn-N1-p18929279	Bn-chrA01-p15793013
Bn-A01-p18306617	0.983471074	0.983471074	Bn-N1-p18954741	Bn-chrA01-p15815977
Bn-A01-p18327940	0.94214876	0.950413223	Bn-N1-p18982111	Bn-chrA01-p15844590
Bn-A01-p18329609	0.983471074	0.991735537	Bn-N1-p18984031	Bn-chrA01-p15846466
Bn-A01-p18329689	0.991735537	0.983471074	Bn-N1-p18984111	Bn-chrA01-p15846546
Bn-A01-p18329796	0.991735537	1	Bn-N1-p18984218	Bn-chrA01-p15846653

Bn-A01-p18330598	0.985074627	0.990049751	Bn-N1-p18985019	Bn-chrA01-p15847455
Bn-A01-p18367467	0.975206612	0.975206612	Bn-N1-p19021045	Bn-chrA01-p15876634
Bn-A01-p18383994	0.991735537	0.983471074	Bn-N1-p19034080	Bn-chrA01-p15896211
Bn-A01-p18391341	0.975206612	0.966942149	Bn-N1-p19043009	Bn-chrA01-p15903771
Bn-A01-p18435876	0.985074627	0.990049751	Bn-N1-p19082054	Bn-chrA01-p15938324
Bn-A01-p18438154	0.980099502	0.980099502	Bn-N1-p19084332	Bn-chrA01-p15940603
Bn-A01-p18612031	0.768595041	0.760330579	Bn-N10-p5697395	Bn-chrA10_random-p615981
Bn-A01-p18635245	0.983471074	0.991735537	Bn-N10-p5679261	Bn-chrA10_random-p132673
Bn-A01-p18635275	0.991735537	0.983471074	Bn-N10-p5679231	Bn-chrA10_random-p132676
Bn-A01-p18635430	1	0.991735537	Bn-N10-p5679076	Bn-chrA10_random-p132691
Bn-A01-p18635517	0.983471074	0.991735537	Bn-N10-p5678989	Bn-chrA10_random-p132700
Bn-A01-p18636804	0.970099668	0.73089701	Bn-N10-p5677577	Bn-chrA07-p8728017
Bn-A01-p18639615	0.983471074	0.983471074	Bn-N10-p5674737	Bn-chrAnn_random-p484414
Bn-A01-p18662925	0.991735537	0.983471074	Bn-N10-p5651690	Bn-chrA10-p5819587
Bn-A01-p18672984	0.991735537	0.983471074	Bn-N10-p5641608	Bn-chrA10-p5829657
Bn-A01-p18673020	0.983471074	0.991735537	Bn-N10-p5641572	Bn-chrA10-p5829693
Bn-A01-p18676639	1	0.991735537	Bn-N10-p5637868	Bn-chrA10-p5833312
Bn-A01-p18691000	1	0.991735537	Bn-N10-p5623634	Bn-chrA10-p5864939
Bn-A01-p18691142	0.983471074	0.975206612	Bn-N10-p5623491	Bn-chrA10-p5865083
Bn-A01-p18692022	0.975206612	0.966942149	Bn-N10-p5622611	Bn-chrA10-p5865948
Bn-A01-p18692229	0.991735537	0.983471074	Bn-N10-p5622404	Bn-chrA10-p5866155
Bn-A01-p18692686	0.975206612	0.983471074	Bn-N10-p5621947	Bn-chrA10-p5866612
Bn-A01-p18694130	0.966942149	0.958677686	Bn-N10-p5618413	Bn-chrA10-p5868054
Bn-A01-p18702163	1	0.993355482	Bn-N10-p5613228	Bn-chrA10-p5874667
Bn-A01-p18703183	0.991735537	1	Bn-N10-p5612208	Bn-chrA10-p5875688
Bn-A01-p18703922	0.991735537	1	Bn-N10-p5611469	Bn-chrA10-p5876427
Bn-A01-p18704205	0.991735537	0.983471074	Bn-N10-p5611186	Bn-chrA10-p5876710
Bn-A01-p18704834	0.991735537	1	Bn-N10-p5610557	Bn-chrA10-p5877343
Bn-A01-p18705556	1	1	Bn-N10-p5609834	Bn-chrA10-p5878042
Bn-A01-p18706337	0.996677741	0.993355482	Bn-N10-p5609053	Bn-chrA10-p5878848
Bn-A01-p18711867	1	0.991735537	Bn-N10-p5603551	Bn-chrA10-p5884661
Bn-A01-p18714050	0.985074627	0.985074627	Bn-Scaffold02078-p2	Bn-chrA10-p5886844
Bn-A01-p18714119	0.980099502	0.980099502	Bn-Scaffold02078-p2	Bn-chrA10-p5886913
Bn-A01-p18714182	0.980099502	0.980099502	Bn-Scaffold02078-p2	Bn-chrA10-p5886976
Bn-A01-p18715909	0.990049751	0.995024876	Bn-Scaffold02078-p3	Bn-chrA10-p5888703
Bn-A01-p18716258	0.970149254	0.965174129	Bn-Scaffold02078-p4	Bn-chrA10-p5889052
Bn-A01-p18727458	0.996677741	0.993355482	Bn-N10-p5577423	Bn-chrA10-p5901756
Bn-A01-p18734383	0.983471074	0.975206612	Bn-N10-p5568380	Bn-chrA10-p5913562
Bn-A01-p18739675	0.975206612	0.983471074	Bn-N10-p5566002	Bn-chrA10-p5917362
Bn-A01-p18813615	0.985074627	0.980099502	Bn-N10-p5489130	Bn-chrA10-p6013210
Bn-A01-p18813849	0.990033223	0.946843854	Bn-N10-p5488896	Bn-chrA10-p6013444
Bn-A01-p18826838	0.991735537	0.983471074	Bn-N10-p5480344	Bn-chrA03-p20772999
Bn-A01-p18826928	0.991735537	0.991735537	Bn-N3-p22245280	Bn-chrA03-p20773089
Bn-A01-p18827383	0.983471074	0.983471074	Bn-Scaffold01701-p3	Bn-chrA03-p20773543
Bn-A01-p18828028	0.975206612	0.975206612	Bn-Scaffold01701-p1	Bn-chrA03-p20774188
Bn-A01-p18828081	0.975206612	0.975206612	Bn-Scaffold01701-p1	Bn-chrA03-p20774241

Bn-A01-p18828505	0.983471074	0.983471074	Bn-Scaffold01701-p1	Bn-chrA03-p20774666
Bn-A01-p18835025	0.966942149	0.975206612	Bn-N10-p5472050	Bn-chrA10-p6024505
Bn-A01-p18837660	0.983471074	0.975206612	Bn-N10-p5469414	Bn-chrA10-p6027254
Bn-A01-p18850969	1	0.991735537	Bn-N10-p5455380	Bn-chrA10-p6039064
Bn-A01-p18866149	0.94214876	0.950413223	Bn-N10-p5439159	Bn-chrA10-p6059853
Bn-A01-p18870174	0.983471074	0.743801653	Bn-N10-p5435069	Bn-chrA10-p6068615
Bn-A01-p18876940	0.990049751	0.990049751	Bn-N10-p5429018	Bn-chrA10-p6075860
Bn-A01-p18881723	0.991735537	1	Bn-N10-p5423154	Bn-chrA10-p6084566
Bn-A01-p18882626	1	0.752066116	Bn-N10-p5422236	Bn-chrC09-p30566941
Bn-A01-p18890238	0.975206612	0.983471074	Bn-N10-p5414239	Bn-chrA10-p6093820
Bn-A01-p18894627	0.983471074	0.975206612	Bn-N10-p5409861	Bn-chrA10-p6096985
Bn-A01-p18896245	0.983471074	0.975206612	Bn-N10-p5408243	Bn-chrA10-p6098602
Bn-A01-p18902391	1	0.991735537	Bn-N10-p5405133	Bn-chrA10-p6103617
Bn-A01-p18914107	0.985074627	0.985074627	Bn-Scaffold01609-p5	Bn-chrA07_random-p736354
Bn-A01-p18914763	0.99137931	0.99137931	Bn-Scaffold01609-p5	Bn-chrA07_random-p735783
Bn-A01-p18917698	1	0.991735537	Bn-N10-p5395574	Bn-chrA10-p6113454
Bn-A01-p19097875	0.980066445	0.986710963	Bn-N10-p5191978	Bn-chrA10-p6309756
Bn-A01-p19098195	0.983471074	0.975206612	Bn-N10-p5191658	Bn-chrA10-p6310076
Bn-A01-p19098776	1	0.884297521	Bn-N10-p5191096	Bn-chrA10-p6311028
Bn-A01-p19107003	0.983471074	0.991735537	Bn-N10-p5183002	Bn-chrA10-p6319979
Bn-A01-p19107198	0.991735537	0.983471074	Bn-N10-p5182806	Bn-chrA10-p6320176
Bn-A01-p19107773	0.991735537	0.958677686	Bn-N10-p5182232	Bn-chrA10-p6320740
Bn-A01-p19108439	0.983471074	0.975206612	Bn-N10-p5181566	Bn-chrA10-p6323768
Bn-A01-p19108743	0.991735537	0.975206612	Bn-N10-p5181262	Bn-chrA10-p6324073
Bn-A01-p19108849	0.958677686	0.966942149	Bn-N10-p5181156	Bn-chrA10-p6324178
Bn-A01-p19110384	0.983471074	0.991735537	Bn-N10-p5179621	Bn-chrA10-p6329499
Bn-A01-p19110508	0.983471074	0.809917355	Bn-N10-p5179497	Bn-chrA10-p6329623
Bn-A01-p19110596	0.991735537	0.619834711	Bn-N10-p5179409	Bn-chrA10-p6329667
Bn-A01-p19111246	0.966942149	0.975206612	Bn-N10-p5178759	Bn-chrA10-p6330317
Bn-A01-p19111305	0.958677686	0.966942149	Bn-N10-p5178700	Bn-chrA10-p6330376
Bn-A01-p19114969	0.966942149	0.958677686	Bn-N10-p5175044	Bn-chrA10-p6333406
Bn-A01-p19115021	0.975206612	0.966942149	Bn-N10-p5174992	Bn-chrA10-p6333458
Bn-A01-p19115242	0.975206612	0.983471074	Bn-N10-p5174771	Bn-chrA10-p6333679
Bn-A01-p19115530	0.958677686	0.966942149	Bn-N10-p5174483	Bn-chrA10-p6333966
Bn-A01-p19115872	0.991735537	0.983471074	Bn-N10-p5174141	Bn-chrA10-p6334307
Bn-A01-p19116006	0.958677686	0.909090909	Bn-N10-p5174007	Bn-chrA10-p6334441
Bn-A01-p19116819	1	0.991735537	Bn-N10-p5173194	Bn-chrA10-p6335256
Bn-A01-p19122048	0.975206612	0.966942149	Bn-N10-p5167876	Bn-chrA10-p6341372
Bn-A01-p19125668	0.991735537	0.595041322	Bn-N10-p5164097	Bn-chrAnn_random-p384721
Bn-A01-p19132144	0.966942149	0.975206612	Bn-N10-p5157599	Bn-chrA10-p6351199
Bn-A01-p19139637	1	0.991735537	Bn-N10-p5144623	Bn-chrA10-p6360602
Bn-A01-p19150923	0.990049751	0.985074627	Bn-N10-p5131193	Bn-chrA10-p6373479
Bn-A01-p19153919	1	0.991735537	Bn-N10-p5124793	Bn-chrA10-p6376473
Bn-A01-p19156177	0.975206612	0.983471074	Bn-N10-p5122536	Bn-chrA10-p6378730
Bn-A01-p19158271	1	0.991735537	Bn-N10-p5120442	Bn-chrA10-p6380825
Bn-A01-p19158362	0.991735537	0.983471074	Bn-N10-p5120351	Bn-chrA10-p6380916

Bn-A01-p19162993	0.980066445	0.993355482	Bn-N10-p5115709	Bn-chrA10-p6385782
Bn-A01-p19163275	0.991735537	0.983471074	Bn-N10-p5115427	Bn-chrA10-p6386064
Bn-A01-p19170760	0.983471074	0.991735537	Bn-N10-p5108815	Bn-chrA10-p6395283
Bn-A01-p19174115	0.991735537	0.983471074	Bn-N10-p5105446	Bn-chrA10-p6398654
Bn-A01-p19185181	1	0.991735537	Bn-N10-p5093953	Bn-chrA10-p6410390
Bn-A01-p19220693	0.996677741	0.996677741	Bn-N10-p5057112	Bn-chrA10-p6449364
Bn-A01-p19283108	0.986710963	0.990033223	Bn-N10-p4986548	Bn-chrA10-p6521131
Bn-A01-p19340658	0.995024876	1	Bn-N1-p19330175	Bn-chrA01-p16187034
Bn-A01-p19355180	0.989583333	0.989583333	Bn-N1-p19347053	Bn-chrAnn_random-p326036
Bn-A01-p19446599	0.991735537	0.991735537	Bn-N1-p19441811	Bn-chrA01-p16300597
Bn-A01-p19447794	0.983471074	0.991735537	Bn-N1-p19443005	Bn-chrCnn_random-p509442
Bn-A01-p19449612	0.970149254	0.975124378	Bn-N1-p19444839	Bn-chrA01-p16303620
Bn-A01-p19498222	0.966942149	0.958677686	Bn-N1-p19492902	Bn-chrA01-p16338589
Bn-A01-p19512499	0.983471074	0.991735537	Bn-N1-p19508482	Bn-chrA01-p16375685
Bn-A01-p19525594	0.94214876	0.950413223	Bn-N1-p19520482	Bn-chrA01-p16388180
Bn-A01-p19527588	0.991735537	0.991735537	Bn-N1-p19522512	Bn-chrA01-p16390210
Bn-A01-p19528388	0.983471074	0.983471074	Bn-N1-p19523317	Bn-chrA01-p16391875
Bn-A01-p19533568	0.953488372	0.953488372	Bn-N1-p19528615	Bn-chrA01-p16397173
Bn-A01-p19535456	0.983471074	0.983471074	Bn-N1-p19532008	Bn-chrA01-p16400565
Bn-A01-p19540960	0.966942149	0.966942149	Bn-N1-p19532984	Bn-chrA01-p16401540
Bn-A01-p19562202	0.94214876	0.94214876	Bn-N1-p19542031	Bn-chrA01-p16410725
Bn-A01-p19564015	0.983471074	0.983471074	Bn-N1-p19542427	Bn-chrA01-p16411121
Bn-A01-p19567680	0.975206612	0.975206612	Bn-N1-p19547008	Bn-chrA01-p16417064
Bn-A01-p19577901	0.933884298	0.933884298	Bn-N1-p19550374	Bn-chrA01-p16420427
Bn-A01-p19579243	0.867768595	0.867768595	Bn-N1-p19551786	Bn-chrA01-p16421837
Bn-A01-p19579412	0.975206612	0.975206612	Bn-N1-p19553322	Bn-chrA01-p16422006
Bn-A01-p19581862	0.991735537	0.991735537	Bn-N1-p19554096	Bn-chrA01-p16422616
Bn-A01-p19582940	0.900826446	0.900826446	Bn-N1-p19555086	Bn-chrA01-p16423606
Bn-A01-p19583367	0.983471074	0.983471074	Bn-N1-p19555518	Bn-chrA01-p16424038
Bn-A01-p19583736	0.991735537	0.991735537	Bn-N1-p19556571	Bn-chrA01_random-p199355
Bn-A01-p19589837	0.991735537	0.991735537	Bn-N1-p19565008	Bn-chrA01-p16429901
Bn-A01-p19589921	1	1	Bn-N1-p19565092	Bn-chrA01-p16429985
Bn-A01-p19592804	1	1	Bn-N1-p19567964	Bn-chrA01-p16433025
Bn-A01-p19593638	0.983471074	0.983471074	Bn-N1-p19568797	Bn-chrA01-p16433859
Bn-A01-p19593702	0.975206612	0.975206612	Bn-N1-p19568892	Bn-chrA01-p16433923
Bn-A01-p19594423	0.958677686	0.958677686	Bn-N1-p19569615	Bn-chrA01-p16434646
Bn-A01-p19598162	0.966942149	0.966942149	Bn-N1-p19573397	Bn-chrA01-p16438428
Bn-A01-p19599270	0.983471074	0.983471074	Bn-N1-p19574507	Bn-chrA01-p16439537
Bn-A01-p19600244	1	1	Bn-N1-p19575482	Bn-chrA01-p16440512
Bn-A01-p19601137	0.983471074	0.983471074	Bn-N1-p19576380	Bn-chrA01-p16441102
Bn-A01-p19601542	0.991735537	0.991735537	Bn-N1-p19576788	Bn-chrA01-p16441510
Bn-A01-p19602112	0.991735537	0.991735537	Bn-N1-p19577350	Bn-chrA01-p16442072
Bn-A01-p19605587	1	1	Bn-N1-p19581411	Bn-chrA01-p16446140
Bn-A01-p19607256	0.983471074	0.983471074	Bn-N1-p19583059	Bn-chrA01-p16447786
Bn-A01-p19617963	0.991735537	0.991735537	Bn-N1-p19589887	Bn-chrA01-p16454913
Bn-A01-p19623880	0.975206612	0.975206612	Bn-N1-p19594827	Bn-chrA01-p16459849

Bn-A01-p19623985	1	1	Bn-N1-p19594932	Bn-chrA01-p16459954
Bn-A01-p19624123	0.976744186	0.976744186	Bn-N1-p19595070	Bn-chrA01-p16460092
Bn-A01-p19624216	0.958677686	0.958677686	Bn-N1-p19595163	Bn-chrA01-p16460185
Bn-A01-p19633815	0.966942149	0.966942149	Bn-N1-p19597950	Bn-chrA01-p16462971
Bn-A01-p19650666	0.990033223	0.990033223	Bn-N1-p19605856	Bn-chrA01-p16469624
Bn-A01-p19661033	0.909090909	0.909090909	Bn-N1-p19607220	Bn-chrA01-p16470988
Bn-A01-p19700004	0.983471074	0.983471074	Bn-N1-p19644864	Bn-chrA01-p16508776
Bn-A01-p19712396	0.975206612	0.975206612	Bn-N1-p19651866	Bn-chrA01-p16518531
Bn-A01-p19722138	0.983471074	0.983471074	Bn-N1-p19655672	Bn-chrA01-p16522087
Bn-A01-p19731678	0.991735537	0.991735537	Bn-N1-p19662300	Bn-chrA01-p16530724
Bn-A01-p19762477	0.975206612	0.975206612	Bn-N1-p19691017	Bn-chrA01-p16559791
Bn-A01-p19764240	0.975206612	0.975206612	Bn-N1-p19692776	Bn-chrA01-p16561549
Bn-A01-p19803988	0.983471074	0.983471074	Bn-N1-p19720010	Bn-chrA01-p16591763
Bn-A01-p19813138	1	1	Bn-N1-p19729500	Bn-chrA01-p16601252
Bn-A01-p19840597	0.975206612	0.975206612	Bn-N1-p19769447	Bn-chrA01-p16647337
Bn-A01-p19855343	0.84079602	0.84079602	Bn-N11-p45699223	Bn-chrC01-p38392202
Bn-A01-p19858735	0.950413223	0.950413223	Bn-N1-p19775531	Bn-chrA01-p16652862
Bn-A01-p19879925	0.983471074	0.983471074	Bn-N1-p19794267	Bn-chrA01-p16671599
Bn-A01-p19929280	0.983471074	0.991735537	Bn-N1-p19844885	Bn-chrA01-p16718108
Bn-A01-p19931030	1	1	Bn-N1-p19847972	Bn-chrA01-p16720891
Bn-A01-p19931596	1	1	Bn-N1-p19848537	Bn-chrA01-p16721456
Bn-A01-p19946190	0.983471074	0.975206612	Bn-N1-p19866994	Bn-chrA01-p16738165
Bn-A01-p19955264	0.991735537	0.983471074	Bn-N1-p19878748	Bn-chrA01-p16752251
Bn-A01-p19961176	0.917355372	0.917355372	Bn-N1-p19884679	Bn-chrA01-p16758190
Bn-A01-p19983739	0.823920266	0.827242525	Bn-N1-p19905997	Bn-chrA01-p16784419
Bn-A01-p19993388	0.995024876	0.995024876	Bn-N1-p19915857	Bn-chrA01-p16794271
Bn-A01-p20133687	0.983471074	0.983471074	Bn-N1-p20042069	Bn-chrCnn_random-p565782
Bn-A01-p20139530	0.966942149	0.966942149	Bn-N1-p20044897	Bn-chrAnn_random-p371162
Bn-A01-p20144346	0.940298507	0.940298507	Bn-N1-p20058240	Bn-chrA01_random-p204582
Bn-A01-p20144596	0.975206612	0.975206612	Bn-N1-p20058490	Bn-chrA01_random-p204607
Bn-A01-p20147405	1	1	Bn-N1-p20062658	Bn-chrA01_random-p205024
Bn-A01-p20159960	0.991735537	0.991735537	Bn-N1-p20076099	Bn-chrAnn_random-p368959
Bn-A01-p20166016	0.991735537	0.991735537	Bn-N1-p20085773	Bn-chrA01-p16946565
Bn-A01-p20167230	0.966942149	0.966942149	Bn-N1-p20086990	Bn-chrA01-p16947782
Bn-A01-p20181046	0.975206612	0.975206612	Bn-N1-p20096054	Bn-chrA01-p16956878
Bn-A01-p20181346	0.983471074	0.975206612	Bn-N1-p20096357	Bn-chrA01-p16957181
Bn-A01-p20196813	0.975206612	0.966942149	Bn-N1-p20110151	Bn-chrA01-p16985421
Bn-A01-p20207440	0.983471074	0.991735537	Bn-N1-p20120467	Bn-chrA01-p17000139
Bn-A01-p20211329	0.958677686	0.958677686	Bn-N1-p20124349	Bn-chrA01-p17004019
Bn-A01-p20211387	0.975206612	0.975206612	Bn-N1-p20124407	Bn-chrA01-p17004077
Bn-A01-p20233113	0.92039801	0.860696517	Bn-N1-p20144558	Bn-chrC01-p30432188
Bn-A01-p20240168	0.975206612	0.983471074	Bn-N1-p20151300	Bn-chrA01-p17049580
Bn-A01-p20240780	0.966942149	0.975206612	Bn-N1-p20151912	Bn-chrAnn_random-p427686
Bn-A01-p20246564	0.975206612	0.966942149	Bn-N1-p20157829	Bn-chrA01_random-p215192
Bn-A01-p20246840	0.991735537	1	Bn-N1-p20158105	Bn-chrA01_random-p215165
Bn-A01-p20248379	1	0.991735537	Bn-N1-p20159644	Bn-chrA01_random-p215011

Bn-A01-p20259868	0.958677686	0.966942149	Bn-N1-p20169042	Bn-chrC01-p30480327
Bn-A01-p20282705	0.933884298	0.776859504	Bn-N1-p20190948	Bn-chrAnn_random-p409397
Bn-A01-p20306930	0.834710744	0.752066116	Bn-Scaffold18786-p4	Bn-chrC01-p30511192
Bn-A01-p20323059	0.980099502	0.985074627	Bn-N1-p20216069	Bn-chrA01-p17126381
Bn-A01-p20393111	0.958677686	1	Bn-N11-p34919884	Bn-chrA01-p17192852
Bn-A01-p20397807	0.991735537	1	Bn-N1-p20284986	Bn-chrA01-p17194080
Bn-A01-p20406782	0.657807309	0.657807309	Bn-N1-p20292023	Bn-chrA01-p17202603
Bn-A01-p20430407	1	1	Bn-N1-p20324743	Bn-chrA01-p17232920
Bn-A01-p20431228	0.970099668	0.970099668	Bn-N1-p20330193	Bn-chrA01-p17238370
Bn-A01-p20433111	0.94214876	0.94214876	Bn-N11-p35064282	Bn-chrC01-p30602470
Bn-A01-p20442457	0.958677686	0.958677686	Bn-N1-p20341020	Bn-chrA01-p17249064
Bn-A01-p20471502	0.983471074	0.950413223	Bn-N1-p20355727	Bn-chrA01-p17258936
Bn-A01-p20482459	0.983388704	0.980066445	Bn-N1-p20367448	Bn-chrA01_random-p211658
Bn-A01-p20483938	0.983471074	0.991735537	Bn-N1-p20368960	Bn-chrA01_random-p211510
Bn-A01-p20497397	0.975206612	0.966942149	Bn-N1-p20396722	Bn-chrA01_random-p210786
Bn-A01-p20564016	0.936877076	0.933554817	Bn-N1-p20461472	Bn-chrA01-p17369813
Bn-A01-p20679722	1	1	Bn-N11-p35510898	Bn-chrC01-p31024332
Bn-A01-p20692707	1	0.909090909	Bn-N1-p20586894	Bn-chrA03-p23555190
Bn-A01-p20696971	0.993355482	0.96345515	Bn-N1-p20591235	Bn-chrA01-p17490376
Bn-A01-p20745330	0.996677741	0.996677741	Bn-Scaffold05610-p3	Bn-chrA01-p17545013
Bn-A01-p20745820	0.873754153	0.986710963	Bn-N18-p34690221	Bn-chrA01-p17545510
Bn-A01-p20753932	0.966942149	0.966942149	Bn-N1-p20664815	Bn-chrA01-p17554037
Bn-A01-p20761933	0.970099668	0.966777409	Bn-N1-p20670011	Bn-chrA01-p17560255
Bn-A01-p20782963	0.646766169	0.646766169	Bn-N18-p19189424	Bn-chrC03-p30114875
Bn-A01-p20783867	0.980099502	0.980099502	Bn-Scaffold58147-p1	Bn-chrA02-p16816835
Bn-A01-p20784377	0.968911917	1	Bn-N13-p54157433	Bn-chrA02-p16816333
Bn-A01-p20789828	0.975206612	0.966942149	Bn-N1-p20704408	Bn-chrA01_random-p203085
Bn-A01-p20805506	0.991735537	0.991735537	Bn-N1-p20714527	Bn-chrA01-p17597616
Bn-A01-p20805613	0.983471074	0.983471074	Bn-N1-p20714634	Bn-chrA01-p17597723
Bn-A01-p20814699	0.975124378	0.975124378	Bn-N1-p20724001	Bn-chrA01-p17621547
Bn-A01-p20836664	0.983471074	0.991735537	Bn-N1-p20744534	Bn-chrA01-p17641031
Bn-A01-p20839901	0.893687708	0.890365449	Bn-N1-p20747772	Bn-chrA01-p17647106
Bn-A01-p20843887	0.628099174	0.958677686	Bn-N1-p7884699	Bn-chrA01-p17651108
Bn-A01-p20866022	0.950413223	0.966942149	Bn-N1-p20768390	Bn-chrA01-p17675721
Bn-A01-p20866518	0.975206612	0.983471074	Bn-N1-p20768885	Bn-chrA01-p17676218
Bn-A01-p20873189	0.966942149	0.975206612	Bn-N1-p20774852	Bn-chrA01-p17681212
Bn-A01-p20873657	0.991735537	1	Bn-N1-p20775320	Bn-chrA01-p17681680
Bn-A01-p20879747	0.94214876	0.94214876	Bn-N1-p20781437	Bn-chrA01-p17687829
Bn-A01-p20890858	0.983471074	0.991735537	Bn-N1-p20789312	Bn-chrA01-p17693741
Bn-A01-p20892080	0.966942149	0.958677686	Bn-N1-p20790535	Bn-chrA01-p17694970
Bn-A01-p20892145	0.983471074	0.975206612	Bn-N1-p20790600	Bn-chrA01-p17695035
Bn-A01-p20899472	0.970149254	0.985074627	Bn-N1-p20797508	Bn-chrA01-p17701537
Bn-A01-p20909649	0.985074627	0.980099502	Bn-N1-p20809797	Bn-chrC01-p31341422
Bn-A01-p20909816	0.960199005	0.965174129	Bn-N1-p20809964	Bn-chrA01-p17717315
Bn-A01-p20924050	0.980099502	0.930348259	Bn-N1-p20816306	Bn-chrC01-p31354720
Bn-A01-p20933189	0.991735537	1	Bn-Scaffold07211-p7	Bn-chrA01-p17737678

Bn-A01-p20987149	0.996677741	0.996677741	Bn-N1-p20870215	Bn-chrA01-p17792510
Bn-A01-p20988977	0.991735537	0.991735537	Bn-N1-p20872195	Bn-chrA01-p17794338
Bn-A01-p20990218	0.966942149	0.991735537	Bn-N1-p20874310	Bn-chrA01-p17795583
Bn-A01-p21018746	0.983471074	0.975206612	Bn-N1-p20900937	Bn-chrA01_random-p227118
Bn-A01-p21035670	0.991735537	0.991735537	Bn-N1-p20906257	Bn-chrA01_random-p226665
Bn-A01-p21038677	1	0.975206612	Bn-N1-p20913951	Bn-chrC01-p31606859
Bn-A01-p21075183	0.946843854	0.983388704	Bn-N1-p20952653	Bn-chrA01_random-p228949
Bn-A01-p21079129	0.983471074	0.991735537	Bn-N1-p20954233	Bn-chrA01_random-p229106
Bn-A01-p21079348	1	0.991735537	Bn-N1-p20954455	Bn-chrA01_random-p229128
Bn-A01-p21079774	0.991735537	1	Bn-N1-p20954885	Bn-chrA01_random-p229171
Bn-A01-p21079909	0.983471074	0.975206612	Bn-N1-p20955020	Bn-chrA01_random-p229184
Bn-A01-p21103014	0.975206612	0.975206612	Bn-N1-p20972501	Bn-chrA01_random-p230772
Bn-A01-p21106413	0.975206612	0.975206612	Bn-N1-p20975775	Bn-chrA01_random-p231100
Bn-A01-p21106687	0.966942149	0.966942149	Bn-N1-p20976049	Bn-chrA01_random-p231127
Bn-A01-p21107496	0.950413223	0.950413223	Bn-N1-p20978138	Bn-chrA01_random-p231336
Bn-A01-p21108496	0.993355482	0.993355482	Bn-N1-p20979139	Bn-chrA01_random-p231436
Bn-A01-p21135145	1	1	Bn-N1-p21004189	Bn-chrA01_random-p221745
Bn-A01-p21136052	0.991735537	0.983471074	Bn-N1-p21005096	Bn-chrA01_random-p221834
Bn-A01-p21141514	0.993355482	0.996677741	Bn-N1-p21010571	Bn-chrA01-p17820642
Bn-A01-p21145008	0.966942149	0.966942149	Bn-N1-p21014074	Bn-chrA01-p17823872
Bn-A01-p21219526	0.975206612	0.975206612	Bn-N1-p21094358	Bn-chrA01_random-p196022
Bn-A01-p21224647	0.993355482	0.993355482	Bn-Scaffold36130-p2	Bn-chrA01_random-p196556
Bn-A01-p21236322	0.983471074	0.983471074	Bn-N1-p21104169	Bn-chrA01_random-p197596
Bn-A01-p21244027	0.990049751	0.970149254	Bn-Scaffold28338-p2	Bn-chrC01-p31853319
Bn-A01-p21277841	0.915422886	0.895522388	Bn-N11-p36781364	Bn-chrC01-p31949019
Bn-A01-p21309566	1	0.991735537	Bn-N1-p21283540	Bn-chrA01-p17922735
Bn-A01-p21329900	0.996677741	0.996677741	Bn-N1-p21305306	Bn-chrAnn_random-p468556
Bn-A01-p21393260	0.993355482	0.993355482	Bn-N1-p21381690	Bn-chrA01-p18016584
Bn-A01-p21394483	0.975206612	0.966942149	Bn-N1-p21383096	Bn-chrA01-p18017804
Bn-A01-p21430708	0.946843854	0.920265781	Bn-N1-p21421234	Bn-chrA01-p18057587
Bn-A01-p21444041	0.991735537	0.983471074	Bn-N1-p21434496	Bn-chrA01-p18070953
Bn-A01-p21473161	0.993355482	0.986710963	Bn-N1-p21471174	Bn-chrA01-p18103656
Bn-A01-p21479969	0.991735537	1	Bn-N1-p21487614	Bn-chrA01-p18110870
Bn-A01-p21484088	0.983471074	0.975206612	Bn-N1-p21491736	Bn-chrA01-p18114985
Bn-A01-p21484875	0.983471074	0.975206612	Bn-N1-p21492523	Bn-chrA01-p18116980
Bn-A01-p21499792	0.975206612	0.966942149	Bn-N1-p21508099	Bn-chrA01-p18124658
Bn-A01-p21500509	0.975206612	0.983471074	Bn-N1-p21508826	Bn-chrA01-p18125374
Bn-A01-p21500631	0.991735537	0.446280992	Bn-N1-p21508948	Bn-chrAnn_random-p245056
Bn-A01-p21512266	0.975206612	0.966942149	Bn-N1-p21510950	Bn-chrA01-p18129923
Bn-A01-p21512514	0.966942149	0.975206612	Bn-N1-p21511198	Bn-chrA01-p18130172
Bn-A01-p21512663	0.991735537	1	Bn-N1-p21511347	Bn-chrA01-p18130323
Bn-A01-p21513096	0.991735537	0.983471074	Bn-N1-p21511780	Bn-chrA01-p18130755
Bn-A01-p21517353	0.983471074	0.975206612	Bn-N1-p21512030	Bn-chrA01-p18131006
Bn-A01-p21519780	0.983471074	0.991735537	Bn-N1-p21514264	Bn-chrA01-p18133212
Bn-A01-p21524150	0.966942149	0.958677686	Bn-N1-p21519435	Bn-chrA01-p18138306
Bn-A01-p21527187	0.980099502	0.980099502	Bn-N1-p21522465	Bn-chrA01-p18141319

Bn-A01-p21532512	0.966942149	0.966942149	Bn-N1-p21526893	Bn-chrA01-p18144224
Bn-A01-p21556957	0.995024876	0.995024876	Bn-N1-p21548844	Bn-chrAnn_random-p333614
Bn-A01-p21605865	0.983471074	0.983471074	Bn-N1-p21604285	Bn-chrA01-p18199826
Bn-A01-p21633202	0.980066445	0.973421927	Bn-N16-p15842318	Bn-chrA01-p18220542
Bn-A01-p21634024	0.980066445	0.983388704	Bn-N16-p15843140	Bn-chrA01-p18221364
Bn-A01-p21634235	0.332225914	0.986710963	Unknown	Bn-chrA01-p18221575
Bn-A01-p21634265	0.235880399	0.983388704	Unknown	Bn-chrA01-p18221605
Bn-A01-p21634646	0.347107438	0.991735537	Bn-N7-p2341755	Bn-chrA01-p18221986
Bn-A01-p21635000	0.634551495	0.993355482	Bn-N11-p5803872	Bn-chrA01-p18222340
Bn-A01-p21635137	0.181818182	0.991735537	Unknown	Bn-chrA01-p18222477
Bn-A01-p21635270	0.588039867	0.986710963	Bn-N2-p10643857	Bn-chrA01-p18222610
Bn-A01-p21636048	0.305785124	0.991735537	Bn-N5-p14625198	Bn-chrA01-p18223388
Bn-A01-p21638516	0.624584718	0.817275748	Bn-N1-p21622853	Bn-chrA01-p18225983
Bn-A01-p21643354	0.983471074	0.975206612	Bn-N1-p21629791	Bn-chrA01-p18230557
Bn-A01-p21644756	0.983471074	0.975206612	Bn-N1-p21631172	Bn-chrA01-p18231943
Bn-A01-p21651835	0.983471074	0.991735537	Bn-N1-p21636899	Bn-chrA01-p18237974
Bn-A01-p21663900	0.950413223	0.925619835	Bn-N1-p21649843	Bn-chrA01-p18248427
Bn-A01-p21708353	0.966942149	0.975206612	Bn-N1-p21688436	Bn-chrA01-p18293960
Bn-A01-p21709916	0.975206612	0.975206612	Bn-N1-p21689831	Bn-chrA01-p18295242
Bn-A01-p21715162	0.993355482	0.996677741	Bn-N1-p21695724	Bn-chrA01-p18303706
Bn-A01-p21724511	0.991735537	0.991735537	Bn-N1-p21704132	Bn-chrA01-p18312329
Bn-A01-p21758046	0.995024876	0.995024876	Bn-N1-p21744520	Bn-chrA01-p18351464
Bn-A01-p21776016	0.980099502	0.980099502	Bn-N1-p21764346	Bn-chrA01-p18386096
Bn-A01-p21776083	0.990049751	0.990049751	Bn-N1-p21764413	Bn-chrA01-p18386163
Bn-A01-p21776155	0.983471074	0.983471074	Bn-N1-p21764485	Bn-chrA01-p18386235
Bn-A01-p21778610	0.990049751	0.990049751	Bn-N1-p21766939	Bn-chrA01-p18388685
Bn-A01-p21791128	0.996677741	0.993355482	Bn-N1-p21779230	Bn-chrA01-p18399558
Bn-A01-p21797129	0.812030075	0.939849624	Bn-N11-p37677475	Bn-chrC01-p32889677
Bn-A01-p21812098	0.903654485	0.887043189	Bn-N1-p21791150	Bn-chrC01-p32899015
Bn-A01-p21846342	0.991735537	1	Bn-N1-p21821902	Bn-chrA01-p18427009
Bn-A01-p21855770	0.966942149	0.950413223	Bn-N1-p21832963	Bn-chrA01-p18441171
Bn-A01-p21856899	0.990033223	0.986710963	Bn-N1-p21834147	Bn-chrA01-p18442344
Bn-A01-p21860162	0.990033223	0.990033223	Bn-N1-p21837377	Bn-chrA01-p18450411
Bn-A01-p21869384	0.966942149	0.966942149	Bn-N1-p21844487	Bn-chrA01-p18457520
Bn-A01-p21902788	0.960199005	0.965174129	Bn-N1-p21875787	Bn-chrA01-p18510573
Bn-A01-p21923141	0.983471074	0.991735537	Bn-N1-p21900463	Bn-chrA01-p18533732
Bn-A01-p21926431	0.983471074	0.991735537	Bn-N1-p21901887	Bn-chrA01-p18535156
Bn-A01-p21928497	0.986710963	0.990033223	Bn-N1-p21903951	Bn-chrA01-p18537219
Bn-A01-p21928531	0.991735537	0.983471074	Bn-N1-p21903985	Bn-chrA01-p18537253
Bn-A01-p21928927	0.996677741	1	Bn-N1-p21904381	Bn-chrA01-p18537649
Bn-A01-p21930611	1	0.991735537	Bn-N1-p21906045	Bn-chrA01-p18539333
Bn-A01-p21930738	1	0.991735537	Bn-N1-p21906172	Bn-chrA01-p18539460
Bn-A01-p21930931	0.979166667	0.979166667	Bn-N1-p21906365	Bn-chrA01-p18539653
Bn-A01-p21931228	0.995024876	1	Bn-N1-p21906662	Bn-chrA01-p18539950
Bn-A01-p21931498	0.983471074	0.975206612	Bn-N1-p21906932	Bn-chrA01-p18540220
Bn-A01-p21931622	0.990049751	0.990049751	Bn-N1-p21907056	Bn-chrA01-p18540344

Bn-A01-p21931853	1	0.991735537	Bn-N1-p21907287	Bn-chrA01-p18540575
Bn-A01-p21937985	0.983471074	0.975206612	Bn-N1-p21914912	Bn-chrA01-p18546682
Bn-A01-p21938751	0.985074627	0.990049751	Bn-N1-p21915672	Bn-chrA01-p18547450
Bn-A01-p21960901	0.991735537	1	Bn-N11-p37908007	Bn-chrA01-p18572100
Bn-A01-p21969405	0.983471074	0.975206612	Bn-N1-p21946464	Bn-chrA01-p18579415
Bn-A01-p21980567	0.865671642	0.865671642	Bn-N1-p21959787	Bn-chrA01-p18589945
Bn-A01-p21997686	0.991735537	0.58677686	Bn-N1-p21978487	Bn-chrC01_random-p357512
Bn-A01-p21998517	0.991735537	0.933884298	Bn-N1-p21979319	Bn-chrC01_random-p356990
Bn-A01-p22014179	0.975124378	0.975124378	Bn-N1-p22001815	Bn-chrA01-p18643379
Bn-A01-p22014707	0.980099502	0.980099502	Bn-N1-p22002341	Bn-chrA01-p18643906
Bn-A01-p22016353	0.970149254	0.970149254	Bn-N1-p22003987	Bn-chrA01-p18645552
Bn-A01-p22018616	0.975206612	0.983471074	Bn-N1-p22010652	Bn-chrA01-p18649378
Bn-A01-p22022023	0.626865672	0.950248756	Bn-N1-p22014059	Bn-chrA01-p18652851
Bn-A01-p22038749	0.983471074	0.991735537	Bn-N1-p22027144	Bn-chrA01-p18656154
Bn-A01-p22038776	0.991735537	0.983471074	Bn-N1-p22027171	Bn-chrA01-p18656181
Bn-A01-p22051021	0.983471074	0.983471074	Bn-N1-p22039403	Bn-chrA01-p18668333
Bn-A01-p22051502	0.985074627	0.985074627	Bn-N1-p22039884	Bn-chrA01-p18668814
Bn-A01-p22051510	0.985074627	0.985074627	Bn-N1-p22039892	Bn-chrA01-p18668822
Bn-A01-p22085117	0.989690722	0.989690722	Bn-N1-p22087529	Bn-chrA01-p18711174
Bn-A01-p22086244	1	1	Bn-N1-p22088656	Bn-chrA01-p18712301
Bn-A01-p22087364	0.970149254	0.970149254	Bn-N1-p22089776	Bn-chrA01-p18713421
Bn-A01-p22087629	0.995024876	0.995024876	Bn-N1-p22090041	Bn-chrA01-p18713686
Bn-A01-p22156169	0.621262458	0.996677741	Bn-N15-p38746904	Bn-chrA01-p18772461
Bn-A01-p22182442	0.970149254	0.970149254	Bn-N1-p22215004	Bn-chrAnn_random-p251059
Bn-A01-p22257309	1	0.996677741	Bn-N3-p34728912	Bn-chrA03_random-p555693
Bn-A01-p22265175	0.993355482	0.990033223	Bn-N3-p34720786	Bn-chrA03_random-p556504
Bn-A01-p22363809	1	0.996677741	Bn-N3-p34474047	Bn-chrCnn_random-p393111
Bn-A01-p22602330	0.990033223	0.990033223	Bn-N3-p34356507	Bn-chrA03_random-p376496
Bn-A01-p22681496	0.983471074	0.991735537	Bn-N1-p22242804	Bn-chrA01-p18831032
Bn-A01-p22684301	0.975206612	0.983471074	Bn-N1-p22245609	Bn-chrA01-p18833829
Bn-A01-p22685756	0.909090909	0.933884298	Bn-N1-p22247100	Bn-chrA01-p18835321
Bn-A01-p22685858	0.94214876	0.958677686	Bn-N1-p22247202	Bn-chrA01-p18835423
Bn-A01-p22739887	0.990049751	0.990049751	Bn-N8-p4094527	Bn-chrA08_random-p470807
Bn-A01-p22741943	0.990049751	0.985074627	Bn-N1-p22296467	Bn-chrA01-p18901219
Bn-A01-p22743403	1	1	Bn-N11-p38467940	Bn-chrA01-p18902679
Bn-A01-p22768523	0.973421927	0.973421927	Bn-N1-p22311096	Bn-chrA01-p18948285
Bn-A01-p22777288	0.993355482	0.993355482	Bn-N1-p22321005	Bn-chrA01-p18957028
Bn-A01-p22841111	1	0.980099502	Bn-N1-p22377873	Bn-chrA01_random-p235567
Bn-A01-p22841314	0.980099502	0.975124378	Bn-N1-p22378076	Bn-chrA01_random-p235547
Bn-A01-p22930245	1	1	Bn-N1-p22438227	Bn-chrA01-p19423613
Bn-A01-p22937843	0.890547264	0.641791045	Bn-Scaffold43347-p8	Bn-chrA05-p8015765
Bn-A01-p22948925	0.985074627	0.985074627	Bn-N1-p22452695	Bn-chrA01-p19438918
Bn-A01-p22949106	0.966942149	0.966942149	Bn-N1-p22452876	Bn-chrA01-p19439099
Bn-A01-p22982065	0.991735537	1	Bn-N11-p38881191	Bn-chrA01-p19472493
Bn-A01-p22983721	0.906976744	0.88372093	Bn-N1-p22482769	Bn-chrA01-p19474151
Bn-A01-p22999151	1	0.995024876	Bn-N1-p22502164	Bn-chrA01-p19494342

Bn-A01-p23020726	0.983471074	0.991735537	Bn-N1-p22522332	Bn-chrA01-p19513183
Bn-A01-p23078554	0.983471074	0.975206612	Bn-N1-p22579592	Bn-chrA01_random-p241195
Bn-A01-p23080525	0.953488372	0.990033223	Bn-N1-p22581568	Bn-chrA01_random-p241001
Bn-A01-p23085796	0.991735537	0.983471074	Bn-N1-p22587691	Bn-chrA01_random-p240304
Bn-A01-p23086253	0.983471074	0.933884298	Bn-N1-p22588148	Bn-chrA01_random-p240259
Bn-A01-p23087044	0.991735537	0.983471074	Bn-N1-p22588939	Bn-chrA01_random-p240190
Bn-A01-p23087707	0.950413223	0.958677686	Bn-N1-p22589602	Bn-chrA01_random-p240124
Bn-A01-p23095567	0.975206612	0.983471074	Bn-N1-p22597454	Bn-chrA01_random-p239262
Bn-A01-p23096147	1	0.985074627	Bn-N1-p22598034	Bn-chrA01_random-p239204
Bn-A01-p23097693	0.958677686	0.950413223	Bn-N1-p22599580	Bn-chrA01_random-p239053
Bn-A01-p23103120	0.975206612	0.983471074	Bn-N1-p22603336	Bn-chrA01_random-p238931
Bn-A01-p23111844	0.983471074	0.983471074	Bn-N1-p22607387	Bn-chrA01_random-p238460
Bn-A01-p23120368	0.995024876	0.990049751	Bn-N11-p39063578	Bn-chrCnn_random-p157293
Bn-A01-p23122066	0.985074627	0.975124378	Bn-N1-p22611877	Bn-chrCnn_random-p157274
Bn-A01-p23169607	0.993355482	0.993355482	Bn-N1-p22645941	Bn-chrA01-p19016490
Bn-A01-p23175111	0.950413223	0.958677686	Bn-N1-p22651446	Bn-chrA01-p19022504
Bn-A01-p23181293	1	0.991735537	Bn-N1-p22658800	Bn-chrAnn_random-p456925
Bn-A01-p23191627	0.996677741	0.996677741	Bn-N1-p22665328	Bn-chrC02-p44156623
Bn-A01-p23195492	0.993355482	0.993355482	Bn-N1-p22672365	Bn-chrA01-p19065022
Bn-A01-p23196022	0.983471074	0.975206612	Bn-N1-p22672895	Bn-chrA01-p19064492
Bn-A01-p23253678	0.986710963	0.973421927	Bn-N1-p22729837	Bn-chrA01-p19111084
Bn-A01-p23259034	0.993355482	0.993355482	Bn-N1-p22735485	Bn-chrA01-p19116586
Bn-A01-p23259741	0.986710963	0.986710963	Bn-N1-p22736190	Bn-chrA01-p19117291
Bn-A01-p23279247	0.990033223	0.990033223	Bn-Scaffold01970-p1	Bn-chrA01-p19144803
Bn-A01-p23280094	0.993355482	0.993355482	Bn-Scaffold01970-p1	Bn-chrA01-p19145650
Bn-A01-p23281709	1	1	Bn-Scaffold01970-p3	Bn-chrA01-p19147255
Bn-A01-p23281878	0.993355482	0.993355482	Bn-Scaffold01970-p3	Bn-chrA01-p19147424
Bn-A01-p23282001	0.990033223	0.990033223	Bn-Scaffold01970-p3	Bn-chrA01-p19147547
Bn-A01-p23288238	1	1	Bn-N1-p22766416	Bn-chrA01-p19158253
Bn-A01-p23303284	0.958677686	0.958677686	Bn-N1-p22787349	Bn-chrA01-p19173222
Bn-A01-p23321507	0.986710963	0.996677741	Bn-N1-p22813949	Bn-chrA01-p19192466
Bn-A01-p23321771	0.983388704	0.990033223	Bn-N1-p22814213	Bn-chrA01-p19192730
Bn-A01-p23322377	0.975206612	0.966942149	Bn-N1-p22814819	Bn-chrA01-p19193318
Bn-A01-p23322438	0.983471074	0.991735537	Bn-N1-p22814880	Bn-chrA01-p19193379
Bn-A01-p23330944	0.980099502	0.990049751	Bn-N1-p22823086	Bn-chrA01-p19203801
Bn-A01-p23370802	1	1	Bn-N1-p22868765	Bn-chrA01-p19243847
Bn-A01-p23448661	0.993355482	0.993355482	Bn-N1-p22954653	Bn-chrA01-p19317004
Bn-A01-p23472380	0.985074627	0.980099502	Bn-N1-p22980942	Bn-chrA01-p19346196
Bn-A01-p23472646	0.990033223	0.976744186	Bn-N1-p22981209	Bn-chrA01-p19346461
Bn-A01-p23473514	0.975206612	0.818181818	Bn-N1-p22982035	Bn-chrA01-p19347317
Bn-A01-p23477194	0.996677741	1	Bn-N1-p22984780	Bn-chrA01-p19350103
Bn-A01-p23478530	0.958677686	0.966942149	Bn-N1-p22986116	Bn-chrC01-p34507078
Bn-A01-p23479473	0.990033223	0.983388704	Bn-N1-p22987059	Bn-chrA01-p19352382
Bn-A01-p23486950	0.986710963	0.96013289	Bn-N1-p22994522	Bn-chrA01-p19359827
Bn-A01-p23493920	0.993355482	0.996677741	Bn-N1-p23001301	Bn-chrA01-p19366797
Bn-A01-p23536634	1	1	Bn-N1-p23036989	Bn-chrAnn_random-p312528

Bn-A01-p23538267	0.993355482	0.774086379	Bn-N1-p23038622	Bn-chrC01-p34626593
Bn-A01-p23538856	0.991735537	0.214876033	Bn-N1-p23039208	Bn-chrC01-p6382651
Bn-A01-p23539305	0.983471074	0.363636364	Bn-N1-p23039656	Unknown
Bn-A01-p23560747	0.991735537	0.983471074	Bn-N1-p23066223	Bn-chrA01-p19595939
Bn-A01-p23568963	0.970099668	0.966777409	Bn-N1-p23075763	Bn-chrA01-p19604155
Bn-A01-p23582571	0.980066445	0.980066445	Bn-N1-p23086314	Bn-chrA01-p19614667
Bn-A01-p23586266	0.958677686	0.958677686	Bn-N1-p23089995	Bn-chrA01-p19618347
Bn-A01-p23600621	0.991735537	0.991735537	Bn-N1-p23104997	Bn-chrA01-p19633032
Bn-A01-p23602361	0.996677741	0.996677741	Bn-N1-p23106768	Bn-chrA01-p19634803
Bn-A01-p23613283	0.970149254	0.970149254	Bn-N1-p23130723	Bn-chrA01-p19661191
Bn-A01-p23614494	0.975206612	0.975206612	Bn-N1-p23132864	Bn-chrA01-p19663330
Bn-A01-p23614887	0.983471074	0.983471074	Bn-N1-p23133257	Bn-chrA01-p19663723
Bn-A01-p23616839	0.94214876	0.94214876	Bn-N1-p23135594	Bn-chrA01-p19666059
Bn-A01-p23627526	0.80730897	0.813953488	Bn-N1-p23147138	Bn-chrC01-p34832390
Bn-A01-p23634753	0.94214876	0.743801653	Bn-N1-p23155057	Bn-chrA01-p19684626
Bn-A01-p23638070	0.966942149	0.966942149	Bn-N1-p23159872	Bn-chrA01-p19687949
Bn-A01-p23638107	0.966942149	0.975206612	Bn-N1-p23159907	Bn-chrA01-p19687984
Bn-A01-p23640103	1	1	Bn-N1-p23161915	Bn-chrA01-p19689928
Bn-A01-p23642494	1	1	Bn-N11-p39967820	Bn-chrC01-p34893174
Bn-A01-p23658023	0.983471074	0.975206612	Bn-N1-p23176240	Bn-chrA01-p19704657
Bn-A01-p23672301	0.980099502	0.990049751	Bn-N1-p23189858	Bn-chrA01-p19724991
Bn-A01-p23685466	0.996677741	0.996677741	Bn-N1-p23199298	Bn-chrA01-p19738170
Bn-A01-p23685978	0.880398671	0.880398671	Bn-N1-p23199810	Bn-chrA01-p19738682
Bn-A01-p23694782	0.996677741	1	Bn-N1-p23209338	Bn-chrA01-p19747486
Bn-A01-p23718726	0.950166113	1	Bn-N11-p40097101	Bn-chrA01-p19770373
Bn-A01-p23719321	0.975206612	0.966942149	Bn-N1-p23232558	Bn-chrA01-p19770968
Bn-A01-p23728095	0.985074627	0.940298507	Bn-N9-p9366899	Bn-chrC04-p44834893
Bn-A01-p23730610	0.983471074	0.983471074	Bn-N1-p23241673	Bn-chrA01-p19782923
Bn-A01-p23736208	0.991735537	0.991735537	Bn-N1-p23247097	Bn-chrAnn_random-p31609[illegible]
Bn-A01-p23739022	0.975206612	0.975206612	Bn-N1-p23249921	Bn-chrAnn_random-p31611[illegible]
Bn-A01-p23763782	0.975206612	0.966942149	Bn-Scaffold20519-p1	Bn-chrA01-p19825340
Bn-A01-p23772904	0.063122924	0.986710963	Bn-N16-p21888348	Bn-chrA01-p19834611
Bn-A01-p23785561	0.986710963	0.950166113	Bn-N1-p23307739	Bn-chrA01-p19858307
Bn-A01-p23809071	0.986710963	0.986710963	Bn-N1-p23325271	Bn-chrA01-p19872813
Bn-A01-p23810423	1	1	Bn-N1-p23326638	Bn-chrA01-p19874179
Bn-A01-p23813837	0.993355482	0.993355482	Bn-N1-p23330051	Bn-chrA01-p19877499
Bn-A01-p23815268	0.991735537	0.991735537	Bn-N1-p23331482	Bn-chrA01-p19878930
Bn-A01-p23819116	0.991735537	0.991735537	Bn-N1-p23335330	Bn-chrA01-p19882778
Bn-A01-p23842865	0.990049751	0.960199005	Bn-N1-p23361899	Bn-chrC01-p35265662
Bn-A01-p23843030	0.980099502	0.980099502	Bn-N1-p23362064	Bn-chrA01-p19908689
Bn-A01-p23843222	0.990033223	0.990033223	Bn-N1-p23362256	Bn-chrA01-p19908881
Bn-A01-p23859890	0.766169154	0.766169154	Bn-N11-p41132070	Bn-chrA01-p20353510
Bn-A01-p23873720	0.991735537	0.983471074	Bn-N1-p23391199	Bn-chrA01-p19936465
Bn-A01-p23876801	0.950413223	0.958677686	Bn-N1-p23394957	Bn-chrA01-p19939546
Bn-A01-p23886020	0.991735537	0.983471074	Bn-N1-p23400298	Bn-chrA01-p19945912
Bn-A01-p23905191	0.989583333	0.989583333	Bn-N1-p23420845	Bn-chrA01-p20068468

Bn-A01-p23935076	0.720930233	0.993355482	Bn-N1-p23449094	Bn-chrA01-p20041723
Bn-A01-p23938571	0.970099668	0.986710963	Bn-N1-p23451987	Bn-chrA01-p20038256
Bn-A01-p23943508	0.973421927	0.996677741	Bn-N1-p23453129	Bn-chrA01-p20037117
Bn-A01-p23948008	0.986710963	0.983388704	Bn-N1-p23458508	Bn-chrA01-p20032263
Bn-A01-p23950559	0.991735537	0.991735537	Bn-N1-p23460955	Bn-chrA01-p20029828
Bn-A01-p23961368	0.991735537	0.983471074	Bn-N1-p23476953	Bn-chrA01-p20018933
Bn-A01-p23965220	0.966942149	0.966942149	Bn-N1-p23480933	Bn-chrA01-p20014955
Bn-A01-p23966537	0.985074627	0.955223881	Bn-N1-p23482250	Bn-chrC01-p35491043
Bn-A01-p23972100	0.990033223	0.993355482	Bn-N1-p23487811	Bn-chrA01-p20008055
Bn-A01-p24006789	0.909090909	0.909090909	Bn-N1-p23522034	Bn-chrC01-p35614031
Bn-A01-p24020373	0.993355482	0.996677741	Bn-N1-p23526404	Bn-chrA01-p20087336
Bn-A01-p24020451	0.990033223	0.986710963	Bn-N1-p23526482	Bn-chrA01-p20087414
Bn-A01-p24057724	0.983471074	0.983471074	Bn-N6-p16372986	Bn-chrA06-p11539061
Bn-A01-p24070704	0.983471074	0.983471074	Bn-N6-p16384895	Bn-chrA06-p11550335
Bn-A01-p24107515	0.995024876	0.995024876	Bn-N6-p16410370	Bn-chrA06-p11576455
Bn-A01-p24145113	0.985074627	0.985074627	Bn-N17-p7038322	Bn-chrC07-p9155958
Bn-A01-p24226107	0.975124378	0.975124378	Bn-N6-p16505682	Bn-chrA06-p11708106
Bn-A01-p24229645	0.985074627	0.980099502	Bn-N6-p16509218	Bn-chrA06-p11711642
Bn-A01-p24308273	0.991735537	0.983471074	Bn-N1-p23543130	Bn-chrC01-p35651986
Bn-A01-p24308957	0.94214876	0.975206612	Bn-N1-p23543815	Bn-chrA01-p20103193
Bn-A01-p24309386	0.983471074	0.975206612	Bn-N1-p23544244	Bn-chrA01-p20103621
Bn-A01-p24310993	0.973421927	0.986710963	Bn-N1-p23545942	Bn-chrA01-p20105734
Bn-A01-p24319792	0.239669421	0.983471074	Bn-N3-p4535279	Bn-chrA01-p20114893
Bn-A01-p24320794	0.421487603	0.966942149	Unknown	Bn-chrA01-p20115086
Bn-A01-p24346952	0.884297521	0.867768595	Bn-N1-p23576534	Bn-chrA01-p20130357
Bn-A01-p24360206	0.996677741	0.996677741	Bn-N1-p23592016	Bn-chrA01-p20142663
Bn-A01-p24369239	0.983471074	0.917355372	Bn-N1-p23599350	Bn-chrC01-p35761380
Bn-A01-p24369455	0.983471074	0.983471074	Bn-N1-p23599566	Bn-chrA01-p20147090
Bn-A01-p24372608	0.88372093	0.96345515	Bn-N11-p40769975	Bn-chrA01-p20160302
Bn-A01-p24390991	0.930348259	0.985074627	Bn-N1-p23626240	Bn-chrA01-p20186023
Bn-A01-p24394537	0.975124378	0.975124378	Bn-N1-p23629804	Bn-chrA01-p20189590
Bn-A01-p24395406	0.900826446	0.801652893	Bn-N1-p23631005	Bn-chrC01-p35817847
Bn-A01-p24396906	0.983471074	0.975206612	Bn-N1-p23631798	Bn-chrA01-p20191020
Bn-A01-p24408637	0.991735537	0.983471074	Bn-N1-p23650798	Bn-chrA01-p20206623
Bn-A01-p24416660	0.986710963	0.993355482	Bn-N1-p23661775	Bn-chrA01-p20214742
Bn-A01-p24425084	0.980099502	0.975124378	Bn-N11-p40896524	Bn-chrC01-p35863052
Bn-A01-p24425211	0.995024876	0.995024876	Bn-N1-p23670004	Bn-chrA01-p20223239
Bn-A01-p24425829	0.995024876	1	Bn-N1-p23670623	Bn-chrC01-p35863806
Bn-A01-p24429653	0.965174129	0.975124378	Bn-N1-p23674556	Bn-chrUnn_random-p59821[illegible]
Bn-A01-p24431478	0.990033223	0.990033223	Bn-N1-p23676567	Bn-chrA01-p20229833
Bn-A01-p24435452	0.970099668	0.970099668	Bn-N1-p23680494	Bn-chrA01-p20237873
Bn-A01-p24487852	0.993355482	0.993355482	Bn-N1-p23728227	Bn-chrA01-p20287051
Bn-A01-p24504495	0.933884298	0.933884298	Bn-N1-p23749741	Bn-chrA01-p20308731
Bn-A01-p24504575	0.991735537	1	Bn-N1-p23749821	Bn-chrA01-p20308811
Bn-A01-p24507466	0.983471074	0.975206612	Bn-N1-p23752586	Bn-chrA01-p20311700
Bn-A01-p24510656	0.983471074	0.132231405	Bn-N1-p23755791	Bn-chrA09-p19433262

Bn-A01-p24520244	0.975206612	0.975206612	Bn-N1-p23767382	Bn-chrA01-p20325349
Bn-A01-p24525303	0.990049751	0.985074627	Bn-N1-p23773086	Bn-chrA01-p20330357
Bn-A01-p24526982	0.986710963	0.993355482	Bn-N1-p23774769	Bn-chrA01-p20332036
Bn-A01-p24529391	0.966942149	0.975206612	Bn-N1-p23777343	Bn-chrA01-p20334462
Bn-A01-p24536432	0.892561983	0.661157025	Bn-N1-p23781744	Bn-chrA01-p20341076
Bn-A01-p24541095	0.958677686	0.950413223	Bn-N1-p23786611	Bn-chrA01-p20344118
Bn-A01-p24556230	0.990049751	0.990049751	Bn-N1-p23799383	Bn-chrA01-p20356854
Bn-A01-p24565084	0.983471074	0.983471074	Bn-N1-p23806529	Bn-chrA01-p20363718
Bn-A01-p24565311	0.991735537	0.991735537	Bn-N1-p23806756	Bn-chrA01-p20363945
Bn-A01-p24570589	0.710743802	0.710743802	Bn-N1-p23808877	Bn-chrA01-p20366065
Bn-A01-p24575577	0.991735537	0.991735537	Bn-N1-p23820457	Bn-chrA01-p20378108
Bn-A01-p24575679	0.983471074	0.983471074	Bn-N1-p23820559	Bn-chrA01-p20378210
Bn-A01-p24578644	0.991735537	0.685950413	Bn-N1-p23824942	Bn-chrC01-p36154617
Bn-A01-p24634303	0.975206612	0.975206612	Bn-N1-p23873357	Bn-chrA01_random-p246106
Bn-A01-p24635378	0.991735537	0.991735537	Bn-N1-p23879100	Bn-chrA01_random-p245521
Bn-A01-p24636537	0.996677741	0.996677741	Bn-N1-p23880517	Bn-chrA01_random-p245379
Bn-A01-p24646334	0.990033223	0.990033223	Bn-N1-p23890376	Bn-chrA01_random-p244236
Bn-A01-p24655110	0.993355482	0.993355482	Bn-N1-p23899338	Bn-chrA01_random-p249019
Bn-A01-p24664331	0.993355482	0.993355482	Bn-N1-p23910662	Bn-chrA01-p20435110
Bn-A01-p24696367	0.996677741	0.996677741	Bn-N1-p23943419	Bn-chrA01-p20464355
Bn-A01-p24697185	0.966942149	0.983471074	Bn-N1-p23944238	Bn-chrA01-p20465173
Bn-A01-p24701885	0.975206612	0.983471074	Bn-N1-p23949100	Bn-chrA01-p20470021
Bn-A01-p24703150	0.966942149	0.975206612	Bn-N1-p23950352	Bn-chrA01-p20471289
Bn-A01-p24705790	0.190082645	0.975206612	Unknown	Bn-chrA01-p20473961
Bn-A01-p24706378	0.958677686	0.966942149	Bn-N1-p23953508	Bn-chrA01-p20474549
Bn-A01-p24707425	0.986710963	0.990033223	Bn-N1-p23954561	Bn-chrA01-p20475601
Bn-A01-p24707727	1	0.991735537	Bn-N1-p23954863	Bn-chrA01-p20475903
Bn-A01-p24720378	0.991735537	0.983471074	Bn-N1-p23962762	Bn-chrA01-p20482216
Bn-A01-p24737418	0.990033223	0.990033223	Bn-N1-p23977024	Bn-chrA01-p20493260
Bn-A01-p24756888	0.96345515	0.96345515	Bn-N1-p23996711	Bn-chrA01-p20511569
Bn-A01-p24811222	0.980099502	0.980099502	Bn-N1-p24028871	Bn-chrA01-p20548291
Bn-A01-p24812396	0.976744186	0.976744186	Bn-N13-p26104669	Bn-chrC03-p22910527
Bn-A01-p24813515	0.92039801	0.92039801	Bn-N1-p24029810	Bn-chrA01-p20549230
Bn-A01-p24828081	0.933884298	0.966942149	Bn-N11-p41574060	Bn-chrA01-p20559818
Bn-A01-p24829245	0.991735537	0.983471074	Bn-N1-p24039411	Bn-chrA01-p20560981
Bn-A01-p24829988	0.983471074	0.975206612	Bn-N1-p24040154	Bn-chrA01-p20561724
Bn-A01-p24830111	0.966942149	0.975206612	Bn-N1-p24040277	Bn-chrA01-p20561847
Bn-A01-p24830366	1	0.991735537	Bn-N1-p24040530	Bn-chrA01-p20562102
Bn-A01-p24835306	0.991735537	0.983471074	Bn-N1-p24045799	Bn-chrA01-p20567057
Bn-A01-p24835449	0.966942149	0.983471074	Bn-N1-p24045942	Bn-chrA01-p20567200
Bn-A01-p24839451	0.826446281	0.983471074	Bn-N1-p24047937	Bn-chrA01-p20571173
Bn-A01-p24839905	0.966942149	0.983471074	Bn-N1-p24048417	Bn-chrA01-p20571631
Bn-A01-p24840306	0.909090909	0.983471074	Bn-N1-p24048818	Bn-chrA01-p20572032
Bn-A01-p24848629	0.983471074	0.991735537	Bn-N1-p24056843	Bn-chrA01-p20581194
Bn-A01-p24849993	0.990033223	0.990033223	Bn-N1-p24058207	Bn-chrA01-p20582558
Bn-A01-p24858036	0.995024876	0.995024876	Bn-N11-p41632850	Bn-chrC01-p36526758

Bn-A01-p24862401	0.975206612	0.966942149	Bn-N1-p24070838	Bn-chrAnn_random-p377085
Bn-A01-p24880356	0.996677741	0.996677741	Bn-N1-p24091067	Bn-chrA01_random-p256694
Bn-A01-p24915384	0.996677741	0.990033223	Bn-N1-p24129893	Bn-chrA01_random-p252970
Bn-A01-p24920662	0.975206612	0.966942149	Bn-N1-p24141141	Bn-chrA01_random-p252044
Bn-A01-p24921162	0.983471074	0.933884298	Bn-N1-p24141641	Bn-chrA01_random-p251947
Bn-A01-p24921355	0.991735537	1	Bn-N1-p24141834	Bn-chrA01_random-p251928
Bn-A01-p24950985	0.990033223	0.990033223	Bn-N1-p24161399	Bn-chrA01_random-p249937
Bn-A01-p24974533	0.983471074	0.975206612	Bn-N1-p24186494	Bn-chrA01_random-p260362
Bn-A01-p24976705	0.970099668	1	Bn-N1-p24188589	Bn-chrA01_random-p260576
Bn-A01-p24981413	0.958677686	0.950413223	Bn-N1-p24192961	Bn-chrA01_random-p260925
Bn-A01-p25007130	0.983471074	0.975206612	Bn-N1-p24213137	Bn-chrAnn_random-p356690
Bn-A01-p25032772	0.991735537	0.983471074	Bn-Scaffold01260-p4	Bn-chrA01-p22586949
Bn-A01-p25040577	1	0.996677741	Bn-N1-p27020484	Bn-chrCnn_random-p649231
Bn-A01-p25050109	0.190082645	0.983471074	Bn-N19-p1337021	Bn-chrA01-p22564442
Bn-A01-p25061137	0.973421927	0.966777409	Bn-N1-p26997912	Bn-chrA01-p22553564
Bn-A01-p25070443	0.900826446	0.900826446	Bn-N1-p26987882	Bn-chrA02-p3937984
Bn-A01-p25072800	0.973421927	0.996677741	Bn-N1-p26985780	Bn-chrA01-p22535817
Bn-A01-p25079412	0.983471074	0.983471074	Bn-N11-p46553829	Bn-chrA01-p22530281
Bn-A01-p25079505	0.976744186	0.973421927	Bn-N1-p26979908	Bn-chrA01-p22530188
Bn-A01-p25099980	0.966942149	0.198347107	Bn-N1-p26949379	Unknown
Bn-A01-p25100490	0.991735537	0.495867769	Bn-N1-p26948871	Unknown
Bn-A01-p25101023	0.973421927	0.993355482	Bn-N1-p26948338	Bn-chrA01-p22380023
Bn-A01-p25117730	0.993355482	0.986710963	Bn-Scaffold49704-p1	Bn-chrA01-p22357285
Bn-A01-p25143334	0.958677686	0.958677686	Bn-N1-p26906891	Bn-chrAnn_random-p436495
Bn-A01-p25171870	0.965174129	0.960199005	Bn-N4-p21066278	Bn-chrA04_random-p144345
Bn-A01-p25175675	0.933884298	0.983471074	Bn-N4-p21062470	Bn-chrA04_random-p137943
Bn-A01-p25183707	0.991735537	1	Bn-N4-p21054418	Bn-chrA04_random-p138733
Bn-A01-p25196502	0.991735537	0.991735537	Bn-N4-p21039730	Bn-chrA04_random-p140527
Bn-A01-p25199395	0.991735537	0.983471074	Bn-N4-p21036845	Bn-chrA04_random-p140943
Bn-A01-p25200386	0.991735537	1	Bn-N4-p21035855	Bn-chrA04_random-p141045
Bn-A01-p25235998	0.995024876	0.990049751	Bn-N1-p26886005	Bn-chrA01-p22206203
Bn-A01-p25255567	1	0.991735537	Bn-N1-p26866822	Bn-chrA01-p22188185
Bn-A01-p25258657	0.983471074	0.305785124	Bn-N1-p26863733	Unknown
Bn-A01-p25259103	0.983471074	0.991735537	Bn-N1-p26863282	Bn-chrAnn_random-p299270
Bn-A01-p25259292	0.950413223	0.950413223	Bn-N1-p26863067	Bn-chrAnn_random-p300016
Bn-A01-p25260327	0.966942149	0.975206612	Bn-N1-p26862107	Bn-chrAnn_random-p299282
Bn-A01-p25260589	0.983471074	0.991735537	Bn-N1-p26861843	Bn-chrAnn_random-p299285
Bn-A01-p25260919	1	1	Bn-N1-p26861513	Bn-chrAnn_random-p300031
Bn-A01-p25267377	0.92358804	0.887043189	Bn-N1-p26854811	Bn-chrAnn_random-p300079
Bn-A01-p25279161	0.950413223	0.603305785	Bn-N1-p26840028	Bn-chrAnn_random-p136074
Bn-A01-p25311527	0.843853821	0.84717608	Bn-N1-p26815508	Bn-chrCnn_random-p567051
Bn-A01-p25326722	0.991735537	0.983471074	Bn-N1-p26804360	Bn-chrAnn_random-p136536
Bn-A01-p25327241	0.990049751	0.990049751	Bn-N1-p26803842	Bn-chrAnn_random-p136541
Bn-A01-p25349875	0.966101695	0.974576271	Bn-N5-p19550526	Bn-chrA05-p16885598
Bn-A01-p25350397	0.958677686	0.950413223	Bn-Scaffold00895-p3	Bn-chrA04-p19103329
Bn-A01-p25351684	0.990049751	0.985074627	Bn-Scaffold00895-p3	Bn-chrA04-p19102048

Bn-A01-p25352753	0.925619835	0.917355372	Bn-Scaffold00895-p3	Bn-chrA04-p19100981
Bn-A01-p25363566	0.966942149	0.975206612	Bn-Scaffold00895-p5	Bn-chrA04-p19086725
Bn-A01-p25364218	0.966942149	0.917355372	Bn-Scaffold00895-p5	Bn-chrA04-p19086072
Bn-A01-p25377739	0.983388704	0.990033223	Bn-Scaffold00895-p6	Bn-chrA04-p19075514
Bn-A01-p25389497	1	0.743801653	Bn-Scaffold00895-p7	Bn-chrC04-p48636469
Bn-A01-p25391997	0.991735537	0.983471074	Bn-Scaffold00895-p7	Bn-chrA04-p19060503
Bn-A01-p25397605	0.980066445	0.980066445	Bn-Scaffold00895-p8	Bn-chrA04-p19054876
Bn-A01-p25405698	0.983388704	0.990033223	Bn-Scaffold00895-p8	Bn-chrA04-p19050660
Bn-A01-p25428278	0.991735537	0.983471074	Bn-N9-p34605210	Bn-chrA04-p19025857
Bn-A01-p25429100	1	0.991735537	Bn-N9-p34604389	Bn-chrA04-p19025040
Bn-A01-p25444722	0.991735537	0.991735537	Bn-N1-p26781277	Bn-chrAnn_random-p16361[illegible]
Bn-A01-p25453874	0.966942149	0.975206612	Bn-N1-p26770166	Bn-chrAnn_random-p163464
Bn-A01-p25462155	0.983471074	0.983471074	Bn-N1-p26762470	Bn-chrAnn_random-p163394
Bn-A01-p25470470	0.975206612	0.966942149	Bn-N1-p26759523	Bn-chrAnn_random-p163332
Bn-A01-p25480187	0.975206612	0.983471074	Bn-N1-p26744990	Bn-chrAnn_random-p163205
Bn-A01-p25480397	0.975206612	0.966942149	Bn-N1-p26744780	Bn-chrAnn_random-p163203
Bn-A01-p25509783	0.991735537	0.983471074	Bn-N1-p26711185	Bn-chrC04-p47793277
Bn-A01-p25512934	0.975206612	0.966942149	Bn-N1-p26702479	Bn-chrC04-p47786358
Bn-A01-p25513710	0.983471074	0.991735537	Bn-N1-p26701702	Bn-chrC04-p47785592
Bn-A01-p25517197	0.970149254	0.960199005	Bn-N1-p26698258	Bn-chrC04-p47782102
Bn-A01-p25517270	0.975124378	0.970149254	Bn-N1-p26698183	Bn-chrC04-p47782029
Bn-A01-p25518745	0.960199005	0.960199005	Bn-N1-p26696571	Bn-chrC04-p47780553
Bn-A01-p25555508	0.917355372	0.47107438	Bn-N1-p26660503	Bn-chrCnn_random-p171182
Bn-A01-p25561204	0.983471074	0.991735537	Bn-N1-p26655514	Bn-chrA01-p23243108
Bn-A01-p25562652	0.975206612	0.991735537	Bn-N11-p46146749	Bn-chrA01-p23241660
Bn-A01-p25570701	0.975206612	0.966942149	Bn-N1-p26644766	Bn-chrA01-p23229594
Bn-A01-p25572254	0.966942149	0.958677686	Bn-N1-p26643152	Bn-chrA01-p23227992
Bn-A01-p25577348	0.983471074	0.94214876	Bn-N1-p26638166	Bn-chrA01-p23222897
Bn-A01-p25580651	0.958677686	0.950413223	Bn-N1-p26634866	Bn-chrA01-p23213416
Bn-A01-p25591508	0.975206612	0.983471074	Bn-N1-p26623720	Bn-chrA01-p23202008
Bn-A01-p25750911	1	0.996677741	Bn-Scaffold01523-p1	Bn-chrA01_random-p127661
Bn-A01-p25898606	0.995024876	0.975124378	Bn-N16-p16194834	Bn-chrA06-p8573793
Bn-A01-p26010353	1	0.996677741	Bn-N1-p15304911	Bn-chrCnn_random-p112067
Bn-A01-p26064479	0.958677686	0.958677686	Bn-N1-p4486639	Bn-chrA01-p4075176
Bn-A01-p26366791	0.990033223	0.986710963	Bn-N1-p24497795	Bn-chrA01-p20889211
Bn-A01-p26369426	0.926910299	0.990033223	Bn-N11-p42649504	Bn-chrA01-p20892840
Bn-A01-p26369651	0.76744186	0.986710963	Bn-N1-p24501624	Bn-chrA01-p20893065
Bn-A01-p26380200	0.966942149	0.958677686	Bn-N1-p24509188	Bn-chrA01-p20906294
Bn-A01-p26384777	0.993355482	0.986710963	Bn-N1-p24513977	Bn-chrA01-p20910893
Bn-A01-p26400154	0.991735537	0.991735537	Bn-N1-p24527640	Bn-chrA01-p20928913
Bn-A01-p26423587	0.94214876	0.94214876	Bn-N1-p24541048	Bn-chrA01-p20942371
Bn-A01-p26425810	0.991735537	0.991735537	Bn-N1-p24543088	Bn-chrA01-p20944411
Bn-A01-p26426004	0.925619835	0.925619835	Bn-N11-p42743962	Bn-chrC01-p37479342
Bn-A01-p26447438	0.950413223	0.950413223	Bn-N1-p24568625	Bn-chrA01-p20969346
Bn-A01-p26461873	0.990033223	0.990033223	Bn-N1-p24582765	Bn-chrA01-p20983732
Bn-A01-p26515072	0.820598007	0.96013289	Bn-N1-p24635752	Bn-chrA01-p21039650

Bn-A01-p26516918	0.995024876	0.960199005	Bn-N1-p24637603	Bn-chrA01-p21041296
Bn-A01-p26518304	0.995024876	1	Bn-Scaffold67684-p1	Bn-chrA01-p21042682
Bn-A01-p26519047	0.995024876	0.995024876	Bn-N1-p24640051	Bn-chrA01-p21043425
Bn-A01-p26541761	0.996677741	0.996677741	Bn-N1-p24672820	Bn-chrAnn_random-p989132
Bn-A01-p26543758	0.990033223	0.990033223	Bn-N1-p24674817	Bn-chrAnn_random-p989331
Bn-A01-p26568738	1	1	Bn-N1-p24698643	Bn-chrAnn_random-p991629
Bn-A01-p26571596	0.830845771	0.980099502	Bn-N5-p22509511	Bn-chrAnn_random-p371732
Bn-A01-p26572331	0.975206612	0.983471074	Bn-N1-p24702848	Bn-chrAnn_random-p371739
Bn-A01-p26575652	0.983388704	0.983388704	Bn-N1-p24707206	Bn-chrAnn_random-p992474
Bn-A01-p26589959	0.975124378	0.975124378	Bn-N1-p24718839	Bn-chrAnn_random-p993841
Bn-A01-p26592021	0.975206612	0.983471074	Bn-N1-p24721846	Bn-chrAnn_random-p272479
Bn-A01-p26594603	0.991735537	0.991735537	Bn-N1-p24724459	Bn-chrAnn_random-p994430
Bn-A01-p26604864	0.800664452	0.800664452	Bn-N1-p24735170	Bn-chrAnn_random-p996090
Bn-A01-p26614232	0.975206612	0.975206612	Bn-N1-p24744652	Bn-chrAnn_random-p997038
Bn-A01-p26624315	0.966942149	0.58677686	Bn-N1-p24754935	Bn-chrA03-p15447508
Bn-A01-p26627091	0.975206612	0.975206612	Bn-N1-p24757734	Bn-chrAnn_random-p998377
Bn-A01-p26658327	0.983471074	0.280991736	Bn-N1-p24790368	Unknown
Bn-A01-p26685015	0.986710963	0.089700997	Bn-N1-p24826505	Unknown
Bn-A01-p26685077	0.983388704	0.119601329	Bn-N1-p24826567	Bn-chrC02_random-p527461
Bn-A01-p26688333	0.993355482	0.415282392	Bn-N1-p24829825	Unknown
Bn-A01-p26713360	0.966942149	0.289256198	Bn-N1-p24851501	Unknown
Bn-A01-p26715665	0.985074627	0.542288557	Bn-N1-p24855239	Bn-chrA05-p19785463
Bn-A01-p26716060	0.996677741	0.073089701	Bn-N1-p24855634	Bn-chrA06-p5535215
Bn-A01-p26722319	0.980099502	0.980099502	Bn-N1-p24860523	Bn-chrAnn_random-p370898
Bn-A01-p26759778	0.966942149	0.966942149	Bn-N9-p1224212	Bn-chrA09_random-p341054
Bn-A01-p26760158	0.958677686	0.958677686	Bn-N9-p1223832	Bn-chrA09_random-p341434
Bn-A01-p26767819	1	0.991735537	Bn-N9-p1216864	Bn-chrAnn_random-p153421
Bn-A01-p26829665	0.980066445	0.980066445	Bn-N19-p1641358	Bn-chrC09-p520163
Bn-A01-p26837207	0.917355372	0.917355372	Bn-N9-p1150435	Bn-chrA09-p916331
Bn-A01-p26853670	0.801652893	0.801652893	Bn-N9-p1128673	Bn-chrA09-p902516
Bn-A01-p26866541	0.958677686	0.958677686	Bn-N9-p1121240	Bn-chrA09-p896361
Bn-A01-p26910983	0.94214876	0.933884298	Bn-N9-p1093649	Bn-chrA09-p870343
Bn-A01-p26914512	0.983471074	0.983471074	Bn-N9-p1090077	Bn-chrA09-p866809
Bn-A01-p26914851	0.887043189	0.880398671	Bn-N9-p1089739	Bn-chrA09-p866469
Bn-A01-p26921219	0.965174129	0.960199005	Bn-N9-p1082272	Bn-chrA09-p861964
Bn-A01-p26921609	0.990049751	0.990049751	Bn-N9-p1081882	Bn-chrA09-p861574
Bn-A01-p26940404	0.983471074	0.983471074	Bn-N9-p1060080	Bn-chrA09-p843615
Bn-A01-p26945726	0.980099502	0.985074627	Bn-N9-p1053858	Bn-chrA09-p837387
Bn-A01-p26946254	0.990049751	0.990049751	Bn-N9-p1053330	Bn-chrA09-p836859
Bn-A01-p26956455	0.94214876	0.933884298	Bn-N9-p1023417	Bn-chrA09-p807768
Bn-A01-p26965229	0.983471074	0.975206612	Bn-N9-p1018791	Bn-chrA09-p803110
Bn-A01-p26966407	0.975206612	0.966942149	Bn-N9-p1017642	Bn-chrA09-p801961
Bn-A01-p26968952	0.975206612	0.983471074	Bn-N9-p1015065	Bn-chrA09-p799287
Bn-A01-p26969210	0.991735537	0.983471074	Bn-N9-p1014810	Bn-chrA09-p799032
Bn-A01-p26973178	0.983388704	0.990033223	Bn-N9-p1009144	Bn-chrA09-p794380
Bn-A01-p26985090	0.995024876	0.990049751	Bn-N9-p996367	Bn-chrA09-p775294

Bn-A01-p26990131	0.983471074	0.975206612	Bn-N9-p992054	Bn-chrA09-p761644
Bn-A01-p26996900	0.920265781	0.920265781	Bn-N9-p985581	Bn-chrC09-p280108
Bn-A01-p26998962	0.983471074	0.991735537	Bn-N9-p980814	Bn-chrA09-p753105
Bn-A01-p27004740	0.401993355	0.996677741	Bn-N9-p1152969	Bn-chrA09-p745841
Bn-A01-p27012139	0.991735537	1	Bn-N9-p960082	Bn-chrA09-p738594
Bn-A01-p27017324	0.958677686	0.966942149	Bn-N9-p954924	Bn-chrA09-p733456
Bn-A01-p27017651	0.933884298	0.925619835	Bn-N9-p954597	Bn-chrA09-p733129
Bn-A01-p27019745	0.991735537	0.983471074	Bn-N9-p952448	Bn-chrA09-p730970
Bn-A01-p27029107	0.933884298	0.94214876	Bn-N9-p943236	Bn-chrA09-p719919
Bn-A01-p27029254	0.966942149	0.975206612	Bn-N9-p943089	Bn-chrA09-p719772
Bn-A01-p27037472	0.94214876	0.909090909	Bn-N9-p934832	Bn-chrA09-p711466
Bn-A01-p27050083	0.993355482	0.993355482	Bn-N9-p922240	Bn-chrA09-p696142
Bn-A01-p27060364	0.983471074	0.983471074	Bn-N9-p912273	Bn-chrA09-p686870
Bn-A01-p27061075	0.975206612	0.975206612	Bn-N9-p911561	Bn-chrA09-p686196
Bn-A01-p27079797	0.990033223	0.970099668	Bn-N1-p24947887	Bn-chrA01-p21078344
Bn-A01-p27101289	0.993355482	0.757475083	Bn-N1-p24969557	Bn-chrC05-p39536638
Bn-A01-p27107081	0.975124378	0.975124378	Bn-N1-p24973721	Bn-chrA01-p21180925
Bn-A01-p27115455	0.993355482	0.996677741	Bn-N1-p24985491	Bn-chrA01-p21190485
Bn-A01-p27117954	0.990033223	0.990033223	Bn-N1-p24987974	Bn-chrA01-p21192968
Bn-A01-p27119250	0.990033223	0.930232558	Bn-N1-p24989268	Bn-chrC03-p22434338
Bn-A01-p27125649	0.975124378	0.960199005	Bn-N11-p43819864	Bn-chrA01-p21228068
Bn-A01-p27147782	0.776119403	0.995024876	Bn-N11-p43775094	Bn-chrA01-p21236289
Bn-A01-p27202461	0.990049751	1	Bn-N11-p43508696	Bn-chrA01-p21360798
Bn-A01-p27220075	0.975124378	0.985074627	Bn-N1-p25101065	Bn-chrA01-p21365293
Bn-A01-p27225628	0.975124378	0.945273632	Bn-N11-p43521596	Bn-chrA01-p21367478
Bn-A01-p27263558	1	1	Bn-N1-p25144147	Bn-chrA01-p21414767
Bn-A01-p27272066	0.990033223	0.990033223	Bn-N1-p25157507	Bn-chrA01-p21423280
Bn-A01-p27280214	0.983471074	0.983471074	Bn-N1-p25167408	Bn-chrA01-p21433237
Bn-A01-p27293125	0.975206612	0.975206612	Bn-N1-p25178686	Bn-chrAnn_random-p400468
Bn-A01-p27299339	0.859504132	0.867768595	Bn-N1-p25185733	Bn-chrAnn_random-p448399
Bn-A01-p27301264	0.859504132	0.239669421	Bn-N11-p43736360	Bn-chrA05-p18345132
Bn-A01-p27301637	0.991735537	0.148760331	Bn-N1-p25190903	Bn-chrC02-p22401276
Bn-A01-p27301980	0.991735537	0.231404959	Bn-N1-p25191246	Bn-chrCnn_random-p553495
Bn-A01-p27302144	0.958677686	0.173553719	Bn-N1-p25191410	Unknown
Bn-A01-p27318082	0.990049751	0.990049751	Bn-N1-p25204446	Bn-chrA01-p21450096
Bn-A01-p27320515	0.983471074	0.983471074	Bn-N1-p25205247	Bn-chrA01-p21450897
Bn-A01-p27331467	1	0.933884298	Bn-N1-p25216255	Bn-chrAnn_random-p307859
Bn-A01-p27336799	0.958677686	0.94214876	Bn-N1-p25221948	Bn-chrA01-p21466120
Bn-A01-p27348128	0.975206612	0.966942149	Bn-N1-p25223373	Bn-chrA01-p21468568
Bn-A01-p27360503	1	0.993355482	Bn-N1-p25235735	Bn-chrA01-p21484318
Bn-A01-p27361855	0.986710963	0.990033223	Bn-N1-p25237072	Bn-chrA01-p21485655
Bn-A01-p27381111	0.920265781	0.930232558	Bn-N1-p25257937	Bn-chrA01-p21505461
Bn-A01-p27389703	0.990033223	0.990033223	Bn-N1-p25263989	Bn-chrA01-p21522102
Bn-A01-p27392534	0.976744186	0.976744186	Bn-Scaffold03168-p7	Bn-chrA01-p21524914
Bn-A01-p27417163	0.913621262	0.983388704	Bn-N1-p25308592	Bn-chrA01-p21558873
Bn-A01-p27463135	0.955223881	0.955223881	Bn-N1-p25347145	Bn-chrA01-p21592139

Bn-A01-p27522299	0.950248756	0.950248756	Bn-N1-p25411408	Bn-chrA01-p21658169
Bn-A01-p27570336	0.966942149	0.975206612	Bn-N1-p25456225	Bn-chrC01-p37843145
Bn-A01-p27570489	0.785123967	0.917355372	Bn-N1-p25456354	Bn-chrC01-p37843298
Bn-A01-p27573404	0.991735537	0.983471074	Bn-N1-p25459315	Bn-chrA01-p21708896
Bn-A01-p27580681	0.991735537	0.900826446	Bn-N1-p25466344	Bn-chrA01-p21719644
Bn-A01-p27581056	0.966942149	0.818181818	Bn-N1-p25466719	Bn-chrCnn_random-p705162
Bn-A01-p27589456	0.995024876	1	Bn-N1-p25475203	Bn-chrAnn_random-p140841
Bn-A01-p27601050	0.975206612	0.966942149	Bn-N1-p25484873	Bn-chrC01_random-p443731
Bn-A01-p27622120	0.966942149	0.975206612	Bn-N1-p25513141	Bn-chrC01-p37885645
Bn-A01-p27622168	0.975206612	0.966942149	Bn-N1-p25513189	Bn-chrC01-p37885693
Bn-A01-p27623686	0.983471074	0.991735537	Bn-N1-p25514740	Bn-chrC01-p37887244
Bn-A01-p27668070	0.996677741	1	Bn-N1-p25547775	Bn-chrUnn_random-p590156
Bn-A01-p27673018	0.925619835	0.900826446	Bn-N1-p25558340	Bn-chrAnn_random-p283385
Bn-A01-p27722989	0.975124378	0.975124378	Bn-N1-p25619480	Bn-chrA01-p21730692
Bn-A01-p27774124	0.980099502	0.980099502	Bn-N1-p25700994	Bn-chrC01-p38105099
Bn-A01-p27774386	0.985074627	0.985074627	Bn-N1-p25701256	Bn-chrC01-p38105361
Bn-A01-p27774666	0.955223881	0.955223881	Bn-N1-p25701534	Bn-chrC01-p38105639
Bn-A01-p27777763	0.958677686	0.933884298	Bn-N1-p25704489	Bn-chrC01-p38108594
Bn-A01-p27796877	0.976744186	0.980066445	Bn-N1-p25722543	Bn-chrC01-p38129852
Bn-A01-p27797076	0.980066445	0.980066445	Bn-N1-p25722742	Bn-chrC01-p38130051
Bn-A01-p27810962	0.996677741	0.996677741	Bn-N1-p25740220	Bn-chrC01-p38139215
Bn-A01-p27822000	0.980066445	0.983388704	Bn-N1-p25755518	Bn-chrC01-p38216269
Bn-A01-p27822790	0.980099502	0.980099502	Bn-N1-p25756308	Bn-chrC01-p38217059
Bn-A01-p27830372	0.975206612	0.983471074	Bn-N1-p25763891	Bn-chrC01-p38224635
Bn-A01-p27831569	0.980066445	1	Bn-N1-p25765086	Bn-chrC01-p38225832
Bn-A01-p27834392	0.933884298	0.958677686	Bn-N1-p25768895	Bn-chrC01-p38231402
Bn-A01-p27834452	0.94214876	0.950413223	Bn-N1-p25768956	Bn-chrC01-p38231463
Bn-A01-p27843242	0.975206612	0.983471074	Bn-N1-p25778161	Bn-chrC05-p8949079
Bn-A01-p27845673	0.975206612	0.966942149	Bn-N1-p25779974	Bn-chrC01-p38247871
Bn-A01-p27846002	0.966942149	0.950413223	Bn-N1-p25780303	Bn-chrC01-p38248200
Bn-A01-p27846227	0.983471074	0.991735537	Bn-N1-p25780528	Bn-chrC01-p38248425
Bn-A01-p27846544	1	0.991735537	Bn-N1-p25780845	Bn-chrC01-p38248743
Bn-A01-p27854949	0.980066445	0.983388704	Bn-N11-p44690191	Bn-chrC01-p38257289
Bn-A01-p27866419	0.986710963	0.92358804	Bn-N1-p25801701	Bn-chrC01-p38268790
Bn-A01-p27877194	0.986710963	0.986710963	Bn-N1-p25814170	Bn-chrC01-p38279650
Bn-A01-p27893933	0.966942149	1	Bn-N11-p44735346	Bn-chrC01-p38295170
Bn-A01-p27905344	1	1	Bn-N1-p25842455	Bn-chrC03-p19259057
Bn-A01-p27907325	0.996677741	0.996677741	Bn-N1-p25844436	Bn-chrC03-p19261038
Bn-A01-p27907422	0.993355482	0.993355482	Bn-N1-p25844533	Bn-chrC03-p19261135
Bn-A01-p27926074	0.991735537	0.289256198	Bn-N1-p25872590	Unknown
Bn-A01-p27926195	0.916943522	0.136212625	Bn-N1-p25872711	Bn-chrAnn_random-p290546
Bn-A01-p27926396	0.920265781	0.109634551	Bn-N1-p25872909	Bn-chrA05_random-p101636
Bn-A01-p27926574	0.996677741	0.42192691	Bn-N1-p25873087	Bn-chrC03-p21474261
Bn-A01-p27926866	1	0.727272727	Bn-N1-p25873379	Bn-chrC03-p21473969
Bn-A01-p27928188	0.975206612	0.983471074	Bn-N1-p25874695	Bn-chrAnn_random-p181962
Bn-A01-p27928239	0.975206612	0.983471074	Bn-N1-p25874746	Bn-chrAnn_random-p181963

Bn-A01-p27936360	0.935323383	0.119402985	Bn-N11-p44770470	Unknown
Bn-A01-p27937532	0.975206612	0.256198347	Bn-N1-p25889430	Unknown
Bn-A01-p27942610	0.993355482	0.993355482	Bn-N11-p44785006	Bn-chrAnn_random-p182136
Bn-A01-p27947381	0.958677686	0.966942149	Bn-N1-p25904401	Bn-chrA01-p21788850
Bn-A01-p27948361	0.966942149	0.190082645	Bn-N1-p25905381	Bn-chrC03-p59540325
Bn-A01-p27953178	0.975206612	0.983471074	Bn-N1-p25910351	Bn-chrA01-p21798996
Bn-A01-p27953261	0.983471074	0.983471074	Bn-N1-p25910434	Bn-chrAnn_random-p182245
Bn-A01-p27954131	0.975206612	0.975206612	Bn-N1-p25913876	Bn-chrAnn_random-p182282
Bn-A01-p27954294	0.983471074	0.991735537	Bn-N11-p44818286	Bn-chrA01-p21800106
Bn-A01-p27956374	0.983471074	0.983471074	Bn-N1-p25916115	Bn-chrAnn_random-p182309
Bn-A01-p27965742	0.970099668	0.983388704	Bn-N1-p25938114	Bn-chrA01-p21812476
Bn-A01-p27968584	0.943521595	0.88372093	Bn-N1-p25940954	Bn-chrA01-p21815278
Bn-A01-p27988223	0.990033223	0.993355482	Bn-N1-p25965645	Bn-chrA01-p21842249
Bn-A01-p27989439	0.936877076	0.993355482	Bn-N11-p44875582	Bn-chrA01-p21843466
Bn-A01-p28047872	0.851239669	0.991735537	Bn-N1-p26028590	Bn-chrAnn_random-p469026
Bn-A01-p28049872	0.933554817	0.996677741	Bn-N1-p26030653	Bn-chrA01-p21963727
Bn-A01-p28089456	0.980066445	0.827242525	Bn-N11-p45003526	Bn-chrA01-p21998996
Bn-A01-p28102080	0.983471074	0.247933884	Bn-N1-p26094193	Bn-chrA04-p16657581
Bn-A01-p28110393	0.96345515	0.990033223	Bn-N1-p26103627	Bn-chrUnn_random-p618215
Bn-A01-p28147757	0.975206612	0.983471074	Bn-N1-p26138783	Bn-chrA01-p22146985
Bn-A01-p28161207	0.975206612	0.966942149	Bn-N1-p26154132	Bn-chrA01-p22162781
Bn-A01-p28186318	0.950413223	0.958677686	Bn-Scaffold01504-p6	Bn-chrAnn_random-p266161
Bn-A01-p28193774	0.991735537	1	Bn-Scaffold01107-p3	Bn-chrAnn_random-p265259
Bn-A01-p28205769	0.983471074	0.991735537	Bn-Scaffold01107-p2	Bn-chrAnn_random-p263769
Bn-A01-p28207450	0.958677686	0.950413223	Bn-Scaffold01107-p1	Bn-chrAnn_random-p263624
Bn-A01-p28217427	0.966942149	0.983471074	Bn-Scaffold01107-p1	Bn-chrAnn_random-p262949
Bn-A01-p28220430	0.991735537	1	Bn-Scaffold01107-p9	Bn-chrAnn_random-p262648
Bn-A01-p28222170	0.570247934	0.578512397	Bn-Scaffold01107-p8	Bn-chrAnn_random-p262474
Bn-A01-p28245023	0.983471074	0.975206612	Bn-N11-p45276246	Bn-chrAnn_random-p248064
Bn-A01-p28249918	0.975206612	0.975206612	Bn-Scaffold01134-p7	Bn-chrAnn_random-p247554
Bn-A01-p28250936	0.272425249	1	Unknown	Bn-chrAnn_random-p247452
Bn-A01-p28261145	0.96013289	0.990033223	Bn-N11-p45297311	Bn-chrAnn_random-p246327
Bn-A01-p28278192	0.685950413	0.867768595	Bn-N11-p45319575	Bn-chrAnn_random-p244544
Bn-A01-p28285580	0.853820598	0.983388704	Bn-N11-p45327077	Bn-chrAnn_random-p243811
Bn-A01-p28326025	0.966942149	0.975206612	Bn-N11-p45367369	Bn-chrAnn_random-p240542
Bn-A01-p28333226	0.644628099	0.975206612	Bn-N11-p45375533	Bn-chrAnn_random-p239498
Bn-A01-p28336382	0.760330579	0.958677686	Bn-N11-p45379417	Bn-chrAnn_random-p239178
Bn-A01-p28336754	0.859504132	0.966942149	Bn-N11-p45380100	Bn-chrAnn_random-p239140
Bn-A01-p28346931	0.993355482	0.993355482	Bn-N1-p26178309	Bn-chrA01-p22599213
Bn-A01-p28360889	0.850498339	0.990033223	Bn-N1-p26191337	Bn-chrA01-p22615340
Bn-A01-p28364600	0.980066445	0.980066445	Bn-N1-p26195825	Bn-chrA01-p22619151
Bn-A01-p28380414	0.966942149	0.958677686	Bn-N1-p26210126	Bn-chrA01-p22637962
Bn-A01-p28385501	0.972972973	0.978378378	Bn-N1-p26215591	Bn-chrA01-p22643051
Bn-A01-p28403438	0.991735537	0.983471074	Bn-N1-p26227824	Bn-chrA01-p22656074
Bn-A01-p28426820	0.983388704	0.970099668	Bn-N1-p26248753	Bn-chrA01-p22710053
Bn-A01-p28447868	0.975206612	0.991735537	Bn-N11-p45750530	Bn-chrA01-p22733537

Bn-A01-p28462071	1	0.995024876	Bn-N1-p26290806	Bn-chrA01-p22750284
Bn-A01-p28497362	0.991735537	0.983471074	Bn-N1-p26332052	Bn-chrA01-p22790563
Bn-A01-p28497900	0.975206612	0.966942149	Bn-N1-p26332590	Bn-chrA01-p22791098
Bn-A01-p28500378	0.996677741	1	Bn-N1-p26335066	Bn-chrA01-p22793573
Bn-A01-p28502173	1	0.991735537	Bn-N1-p26336864	Bn-chrA01-p22795371
Bn-A01-p28534093	0.876033058	0.983471074	Bn-Scaffold00891-p1	Bn-chrCnn_random-p526978
Bn-A01-p28551714	0.966942149	0.958677686	Bn-Scaffold00891-p2	Bn-chrCnn_random-p528741
Bn-A01-p28571117	0.958677686	0.958677686	Bn-Scaffold00891-p7	Bn-chrCnn_random-p533344
Bn-A01-p28577026	0.975206612	0.983471074	Bn-Scaffold00891-p8	Bn-chrCnn_random-p533935
Bn-A01-p28578023	0.966942149	0.966942149	Bn-Scaffold00891-p8	Bn-chrCnn_random-p534034
Bn-A01-p28592050	1	1	Bn-Scaffold00891-p9	Bn-chrCnn_random-p535110
Bn-A02-p1198	0.993355482	0.993355482	Bn-N7-p12867764	Bn-chrA07-p12619483
Bn-A02-p3174	0.986710963	0.986710963	Bn-N7-p12869742	Bn-chrA07-p12617506
Bn-A02-p6233	0.960199005	0.960199005	Bn-N7-p12871398	Bn-chrA07-p12615850
Bn-A02-p6633	0.955223881	0.955223881	Bn-N7-p12871798	Bn-chrA07-p12615450
Bn-A02-p6813	0.970149254	0.970149254	Bn-N7-p12871978	Bn-chrA07-p12615270
Bn-A02-p20398	0.975206612	0.975206612	Bn-N7-p12886830	Bn-chrA07-p12600418
Bn-A02-p21468	0.975124378	0.975124378	Bn-N7-p12887903	Bn-chrA07-p12599345
Bn-A02-p27144	0.909090909	0.950413223	Bn-N14-p14658789	Bn-chrC04-p13026042
Bn-A02-p38907	0.983471074	0.966942149	Bn-N7-p12902701	Bn-chrA07-p12581962
Bn-A02-p39778	0.958677686	0.966942149	Bn-N7-p12903572	Bn-chrA07-p12581091
Bn-A02-p39833	0.983471074	0.966942149	Bn-N7-p12903627	Bn-chrA07-p12581036
Bn-A02-p61866	0.958677686	0.958677686	Bn-N7-p12930362	Bn-chrA07_random-p117818
Bn-A02-p65078	0.970099668	0.84717608	Bn-N7-p12935653	Bn-chrC06-p14739280
Bn-A02-p78265	0.900826446	0.900826446	Bn-N7-p12946735	Bn-chrA07_random-p114486
Bn-A02-p85725	0.94214876	0.94214876	Bn-N7-p12962144	Bn-chrA07-p12724320
Bn-A02-p99883	0.990033223	0.990033223	Bn-N7-p12969397	Bn-chrA07-p12732645
Bn-A02-p108936	0.995024876	0.995024876	Bn-N7-p12980370	Bn-chrA07-p12743716
Bn-A02-p126006	0.995024876	1	Bn-N7-p13000679	Bn-chrA07-p12758854
Bn-A02-p159653	0.983471074	0.983471074	Bn-N7-p13027658	Bn-chrCnn_random-p407794
Bn-A02-p174249	0.993355482	0.990033223	Bn-N7-p13043612	Bn-chrA07-p12796815
Bn-A02-p175605	0.983471074	0.975206612	Bn-N7-p13044968	Bn-chrA07-p12798171
Bn-A02-p179557	0.983471074	0.983471074	Bn-N7-p13049089	Bn-chrA07-p12802939
Bn-A02-p182447	0.996677741	0.993355482	Bn-N7-p13051977	Bn-chrA07-p12805833
Bn-A02-p182618	0.990033223	0.990033223	Bn-N7-p13052148	Bn-chrA07-p12806004
Bn-A02-p183438	0.983388704	0.986710963	Bn-N7-p13052996	Bn-chrA07-p12806825
Bn-A02-p183586	0.991735537	0.991735537	Bn-N7-p13053144	Bn-chrA07-p12806973
Bn-A02-p183840	0.991735537	0.983471074	Bn-N7-p13053398	Bn-chrA07-p12807227
Bn-A02-p185917	0.558139535	0.953488372	Bn-N19-p32747479	Bn-chrA07-p12808960
Bn-A02-p187256	0.495867769	0.917355372	Bn-N14-p22781634	Bn-chrA07-p12811971
Bn-A02-p188268	0.094527363	0.985074627	Unknown	Bn-chrA07-p12812977
Bn-A02-p188454	0.086378738	0.973421927	Bn-N7-p12721004	Bn-chrA07-p12813163
Bn-A02-p201968	0.975206612	0.966942149	Bn-N7-p13061937	Bn-chrA07-p12824434
Bn-A02-p202316	0.986710963	0.830564784	Bn-N7-p13062919	Bn-chrA07-p12824782
Bn-A02-p202493	0.996677741	0.976744186	Bn-N7-p13063095	Bn-chrC06-p14895200
Bn-A02-p202819	0.973421927	0.980066445	Bn-N7-p13063423	Bn-chrA07-p12825285

Bn-A02-p209474	0.958677686	0.958677686	Bn-N7-p13069652	Bn-chrA07-p12831558
Bn-A02-p212959	0.993355482	0.996677741	Bn-N7-p13072225	Bn-chrA07-p12834126
Bn-A02-p251558	0.996677741	0.943521595	Bn-N7-p13098326	Bn-chrA07-p12862429
Bn-A02-p266070	0.933884298	0.933884298	Bn-N7-p13133534	Bn-chrA07-p12889905
Bn-A02-p266121	0.94214876	0.94214876	Bn-N7-p13133586	Bn-chrA07-p12889957
Bn-A02-p276572	0.980066445	0.983388704	Bn-Scaffold12647-p2	Bn-chrA07-p12906262
Bn-A02-p284968	0.983471074	0.983471074	Bn-N7-p13154675	Bn-chrA07-p12910550
Bn-A02-p293860	0.990033223	0.983388704	Bn-N7-p13165342	Bn-chrA07-p12919536
Bn-A02-p294984	0.990049751	0.930348259	Bn-N7-p13166456	Bn-chrA07-p12920659
Bn-A02-p298761	0.611570248	0.991735537	Bn-N7-p13182408	Bn-chrA07-p12924434
Bn-A02-p300727	0.991735537	0.983471074	Bn-N7-p13184963	Bn-chrA07-p12926400
Bn-A02-p302582	0.966942149	0.975206612	Bn-N7-p13186813	Bn-chrA07-p12928254
Bn-A02-p303085	0.950413223	0.94214876	Bn-N7-p13187310	Bn-chrA07-p12928757
Bn-A02-p303243	0.973421927	0.986710963	Bn-N7-p13187468	Bn-chrA07-p12928915
Bn-A02-p303330	0.980066445	0.983388704	Bn-N7-p13187560	Bn-chrA07-p12929002
Bn-A02-p303375	0.980066445	0.983388704	Bn-N7-p13187605	Bn-chrA07-p12929047
Bn-A02-p303515	0.983471074	0.991735537	Bn-N7-p13187745	Bn-chrA07-p12929187
Bn-A02-p304056	0.990033223	0.993355482	Bn-N7-p13188692	Bn-chrA07-p12929728
Bn-A02-p305007	0.991735537	0.991735537	Bn-N7-p13189642	Bn-chrA07-p12930679
Bn-A02-p307342	0.983388704	0.943521595	Bn-N7-p13196459	Bn-chrC06-p15085122
Bn-A02-p307657	0.966942149	0.504132231	Bn-N7-p13196779	Bn-chrC08-p19579285
Bn-A02-p308370	0.826446281	1	Bn-N7-p13197088	Bn-chrA07-p12938151
Bn-A02-p319267	0.955223881	0.955223881	Bn-N7-p13207479	Bn-chrA07-p12954845
Bn-A02-p325065	0.991735537	1	Bn-N7-p13214023	Bn-chrA07-p12958937
Bn-A02-p325788	0.996677741	1	Bn-N7-p13214746	Bn-chrA07-p12959662
Bn-A02-p335197	0.986710963	0.986710963	Bn-N7-p13224385	Bn-chrA07-p12968698
Bn-A02-p337110	1	1	Bn-N7-p13226440	Bn-chrA07-p12970752
Bn-A02-p337457	0.996677741	0.996677741	Bn-N7-p13226787	Bn-chrA07-p12971099
Bn-A02-p379918	0.996677741	0.996677741	Bn-N7-p13266570	Bn-chrA07-p13011107
Bn-A02-p393636	0.958677686	0.950413223	Bn-N7-p13282682	Bn-chrA07-p13021849
Bn-A02-p416231	0.983471074	0.966942149	Bn-N7-p13296926	Bn-chrC06-p15583011
Bn-A02-p425861	0.893687708	0.970099668	Bn-N7-p13305191	Bn-chrA07-p13048252
Bn-A02-p441751	0.991735537	0.991735537	Bn-N7-p13316291	Bn-chrA07-p13060264
Bn-A02-p459981	0.983471074	0.983471074	Bn-N7-p13338835	Bn-chrA07-p13077278
Bn-A02-p463348	0.966942149	0.966942149	Bn-N7-p13346807	Bn-chrA07-p13089257
Bn-A02-p474724	0.990033223	0.990033223	Bn-N7-p13356720	Bn-chrA07-p13099130
Bn-A02-p476011	0.983471074	0.983471074	Bn-N7-p13358348	Bn-chrA07-p13100758
Bn-A02-p488160	0.973421927	0.973421927	Bn-N7-p13371113	Bn-chrA07-p13114093
Bn-A02-p490065	0.980066445	0.980066445	Bn-N7-p13373789	Bn-chrA07-p13115998
Bn-A02-p500418	0.996677741	0.996677741	Bn-N7-p13383260	Bn-chrA07-p13125469
Bn-A02-p501869	0.950413223	0.950413223	Bn-N7-p13389583	Bn-chrA07-p13130371
Bn-A02-p510727	0.92039801	0.92039801	Bn-N7-p13412718	Bn-chrA07_random-p119401
Bn-A02-p518958	0.991735537	0.991735537	Bn-N7-p13423790	Bn-chrA07-p13147011
Bn-A02-p545783	0.966942149	0.966942149	Bn-N7-p13444606	Bn-chrA07-p13168351
Bn-A02-p548469	0.993355482	0.993355482	Bn-N7-p13447251	Bn-chrA07-p13170996
Bn-A02-p551481	0.975206612	0.975206612	Bn-N7-p13449978	Bn-chrA07-p13173722

Bn-A02-p552098	0.958677686	0.958677686	Bn-N7-p13450598	Bn-chrA07-p13174342
Bn-A02-p557523	0.933884298	0.925619835	Bn-N7-p13456681	Bn-chrA07-p13179913
Bn-A02-p561309	0.950413223	0.950413223	Bn-N7-p13460565	Bn-chrA07-p13183710
Bn-A02-p562671	0.958677686	0.958677686	Bn-N7-p13461884	Bn-chrA07-p13185031
Bn-A02-p576204	0.983471074	0.983471074	Bn-N7-p13476565	Bn-chrA07-p13197620
Bn-A02-p584200	0.973421927	0.976744186	Bn-N7-p13489571	Bn-chrA07-p13214097
Bn-A02-p588093	0.975206612	0.975206612	Bn-N7-p13493436	Bn-chrA07-p13217992
Bn-A02-p594109	0.975206612	0.975206612	Bn-N7-p13499429	Bn-chrA07-p13226074
Bn-A02-p594700	0.975124378	0.975124378	Bn-N7-p13500020	Bn-chrA07-p13226665
Bn-A02-p615312	0.993355482	0.993355482	Bn-N7-p13513016	Bn-chrA07-p13237697
Bn-A02-p616247	0.986710963	0.986710963	Bn-N7-p13513951	Bn-chrA07-p13238632
Bn-A02-p624595	0.985074627	0.985074627	Bn-N7-p13523552	Bn-chrC06-p16155852
Bn-A02-p631297	0.975206612	0.975206612	Bn-N7-p13530951	Bn-chrA07-p13251973
Bn-A02-p634256	0.975206612	0.975206612	Bn-N7-p13533959	Bn-chrA07-p13254981
Bn-A02-p636428	0.876033058	0.876033058	Bn-N7-p13536098	Bn-chrA07-p13257120
Bn-A02-p647024	0.958677686	0.958677686	Bn-N7-p13548076	Bn-chrA07-p13271226
Bn-A02-p647706	0.910299003	0.880398671	Bn-N16-p16301190	Bn-chrA07-p13271902
Bn-A02-p652623	0.991735537	0.991735537	Bn-N7-p13551729	Bn-chrA07-p13274879
Bn-A02-p655765	0.958677686	0.958677686	Bn-N7-p13556789	Bn-chrA07-p13279939
Bn-A02-p676719	0.975206612	0.975206612	Bn-N7-p13572008	Bn-chrA07-p13296600
Bn-A02-p677501	1	1	Bn-N7-p13572791	Bn-chrA07-p13297383
Bn-A02-p709952	0.966942149	0.900826446	Bn-N7-p13609162	Bn-chrC06-p16266278
Bn-A02-p714791	0.993355482	0.993355482	Bn-N7-p13615770	Bn-chrA07-p13339754
Bn-A02-p725112	0.953488372	0.950166113	Bn-N7-p13632028	Bn-chrA07-p13354994
Bn-A02-p727584	0.996677741	0.996677741	Bn-N7-p13634500	Bn-chrA07-p13357466
Bn-A02-p745468	0.990033223	0.990033223	Bn-N7-p13652748	Bn-chrA07-p13377023
Bn-A02-p759031	0.985074627	0.985074627	Bn-N7-p13662371	Bn-chrA07-p13387826
Bn-A02-p761524	0.834710744	0.991735537	Bn-N16-p16873742	Bn-chrA07-p13390323
Bn-A02-p770821	0.990049751	0.995024876	Bn-N7-p13675563	Bn-chrA07-p13399759
Bn-A02-p771019	0.990033223	0.986710963	Bn-N7-p13675761	Bn-chrA07-p13399957
Bn-A02-p771313	0.996677741	1	Bn-N7-p13676055	Bn-chrA07-p13400251
Bn-A02-p771951	0.995024876	0.995024876	Bn-N7-p13676693	Bn-chrA07-p13400889
Bn-A02-p785930	0.995024876	0.990049751	Bn-N7-p13691366	Bn-chrA07-p13409810
Bn-A02-p799930	0.966942149	0.966942149	Bn-N7-p13707097	Bn-chrA07-p13428544
Bn-A02-p801257	0.752066116	0.966942149	Bn-N7-p13708328	Bn-chrA07-p13429775
Bn-A02-p806025	0.975206612	0.966942149	Bn-N7-p13712824	Bn-chrA07-p13434530
Bn-A02-p806949	0.980099502	0.980099502	Bn-N7-p13713751	Bn-chrA07-p13435454
Bn-A02-p808711	0.975206612	0.975206612	Bn-N7-p13715521	Bn-chrA07-p13437224
Bn-A02-p809627	0.94214876	0.94214876	Bn-N7-p13718122	Bn-chrA07-p13439820
Bn-A02-p823586	0.94214876	0.94214876	Bn-N7-p13740475	Bn-chrA07-p13457394
Bn-A02-p839171	0.985074627	0.985074627	Bn-N7-p13760257	Bn-chrA07-p13470293
Bn-A02-p847638	0.970099668	0.970099668	Bn-N7-p13768733	Bn-chrA07-p13478768
Bn-A02-p848731	0.94214876	0.94214876	Bn-N7-p13769807	Bn-chrA07-p13479842
Bn-A02-p849524	0.760330579	0.760330579	Bn-N7-p13774662	Bn-chrA07-p13483996
Bn-A02-p849709	0.727272727	0.727272727	Bn-N7-p13774847	Bn-chrA07-p13484181
Bn-A02-p852096	0.73553719	0.73553719	Bn-N7-p13776977	Bn-chrA07-p13486311

Bn-A02-p859079	1	1	Bn-N7-p13781552	Bn-chrA07-p13490886
Bn-A02-p870494	0.958677686	0.958677686	Bn-N7-p13795116	Bn-chrA07-p15792359
Bn-A02-p873656	0.958677686	0.958677686	Bn-N7-p13796203	Bn-chrA07-p13504874
Bn-A02-p873770	0.975206612	0.975206612	Bn-N7-p13796317	Bn-chrA07-p13504988
Bn-A02-p892640	0.983471074	0.983471074	Bn-N7-p13808846	Bn-chrA07-p13519937
Bn-A02-p918384	0.983471074	0.983471074	Bn-N7-p13833514	Bn-chrA07-p13543770
Bn-A02-p925207	0.950413223	0.950413223	Bn-N7-p13838353	Bn-chrA07-p13548749
Bn-A02-p972896	0.950413223	0.950413223	Bn-N7-p13889496	Bn-chrA07-p13601067
Bn-A02-p975209	0.975206612	0.975206612	Bn-N7-p13889911	Bn-chrA07-p13601482
Bn-A02-p978741	0.975206612	0.975206612	Bn-N7-p13899770	Bn-chrA07-p13611860
Bn-A02-p980390	0.958677686	0.958677686	Bn-N7-p13902795	Bn-chrA07-p13614885
Bn-A02-p985366	0.94214876	0.94214876	Bn-N7-p13907779	Bn-chrA07-p13619867
Bn-A02-p1020806	0.991735537	0.991735537	Bn-N7-p13945724	Bn-chrA07-p13652568
Bn-A02-p1025518	0.809917355	0.809917355	Bn-N7-p13950938	Bn-chrA07-p13657778
Bn-A02-p1027570	0.933884298	0.933884298	Bn-N7-p13952977	Bn-chrA07-p13659817
Bn-A02-p1028349	0.983471074	0.983471074	Bn-N7-p13953747	Bn-chrA07-p13660587
Bn-A02-p1068542	0.991735537	1	Bn-N2-p1812897	Bn-chrA02-p292698
Bn-A02-p1075920	0.991735537	0.983471074	Bn-N2-p1806142	Bn-chrA02-p278433
Bn-A02-p1085455	0.892561983	0.892561983	Bn-N12-p1880782	Bn-chrC02-p13584
Bn-A02-p1091779	0.950413223	0.950413223	Bn-N2-p1790897	Bn-chrA02-p263186
Bn-A02-p1092146	0.958677686	0.958677686	Bn-N2-p1790534	Bn-chrA02-p262823
Bn-A02-p1096082	0.94214876	0.94214876	Bn-N2-p1786578	Bn-chrA02-p258885
Bn-A02-p1135078	0.925619835	0.950413223	Bn-N2-p1738606	Bn-chrA02-p234868
Bn-A02-p1155656	0.960199005	0.965174129	Bn-N2-p1716507	Bn-chrC02-p99721
Bn-A02-p1157373	0.980099502	0.980099502	Bn-N2-p1714788	Bn-chrA02-p216127
Bn-A02-p1173704	0.966777409	0.966777409	Bn-N2-p1697068	Bn-chrA02-p195541
Bn-A02-p1188292	0.983471074	0.975206612	Bn-N2-p1682456	Bn-chrA02-p180695
Bn-A02-p1220275	0.983471074	0.983471074	Bn-N2-p1659106	Bn-chrA02-p159507
Bn-A02-p1232964	0.975206612	0.975206612	Bn-N2-p1648567	Bn-chrA02-p147990
Bn-A02-p1235480	0.991735537	1	Bn-N2-p1646053	Bn-chrA02-p145474
Bn-A02-p1250673	0.990033223	0.990033223	Bn-N2-p1628726	Bn-chrA02-p130240
Bn-A02-p1259929	0.975206612	0.975206612	Bn-N2-p1619108	Bn-chrA02-p120960
Bn-A02-p1264906	0.958677686	0.966942149	Bn-N2-p1612584	Bn-chrA02-p114981
Bn-A02-p1265949	0.983471074	0.983471074	Bn-N2-p1611542	Bn-chrA02-p113939
Bn-A02-p1276717	0.73553719	0.801652893	Bn-N2-p1601054	Bn-chrA02-p101762
Bn-A02-p1276995	0.950413223	0.917355372	Bn-N2-p1600747	Bn-chrC02-p246459
Bn-A02-p1290847	1	0.995024876	Bn-N2-p1587146	Bn-chrA02-p87694
Bn-A02-p1292023	0.991735537	0.983471074	Bn-N2-p1585968	Bn-chrA02-p86519
Bn-A02-p1319039	0.966942149	0.94214876	Bn-N2-p1559038	Bn-chrA02-p65681
Bn-A02-p1321904	0.958677686	0.966942149	Bn-N2-p1556172	Bn-chrA02-p62987
Bn-A02-p1324368	0.991735537	1	Bn-N2-p1553708	Bn-chrA02-p60136
Bn-A02-p1325213	0.975206612	0.975206612	Bn-N2-p1552862	Bn-chrA02-p59290
Bn-A02-p1326013	0.983471074	0.983471074	Bn-N2-p1552062	Bn-chrA02-p58490
Bn-A02-p1327787	0.983471074	0.983471074	Bn-N2-p1550342	Bn-chrA02-p56770
Bn-A02-p1335862	0.991735537	0.991735537	Bn-N2-p1542202	Bn-chrA02-p48621
Bn-A02-p1337706	0.991735537	0.991735537	Bn-N2-p1540553	Bn-chrA02-p40455

Bn-A02-p1341101	0.975206612	0.239669421	Bn-N2-p1537227	Bn-chrC08-p24825913
Bn-A02-p1344870	0.96013289	0.946843854	Bn-N12-p1537049	Bn-chrCnn_random-p443606
Bn-A02-p1370581	0.726368159	0.980099502	Bn-N2-p1508906	Bn-chrC02-p321485
Bn-A02-p1372727	1	1	Bn-N2-p1506659	Bn-chrA02-p12296
Bn-A02-p1396892	1	1	Bn-N2-p15550	Bn-chrAnn_random-p15273
Bn-A02-p1402685	0.966942149	0.966942149	Bn-N2-p23190	Bn-chrAnn_random-p22913
Bn-A02-p1411508	0.975206612	0.983471074	Bn-N2-p34829	Bn-chrAnn_random-p31933
Bn-A02-p1485915	0.965174129	0.965174129	Bn-N2-p142072	Bn-chrAnn_random-p16748C
Bn-A02-p1506579	0.991735537	0.991735537	Bn-N2-p169311	Bn-chrAnn_random-p189549
Bn-A02-p1517113	0.966942149	0.966942149	Bn-N2-p184982	Bn-chrAnn_random-p200031
Bn-A02-p1528341	0.983471074	0.983471074	Bn-N2-p204354	Bn-chrAnn_random-p149872
Bn-A02-p1530996	0.990033223	0.990033223	Bn-N2-p207019	Bn-chrAnn_random-p149898
Bn-A02-p1552305	1	1	Bn-N2-p230194	Bn-chrAnn_random-p150138
Bn-A02-p1576350	0.970149254	0.970149254	Bn-N2-p249451	Bn-chrAnn_random-p150337
Bn-A02-p1629448	0.990033223	0.990033223	Bn-N2-p304853	Bn-chrAnn_random-p306861
Bn-A02-p1671689	0.975206612	0.975206612	Bn-N2-p337117	Bn-chrAnn_random-p348259
Bn-A02-p1684625	0.983471074	0.991735537	Bn-N2-p368153	Bn-chrAnn_random-p361794
Bn-A02-p1687398	1	0.983471074	Bn-N2-p372010	Bn-chrAnn_random-p418514
Bn-A02-p1698015	0.950413223	0.966942149	Bn-N2-p379523	Bn-chrAnn_random-p426099
Bn-A02-p1705187	1	0.995024876	Bn-N12-p91942	Bn-chrC02-p1666977
Bn-A02-p1707420	0.966942149	0.966942149	Bn-N2-p392255	Bn-chrAnn_random-p438038
Bn-A02-p1761218	0.995024876	0.995024876	Bn-N2-p461302	Bn-chrAnn_random-p196442
Bn-A02-p1788566	0.993355482	0.993355482	Bn-N2-p498902	Bn-chrAnn_random-p196136
Bn-A02-p1890913	0.980099502	0.980099502	Bn-N2-p590652	Bn-chrAnn_random-p573917
Bn-A02-p1894727	0.981651376	0.981651376	Bn-Scaffold55294-p2	Bn-chrAnn_random-p576373
Bn-A02-p1997812	0.970149254	0.930348259	Bn-N2-p705902	Bn-chrAnn_random-p18727C
Bn-A02-p2075762	1	0.980099502	Bn-N2-p775778	Bn-chrAnn_random-p70138C
Bn-A02-p2105937	0.958677686	0.958677686	Bn-N2-p808758	Bn-chrAnn_random-p735527
Bn-A02-p2121360	0.909090909	0.909090909	Bn-N2-p822620	Bn-chrAnn_random-p748984
Bn-A02-p2122414	0.975206612	0.975206612	Bn-N2-p823830	Bn-chrAnn_random-p750194
Bn-A02-p2122850	1	1	Bn-N2-p824267	Bn-chrAnn_random-p750631
Bn-A02-p2124245	0.685950413	0.685950413	Bn-N2-p825479	Bn-chrAnn_random-p751843
Bn-A02-p2124328	0.975206612	0.975206612	Bn-N2-p825559	Bn-chrAnn_random-p751923
Bn-A02-p2124421	0.719008264	0.719008264	Bn-N2-p825652	Bn-chrAnn_random-p752016
Bn-A02-p2128775	0.966942149	0.966942149	Bn-N2-p828865	Bn-chrAnn_random-p755228
Bn-A02-p2130611	0.892561983	0.892561983	Bn-N2-p830767	Bn-chrAnn_random-p75713C
Bn-A02-p2130925	0.958677686	0.958677686	Bn-N2-p831071	Bn-chrAnn_random-p757434
Bn-A02-p2134112	0.991735537	0.991735537	Bn-N2-p834279	Bn-chrAnn_random-p760642
Bn-A02-p2135053	0.958677686	0.958677686	Bn-N2-p835204	Bn-chrAnn_random-p761567
Bn-A02-p2136039	0.983471074	0.983471074	Bn-N2-p836188	Bn-chrAnn_random-p762551
Bn-A02-p2138408	0.958677686	0.958677686	Bn-N2-p839451	Bn-chrAnn_random-p764905
Bn-A02-p2146210	0.973421927	0.305647841	Bn-N2-p847432	Bn-chrC02-p1068514
Bn-A02-p2154644	0.966942149	0.966942149	Bn-N2-p856400	Bn-chrAnn_random-p784611
Bn-A02-p2165239	0.966942149	0.966942149	Bn-N2-p861862	Bn-chrAnn_random-p791056
Bn-A02-p2182742	1	0.975206612	Bn-N2-p881955	Bn-chrAnn_random-p812032
Bn-A02-p2258714	0.980099502	0.980099502	Bn-N2-p952606	Bn-chrAnn_random-p879181

Bn-A02-p2274901	0.985074627	0.985074627	Bn-N2-p974551	Bn-chrAnn_random-p89805C
Bn-A02-p2277616	0.993670886	1	Bn-Scaffold33561-p2	Bn-chrC02-p941739
Bn-A02-p2287712	0.990049751	0.990049751	Bn-N2-p991484	Bn-chrAnn_random-p914983
Bn-A02-p2288207	0.985074627	0.985074627	Bn-N2-p991976	Bn-chrAnn_random-p915475
Bn-A02-p2288216	0.84079602	0.84079602	Bn-N2-p991985	Bn-chrAnn_random-p915484
Bn-A02-p2292871	0.826446281	0.826446281	Bn-N2-p1000576	Bn-chrAnn_random-p921242
Bn-A02-p2341934	0.966777409	0.966777409	Bn-N2-p1046388	Bn-chrAnn_random-p967762
Bn-A02-p2364069	0.975124378	0.975124378	Bn-N2-p1068047	Bn-chrAnn_random-p989416
Bn-A02-p2364479	0.925619835	0.925619835	Bn-N2-p1068443	Bn-chrAnn_random-p989812
Bn-A02-p2389418	0.785123967	0.975206612	Bn-N12-p1058077	Bn-chrAnn_random-p101416
Bn-A02-p2421888	0.628099174	0.628099174	Bn-Scaffold11307-p1	Bn-chrAnn_random-p104846
Bn-A02-p2434462	0.950413223	0.950413223	Bn-N12-p1099762	Bn-chrC02-p762317
Bn-A02-p2443019	0.950413223	0.950413223	Bn-N2-p1139195	Bn-chrAnn_random-p106073
Bn-A02-p2443275	0.975206612	0.975206612	Bn-N2-p1139491	Bn-chrAnn_random-p106106
Bn-A02-p2451470	0.991735537	0.991735537	Bn-N2-p1147712	Bn-chrAnn_random-p106928
Bn-A02-p2466355	0.983471074	0.983471074	Bn-N2-p1163220	Bn-chrAnn_random-p146086
Bn-A02-p2475208	0.983471074	0.983471074	Bn-N2-p1172087	Bn-chrAnn_random-p146174
Bn-A02-p2480400	0.975206612	0.975206612	Bn-N2-p1176772	Bn-chrAnn_random-p146239
Bn-A02-p2489530	0.970149254	0.970149254	Bn-N2-p1187700	Bn-chrAnn_random-p146328
Bn-A02-p2490293	0.995024876	0.995024876	Bn-N2-p1188463	Bn-chrAnn_random-p146336
Bn-A02-p2502950	0.975206612	0.975206612	Bn-N2-p1201098	Bn-chrAnn_random-p146463
Bn-A02-p2509802	0.983471074	0.983471074	Bn-N2-p1207897	Bn-chrAnn_random-p146535
Bn-A02-p2520421	0.991735537	0.991735537	Bn-N2-p1218503	Bn-chrAnn_random-p146639
Bn-A02-p2527082	0.991735537	0.991735537	Bn-N2-p1226262	Bn-chrAnn_random-p146703
Bn-A02-p2527735	0.983471074	0.231404959	Bn-N2-p1230297	Unknown
Bn-A02-p2535232	1	1	Bn-N2-p1237798	Bn-chrAnn_random-p628589
Bn-A02-p2536779	0.996677741	0.996677741	Bn-N2-p1239345	Bn-chrAnn_random-p628434
Bn-A02-p2542065	0.975206612	0.975206612	Bn-N2-p1244624	Bn-chrC02-p618758
Bn-A02-p2544070	0.983471074	0.983471074	Bn-N2-p1246634	Bn-chrAnn_random-p627705
Bn-A02-p2549141	0.966942149	0.966942149	Bn-N2-p1250691	Bn-chrAnn_random-p627294
Bn-A02-p2551178	0.966942149	0.966942149	Bn-N2-p1252896	Bn-chrAnn_random-p627074
Bn-A02-p2557799	0.990049751	0.990049751	Bn-N2-p1259549	Bn-chrAnn_random-p33527C
Bn-A02-p2568989	0.993355482	0.990033223	Bn-N2-p1270380	Bn-chrAnn_random-p625418
Bn-A02-p2572194	0.983471074	0.983471074	Bn-N2-p1273579	Bn-chrAnn_random-p625098
Bn-A02-p2572625	0.983471074	0.983471074	Bn-N2-p1274020	Bn-chrAnn_random-p625054
Bn-A02-p2582999	0.976744186	0.976744186	Bn-N2-p1284147	Bn-chrAnn_random-p624041
Bn-A02-p2608125	0.991735537	0.991735537	Bn-N2-p1305765	Bn-chrAnn_random-p62188C
Bn-A02-p2615923	0.991735537	1	Bn-N2-p1313171	Bn-chrAnn_random-p621125
Bn-A02-p2617701	0.991735537	1	Bn-N2-p1314947	Bn-chrAnn_random-p620948
Bn-A02-p2617781	0.983471074	0.991735537	Bn-N2-p1315027	Bn-chrAnn_random-p62094C
Bn-A02-p2620397	0.983471074	0.983471074	Bn-N2-p1317615	Bn-chrAnn_random-p620678
Bn-A02-p2624375	0.970149254	0.975124378	Bn-N2-p1321568	Bn-chrAnn_random-p620281
Bn-A02-p2638031	0.990033223	0.986710963	Bn-N2-p1336947	Bn-chrAnn_random-p618877
Bn-A02-p2642904	0.884297521	0.991735537	Bn-N2-p1341829	Bn-chrAnn_random-p61839C
Bn-A02-p2643078	0.966942149	0.991735537	Bn-N2-p1341995	Bn-chrAnn_random-p618373
Bn-A02-p2646929	0.842975207	0.842975207	Bn-N12-p1359174	Bn-chrC02-p500138

Bn-A02-p2654991	0.983471074	0.991735537	Bn-N2-p1355422	Bn-chrAnn_random-p617182
Bn-A02-p2673795	0.970149254	0.930348259	Bn-N2-p1372334	Bn-chrAnn_random-p615467
Bn-A02-p2692116	0.983471074	0.966942149	Bn-N2-p1390902	Bn-chrAnn_random-p614146
Bn-A02-p2695870	0.925619835	0.925619835	Bn-N2-p1394706	Bn-chrAnn_random-p613766
Bn-A02-p2698217	0.958677686	0.958677686	Bn-N2-p1397046	Bn-chrAnn_random-p613532
Bn-A02-p2707979	0.20661157	0.958677686	Unknown	Bn-chrAnn_random-p612505
Bn-A02-p2708161	0.396694215	0.983471074	Bn-N2-p1408616	Bn-chrAnn_random-p612487
Bn-A02-p2718786	0.983471074	0.991735537	Bn-N2-p1426942	Bn-chrAnn_random-p611512
Bn-A02-p2727560	0.950248756	0.975124378	Bn-N12-p1445138	Bn-chrAnn_random-p610603
Bn-A02-p2737068	0.892561983	0.20661157	Bn-Scaffold75598-p1	Bn-chrC09-p26490698
Bn-A02-p2738923	0.958677686	0.958677686	Bn-N2-p1452300	Bn-chrAnn_random-p609954
Bn-A02-p2739888	0.958677686	0.966942149	Bn-N2-p1453265	Bn-chrAnn_random-p609857
Bn-A02-p2753403	0.991735537	0.991735537	Bn-N2-p1465935	Bn-chrAnn_random-p608202
Bn-A02-p2753972	0.991735537	0.991735537	Bn-N2-p1466504	Bn-chrAnn_random-p608146
Bn-A02-p2776432	1	1	Bn-N2-p1849498	Bn-chrA02-p327619
Bn-A02-p2776634	0.980099502	0.980099502	Bn-N2-p1849700	Bn-chrA02-p327821
Bn-A02-p2782176	0.985074627	0.985074627	Bn-N2-p1855987	Bn-chrA02-p334127
Bn-A02-p2784335	0.96013289	0.96013289	Bn-N2-p1858150	Bn-chrA02-p336290
Bn-A02-p2785185	1	1	Bn-N2-p1859025	Bn-chrA02-p337165
Bn-A02-p2788144	0.986710963	0.986710963	Bn-N2-p1865260	Bn-chrA02-p340201
Bn-A02-p2799146	0.995024876	0.995024876	Bn-N2-p1874242	Bn-chrA02-p349183
Bn-A02-p2800227	0.985074627	0.985074627	Bn-N2-p1875323	Bn-chrA02-p350292
Bn-A02-p2800579	0.970149254	0.970149254	Bn-N2-p1875675	Bn-chrA02-p350644
Bn-A02-p2800698	0.980099502	0.980099502	Bn-N2-p1875794	Bn-chrA02-p350763
Bn-A02-p2817281	0.96013289	0.96013289	Bn-Scaffold16598-p1	Bn-chrA02-p362847
Bn-A02-p2817294	0.966942149	0.966942149	Bn-Scaffold16598-p1	Bn-chrA02-p362860
Bn-A02-p2818741	0.909090909	0.909090909	Bn-N2-p1889272	Bn-chrA02-p364238
Bn-A02-p2851231	1	1	Bn-N2-p1920922	Bn-chrA02-p393886
Bn-A02-p2867545	0.976744186	0.976744186	Bn-N2-p1943312	Bn-chrA02-p415097
Bn-A02-p2877222	1	1	Bn-N2-p1952858	Bn-chrA02-p426935
Bn-A02-p2923889	0.966942149	0.966942149	Bn-N2-p1983351	Bn-chrA02_random-p24828
Bn-A02-p2927357	0.958677686	0.958677686	Bn-N2-p1987002	Bn-chrA02_random-p21177
Bn-A02-p2931725	0.966942149	0.966942149	Bn-N2-p1991401	Bn-chrA02_random-p14587
Bn-A02-p2962298	0.958677686	0.958677686	Bn-N2-p2021013	Bn-chrAnn_random-p235527
Bn-A02-p3011103	0.980066445	0.980066445	Bn-N2-p2069982	Bn-chrA02-p485659
Bn-A02-p3033620	0.983471074	0.983471074	Bn-N2-p2091947	Bn-chrA02-p508891
Bn-A02-p3035603	0.818181818	0.818181818	Bn-N2-p2093978	Bn-chrA02-p510921
Bn-A02-p3050150	0.958677686	0.958677686	Bn-N2-p2115368	Bn-chrA02-p529169
Bn-A02-p3072415	0.985074627	0.985074627	Bn-N2-p2149616	Bn-chrA02-p547046
Bn-A02-p3074016	0.958677686	0.950413223	Bn-N2-p2151215	Bn-chrA02-p552308
Bn-A02-p3078017	0.995024876	0.995024876	Bn-N2-p2155233	Bn-chrA02-p556330
Bn-A02-p3080150	0.990049751	0.990049751	Bn-N2-p2157365	Bn-chrA02-p558462
Bn-A02-p3085246	0.94214876	0.925619835	Bn-N2-p2162460	Bn-chrA02-p563508
Bn-A02-p3092363	0.980099502	0.980099502	Bn-N9-p19809154	Bn-chrA02-p570893
Bn-A02-p3092949	0.995024876	0.995024876	Bn-N9-p19809740	Bn-chrA02-p571479
Bn-A02-p3096890	0.975206612	0.975206612	Bn-N2-p2173479	Bn-chrA02-p575414

Bn-A02-p3121742	0.975124378	0.975124378	Bn-N2-p2196718	Bn-chrA02-p605811
Bn-A02-p3138043	0.950413223	0.925619835	Bn-N2-p2215787	Bn-chrC02-p2335263
Bn-A02-p3142416	0.966942149	0.975206612	Bn-N2-p2220109	Bn-chrA02-p626349
Bn-A02-p3145923	0.980099502	0.985074627	Bn-N2-p2223558	Bn-chrA02-p629856
Bn-A02-p3148346	0.933884298	0.851239669	Bn-N2-p2225846	Bn-chrA02-p632118
Bn-A02-p3150860	0.986710963	0.601328904	Bn-N2-p2228465	Bn-chrA02-p634516
Bn-A02-p3160461	0.996677741	0.990033223	Bn-N2-p2238075	Bn-chrA02-p643938
Bn-A02-p3167622	0.958677686	0.966942149	Bn-N2-p2245412	Bn-chrA02-p651222
Bn-A02-p3167673	0.991735537	0.983471074	Bn-N2-p2245463	Bn-chrA02-p651273
Bn-A02-p3175610	0.983471074	0.991735537	Bn-N2-p2256921	Bn-chrA02-p658779
Bn-A02-p3180162	0.983471074	0.975206612	Bn-N2-p2261789	Bn-chrA02-p660538
Bn-A02-p3181443	0.975206612	0.958677686	Bn-N2-p2263070	Bn-chrA02-p667828
Bn-A02-p3183179	0.975206612	0.917355372	Bn-N2-p2264806	Bn-chrC02-p2393757
Bn-A02-p3190650	0.975206612	0.809917355	Bn-N2-p2269507	Bn-chrC06-p11017259
Bn-A02-p3207085	0.966942149	0.975206612	Bn-N2-p2278952	Bn-chrA02-p695308
Bn-A02-p3208275	0.958677686	0.966942149	Bn-N2-p2280143	Bn-chrA02-p700390
Bn-A02-p3214095	0.983471074	0.983471074	Bn-N2-p2285964	Bn-chrA02-p708874
Bn-A02-p3214903	1	0.989583333	Bn-N2-p2286772	Bn-chrA02-p709680
Bn-A02-p3221060	0.975206612	0.975206612	Bn-N2-p2292937	Bn-chrA02-p715841
Bn-A02-p3221634	1	1	Bn-N2-p2293511	Bn-chrA02-p716411
Bn-A02-p3233816	0.975206612	0.991735537	Bn-N2-p2304775	Bn-chrA02-p729679
Bn-A02-p3260536	0.970149254	0.975124378	Bn-N2-p2345136	Bn-chrA02-p743913
Bn-A02-p3281338	0.983471074	0.975206612	Bn-N2-p2367290	Bn-chrA02-p764105
Bn-A02-p3281452	0.970149254	0.985074627	Bn-N2-p2367418	Bn-chrA02-p764233
Bn-A02-p3294682	0.975206612	0.966942149	Bn-N2-p2380926	Bn-chrA02-p784415
Bn-A02-p3295898	0.975206612	0.983471074	Bn-N2-p2382134	Bn-chrA02-p786193
Bn-A02-p3297592	0.966942149	0.958677686	Bn-N2-p2383804	Bn-chrA02-p787627
Bn-A02-p3299206	0.950413223	0.950413223	Bn-N2-p2385418	Bn-chrA02-p789246
Bn-A02-p3300731	0.975124378	0.950248756	Bn-N2-p2387091	Bn-chrA02-p790766
Bn-A02-p3302725	0.991735537	0.983471074	Bn-N2-p2389079	Bn-chrA02-p792753
Bn-A02-p3306076	0.990049751	0.995024876	Bn-N2-p2392704	Bn-chrA02-p796079
Bn-A02-p3344176	1	0.966942149	Bn-N2-p2434012	Bn-chrA02-p834150
Bn-A02-p3349026	0.884297521	0.892561983	Bn-N2-p2438776	Bn-chrA02-p839267
Bn-A02-p3352863	0.965174129	0.985074627	Bn-N2-p2442666	Bn-chrA02-p843197
Bn-A02-p3354301	0.96345515	1	Bn-N12-p2756360	Bn-chrA02-p844704
Bn-A02-p3361391	0.975206612	0.966942149	Bn-N2-p2445850	Bn-chrA02-p849106
Bn-A02-p3374678	0.966942149	0.975206612	Bn-N2-p2458886	Bn-chrA02-p863953
Bn-A02-p3380510	0.966942149	0.958677686	Bn-N2-p2464770	Bn-chrA02-p869834
Bn-A02-p3398506	0.958677686	0.966942149	Bn-N2-p2482742	Bn-chrA02-p891094
Bn-A02-p3398710	0.975206612	0.966942149	Bn-N2-p2482946	Bn-chrA02-p891298
Bn-A02-p3422005	0.990033223	0.993355482	Bn-N2-p2516226	Bn-chrA02-p911462
Bn-A02-p3423823	0.966942149	0.966942149	Bn-N2-p2518081	Bn-chrA02-p913182
Bn-A02-p3427640	0.975124378	0.980099502	Bn-N2-p2524901	Bn-chrA02-p917066
Bn-A02-p3430069	0.975206612	0.809917355	Bn-N2-p2527335	Bn-chrC02-p2727155
Bn-A02-p3446017	0.933884298	0.966942149	Bn-N2-p2538053	Bn-chrA02-p931441
Bn-A02-p3458391	0.953488372	0.953488372	Bn-N11-p2235217	Bn-chrC01-p897626

Bn-A02-p3478450	1	1	Bn-N12-p2919447	Bn-chrC02-p2776957
Bn-A02-p3488996	0.983471074	0.925619835	Bn-N2-p2566292	Bn-chrUnn_random-p52248[illegible]
Bn-A02-p3489974	0.975206612	0.94214876	Bn-N2-p2567267	Bn-chrAnn_random-p31175[illegible]
Bn-A02-p3493190	0.980099502	0.865671642	Bn-N2-p2570456	Bn-chrC02-p2790280
Bn-A02-p3503647	1	0.991735537	Bn-N2-p2580923	Bn-chrAnn_random-p30285[illegible]
Bn-A02-p3507057	0.990049751	0.990049751	Bn-N12-p2959247	Bn-chrC02-p2837509
Bn-A02-p3530703	0.909090909	0.925619835	Bn-N2-p2605586	Bn-chrA09-p29317758
Bn-A02-p3534963	0.983471074	0.892561983	Bn-N2-p2609796	Bn-chrC02-p2918179
Bn-A02-p3535142	0.975206612	0.966942149	Bn-N2-p2609975	Bn-chrA02-p970285
Bn-A02-p3539297	0.991735537	0.983471074	Bn-N2-p2614197	Bn-chrA02-p974316
Bn-A02-p3539377	0.975206612	0.983471074	Bn-N2-p2614277	Bn-chrA02-p974396
Bn-A02-p3542024	0.983471074	0.991735537	Bn-N2-p2616924	Bn-chrA02-p977032
Bn-A02-p3543810	0.983471074	0.991735537	Bn-N2-p2618710	Bn-chrA02-p978818
Bn-A02-p3558502	1	0.991735537	Bn-N2-p2633436	Bn-chrA02-p993808
Bn-A02-p3571230	0.991735537	0.983471074	Bn-N2-p2646280	Bn-chrA02-p1007316
Bn-A02-p3597721	0.958677686	0.975206612	Bn-N2-p2670223	Bn-chrA02-p1032705
Bn-A02-p3606635	0.983471074	0.991735537	Bn-N2-p2679040	Bn-chrA02-p1041673
Bn-A02-p3607564	0.990033223	0.96013289	Bn-N2-p2679970	Bn-chrA02-p1042619
Bn-A02-p3614673	1	0.991735537	Bn-N2-p2685815	Bn-chrA02-p1048358
Bn-A02-p3627856	0.966942149	0.958677686	Bn-N2-p2699130	Bn-chrA02-p1063499
Bn-A02-p3633790	0.958677686	0.950413223	Bn-N2-p2708860	Bn-chrA02-p1069095
Bn-A02-p3635114	0.950413223	0.966942149	Bn-N2-p2712160	Bn-chrA02-p1070417
Bn-A02-p3640490	0.995024876	0.965174129	Bn-N2-p2717536	Bn-chrA02-p1075785
Bn-A02-p3656969	0.958677686	0.966942149	Bn-N2-p2735974	Bn-chrA02-p1092724
Bn-A02-p3658139	0.958677686	0.595041322	Bn-N2-p2737143	Bn-chrA02-p1093648
Bn-A02-p3679028	0.991735537	1	Bn-N2-p2757467	Bn-chrA02-p1118229
Bn-A02-p3708263	0.991735537	0.983471074	Bn-N2-p2779778	Bn-chrA02-p1134676
Bn-A02-p3711684	1	0.996677741	Bn-N2-p2781849	Bn-chrA02-p1136747
Bn-A02-p3716898	0.990033223	0.993355482	Bn-N2-p2787089	Bn-chrA02-p1141998
Bn-A02-p3717426	0.983471074	0.975206612	Bn-N2-p2787615	Bn-chrA02-p1142526
Bn-A02-p3718340	0.985074627	0.995024876	Bn-N2-p2788609	Bn-chrA02-p1143504
Bn-A02-p3728882	0.966942149	0.958677686	Bn-N2-p2799086	Bn-chrA02-p1154084
Bn-A02-p3730794	0.983388704	0.970099668	Bn-N2-p2801052	Bn-chrA02-p1156014
Bn-A02-p3735101	0.956810631	0.956810631	Bn-N2-p2805334	Bn-chrA02-p1160494
Bn-A02-p3742509	0.950413223	0.94214876	Bn-N2-p2812081	Bn-chrA02-p1167211
Bn-A02-p3759462	0.970149254	0.990049751	Bn-N2-p2826887	Bn-chrA02-p1182064
Bn-A02-p3760177	0.975206612	0.983471074	Bn-N2-p2827602	Bn-chrA02-p1182799
Bn-A02-p3760247	0.986710963	0.986710963	Bn-N2-p2827672	Bn-chrA02-p1182869
Bn-A02-p3774623	0.993355482	0.993355482	Bn-N2-p2842035	Bn-chrA02-p1197968
Bn-A02-p3781878	0.990033223	0.990033223	Bn-N2-p2849282	Bn-chrA02-p1205225
Bn-A02-p3792136	0.996677741	0.996677741	Bn-N2-p2859571	Bn-chrA02-p1215513
Bn-A02-p3796695	0.94214876	0.950413223	Bn-N2-p2864109	Bn-chrA02-p1220051
Bn-A02-p3801428	0.990049751	0.995024876	Bn-N2-p2866053	Bn-chrA02-p1221772
Bn-A02-p3803198	0.900826446	0.966942149	Bn-N2-p2867828	Bn-chrA02-p1223592
Bn-A02-p3803477	0.975206612	0.966942149	Bn-N2-p2868097	Bn-chrA02-p1223870
Bn-A02-p3807577	0.983471074	0.157024793	Bn-N2-p2875058	Unknown

Bn-A02-p3809970	0.990049751	0.990049751	Bn-N2-p2877458	Bn-chrA02-p1230201
Bn-A02-p3810028	0.990049751	0.990049751	Bn-N2-p2877516	Bn-chrA02-p1230259
Bn-A02-p3810078	0.990049751	0.990049751	Bn-N2-p2877566	Bn-chrA02-p1230309
Bn-A02-p3821814	0.970149254	0.970149254	Bn-N2-p2890915	Bn-chrA02-p1242248
Bn-A02-p3821944	0.983388704	0.983388704	Bn-N2-p2891045	Bn-chrA02-p1242378
Bn-A02-p3822985	0.980099502	0.980099502	Bn-N2-p2892084	Bn-chrA02-p1243417
Bn-A02-p3859399	0.983388704	0.983388704	Bn-N2-p2924938	Bn-chrA02-p1278949
Bn-A02-p3860483	1	1	Bn-N2-p2926014	Bn-chrA02-p1280025
Bn-A02-p3897213	0.970149254	0.970149254	Bn-N2-p2960517	Bn-chrA02-p1313856
Bn-A02-p3897546	0.996677741	0.996677741	Bn-N2-p2960857	Bn-chrA02-p1314196
Bn-A02-p3898766	1	1	Bn-N2-p2962079	Bn-chrA02-p1315418
Bn-A02-p3914269	0.970099668	0.970099668	Bn-N2-p2975056	Bn-chrA02-p1327742
Bn-A02-p3934729	0.916943522	0.916943522	Bn-Scaffold03677-p1	Bn-chrA02-p1340392
Bn-A02-p3950581	1	1	Bn-N2-p3016075	Bn-chrA02_random-p44198
Bn-A02-p3965286	0.993355482	0.993355482	Bn-N2-p3046915	Bn-chrA02-p1363002
Bn-A02-p3978921	0.993355482	0.993355482	Bn-N2-p3062303	Bn-chrA02-p1375957
Bn-A02-p4035808	1	0.991735537	Bn-N2-p3119297	Bn-chrA02-p1427784
Bn-A02-p4037028	0.991735537	1	Bn-N2-p3120499	Bn-chrA02-p1429004
Bn-A02-p4038224	0.975206612	0.983471074	Bn-N2-p3121692	Bn-chrA02-p1430200
Bn-A02-p4044933	0.94214876	0.950413223	Bn-N2-p3125736	Bn-chrA02-p1433197
Bn-A02-p4045217	0.958677686	0.966942149	Bn-N2-p3126020	Bn-chrA02-p1433481
Bn-A02-p4046982	1	0.991735537	Bn-N8-p18473520	Bn-chrA02-p1435178
Bn-A02-p4065755	0.976744186	0.953488372	Bn-N2-p3142609	Bn-chrC02-p3676696
Bn-A02-p4102727	0.915422886	0.995024876	Bn-N2-p3171759	Bn-chrA02-p1479936
Bn-A02-p4103096	0.960199005	0.965174129	Bn-N2-p3172123	Bn-chrA02-p1480305
Bn-A02-p4103158	0.980099502	0.975124378	Bn-N2-p3172185	Bn-chrA02-p1480367
Bn-A02-p4103543	0.991735537	0.94214876	Bn-N2-p3172570	Bn-chrC02-p3710072
Bn-A02-p4104054	0.96345515	0.96345515	Bn-N2-p3173081	Bn-chrA02-p1481263
Bn-A02-p4110181	0.993355482	0.933554817	Bn-N2-p3178749	Bn-chrA02-p1491621
Bn-A02-p4110757	0.933884298	0.94214876	Bn-N2-p3179343	Bn-chrA02-p1492196
Bn-A02-p4118946	0.953488372	0.996677741	Bn-N12-p3879435	Bn-chrA02-p1500794
Bn-A02-p4151292	0.990049751	0.990049751	Bn-N2-p3222603	Bn-chrA02-p1532562
Bn-A02-p4174594	0.96345515	0.983388704	Bn-N2-p3240276	Bn-chrA02-p1550057
Bn-A02-p4183553	0.966942149	0.958677686	Bn-N2-p3249801	Bn-chrA02-p1559029
Bn-A02-p4199792	0.976744186	0.96345515	Bn-N2-p3261807	Bn-chrA02-p1575499
Bn-A02-p4201122	0.67768595	0.991735537	Bn-N2-p3263201	Bn-chrA02-p1576900
Bn-A02-p4210188	0.809917355	0.818181818	Bn-N2-p3283326	Bn-chrA02-p1585369
Bn-A02-p4210810	0.975124378	0.975124378	Bn-N2-p3283944	Bn-chrA02-p1585986
Bn-A02-p4220181	0.958677686	0.958677686	Bn-N2-p3292631	Bn-chrA02-p1597354
Bn-A02-p4232093	0.913621262	1	Bn-N2-p3301771	Bn-chrA02-p1603644
Bn-A02-p4237047	0.935323383	0.990049751	Bn-N2-p3309912	Bn-chrA02-p1607529
Bn-A02-p4241100	0.996677741	0.996677741	Bn-N2-p3314254	Bn-chrA02-p1611886
Bn-A02-p4243717	0.993355482	0.993355482	Bn-N2-p3316876	Bn-chrA02-p1614508
Bn-A02-p4259381	0.900497512	0.995024876	Bn-N2-p3345856	Bn-chrAnn_random-p169163
Bn-A02-p4262796	0.933884298	0.94214876	Bn-N2-p3349263	Bn-chrAnn_random-p169197
Bn-A02-p4262907	0.983471074	0.991735537	Bn-N2-p3349374	Bn-chrAnn_random-p169198

Bn-A02-p4277649	0.983471074	0.983471074	Bn-N2-p3355557	Bn-chrAnn_random-p16926[illegible]
Bn-A02-p4290375	0.958677686	0.958677686	Bn-N2-p3371689	Bn-chrAnn_random-p169423
Bn-A02-p4291278	0.975206612	0.975206612	Bn-N2-p3372592	Bn-chrAnn_random-p169432
Bn-A02-p4295967	0.980066445	0.980066445	Bn-N2-p3377254	Bn-chrAnn_random-p169478
Bn-A02-p4298976	0.966942149	0.966942149	Bn-N2-p3380125	Bn-chrAnn_random-p169507
Bn-A02-p4299085	0.975206612	0.975206612	Bn-N2-p3380234	Bn-chrAnn_random-p169508
Bn-A02-p4299411	0.975206612	0.975206612	Bn-N2-p3380557	Bn-chrAnn_random-p169511
Bn-A02-p4306196	0.940298507	0.940298507	Bn-N2-p3387309	Bn-chrAnn_random-p16957[illegible]
Bn-A02-p4308819	0.917355372	0.917355372	Bn-N2-p3389909	Bn-chrAnn_random-p169598
Bn-A02-p4343365	0.985074627	0.985074627	Bn-N2-p3424828	Bn-chrC02-p4126873
Bn-A02-p4345046	0.950413223	0.950413223	Bn-N2-p3426496	Bn-chrA02-p1722398
Bn-A02-p4345098	0.900497512	0.900497512	Bn-N2-p3426548	Bn-chrA02-p1722450
Bn-A02-p4351893	0.985074627	0.985074627	Bn-N12-p4250451	Bn-chrC02-p4145053
Bn-A02-p4352396	0.966942149	0.966942149	Bn-N2-p3433468	Bn-chrA02-p1729368
Bn-A02-p4374253	0.983388704	0.983388704	Bn-N2-p3456000	Bn-chrC02-p4185335
Bn-A02-p4488937	0.940199336	0.940199336	Bn-N2-p3564240	Bn-chrA02_random-p92810
Bn-A02-p4567454	0.960199005	0.960199005	Bn-N2-p3648566	Bn-chrAnn_random-p90385[illegible]
Bn-A02-p4568023	0.950248756	0.950248756	Bn-N2-p3649128	Bn-chrAnn_random-p90391[illegible]
Bn-A02-p4611221	0.995024876	0.995024876	Bn-N12-p4665605	Bn-chrC02-p4596471
Bn-A02-p4681731	0.991735537	0.991735537	Bn-N2-p3759227	Bn-chrAnn_random-p914585
Bn-A02-p4698141	0.950413223	0.950413223	Bn-N2-p3778378	Bn-chrAnn_random-p916561
Bn-A02-p4704233	0.966942149	0.975206612	Bn-N2-p3784286	Bn-chrAnn_random-p917152
Bn-A02-p4712683	0.991735537	0.661157025	Bn-N2-p3790849	Bn-chrAnn_random-p917584
Bn-A02-p4716855	0.991735537	0.983471074	Bn-N2-p3794718	Bn-chrAnn_random-p917968
Bn-A02-p4721726	0.845771144	0.970149254	Bn-N12-p4865923	Bn-chrAnn_random-p91847[illegible]
Bn-A02-p4729892	0.991735537	0.975206612	Bn-N2-p3815886	Bn-chrC02-p4848898
Bn-A02-p4744168	0.995024876	0.995024876	Bn-N2-p3833121	Bn-chrC02-p6665299
Bn-A02-p4747805	0.965174129	0.940298507	Bn-N2-p3836082	Bn-chrA02-p1877005
Bn-A02-p4761483	0.990033223	0.993355482	Bn-N2-p3853545	Bn-chrA02-p1887996
Bn-A02-p4773307	0.955223881	0.960199005	Bn-N2-p3865572	Bn-chrA02-p1895670
Bn-A02-p4774095	0.990049751	0.980099502	Bn-N2-p3866360	Bn-chrA02-p1896455
Bn-A02-p4774099	0.990049751	0.980099502	Bn-N2-p3866363	Bn-chrA02-p1896458
Bn-A02-p4777053	0.975206612	0.900826446	Bn-N2-p3872871	Bn-chrC02-p4881273
Bn-A02-p4779271	0.933884298	0.94214876	Bn-N2-p3875092	Bn-chrA02-p1901661
Bn-A02-p4783596	0.990049751	0.990049751	Bn-N2-p3879029	Bn-chrA02-p1908595
Bn-A02-p4881323	0.985074627	0.985074627	Bn-N2-p3980652	Bn-chrA02-p2011936
Bn-A02-p4913845	0.995024876	0.995024876	Bn-N2-p21431451	Bn-chrA01-p1544767
Bn-A02-p4928982	0.990049751	0.990049751	Bn-N2-p4014476	Bn-chrA02-p2045538
Bn-A02-p5040691	0.975124378	0.985074627	Bn-N2-p4122354	Bn-chrA02-p2155909
Bn-A02-p5063790	0.985074627	0.975124378	Bn-N12-p5445372	Bn-chrCnn_random-p510708
Bn-A02-p5144074	0.993355482	0.993355482	Bn-N2-p4229173	Bn-chrA02-p2252722
Bn-A02-p5166772	1	1	Bn-N2-p4257512	Bn-chrA02-p2283134
Bn-A02-p5201568	0.960199005	0.960199005	Bn-N2-p4286646	Bn-chrA02-p2303232
Bn-A02-p5242706	0.995024876	0.990049751	Bn-Scaffold25363-p2	Bn-chrA02-p2334018
Bn-A02-p5271340	0.973421927	0.970099668	Bn-N12-p5884684	Bn-chrAnn_random-p318781
Bn-A02-p5304300	0.966942149	0.975206612	Bn-N2-p4438281	Bn-chrA02-p2424379

Bn-A02-p5308168	0.983471074	0.991735537	Bn-N2-p4442151	Bn-chrA02-p2428246
Bn-A02-p5308657	0.801652893	0.991735537	Bn-N2-p4442635	Bn-chrA02-p2428734
Bn-A02-p5325556	0.990049751	0.990049751	Bn-N2-p4460553	Bn-chrA02-p2445960
Bn-A02-p5326021	0.985074627	0.985074627	Bn-N2-p4461018	Bn-chrA02-p2446425
Bn-A02-p5326222	0.958677686	0.966942149	Bn-N2-p4461220	Bn-chrA02-p2446625
Bn-A02-p5327209	0.975124378	0.975124378	Bn-N2-p4462207	Bn-chrA02-p2447612
Bn-A02-p5360273	0.94214876	0.950413223	Bn-N2-p4487323	Bn-chrA02-p2471405
Bn-A02-p5394958	0.966942149	0.94214876	Bn-N12-p6060989	Bn-chrA02-p2505474
Bn-A02-p5429003	0.989583333	0.989583333	Bn-N2-p4567982	Bn-chrA02-p2541557
Bn-A02-p5436943	0.925619835	0.925619835	Bn-N12-p6121538	Bn-chrC02-p5176773
Bn-A02-p5439746	0.995024876	0.995024876	Bn-N2-p4582902	Bn-chrA02-p2555159
Bn-A02-p5442618	0.985074627	0.985074627	Bn-N2-p4585736	Bn-chrA02-p2557993
Bn-A02-p5485978	0.983471074	0.975206612	Bn-N2-p4642849	Bn-chrA02-p2601982
Bn-A02-p5500107	0.985074627	0.990049751	Bn-N2-p4652864	Bn-chrA02-p2619870
Bn-A02-p5501733	0.985074627	0.980099502	Bn-N2-p4654749	Bn-chrA02-p2621499
Bn-A02-p5507193	0.991735537	0.983471074	Bn-N2-p4660762	Bn-chrA02-p2627083
Bn-A02-p5507530	0.991735537	0.983471074	Bn-N2-p4661101	Bn-chrA02-p2627420
Bn-A02-p5516551	0.966942149	0.975206612	Bn-N2-p4668207	Bn-chrA02-p2642352
Bn-A02-p5517878	0.727272727	1	Bn-N12-p6289690	Bn-chrA02-p2643679
Bn-A02-p5519388	0.727272727	0.983471074	Bn-N2-p4674659	Bn-chrA02-p2645187
Bn-A02-p5519417	0.966942149	0.983471074	Bn-N2-p4674689	Bn-chrA02-p2645216
Bn-A02-p5541204	0.966942149	0.983471074	Bn-N2-p4689717	Bn-chrA02-p2662343
Bn-A02-p5551332	0.975206612	0.991735537	Bn-N2-p4692584	Bn-chrA02-p2670493
Bn-A02-p5553077	0.975206612	0.966942149	Bn-N2-p4694318	Bn-chrA02-p2672239
Bn-A02-p5553663	0.975206612	0.991735537	Bn-N2-p4694906	Bn-chrA02-p2672825
Bn-A02-p5561369	0.958677686	0.950413223	Bn-N2-p4702104	Bn-chrA02-p2680531
Bn-A02-p5570144	0.996677741	0.950166113	Bn-N2-p4720861	Bn-chrA02-p2688485
Bn-A02-p5571981	0.975124378	0.975124378	Bn-N2-p4722669	Bn-chrA02-p2690319
Bn-A02-p5574727	0.950413223	0.958677686	Bn-N2-p4725469	Bn-chrA02-p2693065
Bn-A02-p5582861	0.983471074	0.975206612	Bn-N2-p4733516	Bn-chrA02-p2712565
Bn-A02-p5592678	0.980066445	0.980066445	Bn-N2-p4755097	Bn-chrA02-p2720504
Bn-A02-p5601991	0.990033223	0.986710963	Bn-N2-p4769587	Bn-chrA02-p2728256
Bn-A02-p5602271	0.985074627	0.980099502	Bn-N2-p4769867	Bn-chrA02-p2728536
Bn-A02-p5604926	0.454545455	0.776859504	Bn-N2-p4778603	Bn-chrA02-p2733365
Bn-A02-p5612147	1	1	Bn-N2-p4789072	Bn-chrA02-p2750379
Bn-A02-p5625172	0.975206612	0.966942149	Bn-N2-p4804229	Bn-chrA02-p2764942
Bn-A02-p5628094	0.995024876	0.990049751	Bn-N2-p4807150	Bn-chrA02-p2767864
Bn-A02-p5628473	0.980099502	0.980099502	Bn-N2-p4807529	Bn-chrA02-p2768243
Bn-A02-p5628535	0.985074627	0.985074627	Bn-N2-p4807591	Bn-chrA02-p2768305
Bn-A02-p5640408	1	0.996677741	Bn-Scaffold01856-p3	Bn-chrUnn_random-p263195
Bn-A02-p5644858	0.809917355	1	Bn-N15-p49079592	Bn-chrA02-p2783743
Bn-A02-p5651799	1	0.991735537	Bn-N2-p4828913	Bn-chrA02-p2791605
Bn-A02-p5660188	0.926910299	0.996677741	Bn-N2-p4837452	Bn-chrA02-p2800053
Bn-A02-p5661524	0.743801653	0.983471074	Bn-N2-p4838533	Bn-chrA02-p2801391
Bn-A02-p5664842	0.986710963	0.986710963	Bn-N2-p4841926	Bn-chrA02-p2804707
Bn-A02-p5666475	0.966942149	0.975206612	Bn-N2-p4845121	Bn-chrA02-p2806340

Bn-A02-p5694092	0.991735537	0.983471074	Bn-N2-p4880311	Bn-chrA02-p2835345
Bn-A02-p5705619	0.975206612	0.983471074	Bn-N2-p4894207	Bn-chrA03-p15368250
Bn-A02-p5716304	0.958677686	0.966942149	Bn-N2-p4905448	Bn-chrA02-p2858835
Bn-A02-p5722550	0.975206612	0.231404959	Bn-N2-p4911936	Bn-chrCnn_random-p104711
Bn-A02-p5731499	0.958677686	0.966942149	Bn-N2-p4924739	Bn-chrA02-p2878346
Bn-A02-p5743951	1	0.073089701	Bn-N2-p4938229	Bn-chrC09-p35770575
Bn-A02-p5765840	0.996677741	0.933554817	Bn-N2-p4953417	Bn-chrA02-p2912270
Bn-A02-p5772588	0.950413223	0.537190083	Bn-N2-p4954006	Bn-chrA02-p12858540
Bn-A02-p5772793	0.966942149	0.644628099	Bn-N2-p4954211	Bn-chrC09-p32583640
Bn-A02-p5773812	0.991735537	0.198347107	Bn-N2-p4956215	Bn-chrA03-p7942417
Bn-A02-p5774569	0.991735537	0.198347107	Bn-N2-p4956973	Bn-chrA07-p10762144
Bn-A02-p5797724	0.975206612	0.975206612	Bn-N2-p4977779	Bn-chrA02-p2968419
Bn-A02-p5799113	0.975206612	0.983471074	Bn-N2-p4979108	Bn-chrA02-p2969755
Bn-A02-p5802612	0.966942149	0.958677686	Bn-N2-p4983561	Bn-chrA02-p2974269
Bn-A02-p5802772	0.975206612	0.983471074	Bn-N2-p4983721	Bn-chrA02-p2974429
Bn-A02-p5803164	0.933884298	0.975206612	Bn-N2-p4984177	Bn-chrA02-p2974821
Bn-A02-p5803432	0.975206612	0.983471074	Bn-N2-p4984444	Bn-chrA02-p2975089
Bn-A02-p5807792	0.975124378	0.975124378	Bn-N2-p5248818	Bn-chrAnn_random-p259367
Bn-A02-p5812506	0.995024876	1	Bn-N2-p4989347	Bn-chrA02-p2983005
Bn-A02-p5814583	0.975124378	0.975124378	Bn-N2-p4991408	Bn-chrA02-p2988379
Bn-A02-p5826306	0.952631579	0.952631579	Bn-N2-p5006941	Bn-chrA02-p3002570
Bn-A02-p5826505	0.990049751	0.990049751	Bn-Scaffold16162-p5	Bn-chrAnn_random-p425354
Bn-A02-p5826541	0.990049751	0.990049751	Bn-Scaffold16162-p5	Bn-chrAnn_random-p425355
Bn-A02-p5832870	0.980099502	0.980099502	Bn-N2-p5019782	Bn-chrA02-p3015073
Bn-A02-p5859447	0.867768595	0.867768595	Bn-N2-p5035622	Bn-chrA02-p3030378
Bn-A02-p5866325	0.993355482	0.990033223	Bn-N2-p5045512	Bn-chrA02-p3041752
Bn-A02-p5867250	0.208333333	1	Bn-N9-p9105863	Bn-chrA05-p18234072
Bn-A02-p5882799	0.966942149	0.966942149	Bn-N2-p5065792	Bn-chrA02-p3051556
Bn-A02-p5907701	0.925619835	0.933884298	Bn-N2-p5101425	Bn-chrA02-p3096806
Bn-A02-p5917045	0.966942149	0.975206612	Bn-N2-p5113151	Bn-chrA02-p3104382
Bn-A02-p5923794	0.958677686	0.966942149	Bn-N2-p5119910	Bn-chrA02-p3111261
Bn-A02-p5923878	0.991735537	1	Bn-N2-p5119994	Bn-chrA02-p3111543
Bn-A02-p5924483	0.791855204	0.787330317	Bn-N2-p5120600	Bn-chrA02-p3112151
Bn-A02-p5925648	0.991735537	0.950413223	Bn-N2-p5121765	Bn-chrA02-p3113306
Bn-A02-p5925715	0.958677686	0.933884298	Bn-N2-p5121831	Bn-chrA02-p3113375
Bn-A02-p5925766	0.94214876	0.933884298	Bn-N2-p5121882	Bn-chrA02-p3113426
Bn-A02-p5933593	0.991735537	0.983471074	Bn-N2-p5129628	Bn-chrA02-p3129327
Bn-A02-p5938385	0.983388704	0.993355482	Bn-N2-p5134791	Bn-chrA02-p3134121
Bn-A02-p5958923	0.993355482	0.993355482	Bn-N2-p5155876	Bn-chrA02-p3153345
Bn-A02-p5985314	0.983471074	0.991735537	Bn-N2-p5179695	Bn-chrA02-p3197992
Bn-A02-p5985692	0.983471074	0.991735537	Bn-N2-p5180066	Bn-chrA02-p3198367
Bn-A02-p5991465	0.96345515	0.973421927	Bn-N2-p5185611	Bn-chrA02-p3207170
Bn-A02-p6039769	0.975206612	0.239669421	Bn-N2-p5241431	Unknown
Bn-A02-p6084757	0.991735537	0.983471074	Bn-N2-p5291239	Bn-chrA02-p3274541
Bn-A02-p6097441	0.990033223	0.993355482	Bn-N2-p5303980	Bn-chrA02-p3290349
Bn-A02-p6099790	0.955223881	0.980099502	Bn-N12-p7413031	Bn-chrA02-p3292585

Bn-A02-p6100412	0.870431894	0.906976744	Bn-N12-p7413649	Bn-chrA02-p3293207
Bn-A02-p6105931	0.817275748	0.893687708	Bn-N2-p5337505	Bn-chrC02-p12419558
Bn-A02-p6147730	1	0.991735537	Bn-N2-p5355448	Bn-chrA02-p3316152
Bn-A02-p6147823	0.966942149	0.975206612	Bn-N2-p5355541	Bn-chrA02-p3316245
Bn-A02-p6149358	0.990049751	0.995024876	Bn-N2-p5357074	Bn-chrA02-p3317755
Bn-A02-p6156818	0.983471074	0.991735537	Bn-N2-p5359276	Bn-chrA02-p3319913
Bn-A02-p6164030	0.94214876	0.991735537	Bn-N12-p7463514	Bn-chrA02-p3322588
Bn-A02-p6166043	0.975206612	0.966942149	Bn-N2-p5363962	Bn-chrA02-p3330201
Bn-A02-p6166584	0.876033058	0.958677686	Bn-N12-p7467056	Bn-chrA02-p3330739
Bn-A02-p6167491	0.991735537	0.983471074	Bn-N2-p5365660	Bn-chrA02-p3331734
Bn-A02-p6171263	0.980099502	0.975124378	Bn-N2-p5369382	Bn-chrA02-p3335711
Bn-A02-p6181965	0.94214876	0.826446281	Bn-N2-p5380752	Bn-chrA02-p3349206
Bn-A02-p6182038	0.975206612	0.842975207	Bn-N2-p5380825	Bn-chrA02-p3349272
Bn-A02-p6182486	0.933884298	0.966942149	Bn-N2-p5381267	Bn-chrA02-p3353852
Bn-A02-p6202881	0.966942149	0.958677686	Bn-N2-p5401114	Bn-chrA02-p3373200
Bn-A02-p6203059	0.975206612	0.983471074	Bn-N2-p5401292	Bn-chrA02-p3373378
Bn-A02-p6206533	0.950413223	0.950413223	Bn-N2-p5404859	Bn-chrA02-p3376403
Bn-A02-p6206785	0.966942149	0.975206612	Bn-N2-p5405111	Bn-chrA02-p3376658
Bn-A02-p6209563	0.983471074	0.991735537	Bn-N2-p5408093	Bn-chrA02-p3378393
Bn-A02-p6237474	0.863787375	0.926910299	Bn-N2-p5444958	Bn-chrA02-p3407873
Bn-A02-p6244013	0.933884298	0.975206612	Bn-N2-p5458662	Bn-chrA02-p3419544
Bn-A02-p6252196	0.986710963	0.993355482	Bn-N2-p5465834	Bn-chrA02-p3425100
Bn-A02-p6254332	0.975206612	0.983471074	Bn-N2-p5472544	Bn-chrA02-p3427331
Bn-A02-p6256499	0.991735537	1	Bn-N2-p5474711	Bn-chrA02-p3431650
Bn-A02-p6265274	0.983471074	0.975206612	Bn-N2-p5483616	Bn-chrA02-p3438540
Bn-A02-p6267188	0.975206612	0.983471074	Bn-N2-p5485530	Bn-chrA02-p3440483
Bn-A02-p6267300	0.966942149	0.975206612	Bn-N2-p5485642	Bn-chrA02-p3440595
Bn-A02-p6274305	0.975206612	0.991735537	Bn-N2-p5492381	Bn-chrA02-p3447605
Bn-A02-p6277812	0.950413223	0.94214876	Bn-N2-p5496117	Bn-chrA02-p3451111
Bn-A02-p6280517	0.983388704	0.976744186	Bn-N2-p5498804	Bn-chrA02-p3453816
Bn-A02-p6285260	0.94214876	0.867768595	Bn-N12-p7715437	Bn-chrC02-p5844612
Bn-A02-p6300284	0.966942149	0.983471074	Bn-N2-p5520354	Bn-chrA02-p3473319
Bn-A02-p6310054	0.975206612	0.983471074	Bn-N2-p5524704	Bn-chrA02-p3477663
Bn-A02-p6315194	0.965174129	0.990049751	Bn-N2-p5530606	Bn-chrA02-p3483765
Bn-A02-p6315978	0.985074627	0.980099502	Bn-N2-p5531394	Bn-chrA02-p3484553
Bn-A02-p6360867	0.971830986	0.985915493	Bn-N2-p5570408	Bn-chrA02-p3534368
Bn-A02-p6366262	0.975206612	0.966942149	Bn-N2-p5578109	Bn-chrA02-p3541031
Bn-A02-p6373647	0.867109635	0.867109635	Bn-N2-p5592862	Bn-chrA02-p3548476
Bn-A02-p6375486	0.996677741	0.993355482	Bn-N2-p5598295	Bn-chrA02-p3554718
Bn-A02-p6384663	0.995024876	0.995024876	Bn-N2-p5610536	Bn-chrA02-p3572646
Bn-A02-p6390043	0.966942149	0.950413223	Bn-N2-p5617114	Bn-chrA02-p3578109
Bn-A02-p6391751	0.884297521	0.958677686	Bn-N2-p5618792	Bn-chrA02-p3579819
Bn-A02-p6392450	0.895522388	0.975124378	Bn-N2-p5619470	Bn-chrA02-p3580506
Bn-A02-p6399820	0.975124378	0.970149254	Bn-N2-p5627536	Bn-chrA02-p3593395
Bn-A02-p6439295	0.809917355	0.884297521	Bn-N2-p5672197	Bn-chrA02-p3636636
Bn-A02-p6439449	0.818181818	0.966942149	Bn-N2-p5672451	Bn-chrA02-p3636790

Bn-A02-p6440070	0.776859504	0.975206612	Bn-N2-p5673046	Bn-chrA02-p3637373
Bn-A02-p6441318	0.247933884	0.966942149	Bn-N7-p12126946	Bn-chrA02-p3638622
Bn-A02-p6461773	0.980099502	0.970149254	Bn-N2-p5695102	Bn-chrA02-p3675731
Bn-A02-p6473898	0.985074627	0.990049751	Bn-N2-p5707926	Bn-chrA02-p3687860
Bn-A02-p6474102	0.991735537	0.983471074	Bn-N2-p5708131	Bn-chrA02-p3688064
Bn-A02-p6476748	0.950248756	0.975124378	Bn-N12-p8133121	Bn-chrA02-p3690613
Bn-A02-p6478309	0.966942149	0.975206612	Bn-N2-p5711970	Bn-chrA02-p3692134
Bn-A02-p6483805	0.973421927	0.980066445	Bn-N2-p5717447	Bn-chrA02-p3699509
Bn-A02-p6500009	0.826446281	0.900826446	Bn-N2-p5742328	Bn-chrC02-p6343398
Bn-A02-p6523411	0.950413223	0.892561983	Bn-N2-p5774235	Bn-chrC02-p6359311
Bn-A02-p6523609	0.993355482	0.893687708	Bn-N2-p5774433	Bn-chrA02-p3735798
Bn-A02-p6555729	0.950413223	0.950413223	Bn-N2-p5808783	Bn-chrA02-p3775676
Bn-A02-p6564856	0.990049751	0.915422886	Bn-N2-p5818153	Bn-chrA02-p3784976
Bn-A02-p6564861	0.990049751	0.925373134	Bn-N2-p5818158	Bn-chrA02-p3784981
Bn-A02-p6570881	0.996677741	1	Bn-N2-p5824926	Bn-chrA02-p3792402
Bn-A02-p6576389	0.996677741	0.996677741	Bn-N2-p5830462	Bn-chrA02-p3797869
Bn-A02-p6576872	0.990033223	0.993355482	Bn-N2-p5830945	Bn-chrA02-p3798352
Bn-A02-p6577065	0.983471074	0.991735537	Bn-N2-p5831138	Bn-chrA02-p3798545
Bn-A02-p6578877	0.991735537	0.991735537	Bn-N2-p5832950	Bn-chrA02-p3800357
Bn-A02-p6584506	0.996677741	0.996677741	Bn-N2-p5838571	Bn-chrA02-p3805972
Bn-A02-p6587201	0.925619835	0.925619835	Bn-N12-p8313110	Bn-chrC02-p6442876
Bn-A02-p6615821	0.925619835	0.925619835	Bn-N2-p5870990	Bn-chrCnn_random-p781334
Bn-A02-p6627382	0.887043189	0.843853821	Bn-N2-p5881656	Bn-chrC02-p6550071
Bn-A02-p6653554	0.983388704	0.986710963	Bn-N2-p5914507	Bn-chrA02_random-p110929
Bn-A02-p6658392	0.950413223	0.94214876	Bn-N2-p5919336	Bn-chrA02_random-p115494
Bn-A02-p6689427	0.991735537	0.975206612	Bn-N2-p5945858	Bn-chrA02_random-p141243
Bn-A02-p6692347	0.983471074	0.975206612	Bn-N2-p5948780	Bn-chrA02_random-p144173
Bn-A02-p6697045	1	0.991735537	Bn-N2-p5953460	Bn-chrA02_random-p148852
Bn-A02-p6711579	0.900497512	0.975124378	Bn-N2-p5970390	Bn-chrCnn_random-p539797
Bn-A02-p6711900	0.981927711	0.987951807	Bn-N2-p5970711	Bn-chrCnn_random-p539794
Bn-A02-p6751482	0.950413223	0.950413223	Bn-N2-p6001345	Bn-chrA02_random-p188202
Bn-A02-p6751605	0.950413223	0.950413223	Bn-N2-p6001467	Bn-chrA02_random-p188324
Bn-A02-p6764001	1	1	Bn-N2-p6016991	Bn-chrA02_random-p201378
Bn-A02-p6766615	0.993355482	0.993355482	Bn-N2-p6019793	Bn-chrA02_random-p204182
Bn-A02-p6770000	0.990049751	0.990049751	Bn-N2-p6023835	Bn-chrAnn_random-p447967
Bn-A02-p6794013	0.966942149	0.966942149	Bn-N2-p6042570	Bn-chrA02-p3828515
Bn-A02-p6816192	0.966942149	0.966942149	Bn-N2-p6068556	Bn-chrA02-p3859491
Bn-A02-p6816262	0.966942149	0.966942149	Bn-N2-p6068623	Bn-chrA02-p3859558
Bn-A02-p6820464	0.884297521	0.884297521	Bn-N2-p6070759	Bn-chrA02-p3861694
Bn-A02-p6839427	1	0.991735537	Bn-N2-p6093808	Bn-chrA02-p3881379
Bn-A02-p6843407	0.983471074	0.991735537	Bn-N2-p6097892	Bn-chrA02-p3885362
Bn-A02-p6844873	0.991735537	0.983471074	Bn-N2-p6099358	Bn-chrA02-p3886828
Bn-A02-p6844903	0.983471074	0.991735537	Bn-N2-p6099388	Bn-chrA02-p3886858
Bn-A02-p6845049	0.991735537	0.983471074	Bn-N2-p6099534	Bn-chrA02-p3887004
Bn-A02-p6845122	0.983471074	0.991735537	Bn-N2-p6099607	Bn-chrA02-p3887077
Bn-A02-p6845953	0.993355482	0.993355482	Bn-N2-p6100438	Bn-chrA02-p3887908

Bn-A02-p6845958	0.990033223	0.993355482	Bn-N2-p6100443	Bn-chrA02-p3887913
Bn-A02-p6852756	1	0.983471074	Bn-N2-p6108134	Bn-chrA02-p3894723
Bn-A02-p6859259	0.980066445	0.983388704	Bn-N2-p6114654	Bn-chrA02-p3901227
Bn-A02-p6862619	0.843853821	0.993355482	Bn-N2-p6118405	Bn-chrA02-p3904725
Bn-A02-p6868939	0.966942149	0.958677686	Bn-N2-p6123708	Bn-chrA02-p3910050
Bn-A02-p6871477	0.989583333	0.989583333	Bn-N2-p6126225	Bn-chrA02-p3912790
Bn-A02-p6898425	0.897009967	0.897009967	Bn-N2-p6160240	Bn-chrA02-p3953098
Bn-A02-p6901814	0.991735537	0.991735537	Bn-Scaffold01277-p1	Bn-chrCnn_random-p949657
Bn-A02-p6917044	0.991735537	0.983471074	Bn-Scaffold10158-p2	Bn-chrA02-p3967273
Bn-A02-p6930285	0.983471074	0.975206612	Bn-N2-p6205759	Bn-chrA02_random-p240848
Bn-A02-p6995796	0.991735537	0.983471074	Bn-N2-p6267087	Bn-chrA02_random-p246689
Bn-A02-p7004091	0.991735537	0.991735537	Bn-N2-p6282362	Bn-chrA02-p4037640
Bn-A02-p7012860	0.991735537	0.991735537	Bn-N2-p6291143	Bn-chrA02-p4047981
Bn-A02-p7014257	0.910299003	0.980066445	Bn-N12-p8758997	Bn-chrA02_random-p248046
Bn-A02-p7029950	0.933884298	0.933884298	Bn-N2-p6305274	Bn-chrA02-p4062818
Bn-A02-p7041297	0.975124378	0.980099502	Bn-N2-p6314803	Bn-chrA02-p4077631
Bn-A02-p7043311	0.991735537	0.991735537	Bn-N2-p6316820	Bn-chrA02-p4079677
Bn-A02-p7043901	0.995024876	1	Bn-N2-p6317407	Bn-chrA02-p4080264
Bn-A02-p7056194	0.966942149	0.966942149	Bn-N2-p6326082	Bn-chrA02-p4094997
Bn-A02-p7057571	0.966942149	0.966942149	Bn-N2-p6327490	Bn-chrC02-p7382758
Bn-A02-p7067463	0.966942149	0.983471074	Bn-N2-p6336359	Bn-chrC02-p7403266
Bn-A02-p7104549	0.975206612	0.975206612	Bn-N2-p6375564	Bn-chrA02-p4149400
Bn-A02-p7105435	0.936877076	0.936877076	Bn-N2-p6376451	Bn-chrA02-p4150287
Bn-A02-p7138093	1	0.991735537	Bn-N2-p6416994	Bn-chrA02-p4183535
Bn-A02-p7138191	0.975206612	0.983471074	Bn-N2-p6417092	Bn-chrA02-p4183633
Bn-A02-p7139447	0.983471074	0.975206612	Bn-N2-p6418320	Bn-chrA02-p4184887
Bn-A02-p7147282	0.995024876	0.995024876	Bn-N2-p6425973	Bn-chrA02-p4192779
Bn-A02-p7183200	0.975124378	0.975124378	Bn-N2-p6456448	Bn-chrA02-p4222064
Bn-A02-p7207945	0.983471074	0.991735537	Bn-N2-p6480050	Bn-chrA02-p4247668
Bn-A02-p7236427	0.955223881	0.880597015	Bn-N2-p6511735	Bn-chrC02-p7761222
Bn-A02-p7242957	0.975206612	0.983471074	Bn-N2-p6519240	Bn-chrA02-p4292571
Bn-A02-p7243236	0.983471074	0.975206612	Bn-N2-p6519497	Bn-chrA02-p4292850
Bn-A02-p7245587	0.983388704	0.990033223	Bn-N2-p6521847	Bn-chrA02-p4297329
Bn-A02-p7245763	0.991735537	0.958677686	Bn-N2-p6522023	Bn-chrA02-p4297505
Bn-A02-p7250607	0.975206612	0.983471074	Bn-N2-p6526880	Bn-chrA02-p4302386
Bn-A02-p7260577	0.975124378	0.985074627	Bn-N2-p6537031	Bn-chrA02-p4316388
Bn-A02-p7260946	1	0.991735537	Bn-N2-p6537400	Bn-chrA02-p4316762
Bn-A02-p7264855	0.933884298	0.900826446	Bn-Scaffold50752-p2	Bn-chrC02-p7812184
Bn-A02-p7278213	0.975206612	0.925619835	Bn-N2-p6552902	Bn-chrC02-p7822078
Bn-A02-p7278330	0.975206612	0.966942149	Bn-N2-p6553019	Bn-chrA02-p4328669
Bn-A02-p7279011	0.975206612	0.950413223	Bn-N2-p6553700	Bn-chrA02-p4329386
Bn-A02-p7281627	0.975206612	0.966942149	Bn-N2-p6556606	Bn-chrA02-p4330822
Bn-A02-p7282115	0.966942149	0.975206612	Bn-N2-p6557094	Bn-chrA02-p4331320
Bn-A02-p7297184	0.966942149	0.958677686	Bn-N2-p6572613	Bn-chrA02-p4351067
Bn-A02-p7306278	0.966942149	0.975206612	Bn-N2-p6581856	Bn-chrA02-p4358595
Bn-A02-p7319800	0.966942149	0.950413223	Bn-N2-p6595573	Bn-chrAnn_random-p233448

Bn-A02-p7324958	1	0.991735537	Bn-N2-p6600086	Bn-chrAnn_random-p233403
Bn-A02-p7349063	0.983471074	0.975206612	Bn-N2-p6624910	Bn-chrA02_random-p272619
Bn-A02-p7350064	0.983471074	0.975206612	Bn-N2-p6625937	Bn-chrA02_random-p271618
Bn-A02-p7350199	0.983471074	0.991735537	Bn-N2-p6626072	Bn-chrA02_random-p271483
Bn-A02-p7351109	0.983471074	0.991735537	Bn-N2-p6626975	Bn-chrA02_random-p270573
Bn-A02-p7351619	1	0.991735537	Bn-N2-p6627478	Bn-chrA02_random-p270063
Bn-A02-p7351746	0.94214876	1	Bn-N2-p6627657	Bn-chrA02_random-p269936
Bn-A02-p7361744	1	0.990049751	Bn-N2-p6641973	Bn-chrA02_random-p260018
Bn-A02-p7363071	0.917355372	0.950413223	Bn-N2-p6643287	Bn-chrA02_random-p258691
Bn-A02-p7368167	0.975206612	0.958677686	Bn-N2-p6648450	Bn-chrC02-p8104773
Bn-A02-p7371108	0.991735537	0.975206612	Bn-N2-p6651385	Bn-chrAnn_random-p299703
Bn-A02-p7396082	1	0.970099668	Bn-N2-p6678856	Bn-chrA02-p4418694
Bn-A02-p7396400	0.983471074	0.991735537	Bn-N2-p6679169	Bn-chrA02-p4418976
Bn-A02-p7409699	0.84079602	0.84079602	Bn-N12-p9399431	Bn-chrC02-p8168225
Bn-A02-p7439205	0.965174129	0.980099502	Bn-N12-p9495789	Bn-chrA02-p4460530
Bn-A02-p7453258	0.958677686	0.958677686	Bn-N2-p6746526	Bn-chrA02-p4483136
Bn-A02-p7463965	0.975206612	0.94214876	Bn-N2-p6762423	Bn-chrA02-p4497411
Bn-A02-p7464840	0.983471074	0.884297521	Bn-N2-p6763298	Bn-chrC02-p14834282
Bn-A02-p7464997	0.991735537	0.983471074	Bn-N2-p6763455	Bn-chrA02-p4498418
Bn-A02-p7475028	0.983471074	0.652892562	Bn-N2-p6773762	Bn-chrC02-p8338246
Bn-A02-p7476709	0.973421927	0.940199336	Bn-N2-p6775277	Bn-chrA02-p4509076
Bn-A02-p7482915	0.983471074	0.991735537	Bn-N2-p6777934	Bn-chrA02-p4511692
Bn-A02-p7483107	0.991735537	1	Bn-N2-p6778126	Bn-chrA02-p4511884
Bn-A02-p7483376	0.975206612	0.925619835	Bn-N2-p6778395	Bn-chrC02-p8414528
Bn-A02-p7498192	0.983471074	0.983471074	Bn-N2-p6796960	Bn-chrA02-p4523745
Bn-A02-p7510882	0.996677741	0.996677741	Bn-N2-p6805120	Bn-chrA02-p4531434
Bn-A02-p7514338	1	0.956810631	Bn-N2-p6808561	Bn-chrC02-p8459929
Bn-A02-p7516226	0.950248756	0.945273632	Bn-N2-p6810596	Bn-chrA02-p4536466
Bn-A02-p7516732	0.990049751	0.990049751	Bn-N2-p6811103	Bn-chrA02-p4536979
Bn-A02-p7520783	0.996677741	0.903654485	Bn-N2-p6815147	Bn-chrC02-p8479272
Bn-A02-p7525472	0.958677686	0.661157025	Bn-N2-p6819958	Bn-chrA02-p4546175
Bn-A02-p7531907	0.975124378	0.786069652	Bn-N2-p6825182	Bn-chrC02-p8502943
Bn-A02-p7534591	0.958677686	0.966942149	Bn-N2-p6827866	Bn-chrA02-p4563647
Bn-A02-p7575736	0.975206612	0.975206612	Bn-N2-p6865368	Bn-chrA02-p4597430
Bn-A02-p7589454	0.966942149	0.966942149	Bn-N2-p6879404	Bn-chrA02-p4611026
Bn-A02-p7595911	0.991735537	0.991735537	Bn-N2-p6885350	Bn-chrA02-p4616972
Bn-A02-p7597314	0.991735537	0.991735537	Bn-N2-p6886805	Bn-chrA02-p4618427
Bn-A02-p7647776	0.92358804	0.926910299	Bn-N2-p6934143	Bn-chrA02-p4663757
Bn-A02-p7675384	0.181818182	1	Unknown	Bn-chrA02-p4692035
Bn-A02-p7675505	0.743801653	0.966942149	Bn-N12-p9988211	Bn-chrA02-p4692156
Bn-A02-p7712926	1	1	Bn-N2-p7010305	Bn-chrA02-p4734140
Bn-A02-p7736604	0.181818182	0.966942149	Unknown	Bn-chrCnn_random-p415549
Bn-A02-p7738248	0.157024793	0.966942149	Bn-N3-p35398375	Bn-chrCnn_random-p415570
Bn-A02-p7750368	0.993355482	0.970099668	Bn-N2-p7040922	Bn-chrCnn_random-p415679
Bn-A02-p7753579	0.842975207	0.94214876	Bn-N2-p7044394	Bn-chrCnn_random-p415706
Bn-A02-p7756308	0.943521595	0.996677741	Bn-N17-p44448751	Bn-chrCnn_random-p415737

Bn-A02-p7775332	0.983471074	0.983471074	Bn-N2-p7074093	Bn-chrAnn_random-p296778
Bn-A02-p7784914	0.855721393	0.925373134	Bn-N12-p10169815	Bn-chrA02-p4806925
Bn-A02-p7786183	0.975206612	0.975206612	Bn-N2-p7085204	Bn-chrA02-p4808193
Bn-A02-p7788379	0.966942149	0.975206612	Bn-N2-p7091643	Bn-chrA02-p4810385
Bn-A02-p7788840	0.993355482	0.996677741	Bn-N2-p7092110	Bn-chrA02-p4810852
Bn-A02-p7801111	0.966942149	0.975206612	Bn-N2-p7104651	Bn-chrA02-p4824876
Bn-A02-p7804291	0.909090909	0.933884298	Bn-N2-p7107216	Bn-chrA02-p4827435
Bn-A02-p7812681	0.985074627	0.985074627	Bn-N2-p7115449	Bn-chrA02-p4834598
Bn-A02-p7831145	0.990049751	0.995024876	Bn-N2-p7134497	Bn-chrAnn_random-p240842
Bn-A02-p7840077	0.986710963	0.990033223	Bn-N2-p7145230	Bn-chrAnn_random-p240960
Bn-A02-p7848143	0.958677686	0.958677686	Bn-N2-p7156963	Bn-chrAnn_random-p390315
Bn-A02-p7853194	0.991735537	0.991735537	Bn-N2-p7161904	Bn-chrA02_random-p282574
Bn-A02-p7862412	0.966942149	0.958677686	Bn-N2-p7167173	Bn-chrC02_random-p432638
Bn-A02-p7878229	0.993355482	1	Bn-N2-p7182173	Bn-chrA02-p4863817
Bn-A02-p7882027	0.893687708	0.983388704	Bn-N2-p7188525	Bn-chrA02-p4867614
Bn-A02-p7885731	0.940199336	0.986710963	Bn-N12-p10418828	Bn-chrA02-p4871535
Bn-A02-p7886882	0.976744186	0.970099668	Bn-N2-p7192865	Bn-chrA02-p4872713
Bn-A02-p7892272	0.950413223	0.950413223	Bn-N2-p7198690	Bn-chrA02-p4878508
Bn-A02-p7892561	0.892561983	0.892561983	Bn-N2-p7198972	Bn-chrA02-p4878791
Bn-A02-p7893025	0.983471074	0.983471074	Bn-N2-p7199438	Bn-chrA02-p4879257
Bn-A02-p7893461	0.983471074	0.983471074	Bn-N2-p7199878	Bn-chrA02-p4879696
Bn-A02-p7893901	0.983471074	0.983471074	Bn-N2-p7200317	Bn-chrA02-p4880135
Bn-A02-p7894216	0.983471074	0.983471074	Bn-N2-p7200631	Bn-chrA02-p4880449
Bn-A02-p7894339	0.991735537	0.991735537	Bn-N2-p7200760	Bn-chrA02-p4880578
Bn-A02-p7896923	0.983471074	0.983471074	Bn-N2-p7203033	Bn-chrA02-p4882955
Bn-A02-p7904444	0.993355482	0.687707641	Bn-N2-p7208861	Bn-chrC02-p8956596
Bn-A02-p7926823	0.980099502	0.980099502	Bn-N2-p7222438	Bn-chrA02-p4907941
Bn-A02-p7935221	0.991735537	0.983471074	Bn-N2-p7230944	Bn-chrA02-p4916196
Bn-A02-p7947982	0.975206612	0.983471074	Bn-N2-p7243894	Bn-chrA02-p4929240
Bn-A02-p7952281	0.976744186	0.976744186	Bn-N2-p7248193	Bn-chrA02-p4933540
Bn-A02-p7956983	0.94214876	0.94214876	Bn-N2-p7253614	Bn-chrA02-p4938956
Bn-A02-p7958421	0.983471074	0.983471074	Bn-N2-p7255267	Bn-chrA02-p4940609
Bn-A02-p7970283	0.776859504	0.975206612	Bn-N2-p7269635	Bn-chrA02-p4952352
Bn-A02-p7975165	0.991735537	0.991735537	Bn-Scaffold39323-p2	Bn-chrA02-p4957081
Bn-A02-p7976278	0.975124378	0.970149254	Bn-N2-p7275411	Bn-chrA02-p4958194
Bn-A02-p7978230	0.975206612	0.975206612	Bn-N2-p7277351	Bn-chrA02-p4960146
Bn-A02-p8011131	0.983388704	0.973421927	Bn-N2-p7311587	Bn-chrA02-p4992812
Bn-A02-p8015745	0.272727273	1	Bn-N12-p10245742	Bn-chrA02-p18136475
Bn-A02-p8024157	0.991735537	0.991735537	Bn-N2-p7325379	Bn-chrA02-p5008644
Bn-A02-p8024784	0.983388704	0.983388704	Bn-N2-p7326007	Bn-chrA02-p5009273
Bn-A02-p8026465	0.790697674	0.790697674	Bn-N2-p7329308	Bn-chrA02-p5010952
Bn-A02-p8057234	0.986710963	0.980066445	Bn-N2-p7376125	Bn-chrA02-p5042256
Bn-A02-p8084362	0.993355482	0.993355482	Bn-N2-p7416013	Bn-chrAnn_random-p336735
Bn-A02-p8084605	0.996677741	0.996677741	Bn-N2-p7416256	Bn-chrAnn_random-p336732
Bn-A02-p8090196	0.966942149	0.966942149	Bn-N2-p7418858	Bn-chrAnn_random-p336706
Bn-A02-p8111245	0.991735537	0.991735537	Bn-N2-p7437059	Bn-chrA02-p5094911

Bn-A02-p8140997	0.993355482	0.993355482	Bn-N2-p7467376	Bn-chrA02-p5131911
Bn-A02-p8141909	0.996677741	0.996677741	Bn-N2-p7468288	Bn-chrA02-p5132823
Bn-A02-p8145676	1	1	Bn-N2-p7472055	Bn-chrA02-p5136802
Bn-A02-p8160522	0.993355482	0.953488372	Bn-N2-p7486923	Bn-chrC02_random-p574753
Bn-A02-p8169424	0.975206612	0.983471074	Bn-N2-p7495098	Bn-chrA02-p5160109
Bn-A02-p8169665	0.983471074	0.975206612	Bn-N2-p7494857	Bn-chrA02-p5159868
Bn-A02-p8176181	0.966942149	0.958677686	Bn-N2-p7501025	Bn-chrA02-p5167983
Bn-A02-p8177809	0.990033223	0.993355482	Bn-N2-p7502021	Bn-chrA02-p5168980
Bn-A02-p8179492	0.212624585	0.996677741	Bn-N5-p19811993	Bn-chrA02-p5170938
Bn-A02-p8190375	0.960199005	0.960199005	Bn-N2-p7510164	Bn-chrA02-p5179795
Bn-A02-p8191099	0.975206612	0.975206612	Bn-N2-p7510888	Bn-chrA02-p5180519
Bn-A02-p8199891	0.983471074	0.975206612	Bn-N2-p7521421	Bn-chrA02-p5191339
Bn-A02-p8200381	0.950413223	0.834710744	Bn-N2-p7521911	Bn-chrA02-p5191829
Bn-A02-p8204490	0.983471074	0.991735537	Bn-N2-p7526230	Bn-chrAnn_random-p354236
Bn-A02-p8208117	0.980099502	0.960199005	Bn-N2-p7529846	Bn-chrC04_random-p910583
Bn-A02-p8215285	0.983471074	0.983471074	Bn-N2-p7542048	Bn-chrA02-p5196239
Bn-A02-p8234333	1	0.991735537	Bn-N2-p7559860	Bn-chrA02-p5214369
Bn-A02-p8234485	0.958677686	0.966942149	Bn-N2-p7560012	Bn-chrA02-p5214521
Bn-A02-p8234978	0.975206612	0.983471074	Bn-N2-p7560389	Bn-chrA02-p5215016
Bn-A02-p8235110	1	0.991735537	Bn-N2-p7560521	Bn-chrA02-p5215148
Bn-A02-p8243789	0.991735537	1	Bn-N2-p7569475	Bn-chrA02-p5223207
Bn-A02-p8249838	1	1	Bn-N2-p7574965	Bn-chrA02-p5231714
Bn-A02-p8250856	0.991735537	0.991735537	Bn-N2-p7576288	Bn-chrA02-p5232732
Bn-A02-p8251258	0.975206612	0.975206612	Bn-N2-p7576690	Bn-chrA02-p5233135
Bn-A02-p8264334	0.958677686	0.958677686	Bn-N2-p7585150	Bn-chrA02-p5240895
Bn-A02-p8273655	0.975206612	0.975206612	Bn-N2-p7590109	Bn-chrA02-p5245628
Bn-A02-p8277939	0.966942149	0.966942149	Bn-N2-p7595149	Bn-chrA02-p5250664
Bn-A02-p8284992	0.955223881	0.860696517	Bn-N2-p7603576	Bn-chrA02-p5257697
Bn-A02-p8285151	0.96345515	0.940199336	Bn-N2-p7603734	Bn-chrA02-p5257855
Bn-A02-p8289331	0.983388704	0.983388704	Bn-N2-p7610024	Bn-chrA02-p5261875
Bn-A02-p8294934	0.993355482	0.993355482	Bn-N2-p7611745	Bn-chrA02-p5263594
Bn-A02-p8297807	0.975206612	0.966942149	Bn-N2-p7614375	Bn-chrA02-p5267724
Bn-A02-p8313782	0.991735537	0.983471074	Bn-N2-p7627929	Bn-chrA02-p5280749
Bn-A02-p8323616	0.975206612	0.958677686	Bn-N2-p7636096	Bn-chrA02-p5287814
Bn-A02-p8324359	0.94214876	0.950413223	Bn-N2-p7636811	Bn-chrA02-p5288530
Bn-A02-p8335312	0.867768595	0.867768595	Bn-N2-p7654564	Bn-chrA02-p5300202
Bn-A02-p8336294	0.966942149	0.966942149	Bn-N2-p7662364	Bn-chrA02-p5301184
Bn-A02-p8338189	0.958677686	0.966942149	Bn-N2-p7664260	Bn-chrA02-p5303090
Bn-A02-p8344540	1	1	Bn-N2-p7671572	Bn-chrA02-p5309350
Bn-A02-p8391383	0.975124378	0.975124378	Bn-N2-p7717859	Bn-chrA02-p5341710
Bn-A02-p8391574	0.990033223	0.990033223	Bn-N2-p7718050	Bn-chrA02-p5341901
Bn-A02-p8396864	0.818181818	0.818181818	Bn-N10-p6196801	Bn-chrA10-p4971773
Bn-A02-p8404663	0.996677741	0.990033223	Bn-N2-p7740881	Bn-chrA02-p5356635
Bn-A02-p8412911	0.876033058	0.876033058	Bn-N2-p7748607	Bn-chrA02-p5364633
Bn-A02-p8413531	0.933884298	0.925619835	Bn-N2-p7749409	Bn-chrA02-p5365262
Bn-A02-p8440451	0.980099502	0.985074627	Bn-N2-p7767730	Bn-chrA02-p5381248

Bn-A02-p8443355	0.966942149	0.975206612	Bn-N2-p7770939	Bn-chrA02-p5384170
Bn-A02-p8444558	0.966942149	0.958677686	Bn-N2-p7771866	Bn-chrA02-p5385059
Bn-A02-p8453414	1	0.991735537	Bn-N2-p7787059	Bn-chrA02-p5393916
Bn-A02-p8453695	0.834710744	0.983471074	Bn-N2-p7787295	Bn-chrA02-p5394197
Bn-A02-p8466214	1	0.958677686	Bn-N2-p7794161	Bn-chrA02-p5401230
Bn-A02-p8471639	0.966942149	0.975206612	Bn-N2-p7799584	Bn-chrA02-p5407129
Bn-A02-p8472166	0.996677741	0.986710963	Bn-N2-p7800112	Bn-chrA02-p5407656
Bn-A02-p8473119	0.983471074	0.975206612	Bn-N2-p7801066	Bn-chrA02-p5408612
Bn-A02-p8481759	0.975206612	0.983471074	Bn-N2-p7810711	Bn-chrA02-p5418060
Bn-A02-p8501267	0.94214876	0.933884298	Bn-N2-p7866337	Bn-chrCnn_random-p354436
Bn-A02-p8509407	0.983471074	0.991735537	Bn-N2-p7872796	Bn-chrCnn_random-p354329
Bn-A02-p8509694	0.940199336	0.983388704	Bn-N2-p7873083	Bn-chrCnn_random-p354326
Bn-A02-p8513186	0.991735537	0.991735537	Bn-N2-p7876770	Bn-chrCnn_random-p354291
Bn-A02-p8517135	0.73553719	0.975206612	Bn-N2-p7880377	Bn-chrCnn_random-p354222
Bn-A02-p8527757	0.960199005	0.965174129	Bn-N2-p7894091	Bn-chrA02-p5495652
Bn-A02-p8530142	0.975206612	0.975206612	Bn-N2-p7896474	Bn-chrA02-p5498038
Bn-A02-p8532901	0.940298507	0.985074627	Bn-N2-p7899040	Bn-chrA02-p5501092
Bn-A02-p8538437	0.983471074	0.983471074	Bn-N2-p7904695	Bn-chrA02-p5506755
Bn-A02-p8541841	0.993355482	0.993355482	Bn-N2-p7909830	Bn-chrA02-p5510161
Bn-A02-p8549373	0.958677686	0.975206612	Bn-N2-p7921645	Bn-chrA02-p5516591
Bn-A02-p8549867	0.933884298	0.975206612	Bn-N12-p11602434	Bn-chrA02-p5517109
Bn-A02-p8554870	0.966942149	0.958677686	Bn-N2-p7927221	Bn-chrA02-p5523058
Bn-A02-p8559435	0.991735537	0.991735537	Bn-N2-p7937566	Bn-chrA02-p5533667
Bn-A02-p8560119	0.983471074	0.983471074	Bn-N2-p7936884	Bn-chrA02-p5532985
Bn-A02-p8561027	0.983471074	0.983471074	Bn-N2-p7935979	Bn-chrA02-p5532080
Bn-A02-p8561181	0.925619835	0.925619835	Bn-N2-p7935825	Bn-chrA02-p5531926
Bn-A02-p8567245	0.993355482	0.993355482	Bn-N2-p7932553	Bn-chrA02-p5528652
Bn-A02-p8570729	0.991735537	0.991735537	Bn-N2-p7938977	Bn-chrA02-p5535078
Bn-A02-p8571485	0.975206612	0.975206612	Bn-N2-p7939740	Bn-chrA02-p5537223
Bn-A02-p8571577	0.983471074	0.983471074	Bn-N2-p7939832	Bn-chrA02-p5537315
Bn-A02-p8575325	0.991735537	0.991735537	Bn-N2-p7943615	Bn-chrA02-p5541098
Bn-A02-p8621264	0.980099502	0.980099502	Bn-N2-p7979682	Bn-chrA02-p5571363
Bn-A02-p8624200	0.985074627	0.995024876	Bn-N2-p7982625	Bn-chrA02-p5574301
Bn-A02-p8625851	0.983388704	0.990033223	Bn-N2-p7984224	Bn-chrA02-p5575897
Bn-A02-p8633119	0.950413223	0.950413223	Bn-N2-p7991268	Bn-chrA02-p5583197
Bn-A02-p8634642	0.975206612	0.975206612	Bn-N2-p7992802	Bn-chrA02-p5584729
Bn-A02-p8637915	0.975206612	0.975206612	Bn-N2-p7995626	Bn-chrA02-p5587554
Bn-A02-p8660234	0.983471074	0.975206612	Bn-N2-p8016026	Bn-chrA02-p5621879
Bn-A02-p8660285	0.983471074	0.991735537	Bn-N2-p8016077	Bn-chrA02-p5621937
Bn-A02-p8660632	0.983471074	0.983471074	Bn-N2-p8016424	Bn-chrA02-p5622288
Bn-A02-p8663297	0.966777409	0.96345515	Bn-N2-p8017864	Bn-chrA02-p5615113
Bn-A02-p8667676	0.986710963	0.966777409	Bn-N2-p8025321	Bn-chrA02-p5636621
Bn-A02-p8676211	0.986710963	0.986710963	Bn-N2-p8031461	Bn-chrA02-p5642761
Bn-A02-p8678600	0.975206612	0.975206612	Bn-N2-p8033016	Bn-chrA02-p5644316
Bn-A02-p8682290	0.966942149	0.966942149	Bn-N2-p8036705	Bn-chrA02-p5648007
Bn-A02-p8688298	0.975206612	0.975206612	Bn-Scaffold37590-p7	Bn-chrC02-p10633187

Bn-A02-p8702373	0.991735537	0.991735537	Bn-N2-p8052935	Bn-chrA02-p5669525
Bn-A02-p8703446	0.996677741	0.996677741	Bn-N2-p8055992	Bn-chrA02-p5672321
Bn-A02-p8711609	0.990033223	0.990033223	Bn-N2-p8069422	Bn-chrA02-p5680468
Bn-A02-p8720739	1	1	Bn-N2-p8075766	Bn-chrA02-p5686813
Bn-A02-p8724443	0.993355482	0.993355482	Bn-N2-p8079259	Bn-chrA02-p5690306
Bn-A02-p8726802	0.986710963	0.986710963	Bn-N2-p8081617	Bn-chrA02-p5694684
Bn-A02-p8732961	0.966942149	0.966942149	Bn-N2-p8087358	Bn-chrA02-p5700431
Bn-A02-p8735305	0.993355482	0.990033223	Bn-N2-p8089962	Bn-chrA02-p5702791
Bn-A02-p8747010	0.983471074	0.975206612	Bn-N2-p8103847	Bn-chrA02-p5715008
Bn-A02-p8754935	0.983471074	0.966942149	Bn-N2-p8116569	Bn-chrC02-p10840369
Bn-A02-p8767145	0.983471074	0.983471074	Bn-N2-p8129832	Bn-chrA02-p5739739
Bn-A02-p8777846	0.933884298	0.933884298	Bn-N2-p8138197	Bn-chrA02-p5746571
Bn-A02-p8799151	0.983471074	0.991735537	Bn-N2-p8166488	Bn-chrA02-p5770044
Bn-A02-p8802671	0.975206612	0.983471074	Bn-N2-p8170001	Bn-chrA02-p5773559
Bn-A02-p8804225	0.975206612	0.975206612	Bn-N2-p8171538	Bn-chrA02-p5776089
Bn-A02-p8827347	0.958677686	0.966942149	Bn-N2-p8174872	Bn-chrA02-p5781162
Bn-A02-p8833760	0.991735537	0.983471074	Bn-N2-p8182245	Bn-chrA02-p5787580
Bn-A02-p8834915	0.986710963	0.983388704	Bn-N2-p8183400	Bn-chrA02-p5788735
Bn-A02-p8836098	0.991735537	1	Bn-N2-p8184583	Bn-chrA02-p5789918
Bn-A02-p8849037	0.966942149	0.966942149	Bn-N2-p8196394	Bn-chrA02-p5806668
Bn-A02-p8865764	0.991735537	0.983471074	Bn-N2-p8215157	Bn-chrA02-p5830093
Bn-A02-p8865968	0.975206612	0.983471074	Bn-N2-p8215361	Bn-chrA02-p5830299
Bn-A02-p8867093	1	0.991735537	Bn-N2-p8216486	Bn-chrA02-p5831486
Bn-A02-p8867507	0.983471074	0.991735537	Bn-N2-p8216900	Bn-chrA02-p5831899
Bn-A02-p8867662	0.983471074	0.975206612	Bn-N2-p8217055	Bn-chrA02-p5832054
Bn-A02-p8872271	0.983471074	0.958677686	Bn-N2-p8221887	Bn-chrA02-p5834694
Bn-A02-p8873630	1	0.990049751	Bn-N2-p8223245	Bn-chrA02-p5836050
Bn-A02-p8873864	0.986710963	0.986710963	Bn-N2-p8223479	Bn-chrA02-p5836308
Bn-A02-p8873993	0.991735537	0.983471074	Bn-N2-p8223608	Bn-chrA02-p5836438
Bn-A02-p8874016	0.983471074	0.991735537	Bn-N2-p8223631	Bn-chrA02-p5836461
Bn-A02-p8874086	1	0.991735537	Bn-N2-p8223701	Bn-chrA02-p5836531
Bn-A02-p8874299	0.991735537	0.983471074	Bn-N2-p8223914	Bn-chrA02-p5836756
Bn-A02-p8874380	0.966942149	0.975206612	Bn-N2-p8223995	Bn-chrA02-p5836843
Bn-A02-p8876332	0.990033223	0.986710963	Bn-N2-p8225950	Bn-chrA02-p5838535
Bn-A02-p8877059	0.983471074	0.950413223	Bn-N2-p8226678	Bn-chrA02-p5839256
Bn-A02-p8877587	0.975206612	0.966942149	Bn-N2-p8227206	Bn-chrA02-p5839795
Bn-A02-p8877788	0.966942149	0.958677686	Bn-N2-p8227406	Bn-chrA02-p5839998
Bn-A02-p8878326	1	0.776859504	Bn-N2-p8227944	Bn-chrA02-p5840540
Bn-A02-p8880230	0.991735537	0.983471074	Bn-N2-p8229849	Bn-chrA02-p5842446
Bn-A02-p8880464	0.991735537	0.975206612	Bn-N2-p8230083	Bn-chrA02-p5842680
Bn-A02-p8881070	0.975206612	0.983471074	Bn-N2-p8230689	Bn-chrA02-p5843295
Bn-A02-p8881251	0.983471074	0.983471074	Bn-N2-p8230870	Bn-chrA02-p5843473
Bn-A02-p8883792	0.975206612	0.983471074	Bn-N2-p8231867	Bn-chrA02-p5844467
Bn-A02-p8884242	1	0.991735537	Bn-N2-p8232317	Bn-chrA02-p5844925
Bn-A02-p8884712	0.973421927	0.990033223	Bn-N2-p8232787	Bn-chrA02-p5845397
Bn-A02-p8885004	0.958677686	0.950413223	Bn-N2-p8233079	Bn-chrA02-p5845689

Bn-A02-p8885082	0.966942149	0.950413223	Bn-N2-p8233157	Bn-chrA02-p5845766
Bn-A02-p8885209	0.958677686	0.950413223	Bn-N2-p8233284	Bn-chrA02-p5845893
Bn-A02-p8885908	0.975206612	0.983471074	Bn-N2-p8233983	Bn-chrA02-p5846590
Bn-A02-p8886238	0.983471074	0.991735537	Bn-N2-p8234313	Bn-chrA02-p5846921
Bn-A02-p8891703	0.933884298	0.94214876	Bn-N2-p8235574	Bn-chrA02-p5848214
Bn-A02-p8893045	0.975206612	0.966942149	Bn-N2-p8236916	Bn-chrA02-p5849543
Bn-A02-p8893096	0.983471074	0.991735537	Bn-N2-p8236967	Bn-chrA02-p5849594
Bn-A02-p8895388	0.983471074	0.991735537	Bn-N2-p8239496	Bn-chrA02-p5850987
Bn-A02-p8899305	0.884297521	0.884297521	Bn-N2-p8243784	Bn-chrA02-p5855293
Bn-A02-p8912747	0.960199005	0.960199005	Bn-N2-p8256577	Bn-chrA02-p5869206
Bn-A02-p8915005	0.991735537	0.983471074	Bn-N2-p8258840	Bn-chrA02-p5871468
Bn-A02-p8924447	0.983471074	0.983471074	Bn-N2-p8272990	Bn-chrAnn_random-p866045
Bn-A02-p8925092	0.975206612	0.983471074	Bn-N2-p8273633	Bn-chrAnn_random-p866109
Bn-A02-p8934314	0.940298507	0.960199005	Bn-N2-p8277914	Bn-chrAnn_random-p866463
Bn-A02-p8934537	0.980099502	0.995024876	Bn-N2-p8278210	Bn-chrAnn_random-p866485
Bn-A02-p8937580	0.976744186	0.980066445	Bn-N2-p8281271	Bn-chrAnn_random-p866791
Bn-A02-p8949465	0.976744186	0.973421927	Bn-N2-p8293065	Bn-chrAnn_random-p867980
Bn-A02-p8950348	0.986710963	0.990033223	Bn-N2-p8293937	Bn-chrAnn_random-p868068
Bn-A02-p8954851	0.991735537	1	Bn-N2-p8300842	Bn-chrAnn_random-p868509
Bn-A02-p8955992	0.991735537	0.983471074	Bn-N2-p8301981	Bn-chrAnn_random-p868623
Bn-A02-p8967830	0.975124378	0.368159204	Bn-N14-p3663301	Bn-chrA10-p2841652
Bn-A02-p8999771	0.996677741	0.996677741	Bn-N2-p8354332	Bn-chrAnn_random-p878781
Bn-A02-p9000565	0.993355482	0.993355482	Bn-N2-p8355126	Bn-chrAnn_random-p878860
Bn-A02-p9000921	0.985074627	0.985074627	Bn-N2-p8355482	Bn-chrAnn_random-p878896
Bn-A02-p9000998	0.990049751	0.990049751	Bn-N2-p8355559	Bn-chrAnn_random-p878903
Bn-A02-p9006677	0.991735537	0.983471074	Bn-N2-p8361542	Bn-chrAnn_random-p879472
Bn-A02-p9022920	0.995024876	0.995024876	Bn-N2-p8374548	Bn-chrAnn_random-p880888
Bn-A02-p9051321	0.979166667	1	Bn-N2-p8404290	Bn-chrAnn_random-p883525
Bn-A02-p9064726	0.996677741	1	Bn-N2-p8418126	Bn-chrA02-p5883428
Bn-A02-p9071076	0.993355482	0.996677741	Bn-N2-p8432935	Bn-chrA02-p5888805
Bn-A02-p9080423	0.950248756	0.930348259	Bn-N2-p8438742	Bn-chrA02-p5896848
Bn-A02-p9108719	0.980066445	0.986710963	Bn-Scaffold16068-p4	Bn-chrA02-p5914199
Bn-A02-p9135710	0.915422886	0.995024876	Bn-N14-p14821455	Bn-chrA02-p5933199
Bn-A02-p9140734	0.983471074	0.983471074	Bn-N2-p8490773	Bn-chrA02-p5939732
Bn-A02-p9143216	0.973421927	0.970099668	Bn-N2-p8493277	Bn-chrA02-p5942226
Bn-A02-p9148801	0.876033058	0.966942149	Bn-N2-p8495930	Bn-chrA02-p5946110
Bn-A02-p9160227	0.966942149	0.991735537	Bn-N12-p12566891	Bn-chrA02-p5955889
Bn-A02-p9161498	0.950413223	0.983471074	Bn-N12-p12568136	Bn-chrA02-p5957869
Bn-A02-p9183872	0.966942149	0.966942149	Bn-N2-p8537091	Bn-chrC02-p11587568
Bn-A02-p9187768	0.980099502	0.985074627	Bn-N2-p8539183	Bn-chrA02-p5993442
Bn-A02-p9195997	0.986710963	0.986710963	Bn-N2-p8558461	Bn-chrA02-p6002764
Bn-A02-p9206062	0.970149254	0.970149254	Bn-N8-p3485028	Bn-chrAnn_random-p470655
Bn-A02-p9206254	0.980099502	0.980099502	Bn-N8-p3485220	Bn-chrA08-p15599652
Bn-A02-p9206548	0.975124378	0.970149254	Bn-N8-p17886683	Bn-chrA08-p15600117
Bn-A02-p9223722	0.996677741	0.996677741	Bn-N2-p8573754	Bn-chrA02-p6027264
Bn-A02-p9224016	1	0.996677741	Bn-N2-p8574048	Bn-chrA02-p6027558

Bn-A02-p9225798	0.990033223	0.990033223	Bn-N2-p8575828	Bn-chrA02-p6029341
Bn-A02-p9229113	0.980099502	0.980099502	Bn-N2-p8579132	Bn-chrA02-p6032647
Bn-A02-p9231115	0.983471074	0.975206612	Bn-N2-p8581134	Bn-chrA02-p6034649
Bn-A02-p9239879	0.991735537	0.983471074	Bn-N2-p8587246	Bn-chrA02-p6043246
Bn-A02-p9240182	0.990033223	0.976744186	Bn-N2-p8587549	Bn-chrA02-p6043549
Bn-A02-p9242308	0.990033223	0.986710963	Bn-N2-p8589675	Bn-chrA02-p6045674
Bn-A02-p9246552	0.996677741	0.980066445	Bn-N2-p8596030	Bn-chrA02-p6049923
Bn-A02-p9254995	0.983471074	0.983471074	Bn-N2-p8600433	Bn-chrA02-p6054912
Bn-A02-p9255686	0.991735537	0.991735537	Bn-N2-p8601123	Bn-chrA02-p6055602
Bn-A02-p9256040	0.983471074	0.975206612	Bn-N2-p8601478	Bn-chrA02-p6055956
Bn-A02-p9258117	0.983471074	0.983471074	Bn-N2-p8603565	Bn-chrA02-p6058043
Bn-A02-p9259325	0.983471074	0.983471074	Bn-N2-p8604770	Bn-chrA02-p6059249
Bn-A02-p9265512	0.991735537	0.991735537	Bn-N2-p8612836	Bn-chrA02-p6067953
Bn-A02-p9274719	0.946843854	0.990033223	Bn-N2-p8622494	Bn-chrA02-p6079618
Bn-A02-p9281731	0.993355482	0.993355482	Bn-N2-p8629774	Bn-chrA02-p6089039
Bn-A02-p9308233	0.958677686	0.578512397	Bn-N2-p8656021	Bn-chrCnn_random-p230801
Bn-A02-p9319210	0.995024876	0.995024876	Bn-N12-p12753183	Bn-chrC02-p11760765
Bn-A02-p9322468	0.966942149	0.834710744	Bn-N2-p8674703	Bn-chrA02-p6134682
Bn-A02-p9339288	0.993355482	0.993355482	Bn-N2-p8688243	Bn-chrA02-p6144740
Bn-A02-p9339514	0.950413223	0.950413223	Bn-N2-p8688469	Bn-chrA02-p6144966
Bn-A02-p9340002	0.960199005	0.970149254	Bn-N2-p8688957	Bn-chrA02-p6145454
Bn-A02-p9341487	1	1	Bn-N2-p8691192	Bn-chrA02-p6146939
Bn-A02-p9346140	0.996677741	0.996677741	Bn-N2-p8695857	Bn-chrA02-p6157567
Bn-A02-p9351376	0.96345515	0.96345515	Bn-N12-p4339977	Bn-chrC02-p4242582
Bn-A02-p9353942	0.990049751	0.965174129	Bn-N2-p8701320	Bn-chrC02-p11827261
Bn-A02-p9361967	0.909090909	0.859504132	Bn-N2-p8709878	Bn-chrA02-p6183124
Bn-A02-p9371363	0.970149254	0.975124378	Bn-N2-p8724946	Bn-chrA02-p6192452
Bn-A02-p9371633	0.983471074	0.991735537	Bn-N2-p8725216	Bn-chrA02-p6192723
Bn-A02-p9371841	1	0.933884298	Bn-N2-p8725424	Bn-chrA02-p6192931
Bn-A02-p9371930	0.983471074	0.950413223	Bn-N2-p8725513	Bn-chrA02-p6193017
Bn-A02-p9371948	0.991735537	0.933884298	Bn-N2-p8725531	Bn-chrA02-p6193035
Bn-A02-p9396655	0.157024793	0.975206612	Unknown	Bn-chrA02-p6215984
Bn-A02-p9408651	0.94214876	0.94214876	Bn-N2-p8769849	Bn-chrA02-p6227093
Bn-A02-p9409799	0.94214876	0.94214876	Bn-N2-p8770998	Bn-chrA02-p6228243
Bn-A02-p9415380	0.975124378	0.980099502	Bn-N2-p8777642	Bn-chrA02-p6232680
Bn-A02-p9415636	0.983471074	0.983471074	Bn-N2-p8777898	Bn-chrA02-p6232936
Bn-A02-p9415901	0.965174129	0.965174129	Bn-N2-p8778163	Bn-chrA02-p6233201
Bn-A02-p9416095	0.965174129	0.965174129	Bn-N2-p8778357	Bn-chrA02-p6233395
Bn-A02-p9420837	0.884297521	0.884297521	Bn-N2-p8785290	Bn-chrA02-p6240351
Bn-A02-p9422112	1	0.970099668	Bn-N2-p8786433	Bn-chrA02-p6241451
Bn-A02-p9425125	0.986710963	0.990033223	Bn-N2-p8789447	Bn-chrA02-p6249307
Bn-A02-p9434869	0.943521595	0.940199336	Bn-N12-p12923931	Bn-chrC02-p11965441
Bn-A02-p9464293	0.990033223	0.973421927	Bn-N2-p8815383	Bn-chrA02-p6271472
Bn-A02-p9465668	0.993355482	0.996677741	Bn-N2-p8816758	Bn-chrA02-p6272847
Bn-A02-p9480465	0.983471074	0.983471074	Bn-N2-p8831634	Bn-chrA02-p6286899
Bn-A02-p9497598	0.719008264	0.983471074	Bn-N3-p27918313	Bn-chrA02-p6311853

Bn-A02-p9505646	0.970149254	0.970149254	Bn-N2-p8859739	Bn-chrA02-p6319940
Bn-A02-p9506103	1	1	Bn-N2-p8860196	Bn-chrA02-p6320397
Bn-A02-p9513250	0.933884298	0.94214876	Bn-N2-p8867444	Bn-chrA02-p6327657
Bn-A02-p9519366	0.986710963	0.983388704	Bn-N2-p8873538	Bn-chrA02-p6333770
Bn-A02-p9526960	0.980066445	0.993355482	Bn-N2-p8884509	Bn-chrAnn_random-p310064
Bn-A02-p9558338	0.996677741	0.986710963	Bn-N2-p8898650	Bn-chrA02-p6363667
Bn-A02-p9558523	0.84717608	0.863787375	Bn-N12-p13093775	Bn-chrC02_random-p102712
Bn-A02-p9559304	0.900332226	0.6910299	Bn-N12-p13094435	Bn-chrA02-p6364642
Bn-A02-p9560736	0.975206612	0.966942149	Bn-N2-p8901051	Bn-chrA02-p6367487
Bn-A02-p9566560	1	0.991735537	Bn-N2-p8906874	Bn-chrC02_random-p988225
Bn-A02-p9599263	0.851239669	0.73553719	Bn-N2-p8927884	Bn-chrA02-p6401431
Bn-A02-p9607043	0.892561983	0.157024793	Bn-N2-p8936030	Bn-chrA01-p10169177
Bn-A02-p9607756	0.975206612	0.983471074	Bn-N2-p8937277	Bn-chrA02-p6411341
Bn-A02-p9608319	0.94214876	0.933884298	Bn-N2-p8937858	Bn-chrA02-p6411902
Bn-A02-p9608730	0.975206612	0.975206612	Bn-N2-p8938267	Bn-chrA02-p6412312
Bn-A02-p9610453	0.991735537	0.983471074	Bn-N2-p8940073	Bn-chrA02-p6418418
Bn-A02-p9622161	1	0.991735537	Bn-N2-p8955292	Bn-chrA02-p6432120
Bn-A02-p9625502	0.975206612	0.983471074	Bn-N2-p8958633	Bn-chrA02-p6435462
Bn-A02-p9635795	1	0.991735537	Bn-N2-p8969710	Bn-chrA02-p6445341
Bn-A02-p9635953	0.991735537	0.983471074	Bn-N2-p8969868	Bn-chrA02-p6445499
Bn-A02-p9636219	1	0.983471074	Bn-N2-p8975298	Bn-chrA02-p6445766
Bn-A02-p9640434	0.975206612	0.983471074	Bn-N2-p8979499	Bn-chrAnn_random-p397113
Bn-A02-p9643734	1	0.995024876	Bn-N2-p8982954	Bn-chrA02-p6452921
Bn-A02-p9646068	0.980066445	0.96345515	Bn-N2-p8985287	Bn-chrCnn_random-p133973
Bn-A02-p9648484	0.966777409	0.986710963	Bn-N2-p8987694	Bn-chrA02-p6461108
Bn-A02-p9664226	0.993355482	0.993355482	Bn-N2-p9002188	Bn-chrA02-p6477527
Bn-A02-p9684927	0.966777409	0.996677741	Bn-N2-p9022328	Bn-chrA02-p6491641
Bn-A02-p9703128	0.935323383	0.935323383	Bn-N2-p9038746	Bn-chrA02-p6506865
Bn-A02-p9706613	0.986710963	0.986710963	Bn-N2-p9043636	Bn-chrA02-p6511415
Bn-A02-p9707309	0.991735537	0.991735537	Bn-N2-p9044332	Bn-chrA02-p6512111
Bn-A02-p9719196	0.946843854	0.96345515	Bn-N12-p13314508	Bn-chrA02-p6525302
Bn-A02-p9722276	0.983388704	0.980066445	Bn-N2-p9059358	Bn-chrA02-p6528353
Bn-A02-p9726742	0.958677686	0.950413223	Bn-N2-p9062262	Bn-chrA02-p6531295
Bn-A02-p9740120	0.996677741	0.996677741	Bn-N2-p9080598	Bn-chrAnn_random-p22601C
Bn-A02-p9778318	0.980099502	0.975124378	Bn-N2-p9132674	Bn-chrA02-p6627160
Bn-A02-p9829719	0.980066445	0.980066445	Bn-N2-p9203173	Bn-chrA02-p6702012
Bn-A02-p9846691	0.991735537	0.991735537	Bn-N2-p9219826	Bn-chrA02_random-p306254
Bn-A02-p9846899	0.991735537	0.991735537	Bn-N2-p9220034	Bn-chrA02_random-p306462
Bn-A02-p9885351	0.973421927	0.973421927	Bn-N2-p9298098	Bn-chrA02-p6730348
Bn-A02-p9886564	0.966777409	0.966777409	Bn-N2-p9299311	Bn-chrA02-p6731561
Bn-A02-p9888208	0.983388704	0.983388704	Bn-N2-p9300950	Bn-chrA02-p6733198
Bn-A02-p9891733	0.996677741	0.996677741	Bn-N2-p9304477	Bn-chrA02-p6736725
Bn-A02-p9927808	0.983471074	0.983471074	Bn-N2-p9336590	Bn-chrA02-p6770451
Bn-A02-p9959992	0.983471074	0.966942149	Bn-N2-p9379647	Bn-chrA02-p6808609
Bn-A02-p9961743	0.991735537	0.983471074	Bn-N2-p9381534	Bn-chrA02-p6810318
Bn-A02-p9970625	0.970149254	0.970149254	Bn-N2-p9391132	Bn-chrA02-p6818344

Bn-A02-p9970848	0.996677741	0.993355482	Bn-N2-p9391355	Bn-chrA02-p6818567
Bn-A02-p9973775	0.965174129	0.975124378	Bn-N12-p13756119	Bn-chrA02-p6821494
Bn-A02-p9973927	0.965174129	0.975124378	Bn-N12-p13756282	Bn-chrA02-p6821646
Bn-A02-p9990613	0.983388704	0.973421927	Bn-N2-p9406491	Bn-chrC02_random-p104311
Bn-A02-p9993945	1	0.991735537	Bn-N2-p9410434	Bn-chrA02-p6839809
Bn-A02-p9996703	0.983471074	0.925619835	Bn-N2-p9413235	Bn-chrA02-p6842529
Bn-A02-p9997237	1	0.996677741	Bn-N2-p9413769	Bn-chrA02-p6843063
Bn-A02-p10019636	0.966942149	0.966942149	Bn-N2-p9430743	Bn-chrA02-p6858172
Bn-A02-p10020231	0.966942149	0.958677686	Bn-N2-p9431338	Bn-chrA02-p6858767
Bn-A02-p10067386	0.985074627	0.980099502	Bn-N2-p9467968	Bn-chrA02-p6895777
Bn-A02-p10078171	0.996677741	0.993355482	Bn-N2-p9479375	Bn-chrA02-p6906720
Bn-A02-p10092191	0.991735537	0.983471074	Bn-N2-p9495098	Bn-chrA02-p6919285
Bn-A02-p10096185	0.993355482	0.996677741	Bn-N2-p9499093	Bn-chrA02-p6922499
Bn-A02-p10105701	1	0.993355482	Bn-N2-p9508609	Bn-chrA02-p6931496
Bn-A02-p10107504	1	0.996677741	Bn-N2-p9510412	Bn-chrA02-p6933305
Bn-A02-p10110769	1	0.990033223	Bn-N2-p9513677	Bn-chrA02-p6936917
Bn-A02-p10111305	0.996677741	0.990033223	Bn-N2-p9514213	Bn-chrA02-p6937452
Bn-A02-p10126530	1	0.991735537	Bn-N2-p9546173	Bn-chrC02-p12673269
Bn-A02-p10129605	0.955223881	0.950248756	Bn-N2-p9549248	Bn-chrA02-p6976202
Bn-A02-p10133353	0.996677741	0.996677741	Bn-N2-p9553016	Bn-chrA02-p6979863
Bn-A02-p10138982	1	0.995024876	Bn-N2-p9559614	Bn-chrA02-p6985492
Bn-A02-p10140065	0.980099502	0.985074627	Bn-N2-p9560595	Bn-chrC02-p12691094
Bn-A02-p10153359	0.985074627	0.985074627	Bn-N2-p9575002	Bn-chrA02-p6999778
Bn-A02-p10156025	0.983471074	0.983471074	Bn-N2-p9577668	Bn-chrA02-p7002443
Bn-A02-p10176579	0.966942149	0.975206612	Bn-N2-p9596994	Bn-chrA02-p7019192
Bn-A02-p10176749	0.966942149	0.975206612	Bn-N2-p9597164	Bn-chrA02-p7019373
Bn-A02-p10181448	1	0.991735537	Bn-N2-p9601848	Bn-chrAnn_random-p478355
Bn-A02-p10184110	1	0.983471074	Bn-N2-p9604510	Bn-chrA02-p7030644
Bn-A02-p10186244	0.991735537	0.809917355	Bn-N2-p9608767	Bn-chrA02-p7032774
Bn-A02-p10186594	0.975206612	0.966942149	Bn-N2-p9609117	Bn-chrA02-p7033164
Bn-A02-p10193709	0.993355482	0.993355482	Bn-N2-p9620480	Bn-chrA02-p7040665
Bn-A02-p10218812	0.980066445	0.976744186	Bn-N2-p9642839	Bn-chrA02-p7067099
Bn-A02-p10227986	0.991735537	0.553719008	Bn-N2-p9652004	Bn-chrC02-p12856760
Bn-A02-p10228189	0.817275748	0.554817276	Bn-N12-p14167805	Bn-chrC02-p12856932
Bn-A02-p10231298	0.986710963	0.983388704	Bn-N2-p9655300	Bn-chrC02-p32752977
Bn-A02-p10247756	0.933884298	0.933884298	Bn-N2-p9675879	Bn-chrA02-p7101802
Bn-A02-p10255955	0.855721393	0.855721393	Bn-N2-p9684278	Bn-chrA02-p7109995
Bn-A02-p10259746	0.975206612	0.975206612	Bn-N2-p9688844	Bn-chrA02-p7113798
Bn-A02-p10289331	0.990049751	0.985074627	Bn-N2-p9711669	Bn-chrA02-p7134213
Bn-A02-p10323998	0.966942149	0.975206612	Bn-Scaffold23806-p1	Bn-chrA02-p7170926
Bn-A02-p10325260	0.970099668	0.980066445	Bn-N2-p9739682	Bn-chrA02-p7172671
Bn-A02-p10332863	0.920265781	0.980066445	Bn-N2-p9746334	Bn-chrA02-p7186251
Bn-A02-p10338350	0.990049751	0.990049751	Bn-N2-p9750165	Bn-chrA02-p7202351
Bn-A02-p10377179	0.983471074	0.983471074	Bn-N2-p9789893	Bn-chrA02-p7237988
Bn-A02-p10395108	0.966942149	0.966942149	Bn-N2-p9812760	Bn-chrC03-p23088214
Bn-A02-p10396907	0.94214876	0.950413223	Bn-N2-p9814560	Bn-chrA02-p7267790

Bn-A02-p10404084	0.960199005	0.975124378	Bn-N12-p14438175	Bn-chrA02-p7273536
Bn-A02-p10409103	0.991735537	0.867768595	Bn-N2-p9840386	Bn-chrC02-p12910497
Bn-A02-p10418131	0.877076412	0.877076412	Bn-N2-p9854027	Bn-chrA02-p7293774
Bn-A02-p10444138	0.990049751	0.985074627	Bn-N2-p9877373	Bn-chrA02-p7312558
Bn-A02-p10452854	0.991735537	0.818181818	Bn-N2-p9885618	Bn-chrC02-p13080375
Bn-A02-p10477549	0.991735537	0.991735537	Bn-N2-p9900258	Bn-chrA02-p7337508
Bn-A02-p10485644	0.892561983	0.900826446	Bn-N2-p9908997	Bn-chrA02-p7344509
Bn-A02-p10493685	1	0.991735537	Bn-N2-p9916731	Bn-chrA02-p7351405
Bn-A02-p10507801	0.991735537	0.975206612	Bn-N2-p9930276	Bn-chrA02-p7365234
Bn-A02-p10507864	0.958677686	0.966942149	Bn-N2-p9930339	Bn-chrA02-p7365297
Bn-A02-p10508075	0.983471074	0.991735537	Bn-N2-p9930550	Bn-chrA02-p7365508
Bn-A02-p10510996	1	0.991735537	Bn-N2-p9937231	Bn-chrA02-p7371529
Bn-A02-p10515029	0.983471074	0.991735537	Bn-N2-p9946330	Bn-chrA02-p7376035
Bn-A02-p10521287	0.983471074	0.991735537	Bn-N2-p9952503	Bn-chrC02-p13252633
Bn-A02-p10524842	1	0.991735537	Bn-N2-p9961771	Bn-chrA02-p7382074
Bn-A02-p10527075	1	0.991735537	Bn-N2-p9964003	Bn-chrA02-p7384306
Bn-A02-p10528586	1	0.991735537	Bn-N2-p9965542	Bn-chrA02-p7385821
Bn-A02-p10530978	1	0.991735537	Bn-N2-p9968278	Bn-chrA02-p7389046
Bn-A02-p10533300	0.983471074	0.991735537	Bn-N2-p9971336	Bn-chrA02-p7391368
Bn-A02-p10540052	0.995024876	0.980099502	Bn-N2-p9980461	Bn-chrA02-p7396494
Bn-A02-p10540148	0.975206612	0.966942149	Bn-N2-p9980557	Bn-chrA02-p7396590
Bn-A02-p10556758	0.966942149	0.958677686	Bn-N2-p9991356	Bn-chrA02-p7420795
Bn-A02-p10568947	0.983471074	0.991735537	Bn-N2-p10002325	Bn-chrA02-p7437134
Bn-A02-p10574526	0.991735537	1	Bn-N2-p10010255	Bn-chrA02-p7443188
Bn-A02-p10587170	0.94214876	0.950413223	Bn-N2-p10020924	Bn-chrA02-p7453082
Bn-A02-p10587232	0.983471074	0.991735537	Bn-N2-p10020986	Bn-chrA02-p7453144
Bn-A02-p10587536	0.94214876	0.983471074	Bn-Scaffold01435-p1	Bn-chrA02-p7453448
Bn-A02-p10591779	0.985074627	0.985074627	Bn-Scaffold01435-p4	Bn-chrA02-p7458918
Bn-A02-p10614602	0.983471074	0.975206612	Bn-N2-p10054527	Bn-chrC02-p13409353
Bn-A02-p10616871	0.983471074	0.975206612	Bn-N2-p10056794	Bn-chrA02-p7481917
Bn-A02-p10621504	0.991735537	1	Bn-N2-p10061579	Bn-chrA02-p7486392
Bn-A02-p10622338	0.966942149	0.966942149	Bn-N2-p10062423	Bn-chrA02-p7487226
Bn-A02-p10623838	1	0.991735537	Bn-N2-p10063922	Bn-chrA02-p7488726
Bn-A02-p10624373	0.993355482	0.996677741	Bn-N2-p10064457	Bn-chrA02-p7489261
Bn-A02-p10625720	0.991735537	0.983471074	Bn-N2-p10065821	Bn-chrA02-p7490608
Bn-A02-p10626782	0.996677741	0.996677741	Bn-N2-p10066883	Bn-chrA02-p7491670
Bn-A02-p10629886	0.950413223	0.950413223	Bn-N2-p10069986	Bn-chrA02-p7494729
Bn-A02-p10637202	0.975206612	0.983471074	Bn-N2-p10075194	Bn-chrA02-p7505910
Bn-A02-p10638989	0.933884298	0.958677686	Bn-N2-p10078720	Bn-chrA02-p7507698
Bn-A02-p10641032	0.983471074	0.983471074	Bn-N2-p10080772	Bn-chrA02-p7509740
Bn-A02-p10652585	0.975206612	0.983471074	Bn-N2-p10091465	Bn-chrA02-p7522549
Bn-A02-p10668400	0.983471074	0.991735537	Bn-N2-p10100731	Bn-chrA02-p7539305
Bn-A02-p10681015	0.94214876	0.950413223	Bn-N2-p10108373	Bn-chrA02-p7547375
Bn-A02-p10690394	0.991735537	0.991735537	Bn-N2-p10119103	Bn-chrA02-p7557464
Bn-A02-p10690477	0.975206612	0.975206612	Bn-N2-p10119184	Bn-chrA02-p7557546
Bn-A02-p10699604	0.958677686	0.917355372	Bn-N2-p10129043	Bn-chrA02-p7567981

Bn-A02-p10700544	0.983471074	0.975206612	Bn-N2-p10129980	Bn-chrA02-p7568908
Bn-A02-p10711353	0.993355482	0.990033223	Bn-N2-p10136517	Bn-chrC02_random-p143248
Bn-A02-p10711442	0.991735537	1	Bn-N2-p10136606	Bn-chrC02_random-p143239
Bn-A02-p10711565	0.983471074	0.743801653	Bn-N2-p10136729	Bn-chrCnn_random-p537712
Bn-A02-p10719296	0.989583333	0.96875	Bn-Scaffold79427-p4	Bn-chrC02-p13849225
Bn-A02-p10742809	0.933884298	0.983471074	Bn-N2-p10156665	Bn-chrCnn_random-p569706
Bn-A02-p10745711	0.990049751	0.985074627	Bn-N2-p10159573	Bn-chrC02-p13869736
Bn-A02-p10746556	0.966942149	0.991735537	Bn-N2-p10160429	Bn-chrA02-p7575040
Bn-A02-p10753552	0.975206612	0.983471074	Bn-N2-p10166906	Bn-chrA02-p7580621
Bn-A02-p10763116	0.983471074	0.991735537	Bn-N2-p10175805	Bn-chrA02-p7592763
Bn-A02-p10775283	0.985074627	0.985074627	Bn-N12-p14880222	Bn-chrC02-p13931119
Bn-A02-p10780709	0.983471074	0.991735537	Bn-N2-p10288977	Bn-chrA02-p7607719
Bn-A02-p10781906	1	0.991735537	Bn-N2-p10290173	Bn-chrA02-p7608916
Bn-A02-p10786230	0.983471074	0.983471074	Bn-N2-p10294498	Bn-chrA02-p7613241
Bn-A02-p10787104	0.975206612	0.983471074	Bn-N2-p10295372	Bn-chrA02-p7614115
Bn-A02-p10787380	0.958677686	0.966942149	Bn-N2-p10295648	Bn-chrA02-p7614391
Bn-A02-p10788285	0.975124378	0.965174129	Bn-N2-p10297213	Bn-chrA02-p7615157
Bn-A02-p10790263	0.991735537	0.983471074	Bn-N2-p10298620	Bn-chrA02-p7616861
Bn-A02-p10793326	0.990049751	0.885572139	Bn-N2-p10302101	Bn-chrA02-p7618996
Bn-A02-p10796317	0.975206612	0.983471074	Bn-N2-p10305093	Bn-chrA02-p7624164
Bn-A02-p10796462	0.993355482	0.986710963	Bn-N2-p10305238	Bn-chrA02-p7624309
Bn-A02-p10806477	0.975124378	0.970149254	Bn-N2-p10313296	Bn-chrA02-p7631301
Bn-A02-p10811918	0.970149254	0.955223881	Bn-N2-p10319142	Bn-chrA02-p7638574
Bn-A02-p10815211	0.965174129	0.960199005	Bn-N2-p10323540	Bn-chrA02-p7641799
Bn-A02-p10815571	0.966777409	0.990033223	Bn-N2-p10323900	Bn-chrA02-p7642159
Bn-A02-p10815969	0.975206612	0.975206612	Bn-N2-p10324651	Bn-chrA02-p7642557
Bn-A02-p10818801	0.892561983	0.950413223	Bn-N2-p10328736	Bn-chrA02-p7646443
Bn-A02-p10822102	0.892561983	0.909090909	Bn-N2-p10331231	Bn-chrA02-p7655572
Bn-A02-p10839131	0.991735537	1	Bn-N2-p10342502	Bn-chrA02-p7660240
Bn-A02-p10850012	0.975206612	0.983471074	Bn-N2-p10364056	Bn-chrA02-p7665678
Bn-A02-p10850323	0.996677741	1	Bn-N2-p10364367	Bn-chrA02-p7665989
Bn-A02-p10857857	0.958677686	0.966942149	Bn-N2-p10376106	Bn-chrA02-p7674566
Bn-A02-p10858522	0.966942149	0.975206612	Bn-N2-p10376771	Bn-chrA02-p7675232
Bn-A02-p10861090	0.983471074	0.975206612	Bn-N2-p10379871	Bn-chrA02-p7678208
Bn-A02-p10866642	0.990033223	0.993355482	Bn-N2-p10385347	Bn-chrA02-p7696842
Bn-A02-p10869120	0.909090909	0.917355372	Bn-N2-p10387849	Bn-chrA02-p7699319
Bn-A02-p10879119	0.996677741	1	Bn-N2-p10390357	Bn-chrA02-p7701827
Bn-A02-p10895698	0.991735537	0.983471074	Bn-N2-p10412796	Bn-chrA02-p7741148
Bn-A02-p10906851	0.996677741	1	Bn-N2-p10418115	Bn-chrA02-p7745791
Bn-A02-p10930249	0.652892562	0.983471074	Bn-N2-p10435256	Bn-chrAnn_random-p485796
Bn-A02-p10949322	0.975206612	0.983471074	Bn-N2-p10473667	Bn-chrCnn_random-p632012
Bn-A02-p10956599	1	0.993355482	Bn-N2-p10482187	Bn-chrA02_random-p465221
Bn-A02-p10959141	1	0.966942149	Bn-N2-p10486496	Bn-chrCnn_random-p256524
Bn-A02-p10983922	0.980066445	0.980066445	Bn-N2-p10505168	Bn-chrA02_random-p444441
Bn-A02-p10992266	0.983471074	0.884297521	Bn-N2-p10513227	Bn-chrA02_random-p435635
Bn-A02-p10992866	0.991735537	0.983471074	Bn-N2-p10516209	Bn-chrA02_random-p435081

Bn-A02-p10996151	1	1	Bn-N2-p10519407	Bn-chrA02_random-p431712
Bn-A02-p11014885	0.993355482	0.717607973	Bn-N2-p10551592	Bn-chrA08_random-p799827
Bn-A02-p11038401	0.990033223	0.993355482	Bn-N2-p10576393	Bn-chrA02-p7801099
Bn-A02-p11039119	0.950248756	0.965174129	Bn-N2-p10577070	Bn-chrA02-p7801777
Bn-A02-p11041638	0.986710963	0.983388704	Bn-N2-p10579589	Bn-chrA02-p7804296
Bn-A02-p11041726	0.990033223	0.993355482	Bn-N2-p10579677	Bn-chrA02-p7804384
Bn-A02-p11053817	0.983471074	0.975206612	Bn-N2-p10593161	Bn-chrA02-p7814610
Bn-A02-p11060975	0.890365449	0.996677741	Bn-N2-p10602102	Bn-chrA02-p7821734
Bn-A02-p11062457	0.958677686	0.966942149	Bn-N2-p10604255	Bn-chrA02-p7823235
Bn-A02-p11068431	0.92358804	0.996677741	Bn-N2-p10608743	Bn-chrA02-p7829943
Bn-A02-p11070906	0.990033223	0.990033223	Bn-N2-p10611025	Bn-chrA02-p7833234
Bn-A02-p11073162	0.950413223	0.966942149	Bn-N2-p10613271	Bn-chrA02-p7835707
Bn-A02-p11073326	0.955223881	0.975124378	Bn-N2-p10613433	Bn-chrA02-p7835870
Bn-A02-p11080706	0.986710963	1	Bn-N2-p10619539	Bn-chrA02-p7842510
Bn-A02-p11086855	0.995024876	0.995024876	Bn-N2-p10621790	Bn-chrA02-p7845660
Bn-A02-p11087046	0.970149254	0.970149254	Bn-N2-p10621981	Bn-chrA02-p7845851
Bn-A02-p11087388	0.995024876	0.995024876	Bn-N2-p10622333	Bn-chrA02-p7846203
Bn-A02-p11096221	0.990049751	0.985074627	Bn-N2-p10631489	Bn-chrA02-p7861565
Bn-A02-p11153317	0.991735537	1	Bn-N2-p10684688	Bn-chrA02-p7926659
Bn-A02-p11155686	0.990033223	0.986710963	Bn-N2-p10688424	Bn-chrA02-p7929034
Bn-A02-p11156474	0.980099502	0.975124378	Bn-N2-p10689212	Bn-chrA02-p7929822
Bn-A02-p11159353	0.990033223	0.993355482	Bn-N2-p10692160	Bn-chrA02-p7932769
Bn-A02-p11160726	0.983471074	0.975206612	Bn-N2-p10693526	Bn-chrA02-p7934142
Bn-A02-p11161716	0.990033223	0.986710963	Bn-N2-p10694521	Bn-chrA02-p7935132
Bn-A02-p11170230	0.958677686	0.950413223	Bn-N2-p10703024	Bn-chrA02-p7959705
Bn-A02-p11182703	0.993355482	0.890365449	Bn-N2-p10715981	Bn-chrC02-p15030519
Bn-A02-p11187572	0.581395349	0.990033223	Bn-N2-p10736093	Bn-chrA02-p7978221
Bn-A02-p11192451	0.966942149	0.867768595	Bn-N2-p10738485	Bn-chrC02-p15062023
Bn-A02-p11195341	0.314049587	0.975206612	Unknown	Bn-chrA02-p7984831
Bn-A02-p11200305	0.965174129	0.925373134	Bn-N2-p10747278	Bn-chrA02-p7989942
Bn-A02-p11209194	0.983471074	0.991735537	Bn-N2-p10759101	Bn-chrA02-p7998403
Bn-A02-p11223862	0.983471074	0.991735537	Bn-N2-p10776960	Bn-chrA02_random-p536526
Bn-A02-p11227845	1	0.991735537	Bn-Scaffold22845-p8	Bn-chrA02_random-p540506
Bn-A02-p11235969	0.933884298	0.950413223	Bn-N2-p10796763	Bn-chrA02_random-p548630
Bn-A02-p11237912	1	0.173553719	Bn-N2-p10799509	Unknown
Bn-A02-p11238010	0.826446281	0.280991736	Bn-N2-p10799608	Bn-chrC09-p38601396
Bn-A02-p11238896	0.983471074	0.173553719	Bn-N2-p10799926	Bn-chrA06-p998487
Bn-A02-p11248280	0.983471074	0.991735537	Bn-N2-p10800576	Bn-chrA02-p8255337
Bn-A02-p11248642	0.996677741	0.980066445	Bn-N2-p10800937	Bn-chrA02-p8255699
Bn-A02-p11248742	0.983471074	0.991735537	Bn-N2-p10801037	Bn-chrA02-p8255799
Bn-A02-p11248828	0.975206612	0.983471074	Bn-N2-p10801123	Bn-chrA02-p8255885
Bn-A02-p11259297	0.996677741	0.993355482	Bn-N2-p10810899	Bn-chrA02-p8266354
Bn-A02-p11262178	0.980099502	0.945273632	Bn-N2-p10813874	Bn-chrA02-p8269389
Bn-A02-p11265573	0.925619835	0.975206612	Bn-N2-p10825410	Bn-chrA02-p8272778
Bn-A02-p11278529	0.970149254	0.970149254	Bn-N2-p10841741	Bn-chrA02-p8285472
Bn-A02-p11284285	0.996677741	0.996677741	Bn-N2-p10848744	Bn-chrA02-p8292887

Bn-A02-p11284503	0.990033223	0.990033223	Bn-N2-p10848961	Bn-chrA02-p8293104
Bn-A02-p11291606	0.975206612	0.975206612	Bn-N2-p10856085	Bn-chrA02-p8302443
Bn-A02-p11293986	0.985074627	0.985074627	Bn-N2-p10858464	Bn-chrA02-p8304822
Bn-A02-p11320789	0.958677686	0.966942149	Bn-N2-p10881716	Bn-chrA02-p8327502
Bn-A02-p11328718	0.975206612	0.983471074	Bn-N2-p10889293	Bn-chrA02-p8335186
Bn-A02-p11342711	1	0.975206612	Bn-N2-p10903083	Bn-chrC02-p15394798
Bn-A02-p11345557	0.958677686	0.950413223	Bn-N2-p10906282	Bn-chrA02-p8004860
Bn-A02-p11346389	0.975206612	0.966942149	Bn-N2-p10907135	Bn-chrA02-p8005693
Bn-A02-p11355276	0.945273632	0.980099502	Bn-N2-p10915864	Bn-chrA02-p8014335
Bn-A02-p11356944	0.975206612	0.991735537	Bn-N2-p10917650	Bn-chrA02-p8016134
Bn-A02-p11365167	0.930348259	0.930348259	Bn-N2-p10926253	Bn-chrA02-p8024981
Bn-A02-p11415015	0.989583333	0.989583333	Bn-N2-p11118270	Bn-chrA02-p8123200
Bn-A02-p11439590	0.966942149	0.966942149	Bn-N2-p11144428	Bn-chrA02-p8150371
Bn-A02-p11440895	0.958677686	0.958677686	Bn-N2-p11145734	Bn-chrA02-p8151677
Bn-A02-p11441711	0.966942149	0.966942149	Bn-N2-p11146551	Bn-chrA02-p8152495
Bn-A02-p11449348	0.774086379	0.774086379	Bn-N2-p11155737	Bn-chrA02-p8161183
Bn-A02-p11449488	0.958677686	0.975206612	Bn-N2-p11155877	Bn-chrA02-p8161323
Bn-A02-p11450032	0.933884298	0.983471074	Bn-N2-p11158196	Bn-chrA02-p8161867
Bn-A02-p11450463	0.991735537	0.983471074	Bn-N2-p11158652	Bn-chrA02-p8162298
Bn-A02-p11451035	0.975206612	0.975206612	Bn-N2-p11159233	Bn-chrA02-p8162870
Bn-A02-p11451261	0.561983471	0.991735537	Bn-N2-p11159424	Bn-chrA02-p8163096
Bn-A02-p11454010	0.991735537	0.991735537	Bn-N2-p11162244	Bn-chrA02-p8166003
Bn-A02-p11454573	0.993355482	0.96345515	Bn-N2-p11166930	Bn-chrA02-p8166563
Bn-A02-p11454658	0.975206612	0.975206612	Bn-N2-p11167023	Bn-chrA02-p8166651
Bn-A02-p11454746	0.983388704	0.96345515	Bn-N2-p11167111	Bn-chrA02-p8166739
Bn-A02-p11455068	0.976744186	0.980066445	Bn-N2-p11167436	Bn-chrA02-p8167061
Bn-A02-p11455310	0.975206612	0.983471074	Bn-N2-p11167691	Bn-chrA02-p8167303
Bn-A02-p11455369	0.993355482	0.976744186	Bn-N2-p11167750	Bn-chrA02-p8167362
Bn-A02-p11455659	0.996677741	0.993355482	Bn-N2-p11168040	Bn-chrA02-p8167652
Bn-A02-p11457727	0.983471074	0.991735537	Bn-N2-p11171997	Bn-chrA02-p8169719
Bn-A02-p11463288	0.991735537	0.983471074	Bn-N2-p11177981	Bn-chrA02-p8175280
Bn-A02-p11479169	0.983471074	0.983471074	Bn-N2-p11194498	Bn-chrA02-p8194213
Bn-A02-p11479678	0.991735537	0.991735537	Bn-N2-p11195020	Bn-chrA02-p8194720
Bn-A02-p11492320	0.990033223	0.990033223	Bn-N2-p11204513	Bn-chrA02-p8209172
Bn-A02-p11513420	0.991735537	0.983471074	Bn-N2-p11226065	Bn-chrA02-p8230630
Bn-A02-p11517785	0.991735537	0.991735537	Bn-N2-p11232829	Bn-chrA02-p8233311
Bn-A02-p11521800	0.991735537	0.991735537	Bn-N2-p11238273	Bn-chrA02-p8236244
Bn-A02-p11535570	0.983471074	0.983471074	Bn-N2-p11254616	Bn-chrA02-p8242263
Bn-A02-p11550877	0.991735537	0.983471074	Bn-N2-p11267851	Bn-chrAnn_random-p169674
Bn-A02-p11585751	0.965174129	0.965174129	Bn-N2-p11299726	Bn-chrA02_random-p484336
Bn-A02-p11616676	0.990049751	0.990049751	Bn-N2-p11344990	Bn-chrA02_random-p523698
Bn-A02-p11646535	0.983471074	0.983471074	Bn-N2-p11375694	Bn-chrA02-p8370336
Bn-A02-p11648522	0.619834711	0.619834711	Bn-N2-p11377722	Bn-chrA02-p8372363
Bn-A02-p11660867	0.996677741	1	Bn-N2-p11388027	Bn-chrA02-p8378628
Bn-A02-p11666688	1	0.996677741	Bn-N2-p11393857	Bn-chrA02-p8384458
Bn-A02-p11673455	0.980099502	0.990049751	Bn-N2-p11406038	Bn-chrA02-p8394196

Bn-A02-p11682974	0.965174129	0.965174129	Bn-N2-p11415737	Bn-chrA02-p8405496
Bn-A02-p11683024	0.975124378	0.975124378	Bn-N2-p11415787	Bn-chrA02-p8405546
Bn-A02-p11683481	0.989583333	0.989583333	Bn-N2-p11416244	Bn-chrA02-p8406003
Bn-A02-p11703005	0.943521595	0.990033223	Bn-N2-p11429219	Bn-chrA02-p8418373
Bn-A02-p11713324	0.634551495	0.980066445	Bn-Scaffold42609-p2	Bn-chrA02_random-p552502
Bn-A02-p11713499	0.955223881	0.965174129	Bn-N2-p11442395	Bn-chrA02_random-p552725
Bn-A02-p11723698	0.986710963	0.986710963	Bn-N2-p11453116	Bn-chrAnn_random-p271499
Bn-A02-p11737945	0.991735537	0.983471074	Bn-N2-p11467352	Bn-chrA02-p8450512
Bn-A02-p11738896	0.983471074	0.975206612	Bn-N2-p11468303	Bn-chrA02-p8451463
Bn-A02-p11746891	0.985074627	0.980099502	Bn-N2-p11476302	Bn-chrA02-p8457619
Bn-A02-p11748602	0.993355482	0.990033223	Bn-N2-p11478014	Bn-chrA02-p8459328
Bn-A02-p11750740	0.990033223	0.993355482	Bn-N2-p11480152	Bn-chrA02-p8461462
Bn-A02-p11759281	0.975124378	0.975124378	Bn-N2-p11485420	Bn-chrA02-p8466681
Bn-A02-p11765114	0.975206612	0.975206612	Bn-N2-p11491234	Bn-chrA02-p8472496
Bn-A02-p11772983	0.94214876	0.966942149	Bn-N12-p16654539	Bn-chrA02-p8480672
Bn-A02-p11786253	0.818181818	0.983471074	Bn-N12-p16663448	Bn-chrA02-p8490043
Bn-A02-p11786572	0.925619835	0.958677686	Bn-N2-p11519176	Bn-chrA02-p8490362
Bn-A02-p11789023	0.983471074	0.991735537	Bn-N2-p11521647	Bn-chrA02-p8492823
Bn-A02-p11793499	0.980066445	0.986710963	Bn-N2-p11526101	Bn-chrA02-p8497276
Bn-A02-p11816790	0.970149254	0.970149254	Bn-N2-p11560901	Bn-chrA02-p8522674
Bn-A02-p11817742	0.973421927	0.976744186	Bn-N2-p11561943	Bn-chrA02-p8524524
Bn-A02-p11829280	1	0.917355372	Bn-N2-p11573584	Bn-chrC02-p16209234
Bn-A02-p11842725	0.975124378	0.980099502	Bn-N2-p11585881	Bn-chrA02-p8550776
Bn-A02-p11847986	0.983471074	0.991735537	Bn-N2-p11591140	Bn-chrA02-p8558537
Bn-A02-p11849409	0.96345515	1	Bn-N2-p11592565	Bn-chrA02-p8560451
Bn-A02-p11849571	0.953488372	0.993355482	Bn-N2-p11592727	Bn-chrA02-p8560613
Bn-A02-p11859430	0.983388704	0.976744186	Bn-N2-p11603565	Bn-chrA02-p8570657
Bn-A02-p11863349	0.983471074	0.966942149	Bn-N2-p11607484	Bn-chrA02-p8574522
Bn-A02-p11863681	0.983471074	0.975206612	Bn-N2-p11607816	Bn-chrA02-p8574855
Bn-A02-p11870177	0.991735537	1	Bn-N2-p11614129	Bn-chrA02-p8579417
Bn-A02-p11896294	0.983471074	0.991735537	Bn-N2-p11638646	Bn-chrA02-p8599202
Bn-A02-p11896538	0.983471074	0.975206612	Bn-N2-p11638890	Bn-chrA02-p8599446
Bn-A02-p11901091	0.991735537	1	Bn-N2-p11643449	Bn-chrA02-p8610536
Bn-A02-p11906704	0.966942149	0.958677686	Bn-N2-p11649060	Bn-chrA02-p8616153
Bn-A02-p11909870	0.991735537	0.983471074	Bn-N2-p11652224	Bn-chrA02-p8619317
Bn-A02-p11918483	0.995024876	0.995024876	Bn-N2-p11657722	Bn-chrA02-p8624814
Bn-A02-p11929538	0.975206612	0.975206612	Bn-N2-p11673849	Bn-chrA02-p8641910
Bn-A02-p11935002	0.970149254	0.860696517	Bn-N2-p11679355	Bn-chrA02-p8647876
Bn-A02-p11936177	0.993355482	0.990033223	Bn-N2-p11680540	Bn-chrA02-p8649040
Bn-A02-p11937221	0.990033223	0.993355482	Bn-N2-p11681573	Bn-chrA02-p8650073
Bn-A02-p11939715	0.966942149	0.975206612	Bn-N2-p11684066	Bn-chrA02-p8652573
Bn-A02-p11939983	0.983471074	0.975206612	Bn-N2-p11684352	Bn-chrA02-p8652840
Bn-A02-p11960741	0.980099502	0.990049751	Bn-N2-p11707244	Bn-chrA02-p8674144
Bn-A02-p11961581	0.983471074	0.991735537	Bn-N2-p11708084	Bn-chrA02-p8674984
Bn-A02-p11986866	0.990049751	0.990049751	Bn-N12-p17034516	Bn-chrC02-p16526368
Bn-A02-p11994999	0.985074627	0.985074627	Bn-N2-p11748445	Bn-chrA02-p8701636

Bn-A02-p11995673	0.966777409	0.986710963	Bn-N2-p11749120	Bn-chrA02-p8702311
Bn-A02-p11996587	0.973421927	0.993355482	Bn-N2-p11750602	Bn-chrA02-p8703225
Bn-A02-p11998345	0.950413223	0.884297521	Bn-N2-p11752386	Bn-chrA02-p8704975
Bn-A02-p11998540	0.966942149	0.966942149	Bn-N2-p11752579	Bn-chrA02-p8705170
Bn-A02-p11998921	0.980066445	0.983388704	Bn-N2-p11752962	Bn-chrA02-p8705552
Bn-A02-p12003125	0.991735537	0.991735537	Bn-N2-p11757214	Bn-chrA02-p8709826
Bn-A02-p12003217	0.950413223	0.776859504	Bn-N2-p11757306	Bn-chrC02-p16610107
Bn-A02-p12028821	0.94214876	0.94214876	Bn-N2-p11781061	Bn-chrA02-p8733655
Bn-A02-p12044265	0.990049751	0.995024876	Bn-N2-p11820483	Bn-chrA02-p8771983
Bn-A02-p12061350	0.958677686	0.966942149	Bn-N2-p11825220	Bn-chrA02-p8776717
Bn-A02-p12081581	0.975124378	0.970149254	Bn-N2-p11848712	Bn-chrA02-p8800956
Bn-A02-p12105374	0.915422886	0.980099502	Bn-N12-p17160882	Bn-chrA02-p8823146
Bn-A02-p12106636	0.975124378	0.975124378	Bn-N2-p11875156	Bn-chrA02-p8824404
Bn-A02-p12107746	0.863787375	0.870431894	Bn-N2-p11876266	Bn-chrA02-p8825512
Bn-A02-p12107868	0.96345515	0.976744186	Bn-N2-p11876388	Bn-chrA02-p8825634
Bn-A02-p12145607	0.983471074	0.900826446	Bn-N2-p11905764	Bn-chrC02-p17110998
Bn-A02-p12149042	0.995024876	0.995024876	Bn-N2-p11913121	Bn-chrAnn_random-p297581
Bn-A02-p12150733	0.933884298	0.933884298	Bn-N2-p11914823	Bn-chrAnn_random-p297598
Bn-A02-p12157799	0.966942149	0.958677686	Bn-N2-p11921905	Bn-chrA02_random-p374460
Bn-A02-p12164268	0.94214876	0.859504132	Bn-N2-p11928376	Bn-chrA02-p8872498
Bn-A02-p12169696	0.975206612	0.983471074	Bn-N2-p11933832	Bn-chrA02-p8878210
Bn-A02-p12169776	0.966942149	0.909090909	Bn-N2-p11933912	Bn-chrA02-p8878292
Bn-A02-p12179107	0.780730897	0.910299003	Bn-N2-p11942219	Bn-chrA02-p8887638
Bn-A02-p12179579	0.966942149	0.958677686	Bn-N2-p11943000	Bn-chrA02-p8888429
Bn-A02-p12192921	0.991735537	1	Bn-N2-p11955321	Bn-chrA02-p8898782
Bn-A02-p12196899	0.958677686	0.966942149	Bn-N2-p11960345	Bn-chrA02-p8903856
Bn-A02-p12198897	0.975206612	0.975206612	Bn-N2-p11962322	Bn-chrA02-p8906043
Bn-A02-p12219666	0.970149254	0.985074627	Bn-N2-p11975360	Bn-chrA02-p8929349
Bn-A02-p12220436	0.991735537	0.983471074	Bn-N2-p11976144	Bn-chrA02-p8930119
Bn-A02-p12241488	0.983471074	0.983471074	Bn-N4-p12424386	Bn-chrC02-p17232050
Bn-A02-p12269431	0.983471074	0.991735537	Bn-N2-p12021186	Bn-chrA02-p8983911
Bn-A02-p12278577	0.986710963	0.983388704	Bn-N2-p12031940	Bn-chrA02-p8993676
Bn-A02-p12284078	0.965174129	0.965174129	Bn-N2-p12037386	Bn-chrA02-p9000296
Bn-A02-p12284671	0.985074627	0.990049751	Bn-N2-p12037981	Bn-chrA02-p9000889
Bn-A02-p12284882	0.985074627	0.980099502	Bn-N2-p12038192	Bn-chrA02-p9001100
Bn-A02-p12287615	0.946843854	0.946843854	Bn-N12-p17242648	Bn-chrC02-p17378963
Bn-A02-p12292026	0.991735537	1	Bn-N2-p12045225	Bn-chrC02-p17383211
Bn-A02-p12292768	1	0.991735537	Bn-N2-p12045967	Bn-chrC02-p17383953
Bn-A02-p12293388	1	0.995024876	Bn-N2-p12046587	Bn-chrC02-p17384573
Bn-A02-p12295281	0.983471074	0.983471074	Bn-N2-p12048480	Bn-chrA02-p9011503
Bn-A02-p12295682	0.966942149	0.966942149	Bn-N2-p12048881	Bn-chrA02-p9011903
Bn-A02-p12297473	1	1	Bn-N2-p12055344	Bn-chrA02-p9013718
Bn-A02-p12314542	0.953488372	0.953488372	Bn-N2-p12065913	Bn-chrA02-p9024745
Bn-A02-p12336037	0.996677741	0.996677741	Bn-N2-p12094985	Bn-chrA02-p9050049
Bn-A02-p12342256	0.993355482	0.993355482	Bn-N2-p12104783	Bn-chrA02-p9056392
Bn-A02-p12363140	0.96345515	0.956810631	Bn-N2-p12123041	Bn-chrA02-p9075881

Bn-A02-p12393535	0.993355482	0.993355482	Bn-N2-p12223211	Bn-chrA02-p9107414
Bn-A02-p12393617	0.983471074	0.991735537	Bn-N2-p12223294	Bn-chrA02-p9107500
Bn-A02-p12397326	0.990049751	0.985074627	Bn-N2-p12226976	Bn-chrA02-p9111180
Bn-A02-p12397585	0.983471074	0.975206612	Bn-N2-p12227235	Bn-chrA02-p9111439
Bn-A02-p12415095	0.983471074	0.983471074	Bn-N2-p12247905	Bn-chrA02-p9130270
Bn-A02-p12443767	0.958677686	0.983471074	Bn-N2-p12277105	Bn-chrA02-p9154355
Bn-A02-p12449263	0.890365449	0.893687708	Bn-N2-p12282196	Bn-chrA02-p9158471
Bn-A02-p12452799	0.993355482	0.996677741	Bn-N2-p12286817	Bn-chrA02-p9161987
Bn-A02-p12460628	0.990033223	0.993355482	Bn-N2-p12297288	Bn-chrA02-p9174379
Bn-A02-p12508852	0.975206612	0.975206612	Bn-N2-p12347902	Bn-chrA02-p9227327
Bn-A02-p12512160	1	1	Bn-N2-p12350935	Bn-chrA02-p9230359
Bn-A02-p12512328	0.993355482	0.993355482	Bn-N2-p12351103	Bn-chrA02-p9230527
Bn-A02-p12512533	0.991735537	0.991735537	Bn-N2-p12351308	Bn-chrA02-p9230732
Bn-A02-p12514179	1	1	Bn-N2-p12352956	Bn-chrA02-p9232377
Bn-A02-p12516859	1	1	Bn-N2-p12357618	Bn-chrA02-p9237603
Bn-A02-p12520861	1	1	Bn-N2-p12364780	Bn-chrA02-p9247393
Bn-A02-p12521545	0.991735537	0.991735537	Bn-N2-p12365464	Bn-chrA02-p9248077
Bn-A02-p12524852	0.996677741	0.996677741	Bn-N2-p12368772	Bn-chrA02-p9251385
Bn-A02-p12534375	0.986710963	0.986710963	Bn-N2-p12378787	Bn-chrA02-p9261672
Bn-A02-p12550795	1	1	Bn-N2-p12394571	Bn-chrA02-p9277456
Bn-A02-p12552323	0.990033223	0.990033223	Bn-N2-p12396099	Bn-chrA02-p9278984
Bn-A02-p12554865	0.958677686	0.958677686	Bn-N2-p12398641	Bn-chrA02-p9281526
Bn-A02-p12559982	0.995024876	0.995024876	Bn-N2-p12403756	Bn-chrA02-p9286640
Bn-A02-p12581342	1	1	Bn-N2-p12433892	Bn-chrA02_random-p399822
Bn-A02-p12585867	0.990033223	0.990033223	Bn-N2-p12441778	Bn-chrA02_random-p387006
Bn-A02-p12585930	0.843853821	0.877076412	Bn-N2-p12441841	Bn-chrA02_random-p386943
Bn-A02-p12589304	0.983388704	0.983388704	Bn-N2-p12445215	Bn-chrA02_random-p383569
Bn-A02-p12595278	0.990049751	0.990049751	Bn-N2-p12451189	Bn-chrA02_random-p377596
Bn-A02-p12597733	0.94214876	0.94214876	Bn-N2-p12451853	Bn-chrA02_random-p376932
Bn-A02-p12608089	0.96345515	0.96345515	Bn-N2-p12466634	Bn-chrA02-p9345489
Bn-A02-p12627047	0.909090909	0.909090909	Bn-N12-p17699543	Bn-chrUnn_random-p327525
Bn-A02-p12628141	0.917355372	0.917355372	Bn-N2-p12498200	Bn-chrA02-p9397369
Bn-A02-p12632166	0.917355372	0.917355372	Bn-N2-p12501085	Bn-chrA02-p9400253
Bn-A02-p12637974	0.983471074	0.983471074	Bn-N2-p12506479	Bn-chrA02-p9405645
Bn-A02-p12642708	0.950413223	0.950413223	Bn-N2-p12507345	Bn-chrA02-p9406511
Bn-A02-p12666196	0.993355482	0.993355482	Bn-N2-p12536921	Bn-chrA02-p9429966
Bn-A02-p12678353	0.985074627	0.985074627	Bn-N2-p12552613	Bn-chrA02-p9443176
Bn-A02-p12680940	0.960199005	0.960199005	Bn-N2-p12555310	Bn-chrA02-p9445873
Bn-A02-p12681201	0.950413223	0.950413223	Bn-N2-p12555566	Bn-chrA02-p9446129
Bn-A02-p12681247	0.991735537	0.991735537	Bn-N2-p12555614	Bn-chrA02-p9446177
Bn-A02-p12686417	0.975124378	0.975124378	Bn-N2-p12560760	Bn-chrA02-p9451322
Bn-A02-p12686781	0.990049751	0.990049751	Bn-N2-p12561124	Bn-chrA02-p9451686
Bn-A02-p12688495	0.975206612	0.975206612	Bn-N2-p12562841	Bn-chrA02-p9453402
Bn-A02-p12688906	0.993355482	0.993355482	Bn-N2-p12563252	Bn-chrA02-p9453813
Bn-A02-p12693516	0.970149254	0.970149254	Bn-N2-p12569885	Bn-chrA02-p9460659
Bn-A02-p12739678	1	1	Bn-N2-p12597648	Bn-chrC02_random-p214863

Bn-A02-p12752915	0.990049751	0.990049751	Bn-N2-p12636981	Bn-chrC02-p18353044
Bn-A02-p12756887	0.980066445	0.980066445	Bn-N2-p12640956	Bn-chrA02-p9490339
Bn-A02-p12801838	0.985074627	0.985074627	Bn-N12-p17933424	Bn-chrC02-p18454397
Bn-A02-p12809029	0.611570248	0.611570248	Bn-N12-p17959405	Bn-chrC02_random-p208634
Bn-A02-p12810041	0.990033223	0.990033223	Bn-N2-p12703314	Bn-chrA02-p9560840
Bn-A02-p12815817	0.975124378	0.980099502	Bn-N2-p12709133	Bn-chrA02-p9566657
Bn-A02-p12819772	0.970149254	1	Bn-N12-p17987100	Bn-chrA02-p9570321
Bn-A02-p12821038	0.990033223	0.96345515	Bn-N2-p12728874	Bn-chrA02-p9571586
Bn-A02-p12822311	0.990049751	0.990049751	Bn-N12-p17989621	Bn-chrA02-p9572852
Bn-A02-p12822315	0.990049751	0.990049751	Bn-N2-p12730149	Bn-chrA02-p9572858
Bn-A02-p12848961	0.995024876	1	Bn-N2-p12766232	Bn-chrC07-p7198524
Bn-A02-p12851818	0.892561983	0.776859504	Bn-N2-p12769090	Bn-chrA02-p9607188
Bn-A02-p12939509	1	0.833887043	Bn-N2-p12812391	Bn-chrC02-p18598713
Bn-A02-p12943042	0.980099502	0.31840796	Bn-N2-p12805548	Bn-chrAnn_random-p288833
Bn-A02-p12945561	0.980066445	0.53820598	Bn-N2-p12809032	Bn-chrA02-p9656731
Bn-A02-p12962845	0.991735537	0.983471074	Bn-N2-p12822134	Bn-chrA02-p9674280
Bn-A02-p12971726	0.983471074	0.950413223	Bn-N2-p12831014	Bn-chrA02-p9683713
Bn-A02-p12972954	0.991735537	1	Bn-N2-p12832242	Bn-chrA02-p9684935
Bn-A02-p12976547	0.890365449	0.990033223	Bn-N2-p12836031	Bn-chrAnn_random-p348466
Bn-A02-p12986045	1	0.991735537	Bn-N2-p12845580	Bn-chrA02-p9694333
Bn-A02-p12996998	0.975206612	0.975206612	Bn-N2-p12856198	Bn-chrA02-p9705856
Bn-A02-p12997693	0.975206612	0.975206612	Bn-N2-p12858603	Bn-chrA02-p9707889
Bn-A02-p12999856	0.993355482	0.993355482	Bn-N2-p12861351	Bn-chrA02-p9710637
Bn-A02-p13000494	0.970099668	0.970099668	Bn-N2-p12861986	Bn-chrA02-p9711308
Bn-A02-p13016695	0.991735537	0.983471074	Bn-N2-p12874760	Bn-chrC02-p18792198
Bn-A02-p13019236	0.986710963	0.986710963	Bn-N2-p12877301	Bn-chrA02-p9728637
Bn-A02-p13067449	0.595041322	0.603305785	Bn-N2-p12914411	Bn-chrA02_random-p573460
Bn-A02-p13068355	0.958677686	0.983471074	Bn-N2-p12912891	Bn-chrA02_random-p574015
Bn-A02-p13091073	0.965174129	0.965174129	Bn-N12-p3034369	Bn-chrCnn_random-p456012
Bn-A02-p13118529	1	0.991735537	Bn-N2-p12981704	Bn-chrA02-p9783806
Bn-A02-p13121161	0.966942149	0.975206612	Bn-N2-p12982922	Bn-chrA02-p9784471
Bn-A02-p13123802	0.991735537	0.983471074	Bn-N2-p12985561	Bn-chrA02-p9785309
Bn-A02-p13124058	0.995024876	0.900497512	Bn-N2-p12985817	Bn-chrA02_random-p571039
Bn-A02-p13130726	0.991735537	1	Bn-N2-p12992463	Bn-chrA02-p9795096
Bn-A02-p13153436	1	1	Bn-N2-p13034427	Bn-chrA02-p9833852
Bn-A02-p13167989	0.986710963	0.990033223	Bn-N12-p18532897	Bn-chrA02-p9855671
Bn-A02-p13195248	0.990033223	0.980066445	Bn-N2-p13093965	Bn-chrA02-p9883961
Bn-A02-p13197154	0.933884298	0.94214876	Bn-N2-p13095850	Bn-chrA02-p9885867
Bn-A02-p13212386	0.884297521	0.876033058	Bn-N2-p13103660	Bn-chrA02-p9894440
Bn-A02-p13252027	0.975124378	0.975124378	Bn-N1-p22788462	Bn-chrAnn_random-p382481
Bn-A02-p13252137	0.945273632	0.950248756	Bn-N1-p22788352	Bn-chrAnn_random-p382480
Bn-A02-p13252354	0.950248756	0.950248756	Bn-N1-p22788137	Bn-chrAnn_random-p382477
Bn-A02-p13252652	0.960199005	0.960199005	Bn-N1-p22787838	Bn-chrAnn_random-p382474
Bn-A02-p13382591	1	1	Bn-N2-p13258690	Bn-chrA02-p10022968
Bn-A02-p13411303	0.958677686	0.958677686	Bn-N2-p13305246	Bn-chrA02-p10064603
Bn-A02-p13498319	0.970149254	0.870646766	Bn-N9-p22403153	Bn-chrC09-p23117416

Bn-A02-p13556392	0.976744186	0.976744186	Bn-Scaffold00856b-p	Bn-chrA02-p10230487
Bn-A02-p13556497	0.950413223	0.950413223	Bn-Scaffold00856b-p	Bn-chrA02-p10230593
Bn-A02-p13570742	0.983471074	0.644628099	Bn-N2-p13359983	Bn-chrA02-p10245371
Bn-A02-p13572191	1	1	Bn-N2-p13366263	Bn-chrA02-p10251208
Bn-A02-p13593944	0.991735537	0.991735537	Bn-N2-p13386108	Bn-chrA02-p10271051
Bn-A02-p13598238	0.909090909	0.909090909	Bn-N2-p13390510	Bn-chrA02-p10275452
Bn-A02-p13625461	0.975206612	0.975206612	Bn-N2-p13424875	Bn-chrA02_random-p606001
Bn-A02-p13626431	0.917355372	0.917355372	Bn-N2-p13425886	Bn-chrA02_random-p604991
Bn-A02-p13627045	0.996677741	0.996677741	Bn-N2-p13426522	Bn-chrA02_random-p604355
Bn-A02-p13637436	0.983471074	0.983471074	Bn-N2-p13438528	Bn-chrA02_random-p592544
Bn-A02-p13662225	0.991735537	0.991735537	Bn-N2-p13470531	Bn-chrA02_random-p645506
Bn-A02-p13751737	0.990049751	0.990049751	Bn-N2-p13585870	Bn-chrA02-p10372417
Bn-A02-p13782884	0.989583333	0.989583333	Bn-N2-p13613868	Bn-chrA02-p10402649
Bn-A02-p13794668	1	1	Bn-N2-p13632476	Bn-chrA02-p10424362
Bn-A02-p13798655	0.983471074	0.983471074	Bn-N2-p13634711	Bn-chrA02-p10426597
Bn-A02-p13803383	0.996677741	0.996677741	Bn-N2-p13640071	Bn-chrA02-p10431957
Bn-A02-p13803435	0.993355482	0.993355482	Bn-N2-p13640123	Bn-chrA02-p10432009
Bn-A02-p13822105	0.996677741	0.996677741	Bn-N2-p13676706	Bn-chrA02-p10467920
Bn-A02-p13874627	0.940298507	0.940298507	Bn-N2-p13723961	Bn-chrA02-p10522703
Bn-A02-p13875459	0.991735537	0.991735537	Bn-N2-p13724898	Bn-chrA02-p10523640
Bn-A02-p13951646	0.990033223	0.986710963	Bn-N2-p13788440	Bn-chrA02-p10587095
Bn-A02-p13958436	0.991735537	0.991735537	Bn-N2-p13795239	Bn-chrA02-p10593890
Bn-A02-p13972998	0.990033223	0.990033223	Bn-N2-p13805885	Bn-chrA02-p10603687
Bn-A02-p13999424	0.986710963	0.986710963	Bn-N2-p13832071	Bn-chrAnn_random-p237328
Bn-A02-p14024462	0.991735537	0.991735537	Bn-N2-p13863918	Bn-chrA02_random-p673784
Bn-A02-p14025825	0.966942149	0.966942149	Bn-N2-p13865264	Bn-chrA02_random-p672438
Bn-A02-p14026570	0.991735537	0.991735537	Bn-N2-p13866011	Bn-chrA02_random-p671691
Bn-A02-p14028000	0.983471074	0.983471074	Bn-N2-p13866808	Bn-chrA02_random-p670894
Bn-A02-p14028770	0.950413223	0.950413223	Bn-N2-p13867560	Bn-chrA02_random-p670142
Bn-A02-p14040220	0.975206612	0.975206612	Bn-N2-p13879570	Bn-chrA02-p10824618
Bn-A02-p14053180	0.989583333	0.989583333	Bn-N2-p13893815	Bn-chrCnn_random-p186916
Bn-A02-p14078497	0.953488372	0.953488372	Bn-N2-p13922267	Bn-chrA02-p10868523
Bn-A02-p14103759	1	1	Bn-N2-p13943803	Bn-chrC02-p21220900
Bn-A02-p14104250	0.996677741	0.996677741	Bn-N2-p13944327	Bn-chrA02-p10893306
Bn-A02-p14127042	0.983471074	0.983471074	Bn-N2-p13969128	Bn-chrA02-p10916064
Bn-A02-p14130758	0.983471074	0.983471074	Bn-N2-p13973454	Bn-chrA02-p10920390
Bn-A02-p14134708	0.993355482	0.993355482	Bn-N2-p13977379	Bn-chrAnn_random-p387830
Bn-A02-p14145586	0.991735537	0.991735537	Bn-N2-p13985787	Bn-chrA02-p10933577
Bn-A02-p14148076	1	1	Bn-N2-p13988837	Bn-chrA02-p10936651
Bn-A02-p14151274	0.966942149	0.966942149	Bn-N2-p13993986	Bn-chrA02-p10941802
Bn-A02-p14162326	0.917355372	0.917355372	Bn-N2-p13997769	Bn-chrC02-p21285062
Bn-A02-p14167623	0.965174129	0.965174129	Bn-N2-p14003361	Bn-chrA02-p10951392
Bn-A02-p14170476	0.991735537	0.991735537	Bn-N2-p14009194	Bn-chrA02-p10953896
Bn-A02-p14174686	0.983471074	0.983471074	Bn-N2-p14013805	Bn-chrA02-p10958507
Bn-A02-p14175730	0.991735537	0.991735537	Bn-N2-p14015982	Bn-chrAnn_random-p357924
Bn-A02-p14177785	0.983471074	0.661157025	Bn-N2-p14023369	Bn-chrAnn_random-p419803

Bn-A02-p14178166	0.975206612	0.975206612	Bn-N2-p14023752	Bn-chrAnn_random-p419806
Bn-A02-p14208801	0.966942149	0.966942149	Bn-N2-p14055432	Bn-chrA02-p10992036
Bn-A02-p14215327	0.926910299	0.926910299	Bn-Scaffold21546-p4	Bn-chrA02-p10998968
Bn-A02-p14227678	0.983471074	0.983471074	Bn-N2-p14075845	Bn-chrA02-p11013261
Bn-A02-p14229894	0.925619835	0.925619835	Bn-N2-p14078284	Bn-chrA02-p11015558
Bn-A02-p14232495	0.983471074	0.983471074	Bn-N2-p14082064	Bn-chrA02-p11019157
Bn-A02-p14287347	0.991735537	0.991735537	Bn-N2-p14133767	Bn-chrA02-p10659885
Bn-A02-p14287428	0.933884298	0.933884298	Bn-N2-p14133848	Bn-chrA02-p10659966
Bn-A02-p14293315	1	1	Bn-N2-p14137752	Bn-chrA02-p10663869
Bn-A02-p14328410	0.983388704	0.983388704	Bn-N2-p14160230	Bn-chrA02-p10685090
Bn-A02-p14500945	0.991735537	0.801652893	Bn-N2-p14390617	Bn-chrC03-p52870552
Bn-A02-p14526876	1	1	Bn-N2-p14408299	Bn-chrA02-p11236459
Bn-A02-p14526990	0.975206612	0.975206612	Bn-N2-p14408413	Bn-chrA02-p11236345
Bn-A02-p14527781	1	1	Bn-N2-p14409203	Bn-chrA02-p11235555
Bn-A02-p14529778	0.991735537	0.991735537	Bn-N2-p14411210	Bn-chrA02-p11233548
Bn-A02-p14530693	0.983471074	0.983471074	Bn-N2-p14412131	Bn-chrA02-p11232627
Bn-A02-p14540346	0.980099502	0.980099502	Bn-N2-p14419216	Bn-chrA02-p11223538
Bn-A02-p14564880	0.966942149	0.933884298	Bn-N2-p14437752	Bn-chrCnn_random-p878089
Bn-A02-p14575017	0.976744186	0.976744186	Bn-N2-p14442544	Bn-chrA02-p11197038
Bn-A02-p14576479	0.991735537	0.991735537	Bn-N2-p14443818	Bn-chrA02-p11195764
Bn-A02-p14579685	0.94214876	0.94214876	Bn-N2-p14448487	Bn-chrA02-p11191966
Bn-A02-p14592602	1	1	Bn-N2-p14456030	Bn-chrA02-p11183886
Bn-A02-p14605944	0.996677741	0.996677741	Bn-N2-p14466194	Bn-chrA02-p11171746
Bn-A02-p14608076	0.985074627	0.985074627	Bn-N2-p14469002	Bn-chrA02-p11168938
Bn-A02-p14612382	0.976744186	0.976744186	Bn-N2-p14473251	Bn-chrA02-p11164689
Bn-A02-p14615703	0.950166113	0.950166113	Bn-N2-p14476576	Bn-chrA02-p11161365
Bn-A02-p14650284	0.985074627	0.985074627	Bn-N2-p14524253	Bn-chrA02-p11107656
Bn-A02-p14653729	0.975206612	0.975206612	Bn-N2-p14535027	Bn-chrAnn_random-p365423
Bn-A02-p14695760	0.983471074	0.983471074	Bn-N2-p14576541	Bn-chrA02-p11056747
Bn-A02-p14707749	0.990049751	0.990049751	Bn-N7-p9701975	Bn-chrA07-p9584113
Bn-A02-p14708741	0.995024876	0.995024876	Bn-N7-p9700983	Bn-chrA07-p9583121
Bn-A02-p14736669	0.92039801	0.925373134	Bn-N15-p44102677	Bn-chrA02_random-p704090
Bn-A02-p14775699	0.975206612	0.975206612	Bn-N2-p14666326	Bn-chrAnn_random-p432276
Bn-A02-p14781371	0.975206612	0.975206612	Bn-N2-p14671951	Bn-chrA02-p11371513
Bn-A02-p14886507	1	1	Bn-N2-p14799403	Bn-chrA02-p11496460
Bn-A02-p14896274	0.983471074	0.983471074	Bn-N2-p14809326	Bn-chrA02-p11510832
Bn-A02-p14898010	0.991735537	0.991735537	Bn-N2-p14811062	Bn-chrA02-p11512568
Bn-A02-p14902806	0.985074627	0.985074627	Bn-N2-p14815810	Bn-chrA02-p11517523
Bn-A02-p14903483	0.993355482	0.993355482	Bn-N2-p14816487	Bn-chrA02-p11518200
Bn-A02-p14903643	0.991735537	0.991735537	Bn-N2-p14816647	Bn-chrA02-p11518360
Bn-A02-p14905419	0.983471074	0.983471074	Bn-N2-p14818427	Bn-chrA02-p11520140
Bn-A02-p14925274	1	1	Bn-N2-p14836652	Bn-chrA02-p11534790
Bn-A02-p14926973	0.983471074	0.983471074	Bn-N2-p14838349	Bn-chrA02-p11536486
Bn-A02-p14938768	0.975206612	0.975206612	Bn-N6-p8774529	Bn-chrA06-p8581113
Bn-A02-p14938941	0.991735537	0.991735537	Bn-N6-p8774702	Bn-chrA06-p8581286
Bn-A02-p14939071	0.991735537	0.991735537	Bn-N6-p8774832	Bn-chrA06-p8581416

Bn-A02-p14939204	0.966942149	0.966942149	Bn-N6-p8774966	Bn-chrA06-p8581550
Bn-A02-p14939483	0.983471074	0.983471074	Bn-N6-p8775245	Bn-chrA06-p8581829
Bn-A02-p14940026	0.983471074	0.983471074	Bn-N6-p8775788	Bn-chrA06-p8582372
Bn-A02-p14940029	0.991735537	0.991735537	Bn-N6-p8775791	Bn-chrA06-p8582375
Bn-A02-p14940149	0.991735537	0.991735537	Bn-N6-p8775911	Bn-chrA06-p8582495
Bn-A02-p14940218	0.983471074	0.983471074	Bn-N6-p8775980	Bn-chrA06-p8582564
Bn-A02-p14941541	0.950413223	0.950413223	Bn-N6-p8777303	Bn-chrA06-p8583887
Bn-A02-p14941595	0.983471074	0.983471074	Bn-N6-p8777357	Bn-chrA06-p8583941
Bn-A02-p14941620	0.991735537	0.991735537	Bn-N6-p8777382	Bn-chrA06-p8583966
Bn-A02-p14942048	0.991735537	0.991735537	Bn-N6-p8777810	Bn-chrA06-p8584394
Bn-A02-p14944551	0.966942149	0.966942149	Bn-N2-p14850664	Bn-chrA02-p11545321
Bn-A02-p14946150	0.917355372	0.975206612	Bn-N12-p20671307	Bn-chrA02-p11546905
Bn-A02-p14956864	0.983471074	0.983471074	Bn-N2-p14875554	Bn-chrA02-p11567065
Bn-A02-p14992706	0.983471074	0.983471074	Bn-N2-p14911092	Bn-chrA02-p11602325
Bn-A02-p14992781	0.991735537	0.991735537	Bn-N2-p14911167	Bn-chrA02-p11602400
Bn-A02-p14992802	0.958677686	0.958677686	Bn-N2-p14911188	Bn-chrA02-p11602421
Bn-A02-p14996107	0.94214876	0.94214876	Bn-N2-p14918578	Bn-chrA02-p11608766
Bn-A02-p15184172	0.995024876	0.995024876	Bn-N2-p15064754	Bn-chrA02-p11750712
Bn-A02-p15322404	0.990033223	0.990033223	Bn-N2-p15227760	Bn-chrA02-p11868322
Bn-A02-p15323930	0.980099502	0.980099502	Bn-N2-p15229760	Bn-chrA02-p11869848
Bn-A02-p15323936	0.985074627	0.985074627	Bn-N2-p15229766	Bn-chrA02-p11869854
Bn-A02-p15457681	0.552238806	0.965174129	Bn-N18-p23083283	Bn-chrA09-p5141698
Bn-A02-p15569271	0.993355482	0.990033223	Bn-N2-p15445555	Bn-chrA02-p11995191
Bn-A02-p15628202	0.965174129	0.965174129	Bn-N7-p25365031	Bn-chrA07-p23924890
Bn-A02-p15637932	0.991735537	0.991735537	Bn-N2-p15525546	Bn-chrA02_random-p800459
Bn-A02-p15693192	0.958677686	0.958677686	Bn-N2-p15585036	Bn-chrA02-p12089164
Bn-A02-p15717143	1	0.983471074	Bn-N2-p15605193	Bn-chrA02-p12120802
Bn-A02-p15745628	1	0.991735537	Bn-N2-p15638511	Bn-chrA02_random-p836901
Bn-A02-p15750257	1	0.991735537	Bn-N2-p15643140	Bn-chrA02-p12168179
Bn-A02-p15756475	0.991735537	0.983471074	Bn-N2-p15649360	Bn-chrA09-p17593709
Bn-A02-p15758928	0.986710963	0.990033223	Bn-N2-p15651816	Bn-chrAnn_random-p433812
Bn-A02-p15760465	0.975206612	0.966942149	Bn-N2-p15653353	Bn-chrAnn_random-p433828
Bn-A02-p15764198	0.785123967	0.79338843	Bn-N2-p15655290	Bn-chrA02-p12180723
Bn-A02-p15766649	0.991735537	0.975206612	Bn-N2-p15656768	Bn-chrC02-p19975153
Bn-A02-p15789088	0.990033223	0.986710963	Bn-N2-p15667274	Bn-chrA02-p12191341
Bn-A02-p15806083	0.991735537	1	Bn-N2-p15685504	Bn-chrA02-p12208639
Bn-A02-p15820017	0.991735537	0.809917355	Bn-N2-p15705012	Bn-chrC02-p20086787
Bn-A02-p15832342	0.988888889	0.988888889	Bn-N2-p15718293	Bn-chrC02-p20113637
Bn-A02-p15870327	0.990033223	0.983388704	Bn-N2-p15740491	Bn-chrA02-p12249503
Bn-A02-p15884717	0.991735537	0.925619835	Bn-N2-p15752161	Bn-chrA02-p12262242
Bn-A02-p15898839	0.983471074	0.983471074	Bn-N2-p15766343	Bn-chrA02-p12270588
Bn-A02-p15912199	0.950413223	0.975206612	Bn-N2-p15783884	Bn-chrA02-p12286150
Bn-A02-p15928585	0.975124378	0.970149254	Bn-N7-p6232280	Bn-chrAnn_random-p303287
Bn-A02-p16058769	0.983388704	0.983388704	Bn-N2-p15945767	Bn-chrA02-p12493301
Bn-A02-p16083197	0.980099502	0.980099502	Bn-N2-p15961807	Bn-chrA02-p12476538
Bn-A02-p16209439	0.993355482	0.990033223	Bn-N2-p16108396	Bn-chrA02_random-p960462

Bn-A02-p16220163	0.966942149	0.809917355	Bn-N2-p16122068	Bn-chrA02_random-p978250
Bn-A02-p16223740	0.909090909	0.917355372	Bn-N2-p16125170	Bn-chrA02_random-p981436
Bn-A02-p16259084	0.986710963	0.990033223	Bn-N2-p16160231	Bn-chrA02_random-p884460
Bn-A02-p16259298	0.976744186	0.986710963	Bn-N2-p16160445	Bn-chrA02_random-p884674
Bn-A02-p16311535	0.991735537	0.983471074	Bn-N2-p16217228	Bn-chrA02-p12542915
Bn-A02-p16339698	0.996677741	0.996677741	Bn-N2-p16248137	Bn-chrA02-p12566827
Bn-A02-p16428766	0.993355482	0.993355482	Bn-N2-p16333048	Bn-chrC02-p35923963
Bn-A02-p16486966	0.996677741	0.996677741	Bn-N2-p16395868	Bn-chrA02-p12704853
Bn-A02-p16520874	0.991735537	0.991735537	Bn-N2-p16432374	Bn-chrA02-p12741048
Bn-A02-p16521871	0.991735537	0.991735537	Bn-N2-p16433371	Bn-chrA02-p12742045
Bn-A02-p16526928	0.995024876	0.995024876	Bn-N2-p16438428	Bn-chrA02-p12747102
Bn-A02-p16528486	0.991735537	0.991735537	Bn-N2-p16440230	Bn-chrA02-p12748662
Bn-A02-p16529092	0.985074627	0.985074627	Bn-N2-p16440836	Bn-chrA02-p12749268
Bn-A02-p16571709	0.983471074	0.983471074	Bn-N2-p16481172	Bn-chrA02-p12788306
Bn-A02-p16572757	0.958677686	0.958677686	Bn-N2-p16482186	Bn-chrA02-p12789323
Bn-A02-p16666866	0.983471074	0.983471074	Bn-N2-p17563229	Bn-chrA02-p13863707
Bn-A02-p17012938	0.996677741	0.996677741	Bn-N2-p17923893	Bn-chrA02-p14190976
Bn-A02-p17112647	1	1	Bn-N2-p18013574	Bn-chrA02-p14284423
Bn-A02-p17156661	0.995024876	0.995024876	Bn-N2-p18059115	Bn-chrA02-p14324775
Bn-A02-p17189095	0.965174129	0.965174129	Bn-N2-p18091658	Bn-chrA02-p14357315
Bn-A02-p17189164	0.985074627	0.985074627	Bn-N2-p18091727	Bn-chrA02-p14357384
Bn-A02-p17224721	0.990049751	0.990049751	Bn-N2-p18127024	Bn-chrA02-p14386439
Bn-A02-p17229398	1	1	Bn-N2-p18131701	Bn-chrA02-p14391116
Bn-A02-p17333549	1	1	Bn-N18-p3847174	Bn-chrC08-p3080059
Bn-A02-p17381818	0.980099502	0.985074627	Bn-N2-p18281250	Bn-chrA02-p14550606
Bn-A02-p17532943	0.970149254	0.970149254	Bn-N2-p18438009	Bn-chrA02-p16676156
Bn-A02-p17910994	0.989583333	0.989583333	Bn-N2-p20510858	Bn-chrA02-p17160234
Bn-A02-p17938666	0.993355482	0.986710963	Bn-N2-p7355591	Bn-chrAnn_random-p309824
Bn-A02-p17996122	0.996677741	0.996677741	Bn-N2-p20603259	Bn-chrA02_random-p109805
Bn-A02-p18076499	0.993355482	0.993355482	Bn-N2-p20645895	Bn-chrA02-p17244427
Bn-A02-p18101171	0.993355482	0.990033223	Bn-N2-p20662672	Bn-chrA02-p17261239
Bn-A02-p18310675	0.980099502	0.980099502	Bn-N2-p20857052	Bn-chrA09-p26971640
Bn-A02-p18438691	0.996677741	1	Bn-N2-p20982980	Bn-chrA02_random-p114051
Bn-A02-p18571872	0.73553719	0.73553719	Bn-N10-p4896660	Bn-chrA07-p14547197
Bn-A02-p18599005	0.97752809	0.97752809	Bn-N2-p21124875	Bn-chrA02-p17701446
Bn-A02-p18917772	0.975206612	0.966942149	Bn-N2-p21457111	Bn-chrA02_random-p142756
Bn-A02-p18949958	0.991735537	0.983471074	Bn-N2-p21484515	Bn-chrA02-p17954962
Bn-A02-p18966918	0.983471074	0.991735537	Bn-N2-p21499463	Bn-chrA02-p17971039
Bn-A02-p18974322	0.991735537	0.983471074	Bn-N2-p21506168	Bn-chrA02-p17985162
Bn-A02-p18979982	0.950413223	0.958677686	Bn-N2-p21513905	Bn-chrA02-p17986061
Bn-A02-p18984484	0.975206612	0.966942149	Bn-N2-p21515507	Bn-chrA02-p17989174
Bn-A02-p18985754	0.975206612	0.983471074	Bn-N2-p21516778	Bn-chrA02-p17990432
Bn-A02-p18989840	0.991735537	0.958677686	Bn-N2-p21518103	Bn-chrC02-p35264932
Bn-A02-p18994242	0.975206612	0.975206612	Bn-N2-p21524346	Bn-chrA02-p17997627
Bn-A02-p18994312	0.975206612	0.966942149	Bn-N2-p21524415	Bn-chrA02-p17997697
Bn-A02-p18994472	0.991735537	0.975206612	Bn-N2-p21524575	Bn-chrA02-p17997856

Bn-A02-p18998627	0.975206612	0.950413223	Bn-N2-p21528916	Bn-chrA02-p17998925
Bn-A02-p19003944	0.983471074	0.991735537	Bn-N2-p21534227	Bn-chrA02-p18003185
Bn-A02-p19045730	0.990033223	0.471760797	Bn-N2-p21584042	Bn-chrAnn_random-p195586
Bn-A02-p19049506	0.975206612	0.983471074	Bn-N2-p21587820	Bn-chrA02-p18088762
Bn-A02-p19049989	0.975206612	0.983471074	Bn-N2-p21588455	Bn-chrA02-p18089223
Bn-A02-p19052873	0.958677686	0.917355372	Bn-N2-p21591340	Bn-chrA02-p18091938
Bn-A02-p19053770	0.966942149	0.966942149	Bn-N2-p21592229	Bn-chrA02-p18092867
Bn-A02-p19058136	0.990049751	0.990049751	Bn-N2-p21596996	Bn-chrA02-p18099263
Bn-A02-p19070958	0.950413223	0.958677686	Bn-N2-p21607674	Bn-chrA02-p18106291
Bn-A02-p19123194	0.990033223	0.986710963	Bn-N2-p21695814	Bn-chrA02-p18168189
Bn-A02-p19125023	0.996677741	0.996677741	Bn-N2-p21697642	Bn-chrA02-p18170018
Bn-A02-p19160671	0.975206612	0.966942149	Bn-N2-p21729736	Bn-chrAnn_random-p417469
Bn-A02-p19170689	0.983471074	0.991735537	Bn-N2-p21739691	Bn-chrA02-p18208657
Bn-A02-p19174964	0.991735537	0.983471074	Bn-N2-p21743923	Bn-chrA02-p18212940
Bn-A02-p19175402	0.975206612	0.966942149	Bn-N2-p21744372	Bn-chrA02-p18213378
Bn-A02-p19190669	0.710743802	1	Bn-N2-p21750802	Bn-chrCnn_random-p249789
Bn-A02-p19201685	0.950413223	0.94214876	Bn-N2-p21755205	Bn-chrA02-p18243835
Bn-A02-p19207431	0.980099502	0.985074627	Bn-N2-p21760905	Bn-chrA02-p18249529
Bn-A02-p19209964	0.975206612	0.983471074	Bn-N2-p21763435	Bn-chrA02-p18252063
Bn-A02-p19212766	0.983471074	0.991735537	Bn-N2-p21773179	Bn-chrA02-p18255015
Bn-A02-p19218108	1	1	Bn-N2-p21778766	Bn-chrA02-p18260586
Bn-A02-p19244695	0.966942149	0.975206612	Bn-N2-p21805259	Bn-chrA02-p18286434
Bn-A02-p19260818	0.980066445	0.980066445	Bn-N2-p21821497	Bn-chrA02-p18304225
Bn-A02-p19261853	0.993355482	0.993355482	Bn-N2-p21822532	Bn-chrA02-p18305260
Bn-A02-p19285211	0.930348259	0.92039801	Bn-N2-p21834040	Bn-chrUnn_random-p471584
Bn-A02-p19294461	0.983471074	0.975206612	Bn-N2-p21838446	Bn-chrAnn_random-p237690
Bn-A02-p19299222	0.991735537	0.983471074	Bn-N2-p21843849	Bn-chrAnn_random-p237738
Bn-A02-p19299455	0.983471074	0.991735537	Bn-N2-p21844083	Bn-chrAnn_random-p237740
Bn-A02-p19316657	0.960199005	0.960199005	Bn-N2-p21856271	Bn-chrAnn_random-p483165
Bn-A02-p19317643	0.986710963	0.986710963	Bn-N2-p21857257	Bn-chrAnn_random-p483155
Bn-A02-p19343485	1	1	Bn-N2-p21891969	Bn-chrA02-p18320838
Bn-A02-p19348348	0.983471074	0.983471074	Bn-N2-p21897001	Bn-chrA02-p18325487
Bn-A02-p19373757	0.996677741	0.996677741	Bn-N2-p21907037	Bn-chrA02-p18335522
Bn-A02-p19373825	0.975206612	0.975206612	Bn-N2-p21907105	Bn-chrA02-p18335590
Bn-A02-p19374782	0.983471074	0.983471074	Bn-N2-p21908063	Bn-chrA02-p18336548
Bn-A02-p19375860	1	1	Bn-N2-p21909141	Bn-chrA02-p18337626
Bn-A02-p19385896	0.986710963	0.986710963	Bn-N2-p21910271	Bn-chrA02-p18338756
Bn-A02-p19385986	0.993355482	0.993355482	Bn-N2-p21910361	Bn-chrA02-p18338846
Bn-A02-p19395795	0.991735537	0.991735537	Bn-N2-p21920826	Bn-chrA02-p18348727
Bn-A02-p19401844	0.996677741	0.996677741	Bn-N2-p21926877	Bn-chrA02-p18354778
Bn-A02-p19402089	0.993355482	0.993355482	Bn-N2-p21927122	Bn-chrA02-p18355023
Bn-A02-p19408740	0.983471074	0.983471074	Bn-N2-p21935520	Bn-chrA02-p18363201
Bn-A02-p19410459	0.983471074	0.983471074	Bn-N2-p21937682	Bn-chrA02-p18365364
Bn-A02-p19450017	0.993355482	0.993355482	Bn-N2-p21979947	Bn-chrA02-p18408223
Bn-A02-p19450418	0.990033223	0.990033223	Bn-N2-p21980348	Bn-chrA02-p18408624
Bn-A02-p19451380	0.993355482	0.993355482	Bn-N2-p21981310	Bn-chrA02-p18409586

Bn-A02-p19460299	0.990049751	0.990049751	Bn-N2-p21987946	Bn-chrA02-p18416228
Bn-A02-p19521910	1	1	Bn-N2-p22234936	Bn-chrA02-p18643025
Bn-A02-p19534217	0.993355482	0.598006645	Bn-N2-p22245724	Bn-chrA02-p18626360
Bn-A02-p19549587	0.983471074	0.975206612	Bn-N2-p22266105	Bn-chrA02-p18660663
Bn-A02-p19566951	0.94214876	0.818181818	Bn-N2-p22275103	Bn-chrA02-p18669694
Bn-A02-p19573867	0.983388704	0.980066445	Bn-N2-p22285981	Bn-chrA02-p18678436
Bn-A02-p19575587	0.970099668	0.983388704	Bn-N2-p22287665	Bn-chrA02-p18680150
Bn-A02-p19587474	0.976744186	0.986710963	Bn-N2-p22291612	Bn-chrA02-p18698241
Bn-A02-p19587812	0.990033223	0.986710963	Bn-N2-p22291950	Bn-chrA02-p18698579
Bn-A02-p19589605	0.109634551	1	Unknown	Bn-chrA02-p18700486
Bn-A02-p19602796	0.512437811	0.995024876	Bn-N17-p18413201	Bn-chrA02-p18714613
Bn-A02-p19619823	0.958677686	0.933884298	Bn-N2-p22315365	Bn-chrA02-p18733031
Bn-A02-p19630208	0.966942149	0.975206612	Bn-N2-p22326488	Bn-chrA02-p18739832
Bn-A02-p19645773	0.960199005	0.955223881	Bn-N2-p22331062	Bn-chrA02-p18744108
Bn-A02-p19662482	0.975206612	0.966942149	Bn-N2-p22355660	Bn-chrA02-p18769193
Bn-A02-p19674831	1	0.991735537	Bn-N2-p22368745	Bn-chrA02-p18784335
Bn-A02-p19682273	0.996677741	0.996677741	Bn-N2-p22376166	Bn-chrA02-p18791802
Bn-A02-p19682984	0.993355482	0.993355482	Bn-N2-p22376878	Bn-chrA02-p18792514
Bn-A02-p19683093	0.990033223	0.990033223	Bn-N2-p22376987	Bn-chrA02-p18792623
Bn-A02-p19702955	0.983388704	0.983388704	Bn-N2-p22414997	Bn-chrA02-p18832645
Bn-A02-p19704677	0.991735537	0.991735537	Bn-N2-p22416723	Bn-chrA02-p18834371
Bn-A02-p19709973	0.995024876	0.995024876	Bn-N12-p34084308	Bn-chrCnn_random-p471794
Bn-A02-p19717843	0.985074627	0.134328358	Bn-N12-p34143722	Bn-chrA05-p5366950
Bn-A02-p19731316	0.980099502	0.985074627	Bn-N2-p24147282	Bn-chrA02-p20407113
Bn-A02-p19756499	0.975206612	0.983471074	Bn-N2-p24122314	Bn-chrC02-p38410482
Bn-A02-p19872956	0.925619835	0.94214876	Bn-N2-p24032897	Bn-chrA02-p20278674
Bn-A02-p19874478	0.867768595	0.983471074	Bn-Scaffold41690-p2	Bn-chrA02-p20277521
Bn-A02-p19875345	1	0.892561983	Bn-N2-p24025114	Bn-chrC07-p27491909
Bn-A02-p19886585	0.834710744	0.842975207	Bn-N2-p24021449	Bn-chrA02-p20270893
Bn-A02-p19889192	0.991735537	1	Bn-N2-p24017850	Bn-chrA02-p20262202
Bn-A02-p19892433	0.983471074	0.991735537	Bn-N2-p24012090	Bn-chrA02-p20258615
Bn-A02-p19892632	0.975206612	0.983471074	Bn-N2-p24011891	Bn-chrA02-p20258416
Bn-A02-p19892839	0.975206612	0.966942149	Bn-N2-p24011684	Bn-chrA02-p20258209
Bn-A02-p19927952	0.983471074	0.991735537	Bn-N2-p23977051	Bn-chrA02-p20223345
Bn-A02-p20055831	0.239669421	0.239669421	Unknown	Bn-chrCnn_random-p724778
Bn-A02-p20060311	0.990049751	0.990049751	Bn-N2-p23873981	Bn-chrA02-p20140521
Bn-A02-p20062461	0.990049751	0.990049751	Bn-N2-p23871830	Bn-chrA02-p20138370
Bn-A02-p20062658	0.985074627	0.990049751	Bn-N19-p39038724	Bn-chrC01-p29419925
Bn-A02-p20062733	0.985074627	0.985074627	Bn-N19-p39038648	Bn-chrC09-p34024954
Bn-A02-p20063202	0.985074627	0.985074627	Bn-N2-p23871041	Bn-chrA02-p20137581
Bn-A02-p20157985	0.933884298	0.925619835	Bn-N2-p23769071	Bn-chrA02_random-p149935
Bn-A02-p20158101	0.925619835	0.94214876	Bn-N2-p23768956	Bn-chrA02_random-p149923
Bn-A02-p20159139	0.983471074	0.975206612	Bn-N2-p23767956	Bn-chrA02_random-p149820
Bn-A02-p20166067	0.991735537	0.983471074	Bn-N2-p23761268	Bn-chrA02_random-p149000
Bn-A02-p20196208	0.975206612	0.983471074	Bn-N2-p23738770	Bn-chrA02_random-p147083
Bn-A02-p20200723	0.966942149	0.826446281	Bn-N2-p23729895	Bn-chrA02_random-p146080

Bn-A02-p20201241	0.983471074	0.991735537	Bn-N2-p23729377	Bn-chrA02_random-p146031
Bn-A02-p20203160	0.975206612	0.966942149	Bn-N2-p23727429	Bn-chrA02_random-p145912
Bn-A02-p20207870	0.958677686	0.958677686	Bn-N2-p23716622	Bn-chrA02-p19776681
Bn-A02-p20242753	0.990033223	0.990033223	Bn-N2-p23685668	Bn-chrA02-p19738620
Bn-A02-p20363776	0.966942149	0.966942149	Bn-N2-p23598377	Bn-chrA02-p19650587
Bn-A02-p20371730	0.990049751	0.990049751	Bn-N2-p23584575	Bn-chrA02-p19635243
Bn-A02-p20371774	0.990049751	0.990049751	Bn-N2-p23584531	Bn-chrA02-p19635199
Bn-A02-p20396880	0.96345515	0.96345515	Bn-N2-p23560271	Bn-chrA02-p19611038
Bn-A02-p20461477	0.993355482	0.993355482	Bn-N2-p23499487	Bn-chrA02-p19549521
Bn-A02-p20462487	0.993355482	0.993355482	Bn-N2-p23498494	Bn-chrA02-p19548528
Bn-A02-p20526431	0.996677741	0.996677741	Bn-N2-p23437887	Bn-chrA02-p19487025
Bn-A02-p20536885	0.990033223	0.990033223	Bn-N2-p23428442	Bn-chrA02-p19476903
Bn-A02-p20561132	0.983388704	0.983388704	Bn-N2-p23404761	Bn-chrA02-p19448679
Bn-A02-p20568585	0.996677741	0.996677741	Bn-N2-p23402078	Bn-chrA02-p19445996
Bn-A02-p20571280	0.993355482	0.993355482	Bn-N2-p23400250	Bn-chrA02-p19444168
Bn-A02-p20580541	0.165289256	0.975206612	Bn-N11-p27168690	Bn-chrA02-p23299518
Bn-A02-p20634433	0.985074627	0.985074627	Bn-N2-p23331680	Bn-chrA02-p19375269
Bn-A02-p20678898	0.996677741	0.996677741	Bn-N2-p23284784	Bn-chrA02-p19312319
Bn-A02-p20700463	1	1	Bn-N2-p23267751	Bn-chrA02-p19294030
Bn-A02-p20718571	0.986710963	0.986710963	Bn-N2-p23249911	Bn-chrA02-p19272096
Bn-A02-p20718651	0.990033223	0.990033223	Bn-N2-p23249831	Bn-chrA02-p19272016
Bn-A02-p20735027	0.996677741	0.996677741	Bn-N2-p23238672	Bn-chrA02-p19259520
Bn-A02-p20746287	0.993355482	0.990033223	Bn-N2-p23228820	Bn-chrA02-p19248437
Bn-A02-p20762322	1	1	Bn-N2-p23216999	Bn-chrA02-p19237259
Bn-A02-p20776266	0.993355482	0.993355482	Bn-N2-p23202762	Bn-chrA02-p19223070
Bn-A02-p20832528	0.993355482	0.993355482	Bn-N2-p23155219	Bn-chrA02-p19176472
Bn-A02-p20845905	0.993355482	0.993355482	Bn-N2-p23134950	Bn-chrA02-p19157533
Bn-A02-p20846941	0.966777409	0.966777409	Bn-N2-p23133914	Bn-chrA02-p19156497
Bn-A02-p20882001	0.996677741	0.996677741	Bn-N2-p23092851	Bn-chrA02-p19117118
Bn-A02-p20882153	1	1	Bn-N2-p23092699	Bn-chrA02-p19116966
Bn-A02-p21009288	0.933554817	0.933554817	Bn-N12-p34848776	Bn-chrC02_random-p382346
Bn-A02-p21023727	1	0.966942149	Bn-N2-p22923264	Bn-chrUnn_random-p789115
Bn-A02-p21032956	0.993355482	0.990033223	Bn-N2-p22913975	Bn-chrA02-p18936128
Bn-A02-p21061002	1	1	Bn-N2-p22881947	Bn-chrA02-p18906337
Bn-A02-p21062567	0.995024876	0.995024876	Bn-N2-p22880382	Bn-chrA02-p18904772
Bn-A02-p21062857	0.985074627	0.980099502	Bn-N2-p22880092	Bn-chrA02-p18904482
Bn-A02-p21073755	0.993355482	0.993355482	Bn-N2-p22873663	Bn-chrA02-p18897627
Bn-A02-p21207148	0.986710963	0.986710963	Bn-N2-p22699171	Bn-chrA02-p19823199
Bn-A02-p21211198	0.980066445	0.980066445	Bn-N2-p22695143	Bn-chrA02-p19827227
Bn-A02-p21256748	0.996677741	0.996677741	Bn-N2-p22665710	Bn-chrA02-p19856603
Bn-A02-p21263855	0.990033223	0.973421927	Bn-N2-p22659971	Bn-chrA02-p19862342
Bn-A02-p21322148	0.980099502	0.980099502	Bn-N2-p22612406	Bn-chrA02-p19910790
Bn-A02-p21324539	0.986710963	0.986710963	Bn-N2-p22603538	Bn-chrA02-p19917424
Bn-A02-p21325110	0.976744186	0.976744186	Bn-N2-p22602967	Bn-chrA02-p19917995
Bn-A02-p21378450	0.991735537	0.983471074	Bn-N2-p25492115	Bn-chrA02-p21741967
Bn-A02-p21379810	0.991735537	0.983471074	Bn-N2-p25490754	Bn-chrA02-p21740606

Bn-A02-p21409149	0.965174129	0.965174129	Bn-N2-p25461292	Bn-chrA02-p21710473
Bn-A02-p21409508	1	1	Bn-N2-p25460933	Bn-chrA02-p21710114
Bn-A02-p21410424	0.970149254	0.975124378	Bn-N2-p25460024	Bn-chrA02-p21709205
Bn-A02-p21467101	0.996677741	0.996677741	Bn-N2-p25412797	Bn-chrA02-p21658109
Bn-A02-p21489620	0.990033223	0.990033223	Bn-N2-p25392820	Bn-chrA02-p21636642
Bn-A02-p21490663	0.990049751	0.990049751	Bn-N2-p25391783	Bn-chrA02-p21635605
Bn-A02-p21491139	0.990033223	0.946843854	Bn-N2-p25391305	Bn-chrA02-p21635127
Bn-A02-p21510968	0.990033223	0.990033223	Bn-N2-p25366085	Bn-chrA02-p21610136
Bn-A02-p21544735	0.986710963	0.986710963	Bn-N2-p25325581	Bn-chrA02-p21565848
Bn-A02-p21563911	0.975124378	0.980099502	Bn-N2-p25304341	Bn-chrA02-p21544875
Bn-A02-p21570784	0.930232558	0.930232558	Bn-N12-p39417242	Bn-chrC02-p40535785
Bn-A02-p21595382	0.993355482	0.993355482	Bn-N2-p25269719	Bn-chrA02-p21514694
Bn-A02-p21598341	0.993355482	0.993355482	Bn-N2-p25266770	Bn-chrA02-p21511745
Bn-A02-p21616494	0.990033223	0.990033223	Bn-N2-p25248893	Bn-chrA02-p21495887
Bn-A02-p21625125	0.996677741	0.996677741	Bn-N2-p25238071	Bn-chrA02-p21487377
Bn-A02-p21639869	0.945273632	0.945273632	Bn-N2-p25226448	Bn-chrA02-p21475754
Bn-A02-p21648504	0.990033223	0.990033223	Bn-N2-p25217692	Bn-chrA02-p21467098
Bn-A02-p21701259	0.990049751	0.990049751	Bn-N19-p28927263	Bn-chrUnn_random-p28770[illegible]
Bn-A02-p21713756	0.980066445	0.980066445	Bn-N12-p39217463	Bn-chrC02-p40255672
Bn-A02-p21713818	0.915422886	0.915422886	Bn-N12-p39217401	Bn-chrC02-p40255610
Bn-A02-p21736387	0.986710963	0.986710963	Bn-N2-p25126329	Bn-chrA02-p21372904
Bn-A02-p21737275	0.980066445	0.980066445	Bn-N2-p25125441	Bn-chrA02-p21372016
Bn-A02-p21891973	0.995024876	0.995024876	Bn-N2-p24954183	Bn-chrCnn_random-p513511
Bn-A02-p21930156	0.826446281	0.983471074	Bn-N2-p24931155	Bn-chrA02-p21226298
Bn-A02-p22296426	0.94214876	0.958677686	Bn-N2-p24561969	Bn-chrA02-p20862994
Bn-A02-p22332961	0.859504132	0.991735537	Bn-N2-p24521736	Bn-chrA02-p20818388
Bn-A02-p22351644	0.996677741	0.843853821	Bn-N2-p24509208	Bn-chrA02-p20807683
Bn-A02-p22352518	0.991735537	0.859504132	Bn-N2-p24508335	Bn-chrCnn_random-p552174
Bn-A02-p22357265	0.996677741	0.956810631	Bn-N2-p24503527	Bn-chrC02-p39389505
Bn-A02-p22365337	0.966942149	0.975206612	Bn-Scaffold05113-p1	Bn-chrA02-p20793044
Bn-A02-p22371956	0.991735537	0.983471074	Bn-N2-p24492412	Bn-chrA02-p20787695
Bn-A02-p22375400	0.983471074	0.975206612	Bn-N2-p24489078	Bn-chrA02-p20784371
Bn-A02-p22375887	0.958677686	0.94214876	Bn-N2-p24488586	Bn-chrA02-p20783879
Bn-A02-p22376180	0.991735537	0.925619835	Bn-N2-p24488293	Bn-chrA02-p20783556
Bn-A02-p22383771	0.966942149	0.950413223	Bn-N2-p24482469	Bn-chrA02-p20778246
Bn-A02-p22386317	0.983471074	0.892561983	Bn-N2-p24479923	Bn-chrA02-p20775744
Bn-A02-p22388092	0.991735537	0.380165289	Bn-N2-p24478147	Unknown
Bn-A02-p22394284	0.983471074	0.975206612	Bn-N2-p24463521	Bn-chrA02-p20767215
Bn-A02-p22394632	0.996677741	0.996677741	Bn-N2-p24463173	Bn-chrA02-p20766867
Bn-A02-p22395060	0.986710963	0.990033223	Bn-N2-p24462745	Bn-chrA02-p20766439
Bn-A02-p22398620	0.983471074	0.991735537	Bn-N2-p24459187	Bn-chrA02-p20762905
Bn-A02-p22409779	0.991735537	0.983471074	Bn-N2-p24452906	Bn-chrA02-p20756474
Bn-A02-p22426466	0.966942149	0.975206612	Bn-N2-p24435716	Bn-chrA02-p20744184
Bn-A02-p22427387	0.966942149	0.900826446	Bn-N2-p24433587	Bn-chrA02-p20743276
Bn-A02-p22429308	1	0.990033223	Bn-N2-p24431116	Bn-chrA02-p20741354
Bn-A02-p22429563	0.94214876	0.983471074	Bn-N12-p38144741	Bn-chrA02-p20741099

Bn-A02-p22429871	0.975206612	0.975206612	Bn-N2-p24430582	Bn-chrA02-p20740796
Bn-A02-p22433753	0.900497512	0.900497512	Bn-N2-p24426719	Bn-chrA02-p20736861
Bn-A02-p22443535	0.958677686	0.958677686	Bn-N2-p24416123	Bn-chrA02-p20723010
Bn-A02-p22460758	0.965174129	0.960199005	Bn-Scaffold05962-p1	Bn-chrAnn_random-p468222
Bn-A02-p22478377	0.966942149	0.966942149	Bn-N10-p7103511	Bn-chrA10_random-p547489
Bn-A02-p22479272	0.991735537	0.991735537	Bn-N10-p7102617	Bn-chrA10_random-p546594
Bn-A02-p22485435	0.991735537	0.991735537	Bn-N10-p7095750	Bn-chrA10_random-p540450
Bn-A02-p22485606	0.983471074	0.983471074	Bn-N10-p7095579	Bn-chrA10_random-p540279
Bn-A02-p22487030	0.993355482	0.993355482	Bn-N10-p7094155	Bn-chrA10_random-p538855
Bn-A02-p22492507	0.991735537	0.991735537	Bn-N10-p7088678	Bn-chrA10_random-p533377
Bn-A02-p22493338	0.970149254	0.970149254	Bn-N10-p7087848	Bn-chrA10_random-p532547
Bn-A02-p22501359	1	1	Bn-N10-p7079771	Bn-chrA10_random-p524406
Bn-A02-p22507142	0.975206612	0.975206612	Bn-N10-p7073660	Bn-chrA10_random-p513506
Bn-A02-p22508460	0.958677686	0.958677686	Bn-N10-p7071832	Bn-chrA10_random-p512086
Bn-A02-p22508870	1	1	Bn-N10-p7071423	Bn-chrA10_random-p511677
Bn-A02-p22509521	0.545454545	0.545454545	Bn-N10-p7070937	Bn-chrA10_random-p511191
Bn-A02-p22515745	1	1	Bn-N10-p7070562	Bn-chrA10_random-p510814
Bn-A02-p22518385	0.966942149	0.966942149	Bn-N10-p7068076	Bn-chrA10_random-p508328
Bn-A02-p22519689	0.991735537	0.991735537	Bn-N10-p7066783	Bn-chrA10_random-p507035
Bn-A02-p22520110	0.991735537	0.991735537	Bn-N10-p7066362	Bn-chrA10_random-p506614
Bn-A02-p22520724	0.983471074	0.983471074	Bn-N10-p7065747	Bn-chrA10_random-p505999
Bn-A02-p22521362	1	1	Bn-N10-p7065103	Bn-chrA10_random-p505355
Bn-A02-p22521802	0.975206612	0.966942149	Bn-N10-p7064588	Bn-chrA10_random-p504901
Bn-A02-p22528701	0.985074627	0.985074627	Bn-N10-p7057581	Bn-chrA10_random-p498869
Bn-A02-p22534152	1	1	Bn-N10-p7050259	Bn-chrA10_random-p490772
Bn-A02-p22534735	0.991735537	0.991735537	Bn-N10-p7049667	Bn-chrA10_random-p490180
Bn-A02-p22543683	0.993355482	0.993355482	Bn-N10-p7041688	Bn-chrA10_random-p480738
Bn-A02-p22550542	1	1	Bn-N10-p7034603	Bn-chrA10_random-p473654
Bn-A02-p22552916	0.991735537	0.991735537	Bn-N10-p7032225	Bn-chrA10_random-p471276
Bn-A02-p22557981	1	1	Bn-N10-p7027206	Bn-chrA10_random-p466177
Bn-A02-p22558530	1	1	Bn-N10-p7026657	Bn-chrA10_random-p465628
Bn-A02-p22560577	0.983471074	0.983471074	Bn-N10-p7024617	Bn-chrA10_random-p463588
Bn-A02-p22563469	1	1	Bn-N10-p7021724	Bn-chrA10_random-p460695
Bn-A02-p22564964	0.993355482	0.993355482	Bn-N10-p7020229	Bn-chrA10_random-p459200
Bn-A02-p22565755	0.966942149	0.958677686	Bn-N10-p7019439	Bn-chrA10_random-p458411
Bn-A02-p22570273	0.976744186	0.976744186	Bn-N10-p7014936	Bn-chrA10_random-p453908
Bn-A02-p22571187	1	1	Bn-N10-p7014022	Bn-chrA10_random-p452994
Bn-A02-p22571726	0.991735537	0.991735537	Bn-N10-p7013485	Bn-chrA10_random-p452457
Bn-A02-p22576112	0.966942149	0.966942149	Bn-N10-p7008947	Bn-chrA10_random-p448072
Bn-A02-p22576511	0.991735537	0.991735537	Bn-N10-p7008548	Bn-chrA10_random-p447673
Bn-A02-p22577127	0.991735537	0.991735537	Bn-N10-p7007931	Bn-chrA10_random-p447056
Bn-A02-p22581557	0.991735537	0.991735537	Bn-N10-p7003500	Bn-chrA10_random-p442626
Bn-A02-p22590101	0.950248756	0.950248756	Bn-N19-p27206514	Bn-chrA04-p6408558
Bn-A02-p22596234	0.991735537	0.991735537	Bn-N10-p7000000	Bn-chrA10_random-p439126
Bn-A02-p22596523	1	1	Bn-N10-p6999711	Bn-chrA10_random-p438837
Bn-A02-p22597014	0.991735537	0.991735537	Bn-N10-p6999220	Bn-chrA10_random-p438346

Bn-A02-p22599833	0.983471074	0.983471074	Bn-N10-p6996400	Bn-chrA10_random-p435528
Bn-A02-p22601555	0.983471074	0.983471074	Bn-N10-p6994289	Bn-chrA10_random-p432971
Bn-A02-p22602317	0.983471074	0.983471074	Bn-N10-p6993526	Bn-chrA10_random-p432208
Bn-A02-p22637438	1	1	Bn-N10-p6974411	Bn-chrA10_random-p778757
Bn-A02-p22637504	0.983471074	0.983471074	Bn-N10-p6974345	Bn-chrA10_random-p778823
Bn-A02-p22638512	1	1	Bn-N10-p6973336	Bn-chrA10_random-p783401
Bn-A02-p22641483	0.991735537	0.991735537	Bn-N10-p6970365	Bn-chrA10_random-p786372
Bn-A02-p22642101	0.991735537	0.991735537	Bn-N10-p6969753	Bn-chrA10_random-p786983
Bn-A02-p22650896	1	1	Bn-N10-p6960950	Bn-chrA10_random-p925428
Bn-A02-p22651355	0.991735537	0.991735537	Bn-N10-p6960491	Bn-chrA10_random-p925887
Bn-A02-p22654465	0.966942149	0.966942149	Bn-N10-p6957379	Bn-chrA10_random-p800562
Bn-A02-p22670299	0.983471074	0.983471074	Bn-N10-p6943128	Bn-chrA10_random-p817567
Bn-A02-p22682931	0.993355482	0.993355482	Bn-N10-p6933483	Bn-chrA10_random-p830155
Bn-A02-p22693351	0.996677741	0.993355482	Bn-N10-p6922851	Bn-chrA10_random-p840594
Bn-A02-p22697509	0.990033223	0.993355482	Bn-N10-p6918692	Bn-chrA10_random-p844752
Bn-A02-p22702484	1	1	Bn-N10-p6914189	Bn-chrAnn_random-p416420
Bn-A02-p22741797	0.990049751	0.990049751	Bn-N2-p24315106	Bn-chrA02-p20575859
Bn-A02-p22757902	0.991735537	0.991735537	Bn-N2-p24299028	Bn-chrAnn_random-p442032
Bn-A02-p22787085	0.933884298	0.933884298	Bn-N2-p24276200	Bn-chrA02-p20538434
Bn-A02-p22798107	1	1	Bn-N2-p24273663	Bn-chrA02-p20535897
Bn-A02-p22811512	0.950413223	0.950413223	Bn-N2-p24256346	Bn-chrA02-p20519620
Bn-A02-p22843446	0.958677686	0.966942149	Bn-N2-p24221591	Bn-chrA02-p20489037
Bn-A02-p22847040	0.975206612	0.966942149	Bn-N2-p24218772	Bn-chrA02-p20486025
Bn-A02-p22847472	0.983471074	0.991735537	Bn-N2-p24218337	Bn-chrA02-p20485591
Bn-A02-p22863292	0.572139303	0.990049751	Bn-N10-p3243689	Bn-chrA02-p20469898
Bn-A02-p22863757	0.383084577	1	Bn-N17-p1435043	Bn-chrA02-p20469433
Bn-A02-p22863981	0.432835821	0.985074627	Bn-N15-p25032831	Bn-chrA02-p20469209
Bn-A02-p22872607	0.991735537	0.983471074	Bn-N2-p24201000	Bn-chrA02-p20460583
Bn-A02-p22875408	0.991735537	0.983471074	Bn-N2-p24197608	Bn-chrA02-p20457797
Bn-A02-p22899292	0.991735537	0.983471074	Bn-N2-p24172355	Bn-chrA02-p20435480
Bn-A02-p22899854	0.933884298	0.991735537	Bn-N2-p24169582	Bn-chrA02-p20434918
Bn-A02-p22906452	0.636363636	0.983471074	Bn-N2-p24160484	Bn-chrA02-p20428321
Bn-A02-p22906797	1	0.991735537	Bn-N2-p24160137	Bn-chrA02-p20427976
Bn-A02-p23092476	0.958677686	0.884297521	Bn-N5-p24537998	Bn-chrC05-p41622097
Bn-A02-p23095572	0.985074627	0.985074627	Bn-N5-p24533762	Bn-chrA05-p21629396
Bn-A02-p23098985	0.966942149	0.966942149	Bn-N5-p24527899	Bn-chrA05-p21622101
Bn-A02-p23099281	0.966942149	0.966942149	Bn-N5-p24527619	Bn-chrA05-p21621821
Bn-A02-p23101889	0.79338843	0.79338843	Bn-N5-p24525129	Bn-chrA05-p21619331
Bn-A02-p23108517	0.950413223	0.958677686	Bn-N5-p24518497	Bn-chrC05-p41610268
Bn-A02-p23110903	0.958677686	0.958677686	Bn-N5-p24515867	Bn-chrA05-p21610069
Bn-A02-p23112746	0.990033223	0.990033223	Bn-N5-p24514024	Bn-chrA05-p21608226
Bn-A02-p23128571	0.958677686	0.958677686	Bn-N5-p24499063	Bn-chrA05-p21593900
Bn-A02-p23135500	0.980099502	0.980099502	Bn-N5-p24491803	Bn-chrA05-p21586581
Bn-A02-p23161914	0.991735537	0.991735537	Bn-N5-p24463861	Bn-chrA05-p21559341
Bn-A02-p23163100	0.983471074	0.983471074	Bn-N5-p24461823	Bn-chrA05-p21557303
Bn-A02-p23168213	0.990033223	0.990033223	Bn-N5-p24457133	Bn-chrA05-p21552613

Bn-A02-p23237139	0.996677741	0.996677741	Bn-N5-p24376662	Bn-chrA05-p21483053
Bn-A02-p23251431	0.996677741	0.996677741	Bn-N5-p24360162	Bn-chrA05-p21465193
Bn-A02-p23257505	0.975206612	0.975206612	Bn-N5-p24358820	Bn-chrA05-p21463851
Bn-A02-p23277919	0.96345515	0.96345515	Bn-N5-p24340494	Bn-chrA05-p21446878
Bn-A02-p23279287	0.983471074	0.983471074	Bn-N5-p24339131	Bn-chrA05-p21445514
Bn-A02-p23280472	1	1	Bn-N5-p24337931	Bn-chrA05-p21444314
Bn-A02-p23280665	0.995024876	0.985074627	Bn-N5-p24337738	Bn-chrA05-p21444121
Bn-A02-p23280869	0.985074627	0.985074627	Bn-N5-p24337534	Bn-chrA05-p21443919
Bn-A02-p23295254	0.991735537	0.983471074	Bn-N5-p24327677	Bn-chrA05-p21434153
Bn-A02-p23297690	0.860465116	0.996677741	Bn-N15-p46318587	Bn-chrA05-p21431716
Bn-A02-p23297789	0.88372093	0.986710963	Bn-N15-p46318493	Bn-chrA05-p21431617
Bn-A02-p23309610	0.986710963	0.986710963	Bn-N5-p24312531	Bn-chrA05-p21418682
Bn-A02-p23362842	0.993355482	0.996677741	Bn-N2-p25780097	Bn-chrAnn_random-p447152
Bn-A02-p23408870	0.983471074	0.991735537	Bn-N2-p25826230	Bn-chrAnn_random-p794863
Bn-A02-p23411852	0.983471074	0.991735537	Bn-N2-p25829195	Bn-chrAnn_random-p795162
Bn-A02-p23414384	1	0.991735537	Bn-N2-p25834241	Bn-chrAnn_random-p795411
Bn-A02-p23414610	0.983471074	0.752066116	Bn-N2-p25834467	Bn-chrAnn_random-p795431
Bn-A02-p23414614	0.983471074	0.785123967	Bn-N2-p25834471	Bn-chrAnn_random-p795431
Bn-A02-p23423377	0.983471074	0.966942149	Bn-N2-p25841676	Bn-chrAnn_random-p795668
Bn-A02-p23425517	0.966942149	0.958677686	Bn-N2-p25843437	Bn-chrAnn_random-p796433
Bn-A02-p23428461	0.983471074	0.975206612	Bn-N2-p25846391	Bn-chrC01-p34824627
Bn-A02-p23432823	0.991735537	0.983471074	Bn-N2-p25852350	Bn-chrAnn_random-p797761
Bn-A02-p23436829	0.958677686	0.966942149	Bn-N2-p25856614	Bn-chrAnn_random-p798174
Bn-A02-p23448271	0.983471074	0.975206612	Bn-N2-p25867943	Bn-chrAnn_random-p799157
Bn-A02-p23454935	0.993355482	0.850498339	Bn-N2-p25872381	Bn-chrAnn_random-p799752
Bn-A02-p23456103	0.991735537	0.983471074	Bn-N2-p25873549	Bn-chrAnn_random-p799868
Bn-A02-p23472944	0.983471074	0.975206612	Bn-N2-p25888075	Bn-chrAnn_random-p801768
Bn-A02-p23473807	0.975206612	0.842975207	Bn-N2-p25888938	Bn-chrAnn_random-p801854
Bn-A02-p23474309	0.958677686	0.950413223	Bn-N2-p25889440	Bn-chrAnn_random-p801904
Bn-A02-p23477774	1	0.991735537	Bn-N2-p25895492	Bn-chrAnn_random-p802326
Bn-A02-p23477968	0.990033223	0.993355482	Bn-N2-p25895693	Bn-chrAnn_random-p802346
Bn-A02-p23479432	0.990049751	0.990049751	Bn-N12-p40120329	Bn-chrC02-p41257995
Bn-A02-p23482499	0.991735537	0.983471074	Bn-N2-p25900190	Bn-chrAnn_random-p802813
Bn-A02-p23486694	0.970149254	0.985074627	Bn-N2-p25904143	Bn-chrAnn_random-p803719
Bn-A02-p23488731	0.991735537	1	Bn-N2-p25906177	Bn-chrAnn_random-p803923
Bn-A02-p23491463	0.990049751	0.995024876	Bn-N2-p6644613	Bn-chrA02_random-p257320
Bn-A02-p23494185	0.892561983	0.991735537	Bn-N2-p25911582	Bn-chrAnn_random-p804860
Bn-A02-p23495061	0.876033058	0.983471074	Bn-N2-p25912435	Bn-chrAnn_random-p804948
Bn-A02-p23497111	0.966942149	1	Bn-N2-p25914511	Bn-chrAnn_random-p805153
Bn-A02-p23509687	0.950413223	0.94214876	Bn-N2-p25929865	Bn-chrAnn_random-p806267
Bn-A02-p23509986	0.958677686	0.950413223	Bn-N2-p25930169	Bn-chrAnn_random-p806298
Bn-A02-p23510178	0.983471074	0.991735537	Bn-N2-p25930361	Bn-chrAnn_random-p806317
Bn-A02-p23523709	0.975206612	0.983471074	Bn-N2-p25944732	Bn-chrAnn_random-p807746
Bn-A02-p23668607	0.955223881	0.980099502	Bn-N2-p26087020	Bn-chrA02-p21869211
Bn-A02-p23669898	0.966942149	0.983471074	Bn-N2-p26088247	Bn-chrA02-p21870442
Bn-A02-p23685036	0.975206612	0.966942149	Bn-N2-p26099373	Bn-chrA02-p21882531

Bn-A02-p23686560	0.991735537	0.983471074	Bn-N2-p26100891	Bn-chrA02-p21884055
Bn-A02-p23700858	0.930232558	0.996677741	Bn-N12-p40444818	Bn-chrA02-p21892563
Bn-A02-p23708117	0.247933884	0.58677686	Unknown	Bn-chrA02-p21917440
Bn-A02-p23708299	0.603305785	0.991735537	Bn-N17-p35241902	Bn-chrA02-p21917622
Bn-A02-p23708516	0.148760331	0.991735537	Bn-N10-p9972797	Bn-chrA02-p21917839
Bn-A02-p23708678	0.32231405	0.991735537	Unknown	Bn-chrA02-p21918001
Bn-A02-p23726200	0.636363636	0.983471074	Bn-N12-p40512125	Bn-chrAnn_random-p303933
Bn-A02-p23728173	0.880597015	0.940298507	Bn-N2-p26116086	Bn-chrAnn_random-p303899
Bn-A02-p23729046	0.606965174	0.985074627	Bn-N11-p18438566	Bn-chrAnn_random-p303891
Bn-A02-p23730904	0.859504132	0.958677686	Bn-N12-p40526823	Bn-chrA02-p21933102
Bn-A02-p23743267	0.943521595	0.980066445	Bn-N2-p26126049	Bn-chrA02-p21956078
Bn-A02-p23757876	0.970099668	0.96345515	Bn-N2-p26139876	Bn-chrA02-p21986982
Bn-A02-p23788301	1	0.996677741	Bn-N2-p26176003	Bn-chrA02-p22014556
Bn-A02-p23792703	0.986710963	0.986710963	Bn-N2-p26180452	Bn-chrA02-p22019005
Bn-A02-p23819701	0.975206612	0.983471074	Bn-N2-p26207834	Bn-chrA02-p22047795
Bn-A02-p23819797	0.965174129	0.965174129	Bn-N2-p26207929	Bn-chrA02-p22047890
Bn-A02-p23824343	0.834710744	0.983471074	Bn-N2-p26213639	Bn-chrA02-p22051853
Bn-A02-p23826324	1	0.991735537	Bn-N2-p26215628	Bn-chrA02-p22053834
Bn-A02-p23831173	0.966942149	0.966942149	Bn-N2-p26219071	Bn-chrA02-p22058109
Bn-A02-p23832547	0.991735537	1	Bn-N2-p26220424	Bn-chrA02-p22066027
Bn-A02-p23835683	0.966942149	0.975206612	Bn-N2-p26223574	Bn-chrA02-p22068083
Bn-A02-p23836232	0.966942149	0.975206612	Bn-N2-p26224099	Bn-chrA02-p22068630
Bn-A02-p23842103	0.983471074	0.975206612	Bn-N2-p26229880	Bn-chrA02-p22074451
Bn-A02-p23842763	0.991735537	0.983471074	Bn-N2-p26230540	Bn-chrA02-p22075112
Bn-A02-p23856500	1	0.991735537	Bn-N2-p26249411	Bn-chrA02-p22090286
Bn-A02-p23857384	0.991735537	0.983471074	Bn-N2-p26250302	Bn-chrA02-p22091170
Bn-A02-p23875682	0.983471074	0.991735537	Bn-N2-p26268225	Bn-chrA02-p22108463
Bn-A02-p23889188	0.958677686	0.966942149	Bn-N2-p26279361	Bn-chrA02-p22120695
Bn-A02-p23897014	0.983471074	0.991735537	Bn-N2-p26287727	Bn-chrA02-p22129177
Bn-A02-p23910066	0.991735537	0.991735537	Bn-N1-p25931195	Bn-chrAnn_random-p338879
Bn-A02-p23920155	0.986710963	0.973421927	Bn-N10-p2032226	Bn-chrA10-p1894584
Bn-A02-p23925334	1	0.993355482	Bn-N10-p2027091	Bn-chrA10-p1889430
Bn-A02-p24015556	0.996677741	1	Bn-N2-p26386995	Bn-chrA02-p22226481
Bn-A02-p24178094	0.970149254	0.970149254	Bn-N2-p26534947	Bn-chrA02-p22406630
Bn-A02-p24216905	0.965174129	0.970149254	Bn-N2-p26572947	Bn-chrAnn_random-p125094
Bn-A02-p24237359	0.996677741	0.996677741	Bn-N2-p26593264	Bn-chrAnn_random-p124901
Bn-A02-p24268496	0.990033223	0.990033223	Bn-N2-p26620206	Bn-chrAnn_random-p124648
Bn-A02-p24269435	0.996677741	0.996677741	Bn-N2-p26621146	Bn-chrAnn_random-p124638
Bn-A02-p24316498	0.991735537	0.991735537	Bn-N2-p26676979	Bn-chrA02-p22435606
Bn-A02-p24317870	0.995024876	0.990049751	Bn-N2-p26678351	Bn-chrA02-p22436977
Bn-A02-p24345550	1	0.991735537	Bn-N2-p26695494	Bn-chrA02-p22472050
Bn-A02-p24345838	0.975124378	0.980099502	Bn-N2-p26695782	Bn-chrA02-p22472338
Bn-A02-p24351316	0.983471074	0.975206612	Bn-N2-p26698268	Bn-chrA02-p22474819
Bn-A02-p24353955	0.975206612	0.983471074	Bn-N2-p26701541	Bn-chrA02-p22477573
Bn-A02-p24353997	0.983471074	0.991735537	Bn-N2-p26701583	Bn-chrA02-p22477615
Bn-A02-p24354876	0.991735537	1	Bn-N2-p26702462	Bn-chrA02-p22478494

Bn-A02-p24359370	0.975206612	0.983471074	Bn-N2-p26707970	Bn-chrC02-p42606254
Bn-A02-p24366221	1	1	Bn-N2-p26715049	Bn-chrA02-p22490280
Bn-A02-p24378297	0.995024876	1	Bn-N2-p26731687	Bn-chrA02-p22502529
Bn-A02-p24386848	0.991735537	0.404958678	Bn-N2-p26740390	Bn-chrA03_random-p529855
Bn-A02-p24399308	0.986710963	0.986710963	Bn-N2-p26752657	Bn-chrA02-p22525673
Bn-A02-p24401267	0.925619835	0.991735537	Bn-N12-p41622271	Bn-chrA02-p22527532
Bn-A02-p24402133	0.983471074	0.966942149	Bn-N2-p26755405	Bn-chrA02-p22528403
Bn-A02-p24415454	0.983471074	0.975206612	Bn-N2-p26761096	Bn-chrA02-p22534260
Bn-A02-p24417020	0.983388704	0.983388704	Bn-N2-p26762687	Bn-chrA02-p22535851
Bn-A02-p24438115	1	1	Bn-N2-p26785340	Bn-chrA02-p22556598
Bn-A02-p24449690	0.983388704	0.983388704	Bn-N2-p26799071	Bn-chrA02-p22569686
Bn-A02-p24462762	0.973421927	0.973421927	Bn-N2-p26817344	Bn-chrA02-p22582493
Bn-A02-p24477743	0.983471074	0.809917355	Bn-N2-p26831011	Bn-chrC02-p42774199
Bn-A02-p24505252	0.94214876	0.925619835	Bn-N2-p26856037	Bn-chrA02-p22627528
Bn-A02-p24508488	0.965174129	0.990049751	Bn-N2-p26859241	Bn-chrA02-p22630376
Bn-A02-p24522983	0.958677686	0.94214876	Bn-N2-p26882288	Bn-chrA02-p22654494
Bn-A02-p24532892	0.780730897	0.993355482	Bn-N2-p26899397	Bn-chrA02-p22663376
Bn-A02-p24533751	0.909090909	0.933884298	Bn-N2-p26907160	Bn-chrA02-p22665182
Bn-A02-p24543172	0.966942149	0.975206612	Bn-N2-p26913794	Bn-chrA02-p22671040
Bn-A02-p24548194	1	0.991735537	Bn-N2-p26918705	Bn-chrA02-p22675944
Bn-A02-p24552203	0.991735537	1	Bn-N2-p26923562	Bn-chrA02-p22692082
Bn-A02-p24552476	0.966942149	0.975206612	Bn-N2-p26923836	Bn-chrA02-p22692355
Bn-A02-p24552542	1	0.991735537	Bn-N2-p26923902	Bn-chrA02-p22692421
Bn-A02-p24552639	0.975206612	0.983471074	Bn-N2-p26924001	Bn-chrA02-p22692518
Bn-A02-p24552721	0.950413223	0.983471074	Bn-N2-p26924084	Bn-chrA02-p22692600
Bn-A02-p24552840	0.975206612	0.983471074	Bn-N2-p26924199	Bn-chrA02-p22692719
Bn-A02-p24552843	0.983471074	0.975206612	Bn-N2-p26924202	Bn-chrA02-p22692722
Bn-A02-p24559792	0.990049751	0.990049751	Bn-N2-p26931265	Bn-chrA02-p22699841
Bn-A02-p24575998	0.710963455	0.996677741	Bn-N2-p26942553	Bn-chrA02-p22711756
Bn-A02-p24577932	0.983471074	0.991735537	Bn-N2-p26944486	Bn-chrA02-p22713691
Bn-A02-p24584058	0.993355482	0.993355482	Bn-N2-p26950533	Bn-chrA02-p22719813
Bn-A02-p24675182	0.986710963	0.986710963	Bn-N2-p27027234	Bn-chrA02-p22769251
Bn-A02-p24687309	0.996677741	0.996677741	Bn-N2-p27039826	Bn-chrA02-p22781275
Bn-A02-p24688870	0.996677741	0.996677741	Bn-N2-p27041388	Bn-chrA02-p22782837
Bn-A02-p24700081	0.995024876	0.995024876	Bn-N2-p27052644	Bn-chrC06-p36950115
Bn-A02-p24700485	0.990049751	0.985074627	Bn-Scaffold02307-p3	Bn-chrA02-p3586349
Bn-A02-p24700670	0.975124378	0.970149254	Bn-Scaffold02307-p3	Bn-chrA02-p3586534
Bn-A02-p24700750	0.960199005	0.955223881	Bn-Scaffold02307-p3	Bn-chrA02-p3586614
Bn-A02-p24702518	0.950248756	0.950248756	Bn-Scaffold02307-p1	Bn-chrA02-p22793839
Bn-A02-p24702986	0.945273632	0.945273632	Bn-Scaffold02307-p8	Bn-chrA02-p3588850
Bn-A02-p24703050	0.950248756	0.950248756	Bn-Scaffold02307-p8	Bn-chrA02-p22794371
Bn-A02-p24706646	0.993355482	0.993355482	Bn-N2-p27057373	Bn-chrA02-p22797968
Bn-A02-p24708197	0.920265781	0.920265781	Bn-N2-p27058924	Bn-chrA02-p22799519
Bn-A02-p24718110	0.983471074	0.983471074	Bn-Scaffold13473-p3	Bn-chrA02-p22809620
Bn-A02-p24722684	0.983471074	0.975206612	Bn-N2-p27070634	Bn-chrA02-p22810424
Bn-A02-p24731379	0.991735537	0.975206612	Bn-N2-p27078849	Bn-chrA02-p22820859

Bn-A02-p24809526	0.996677741	0.996677741	Bn-N2-p27155988	Bn-chrA02-p22898350
Bn-A02-p24810609	0.965174129	0.965174129	Bn-N2-p27157071	Bn-chrA02-p22899433
Bn-A02-p24827362	0.993355482	0.973421927	Bn-N2-p27181943	Bn-chrC02-p43395534
Bn-A02-p24844291	1	0.991735537	Bn-N2-p27195801	Bn-chrA02-p22934813
Bn-A02-p24851624	0.983471074	0.991735537	Bn-N2-p27203225	Bn-chrA02-p22942172
Bn-A02-p24878657	0.966777409	0.990033223	Bn-N2-p27227551	Bn-chrA02-p22969033
Bn-A02-p24909771	0.975124378	0.985074627	Bn-N2-p27260683	Bn-chrA02-p23001197
Bn-A02-p24911505	0.993355482	0.996677741	Bn-N2-p27262414	Bn-chrA02-p23002931
Bn-A02-p24912792	0.993355482	0.993355482	Bn-N2-p27263700	Bn-chrA02-p23004218
Bn-A02-p24914083	0.958677686	0.991735537	Bn-N2-p27264991	Bn-chrA02-p23005509
Bn-A02-p24919366	0.950413223	0.925619835	Bn-N2-p27271338	Bn-chrA02-p23011843
Bn-A02-p24928201	0.983471074	0.975206612	Bn-N2-p27283141	Bn-chrA02-p23021054
Bn-A02-p24928311	0.94214876	0.975206612	Bn-N2-p27283251	Bn-chrA02-p23021164
Bn-A02-p24945785	0.983471074	0.975206612	Bn-N2-p27306231	Bn-chrAnn_random-p387516
Bn-A02-p24948938	0.983471074	0.479338843	Bn-N2-p27309412	Unknown
Bn-A02-p24967958	0.956810631	0.953488372	Bn-N2-p27323065	Bn-chrAnn_random-p392595
Bn-A02-p24971607	0.975206612	0.983471074	Bn-N2-p27330481	Bn-chrA02-p23067298
Bn-A02-p25022333	0.975206612	0.975206612	Bn-N12-p42692644	Bn-chrC02-p43782014
Bn-A02-p25022921	0.96345515	0.96345515	Bn-N2-p27354623	Bn-chrA02-p23096021
Bn-A02-p25027576	0.990049751	0.990049751	Bn-N12-p42698621	Bn-chrC02-p43787990
Bn-A02-p25079431	0.975124378	0.975124378	Bn-N2-p27414812	Bn-chrA02-p23154160
Bn-A02-p25081911	0.986710963	0.986710963	Bn-N2-p27417291	Bn-chrA02-p23156639
Bn-A02-p25081916	0.983388704	0.983388704	Bn-N2-p27417296	Bn-chrA02-p23156644
Bn-A02-p25100451	0.991735537	0.991735537	Bn-N2-p27435756	Bn-chrA02-p23175159
Bn-A02-p25105527	0.980066445	0.980066445	Bn-N2-p27441225	Bn-chrA02-p23180627
Bn-A02-p25109769	1	1	Bn-N2-p27445704	Bn-chrA02-p23183973
Bn-A02-p25118816	0.754152824	0.930232558	Bn-N3-p20735120	Bn-chrA02-p23197224
Bn-A02-p25174583	0.850746269	0.950248756	Bn-N6-p19184266	Bn-chrA02-p23256874
Bn-A02-p25180996	0.995024876	1	Bn-N2-p27515009	Bn-chrA02-p23261400
Bn-A02-p25181474	0.985074627	1	Bn-N2-p27515640	Bn-chrA02-p23261878
Bn-A02-p25181726	0.993355482	0.993355482	Bn-N2-p27515892	Bn-chrA02-p23262130
Bn-A02-p25188041	0.818181818	0.694214876	Bn-N2-p27518281	Bn-chrCnn_random-p770128
Bn-A02-p25190854	0.975206612	0.983471074	Bn-N2-p27521196	Bn-chrA02-p23272891
Bn-A02-p25199281	0.820598007	0.903654485	Bn-N12-p42970521	Bn-chrA02-p23279002
Bn-A02-p25201372	0.986710963	0.990033223	Bn-N2-p27529744	Bn-chrA02-p23281113
Bn-A02-p25205468	0.990033223	0.993355482	Bn-N2-p27537494	Bn-chrA02-p23285902
Bn-A02-p25206495	0.991735537	0.991735537	Bn-N2-p27538529	Bn-chrA02-p23295977
Bn-A02-p25206605	0.995024876	1	Bn-N2-p27538639	Bn-chrA02-p23296086
Bn-A02-p25216530	0.983471074	0.991735537	Bn-N2-p27548047	Bn-chrA02-p23306148
Bn-A02-p25218817	0.970149254	0.965174129	Bn-N2-p27550327	Bn-chrA02-p23306896
Bn-A02-p25260066	0.976744186	0.983388704	Bn-N2-p27602638	Bn-chrA02-p23337158
Bn-A02-p25285941	0.940298507	0.980099502	Bn-N16-p34442110	Bn-chrA02-p23362460
Bn-A02-p25286323	0.950248756	0.975124378	Bn-N16-p34442492	Bn-chrA02-p23362842
Bn-A02-p25286373	0.935323383	0.970149254	Bn-N16-p34442542	Bn-chrA10_random-p589435
Bn-A02-p25332661	0.950413223	0.950413223	Bn-N2-p27634976	Bn-chrA02-p23410308
Bn-A02-p25334100	0.996677741	0.996677741	Bn-N2-p27636469	Bn-chrA02-p23411799

Bn-A02-p25334266	0.975206612	0.975206612	Bn-N2-p27636635	Bn-chrA02-p23411965
Bn-A02-p25334271	0.975206612	0.975206612	Bn-N2-p27636640	Bn-chrA02-p23411970
Bn-A02-p25350701	0.993355482	0.986710963	Bn-N2-p27653249	Bn-chrA02-p23427763
Bn-A02-p25367886	0.985074627	0.985074627	Bn-N12-p43148129	Bn-chrA02_random-p158560
Bn-A02-p25375825	0.980099502	0.985074627	Bn-N2-p27670381	Bn-chrAnn_random-p378785
Bn-A02-p25389902	0.975206612	0.983471074	Bn-N2-p27681881	Bn-chrA02_random-p160153
Bn-A02-p25400515	0.958677686	0.966942149	Bn-N2-p27686740	Bn-chrA02_random-p161465
Bn-A02-p25400591	0.950413223	0.975206612	Bn-N2-p27686816	Bn-chrA02_random-p161473
Bn-A02-p25401060	0.983471074	0.991735537	Bn-N2-p27687274	Bn-chrA02_random-p161520
Bn-A02-p25402286	0.950413223	0.958677686	Bn-N2-p27687764	Bn-chrA02_random-p161642
Bn-A02-p25402971	0.991735537	1	Bn-N2-p27689228	Bn-chrA02_random-p161711
Bn-A02-p25413153	0.991735537	0.983471074	Bn-N2-p27698334	Bn-chrA02_random-p161983
Bn-A02-p25419536	0.991735537	0.983471074	Bn-N2-p27704681	Bn-chrA02_random-p157848
Bn-A02-p25429624	1	0.73553719	Bn-N2-p27705618	Bn-chrC02-p44270519
Bn-A02-p25429837	0.985074627	0.970149254	Bn-N2-p27705832	Bn-chrA02_random-p157251
Bn-A02-p25436510	0.960199005	0.985074627	Bn-N2-p27714267	Bn-chrA02_random-p156579
Bn-A02-p25438684	0.966942149	0.975206612	Bn-N2-p27715822	Bn-chrA02_random-p156362
Bn-A02-p25445157	0.983471074	0.975206612	Bn-N2-p27722794	Bn-chrA02_random-p155547
Bn-A02-p25446521	0.983471074	1	Bn-N2-p27724158	Bn-chrA02_random-p155411
Bn-A02-p25448092	0.983471074	0.991735537	Bn-N2-p27725728	Bn-chrA02_random-p155253
Bn-A02-p25453070	0.975206612	0.991735537	Bn-N2-p27730776	Bn-chrA02_random-p154748
Bn-A02-p25453447	0.958677686	0.950413223	Bn-N2-p27731154	Bn-chrA02_random-p154711
Bn-A02-p25454242	0.991735537	0.983471074	Bn-N2-p27732531	Bn-chrA02_random-p154631
Bn-A02-p25454499	0.975206612	0.983471074	Bn-N2-p27732788	Bn-chrA02_random-p154605
Bn-A02-p25454882	0.975206612	0.983471074	Bn-N2-p27733184	Bn-chrA02_random-p154566
Bn-A02-p25456985	0.983471074	0.975206612	Bn-N2-p27735438	Bn-chrA02_random-p154329
Bn-A02-p25457045	0.966942149	0.958677686	Bn-N2-p27735498	Bn-chrA02_random-p154323
Bn-A02-p25457843	0.884297521	0.975206612	Bn-N2-p27735941	Bn-chrA02_random-p154243
Bn-A02-p25463582	0.94214876	0.950413223	Bn-N2-p27740104	Bn-chrA02-p23491943
Bn-A02-p25465508	1	0.991735537	Bn-N2-p27741906	Bn-chrA02-p23493853
Bn-A02-p25465589	0.983471074	0.991735537	Bn-N2-p27741985	Bn-chrA02-p23493933
Bn-A02-p25477417	0.983471074	0.991735537	Bn-N2-p27756740	Bn-chrA02-p23505814
Bn-A02-p25479893	0.975206612	0.983471074	Bn-N2-p27760434	Bn-chrA02-p23508290
Bn-A02-p25484876	0.980099502	0.975124378	Bn-N2-p27765188	Bn-chrC02-p44381410
Bn-A02-p25485627	0.991735537	0.94214876	Bn-N2-p27765939	Bn-chrC02-p44382175
Bn-A02-p25488435	0.996677741	0.983388704	Bn-N2-p27768345	Bn-chrC02-p44384556
Bn-A02-p25497743	0.983471074	0.991735537	Bn-N2-p27774409	Bn-chrA02-p23529563
Bn-A02-p25530111	0.966942149	0.975206612	Bn-N2-p27786711	Bn-chrA02-p23545945
Bn-A02-p25530994	0.958677686	0.966942149	Bn-N2-p27787594	Bn-chrA02-p23546829
Bn-A02-p25554878	0.966942149	0.975206612	Bn-N2-p27810682	Bn-chrA02-p23576369
Bn-A02-p25564792	0.975206612	0.975206612	Bn-N2-p27816660	Bn-chrA02-p23582275
Bn-A02-p25572877	0.990049751	0.970149254	Bn-N2-p27824731	Bn-chrA02-p23593481
Bn-A02-p25612808	0.996677741	0.976744186	Bn-N2-p27880540	Bn-chrA02-p23642309
Bn-A02-p25615285	0.958677686	0.950413223	Bn-N2-p27883015	Bn-chrA02-p23644817
Bn-A02-p25625124	0.950413223	0.876033058	Bn-N2-p27892772	Bn-chrA02-p23654348
Bn-A02-p25625846	0.993355482	0.893687708	Bn-N2-p27893501	Bn-chrC02-p44529636

Bn-A02-p25626500	0.980099502	0.910447761	Bn-N2-p27894166	Bn-chrC02-p44530305
Bn-A02-p25630745	0.993355482	0.50166113	Bn-N2-p27898241	Bn-chrA10-p792403
Bn-A02-p25631431	0.993355482	0.558139535	Bn-N2-p27898927	Bn-chrA08-p10526267
Bn-A02-p25632681	0.993355482	0.312292359	Bn-N2-p27900178	Bn-chrC02-p44531403
Bn-A02-p25636038	0.991735537	0.314049587	Bn-N2-p27903643	Bn-chrA10-p17390203
Bn-A02-p25636136	0.983388704	0.544850498	Bn-N2-p27903741	Bn-chrC05-p12949302
Bn-A02-p25644999	0.970099668	0.966777409	Bn-N2-p27912524	Bn-chrC02-p44541182
Bn-A02-p25652516	0.958677686	0.950413223	Bn-N2-p27919040	Bn-chrAnn_random-p38854C
Bn-A02-p25655528	0.966942149	0.958677686	Bn-N2-p27921272	Bn-chrAnn_random-p420881
Bn-A02-p25670193	0.991735537	0.983471074	Bn-N2-p27937138	Bn-chrAnn_random-p666445
Bn-A02-p25678882	0.958677686	0.966942149	Bn-N2-p27945749	Bn-chrAnn_random-p66732C
Bn-A02-p25681480	0.975206612	0.760330579	Bn-N2-p27948371	Bn-chrA05-p17245400
Bn-A02-p25687470	0.983471074	0.966942149	Bn-N2-p27956264	Bn-chrC02-p44637994
Bn-A02-p25734367	0.975206612	0.983471074	Bn-N2-p28013164	Bn-chrAnn_random-p673001
Bn-A02-p25735700	0.990033223	0.943521595	Bn-N2-p28014498	Bn-chrAnn_random-p673134
Bn-A02-p25736307	1	0.986710963	Bn-N2-p28015128	Bn-chrAnn_random-p673195
Bn-A02-p25739024	0.996677741	0.996677741	Bn-N12-p43787425	Bn-chrC02-p44723373
Bn-A02-p25741878	0.990033223	0.990033223	Bn-N2-p28025732	Bn-chrAnn_random-p673749
Bn-A02-p25746504	0.96345515	0.973421927	Bn-N2-p28033328	Bn-chrAnn_random-p674208
Bn-A02-p25747937	0.950248756	0.950248756	Bn-N12-p43814306	Bn-chrC02-p44751543
Bn-A02-p25762081	0.826446281	0.826446281	Bn-N2-p28047144	Bn-chrAnn_random-p67606C
Bn-A02-p25795588	0.958677686	0.958677686	Bn-N2-p28058120	Bn-chrAnn_random-p677535
Bn-A02-p25814719	0.973421927	0.973421927	Bn-N12-p43888045	Bn-chrC02-p44836079
Bn-A02-p25820450	0.983471074	0.983471074	Bn-N2-p28086130	Bn-chrAnn_random-p679799
Bn-A02-p25821750	0.975206612	0.975206612	Bn-N2-p28087430	Bn-chrAnn_random-p679929
Bn-A02-p25826690	0.950166113	0.950166113	Bn-N2-p28092398	Bn-chrAnn_random-p680504
Bn-A02-p25827208	1	1	Bn-N2-p28092916	Bn-chrAnn_random-p680556
Bn-A02-p25841130	0.975206612	0.975206612	Bn-N2-p28101502	Bn-chrAnn_random-p681699
Bn-A02-p25841252	0.983471074	0.975206612	Bn-N2-p28101630	Bn-chrAnn_random-p681711
Bn-A02-p25841382	0.966942149	0.966942149	Bn-N2-p28101760	Bn-chrAnn_random-p681724
Bn-A02-p25841722	1	0.991735537	Bn-N2-p28102100	Bn-chrAnn_random-p681758
Bn-A02-p25842875	0.983471074	0.975206612	Bn-N2-p28103234	Bn-chrAnn_random-p681878
Bn-A02-p25844300	0.975206612	0.991735537	Bn-N2-p28107422	Bn-chrAnn_random-p682091
Bn-A02-p25845339	0.958677686	0.975206612	Bn-N2-p28108452	Bn-chrAnn_random-p682195
Bn-A02-p25857471	0.983471074	0.925619835	Bn-N2-p28118552	Bn-chrC02-p44883995
Bn-A02-p25864051	0.975206612	0.975206612	Bn-N2-p28124124	Bn-chrAnn_random-p683725
Bn-A02-p25864853	0.986710963	0.973421927	Bn-N2-p28124925	Bn-chrAnn_random-p684014
Bn-A02-p25865360	0.973421927	0.96013289	Bn-N2-p28125432	Bn-chrAnn_random-p684065
Bn-A02-p25876712	0.990033223	0.993355482	Bn-N2-p28136615	Bn-chrAnn_random-p684952
Bn-A02-p25881031	0.915422886	0.940298507	Bn-N2-p28144606	Bn-chrAnn_random-p685874
Bn-A02-p25881408	0.983471074	0.991735537	Bn-N2-p28144982	Bn-chrAnn_random-p685912
Bn-A02-p25883567	0.983471074	0.975206612	Bn-N2-p28147165	Bn-chrAnn_random-p68613C
Bn-A02-p25966536	0.985074627	0.985074627	Bn-Scaffold00372b-p	Bn-chrAnn_random-p691454
Bn-A02-p26013503	0.636363636	0.280991736	Bn-N2-p28212500	Unknown
Bn-A02-p26154897	0.880398671	0.880398671	Bn-N2-p28334294	Bn-chrA02-p23778761
Bn-A02-p26154951	0.955223881	0.955223881	Bn-N2-p28334348	Bn-chrA02-p23778815

Bn-A02-p26193572	0.975206612	0.975206612	Bn-N2-p28398142	Bn-chrAnn_random-p394318
Bn-A02-p26214379	0.980066445	0.980066445	Bn-N2-p28414971	Bn-chrAnn_random-p420233
Bn-A02-p26304354	0.989583333	1	Bn-N2-p28494437	Bn-chrAnn_random-p243607
Bn-A02-p26307761	0.991735537	0.966942149	Bn-N2-p28497831	Bn-chrCnn_random-p703064
Bn-A02-p26322916	0.94214876	0.876033058	Bn-N2-p28512399	Bn-chrC02-p44927610
Bn-A02-p26335941	0.975206612	0.975206612	Bn-N2-p28526343	Bn-chrA02-p24055586
Bn-A02-p26345558	0.92039801	0.980099502	Bn-N12-p44049052	Bn-chrA02-p24065091
Bn-A02-p26373377	0.958677686	0.966942149	Bn-N2-p28564456	Bn-chrA02-p24089096
Bn-A02-p26380384	0.975206612	0.983471074	Bn-N2-p28575801	Bn-chrA02-p24093022
Bn-A02-p26398002	0.983471074	0.983471074	Bn-N2-p28591852	Bn-chrA02-p24112931
Bn-A02-p26411585	0.980099502	0.985074627	Bn-N2-p28604732	Bn-chrA02-p24128650
Bn-A02-p26412693	0.995024876	0.985074627	Bn-Scaffold45799-p2	Bn-chrC02-p45060740
Bn-A02-p26414199	0.990033223	0.990033223	Bn-N2-p28607296	Bn-chrA02-p24131188
Bn-A02-p26419529	0.991735537	0.768595041	Bn-N2-p28614877	Bn-chrA02-p24139624
Bn-A02-p26421790	0.94214876	0.859504132	Bn-N2-p28617133	Bn-chrA02-p24150768
Bn-A02-p26425347	0.479338843	0.842975207	Bn-N2-p28620666	Bn-chrA02-p24155641
Bn-A02-p26431587	0.975206612	0.983471074	Bn-N2-p28624949	Bn-chrA02-p24159646
Bn-A02-p26435743	0.983471074	0.975206612	Bn-N2-p28630812	Bn-chrA02-p24164044
Bn-A02-p26446178	0.983471074	0.950413223	Bn-N2-p28645323	Bn-chrUnn_random-p126667
Bn-A02-p26512828	0.966942149	0.975206612	Bn-N2-p28694702	Bn-chrA02-p24210490
Bn-A02-p26512923	0.958677686	0.975206612	Bn-N2-p28694793	Bn-chrAnn_random-p354768
Bn-A02-p26514827	0.980099502	0.865671642	Bn-N2-p28696696	Bn-chrAnn_random-p354749
Bn-A02-p26535192	0.985074627	0.985074627	Bn-N8-p8572324	Bn-chrA08-p5347147
Bn-A02-p26539305	0.983471074	0.983471074	Bn-N2-p28710720	Bn-chrA02-p24229018
Bn-A02-p26547428	0.94214876	0.933884298	Bn-N2-p28720289	Bn-chrA02-p24237428
Bn-A02-p26554539	0.965174129	0.990049751	Bn-N12-p44317017	Bn-chrA02-p24243807
Bn-A02-p26564337	0.975206612	0.892561983	Bn-N2-p28737357	Bn-chrA02-p24255389
Bn-A02-p26567426	0.975206612	0.966942149	Bn-N2-p28741025	Bn-chrA02-p24262940
Bn-A02-p26571107	0.991735537	0.983471074	Bn-N2-p28744664	Bn-chrA02-p24266598
Bn-A02-p26620662	0.94214876	0.94214876	Bn-N5-p25381428	Bn-chrA05-p22112152
Bn-A02-p26621160	0.867768595	0.867768595	Bn-N5-p25381220	Bn-chrA05-p22112360
Bn-A02-p26625153	0.958677686	0.958677686	Bn-N5-p25377236	Bn-chrA05-p22118239
Bn-A02-p26625696	0.983471074	0.983471074	Bn-N5-p25376687	Bn-chrA05-p22118788
Bn-A02-p26630032	0.933884298	0.933884298	Bn-N5-p25371876	Bn-chrA05-p22123074
Bn-A02-p26640222	0.980066445	0.983388704	Bn-N5-p25361600	Bn-chrA05-p22133249
Bn-A02-p26641996	0.975206612	0.950413223	Bn-N5-p25359883	Bn-chrA05-p22135016
Bn-A02-p26644297	0.980066445	0.996677741	Bn-N5-p25357590	Bn-chrA05-p22137234
Bn-A02-p26645956	0.94214876	0.975206612	Bn-N5-p25355940	Bn-chrA05-p22138887
Bn-A02-p26646040	0.975206612	0.975206612	Bn-N5-p25355861	Bn-chrA05-p22138970
Bn-A02-p26646436	0.991735537	0.851239669	Bn-N5-p25355466	Bn-chrCnn_random-p557894
Bn-A02-p26651278	0.990033223	0.993355482	Bn-N5-p25350622	Bn-chrA05-p22144090
Bn-A02-p26653626	0.983471074	0.991735537	Bn-N5-p25348367	Bn-chrA05-p22146362
Bn-A02-p26654006	0.991735537	1	Bn-N5-p25347987	Bn-chrA05-p22146742
Bn-A02-p26656067	0.991735537	0.983471074	Bn-N5-p25345927	Bn-chrA05-p22148785
Bn-A02-p26656981	0.983471074	0.983471074	Bn-N5-p25344966	Bn-chrA05-p22149700
Bn-A02-p26658350	0.958677686	0.950413223	Bn-N5-p25343597	Bn-chrA05-p22151069

Bn-A02-p26665282	0.958677686	0.975206612	Bn-N5-p25336681	Bn-chrA05-p22158012
Bn-A02-p26668289	0.985074627	0.990049751	Bn-N5-p25333670	Bn-chrA05-p22167053
Bn-A02-p26669888	0.975124378	0.970149254	Bn-N5-p25332080	Bn-chrA05-p22168650
Bn-A02-p26686239	0.950166113	0.956810631	Bn-N5-p25307307	Bn-chrA05-p22275439
Bn-A02-p26689319	0.966942149	0.975206612	Bn-N5-p25304193	Bn-chrA05-p22278551
Bn-A02-p26710300	0.985074627	0.985074627	Bn-N5-p25282988	Bn-chrA05-p22302246
Bn-A02-p26713316	0.958677686	0.958677686	Bn-N5-p25279974	Bn-chrA05-p22305260
Bn-A02-p26733511	0.79338843	0.79338843	Bn-N5-p25264790	Bn-chrA05-p22319201
Bn-A02-p26792851	0.986710963	0.983388704	Bn-Scaffold00893-p9	Bn-chrA05-p22447438
Bn-A02-p26803902	0.800664452	0.900332226	Bn-Scaffold00893-p7	Bn-chrC05-p42781110
Bn-A02-p26804091	0.958677686	0.958677686	Bn-Scaffold00893-p7	Bn-chrA05-p22434480
Bn-A02-p26816101	0.958677686	0.958677686	Bn-Scaffold00893-p6	Bn-chrA05-p22421079
Bn-A02-p26816506	0.983471074	0.983471074	Bn-Scaffold00893-p6	Bn-chrA05-p22420679
Bn-A02-p26819130	0.991735537	0.991735537	Bn-Scaffold00893-p6	Bn-chrA05-p22418055
Bn-A02-p26819608	1	1	Bn-Scaffold00893-p6	Bn-chrA05-p22417577
Bn-A02-p26824239	0.983471074	0.983471074	Bn-Scaffold00893-p5	Bn-chrA05-p22412985
Bn-A02-p26824300	0.991735537	0.991735537	Bn-Scaffold00893-p5	Bn-chrA05-p22412924
Bn-A02-p26824823	0.950413223	0.950413223	Bn-Scaffold00893-p5	Bn-chrA05-p22412401
Bn-A02-p26832454	0.983471074	0.983471074	Bn-Scaffold00893-p4	Bn-chrA05-p22405036
Bn-A02-p26845132	1	1	Bn-Scaffold00893-p3	Bn-chrA05-p22391834
Bn-A02-p26853555	0.975206612	0.983471074	Bn-Scaffold00893-p2	Bn-chrA05-p22383270
Bn-A02-p26854880	0.983388704	0.973421927	Bn-Scaffold00893-p2	Bn-chrA05-p22381945
Bn-A02-p26854933	0.983471074	0.983471074	Bn-Scaffold00893-p2	Bn-chrA05-p22381892
Bn-A02-p26857230	0.950413223	0.958677686	Bn-Scaffold00893-p1	Bn-chrA05-p22378381
Bn-A02-p26883269	0.991735537	0.991735537	Bn-N5-p25199646	Bn-chrA05_random-p291206
Bn-A02-p26887073	0.986710963	0.986710963	Bn-N5-p25195870	Bn-chrA05_random-p291583
Bn-A02-p26890561	0.980099502	0.980099502	Bn-N5-p25192390	Bn-chrA05_random-p291932
Bn-A02-p26892575	0.975206612	0.975206612	Bn-N5-p25190331	Bn-chrA05_random-p292138
Bn-A02-p26900937	0.991735537	0.991735537	Bn-N5-p25180545	Bn-chrA05_random-p293571
Bn-A02-p26901243	0.975206612	0.975206612	Bn-N5-p25180238	Bn-chrA05_random-p293602
Bn-A02-p26921384	0.996677741	0.996677741	Bn-N5-p25156493	Bn-chrA05_random-p295296
Bn-A02-p26946005	0.990033223	0.990033223	Bn-N5-p25132913	Bn-chrA05_random-p297684
Bn-A02-p26947254	0.991735537	1	Bn-N5-p25131677	Bn-chrA05_random-p297809
Bn-A02-p26975795	1	1	Bn-N5-p25106785	Bn-chrA05_random-p300403
Bn-A02-p26982864	0.925619835	0.925619835	Bn-N2-p28771949	Bn-chrA02-p24298178
Bn-A02-p26986319	0.990033223	0.983388704	Bn-N12-p44380576	Bn-chrA02-p24301628
Bn-A02-p26987864	0.983471074	0.983471074	Bn-N2-p28776996	Bn-chrA02-p24303225
Bn-A02-p27022909	0.975206612	0.94214876	Bn-N2-p28804745	Bn-chrA02-p24330966
Bn-A02-p27024443	0.893687708	0.890365449	Bn-N2-p28806279	Bn-chrA02-p24332799
Bn-A02-p27030941	0.94214876	0.950413223	Bn-N2-p28812771	Bn-chrA02-p24339377
Bn-A02-p27035366	0.983471074	0.975206612	Bn-N2-p28817355	Bn-chrA02-p24342879
Bn-A02-p27035425	0.975206612	0.983471074	Bn-N2-p28817414	Bn-chrA02-p24342938
Bn-A02-p27042108	0.975206612	0.785123967	Bn-N2-p28825769	Bn-chrA02-p24352496
Bn-A02-p27043712	0.983471074	0.975206612	Bn-N2-p28827375	Bn-chrA02-p24357153
Bn-A02-p27051969	0.983471074	0.636363636	Bn-N2-p28834782	Bn-chrA02-p24373600
Bn-A02-p27052532	0.975206612	0.966942149	Bn-N2-p28835599	Bn-chrA02-p24374383

Bn-A02-p27052742	0.991735537	0.991735537	Bn-N2-p28835810	Bn-chrA02-p24374599
Bn-A02-p27055844	0.950413223	0.950413223	Bn-N2-p28841236	Bn-chrA02-p24377661
Bn-A02-p27055872	0.950413223	0.950413223	Bn-N2-p28841264	Bn-chrA02-p24377689
Bn-A02-p27109424	0.991735537	0.983471074	Bn-N2-p28892405	Bn-chrA02-p24434138
Bn-A02-p27109713	0.975206612	0.983471074	Bn-N2-p28894210	Bn-chrA02-p24434420
Bn-A02-p27109858	0.983471074	0.991735537	Bn-N2-p28894355	Bn-chrA02-p24434565
Bn-A02-p27110006	0.975206612	0.966942149	Bn-N2-p28894503	Bn-chrA02-p24434713
Bn-A02-p27110104	0.79338843	0.917355372	Bn-N2-p28894601	Bn-chrA02-p24434811
Bn-A02-p27111886	0.991735537	1	Bn-N2-p28895594	Bn-chrA02-p24435193
Bn-A02-p27111963	0.983471074	1	Bn-N2-p28895671	Bn-chrA02-p24435270
Bn-A02-p27121126	0.991735537	0.983471074	Bn-N2-p28903046	Bn-chrA02-p24443866
Bn-A02-p27122935	0.462809917	1	Unknown	Bn-chrA02-p24446240
Bn-A02-p27124090	0.958677686	0.950413223	Bn-N2-p28905991	Bn-chrA02-p24447383
Bn-A02-p27125319	0.958677686	0.950413223	Bn-N2-p28907220	Bn-chrA02-p24448470
Bn-A02-p27134251	0.966942149	0.950413223	Bn-N2-p28917373	Bn-chrC02-p45471633
Bn-A02-p27149778	0.950413223	0.94214876	Bn-N2-p28929800	Bn-chrA02-p24468464
Bn-A02-p27149888	0.990049751	0.985074627	Bn-N2-p28929911	Bn-chrA02-p24469045
Bn-A02-p27158239	0.975206612	0.975206612	Bn-N2-p28936385	Bn-chrC02-p45504191
Bn-A02-p27158781	0.975206612	0.975206612	Bn-N2-p28936927	Bn-chrA02-p24475151
Bn-A02-p27171199	0.840531561	0.850498339	Bn-Scaffold06017-p9	Bn-chrA02-p24486847
Bn-A02-p27172338	0.801652893	0.958677686	Bn-N2-p28949788	Bn-chrA02-p24487985
Bn-A02-p27179350	0.975206612	0.983471074	Bn-N2-p28957063	Bn-chrA02-p24495095
Bn-A02-p27180049	0.983471074	0.975206612	Bn-N2-p28957777	Bn-chrA02-p24495808
Bn-A02-p27187948	0.966942149	0.925619835	Bn-N2-p28965437	Bn-chrC02-p45544544
Bn-A02-p27203061	0.983471074	0.991735537	Bn-N2-p28990408	Bn-chrA02-p24526396
Bn-A02-p27215745	0.976744186	0.996677741	Bn-N12-p44751216	Bn-chrA02-p24541029
Bn-A02-p27220702	0.842975207	0.966942149	Bn-N12-p44757887	Bn-chrA02-p24545988
Bn-A02-p27226579	0.925619835	0.925619835	Bn-N2-p29014558	Bn-chrA02-p24551863
Bn-A02-p27245861	0.975206612	0.966942149	Bn-N2-p29032604	Bn-chrA02-p24567493
Bn-A02-p27246186	0.747508306	0.634551495	Bn-N2-p29032929	Bn-chrC02-p45618439
Bn-A02-p27255197	0.96345515	0.970099668	Bn-N12-p44825351	Bn-chrAnn_random-p423736
Bn-A02-p27262588	0.958677686	0.67768595	Bn-N2-p29061532	Bn-chrA06-p487133
Bn-A02-p27286923	0.983471074	0.983471074	Bn-N2-p29080099	Bn-chrA02-p24599211
Bn-A02-p27288011	0.950413223	0.950413223	Bn-N2-p29084819	Bn-chrA02-p24605400
Bn-A02-p27288044	0.975206612	0.975206612	Bn-N2-p29084852	Bn-chrA02-p24605433
Bn-A02-p27288121	0.991735537	0.991735537	Bn-N2-p29084929	Bn-chrA02-p24605510
Bn-A02-p27303569	0.743801653	0.743801653	Bn-N2-p29090575	Bn-chrA02-p24611154
Bn-A02-p27304105	0.958677686	0.958677686	Bn-N2-p29090784	Bn-chrA02-p24611363
Bn-A02-p27321599	0.983388704	0.983388704	Bn-N2-p29101998	Bn-chrA02-p24621856
Bn-A02-p27327804	0.985074627	0.985074627	Bn-N2-p29103428	Bn-chrA02-p24623285
Bn-A02-p27336483	0.966942149	0.966942149	Bn-N2-p29115133	Bn-chrA02-p24633305
Bn-A02-p27358734	0.991735537	0.991735537	Bn-N2-p29138890	Bn-chrAnn_random-p540054
Bn-A02-p27360365	0.983471074	0.983471074	Bn-N2-p29140519	Bn-chrAnn_random-p540217
Bn-A02-p27399448	0.966942149	0.966942149	Bn-N2-p29187227	Bn-chrAnn_random-p544396
Bn-A02-p27403121	0.776859504	0.727272727	Bn-N2-p29188807	Bn-chrC02-p45846359
Bn-A02-p27405658	0.996677741	0.983388704	Bn-N2-p29191169	Bn-chrAnn_random-p544913

Bn-A02-p27408435	0.990033223	0.956810631	Bn-N2-p29193375	Bn-chrAnn_random-p545131
Bn-A02-p27414529	0.752066116	0.975206612	Bn-N2-p29198441	Bn-chrAnn_random-p545607
Bn-A02-p27414794	0.983471074	0.991735537	Bn-N2-p29198706	Bn-chrAnn_random-p545634
Bn-A02-p27420743	0.970149254	0.965174129	Bn-N2-p29206182	Bn-chrAnn_random-p546226
Bn-A02-p27420799	0.990033223	0.986710963	Bn-N2-p29206238	Bn-chrAnn_random-p546232
Bn-A02-p27427939	0.991735537	0.991735537	Bn-N2-p29214106	Bn-chrAnn_random-p546990
Bn-A02-p27433275	0.991735537	0.991735537	Bn-N2-p29219482	Bn-chrAnn_random-p547543
Bn-A02-p27450764	0.966942149	0.966942149	Bn-N2-p29238960	Bn-chrAnn_random-p426966
Bn-A02-p27490396	0.983471074	0.983471074	Bn-N2-p29279740	Bn-chrAnn_random-p554076
Bn-A02-p27503846	0.950413223	0.950413223	Bn-N2-p29291919	Bn-chrAnn_random-p555293
Bn-A02-p27504018	0.975206612	0.975206612	Bn-N2-p29292091	Bn-chrAnn_random-p555310
Bn-A02-p27514432	0.983471074	0.983471074	Bn-N2-p29304951	Bn-chrAnn_random-p556839
Bn-A02-p27514988	0.884297521	0.884297521	Bn-N2-p29306185	Bn-chrAnn_random-p556962
Bn-A02-p27515530	0.991735537	0.991735537	Bn-N2-p29306724	Bn-chrAnn_random-p557016
Bn-A02-p27525531	0.991735537	0.991735537	Bn-N2-p29316220	Bn-chrAnn_random-p558217
Bn-A02-p27565132	0.958677686	0.958677686	Bn-N2-p29356886	Bn-chrAnn_random-p563638
Bn-A02-p27567204	0.983471074	0.983471074	Bn-N2-p29358375	Bn-chrAnn_random-p564577
Bn-A02-p27567264	0.975206612	0.975206612	Bn-N2-p29358435	Bn-chrAnn_random-p564583
Bn-A02-p27569069	0.991735537	0.991735537	Bn-N2-p29360081	Bn-chrAnn_random-p564747
Bn-A02-p27613710	0.973421927	0.973421927	Bn-N2-p29402065	Bn-chrAnn_random-p569821
Bn-A02-p27623430	0.991735537	0.991735537	Bn-N2-p29403624	Bn-chrAnn_random-p569976
Bn-A02-p27625682	0.991735537	0.991735537	Bn-N2-p29405875	Bn-chrAnn_random-p570202
Bn-A02-p27633795	1	1	Bn-N2-p29412373	Bn-chrAnn_random-p570851
Bn-A02-p27645672	1	1	Bn-N2-p29416753	Bn-chrAnn_random-p571289
Bn-A02-p27651489	1	1	Bn-N2-p29424569	Bn-chrAnn_random-p572071
Bn-A02-p27653095	0.991735537	0.991735537	Bn-N2-p29426175	Bn-chrAnn_random-p572231
Bn-A02-p27653419	0.983471074	0.983471074	Bn-N2-p29426499	Bn-chrAnn_random-p572264
Bn-A02-p27679891	0.990049751	0.985074627	Bn-N2-p29465907	Bn-chrA02-p24657595
Bn-A02-p27679897	0.985074627	0.985074627	Bn-N2-p29465913	Bn-chrA02-p24657601
Bn-A02-p27680472	0.974226804	0.974226804	Bn-N2-p29466488	Bn-chrA02-p24658176
Bn-A02-p27682762	0.877076412	0.877076412	Bn-N2-p29468717	Bn-chrA02-p24660225
Bn-A02-p27702997	0.991735537	0.991735537	Bn-N2-p29488078	Bn-chrAnn_random-p355408
Bn-A02-p27714758	0.991735537	0.991735537	Bn-N2-p29502888	Bn-chrA02-p24693607
Bn-A02-p27743910	1	1	Bn-N2-p29533207	Bn-chrA02-p24724645
Bn-A02-p27749719	0.991735537	0.991735537	Bn-N2-p29537328	Bn-chrA02-p24728764
Bn-A02-p27755843	0.991735537	0.991735537	Bn-N2-p29544132	Bn-chrAnn_random-p444005
Bn-A02-p27781100	0.980099502	0.980099502	Bn-N2-p29564345	Bn-chrA02-p24735083
Bn-A02-p27786739	0.983471074	0.983471074	Bn-N2-p29574107	Bn-chrA02-p24744845
Bn-A02-p27792444	0.983471074	0.983471074	Bn-N2-p29580870	Bn-chrA02-p24751608
Bn-A02-p27799727	0.983471074	0.983471074	Bn-N2-p29585852	Bn-chrA02-p24756589
Bn-A02-p27831174	0.989583333	0.989583333	Bn-N2-p29609872	Bn-chrA02-p24780606
Bn-A02-p27833071	0.925619835	0.925619835	Bn-N2-p29611851	Bn-chrA02-p24782585
Bn-A02-p27834072	0.966942149	0.966942149	Bn-N2-p29612855	Bn-chrA02-p24783589
Bn-A02-p27845279	1	1	Bn-N2-p29621344	Bn-chrA02-p24791487
Bn-A03-p12026	0.989583333	0.989583333	Bn-N3-p1810	Bn-chrA03-p1827
Bn-A03-p15732	0.953488372	0.953488372	Bn-N14-p34435857	Bn-chrC04-p30274981

Bn-A03-p16309	0.940199336	0.940199336	Bn-N14-p34435274	Bn-chrC04-p30274398
Bn-A03-p17588	0.930232558	0.930232558	Bn-N14-p34433995	Bn-chrC04-p30273119
Bn-A03-p22729	0.975206612	0.975206612	Bn-N3-p5543	Bn-chrA03-p5560
Bn-A03-p23899	0.966942149	0.94214876	Bn-N3-p6712	Bn-chrA03-p6731
Bn-A03-p29938	0.958677686	0.958677686	Bn-N3-p14251	Bn-chrA03-p13579
Bn-A03-p49020	0.966942149	0.545454545	Bn-N3-p42350	Bn-chrC01-p28560214
Bn-A03-p72933	0.966942149	0.966942149	Bn-N3-p72904	Bn-chrA03-p81487
Bn-A03-p77099	0.975206612	0.975206612	Bn-N3-p80627	Bn-chrA03-p86160
Bn-A03-p78559	0.958677686	0.958677686	Bn-N3-p82106	Bn-chrA03-p87637
Bn-A03-p90436	0.975206612	0.975206612	Bn-N3-p102637	Bn-chrA03-p99592
Bn-A03-p135145	0.784053156	0.897009967	Bn-N3-p145541	Bn-chrC03-p155819
Bn-A03-p141681	1	1	Bn-N3-p152180	Bn-chrA03-p148643
Bn-A03-p162225	0.910299003	0.910299003	Bn-N3-p173341	Bn-chrA03-p167709
Bn-A03-p166292	0.983471074	0.983471074	Bn-N3-p177374	Bn-chrA03-p171742
Bn-A03-p166402	0.975206612	0.975206612	Bn-N3-p177484	Bn-chrA03-p171852
Bn-A03-p187017	0.993355482	0.993355482	Bn-N3-p198287	Bn-chrA03_random-p379
Bn-A03-p187972	1	1	Bn-N3-p199219	Bn-chrA03_random-p1311
Bn-A03-p196636	0.991735537	0.991735537	Bn-N3-p210139	Bn-chrA03_random-p71364
Bn-A03-p198822	0.975206612	0.975206612	Bn-N3-p211867	Bn-chrA03_random-p73092
Bn-A03-p201146	0.996677741	0.996677741	Bn-N3-p213799	Bn-chrA03_random-p75024
Bn-A03-p203000	0.993355482	0.993355482	Bn-N3-p215656	Bn-chrA03_random-p76881
Bn-A03-p207748	0.990049751	0.990049751	Bn-N3-p226168	Bn-chrA03_random-p85903
Bn-A03-p221207	0.991735537	0.991735537	Bn-N3-p238282	Bn-chrA03_random-p102201
Bn-A03-p235039	0.983388704	0.983388704	Bn-N3-p253211	Bn-chrA03_random-p117127
Bn-A03-p262793	0.983388704	0.983388704	Bn-N3-p288657	Bn-chrA03_random-p51571
Bn-A03-p288544	0.991735537	0.991735537	Bn-Scaffold13624-p6	Bn-chrAnn_random-p337126
Bn-A03-p298309	0.986710963	0.986710963	Bn-N3-p314838	Bn-chrA03_random-p28360
Bn-A03-p306814	0.983471074	0.983471074	Bn-N3-p301911	Bn-chrA03_random-p16619
Bn-A03-p312762	0.975206612	0.975206612	Bn-N3-p330552	Bn-chrA03_random-p11843
Bn-A03-p319304	0.990049751	0.990049751	Bn-N3-p336597	Bn-chrA03_random-p5799
Bn-A03-p334010	0.993355482	0.993355482	Bn-N3-p351696	Bn-chrA03-p255421
Bn-A03-p360880	0.900826446	0.892561983	Bn-N3-p447326	Bn-chrA03-p351391
Bn-A03-p373134	0.94214876	0.991735537	Bn-N3-p439036	Bn-chrA03-p342825
Bn-A03-p373153	0.859504132	0.991735537	Bn-N3-p439017	Bn-chrA03-p342806
Bn-A03-p386542	0.985074627	0.985074627	Bn-N3-p431647	Bn-chrA03-p337886
Bn-A03-p403559	0.991735537	1	Bn-N3-p417823	Bn-chrA03-p321277
Bn-A03-p419250	0.983388704	0.983388704	Bn-N3-p397357	Bn-chrA03-p302874
Bn-A03-p420642	0.986710963	0.986710963	Bn-N3-p395965	Bn-chrA03-p301482
Bn-A03-p471570	0.966942149	0.966942149	Bn-N3-p471270	Bn-chrA03-p373818
Bn-A03-p478702	0.909090909	0.917355372	Bn-N3-p479111	Bn-chrA03-p383119
Bn-A03-p487666	0.993355482	0.996677741	Bn-N4-p19088456	Bn-chrA08-p15294017
Bn-A03-p493766	0.980066445	0.990033223	Bn-N3-p489352	Bn-chrA03-p393263
Bn-A03-p494427	0.96013289	0.990033223	Bn-N3-p490017	Bn-chrA03-p393924
Bn-A03-p498320	0.724252492	0.740863787	Bn-N3-p494001	Bn-chrA03-p397786
Bn-A03-p528276	0.975206612	0.975206612	Bn-N3-p517505	Bn-chrA03-p419937
Bn-A03-p532166	0.966942149	0.198347107	Bn-N3-p523511	Unknown

Bn-A03-p537905	0.975206612	0.958677686	Bn-N3-p526805	Bn-chrA03-p430986
Bn-A03-p560769	0.975206612	0.983471074	Bn-N3-p545303	Bn-chrA03-p445706
Bn-A03-p560941	0.958677686	0.950413223	Bn-N3-p545475	Bn-chrA03-p445878
Bn-A03-p563787	0.966942149	0.801652893	Bn-N3-p548319	Bn-chrC03-p632242
Bn-A03-p565187	0.945273632	0.945273632	Bn-N3-p549719	Bn-chrA03-p450094
Bn-A03-p565363	0.995024876	0.990049751	Bn-N3-p549895	Bn-chrA03-p450270
Bn-A03-p565637	0.975124378	0.970149254	Bn-N3-p550169	Bn-chrA03-p450545
Bn-A03-p623390	0.993355482	0.993355482	Bn-N3-p617378	Bn-chrA03-p545947
Bn-A03-p630979	0.991735537	0.983471074	Bn-N3-p624785	Bn-chrA03-p553192
Bn-A03-p641927	0.975206612	0.983471074	Bn-N3-p643986	Bn-chrA03-p557029
Bn-A03-p654363	0.991735537	0.983471074	Bn-N3-p658962	Bn-chrA03_random-p130706
Bn-A03-p657593	0.925619835	0.900826446	Bn-N3-p662130	Bn-chrA03_random-p133919
Bn-A03-p671279	0.983388704	0.850498339	Bn-N3-p671635	Bn-chrC03-p724255
Bn-A03-p708787	0.859504132	0.867768595	Bn-N3-p712599	Bn-chrA03-p573320
Bn-A03-p709005	0.983471074	0.991735537	Bn-N3-p712817	Bn-chrA03-p573538
Bn-A03-p709332	0.719008264	0.94214876	Bn-N3-p713087	Bn-chrA03-p573865
Bn-A03-p711408	0.990033223	0.990033223	Bn-N3-p715159	Bn-chrA03-p575938
Bn-A03-p712308	0.990033223	0.990033223	Bn-N3-p716059	Bn-chrA03-p576838
Bn-A03-p726812	0.985074627	0.985074627	Bn-N13-p892838	Bn-chrC03_random-p37921
Bn-A03-p731558	0.995024876	0.935323383	Bn-N3-p738745	Bn-chrC03_random-p43024
Bn-A03-p755100	0.950413223	0.94214876	Bn-N3-p761932	Bn-chrA03-p623349
Bn-A03-p757592	0.966942149	0.983471074	Bn-N3-p764664	Bn-chrA03-p625846
Bn-A03-p764274	0.991735537	0.983471074	Bn-N3-p771325	Bn-chrA03-p632525
Bn-A03-p765077	0.94214876	0.933884298	Bn-N3-p772129	Bn-chrA03-p633328
Bn-A03-p766322	0.996677741	0.710963455	Bn-N3-p773472	Bn-chrA03-p634572
Bn-A03-p830116	0.990033223	0.993355482	Bn-N3-p837687	Bn-chrA03-p707629
Bn-A03-p830487	0.995024876	0.990049751	Bn-N3-p838058	Bn-chrA03-p708000
Bn-A03-p831433	1	0.991735537	Bn-N3-p839004	Bn-chrA03-p708946
Bn-A03-p832473	0.991735537	0.983471074	Bn-N3-p843464	Bn-chrA03-p709905
Bn-A03-p834379	0.958677686	0.950413223	Bn-N3-p843794	Bn-chrA03-p710234
Bn-A03-p835162	0.958677686	0.991735537	Bn-N3-p844578	Bn-chrA03-p711017
Bn-A03-p835987	0.826446281	0.983471074	Bn-N3-p845609	Bn-chrA03-p711842
Bn-A03-p836873	0.991735537	1	Bn-N3-p846459	Bn-chrA03-p712721
Bn-A03-p858910	0.990033223	0.990033223	Bn-N3-p873123	Bn-chrA03_random-p162597
Bn-A03-p898067	0.96345515	0.983388704	Bn-N3-p910599	Bn-chrA03_random-p200291
Bn-A03-p912314	0.975124378	0.975124378	Bn-N3-p924972	Bn-chrA03_random-p216205
Bn-A03-p924676	0.975206612	0.966942149	Bn-N3-p935672	Bn-chrA03_random-p229176
Bn-A03-p924780	0.958677686	0.966942149	Bn-N3-p935776	Bn-chrA03_random-p229280
Bn-A03-p927517	0.813953488	0.817275748	Bn-N3-p937636	Bn-chrA03_random-p231067
Bn-A03-p928009	0.950413223	0.950413223	Bn-N3-p938126	Bn-chrA03_random-p231559
Bn-A03-p928274	0.884297521	0.867768595	Bn-N3-p938432	Bn-chrA03_random-p231866
Bn-A03-p943531	0.975206612	0.214876033	Bn-N3-p960953	Bn-chrAnn_random-p593646
Bn-A03-p950999	0.980066445	0.980066445	Bn-N3-p968276	Bn-chrA03-p785590
Bn-A03-p951365	0.991735537	0.983471074	Bn-N3-p968642	Bn-chrA03-p785956
Bn-A03-p952091	0.950413223	0.958677686	Bn-N3-p969367	Bn-chrA03-p786682
Bn-A03-p955176	0.975206612	0.966942149	Bn-N3-p972434	Bn-chrA03-p789737

Bn-A03-p974894	0.297520661	0.991735537	Unknown	Bn-chrA03-p811834
Bn-A03-p991949	0.991735537	0.991735537	Bn-N3-p1010731	Bn-chrA03-p830738
Bn-A03-p1004918	0.833887043	0.837209302	Bn-N3-p1022213	Bn-chrA03-p840821
Bn-A03-p1008445	0.983471074	0.975206612	Bn-N3-p1025736	Bn-chrA03-p845125
Bn-A03-p1013445	0.975206612	0.983471074	Bn-N3-p1032818	Bn-chrA03-p849754
Bn-A03-p1046864	0.993355482	0.993355482	Bn-N3-p1077426	Bn-chrA03-p879278
Bn-A03-p1050437	0.983471074	1	Bn-N3-p1080752	Bn-chrA03-p882829
Bn-A03-p1050893	0.983471074	0.991735537	Bn-N3-p1081207	Bn-chrA03-p883287
Bn-A03-p1051774	0.966942149	0.826446281	Bn-N3-p1082089	Bn-chrC03-p1188113
Bn-A03-p1065055	1	0.925619835	Bn-N3-p1092881	Bn-chrC03-p1196592
Bn-A03-p1067313	0.958677686	0.983471074	Bn-Scaffold03750-p4	Bn-chrA02-p21524211
Bn-A03-p1068165	0.991735537	0.983471074	Bn-Scaffold03750-p1	Bn-chrA02-p21523354
Bn-A03-p1070680	0.94214876	0.958677686	Bn-N3-p1098950	Bn-chrA02-p21520725
Bn-A03-p1075837	0.966942149	0.826446281	Bn-N3-p1104084	Bn-chrC03-p1215047
Bn-A03-p1084819	0.966942149	0.975206612	Bn-N3-p1107969	Bn-chrA03_random-p251174
Bn-A03-p1089875	0.983471074	0.958677686	Bn-N3-p1114121	Bn-chrA03_random-p256176
Bn-A03-p1098531	0.990033223	0.990033223	Bn-N3-p1123511	Bn-chrA03_random-p265167
Bn-A03-p1194072	0.965174129	0.965174129	Bn-N3-p1196191	Bn-chrA03_random-p379819
Bn-A03-p1195712	0.96345515	0.993355482	Bn-N13-p1532292	Bn-chrA03_random-p381469
Bn-A03-p1218291	0.975206612	0.983471074	Bn-N3-p1220578	Bn-chrA03_random-p249013
Bn-A03-p1238329	0.958677686	0.983471074	Bn-N3-p1250119	Bn-chrA03_random-p326598
Bn-A03-p1254871	1	0.991735537	Bn-N3-p1268209	Bn-chrAnn_random-p354862
Bn-A03-p1297873	0.966942149	0.975206612	Bn-N3-p1311437	Bn-chrA03-p929808
Bn-A03-p1308820	0.986710963	0.986710963	Bn-N3-p1322771	Bn-chrA03-p945040
Bn-A03-p1352996	1	0.991735537	Bn-N3-p1362960	Bn-chrA03-p986708
Bn-A03-p1353309	0.975206612	0.983471074	Bn-N3-p1363269	Bn-chrA03-p987017
Bn-A03-p1354206	0.958677686	0.975206612	Bn-N3-p1364248	Bn-chrA03-p987918
Bn-A03-p1354375	1	0.991735537	Bn-N3-p1364418	Bn-chrA03-p988087
Bn-A03-p1373885	0.980099502	0.980099502	Bn-N13-p1752270	Bn-chrC03-p1556553
Bn-A03-p1435886	0.991735537	0.991735537	Bn-N3-p1446295	Bn-chrA03-p1066350
Bn-A03-p1461633	0.980099502	0.980099502	Bn-N3-p1475985	Bn-chrAnn_random-p295073
Bn-A03-p1466010	0.980099502	0.980099502	Bn-N3-p1480002	Bn-chrAnn_random-p295111
Bn-A03-p1475495	1	0.991735537	Bn-N3-p1485762	Bn-chrA03-p1100942
Bn-A03-p1488306	0.996677741	1	Bn-N3-p1499294	Bn-chrA03-p1115031
Bn-A03-p1503462	0.993355482	0.990033223	Bn-N3-p1513506	Bn-chrA03-p1127892
Bn-A03-p1506511	0.983471074	0.983471074	Bn-N3-p1516694	Bn-chrA03-p1131071
Bn-A03-p1507769	0.983471074	0.991735537	Bn-N3-p1517990	Bn-chrA03-p1132330
Bn-A03-p1514927	0.933884298	1	Bn-N3-p1524562	Bn-chrA03_random-p384402
Bn-A03-p1532114	0.96013289	0.990033223	Bn-N3-p1543888	Bn-chrA03_random-p402048
Bn-A03-p1538555	0.933884298	0.933884298	Bn-N3-p1550326	Bn-chrA03_random-p408493
Bn-A03-p1559742	1	0.991735537	Bn-N3-p1568936	Bn-chrA03-p1181170
Bn-A03-p1562119	0.991735537	0.983471074	Bn-N3-p1571317	Bn-chrA03-p1183518
Bn-A03-p1563613	1	0.991735537	Bn-N3-p1572813	Bn-chrA03-p1185012
Bn-A03-p1577561	0.990033223	0.986710963	Bn-N3-p1585403	Bn-chrA03-p1197534
Bn-A03-p1588735	0.980066445	0.983388704	Bn-N3-p1596648	Bn-chrA03-p1208754
Bn-A03-p1596091	0.96013289	0.973421927	Bn-N3-p1607870	Bn-chrA03-p1216023

Bn-A03-p1638326	0.975206612	0.958677686	Bn-N3-p1664185	Bn-chrC03-p1832548
Bn-A03-p1639446	0.857142857	0.850498339	Bn-N3-p1665303	Bn-chrA03-p1256811
Bn-A03-p1652490	0.900826446	0.966942149	Bn-N3-p1683017	Bn-chrA03_random-p438747
Bn-A03-p1687166	0.993355482	0.993355482	Bn-N3-p1724606	Bn-chrA03_random-p467280
Bn-A03-p1698016	0.983471074	0.991735537	Bn-N3-p1740688	Bn-chrA03_random-p456456
Bn-A03-p1711856	0.993355482	0.993355482	Bn-N3-p1755477	Bn-chrA03_random-p442728
Bn-A03-p1716217	0.991735537	0.94214876	Bn-N3-p1759871	Bn-chrC03-p1942387
Bn-A03-p1719791	0.958677686	0.561983471	Bn-N3-p1761406	Bn-chrAnn_random-p337976
Bn-A03-p1741556	0.958677686	0.958677686	Bn-N3-p1787910	Bn-chrA03-p1341696
Bn-A03-p1755651	1	1	Bn-N3-p1797273	Bn-chrA03-p1354996
Bn-A03-p1762980	0.980066445	0.980066445	Bn-N3-p1802282	Bn-chrA03-p1359390
Bn-A03-p1777701	0.970149254	0.970149254	Bn-N3-p1824146	Bn-chrA03-p1377979
Bn-A03-p1805828	0.965174129	0.970149254	Bn-N3-p1857444	Bn-chrA03-p1405458
Bn-A03-p1813564	0.996677741	0.966777409	Bn-N3-p1869293	Bn-chrC03-p2070023
Bn-A03-p1817556	0.837209302	0.853820598	Bn-N3-p1873254	Bn-chrA03-p1417854
Bn-A03-p1831525	0.983471074	0.991735537	Bn-N3-p1898249	Bn-chrA03-p1436634
Bn-A03-p1839932	0.930232558	0.936877076	Bn-N13-p2372489	Bn-chrA03-p1448487
Bn-A03-p1869248	0.950413223	0.892561983	Bn-N3-p1957068	Bn-chrC03-p2135056
Bn-A03-p1871012	0.966942149	0.966942149	Bn-N3-p1958934	Bn-chrA03-p1478404
Bn-A03-p1894119	0.975206612	0.975206612	Bn-N3-p1980524	Bn-chrA03-p1506532
Bn-A03-p1899032	1	0.989583333	Bn-N3-p1989483	Bn-chrA03-p1511508
Bn-A03-p1904916	0.986710963	0.986710963	Bn-N3-p1995360	Bn-chrA03-p1518455
Bn-A03-p1918536	0.986710963	0.986710963	Bn-N3-p2012527	Bn-chrA03-p1536895
Bn-A03-p1922712	0.983471074	0.983471074	Bn-N3-p2016565	Bn-chrA03-p1540693
Bn-A03-p1923025	0.966942149	0.966942149	Bn-N3-p2016876	Bn-chrA03-p1541004
Bn-A03-p1926809	0.983471074	0.983471074	Bn-N3-p2019723	Bn-chrA03-p1543644
Bn-A03-p1951453	1	1	Bn-N3-p2051958	Bn-chrA03-p1577628
Bn-A03-p1954639	0.953488372	0.953488372	Bn-N3-p2055094	Bn-chrA03-p1580764
Bn-A03-p1959964	0.983471074	0.983471074	Bn-N3-p2060235	Bn-chrA03-p1585906
Bn-A03-p1960313	0.991735537	0.991735537	Bn-N3-p2060586	Bn-chrA03-p1586257
Bn-A03-p1960694	0.859504132	0.859504132	Bn-N3-p2060923	Bn-chrA03-p1586594
Bn-A03-p1990974	0.926910299	0.926910299	Bn-N13-p2605446	Bn-chrC03-p2315899
Bn-A03-p2003715	0.995024876	0.995024876	Bn-N3-p2098853	Bn-chrA03-p1624410
Bn-A03-p2004585	0.995024876	0.995024876	Bn-N3-p2099723	Bn-chrA03-p1625280
Bn-A03-p2005352	0.993355482	0.993355482	Bn-N3-p2100490	Bn-chrA03-p1626047
Bn-A03-p2018577	0.975206612	0.983471074	Bn-N3-p2113623	Bn-chrAnn_random-p307448
Bn-A03-p2030676	0.990049751	0.930348259	Bn-Scaffold10148-p5	Bn-chrA03-p1649707
Bn-A03-p2054412	0.993355482	0.996677741	Bn-N3-p2159108	Bn-chrA03-p1672759
Bn-A03-p2055179	0.966942149	0.975206612	Bn-N3-p2159875	Bn-chrA03-p1673526
Bn-A03-p2071494	0.993355482	0.993355482	Bn-N3-p2173818	Bn-chrA03-p1686562
Bn-A03-p2073180	0.990033223	0.990033223	Bn-N3-p2175513	Bn-chrA03-p1688248
Bn-A03-p2074890	1	1	Bn-N3-p2177223	Bn-chrA03-p1689959
Bn-A03-p2100226	0.983471074	0.983471074	Bn-N3-p2202584	Bn-chrA03-p1709434
Bn-A03-p2130281	0.975206612	0.975206612	Bn-N3-p2226318	Bn-chrA03-p1731306
Bn-A03-p2140542	0.993355482	0.996677741	Bn-N3-p2233968	Bn-chrA03-p1739896
Bn-A03-p2159699	0.991735537	0.991735537	Bn-N3-p2247859	Bn-chrA03-p1754313

Bn-A03-p2171944	0.975206612	0.933884298	Bn-N3-p2259036	Bn-chrA03-p1762765
Bn-A03-p2190638	0.975206612	0.983471074	Bn-N3-p2278303	Bn-chrA03-p1777226
Bn-A03-p2203942	0.909090909	0.991735537	Bn-N3-p2289223	Bn-chrA03-p1791352
Bn-A03-p2206256	0.985074627	0.995024876	Bn-N13-p2851955	Bn-chrC03-p2573048
Bn-A03-p2228813	0.993355482	0.993355482	Bn-N3-p2307358	Bn-chrA03-p1810188
Bn-A03-p2230320	0.950413223	0.950413223	Bn-N3-p2308849	Bn-chrA03-p1811687
Bn-A03-p2262214	0.983471074	0.983471074	Bn-N3-p2332621	Bn-chrA03-p1832035
Bn-A03-p2271873	0.966942149	0.975206612	Bn-N3-p2339537	Bn-chrC03-p2655790
Bn-A03-p2297079	0.975124378	0.975124378	Bn-N13-p2973868	Bn-chrC03-p2703295
Bn-A03-p2297145	0.980099502	0.985074627	Bn-N3-p2372424	Bn-chrA03-p1863891
Bn-A03-p2311607	0.991735537	0.991735537	Bn-N3-p2387210	Bn-chrA03-p1879700
Bn-A03-p2313208	0.925373134	0.930348259	Bn-N3-p2388820	Bn-chrA03-p1881300
Bn-A03-p2342965	0.975206612	0.966942149	Bn-N3-p2425829	Bn-chrA03-p1916284
Bn-A03-p2344066	0.975206612	0.983471074	Bn-N13-p3023239	Bn-chrA03-p1917385
Bn-A03-p2366510	0.983471074	0.991735537	Bn-N3-p2441506	Bn-chrCnn_random-p270437
Bn-A03-p2402265	1	0.991735537	Bn-N3-p2489754	Bn-chrA03-p1963409
Bn-A03-p2405726	0.983471074	0.991735537	Bn-Scaffold08629-p8	Bn-chrA03-p1965083
Bn-A03-p2412263	1	0.996677741	Bn-N3-p2505310	Bn-chrA03-p1971697
Bn-A03-p2423548	0.965174129	0.975124378	Bn-N3-p2512924	Bn-chrA03-p1978091
Bn-A03-p2427713	0.58677686	0.991735537	Bn-Scaffold82560-p4	Bn-chrA09-p13520602
Bn-A03-p2431404	0.925619835	0.917355372	Bn-N3-p2525430	Bn-chrA03-p1982127
Bn-A03-p2449290	0.990049751	0.985074627	Bn-N3-p2541663	Bn-chrA03-p1987475
Bn-A03-p2453901	0.991735537	0.991735537	Bn-N3-p2552556	Bn-chrA03-p1991989
Bn-A03-p2457869	0.983471074	0.991735537	Bn-N3-p2556456	Bn-chrA03-p1995885
Bn-A03-p2457934	0.950413223	0.958677686	Bn-N3-p2556520	Bn-chrA03-p1995949
Bn-A03-p2460475	0.611570248	0.611570248	Bn-N3-p2558606	Bn-chrA03-p1998150
Bn-A03-p2468983	0.990049751	1	Bn-N3-p2567726	Bn-chrA03-p2006936
Bn-A03-p2469223	0.991735537	0.991735537	Bn-Scaffold62262-p1	Bn-chrA03-p2007177
Bn-A03-p2479213	0.752066116	0.958677686	Bn-N3-p2579332	Bn-chrA03-p2019584
Bn-A03-p2483644	0.983471074	0.983471074	Bn-N3-p2583835	Bn-chrA03-p2024064
Bn-A03-p2491346	0.993355482	0.996677741	Bn-N3-p2590934	Bn-chrA03-p2036113
Bn-A03-p2493541	0.900826446	0.900826446	Bn-N13-p3174052	Bn-chrC03-p2864653
Bn-A03-p2511868	0.983388704	0.983388704	Bn-N3-p2621627	Bn-chrA03-p2057151
Bn-A03-p2520008	0.986710963	0.980066445	Bn-N3-p2632049	Bn-chrA03-p2073564
Bn-A03-p2521128	0.983471074	0.983471074	Bn-N3-p2633204	Bn-chrA03-p2074683
Bn-A03-p2532699	0.983471074	0.983471074	Bn-N3-p2645979	Bn-chrA03-p2086314
Bn-A03-p2548187	0.737541528	0.737541528	Bn-N3-p2657992	Bn-chrA03-p2098331
Bn-A03-p2557607	0.983471074	0.983471074	Bn-N3-p2669732	Bn-chrA03-p2107785
Bn-A03-p2579866	0.960199005	0.960199005	Bn-N3-p2679025	Bn-chrA03-p2118170
Bn-A03-p2589077	0.851239669	0.851239669	Bn-N3-p2687867	Bn-chrA03-p2132227
Bn-A03-p2596410	0.991735537	0.991735537	Bn-N3-p2695450	Bn-chrA03-p2139222
Bn-A03-p2606192	0.980066445	0.946843854	Bn-N3-p2705395	Bn-chrC03-p2975665
Bn-A03-p2616068	0.990049751	0.990049751	Bn-N3-p2715484	Bn-chrA03-p2159160
Bn-A03-p2621677	0.94214876	0.975206612	Bn-N3-p2721101	Bn-chrA03-p2164113
Bn-A03-p2627552	0.940298507	0.965174129	Bn-N3-p2724321	Bn-chrA03-p2173075
Bn-A03-p2638510	0.980066445	0.980066445	Bn-N3-p2735170	Bn-chrA03-p2184763

Bn-A03-p2643134	0.960199005	0.975124378	Bn-N3-p2739794	Bn-chrA03-p2190785
Bn-A03-p2645391	0.993355482	0.996677741	Bn-N3-p2742051	Bn-chrA03-p2193069
Bn-A03-p2662675	0.94214876	0.975206612	Bn-N13-p3373305	Bn-chrA03-p2215366
Bn-A03-p2665731	0.834710744	0.966942149	Bn-N3-p2765209	Bn-chrA03-p2218213
Bn-A03-p2666271	0.92039801	0.791044776	Bn-N3-p2765749	Bn-chrA03-p2218753
Bn-A03-p2667726	0.966942149	0.966942149	Bn-N3-p2767202	Bn-chrA03-p2220207
Bn-A03-p2668128	0.966942149	0.966942149	Bn-N3-p2767611	Bn-chrA03-p2220616
Bn-A03-p2683534	0.966942149	0.958677686	Bn-N3-p2782003	Bn-chrA03-p2235843
Bn-A03-p2691383	1	0.996677741	Bn-N3-p2789855	Bn-chrA03-p2243694
Bn-A03-p2695311	0.933884298	0.917355372	Bn-N3-p2793803	Bn-chrA03-p2247622
Bn-A03-p2704795	0.990049751	0.990049751	Bn-N3-p2807568	Bn-chrA03-p2258794
Bn-A03-p2714251	0.986710963	0.993355482	Bn-N3-p2816954	Bn-chrA03-p2268379
Bn-A03-p2719384	0.975124378	0.985074627	Bn-N3-p2823603	Bn-chrA03-p2273905
Bn-A03-p2727877	0.991735537	0.983471074	Bn-N3-p2832338	Bn-chrA03-p2282894
Bn-A03-p2731426	0.71641791	0.71641791	Bn-N3-p2835990	Bn-chrA03-p2286433
Bn-A03-p2736960	0.985074627	0.985074627	Bn-N3-p2839213	Bn-chrA03-p2289654
Bn-A03-p2737116	0.985074627	0.985074627	Bn-N3-p2839369	Bn-chrA03-p2289810
Bn-A03-p2737190	0.985074627	0.985074627	Bn-N3-p2839443	Bn-chrA03-p2289884
Bn-A03-p2737338	0.990033223	0.990033223	Bn-N3-p2839591	Bn-chrA03-p2290032
Bn-A03-p2748923	0.950413223	0.94214876	Bn-N3-p2852910	Bn-chrA03-p2305470
Bn-A03-p2789113	0.667774086	0.6910299	Bn-N13-p3541265	Bn-chrA03-p2361246
Bn-A03-p2793288	0.983471074	0.983471074	Bn-N3-p2892489	Bn-chrA03-p2365423
Bn-A03-p2834684	0.993355482	0.993355482	Bn-N3-p2918902	Bn-chrA03-p2404887
Bn-A03-p2843108	0.991735537	0.983471074	Bn-N3-p2933974	Bn-chrA03-p2419830
Bn-A03-p2856933	0.966942149	0.966942149	Bn-N3-p2939023	Bn-chrA03-p2424876
Bn-A03-p2858882	0.933884298	0.900826446	Bn-N3-p2940916	Bn-chrC03-p3245230
Bn-A03-p2858972	0.975124378	0.975124378	Bn-N3-p2941090	Bn-chrA03-p2426943
Bn-A03-p2860489	0.983471074	0.983471074	Bn-N3-p2942605	Bn-chrA03-p2428458
Bn-A03-p2862687	0.983471074	0.983471074	Bn-N3-p2945138	Bn-chrC02-p3100456
Bn-A03-p2872671	1	1	Bn-N3-p2948138	Bn-chrC02-p3103456
Bn-A03-p2879773	0.990033223	0.993355482	Bn-N3-p2954702	Bn-chrA03-p2440092
Bn-A03-p2891056	0.980066445	0.980066445	Bn-N3-p2966126	Bn-chrA03-p2451234
Bn-A03-p2891934	0.983471074	0.983471074	Bn-N3-p2966999	Bn-chrA03-p2452107
Bn-A03-p2897610	0.991735537	0.991735537	Bn-N3-p2972633	Bn-chrA03-p2457741
Bn-A03-p2898043	0.860465116	0.897009967	Bn-N3-p2973544	Bn-chrA03-p2458180
Bn-A03-p2914046	0.983471074	0.983471074	Bn-N3-p2987792	Bn-chrA03-p2472441
Bn-A03-p2917477	0.983471074	0.983471074	Bn-N3-p2991771	Bn-chrA03-p2476437
Bn-A03-p2929711	0.79338843	0.79338843	Bn-N3-p3005203	Bn-chrA03-p2489868
Bn-A03-p2946358	0.996677741	0.996677741	Bn-N3-p3023164	Bn-chrA03-p2508153
Bn-A03-p2948394	0.893687708	0.893687708	Bn-N3-p3025293	Bn-chrA03-p2510282
Bn-A03-p2948695	0.983471074	0.983471074	Bn-N3-p3025594	Bn-chrA03-p2510583
Bn-A03-p2971192	0.980099502	0.980099502	Bn-N3-p3047863	Bn-chrA03-p2532550
Bn-A03-p2976496	0.991735537	0.991735537	Bn-N3-p3053166	Bn-chrA03-p2537850
Bn-A03-p2978588	0.975206612	0.975206612	Bn-N3-p3055256	Bn-chrA03-p2539940
Bn-A03-p2997270	0.958677686	0.958677686	Bn-N3-p3074368	Bn-chrA03-p2559261
Bn-A03-p3001467	0.989583333	0.989583333	Bn-N3-p3078563	Bn-chrA03-p2563456

Bn-A03-p3036102	0.975124378	0.975124378	Bn-N3-p3110843	Bn-chrA03-p2595208
Bn-A03-p3036446	0.990049751	0.990049751	Bn-N3-p3111187	Bn-chrA03-p2595552
Bn-A03-p3052882	0.92358804	0.92358804	Bn-N3-p3129308	Bn-chrA03-p2612655
Bn-A03-p3053702	0.980099502	0.980099502	Bn-N3-p3130128	Bn-chrA03-p2613475
Bn-A03-p3073797	0.970149254	0.970149254	Bn-N3-p3156265	Bn-chrA03-p2638664
Bn-A03-p3074927	0.975206612	0.975206612	Bn-N3-p3157400	Bn-chrA03-p2639799
Bn-A03-p3075427	0.950413223	0.950413223	Bn-N3-p3157903	Bn-chrA03-p2640302
Bn-A03-p3084593	0.975206612	0.975206612	Bn-N3-p3167245	Bn-chrA03-p2649285
Bn-A03-p3084866	0.958677686	0.958677686	Bn-N3-p3167518	Bn-chrA03-p2649558
Bn-A03-p3091011	0.958677686	0.958677686	Bn-N3-p3172467	Bn-chrA03-p2654507
Bn-A03-p3097703	0.983471074	0.966942149	Bn-N3-p3181533	Bn-chrA03-p2662458
Bn-A03-p3098147	0.996677741	0.933554817	Bn-N3-p3181977	Bn-chrC03-p3575869
Bn-A03-p3098813	0.980099502	0.975124378	Bn-N3-p3182643	Bn-chrA03-p2663571
Bn-A03-p3099901	0.983471074	0.975206612	Bn-N3-p3183725	Bn-chrA03-p2665942
Bn-A03-p3115732	0.975206612	0.958677686	Bn-N3-p3206113	Bn-chrA03-p2686791
Bn-A03-p3116829	0.991735537	0.983471074	Bn-N3-p3207213	Bn-chrA03-p2687894
Bn-A03-p3131450	0.965174129	0.965174129	Bn-N3-p3221824	Bn-chrA03-p2702503
Bn-A03-p3156872	0.903654485	0.993355482	Bn-N13-p4106390	Bn-chrA03-p2736702
Bn-A03-p3189093	0.975206612	0.975206612	Bn-N3-p3292006	Bn-chrA03-p2764180
Bn-A03-p3189333	0.983471074	0.983471074	Bn-N3-p3292246	Bn-chrA03-p2764420
Bn-A03-p3219484	0.991735537	0.983471074	Bn-N3-p3317486	Bn-chrA03-p2789476
Bn-A03-p3272346	0.991735537	0.991735537	Bn-N3-p3357744	Bn-chrA03-p2830857
Bn-A03-p3273629	0.990033223	0.990033223	Bn-N3-p3359027	Bn-chrA03-p2832140
Bn-A03-p3319357	1	0.996677741	Bn-N3-p3406926	Bn-chrA03-p2877318
Bn-A03-p3381122	0.950166113	0.993355482	Bn-N13-p4396027	Bn-chrA03-p2951212
Bn-A03-p3392103	0.986710963	0.986710963	Bn-N3-p3477614	Bn-chrA03-p2962394
Bn-A03-p3476060	0.976744186	0.976744186	Bn-N3-p3545270	Bn-chrA03-p3035810
Bn-A03-p3476410	0.950166113	0.92358804	Bn-N13-p4496684	Bn-chrA03-p3036160
Bn-A03-p3489437	0.991735537	0.991735537	Bn-N3-p3558640	Bn-chrA03-p3049179
Bn-A03-p3492107	0.925619835	0.925619835	Bn-N3-p3561319	Bn-chrA03-p3051858
Bn-A03-p3507222	0.975206612	0.975206612	Bn-N3-p3596471	Bn-chrA03-p3073642
Bn-A03-p3517306	0.985074627	0.985074627	Bn-N3-p3606622	Bn-chrA03-p3083790
Bn-A03-p3544165	0.966942149	0.966942149	Bn-N3-p3632093	Bn-chrA03-p3108910
Bn-A03-p3544245	0.983471074	0.983471074	Bn-N3-p3632173	Bn-chrA03-p3108990
Bn-A03-p3561270	0.995024876	0.995024876	Bn-N3-p3648874	Bn-chrA03-p3125691
Bn-A03-p3571622	0.917355372	0.917355372	Bn-N13-p4633364	Bn-chrC03-p4255266
Bn-A03-p3571859	0.990033223	0.990033223	Bn-N13-p4633600	Bn-chrC03-p4255502
Bn-A03-p3674265	0.562189055	0.985074627	Bn-N13-p5303096	Bn-chrA03-p3235220
Bn-A03-p3692048	0.993355482	0.996677741	Bn-N3-p3772359	Bn-chrA03-p3249677
Bn-A03-p3698620	0.965174129	0.970149254	Bn-N3-p3778772	Bn-chrA03-p3256206
Bn-A03-p3795299	0.983471074	0.900826446	Bn-N3-p3885826	Bn-chrA03-p3348560
Bn-A03-p3795523	0.983471074	0.950413223	Bn-N3-p3886050	Bn-chrA03-p3348777
Bn-A03-p3796394	0.966942149	0.933884298	Bn-N3-p3886921	Bn-chrA03-p3349643
Bn-A03-p3796557	0.983471074	0.975206612	Bn-N3-p3887084	Bn-chrA03-p3349809
Bn-A03-p3796624	0.983471074	0.966942149	Bn-N3-p3887151	Bn-chrA03-p3349876
Bn-A03-p3799804	0.991735537	0.983471074	Bn-N3-p3890453	Bn-chrA03-p3354759

Bn-A03-p3801626	1	1	Bn-N3-p3892311	Bn-chrA03-p3356584
Bn-A03-p3808441	0.983471074	0.983471074	Bn-Scaffold01563-p6	Bn-chrA03-p3364365
Bn-A03-p3819549	0.983471074	0.983471074	Bn-N3-p3910025	Bn-chrA03-p3374434
Bn-A03-p3823071	0.966942149	0.966942149	Bn-N3-p3913548	Bn-chrA03-p3377957
Bn-A03-p3839084	0.710743802	0.710743802	Bn-N3-p3931501	Bn-chrA03-p3397107
Bn-A03-p3851195	0.991735537	0.991735537	Bn-N3-p3941404	Bn-chrA03-p3407003
Bn-A03-p3857752	0.983471074	0.983471074	Bn-N3-p3948397	Bn-chrA03-p3414261
Bn-A03-p3861991	0.991735537	0.991735537	Bn-N3-p3952637	Bn-chrA03-p3418501
Bn-A03-p3862302	0.950413223	0.950413223	Bn-N3-p3952950	Bn-chrA03-p3418814
Bn-A03-p3862406	0.983471074	0.983471074	Bn-N3-p3953975	Bn-chrA03-p3421750
Bn-A03-p3862928	0.991735537	0.991735537	Bn-N3-p3954498	Bn-chrA03-p3422273
Bn-A03-p3877500	0.983471074	0.983471074	Bn-N3-p3969148	Bn-chrA03-p3436843
Bn-A03-p3877572	0.966942149	0.966942149	Bn-N3-p3969220	Bn-chrA03-p3436915
Bn-A03-p3951623	0.983471074	0.975206612	Bn-N3-p4037875	Bn-chrA03-p3513216
Bn-A03-p3951816	0.925619835	0.983471074	Bn-N3-p4039061	Bn-chrA03-p3513409
Bn-A03-p3969037	0.933884298	1	Bn-N3-p4057117	Bn-chrA03-p3529935
Bn-A03-p3969278	0.983471074	0.975206612	Bn-N3-p4065533	Bn-chrA03-p3530166
Bn-A03-p3984795	0.940298507	0.940298507	Bn-N3-p4079139	Bn-chrC03-p4792855
Bn-A03-p3994857	0.993355482	0.993355482	Bn-N3-p4088002	Bn-chrA03-p3556523
Bn-A03-p4008078	0.975206612	0.975206612	Bn-N3-p4100924	Bn-chrA03-p3570498
Bn-A03-p4036118	0.950413223	0.958677686	Bn-N3-p4132848	Bn-chrA03_random-p588971
Bn-A03-p4036196	0.950413223	0.950413223	Bn-N3-p4132930	Bn-chrA03_random-p588892
Bn-A03-p4050929	0.165289256	0.975206612	Bn-Scaffold01372-p1	Bn-chrC03-p4868479
Bn-A03-p4092312	0.983471074	0.983471074	Bn-N3-p4180308	Bn-chrA03_random-p527334
Bn-A03-p4096019	0.975206612	0.983471074	Bn-N3-p4184014	Bn-chrA03_random-p523629
Bn-A03-p4100027	0.991735537	0.983471074	Bn-N3-p4188260	Bn-chrA03_random-p604886
Bn-A03-p4116886	0.991735537	0.991735537	Bn-N3-p4210706	Bn-chrA03-p3645853
Bn-A03-p4118881	0.983471074	0.983471074	Bn-N3-p4225257	Bn-chrA03-p3647518
Bn-A03-p4123164	0.991735537	0.983471074	Bn-N3-p4229217	Bn-chrA03-p3651477
Bn-A03-p4123209	0.983471074	0.991735537	Bn-N3-p4229262	Bn-chrA03-p3651522
Bn-A03-p4130585	1	0.991735537	Bn-N3-p4235090	Bn-chrAnn_random-p359221
Bn-A03-p4138735	0.966942149	0.983471074	Bn-N3-p4244632	Bn-chrA03-p3663133
Bn-A03-p4153697	0.975206612	0.966942149	Bn-N3-p4266872	Bn-chrA03-p3686185
Bn-A03-p4167644	0.966942149	0.73553719	Bn-N3-p4287809	Bn-chrA03-p3705364
Bn-A03-p4177118	0.976744186	0.976744186	Bn-N3-p4297206	Bn-chrA03-p3714773
Bn-A03-p4180932	0.980099502	0.980099502	Bn-N13-p5989045	Bn-chrC03-p5016999
Bn-A03-p4183056	0.986710963	0.986710963	Bn-N3-p4303170	Bn-chrA03-p3720446
Bn-A03-p4193022	0.603305785	0.991735537	Bn-N3-p4314795	Bn-chrA03-p3732230
Bn-A03-p4196989	0.966942149	0.958677686	Bn-N3-p4315303	Bn-chrA03-p3735422
Bn-A03-p4202242	0.991735537	1	Bn-N3-p4321291	Bn-chrA03-p3741155
Bn-A03-p4205159	0.991735537	0.991735537	Bn-N3-p4324200	Bn-chrA03-p3744069
Bn-A03-p4211072	0.983471074	0.983471074	Bn-N3-p4330485	Bn-chrA03-p3749930
Bn-A03-p4215720	1	0.884297521	Bn-N3-p4336492	Bn-chrC06-p13850934
Bn-A03-p4216444	0.983471074	0.983471074	Bn-N3-p4337216	Bn-chrA03-p3755339
Bn-A03-p4232493	0.996677741	0.993355482	Bn-N3-p4352276	Bn-chrA03-p3769952
Bn-A03-p4235658	0.877076412	0.910299003	Bn-N3-p4355441	Bn-chrA03-p3773108

Bn-A03-p4244641	0.966942149	0.975206612	Bn-N3-p4365825	Bn-chrA03-p3782213
Bn-A03-p4256940	1	0.996677741	Bn-N3-p4379526	Bn-chrA03-p3794794
Bn-A03-p4279693	0.950413223	0.94214876	Bn-N3-p4390447	Bn-chrA03-p3808738
Bn-A03-p4279836	0.991735537	0.975206612	Bn-N3-p4390590	Bn-chrA03-p3808880
Bn-A03-p4292459	0.996677741	0.996677741	Bn-N3-p4403664	Bn-chrA03-p3821603
Bn-A03-p4295145	0.980099502	0.985074627	Bn-N3-p4406351	Bn-chrA03-p3824287
Bn-A03-p4309562	0.983471074	0.983471074	Bn-N3-p4421100	Bn-chrA03-p3838530
Bn-A03-p4335536	0.958677686	0.958677686	Bn-N3-p4445059	Bn-chrA03-p3867262
Bn-A03-p4339301	0.958677686	0.958677686	Bn-N3-p4448837	Bn-chrA03-p3871042
Bn-A03-p4342338	0.966942149	0.966942149	Bn-N3-p4451981	Bn-chrA03-p3874203
Bn-A03-p4348997	0.975206612	0.975206612	Bn-N3-p4458513	Bn-chrA03-p3880737
Bn-A03-p4352837	0.975206612	0.975206612	Bn-N3-p4462956	Bn-chrA03-p3885180
Bn-A03-p4378124	0.983471074	0.975206612	Bn-N3-p4480524	Bn-chrA03-p3908268
Bn-A03-p4382093	0.983471074	0.991735537	Bn-N3-p4484494	Bn-chrA03-p3912230
Bn-A03-p4387230	0.991735537	0.991735537	Bn-N3-p4491101	Bn-chrA03-p3919582
Bn-A03-p4399285	1	1	Bn-N3-p4500151	Bn-chrA03-p3929928
Bn-A03-p4407987	0.991735537	0.983471074	Bn-N3-p4510032	Bn-chrA03-p3938008
Bn-A03-p4408006	0.983471074	0.991735537	Bn-N3-p4510051	Bn-chrA03-p3938027
Bn-A03-p4408895	0.975206612	0.983471074	Bn-N3-p4510940	Bn-chrA03-p3938915
Bn-A03-p4409606	0.983471074	0.983471074	Bn-N3-p4511651	Bn-chrA03-p3939596
Bn-A03-p4409970	0.983471074	0.991735537	Bn-N3-p4512015	Bn-chrA03-p3939960
Bn-A03-p4416817	0.975206612	0.983471074	Bn-N3-p4518862	Bn-chrA03-p3943674
Bn-A03-p4423212	0.940298507	0.935323383	Bn-N3-p4529466	Bn-chrA03-p3955145
Bn-A03-p4423604	0.966942149	0.975206612	Bn-N3-p4529861	Bn-chrA03-p3955534
Bn-A03-p4425566	0.958677686	0.958677686	Bn-N3-p4531826	Bn-chrA03-p3957506
Bn-A03-p4425819	0.983471074	0.983471074	Bn-N3-p4532079	Bn-chrA03-p3957759
Bn-A03-p4437907	0.991735537	0.991735537	Bn-N3-p4540615	Bn-chrA03-p3964748
Bn-A03-p4473205	0.983471074	0.991735537	Bn-N3-p4577573	Bn-chrA03-p4004842
Bn-A03-p4488639	0.958677686	0.966942149	Bn-N3-p4585484	Bn-chrA03-p4014745
Bn-A03-p4498766	0.985074627	0.980099502	Bn-N3-p4593459	Bn-chrA03-p4023443
Bn-A03-p4507718	0.983471074	0.983471074	Bn-N3-p4602316	Bn-chrA03-p4030660
Bn-A03-p4509102	0.975206612	0.983471074	Bn-N3-p4604576	Bn-chrA03-p4036726
Bn-A03-p4548924	0.991735537	0.983471074	Bn-Scaffold20404-p4	Bn-chrA03-p4069908
Bn-A03-p4549344	0.975206612	0.983471074	Bn-N3-p4647289	Bn-chrA03-p4070331
Bn-A03-p4550270	0.991735537	0.991735537	Bn-N3-p4648215	Bn-chrA03-p4071257
Bn-A03-p4560576	0.983471074	0.983471074	Bn-N3-p4663300	Bn-chrA03-p4081451
Bn-A03-p4567578	0.990049751	0.990049751	Bn-N3-p4670615	Bn-chrA03-p4088458
Bn-A03-p4572110	0.991735537	1	Bn-N3-p4675147	Bn-chrA03-p4092990
Bn-A03-p4575317	0.983471074	0.983471074	Bn-N3-p4678354	Bn-chrA03-p4096198
Bn-A03-p4590174	0.975206612	0.975206612	Bn-N3-p4684505	Bn-chrA03-p4102348
Bn-A03-p4593298	0.975206612	0.190082645	Bn-N3-p4687569	Bn-chrAnn_random-p462164
Bn-A03-p4593331	0.966942149	0.305785124	Bn-N3-p4687602	Bn-chrC03-p5546606
Bn-A03-p4594763	0.996677741	0.857142857	Bn-N3-p4689034	Bn-chrA08-p7923798
Bn-A03-p4605838	0.991735537	0.991735537	Bn-N3-p4700097	Bn-chrA03-p4108933
Bn-A03-p4606213	0.983471074	0.983471074	Bn-N3-p4700472	Bn-chrA03-p4109308
Bn-A03-p4607120	0.983471074	0.983471074	Bn-N3-p4701379	Bn-chrA03-p4110215

Bn-A03-p4607947	0.975206612	0.975206612	Bn-N3-p4702206	Bn-chrA03-p4111042
Bn-A03-p4620459	0.960199005	0.965174129	Bn-N3-p4715690	Bn-chrA03-p4126291
Bn-A03-p4638893	0.991735537	0.975206612	Bn-N3-p4730296	Bn-chrA03-p4143209
Bn-A03-p4659068	1	0.991735537	Bn-N3-p4744028	Bn-chrA03-p4158468
Bn-A03-p4673503	0.983471074	0.991735537	Bn-N3-p4759424	Bn-chrA03-p4171668
Bn-A03-p4673508	0.970149254	0.970149254	Bn-N3-p4759429	Bn-chrA03-p4171673
Bn-A03-p4682207	0.983388704	0.976744186	Bn-N13-p6630129	Bn-chrC03-p5656675
Bn-A03-p4753001	0.933884298	0.94214876	Bn-N3-p4805875	Bn-chrA03-p4233585
Bn-A03-p4769653	0.989583333	1	Bn-N3-p4817421	Bn-chrA03-p4254779
Bn-A03-p4783485	0.933884298	0.785123967	Bn-N3-p4831677	Bn-chrA03-p4262371
Bn-A03-p4798485	0.991735537	1	Bn-N3-p4851110	Bn-chrA03-p4284073
Bn-A03-p4799142	1	0.991735537	Bn-N3-p4851767	Bn-chrA03-p4284730
Bn-A03-p4809473	0.991735537	0.991735537	Bn-N3-p4863779	Bn-chrA03-p4295004
Bn-A03-p4827846	0.909090909	0.991735537	Bn-N3-p4880681	Bn-chrA03-p4312981
Bn-A03-p4829139	0.975206612	0.983471074	Bn-N3-p4881956	Bn-chrA03-p4314270
Bn-A03-p4861136	0.996677741	0.943521595	Bn-N3-p4910235	Bn-chrC03-p5944345
Bn-A03-p4871103	0.990033223	0.993355482	Bn-N3-p4919913	Bn-chrAnn_random-p184116
Bn-A03-p4879812	0.965174129	0.960199005	Bn-N3-p4928778	Bn-chrAnn_random-p184203
Bn-A03-p4881229	0.94214876	1	Bn-N3-p4930160	Bn-chrAnn_random-p184217
Bn-A03-p4909233	0.966942149	0.958677686	Bn-N3-p4958930	Bn-chrAnn_random-p184463
Bn-A03-p4909313	0.933884298	0.958677686	Bn-N3-p4959008	Bn-chrAnn_random-p184463
Bn-A03-p4909522	0.960199005	0.965174129	Bn-N3-p4959215	Bn-chrA03_random-p607989
Bn-A03-p4916224	0.975206612	1	Bn-N3-p4964782	Bn-chrA03_random-p615964
Bn-A03-p4934887	0.991735537	0.190082645	Bn-N3-p4977006	Unknown
Bn-A03-p4944913	0.995024876	0.124378109	Bn-N3-p4985911	Unknown
Bn-A03-p4989769	0.991735537	0.94214876	Bn-N3-p5023410	Bn-chrA03-p4423261
Bn-A03-p4992039	0.975206612	0.975206612	Bn-N3-p5025754	Bn-chrA03-p4425428
Bn-A03-p4997351	0.950166113	0.92358804	Bn-N3-p5030697	Bn-chrC03-p6006826
Bn-A03-p4999662	0.990033223	0.983388704	Bn-N3-p5033013	Bn-chrA03-p4432537
Bn-A03-p5006544	0.983388704	0.983388704	Bn-N3-p5040509	Bn-chrA03-p4439288
Bn-A03-p5023169	1	0.302325581	Bn-N3-p5057209	Unknown
Bn-A03-p5025156	0.986710963	0.990033223	Bn-N3-p5059196	Bn-chrA03-p4457676
Bn-A03-p5038403	0.776859504	0.776859504	Bn-N10-p9907929	Bn-chrA10_random-p163424
Bn-A03-p5039235	0.983471074	0.983471074	Bn-N3-p5072378	Bn-chrA03-p4472290
Bn-A03-p5039586	0.983471074	0.983471074	Bn-N3-p5073203	Bn-chrA03-p4473113
Bn-A03-p5042719	0.950166113	0.986710963	Bn-N3-p5076308	Bn-chrC03-p6066057
Bn-A03-p5045095	1	1	Bn-N3-p5078686	Bn-chrA03-p4478595
Bn-A03-p5052289	0.438016529	0.438016529	Bn-Scaffold08851-p7	Bn-chrC03-p6075568
Bn-A03-p5067556	0.993355482	0.993355482	Bn-N3-p5101194	Bn-chrA03-p4501180
Bn-A03-p5072729	0.990033223	0.986710963	Bn-N3-p5106348	Bn-chrA03-p4506334
Bn-A03-p5078310	0.991735537	0.983471074	Bn-N3-p5111964	Bn-chrA03-p4511921
Bn-A03-p5083198	0.986710963	0.990033223	Bn-N3-p5115640	Bn-chrA03-p4515915
Bn-A03-p5083386	0.975206612	0.983471074	Bn-N3-p5115828	Bn-chrA03-p4516103
Bn-A03-p5083430	0.975206612	0.983471074	Bn-N3-p5115872	Bn-chrA03-p4516147
Bn-A03-p5111446	0.936877076	0.986710963	Bn-N3-p5149867	Bn-chrA03-p4544230
Bn-A03-p5115321	0.975206612	0.983471074	Bn-N3-p5153991	Bn-chrA03-p4548249

Bn-A03-p5131282	0.986710963	0.993355482	Bn-N3-p5161707	Bn-chrA03-p4554529
Bn-A03-p5131371	0.983471074	0.991735537	Bn-N3-p5161796	Bn-chrA03-p4554618
Bn-A03-p5133977	0.983471074	0.991735537	Bn-N3-p5164414	Bn-chrA03-p4558145
Bn-A03-p5135902	0.993355482	0.973421927	Bn-N3-p5167884	Bn-chrC03-p6149372
Bn-A03-p5140330	0.991735537	0.842975207	Bn-N3-p5172647	Bn-chrC03-p6154247
Bn-A03-p5140947	0.991735537	1	Bn-N3-p5173270	Bn-chrA03-p4568578
Bn-A03-p5144171	0.975124378	0.995024876	Bn-N3-p5176740	Bn-chrA03-p4573108
Bn-A03-p5153735	0.980099502	0.975124378	Bn-N3-p5185451	Bn-chrA03-p4581101
Bn-A03-p5159930	0.990033223	0.950166113	Bn-N3-p5191753	Bn-chrA03-p4587152
Bn-A03-p5160088	0.983471074	0.958677686	Bn-N3-p5191911	Bn-chrA03-p4587309
Bn-A03-p5163056	0.991735537	1	Bn-N3-p5194879	Bn-chrA03-p4590302
Bn-A03-p5170706	0.983471074	0.198347107	Bn-N3-p5202612	Bn-chrC09-p8166746
Bn-A03-p5177830	0.983471074	0.975206612	Bn-N3-p5211714	Bn-chrA03-p4604524
Bn-A03-p5180655	0.993355482	0.956810631	Bn-N3-p5214539	Bn-chrA03-p4607335
Bn-A03-p5182850	0.986710963	0.986710963	Bn-N3-p5216734	Bn-chrA03-p4609673
Bn-A03-p5222291	0.936877076	0.986710963	Bn-N13-p7360760	Bn-chrA03-p4651899
Bn-A03-p5227700	0.991735537	0.991735537	Bn-N3-p5261164	Bn-chrA03-p4657049
Bn-A03-p5230726	0.985074627	0.990049751	Bn-N3-p5264185	Bn-chrA03-p4660636
Bn-A03-p5263828	0.983471074	0.991735537	Bn-N3-p5296789	Bn-chrA03-p4692171
Bn-A03-p5268272	0.993355482	0.996677741	Bn-N3-p5300453	Bn-chrA03-p4696470
Bn-A03-p5273046	0.986710963	0.890365449	Bn-N3-p5306760	Bn-chrA03-p4701251
Bn-A03-p5333699	0.976744186	0.980066445	Bn-N3-p5352536	Bn-chrAnn_random-p348913
Bn-A03-p5341139	0.958677686	0.975206612	Bn-N3-p5360784	Bn-chrA03-p4754949
Bn-A03-p5357737	0.990033223	0.986710963	Bn-N3-p5379420	Bn-chrCnn_random-p465502
Bn-A03-p5360744	0.991735537	0.983471074	Bn-N3-p5382441	Bn-chrC03-p6425903
Bn-A03-p5374417	1	0.991735537	Bn-N3-p5394308	Bn-chrA03-p4791305
Bn-A03-p5380857	0.983471074	0.983471074	Bn-N3-p5400514	Bn-chrA03-p4797861
Bn-A03-p5408678	0.983471074	0.958677686	Bn-N13-p7615820	Bn-chrA03-p4826009
Bn-A03-p5414946	0.996677741	0.996677741	Bn-N3-p5434725	Bn-chrA03-p4832274
Bn-A03-p5423318	0.996677741	0.996677741	Bn-N3-p5447066	Bn-chrA03-p4842974
Bn-A03-p5434265	0.950413223	0.950413223	Bn-N3-p5456465	Bn-chrA03-p4853120
Bn-A03-p5439400	0.993355482	0.993355482	Bn-N3-p5461030	Bn-chrA03-p4857685
Bn-A03-p5454100	0.990033223	0.990033223	Bn-N3-p5475261	Bn-chrA03-p4871915
Bn-A03-p5455069	0.958677686	0.958677686	Bn-N3-p5476226	Bn-chrA03-p4872880
Bn-A03-p5462418	0.966942149	0.966942149	Bn-N3-p5480987	Bn-chrA03-p4877634
Bn-A03-p5467712	0.975206612	0.975206612	Bn-N3-p5487249	Bn-chrA03-p4883895
Bn-A03-p5468447	0.94214876	0.94214876	Bn-N3-p5490652	Bn-chrA03-p4887277
Bn-A03-p5471743	0.734219269	0.734219269	Bn-N3-p5493961	Bn-chrA03-p4890586
Bn-A03-p5502308	0.980066445	0.980066445	Bn-N3-p5535586	Bn-chrA03-p4931843
Bn-A03-p5503857	0.993355482	0.993355482	Bn-N3-p5537135	Bn-chrA03-p4933392
Bn-A03-p5523239	0.996677741	0.996677741	Bn-N3-p5558176	Bn-chrA03-p4954768
Bn-A03-p5523813	0.986710963	0.986710963	Bn-N3-p5558750	Bn-chrA03-p4955342
Bn-A03-p5523943	0.993355482	0.993355482	Bn-N3-p5558880	Bn-chrA03-p4955472
Bn-A03-p5532043	0.996677741	0.996677741	Bn-N3-p5567045	Bn-chrA03-p4963639
Bn-A03-p5548413	0.983471074	0.983471074	Bn-Scaffold05514-p1	Bn-chrA03-p4979561
Bn-A03-p5551018	0.801652893	0.801652893	Bn-N3-p5586379	Bn-chrA03-p4985664

Bn-A03-p5563280	0.975206612	0.966942149	Bn-N13-p7833695	Bn-chrA03-p4997704
Bn-A03-p5564849	0.958677686	0.958677686	Bn-N3-p5599272	Bn-chrA03-p5004361
Bn-A03-p5578777	0.809917355	0.809917355	Bn-N3-p5607743	Bn-chrA03-p5012667
Bn-A03-p5597551	0.975206612	0.966942149	Bn-N3-p5628250	Bn-chrA03-p5031002
Bn-A03-p5608927	0.976744186	0.980066445	Bn-N3-p5639957	Bn-chrA03-p5043286
Bn-A03-p5611940	0.991735537	0.991735537	Bn-N3-p5649430	Bn-chrA03-p5046056
Bn-A03-p5612769	0.991735537	0.983471074	Bn-N3-p5650259	Bn-chrA03-p5046885
Bn-A03-p5613665	0.983471074	0.975206612	Bn-N3-p5651152	Bn-chrA03-p5047778
Bn-A03-p5614097	0.94214876	0.94214876	Bn-N3-p5651581	Bn-chrA03-p5048241
Bn-A03-p5626575	0.960199005	0.995024876	Bn-N3-p5668974	Bn-chrA03-p5065142
Bn-A03-p5626694	0.970149254	0.975124378	Bn-N3-p5669092	Bn-chrA03-p5065261
Bn-A03-p5627074	0.980066445	0.983388704	Bn-N3-p5669472	Bn-chrA03-p5065644
Bn-A03-p5627585	0.990049751	0.990049751	Bn-N3-p5669983	Bn-chrA03-p5066155
Bn-A03-p5643775	1	0.991735537	Bn-N3-p5677021	Bn-chrA03-p5068643
Bn-A03-p5649178	0.983388704	0.990033223	Bn-N3-p5682437	Bn-chrA03-p5074049
Bn-A03-p5654220	0.966942149	0.900826446	Bn-N3-p5688103	Bn-chrC03-p6791980
Bn-A03-p5669661	0.990049751	0.990049751	Bn-N3-p5699703	Bn-chrA03-p5097185
Bn-A03-p5682911	0.958677686	0.950413223	Bn-N3-p5710737	Bn-chrA03-p5108311
Bn-A03-p5694401	0.983471074	0.983471074	Bn-N3-p5723046	Bn-chrA03-p5119830
Bn-A03-p5696470	0.950413223	0.950413223	Bn-N3-p5727160	Bn-chrA03-p5121853
Bn-A03-p5711554	0.993355482	0.993355482	Bn-N3-p5740667	Bn-chrA03-p5139563
Bn-A03-p5711923	0.991735537	0.991735537	Bn-N3-p5741036	Bn-chrA03-p5139932
Bn-A03-p5713023	0.834710744	0.834710744	Bn-N3-p5742221	Bn-chrA03-p5141366
Bn-A03-p5721171	0.94214876	0.94214876	Bn-Scaffold01911-p2	Bn-chrA03-p5149621
Bn-A03-p5721231	0.966942149	0.966942149	Bn-Scaffold01911-p2	Bn-chrA03-p5149681
Bn-A03-p5721443	0.983471074	0.983471074	Bn-Scaffold01911-p2	Bn-chrA03-p5149893
Bn-A03-p5721617	0.958677686	0.958677686	Bn-Scaffold01911-p3	Bn-chrA03-p5150067
Bn-A03-p5722009	0.958677686	0.958677686	Bn-Scaffold01911-p3	Bn-chrA03-p5150461
Bn-A03-p5722579	0.958677686	0.958677686	Bn-Scaffold01911-p4	Bn-chrA03-p5151031
Bn-A03-p5741340	0.975206612	0.975206612	Bn-N3-p5775835	Bn-chrA03-p5170474
Bn-A03-p5741875	0.966942149	0.966942149	Bn-N3-p5776380	Bn-chrA03-p5171019
Bn-A03-p5742880	0.966942149	0.966942149	Bn-N3-p5777953	Bn-chrA03-p5172025
Bn-A03-p5742916	0.966942149	0.966942149	Bn-N3-p5777989	Bn-chrA03-p5172061
Bn-A03-p5743020	0.991735537	0.991735537	Bn-N3-p5778093	Bn-chrA03-p5172165
Bn-A03-p5744242	0.983471074	0.983471074	Bn-N3-p5782157	Bn-chrA03-p5177705
Bn-A03-p5744750	0.975206612	0.975206612	Bn-N3-p5782662	Bn-chrA03-p5178211
Bn-A03-p5745392	0.975206612	0.975206612	Bn-N3-p5783297	Bn-chrA03-p5178846
Bn-A03-p5745449	0.966942149	0.966942149	Bn-N3-p5783354	Bn-chrA03-p5178903
Bn-A03-p5746114	0.983471074	0.983471074	Bn-N3-p5784019	Bn-chrA03-p5179568
Bn-A03-p5748366	1	1	Bn-N3-p5786322	Bn-chrA03-p5181854
Bn-A03-p5748775	1	1	Bn-N3-p5786731	Bn-chrA03-p5182263
Bn-A03-p5748884	0.991735537	0.991735537	Bn-N3-p5786840	Bn-chrA03-p5182372
Bn-A03-p5749121	0.991735537	0.991735537	Bn-N3-p5787077	Bn-chrA03-p5182609
Bn-A03-p5749213	0.983471074	0.983471074	Bn-N3-p5787169	Bn-chrA03-p5182701
Bn-A03-p5749663	0.983471074	0.983471074	Bn-N3-p5787619	Bn-chrA03-p5183151
Bn-A03-p5749832	1	1	Bn-N3-p5787788	Bn-chrA03-p5183320

Bn-A03-p5750321	0.991735537	0.991735537	Bn-N3-p5788277	Bn-chrA03-p5183809
Bn-A03-p5751160	0.991735537	0.991735537	Bn-N3-p5789116	Bn-chrA03-p5185003
Bn-A03-p5751992	0.983471074	0.983471074	Bn-N3-p5789946	Bn-chrA03-p5185833
Bn-A03-p5752052	0.983471074	0.983471074	Bn-N3-p5790006	Bn-chrA03-p5185893
Bn-A03-p5752371	1	1	Bn-N3-p5790325	Bn-chrA03-p5186212
Bn-A03-p5762915	0.986710963	0.986710963	Bn-N3-p5800914	Bn-chrA03-p5201470
Bn-A03-p5770833	0.980066445	0.980066445	Bn-N13-p8100180	Bn-chrC03-p6933518
Bn-A03-p5784532	0.983471074	0.983471074	Bn-N3-p5820502	Bn-chrA03-p5215835
Bn-A03-p5796033	0.993355482	0.993355482	Bn-N3-p5834914	Bn-chrA03-p5227228
Bn-A03-p5800566	1	1	Bn-N3-p5839462	Bn-chrA03-p5231777
Bn-A03-p5809786	0.976744186	0.983388704	Bn-N3-p5849472	Bn-chrA03-p5241553
Bn-A03-p5809860	0.983471074	0.983471074	Bn-N3-p5849546	Bn-chrA03-p5241627
Bn-A03-p5814447	0.983471074	0.991735537	Bn-N3-p5854130	Bn-chrA03-p5245778
Bn-A03-p5866896	0.950413223	0.950413223	Bn-N3-p5900253	Bn-chrA03-p5292620
Bn-A03-p5892557	0.993355482	0.843853821	Bn-N3-p5921392	Bn-chrC03-p6993437
Bn-A03-p5893002	0.988188976	0.988188976	Bn-N3-p5921790	Bn-chrAnn_random-p473182
Bn-A03-p5903435	0.975206612	0.975206612	Bn-N3-p5931743	Bn-chrA03-p5333777
Bn-A03-p5912430	1	1	Bn-N7-p10359233	Bn-chrC05-p42652557
Bn-A03-p5952923	0.983471074	0.991735537	Bn-N3-p5980466	Bn-chrA03-p5366394
Bn-A03-p5956851	0.955223881	0.960199005	Bn-N3-p5984115	Bn-chrA03-p5370322
Bn-A03-p5956934	0.991735537	0.991735537	Bn-N3-p5984198	Bn-chrA03-p5370405
Bn-A03-p5984123	0.991735537	0.991735537	Bn-N3-p6020429	Bn-chrA03-p5399751
Bn-A03-p6036521	0.991735537	0.991735537	Bn-N3-p6077412	Bn-chrA03-p5448828
Bn-A03-p6039029	0.965174129	0.965174129	Bn-N3-p6079930	Bn-chrA03-p5451347
Bn-A03-p6057369	0.966777409	0.990033223	Bn-N3-p6097929	Bn-chrA03-p5472575
Bn-A03-p6126012	0.980066445	0.980066445	Bn-N3-p6172559	Bn-chrA03-p5497351
Bn-A03-p6132626	1	1	Bn-N3-p6179333	Bn-chrA03-p5503965
Bn-A03-p6136484	0.983471074	0.991735537	Bn-N3-p6183478	Bn-chrA03-p5507282
Bn-A03-p6138644	0.980099502	0.990049751	Bn-N3-p6185740	Bn-chrA03-p5510965
Bn-A03-p6149485	0.966942149	0.966942149	Bn-N3-p6196755	Bn-chrA03-p5519296
Bn-A03-p6157864	0.983471074	0.983471074	Bn-N3-p6205135	Bn-chrA03-p5527677
Bn-A03-p6158211	0.966942149	0.966942149	Bn-N3-p6205483	Bn-chrA03-p5528025
Bn-A03-p6182289	0.960199005	0.980099502	Bn-N3-p6227250	Bn-chrA03-p5549660
Bn-A03-p6204498	0.990033223	0.990033223	Bn-N3-p6249247	Bn-chrA03-p5567585
Bn-A03-p6224340	1	0.710743802	Bn-N3-p6266691	Bn-chrA03-p5590560
Bn-A03-p6228570	0.975206612	0.983471074	Bn-N3-p6270365	Bn-chrA03-p5594710
Bn-A03-p6265064	0.993355482	0.980066445	Bn-N2-p6270721	Bn-chrA02-p4033583
Bn-A03-p6274828	0.936877076	0.936877076	Bn-N3-p6308534	Bn-chrA03-p5625604
Bn-A03-p6276385	0.989583333	0.989583333	Bn-N3-p6310134	Bn-chrA03-p5627203
Bn-A03-p6286363	0.996677741	0.996677741	Bn-N3-p6317373	Bn-chrA03-p5636296
Bn-A03-p6288173	0.991735537	0.991735537	Bn-N3-p6319183	Bn-chrA03-p5638107
Bn-A03-p6299523	0.631840796	0.63681592	Bn-N3-p6330787	Bn-chrA03-p5649456
Bn-A03-p6317002	0.966942149	0.983471074	Bn-N3-p6356236	Bn-chrA03-p5664096
Bn-A03-p6319963	0.993355482	0.980066445	Bn-N3-p6359197	Bn-chrA03-p5667033
Bn-A03-p6324760	0.940199336	0.940199336	Bn-N3-p6363731	Bn-chrA03-p5671616
Bn-A03-p6332797	0.983471074	0.975206612	Bn-N3-p6374184	Bn-chrA03-p5681359

Bn-A03-p6335597	0.960199005	0.965174129	Bn-N3-p6376681	Bn-chrA03-p5683896
Bn-A03-p6338496	0.983471074	0.975206612	Bn-N3-p6379605	Bn-chrAnn_random-p371398
Bn-A03-p6354338	0.991735537	0.991735537	Bn-N3-p6406637	Bn-chrA03_random-p654102
Bn-A03-p6358993	0.985074627	0.980099502	Bn-N3-p6411293	Bn-chrA03_random-p660352
Bn-A03-p6384649	1	1	Bn-N3-p6443970	Bn-chrA03-p5707699
Bn-A03-p6385958	0.993355482	0.990033223	Bn-N3-p6445278	Bn-chrA03-p5710065
Bn-A03-p6387774	0.975206612	0.991735537	Bn-N3-p6446504	Bn-chrA03-p5711309
Bn-A03-p6388604	0.990049751	0.995024876	Bn-N3-p6447333	Bn-chrA03-p5712139
Bn-A03-p6404659	0.960199005	0.950248756	Bn-N3-p6461195	Bn-chrA03-p5726779
Bn-A03-p6404903	0.975206612	0.975206612	Bn-N3-p6461439	Bn-chrA03-p5727023
Bn-A03-p6408156	0.752066116	0.743801653	Bn-N13-p8999465	Bn-chrC03-p7704082
Bn-A03-p6420355	1	0.991735537	Bn-N3-p6475470	Bn-chrA03-p5740679
Bn-A03-p6423049	0.991735537	1	Bn-N3-p6478165	Bn-chrA03-p5743626
Bn-A03-p6423198	0.991735537	0.991735537	Bn-N3-p6478314	Bn-chrA03-p5743775
Bn-A03-p6423795	0.975206612	0.975206612	Bn-N3-p6478911	Bn-chrA03-p25063305
Bn-A03-p6424980	0.995024876	1	Bn-N3-p6480096	Bn-chrAnn_random-p369359
Bn-A03-p6425315	1	1	Bn-N3-p6480430	Bn-chrAnn_random-p369362
Bn-A03-p6425858	1	0.995024876	Bn-N3-p6480974	Bn-chrAnn_random-p369368
Bn-A03-p6427905	0.990033223	0.953488372	Bn-N3-p6487560	Bn-chrCnn_random-p574532
Bn-A03-p6428220	0.991735537	0.801652893	Bn-N3-p6487875	Bn-chrC03-p7736325
Bn-A03-p6439432	0.986710963	0.983388704	Bn-N3-p6497920	Bn-chrA03-p5754736
Bn-A03-p6456584	0.950413223	0.950413223	Bn-Scaffold67092-p1	Bn-chrA03-p5770337
Bn-A03-p6484137	0.966942149	0.975206612	Bn-N3-p6522588	Bn-chrA03-p5777414
Bn-A03-p6498305	0.991735537	1	Bn-N3-p6535515	Bn-chrA03-p5791093
Bn-A03-p6502463	0.67768595	0.851239669	Bn-N3-p6539671	Bn-chrC03-p7796444
Bn-A03-p6514858	0.985074627	0.990049751	Bn-N3-p6554535	Bn-chrA03-p5804395
Bn-A03-p6522454	0.983388704	0.980066445	Bn-N3-p6560772	Bn-chrA03-p5813279
Bn-A03-p6528914	1	1	Bn-N3-p6568754	Bn-chrA03-p5819772
Bn-A03-p6556647	0.980099502	0.985074627	Bn-N3-p6593693	Bn-chrA03-p5848921
Bn-A03-p6576575	0.991735537	1	Bn-N3-p6618284	Bn-chrA03-p5874703
Bn-A03-p6577213	0.985074627	0.910447761	Bn-N3-p6618922	Bn-chrC03-p7941563
Bn-A03-p6578752	0.990033223	0.990033223	Bn-N3-p6620459	Bn-chrA03-p5876847
Bn-A03-p6583208	0.986710963	0.990033223	Bn-N3-p6624914	Bn-chrA03-p5881302
Bn-A03-p6585645	0.983471074	0.975206612	Bn-N3-p6629360	Bn-chrA03-p5883732
Bn-A03-p6586436	1	0.993355482	Bn-N3-p6630151	Bn-chrA03-p5884508
Bn-A03-p6590051	0.975206612	0.983471074	Bn-N3-p6633766	Bn-chrA03-p5886732
Bn-A03-p6591745	1	0.973421927	Bn-N3-p6635460	Bn-chrC03-p7954456
Bn-A03-p6602787	0.966942149	0.975206612	Bn-N3-p6650828	Bn-chrA03-p5896305
Bn-A03-p6602851	0.983471074	0.975206612	Bn-N3-p6650892	Bn-chrA03-p5896369
Bn-A03-p6636780	0.983471074	0.991735537	Bn-N3-p6679319	Bn-chrA03-p5928259
Bn-A03-p6646094	0.980066445	0.980066445	Bn-N3-p6682601	Bn-chrA03-p5931531
Bn-A03-p6668218	0.985074627	0.985074627	Bn-N3-p6704487	Bn-chrA03-p5952393
Bn-A03-p6668259	1	1	Bn-N3-p6704528	Bn-chrA03-p5952434
Bn-A03-p6672024	0.983471074	0.991735537	Bn-N3-p6708373	Bn-chrA03-p5956088
Bn-A03-p6677819	0.991735537	1	Bn-N3-p6710985	Bn-chrA03-p5958708
Bn-A03-p6681953	0.991735537	0.983471074	Bn-N3-p6715108	Bn-chrA03-p5962825

Bn-A03-p6706586	0.958677686	0.710743802	Bn-N3-p6734445	Bn-chrA03-p5981987
Bn-A03-p6708217	0.986710963	0.986710963	Bn-N3-p6736061	Bn-chrA03-p5983602
Bn-A03-p6708475	0.983471074	0.975206612	Bn-N3-p6736318	Bn-chrA03-p5983865
Bn-A03-p6710409	1	0.991735537	Bn-N3-p6737400	Bn-chrA03-p5985980
Bn-A03-p6710651	0.976744186	0.980066445	Bn-N3-p6737642	Bn-chrA03-p5986222
Bn-A03-p6717546	0.990033223	0.990033223	Bn-N3-p6745534	Bn-chrA03-p5993115
Bn-A03-p6717785	0.996677741	0.996677741	Bn-N3-p6745773	Bn-chrA03-p5993354
Bn-A03-p6744274	0.983471074	0.975206612	Bn-N3-p6770920	Bn-chrA03-p6017737
Bn-A03-p6744344	0.983471074	0.983471074	Bn-N3-p6770990	Bn-chrA03-p6017807
Bn-A03-p6760051	0.976744186	0.993355482	Bn-N3-p6790950	Bn-chrA03-p6040221
Bn-A03-p6765024	0.996677741	1	Bn-N3-p6795796	Bn-chrA03-p6045182
Bn-A03-p6771876	0.991735537	1	Bn-N3-p6803630	Bn-chrA03-p6052034
Bn-A03-p6772326	0.958677686	0.958677686	Bn-N3-p6804089	Bn-chrA03-p6052481
Bn-A03-p6781156	0.975206612	0.958677686	Bn-N3-p6805943	Bn-chrA03-p6054337
Bn-A03-p6782910	0.975206612	0.966942149	Bn-N3-p6807676	Bn-chrA03-p6056127
Bn-A03-p6782961	0.958677686	0.966942149	Bn-N3-p6807728	Bn-chrA03-p6056178
Bn-A03-p6783685	0.983471074	0.975206612	Bn-N3-p6809695	Bn-chrA03-p6056898
Bn-A03-p6789784	0.996677741	0.996677741	Bn-N3-p6815796	Bn-chrA03-p6062886
Bn-A03-p6791851	0.980099502	0.980099502	Bn-N3-p6817864	Bn-chrA03-p6064954
Bn-A03-p6804536	0.958677686	0.966942149	Bn-N3-p6834962	Bn-chrA03-p6077480
Bn-A03-p6804857	0.966942149	0.975206612	Bn-N3-p6835283	Bn-chrA03-p6077800
Bn-A03-p6807075	1	0.991735537	Bn-N3-p6837516	Bn-chrA03-p6080014
Bn-A03-p6834343	0.996677741	0.996677741	Bn-N3-p6863617	Bn-chrA03-p6109282
Bn-A03-p6845546	0.975206612	0.975206612	Bn-N3-p6875205	Bn-chrA03-p6121068
Bn-A03-p6849607	0.983471074	0.966942149	Bn-Scaffold18623-p5	Bn-chrA03-p6139159
Bn-A03-p6888670	0.958677686	1	Bn-N13-p9632862	Bn-chrA03-p6172590
Bn-A03-p6895946	0.991735537	0.983471074	Bn-N3-p6934255	Bn-chrAnn_random-p315359
Bn-A03-p6897034	0.983471074	0.991735537	Bn-N3-p6935343	Bn-chrAnn_random-p315369
Bn-A03-p6898220	0.975124378	0.975124378	Bn-N18-p17540552	Bn-chrA03-p6179649
Bn-A03-p6907743	1	1	Bn-N18-p17531568	Bn-chrA03-p6195000
Bn-A03-p6909237	0.991735537	0.991735537	Bn-N18-p17530072	Bn-chrA03-p6196496
Bn-A03-p6910492	0.991735537	0.983471074	Bn-N18-p17528815	Bn-chrA03-p6198036
Bn-A03-p6919500	0.993355482	0.358803987	Bn-N3-p6953180	Bn-chrA03-p6206719
Bn-A03-p6934438	0.983471074	0.991735537	Bn-N18-p17523000	Bn-chrAnn_random-p326083
Bn-A03-p6934751	0.991735537	1	Bn-N18-p17522687	Bn-chrAnn_random-p326080
Bn-A03-p6937615	0.991735537	0.925619835	Bn-N3-p6960890	Bn-chrCnn_random-p680996
Bn-A03-p6967307	1	1	Bn-N3-p6989417	Bn-chrA03-p6245357
Bn-A03-p6984742	0.993355482	0.993355482	Bn-N3-p7005668	Bn-chrA03-p6265677
Bn-A03-p6994294	1	0.991735537	Bn-Scaffold86870-p1	Bn-chrA03-p6278471
Bn-A03-p7007376	0.996677741	0.671096346	Bn-N3-p7039005	Bn-chrA03-p6291347
Bn-A03-p7007377	0.94214876	0.991735537	Bn-N3-p7039006	Bn-chrA03-p6291348
Bn-A03-p7011698	0.995024876	0.995024876	Bn-N3-p7044416	Bn-chrA03-p6295690
Bn-A03-p7011746	0.990049751	0.990049751	Bn-N3-p7044464	Bn-chrA03-p6295738
Bn-A03-p7012232	1	1	Bn-N3-p7044950	Bn-chrA03-p6296224
Bn-A03-p7015536	0.983471074	0.975206612	Bn-N3-p7049530	Bn-chrC03-p8467183
Bn-A03-p7049396	1	0.983471074	Bn-N3-p7100631	Bn-chrA03-p6334089

Bn-A03-p7054553	0.993355482	0.990033223	Bn-N3-p7109282	Bn-chrA03-p6340997
Bn-A03-p7058001	0.983471074	0.991735537	Bn-N3-p7112725	Bn-chrA03-p6344405
Bn-A03-p7067563	0.975206612	0.983471074	Bn-N3-p7118530	Bn-chrA03-p6350183
Bn-A03-p7074078	0.960199005	0.960199005	Bn-N3-p7124947	Bn-chrA03-p6359471
Bn-A03-p7075595	0.986710963	0.986710963	Bn-N3-p7126465	Bn-chrA03-p6360991
Bn-A03-p7079712	0.995024876	0.990049751	Bn-N3-p7130834	Bn-chrA03-p6365302
Bn-A03-p7085943	0.996677741	1	Bn-N3-p7136602	Bn-chrA03-p6368231
Bn-A03-p7088375	0.917355372	0.94214876	Bn-N3-p7138714	Bn-chrC03-p8569346
Bn-A03-p7090745	0.991735537	0.983471074	Bn-N3-p7148150	Bn-chrA03-p6372728
Bn-A03-p7094698	0.993355482	0.996677741	Bn-N3-p7152066	Bn-chrA03-p6380016
Bn-A03-p7110332	0.991735537	0.983471074	Bn-N3-p7166209	Bn-chrA03-p6388229
Bn-A03-p7157714	0.993355482	0.990033223	Bn-N3-p7213961	Bn-chrA03-p6433692
Bn-A03-p7157782	0.990033223	0.993355482	Bn-N3-p7214029	Bn-chrA03-p6433760
Bn-A03-p7159916	0.991735537	0.991735537	Bn-N3-p7216160	Bn-chrA03-p6435890
Bn-A03-p7167442	0.94214876	0.94214876	Bn-N3-p7228475	Bn-chrA03-p6443993
Bn-A03-p7177161	0.900826446	0.925619835	Bn-N3-p7233966	Bn-chrA03-p6449447
Bn-A03-p7177504	0.958677686	0.966942149	Bn-N3-p7234314	Bn-chrA03-p6450533
Bn-A03-p7177776	0.900826446	0.909090909	Bn-N3-p7234546	Bn-chrA03-p6450763
Bn-A03-p7178551	0.986710963	0.983388704	Bn-N3-p7235355	Bn-chrA03-p6451571
Bn-A03-p7178650	0.986710963	0.986710963	Bn-N3-p7235454	Bn-chrA03-p6451670
Bn-A03-p7178917	0.966942149	0.975206612	Bn-N3-p7235721	Bn-chrA03-p6451937
Bn-A03-p7180288	0.980099502	0.970149254	Bn-N3-p7237090	Bn-chrA03-p6453308
Bn-A03-p7186958	0.990033223	0.990033223	Bn-N3-p7242704	Bn-chrA03-p6458921
Bn-A03-p7187473	0.996677741	0.993355482	Bn-N3-p7243219	Bn-chrA03-p6459436
Bn-A03-p7218582	0.975206612	0.975206612	Bn-N3-p7276303	Bn-chrA03-p6493980
Bn-A03-p7224854	0.893687708	0.88372093	Bn-N3-p7284957	Bn-chrA03-p6500076
Bn-A03-p7228211	0.991735537	0.991735537	Bn-N3-p7292340	Bn-chrA03-p6502944
Bn-A03-p7228783	0.983471074	0.975206612	Bn-N3-p7292927	Bn-chrA03-p6503531
Bn-A03-p7234862	0.975206612	0.983471074	Bn-N3-p7299113	Bn-chrA03-p6510685
Bn-A03-p7234988	0.991735537	0.991735537	Bn-N3-p7299238	Bn-chrA03-p6510811
Bn-A03-p7235826	0.983471074	0.991735537	Bn-N3-p7300243	Bn-chrA03-p6511649
Bn-A03-p7236103	0.933884298	0.950413223	Bn-N3-p7300520	Bn-chrA03-p6511926
Bn-A03-p7239205	0.966942149	0.975206612	Bn-N3-p7307021	Bn-chrA03-p6515029
Bn-A03-p7239260	0.983471074	0.991735537	Bn-N3-p7307076	Bn-chrA03-p6515085
Bn-A03-p7248970	0.958677686	0.958677686	Bn-N3-p7313541	Bn-chrA03-p6521634
Bn-A03-p7253298	1	0.991735537	Bn-Scaffold02377-p2	Bn-chrA03-p6526359
Bn-A03-p7281096	0.950166113	0.926910299	Bn-N3-p7343067	Bn-chrC03-p8810423
Bn-A03-p7284090	0.990033223	0.986710963	Bn-N3-p7346059	Bn-chrA03-p6561996
Bn-A03-p7295912	0.993355482	0.983388704	Bn-N3-p7359855	Bn-chrA03-p6574970
Bn-A03-p7319235	0.950413223	0.94214876	Bn-N3-p7380779	Bn-chrA03-p6598639
Bn-A03-p7326202	0.983471074	0.983471074	Bn-N3-p7388837	Bn-chrA03-p6605442
Bn-A03-p7331109	0.960199005	0.975124378	Bn-N3-p7394719	Bn-chrA03-p6610372
Bn-A03-p7331517	0.983471074	0.991735537	Bn-N3-p7395122	Bn-chrA03-p6610774
Bn-A03-p7356882	0.983471074	0.884297521	Bn-N3-p7421765	Bn-chrA03-p6648398
Bn-A03-p7357257	0.776859504	0.644628099	Bn-N3-p7422243	Bn-chrC03-p8939448
Bn-A03-p7366616	0.975206612	0.983471074	Bn-N3-p7435689	Bn-chrA03-p6663506

Bn-A03-p7366783	0.876033058	0.884297521	Bn-N3-p7435856	Bn-chrA03-p6663673
Bn-A03-p7378514	0.983471074	0.975206612	Bn-N3-p7447903	Bn-chrA03-p6674928
Bn-A03-p7382887	0.975124378	0.975124378	Bn-N3-p7452614	Bn-chrA03-p6681140
Bn-A03-p7411648	0.94214876	0.933884298	Bn-N3-p7478253	Bn-chrA03-p6704355
Bn-A03-p7420835	1	0.371900826	Bn-N3-p7487460	Bn-chrC06-p29299153
Bn-A03-p7421178	0.950413223	0.247933884	Bn-N3-p7487804	Bn-chrA05-p6758251
Bn-A03-p7428803	0.985074627	0.995024876	Bn-N13-p10438134	Bn-chrA03-p6721581
Bn-A03-p7434488	0.983471074	0.983471074	Bn-N3-p7501240	Bn-chrA03-p6726812
Bn-A03-p7434950	1	0.996677741	Bn-N3-p7501710	Bn-chrA03-p6727279
Bn-A03-p7442744	1	0.995024876	Bn-N3-p7509067	Bn-chrA03-p6735118
Bn-A03-p7445206	0.966942149	0.966942149	Bn-N3-p7511807	Bn-chrA03-p6741092
Bn-A03-p7458972	0.958677686	0.975206612	Bn-N3-p7523983	Bn-chrA03-p6757068
Bn-A03-p7460244	0.905472637	0.910447761	Bn-N3-p7525249	Bn-chrA03-p6758340
Bn-A03-p7467515	0.991735537	0.983471074	Bn-N3-p7531661	Bn-chrA03-p6765570
Bn-A03-p7478438	0.958677686	0.966942149	Bn-N3-p7541615	Bn-chrA10-p10535582
Bn-A03-p7480105	0.980066445	0.983388704	Bn-N3-p7543419	Bn-chrA03-p6782724
Bn-A03-p7482812	0.950413223	0.958677686	Bn-N3-p7546148	Bn-chrA03-p6785431
Bn-A03-p7485450	0.986710963	0.986710963	Bn-N3-p7548786	Bn-chrA03-p6788069
Bn-A03-p7491211	0.983471074	0.991735537	Bn-N3-p7550312	Bn-chrA03-p6789397
Bn-A03-p7493664	0.884297521	0.983471074	Bn-N3-p7552746	Bn-chrA03-p6791842
Bn-A03-p7500107	0.993355482	0.993355482	Bn-N3-p7559200	Bn-chrA03-p6798399
Bn-A03-p7501352	0.996677741	0.970099668	Bn-N3-p7560441	Bn-chrA03-p6799644
Bn-A03-p7505194	0.990049751	0.800995025	Bn-N3-p7564337	Bn-chrA03-p6802823
Bn-A03-p7506855	0.965174129	0.960199005	Bn-N3-p7565998	Bn-chrA03-p6804482
Bn-A03-p7514571	0.991735537	0.966942149	Bn-N3-p7573714	Bn-chrA03-p6810753
Bn-A03-p7514678	1	0.991735537	Bn-N3-p7573821	Bn-chrA03-p6810871
Bn-A03-p7514862	0.983471074	0.975206612	Bn-N3-p7574005	Bn-chrA03-p6811058
Bn-A03-p7521958	0.986710963	0.990033223	Bn-N3-p7580658	Bn-chrA03-p6814477
Bn-A03-p7528981	0.990033223	0.926910299	Bn-N3-p7587681	Bn-chrA03-p6825036
Bn-A03-p7532415	0.955223881	0.890547264	Bn-N3-p7590305	Bn-chrA03-p6827615
Bn-A03-p7543362	0.976744186	0.956810631	Bn-N3-p7601180	Bn-chrA03-p6848465
Bn-A03-p7551518	0.993355482	0.990033223	Bn-N3-p7608265	Bn-chrA03-p6857245
Bn-A03-p7555264	0.966942149	0.975206612	Bn-N3-p7612737	Bn-chrA03-p6861108
Bn-A03-p7571364	0.996677741	0.980066445	Bn-N3-p7630005	Bn-chrA03-p6881610
Bn-A03-p7572109	0.710743802	0.975206612	Bn-N3-p7630729	Bn-chrA03-p6882350
Bn-A03-p7572128	0.561983471	0.958677686	Bn-N3-p7630748	Bn-chrA03-p6882369
Bn-A03-p7575620	0.983471074	0.975206612	Bn-N3-p7634144	Bn-chrCnn_random-p514698
Bn-A03-p7581383	1	1	Bn-N3-p7640637	Bn-chrAnn_random-p319821
Bn-A03-p7582235	0.983471074	0.867768595	Bn-N3-p7641496	Bn-chrAnn_random-p319829
Bn-A03-p7583703	0.983471074	0.983471074	Bn-N3-p7642964	Bn-chrAnn_random-p319844
Bn-A03-p7588123	0.983471074	0.975206612	Bn-N3-p7647385	Bn-chrA03_random-p678616
Bn-A03-p7595626	1	1	Bn-N3-p7653957	Bn-chrA03_random-p685224
Bn-A03-p7597598	0.983388704	0.983388704	Bn-N3-p7655980	Bn-chrA03_random-p687247
Bn-A03-p7613494	1	0.996677741	Bn-N3-p7669799	Bn-chrA03-p6909901
Bn-A03-p7614596	0.970149254	0.970149254	Bn-N3-p7670901	Bn-chrA03-p6911003
Bn-A03-p7618555	0.975206612	0.983471074	Bn-N3-p7674859	Bn-chrA03-p6914964

Bn-A03-p7627590	0.990033223	0.950166113	Bn-N3-p7684049	Bn-chrA03-p6928723
Bn-A03-p7628749	0.970149254	0.965174129	Bn-N3-p7685208	Bn-chrA03-p6929889
Bn-A03-p7628905	0.995024876	1	Bn-N3-p7685364	Bn-chrA03-p6930045
Bn-A03-p7629219	0.940298507	0.995024876	Bn-N13-p10704969	Bn-chrA03-p6930358
Bn-A03-p7630376	0.950248756	0.990049751	Bn-N13-p10706102	Bn-chrA03-p6931515
Bn-A03-p7630628	0.975206612	0.975206612	Bn-N3-p7687041	Bn-chrA03-p6931767
Bn-A03-p7631083	0.985074627	0.985074627	Bn-N3-p7687496	Bn-chrA03-p6932222
Bn-A03-p7631177	0.970149254	0.975124378	Bn-N3-p7687592	Bn-chrA03-p6932318
Bn-A03-p7641673	0.975206612	0.975206612	Bn-N3-p7698151	Bn-chrA03-p6942893
Bn-A03-p7642519	0.975206612	0.975206612	Bn-N3-p7698626	Bn-chrA03-p6943367
Bn-A03-p7642692	1	1	Bn-N3-p7698799	Bn-chrA03-p6943540
Bn-A03-p7643795	0.985074627	0.980099502	Bn-N3-p7699902	Bn-chrA03-p6944643
Bn-A03-p7652326	0.991735537	0.991735537	Bn-N3-p7708687	Bn-chrA03-p6953399
Bn-A03-p7672403	0.975206612	0.975206612	Bn-N3-p7728895	Bn-chrA03-p6972869
Bn-A03-p7675340	0.970149254	0.990049751	Bn-N3-p7731832	Bn-chrA03-p6975802
Bn-A03-p7678159	0.983388704	0.996677741	Bn-N13-p10765274	Bn-chrA03-p6980756
Bn-A03-p7685279	0.940298507	0.975124378	Bn-N3-p7743233	Bn-chrA03-p6983861
Bn-A03-p7688578	0.993355482	0.966777409	Bn-N3-p7746543	Bn-chrA03-p6987172
Bn-A03-p7692467	0.983471074	0.983471074	Bn-N9-p5856099	Bn-chrA09_random-p721815
Bn-A03-p7738300	0.930348259	0.980099502	Bn-N3-p7781093	Bn-chrA03-p6998307
Bn-A03-p7757095	0.965174129	0.950248756	Bn-N3-p7800636	Bn-chrA03-p7020782
Bn-A03-p7760102	1	1	Bn-N3-p7803643	Bn-chrA03-p7033778
Bn-A03-p7761061	1	0.165289256	Bn-N3-p7804700	Bn-chrC05-p12256386
Bn-A03-p7766116	0.990033223	0.986710963	Bn-N3-p7820469	Bn-chrA03-p7053262
Bn-A03-p7773812	0.925619835	0.94214876	Bn-N3-p7828164	Bn-chrA03-p7063566
Bn-A03-p7788800	0.975206612	0.975206612	Bn-N3-p7843561	Bn-chrA03-p7081584
Bn-A03-p7789818	0.991735537	0.991735537	Bn-N3-p7844754	Bn-chrA03-p7082652
Bn-A03-p7793076	0.973421927	0.970099668	Bn-N3-p7847636	Bn-chrA03-p7085843
Bn-A03-p7799483	0.986710963	0.993355482	Bn-N3-p7854102	Bn-chrA03-p7092274
Bn-A03-p7806058	0.975124378	0.970149254	Bn-N3-p7860761	Bn-chrA03-p7098942
Bn-A03-p7814324	0.966942149	0.966942149	Bn-N3-p7866417	Bn-chrA03-p7104584
Bn-A03-p7818081	0.983471074	0.983471074	Bn-N3-p7870422	Bn-chrA03-p7108589
Bn-A03-p7818503	0.975206612	0.983471074	Bn-N3-p7870844	Bn-chrA03-p7109011
Bn-A03-p7823490	0.990033223	0.980066445	Bn-N3-p7879240	Bn-chrA03-p7115516
Bn-A03-p7835836	0.993355482	0.710963455	Bn-N3-p7891520	Bn-chrC09-p12327345
Bn-A03-p7838070	0.966942149	0.991735537	Bn-N13-p10974843	Bn-chrA03-p7130137
Bn-A03-p7840758	0.991735537	0.983471074	Bn-N3-p7901905	Bn-chrA03-p7132811
Bn-A03-p7851577	0.983388704	0.983388704	Bn-N3-p7907076	Bn-chrA03-p7139858
Bn-A03-p7857447	0.976744186	0.970099668	Bn-N3-p7917659	Bn-chrA03-p7145720
Bn-A03-p7870307	0.983471074	0.975206612	Bn-N3-p7929566	Bn-chrA03-p7158362
Bn-A03-p7870541	0.958677686	0.966942149	Bn-N3-p7929800	Bn-chrA03-p7158596
Bn-A03-p7873426	0.975206612	0.975206612	Bn-N3-p7932668	Bn-chrA03-p7161482
Bn-A03-p7878888	0.975206612	0.983471074	Bn-N3-p7939377	Bn-chrA03-p7166944
Bn-A03-p7883846	0.897009967	0.897009967	Bn-N3-p7944789	Bn-chrA03_random-p688944
Bn-A03-p7887578	0.991735537	0.983471074	Bn-N3-p7948958	Bn-chrA03-p7175607
Bn-A03-p7897822	0.983471074	0.94214876	Bn-N3-p7961090	Bn-chrC03-p9605086

Bn-A03-p7902457	0.996677741	0.990033223	Bn-N3-p7965564	Bn-chrA03-p7190210
Bn-A03-p7919195	0.958677686	0.958677686	Bn-N3-p7975701	Bn-chrA03-p7200377
Bn-A03-p7919322	0.991735537	0.991735537	Bn-N3-p7975828	Bn-chrA03-p7200504
Bn-A03-p7920650	0.973421927	0.976744186	Bn-N3-p7977117	Bn-chrA03-p7201792
Bn-A03-p7939679	0.996677741	0.996677741	Bn-N3-p7994167	Bn-chrA03-p7219615
Bn-A03-p7940082	0.993355482	0.993355482	Bn-N3-p7994570	Bn-chrA03-p7220018
Bn-A03-p7943450	0.975206612	0.975206612	Bn-N3-p8003151	Bn-chrA03-p7227345
Bn-A03-p7946505	0.94214876	0.94214876	Bn-N3-p8006382	Bn-chrA03-p7230576
Bn-A03-p7949241	0.94214876	0.94214876	Bn-N3-p8009117	Bn-chrA03-p7233311
Bn-A03-p7961581	0.966942149	0.966942149	Bn-N3-p8020095	Bn-chrA03-p7243065
Bn-A03-p7963101	0.950413223	0.950413223	Bn-N3-p8021615	Bn-chrA03-p7244585
Bn-A03-p7964361	0.950413223	0.950413223	Bn-N3-p8022914	Bn-chrA03-p7245884
Bn-A03-p7964519	0.966942149	0.966942149	Bn-N3-p8023072	Bn-chrA03-p7246042
Bn-A03-p7964789	0.966942149	0.966942149	Bn-N3-p8023341	Bn-chrA03-p7246311
Bn-A03-p7965237	0.860696517	0.860696517	Bn-N3-p8023787	Bn-chrA03-p7246757
Bn-A03-p7966805	0.983471074	0.983471074	Bn-N3-p8026106	Bn-chrA03-p7249076
Bn-A03-p7969685	0.917355372	1	Bn-N3-p8029269	Bn-chrA03-p7251914
Bn-A03-p7974602	0.975206612	0.966942149	Bn-N3-p8035716	Bn-chrA03-p7256732
Bn-A03-p7983071	0.970149254	0.970149254	Bn-N13-p11170486	Bn-chrC03-p9733467
Bn-A03-p7985158	0.966942149	0.966942149	Bn-N3-p8045443	Bn-chrA03-p7266460
Bn-A03-p7988522	0.991735537	1	Bn-N3-p8049152	Bn-chrA03-p7273500
Bn-A03-p7988667	0.975206612	0.983471074	Bn-N3-p8049297	Bn-chrA03-p7273645
Bn-A03-p7988820	0.991735537	0.983471074	Bn-N3-p8049450	Bn-chrA03-p7273798
Bn-A03-p8008867	0.996677741	0.996677741	Bn-N3-p8069505	Bn-chrA03-p7292373
Bn-A03-p8022406	0.958677686	0.958677686	Bn-N3-p8084828	Bn-chrA03-p7311327
Bn-A03-p8032849	0.983471074	0.983471074	Bn-N3-p8096883	Bn-chrA03-p7321711
Bn-A03-p8033095	1	0.991735537	Bn-N3-p8097129	Bn-chrA03-p7321957
Bn-A03-p8038504	0.975206612	0.975206612	Bn-N3-p8102537	Bn-chrA03-p7327372
Bn-A03-p8039401	0.991735537	0.991735537	Bn-N3-p8103434	Bn-chrA03-p7328269
Bn-A03-p8043620	0.983388704	0.990033223	Bn-N3-p8107615	Bn-chrA03-p7332442
Bn-A03-p8043983	0.965174129	0.955223881	Bn-N3-p8107978	Bn-chrA03-p7332805
Bn-A03-p8059035	0.993355482	0.993355482	Bn-N3-p8120685	Bn-chrA03-p7351030
Bn-A03-p8070883	0.958677686	0.958677686	Bn-N3-p8129882	Bn-chrA03-p7363170
Bn-A03-p8075228	0.940298507	0.945273632	Bn-N3-p8133907	Bn-chrA03-p7367442
Bn-A03-p8080680	0.958677686	0.983471074	Bn-N3-p8137197	Bn-chrA03-p7370739
Bn-A03-p8083083	0.960199005	0.985074627	Bn-N3-p8139630	Bn-chrA03-p7373141
Bn-A03-p8084586	0.983471074	0.975206612	Bn-N3-p8145108	Bn-chrA03-p7374699
Bn-A03-p8095796	0.990033223	0.986710963	Bn-N3-p8156219	Bn-chrA03-p7385908
Bn-A03-p8103457	0.94214876	0.900826446	Bn-N3-p8163884	Bn-chrA03-p7394688
Bn-A03-p8109045	0.936877076	0.936877076	Bn-N13-p11370963	Bn-chrC03-p9896289
Bn-A03-p8112016	0.956810631	0.956810631	Bn-N13-p11373962	Bn-chrC03-p9899288
Bn-A03-p8122222	0.983471074	0.983471074	Bn-N3-p8178415	Bn-chrA03-p7408962
Bn-A03-p8133096	0.909090909	0.917355372	Bn-N3-p8190537	Bn-chrAnn_random-p423552
Bn-A03-p8148926	0.950166113	0.983388704	Bn-N17-p19243259	Bn-chrA03-p7444686
Bn-A03-p8152842	0.990033223	0.993355482	Bn-N3-p8195522	Bn-chrA03-p7448608
Bn-A03-p8158265	0.990049751	0.990049751	Bn-N3-p8201678	Bn-chrA03-p7455575

Bn-A03-p8169179	0.983471074	0.991735537	Bn-N3-p8212799	Bn-chrA03-p7466103
Bn-A03-p8170734	0.958677686	0.94214876	Bn-N3-p8214576	Bn-chrA03-p7467173
Bn-A03-p8171211	0.958677686	0.900826446	Bn-N3-p8215053	Bn-chrA03-p7467639
Bn-A03-p8171248	0.784053156	0.774086379	Bn-N3-p8215090	Bn-chrA03-p7467676
Bn-A03-p8177644	0.933884298	0.966942149	Bn-N3-p8221662	Bn-chrA03-p7472583
Bn-A03-p8177695	0.94214876	0.966942149	Bn-N3-p8221709	Bn-chrA03-p7472634
Bn-A03-p8181125	0.983471074	0.983471074	Bn-N3-p8226462	Bn-chrA03-p7488406
Bn-A03-p8181187	0.970149254	0.970149254	Bn-N3-p8226524	Bn-chrA03-p7488468
Bn-A03-p8181778	0.991735537	0.991735537	Bn-N3-p8227115	Bn-chrA03-p7489059
Bn-A03-p8190847	0.991735537	1	Bn-N3-p8237866	Bn-chrA03-p7501531
Bn-A03-p8204479	0.980066445	0.910299003	Bn-N3-p8246931	Bn-chrA03_random-p717540
Bn-A03-p8206961	0.884297521	0.884297521	Bn-N3-p8249527	Bn-chrA03_random-p715042
Bn-A03-p8217276	0.950413223	0.950413223	Bn-N3-p8260182	Bn-chrA03_random-p704494
Bn-A03-p8217358	0.958677686	0.958677686	Bn-N3-p8260263	Bn-chrA03_random-p704413
Bn-A03-p8218946	0.993355482	0.993355482	Bn-N3-p8261863	Bn-chrA03_random-p702813
Bn-A03-p8260551	0.966942149	0.983471074	Bn-Scaffold17470-p1	Bn-chrA03-p7575542
Bn-A03-p8279849	0.966942149	0.966942149	Bn-N3-p8324319	Bn-chrA03-p7589945
Bn-A03-p8282545	0.966942149	0.966942149	Bn-N3-p8327365	Bn-chrA03-p7593171
Bn-A03-p8285385	0.986710963	0.986710963	Bn-N3-p8330205	Bn-chrA03-p7596010
Bn-A03-p8288895	0.970149254	0.970149254	Bn-N3-p8332322	Bn-chrA03-p7598125
Bn-A03-p8298067	0.996677741	0.990033223	Bn-N3-p8341560	Bn-chrAnn_random-p359741
Bn-A03-p8298336	0.983388704	0.983388704	Bn-N3-p8341863	Bn-chrAnn_random-p359738
Bn-A03-p8308938	0.993355482	0.993355482	Bn-N3-p8353930	Bn-chrA03-p7616979
Bn-A03-p8314005	0.996677741	1	Bn-N3-p8359018	Bn-chrA03-p7622066
Bn-A03-p8329291	0.975206612	0.975206612	Bn-N3-p8379755	Bn-chrA03-p7638396
Bn-A03-p8333536	0.94214876	0.94214876	Bn-N3-p8383999	Bn-chrA03-p7642640
Bn-A03-p8337135	0.985074627	0.975124378	Bn-N3-p8387612	Bn-chrA03-p7646245
Bn-A03-p8337378	0.983471074	0.983471074	Bn-N3-p8388118	Bn-chrA03-p7646488
Bn-A03-p8347370	0.950413223	0.900826446	Bn-N3-p8402995	Bn-chrA03-p7652078
Bn-A03-p8359092	0.976744186	0.983388704	Bn-N3-p8414225	Bn-chrA03-p7662887
Bn-A03-p8360894	0.083056478	0.996677741	Bn-N13-p41521362	Bn-chrA03-p7664689
Bn-A03-p8372559	0.986710963	0.983388704	Bn-N3-p8424764	Bn-chrA03-p7679870
Bn-A03-p8380550	0.810631229	0.986710963	Bn-N13-p11885346	Bn-chrA03-p7692658
Bn-A03-p8381012	0.983471074	0.991735537	Bn-N3-p8432862	Bn-chrA03-p7693119
Bn-A03-p8394184	0.976744186	0.990033223	Bn-N3-p8446939	Bn-chrA03-p7707386
Bn-A03-p8395621	0.966942149	0.991735537	Bn-N3-p8453706	Bn-chrA03-p7708793
Bn-A03-p8396525	0.926910299	0.993355482	Bn-N3-p8454576	Bn-chrA03-p7709679
Bn-A03-p8398790	0.980099502	0.975124378	Bn-N3-p8456836	Bn-chrA03-p7711874
Bn-A03-p8403705	0.983471074	0.983471074	Bn-N3-p8460384	Bn-chrA03-p7715418
Bn-A03-p8406930	0.955223881	0.970149254	Bn-N3-p8463587	Bn-chrA03-p7718606
Bn-A03-p8407999	0.975206612	0.975206612	Bn-N3-p8464679	Bn-chrA03-p7719700
Bn-A03-p8408815	0.990049751	0.985074627	Bn-N3-p8465479	Bn-chrC03-p10422757
Bn-A03-p8420805	0.809917355	0.834710744	Bn-N13-p11968682	Bn-chrA03-p7727177
Bn-A03-p8439865	0.884297521	0.884297521	Bn-N3-p8507886	Bn-chrA03-p7744339
Bn-A03-p8454293	0.966942149	0.966942149	Bn-N3-p8537978	Bn-chrA03-p7761242
Bn-A03-p8467104	0.966942149	0.966942149	Bn-N3-p8551517	Bn-chrA03-p7770330

Bn-A03-p8475614	0.990033223	0.990033223	Bn-N3-p8559885	Bn-chrA03-p7780069
Bn-A03-p8479803	0.990049751	0.990049751	Bn-N3-p8564064	Bn-chrA03-p7784258
Bn-A03-p8480669	0.985074627	0.995024876	Bn-N3-p8564933	Bn-chrA03-p7785121
Bn-A03-p8483706	0.996677741	0.996677741	Bn-N3-p8567970	Bn-chrA03-p7788161
Bn-A03-p8497543	0.970149254	0.970149254	Bn-N3-p8582113	Bn-chrA03_random-p720349
Bn-A03-p8554739	0.958677686	0.950413223	Bn-N3-p8635007	Bn-chrA03-p7848625
Bn-A03-p8559617	0.983471074	0.983471074	Bn-N3-p8645677	Bn-chrA03-p7861264
Bn-A03-p8559794	0.991735537	0.991735537	Bn-N3-p8645854	Bn-chrA03-p7861441
Bn-A03-p8585196	0.955223881	0.960199005	Bn-N3-p8668688	Bn-chrA03_random-p695062
Bn-A03-p8620584	0.990033223	0.996677741	Bn-N3-p8700827	Bn-chrA03-p7909908
Bn-A03-p8629992	0.990033223	0.990033223	Bn-N3-p8710182	Bn-chrA03-p7919667
Bn-A03-p8631833	0.867768595	0.867768595	Bn-N3-p8713343	Bn-chrA03-p7921467
Bn-A03-p8632710	0.801652893	0.479338843	Bn-N13-p12313034	Unknown
Bn-A03-p8637914	0.975206612	0.975206612	Bn-N3-p8718378	Bn-chrA03-p7927540
Bn-A03-p8638217	0.975206612	0.975206612	Bn-N3-p8718723	Bn-chrA03-p7927885
Bn-A03-p8642052	0.991735537	0.991735537	Bn-N3-p8725666	Bn-chrA03-p7933438
Bn-A03-p8645654	0.991735537	0.991735537	Bn-N3-p8728661	Bn-chrA03-p7936433
Bn-A03-p8654614	0.983471074	0.983471074	Bn-N3-p8737028	Bn-chrA03-p7944919
Bn-A03-p8663803	0.990033223	0.990033223	Bn-N3-p8747803	Bn-chrA03-p7955567
Bn-A03-p8665794	0.950413223	0.950413223	Bn-N3-p8749787	Bn-chrA03-p7957551
Bn-A03-p8671468	0.96345515	0.986710963	Bn-N3-p8755325	Bn-chrA03-p7962824
Bn-A03-p8671697	0.990033223	0.996677741	Bn-N3-p8755556	Bn-chrA03-p7963059
Bn-A03-p8681690	0.970149254	0.980099502	Bn-N3-p8762847	Bn-chrA03-p7968929
Bn-A03-p8733974	0.996677741	0.993355482	Bn-N3-p8831928	Bn-chrA03-p8023114
Bn-A03-p8735039	0.975124378	0.990049751	Bn-N3-p8832993	Bn-chrA03-p8024179
Bn-A03-p8741978	0.966942149	0.966942149	Bn-N3-p8842895	Bn-chrA03-p8035706
Bn-A03-p8741996	0.966942149	0.966942149	Bn-N3-p8842913	Bn-chrA03-p8035724
Bn-A03-p8745839	0.975124378	0.950248756	Bn-N3-p8846748	Bn-chrC03-p10967868
Bn-A03-p8756590	0.983471074	0.983471074	Bn-N3-p8857864	Bn-chrA03-p8052565
Bn-A03-p8764251	0.950413223	0.950413223	Bn-N3-p8872133	Bn-chrA03-p8060875
Bn-A03-p8764481	1	1	Bn-N3-p8872358	Bn-chrA03-p8061100
Bn-A03-p8769576	0.94214876	0.94214876	Bn-N3-p8876989	Bn-chrA03-p8065497
Bn-A03-p8802360	0.980099502	0.985074627	Bn-N3-p8929217	Bn-chrA03-p8105327
Bn-A03-p8805441	0.983471074	0.983471074	Bn-N3-p8932262	Bn-chrA03-p8108373
Bn-A03-p8812807	0.958677686	0.958677686	Bn-N3-p8939877	Bn-chrA03-p8116341
Bn-A03-p8822734	0.995024876	0.995024876	Bn-N3-p8949754	Bn-chrA03-p8127205
Bn-A03-p8823042	0.955223881	0.960199005	Bn-N3-p8950062	Bn-chrA03-p8127513
Bn-A03-p8833016	0.996677741	1	Bn-N3-p8961102	Bn-chrA03-p8137479
Bn-A03-p8836751	0.980099502	0.990049751	Bn-N3-p8965635	Bn-chrA03-p8139994
Bn-A03-p8842546	1	1	Bn-N3-p8971444	Bn-chrA03-p8146927
Bn-A03-p8850004	0.983471074	0.983471074	Bn-N3-p8978988	Bn-chrA03-p8154426
Bn-A03-p8850377	0.893687708	0.996677741	Bn-N3-p8980740	Bn-chrA03-p8156041
Bn-A03-p8851043	0.985074627	0.980099502	Bn-N3-p8980899	Bn-chrA03-p8156200
Bn-A03-p8851142	0.983471074	0.983471074	Bn-N3-p8980998	Bn-chrA03-p8156299
Bn-A03-p8860638	0.991735537	1	Bn-N3-p8990569	Bn-chrA03-p8164402
Bn-A03-p8861495	0.991735537	0.991735537	Bn-N3-p8991426	Bn-chrA03-p8165263

Bn-A03-p8881377	0.983471074	0.983471074	Bn-N3-p9011072	Bn-chrA03-p8182097
Bn-A03-p8881585	0.991735537	0.991735537	Bn-N3-p9011283	Bn-chrA03-p8182308
Bn-A03-p8883782	0.975206612	0.975206612	Bn-N3-p9013572	Bn-chrA03-p8184602
Bn-A03-p8883841	0.993355482	0.993355482	Bn-N3-p9013631	Bn-chrA03-p8184661
Bn-A03-p8897362	0.909090909	0.909090909	Bn-N3-p9029427	Bn-chrA03-p8206306
Bn-A03-p8906751	0.993355482	0.990033223	Bn-N3-p9037372	Bn-chrA03-p8214602
Bn-A03-p8915240	0.986710963	0.986710963	Bn-N3-p9047686	Bn-chrA03-p8223735
Bn-A03-p8924852	0.991735537	0.991735537	Bn-N3-p9057017	Bn-chrA03-p8233060
Bn-A03-p8952053	0.990033223	0.990033223	Bn-N3-p9081014	Bn-chrA03-p8256291
Bn-A03-p8956355	0.958677686	0.958677686	Bn-N3-p9086304	Bn-chrA03-p8260593
Bn-A03-p8957734	0.991735537	0.991735537	Bn-N3-p9087684	Bn-chrA03-p8262044
Bn-A03-p8960332	0.991735537	0.991735537	Bn-N3-p9090488	Bn-chrA03-p8264839
Bn-A03-p8973137	0.94214876	0.958677686	Bn-N3-p9103360	Bn-chrA03-p8279673
Bn-A03-p8978761	0.752066116	1	Bn-N15-p30777173	Bn-chrA03-p8285339
Bn-A03-p8983947	0.495867769	1	Bn-N4-p10812845	Bn-chrA03-p8290524
Bn-A03-p8987098	0.983471074	0.983471074	Bn-N3-p9118825	Bn-chrA03-p8293649
Bn-A03-p8987269	0.958677686	0.958677686	Bn-N3-p9118996	Bn-chrA03-p8293820
Bn-A03-p8987820	0.834710744	0.834710744	Bn-N3-p9119547	Bn-chrA03-p8294371
Bn-A03-p9011801	0.975206612	0.975206612	Bn-N3-p9139197	Bn-chrA03-p8308915
Bn-A03-p9011966	0.958677686	0.958677686	Bn-N3-p9139360	Bn-chrA03-p8309078
Bn-A03-p9020167	0.983471074	0.983471074	Bn-N3-p9149097	Bn-chrA03-p8317529
Bn-A03-p9020291	0.975206612	0.975206612	Bn-N3-p9149221	Bn-chrA03-p8317653
Bn-A03-p9047380	0.983471074	0.983471074	Bn-N3-p9183454	Bn-chrA03-p8342563
Bn-A03-p9093699	0.785123967	0.975206612	Bn-N3-p9226323	Bn-chrA03-p8392565
Bn-A03-p9098576	0.909090909	0.909090909	Bn-N3-p9230623	Bn-chrA03-p8405244
Bn-A03-p9098773	0.958677686	0.958677686	Bn-N3-p9230818	Bn-chrA03-p8405439
Bn-A03-p9100699	0.966942149	0.966942149	Bn-N3-p9232703	Bn-chrA03-p8407315
Bn-A03-p9115020	0.983471074	0.983471074	Bn-N3-p9247475	Bn-chrA03_random-p740134
Bn-A03-p9123542	0.993355482	0.993355482	Bn-N3-p9264008	Bn-chrA03_random-p729816
Bn-A03-p9124799	0.975124378	0.975124378	Bn-N3-p9265281	Bn-chrA03_random-p728544
Bn-A03-p9129107	0.986710963	0.990033223	Bn-N3-p9271592	Bn-chrA03-p8414875
Bn-A03-p9143356	0.975206612	0.983471074	Bn-N3-p9294189	Bn-chrA03-p8429021
Bn-A03-p9148822	0.993355482	0.936877076	Bn-N3-p9302770	Bn-chrC03-p11571642
Bn-A03-p9170934	0.958677686	0.917355372	Bn-N3-p9324364	Bn-chrA03-p8454033
Bn-A03-p9179284	0.756218905	0.756218905	Bn-N3-p9332586	Bn-chrA03-p8462282
Bn-A03-p9179896	0.985074627	0.985074627	Bn-N3-p9333198	Bn-chrA03-p8462894
Bn-A03-p9186605	0.990033223	0.990033223	Bn-N3-p9340174	Bn-chrA03-p8468062
Bn-A03-p9191787	0.925619835	0.925619835	Bn-N13-p13262973	Bn-chrC03_random-p551327
Bn-A03-p9203389	0.991735537	0.636363636	Bn-N3-p9356784	Bn-chrA04-p1822384
Bn-A03-p9203536	0.991735537	0.297520661	Bn-N3-p9356933	Unknown
Bn-A03-p9203834	1	0.867768595	Bn-N3-p9357231	Bn-chrA04-p13210575
Bn-A03-p9203992	0.991735537	0.280991736	Bn-N3-p9357389	Bn-chrCnn_random-p135110
Bn-A03-p9203999	0.991735537	0.198347107	Bn-N3-p9357396	Unknown
Bn-A03-p9208735	0.975124378	0.955223881	Bn-N3-p9362802	Bn-chrA03-p8487655
Bn-A03-p9233645	0.991735537	0.991735537	Bn-N3-p9386412	Bn-chrA03-p8515188
Bn-A03-p9243701	0.880398671	1	Bn-N3-p9397787	Bn-chrA03-p8525585

Bn-A03-p9244195	0.94214876	0.950413223	Bn-N3-p9398282	Bn-chrA03-p8526078
Bn-A03-p9245937	0.980099502	0.980099502	Bn-N3-p9400025	Bn-chrA03-p8527812
Bn-A03-p9246222	0.985074627	0.990049751	Bn-N3-p9400309	Bn-chrA03-p8528095
Bn-A03-p9247666	0.966942149	0.950413223	Bn-N3-p9401676	Bn-chrA03-p8529604
Bn-A03-p9247798	0.991735537	0.983471074	Bn-N3-p9401808	Bn-chrA03-p8529741
Bn-A03-p9250832	0.933554817	0.986710963	Bn-N3-p9405991	Bn-chrA03-p8532801
Bn-A03-p9255139	0.976744186	0.993355482	Bn-N3-p9410111	Bn-chrA03-p8536834
Bn-A03-p9265554	0.983471074	0.991735537	Bn-N3-p9422013	Bn-chrA03-p8547292
Bn-A03-p9266328	0.993355482	0.993355482	Bn-N3-p9422787	Bn-chrA03-p8548066
Bn-A03-p9300575	0.983471074	0.983471074	Bn-N3-p9461892	Bn-chrA03-p8581203
Bn-A03-p9301541	0.950413223	0.950413223	Bn-N3-p9464474	Bn-chrA03-p8583785
Bn-A03-p9303850	0.94214876	0.94214876	Bn-N18-p36207298	Bn-chrC08-p31058611
Bn-A03-p9304563	0.991735537	0.991735537	Bn-N3-p9466679	Bn-chrA03-p8585990
Bn-A03-p9304885	0.592039801	0.502487562	Bn-N13-p13508556	Bn-chrC03-p11843321
Bn-A03-p9324653	0.990033223	0.986710963	Bn-N3-p9485997	Bn-chrA03-p8604278
Bn-A03-p9328470	0.990033223	0.973421927	Bn-N3-p9496324	Bn-chrA03-p8609824
Bn-A03-p9338698	1	1	Bn-N3-p9507021	Bn-chrA03-p8622360
Bn-A03-p9343295	0.996677741	0.996677741	Bn-N3-p9511776	Bn-chrA03-p8626946
Bn-A03-p9343716	0.993355482	0.993355482	Bn-N3-p9512197	Bn-chrA03-p8627367
Bn-A03-p9357075	1	1	Bn-N3-p9518565	Bn-chrA03-p8633734
Bn-A03-p9361280	0.983471074	0.983471074	Bn-N3-p9524261	Bn-chrA03-p8637939
Bn-A03-p9382281	0.996677741	1	Bn-N3-p9548800	Bn-chrA03-p8659161
Bn-A03-p9383602	0.975124378	0.975124378	Bn-N13-p13601842	Bn-chrC03-p11938149
Bn-A03-p9429437	0.991735537	1	Bn-N3-p9610692	Bn-chrA03-p8702777
Bn-A03-p9439354	0.991735537	0.975206612	Bn-N3-p9621591	Bn-chrA03-p8712686
Bn-A03-p9439893	0.991735537	1	Bn-N3-p9622130	Bn-chrA03-p8713235
Bn-A03-p9448206	0.982142857	0.976190476	Bn-N3-p9630571	Bn-chrA03-p8722237
Bn-A03-p9449316	0.993355482	0.986710963	Bn-N3-p9631681	Bn-chrA03-p8723347
Bn-A03-p9468025	0.966942149	0.975206612	Bn-N3-p9650408	Bn-chrA03-p8742060
Bn-A03-p9476053	0.996677741	0.996677741	Bn-N3-p9659236	Bn-chrA03-p8750098
Bn-A03-p9480203	0.991735537	0.991735537	Bn-N3-p9666732	Bn-chrA03-p8754199
Bn-A03-p9485365	0.983471074	0.983471074	Bn-N3-p9671897	Bn-chrA03-p8759360
Bn-A03-p9488435	0.960199005	0.970149254	Bn-N3-p9675203	Bn-chrA03-p8764345
Bn-A03-p9497697	0.92039801	0.960199005	Bn-N3-p9679562	Bn-chrA03-p8767274
Bn-A03-p9497821	0.970149254	0.985074627	Bn-N3-p9679686	Bn-chrA03-p8767398
Bn-A03-p9522072	0.950413223	0.958677686	Bn-N13-p13808871	Bn-chrCnn_random-p641831
Bn-A03-p9525820	0.965174129	0.970149254	Bn-N3-p9697617	Bn-chrA03-p8797160
Bn-A03-p9537155	0.983471074	0.975206612	Bn-N3-p9706667	Bn-chrA03-p8807186
Bn-A03-p9537618	0.995024876	0.985074627	Bn-N3-p9707126	Bn-chrA03-p8807649
Bn-A03-p9564515	0.990049751	0.990049751	Bn-N13-p13849965	Bn-chrC03_random-p868024
Bn-A03-p9567767	0.975206612	0.975206612	Bn-N3-p9735568	Bn-chrA03-p8832145
Bn-A03-p9601581	0.965174129	0.970149254	Bn-N3-p9768372	Bn-chrA03_random-p791185
Bn-A03-p9602074	0.479338843	0.975206612	Unknown	Bn-chrA03_random-p790692
Bn-A03-p9605319	0.975124378	0.975124378	Bn-N3-p9772136	Bn-chrA03_random-p787447
Bn-A03-p9613933	0.986710963	0.986710963	Bn-N3-p9778512	Bn-chrA03_random-p778887
Bn-A03-p9615126	0.970149254	0.990049751	Bn-N3-p9779701	Bn-chrC03-p12278812

Bn-A03-p9618630	0.785123967	0.785123967	Bn-N3-p9783356	Bn-chrA03_random-p774108
Bn-A03-p9626063	0.983471074	0.975206612	Bn-N3-p9787435	Bn-chrA03_random-p770020
Bn-A03-p9627365	0.990033223	0.950166113	Bn-N3-p9788737	Bn-chrA03_random-p766116
Bn-A03-p9637487	0.909090909	0.909090909	Bn-N3-p9809191	Bn-chrA03-p8872075
Bn-A03-p9638708	0.960199005	0.955223881	Bn-N3-p9810417	Bn-chrA03-p8873301
Bn-A03-p9638726	0.975206612	0.983471074	Bn-N3-p9810435	Bn-chrA03-p8873319
Bn-A03-p9641671	0.993355482	0.993355482	Bn-N3-p9813360	Bn-chrA03-p8876213
Bn-A03-p9651526	0.950413223	0.950413223	Bn-N3-p9829080	Bn-chrA03-p8886077
Bn-A03-p9657864	0.980066445	0.986710963	Bn-N3-p9835434	Bn-chrA03-p8892369
Bn-A03-p9660869	0.94214876	0.933884298	Bn-N3-p9838370	Bn-chrA03-p8895333
Bn-A03-p9677969	0.975206612	0.975206612	Bn-N3-p9845555	Bn-chrA03-p8902583
Bn-A03-p9678981	0.966942149	0.966942149	Bn-N3-p9846554	Bn-chrA03-p8903582
Bn-A03-p9680882	0.966942149	0.966942149	Bn-N3-p9847047	Bn-chrA03-p8904075
Bn-A03-p9705481	0.991735537	0.991735537	Bn-N3-p9870689	Bn-chrA03-p8930569
Bn-A03-p9706640	0.991735537	0.991735537	Bn-N3-p9871849	Bn-chrA03-p8931729
Bn-A03-p9734588	0.983471074	0.991735537	Bn-N3-p9918198	Bn-chrA03-p8962400
Bn-A03-p9747710	1	0.991735537	Bn-N3-p9938952	Bn-chrA03-p8977621
Bn-A03-p9747824	0.991735537	0.983471074	Bn-N3-p9939066	Bn-chrA03-p8977735
Bn-A03-p9751621	0.991735537	1	Bn-N3-p9943410	Bn-chrA03-p8982668
Bn-A03-p9758514	0.991735537	1	Bn-N3-p9950899	Bn-chrA03-p8989546
Bn-A03-p9764680	0.94214876	0.94214876	Bn-N3-p9956468	Bn-chrA03-p8995708
Bn-A03-p9765420	0.991735537	0.991735537	Bn-N3-p9957200	Bn-chrA03-p8996440
Bn-A03-p9765623	0.94214876	0.94214876	Bn-N3-p9957420	Bn-chrA03-p8996660
Bn-A03-p9818699	0.976744186	0.973421927	Bn-N3-p10013767	Bn-chrA03_random-p756037
Bn-A03-p9819365	0.975206612	0.975206612	Bn-N3-p10014432	Bn-chrA03_random-p755372
Bn-A03-p9819389	0.950248756	0.950248756	Bn-N3-p10014456	Bn-chrA03_random-p755348
Bn-A03-p9823618	0.980099502	0.134328358	Bn-N3-p10018687	Bn-chrA10-p14970553
Bn-A03-p9825032	0.950166113	0.764119601	Bn-N3-p10020110	Bn-chrC03-p12604992
Bn-A03-p9833648	0.995024876	0.995024876	Bn-N3-p10026056	Bn-chrA03-p9054087
Bn-A03-p9836757	0.966942149	0.975206612	Bn-N3-p10029065	Bn-chrA03-p9057095
Bn-A03-p9839212	0.983388704	0.983388704	Bn-N3-p10031520	Bn-chrA03-p9059553
Bn-A03-p9847680	0.990033223	0.996677741	Bn-N3-p10040187	Bn-chrA03-p9067103
Bn-A03-p9856465	0.958677686	0.950413223	Bn-N3-p10048973	Bn-chrA03-p9075597
Bn-A03-p9856923	0.903654485	0.990033223	Bn-N3-p10049431	Bn-chrA03-p9076055
Bn-A03-p9863138	1	0.991735537	Bn-N3-p10056625	Bn-chrA03-p9079560
Bn-A03-p9863553	0.975206612	0.983471074	Bn-N3-p10057039	Bn-chrA03-p9079974
Bn-A03-p9863600	0.983471074	0.975206612	Bn-N3-p10057086	Bn-chrA03-p9080021
Bn-A03-p9869156	0.986710963	0.986710963	Bn-N3-p10063572	Bn-chrA03-p9085530
Bn-A03-p9869182	0.983388704	0.983388704	Bn-N3-p10063598	Bn-chrA03-p9085556
Bn-A03-p9873327	0.983471074	0.991735537	Bn-N3-p10066296	Bn-chrA03-p9088253
Bn-A03-p9873384	0.966942149	0.958677686	Bn-N3-p10066353	Bn-chrA03-p9088310
Bn-A03-p9876378	0.986710963	0.990033223	Bn-N3-p10069357	Bn-chrA03-p9091356
Bn-A03-p9883544	0.983471074	0.991735537	Bn-N3-p10076547	Bn-chrA03-p9098600
Bn-A03-p9899417	0.966942149	0.975206612	Bn-N3-p10086814	Bn-chrA03-p9108751
Bn-A03-p9920847	0.983471074	0.983471074	Bn-N3-p10103147	Bn-chrA03-p9122692
Bn-A03-p9921379	0.925619835	0.925619835	Bn-N3-p10103741	Bn-chrA03-p9123286

Bn-A03-p9921928	0.991735537	0.991735537	Bn-N3-p10104621	Bn-chrA03-p9124166
Bn-A03-p9924047	0.993355482	0.993355482	Bn-N3-p10106412	Bn-chrA03-p9125957
Bn-A03-p9925289	0.983471074	0.983471074	Bn-N3-p10107666	Bn-chrA03-p9127209
Bn-A03-p9925550	0.768595041	0.561983471	Bn-N3-p10107923	Bn-chrA07-p14509328
Bn-A03-p9926783	0.983471074	0.983471074	Bn-N3-p10109149	Bn-chrA03-p9128691
Bn-A03-p9939018	0.900826446	0.975206612	Bn-N3-p10122165	Bn-chrA03-p9139698
Bn-A03-p9966625	0.986710963	0.990033223	Bn-N3-p10150694	Bn-chrA03-p9165370
Bn-A03-p9969989	0.970149254	0.970149254	Bn-N3-p10154725	Bn-chrA03-p9168734
Bn-A03-p10002791	0.983388704	0.980066445	Bn-N3-p10187421	Bn-chrA03-p9197326
Bn-A03-p10008905	0.870431894	0.990033223	Bn-N13-p14663572	Bn-chrA03-p9203392
Bn-A03-p10010569	0.983471074	0.884297521	Bn-N3-p10203419	Bn-chrA03-p9204998
Bn-A03-p10011955	0.990033223	0.990033223	Bn-N3-p10204804	Bn-chrA03-p9206397
Bn-A03-p10023639	0.996677741	0.996677741	Bn-N3-p10212875	Bn-chrA03-p9214456
Bn-A03-p10027020	0.996677741	0.996677741	Bn-N3-p10215077	Bn-chrA03-p9216657
Bn-A03-p10045527	0.990049751	0.990049751	Bn-N3-p10233346	Bn-chrC03-p13022208
Bn-A03-p10047160	0.990033223	0.990033223	Bn-N3-p10235963	Bn-chrA03-p9236571
Bn-A03-p10047998	0.990033223	0.990033223	Bn-N3-p10236800	Bn-chrA03-p9237408
Bn-A03-p10062838	1	0.993355482	Bn-N3-p10256288	Bn-chrA03-p9260512
Bn-A03-p10076616	0.986710963	0.986710963	Bn-N3-p10270934	Bn-chrA03-p9274340
Bn-A03-p10081359	0.975206612	0.966942149	Bn-N13-p14764453	Bn-chrA03-p9279083
Bn-A03-p10084501	0.827242525	0.827242525	Bn-N3-p10278812	Bn-chrA03-p9282217
Bn-A03-p10086496	0.950248756	0.955223881	Bn-N13-p14770283	Bn-chrC03-p13089209
Bn-A03-p10087723	0.644628099	0.644628099	Bn-N3-p10282080	Bn-chrA03-p9285484
Bn-A03-p10092390	0.980099502	0.980099502	Bn-N3-p10286701	Bn-chrA03-p9290103
Bn-A03-p10092885	0.990049751	0.990049751	Bn-N3-p10287196	Bn-chrA03-p9290598
Bn-A03-p10110586	0.980099502	0.980099502	Bn-N3-p10306623	Bn-chrA03-p9309350
Bn-A03-p10113984	0.876033058	0.876033058	Bn-N3-p10309992	Bn-chrA03-p9312719
Bn-A03-p10117543	0.950413223	0.950413223	Bn-N3-p10314881	Bn-chrA03-p9317607
Bn-A03-p10129836	0.996677741	0.996677741	Bn-N3-p10327525	Bn-chrA03-p9330550
Bn-A03-p10130816	0.986710963	0.986710963	Bn-N3-p10328499	Bn-chrA03-p9331524
Bn-A03-p10218040	0.995024876	0.995024876	Bn-N13-p14933641	Bn-chrC03-p13244376
Bn-A03-p10248162	0.966942149	0.975206612	Bn-N3-p10464780	Bn-chrA03_random-p839735
Bn-A03-p10273355	1	0.991735537	Bn-N3-p10500391	Bn-chrA03_random-p813238
Bn-A03-p10285809	0.975124378	0.980099502	Bn-N3-p10513379	Bn-chrA03-p9459111
Bn-A03-p10288202	0.950413223	0.966942149	Bn-N3-p10518229	Bn-chrA03-p9461503
Bn-A03-p10293071	0.991735537	1	Bn-N3-p10521511	Bn-chrA03-p9464800
Bn-A03-p10296969	0.995024876	1	Bn-N3-p10525409	Bn-chrA03-p9468698
Bn-A03-p10313368	0.990033223	0.990033223	Bn-N3-p10546533	Bn-chrA03-p9485354
Bn-A03-p10321263	0.980099502	0.815920398	Bn-N3-p10555810	Bn-chrC03-p13406107
Bn-A03-p10321416	0.975124378	0.975124378	Bn-N3-p10555964	Bn-chrA03-p9493437
Bn-A03-p10342088	0.983471074	0.983471074	Bn-N3-p10576064	Bn-chrA03-p9513161
Bn-A03-p10350207	0.983388704	0.983388704	Bn-N3-p10585533	Bn-chrA03-p9525405
Bn-A03-p10355167	0.975206612	0.975206612	Bn-N3-p10591650	Bn-chrA03-p9530262
Bn-A03-p10376862	0.991735537	1	Bn-N3-p10611530	Bn-chrA04-p12608226
Bn-A03-p10388827	0.986710963	0.986710963	Bn-N3-p10628159	Bn-chrA03-p9573486
Bn-A03-p10393461	0.975206612	0.975206612	Bn-N3-p10632845	Bn-chrA03-p9578174

Bn-A03-p10400341	0.993355482	0.993355482	Bn-N3-p10639501	Bn-chrCnn_random-p300224
Bn-A03-p10402639	0.976744186	0.976744186	Bn-N3-p10641727	Bn-chrA03-p9586963
Bn-A03-p10414176	1	1	Bn-N3-p10653298	Bn-chrA03-p9598534
Bn-A03-p10445051	0.990033223	0.993355482	Bn-N3-p10694479	Bn-chrA03-p9629102
Bn-A03-p10448318	0.970149254	0.970149254	Bn-N3-p10697768	Bn-chrA03-p9632422
Bn-A03-p10449972	1	1	Bn-N3-p10699422	Bn-chrA03-p9634076
Bn-A03-p10458923	0.993355482	0.993355482	Bn-N3-p10708311	Bn-chrA03-p9642962
Bn-A03-p10464551	0.993355482	0.993355482	Bn-N3-p10704304	Bn-chrA03-p9638955
Bn-A03-p10466633	0.996677741	0.996677741	Bn-N3-p10705333	Bn-chrA03-p9639984
Bn-A03-p10482765	1	1	Bn-N3-p10719464	Bn-chrA03-p9654114
Bn-A03-p10487754	1	1	Bn-N3-p10724531	Bn-chrA03-p9658677
Bn-A03-p10517056	1	1	Bn-N3-p10757283	Bn-chrA03-p9693451
Bn-A03-p10517505	0.983388704	0.983388704	Bn-N3-p10757732	Bn-chrA03-p9693900
Bn-A03-p10533775	0.986710963	0.986710963	Bn-N3-p10779956	Bn-chrA03-p9713101
Bn-A03-p10547280	0.980066445	0.980066445	Bn-N3-p10795852	Bn-chrA03-p9728137
Bn-A03-p10562274	0.817275748	0.820598007	Bn-N3-p10812473	Bn-chrA03-p9742807
Bn-A03-p10565738	0.966942149	0.966942149	Bn-N3-p10815943	Bn-chrA03-p9746276
Bn-A03-p10566503	0.975206612	0.975206612	Bn-N3-p10816703	Bn-chrA03-p9747036
Bn-A03-p10573912	0.996677741	0.996677741	Bn-N3-p10824349	Bn-chrCnn_random-p559961
Bn-A03-p10583411	1	1	Bn-N3-p10836704	Bn-chrAnn_random-p210753
Bn-A03-p10584028	0.975206612	0.975206612	Bn-N3-p10837322	Bn-chrAnn_random-p210747
Bn-A03-p10587547	0.975206612	0.975206612	Bn-N3-p10840760	Bn-chrAnn_random-p483122
Bn-A03-p10600103	0.966942149	0.966942149	Bn-N3-p10850182	Bn-chrAnn_random-p210599
Bn-A03-p10600567	0.990049751	0.990049751	Bn-N3-p10850644	Bn-chrAnn_random-p210594
Bn-A03-p10607288	0.975206612	0.917355372	Bn-N3-p10864571	Bn-chrAnn_random-p210497
Bn-A03-p10608519	0.990033223	0.96013289	Bn-N3-p10866539	Bn-chrAnn_random-p427498
Bn-A03-p10617866	0.975206612	0.975206612	Bn-N3-p10875529	Bn-chrA03-p9772658
Bn-A03-p10623167	0.801652893	0.801652893	Bn-N3-p10883047	Bn-chrA03-p9777950
Bn-A03-p10640254	0.94214876	0.818181818	Bn-N3-p10898911	Bn-chrA03-p9793874
Bn-A03-p10640643	0.94214876	0.892561983	Bn-N3-p10900706	Bn-chrA03-p9794240
Bn-A03-p10641561	0.958677686	0.801652893	Bn-N3-p10901631	Bn-chrA03-p9795154
Bn-A03-p10641836	0.917355372	0.991735537	Bn-N3-p10901909	Bn-chrA03-p9795429
Bn-A03-p10649410	0.991735537	0.991735537	Bn-N3-p10913353	Bn-chrA03-p9805971
Bn-A03-p10651420	0.970149254	0.975124378	Bn-N3-p10915390	Bn-chrA03-p9808008
Bn-A03-p10664726	0.983471074	0.975206612	Bn-N3-p10936357	Bn-chrA03-p9821351
Bn-A03-p10676505	0.970149254	0.965174129	Bn-N3-p10945231	Bn-chrC03-p13915070
Bn-A03-p10680539	0.933884298	0.950413223	Bn-N3-p10949207	Bn-chrA03-p9833573
Bn-A03-p10682159	0.933884298	0.94214876	Bn-N3-p10951479	Bn-chrA03-p9835193
Bn-A03-p10683528	0.966942149	0.991735537	Bn-N3-p10952808	Bn-chrA03-p9836586
Bn-A03-p10692514	0.991735537	1	Bn-N3-p10961822	Bn-chrA03-p9850617
Bn-A03-p10703085	0.975206612	0.975206612	Bn-N3-p10967854	Bn-chrA03-p9856654
Bn-A03-p10714724	0.966942149	0.892561983	Bn-N3-p10980676	Bn-chrA03-p9868320
Bn-A03-p10723392	0.991735537	0.272727273	Bn-N3-p10989270	Unknown
Bn-A03-p10723484	0.975206612	0.79338843	Bn-N3-p10989362	Bn-chrA03-p9876796
Bn-A03-p10732934	0.991735537	0.983471074	Bn-N3-p10999696	Bn-chrA03-p9891804
Bn-A03-p10749391	0.990033223	0.956810631	Bn-N3-p11020846	Bn-chrA03-p9910331

Bn-A03-p10757525	1	1	Bn-N3-p11028842	Bn-chrA03-p9918346
Bn-A03-p10758970	0.975206612	0.975206612	Bn-N3-p11030285	Bn-chrA03-p9919789
Bn-A03-p10761386	0.986710963	0.986710963	Bn-N3-p11032703	Bn-chrA03-p9925979
Bn-A03-p10761460	0.993355482	0.993355482	Bn-N3-p11032777	Bn-chrA03-p9926053
Bn-A03-p10773321	0.986710963	0.986710963	Bn-N3-p11054307	Bn-chrA03-p9945815
Bn-A03-p10773339	0.983388704	0.983388704	Bn-N3-p11054325	Bn-chrA03-p9945833
Bn-A03-p10794513	0.966777409	0.966777409	Bn-N3-p11072359	Bn-chrA03-p9964554
Bn-A03-p10794985	0.975206612	0.975206612	Bn-N3-p11072816	Bn-chrA03-p9965011
Bn-A03-p10797405	0.958677686	0.958677686	Bn-N3-p11075281	Bn-chrA03-p9967476
Bn-A03-p10802728	0.966942149	0.966942149	Bn-N3-p11080901	Bn-chrA03-p9973103
Bn-A03-p10803927	0.946843854	0.946843854	Bn-N3-p11082099	Bn-chrA03-p9974301
Bn-A03-p10814591	0.983471074	0.983471074	Bn-N3-p11092942	Bn-chrA03-p9985144
Bn-A03-p10819432	0.983471074	0.983471074	Bn-N3-p11097787	Bn-chrA03-p9989989
Bn-A03-p10823720	0.993355482	0.993355482	Bn-N3-p11103919	Bn-chrA03-p9994797
Bn-A03-p10830360	0.986710963	0.986710963	Bn-N3-p11109054	Bn-chrA03-p9999932
Bn-A03-p10833240	1	1	Bn-N3-p11111934	Bn-chrA03-p10002812
Bn-A03-p10853616	0.991735537	0.743801653	Bn-N3-p11135424	Bn-chrC03-p14182343
Bn-A03-p10855422	0.983388704	0.983388704	Bn-N3-p11138274	Bn-chrAnn_random-p313663
Bn-A03-p10862251	0.966942149	0.966942149	Bn-N3-p11145180	Bn-chrA03_random-p866872
Bn-A03-p10873434	0.991735537	0.991735537	Bn-N3-p11156706	Bn-chrA03_random-p875773
Bn-A03-p10883930	0.980099502	0.980099502	Bn-N3-p11171589	Bn-chrA03_random-p888654
Bn-A03-p10889554	0.96345515	0.96345515	Bn-N3-p11176825	Bn-chrA03_random-p894732
Bn-A03-p10897572	0.985074627	0.985074627	Bn-N3-p11183158	Bn-chrA03_random-p901059
Bn-A03-p10898289	0.813953488	0.96345515	Bn-N3-p11183810	Bn-chrA03_random-p901710
Bn-A03-p10900301	1	1	Bn-N3-p11185817	Bn-chrA03_random-p903717
Bn-A03-p10900813	1	1	Bn-N3-p11186329	Bn-chrA03_random-p904229
Bn-A03-p10907793	1	1	Bn-N3-p11191377	Bn-chrA03_random-p909277
Bn-A03-p10912453	0.993355482	0.993355482	Bn-N3-p11195905	Bn-chrA03_random-p913767
Bn-A03-p10912815	0.966942149	0.966942149	Bn-N3-p11197689	Bn-chrA03_random-p914130
Bn-A03-p10928669	0.884297521	0.884297521	Bn-N3-p11206326	Bn-chrA03_random-p922759
Bn-A03-p10928990	0.818181818	0.818181818	Bn-N3-p11206647	Bn-chrA03_random-p923080
Bn-A03-p10930541	0.980066445	0.980066445	Bn-N3-p11208153	Bn-chrA03_random-p924586
Bn-A03-p10947710	0.933884298	0.933884298	Bn-N3-p11229800	Bn-chrC03-p14302676
Bn-A03-p10952575	0.995024876	0.995024876	Bn-N3-p11234959	Bn-chrAnn_random-p306312
Bn-A03-p10958194	0.993355482	0.993355482	Bn-N3-p11239704	Bn-chrA03-p10074192
Bn-A03-p10959387	0.966942149	0.966942149	Bn-N3-p11240899	Bn-chrA03-p10075387
Bn-A03-p10959439	0.975206612	0.975206612	Bn-N3-p11240951	Bn-chrA03-p10075439
Bn-A03-p10962307	0.983471074	0.983471074	Bn-N3-p11242269	Bn-chrA03-p10076757
Bn-A03-p10962645	1	1	Bn-N3-p11242606	Bn-chrA03-p10077094
Bn-A03-p10962969	0.983471074	0.983471074	Bn-N3-p11242930	Bn-chrA03-p10077418
Bn-A03-p10963046	0.970149254	0.970149254	Bn-N3-p11243007	Bn-chrA03-p10077495
Bn-A03-p10964394	0.983471074	0.983471074	Bn-N3-p11244349	Bn-chrA03-p10078837
Bn-A03-p10964511	0.975206612	0.975206612	Bn-N3-p11244466	Bn-chrA03-p10078954
Bn-A03-p10964682	0.966942149	0.966942149	Bn-N3-p11244639	Bn-chrA03-p10079127
Bn-A03-p10967268	0.909090909	0.950413223	Bn-N3-p11247217	Bn-chrC03-p14333736
Bn-A03-p10969109	0.993355482	0.993355482	Bn-N3-p11249111	Bn-chrA03-p10083597

Bn-A03-p10993904	1	1	Bn-N3-p11276586	Bn-chrA03-p10104537
Bn-A03-p11027825	0.943521595	0.943521595	Bn-N3-p11316868	Bn-chrA03-p10142658
Bn-A03-p11051970	0.996677741	0.996677741	Bn-N3-p11346446	Bn-chrA03-p10170381
Bn-A03-p11053758	0.975206612	0.975206612	Bn-N3-p11348227	Bn-chrA03-p10172163
Bn-A03-p11053857	0.933884298	0.933884298	Bn-N3-p11348345	Bn-chrA03-p10172281
Bn-A03-p11065674	0.975206612	0.983471074	Bn-N3-p11361404	Bn-chrA03-p10192260
Bn-A03-p11070576	1	0.990033223	Bn-N3-p11366306	Bn-chrAnn_random-p368184
Bn-A03-p11070958	0.863787375	0.877076412	Bn-N3-p11366690	Bn-chrAnn_random-p36818C
Bn-A03-p11071010	0.966942149	0.958677686	Bn-N3-p11366742	Bn-chrA03-p10197586
Bn-A03-p11071320	0.983471074	0.975206612	Bn-N3-p11367052	Bn-chrA03-p10197966
Bn-A03-p11071640	0.975206612	0.966942149	Bn-N3-p11367372	Bn-chrA03-p10198286
Bn-A03-p11086751	0.94214876	0.950413223	Bn-N3-p11373461	Bn-chrA03-p10203617
Bn-A03-p11087692	0.958677686	0.950413223	Bn-N3-p11376031	Bn-chrA03-p10204558
Bn-A03-p11122273	0.933884298	0.94214876	Bn-N13-p51226502	Bn-chrC07-p41947272
Bn-A03-p11133743	1	1	Bn-N3-p11413127	Bn-chrA03-p10238111
Bn-A03-p11138137	0.958677686	0.958677686	Bn-N13-p16498143	Bn-chrC03_random-p724562
Bn-A03-p11143696	0.955223881	0.950248756	Bn-N3-p11425938	Bn-chrA03-p10248404
Bn-A03-p11156293	0.983471074	0.983471074	Bn-N3-p11438941	Bn-chrA03-p10261429
Bn-A03-p11171067	0.958677686	0.966942149	Bn-N3-p11447645	Bn-chrA03-p10269510
Bn-A03-p11177892	0.990033223	0.990033223	Bn-N3-p11458348	Bn-chrA03-p10280270
Bn-A03-p11188500	0.975206612	0.975206612	Bn-N3-p11468882	Bn-chrA03-p10290867
Bn-A03-p11191805	1	1	Bn-N3-p11472341	Bn-chrA03-p10294325
Bn-A03-p11202719	0.983388704	0.980066445	Bn-N3-p11485348	Bn-chrA03-p10319617
Bn-A03-p11217606	0.950413223	0.958677686	Bn-N3-p11500395	Bn-chrA03-p10339936
Bn-A03-p11218468	0.990033223	0.990033223	Bn-N3-p11501278	Bn-chrA03-p10340819
Bn-A03-p11227755	0.983471074	0.975206612	Bn-N3-p11510580	Bn-chrA03-p10352316
Bn-A03-p11232586	0.991735537	0.983471074	Bn-N3-p11515431	Bn-chrA03-p10358437
Bn-A03-p11247615	0.917355372	0.958677686	Bn-N3-p11530218	Bn-chrA03-p10372840
Bn-A03-p11256138	0.983471074	0.983471074	Bn-N3-p11538775	Bn-chrA03-p10381372
Bn-A03-p11256392	0.975206612	0.975206612	Bn-N3-p11539029	Bn-chrA03-p10381626
Bn-A03-p11256664	0.975206612	0.975206612	Bn-N3-p11539306	Bn-chrA03-p10381903
Bn-A03-p11257708	0.966942149	0.966942149	Bn-N3-p11540371	Bn-chrA03-p10382967
Bn-A03-p11294370	0.990049751	0.970149254	Bn-N3-p11581195	Bn-chrA03-p10411223
Bn-A03-p11294858	0.975124378	0.975124378	Bn-N3-p11581684	Bn-chrA03-p10411711
Bn-A03-p11319722	0.996677741	1	Bn-N3-p11599250	Bn-chrA03-p10434294
Bn-A03-p11319901	1	0.991735537	Bn-N3-p11599429	Bn-chrA03-p10434473
Bn-A03-p11325897	0.990049751	0.985074627	Bn-N3-p11604325	Bn-chrA03-p10441124
Bn-A03-p11345845	0.991735537	0.983471074	Bn-N3-p11621724	Bn-chrA03-p10458203
Bn-A03-p11347564	0.975206612	0.983471074	Bn-N11-p33676166	Bn-chrA03_random-p629378
Bn-A03-p11356180	1	0.996677741	Bn-N3-p11629216	Bn-chrA03-p10465750
Bn-A03-p11364656	0.993355482	0.996677741	Bn-N3-p11640091	Bn-chrA03-p10475039
Bn-A03-p11381125	1	1	Bn-N3-p11653289	Bn-chrA03-p10496225
Bn-A03-p11383138	0.975206612	0.975206612	Bn-N3-p11658215	Bn-chrA03-p10501143
Bn-A03-p11391930	0.966942149	0.933884298	Bn-N3-p11668666	Bn-chrA03-p10509900
Bn-A03-p11392385	0.983471074	0.975206612	Bn-N3-p11669121	Bn-chrA03-p10510354
Bn-A03-p11399915	0.58677686	0.925619835	Bn-N3-p11685041	Bn-chrA03-p10522683

Bn-A03-p11402714	0.958677686	0.958677686	Bn-N3-p11687825	Bn-chrA03-p10525662
Bn-A03-p11418849	0.993355482	0.983388704	Bn-N3-p11699513	Bn-chrA03-p10539314
Bn-A03-p11421511	0.980099502	0.965174129	Bn-N3-p11702179	Bn-chrA03-p10543690
Bn-A03-p11423310	0.975206612	0.884297521	Bn-N3-p11703983	Bn-chrA03-p10545527
Bn-A03-p11429520	0.975206612	0.983471074	Bn-N3-p11711835	Bn-chrA03-p10551863
Bn-A03-p11444081	0.975206612	0.966942149	Bn-N3-p11733750	Bn-chrAnn_random-p328579
Bn-A03-p11444389	0.983471074	0.975206612	Bn-N3-p11734058	Bn-chrAnn_random-p328582
Bn-A03-p11456115	0.991735537	0.983471074	Bn-N3-p11746601	Bn-chrA03-p10586577
Bn-A03-p11468741	0.991735537	0.983471074	Bn-N3-p11761527	Bn-chrA03-p10604140
Bn-A03-p11499523	0.985074627	0.985074627	Bn-N3-p11784223	Bn-chrA03-p10631745
Bn-A03-p11505658	0.975206612	0.983471074	Bn-N3-p11789875	Bn-chrA03-p10637270
Bn-A03-p11515810	0.995024876	0.995024876	Bn-N3-p11801722	Bn-chrA03-p10647480
Bn-A03-p11517597	1	0.933884298	Bn-N3-p11803507	Bn-chrA03-p10650375
Bn-A03-p11519012	0.991735537	0.991735537	Bn-N3-p11804914	Bn-chrA03-p10651781
Bn-A03-p11519264	0.975124378	0.985074627	Bn-N3-p11805166	Bn-chrA03-p10652033
Bn-A03-p11520156	0.983471074	0.975206612	Bn-N3-p11806708	Bn-chrA03-p10652933
Bn-A03-p11534030	1	0.996677741	Bn-N3-p11825806	Bn-chrA03-p10666064
Bn-A03-p11544253	1	0.983471074	Bn-N3-p11832049	Bn-chrC03-p32231416
Bn-A03-p11546520	1	0.991735537	Bn-N3-p11834318	Bn-chrA03-p10672208
Bn-A03-p11549018	0.975206612	0.983471074	Bn-N3-p11836813	Bn-chrA03-p10676837
Bn-A03-p11549288	0.985074627	0.980099502	Bn-N3-p11837085	Bn-chrA03-p10677109
Bn-A03-p11572347	0.975206612	0.975206612	Bn-N3-p11852703	Bn-chrA03-p10694785
Bn-A03-p11578563	0.980066445	0.976744186	Bn-N3-p11866652	Bn-chrA03-p10701503
Bn-A03-p11583086	0.995024876	0.990049751	Bn-N3-p11883870	Bn-chrA03-p10705960
Bn-A03-p11590315	0.933884298	0.933884298	Bn-N3-p11892910	Bn-chrA03-p10715094
Bn-A03-p11605644	0.991735537	0.983471074	Bn-N3-p11919294	Bn-chrA03-p10739526
Bn-A03-p11613167	0.965174129	0.955223881	Bn-N3-p11925893	Bn-chrA03-p10746041
Bn-A03-p11613865	0.990049751	0.975124378	Bn-N3-p11926590	Bn-chrA03-p10746673
Bn-A03-p11617534	0.965174129	0.965174129	Bn-N3-p11934038	Bn-chrA03-p10749329
Bn-A03-p11618241	0.980099502	0.980099502	Bn-N3-p11934741	Bn-chrA03-p10750032
Bn-A03-p11621375	0.958677686	0.958677686	Bn-N3-p11937837	Bn-chrC03-p15212931
Bn-A03-p11623083	0.983471074	0.983471074	Bn-N3-p11939547	Bn-chrA03-p10754838
Bn-A03-p11624578	0.995024876	0.990049751	Bn-N3-p11941042	Bn-chrA03-p10756332
Bn-A03-p11626015	0.966942149	0.966942149	Bn-N3-p11942485	Bn-chrA03-p10757774
Bn-A03-p11626279	0.990033223	0.990033223	Bn-N3-p11942749	Bn-chrA03-p10758038
Bn-A03-p11633959	0.960199005	0.960199005	Bn-N7-p9599253	Bn-chrA07-p9471441
Bn-A03-p11640168	0.966942149	0.966942149	Bn-N3-p11957456	Bn-chrA03-p10774029
Bn-A03-p11646564	0.993355482	0.993355482	Bn-N3-p11963787	Bn-chrA03-p10780997
Bn-A03-p11655161	1	1	Bn-N3-p11973191	Bn-chrA03-p10790390
Bn-A03-p11655433	0.950413223	0.950413223	Bn-N3-p11973725	Bn-chrA03-p10790924
Bn-A03-p11655749	0.983471074	0.983471074	Bn-N3-p11974044	Bn-chrA03-p10791243
Bn-A03-p11656333	0.935323383	0.935323383	Bn-N3-p11974609	Bn-chrA09-p7627646
Bn-A03-p11683045	0.976744186	0.996677741	Bn-N13-p17776730	Bn-chrA03-p10809866
Bn-A03-p11684352	0.970149254	0.865671642	Bn-N3-p11994008	Bn-chrA03-p10811153
Bn-A03-p11687251	0.958677686	0.950413223	Bn-N3-p11996908	Bn-chrA03-p10814054
Bn-A03-p11704125	0.955223881	0.950248756	Bn-N3-p12014773	Bn-chrA03-p10833039

Bn-A03-p11704711	1	0.991735537	Bn-N3-p12015363	Bn-chrA03-p10833620
Bn-A03-p11708366	0.986710963	0.983388704	Bn-N3-p12019703	Bn-chrA03-p10837299
Bn-A03-p11739768	0.950248756	0.950248756	Bn-N3-p12055781	Bn-chrA03-p10873333
Bn-A03-p11744082	0.991735537	0.669421488	Bn-N3-p12061079	Bn-chrA03-p10877567
Bn-A03-p11751694	0.991735537	1	Bn-N3-p12075423	Bn-chrA03-p10887036
Bn-A03-p11765203	0.995024876	0.995024876	Bn-N11-p37351350	Bn-chrC01-p32558895
Bn-A03-p11771139	0.960199005	0.960199005	Bn-N3-p12093780	Bn-chrA03-p10904469
Bn-A03-p11772387	0.963350785	0.97382199	Bn-N3-p12095031	Bn-chrC03-p15500353
Bn-A03-p11785159	0.983388704	0.983388704	Bn-N3-p12115162	Bn-chrA03-p10925697
Bn-A03-p11792328	0.975124378	0.975124378	Bn-N3-p12122804	Bn-chrC03-p15562512
Bn-A03-p11798309	0.991735537	0.991735537	Bn-N3-p12129486	Bn-chrA03-p10937286
Bn-A03-p11822847	0.909090909	0.909090909	Bn-N3-p12151785	Bn-chrA03-p10959038
Bn-A03-p11831815	0.990033223	0.986710963	Bn-N3-p12165148	Bn-chrA03-p10970043
Bn-A03-p11883572	1	0.989583333	Bn-N3-p12215088	Bn-chrA03-p11016206
Bn-A03-p12008511	0.980099502	0.980099502	Bn-N3-p12346608	Bn-chrA03-p11149331
Bn-A03-p12065947	0.955223881	0.955223881	Bn-N3-p12412283	Bn-chrA03_random-p955883
Bn-A03-p12076661	0.980066445	0.980066445	Bn-N3-p12426160	Bn-chrA03-p11192971
Bn-A03-p12186958	0.995024876	0.995024876	Bn-N3-p12537961	Bn-chrA03-p11303687
Bn-A03-p12201775	0.958677686	0.958677686	Bn-N3-p12553115	Bn-chrA03-p11318867
Bn-A03-p12213267	0.950248756	0.950248756	Bn-N3-p12560738	Bn-chrA03-p11326490
Bn-A03-p12219523	0.986710963	0.986710963	Bn-N3-p12575097	Bn-chrA03-p11331898
Bn-A03-p12250401	0.991735537	0.991735537	Bn-N3-p12612221	Bn-chrA03-p11367399
Bn-A03-p12279241	0.983471074	0.983471074	Bn-N3-p12629150	Bn-chrA03_random-p986505
Bn-A03-p12279706	0.975206612	0.975206612	Bn-N3-p12629615	Bn-chrA03_random-p986970
Bn-A03-p12286718	0.983471074	0.983471074	Bn-N3-p12639971	Bn-chrA03_random-p996716
Bn-A03-p12287907	0.991735537	0.991735537	Bn-N3-p12641160	Bn-chrA03_random-p997905
Bn-A03-p12287911	0.991735537	0.991735537	Bn-N3-p12641164	Bn-chrA03_random-p997909
Bn-A03-p12296442	0.983471074	0.983471074	Bn-N3-p12643784	Bn-chrAnn_random-p385541
Bn-A03-p12296651	1	1	Bn-N3-p12643575	Bn-chrAnn_random-p385539
Bn-A03-p12302996	0.983471074	0.892561983	Bn-N3-p12648685	Bn-chrC03-p16540162
Bn-A03-p12308046	1	1	Bn-N3-p12653513	Bn-chrA03_random-p100183
Bn-A03-p12308158	0.975206612	0.975206612	Bn-N3-p12653627	Bn-chrA03_random-p100195
Bn-A03-p12308276	0.958677686	0.958677686	Bn-N3-p12653745	Bn-chrA03_random-p100206
Bn-A03-p12308684	0.958677686	0.958677686	Bn-N3-p12654117	Bn-chrA03_random-p100244
Bn-A03-p12308737	0.983471074	0.983471074	Bn-N3-p12654170	Bn-chrA03_random-p100249
Bn-A03-p12309478	0.917355372	0.94214876	Bn-N3-p12654906	Bn-chrC09-p30312019
Bn-A03-p12311830	0.991735537	0.991735537	Bn-N3-p12658442	Bn-chrA03-p11372105
Bn-A03-p12313036	1	1	Bn-N3-p12660008	Bn-chrA03-p11373488
Bn-A03-p12313330	0.966942149	0.966942149	Bn-N3-p12660302	Bn-chrA03-p11373782
Bn-A03-p12319647	0.983471074	0.983471074	Bn-N3-p12668118	Bn-chrA09-p17656372
Bn-A03-p12322277	0.983471074	0.975206612	Bn-N3-p12670776	Bn-chrA03-p11386559
Bn-A03-p12331321	0.983471074	0.991735537	Bn-N3-p12679790	Bn-chrA03-p11395550
Bn-A03-p12331460	0.966942149	0.975206612	Bn-N3-p12679929	Bn-chrA03-p11395689
Bn-A03-p12331575	0.983471074	0.975206612	Bn-N3-p12680044	Bn-chrA03-p11395804
Bn-A03-p12332552	0.966942149	0.933884298	Bn-N3-p12680815	Bn-chrA03-p11399553
Bn-A03-p12342812	0.975206612	0.975206612	Bn-N3-p12695564	Bn-chrA03-p11418589

Bn-A03-p12343742	0.975206612	0.975206612	Bn-N3-p12696451	Bn-chrA03-p11419476
Bn-A03-p12353218	0.983471074	0.983471074	Bn-N3-p12710315	Bn-chrA03-p11435593
Bn-A03-p12353370	0.975206612	0.975206612	Bn-N3-p12710471	Bn-chrA03-p11435749
Bn-A03-p12359936	0.966942149	0.966942149	Bn-N3-p12716589	Bn-chrA03-p11441864
Bn-A03-p12393626	0.970149254	0.970149254	Bn-N3-p12750656	Bn-chrA03-p11476997
Bn-A03-p12393711	0.960199005	0.960199005	Bn-N3-p12750742	Bn-chrA03-p11477083
Bn-A03-p12402349	1	1	Bn-N3-p12759891	Bn-chrA03-p11486592
Bn-A03-p12411362	0.983471074	0.983471074	Bn-N3-p12781741	Bn-chrA03-p11508362
Bn-A03-p12426461	0.958677686	0.958677686	Bn-N3-p12802514	Bn-chrA03-p11534668
Bn-A03-p12429205	0.94214876	0.94214876	Bn-N3-p12805622	Bn-chrA03-p11537772
Bn-A03-p12431376	0.966942149	0.966942149	Bn-N3-p12807491	Bn-chrA03-p11539641
Bn-A03-p12439824	0.993355482	0.976744186	Bn-N12-p9898511	Bn-chrC05-p13972486
Bn-A03-p12444739	0.950413223	0.950413223	Bn-N3-p12818757	Bn-chrA03_random-p101241
Bn-A03-p12453877	0.950413223	0.950413223	Bn-N3-p12830096	Bn-chrA03-p11566750
Bn-A03-p12457385	0.960199005	0.960199005	Bn-N3-p12833459	Bn-chrA03-p11570113
Bn-A03-p12457751	0.975124378	0.975124378	Bn-N3-p12833828	Bn-chrC03-p16925911
Bn-A03-p12471513	0.965174129	0.965174129	Bn-N3-p12849878	Bn-chrA03-p11587322
Bn-A03-p12477334	0.966292135	0.966292135	Bn-N3-p12856222	Bn-chrA03-p11593666
Bn-A03-p12482349	0.983471074	0.983471074	Bn-N3-p12861201	Bn-chrA03-p11598645
Bn-A03-p12511746	0.993355482	0.993355482	Bn-N3-p12892890	Bn-chrA03-p11630658
Bn-A03-p12512134	0.980099502	0.980099502	Bn-N3-p12893278	Bn-chrA03-p11631046
Bn-A03-p12518314	0.991735537	0.991735537	Bn-N3-p12898989	Bn-chrA03-p11636637
Bn-A03-p12532686	0.991735537	0.991735537	Bn-N3-p12912343	Bn-chrA03-p11650968
Bn-A03-p12541037	0.966777409	0.966777409	Bn-N3-p12920844	Bn-chrA03-p11660773
Bn-A03-p12541207	0.990049751	0.990049751	Bn-N3-p12921014	Bn-chrA03-p11660943
Bn-A03-p12541337	0.965174129	0.965174129	Bn-N3-p12921144	Bn-chrA03-p11661073
Bn-A03-p12622050	0.983388704	0.986710963	Bn-N3-p13009288	Bn-chrA03-p11752266
Bn-A03-p12623286	0.996677741	0.950166113	Bn-N3-p13010524	Bn-chrA03-p11753482
Bn-A03-p12628781	0.996677741	0.950166113	Bn-N3-p13016117	Bn-chrC03-p17298691
Bn-A03-p12631124	0.980066445	0.980066445	Bn-N3-p13019250	Bn-chrA03-p11765524
Bn-A03-p12638944	0.991735537	1	Bn-N3-p13028489	Bn-chrA03-p11774210
Bn-A03-p12639200	0.860465116	0.890365449	Bn-N3-p13028750	Bn-chrA03-p11774456
Bn-A03-p12639319	1	0.991735537	Bn-N3-p13028869	Bn-chrA03-p11774575
Bn-A03-p12737336	0.990049751	0.995024876	Bn-N13-p20002801	Bn-chrC03-p17495656
Bn-A03-p12783093	0.991735537	0.983471074	Bn-N3-p13174915	Bn-chrA03-p11921855
Bn-A03-p12784925	0.966942149	0.975206612	Bn-N3-p13176749	Bn-chrA03-p11923689
Bn-A03-p12820905	0.991735537	0.983471074	Bn-N3-p13217701	Bn-chrA03-p11960607
Bn-A03-p12826949	0.975206612	0.975206612	Bn-Scaffold01631-p5	Bn-chrA03-p11964447
Bn-A03-p12859516	1	0.991735537	Bn-N3-p13257421	Bn-chrA03-p11999994
Bn-A03-p12863264	0.96345515	0.96345515	Bn-N3-p13260650	Bn-chrA03-p12003214
Bn-A03-p12863642	0.983471074	0.991735537	Bn-N3-p13261028	Bn-chrA03-p12003592
Bn-A03-p12863684	0.993355482	0.990033223	Bn-N3-p13261070	Bn-chrA03-p12003634
Bn-A03-p12869911	0.966942149	0.966942149	Bn-N3-p13261481	Bn-chrA03-p12004006
Bn-A03-p12903591	0.983471074	0.975206612	Bn-N3-p13294028	Bn-chrA03-p12032631
Bn-A03-p12932399	0.933554817	0.990033223	Bn-N3-p13323285	Bn-chrA03-p12063555
Bn-A03-p12932760	0.940199336	0.993355482	Bn-N3-p13323628	Bn-chrA03-p12063916

Bn-A03-p12933099	0.983388704	0.990033223	Bn-N3-p13323965	Bn-chrA03-p12064253
Bn-A03-p12935447	0.983471074	0.991735537	Bn-N3-p13326359	Bn-chrAnn_random-p200268
Bn-A03-p12941404	0.975206612	0.983471074	Bn-N3-p13331957	Bn-chrA03-p12071687
Bn-A03-p12945091	0.975206612	0.975206612	Bn-N3-p13340397	Bn-chrA03-p12075427
Bn-A03-p12973462	0.996677741	0.996677741	Bn-Scaffold01261-p1	Bn-chrA03-p12105737
Bn-A03-p12981348	1	0.991735537	Bn-N3-p13375979	Bn-chrA03-p12113814
Bn-A03-p12990610	1	0.996677741	Bn-N3-p13385371	Bn-chrA03-p12123206
Bn-A03-p12993600	0.975206612	0.958677686	Bn-N3-p13388486	Bn-chrA03-p12126343
Bn-A03-p12994885	0.983471074	0.991735537	Bn-N3-p13389764	Bn-chrC03-p17698667
Bn-A03-p13016324	0.983471074	0.851239669	Bn-N3-p13418691	Bn-chrC09-p25654024
Bn-A03-p13017512	0.966942149	0.975206612	Bn-N3-p13419879	Bn-chrA03-p12148126
Bn-A03-p13017704	1	0.991735537	Bn-N3-p13420071	Bn-chrA03-p12148318
Bn-A03-p13018172	0.958677686	0.966942149	Bn-N3-p13420538	Bn-chrA03-p12148788
Bn-A03-p13032037	0.966942149	0.975206612	Bn-N3-p13428894	Bn-chrA03-p12160004
Bn-A03-p13032092	0.966942149	0.975206612	Bn-N3-p13428949	Bn-chrA03-p12160059
Bn-A03-p13037975	1	0.991735537	Bn-N3-p13434458	Bn-chrA03-p12165938
Bn-A03-p13039000	0.991735537	0.983471074	Bn-N3-p13435486	Bn-chrA03-p12166966
Bn-A03-p13039285	0.976744186	0.976744186	Bn-N3-p13435771	Bn-chrA03-p12167251
Bn-A03-p13043164	0.983471074	0.975206612	Bn-N3-p13438127	Bn-chrA03-p12169605
Bn-A03-p13044950	0.950413223	0.94214876	Bn-N3-p13439822	Bn-chrA03-p12171393
Bn-A03-p13050570	0.983471074	0.991735537	Bn-N3-p13445194	Bn-chrA03-p12176797
Bn-A03-p13058729	0.975206612	0.991735537	Bn-N3-p13455084	Bn-chrA03-p12185351
Bn-A03-p13061709	0.990033223	0.986710963	Bn-N3-p13458036	Bn-chrA03-p12188326
Bn-A03-p13094043	0.980099502	0.980099502	Bn-N3-p13480093	Bn-chrA03-p12222543
Bn-A03-p13099476	0.966942149	0.975206612	Bn-N3-p13485759	Bn-chrA03-p12228208
Bn-A03-p13104283	0.776859504	0.776859504	Bn-N3-p13489967	Bn-chrA03-p12232416
Bn-A03-p13107268	0.823920266	0.827242525	Bn-N3-p13501411	Bn-chrA03-p12241773
Bn-A03-p13109267	0.833887043	0.803986711	Bn-N3-p13503467	Bn-chrC03-p17925034
Bn-A03-p13131453	0.983388704	0.996677741	Bn-N3-p13512412	Bn-chrA03-p12251462
Bn-A03-p13145923	0.970149254	0.965174129	Bn-N3-p13529675	Bn-chrA03-p12283419
Bn-A03-p13160528	0.983388704	0.986710963	Bn-N3-p13547494	Bn-chrA03-p12296424
Bn-A03-p13169138	0.975206612	0.958677686	Bn-N3-p13558157	Bn-chrA03-p12311209
Bn-A03-p13169346	0.950413223	0.958677686	Bn-N3-p13558365	Bn-chrA03-p12311417
Bn-A03-p13177232	0.983471074	0.26446281	Bn-N3-p13566400	Bn-chrC09-p47061263
Bn-A03-p13189171	1	0.983471074	Bn-N3-p13579099	Bn-chrA03-p12371518
Bn-A03-p13216785	0.975206612	0.900826446	Bn-N3-p13605162	Bn-chrC03-p18056467
Bn-A03-p13218451	1	0.876033058	Bn-N3-p13606828	Bn-chrC03-p18057902
Bn-A03-p13219815	0.985074627	0.348258706	Bn-N3-p13608192	Bn-chrA03-p17259686
Bn-A03-p13221584	0.991735537	0.20661157	Bn-N3-p13609961	Bn-chrC04-p22237128
Bn-A03-p13223204	0.983471074	0.239669421	Bn-N3-p13614309	Unknown
Bn-A03-p13236840	0.956810631	0.996677741	Bn-N13-p20803975	Bn-chrA03-p12404979
Bn-A03-p13253155	0.970099668	0.993355482	Bn-N13-p20830258	Bn-chrA03-p12419180
Bn-A03-p13254564	0.950413223	0.975206612	Bn-N13-p20831776	Bn-chrA03-p12420557
Bn-A03-p13266036	0.975206612	0.958677686	Bn-Scaffold20951-p1	Bn-chrA03-p12427916
Bn-A03-p13286574	0.991735537	0.991735537	Bn-N3-p13645159	Bn-chrA03-p12444405
Bn-A03-p13287670	0.991735537	0.991735537	Bn-N3-p13646257	Bn-chrA03-p12445503

Bn-A03-p13288429	0.975206612	0.975206612	Bn-N3-p13647015	Bn-chrA03-p12446261
Bn-A03-p13291935	0.94214876	0.94214876	Bn-N3-p13650548	Bn-chrA03-p12449797
Bn-A03-p13298734	0.953488372	0.953488372	Bn-N3-p13654777	Bn-chrA03-p12454026
Bn-A03-p13302773	0.909090909	0.909090909	Bn-N3-p13659565	Bn-chrA03-p12458846
Bn-A03-p13302882	0.820895522	0.815920398	Bn-N13-p20897815	Bn-chrA03-p12458937
Bn-A03-p13306819	0.966942149	0.966942149	Bn-N3-p13663950	Bn-chrCnn_random-p638955
Bn-A03-p13324647	0.173553719	0.148760331	Unknown	Bn-chrC03_random-p363007
Bn-A03-p13325522	0.198347107	0.190082645	Bn-N18-p40161473	Unknown
Bn-A03-p13335275	0.991735537	0.975206612	Bn-N3-p13687875	Bn-chrA03_random-p103506
Bn-A03-p13335946	0.975206612	0.966942149	Bn-N13-p20967497	Bn-chrA03_random-p103438
Bn-A03-p13365712	0.996677741	0.990033223	Bn-N3-p13707451	Bn-chrA03-p12501630
Bn-A03-p13421039	0.965174129	0.965174129	Bn-N3-p13744415	Bn-chrA03-p12540906
Bn-A03-p13440035	0.983471074	0.983471074	Bn-N3-p13762395	Bn-chrA03-p12558881
Bn-A03-p13451851	0.975124378	0.975124378	Bn-N3-p13773397	Bn-chrA03-p12570027
Bn-A03-p13465062	0.983471074	0.983471074	Bn-N3-p13787184	Bn-chrA03-p12584099
Bn-A03-p13469770	0.980099502	0.980099502	Bn-N3-p13802351	Bn-chrA03-p12594419
Bn-A03-p13492986	0.993355482	0.990033223	Bn-N3-p13823753	Bn-chrA03-p12632977
Bn-A03-p13502336	0.993355482	0.986710963	Bn-N3-p13834517	Bn-chrA03-p12642630
Bn-A03-p13502830	0.975206612	0.983471074	Bn-N3-p13835018	Bn-chrA03-p12643131
Bn-A03-p13514947	0.946843854	0.986710963	Bn-N13-p21438071	Bn-chrA03-p12656534
Bn-A03-p13523343	0.950413223	0.950413223	Bn-N3-p13853217	Bn-chrA03-p12664782
Bn-A03-p13533904	0.991735537	0.991735537	Bn-N3-p13865572	Bn-chrA03-p12677384
Bn-A03-p13545223	0.983471074	0.975206612	Bn-N3-p13886490	Bn-chrA03-p12691986
Bn-A03-p13546174	0.980099502	0.980099502	Bn-N3-p13887442	Bn-chrA03-p12692939
Bn-A03-p13567559	0.966942149	0.966942149	Bn-N3-p13920556	Bn-chrA03-p12722258
Bn-A03-p13572495	0.975206612	0.975206612	Bn-N3-p13926123	Bn-chrA03-p12727748
Bn-A03-p13585261	0.991735537	1	Bn-N3-p13940936	Bn-chrA03_random-p107561
Bn-A03-p13605344	0.983471074	0.190082645	Bn-N3-p13961114	Bn-chrC04-p29078988
Bn-A03-p13610858	0.983471074	0.983471074	Bn-N3-p13966548	Bn-chrCnn_random-p681582
Bn-A03-p13616280	0.990033223	0.990033223	Bn-Scaffold29317-p1	Bn-chrA03_random-p109701
Bn-A03-p13624310	0.943521595	0.943521595	Bn-N3-p13985853	Bn-chrA03-p12772928
Bn-A03-p13630048	0.619834711	0.619834711	Bn-N3-p13991377	Bn-chrA03-p12778452
Bn-A03-p13640859	0.94214876	0.94214876	Bn-N3-p14003635	Bn-chrA03-p12790150
Bn-A03-p13653603	0.900826446	0.900826446	Bn-N13-p21701987	Bn-chrC03-p18831418
Bn-A03-p13658460	0.860465116	0.860465116	Bn-N3-p14017333	Bn-chrA03-p12803848
Bn-A03-p13664500	0.925619835	0.925619835	Bn-N3-p14023026	Bn-chrA03-p12809539
Bn-A03-p13664657	0.966942149	0.966942149	Bn-N3-p14023187	Bn-chrA03-p12809700
Bn-A03-p13678735	0.966777409	0.970099668	Bn-N13-p21744521	Bn-chrA03-p12824363
Bn-A03-p13679854	0.986710963	0.983388704	Bn-N3-p14037826	Bn-chrA03-p12825482
Bn-A03-p13746713	0.991735537	1	Bn-N3-p14077688	Bn-chrA03-p12863966
Bn-A03-p13747074	0.980099502	0.975124378	Bn-N3-p14078069	Bn-chrA03-p12864327
Bn-A03-p13755726	0.958677686	0.966942149	Bn-N3-p14110560	Bn-chrA03-p12890871
Bn-A03-p13758392	0.985074627	0.990049751	Bn-N3-p14117336	Bn-chrA03-p12893544
Bn-A03-p13773583	0.88372093	0.877076412	Bn-N3-p14130625	Bn-chrA03-p12904203
Bn-A03-p13777203	0.975206612	0.983471074	Bn-N3-p14134241	Bn-chrA03-p12907798
Bn-A03-p13778786	0.94214876	0.94214876	Bn-N3-p14135815	Bn-chrA03-p12909373

Bn-A03-p13783263	0.993355482	0.906976744	Bn-N3-p14140217	Bn-chrA03-p12913724
Bn-A03-p13802162	0.925373134	0.985074627	Bn-N3-p14145741	Bn-chrA03-p12919118
Bn-A03-p13810458	0.818181818	0.933884298	Bn-N13-p21976769	Bn-chrA03-p12928563
Bn-A03-p13814543	0.935323383	0.965174129	Bn-N13-p21981294	Bn-chrA03-p12933336
Bn-A03-p13815665	0.940298507	0.940298507	Bn-N3-p14159620	Bn-chrA03-p12934458
Bn-A03-p13853558	0.958677686	0.958677686	Bn-N3-p14175996	Bn-chrA03-p12954683
Bn-A03-p13859927	0.975206612	0.94214876	Bn-Scaffold17169-p1	Bn-chrA03-p12963667
Bn-A03-p13861679	0.950413223	0.909090909	Bn-N3-p14182950	Bn-chrA03-p12965414
Bn-A03-p13861749	0.760330579	0.966942149	Bn-N3-p14183025	Bn-chrA03-p12965486
Bn-A03-p13863828	0.925619835	0.983471074	Bn-N13-p22045080	Bn-chrA03-p12967639
Bn-A03-p13883754	0.751243781	0.985074627	Bn-N3-p14210407	Bn-chrA03-p12992647
Bn-A03-p13906492	0.818181818	0.818181818	Bn-N3-p14232208	Bn-chrA03-p13026717
Bn-A03-p13908787	0.991735537	0.991735537	Bn-N3-p14234295	Bn-chrA03-p13015172
Bn-A03-p13912476	0.990033223	0.983388704	Bn-N3-p14236228	Bn-chrA03-p13017105
Bn-A03-p13922223	1	0.991735537	Bn-N3-p14248562	Bn-chrA03-p13047816
Bn-A03-p13922405	0.975206612	0.983471074	Bn-N3-p14248744	Bn-chrA03-p13047998
Bn-A03-p13925731	0.975206612	0.983471074	Bn-N3-p14253184	Bn-chrA03-p13055174
Bn-A03-p13943992	0.945273632	0.960199005	Bn-N3-p14273849	Bn-chrA03-p13075087
Bn-A03-p13968921	0.991735537	0.991735537	Bn-N3-p14303293	Bn-chrA03-p13105498
Bn-A03-p13970114	0.702479339	0.933884298	Bn-N3-p14304481	Bn-chrA03-p13106624
Bn-A03-p13970168	0.809917355	0.983471074	Bn-N3-p14304515	Bn-chrA03-p13106678
Bn-A03-p13974725	0.94214876	0.950413223	Bn-N3-p14309064	Bn-chrA03-p13116790
Bn-A03-p13975945	0.96345515	1	Bn-N3-p14310286	Bn-chrA03-p13117998
Bn-A03-p13977740	0.991735537	0.983471074	Bn-N3-p14311970	Bn-chrA03-p13120411
Bn-A03-p13987679	0.983471074	0.842975207	Bn-N3-p14315118	Bn-chrA03-p13121643
Bn-A03-p13996014	0.990033223	0.993355482	Bn-N3-p14323705	Bn-chrA03-p13139772
Bn-A03-p14003701	0.983471074	0.909090909	Bn-N3-p14331357	Bn-chrA03-p13147806
Bn-A03-p14009871	0.993355482	0.993355482	Bn-N3-p14338096	Bn-chrA03-p13157670
Bn-A03-p14015779	0.970099668	0.970099668	Bn-N3-p14341517	Bn-chrA03-p13161092
Bn-A03-p14023392	0.993355482	0.990033223	Bn-N3-p14348717	Bn-chrA03-p13168253
Bn-A03-p14024778	0.943521595	0.996677741	Bn-N3-p14350104	Bn-chrA03-p13169640
Bn-A03-p14026999	0.720930233	0.996677741	Bn-N3-p14352324	Bn-chrA03-p13171802
Bn-A03-p14031468	1	0.991735537	Bn-N3-p14356474	Bn-chrA03-p13175871
Bn-A03-p14034228	0.996677741	1	Bn-N3-p14362485	Bn-chrA03-p13180130
Bn-A03-p14037892	0.983388704	0.983388704	Bn-N3-p14366149	Bn-chrA03-p13183870
Bn-A03-p14041361	0.991735537	0.991735537	Bn-N3-p14370593	Bn-chrA03-p13187390
Bn-A03-p14041931	1	0.996677741	Bn-N3-p14371157	Bn-chrA03-p13187954
Bn-A03-p14092692	0.983471074	0.983471074	Bn-N3-p14418412	Bn-chrA03-p13276830
Bn-A03-p14095948	0.975206612	0.975206612	Bn-N3-p14422444	Bn-chrA03-p13280865
Bn-A03-p14112910	0.958677686	0.958677686	Bn-N3-p14438774	Bn-chrA03-p13297288
Bn-A03-p14113489	0.975206612	0.975206612	Bn-N3-p14439353	Bn-chrA03-p13297867
Bn-A03-p14121492	0.991735537	0.991735537	Bn-N3-p14447394	Bn-chrA03-p13305961
Bn-A03-p14127649	0.996677741	0.996677741	Bn-N3-p14453580	Bn-chrA03-p13314548
Bn-A03-p14128333	0.996677741	0.996677741	Bn-N3-p14454264	Bn-chrA03-p13315232
Bn-A03-p14153283	0.991735537	0.975206612	Bn-N3-p14479202	Bn-chrA03-p13346806
Bn-A03-p14153306	0.983471074	0.950413223	Bn-N3-p14479225	Bn-chrA03-p13346829

Bn-A03-p14153376	0.983471074	0.975206612	Bn-N3-p14479295	Bn-chrA03-p13346905
Bn-A03-p14154555	0.950413223	0.958677686	Bn-N3-p14483678	Bn-chrA03-p13348071
Bn-A03-p14154912	0.966942149	0.958677686	Bn-N3-p14484035	Bn-chrA03-p13348451
Bn-A03-p14155274	0.983471074	0.94214876	Bn-N3-p14484391	Bn-chrA03-p13348803
Bn-A03-p14175904	1	1	Bn-N3-p14508983	Bn-chrA03-p13373240
Bn-A03-p14176469	0.995024876	1	Bn-N3-p14509547	Bn-chrA03-p13373806
Bn-A03-p14196859	0.388059701	0.567164179	Bn-N13-p22633960	Bn-chrA03-p13382003
Bn-A03-p14197151	0.958677686	0.966942149	Bn-N3-p14531428	Bn-chrA03-p13382300
Bn-A03-p14201146	0.644628099	0.991735537	Bn-N3-p14535159	Bn-chrA03-p13391159
Bn-A03-p14209971	0.983471074	0.991735537	Bn-N3-p14543573	Bn-chrA03_random-p108878
Bn-A03-p14212196	0.991735537	1	Bn-N3-p14544098	Bn-chrA03_random-p108931
Bn-A03-p14251137	1	0.990049751	Bn-N3-p14584738	Bn-chrA03-p13444423
Bn-A03-p14261914	0.996677741	0.980066445	Bn-N3-p14599964	Bn-chrA03-p13448735
Bn-A03-p14262103	1	1	Bn-N3-p14600153	Bn-chrA03-p13450374
Bn-A03-p14273821	0.966942149	0.975206612	Bn-N3-p14609027	Bn-chrA03-p13456918
Bn-A03-p14276328	0.966942149	0.975206612	Bn-N3-p14611514	Bn-chrA03-p13459403
Bn-A03-p14282460	0.980099502	0.975124378	Bn-N3-p14617650	Bn-chrA03-p13465551
Bn-A03-p14286873	0.983471074	0.966942149	Bn-N3-p14622130	Bn-chrA03-p13469904
Bn-A03-p14290741	0.991735537	0.983471074	Bn-N3-p14626122	Bn-chrA03-p13473758
Bn-A03-p14291952	0.986710963	0.970099668	Bn-N3-p14627320	Bn-chrA03-p13474966
Bn-A03-p14295089	0.983471074	0.975206612	Bn-N3-p14630481	Bn-chrA03-p13478102
Bn-A03-p14295396	0.96345515	0.980066445	Bn-N3-p14630797	Bn-chrA03-p13478402
Bn-A03-p14306440	0.990033223	0.993355482	Bn-N3-p14641501	Bn-chrA03-p13490848
Bn-A03-p14309803	0.983471074	0.975206612	Bn-N3-p14644864	Bn-chrA03-p13494198
Bn-A03-p14310386	0.983471074	0.991735537	Bn-N3-p14645446	Bn-chrA03-p13494780
Bn-A03-p14310805	0.991735537	0.925619835	Bn-N3-p14645865	Bn-chrA05-p22795993
Bn-A03-p14330609	0.96345515	0.936877076	Bn-N13-p22841833	Bn-chrA03-p13516748
Bn-A03-p14353080	0.983471074	0.719008264	Bn-N3-p14687684	Bn-chrA03-p13528820
Bn-A03-p14355646	0.966942149	0.975206612	Bn-N3-p14688286	Bn-chrA03-p13533972
Bn-A03-p14363525	0.985074627	0.945273632	Bn-N8-p16415037	Bn-chrC06-p11104546
Bn-A03-p14369287	0.966942149	0.966942149	Bn-N3-p14693565	Bn-chrA03-p13539250
Bn-A03-p14385006	0.975206612	0.975206612	Bn-N3-p14711636	Bn-chrA03-p13553439
Bn-A03-p14387177	0.958677686	0.958677686	Bn-N3-p14713821	Bn-chrA03-p13555624
Bn-A03-p14398744	0.975206612	0.975206612	Bn-N3-p14728197	Bn-chrA03-p13569745
Bn-A03-p14399972	0.909090909	0.909090909	Bn-N3-p14729444	Bn-chrA03-p13571282
Bn-A03-p14400020	0.909090909	0.909090909	Bn-N3-p14729492	Bn-chrA03-p13571330
Bn-A03-p14402509	0.995024876	0.995024876	Bn-N3-p14732311	Bn-chrA03-p13574149
Bn-A03-p14413451	0.983471074	0.983471074	Bn-N3-p14738917	Bn-chrA03-p13580378
Bn-A03-p14418556	0.985074627	0.985074627	Bn-N13-p22939644	Bn-chrC03-p20021286
Bn-A03-p14418650	0.970149254	0.970149254	Bn-N3-p14747284	Bn-chrA03-p13589342
Bn-A03-p14423477	0.983471074	0.983471074	Bn-N3-p14752111	Bn-chrA03-p13594169
Bn-A03-p14434665	0.966942149	0.966942149	Bn-N3-p14764950	Bn-chrA03-p13603483
Bn-A03-p14447238	0.983471074	0.983471074	Bn-N3-p14777622	Bn-chrA03-p13616214
Bn-A03-p14458139	0.990033223	0.990033223	Bn-N3-p14791402	Bn-chrA03-p13629485
Bn-A03-p14461008	0.925619835	0.925619835	Bn-N3-p14792796	Bn-chrA03-p13630879
Bn-A03-p14462148	0.991735537	0.991735537	Bn-N3-p14793905	Bn-chrA03-p13631987

Bn-A03-p14462570	0.980066445	0.980066445	Bn-N3-p14794327	Bn-chrA03-p13632409
Bn-A03-p14472406	0.980099502	0.980099502	Bn-N3-p14804413	Bn-chrA03-p13642217
Bn-A03-p14473184	0.955223881	0.950248756	Bn-N3-p14805191	Bn-chrA03-p13642997
Bn-A03-p14473296	0.945273632	0.935323383	Bn-N3-p14805303	Bn-chrA03-p13643109
Bn-A03-p14474478	0.983471074	0.975206612	Bn-N3-p14806463	Bn-chrA03-p13644281
Bn-A03-p14511431	0.991735537	0.991735537	Bn-N3-p14845698	Bn-chrA03-p13673373
Bn-A03-p14552086	0.975206612	0.966942149	Bn-N3-p14890862	Bn-chrA03-p13723717
Bn-A03-p14557663	0.986710963	0.986710963	Bn-N3-p14896577	Bn-chrA03-p13729386
Bn-A03-p14577690	0.966942149	0.958677686	Bn-N3-p14922691	Bn-chrC03-p20254365
Bn-A03-p14583041	0.958677686	0.950413223	Bn-N3-p14927519	Bn-chrA03-p13754461
Bn-A03-p14585799	0.958677686	0.958677686	Bn-N3-p14930339	Bn-chrA03-p13757231
Bn-A03-p14586392	0.975206612	0.975206612	Bn-N3-p14931181	Bn-chrA03-p13757825
Bn-A03-p14598476	0.993355482	0.993355482	Bn-N3-p14940380	Bn-chrA03-p13767314
Bn-A03-p14611641	0.909090909	0.975206612	Bn-N3-p14954238	Bn-chrA03-p13781418
Bn-A03-p14684924	0.870431894	0.873754153	Bn-N3-p15060573	Bn-chrA03-p13875212
Bn-A03-p14685500	0.661157025	0.933884298	Bn-N3-p15061007	Bn-chrA03-p13875792
Bn-A03-p14692579	0.920265781	0.50166113	Bn-N3-p15067937	Bn-chrA03-p13882706
Bn-A03-p14710981	0.996677741	0.674418605	Bn-N3-p15077833	Bn-chrC03-p20454439
Bn-A03-p14735831	0.991735537	0.983471074	Bn-N3-p15096208	Bn-chrA03-p13912968
Bn-A03-p14736161	0.991735537	1	Bn-N3-p15096538	Bn-chrA03-p13913299
Bn-A03-p14742926	0.950413223	0.900826446	Bn-N3-p15098351	Bn-chrA03_random-p110085
Bn-A03-p14747263	0.958677686	0.950413223	Bn-N3-p15102947	Bn-chrA03-p13916047
Bn-A03-p14758285	0.933884298	0.933884298	Bn-N3-p15112267	Bn-chrA03-p13927475
Bn-A03-p14759764	0.983471074	0.991735537	Bn-N3-p15114011	Bn-chrA03-p13929211
Bn-A03-p14760518	0.603305785	0.595041322	Bn-N3-p15114831	Bn-chrA03-p13929949
Bn-A03-p14784764	0.991735537	0.991735537	Bn-N3-p15133628	Bn-chrA03-p13948546
Bn-A03-p14792548	0.958677686	0.958677686	Bn-N3-p15145912	Bn-chrA03-p13957329
Bn-A03-p14803521	0.958677686	0.958677686	Bn-N3-p15176806	Bn-chrA03-p13984699
Bn-A03-p14811204	0.975206612	0.983471074	Bn-Scaffold01345-p1	Bn-chrA03-p13977041
Bn-A03-p14811653	0.983388704	0.990033223	Bn-N3-p15168702	Bn-chrA03-p13976592
Bn-A03-p14816541	0.940298507	0.940298507	Bn-N3-p15164082	Bn-chrA03-p13972274
Bn-A03-p14839497	0.945273632	0.940298507	Bn-N3-p15188072	Bn-chrA03-p13995704
Bn-A03-p14839583	0.983471074	0.991735537	Bn-N3-p15188158	Bn-chrA03-p13995790
Bn-A03-p14848117	0.950413223	0.958677686	Bn-N3-p15196753	Bn-chrA03-p14003178
Bn-A03-p14852785	0.995024876	0.995024876	Bn-N3-p15208068	Bn-chrA03-p14007844
Bn-A03-p14870270	0.970149254	0.965174129	Bn-N3-p15234864	Bn-chrA03-p14024293
Bn-A03-p14870763	0.990049751	0.990049751	Bn-N3-p15235357	Bn-chrC03-p20625105
Bn-A03-p14871005	0.950248756	0.990049751	Bn-N13-p23681566	Bn-chrC03-p20625347
Bn-A03-p14885241	0.983471074	0.975206612	Bn-N3-p15250253	Bn-chrA03-p14038821
Bn-A03-p14888403	0.950166113	0.996677741	Bn-N3-p15253419	Bn-chrA03-p14043106
Bn-A03-p14903814	0.990049751	0.990049751	Bn-N3-p15262450	Bn-chrA03-p14052102
Bn-A03-p14927037	0.983388704	0.986710963	Bn-N3-p15285911	Bn-chrA03-p14075178
Bn-A03-p14949812	0.995024876	0.995024876	Bn-N3-p15309330	Bn-chrAnn_random-p251486
Bn-A03-p14962288	0.975206612	0.983471074	Bn-N3-p15323141	Bn-chrAnn_random-p264870
Bn-A03-p14968153	0.991735537	0.991735537	Bn-N3-p15329045	Bn-chrAnn_random-p264929
Bn-A03-p15002477	0.975124378	0.980099502	Bn-N3-p15359664	Bn-chrA03-p14132364

Bn-A03-p15004059	0.975206612	0.933884298	Bn-N3-p15361204	Bn-chrA03-p14133891
Bn-A03-p15015706	0.933884298	0.925619835	Bn-N3-p15373470	Bn-chrA03-p14145284
Bn-A03-p15038774	0.875621891	0.945273632	Bn-N3-p15403139	Bn-chrC05-p12685864
Bn-A03-p15044158	0.983471074	0.991735537	Bn-N3-p15409110	Bn-chrA03-p14164839
Bn-A03-p15070818	0.104477612	0.104477612	Bn-N17-p12930150	Bn-chrC07-p11399753
Bn-A03-p15077006	0.966942149	0.966942149	Bn-N3-p15430341	Bn-chrA03-p14182303
Bn-A03-p15102212	0.975206612	0.958677686	Bn-N3-p15455360	Bn-chrA03-p14213467
Bn-A03-p15103575	0.966942149	0.966942149	Bn-N3-p15456683	Bn-chrA03-p14215052
Bn-A03-p15107284	0.975124378	0.975124378	Bn-N3-p15460229	Bn-chrA03-p14216824
Bn-A03-p15120410	0.94214876	0.966942149	Bn-N3-p15474549	Bn-chrA03-p14229116
Bn-A03-p15139190	0.975124378	0.975124378	Bn-N3-p15493124	Bn-chrA03-p14247508
Bn-A03-p15139484	0.995024876	0.895522388	Bn-N3-p15493418	Bn-chrC03-p20904252
Bn-A03-p15141821	0.985074627	0.870646766	Bn-N3-p15495724	Bn-chrA03-p14250181
Bn-A03-p15149454	0.958677686	0.958677686	Bn-N3-p15498813	Bn-chrA03-p14253189
Bn-A03-p15156393	0.991735537	0.991735537	Bn-N3-p15508378	Bn-chrA03_random-p110608
Bn-A03-p15174986	0.96345515	0.956810631	Bn-N3-p15533333	Bn-chrC03-p20952211
Bn-A03-p15184379	0.925619835	0.925619835	Bn-N3-p15537761	Bn-chrA03_random-p113113
Bn-A03-p15200901	0.726368159	0.726368159	Bn-N3-p15559804	Bn-chrA03_random-p114891
Bn-A03-p15201279	0.993355482	0.990033223	Bn-N3-p15560182	Bn-chrA03_random-p114929
Bn-A03-p15207594	0.966942149	0.958677686	Bn-N3-p15570064	Bn-chrAnn_random-p335991
Bn-A03-p15229988	0.96345515	0.96345515	Bn-N5-p18784285	Bn-chrA08-p17065528
Bn-A03-p15233860	0.966942149	0.950413223	Bn-N3-p15592124	Bn-chrA03-p14291858
Bn-A03-p15237693	0.983471074	0.991735537	Bn-N3-p15595799	Bn-chrA03-p14294154
Bn-A03-p15240794	0.993355482	0.990033223	Bn-N3-p15598875	Bn-chrA03-p14297289
Bn-A03-p15240992	0.983388704	0.986710963	Bn-N3-p15599073	Bn-chrA03-p14297487
Bn-A03-p15245600	0.721393035	0.960199005	Bn-Scaffold01107-p1	Bn-chrA03-p14302116
Bn-A03-p15265791	0.958677686	0.958677686	Bn-N3-p15623742	Bn-chrA03-p14319683
Bn-A03-p15266403	0.975124378	0.985074627	Bn-N3-p15624345	Bn-chrA03-p14320292
Bn-A03-p15300653	0.975206612	0.975206612	Bn-N3-p15667081	Bn-chrA03-p14355865
Bn-A03-p15326805	0.96013289	0.986710963	Bn-N3-p15677762	Bn-chrA03-p14366473
Bn-A03-p15327817	0.983388704	0.986710963	Bn-N3-p15678774	Bn-chrA03-p14367469
Bn-A03-p15327921	0.983388704	0.980066445	Bn-N3-p15678878	Bn-chrA03-p14367573
Bn-A03-p15337088	0.991735537	1	Bn-N3-p15688638	Bn-chrA03-p14377308
Bn-A03-p15346153	0.983471074	0.975206612	Bn-N3-p15692669	Bn-chrA03-p14383303
Bn-A03-p15347967	0.990033223	0.990033223	Bn-N3-p15694477	Bn-chrA03-p14385109
Bn-A03-p15349925	0.980066445	0.983388704	Bn-N3-p15696427	Bn-chrA03-p14387066
Bn-A03-p15351982	0.925619835	0.925619835	Bn-N3-p15698667	Bn-chrA03-p14389287
Bn-A03-p15359867	0.983471074	0.983471074	Bn-N3-p15710902	Bn-chrA03-p14401493
Bn-A03-p15363919	0.990033223	0.990033223	Bn-N3-p15714953	Bn-chrA03-p14405544
Bn-A03-p15365259	0.958677686	0.900826446	Bn-N3-p15718306	Bn-chrA03-p14406862
Bn-A03-p15368328	0.970149254	0.980099502	Bn-N3-p15727046	Bn-chrA03-p14422152
Bn-A03-p15397187	0.958677686	0.966942149	Bn-N3-p15746620	Bn-chrA03-p14446605
Bn-A03-p15404736	0.983471074	0.710743802	Bn-N3-p15754783	Bn-chrA03-p14454534
Bn-A03-p15405498	0.975206612	0.983471074	Bn-N3-p15758953	Bn-chrA03-p14455341
Bn-A03-p15406047	0.915422886	0.92039801	Bn-N3-p15759493	Bn-chrA03-p14455888
Bn-A03-p15416728	0.953488372	0.986710963	Bn-N3-p15771428	Bn-chrA03-p14467832

Bn-A03-p15417676	0.980066445	0.983388704	Bn-N13-p24436448	Bn-chrC03-p21252094
Bn-A03-p15424536	0.776859504	0.966942149	Bn-N3-p15779216	Bn-chrA03-p14475327
Bn-A03-p15435114	0.995024876	0.990049751	Bn-N3-p15791833	Bn-chrA03-p14485569
Bn-A03-p15435174	0.990049751	0.995024876	Bn-N3-p15791893	Bn-chrA03-p14485629
Bn-A03-p15435442	0.955223881	0.985074627	Bn-N3-p15792170	Bn-chrA03-p14485897
Bn-A03-p15481319	1	1	Bn-N3-p15854768	Bn-chrA03-p14542163
Bn-A03-p15481682	0.857142857	0.857142857	Bn-N3-p15855146	Bn-chrA03-p14542541
Bn-A03-p15496608	0.991735537	1	Bn-N3-p15876140	Bn-chrA03-p14558144
Bn-A03-p15496962	0.966942149	0.975206612	Bn-N3-p15876498	Bn-chrA03-p14558509
Bn-A03-p15499288	0.980099502	0.980099502	Bn-N3-p15878828	Bn-chrA03-p14560809
Bn-A03-p15546590	0.917355372	0.917355372	Bn-N3-p15918295	Bn-chrA03-p14598664
Bn-A03-p15548506	0.995024876	0.995024876	Bn-N3-p15921837	Bn-chrA03-p14602206
Bn-A03-p15548953	0.990033223	0.990033223	Bn-N3-p15922286	Bn-chrA03-p14602655
Bn-A03-p15559809	0.991735537	0.991735537	Bn-N3-p15934827	Bn-chrA07-p108291
Bn-A03-p15576074	0.996677741	0.996677741	Bn-N3-p15954172	Bn-chrA03-p14635823
Bn-A03-p15577849	0.980066445	0.980066445	Bn-N3-p15957400	Bn-chrA03-p14638235
Bn-A03-p15593483	1	1	Bn-N3-p15973724	Bn-chrA03-p14651400
Bn-A03-p15605355	0.815920398	0.980099502	Bn-N3-p15980808	Bn-chrA03-p14667295
Bn-A03-p15615278	0.966942149	0.975206612	Bn-N3-p15989848	Bn-chrA03-p14676316
Bn-A03-p15622267	1	0.991735537	Bn-N3-p15997734	Bn-chrA03-p14683281
Bn-A03-p15624030	0.950413223	0.958677686	Bn-N3-p15999408	Bn-chrA03-p14692101
Bn-A03-p15624379	0.966942149	0.983471074	Bn-N3-p15999767	Bn-chrA03-p14692459
Bn-A03-p15627698	0.983471074	0.983471074	Bn-Scaffold48530-p2	Bn-chrA03-p14695725
Bn-A03-p15634432	0.903654485	0.993355482	Bn-N3-p16009808	Bn-chrA03-p14702459
Bn-A03-p15675871	0.980066445	0.993355482	Bn-N13-p24799629	Bn-chrA03-p14735141
Bn-A03-p15689225	0.980099502	0.980099502	Bn-N3-p16036365	Bn-chrA03-p14748720
Bn-A03-p15702943	0.993355482	0.993355482	Bn-N3-p16050732	Bn-chrA03-p14763107
Bn-A03-p15704793	0.970149254	0.980099502	Bn-N3-p16052583	Bn-chrA03-p14764958
Bn-A03-p15704830	0.980099502	0.980099502	Bn-N3-p16052620	Bn-chrA03-p14764995
Bn-A03-p15705021	0.985074627	0.980099502	Bn-N3-p16052811	Bn-chrA03-p14765186
Bn-A03-p15708192	0.990033223	0.986710963	Bn-N3-p16057209	Bn-chrA03-p14767940
Bn-A03-p15708289	1	1	Bn-N3-p16057306	Bn-chrA03-p14768037
Bn-A03-p15725925	0.983471074	0.983471074	Bn-N3-p16075709	Bn-chrA03-p14790995
Bn-A03-p15739646	0.996677741	1	Bn-N3-p16090079	Bn-chrA03-p14810068
Bn-A03-p15744983	1	0.991735537	Bn-N3-p16097061	Bn-chrA03-p14818508
Bn-A03-p15748819	0.930232558	0.993355482	Bn-N3-p16098680	Bn-chrA03-p14820700
Bn-A03-p15749083	0.983471074	0.983471074	Bn-N3-p16098951	Bn-chrA03-p14820971
Bn-A03-p15766386	0.970099668	0.973421927	Bn-N3-p16118711	Bn-chrA03-p14839068
Bn-A03-p15768536	0.966942149	0.975206612	Bn-N3-p16121133	Bn-chrA03-p14841203
Bn-A03-p15776521	0.965174129	0.965174129	Bn-N3-p16129121	Bn-chrA03-p14849189
Bn-A03-p15776756	0.960199005	0.980099502	Bn-N3-p16129355	Bn-chrA03-p14849424
Bn-A03-p15801208	0.975206612	0.983471074	Bn-N3-p16156278	Bn-chrA03-p14875540
Bn-A03-p15811901	0.970149254	0.92039801	Bn-N3-p16168657	Bn-chrA03-p14886258
Bn-A03-p15815965	0.975206612	0.975206612	Bn-N3-p16173526	Bn-chrA03-p14892225
Bn-A03-p15818896	0.990033223	0.986710963	Bn-N3-p16176575	Bn-chrA03-p14895228
Bn-A03-p15824796	0.975206612	0.983471074	Bn-N3-p16183335	Bn-chrA03-p14904247

Bn-A03-p15827113	0.966942149	0.966942149	Bn-N3-p16185502	Bn-chrA03-p14906392
Bn-A03-p15828397	0.975206612	0.975206612	Bn-N3-p16186372	Bn-chrA03-p14911926
Bn-A03-p15852439	0.958677686	0.94214876	Bn-N3-p16210478	Bn-chrC03-p21877502
Bn-A03-p15853765	0.983471074	0.983471074	Bn-N3-p16211799	Bn-chrA03-p14939064
Bn-A03-p15871284	0.991735537	0.983471074	Bn-N3-p16228427	Bn-chrA03-p14958234
Bn-A03-p15875404	0.991735537	0.983471074	Bn-N3-p16232548	Bn-chrA03-p14967684
Bn-A03-p15892296	0.820895522	0.965174129	Bn-N3-p16242827	Bn-chrA03-p14976419
Bn-A03-p15893952	0.966942149	0.991735537	Bn-N3-p16244534	Bn-chrA03-p14978075
Bn-A03-p15894013	0.958677686	0.966942149	Bn-N3-p16244593	Bn-chrA03-p14978136
Bn-A03-p15900189	0.958677686	0.958677686	Bn-N3-p16247749	Bn-chrA03-p14981287
Bn-A03-p15906810	0.966942149	0.991735537	Bn-N3-p16254855	Bn-chrA03-p14987907
Bn-A03-p15916054	0.991735537	0.991735537	Bn-N3-p16264170	Bn-chrA03-p14995997
Bn-A03-p15916258	0.983471074	0.975206612	Bn-N3-p16265739	Bn-chrA03-p14996201
Bn-A03-p15923626	0.933884298	0.94214876	Bn-N13-p25175440	Bn-chrC03-p21953935
Bn-A03-p15936354	0.975206612	0.917355372	Bn-N3-p16290028	Bn-chrC03-p21987876
Bn-A03-p15954297	0.975206612	0.975206612	Bn-N3-p16309095	Bn-chrA03-p15032340
Bn-A03-p15957657	0.980066445	0.980066445	Bn-N3-p16312745	Bn-chrA03-p15038093
Bn-A03-p15958035	0.986710963	0.950166113	Bn-N3-p16313138	Bn-chrA03-p15038486
Bn-A03-p15960001	0.851239669	0.909090909	Bn-N3-p16314717	Bn-chrA03-p15040275
Bn-A03-p15960376	0.94214876	0.933884298	Bn-N3-p16315082	Bn-chrA03-p15040656
Bn-A03-p15968189	0.917355372	0.909090909	Bn-N3-p16323237	Bn-chrA03-p15048544
Bn-A03-p15973005	0.94214876	0.950413223	Bn-N3-p16328200	Bn-chrA03-p15053001
Bn-A03-p15974991	0.980066445	0.970099668	Bn-N3-p16330280	Bn-chrC03-p22601568
Bn-A03-p15979252	0.983471074	0.826446281	Bn-N3-p16334534	Bn-chrC05-p39962947
Bn-A03-p15983019	0.94214876	0.950413223	Bn-N3-p16338309	Bn-chrA03-p15063006
Bn-A03-p16000194	0.84717608	0.84717608	Bn-N13-p25301116	Bn-chrC03-p22070063
Bn-A03-p16017186	0.983471074	0.991735537	Bn-N3-p16376636	Bn-chrA03-p15100308
Bn-A03-p16038553	0.958677686	0.950413223	Bn-N3-p16401172	Bn-chrA03-p15121067
Bn-A03-p16051661	0.976744186	0.973421927	Bn-N3-p16417695	Bn-chrA03-p15132985
Bn-A03-p16055930	0.865671642	0.870646766	Bn-N3-p16422048	Bn-chrA03-p15137247
Bn-A03-p16056039	0.857142857	0.986710963	Bn-N13-p25412548	Bn-chrA03-p15137356
Bn-A03-p16056249	0.885572139	0.92039801	Bn-N3-p16422373	Bn-chrA03-p15137559
Bn-A03-p16056460	0.950248756	0.945273632	Bn-N3-p16422587	Bn-chrA03-p15137774
Bn-A03-p16056621	0.996677741	0.996677741	Bn-N3-p16422754	Bn-chrA03-p15137941
Bn-A03-p16070543	0.917355372	0.917355372	Bn-N3-p16436635	Bn-chrA03-p15158606
Bn-A03-p16079386	1	0.991735537	Bn-N3-p16445149	Bn-chrA03-p15178485
Bn-A03-p16081434	0.933884298	0.94214876	Bn-N3-p16447196	Bn-chrA03-p15180531
Bn-A03-p16084302	0.991735537	0.983471074	Bn-N3-p16450065	Bn-chrA03-p15185381
Bn-A03-p16098207	0.980099502	0.980099502	Bn-N3-p16463667	Bn-chrA03_random-p115497
Bn-A03-p16103891	0.876033058	0.884297521	Bn-N3-p16469347	Bn-chrA03-p15206003
Bn-A03-p16123758	0.975206612	0.966942149	Bn-N3-p16484258	Bn-chrA03-p15217517
Bn-A03-p16126013	0.975206612	0.966942149	Bn-N3-p16486504	Bn-chrA03-p15219771
Bn-A03-p16134415	0.983471074	0.991735537	Bn-N3-p16494416	Bn-chrA03-p15228244
Bn-A03-p16158248	0.995024876	1	Bn-N3-p16525656	Bn-chrA03-p15252805
Bn-A03-p16162908	0.935323383	0.935323383	Bn-N3-p16530314	Bn-chrA03-p15257415
Bn-A03-p16167780	0.975124378	0.970149254	Bn-N11-p27925414	Bn-chrCnn_random-p575252

Bn-A03-p16170389	0.995024876	0.995024876	Bn-N7-p21285723	Bn-chrCnn_random-p575278
Bn-A03-p16185537	0.990033223	0.990033223	Bn-N3-p16558996	Bn-chrA03-p15274207
Bn-A03-p16190643	0.991735537	0.983471074	Bn-N3-p16565378	Bn-chrA03-p15279515
Bn-A03-p16211692	0.986710963	0.986710963	Bn-N3-p16583768	Bn-chrA03-p15297400
Bn-A03-p16229294	1	1	Bn-N3-p16606777	Bn-chrA03-p15317746
Bn-A03-p16262184	0.985074627	0.985074627	Bn-N3-p16633307	Bn-chrA03-p15358577
Bn-A03-p16269745	0.826446281	0.966942149	Bn-N3-p16649156	Bn-chrA03-p15401357
Bn-A03-p16275650	0.960199005	0.955223881	Bn-N3-p16651582	Bn-chrA03-p15403873
Bn-A03-p16278251	0.991735537	0.991735537	Bn-N3-p16653377	Bn-chrA03-p15405667
Bn-A03-p16284595	0.983471074	0.991735537	Bn-N3-p16660095	Bn-chrA03-p15412046
Bn-A03-p16289300	0.995024876	0.995024876	Bn-N11-p37350963	Bn-chrC01-p32558508
Bn-A03-p16301988	0.970149254	0.965174129	Bn-N3-p16677585	Bn-chrA03-p15428378
Bn-A03-p16303181	0.975124378	0.950248756	Bn-N3-p16680216	Bn-chrA03-p15429784
Bn-A03-p16303780	0.983471074	0.991735537	Bn-N3-p16680815	Bn-chrA03-p15430383
Bn-A03-p16342771	0.975124378	0.995024876	Bn-N3-p16718081	Bn-chrA03-p15465411
Bn-A03-p16355867	0.996677741	0.996677741	Bn-N3-p16731159	Bn-chrA03-p15484314
Bn-A03-p16371467	0.966942149	0.975206612	Bn-N3-p16745660	Bn-chrA03-p15498700
Bn-A03-p16378233	0.975206612	0.975206612	Bn-N3-p16753163	Bn-chrA03-p15506566
Bn-A03-p16379045	0.991735537	0.991735537	Bn-N3-p16753948	Bn-chrA03-p15507351
Bn-A03-p16380304	0.970149254	0.940298507	Bn-N3-p16755208	Bn-chrA03-p15508568
Bn-A03-p16404390	0.990049751	0.995024876	Bn-N3-p16774110	Bn-chrA03-p15546010
Bn-A03-p16404538	0.980099502	0.985074627	Bn-N3-p16774256	Bn-chrA03-p15546156
Bn-A03-p16404871	1	1	Bn-N3-p16774587	Bn-chrA03-p15546489
Bn-A03-p16405269	0.983471074	0.991735537	Bn-N3-p16774984	Bn-chrA03-p15546889
Bn-A03-p16409353	0.975206612	0.966942149	Bn-N3-p16781840	Bn-chrA03-p15552252
Bn-A03-p16416948	0.813953488	0.790697674	Bn-N3-p16792476	Bn-chrC03-p22981953
Bn-A03-p16422604	0.985074627	0.990049751	Bn-N3-p16798020	Bn-chrA03-p15564954
Bn-A03-p16423239	0.950248756	0.955223881	Bn-N3-p16798655	Bn-chrA03-p15565589
Bn-A03-p16431100	0.950413223	0.958677686	Bn-N3-p16801093	Bn-chrA03-p15568261
Bn-A03-p16437979	0.950413223	0.958677686	Bn-N3-p16805278	Bn-chrA03-p15573072
Bn-A03-p16444510	0.958677686	0.983471074	Bn-N3-p16812048	Bn-chrA03-p15580412
Bn-A03-p16454331	0.958677686	0.966942149	Bn-N3-p16821698	Bn-chrA03-p15594658
Bn-A03-p16481995	0.996677741	0.996677741	Bn-N3-p16847571	Bn-chrA03-p15619316
Bn-A03-p16482731	0.993355482	0.990033223	Bn-N3-p16848307	Bn-chrA03-p15620052
Bn-A03-p16501946	1	0.996677741	Bn-N3-p16862460	Bn-chrA03-p15633643
Bn-A03-p16530135	0.966777409	0.980066445	Bn-N3-p16901349	Bn-chrA03-p15661187
Bn-A03-p16565487	0.970099668	0.973421927	Bn-N3-p16933963	Bn-chrA03-p15689356
Bn-A03-p16607714	0.990033223	0.986710963	Bn-N3-p16961243	Bn-chrA03-p15721886
Bn-A03-p16610235	0.970149254	0.975124378	Bn-N3-p16963783	Bn-chrA03-p15724439
Bn-A03-p16611349	0.92039801	0.925373134	Bn-N3-p16964899	Bn-chrA03-p15725556
Bn-A03-p16613382	0.985074627	0.985074627	Bn-N3-p16966918	Bn-chrA03-p15727577
Bn-A03-p16742011	0.980099502	0.980099502	Bn-N3-p17112362	Bn-chrA03-p15873548
Bn-A03-p16758311	1	0.995024876	Bn-N13-p26698823	Bn-chrC03-p23288144
Bn-A03-p16759411	0.990049751	0.995024876	Bn-N3-p17131381	Bn-chrA03-p15887505
Bn-A03-p16769908	0.966942149	0.975206612	Bn-N3-p17141747	Bn-chrC03-p23304754
Bn-A03-p16800145	0.859504132	0.933884298	Bn-N3-p6008010	Bn-chrA03-p5386822

Bn-A03-p16811562	0.975206612	0.983471074	Bn-N3-p17184568	Bn-chrA03-p15967292
Bn-A03-p16812953	0.995024876	0.990049751	Bn-N3-p17185957	Bn-chrC03-p23358259
Bn-A03-p16852209	0.970149254	0.975124378	Bn-N3-p17230620	Bn-chrA03-p16012380
Bn-A03-p16901041	0.983471074	0.991735537	Bn-N3-p17289286	Bn-chrA03-p16074819
Bn-A03-p16915803	0.983471074	0.991735537	Bn-N3-p17304476	Bn-chrAnn_random-p354377
Bn-A03-p16915877	0.983471074	0.975206612	Bn-N3-p17304550	Bn-chrAnn_random-p354377
Bn-A03-p16955732	0.996677741	0.996677741	Bn-N3-p17352306	Bn-chrA03_random-p117560
Bn-A03-p16969084	0.993355482	0.993355482	Bn-N3-p17368189	Bn-chrA03_random-p116183
Bn-A03-p16976391	0.980099502	0.980099502	Bn-N3-p17376828	Bn-chrA03-p16128690
Bn-A03-p16981919	0.975124378	0.975124378	Bn-N3-p17381095	Bn-chrA03-p16132957
Bn-A03-p16989353	0.993355482	0.993355482	Bn-N3-p17388681	Bn-chrC09-p42015689
Bn-A03-p16990947	0.996677741	0.996677741	Bn-N3-p17390270	Bn-chrA03-p16144307
Bn-A03-p17019251	0.99270073	0.99270073	Bn-N3-p17416648	Bn-chrA03-p16172981
Bn-A03-p17038064	0.990049751	0.990049751	Bn-N3-p17438283	Bn-chrA03-p16190272
Bn-A03-p17038887	0.955223881	0.955223881	Bn-N3-p17439104	Bn-chrA03-p16191093
Bn-A03-p17038965	0.933884298	0.933884298	Bn-N3-p17439181	Bn-chrA03-p16191170
Bn-A03-p17056395	0.991735537	0.991735537	Bn-N3-p17455648	Bn-chrA03-p16208595
Bn-A03-p17125715	0.97752809	0.97752809	Bn-N13-p27358796	Bn-chrC03-p23897144
Bn-A03-p17139931	0.991735537	0.991735537	Bn-N3-p17542662	Bn-chrA03-p16286257
Bn-A03-p17176782	0.950413223	0.669421488	Bn-N3-p17578953	Bn-chrA03-p16322552
Bn-A03-p17182494	0.983471074	0.975206612	Bn-N3-p17584688	Bn-chrA03-p16329522
Bn-A03-p17183616	0.979166667	1	Bn-N3-p17585847	Bn-chrA03-p16330681
Bn-A03-p17184441	0.94214876	0.94214876	Bn-N3-p17586634	Bn-chrA03-p16331471
Bn-A03-p17190733	0.669421488	0.661157025	Bn-N3-p17587615	Bn-chrA03-p16332814
Bn-A03-p17194139	0.983388704	0.983388704	Bn-N3-p17590948	Bn-chrA03-p16336164
Bn-A03-p17207547	0.859504132	0.859504132	Bn-N3-p17607592	Bn-chrA03-p16347396
Bn-A03-p17213825	1	1	Bn-N3-p17614336	Bn-chrA03-p16354140
Bn-A03-p17217528	0.975124378	0.975124378	Bn-N3-p17618294	Bn-chrA03-p16357981
Bn-A03-p17281617	0.980099502	0.980099502	Bn-N3-p17683591	Bn-chrA03-p16423339
Bn-A03-p17334423	0.995024876	0.995024876	Bn-N11-p17299239	Bn-chrC09-p42521591
Bn-A03-p17336041	0.995024876	0.985074627	Bn-N11-p17297622	Bn-chrA08_random-p276685
Bn-A03-p17532322	1	1	Bn-N3-p17889546	Bn-chrA03-p16603219
Bn-A03-p17782292	1	1	Bn-N3-p18120292	Bn-chrA03-p16830998
Bn-A03-p17790084	0.993355482	0.993355482	Bn-N3-p18124358	Bn-chrA03-p16839143
Bn-A03-p17921239	0.946843854	0.950166113	Bn-N3-p18249832	Bn-chrAnn_random-p121665
Bn-A03-p17926123	0.986710963	0.96013289	Bn-N3-p18254716	Bn-chrAnn_random-p121714
Bn-A03-p17985410	0.991735537	1	Bn-N3-p18334835	Bn-chrA03-p16992552
Bn-A03-p17986611	0.975206612	0.983471074	Bn-N3-p18336036	Bn-chrA03-p16993754
Bn-A03-p18014273	0.983388704	0.983388704	Bn-N13-p28957446	Bn-chrC03-p25498760
Bn-A03-p18019822	0.993355482	0.993355482	Bn-N3-p18370833	Bn-chrA03-p17025696
Bn-A03-p18025565	0.975206612	0.975206612	Bn-N3-p18376463	Bn-chrA03-p17031139
Bn-A03-p18025753	0.983471074	0.983471074	Bn-N3-p18376651	Bn-chrA03-p17031327
Bn-A03-p18120938	0.993355482	1	Bn-N3-p18475063	Bn-chrA03-p17121293
Bn-A03-p18183550	0.960199005	0.965174129	Bn-N3-p18538515	Bn-chrA03-p17180232
Bn-A03-p18198495	0.972413793	0.993103448	Bn-N3-p18559964	Bn-chrA03-p17194059
Bn-A03-p18208425	0.990033223	0.996677741	Bn-N3-p18571426	Bn-chrA03-p17203873

Bn-A03-p18376493	0.993355482	0.990033223	Bn-N3-p18740982	Bn-chrA03-p17376478
Bn-A03-p18383780	0.892561983	0.958677686	Bn-N16-p11506053	Bn-chrAnn_random-p369448
Bn-A03-p18405869	0.955223881	0.955223881	Bn-N3-p18771675	Bn-chrA03-p17402330
Bn-A03-p18414643	0.983471074	0.983471074	Bn-N3-p18780518	Bn-chrA03-p17411173
Bn-A03-p18451316	0.940298507	0.940298507	Bn-N3-p18813514	Bn-chrA03-p17444341
Bn-A03-p18455020	0.991735537	0.991735537	Bn-N3-p18817217	Bn-chrA03-p17448044
Bn-A03-p18455932	0.983471074	0.983471074	Bn-N3-p18818191	Bn-chrA03-p17449059
Bn-A03-p18461597	0.545454545	1	Bn-N3-p18828574	Bn-chrA03-p17459850
Bn-A03-p18461778	0.983471074	0.975206612	Bn-N3-p18828755	Bn-chrA03-p17460031
Bn-A03-p18462261	0.975206612	0.966942149	Bn-N3-p18829258	Bn-chrA03-p17460514
Bn-A03-p18468633	0.958677686	0.950413223	Bn-N3-p18835812	Bn-chrA03-p17470248
Bn-A03-p18471820	0.991735537	1	Bn-N3-p18839043	Bn-chrA03-p17473443
Bn-A03-p18570046	0.975206612	0.975206612	Bn-N3-p18949264	Bn-chrA03-p17576929
Bn-A03-p18570852	0.993355482	0.993355482	Bn-N3-p18950054	Bn-chrA03-p17577719
Bn-A03-p18571065	0.993355482	0.993355482	Bn-N3-p18950267	Bn-chrA03-p17577932
Bn-A03-p18579721	0.993355482	0.993355482	Bn-N3-p18959014	Bn-chrAnn_random-p314119
Bn-A03-p18592597	0.965174129	0.965174129	Bn-N3-p18977129	Bn-chrA03-p17606519
Bn-A03-p18593371	0.983471074	0.983471074	Bn-N3-p18977923	Bn-chrA03-p17607314
Bn-A03-p18593905	0.973421927	0.973421927	Bn-N3-p18978456	Bn-chrA03-p17607846
Bn-A03-p18594273	0.960199005	0.965174129	Bn-N3-p18978824	Bn-chrA03-p17608214
Bn-A03-p18595122	0.965174129	0.965174129	Bn-N3-p18979675	Bn-chrA03-p17609334
Bn-A03-p18602597	0.983471074	0.991735537	Bn-N3-p18986720	Bn-chrA03-p17615946
Bn-A03-p18606297	0.925619835	0.727272727	Bn-N3-p18990492	Bn-chrA03-p17620342
Bn-A03-p18618117	0.995024876	0.990049751	Bn-N3-p19000422	Bn-chrA03-p17635152
Bn-A03-p18618990	0.936877076	0.980066445	Bn-N3-p19001295	Bn-chrA03-p17636069
Bn-A03-p18622329	0.991735537	0.983471074	Bn-N3-p19005575	Bn-chrA03-p17640439
Bn-A03-p18638146	0.958677686	0.950413223	Bn-N3-p19022428	Bn-chrA03-p17661656
Bn-A03-p18668408	0.990033223	0.990033223	Bn-N3-p19056204	Bn-chrA03-p17695093
Bn-A03-p18682521	0.975206612	0.983471074	Bn-N3-p19069163	Bn-chrA03-p17710827
Bn-A03-p18692928	0.986710963	0.983388704	Bn-N3-p19078178	Bn-chrA03-p17720501
Bn-A03-p18706654	0.958677686	0.966942149	Bn-N3-p19089500	Bn-chrA03-p17732692
Bn-A03-p18737967	0.983471074	0.983471074	Bn-N3-p19109711	Bn-chrA03-p17753620
Bn-A03-p18744023	0.983471074	0.975206612	Bn-N3-p19114714	Bn-chrA03-p17758406
Bn-A03-p18744159	1	0.983471074	Bn-N3-p19114850	Bn-chrA03-p17758542
Bn-A03-p18745119	0.958677686	0.809917355	Bn-N3-p19115807	Bn-chrA03-p17761731
Bn-A03-p18746523	0.983471074	0.991735537	Bn-N3-p19117217	Bn-chrA03-p17763832
Bn-A03-p18746612	0.859504132	0.851239669	Bn-N3-p19117306	Bn-chrA03-p17763921
Bn-A03-p18746881	0.966942149	0.975206612	Bn-N3-p19117518	Bn-chrA03-p17764134
Bn-A03-p18747847	0.966942149	0.958677686	Bn-N3-p19118481	Bn-chrA03-p17768362
Bn-A03-p18749943	0.991735537	0.983471074	Bn-N3-p19120540	Bn-chrA03-p17771068
Bn-A03-p18749952	0.991735537	0.983471074	Bn-N3-p19120549	Bn-chrA03-p17771077
Bn-A03-p18750371	0.991735537	0.983471074	Bn-N3-p19120972	Bn-chrA03-p17771534
Bn-A03-p18752710	1	0.991735537	Bn-N3-p19123010	Bn-chrA03-p17773688
Bn-A03-p18753036	0.966942149	0.975206612	Bn-N3-p19123337	Bn-chrA03-p17778986
Bn-A03-p18762440	0.983471074	0.991735537	Bn-N3-p19125889	Bn-chrA03-p17779754
Bn-A03-p18762453	0.983471074	0.991735537	Bn-N3-p19125902	Bn-chrA03-p17779767

Bn-A03-p18762984	0.966942149	0.966942149	Bn-N3-p19126433	Bn-chrA03-p17780298
Bn-A03-p18764384	0.933884298	0.975206612	Bn-N3-p19133723	Bn-chrA03-p17782841
Bn-A03-p18765971	0.94214876	0.950413223	Bn-N3-p19135030	Bn-chrA03-p17784426
Bn-A03-p18766028	0.983471074	0.983471074	Bn-N3-p19135086	Bn-chrA03-p17784483
Bn-A03-p18768321	1	0.991735537	Bn-N3-p19142067	Bn-chrA03-p17786776
Bn-A03-p18770617	0.975206612	0.966942149	Bn-N3-p19144137	Bn-chrA03-p17789072
Bn-A03-p18770982	0.975206612	0.983471074	Bn-N3-p19144502	Bn-chrA03-p17789437
Bn-A03-p18775919	0.983388704	0.996677741	Bn-N3-p19147826	Bn-chrA03-p17795540
Bn-A03-p18776071	0.975206612	0.966942149	Bn-N3-p19147979	Bn-chrA03-p17795692
Bn-A03-p18782352	0.991735537	1	Bn-N3-p19154466	Bn-chrA03-p17802207
Bn-A03-p18782416	1	0.991735537	Bn-N3-p19154530	Bn-chrA03-p17802271
Bn-A03-p18788929	0.975206612	0.842975207	Bn-N3-p19161041	Bn-chrAnn_random-p27757[illegible]
Bn-A03-p18803630	0.983471074	0.983471074	Bn-N3-p19175851	Bn-chrA03-p17818297
Bn-A03-p18807150	0.983471074	0.991735537	Bn-N3-p19184017	Bn-chrA03-p17819960
Bn-A03-p18810251	0.983471074	0.991735537	Bn-N3-p19187118	Bn-chrA03-p17825134
Bn-A03-p18811487	0.991735537	0.991735537	Bn-N3-p19188356	Bn-chrA03-p17826372
Bn-A03-p18816507	0.991735537	1	Bn-N3-p19190361	Bn-chrA03-p17828374
Bn-A03-p18818925	0.983471074	0.991735537	Bn-N3-p19192932	Bn-chrA03-p17831392
Bn-A03-p18819844	1	0.991735537	Bn-N3-p19193851	Bn-chrA03-p17833894
Bn-A03-p18820592	0.975206612	0.966942149	Bn-N3-p19194595	Bn-chrA03-p17834645
Bn-A03-p18824580	0.950413223	0.826446281	Bn-N3-p19198936	Bn-chrC03-p27079542
Bn-A03-p18826478	0.991735537	0.950413223	Bn-N3-p19200841	Bn-chrC03-p27085140
Bn-A03-p18828645	0.991735537	0.991735537	Bn-N3-p19203009	Bn-chrA03-p17842421
Bn-A03-p18830716	0.993355482	0.42192691	Bn-N3-p19204262	Bn-chrA03-p17843980
Bn-A03-p18834464	0.993355482	0.986710963	Bn-N3-p19214241	Bn-chrA03-p17848899
Bn-A03-p18836509	0.970099668	0.970099668	Bn-N3-p19216285	Bn-chrA03-p17850943
Bn-A03-p18846268	0.983471074	0.983471074	Bn-N3-p19222659	Bn-chrA03-p17857317
Bn-A03-p18848732	0.976744186	0.797342193	Bn-N3-p19225129	Bn-chrC03-p27168823
Bn-A03-p18855689	0.991735537	0.991735537	Bn-N3-p19227935	Bn-chrA03-p17870301
Bn-A03-p18859928	0.983471074	0.983471074	Bn-N3-p19234769	Bn-chrA03-p17873865
Bn-A03-p18873704	0.996677741	0.990033223	Bn-N3-p19244794	Bn-chrA03-p17888587
Bn-A03-p18883097	0.965174129	0.965174129	Bn-N12-p3103504	Bn-chrC02-p3003465
Bn-A03-p18883151	0.960199005	0.960199005	Bn-N12-p3103558	Bn-chrC02-p3003519
Bn-A03-p18886205	0.140495868	0.991735537	Bn-N13-p52649385	Bn-chrC02-p4135981
Bn-A03-p18899251	0.966942149	0.966942149	Bn-N3-p19265231	Bn-chrA03-p17922920
Bn-A03-p18899284	0.966942149	0.966942149	Bn-N3-p19265264	Bn-chrA03-p17922953
Bn-A03-p19045801	0.991735537	0.991735537	Bn-N3-p19315281	Bn-chrA05-p11150711
Bn-A03-p19046345	0.983471074	0.958677686	Bn-N3-p19316901	Bn-chrA03-p17987159
Bn-A03-p19047537	0.991735537	0.991735537	Bn-N3-p19318093	Bn-chrA03-p17988350
Bn-A03-p19065774	0.983471074	0.983471074	Bn-N3-p19331196	Bn-chrA03-p18006818
Bn-A03-p19066579	0.983471074	0.983471074	Bn-N3-p19332613	Bn-chrA03-p18008235
Bn-A03-p19068030	0.983471074	0.983471074	Bn-N3-p19334061	Bn-chrA03-p18009682
Bn-A03-p19071304	0.950413223	0.950413223	Bn-N3-p19337910	Bn-chrA03-p18013530
Bn-A03-p19071572	1	1	Bn-N3-p19338172	Bn-chrA03-p18013792
Bn-A03-p19078721	0.991735537	0.983471074	Bn-N3-p19349248	Bn-chrA03-p18021097
Bn-A03-p19091667	0.993355482	0.996677741	Bn-N3-p19366750	Bn-chrA03-p18034035

Bn-A03-p19094554	0.940199336	0.96013289	Bn-N13-p31123378	Bn-chrA03-p18036907
Bn-A03-p19129006	0.983471074	0.983471074	Bn-N3-p19402033	Bn-chrA03-p18067902
Bn-A03-p19132431	1	1	Bn-N3-p19405475	Bn-chrA03-p18074556
Bn-A03-p19147644	0.96013289	0.990033223	Bn-N3-p19431402	Bn-chrA03-p18090446
Bn-A03-p19160298	0.983471074	0.983471074	Bn-N3-p19449949	Bn-chrA03-p18107297
Bn-A03-p19166062	0.983471074	0.983471074	Bn-N3-p19455741	Bn-chrA03-p18113081
Bn-A03-p19189483	0.925619835	0.925619835	Bn-N3-p19480829	Bn-chrA03-p18136329
Bn-A03-p19191616	1	0.991735537	Bn-N3-p19482961	Bn-chrA03-p18139695
Bn-A03-p19198939	0.983471074	0.991735537	Bn-N3-p19490620	Bn-chrA03-p18147813
Bn-A03-p19202899	0.991735537	1	Bn-N3-p19493925	Bn-chrA03-p18151773
Bn-A03-p19203494	0.983471074	0.975206612	Bn-N3-p19494521	Bn-chrA03-p18152368
Bn-A03-p19204483	0.991735537	1	Bn-N3-p19495568	Bn-chrA03-p18153357
Bn-A03-p19210113	1	0.991735537	Bn-N3-p19501669	Bn-chrA03-p18163211
Bn-A03-p19243266	0.950413223	0.94214876	Bn-N3-p19532856	Bn-chrA03-p18202352
Bn-A03-p19252196	0.983471074	0.975206612	Bn-N3-p19541020	Bn-chrA03-p18215419
Bn-A03-p19254792	1	0.991735537	Bn-N3-p19545827	Bn-chrA03-p18217997
Bn-A03-p19274997	0.991735537	1	Bn-N3-p19566361	Bn-chrA03_random-p123918
Bn-A03-p19323656	0.990033223	0.920265781	Bn-N3-p19616931	Bn-chrA03-p18289236
Bn-A03-p19333794	0.96345515	0.946843854	Bn-N3-p19627079	Bn-chrA03-p18299416
Bn-A03-p19344007	0.995024876	0.995024876	Bn-N3-p19638274	Bn-chrA03_random-p123498
Bn-A03-p19359327	1	0.983471074	Bn-N3-p19653242	Bn-chrAnn_random-p400360
Bn-A03-p19366010	0.975206612	0.983471074	Bn-N3-p19659776	Bn-chrA03-p18347634
Bn-A03-p19375820	0.991735537	0.933884298	Bn-N3-p19669593	Bn-chrA03-p18356183
Bn-A03-p19380485	0.996677741	0.993355482	Bn-N3-p19674387	Bn-chrA03-p18359770
Bn-A03-p19380801	0.983471074	0.983471074	Bn-N3-p19674703	Bn-chrA03-p18360083
Bn-A03-p19389076	0.909090909	0.909090909	Bn-N3-p19683020	Bn-chrA03-p18368332
Bn-A03-p19408999	0.996677741	0.996677741	Bn-N3-p19703464	Bn-chrAnn_random-p270688
Bn-A03-p19438796	0.983471074	0.983471074	Bn-N3-p19733694	Bn-chrA03-p18417565
Bn-A03-p19441544	0.991735537	0.991735537	Bn-N3-p19736425	Bn-chrA03-p18420321
Bn-A03-p19441620	0.975206612	0.983471074	Bn-N3-p19736501	Bn-chrA03-p18420397
Bn-A03-p19442945	0.986710963	0.986710963	Bn-N3-p19737829	Bn-chrA03-p18421722
Bn-A03-p19462036	0.986710963	0.996677741	Bn-N3-p19752040	Bn-chrA03-p18437254
Bn-A03-p19475069	0.966942149	0.966942149	Bn-N3-p19766652	Bn-chrC03-p27134508
Bn-A03-p19501529	0.983471074	0.851239669	Bn-N3-p19795423	Bn-chrA03-p18476014
Bn-A03-p19510486	0.975206612	0.966942149	Bn-N3-p19814265	Bn-chrA03-p18485019
Bn-A03-p19512402	0.479338843	0.776859504	Bn-N3-p19817951	Bn-chrA03-p18486688
Bn-A03-p19519674	0.980099502	0.975124378	Bn-N3-p19820402	Bn-chrC03-p28829451
Bn-A03-p19526788	0.991735537	0.983471074	Bn-N3-p19828634	Bn-chrAnn_random-p408879
Bn-A03-p19527199	0.966942149	0.975206612	Bn-N3-p19829045	Bn-chrAnn_random-p408883
Bn-A03-p19536775	1	0.991735537	Bn-N3-p19837268	Bn-chrA03-p18513798
Bn-A03-p19542946	0.979166667	0.989583333	Bn-N3-p19844593	Bn-chrA03_random-p138251
Bn-A03-p19559007	0.983471074	0.983471074	Bn-N3-p19855655	Bn-chrA03-p18521138
Bn-A03-p19560497	0.983471074	0.983471074	Bn-N3-p19857157	Bn-chrA03-p18522640
Bn-A03-p19560700	0.966942149	0.966942149	Bn-N3-p19857360	Bn-chrA03-p18522843
Bn-A03-p19564317	0.958677686	0.958677686	Bn-N3-p19861149	Bn-chrAnn_random-p416800
Bn-A03-p19564333	0.958677686	0.958677686	Bn-N3-p19861165	Bn-chrAnn_random-p416800

Bn-A03-p19573902	0.995024876	0.995024876	Bn-N3-p19870417	Bn-chrA03-p18536383
Bn-A03-p19582066	0.966942149	0.966942149	Bn-N3-p19877863	Bn-chrA03-p18543830
Bn-A03-p19727558	0.966942149	0.958677686	Bn-N3-p20009420	Bn-chrA03-p18670129
Bn-A03-p19740654	0.996677741	0.887043189	Bn-N3-p20028569	Bn-chrC03_random-p212865
Bn-A03-p19751864	0.905472637	0.905472637	Bn-N13-p32798196	Bn-chrC03_random-p214953
Bn-A03-p19752316	0.965174129	0.965174129	Bn-N3-p20057021	Bn-chrA03-p18717287
Bn-A03-p19753364	0.980099502	0.980099502	Bn-N3-p20058074	Bn-chrA03-p18718340
Bn-A03-p19753436	0.970149254	0.970149254	Bn-N3-p20058146	Bn-chrA03-p18718412
Bn-A03-p19753470	0.994764398	0.989528796	Bn-N3-p20058180	Bn-chrA03-p18718446
Bn-A03-p19766214	0.980066445	0.996677741	Bn-N3-p20070444	Bn-chrA03_random-p128529
Bn-A03-p19768447	0.983388704	1	Bn-N3-p20073818	Bn-chrA03_random-p129109
Bn-A03-p19769998	0.966942149	0.966942149	Bn-N3-p20075833	Bn-chrA03_random-p129264
Bn-A03-p19772056	0.985074627	0.980099502	Bn-N3-p20078266	Bn-chrC03-p29731278
Bn-A03-p19776651	0.958677686	0.950413223	Bn-N3-p20082865	Bn-chrA03_random-p129934
Bn-A03-p19793770	0.975206612	0.983471074	Bn-N3-p20092686	Bn-chrA03_random-p131305
Bn-A03-p19796541	0.983471074	0.975206612	Bn-N3-p20095506	Bn-chrA03_random-p131847
Bn-A03-p19798208	0.950413223	0.966942149	Bn-N3-p20097172	Bn-chrA03_random-p132524
Bn-A03-p19805933	0.976744186	0.976744186	Bn-N3-p20104897	Bn-chrA03_random-p133308
Bn-A03-p19806018	0.965174129	0.965174129	Bn-N3-p20104982	Bn-chrA03_random-p133316
Bn-A03-p19819975	0.996677741	0.996677741	Bn-N3-p20116646	Bn-chrA03_random-p134341
Bn-A03-p19832980	0.966942149	0.900826446	Bn-N3-p20133234	Bn-chrA03_random-p135935
Bn-A03-p19844661	0.955223881	0.955223881	Bn-N3-p20151796	Bn-chrA03_random-p126070
Bn-A03-p19850198	0.990033223	0.993355482	Bn-N3-p20157427	Bn-chrA03_random-p126633
Bn-A03-p19851089	0.975206612	0.966942149	Bn-N3-p20158318	Bn-chrA03_random-p126722
Bn-A03-p19868485	0.993355482	0.877076412	Bn-N3-p20178063	Bn-chrA03_random-p137381
Bn-A03-p19874058	0.983471074	0.983471074	Bn-N3-p20187358	Bn-chrA03_random-p136714
Bn-A03-p19880918	0.993355482	0.993355482	Bn-N3-p20192016	Bn-chrA03_random-p136226
Bn-A03-p19896843	0.628099174	0.966942149	Bn-N13-p33240254	Bn-chrA03-p18785981
Bn-A03-p19897251	0.996677741	0.96345515	Bn-N3-p20214153	Bn-chrA03-p18786389
Bn-A03-p19902796	0.983388704	0.983388704	Bn-N3-p20219485	Bn-chrA03-p18791928
Bn-A03-p19916449	0.983471074	0.975206612	Bn-N3-p20231441	Bn-chrA03-p18806560
Bn-A03-p19925593	0.993355482	0.990033223	Bn-N3-p20240617	Bn-chrA03-p18815285
Bn-A03-p19928269	0.970149254	0.970149254	Bn-N3-p20244850	Bn-chrA03-p18818515
Bn-A03-p19935973	0.991735537	0.983471074	Bn-N3-p20247677	Bn-chrA03-p18822319
Bn-A03-p19942172	0.983471074	0.991735537	Bn-N3-p20254056	Bn-chrA03-p18826998
Bn-A03-p19951011	0.958677686	0.966942149	Bn-N3-p20257694	Bn-chrA03-p18834424
Bn-A03-p19953514	0.980066445	0.966777409	Bn-N3-p20259977	Bn-chrA03-p18836927
Bn-A03-p19956358	0.973421927	0.973421927	Bn-N3-p20262720	Bn-chrA03-p18839740
Bn-A03-p19973423	1	1	Bn-N3-p20283079	Bn-chrA03-p18856865
Bn-A03-p19974784	0.980066445	0.976744186	Bn-N3-p20284442	Bn-chrA03-p18858226
Bn-A03-p19975539	0.950413223	0.950413223	Bn-N3-p20285197	Bn-chrA03-p18858981
Bn-A03-p20000930	0.991735537	0.991735537	Bn-N3-p20301366	Bn-chrA03-p18872717
Bn-A03-p20005216	0.990033223	0.976744186	Bn-N3-p20304899	Bn-chrA03-p18876742
Bn-A03-p20007146	0.980099502	0.995024876	Bn-N3-p20306827	Bn-chrA03-p18878672
Bn-A03-p20008868	0.980066445	0.980066445	Bn-N3-p20308557	Bn-chrA03-p18880393
Bn-A03-p20025367	0.990033223	0.990033223	Bn-N3-p20323351	Bn-chrA03-p18896888

Bn-A03-p20033408	0.965174129	0.970149254	Bn-N3-p20329049	Bn-chrA03-p18902489
Bn-A03-p20044499	1	1	Bn-N3-p20342349	Bn-chrA03-p18919847
Bn-A03-p20079160	0.975206612	0.966942149	Bn-N3-p20371279	Bn-chrA03-p18944654
Bn-A03-p20079452	0.958677686	0.94214876	Bn-N3-p20371571	Bn-chrA03-p18944946
Bn-A03-p20080800	0.993355482	0.990033223	Bn-N3-p20374281	Bn-chrA03-p18946293
Bn-A03-p20086767	0.991735537	0.983471074	Bn-N3-p20380413	Bn-chrA03-p18952375
Bn-A03-p20100490	0.983471074	0.983471074	Bn-N3-p20394143	Bn-chrA03-p18967299
Bn-A03-p20114910	0.936877076	0.887043189	Bn-N3-p20408835	Bn-chrC03-p30075856
Bn-A03-p20122616	1	1	Bn-N3-p20417490	Bn-chrA03-p18991810
Bn-A03-p20125361	0.892561983	0.991735537	Bn-N3-p20420513	Bn-chrA03-p18994551
Bn-A03-p20145024	0.991735537	0.983471074	Bn-N3-p20439108	Bn-chrA03-p19008704
Bn-A03-p20150479	1	1	Bn-N3-p20444921	Bn-chrA03-p19014167
Bn-A03-p20151244	0.958677686	0.966942149	Bn-N3-p20445824	Bn-chrA03-p19014932
Bn-A03-p20158806	0.966942149	0.975206612	Bn-N3-p20453522	Bn-chrA03-p19023816
Bn-A03-p20164751	0.966942149	0.975206612	Bn-N3-p20459489	Bn-chrA03-p19029743
Bn-A03-p20180463	0.966942149	0.966942149	Bn-N3-p20469807	Bn-chrA03-p19041723
Bn-A03-p20186336	0.975206612	0.975206612	Bn-N3-p20476750	Bn-chrA03-p19047588
Bn-A03-p20213689	0.983471074	0.975206612	Bn-N3-p20500455	Bn-chrA03-p19072481
Bn-A03-p20216550	0.966942149	0.958677686	Bn-N3-p20503412	Bn-chrA03-p19075437
Bn-A03-p20221017	0.743801653	0.950413223	Bn-N4-p3960452	Bn-chrA03-p19083192
Bn-A03-p20225335	0.986710963	0.986710963	Bn-N3-p20518496	Bn-chrA03-p19086285
Bn-A03-p20237681	1	1	Bn-N3-p20532008	Bn-chrA03-p19098609
Bn-A03-p20262635	0.985074627	0.985074627	Bn-N3-p20559209	Bn-chrA03-p19122036
Bn-A03-p20269025	0.989208633	0.989208633	Bn-N3-p20568388	Bn-chrA03-p19131214
Bn-A03-p20286113	0.975206612	0.975206612	Bn-N3-p20584427	Bn-chrA03-p19147254
Bn-A03-p20318892	0.933884298	0.933884298	Bn-N3-p20624118	Bn-chrA03-p19186406
Bn-A03-p20319347	0.991735537	0.991735537	Bn-N3-p20624573	Bn-chrA03-p19186861
Bn-A03-p20324951	0.945273632	0.945273632	Bn-N3-p20630056	Bn-chrA03-p19192489
Bn-A03-p20350690	0.960199005	0.960199005	Bn-N3-p20662632	Bn-chrA03-p19217416
Bn-A03-p20358050	0.958677686	0.958677686	Bn-N3-p20670177	Bn-chrA03-p19224959
Bn-A03-p20361721	0.996677741	0.996677741	Bn-N3-p20676335	Bn-chrA03-p19233392
Bn-A03-p20369417	0.953488372	0.953488372	Bn-N3-p20684551	Bn-chrA03-p19241450
Bn-A03-p20369521	0.980066445	0.980066445	Bn-N3-p20684656	Bn-chrA03-p19241555
Bn-A03-p20375108	0.990049751	0.990049751	Bn-N13-p34174138	Bn-chrC03-p30597076
Bn-A03-p20375514	0.991735537	1	Bn-N3-p20690914	Bn-chrA03-p19247586
Bn-A03-p20383979	0.950413223	0.950413223	Bn-N3-p20702073	Bn-chrA03-p19258847
Bn-A03-p20395531	0.94214876	0.94214876	Bn-N3-p20706806	Bn-chrA03-p19263686
Bn-A03-p20395584	0.933884298	0.933884298	Bn-N3-p20706859	Bn-chrA03-p19263739
Bn-A03-p20398340	0.975206612	0.975206612	Bn-N3-p20710100	Bn-chrA03-p19267907
Bn-A03-p20398711	0.991735537	0.991735537	Bn-N3-p20710471	Bn-chrA03-p19268278
Bn-A03-p20409233	0.983471074	0.983471074	Bn-N3-p20717493	Bn-chrA03-p19275299
Bn-A03-p20413103	1	1	Bn-N3-p20721361	Bn-chrA03-p19279166
Bn-A03-p20417630	0.993355482	0.993355482	Bn-N3-p20726042	Bn-chrA03-p19283847
Bn-A03-p20424880	1	1	Bn-N3-p20742143	Bn-chrA03-p19296130
Bn-A03-p20427554	0.996677741	0.996677741	Bn-N3-p20744331	Bn-chrA03-p19298186
Bn-A03-p20429794	0.996677741	0.996677741	Bn-N3-p20746570	Bn-chrA03-p19300425

Bn-A03-p20442807	0.917355372	0.917355372	Bn-N13-p34300822	Bn-chrC03-p30731308
Bn-A03-p20477726	0.996677741	0.996677741	Bn-N3-p20787211	Bn-chrAnn_random-p327977
Bn-A03-p20495253	0.975124378	0.975124378	Bn-N3-p20805694	Bn-chrA03-p19314986
Bn-A03-p20495700	0.975206612	0.975206612	Bn-N3-p20806147	Bn-chrA03-p19315439
Bn-A03-p20514460	0.966942149	0.966942149	Bn-N3-p20824884	Bn-chrA03-p19333900
Bn-A03-p20518761	0.966942149	0.966942149	Bn-N3-p20829184	Bn-chrA03-p19338200
Bn-A03-p20523474	0.970149254	0.970149254	Bn-N3-p20834069	Bn-chrA03-p19342919
Bn-A03-p20523585	0.990049751	0.990049751	Bn-N3-p20834180	Bn-chrA03-p19343030
Bn-A03-p20524035	0.960199005	0.960199005	Bn-N3-p20834644	Bn-chrA03-p19343494
Bn-A03-p20525474	0.892561983	0.892561983	Bn-N3-p20836118	Bn-chrA03-p19344968
Bn-A03-p20525591	0.958677686	0.958677686	Bn-N3-p20836235	Bn-chrA03-p19345085
Bn-A03-p20550791	0.950413223	0.950413223	Bn-N3-p20874537	Bn-chrA03-p19385492
Bn-A03-p20551216	0.991735537	0.991735537	Bn-N3-p20875899	Bn-chrA03-p19385881
Bn-A03-p20560569	0.996677741	0.990033223	Bn-N3-p20885422	Bn-chrA03-p19395239
Bn-A03-p20568764	0.975124378	0.985074627	Bn-N3-p20893626	Bn-chrA03-p19403427
Bn-A03-p20584351	0.983471074	0.975206612	Bn-N3-p20899843	Bn-chrA03-p19412817
Bn-A03-p20604949	0.986710963	0.986710963	Bn-N3-p20913968	Bn-chrA03-p19432686
Bn-A03-p20609910	0.966942149	0.975206612	Bn-N3-p20919598	Bn-chrA03-p19437654
Bn-A03-p20627333	0.980099502	0.975124378	Bn-N3-p20929632	Bn-chrA03-p19449512
Bn-A03-p20656717	0.991735537	1	Bn-N3-p20943128	Bn-chrA03-p19481532
Bn-A03-p20662048	0.983471074	0.975206612	Bn-N3-p20950682	Bn-chrA03-p19487582
Bn-A03-p20664670	0.996677741	0.993355482	Bn-N3-p20953283	Bn-chrA03-p19488641
Bn-A03-p20670561	0.965174129	0.955223881	Bn-N3-p20960556	Bn-chrA03-p19494699
Bn-A03-p20673486	0.983471074	0.991735537	Bn-N3-p20963481	Bn-chrA03-p19497619
Bn-A03-p20678223	0.996677741	0.996677741	Bn-N3-p20968522	Bn-chrA03-p19502658
Bn-A03-p20678571	0.983471074	0.983471074	Bn-N3-p20968870	Bn-chrA03-p19503006
Bn-A03-p20691359	1	1	Bn-N3-p20976275	Bn-chrA03-p19510294
Bn-A03-p20693752	0.910447761	0.910447761	Bn-N3-p20978666	Bn-chrA03-p19512685
Bn-A03-p20701991	0.991735537	0.991735537	Bn-N3-p20988449	Bn-chrA06-p10170617
Bn-A03-p20702469	0.996677741	0.996677741	Bn-N3-p20988927	Bn-chrA03-p19519856
Bn-A03-p20727133	0.970149254	0.970149254	Bn-N3-p21005324	Bn-chrA03-p19537073
Bn-A03-p20730830	1	1	Bn-N3-p21008614	Bn-chrA03-p19543176
Bn-A03-p20736965	0.975206612	0.975206612	Bn-N3-p21017357	Bn-chrA03-p19554479
Bn-A03-p20749815	1	1	Bn-N3-p21024958	Bn-chrA03-p19562439
Bn-A03-p20762376	1	1	Bn-N3-p21035723	Bn-chrA03-p19574024
Bn-A03-p20786060	0.958677686	0.958677686	Bn-N3-p21059443	Bn-chrA03-p19597689
Bn-A03-p20848887	0.820598007	0.820598007	Bn-N3-p21107678	Bn-chrAnn_random-p276594
Bn-A03-p20909567	0.990033223	0.993355482	Bn-N3-p21159284	Bn-chrA03-p19683897
Bn-A03-p20937268	0.980099502	0.980099502	Bn-N3-p21187651	Bn-chrA03-p19709603
Bn-A03-p20983297	0.991735537	0.909090909	Bn-N3-p21231831	Bn-chrA03-p19750034
Bn-A03-p20983398	1	0.925619835	Bn-N3-p21231932	Bn-chrA03-p19750133
Bn-A03-p21075664	0.985074627	0.985074627	Bn-N3-p21310390	Bn-chrA03-p19826658
Bn-A03-p21076805	0.970149254	0.970149254	Bn-N17-p38456629	Bn-chrCnn_random-p198736
Bn-A03-p21155851	0.990049751	0.990049751	Bn-N17-p38567519	Bn-chrC07_random-p221301
Bn-A03-p21199018	0.986710963	0.986710963	Bn-N17-p38615424	Bn-chrC07-p35243939
Bn-A03-p21205471	0.96345515	0.983388704	Bn-N3-p21448155	Bn-chrA03-p19955868

Bn-A03-p21210189	0.936877076	0.996677741	Bn-N3-p21451794	Bn-chrA03-p19959524
Bn-A03-p21225846	0.909090909	0.966942149	Bn-N3-p21474863	Bn-chrA03-p19974521
Bn-A03-p21226758	0.958677686	0.966942149	Bn-N3-p21486750	Bn-chrA03-p19990668
Bn-A03-p21234281	0.965174129	0.960199005	Bn-N3-p21496081	Bn-chrA03-p19999557
Bn-A03-p21237671	0.950248756	0.950248756	Bn-N3-p21499493	Bn-chrA03-p20002967
Bn-A03-p21238466	0.970099668	0.970099668	Bn-N3-p21500288	Bn-chrA03-p20003762
Bn-A03-p21241827	0.996677741	0.996677741	Bn-N3-p21503657	Bn-chrA03-p20007131
Bn-A03-p21252366	0.996677741	0.996677741	Bn-N3-p21524087	Bn-chrA03-p20020089
Bn-A03-p21259337	0.993355482	0.993355482	Bn-N3-p21530752	Bn-chrA03-p20028204
Bn-A03-p21271941	0.966777409	0.966777409	Bn-N3-p21543364	Bn-chrA03-p20040918
Bn-A03-p21283570	0.993355482	0.993355482	Bn-N3-p21561811	Bn-chrA03-p20052669
Bn-A03-p21284312	1	1	Bn-N3-p21562554	Bn-chrA03-p20053412
Bn-A03-p21292773	0.965174129	0.965174129	Bn-N3-p21568955	Bn-chrA03-p20059088
Bn-A03-p21294836	0.983471074	0.991735537	Bn-N3-p21572010	Bn-chrA03-p20061150
Bn-A03-p21298611	0.990049751	0.985074627	Bn-N3-p21579109	Bn-chrA03-p20064926
Bn-A03-p21329715	0.996677741	0.996677741	Bn-N3-p21616626	Bn-chrA03-p20095857
Bn-A03-p21344477	0.966942149	0.966942149	Bn-N3-p21637085	Bn-chrA03-p20116086
Bn-A03-p21353686	0.993355482	0.993355482	Bn-N3-p21646759	Bn-chrA03-p20127475
Bn-A03-p21357849	0.991735537	0.991735537	Bn-N3-p21653489	Bn-chrA03-p20131640
Bn-A03-p21375969	0.834710744	0.834710744	Bn-N3-p21667099	Bn-chrA03-p20145255
Bn-A03-p21377430	0.980099502	0.980099502	Bn-N3-p21668568	Bn-chrA03-p20146724
Bn-A03-p21414316	0.983471074	0.983471074	Bn-N3-p21705306	Bn-chrA03-p20182595
Bn-A03-p21422696	0.975206612	0.975206612	Bn-N3-p21713680	Bn-chrA03-p20189818
Bn-A03-p21428952	0.975206612	0.975206612	Bn-N3-p21720667	Bn-chrA03-p20196337
Bn-A03-p21436299	0.975206612	0.975206612	Bn-N3-p21728055	Bn-chrA03-p20203724
Bn-A03-p21478889	1	0.991735537	Bn-N3-p21745571	Bn-chrA03-p20250602
Bn-A03-p21487106	0.983471074	0.975206612	Bn-N3-p21756700	Bn-chrA03-p20263316
Bn-A03-p21503102	0.636363636	0.991735537	Bn-N13-p55033728	Bn-chrA03-p20291941
Bn-A03-p21504871	0.747508306	0.747508306	Bn-N17-p39038160	Bn-chrC07-p35598050
Bn-A03-p21505363	0.991735537	0.991735537	Bn-N3-p21779136	Bn-chrA03-p20289680
Bn-A03-p21505431	0.983471074	0.983471074	Bn-N3-p21779068	Bn-chrA03-p20289612
Bn-A03-p21505440	0.983471074	0.983471074	Bn-N3-p21779059	Bn-chrA03-p20289603
Bn-A03-p21505530	0.991735537	0.991735537	Bn-N3-p21778969	Bn-chrA03-p20289513
Bn-A03-p21507834	0.990033223	0.986710963	Bn-N3-p21776664	Bn-chrA03-p20287209
Bn-A03-p21507915	0.983471074	0.975206612	Bn-N3-p21776583	Bn-chrA03-p20287128
Bn-A03-p21508594	0.975206612	0.966942149	Bn-N3-p21775904	Bn-chrA03-p20286449
Bn-A03-p21536745	0.966942149	0.966942149	Bn-N3-p21807639	Bn-chrA03-p20318747
Bn-A03-p21540310	0.696517413	0.696517413	Bn-N3-p21808832	Bn-chrA03-p20319940
Bn-A03-p21570061	0.966942149	0.975206612	Bn-N3-p21837255	Bn-chrA03-p20352154
Bn-A03-p21614714	0.983471074	0.983471074	Bn-N3-p21884755	Bn-chrA03-p20403566
Bn-A03-p21615990	0.991735537	0.991735537	Bn-N3-p21886031	Bn-chrA03-p20404842
Bn-A03-p21619999	0.975206612	0.975206612	Bn-N3-p21886667	Bn-chrA03-p20405477
Bn-A03-p21632587	0.975206612	0.983471074	Bn-N3-p21900335	Bn-chrA03-p20419327
Bn-A03-p21660490	0.842975207	0.842975207	Bn-N3-p21928563	Bn-chrA03-p20447526
Bn-A03-p21666750	0.694214876	0.694214876	Bn-N3-p21930097	Bn-chrA03-p20449060
Bn-A03-p21666983	0.983471074	0.983471074	Bn-N3-p21930330	Bn-chrA03-p20449293

Bn-A03-p21669774	0.925619835	0.925619835	Bn-N3-p21934136	Bn-chrA03-p20452811
Bn-A03-p21675680	0.900826446	0.892561983	Bn-N3-p21940995	Bn-chrA03-p20459778
Bn-A03-p21676845	0.958677686	0.958677686	Bn-N3-p21941960	Bn-chrA03-p20460743
Bn-A03-p21685480	0.975206612	0.975206612	Bn-N3-p21947903	Bn-chrA03-p20466685
Bn-A03-p21744993	0.983471074	0.975206612	Bn-N3-p22019268	Bn-chrA03-p20527712
Bn-A03-p21750559	0.983471074	0.975206612	Bn-N3-p22024535	Bn-chrC02-p18048192
Bn-A03-p21750922	0.983471074	0.975206612	Bn-N3-p22024898	Bn-chrC02-p18047829
Bn-A03-p21752115	0.975206612	0.983471074	Bn-Scaffold07531-p7	Bn-chrC02-p18046685
Bn-A03-p21752426	0.966942149	0.958677686	Bn-Scaffold07531-p4	Bn-chrC02-p18046375
Bn-A03-p21770898	0.975206612	0.966942149	Bn-N3-p22030426	Bn-chrA03-p20553117
Bn-A03-p21771317	0.983471074	0.975206612	Bn-N3-p22031904	Bn-chrA03-p20553537
Bn-A03-p21782249	0.983471074	0.991735537	Bn-N3-p22041349	Bn-chrA03-p20564490
Bn-A03-p21783015	0.975206612	0.983471074	Bn-N3-p22042117	Bn-chrA03-p20565294
Bn-A03-p21786943	0.975206612	0.909090909	Bn-N3-p22045211	Bn-chrC07-p35846713
Bn-A03-p21787407	0.991735537	1	Bn-N3-p22047478	Bn-chrA03-p20570287
Bn-A03-p21787813	0.842975207	0.975206612	Bn-N3-p22047905	Bn-chrA03-p20570693
Bn-A03-p21798303	0.933554817	0.950166113	Bn-N3-p22053427	Bn-chrA03-p20581347
Bn-A03-p21813117	1	0.991735537	Bn-N3-p22063994	Bn-chrA03-p20591588
Bn-A03-p21820847	0.966942149	0.966942149	Bn-N3-p22073156	Bn-chrA03-p20604725
Bn-A03-p21830330	0.975124378	0.990049751	Bn-N3-p22082635	Bn-chrA03-p20615561
Bn-A03-p21832912	0.983471074	0.975206612	Bn-N3-p22085294	Bn-chrA03-p20618213
Bn-A03-p21839200	0.950413223	0.958677686	Bn-N3-p22091569	Bn-chrA03-p20624435
Bn-A03-p21841965	0.991735537	0.983471074	Bn-N3-p22094376	Bn-chrA03-p20627151
Bn-A03-p21846413	0.917355372	0.975206612	Bn-N3-p22104485	Bn-chrA03-p20631563
Bn-A03-p21860400	0.958677686	0.917355372	Bn-N3-p22117895	Bn-chrA03-p20650444
Bn-A03-p21862988	0.940298507	0.940298507	Bn-N3-p22120476	Bn-chrA03-p20653028
Bn-A03-p21864294	0.980099502	0.985074627	Bn-N3-p22121774	Bn-chrA03-p20654324
Bn-A03-p21904842	0.991735537	0.991735537	Bn-N3-p22166287	Bn-chrA03-p20689461
Bn-A03-p21907151	0.975206612	0.983471074	Bn-N3-p22170891	Bn-chrA03-p20691784
Bn-A03-p21909917	0.975206612	0.975206612	Bn-N3-p22173162	Bn-chrA03-p20694065
Bn-A03-p21921653	0.993355482	0.993355482	Bn-N3-p22190031	Bn-chrA03-p20715719
Bn-A03-p21927724	0.995024876	0.995024876	Bn-N3-p22195846	Bn-chrA03-p20721533
Bn-A03-p21937030	0.975206612	0.975206612	Bn-N3-p22206595	Bn-chrA03-p20731195
Bn-A03-p21941473	0.966942149	0.966942149	Bn-N3-p22214643	Bn-chrA03-p20740672
Bn-A03-p21942870	0.991735537	0.991735537	Bn-N3-p22215886	Bn-chrA03-p20741915
Bn-A03-p21945987	0.975206612	0.975206612	Bn-N3-p22219002	Bn-chrA03-p20745031
Bn-A03-p21964085	0.966942149	0.966942149	Bn-N3-p22234670	Bn-chrA03-p20761367
Bn-A03-p21967946	0.94214876	0.933884298	Bn-N3-p22241682	Bn-chrC07-p36077540
Bn-A03-p21979477	0.970149254	0.970149254	Bn-N3-p22258835	Bn-chrA03-p20795987
Bn-A03-p21980336	0.955223881	0.955223881	Bn-N3-p22259691	Bn-chrA03-p20796843
Bn-A03-p22002284	0.986710963	0.986710963	Bn-N3-p22286259	Bn-chrA03-p20829304
Bn-A03-p22003165	0.991735537	0.983471074	Bn-N3-p22287140	Bn-chrA03-p20830211
Bn-A03-p22012070	0.975206612	0.983471074	Bn-N3-p22296381	Bn-chrA03-p20837107
Bn-A03-p22014622	0.983471074	0.983471074	Bn-N3-p22297575	Bn-chrA03-p20838305
Bn-A03-p22028778	0.996677741	0.996677741	Bn-N3-p22308052	Bn-chrA03-p20848772
Bn-A03-p22065637	0.996677741	0.996677741	Bn-N3-p22341029	Bn-chrA03_random-p974908

Bn-A03-p22072036	1	0.991735537	Bn-N3-p22348081	Bn-chrA03-p20889934
Bn-A03-p22088201	0.975206612	0.966942149	Bn-N3-p22360867	Bn-chrC07-p36224441
Bn-A03-p22090414	1	0.990033223	Bn-N3-p22363065	Bn-chrA03-p20922633
Bn-A03-p22098429	0.976744186	0.993355482	Bn-N3-p22371208	Bn-chrA03-p20929981
Bn-A03-p22102124	0.996677741	0.990033223	Bn-N3-p22374903	Bn-chrA03-p20933676
Bn-A03-p22112518	0.915422886	0.980099502	Bn-N3-p22385031	Bn-chrC07-p36246860
Bn-A03-p22121980	0.966942149	0.966942149	Bn-N3-p22393687	Bn-chrAnn_random-p421805
Bn-A03-p22125583	0.966942149	0.966942149	Bn-N3-p22398362	Bn-chrA03-p20956967
Bn-A03-p22132164	0.933884298	0.933884298	Bn-N3-p22403764	Bn-chrA03-p20962368
Bn-A03-p22132555	0.991735537	0.991735537	Bn-N3-p22404155	Bn-chrA03-p20962759
Bn-A03-p22132662	0.983471074	0.983471074	Bn-N3-p22404262	Bn-chrA03-p20962866
Bn-A03-p22138059	0.983471074	0.983471074	Bn-N3-p22410574	Bn-chrA03-p20968376
Bn-A03-p22145945	0.975206612	0.975206612	Bn-N3-p22417163	Bn-chrA03-p20974965
Bn-A03-p22149000	0.991735537	0.991735537	Bn-N3-p22420219	Bn-chrA03-p20976730
Bn-A03-p22164764	0.983471074	0.983471074	Bn-N3-p22439014	Bn-chrA03-p20995340
Bn-A03-p22183905	0.993355482	0.993355482	Bn-N3-p22469046	Bn-chrCnn_random-p601164
Bn-A03-p22227279	0.950413223	0.760330579	Bn-N3-p22513871	Bn-chrAnn_random-p414686
Bn-A03-p22237846	0.983471074	0.983471074	Bn-N3-p22526046	Bn-chrA03_random-p146393
Bn-A03-p22238801	0.991735537	0.991735537	Bn-N3-p22527001	Bn-chrA03_random-p146297
Bn-A03-p22272850	0.991735537	0.983471074	Bn-N3-p22554910	Bn-chrA03-p21056512
Bn-A03-p22285105	0.983388704	0.950166113	Bn-N3-p22561791	Bn-chrC07-p36382659
Bn-A03-p22299476	0.991735537	1	Bn-N3-p22576660	Bn-chrA03-p21073992
Bn-A03-p22300372	0.993355482	0.990033223	Bn-N3-p22577562	Bn-chrA03-p21074893
Bn-A03-p22304555	0.993355482	0.993355482	Bn-N3-p22580370	Bn-chrA03-p21079049
Bn-A03-p22306212	0.975206612	0.975206612	Bn-N3-p22582859	Bn-chrA03-p21081539
Bn-A03-p22306743	0.995024876	0.995024876	Bn-N3-p22583395	Bn-chrA03-p21082075
Bn-A03-p22313737	1	0.990033223	Bn-N3-p22591655	Bn-chrA03-p21088716
Bn-A03-p22323976	0.966942149	0.991735537	Bn-N3-p22603974	Bn-chrA03-p21101718
Bn-A03-p22339476	0.958677686	0.966942149	Bn-N3-p22622633	Bn-chrA03-p21115201
Bn-A03-p22339742	0.975206612	0.958677686	Bn-N3-p22623918	Bn-chrA03-p21115465
Bn-A03-p22349705	0.975206612	0.966942149	Bn-N3-p22635786	Bn-chrA03-p21129669
Bn-A03-p22369823	0.897009967	0.897009967	Bn-N3-p22656279	Bn-chrA03-p21148581
Bn-A03-p22378361	0.975206612	0.966942149	Bn-N3-p22664130	Bn-chrA03-p21155869
Bn-A03-p22382259	0.985074627	0.980099502	Bn-N3-p22667980	Bn-chrA03-p21160052
Bn-A03-p22382828	0.985074627	0.990049751	Bn-N3-p22668550	Bn-chrA03-p21160622
Bn-A03-p22385937	1	1	Bn-N3-p22671584	Bn-chrA03-p21163249
Bn-A03-p22398141	0.993355482	0.986710963	Bn-N3-p22691366	Bn-chrA03-p21175751
Bn-A03-p22465067	0.950413223	0.94214876	Bn-N3-p22764523	Bn-chrA03-p21249639
Bn-A03-p22465690	0.983471074	0.991735537	Bn-N3-p22765146	Bn-chrA03-p21250261
Bn-A03-p22465764	0.950413223	0.94214876	Bn-N3-p22765220	Bn-chrA03-p21250335
Bn-A03-p22506637	0.950248756	0.950248756	Bn-N3-p22799247	Bn-chrA03-p21284156
Bn-A03-p22556722	0.958677686	0.966942149	Bn-N3-p22848336	Bn-chrA03-p21324726
Bn-A03-p22574983	0.975206612	0.966942149	Bn-N3-p22867361	Bn-chrA03-p21347106
Bn-A03-p22582234	0.991735537	0.983471074	Bn-Scaffold61537-p1	Bn-chrA03-p21359730
Bn-A03-p22582673	0.975206612	0.966942149	Bn-N3-p22880397	Bn-chrA03-p21360130
Bn-A03-p22582862	0.966942149	0.958677686	Bn-N3-p22880586	Bn-chrA03-p21360319

Bn-A03-p22588041	0.960199005	0.960199005	Bn-N3-p22886076	Bn-chrA03-p21365763
Bn-A03-p22590442	0.983471074	0.975206612	Bn-N3-p22888749	Bn-chrA03-p21368492
Bn-A03-p22595213	0.966942149	0.975206612	Bn-N3-p22893279	Bn-chrA03-p21388093
Bn-A03-p22610222	0.975206612	0.983471074	Bn-N3-p22902373	Bn-chrA03-p21396964
Bn-A03-p22614031	0.985074627	0.985074627	Bn-N19-p51968890	Bn-chrC09-p44895165
Bn-A03-p22619997	0.975206612	0.966942149	Bn-N3-p22908167	Bn-chrA03-p21405747
Bn-A03-p22626240	0.975206612	0.983471074	Bn-N3-p22918251	Bn-chrA03-p21413361
Bn-A03-p22627731	0.975124378	0.980099502	Bn-N3-p22919687	Bn-chrA03-p21415615
Bn-A03-p22628435	0.970149254	0.975124378	Bn-N3-p22920391	Bn-chrA03-p21416319
Bn-A03-p22630782	0.985074627	0.990049751	Bn-N3-p22922739	Bn-chrA03-p21418664
Bn-A03-p22633445	1	0.983471074	Bn-N3-p22925476	Bn-chrA03-p21425476
Bn-A03-p22635426	0.966942149	0.884297521	Bn-N3-p22927684	Bn-chrA03-p21426037
Bn-A03-p22643433	0.975206612	0.983471074	Bn-N3-p22936404	Bn-chrA03-p21435516
Bn-A03-p22651625	0.958677686	0.975206612	Bn-N3-p22938039	Bn-chrA03-p21436946
Bn-A03-p22655575	0.980099502	0.980099502	Bn-N3-p22944155	Bn-chrA03-p21441167
Bn-A03-p22679507	0.996677741	0.990033223	Bn-N3-p22977266	Bn-chrA03-p21476978
Bn-A03-p22708175	0.989583333	0.979166667	Bn-N3-p23017141	Bn-chrA03-p21509537
Bn-A03-p22730208	1	1	Bn-N17-p40622325	Bn-chrC07-p36964276
Bn-A03-p22737711	0.975206612	0.809917355	Bn-N3-p23047591	Bn-chrA03-p21527219
Bn-A03-p22739253	0.990049751	0.985074627	Bn-N3-p23049133	Bn-chrA03-p21528867
Bn-A03-p22739529	1	0.995024876	Bn-N3-p23049409	Bn-chrA03-p21529143
Bn-A03-p22744766	0.983471074	0.958677686	Bn-N3-p23054682	Bn-chrA03_random-p150610
Bn-A03-p22750972	0.991735537	1	Bn-N3-p23059076	Bn-chrA03_random-p148543
Bn-A03-p22752812	0.983471074	0.975206612	Bn-N3-p23060917	Bn-chrA03_random-p148357
Bn-A03-p22752871	0.975206612	0.958677686	Bn-N3-p23060976	Bn-chrA03_random-p148351
Bn-A03-p22776355	0.966942149	0.975206612	Bn-N3-p23085025	Bn-chrA03-p21550469
Bn-A03-p22779259	0.991735537	0.79338843	Bn-N3-p23087925	Bn-chrA03-p21552855
Bn-A03-p22816593	0.231404959	0.983471074	Unknown	Bn-chrA03-p21569717
Bn-A03-p22816713	0.20661157	0.950413223	Unknown	Bn-chrA03-p21569837
Bn-A03-p22816724	0.173553719	0.966942149	Unknown	Bn-chrA03-p21569848
Bn-A03-p22816854	0.272727273	0.958677686	Bn-N17-p17253028	Bn-chrA03-p21569975
Bn-A03-p22818464	0.991735537	0.991735537	Bn-N10-p594419	Bn-chrA10-p567708
Bn-A03-p22820800	0.727272727	0.975206612	Bn-N3-p23119525	Bn-chrA03-p21570084
Bn-A03-p22820867	0.94214876	0.975206612	Bn-N3-p23119593	Bn-chrA03-p21570151
Bn-A03-p22820943	0.925619835	0.94214876	Bn-N3-p23119669	Bn-chrA03-p21570227
Bn-A03-p22820996	0.94214876	0.933884298	Bn-N3-p23119722	Bn-chrA03-p21570280
Bn-A03-p22821346	0.983471074	0.975206612	Bn-N3-p23120072	Bn-chrA03-p21570632
Bn-A03-p22838495	0.958677686	0.925619835	Bn-N3-p23140811	Bn-chrA03-p21589296
Bn-A03-p22841929	0.945273632	0.980099502	Bn-N3-p23144235	Bn-chrA03-p21592749
Bn-A03-p22858823	0.958677686	1	Bn-N3-p23162006	Bn-chrA03-p21609255
Bn-A03-p22882585	0.94214876	0.900826446	Bn-N3-p23181434	Bn-chrC07-p37188477
Bn-A03-p22886478	1	0.991735537	Bn-N3-p23187226	Bn-chrA03-p21634473
Bn-A03-p22974798	0.970149254	0.980099502	Bn-N3-p23289690	Bn-chrA03-p21755401
Bn-A03-p22975377	0.980099502	0.980099502	Bn-N3-p23290269	Bn-chrA03-p21755980
Bn-A03-p22976259	0.966942149	0.966942149	Bn-N3-p23291222	Bn-chrA03-p21756933
Bn-A03-p22976648	0.995024876	0.995024876	Bn-N3-p23291624	Bn-chrA03-p21757335

Bn-A03-p23017354	0.989583333	0.989583333	Bn-N3-p23342059	Bn-chrA03-p21804445
Bn-A03-p23020431	0.990033223	0.990033223	Bn-N3-p23345136	Bn-chrA03-p21807520
Bn-A03-p23023607	0.970149254	0.975124378	Bn-N3-p23348315	Bn-chrA03-p21810691
Bn-A03-p23023930	0.991735537	0.983471074	Bn-N3-p23348637	Bn-chrA03-p21811014
Bn-A03-p23025381	0.983471074	0.991735537	Bn-N3-p23350100	Bn-chrA03-p21812465
Bn-A03-p23026734	0.96345515	0.993355482	Bn-N3-p23351456	Bn-chrA03-p21813819
Bn-A03-p23027465	0.851239669	0.859504132	Bn-N3-p23352211	Bn-chrA03-p21814587
Bn-A03-p23036027	0.727272727	0.727272727	Bn-N3-p23360916	Bn-chrA03-p21826154
Bn-A03-p23039421	1	1	Bn-N3-p23361108	Bn-chrA03-p21826346
Bn-A03-p23041622	0.983388704	0.990033223	Bn-N3-p23363312	Bn-chrA03-p21828549
Bn-A03-p23041853	0.966942149	0.966942149	Bn-N3-p23363543	Bn-chrA03-p21828780
Bn-A03-p23043608	0.983471074	0.983471074	Bn-N3-p23365036	Bn-chrA03-p21830276
Bn-A03-p23046592	0.966942149	0.983471074	Bn-N3-p23369171	Bn-chrA03-p21836049
Bn-A03-p23047749	0.991735537	0.983471074	Bn-N3-p23370355	Bn-chrA03-p21837220
Bn-A03-p23061800	0.990033223	0.990033223	Bn-N3-p23382997	Bn-chrA03-p21846864
Bn-A03-p23065216	0.983471074	0.983471074	Bn-N3-p23387233	Bn-chrA03-p21851100
Bn-A03-p23065321	0.983471074	0.983471074	Bn-N3-p23387338	Bn-chrA03-p21851205
Bn-A03-p23077236	0.785123967	1	Bn-N17-p41072072	Bn-chrA03-p21860333
Bn-A03-p23080487	0.991735537	1	Bn-N3-p23402061	Bn-chrA03-p21867238
Bn-A03-p23086442	0.603305785	0.991735537	Bn-N17-p41083571	Bn-chrA03-p21873194
Bn-A03-p23088716	0.986710963	0.990033223	Bn-N3-p23411579	Bn-chrA03-p21882645
Bn-A03-p23123050	0.975206612	0.975206612	Bn-N3-p23429714	Bn-chrAnn_random-p484243
Bn-A03-p23123128	0.993355482	0.993355482	Bn-N3-p23429793	Bn-chrAnn_random-p484244
Bn-A03-p23127658	0.996677741	0.990033223	Bn-N3-p23435010	Bn-chrA03-p21914225
Bn-A03-p23129458	0.991735537	1	Bn-N3-p23436824	Bn-chrA03-p21916024
Bn-A03-p23130971	0.975206612	0.975206612	Bn-N3-p23439922	Bn-chrA03-p21917686
Bn-A03-p23143134	0.983471074	0.975206612	Bn-N3-p23445947	Bn-chrA03-p21924071
Bn-A03-p23156409	0.950413223	0.950413223	Bn-N3-p23464092	Bn-chrA03-p21934819
Bn-A03-p23156512	0.975206612	0.983471074	Bn-N3-p23464194	Bn-chrA03-p21934922
Bn-A03-p23157775	0.958677686	0.958677686	Bn-N3-p23465419	Bn-chrA03-p21936147
Bn-A03-p23159350	0.983471074	0.983471074	Bn-N3-p23466996	Bn-chrA03-p21937720
Bn-A03-p23160671	0.996677741	0.996677741	Bn-N3-p23468315	Bn-chrA03-p21939038
Bn-A03-p23170401	1	0.991735537	Bn-N3-p23473792	Bn-chrA03-p21944734
Bn-A03-p23181984	0.946843854	0.950166113	Bn-N3-p23484563	Bn-chrC07-p37592381
Bn-A03-p23185327	0.933554817	0.936877076	Bn-N17-p41198220	Bn-chrC07-p37596287
Bn-A03-p23193342	0.991735537	0.983471074	Bn-N3-p23495929	Bn-chrA03-p21966789
Bn-A03-p23205791	0.996677741	0.996677741	Bn-N3-p23506348	Bn-chrA03-p21977810
Bn-A03-p23218296	0.991735537	0.991735537	Bn-N3-p23518958	Bn-chrA03-p22003574
Bn-A03-p23222657	0.833887043	0.837209302	Bn-N3-p23523310	Bn-chrA03-p22008091
Bn-A03-p23240923	0.970149254	0.970149254	Bn-N3-p23545175	Bn-chrA03-p22025982
Bn-A03-p23243420	0.975206612	0.975206612	Bn-N3-p23547636	Bn-chrA03-p22028443
Bn-A03-p23253636	0.983388704	0.983388704	Bn-N3-p23557909	Bn-chrA03-p22038478
Bn-A03-p23286977	0.991735537	0.991735537	Bn-N3-p23594045	Bn-chrA03_random-p160642
Bn-A03-p23303062	1	1	Bn-N3-p23613109	Bn-chrA03_random-p162615
Bn-A03-p23318910	0.975124378	0.975124378	Bn-N3-p23628736	Bn-chrA03_random-p164130
Bn-A03-p23319135	0.958677686	0.958677686	Bn-N3-p23628961	Bn-chrA03_random-p164153

Bn-A03-p23396260	0.980099502	0.980099502	Bn-N3-p23695516	Bn-chrA03_random-p157642
Bn-A03-p23414886	0.996677741	0.996677741	Bn-N3-p23711873	Bn-chrAnn_random-p388816
Bn-A03-p23426911	1	1	Bn-N3-p23719777	Bn-chrCnn_random-p673255
Bn-A03-p23432067	0.989583333	0.989583333	Bn-N3-p23724933	Bn-chrA03_random-p165376
Bn-A03-p23454434	0.980099502	0.980099502	Bn-N15-p38211658	Bn-chrA04-p14730188
Bn-A03-p23485055	0.983471074	0.983471074	Bn-N3-p23782520	Bn-chrA03-p22043912
Bn-A03-p23485435	0.933884298	0.933884298	Bn-N3-p23782898	Bn-chrA03-p22044290
Bn-A03-p23503118	0.883018868	0.883018868	Bn-N3-p23799741	Bn-chrA03-p22063684
Bn-A03-p23522509	0.983471074	0.983471074	Bn-N3-p23810698	Bn-chrA03-p22072593
Bn-A03-p23526338	0.933884298	0.933884298	Bn-N3-p23815034	Bn-chrA03-p22077114
Bn-A03-p23526756	0.983388704	0.983388704	Bn-N3-p23815448	Bn-chrA03-p22077528
Bn-A03-p23526967	0.996677741	0.996677741	Bn-N3-p23815659	Bn-chrA03-p22077739
Bn-A03-p23528989	0.991735537	0.991735537	Bn-N3-p23817639	Bn-chrA03-p22079720
Bn-A03-p23529225	0.991735537	0.991735537	Bn-N3-p23817882	Bn-chrA03-p22079963
Bn-A03-p23550322	0.991735537	1	Bn-N3-p23845219	Bn-chrA03-p22101248
Bn-A03-p23552464	0.983471074	0.991735537	Bn-N3-p23847416	Bn-chrA03-p22103390
Bn-A03-p23552601	0.991735537	0.983471074	Bn-N3-p23847553	Bn-chrA03-p22103527
Bn-A03-p23562008	0.986710963	0.990033223	Bn-N3-p23857806	Bn-chrA03-p22112847
Bn-A03-p23596892	0.993355482	0.910299003	Bn-N3-p23896402	Bn-chrAnn_random-p405950
Bn-A03-p23599149	1	0.996677741	Bn-N3-p23898664	Bn-chrAnn_random-p472363
Bn-A03-p23607037	0.975124378	0.343283582	Bn-N3-p23910495	Bn-chrA09_random-p174625
Bn-A03-p23608564	0.897009967	0.897009967	Bn-N3-p23911776	Bn-chrA03-p22176399
Bn-A03-p23609377	0.980099502	0.980099502	Bn-N3-p23912591	Bn-chrA03-p22177216
Bn-A03-p23609934	0.958677686	0.966942149	Bn-N3-p23913149	Bn-chrA03-p22177774
Bn-A03-p23621455	1	0.991735537	Bn-N3-p23926412	Bn-chrA03-p22187341
Bn-A03-p23634723	0.774086379	0.986710963	Bn-N15-p6395984	Bn-chrA03-p22200697
Bn-A03-p23635020	0.209302326	0.996677741	Unknown	Bn-chrA03-p22200994
Bn-A03-p23663605	0.975206612	0.966942149	Bn-N3-p23966240	Bn-chrA03-p22228173
Bn-A03-p23666221	0.950413223	0.94214876	Bn-N3-p23968460	Bn-chrA03-p22236382
Bn-A03-p23672735	0.975206612	0.966942149	Bn-N3-p23971844	Bn-chrA03-p22240592
Bn-A03-p23674889	0.983471074	0.991735537	Bn-N3-p23974210	Bn-chrA03-p22242712
Bn-A03-p23685798	0.996677741	0.996677741	Bn-N3-p23985120	Bn-chrA03-p22255541
Bn-A03-p23696737	0.983471074	0.94214876	Bn-N3-p23998720	Bn-chrC07-p38185332
Bn-A03-p23704255	0.930348259	0.950248756	Bn-N17-p41798847	Bn-chrA03-p22276871
Bn-A03-p23708487	0.996677741	0.943521595	Bn-N3-p24009643	Bn-chrC07-p38196084
Bn-A03-p23711362	0.991735537	1	Bn-N3-p24012518	Bn-chrA03-p22283834
Bn-A03-p23715660	0.991735537	0.94214876	Bn-N3-p24016045	Bn-chrA03-p22286906
Bn-A03-p23719771	0.990033223	0.910299003	Bn-N3-p24019665	Bn-chrA03-p22290997
Bn-A03-p23735715	0.933884298	0.933884298	Bn-N3-p24031518	Bn-chrC07-p38286199
Bn-A03-p23736096	0.985074627	0.980099502	Bn-N3-p24031897	Bn-chrA03-p22299634
Bn-A03-p23736392	0.826446281	0.818181818	Bn-N17-p41911009	Bn-chrC07-p38286873
Bn-A03-p23760564	0.996677741	0.996677741	Bn-N3-p24056148	Bn-chrA03-p22321686
Bn-A03-p23798235	0.996677741	0.980066445	Bn-N3-p24090508	Bn-chrA03-p22475915
Bn-A03-p23799968	0.983471074	0.991735537	Bn-N3-p24092242	Bn-chrA03-p22477652
Bn-A03-p23801324	0.983471074	0.958677686	Bn-N3-p24093598	Bn-chrA03-p22482107
Bn-A03-p23809317	0.975206612	0.975206612	Bn-N3-p24100413	Bn-chrA03-p22488033

Bn-A03-p23809595	0.986710963	0.980066445	Bn-N3-p24100691	Bn-chrA03-p22488311
Bn-A03-p23810014	0.975206612	0.966942149	Bn-N3-p24101110	Bn-chrA03-p22488741
Bn-A03-p23822093	0.966942149	0.925619835	Bn-N3-p24105409	Bn-chrA03-p22491338
Bn-A03-p23853396	0.94214876	0.950413223	Bn-N2-p12919973	Bn-chrC01-p19798405
Bn-A03-p23860825	0.983471074	0.991735537	Bn-N3-p24126497	Bn-chrA03-p22514591
Bn-A03-p23861481	0.983471074	0.983471074	Bn-N3-p24127148	Bn-chrA03-p22515807
Bn-A03-p23862018	0.975206612	0.975206612	Bn-N3-p24127685	Bn-chrA03-p22516342
Bn-A03-p23862683	0.983471074	0.983471074	Bn-N3-p24128350	Bn-chrA03-p22516997
Bn-A03-p23868030	0.975206612	0.966942149	Bn-N3-p24133071	Bn-chrA03-p22523377
Bn-A03-p23887373	0.993355482	0.893687708	Bn-N3-p24152415	Bn-chrA03-p22540227
Bn-A03-p23890040	0.94214876	0.909090909	Bn-N3-p24155083	Bn-chrA03-p22542813
Bn-A03-p23917701	0.975206612	0.925619835	Bn-N3-p24186212	Bn-chrA03-p22573239
Bn-A03-p23921932	0.991735537	0.958677686	Bn-N3-p24192294	Bn-chrA03-p22582425
Bn-A03-p23973475	0.983471074	0.983471074	Bn-N3-p24237586	Bn-chrA03-p22613859
Bn-A03-p23980725	0.985074627	0.975124378	Bn-N3-p24244876	Bn-chrA03-p22622258
Bn-A03-p23980912	0.985074627	0.995024876	Bn-N3-p24245064	Bn-chrA03-p22622446
Bn-A03-p23984129	0.561983471	0.983471074	Bn-N3-p24247463	Bn-chrA03-p22625843
Bn-A03-p23992075	0.958677686	0.884297521	Bn-N3-p24259165	Bn-chrA03-p22635923
Bn-A03-p23996628	0.983471074	0.909090909	Bn-N3-p24263791	Bn-chrA03-p22640491
Bn-A03-p24002443	1	0.900826446	Bn-N3-p24269601	Bn-chrA03-p22644422
Bn-A03-p24016551	0.965174129	0.965174129	Bn-N3-p24283710	Bn-chrA03-p22659088
Bn-A03-p24017311	0.990049751	0.975124378	Bn-N3-p24284469	Bn-chrA03-p22659846
Bn-A03-p24017859	0.973262032	0.967914439	Bn-N3-p24285016	Bn-chrA03-p22660396
Bn-A03-p24017924	0.975206612	0.975206612	Bn-N3-p24285082	Bn-chrA03-p22660462
Bn-A03-p24022648	0.983471074	0.983471074	Bn-N3-p24289836	Bn-chrA03-p22664683
Bn-A03-p24032564	0.991735537	0.983471074	Bn-N3-p24315585	Bn-chrA03_random-p174302
Bn-A03-p24046319	1	0.983471074	Bn-N3-p24323586	Bn-chrA03_random-p173442
Bn-A03-p24046452	0.991735537	1	Bn-N3-p24323719	Bn-chrA03_random-p173429
Bn-A03-p24048132	0.983471074	0.991735537	Bn-N3-p24324039	Bn-chrA03_random-p173396
Bn-A03-p24059470	0.980099502	0.985074627	Bn-N3-p24342623	Bn-chrA03_random-p172184
Bn-A03-p24090020	0.983471074	0.991735537	Bn-N3-p24358747	Bn-chrA03_random-p170646
Bn-A03-p24092986	0.917355372	0.975206612	Bn-N3-p24362517	Bn-chrA03_random-p170204
Bn-A03-p24104930	0.983471074	0.983471074	Bn-N3-p24373519	Bn-chrA03_random-p168714
Bn-A03-p24117554	0.975206612	0.966942149	Bn-N3-p24385535	Bn-chrA03-p22712215
Bn-A03-p24124318	0.917355372	0.966942149	Bn-N16-p11147856	Bn-chrA09-p15645058
Bn-A03-p24134454	0.970149254	0.970149254	Bn-N17-p42241788	Bn-chrC07-p38670001
Bn-A03-p24154359	0.909090909	0.94214876	Bn-N3-p24403767	Bn-chrA03-p22730500
Bn-A03-p24169327	0.983471074	0.958677686	Bn-N3-p24422726	Bn-chrA03-p22744880
Bn-A03-p24175395	0.983471074	0.975206612	Bn-N3-p24430883	Bn-chrA03-p22751149
Bn-A03-p24176332	0.909090909	0.983471074	Bn-N3-p24431801	Bn-chrA03-p22752070
Bn-A03-p24179750	0.983471074	0.991735537	Bn-N3-p24446072	Bn-chrA03-p22755262
Bn-A03-p24181488	0.983471074	0.950413223	Bn-N3-p24447077	Bn-chrA03-p22756254
Bn-A03-p24181569	0.991735537	0.983471074	Bn-N3-p24447158	Bn-chrA03-p22756335
Bn-A03-p24218520	0.752066116	0.752066116	Bn-N17-p42320329	Bn-chrC07-p38756891
Bn-A03-p24249333	0.925619835	0.933884298	Bn-N3-p24515175	Bn-chrA03-p22809885
Bn-A03-p24255868	0.983471074	0.975206612	Bn-N3-p24521572	Bn-chrA03-p22816401

Bn-A03-p24294571	0.990033223	0.976744186	Bn-N3-p24553962	Bn-chrA03-p22843298
Bn-A03-p24298999	0.958677686	0.917355372	Bn-N3-p24559224	Bn-chrA03-p22847090
Bn-A03-p24345237	0.950413223	0.950413223	Bn-N3-p24599088	Bn-chrA03-p22879256
Bn-A03-p24349898	0.975206612	0.975206612	Bn-N3-p24608085	Bn-chrA03-p22886971
Bn-A03-p24355827	0.94214876	0.94214876	Bn-N3-p24614035	Bn-chrA03-p22892917
Bn-A03-p24355878	0.950413223	0.950413223	Bn-N3-p24614086	Bn-chrA03-p22892968
Bn-A03-p24363260	0.970149254	0.970149254	Bn-N3-p24621501	Bn-chrA03-p22900381
Bn-A03-p24363811	0.983471074	0.983471074	Bn-N3-p24622052	Bn-chrA03-p22900932
Bn-A03-p24365383	0.980066445	0.980066445	Bn-N3-p24623582	Bn-chrA03-p22902462
Bn-A03-p24392068	0.975206612	0.975206612	Bn-N3-p24630045	Bn-chrA03-p22908925
Bn-A03-p24394796	0.983471074	0.983471074	Bn-N3-p24631719	Bn-chrA03-p22910599
Bn-A03-p24395668	0.983471074	0.983471074	Bn-N3-p24632590	Bn-chrA03-p22911470
Bn-A03-p24396004	1	1	Bn-N3-p24632888	Bn-chrA03-p22911768
Bn-A03-p24397912	0.991735537	0.991735537	Bn-N3-p24634841	Bn-chrA03-p22913721
Bn-A03-p24397916	0.983471074	0.983471074	Bn-N3-p24634845	Bn-chrA03-p22913725
Bn-A03-p24398419	0.991735537	0.991735537	Bn-N3-p24635348	Bn-chrA03-p22914228
Bn-A03-p24400338	0.983471074	0.983471074	Bn-N3-p24635504	Bn-chrA03-p22914384
Bn-A03-p24405388	0.925619835	0.925619835	Bn-N3-p29312992	Bn-chrA03-p27310833
Bn-A03-p24410521	0.983471074	0.983471074	Bn-N3-p24637392	Bn-chrA03_random-p174655
Bn-A03-p24411222	0.884297521	0.884297521	Bn-N3-p24639573	Bn-chrC05-p35511539
Bn-A03-p24415246	0.991735537	0.991735537	Bn-N3-p24641971	Bn-chrA03_random-p179084
Bn-A03-p24417118	0.958677686	0.991735537	Bn-N3-p24643877	Bn-chrA03_random-p178893
Bn-A03-p24419229	0.983471074	0.983471074	Bn-N3-p24646980	Bn-chrA03_random-p177998
Bn-A03-p24420093	0.991735537	0.991735537	Bn-N3-p24650940	Bn-chrA03_random-p178373
Bn-A03-p24420842	0.553719008	0.975206612	Bn-N3-p24651687	Bn-chrA03_random-p178448
Bn-A03-p24422801	0.520661157	0.495867769	Bn-N4-p19627187	Bn-chrA03_random-p178515
Bn-A03-p24423536	0.966942149	0.966942149	Bn-N3-p24653094	Bn-chrA03_random-p178589
Bn-A03-p24423762	0.950413223	0.950413223	Bn-N3-p24653317	Bn-chrA03_random-p178611
Bn-A03-p24423797	0.983471074	0.983471074	Bn-N3-p24653352	Bn-chrA03_random-p178615
Bn-A03-p24424174	0.991735537	0.991735537	Bn-N3-p24653729	Bn-chrA03_random-p178652
Bn-A03-p24424231	0.983471074	0.983471074	Bn-N3-p24653786	Bn-chrA03_random-p178658
Bn-A03-p24424286	0.983471074	0.983471074	Bn-N3-p24653841	Bn-chrA03_random-p178663
Bn-A03-p24424807	0.983471074	0.983471074	Bn-N3-p24654359	Bn-chrA03_random-p178715
Bn-A03-p24426088	0.991735537	0.991735537	Bn-N3-p24655654	Bn-chrAnn_random-p438628
Bn-A03-p24445966	0.950413223	0.950413223	Bn-N3-p24678709	Bn-chrA03-p22943040
Bn-A03-p24482649	0.94214876	0.94214876	Bn-N3-p24721128	Bn-chrA03-p22991998
Bn-A03-p24486881	0.867768595	0.917355372	Bn-N3-p24724768	Bn-chrC07-p39001273
Bn-A03-p24494026	0.826446281	0.826446281	Bn-N3-p24728771	Bn-chrA03-p22999641
Bn-A03-p24494224	0.79338843	0.79338843	Bn-N3-p24728955	Bn-chrA03-p22999825
Bn-A03-p24497262	0.960199005	0.960199005	Bn-N3-p24731628	Bn-chrA03-p23002498
Bn-A03-p24512946	0.966942149	0.966942149	Bn-N3-p24751925	Bn-chrA03-p23017117
Bn-A03-p24526574	0.945273632	0.945273632	Bn-N3-p24770429	Bn-chrA03-p23035508
Bn-A03-p24527313	0.909090909	0.909090909	Bn-N3-p24771219	Bn-chrA03-p23036298
Bn-A03-p24537588	0.966942149	0.966942149	Bn-N3-p24774096	Bn-chrA03-p23039195
Bn-A03-p24544179	0.966942149	0.966942149	Bn-N3-p24780577	Bn-chrA03-p23045676
Bn-A03-p24565421	1	1	Bn-N3-p24794707	Bn-chrA03-p23063728

Bn-A03-p24576670	1	1	Bn-N3-p24806668	Bn-chrA03-p23076876
Bn-A03-p24581655	0.993355482	0.993355482	Bn-N3-p24810883	Bn-chrA03-p23081091
Bn-A03-p24588576	0.975206612	0.975206612	Bn-N3-p24825689	Bn-chrA03-p23095823
Bn-A03-p24596982	0.983471074	0.983471074	Bn-N3-p24833015	Bn-chrA03-p23103148
Bn-A03-p24606883	0.553719008	0.553719008	Bn-N11-p31790301	Bn-chrC01-p28171309
Bn-A03-p24612593	0.975206612	0.975206612	Bn-N3-p24842485	Bn-chrA03-p23112618
Bn-A03-p24613172	0.990033223	0.966777409	Bn-N3-p24843059	Bn-chrA03-p23113192
Bn-A03-p24626484	0.925619835	0.925619835	Bn-N3-p24847100	Bn-chrA03-p23117574
Bn-A03-p24627047	0.993355482	0.993355482	Bn-N3-p24847663	Bn-chrA03-p23118137
Bn-A03-p24650359	0.975206612	0.975206612	Bn-N3-p24866051	Bn-chrA03_random-p175172
Bn-A03-p24669270	0.380165289	0.702479339	Unknown	Bn-chrA03_random-p203844
Bn-A03-p24688180	0.71641791	0.870646766	Bn-N3-p24910174	Bn-chrA03-p23155012
Bn-A03-p24690789	0.966942149	0.966942149	Bn-N3-p24912822	Bn-chrA03-p23157660
Bn-A03-p24704126	0.983471074	0.983471074	Bn-N3-p24919591	Bn-chrA03-p23164428
Bn-A03-p24710055	0.980099502	0.980099502	Bn-N3-p24925103	Bn-chrA03-p23169940
Bn-A03-p24739525	0.975206612	0.975206612	Bn-N3-p24949959	Bn-chrA03-p23193999
Bn-A03-p24745951	0.966942149	0.966942149	Bn-N3-p24956620	Bn-chrA03-p23200436
Bn-A03-p24746007	0.975206612	0.975206612	Bn-N3-p24956676	Bn-chrA03-p23200492
Bn-A03-p24748004	0.973421927	0.973421927	Bn-N3-p24957877	Bn-chrA03-p23201693
Bn-A03-p24753667	0.975206612	0.975206612	Bn-N3-p24963556	Bn-chrA03-p23207372
Bn-A03-p24753829	0.991735537	0.991735537	Bn-N3-p24963718	Bn-chrA03-p23207534
Bn-A03-p24763656	0.991735537	0.884297521	Bn-N3-p24973487	Bn-chrC07-p39329319
Bn-A03-p24773584	0.966942149	0.966942149	Bn-N3-p24984578	Bn-chrAnn_random-p433518
Bn-A03-p24780067	0.853820598	0.797342193	Bn-N17-p42904503	Bn-chrA03-p23230738
Bn-A03-p24800655	0.966777409	0.970099668	Bn-N3-p25012870	Bn-chrA03-p23252117
Bn-A03-p24805476	0.991735537	0.991735537	Bn-N3-p25019865	Bn-chrA03-p23261157
Bn-A03-p24809290	0.975206612	0.975206612	Bn-N3-p25023698	Bn-chrA03-p23264989
Bn-A03-p24811187	0.966942149	0.966942149	Bn-N3-p25025579	Bn-chrA03-p23266870
Bn-A03-p24823015	0.991735537	0.983471074	Bn-N3-p25044953	Bn-chrA03-p23290872
Bn-A03-p24824357	0.975206612	0.975206612	Bn-N3-p25046301	Bn-chrA03-p23292186
Bn-A03-p24827646	0.930232558	0.926910299	Bn-N3-p25048900	Bn-chrA03-p23294784
Bn-A03-p24829281	0.950413223	0.958677686	Bn-N17-p42963272	Bn-chrC07-p39405797
Bn-A03-p24842021	0.859504132	0.669421488	Bn-N3-p25066947	Bn-chrA03-p23322997
Bn-A03-p24864494	0.875621891	0.273631841	Bn-N3-p25095312	Unknown
Bn-A03-p24896477	0.297520661	0.975206612	Bn-N8-p8225339	Bn-chrA03_random-p181966
Bn-A03-p24896531	0.297520661	0.983471074	Unknown	Bn-chrA03_random-p181971
Bn-A03-p24896769	0.231404959	0.975206612	Unknown	Bn-chrA03_random-p181995
Bn-A03-p24896884	0.190082645	0.991735537	Unknown	Bn-chrA03_random-p182006
Bn-A03-p24897111	0.165289256	0.983471074	Bn-N12-p13991406	Bn-chrA03_random-p182236
Bn-A03-p24897118	0.140495868	0.983471074	Unknown	Bn-chrA03_random-p182237
Bn-A03-p24897286	0.132231405	0.94214876	Unknown	Bn-chrA03_random-p182255
Bn-A03-p24900647	0.272727273	0.743801653	Unknown	Bn-chrA03_random-p182373
Bn-A03-p24902152	0.214876033	0.983471074	Bn-N19-p52550305	Bn-chrA03_random-p182387
Bn-A03-p24902631	0.181818182	0.181818182	Bn-N3-p11952122	Bn-chrA03-p10767412
Bn-A03-p24906226	0.991735537	1	Bn-N3-p25124773	Bn-chrA03_random-p183046
Bn-A03-p24920415	0.983471074	0.983471074	Bn-N3-p25142379	Bn-chrA03-p23344303

Bn-A03-p24928989	0.991735537	0.991735537	Bn-N3-p25151296	Bn-chrA03-p23353375
Bn-A03-p24965131	0.741293532	0.940298507	Bn-N3-p25178738	Bn-chrC07-p39492824
Bn-A03-p24971161	1	1	Bn-N3-p25183358	Bn-chrA03-p23385545
Bn-A03-p24971360	0.975206612	0.975206612	Bn-N3-p25183557	Bn-chrA03-p23385744
Bn-A03-p24985700	0.568106312	0.810631229	Bn-N3-p25197464	Bn-chrA03-p23399835
Bn-A03-p24986472	0.976744186	0.976744186	Bn-N3-p25198208	Bn-chrA03-p23400579
Bn-A03-p24990059	0.983388704	0.983388704	Bn-N3-p25201832	Bn-chrA03-p23404202
Bn-A03-p24998976	0.818181818	0.818181818	Bn-N17-p43120499	Bn-chrC07-p39520391
Bn-A03-p24999716	0.666666667	0.741293532	Bn-N17-p43120844	Bn-chrC07-p39520748
Bn-A03-p25008908	0.983388704	0.983388704	Bn-Scaffold01419-p7	Bn-chrA03-p23408712
Bn-A03-p25009736	0.983471074	0.983471074	Bn-N3-p25207170	Bn-chrA03-p23409540
Bn-A03-p25009919	0.983471074	0.983471074	Bn-N3-p25207353	Bn-chrA03-p23409723
Bn-A03-p25009925	0.983471074	0.983471074	Bn-N3-p25207359	Bn-chrA03-p23409729
Bn-A03-p25024647	0.94214876	0.94214876	Bn-N3-p25214769	Bn-chrA03-p23417138
Bn-A03-p25024749	0.991735537	0.991735537	Bn-N3-p25214871	Bn-chrA03-p23417240
Bn-A03-p25024928	0.991735537	0.991735537	Bn-N3-p25215047	Bn-chrA03-p23417416
Bn-A03-p25025360	0.975206612	0.975206612	Bn-N3-p25215488	Bn-chrA03-p23417857
Bn-A03-p25032014	0.508305648	0.508305648	Bn-N17-p43143511	Bn-chrC07-p39542481
Bn-A03-p25033468	0.664451827	0.661129568	Bn-N17-p43145031	Bn-chrC07-p39544001
Bn-A03-p25037423	0.966942149	0.966942149	Bn-N3-p25222063	Bn-chrA03-p23424432
Bn-A03-p25043466	0.980066445	0.966777409	Bn-N3-p25228096	Bn-chrA03-p23430465
Bn-A03-p25046120	0.991735537	0.991735537	Bn-N3-p25230750	Bn-chrA03-p23433079
Bn-A03-p25047750	0.996677741	0.993355482	Bn-N3-p25232380	Bn-chrA03-p23434704
Bn-A03-p25055647	0.983471074	0.991735537	Bn-N3-p25240192	Bn-chrA03-p23442502
Bn-A03-p25071259	0.958677686	0.958677686	Bn-N3-p25256225	Bn-chrA03-p23458534
Bn-A03-p25073363	0.996677741	0.996677741	Bn-N3-p25258404	Bn-chrA03-p23460713
Bn-A03-p25082044	0.986710963	0.986710963	Bn-N3-p25266974	Bn-chrA03_random-p186574
Bn-A03-p25111028	0.991735537	0.991735537	Bn-N3-p25316392	Bn-chrA03-p23502093
Bn-A03-p25111171	0.983471074	0.983471074	Bn-N3-p25316535	Bn-chrA03-p23502236
Bn-A03-p25119771	1	1	Bn-N3-p25323805	Bn-chrA03-p23509305
Bn-A03-p25265364	0.985074627	0.985074627	Bn-N3-p25434700	Bn-chrA03-p23628445
Bn-A03-p25296332	0.985074627	0.985074627	Bn-N3-p25475054	Bn-chrA03_random-p187355
Bn-A03-p25314490	0.991735537	1	Bn-N3-p25499702	Bn-chrA03-p23669134
Bn-A03-p25314570	0.975206612	0.991735537	Bn-N3-p25499782	Bn-chrA03-p23669214
Bn-A03-p25330901	1	1	Bn-N3-p25516345	Bn-chrA03-p23687986
Bn-A03-p25337594	0.991735537	1	Bn-N3-p25524837	Bn-chrA03-p23695451
Bn-A03-p25342507	0.958677686	0.966942149	Bn-N3-p25529747	Bn-chrA03-p23700354
Bn-A03-p25343812	0.966942149	0.966942149	Bn-N3-p25531054	Bn-chrA03-p23703121
Bn-A03-p25361950	0.826446281	0.826446281	Bn-N3-p25545269	Bn-chrA03-p23717336
Bn-A03-p25362364	0.925619835	0.925619835	Bn-N3-p25545643	Bn-chrA03-p23717710
Bn-A03-p25365003	0.980066445	0.980066445	Bn-N3-p25548814	Bn-chrA03-p23720740
Bn-A03-p25393494	0.991735537	0.991735537	Bn-N3-p25594093	Bn-chrA03-p23763377
Bn-A03-p25402301	0.966942149	0.966942149	Bn-N3-p25600454	Bn-chrA03-p23770762
Bn-A03-p25402717	0.983471074	0.983471074	Bn-N3-p25600870	Bn-chrA03-p23771178
Bn-A03-p25403786	0.983471074	0.983471074	Bn-N3-p25601946	Bn-chrA03-p23772254
Bn-A03-p25408779	1	1	Bn-N3-p25605061	Bn-chrA03-p23775369

Bn-A03-p25423612	1	0.991735537	Bn-N3-p25624074	Bn-chrA03-p23801861
Bn-A03-p25437069	0.993355482	0.993355482	Bn-N3-p25637986	Bn-chrA03-p23820330
Bn-A03-p25471848	1	1	Bn-N3-p25668602	Bn-chrA08-p9981263
Bn-A03-p25473832	0.996677741	0.996677741	Bn-N3-p25670586	Bn-chrA03-p23841764
Bn-A03-p25474850	0.993355482	0.993355482	Bn-N3-p25671604	Bn-chrA03-p23842782
Bn-A03-p25489551	0.983388704	0.983388704	Bn-N3-p25688788	Bn-chrA03-p23858622
Bn-A03-p25494539	0.991735537	1	Bn-N3-p25693625	Bn-chrA03-p23863290
Bn-A03-p25511978	0.993355482	0.993355482	Bn-N3-p25710834	Bn-chrA03-p23878885
Bn-A03-p25521989	0.975206612	0.975206612	Bn-N3-p25721078	Bn-chrA03-p23888890
Bn-A03-p25525612	0.983471074	0.983471074	Bn-N3-p25724701	Bn-chrA03-p23892513
Bn-A03-p25555423	0.990049751	0.990049751	Bn-N3-p25756341	Bn-chrA03-p23920509
Bn-A03-p25582691	1	1	Bn-N3-p25795613	Bn-chrA03-p23947676
Bn-A03-p25584526	0.973421927	0.973421927	Bn-N3-p25797448	Bn-chrA03-p23949511
Bn-A03-p25591818	0.983471074	0.983471074	Bn-N3-p25804790	Bn-chrA03-p23956800
Bn-A03-p25599340	0.975206612	0.975206612	Bn-N3-p25812989	Bn-chrA03-p23964328
Bn-A03-p25604179	0.975206612	0.975206612	Bn-N3-p25818519	Bn-chrA03-p23969586
Bn-A03-p25606900	0.996677741	0.996677741	Bn-N3-p25820859	Bn-chrA03-p23971927
Bn-A03-p25608893	0.754152824	0.757475083	Bn-N3-p25822853	Bn-chrA03-p23973921
Bn-A03-p25614406	0.996677741	0.950166113	Bn-N3-p25828301	Bn-chrC07-p40091561
Bn-A03-p25625732	0.990049751	0.990049751	Bn-N3-p25839627	Bn-chrA03-p23990720
Bn-A03-p25629067	0.993355482	0.993355482	Bn-N3-p25842962	Bn-chrA03-p23994054
Bn-A03-p25631316	0.925619835	0.545454545	Bn-N3-p25845246	Bn-chrC07-p40113228
Bn-A03-p25636300	0.976744186	0.873754153	Bn-N3-p25849712	Bn-chrA03-p23999963
Bn-A03-p25637279	0.958677686	0.909090909	Bn-N3-p25850691	Bn-chrC07-p40118928
Bn-A03-p25640844	0.958677686	0.966942149	Bn-N3-p25855762	Bn-chrA03-p24005638
Bn-A03-p25668597	0.986710963	0.986710963	Bn-N3-p25885248	Bn-chrA03-p24029162
Bn-A03-p25671744	0.994413408	0.994413408	Bn-Scaffold04006-p2	Bn-chrA03-p24032305
Bn-A03-p25679775	0.933884298	0.694214876	Bn-N3-p25904465	Bn-chrC07-p40164231
Bn-A03-p25681343	0.983471074	0.983471074	Bn-N3-p25908866	Bn-chrA03-p24048162
Bn-A03-p25683964	0.785123967	0.785123967	Bn-N17-p43759613	Bn-chrC07-p40169291
Bn-A03-p25695855	0.966942149	0.966942149	Bn-N3-p25927123	Bn-chrA03-p24064275
Bn-A03-p25704793	0.973421927	0.973421927	Bn-N3-p25936050	Bn-chrA03-p24073202
Bn-A03-p25708944	0.950413223	0.950413223	Bn-N3-p25951642	Bn-chrA03-p24090075
Bn-A03-p25715049	0.966942149	0.966942149	Bn-N3-p25957521	Bn-chrA03-p24095954
Bn-A03-p25718518	0.980099502	0.980099502	Bn-N3-p25965615	Bn-chrA03-p24103311
Bn-A03-p25718716	0.975124378	0.975124378	Bn-N3-p25965813	Bn-chrA03-p24103509
Bn-A03-p25729227	1	0.991735537	Bn-N3-p25982788	Bn-chrA03-p24123601
Bn-A03-p25729305	0.983471074	0.975206612	Bn-N3-p25982866	Bn-chrA03-p24123679
Bn-A03-p25734907	0.966942149	0.975206612	Bn-N3-p25988524	Bn-chrA03-p24129001
Bn-A03-p25751759	0.983471074	0.983471074	Bn-N3-p26001616	Bn-chrA03-p24142102
Bn-A03-p25756310	1	1	Bn-N3-p26003524	Bn-chrA03-p24144009
Bn-A03-p25765687	0.991735537	0.991735537	Bn-N3-p26007155	Bn-chrA03-p24147624
Bn-A03-p25774773	0.983471074	0.975206612	Bn-N3-p26015607	Bn-chrA03-p24154165
Bn-A03-p25774827	0.975206612	0.975206612	Bn-N3-p26015661	Bn-chrA03-p24154219
Bn-A03-p25779411	0.960199005	0.980099502	Bn-N3-p26020241	Bn-chrA03-p24158802
Bn-A03-p25785875	0.991735537	1	Bn-N3-p26026651	Bn-chrA03-p24165209

Bn-A03-p25801783	0.983471074	0.975206612	Bn-N3-p26044405	Bn-chrA03-p24183050
Bn-A03-p25805632	0.965174129	0.980099502	Bn-N3-p26048265	Bn-chrA03-p24186896
Bn-A03-p25810585	0.970149254	0.970149254	Bn-N3-p26055225	Bn-chrA03-p24189941
Bn-A03-p25819955	0.980066445	0.953488372	Bn-N3-p26062430	Bn-chrC07-p40286469
Bn-A03-p25825392	0.983471074	0.975206612	Bn-N3-p26071288	Bn-chrA03-p24204645
Bn-A03-p25826578	0.719008264	0.991735537	Bn-N3-p26072457	Bn-chrA03-p24205810
Bn-A03-p25827210	0.996677741	0.993355482	Bn-N3-p26073088	Bn-chrA03-p24206442
Bn-A03-p25827833	0.917355372	0.925619835	Bn-N3-p26073710	Bn-chrA03-p24207065
Bn-A03-p25828479	0.859504132	0.975206612	Bn-N3-p26074364	Bn-chrA03-p24207715
Bn-A03-p25839360	0.983471074	0.975206612	Bn-N3-p26085311	Bn-chrA03-p24214079
Bn-A03-p25841013	0.976744186	0.973421927	Bn-N3-p26086965	Bn-chrA03-p24215709
Bn-A03-p25841615	0.983471074	0.975206612	Bn-N3-p26087567	Bn-chrA03-p24216311
Bn-A03-p25843937	0.996677741	0.990033223	Bn-N3-p26089889	Bn-chrA03-p24217896
Bn-A03-p25849429	0.975124378	0.975124378	Bn-N3-p26092619	Bn-chrA03-p24220608
Bn-A03-p25849463	0.975124378	0.975124378	Bn-N3-p26092653	Bn-chrA03-p24220642
Bn-A03-p25850146	0.970149254	0.975124378	Bn-N3-p26093336	Bn-chrA03-p24221325
Bn-A03-p25851789	1	1	Bn-N3-p26094977	Bn-chrA03-p24222968
Bn-A03-p25858075	1	0.936877076	Bn-N3-p26101261	Bn-chrA02_random-p132544
Bn-A03-p25868537	0.990049751	0.990049751	Bn-N3-p26109416	Bn-chrA03-p24234727
Bn-A03-p25879876	0.986710963	0.976744186	Bn-N3-p26119211	Bn-chrA03-p24244585
Bn-A03-p25885330	0.950413223	0.958677686	Bn-N3-p26124817	Bn-chrA03-p24250210
Bn-A03-p25949532	0.990033223	0.993355482	Bn-N3-p26180052	Bn-chrA03-p24308348
Bn-A03-p25949676	0.940199336	0.956810631	Bn-N3-p26180196	Bn-chrA03-p24308492
Bn-A03-p25951191	0.990033223	0.993355482	Bn-N3-p26181711	Bn-chrA03-p24310007
Bn-A03-p25952725	0.996677741	0.996677741	Bn-N3-p26183591	Bn-chrA03-p24311391
Bn-A03-p25957465	0.996677741	0.996677741	Bn-N3-p26188816	Bn-chrA03-p24317639
Bn-A03-p25993601	1	0.991735537	Bn-N3-p26231379	Bn-chrA03-p24356881
Bn-A03-p26001258	0.975206612	0.983471074	Bn-N3-p26239055	Bn-chrA03-p24364511
Bn-A03-p26004171	0.636363636	0.636363636	Bn-N3-p26244407	Bn-chrA03-p24368061
Bn-A03-p26013651	0.933884298	0.933884298	Bn-N17-p44067603	Bn-chrC07-p40458388
Bn-A03-p26014745	0.877076412	0.893687708	Bn-N3-p26249043	Bn-chrA03-p24372552
Bn-A03-p26021585	0.983471074	0.975206612	Bn-N3-p26255287	Bn-chrA03-p24378568
Bn-A03-p26055806	0.991735537	0.991735537	Bn-N3-p26279174	Bn-chrA03-p24406111
Bn-A03-p26066930	0.975206612	0.983471074	Bn-N3-p26287335	Bn-chrA03-p24418801
Bn-A03-p26071059	0.94214876	0.966942149	Bn-N3-p26292836	Bn-chrA03-p24423053
Bn-A03-p26071126	0.975206612	0.975206612	Bn-N3-p26292904	Bn-chrA03-p24423121
Bn-A03-p26075478	0.983471074	0.983471074	Bn-N3-p26295154	Bn-chrA03-p24423989
Bn-A03-p26075535	0.983471074	0.983471074	Bn-N3-p26295211	Bn-chrA03-p24424046
Bn-A03-p26079114	0.993355482	0.990033223	Bn-N3-p26298801	Bn-chrA03-p24427624
Bn-A03-p26081548	0.960199005	0.965174129	Bn-N3-p26301236	Bn-chrA03-p24430059
Bn-A03-p26090388	0.996677741	1	Bn-N3-p26318287	Bn-chrA03-p24438883
Bn-A03-p26100548	0.991735537	0.991735537	Bn-N3-p26325852	Bn-chrA03-p24450368
Bn-A03-p26100814	0.975206612	0.975206612	Bn-N3-p26326118	Bn-chrA03-p24450634
Bn-A03-p26116027	0.980066445	0.976744186	Bn-N3-p26344598	Bn-chrA03-p24461564
Bn-A03-p26117920	0.966777409	0.966777409	Bn-N3-p26346507	Bn-chrA03-p24463474
Bn-A03-p26119193	0.983471074	0.991735537	Bn-N3-p26347769	Bn-chrA03-p24464736

Bn-A03-p26121502	0.966942149	0.958677686	Bn-N3-p26350023	Bn-chrAnn_random-p474992
Bn-A03-p26131473	0.933884298	0.917355372	Bn-N3-p26354110	Bn-chrA03-p24473446
Bn-A03-p26132481	0.94214876	0.975206612	Bn-N3-p26355096	Bn-chrA03-p24474453
Bn-A03-p26133142	0.983471074	0.991735537	Bn-N3-p26355757	Bn-chrA03-p24475114
Bn-A03-p26133346	0.975206612	0.991735537	Bn-N3-p26355960	Bn-chrA03-p24475318
Bn-A03-p26148876	0.685950413	0.801652893	Bn-N3-p26368182	Bn-chrC07-p40566247
Bn-A03-p26167847	0.970149254	0.970149254	Bn-N3-p26383967	Bn-chrA03-p24516667
Bn-A03-p26167992	0.860696517	0.860696517	Bn-N3-p26384112	Bn-chrA03-p24516812
Bn-A03-p26168881	0.990049751	0.990049751	Bn-N3-p26385056	Bn-chrA03-p24517756
Bn-A03-p26195875	0.975206612	0.975206612	Bn-N3-p26407204	Bn-chrA03-p24539574
Bn-A03-p26197815	0.925619835	0.925619835	Bn-N3-p26409275	Bn-chrA03-p24541664
Bn-A03-p26202244	0.818181818	0.694214876	Bn-N3-p26413504	Bn-chrC07-p40622619
Bn-A03-p26216950	0.966942149	0.867768595	Bn-N3-p26430784	Bn-chrA03-p24558414
Bn-A03-p26232681	0.966777409	0.970099668	Bn-N3-p26447546	Bn-chrA03-p24575190
Bn-A03-p26248671	0.980099502	0.975124378	Bn-N3-p26464453	Bn-chrA03-p24602386
Bn-A03-p26264242	0.983471074	0.991735537	Bn-N3-p26479359	Bn-chrA03-p24636787
Bn-A03-p26272560	0.983388704	0.966777409	Bn-N3-p26505748	Bn-chrA03-p24660653
Bn-A03-p26275444	0.975206612	0.966942149	Bn-N3-p26508632	Bn-chrA03-p24663591
Bn-A03-p26275615	0.983471074	0.975206612	Bn-N3-p26508803	Bn-chrA03-p24663762
Bn-A03-p26276349	0.983471074	0.991735537	Bn-N3-p26509537	Bn-chrA03-p24664516
Bn-A03-p26284129	0.976744186	0.976744186	Bn-N3-p26516781	Bn-chrA03-p24675623
Bn-A03-p26284449	0.976744186	0.980066445	Bn-N3-p26517101	Bn-chrA03-p24675943
Bn-A03-p26284882	0.996677741	0.990033223	Bn-N3-p26517534	Bn-chrA03-p24676376
Bn-A03-p26286301	0.991735537	0.983471074	Bn-N3-p26518958	Bn-chrA03-p24677587
Bn-A03-p26288769	0.985074627	0.985074627	Bn-N3-p26521343	Bn-chrA03-p24679994
Bn-A03-p26309336	0.975206612	0.983471074	Bn-N3-p26551674	Bn-chrA03-p24699273
Bn-A03-p26309550	0.950413223	0.933884298	Bn-N3-p26551888	Bn-chrA03-p24699486
Bn-A03-p26310351	0.985074627	0.985074627	Bn-N3-p26552691	Bn-chrA03-p24700288
Bn-A03-p26311193	0.975206612	0.966942149	Bn-N3-p26570714	Bn-chrA03-p24704911
Bn-A03-p26319168	0.989583333	0.989583333	Bn-N3-p26584329	Bn-chrA03-p24711969
Bn-A03-p26359851	0.975206612	0.991735537	Bn-N3-p26621508	Bn-chrA03-p24749109
Bn-A03-p26405367	0.975206612	0.917355372	Bn-N3-p26663953	Bn-chrA03-p24797250
Bn-A03-p26406372	0.991735537	0.214876033	Bn-N3-p26664961	Unknown
Bn-A03-p26433672	0.975206612	0.983471074	Bn-N3-p26687748	Bn-chrA03-p24825262
Bn-A03-p26433849	0.983471074	0.983471074	Bn-N3-p26687923	Bn-chrA03-p24825439
Bn-A03-p26433912	0.958677686	0.950413223	Bn-N3-p26687986	Bn-chrA03-p24825502
Bn-A03-p26433948	0.94214876	0.950413223	Bn-N3-p26688024	Bn-chrA03-p24825537
Bn-A03-p26437565	0.991735537	0.991735537	Bn-N3-p26692066	Bn-chrA03-p24829501
Bn-A03-p26437680	0.983471074	0.983471074	Bn-N3-p26692181	Bn-chrA03-p24829616
Bn-A03-p26437883	0.983388704	0.983388704	Bn-N3-p26692384	Bn-chrA03-p24829819
Bn-A03-p26439962	0.983471074	0.983471074	Bn-N3-p26698287	Bn-chrA03-p24837576
Bn-A03-p26444464	0.985074627	0.985074627	Bn-N3-p26702481	Bn-chrA03-p24841770
Bn-A03-p26444945	0.985074627	0.985074627	Bn-N3-p26702962	Bn-chrA03-p24842251
Bn-A03-p26445028	0.975124378	0.975124378	Bn-N3-p26703045	Bn-chrA03-p24842334
Bn-A03-p26477533	0.991735537	0.991735537	Bn-N3-p26736040	Bn-chrA03-p24875311
Bn-A03-p26478842	1	1	Bn-N3-p26737349	Bn-chrA03-p24876620

Bn-A03-p26506472	0.995024876	0.995024876	Bn-N17-p44591616	Bn-chrC07-p40929736
Bn-A03-p26510093	0.993355482	0.993355482	Bn-N3-p26768650	Bn-chrA03-p24908504
Bn-A03-p26528958	0.970149254	0.970149254	Bn-N3-p26796689	Bn-chrA03_random-p190764
Bn-A03-p26546744	0.977272727	0.920454545	Bn-N17-p44634091	Bn-chrAnn_random-p363972
Bn-A03-p26560194	0.960199005	0.960199005	Bn-N3-p26828341	Bn-chrA03-p24922932
Bn-A03-p26567111	0.983388704	0.983388704	Bn-N3-p26835139	Bn-chrA03-p24929727
Bn-A03-p26567993	1	1	Bn-N3-p26836021	Bn-chrA03-p24930609
Bn-A03-p26571083	0.983388704	0.983388704	Bn-N3-p26840399	Bn-chrA03-p24934987
Bn-A03-p26572622	0.975206612	0.975206612	Bn-N3-p26841937	Bn-chrA03-p24936524
Bn-A03-p26577952	0.991735537	0.991735537	Bn-N3-p26847256	Bn-chrA03-p24941902
Bn-A03-p26603077	1	1	Bn-N3-p26873097	Bn-chrA03-p24967814
Bn-A03-p26604097	0.950413223	0.950413223	Bn-N3-p26874117	Bn-chrA03-p24968834
Bn-A03-p26604578	0.983471074	0.983471074	Bn-N3-p26874599	Bn-chrA03-p24969316
Bn-A03-p26607921	0.986710963	0.986710963	Bn-N3-p26878086	Bn-chrA03-p24972803
Bn-A03-p26740723	0.979166667	0.989583333	Bn-N3-p27032084	Bn-chrA03-p25162635
Bn-A03-p26742006	0.991735537	0.983471074	Bn-N3-p27033418	Bn-chrA03-p25163969
Bn-A03-p26756701	0.96345515	0.943521595	Bn-N17-p44816015	Bn-chrA03-p25168967
Bn-A03-p26763577	0.975206612	0.983471074	Bn-N3-p27047775	Bn-chrA03-p25175834
Bn-A03-p26764606	0.991735537	0.983471074	Bn-N3-p27048805	Bn-chrA03-p25176863
Bn-A03-p26771482	0.991735537	0.983471074	Bn-N3-p27058874	Bn-chrA03-p25183724
Bn-A03-p26775994	1	0.991735537	Bn-N3-p27060036	Bn-chrA03-p25184886
Bn-A03-p26786549	0.991735537	0.983471074	Bn-N3-p27070755	Bn-chrA03-p25193954
Bn-A03-p26788875	0.966942149	0.975206612	Bn-N3-p27073110	Bn-chrA03-p25196304
Bn-A03-p26789755	0.966942149	0.975206612	Bn-N3-p27073988	Bn-chrA03-p25197174
Bn-A03-p26798652	0.720930233	0.720930233	Bn-N3-p27083185	Bn-chrA03-p25206364
Bn-A03-p26800384	0.991735537	0.991735537	Bn-N3-p27085064	Bn-chrA03-p25214762
Bn-A03-p26803262	0.950166113	0.953488372	Bn-N3-p27088796	Bn-chrA03-p25217661
Bn-A03-p26809706	0.966942149	0.966942149	Bn-N3-p27095429	Bn-chrA03-p25224263
Bn-A03-p26810924	0.993355482	0.993355482	Bn-N3-p27096649	Bn-chrA03-p25225483
Bn-A03-p26811025	1	1	Bn-N3-p27096750	Bn-chrA03-p25225584
Bn-A03-p26811385	0.958677686	0.958677686	Bn-N3-p27097110	Bn-chrA03-p25225944
Bn-A03-p26815896	0.990033223	0.990033223	Bn-N3-p27114764	Bn-chrA03-p25244510
Bn-A03-p26822285	0.958677686	0.958677686	Bn-N3-p27123653	Bn-chrA03-p25252545
Bn-A03-p26822384	0.991735537	0.991735537	Bn-N3-p27123748	Bn-chrA03-p25252640
Bn-A03-p26824343	1	1	Bn-N3-p27128299	Bn-chrA03-p25257196
Bn-A03-p26833555	0.910447761	0.995024876	Bn-N3-p27137550	Bn-chrA03-p25271472
Bn-A03-p26833841	0.985074627	0.980099502	Bn-N3-p27137830	Bn-chrA03-p25271758
Bn-A03-p26839758	0.926910299	0.996677741	Bn-N3-p27142046	Bn-chrA03_random-p192373
Bn-A03-p26840269	0.965174129	0.965174129	Bn-N3-p27146293	Bn-chrA03_random-p192322
Bn-A03-p26848326	0.993355482	0.993355482	Bn-N3-p27158857	Bn-chrA03-p25300085
Bn-A03-p26852795	0.996677741	0.996677741	Bn-N3-p27163563	Bn-chrA03-p25304602
Bn-A03-p26931199	0.985074627	0.985074627	Bn-N3-p27246627	Bn-chrA03-p25395191
Bn-A03-p27002187	0.973421927	0.976744186	Bn-N3-p27314431	Bn-chrC07-p41302548
Bn-A03-p27006222	0.933884298	0.991735537	Bn-N3-p27319051	Bn-chrA03_random-p201091
Bn-A03-p27008420	0.851239669	0.983471074	Bn-N3-p27319196	Bn-chrA03_random-p201069
Bn-A03-p27028300	0.900826446	0.900826446	Bn-N3-p27340263	Bn-chrA03_random-p198787

Bn-A03-p27033682	0.578512397	0.94214876	Bn-N17-p45066037	Bn-chrAnn_random-p375856
Bn-A03-p27066000	0.976744186	0.993355482	Bn-N3-p27367099	Bn-chrA03_random-p207946
Bn-A03-p27070872	0.966942149	0.966942149	Bn-N3-p27371835	Bn-chrA03_random-p208419
Bn-A03-p27101177	0.94214876	0.933884298	Bn-N3-p27401411	Bn-chrA03_random-p211255
Bn-A03-p27104733	0.990033223	0.990033223	Bn-N3-p27404993	Bn-chrA03_random-p211611
Bn-A03-p27111541	0.975206612	0.983471074	Bn-N3-p27410908	Bn-chrA03_random-p212303
Bn-A03-p27112789	0.966942149	0.975206612	Bn-N3-p27412155	Bn-chrA03_random-p212428
Bn-A03-p27146568	1	1	Bn-Scaffold43562-p2	Bn-chrCnn_random-p622427
Bn-A03-p27156983	0.993355482	0.993355482	Bn-N3-p27503944	Bn-chrA03_random-p194783
Bn-A03-p27164178	0.983471074	0.983471074	Bn-N3-p27508522	Bn-chrA03_random-p195241
Bn-A03-p27168311	0.991735537	0.991735537	Bn-N3-p27512703	Bn-chrA03_random-p195659
Bn-A03-p27171824	1	1	Bn-N3-p27516373	Bn-chrA03_random-p196026
Bn-A03-p27172113	0.996677741	0.996677741	Bn-N3-p27516662	Bn-chrA03_random-p196055
Bn-A03-p27172168	0.996677741	0.996677741	Bn-N3-p27516717	Bn-chrA03_random-p196060
Bn-A03-p27179255	0.975206612	0.975206612	Bn-N3-p27522943	Bn-chrA03_random-p196964
Bn-A03-p27179357	0.986710963	0.986710963	Bn-N3-p27523045	Bn-chrA03_random-p196975
Bn-A03-p27181751	0.993355482	0.993355482	Bn-N3-p27525442	Bn-chrA03_random-p197214
Bn-A03-p27182289	0.975206612	0.975206612	Bn-N3-p27525980	Bn-chrA03_random-p197268
Bn-A03-p27192830	0.980099502	0.975124378	Bn-N3-p27536312	Bn-chrA03_random-p207221
Bn-A03-p27193587	0.990033223	0.139534884	Bn-N3-p27537735	Bn-chrA07-p10154315
Bn-A03-p27218655	0.975206612	0.975206612	Bn-N3-p27573058	Bn-chrA03-p25455931
Bn-A03-p27253035	0.983471074	0.975206612	Bn-N3-p27602495	Bn-chrA03-p25482544
Bn-A03-p27253678	0.983471074	0.991735537	Bn-N3-p27603138	Bn-chrA03-p25483186
Bn-A03-p27255790	0.983471074	0.983471074	Bn-N3-p27605270	Bn-chrA03-p25485297
Bn-A03-p27256355	0.925373134	0.970149254	Bn-N3-p27605832	Bn-chrA03-p25485861
Bn-A03-p27257933	0.933884298	0.958677686	Bn-N3-p27610000	Bn-chrA03-p25487433
Bn-A03-p27269401	0.975206612	0.966942149	Bn-N3-p27642984	Bn-chrA03-p25517505
Bn-A03-p27288521	0.996677741	0.996677741	Bn-N3-p27649606	Bn-chrA03-p25524140
Bn-A03-p27289437	0.996677741	0.990033223	Bn-N3-p27650525	Bn-chrA03-p25525060
Bn-A03-p27298810	0.991735537	0.983471074	Bn-N3-p27661207	Bn-chrA03-p25539528
Bn-A03-p27304692	1	0.983471074	Bn-N3-p27667098	Bn-chrA03-p25546732
Bn-A03-p27337536	0.990033223	0.990033223	Bn-N3-p27696860	Bn-chrA03-p25579665
Bn-A03-p27339890	0.96013289	0.96013289	Bn-N3-p27699176	Bn-chrA03-p25581981
Bn-A03-p27354494	0.975206612	0.966942149	Bn-Scaffold06285-p1	Bn-chrA03-p25612901
Bn-A03-p27356417	0.975206612	0.966942149	Bn-N3-p27726351	Bn-chrA03-p25614824
Bn-A03-p27356726	0.958677686	0.975206612	Bn-N3-p27726645	Bn-chrA03-p25615133
Bn-A03-p27357082	0.884297521	0.983471074	Bn-N3-p27727003	Bn-chrA03-p25615489
Bn-A03-p27358390	0.537190083	0.743801653	Bn-N3-p27728277	Bn-chrA03-p25616794
Bn-A03-p27360321	0.983471074	0.991735537	Bn-N3-p27728417	Bn-chrA03-p25616935
Bn-A03-p27361855	0.983471074	0.991735537	Bn-N3-p27729943	Bn-chrA03-p25618473
Bn-A03-p27365971	0.990033223	0.990033223	Bn-N3-p27731724	Bn-chrA03-p25623492
Bn-A03-p27369889	0.991735537	0.991735537	Bn-N3-p27737094	Bn-chrA03-p25627415
Bn-A03-p27372281	0.983471074	0.975206612	Bn-N3-p27744103	Bn-chrA03-p25629837
Bn-A03-p27376850	0.986710963	0.990033223	Bn-N3-p27748638	Bn-chrA03-p25634836
Bn-A03-p27377729	0.983388704	0.980066445	Bn-Scaffold01974-p9	Bn-chrA03-p25635715
Bn-A03-p27377839	0.980066445	0.983388704	Bn-Scaffold01974-p1	Bn-chrA03-p25635825

Bn-A03-p27377892	0.990033223	0.986710963	Bn-Scaffold01974-p1	Bn-chrA03-p25635878
Bn-A03-p27378026	0.986710963	0.980066445	Bn-Scaffold01974-p1	Bn-chrA03-p25636012
Bn-A03-p27378198	0.983471074	0.975206612	Bn-Scaffold01974-p1	Bn-chrA03-p25636184
Bn-A03-p27378353	0.966942149	0.925619835	Bn-Scaffold01974-p1	Bn-chrA03-p25636333
Bn-A03-p27379193	0.975206612	0.983471074	Bn-Scaffold01974-p2	Bn-chrA03-p25637173
Bn-A03-p27379303	0.991735537	0.983471074	Bn-Scaffold01974-p2	Bn-chrA03-p25637283
Bn-A03-p27379570	0.993355482	0.990033223	Bn-Scaffold01974-p2	Bn-chrA03-p25637550
Bn-A03-p27380908	0.983388704	0.986710963	Bn-Scaffold01974-p4	Bn-chrA03-p25638888
Bn-A03-p27381126	0.991735537	0.983471074	Bn-Scaffold01974-p4	Bn-chrA03-p25639106
Bn-A03-p27381378	1	0.991735537	Bn-Scaffold01974-p4	Bn-chrA03-p25639358
Bn-A03-p27385155	0.996677741	0.993355482	Bn-N3-p27755002	Bn-chrA03-p25642454
Bn-A03-p27387912	0.991735537	0.975206612	Bn-N3-p27757903	Bn-chrA03-p25644818
Bn-A03-p27397690	0.950413223	0.958677686	Bn-N3-p27768319	Bn-chrA03-p25670536
Bn-A03-p27398341	0.991735537	0.983471074	Bn-N3-p27768970	Bn-chrA03-p25671195
Bn-A03-p27398352	0.975206612	0.983471074	Bn-N3-p27768981	Bn-chrA03-p25671206
Bn-A03-p27398935	0.996677741	0.926910299	Bn-N3-p27769564	Bn-chrA03-p25671780
Bn-A03-p27399719	1	0.966942149	Bn-N3-p27770145	Bn-chrA03-p25672515
Bn-A03-p27406806	0.991735537	0.991735537	Bn-N3-p27778502	Bn-chrA03-p25684335
Bn-A03-p27416815	0.983471074	0.983471074	Bn-N3-p27789758	Bn-chrA03-p25694462
Bn-A03-p27419226	0.925619835	0.925619835	Bn-N3-p27792635	Bn-chrAnn_random-p461466
Bn-A03-p27419285	0.975206612	0.975206612	Bn-N3-p27792693	Bn-chrAnn_random-p461466
Bn-A03-p27420794	0.958677686	0.958677686	Bn-N3-p27793922	Bn-chrA03-p25698736
Bn-A03-p27423379	0.993355482	0.990033223	Bn-N3-p27796507	Bn-chrA03-p25701321
Bn-A03-p27438081	1	0.991735537	Bn-N3-p27810899	Bn-chrA03-p25716274
Bn-A03-p27445819	0.990033223	0.986710963	Bn-N3-p27818670	Bn-chrA03-p25729372
Bn-A03-p27454930	0.953488372	1	Bn-N3-p27826872	Bn-chrA03-p25737596
Bn-A03-p27455156	0.991735537	0.983471074	Bn-N3-p27827095	Bn-chrA03-p25737822
Bn-A03-p27484868	0.983471074	0.983471074	Bn-N3-p27857151	Bn-chrA03-p25782888
Bn-A03-p27489686	0.991735537	0.991735537	Bn-N3-p27861973	Bn-chrA03-p25787710
Bn-A03-p27516492	0.996677741	0.996677741	Bn-N3-p27891957	Bn-chrA03-p25815769
Bn-A03-p27541115	0.991735537	0.991735537	Bn-N3-p27914811	Bn-chrA03-p25838259
Bn-A03-p27546971	0.983471074	0.983471074	Bn-N3-p27923490	Bn-chrA03-p25867237
Bn-A03-p27547270	0.983471074	0.983471074	Bn-N3-p27923776	Bn-chrA03-p25867523
Bn-A03-p27564692	0.983471074	0.983471074	Bn-N3-p27940257	Bn-chrA03-p25884950
Bn-A03-p27568225	0.983471074	0.983471074	Bn-N3-p27946080	Bn-chrA03-p25889340
Bn-A03-p27568580	0.983471074	0.983471074	Bn-N3-p27947509	Bn-chrA03-p25889695
Bn-A03-p27581773	0.986710963	0.986710963	Bn-N3-p27957944	Bn-chrA03-p25898535
Bn-A03-p27582705	0.958677686	0.958677686	Bn-N3-p27958875	Bn-chrA03-p25899466
Bn-A03-p27596068	0.966942149	0.966942149	Bn-N3-p27988913	Bn-chrAnn_random-p120252
Bn-A03-p27600547	0.909090909	0.909090909	Bn-N3-p27993022	Bn-chrAnn_random-p120293
Bn-A03-p27607709	0.975206612	0.975206612	Bn-N3-p28000064	Bn-chrAnn_random-p120363
Bn-A03-p27612791	0.966777409	0.966777409	Bn-N3-p28005407	Bn-chrAnn_random-p120413
Bn-A03-p27616297	0.966942149	0.966942149	Bn-N3-p28008656	Bn-chrAnn_random-p120445
Bn-A03-p27629415	0.966942149	0.966942149	Bn-N3-p28022921	Bn-chrAnn_random-p120591
Bn-A03-p27650553	0.966942149	0.966942149	Bn-N3-p28035188	Bn-chrAnn_random-p120732
Bn-A03-p27662317	0.983471074	0.983471074	Bn-N3-p28044112	Bn-chrAnn_random-p120821

Bn-A03-p27663888	0.975206612	0.975206612	Bn-N3-p28044630	Bn-chrAnn_random-p120826
Bn-A03-p27665971	0.980099502	0.980099502	Bn-Scaffold23998-p1	Bn-chrAnn_random-p120847
Bn-A03-p27683723	0.975206612	0.975206612	Bn-N3-p28068549	Bn-chrAnn_random-p121026
Bn-A03-p27683903	0.950413223	0.950413223	Bn-N3-p28068732	Bn-chrAnn_random-p121028
Bn-A03-p27685078	0.975206612	0.975206612	Bn-N3-p28069814	Bn-chrAnn_random-p121039
Bn-A03-p27686257	0.892561983	0.892561983	Bn-N3-p28071097	Bn-chrAnn_random-p351696
Bn-A03-p27686487	0.900826446	0.900826446	Bn-N3-p28071325	Bn-chrAnn_random-p351694
Bn-A03-p27686879	0.603305785	0.570247934	Bn-N3-p28091486	Bn-chrC07-p41894319
Bn-A03-p27712521	1	1	Bn-N3-p28135958	Bn-chrA03-p25938526
Bn-A03-p27713307	0.991735537	0.991735537	Bn-N3-p28136742	Bn-chrA03-p25939310
Bn-A03-p27713975	1	1	Bn-N3-p28137408	Bn-chrA03-p25939976
Bn-A03-p27715593	0.975206612	0.975206612	Bn-N3-p28139016	Bn-chrA03-p25941584
Bn-A03-p27719757	0.990049751	0.990049751	Bn-N3-p28143273	Bn-chrA03-p25945786
Bn-A03-p27720045	0.985074627	0.985074627	Bn-N3-p28143561	Bn-chrA03-p25946074
Bn-A03-p27741110	0.958677686	0.958677686	Bn-N3-p28157816	Bn-chrA03-p25960622
Bn-A03-p27742950	0.975206612	0.966942149	Bn-N3-p28159664	Bn-chrC07-p41965585
Bn-A03-p27746955	0.955223881	0.955223881	Bn-N3-p28163911	Bn-chrA03-p25966065
Bn-A03-p27747178	0.960199005	0.960199005	Bn-N3-p28164136	Bn-chrA03-p25966290
Bn-A03-p27747229	0.950248756	0.950248756	Bn-N3-p28164187	Bn-chrA03-p25966341
Bn-A03-p27752076	0.975206612	0.975206612	Bn-N3-p28169047	Bn-chrA03-p25971199
Bn-A03-p27752857	1	1	Bn-N3-p28169828	Bn-chrA03-p25971980
Bn-A03-p27791357	0.983471074	0.983471074	Bn-N3-p28198375	Bn-chrA03-p26001485
Bn-A03-p27791437	0.975206612	0.975206612	Bn-N3-p28198455	Bn-chrA03-p26001565
Bn-A03-p27793125	0.991735537	0.991735537	Bn-N3-p28201195	Bn-chrA03-p26003253
Bn-A03-p27794041	0.975206612	0.975206612	Bn-N3-p28202111	Bn-chrA03-p26004169
Bn-A03-p27794263	1	1	Bn-N3-p28202333	Bn-chrA03-p26004391
Bn-A03-p27794657	0.991735537	0.991735537	Bn-N3-p28202727	Bn-chrA03-p26004785
Bn-A03-p27794772	0.983471074	0.983471074	Bn-N3-p28202842	Bn-chrA03-p26004900
Bn-A03-p27794775	0.983471074	0.983471074	Bn-N3-p28202845	Bn-chrA03-p26004903
Bn-A03-p27794848	0.966942149	0.966942149	Bn-N3-p28202918	Bn-chrA03-p26004976
Bn-A03-p27796940	0.966942149	0.966942149	Bn-N3-p28205010	Bn-chrA03-p26007068
Bn-A03-p27805759	0.966942149	0.966942149	Bn-N3-p28213830	Bn-chrA03-p26015888
Bn-A03-p27824257	1	1	Bn-N3-p28231927	Bn-chrA03-p26038200
Bn-A03-p27825739	0.975206612	0.975206612	Bn-N3-p28233409	Bn-chrA03-p26039682
Bn-A03-p27828889	0.991735537	0.991735537	Bn-N3-p28237421	Bn-chrA03-p26043844
Bn-A03-p27838177	0.983471074	0.983471074	Bn-N3-p28247743	Bn-chrC07-p42062259
Bn-A03-p27840169	0.995024876	0.995024876	Bn-N3-p28249727	Bn-chrA03-p26059268
Bn-A03-p27841728	0.975206612	0.975206612	Bn-N3-p28251286	Bn-chrA03-p26060827
Bn-A03-p27853312	0.94214876	0.94214876	Bn-N3-p28262385	Bn-chrA03-p26071962
Bn-A03-p27866782	0.966942149	0.966942149	Bn-N3-p28280474	Bn-chrA03-p26089340
Bn-A03-p27906760	0.983471074	0.983471074	Bn-N3-p28341690	Bn-chrA10-p5489567
Bn-A03-p27917455	0.983471074	0.991735537	Bn-N3-p28356341	Bn-chrA03-p26153065
Bn-A03-p27927967	0.966942149	0.966942149	Bn-N3-p28361268	Bn-chrA03-p26157992
Bn-A03-p27928232	0.933884298	0.933884298	Bn-N3-p28361483	Bn-chrA03-p26158207
Bn-A03-p27951283	0.993355482	0.993355482	Bn-N3-p28379354	Bn-chrA03-p26177318
Bn-A03-p27951979	0.950413223	0.950413223	Bn-N3-p28380203	Bn-chrA03-p26178063

Bn-A03-p27972408	0.935323383	0.935323383	Bn-N3-p28386690	Bn-chrA03-p26184522
Bn-A03-p27972738	0.983471074	0.983471074	Bn-N3-p28387020	Bn-chrA03-p26184852
Bn-A03-p28036367	0.991735537	0.991735537	Bn-N3-p28465332	Bn-chrA03-p26497854
Bn-A03-p28039749	0.966942149	0.966942149	Bn-N3-p28469456	Bn-chrA03-p26501977
Bn-A03-p28043216	0.933884298	0.933884298	Bn-N3-p28478610	Bn-chrA03-p26511637
Bn-A03-p28046101	0.958677686	0.958677686	Bn-N3-p28480905	Bn-chrA03-p26513932
Bn-A03-p28091909	0.611570248	0.611570248	Bn-N3-p28530811	Bn-chrA03-p26561186
Bn-A03-p28097052	0.993355482	0.993355482	Bn-N3-p28537367	Bn-chrA03-p26568361
Bn-A03-p28109103	0.933884298	0.933884298	Bn-N3-p28549660	Bn-chrA03-p26580117
Bn-A03-p28117836	0.993355482	0.993355482	Bn-N3-p28558388	Bn-chrA03-p26588844
Bn-A03-p28126287	0.990049751	0.990049751	Bn-N3-p28566839	Bn-chrA03-p26597295
Bn-A03-p28127216	1	1	Bn-N3-p28567770	Bn-chrA03-p26598226
Bn-A03-p28153736	0.985074627	0.985074627	Bn-N3-p28574150	Bn-chrA03-p26604606
Bn-A03-p28153797	0.975124378	0.975124378	Bn-N3-p28574211	Bn-chrA03-p26604667
Bn-A03-p28154053	0.995024876	0.995024876	Bn-N3-p28574467	Bn-chrA03-p26604923
Bn-A03-p28154830	0.975206612	0.975206612	Bn-N3-p28575244	Bn-chrA03-p26605700
Bn-A03-p28154970	0.710743802	0.710743802	Bn-N17-p46115361	Bn-chrC07-p42312151
Bn-A03-p28155412	0.975206612	0.975206612	Bn-N3-p28580424	Bn-chrA03-p26613018
Bn-A03-p28184972	0.995024876	0.995024876	Bn-N3-p28609414	Bn-chrA03-p26641783
Bn-A03-p28202050	0.96875	0.96875	Bn-N3-p28631422	Bn-chrA03-p26668797
Bn-A03-p28207594	0.966942149	0.966942149	Bn-N3-p28636899	Bn-chrA03-p26674274
Bn-A03-p28221433	0.966942149	0.966942149	Bn-N3-p28650499	Bn-chrA03-p26687873
Bn-A03-p28222096	0.950166113	0.950166113	Bn-N3-p28651162	Bn-chrA03-p26688536
Bn-A03-p28227026	0.983471074	0.983471074	Bn-N3-p28656504	Bn-chrA03-p26693421
Bn-A03-p28229360	0.966942149	0.966942149	Bn-N3-p28658838	Bn-chrA03-p26695755
Bn-A03-p28232863	0.983471074	0.983471074	Bn-N3-p28662341	Bn-chrA03-p26699258
Bn-A03-p28233201	0.966942149	0.966942149	Bn-N3-p28662679	Bn-chrA03-p26699596
Bn-A03-p28233701	0.950413223	0.950413223	Bn-N3-p28663179	Bn-chrA03-p26700096
Bn-A03-p28234166	1	1	Bn-N3-p28663645	Bn-chrA03-p26700562
Bn-A03-p28234586	0.975206612	0.975206612	Bn-N3-p28664065	Bn-chrA03-p26700982
Bn-A03-p28242905	0.975206612	0.975206612	Bn-N3-p28672391	Bn-chrA03-p26709305
Bn-A03-p28243306	0.991735537	0.991735537	Bn-N3-p28672792	Bn-chrA03-p26709706
Bn-A03-p28256169	0.975206612	0.975206612	Bn-N3-p28692893	Bn-chrA03-p26731630
Bn-A03-p28265602	0.756218905	0.726368159	Bn-N3-p28713559	Bn-chrA03-p26744397
Bn-A03-p28276873	0.975206612	0.975206612	Bn-N3-p28721679	Bn-chrA03-p26753910
Bn-A03-p28279796	0.958677686	0.958677686	Bn-N3-p28724592	Bn-chrA03-p26756822
Bn-A03-p28297407	0.991735537	0.991735537	Bn-N3-p28747350	Bn-chrA03-p26779637
Bn-A03-p28302949	0.958677686	0.958677686	Bn-N3-p28752921	Bn-chrA03-p26785208
Bn-A03-p28360281	1	1	Bn-N3-p28798265	Bn-chrA03-p26829810
Bn-A03-p28389539	0.990033223	0.96345515	Bn-Scaffold12025-p3	Bn-chrA03-p26848009
Bn-A03-p28464995	0.980099502	0.975124378	Bn-N3-p28886686	Bn-chrA03-p26915891
Bn-A03-p28468317	0.985074627	0.985074627	Bn-N3-p28890008	Bn-chrA03-p26919213
Bn-A03-p28469194	0.995024876	0.990049751	Bn-N3-p28890890	Bn-chrA03-p26920090
Bn-A03-p28491294	1	0.991735537	Bn-N3-p28916350	Bn-chrA03-p26941594
Bn-A03-p28491779	0.991735537	1	Bn-N3-p28916833	Bn-chrA03-p26942079
Bn-A03-p28498617	0.990049751	0.990049751	Bn-N3-p28933444	Bn-chrA03-p26949163

Bn-A03-p28498755	0.990049751	0.990049751	Bn-N3-p28933582	Bn-chrA03-p26949301
Bn-A03-p28499049	0.996677741	0.996677741	Bn-N3-p28933876	Bn-chrA03-p26949595
Bn-A03-p28499141	0.995024876	0.995024876	Bn-N3-p28933967	Bn-chrA03-p26949686
Bn-A03-p28535638	0.983471074	0.983471074	Bn-N3-p28971341	Bn-chrA03-p26990079
Bn-A03-p28541411	1	1	Bn-N3-p28984296	Bn-chrA03-p27003051
Bn-A03-p28551517	0.975206612	0.983471074	Bn-N3-p29002208	Bn-chrA03-p27013195
Bn-A03-p28551626	0.950413223	0.983471074	Bn-N3-p29002313	Bn-chrA03-p27013304
Bn-A03-p28551775	0.958677686	0.966942149	Bn-N3-p29002459	Bn-chrA03-p27013453
Bn-A03-p28560125	1	0.989583333	Bn-N3-p29014512	Bn-chrA03-p27022371
Bn-A03-p28560659	0.950413223	0.975206612	Bn-N17-p46458977	Bn-chrA03-p27022905
Bn-A03-p28570053	0.909090909	0.958677686	Bn-N3-p29028377	Bn-chrA03-p27041863
Bn-A03-p28583587	0.983471074	0.975206612	Bn-N3-p29038514	Bn-chrA03-p27051947
Bn-A03-p28583685	0.958677686	0.958677686	Bn-N3-p29038612	Bn-chrA03-p27052045
Bn-A03-p28588701	0.953488372	0.953488372	Bn-N3-p29043829	Bn-chrA03-p27058837
Bn-A03-p28590688	0.985074627	0.985074627	Bn-N3-p29045816	Bn-chrA03-p27060824
Bn-A03-p28590746	0.990033223	0.990033223	Bn-N3-p29045874	Bn-chrA03-p27060882
Bn-A03-p28594732	0.991735537	0.991735537	Bn-N3-p29050212	Bn-chrA03-p27062582
Bn-A03-p28616355	0.975206612	0.958677686	Bn-N3-p29071613	Bn-chrC07-p42662257
Bn-A03-p28698732	0.965174129	0.965174129	Bn-N17-p46544136	Bn-chrC07-p42696837
Bn-A03-p28808705	0.983471074	0.983471074	Bn-N3-p29254604	Bn-chrA03-p27252822
Bn-A03-p28840414	0.991735537	0.991735537	Bn-N3-p29280162	Bn-chrA03-p27277900
Bn-A03-p28843588	0.991735537	0.991735537	Bn-N3-p29283295	Bn-chrA03-p27281256
Bn-A03-p28847903	0.950413223	0.950413223	Bn-N3-p29286039	Bn-chrA03-p27284000
Bn-A03-p28851691	0.983471074	0.983471074	Bn-N3-p29293424	Bn-chrA03-p27291411
Bn-A03-p28873781	0.965174129	0.965174129	Bn-N1-p7410314	Bn-chrAnn_random-p276491
Bn-A03-p28877626	0.975206612	0.975206612	Bn-N3-p29314459	Bn-chrA03-p27312300
Bn-A03-p28877719	0.991735537	0.991735537	Bn-N3-p29314552	Bn-chrA03-p27312393
Bn-A03-p28878501	0.993355482	0.993355482	Bn-N3-p29315338	Bn-chrA03-p27313179
Bn-A03-p28878698	0.983471074	0.983471074	Bn-N3-p29315535	Bn-chrA03-p27313376
Bn-A03-p28878703	0.991735537	0.991735537	Bn-N3-p29315540	Bn-chrA03-p27313381
Bn-A03-p28879610	0.966942149	0.966942149	Bn-N3-p29316444	Bn-chrA03-p27314285
Bn-A03-p28882585	0.965174129	0.965174129	Bn-N3-p29319418	Bn-chrA03-p27317259
Bn-A03-p28883986	0.975206612	0.975206612	Bn-N3-p29320816	Bn-chrA03-p27318657
Bn-A03-p28910384	0.669421488	0.669421488	Bn-N17-p46685646	Bn-chrC07-p42831690
Bn-A03-p28910430	0.958677686	0.363636364	Bn-Scaffold12474-p7	Bn-chrC07-p42831735
Bn-A03-p28924123	0.980066445	0.980066445	Bn-N3-p29356139	Bn-chrA03-p27337192
Bn-A03-p28926445	0.933554817	0.933554817	Bn-N3-p29358461	Bn-chrA03-p27339514
Bn-A03-p28928570	0.983471074	0.983471074	Bn-N3-p29360580	Bn-chrA03-p27341633
Bn-A03-p28948799	0.991735537	0.991735537	Bn-N3-p29379477	Bn-chrA03-p27358861
Bn-A03-p28963072	0.993355482	0.993355482	Bn-N3-p29389977	Bn-chrA03-p27373344
Bn-A03-p28977058	0.975206612	0.983471074	Bn-N3-p29404545	Bn-chrA03-p27387330
Bn-A03-p28981296	0.991735537	1	Bn-N3-p29408925	Bn-chrA03-p27391710
Bn-A03-p28982546	0.975206612	0.966942149	Bn-N3-p29410176	Bn-chrA03-p27392984
Bn-A03-p28995612	0.985074627	0.985074627	Bn-N17-p46747013	Bn-chrC07-p42892718
Bn-A03-p28996082	0.975124378	0.975124378	Bn-N3-p29425146	Bn-chrA03-p27408885
Bn-A03-p29002618	0.94214876	0.94214876	Bn-N3-p29438003	Bn-chrA03-p27421311

Bn-A03-p29005979	0.94214876	0.94214876	Bn-N3-p29441263	Bn-chrA03-p27424707
Bn-A03-p29007606	0.925619835	0.925619835	Bn-N3-p29444325	Bn-chrA03-p27427769
Bn-A03-p29009401	0.950413223	0.950413223	Bn-N3-p29446118	Bn-chrA03-p27429561
Bn-A03-p29046284	0.975206612	0.983471074	Bn-N3-p29475909	Bn-chrA03-p27461837
Bn-A03-p29050002	0.975206612	0.975206612	Bn-N3-p29476944	Bn-chrA03-p27462883
Bn-A03-p29053385	0.983471074	0.925619835	Bn-N3-p29480332	Bn-chrA03-p27466277
Bn-A03-p29060027	0.991735537	1	Bn-N3-p29486973	Bn-chrA03-p27472882
Bn-A03-p29060253	0.983471074	0.975206612	Bn-N3-p29487199	Bn-chrA03-p27473108
Bn-A03-p29065314	0.985074627	0.990049751	Bn-N3-p29492260	Bn-chrA03-p27478171
Bn-A03-p29066510	0.983471074	0.975206612	Bn-N3-p29493456	Bn-chrA03-p27483969
Bn-A03-p29067657	0.983471074	0.991735537	Bn-N3-p29494603	Bn-chrA03-p27485116
Bn-A03-p29068059	1	0.991735537	Bn-N3-p29495005	Bn-chrA03-p27485518
Bn-A03-p29068198	0.975206612	0.983471074	Bn-N3-p29495144	Bn-chrA03-p27485657
Bn-A03-p29075333	0.991735537	1	Bn-N3-p29502262	Bn-chrA03-p27494361
Bn-A03-p29081614	0.991735537	0.991735537	Bn-N3-p29513622	Bn-chrA03-p27510984
Bn-A03-p29097772	0.990049751	0.985074627	Bn-N3-p29528749	Bn-chrAnn_random-p344251
Bn-A03-p29102865	0.960199005	0.965174129	Bn-N3-p29533842	Bn-chrA03-p26240559
Bn-A03-p29118954	0.966942149	0.966942149	Bn-N3-p29549720	Bn-chrA03-p26262031
Bn-A03-p29198982	0.983471074	0.991735537	Bn-N3-p29679770	Bn-chrA03-p26320510
Bn-A03-p29212835	0.975206612	0.966942149	Bn-N3-p29692947	Bn-chrA03-p26300410
Bn-A03-p29216506	0.950413223	0.909090909	Bn-N3-p29696617	Bn-chrA03-p26291286
Bn-A03-p29216612	0.966942149	0.975206612	Bn-N3-p29696722	Bn-chrA03-p26291179
Bn-A03-p29234677	0.950413223	0.94214876	Bn-N3-p29751828	Bn-chrA03-p26351125
Bn-A03-p29264946	0.966942149	0.925619835	Bn-N3-p29715620	Bn-chrA03-p26289639
Bn-A03-p29269385	0.966942149	0.958677686	Bn-N3-p29720055	Bn-chrC07-p43038094
Bn-A03-p29271424	0.966942149	0.975206612	Bn-N3-p29722078	Bn-chrA03-p26286136
Bn-A03-p29271737	0.991735537	0.983471074	Bn-N3-p29722391	Bn-chrA03-p26285835
Bn-A03-p29274532	0.966942149	0.876033058	Bn-N3-p29726484	Bn-chrC07-p43030684
Bn-A03-p29279909	0.991735537	0.983471074	Bn-N3-p29731867	Bn-chrA03-p26274082
Bn-A03-p29326525	0.966942149	0.958677686	Bn-N3-p29785986	Bn-chrA03-p26362505
Bn-A03-p29335920	1	0.991735537	Bn-N3-p29796029	Bn-chrA03-p26365755
Bn-A03-p29338454	1	0.991735537	Bn-N3-p29798764	Bn-chrA03-p26366480
Bn-A03-p29342929	0.990049751	0.980099502	Bn-N3-p29803578	Bn-chrA03-p26371803
Bn-A03-p29346125	0.990033223	0.996677741	Bn-N3-p29808039	Bn-chrA03-p26377945
Bn-A03-p29347368	1	0.983471074	Bn-N3-p29809280	Bn-chrA03-p26378874
Bn-A03-p29364596	0.975206612	0.983471074	Bn-N3-p29824338	Bn-chrA03-p26381263
Bn-A03-p29367826	0.991735537	1	Bn-N3-p29827582	Bn-chrA03-p26384540
Bn-A03-p29390022	0.826446281	0.834710744	Bn-N3-p29843135	Bn-chrA03-p26398267
Bn-A03-p29391849	0.975206612	0.983471074	Bn-N3-p29844867	Bn-chrA03-p26400093
Bn-A03-p29564970	0.975206612	0.983471074	Bn-N3-p30007309	Bn-chrA03-p27595162
Bn-A03-p29565087	0.975206612	0.933884298	Bn-N3-p30007426	Bn-chrC07-p43351819
Bn-A03-p29566168	0.985074627	0.985074627	Bn-N3-p30008507	Bn-chrA03-p27596644
Bn-A03-p29571938	0.996677741	0.996677741	Bn-N3-p23667615	Bn-chrA03_random-p154684
Bn-A03-p29573204	0.966942149	0.958677686	Bn-N3-p30016471	Bn-chrA03-p27608960
Bn-A03-p29583640	0.958677686	0.950413223	Bn-N3-p30022628	Bn-chrA03-p27619653
Bn-A03-p29587388	0.991735537	1	Bn-N3-p30026567	Bn-chrA03-p27622920

Bn-A03-p29587945	0.975206612	0.966942149	Bn-N3-p30027124	Bn-chrA03-p27623477
Bn-A03-p29590349	0.991735537	1	Bn-N3-p30028438	Bn-chrA03-p27624791
Bn-A03-p29593434	0.983471074	0.991735537	Bn-N3-p30031524	Bn-chrA03-p27627878
Bn-A03-p29595276	0.983471074	0.975206612	Bn-N3-p30033367	Bn-chrA03-p27629723
Bn-A03-p29598801	0.975206612	0.983471074	Bn-N3-p30037033	Bn-chrA03-p27632871
Bn-A03-p29606245	0.966942149	0.958677686	Bn-N3-p30045072	Bn-chrA03-p27637884
Bn-A03-p29614729	0.991735537	0.595041322	Bn-N3-p30055629	Bn-chrC07-p28136954
Bn-A03-p29629982	0.975206612	0.983471074	Bn-N3-p30096660	Bn-chrA03-p27665153
Bn-A03-p29632526	0.925373134	0.910447761	Bn-N3-p30099193	Bn-chrA03-p27667701
Bn-A03-p29656801	0.909090909	0.975206612	Bn-N3-p30118965	Bn-chrA03-p27697052
Bn-A03-p29661431	0.980099502	0.985074627	Bn-N3-p30123893	Bn-chrA03-p27705738
Bn-A03-p29764052	0.991735537	0.983471074	Bn-N3-p30210760	Bn-chrA03-p27784537
Bn-A03-p29776900	0.983471074	0.975206612	Bn-N3-p30223749	Bn-chrA03-p27796815
Bn-A03-p29780293	0.983471074	0.975206612	Bn-N3-p30227139	Bn-chrA03-p27800213
Bn-A03-p29781065	1	0.991735537	Bn-N3-p30227911	Bn-chrA03-p27800985
Bn-A03-p29784941	0.991735537	0.776859504	Bn-N3-p30231466	Bn-chrC01_random-p278529
Bn-A03-p29790183	1	0.991735537	Bn-N3-p30235734	Bn-chrA03-p27806800
Bn-A03-p29802913	0.991735537	0.983471074	Bn-N3-p30254166	Bn-chrA03-p27824479
Bn-A03-p29803241	1	0.991735537	Bn-N3-p30254494	Bn-chrA03-p27824809
Bn-A03-p29803351	0.975206612	0.966942149	Bn-N3-p30254604	Bn-chrA03-p27824920
Bn-A03-p29805598	1	0.991735537	Bn-N3-p30256851	Bn-chrA03-p27827164
Bn-A03-p29806105	1	0.991735537	Bn-N3-p30257358	Bn-chrA03-p27827671
Bn-A03-p29816785	0.991735537	0.983471074	Bn-N3-p30274600	Bn-chrA03_random-p220738
Bn-A03-p29823747	0.983471074	0.975206612	Bn-N3-p30284461	Bn-chrA03_random-p221619
Bn-A03-p29834625	0.983471074	0.991735537	Bn-N3-p30295442	Bn-chrA03_random-p218614
Bn-A03-p29840518	0.975206612	0.983471074	Bn-N3-p30300511	Bn-chrAnn_random-p478788
Bn-A03-p29849117	0.983471074	0.975206612	Bn-N3-p30309239	Bn-chrA03-p27894765
Bn-A03-p29849191	0.930232558	0.953488372	Bn-N3-p30309313	Bn-chrA03-p27894839
Bn-A03-p29855378	0.991735537	0.983471074	Bn-N3-p30314104	Bn-chrA03-p27900255
Bn-A03-p29866453	0.983471074	0.991735537	Bn-N3-p30323016	Bn-chrA03-p27908788
Bn-A03-p29868254	0.950413223	0.958677686	Bn-N3-p30324817	Bn-chrA03-p27910590
Bn-A03-p29873919	1	0.991735537	Bn-N3-p30330320	Bn-chrA03-p27911918
Bn-A03-p29874052	1	0.991735537	Bn-N3-p30330453	Bn-chrA03-p27912051
Bn-A03-p29883989	0.966942149	0.52892562	Bn-N3-p30338850	Bn-chrCnn_random-p203423
Bn-A03-p29896540	0.996677741	0.993355482	Bn-N3-p30351399	Bn-chrA03-p27935049
Bn-A03-p29899721	0.983471074	0.975206612	Bn-N3-p30358187	Bn-chrA03-p27938230
Bn-A03-p29900824	0.950413223	0.966942149	Bn-N17-p47410859	Bn-chrA03-p27939333
Bn-A03-p29926117	0.975206612	0.983471074	Bn-N3-p30377785	Bn-chrA03-p27966773
Bn-A03-p29956906	0.966942149	0.991735537	Bn-N3-p30399898	Bn-chrA03-p27989280
Bn-A03-p29964330	0.975206612	0.983471074	Bn-N3-p30407919	Bn-chrA03-p27995742
Bn-A03-p29998247	0.993355482	0.993355482	Bn-N3-p30445825	Bn-chrA03-p28031200
Bn-A03-p29999738	1	1	Bn-N3-p30447312	Bn-chrA03-p28032687
Bn-A03-p30029704	0.980099502	0.985074627	Bn-N3-p30463369	Bn-chrAnn_random-p273184
Bn-A03-p30052524	0.983471074	0.652892562	Bn-N3-p30486960	Bn-chrAnn_random-p884893
Bn-A03-p30057461	0.965174129	0.970149254	Bn-N3-p30492171	Bn-chrAnn_random-p884963
Bn-A03-p30170124	0.990049751	0.990049751	Bn-N3-p30585268	Bn-chrAnn_random-p895491

Bn-A03-p30183760	0.990049751	0.990049751	Bn-N3-p30604879	Bn-chrAnn_random-p897469
Bn-A03-p30183937	0.774086379	0.774086379	Bn-N3-p30605056	Bn-chrAnn_random-p897486
Bn-A03-p30243799	0.817275748	0.823920266	Bn-N3-p30665384	Bn-chrA03_random-p222459
Bn-A03-p30291719	0.993355482	0.993355482	Bn-N3-p30731942	Bn-chrAnn_random-p216416
Bn-A03-p30351345	0.970149254	0.970149254	Bn-N3-p30825934	Bn-chrA03-p28102692
Bn-A03-p30358487	0.970149254	0.970149254	Bn-N3-p30833534	Bn-chrC09-p7601439
Bn-A03-p30564852	0.983471074	0.975206612	Bn-N3-p31074167	Bn-chrA03-p28308848
Bn-A03-p30658785	0.986710963	0.990033223	Bn-N3-p31198615	Bn-chrA03-p28438798
Bn-A03-p30663365	1	0.991735537	Bn-N3-p31203195	Bn-chrA03-p28439822
Bn-A03-p30667694	0.991735537	0.983471074	Bn-N3-p31207684	Bn-chrA03-p28444334
Bn-A03-p30693412	0.991735537	0.983471074	Bn-N3-p31246596	Bn-chrA03-p28473978
Bn-A03-p30831188	1	0.991735537	Bn-N3-p31375211	Bn-chrA03-p28618994
Bn-A03-p30852922	0.996677741	0.996677741	Bn-N3-p31409363	Bn-chrA03-p28640932
Bn-A03-p30873818	0.975124378	0.975124378	Bn-N3-p31430489	Bn-chrA03-p28666591
Bn-A03-p30873956	0.985074627	0.985074627	Bn-N3-p31430627	Bn-chrA03-p28666729
Bn-A03-p30874086	0.970149254	0.975124378	Bn-N3-p31430757	Bn-chrA03-p28666859
Bn-A03-p30876909	0.983471074	0.975206612	Bn-N3-p31433263	Bn-chrA03-p28669682
Bn-A03-p30879004	0.94214876	0.983471074	Bn-N3-p31436223	Bn-chrA03-p28671764
Bn-A03-p30879164	0.975206612	0.983471074	Bn-N3-p31437667	Bn-chrA03-p28671924
Bn-A03-p30888263	0.991735537	0.983471074	Bn-N3-p31446102	Bn-chrA10-p11782592
Bn-A03-p30897153	0.983471074	0.801652893	Bn-N3-p31462276	Bn-chrC07-p44200209
Bn-A03-p30905429	0.94214876	0.94214876	Bn-N3-p31465405	Bn-chrA03-p28699941
Bn-A03-p30906755	0.975206612	0.975206612	Bn-N3-p31466728	Bn-chrA03-p28701264
Bn-A03-p30910234	0.983471074	0.983471074	Bn-N3-p31470426	Bn-chrA03-p28704962
Bn-A03-p30920789	0.991735537	0.966942149	Bn-Scaffold03441-p4	Bn-chrC07-p44226069
Bn-A03-p30925102	0.983471074	0.983471074	Bn-N3-p31490325	Bn-chrA03-p28728947
Bn-A03-p30951542	0.985074627	0.975124378	Bn-N3-p31523807	Bn-chrA03_random-p365105
Bn-A03-p30974143	1	1	Bn-N3-p31587139	Bn-chrA03-p28807657
Bn-A03-p30974251	0.979166667	0.979166667	Bn-N3-p31587188	Bn-chrA03-p28807706
Bn-A03-p31030338	0.991735537	0.991735537	Bn-N3-p31661304	Bn-chrC07-p44305923
Bn-A03-p31049094	0.991735537	1	Bn-N3-p31683892	Bn-chrA03-p28898756
Bn-A03-p31076742	0.991735537	0.991735537	Bn-N3-p31714422	Bn-chrA03-p28971163
Bn-A03-p31076904	0.985074627	0.985074627	Bn-N3-p31714586	Bn-chrA03-p28971327
Bn-A03-p31077034	0.985074627	0.990049751	Bn-N3-p31714734	Bn-chrA03-p28971475
Bn-A03-p31078490	0.955357143	0.991071429	Bn-N3-p31716605	Bn-chrA03-p28972876
Bn-A03-p31088060	0.991735537	1	Bn-Scaffold02077-p2	Bn-chrA03-p28984662
Bn-A03-p31088264	0.975124378	0.970149254	Bn-Scaffold02077-p2	Bn-chrA03-p28984866
Bn-A03-p31091148	0.983471074	0.975206612	Bn-N3-p31728620	Bn-chrA03-p28987753
Bn-A03-p31093169	0.980099502	0.980099502	Bn-N3-p31730813	Bn-chrA03-p28989948
Bn-A03-p31095221	0.995024876	0.990049751	Bn-N3-p31733173	Bn-chrA03-p28992308
Bn-A03-p31113968	0.990049751	0.995024876	Bn-N3-p31751578	Bn-chrA03-p29008514
Bn-A03-p31147953	0.975124378	0.970149254	Bn-N3-p32213844	Bn-chrA03_random-p316773
Bn-A03-p31160337	0.913621262	0.913621262	Bn-N2-p12784484	Bn-chrA02-p9630993
Bn-A03-p31179815	0.991735537	1	Bn-N3-p31785347	Bn-chrA03-p29049025
Bn-A03-p31212393	0.975206612	0.966942149	Bn-N3-p31819445	Bn-chrA03-p29098027
Bn-A03-p31242297	1	1	Bn-N9-p28670345	Bn-chrAnn_random-p454904

Bn-A03-p31242854	0.995024876	0.995024876	Bn-N9-p28670902	Bn-chrAnn_random-p45491C
Bn-A03-p31246266	0.995024876	1	Bn-N9-p28674315	Bn-chrC04-p34959798
Bn-A03-p31327366	1	0.996677741	Bn-N3-p31953292	Bn-chrA03-p29263607
Bn-A03-p31535298	0.990033223	0.986710963	Bn-N3-p32128341	Bn-chrA03_random-p326119
Bn-A03-p31540985	0.925619835	0.933884298	Bn-N3-p32132964	Bn-chrA03_random-p325657
Bn-A03-p31546901	0.965174129	0.970149254	Bn-N1-p16898334	Bn-chrC04-p18413515
Bn-A03-p31579593	0.989583333	0.989583333	Bn-N3-p32159871	Bn-chrA03_random-p322961
Bn-A03-p31651142	0.885572139	0.895522388	Bn-Scaffold42408-p1	Bn-chrAnn_random-p32931C
Bn-A03-p31654131	0.726368159	0.731343284	Bn-N3-p32384703	Bn-chrAnn_random-p347389
Bn-A04-p24368	0.958677686	0.958677686	Bn-N4-p133409	Bn-chrA04-p42776
Bn-A04-p24411	0.975206612	0.975206612	Bn-N4-p133366	Bn-chrA04-p42733
Bn-A04-p113641	0.983471074	0.983471074	Bn-N4-p204073	Bn-chrA04-p120141
Bn-A04-p122280	0.993355482	0.993355482	Bn-N4-p193375	Bn-chrA04-p104399
Bn-A04-p149787	0.993355482	0.993355482	Bn-N14-p25447181	Bn-chrC04-p21927688
Bn-A04-p223001	0.94214876	0.94214876	Bn-N4-p227020	Bn-chrA04-p157674
Bn-A04-p225323	0.983471074	0.983471074	Bn-N4-p229474	Bn-chrA04-p159996
Bn-A04-p233588	1	1	Bn-N4-p239356	Bn-chrA04-p171605
Bn-A04-p234664	0.867768595	0.867768595	Bn-N4-p240436	Bn-chrA04-p172685
Bn-A04-p248884	0.94214876	0.94214876	Bn-N4-p249828	Bn-chrA04-p181949
Bn-A04-p251383	1	1	Bn-N4-p252312	Bn-chrA04-p184433
Bn-A04-p261340	0.975206612	0.975206612	Bn-N4-p263953	Bn-chrA04-p194583
Bn-A04-p262404	0.983471074	0.983471074	Bn-N4-p265017	Bn-chrA04-p195647
Bn-A04-p267294	0.983471074	0.983471074	Bn-N4-p269934	Bn-chrA04-p200564
Bn-A04-p288906	0.950413223	0.950413223	Bn-N4-p298174	Bn-chrA04-p228278
Bn-A04-p289974	0.958677686	0.958677686	Bn-N4-p299261	Bn-chrA04-p229365
Bn-A04-p312324	0.991735537	0.983471074	Bn-N4-p327652	Bn-chrA04-p256249
Bn-A04-p313091	0.975206612	0.975206612	Bn-N4-p328419	Bn-chrA04-p257015
Bn-A04-p313410	0.983471074	0.983471074	Bn-N4-p328738	Bn-chrA04-p257334
Bn-A04-p313935	0.984496124	0.984496124	Bn-N4-p329263	Bn-chrA04-p257859
Bn-A04-p314440	0.983471074	0.983471074	Bn-N4-p329768	Bn-chrA04-p258364
Bn-A04-p314536	0.990049751	0.990049751	Bn-N4-p329864	Bn-chrA04-p258460
Bn-A04-p314872	0.983471074	0.983471074	Bn-N4-p330200	Bn-chrA04-p258796
Bn-A04-p320972	0.995024876	0.995024876	Bn-N4-p334926	Bn-chrA04-p263519
Bn-A04-p322820	0.975206612	0.975206612	Bn-N4-p336773	Bn-chrA04-p265366
Bn-A04-p324144	0.966942149	0.966942149	Bn-N4-p338158	Bn-chrA04-p268118
Bn-A04-p325019	0.990033223	0.990033223	Bn-N4-p338730	Bn-chrA04-p268690
Bn-A04-p326007	0.892561983	0.892561983	Bn-N4-p339717	Bn-chrA04-p269677
Bn-A04-p330341	0.956810631	0.996677741	Bn-N4-p344068	Bn-chrA04-p274028
Bn-A04-p354688	0.991735537	0.991735537	Bn-N4-p367121	Bn-chrA04_random-p17549
Bn-A04-p356570	0.983471074	0.983471074	Bn-N4-p369005	Bn-chrA04_random-p15665
Bn-A04-p357272	0.991735537	0.991735537	Bn-N4-p369707	Bn-chrA04_random-p14963
Bn-A04-p370297	0.979166667	0.979166667	Bn-N4-p382749	Bn-chrAnn_random-p32872C
Bn-A04-p375224	0.975206612	0.975206612	Bn-N4-p387989	Bn-chrA04_random-p88490
Bn-A04-p375518	0.966942149	0.966942149	Bn-N4-p388282	Bn-chrA04_random-p88783
Bn-A04-p397523	0.966942149	0.966942149	Bn-N4-p397170	Bn-chrA04_random-p97669
Bn-A04-p398807	0.973421927	0.973421927	Bn-N4-p398455	Bn-chrA04_random-p98954

Bn-A04-p404042	0.990049751	0.990049751	Bn-N1-p3955833	Bn-chrA01-p3533933
Bn-A04-p405060	0.975206612	0.975206612	Bn-N4-p404783	Bn-chrA04_random-p105178
Bn-A04-p455869	0.991735537	0.991735537	Bn-N4-p463086	Bn-chrAnn_random-p419232
Bn-A04-p459395	0.958677686	0.975206612	Bn-N12-p44797245	Bn-chrA05-p11319603
Bn-A04-p461679	0.900826446	1	Bn-N14-p23384035	Bn-chrA05-p11303026
Bn-A04-p462219	0.991735537	1	Bn-N12-p44797489	Bn-chrA05-p11302486
Bn-A04-p462966	0.986710963	0.986710963	Bn-N3-p20521908	Bn-chrA03-p19089697
Bn-A04-p463864	0.975206612	0.975206612	Bn-N4-p473013	Bn-chrA04-p361418
Bn-A04-p487513	0.983471074	0.983471074	Bn-N4-p494605	Bn-chrA04-p383031
Bn-A04-p490967	0.710743802	0.752066116	Bn-N14-p23794847	Bn-chrC03-p20694353
Bn-A04-p534430	0.966942149	0.884297521	Bn-N14-p26057912	Bn-chrA04-p421938
Bn-A04-p534845	0.859504132	0.859504132	Bn-N4-p534732	Bn-chrA04-p422536
Bn-A04-p538802	1	1	Bn-N4-p535101	Bn-chrA04-p422905
Bn-A04-p541336	0.975206612	0.975206612	Bn-N4-p537891	Bn-chrA04-p425733
Bn-A04-p556991	0.991735537	0.991735537	Bn-N4-p550021	Bn-chrA04-p437301
Bn-A04-p557097	0.983471074	0.983471074	Bn-N4-p550134	Bn-chrA04-p437414
Bn-A04-p560622	0.983471074	0.983471074	Bn-N4-p554487	Bn-chrA04-p441599
Bn-A04-p570250	1	1	Bn-N4-p564486	Bn-chrA04-p451708
Bn-A04-p581704	0.966942149	0.966942149	Bn-N4-p574501	Bn-chrA04-p461639
Bn-A04-p582626	0.966942149	0.966942149	Bn-N4-p575467	Bn-chrA04-p462605
Bn-A04-p582979	0.958677686	0.958677686	Bn-N4-p575660	Bn-chrA04-p462798
Bn-A04-p583036	0.983471074	0.983471074	Bn-N4-p575717	Bn-chrA04-p462855
Bn-A04-p583242	0.983471074	0.983471074	Bn-N4-p575923	Bn-chrA04-p463061
Bn-A04-p583254	0.983471074	0.983471074	Bn-N4-p575935	Bn-chrA04-p463073
Bn-A04-p583798	0.950413223	0.950413223	Bn-N4-p577190	Bn-chrA04-p464554
Bn-A04-p597296	1	1	Bn-N4-p608275	Bn-chrA04-p478046
Bn-A04-p599845	0.983471074	0.983471074	Bn-N4-p610824	Bn-chrA04-p480595
Bn-A04-p600223	0.973421927	0.973421927	Bn-N4-p611202	Bn-chrA04-p480973
Bn-A04-p619001	1	1	Bn-N4-p627886	Bn-chrA04-p498280
Bn-A04-p637683	0.990049751	0.990049751	Bn-N4-p648894	Bn-chrA04-p519452
Bn-A04-p641998	1	1	Bn-N4-p653373	Bn-chrA04-p523924
Bn-A04-p643876	0.991735537	0.991735537	Bn-N4-p655223	Bn-chrA04-p525774
Bn-A04-p645702	0.983388704	0.986710963	Bn-N4-p657044	Bn-chrA04-p527595
Bn-A04-p646064	0.985074627	0.985074627	Bn-N4-p657427	Bn-chrA04-p527978
Bn-A04-p646105	0.980099502	0.980099502	Bn-N4-p657447	Bn-chrA04-p527998
Bn-A04-p648152	1	1	Bn-N4-p659515	Bn-chrA04-p530066
Bn-A04-p652225	0.975206612	0.975206612	Bn-N4-p664538	Bn-chrA04-p535085
Bn-A04-p664994	0.940298507	0.940298507	Bn-N4-p669872	Bn-chrA04-p540419
Bn-A04-p665754	0.990049751	0.990049751	Bn-N4-p671632	Bn-chrA04-p542167
Bn-A04-p665932	0.985074627	0.985074627	Bn-N4-p671810	Bn-chrA04-p542345
Bn-A04-p665967	0.980099502	0.980099502	Bn-N4-p671845	Bn-chrA04-p542380
Bn-A04-p668378	0.975206612	0.975206612	Bn-N4-p674606	Bn-chrA04-p545140
Bn-A04-p669182	0.966942149	0.966942149	Bn-N4-p675443	Bn-chrA04-p545975
Bn-A04-p716889	1	1	Bn-N4-p728620	Bn-chrA04-p601033
Bn-A04-p726356	0.975206612	0.975206612	Bn-N4-p730447	Bn-chrA04-p602860
Bn-A04-p741528	0.986710963	0.986710963	Bn-N4-p745025	Bn-chrA04-p617434

Bn-A04-p745024	0.993355482	0.993355482	Bn-N4-p748522	Bn-chrA04-p620930
Bn-A04-p757668	0.975206612	0.975206612	Bn-N4-p756721	Bn-chrA04-p628867
Bn-A04-p761383	0.958677686	0.950413223	Bn-N4-p759440	Bn-chrC04-p23112830
Bn-A04-p783339	0.983471074	0.983471074	Bn-N4-p781449	Bn-chrA04-p654736
Bn-A04-p784708	0.983471074	0.983471074	Bn-N4-p782818	Bn-chrA04-p656105
Bn-A04-p784894	0.995024876	0.995024876	Bn-N4-p783004	Bn-chrA04-p656291
Bn-A04-p785628	0.990049751	0.990049751	Bn-N4-p783742	Bn-chrA04-p657029
Bn-A04-p786588	0.950248756	0.950248756	Bn-N4-p784701	Bn-chrA04-p657988
Bn-A04-p792399	0.995024876	0.995024876	Bn-N4-p790541	Bn-chrA04-p663828
Bn-A04-p803315	0.970149254	0.970149254	Bn-N4-p801225	Bn-chrA04-p676921
Bn-A04-p808824	0.975206612	0.975206612	Bn-N4-p806747	Bn-chrC07-p20976962
Bn-A04-p808834	0.983471074	0.983471074	Bn-N4-p806757	Bn-chrC07-p20976972
Bn-A04-p809984	0.975206612	0.975206612	Bn-N4-p810348	Bn-chrA04-p686044
Bn-A04-p810039	0.975206612	0.975206612	Bn-N4-p810403	Bn-chrA04-p686099
Bn-A04-p810166	0.983471074	0.983471074	Bn-N4-p810530	Bn-chrA04-p686226
Bn-A04-p810935	0.983471074	0.983471074	Bn-N4-p811294	Bn-chrA04-p686983
Bn-A04-p813173	0.991735537	0.991735537	Bn-N4-p813484	Bn-chrA04-p689173
Bn-A04-p814033	0.983471074	0.983471074	Bn-N4-p814343	Bn-chrA04-p690032
Bn-A04-p831099	0.980099502	0.980099502	Bn-N4-p830188	Bn-chrA04-p705668
Bn-A04-p860212	0.975206612	0.975206612	Bn-N4-p855702	Bn-chrA04-p732051
Bn-A04-p865058	0.991735537	0.991735537	Bn-N4-p859905	Bn-chrA04-p736254
Bn-A04-p869491	0.991735537	0.991735537	Bn-N4-p864354	Bn-chrA08-p7511387
Bn-A04-p870365	1	1	Bn-N4-p865228	Bn-chrA04-p740504
Bn-A04-p874008	0.983471074	0.983471074	Bn-N4-p869997	Bn-chrA04-p750452
Bn-A04-p887081	0.991666667	0.991666667	Bn-N4-p881316	Bn-chrA04-p764819
Bn-A04-p922828	0.975124378	0.975124378	Bn-N4-p912024	Bn-chrA04-p792969
Bn-A04-p926916	0.958677686	0.958677686	Bn-N4-p915890	Bn-chrA04-p796835
Bn-A04-p928350	0.986710963	0.986710963	Bn-N4-p917323	Bn-chrA04-p798300
Bn-A04-p932865	0.996677741	0.996677741	Bn-N4-p921851	Bn-chrAnn_random-p392007
Bn-A04-p934150	0.94214876	0.94214876	Bn-N4-p923006	Bn-chrAnn_random-p392019
Bn-A04-p980952	0.966942149	0.966942149	Bn-N4-p930085	Bn-chrAnn_random-p479260
Bn-A04-p982068	0.983471074	0.983471074	Bn-N4-p931248	Bn-chrA04_random-p81320
Bn-A04-p1001237	0.966942149	0.966942149	Bn-N4-p932584	Bn-chrA04_random-p79984
Bn-A04-p1007534	0.958677686	0.958677686	Bn-N4-p942388	Bn-chrA04_random-p71721
Bn-A04-p1009976	0.983471074	0.983471074	Bn-N4-p944820	Bn-chrA04_random-p69289
Bn-A04-p1018807	0.950248756	0.950248756	Bn-N4-p954367	Bn-chrA04_random-p59678
Bn-A04-p1021879	0.980066445	0.980066445	Bn-N4-p957845	Bn-chrA04_random-p55528
Bn-A04-p1032336	0.991735537	0.991735537	Bn-N4-p965926	Bn-chrA04_random-p47447
Bn-A04-p1036308	0.991735537	0.991735537	Bn-N4-p969833	Bn-chrA04_random-p43540
Bn-A04-p1040916	1	1	Bn-N4-p975439	Bn-chrA04_random-p37941
Bn-A04-p1042273	0.985074627	0.985074627	Bn-N4-p975963	Bn-chrA04_random-p37417
Bn-A04-p1043111	0.970149254	0.970149254	Bn-N4-p976801	Bn-chrA04_random-p36579
Bn-A04-p1048288	0.958677686	0.958677686	Bn-N4-p982842	Bn-chrA04_random-p30363
Bn-A04-p1062119	0.975206612	0.975206612	Bn-N4-p1003047	Bn-chrA04-p808676
Bn-A04-p1065480	0.996677741	0.996677741	Bn-N4-p1006422	Bn-chrA04-p812051
Bn-A04-p1070815	0.958677686	0.958677686	Bn-N4-p1011727	Bn-chrA04-p817356

Bn-A04-p1077522	0.917355372	0.917355372	Bn-N4-p1018128	Bn-chrA04-p823554
Bn-A04-p1091727	0.991735537	0.991735537	Bn-N4-p1036358	Bn-chrA04-p839926
Bn-A04-p1104385	0.996677741	0.996677741	Bn-N4-p1053356	Bn-chrA04-p854149
Bn-A04-p1145007	0.991735537	0.495867769	Bn-N4-p1078166	Bn-chrC04-p23491406
Bn-A04-p1156390	0.983471074	0.983471074	Bn-N4-p1083852	Bn-chrA04-p901857
Bn-A04-p1159662	0.991735537	0.991735537	Bn-N4-p1093634	Bn-chrA04-p911577
Bn-A04-p1159924	0.925619835	0.925619835	Bn-N4-p1093896	Bn-chrA04-p911839
Bn-A04-p1161563	0.991735537	0.991735537	Bn-N4-p1095696	Bn-chrA04-p913639
Bn-A04-p1176453	0.958677686	0.958677686	Bn-N4-p1110879	Bn-chrA04-p927419
Bn-A04-p1187598	0.897009967	0.900332226	Bn-N4-p1121540	Bn-chrA04-p938078
Bn-A04-p1212589	0.993355482	0.993355482	Bn-N4-p1147464	Bn-chrA04-p963343
Bn-A04-p1213196	0.970099668	0.970099668	Bn-N4-p1148070	Bn-chrA04-p963949
Bn-A04-p1215863	0.993355482	0.993355482	Bn-N4-p1150735	Bn-chrA04-p966614
Bn-A04-p1231047	0.983471074	0.983471074	Bn-N4-p1164595	Bn-chrA04-p980472
Bn-A04-p1231127	0.975206612	0.975206612	Bn-N4-p1164675	Bn-chrA04-p980552
Bn-A04-p1239789	0.991735537	0.991735537	Bn-N4-p1170671	Bn-chrA04-p988396
Bn-A04-p1240196	0.975206612	0.975206612	Bn-N4-p1171078	Bn-chrA04-p988803
Bn-A04-p1244890	0.966777409	0.966777409	Bn-N4-p1175970	Bn-chrA04-p993695
Bn-A04-p1274596	0.983471074	0.983471074	Bn-N4-p1214158	Bn-chrA04-p1036244
Bn-A04-p1276308	0.983471074	0.983471074	Bn-N4-p1215871	Bn-chrA04-p1037957
Bn-A04-p1279752	0.980066445	0.980066445	Bn-N4-p1219316	Bn-chrA04-p1041276
Bn-A04-p1282528	0.976744186	0.96013289	Bn-N4-p1223168	Bn-chrA04-p1044306
Bn-A04-p1291618	0.990049751	0.990049751	Bn-N4-p1233246	Bn-chrA04-p1054384
Bn-A04-p1311487	0.986710963	0.986710963	Bn-N4-p1247303	Bn-chrA04-p1070569
Bn-A04-p1311567	0.983471074	0.983471074	Bn-N4-p1247383	Bn-chrA04-p1070649
Bn-A04-p1317446	0.925619835	0.925619835	Bn-N4-p1252454	Bn-chrA04-p1075719
Bn-A04-p1318613	0.966942149	0.966942149	Bn-N4-p1253627	Bn-chrA04-p1076892
Bn-A04-p1322758	0.933884298	0.933884298	Bn-N4-p1263125	Bn-chrA04-p1086001
Bn-A04-p1323611	0.975206612	0.975206612	Bn-N4-p1264243	Bn-chrA04-p1087119
Bn-A04-p1324549	0.950413223	0.950413223	Bn-N4-p1266134	Bn-chrA04-p1089009
Bn-A04-p1329352	0.876033058	0.876033058	Bn-N14-p28062805	Bn-chrC04-p24229336
Bn-A04-p1329438	0.991735537	0.991735537	Bn-N4-p1271104	Bn-chrA04-p1093979
Bn-A04-p1330321	0.975206612	0.975206612	Bn-N4-p1271987	Bn-chrA04-p1094862
Bn-A04-p1346738	0.983471074	0.983471074	Bn-N4-p1274801	Bn-chrA04-p1097676
Bn-A04-p1349255	0.975206612	0.975206612	Bn-N4-p1277334	Bn-chrA04-p1100209
Bn-A04-p1351337	0.983471074	0.983471074	Bn-N4-p1279432	Bn-chrA04-p1102307
Bn-A04-p1353175	0.983471074	0.983471074	Bn-N4-p1282151	Bn-chrA04-p1105025
Bn-A04-p1353238	0.975206612	0.975206612	Bn-N4-p1282214	Bn-chrA04-p1105088
Bn-A04-p1355502	0.975206612	0.975206612	Bn-N4-p1284481	Bn-chrA04-p1107354
Bn-A04-p1356122	0.950413223	0.950413223	Bn-N4-p1285102	Bn-chrA04-p1107975
Bn-A04-p1377226	0.996677741	0.996677741	Bn-N4-p1305593	Bn-chrA04-p1129021
Bn-A04-p1390449	0.975206612	0.975206612	Bn-N4-p1324713	Bn-chrCnn_random-p568021
Bn-A04-p1416287	0.996677741	0.996677741	Bn-N4-p1354082	Bn-chrA04-p1171662
Bn-A04-p1417481	0.966942149	0.966942149	Bn-N4-p1355252	Bn-chrA04-p1172832
Bn-A04-p1429850	0.996677741	0.996677741	Bn-N4-p1370880	Bn-chrAnn_random-p454052
Bn-A04-p1451594	0.985074627	0.985074627	Bn-N4-p1389366	Bn-chrA04-p1203872

Bn-A04-p1454434	0.966942149	0.966942149	Bn-N4-p1392198	Bn-chrA04-p1206704
Bn-A04-p1465207	0.983471074	0.983471074	Bn-N4-p1406471	Bn-chrA04-p1216625
Bn-A04-p1470844	0.991735537	0.991735537	Bn-N4-p1412206	Bn-chrA04-p1221820
Bn-A04-p1471607	0.993355482	0.993355482	Bn-N4-p1412969	Bn-chrA04-p1222583
Bn-A04-p1471982	0.983388704	0.986710963	Bn-N4-p1413344	Bn-chrA04-p1222958
Bn-A04-p1472416	1	0.991735537	Bn-N4-p1413778	Bn-chrA04-p1223392
Bn-A04-p1472739	0.983388704	0.990033223	Bn-N4-p1414101	Bn-chrA04-p1223715
Bn-A04-p1479491	0.970930233	0.970930233	Bn-N4-p1430349	Bn-chrA04-p1224418
Bn-A04-p1480226	0.980099502	0.980099502	Bn-N4-p1431084	Bn-chrA04-p1225154
Bn-A04-p1481110	0.990049751	0.975124378	Bn-N4-p1431968	Bn-chrA04-p1226034
Bn-A04-p1490485	0.975206612	0.975206612	Bn-N4-p1439997	Bn-chrA04-p1235344
Bn-A04-p1576564	0.996677741	0.996677741	Bn-N4-p1537124	Bn-chrA04-p1329349
Bn-A04-p1585447	0.993355482	0.983388704	Bn-N4-p1549515	Bn-chrA04-p1338227
Bn-A04-p1589006	1	0.991735537	Bn-N4-p1553265	Bn-chrA04-p1341438
Bn-A04-p1590169	0.983471074	0.991735537	Bn-N4-p1554428	Bn-chrA04-p1342543
Bn-A04-p1590669	0.983471074	0.983471074	Bn-N4-p1554928	Bn-chrA04-p1343044
Bn-A04-p1590931	0.52892562	0.966942149	Bn-N4-p1555193	Bn-chrA04-p1343310
Bn-A04-p1623151	1	0.991735537	Bn-N4-p1567651	Bn-chrA04-p1349521
Bn-A04-p1633159	0.986710963	0.986710963	Bn-N4-p1574224	Bn-chrA04-p1356074
Bn-A04-p1640389	0.495867769	0.760330579	Bn-N14-p28731063	Bn-chrAnn_random-p431615
Bn-A04-p1655416	0.975206612	0.975206612	Bn-N4-p1589334	Bn-chrA04_random-p169016
Bn-A04-p1674763	0.991735537	0.975206612	Bn-N4-p1602173	Bn-chrA04_random-p156084
Bn-A04-p1680506	0.909090909	0.900826446	Bn-N4-p1607740	Bn-chrA04_random-p150749
Bn-A04-p1680518	0.917355372	0.909090909	Bn-N4-p1607752	Bn-chrA04_random-p150737
Bn-A04-p1680673	0.900826446	0.892561983	Bn-N4-p1607903	Bn-chrA04_random-p150576
Bn-A04-p1683090	0.965174129	0.965174129	Bn-N4-p1610304	Bn-chrA04_random-p148155
Bn-A04-p1684695	0.966942149	0.917355372	Bn-N4-p1612010	Bn-chrC04-p25032285
Bn-A04-p1718231	0.747508306	0.747508306	Bn-N4-p1659171	Bn-chrA04-p1443159
Bn-A04-p1718248	0.813953488	0.813953488	Bn-N4-p1659191	Bn-chrA04-p1443179
Bn-A04-p1722346	0.966777409	0.966777409	Bn-N4-p1663325	Bn-chrA04-p1447313
Bn-A04-p1738118	0.966942149	0.966942149	Bn-N4-p1680157	Bn-chrA04-p1464831
Bn-A04-p1738861	0.958677686	0.958677686	Bn-N4-p1680900	Bn-chrA04-p1465574
Bn-A04-p1745832	0.958677686	0.958677686	Bn-N4-p1686812	Bn-chrA04-p1471486
Bn-A04-p1756591	1	0.989583333	Bn-N1-p19472011	Bn-chrA01-p16317351
Bn-A04-p1758570	0.991735537	0.991735537	Bn-N4-p1699768	Bn-chrA04-p1484445
Bn-A04-p1759257	0.983471074	0.983471074	Bn-N4-p1700455	Bn-chrA04-p1485132
Bn-A04-p1764916	0.991735537	0.991735537	Bn-N4-p1706479	Bn-chrA04-p1491137
Bn-A04-p1765163	1	1	Bn-N4-p1706726	Bn-chrA04-p1491384
Bn-A04-p1773333	0.983471074	0.983471074	Bn-N4-p1721944	Bn-chrA04-p1509564
Bn-A04-p1774031	0.983471074	0.983471074	Bn-N4-p1722642	Bn-chrA04-p1510262
Bn-A04-p1774926	0.950413223	0.950413223	Bn-N4-p1723549	Bn-chrA04-p1511169
Bn-A04-p1775155	0.983471074	0.983471074	Bn-N4-p1723774	Bn-chrA04-p1511394
Bn-A04-p1777948	0.950248756	0.950248756	Bn-N4-p1725544	Bn-chrA04-p1513164
Bn-A04-p1780149	0.996677741	0.996677741	Bn-N4-p1728952	Bn-chrA04-p1516572
Bn-A04-p1783485	0.975206612	0.975206612	Bn-N4-p1732273	Bn-chrA04-p1519892
Bn-A04-p1815404	0.950413223	0.950413223	Bn-N4-p1753561	Bn-chrA04-p1540119

Bn-A04-p1824456	0.989583333	0.989583333	Bn-N4-p1770094	Bn-chrA04-p1553346
Bn-A04-p1827230	0.950413223	0.950413223	Bn-N4-p1777273	Bn-chrA04-p1560068
Bn-A04-p1829219	0.950413223	0.950413223	Bn-N4-p1779273	Bn-chrA04-p1562068
Bn-A04-p1837535	0.983471074	0.983471074	Bn-N4-p1787089	Bn-chrA04-p1569774
Bn-A04-p1854718	0.966942149	0.966942149	Bn-N4-p1799718	Bn-chrA04-p1583043
Bn-A04-p1858140	0.983471074	0.983471074	Bn-N4-p1802523	Bn-chrA04-p1585918
Bn-A04-p1865434	0.975124378	0.975124378	Bn-N4-p1805109	Bn-chrA04-p1588506
Bn-A04-p1867423	0.975206612	0.975206612	Bn-N4-p1807140	Bn-chrA04-p1590536
Bn-A04-p1869508	0.94214876	0.94214876	Bn-N4-p1821292	Bn-chrAnn_random-p467001
Bn-A04-p1880654	0.950413223	0.950413223	Bn-N4-p1836032	Bn-chrA04-p1618218
Bn-A04-p1882240	0.983471074	0.983471074	Bn-N4-p1837609	Bn-chrA04-p1622487
Bn-A04-p1893968	0.975206612	0.975206612	Bn-N4-p1847854	Bn-chrA04-p1632730
Bn-A04-p1895018	0.431893688	0.465116279	Bn-N4-p1849721	Bn-chrCnn_random-p570836
Bn-A04-p1897541	0.966942149	0.966942149	Bn-N4-p1852249	Bn-chrA04-p1637150
Bn-A04-p1900422	0.96013289	0.96013289	Bn-N4-p1855279	Bn-chrA04-p1640180
Bn-A04-p1913537	0.991735537	0.991735537	Bn-N4-p1868652	Bn-chrA04-p1645478
Bn-A04-p1927138	0.983471074	0.983471074	Bn-N4-p1882542	Bn-chrA04-p1659368
Bn-A04-p1927832	0.990033223	0.990033223	Bn-N4-p1883249	Bn-chrA04-p1660161
Bn-A04-p1928290	0.983471074	0.983471074	Bn-N4-p1883711	Bn-chrA04-p1660623
Bn-A04-p1931502	0.661129568	0.747508306	Bn-N4-p1871339	Bn-chrA04-p1648165
Bn-A04-p1932300	0.983471074	0.983471074	Bn-N4-p2262491	Bn-chrA04-p2045317
Bn-A04-p1932352	0.966942149	0.966942149	Bn-N4-p2262439	Bn-chrA04-p2045265
Bn-A04-p1935147	0.727272727	0.727272727	Bn-N4-p2259968	Bn-chrA04-p2042794
Bn-A04-p1938332	0.991735537	0.991735537	Bn-N4-p2256529	Bn-chrA04-p2039343
Bn-A04-p1941188	0.966777409	0.966777409	Bn-N4-p2253675	Bn-chrA04-p2036489
Bn-A04-p1943749	0.996677741	0.996677741	Bn-N4-p2251056	Bn-chrA04-p2033870
Bn-A04-p1948034	0.983471074	0.983471074	Bn-N4-p2246771	Bn-chrA04-p2029585
Bn-A04-p1950544	0.933884298	0.933884298	Bn-N14-p29487619	Bn-chrC04-p25456509
Bn-A04-p1962888	0.990049751	0.990049751	Bn-N4-p2230820	Bn-chrA04-p2014574
Bn-A04-p1971842	0.975206612	0.975206612	Bn-N4-p2224803	Bn-chrA04-p2008557
Bn-A04-p1976999	0.325581395	0.325581395	Unknown	Unknown
Bn-A04-p1978703	0.983471074	0.983471074	Bn-N4-p2221275	Bn-chrC02-p41197251
Bn-A04-p1979834	1	1	Bn-N4-p2220145	Bn-chrA04-p2002973
Bn-A04-p1983164	0.990033223	0.990033223	Bn-N4-p2214962	Bn-chrA04-p1998506
Bn-A04-p1991843	0.983388704	0.983388704	Bn-N4-p2207545	Bn-chrA04-p1991984
Bn-A04-p1993598	0.975206612	0.975206612	Bn-N4-p2205665	Bn-chrA04-p1990227
Bn-A04-p2000268	0.966942149	0.966942149	Bn-N4-p2198222	Bn-chrA04-p1983503
Bn-A04-p2009476	1	1	Bn-N4-p2188750	Bn-chrA04-p1974047
Bn-A04-p2010971	1	1	Bn-N4-p2187255	Bn-chrA04-p1972552
Bn-A04-p2029030	0.975206612	0.975206612	Bn-N4-p2172423	Bn-chrA04-p1956208
Bn-A04-p2037077	0.990049751	0.990049751	Bn-N4-p2161110	Bn-chrA04-p1944267
Bn-A04-p2048587	0.996677741	0.996677741	Bn-N4-p2149677	Bn-chrA04-p1932834
Bn-A04-p2094469	0.975206612	0.975206612	Bn-N4-p2104569	Bn-chrA04-p1880805
Bn-A04-p2094818	0.917355372	0.917355372	Bn-N4-p2103753	Bn-chrA04-p1879844
Bn-A04-p2104072	0.991735537	0.991735537	Bn-N4-p2096227	Bn-chrA04-p1872573
Bn-A04-p2111647	0.818181818	0.818181818	Bn-N4-p2089154	Bn-chrA04-p1865500

Bn-A04-p2122324	0.996677741	0.996677741	Bn-N4-p2075647	Bn-chrA04-p1852219
Bn-A04-p2124879	0.876033058	0.876033058	Bn-N4-p2070320	Bn-chrA04-p1847144
Bn-A04-p2126232	0.991735537	0.991735537	Bn-N4-p2068964	Bn-chrA04-p1845788
Bn-A04-p2126294	0.991735537	0.991735537	Bn-N4-p2068901	Bn-chrA04-p1845725
Bn-A04-p2135204	0.991735537	0.991735537	Bn-N4-p2065538	Bn-chrA04-p1842365
Bn-A04-p2137317	0.991735537	0.991735537	Bn-N4-p2063013	Bn-chrA04-p1839840
Bn-A04-p2146459	0.909090909	0.909090909	Bn-N4-p2059248	Bn-chrA04-p1836075
Bn-A04-p2149134	0.895522388	0.895522388	Bn-N4-p2056362	Bn-chrA04-p1833189
Bn-A04-p2149214	0.950248756	0.950248756	Bn-N4-p2056282	Bn-chrA04-p1833109
Bn-A04-p2151567	0.966942149	0.966942149	Bn-N4-p2053977	Bn-chrA04-p1830804
Bn-A04-p2152171	0.958677686	0.958677686	Bn-N4-p2053388	Bn-chrA04-p1830215
Bn-A04-p2152528	0.933884298	0.933884298	Bn-N4-p2053034	Bn-chrA04-p1829861
Bn-A04-p2178146	0.991735537	0.991735537	Bn-N4-p2030254	Bn-chrA04-p1807148
Bn-A04-p2191612	0.996677741	0.996677741	Bn-N4-p2016910	Bn-chrA04-p1793416
Bn-A04-p2192192	0.975206612	0.975206612	Bn-N4-p2016330	Bn-chrA04-p1792836
Bn-A04-p2200043	0.96013289	0.953488372	Bn-N14-p29877257	Bn-chrA02-p23194956
Bn-A04-p2213805	0.996677741	0.996677741	Bn-N4-p1988763	Bn-chrA04-p1763497
Bn-A04-p2215376	0.980099502	0.980099502	Bn-N4-p1987197	Bn-chrA04-p1761931
Bn-A04-p2215559	0.995024876	0.995024876	Bn-N4-p1987014	Bn-chrA04-p1761748
Bn-A04-p2218115	0.983471074	0.983471074	Bn-N4-p1984461	Bn-chrA04-p1759195
Bn-A04-p2222852	0.983471074	0.983471074	Bn-N4-p1977753	Bn-chrA04-p1752488
Bn-A04-p2270213	0.900497512	0.900497512	Bn-N4-p1946962	Bn-chrA04-p1730069
Bn-A04-p2282199	0.983388704	0.983388704	Bn-N4-p1935826	Bn-chrA04-p1718920
Bn-A04-p2288114	1	1	Bn-N4-p1930836	Bn-chrA04-p1713891
Bn-A04-p2309625	0.970099668	0.970099668	Bn-N4-p1905792	Bn-chrA04-p1681766
Bn-A04-p2325988	0.991735537	0.991735537	Bn-N4-p1896955	Bn-chrA04-p1672929
Bn-A04-p2330084	0.983471074	0.983471074	Bn-N4-p1892856	Bn-chrA04-p1668830
Bn-A04-p2334920	0.958677686	0.958677686	Bn-N4-p1888005	Bn-chrAnn_random-p379091
Bn-A04-p2342131	0.837209302	0.986710963	Bn-N4-p2271964	Bn-chrA04-p2059029
Bn-A04-p2353879	0.993355482	0.993355482	Bn-N4-p2283037	Bn-chrA04-p2069286
Bn-A04-p2367374	0.991735537	0.991735537	Bn-N4-p2294513	Bn-chrA04-p2082164
Bn-A04-p2401413	0.991735537	0.991735537	Bn-N4-p2305853	Bn-chrA04-p2093504
Bn-A04-p2411039	1	1	Bn-N4-p6128312	Bn-chrA04-p5850119
Bn-A04-p2415169	0.936877076	0.933554817	Bn-N1-p21279888	Bn-chrA01-p17918186
Bn-A04-p2429853	0.975206612	0.975206612	Bn-N4-p2332536	Bn-chrA04-p2118533
Bn-A04-p2434674	0.983471074	0.983471074	Bn-N4-p2334217	Bn-chrA04-p2120214
Bn-A04-p2439163	1	1	Bn-N4-p2340231	Bn-chrA04-p2126136
Bn-A04-p2448580	0.983471074	0.983471074	Bn-N4-p2347721	Bn-chrA04-p2133626
Bn-A04-p2456200	0.970099668	0.970099668	Bn-N4-p2354532	Bn-chrA04-p2140437
Bn-A04-p2475793	0.965174129	0.965174129	Bn-N4-p2373181	Bn-chrA04-p2157752
Bn-A04-p2498254	0.990033223	0.990033223	Bn-N4-p2415593	Bn-chrA04-p2212059
Bn-A04-p2537756	0.991735537	0.991735537	Bn-N4-p2460369	Bn-chrA04-p2257490
Bn-A04-p2542000	0.958677686	0.958677686	Bn-N4-p2464595	Bn-chrA04-p2260566
Bn-A04-p2552293	1	1	Bn-N4-p2476395	Bn-chrA04-p2271081
Bn-A04-p2562005	0.983388704	0.983388704	Bn-N4-p2479855	Bn-chrA04-p2274397
Bn-A04-p2566546	0.990033223	0.990033223	Bn-N4-p2482817	Bn-chrA04-p2277362

Bn-A04-p2568394	0.991735537	0.991735537	Bn-N4-p2484663	Bn-chrA04-p2279208
Bn-A04-p2592215	0.958677686	0.958677686	Bn-N4-p2505134	Bn-chrAnn_random-p440156
Bn-A04-p2593658	0.990033223	0.990033223	Bn-N4-p2506550	Bn-chrA04-p2299048
Bn-A04-p2603671	1	1	Bn-N4-p2515668	Bn-chrA04-p2308728
Bn-A04-p2611718	0.993355482	0.993355482	Bn-N4-p2523754	Bn-chrA04-p2316812
Bn-A04-p2616582	0.991735537	0.991735537	Bn-N4-p2528793	Bn-chrA04-p2321801
Bn-A04-p2623116	1	1	Bn-N4-p2533034	Bn-chrA04-p2326041
Bn-A04-p2648823	0.986710963	0.986710963	Bn-N4-p2562922	Bn-chrA04-p2353171
Bn-A04-p2650427	0.996677741	0.996677741	Bn-N4-p2564526	Bn-chrA04-p2354775
Bn-A04-p2651171	0.990033223	0.990033223	Bn-N4-p2565270	Bn-chrA04-p2355519
Bn-A04-p2657695	0.958677686	0.958677686	Bn-N4-p2571351	Bn-chrA04-p2361599
Bn-A04-p2658023	0.983471074	0.983471074	Bn-N4-p2571679	Bn-chrA04-p2361927
Bn-A04-p2660465	0.965174129	0.965174129	Bn-N4-p2574324	Bn-chrA04-p2364572
Bn-A04-p2662208	1	1	Bn-N4-p2576067	Bn-chrA04-p2366315
Bn-A04-p2664124	0.975206612	0.975206612	Bn-N4-p2577983	Bn-chrA04-p2368231
Bn-A04-p2665421	0.975206612	0.975206612	Bn-N4-p2579461	Bn-chrA04-p2369709
Bn-A04-p2666754	0.958677686	0.958677686	Bn-N4-p2580794	Bn-chrA04-p2371042
Bn-A04-p2668053	0.991735537	0.991735537	Bn-N4-p2582248	Bn-chrA04-p2372496
Bn-A04-p2669971	0.991735537	0.991735537	Bn-N4-p2584166	Bn-chrA04-p2374414
Bn-A04-p2670078	0.983471074	0.983471074	Bn-N4-p2584273	Bn-chrA04-p2374521
Bn-A04-p2670318	0.975206612	0.975206612	Bn-N4-p2584513	Bn-chrA04-p2374761
Bn-A04-p2671273	0.983471074	0.983471074	Bn-N4-p2585468	Bn-chrA04-p2375716
Bn-A04-p2680732	1	1	Bn-N4-p2593065	Bn-chrA04-p2385777
Bn-A04-p2689124	0.983388704	0.983388704	Bn-N4-p2604056	Bn-chrA04-p2395569
Bn-A04-p2692131	0.958677686	0.958677686	Bn-Scaffold07981-p8	Bn-chrA04-p2403979
Bn-A04-p2713517	0.983471074	0.983471074	Bn-N4-p2623471	Bn-chrA04-p2419857
Bn-A04-p2713564	0.983471074	0.983471074	Bn-N4-p2623518	Bn-chrA04-p2419904
Bn-A04-p2723787	0.996677741	0.996677741	Bn-N4-p2632977	Bn-chrA04-p2428107
Bn-A04-p2730690	0.991735537	0.991735537	Bn-N4-p2641392	Bn-chrA04-p2436520
Bn-A04-p2748876	0.438016529	0.438016529	Bn-N14-p30928787	Bn-chrC04-p26618343
Bn-A04-p2750401	1	1	Bn-N4-p2658421	Bn-chrA04-p2453549
Bn-A04-p2751097	0.975206612	0.975206612	Bn-N4-p2659118	Bn-chrA04-p2454246
Bn-A04-p2753796	0.950413223	0.950413223	Bn-N4-p2661812	Bn-chrA04-p2456940
Bn-A04-p2753824	0.986710963	0.986710963	Bn-N4-p2661840	Bn-chrA04-p2456968
Bn-A04-p2760477	0.950413223	0.950413223	Bn-N4-p2666235	Bn-chrA04-p2461363
Bn-A04-p2761067	0.958677686	0.958677686	Bn-N4-p2666824	Bn-chrA04-p2461952
Bn-A04-p2762891	0.983471074	0.983471074	Bn-N4-p2668661	Bn-chrA04-p2463789
Bn-A04-p2763423	0.925619835	0.925619835	Bn-N4-p2669192	Bn-chrA04-p2464320
Bn-A04-p2765547	1	1	Bn-N4-p2671264	Bn-chrA04-p2466392
Bn-A04-p2780974	0.958677686	0.958677686	Bn-N4-p2687264	Bn-chrA04-p2480977
Bn-A04-p2781853	0.958677686	0.958677686	Bn-N4-p2688141	Bn-chrA04-p2481854
Bn-A04-p2782258	0.983471074	0.983471074	Bn-N4-p2688543	Bn-chrA04-p2482256
Bn-A04-p2783207	0.991735537	0.991735537	Bn-N4-p2689459	Bn-chrA04-p2483172
Bn-A04-p2789250	0.991735537	0.991735537	Bn-N4-p2692610	Bn-chrA04-p2486232
Bn-A04-p2793807	0.975206612	0.975206612	Bn-N4-p2696256	Bn-chrAnn_random-p481022
Bn-A04-p2802411	0.991735537	0.991735537	Bn-N4-p2705775	Bn-chrA04-p2498530

Bn-A04-p2807465	1	1	Bn-N4-p2710847	Bn-chrA04-p2503602
Bn-A04-p2817722	0.991735537	0.991735537	Bn-N4-p2719497	Bn-chrA04-p2512168
Bn-A04-p2818095	0.958677686	0.958677686	Bn-N4-p2719870	Bn-chrA04-p2512541
Bn-A04-p2823217	0.975124378	0.771144279	Bn-Scaffold01988-p9	Bn-chrC02-p33525316
Bn-A04-p2825294	0.980066445	0.980066445	Bn-N4-p2721433	Bn-chrA04-p2514118
Bn-A04-p2825362	0.980099502	0.980099502	Bn-N4-p2721501	Bn-chrA04-p2514186
Bn-A04-p2833736	0.991735537	0.991735537	Bn-N4-p2729937	Bn-chrC04-p47121776
Bn-A04-p2841305	0.925619835	0.925619835	Bn-N14-p31134719	Bn-chrCnn_random-p459268
Bn-A04-p2841433	0.958677686	0.958677686	Bn-N4-p2737553	Bn-chrA04-p2529955
Bn-A04-p2843802	0.966942149	0.966942149	Bn-N4-p2740497	Bn-chrA04-p2532899
Bn-A04-p2845855	0.79338843	0.79338843	Bn-N4-p2742534	Bn-chrA04-p2534936
Bn-A04-p2847914	0.975206612	0.975206612	Bn-N4-p2747567	Bn-chrA02-p1977941
Bn-A04-p2881354	1	1	Bn-N1-p20338159	Bn-chrA01-p17246203
Bn-A04-p2889239	0.990049751	0.990049751	Bn-N4-p2767110	Bn-chrA04-p2555909
Bn-A04-p2892557	0.727272727	0.727272727	Bn-N4-p2770374	Bn-chrA04-p2559173
Bn-A04-p2892722	0.993355482	0.993355482	Bn-N4-p2770540	Bn-chrA04-p2559339
Bn-A04-p2892985	0.993355482	0.993355482	Bn-N4-p2770803	Bn-chrA04-p2559602
Bn-A04-p2893053	0.990033223	0.990033223	Bn-N4-p2770871	Bn-chrA04-p2559670
Bn-A04-p2903530	0.990049751	0.990049751	Bn-N4-p2778620	Bn-chrA04-p2567027
Bn-A04-p2906681	1	1	Bn-Scaffold02395-p3	Bn-chrA01-p13092113
Bn-A04-p2909242	1	1	Bn-Scaffold02395-p8	Bn-chrA01-p13094606
Bn-A04-p2913939	0.966942149	0.966942149	Bn-N4-p2783770	Bn-chrA04-p2572063
Bn-A04-p2914409	0.958677686	0.958677686	Bn-N4-p2784240	Bn-chrA04-p2572533
Bn-A04-p2921313	0.975206612	0.975206612	Bn-N4-p2787268	Bn-chrA04-p2575559
Bn-A04-p2921682	0.991735537	0.991735537	Bn-N4-p2787637	Bn-chrA04-p2575928
Bn-A04-p2921832	1	1	Bn-N4-p2787787	Bn-chrA04-p2576078
Bn-A04-p2923396	0.966942149	0.966942149	Bn-N4-p2789585	Bn-chrA04-p2577875
Bn-A04-p2925273	0.991735537	0.991735537	Bn-N4-p2797228	Bn-chrA04-p2587209
Bn-A04-p2932086	0.966942149	0.966942149	Bn-N4-p2803713	Bn-chrA04-p2593693
Bn-A04-p2933140	0.843853821	0.843853821	Bn-N4-p2810217	Bn-chrA04-p2599449
Bn-A04-p2941201	0.990049751	0.990049751	Bn-N4-p2813079	Bn-chrA04-p2602310
Bn-A04-p2944723	0.975206612	0.975206612	Bn-N4-p2816602	Bn-chrA04-p2605833
Bn-A04-p2947993	0.990049751	0.990049751	Bn-N4-p2819871	Bn-chrA04-p2609102
Bn-A04-p2948936	0.990049751	0.990049751	Bn-N4-p2821197	Bn-chrA04-p2610081
Bn-A04-p2949084	0.985074627	0.985074627	Bn-N4-p2821345	Bn-chrA04-p2610229
Bn-A04-p2949339	0.756218905	0.756218905	Bn-N4-p2821600	Bn-chrA04-p2610484
Bn-A04-p2954791	0.990049751	0.960199005	Bn-N4-p2825854	Bn-chrA04-p2614802
Bn-A04-p2954824	0.990049751	0.960199005	Bn-N4-p2825887	Bn-chrA04-p2614835
Bn-A04-p2957928	0.930232558	0.930232558	Bn-N4-p2855311	Bn-chrA04_random-p245270
Bn-A04-p2957984	0.96345515	0.96345515	Bn-N4-p2855367	Bn-chrA04_random-p245326
Bn-A04-p2960723	0.970099668	0.970099668	Bn-N4-p2834240	Bn-chrA04-p2623118
Bn-A04-p2963387	0.996677741	0.996677741	Bn-N4-p2873842	Bn-chrA04_random-p199198
Bn-A04-p2969081	0.996677741	0.996677741	Bn-N4-p2879542	Bn-chrA04_random-p204898
Bn-A04-p2983187	0.985074627	0.985074627	Bn-N4-p2887116	Bn-chrAnn_random-p320548
Bn-A04-p2999394	0.933884298	0.933884298	Bn-N4-p2897148	Bn-chrA04_random-p212801
Bn-A04-p3002670	0.976744186	0.976744186	Bn-N4-p2905453	Bn-chrA04-p2722741

Bn-A04-p3004068	0.980066445	0.980066445	Bn-N4-p2906851	Bn-chrC04-p27035448
Bn-A04-p3016121	0.966942149	0.966942149	Bn-N4-p2913345	Bn-chrA04-p2729676
Bn-A04-p3021710	0.958677686	0.958677686	Bn-N4-p2914174	Bn-chrA04-p2730504
Bn-A04-p3022681	0.983471074	0.983471074	Bn-N4-p2915145	Bn-chrA04-p2731475
Bn-A04-p3095652	0.975206612	0.975206612	Bn-N4-p2990898	Bn-chrCnn_random-p367150
Bn-A04-p3116993	0.990049751	0.985074627	Bn-N4-p3015495	Bn-chrCnn_random-p367375
Bn-A04-p3118075	1	1	Bn-N4-p3016832	Bn-chrCnn_random-p367386
Bn-A04-p3126855	0.986710963	0.986710963	Bn-N4-p3021601	Bn-chrCnn_random-p367433
Bn-A04-p3128038	1	1	Bn-N4-p3022766	Bn-chrCnn_random-p367445
Bn-A04-p3135512	0.958677686	0.958677686	Bn-N4-p3035491	Bn-chrCnn_random-p528536
Bn-A04-p3135978	0.970149254	0.970149254	Bn-N4-p3035948	Bn-chrCnn_random-p528531
Bn-A04-p3138111	0.94214876	0.94214876	Bn-N4-p3038085	Bn-chrCnn_random-p528510
Bn-A04-p3138462	0.958677686	0.958677686	Bn-N4-p3038906	Bn-chrA04_random-p214056
Bn-A04-p3140726	0.966942149	0.966942149	Bn-N4-p3040988	Bn-chrA04_random-p216138
Bn-A04-p3144839	1	1	Bn-N4-p3045094	Bn-chrA04_random-p220244
Bn-A04-p3152494	0.983388704	0.983388704	Bn-N4-p3052809	Bn-chrA04_random-p227099
Bn-A04-p3152554	0.970149254	0.970149254	Bn-N4-p3052869	Bn-chrA04_random-p227159
Bn-A04-p3172854	0.876033058	0.876033058	Bn-N4-p3075892	Bn-chrAnn_random-p298214
Bn-A04-p3181336	0.933884298	0.933884298	Bn-N4-p3088237	Bn-chrA04-p2857066
Bn-A04-p3182956	0.991735537	0.991735537	Bn-N4-p3089856	Bn-chrA04-p2858685
Bn-A04-p3190463	0.619834711	0.619834711	Bn-N4-p3096077	Bn-chrA04-p2864903
Bn-A04-p3219148	0.975206612	0.975206612	Bn-N4-p3123031	Bn-chrA04-p2891079
Bn-A04-p3220424	0.956810631	0.956810631	Bn-N4-p3124308	Bn-chrA04-p2892356
Bn-A04-p3220819	0.966850829	0.966850829	Bn-N4-p3124704	Bn-chrA04-p2892752
Bn-A04-p3225119	0.950166113	0.950166113	Bn-N4-p3129508	Bn-chrA04-p2897556
Bn-A04-p3229572	0.983471074	0.917355372	Bn-N4-p8456270	Bn-chrC08-p12005479
Bn-A04-p3235336	0.980099502	0.980099502	Bn-N4-p3138888	Bn-chrA04-p2906584
Bn-A04-p3254177	0.985074627	0.985074627	Bn-N4-p3160241	Bn-chrA04_random-p281363
Bn-A04-p3255140	0.995024876	0.995024876	Bn-N4-p3161205	Bn-chrA04_random-p276858
Bn-A04-p3255172	0.990049751	0.990049751	Bn-N4-p3161237	Bn-chrA04_random-p276826
Bn-A04-p3262432	0.84079602	0.84079602	Bn-N4-p3170115	Bn-chrA04_random-p269379
Bn-A04-p3263085	0.975124378	0.975124378	Bn-N4-p3170762	Bn-chrA04_random-p268732
Bn-A04-p3267803	1	1	Bn-N4-p3175665	Bn-chrA04_random-p263828
Bn-A04-p3272740	0.980066445	0.980066445	Bn-N4-p3187032	Bn-chrA04_random-p285919
Bn-A04-p3273187	0.975206612	0.975206612	Bn-N4-p3187479	Bn-chrA04_random-p286366
Bn-A04-p3274507	1	1	Bn-N4-p3188799	Bn-chrA04_random-p287686
Bn-A04-p3283282	0.983471074	0.983471074	Bn-N4-p3190776	Bn-chrA04_random-p289665
Bn-A04-p3294004	0.966942149	0.966942149	Bn-N4-p3202561	Bn-chrA04_random-p302410
Bn-A04-p3297115	0.960199005	0.960199005	Bn-N4-p3205483	Bn-chrA04_random-p305332
Bn-A04-p3298487	0.985074627	0.985074627	Bn-N4-p3206903	Bn-chrA04_random-p306752
Bn-A04-p3306253	0.930348259	0.930348259	Bn-N4-p3213879	Bn-chrA04_random-p313620
Bn-A04-p3308299	0.975124378	0.975124378	Bn-N4-p3215925	Bn-chrA04_random-p315666
Bn-A04-p3331930	0.975206612	0.975206612	Bn-N4-p3246584	Bn-chrA04-p2997699
Bn-A04-p3331969	0.958677686	0.958677686	Bn-N4-p3246623	Bn-chrA04-p2997738
Bn-A04-p3332039	0.991735537	0.991735537	Bn-N4-p3246696	Bn-chrA04-p2997811
Bn-A04-p3332042	0.983471074	0.983471074	Bn-N4-p3246699	Bn-chrA04-p2997814

Bn-A04-p3345530	0.970099668	0.970099668	Bn-N4-p3261325	Bn-chrA04-p3011569
Bn-A04-p3351067	0.991735537	0.991735537	Bn-N4-p3266318	Bn-chrA04-p3016756
Bn-A04-p3351150	0.966942149	0.966942149	Bn-N4-p3266401	Bn-chrA04-p3016839
Bn-A04-p3357098	0.986710963	0.986710963	Bn-N4-p3272380	Bn-chrA04-p3022818
Bn-A04-p3357519	0.966942149	0.966942149	Bn-N4-p3272801	Bn-chrA04-p3023239
Bn-A04-p3357700	0.966942149	0.966942149	Bn-N4-p3272981	Bn-chrA04-p3023419
Bn-A04-p3358441	0.975206612	0.975206612	Bn-N4-p3273734	Bn-chrA04-p3024172
Bn-A04-p3363190	0.983388704	0.983388704	Bn-N4-p3278771	Bn-chrA04-p3029209
Bn-A04-p3379410	0.983471074	0.975206612	Bn-N4-p3299665	Bn-chrA04-p3049836
Bn-A04-p3390331	0.991735537	0.983471074	Bn-N4-p3310984	Bn-chrA04-p3055454
Bn-A04-p3395103	1	0.995024876	Bn-N4-p3317226	Bn-chrA04-p3060018
Bn-A04-p3413548	0.990049751	0.990049751	Bn-N4-p3332043	Bn-chrA04-p3076159
Bn-A04-p3414140	0.966942149	0.966942149	Bn-N4-p3332635	Bn-chrA04-p3076751
Bn-A04-p3414232	0.966942149	0.966942149	Bn-N4-p3332727	Bn-chrA04-p3076843
Bn-A04-p3414449	0.990049751	0.990049751	Bn-N4-p3332942	Bn-chrA04-p3077058
Bn-A04-p3420532	0.603305785	0.603305785	Bn-N4-p3334529	Bn-chrA04-p3078645
Bn-A04-p3421117	0.975206612	0.975206612	Bn-N4-p3335114	Bn-chrA04-p3079230
Bn-A04-p3448682	1	1	Bn-N4-p3361874	Bn-chrA04-p3103903
Bn-A04-p3449764	0.986710963	0.986710963	Bn-N4-p3362953	Bn-chrA04-p3104982
Bn-A04-p3457753	0.996677741	0.996677741	Bn-N4-p3370906	Bn-chrAnn_random-p388068
Bn-A04-p3462758	0.985074627	0.985074627	Bn-N4-p3376272	Bn-chrA04-p3116376
Bn-A04-p3463785	0.990033223	0.990033223	Bn-N4-p3377300	Bn-chrA04-p3117404
Bn-A04-p3471699	0.983471074	0.983471074	Bn-N4-p3386454	Bn-chrA04-p3126895
Bn-A04-p3473990	0.975206612	0.975206612	Bn-N4-p3388770	Bn-chrA04-p3129209
Bn-A04-p3483694	0.965174129	0.965174129	Bn-N4-p3392904	Bn-chrA04-p3133100
Bn-A04-p3483780	0.975206612	0.975206612	Bn-N4-p3392990	Bn-chrA04-p3133186
Bn-A04-p3485611	0.996677741	0.996677741	Bn-N4-p3394821	Bn-chrA04-p3135017
Bn-A04-p3509049	0.991735537	0.991735537	Bn-N4-p3414733	Bn-chrA04-p3155219
Bn-A04-p3528213	0.989583333	0.989583333	Bn-N4-p3440624	Bn-chrA04-p3192476
Bn-A04-p3540163	0.993355482	0.993355482	Bn-N4-p3458305	Bn-chrA04-p3211887
Bn-A04-p3551969	0.975206612	0.975206612	Bn-N4-p3469581	Bn-chrA04-p3223164
Bn-A04-p3563096	0.990033223	0.990033223	Bn-N4-p3484186	Bn-chrA04-p3239016
Bn-A04-p3571259	0.983471074	0.983471074	Bn-N4-p3486900	Bn-chrA04-p3241577
Bn-A04-p3572717	0.966942149	0.966942149	Bn-N4-p3488326	Bn-chrA04-p3243003
Bn-A04-p3572777	0.933884298	0.933884298	Bn-N4-p3488386	Bn-chrA04-p3243063
Bn-A04-p3573078	0.958677686	0.958677686	Bn-N4-p3488718	Bn-chrA04-p3243395
Bn-A04-p3583216	0.983471074	0.975206612	Bn-N4-p3498786	Bn-chrA04-p3253401
Bn-A04-p3589494	0.983388704	0.990033223	Bn-N4-p3505048	Bn-chrA04-p3259661
Bn-A04-p3602299	0.900826446	0.909090909	Bn-N4-p3518560	Bn-chrA04-p3271347
Bn-A04-p3615799	1	1	Bn-N4-p3531693	Bn-chrA04-p3284475
Bn-A04-p3618090	0.996677741	0.926910299	Bn-N4-p3534145	Bn-chrA04-p3286925
Bn-A04-p3628682	0.996677741	0.983388704	Bn-N4-p3544790	Bn-chrA04-p3297129
Bn-A04-p3630793	0.966777409	0.966777409	Bn-N4-p3546895	Bn-chrA04-p3299231
Bn-A04-p3631563	0.950413223	0.958677686	Bn-N4-p3547667	Bn-chrA04-p3300001
Bn-A04-p3632226	0.991735537	0.991735537	Bn-N4-p3548333	Bn-chrA04-p3300664
Bn-A04-p3632611	0.991735537	0.983471074	Bn-N4-p3548718	Bn-chrA04-p3301049

Bn-A04-p3651589	0.993355482	0.993355482	Bn-N4-p3565731	Bn-chrA04-p3315323
Bn-A04-p3668627	0.958677686	0.958677686	Bn-N4-p3583041	Bn-chrA04-p3332632
Bn-A04-p3690435	0.554817276	0.554817276	Bn-N14-p32588755	Bn-chrC04-p28232055
Bn-A04-p3695547	0.691542289	0.691542289	Bn-N4-p3600407	Bn-chrA04-p3348638
Bn-A04-p3702357	0.991735537	0.991735537	Bn-N4-p3609230	Bn-chrA04_random-p341559
Bn-A04-p3702896	0.975124378	0.975124378	Bn-N4-p3609768	Bn-chrA04_random-p342097
Bn-A04-p3719381	1	1	Bn-N4-p3621830	Bn-chrA04-p3367122
Bn-A04-p3721587	0.985074627	0.985074627	Bn-N4-p3624036	Bn-chrA04-p3369328
Bn-A04-p3724573	0.880398671	0.873754153	Bn-N4-p3627001	Bn-chrA04-p3372293
Bn-A04-p3743549	1	1	Bn-N4-p3642885	Bn-chrA04-p3388176
Bn-A04-p3750975	0.975206612	0.975206612	Bn-N4-p3653112	Bn-chrA04-p3396550
Bn-A04-p3757486	0.996677741	0.996677741	Bn-N4-p3659836	Bn-chrA04-p3403851
Bn-A04-p3761523	0.983471074	0.983471074	Bn-N4-p3662467	Bn-chrA04-p3406482
Bn-A04-p3820381	0.966942149	0.966942149	Bn-N4-p3700659	Bn-chrA04-p3446783
Bn-A04-p3851969	0.975206612	0.975206612	Bn-Scaffold04520-p1	Bn-chrC05-p1303556
Bn-A04-p3852124	1	1	Bn-Scaffold04520-p3	Bn-chrC05-p1303711
Bn-A04-p3853621	0.966942149	0.966942149	Bn-Scaffold04520-p1	Bn-chrC05-p1305483
Bn-A04-p3875034	0.990033223	0.990033223	Bn-N4-p3744805	Bn-chrA04-p3492186
Bn-A04-p3915978	0.973421927	0.903654485	Bn-N4-p3773355	Bn-chrC04-p28556494
Bn-A04-p3923982	0.961685824	0.996168582	Bn-N12-p38098617	Bn-chrAnn_random-p322402
Bn-A04-p3933714	1	0.991735537	Bn-N4-p3786133	Bn-chrA04-p3540219
Bn-A04-p3934671	0.966942149	0.975206612	Bn-N4-p3787097	Bn-chrA04-p3541177
Bn-A04-p3990611	0.975206612	0.975206612	Bn-N4-p3839135	Bn-chrA04-p3587905
Bn-A04-p3990828	0.991735537	0.991735537	Bn-N4-p3839352	Bn-chrA04-p3588122
Bn-A04-p3991261	1	1	Bn-N4-p3839784	Bn-chrA04-p3588554
Bn-A04-p4002585	0.975206612	0.975206612	Bn-N14-p32977648	Bn-chrC04-p28921645
Bn-A04-p4034426	0.993355482	0.993355482	Bn-N4-p4272227	Bn-chrA04-p4102327
Bn-A04-p4035141	0.980066445	0.980066445	Bn-N4-p4272942	Bn-chrA04-p4103042
Bn-A04-p4056517	1	1	Bn-N4-p4294633	Bn-chrA04-p4124401
Bn-A04-p4060806	0.966942149	0.966942149	Bn-N4-p4302052	Bn-chrA04-p4131614
Bn-A04-p4074166	1	1	Bn-N4-p4315492	Bn-chrA04-p4143658
Bn-A04-p4074703	1	1	Bn-N4-p4315968	Bn-chrA04-p4144134
Bn-A04-p4082416	1	1	Bn-N4-p4326034	Bn-chrA04-p4151844
Bn-A04-p4087475	0.975124378	0.990049751	Bn-N4-p4332113	Bn-chrA04-p4156903
Bn-A04-p4090389	0.993355482	0.993355482	Bn-N4-p4335027	Bn-chrA04-p4159816
Bn-A04-p4097272	0.983471074	0.983471074	Bn-N4-p4342053	Bn-chrA04-p4167158
Bn-A04-p4099958	0.996677741	0.996677741	Bn-N4-p4344705	Bn-chrA04-p4169810
Bn-A04-p4101266	0.983471074	0.983471074	Bn-N4-p4346013	Bn-chrA04-p4171118
Bn-A04-p4101473	0.966942149	0.966942149	Bn-N4-p4346220	Bn-chrA04-p4171325
Bn-A04-p4102094	0.986710963	0.986710963	Bn-N4-p4346841	Bn-chrA04-p4171946
Bn-A04-p4107072	0.983471074	0.983471074	Bn-N4-p4351823	Bn-chrA04-p4176928
Bn-A04-p4112460	0.996677741	0.996677741	Bn-N4-p4357591	Bn-chrA04-p4182149
Bn-A04-p4117100	0.996677741	0.996677741	Bn-N4-p4362433	Bn-chrAnn_random-p472009
Bn-A04-p4121578	0.990033223	0.990033223	Bn-N4-p4367118	Bn-chrA04-p4189807
Bn-A04-p4124082	0.986710963	0.986710963	Bn-N4-p4369602	Bn-chrA04-p4192128
Bn-A04-p4124240	0.991735537	0.991735537	Bn-N4-p4369760	Bn-chrA04-p4192286

Bn-A04-p4131451	0.990049751	0.990049751	Bn-N4-p4377084	Bn-chrA04-p4199497
Bn-A04-p4150340	0.990033223	0.990033223	Bn-N4-p4400399	Bn-chrA04-p4203006
Bn-A04-p4152511	0.990033223	0.990033223	Bn-N4-p4402570	Bn-chrA04-p4205177
Bn-A04-p4169131	0.983471074	0.983471074	Bn-N4-p4415466	Bn-chrA04-p4219108
Bn-A04-p4179724	0.985074627	0.985074627	Bn-N4-p4426085	Bn-chrA04-p4229726
Bn-A04-p4179915	0.980099502	0.980099502	Bn-N4-p4426276	Bn-chrA04-p4229917
Bn-A04-p4200354	0.950413223	0.950413223	Bn-N4-p4447171	Bn-chrA04-p4250392
Bn-A04-p4203564	0.983471074	0.983471074	Bn-N4-p4452107	Bn-chrA04-p4255079
Bn-A04-p4204119	0.966942149	0.966942149	Bn-N4-p4454024	Bn-chrA04-p4256883
Bn-A04-p4204775	0.966942149	0.966942149	Bn-N4-p4454683	Bn-chrA04-p4257542
Bn-A04-p4207198	0.975206612	0.975206612	Bn-N4-p4457320	Bn-chrA04-p4260179
Bn-A04-p4210431	0.983388704	0.983388704	Bn-N4-p4475571	Bn-chrA04-p4278778
Bn-A04-p4217227	1	1	Bn-N4-p4481880	Bn-chrA04-p4292602
Bn-A04-p4221603	0.995024876	0.995024876	Bn-N4-p4491938	Bn-chrA04-p4302659
Bn-A04-p4222491	0.983388704	0.983388704	Bn-N4-p4492827	Bn-chrA04-p4303548
Bn-A04-p4223859	0.996677741	0.996677741	Bn-N4-p4494197	Bn-chrA04-p4304918
Bn-A04-p4224646	0.991735537	0.991735537	Bn-N4-p4494988	Bn-chrA04-p4305709
Bn-A04-p4224705	0.983471074	0.983471074	Bn-N4-p4495047	Bn-chrA04-p4305768
Bn-A04-p4244824	0.993355482	0.993355482	Bn-N4-p4517640	Bn-chrA04-p4330279
Bn-A04-p4249132	0.976744186	0.976744186	Bn-N4-p4523660	Bn-chrA04-p4336299
Bn-A04-p4254366	0.890547264	0.92039801	Bn-N15-p10724900	Bn-chrA06-p11950078
Bn-A04-p4265054	0.996677741	0.996677741	Bn-N4-p4532069	Bn-chrA04-p4344708
Bn-A04-p4265606	0.986710963	0.986710963	Bn-N4-p4532621	Bn-chrA04-p4345260
Bn-A04-p4276334	0.993355482	0.993355482	Bn-N4-p4547759	Bn-chrA04-p4364522
Bn-A04-p4286343	0.966942149	0.966942149	Bn-N4-p4577694	Bn-chrA04-p4395254
Bn-A04-p4294569	0.983388704	0.983388704	Bn-N4-p4585331	Bn-chrA04-p4402891
Bn-A04-p4307559	0.983388704	0.983388704	Bn-N4-p4600850	Bn-chrA04-p4418981
Bn-A04-p4319736	0.985074627	0.985074627	Bn-N4-p4613586	Bn-chrA04-p4431128
Bn-A04-p4325390	0.995024876	0.995024876	Bn-N4-p4620668	Bn-chrA04-p4443618
Bn-A04-p4330419	0.996677741	0.996677741	Bn-N4-p4625457	Bn-chrA04-p4447529
Bn-A04-p4332836	0.990033223	0.990033223	Bn-N4-p4627873	Bn-chrA04-p4449945
Bn-A04-p4346492	0.975124378	0.975124378	Bn-N4-p4641714	Bn-chrA04-p4465542
Bn-A04-p4349715	0.991735537	0.991735537	Bn-N4-p4644935	Bn-chrA04-p4468763
Bn-A04-p4350372	0.991735537	0.991735537	Bn-N4-p4649324	Bn-chrA04-p4474536
Bn-A04-p4350555	0.834710744	0.834710744	Bn-N4-p4649459	Bn-chrA04-p4474671
Bn-A04-p4382202	1	1	Bn-N4-p4679839	Bn-chrA04-p4503020
Bn-A04-p4392113	0.991735537	0.991735537	Bn-N4-p4689011	Bn-chrA04-p4512265
Bn-A04-p4409348	0.991735537	0.991735537	Bn-N4-p4706883	Bn-chrA04-p4529719
Bn-A04-p4409463	0.867768595	0.867768595	Bn-N4-p4707005	Bn-chrA04-p4529841
Bn-A04-p4409752	0.991735537	0.991735537	Bn-N4-p4707284	Bn-chrA04-p4530120
Bn-A04-p4410113	0.975206612	0.975206612	Bn-N4-p4709874	Bn-chrA04-p4532710
Bn-A04-p4410144	0.966942149	0.966942149	Bn-N4-p4709905	Bn-chrA04-p4532741
Bn-A04-p4410196	0.958677686	0.958677686	Bn-N4-p4709957	Bn-chrA04-p4532793
Bn-A04-p4418670	0.950413223	0.950413223	Bn-N4-p4719841	Bn-chrA04-p4543027
Bn-A04-p4418715	0.933884298	0.933884298	Bn-N4-p4719886	Bn-chrA04-p4543072
Bn-A04-p4529360	0.980099502	0.980099502	Bn-N4-p4806174	Bn-chrA04-p4625893

Bn-A04-p4531566	0.991735537	0.933884298	Bn-Scaffold14836-p6	Bn-chrC04-p37057048
Bn-A04-p4532802	0.94214876	0.925619835	Bn-Scaffold29045-p3	Bn-chrC04-p37056179
Bn-A04-p4544600	0.991735537	1	Bn-N3-p21766023	Bn-chrA03-p20272639
Bn-A04-p4598738	0.976744186	0.448504983	Bn-Scaffold02085-p5	Bn-chrC05-p24719107
Bn-A04-p4598943	0.970099668	0.531561462	Bn-Scaffold02085-p7	Bn-chrC03-p19348706
Bn-A04-p4599093	0.96013289	0.558139535	Bn-Scaffold02085-p8	Bn-chrC03-p29081769
Bn-A04-p4599664	0.990033223	0.438538206	Bn-Scaffold02085-p1	Bn-chrCnn_random-p670114
Bn-A04-p4599814	0.975124378	0.741293532	Bn-Scaffold02085-p1	Bn-chrAnn_random-p123223
Bn-A04-p4599916	0.96013289	0.528239203	Bn-Scaffold02085-p1	Bn-chrA08-p1945019
Bn-A04-p4599988	0.960199005	0.577114428	Bn-Scaffold02085-p1	Bn-chrA04-p12366241
Bn-A04-p4600223	0.965174129	0.462686567	Bn-Scaffold02085-p2	Bn-chrA02-p10680751
Bn-A04-p4601980	0.966777409	0.671096346	Bn-Scaffold02085-p3	Bn-chrA04-p12364246
Bn-A04-p4602694	0.910299003	0.568106312	Bn-Scaffold02085-p4	Bn-chrAnn_random-p123252
Bn-A04-p4693254	0.990049751	0.980099502	Bn-N4-p5013040	Bn-chrA04-p4676285
Bn-A04-p4717909	0.966942149	0.966942149	Bn-N4-p5041861	Bn-chrA04-p4707504
Bn-A04-p4730302	0.785123967	0.785123967	Bn-N4-p5062253	Bn-chrA04-p4734269
Bn-A04-p4736023	0.983471074	0.975206612	Bn-N4-p5076254	Bn-chrA04-p4749995
Bn-A04-p4742377	0.933884298	0.925619835	Bn-N4-p5091187	Bn-chrA04-p4763246
Bn-A04-p4742432	0.909090909	0.909090909	Bn-N4-p5091242	Bn-chrA04-p4763301
Bn-A04-p4823556	0.996677741	0.993355482	Bn-N4-p5186650	Bn-chrA04-p4860848
Bn-A04-p4825669	0.958677686	0.966942149	Bn-N4-p5188763	Bn-chrA04-p4862946
Bn-A04-p4834943	0.966942149	0.975206612	Bn-N4-p5205440	Bn-chrA04-p4871834
Bn-A04-p4835096	0.983471074	0.975206612	Bn-N4-p5205593	Bn-chrA04-p4871987
Bn-A04-p4835244	0.958677686	0.950413223	Bn-N4-p5205743	Bn-chrA04-p4872135
Bn-A04-p4835883	0.958677686	0.966942149	Bn-N4-p5206381	Bn-chrA04-p4872775
Bn-A04-p4836499	0.991735537	1	Bn-N4-p5206944	Bn-chrA04-p4873389
Bn-A04-p4848743	0.983471074	0.975206612	Bn-N4-p5214354	Bn-chrA04-p4889608
Bn-A04-p4864900	0.975206612	0.966942149	Bn-N4-p5219184	Bn-chrA04-p4904094
Bn-A04-p4866786	0.983471074	0.975206612	Bn-N4-p5221092	Bn-chrA04-p4905979
Bn-A04-p4872836	0.983471074	0.991735537	Bn-N4-p5229923	Bn-chrA04-p4911058
Bn-A04-p4874261	0.966942149	0.958677686	Bn-N4-p5231353	Bn-chrA04-p4912483
Bn-A04-p4874840	0.983471074	0.991735537	Bn-N4-p5232333	Bn-chrA04-p4913059
Bn-A04-p4879262	0.975206612	0.983471074	Bn-N4-p5236813	Bn-chrA04-p4919425
Bn-A04-p4880759	0.970149254	0.960199005	Bn-N4-p5238319	Bn-chrA04-p4920941
Bn-A04-p4881168	0.985074627	0.990049751	Bn-N4-p5238741	Bn-chrA04-p4921362
Bn-A04-p4881807	0.965174129	0.985074627	Bn-N14-p34631291	Bn-chrA04-p4922028
Bn-A04-p4891169	0.975206612	0.512396694	Bn-N4-p5310615	Bn-chrA06-p14969726
Bn-A04-p4897489	0.950413223	0.958677686	Bn-N4-p5314588	Bn-chrA04-p5006726
Bn-A04-p4909402	0.989583333	1	Bn-N4-p5326434	Bn-chrA04-p5023594
Bn-A04-p4910033	0.995024876	1	Bn-N4-p5327114	Bn-chrA04-p5024274
Bn-A04-p4932716	0.990033223	0.983388704	Bn-N4-p5345059	Bn-chrA04-p5050514
Bn-A04-p4976932	0.983471074	0.991735537	Bn-N4-p5380466	Bn-chrA04-p5115640
Bn-A04-p4977488	0.991735537	0.991735537	Bn-N4-p5381022	Bn-chrA04-p5116196
Bn-A04-p4978478	0.983471074	0.950413223	Bn-N4-p5382012	Bn-chrA04-p5117186
Bn-A04-p4978492	0.991735537	0.975206612	Bn-N4-p5382026	Bn-chrA04-p5117200
Bn-A04-p4978780	0.721393035	0.965174129	Bn-N4-p5382314	Bn-chrA04-p5117487

Bn-A04-p5133653	0.980099502	0.960199005	Bn-N4-p5565820	Bn-chrA04-p5305611
Bn-A04-p5137321	1	0.936877076	Bn-N4-p5569488	Bn-chrC04_random-p340638
Bn-A04-p5183306	0.958677686	0.958677686	Bn-N4-p5614929	Bn-chrA04-p5349947
Bn-A04-p5223849	0.991735537	0.991735537	Bn-N4-p5665588	Bn-chrA04-p5401756
Bn-A04-p5270881	0.980066445	0.980066445	Bn-N4-p5713624	Bn-chrA04-p5451673
Bn-A04-p5273199	0.975206612	0.975206612	Bn-N4-p5715924	Bn-chrA04-p5453973
Bn-A04-p5378408	0.940298507	0.940298507	Bn-N4-p5827825	Bn-chrA04-p5576124
Bn-A04-p5398352	0.995024876	0.995024876	Bn-N4-p5946136	Bn-chrA04-p5687201
Bn-A04-p5501342	0.980099502	0.990049751	Bn-N4-p6053818	Bn-chrA04-p5797319
Bn-A04-p5501870	0.96969697	0.96969697	Bn-N4-p6054346	Bn-chrA02_random-p632666
Bn-A04-p5553976	1	1	Bn-Scaffold05090-p1	Bn-chrA03-p3304159
Bn-A04-p5554444	0.995024876	0.995024876	Bn-N4-p6108204	Bn-chrA03-p3304627
Bn-A04-p5576673	0.990049751	0.990049751	Bn-N4-p6130608	Bn-chrA04-p5852415
Bn-A04-p5646833	0.995024876	0.995024876	Bn-N13-p46492901	Bn-chrC03-p41039236
Bn-A04-p5715780	0.989583333	0.989583333	Bn-N4-p6269396	Bn-chrA04-p5988377
Bn-A04-p5788908	0.991735537	0.991735537	Bn-N4-p7409965	Bn-chrA04-p6929649
Bn-A04-p5809264	0.940298507	0.940298507	Bn-N4-p7427323	Bn-chrA04-p6947001
Bn-A04-p5827901	0.966942149	0.966942149	Bn-N4-p7449869	Bn-chrA04-p6969037
Bn-A04-p5843077	0.94214876	0.94214876	Bn-N4-p7454019	Bn-chrA04-p6973187
Bn-A04-p5852413	0.975206612	0.975206612	Bn-N4-p7460179	Bn-chrA04-p6978751
Bn-A04-p5853514	0.980099502	0.980099502	Bn-N4-p7457881	Bn-chrA04-p6976452
Bn-A04-p5872564	0.975206612	0.975206612	Bn-N4-p7480361	Bn-chrA04-p6999270
Bn-A04-p5873190	0.900826446	0.900826446	Bn-N4-p7481020	Bn-chrA04-p6999929
Bn-A04-p5880992	0.983471074	0.983471074	Bn-N4-p7489397	Bn-chrA04-p7008006
Bn-A04-p5885917	0.950413223	0.950413223	Bn-N14-p36769077	Bn-chrC04-p31960267
Bn-A04-p5919648	0.996677741	0.996677741	Bn-N4-p7563178	Bn-chrA04-p7097364
Bn-A04-p5938125	0.985074627	0.985074627	Bn-N4-p7586749	Bn-chrA04-p7125008
Bn-A04-p5938245	0.915422886	0.915422886	Bn-N4-p7586869	Bn-chrA04-p7125128
Bn-A04-p5959753	0.991735537	0.991735537	Bn-N4-p7608030	Bn-chrA04-p7148096
Bn-A04-p5964175	0.975206612	0.975206612	Bn-N4-p7612487	Bn-chrA04-p7152553
Bn-A04-p5965590	0.980099502	0.980099502	Bn-N4-p7613904	Bn-chrA04-p7153970
Bn-A04-p5967828	0.661157025	0.983471074	Bn-N4-p7621193	Bn-chrAnn_random-p442614
Bn-A04-p5971620	0.991735537	0.991735537	Bn-N4-p7629728	Bn-chrA04-p7166335
Bn-A04-p5973348	0.975206612	0.975206612	Bn-N4-p7631623	Bn-chrA04-p7168230
Bn-A04-p5973919	0.991735537	0.991735537	Bn-N4-p7632194	Bn-chrA04-p7168801
Bn-A04-p5974195	0.991735537	0.991735537	Bn-N4-p7632470	Bn-chrA04-p7169077
Bn-A04-p5979118	0.933884298	0.933884298	Bn-N4-p7637415	Bn-chrA04-p7174022
Bn-A04-p5982123	0.980066445	0.980066445	Bn-N4-p7640512	Bn-chrA04-p7177075
Bn-A04-p5983577	0.826446281	0.826446281	Bn-N4-p7641981	Bn-chrA04-p7178544
Bn-A04-p5985513	0.986710963	0.976744186	Bn-N4-p7643273	Bn-chrA04-p7179836
Bn-A04-p5987463	0.958677686	0.958677686	Bn-N4-p7645250	Bn-chrA04-p7181813
Bn-A04-p5999405	0.983471074	0.983471074	Bn-N4-p7654373	Bn-chrA04-p7190935
Bn-A04-p6004690	0.975206612	0.975206612	Bn-N4-p7660789	Bn-chrA04-p7197880
Bn-A04-p6008358	0.94214876	0.94214876	Bn-N4-p7665419	Bn-chrA04-p7202507
Bn-A04-p6008778	0.966942149	0.966942149	Bn-N4-p7665838	Bn-chrA04-p7202926
Bn-A04-p6017218	0.933884298	0.933884298	Bn-N4-p7675141	Bn-chrA04-p7213347

Bn-A04-p6017599	0.991735537	0.991735537	Bn-N4-p7675514	Bn-chrA04-p7213720
Bn-A04-p6023914	0.900332226	0.900332226	Bn-N4-p7681393	Bn-chrA04-p7219761
Bn-A04-p6027878	0.94214876	0.727272727	Bn-N4-p7685350	Bn-chrC04-p32141340
Bn-A04-p6028097	0.983471074	0.983471074	Bn-N4-p7686443	Bn-chrA04-p7224865
Bn-A04-p6028334	0.983471074	0.983471074	Bn-N4-p7686677	Bn-chrA04-p7225099
Bn-A04-p6029797	0.950413223	0.950413223	Bn-N4-p7688234	Bn-chrA04-p7226656
Bn-A04-p6030108	0.909090909	0.909090909	Bn-N4-p7688546	Bn-chrA04-p7226968
Bn-A04-p6042013	0.983471074	0.983471074	Bn-N4-p7699147	Bn-chrA04-p7239236
Bn-A04-p6047600	0.983471074	0.983471074	Bn-N4-p7704798	Bn-chrA04-p7251703
Bn-A04-p6050616	0.920265781	0.920265781	Bn-N4-p7707855	Bn-chrA04-p7254762
Bn-A04-p6052986	0.950413223	0.950413223	Bn-Scaffold02245-p3	Bn-chrAnn_random-p181132
Bn-A04-p6053997	0.966942149	0.966942149	Bn-Scaffold02245-p2	Bn-chrAnn_random-p181142
Bn-A04-p6063409	0.970149254	0.970149254	Bn-N4-p7717172	Bn-chrAnn_random-p181401
Bn-A04-p6100884	0.975206612	0.975206612	Bn-N4-p7751442	Bn-chrA04-p7301833
Bn-A04-p6165743	0.958677686	0.958677686	Bn-N4-p7830973	Bn-chrA04-p7370547
Bn-A04-p6228211	0.986710963	0.986710963	Bn-N14-p37220173	Bn-chrC04-p32430403
Bn-A04-p6240855	0.958677686	0.958677686	Bn-N4-p7917200	Bn-chrC04-p32496458
Bn-A04-p6255997	0.991735537	0.991735537	Bn-N4-p7926047	Bn-chrA04-p7470364
Bn-A04-p6263702	0.975206612	0.975206612	Bn-N4-p7931828	Bn-chrA04-p7476310
Bn-A04-p6264043	0.983471074	0.983471074	Bn-N4-p7932174	Bn-chrA04-p7476656
Bn-A04-p6277634	0.975206612	0.975206612	Bn-N4-p7970235	Bn-chrA04-p7495579
Bn-A04-p6284377	0.975206612	0.975206612	Bn-N4-p7977624	Bn-chrA04-p7504472
Bn-A04-p6296526	0.958677686	0.958677686	Bn-N4-p7992709	Bn-chrA04-p7519557
Bn-A04-p6319430	0.995024876	0.995024876	Bn-N4-p8014387	Bn-chrA04-p7548823
Bn-A04-p6340854	0.983471074	0.983471074	Bn-N4-p8036353	Bn-chrA04-p7570612
Bn-A04-p6341516	0.983471074	0.983471074	Bn-N4-p8037015	Bn-chrA04-p7571274
Bn-A04-p6376472	0.913621262	0.913621262	Bn-N14-p37507550	Bn-chrA04-p7581032
Bn-A04-p6389136	0.990049751	0.990049751	Bn-N4-p8085537	Bn-chrA04-p7593915
Bn-A04-p6395765	0.965174129	0.965174129	Bn-N4-p8092387	Bn-chrA04-p7600765
Bn-A04-p6402808	0.990049751	0.990049751	Bn-N4-p8099707	Bn-chrA04-p7607775
Bn-A04-p6422450	0.958677686	0.958677686	Bn-N4-p8119240	Bn-chrA04-p7624947
Bn-A04-p6428686	0.983471074	0.983471074	Bn-N4-p8125466	Bn-chrC04-p32749443
Bn-A04-p6445083	0.975206612	0.975206612	Bn-Scaffold01456-p1	Bn-chrA04-p7646294
Bn-A04-p6448172	0.983471074	0.983471074	Bn-N4-p8134730	Bn-chrA04-p7650660
Bn-A04-p6453979	0.983471074	0.983471074	Bn-N4-p8140686	Bn-chrA04-p7656617
Bn-A04-p6454976	1	1	Bn-N4-p8141683	Bn-chrA04-p7657614
Bn-A04-p6455109	0.958677686	0.958677686	Bn-N4-p8141816	Bn-chrA04-p7657747
Bn-A04-p6456456	0.966942149	0.966942149	Bn-N4-p8143162	Bn-chrA04-p7659093
Bn-A04-p6463878	0.991735537	0.991735537	Bn-N4-p8150581	Bn-chrA04-p7666512
Bn-A04-p6465442	0.704318937	0.704318937	Bn-N14-p37562608	Bn-chrC04-p32787382
Bn-A04-p6486540	0.743801653	0.743801653	Bn-N4-p8180888	Bn-chrA04-p7696040
Bn-A04-p6487804	0.991735537	0.991735537	Bn-N4-p8180990	Bn-chrA04-p7696142
Bn-A04-p6533593	0.950413223	0.966942149	Bn-N14-p37652911	Bn-chrA04-p7743922
Bn-A04-p6550369	0.975206612	0.975206612	Bn-N4-p8242241	Bn-chrA04-p7754487
Bn-A04-p6550757	0.966777409	0.966777409	Bn-N4-p8242628	Bn-chrA04-p7754874
Bn-A04-p6555913	0.966942149	0.966942149	Bn-N4-p8247861	Bn-chrA04-p7760060

Bn-A04-p6556405	0.966942149	0.966942149	Bn-N4-p8248353	Bn-chrA04-p7760552
Bn-A04-p6563107	0.975124378	0.975124378	Bn-N4-p8255950	Bn-chrA04-p7768149
Bn-A04-p6563200	0.990049751	0.990049751	Bn-N4-p8256043	Bn-chrA04-p7768242
Bn-A04-p6588935	0.975206612	0.975206612	Bn-N4-p8296575	Bn-chrA04-p7808884
Bn-A04-p6632126	0.966942149	0.966942149	Bn-N4-p8348683	Bn-chrA04-p7864820
Bn-A04-p6637458	0.94214876	0.94214876	Bn-N4-p8357278	Bn-chrA04-p7870252
Bn-A04-p6640990	0.991735537	0.991735537	Bn-N4-p8360809	Bn-chrA04-p7873783
Bn-A04-p6646210	0.975206612	0.975206612	Bn-N4-p8366049	Bn-chrA04-p7879170
Bn-A04-p6653508	0.991735537	0.991735537	Bn-N4-p8373347	Bn-chrA04-p7885464
Bn-A04-p6657342	0.983471074	0.983471074	Bn-N4-p8377182	Bn-chrA04-p7889298
Bn-A04-p6673120	0.975206612	0.950413223	Bn-N4-p8391311	Bn-chrC04-p33005162
Bn-A04-p6678797	0.992	0.992	Bn-N4-p8396236	Bn-chrA04-p7908248
Bn-A04-p6679263	0.995024876	0.995024876	Bn-N4-p8396702	Bn-chrA04-p7908714
Bn-A04-p6679568	0.940298507	0.940298507	Bn-N4-p8397006	Bn-chrA04-p7909018
Bn-A04-p6682658	0.966942149	0.966942149	Bn-N4-p8400097	Bn-chrA04-p7912109
Bn-A04-p6682728	0.966942149	0.966942149	Bn-N4-p8400167	Bn-chrA04-p7912179
Bn-A04-p6683009	0.983471074	0.983471074	Bn-N4-p8400448	Bn-chrA04-p7912460
Bn-A04-p6684054	0.983471074	0.983471074	Bn-N4-p8401493	Bn-chrA04-p7913505
Bn-A04-p6685027	0.983471074	0.983471074	Bn-N4-p8406418	Bn-chrA04-p7918429
Bn-A04-p6685062	0.966942149	0.966942149	Bn-N4-p8406453	Bn-chrA04-p7918464
Bn-A04-p6685294	1	1	Bn-N4-p8406685	Bn-chrA04-p7918696
Bn-A04-p6685371	0.983471074	0.983471074	Bn-N4-p8406762	Bn-chrA04-p7918773
Bn-A04-p6697580	0.958677686	0.958677686	Bn-N4-p8417976	Bn-chrA04-p7929147
Bn-A04-p6718464	0.900826446	0.900826446	Bn-N4-p8437700	Bn-chrA04-p7950634
Bn-A04-p6719949	0.996677741	0.996677741	Bn-N4-p8439218	Bn-chrA04-p7952152
Bn-A04-p6747807	1	1	Bn-N4-p8481482	Bn-chrA04-p8000432
Bn-A04-p6748822	0.983471074	0.983471074	Bn-N4-p8482498	Bn-chrA04-p8001447
Bn-A04-p6749073	0.991735537	0.991735537	Bn-N4-p8482749	Bn-chrA04-p8001698
Bn-A04-p6749114	0.983471074	0.983471074	Bn-N4-p8482790	Bn-chrA04-p8001739
Bn-A04-p6749322	0.925619835	0.925619835	Bn-N4-p8483047	Bn-chrA04-p8002326
Bn-A04-p6752299	0.991735537	0.991735537	Bn-N4-p8486515	Bn-chrA04-p8005567
Bn-A04-p6752979	0.983471074	0.983471074	Bn-N4-p8487195	Bn-chrA04-p8006247
Bn-A04-p6760294	0.975206612	0.388429752	Bn-N4-p8494359	Bn-chrC07-p33725145
Bn-A04-p6764233	0.983471074	0.983471074	Bn-N4-p8500149	Bn-chrA04-p8018477
Bn-A04-p6765045	0.983471074	0.983471074	Bn-N4-p8500961	Bn-chrA04-p8019289
Bn-A04-p6771264	0.991735537	0.991735537	Bn-N4-p8507147	Bn-chrA04-p8025475
Bn-A04-p6773984	0.983471074	0.983471074	Bn-N4-p8509867	Bn-chrA04-p8028195
Bn-A04-p6775008	0.975206612	0.975206612	Bn-N4-p8510915	Bn-chrA04-p8029243
Bn-A04-p6776597	0.983471074	0.983471074	Bn-N4-p8512504	Bn-chrA04-p8030832
Bn-A04-p6776786	0.991735537	0.991735537	Bn-N4-p8512693	Bn-chrA04-p8031021
Bn-A04-p6778593	0.41322314	1	Bn-N4-p8514107	Bn-chrA04-p8032829
Bn-A04-p6780163	0.994382022	0.994382022	Bn-N4-p8515677	Bn-chrA04-p8034399
Bn-A04-p6783717	0.991735537	0.991735537	Bn-N4-p8519231	Bn-chrA04-p8037953
Bn-A04-p6790373	0.975206612	0.975206612	Bn-N4-p8525888	Bn-chrA04-p8044609
Bn-A04-p6929056	0.980099502	0.980099502	Bn-N4-p8682719	Bn-chrA04-p8211191
Bn-A04-p6953674	0.96013289	0.96013289	Bn-N4-p8706711	Bn-chrA04-p8235852

Bn-A04-p6958157	0.983471074	0.983471074	Bn-N4-p8711151	Bn-chrA04-p8240292
Bn-A04-p6966154	0.990049751	0.990049751	Bn-N4-p8719557	Bn-chrA04-p8249306
Bn-A04-p6966782	0.986710963	0.986710963	Bn-N4-p8720182	Bn-chrA04-p8249931
Bn-A04-p6968899	0.983471074	0.983471074	Bn-N4-p8724710	Bn-chrA04-p8255422
Bn-A04-p6973605	0.975206612	0.975206612	Bn-N4-p8729888	Bn-chrA04-p8260506
Bn-A04-p6973712	1	1	Bn-N4-p8729995	Bn-chrA04-p8260613
Bn-A04-p6974206	0.991735537	0.991735537	Bn-N4-p8730489	Bn-chrA04-p8261107
Bn-A04-p6975646	0.983471074	0.983471074	Bn-N4-p8731938	Bn-chrA04-p8262556
Bn-A04-p6978108	0.975206612	0.975206612	Bn-N4-p8734411	Bn-chrA04-p8265031
Bn-A04-p6978163	0.983471074	0.983471074	Bn-N4-p8734466	Bn-chrA04-p8265086
Bn-A04-p6979129	0.983471074	0.983471074	Bn-N4-p8735429	Bn-chrA04-p8266048
Bn-A04-p6980459	0.958677686	0.958677686	Bn-N4-p8736901	Bn-chrA04-p8267520
Bn-A04-p7003715	0.973421927	0.973421927	Bn-N4-p8751132	Bn-chrA04-p8283228
Bn-A04-p7026999	0.970149254	0.965174129	Bn-N16-p34123106	Bn-chrCnn_random-p335269
Bn-A04-p7027225	0.970149254	0.865671642	Bn-N16-p34122880	Bn-chrC09-p25162729
Bn-A04-p7027307	0.970149254	0.925373134	Bn-N16-p34122798	Bn-chrCnn_random-p335267
Bn-A04-p7044446	0.980099502	0.980099502	Bn-N14-p38133852	Bn-chrC04-p33343528
Bn-A04-p7066181	0.950248756	0.950248756	Bn-N4-p8794886	Bn-chrA04-p8336302
Bn-A04-p7066595	0.943521595	0.943521595	Bn-N14-p38166443	Bn-chrC04-p42529658
Bn-A04-p7068079	0.980099502	0.980099502	Bn-N4-p8797594	Bn-chrA04-p8339010
Bn-A04-p7077586	0.990033223	0.990033223	Bn-N4-p8807525	Bn-chrA04-p8348735
Bn-A04-p7088336	0.991735537	0.991735537	Bn-N4-p8819355	Bn-chrA04-p8360446
Bn-A04-p7090997	0.965174129	0.965174129	Bn-N4-p8822364	Bn-chrA04-p8363453
Bn-A04-p7093491	0.996677741	0.996677741	Bn-N4-p8824849	Bn-chrA04-p8365938
Bn-A04-p7107858	0.983471074	0.983471074	Bn-N4-p8849472	Bn-chrA04-p8384947
Bn-A04-p7109346	1	1	Bn-N4-p8850965	Bn-chrA04-p8386440
Bn-A04-p7109625	0.94214876	0.94214876	Bn-N4-p8851142	Bn-chrA04-p8386617
Bn-A04-p7112803	0.975206612	0.975206612	Bn-N4-p8853018	Bn-chrA04-p8388493
Bn-A04-p7113045	0.966942149	0.966942149	Bn-N4-p8853259	Bn-chrA04-p8388734
Bn-A04-p7113156	0.991735537	0.991735537	Bn-N4-p8853369	Bn-chrA04-p8388844
Bn-A04-p7113281	1	1	Bn-N4-p8853496	Bn-chrA04-p8388971
Bn-A04-p7113498	0.991735537	0.991735537	Bn-N4-p8853714	Bn-chrA04-p8389189
Bn-A04-p7113584	0.983471074	0.983471074	Bn-N4-p8853800	Bn-chrA04-p8389275
Bn-A04-p7133952	0.975206612	0.975206612	Bn-N4-p8856051	Bn-chrA04-p8391526
Bn-A04-p7134022	0.991735537	0.991735537	Bn-N4-p8856121	Bn-chrA04-p8391596
Bn-A04-p7139140	0.710743802	0.710743802	Bn-N4-p8862101	Bn-chrA04-p8395702
Bn-A04-p7141171	0.980099502	0.980099502	Bn-N4-p8866013	Bn-chrA04-p8399614
Bn-A04-p7142597	0.958677686	0.958677686	Bn-N4-p8867424	Bn-chrA04-p8401025
Bn-A04-p7143891	0.966942149	0.966942149	Bn-N4-p8868696	Bn-chrA04-p8402297
Bn-A04-p7149396	0.991735537	0.991735537	Bn-N4-p8873308	Bn-chrA04-p8406909
Bn-A04-p7150064	0.975206612	0.975206612	Bn-N4-p8873974	Bn-chrA04-p8407575
Bn-A04-p7150104	0.975206612	0.975206612	Bn-N4-p8874014	Bn-chrA04-p8407615
Bn-A04-p7150329	0.950413223	0.950413223	Bn-N4-p8874239	Bn-chrA04-p8407840
Bn-A04-p7157859	0.985074627	0.985074627	Bn-N4-p8893629	Bn-chrA04-p8426083
Bn-A04-p7161326	0.983471074	0.983471074	Bn-N4-p8897087	Bn-chrA04-p8429541
Bn-A04-p7162690	1	1	Bn-N4-p8898451	Bn-chrA04-p8430905

Bn-A04-p7212436	1	1	Bn-N4-p8940673	Bn-chrA04-p8477846
Bn-A04-p7233978	1	1	Bn-N4-p8978598	Bn-chrA04-p8521887
Bn-A04-p7251871	0.983471074	0.983471074	Bn-N4-p8999628	Bn-chrA04-p8539876
Bn-A04-p7275318	0.980066445	0.980066445	Bn-N4-p9024078	Bn-chrA04-p8562276
Bn-A04-p7297317	0.820895522	0.820895522	Bn-Scaffold27301-p3	Bn-chrA04-p8597603
Bn-A04-p7322000	0.990033223	0.976744186	Bn-N4-p9083388	Bn-chrC04-p33629786
Bn-A04-p7322497	0.996677741	0.996677741	Bn-N4-p9083885	Bn-chrA04-p8621857
Bn-A04-p7323275	0.975124378	0.975124378	Bn-N4-p9084663	Bn-chrA04-p8622635
Bn-A04-p7349115	0.983471074	0.983471074	Bn-N4-p9160821	Bn-chrA04_random-p377172
Bn-A04-p7349794	0.985074627	0.985074627	Bn-N4-p9161498	Bn-chrA04_random-p376495
Bn-A04-p7350870	0.826446281	0.826446281	Bn-N4-p9162577	Bn-chrA04_random-p375416
Bn-A04-p7373248	0.975206612	0.975206612	Bn-N4-p9185157	Bn-chrA04-p8697382
Bn-A04-p7389149	0.991735537	0.991735537	Bn-N4-p9203787	Bn-chrA04-p8718487
Bn-A04-p7407657	0.950413223	0.958677686	Bn-N4-p9228932	Bn-chrA04-p8742173
Bn-A04-p7410699	0.94214876	0.94214876	Bn-N4-p9232963	Bn-chrA04-p8746198
Bn-A04-p7422354	0.975206612	0.975206612	Bn-N4-p9245171	Bn-chrA04-p8759869
Bn-A04-p7425311	0.975206612	0.975206612	Bn-N4-p9247191	Bn-chrA04-p8761888
Bn-A04-p7425359	0.975206612	0.975206612	Bn-N4-p9247238	Bn-chrA04-p8761935
Bn-A04-p7425697	0.950413223	0.950413223	Bn-N4-p9247576	Bn-chrA04-p8762273
Bn-A04-p7426527	0.983471074	0.983471074	Bn-N4-p9248406	Bn-chrA04-p8763103
Bn-A04-p7431245	1	1	Bn-N4-p9252792	Bn-chrA04-p8766523
Bn-A04-p7431816	0.993355482	0.993355482	Bn-N4-p9253344	Bn-chrA04-p8767075
Bn-A04-p7441018	0.991735537	0.991735537	Bn-N4-p9256354	Bn-chrA04-p8770085
Bn-A04-p7441659	0.991735537	0.991735537	Bn-N4-p9256990	Bn-chrA04-p8770721
Bn-A04-p7442353	0.958677686	0.958677686	Bn-N4-p9257693	Bn-chrA04-p8771424
Bn-A04-p7442886	0.991735537	0.991735537	Bn-N4-p9258226	Bn-chrA04-p8771957
Bn-A04-p7443395	0.975206612	0.975206612	Bn-N4-p9258735	Bn-chrA04-p8772466
Bn-A04-p7446849	0.986710963	0.986710963	Bn-N3-p28289535	Bn-chrA03_random-p217462
Bn-A04-p7450678	0.980066445	0.980066445	Bn-N4-p9273747	Bn-chrA04-p8787826
Bn-A04-p7471870	0.975206612	0.975206612	Bn-N4-p9311436	Bn-chrC04-p28166678
Bn-A04-p7474589	0.933884298	0.933884298	Bn-N4-p9314153	Bn-chrC04-p28163961
Bn-A04-p7482491	0.975206612	0.975206612	Bn-N4-p9342420	Bn-chrA04-p8805675
Bn-A04-p7488675	1	1	Bn-N4-p9348797	Bn-chrA04-p8811489
Bn-A04-p7490390	0.975206612	0.975206612	Bn-N14-p38764191	Bn-chrC04-p33857096
Bn-A04-p7493966	0.975206612	0.975206612	Bn-N4-p9354869	Bn-chrA04-p8816969
Bn-A04-p7500685	0.983471074	0.983471074	Bn-N4-p9362194	Bn-chrA04-p8824192
Bn-A04-p7503042	0.958677686	0.958677686	Bn-N4-p9364769	Bn-chrA04-p8826779
Bn-A04-p7519541	0.983471074	0.933884298	Bn-N4-p9382640	Bn-chrCnn_random-p607723
Bn-A04-p7520330	0.980066445	0.681063123	Bn-N4-p9383429	Bn-chrA04-p8700153
Bn-A04-p7540834	0.975206612	0.975206612	Bn-N4-p9413950	Bn-chrA04-p8885015
Bn-A04-p7541325	0.966942149	0.966942149	Bn-N4-p9414441	Bn-chrA04-p8885506
Bn-A04-p7541762	0.983471074	0.983471074	Bn-N4-p9414878	Bn-chrA04-p8885943
Bn-A04-p7542594	0.991735537	0.991735537	Bn-N4-p9415709	Bn-chrA04-p8886774
Bn-A04-p7556327	0.925619835	0.925619835	Bn-N14-p38899372	Bn-chrCnn_random-p228533
Bn-A04-p7582986	0.946843854	0.96013289	Bn-N4-p9451011	Bn-chrA04-p8921477
Bn-A04-p7584788	0.933884298	0.685950413	Bn-N4-p9452812	Bn-chrA04-p8923283

Bn-A04-p7586476	0.973421927	0.990033223	Bn-N4-p9454517	Bn-chrA04-p8924960
Bn-A04-p7588820	0.990033223	0.993355482	Bn-N4-p9456872	Bn-chrA04-p8927301
Bn-A04-p7596556	0.958677686	0.950413223	Bn-N4-p9467566	Bn-chrA04-p8940838
Bn-A04-p7598934	0.983471074	0.983471074	Bn-N4-p9469966	Bn-chrA04-p8943515
Bn-A04-p7601638	0.983471074	0.975206612	Bn-N4-p9472834	Bn-chrA04-p8945428
Bn-A04-p7601731	0.983471074	0.975206612	Bn-N4-p9472927	Bn-chrA04-p8945521
Bn-A04-p7616574	0.950413223	0.752066116	Bn-N4-p9488385	Bn-chrA04-p8950526
Bn-A04-p7617794	0.983471074	0.975206612	Bn-N4-p9489640	Bn-chrA04-p8951739
Bn-A04-p7625503	0.983388704	0.986710963	Bn-N4-p9498896	Bn-chrA04-p8959165
Bn-A04-p7629926	0.603305785	0.966942149	Bn-N4-p9503381	Bn-chrA04-p8963653
Bn-A04-p7635112	0.950413223	0.958677686	Bn-N4-p9510178	Bn-chrA04-p8969079
Bn-A04-p7643584	0.993355482	0.996677741	Bn-N4-p9520039	Bn-chrA04-p8977340
Bn-A04-p7656041	0.958677686	0.958677686	Bn-N4-p9532655	Bn-chrA04-p8990560
Bn-A04-p7666812	0.991735537	0.975206612	Bn-N4-p9540723	Bn-chrA04-p8998673
Bn-A04-p7666981	0.991735537	0.983471074	Bn-N4-p9540892	Bn-chrA04-p8998842
Bn-A04-p7670170	0.94214876	0.950413223	Bn-N4-p9544207	Bn-chrA04-p9002615
Bn-A04-p7670852	0.975206612	0.975206612	Bn-N4-p9544942	Bn-chrA04-p9003349
Bn-A04-p7671731	0.950413223	0.94214876	Bn-N4-p9545821	Bn-chrA04-p9004227
Bn-A04-p7672240	0.983471074	0.479338843	Bn-N4-p9546315	Bn-chrC04-p34113324
Bn-A04-p7680446	0.950413223	0.950413223	Bn-N4-p9549182	Bn-chrA04_random-p386120
Bn-A04-p7681107	0.993355482	1	Bn-N4-p9549843	Bn-chrA04_random-p386781
Bn-A04-p7681563	0.983471074	0.983471074	Bn-N4-p9550299	Bn-chrA04-p9010913
Bn-A04-p7681694	0.970149254	0.975124378	Bn-N4-p9550424	Bn-chrA04-p9011038
Bn-A04-p7683677	0.933884298	0.966942149	Bn-N4-p9552405	Bn-chrC03-p31852125
Bn-A04-p7684111	1	1	Bn-N4-p9552850	Bn-chrC03-p31852559
Bn-A04-p7685115	0.991735537	0.983471074	Bn-N4-p9554937	Bn-chrC03-p31853247
Bn-A04-p7694288	0.991735537	0.983471074	Bn-N4-p9562834	Bn-chrA04-p9023644
Bn-A04-p7760488	0.983471074	0.983471074	Bn-N4-p9647741	Bn-chrA04-p9078490
Bn-A04-p7760541	0.975206612	0.975206612	Bn-N4-p9647794	Bn-chrA04-p9078543
Bn-A04-p7769990	0.991735537	0.991735537	Bn-N4-p9659964	Bn-chrA04-p9093670
Bn-A04-p7770904	0.991735537	0.991735537	Bn-N4-p9660892	Bn-chrA04-p9094588
Bn-A04-p7776319	0.991735537	0.991735537	Bn-N4-p9666609	Bn-chrA04-p9100306
Bn-A04-p7790919	0.966942149	0.966942149	Bn-N4-p9684201	Bn-chrA04-p9112865
Bn-A04-p7791214	0.909090909	0.909090909	Bn-N4-p9684497	Bn-chrA04-p9113161
Bn-A04-p7797300	0.933884298	0.933884298	Bn-N4-p9689667	Bn-chrA04-p9118370
Bn-A04-p7808804	0.958677686	0.958677686	Bn-N4-p9701242	Bn-chrA04-p9129999
Bn-A04-p7813672	0.900826446	0.900826446	Bn-N4-p9706103	Bn-chrA04-p9134860
Bn-A04-p7818700	0.975206612	0.975206612	Bn-N4-p9709599	Bn-chrA04-p9138355
Bn-A04-p7819391	0.809917355	0.809917355	Bn-N4-p9710288	Bn-chrA04-p9139044
Bn-A04-p7819525	0.991735537	0.991735537	Bn-N4-p9710410	Bn-chrA04-p9139166
Bn-A04-p7822519	0.991735537	0.991735537	Bn-N4-p9713393	Bn-chrA04-p9142149
Bn-A04-p7822585	1	1	Bn-N4-p9713459	Bn-chrA04-p9142215
Bn-A04-p7857679	0.990049751	0.990049751	Bn-N4-p9747560	Bn-chrA04-p9176216
Bn-A04-p7857722	0.985074627	0.985074627	Bn-N4-p9747603	Bn-chrA04-p9176259
Bn-A04-p7858224	0.995024876	0.995024876	Bn-N4-p9748105	Bn-chrA04-p9176761
Bn-A04-p7870001	0.983471074	0.983471074	Bn-N4-p9761069	Bn-chrA04-p9188825

Bn-A04-p7883120	1	1	Bn-N4-p9775987	Bn-chrA04-p9208052
Bn-A04-p7886817	0.909090909	0.909090909	Bn-N4-p9778175	Bn-chrA04-p9210240
Bn-A04-p7887561	0.925619835	0.925619835	Bn-N4-p9778920	Bn-chrA04-p9210985
Bn-A04-p7888986	0.975206612	0.975206612	Bn-N4-p9781792	Bn-chrA04-p9213857
Bn-A04-p7889763	0.94214876	0.94214876	Bn-N4-p9782736	Bn-chrA04-p9214801
Bn-A04-p7897465	0.983471074	0.983471074	Bn-N4-p9784019	Bn-chrA04-p9216084
Bn-A04-p7914869	0.985074627	0.985074627	Bn-N4-p9807029	Bn-chrA04-p9237296
Bn-A04-p7930087	0.966942149	0.958677686	Bn-N4-p9821461	Bn-chrA04-p9256181
Bn-A04-p7931854	0.975206612	0.983471074	Bn-N4-p9822880	Bn-chrA04-p9257589
Bn-A04-p7933628	0.966942149	0.958677686	Bn-N4-p9829715	Bn-chrA04-p9261697
Bn-A04-p7955059	1	1	Bn-N4-p9855100	Bn-chrA04-p9283379
Bn-A04-p7965197	0.966942149	0.966942149	Bn-N4-p9857292	Bn-chrA04-p9285571
Bn-A04-p7984004	0.975206612	0.975206612	Bn-N4-p9888171	Bn-chrA04-p9313972
Bn-A04-p7990037	0.94214876	0.94214876	Bn-N4-p9893771	Bn-chrA04-p9319572
Bn-A04-p7990273	0.991735537	0.991735537	Bn-N4-p9894013	Bn-chrA04-p9319814
Bn-A04-p8004085	0.958677686	0.958677686	Bn-N4-p9903148	Bn-chrAnn_random-p378678
Bn-A04-p8009543	0.991735537	0.991735537	Bn-N4-p9910564	Bn-chrA04-p9332737
Bn-A04-p8017096	0.983471074	0.983471074	Bn-N4-p9914257	Bn-chrA04-p9336430
Bn-A04-p8114074	0.983471074	0.975206612	Bn-N4-p9982801	Bn-chrC04-p34886495
Bn-A04-p8114079	0.991735537	0.966942149	Bn-N4-p9982806	Bn-chrC04-p34886490
Bn-A04-p8116942	0.79338843	0.966942149	Bn-N4-p9985687	Bn-chrA04-p9402187
Bn-A04-p8117244	0.991735537	0.983471074	Bn-N4-p9986143	Bn-chrA04-p9402483
Bn-A04-p8178053	0.96345515	0.96345515	Bn-N14-p39839969	Bn-chrC04-p34768906
Bn-A04-p8178997	0.976744186	0.976744186	Bn-N4-p10061305	Bn-chrA04-p9481639
Bn-A04-p8186888	0.983471074	0.991735537	Bn-N4-p10069080	Bn-chrA07-p14149785
Bn-A04-p8208799	0.991735537	0.966942149	Bn-N4-p10084261	Bn-chrA04-p9504530
Bn-A04-p8285381	0.578512397	0.578512397	Bn-N4-p10183750	Bn-chrA04-p9604801
Bn-A04-p8287776	0.991735537	0.991735537	Bn-N4-p10186151	Bn-chrA04-p9607197
Bn-A04-p8287906	0.975206612	0.975206612	Bn-N4-p10186282	Bn-chrA04-p9607328
Bn-A04-p8292499	0.956810631	0.956810631	Bn-N4-p10191316	Bn-chrA04-p9612361
Bn-A04-p8299391	0.950413223	0.950413223	Bn-N4-p10197873	Bn-chrA04_random-p414514
Bn-A04-p8310242	0.983471074	0.983471074	Bn-N4-p10209082	Bn-chrA04-p9628697
Bn-A04-p8310328	0.958677686	0.958677686	Bn-N4-p10209166	Bn-chrA04-p9628781
Bn-A04-p8312347	0.94214876	0.975206612	Bn-Scaffold78125-p1	Bn-chrA04-p9630961
Bn-A04-p8328216	0.991735537	0.991735537	Bn-N4-p10213376	Bn-chrA04-p9637750
Bn-A04-p8329549	0.983471074	0.983471074	Bn-N4-p10214706	Bn-chrA04-p9639078
Bn-A04-p8344866	0.983471074	0.983471074	Bn-N4-p10228066	Bn-chrAnn_random-p426625
Bn-A04-p8345666	0.950413223	0.950413223	Bn-N4-p10230334	Bn-chrA04-p9651758
Bn-A04-p8348145	0.935323383	0.935323383	Bn-N4-p10238186	Bn-chrA04-p9654246
Bn-A04-p8348750	0.958677686	0.958677686	Bn-N4-p10238804	Bn-chrA04-p9654864
Bn-A04-p8348806	0.958677686	0.958677686	Bn-N4-p10238860	Bn-chrA04-p9654920
Bn-A04-p8403728	0.975206612	0.966942149	Bn-N4-p10325716	Bn-chrA04-p9736333
Bn-A04-p8407945	0.960199005	0.955223881	Bn-N4-p10327890	Bn-chrA04-p9740380
Bn-A04-p8441932	0.983471074	0.975206612	Bn-N4-p10359241	Bn-chrA04-p9773044
Bn-A04-p8473768	0.983471074	0.991735537	Bn-N4-p10393619	Bn-chrA04-p9809214
Bn-A04-p8481222	0.991735537	1	Bn-N4-p10401209	Bn-chrA04-p9817216

Bn-A04-p8555881	0.985074627	0.992537313	Bn-N4-p10457792	Bn-chrA04-p9866504
Bn-A04-p8559580	0.990033223	0.986710963	Bn-N4-p10461522	Bn-chrA04-p9870208
Bn-A04-p8564072	0.950413223	0.950413223	Bn-N4-p10466045	Bn-chrA04-p9874714
Bn-A04-p8575908	0.950413223	0.958677686	Bn-N4-p10474725	Bn-chrA04-p9886529
Bn-A04-p8575962	0.975206612	0.975206612	Bn-N4-p10474779	Bn-chrA04-p9886583
Bn-A04-p8587019	0.94214876	0.950413223	Bn-N4-p10484006	Bn-chrA04-p9899905
Bn-A04-p8595035	0.975124378	0.975124378	Bn-N7-p13676973	Bn-chrA07-p13401169
Bn-A04-p8609799	0.966777409	0.966777409	Bn-N14-p40467836	Bn-chrUnn_random-p29539[illegible]
Bn-A04-p8612311	0.983388704	0.973421927	Bn-N4-p10512694	Bn-chrUnn_random-p29288[illegible]
Bn-A04-p8615093	0.900826446	0.900826446	Bn-N14-p40473985	Bn-chrUnn_random-p29011[illegible]
Bn-A04-p8686116	0.975206612	0.975206612	Bn-N4-p10588664	Bn-chrUnn_random-p57755
Bn-A04-p8686934	0.975206612	0.975206612	Bn-N4-p10589490	Bn-chrUnn_random-p56939
Bn-A04-p8700128	0.950413223	0.867768595	Bn-N4-p10603635	Bn-chrUnn_random-p44709
Bn-A04-p8705840	0.983471074	0.958677686	Bn-N4-p10610117	Bn-chrUnn_random-p39168
Bn-A04-p8706392	0.991735537	1	Bn-N4-p10610676	Bn-chrUnn_random-p38636
Bn-A04-p8709391	0.950413223	0.958677686	Bn-N4-p10615064	Bn-chrUnn_random-p35332
Bn-A04-p8713733	0.975206612	0.983471074	Bn-N4-p10619739	Bn-chrUnn_random-p29686
Bn-A04-p8718115	0.970099668	0.983388704	Bn-N4-p10627001	Bn-chrUnn_random-p25100
Bn-A04-p8729788	0.966942149	0.925619835	Bn-N4-p10645233	Bn-chrC04-p36075757
Bn-A04-p8761898	0.985074627	0.985074627	Bn-N4-p10686169	Bn-chrA04_random-p490263
Bn-A04-p8773241	0.933884298	0.933884298	Bn-N4-p10690583	Bn-chrA04_random-p485849
Bn-A04-p8773286	0.991735537	0.991735537	Bn-N4-p10690628	Bn-chrA04_random-p485804
Bn-A04-p8782104	0.975206612	0.975206612	Bn-N4-p10697170	Bn-chrA04_random-p478258
Bn-A04-p8786765	0.950413223	0.950413223	Bn-N4-p10719312	Bn-chrA04_random-p465962
Bn-A04-p8788445	0.96969697	0.96969697	Bn-N4-p10721042	Bn-chrA04_random-p464195
Bn-A04-p8790105	0.991735537	0.991735537	Bn-N4-p10722959	Bn-chrA04_random-p462315
Bn-A04-p8790433	0.980099502	0.980099502	Bn-N4-p10723288	Bn-chrA04_random-p461986
Bn-A04-p8796688	0.983471074	0.983471074	Bn-N4-p10741775	Bn-chrA04_random-p445172
Bn-A04-p8796706	0.975206612	0.975206612	Bn-N4-p10741793	Bn-chrA04_random-p445154
Bn-A04-p8803630	0.991735537	0.991735537	Bn-N4-p10744229	Bn-chrC04-p35968021
Bn-A04-p8807708	0.975206612	0.842975207	Bn-N4-p10747215	Bn-chrC04-p35966749
Bn-A04-p8811740	0.975206612	0.975206612	Bn-N4-p10751522	Bn-chrA04_random-p436128
Bn-A04-p8813038	0.966942149	0.966942149	Bn-N4-p10752832	Bn-chrA04_random-p434818
Bn-A04-p8817223	0.595041322	0.41322314	Bn-N4-p10773879	Unknown
Bn-A04-p8817780	0.892561983	0.884297521	Bn-N4-p10760090	Bn-chrA04_random-p426422
Bn-A04-p8824695	0.975206612	0.595041322	Bn-N4-p10776079	Bn-chrC04-p35897764
Bn-A04-p8828395	0.995024876	0.995024876	Bn-N4-p10779021	Bn-chrA04-p9941507
Bn-A04-p8828462	0.980099502	0.980099502	Bn-N4-p10779088	Bn-chrA04-p9941574
Bn-A04-p8830454	0.983471074	0.983471074	Bn-N4-p10781189	Bn-chrA04-p9943675
Bn-A04-p8834077	0.966942149	0.966942149	Bn-N4-p10784937	Bn-chrA04-p9947700
Bn-A04-p8834259	0.983471074	0.983471074	Bn-N4-p10785120	Bn-chrA04-p9947883
Bn-A04-p8840146	0.933884298	0.933884298	Bn-N4-p10791067	Bn-chrA04-p9953829
Bn-A04-p8847485	0.983471074	0.983471074	Bn-N4-p10799340	Bn-chrA04-p9961766
Bn-A04-p8850036	0.983471074	0.983471074	Bn-N4-p10802012	Bn-chrA04-p9964438
Bn-A04-p8865894	0.958677686	0.438016529	Bn-N4-p10818166	Unknown
Bn-A04-p8888969	0.993355482	0.993355482	Bn-N4-p10843538	Bn-chrA04-p10000868

Bn-A04-p8889253	0.983388704	0.983388704	Bn-N4-p10843822	Bn-chrA04-p10001152
Bn-A04-p8892416	0.983471074	0.983471074	Bn-N4-p10846981	Bn-chrA04-p10004311
Bn-A04-p8906594	0.990033223	0.990033223	Bn-N4-p10861544	Bn-chrA04-p10018903
Bn-A04-p8974361	0.966942149	0.966942149	Bn-N4-p10922129	Bn-chrA04-p10076079
Bn-A04-p9024009	0.986710963	0.986710963	Bn-N4-p10964749	Bn-chrA04-p10119446
Bn-A04-p9086923	0.983471074	0.983471074	Bn-N4-p11036134	Bn-chrA04-p10179623
Bn-A04-p9088043	0.983471074	0.983471074	Bn-N4-p11037233	Bn-chrA04-p10180722
Bn-A04-p9088760	0.966942149	0.966942149	Bn-N4-p11037947	Bn-chrA04-p10181436
Bn-A04-p9097344	1	1	Bn-N4-p11046720	Bn-chrA04-p10189187
Bn-A04-p9098709	0.995024876	0.995024876	Bn-N4-p11048084	Bn-chrA04-p10190551
Bn-A04-p9099209	0.985074627	0.985074627	Bn-N4-p11048585	Bn-chrA04-p10191052
Bn-A04-p9107147	0.975206612	0.975206612	Bn-N4-p11056629	Bn-chrA04-p10199088
Bn-A04-p9114826	0.975206612	0.975206612	Bn-N4-p11067100	Bn-chrA04-p10207692
Bn-A04-p9119079	0.983471074	0.983471074	Bn-N4-p11071258	Bn-chrA04-p10211850
Bn-A04-p9119142	0.991735537	0.991735537	Bn-N4-p11071321	Bn-chrA04-p10211913
Bn-A04-p9119675	0.989583333	0.989583333	Bn-N4-p11071903	Bn-chrA04-p10212495
Bn-A04-p9119867	0.983471074	0.983471074	Bn-N4-p11072046	Bn-chrA04-p10212638
Bn-A04-p9174273	0.990033223	0.990033223	Bn-N4-p11126727	Bn-chrA04-p10268881
Bn-A04-p9231842	0.880597015	0.850746269	Bn-N4-p11168852	Bn-chrA04-p10346730
Bn-A04-p9232024	0.910447761	0.915422886	Bn-N4-p11169034	Bn-chrA04-p10346910
Bn-A04-p9232365	0.990049751	0.990049751	Bn-N4-p11169375	Bn-chrA04-p10347251
Bn-A04-p9232828	0.975206612	0.975206612	Bn-N4-p11169849	Bn-chrA04-p10347716
Bn-A04-p9245980	0.970149254	0.965174129	Bn-N15-p33708058	Bn-chrAnn_random-p291024
Bn-A04-p9296314	0.991735537	0.983471074	Bn-N4-p11235730	Bn-chrA04-p10415680
Bn-A04-p9296465	0.950413223	0.966942149	Bn-N14-p41597682	Bn-chrA04-p10415831
Bn-A04-p9309213	0.983471074	0.991735537	Bn-N4-p11252760	Bn-chrA04-p10427688
Bn-A04-p9317798	0.991735537	0.991735537	Bn-N4-p11260262	Bn-chrA04-p10434969
Bn-A04-p9322160	0.933884298	0.94214876	Bn-N4-p11264561	Bn-chrA04-p10440240
Bn-A04-p9331682	0.990049751	0.990049751	Bn-N8-p7650791	Bn-chrA08-p7267860
Bn-A04-p9337637	0.990049751	0.542288557	Bn-N6-p5007440	Bn-chrA08-p10927964
Bn-A04-p9339599	0.970149254	0.970149254	Bn-N6-p5005346	Bn-chrA04-p10456997
Bn-A04-p9340809	0.980099502	0.980099502	Bn-N6-p5004136	Bn-chrAnn_random-p474755
Bn-A04-p9345530	0.966942149	0.958677686	Bn-N4-p11277937	Bn-chrA04-p10462928
Bn-A04-p9355248	0.995024876	0.995024876	Bn-N4-p11290169	Bn-chrA04-p10474130
Bn-A04-p9363550	0.983471074	0.975206612	Bn-N4-p11302129	Bn-chrA04-p10481803
Bn-A04-p9379937	0.989583333	1	Bn-N4-p11330582	Bn-chrA04-p10503409
Bn-A04-p9392577	0.991735537	0.983471074	Bn-N4-p11349375	Bn-chrA04-p10515356
Bn-A04-p9400393	0.983471074	0.991735537	Bn-N4-p11364987	Bn-chrA04-p10521765
Bn-A04-p9422027	0.975206612	0.958677686	Bn-N4-p11398821	Bn-chrA04-p10542933
Bn-A04-p9426523	0.909090909	0.801652893	Bn-N4-p11403256	Bn-chrA04-p10547377
Bn-A04-p9450472	0.975124378	0.980099502	Bn-N16-p7681702	Bn-chrAnn_random-p409139
Bn-A04-p9484747	0.993355482	0.993355482	Bn-N4-p11472813	Bn-chrA04_random-p586159
Bn-A04-p9505012	0.995024876	0.995024876	Bn-N4-p11485501	Bn-chrA04-p10638153
Bn-A04-p9508676	0.983471074	0.983471074	Bn-N4-p11489190	Bn-chrA04-p10641842
Bn-A04-p9509486	0.991735537	0.991735537	Bn-N4-p11490001	Bn-chrA04-p10642653
Bn-A04-p9510223	0.975206612	0.975206612	Bn-N4-p11490738	Bn-chrA04-p10643390

Bn-A04-p9510750	0.975206612	0.975206612	Bn-N4-p11491266	Bn-chrA04-p10643918
Bn-A04-p9510764	0.983471074	0.983471074	Bn-N4-p11491280	Bn-chrA04-p10643932
Bn-A04-p9511386	0.933884298	0.933884298	Bn-N4-p11491921	Bn-chrA04-p10644573
Bn-A04-p9522781	0.983471074	0.983471074	Bn-N4-p11511622	Bn-chrA04-p10665228
Bn-A04-p9524800	0.991735537	0.991735537	Bn-N4-p11513641	Bn-chrA04-p10667247
Bn-A04-p9532027	0.980066445	0.980066445	Bn-N4-p11520834	Bn-chrA04-p10672871
Bn-A04-p9532255	0.966942149	0.966942149	Bn-N4-p11521063	Bn-chrA04-p10673100
Bn-A04-p9543062	0.991735537	0.983471074	Bn-N4-p11529941	Bn-chrA04-p10681974
Bn-A04-p9543969	0.983388704	0.983388704	Bn-N4-p11530852	Bn-chrA04-p10682884
Bn-A04-p9566708	0.996677741	0.996677741	Bn-N4-p11552797	Bn-chrA04-p10720168
Bn-A04-p9582109	0.990033223	0.910299003	Bn-N4-p11569124	Bn-chrCnn_random-p410557
Bn-A04-p9606190	0.996677741	0.986710963	Bn-N4-p11603803	Bn-chrA04-p10742478
Bn-A04-p9623505	0.975124378	0.970149254	Bn-N4-p11612168	Bn-chrA04-p10751282
Bn-A04-p9623913	0.990033223	0.990033223	Bn-N4-p11612576	Bn-chrA04-p10751690
Bn-A04-p9632786	0.958677686	0.958677686	Bn-N4-p11625156	Bn-chrA04-p10764117
Bn-A04-p9639648	0.966777409	0.966777409	Bn-N4-p11631956	Bn-chrA04-p10771237
Bn-A04-p9658559	1	1	Bn-N4-p11656499	Bn-chrA04-p10803106
Bn-A04-p9670525	0.966942149	0.966942149	Bn-N4-p11664333	Bn-chrA04-p10809512
Bn-A04-p9673337	0.94214876	0.94214876	Bn-N4-p11667162	Bn-chrA04-p10812341
Bn-A04-p9675790	0.983471074	0.983471074	Bn-N4-p11669634	Bn-chrA04-p10814813
Bn-A04-p9716870	0.989583333	0.989583333	Bn-Scaffold86587-p1	Bn-chrA04-p10860632
Bn-A04-p9746407	0.940199336	0.940199336	Bn-N4-p11733588	Bn-chrA04-p10880912
Bn-A04-p9802131	0.975124378	0.975124378	Bn-N4-p11785129	Bn-chrA04-p10931778
Bn-A04-p9808875	0.892561983	0.892561983	Bn-N4-p11790336	Bn-chrA04-p10937221
Bn-A04-p9811506	0.995024876	0.995024876	Bn-N4-p11792965	Bn-chrA04-p10939849
Bn-A04-p9814236	0.985074627	0.985074627	Bn-N4-p11795696	Bn-chrA04-p10942580
Bn-A04-p9814284	0.955223881	0.955223881	Bn-N4-p11795744	Bn-chrA04-p10942628
Bn-A04-p9815052	0.990049751	0.990049751	Bn-N4-p11796512	Bn-chrA04-p10943396
Bn-A04-p9819724	0.975206612	0.975206612	Bn-N4-p11801084	Bn-chrA04-p10947968
Bn-A04-p9819885	0.958677686	0.958677686	Bn-N4-p11801243	Bn-chrA04-p10948127
Bn-A04-p9830766	0.983388704	0.983388704	Bn-N4-p11814273	Bn-chrA04-p10962828
Bn-A04-p9841094	0.94214876	0.94214876	Bn-N4-p11823878	Bn-chrA04-p10972875
Bn-A04-p9844020	0.950413223	0.950413223	Bn-N4-p11826893	Bn-chrA04-p10975889
Bn-A04-p9847875	0.993355482	0.993355482	Bn-N4-p11830458	Bn-chrA04-p10979452
Bn-A04-p9848849	0.958677686	0.958677686	Bn-N4-p11831435	Bn-chrA04-p10980429
Bn-A04-p9861037	0.94214876	0.94214876	Bn-N4-p11843496	Bn-chrA04-p10990759
Bn-A04-p9863329	0.975206612	0.975206612	Bn-N4-p11845785	Bn-chrA04-p10993048
Bn-A04-p9864811	0.983471074	0.983471074	Bn-N4-p11847897	Bn-chrA04-p10995159
Bn-A04-p9864983	0.991735537	0.991735537	Bn-N4-p11852935	Bn-chrA04-p11002953
Bn-A04-p9865629	0.983471074	0.983471074	Bn-N4-p11853578	Bn-chrA04-p11003596
Bn-A04-p9865712	0.966942149	0.966942149	Bn-N4-p11853659	Bn-chrA04-p11003677
Bn-A04-p9866201	0.985074627	0.985074627	Bn-N4-p11854144	Bn-chrA04-p11004162
Bn-A04-p9868258	0.966942149	0.966942149	Bn-N4-p11856220	Bn-chrA04-p11006359
Bn-A04-p9870167	0.983471074	0.983471074	Bn-N4-p11858133	Bn-chrA04-p11008272
Bn-A04-p9870879	0.975206612	0.975206612	Bn-N4-p11859341	Bn-chrA04-p11009480
Bn-A04-p9878192	0.983471074	0.983471074	Bn-N4-p11866661	Bn-chrA04-p11017472

Bn-A04-p9878444	0.958677686	0.958677686	Bn-N4-p11866946	Bn-chrA04-p11017757
Bn-A04-p9879436	0.983471074	0.983471074	Bn-N4-p11867958	Bn-chrA04-p11018769
Bn-A04-p9920827	0.983471074	0.983471074	Bn-N4-p11910916	Bn-chrA04-p11056585
Bn-A04-p9920873	0.975206612	0.975206612	Bn-N4-p11910962	Bn-chrA04-p11056631
Bn-A04-p9921876	0.975206612	0.975206612	Bn-N4-p11911966	Bn-chrA04-p11057635
Bn-A04-p9930371	0.993355482	0.993355482	Bn-N4-p11920065	Bn-chrA04-p11066184
Bn-A04-p9932606	0.983471074	0.983471074	Bn-N4-p11922280	Bn-chrA04-p11068399
Bn-A04-p9983165	0.975206612	0.975206612	Bn-N4-p11965185	Bn-chrA04-p11110080
Bn-A04-p9991606	0.933884298	0.933884298	Bn-N4-p11983093	Bn-chrA04-p11125580
Bn-A04-p10016854	0.975124378	0.975124378	Bn-N4-p12005681	Bn-chrA04-p11146862
Bn-A04-p10043859	0.966942149	0.966942149	Bn-N4-p12029146	Bn-chrA04-p11169519
Bn-A04-p10049865	0.983471074	0.983471074	Bn-N4-p12035781	Bn-chrA04-p11176154
Bn-A04-p10049966	0.950413223	0.950413223	Bn-N4-p12035882	Bn-chrA04-p11176255
Bn-A04-p10054968	0.970149254	0.970149254	Bn-N4-p12040881	Bn-chrA04-p11181254
Bn-A04-p10056980	0.975206612	0.975206612	Bn-N4-p12042042	Bn-chrA04-p11182415
Bn-A04-p10058358	0.966942149	0.966942149	Bn-N4-p12043396	Bn-chrA04-p11183769
Bn-A04-p10059165	0.991735537	0.991735537	Bn-N4-p12044208	Bn-chrA04-p11184581
Bn-A04-p10069116	0.991735537	0.991735537	Bn-N4-p12068797	Bn-chrA04-p11208735
Bn-A04-p10071085	0.933884298	0.644628099	Bn-N4-p12073769	Bn-chrA05-p11119641
Bn-A04-p10085135	1	1	Bn-N4-p12090675	Bn-chrA04-p11227196
Bn-A04-p10085844	0.958677686	0.958677686	Bn-N4-p12091455	Bn-chrA04-p11227976
Bn-A04-p10087754	0.975206612	0.975206612	Bn-N4-p12092896	Bn-chrA04-p11229417
Bn-A04-p10088142	0.991735537	0.991735537	Bn-N4-p12093284	Bn-chrA04-p11229805
Bn-A04-p10088385	0.966942149	0.966942149	Bn-N4-p12093528	Bn-chrA04-p11230049
Bn-A04-p10088445	0.966942149	0.966942149	Bn-N4-p12093588	Bn-chrA04-p11230109
Bn-A04-p10088570	0.983471074	0.983471074	Bn-N4-p12093713	Bn-chrA04-p11230234
Bn-A04-p10089027	0.933884298	0.933884298	Bn-N4-p12094170	Bn-chrA04-p11230691
Bn-A04-p10089238	0.991735537	0.991735537	Bn-N4-p12094380	Bn-chrA04-p11230901
Bn-A04-p10089301	0.950413223	0.950413223	Bn-N4-p12094443	Bn-chrA04-p11230964
Bn-A04-p10089384	0.958677686	0.958677686	Bn-N4-p12094526	Bn-chrA04-p11231047
Bn-A04-p10089680	0.966942149	0.966942149	Bn-N4-p12094821	Bn-chrA04-p11231342
Bn-A04-p10089715	0.966942149	0.966942149	Bn-N4-p12094856	Bn-chrA04-p11231377
Bn-A04-p10090470	0.976744186	0.976744186	Bn-N4-p12095610	Bn-chrA04-p11232131
Bn-A04-p10093618	0.975206612	0.975206612	Bn-N4-p12105517	Bn-chrA04-p11238685
Bn-A04-p10094831	0.975206612	0.975206612	Bn-N4-p12106732	Bn-chrA04-p11239900
Bn-A04-p10099096	1	1	Bn-N4-p12112331	Bn-chrA04-p11248648
Bn-A04-p10101293	0.991735537	0.991735537	Bn-N4-p12114669	Bn-chrA04-p11250984
Bn-A04-p10103888	0.975206612	0.975206612	Bn-N4-p12116110	Bn-chrAnn_random-p288454
Bn-A04-p10104666	1	1	Bn-N4-p12116889	Bn-chrAnn_random-p288446
Bn-A04-p10108610	0.983471074	0.983471074	Bn-N4-p12122593	Bn-chrA04-p11258745
Bn-A04-p10113831	0.983471074	0.983471074	Bn-N4-p12128700	Bn-chrA04-p11263934
Bn-A04-p10113969	0.809917355	0.809917355	Bn-N4-p12128838	Bn-chrA04-p11264072
Bn-A04-p10129538	0.983471074	0.983471074	Bn-N4-p12141458	Bn-chrA04-p11276750
Bn-A04-p10154400	0.991735537	0.991735537	Bn-N4-p12164658	Bn-chrA04-p11300081
Bn-A04-p10155907	0.993355482	0.993355482	Bn-N4-p12166165	Bn-chrA04-p11301588
Bn-A04-p10176387	0.991735537	0.991735537	Bn-N4-p12196262	Bn-chrA04-p11321957

Bn-A04-p10182453	0.880597015	0.880597015	Bn-N4-p12202408	Bn-chrA04-p11328103
Bn-A04-p10188827	0.996677741	0.996677741	Bn-N4-p12207503	Bn-chrA04-p11333029
Bn-A04-p10196289	0.993355482	0.993355482	Bn-N4-p12216738	Bn-chrA04-p11344285
Bn-A04-p10250633	0.983471074	0.983471074	Bn-N4-p12274665	Bn-chrA04-p11398098
Bn-A04-p10261874	1	1	Bn-N4-p12283635	Bn-chrA04-p11407828
Bn-A04-p10286035	0.956810631	0.956810631	Bn-N4-p12307969	Bn-chrA04-p11429972
Bn-A04-p10288476	0.905472637	0.945273632	Bn-N4-p12310391	Bn-chrCnn_random-p776594
Bn-A04-p10372116	0.880597015	0.880597015	Bn-N4-p12627041	Bn-chrA04-p11515915
Bn-A04-p10383434	0.996677741	0.993355482	Bn-N4-p12637478	Bn-chrA04-p11525225
Bn-A04-p10387804	0.983471074	0.975206612	Bn-N4-p12641858	Bn-chrA04-p11529656
Bn-A04-p10393460	0.975206612	0.975206612	Bn-N4-p12647481	Bn-chrA04-p11537744
Bn-A04-p10434805	0.966942149	0.859504132	Bn-N4-p12697821	Bn-chrA04-p11594670
Bn-A04-p10458946	0.983471074	0.991735537	Bn-N4-p12731491	Bn-chrA04-p11619110
Bn-A04-p10464942	0.996677741	0.996677741	Bn-N4-p12728711	Bn-chrA04-p11622669
Bn-A04-p10490223	0.958677686	0.966942149	Bn-N4-p12769461	Bn-chrA04_random-p594435
Bn-A04-p10490380	0.940298507	0.945273632	Bn-N4-p12769618	Bn-chrA04_random-p594278
Bn-A04-p10494765	0.991735537	1	Bn-N4-p12772709	Bn-chrA07-p16691607
Bn-A04-p10502015	0.983471074	0.975206612	Bn-N4-p12778919	Bn-chrA04-p11645689
Bn-A04-p10503082	0.983471074	0.983471074	Bn-N4-p12779988	Bn-chrA04-p11646744
Bn-A04-p10515374	0.996677741	0.996677741	Bn-N4-p12793100	Bn-chrA04-p11660449
Bn-A04-p10541268	0.983471074	0.983471074	Bn-N4-p12819154	Bn-chrA04-p11685649
Bn-A04-p10555408	1	1	Bn-N4-p12830886	Bn-chrA04-p11697380
Bn-A04-p10559481	0.991735537	0.991735537	Bn-N4-p12835744	Bn-chrA04-p11702237
Bn-A04-p10560896	0.991735537	0.991735537	Bn-N4-p12837177	Bn-chrA04-p11703670
Bn-A04-p10566018	0.983388704	0.983388704	Bn-N4-p12842304	Bn-chrA04-p11708799
Bn-A04-p10592914	0.983471074	0.983471074	Bn-N4-p12867235	Bn-chrA04-p11740585
Bn-A04-p10600455	0.913621262	0.916943522	Bn-N4-p12872786	Bn-chrA04-p11745881
Bn-A04-p10602815	0.965174129	0.995024876	Bn-N14-p43716734	Bn-chrA04-p11748234
Bn-A04-p10608772	0.991735537	0.826446281	Bn-N4-p12882182	Bn-chrC07-p11529164
Bn-A04-p10628643	0.702479339	0.950413223	Bn-N4-p12920466	Bn-chrA04-p11766578
Bn-A04-p10628977	0.991735537	1	Bn-N4-p12893052	Bn-chrA04-p11766912
Bn-A04-p10630934	0.975206612	0.983471074	Bn-N4-p12895140	Bn-chrA04-p11775918
Bn-A04-p10649540	0.985074627	0.970149254	Bn-N4-p12913755	Bn-chrA04-p11794984
Bn-A04-p10661890	0.983471074	0.975206612	Bn-N4-p12928183	Bn-chrA04-p11806345
Bn-A04-p10661931	0.983471074	0.975206612	Bn-N4-p12928224	Bn-chrA04-p11806386
Bn-A04-p10662404	0.991735537	0.983471074	Bn-N4-p12928696	Bn-chrA04-p11806859
Bn-A04-p10671164	0.900826446	0.94214876	Bn-N4-p12936968	Bn-chrA04-p11819112
Bn-A04-p10671721	0.991735537	0.983471074	Bn-N4-p12937505	Bn-chrA04-p11819673
Bn-A04-p10680523	0.983471074	0.983471074	Bn-N4-p12946669	Bn-chrA04-p11830026
Bn-A04-p10688504	0.966942149	0.958677686	Bn-N4-p12960006	Bn-chrA04-p11838388
Bn-A04-p10718521	1	1	Bn-N4-p12980877	Bn-chrA04-p11860002
Bn-A04-p10719274	0.975206612	0.975206612	Bn-N4-p12981626	Bn-chrA04-p11860751
Bn-A04-p10719331	0.958677686	0.958677686	Bn-N4-p12981683	Bn-chrA04-p11860808
Bn-A04-p10744003	0.94214876	0.94214876	Bn-N4-p13003736	Bn-chrA04-p11885058
Bn-A04-p10748559	0.925619835	0.925619835	Bn-N4-p13009208	Bn-chrA04-p11890530
Bn-A04-p10759605	0.983471074	0.983471074	Bn-N4-p13016964	Bn-chrA04-p11898272

Bn-A04-p10759783	0.991735537	0.991735537	Bn-N4-p13017142	Bn-chrA04-p11898450
Bn-A04-p10760138	0.966942149	0.966942149	Bn-N4-p13017496	Bn-chrA04-p11898804
Bn-A04-p10760352	0.975206612	0.975206612	Bn-N4-p13017710	Bn-chrA04-p11899018
Bn-A04-p10760790	0.975206612	0.975206612	Bn-N4-p13018148	Bn-chrA04-p11899456
Bn-A04-p10769811	1	1	Bn-N4-p13027215	Bn-chrA04-p11908523
Bn-A04-p10774641	0.983471074	0.983471074	Bn-N4-p13032331	Bn-chrA04-p11913668
Bn-A04-p10786704	0.958677686	0.958677686	Bn-N4-p13035554	Bn-chrA04-p11917921
Bn-A04-p10792201	0.986710963	0.986710963	Bn-N4-p13041412	Bn-chrA04-p11924915
Bn-A04-p10808508	0.986710963	0.986710963	Bn-N4-p13064299	Bn-chrA04-p11947003
Bn-A04-p10808805	0.993355482	0.993355482	Bn-N4-p13064596	Bn-chrA04-p11947300
Bn-A04-p10809637	0.955223881	0.965174129	Bn-N4-p13065428	Bn-chrA04-p11948132
Bn-A04-p10833713	0.983471074	0.983471074	Bn-N4-p13110030	Bn-chrA04-p11988012
Bn-A04-p10841936	0.966942149	0.975206612	Bn-N4-p13124685	Bn-chrA04-p12001772
Bn-A04-p10852032	1	0.983471074	Bn-N4-p13137303	Bn-chrA04-p12012453
Bn-A04-p10861826	0.966942149	0.958677686	Bn-N4-p13148883	Bn-chrA04-p12021822
Bn-A04-p10867512	0.990033223	0.973421927	Bn-N4-p13153794	Bn-chrA04-p12028144
Bn-A04-p10870734	0.975206612	0.537190083	Bn-N4-p13157034	Bn-chrA04-p12031374
Bn-A04-p10871341	0.975206612	0.818181818	Bn-N4-p13157625	Bn-chrA04-p12032502
Bn-A04-p10871494	0.966942149	0.958677686	Bn-N4-p13157782	Bn-chrA04-p12031680
Bn-A04-p10872405	0.933884298	0.983471074	Bn-N4-p13158750	Bn-chrA04-p12032645
Bn-A04-p10881502	0.991735537	0.983471074	Bn-N4-p13172647	Bn-chrA04-p12041691
Bn-A04-p10902724	0.983471074	0.991735537	Bn-N4-p13187409	Bn-chrA04-p12064304
Bn-A04-p10903353	0.966777409	0.966777409	Bn-N14-p44310234	Bn-chrC04-p37955631
Bn-A04-p10912425	0.973421927	0.986710963	Bn-N4-p13198905	Bn-chrA04-p12074005
Bn-A04-p10914108	0.983471074	0.975206612	Bn-N4-p13200712	Bn-chrA04-p12075691
Bn-A04-p10975832	0.991735537	0.958677686	Bn-N4-p13271446	Bn-chrA04-p12154977
Bn-A04-p10976023	0.943521595	0.943521595	Bn-N4-p13271637	Bn-chrA04-p12155164
Bn-A04-p10980342	0.983471074	0.983471074	Bn-N4-p13291720	Bn-chrA04-p12172483
Bn-A04-p10983805	0.876033058	0.925619835	Bn-N4-p13295084	Bn-chrA04-p12175847
Bn-A04-p11015443	0.817275748	0.810631229	Bn-N4-p13326516	Bn-chrA04-p12211710
Bn-A04-p11017537	0.917355372	0.975206612	Bn-N4-p13328613	Bn-chrA04-p12213828
Bn-A04-p11018682	0.991735537	0.991735537	Bn-N4-p13329448	Bn-chrA04-p12214972
Bn-A04-p11020444	1	0.991735537	Bn-N4-p13331211	Bn-chrA04-p12216734
Bn-A04-p11026664	0.991735537	0.991735537	Bn-N4-p13340907	Bn-chrA04-p12223051
Bn-A04-p11058870	1	0.975206612	Bn-N4-p13372031	Bn-chrA04_random-p684501
Bn-A04-p11119216	0.975206612	0.966942149	Bn-N4-p13412903	Bn-chrA04_random-p726215
Bn-A04-p11119582	0.983471074	0.94214876	Bn-N4-p13413271	Bn-chrA04_random-p726534
Bn-A04-p11164883	0.760330579	0.966942149	Bn-N4-p13449277	Bn-chrA04-p12315824
Bn-A04-p11191754	0.94214876	0.950413223	Bn-N4-p13482331	Bn-chrA04-p12343654
Bn-A04-p11192862	0.991735537	1	Bn-N4-p13483986	Bn-chrA04-p12344762
Bn-A04-p11197346	1	0.991735537	Bn-N4-p13491897	Bn-chrA04-p12349427
Bn-A04-p11197707	0.955223881	0.950248756	Bn-N4-p13492258	Bn-chrA04-p12349788
Bn-A04-p11203305	0.958677686	0.958677686	Bn-N4-p13507381	Bn-chrA04-p12369551
Bn-A04-p11209663	0.910447761	0.915422886	Bn-N4-p13515177	Bn-chrA04_random-p758201
Bn-A04-p11209773	0.935323383	0.930348259	Bn-N4-p13515286	Bn-chrA04_random-p758092
Bn-A04-p11209827	0.925619835	0.933884298	Bn-N4-p13515340	Bn-chrA04_random-p758038

Bn-A04-p11213108	0.851239669	0.991735537	Bn-N4-p13518558	Bn-chrA04_random-p754785
Bn-A04-p11243983	0.966942149	0.966942149	Bn-N4-p13553512	Bn-chrA04_random-p629374
Bn-A04-p11243994	0.980099502	0.980099502	Bn-N4-p13553523	Bn-chrA04_random-p629385
Bn-A04-p11244643	0.960199005	0.960199005	Bn-N4-p13554202	Bn-chrA04_random-p630064
Bn-A04-p11244673	0.960199005	0.960199005	Bn-N4-p13554232	Bn-chrA04_random-p630094
Bn-A04-p11245906	0.985074627	0.985074627	Bn-N4-p13555465	Bn-chrA04_random-p631326
Bn-A04-p11247206	0.991735537	1	Bn-N4-p13556851	Bn-chrA04_random-p633544
Bn-A04-p11250035	0.983471074	0.991735537	Bn-N4-p13559742	Bn-chrA04_random-p636396
Bn-A04-p11250141	0.975206612	0.983471074	Bn-N4-p13559848	Bn-chrA04_random-p636502
Bn-A04-p11256083	0.603305785	0.595041322	Bn-N4-p13563883	Bn-chrA04_random-p638730
Bn-A04-p11274255	0.975206612	0.966942149	Bn-N4-p13575212	Bn-chrA04_random-p656243
Bn-A04-p11291596	0.975206612	0.966942149	Bn-N4-p13584873	Bn-chrA04_random-p668661
Bn-A04-p11293892	0.983471074	0.991735537	Bn-N4-p13588095	Bn-chrA04_random-p670963
Bn-A04-p11319703	0.991735537	0.504132231	Bn-N4-p13621425	Bn-chrA04-p12376726
Bn-A04-p11320045	0.917355372	0.752066116	Bn-N4-p13621735	Bn-chrC04-p38895065
Bn-A04-p11325008	0.958677686	0.94214876	Bn-N4-p13625199	Bn-chrC04-p38906149
Bn-A04-p11329756	1	0.94214876	Bn-N4-p13636377	Bn-chrA04-p12387665
Bn-A04-p11352544	0.966942149	0.958677686	Bn-N4-p13660870	Bn-chrA04-p12406072
Bn-A04-p11359006	0.940298507	0.940298507	Bn-N4-p13667364	Bn-chrA04-p12412863
Bn-A04-p11364790	0.983471074	0.991735537	Bn-N4-p13679025	Bn-chrA04-p12418536
Bn-A04-p11376488	1	1	Bn-N4-p13688766	Bn-chrA04-p12432998
Bn-A04-p11377702	0.983471074	0.983471074	Bn-N4-p13690012	Bn-chrA04-p12434244
Bn-A04-p11377775	0.983471074	0.983471074	Bn-N4-p13690084	Bn-chrA04-p12434316
Bn-A04-p11386737	0.980099502	0.980099502	Bn-N4-p13701647	Bn-chrA04-p12443666
Bn-A04-p11402649	0.990033223	0.990033223	Bn-N4-p13716359	Bn-chrA04-p12460806
Bn-A04-p11413704	0.966942149	0.966942149	Bn-N4-p13728544	Bn-chrC04-p39032676
Bn-A04-p11420466	0.983471074	0.975206612	Bn-N4-p13744208	Bn-chrA04-p12471834
Bn-A04-p11425304	0.995024876	1	Bn-N4-p13749037	Bn-chrA04-p12479834
Bn-A04-p11428085	0.958677686	0.958677686	Bn-N4-p13751811	Bn-chrA04-p12482606
Bn-A04-p11437131	0.996677741	0.996677741	Bn-N4-p13759745	Bn-chrA04-p12490540
Bn-A04-p11449464	1	1	Bn-N4-p13775526	Bn-chrA04-p12506320
Bn-A04-p11450339	0.996677741	0.996677741	Bn-N4-p13776401	Bn-chrA04-p12507195
Bn-A04-p11452549	0.925619835	0.925619835	Bn-N4-p13778611	Bn-chrA04-p12509403
Bn-A04-p11453319	0.983471074	0.983471074	Bn-N4-p13779386	Bn-chrA04-p12510178
Bn-A04-p11463316	0.993355482	0.993355482	Bn-N4-p13788185	Bn-chrA04-p12518836
Bn-A04-p11465588	1	1	Bn-N4-p13789033	Bn-chrA04-p12519684
Bn-A04-p11503303	0.975206612	0.975206612	Bn-N4-p13828317	Bn-chrA04-p12559493
Bn-A04-p11509775	0.990049751	0.990049751	Bn-N4-p13835033	Bn-chrA04-p12566209
Bn-A04-p11510797	0.975124378	0.975124378	Bn-N4-p13836054	Bn-chrA04-p12567230
Bn-A04-p11512379	0.996677741	0.996677741	Bn-N4-p13837635	Bn-chrA04-p12568810
Bn-A04-p11513352	0.973421927	0.973421927	Bn-N4-p13838707	Bn-chrA04-p12569882
Bn-A04-p11585127	0.946843854	0.946843854	Bn-N4-p13918194	Bn-chrA04-p12640632
Bn-A04-p11586565	0.993355482	0.993355482	Bn-N4-p13919632	Bn-chrA04-p12642070
Bn-A04-p11588227	0.980066445	0.980066445	Bn-N4-p13921295	Bn-chrA04-p12643733
Bn-A04-p11595428	0.887043189	0.946843854	Bn-N14-p45448306	Bn-chrC04-p39425187
Bn-A04-p11633209	0.995024876	0.995024876	Bn-N4-p13967615	Bn-chrA04-p12690915

Bn-A04-p11633439	0.990049751	0.990049751	Bn-N4-p13967845	Bn-chrA04-p12691145
Bn-A04-p11693521	0.983471074	0.991735537	Bn-N4-p14007192	Bn-chrAnn_random-p479723
Bn-A04-p11734372	0.915422886	0.955223881	Bn-N4-p14044443	Bn-chrAnn_random-p441135
Bn-A04-p11765380	0.991735537	0.991735537	Bn-N4-p14069590	Bn-chrA04-p12780295
Bn-A04-p11791366	0.966942149	0.966942149	Bn-N4-p14083506	Bn-chrA04-p12792684
Bn-A04-p11794986	0.975206612	0.983471074	Bn-N4-p14087583	Bn-chrA04-p12796144
Bn-A04-p11795779	0.973421927	0.906976744	Bn-N4-p14088376	Bn-chrA04-p12796936
Bn-A04-p11802638	1	0.991735537	Bn-N4-p14095215	Bn-chrA04-p12807038
Bn-A04-p11810574	0.975124378	0.970149254	Bn-N4-p14106202	Bn-chrA04-p12814483
Bn-A04-p11818064	0.975206612	0.966942149	Bn-N4-p14114079	Bn-chrA04-p12822987
Bn-A04-p11818085	0.966942149	0.958677686	Bn-N4-p14114100	Bn-chrA04-p12823008
Bn-A04-p11865854	0.970099668	0.973421927	Bn-N4-p14170993	Bn-chrA04-p12885147
Bn-A04-p11888044	0.990049751	0.990049751	Bn-N4-p14211428	Bn-chrA04-p12910864
Bn-A04-p11890416	0.991735537	1	Bn-N4-p14213799	Bn-chrA04-p12913211
Bn-A04-p11896581	0.965174129	0.965174129	Bn-N4-p14225042	Bn-chrA04-p12919376
Bn-A04-p11896696	0.950248756	0.950248756	Bn-N4-p14225154	Bn-chrA04-p12919488
Bn-A04-p11897079	0.990049751	0.990049751	Bn-N4-p14225546	Bn-chrA04-p12919880
Bn-A04-p11900571	0.520661157	0.94214876	Bn-N4-p14229076	Bn-chrAnn_random-p156869
Bn-A04-p11902113	0.458471761	0.956810631	Bn-N2-p7187933	Bn-chrAnn_random-p156884
Bn-A04-p11907565	0.958677686	0.966942149	Bn-N4-p14236714	Bn-chrAnn_random-p156938
Bn-A04-p11922910	0.975206612	0.975206612	Bn-N4-p14244731	Bn-chrAnn_random-p157019
Bn-A04-p11923310	0.990049751	0.990049751	Bn-N4-p14245131	Bn-chrAnn_random-p157023
Bn-A04-p11923511	0.980099502	0.980099502	Bn-N4-p14245332	Bn-chrAnn_random-p157025
Bn-A04-p11924219	0.991735537	0.991735537	Bn-N4-p14246038	Bn-chrAnn_random-p157032
Bn-A04-p11957518	0.983388704	0.986710963	Bn-N4-p14285273	Bn-chrA04-p12923275
Bn-A04-p11958047	1	0.996677741	Bn-N4-p14285802	Bn-chrA04-p12923804
Bn-A04-p11961175	0.993355482	0.996677741	Bn-N4-p14293805	Bn-chrA04-p12926925
Bn-A04-p11988057	0.991735537	0.983471074	Bn-Scaffold07227-p2	Bn-chrA04-p12945285
Bn-A04-p12010066	0.925619835	0.917355372	Bn-N4-p14342001	Bn-chrA04-p12970481
Bn-A04-p12011281	0.980099502	0.980099502	Bn-N4-p14343231	Bn-chrA04-p12971690
Bn-A04-p12037515	0.983471074	0.991735537	Bn-N4-p14372100	Bn-chrA04-p13015334
Bn-A04-p12051277	0.991735537	0.983471074	Bn-N4-p14385352	Bn-chrA04-p13028972
Bn-A04-p12084855	0.991735537	0.991735537	Bn-N4-p14424587	Bn-chrA04-p13058426
Bn-A04-p12087644	0.980099502	0.820895522	Bn-N4-p14427376	Bn-chrA04-p13065398
Bn-A04-p12112148	1	0.991735537	Bn-N4-p14449321	Bn-chrA04-p13103219
Bn-A04-p12112787	0.991735537	0.991735537	Bn-N4-p14449960	Bn-chrA04-p13103858
Bn-A04-p12119805	0.985074627	0.990049751	Bn-N4-p14456432	Bn-chrA04-p13108902
Bn-A04-p12151317	0.996677741	0.996677741	Bn-N4-p14483050	Bn-chrA04-p13137660
Bn-A04-p12159795	0.975206612	0.900826446	Bn-N4-p14488662	Bn-chrA04-p13143351
Bn-A04-p12163578	0.950248756	0.955223881	Bn-N4-p14496571	Bn-chrA04-p13146945
Bn-A04-p12163967	0.980099502	0.990049751	Bn-N4-p14496966	Bn-chrA04-p13147340
Bn-A04-p12170206	0.710743802	0.958677686	Bn-N4-p14501990	Bn-chrA04-p13152470
Bn-A04-p12204775	0.983471074	0.991735537	Bn-N4-p14540890	Bn-chrA04-p13192738
Bn-A04-p12205378	0.983388704	0.980066445	Bn-N4-p14541493	Bn-chrA04-p13193341
Bn-A04-p12210199	0.995024876	0.990049751	Bn-N4-p14546314	Bn-chrA04-p13198155
Bn-A04-p12211993	1	0.995024876	Bn-N4-p14548108	Bn-chrA04-p13201091

Bn-A04-p12236240	1	1	Bn-N4-p14573838	Bn-chrA04-p13224285
Bn-A04-p12243311	0.986710963	0.986710963	Bn-N4-p14580040	Bn-chrA04-p13231046
Bn-A04-p12251256	0.983471074	0.950413223	Bn-N4-p14587984	Bn-chrA04-p13237879
Bn-A04-p12256481	0.975206612	0.983471074	Bn-N4-p14593454	Bn-chrA04-p13245213
Bn-A04-p12256574	1	0.991735537	Bn-N4-p14593547	Bn-chrA04-p13245306
Bn-A04-p12256636	0.975206612	0.966942149	Bn-N4-p14593609	Bn-chrA04-p13245368
Bn-A04-p12256681	0.966942149	0.975206612	Bn-N4-p14593654	Bn-chrA04-p13245413
Bn-A04-p12259499	0.980099502	0.980099502	Bn-N4-p14596496	Bn-chrA04-p13248239
Bn-A04-p12263920	0.975206612	0.975206612	Bn-N4-p14600964	Bn-chrA04-p13252707
Bn-A04-p12283561	0.995024876	0.995024876	Bn-N4-p14619009	Bn-chrA04-p13272610
Bn-A04-p12286484	0.995024876	0.995024876	Bn-N4-p14621921	Bn-chrA04-p13275522
Bn-A04-p12288635	0.958677686	0.958677686	Bn-N4-p14624049	Bn-chrA04-p13277649
Bn-A04-p12314901	0.966942149	0.966942149	Bn-N4-p14652564	Bn-chrA04-p13306235
Bn-A04-p12314909	0.950413223	0.950413223	Bn-N4-p14652572	Bn-chrA04-p13306243
Bn-A04-p12328218	0.975206612	0.966942149	Bn-Scaffold07137-p2	Bn-chrA04-p13313147
Bn-A04-p12333381	0.958677686	0.958677686	Bn-N4-p14660450	Bn-chrA04-p13316048
Bn-A04-p12341429	0.970149254	0.975124378	Bn-N4-p14668576	Bn-chrA04-p13324249
Bn-A04-p12344157	0.991735537	0.983471074	Bn-N4-p14671293	Bn-chrA04-p13326977
Bn-A04-p12344342	0.983471074	0.991735537	Bn-N4-p14671478	Bn-chrA04-p13327162
Bn-A04-p12344614	0.983388704	0.983388704	Bn-N4-p14671750	Bn-chrA04-p13327434
Bn-A04-p12348826	0.983471074	0.991735537	Bn-N4-p14681626	Bn-chrA04-p13331645
Bn-A04-p12350578	0.991735537	1	Bn-N4-p14683376	Bn-chrA04-p13333397
Bn-A04-p12360869	0.991735537	0.933884298	Bn-N4-p14697921	Bn-chrA04_random-p780307
Bn-A04-p12365264	0.985074627	0.985074627	Bn-N4-p14702559	Bn-chrA04-p13349174
Bn-A04-p12372810	1	0.991735537	Bn-N4-p14711545	Bn-chrA04-p13355491
Bn-A04-p12411454	0.955223881	0.980099502	Bn-N1-p9095034	Bn-chrA10-p3938098
Bn-A04-p12452535	0.990033223	0.983388704	Bn-N10-p4769041	Bn-chrA10-p3969446
Bn-A04-p12452755	0.965174129	0.960199005	Bn-N10-p4768821	Bn-chrA10-p3969666
Bn-A04-p12509027	0.990033223	0.990033223	Bn-N10-p4733803	Bn-chrA10-p4000649
Bn-A04-p12526741	1	0.989583333	Bn-N10-p4716216	Bn-chrA10-p4019222
Bn-A04-p12527154	0.996677741	0.996677741	Bn-N10-p4715870	Bn-chrA10-p4019635
Bn-A04-p12540627	0.933884298	0.801652893	Bn-N10-p4689414	Bn-chrA10-p4046074
Bn-A04-p12541660	0.996677741	0.996677741	Bn-N10-p4688367	Bn-chrA10-p4047121
Bn-A04-p12542184	0.991735537	0.991735537	Bn-N10-p4687831	Bn-chrA10-p4047657
Bn-A04-p12547910	0.958677686	0.958677686	Bn-N10-p4676521	Bn-chrA10-p4058964
Bn-A04-p12550756	0.983471074	0.983471074	Bn-N10-p4673693	Bn-chrA10-p4061795
Bn-A04-p12551118	0.966942149	0.966942149	Bn-N10-p4673331	Bn-chrA10-p4062157
Bn-A04-p12555320	0.958677686	0.958677686	Bn-N10-p4667753	Bn-chrA10-p4066390
Bn-A04-p12572164	0.933884298	0.925619835	Bn-N10-p4627837	Bn-chrA10-p4111779
Bn-A04-p12572310	0.991735537	0.991735537	Bn-N10-p4627690	Bn-chrA10-p4111926
Bn-A04-p12572376	0.719008264	0.719008264	Bn-N10-p4627624	Bn-chrA10-p4111992
Bn-A04-p12572577	0.983471074	0.991735537	Bn-N10-p4627461	Bn-chrA10-p4112155
Bn-A04-p12575050	0.983471074	0.983471074	Bn-N10-p4625825	Bn-chrA10-p4113789
Bn-A04-p12575115	0.975206612	0.966942149	Bn-N10-p4625760	Bn-chrA10-p4113854
Bn-A04-p12578822	0.6910299	0.976744186	Bn-N10-p4622068	Bn-chrA10-p4126600
Bn-A04-p12580768	0.933884298	0.933884298	Bn-N10-p4620443	Bn-chrA10-p4129947

Bn-A04-p12584051	0.94214876	0.950413223	Bn-N19-p33271102	Bn-chrA10-p4133253
Bn-A04-p12586651	0.958677686	0.958677686	Bn-N10-p4614009	Bn-chrA10-p4136378
Bn-A04-p12599627	0.976744186	0.996677741	Bn-N10-p4608812	Bn-chrA10-p4139826
Bn-A04-p12599843	0.966942149	0.966942149	Bn-N10-p4608596	Bn-chrA10-p4140028
Bn-A04-p12600635	0.859504132	0.909090909	Bn-N10-p4607805	Bn-chrA10-p4140819
Bn-A04-p12631523	0.975206612	0.983471074	Bn-N10-p4580182	Bn-chrA10-p4161955
Bn-A04-p12634330	0.975206612	0.966942149	Bn-N10-p4577391	Bn-chrA10-p4164773
Bn-A04-p12634856	1	0.991735537	Bn-N10-p4576865	Bn-chrA10-p4165299
Bn-A04-p12635106	0.986710963	0.990033223	Bn-N10-p4576615	Bn-chrA10-p4165549
Bn-A04-p12635986	0.983471074	0.991735537	Bn-N10-p4575735	Bn-chrA10-p4166429
Bn-A04-p12639105	0.996677741	0.993355482	Bn-N10-p4572601	Bn-chrA10-p4169563
Bn-A04-p12651819	0.685950413	0.933884298	Bn-N14-p46551005	Bn-chrA04-p13367498
Bn-A04-p12655669	1	0.991735537	Bn-N4-p14722671	Bn-chrA04-p13373665
Bn-A04-p12659254	0.958677686	0.966942149	Bn-N4-p14726255	Bn-chrA04-p13377303
Bn-A04-p12659402	0.958677686	0.966942149	Bn-N4-p14726403	Bn-chrA04-p13377452
Bn-A04-p12670129	0.94214876	0.94214876	Bn-N4-p14745871	Bn-chrC04-p40643441
Bn-A04-p12671369	0.966942149	0.958677686	Bn-N4-p14747087	Bn-chrA04-p13396012
Bn-A04-p12675088	0.920265781	0.926910299	Bn-N4-p14751019	Bn-chrA04-p13399706
Bn-A04-p12677961	0.983471074	0.975206612	Bn-N4-p14753696	Bn-chrA04-p13402558
Bn-A04-p12680156	0.966942149	0.958677686	Bn-N4-p14758361	Bn-chrA04-p13404762
Bn-A04-p12687510	0.966942149	0.958677686	Bn-N4-p14763421	Bn-chrA04-p13415106
Bn-A04-p12690759	0.958677686	0.983471074	Bn-N4-p14766672	Bn-chrA04_random-p767148
Bn-A04-p12691245	0.94214876	0.950413223	Bn-N4-p14767153	Bn-chrA04_random-p767632
Bn-A04-p12712772	0.940199336	0.96013289	Bn-N4-p14778835	Bn-chrA04_random-p806915
Bn-A04-p12715859	0.958677686	0.966942149	Bn-N4-p14781922	Bn-chrA04_random-p803966
Bn-A04-p12717815	0.983471074	0.991735537	Bn-N4-p14783877	Bn-chrA04_random-p802003
Bn-A04-p12719682	0.966942149	0.966942149	Bn-N4-p14785933	Bn-chrA04_random-p800134
Bn-A04-p12724637	1	0.991735537	Bn-N4-p14794411	Bn-chrA04_random-p795908
Bn-A04-p12725596	0.993355482	0.990033223	Bn-N4-p14795370	Bn-chrA04_random-p794950
Bn-A04-p12726067	0.975206612	0.983471074	Bn-N4-p14795841	Bn-chrA04_random-p794479
Bn-A04-p12726676	0.970099668	0.970099668	Bn-N4-p14796450	Bn-chrA04_random-p793870
Bn-A04-p12728362	0.983471074	0.991735537	Bn-N4-p14798136	Bn-chrA04_random-p792457
Bn-A04-p12734346	0.991735537	0.983471074	Bn-N4-p14804042	Bn-chrA04_random-p783711
Bn-A04-p12735594	0.991735537	1	Bn-N4-p14808644	Bn-chrA04_random-p782508
Bn-A04-p12749707	0.983471074	0.966942149	Bn-N4-p14815362	Bn-chrA04-p13419164
Bn-A04-p12751774	0.975206612	0.966942149	Bn-N4-p14817423	Bn-chrA04-p13421063
Bn-A04-p12756666	0.983471074	0.975206612	Bn-N4-p14822272	Bn-chrA04-p13425915
Bn-A04-p12759219	1	0.991735537	Bn-N4-p14825038	Bn-chrA04-p13429362
Bn-A04-p12759499	0.975206612	0.983471074	Bn-N4-p14825318	Bn-chrA04-p13429642
Bn-A04-p12764046	0.966942149	0.975206612	Bn-N4-p14829864	Bn-chrA04-p13435881
Bn-A04-p12769407	0.975206612	0.966942149	Bn-N4-p14835031	Bn-chrA04-p13445294
Bn-A04-p12772506	0.950413223	0.694214876	Bn-N4-p14837837	Bn-chrA04-p13451925
Bn-A04-p12782652	1	0.139534884	Bn-N6-p25885189	Bn-chrC07-p21388875
Bn-A04-p12793333	0.991735537	0.975206612	Bn-N4-p14849520	Bn-chrA04-p13460729
Bn-A04-p12793456	0.985074627	0.990049751	Bn-N4-p14849643	Bn-chrA04-p13460855
Bn-A04-p12793487	0.990049751	0.985074627	Bn-N4-p14849674	Bn-chrA04-p13460886

Bn-A04-p12798976	0.975124378	0.980099502	Bn-N4-p14855183	Bn-chrA04_random-p772390
Bn-A04-p12802914	0.991735537	1	Bn-N4-p14859732	Bn-chrA04_random-p768378
Bn-A04-p12818353	1	0.991735537	Bn-N4-p14870705	Bn-chrA04-p13483012
Bn-A04-p12818639	0.867768595	0.983471074	Bn-N4-p14871244	Bn-chrA04-p13483295
Bn-A04-p12819355	0.876033058	0.983471074	Bn-N4-p14871956	Bn-chrA04-p13484011
Bn-A04-p12820145	0.991735537	0.991735537	Bn-N4-p14872803	Bn-chrA04-p13484820
Bn-A04-p12822929	0.958677686	0.983471074	Bn-N4-p14874834	Bn-chrA04-p13487613
Bn-A04-p12824172	0.983471074	0.991735537	Bn-N4-p14876272	Bn-chrA04-p13490160
Bn-A04-p12825295	0.983471074	0.975206612	Bn-N4-p14877397	Bn-chrA04-p13491287
Bn-A04-p12826986	0.958677686	0.20661157	Bn-N4-p14879012	Unknown
Bn-A04-p12827056	1	0.214876033	Bn-N4-p14879083	Unknown
Bn-A04-p12827460	0.991735537	0.26446281	Bn-N4-p14879487	Unknown
Bn-A04-p12829159	0.909090909	0.983471074	Bn-N4-p14881219	Bn-chrA04-p13507081
Bn-A04-p12829470	0.966942149	0.975206612	Bn-N4-p14881530	Bn-chrA04-p13507392
Bn-A04-p12840183	0.996677741	0.996677741	Bn-N4-p14892410	Bn-chrA04-p13514049
Bn-A04-p12857210	0.975206612	0.983471074	Bn-N4-p14911581	Bn-chrA04-p13528823
Bn-A04-p12857974	0.975206612	0.966942149	Bn-N4-p14912325	Bn-chrA04-p13531886
Bn-A04-p12868057	0.950413223	0.958677686	Bn-N4-p14921042	Bn-chrAnn_random-p448809
Bn-A04-p12877134	0.975206612	0.991735537	Bn-N4-p14930735	Bn-chrA04-p13556759
Bn-A04-p12888388	0.990033223	0.986710963	Bn-N4-p14942933	Bn-chrAnn_random-p429617
Bn-A04-p12891881	0.842975207	0.94214876	Bn-N4-p14949497	Bn-chrA04-p13577906
Bn-A04-p12893331	0.991735537	0.983471074	Bn-N4-p14950941	Bn-chrA04-p13579345
Bn-A04-p12893630	0.925619835	0.917355372	Bn-N4-p14951248	Bn-chrA04-p13579644
Bn-A04-p12893753	0.983471074	0.975206612	Bn-N4-p14952600	Bn-chrA04-p13579835
Bn-A04-p12894555	0.955223881	0.965174129	Bn-N4-p14954037	Bn-chrA04-p13580639
Bn-A04-p12897769	0.966942149	0.487603306	Bn-N4-p14959274	Bn-chrA04-p13584161
Bn-A04-p12899754	0.94214876	0.94214876	Bn-N4-p14961354	Bn-chrA04-p13586126
Bn-A04-p12899869	0.991735537	0.983471074	Bn-N4-p14961470	Bn-chrA04-p13586243
Bn-A04-p12901227	0.958677686	0.975206612	Bn-N4-p14962798	Bn-chrA04-p13587599
Bn-A04-p12917949	0.975124378	0.975124378	Bn-N4-p14969070	Bn-chrA04-p13595304
Bn-A04-p12942400	0.985074627	0.985074627	Bn-N4-p14986900	Bn-chrA04-p13629318
Bn-A04-p12953418	0.983388704	0.96345515	Bn-N4-p14998960	Bn-chrA04-p13642239
Bn-A04-p12959668	0.966942149	0.975206612	Bn-N4-p15005514	Bn-chrA04-p13648408
Bn-A04-p12985030	0.970149254	0.970149254	Bn-N8-p11108759	Bn-chrC04-p3994481
Bn-A04-p12987331	0.990033223	0.990033223	Bn-Scaffold22137-p1	Bn-chrAnn_random-p352194
Bn-A04-p12994676	0.975124378	0.975124378	Bn-N4-p15036643	Bn-chrA04-p13684887
Bn-A04-p13009893	0.965174129	0.965174129	Bn-N4-p15054322	Bn-chrA04-p13700434
Bn-A04-p13050240	0.983471074	0.991735537	Bn-N4-p15092086	Bn-chrA04-p13737905
Bn-A04-p13052013	0.983471074	0.975206612	Bn-N4-p15093867	Bn-chrC04-p41199813
Bn-A04-p13056296	0.834710744	0.983471074	Bn-N4-p15102871	Bn-chrA04-p13743977
Bn-A04-p13056382	0.950413223	0.950413223	Bn-N4-p15102956	Bn-chrA04-p13744063
Bn-A04-p13056438	0.719008264	0.925619835	Bn-N4-p15103012	Bn-chrA04-p13744119
Bn-A04-p13059166	0.965174129	0.955223881	Bn-N4-p15105714	Bn-chrA04-p13747927
Bn-A04-p13059485	0.958677686	0.950413223	Bn-N4-p15106045	Bn-chrA04-p13748246
Bn-A04-p13061139	0.966942149	0.950413223	Bn-N4-p15107691	Bn-chrA04-p13749892
Bn-A04-p13062081	0.966942149	0.975206612	Bn-N4-p15110146	Bn-chrA04-p13752672

Bn-A04-p13063145	0.983471074	0.975206612	Bn-N4-p15111227	Bn-chrA04-p13753748
Bn-A04-p13068545	0.966942149	0.975206612	Bn-N4-p15114834	Bn-chrA04-p13761679
Bn-A04-p13069741	0.973421927	0.973421927	Bn-N4-p15116045	Bn-chrA04-p13762887
Bn-A04-p13078078	0.970149254	0.995024876	Bn-N4-p15122228	Bn-chrA04-p13769067
Bn-A04-p13091624	1	0.991735537	Bn-N4-p15174842	Bn-chrA04-p13850812
Bn-A04-p13096878	0.975206612	0.983471074	Bn-N4-p15180049	Bn-chrA04-p13855175
Bn-A04-p13107522	0.986710963	0.996677741	Bn-Scaffold04937-p8	Bn-chrA04-p13865793
Bn-A04-p13112913	0.983388704	0.943521595	Bn-N4-p15196249	Bn-chrA04-p13871201
Bn-A04-p13116453	0.990049751	0.995024876	Bn-N4-p15199773	Bn-chrA04-p13874737
Bn-A04-p13118144	0.975206612	0.966942149	Bn-N4-p15201464	Bn-chrA04-p13876447
Bn-A04-p13119516	0.966942149	0.966942149	Bn-N4-p15202848	Bn-chrA04-p13877734
Bn-A04-p13122589	0.958677686	0.950413223	Bn-N4-p15205829	Bn-chrA04-p13880754
Bn-A04-p13125820	0.975206612	0.966942149	Bn-N4-p15208727	Bn-chrA04-p13889986
Bn-A04-p13128840	0.855721393	0.945273632	Bn-N4-p15212940	Bn-chrA04-p13894566
Bn-A04-p13129093	0.983471074	0.991735537	Bn-N4-p15213192	Bn-chrA04-p13894819
Bn-A04-p13129306	1	0.991735537	Bn-N4-p15213405	Bn-chrA04-p13895032
Bn-A04-p13129601	0.975206612	0.991735537	Bn-N4-p15213699	Bn-chrA04-p13895327
Bn-A04-p13129741	0.996677741	0.993355482	Bn-N4-p15213841	Bn-chrA04-p13895467
Bn-A04-p13140266	0.975206612	0.983471074	Bn-N4-p15227803	Bn-chrA04-p13905031
Bn-A04-p13145539	1	0.996677741	Bn-N4-p15233023	Bn-chrA04-p13913101
Bn-A04-p13173919	0.603305785	0.991735537	Bn-N4-p15251270	Bn-chrA04-p13935740
Bn-A04-p13196638	0.980099502	0.980099502	Bn-N4-p15270737	Bn-chrA04-p13956721
Bn-A04-p13206432	0.983471074	0.983471074	Bn-N4-p15287831	Bn-chrA04_random-p902224
Bn-A04-p13207715	0.943521595	0.940199336	Bn-N4-p15289091	Bn-chrA04_random-p900967
Bn-A04-p13208442	0.986710963	0.986710963	Bn-N4-p15289638	Bn-chrA04_random-p900420
Bn-A04-p13215929	0.965174129	0.970149254	Bn-N4-p15296419	Bn-chrA04_random-p888787
Bn-A04-p13216114	0.996677741	0.993355482	Bn-N4-p15296604	Bn-chrA04_random-p888602
Bn-A04-p13235708	0.958677686	0.958677686	Bn-N4-p15316208	Bn-chrA04_random-p870085
Bn-A04-p13241959	0.996677741	0.92358804	Bn-N4-p15322956	Bn-chrC04-p41504291
Bn-A04-p13245778	0.925373134	0.985074627	Bn-N4-p15328477	Bn-chrA04_random-p859817
Bn-A04-p13246454	0.925619835	0.975206612	Bn-N14-p47548179	Bn-chrA04_random-p859141
Bn-A04-p13247108	0.917355372	1	Bn-N4-p15329791	Bn-chrA04_random-p858487
Bn-A04-p13249011	0.983471074	0.983471074	Bn-N4-p15332454	Bn-chrA04_random-p856539
Bn-A04-p13269135	0.884297521	0.966942149	Bn-N4-p15337468	Bn-chrA04_random-p848843
Bn-A04-p13286494	1	0.936877076	Bn-N4-p15349049	Bn-chrC04-p41551229
Bn-A04-p13293545	0.996677741	0.996677741	Bn-N4-p15352692	Bn-chrA04_random-p834554
Bn-A04-p13293640	0.990033223	0.990033223	Bn-N4-p15352787	Bn-chrA04_random-p834459
Bn-A04-p13294322	0.991735537	1	Bn-N4-p15353469	Bn-chrA04_random-p833771
Bn-A04-p13302511	1	0.900826446	Bn-N4-p15370445	Bn-chrC04_random-p414267
Bn-A04-p13306369	0.975124378	0.970149254	Bn-N4-p15377894	Bn-chrA04-p13968724
Bn-A04-p13330392	0.983471074	0.950413223	Bn-N4-p15400722	Bn-chrC04-p41601971
Bn-A04-p13336924	0.991735537	0.983471074	Bn-N4-p15405333	Bn-chrA04-p14001096
Bn-A04-p13368998	0.983471074	0.966942149	Bn-N4-p15435930	Bn-chrA04-p14037595
Bn-A04-p13370433	0.983471074	0.983471074	Bn-N4-p15437344	Bn-chrA04-p14038989
Bn-A04-p13372886	0.933884298	0.933884298	Bn-N4-p15439797	Bn-chrA04-p14041474
Bn-A04-p13384606	0.993355482	0.996677741	Bn-N4-p15444121	Bn-chrA04-p14046633

Bn-A04-p13389366	1	0.991735537	Bn-N4-p15444250	Bn-chrA04-p14046762
Bn-A04-p13389622	0.996677741	0.993355482	Bn-N4-p15444506	Bn-chrA04-p14047018
Bn-A04-p13389743	0.966942149	0.966942149	Bn-N4-p15444627	Bn-chrA04-p14047139
Bn-A04-p13389784	0.950413223	0.966942149	Bn-N4-p15445039	Bn-chrA04-p14047180
Bn-A04-p13390838	0.983471074	0.983471074	Bn-N4-p15446334	Bn-chrA04-p14048232
Bn-A04-p13394479	0.983471074	0.983471074	Bn-N4-p15449389	Bn-chrA04-p14051242
Bn-A04-p13398568	0.909090909	0.909090909	Bn-N4-p15456214	Bn-chrA04-p14058070
Bn-A04-p13418563	0.983471074	0.537190083	Bn-N4-p15477342	Bn-chrA05-p7036787
Bn-A04-p13428137	0.991735537	1	Bn-N4-p15486538	Bn-chrA04-p14082609
Bn-A04-p13428243	0.991735537	0.991735537	Bn-N4-p15486644	Bn-chrA04-p14082715
Bn-A04-p13428324	0.983471074	0.983471074	Bn-N4-p15486725	Bn-chrA04-p14082796
Bn-A04-p13429557	0.990033223	0.986710963	Bn-N4-p15487928	Bn-chrA04-p14084029
Bn-A04-p13430155	0.983471074	0.983471074	Bn-N4-p15488520	Bn-chrA04-p14084627
Bn-A04-p13430376	0.991735537	1	Bn-N4-p15488741	Bn-chrA04-p14084848
Bn-A04-p13430818	0.983471074	0.975206612	Bn-N4-p15489185	Bn-chrA04-p14085290
Bn-A04-p13431005	1	1	Bn-N4-p15489372	Bn-chrA04-p14085477
Bn-A04-p13461501	0.983471074	0.975206612	Bn-N4-p15496412	Bn-chrA04-p14111824
Bn-A04-p13490747	0.986710963	0.993355482	Bn-N4-p15520771	Bn-chrA04-p14152872
Bn-A04-p13493883	0.73553719	0.94214876	Bn-N4-p15523423	Bn-chrA04-p14157286
Bn-A04-p13497608	0.958677686	0.966942149	Bn-N4-p15527167	Bn-chrA04-p14160938
Bn-A04-p13501943	0.991735537	0.991735537	Bn-N4-p15531244	Bn-chrA04-p14165250
Bn-A04-p13504670	0.975206612	0.983471074	Bn-N4-p15533186	Bn-chrA04-p14168234
Bn-A04-p13505017	0.983471074	0.983471074	Bn-N4-p15533527	Bn-chrA04-p14168579
Bn-A04-p13510301	0.983471074	0.983471074	Bn-N4-p15535912	Bn-chrA04-p14173113
Bn-A04-p13516223	0.983471074	0.983471074	Bn-N4-p15540324	Bn-chrA04-p14181823
Bn-A04-p13516274	0.983471074	0.983471074	Bn-N4-p15540375	Bn-chrA04-p14181874
Bn-A04-p13516285	0.983471074	0.983471074	Bn-N4-p15540386	Bn-chrA04-p14181885
Bn-A04-p13516411	0.991735537	0.991735537	Bn-N4-p15546886	Bn-chrA04-p14182011
Bn-A04-p13517158	0.958677686	0.958677686	Bn-N4-p15547601	Bn-chrA04-p14185771
Bn-A04-p13520736	0.752066116	0.760330579	Bn-N4-p15547838	Bn-chrA04-p14186008
Bn-A04-p13521376	0.975206612	0.975206612	Bn-N4-p15548809	Bn-chrA04-p14186649
Bn-A04-p13521405	0.990033223	0.993355482	Bn-N4-p15548838	Bn-chrA04-p14186678
Bn-A04-p13532829	0.966942149	0.958677686	Bn-N4-p15554194	Bn-chrA04-p14212583
Bn-A04-p13534631	0.280991736	0.975206612	Unknown	Bn-chrA04-p14208949
Bn-A04-p13541465	0.991735537	0.983471074	Bn-N4-p15561324	Bn-chrA04-p14204157
Bn-A04-p13552122	0.983388704	0.983388704	Bn-N4-p15580400	Bn-chrA04-p14228568
Bn-A04-p13562332	0.975206612	0.975206612	Bn-N4-p15592185	Bn-chrA04-p14240152
Bn-A04-p13572341	0.94214876	0.94214876	Bn-N4-p15595532	Bn-chrA04-p14244619
Bn-A04-p13578411	0.876033058	0.900826446	Bn-N4-p15601075	Bn-chrA04-p14249886
Bn-A04-p13583325	0.960199005	0.960199005	Bn-N4-p15605130	Bn-chrA04-p14254894
Bn-A04-p13589498	0.991735537	1	Bn-N4-p15608137	Bn-chrA04-p14259534
Bn-A04-p13600415	0.966942149	0.958677686	Bn-N4-p15622445	Bn-chrA04-p14270151
Bn-A04-p13615482	0.965174129	0.965174129	Bn-N4-p15654000	Bn-chrA04-p14280642
Bn-A04-p13645293	0.990049751	0.990049751	Bn-N4-p15663880	Bn-chrA04-p14288751
Bn-A04-p13655958	0.975206612	0.975206612	Bn-N4-p15674600	Bn-chrA04-p14299471
Bn-A04-p13659690	0.991735537	0.991735537	Bn-N4-p15678260	Bn-chrA04-p14303131

Bn-A04-p13665489	0.990033223	0.990033223	Bn-N4-p15684044	Bn-chrA04-p14308915
Bn-A04-p13670045	0.96013289	0.96013289	Bn-N4-p15688616	Bn-chrA04-p14313487
Bn-A04-p13685561	0.94214876	0.94214876	Bn-N4-p15701910	Bn-chrA04-p14331342
Bn-A04-p13689816	0.991735537	0.991735537	Bn-N4-p15707430	Bn-chrA04-p14335562
Bn-A04-p13705410	0.975206612	0.966942149	Bn-N4-p15714656	Bn-chrA04-p14344443
Bn-A04-p13705548	0.965174129	0.985074627	Bn-N4-p15714794	Bn-chrA04-p14344581
Bn-A04-p13705636	0.966942149	0.958677686	Bn-N4-p15714880	Bn-chrA04-p14344669
Bn-A04-p13705760	0.950413223	0.94214876	Bn-N4-p15715005	Bn-chrA04-p14344793
Bn-A04-p13711282	0.966942149	0.975206612	Bn-N4-p15722446	Bn-chrA04-p14351459
Bn-A04-p13726056	0.890365449	0.96013289	Bn-N4-p15738093	Bn-chrA04-p14368416
Bn-A04-p13738279	0.966942149	0.966942149	Bn-N4-p15750765	Bn-chrA04-p14381083
Bn-A04-p13761897	0.975206612	0.975206612	Bn-N4-p15771314	Bn-chrA04-p14401988
Bn-A04-p13767868	0.785123967	0.785123967	Bn-N4-p15777042	Bn-chrA04-p14407716
Bn-A04-p13856134	0.976744186	0.993355482	Bn-N4-p15881188	Bn-chrA04_random-p944132
Bn-A04-p13859935	0.966942149	0.975206612	Bn-N4-p15886186	Bn-chrA04_random-p949141
Bn-A04-p13866861	0.966942149	0.966942149	Bn-N4-p15894606	Bn-chrA04_random-p956151
Bn-A04-p13899576	0.983471074	0.975206612	Bn-N4-p15920125	Bn-chrA04-p14487876
Bn-A04-p13900963	0.991735537	0.983471074	Bn-N4-p15921512	Bn-chrA04-p14489331
Bn-A04-p13901008	0.975206612	0.983471074	Bn-N4-p15921557	Bn-chrA04-p14489376
Bn-A04-p13910168	0.983471074	0.991735537	Bn-N4-p15934714	Bn-chrA04_random-p974698
Bn-A04-p13910822	0.983471074	0.975206612	Bn-N4-p15935376	Bn-chrA04_random-p975383
Bn-A04-p13927295	0.876033058	0.94214876	Bn-N4-p15951728	Bn-chrA04-p14518985
Bn-A04-p13930713	0.826446281	0.826446281	Bn-N4-p15955034	Bn-chrA04-p14532467
Bn-A04-p13992889	0.986710963	0.980066445	Bn-N4-p15997563	Bn-chrA04_random-p985240
Bn-A04-p13993666	0.983471074	0.975206612	Bn-N4-p15998339	Bn-chrA04_random-p986206
Bn-A04-p13993683	0.983471074	0.975206612	Bn-N4-p15998356	Bn-chrA04_random-p986223
Bn-A04-p13995098	0.958677686	0.950413223	Bn-N4-p16001193	Bn-chrA04_random-p988447
Bn-A04-p14008077	0.986710963	0.986710963	Bn-N4-p16014935	Bn-chrA04_random-p100620
Bn-A04-p14018867	0.975206612	0.983471074	Bn-N4-p16024070	Bn-chrA04_random-p101720
Bn-A04-p14020738	0.990049751	0.990049751	Bn-N4-p16025942	Bn-chrA04_random-p101907
Bn-A04-p14020855	0.983471074	0.867768595	Bn-N4-p16026059	Bn-chrA04_random-p101919
Bn-A04-p14023419	0.893687708	0.983388704	Bn-N4-p16028620	Bn-chrA04_random-p102181
Bn-A04-p14026143	0.995024876	0.995024876	Bn-N4-p16031244	Bn-chrA04_random-p102608
Bn-A04-p14060096	0.958677686	0.958677686	Bn-N4-p16068662	Bn-chrA04-p14601024
Bn-A04-p14092385	0.980099502	0.980099502	Bn-N4-p16096135	Bn-chrA04-p14628642
Bn-A04-p14156506	0.727272727	0.991735537	Bn-N14-p48976254	Bn-chrA04-p14687908
Bn-A04-p14159293	0.990049751	0.990049751	Bn-N4-p16160739	Bn-chrA04-p14690701
Bn-A04-p14159494	1	0.990049751	Bn-N4-p16160940	Bn-chrA04-p14690902
Bn-A04-p14159558	0.970149254	0.980099502	Bn-N4-p16161003	Bn-chrA04-p14690966
Bn-A04-p14226310	0.995024876	0.945273632	Bn-N4-p16235932	Bn-chrA04-p14756280
Bn-A04-p14255771	0.958677686	0.966942149	Bn-N4-p16255878	Bn-chrA04-p14766239
Bn-A04-p14271109	0.966942149	0.768595041	Bn-N4-p16258546	Bn-chrA04-p14767382
Bn-A04-p14271110	0.966942149	0.776859504	Bn-N4-p16258547	Bn-chrA04-p14767383
Bn-A04-p14271161	0.958677686	0.966942149	Bn-N4-p16258598	Bn-chrA04-p14767434
Bn-A04-p14289058	0.936877076	0.803986711	Bn-N4-p16277785	Bn-chrCnn_random-p315068
Bn-A04-p14312019	0.986710963	0.986710963	Bn-N4-p16304417	Bn-chrA04-p14808525

Bn-A04-p14315336	0.991735537	1	Bn-N4-p16307392	Bn-chrA04-p14811841
Bn-A04-p14332759	0.983471074	0.983471074	Bn-N4-p16322233	Bn-chrA04_random-p108299
Bn-A04-p14333001	0.986710963	0.990033223	Bn-N4-p16322475	Bn-chrA04_random-p108275
Bn-A04-p14353463	0.970149254	0.975124378	Bn-N4-p16341393	Bn-chrA04_random-p106512
Bn-A04-p14357191	0.966942149	0.983471074	Bn-N4-p16345119	Bn-chrA04_random-p106139
Bn-A04-p14357445	0.958677686	0.966942149	Bn-N4-p16347279	Bn-chrA04_random-p106114
Bn-A04-p14360385	0.975206612	0.983471074	Bn-N4-p16350269	Bn-chrA04_random-p105820
Bn-A04-p14360799	0.983471074	0.975206612	Bn-N4-p16350682	Bn-chrA04_random-p105779
Bn-A04-p14365868	0.983471074	0.991735537	Bn-N4-p16358635	Bn-chrA04_random-p105260
Bn-A04-p14380140	0.966942149	0.966942149	Bn-N4-p16378096	Bn-chrA04-p14853313
Bn-A04-p14397957	0.990033223	0.986710963	Bn-N4-p16401198	Bn-chrA04-p14873132
Bn-A04-p14398335	0.996677741	1	Bn-N4-p16401576	Bn-chrA04-p14873510
Bn-A04-p14410667	0.983471074	0.983471074	Bn-N4-p16409373	Bn-chrA04-p14881039
Bn-A04-p14478925	0.966942149	0.975206612	Bn-N4-p16451168	Bn-chrA04-p14929903
Bn-A04-p14479867	0.958677686	0.975206612	Bn-N4-p16452111	Bn-chrA04-p14930847
Bn-A04-p14483727	0.876033058	0.892561983	Bn-N4-p16454593	Bn-chrA04-p14933173
Bn-A04-p14528871	0.958677686	0.975206612	Bn-N4-p16492142	Bn-chrA04-p14976156
Bn-A04-p14532353	0.995024876	0.995024876	Bn-N4-p16495684	Bn-chrA04-p14979637
Bn-A04-p14559897	0.983471074	0.983471074	Bn-N4-p16518169	Bn-chrA04-p15000508
Bn-A04-p14563007	0.94214876	1	Bn-N14-p49631250	Bn-chrA04-p15003618
Bn-A04-p14565138	0.975206612	0.991735537	Bn-N4-p16523294	Bn-chrA04-p15005731
Bn-A04-p14565883	0.925619835	0.925619835	Bn-N4-p16524041	Bn-chrA04-p15006478
Bn-A04-p14572391	0.958677686	0.975206612	Bn-N14-p49640547	Bn-chrA04-p15011438
Bn-A04-p14582431	0.996677741	0.966777409	Bn-N4-p16548395	Bn-chrA04-p15024595
Bn-A04-p14582584	0.996677741	0.976744186	Bn-N4-p16548548	Bn-chrA04-p15024748
Bn-A04-p14586235	0.980099502	0.965174129	Bn-N4-p16553035	Bn-chrA04-p15027146
Bn-A04-p14588183	0.983388704	0.986710963	Bn-N4-p16554984	Bn-chrA04-p15029135
Bn-A04-p14622218	1	1	Bn-N4-p16573987	Bn-chrA04-p15094182
Bn-A04-p14630533	0.980099502	0.980099502	Bn-N14-p49752851	Bn-chrC04-p43620841
Bn-A04-p14630759	0.975124378	0.975124378	Bn-N14-p49753070	Bn-chrC04-p43621060
Bn-A04-p14631343	0.985074627	0.985074627	Bn-N14-p49753696	Bn-chrC04-p43621686
Bn-A04-p14631586	0.857142857	0.986710963	Bn-N4-p16585003	Bn-chrA04-p15102829
Bn-A04-p14640007	0.990033223	0.986710963	Bn-N4-p16594072	Bn-chrA04-p15110569
Bn-A04-p14642152	0.975124378	0.975124378	Bn-N4-p16596219	Bn-chrA04-p15112716
Bn-A04-p14652223	0.975206612	0.975206612	Bn-N4-p16606295	Bn-chrA04-p15124491
Bn-A04-p14661285	1	0.88372093	Bn-N4-p16617010	Bn-chrA04-p15132373
Bn-A04-p14662924	1	0.970149254	Bn-N4-p16618649	Bn-chrC04-p43674629
Bn-A04-p14670237	0.983471074	0.975206612	Bn-N4-p16625955	Bn-chrA04-p15141564
Bn-A04-p14670992	1	0.991735537	Bn-N4-p16626708	Bn-chrA04-p15142319
Bn-A04-p14673783	0.983471074	0.975206612	Bn-N4-p16627428	Bn-chrA04-p15145097
Bn-A04-p14687930	0.995024876	0.995024876	Bn-N4-p16642399	Bn-chrA04-p15158347
Bn-A04-p14742779	0.785123967	0.958677686	Bn-N4-p16697370	Bn-chrA04-p15190620
Bn-A04-p14756001	0.966942149	0.966942149	Bn-N4-p16711540	Bn-chrA04-p15205214
Bn-A04-p14756394	0.247933884	0.991735537	Bn-N16-p29869414	Bn-chrA04-p15205607
Bn-A04-p14777980	0.975206612	0.983471074	Bn-N4-p16731176	Bn-chrA04-p15228895
Bn-A04-p14778146	0.991735537	0.983471074	Bn-N4-p16731342	Bn-chrA04-p15229061

Bn-A04-p14807002	0.913621262	0.993355482	Bn-N4-p16759991	Bn-chrA04-p15257549
Bn-A04-p14807150	0.955223881	0.965174129	Bn-N14-p50102197	Bn-chrA04-p15257697
Bn-A04-p14807561	0.975124378	0.975124378	Bn-N4-p16760524	Bn-chrA04-p15258108
Bn-A04-p14808819	0.966942149	0.966942149	Bn-N4-p16761776	Bn-chrA04-p15259363
Bn-A04-p14815251	0.983388704	0.990033223	Bn-N4-p16768147	Bn-chrA04-p15265728
Bn-A04-p14822521	0.92039801	0.965174129	Bn-N4-p16777601	Bn-chrA04-p15274859
Bn-A04-p14826595	0.991735537	0.975206612	Bn-N4-p16789347	Bn-chrA04-p15278962
Bn-A04-p14829686	0.955223881	0.950248756	Bn-N4-p16792438	Bn-chrA04-p15282049
Bn-A04-p14867427	0.960199005	0.940298507	Bn-N4-p16836321	Bn-chrA04-p15315368
Bn-A04-p14877869	0.991735537	0.991735537	Bn-N4-p16852137	Bn-chrA04-p15333203
Bn-A04-p14893870	0.950413223	0.983471074	Bn-N4-p16865247	Bn-chrA04-p15347511
Bn-A04-p14908542	0.985074627	0.985074627	Bn-N4-p16876933	Bn-chrA04-p15354909
Bn-A04-p14917114	0.991735537	0.983471074	Bn-N4-p16884111	Bn-chrA04-p15361966
Bn-A04-p14922562	0.965174129	0.970149254	Bn-N4-p16889546	Bn-chrA04-p15368678
Bn-A04-p14923168	0.975124378	0.985074627	Bn-N4-p16890152	Bn-chrA04-p15369284
Bn-A04-p14944336	0.966942149	0.966942149	Bn-N4-p16911000	Bn-chrA04-p15393238
Bn-A04-p14944421	0.976744186	0.976744186	Bn-N4-p16911085	Bn-chrA04-p15393323
Bn-A04-p14954537	0.827242525	0.548172757	Bn-N4-p16913255	Bn-chrC04-p43115777
Bn-A04-p14961814	0.983388704	0.983388704	Bn-N4-p16924672	Bn-chrA04-p15412721
Bn-A04-p14965926	0.966942149	0.966942149	Bn-N4-p16929784	Bn-chrA04-p15418128
Bn-A04-p14972825	0.950413223	0.94214876	Bn-N4-p16942573	Bn-chrA04-p15424817
Bn-A04-p14986920	0.975206612	0.975206612	Bn-N14-p50385352	Bn-chrC04-p44290271
Bn-A04-p14987934	0.950413223	0.950413223	Bn-N4-p16954493	Bn-chrA04-p15436750
Bn-A04-p14988304	0.983471074	0.983471074	Bn-N4-p16954861	Bn-chrA04-p15437118
Bn-A04-p14999714	0.966942149	0.966942149	Bn-N4-p16963218	Bn-chrA04-p15445316
Bn-A04-p14999768	0.636363636	0.636363636	Bn-N4-p16963272	Bn-chrA04-p15445370
Bn-A04-p15001710	0.996677741	0.996677741	Bn-N4-p16963400	Bn-chrA04-p15445498
Bn-A04-p15007146	0.983388704	0.983388704	Bn-N4-p16965424	Bn-chrA04-p15447522
Bn-A04-p15009639	0.966942149	0.966942149	Bn-N4-p16967420	Bn-chrA04-p15449482
Bn-A04-p15011193	0.966942149	0.966942149	Bn-N4-p16968929	Bn-chrA04-p15450991
Bn-A04-p15030355	0.995024876	0.970149254	Bn-N4-p16985302	Bn-chrC04-p44361030
Bn-A04-p15030899	0.980066445	0.970099668	Bn-N4-p16985846	Bn-chrA04-p15468054
Bn-A04-p15030963	0.983388704	0.966777409	Bn-N4-p16985910	Bn-chrA04-p15468118
Bn-A04-p15041769	0.973421927	0.973421927	Bn-Scaffold00208b-p	Bn-chrC04-p44365372
Bn-A04-p15048204	0.950248756	0.945273632	Bn-N4-p17002317	Bn-chrA04-p15484526
Bn-A04-p15066563	0.980099502	0.975124378	Bn-N4-p17012663	Bn-chrA04-p15494654
Bn-A04-p15070651	0.991735537	0.223140496	Bn-Scaffold50163-p7	Unknown
Bn-A04-p15108913	0.946843854	0.946843854	Bn-N4-p17051397	Bn-chrA04-p15545720
Bn-A04-p15122412	0.985074627	0.985074627	Bn-Scaffold00208b-p	Bn-chrA04-p15554769
Bn-A04-p15134578	0.644628099	0.652892562	Bn-Scaffold00208b-p	Bn-chrCnn_random-p775416
Bn-A04-p15150564	0.980099502	0.686567164	Bn-Scaffold00208b-p	Bn-chrC04-p7752968
Bn-A04-p15152016	0.983471074	0.983471074	Bn-N4-p17082281	Bn-chrA04-p15574929
Bn-A04-p15164920	0.983471074	0.958677686	Bn-N4-p17088020	Bn-chrA04-p15589708
Bn-A04-p15168889	0.980066445	0.976744186	Bn-Scaffold03862-p1	Bn-chrA04-p15593758
Bn-A04-p15184744	0.906976744	0.906976744	Bn-N4-p17114354	Bn-chrA04-p15611623
Bn-A04-p15190648	0.983471074	0.983471074	Bn-N4-p17130182	Bn-chrA04-p15625263

Bn-A04-p15195920	0.983471074	0.983471074	Bn-N4-p17137351	Bn-chrA04-p15629101
Bn-A04-p15207252	0.983471074	0.991735537	Bn-N4-p17152856	Bn-chrA04-p15651319
Bn-A04-p15244369	0.687707641	0.996677741	Bn-Scaffold85755-p7	Bn-chrA04-p15693454
Bn-A04-p15287200	0.950413223	0.94214876	Bn-N2-p8682855	Bn-chrA04-p15748927
Bn-A04-p15296217	0.996677741	0.993355482	Bn-N4-p17269452	Bn-chrA04-p15753637
Bn-A04-p15298740	0.989583333	0.989583333	Bn-N4-p17271998	Bn-chrA04-p15756179
Bn-A04-p15310116	0.980099502	0.980099502	Bn-Scaffold12290-p6	Bn-chrAnn_random-p418585
Bn-A04-p15312437	0.570247934	0.859504132	Bn-N4-p17253809	Bn-chrA04-p15774667
Bn-A04-p15315594	0.946843854	0.983388704	Bn-N4-p17297494	Bn-chrAnn_random-p211476
Bn-A04-p15341169	0.983471074	0.983471074	Bn-Scaffold03852-p1	Bn-chrAnn_random-p167133
Bn-A04-p15341244	0.975206612	0.975206612	Bn-Scaffold03852-p1	Bn-chrAnn_random-p167132
Bn-A04-p15367046	0.970099668	0.983388704	Bn-N4-p17334192	Bn-chrCnn_random-p538425
Bn-A04-p15377693	0.991735537	0.991735537	Bn-Scaffold02382-p2	Bn-chrAnn_random-p303422
Bn-A04-p15378781	0.966942149	0.966942149	Bn-Scaffold01556-p1	Bn-chrAnn_random-p166732
Bn-A04-p15399118	0.983471074	0.983471074	Bn-N4-p17361369	Bn-chrA04-p15791897
Bn-A04-p15404568	0.975206612	0.975206612	Bn-N4-p17369430	Bn-chrA04-p15797351
Bn-A04-p15409836	0.950413223	0.950413223	Bn-N4-p17378738	Bn-chrA04-p15808130
Bn-A04-p15411699	0.993355482	0.993355482	Bn-N4-p17380605	Bn-chrA04-p15809998
Bn-A04-p15413213	0.991735537	0.991735537	Bn-N4-p17382119	Bn-chrA04-p15811512
Bn-A04-p15414343	0.79338843	0.79338843	Bn-N4-p17383214	Bn-chrA04-p15812608
Bn-A04-p15420126	0.925373134	0.975124378	Bn-N4-p17387416	Bn-chrA04-p15817016
Bn-A04-p15429891	0.983388704	0.976744186	Bn-N4-p17395095	Bn-chrA04-p15824373
Bn-A04-p15436139	0.980099502	0.985074627	Bn-N4-p17402396	Bn-chrA04-p15830648
Bn-A04-p15441336	1	0.979166667	Bn-N4-p17407590	Bn-chrA04-p15852496
Bn-A04-p15456188	0.995024876	0.995024876	Bn-N14-p50600923	Bn-chrC04-p44583868
Bn-A04-p15466343	0.958677686	0.983471074	Bn-N4-p17437715	Bn-chrA04-p15881197
Bn-A04-p15471964	0.983471074	0.975206612	Bn-N4-p17447676	Bn-chrAnn_random-p478247
Bn-A04-p15475759	0.966942149	0.975206612	Bn-N4-p17450818	Bn-chrA04-p15889281
Bn-A04-p15485629	0.975206612	0.983471074	Bn-N4-p17459433	Bn-chrA04-p15906798
Bn-A04-p15492182	0.983471074	0.975206612	Bn-N4-p17465788	Bn-chrA04-p15913314
Bn-A04-p15498393	0.975206612	0.983471074	Bn-N4-p17476425	Bn-chrA04-p15920275
Bn-A04-p15500804	0.917355372	0.991735537	Bn-N4-p17478812	Bn-chrA04-p15922843
Bn-A04-p15514869	0.983471074	0.983471074	Bn-N4-p17499224	Bn-chrA04-p15938732
Bn-A04-p15519877	0.956810631	0.96345515	Bn-N4-p17504210	Bn-chrA04-p15943750
Bn-A04-p15530059	0.933884298	0.94214876	Bn-N4-p17513547	Bn-chrA04-p15952234
Bn-A04-p15530609	0.966942149	0.966942149	Bn-N4-p17514102	Bn-chrA04-p15952786
Bn-A04-p15530625	0.980099502	0.975124378	Bn-N4-p17514118	Bn-chrA04-p15952802
Bn-A04-p15534626	0.991735537	0.991735537	Bn-N4-p17518121	Bn-chrA04-p15957020
Bn-A04-p15534921	0.925619835	0.925619835	Bn-N4-p17518408	Bn-chrA04-p15957307
Bn-A04-p15535011	0.983471074	0.983471074	Bn-N4-p17518498	Bn-chrA04-p15957397
Bn-A04-p15555443	0.940298507	0.92039801	Bn-N4-p17547137	Bn-chrC04-p44730512
Bn-A04-p15556246	0.94214876	0.933884298	Bn-N4-p17547950	Bn-chrAnn_random-p442667
Bn-A04-p15559138	0.975206612	0.966942149	Bn-N4-p17550829	Bn-chrA04-p15989896
Bn-A04-p15597988	0.991735537	0.983471074	Bn-N4-p17587624	Bn-chrA04-p16043837
Bn-A04-p15600337	0.983471074	0.983471074	Bn-N4-p17593159	Bn-chrA04-p16046150
Bn-A04-p15617379	0.975206612	0.983471074	Bn-N4-p17609593	Bn-chrA04-p16061357

Bn-A04-p15619897	1	0.991735537	Bn-N4-p17613099	Bn-chrA04-p16063877
Bn-A04-p15630537	0.990049751	0.995024876	Bn-N4-p17616595	Bn-chrA04-p16069034
Bn-A04-p15631388	0.975206612	0.983471074	Bn-N4-p17617446	Bn-chrA04-p16069880
Bn-A04-p15643031	0.996677741	0.976744186	Bn-N4-p17628938	Bn-chrA04-p16084490
Bn-A04-p15655213	0.990033223	0.993355482	Bn-N4-p17648503	Bn-chrAnn_random-p743005
Bn-A04-p15658143	0.900497512	0.995024876	Bn-N4-p17651457	Bn-chrAnn_random-p742708
Bn-A04-p15661972	0.991735537	0.983471074	Bn-N4-p17655589	Bn-chrAnn_random-p742428
Bn-A04-p15662133	0.975206612	0.966942149	Bn-N4-p17655750	Bn-chrAnn_random-p742412
Bn-A04-p15670183	0.991735537	0.983471074	Bn-N4-p17667902	Bn-chrAnn_random-p741036
Bn-A04-p15670197	0.983471074	0.991735537	Bn-N4-p17667916	Bn-chrAnn_random-p741034
Bn-A04-p15701924	0.851239669	0.834710744	Bn-N14-p50948845	Bn-chrAnn_random-p737679
Bn-A04-p15707649	0.73553719	0.966942149	Bn-N4-p17703045	Bn-chrAnn_random-p737144
Bn-A04-p15710186	0.983471074	0.991735537	Bn-N4-p17705566	Bn-chrA03-p638518
Bn-A04-p15713429	0.991735537	0.983471074	Bn-N4-p17708896	Bn-chrAnn_random-p736372
Bn-A04-p15727549	1	0.991735537	Bn-N4-p17722846	Bn-chrAnn_random-p734789
Bn-A04-p15731996	0.983471074	0.975206612	Bn-N4-p17729799	Bn-chrUnn_random-p464593
Bn-A04-p15742373	0.993355482	0.996677741	Bn-N4-p17738871	Bn-chrAnn_random-p732702
Bn-A04-p15745275	0.975206612	0.975206612	Bn-N4-p17741874	Bn-chrAnn_random-p732402
Bn-A04-p15835310	0.991735537	0.991735537	Bn-Scaffold31010-p3	Bn-chrAnn_random-p721450
Bn-A04-p15840589	0.991735537	0.991735537	Bn-Scaffold00305b-p	Bn-chrAnn_random-p721046
Bn-A04-p15870508	0.991735537	0.991735537	Bn-Scaffold01341-p3	Bn-chrAnn_random-p235286
Bn-A04-p15877051	0.980099502	0.517412935	Bn-Scaffold00305b-p	Bn-chrAnn_random-p235350
Bn-A04-p15885427	0.976744186	0.993355482	Bn-Scaffold00305b-p	Bn-chrAnn_random-p235482
Bn-A04-p15893499	0.784053156	0.448504983	Bn-Scaffold00305b-p	Bn-chrA05-p4169279
Bn-A04-p15895339	0.132231405	0.991735537	Bn-N7-p15002311	Bn-chrA04-p16092342
Bn-A04-p15895510	0.365448505	0.996677741	Bn-Scaffold00305b-p	Bn-chrA04-p16092513
Bn-A04-p15895735	0.094527363	0.965174129	Unknown	Bn-chrA04-p16092738
Bn-A04-p15906747	0.983471074	0.991735537	Bn-Scaffold06701-p9	Bn-chrA04-p16102942
Bn-A04-p15933265	0.985074627	0.194029851	Bn-Scaffold00305b-p	Unknown
Bn-A04-p15939956	0.983388704	0.857142857	Bn-Scaffold00305b-p	Bn-chrAnn_random-p380370
Bn-A04-p15942668	1	1	Bn-Scaffold00305b-p	Bn-chrAnn_random-p278086
Bn-A04-p15951138	0.903654485	0.993355482	Bn-N5-p4477991	Bn-chrCnn_random-p544257
Bn-A04-p15953263	0.983471074	0.148760331	Bn-N4-p17809614	Bn-chrA01-p883599
Bn-A04-p15970355	1	1	Bn-N4-p17899682	Bn-chrA04-p16138399
Bn-A04-p16000929	0.985074627	0.985074627	Bn-N4-p17918083	Bn-chrC09-p3974867
Bn-A04-p16014198	0.990033223	0.990033223	Bn-N4-p17929694	Bn-chrA04-p16166294
Bn-A04-p16022528	0.975206612	0.785123967	Bn-N4-p17939671	Bn-chrA04-p16180581
Bn-A04-p16025591	0.970149254	0.935323383	Bn-N4-p17942713	Bn-chrA04-p16183667
Bn-A04-p16056186	0.970099668	0.970099668	Bn-N4-p17983696	Bn-chrA04_random-p124458
Bn-A04-p16074808	0.991735537	0.991735537	Bn-N4-p18005332	Bn-chrAnn_random-p371770
Bn-A04-p16090875	0.983471074	0.983471074	Bn-N4-p18045765	Bn-chrA04-p16283277
Bn-A04-p16104213	0.975206612	0.966942149	Bn-N4-p18058956	Bn-chrA04-p16299500
Bn-A04-p16104616	0.975206612	0.975206612	Bn-N4-p18059359	Bn-chrA04-p16299897
Bn-A04-p16107844	0.877952756	0.877952756	Bn-N14-p51103546	Bn-chrC04-p44900016
Bn-A04-p16109558	0.970149254	0.970149254	Bn-N14-p51105249	Bn-chrC04-p44901718
Bn-A04-p16119082	0.985074627	0.985074627	Bn-N14-p51119553	Bn-chrC04-p44916145

Bn-A04-p16120092	0.980099502	0.980099502	Bn-N14-p51120555	Bn-chrC04-p44917147
Bn-A04-p16123695	0.975206612	0.966942149	Bn-N4-p18078529	Bn-chrA04-p16318675
Bn-A04-p16131108	1	0.991735537	Bn-N4-p18090141	Bn-chrA04-p16326677
Bn-A04-p16137916	0.983471074	0.975206612	Bn-N4-p18096987	Bn-chrA04-p16333557
Bn-A04-p16143324	0.990033223	0.990033223	Bn-N4-p18101859	Bn-chrA04-p16344924
Bn-A04-p16155604	0.975206612	0.983471074	Bn-N4-p18112774	Bn-chrA04-p16355822
Bn-A04-p16156157	0.983471074	0.983471074	Bn-N4-p18113327	Bn-chrA04-p16356433
Bn-A04-p16157904	0.990033223	0.993355482	Bn-N4-p18116654	Bn-chrA04-p16359924
Bn-A04-p16166839	0.909090909	0.909090909	Bn-N4-p18132806	Bn-chrA04-p16368214
Bn-A04-p16224941	0.993355482	0.993355482	Bn-N4-p18190479	Bn-chrA04_random-p114791
Bn-A04-p16233024	0.986710963	0.986710963	Bn-N4-p18198567	Bn-chrA04_random-p113983
Bn-A04-p16233377	0.990033223	0.990033223	Bn-N4-p18198920	Bn-chrA04_random-p113947
Bn-A04-p16237245	0.940298507	0.940298507	Bn-N4-p18202785	Bn-chrA04_random-p113570
Bn-A04-p16259439	1	0.991735537	Bn-N4-p18232627	Bn-chrA04_random-p118889
Bn-A04-p16264494	0.966942149	0.958677686	Bn-N4-p18235505	Bn-chrA04_random-p119172
Bn-A04-p16268792	0.975206612	0.983471074	Bn-N4-p18239986	Bn-chrA04_random-p119678
Bn-A04-p16275472	0.991735537	1	Bn-N4-p18246343	Bn-chrA04_random-p120340
Bn-A04-p16275979	0.991735537	1	Bn-N4-p18246849	Bn-chrA04_random-p120391
Bn-A04-p16276156	0.991735537	1	Bn-N4-p18247026	Bn-chrA04_random-p120409
Bn-A04-p16276464	1	0.991735537	Bn-N4-p18247334	Bn-chrA04_random-p120439
Bn-A04-p16277340	0.893687708	0.897009967	Bn-N4-p18248210	Bn-chrA04_random-p120528
Bn-A04-p16280279	0.991735537	1	Bn-N4-p18251300	Bn-chrA04_random-p121217
Bn-A04-p16282039	0.991735537	1	Bn-N4-p18253060	Bn-chrA04_random-p121394
Bn-A04-p16283972	0.991735537	1	Bn-N4-p18254993	Bn-chrA04_random-p121563
Bn-A04-p16287850	0.991735537	1	Bn-N4-p18258871	Bn-chrA04_random-p121951
Bn-A04-p16288681	1	0.314049587	Bn-N4-p18259831	Bn-chrC01-p32672928
Bn-A04-p16303678	0.983388704	0.990033223	Bn-N4-p18275338	Bn-chrA04-p16484408
Bn-A04-p16303728	0.975206612	0.983471074	Bn-N4-p18275388	Bn-chrA04-p16484458
Bn-A04-p16313477	0.975206612	0.975206612	Bn-N4-p18286010	Bn-chrA04-p16495267
Bn-A04-p16320671	0.825870647	0.825870647	Bn-N14-p51382876	Bn-chrC04-p45199496
Bn-A04-p16336301	0.983471074	0.983471074	Bn-N4-p18308270	Bn-chrA04-p16518830
Bn-A04-p16337105	0.966942149	0.966942149	Bn-N4-p18308983	Bn-chrA04-p16519542
Bn-A04-p16365226	0.966777409	0.983388704	Bn-N4-p18338936	Bn-chrA04-p16540801
Bn-A04-p16368407	0.975206612	0.966942149	Bn-N4-p18342818	Bn-chrA04-p16545883
Bn-A04-p16368852	0.983471074	0.991735537	Bn-N4-p18343289	Bn-chrA04-p16546328
Bn-A04-p16382491	0.991735537	0.958677686	Bn-N4-p18356446	Bn-chrA04-p16563269
Bn-A04-p16394755	0.991735537	1	Bn-N4-p18368736	Bn-chrAnn_random-p358209
Bn-A04-p16403131	0.996677741	1	Bn-N4-p18382750	Bn-chrA04-p16580390
Bn-A04-p16405335	1	1	Bn-N4-p18384300	Bn-chrA04-p16581942
Bn-A04-p16416104	0.993355482	0.993355482	Bn-N4-p18394427	Bn-chrA04-p16594832
Bn-A04-p16427350	0.991735537	0.983471074	Bn-N4-p18408150	Bn-chrA04-p16606997
Bn-A04-p16431550	0.966942149	0.966942149	Bn-N4-p18412147	Bn-chrA04-p16610941
Bn-A04-p16445225	0.983471074	0.983471074	Bn-N4-p18420558	Bn-chrA04-p16618792
Bn-A04-p16445602	0.991735537	0.991735537	Bn-N4-p18420961	Bn-chrA04-p16619169
Bn-A04-p16445675	0.991735537	1	Bn-N4-p18421037	Bn-chrA04-p16619242
Bn-A04-p16446976	0.991735537	0.991735537	Bn-N4-p18422343	Bn-chrA04-p16620543

Bn-A04-p16447217	0.991735537	0.991735537	Bn-N4-p18422584	Bn-chrA04-p16620784
Bn-A04-p16451992	0.983388704	0.986710963	Bn-N4-p18427361	Bn-chrA04-p16625563
Bn-A04-p16452098	0.991735537	0.991735537	Bn-N4-p18427467	Bn-chrA04-p16625669
Bn-A04-p16459969	0.925619835	0.933884298	Bn-N4-p18435319	Bn-chrC04_random-p426359
Bn-A04-p16477853	1	0.979166667	Bn-N4-p18442566	Bn-chrA04-p16647831
Bn-A04-p16479236	0.996677741	0.996677741	Bn-N4-p18443986	Bn-chrA04-p16649437
Bn-A04-p16486340	0.950166113	0.996677741	Bn-N4-p18447576	Bn-chrA04-p16652922
Bn-A04-p16503326	0.983388704	0.996677741	Bn-N4-p18460684	Bn-chrA04-p16668129
Bn-A04-p16506148	0.975206612	0.991735537	Bn-N4-p18463613	Bn-chrA04-p16671009
Bn-A04-p16508914	0.975124378	0.980099502	Bn-N4-p18466378	Bn-chrC04_random-p423090
Bn-A04-p16514488	0.966942149	0.966942149	Bn-N4-p18470743	Bn-chrA04-p16678139
Bn-A04-p16517396	0.925619835	0.925619835	Bn-N4-p18473053	Bn-chrA04-p16680447
Bn-A04-p16523010	0.991735537	0.991735537	Bn-N4-p18477898	Bn-chrA04-p16684039
Bn-A04-p16523116	0.973421927	0.980066445	Bn-N4-p18478004	Bn-chrA04-p16684145
Bn-A04-p16528010	0.900826446	0.958677686	Bn-N4-p18485408	Bn-chrA04-p16689082
Bn-A04-p16528107	0.917355372	0.966942149	Bn-N4-p18485498	Bn-chrA04-p16689179
Bn-A04-p16540340	0.983388704	0.983388704	Bn-N4-p18493849	Bn-chrA04-p16700726
Bn-A04-p16545584	0.983471074	0.983471074	Bn-N4-p18497475	Bn-chrA04-p16704355
Bn-A04-p16552002	1	1	Bn-N4-p18504562	Bn-chrA04-p16710235
Bn-A04-p16553214	0.991735537	0.991735537	Bn-N4-p18505413	Bn-chrA04-p16711259
Bn-A04-p16553276	0.991735537	0.983471074	Bn-N4-p18505475	Bn-chrA04-p16711321
Bn-A04-p16555982	0.980099502	0.990049751	Bn-N4-p18508172	Bn-chrA04-p16714029
Bn-A04-p16556868	0.991735537	0.991735537	Bn-N4-p18509099	Bn-chrA04-p16714915
Bn-A04-p16558888	0.996677741	0.953488372	Bn-N4-p18511089	Bn-chrA04-p16716889
Bn-A04-p16563543	0.996677741	0.993355482	Bn-N4-p18512244	Bn-chrA04-p16718051
Bn-A04-p16565696	0.909090909	0.909090909	Bn-N4-p18514298	Bn-chrA04-p16721692
Bn-A04-p16579551	0.991735537	0.983471074	Bn-N4-p18526375	Bn-chrA04-p16733534
Bn-A04-p16584084	0.940199336	0.940199336	Bn-N14-p51708235	Bn-chrC04-p45498974
Bn-A04-p16595064	0.991735537	0.950413223	Bn-N4-p18534997	Bn-chrA04-p16751565
Bn-A04-p16598514	0.983388704	0.986710963	Bn-N4-p18538501	Bn-chrA04-p16755284
Bn-A04-p16605277	0.985074627	0.990049751	Bn-N4-p18544659	Bn-chrA04-p16762101
Bn-A04-p16607433	0.975206612	0.966942149	Bn-N4-p18547436	Bn-chrA04-p16764252
Bn-A04-p16608584	0.975206612	0.983471074	Bn-N4-p18548589	Bn-chrA04-p16765402
Bn-A04-p16621448	0.986710963	0.976744186	Bn-N4-p18562429	Bn-chrA04-p16771661
Bn-A04-p16627363	1	0.966942149	Bn-N4-p18566978	Bn-chrC04-p45535992
Bn-A04-p16651306	0.995024876	0.995024876	Bn-N4-p18603492	Bn-chrA04-p16823695
Bn-A04-p16663552	0.975206612	0.975206612	Bn-N4-p18620726	Bn-chrA04-p16843246
Bn-A04-p16677612	0.996677741	0.996677741	Bn-N4-p18639670	Bn-chrA04-p16856832
Bn-A04-p16678843	0.996677741	0.996677741	Bn-N4-p18640901	Bn-chrA04-p16858063
Bn-A04-p16684288	1	1	Bn-N4-p18646102	Bn-chrA04-p16863198
Bn-A04-p16711900	0.980066445	0.980066445	Bn-N4-p18671463	Bn-chrA04-p16888721
Bn-A04-p16719721	0.993355482	0.993355482	Bn-N4-p18678685	Bn-chrA04-p16895943
Bn-A04-p16719955	0.996677741	0.996677741	Bn-N4-p18678919	Bn-chrA04-p16896177
Bn-A04-p16743976	0.991735537	0.991735537	Bn-N4-p19028528	Bn-chrA04-p17241586
Bn-A04-p16744035	0.991735537	0.991735537	Bn-N4-p19028469	Bn-chrA04-p17241527
Bn-A04-p16745356	1	1	Bn-N4-p19027130	Bn-chrA04-p17240188

Bn-A04-p16748162	0.991735537	0.991735537	Bn-N4-p19024648	Bn-chrA04-p17237706
Bn-A04-p16751677	0.991735537	0.991735537	Bn-N4-p19021064	Bn-chrA04-p17230969
Bn-A04-p16791082	0.956810631	0.956810631	Bn-N4-p18980996	Bn-chrA04-p17193728
Bn-A04-p16798852	0.975124378	0.975124378	Bn-N4-p18973231	Bn-chrA04-p17185964
Bn-A04-p16805391	0.993355482	0.993355482	Bn-N4-p18969850	Bn-chrA04-p17182583
Bn-A04-p16819445	0.995024876	0.995024876	Bn-N4-p18957489	Bn-chrA04-p17170308
Bn-A04-p16821442	0.980099502	0.980099502	Bn-N4-p18955629	Bn-chrA04-p17168448
Bn-A04-p16830389	0.975124378	0.975124378	Bn-N4-p18946420	Bn-chrA04-p17159240
Bn-A04-p16832545	0.986710963	0.986710963	Bn-N4-p18943797	Bn-chrA04-p17156618
Bn-A04-p16841462	0.985074627	0.985074627	Bn-N4-p18935470	Bn-chrA04-p17148291
Bn-A04-p16892915	1	1	Bn-N4-p18888967	Bn-chrA04-p17100330
Bn-A04-p16919648	0.990049751	0.990049751	Bn-N4-p18859650	Bn-chrA04-p17069478
Bn-A04-p16919672	0.990049751	0.990049751	Bn-N4-p18859626	Bn-chrA04-p17069454
Bn-A04-p16946306	0.973421927	0.96345515	Bn-N14-p52115668	Bn-chrC04-p45917366
Bn-A04-p16980928	0.966777409	0.966777409	Bn-N4-p18808278	Bn-chrA04-p17024681
Bn-A04-p16998111	0.548172757	0.548172757	Bn-N4-p18791320	Bn-chrA04-p17007520
Bn-A04-p17003395	0.993355482	0.993355482	Bn-N4-p18788355	Bn-chrA04-p17004555
Bn-A04-p17019529	0.986710963	0.986710963	Bn-N4-p18775521	Bn-chrA04-p16992222
Bn-A04-p17044228	0.786069652	0.786069652	Bn-N4-p18752848	Bn-chrA04-p16968406
Bn-A04-p17045348	0.96013289	0.96013289	Bn-N4-p18751707	Bn-chrA04-p16967265
Bn-A04-p17046250	0.990033223	0.990033223	Bn-N4-p18749074	Bn-chrA04-p16964819
Bn-A04-p17047140	0.990033223	0.990033223	Bn-N4-p18748160	Bn-chrA04-p16963905
Bn-A04-p17052071	0.990033223	0.990033223	Bn-N4-p18743497	Bn-chrA04-p16959242
Bn-A04-p17058367	0.990033223	0.990033223	Bn-N4-p18737197	Bn-chrA04-p16953157
Bn-A04-p17105440	0.985074627	0.990049751	Bn-N4-p18698782	Bn-chrA04-p16916617
Bn-A04-p17106064	0.993355482	0.993355482	Bn-N4-p18698148	Bn-chrA04-p16915983
Bn-A04-p17107045	0.975124378	0.975124378	Bn-N4-p18697146	Bn-chrA04-p16914981
Bn-A04-p17125513	0.989583333	0.989583333	Bn-N4-p19050827	Bn-chrA04-p17263827
Bn-A04-p17142570	0.970149254	0.970149254	Bn-N4-p19070786	Bn-chrA04_random-p130063
Bn-A04-p17155901	0.983471074	0.983471074	Bn-N4-p19083440	Bn-chrA04_random-p131328
Bn-A04-p17157304	0.990033223	0.990033223	Bn-N4-p19084843	Bn-chrA04_random-p131469
Bn-A04-p17170974	0.995024876	0.995024876	Bn-N4-p19106540	Bn-chrA04_random-p133550
Bn-A04-p17171349	0.991735537	0.991735537	Bn-N4-p19106930	Bn-chrA04_random-p133589
Bn-A04-p17172246	0.966942149	0.966942149	Bn-N4-p19107894	Bn-chrA04_random-p133685
Bn-A04-p17174846	0.993355482	0.993355482	Bn-N4-p19110260	Bn-chrA04_random-p133922
Bn-A04-p17179870	0.986710963	0.986710963	Bn-N4-p19115278	Bn-chrA04_random-p134424
Bn-A04-p17212731	0.993355482	0.993355482	Bn-N4-p19152134	Bn-chrA04-p17334126
Bn-A04-p17217840	0.897959184	0.897959184	Bn-N4-p19156987	Bn-chrA04-p17338628
Bn-A04-p17219361	0.975206612	0.975206612	Bn-N4-p19160623	Bn-chrA04-p17340116
Bn-A04-p17219743	0.991735537	0.991735537	Bn-N4-p19161005	Bn-chrA04-p17340498
Bn-A04-p17220068	0.917355372	0.917355372	Bn-N4-p19161330	Bn-chrA04-p17340823
Bn-A04-p17221332	0.950413223	0.950413223	Bn-N4-p19162594	Bn-chrA04-p17342087
Bn-A04-p17223885	0.983471074	0.983471074	Bn-N4-p19165147	Bn-chrA04-p17344640
Bn-A04-p17231009	0.975206612	0.975206612	Bn-N4-p19172243	Bn-chrA04-p17351736
Bn-A04-p17238605	0.958677686	0.958677686	Bn-N4-p19179838	Bn-chrA04-p17359331
Bn-A04-p17239071	0.991735537	0.991735537	Bn-N4-p19180304	Bn-chrA04-p17359797

Bn-A04-p17239509	0.966942149	0.966942149	Bn-N4-p19180742	Bn-chrA04-p17360235
Bn-A04-p17273076	0.996677741	0.996677741	Bn-N4-p19212267	Bn-chrA04-p17389139
Bn-A04-p17277010	0.975124378	0.975124378	Bn-N14-p52493332	Bn-chrC04-p46328768
Bn-A04-p17278229	0.986710963	0.986710963	Bn-N4-p19217525	Bn-chrA04-p17394397
Bn-A04-p17281577	0.975124378	0.970149254	Bn-N4-p19221228	Bn-chrA04-p17398099
Bn-A04-p17295665	0.991735537	0.892561983	Bn-N4-p19237849	Bn-chrC04-p46347810
Bn-A04-p17300068	0.985074627	0.970149254	Bn-N4-p19242580	Bn-chrA04-p17424310
Bn-A04-p17312041	0.983388704	0.983388704	Bn-N4-p19254592	Bn-chrA04-p17437078
Bn-A04-p17320026	0.991735537	0.991735537	Bn-N4-p19258714	Bn-chrA04-p17441158
Bn-A04-p17322241	0.990049751	0.995024876	Bn-N4-p19262174	Bn-chrA04-p17443692
Bn-A04-p17331915	0.993355482	0.993355482	Bn-N4-p19271681	Bn-chrA04-p17453333
Bn-A04-p17358519	0.991735537	0.966942149	Bn-N4-p19307982	Bn-chrC04-p46402392
Bn-A04-p17372718	0.993355482	0.990033223	Bn-N4-p19322488	Bn-chrA04-p17480240
Bn-A04-p17383739	0.950413223	0.966942149	Bn-N4-p19332706	Bn-chrC04-p46445133
Bn-A04-p17391232	0.975206612	0.975206612	Bn-N4-p19341607	Bn-chrAnn_random-p417863
Bn-A04-p17391802	0.990033223	0.983388704	Bn-N4-p19342177	Bn-chrAnn_random-p417868
Bn-A04-p17394552	0.970149254	0.965174129	Bn-N4-p19346776	Bn-chrA04-p17492227
Bn-A04-p17394736	0.990049751	0.995024876	Bn-N4-p19346960	Bn-chrA04-p17492411
Bn-A04-p17401632	0.966942149	0.958677686	Bn-N4-p19353857	Bn-chrA04-p17499319
Bn-A04-p17412823	0.945273632	0.990049751	Bn-N4-p19364932	Bn-chrA04-p17510910
Bn-A04-p17416243	0.980099502	0.970149254	Bn-N4-p19368191	Bn-chrA04-p17512833
Bn-A04-p17417142	0.995024876	0.980099502	Bn-N4-p19369086	Bn-chrA04-p17513732
Bn-A04-p17422800	0.975206612	0.966942149	Bn-N4-p19374651	Bn-chrA04-p17519980
Bn-A04-p17425190	0.983471074	0.975206612	Bn-N4-p19377040	Bn-chrC04_random-p437180
Bn-A04-p17439839	0.966942149	0.975206612	Bn-N4-p19388754	Bn-chrA04-p17534681
Bn-A04-p17475728	0.996677741	0.996677741	Bn-N4-p19429574	Bn-chrA04-p17567821
Bn-A04-p17487757	0.993355482	0.996677741	Bn-N4-p19437505	Bn-chrA04-p17575752
Bn-A04-p17489063	0.933884298	0.950413223	Bn-N4-p19438815	Bn-chrA04-p17577055
Bn-A04-p17490425	0.958677686	0.958677686	Bn-N4-p19440153	Bn-chrA04-p17578417
Bn-A04-p17496899	0.989583333	0.989583333	Bn-N4-p19446545	Bn-chrA04-p17584809
Bn-A04-p17530621	0.955223881	0.955223881	Bn-N4-p19479475	Bn-chrA04-p17632597
Bn-A04-p17535104	0.975206612	0.975206612	Bn-N4-p19490724	Bn-chrA04-p17642161
Bn-A04-p17541500	0.958677686	0.958677686	Bn-N4-p19497129	Bn-chrA04-p17648565
Bn-A04-p17541623	0.996677741	0.996677741	Bn-N4-p19497252	Bn-chrA04-p17648688
Bn-A04-p17550209	0.925619835	0.925619835	Bn-Scaffold51770-p1	Bn-chrA04-p17658173
Bn-A04-p17574162	0.966942149	0.966942149	Bn-N4-p19542103	Bn-chrA04-p17686221
Bn-A04-p17576444	0.975124378	0.975124378	Bn-N4-p19544316	Bn-chrA04-p17688434
Bn-A04-p17577700	0.980066445	0.980066445	Bn-N4-p19545572	Bn-chrA04-p17689690
Bn-A04-p17578444	0.983471074	0.73553719	Bn-N4-p19546312	Bn-chrA04-p17690434
Bn-A04-p17580804	0.917355372	0.950413223	Bn-N4-p19548845	Bn-chrA04-p17692401
Bn-A04-p17613869	0.975206612	0.983471074	Bn-N4-p19579676	Bn-chrA04-p17725003
Bn-A04-p17614504	0.990049751	0.985074627	Bn-N4-p19580311	Bn-chrA04-p17725638
Bn-A04-p17615088	0.980099502	0.980099502	Bn-N4-p19580895	Bn-chrA04-p17726222
Bn-A04-p17623914	0.986710963	0.986710963	Bn-N4-p19587713	Bn-chrA04-p17733411
Bn-A04-p17624469	0.985074627	0.985074627	Bn-N4-p19588268	Bn-chrA04-p17733966
Bn-A04-p17624526	0.990049751	0.990049751	Bn-N4-p19588325	Bn-chrA04-p17734023

Bn-A04-p17632009	0.955223881	0.955223881	Bn-N4-p19595954	Bn-chrA04-p17741611
Bn-A04-p17634469	0.970099668	0.970099668	Bn-N4-p19598402	Bn-chrA04-p17744092
Bn-A04-p17658206	0.59375	1	Bn-N14-p53017807	Bn-chrA04-p17773140
Bn-A04-p17685210	0.975206612	0.966942149	Bn-N4-p19648488	Bn-chrA04-p17793931
Bn-A04-p17686228	0.975206612	0.983471074	Bn-N4-p19649505	Bn-chrA04-p17794982
Bn-A04-p17689002	0.980099502	0.985074627	Bn-N4-p19652081	Bn-chrA04-p17797759
Bn-A04-p17689324	0.995024876	0.995024876	Bn-N4-p19652403	Bn-chrA04-p17798081
Bn-A04-p17692138	1	0.996677741	Bn-N4-p19655215	Bn-chrA04-p17801901
Bn-A04-p17698780	0.991735537	0.991735537	Bn-N4-p19662384	Bn-chrA04-p17808940
Bn-A04-p17704458	0.983471074	0.983471074	Bn-N4-p19668024	Bn-chrA04-p17814614
Bn-A04-p17706333	0.950413223	0.950413223	Bn-N4-p19669966	Bn-chrA04-p17816556
Bn-A04-p17712184	0.958677686	0.958677686	Bn-N4-p19675646	Bn-chrA04-p17822235
Bn-A04-p17736387	0.990033223	0.993355482	Bn-N4-p19701055	Bn-chrA04-p17846201
Bn-A04-p17736770	0.966942149	0.975206612	Bn-N4-p19701438	Bn-chrA04-p17846584
Bn-A04-p17738097	1	1	Bn-N4-p19702768	Bn-chrA04-p17847919
Bn-A04-p17740663	0.900497512	0.975124378	Bn-N4-p19705522	Bn-chrA04-p17850498
Bn-A04-p17742799	0.958677686	0.966942149	Bn-N4-p19707735	Bn-chrA04-p17852708
Bn-A04-p17756059	0.990049751	0.990049751	Bn-N4-p19720981	Bn-chrA04-p17865768
Bn-A04-p17760280	0.990049751	0.980099502	Bn-N4-p19725124	Bn-chrA04-p17869910
Bn-A04-p17760869	0.996677741	1	Bn-N4-p19725713	Bn-chrA04-p17870499
Bn-A04-p17766173	0.983471074	0.975206612	Bn-N4-p19730522	Bn-chrC04-p46951053
Bn-A04-p17768404	0.983388704	0.980066445	Bn-N4-p19732754	Bn-chrA04-p17877587
Bn-A04-p17770281	0.983388704	0.980066445	Bn-N4-p19734627	Bn-chrA04-p17879459
Bn-A04-p17790228	0.950413223	0.958677686	Bn-N4-p19753540	Bn-chrA04-p17895980
Bn-A04-p17811740	0.880597015	0.995024876	Bn-N4-p19775221	Bn-chrA04-p17917725
Bn-A04-p17814849	0.983471074	0.983471074	Bn-N4-p19778379	Bn-chrA04-p17921052
Bn-A04-p17816796	0.933884298	0.933884298	Bn-N4-p19780403	Bn-chrA04-p17923085
Bn-A04-p17816969	0.820598007	0.820598007	Bn-N4-p19780571	Bn-chrA04-p17923253
Bn-A04-p17820683	0.995024876	0.995024876	Bn-N4-p19784349	Bn-chrA04-p17926940
Bn-A04-p17832455	0.990049751	0.990049751	Bn-N4-p19794435	Bn-chrA04-p17936912
Bn-A04-p17845366	0.995024876	0.995024876	Bn-N4-p19806470	Bn-chrA04-p17948898
Bn-A04-p17858149	0.975206612	0.975206612	Bn-N4-p19821474	Bn-chrA04-p17966721
Bn-A04-p17864572	0.975206612	0.983471074	Bn-N4-p19827880	Bn-chrA04-p17973221
Bn-A04-p17864879	0.983471074	0.983471074	Bn-N4-p19828476	Bn-chrA04-p17973834
Bn-A04-p17870056	0.975206612	0.950413223	Bn-N4-p19832166	Bn-chrA04-p17977446
Bn-A04-p17871505	0.950166113	0.990033223	Bn-N4-p19834650	Bn-chrA04-p17978824
Bn-A04-p17885659	0.996677741	0.996677741	Bn-N4-p19849172	Bn-chrA04-p17995307
Bn-A04-p17896333	0.873754153	0.986710963	Bn-N14-p53373300	Bn-chrC06-p12583315
Bn-A04-p17910578	0.973421927	0.986710963	Bn-N4-p19885124	Bn-chrA04-p18011681
Bn-A04-p17934648	0.985074627	0.985074627	Bn-N4-p19909024	Bn-chrA04-p18036910
Bn-A04-p17940697	0.983471074	0.983471074	Bn-N4-p19914663	Bn-chrA04-p18042553
Bn-A04-p17940767	0.492537313	0.492537313	Unknown	Unknown
Bn-A04-p17968847	1	1	Bn-N4-p19931341	Bn-chrA04-p18048099
Bn-A04-p17969838	0.983471074	0.983471074	Bn-N4-p19932331	Bn-chrA04-p18049089
Bn-A04-p17976746	0.993355482	0.996677741	Bn-N4-p19941543	Bn-chrA04-p18056886
Bn-A04-p18003170	0.983471074	0.983471074	Bn-N4-p19970316	Bn-chrA04-p18079803

Bn-A04-p18003813	0.995024876	0.995024876	Bn-N4-p19970959	Bn-chrA04-p18080446
Bn-A04-p18007716	0.983471074	0.983471074	Bn-N4-p19975008	Bn-chrA04-p18086577
Bn-A04-p18009131	0.983471074	0.983471074	Bn-N4-p19976423	Bn-chrA04-p18087992
Bn-A04-p18012102	0.983471074	0.983471074	Bn-N4-p19979393	Bn-chrA04-p18090962
Bn-A04-p18013923	0.975206612	0.975206612	Bn-N4-p19981213	Bn-chrA04-p18092783
Bn-A04-p18015189	0.991735537	0.991735537	Bn-N4-p19982479	Bn-chrA04-p18094049
Bn-A04-p18027570	0.991735537	0.975206612	Bn-N4-p19995455	Bn-chrA04-p18105473
Bn-A04-p18031439	0.975124378	0.975124378	Bn-N4-p20005862	Bn-chrA04-p18111930
Bn-A04-p18038306	1	1	Bn-N4-p20012824	Bn-chrA04-p18118804
Bn-A04-p18047327	1	0.611570248	Bn-N4-p20021655	Bn-chrC04-p2010930
Bn-A04-p18047606	0.996677741	0.488372093	Bn-N4-p20021934	Bn-chrA04-p18121114
Bn-A04-p18055770	0.996677741	1	Bn-N4-p20029924	Bn-chrA04-p18136835
Bn-A04-p18062365	0.92358804	0.926910299	Bn-N4-p20036288	Bn-chrA04-p18143560
Bn-A04-p18065518	0.950413223	0.958677686	Bn-N4-p20040802	Bn-chrA04-p18146749
Bn-A04-p18065896	0.975206612	0.933884298	Bn-N4-p20041181	Bn-chrA04-p18147127
Bn-A04-p18071582	0.983471074	0.603305785	Bn-N4-p20047890	Bn-chrA04-p18153890
Bn-A04-p18071791	0.958677686	0.966942149	Bn-N4-p20048099	Bn-chrA04-p18154098
Bn-A04-p18075680	0.958677686	0.950413223	Bn-N4-p20051903	Bn-chrA04-p18157869
Bn-A04-p18081163	0.917355372	0.917355372	Bn-N4-p20056989	Bn-chrA04-p18164000
Bn-A04-p18083568	0.985074627	0.985074627	Bn-N4-p20063171	Bn-chrA04-p18170543
Bn-A04-p18085734	0.958677686	0.958677686	Bn-N4-p20065336	Bn-chrA04-p18172712
Bn-A04-p18086224	0.950413223	0.950413223	Bn-N4-p20065825	Bn-chrA04-p18173201
Bn-A04-p18087342	0.983471074	0.983471074	Bn-N4-p20066943	Bn-chrA04-p18174319
Bn-A04-p18087738	0.983471074	0.983471074	Bn-N4-p20067340	Bn-chrA04-p18174717
Bn-A04-p18089854	0.991735537	0.983471074	Bn-N4-p20069478	Bn-chrA04-p18176833
Bn-A04-p18090397	0.991735537	1	Bn-N4-p20070015	Bn-chrA04-p18177376
Bn-A04-p18097103	0.990033223	0.900332226	Bn-N4-p20080551	Bn-chrA04-p18184060
Bn-A04-p18097421	0.895522388	1	Bn-N4-p20080869	Bn-chrC04-p47322719
Bn-A04-p18112711	0.983471074	0.975206612	Bn-N4-p20095247	Bn-chrA04-p18198460
Bn-A04-p18124053	0.926910299	0.92358804	Bn-N14-p53653942	Bn-chrC04-p47369621
Bn-A04-p18125528	0.983388704	0.986710963	Bn-N4-p20114547	Bn-chrA04-p18210964
Bn-A04-p18125679	0.991735537	0.983471074	Bn-N4-p20114698	Bn-chrA04-p18211115
Bn-A04-p18129552	0.983471074	0.991735537	Bn-N4-p20119480	Bn-chrA04-p18214890
Bn-A04-p18136301	0.985074627	0.975124378	Bn-N4-p20125262	Bn-chrA04-p18219328
Bn-A04-p18152854	0.990033223	0.983388704	Bn-N4-p20141390	Bn-chrA04-p18236815
Bn-A04-p18156458	0.990033223	0.990033223	Bn-N4-p20144992	Bn-chrA04-p18240401
Bn-A04-p18167535	0.975206612	0.975206612	Bn-N4-p20156162	Bn-chrA04-p18251467
Bn-A04-p18167614	0.966942149	0.966942149	Bn-N4-p20156241	Bn-chrA04-p18251546
Bn-A04-p18168733	0.900332226	0.990033223	Bn-N14-p53703397	Bn-chrA04-p18252630
Bn-A04-p18178609	0.993355482	0.990033223	Bn-N4-p20166867	Bn-chrA04-p18262474
Bn-A04-p18180777	0.996677741	1	Bn-N4-p20169038	Bn-chrA04-p18264642
Bn-A04-p18252317	0.966942149	0.975206612	Bn-N4-p20232640	Bn-chrA04-p18326726
Bn-A04-p18256793	0.776119403	0.701492537	Bn-N19-p37320797	Bn-chrC02-p5605293
Bn-A04-p18273048	0.975206612	0.983471074	Bn-N4-p20260734	Bn-chrA04-p18342595
Bn-A04-p18354325	1	1	Bn-N4-p20339256	Bn-chrA04-p18424204
Bn-A04-p18358463	0.996677741	0.990033223	Bn-N3-p26291846	Bn-chrA08-p15589932

Bn-A04-p18377757	0.950248756	0.995024876	Bn-N4-p20357803	Bn-chrA04-p18444869
Bn-A04-p18390795	0.950413223	0.975206612	Bn-N4-p20372271	Bn-chrA07-p21467328
Bn-A04-p18409410	0.830564784	0.830564784	Bn-N4-p20394704	Bn-chrA04-p18481814
Bn-A04-p18417786	0.991735537	1	Bn-Scaffold15937-p3	Bn-chrA04-p18488076
Bn-A04-p18429573	1	1	Bn-N4-p20419859	Bn-chrA04-p18512174
Bn-A04-p18455291	0.975124378	0.975124378	Bn-N4-p20447374	Bn-chrA04-p18544286
Bn-A04-p18457550	0.958677686	0.975206612	Bn-N14-p54066608	Bn-chrA04-p18546546
Bn-A04-p18458146	0.975124378	0.985074627	Bn-N4-p20450574	Bn-chrA04-p18547142
Bn-A04-p18467235	0.983471074	0.975206612	Bn-N4-p20459449	Bn-chrA04-p18556566
Bn-A04-p18471424	0.996677741	1	Bn-N4-p20465726	Bn-chrA04-p18560897
Bn-A04-p18472857	0.983471074	0.991735537	Bn-N4-p20467160	Bn-chrA04-p18562330
Bn-A04-p18487715	0.890547264	0.970149254	Bn-N4-p20483625	Bn-chrA04-p18578184
Bn-A04-p18499666	0.996677741	0.990033223	Bn-N4-p20494822	Bn-chrA04-p18591968
Bn-A04-p18502854	0.975206612	0.975206612	Bn-N4-p20498001	Bn-chrAnn_random-p349146
Bn-A04-p18515323	0.966942149	0.975206612	Bn-N4-p20512592	Bn-chrA04-p18605201
Bn-A04-p18522691	0.975206612	0.975206612	Bn-N4-p20520720	Bn-chrA04-p18612644
Bn-A04-p18530009	0.975206612	0.983471074	Bn-N4-p20529065	Bn-chrA04-p18621616
Bn-A04-p18552075	0.996677741	0.996677741	Bn-N4-p20552094	Bn-chrA04-p18678331
Bn-A04-p18562244	0.996677741	0.996677741	Bn-N4-p20563734	Bn-chrA04-p18693930
Bn-A04-p18572841	0.995024876	0.995024876	Bn-N4-p20574835	Bn-chrA04-p18704383
Bn-A04-p18573606	0.958677686	0.933884298	Bn-N4-p20575602	Bn-chrA04-p18705148
Bn-A04-p18577466	0.980066445	0.993355482	Bn-N4-p20579460	Bn-chrA04-p18709025
Bn-A04-p18585603	0.996677741	1	Bn-N4-p20585798	Bn-chrA04-p18713463
Bn-A04-p18595858	0.983471074	0.975206612	Bn-N4-p20602010	Bn-chrA04-p18723762
Bn-A04-p18606660	0.990033223	0.990033223	Bn-N4-p20612582	Bn-chrA04-p18734282
Bn-A04-p18613344	0.990033223	0.986710963	Bn-N4-p20621135	Bn-chrA04-p18741076
Bn-A04-p18617343	0.975206612	0.975206612	Bn-N4-p20625106	Bn-chrA04-p18745068
Bn-A04-p18620226	0.983388704	0.983388704	Bn-N4-p20626993	Bn-chrA04-p18746955
Bn-A04-p18621492	0.958677686	0.950413223	Bn-N4-p20628250	Bn-chrA04-p18748210
Bn-A04-p18639840	0.973421927	0.986710963	Bn-N4-p20641939	Bn-chrA04-p18762293
Bn-A04-p18639922	0.926910299	0.993355482	Bn-N14-p54330912	Bn-chrA04-p18762375
Bn-A04-p18673015	0.980099502	0.980099502	Bn-N4-p20670875	Bn-chrA04-p18791935
Bn-A04-p18693211	0.983471074	0.983471074	Bn-N4-p20687712	Bn-chrA04-p18808770
Bn-A04-p18694989	0.986928105	0.986928105	Bn-N4-p20689447	Bn-chrA04-p18810504
Bn-A04-p18702920	0.990049751	0.990049751	Bn-N4-p20696885	Bn-chrA04-p18817942
Bn-A04-p18731541	0.990049751	0.990049751	Bn-N4-p20731935	Bn-chrA04-p18856228
Bn-A04-p18763435	1	1	Bn-N4-p20778423	Bn-chrA04_random-p146933
Bn-A04-p18789871	0.950248756	0.950248756	Bn-N4-p20817952	Bn-chrA04-p19140092
Bn-A04-p18806834	0.980099502	0.980099502	Bn-N4-p20835363	Bn-chrA04-p19121967
Bn-A04-p18867216	1	0.991735537	Bn-N4-p20899595	Bn-chrA04-p18900936
Bn-A04-p18913495	0.990033223	1	Bn-N4-p20955081	Bn-chrA04-p18961269
Bn-A04-p18918997	0.975124378	0.970149254	Bn-N14-p54659166	Bn-chrC04-p48844038
Bn-A04-p18943386	0.933884298	0.991735537	Bn-N4-p20988227	Bn-chrA04-p18992064
Bn-A04-p18946513	0.980099502	0.985074627	Bn-N4-p20990324	Bn-chrA04-p19001842
Bn-A04-p18960154	0.983471074	0.991735537	Bn-N4-p21002090	Bn-chrA04-p19012468
Bn-A04-p18961456	1	0.991735537	Bn-N4-p21004100	Bn-chrA04-p19013770

Bn-A04-p18961945	0.980099502	0.980099502	Bn-N4-p21004588	Bn-chrA04-p19014259
Bn-A04-p18961947	0.980099502	0.980099502	Bn-N4-p21004590	Bn-chrA04-p19014261
Bn-A04-p18962305	0.990049751	0.990049751	Bn-N4-p21004948	Bn-chrA04-p19014619
Bn-A04-p18962378	0.980099502	0.980099502	Bn-N4-p21005021	Bn-chrA04-p19014692
Bn-A04-p18962442	0.985074627	0.985074627	Bn-N4-p21005085	Bn-chrA04-p19014756
Bn-A05-p25416	0.991735537	0.768595041	Bn-N5-p29775	Bn-chrA05-p37849
Bn-A05-p25420	0.991735537	0.801652893	Bn-N5-p29779	Bn-chrA05-p37853
Bn-A05-p44690	0.993355482	0.990033223	Bn-N5-p49148	Bn-chrAnn_random-p446894
Bn-A05-p58992	0.966942149	0.975206612	Bn-N5-p78052	Bn-chrA05-p76673
Bn-A05-p59055	0.980066445	0.976744186	Bn-N5-p78115	Bn-chrA05-p76736
Bn-A05-p59178	0.991735537	0.983471074	Bn-N5-p78238	Bn-chrA05-p76859
Bn-A05-p61233	0.991735537	0.991735537	Bn-N5-p80291	Bn-chrA05-p78910
Bn-A05-p76428	0.991735537	1	Bn-N5-p93221	Bn-chrA05-p91392
Bn-A05-p81414	0.993355482	0.996677741	Bn-N5-p98365	Bn-chrA05-p96506
Bn-A05-p95346	0.975206612	0.991735537	Bn-N5-p118043	Bn-chrA05-p110986
Bn-A05-p103817	0.960199005	0.985074627	Bn-N5-p123677	Bn-chrA05-p118482
Bn-A05-p108510	0.966942149	0.975206612	Bn-N5-p127460	Bn-chrA05-p122907
Bn-A05-p109998	0.975206612	0.983471074	Bn-N5-p128987	Bn-chrA05-p124406
Bn-A05-p114598	1	0.996677741	Bn-N5-p133603	Bn-chrA05-p128996
Bn-A05-p137314	0.991735537	0.991735537	Bn-N5-p169816	Bn-chrA05-p167249
Bn-A05-p140798	0.990033223	0.993355482	Bn-N5-p173295	Bn-chrA05-p170733
Bn-A05-p143572	0.991735537	0.983471074	Bn-N5-p176112	Bn-chrA05-p174585
Bn-A05-p182664	0.801652893	0.975206612	Bn-N5-p215149	Bn-chrA05-p216972
Bn-A05-p200254	0.975206612	0.966942149	Bn-N5-p226496	Bn-chrA05-p227961
Bn-A05-p221189	0.966942149	0.545454545	Bn-N5-p252763	Bn-chrC04_random-p336836
Bn-A05-p228555	0.966942149	0.925619835	Bn-N5-p260129	Bn-chrA05-p249685
Bn-A05-p244051	0.996677741	1	Bn-N5-p272544	Bn-chrA05-p261146
Bn-A05-p249420	0.966942149	0.876033058	Bn-N5-p278177	Bn-chrA05-p267448
Bn-A05-p253584	0.940199336	0.936877076	Bn-N5-p281613	Bn-chrA05-p270865
Bn-A05-p262934	0.983388704	0.96013289	Bn-N5-p291486	Bn-chrC04-p108840
Bn-A05-p267915	0.975206612	0.975206612	Bn-N5-p296316	Bn-chrA05-p285069
Bn-A05-p271108	0.820598007	0.813953488	Bn-N5-p301133	Bn-chrA05-p290073
Bn-A05-p280800	0.975206612	0.975206612	Bn-N5-p312603	Bn-chrAnn_random-p395022
Bn-A05-p342596	0.990033223	0.830564784	Bn-N5-p382767	Bn-chrA05-p424522
Bn-A05-p346822	0.993355482	0.990033223	Bn-N5-p384683	Bn-chrA05-p426370
Bn-A05-p347486	0.960199005	0.965174129	Bn-N5-p385347	Bn-chrA05-p427034
Bn-A05-p355200	0.975206612	0.975206612	Bn-N5-p410256	Bn-chrA05-p463821
Bn-A05-p363138	0.966942149	0.958677686	Bn-N5-p419017	Bn-chrA05-p477324
Bn-A05-p377542	0.983471074	0.975206612	Bn-N5-p434394	Bn-chrA05-p492376
Bn-A05-p389230	1	1	Bn-Scaffold11756-p5	Bn-chrA05-p506405
Bn-A05-p392053	0.965174129	0.965174129	Bn-N5-p448974	Bn-chrA05-p509226
Bn-A05-p449086	0.966942149	0.966942149	Bn-N5-p524531	Bn-chrA05-p558948
Bn-A05-p452939	0.958677686	0.958677686	Bn-N5-p528432	Bn-chrA05-p562841
Bn-A05-p458357	0.936877076	0.936877076	Bn-N5-p533849	Bn-chrA05-p568244
Bn-A05-p461633	0.975124378	0.975124378	Bn-N5-p537130	Bn-chrA05-p571526
Bn-A05-p468551	0.990049751	0.990049751	Bn-N5-p544120	Bn-chrA05-p579945

Bn-A05-p472271	0.975124378	0.975124378	Bn-N5-p547781	Bn-chrA05-p583643
Bn-A05-p474257	0.983471074	0.991735537	Bn-N5-p549766	Bn-chrA05-p585628
Bn-A05-p482473	0.976744186	0.983388704	Bn-N5-p561498	Bn-chrA05-p594599
Bn-A05-p484591	0.990033223	0.986710963	Bn-N5-p564912	Bn-chrA05-p596701
Bn-A05-p518923	1	0.991735537	Bn-N5-p593059	Bn-chrA05-p632623
Bn-A05-p529716	0.950413223	0.958677686	Bn-N5-p604085	Bn-chrA05-p647900
Bn-A05-p541565	0.823920266	0.830564784	Bn-N11-p1777591	Bn-chrUnn_random-p591562
Bn-A05-p547582	1	1	Bn-N5-p622657	Bn-chrA05-p668891
Bn-A05-p550807	0.991735537	0.991735537	Bn-N5-p625889	Bn-chrA05-p672231
Bn-A05-p556585	0.991735537	1	Bn-N5-p634308	Bn-chrA05-p675759
Bn-A05-p559597	1	1	Bn-N5-p637306	Bn-chrA05-p678757
Bn-A05-p565719	0.991735537	0.991735537	Bn-N5-p643973	Bn-chrA05-p687710
Bn-A05-p575873	0.991735537	0.991735537	Bn-N5-p654857	Bn-chrA05-p698065
Bn-A05-p579882	0.975206612	0.975206612	Bn-N5-p663919	Bn-chrA05-p702059
Bn-A05-p583972	1	1	Bn-N5-p666924	Bn-chrA04-p18683664
Bn-A05-p589658	0.991735537	0.991735537	Bn-N5-p673281	Bn-chrA05-p711827
Bn-A05-p600196	0.983471074	0.983471074	Bn-N5-p684459	Bn-chrA05-p720802
Bn-A05-p619336	0.975206612	0.966942149	Bn-N5-p701378	Bn-chrA05-p737606
Bn-A05-p621136	0.96345515	0.96345515	Bn-Scaffold04817-p1	Bn-chrA05-p739407
Bn-A05-p652475	0.995024876	1	Bn-N14-p866724	Bn-chrC04_random-p238612
Bn-A05-p653606	0.727272727	0.983471074	Bn-N5-p738760	Bn-chrA05-p776011
Bn-A05-p671910	0.990049751	0.990049751	Bn-N5-p748023	Bn-chrA05-p793277
Bn-A05-p673050	0.991735537	0.983471074	Bn-N5-p749162	Bn-chrA05-p794410
Bn-A05-p677619	1	0.996677741	Bn-N5-p757125	Bn-chrA05-p799088
Bn-A05-p679803	0.980099502	0.965174129	Bn-N5-p759307	Bn-chrA05-p801268
Bn-A05-p686912	0.991735537	1	Bn-N5-p766534	Bn-chrA05-p808366
Bn-A05-p687790	1	0.995024876	Bn-N5-p767412	Bn-chrA05-p809244
Bn-A05-p701389	0.975206612	0.966942149	Bn-N5-p773928	Bn-chrA05-p815814
Bn-A05-p717416	0.809917355	0.966942149	Bn-N5-p790244	Bn-chrA05-p829989
Bn-A05-p726481	0.966942149	0.975206612	Bn-N5-p802595	Bn-chrA05-p844998
Bn-A05-p757624	0.975206612	0.975206612	Bn-N5-p827007	Bn-chrA05-p867973
Bn-A05-p761283	0.995024876	0.995024876	Bn-N5-p831536	Bn-chrA05-p871689
Bn-A05-p761376	0.994186047	0.994186047	Bn-N5-p831630	Bn-chrA05-p871783
Bn-A05-p771943	0.966942149	0.966942149	Bn-N5-p871128	Bn-chrA05-p901575
Bn-A05-p772777	0.983471074	0.983471074	Bn-N5-p871952	Bn-chrA05-p902399
Bn-A05-p779415	0.727272727	0.727272727	Bn-N5-p878821	Bn-chrA05-p911922
Bn-A05-p780844	0.975124378	0.975124378	Bn-N5-p880252	Bn-chrA05-p913353
Bn-A05-p784331	0.993355482	0.993355482	Bn-N5-p883877	Bn-chrA05-p916977
Bn-A05-p794760	0.991735537	0.983471074	Bn-N5-p894417	Bn-chrA05-p926960
Bn-A05-p800515	0.975206612	0.595041322	Bn-N5-p902155	Bn-chrC04-p783707
Bn-A05-p800549	0.991735537	0.512396694	Bn-N5-p902189	Bn-chrA05-p927126
Bn-A05-p802111	0.975206612	0.719008264	Bn-N5-p903818	Bn-chrC04-p782069
Bn-A05-p815322	0.991735537	0.991735537	Bn-N5-p916272	Bn-chrA05-p948832
Bn-A05-p816963	0.983471074	0.983471074	Bn-N5-p916761	Bn-chrA05-p949321
Bn-A05-p817036	0.983471074	0.983471074	Bn-N5-p916834	Bn-chrA05-p949394
Bn-A05-p822087	0.975124378	0.975124378	Bn-N5-p923155	Bn-chrA05-p957669

Bn-A05-p822597	0.965174129	0.965174129	Bn-N5-p923666	Bn-chrA05-p958180
Bn-A05-p825088	0.991735537	0.991735537	Bn-N5-p927351	Bn-chrA05-p960545
Bn-A05-p825915	0.991735537	0.991735537	Bn-N5-p928178	Bn-chrA05-p961372
Bn-A05-p837013	1	1	Bn-N5-p945099	Bn-chrA05-p980262
Bn-A05-p849712	0.991735537	0.991735537	Bn-N5-p951999	Bn-chrA05_random-p9191
Bn-A05-p853364	0.991735537	0.991735537	Bn-N5-p955303	Bn-chrA05_random-p12495
Bn-A05-p889343	0.958677686	0.958677686	Bn-N5-p997791	Bn-chrA05-p1022777
Bn-A05-p892520	0.991735537	0.991735537	Bn-N5-p1000999	Bn-chrA05-p1025982
Bn-A05-p894768	1	1	Bn-N14-p1266048	Bn-chrC04-p1113739
Bn-A05-p895276	0.965174129	0.965174129	Bn-N5-p1003703	Bn-chrA05-p1028686
Bn-A05-p895522	0.975124378	0.975124378	Bn-N5-p1003937	Bn-chrA05-p1028920
Bn-A05-p904751	0.958677686	0.958677686	Bn-N5-p1012825	Bn-chrA05-p1037808
Bn-A05-p907258	0.995024876	0.995024876	Bn-N5-p1015308	Bn-chrA05-p1040291
Bn-A05-p910039	1	1	Bn-N14-p1292630	Bn-chrA05-p1043217
Bn-A05-p917769	0.983471074	0.975206612	Bn-N5-p1025845	Bn-chrA05-p1050739
Bn-A05-p922813	0.950413223	0.950413223	Bn-N5-p1030016	Bn-chrA05-p1055249
Bn-A05-p926361	0.94214876	0.94214876	Bn-N5-p1033381	Bn-chrA05-p1058614
Bn-A05-p933536	0.983471074	0.983471074	Bn-N5-p1046398	Bn-chrA05-p1070605
Bn-A05-p934600	0.966942149	0.966942149	Bn-N5-p1047457	Bn-chrA05-p1071664
Bn-A05-p956298	1	1	Bn-N5-p1066806	Bn-chrA05-p1091785
Bn-A05-p958740	0.94214876	0.94214876	Bn-N5-p1068268	Bn-chrA05-p1093247
Bn-A05-p966483	0.96345515	0.96345515	Bn-N5-p1076071	Bn-chrA05-p1101052
Bn-A05-p967131	0.991735537	0.991735537	Bn-N5-p1076788	Bn-chrA05-p1101769
Bn-A05-p967575	0.933884298	0.933884298	Bn-N5-p1077255	Bn-chrA05-p1102236
Bn-A05-p970816	1	1	Bn-N5-p1080492	Bn-chrA05-p1105473
Bn-A05-p983618	0.958677686	0.966942149	Bn-N5-p1093388	Bn-chrA05-p1118256
Bn-A05-p996188	0.975206612	0.983471074	Bn-N5-p1105629	Bn-chrA05-p1135999
Bn-A05-p1006754	0.986710963	0.983388704	Bn-N5-p1117066	Bn-chrA05-p1151799
Bn-A05-p1030058	0.950413223	0.950413223	Bn-N5-p1135627	Bn-chrA05-p1168923
Bn-A05-p1035167	1	0.995024876	Bn-N5-p1142967	Bn-chrA05-p1176267
Bn-A05-p1071371	0.991735537	1	Bn-N5-p1174032	Bn-chrA05-p1204298
Bn-A05-p1074864	0.884297521	0.925619835	Bn-N5-p1182966	Bn-chrA05-p1207497
Bn-A05-p1084980	0.991735537	1	Bn-N5-p1194325	Bn-chrA05-p1222101
Bn-A05-p1096935	0.991735537	0.983471074	Bn-N5-p1205324	Bn-chrC04-p1464667
Bn-A05-p1097088	0.966942149	0.975206612	Bn-N5-p1205478	Bn-chrA05-p1234154
Bn-A05-p1107888	0.983471074	0.975206612	Bn-N5-p1216224	Bn-chrA05-p1243846
Bn-A05-p1134104	0.945273632	0.985074627	Bn-N5-p1245455	Bn-chrA05-p1271943
Bn-A05-p1192706	0.900826446	0.809917355	Bn-N5-p1281106	Bn-chrA05-p1304538
Bn-A05-p1196947	0.983471074	0.884297521	Bn-N5-p1284750	Bn-chrC04-p1591412
Bn-A05-p1204987	0.983388704	0.983388704	Bn-N5-p1293109	Bn-chrA05-p1327000
Bn-A05-p1214053	0.940298507	0.985074627	Bn-N14-p1794245	Bn-chrA05-p1336116
Bn-A05-p1216828	0.975124378	0.980099502	Bn-N5-p1309989	Bn-chrA05-p1338821
Bn-A05-p1220485	0.980099502	0.980099502	Bn-N5-p1313649	Bn-chrA05-p1342481
Bn-A05-p1237296	0.980099502	0.985074627	Bn-N5-p1331338	Bn-chrA05-p1353702
Bn-A05-p1254185	0.991735537	0.991735537	Bn-N5-p1355423	Bn-chrA05-p1370445
Bn-A05-p1259869	0.993355482	0.996677741	Bn-N5-p1362258	Bn-chrA05-p1376158

Bn-A05-p1260228	0.991735537	0.983471074	Bn-N5-p1362617	Bn-chrA05-p1376516
Bn-A05-p1260305	0.991735537	1	Bn-N5-p1362694	Bn-chrA05-p1376593
Bn-A05-p1260565	0.991735537	0.983471074	Bn-N5-p1362954	Bn-chrA05-p1376853
Bn-A05-p1267683	0.975206612	0.983471074	Bn-N5-p1371434	Bn-chrA05-p1383954
Bn-A05-p1279304	0.993355482	0.990033223	Bn-N5-p1383086	Bn-chrA05-p1395561
Bn-A05-p1281138	0.892561983	0.975206612	Bn-N5-p1384962	Bn-chrA05-p1397395
Bn-A05-p1299570	0.995024876	0.995024876	Bn-N5-p1401301	Bn-chrA05-p1414124
Bn-A05-p1308471	0.991735537	0.991735537	Bn-N5-p1410753	Bn-chrA05-p1423575
Bn-A05-p1310329	0.983471074	0.983471074	Bn-Scaffold03131-p1	Bn-chrA05-p1425448
Bn-A05-p1311652	0.983471074	0.983471074	Bn-Scaffold03131-p2	Bn-chrA05-p1426794
Bn-A05-p1335771	0.983471074	0.991735537	Bn-N5-p1436231	Bn-chrA05-p1453229
Bn-A05-p1347246	0.990049751	0.995024876	Bn-N5-p1451685	Bn-chrA05-p1464867
Bn-A05-p1349520	0.975206612	0.983471074	Bn-N5-p1453955	Bn-chrA05-p1467506
Bn-A05-p1350976	0.966942149	0.958677686	Bn-N5-p1455247	Bn-chrA05-p1468817
Bn-A05-p1353104	0.936877076	0.996677741	Bn-N14-p2141728	Bn-chrA05-p1470946
Bn-A05-p1356378	0.966942149	0.975206612	Bn-N5-p1459257	Bn-chrA05-p1474158
Bn-A05-p1368115	0.933884298	0.94214876	Bn-N5-p1475864	Bn-chrA05-p1494468
Bn-A05-p1372057	0.993355482	0.993355482	Bn-N5-p1480737	Bn-chrA05-p1509794
Bn-A05-p1384000	0.983471074	0.991735537	Bn-N5-p1493281	Bn-chrA05-p1526023
Bn-A05-p1384677	0.991735537	0.991735537	Bn-N5-p1493971	Bn-chrA05-p1526861
Bn-A05-p1385188	0.975206612	0.966942149	Bn-N5-p1494482	Bn-chrA05-p1527341
Bn-A05-p1396162	0.975206612	0.983471074	Bn-N5-p1505543	Bn-chrA05-p1538939
Bn-A05-p1396567	0.975206612	0.966942149	Bn-N5-p1505947	Bn-chrA05-p1539344
Bn-A05-p1396959	0.702479339	0.710743802	Bn-N5-p1506339	Bn-chrA05-p1539740
Bn-A05-p1434959	0.991735537	0.991735537	Bn-N5-p1565456	Bn-chrA05_random-p59646
Bn-A05-p1436011	0.950413223	0.950413223	Bn-N5-p1568006	Bn-chrC04-p1817192
Bn-A05-p1451750	0.990033223	0.990033223	Bn-N5-p1577981	Bn-chrA05_random-p48255
Bn-A05-p1457058	0.989583333	0.989583333	Bn-N5-p1583214	Bn-chrA05_random-p42941
Bn-A05-p1462945	0.983471074	0.983471074	Bn-N5-p1588549	Bn-chrA05_random-p37862
Bn-A05-p1473472	0.940298507	0.895522388	Bn-Scaffold43029-p9	Bn-chrA05_random-p25821
Bn-A05-p1475369	0.983471074	0.983471074	Bn-N5-p1602173	Bn-chrC04_random-p499550
Bn-A05-p1479325	0.991735537	0.991735537	Bn-N5-p1605466	Bn-chrAnn_random-p32315C
Bn-A05-p1492327	0.986710963	0.986710963	Bn-N5-p1619320	Bn-chrA05-p1615867
Bn-A05-p1496037	1	1	Bn-N5-p1623592	Bn-chrA05-p1619592
Bn-A05-p1499192	0.930348259	0.935323383	Bn-N5-p1626797	Bn-chrA05-p1622795
Bn-A05-p1554943	0.970149254	0.970149254	Bn-N5-p1722333	Bn-chrA05-p1719644
Bn-A05-p1561518	0.983388704	0.983388704	Bn-N5-p1728875	Bn-chrA05-p1726185
Bn-A05-p1566005	0.983471074	0.983471074	Bn-N5-p1739300	Bn-chrA05-p1731325
Bn-A05-p1578074	1	1	Bn-N5-p1753871	Bn-chrA05_random-p101494
Bn-A05-p1579155	0.983471074	0.983471074	Bn-N5-p1754900	Bn-chrA05_random-p102523
Bn-A05-p1579208	0.965174129	0.965174129	Bn-N5-p1754953	Bn-chrA05_random-p102576
Bn-A05-p1580246	0.985074627	0.985074627	Bn-N5-p1755984	Bn-chrA05_random-p103607
Bn-A05-p1630204	1	1	Bn-N5-p1805167	Bn-chrAnn_random-p372972
Bn-A05-p1671823	0.993355482	0.993355482	Bn-N5-p1870680	Bn-chrA05-p1815006
Bn-A05-p1676024	0.958677686	0.958677686	Bn-N5-p1879670	Bn-chrA05-p1823545
Bn-A05-p1679589	0.990033223	0.990033223	Bn-N5-p1885398	Bn-chrA05-p1829273

Bn-A05-p1689277	0.859504132	0.859504132	Bn-N5-p1890245	Bn-chrA05-p1834120
Bn-A05-p1690598	0.980099502	0.980099502	Bn-N5-p1891553	Bn-chrA05-p1835428
Bn-A05-p1692254	0.991735537	0.958677686	Bn-N5-p1893365	Bn-chrA05-p1837237
Bn-A05-p1692692	0.975206612	0.966942149	Bn-N5-p1893801	Bn-chrA05-p1837677
Bn-A05-p1693000	0.950413223	0.975206612	Bn-N5-p1894188	Bn-chrA05-p1837985
Bn-A05-p1693196	0.785123967	0.909090909	Bn-N5-p1894384	Bn-chrA05-p1838182
Bn-A05-p1695394	0.909090909	0.983471074	Bn-N5-p1894784	Bn-chrA05-p1838590
Bn-A05-p1695552	0.966942149	0.983471074	Bn-N5-p1894938	Bn-chrA05-p1838748
Bn-A05-p1696330	0.966942149	0.975206612	Bn-N5-p1895717	Bn-chrA05-p1839526
Bn-A05-p1696411	0.975206612	0.983471074	Bn-N5-p1895799	Bn-chrA05-p1839608
Bn-A05-p1696613	0.966942149	0.966942149	Bn-N5-p1896002	Bn-chrA05-p1839810
Bn-A05-p1697018	0.975206612	0.983471074	Bn-N5-p1896407	Bn-chrA05-p1840214
Bn-A05-p1697401	0.993355482	0.973421927	Bn-N5-p1896787	Bn-chrA05-p1840597
Bn-A05-p1701030	0.958677686	0.809917355	Bn-N5-p1901300	Bn-chrC04-p2062213
Bn-A05-p1744534	0.991735537	0.991735537	Bn-N5-p1944931	Bn-chrA05-p1887134
Bn-A05-p1761473	0.975206612	0.983471074	Bn-N5-p1949635	Bn-chrA05-p1890782
Bn-A05-p1762229	0.743801653	0.776859504	Bn-N5-p1949899	Bn-chrA05-p1891052
Bn-A05-p1764530	0.970149254	0.975124378	Bn-N5-p1953359	Bn-chrA05-p1893353
Bn-A05-p1765880	0.983471074	0.975206612	Bn-N5-p1954629	Bn-chrA05-p1894703
Bn-A05-p1766223	0.958677686	0.991735537	Bn-N5-p1955178	Bn-chrA05-p1895046
Bn-A05-p1775540	0.995024876	0.990049751	Bn-N5-p1963478	Bn-chrA05-p1904488
Bn-A05-p1806574	0.986710963	0.966777409	Bn-N5-p1985171	Bn-chrA05-p1928896
Bn-A05-p1818846	0.980066445	0.990033223	Bn-N5-p1997605	Bn-chrA05-p1941091
Bn-A05-p1854510	0.975206612	0.983471074	Bn-N5-p2036895	Bn-chrA05-p1973090
Bn-A05-p1857946	0.970149254	0.970149254	Bn-N5-p2039192	Bn-chrA05-p1975387
Bn-A05-p1893613	0.975206612	0.975206612	Bn-N5-p2071802	Bn-chrA05-p2002096
Bn-A05-p1898273	0.975206612	0.983471074	Bn-N5-p2075840	Bn-chrA05-p2006072
Bn-A05-p1919060	0.975206612	0.917355372	Bn-N5-p2097213	Bn-chrA05-p2024603
Bn-A05-p1942242	0.990049751	0.995024876	Bn-N5-p2132498	Bn-chrA05-p2053951
Bn-A05-p1943093	0.966942149	0.975206612	Bn-N5-p2132766	Bn-chrA05-p2054218
Bn-A05-p1974178	0.980066445	0.980066445	Bn-N5-p2162152	Bn-chrC04-p2554990
Bn-A05-p1975785	0.958677686	0.966942149	Bn-N5-p2163761	Bn-chrAnn_random-p207636
Bn-A05-p1978957	0.760330579	0.752066116	Bn-N5-p2166643	Bn-chrAnn_random-p207667
Bn-A05-p1981647	0.975206612	0.975206612	Bn-N5-p2168874	Bn-chrAnn_random-p207692
Bn-A05-p2024975	0.966942149	0.975206612	Bn-N5-p2214915	Bn-chrA05-p2153370
Bn-A05-p2030789	0.991735537	1	Bn-N5-p2220726	Bn-chrA05-p2163606
Bn-A05-p2039690	0.966942149	0.975206612	Bn-N5-p2230561	Bn-chrA05_random-p149865
Bn-A05-p2062917	0.983471074	0.975206612	Bn-N5-p2252597	Bn-chrA05-p2194374
Bn-A05-p2067722	0.991735537	1	Bn-N5-p2257407	Bn-chrA05-p2199180
Bn-A05-p2091308	0.990033223	0.993355482	Bn-N5-p2283419	Bn-chrA05-p2222853
Bn-A05-p2102634	0.983471074	0.975206612	Bn-N5-p2295512	Bn-chrA05-p2232259
Bn-A05-p2106680	0.570247934	0.983471074	Bn-N5-p2298341	Bn-chrA05-p2236353
Bn-A05-p2107808	0.983471074	0.991735537	Bn-N5-p2299339	Bn-chrA05-p2237480
Bn-A05-p2108713	0.976744186	0.976744186	Bn-N5-p2300244	Bn-chrA05-p2244524
Bn-A05-p2109546	0.837209302	0.604651163	Bn-N5-p2301077	Bn-chrA05-p2239218
Bn-A05-p2110255	0.694352159	0.976744186	Bn-N5-p2302923	Bn-chrA05-p2239926

Bn-A05-p2110344	0.970099668	0.983388704	Bn-N5-p2303012	Bn-chrA05-p2240015
Bn-A05-p2111165	0.991735537	0.983471074	Bn-N5-p2303825	Bn-chrA05-p2240815
Bn-A05-p2138342	0.935323383	0.960199005	Bn-N5-p2335324	Bn-chrA05-p2269481
Bn-A05-p2139529	0.986710963	0.983388704	Bn-N5-p2336512	Bn-chrA05-p2270668
Bn-A05-p2142102	0.933884298	0.685950413	Bn-N5-p2339828	Bn-chrA05-p2273504
Bn-A05-p2143241	0.958677686	0.595041322	Bn-N5-p2340764	Bn-chrA04-p18442758
Bn-A05-p2143292	0.859504132	0.785123967	Bn-N5-p2340815	Bn-chrA05-p2274889
Bn-A05-p2151202	1	0.989583333	Bn-N5-p2353516	Bn-chrA05_random-p152162
Bn-A05-p2155466	0.975206612	0.983471074	Bn-N5-p2357631	Bn-chrA05-p2287415
Bn-A05-p2166681	0.958677686	0.966942149	Bn-N5-p2368827	Bn-chrA05-p2301582
Bn-A05-p2167188	0.975206612	0.966942149	Bn-N5-p2369335	Bn-chrA05-p2302095
Bn-A05-p2167269	0.958677686	0.966942149	Bn-N5-p2369416	Bn-chrA05-p2302184
Bn-A05-p2167540	0.985074627	0.950248756	Bn-N5-p2369687	Bn-chrA05-p2302454
Bn-A05-p2172320	1	0.876033058	Bn-N5-p2374463	Bn-chrC04-p3657413
Bn-A05-p2208960	0.960199005	0.960199005	Bn-N5-p2407963	Bn-chrA05-p2335696
Bn-A05-p2227024	1	0.991735537	Bn-N14-p3660610	Bn-chrA05-p2352372
Bn-A05-p2233637	0.983471074	0.983471074	Bn-N5-p2431088	Bn-chrA05-p2357535
Bn-A05-p2233700	0.958677686	0.958677686	Bn-N5-p2431151	Bn-chrA05-p2357598
Bn-A05-p2234082	0.809917355	0.809917355	Bn-N5-p2431541	Bn-chrA05-p2357988
Bn-A05-p2246038	0.996677741	0.996677741	Bn-N5-p2440678	Bn-chrA05-p2366837
Bn-A05-p2254100	0.983471074	0.983471074	Bn-N5-p2449849	Bn-chrA05-p2375878
Bn-A05-p2261738	1	1	Bn-N5-p2458126	Bn-chrA05-p2384152
Bn-A05-p2262914	1	1	Bn-N14-p3739605	Bn-chrC04-p2867699
Bn-A05-p2272518	0.990033223	0.990033223	Bn-N5-p2468787	Bn-chrA05-p2394444
Bn-A05-p2273196	0.980099502	0.980099502	Bn-N5-p2469464	Bn-chrA05-p2395121
Bn-A05-p2274301	0.970149254	0.970149254	Bn-N5-p2470569	Bn-chrA05-p2396226
Bn-A05-p2280549	0.986710963	0.986710963	Bn-N5-p2476665	Bn-chrA05-p2402352
Bn-A05-p2285903	0.970099668	0.970099668	Bn-N5-p2481355	Bn-chrA05-p2407040
Bn-A05-p2287815	0.966777409	0.990033223	Bn-N5-p2483134	Bn-chrA05-p2408983
Bn-A05-p2293577	0.990033223	0.897009967	Bn-N5-p2490392	Bn-chrC04-p2905352
Bn-A05-p2296307	0.975206612	0.966942149	Bn-N5-p2493077	Bn-chrA05-p2416391
Bn-A05-p2297395	0.809917355	0.966942149	Bn-N5-p2494164	Bn-chrA05-p2417482
Bn-A05-p2299167	0.933884298	0.94214876	Bn-N5-p2500849	Bn-chrA05-p2419266
Bn-A05-p2299442	1	0.991735537	Bn-N5-p2501123	Bn-chrA05-p2419541
Bn-A05-p2300076	0.983471074	0.900826446	Bn-N5-p2501765	Bn-chrA05-p2420414
Bn-A05-p2301167	0.646766169	0.930348259	Bn-N16-p6043564	Bn-chrA05-p2425252
Bn-A05-p2303112	0.975206612	0.983471074	Bn-N5-p2509507	Bn-chrA05-p2427197
Bn-A05-p2329965	0.983388704	0.983388704	Bn-N16-p32629114	Bn-chrC06-p30487015
Bn-A05-p2337684	0.975206612	0.983471074	Bn-N5-p2530906	Bn-chrA05-p2459059
Bn-A05-p2340565	0.990033223	0.980066445	Bn-N5-p2538482	Bn-chrAnn_random-p406337
Bn-A05-p2345233	0.760330579	0.958677686	Bn-N14-p3918239	Bn-chrA05-p2468112
Bn-A05-p2345849	0.920265781	0.986710963	Bn-N14-p3918856	Bn-chrA05-p2468716
Bn-A05-p2350131	0.990049751	1	Bn-N5-p2546124	Bn-chrA05-p2473367
Bn-A05-p2351545	0.993355482	0.990033223	Bn-N5-p2547531	Bn-chrA05-p2474788
Bn-A05-p2353656	0.975206612	0.983471074	Bn-N5-p2549650	Bn-chrA05-p2476235
Bn-A05-p2367876	0.909090909	0.909090909	Bn-N5-p2562704	Bn-chrA05-p2490459

Bn-A05-p2376801	0.975124378	0.980099502	Bn-N5-p2572299	Bn-chrA05-p2501864
Bn-A05-p2393098	0.983471074	0.983471074	Bn-N5-p2585650	Bn-chrA05-p2515721
Bn-A05-p2394919	0.966942149	0.958677686	Bn-N5-p2587474	Bn-chrA05-p2517523
Bn-A05-p2416806	0.995024876	0.995024876	Bn-N5-p2612057	Bn-chrA05-p2535587
Bn-A05-p2422387	0.983471074	0.991735537	Bn-N5-p2616135	Bn-chrA05-p2540404
Bn-A05-p2423005	0.966942149	0.958677686	Bn-N5-p2616753	Bn-chrA05-p2541017
Bn-A05-p2431338	1	0.991735537	Bn-N5-p2623766	Bn-chrA05-p2546514
Bn-A05-p2434176	0.983471074	0.975206612	Bn-N5-p2626449	Bn-chrAnn_random-p364377
Bn-A05-p2440512	0.950413223	0.94214876	Bn-N5-p2633361	Bn-chrA05-p2554522
Bn-A05-p2460244	0.958677686	0.966942149	Bn-N5-p2649995	Bn-chrA05-p2567978
Bn-A05-p2469338	0.958677686	0.983471074	Bn-N5-p2658699	Bn-chrA05-p2577251
Bn-A05-p2472050	0.945273632	0.935323383	Bn-N5-p2661410	Bn-chrA05-p2579957
Bn-A05-p2472584	0.986710963	0.946843854	Bn-N5-p2661943	Bn-chrA05-p2580486
Bn-A05-p2488952	1	1	Bn-N5-p2675301	Bn-chrA05-p2594852
Bn-A05-p2489101	0.995024876	0.995024876	Bn-N5-p2675450	Bn-chrA05-p2595001
Bn-A05-p2492451	0.980099502	0.980099502	Bn-N5-p2676866	Bn-chrA05-p2596417
Bn-A05-p2494204	0.995024876	0.995024876	Bn-N5-p2678611	Bn-chrA05-p2598162
Bn-A05-p2495066	0.940298507	0.940298507	Bn-N5-p2679473	Bn-chrA05-p2599024
Bn-A05-p2495419	0.975124378	0.980099502	Bn-N5-p2679827	Bn-chrA05-p2599378
Bn-A05-p2495697	0.995024876	0.995024876	Bn-N5-p2680108	Bn-chrA05-p2599659
Bn-A05-p2497466	0.980066445	0.990033223	Bn-N5-p2681879	Bn-chrA05-p2601429
Bn-A05-p2501988	0.983471074	0.983471074	Bn-N5-p2686383	Bn-chrA05-p2605937
Bn-A05-p2523070	0.975206612	0.966942149	Bn-N5-p2698754	Bn-chrA05-p2619040
Bn-A05-p2523179	0.983471074	0.859504132	Bn-N5-p2698863	Bn-chrA05-p2619162
Bn-A05-p2525969	0.983471074	0.983471074	Bn-N5-p2702065	Bn-chrC04-p3258371
Bn-A05-p2534219	0.990033223	0.986710963	Bn-N5-p2710880	Bn-chrA05-p2631231
Bn-A05-p2555885	0.983471074	0.983471074	Bn-Scaffold15004-p1	Bn-chrA05-p2664076
Bn-A05-p2563320	0.975206612	0.983471074	Bn-N5-p2745504	Bn-chrA05-p2672991
Bn-A05-p2565185	0.996677741	0.983388704	Bn-N5-p2748760	Bn-chrA05-p2674852
Bn-A05-p2565586	0.983471074	0.991735537	Bn-N5-p2749161	Bn-chrA05-p2675253
Bn-A05-p2565706	0.983471074	0.975206612	Bn-N5-p2749281	Bn-chrA05-p2675377
Bn-A05-p2565761	0.983471074	0.975206612	Bn-N5-p2749336	Bn-chrA05-p2675432
Bn-A05-p2582423	0.983471074	0.958677686	Bn-N5-p2769101	Bn-chrA05-p2692141
Bn-A05-p2592847	0.96013289	0.986710963	Bn-N5-p2778500	Bn-chrA05-p2702494
Bn-A05-p2600118	0.966942149	0.958677686	Bn-N5-p2791865	Bn-chrA05-p2710837
Bn-A05-p2604531	0.979674797	0.967479675	Bn-N5-p2794879	Bn-chrA05-p2715163
Bn-A05-p2604957	0.958677686	0.644628099	Bn-N5-p2795396	Bn-chrC04-p3411388
Bn-A05-p2606660	0.975206612	0.975206612	Bn-N5-p2797098	Bn-chrA05-p2717407
Bn-A05-p2610006	0.995024876	0.995024876	Bn-N5-p2816986	Bn-chrA05-p2720763
Bn-A05-p2641800	0.983471074	0.512396694	Bn-N5-p2836831	Bn-chrA04-p18387031
Bn-A05-p2645307	0.746268657	0.965174129	Bn-N5-p2830757	Bn-chrA05-p2749128
Bn-A05-p2645365	0.850746269	0.960199005	Bn-N5-p2830821	Bn-chrA05-p2749186
Bn-A05-p2648679	0.885572139	0.885572139	Bn-N5-p2843690	Bn-chrA05-p2752416
Bn-A05-p2653139	0.966942149	0.966942149	Bn-N5-p2848406	Bn-chrA05-p2757129
Bn-A05-p2656042	0.980066445	0.980066445	Bn-N5-p2851329	Bn-chrA05-p2760052
Bn-A05-p2671921	0.986710963	0.986710963	Bn-N5-p2870666	Bn-chrA05-p2781175

Bn-A05-p2688101	0.935323383	0.84079602	Bn-N5-p2893352	Bn-chrC04-p3526303
Bn-A05-p2711007	0.884297521	0.884297521	Bn-N5-p2902787	Bn-chrA05-p2808436
Bn-A05-p2711208	0.975206612	0.975206612	Bn-N5-p2902988	Bn-chrA05-p2808637
Bn-A05-p2718015	0.990033223	0.990033223	Bn-N5-p2912485	Bn-chrA05-p2815546
Bn-A05-p2726301	0.966942149	0.966942149	Bn-N5-p2920992	Bn-chrA05-p2824113
Bn-A05-p2732409	0.990033223	0.990033223	Bn-N5-p2927711	Bn-chrA05-p2830126
Bn-A05-p2735134	1	1	Bn-N5-p2930430	Bn-chrA05-p2832845
Bn-A05-p2746921	0.592039801	0.592039801	Bn-N5-p2942154	Bn-chrAnn_random-p23646[illegible]
Bn-A05-p2751864	0.986710963	0.986710963	Bn-N5-p2947094	Bn-chrAnn_random-p23650[illegible]
Bn-A05-p2765988	1	1	Bn-N5-p2961556	Bn-chrA05_random-p164812
Bn-A05-p2773820	0.991735537	0.991735537	Bn-N5-p2970653	Bn-chrA05_random-p177608
Bn-A05-p2777110	0.958677686	0.966942149	Bn-N5-p2973901	Bn-chrA05_random-p182541
Bn-A05-p2777824	0.983471074	0.975206612	Bn-N5-p2974613	Bn-chrA05_random-p183257
Bn-A05-p2777912	0.983471074	0.975206612	Bn-N5-p2974701	Bn-chrA05_random-p183345
Bn-A05-p2778040	0.975206612	0.983471074	Bn-N5-p2974829	Bn-chrA05_random-p183473
Bn-A05-p2778230	0.958677686	0.966942149	Bn-N5-p2975016	Bn-chrA05_random-p183661
Bn-A05-p2778577	0.983471074	0.991735537	Bn-N5-p2975363	Bn-chrA05_random-p184014
Bn-A05-p2778679	0.983471074	0.975206612	Bn-N5-p2975465	Bn-chrA05_random-p184116
Bn-A05-p2778737	0.975206612	0.975206612	Bn-N5-p2975523	Bn-chrA05_random-p184174
Bn-A05-p2779142	0.966942149	0.966942149	Bn-N5-p2975937	Bn-chrA05_random-p184585
Bn-A05-p2791353	0.925619835	0.371900826	Bn-N5-p2981394	Bn-chrCnn_random-p265230
Bn-A05-p2794364	0.993355482	0.973421927	Bn-N5-p2983234	Bn-chrA05_random-p192155
Bn-A05-p2804703	0.986710963	0.986710963	Bn-N5-p2992931	Bn-chrA05_random-p211863
Bn-A05-p2830103	0.986710963	0.986710963	Bn-N5-p3017274	Bn-chrCnn_random-p479144
Bn-A05-p2839985	0.875621891	0.875621891	Bn-N5-p3028806	Bn-chrA05-p2900906
Bn-A05-p2840537	0.980099502	0.980099502	Bn-N5-p3029498	Bn-chrA05-p2901464
Bn-A05-p2840734	0.980099502	0.980099502	Bn-N5-p3029695	Bn-chrA05-p2901661
Bn-A05-p2841024	1	1	Bn-N5-p3029985	Bn-chrA05-p2901951
Bn-A05-p2843347	0.990049751	0.985074627	Bn-N5-p3032379	Bn-chrA05-p2904020
Bn-A05-p2844059	0.983471074	0.991735537	Bn-N5-p3033090	Bn-chrA05-p2904734
Bn-A05-p2849284	0.966942149	0.966942149	Bn-N5-p3038328	Bn-chrA05-p2909955
Bn-A05-p2852544	0.983471074	0.975206612	Bn-N5-p3043378	Bn-chrA05-p2912365
Bn-A05-p2860757	0.983471074	0.983471074	Bn-N5-p3053056	Bn-chrA05-p2921970
Bn-A05-p2863301	0.983471074	0.983471074	Bn-N5-p3055382	Bn-chrA05-p2924296
Bn-A05-p2863674	0.950413223	0.950413223	Bn-N5-p3056016	Bn-chrA05-p2924930
Bn-A05-p2864686	0.966942149	0.966942149	Bn-N5-p3056777	Bn-chrA05-p2925691
Bn-A05-p2865380	0.966942149	0.966942149	Bn-N5-p3057390	Bn-chrA05-p2926304
Bn-A05-p2865634	0.983471074	0.983471074	Bn-N5-p3057889	Bn-chrA05-p2926632
Bn-A05-p2865716	0.900826446	0.900826446	Bn-N5-p3057960	Bn-chrA05-p2926703
Bn-A05-p2868976	0.975206612	0.975206612	Bn-N5-p3061215	Bn-chrA05-p2929958
Bn-A05-p2898061	0.990033223	0.990033223	Bn-N14-p4774324	Bn-chrC04-p3849267
Bn-A05-p2924872	0.966942149	0.991735537	Bn-N5-p3142611	Bn-chrA05-p2981825
Bn-A05-p2925195	0.975206612	0.983471074	Bn-N5-p3144077	Bn-chrA05-p2982148
Bn-A05-p2929698	0.966942149	0.983471074	Bn-N5-p3152646	Bn-chrA05-p2986695
Bn-A05-p2937826	0.970149254	0.970149254	Bn-N5-p3162058	Bn-chrA05-p2994373
Bn-A05-p2937829	0.970149254	0.970149254	Bn-N5-p3162061	Bn-chrA05-p2994376

Bn-A05-p2940969	0.991735537	0.991735537	Bn-N5-p3165178	Bn-chrA05-p2997485
Bn-A05-p2984656	0.990049751	0.990049751	Bn-N5-p3208071	Bn-chrC04-p3997824
Bn-A05-p3002824	0.809917355	0.966942149	Bn-N14-p4923992	Bn-chrA05-p3060226
Bn-A05-p3005109	0.983471074	0.983471074	Bn-N5-p3236393	Bn-chrA05-p3062514
Bn-A05-p3019980	0.975124378	0.975124378	Bn-N5-p3249549	Bn-chrA05-p3082710
Bn-A05-p3021668	0.950413223	0.958677686	Bn-N5-p3251235	Bn-chrA05-p3084383
Bn-A05-p3042172	0.983471074	0.983471074	Bn-N5-p3274081	Bn-chrA05-p3106088
Bn-A05-p3042173	0.975206612	0.975206612	Bn-N5-p3274082	Bn-chrA05-p3106089
Bn-A05-p3043365	0.991735537	0.991735537	Bn-N5-p3275694	Bn-chrA05-p3107701
Bn-A05-p3045689	1	0.991735537	Bn-N5-p3278028	Bn-chrA05-p3110035
Bn-A05-p3053025	0.958677686	0.958677686	Bn-N5-p3284873	Bn-chrA05_random-p304232
Bn-A05-p3053859	0.983471074	0.983471074	Bn-N5-p3285663	Bn-chrA05_random-p303442
Bn-A05-p3061052	0.980099502	0.980099502	Bn-N5-p3291517	Bn-chrA05_random-p295392
Bn-A05-p3068418	0.867768595	0.867768595	Bn-N5-p3301253	Bn-chrA05_random-p288081
Bn-A05-p3071868	0.991735537	0.991735537	Bn-N5-p3305012	Bn-chrA05_random-p284322
Bn-A05-p3099878	0.909090909	0.966942149	Bn-N5-p3333556	Bn-chrA05_random-p254664
Bn-A05-p3112401	0.975206612	0.983471074	Bn-N5-p3351588	Bn-chrA05_random-p242227
Bn-A05-p3117520	0.991735537	0.950413223	Bn-N5-p3361502	Bn-chrAnn_random-p410185
Bn-A05-p3129492	0.966942149	0.975206612	Bn-N5-p3366223	Bn-chrA05-p3155661
Bn-A05-p3130062	0.94214876	0.958677686	Bn-N5-p3366793	Bn-chrA05-p3156207
Bn-A05-p3135130	0.659663866	0.659663866	Bn-N14-p5255038	Bn-chrCnn_random-p435014
Bn-A05-p3135213	0.983471074	0.991735537	Bn-N5-p3372511	Bn-chrA05-p3161341
Bn-A05-p3163568	0.958677686	0.867768595	Bn-N5-p3420861	Bn-chrC04-p4434178
Bn-A05-p3166433	0.966942149	0.975206612	Bn-N5-p3423714	Bn-chrA05-p3195183
Bn-A05-p3188172	0.94214876	0.933884298	Bn-N5-p3436050	Bn-chrC04-p4454812
Bn-A05-p3188714	0.975206612	1	Bn-N14-p5324758	Bn-chrA05-p3207114
Bn-A05-p3191390	0.975206612	0.983471074	Bn-N5-p3439087	Bn-chrA05-p3209563
Bn-A05-p3198955	0.966942149	0.958677686	Bn-N5-p3445947	Bn-chrA05-p3222565
Bn-A05-p3242299	1	0.991735537	Bn-N5-p3492083	Bn-chrA05-p3271162
Bn-A05-p3246311	0.993355482	0.950166113	Bn-N5-p3496090	Bn-chrC04-p4548731
Bn-A05-p3248878	0.965174129	0.965174129	Bn-N5-p3498660	Bn-chrA05-p3277719
Bn-A05-p3251117	0.537190083	0.991735537	Bn-N9-p31248194	Bn-chrA05-p3280144
Bn-A05-p3258566	0.966942149	0.958677686	Bn-N5-p3509635	Bn-chrA05-p3287604
Bn-A05-p3264129	0.996677741	0.956810631	Bn-N5-p3515043	Bn-chrA05-p3293234
Bn-A05-p3275289	0.983471074	0.975206612	Bn-N5-p3520163	Bn-chrA05-p3298354
Bn-A05-p3278023	0.975124378	0.975124378	Bn-N5-p3523252	Bn-chrA05-p3301106
Bn-A05-p3278514	0.925619835	0.991735537	Bn-N5-p3523738	Bn-chrA05-p3301597
Bn-A05-p3280592	0.975206612	0.975206612	Bn-N5-p3525724	Bn-chrA05-p3303675
Bn-A05-p3282089	0.980099502	0.995024876	Bn-N5-p3526947	Bn-chrA05-p3305180
Bn-A05-p3300112	0.960199005	0.965174129	Bn-N5-p3545250	Bn-chrA05-p3322411
Bn-A05-p3307736	0.975206612	0.966942149	Bn-N5-p3553066	Bn-chrA05-p3330594
Bn-A05-p3311786	0.985074627	0.985074627	Bn-N5-p3557125	Bn-chrA05-p3334647
Bn-A05-p3311869	0.985074627	0.985074627	Bn-N5-p3557209	Bn-chrA05-p3334731
Bn-A05-p3312679	0.975124378	0.980099502	Bn-N5-p3558019	Bn-chrA05-p3335541
Bn-A05-p3312786	0.970149254	0.985074627	Bn-N5-p3558126	Bn-chrA05-p3335648
Bn-A05-p3320783	0.945273632	0.980099502	Bn-N5-p3569934	Bn-chrA05-p3346543

Bn-A05-p3322798	0.983471074	0.991735537	Bn-N5-p3572308	Bn-chrA05-p3348552
Bn-A05-p3323878	0.63681592	0.63681592	Bn-N14-p5588941	Bn-chrC04-p4751023
Bn-A05-p3330640	0.983471074	0.991735537	Bn-N5-p3581911	Bn-chrA05-p3356331
Bn-A05-p3361741	0.983471074	0.983471074	Bn-N5-p3602767	Bn-chrA05-p3372274
Bn-A05-p3369681	0.983471074	0.975206612	Bn-N5-p3609983	Bn-chrA05-p3379499
Bn-A05-p3394311	0.966942149	0.909090909	Bn-N5-p3631412	Bn-chrA05_random-p340101
Bn-A05-p3401393	0.983471074	0.991735537	Bn-N5-p3638534	Bn-chrA05_random-p333059
Bn-A05-p3406573	0.985074627	0.985074627	Bn-N5-p3644816	Bn-chrA05_random-p327881
Bn-A05-p3414540	0.958677686	0.966942149	Bn-N5-p3661117	Bn-chrAnn_random-p31530C
Bn-A05-p3421628	0.991735537	0.991735537	Bn-N5-p3666804	Bn-chrA05_random-p315473
Bn-A05-p3427883	0.958677686	0.958677686	Bn-N5-p3671044	Bn-chrA05_random-p311234
Bn-A05-p3427993	1	1	Bn-N5-p3671154	Bn-chrA05_random-p311124
Bn-A05-p3428986	0.991735537	0.983471074	Bn-N1-p2711010	Bn-chrA01-p14050097
Bn-A05-p3429612	0.975206612	0.966942149	Bn-N1-p2711636	Bn-chrA01-p14050719
Bn-A05-p3450929	0.980066445	0.983388704	Bn-N5-p3692287	Bn-chrA05_random-p355245
Bn-A05-p3454754	0.950413223	0.950413223	Bn-N5-p3693963	Bn-chrA05_random-p353568
Bn-A05-p3456024	0.950413223	0.950413223	Bn-N5-p3695213	Bn-chrA05_random-p352328
Bn-A05-p3456661	0.950413223	0.966942149	Bn-N5-p3695850	Bn-chrA05_random-p351690
Bn-A05-p3467036	0.991735537	0.983471074	Bn-N5-p3707907	Bn-chrAnn_random-p142154
Bn-A05-p3467060	0.983471074	0.991735537	Bn-N5-p3707931	Bn-chrAnn_random-p142154
Bn-A05-p3467688	0.966942149	0.809917355	Bn-N5-p3708559	Bn-chrAnn_random-p142145
Bn-A05-p3468129	0.975206612	0.983471074	Bn-N5-p3709000	Bn-chrAnn_random-p142141
Bn-A05-p3468204	0.991735537	0.983471074	Bn-N5-p3709075	Bn-chrAnn_random-p14214C
Bn-A05-p3469996	0.958677686	0.966942149	Bn-N5-p3710345	Bn-chrAnn_random-p142129
Bn-A05-p3481481	0.983471074	0.975206612	Bn-N5-p3726270	Bn-chrAnn_random-p141995
Bn-A05-p3481611	0.991735537	0.966942149	Bn-N5-p3726403	Bn-chrAnn_random-p141994
Bn-A05-p3482813	0.945273632	0.950248756	Bn-N5-p3729580	Bn-chrAnn_random-p141915
Bn-A05-p3494729	0.958677686	0.958677686	Bn-N5-p3740258	Bn-chrAnn_random-p141806
Bn-A05-p3507831	0.975206612	0.94214876	Bn-N5-p3749456	Bn-chrAnn_random-p141722
Bn-A05-p3516602	0.966942149	0.966942149	Bn-N5-p3752902	Bn-chrAnn_random-p141671
Bn-A05-p3518253	0.975124378	0.975124378	Bn-N5-p3755219	Bn-chrAnn_random-p141655
Bn-A05-p3548736	0.966942149	0.983471074	Bn-N5-p3785693	Bn-chrA05-p3465156
Bn-A05-p3550203	0.940199336	0.996677741	Bn-N5-p3787183	Bn-chrA05-p3466662
Bn-A05-p3557797	0.975124378	0.980099502	Bn-N5-p3794933	Bn-chrA05-p3475684
Bn-A05-p3598362	0.890365449	0.996677741	Bn-N5-p3839844	Bn-chrA05-p3517858
Bn-A05-p3598758	0.966777409	0.990033223	Bn-N5-p3840232	Bn-chrA05-p3518254
Bn-A05-p3599315	0.945273632	0.950248756	Bn-N5-p3840791	Bn-chrA05-p3518811
Bn-A05-p3605605	0.976744186	0.993355482	Bn-N5-p3846640	Bn-chrA05-p3525752
Bn-A05-p3610111	0.574750831	0.993355482	Bn-N5-p3853841	Bn-chrA05-p3530050
Bn-A05-p3611878	0.604651163	0.990033223	Bn-N5-p3857996	Bn-chrA05-p3531817
Bn-A05-p3617320	0.909090909	0.909090909	Bn-N5-p3872235	Bn-chrA05-p3537247
Bn-A05-p3618356	0.956810631	0.966777409	Bn-N5-p3873260	Bn-chrA05-p3538269
Bn-A05-p3629424	0.950413223	0.991735537	Bn-N5-p3890098	Bn-chrA05-p3550622
Bn-A05-p3649985	1	0.991735537	Bn-N5-p3920087	Bn-chrAnn_random-p367166
Bn-A05-p3650066	1	0.991735537	Bn-N5-p3920168	Bn-chrAnn_random-p367165
Bn-A05-p3659292	0.975206612	0.966942149	Bn-N5-p3931406	Bn-chrA05-p3587696

Bn-A05-p3659796	0.975206612	0.983471074	Bn-N5-p3931910	Bn-chrA05-p3588200
Bn-A05-p3670158	0.983471074	0.975206612	Bn-N5-p3943686	Bn-chrA05-p3598547
Bn-A05-p3681637	0.991735537	0.991735537	Bn-N5-p3957300	Bn-chrA05-p3606806
Bn-A05-p3684668	0.810631229	0.817275748	Bn-N5-p3963432	Bn-chrA05-p3613085
Bn-A05-p3703643	0.975206612	0.983471074	Bn-N5-p3970509	Bn-chrA05-p3621932
Bn-A05-p3719423	0.991735537	1	Bn-N5-p3984819	Bn-chrA05-p3637669
Bn-A05-p3754543	0.975206612	0.966942149	Bn-N5-p4022315	Bn-chrA05-p3670692
Bn-A05-p3759320	0.975206612	0.958677686	Bn-N5-p4027117	Bn-chrA05-p3675545
Bn-A05-p3781544	0.990049751	0.990049751	Bn-N5-p4056950	Bn-chrA05_random-p391761
Bn-A05-p3796206	1	1	Bn-Scaffold01568-p1	Bn-chrUnn_random-p231699
Bn-A05-p3808836	0.993355482	0.993355482	Bn-N5-p4082618	Bn-chrA05-p3706172
Bn-A05-p3812776	0.955223881	0.955223881	Bn-N5-p4085888	Bn-chrA05-p3709442
Bn-A05-p3833073	0.958677686	0.966942149	Bn-N5-p4103596	Bn-chrA05-p3726647
Bn-A05-p3833308	0.970149254	0.975124378	Bn-N14-p6569553	Bn-chrC04-p5714791
Bn-A05-p3834445	0.94214876	0.933884298	Bn-N5-p4104667	Bn-chrA05-p3727720
Bn-A05-p3834639	0.975206612	0.983471074	Bn-N5-p4104862	Bn-chrA05-p3727918
Bn-A05-p3846005	0.975206612	0.966942149	Bn-N5-p4115183	Bn-chrA05-p3736494
Bn-A05-p3847996	0.975206612	0.983471074	Bn-N5-p4117275	Bn-chrA05-p3738075
Bn-A05-p3871998	0.966777409	0.966777409	Bn-N5-p4138868	Bn-chrA05-p3762765
Bn-A05-p3882826	0.966777409	0.966777409	Bn-N5-p4150021	Bn-chrA05-p3773918
Bn-A05-p3901565	0.958677686	0.958677686	Bn-N5-p4166065	Bn-chrA05-p3789409
Bn-A05-p3919804	0.966942149	0.958677686	Bn-N5-p4178615	Bn-chrA04-p9679249
Bn-A05-p3920198	0.991735537	0.991735537	Bn-N4-p10269549	Bn-chrA04-p9678854
Bn-A05-p3923692	1	0.991735537	Bn-N5-p4182501	Bn-chrA05-p3798434
Bn-A05-p3923814	0.993355482	0.986710963	Bn-N5-p4182623	Bn-chrA05-p3798556
Bn-A05-p3939102	0.975206612	0.975206612	Bn-N5-p4203438	Bn-chrA05-p3812575
Bn-A05-p3953066	0.966942149	0.966942149	Bn-N5-p4214245	Bn-chrA05-p3821303
Bn-A05-p3963790	0.966942149	0.983471074	Bn-N5-p4225988	Bn-chrA05-p3831408
Bn-A05-p3966848	0.966942149	0.966942149	Bn-N5-p4230168	Bn-chrA05-p3834211
Bn-A05-p3988218	0.966777409	0.966777409	Bn-N5-p4249789	Bn-chrA05-p3853825
Bn-A05-p3988279	0.966942149	0.966942149	Bn-N5-p4249850	Bn-chrA05-p3853886
Bn-A05-p3988958	0.986710963	0.980066445	Bn-N5-p4250529	Bn-chrA05-p3854565
Bn-A05-p3989555	0.983471074	0.983471074	Bn-N5-p4251126	Bn-chrA05-p3854878
Bn-A05-p4006770	0.983388704	1	Bn-N5-p4268889	Bn-chrA05-p3870713
Bn-A05-p4016172	0.900332226	0.897009967	Bn-N5-p4278430	Bn-chrC04-p6009070
Bn-A05-p4017270	0.913621262	0.913621262	Bn-N5-p4279532	Bn-chrA05-p3882299
Bn-A05-p4022440	0.96345515	0.983388704	Bn-N5-p4284420	Bn-chrA05-p3887773
Bn-A05-p4023464	0.909090909	0.975206612	Bn-N5-p4285459	Bn-chrA05-p3888812
Bn-A05-p4036806	0.990033223	0.890365449	Bn-N5-p4292309	Bn-chrC04-p6028318
Bn-A05-p4043474	0.983471074	0.975206612	Bn-N5-p4302708	Bn-chrA05-p3903352
Bn-A05-p4046605	1	0.996677741	Bn-N5-p4305842	Bn-chrA05-p3906483
Bn-A05-p4053502	0.993355482	0.996677741	Bn-N5-p4312704	Bn-chrA05-p3909610
Bn-A05-p4054823	0.960199005	0.970149254	Bn-N5-p4314001	Bn-chrA05-p3910931
Bn-A05-p4055656	0.985074627	0.990049751	Bn-N5-p4314837	Bn-chrA05-p3911764
Bn-A05-p4055839	0.985074627	0.980099502	Bn-N5-p4315020	Bn-chrA05-p3911947
Bn-A05-p4059534	0.975206612	0.991735537	Bn-N5-p4323014	Bn-chrA05-p3915640

Bn-A05-p4061730	0.966942149	0.958677686	Bn-N5-p4325214	Bn-chrA05-p3917837
Bn-A05-p4061978	0.983471074	0.975206612	Bn-N5-p4325462	Bn-chrA05-p3918085
Bn-A05-p4062134	0.970149254	0.995024876	Bn-N5-p4325616	Bn-chrA05-p3918241
Bn-A05-p4068277	0.980099502	0.975124378	Bn-N5-p4328257	Bn-chrA05-p3929212
Bn-A05-p4069460	0.990049751	0.995024876	Bn-N5-p4329441	Bn-chrA05-p3930395
Bn-A05-p4073230	0.975206612	0.983471074	Bn-N5-p4333154	Bn-chrA05-p3934234
Bn-A05-p4073997	1	0.996677741	Bn-N5-p4333921	Bn-chrA05-p3939575
Bn-A05-p4078496	0.966942149	0.975206612	Bn-N5-p4338418	Bn-chrA05-p3945171
Bn-A05-p4080798	0.867768595	0.958677686	Bn-N5-p4341437	Bn-chrA05-p3947424
Bn-A05-p4085121	0.991735537	1	Bn-N5-p4345906	Bn-chrA05-p3951562
Bn-A05-p4086144	0.991735537	0.975206612	Bn-N5-p4346940	Bn-chrA05-p3952584
Bn-A05-p4093306	0.993355482	0.993355482	Bn-N5-p4355033	Bn-chrA05-p3959827
Bn-A05-p4102906	0.900332226	0.973421927	Bn-N5-p4365753	Bn-chrA05-p3978852
Bn-A05-p4104070	0.975206612	0.983471074	Bn-N5-p4366930	Bn-chrA05-p3980014
Bn-A05-p4108818	0.973421927	0.943521595	Bn-N5-p4372981	Bn-chrA05-p3986064
Bn-A05-p4110015	0.993355482	0.993355482	Bn-N5-p4374186	Bn-chrA05-p3987268
Bn-A05-p4121158	0.983388704	0.983388704	Bn-N5-p4386273	Bn-chrA05-p4000089
Bn-A05-p4176921	0.990049751	0.990049751	Bn-N5-p4433574	Bn-chrA05-p4047718
Bn-A05-p4196214	0.887043189	0.887043189	Bn-N5-p4450386	Bn-chrA05-p4065036
Bn-A05-p4197697	0.983471074	0.975206612	Bn-N5-p4451869	Bn-chrA05-p4066519
Bn-A05-p4212213	0.993355482	0.993355482	Bn-N5-p4463509	Bn-chrA05-p4079456
Bn-A05-p4230422	0.996677741	0.996677741	Bn-N5-p4488675	Bn-chrA05-p4106600
Bn-A05-p4290068	0.628099174	0.991735537	Bn-N5-p4557642	Bn-chrC04-p6432011
Bn-A05-p4304172	0.909090909	0.966942149	Bn-N14-p7388271	Bn-chrC04-p6449454
Bn-A05-p4307316	0.94214876	0.950413223	Bn-N5-p4533286	Bn-chrC04-p6451985
Bn-A05-p4316642	0.94214876	0.933884298	Bn-N5-p4565661	Bn-chrA05-p4190636
Bn-A05-p4316943	0.909090909	0.909090909	Bn-N5-p4565962	Bn-chrA05-p4190926
Bn-A05-p4333409	0.958677686	0.966942149	Bn-N5-p4593820	Bn-chrA05-p4207579
Bn-A05-p4336667	0.958677686	0.966942149	Bn-N5-p4597112	Bn-chrA05-p4210705
Bn-A05-p4353454	0.995024876	0.990049751	Bn-N5-p4607614	Bn-chrA05-p4224193
Bn-A05-p4354442	0.970149254	0.965174129	Bn-N5-p4608601	Bn-chrA05-p4225180
Bn-A05-p4355047	0.980099502	0.980099502	Bn-N5-p4609263	Bn-chrA05-p4225785
Bn-A05-p4373694	0.986710963	0.986710963	Bn-N5-p4623279	Bn-chrA05-p4243236
Bn-A05-p4376029	0.966942149	0.975206612	Bn-N5-p4625600	Bn-chrA05-p4245604
Bn-A05-p4398134	0.966942149	0.975206612	Bn-N5-p4640100	Bn-chrA05-p4263897
Bn-A05-p4399949	0.950413223	0.950413223	Bn-N5-p4641889	Bn-chrA05-p4265716
Bn-A05-p4418703	0.96013289	0.996677741	Bn-N5-p4658704	Bn-chrA05-p4287514
Bn-A05-p4422786	0.990049751	0.990049751	Bn-N5-p4663810	Bn-chrA05-p4295731
Bn-A05-p4498290	0.995024876	0.995024876	Bn-N5-p4720302	Bn-chrA05-p4354451
Bn-A05-p4498398	0.935323383	0.995024876	Bn-N5-p4720410	Bn-chrA05-p4354559
Bn-A05-p4516526	0.950248756	0.950248756	Bn-N5-p4775030	Bn-chrA05-p4373237
Bn-A05-p4516748	0.825870647	0.825870647	Bn-N14-p7637596	Bn-chrC04-p6701328
Bn-A05-p4520844	0.965517241	0.982758621	Bn-N14-p7641133	Bn-chrA05-p4377072
Bn-A05-p4524286	0.854166667	0.989583333	Bn-N5-p4783008	Bn-chrA05-p4385975
Bn-A05-p4525705	0.801652893	0.892561983	Bn-N5-p4784405	Bn-chrA05-p4387443
Bn-A05-p4542257	0.983471074	0.975206612	Bn-N5-p4804774	Bn-chrA05-p4397501

Bn-A05-p4549959	0.980066445	0.986710963	Bn-N5-p4812406	Bn-chrA05-p4405188
Bn-A05-p4550465	0.983471074	0.991735537	Bn-N5-p4812912	Bn-chrA05-p4405704
Bn-A05-p4569670	0.983471074	0.983471074	Bn-N5-p4831145	Bn-chrA05-p4422450
Bn-A05-p4590055	0.983471074	0.991735537	Bn-N5-p4845175	Bn-chrA05-p4433823
Bn-A05-p4590326	0.94214876	0.958677686	Bn-N5-p4845447	Bn-chrA05-p4434097
Bn-A05-p4593001	0.986710963	0.990033223	Bn-N5-p4848123	Bn-chrA05-p4436773
Bn-A05-p4612911	0.966942149	0.925619835	Bn-N5-p4863246	Bn-chrA05-p4456050
Bn-A05-p4616151	0.958677686	0.958677686	Bn-N5-p4866381	Bn-chrA05-p4459237
Bn-A05-p4618158	0.993355482	0.993355482	Bn-N5-p4868223	Bn-chrA05-p4461079
Bn-A05-p4619303	1	1	Bn-N5-p4869368	Bn-chrA05-p4462224
Bn-A05-p4621087	0.975206612	0.975206612	Bn-N5-p4871145	Bn-chrA05-p4464000
Bn-A05-p4621949	0.996677741	0.996677741	Bn-N5-p4872017	Bn-chrA05-p4464872
Bn-A05-p4625019	0.991735537	0.991735537	Bn-N5-p4875635	Bn-chrA05-p4468491
Bn-A05-p4632407	0.958677686	0.958677686	Bn-N5-p4885817	Bn-chrA05-p4478433
Bn-A05-p4644984	1	1	Bn-N5-p4902267	Bn-chrA05-p4494882
Bn-A05-p4653714	0.737541528	0.727574751	Bn-N1-p19415948	Bn-chrC03-p12988811
Bn-A05-p4655845	0.684385382	0.684385382	Bn-N5-p4924500	Bn-chrA05-p4518640
Bn-A05-p4665099	1	1	Bn-N5-p4932628	Bn-chrA05-p4526768
Bn-A05-p4679663	0.986710963	0.986710963	Bn-N5-p4945932	Bn-chrA05-p4539949
Bn-A05-p4696360	0.966942149	0.966942149	Bn-N5-p4959227	Bn-chrA05-p4553313
Bn-A05-p4715599	0.983471074	0.983471074	Bn-N5-p4975572	Bn-chrA05-p4572583
Bn-A05-p4717725	0.958677686	0.958677686	Bn-N5-p4977698	Bn-chrA05-p4574709
Bn-A05-p4717815	0.933884298	0.933884298	Bn-N5-p4977788	Bn-chrA05-p4574799
Bn-A05-p4728882	0.975206612	0.975206612	Bn-N5-p4989909	Bn-chrA05-p4592802
Bn-A05-p4729566	0.983471074	0.983471074	Bn-N5-p4990593	Bn-chrA05-p4593486
Bn-A05-p4732914	0.950413223	0.950413223	Bn-N5-p5007390	Bn-chrAnn_random-p230681
Bn-A05-p4736340	0.975206612	0.181818182	Bn-N5-p5010815	Bn-chrA05-p16600876
Bn-A05-p4737634	0.991735537	0.991735537	Bn-N5-p5012107	Bn-chrAnn_random-p230739
Bn-A05-p4749718	0.983471074	0.983471074	Bn-N5-p5026641	Bn-chrAnn_random-p372702
Bn-A05-p4762044	0.985074627	0.985074627	Bn-N14-p8085793	Bn-chrC04-p7144272
Bn-A05-p4772468	0.975206612	0.975206612	Bn-N5-p5046659	Bn-chrA05-p4624691
Bn-A05-p4792988	0.950413223	0.950413223	Bn-N5-p5070399	Bn-chrA05-p4647061
Bn-A05-p4793033	0.966942149	0.966942149	Bn-N5-p5070444	Bn-chrA05-p4647106
Bn-A05-p4794141	0.995024876	0.990049751	Bn-N5-p5071552	Bn-chrA05-p4648214
Bn-A05-p4820365	0.966942149	0.966942149	Bn-N5-p5097227	Bn-chrA05-p4673685
Bn-A05-p4871527	0.991735537	0.991735537	Bn-N5-p5145381	Bn-chrAnn_random-p468118
Bn-A05-p4904405	0.983471074	0.983471074	Bn-N5-p5164920	Bn-chrA05-p4770325
Bn-A05-p4915516	0.966942149	0.966942149	Bn-N5-p5171809	Bn-chrA05-p4777240
Bn-A05-p4919899	1	1	Bn-N5-p5176166	Bn-chrA05-p4781592
Bn-A05-p4924856	0.970099668	0.92358804	Bn-N5-p5181567	Bn-chrC04-p7371721
Bn-A05-p4937055	0.975206612	0.975206612	Bn-N5-p5190282	Bn-chrA05-p4796651
Bn-A05-p4940676	0.975206612	0.975206612	Bn-N5-p5194031	Bn-chrA05-p4800399
Bn-A05-p4940713	0.975206612	0.975206612	Bn-N5-p5194068	Bn-chrA05-p4800436
Bn-A05-p4940779	0.983471074	0.983471074	Bn-N5-p5194134	Bn-chrA05-p4800502
Bn-A05-p4941234	0.966942149	0.966942149	Bn-N5-p5194586	Bn-chrA05-p4800954
Bn-A05-p4959282	0.985074627	0.985074627	Bn-N5-p5206299	Bn-chrA05_random-p426152

Bn-A05-p4961402	0.966942149	0.966942149	Bn-N5-p5216947	Bn-chrA05_random-p415191
Bn-A05-p4973912	0.958677686	0.966942149	Bn-N5-p5225439	Bn-chrA05-p4808641
Bn-A05-p4984837	0.991735537	0.983471074	Bn-N5-p5233035	Bn-chrA05-p4815645
Bn-A05-p5001194	0.983471074	0.975206612	Bn-Scaffold09271-p2	Bn-chrA04-p94839
Bn-A05-p5002988	0.983471074	0.966942149	Bn-N5-p5244900	Bn-chrA05-p4819987
Bn-A05-p5004187	0.983471074	0.950413223	Bn-N5-p5246207	Bn-chrC04-p7435747
Bn-A05-p5020058	1	1	Bn-N5-p5276623	Bn-chrA05-p4841157
Bn-A05-p5026139	0.983471074	0.975206612	Bn-N5-p5284473	Bn-chrA05-p4847212
Bn-A05-p5037839	0.983471074	0.975206612	Bn-N5-p5295656	Bn-chrA05-p4858600
Bn-A05-p5038148	0.995024876	1	Bn-N5-p5295965	Bn-chrA05-p4858909
Bn-A05-p5041159	0.966942149	0.851239669	Bn-N5-p5298976	Bn-chrA05-p4861923
Bn-A05-p5043499	1	0.926910299	Bn-N5-p5301311	Bn-chrA05-p4864632
Bn-A05-p5057096	0.975124378	0.975124378	Bn-N5-p5316568	Bn-chrA05-p4876370
Bn-A05-p5057141	0.985074627	0.985074627	Bn-N5-p5316613	Bn-chrA05-p4876415
Bn-A05-p5058143	1	1	Bn-N5-p5317614	Bn-chrA05-p4877416
Bn-A05-p5093548	0.94214876	0.950413223	Bn-N5-p5361751	Bn-chrA05-p4913129
Bn-A05-p5100352	0.970149254	0.965174129	Bn-N5-p5368829	Bn-chrA05-p4920149
Bn-A05-p5102120	0.983471074	0.983471074	Bn-N5-p5370597	Bn-chrA05-p4921917
Bn-A05-p5102147	0.991735537	0.991735537	Bn-N5-p5370624	Bn-chrA05-p4921944
Bn-A05-p5104821	0.975124378	0.965174129	Bn-N5-p5376501	Bn-chrA05-p4924675
Bn-A05-p5133417	0.996677741	1	Bn-N5-p5401575	Bn-chrA05-p4947944
Bn-A05-p5135426	0.975206612	0.983471074	Bn-N5-p5403580	Bn-chrA05-p4949946
Bn-A05-p5251613	0.975206612	0.966942149	Bn-N5-p5501272	Bn-chrA05-p5036103
Bn-A05-p5252542	0.991735537	0.983471074	Bn-N5-p5502201	Bn-chrA05-p5043023
Bn-A05-p5264904	0.94214876	0.983471074	Bn-N5-p5540139	Bn-chrA05-p5052125
Bn-A05-p5279229	0.983388704	0.980066445	Bn-N5-p5552874	Bn-chrA05-p5066964
Bn-A05-p5280016	0.993355482	0.990033223	Bn-N5-p5553661	Bn-chrA05-p5067751
Bn-A05-p5281478	0.976744186	0.976744186	Bn-N5-p5555132	Bn-chrA05-p5069254
Bn-A05-p5283304	0.752066116	0.983471074	Bn-N14-p9128434	Bn-chrA05-p5071100
Bn-A05-p5328959	0.993355482	0.996677741	Bn-N5-p5602569	Bn-chrA05-p5103067
Bn-A05-p5337620	1	0.991735537	Bn-N5-p5611393	Bn-chrA05-p5118454
Bn-A05-p5344859	0.991735537	0.983471074	Bn-N5-p5612892	Bn-chrA05-p5120232
Bn-A05-p5349944	0.983471074	0.975206612	Bn-N5-p5617991	Bn-chrA05-p5123897
Bn-A05-p5350567	1	0.991735537	Bn-N5-p5618612	Bn-chrA05-p5126827
Bn-A05-p5350892	0.743801653	0.991735537	Bn-N5-p5618923	Bn-chrA05-p5127153
Bn-A05-p5372015	0.983471074	0.983471074	Bn-N5-p5652046	Bn-chrA05-p5159967
Bn-A05-p5374000	0.975206612	0.975206612	Bn-N5-p5654806	Bn-chrA05-p5162727
Bn-A05-p5391937	0.983471074	0.983471074	Bn-N5-p5672976	Bn-chrA05-p5183157
Bn-A05-p5437257	0.975206612	0.966942149	Bn-N5-p5711890	Bn-chrC04-p8416340
Bn-A05-p5445252	0.966942149	0.958677686	Bn-N5-p5722369	Bn-chrA05-p5230560
Bn-A05-p5446122	0.975206612	0.950413223	Bn-N5-p5722728	Bn-chrA05-p5230918
Bn-A05-p5457877	0.958677686	0.876033058	Bn-N5-p5731679	Bn-chrA05-p5239534
Bn-A05-p5475992	0.970149254	0.960199005	Bn-N5-p5746359	Bn-chrA05-p5254455
Bn-A05-p5483612	0.996677741	0.993355482	Bn-N5-p5754505	Bn-chrA05-p5260443
Bn-A05-p5492491	0.966942149	0.966942149	Bn-N5-p5763276	Bn-chrA05-p5269333
Bn-A05-p5519729	0.94214876	0.94214876	Bn-N5-p5788080	Bn-chrAnn_random-p289391

Bn-A05-p5521242	0.958677686	0.958677686	Bn-N5-p5789631	Bn-chrAnn_random-p289407
Bn-A05-p5522210	0.970149254	0.970149254	Bn-N5-p5790600	Bn-chrAnn_random-p289416
Bn-A05-p5533987	0.966942149	0.966942149	Bn-N5-p5799452	Bn-chrA05-p5283814
Bn-A05-p5535142	0.94214876	0.94214876	Bn-N5-p5800628	Bn-chrA05-p5284990
Bn-A05-p5537385	0.958677686	0.958677686	Bn-N5-p5802853	Bn-chrA05-p5287460
Bn-A05-p5537609	0.991735537	0.991735537	Bn-N5-p5803050	Bn-chrA05-p5287657
Bn-A05-p5537751	0.991735537	0.991735537	Bn-N5-p5803193	Bn-chrA05-p5287800
Bn-A05-p5541232	0.983471074	0.991735537	Bn-N5-p5806607	Bn-chrA05-p5291212
Bn-A05-p5542434	1	0.991735537	Bn-N5-p5807936	Bn-chrA05-p5292541
Bn-A05-p5552246	0.950413223	0.94214876	Bn-N5-p5817607	Bn-chrA05-p5306929
Bn-A05-p5565945	0.993355482	0.990033223	Bn-N5-p5831310	Bn-chrA05-p5320688
Bn-A05-p5566193	0.990033223	0.990033223	Bn-N5-p5831558	Bn-chrA05-p5320939
Bn-A05-p5575695	0.958677686	0.950413223	Bn-N5-p5841821	Bn-chrA05-p5325972
Bn-A05-p5586839	0.834710744	0.760330579	Bn-N5-p5852889	Bn-chrA05-p5333851
Bn-A05-p5588271	0.950413223	0.94214876	Bn-N5-p5854323	Bn-chrA05-p5335252
Bn-A05-p5595403	0.950413223	0.950413223	Bn-N5-p5861454	Bn-chrA05-p5343605
Bn-A05-p5595578	0.980099502	0.985074627	Bn-N5-p5861629	Bn-chrA05-p5343779
Bn-A05-p5614651	0.970149254	0.970149254	Bn-N5-p5885217	Bn-chrA05-p5360638
Bn-A05-p5615444	0.995024876	0.995024876	Bn-N5-p5886010	Bn-chrA05-p5361431
Bn-A05-p5627413	0.975206612	0.966942149	Bn-N5-p5900102	Bn-chrA05-p5376033
Bn-A05-p5644210	0.930232558	0.930232558	Bn-N5-p5916356	Bn-chrA05-p5398350
Bn-A05-p5645000	0.993355482	0.993355482	Bn-N5-p5917146	Bn-chrA05-p5399140
Bn-A05-p5647262	0.996677741	0.996677741	Bn-N5-p5919408	Bn-chrA05-p5401402
Bn-A05-p5657708	0.925619835	0.933884298	Bn-N5-p5929818	Bn-chrA05-p5411907
Bn-A05-p5661136	0.94214876	0.950413223	Bn-N5-p5933631	Bn-chrA05-p5418568
Bn-A05-p5662601	0.995024876	1	Bn-N5-p5935093	Bn-chrA05-p5420021
Bn-A05-p5664658	0.991735537	0.983471074	Bn-N5-p5937171	Bn-chrA05-p5422098
Bn-A05-p5670181	0.945273632	0.945273632	Bn-N5-p5941716	Bn-chrA05-p5426727
Bn-A05-p5684860	0.975206612	0.966942149	Bn-N5-p5948811	Bn-chrA05-p5433909
Bn-A05-p5687833	0.966942149	0.975206612	Bn-N5-p5951784	Bn-chrA05-p5436878
Bn-A05-p5689705	0.933884298	0.94214876	Bn-N5-p5953656	Bn-chrA05-p5440012
Bn-A05-p5691374	0.975206612	0.966942149	Bn-N5-p5955278	Bn-chrA05-p5441662
Bn-A05-p5693743	0.966942149	0.917355372	Bn-N5-p5957647	Bn-chrA05-p5444015
Bn-A05-p5700216	0.933884298	0.785123967	Bn-N5-p5962932	Bn-chrA05-p5444600
Bn-A05-p5700891	0.966942149	0.818181818	Bn-N5-p5963607	Bn-chrA05-p5445274
Bn-A05-p5714702	0.985074627	0.985074627	Bn-N5-p5978793	Bn-chrA05-p5459849
Bn-A05-p5714756	0.980099502	0.980099502	Bn-N5-p5978847	Bn-chrA05-p5459903
Bn-A05-p5714836	0.975206612	0.983471074	Bn-N5-p5978927	Bn-chrA05-p5459983
Bn-A05-p5715403	0.980099502	0.980099502	Bn-N5-p5979494	Bn-chrA05-p5460551
Bn-A05-p5764156	0.94214876	0.966942149	Bn-N5-p6030857	Bn-chrA05-p5494720
Bn-A05-p5770114	0.983471074	0.975206612	Bn-N5-p6038169	Bn-chrA05-p5501520
Bn-A05-p5778158	0.993355482	0.990033223	Bn-N5-p6046199	Bn-chrA05-p5509573
Bn-A05-p5790313	0.995024876	0.995024876	Bn-N5-p6064158	Bn-chrA09-p26594641
Bn-A05-p5796678	0.909090909	0.909090909	Bn-N5-p6070566	Bn-chrA05-p5531033
Bn-A05-p5805445	0.966942149	0.966942149	Bn-N5-p6077945	Bn-chrA05-p5538406
Bn-A05-p5808654	0.966942149	0.958677686	Bn-N5-p6081172	Bn-chrA05-p5541660

Bn-A05-p5808895	0.980099502	0.945273632	Bn-N5-p6081413	Bn-chrC04-p8549446
Bn-A05-p5809043	0.985074627	0.985074627	Bn-N5-p6081561	Bn-chrA05-p5542058
Bn-A05-p5812537	0.933884298	0.950413223	Bn-N5-p6084998	Bn-chrA05-p5545506
Bn-A05-p5817231	0.966942149	0.975206612	Bn-N5-p6087991	Bn-chrA05-p5548397
Bn-A05-p5831673	0.990049751	0.990049751	Bn-N5-p6099375	Bn-chrA05-p5558562
Bn-A05-p5838524	0.975206612	0.966942149	Bn-N5-p6106417	Bn-chrA05-p5563282
Bn-A05-p5839222	0.983471074	0.991735537	Bn-N5-p6109754	Bn-chrC04-p8598592
Bn-A05-p5839438	0.983471074	0.991735537	Bn-N5-p6109970	Bn-chrC04-p8598813
Bn-A05-p5842980	0.975206612	0.892561983	Bn-N5-p6113510	Bn-chrC04-p8612852
Bn-A05-p5865143	0.975206612	0.966942149	Bn-N5-p6136618	Bn-chrA05-p5592092
Bn-A05-p5878329	0.975206612	0.983471074	Bn-N5-p6148874	Bn-chrA05-p5606124
Bn-A05-p5897513	0.926910299	0.913621262	Bn-N5-p6167768	Bn-chrA05-p5622788
Bn-A05-p5924319	0.966942149	0.975206612	Bn-N5-p6195750	Bn-chrA05-p5661405
Bn-A05-p5926631	0.991735537	0.975206612	Bn-N5-p6198075	Bn-chrA05-p5664439
Bn-A05-p5936565	0.940298507	0.940298507	Bn-Scaffold39872-p2	Bn-chrA01-p2607674
Bn-A05-p5970916	0.975206612	0.983471074	Bn-N5-p6250576	Bn-chrA05-p5708838
Bn-A05-p5973528	1	1	Bn-N5-p6252907	Bn-chrA05-p5711169
Bn-A05-p5973876	0.993355482	0.993355482	Bn-N5-p6253255	Bn-chrA05-p5711517
Bn-A05-p5992612	0.983388704	0.983388704	Bn-N5-p6282683	Bn-chrAnn_random-p321753
Bn-A05-p6004356	0.993355482	0.980066445	Bn-N5-p6293193	Bn-chrA05-p5744967
Bn-A05-p6020205	0.993355482	0.993355482	Bn-N5-p6308665	Bn-chrA05-p5766873
Bn-A05-p6021593	0.970149254	0.965174129	Bn-N5-p6310052	Bn-chrA05-p5768260
Bn-A05-p6055523	0.983471074	0.933884298	Bn-N5-p6334030	Bn-chrCnn_random-p787683
Bn-A05-p6061277	0.995024876	0.980099502	Bn-N6-p7011534	Bn-chrA09-p17269474
Bn-A05-p6063715	0.990049751	0.985074627	Bn-N5-p6337619	Bn-chrA05-p5808915
Bn-A05-p6074427	0.996677741	0.996677741	Bn-N5-p6347876	Bn-chrA05-p5820354
Bn-A05-p6154871	0.917355372	0.925619835	Bn-N5-p6434195	Bn-chrA05_random-p545526
Bn-A05-p6155799	0.983471074	0.975206612	Bn-N5-p6435123	Bn-chrA05_random-p546454
Bn-A05-p6155901	0.985074627	0.985074627	Bn-N5-p6435225	Bn-chrA05_random-p546556
Bn-A05-p6176943	0.975124378	0.975124378	Bn-N5-p6457548	Bn-chrA05_random-p569162
Bn-A05-p6236807	1	1	Bn-N5-p6514458	Bn-chrAnn_random-p180587
Bn-A05-p6261796	0.970149254	0.975124378	Bn-N5-p6540668	Bn-chrA05_random-p582953
Bn-A05-p6269991	0.993355482	0.993355482	Bn-N5-p6552395	Bn-chrAnn_random-p172361
Bn-A05-p6271443	0.970149254	0.990049751	Bn-N5-p6553847	Bn-chrAnn_random-p172375
Bn-A05-p6284593	0.975206612	0.966942149	Bn-N5-p6572084	Bn-chrA05-p5844448
Bn-A05-p6288330	0.983471074	0.991735537	Bn-N5-p6575827	Bn-chrA05-p5848176
Bn-A05-p6308471	0.950413223	0.950413223	Bn-N5-p6599347	Bn-chrA05-p5867212
Bn-A05-p6308543	0.991735537	0.991735537	Bn-N5-p6599419	Bn-chrA05-p5867284
Bn-A05-p6335652	0.94214876	0.94214876	Bn-N5-p6622776	Bn-chrA05-p5896227
Bn-A05-p6337412	0.996677741	0.996677741	Bn-N5-p6626825	Bn-chrA05-p5900411
Bn-A05-p6402777	0.966942149	0.966942149	Bn-N5-p6686533	Bn-chrA05-p5954217
Bn-A05-p6404424	0.975124378	0.975124378	Bn-N5-p6688182	Bn-chrA05-p5955866
Bn-A05-p6404595	0.985074627	0.980099502	Bn-N5-p6688353	Bn-chrA05-p5956037
Bn-A05-p6404775	0.945273632	0.950248756	Bn-N5-p6688533	Bn-chrA05-p5956217
Bn-A05-p6405021	0.970149254	0.960199005	Bn-N5-p6688779	Bn-chrA05-p5956466
Bn-A05-p6405095	0.970149254	0.975124378	Bn-N5-p6688853	Bn-chrA05-p5956541

Bn-A05-p6405336	0.970149254	0.975124378	Bn-N5-p6689094	Bn-chrA05-p5956781
Bn-A05-p6405415	0.990049751	0.995024876	Bn-N5-p6689173	Bn-chrA05-p5956860
Bn-A05-p6412530	0.966942149	0.958677686	Bn-N5-p6696504	Bn-chrA05-p5958804
Bn-A05-p6415983	1	0.991735537	Bn-N5-p6699957	Bn-chrA05-p5964030
Bn-A05-p6416604	0.975206612	0.603305785	Bn-N5-p6700578	Bn-chrAnn_random-p145092
Bn-A05-p6423355	0.983471074	0.991735537	Bn-N5-p6707943	Bn-chrA05-p5972003
Bn-A05-p6423710	0.966942149	0.950413223	Bn-N5-p6708298	Bn-chrA05-p5972314
Bn-A05-p6438367	0.975206612	0.983471074	Bn-N5-p6717911	Bn-chrA05-p5983061
Bn-A05-p6441813	0.983471074	0.991735537	Bn-N5-p6721334	Bn-chrA05-p5985905
Bn-A05-p6457465	1	0.991735537	Bn-N5-p6739622	Bn-chrA05-p5999019
Bn-A05-p6473296	0.993355482	0.986710963	Bn-N5-p6767318	Bn-chrA05_random-p625422
Bn-A05-p6479603	1	1	Bn-N5-p6773398	Bn-chrA05_random-p619522
Bn-A05-p6480168	0.966942149	0.950413223	Bn-N5-p6773964	Bn-chrA05_random-p619051
Bn-A05-p6480907	0.966942149	0.958677686	Bn-N5-p6774703	Bn-chrA05_random-p618312
Bn-A05-p6480981	0.991735537	0.983471074	Bn-N5-p6774777	Bn-chrA05_random-p618238
Bn-A05-p6494313	0.945273632	0.945273632	Bn-N5-p6784054	Bn-chrA05_random-p610652
Bn-A05-p6534544	0.966942149	0.966942149	Bn-N5-p6830606	Bn-chrA05_random-p675326
Bn-A05-p6535233	0.985074627	0.985074627	Bn-N5-p6831295	Bn-chrA05_random-p676015
Bn-A05-p6539730	0.966942149	0.975206612	Bn-N5-p6968830	Bn-chrA05-p6207592
Bn-A05-p6541429	0.966942149	0.975206612	Bn-N5-p6966977	Bn-chrA05-p6205892
Bn-A05-p6542040	0.991735537	1	Bn-N5-p6966372	Bn-chrA05-p6205281
Bn-A05-p6558171	0.991735537	0.991735537	Bn-N5-p6945237	Bn-chrA05-p6184485
Bn-A05-p6562415	1	0.991735537	Bn-N5-p6940989	Bn-chrA05-p6180237
Bn-A05-p6571327	0.980099502	0.975124378	Bn-N5-p6932082	Bn-chrA05-p6171320
Bn-A05-p6571330	0.975124378	0.980099502	Bn-N5-p6932079	Bn-chrA05-p6171317
Bn-A05-p6654285	0.991735537	0.991735537	Bn-N5-p6835828	Bn-chrCnn_random-p707473
Bn-A05-p6654419	0.966942149	0.966942149	Bn-N5-p6835694	Bn-chrCnn_random-p707474
Bn-A05-p6672638	0.993355482	0.993355482	Bn-N8-p18042252	Bn-chrA08-p15754074
Bn-A05-p6674758	0.991735537	0.975206612	Bn-N5-p6976079	Bn-chrA05-p6215089
Bn-A05-p6682796	0.975206612	0.975206612	Bn-N5-p6984110	Bn-chrA05-p6228433
Bn-A05-p6688695	0.917355372	0.917355372	Bn-N5-p6991428	Bn-chrA05-p6235752
Bn-A05-p6739093	0.958677686	0.958677686	Bn-N5-p7031142	Bn-chrAnn_random-p399721
Bn-A05-p6739341	0.991735537	0.991735537	Bn-N5-p7031390	Bn-chrAnn_random-p399718
Bn-A05-p6748150	0.996677741	0.996677741	Bn-N5-p7046074	Bn-chrA10-p10431924
Bn-A05-p6750239	0.983471074	0.983471074	Bn-N5-p7048162	Bn-chrA10-p10434013
Bn-A05-p6750458	0.975206612	0.975206612	Bn-N5-p7048381	Bn-chrA10-p10434232
Bn-A05-p6751337	1	1	Bn-N5-p7049262	Bn-chrA10-p10435111
Bn-A05-p6751841	0.991735537	0.991735537	Bn-N5-p7049766	Bn-chrA10-p10435615
Bn-A05-p6757087	0.989583333	0.989583333	Bn-N5-p7055675	Bn-chrA05_random-p699443
Bn-A05-p6774249	0.983471074	0.983471074	Bn-N5-p7074505	Bn-chrA05_random-p717765
Bn-A05-p6775737	0.985074627	0.985074627	Bn-N5-p7075993	Bn-chrA05_random-p719253
Bn-A05-p6776954	0.991735537	0.991735537	Bn-N5-p7077214	Bn-chrA05_random-p720470
Bn-A05-p6786862	1	0.996677741	Bn-N5-p7081718	Bn-chrA05_random-p724973
Bn-A05-p6787164	1	1	Bn-N5-p7082020	Bn-chrA05_random-p725275
Bn-A05-p6822861	0.987730061	0.987730061	Bn-N9-p21546650	Bn-chrA09_random-p230273
Bn-A05-p6845189	0.991735537	0.991735537	Bn-N5-p7137663	Bn-chrA05-p6293524

Bn-A05-p6867531	0.996677741	0.96013289	Bn-N5-p7163028	Bn-chrA05-p6316616
Bn-A05-p6877058	0.975206612	0.975206612	Bn-N5-p7170758	Bn-chrA05-p6326688
Bn-A05-p6878538	0.980066445	0.976744186	Bn-N5-p7172426	Bn-chrCnn_random-p560474
Bn-A05-p6882852	0.986710963	0.956810631	Bn-N5-p23052074	Bn-chrA05-p6329875
Bn-A05-p6897871	1	0.991735537	Bn-N5-p7188085	Bn-chrA05-p6348866
Bn-A05-p6898696	0.885572139	0.975124378	Bn-N14-p12313445	Bn-chrA05-p6349707
Bn-A05-p6910402	0.995024876	0.995024876	Bn-N5-p7201619	Bn-chrCnn_random-p292129
Bn-A05-p6918694	0.982352941	0.994117647	Bn-N3-p17029747	Bn-chrA07-p15012567
Bn-A05-p6951707	0.975124378	0.990049751	Bn-N5-p7239361	Bn-chrA05-p6377916
Bn-A05-p6955996	0.983388704	0.983388704	Bn-Scaffold09921-p2	Bn-chrA05-p11918382
Bn-A05-p6957948	0.980099502	0.990049751	Bn-N5-p7245977	Bn-chrA05-p6385713
Bn-A05-p6975353	0.995024876	0.995024876	Bn-N5-p7267602	Bn-chrA05-p6404051
Bn-A05-p6984362	0.983471074	0.983471074	Bn-N5-p7276887	Bn-chrA05-p6411956
Bn-A05-p6995896	0.986710963	0.983388704	Bn-N5-p7292023	Bn-chrA05-p6423488
Bn-A05-p7011916	0.991735537	0.991735537	Bn-N5-p7304200	Bn-chrA05-p6439431
Bn-A05-p7031053	0.966942149	0.966942149	Bn-N5-p7323697	Bn-chrA05-p6457049
Bn-A05-p7032286	0.991735537	0.991735537	Bn-N5-p7324930	Bn-chrA05-p6458282
Bn-A05-p7038463	0.983471074	0.983471074	Bn-N5-p7331107	Bn-chrA05-p6464294
Bn-A05-p7048173	0.983471074	0.983471074	Bn-N5-p7341118	Bn-chrA05-p6475391
Bn-A05-p7052463	0.975124378	0.975124378	Bn-N5-p7345636	Bn-chrA05-p6479726
Bn-A05-p7059671	0.975206612	0.975206612	Bn-N5-p7375796	Bn-chrA05-p6508466
Bn-A05-p7062575	0.983471074	0.983471074	Bn-N5-p7378703	Bn-chrA05-p6511373
Bn-A05-p7062784	0.990049751	0.990049751	Bn-N5-p7378912	Bn-chrA05-p6511582
Bn-A05-p7076442	0.975206612	0.975206612	Bn-N5-p7398390	Bn-chrA05-p6528084
Bn-A05-p7098949	0.950413223	0.950413223	Bn-N5-p7418100	Bn-chrA05-p6546166
Bn-A05-p7105221	0.975206612	0.975206612	Bn-N5-p7424376	Bn-chrA05-p6552442
Bn-A05-p7119719	0.950248756	0.950248756	Bn-N5-p7434666	Bn-chrA05-p6562696
Bn-A05-p7125379	0.859504132	0.884297521	Bn-N12-p32151704	Bn-chrA04-p13325489
Bn-A05-p7137614	0.991735537	0.991735537	Bn-N5-p7446413	Bn-chrA05-p6574447
Bn-A05-p7140511	0.993355482	0.993355482	Bn-N5-p7449294	Bn-chrA05-p6580064
Bn-A05-p7141123	0.996677741	0.996677741	Bn-N5-p7449901	Bn-chrA05-p6580670
Bn-A05-p7154689	1	1	Bn-N5-p7457067	Bn-chrA05-p6591784
Bn-A05-p7187425	0.966942149	0.966942149	Bn-N5-p7485167	Bn-chrA05-p6615357
Bn-A05-p7222795	0.975206612	0.975206612	Bn-N5-p7509876	Bn-chrA05-p6641835
Bn-A05-p7224750	0.950413223	0.950413223	Bn-N5-p7518084	Bn-chrA05-p6643784
Bn-A05-p7227877	0.991735537	0.983471074	Bn-N5-p7521211	Bn-chrA05-p6651271
Bn-A05-p7231369	0.983471074	0.975206612	Bn-N5-p7525358	Bn-chrA05-p6655356
Bn-A05-p7246705	0.980066445	0.973421927	Bn-N5-p7540642	Bn-chrA05-p6670898
Bn-A05-p7255746	0.754152824	0.740863787	Bn-N5-p7549889	Bn-chrA05-p6687391
Bn-A05-p7346478	0.966942149	0.966942149	Bn-N5-p7631525	Bn-chrA05-p6771822
Bn-A05-p7352554	1	1	Bn-N5-p7638503	Bn-chrA05-p6778799
Bn-A05-p7370869	0.985074627	0.562189055	Bn-N5-p7650848	Bn-chrA09-p5235427
Bn-A05-p7434500	0.880398671	0.940199336	Bn-N15-p18274669	Bn-chrA07-p1771993
Bn-A05-p7472918	0.983471074	0.983471074	Bn-N5-p7806657	Bn-chrAnn_random-p430792
Bn-A05-p7491426	0.94214876	0.925619835	Bn-N14-p13447694	Bn-chrA05-p6997488
Bn-A05-p7496554	0.980099502	0.980099502	Bn-N5-p7850202	Bn-chrA05-p7006248

Bn-A05-p7552614	0.991735537	1	Bn-N5-p7907553	Bn-chrA05-p7060766
Bn-A05-p7558647	0.933884298	0.933884298	Bn-N14-p13605529	Bn-chrC04-p12082740
Bn-A05-p7589125	0.950248756	0.965174129	Bn-N5-p7948967	Bn-chrA05-p7095554
Bn-A05-p7589257	0.965174129	0.970149254	Bn-N5-p7949099	Bn-chrA05-p7095686
Bn-A05-p7591501	0.966942149	0.79338843	Bn-N5-p7951338	Bn-chrC04-p12183798
Bn-A05-p7613069	0.996677741	1	Bn-N5-p7971834	Bn-chrA05-p7115882
Bn-A05-p7618158	0.993355482	0.990033223	Bn-N5-p7976875	Bn-chrA05-p7120923
Bn-A05-p7632429	1	1	Bn-N5-p7985206	Bn-chrA05-p7128539
Bn-A05-p7651411	0.993355482	0.996677741	Bn-N5-p8005891	Bn-chrA05-p7147826
Bn-A05-p7662456	0.990049751	0.990049751	Bn-N1-p14783310	Bn-chrAnn_random-p188082
Bn-A05-p7705565	1	0.172757475	Bn-N5-p8060660	Unknown
Bn-A05-p7729367	0.983388704	0.877076412	Bn-N5-p8083716	Bn-chrAnn_random-p166577
Bn-A05-p7741244	0.985074627	0.990049751	Bn-N5-p8094580	Bn-chrAnn_random-p372857
Bn-A05-p7778186	0.966942149	0.933884298	Bn-N5-p8128096	Bn-chrC04-p12477109
Bn-A05-p7781811	0.983388704	0.980066445	Bn-N5-p8137605	Bn-chrA05_random-p836878
Bn-A05-p7783330	0.983471074	0.876033058	Bn-N5-p8139124	Bn-chrC04-p12489360
Bn-A05-p7791550	0.995024876	1	Bn-N5-p8153993	Bn-chrA05_random-p848353
Bn-A05-p7809870	0.990033223	0.990033223	Bn-N5-p8178085	Bn-chrA05-p7192788
Bn-A05-p7811409	0.993355482	0.993355482	Bn-N5-p8179626	Bn-chrA05-p7194328
Bn-A05-p7826230	0.958677686	0.958677686	Bn-N5-p8192101	Bn-chrA05-p7206803
Bn-A05-p7840300	0.983471074	0.991735537	Bn-N5-p8202968	Bn-chrA05-p7217944
Bn-A05-p7841073	0.996677741	0.996677741	Bn-N5-p8203748	Bn-chrA05-p7218724
Bn-A05-p7844876	1	0.991735537	Bn-N5-p8207484	Bn-chrA05-p7222412
Bn-A05-p7855766	1	1	Bn-N5-p8218313	Bn-chrA05-p7233238
Bn-A05-p7873080	0.975124378	0.965174129	Bn-N5-p8228964	Bn-chrA05-p7243975
Bn-A05-p7874994	0.990049751	0.980099502	Bn-N5-p8230878	Bn-chrA05-p7245889
Bn-A05-p7901866	0.986710963	0.96345515	Bn-N5-p8250121	Bn-chrA05-p7279178
Bn-A05-p7903619	0.975206612	0.983471074	Bn-N5-p8251744	Bn-chrAnn_random-p289362
Bn-A05-p7911054	0.985074627	0.980099502	Bn-N5-p8260953	Bn-chrA05-p7303400
Bn-A05-p7926510	0.991735537	1	Bn-N5-p8278735	Bn-chrA05-p7315853
Bn-A05-p8120795	0.985074627	0.985074627	Bn-N8-p20095914	Bn-chrA08-p17543710
Bn-A05-p8120997	0.975124378	0.975124378	Bn-N8-p20096116	Bn-chrA08-p17543912
Bn-A05-p8135828	0.970149254	0.970149254	Bn-N8-p20110687	Bn-chrA08-p17558482
Bn-A05-p8165715	0.953488372	0.953488372	Bn-N8-p19810376	Bn-chrA08-p17293999
Bn-A05-p8202536	1	1	Bn-N8-p20169554	Bn-chrA08-p17616366
Bn-A05-p8206349	0.588039867	0.588039867	Bn-N8-p20173578	Bn-chrA08-p17620390
Bn-A05-p8210067	0.920265781	0.920265781	Bn-N18-p21801715	Bn-chrC08-p19150608
Bn-A05-p8222879	0.96345515	0.96345515	Bn-N8-p20191243	Bn-chrC03_random-p791402
Bn-A05-p8245454	0.993355482	0.993355482	Bn-N8-p20224691	Bn-chrA08-p17667611
Bn-A05-p8261872	0.996677741	0.996677741	Bn-N8-p20236998	Bn-chrA08-p17680771
Bn-A05-p8290002	0.970099668	0.970099668	Bn-N8-p20268763	Bn-chrA08-p17699404
Bn-A05-p8297352	0.940298507	0.940298507	Bn-N8-p20277216	Bn-chrA08-p17706753
Bn-A05-p8297563	0.975124378	0.975124378	Bn-N8-p20277427	Bn-chrA08-p17706964
Bn-A05-p8318433	0.933554817	0.933554817	Bn-N8-p20299320	Bn-chrA08-p17725701
Bn-A05-p8320167	0.986710963	0.986710963	Bn-N8-p20301042	Bn-chrA08-p17727422
Bn-A05-p8322435	0.990033223	0.990033223	Bn-N8-p20303309	Bn-chrA08-p17729689

Bn-A05-p8327216	0.955223881	0.955223881	Bn-N8-p20308092	Bn-chrA08-p17734472
Bn-A05-p8329506	0.965174129	0.965174129	Bn-N8-p20311039	Bn-chrA08-p17736342
Bn-A05-p8376865	0.993355482	0.990033223	Bn-N2-p17479874	Bn-chrA02-p13790524
Bn-A05-p8511229	0.995024876	0.995024876	Bn-N2-p17324082	Bn-chrA02-p13624226
Bn-A05-p8522584	0.985074627	0.985074627	Bn-N2-p17307307	Bn-chrC09-p22569530
Bn-A05-p8542569	0.985074627	0.985074627	Bn-N2-p17286059	Bn-chrAnn_random-p335936
Bn-A05-p8786891	0.990033223	0.990033223	Bn-N2-p17049997	Bn-chrA02-p13315928
Bn-A05-p8798628	1	1	Bn-N2-p17038049	Bn-chrA02-p13302963
Bn-A05-p8844881	0.945273632	0.940298507	Bn-N2-p16991247	Bn-chrA02-p13258228
Bn-A05-p8851115	0.996677741	0.996677741	Bn-N2-p16985012	Bn-chrA02-p13251993
Bn-A05-p8855134	0.996677741	0.996677741	Bn-N2-p16980993	Bn-chrA02-p13247974
Bn-A05-p8865637	0.975124378	0.975124378	Bn-N2-p16978911	Bn-chrA02-p13245890
Bn-A05-p8892295	1	1	Bn-N2-p16950420	Bn-chrA02-p13218637
Bn-A05-p8943009	0.980099502	0.975124378	Bn-N2-p16891506	Bn-chrA02-p13166708
Bn-A05-p9051665	0.975206612	0.950413223	Bn-N2-p16779672	Bn-chrC02-p25004343
Bn-A05-p9058906	1	0.991735537	Bn-N2-p16771429	Bn-chrA02-p13034620
Bn-A05-p9063726	0.990033223	0.996677741	Bn-N2-p16762042	Bn-chrA02-p13031065
Bn-A05-p9076153	0.993355482	0.993355482	Bn-N2-p16743869	Bn-chrA02-p13017024
Bn-A05-p9076704	0.991735537	0.983471074	Bn-N2-p16743318	Bn-chrA02-p13016473
Bn-A05-p9077059	0.983471074	0.975206612	Bn-N2-p16742963	Bn-chrA02-p13016118
Bn-A05-p9078281	0.983471074	0.991735537	Bn-N2-p16741741	Bn-chrA02-p13014896
Bn-A05-p9078313	0.991735537	0.983471074	Bn-N2-p16741709	Bn-chrA02-p13014864
Bn-A05-p9078783	0.975206612	0.983471074	Bn-N2-p16741239	Bn-chrA02-p13014394
Bn-A05-p9079126	0.993355482	0.996677741	Bn-N2-p16740896	Bn-chrA02-p13014051
Bn-A05-p9079762	0.983471074	0.975206612	Bn-N2-p16740260	Bn-chrA02-p13013414
Bn-A05-p9080340	0.993355482	0.990033223	Bn-N2-p16739682	Bn-chrA02-p13012836
Bn-A05-p9116236	0.991735537	1	Bn-N2-p16703689	Bn-chrA02-p12983735
Bn-A05-p9118240	0.983471074	0.975206612	Bn-N2-p16701742	Bn-chrA02-p12981776
Bn-A05-p9209378	0.996677741	0.996677741	Bn-N2-p16611823	Bn-chrA02_random-p101825
Bn-A05-p9224719	0.925619835	0.933884298	Bn-N2-p16590234	Bn-chrC02-p26446430
Bn-A05-p9225459	0.966942149	0.966942149	Bn-N2-p16589487	Bn-chrA02_random-p996727
Bn-A05-p9226284	0.909090909	0.909090909	Bn-N2-p16588583	Bn-chrA02_random-p995823
Bn-A05-p9231059	0.851239669	0.975206612	Bn-N2-p16580539	Bn-chrC02-p26442755
Bn-A05-p9247748	0.96345515	0.966777409	Bn-N12-p23343652	Bn-chrA02-p12872269
Bn-A05-p9250358	0.966942149	0.966942149	Bn-N2-p16562964	Bn-chrA02-p12869710
Bn-A05-p9253661	0.950413223	0.950413223	Bn-N2-p16556803	Bn-chrA02-p12864218
Bn-A05-p9266289	0.975206612	0.975206612	Bn-N2-p16544231	Bn-chrA02-p12851647
Bn-A05-p9277011	0.966942149	0.966942149	Bn-Scaffold02521-p2	Bn-chrA02-p12840852
Bn-A05-p9286929	0.94214876	0.94214876	Bn-Scaffold83959-p1	Bn-chrC02-p26300849
Bn-A05-p9319851	0.983471074	0.983471074	Bn-N5-p8702877	Bn-chrC06-p13991979
Bn-A05-p9347113	0.990033223	0.990033223	Bn-N5-p8672485	Bn-chrA05-p7403358
Bn-A05-p9364170	0.966942149	0.966942149	Bn-N5-p8656664	Bn-chrA05-p7417914
Bn-A05-p9370169	0.990049751	0.990049751	Bn-N9-p26375634	Bn-chrA01-p12919740
Bn-A05-p9376879	0.991735537	0.991735537	Bn-N5-p8652776	Bn-chrA05-p7421802
Bn-A05-p9384270	0.809917355	0.809917355	Bn-N5-p8645321	Bn-chrA05-p7429257
Bn-A05-p9385617	0.966942149	0.966942149	Bn-N5-p8644809	Bn-chrA05-p7429768

Bn-A05-p9389251	0.710743802	0.884297521	Bn-N5-p8636774	Bn-chrA05-p7435757
Bn-A05-p9408634	0.990049751	0.990049751	Bn-N5-p8624900	Bn-chrA05-p7447631
Bn-A05-p9409136	0.983471074	0.983471074	Bn-N5-p8624398	Bn-chrA05-p7480216
Bn-A05-p9431593	0.94214876	0.94214876	Bn-N5-p8606637	Bn-chrA05-p7467337
Bn-A05-p9451391	0.975206612	0.975206612	Bn-N5-p8588358	Bn-chrA05-p7485381
Bn-A05-p9455372	0.966942149	0.966942149	Bn-N5-p8584357	Bn-chrAnn_random-p314311
Bn-A05-p9472646	0.975124378	0.975124378	Bn-N5-p8564641	Bn-chrA05-p7516063
Bn-A05-p9472820	0.985074627	0.985074627	Bn-N5-p8564467	Bn-chrA05-p7516237
Bn-A05-p9495579	0.991735537	0.991735537	Bn-N5-p8545241	Bn-chrA05-p7536754
Bn-A05-p9502471	0.975124378	0.975124378	Bn-N5-p8538325	Bn-chrA05-p7543670
Bn-A05-p9502893	0.982857143	0.982857143	Bn-N5-p8537929	Bn-chrA05-p7544092
Bn-A05-p9555189	0.966942149	0.966942149	Bn-N5-p8494946	Bn-chrA05-p7595611
Bn-A05-p9602710	0.989583333	0.989583333	Bn-N5-p8778514	Bn-chrA05_random-p969448
Bn-A05-p9602897	0.950413223	0.950413223	Bn-N5-p8778701	Bn-chrA05_random-p969296
Bn-A05-p9623131	0.983471074	0.983471074	Bn-N5-p8791583	Bn-chrA05-p7727618
Bn-A05-p9638089	0.975206612	0.975206612	Bn-N5-p8804262	Bn-chrA05-p7740296
Bn-A05-p9638802	0.975206612	0.975206612	Bn-N5-p8807023	Bn-chrA05-p7743268
Bn-A05-p9646716	0.980079681	0.980079681	Bn-N5-p8813919	Bn-chrA05-p7750162
Bn-A05-p9660152	0.975206612	0.975206612	Bn-N5-p8832703	Bn-chrA05-p7768797
Bn-A05-p9668222	0.983471074	0.983471074	Bn-N5-p8856168	Bn-chrA05-p7855250
Bn-A05-p9758517	0.983471074	0.983471074	Bn-N5-p8936796	Bn-chrA05-p7950815
Bn-A05-p9765872	0.983471074	0.983471074	Bn-N5-p8944491	Bn-chrA05-p7958287
Bn-A05-p9766332	0.892561983	0.892561983	Bn-N5-p8944953	Bn-chrA05-p7958749
Bn-A05-p9766628	0.950413223	0.950413223	Bn-N5-p8945238	Bn-chrA05-p7959034
Bn-A05-p9773183	0.991735537	0.991735537	Bn-N5-p8951234	Bn-chrA05-p7965030
Bn-A05-p9777066	0.986710963	0.986710963	Bn-N5-p8962297	Bn-chrA05-p7976959
Bn-A05-p9777250	0.956810631	0.956810631	Bn-N5-p8962481	Bn-chrA05-p7977143
Bn-A05-p9849049	0.991735537	0.991735537	Bn-N5-p9030784	Bn-chrA05-p8051354
Bn-A05-p9849822	0.993355482	0.993355482	Bn-N5-p9031562	Bn-chrA05-p8052132
Bn-A05-p9849941	0.958677686	0.958677686	Bn-N16-p13514935	Bn-chrC06-p13375015
Bn-A05-p9860991	0.920265781	0.916943522	Bn-N2-p27253127	Bn-chrA02-p22993722
Bn-A05-p9877872	0.986710963	0.986710963	Bn-N5-p9093677	Bn-chrA05_random-p778179
Bn-A05-p9890807	0.983471074	0.983471074	Bn-N5-p9103213	Bn-chrA05_random-p767971
Bn-A05-p9900987	0.966777409	0.966777409	Bn-N5-p9112819	Bn-chrA05_random-p757923
Bn-A05-p9937099	0.990033223	0.993355482	Bn-N5-p9135367	Bn-chrA05-p8134729
Bn-A05-p10009197	0.975206612	0.975206612	Bn-N5-p9213866	Bn-chrA05-p8199006
Bn-A05-p10009485	0.96345515	0.96345515	Bn-N5-p9214153	Bn-chrA05-p8199293
Bn-A05-p10101040	1	1	Bn-N5-p9310656	Bn-chrA05-p8292724
Bn-A05-p10103295	1	1	Bn-N5-p9312335	Bn-chrA05-p8294403
Bn-A05-p10171248	1	0.991735537	Bn-N5-p9722800	Bn-chrA05-p8715991
Bn-A05-p10179640	1	0.991735537	Bn-N5-p9731217	Bn-chrA05-p8724356
Bn-A05-p10181096	0.991735537	0.983471074	Bn-N5-p9732665	Bn-chrA05-p8725814
Bn-A05-p10214000	0.958677686	0.966942149	Bn-N5-p9780360	Bn-chrA05-p8775114
Bn-A05-p10214398	0.94214876	0.933884298	Bn-N5-p9780758	Bn-chrA05-p8775512
Bn-A05-p10241814	0.996677741	0.993355482	Bn-N5-p9805761	Bn-chrA05-p8804801
Bn-A05-p10264427	1	0.991735537	Bn-N5-p9825576	Bn-chrA05-p8829222

Bn-A05-p10264494	1	0.991735537	Bn-N5-p9825643	Bn-chrA05-p8829289
Bn-A05-p10267278	0.991735537	0.983471074	Bn-N5-p9828613	Bn-chrA05-p8835272
Bn-A05-p10269046	0.991735537	0.991735537	Bn-N5-p9830494	Bn-chrA05-p8837153
Bn-A05-p10278245	0.991735537	0.991735537	Bn-N5-p9842754	Bn-chrA05-p8854658
Bn-A05-p10281337	0.983471074	0.983471074	Bn-N5-p9845857	Bn-chrA05-p8857758
Bn-A05-p10292778	0.986710963	0.986710963	Bn-N5-p9856309	Bn-chrA05-p8873193
Bn-A05-p10296509	0.991735537	0.991735537	Bn-N5-p9866091	Bn-chrA05-p8877163
Bn-A05-p10305352	0.986710963	0.986710963	Bn-N5-p9880048	Bn-chrA05-p8895622
Bn-A05-p10320143	0.996677741	0.996677741	Bn-N5-p9895872	Bn-chrA05-p8911547
Bn-A05-p10320501	0.990049751	0.990049751	Bn-N5-p9896230	Bn-chrA05-p8911905
Bn-A05-p10321304	0.995024876	0.995024876	Bn-N5-p9897033	Bn-chrA05-p8912708
Bn-A05-p10321580	0.980099502	0.980099502	Bn-N5-p9897309	Bn-chrA05-p8912984
Bn-A05-p10331539	0.975206612	0.975206612	Bn-N5-p9907280	Bn-chrA05-p8922941
Bn-A05-p10349551	1	1	Bn-N5-p9924616	Bn-chrA05-p8942518
Bn-A05-p10357316	0.991735537	0.991735537	Bn-N5-p9932386	Bn-chrA05-p8950286
Bn-A05-p10364013	0.993355482	0.993355482	Bn-N5-p9942033	Bn-chrA05-p8958027
Bn-A05-p10365857	0.975206612	0.611570248	Bn-N5-p9944757	Bn-chrCnn_random-p199805
Bn-A05-p10367279	0.990033223	0.990033223	Bn-N5-p9948397	Bn-chrA05-p8968485
Bn-A05-p10368686	1	1	Bn-N5-p9949984	Bn-chrA05-p8970072
Bn-A05-p10403917	0.669421488	0.669421488	Bn-N5-p10010140	Bn-chrA05-p9043396
Bn-A05-p10413113	0.966942149	0.966942149	Bn-N5-p10011937	Bn-chrA05-p9045193
Bn-A05-p10413574	0.975206612	0.975206612	Bn-N5-p10012395	Bn-chrA05-p9045651
Bn-A05-p10423564	0.980099502	0.980099502	Bn-N5-p10023951	Bn-chrA05-p9054965
Bn-A05-p10425198	0.985074627	0.985074627	Bn-N5-p10025587	Bn-chrA05-p9056601
Bn-A05-p10426347	0.970149254	0.970149254	Bn-N5-p10026726	Bn-chrA05-p9057740
Bn-A05-p10426618	0.990049751	0.995024876	Bn-N5-p10027013	Bn-chrA05-p9058027
Bn-A05-p10437937	0.983471074	0.983471074	Bn-N5-p10038696	Bn-chrA05-p9068567
Bn-A05-p10452570	0.990049751	0.990049751	Bn-N5-p10057051	Bn-chrA05-p9087403
Bn-A05-p10622526	1	1	Bn-N5-p10251993	Bn-chrA05_random-p110481
Bn-A05-p10624916	0.983471074	0.983471074	Bn-N5-p10254339	Bn-chrA05_random-p110715
Bn-A05-p10625034	0.995024876	0.995024876	Bn-N5-p10254457	Bn-chrA05_random-p110727
Bn-A05-p10625215	0.975206612	0.975206612	Bn-N5-p10254638	Bn-chrA05_random-p110745
Bn-A05-p10625236	0.965174129	0.965174129	Bn-N5-p10254659	Bn-chrA05_random-p110747
Bn-A05-p10665044	0.96345515	0.96345515	Bn-N5-p10283957	Bn-chrA05_random-p116600
Bn-A05-p10694572	0.973421927	0.973421927	Bn-N5-p10318059	Bn-chrA05_random-p113515
Bn-A05-p10697862	0.993355482	0.993355482	Bn-N5-p10321363	Bn-chrA05_random-p113185
Bn-A05-p10719967	1	1	Bn-N5-p10361634	Bn-chrA05-p9301001
Bn-A05-p10734565	0.995024876	0.995024876	Bn-N5-p10376340	Bn-chrA05-p9315601
Bn-A05-p10752346	0.991735537	0.991735537	Bn-N5-p10393746	Bn-chrA05-p9333154
Bn-A05-p10760570	0.890365449	0.890365449	Bn-N5-p10401975	Bn-chrA05-p9341113
Bn-A05-p10778859	0.94214876	0.94214876	Bn-N16-p9311278	Bn-chrC06-p11502763
Bn-A05-p10780480	0.917355372	0.917355372	Bn-N5-p10416785	Bn-chrA05-p9357169
Bn-A05-p10783057	1	1	Bn-N5-p10419340	Bn-chrA05-p9359723
Bn-A05-p10798961	1	1	Bn-N5-p10436332	Bn-chrA05-p9378537
Bn-A05-p10804278	0.983471074	0.983471074	Bn-N5-p10441834	Bn-chrA05-p9382729
Bn-A05-p10820194	0.991735537	0.991735537	Bn-N5-p10453043	Bn-chrA05-p9393938

Bn-A05-p10840480	0.800664452	0.794019934	Bn-N1-p7572556	Bn-chrA01-p8601093
Bn-A05-p10868044	0.983471074	0.983471074	Bn-N5-p10524003	Bn-chrA05-p9455182
Bn-A05-p10922835	0.983471074	0.983471074	Bn-N5-p10577272	Bn-chrA05-p9511396
Bn-A05-p10924105	0.975206612	0.975206612	Bn-N5-p10578539	Bn-chrA05-p9512663
Bn-A05-p10930228	0.996677741	0.996677741	Bn-N5-p10584837	Bn-chrA05-p9518960
Bn-A05-p10939686	0.980066445	0.980066445	Bn-N5-p10599417	Bn-chrA05-p9532515
Bn-A05-p10939740	0.983388704	0.983388704	Bn-N5-p10599471	Bn-chrA05-p9532569
Bn-A05-p10940401	0.986710963	0.986710963	Bn-N5-p10600132	Bn-chrA05-p9533230
Bn-A05-p10945933	0.983388704	0.983388704	Bn-N5-p10605719	Bn-chrA05-p9538817
Bn-A05-p10946327	0.973421927	0.973421927	Bn-N5-p10606113	Bn-chrA05-p9539211
Bn-A05-p10947783	0.980066445	0.980066445	Bn-N5-p10607584	Bn-chrA05-p9540682
Bn-A05-p10948934	0.965174129	0.965174129	Bn-N5-p10608734	Bn-chrA05-p9541832
Bn-A05-p10951155	0.973421927	0.973421927	Bn-N5-p10610958	Bn-chrA05-p9544056
Bn-A05-p10951975	0.990033223	0.990033223	Bn-N5-p10611771	Bn-chrA05-p9544869
Bn-A05-p10964994	0.976744186	0.976744186	Bn-N5-p10627144	Bn-chrA05-p9560251
Bn-A05-p10966950	0.989583333	0.989583333	Bn-N5-p10631100	Bn-chrA05-p9564844
Bn-A05-p10981427	0.975206612	0.975206612	Bn-N5-p10648004	Bn-chrA05-p9591238
Bn-A05-p10999977	0.985074627	0.985074627	Bn-N5-p10656438	Bn-chrA05-p9599669
Bn-A05-p11000036	0.983471074	0.983471074	Bn-N5-p10656497	Bn-chrA05-p9599728
Bn-A05-p11000261	0.960199005	0.960199005	Bn-N5-p10656719	Bn-chrA05-p9599950
Bn-A05-p11012409	0.694214876	0.694214876	Bn-N5-p10662213	Bn-chrA05-p9606922
Bn-A05-p11013933	0.966942149	0.966942149	Bn-N5-p10663727	Bn-chrA05-p9608436
Bn-A05-p11013981	0.983388704	0.983388704	Bn-N5-p10663775	Bn-chrA05-p9608484
Bn-A05-p11044537	0.983471074	0.983471074	Bn-N5-p10674781	Bn-chrA05_random-p138217
Bn-A05-p11048761	0.895522388	0.985074627	Bn-N5-p10678977	Bn-chrA05_random-p137797
Bn-A05-p11056573	0.991735537	0.991735537	Bn-N5-p10686874	Bn-chrA05-p9853274
Bn-A05-p11057820	0.996677741	0.996677741	Bn-N5-p10688058	Bn-chrA05-p9852091
Bn-A05-p11068866	0.975206612	0.991735537	Bn-N5-p10699281	Bn-chrA05-p9840925
Bn-A05-p11084469	0.983471074	0.991735537	Bn-N5-p10708377	Bn-chrA05-p9824187
Bn-A05-p11088814	0.993355482	0.990033223	Bn-N5-p10712086	Bn-chrA05-p9819654
Bn-A05-p11120195	0.983471074	0.975206612	Bn-N5-p10755775	Bn-chrA05-p9793715
Bn-A05-p11120274	0.966942149	0.975206612	Bn-N5-p10755854	Bn-chrA05-p9793636
Bn-A05-p11143078	0.950413223	0.94214876	Bn-N5-p10776513	Bn-chrA05-p9772566
Bn-A05-p11146315	0.975206612	0.991735537	Bn-N5-p10779764	Bn-chrA05-p9769373
Bn-A05-p11146571	0.995024876	0.975124378	Bn-N5-p10780019	Bn-chrA05-p9769117
Bn-A05-p11151246	0.876033058	0.94214876	Bn-N5-p10788978	Bn-chrA05-p9762134
Bn-A05-p11164954	0.975206612	0.975206612	Bn-N5-p10798655	Bn-chrA05-p9747779
Bn-A05-p11168272	0.96345515	0.946843854	Bn-N5-p10801969	Bn-chrC06-p10538671
Bn-A05-p11168423	0.94214876	0.933884298	Bn-N5-p10802109	Bn-chrC06-p10538519
Bn-A05-p11225095	0.993355482	0.993355482	Bn-N5-p10868110	Bn-chrA05-p9675913
Bn-A05-p11226462	0.993355482	0.993355482	Bn-N5-p10869483	Bn-chrA05-p9674529
Bn-A05-p11254494	0.970099668	0.970099668	Bn-N5-p10888811	Bn-chrA05-p9653985
Bn-A05-p11255103	0.973421927	0.797342193	Bn-N5-p10889420	Bn-chrA05-p9653376
Bn-A05-p11279325	0.990033223	0.990033223	Bn-N5-p10912453	Bn-chrA05-p9631931
Bn-A05-p11279669	0.993355482	0.993355482	Bn-N5-p10912797	Bn-chrA05-p9631587
Bn-A05-p11283530	0.990033223	0.990033223	Bn-N5-p10916656	Bn-chrA05-p9627728

Bn-A05-p11297100	0.943521595	0.943521595	Bn-N5-p10930502	Bn-chrA05-p9614156
Bn-A05-p11372809	0.958677686	0.958677686	Bn-N5-p11016919	Bn-chrA05-p9891126
Bn-A05-p11377111	0.995024876	0.995024876	Bn-N5-p11021231	Bn-chrA05-p9895438
Bn-A05-p11384651	0.976744186	0.986710963	Bn-N5-p11029410	Bn-chrA05-p9903618
Bn-A05-p11426194	1	1	Bn-N5-p11073866	Bn-chrA05-p9952192
Bn-A05-p11429310	0.995024876	0.995024876	Bn-N5-p11076942	Bn-chrA05-p9955269
Bn-A05-p11514604	0.986710963	0.993355482	Bn-N5-p11144951	Bn-chrA05-p10032289
Bn-A05-p11562034	0.970099668	0.970099668	Bn-N5-p11189171	Bn-chrA05-p10082192
Bn-A05-p11593933	0.956810631	0.956810631	Bn-N15-p25890644	Bn-chrC05-p23478944
Bn-A05-p11606554	0.980099502	0.980099502	Bn-N5-p11228759	Bn-chrA05-p10139803
Bn-A05-p11607344	1	1	Bn-N5-p11229549	Bn-chrA05-p10140593
Bn-A05-p11626174	0.993355482	0.993355482	Bn-N5-p11248723	Bn-chrA05-p10157901
Bn-A05-p11686214	0.983388704	0.983388704	Bn-N5-p11297083	Bn-chrA05-p10218074
Bn-A05-p11687380	0.926910299	0.940199336	Bn-N15-p25498290	Bn-chrC05_random-p245999
Bn-A05-p11689852	0.986710963	0.986710963	Bn-N5-p11300721	Bn-chrA05-p10221712
Bn-A05-p11690317	0.990033223	0.990033223	Bn-N5-p11301186	Bn-chrA05-p10222177
Bn-A05-p11702049	0.986710963	0.993355482	Bn-N5-p11314492	Bn-chrA08_random-p88915
Bn-A05-p11735505	0.966777409	0.966777409	Bn-N5-p11348616	Bn-chrA05-p10272519
Bn-A05-p11736698	0.985074627	0.985074627	Bn-N5-p11349809	Bn-chrA05-p10273712
Bn-A05-p11737404	0.966942149	0.966942149	Bn-N5-p11350515	Bn-chrA05-p10274418
Bn-A05-p11738337	0.946843854	0.946843854	Bn-N5-p11351448	Bn-chrA05-p10275351
Bn-A05-p11740536	0.916943522	0.920265781	Bn-N5-p11353647	Bn-chrA05-p10277550
Bn-A05-p11751401	0.966942149	0.966942149	Bn-N5-p11364647	Bn-chrA05-p10291687
Bn-A05-p11767399	0.985074627	0.985074627	Bn-N5-p11381382	Bn-chrA05-p10306866
Bn-A05-p11776888	0.985074627	0.985074627	Bn-N5-p11385605	Bn-chrA05-p10311089
Bn-A05-p11781784	0.940298507	0.940298507	Bn-N5-p11390633	Bn-chrA05-p10315942
Bn-A05-p11785796	0.990033223	0.990033223	Bn-N5-p11394645	Bn-chrA05-p10319954
Bn-A05-p11786537	0.983471074	0.983471074	Bn-N5-p11395386	Bn-chrA05-p10320695
Bn-A05-p11812178	1	1	Bn-N5-p11423033	Bn-chrA05-p10346550
Bn-A05-p11812265	0.990033223	0.990033223	Bn-N5-p11423120	Bn-chrA05-p10346637
Bn-A05-p11818095	0.975206612	0.975206612	Bn-N5-p11429084	Bn-chrA05-p10352601
Bn-A05-p11840240	0.996677741	0.996677741	Bn-N5-p11445225	Bn-chrA05-p10368236
Bn-A05-p11856345	0.976744186	0.976744186	Bn-N5-p11464718	Bn-chrA05-p10377052
Bn-A05-p11910270	0.429752066	0.512396694	Bn-N6-p10829928	Bn-chrC04-p31890055
Bn-A05-p11926097	0.933884298	0.933884298	Bn-N5-p11862638	Bn-chrA05-p10742079
Bn-A05-p11927100	0.958677686	0.958677686	Bn-N5-p11864007	Bn-chrA05-p10743116
Bn-A05-p11927159	0.983471074	0.983471074	Bn-N5-p11864066	Bn-chrA05-p10743175
Bn-A05-p11927165	0.983471074	0.983471074	Bn-N5-p11864072	Bn-chrA05-p10743181
Bn-A05-p11942356	0.983471074	0.983471074	Bn-N5-p11874014	Bn-chrA05-p10751530
Bn-A05-p11961924	0.991735537	0.991735537	Bn-N5-p11897495	Bn-chrA05-p10772808
Bn-A05-p11966222	0.990033223	0.990033223	Bn-N5-p11902010	Bn-chrA05-p10777107
Bn-A05-p11975840	0.986710963	0.986710963	Bn-N5-p11911662	Bn-chrA05-p10786760
Bn-A05-p11990369	0.991735537	0.991735537	Bn-N5-p11929939	Bn-chrA05-p10799862
Bn-A05-p12002033	0.966942149	0.966942149	Bn-N5-p11942848	Bn-chrA05-p10812843
Bn-A05-p12018989	0.976744186	0.976744186	Bn-N5-p11960281	Bn-chrA05-p10829896
Bn-A05-p12019087	0.983388704	0.983388704	Bn-N5-p11960379	Bn-chrA05-p10829994

Bn-A05-p12019708	0.985074627	0.985074627	Bn-N5-p11961000	Bn-chrA05-p10830615
Bn-A05-p12021730	0.993355482	0.993355482	Bn-N5-p11963021	Bn-chrA05-p10832635
Bn-A05-p12021976	1	1	Bn-N5-p11963267	Bn-chrA05-p10832881
Bn-A05-p12022144	0.996677741	0.996677741	Bn-N5-p11963435	Bn-chrA05-p10833049
Bn-A05-p12022540	0.996677741	0.996677741	Bn-N5-p11963831	Bn-chrA05-p10833445
Bn-A05-p12024011	0.986710963	0.986710963	Bn-N5-p11965302	Bn-chrA05-p10834916
Bn-A05-p12026117	0.996677741	0.996677741	Bn-N5-p11967408	Bn-chrA05-p10837022
Bn-A05-p12032938	0.990033223	0.990033223	Bn-N5-p11974229	Bn-chrA05-p10843843
Bn-A05-p12046650	0.996677741	0.996677741	Bn-N5-p11984567	Bn-chrCnn_random-p533488
Bn-A05-p12052571	0.990033223	0.990033223	Bn-N5-p11990675	Bn-chrA05-p10860605
Bn-A05-p12056452	0.990033223	0.990033223	Bn-N5-p11994559	Bn-chrA05-p10864491
Bn-A05-p12059250	0.991735537	0.991735537	Bn-N5-p11997358	Bn-chrA05-p10867290
Bn-A05-p12067093	0.975206612	0.975206612	Bn-N5-p12005202	Bn-chrA05-p10875134
Bn-A05-p12069010	0.996677741	0.996677741	Bn-N5-p12007119	Bn-chrA05-p10877051
Bn-A05-p12102885	0.996677741	0.996677741	Bn-N5-p12042428	Bn-chrA05_random-p250747
Bn-A05-p12103432	0.966942149	0.966942149	Bn-N5-p12042975	Bn-chrA05_random-p250692
Bn-A05-p12104940	0.966942149	0.966942149	Bn-N5-p12044483	Bn-chrA05_random-p250542
Bn-A05-p12105780	0.986710963	0.986710963	Bn-N5-p12045323	Bn-chrA05_random-p250458
Bn-A05-p12115583	0.975206612	0.975206612	Bn-N5-p12064364	Bn-chrA05_random-p238129
Bn-A05-p12119364	0.993355482	0.993355482	Bn-N5-p12068040	Bn-chrA05_random-p237761
Bn-A05-p12120855	0.996677741	0.996677741	Bn-N5-p12069531	Bn-chrA05_random-p237612
Bn-A05-p12131090	0.983471074	0.983471074	Bn-N5-p12079902	Bn-chrA05_random-p236575
Bn-A05-p12141401	0.983471074	0.983471074	Bn-N5-p12089944	Bn-chrAnn_random-p350559
Bn-A05-p12158169	0.983471074	0.983471074	Bn-N5-p12100389	Bn-chrA05_random-p234350
Bn-A05-p12170375	0.966942149	0.966942149	Bn-N5-p12109308	Bn-chrAnn_random-p463267
Bn-A05-p12193915	0.966942149	0.966942149	Bn-N5-p12138543	Bn-chrA05_random-p239044
Bn-A05-p12199879	0.983471074	0.983471074	Bn-N5-p12144089	Bn-chrA05_random-p239639
Bn-A05-p12205591	0.966942149	0.966942149	Bn-N5-p12149801	Bn-chrA05_random-p240218
Bn-A05-p12207497	0.958677686	0.958677686	Bn-N5-p12151707	Bn-chrA05_random-p240409
Bn-A05-p12208604	0.903654485	0.903654485	Bn-N5-p12152813	Bn-chrA05_random-p240520
Bn-A05-p12208728	0.983471074	0.983471074	Bn-N5-p12152937	Bn-chrA05_random-p240532
Bn-A05-p12208840	0.991735537	0.991735537	Bn-N5-p12153049	Bn-chrA05_random-p240543
Bn-A05-p12209129	0.966942149	0.966942149	Bn-N5-p12153338	Bn-chrA05_random-p240572
Bn-A05-p12209466	0.983388704	0.983388704	Bn-N5-p12153675	Bn-chrA05_random-p240606
Bn-A05-p12216620	0.975206612	0.975206612	Bn-N5-p12160934	Bn-chrA05_random-p241331
Bn-A05-p12246566	0.966942149	0.966942149	Bn-N5-p12190446	Bn-chrA05-p12196344
Bn-A05-p12248987	0.94214876	0.94214876	Bn-N5-p12193051	Bn-chrA05-p12198950
Bn-A05-p12298408	0.983471074	0.983471074	Bn-N5-p12243956	Bn-chrA05-p12290402
Bn-A05-p12321766	0.983471074	0.983471074	Bn-N5-p12270261	Bn-chrA05-p12314173
Bn-A05-p12362411	1	1	Bn-N5-p12311564	Bn-chrA05-p12353642
Bn-A05-p12378382	0.991735537	0.991735537	Bn-N5-p12329634	Bn-chrA05-p12369551
Bn-A05-p12395933	0.975206612	0.975206612	Bn-N5-p12347420	Bn-chrA05-p12387344
Bn-A05-p12408504	1	1	Bn-N5-p12360876	Bn-chrA05-p12400082
Bn-A05-p12418607	0.991735537	0.991735537	Bn-N5-p12372311	Bn-chrA05-p12409339
Bn-A05-p12418761	0.983471074	0.983471074	Bn-N5-p12372465	Bn-chrA05-p12409493
Bn-A05-p12418828	0.991735537	0.983471074	Bn-N5-p12372532	Bn-chrA05-p12409560

Bn-A05-p12436060	0.975206612	0.975206612	Bn-N5-p12379038	Bn-chrA05-p12427305
Bn-A05-p12447042	0.990049751	0.990049751	Bn-N5-p12388738	Bn-chrA05-p12438921
Bn-A05-p12447170	0.986710963	0.986710963	Bn-N5-p12388866	Bn-chrA05-p12439049
Bn-A05-p12450816	0.983471074	0.991735537	Bn-N5-p12392526	Bn-chrA05-p12453328
Bn-A05-p12453362	0.991735537	0.991735537	Bn-N5-p12394037	Bn-chrA05-p12456140
Bn-A05-p12457036	0.975206612	0.975206612	Bn-N5-p12397724	Bn-chrA05-p12459812
Bn-A05-p12457173	0.980099502	0.980099502	Bn-N5-p12397861	Bn-chrA05-p12459949
Bn-A05-p12457670	0.993355482	0.990033223	Bn-N5-p12398358	Bn-chrA05-p12460446
Bn-A05-p12489885	0.958677686	0.958677686	Bn-N5-p12419312	Bn-chrA05-p12486491
Bn-A05-p12493072	0.975206612	0.975206612	Bn-N5-p12422493	Bn-chrA05-p12489682
Bn-A05-p12497800	0.231404959	0.975206612	Bn-N5-p12426980	Bn-chrA05-p12494410
Bn-A05-p12507206	0.966942149	0.966942149	Bn-N5-p12436378	Bn-chrA05-p12503816
Bn-A05-p12511706	0.958677686	0.958677686	Bn-N5-p12442262	Bn-chrA05-p12508364
Bn-A05-p12519886	0.991735537	0.991735537	Bn-N5-p12450659	Bn-chrA05-p12516874
Bn-A05-p12529959	0.985074627	0.990049751	Bn-N5-p12460008	Bn-chrA05-p12526948
Bn-A05-p12546760	0.983471074	0.983471074	Bn-N5-p12474386	Bn-chrA05-p12542123
Bn-A05-p12553495	0.966942149	0.966942149	Bn-N5-p12481361	Bn-chrA05-p12549056
Bn-A05-p12553851	0.983471074	0.983471074	Bn-N5-p12481715	Bn-chrA05-p12549412
Bn-A05-p12554299	0.991735537	0.991735537	Bn-N5-p12482163	Bn-chrA05-p12549860
Bn-A05-p12573416	1	1	Bn-N5-p12503407	Bn-chrA05-p12576139
Bn-A05-p12592066	0.983471074	0.983471074	Bn-N5-p12513646	Bn-chrA05_random-p249612
Bn-A05-p12597194	1	1	Bn-N5-p12521345	Bn-chrA05_random-p249087
Bn-A05-p12597426	0.991735537	0.991735537	Bn-N5-p12521577	Bn-chrA05_random-p249064
Bn-A05-p12608926	0.975206612	0.975206612	Bn-N5-p12531133	Bn-chrA05_random-p248064
Bn-A05-p12643056	0.983471074	0.983471074	Bn-N5-p12558838	Bn-chrA05-p12624990
Bn-A05-p12684736	1	1	Bn-N5-p12597351	Bn-chrA05-p12663103
Bn-A05-p12734993	0.975206612	0.975206612	Bn-N5-p12647061	Bn-chrA05-p12714624
Bn-A05-p12821101	0.991735537	0.991735537	Bn-N5-p12734487	Bn-chrA05-p12803012
Bn-A05-p12821172	0.991735537	0.991735537	Bn-N5-p12734558	Bn-chrA05-p12803083
Bn-A05-p12838668	0.990033223	0.986710963	Bn-N5-p12750888	Bn-chrA05-p12820181
Bn-A05-p12845300	0.966942149	0.958677686	Bn-N5-p12782155	Bn-chrA05-p13937898
Bn-A05-p12867516	0.983471074	0.983471074	Bn-N5-p12804242	Bn-chrA05-p13960310
Bn-A05-p12868006	0.993355482	0.980066445	Bn-N5-p12804732	Bn-chrA05-p13960800
Bn-A05-p12878356	0.975206612	0.975206612	Bn-N5-p12813252	Bn-chrAnn_random-p381083
Bn-A05-p12888273	0.993355482	0.986710963	Bn-N5-p12828700	Bn-chrA05-p13980412
Bn-A05-p12888422	0.993355482	0.986710963	Bn-N5-p12828849	Bn-chrA05-p13980561
Bn-A05-p12889434	0.990033223	0.986710963	Bn-N5-p12829861	Bn-chrA05-p13981573
Bn-A05-p12890298	0.993355482	0.993355482	Bn-N12-p4814158	Bn-chrA05-p13982437
Bn-A05-p12890362	0.990033223	0.990033223	Bn-N12-p4814094	Bn-chrA05-p13982501
Bn-A05-p12891251	0.993355482	0.990033223	Bn-N12-p4813205	Bn-chrA05-p13983390
Bn-A05-p12891987	0.973421927	0.976744186	Bn-N12-p4812469	Bn-chrA05-p13984126
Bn-A05-p12895483	0.991735537	0.991735537	Bn-N5-p12835930	Bn-chrA05-p13987622
Bn-A05-p12911399	0.975206612	0.752066116	Bn-N5-p12850937	Bn-chrC05-p20256464
Bn-A05-p12930251	0.993355482	1	Bn-N5-p12867251	Bn-chrA05-p14020745
Bn-A05-p12952248	0.983471074	0.991735537	Bn-N15-p22372553	Bn-chrC05-p20167788
Bn-A05-p13000538	0.980099502	0.975124378	Bn-N5-p12940124	Bn-chrA05-p14111802

Bn-A05-p13013349	0.991735537	1	Bn-N5-p12952826	Bn-chrA05-p14124606
Bn-A05-p13094470	0.966942149	0.966942149	Bn-N5-p13048977	Bn-chrAnn_random-p477858
Bn-A05-p13098758	0.909090909	0.983471074	Bn-N5-p13052478	Bn-chrA03-p16674673
Bn-A05-p13119450	0.933884298	0.933884298	Bn-N5-p11820809	Bn-chrA05-p10699158
Bn-A05-p13126937	0.975206612	0.975206612	Bn-N5-p11813560	Bn-chrA05-p10691742
Bn-A05-p13165406	0.975124378	0.975124378	Bn-N5-p11787351	Bn-chrC09-p14281251
Bn-A05-p13168139	0.983471074	0.983471074	Bn-N5-p11780191	Bn-chrA05-p10659927
Bn-A05-p13175224	0.983471074	0.983471074	Bn-N5-p11770044	Bn-chrA05-p10650344
Bn-A05-p13178934	0.983471074	0.983471074	Bn-N5-p11768861	Bn-chrA05-p10649160
Bn-A05-p13178955	0.975206612	0.975206612	Bn-N5-p11768840	Bn-chrA05-p10649139
Bn-A05-p13179258	0.966942149	0.966942149	Bn-N5-p11768537	Bn-chrA05-p10648836
Bn-A05-p13184792	0.975206612	0.975206612	Bn-N5-p11762470	Bn-chrA05-p10642771
Bn-A05-p13187401	0.958677686	0.958677686	Bn-N5-p17863815	Bn-chrA05_random-p217592
Bn-A05-p13190945	0.933884298	0.958677686	Bn-N5-p17871658	Bn-chrA05_random-p217237
Bn-A05-p13191000	0.950413223	0.958677686	Bn-N5-p17871713	Bn-chrA05_random-p217232
Bn-A05-p13224690	0.993355482	0.986710963	Bn-N5-p17912096	Bn-chrA05_random-p214200
Bn-A05-p13227178	0.990033223	0.990033223	Bn-N5-p17914574	Bn-chrA05_random-p213951
Bn-A05-p13282990	0.966942149	0.966942149	Bn-N5-p17988601	Bn-chrA05_random-p208659
Bn-A05-p13301511	0.861209964	0.964412811	Bn-N5-p18016716	Bn-chrAnn_random-p452893
Bn-A05-p13372155	1	0.991735537	Bn-N5-p17698898	Bn-chrAnn_random-p478677
Bn-A05-p13453538	0.983471074	0.983471074	Bn-N5-p13153051	Bn-chrAnn_random-p520912
Bn-A05-p13460617	0.983471074	0.983471074	Bn-N5-p13161586	Bn-chrAnn_random-p521620
Bn-A05-p13482596	0.975206612	0.975206612	Bn-N5-p13165597	Bn-chrAnn_random-p523989
Bn-A05-p13483874	0.950413223	0.958677686	Bn-N5-p13166877	Bn-chrAnn_random-p524117
Bn-A05-p13483931	0.986710963	0.966777409	Bn-N5-p13166934	Bn-chrAnn_random-p524122
Bn-A05-p13488514	0.975206612	0.975206612	Bn-N5-p13172263	Bn-chrAnn_random-p524580
Bn-A05-p13488725	0.975206612	0.975206612	Bn-N5-p13172474	Bn-chrAnn_random-p524601
Bn-A05-p13489002	0.983471074	0.983471074	Bn-N5-p13172751	Bn-chrAnn_random-p524629
Bn-A05-p13493260	0.966942149	0.975206612	Bn-N5-p13182599	Bn-chrAnn_random-p525050
Bn-A05-p13493392	0.991735537	0.991735537	Bn-N5-p13182731	Bn-chrAnn_random-p525063
Bn-A05-p13493704	0.983471074	0.983471074	Bn-N5-p13183043	Bn-chrAnn_random-p525094
Bn-A05-p13493781	0.983471074	0.983471074	Bn-N5-p13183120	Bn-chrAnn_random-p525102
Bn-A05-p13493836	0.975206612	0.975206612	Bn-N5-p13183175	Bn-chrAnn_random-p525108
Bn-A05-p13494389	0.991735537	0.983471074	Bn-N5-p13183727	Bn-chrAnn_random-p525163
Bn-A05-p13494414	0.991735537	0.991735537	Bn-N5-p13183752	Bn-chrAnn_random-p525165
Bn-A05-p13504387	0.991735537	0.991735537	Bn-N5-p13190046	Bn-chrAnn_random-p526424
Bn-A05-p13514214	0.975206612	0.975206612	Bn-N5-p13200321	Bn-chrAnn_random-p527421
Bn-A05-p13526183	0.983471074	0.983471074	Bn-N5-p13212338	Bn-chrAnn_random-p528629
Bn-A05-p13528032	0.611570248	0.983471074	Bn-N12-p11291442	Bn-chrAnn_random-p528814
Bn-A05-p13532974	0.975206612	0.975206612	Bn-N5-p13214691	Bn-chrAnn_random-p529326
Bn-A05-p13533202	1	1	Bn-N5-p13214919	Bn-chrAnn_random-p529349
Bn-A05-p13533449	0.975206612	0.983471074	Bn-N5-p13215166	Bn-chrAnn_random-p529374
Bn-A05-p13541711	0.983471074	0.975206612	Bn-N5-p13223459	Bn-chrAnn_random-p530203
Bn-A05-p13552586	0.983471074	0.983471074	Bn-N5-p13229562	Bn-chrAnn_random-p531299
Bn-A05-p13553481	0.958677686	0.958677686	Bn-N5-p13230476	Bn-chrAnn_random-p531388
Bn-A05-p13553743	0.966942149	0.966942149	Bn-N5-p13230738	Bn-chrAnn_random-p531415

Bn-A05-p13554854	0.966942149	0.966942149	Bn-N5-p13231849	Bn-chrAnn_random-p531526
Bn-A05-p13565564	0.975206612	0.983471074	Bn-N5-p13247076	Bn-chrAnn_random-p532563
Bn-A05-p13687507	0.991735537	0.140495868	Bn-Scaffold03512-p2	Unknown
Bn-A05-p13688426	0.958677686	0.958677686	Bn-N5-p13343674	Bn-chrA05-p13401680
Bn-A05-p13688982	0.995024876	0.995024876	Bn-N5-p13344230	Bn-chrA05-p13402236
Bn-A05-p13690413	0.983471074	1	Bn-N5-p13345661	Bn-chrA05-p13403667
Bn-A05-p13751450	0.966942149	0.966942149	Bn-N5-p13415953	Bn-chrA05-p13492106
Bn-A05-p13760191	0.910447761	0.965174129	Bn-N15-p28476724	Bn-chrA05-p13500302
Bn-A05-p13760281	0.990049751	0.995024876	Bn-N5-p13423511	Bn-chrA05-p13500392
Bn-A05-p13766174	0.958677686	0.983471074	Bn-Scaffold03828-p2	Bn-chrA05-p13506458
Bn-A05-p13774122	0.965174129	0.970149254	Bn-N5-p10894358	Bn-chrA05-p13514408
Bn-A05-p13774324	0.950248756	0.950248756	Bn-N5-p10894559	Bn-chrA05-p13514609
Bn-A05-p13778123	0.272727273	0.983471074	Bn-N13-p36788938	Bn-chrA05-p13518401
Bn-A05-p13778371	0.157024793	0.991735537	Unknown	Bn-chrA05-p13518649
Bn-A05-p13778538	0.280991736	1	Bn-N17-p6935980	Bn-chrA05-p13518816
Bn-A05-p13809267	0.991735537	0.983471074	Bn-N5-p13478231	Bn-chrA05-p13549453
Bn-A05-p13811322	0.842975207	0.975206612	Bn-N15-p28604572	Bn-chrA05-p13551562
Bn-A05-p13811525	0.983471074	0.983471074	Bn-N5-p13488373	Bn-chrA05-p13551765
Bn-A05-p13813146	0.991735537	0.991735537	Bn-N5-p13489952	Bn-chrA05-p13553386
Bn-A05-p13816756	0.966942149	0.966942149	Bn-N5-p13493842	Bn-chrA05-p13556996
Bn-A05-p13816826	0.975206612	0.975206612	Bn-N5-p13493912	Bn-chrA05-p13557066
Bn-A05-p13816842	0.966942149	0.966942149	Bn-N5-p13493928	Bn-chrA05-p13557082
Bn-A05-p13817153	0.966942149	0.966942149	Bn-N5-p13494239	Bn-chrA05-p13557393
Bn-A05-p13817451	0.983471074	0.975206612	Bn-N5-p13494537	Bn-chrA05-p13557691
Bn-A05-p13822414	1	1	Bn-N5-p13499507	Bn-chrA05-p13562654
Bn-A05-p13822616	0.983471074	0.983471074	Bn-N5-p13499709	Bn-chrA05-p13562856
Bn-A05-p13823408	1	0.991735537	Bn-N5-p13500501	Bn-chrA05-p13563648
Bn-A05-p13825402	1	0.94214876	Bn-N5-p13502511	Bn-chrC05-p25323207
Bn-A05-p13826013	0.983471074	0.859504132	Bn-N5-p13503120	Bn-chrC05-p25337877
Bn-A05-p13834175	0.884297521	0.933884298	Bn-N5-p13503940	Bn-chrA05-p13572217
Bn-A05-p13834240	0.991735537	0.991735537	Bn-N5-p13504005	Bn-chrA05-p13572282
Bn-A05-p13844605	0.990033223	0.990033223	Bn-N5-p13513478	Bn-chrA05-p13583401
Bn-A05-p13845255	0.991735537	0.991735537	Bn-N5-p13514127	Bn-chrA05-p13584051
Bn-A05-p13852930	0.983471074	0.991735537	Bn-N5-p13542541	Bn-chrA05-p13590869
Bn-A05-p13853329	0.834710744	0.991735537	Bn-N16-p27937442	Bn-chrA05-p13591268
Bn-A05-p13856683	0.94214876	0.950413223	Bn-N5-p13544202	Bn-chrA05-p13594625
Bn-A05-p13863624	0.975206612	0.975206612	Bn-N5-p13551129	Bn-chrA05-p13601568
Bn-A05-p13863673	0.983471074	0.983471074	Bn-N5-p13551178	Bn-chrA05-p13601617
Bn-A05-p13863975	0.983471074	0.983471074	Bn-N5-p13551480	Bn-chrA05-p13601919
Bn-A05-p13864064	1	1	Bn-N5-p13551569	Bn-chrA05-p13602008
Bn-A05-p13864133	0.983471074	0.983471074	Bn-N5-p13551638	Bn-chrA05-p13602077
Bn-A05-p13864467	0.986710963	0.980066445	Bn-N5-p13551973	Bn-chrA05-p13602411
Bn-A05-p13869168	0.975206612	0.966942149	Bn-N5-p13556825	Bn-chrA05-p13607112
Bn-A05-p13876782	0.983471074	0.842975207	Bn-N5-p13558528	Bn-chrC05-p25360934
Bn-A05-p13880377	0.973421927	0.956810631	Bn-N5-p13562138	Bn-chrA05-p13614718
Bn-A05-p13882697	0.986710963	0.983388704	Bn-N5-p13564460	Bn-chrA05-p13617038

Bn-A05-p13883109	0.995024876	0.995024876	Bn-N5-p13564871	Bn-chrA05-p13617450
Bn-A05-p13886960	1	1	Bn-N5-p13568722	Bn-chrA05-p13621301
Bn-A05-p13891848	0.991735537	0.983471074	Bn-N5-p13573590	Bn-chrA05-p13626320
Bn-A05-p13895100	0.966942149	0.966942149	Bn-N5-p13576841	Bn-chrA05-p13629487
Bn-A05-p13895153	0.950413223	0.950413223	Bn-N5-p13576894	Bn-chrA05-p13629540
Bn-A05-p13896924	0.966942149	0.966942149	Bn-N5-p13578663	Bn-chrA05-p13631310
Bn-A05-p13897888	0.991735537	0.991735537	Bn-N5-p13579644	Bn-chrA05-p13632274
Bn-A05-p13900766	0.975206612	0.991735537	Bn-N5-p13582475	Bn-chrA05-p13635152
Bn-A05-p13907424	0.996677741	0.651162791	Bn-N5-p13593274	Bn-chrCnn_random-p262450
Bn-A05-p13908499	0.980066445	0.953488372	Bn-N5-p13593939	Bn-chrA05-p13643357
Bn-A05-p13931810	0.958677686	0.975206612	Bn-N5-p13648379	Bn-chrA05-p13669018
Bn-A05-p13948854	0.892561983	0.991735537	Bn-N5-p13668111	Bn-chrA05-p13685568
Bn-A05-p13962356	0.983471074	0.975206612	Bn-N5-p13680824	Bn-chrA05-p13698829
Bn-A05-p13963172	0.991735537	0.983471074	Bn-N5-p13681641	Bn-chrA05-p13699927
Bn-A05-p13965323	0.983388704	0.966777409	Bn-N5-p13683744	Bn-chrA05-p13702078
Bn-A05-p13968337	0.958677686	0.950413223	Bn-N5-p13686702	Bn-chrA05-p13705092
Bn-A05-p13969222	0.983471074	0.975206612	Bn-N5-p13687574	Bn-chrA05-p13705977
Bn-A05-p13973628	1	0.980066445	Bn-N5-p13690810	Bn-chrA05-p13710382
Bn-A05-p13975657	0.966942149	0.966942149	Bn-N5-p13692766	Bn-chrA05-p13712326
Bn-A05-p14021272	0.983471074	0.975206612	Bn-N5-p13733671	Bn-chrA05-p13759090
Bn-A05-p14029097	0.990033223	0.205980066	Bn-N5-p13747858	Bn-chrC07-p38802225
Bn-A05-p14029214	0.96345515	0.561461794	Bn-N5-p13747975	Bn-chrUnn_random-p377815
Bn-A05-p14040257	0.989583333	1	Bn-N9-p13807219	Bn-chrA09-p12002436
Bn-A05-p14040907	1	0.993355482	Bn-N5-p13760234	Bn-chrA05-p13781509
Bn-A05-p14042687	0.996677741	0.993355482	Bn-N5-p13761427	Bn-chrA05-p13783289
Bn-A05-p14104827	0.985074627	0.985074627	Bn-N5-p13828339	Bn-chrA05-p13847589
Bn-A05-p14106384	0.980066445	0.983388704	Bn-N5-p13829896	Bn-chrA05-p13849145
Bn-A05-p14121401	0.993355482	0.996677741	Bn-N5-p13864056	Bn-chrA05-p13865407
Bn-A05-p14131155	0.989583333	0.979166667	Bn-N5-p13873874	Bn-chrA05-p13875469
Bn-A05-p14144406	0.996677741	0.970099668	Bn-N5-p13890086	Bn-chrA05-p13888653
Bn-A05-p14146305	0.973421927	0.996677741	Bn-N5-p13891987	Bn-chrA05-p13890552
Bn-A05-p14175152	1	0.989583333	Bn-N5-p13911938	Bn-chrA05-p13919873
Bn-A05-p14198626	0.989583333	1	Bn-N5-p13959974	Bn-chrA05-p16022566
Bn-A05-p14202849	0.990033223	0.986710963	Bn-N5-p13964134	Bn-chrA05-p16026745
Bn-A05-p14205845	0.975206612	0.966942149	Bn-N5-p13975113	Bn-chrA05-p16029741
Bn-A05-p14206065	0.966942149	0.975206612	Bn-N5-p13975333	Bn-chrA05-p16029961
Bn-A05-p14206169	0.983388704	0.983388704	Bn-N5-p13975437	Bn-chrA05-p16030065
Bn-A05-p14206731	0.986710963	0.986710963	Bn-N5-p13975999	Bn-chrA05-p16030627
Bn-A05-p14207371	0.983388704	0.980066445	Bn-N5-p13976640	Bn-chrA05-p16031267
Bn-A05-p14207788	0.966777409	0.986710963	Bn-N5-p13977056	Bn-chrA05-p16031684
Bn-A05-p14208091	0.986710963	0.986710963	Bn-N5-p13977354	Bn-chrA05-p16031987
Bn-A05-p14208624	0.983388704	0.983388704	Bn-N5-p13977887	Bn-chrA05-p16032520
Bn-A05-p14208906	0.983471074	0.975206612	Bn-N5-p13978169	Bn-chrA05-p16032802
Bn-A05-p14213049	0.993355482	0.990033223	Bn-N5-p13982304	Bn-chrA05-p16036945
Bn-A05-p14253804	0.995024876	0.995024876	Bn-N15-p29351053	Bn-chrC05-p25980219
Bn-A05-p14255879	0.995024876	0.995024876	Bn-N15-p29353019	Bn-chrC05-p25982184

Bn-A05-p14256951	0.993355482	0.990033223	Bn-N5-p14031409	Bn-chrA05-p16083971
Bn-A05-p14285804	0.985074627	0.985074627	Bn-N5-p14803984	Bn-chrA05-p10905393
Bn-A05-p14285873	0.980099502	0.980099502	Bn-N5-p14804053	Bn-chrA05-p10905462
Bn-A05-p14313107	0.983388704	0.983388704	Bn-N5-p14830336	Bn-chrA05-p10931271
Bn-A05-p14325043	0.996677741	1	Bn-N5-p14842237	Bn-chrA05-p10943171
Bn-A05-p14332800	0.990033223	0.990033223	Bn-N5-p14860009	Bn-chrA05-p10950996
Bn-A05-p14336877	0.993355482	0.993355482	Bn-N5-p14864086	Bn-chrA05-p10955073
Bn-A05-p14338060	0.980099502	0.980099502	Bn-N5-p14865277	Bn-chrA05-p10956264
Bn-A05-p14347292	1	1	Bn-N5-p14880812	Bn-chrA05-p10969117
Bn-A05-p14347603	0.983471074	0.983471074	Bn-N5-p14881123	Bn-chrA05-p10969428
Bn-A05-p14347674	0.986710963	0.986710963	Bn-N5-p14881194	Bn-chrA05-p10969499
Bn-A05-p14355487	0.996677741	0.996677741	Bn-N5-p14888908	Bn-chrA05-p10977468
Bn-A05-p14364345	0.995024876	0.995024876	Bn-N5-p14902272	Bn-chrA05-p10990826
Bn-A05-p14364568	0.990049751	0.995024876	Bn-N5-p14902495	Bn-chrA06-p3935812
Bn-A05-p14372759	0.991735537	0.991735537	Bn-N5-p14909937	Bn-chrA05-p10998465
Bn-A05-p14374199	0.892561983	0.892561983	Bn-N5-p14911377	Bn-chrA05-p10999905
Bn-A05-p14374632	0.950413223	0.950413223	Bn-N5-p14911806	Bn-chrA05-p11000334
Bn-A05-p14380049	0.892561983	0.892561983	Bn-N5-p14919338	Bn-chrAnn_random-p294436
Bn-A05-p14394157	0.966777409	0.966777409	Bn-N5-p14931750	Bn-chrA05-p11021745
Bn-A05-p14394895	0.950413223	0.950413223	Bn-N5-p14932487	Bn-chrA05-p11022482
Bn-A05-p14402155	0.958677686	0.958677686	Bn-N5-p14938829	Bn-chrA05-p11030339
Bn-A05-p14426042	0.975206612	0.975206612	Bn-N5-p14948520	Bn-chrA05-p11039056
Bn-A05-p14426121	0.958677686	0.950413223	Bn-N5-p14948599	Bn-chrA05-p11039135
Bn-A05-p14435461	0.986710963	0.986710963	Bn-N5-p14958613	Bn-chrA05-p11049151
Bn-A05-p14443915	0.993355482	0.996677741	Bn-N5-p14967024	Bn-chrA05_random-p132873
Bn-A05-p14444179	0.980066445	0.980066445	Bn-N5-p14967288	Bn-chrA05_random-p132899
Bn-A05-p14447476	0.983471074	0.983471074	Bn-N5-p14970698	Bn-chrA05-p11063538
Bn-A05-p14464244	0.990033223	0.990033223	Bn-N5-p14984686	Bn-chrA05-p11071159
Bn-A05-p14464476	0.980066445	0.980066445	Bn-N5-p14984918	Bn-chrA05-p11071391
Bn-A05-p14477462	0.991735537	0.991735537	Bn-N5-p14992271	Bn-chrA05-p11078149
Bn-A05-p14480752	0.867768595	0.867768595	Bn-N5-p15002168	Bn-chrA05-p11086494
Bn-A05-p14482654	0.975206612	0.975206612	Bn-N5-p15012358	Bn-chrA05-p11100403
Bn-A05-p14482858	0.826446281	0.826446281	Bn-N5-p15012548	Bn-chrA05-p11100593
Bn-A05-p14483099	0.94214876	0.94214876	Bn-N5-p15012795	Bn-chrA05-p11100840
Bn-A05-p14483962	0.983471074	0.983471074	Bn-N5-p15020078	Bn-chrA05-p11115349
Bn-A05-p14496771	0.884297521	0.884297521	Bn-N5-p15021872	Bn-chrA05-p11117143
Bn-A05-p14505946	0.958677686	0.421487603	Bn-N5-p15026050	Bn-chrC05-p27633888
Bn-A05-p14516105	0.966942149	0.256198347	Bn-N5-p15035059	Bn-chrA04-p378340
Bn-A05-p14521315	0.996677741	0.996677741	Bn-N5-p15036982	Bn-chrA05-p11131143
Bn-A05-p14525918	0.181818182	0.181818182	Bn-N1-p2911278	Bn-chrA01-p2359439
Bn-A05-p14529716	1	1	Bn-N5-p15044093	Bn-chrA05-p11138254
Bn-A05-p14532593	0.884297521	0.884297521	Bn-N17-p873691	Bn-chrC07-p1046427
Bn-A05-p14533489	0.917355372	0.917355372	Bn-N17-p872796	Bn-chrC07-p1045532
Bn-A05-p14537104	0.727272727	0.52892562	Bn-N15-p31120570	Bn-chrC05-p27663505
Bn-A05-p14546189	0.867768595	0.867768595	Bn-N15-p31121844	Bn-chrC05-p27664779
Bn-A05-p14547107	0.297520661	0.487603306	Unknown	Bn-chrA04-p15888563

Bn-A05-p14555358	0.52892562	0.462809917	Bn-N15-p31198686	Bn-chrUnn_random-p12017[illegible]
Bn-A05-p14557992	0.851239669	0.851239669	Bn-N15-p31200429	Bn-chrUnn_random-p12004[illegible]
Bn-A05-p14558091	0.909090909	0.909090909	Bn-N15-p31200528	Bn-chrUnn_random-p12003[illegible]
Bn-A05-p14558121	0.851239669	0.851239669	Bn-N15-p31200558	Bn-chrUnn_random-p12003[illegible]
Bn-A05-p14579391	0.966942149	0.966942149	Bn-N5-p15079447	Bn-chrA05-p11172736
Bn-A05-p14580151	0.983471074	0.983471074	Bn-N5-p15080209	Bn-chrA05-p11173498
Bn-A05-p14588431	0.966942149	0.966942149	Bn-N5-p15088228	Bn-chrA05-p11181517
Bn-A05-p14591985	0.876033058	0.876033058	Bn-N5-p15092454	Bn-chrA09-p17463441
Bn-A05-p14592407	0.975206612	0.975206612	Bn-N5-p15097493	Bn-chrA05-p11189714
Bn-A05-p14599830	0.834710744	0.975206612	Bn-N5-p15107306	Bn-chrA05-p11197664
Bn-A05-p14600386	0.991735537	0.991735537	Bn-N5-p15107862	Bn-chrA05-p11198221
Bn-A05-p14602709	0.975206612	0.975206612	Bn-N5-p15110184	Bn-chrA05-p11200543
Bn-A05-p14602869	0.975206612	0.975206612	Bn-N5-p15110344	Bn-chrA05-p11200703
Bn-A05-p14603280	0.991735537	0.991735537	Bn-N5-p15110755	Bn-chrA05-p11201114
Bn-A05-p14612446	0.991735537	0.991735537	Bn-N5-p15115218	Bn-chrA05-p11205398
Bn-A05-p14620205	0.991735537	0.991735537	Bn-N5-p15122975	Bn-chrA05-p11213156
Bn-A05-p14624727	0.966942149	0.966942149	Bn-N5-p15127575	Bn-chrA05-p11217665
Bn-A05-p14624784	0.975206612	0.975206612	Bn-N5-p15127632	Bn-chrA05-p11217722
Bn-A05-p14636717	1	1	Bn-N5-p15139750	Bn-chrA05-p11242320
Bn-A05-p14662656	0.975206612	0.966942149	Bn-N5-p15173819	Bn-chrA05-p11276588
Bn-A05-p14663154	0.925619835	0.925619835	Bn-N15-p31441363	Bn-chrC05-p27976240
Bn-A05-p14673407	0.983471074	0.983471074	Bn-N5-p15189140	Bn-chrAnn_random-p292048
Bn-A05-p14709014	0.983471074	0.983471074	Bn-N5-p15216588	Bn-chrA05_random-p155567
Bn-A05-p14709370	1	1	Bn-N5-p15216944	Bn-chrA05_random-p155532
Bn-A05-p14712935	0.983471074	0.983471074	Bn-N5-p15218846	Bn-chrA05_random-p155341
Bn-A05-p14715097	0.983471074	0.983471074	Bn-N5-p15221014	Bn-chrA05_random-p186912
Bn-A05-p14723523	0.991735537	0.991735537	Bn-N5-p15229344	Bn-chrA05_random-p154414
Bn-A05-p14726545	0.983471074	0.983471074	Bn-N5-p15238883	Bn-chrA05_random-p153485
Bn-A05-p14726690	0.983471074	0.983471074	Bn-N5-p15239028	Bn-chrA05_random-p153471
Bn-A05-p14734937	0.975206612	0.975206612	Bn-N5-p15247403	Bn-chrA05_random-p152633
Bn-A05-p14739008	0.975206612	0.975206612	Bn-N5-p15251706	Bn-chrA05_random-p151910
Bn-A05-p14739080	0.966942149	0.966942149	Bn-N5-p15251778	Bn-chrA05_random-p151903
Bn-A05-p14740992	0.877076412	0.877076412	Bn-N5-p15253702	Bn-chrA05_random-p151711
Bn-A05-p14742715	0.990033223	0.990033223	Bn-N5-p15255426	Bn-chrA05_random-p151538
Bn-A05-p14743027	0.991735537	0.991735537	Bn-N5-p15255738	Bn-chrA05_random-p151507
Bn-A05-p14747774	0.958677686	0.958677686	Bn-N5-p15260421	Bn-chrC04-p5257748
Bn-A05-p14755242	0.983471074	0.983471074	Bn-N5-p15272447	Bn-chrA05_random-p149836
Bn-A05-p14764932	0.289256198	0.190082645	Bn-N11-p45880799	Bn-chrC09-p3832877
Bn-A05-p14786195	0.950413223	0.958677686	Bn-N5-p15325028	Bn-chrA05-p11320722
Bn-A05-p14787361	0.975206612	0.975206612	Bn-N5-p15326031	Bn-chrA05-p11321727
Bn-A05-p14801451	0.983471074	0.983471074	Bn-N5-p15342813	Bn-chrA05-p11331615
Bn-A05-p14813049	0.966942149	0.950413223	Bn-N5-p15370917	Bn-chrA05-p11354446
Bn-A05-p14813106	0.909090909	0.900826446	Bn-N5-p15370974	Bn-chrA05-p11354503
Bn-A05-p14821280	0.966942149	0.966942149	Bn-N5-p15379851	Bn-chrA05-p11362932
Bn-A05-p14821998	0.966942149	0.966942149	Bn-N5-p15380563	Bn-chrA05-p11363645
Bn-A05-p14830384	0.958677686	0.958677686	Bn-N5-p15384688	Bn-chrA05-p11367768

Bn-A05-p14840113	0.991735537	0.991735537	Bn-N5-p15391547	Bn-chrA05-p11379233
Bn-A05-p14844746	0.983471074	0.983471074	Bn-N5-p15396495	Bn-chrA05-p11383917
Bn-A05-p14882151	0.917355372	0.917355372	Bn-N5-p15415387	Bn-chrA05-p11407742
Bn-A05-p14890882	0.966942149	0.876033058	Bn-N5-p15429961	Bn-chrA05-p11422312
Bn-A05-p14899711	0.996677741	0.993355482	Bn-N5-p15439571	Bn-chrA05-p11432208
Bn-A05-p14906734	0.966942149	0.975206612	Bn-N5-p15448229	Bn-chrA05-p11442551
Bn-A05-p14951722	0.917355372	0.925619835	Bn-N5-p15501739	Bn-chrA05-p11477329
Bn-A05-p14959506	0.685950413	0.983471074	Bn-N15-p37409471	Bn-chrA05-p11484907
Bn-A05-p14960328	0.867768595	0.983471074	Bn-N11-p22136508	Bn-chrA05-p11485731
Bn-A05-p14991084	0.94214876	0.975206612	Bn-N15-p32031213	Bn-chrAnn_random-p354345
Bn-A05-p15004033	0.991735537	0.975206612	Bn-N5-p15551040	Bn-chrC05-p28408361
Bn-A05-p15013585	0.993355482	0.950166113	Bn-N5-p15560869	Bn-chrA05-p11539343
Bn-A05-p15015962	0.966942149	0.975206612	Bn-N5-p15563246	Bn-chrA05-p11542419
Bn-A05-p15016066	0.983471074	0.991735537	Bn-N5-p15563350	Bn-chrA05-p11542523
Bn-A05-p15035058	0.983471074	0.983471074	Bn-N5-p15577934	Bn-chrA05-p11553131
Bn-A05-p15082396	0.975206612	0.966942149	Bn-N5-p15617739	Bn-chrA05-p11565608
Bn-A05-p15086331	1	0.990033223	Bn-N5-p15621829	Bn-chrA05-p11569544
Bn-A05-p15099108	1	0.975206612	Bn-N5-p15634273	Bn-chrA05-p11576759
Bn-A05-p15103134	0.975206612	0.983471074	Bn-N5-p15638299	Bn-chrA05-p11580786
Bn-A05-p15107406	0.950413223	0.958677686	Bn-N5-p15642723	Bn-chrA05-p11584772
Bn-A05-p15108699	0.611570248	0.975206612	Bn-N5-p15644015	Bn-chrA05-p11589718
Bn-A05-p15113044	0.94214876	0.966942149	Bn-N5-p15645622	Bn-chrA05-p11594072
Bn-A05-p15113541	0.991735537	0.983471074	Bn-N5-p15646122	Bn-chrA05-p11594569
Bn-A05-p15113570	0.983471074	0.991735537	Bn-N5-p15646151	Bn-chrA05-p11594598
Bn-A05-p15113653	0.975206612	0.983471074	Bn-N5-p15646234	Bn-chrA05-p11594681
Bn-A05-p15115420	0.892561983	0.991735537	Bn-N5-p15646653	Bn-chrA05-p11597421
Bn-A05-p15116155	0.975206612	0.966942149	Bn-N5-p15647388	Bn-chrA05-p11598168
Bn-A05-p15116220	0.983471074	0.975206612	Bn-N5-p15647453	Bn-chrA05-p11598233
Bn-A05-p15126127	0.975206612	0.966942149	Bn-N5-p15684555	Bn-chrA05-p11608015
Bn-A05-p15136372	0.94214876	0.966942149	Bn-N15-p32124661	Bn-chrA05-p11618247
Bn-A05-p15157665	0.958677686	0.958677686	Bn-N5-p15701584	Bn-chrA05-p11637881
Bn-A05-p15160819	0.421487603	1	Bn-Scaffold58037-p2	Bn-chrA05-p11641878
Bn-A05-p15169260	0.993355482	0.980066445	Bn-N5-p15704485	Bn-chrA05-p11656597
Bn-A05-p15169361	0.991735537	0.983471074	Bn-N5-p15704586	Bn-chrA05-p11656698
Bn-A05-p15187671	0.996677741	1	Bn-N5-p15717540	Bn-chrA05-p11671949
Bn-A05-p15218436	1	0.983471074	Bn-N5-p15725759	Bn-chrA05-p11697028
Bn-A05-p15224458	0.990049751	0.990049751	Bn-N5-p15731705	Bn-chrA05-p11702998
Bn-A05-p15227777	0.991735537	0.983471074	Bn-N5-p15735023	Bn-chrA05-p11706313
Bn-A05-p15232488	0.910299003	0.983388704	Bn-N5-p15734084	Bn-chrA05-p11709787
Bn-A05-p15236669	0.950413223	0.950413223	Bn-N5-p15752905	Bn-chrA05-p11713915
Bn-A05-p15250834	0.94214876	0.94214876	Bn-N5-p15761117	Bn-chrA05-p11722125
Bn-A05-p15281306	0.966942149	0.561983471	Bn-N5-p15770182	Bn-chrA05-p11728711
Bn-A05-p15281766	0.975206612	0.975206612	Bn-N5-p15770643	Bn-chrA05-p11732097
Bn-A05-p15282156	0.950413223	0.950413223	Bn-N5-p15771033	Bn-chrA05-p11732487
Bn-A05-p15290524	0.991735537	0.991735537	Bn-N5-p15778804	Bn-chrA05-p11740258
Bn-A05-p15297460	0.966942149	0.966942149	Bn-N5-p15782664	Bn-chrA05-p11744118

Bn-A05-p15320768	0.933884298	0.933884298	Bn-N5-p15801621	Bn-chrA05-p11758016
Bn-A05-p15322236	0.983471074	0.983471074	Bn-N5-p15803039	Bn-chrA05-p11759434
Bn-A05-p15343768	0.966942149	0.958677686	Bn-N5-p15832100	Bn-chrA05-p11832742
Bn-A05-p15351341	0.965174129	0.935323383	Bn-N5-p15832847	Bn-chrA05-p11833491
Bn-A05-p15369031	0.975206612	0.983471074	Bn-N5-p15853877	Bn-chrA05-p11840238
Bn-A05-p15374255	0.991735537	0.983471074	Bn-N5-p15858833	Bn-chrA05-p11851466
Bn-A05-p15375132	0.975206612	0.975206612	Bn-N5-p15859718	Bn-chrA05-p11852328
Bn-A05-p15383133	0.993355482	0.860465116	Bn-N5-p15862879	Bn-chrA05-p11858095
Bn-A05-p15383563	0.983471074	0.966942149	Bn-N5-p15863310	Bn-chrA05-p11858527
Bn-A05-p15385848	0.985074627	0.955223881	Bn-N5-p15865609	Bn-chrA05-p11860839
Bn-A05-p15387386	0.801652893	0.933884298	Bn-N5-p15867142	Bn-chrA05-p11862334
Bn-A05-p15397425	0.986710963	0.431893688	Bn-N5-p15877196	Bn-chrA03_random-p270041
Bn-A05-p15426487	0.991735537	0.991735537	Bn-N5-p15911825	Bn-chrA05-p11907488
Bn-A05-p15443959	0.991735537	0.991735537	Bn-N5-p15937246	Bn-chrA05-p11930491
Bn-A05-p15445826	0.975206612	0.975206612	Bn-N5-p15939016	Bn-chrA05-p11932261
Bn-A05-p15446452	0.991735537	0.991735537	Bn-N5-p15939643	Bn-chrA05-p11932888
Bn-A05-p15446585	1	1	Bn-N5-p15939776	Bn-chrA05-p11933021
Bn-A05-p15446739	0.975206612	0.975206612	Bn-N5-p15939930	Bn-chrA05-p11933175
Bn-A05-p15447107	1	1	Bn-N5-p15940303	Bn-chrA05-p11933548
Bn-A05-p15447772	0.991735537	0.991735537	Bn-N5-p15940968	Bn-chrA05-p11934213
Bn-A05-p15459231	0.975206612	0.975206612	Bn-N5-p15943543	Bn-chrA05-p11936788
Bn-A05-p15459604	0.991735537	0.991735537	Bn-N5-p15943916	Bn-chrA05-p11937161
Bn-A05-p15459993	0.975206612	0.975206612	Bn-N5-p15944305	Bn-chrA05-p11937550
Bn-A05-p15460116	0.966942149	0.966942149	Bn-N5-p15944427	Bn-chrA05-p11937672
Bn-A05-p15460178	0.991735537	0.991735537	Bn-N5-p15944489	Bn-chrA05-p11937734
Bn-A05-p15460626	0.94214876	0.94214876	Bn-N5-p15944935	Bn-chrA05-p11938180
Bn-A05-p15460677	0.983471074	0.983471074	Bn-N5-p15944986	Bn-chrA05-p11938231
Bn-A05-p15461137	0.975206612	0.975206612	Bn-N5-p15945430	Bn-chrA05-p11938675
Bn-A05-p15463933	0.950413223	0.950413223	Bn-N5-p15948398	Bn-chrA05-p11941643
Bn-A05-p15464720	0.966942149	0.966942149	Bn-N5-p15949198	Bn-chrA05-p11942443
Bn-A05-p15465648	0.727272727	0.966942149	Bn-N5-p15951195	Bn-chrA05-p11943560
Bn-A05-p15466228	0.983471074	0.983471074	Bn-N5-p15951776	Bn-chrC05-p29211507
Bn-A05-p15472992	0.991735537	0.991735537	Bn-N5-p15960968	Bn-chrA05-p11953078
Bn-A05-p15475096	0.983471074	0.983471074	Bn-N5-p15963057	Bn-chrA05-p11955390
Bn-A05-p15478674	0.983471074	0.983471074	Bn-N5-p15964864	Bn-chrA05-p11957197
Bn-A05-p15478769	0.983471074	0.983471074	Bn-N5-p15964959	Bn-chrA05-p11957292
Bn-A05-p15491528	0.975206612	0.975206612	Bn-N5-p15975442	Bn-chrAnn_random-p348059
Bn-A05-p15510479	0.993355482	0.993355482	Bn-N5-p15991734	Bn-chrA05-p11978405
Bn-A05-p15511775	0.996677741	0.996677741	Bn-N5-p15993030	Bn-chrA05-p11979701
Bn-A05-p15521397	0.991735537	0.991735537	Bn-N5-p16001772	Bn-chrA05-p11988934
Bn-A05-p15532294	0.991735537	0.991735537	Bn-N5-p16022349	Bn-chrA05-p12011647
Bn-A05-p15551715	0.980066445	0.950166113	Bn-N9-p27210266	Bn-chrC05-p11478118
Bn-A05-p15663987	1	1	Bn-N9-p27325278	Bn-chrA09-p22924262
Bn-A05-p15670035	1	1	Bn-N9-p27330449	Bn-chrA09-p22929433
Bn-A05-p15676718	0.996677741	0.996677741	Bn-N9-p27338950	Bn-chrA09-p22938196
Bn-A05-p15679327	1	1	Bn-N9-p27341559	Bn-chrA09-p22940805

Bn-A05-p15702744	0.975206612	0.975206612	Bn-N9-p27370542	Bn-chrA09-p22969295
Bn-A05-p15780983	0.986710963	0.980066445	Bn-N9-p27461615	Bn-chrAnn_random-p41858[illegible]
Bn-A05-p15789974	1	0.996677741	Bn-N9-p27478548	Bn-chrAnn_random-p417554
Bn-A05-p15797839	0.991735537	0.983471074	Bn-N9-p27486403	Bn-chrAnn_random-p416352
Bn-A05-p15802321	0.958677686	0.966942149	Bn-N9-p27491742	Bn-chrAnn_random-p415707
Bn-A05-p15805806	0.993355482	0.990033223	Bn-N9-p27495421	Bn-chrAnn_random-p415334
Bn-A05-p15805908	0.983471074	0.975206612	Bn-N9-p27495523	Bn-chrAnn_random-p415324
Bn-A05-p15808942	0.991735537	0.983471074	Bn-N9-p27504187	Bn-chrAnn_random-p41472[illegible]
Bn-A05-p15812933	0.983471074	0.991735537	Bn-N9-p27510150	Bn-chrAnn_random-p414264
Bn-A05-p15859690	0.980099502	0.980099502	Bn-N5-p16065172	Bn-chrA05-p12055338
Bn-A05-p15867343	0.983471074	0.983471074	Bn-N5-p16081347	Bn-chrA05_random-p183191
Bn-A05-p15868478	0.991735537	0.991735537	Bn-N5-p16082482	Bn-chrA05_random-p183305
Bn-A05-p15894915	0.991735537	0.991735537	Bn-N5-p16112613	Bn-chrA05-p12140359
Bn-A05-p15905187	0.983471074	0.983471074	Bn-N5-p16123051	Bn-chrA05-p12150797
Bn-A05-p15906659	0.991735537	0.991735537	Bn-N5-p16124527	Bn-chrA05-p12152270
Bn-A05-p15938967	0.909090909	0.909090909	Bn-N5-p16147503	Bn-chrA05-p12173689
Bn-A05-p15940232	0.991735537	0.991735537	Bn-N5-p16148773	Bn-chrA05-p12174958
Bn-A05-p15948690	0.986710963	0.986710963	Bn-N5-p16159482	Bn-chrA05-p12188018
Bn-A05-p16026452	0.983471074	0.975206612	Bn-N5-p16271909	Bn-chrA01-p2458258
Bn-A05-p16030815	0.991735537	0.909090909	Bn-N5-p16277736	Bn-chrCnn_random-p203696
Bn-A05-p16039471	0.991735537	0.991735537	Bn-N5-p16292214	Bn-chrCnn_random-p287464
Bn-A05-p16051992	0.966942149	0.975206612	Bn-N5-p16315040	Bn-chrCnn_random-p287265
Bn-A05-p16054367	0.975206612	0.975206612	Bn-N5-p16317700	Bn-chrCnn_random-p287238
Bn-A05-p16092993	0.991735537	0.991735537	Bn-N5-p16370464	Bn-chrA05-p14800219
Bn-A05-p16098564	0.975206612	0.975206612	Bn-N5-p16376272	Bn-chrA05-p14806060
Bn-A05-p16099943	0.975206612	0.975206612	Bn-N5-p16377651	Bn-chrA05-p14807439
Bn-A05-p16099945	0.975206612	0.975206612	Bn-N5-p16377653	Bn-chrA05-p14807441
Bn-A05-p16106702	0.925619835	0.925619835	Bn-N5-p16389529	Bn-chrA05-p14819395
Bn-A05-p16106977	0.975206612	0.975206612	Bn-N5-p16389794	Bn-chrA05-p14819660
Bn-A05-p16109920	0.950413223	0.950413223	Bn-N5-p16392905	Bn-chrA05-p14822773
Bn-A05-p16167725	0.966942149	0.966942149	Bn-N5-p16450714	Bn-chrA05-p14879858
Bn-A05-p16183886	0.975206612	0.975206612	Bn-N5-p16465968	Bn-chrA05-p14892502
Bn-A05-p16186321	0.983471074	0.983471074	Bn-N5-p16472430	Bn-chrAnn_random-p454343
Bn-A05-p16195531	0.990049751	0.990049751	Bn-N5-p16479473	Bn-chrA05-p14906187
Bn-A05-p16196113	1	1	Bn-N5-p16480057	Bn-chrA05-p14906771
Bn-A05-p16209763	0.958677686	0.958677686	Bn-N5-p16494801	Bn-chrA05-p14922875
Bn-A05-p16224115	0.983471074	0.983471074	Bn-N5-p16512265	Bn-chrAnn_random-p337274
Bn-A05-p16224273	0.975206612	0.975206612	Bn-N5-p16512423	Bn-chrAnn_random-p337275
Bn-A05-p16225090	0.975206612	0.975206612	Bn-N5-p16513246	Bn-chrAnn_random-p337284
Bn-A05-p16230123	0.983471074	0.983471074	Bn-N5-p16517657	Bn-chrA05-p14945252
Bn-A05-p16234543	0.975206612	0.975206612	Bn-N5-p16521414	Bn-chrA05-p14949009
Bn-A05-p16240970	0.950413223	0.958677686	Bn-N5-p16527815	Bn-chrAnn_random-p408213
Bn-A05-p16244846	0.983471074	0.983471074	Bn-N5-p16534878	Bn-chrA05-p14962076
Bn-A05-p16245038	0.989583333	0.989583333	Bn-N5-p16535070	Bn-chrA05-p14962268
Bn-A05-p16260356	0.975206612	0.975206612	Bn-N5-p16545400	Bn-chrA05-p14975235
Bn-A05-p16260894	0.958677686	0.958677686	Bn-N5-p16546806	Bn-chrA05-p14976641

Bn-A05-p16272686	0.960199005	0.960199005	Bn-N5-p16556180	Bn-chrA05-p14985581
Bn-A05-p16274076	0.983471074	0.983471074	Bn-N5-p16557571	Bn-chrA05-p14986972
Bn-A05-p16305594	0.915422886	0.990049751	Bn-Scaffold02638-p1	Bn-chrC02-p17766794
Bn-A05-p16338133	0.989583333	0.989583333	Bn-N5-p16613633	Bn-chrA05-p15041844
Bn-A05-p16359545	1	1	Bn-N5-p16652852	Bn-chrA05-p15081650
Bn-A05-p16392699	0.983471074	0.983471074	Bn-N5-p16690718	Bn-chrA05_random-p255943
Bn-A05-p16406388	0.996677741	0.996677741	Bn-N5-p16707710	Bn-chrA05_random-p257643
Bn-A05-p16422457	1	1	Bn-N5-p16724208	Bn-chrA05_random-p259438
Bn-A05-p16460167	0.991735537	0.991735537	Bn-N5-p16762643	Bn-chrA05_random-p263539
Bn-A05-p16468152	0.994219653	0.994219653	Bn-N4-p1024552	Bn-chrC08-p29886363
Bn-A05-p16471616	0.958677686	0.950413223	Bn-N15-p34242973	Bn-chrA05_random-p264227
Bn-A05-p16477848	0.983471074	0.983471074	Bn-N5-p16779461	Bn-chrAnn_random-p46370C
Bn-A05-p16489462	0.950413223	0.950413223	Bn-N5-p16785564	Bn-chrA05-p15113664
Bn-A05-p16490679	0.983471074	0.983471074	Bn-N5-p16786907	Bn-chrA05-p15115007
Bn-A05-p16506888	0.991735537	0.991735537	Bn-N5-p16801315	Bn-chrA05-p15130003
Bn-A05-p16507044	0.958677686	0.958677686	Bn-N5-p16801471	Bn-chrA05-p15130159
Bn-A05-p16507306	0.991735537	0.991735537	Bn-N5-p16801749	Bn-chrA05-p15130437
Bn-A05-p16509283	0.983471074	0.983471074	Bn-N5-p16808652	Bn-chrA05-p15139738
Bn-A05-p16538605	0.995024876	0.995024876	Bn-N5-p16825020	Bn-chrA05-p15158392
Bn-A05-p16544454	0.983471074	0.983471074	Bn-N5-p16828605	Bn-chrC05-p29973768
Bn-A05-p16545436	0.991735537	0.685950413	Bn-N5-p16829597	Bn-chrC05-p30012531
Bn-A05-p16546416	0.966942149	0.966942149	Bn-N5-p16830842	Bn-chrA05-p15163621
Bn-A05-p16553141	0.991735537	0.991735537	Bn-N5-p16843913	Bn-chrA05-p15176783
Bn-A05-p16561237	1	1	Bn-N5-p16852482	Bn-chrA05-p15185822
Bn-A05-p16581289	0.983471074	0.983471074	Bn-N5-p16871928	Bn-chrA05-p15205268
Bn-A05-p16588569	0.933884298	0.933884298	Bn-N5-p16883842	Bn-chrA05-p15219009
Bn-A05-p16590105	0.983471074	0.983471074	Bn-N5-p16888750	Bn-chrA05_random-p255423
Bn-A05-p16625783	0.983471074	0.983471074	Bn-N5-p16955195	Bn-chrA05-p15295466
Bn-A05-p16637476	0.966942149	0.966942149	Bn-N5-p16965358	Bn-chrA05-p15305626
Bn-A05-p16648406	0.94214876	0.94214876	Bn-N5-p16981476	Bn-chrA05-p15321968
Bn-A05-p16651858	0.818181818	0.818181818	Bn-N5-p16982177	Bn-chrA05-p15322669
Bn-A05-p16652401	0.966942149	0.966942149	Bn-N5-p16995647	Bn-chrA05-p15332973
Bn-A05-p16664411	0.975206612	0.975206612	Bn-N5-p17005160	Bn-chrA05-p15341846
Bn-A05-p16671522	0.975206612	0.975206612	Bn-N5-p17012472	Bn-chrA05-p15349562
Bn-A05-p16735186	0.970149254	0.970149254	Bn-N5-p17072997	Bn-chrA05-p15422994
Bn-A05-p16738292	0.983471074	0.983471074	Bn-N5-p17074249	Bn-chrA05-p15424128
Bn-A05-p16738871	0.950413223	0.950413223	Bn-N5-p17074827	Bn-chrA05-p15424706
Bn-A05-p16747703	0.966942149	0.966942149	Bn-N5-p17084028	Bn-chrA05-p15433691
Bn-A05-p16747797	0.966942149	0.966942149	Bn-N5-p17084123	Bn-chrA05-p15433786
Bn-A05-p16754780	0.892561983	0.892561983	Bn-N5-p17096806	Bn-chrA05-p15446861
Bn-A05-p16755625	0.925619835	0.925619835	Bn-N5-p17098424	Bn-chrA05-p15448479
Bn-A05-p16775295	0.958677686	0.958677686	Bn-N5-p17111656	Bn-chrC05-p30732515
Bn-A05-p16789633	1	1	Bn-N5-p17126736	Bn-chrCnn_random-p706723
Bn-A05-p16789808	0.991735537	0.933884298	Bn-N5-p17126911	Bn-chrCnn_random-p706722
Bn-A05-p16801582	0.983471074	0.983471074	Bn-N5-p17143068	Bn-chrA05-p15493812
Bn-A05-p16811156	1	1	Bn-N5-p17150885	Bn-chrA05-p15501786

Bn-A05-p16833526	0.966777409	0.966777409	Bn-N5-p17171712	Bn-chrA05-p15523511
Bn-A05-p16834136	0.966942149	0.966942149	Bn-N5-p17172322	Bn-chrA05-p15524121
Bn-A05-p16835570	0.993355482	0.993355482	Bn-N5-p17173754	Bn-chrA05-p15525553
Bn-A05-p16836518	0.884297521	0.884297521	Bn-N5-p17174702	Bn-chrA05-p15526501
Bn-A05-p16842656	0.991735537	0.991735537	Bn-N5-p17181358	Bn-chrA05-p15532697
Bn-A05-p16844203	0.996677741	0.996677741	Bn-N5-p17182905	Bn-chrA05-p15534244
Bn-A05-p16844504	0.983471074	0.983471074	Bn-N5-p17183206	Bn-chrA05-p15534545
Bn-A05-p16846880	0.991735537	0.991735537	Bn-N5-p17185581	Bn-chrA05-p15536918
Bn-A05-p16848755	0.990033223	0.990033223	Bn-N5-p17187456	Bn-chrA05-p15538793
Bn-A05-p16862109	0.966942149	0.966942149	Bn-N5-p17213370	Bn-chrA05-p15558136
Bn-A05-p16863502	0.966942149	0.966942149	Bn-N5-p17215616	Bn-chrA05-p15559615
Bn-A05-p16867347	0.975206612	0.975206612	Bn-N5-p17219560	Bn-chrA05-p15563455
Bn-A05-p16892759	0.983471074	0.983471074	Bn-N5-p17224711	Bn-chrA05-p15568606
Bn-A05-p16897600	0.950413223	0.950413223	Bn-N5-p17229653	Bn-chrA05-p15573547
Bn-A05-p16898305	0.980066445	0.980066445	Bn-N5-p17230322	Bn-chrA05-p15574216
Bn-A05-p16900864	0.975206612	0.975206612	Bn-N5-p17232898	Bn-chrA05-p15576792
Bn-A05-p16909798	0.993355482	0.993355482	Bn-N5-p17242220	Bn-chrA05-p15586113
Bn-A05-p16909956	0.990033223	0.990033223	Bn-N5-p17242378	Bn-chrA05-p15586271
Bn-A05-p16935999	0.991735537	0.991735537	Bn-N5-p17260882	Bn-chrA05-p15604265
Bn-A05-p16938263	0.996677741	0.996677741	Bn-N5-p17263146	Bn-chrA05-p15606529
Bn-A05-p16942305	0.990033223	0.990033223	Bn-N5-p17269139	Bn-chrA05-p15610571
Bn-A05-p16953641	0.983471074	0.190082645	Bn-N5-p17282036	Unknown
Bn-A05-p17093469	0.983388704	0.983388704	Bn-N5-p17575043	Bn-chrA05-p15778136
Bn-A05-p17108506	0.993355482	0.993355482	Bn-N5-p17595916	Bn-chrAnn_random-p420195
Bn-A05-p17202622	0.991735537	0.991735537	Bn-N5-p18110103	Bn-chrA05_random-p167015
Bn-A05-p17203695	0.933554817	0.933554817	Bn-N5-p18111176	Bn-chrA05_random-p166908
Bn-A05-p17215153	0.993355482	0.993355482	Bn-N5-p18137448	Bn-chrA05-p14184455
Bn-A05-p17241091	0.983471074	0.983471074	Bn-N5-p18160629	Bn-chrA05-p14207114
Bn-A05-p17243214	0.94214876	0.94214876	Bn-N5-p18167468	Bn-chrA05-p14212082
Bn-A05-p17262653	0.94214876	0.94214876	Bn-N5-p18175902	Bn-chrA05-p14220516
Bn-A05-p17292224	0.986710963	0.983388704	Bn-N5-p18232665	Bn-chrA05-p14285552
Bn-A05-p17330707	0.966942149	0.966942149	Bn-N11-p17875360	Bn-chrA05-p14362117
Bn-A05-p17366544	0.842975207	0.842975207	Bn-N15-p36454254	Bn-chrC05-p32023734
Bn-A05-p17368155	0.958677686	0.958677686	Bn-N5-p18290404	Bn-chrA05-p14411606
Bn-A05-p17392998	1	0.995024876	Bn-N5-p18314195	Bn-chrA05-p14435442
Bn-A05-p17399423	0.975206612	0.966942149	Bn-N5-p18320716	Bn-chrA05-p14449080
Bn-A05-p17402697	0.983471074	0.966942149	Bn-N5-p18327478	Bn-chrA05-p14459410
Bn-A05-p17417422	0.913621262	0.913621262	Bn-N15-p36517662	Bn-chrC05-p32092814
Bn-A05-p17419591	0.983471074	0.991735537	Bn-N5-p18337436	Bn-chrA05-p14468612
Bn-A05-p17420484	0.995024876	0.995024876	Bn-N5-p18338329	Bn-chrA05-p14469505
Bn-A05-p17420613	0.975124378	0.975124378	Bn-N5-p18338458	Bn-chrA05-p14469634
Bn-A05-p17442162	0.985074627	0.985074627	Bn-N15-p36557068	Bn-chrC05-p32133274
Bn-A05-p17445937	0.980066445	0.986710963	Bn-N5-p18353332	Bn-chrA05-p14487522
Bn-A05-p17482579	0.980099502	0.965174129	Bn-N5-p18380269	Bn-chrA05-p14538248
Bn-A05-p17511337	0.990049751	0.990049751	Bn-N5-p18412421	Bn-chrA05-p14562249
Bn-A05-p17514353	0.966942149	0.958677686	Bn-N5-p18413818	Bn-chrA05-p14564199

Bn-A05-p17542638	0.830564784	0.823920266	Bn-N5-p18435862	Bn-chrA05-p14594951
Bn-A05-p17544675	0.895522388	0.990049751	Bn-N5-p18437903	Bn-chrA05-p14596990
Bn-A05-p17564038	0.960199005	0.960199005	Bn-N5-p18571997	Bn-chrA05_random-p265285
Bn-A05-p17566846	0.985074627	0.990049751	Bn-N5-p18574806	Bn-chrA05_random-p265566
Bn-A05-p17567179	0.980066445	0.983388704	Bn-N5-p18575139	Bn-chrA05_random-p265599
Bn-A05-p17568606	0.985074627	0.980099502	Bn-N5-p18576572	Bn-chrA05_random-p265741
Bn-A05-p17587730	0.975206612	0.991735537	Bn-Scaffold04881-p1	Bn-chrA05_random-p270038
Bn-A05-p17614751	0.983471074	0.991735537	Bn-N5-p18607319	Bn-chrA05_random-p271000
Bn-A05-p17615064	0.991735537	0.983471074	Bn-N5-p18607632	Bn-chrA05_random-p271031
Bn-A05-p17616044	0.975206612	0.983471074	Bn-N5-p18608637	Bn-chrA05_random-p271131
Bn-A05-p17616132	0.975206612	0.983471074	Bn-N5-p18608725	Bn-chrA05_random-p271140
Bn-A05-p17617527	0.985074627	0.900497512	Bn-N5-p18610123	Bn-chrA05_random-p271278
Bn-A05-p17617724	0.995024876	0.985074627	Bn-N5-p18740415	Bn-chrA05-p15909981
Bn-A05-p17624348	0.983471074	0.975206612	Bn-N5-p18617333	Bn-chrA05_random-p271961
Bn-A05-p17625934	0.990049751	0.985074627	Bn-N5-p18618920	Bn-chrA05_random-p272457
Bn-A05-p17625943	0.983471074	0.975206612	Bn-N5-p18618929	Bn-chrA05_random-p272458
Bn-A05-p17675829	0.973421927	0.976744186	Bn-N5-p18660925	Bn-chrAnn_random-p446462
Bn-A05-p17708970	1	0.995024876	Bn-N5-p18698170	Bn-chrA05-p15861728
Bn-A05-p17739455	0.975206612	0.975206612	Bn-N5-p18759377	Bn-chrA05-p15930173
Bn-A05-p17750022	0.955223881	0.965174129	Bn-N5-p18765736	Bn-chrA05-p15936964
Bn-A05-p17758084	0.950570342	0.844106464	Bn-N5-p18779379	Bn-chrA05-p15944792
Bn-A05-p17761769	0.96345515	0.983388704	Bn-N5-p18787203	Bn-chrA05-p15948477
Bn-A05-p17770256	0.989583333	1	Bn-N5-p18796106	Bn-chrA05-p15954407
Bn-A05-p17774694	0.990033223	0.973421927	Bn-N5-p18803472	Bn-chrA05-p15959252
Bn-A05-p17799506	0.986710963	0.980066445	Bn-N5-p18828344	Bn-chrA05-p15983300
Bn-A05-p17822322	0.973421927	0.983388704	Bn-N5-p18839244	Bn-chrA05-p15997396
Bn-A05-p17830597	0.986710963	0.990033223	Bn-N5-p18843295	Bn-chrA05-p16001485
Bn-A05-p17835705	0.975124378	0.975124378	Bn-N2-p20810162	Bn-chrA02-p17453298
Bn-A05-p17880874	0.995024876	0.995024876	Bn-N5-p18886242	Bn-chrA05-p16187103
Bn-A05-p17894045	0.966777409	0.950166113	Bn-N5-p18900369	Bn-chrC05-p33067591
Bn-A05-p17895197	0.985074627	0.980099502	Bn-N5-p18901501	Bn-chrC05-p33068792
Bn-A05-p17913760	0.976744186	0.976744186	Bn-N15-p37531187	Bn-chrC05-p33126656
Bn-A05-p17928363	0.986710963	0.986710963	Bn-N5-p18933887	Bn-chrA05-p16241499
Bn-A05-p17931505	0.955223881	0.955223881	Bn-N5-p18936830	Bn-chrA05-p16244441
Bn-A05-p18066693	0.980099502	0.980099502	Bn-N5-p19035144	Bn-chrA05-p16343069
Bn-A05-p18088421	0.983388704	0.986710963	Bn-N5-p19057410	Bn-chrA05-p16365332
Bn-A05-p18101769	0.991735537	0.991735537	Bn-N5-p19071553	Bn-chrA05-p16381142
Bn-A05-p18109527	0.909090909	0.909090909	Bn-N5-p19079699	Bn-chrA05-p16389220
Bn-A05-p18109536	0.925619835	0.925619835	Bn-N5-p19079699	Bn-chrA05-p16389220
Bn-A05-p18112139	0.760330579	0.760330579	Bn-N5-p19080269	Bn-chrA05-p16389790
Bn-A05-p18118640	1	1	Bn-N5-p19091924	Bn-chrA05-p16400750
Bn-A05-p18125663	0.884297521	0.884297521	Bn-N5-p19101504	Bn-chrA05-p16411446
Bn-A05-p18135244	0.975206612	0.975206612	Bn-N5-p19111088	Bn-chrA05-p16421644
Bn-A05-p18137410	0.94214876	0.94214876	Bn-N5-p19113218	Bn-chrA05-p16423774
Bn-A05-p18139526	0.966942149	0.966942149	Bn-N5-p19115298	Bn-chrA05-p16425854
Bn-A05-p18147040	0.631229236	0.990033223	Bn-Scaffold01376-p6	Bn-chrA09-p12040387

Bn-A05-p18161591	0.945273632	0.945273632	Bn-N5-p19136523	Bn-chrA05-p16447216
Bn-A05-p18191062	1	1	Bn-N5-p19155169	Bn-chrA05-p16463463
Bn-A05-p18196942	0.986710963	0.986710963	Bn-N5-p19164488	Bn-chrA05-p16469355
Bn-A05-p18214148	0.991735537	0.991735537	Bn-N5-p19184848	Bn-chrA05-p16490538
Bn-A05-p18223406	0.995024876	0.995024876	Bn-N5-p19199277	Bn-chrA05-p16514072
Bn-A05-p18229790	0.925619835	0.925619835	Bn-N5-p19205661	Bn-chrA05-p16520462
Bn-A05-p18306160	0.991735537	0.983471074	Bn-N5-p19261411	Bn-chrA05-p16576112
Bn-A05-p18309576	0.980066445	0.990033223	Bn-N5-p19266967	Bn-chrA05-p16579521
Bn-A05-p18310658	0.983471074	0.809917355	Bn-N5-p19268047	Bn-chrA05-p16580607
Bn-A05-p18310838	0.94214876	0.983471074	Bn-N5-p19268227	Bn-chrA05-p16580787
Bn-A05-p18349076	0.986710963	0.990033223	Bn-N16-p37002620	Bn-chrCnn_random-p392114
Bn-A05-p18376815	0.969811321	0.969811321	Bn-N5-p19343575	Bn-chrA05-p16652164
Bn-A05-p18391243	0.867109635	0.867109635	Bn-N5-p19350612	Bn-chrA05-p16659330
Bn-A05-p18391581	0.991735537	0.991735537	Bn-N5-p19350950	Bn-chrA05-p16659668
Bn-A05-p18452462	0.950248756	0.950248756	Bn-N5-p19412722	Bn-chrA05-p16713514
Bn-A05-p18452563	0.975206612	0.991735537	Bn-N5-p19412824	Bn-chrA05-p16713616
Bn-A05-p18453140	0.995024876	0.990049751	Bn-N5-p19413401	Bn-chrA05-p16714193
Bn-A05-p18458181	0.950166113	0.950166113	Bn-N5-p19422118	Bn-chrA05-p16722911
Bn-A05-p18475836	0.983388704	0.983388704	Bn-N5-p19443775	Bn-chrA05-p16745164
Bn-A05-p18480152	0.993355482	0.993355482	Bn-N5-p19448069	Bn-chrA05-p16749457
Bn-A05-p18490530	0.958677686	0.966942149	Bn-N5-p19458814	Bn-chrA05-p16760200
Bn-A05-p18496803	0.977777778	0.985185185	Bn-N5-p13828020	Bn-chrA05-p13847271
Bn-A05-p18505935	0.980099502	0.995024876	Bn-N5-p19477927	Bn-chrA05-p16781811
Bn-A05-p18528112	0.993355482	0.990033223	Bn-N5-p19492197	Bn-chrAnn_random-p324562
Bn-A05-p18543237	0.975206612	0.94214876	Bn-N5-p19498317	Bn-chrC05-p34388645
Bn-A05-p18543933	0.975206612	0.454545455	Bn-N5-p19498971	Bn-chrA03-p21246062
Bn-A05-p18547040	0.950413223	0.958677686	Bn-N5-p19501941	Bn-chrA05-p16816257
Bn-A05-p18590326	0.966942149	0.958677686	Bn-N5-p19539682	Bn-chrA05-p16862858
Bn-A05-p18598001	0.983471074	0.892561983	Bn-N5-p19546408	Bn-chrC05-p34499575
Bn-A05-p18615644	0.983471074	0.991735537	Bn-N5-p19559413	Bn-chrA05-p16892951
Bn-A05-p18616260	0.991735537	1	Bn-N5-p19560029	Bn-chrA05-p16893571
Bn-A05-p18627784	0.986710963	0.976744186	Bn-Scaffold00820-p9	Bn-chrC05-p34532869
Bn-A05-p18637087	0.991735537	0.983471074	Bn-N5-p19576156	Bn-chrA05-p16907957
Bn-A05-p18642842	0.958677686	0.842975207	Bn-N5-p19591839	Bn-chrA05-p6666164
Bn-A05-p18643459	0.975206612	0.950413223	Bn-N5-p19592456	Bn-chrA05-p6666785
Bn-A05-p18682729	0.94214876	0.950413223	Bn-N5-p19635519	Bn-chrA05-p16954817
Bn-A05-p18683201	0.991735537	0.983471074	Bn-N5-p19635991	Bn-chrA05-p16955290
Bn-A05-p18687031	0.991735537	0.983471074	Bn-N5-p19639821	Bn-chrA05-p16959093
Bn-A05-p18689306	0.983471074	0.975206612	Bn-N5-p19642096	Bn-chrA05-p16961779
Bn-A05-p18704009	0.966942149	0.958677686	Bn-N5-p19656187	Bn-chrA05-p16969064
Bn-A05-p18704064	0.958677686	0.950413223	Bn-N5-p19656242	Bn-chrA05-p16969119
Bn-A05-p18704349	0.966942149	0.975206612	Bn-N5-p19656527	Bn-chrA05-p16969403
Bn-A05-p18730286	0.983471074	0.983471074	Bn-N5-p19673681	Bn-chrA05-p16983455
Bn-A05-p18744312	0.983471074	0.983471074	Bn-N5-p19704288	Bn-chrA05-p17001945
Bn-A05-p18744372	0.975206612	0.975206612	Bn-N5-p19704348	Bn-chrA05-p17002005
Bn-A05-p18753257	0.950413223	0.950413223	Bn-N5-p19708503	Bn-chrA05-p17006162

Bn-A05-p18759144	0.966942149	0.966942149	Bn-N5-p19718062	Bn-chrA05-p17013555
Bn-A05-p18761878	0.743801653	0.743801653	Bn-N5-p19720494	Bn-chrA05-p17015987
Bn-A05-p18768801	0.94214876	0.94214876	Bn-N5-p19730837	Bn-chrA05-p17025635
Bn-A05-p18770393	0.975124378	0.975124378	Bn-N5-p19732402	Bn-chrA05-p17027200
Bn-A05-p18773930	0.94214876	0.917355372	Bn-N5-p19736037	Bn-chrA05-p17030835
Bn-A05-p18793322	0.990049751	0.990049751	Bn-N5-p19752600	Bn-chrA05-p17048451
Bn-A05-p18807426	0.986710963	0.986710963	Bn-N5-p19772031	Bn-chrA05-p17065821
Bn-A05-p18819337	0.990033223	0.990033223	Bn-N5-p19783771	Bn-chrA05-p17077728
Bn-A05-p18819519	0.993355482	0.993355482	Bn-N5-p19783953	Bn-chrA05-p17077910
Bn-A05-p18819673	0.990033223	0.990033223	Bn-N5-p19784107	Bn-chrA05-p17078064
Bn-A05-p18820057	0.986710963	0.986710963	Bn-N5-p19784491	Bn-chrA05-p17078448
Bn-A05-p18820492	0.993355482	0.993355482	Bn-N5-p19784927	Bn-chrA05-p17078884
Bn-A05-p18828578	0.996677741	0.996677741	Bn-N5-p19793031	Bn-chrA05-p17086988
Bn-A05-p18828589	0.993355482	0.993355482	Bn-N5-p19793042	Bn-chrA05-p17086999
Bn-A05-p18905070	1	1	Bn-N5-p19863530	Bn-chrA05-p17153741
Bn-A05-p18922154	0.991735537	0.900826446	Bn-N5-p19878612	Bn-chrA05-p17167806
Bn-A05-p18958059	0.975206612	0.983471074	Bn-N5-p19908496	Bn-chrA05-p17203691
Bn-A05-p18958083	0.975206612	0.983471074	Bn-N5-p19908520	Bn-chrA05-p17203715
Bn-A05-p19001293	0.983471074	0.991735537	Bn-N5-p19946962	Bn-chrA05-p17234988
Bn-A05-p19017469	0.990049751	0.990049751	Bn-N5-p19964602	Bn-chrA05-p17253663
Bn-A05-p19029505	0.975206612	0.975206612	Bn-N5-p19973781	Bn-chrA05-p17264893
Bn-A05-p19031269	0.980099502	0.980099502	Bn-N5-p19975569	Bn-chrA05-p17266679
Bn-A05-p19032247	0.96013289	0.96013289	Bn-N5-p19976489	Bn-chrA05-p17267599
Bn-A05-p19041695	0.983471074	0.983471074	Bn-N5-p19986389	Bn-chrA05-p17277977
Bn-A05-p19048941	0.991735537	0.991735537	Bn-N5-p19993850	Bn-chrA05-p17285222
Bn-A05-p19064234	0.983471074	0.983471074	Bn-N5-p20005163	Bn-chrA05-p17296257
Bn-A05-p19067421	0.989583333	0.989583333	Bn-N5-p20008350	Bn-chrA05-p17299444
Bn-A05-p19070214	0.958677686	0.958677686	Bn-N5-p20015866	Bn-chrA05-p17302241
Bn-A05-p19070284	0.892561983	0.892561983	Bn-N5-p20015936	Bn-chrA05-p17302311
Bn-A05-p19071118	0.975206612	0.975206612	Bn-N5-p20016769	Bn-chrA05-p17303144
Bn-A05-p19096242	0.960199005	0.960199005	Bn-N5-p20037433	Bn-chrA05-p17323749
Bn-A05-p19105275	0.983471074	0.983471074	Bn-N5-p20043953	Bn-chrA05-p17330159
Bn-A05-p19105857	0.975206612	0.818181818	Bn-N5-p20045143	Bn-chrC05-p35401317
Bn-A05-p19116891	0.958677686	0.958677686	Bn-N5-p20060451	Bn-chrA05_random-p274203
Bn-A05-p19128595	0.991735537	0.991735537	Bn-N5-p20074142	Bn-chrA05-p17359503
Bn-A05-p19130774	0.991735537	0.991735537	Bn-N5-p20076321	Bn-chrA05-p17361682
Bn-A05-p19178139	0.966942149	0.958677686	Bn-N5-p20127318	Bn-chrA05-p17420760
Bn-A05-p19186596	0.983471074	0.991735537	Bn-N5-p20134679	Bn-chrA05-p17432776
Bn-A05-p19187149	1	0.985074627	Bn-N5-p20135232	Bn-chrA05-p17433327
Bn-A05-p19195181	0.965174129	0.965174129	Bn-N5-p20142943	Bn-chrA05-p17441037
Bn-A05-p19207735	0.966942149	0.966942149	Bn-N5-p20153412	Bn-chrA05-p17451512
Bn-A05-p19209866	0.945273632	0.945273632	Bn-N5-p20155541	Bn-chrA05-p17453639
Bn-A05-p19224833	0.980099502	0.980099502	Bn-N5-p20178234	Bn-chrA05-p17470697
Bn-A05-p19238078	0.975206612	0.933884298	Bn-N5-p20191672	Bn-chrA05-p17484134
Bn-A05-p19238134	0.975206612	0.966942149	Bn-N5-p20191728	Bn-chrA05-p17484189
Bn-A05-p19238534	0.983471074	0.983471074	Bn-N5-p20192126	Bn-chrA05-p17484587

Bn-A05-p19242100	0.958677686	0.958677686	Bn-N5-p20194281	Bn-chrA05-p17486742
Bn-A05-p19242397	0.975206612	0.975206612	Bn-N5-p20194584	Bn-chrA05-p17487045
Bn-A05-p19246371	0.958677686	0.958677686	Bn-N5-p20196225	Bn-chrA05-p17488686
Bn-A05-p19250569	0.975206612	0.975206612	Bn-N5-p20199033	Bn-chrA05-p17491338
Bn-A05-p19251131	0.991735537	0.991735537	Bn-N5-p20199574	Bn-chrA05-p17491879
Bn-A05-p19267100	0.950413223	0.950413223	Bn-N5-p20209716	Bn-chrA05-p17502025
Bn-A05-p19267153	0.900826446	0.900826446	Bn-N5-p20209769	Bn-chrA05-p17502078
Bn-A05-p19267475	0.983471074	0.983471074	Bn-N5-p20211259	Bn-chrA05-p17503568
Bn-A05-p19268141	0.991735537	0.991735537	Bn-N5-p20211837	Bn-chrA05-p17504146
Bn-A05-p19278249	0.995024876	0.995024876	Bn-N5-p20230377	Bn-chrA05-p17522686
Bn-A05-p19278438	0.990049751	0.990049751	Bn-N5-p20230566	Bn-chrA05-p17522875
Bn-A05-p19359433	0.975206612	0.983471074	Bn-N5-p20308057	Bn-chrA05-p17605513
Bn-A05-p19359443	0.975206612	0.983471074	Bn-N5-p20308067	Bn-chrA05-p17605523
Bn-A05-p19362820	0.935323383	0.935323383	Bn-Scaffold02025-p3	Bn-chrA08-p1946286
Bn-A05-p19425901	0.991735537	0.983471074	Bn-N5-p20357880	Bn-chrA05-p17650932
Bn-A05-p19436126	0.975206612	0.958677686	Bn-N5-p20363390	Bn-chrA05-p17655794
Bn-A05-p19445367	0.818181818	0.809917355	Bn-N5-p20376284	Bn-chrA05-p17663913
Bn-A05-p19448529	0.993355482	0.993355482	Bn-N5-p20379449	Bn-chrA05-p17670337
Bn-A05-p19473882	0.985074627	0.980099502	Bn-N5-p20426549	Bn-chrA05_random-p276806
Bn-A05-p19514459	0.966942149	0.966942149	Bn-N5-p20467794	Bn-chrA05-p17704669
Bn-A05-p19530568	0.990049751	0.990049751	Bn-N5-p20484395	Bn-chrA05-p17721264
Bn-A05-p19532660	0.993355482	0.993355482	Bn-N5-p20486481	Bn-chrA05-p17723350
Bn-A05-p19539167	0.983471074	0.991735537	Bn-N5-p20494828	Bn-chrA05-p17729599
Bn-A05-p19539235	0.991735537	1	Bn-N5-p20494896	Bn-chrA05-p17729669
Bn-A05-p19539677	0.991735537	0.867768595	Bn-N5-p20495339	Bn-chrA05-p17730094
Bn-A05-p19547378	0.966942149	0.966942149	Bn-N5-p20502885	Bn-chrA05-p17737621
Bn-A05-p19550645	0.986710963	0.986710963	Bn-N5-p20506085	Bn-chrA05-p17740821
Bn-A05-p19554281	0.955223881	0.955223881	Bn-N5-p20509798	Bn-chrA05-p17744534
Bn-A05-p19571798	0.975124378	0.975124378	Bn-N5-p20531407	Bn-chrA05-p17765955
Bn-A05-p19575123	0.995024876	0.970149254	Bn-N5-p20537173	Bn-chrC05-p36304968
Bn-A05-p19583714	0.983471074	0.983471074	Bn-N5-p20543965	Bn-chrA05-p17774643
Bn-A05-p19599390	0.818181818	0.966942149	Bn-Scaffold09660-p2	Bn-chrA05-p17795602
Bn-A05-p19599809	0.975206612	0.975206612	Bn-N5-p20565652	Bn-chrA05-p17799640
Bn-A05-p19612275	0.94214876	0.94214876	Bn-N5-p20578305	Bn-chrA05-p17812242
Bn-A05-p19622826	0.990033223	0.990033223	Bn-N5-p20590239	Bn-chrA05-p17822655
Bn-A05-p19623018	0.983471074	0.983471074	Bn-N5-p20590431	Bn-chrA05-p17822847
Bn-A05-p19650213	0.993355482	0.993355482	Bn-N5-p20653161	Bn-chrA05-p17869976
Bn-A05-p19650278	0.975206612	0.975206612	Bn-N5-p20653226	Bn-chrA05-p17870041
Bn-A05-p19650965	0.966942149	0.966942149	Bn-N5-p20653946	Bn-chrA05-p17870761
Bn-A05-p19653084	0.917355372	0.917355372	Bn-N5-p20656318	Bn-chrA05-p17873133
Bn-A05-p19660820	0.983471074	0.983471074	Bn-N5-p20663333	Bn-chrAnn_random-p478716
Bn-A05-p19666434	0.991735537	0.991735537	Bn-N5-p20668684	Bn-chrA05-p17887982
Bn-A05-p19668317	0.983471074	0.983471074	Bn-N5-p20670585	Bn-chrA05-p17889889
Bn-A05-p19668632	0.983471074	0.983471074	Bn-N5-p20670900	Bn-chrA05-p17890204
Bn-A05-p19728614	0.973421927	0.990033223	Bn-N15-p40940767	Bn-chrA05-p17930951
Bn-A05-p19730291	0.975124378	0.975124378	Bn-Scaffold15556-p5	Bn-chrC05-p36540379

Bn-A05-p19745674	1	1	Bn-N5-p20746277	Bn-chrA05-p17967751
Bn-A05-p19777231	0.955223881	0.955223881	Bn-N5-p20764810	Bn-chrA05-p17985502
Bn-A05-p19777547	0.855721393	0.870646766	Bn-N5-p20765142	Bn-chrC05-p36604364
Bn-A05-p19802577	0.955223881	0.955223881	Bn-N5-p20796236	Bn-chrA05-p18017730
Bn-A05-p19806333	0.975206612	0.975206612	Bn-N5-p20806733	Bn-chrA05-p18021517
Bn-A05-p19846575	0.950413223	0.950413223	Bn-N5-p20843840	Bn-chrA05-p18060391
Bn-A05-p19857351	0.975206612	0.975206612	Bn-N5-p20863496	Bn-chrA05-p18074539
Bn-A05-p19857532	0.990049751	0.990049751	Bn-N15-p41170678	Bn-chrC05-p36919203
Bn-A05-p19873242	0.989583333	0.989583333	Bn-N5-p20880128	Bn-chrA05-p18090398
Bn-A05-p19886554	0.975124378	0.975124378	Bn-N5-p20893009	Bn-chrA05-p18116430
Bn-A05-p19887308	0.996677741	0.996677741	Bn-N5-p20893763	Bn-chrA05-p18117184
Bn-A05-p19897379	0.966942149	0.966942149	Bn-N5-p20901186	Bn-chrA05-p18124607
Bn-A05-p19909249	0.991735537	0.991735537	Bn-N5-p20915043	Bn-chrA05-p18136718
Bn-A05-p19919111	0.983471074	0.991735537	Bn-N5-p20921026	Bn-chrA05-p18142243
Bn-A05-p19919179	0.983471074	0.991735537	Bn-N5-p20921094	Bn-chrA05-p18142311
Bn-A05-p19930910	0.983471074	0.983471074	Bn-N5-p20933033	Bn-chrA05-p18152712
Bn-A05-p19943528	0.502487562	0.497512438	Bn-N15-p41305004	Unknown
Bn-A05-p19946413	0.980099502	0.990049751	Bn-N5-p20947845	Bn-chrA05-p18170942
Bn-A05-p19967151	0.975124378	0.985074627	Bn-N5-p20959304	Bn-chrA05-p18182732
Bn-A05-p19973413	0.990049751	0.985074627	Bn-N5-p20966234	Bn-chrA05-p18188657
Bn-A05-p19978706	0.990049751	0.995024876	Bn-N5-p20971492	Bn-chrA05-p18201609
Bn-A05-p19990020	0.991735537	0.198347107	Bn-N5-p20975583	Bn-chrCnn_random-p575242
Bn-A05-p19990560	0.958677686	0.157024793	Bn-N5-p20976126	Bn-chrUnn_random-p91463C
Bn-A05-p20009925	0.983471074	0.983471074	Bn-N5-p20990105	Bn-chrA05-p18223896
Bn-A05-p20019130	0.94214876	0.94214876	Bn-N5-p20999054	Bn-chrA05-p18232842
Bn-A05-p20020286	0.983471074	0.975206612	Bn-Scaffold16449-p3	Bn-chrC05-p37142470
Bn-A05-p20074144	0.986710963	0.986710963	Bn-N5-p21047030	Bn-chrA05-p18273916
Bn-A05-p20075053	0.966942149	0.966942149	Bn-N5-p21047945	Bn-chrA05-p18274831
Bn-A05-p20100234	0.925619835	0.925619835	Bn-N15-p41508343	Bn-chrCnn_random-p590319
Bn-A05-p20105944	0.983471074	0.983471074	Bn-N5-p21075694	Bn-chrA05-p18303971
Bn-A05-p20109948	0.966942149	0.429752066	Bn-N5-p21081970	Bn-chrC01-p35477337
Bn-A05-p20113630	0.983471074	0.917355372	Bn-N5-p21087722	Bn-chrA05-p18307723
Bn-A05-p20124425	0.818181818	0.818181818	Bn-N5-p21103930	Bn-chrA05-p18322057
Bn-A05-p20125478	0.975206612	0.975206612	Bn-N5-p21107745	Bn-chrA05-p18325978
Bn-A05-p20125982	0.980066445	0.980066445	Bn-N5-p21108248	Bn-chrA05-p18326481
Bn-A05-p20126071	0.966942149	0.966942149	Bn-N5-p21108337	Bn-chrA05-p18326570
Bn-A05-p20127293	0.975124378	0.975124378	Bn-N5-p21109638	Bn-chrA05-p18327871
Bn-A05-p20127547	0.970149254	0.970149254	Bn-N5-p21109891	Bn-chrA05-p18328124
Bn-A05-p20128796	0.958677686	0.958677686	Bn-N5-p21111137	Bn-chrA05-p18329370
Bn-A05-p20129266	0.966942149	0.966942149	Bn-N5-p21112649	Bn-chrA05-p18330893
Bn-A05-p20138451	0.983471074	0.983471074	Bn-N5-p21122742	Bn-chrA05-p18340986
Bn-A05-p20139872	0.996677741	0.996677741	Bn-N5-p21124163	Bn-chrA05-p18342407
Bn-A05-p20141883	1	1	Bn-N5-p21126172	Bn-chrA05-p18344416
Bn-A05-p20142804	0.983471074	0.983471074	Bn-N5-p21127093	Bn-chrA05-p18345337
Bn-A05-p20161210	1	1	Bn-N5-p21148095	Bn-chrA05-p18364819
Bn-A05-p20163118	0.975206612	0.975206612	Bn-N5-p21150003	Bn-chrA05-p18366727

Bn-A05-p20167176	0.975206612	0.975206612	Bn-N5-p21154067	Bn-chrA05-p18370789
Bn-A05-p20168806	0.995024876	0.995024876	Bn-N5-p21155697	Bn-chrA05-p18372419
Bn-A05-p20168909	0.955223881	0.955223881	Bn-N5-p21155799	Bn-chrA05-p18372521
Bn-A05-p20174313	0.976744186	0.976744186	Bn-N5-p21161214	Bn-chrA05-p18377936
Bn-A05-p20178007	1	1	Bn-N5-p21164918	Bn-chrA05-p18381633
Bn-A05-p20179255	0.925619835	0.454545455	Bn-N15-p41591244	Bn-chrC05-p37294162
Bn-A05-p20232989	0.989583333	0.989583333	Bn-N5-p21229250	Bn-chrA05-p18445975
Bn-A05-p20254809	1	1	Bn-N5-p21252649	Bn-chrA05-p18469676
Bn-A05-p20283717	0.991735537	0.991735537	Bn-N5-p21273031	Bn-chrA05-p18489856
Bn-A05-p20283817	0.975206612	0.975206612	Bn-N5-p21273131	Bn-chrA05-p18489956
Bn-A05-p20290091	1	0.809917355	Bn-N5-p21282620	Bn-chrA05-p18500793
Bn-A05-p20292751	0.990049751	0.985074627	Bn-N5-p21285273	Bn-chrA05-p18503267
Bn-A05-p20296253	0.983471074	0.983471074	Bn-N5-p21294877	Bn-chrA05-p18506814
Bn-A05-p20300638	0.983471074	0.966942149	Bn-N5-p21299730	Bn-chrA05-p18511228
Bn-A05-p20302128	0.966942149	0.975206612	Bn-N5-p21301220	Bn-chrA05-p18512804
Bn-A05-p20314128	0.991735537	0.991735537	Bn-N5-p21314748	Bn-chrA05-p18526323
Bn-A05-p20316923	0.991735537	0.991735537	Bn-N5-p21317537	Bn-chrA05-p18529112
Bn-A05-p20319622	0.993355482	0.996677741	Bn-N5-p21320240	Bn-chrA05-p18531816
Bn-A05-p20328249	0.983388704	0.950166113	Bn-N5-p21328859	Bn-chrC05-p37563102
Bn-A05-p20328443	0.973421927	0.96345515	Bn-N6-p12659946	Bn-chrA05-p18540542
Bn-A05-p20337405	0.946843854	0.940199336	Bn-N15-p41803731	Bn-chrA05-p18550347
Bn-A05-p20354606	0.953488372	0.950166113	Bn-N5-p21361008	Bn-chrA05-p18564620
Bn-A05-p20359381	0.917355372	0.917355372	Bn-N5-p21367445	Bn-chrA05-p18569886
Bn-A05-p20359553	0.966942149	0.966942149	Bn-N5-p21367608	Bn-chrA05-p18570049
Bn-A05-p20365953	0.956810631	0.956810631	Bn-N15-p41914828	Bn-chrC05-p37684642
Bn-A05-p20372600	0.976744186	0.976744186	Bn-N5-p21377099	Bn-chrA05-p18579628
Bn-A05-p20375812	1	0.996677741	Bn-N5-p21380281	Bn-chrA05-p18582800
Bn-A05-p20377770	0.975124378	0.950248756	Bn-N5-p21383427	Bn-chrA05-p18584639
Bn-A05-p20379970	0.958677686	0.826446281	Bn-N5-p21387104	Bn-chrC05-p37698448
Bn-A05-p20380629	0.94214876	0.289256198	Bn-N5-p21387764	Bn-chrC05-p37705562
Bn-A05-p20386700	0.993355482	0.990033223	Bn-N5-p21394148	Bn-chrA05-p18600127
Bn-A05-p20390129	0.94214876	0.950413223	Bn-N5-p21397579	Bn-chrA05-p18603507
Bn-A05-p20394162	0.983471074	0.991735537	Bn-N5-p21403931	Bn-chrA05-p18607781
Bn-A05-p20399452	0.940199336	0.936877076	Bn-N5-p21409222	Bn-chrA05-p18613093
Bn-A05-p20404099	0.985074627	0.980099502	Bn-N5-p21413614	Bn-chrA05-p18617472
Bn-A05-p20422081	0.950248756	0.995024876	Bn-N15-p42032342	Bn-chrA05-p18633517
Bn-A05-p20425452	0.995024876	0.995024876	Bn-N5-p21430652	Bn-chrA05-p18636250
Bn-A05-p20425648	0.995024876	0.995024876	Bn-N5-p21430848	Bn-chrA05-p18636446
Bn-A05-p20429403	0.996677741	0.996677741	Bn-N5-p21434617	Bn-chrA05-p18641260
Bn-A05-p20443393	1	1	Bn-N5-p21445934	Bn-chrA05-p18651194
Bn-A05-p20449058	0.991735537	0.991735537	Bn-N5-p21446601	Bn-chrA05-p18651861
Bn-A05-p20458590	0.983471074	0.983471074	Bn-N5-p21454386	Bn-chrA05-p18659644
Bn-A05-p20461416	0.983471074	0.983471074	Bn-N5-p21457459	Bn-chrA05-p18662529
Bn-A05-p20461727	0.991735537	0.991735537	Bn-N5-p21457770	Bn-chrA05-p18662840
Bn-A05-p20467809	0.991735537	0.991735537	Bn-N5-p21463759	Bn-chrA05-p18668829
Bn-A05-p20478486	0.983471074	0.983471074	Bn-N5-p21473270	Bn-chrA05-p18678339

Bn-A05-p20479068	0.983471074	0.983471074	Bn-N5-p21473852	Bn-chrA05-p18678921
Bn-A05-p20485998	0.983471074	0.983471074	Bn-N5-p21481924	Bn-chrA05-p18685868
Bn-A05-p20487983	1	1	Bn-N5-p21488314	Bn-chrA05-p18692257
Bn-A05-p20492525	0.950413223	0.950413223	Bn-N5-p21490939	Bn-chrA05-p18694882
Bn-A05-p20503767	0.985074627	0.985074627	Bn-N5-p21502198	Bn-chrA05-p18706142
Bn-A05-p20534431	0.991735537	0.991735537	Bn-N5-p21536945	Bn-chrA05-p18730739
Bn-A05-p20541690	0.986710963	0.986710963	Bn-N5-p21543485	Bn-chrA05-p18741646
Bn-A05-p20541987	0.965174129	0.965174129	Bn-N5-p21543782	Bn-chrA05-p18741943
Bn-A05-p20549826	0.958677686	0.958677686	Bn-N5-p21551551	Bn-chrA05-p18749707
Bn-A05-p20550694	0.979166667	0.979166667	Bn-N5-p21552451	Bn-chrA05-p18750607
Bn-A05-p20554567	0.966942149	0.966942149	Bn-N5-p21556284	Bn-chrA05-p18754440
Bn-A05-p20555996	0.983471074	0.983471074	Bn-N5-p21557718	Bn-chrA05-p18755874
Bn-A05-p20563653	0.975206612	0.975206612	Bn-N5-p21565316	Bn-chrA05-p18763474
Bn-A05-p20564729	0.980066445	0.980066445	Bn-N5-p21566392	Bn-chrA05-p18764550
Bn-A05-p20591895	0.975206612	0.776859504	Bn-N5-p21589324	Bn-chrAnn_random-p294242
Bn-A05-p20591964	0.991735537	0.884297521	Bn-N5-p21589393	Bn-chrC04_random-p359459
Bn-A05-p20594196	0.990049751	0.990049751	Bn-N5-p21593116	Bn-chrA05-p18791910
Bn-A05-p20597839	0.983471074	0.983471074	Bn-N5-p21596300	Bn-chrA05-p18795555
Bn-A05-p20601139	0.955223881	0.980099502	Bn-N5-p21609623	Bn-chrA05-p18805551
Bn-A05-p20623573	0.983471074	0.991735537	Bn-N5-p21632033	Bn-chrA05-p18827947
Bn-A05-p20626818	0.966777409	0.966777409	Bn-N5-p21634665	Bn-chrA05-p18830588
Bn-A05-p20632153	0.980099502	0.985074627	Bn-N5-p21640251	Bn-chrA05-p18835949
Bn-A05-p20673293	0.966942149	0.966942149	Bn-N5-p21686601	Bn-chrA05-p18882599
Bn-A05-p20676409	0.995024876	0.995024876	Bn-N4-p9291667	Bn-chrC04-p28186145
Bn-A05-p20681871	0.991735537	0.991735537	Bn-N5-p21694862	Bn-chrA05-p18891109
Bn-A05-p20690058	0.950413223	0.950413223	Bn-N5-p21700221	Bn-chrA05-p18896873
Bn-A05-p20692714	0.991735537	0.991735537	Bn-N5-p21702875	Bn-chrA05-p18899527
Bn-A05-p20697145	0.966942149	0.966942149	Bn-N5-p21705718	Bn-chrA05-p18902367
Bn-A05-p20697410	0.958677686	0.958677686	Bn-N5-p21705983	Bn-chrA05-p18902632
Bn-A05-p20697473	0.900826446	0.900826446	Bn-N5-p21706043	Bn-chrA05-p18902692
Bn-A05-p20697824	0.909090909	0.909090909	Bn-N5-p21706401	Bn-chrA05-p18903050
Bn-A05-p20697908	0.892561983	0.892561983	Bn-N5-p21706477	Bn-chrA05-p18903126
Bn-A05-p20698928	0.619834711	0.619834711	Bn-N5-p21707505	Bn-chrA05-p18904154
Bn-A05-p20699671	0.909090909	0.909090909	Bn-N5-p21707895	Bn-chrA05-p18904544
Bn-A05-p20705675	0.975206612	0.975206612	Bn-N5-p21713131	Bn-chrC05-p38002849
Bn-A05-p20712388	0.983471074	0.983471074	Bn-N5-p21721917	Bn-chrA05-p18915603
Bn-A05-p20714207	0.985074627	0.985074627	Bn-N5-p21723709	Bn-chrA05-p18917395
Bn-A05-p20714302	0.975206612	0.975206612	Bn-N5-p21723804	Bn-chrA05-p18917490
Bn-A05-p20714581	0.975206612	0.975206612	Bn-N5-p21724083	Bn-chrA05-p18917769
Bn-A05-p20716500	0.900826446	0.900826446	Bn-N5-p21726006	Bn-chrA05-p18919692
Bn-A05-p20718776	0.966942149	0.966942149	Bn-N5-p21728375	Bn-chrA05-p18922060
Bn-A05-p20719343	0.958677686	0.958677686	Bn-N5-p21728928	Bn-chrA05-p18922613
Bn-A05-p20728232	0.743801653	0.743801653	Bn-N5-p21737810	Bn-chrA05-p18931495
Bn-A05-p20728284	0.809917355	0.809917355	Bn-N15-p42678814	Bn-chrC05-p38029373
Bn-A05-p20734549	0.958677686	0.958677686	Bn-N5-p21749480	Bn-chrA05-p18941779
Bn-A05-p20747913	0.966942149	0.966942149	Bn-N5-p21762829	Bn-chrA05-p18955094

Bn-A05-p20790867	0.975206612	0.966942149	Bn-N5-p21811489	Bn-chrA05-p19000644
Bn-A05-p20791306	0.991735537	0.983471074	Bn-N5-p21812142	Bn-chrC06-p22163637
Bn-A05-p20794306	0.990033223	0.990033223	Bn-N5-p21819292	Bn-chrA05_random-p281566
Bn-A05-p20808964	0.983471074	0.975206612	Bn-N5-p21833178	Bn-chrA05_random-p282794
Bn-A05-p20809217	0.983471074	0.991735537	Bn-N5-p21833431	Bn-chrA05_random-p282819
Bn-A05-p20809327	0.950413223	0.909090909	Bn-N5-p21833541	Bn-chrA05_random-p282830
Bn-A05-p20811510	0.990033223	0.986710963	Bn-N5-p21835724	Bn-chrA05_random-p283106
Bn-A05-p20818194	0.958677686	0.966942149	Bn-N5-p21836982	Bn-chrA05_random-p283232
Bn-A05-p20818989	0.983471074	0.975206612	Bn-N5-p21837774	Bn-chrA05_random-p283422
Bn-A05-p20824936	0.966942149	0.859504132	Bn-N5-p21843939	Bn-chrA05_random-p284021
Bn-A05-p20825280	0.975206612	0.975206612	Bn-N5-p21844288	Bn-chrA05_random-p284055
Bn-A05-p20825449	0.966942149	0.975206612	Bn-N5-p21845569	Bn-chrA05_random-p284072
Bn-A05-p20828693	0.980099502	0.985074627	Bn-N5-p21848115	Bn-chrA05_random-p284581
Bn-A05-p20839044	0.950413223	0.958677686	Bn-N5-p21858583	Bn-chrA05_random-p285258
Bn-A05-p20843453	0.983471074	0.991735537	Bn-N5-p21860833	Bn-chrA05_random-p285481
Bn-A05-p20844496	0.892561983	0.900826446	Bn-N5-p21861878	Bn-chrA05_random-p285586
Bn-A05-p20844938	1	0.991735537	Bn-N5-p21862307	Bn-chrA05_random-p285629
Bn-A05-p20852240	0.983471074	0.975206612	Bn-N5-p21869120	Bn-chrA05-p19003978
Bn-A05-p20888695	0.975206612	0.975206612	Bn-N5-p21899709	Bn-chrA05-p19026135
Bn-A05-p20894465	0.875621891	0.955223881	Bn-N15-p42927017	Bn-chrA05-p19032469
Bn-A05-p20903214	0.950413223	0.950413223	Bn-N5-p21916523	Bn-chrA05-p19038925
Bn-A05-p20903608	0.986710963	0.986710963	Bn-N5-p21916921	Bn-chrA05-p19039321
Bn-A05-p20940971	0.983471074	0.983471074	Bn-N5-p21955512	Bn-chrA05-p19078471
Bn-A05-p20955184	0.975206612	0.975206612	Bn-N5-p21968005	Bn-chrA05-p19093179
Bn-A05-p20972649	0.983471074	0.991735537	Bn-N5-p21986809	Bn-chrA05-p19111527
Bn-A05-p20974054	0.975206612	0.983471074	Bn-N5-p21995827	Bn-chrA05-p19112827
Bn-A05-p20998848	0.966942149	0.983471074	Bn-N5-p22018624	Bn-chrA05-p19159959
Bn-A05-p21000008	0.925619835	0.975206612	Bn-N5-p22021124	Bn-chrA05-p19161119
Bn-A05-p21000614	0.990049751	0.985074627	Bn-N5-p22021619	Bn-chrA05-p19161725
Bn-A05-p21006621	0.975206612	0.975206612	Bn-N5-p22030114	Bn-chrA05-p19166357
Bn-A05-p21028051	0.983471074	0.884297521	Bn-N5-p22049826	Bn-chrA05-p19186057
Bn-A05-p21029850	0.975206612	0.966942149	Bn-N5-p22051769	Bn-chrA05-p19187865
Bn-A05-p21029910	0.958677686	0.958677686	Bn-N5-p22051829	Bn-chrA05-p19187925
Bn-A05-p21036828	0.94214876	0.958677686	Bn-N5-p22058965	Bn-chrA05-p19195323
Bn-A05-p21037865	0.991735537	0.991735537	Bn-N5-p22060030	Bn-chrA05-p19196359
Bn-A05-p21047802	0.966942149	0.876033058	Bn-N5-p22069891	Bn-chrA05-p19204000
Bn-A05-p21052552	0.983471074	0.991735537	Bn-N5-p22075109	Bn-chrA05-p19208767
Bn-A05-p21054101	0.975206612	0.983471074	Bn-N5-p22076654	Bn-chrA05-p19210317
Bn-A05-p21056938	0.983471074	0.966942149	Bn-N5-p22079469	Bn-chrA05-p19214775
Bn-A05-p21057936	0.991735537	0.983471074	Bn-N5-p22080486	Bn-chrA05-p19215767
Bn-A05-p21058156	0.991735537	0.991735537	Bn-N5-p22080734	Bn-chrA05-p19215988
Bn-A05-p21058435	0.966942149	0.958677686	Bn-N5-p22081012	Bn-chrA05-p19216267
Bn-A05-p21064254	0.825870647	0.935323383	Bn-N5-p22086864	Bn-chrA05-p19222138
Bn-A05-p21065087	0.983471074	0.975206612	Bn-N5-p22087896	Bn-chrA05-p19222971
Bn-A05-p21070302	0.996677741	0.996677741	Bn-N5-p22092218	Bn-chrA05-p19227299
Bn-A05-p21070991	0.993355482	0.993355482	Bn-N5-p22092907	Bn-chrA05-p19227988

Bn-A05-p21081145	0.983471074	0.975206612	Bn-N5-p22097194	Bn-chrA05-p19232253
Bn-A05-p21093954	0.991735537	0.991735537	Bn-N5-p22109604	Bn-chrA05-p19244651
Bn-A05-p21106566	1	1	Bn-N5-p22125989	Bn-chrA05-p19255016
Bn-A05-p21107181	0.996677741	0.996677741	Bn-N5-p22126612	Bn-chrA05-p19255639
Bn-A05-p21163226	0.983471074	0.983471074	Bn-N5-p22198721	Bn-chrA05-p19316343
Bn-A05-p21176831	0.989583333	0.989583333	Bn-N5-p22212636	Bn-chrA05-p19330660
Bn-A05-p21182276	1	1	Bn-N5-p22216406	Bn-chrA05-p19334430
Bn-A05-p21188442	1	1	Bn-N5-p22222880	Bn-chrA05-p19340596
Bn-A05-p21193491	0.983471074	0.983471074	Bn-N5-p22228410	Bn-chrA05-p19346126
Bn-A05-p21201908	0.991735537	0.991735537	Bn-N5-p22236093	Bn-chrA05-p19361427
Bn-A05-p21201956	0.990033223	0.990033223	Bn-N5-p22236141	Bn-chrA05-p19361475
Bn-A05-p21208394	0.975206612	0.975206612	Bn-N5-p22242588	Bn-chrA05-p19367922
Bn-A05-p21210078	0.970149254	0.970149254	Bn-N5-p22244271	Bn-chrA05-p19369605
Bn-A05-p21221296	0.966942149	0.966942149	Bn-N5-p22260441	Bn-chrA05-p19386976
Bn-A05-p21233666	0.991735537	0.991735537	Bn-N5-p22267180	Bn-chrA05-p19395677
Bn-A05-p21240761	0.975206612	0.975206612	Bn-N5-p22281026	Bn-chrA05-p19399818
Bn-A05-p21243931	0.94214876	0.94214876	Bn-N5-p22284184	Bn-chrA05-p19402976
Bn-A05-p21248382	0.995024876	0.995024876	Bn-N5-p22288664	Bn-chrA05-p19407517
Bn-A05-p21248720	0.995024876	0.995024876	Bn-N5-p22288997	Bn-chrA05-p19407850
Bn-A05-p21267598	0.991735537	0.991735537	Bn-N5-p22308940	Bn-chrA05-p19427994
Bn-A05-p21299910	0.955223881	0.995024876	Bn-N5-p22345549	Bn-chrA05-p19464223
Bn-A05-p21313618	0.985074627	0.985074627	Bn-N5-p22356167	Bn-chrA05-p19474805
Bn-A05-p21319243	0.985074627	0.985074627	Bn-N5-p22361875	Bn-chrA05-p19480513
Bn-A05-p21325400	0.995024876	0.990049751	Bn-N5-p22364916	Bn-chrA05-p19483554
Bn-A05-p21326091	0.985074627	0.985074627	Bn-N5-p22365600	Bn-chrA05-p19484238
Bn-A05-p21326775	0.955223881	0.940298507	Bn-N5-p22366288	Bn-chrC05-p38825787
Bn-A05-p21345579	0.900497512	0.940298507	Bn-N15-p43570276	Bn-chrAnn_random-p42205[illegible]
Bn-A05-p21347821	0.983471074	0.247933884	Bn-N5-p22392693	Bn-chrA09-p23585417
Bn-A05-p21370435	0.975124378	0.970149254	Bn-N5-p22475280	Bn-chrA05-p19541534
Bn-A05-p21370618	0.990049751	0.995024876	Bn-N5-p22475463	Bn-chrA05-p19541717
Bn-A05-p21375513	0.985074627	0.990049751	Bn-N5-p22477845	Bn-chrA05-p19544098
Bn-A05-p21375514	0.983471074	0.991735537	Bn-N5-p22477845	Bn-chrA05-p19544098
Bn-A05-p21377682	0.950248756	0.950248756	Bn-N5-p22480062	Bn-chrA05-p19546315
Bn-A05-p21381782	0.88372093	0.764119601	Bn-N5-p22483019	Bn-chrC05-p38950267
Bn-A05-p21384657	0.975206612	0.975206612	Bn-N5-p22488648	Bn-chrA05-p19554075
Bn-A05-p21385524	0.930348259	0.995024876	Bn-N5-p22489513	Bn-chrA05-p19554969
Bn-A05-p21463902	0.965174129	0.965174129	Bn-N15-p43799232	Bn-chrC05-p39065656
Bn-A05-p21470614	0.991735537	0.991735537	Bn-N5-p22571580	Bn-chrA05-p19645315
Bn-A05-p21480994	0.983388704	0.983388704	Bn-N5-p22581296	Bn-chrA05-p19658295
Bn-A05-p21484113	0.990033223	0.990033223	Bn-N5-p22584410	Bn-chrA05-p19661408
Bn-A05-p21491115	0.991735537	0.991735537	Bn-N5-p22592447	Bn-chrA05-p19669585
Bn-A05-p21493851	1	0.429752066	Bn-N5-p22595155	Unknown
Bn-A05-p21532839	0.990049751	0.990049751	Bn-N5-p22635384	Bn-chrA05-p19716162
Bn-A05-p21538700	0.983471074	0.983471074	Bn-N5-p22641252	Bn-chrA05-p19722026
Bn-A05-p21579642	0.996677741	0.996677741	Bn-N5-p22686386	Bn-chrA05-p19765674
Bn-A05-p21587317	0.983471074	0.983471074	Bn-N5-p22695386	Bn-chrA05-p19773740

Bn-A05-p21600657	0.975206612	0.975206612	Bn-N5-p22706792	Bn-chrA05-p19784749
Bn-A05-p21600954	0.890365449	0.890365449	Bn-N5-p22707327	Bn-chrA05-p19785284
Bn-A05-p21631586	0.995024876	0.995024876	Bn-N5-p22736616	Bn-chrA05-p19814786
Bn-A05-p21633055	0.950248756	0.950248756	Bn-N5-p22738083	Bn-chrA05-p19816253
Bn-A05-p21638427	0.975206612	0.975206612	Bn-N5-p22743379	Bn-chrA05-p19821532
Bn-A05-p21641604	0.975206612	0.975206612	Bn-N5-p22746726	Bn-chrA05-p19824879
Bn-A05-p21641616	0.975206612	0.975206612	Bn-N5-p22746738	Bn-chrA05-p19824891
Bn-A05-p21646684	0.990033223	0.993355482	Bn-N5-p22751748	Bn-chrA05-p19829926
Bn-A05-p21658509	0.52892562	0.950413223	Bn-N5-p22756394	Bn-chrA05-p19848578
Bn-A05-p21660030	0.975206612	0.966942149	Bn-N5-p22762138	Bn-chrA05-p19850099
Bn-A05-p21660088	0.73553719	0.983471074	Bn-N5-p22762196	Bn-chrA05-p19850157
Bn-A05-p21661113	0.702479339	0.991735537	Bn-N5-p22762393	Bn-chrA05-p19851182
Bn-A05-p21668872	0.990049751	0.990049751	Bn-N5-p22769764	Bn-chrA05-p19858864
Bn-A05-p21698558	0.926910299	0.993355482	Bn-N15-p44190242	Bn-chrA05-p19885812
Bn-A05-p21699314	1	0.993355482	Bn-N5-p22798882	Bn-chrA05-p19887033
Bn-A05-p21806633	0.991735537	0.991735537	Bn-N5-p22895992	Bn-chrA05-p19980932
Bn-A05-p21806801	0.991735537	0.991735537	Bn-N5-p22896160	Bn-chrA05-p19981100
Bn-A05-p21809718	0.991735537	0.991735537	Bn-N5-p22899077	Bn-chrA05-p19984017
Bn-A05-p21814557	0.966942149	0.975206612	Bn-N6-p10711622	Bn-chrA06-p10240474
Bn-A05-p21848003	0.925619835	0.925619835	Bn-N5-p22904785	Bn-chrA05-p19989725
Bn-A05-p21852014	0.966942149	0.966942149	Bn-N5-p22908803	Bn-chrA05-p19993741
Bn-A05-p21852563	0.975206612	0.975206612	Bn-N5-p22909386	Bn-chrA05-p19994324
Bn-A05-p21853739	0.975206612	0.975206612	Bn-N5-p22910586	Bn-chrA05-p19995524
Bn-A05-p21865451	0.933884298	0.933884298	Bn-N5-p22920042	Bn-chrA05_random-p286514
Bn-A05-p21886422	0.983471074	0.991735537	Bn-N5-p22949280	Bn-chrA05-p20034309
Bn-A05-p21887311	0.990049751	0.985074627	Bn-N5-p22950175	Bn-chrA05-p20035198
Bn-A05-p21889289	0.766169154	0.781094527	Bn-N5-p22951199	Bn-chrA05-p20036210
Bn-A05-p21915455	1	1	Bn-Scaffold03647-p2	Bn-chrA05-p20058131
Bn-A05-p21930978	0.991735537	0.991735537	Bn-N5-p22994971	Bn-chrA05-p20074457
Bn-A05-p21973951	0.995024876	0.995024876	Bn-N5-p23033256	Bn-chrA05-p20109429
Bn-A05-p22015412	0.975206612	0.975206612	Bn-N5-p23079653	Bn-chrA05-p20154644
Bn-A05-p22021177	0.991735537	0.991735537	Bn-N5-p23085437	Bn-chrA05-p20160427
Bn-A05-p22027389	0.94214876	0.867768595	Bn-N5-p23093885	Bn-chrC05-p39871526
Bn-A05-p22028401	1	1	Bn-N5-p23094897	Bn-chrA05-p20170345
Bn-A05-p22060825	0.983471074	0.983471074	Bn-N5-p23110211	Bn-chrA05-p20186065
Bn-A05-p22089650	0.983471074	0.983471074	Bn-N5-p23135358	Bn-chrA05-p20211014
Bn-A05-p22101592	0.975206612	0.975206612	Bn-N5-p23143905	Bn-chrA05-p20219561
Bn-A05-p22111286	0.975124378	0.975124378	Bn-N5-p23146885	Bn-chrA05-p20222541
Bn-A05-p22111345	0.995024876	0.995024876	Bn-N5-p23146944	Bn-chrA05-p20222600
Bn-A05-p22114137	0.976744186	0.976744186	Bn-N5-p23149741	Bn-chrA05-p20225397
Bn-A05-p22117809	0.991735537	0.991735537	Bn-N5-p23153978	Bn-chrA05-p20229479
Bn-A05-p22132297	0.958677686	0.966942149	Bn-N5-p23167931	Bn-chrA05-p20243411
Bn-A05-p22135265	0.975206612	0.966942149	Bn-N5-p23170850	Bn-chrA05-p20246364
Bn-A05-p22141046	0.925619835	0.933884298	Bn-N5-p23176773	Bn-chrA05-p20252157
Bn-A05-p22142609	0.975206612	0.966942149	Bn-N5-p23178285	Bn-chrA05-p20253713
Bn-A05-p22145624	0.975206612	0.983471074	Bn-N5-p23181249	Bn-chrA05-p20257041

Bn-A05-p22152482	0.945273632	0.915422886	Bn-N5-p23203724	Bn-chrAnn_random-p17661(
Bn-A05-p22153383	0.985074627	0.985074627	Bn-N5-p23204638	Bn-chrAnn_random-p176602
Bn-A05-p22159613	0.983471074	0.983471074	Bn-N5-p23215538	Bn-chrAnn_random-p176515
Bn-A05-p22169560	0.966942149	0.966942149	Bn-N5-p23229552	Bn-chrAnn_random-p176416
Bn-A05-p22182628	0.950413223	0.983471074	Bn-N5-p23242487	Bn-chrAnn_random-p176286
Bn-A05-p22218776	0.966942149	0.975206612	Bn-N5-p23280486	Bn-chrA05-p20293808
Bn-A05-p22221563	0.983471074	0.991735537	Bn-N5-p23283281	Bn-chrA05-p20296614
Bn-A05-p22224811	0.985074627	0.990049751	Bn-N5-p23285588	Bn-chrA05-p20299773
Bn-A05-p22249401	0.975206612	0.983471074	Bn-N5-p23311561	Bn-chrA05-p20320966
Bn-A05-p22265537	0.983471074	0.925619835	Bn-N5-p23328186	Bn-chrA05-p20332449
Bn-A05-p22266340	0.983471074	0.991735537	Bn-N5-p23328990	Bn-chrA05-p20333159
Bn-A05-p22266933	0.975124378	0.970149254	Bn-N5-p23329583	Bn-chrA05-p20333752
Bn-A05-p22269511	0.975124378	0.980099502	Bn-N5-p23332175	Bn-chrA05-p20336335
Bn-A05-p22276626	0.966942149	1	Bn-N15-p45058666	Bn-chrA05-p20343448
Bn-A05-p22282750	0.561983471	0.983471074	Bn-N5-p23348012	Bn-chrA05-p20349424
Bn-A05-p22282814	0.876033058	0.991735537	Bn-N5-p23348130	Bn-chrA05-p20349488
Bn-A05-p22284501	0.975124378	0.980099502	Bn-N5-p23349971	Bn-chrA05-p20351180
Bn-A05-p22298146	0.965174129	0.965174129	Bn-N5-p21597469	Bn-chrA05-p18796724
Bn-A05-p22299077	0.955223881	0.955223881	Bn-N5-p21598402	Bn-chrA05-p18798632
Bn-A05-p22299214	0.980099502	0.980099502	Bn-N4-p13706187	Bn-chrA05-p18798768
Bn-A05-p22310050	0.975206612	0.975206612	Bn-N5-p23373793	Bn-chrA05-p20374179
Bn-A05-p22318660	0.970149254	0.970149254	Bn-N5-p23381225	Bn-chrA05-p20381611
Bn-A05-p22320477	0.925373134	0.925373134	Bn-N5-p23383035	Bn-chrA05-p20383421
Bn-A05-p22324761	0.956810631	0.956810631	Bn-N5-p23387315	Bn-chrA05-p20387699
Bn-A05-p22350368	0.966942149	0.966942149	Bn-N5-p23418482	Bn-chrA05-p20415027
Bn-A05-p22351135	0.801652893	0.909090909	Bn-N5-p23419218	Bn-chrA05-p20415763
Bn-A05-p22392750	0.991735537	0.991735537	Bn-N5-p23463991	Bn-chrA05-p20459978
Bn-A05-p22406985	0.876033058	0.876033058	Bn-N5-p23484017	Bn-chrA05-p20478033
Bn-A05-p22427412	0.976744186	0.976744186	Bn-N5-p23503111	Bn-chrA05-p20498474
Bn-A05-p22452120	0.983471074	0.983471074	Bn-N5-p23527576	Bn-chrA05-p20521931
Bn-A05-p22454211	0.991735537	0.991735537	Bn-N5-p23529568	Bn-chrA05-p20523923
Bn-A05-p22454557	0.975206612	0.975206612	Bn-N5-p23529914	Bn-chrA05-p20524269
Bn-A05-p22459438	1	1	Bn-N5-p23534788	Bn-chrA05-p20529142
Bn-A05-p22463256	0.975124378	0.975124378	Bn-N5-p23538588	Bn-chrA05-p20533240
Bn-A05-p22476414	0.983471074	0.991735537	Bn-N5-p23551706	Bn-chrA05-p20546379
Bn-A05-p22478538	0.979166667	0.96875	Bn-N5-p23553830	Bn-chrA05-p20548503
Bn-A05-p22480535	0.991735537	1	Bn-N5-p23555829	Bn-chrA05-p20550490
Bn-A05-p22488454	0.933884298	0.94214876	Bn-N5-p23562937	Bn-chrA05_random-p287859
Bn-A05-p22498838	0.958677686	0.950413223	Bn-N5-p23571097	Bn-chrA05_random-p288681
Bn-A05-p22506006	0.975206612	0.983471074	Bn-N5-p23578251	Bn-chrA05_random-p289284
Bn-A05-p22511813	0.669421488	0.67768595	Bn-N5-p23586216	Bn-chrA05_random-p289668
Bn-A05-p22513075	0.958677686	0.950413223	Bn-N5-p23587400	Bn-chrA05_random-p289786
Bn-A05-p22519751	0.991735537	0.983471074	Bn-N5-p23601213	Bn-chrA05-p20558577
Bn-A05-p22519836	0.975206612	0.983471074	Bn-N5-p23601298	Bn-chrA05-p20558662
Bn-A05-p22553631	0.935323383	0.940298507	Bn-N5-p23621952	Bn-chrA05-p20578359
Bn-A05-p22563719	0.960199005	0.955223881	Bn-N5-p23632498	Bn-chrC05-p40486954

Bn-A05-p22585118	0.975206612	0.983471074	Bn-N5-p23645999	Bn-chrA05-p20603965
Bn-A05-p22589874	0.986710963	0.990033223	Bn-N5-p23651326	Bn-chrA05-p20620097
Bn-A05-p22591072	0.933884298	0.933884298	Bn-N5-p23652525	Bn-chrA05-p20621296
Bn-A05-p22591626	0.958677686	0.966942149	Bn-N5-p23653076	Bn-chrA05-p20621851
Bn-A05-p22599241	0.953488372	0.993355482	Bn-N5-p23662711	Bn-chrA05-p20630559
Bn-A05-p22613926	0.884297521	0.966942149	Bn-N5-p23676910	Bn-chrA05-p20644255
Bn-A05-p22622143	0.966942149	0.685950413	Bn-N5-p23685367	Bn-chrA05-p20652769
Bn-A05-p22636722	0.975206612	0.983471074	Bn-N5-p23705220	Bn-chrA05-p20666496
Bn-A05-p22650582	1	0.991735537	Bn-N5-p23716430	Bn-chrA05-p20677467
Bn-A05-p22651145	0.991735537	0.983471074	Bn-N5-p23716993	Bn-chrA05-p20678030
Bn-A05-p22653151	0.983471074	0.975206612	Bn-N5-p23719329	Bn-chrA05-p20680044
Bn-A05-p22657528	1	0.991735537	Bn-N5-p23723629	Bn-chrA05-p20684409
Bn-A05-p22658512	0.996677741	0.993355482	Bn-N5-p23724605	Bn-chrA05-p20685362
Bn-A05-p22660561	0.996677741	0.990033223	Bn-N5-p23726662	Bn-chrA05-p20687559
Bn-A05-p22669409	0.975206612	0.768595041	Bn-N5-p23735880	Bn-chrA01-p10045587
Bn-A05-p22677701	0.958677686	0.950413223	Bn-N5-p23742924	Bn-chrA05-p20747927
Bn-A05-p22677829	0.983471074	0.975206612	Bn-N5-p23743052	Bn-chrA05-p20748063
Bn-A05-p22677937	0.983471074	0.991735537	Bn-N5-p23743160	Bn-chrA05-p20748171
Bn-A05-p22682587	0.966942149	0.975206612	Bn-N5-p23750300	Bn-chrA05-p20760422
Bn-A05-p22682639	0.975206612	0.94214876	Bn-N5-p23750352	Bn-chrA05-p20760474
Bn-A05-p22689848	0.983471074	0.975206612	Bn-N5-p23757583	Bn-chrA05-p20767719
Bn-A05-p22691469	0.975206612	0.966942149	Bn-N5-p23759203	Bn-chrA05-p20769339
Bn-A05-p22706491	0.983471074	0.983471074	Bn-N5-p23769388	Bn-chrA05-p20779536
Bn-A05-p22712783	0.958677686	0.950413223	Bn-N5-p23775665	Bn-chrA05-p20786182
Bn-A05-p22727640	0.966942149	0.958677686	Bn-N5-p23790203	Bn-chrAnn_random-p485364
Bn-A05-p22766713	0.993355482	0.996677741	Bn-N5-p23834808	Bn-chrA05-p20840276
Bn-A05-p22766965	1	0.991735537	Bn-N5-p23835061	Bn-chrA05-p20840529
Bn-A05-p22767027	0.990033223	0.993355482	Bn-N5-p23835122	Bn-chrA05-p20840590
Bn-A05-p22767106	0.983471074	0.991735537	Bn-N5-p23835201	Bn-chrA05-p20840669
Bn-A05-p22771419	0.966942149	0.975206612	Bn-N5-p23839622	Bn-chrA05-p20846879
Bn-A05-p22780601	0.950248756	0.960199005	Bn-N5-p23850449	Bn-chrA05-p20854878
Bn-A05-p22780670	0.966942149	0.991735537	Bn-N5-p23850516	Bn-chrA05-p20854945
Bn-A05-p22786339	0.950413223	0.94214876	Bn-N5-p23856136	Bn-chrA05-p20860607
Bn-A05-p22789344	0.917355372	0.917355372	Bn-N5-p23859137	Bn-chrC05-p40763060
Bn-A05-p22789711	0.991735537	0.991735537	Bn-N5-p23859503	Bn-chrA05-p20863971
Bn-A05-p22789974	0.933884298	0.933884298	Bn-N5-p23859766	Bn-chrA05-p20864234
Bn-A05-p22797664	0.966942149	0.966942149	Bn-N5-p23867710	Bn-chrA05-p20872177
Bn-A05-p22804927	0.991735537	1	Bn-N5-p23874385	Bn-chrA05-p20878855
Bn-A05-p22812957	0.965174129	0.955223881	Bn-N5-p23882504	Bn-chrA05-p20886864
Bn-A05-p22813120	0.985074627	0.930348259	Bn-N5-p23882667	Bn-chrC05-p40788349
Bn-A05-p22813805	0.993355482	0.96345515	Bn-N5-p23883352	Bn-chrA05-p20887734
Bn-A05-p22826924	0.933884298	0.933884298	Bn-N5-p23893742	Bn-chrA05-p20903284
Bn-A05-p22853415	0.892561983	0.925619835	Bn-N5-p23912947	Bn-chrA05-p20926157
Bn-A05-p22863641	0.983471074	0.991735537	Bn-N5-p23926491	Bn-chrA05-p20940620
Bn-A05-p22868682	0.966942149	0.958677686	Bn-N5-p23932304	Bn-chrA05-p20951232
Bn-A05-p22868951	0.983471074	0.966942149	Bn-N5-p23932573	Bn-chrA05-p20951501

Bn-A05-p22874045	0.956810631	0.956810631	Bn-N5-p23937670	Bn-chrA05-p20956602
Bn-A05-p22889788	0.94214876	0.94214876	Bn-N5-p23989852	Bn-chrA05-p21007388
Bn-A05-p22899595	0.983471074	0.975206612	Bn-N5-p23997708	Bn-chrA05-p21015599
Bn-A05-p22911906	0.975206612	0.958677686	Bn-N5-p24009871	Bn-chrA05-p21027834
Bn-A05-p22912544	0.950166113	0.993355482	Bn-N5-p24010509	Bn-chrA05-p21028472
Bn-A05-p22913147	0.983471074	0.983471074	Bn-N5-p24011109	Bn-chrA05-p21029074
Bn-A05-p22913571	0.935323383	0.935323383	Bn-N5-p24011532	Bn-chrA05-p21029497
Bn-A05-p22913874	0.975124378	0.980099502	Bn-N5-p24011823	Bn-chrA05-p21029800
Bn-A05-p22915357	0.995024876	1	Bn-N5-p24013306	Bn-chrA05-p21031284
Bn-A05-p22917436	0.990033223	0.986710963	Bn-N5-p24015388	Bn-chrA05-p21033362
Bn-A05-p22920228	0.985074627	0.995024876	Bn-N5-p24018180	Bn-chrA05-p21036154
Bn-A05-p22934224	0.990033223	0.950166113	Bn-N5-p24027080	Bn-chrA05-p21044642
Bn-A05-p22938228	0.980066445	0.980066445	Bn-N5-p24032689	Bn-chrA05-p21048892
Bn-A05-p22943215	0.990033223	0.986710963	Bn-N5-p24038161	Bn-chrA05-p21053903
Bn-A05-p22946715	0.958677686	0.867768595	Bn-N5-p24041620	Bn-chrC05-p40977389
Bn-A05-p22946858	0.996677741	0.996677741	Bn-N5-p24041763	Bn-chrA05-p21057548
Bn-A05-p22948751	0.995024876	0.990049751	Bn-N5-p24043719	Bn-chrA05-p21059486
Bn-A05-p22969236	0.836065574	0.830601093	Bn-N15-p45959527	Bn-chrC05-p41018606
Bn-A05-p22969550	0.960199005	0.955223881	Bn-N5-p24061206	Bn-chrA05-p21077005
Bn-A05-p22973324	0.983388704	0.96345515	Bn-N5-p24063948	Bn-chrA05-p21079757
Bn-A05-p22976431	0.909090909	0.909090909	Bn-N5-p24070006	Bn-chrA05-p21082875
Bn-A05-p22977061	0.975206612	0.975206612	Bn-N5-p24070632	Bn-chrA05-p21083501
Bn-A05-p22981266	0.917355372	0.900826446	Bn-N5-p24074498	Bn-chrA05-p21087366
Bn-A05-p23019620	0.595041322	0.595041322	Bn-N5-p24114691	Bn-chrA05-p21180556
Bn-A05-p23020463	0.685950413	0.685950413	Bn-N5-p24114724	Bn-chrA05-p21180589
Bn-A05-p23020834	0.837209302	0.837209302	Bn-N5-p24115095	Bn-chrA05-p21180960
Bn-A05-p23027881	0.975206612	0.975206612	Bn-N5-p24122493	Bn-chrA05-p21188358
Bn-A05-p23030143	0.990033223	0.990033223	Bn-N5-p24126846	Bn-chrA05-p21192419
Bn-A05-p23032803	1	1	Bn-N5-p24129511	Bn-chrA05-p21195084
Bn-A05-p23044521	0.966942149	0.991735537	Bn-N5-p24153291	Bn-chrA05-p21207237
Bn-A05-p23079485	0.990033223	0.990033223	Bn-N5-p24168396	Bn-chrA05-p21222332
Bn-A05-p23081875	0.993355482	0.993355482	Bn-N5-p24170788	Bn-chrA05-p21224724
Bn-A05-p23102053	0.985074627	0.995024876	Bn-N5-p24193947	Bn-chrC05-p41149049
Bn-A05-p23126108	0.916943522	0.916943522	Bn-N5-p24227096	Bn-chrA05-p21276092
Bn-A05-p23140205	0.991735537	0.991735537	Bn-N5-p24252004	Bn-chrA05-p21298993
Bn-A05-p23141080	0.991735537	0.991735537	Bn-N5-p24252879	Bn-chrA05-p21299868
Bn-A05-p23143046	0.993355482	0.993355482	Bn-N5-p24254846	Bn-chrA05-p21301835
Bn-A05-p23145678	0.975206612	0.975206612	Bn-N5-p24257477	Bn-chrA05-p21304466
Bn-A05-p23146882	0.980066445	0.980066445	Bn-N5-p24258683	Bn-chrA05-p21305671
Bn-A05-p23154172	0.976744186	0.976744186	Bn-N5-p24265534	Bn-chrA05-p21312522
Bn-A05-p23159292	0.983471074	0.983471074	Bn-N5-p24270374	Bn-chrA05-p21317458
Bn-A05-p23165568	0.983471074	0.983471074	Bn-N5-p24277022	Bn-chrA05-p21324106
Bn-A05-p23166612	0.983471074	0.983471074	Bn-N5-p24278778	Bn-chrA05-p21325862
Bn-A05-p23166708	0.983471074	0.983471074	Bn-N5-p24278874	Bn-chrA05-p21325958
Bn-A05-p23167201	0.991735537	0.991735537	Bn-N5-p24279379	Bn-chrA05-p21326463
Bn-A05-p23172786	1	1	Bn-N5-p24285127	Bn-chrA05-p21332451

Bn-A05-p23179980	0.950413223	0.950413223	Bn-N5-p24290658	Bn-chrA05-p21337114
Bn-A05-p23188259	0.991735537	0.991735537	Bn-N5-p24299145	Bn-chrA05-p21346578
Bn-A05-p23188406	0.897009967	0.897009967	Bn-N5-p24299292	Bn-chrA05-p21346725
Bn-A05-p23196656	0.960199005	0.960199005	Bn-N5-p24302511	Bn-chrA05-p21349944
Bn-A05-p23207708	0.993355482	0.996677741	Bn-N5-p24572506	Bn-chrA05-p21719627
Bn-A05-p23253826	1	0.989583333	Bn-N5-p24618267	Bn-chrA05-p21767296
Bn-A05-p23276053	0.991735537	0.991735537	Bn-N5-p24638929	Bn-chrA05-p21788130
Bn-A05-p23285926	0.980099502	0.980099502	Bn-N5-p24655514	Bn-chrA05-p21798169
Bn-A05-p23288913	0.993355482	0.996677741	Bn-N5-p24657940	Bn-chrA05-p21800590
Bn-A05-p23290863	0.859504132	0.859504132	Bn-N5-p24659891	Bn-chrC05-p41789253
Bn-A05-p23314738	0.991735537	1	Bn-N5-p24678264	Bn-chrA05-p21873212
Bn-A05-p23318038	0.976744186	0.976744186	Bn-N5-p24681647	Bn-chrA05-p21876627
Bn-A05-p23340419	0.966942149	0.975206612	Bn-N5-p24703204	Bn-chrA05-p21899702
Bn-A05-p23358283	0.940298507	0.915422886	Bn-N5-p24720294	Bn-chrA05-p21916788
Bn-A05-p23372051	0.826446281	0.950413223	Bn-N5-p24733993	Bn-chrA05-p21931354
Bn-A05-p23377419	0.996677741	0.993355482	Bn-N5-p24738956	Bn-chrA05-p21936483
Bn-A05-p23397606	0.991735537	0.983471074	Bn-N5-p24761238	Bn-chrA05-p21956333
Bn-A05-p23405117	0.983471074	0.991735537	Bn-N5-p24768396	Bn-chrA05-p21962335
Bn-A05-p23415646	0.991735537	1	Bn-N5-p24772897	Bn-chrA05-p21997053
Bn-A05-p23424482	0.991735537	0.983471074	Bn-N5-p24780757	Bn-chrA05-p22006632
Bn-A05-p23433947	0.975206612	0.966942149	Bn-N15-p47049644	Bn-chrC05-p42034458
Bn-A05-p23445454	0.950413223	0.94214876	Bn-N5-p24808015	Bn-chrA05-p22025641
Bn-A05-p23465599	0.987878788	0.981818182	Bn-N1-p19226027	Bn-chrA01-p16089061
Bn-A05-p23466337	0.965174129	0.970149254	Bn-N1-p19226765	Bn-chrA01-p16089799
Bn-A05-p23478913	0.94214876	0.933884298	Bn-N5-p24842502	Bn-chrA05-p22059040
Bn-A05-p23488683	0.975206612	0.983471074	Bn-N5-p24852039	Bn-chrA05-p22068528
Bn-A05-p23498540	0.966942149	0.975206612	Bn-N5-p24861878	Bn-chrA05-p22078330
Bn-A05-p23502993	0.976744186	0.980066445	Bn-N5-p24865653	Bn-chrA05-p22081867
Bn-A05-p23512396	1	0.991735537	Bn-N5-p24875054	Bn-chrA05-p22090341
Bn-A05-p23512931	0.983471074	0.909090909	Bn-N5-p24875589	Bn-chrA05-p22090877
Bn-A05-p23529753	0.983471074	0.983471074	Bn-N5-p25397621	Bn-chrA05-p22581537
Bn-A05-p23529824	0.983471074	0.983471074	Bn-N5-p25397693	Bn-chrA05-p22581609
Bn-A05-p23529883	0.975206612	0.975206612	Bn-N5-p25397752	Bn-chrA05-p22581668
Bn-A05-p23530018	0.991735537	0.991735537	Bn-N5-p25397887	Bn-chrA05-p22581803
Bn-A05-p23530095	0.991735537	0.991735537	Bn-N5-p25397964	Bn-chrA05-p22581880
Bn-A05-p23531991	0.975206612	0.975206612	Bn-N5-p25399525	Bn-chrA05-p22583440
Bn-A05-p23532126	0.983471074	0.983471074	Bn-N5-p25399660	Bn-chrA05-p22583575
Bn-A05-p23532489	1	1	Bn-N5-p25400023	Bn-chrA05-p22583938
Bn-A05-p23533696	1	1	Bn-N5-p25401230	Bn-chrA05-p22585145
Bn-A05-p23543751	0.983388704	0.983388704	Bn-N5-p25412615	Bn-chrA05-p22596411
Bn-A05-p23547791	0.983388704	0.983388704	Bn-N5-p25416667	Bn-chrA05-p22600463
Bn-A05-p23553029	0.966942149	0.966942149	Bn-N5-p25421902	Bn-chrA05-p22605697
Bn-A05-p23562940	0.970099668	0.976744186	Bn-N5-p25433017	Bn-chrA05-p22616597
Bn-A05-p23587018	0.975206612	0.966942149	Bn-N5-p25458443	Bn-chrA05-p22646119
Bn-A05-p23587547	0.996677741	0.996677741	Bn-N5-p25458971	Bn-chrA05-p22646648
Bn-A05-p23588992	1	1	Bn-N5-p25460397	Bn-chrA05-p22648091

Bn-A05-p23593836	0.975206612	0.975206612	Bn-N5-p25465340	Bn-chrA05-p22652968
Bn-A05-p23598510	0.975206612	0.975206612	Bn-N5-p25471487	Bn-chrA05-p22666504
Bn-A05-p23607588	0.909090909	0.94214876	Bn-N5-p25483004	Bn-chrA05-p22675117
Bn-A05-p23680925	1	1	Bn-N5-p25550142	Bn-chrAnn_random-p178946
Bn-A05-p23691939	0.983471074	0.983471074	Bn-N5-p25561134	Bn-chrAnn_random-p179056
Bn-A05-p23692249	0.975206612	0.975206612	Bn-N5-p25561438	Bn-chrAnn_random-p179059
Bn-A05-p23693572	0.990049751	0.990049751	Bn-N5-p25562767	Bn-chrAnn_random-p179072
Bn-A05-p23699894	0.975206612	0.983471074	Bn-N5-p25569197	Bn-chrAnn_random-p179127
Bn-A05-p23701898	0.991735537	0.404958678	Bn-N5-p25571202	Bn-chrC05-p42896014
Bn-A05-p23702049	0.983471074	0.752066116	Bn-N5-p25571353	Bn-chrC05-p42896136
Bn-A05-p23702275	0.983471074	0.933884298	Bn-N5-p25571579	Bn-chrC05-p42896361
Bn-A05-p23703740	0.925619835	0.438016529	Bn-N5-p25571782	Bn-chrC05-p42897066
Bn-A05-p23704743	0.966942149	0.884297521	Bn-N5-p25572799	Bn-chrC05-p42897712
Bn-A05-p23717457	0.950413223	0.760330579	Bn-N5-p25592237	Bn-chrC05-p42911812
Bn-A05-p23718363	0.975206612	0.272727273	Bn-N5-p25593143	Bn-chrC05-p42912659
Bn-A05-p23726137	0.991735537	1	Bn-N5-p25594795	Bn-chrA05_random-p301605
Bn-A05-p23745136	0.983388704	0.980066445	Bn-N5-p25622925	Bn-chrAnn_random-p395460
Bn-A05-p23776343	1	1	Bn-N5-p25659292	Bn-chrA05-p23024268
Bn-A05-p23776869	0.983471074	0.975206612	Bn-N5-p25659796	Bn-chrA05-p23023764
Bn-A05-p23782123	0.991735537	0.966942149	Bn-N5-p25665315	Bn-chrA05-p23018247
Bn-A05-p23782304	0.958677686	0.958677686	Bn-N5-p25665496	Bn-chrA05-p23018068
Bn-A05-p23790987	0.990033223	0.990033223	Bn-N5-p25672415	Bn-chrA05-p23011205
Bn-A05-p23791467	0.983388704	0.983388704	Bn-N5-p25672898	Bn-chrA05-p23010722
Bn-A05-p23802326	0.996677741	0.996677741	Bn-N5-p25684089	Bn-chrA05-p22999894
Bn-A05-p23838032	0.975206612	0.975206612	Bn-Scaffold01069-p2	Bn-chrA05-p22896350
Bn-A05-p23844016	0.933884298	0.933884298	Bn-Scaffold01069-p2	Bn-chrA05-p22889538
Bn-A05-p23844225	0.917355372	0.917355372	Bn-Scaffold01069-p2	Bn-chrA05-p22889370
Bn-A05-p23848037	0.991735537	0.991735537	Bn-Scaffold01069-p1	Bn-chrA05-p22885557
Bn-A05-p23855991	0.983471074	0.983471074	Bn-Scaffold01727-p1	Bn-chrA05-p22876397
Bn-A05-p23873413	0.991735537	0.991735537	Bn-Scaffold29062-p8	Bn-chrA05-p22850229
Bn-A05-p23876386	1	0.991735537	Bn-Scaffold67398-p8	Bn-chrA05-p22846938
Bn-A05-p23893594	0.975206612	0.975206612	Bn-N2-p11020556	Bn-chrA05-p22828740
Bn-A05-p23899052	0.975206612	0.966942149	Bn-N2-p11026377	Bn-chrA05-p22823205
Bn-A05-p23899654	0.975206612	0.966942149	Bn-N2-p11026979	Bn-chrA05-p22822603
Bn-A05-p23906880	0.996677741	0.996677741	Bn-Scaffold15975-p3	Bn-chrA05-p22815907
Bn-A05-p23909209	0.743801653	0.743801653	Bn-N2-p11036782	Bn-chrA05-p22815106
Bn-A05-p23928643	0.991735537	0.983471074	Bn-N2-p11049107	Bn-chrA05-p22798621
Bn-A06-p15048	0.993355482	0.993355482	Bn-N5-p25726266	Bn-chrA06-p16087
Bn-A06-p18783	0.970099668	0.990033223	Bn-N5-p25729964	Bn-chrA06-p19809
Bn-A06-p49287	0.993355482	0.996677741	Bn-N5-p25750407	Bn-chrA06-p42600
Bn-A06-p49438	1	1	Bn-N5-p25750558	Bn-chrA06-p42751
Bn-A06-p50386	1	0.996677741	Bn-N5-p25751506	Bn-chrA06-p43699
Bn-A06-p53517	0.990033223	0.986710963	Bn-N5-p25754637	Bn-chrA06-p46830
Bn-A06-p54687	0.986710963	0.990033223	Bn-N5-p25755799	Bn-chrA06-p47992
Bn-A06-p55225	1	1	Bn-N5-p25756337	Bn-chrA06-p48530
Bn-A06-p69515	1	1	Bn-N5-p25763328	Bn-chrA06-p55519

Bn-A06-p72383	0.990049751	0.990049751	Bn-N5-p25766270	Bn-chrA06-p58478
Bn-A06-p72878	0.995024876	0.995024876	Bn-N5-p25766765	Bn-chrA06-p58973
Bn-A06-p73924	0.996677741	0.996677741	Bn-N5-p25767811	Bn-chrA06-p60019
Bn-A06-p87536	0.970149254	0.975124378	Bn-N5-p25781557	Bn-chrA06-p73619
Bn-A06-p89131	0.990033223	0.993355482	Bn-Scaffold10358-p2	Bn-chrA06-p75214
Bn-A06-p112339	0.991735537	0.983471074	Bn-N6-p19617	Bn-chrCnn_random-p239921
Bn-A06-p113900	1	0.96875	Bn-N6-p21178	Bn-chrCnn_random-p239936
Bn-A06-p114634	0.743801653	0.743801653	Bn-N6-p21912	Bn-chrAnn_random-p380017
Bn-A06-p115737	0.991735537	0.785123967	Bn-N6-p22970	Bn-chrAnn_random-p380006
Bn-A06-p115948	0.958677686	0.958677686	Bn-N6-p26608	Bn-chrA06-p79969
Bn-A06-p121547	0.975124378	0.975124378	Bn-N6-p32216	Bn-chrA06-p85616
Bn-A06-p137614	0.917355372	0.917355372	Bn-N6-p47470	Bn-chrA06-p100670
Bn-A06-p139033	0.993355482	0.993355482	Bn-N6-p48890	Bn-chrA06-p102090
Bn-A06-p149145	0.983471074	0.983471074	Bn-N6-p63276	Bn-chrA06-p116247
Bn-A06-p163388	0.958677686	0.958677686	Bn-N6-p78822	Bn-chrA06-p133033
Bn-A06-p167382	0.991735537	0.991735537	Bn-N6-p85190	Bn-chrA06-p140837
Bn-A06-p172171	0.983471074	0.983471074	Bn-N6-p88511	Bn-chrA06-p144158
Bn-A06-p172731	0.950413223	0.950413223	Bn-N6-p89072	Bn-chrA06-p144719
Bn-A06-p183811	0.958677686	0.958677686	Bn-N6-p95728	Bn-chrA06-p152706
Bn-A06-p195449	0.958677686	0.958677686	Bn-N6-p108297	Bn-chrA06-p166839
Bn-A06-p197604	1	1	Bn-N6-p110450	Bn-chrA06-p168991
Bn-A06-p205050	0.993355482	0.993355482	Bn-N6-p117217	Bn-chrA06-p175758
Bn-A06-p267765	0.966777409	0.996677741	Bn-N6-p171195	Bn-chrA06-p222377
Bn-A06-p275949	0.983471074	0.975206612	Bn-N6-p179121	Bn-chrA06-p231711
Bn-A06-p279045	0.991735537	0.983471074	Bn-N6-p184034	Bn-chrA06-p232855
Bn-A06-p280351	0.983471074	0.991735537	Bn-N6-p190792	Bn-chrA06-p234221
Bn-A06-p281559	0.983471074	0.933884298	Bn-N6-p192087	Bn-chrA06-p235513
Bn-A06-p281874	0.975206612	0.20661157	Bn-N6-p192402	Unknown
Bn-A06-p282853	1	0.578512397	Bn-N6-p193379	Bn-chrA06-p249660
Bn-A06-p283425	0.975206612	0.966942149	Bn-N6-p193951	Bn-chrA06-p242627
Bn-A06-p283640	0.975206612	0.983471074	Bn-N6-p198857	Bn-chrA06-p242842
Bn-A06-p284408	0.983471074	0.975206612	Bn-N6-p199623	Bn-chrA06-p243608
Bn-A06-p285515	0.991735537	1	Bn-N6-p200730	Bn-chrA06-p244715
Bn-A06-p287053	1	1	Bn-N6-p202154	Bn-chrA06-p246263
Bn-A06-p288377	0.966942149	0.966942149	Bn-N6-p204046	Bn-chrA06-p248123
Bn-A06-p289876	0.983471074	0.983471074	Bn-N6-p205583	Bn-chrA06-p249660
Bn-A06-p290355	0.996677741	1	Bn-N6-p206062	Bn-chrA06-p250139
Bn-A06-p291943	0.933884298	0.94214876	Bn-N6-p207950	Bn-chrA06-p252035
Bn-A06-p301366	0.966942149	0.826446281	Bn-N6-p215298	Bn-chrC06-p7678827
Bn-A06-p307436	0.975206612	0.966942149	Bn-N6-p221356	Bn-chrA06-p266415
Bn-A06-p313319	0.991735537	1	Bn-N6-p227239	Bn-chrA06-p272291
Bn-A06-p313835	0.991735537	0.983471074	Bn-N6-p227755	Bn-chrA06-p272807
Bn-A06-p318331	0.94214876	0.94214876	Bn-N6-p237673	Bn-chrA06-p277014
Bn-A06-p329217	0.685950413	0.966942149	Bn-N6-p249355	Bn-chrA06-p291654
Bn-A06-p330826	0.956810631	0.990033223	Bn-N6-p251006	Bn-chrA06-p293264
Bn-A06-p337583	0.933884298	0.94214876	Bn-N6-p257924	Bn-chrA06-p299826

Bn-A06-p362402	0.958677686	0.966942149	Bn-N6-p294802	Bn-chrA06-p808319
Bn-A06-p364061	0.933884298	0.933884298	Bn-N6-p296469	Bn-chrA06-p809994
Bn-A06-p369746	1	0.991735537	Bn-N6-p303470	Bn-chrA06-p815916
Bn-A06-p370376	0.950413223	0.991735537	Bn-N6-p304106	Bn-chrA06-p816547
Bn-A06-p393328	0.916943522	0.990033223	Bn-N6-p327818	Bn-chrA06-p834620
Bn-A06-p410058	0.996677741	1	Bn-N6-p345216	Bn-chrA06-p847339
Bn-A06-p428400	1	1	Bn-N6-p362804	Bn-chrA06-p860355
Bn-A06-p506366	0.958677686	0.851239669	Bn-N6-p444427	Bn-chrA06-p456329
Bn-A06-p543411	0.818181818	0.900826446	Bn-N6-p479364	Bn-chrA06-p499177
Bn-A06-p556859	0.214876033	0.966942149	Bn-N8-p10329715	Bn-chrA06-p513814
Bn-A06-p557358	0.975206612	0.975206612	Bn-N6-p511158	Bn-chrA06-p589614
Bn-A06-p557754	0.983471074	0.983471074	Bn-N6-p511555	Bn-chrA06-p590011
Bn-A06-p558792	0.983471074	0.983471074	Bn-N6-p512034	Bn-chrA06-p590490
Bn-A06-p558853	0.975206612	0.975206612	Bn-N6-p512095	Bn-chrA06-p590551
Bn-A06-p559060	0.966942149	0.966942149	Bn-N6-p512493	Bn-chrA06-p590949
Bn-A06-p559417	0.983471074	0.983471074	Bn-N6-p512850	Bn-chrA06-p591306
Bn-A06-p568101	0.958677686	0.958677686	Bn-N6-p529120	Bn-chrA06-p605025
Bn-A06-p569582	0.983122363	0.983122363	Bn-N6-p530605	Bn-chrA06-p606510
Bn-A06-p576740	0.975206612	0.975206612	Bn-N6-p537835	Bn-chrA06-p614073
Bn-A06-p590960	0.94214876	0.991735537	Bn-N6-p543157	Bn-chrA06-p618910
Bn-A06-p600029	0.990033223	0.986710963	Bn-N6-p552786	Bn-chrA06-p627923
Bn-A06-p608554	0.991735537	0.983471074	Bn-N6-p561236	Bn-chrA06-p636479
Bn-A06-p608570	0.993355482	0.986710963	Bn-N6-p561252	Bn-chrA06-p636495
Bn-A06-p611589	0.983471074	0.991735537	Bn-N6-p564271	Bn-chrA06-p639455
Bn-A06-p613789	0.983471074	0.991735537	Bn-N6-p566471	Bn-chrA06-p641643
Bn-A06-p621506	0.94214876	0.991735537	Bn-N6-p578530	Bn-chrA06-p648969
Bn-A06-p621695	0.958677686	0.983471074	Bn-N6-p566321	Bn-chrA06-p649158
Bn-A06-p649348	0.960199005	0.965174129	Bn-N6-p608607	Bn-chrA06-p677160
Bn-A06-p650072	0.903654485	0.996677741	Bn-N16-p7142200	Bn-chrA06-p677874
Bn-A06-p650790	0.581395349	0.990033223	Bn-N16-p7141516	Bn-chrA06-p678594
Bn-A06-p659391	0.570247934	0.561983471	Bn-N6-p614057	Bn-chrA06-p682550
Bn-A06-p690460	0.975206612	0.975206612	Bn-N6-p653650	Bn-chrA06-p713553
Bn-A06-p709547	0.975206612	0.975206612	Bn-N6-p668466	Bn-chrA06-p728368
Bn-A06-p781674	0.983471074	0.991735537	Bn-N6-p727954	Bn-chrA06-p787710
Bn-A06-p798821	0.975206612	0.983471074	Bn-N6-p757450	Bn-chrA06-p1018139
Bn-A06-p825900	0.983471074	0.991735537	Bn-N6-p784591	Bn-chrA06-p1045812
Bn-A06-p836912	0.910447761	0.890547264	Bn-N6-p795572	Bn-chrA06-p1060126
Bn-A06-p846095	0.980099502	0.975124378	Bn-N6-p804673	Bn-chrA06_random-p2360
Bn-A06-p853722	0.950413223	0.950413223	Bn-N6-p812208	Bn-chrA06-p1082923
Bn-A06-p853974	0.995024876	0.995024876	Bn-N6-p812460	Bn-chrA06-p1083175
Bn-A06-p860631	0.993355482	0.980066445	Bn-N6-p821079	Bn-chrA06_random-p13027
Bn-A06-p899811	0.916943522	0.916943522	Bn-N6-p863299	Bn-chrA06_random-p6758
Bn-A06-p908271	0.966942149	0.966942149	Bn-N16-p6542105	Bn-chrC06-p6193807
Bn-A06-p918871	0.94214876	0.94214876	Bn-N6-p884932	Bn-chrA06-p1095736
Bn-A06-p938677	0.993355482	0.993355482	Bn-N6-p895249	Bn-chrA06-p1106052
Bn-A06-p945992	0.983471074	0.983471074	Bn-N6-p902740	Bn-chrA06_random-p19642

Bn-A06-p947740	0.975206612	0.975206612	Bn-N6-p904664	Bn-chrA06_random-p24500
Bn-A06-p999044	0.958677686	0.950413223	Bn-N6-p961197	Bn-chrA06-p991164
Bn-A06-p1015217	0.991735537	0.983471074	Bn-N6-p975443	Bn-chrA06-p1004365
Bn-A06-p1016417	0.975206612	0.966942149	Bn-N6-p976643	Bn-chrA06-p1005564
Bn-A06-p1054104	0.983471074	0.991735537	Bn-N6-p1011787	Bn-chrA06-p1129709
Bn-A06-p1071392	0.975206612	1	Bn-N6-p1025434	Bn-chrAnn_random-p335605
Bn-A06-p1082496	1	0.392026578	Bn-N6-p1034756	Bn-chrC02-p4053580
Bn-A06-p1082655	0.983471074	0.900826446	Bn-N6-p1034915	Bn-chrC05-p40099069
Bn-A06-p1083892	0.983471074	0.983471074	Bn-N6-p1036160	Bn-chrA06_random-p31238
Bn-A06-p1094608	1	1	Bn-N9-p36212643	Bn-chrAnn_random-p200655
Bn-A06-p1099469	0.991735537	0.991735537	Bn-N6-p1048700	Bn-chrA06_random-p48926
Bn-A06-p1099939	0.966942149	0.966942149	Bn-N6-p1049170	Bn-chrA06_random-p49396
Bn-A06-p1106605	0.996677741	0.996677741	Bn-N6-p1051110	Bn-chrA06_random-p51337
Bn-A06-p1115978	0.983388704	0.990033223	Bn-N6-p1062441	Bn-chrA06-p1155876
Bn-A06-p1116784	0.973421927	0.980066445	Bn-N6-p1064271	Bn-chrA06-p1156682
Bn-A06-p1116867	0.970099668	0.976744186	Bn-N6-p1064354	Bn-chrA06-p1156765
Bn-A06-p1116930	0.950413223	0.94214876	Bn-N6-p1064417	Bn-chrA06-p1156828
Bn-A06-p1119681	0.958677686	0.958677686	Bn-N6-p1065358	Bn-chrA06-p1159806
Bn-A06-p1119751	0.991735537	0.983471074	Bn-N6-p1065429	Bn-chrA06-p1159876
Bn-A06-p1120102	0.983471074	0.983471074	Bn-N6-p1065780	Bn-chrA06-p1160161
Bn-A06-p1120107	0.983471074	0.991735537	Bn-N6-p1065785	Bn-chrA06-p1160166
Bn-A06-p1135789	0.734219269	0.996677741	Bn-N16-p5825826	Bn-chrA06-p1177346
Bn-A06-p1142557	0.355371901	0.925619835	Bn-N8-p1704686	Bn-chrA06-p1183972
Bn-A06-p1149034	0.975206612	0.966942149	Bn-N6-p1098793	Bn-chrA06-p1190419
Bn-A06-p1154840	0.958677686	0.950413223	Bn-N6-p1106326	Bn-chrA06-p1196233
Bn-A06-p1160984	0.94214876	0.950413223	Bn-N6-p1114975	Bn-chrA06-p1202060
Bn-A06-p1168447	0.996677741	0.996677741	Bn-N6-p1123632	Bn-chrA06-p1208817
Bn-A06-p1173126	0.975206612	0.975206612	Bn-N6-p1124569	Bn-chrA06-p1209754
Bn-A06-p1176062	0.990033223	0.990033223	Bn-N6-p1128866	Bn-chrA06-p1214684
Bn-A06-p1184282	1	1	Bn-N6-p1136666	Bn-chrA06-p1222515
Bn-A06-p1185371	0.986710963	0.990033223	Bn-N6-p1137755	Bn-chrA06-p1223604
Bn-A06-p1187915	0.970149254	0.950248756	Bn-N6-p1140296	Bn-chrC06-p5602914
Bn-A06-p1217543	0.983471074	0.991735537	Bn-N6-p1158451	Bn-chrA06-p1256797
Bn-A06-p1225010	0.995024876	0.995024876	Bn-N16-p5520725	Bn-chrC06-p5394098
Bn-A06-p1232267	0.958677686	0.958677686	Bn-N6-p1172718	Bn-chrA06-p1273936
Bn-A06-p1278419	0.925619835	0.917355372	Bn-N6-p1209909	Bn-chrA06-p1309007
Bn-A06-p1291667	0.975206612	0.966942149	Bn-N6-p1222954	Bn-chrA06-p1322077
Bn-A06-p1292241	0.94214876	0.950413223	Bn-N6-p1223528	Bn-chrA06-p1322652
Bn-A06-p1312619	0.933884298	0.933884298	Bn-N6-p1246912	Bn-chrA06-p1350492
Bn-A06-p1322724	0.991735537	0.991735537	Bn-N6-p1256849	Bn-chrA06-p1360429
Bn-A06-p1330567	0.990033223	0.990033223	Bn-N6-p1264649	Bn-chrA06-p1368819
Bn-A06-p1345642	0.966942149	0.173553719	Bn-N6-p1279393	Bn-chrC03-p13718291
Bn-A06-p1348404	0.975206612	0.900826446	Bn-N6-p1282105	Bn-chrC06_random-p398476
Bn-A06-p1350786	0.983471074	0.975206612	Bn-N6-p1284288	Bn-chrA06-p1385825
Bn-A06-p1358955	0.958677686	0.966942149	Bn-N6-p1292497	Bn-chrA06-p1398385
Bn-A06-p1366713	0.983471074	0.991735537	Bn-N6-p1299195	Bn-chrAnn_random-p45778[illegible]

Bn-A06-p1373016	0.958677686	0.966942149	Bn-N6-p1306727	Bn-chrA06-p1410066
Bn-A06-p1378913	0.990049751	0.995024876	Bn-N6-p1311866	Bn-chrA06-p1416722
Bn-A06-p1383275	0.991735537	0.983471074	Bn-N6-p1316228	Bn-chrA06-p1421084
Bn-A06-p1386474	0.545454545	0.545454545	Bn-N6-p1319359	Bn-chrA06-p1424194
Bn-A06-p1403969	0.933884298	0.933884298	Bn-N6-p1336830	Bn-chrA06-p1441858
Bn-A06-p1404859	0.980099502	0.980099502	Bn-N6-p1337715	Bn-chrA06-p1442743
Bn-A06-p1408273	0.966942149	0.958677686	Bn-N6-p1343345	Bn-chrA06-p1447161
Bn-A06-p1427206	0.991735537	0.991735537	Bn-N6-p1367548	Bn-chrA06-p1463625
Bn-A06-p1432446	0.966942149	0.966942149	Bn-N6-p1372772	Bn-chrA06-p1469265
Bn-A06-p1433021	0.983471074	0.983471074	Bn-N6-p1373346	Bn-chrA06-p1469839
Bn-A06-p1443684	0.966942149	0.966942149	Bn-N6-p1384557	Bn-chrA06-p1480582
Bn-A06-p1450741	0.958677686	0.958677686	Bn-N6-p1400741	Bn-chrC06-p4829956
Bn-A06-p1455751	0.966942149	0.966942149	Bn-N6-p1406092	Bn-chrC06-p4824605
Bn-A06-p1455781	0.975206612	0.975206612	Bn-N6-p1406122	Bn-chrC06-p4824575
Bn-A06-p1456521	0.975206612	0.975206612	Bn-N6-p1406862	Bn-chrC06-p4823835
Bn-A06-p1457087	0.96345515	0.727574751	Bn-N6-p1407441	Bn-chrC06-p4823256
Bn-A06-p1457383	0.996677741	0.996677741	Bn-N6-p1407744	Bn-chrC06-p4822953
Bn-A06-p1460039	1	1	Bn-N6-p1410403	Bn-chrC06-p4820294
Bn-A06-p1481362	0.976744186	0.980066445	Bn-N6-p1422449	Bn-chrC06-p4808251
Bn-A06-p1485525	0.890365449	0.890365449	Bn-N6-p1426600	Bn-chrC06-p4804100
Bn-A06-p1490969	0.990033223	0.990033223	Bn-N6-p1429636	Bn-chrC06-p4801064
Bn-A06-p1507197	0.958677686	0.958677686	Bn-N16-p4752677	Bn-chrC06-p4593682
Bn-A06-p1517263	0.991735537	0.991735537	Bn-N6-p1461594	Bn-chrA06-p1555093
Bn-A06-p1530126	0.980066445	0.980066445	Bn-N6-p1475872	Bn-chrA06-p1568907
Bn-A06-p1612900	0.983471074	0.983471074	Bn-N6-p1568749	Bn-chrA06-p1663684
Bn-A06-p1615726	0.950413223	0.958677686	Bn-N6-p1571469	Bn-chrA06-p1666304
Bn-A06-p1626020	0.993355482	0.993355482	Bn-N6-p1582569	Bn-chrA06-p1675637
Bn-A06-p1636449	0.973421927	0.996677741	Bn-N16-p4442046	Bn-chrA06-p1690982
Bn-A06-p1650453	0.983471074	0.991735537	Bn-N6-p1606387	Bn-chrA06-p1748163
Bn-A06-p1650457	0.991735537	0.983471074	Bn-N6-p1606391	Bn-chrA06-p1748167
Bn-A06-p1650529	0.950413223	0.867768595	Bn-N6-p1606463	Bn-chrA06-p1748240
Bn-A06-p1650786	0.975206612	0.94214876	Bn-N6-p1606720	Bn-chrA06-p1748501
Bn-A06-p1652088	0.986710963	0.933554817	Bn-N6-p1608021	Bn-chrA06-p1749728
Bn-A06-p1658095	0.966942149	0.966942149	Bn-N6-p1614708	Bn-chrA06-p1756502
Bn-A06-p1661477	0.966942149	0.975206612	Bn-N6-p1621360	Bn-chrA06-p1759824
Bn-A06-p1685018	0.991735537	0.79338843	Bn-N6-p1639147	Bn-chrC06-p4062352
Bn-A06-p1685192	0.991735537	0.917355372	Bn-N6-p1639321	Bn-chrA06-p1780626
Bn-A06-p1722627	0.991735537	1	Bn-N6-p1678365	Bn-chrA06_random-p153153
Bn-A06-p1743446	0.983471074	0.983471074	Bn-N6-p1700511	Bn-chrA06_random-p122186
Bn-A06-p1768254	0.975206612	0.975206612	Bn-N6-p1726972	Bn-chrA06_random-p99792
Bn-A06-p1768918	0.991735537	0.991735537	Bn-N6-p1727635	Bn-chrA06_random-p99129
Bn-A06-p1776668	0.983471074	0.991735537	Bn-N6-p1734965	Bn-chrA06_random-p91805
Bn-A06-p1777127	0.950413223	0.958677686	Bn-N6-p1735427	Bn-chrA06_random-p91349
Bn-A06-p1793586	0.993355482	0.996677741	Bn-N6-p1750370	Bn-chrA06_random-p75970
Bn-A06-p1800467	0.355371901	0.355371901	Bn-N6-p1756825	Unknown
Bn-A06-p1802123	0.958677686	0.966942149	Bn-N6-p1758485	Bn-chrA06_random-p67175

Bn-A06-p1834863	0.983388704	0.983388704	Bn-N6-p1814843	Bn-chrA06-p1815064
Bn-A06-p1872484	0.940298507	0.965174129	Bn-N6-p1837548	Bn-chrA06-p1837781
Bn-A06-p1872597	0.958677686	0.950413223	Bn-N6-p1837661	Bn-chrA06-p1837894
Bn-A06-p1874196	0.996677741	0.996677741	Bn-N6-p1839348	Bn-chrA06-p1839761
Bn-A06-p1875896	0.996677741	1	Bn-N6-p1841048	Bn-chrA06-p1841462
Bn-A06-p1892397	0.933554817	0.933554817	Bn-N6-p1847127	Bn-chrA06-p1846400
Bn-A06-p1892464	0.990033223	0.990033223	Bn-N6-p1847194	Bn-chrA06-p1846467
Bn-A06-p1903170	0.980099502	0.980099502	Bn-N6-p1859792	Bn-chrA06-p1860367
Bn-A06-p1903532	0.990049751	0.990049751	Bn-N6-p1860154	Bn-chrA06-p1860729
Bn-A06-p1908583	0.966942149	0.966942149	Bn-N6-p1865204	Bn-chrA06-p1865908
Bn-A06-p1908707	0.958677686	0.958677686	Bn-N6-p1865328	Bn-chrA06-p1866032
Bn-A06-p1909050	0.983471074	0.983471074	Bn-N6-p1865670	Bn-chrA06-p1866374
Bn-A06-p1925858	0.975206612	0.975206612	Bn-N6-p1891554	Bn-chrAnn_random-p38676C
Bn-A06-p1947247	0.975206612	0.975206612	Bn-N6-p1911301	Bn-chrA06-p1904642
Bn-A06-p1961013	0.983471074	0.983471074	Bn-N6-p1926308	Bn-chrA06-p1919748
Bn-A06-p1973105	0.966942149	0.966942149	Bn-N6-p1942080	Bn-chrA06-p1936728
Bn-A06-p2060583	0.996677741	0.996677741	Bn-N6-p2036044	Bn-chrA06-p2028043
Bn-A06-p2065936	0.958677686	0.958677686	Bn-N6-p2052031	Bn-chrA06-p2046303
Bn-A06-p2077171	0.950413223	0.950413223	Bn-N6-p2062066	Bn-chrA06-p2056288
Bn-A06-p2107176	0.975124378	0.975124378	Bn-N6-p2090738	Bn-chrA06-p2109807
Bn-A06-p2107610	0.935323383	0.940298507	Bn-N6-p2091184	Bn-chrA06-p2084581
Bn-A06-p2165351	0.990033223	0.990033223	Bn-N6-p2144614	Bn-chrA06-p2149249
Bn-A06-p2168634	0.983471074	0.983471074	Bn-N6-p2147887	Bn-chrA06-p2152522
Bn-A06-p2185099	0.975206612	0.975206612	Bn-N6-p2166386	Bn-chrA06-p2173842
Bn-A06-p2217578	0.965174129	0.965174129	Bn-N6-p2203877	Bn-chrA06-p2212250
Bn-A06-p2218956	0.996677741	0.980066445	Bn-N6-p2205250	Bn-chrA06-p2213629
Bn-A06-p2222872	0.553719008	0.553719008	Bn-N6-p2209177	Bn-chrA06-p2217550
Bn-A06-p2234696	0.983471074	0.842975207	Bn-N6-p2229916	Bn-chrA06-p2242915
Bn-A06-p2234718	0.983471074	0.925619835	Bn-N6-p2229938	Bn-chrA06-p2242978
Bn-A06-p2251910	0.993355482	0.993355482	Bn-N6-p2243184	Bn-chrA06-p2256316
Bn-A06-p2252815	0.993355482	0.990033223	Bn-N6-p2244072	Bn-chrA06-p2257204
Bn-A06-p2268393	0.910299003	0.910299003	Bn-N6-p2261524	Bn-chrA06-p2274725
Bn-A06-p2274318	0.945273632	0.945273632	Bn-N6-p2267445	Bn-chrA06-p2280645
Bn-A06-p2275754	0.94214876	0.94214876	Bn-N16-p3090573	Bn-chrC01_random-p402424
Bn-A06-p2292578	0.991735537	0.991735537	Bn-N6-p2286327	Bn-chrA06-p2299525
Bn-A06-p2293069	0.950413223	0.950413223	Bn-N6-p2286818	Bn-chrA06-p2300016
Bn-A06-p2300342	0.958677686	0.958677686	Bn-N6-p2299408	Bn-chrA06-p2313780
Bn-A06-p2303914	0.990033223	0.986710963	Bn-N6-p2303718	Bn-chrA06-p2318088
Bn-A06-p2304435	1	1	Bn-N6-p2304239	Bn-chrA06-p2318609
Bn-A06-p2305844	0.842975207	0.900826446	Bn-N6-p2305653	Bn-chrC06-p2728870
Bn-A06-p2306499	0.983388704	0.983388704	Bn-N6-p2306276	Bn-chrA06-p2320646
Bn-A06-p2307291	0.933884298	0.933884298	Bn-N6-p2307066	Bn-chrA06-p2321436
Bn-A06-p2316252	0.990049751	0.990049751	Bn-N6-p2317512	Bn-chrA06_random-p163398
Bn-A06-p2341154	0.983388704	0.983388704	Bn-N6-p2342384	Bn-chrA06-p2356360
Bn-A06-p2345966	1	1	Bn-N6-p2346938	Bn-chrA06-p2360914
Bn-A06-p2347754	0.991735537	0.991735537	Bn-N6-p2348726	Bn-chrA06-p2362702

Bn-A06-p2348428	0.893687708	0.893687708	Bn-N6-p2349397	Bn-chrA06-p2363373
Bn-A06-p2357647	0.950413223	0.950413223	Bn-N6-p2358735	Bn-chrA06-p2372710
Bn-A06-p2365869	0.996677741	0.996677741	Bn-N6-p2367169	Bn-chrA06-p2381259
Bn-A06-p2390609	0.840531561	0.990033223	Bn-N6-p11658526	Bn-chrA06-p2400369
Bn-A06-p2405022	0.990049751	0.990049751	Bn-N16-p2735237	Bn-chrC06-p2493053
Bn-A06-p2407080	0.991735537	0.991735537	Bn-N6-p2407250	Bn-chrA06-p2420107
Bn-A06-p2412998	0.933554817	0.933554817	Bn-N6-p2413163	Bn-chrA06-p2426020
Bn-A06-p2413971	0.933884298	0.933884298	Bn-N6-p2414152	Bn-chrA06-p2427009
Bn-A06-p2434155	0.986710963	0.986710963	Bn-N16-p2680601	Bn-chrC06-p2444177
Bn-A06-p2437284	1	1	Bn-N6-p2439044	Bn-chrA06-p2448813
Bn-A06-p2514723	0.976744186	0.976744186	Bn-N6-p2468768	Bn-chrA06-p2477670
Bn-A06-p2521454	0.987878788	0.987878788	Bn-N6-p2475477	Bn-chrA06-p2484378
Bn-A06-p2521668	0.975124378	0.975124378	Bn-N6-p2475691	Bn-chrA06-p2484592
Bn-A06-p2525037	0.976047904	0.97005988	Bn-N6-p2479080	Bn-chrA06-p2487987
Bn-A06-p2526552	0.990033223	0.986710963	Bn-N6-p2480572	Bn-chrA06-p2489481
Bn-A06-p2542722	0.996677741	0.976744186	Bn-N6-p2492906	Bn-chrA06-p2501015
Bn-A06-p2563025	0.980066445	0.980066445	Bn-N6-p2513183	Bn-chrA06-p2519369
Bn-A06-p2570378	0.809917355	0.809917355	Bn-N6-p2521316	Bn-chrA06-p2527958
Bn-A06-p2582613	0.983471074	0.991735537	Bn-N6-p2539378	Bn-chrA06-p2543044
Bn-A06-p2586825	0.983471074	0.983471074	Bn-N6-p2544103	Bn-chrA06-p2547347
Bn-A06-p2599060	0.900826446	0.900826446	Bn-N6-p2551904	Bn-chrA06-p2555145
Bn-A06-p2599150	0.884297521	0.884297521	Bn-N6-p2551988	Bn-chrA06-p2555229
Bn-A06-p2617079	0.991735537	0.991735537	Bn-N6-p2568453	Bn-chrA06-p2574150
Bn-A06-p2619089	0.966942149	0.966942149	Bn-N6-p2570466	Bn-chrA06-p2576163
Bn-A06-p2630468	0.991735537	0.991735537	Bn-N6-p2587619	Bn-chrA06-p2591877
Bn-A06-p2633224	0.979166667	0.979166667	Bn-N6-p2590382	Bn-chrA06-p2594640
Bn-A06-p2633764	0.983388704	0.983388704	Bn-N6-p2590921	Bn-chrA06-p2595179
Bn-A06-p2643305	0.950248756	0.950248756	Bn-N6-p2598687	Bn-chrA06-p2602949
Bn-A06-p2643564	0.940298507	0.940298507	Bn-N6-p2598946	Bn-chrA06-p2603208
Bn-A06-p2646572	0.991735537	0.991735537	Bn-N6-p2601945	Bn-chrA06-p2606207
Bn-A06-p2650442	0.922222222	0.988888889	Bn-N6-p2605783	Bn-chrA06-p2610035
Bn-A06-p2651708	0.986710963	0.986710963	Bn-N6-p2607079	Bn-chrA06-p2611330
Bn-A06-p2676314	0.96875	1	Bn-N6-p2616610	Bn-chrA06-p2618933
Bn-A06-p2676798	0.966942149	0.975206612	Bn-N6-p2617104	Bn-chrA06-p2619418
Bn-A06-p2682929	0.930232558	0.993355482	Bn-N15-p2990291	Bn-chrA06-p2625420
Bn-A06-p2698067	0.983388704	0.983388704	Bn-N6-p2647009	Bn-chrA06-p2640016
Bn-A06-p2709177	0.983471074	0.983471074	Bn-N6-p2651627	Bn-chrA06-p2644634
Bn-A06-p2714610	0.980066445	0.980066445	Bn-N6-p2657076	Bn-chrA06-p2650083
Bn-A06-p2717967	0.985074627	0.985074627	Bn-N6-p2660431	Bn-chrA06-p2653438
Bn-A06-p2718852	0.985074627	0.985074627	Bn-N6-p2661302	Bn-chrA06-p2654309
Bn-A06-p2729577	0.933884298	0.933884298	Bn-N6-p2671625	Bn-chrA06-p2664631
Bn-A06-p2732731	0.21875	0.989583333	Bn-N6-p15587040	Bn-chrAnn_random-p25497C
Bn-A06-p2743076	1	1	Bn-N6-p2677066	Bn-chrA06-p2670064
Bn-A06-p2748311	1	0.991735537	Bn-Scaffold14409-p6	Bn-chrA06-p2675302
Bn-A06-p2756247	0.991735537	0.983471074	Bn-N6-p2687616	Bn-chrA06-p2678998
Bn-A06-p2756792	0.983388704	0.993355482	Bn-N6-p2688161	Bn-chrA06-p2679549

Bn-A06-p2758495	1	0.966942149	Bn-N6-p2689864	Bn-chrA06-p2681244
Bn-A06-p2778072	0.976744186	0.976744186	Bn-N6-p2705931	Bn-chrA06-p2690612
Bn-A06-p2778829	0.986710963	0.986710963	Bn-N6-p2706688	Bn-chrA06-p2691369
Bn-A06-p2778837	0.986710963	0.986710963	Bn-N6-p2706696	Bn-chrA06-p2691377
Bn-A06-p2779243	0.980066445	0.980066445	Bn-N6-p2707101	Bn-chrA06-p2691782
Bn-A06-p2788585	0.993355482	0.993355482	Bn-N6-p2716511	Bn-chrA06-p2701245
Bn-A06-p2793899	0.990033223	0.990033223	Bn-N6-p2722033	Bn-chrA06-p2706767
Bn-A06-p2801792	0.986710963	0.986710963	Bn-N6-p2729429	Bn-chrA06-p2714171
Bn-A06-p2807759	0.990033223	0.990033223	Bn-N6-p2735396	Bn-chrA06-p2720135
Bn-A06-p2823066	0.975206612	0.966942149	Bn-N6-p2745522	Bn-chrA06-p2730311
Bn-A06-p2824500	0.976744186	0.976744186	Bn-N6-p2746956	Bn-chrA06-p2731745
Bn-A06-p2826315	0.983388704	0.973421927	Bn-N6-p2748786	Bn-chrA06-p2733560
Bn-A06-p2826630	0.983471074	0.975206612	Bn-N6-p2749101	Bn-chrA06-p2733875
Bn-A06-p2842757	0.993355482	0.993355482	Bn-N6-p2761621	Bn-chrA06-p2744207
Bn-A06-p2851834	0.990033223	0.990033223	Bn-N6-p2770699	Bn-chrA06-p2753285
Bn-A06-p2870413	0.983471074	0.983471074	Bn-N6-p2785638	Bn-chrA06-p2768312
Bn-A06-p2874216	0.983471074	0.983471074	Bn-N6-p2789474	Bn-chrC05-p2959592
Bn-A06-p2883231	0.975206612	0.975206612	Bn-N6-p2798444	Bn-chrA06-p2781154
Bn-A06-p2883297	0.983471074	0.983471074	Bn-N6-p2798510	Bn-chrA06-p2781220
Bn-A06-p2890703	0.990033223	0.973421927	Bn-N6-p2815816	Bn-chrA06-p2788973
Bn-A06-p2891175	1	1	Bn-N6-p2816336	Bn-chrA06-p2789459
Bn-A06-p2891290	0.980066445	0.980066445	Bn-N6-p2816451	Bn-chrA06-p2789574
Bn-A06-p2901305	0.917355372	0.818181818	Bn-N6-p2821418	Bn-chrA06-p2794528
Bn-A06-p2903017	0.986710963	0.990033223	Bn-N6-p2823115	Bn-chrA06-p2796354
Bn-A06-p2917807	0.975206612	0.975206612	Bn-N6-p2837478	Bn-chrA06-p2810751
Bn-A06-p2929852	0.983471074	0.983471074	Bn-N6-p2854863	Bn-chrA06-p2832101
Bn-A06-p2930156	0.991735537	0.991735537	Bn-N6-p2855167	Bn-chrA06-p2832405
Bn-A06-p2936438	0.986710963	0.986710963	Bn-N6-p2861174	Bn-chrA06-p2836101
Bn-A06-p2938450	0.993355482	1	Bn-N6-p2863181	Bn-chrA06-p2838106
Bn-A06-p2938890	0.983471074	0.983471074	Bn-N6-p2863623	Bn-chrA06-p2838548
Bn-A06-p2940743	0.966942149	0.94214876	Bn-N6-p2865465	Bn-chrA06-p2840416
Bn-A06-p2949562	0.991735537	0.991735537	Bn-N6-p2872323	Bn-chrA06-p2848002
Bn-A06-p2949781	0.950413223	0.966942149	Bn-N6-p2872542	Bn-chrA06-p2848221
Bn-A06-p2949858	0.991735537	0.983471074	Bn-N6-p2872619	Bn-chrA06-p2848298
Bn-A06-p2954752	0.983471074	0.983471074	Bn-N6-p2876401	Bn-chrA06-p2852078
Bn-A06-p2956891	0.985074627	0.995024876	Bn-N6-p2878542	Bn-chrA06-p2854201
Bn-A06-p2958095	0.980099502	0.980099502	Bn-N6-p2879696	Bn-chrA06-p2855599
Bn-A06-p2966556	0.925619835	0.925619835	Bn-N6-p2885692	Bn-chrA06-p2861595
Bn-A06-p2973074	0.966942149	0.966942149	Bn-N6-p2892469	Bn-chrA06-p2867926
Bn-A06-p2981395	0.986710963	0.990033223	Bn-N6-p2900961	Bn-chrA06-p2876219
Bn-A06-p2984184	0.966942149	0.966942149	Bn-N15-p3364850	Bn-chrC05-p3079312
Bn-A06-p2987202	0.950413223	0.950413223	Bn-N6-p2908047	Bn-chrA06-p2881591
Bn-A06-p2993800	0.983388704	0.990033223	Bn-N6-p2915124	Bn-chrA06-p2887494
Bn-A06-p2995114	0.996677741	0.950166113	Bn-N6-p2917914	Bn-chrA06-p2888813
Bn-A06-p3005047	0.991735537	0.983471074	Bn-N6-p2921751	Bn-chrA06-p2892692
Bn-A06-p3005981	0.966942149	0.958677686	Bn-N6-p2922682	Bn-chrA06-p2893591

Bn-A06-p3009854	0.991735537	0.983471074	Bn-N6-p2925912	Bn-chrA06-p2896587
Bn-A06-p3010221	0.986710963	0.983388704	Bn-N6-p2926280	Bn-chrA06-p2896955
Bn-A06-p3012876	0.966942149	0.966942149	Bn-N6-p2930003	Bn-chrA06-p2900889
Bn-A06-p3013547	0.975206612	0.983471074	Bn-N6-p2930693	Bn-chrA06-p2901629
Bn-A06-p3016186	0.950413223	0.983471074	Bn-N6-p2933241	Bn-chrA06-p2904271
Bn-A06-p3016356	0.809917355	0.975206612	Bn-N6-p2933412	Bn-chrA06-p2904441
Bn-A06-p3031327	0.990033223	0.990033223	Bn-N6-p2949477	Bn-chrA06-p2919668
Bn-A06-p3049884	0.983388704	0.983388704	Bn-N6-p2967649	Bn-chrA06-p2942829
Bn-A06-p3050205	1	0.995024876	Bn-N6-p2967970	Bn-chrA06-p2943150
Bn-A06-p3050747	0.995024876	1	Bn-N6-p2968512	Bn-chrA06-p2943692
Bn-A06-p3051257	0.990033223	0.990033223	Bn-N6-p2969022	Bn-chrA06-p2944202
Bn-A06-p3051799	0.983388704	0.980066445	Bn-N6-p2969566	Bn-chrA06-p2944746
Bn-A06-p3051928	0.991735537	0.991735537	Bn-N6-p2969695	Bn-chrA06-p2944875
Bn-A06-p3065773	0.966942149	0.94214876	Bn-N6-p2984327	Bn-chrA06-p2958563
Bn-A06-p3066038	0.983471074	0.975206612	Bn-N6-p2984592	Bn-chrA06-p2958826
Bn-A06-p3066372	0.993355482	0.993355482	Bn-N6-p2984926	Bn-chrA06-p2959161
Bn-A06-p3072724	0.958677686	0.966942149	Bn-N6-p2991285	Bn-chrA06-p2964436
Bn-A06-p3088460	0.983471074	0.975206612	Bn-N6-p3007343	Bn-chrA06-p2978866
Bn-A06-p3090078	0.990033223	0.993355482	Bn-N6-p3008961	Bn-chrA06-p2980477
Bn-A06-p3093741	0.818181818	0.991735537	Bn-N6-p3020965	Bn-chrA06-p2984136
Bn-A06-p3096813	0.990049751	0.990049751	Bn-N6-p3024061	Bn-chrA06-p2987221
Bn-A06-p3105589	0.958677686	0.958677686	Bn-N6-p3030913	Bn-chrA06-p2996530
Bn-A06-p3114818	0.996677741	1	Bn-N6-p3040061	Bn-chrA06_random-p165035
Bn-A06-p3115038	0.996677741	0.96013289	Bn-N6-p3040281	Bn-chrA06_random-p164815
Bn-A06-p3118627	0.96345515	0.946843854	Bn-N6-p3048479	Bn-chrA06_random-p169127
Bn-A06-p3125473	0.980066445	0.976744186	Bn-N6-p3060169	Bn-chrA06_random-p183389
Bn-A06-p3132058	0.92039801	0.965174129	Bn-N6-p3067033	Bn-chrA06_random-p176527
Bn-A06-p3132860	0.990033223	0.990033223	Bn-N6-p3067839	Bn-chrA06_random-p175721
Bn-A06-p3146367	0.94214876	0.94214876	Bn-N6-p3081425	Bn-chrA06-p3025789
Bn-A06-p3146720	0.958677686	0.958677686	Bn-N6-p3081783	Bn-chrA06-p3026147
Bn-A06-p3167411	0.993355482	0.993355482	Bn-N6-p3098962	Bn-chrA06-p3043132
Bn-A06-p3177392	0.867768595	0.867768595	Bn-N6-p3108185	Bn-chrA06-p3053058
Bn-A06-p3177472	0.990033223	0.990033223	Bn-N6-p3108265	Bn-chrA06-p3053138
Bn-A06-p3177570	0.966942149	0.966942149	Bn-N6-p3108363	Bn-chrA06-p3053236
Bn-A06-p3184395	0.985074627	0.985074627	Bn-N6-p3115162	Bn-chrA06-p3060035
Bn-A06-p3188819	0.975206612	0.975206612	Bn-N6-p3119656	Bn-chrA06-p3064530
Bn-A06-p3189490	0.950413223	0.950413223	Bn-N6-p3120329	Bn-chrA06-p3065203
Bn-A06-p3202098	0.925619835	0.925619835	Bn-N6-p3133254	Bn-chrA06-p3078155
Bn-A06-p3202615	0.983471074	0.983471074	Bn-N6-p3133744	Bn-chrA06-p3078645
Bn-A06-p3206017	0.983471074	0.983471074	Bn-N6-p3138340	Bn-chrA06_random-p196789
Bn-A06-p3207623	0.975206612	0.975206612	Bn-N6-p3139949	Bn-chrA06_random-p228811
Bn-A06-p3210791	0.94214876	0.94214876	Bn-N6-p3144086	Bn-chrA06_random-p226136
Bn-A06-p3226693	0.980066445	0.980066445	Bn-N6-p3150293	Bn-chrA06_random-p219929
Bn-A06-p3227752	0.989583333	0.989583333	Bn-N6-p3151334	Bn-chrA06_random-p218945
Bn-A06-p3230963	0.94214876	0.94214876	Bn-N6-p3154575	Bn-chrA06_random-p215647
Bn-A06-p3239087	0.958677686	0.958677686	Bn-N6-p3157658	Bn-chrA06_random-p212562

Bn-A06-p3255819	0.933554817	0.933554817	Bn-N6-p3168084	Bn-chrA06_random-p202783
Bn-A06-p3256782	0.983471074	0.983471074	Bn-N6-p3168900	Bn-chrA06_random-p201967
Bn-A06-p3257163	0.94214876	0.94214876	Bn-N6-p3169276	Bn-chrA06_random-p201591
Bn-A06-p3269320	0.975206612	0.975206612	Bn-N6-p3178901	Bn-chrA06-p3090575
Bn-A06-p3278682	0.985074627	0.985074627	Bn-N6-p3189492	Bn-chrA06-p3100338
Bn-A06-p3287018	0.669421488	0.669421488	Bn-N16-p35558039	Bn-chrC06-p33616927
Bn-A06-p3291936	0.975206612	0.975206612	Bn-N6-p3201619	Bn-chrA06-p3112464
Bn-A06-p3295431	0.958677686	0.958677686	Bn-N6-p3205347	Bn-chrA06-p3116192
Bn-A06-p3302657	0.966942149	0.32231405	Bn-N6-p3212001	Bn-chrC01-p34042442
Bn-A06-p3308398	0.955223881	0.930348259	Bn-N6-p3219914	Bn-chrA06-p3124023
Bn-A06-p3316040	1	0.256198347	Bn-N6-p3227453	Unknown
Bn-A06-p3316207	0.983471074	0.26446281	Bn-N6-p3227622	Bn-chrC01-p12836135
Bn-A06-p3318611	0.990033223	0.96345515	Bn-N6-p3230026	Bn-chrC05-p3590059
Bn-A06-p3325502	0.983388704	0.983388704	Bn-N6-p3236858	Bn-chrA06-p3143114
Bn-A06-p3325566	0.940199336	0.940199336	Bn-N6-p3236922	Bn-chrA06-p3143178
Bn-A06-p3329211	0.966777409	0.966777409	Bn-N6-p3241930	Bn-chrA06-p3146818
Bn-A06-p3339435	0.669421488	0.966942149	Bn-N15-p3913044	Bn-chrA06-p3156905
Bn-A06-p3353405	0.993355482	0.993355482	Bn-N6-p3266387	Bn-chrA06-p3171170
Bn-A06-p3354754	0.983388704	0.983388704	Bn-N6-p3268390	Bn-chrA06-p3173173
Bn-A06-p3361377	0.975206612	0.975206612	Bn-N6-p3274422	Bn-chrA06-p3179205
Bn-A06-p3363188	0.991735537	0.991735537	Bn-N6-p3276226	Bn-chrA06-p3181009
Bn-A06-p3370349	0.996677741	0.996677741	Bn-N6-p3283380	Bn-chrA06-p3188163
Bn-A06-p3380495	0.975206612	0.975206612	Bn-N6-p3294131	Bn-chrA06-p3198536
Bn-A06-p3399869	0.993355482	0.993355482	Bn-N6-p3312066	Bn-chrA06-p3216471
Bn-A06-p3399973	0.991735537	0.991735537	Bn-N6-p3312170	Bn-chrA06-p3216575
Bn-A06-p3405779	0.970099668	0.970099668	Bn-N6-p3318240	Bn-chrA06-p3222645
Bn-A06-p3406041	0.966942149	0.966942149	Bn-N6-p3318501	Bn-chrA06-p3222906
Bn-A06-p3414128	0.960199005	0.955223881	Bn-N6-p3326604	Bn-chrA06-p3231009
Bn-A06-p3416803	0.975206612	0.975206612	Bn-N6-p3329194	Bn-chrA06-p3232881
Bn-A06-p3428214	0.966942149	0.966942149	Bn-N6-p3343630	Bn-chrA06-p3247125
Bn-A06-p3434498	0.94214876	0.950413223	Bn-N15-p4041812	Bn-chrC05_random-p149336
Bn-A06-p3435985	0.993355482	0.993355482	Bn-N6-p3351477	Bn-chrA06-p3257418
Bn-A06-p3437409	0.983471074	0.983471074	Bn-N6-p3352894	Bn-chrA06-p3258835
Bn-A06-p3442742	0.818181818	0.818181818	Bn-N6-p3362632	Bn-chrA06_random-p280307
Bn-A06-p3444868	0.990033223	0.990033223	Bn-N6-p3363853	Bn-chrA06_random-p279086
Bn-A06-p3446956	0.966942149	0.966942149	Bn-N6-p3365936	Bn-chrA06_random-p277003
Bn-A06-p3447648	0.990049751	0.990049751	Bn-Scaffold25383-p2	Bn-chrA06_random-p276312
Bn-A06-p3452904	0.950413223	0.950413223	Bn-N6-p3369287	Bn-chrA06_random-p273155
Bn-A06-p3468374	0.966942149	0.966942149	Bn-N6-p3384780	Bn-chrA06_random-p259916
Bn-A06-p3483249	0.958677686	0.958677686	Bn-N6-p3399292	Bn-chrA06_random-p241557
Bn-A06-p3483382	0.958677686	0.958677686	Bn-N6-p3399426	Bn-chrA06_random-p241423
Bn-A06-p3491241	0.991735537	0.991735537	Bn-N6-p3411122	Bn-chrA06_random-p232284
Bn-A06-p3492295	0.991735537	0.991735537	Bn-N6-p3412183	Bn-chrA06_random-p231223
Bn-A06-p3496759	0.990033223	0.990033223	Bn-N6-p3418698	Bn-chrA06-p3282873
Bn-A06-p3528559	0.975206612	0.975206612	Bn-N6-p3453095	Bn-chrA06-p3318559
Bn-A06-p3528628	0.975206612	0.975206612	Bn-N6-p3453164	Bn-chrA06-p3318628

Bn-A06-p3532353	0.996677741	0.996677741	Bn-N6-p3456894	Bn-chrA06-p3322358
Bn-A06-p3561531	0.996677741	0.993355482	Bn-N6-p3488276	Bn-chrA06-p3346785
Bn-A06-p3567180	0.996677741	1	Bn-N6-p3494019	Bn-chrA06-p3350988
Bn-A06-p3584489	0.996677741	0.996677741	Bn-N6-p3515997	Bn-chrA06-p3368777
Bn-A06-p3586728	0.986710963	0.983388704	Bn-N6-p3518194	Bn-chrA06-p3371000
Bn-A06-p3587032	0.958677686	0.966942149	Bn-N6-p3518491	Bn-chrA06-p3371304
Bn-A06-p3611806	0.980066445	0.980066445	Bn-N6-p3534417	Bn-chrA06-p3387324
Bn-A06-p3612397	0.993355482	0.993355482	Bn-N6-p3535008	Bn-chrA06-p3387915
Bn-A06-p3625328	0.993355482	0.993355482	Bn-N6-p3546800	Bn-chrA06-p3400358
Bn-A06-p3628159	0.993355482	0.983388704	Bn-N6-p3549760	Bn-chrA06-p3403323
Bn-A06-p3651074	0.965174129	0.965174129	Bn-N15-p4343868	Bn-chrC05-p4112104
Bn-A06-p3651473	0.976744186	0.833887043	Bn-N6-p3577649	Bn-chrC05-p4112395
Bn-A06-p3660997	0.965174129	0.855721393	Bn-N6-p3581683	Bn-chrA06-p3449752
Bn-A06-p3661134	0.965174129	0.970149254	Bn-N6-p3581820	Bn-chrA06-p3424066
Bn-A06-p3661342	0.975124378	0.975124378	Bn-N6-p3582029	Bn-chrA06-p3450097
Bn-A06-p3667300	0.990033223	0.990033223	Bn-N6-p3588340	Bn-chrA06-p3456391
Bn-A06-p3677526	0.940298507	0.781094527	Bn-Scaffold59679-p1	Bn-chrA06-p8672809
Bn-A06-p3691343	0.965174129	0.965174129	Bn-N6-p3600793	Bn-chrA06-p3468833
Bn-A06-p3703307	0.966942149	0.966942149	Bn-N6-p3608896	Bn-chrA06-p3476935
Bn-A06-p3708256	0.956810631	0.956810631	Bn-N11-p43809110	Bn-chrA01-p6911471
Bn-A06-p3712646	0.996677741	0.996677741	Bn-N6-p3615283	Bn-chrA06-p3483322
Bn-A06-p3723340	0.990033223	0.990033223	Bn-N6-p3628074	Bn-chrA06-p3495951
Bn-A06-p3731147	0.990033223	0.990033223	Bn-N6-p3636576	Bn-chrA06-p3504638
Bn-A06-p3759020	0.983388704	0.983388704	Bn-N6-p3658647	Bn-chrA06-p3527778
Bn-A06-p3805018	0.990033223	0.990033223	Bn-N6-p3707767	Bn-chrA06-p3565417
Bn-A06-p3809199	1	1	Bn-N6-p3712054	Bn-chrA06-p3569703
Bn-A06-p3818421	0.850498339	0.970099668	Bn-N15-p4594195	Bn-chrA06-p3577754
Bn-A06-p3818484	0.909090909	0.983471074	Bn-N15-p4594266	Bn-chrA06-p3577817
Bn-A06-p3825492	0.966942149	0.975206612	Bn-N6-p3726652	Bn-chrA06-p3584818
Bn-A06-p3834324	0.950413223	0.958677686	Bn-N6-p3732765	Bn-chrA06-p3590895
Bn-A06-p3837553	0.975206612	0.975206612	Bn-N6-p3742618	Bn-chrA06-p3600210
Bn-A06-p3839293	0.996677741	0.996677741	Bn-N6-p3744356	Bn-chrA06-p3601948
Bn-A06-p3867353	0.919678715	0.919678715	Bn-N6-p3774461	Bn-chrA06-p3634766
Bn-A06-p3871984	0.980066445	0.980066445	Bn-N6-p3779144	Bn-chrA06-p3639448
Bn-A06-p3900404	0.94214876	0.983471074	Bn-N6-p3809412	Bn-chrA06-p3669193
Bn-A06-p3913280	0.996677741	0.993355482	Bn-N6-p3822387	Bn-chrA06-p3684254
Bn-A06-p3928714	0.976744186	0.976744186	Bn-N6-p3843129	Bn-chrA06-p3713135
Bn-A06-p3934890	0.996677741	0.996677741	Bn-N6-p3849250	Bn-chrA06-p3719872
Bn-A06-p3953661	0.973421927	0.973421927	Bn-N6-p3871081	Bn-chrA06-p3743036
Bn-A06-p3970972	0.983471074	0.983471074	Bn-N6-p3893142	Bn-chrA06-p3759027
Bn-A06-p3976759	0.990033223	0.990033223	Bn-N6-p3900880	Bn-chrA06-p3765407
Bn-A06-p3986320	0.983471074	0.975206612	Bn-N6-p3910622	Bn-chrA06-p3774984
Bn-A06-p3987067	0.975206612	0.983471074	Bn-N6-p3911695	Bn-chrA06-p3775795
Bn-A06-p3988352	0.900332226	0.897009967	Bn-N6-p3912970	Bn-chrA06-p3777055
Bn-A06-p3992289	0.801652893	0.950413223	Bn-N6-p3921192	Bn-chrA06-p3781263
Bn-A06-p3997307	1	1	Bn-N6-p3926060	Bn-chrA06-p3785973

Bn-A06-p3998593	1	1	Bn-N6-p3927346	Bn-chrA06-p3787259
Bn-A06-p3999986	0.990049751	0.990049751	Bn-N6-p3928739	Bn-chrA06-p3788652
Bn-A06-p4001378	0.990033223	0.990033223	Bn-N6-p3930131	Bn-chrA06-p3790044
Bn-A06-p4013010	0.851239669	0.851239669	Bn-N6-p3949955	Bn-chrA06-p3812502
Bn-A06-p4016422	0.884297521	0.884297521	Bn-N15-p4844920	Bn-chrCnn_random-p168335
Bn-A06-p4017460	0.975206612	0.983471074	Bn-N6-p3954297	Bn-chrA06-p3816788
Bn-A06-p4019827	0.983471074	0.991735537	Bn-N6-p3958445	Bn-chrA06-p3819163
Bn-A06-p4021930	0.991735537	0.983471074	Bn-N6-p3961778	Bn-chrA06-p3825379
Bn-A06-p4025613	0.983471074	0.975206612	Bn-N6-p3965563	Bn-chrA06-p3828182
Bn-A06-p4026453	0.893687708	0.993355482	Bn-N6-p3966708	Bn-chrA06-p3829021
Bn-A06-p4027051	0.909090909	0.991735537	Bn-N6-p3967285	Bn-chrA06-p3829619
Bn-A06-p4039206	0.995024876	1	Bn-N6-p3985083	Bn-chrA06-p3842367
Bn-A06-p4040976	0.950248756	0.950248756	Bn-N6-p3986853	Bn-chrA06-p3844137
Bn-A06-p4056446	0.983471074	0.975206612	Bn-N6-p4005291	Bn-chrA06-p3866750
Bn-A06-p4060202	0.983388704	0.993355482	Bn-N6-p4010842	Bn-chrA06-p3870458
Bn-A06-p4060310	0.990033223	0.986710963	Bn-N6-p4010950	Bn-chrA06-p3870566
Bn-A06-p4072818	0.97260274	0.98630137	Bn-N6-p4021349	Bn-chrA06-p3882229
Bn-A06-p4098532	0.990033223	0.990033223	Bn-N6-p4046119	Bn-chrA06-p3904637
Bn-A06-p4098800	0.993355482	0.993355482	Bn-N6-p4046387	Bn-chrA06-p3904905
Bn-A06-p4179318	0.976744186	0.993355482	Bn-N6-p4111353	Bn-chrA06-p3978930
Bn-A06-p4195181	1	0.892561983	Bn-N6-p4116659	Bn-chrC05-p4773090
Bn-A06-p4196008	0.958677686	0.966942149	Bn-N6-p4118639	Bn-chrA06-p3990050
Bn-A06-p4211266	0.975206612	0.983471074	Bn-N6-p4139767	Bn-chrA06-p4005067
Bn-A06-p4218801	0.73553719	0.975206612	Bn-N6-p4149689	Bn-chrA06-p4011138
Bn-A06-p4229919	0.96013289	0.996677741	Bn-N6-p4167424	Bn-chrA06-p4021529
Bn-A06-p4315934	0.801652893	0.975206612	Bn-N6-p4253412	Bn-chrA06-p4114569
Bn-A06-p4316905	0.975206612	0.950413223	Bn-N6-p4254384	Bn-chrC05-p4924693
Bn-A06-p4317300	0.996677741	0.996677741	Bn-N6-p4254779	Bn-chrA06-p4115936
Bn-A06-p4331202	0.983471074	0.991735537	Bn-N6-p4268691	Bn-chrA06-p4130940
Bn-A06-p4332149	0.975206612	0.966942149	Bn-N6-p4269638	Bn-chrA06-p4132219
Bn-A06-p4332223	0.975206612	0.983471074	Bn-N6-p4269712	Bn-chrA06-p4132535
Bn-A06-p4333047	0.983471074	0.892561983	Bn-N6-p4272100	Bn-chrA06-p4132720
Bn-A06-p4333144	0.975206612	0.958677686	Bn-N6-p4272197	Bn-chrA06-p4132813
Bn-A06-p4333227	0.983471074	0.958677686	Bn-N6-p4272280	Bn-chrA06-p4132896
Bn-A06-p4337931	0.966942149	0.958677686	Bn-N6-p4275985	Bn-chrA06-p4133518
Bn-A06-p4340434	0.983471074	0.991735537	Bn-N6-p4278232	Bn-chrA06-p4135513
Bn-A06-p4340819	0.94214876	0.933884298	Bn-N6-p4278617	Bn-chrA06-p4144315
Bn-A06-p4361865	0.983471074	0.991735537	Bn-N6-p4289509	Bn-chrA06-p4155524
Bn-A06-p4362245	0.980066445	0.980066445	Bn-N6-p4289889	Bn-chrA06-p4155904
Bn-A06-p4362386	0.983388704	0.983388704	Bn-N6-p4290030	Bn-chrA06-p4156045
Bn-A06-p4362700	0.990033223	0.986710963	Bn-N6-p4290452	Bn-chrA06-p4156359
Bn-A06-p4365836	1	0.995024876	Bn-N6-p4293588	Bn-chrA06-p4159833
Bn-A06-p4365949	1	0.995024876	Bn-N6-p4293701	Bn-chrA06-p4159946
Bn-A06-p4371627	0.966942149	0.983471074	Bn-N6-p4304145	Bn-chrA06-p4174029
Bn-A06-p4409355	0.916943522	0.910299003	Bn-N6-p4334831	Bn-chrA06_random-p320583
Bn-A06-p4410177	0.976744186	0.980066445	Bn-N6-p4335643	Bn-chrA06_random-p319633

Bn-A06-p4418231	0.958677686	0.983471074	Bn-N6-p4341969	Bn-chrA06_random-p310914
Bn-A06-p4419571	0.991735537	0.991735537	Bn-N6-p4343325	Bn-chrA06_random-p309553
Bn-A06-p4420263	0.983471074	0.975206612	Bn-N6-p4344015	Bn-chrA06_random-p308863
Bn-A06-p4421214	0.995024876	0.995024876	Bn-N6-p4344970	Bn-chrA06_random-p307911
Bn-A06-p4428703	0.853820598	0.946843854	Bn-N6-p4352626	Bn-chrA06_random-p301737
Bn-A06-p4435926	0.900826446	0.958677686	Bn-N15-p5380511	Bn-chrA06-p4266045
Bn-A06-p4436168	0.983388704	0.983388704	Bn-N6-p4361910	Bn-chrA06-p4266287
Bn-A06-p4437120	0.834710744	0.983471074	Bn-N6-p4362910	Bn-chrA06-p4267238
Bn-A06-p4452778	0.990033223	0.986710963	Bn-N6-p4376374	Bn-chrA06-p4280288
Bn-A06-p4461241	0.909090909	0.958677686	Bn-N6-p4385362	Bn-chrA06-p4290985
Bn-A06-p4466057	0.993355482	0.993355482	Bn-N7-p1654362	Bn-chrA06_random-p214822
Bn-A06-p4508774	0.991735537	1	Bn-N6-p4418843	Bn-chrA06-p4320835
Bn-A06-p4509926	0.933884298	0.975206612	Bn-N6-p4426080	Bn-chrA06-p4328313
Bn-A06-p4517457	0.966942149	0.975206612	Bn-N6-p4433651	Bn-chrA06-p4335822
Bn-A06-p4558209	0.965174129	0.965174129	Bn-N11-p36461876	Bn-chrC01-p31787196
Bn-A06-p4597746	0.950413223	0.958677686	Bn-N6-p4511003	Bn-chrA06-p4414909
Bn-A06-p4619013	0.958677686	0.966942149	Bn-N6-p4524219	Bn-chrA06-p4430345
Bn-A06-p4623401	0.990049751	0.980099502	Bn-N6-p4528589	Bn-chrAnn_random-p411824
Bn-A06-p4703366	0.983471074	0.975206612	Bn-N6-p4574042	Bn-chrA06_random-p444255
Bn-A06-p4729500	0.990049751	0.985074627	Bn-N6-p4606368	Bn-chrC05-p5446597
Bn-A06-p4731996	1	0.965174129	Bn-N6-p4613334	Bn-chrC05-p5448764
Bn-A06-p4837796	0.983471074	0.983471074	Bn-N2-p9286482	Bn-chrA02-p6717673
Bn-A06-p4838544	0.983388704	0.983388704	Bn-N2-p9285739	Bn-chrA02-p6716929
Bn-A06-p4844167	0.825870647	0.845771144	Bn-N2-p9281257	Bn-chrA02-p6712921
Bn-A06-p4874556	0.727574751	0.724252492	Bn-N6-p4795418	Bn-chrA06-p4748698
Bn-A06-p4881785	0.930232558	0.930232558	Bn-N6-p4802388	Bn-chrA06-p4755665
Bn-A06-p4896084	0.983471074	0.983471074	Bn-N6-p4832997	Bn-chrA06-p4791517
Bn-A06-p4901091	0.909090909	0.909090909	Bn-N6-p4837924	Bn-chrA06-p4796442
Bn-A06-p4901457	0.975206612	0.975206612	Bn-N6-p4838282	Bn-chrA06-p4796800
Bn-A06-p4927344	0.96345515	0.986710963	Bn-N15-p6144435	Bn-chrAnn_random-p349626
Bn-A06-p4929018	0.990033223	0.993355482	Bn-N6-p4874165	Bn-chrA06-p4834289
Bn-A06-p4933932	0.985074627	0.985074627	Bn-N6-p4879063	Bn-chrA06-p4839177
Bn-A06-p4976377	0.950413223	0.950413223	Bn-N6-p4901696	Bn-chrA06_random-p329858
Bn-A06-p5020498	0.966777409	0.966777409	Bn-N6-p4944027	Bn-chrA06_random-p376540
Bn-A06-p5020605	0.950413223	0.950413223	Bn-N6-p4944133	Bn-chrA06_random-p376646
Bn-A06-p5020675	0.955223881	0.955223881	Bn-N6-p4944203	Bn-chrA06_random-p376716
Bn-A06-p5040441	0.983471074	0.983471074	Bn-N6-p4950278	Bn-chrA06_random-p382791
Bn-A06-p5047871	0.975206612	0.975206612	Bn-N6-p4957470	Bn-chrCnn_random-p486695
Bn-A06-p5053748	0.842975207	0.842975207	Bn-N6-p4970334	Bn-chrA06-p4856754
Bn-A06-p5077968	0.975206612	0.975206612	Bn-N6-p4995783	Bn-chrA06-p4881268
Bn-A06-p5091252	0.975206612	0.975206612	Bn-N6-p5017241	Bn-chrA06-p4899658
Bn-A06-p5094790	0.991735537	0.991735537	Bn-N6-p5020316	Bn-chrA06-p4902731
Bn-A06-p5103824	0.950413223	0.950413223	Bn-N6-p5029281	Bn-chrA06-p4911696
Bn-A06-p5114238	0.945273632	0.945273632	Bn-N6-p5040596	Bn-chrA06-p4927258
Bn-A06-p5114770	0.966942149	0.975206612	Bn-N6-p5041218	Bn-chrA06-p4927798
Bn-A06-p5130670	1	1	Bn-N6-p5061916	Bn-chrA06-p4944020

Bn-A06-p5147575	0.975206612	0.975206612	Bn-N6-p5077880	Bn-chrA06-p4964208
Bn-A06-p5151254	0.983471074	0.983471074	Bn-N6-p5081581	Bn-chrA06-p4967878
Bn-A06-p5151337	0.980099502	0.980099502	Bn-N6-p5081664	Bn-chrA06-p4967961
Bn-A06-p5179267	0.909090909	0.909090909	Bn-N4-p4245623	Bn-chrA04-p4053269
Bn-A06-p5182164	0.991735537	0.991735537	Bn-N4-p4243391	Bn-chrA04-p4051037
Bn-A06-p5182253	0.991735537	0.991735537	Bn-N4-p4243302	Bn-chrA04-p4050948
Bn-A06-p5182315	0.983471074	0.983471074	Bn-N4-p4243240	Bn-chrA04-p4050886
Bn-A06-p5183425	0.966942149	0.966942149	Bn-N4-p4241562	Bn-chrA04-p4049207
Bn-A06-p5184830	0.966942149	0.966942149	Bn-N4-p4240171	Bn-chrA04-p4047816
Bn-A06-p5187939	0.983471074	0.983471074	Bn-N4-p4237063	Bn-chrA04-p4044708
Bn-A06-p5188207	0.990033223	0.990033223	Bn-N4-p4236795	Bn-chrA04-p4044440
Bn-A06-p5188627	0.990033223	0.990033223	Bn-N4-p4236375	Bn-chrA04-p4044020
Bn-A06-p5190285	0.975206612	0.975206612	Bn-N1-p23467548	Bn-chrA04-p4042407
Bn-A06-p5190418	0.966942149	0.966942149	Bn-N1-p23467682	Bn-chrA04-p4042273
Bn-A06-p5190514	0.991735537	0.991735537	Bn-N1-p23467778	Bn-chrA04-p4042177
Bn-A06-p5192434	0.983471074	0.983471074	Bn-N4-p4224361	Bn-chrA04-p4033024
Bn-A06-p5197572	0.991735537	0.991735537	Bn-N4-p4219476	Bn-chrA04-p4028140
Bn-A06-p5197898	0.94214876	0.94214876	Bn-N4-p4219149	Bn-chrA04-p4027813
Bn-A06-p5201102	0.983471074	0.983471074	Bn-N4-p4216812	Bn-chrA04-p4025476
Bn-A06-p5217195	0.983471074	0.983471074	Bn-N14-p33298973	Bn-chrCnn_random-p727854
Bn-A06-p5226816	0.983471074	0.983471074	Bn-N4-p4182634	Bn-chrAnn_random-p243384
Bn-A06-p5227178	0.975206612	0.975206612	Bn-N4-p4182270	Bn-chrAnn_random-p243381
Bn-A06-p5227365	0.958677686	0.958677686	Bn-N4-p4182083	Bn-chrAnn_random-p243379
Bn-A06-p5232462	0.983471074	0.983471074	Bn-N4-p4181313	Bn-chrAnn_random-p243371
Bn-A06-p5232488	0.983471074	0.983471074	Bn-N4-p4181287	Bn-chrAnn_random-p243371
Bn-A06-p5232937	0.966942149	0.966942149	Bn-N4-p4180859	Bn-chrAnn_random-p243367
Bn-A06-p5233115	0.991735537	0.991735537	Bn-N4-p4180679	Bn-chrAnn_random-p243365
Bn-A06-p5233226	0.975206612	0.975206612	Bn-N4-p4180569	Bn-chrAnn_random-p243364
Bn-A06-p5235813	0.983471074	0.983471074	Bn-N4-p4178556	Bn-chrAnn_random-p243344
Bn-A06-p5235889	0.925619835	0.925619835	Bn-N4-p4178480	Bn-chrAnn_random-p243343
Bn-A06-p5236016	0.983471074	0.983471074	Bn-N4-p4178354	Bn-chrAnn_random-p243341
Bn-A06-p5236102	0.975206612	0.975206612	Bn-N4-p4178262	Bn-chrAnn_random-p243341
Bn-A06-p5236283	0.983471074	0.983471074	Bn-N4-p4178072	Bn-chrAnn_random-p243339
Bn-A06-p5236645	0.950413223	0.950413223	Bn-N4-p4177710	Bn-chrAnn_random-p243335
Bn-A06-p5246637	0.975206612	0.975206612	Bn-N4-p4156608	Bn-chrA04-p3887456
Bn-A06-p5260549	0.983471074	0.983471074	Bn-N4-p4104332	Bn-chrA04-p3866977
Bn-A06-p5274160	0.983471074	0.983471074	Bn-N4-p4100424	Bn-chrA04-p3864002
Bn-A06-p5275416	0.975206612	0.975206612	Bn-N4-p4099123	Bn-chrA04-p3862701
Bn-A06-p5275803	0.975206612	0.975206612	Bn-N4-p4098735	Bn-chrA04-p3862313
Bn-A06-p5276150	0.983471074	0.983471074	Bn-N4-p4098388	Bn-chrA04-p3861966
Bn-A06-p5276251	0.975206612	0.975206612	Bn-N4-p4098287	Bn-chrA04-p3861865
Bn-A06-p5276641	0.991735537	0.991735537	Bn-N4-p4097897	Bn-chrA04-p3861475
Bn-A06-p5276713	0.966942149	0.966942149	Bn-N4-p4097825	Bn-chrA04-p3861403
Bn-A06-p5276751	0.966942149	0.966942149	Bn-N4-p4097787	Bn-chrA04-p3861365
Bn-A06-p5277548	0.983471074	0.983471074	Bn-N4-p4096909	Bn-chrA04-p3860487
Bn-A06-p5278752	0.993355482	0.993355482	Bn-N4-p4095699	Bn-chrA04-p3859277

Bn-A06-p5279490	0.975206612	0.975206612	Bn-N4-p4094961	Bn-chrA04-p3858539
Bn-A06-p5280041	0.991735537	0.991735537	Bn-N4-p4094410	Bn-chrA04-p3857988
Bn-A06-p5280225	0.983471074	0.983471074	Bn-N4-p4094226	Bn-chrA04-p3857804
Bn-A06-p5284260	0.94214876	0.94214876	Bn-N4-p4090036	Bn-chrC06-p16962144
Bn-A06-p5285976	0.983471074	0.983471074	Bn-N4-p4088273	Bn-chrA04-p3851851
Bn-A06-p5299890	0.975206612	0.975206612	Bn-N4-p4071540	Bn-chrA04-p3833407
Bn-A06-p5300943	1	1	Bn-N4-p4070767	Bn-chrA04-p3832634
Bn-A06-p5301714	0.985074627	0.985074627	Bn-N4-p4069973	Bn-chrA04-p3831840
Bn-A06-p5305727	0.958677686	0.958677686	Bn-N4-p4062435	Bn-chrA04-p3819264
Bn-A06-p5305781	0.917355372	0.917355372	Bn-N4-p4062381	Bn-chrA04-p3819210
Bn-A06-p5306445	0.966942149	0.966942149	Bn-N4-p4061742	Bn-chrA04-p3818570
Bn-A06-p5310499	0.983471074	0.190082645	Bn-N4-p4059076	Bn-chrA04-p14615307
Bn-A06-p5310521	0.975206612	0.173553719	Bn-N4-p4059054	Bn-chrC02-p12405853
Bn-A06-p5310579	0.983471074	0.148760331	Bn-Scaffold85137-p8	Unknown
Bn-A06-p5311852	0.966942149	0.966942149	Bn-N4-p4056190	Bn-chrAnn_random-p470183
Bn-A06-p5314603	0.950413223	0.950413223	Bn-N4-p4055809	Bn-chrAnn_random-p470179
Bn-A06-p5315085	0.958677686	0.958677686	Bn-N4-p4055317	Bn-chrAnn_random-p470174
Bn-A06-p5315140	0.983471074	0.983471074	Bn-N4-p4055262	Bn-chrAnn_random-p470174
Bn-A06-p5320572	0.975206612	0.975206612	Bn-N4-p4049718	Bn-chrA04-p3811676
Bn-A06-p5321651	0.975206612	0.975206612	Bn-N4-p4048597	Bn-chrA04-p3810555
Bn-A06-p5325547	0.991735537	0.991735537	Bn-N4-p4046646	Bn-chrA04-p3808604
Bn-A06-p5331686	0.966942149	0.966942149	Bn-N4-p4042074	Bn-chrA04-p3805004
Bn-A06-p5333246	0.983471074	0.983471074	Bn-N4-p4040503	Bn-chrA04-p3803433
Bn-A06-p5333348	0.983471074	0.983471074	Bn-N4-p4040401	Bn-chrA04-p3803331
Bn-A06-p5335605	0.983471074	0.983471074	Bn-N4-p4038127	Bn-chrA04-p3801059
Bn-A06-p5339433	0.966942149	0.966942149	Bn-N4-p4033677	Bn-chrA04-p3796609
Bn-A06-p5340584	0.990033223	0.990033223	Bn-N4-p4031224	Bn-chrA04-p3794320
Bn-A06-p5344950	0.960199005	0.960199005	Bn-N4-p4024978	Bn-chrA04-p3789080
Bn-A06-p5345401	0.887043189	0.887043189	Bn-N4-p4024527	Bn-chrA04-p3788629
Bn-A06-p5346654	0.736318408	0.736318408	Bn-N14-p33090501	Bn-chrCnn_random-p497817
Bn-A06-p5346849	0.946843854	0.946843854	Bn-N4-p4023079	Bn-chrA04-p3787181
Bn-A06-p5372653	0.966942149	0.966942149	Bn-N4-p4000303	Bn-chrA04-p3761542
Bn-A06-p5384725	1	1	Bn-N4-p3995321	Bn-chrA04-p3756560
Bn-A06-p5386579	0.975206612	0.975206612	Bn-N4-p3993216	Bn-chrAnn_random-p450397
Bn-A06-p5389758	0.991735537	0.991735537	Bn-N4-p3990597	Bn-chrA04-p3751883
Bn-A06-p5390277	1	1	Bn-N4-p3990078	Bn-chrA04-p3751364
Bn-A06-p5393231	0.991735537	0.991735537	Bn-N4-p3987122	Bn-chrA04-p3748408
Bn-A06-p5427889	0.750830565	0.750830565	Bn-N4-p3947979	Bn-chrA04-p3702696
Bn-A06-p5436800	0.958677686	0.958677686	Bn-N4-p3927266	Bn-chrA04-p3679062
Bn-A06-p5438131	0.966942149	0.966942149	Bn-N4-p3926254	Bn-chrA04-p3678050
Bn-A06-p5441991	0.950413223	0.950413223	Bn-N4-p3922972	Bn-chrA04-p3674724
Bn-A06-p5535537	0.975124378	0.985074627	Bn-N6-p5127629	Bn-chrA06-p5007676
Bn-A06-p5540327	0.917355372	0.975206612	Bn-N6-p5130414	Bn-chrA06-p5010462
Bn-A06-p5546411	0.958677686	0.785123967	Bn-N6-p5136130	Bn-chrA06-p5015306
Bn-A06-p5577934	0.983388704	0.970099668	Bn-N6-p5160478	Bn-chrAnn_random-p249475
Bn-A06-p5593050	0.933884298	0.933884298	Bn-N6-p5181681	Bn-chrA06-p5044682

Bn-A06-p5600636	0.983471074	0.983471074	Bn-N6-p5188652	Bn-chrA06-p5051901
Bn-A06-p5604858	0.989583333	0.989583333	Bn-N6-p5192795	Bn-chrA06-p5056044
Bn-A06-p5616164	0.966942149	0.966942149	Bn-N6-p5200409	Bn-chrA06-p5062564
Bn-A06-p5618302	0.995024876	0.995024876	Bn-N6-p5202546	Bn-chrA06-p5064701
Bn-A06-p5641863	0.966942149	0.966942149	Bn-N6-p5228100	Bn-chrA06-p5085974
Bn-A06-p5669960	0.966942149	0.975206612	Bn-N6-p5255428	Bn-chrA06-p5119717
Bn-A06-p5690066	0.983471074	0.975206612	Bn-N6-p5278655	Bn-chrA06-p5138085
Bn-A06-p5693735	0.966942149	0.975206612	Bn-N6-p5412407	Bn-chrA06-p5250207
Bn-A06-p5739682	0.94214876	0.380165289	Bn-N6-p5325363	Bn-chrC05-p6547205
Bn-A06-p5741557	0.966942149	0.966942149	Bn-N6-p5330191	Bn-chrA06-p5182655
Bn-A06-p5747694	0.985074627	0.995024876	Bn-N6-p5341945	Bn-chrA06-p5185964
Bn-A06-p5758576	0.975206612	0.983471074	Bn-N6-p5353764	Bn-chrA06-p5202216
Bn-A06-p5761019	0.983388704	0.986710963	Bn-N6-p5356257	Bn-chrA10-p14239338
Bn-A06-p5761097	0.986710963	0.990033223	Bn-N6-p5356335	Bn-chrA10-p14239416
Bn-A06-p5762475	0.996677741	0.996677741	Bn-N6-p5357713	Bn-chrA10-p14240794
Bn-A06-p5762802	0.996677741	0.96013289	Bn-N6-p5358040	Bn-chrA10-p14241121
Bn-A06-p5767097	0.989583333	1	Bn-N6-p5362304	Bn-chrA06-p5206419
Bn-A06-p5780982	0.991735537	0.991735537	Bn-N6-p5378080	Bn-chrA06-p5218016
Bn-A06-p5785209	0.990049751	0.995024876	Bn-N6-p5382312	Bn-chrA06-p5222247
Bn-A06-p5824546	0.966942149	0.966942149	Bn-N6-p5414690	Bn-chrA06-p5252455
Bn-A06-p5826493	0.721393035	0.985074627	Bn-N19-p41466660	Bn-chrA06-p5254397
Bn-A06-p5830298	0.966942149	0.966942149	Bn-N6-p5420524	Bn-chrA06-p5258210
Bn-A06-p5844403	1	1	Bn-N6-p5428823	Bn-chrA06-p5265993
Bn-A06-p5845542	0.975206612	0.975206612	Bn-N6-p5429962	Bn-chrA06-p5267132
Bn-A06-p5868273	0.983471074	0.983471074	Bn-N6-p5455035	Bn-chrA06-p5289904
Bn-A06-p5878990	0.975206612	0.983471074	Bn-N6-p5465758	Bn-chrA06-p5300782
Bn-A06-p5880980	0.991735537	0.983471074	Bn-N6-p5467747	Bn-chrA06-p5302770
Bn-A06-p5888779	0.966942149	0.958677686	Bn-N6-p5475639	Bn-chrA06-p5310061
Bn-A06-p5904190	0.983471074	0.983471074	Bn-N6-p5495718	Bn-chrA06-p5334805
Bn-A06-p5907449	0.975206612	0.966942149	Bn-N6-p5499009	Bn-chrA06-p5340130
Bn-A06-p5922951	0.973421927	0.976744186	Bn-N6-p5512941	Bn-chrA06-p5354720
Bn-A06-p5930501	0.985074627	0.985074627	Bn-N6-p5524811	Bn-chrA06-p5364428
Bn-A06-p5931022	0.988023952	0.988023952	Bn-N6-p5526117	Bn-chrA06-p5364962
Bn-A06-p5931130	0.995024876	0.995024876	Bn-N6-p5526225	Bn-chrA06-p5368168
Bn-A06-p5944090	0.970149254	0.970149254	Bn-N15-p7306523	Bn-chrC05-p6840873
Bn-A06-p5945068	0.960199005	0.960199005	Bn-N6-p5545254	Bn-chrA06-p5385403
Bn-A06-p5945471	0.960199005	0.960199005	Bn-N6-p5545656	Bn-chrA06-p5385805
Bn-A06-p5947781	0.983471074	0.983471074	Bn-N6-p5547966	Bn-chrA06-p5388115
Bn-A06-p5956274	0.973421927	0.973421927	Bn-N6-p5565642	Bn-chrA06-p5403991
Bn-A06-p5957378	0.990049751	0.990049751	Bn-N6-p5566746	Bn-chrA06-p5405095
Bn-A06-p5967477	0.993355482	0.993355482	Bn-N6-p5579048	Bn-chrA06-p5418147
Bn-A06-p5969087	0.983471074	0.983471074	Bn-N6-p5580655	Bn-chrA06-p5419754
Bn-A06-p5969789	0.991735537	0.991735537	Bn-N6-p5584408	Bn-chrA06-p5423507
Bn-A06-p5973569	1	1	Bn-N6-p5588183	Bn-chrA06-p5427281
Bn-A06-p5974383	0.983471074	0.983471074	Bn-N6-p5589594	Bn-chrA06-p5428692
Bn-A06-p5974957	0.991735537	0.991735537	Bn-N6-p5590193	Bn-chrA06-p5429290

Bn-A06-p5976042	0.975206612	0.975206612	Bn-N6-p5591263	Bn-chrA06-p5430360
Bn-A06-p5978510	0.991735537	0.991735537	Bn-N6-p5591539	Bn-chrA06-p5430636
Bn-A06-p5978667	0.991735537	0.991735537	Bn-N6-p5591696	Bn-chrA06-p5430793
Bn-A06-p5979198	0.983471074	0.983471074	Bn-N6-p5592227	Bn-chrA06-p5431324
Bn-A06-p5979527	0.983471074	0.983471074	Bn-N6-p5592553	Bn-chrA06-p5431650
Bn-A06-p5981335	0.958677686	0.958677686	Bn-N6-p5599833	Bn-chrA06-p5439143
Bn-A06-p5981608	0.975206612	0.975206612	Bn-N6-p5600106	Bn-chrA06-p5439416
Bn-A06-p5982453	0.983471074	0.983471074	Bn-N6-p5600950	Bn-chrA06-p5440260
Bn-A06-p5983201	0.983471074	0.983471074	Bn-N6-p5601734	Bn-chrA06-p5441044
Bn-A06-p5984910	0.991735537	0.991735537	Bn-N6-p5605252	Bn-chrA06-p5444558
Bn-A06-p5990338	0.983471074	0.983471074	Bn-N6-p5605857	Bn-chrA06-p5445163
Bn-A06-p5991117	0.983471074	0.983471074	Bn-N6-p5606633	Bn-chrA06-p5445939
Bn-A06-p5991309	0.991735537	0.991735537	Bn-N6-p5608341	Bn-chrA06-p5446791
Bn-A06-p5993672	0.966942149	0.975206612	Bn-N6-p5611038	Bn-chrA06-p5449486
Bn-A06-p5995778	0.975206612	0.975206612	Bn-N6-p5613149	Bn-chrA06-p5451597
Bn-A06-p5996847	0.990049751	0.985074627	Bn-N6-p5614218	Bn-chrA06-p5452665
Bn-A06-p6006425	0.966942149	0.966942149	Bn-N6-p5616830	Bn-chrA06-p5455276
Bn-A06-p6009064	0.975206612	0.975206612	Bn-N6-p5619468	Bn-chrA06-p5457914
Bn-A06-p6023926	0.983471074	0.983471074	Bn-N6-p5630197	Bn-chrA06-p5468640
Bn-A06-p6047002	0.975206612	0.975206612	Bn-N6-p5656244	Bn-chrA06-p5495055
Bn-A06-p6056844	0.980099502	0.980099502	Bn-N6-p5661359	Bn-chrA06-p5499178
Bn-A06-p6056922	0.996677741	0.996677741	Bn-N6-p5661437	Bn-chrA06-p5499256
Bn-A06-p6057865	0.990033223	0.990033223	Bn-N6-p5662381	Bn-chrA06-p5500200
Bn-A06-p6059527	0.993355482	0.993355482	Bn-N6-p5664049	Bn-chrA06-p5501868
Bn-A06-p6066073	0.993355482	0.993355482	Bn-N6-p5672077	Bn-chrA06-p5510078
Bn-A06-p6091453	0.975206612	0.975206612	Bn-N6-p5701636	Bn-chrA06-p5537039
Bn-A06-p6103844	0.975206612	0.966942149	Bn-N6-p5707075	Bn-chrA06-p5542254
Bn-A06-p6103921	0.955223881	0.955223881	Bn-N6-p5707152	Bn-chrA06-p5542331
Bn-A06-p6118760	0.842975207	0.834710744	Bn-N6-p5725255	Bn-chrA06-p5555541
Bn-A06-p6129072	0.975206612	0.983471074	Bn-N6-p5728504	Bn-chrA06-p5563828
Bn-A06-p6154470	0.958677686	0.966942149	Bn-N6-p5740028	Bn-chrA06-p5583362
Bn-A06-p6182046	0.94214876	0.94214876	Bn-N6-p5760603	Bn-chrA06-p5603282
Bn-A06-p6182593	0.950166113	0.950166113	Bn-N6-p5761153	Bn-chrA06-p5603832
Bn-A06-p6183859	0.980066445	0.980066445	Bn-N6-p5762420	Bn-chrA06-p5605099
Bn-A06-p6184455	0.983471074	0.983471074	Bn-N6-p5763016	Bn-chrA06-p5605695
Bn-A06-p6195732	0.975206612	0.975206612	Bn-N6-p5779813	Bn-chrA06-p5625053
Bn-A06-p6196005	0.991735537	0.991735537	Bn-N6-p5780087	Bn-chrA06-p5625327
Bn-A06-p6198128	0.995024876	0.995024876	Bn-N6-p5782654	Bn-chrA06-p5627892
Bn-A06-p6204652	0.953488372	0.950166113	Bn-N15-p7700309	Bn-chrC05-p7088531
Bn-A06-p6270772	0.975124378	0.975124378	Bn-N6-p5854517	Bn-chrA06-p5700497
Bn-A06-p6308667	0.966942149	0.975206612	Bn-N6-p5896086	Bn-chrA06-p5756087
Bn-A06-p6311231	0.991735537	0.991735537	Bn-N6-p5898614	Bn-chrA06-p5758615
Bn-A06-p6311289	0.958677686	0.958677686	Bn-N6-p5898672	Bn-chrA06-p5758673
Bn-A06-p6319212	0.993355482	0.993355482	Bn-N6-p5908559	Bn-chrA06-p5768576
Bn-A06-p6341389	0.983471074	0.983471074	Bn-N6-p5932216	Bn-chrA06-p5792363
Bn-A06-p6343931	0.983388704	0.983388704	Bn-N6-p5934735	Bn-chrA06-p5794882

Bn-A06-p6356463	0.975206612	0.975206612	Bn-N6-p5946940	Bn-chrA06_random-p512506
Bn-A06-p6358974	0.975206612	0.975206612	Bn-N6-p5949390	Bn-chrA06_random-p514956
Bn-A06-p6359207	0.991735537	0.991735537	Bn-N6-p5949623	Bn-chrA06_random-p515189
Bn-A06-p6363956	0.983471074	0.20661157	Bn-N15-p8021320	Bn-chrA03-p17488893
Bn-A06-p6382773	0.975206612	0.975206612	Bn-N6-p5975051	Bn-chrA06_random-p489853
Bn-A06-p6388760	0.983471074	0.983471074	Bn-N6-p5982104	Bn-chrA06_random-p523455
Bn-A06-p6389318	0.958677686	0.958677686	Bn-N6-p5982669	Bn-chrA06_random-p524020
Bn-A06-p6403382	0.94214876	0.94214876	Bn-N6-p5996788	Bn-chrA06-p5849406
Bn-A06-p6408922	0.945273632	0.945273632	Bn-N15-p8111890	Bn-chrC05-p7423906
Bn-A06-p6432467	0.966942149	0.966942149	Bn-N6-p6020344	Bn-chrA06-p5871900
Bn-A06-p6437068	0.985074627	0.985074627	Bn-N6-p6024972	Bn-chrA06-p5876490
Bn-A06-p6439396	0.970149254	0.975124378	Bn-N6-p6027275	Bn-chrA06-p5878835
Bn-A06-p6446571	0.950413223	0.983471074	Bn-N15-p8149326	Bn-chrA06-p5885970
Bn-A06-p6450368	0.996677741	0.940199336	Bn-N6-p6039371	Bn-chrA06-p5889764
Bn-A06-p6451055	1	0.950413223	Bn-N6-p6040067	Bn-chrA06-p5890452
Bn-A06-p6452160	0.991735537	1	Bn-N6-p6041172	Bn-chrA06-p5891552
Bn-A06-p6457529	0.960199005	0.955223881	Bn-N6-p6046842	Bn-chrA06-p5896857
Bn-A06-p6458266	0.975124378	0.980099502	Bn-N6-p6047598	Bn-chrA06-p5897594
Bn-A06-p6464476	0.966942149	0.958677686	Bn-N6-p6053760	Bn-chrA06-p5903558
Bn-A06-p6477943	0.983471074	0.983471074	Bn-N6-p6067046	Bn-chrA06-p5916879
Bn-A06-p6482307	0.985074627	0.985074627	Bn-N6-p6071232	Bn-chrC05-p7497060
Bn-A06-p6483553	0.965174129	0.965174129	Bn-N6-p6072476	Bn-chrA06-p5922309
Bn-A06-p6486764	0.983471074	0.983471074	Bn-N6-p6074760	Bn-chrA06-p5924593
Bn-A06-p6508162	1	1	Bn-N6-p6089912	Bn-chrA06-p5939740
Bn-A06-p6517823	0.975206612	0.975206612	Bn-N6-p6099643	Bn-chrA06-p5949397
Bn-A06-p6520301	0.973421927	0.973421927	Bn-N6-p6101976	Bn-chrA06-p5951730
Bn-A06-p6532740	0.983471074	0.983471074	Bn-N6-p6118014	Bn-chrA06-p5965745
Bn-A06-p6555456	0.989583333	0.989583333	Bn-N6-p6151640	Bn-chrAnn_random-p259880
Bn-A06-p6563295	1	1	Bn-N6-p6159581	Bn-chrAnn_random-p296456
Bn-A06-p6569468	0.975124378	0.975124378	Bn-N15-p8359521	Bn-chrC05-p7660856
Bn-A06-p6573582	0.991735537	0.991735537	Bn-N6-p6175859	Bn-chrA06_random-p538980
Bn-A06-p6576675	0.975206612	0.975206612	Bn-N11-p5064803	Bn-chrC01-p3548516
Bn-A06-p6588828	0.985074627	0.985074627	Bn-N6-p6185821	Bn-chrA06_random-p548941
Bn-A06-p6591354	0.996677741	0.996677741	Bn-N6-p6188321	Bn-chrA06_random-p551441
Bn-A06-p6617274	0.810631229	0.810631229	Bn-N6-p6216436	Bn-chrA06-p5997343
Bn-A06-p6622319	0.990033223	0.990033223	Bn-N6-p6224488	Bn-chrA06-p6017553
Bn-A06-p6624071	0.966942149	0.966942149	Bn-N6-p6226240	Bn-chrA06-p6019305
Bn-A06-p6624464	0.966942149	0.966942149	Bn-N6-p6226633	Bn-chrA06-p6019698
Bn-A06-p6636202	0.966942149	0.966942149	Bn-N6-p6239300	Bn-chrA06-p6031226
Bn-A06-p6646249	0.991735537	0.991735537	Bn-N6-p6245632	Bn-chrA06-p6037557
Bn-A06-p6647323	0.983471074	0.983471074	Bn-N6-p6246719	Bn-chrA06-p6038644
Bn-A06-p6655047	0.956810631	0.956810631	Bn-N15-p8481600	Bn-chrAnn_random-p254686
Bn-A06-p6661719	0.958677686	0.958677686	Bn-N6-p6260543	Bn-chrC05-p7805089
Bn-A06-p6679810	0.975206612	0.975206612	Bn-N6-p6279236	Bn-chrA06-p6069771
Bn-A06-p6689858	0.976744186	0.976744186	Bn-N6-p6293418	Bn-chrA06-p6085138
Bn-A06-p6691628	0.980066445	0.980066445	Bn-N6-p6295137	Bn-chrA06-p6086857

Bn-A06-p6692851	0.524916944	0.524916944	Bn-N6-p6296354	Bn-chrA06-p6088074
Bn-A06-p6699971	0.993355482	0.993355482	Bn-N6-p6300228	Bn-chrA06-p6091947
Bn-A06-p6705911	0.966942149	0.966942149	Bn-N6-p6306290	Bn-chrA06-p6098009
Bn-A06-p6706067	0.965174129	0.965174129	Bn-N6-p6306446	Bn-chrA06-p6098165
Bn-A06-p6723804	0.983471074	0.983471074	Bn-N6-p6319916	Bn-chrA06-p6111633
Bn-A06-p6744737	0.958677686	0.958677686	Bn-N6-p6340136	Bn-chrA06-p6131728
Bn-A06-p6747460	0.983471074	0.983471074	Bn-N6-p6342582	Bn-chrA06-p6134174
Bn-A06-p6761232	0.966942149	0.966942149	Bn-N6-p6349603	Bn-chrA06-p6141195
Bn-A06-p6761241	0.966942149	0.966942149	Bn-N6-p6349612	Bn-chrA06-p6141204
Bn-A06-p6761331	1	1	Bn-N6-p6349702	Bn-chrA06-p6141294
Bn-A06-p6779990	0.900826446	0.900826446	Bn-N6-p6359912	Bn-chrA06-p6151503
Bn-A06-p6780446	0.958677686	0.628099174	Bn-N6-p6365472	Bn-chrC05_random-p703795
Bn-A06-p6780542	0.991735537	0.752066116	Bn-N6-p6365568	Bn-chrC05_random-p703699
Bn-A06-p6782162	0.976744186	0.976744186	Bn-N6-p6367200	Bn-chrA06-p6152750
Bn-A06-p6782980	0.983471074	0.983471074	Bn-N6-p6368018	Bn-chrA06-p6153568
Bn-A06-p6798408	0.996677741	0.996677741	Bn-N6-p6381959	Bn-chrA06-p6168027
Bn-A06-p6822469	0.892561983	0.892561983	Bn-N6-p6406811	Bn-chrA06-p6195559
Bn-A06-p6843211	0.976744186	0.976744186	Bn-N9-p13997203	Bn-chrAnn_random-p333395
Bn-A06-p6843711	0.990049751	0.990049751	Bn-N9-p13997703	Bn-chrAnn_random-p333400
Bn-A06-p6843808	0.985074627	0.990049751	Bn-N9-p13997800	Bn-chrAnn_random-p333401
Bn-A06-p6844127	1	1	Bn-N9-p13998118	Bn-chrAnn_random-p333404
Bn-A06-p6847261	0.980099502	0.980099502	Bn-N6-p6426590	Bn-chrA06-p6240766
Bn-A06-p6847367	0.900497512	0.900497512	Bn-N6-p6426696	Bn-chrA06-p6240872
Bn-A06-p6864079	0.989583333	0.989583333	Bn-N6-p6443334	Bn-chrA06-p6257508
Bn-A06-p6875144	0.985074627	0.985074627	Bn-N6-p6455339	Bn-chrA06-p6269414
Bn-A06-p6878792	0.973421927	0.973421927	Bn-N6-p6458990	Bn-chrA09-p30191561
Bn-A06-p6879017	0.933554817	0.933554817	Bn-N6-p6459215	Bn-chrA09-p30191786
Bn-A06-p6895399	0.983471074	0.983471074	Bn-N6-p6493669	Bn-chrA06-p6315010
Bn-A06-p6913483	0.983471074	0.983471074	Bn-N6-p6548826	Bn-chrA06-p6369656
Bn-A06-p6919765	0.983471074	0.983471074	Bn-N6-p6553172	Bn-chrA06-p6373996
Bn-A06-p6920925	0.990033223	0.990033223	Bn-N6-p6554332	Bn-chrA06-p6375156
Bn-A06-p6951503	0.983471074	0.983471074	Bn-N6-p6583392	Bn-chrA06-p6406290
Bn-A06-p6955437	0.950413223	0.950413223	Bn-N6-p6587433	Bn-chrA06-p6410167
Bn-A06-p6959460	0.983471074	0.983471074	Bn-N6-p6590676	Bn-chrA06_random-p567873
Bn-A06-p6961069	0.966942149	0.966942149	Bn-N6-p6592285	Bn-chrA06-p6416212
Bn-A06-p6966525	0.980099502	0.980099502	Bn-N6-p6597736	Bn-chrA06-p6421663
Bn-A06-p6970539	0.983471074	0.983471074	Bn-N6-p6601765	Bn-chrA06-p6425691
Bn-A06-p6971737	1	1	Bn-N6-p6602868	Bn-chrA06-p6426794
Bn-A06-p6971900	0.966942149	0.966942149	Bn-N6-p6603126	Bn-chrA06-p6427052
Bn-A06-p6972345	0.983471074	0.983471074	Bn-N6-p6603567	Bn-chrA06-p6427493
Bn-A06-p6980159	0.975206612	0.983471074	Bn-N6-p6615256	Bn-chrA06_random-p559953
Bn-A06-p6985717	0.983471074	0.975206612	Bn-N6-p6621007	Bn-chrA06-p6437251
Bn-A06-p6990496	0.966942149	0.933884298	Bn-N6-p6625781	Bn-chrA06-p6441989
Bn-A06-p6993862	0.975206612	0.966942149	Bn-N6-p6629147	Bn-chrC05-p8116501
Bn-A06-p6995492	0.991735537	0.983471074	Bn-N6-p6630777	Bn-chrA06-p6446982
Bn-A06-p7003598	0.991735537	0.983471074	Bn-N6-p6638576	Bn-chrA06-p6454133

Bn-A06-p7023598	0.958677686	0.958677686	Bn-N6-p6659814	Bn-chrA06-p6472474
Bn-A06-p7023804	0.991735537	0.983471074	Bn-N6-p6660020	Bn-chrA06-p6474875
Bn-A06-p7023818	0.991735537	0.983471074	Bn-N6-p6660034	Bn-chrA06-p6474889
Bn-A06-p7023924	0.983471074	0.975206612	Bn-N6-p6660140	Bn-chrA06-p6474995
Bn-A06-p7083415	0.958677686	0.958677686	Bn-N6-p6711796	Bn-chrA06-p6522967
Bn-A06-p7107840	0.983471074	0.983471074	Bn-N6-p6741338	Bn-chrA06-p6544811
Bn-A06-p7119339	0.975206612	0.975206612	Bn-N6-p6753013	Bn-chrA06-p6556222
Bn-A06-p7134425	0.996677741	0.996677741	Bn-N6-p6779221	Bn-chrA06-p6573492
Bn-A06-p7179419	0.966942149	0.966942149	Bn-N6-p6841031	Bn-chrA06-p6644098
Bn-A06-p7187455	0.936877076	0.936877076	Bn-N6-p6849245	Bn-chrA06-p6652312
Bn-A06-p7230364	0.983471074	0.983471074	Bn-N6-p6896883	Bn-chrA06-p6703622
Bn-A06-p7247810	0.995024876	0.995024876	Bn-N6-p6912944	Bn-chrA06-p6721359
Bn-A06-p7266200	0.990033223	0.990033223	Bn-N6-p6924945	Bn-chrA06-p6733929
Bn-A06-p7279909	0.975206612	0.975206612	Bn-N6-p6942697	Bn-chrA06-p6758391
Bn-A06-p7288881	0.991735537	0.991735537	Bn-N6-p6967901	Bn-chrA06-p6786023
Bn-A06-p7289388	0.966942149	0.966942149	Bn-N6-p6968408	Bn-chrA06-p6786530
Bn-A06-p7290511	0.867768595	0.867768595	Bn-N6-p6969531	Bn-chrA06-p6787653
Bn-A06-p7290893	0.975206612	0.975206612	Bn-N6-p6969852	Bn-chrA06-p6787974
Bn-A06-p7290973	0.809917355	0.809917355	Bn-N6-p6969910	Bn-chrA06-p6788032
Bn-A06-p7302651	0.991735537	0.991735537	Bn-N6-p6977846	Bn-chrA06-p6795965
Bn-A06-p7305466	0.990033223	0.990033223	Bn-N6-p6979779	Bn-chrA06-p6797898
Bn-A06-p7313589	0.975206612	0.975206612	Bn-N6-p6988346	Bn-chrA06-p6806634
Bn-A06-p7314403	0.975206612	0.975206612	Bn-N6-p6989144	Bn-chrA06-p6807432
Bn-A06-p7355866	0.975206612	0.925619835	Bn-N6-p7032204	Bn-chrA06-p6850036
Bn-A06-p7384367	0.958677686	0.958677686	Bn-N15-p9456872	Bn-chrC05-p8613741
Bn-A06-p7393806	0.867768595	0.867768595	Bn-N15-p9479646	Bn-chrC05-p8636295
Bn-A06-p7420466	1	0.991735537	Bn-N6-p7111117	Bn-chrA06-p6926189
Bn-A06-p7432516	1	0.996677741	Bn-N6-p7122777	Bn-chrA06-p6938254
Bn-A06-p7437586	0.975206612	0.966942149	Bn-N6-p7128658	Bn-chrA06-p6943317
Bn-A06-p7439750	0.966777409	0.980066445	Bn-N6-p7135317	Bn-chrA06-p6945484
Bn-A06-p7444483	0.995024876	1	Bn-N6-p7138783	Bn-chrA06-p6948942
Bn-A06-p7459428	0.966942149	0.975206612	Bn-N6-p7154097	Bn-chrA06-p6966982
Bn-A06-p7459843	0.965174129	0.990049751	Bn-Scaffold16442-p4	Bn-chrA06-p6967397
Bn-A06-p7460077	0.983471074	0.975206612	Bn-Scaffold16442-p6	Bn-chrA06-p6967631
Bn-A06-p7499751	0.966942149	0.966942149	Bn-N6-p7197412	Bn-chrA06-p7008147
Bn-A06-p7501594	0.983471074	0.983471074	Bn-N6-p7199257	Bn-chrA06-p7009992
Bn-A06-p7515700	0.990033223	0.96345515	Bn-N6-p7213551	Bn-chrA06-p7023993
Bn-A06-p7553160	0.985074627	0.985074627	Bn-N6-p7251134	Bn-chrA06-p7060298
Bn-A06-p7556709	0.900826446	0.900826446	Bn-N6-p7254556	Bn-chrA06-p7201340
Bn-A06-p7560098	1	1	Bn-N6-p7263744	Bn-chrA06-p7210528
Bn-A06-p7566996	0.983471074	0.983471074	Bn-N6-p7270639	Bn-chrA06-p7217420
Bn-A06-p7583628	1	1	Bn-N6-p7290539	Bn-chrA06_random-p569419
Bn-A06-p7584691	1	0.991735537	Bn-N6-p7291602	Bn-chrA06_random-p570482
Bn-A06-p7589883	0.84717608	0.84717608	Bn-N15-p9848110	Bn-chrA06-p7107396
Bn-A06-p7616430	0.983471074	0.94214876	Bn-N6-p7324192	Bn-chrA06-p7257713
Bn-A06-p7624783	0.975206612	0.983471074	Bn-N6-p7332959	Bn-chrA06-p7266286

Bn-A06-p7626450	0.983471074	0.975206612	Bn-N6-p7334625	Bn-chrA06-p7267954
Bn-A06-p7632651	0.990033223	0.983388704	Bn-N6-p7341481	Bn-chrA06-p7274383
Bn-A06-p7636623	0.983388704	0.983388704	Bn-N6-p7345346	Bn-chrA06-p7278299
Bn-A06-p7636729	0.983471074	0.983471074	Bn-N6-p7345452	Bn-chrA06-p7278405
Bn-A06-p7641474	1	0.991735537	Bn-N6-p7350279	Bn-chrA06-p7280606
Bn-A06-p7653199	0.990033223	0.993355482	Bn-N6-p7363686	Bn-chrA06-p7287236
Bn-A06-p7657281	0.975206612	0.966942149	Bn-N6-p7368033	Bn-chrA06-p7292882
Bn-A06-p7658535	0.983471074	0.975206612	Bn-N6-p7369287	Bn-chrA06-p7294136
Bn-A06-p7665759	0.991735537	0.983471074	Bn-N6-p7376583	Bn-chrA06-p7301443
Bn-A06-p7670615	0.996677741	0.986710963	Bn-N6-p7381857	Bn-chrA06-p7306246
Bn-A06-p7670696	1	0.991735537	Bn-N6-p7381938	Bn-chrA06-p7306328
Bn-A06-p7701069	0.983471074	0.983471074	Bn-N6-p7413359	Bn-chrA06-p7331462
Bn-A06-p7703242	0.975206612	0.975206612	Bn-N6-p7415532	Bn-chrA06-p7333635
Bn-A06-p7707699	0.966942149	0.966942149	Bn-N6-p7419989	Bn-chrA06-p7338092
Bn-A06-p7726350	0.950248756	0.945273632	Bn-N6-p7439399	Bn-chrA06-p7356713
Bn-A06-p7771695	0.980099502	0.985074627	Bn-N6-p7482543	Bn-chrA06-p7398902
Bn-A06-p7857624	0.611295681	0.607973422	Bn-N4-p10052791	Bn-chrAnn_random-p379297
Bn-A06-p7927622	0.991735537	0.991735537	Bn-N6-p7639086	Bn-chrA06-p7556059
Bn-A06-p7927767	0.983471074	0.983471074	Bn-N6-p7639231	Bn-chrA06-p7556204
Bn-A06-p7928380	0.736318408	0.736318408	Bn-N6-p7639845	Bn-chrA06-p7556818
Bn-A06-p7931177	0.991735537	0.991735537	Bn-N6-p7644220	Bn-chrA06-p7560549
Bn-A06-p7949147	0.975206612	0.975206612	Bn-N6-p7658347	Bn-chrA06-p7580680
Bn-A06-p7959476	0.986710963	0.990033223	Bn-N6-p7670105	Bn-chrA06-p7616736
Bn-A06-p7962905	0.975206612	0.975206612	Bn-N6-p7677089	Bn-chrA06-p7623719
Bn-A06-p7965038	0.636363636	0.628099174	Bn-N6-p7679228	Bn-chrAnn_random-p39673C
Bn-A06-p7965415	0.966942149	0.966942149	Bn-N6-p7680265	Bn-chrAnn_random-p39672C
Bn-A06-p7973826	0.983471074	0.983471074	Bn-N6-p7683289	Bn-chrA06-p7629589
Bn-A06-p7974986	0.842975207	0.842975207	Bn-N6-p7684039	Bn-chrA06-p7630339
Bn-A06-p7993829	0.975206612	0.975206612	Bn-N6-p7699586	Bn-chrA06-p7645382
Bn-A06-p8006619	0.991735537	0.991735537	Bn-N6-p7712142	Bn-chrA06-p7658143
Bn-A06-p8010125	0.94214876	0.94214876	Bn-N6-p7715656	Bn-chrA06-p7661656
Bn-A06-p8010345	0.94214876	0.94214876	Bn-N6-p7715881	Bn-chrA06-p7661881
Bn-A06-p8021875	0.818181818	0.818181818	Bn-N6-p7719603	Bn-chrA06-p7665601
Bn-A06-p8022058	0.94214876	0.94214876	Bn-N6-p7719785	Bn-chrA06-p7665783
Bn-A06-p8033701	0.983471074	0.983471074	Bn-N6-p7728912	Bn-chrA06-p7673937
Bn-A06-p8042439	0.991735537	1	Bn-N6-p7737931	Bn-chrA06-p7677436
Bn-A06-p8042901	0.960199005	0.955223881	Bn-N6-p7738385	Bn-chrA06-p7677890
Bn-A06-p8043214	0.983471074	0.975206612	Bn-N6-p7738698	Bn-chrA06-p7678203
Bn-A06-p8047893	0.958677686	0.851239669	Bn-N6-p7743472	Bn-chrA06-p7682880
Bn-A06-p8080390	0.970149254	0.980099502	Bn-N6-p7779052	Bn-chrA06-p7723585
Bn-A06-p8080666	0.983471074	0.991735537	Bn-N6-p7779328	Bn-chrA06-p7723861
Bn-A06-p8090520	0.983471074	0.983471074	Bn-N6-p7789205	Bn-chrCnn_random-p194027
Bn-A06-p8104956	0.884297521	0.892561983	Bn-N6-p7796860	Bn-chrA06-p7741343
Bn-A06-p8109713	0.983471074	0.983471074	Bn-N6-p7801530	Bn-chrA06-p7746045
Bn-A06-p8123266	0.975206612	0.975206612	Bn-N6-p7809365	Bn-chrUnn_random-p336101
Bn-A06-p8180116	0.983471074	0.652892562	Bn-N6-p7866297	Bn-chrUnn_random-p823145

Bn-A06-p8199618	0.884297521	0.884297521	Bn-N6-p7886694	Bn-chrA06-p7779141
Bn-A06-p8200169	0.900826446	0.900826446	Bn-N6-p7887234	Bn-chrA06-p7779681
Bn-A06-p8201495	0.985074627	0.935323383	Bn-N6-p7888552	Bn-chrA06-p7780998
Bn-A06-p8201581	0.958677686	0.842975207	Bn-N6-p7888638	Bn-chrA06-p7781073
Bn-A06-p8221017	0.996677741	0.813953488	Bn-N6-p7909080	Bn-chrC05-p9624873
Bn-A06-p8230834	0.975206612	0.859504132	Bn-N6-p7919164	Bn-chrA06-p7800746
Bn-A06-p8252024	0.940199336	0.943521595	Bn-N6-p7941550	Bn-chrA06-p7814281
Bn-A06-p8253130	0.940199336	0.943521595	Bn-N6-p7942394	Bn-chrA06-p7815155
Bn-A06-p8263325	0.983471074	0.983471074	Bn-N6-p7956675	Bn-chrA06-p7829462
Bn-A06-p8277116	0.94214876	0.94214876	Bn-N6-p7961563	Bn-chrA06-p7834350
Bn-A06-p8356542	0.991735537	0.991735537	Bn-N6-p8033717	Bn-chrA06-p7907761
Bn-A06-p8402978	0.966942149	0.966942149	Bn-N6-p8082983	Bn-chrA06-p7951624
Bn-A06-p8416265	0.975206612	0.975206612	Bn-N6-p8098276	Bn-chrA06-p7966871
Bn-A06-p8426071	0.995024876	0.990049751	Bn-N6-p8109487	Bn-chrA06-p7976724
Bn-A06-p8430642	0.996677741	0.996677741	Bn-N6-p8112214	Bn-chrA06-p7979457
Bn-A06-p8434088	0.973421927	0.976744186	Bn-N6-p8115655	Bn-chrA06-p7982903
Bn-A06-p8446816	0.983471074	0.983471074	Bn-N6-p8125820	Bn-chrA06-p7993144
Bn-A06-p8472457	0.975206612	0.975206612	Bn-N6-p8149405	Bn-chrA06-p8014470
Bn-A06-p8475485	0.983471074	0.94214876	Bn-N6-p8152434	Bn-chrA06-p8018682
Bn-A06-p8491315	0.983471074	0.867768595	Bn-N6-p8164575	Bn-chrC03_random-p636273
Bn-A06-p8527596	0.991735537	0.983471074	Bn-N6-p8188433	Bn-chrA06-p8046270
Bn-A06-p8536023	0.996677741	1	Bn-N6-p8197103	Bn-chrA06-p8054406
Bn-A06-p8539863	0.991735537	0.983471074	Bn-N6-p8200952	Bn-chrA06-p8058246
Bn-A06-p8545758	1	1	Bn-N14-p49714674	Bn-chrC04-p43596728
Bn-A06-p8546149	0.983471074	0.975206612	Bn-N6-p8206489	Bn-chrA06-p8063823
Bn-A06-p8546698	1	0.991735537	Bn-N6-p8207038	Bn-chrA06-p8064372
Bn-A06-p8573607	0.993355482	0.993355482	Bn-N6-p8250658	Bn-chrA06-p8101248
Bn-A06-p8581295	1	1	Bn-N6-p8258438	Bn-chrA06-p8109396
Bn-A06-p8593423	0.985074627	0.985074627	Bn-N6-p8270874	Bn-chrA06-p8121768
Bn-A06-p8600040	1	1	Bn-N6-p8277492	Bn-chrA06-p8128583
Bn-A06-p8601048	0.983471074	0.975206612	Bn-N6-p8278500	Bn-chrA06-p8129591
Bn-A06-p8601089	0.975206612	0.983471074	Bn-N6-p8278541	Bn-chrA06-p8129632
Bn-A06-p8614872	0.958677686	0.958677686	Bn-N6-p8293012	Bn-chrA06-p8146323
Bn-A06-p8615815	0.916943522	0.916943522	Bn-N6-p8293955	Bn-chrA06-p8147266
Bn-A06-p8633786	0.995024876	0.990049751	Bn-N6-p8316471	Bn-chrA06-p8174294
Bn-A06-p8634166	0.985074627	0.980099502	Bn-N6-p8316851	Bn-chrA06-p8174674
Bn-A06-p8634648	0.990049751	0.985074627	Bn-N6-p8317333	Bn-chrA06-p8175155
Bn-A06-p8643665	0.990049751	0.990049751	Bn-N14-p25064073	Bn-chrC04-p21517039
Bn-A06-p8697187	0.966942149	0.966942149	Bn-N6-p8381641	Bn-chrA06-p8229880
Bn-A06-p8697193	0.983471074	0.983471074	Bn-N6-p8381647	Bn-chrA06-p8229886
Bn-A06-p8732612	0.991735537	0.991735537	Bn-N6-p8395518	Bn-chrA06-p8244781
Bn-A06-p8766088	0.985074627	0.990049751	Bn-N6-p8429433	Bn-chrA06_random-p616007
Bn-A06-p8784719	0.996677741	0.993355482	Bn-N6-p8462651	Bn-chrA06-p8279942
Bn-A06-p8791868	0.985074627	0.985074627	Bn-N6-p8470186	Bn-chrA06-p8287091
Bn-A06-p8792429	0.995024876	0.995024876	Bn-N6-p8470747	Bn-chrA06-p8287652
Bn-A06-p8792843	0.960199005	0.960199005	Bn-N6-p8471173	Bn-chrA06-p8288066

Bn-A06-p8809314	0.958677686	0.966942149	Bn-N6-p8494827	Bn-chrA06-p8303303
Bn-A06-p8810900	0.956810631	0.956810631	Bn-N6-p8496421	Bn-chrA06-p8304902
Bn-A06-p8811363	0.966942149	0.966942149	Bn-N6-p8496984	Bn-chrA06-p8305642
Bn-A06-p8825744	0.94214876	0.512396694	Bn-Scaffold02087-p4	Bn-chrC03_random-p160603
Bn-A06-p8833793	0.900826446	0.950413223	Bn-N6-p8516429	Bn-chrA06-p8323343
Bn-A06-p8834741	0.966942149	0.983471074	Bn-N6-p8518553	Bn-chrA06-p8324290
Bn-A06-p8837251	0.983471074	0.975206612	Bn-N6-p8523533	Bn-chrA06-p8326866
Bn-A06-p8837290	0.993355482	0.996677741	Bn-N6-p8523572	Bn-chrA06-p8326905
Bn-A06-p8842980	1	1	Bn-N6-p8530929	Bn-chrA06-p8331824
Bn-A06-p8854250	1	0.996677741	Bn-N6-p8546668	Bn-chrA06-p8347382
Bn-A06-p8864234	0.996677741	0.996677741	Bn-N6-p8558555	Bn-chrA06-p8358352
Bn-A06-p8871997	0.993355482	0.993355482	Bn-N6-p8561878	Bn-chrA06-p8361675
Bn-A06-p8907559	0.950248756	0.985074627	Bn-N6-p8593617	Bn-chrA06-p8388737
Bn-A06-p8923160	0.910299003	0.976744186	Bn-N6-p8609051	Bn-chrA06-p8400591
Bn-A06-p8948420	0.983471074	0.983471074	Bn-N6-p8634880	Bn-chrA06-p8425312
Bn-A06-p8953809	0.991735537	0.991735537	Bn-N6-p8637465	Bn-chrA06-p8428209
Bn-A06-p8968750	0.993355482	0.993355482	Bn-N6-p8652804	Bn-chrA06-p8445225
Bn-A06-p8972533	0.983471074	0.983471074	Bn-N6-p8656510	Bn-chrA06-p8448931
Bn-A06-p8987272	0.958677686	0.94214876	Bn-N6-p8675473	Bn-chrA06-p8459892
Bn-A06-p8987370	0.991735537	0.975206612	Bn-N6-p8675571	Bn-chrA06-p8459987
Bn-A06-p8988728	1	0.991735537	Bn-N6-p8676929	Bn-chrA06-p8463642
Bn-A06-p8988945	0.983471074	0.991735537	Bn-N6-p8677146	Bn-chrA06-p8463860
Bn-A06-p8992364	0.975206612	0.975206612	Bn-N6-p8680322	Bn-chrA06-p8465994
Bn-A06-p8998572	0.983471074	0.975206612	Bn-N6-p8686530	Bn-chrA06-p8477192
Bn-A06-p9009861	0.975206612	0.975206612	Bn-N6-p8695592	Bn-chrA06-p8488097
Bn-A06-p9010952	0.966942149	0.966942149	Bn-N6-p8696705	Bn-chrA06-p8489210
Bn-A06-p9013844	0.966942149	0.966942149	Bn-N6-p8699590	Bn-chrA06-p8492095
Bn-A06-p9020967	0.966942149	0.966942149	Bn-Scaffold01201-p2	Bn-chrA06-p8505345
Bn-A06-p9032267	0.983471074	0.983471074	Bn-N6-p8711009	Bn-chrA06-p8514918
Bn-A06-p9064537	0.870431894	0.870431894	Bn-N6-p8722289	Bn-chrA06-p8526398
Bn-A06-p9065249	0.985074627	0.985074627	Bn-N6-p8723001	Bn-chrA06-p8527110
Bn-A06-p9074653	0.991735537	0.991735537	Bn-N6-p8739420	Bn-chrA06-p8543338
Bn-A06-p9076011	0.980099502	0.980099502	Bn-N6-p8740778	Bn-chrA06-p8544696
Bn-A06-p9087487	0.983388704	0.983388704	Bn-N6-p8752692	Bn-chrA06-p8560587
Bn-A06-p9090817	0.991735537	0.363636364	Bn-N6-p8757728	Unknown
Bn-A06-p9094415	0.991735537	0.991735537	Bn-N6-p8762631	Bn-chrA06-p8566443
Bn-A06-p9094772	0.975206612	0.975206612	Bn-N6-p8762986	Bn-chrA06-p8566798
Bn-A06-p9119114	0.950413223	0.950413223	Bn-N6-p8780803	Bn-chrA06-p8587387
Bn-A06-p9127880	0.958677686	0.958677686	Bn-N6-p8787316	Bn-chrA06-p8593900
Bn-A06-p9128417	0.940298507	0.940298507	Bn-N6-p8787853	Bn-chrA06-p8594437
Bn-A06-p9132494	0.975206612	0.975206612	Bn-N6-p8792817	Bn-chrA06-p8599413
Bn-A06-p9135772	0.980099502	0.980099502	Bn-N6-p8796109	Bn-chrA06-p8602704
Bn-A06-p9248676	0.985074627	0.985074627	Bn-N6-p8913420	Bn-chrA06-p8722911
Bn-A06-p9250298	1	1	Bn-N6-p8915041	Bn-chrA06-p8724532
Bn-A06-p9287101	0.995024876	0.995024876	Bn-N6-p8953040	Bn-chrA06-p8757743
Bn-A06-p9289355	0.983471074	0.983471074	Bn-N6-p8955235	Bn-chrA06-p8759938

Bn-A06-p9291582	0.985074627	0.985074627	Bn-N6-p8956427	Bn-chrA06-p8761130
Bn-A06-p9292257	0.996677741	0.996677741	Bn-N6-p8957102	Bn-chrA06-p8761805
Bn-A06-p9299877	0.983471074	0.983471074	Bn-N6-p8966729	Bn-chrA06-p8771432
Bn-A06-p9371495	0.983388704	0.986710963	Bn-N1-p23082579	Bn-chrA01-p19610932
Bn-A06-p9376280	0.966942149	0.966942149	Bn-N6-p9043641	Bn-chrA06-p8853680
Bn-A06-p9384998	0.986710963	0.986710963	Bn-N6-p9054061	Bn-chrA06-p8864143
Bn-A06-p9390167	0.980099502	0.980099502	Bn-N6-p9059228	Bn-chrA06-p8869310
Bn-A06-p9399854	0.96013289	0.96013289	Bn-N18-p28491976	Bn-chrC08-p23516356
Bn-A06-p9424836	0.958677686	0.958677686	Bn-N6-p9098578	Bn-chrA06-p8905365
Bn-A06-p9452981	1	1	Bn-N6-p9147293	Bn-chrA06-p8960534
Bn-A06-p9465709	0.958677686	0.958677686	Bn-N6-p9159585	Bn-chrA06-p8972198
Bn-A06-p9475900	0.958677686	0.958677686	Bn-N6-p9170777	Bn-chrA06-p8983390
Bn-A06-p9491200	0.903654485	0.887043189	Bn-N18-p8511544	Bn-chrC03_random-p528061
Bn-A06-p9495829	0.975206612	0.975206612	Bn-N6-p9190177	Bn-chrA06-p9003126
Bn-A06-p9517147	0.989583333	0.989583333	Bn-N6-p9200968	Bn-chrA06-p9013916
Bn-A06-p9562200	0.983471074	0.983471074	Bn-N6-p9265150	Bn-chrA06-p9087478
Bn-A06-p9593892	0.985074627	0.985074627	Bn-N6-p9286025	Bn-chrA06_random-p683726
Bn-A06-p9630478	0.970149254	0.970149254	Bn-N6-p9313558	Bn-chrC08-p23256907
Bn-A06-p9665402	1	1	Bn-N6-p9350124	Bn-chrA06_random-p712630
Bn-A06-p9684583	0.991735537	0.611570248	Bn-N6-p9366293	Bn-chrCnn_random-p494236
Bn-A06-p9721031	0.990049751	0.990049751	Bn-N6-p9399172	Bn-chrA06-p9165808
Bn-A06-p9722046	0.995024876	0.995024876	Bn-N6-p9400189	Bn-chrA06-p9166825
Bn-A06-p9753017	0.991735537	0.991735537	Bn-N6-p9436407	Bn-chrA06-p9202555
Bn-A06-p9936409	0.950413223	0.950413223	Bn-N6-p9595488	Bn-chrA03-p24336931
Bn-A06-p9940067	0.991735537	0.991735537	Bn-N6-p9599145	Bn-chrA03-p24333274
Bn-A06-p9957408	1	1	Bn-N6-p9619524	Bn-chrA06-p9361748
Bn-A06-p10005161	0.958677686	0.958677686	Bn-N6-p9678971	Bn-chrA06-p9418495
Bn-A06-p10018992	0.983471074	0.983471074	Bn-N6-p9692002	Bn-chrA06-p9431065
Bn-A06-p10019580	1	1	Bn-N6-p9692591	Bn-chrA06-p9431654
Bn-A06-p10021272	0.975206612	0.975206612	Bn-N6-p9694318	Bn-chrA06-p9433381
Bn-A06-p10021339	0.983471074	0.983471074	Bn-N6-p9694385	Bn-chrA06-p9433448
Bn-A06-p10021393	0.983471074	0.983471074	Bn-N6-p9694439	Bn-chrA06-p9433502
Bn-A06-p10022213	0.991735537	0.991735537	Bn-N6-p9695291	Bn-chrA06-p9434354
Bn-A06-p10022347	0.975206612	0.975206612	Bn-N6-p9695423	Bn-chrA06-p9434486
Bn-A06-p10022751	0.925619835	0.925619835	Bn-N6-p9695826	Bn-chrA06-p9434889
Bn-A06-p10023556	0.917355372	0.925619835	Bn-N6-p9696647	Bn-chrA06-p9435710
Bn-A06-p10031447	1	1	Bn-N6-p9709064	Bn-chrA06-p9448126
Bn-A06-p10031660	0.991735537	0.991735537	Bn-N6-p9709277	Bn-chrA06-p9448339
Bn-A06-p10032901	0.983471074	0.983471074	Bn-N6-p9710715	Bn-chrA06-p9449573
Bn-A06-p10033398	1	1	Bn-N6-p9711212	Bn-chrA06-p9450070
Bn-A06-p10033718	0.991735537	0.991735537	Bn-N6-p9711532	Bn-chrA06-p9450390
Bn-A06-p10033903	1	1	Bn-N6-p9711717	Bn-chrA06-p9450575
Bn-A06-p10040506	0.983471074	0.983471074	Bn-N6-p9717775	Bn-chrA06-p9456633
Bn-A06-p10046575	0.990033223	0.990033223	Bn-N6-p9723846	Bn-chrA06-p9462703
Bn-A06-p10046691	0.990033223	0.990033223	Bn-N6-p9723962	Bn-chrA06-p9462819
Bn-A06-p10053057	0.958677686	0.958677686	Bn-N6-p9730325	Bn-chrA06-p9469299

Bn-A06-p10053801	0.990049751	0.990049751	Bn-N6-p9731068	Bn-chrA06-p9470042
Bn-A06-p10064955	0.966942149	0.966942149	Bn-N6-p9743287	Bn-chrA06-p9482949
Bn-A06-p10068703	0.958677686	0.958677686	Bn-N6-p9747331	Bn-chrA06-p9486993
Bn-A06-p10069150	0.991735537	0.991735537	Bn-N6-p9747779	Bn-chrA06-p9487441
Bn-A06-p10085385	0.983471074	0.983471074	Bn-N6-p9768805	Bn-chrA06-p9506200
Bn-A06-p10085689	0.965174129	0.706467662	Bn-N6-p9769110	Bn-chrA06-p9506505
Bn-A06-p10086667	0.993355482	0.993355482	Bn-N6-p9770089	Bn-chrA06-p9507484
Bn-A06-p10124135	0.975206612	0.975206612	Bn-N6-p9805692	Bn-chrAnn_random-p301613
Bn-A06-p10129250	0.975206612	0.975206612	Bn-N6-p9813845	Bn-chrA06-p9547457
Bn-A06-p10135140	0.980066445	0.980066445	Bn-N6-p9819739	Bn-chrA06-p9553351
Bn-A06-p10146008	0.970149254	0.970149254	Bn-N6-p9832606	Bn-chrA06-p9567484
Bn-A06-p10169231	0.966942149	0.966942149	Bn-N13-p51701167	Bn-chrC03_random-p463953
Bn-A06-p10182944	1	1	Bn-N6-p9866941	Bn-chrA06-p9595710
Bn-A06-p10200920	0.94214876	0.94214876	Bn-N6-p9870439	Bn-chrA06-p9599208
Bn-A06-p10202557	0.991735537	0.991735537	Bn-N6-p9871396	Bn-chrA06-p9600165
Bn-A06-p10210368	0.94214876	0.94214876	Bn-N6-p9884626	Bn-chrA06-p9613395
Bn-A06-p10238741	0.985074627	0.985074627	Bn-N6-p9919737	Bn-chrA06-p9639372
Bn-A06-p10238985	0.933884298	0.933884298	Bn-N6-p9919979	Bn-chrA06-p9639614
Bn-A06-p10240329	0.991735537	0.991735537	Bn-N6-p9921350	Bn-chrA06-p9640985
Bn-A06-p10251899	0.993355482	0.993355482	Bn-N6-p9932924	Bn-chrA06-p9652425
Bn-A06-p10296909	0.925619835	0.859504132	Bn-N6-p9961244	Bn-chrA06-p9686010
Bn-A06-p10297153	0.892561983	0.892561983	Bn-N6-p9961478	Bn-chrA06-p9686244
Bn-A06-p10300664	0.925619835	0.958677686	Bn-N6-p9971045	Bn-chrA06-p9694143
Bn-A06-p10308910	0.991735537	0.983471074	Bn-N6-p9981241	Bn-chrA06-p9702407
Bn-A06-p10320803	0.958677686	0.958677686	Bn-N6-p9990009	Bn-chrA06-p9711073
Bn-A06-p10327909	0.983471074	0.975206612	Bn-N6-p9998566	Bn-chrA06-p9720037
Bn-A06-p10330066	0.991735537	0.991735537	Bn-N6-p10000718	Bn-chrA06-p9722194
Bn-A06-p10349427	0.975206612	0.983471074	Bn-N6-p10020752	Bn-chrA06-p9741112
Bn-A06-p10351604	0.991735537	0.983471074	Bn-N6-p10022930	Bn-chrA06-p9743291
Bn-A06-p10377145	0.925619835	0.991735537	Bn-N12-p16602530	Bn-chrA09-p15348726
Bn-A06-p10391346	0.94214876	0.94214876	Bn-N6-p10059495	Bn-chrC03-p46402583
Bn-A06-p10400171	0.986710963	0.986710963	Bn-N6-p10066592	Bn-chrA06-p9782419
Bn-A06-p10401376	0.976744186	0.976744186	Bn-N13-p51376456	Bn-chrC03-p46376720
Bn-A06-p10403901	0.96345515	0.986710963	Bn-N6-p10070451	Bn-chrA06-p9786119
Bn-A06-p10416354	0.996677741	0.996677741	Bn-N6-p10085348	Bn-chrA06-p9796937
Bn-A06-p10442596	0.983471074	0.983471074	Bn-N6-p10113233	Bn-chrA06-p9824209
Bn-A06-p10443399	0.966942149	0.966942149	Bn-N6-p10114036	Bn-chrA06-p9825012
Bn-A06-p10452758	0.991735537	0.991735537	Bn-N6-p10123573	Bn-chrA06-p9834017
Bn-A06-p10453182	0.983471074	0.983471074	Bn-N6-p10123997	Bn-chrA06-p9834441
Bn-A06-p10457871	0.958677686	0.958677686	Bn-N6-p10128686	Bn-chrA06-p9839130
Bn-A06-p10470779	0.909090909	0.917355372	Bn-N6-p10146132	Bn-chrA06-p9856468
Bn-A06-p10480404	0.975206612	0.975206612	Bn-N6-p10160456	Bn-chrA06-p9868552
Bn-A06-p10499003	0.970099668	0.970099668	Bn-N6-p10184709	Bn-chrA06_random-p665813
Bn-A06-p10574739	1	0.995024876	Bn-N6-p10259845	Bn-chrA06-p9872542
Bn-A06-p10577973	0.990033223	0.990033223	Bn-N6-p10263532	Bn-chrA06-p9875774
Bn-A06-p10578969	0.991735537	0.983471074	Bn-N6-p10264860	Bn-chrA06-p9876770

Bn-A06-p10579615	0.975206612	0.966942149	Bn-N6-p10265506	Bn-chrA06-p9877416
Bn-A06-p10588412	0.950413223	0.958677686	Bn-N6-p10277025	Bn-chrA06-p9887300
Bn-A06-p10594170	1	0.991735537	Bn-N6-p10282763	Bn-chrA06-p9893058
Bn-A06-p10606807	0.975206612	0.958677686	Bn-N6-p10295588	Bn-chrA06-p9900892
Bn-A06-p10609574	0.975124378	0.980099502	Bn-N6-p10298354	Bn-chrA06-p9902591
Bn-A06-p10609641	0.993355482	0.993355482	Bn-N6-p10298421	Bn-chrA06-p9902658
Bn-A06-p10642761	0.983471074	0.991735537	Bn-N6-p10326476	Bn-chrA06-p9933101
Bn-A06-p10781335	0.975206612	0.983471074	Bn-N6-p10470960	Bn-chrA06-p10040143
Bn-A06-p10795156	1	0.995024876	Bn-N6-p10486461	Bn-chrA06-p10054080
Bn-A06-p10818634	0.991735537	0.983471074	Bn-N6-p10510443	Bn-chrA06-p10080154
Bn-A06-p10832056	0.966942149	0.958677686	Bn-N6-p10519745	Bn-chrA06-p10090715
Bn-A06-p10833219	0.975206612	0.983471074	Bn-N6-p10521827	Bn-chrA06-p10091906
Bn-A06-p10836353	0.975206612	0.966942149	Bn-N6-p10525052	Bn-chrAnn_random-p337422
Bn-A06-p10854312	0.975206612	0.975206612	Bn-N6-p10536173	Bn-chrA06-p10102961
Bn-A06-p10859915	0.985074627	0.985074627	Bn-N6-p10543512	Bn-chrA06-p10108522
Bn-A06-p10863673	0.958677686	0.958677686	Bn-N6-p10547446	Bn-chrA06-p10112456
Bn-A06-p10873048	0.991735537	0.991735537	Bn-N6-p10558225	Bn-chrA06-p10119176
Bn-A06-p11021085	0.983471074	0.991735537	Bn-N6-p10683454	Bn-chrA06-p10228344
Bn-A06-p11024576	0.975206612	0.983471074	Bn-N6-p10687225	Bn-chrA06-p10231803
Bn-A06-p11025660	0.980099502	0.980099502	Bn-Scaffold48758-p1	Bn-chrA06-p10232905
Bn-A06-p11165324	0.991735537	0.925619835	Bn-N6-p10867777	Bn-chrA06-p10353936
Bn-A06-p11165375	0.975206612	0.909090909	Bn-N6-p10867828	Bn-chrA06-p10353980
Bn-A06-p11231528	0.933884298	0.917355372	Bn-N6-p10942944	Bn-chrA06-p10410186
Bn-A06-p11239689	0.991735537	0.983471074	Bn-N6-p10949414	Bn-chrA06-p10450560
Bn-A06-p11291712	0.975206612	0.975206612	Bn-N6-p11010799	Bn-chrA06-p10492064
Bn-A06-p11297663	0.983471074	0.975206612	Bn-N6-p11012548	Bn-chrA06-p10493889
Bn-A06-p11301532	0.958677686	0.975206612	Bn-N6-p11016435	Bn-chrA06-p10497831
Bn-A06-p11343875	1	0.991735537	Bn-N6-p11059893	Bn-chrA06-p10536702
Bn-A06-p11344945	0.983471074	0.991735537	Bn-N6-p11060957	Bn-chrA06-p10537734
Bn-A06-p11356047	0.975206612	0.966942149	Bn-N6-p11079778	Bn-chrA06-p10558288
Bn-A06-p11356414	0.966942149	0.958677686	Bn-N6-p11080145	Bn-chrA06-p10558655
Bn-A06-p11356524	0.966942149	0.966942149	Bn-N6-p11080255	Bn-chrA06-p10558764
Bn-A06-p11362290	0.975206612	0.983471074	Bn-N6-p11088528	Bn-chrA06-p10561676
Bn-A06-p11366920	0.930348259	0.930348259	Bn-N4-p3912684	Bn-chrA04-p3664048
Bn-A06-p11368090	0.154228856	0.154228856	Bn-N15-p11244670	Unknown
Bn-A06-p11380334	0.975124378	0.975124378	Bn-N13-p32571682	Bn-chrA06-p10577920
Bn-A06-p11380775	0.970149254	0.990049751	Bn-N13-p32571241	Bn-chrA09-p5148322
Bn-A06-p11381026	0.970149254	0.990049751	Bn-N13-p32570990	Bn-chrA06-p10578612
Bn-A06-p11415584	0.975206612	0.975206612	Bn-N6-p11123038	Bn-chrA06-p10612942
Bn-A06-p11432316	0.983471074	0.991735537	Bn-N9-p20745327	Bn-chrAnn_random-p28489C
Bn-A06-p11455914	1	1	Bn-N6-p11161601	Bn-chrA06-p10650276
Bn-A06-p11500765	0.996677741	0.996677741	Bn-N6-p11224509	Bn-chrA06_random-p880064
Bn-A06-p11588495	0.989583333	0.989583333	Bn-N6-p11286605	Bn-chrA06-p10744573
Bn-A06-p11704401	0.986710963	0.990033223	Bn-N6-p14526584	Bn-chrA06_random-p127250
Bn-A06-p11708819	0.993355482	0.996677741	Bn-N6-p14522882	Bn-chrA06_random-p126872
Bn-A06-p11776560	0.991735537	0.991735537	Bn-N6-p14450010	Bn-chrA06-p14744125

Bn-A06-p11808857	0.990033223	0.990033223	Bn-N6-p14460472	Bn-chrC08-p35923721
Bn-A06-p11819912	0.985074627	0.990049751	Bn-N6-p14395105	Bn-chrA06-p14695054
Bn-A06-p11819954	0.995024876	0.995024876	Bn-N6-p14390646	Bn-chrA06-p14695012
Bn-A06-p11840908	0.876033058	0.545454545	Bn-N6-p14372265	Bn-chrA06-p14662465
Bn-A06-p11848553	0.966942149	0.983471074	Bn-N6-p14360239	Bn-chrA06-p14657393
Bn-A06-p11848767	0.980099502	0.980099502	Bn-N6-p14360023	Bn-chrA06-p14657179
Bn-A06-p11848770	0.980066445	0.996677741	Bn-N6-p14360023	Bn-chrA06-p14657179
Bn-A06-p11919396	0.991735537	0.991735537	Bn-N6-p14278810	Bn-chrA06-p14582212
Bn-A06-p11995250	0.970099668	0.980066445	Bn-N8-p17436616	Bn-chrA08-p15148409
Bn-A06-p12030020	0.979166667	0.979166667	Bn-N6-p14153152	Bn-chrA06_random-p128989
Bn-A06-p12078648	0.106312292	0.209302326	Bn-N14-p16012148	Bn-chrA03_random-p252704
Bn-A06-p12093192	0.958677686	0.958677686	Bn-N6-p14081442	Bn-chrA06-p11438884
Bn-A06-p12135104	0.975124378	0.975124378	Bn-N6-p14037351	Bn-chrA06-p11395489
Bn-A06-p12142438	0.991735537	0.991735537	Bn-N6-p14030066	Bn-chrA06_random-p720160
Bn-A06-p12202203	0.983471074	0.983471074	Bn-N6-p13954665	Bn-chrA06-p11314435
Bn-A06-p12203381	0.975206612	0.975206612	Bn-N6-p13953516	Bn-chrA06-p11313178
Bn-A06-p12205865	1	1	Bn-N6-p13951409	Bn-chrA06-p11311071
Bn-A06-p12213032	0.975206612	0.975206612	Bn-N6-p13946753	Bn-chrA06-p11306413
Bn-A06-p12337022	0.983471074	0.983471074	Bn-N6-p13820546	Bn-chrA06-p11179342
Bn-A06-p12337596	0.991735537	0.991735537	Bn-N6-p13819978	Bn-chrA06-p11178774
Bn-A06-p12337598	0.991735537	0.991735537	Bn-N6-p13819976	Bn-chrA06-p11178772
Bn-A06-p12346760	0.975206612	0.975206612	Bn-N6-p13801918	Bn-chrA06-p11161362
Bn-A06-p12348915	0.975206612	0.975206612	Bn-N6-p13799443	Bn-chrA06-p11158887
Bn-A06-p12354309	0.991735537	0.991735537	Bn-N6-p13795175	Bn-chrA06-p11154619
Bn-A06-p12354524	0.983471074	0.983471074	Bn-N6-p13794960	Bn-chrA06-p11154404
Bn-A06-p12354582	0.983471074	0.983471074	Bn-N6-p13794902	Bn-chrA06-p11154346
Bn-A06-p12355438	0.991735537	0.991735537	Bn-N6-p13794046	Bn-chrA06-p11153490
Bn-A06-p12355762	1	1	Bn-N6-p13793722	Bn-chrA06-p11153166
Bn-A06-p12356277	0.983471074	0.983471074	Bn-N6-p13793207	Bn-chrA06-p11152651
Bn-A06-p12356525	0.975206612	0.975206612	Bn-N6-p13792958	Bn-chrA06-p11152402
Bn-A06-p12356708	0.966942149	0.966942149	Bn-N6-p13792769	Bn-chrA06-p11152213
Bn-A06-p12357497	0.983471074	0.983471074	Bn-N6-p13791979	Bn-chrA06-p11151423
Bn-A06-p12358748	0.991735537	0.991735537	Bn-N6-p13790727	Bn-chrA06-p11150171
Bn-A06-p12362853	0.991735537	0.991735537	Bn-N6-p13787987	Bn-chrA06-p11147431
Bn-A06-p12363034	0.983471074	0.983471074	Bn-N6-p13787806	Bn-chrA06-p11147250
Bn-A06-p12364597	0.991735537	0.760330579	Bn-N6-p13770149	Bn-chrA03-p20545581
Bn-A06-p12364605	0.983471074	0.694214876	Bn-N6-p13770141	Bn-chrA03-p20545573
Bn-A06-p12376674	0.983471074	0.983471074	Bn-N6-p13750275	Bn-chrA06-p14481072
Bn-A06-p12402009	0.991735537	0.991735537	Bn-N6-p13726662	Bn-chrA06-p14457389
Bn-A06-p12404563	0.983471074	0.983471074	Bn-N6-p13724107	Bn-chrA06-p14454834
Bn-A06-p12404570	0.991735537	0.991735537	Bn-N6-p13724100	Bn-chrA06-p14454827
Bn-A06-p12404848	0.983471074	0.983471074	Bn-N6-p13723822	Bn-chrA06-p14454549
Bn-A06-p12436092	0.983471074	0.983471074	Bn-N6-p13696629	Bn-chrA06-p14426548
Bn-A06-p12438791	0.995024876	0.995024876	Bn-N6-p13693924	Bn-chrA06-p14423843
Bn-A06-p12441450	0.975206612	0.983471074	Bn-N6-p13690342	Bn-chrA06-p14421131
Bn-A06-p12451649	0.917355372	0.917355372	Bn-N6-p13681372	Bn-chrA06-p14411990

Bn-A06-p12464905	0.983471074	0.983471074	Bn-N6-p13658396	Bn-chrA06-p14377276
Bn-A06-p12517420	0.991735537	0.991735537	Bn-N6-p13619986	Bn-chrA06-p14339735
Bn-A06-p12534357	0.975206612	0.975206612	Bn-N13-p44084395	Bn-chrC03-p38560697
Bn-A06-p12609046	0.966942149	0.966942149	Bn-N6-p13549964	Bn-chrA06-p14264655
Bn-A06-p12627660	0.991735537	0.991735537	Bn-N6-p13530361	Bn-chrA06-p14246267
Bn-A06-p12632207	0.993355482	0.993355482	Bn-N6-p13525814	Bn-chrA06-p14241720
Bn-A06-p12634012	0.958677686	0.958677686	Bn-N6-p13524009	Bn-chrA06-p14239915
Bn-A06-p12634097	0.983471074	0.983471074	Bn-N6-p13523924	Bn-chrA06-p14239830
Bn-A06-p12641317	0.991735537	0.991735537	Bn-N6-p13519070	Bn-chrA06-p14234976
Bn-A06-p12641905	1	1	Bn-N6-p13518482	Bn-chrA06-p14234388
Bn-A06-p12645785	0.983471074	0.983471074	Bn-N6-p13515974	Bn-chrA06-p14231880
Bn-A06-p12658161	0.79338843	0.79338843	Bn-N6-p13509846	Bn-chrA06-p14221541
Bn-A06-p12696157	0.966942149	0.966942149	Bn-N6-p13492041	Bn-chrA06-p14201197
Bn-A06-p12718659	0.975206612	0.975206612	Bn-N6-p13476531	Bn-chrA06-p14185392
Bn-A06-p12732487	0.975206612	0.975206612	Bn-N6-p13462104	Bn-chrA06-p14170966
Bn-A06-p12734894	0.975206612	0.975206612	Bn-N6-p13459708	Bn-chrA06-p14168570
Bn-A06-p12748821	0.991735537	0.991735537	Bn-N6-p13444498	Bn-chrA06-p14152172
Bn-A06-p12768136	0.991735537	0.991735537	Bn-N6-p13420007	Bn-chrA06-p14126760
Bn-A06-p12775306	0.991735537	0.991735537	Bn-N6-p13415497	Bn-chrA06-p14122251
Bn-A06-p12775616	0.983471074	0.983471074	Bn-N6-p13415187	Bn-chrA06-p14121941
Bn-A06-p12775665	0.983471074	0.983471074	Bn-N6-p13415138	Bn-chrA06-p14121892
Bn-A06-p12775802	0.991735537	0.991735537	Bn-N6-p13415001	Bn-chrA06-p14121755
Bn-A06-p12824031	0.970149254	0.970149254	Bn-Scaffold45955-p2	Bn-chrA10-p3061470
Bn-A06-p12843916	0.966942149	0.966942149	Bn-N6-p13370147	Bn-chrA06-p14069064
Bn-A06-p12846574	1	1	Bn-N6-p13368208	Bn-chrA06-p14067125
Bn-A06-p12868864	0.975124378	0.975124378	Bn-N6-p13343802	Bn-chrA06_random-p848894
Bn-A06-p12876501	0.991735537	0.991735537	Bn-N6-p13310143	Bn-chrA06_random-p806316
Bn-A06-p12880298	0.958677686	0.958677686	Bn-N6-p13306303	Bn-chrA06_random-p810156
Bn-A06-p12884492	1	1	Bn-N6-p13298060	Bn-chrA06_random-p818399
Bn-A06-p12884843	0.96013289	0.96013289	Bn-N6-p13297699	Bn-chrA06_random-p818760
Bn-A06-p12886443	1	1	Bn-N6-p13296075	Bn-chrA06_random-p820384
Bn-A06-p12886553	0.933884298	0.933884298	Bn-N6-p13295965	Bn-chrA06_random-p820494
Bn-A06-p12896905	0.958677686	0.958677686	Bn-N6-p13289964	Bn-chrA06_random-p826709
Bn-A06-p12911577	0.966942149	0.966942149	Bn-N6-p13259225	Bn-chrA06-p14026462
Bn-A06-p12980841	0.892561983	0.983471074	Bn-N12-p25932727	Bn-chrAnn_random-p15333C
Bn-A06-p12986192	0.983471074	0.983471074	Bn-N6-p13194796	Bn-chrA06-p13959074
Bn-A06-p12995133	0.966942149	0.966942149	Bn-N6-p13171366	Bn-chrA06-p13935296
Bn-A06-p13007607	0.975206612	0.975206612	Bn-N6-p13158023	Bn-chrA06-p13922222
Bn-A06-p13008545	0.983471074	0.983471074	Bn-N6-p13157075	Bn-chrA06-p13921274
Bn-A06-p13009409	0.966942149	0.966942149	Bn-N6-p13156204	Bn-chrA06-p13920403
Bn-A06-p13021244	0.983471074	0.983471074	Bn-N6-p13145152	Bn-chrA06-p13909388
Bn-A06-p13105867	0.983471074	0.983471074	Bn-N6-p13122934	Bn-chrA06-p13888187
Bn-A06-p13112819	0.966942149	0.966942149	Bn-N6-p13119725	Bn-chrA06-p13884978
Bn-A06-p13126839	0.950413223	0.950413223	Bn-N6-p13098483	Bn-chrA06-p13863642
Bn-A06-p13144922	0.966942149	0.966942149	Bn-N6-p13073203	Bn-chrA06-p12505912
Bn-A06-p13147955	0.966942149	0.966942149	Bn-N6-p13069775	Bn-chrA06-p12502767

Bn-A06-p13159551	1	1	Bn-N6-p13066677	Bn-chrA06-p12499669
Bn-A06-p13162770	0.966942149	0.966942149	Bn-N6-p13062333	Bn-chrA06-p12496133
Bn-A06-p13190352	0.991735537	0.991735537	Bn-N6-p13048689	Bn-chrA06-p12482489
Bn-A06-p13194586	0.966942149	0.966942149	Bn-N6-p13041309	Bn-chrA06-p12475191
Bn-A06-p13204772	0.983471074	0.983471074	Bn-N6-p13030458	Bn-chrA06-p12465201
Bn-A06-p13230875	1	1	Bn-N6-p13017792	Bn-chrA06-p12452368
Bn-A06-p13230989	0.991735537	0.991735537	Bn-N6-p13017678	Bn-chrA06-p12452254
Bn-A06-p13583056	0.990049751	0.990049751	Bn-N19-p39782829	Bn-chrC09-p34721978
Bn-A06-p13610210	0.985074627	0.980099502	Bn-N6-p12653871	Bn-chrA06-p12105933
Bn-A06-p13726921	0.96013289	0.96013289	Bn-N13-p47843647	Bn-chrC03-p42941358
Bn-A06-p13728321	0.983471074	0.983471074	Bn-N6-p11754905	Bn-chrA06_random-p120051
Bn-A06-p13732668	0.958677686	0.958677686	Bn-N6-p11750499	Bn-chrA06_random-p120492
Bn-A06-p13787740	0.975206612	0.975206612	Bn-N6-p11687808	Bn-chrA06_random-p120909
Bn-A06-p13825200	0.983471074	0.983471074	Bn-N6-p11628025	Bn-chrA06-p11130277
Bn-A06-p13850939	1	1	Bn-N6-p11598615	Bn-chrA06-p11099822
Bn-A06-p13859891	1	1	Bn-N6-p11588034	Bn-chrA06-p11088752
Bn-A06-p13870364	0.986710963	0.986710963	Bn-N6-p11575111	Bn-chrA06-p11077570
Bn-A06-p13872191	0.993355482	0.993355482	Bn-N6-p11573284	Bn-chrA06-p11075743
Bn-A06-p13910548	0.983471074	0.983471074	Bn-N6-p11552740	Bn-chrA06-p11054817
Bn-A06-p13913895	0.966942149	0.966942149	Bn-N6-p11549389	Bn-chrA06-p11051466
Bn-A06-p13958812	1	1	Bn-N6-p11506638	Bn-chrA06-p10999717
Bn-A06-p14121814	0.958677686	0.958677686	Bn-N6-p15462382	Bn-chrC04-p1330363
Bn-A06-p14163715	0.537190083	0.537190083	Bn-N6-p15501716	Bn-chrA06_random-p140977
Bn-A06-p14179943	0.991735537	0.991735537	Bn-N6-p15516387	Bn-chrA06-p15776074
Bn-A06-p14189236	0.720930233	0.720930233	Bn-N6-p15533259	Bn-chrA06-p15790553
Bn-A06-p14192915	0.955223881	0.955223881	Bn-N6-p15536945	Bn-chrA06-p15794239
Bn-A06-p14193381	0.980099502	0.985074627	Bn-N6-p15537409	Bn-chrA06-p15794703
Bn-A06-p14198240	0.873754153	0.873754153	Bn-N6-p15542913	Bn-chrA06-p15800207
Bn-A06-p14207547	0.970149254	0.970149254	Bn-N6-p15550976	Bn-chrA06-p15807772
Bn-A06-p14212186	0.94214876	0.94214876	Bn-N6-p15551621	Bn-chrA06-p15808417
Bn-A06-p14214930	0.950413223	0.950413223	Bn-N6-p15554070	Bn-chrA06-p15810866
Bn-A06-p14232702	0.198347107	0.198347107	Bn-Scaffold00776-p6	Bn-chrC04-p28560634
Bn-A06-p14232703	0.198347107	0.198347107	Bn-Scaffold00776-p6	Bn-chrC04-p28560633
Bn-A06-p14241294	0.950413223	0.950413223	Bn-N6-p15573174	Bn-chrA06-p15828654
Bn-A06-p14266102	0.993355482	0.993355482	Bn-N6-p15591087	Bn-chrA06-p15851914
Bn-A06-p14269763	0.892561983	0.892561983	Bn-N19-p5165096	Bn-chrCnn_random-p148277
Bn-A06-p14281185	1	1	Bn-N6-p15606489	Bn-chrA06_random-p137494
Bn-A06-p14285203	0.983471074	0.983471074	Bn-N6-p15610450	Bn-chrA06_random-p137098
Bn-A06-p14300543	0.983471074	0.94214876	Bn-N6-p15623085	Bn-chrC03-p35254362
Bn-A06-p14300642	0.983471074	0.867768595	Bn-N6-p15623184	Bn-chrC03-p35254263
Bn-A06-p14300919	1	0.867768595	Bn-N6-p15623461	Bn-chrC03-p35253896
Bn-A06-p14316307	0.975206612	0.975206612	Bn-N6-p15643666	Bn-chrAnn_random-p255007
Bn-A06-p14327501	0.983471074	0.983471074	Bn-N6-p15660129	Bn-chrAnn_random-p306836
Bn-A06-p14369166	0.976744186	0.976744186	Bn-N6-p15713533	Bn-chrA06-p15933144
Bn-A06-p14373935	0.980099502	0.980099502	Bn-N6-p15718977	Bn-chrA06-p15938605
Bn-A06-p14375425	0.975206612	0.975206612	Bn-N6-p15720508	Bn-chrA06-p15940136

Bn-A06-p14375498	0.958677686	0.958677686	Bn-N6-p15720581	Bn-chrA06-p15940209
Bn-A06-p14389679	0.983471074	0.983471074	Bn-N6-p15729660	Bn-chrA06-p15949286
Bn-A06-p14391905	0.958677686	0.958677686	Bn-N6-p15731885	Bn-chrA06-p15951511
Bn-A06-p14399524	0.933884298	0.933884298	Bn-N6-p15741968	Bn-chrA06-p15959346
Bn-A06-p14399853	0.809917355	0.809917355	Bn-N6-p15742296	Bn-chrA06-p15959674
Bn-A06-p14402043	0.933884298	0.884297521	Bn-N6-p15750171	Bn-chrA06-p15968447
Bn-A06-p14417437	1	1	Bn-N6-p15771426	Bn-chrA06-p15989191
Bn-A06-p14428186	0.985074627	0.985074627	Bn-N6-p15782391	Bn-chrA06-p15999936
Bn-A06-p14428293	0.983388704	0.983388704	Bn-N6-p15782498	Bn-chrA06-p16000043
Bn-A06-p14439178	0.975206612	0.975206612	Bn-N6-p15797519	Bn-chrA06-p16011929
Bn-A06-p14439400	0.823920266	0.823920266	Bn-N6-p15797741	Bn-chrA06-p16012151
Bn-A06-p14441081	0.930232558	0.92358804	Bn-N13-p38763344	Bn-chrA06-p16013839
Bn-A06-p14457099	0.970099668	0.970099668	Bn-N6-p15814345	Bn-chrA06-p16027694
Bn-A06-p14469354	0.870431894	0.764119601	Bn-N13-p38743146	Bn-chrC03-p35051168
Bn-A06-p14474461	0.996677741	0.996677741	Bn-N6-p15827597	Bn-chrA06-p16038171
Bn-A06-p14495889	0.892561983	0.892561983	Bn-N6-p15852302	Bn-chrA06-p16059292
Bn-A06-p14510328	1	1	Bn-N6-p15868370	Bn-chrA06-p16081713
Bn-A06-p14527757	0.983471074	0.983471074	Bn-N6-p15881475	Bn-chrA06-p16093850
Bn-A06-p14546946	0.976744186	0.976744186	Bn-N6-p15897783	Bn-chrA06-p16108297
Bn-A06-p14576278	0.92358804	0.92358804	Bn-N6-p15919859	Bn-chrA06-p16129555
Bn-A06-p14578177	0.960199005	0.950248756	Bn-N6-p15921749	Bn-chrA06-p16131443
Bn-A06-p14578303	0.983471074	0.983471074	Bn-N6-p15921875	Bn-chrA06-p16131569
Bn-A06-p14588321	0.950413223	0.950413223	Bn-Scaffold26939-p3	Bn-chrA06-p16148089
Bn-A06-p14594965	0.983388704	0.983388704	Bn-N6-p16544357	Bn-chrA06-p16154047
Bn-A06-p14595126	0.983388704	0.983388704	Bn-N6-p16544518	Bn-chrA06-p16154208
Bn-A06-p14616522	0.975206612	0.975206612	Bn-N6-p16565866	Bn-chrA06-p16175555
Bn-A06-p14621640	0.990033223	0.990033223	Bn-N2-p11813392	Bn-chrUnn_random-p71669
Bn-A06-p14664213	1	1	Bn-N6-p16607904	Bn-chrA06-p16219580
Bn-A06-p14676716	1	1	Bn-N6-p16620412	Bn-chrA06-p16232089
Bn-A06-p14690714	0.965174129	0.965174129	Bn-N1-p12037962	Bn-chrA03_random-p452309
Bn-A06-p14737621	0.983471074	0.983471074	Bn-N6-p16667871	Bn-chrA06-p16284450
Bn-A06-p14773179	0.995024876	0.995024876	Bn-N6-p16698540	Bn-chrAnn_random-p161396
Bn-A06-p14809503	1	1	Bn-N6-p16742491	Bn-chrA06-p16294442
Bn-A06-p14813811	0.958677686	0.958677686	Bn-N6-p16746780	Bn-chrA06-p16298731
Bn-A06-p14816143	0.991735537	0.991735537	Bn-N6-p16749183	Bn-chrA06-p16301134
Bn-A06-p14819465	0.983471074	0.983471074	Bn-N6-p16754346	Bn-chrA06-p16306297
Bn-A06-p14829925	0.975206612	0.975206612	Bn-N6-p16757684	Bn-chrA06-p16309635
Bn-A06-p14834424	0.966942149	0.966942149	Bn-N6-p16760737	Bn-chrA06-p16312688
Bn-A06-p14836066	0.983471074	0.983471074	Bn-N6-p16762388	Bn-chrA06-p16314339
Bn-A06-p14836486	0.935323383	0.935323383	Bn-N6-p16762802	Bn-chrA06-p16314753
Bn-A06-p14843109	0.986710963	0.986710963	Bn-N6-p16771370	Bn-chrA06-p16323463
Bn-A06-p14848662	0.991735537	0.991735537	Bn-N6-p16776339	Bn-chrA06-p16328432
Bn-A06-p14850300	0.990049751	0.990049751	Bn-N6-p16778601	Bn-chrA06-p16330765
Bn-A06-p14852091	0.983471074	0.983471074	Bn-N6-p16780381	Bn-chrA06-p16332545
Bn-A06-p14852157	1	1	Bn-N6-p16780447	Bn-chrA06-p16332611
Bn-A06-p14864165	0.966942149	0.966942149	Bn-N6-p16792178	Bn-chrA06-p16344345

Bn-A06-p14878721	0.983471074	0.983471074	Bn-N6-p16806241	Bn-chrA06-p16357783
Bn-A06-p14880259	0.990033223	0.990033223	Bn-N6-p16807775	Bn-chrA06-p16359317
Bn-A06-p14880848	0.965174129	0.965174129	Bn-N6-p16808362	Bn-chrA06-p16359904
Bn-A06-p15006796	0.976744186	0.976744186	Bn-N6-p16935622	Bn-chrA06-p16477378
Bn-A06-p15013369	0.993355482	0.993355482	Bn-N6-p16941792	Bn-chrA06-p16483299
Bn-A06-p15037349	0.991735537	0.983471074	Bn-N6-p16959442	Bn-chrA06-p16498857
Bn-A06-p15059166	0.990033223	0.986710963	Bn-N6-p16980240	Bn-chrA06-p16517888
Bn-A06-p15065078	0.926910299	0.641196013	Bn-N6-p16987915	Bn-chrA06-p16524145
Bn-A06-p15101156	0.983471074	0.975206612	Bn-N6-p17044210	Bn-chrA06-p16576658
Bn-A06-p15112901	0.975124378	0.975124378	Bn-N15-p29562657	Bn-chrC05-p26162305
Bn-A06-p15210596	0.983471074	0.991735537	Bn-N6-p17142669	Bn-chrA06-p16683528
Bn-A06-p15258692	0.991735537	0.983471074	Bn-N6-p17186112	Bn-chrA06-p16731521
Bn-A06-p15262271	0.985663082	0.989247312	Bn-N6-p17189675	Bn-chrA06-p16735058
Bn-A06-p15285770	0.983471074	0.983471074	Bn-N6-p17211209	Bn-chrA06-p16759125
Bn-A06-p15294160	0.884297521	0.884297521	Bn-N6-p17215538	Bn-chrA06-p16763454
Bn-A06-p15339222	0.940298507	0.940298507	Bn-N6-p17253322	Bn-chrA06-p16801220
Bn-A06-p15358902	0.950248756	0.950248756	Bn-N6-p17278626	Bn-chrA06-p16826592
Bn-A06-p15360859	0.94214876	0.94214876	Bn-N6-p17280647	Bn-chrCnn_random-p435506
Bn-A06-p15375170	0.975206612	0.975206612	Bn-N6-p17300409	Bn-chrA06_random-p142934
Bn-A06-p15375395	0.976744186	0.976744186	Bn-N6-p17300634	Bn-chrA06_random-p142956
Bn-A06-p15406244	0.966942149	0.966942149	Bn-N6-p17328641	Bn-chrA06-p16876113
Bn-A06-p15415078	0.983471074	0.983471074	Bn-N6-p17337429	Bn-chrA06-p16884829
Bn-A06-p15420581	0.983471074	0.975206612	Bn-N6-p17342922	Bn-chrA06-p16890366
Bn-A06-p15421020	0.983471074	0.975206612	Bn-N6-p17343361	Bn-chrA06-p16890805
Bn-A06-p15432171	0.958677686	0.685950413	Bn-N6-p17365262	Bn-chrC03-p33727642
Bn-A06-p15434445	0.966942149	0.975206612	Bn-N6-p17369634	Bn-chrA06-p16905017
Bn-A06-p15470965	0.983471074	0.983471074	Bn-N6-p17399688	Bn-chrAnn_random-p27342[illegible]
Bn-A06-p15489475	0.966942149	0.966942149	Bn-N6-p17417175	Bn-chrA06-p16999161
Bn-A06-p15513288	0.991735537	0.991735537	Bn-N6-p17437593	Bn-chrA06-p17018079
Bn-A06-p15515345	0.975206612	0.975206612	Bn-N6-p17439650	Bn-chrA06-p17020136
Bn-A06-p15581849	0.958677686	0.966942149	Bn-N6-p17504631	Bn-chrA06-p17083863
Bn-A06-p15582577	0.980099502	0.980099502	Bn-N6-p17505359	Bn-chrA06-p17084591
Bn-A06-p15582617	0.990049751	0.985074627	Bn-N6-p17505398	Bn-chrA06-p17084630
Bn-A06-p15584456	0.910447761	0.905472637	Bn-N6-p17507238	Bn-chrA06-p17086470
Bn-A06-p15632434	0.935323383	0.985074627	Bn-N6-p17553824	Bn-chrA06-p17132232
Bn-A06-p15649917	0.950413223	0.950413223	Bn-N6-p17574012	Bn-chrA06-p17149711
Bn-A06-p15661345	0.991735537	0.991735537	Bn-N6-p17585697	Bn-chrA06-p17161088
Bn-A06-p15662331	0.983471074	0.983471074	Bn-N6-p17590888	Bn-chrA06-p17166456
Bn-A06-p15691701	0.983471074	0.41322314	Bn-N6-p17626600	Unknown
Bn-A06-p15700525	0.966942149	0.966942149	Bn-N6-p17628829	Bn-chrA06-p17206540
Bn-A06-p15701094	0.966942149	0.966942149	Bn-N6-p17629388	Bn-chrA06-p17207099
Bn-A06-p15703535	0.79338843	0.79338843	Bn-N6-p17630548	Bn-chrA06-p17208259
Bn-A06-p15709579	0.976744186	0.986710963	Bn-N6-p17639162	Bn-chrA06-p17214357
Bn-A06-p15712062	0.993355482	0.990033223	Bn-N6-p17641645	Bn-chrA06-p17216840
Bn-A06-p15743629	0.995024876	0.965174129	Bn-N6-p17673218	Bn-chrA06-p17252531
Bn-A06-p15748291	0.991735537	0.983471074	Bn-N6-p17677887	Bn-chrA06-p17257201

Bn-A06-p15749428	0.966942149	0.966942149	Bn-N6-p17679011	Bn-chrA06-p17258349
Bn-A06-p15760165	0.983471074	0.983471074	Bn-N6-p17692316	Bn-chrA06-p17270047
Bn-A06-p15775625	0.975124378	0.975124378	Bn-N6-p17707009	Bn-chrA06-p17281815
Bn-A06-p15778397	0.983388704	0.983388704	Bn-N6-p17709786	Bn-chrA06-p17284591
Bn-A06-p15791244	0.975206612	0.975206612	Bn-N6-p17724325	Bn-chrA06-p17298251
Bn-A06-p15801373	0.991735537	0.991735537	Bn-N6-p17733531	Bn-chrA06-p17307456
Bn-A06-p15893014	0.983471074	1	Bn-N6-p17826634	Bn-chrA06-p17351478
Bn-A06-p15893221	0.970149254	0.965174129	Bn-N6-p17830879	Bn-chrA06-p17351685
Bn-A06-p15894458	0.958677686	0.983471074	Bn-N13-p36535736	Bn-chrA06-p17352928
Bn-A06-p15894910	0.983471074	1	Bn-N6-p17832537	Bn-chrA06-p17353389
Bn-A06-p15913910	0.990033223	0.993355482	Bn-N6-p17852130	Bn-chrA06-p17373537
Bn-A06-p15921988	0.950413223	0.958677686	Bn-N6-p17858049	Bn-chrA06-p17382425
Bn-A06-p15922683	0.933884298	0.950413223	Bn-N6-p17858737	Bn-chrA06-p17383112
Bn-A06-p15966650	0.983471074	0.983471074	Bn-N6-p17910735	Bn-chrA06-p17448326
Bn-A06-p15967529	0.983471074	0.983471074	Bn-N6-p17911611	Bn-chrA06-p17449202
Bn-A06-p15967682	1	1	Bn-N6-p17911764	Bn-chrA06-p17449355
Bn-A06-p15988623	0.975206612	0.975206612	Bn-N6-p17929879	Bn-chrA06-p17467371
Bn-A06-p15990313	0.950413223	0.950413223	Bn-N6-p17931561	Bn-chrA06-p17469053
Bn-A06-p16000523	0.859504132	0.958677686	Bn-N12-p29752787	Bn-chrAnn_random-p35827[illegible]
Bn-A06-p16009894	0.975124378	0.975124378	Bn-N6-p17949689	Bn-chrA06-p17486947
Bn-A06-p16013571	0.94214876	0.94214876	Bn-N6-p17959283	Bn-chrA06-p17496394
Bn-A06-p16013796	0.983471074	0.983471074	Bn-N6-p17959508	Bn-chrA06-p17496619
Bn-A06-p16014870	0.966942149	0.966942149	Bn-N6-p17960475	Bn-chrA06-p17497586
Bn-A06-p16014941	0.991735537	0.991735537	Bn-N6-p17960546	Bn-chrA06-p17497657
Bn-A06-p16015026	0.983471074	0.983471074	Bn-N6-p17960631	Bn-chrA06-p17497742
Bn-A06-p16015727	0.94214876	0.94214876	Bn-N6-p17961288	Bn-chrA06-p17498399
Bn-A06-p16015795	0.966942149	0.966942149	Bn-N6-p17961356	Bn-chrA06-p17498467
Bn-A06-p16016634	0.958677686	0.958677686	Bn-N6-p17962193	Bn-chrA06-p17499304
Bn-A06-p16025514	0.995024876	0.995024876	Bn-N6-p17973737	Bn-chrA06-p17511854
Bn-A06-p16030994	1	1	Bn-N6-p17979443	Bn-chrA06-p17518549
Bn-A06-p16032028	0.991735537	0.991735537	Bn-N6-p17980477	Bn-chrA06-p17519583
Bn-A06-p16042593	0.983471074	0.983471074	Bn-N6-p17994366	Bn-chrA06-p17533782
Bn-A06-p16064239	0.991735537	0.991735537	Bn-N6-p18007169	Bn-chrA06-p17547128
Bn-A06-p16071214	0.826446281	0.826446281	Bn-N6-p18017612	Bn-chrA06-p17559623
Bn-A06-p16102726	0.917355372	0.917355372	Bn-N6-p18042370	Bn-chrA06-p17583891
Bn-A06-p16106230	0.990033223	0.990033223	Bn-N6-p18045903	Bn-chrA06-p17587424
Bn-A06-p16131586	0.983471074	0.983471074	Bn-N6-p18071287	Bn-chrA06-p17618616
Bn-A06-p16133328	0.991735537	0.991735537	Bn-N6-p18073029	Bn-chrA06-p17620358
Bn-A06-p16134293	0.983471074	0.983471074	Bn-N6-p18073994	Bn-chrA06-p17621323
Bn-A06-p16148736	1	0.995024876	Bn-N6-p18088613	Bn-chrA06-p17636579
Bn-A06-p16149093	1	1	Bn-N6-p18088970	Bn-chrA06-p17636936
Bn-A06-p16150365	1	0.991735537	Bn-N6-p18090242	Bn-chrA06-p17638208
Bn-A06-p16150919	0.985074627	0.980099502	Bn-N6-p18090796	Bn-chrA06-p17638762
Bn-A06-p16166965	0.991735537	0.983471074	Bn-N6-p18111085	Bn-chrA06-p17654359
Bn-A06-p16168931	0.975206612	0.983471074	Bn-N6-p18113051	Bn-chrA06-p17656299
Bn-A06-p16169045	0.983471074	0.975206612	Bn-N6-p18113165	Bn-chrA06-p17656413

Bn-A06-p16169367	0.991735537	1	Bn-N6-p18113487	Bn-chrA06-p17656735
Bn-A06-p16169669	0.966942149	0.975206612	Bn-N6-p18113789	Bn-chrA06-p17657036
Bn-A06-p16170755	0.950413223	0.809917355	Bn-N6-p18115158	Bn-chrA06-p17663087
Bn-A06-p16170813	0.94214876	0.917355372	Bn-N6-p18115216	Bn-chrA06-p17663141
Bn-A06-p16173797	1	0.991735537	Bn-N6-p18117998	Bn-chrA06-p17665216
Bn-A06-p16181242	0.966942149	0.975206612	Bn-N6-p18152112	Bn-chrA06-p17699638
Bn-A06-p16188512	1	0.991735537	Bn-N6-p18159452	Bn-chrA06-p17706976
Bn-A06-p16237929	0.892561983	0.892561983	Bn-N6-p18203493	Bn-chrA06-p17753565
Bn-A06-p16256906	0.983471074	0.975206612	Bn-N6-p18220641	Bn-chrA06-p17780123
Bn-A06-p16262957	0.454545455	0.975206612	Bn-N13-p35942732	Bn-chrA06-p17790502
Bn-A06-p16283909	0.966942149	0.958677686	Bn-N6-p18222666	Bn-chrA06-p17810366
Bn-A06-p16284324	0.958677686	0.966942149	Bn-N6-p18223081	Bn-chrA06-p17810783
Bn-A06-p16284610	0.975206612	0.966942149	Bn-N6-p18223367	Bn-chrA06-p17811069
Bn-A06-p16303805	0.983471074	0.975206612	Bn-N6-p18248307	Bn-chrA06-p17840965
Bn-A06-p16303860	0.975206612	0.975206612	Bn-N6-p18248362	Bn-chrA06-p17841020
Bn-A06-p16308544	0.958677686	0.966942149	Bn-N6-p18253330	Bn-chrA06-p17843786
Bn-A06-p16314064	0.990049751	0.990049751	Bn-N17-p36190094	Bn-chrC07_random-p201750
Bn-A06-p16325882	0.958677686	0.958677686	Bn-N6-p18259899	Bn-chrA06-p17847826
Bn-A06-p16325934	0.975206612	0.892561983	Bn-N6-p18259951	Bn-chrA06-p17847877
Bn-A06-p16330377	0.958677686	0.958677686	Bn-N6-p18269786	Bn-chrA06-p17852150
Bn-A06-p16342269	0.94214876	0.876033058	Bn-N6-p18285012	Bn-chrCnn_random-p719785
Bn-A06-p16362555	0.991735537	0.991735537	Bn-N6-p18320935	Bn-chrA06-p17866196
Bn-A06-p16381299	0.880398671	0.880398671	Bn-N6-p18340438	Bn-chrA06-p17886136
Bn-A06-p16386782	1	1	Bn-N6-p18353094	Bn-chrA06-p17897940
Bn-A06-p16386992	0.958677686	0.958677686	Bn-N6-p18353304	Bn-chrA06-p17898150
Bn-A06-p16406113	0.966942149	0.950413223	Bn-N6-p18374409	Bn-chrA06-p17919298
Bn-A06-p16449642	0.966942149	0.966942149	Bn-N6-p18419519	Bn-chrA06-p17965727
Bn-A06-p16460575	0.970149254	0.985074627	Bn-N6-p18429911	Bn-chrA06-p17976072
Bn-A06-p16461885	0.966942149	0.966942149	Bn-N6-p18431224	Bn-chrA06-p17977383
Bn-A06-p16553412	0.990049751	0.990049751	Bn-N6-p18512157	Bn-chrA06-p18056357
Bn-A06-p16553640	0.995024876	0.995024876	Bn-N6-p18512385	Bn-chrA06-p18056585
Bn-A06-p16554297	0.980099502	0.980099502	Bn-N6-p18513041	Bn-chrA06-p18057241
Bn-A06-p16588120	0.983471074	0.991735537	Bn-N6-p18549378	Bn-chrA06-p18090767
Bn-A06-p16588154	0.991735537	0.983471074	Bn-N6-p18549412	Bn-chrA06-p18090801
Bn-A06-p16613538	0.950413223	0.966942149	Bn-N6-p18575447	Bn-chrA06-p18106719
Bn-A06-p16620858	0.956810631	0.980066445	Bn-N6-p18582061	Bn-chrA06-p18115230
Bn-A06-p16620984	0.980066445	0.993355482	Bn-N6-p18582187	Bn-chrA06-p18115356
Bn-A06-p16621639	0.950413223	0.958677686	Bn-N6-p18582839	Bn-chrA06-p18116017
Bn-A06-p16624574	0.986710963	0.990033223	Bn-N6-p18585322	Bn-chrA06-p18118493
Bn-A06-p16629547	0.975206612	0.983471074	Bn-N6-p18590300	Bn-chrA06-p18123548
Bn-A06-p16645035	0.975206612	0.983471074	Bn-N6-p18613204	Bn-chrA06-p18138282
Bn-A06-p16645140	0.991735537	0.983471074	Bn-N6-p18613309	Bn-chrA06-p18138387
Bn-A06-p16656745	0.975206612	0.966942149	Bn-N6-p18623606	Bn-chrA06-p18150056
Bn-A06-p16657894	0.983471074	0.991735537	Bn-N6-p18624752	Bn-chrA06-p18151210
Bn-A06-p16660065	0.983388704	0.996677741	Bn-N6-p18626916	Bn-chrA06-p18153392
Bn-A06-p16678748	0.991735537	0.991735537	Bn-N6-p18642751	Bn-chrA06-p18166436

Bn-A06-p16682224	0.991735537	0.991735537	Bn-N6-p18646246	Bn-chrA06-p18169931
Bn-A06-p16689717	1	1	Bn-N6-p18653034	Bn-chrA06-p18180088
Bn-A06-p16701773	0.950413223	0.950413223	Bn-N6-p18661738	Bn-chrA06-p18188789
Bn-A06-p16702471	0.993355482	0.993355482	Bn-N6-p18662433	Bn-chrA06-p18189484
Bn-A06-p16703929	0.996677741	1	Bn-N6-p18663885	Bn-chrA06-p18190936
Bn-A06-p16718752	0.975124378	0.975124378	Bn-N6-p18681628	Bn-chrA06-p18208680
Bn-A06-p16728885	0.983471074	0.983471074	Bn-N6-p18685741	Bn-chrA06-p18211834
Bn-A06-p16829292	1	0.995024876	Bn-N6-p18705501	Bn-chrA06-p18231612
Bn-A06-p16840167	0.983471074	0.983471074	Bn-N6-p18716297	Bn-chrA06-p18243074
Bn-A06-p16847178	0.990049751	0.990049751	Bn-N6-p18718987	Bn-chrA06-p18245583
Bn-A06-p16847229	0.980099502	0.980099502	Bn-N6-p18719038	Bn-chrA06-p18245634
Bn-A06-p16875263	0.805970149	0.791044776	Bn-N19-p35134017	Bn-chrA06-p18283396
Bn-A06-p16933880	0.958677686	0.975206612	Bn-N6-p18987149	Bn-chrA06-p18516367
Bn-A06-p16985788	0.996677741	1	Bn-N6-p18861927	Bn-chrA06-p18399507
Bn-A06-p16992951	0.940298507	0.945273632	Bn-N6-p18873563	Bn-chrA06-p18406838
Bn-A06-p16993923	0.993355482	0.990033223	Bn-N6-p18874537	Bn-chrA06-p18407810
Bn-A06-p16994210	0.990033223	0.96013289	Bn-N6-p18874824	Bn-chrA06-p18408097
Bn-A06-p17003685	0.943521595	0.986710963	Bn-N6-p18884318	Bn-chrA06-p18421683
Bn-A06-p17006799	0.991735537	1	Bn-N6-p18887432	Bn-chrA06-p18424796
Bn-A06-p17007775	1	0.991735537	Bn-N6-p18888408	Bn-chrA06-p18425772
Bn-A06-p17010360	0.96013289	0.96013289	Bn-N6-p18891026	Bn-chrA06-p18428358
Bn-A06-p17011382	0.990033223	0.993355482	Bn-N6-p18892039	Bn-chrA06-p18429380
Bn-A06-p17021956	0.990033223	0.980066445	Bn-N6-p18901434	Bn-chrA06-p18438952
Bn-A06-p17023559	0.950166113	0.996677741	Bn-N17-p37802158	Bn-chrA06-p18440570
Bn-A06-p17024179	0.983471074	0.975206612	Bn-N6-p18903462	Bn-chrA06-p18441190
Bn-A06-p17026294	0.933554817	0.976744186	Bn-N6-p18905561	Bn-chrA06-p18443305
Bn-A06-p17026843	0.96013289	0.990033223	Bn-N6-p18906110	Bn-chrA06-p18443854
Bn-A06-p17032583	0.976744186	0.996677741	Bn-N6-p18911850	Bn-chrA06-p18449594
Bn-A06-p17033313	0.991735537	0.983471074	Bn-N6-p18912491	Bn-chrA06-p18450324
Bn-A06-p17037739	0.965174129	0.970149254	Bn-N6-p18917068	Bn-chrA06-p18454361
Bn-A06-p17037912	0.995024876	0.990049751	Bn-N6-p18917241	Bn-chrA06-p18454534
Bn-A06-p17042021	0.958677686	0.991735537	Bn-N6-p18922849	Bn-chrA06-p18458651
Bn-A06-p17042110	0.983471074	0.975206612	Bn-N6-p18922938	Bn-chrA06-p18458740
Bn-A06-p17049401	0.983388704	0.986710963	Bn-N6-p18929906	Bn-chrA06-p18465289
Bn-A06-p17058018	0.975206612	0.983471074	Bn-N6-p18939441	Bn-chrA06_random-p148983
Bn-A06-p17058537	0.983471074	0.991735537	Bn-N6-p18939960	Bn-chrA06_random-p149035
Bn-A06-p17059130	1	0.991735537	Bn-N6-p18940553	Bn-chrA06_random-p149094
Bn-A06-p17059461	0.975206612	0.983471074	Bn-N6-p18940884	Bn-chrA06_random-p149127
Bn-A06-p17065808	0.966942149	0.975206612	Bn-N6-p18947610	Bn-chrA06_random-p148401
Bn-A06-p17093738	0.970099668	0.993355482	Bn-N6-p18969189	Bn-chrA06-p18500290
Bn-A06-p17097734	0.980099502	0.985074627	Bn-N6-p18974642	Bn-chrA06-p18504396
Bn-A06-p17099451	1	0.966942149	Bn-N6-p18976375	Bn-chrA06-p18505961
Bn-A06-p17109583	0.980066445	0.973421927	Bn-N6-p18988963	Bn-chrA06-p18518174
Bn-A06-p17112322	1	0.965174129	Bn-N6-p18991987	Bn-chrA06-p18520037
Bn-A06-p17114458	0.983471074	0.975206612	Bn-N6-p18994122	Bn-chrA06-p18522401
Bn-A06-p17143376	0.966942149	0.239669421	Bn-N6-p19011860	Bn-chrA06-p18539210

Bn-A06-p17156091	0.975206612	0.983471074	Bn-N6-p19024988	Bn-chrA06-p18545405
Bn-A06-p17173552	0.973421927	0.996677741	Bn-N6-p19058882	Bn-chrA06-p18573073
Bn-A06-p17175388	0.916943522	0.930232558	Bn-Scaffold08133-p7	Bn-chrA06-p18574908
Bn-A06-p17176086	0.993355482	0.990033223	Bn-N6-p19061389	Bn-chrA06-p18575606
Bn-A06-p17190074	1	0.991735537	Bn-N6-p19076023	Bn-chrA06-p18590206
Bn-A06-p17208027	0.975206612	0.966942149	Bn-N6-p19092598	Bn-chrA06-p18609914
Bn-A06-p17220153	0.760797342	0.84717608	Bn-N6-p19105195	Bn-chrA06-p18624032
Bn-A06-p17226648	0.975206612	0.975206612	Bn-N6-p19111370	Bn-chrAnn_random-p350114
Bn-A06-p17242102	0.154228856	0.621890547	Bn-N17-p6672556	Bn-chrA06-p18636519
Bn-A06-p17242631	0.149253731	0.805970149	Bn-N3-p2167500	Bn-chrA06-p18636410
Bn-A06-p17260160	0.991735537	1	Bn-N6-p19126196	Bn-chrA06-p18652637
Bn-A06-p17260573	0.983471074	0.991735537	Bn-N6-p19126609	Bn-chrA06-p18653050
Bn-A06-p17262265	0.628099174	0.983471074	Bn-N6-p19132013	Bn-chrA06-p18654742
Bn-A06-p17267787	0.966942149	0.983471074	Bn-N6-p19135261	Bn-chrA06-p18660265
Bn-A06-p17289947	0.975206612	0.966942149	Bn-N6-p19153944	Bn-chrA06-p18680225
Bn-A06-p17291868	1	0.991735537	Bn-N6-p19157485	Bn-chrA06-p18682144
Bn-A06-p17301456	0.975206612	0.983471074	Bn-N6-p19167067	Bn-chrA06-p18691732
Bn-A06-p17313345	0.966942149	0.966942149	Bn-N6-p19178905	Bn-chrA06-p18702434
Bn-A06-p17321987	0.983471074	0.975206612	Bn-N6-p19187566	Bn-chrA06-p18711069
Bn-A06-p17331074	0.950413223	0.58677686	Bn-N6-p19196368	Bn-chrC07-p34422943
Bn-A06-p17332795	0.983471074	0.991735537	Bn-N6-p19198077	Bn-chrA06-p18722606
Bn-A06-p17337842	0.966942149	0.975206612	Bn-N6-p19203092	Bn-chrA06-p18727649
Bn-A06-p17346654	0.966942149	0.983471074	Bn-N6-p19213648	Bn-chrA06-p18739559
Bn-A06-p17375407	1	1	Bn-N6-p19241908	Bn-chrA06-p18768242
Bn-A06-p17375882	0.986710963	0.986710963	Bn-N6-p19242383	Bn-chrA06-p18768717
Bn-A06-p17411706	0.989583333	0.989583333	Bn-N6-p19286818	Bn-chrA06-p18806223
Bn-A06-p17415359	0.970149254	0.970149254	Bn-N6-p19290034	Bn-chrA06-p18809439
Bn-A06-p17430491	0.985074627	0.985074627	Bn-N6-p19300389	Bn-chrA06-p18823783
Bn-A06-p17452087	0.973421927	0.973421927	Bn-N6-p19318924	Bn-chrA06-p18845492
Bn-A06-p17455962	0.966942149	0.933884298	Bn-N6-p19324220	Bn-chrC07-p34304209
Bn-A06-p17548258	0.995024876	0.995024876	Bn-N6-p19419365	Bn-chrA06-p18943930
Bn-A06-p17548894	0.985074627	0.985074627	Bn-N6-p19420003	Bn-chrA06-p18944568
Bn-A06-p17549347	0.975206612	0.975206612	Bn-N6-p19420456	Bn-chrA06-p18945021
Bn-A06-p17549603	0.991735537	0.991735537	Bn-N6-p19420713	Bn-chrA06-p18945278
Bn-A06-p17566259	0.990049751	0.990049751	Bn-N6-p19436445	Bn-chrA06-p18956668
Bn-A06-p17659127	0.975206612	0.966942149	Bn-N6-p19553577	Bn-chrA06-p19093507
Bn-A06-p17671185	0.991735537	0.983471074	Bn-N6-p19565919	Bn-chrA06-p19108570
Bn-A06-p17681092	0.975124378	0.975124378	Bn-Scaffold19869-p4	Bn-chrA08_random-p900042
Bn-A06-p17684443	0.993355482	0.993355482	Bn-Scaffold08939-p2	Bn-chrA06_random-p150308
Bn-A06-p17689034	0.966942149	0.966942149	Bn-N6-p19583050	Bn-chrA06_random-p150635
Bn-A06-p17689415	0.983471074	0.983471074	Bn-N6-p19583431	Bn-chrA06_random-p150866
Bn-A06-p17697154	0.991735537	0.991735537	Bn-N6-p19591170	Bn-chrA06_random-p151639
Bn-A06-p17699971	0.945273632	0.950248756	Bn-N6-p19594610	Bn-chrA06_random-p151922
Bn-A06-p17710201	0.975206612	0.975206612	Bn-N6-p19604831	Bn-chrA06_random-p152999
Bn-A06-p17721678	1	0.991735537	Bn-N6-p19616601	Bn-chrA06-p19159906
Bn-A06-p17729064	1	0.985074627	Bn-N6-p19623986	Bn-chrA06-p19167280

Bn-A06-p17734402	0.975206612	0.983471074	Bn-N6-p19630296	Bn-chrA06-p19172603
Bn-A06-p17734904	0.917355372	0.925619835	Bn-N6-p19630798	Bn-chrA06-p19173105
Bn-A06-p17737199	1	1	Bn-N6-p19633141	Bn-chrA06-p19175400
Bn-A06-p17741868	0.970149254	0.970149254	Bn-N6-p19641317	Bn-chrA06-p19179091
Bn-A06-p17741952	0.970149254	0.970149254	Bn-N6-p19641401	Bn-chrA06-p19179175
Bn-A06-p17744170	0.996677741	0.996677741	Bn-N6-p19643619	Bn-chrA06-p19181393
Bn-A06-p17755205	0.985074627	0.985074627	Bn-N6-p19654389	Bn-chrA06-p19192095
Bn-A06-p17778079	0.990049751	0.990049751	Bn-N6-p19677313	Bn-chrA06-p19214981
Bn-A06-p17778210	1	1	Bn-N6-p19677443	Bn-chrA06-p19215111
Bn-A06-p17785331	0.983471074	0.983471074	Bn-N6-p19684269	Bn-chrA06-p19221937
Bn-A06-p17790290	1	1	Bn-N6-p19689270	Bn-chrA06-p19227047
Bn-A06-p17791496	0.996677741	0.996677741	Bn-N6-p19690501	Bn-chrA06-p19228278
Bn-A06-p17791746	0.976744186	0.980066445	Bn-N6-p19690751	Bn-chrA06-p19228528
Bn-A06-p17823115	0.990033223	0.990033223	Bn-N6-p19706174	Bn-chrA06-p19243070
Bn-A06-p17831661	0.975206612	0.975206612	Bn-N6-p19714495	Bn-chrA06-p19251191
Bn-A06-p17863217	0.990049751	0.990049751	Bn-N17-p522431	Bn-chrAnn_random-p444922
Bn-A06-p17887060	0.966942149	0.966942149	Bn-N6-p19760321	Bn-chrA06-p19302566
Bn-A06-p17888567	0.943521595	0.943521595	Bn-N6-p19761829	Bn-chrA06-p19304074
Bn-A06-p17889311	0.985074627	0.985074627	Bn-N6-p19762621	Bn-chrA06-p19304866
Bn-A06-p17908957	0.975206612	0.975206612	Bn-N6-p19783532	Bn-chrA06_random-p154679
Bn-A06-p17920968	0.975124378	0.975124378	Bn-N6-p19795122	Bn-chrA06-p19340367
Bn-A06-p17929532	0.983471074	0.975206612	Bn-N6-p19802207	Bn-chrA06-p19350021
Bn-A06-p17932959	0.983471074	0.975206612	Bn-N6-p19805639	Bn-chrA06-p19353450
Bn-A06-p17938497	0.843853821	0.853820598	Bn-N6-p19809927	Bn-chrA06-p19357734
Bn-A06-p17939632	0.917355372	0.917355372	Bn-N6-p19816609	Bn-chrA06-p19358867
Bn-A06-p17956497	0.330578512	0.983471074	Bn-N9-p35610103	Bn-chrA06_random-p159929
Bn-A06-p17959919	0.958677686	0.975206612	Bn-Scaffold06875-p1	Bn-chrA06_random-p160271
Bn-A06-p17967505	0.958677686	0.966942149	Bn-N6-p19850690	Bn-chrA06_random-p154904
Bn-A06-p18000403	0.950413223	0.958677686	Bn-N6-p19883329	Bn-chrA06_random-p158545
Bn-A06-p18000461	0.975206612	0.983471074	Bn-N6-p19883387	Bn-chrA06_random-p158551
Bn-A06-p18006686	0.983471074	0.975206612	Bn-N6-p19893702	Bn-chrA06_random-p159329
Bn-A06-p18009980	0.950413223	0.950413223	Bn-N6-p19901068	Bn-chrUnn_random-p528903
Bn-A06-p18022188	0.933884298	0.933884298	Bn-N6-p19920755	Bn-chrA06-p19409442
Bn-A06-p18025751	0.983471074	0.991735537	Bn-N6-p19924757	Bn-chrA06-p19413005
Bn-A06-p18028791	0.925619835	0.925619835	Bn-N6-p19927769	Bn-chrA06-p19416017
Bn-A06-p18028879	0.94214876	0.94214876	Bn-N6-p19927852	Bn-chrA06-p19416100
Bn-A06-p18028921	0.975206612	0.975206612	Bn-N6-p19927894	Bn-chrA06-p19416142
Bn-A06-p18029021	0.991735537	0.991735537	Bn-N6-p19927994	Bn-chrA06-p19416243
Bn-A06-p18029621	0.975206612	0.975206612	Bn-N6-p19928590	Bn-chrA06-p19416839
Bn-A06-p18033006	0.983471074	0.983471074	Bn-N6-p19929931	Bn-chrA06-p19418180
Bn-A06-p18040363	0.975206612	0.983471074	Bn-N6-p19937310	Bn-chrA06-p19425736
Bn-A06-p18040423	0.94214876	0.950413223	Bn-N6-p19937370	Bn-chrA06-p19425796
Bn-A06-p18058671	0.983471074	0.983471074	Bn-N6-p19950769	Bn-chrA06-p19436851
Bn-A06-p18059278	0.983388704	0.993355482	Bn-N6-p19951415	Bn-chrA06-p19437460
Bn-A06-p18059565	0.970149254	0.975124378	Bn-N6-p19951703	Bn-chrA06-p19437747
Bn-A06-p18059678	0.993355482	0.990033223	Bn-N6-p19951816	Bn-chrA06-p19437860

Bn-A06-p18065846	0.983471074	0.983471074	Bn-N6-p19958908	Bn-chrA06-p19443729
Bn-A06-p18089377	0.920265781	0.993355482	Bn-N6-p19994752	Bn-chrA06-p19484736
Bn-A06-p18102164	0.975206612	0.966942149	Bn-N6-p20013669	Bn-chrA06-p19504351
Bn-A06-p18113924	0.975206612	0.983471074	Bn-N6-p20025477	Bn-chrA06-p19516128
Bn-A06-p18115817	0.975206612	0.983471074	Bn-N6-p20027175	Bn-chrAnn_random-p322105
Bn-A06-p18120189	0.980099502	0.980099502	Bn-N5-p11385769	Bn-chrA05-p10311253
Bn-A06-p18120195	0.980099502	0.985074627	Bn-N5-p11390693	Bn-chrA01-p11707926
Bn-A06-p18127830	0.990049751	0.995024876	Bn-N6-p20043057	Bn-chrA06-p19529401
Bn-A06-p18129979	0.975206612	0.983471074	Bn-N6-p20047298	Bn-chrA06-p19531548
Bn-A06-p18131525	0.983471074	0.975206612	Bn-N6-p20052081	Bn-chrA06-p19533121
Bn-A06-p18136235	0.958677686	0.958677686	Bn-N6-p20058827	Bn-chrA06-p19537354
Bn-A06-p18141167	0.983471074	0.983471074	Bn-N6-p20063792	Bn-chrA06-p19542312
Bn-A06-p18144127	1	1	Bn-N6-p20066752	Bn-chrA06-p19545248
Bn-A06-p18152497	0.983471074	0.975206612	Bn-N6-p20075480	Bn-chrA06-p19553709
Bn-A06-p18182395	0.991735537	0.603305785	Bn-N6-p20101071	Bn-chrCnn_random-p181622
Bn-A06-p18188412	1	0.198347107	Bn-N6-p20104547	Bn-chrC08-p29401455
Bn-A06-p18188496	0.991735537	0.272727273	Bn-N6-p20104631	Unknown
Bn-A06-p18207917	0.876033058	0.991735537	Bn-N6-p20127269	Bn-chrAnn_random-p324494
Bn-A06-p18219362	0.983471074	0.983471074	Bn-N6-p20136498	Bn-chrAnn_random-p257926
Bn-A06-p18220327	0.966942149	0.479338843	Bn-N6-p20137469	Bn-chrC07-p33464401
Bn-A06-p18232202	0.975206612	0.975206612	Bn-N6-p20153296	Bn-chrA06-p19619438
Bn-A06-p18235310	0.956810631	0.956810631	Bn-N6-p20156394	Bn-chrA06-p19622534
Bn-A06-p18237512	0.991735537	0.991735537	Bn-N6-p20158797	Bn-chrA06-p19624935
Bn-A06-p18246821	0.983471074	0.983471074	Bn-N6-p20164370	Bn-chrA06-p19630331
Bn-A06-p18247490	0.991735537	0.702479339	Bn-N6-p20165039	Bn-chrC07-p33437139
Bn-A06-p18248917	0.983471074	0.537190083	Bn-N6-p20166468	Bn-chrC02-p42751651
Bn-A06-p18249054	1	0.148760331	Bn-N6-p20166605	Bn-chrC03-p19275946
Bn-A06-p18252711	0.983471074	0.983471074	Bn-N6-p20170308	Bn-chrA06-p19636538
Bn-A06-p18254877	0.975206612	0.975206612	Bn-N6-p20172474	Bn-chrA06-p19638691
Bn-A06-p18260387	0.985074627	0.985074627	Bn-N6-p20177956	Bn-chrA06-p19646994
Bn-A06-p18277724	0.989583333	1	Bn-N6-p20193140	Bn-chrA06-p19663598
Bn-A06-p18277802	0.986710963	0.990033223	Bn-N6-p20193287	Bn-chrA06-p19663745
Bn-A06-p18364834	0.975124378	0.975124378	Bn-N6-p20277325	Bn-chrA06-p19735783
Bn-A06-p18369013	0.991735537	0.991735537	Bn-N6-p20281720	Bn-chrA06-p19740178
Bn-A06-p18380196	0.991735537	0.991735537	Bn-N6-p20293170	Bn-chrA06-p19756096
Bn-A06-p18388041	0.950413223	0.462809917	Bn-N6-p20301015	Bn-chrA06-p19763337
Bn-A06-p18388778	0.990033223	0.990033223	Bn-N6-p20301809	Bn-chrA06-p19764044
Bn-A06-p18390889	0.53820598	0.53820598	Bn-N6-p20303918	Bn-chrA06-p19766154
Bn-A06-p18392463	0.990033223	0.990033223	Bn-N6-p20305492	Bn-chrA06-p19767728
Bn-A06-p18394892	0.986710963	0.986710963	Bn-N6-p20307952	Bn-chrA06-p19770187
Bn-A06-p18397592	0.990049751	0.990049751	Bn-N6-p20310652	Bn-chrA06-p19772887
Bn-A06-p18402713	0.973421927	0.973421927	Bn-N6-p20315760	Bn-chrA06-p19777999
Bn-A06-p18403451	0.966942149	0.966942149	Bn-N6-p20316498	Bn-chrA06-p19778737
Bn-A06-p18408257	0.990033223	0.990033223	Bn-N6-p20321397	Bn-chrA06-p19783636
Bn-A06-p18430792	0.990033223	0.990033223	Bn-N6-p20340671	Bn-chrA06-p19800422
Bn-A06-p18432812	1	1	Bn-N6-p20342677	Bn-chrA06-p19802427

Bn-A06-p18438509	0.950413223	0.950413223	Bn-N6-p20348368	Bn-chrA06-p19808118
Bn-A06-p18445250	0.989583333	0.989583333	Bn-N6-p20355116	Bn-chrA06-p19814864
Bn-A06-p18454864	0.940199336	0.940199336	Bn-N6-p20364712	Bn-chrA06-p19824021
Bn-A06-p18455125	0.983471074	0.983471074	Bn-N6-p20364973	Bn-chrA06-p19824282
Bn-A06-p18500429	0.983388704	0.983388704	Bn-N6-p20405223	Bn-chrA06-p19874275
Bn-A06-p18500596	0.980099502	0.980099502	Bn-N6-p20405390	Bn-chrA06-p19874442
Bn-A06-p18500625	0.990049751	0.990049751	Bn-N6-p20405419	Bn-chrA06-p19874471
Bn-A06-p18502274	1	1	Bn-N6-p20407023	Bn-chrA06-p19876075
Bn-A06-p18502399	0.885572139	0.885572139	Bn-N6-p20407149	Bn-chrA06-p19876201
Bn-A06-p18563686	0.983471074	0.991735537	Bn-N6-p20466441	Bn-chrA06_random-p163132
Bn-A06-p18573338	0.990033223	0.986710963	Bn-N6-p20476134	Bn-chrA06-p19935482
Bn-A06-p18600460	0.989583333	0.90625	Bn-N6-p20504508	Bn-chrA06-p19965312
Bn-A06-p18614110	0.94214876	0.94214876	Bn-N17-p35812600	Bn-chrC07-p32981323
Bn-A06-p18624741	0.995024876	0.955223881	Bn-N6-p20533644	Bn-chrA06-p19988939
Bn-A06-p18634396	0.993355482	0.990033223	Bn-N6-p20543306	Bn-chrA06-p19998593
Bn-A06-p18642085	0.993355482	0.996677741	Bn-N6-p20550993	Bn-chrA06-p20006284
Bn-A06-p18659358	0.991735537	0.983471074	Bn-N6-p20570446	Bn-chrA06-p20023535
Bn-A06-p18692755	0.990049751	0.990049751	Bn-N6-p20591624	Bn-chrA06-p20045817
Bn-A06-p18706102	0.983471074	0.983471074	Bn-N6-p20604957	Bn-chrA06-p20059150
Bn-A06-p18709596	1	0.991735537	Bn-N6-p20608453	Bn-chrA06-p20062646
Bn-A06-p18717323	0.975124378	0.975124378	Bn-N6-p20616335	Bn-chrA06-p20070522
Bn-A06-p18718829	0.990049751	0.990049751	Bn-N6-p20617678	Bn-chrA06-p20071865
Bn-A06-p18729660	0.991735537	0.983471074	Bn-N6-p20630481	Bn-chrA06-p20082675
Bn-A06-p18740524	0.958677686	0.958677686	Bn-N6-p20631917	Bn-chrA06-p20084110
Bn-A06-p18742389	1	0.975206612	Bn-N6-p20634796	Bn-chrA06-p20085952
Bn-A06-p18754657	0.991735537	0.991735537	Bn-N6-p20650321	Bn-chrA06-p20088499
Bn-A06-p18755269	0.970149254	0.970149254	Bn-N17-p35645282	Bn-chrC07-p32823091
Bn-A06-p18755859	1	0.995024876	Bn-N6-p20651519	Bn-chrA06-p20089699
Bn-A06-p18757339	0.661691542	0.975124378	Bn-N6-p20652953	Bn-chrA06-p20091178
Bn-A06-p18776948	0.925619835	0.925619835	Bn-N6-p20675827	Bn-chrA06-p20111194
Bn-A06-p18777001	0.966942149	0.966942149	Bn-N6-p20675880	Bn-chrA06-p20111247
Bn-A06-p18785908	0.991735537	0.79338843	Bn-N6-p20685466	Bn-chrC07-p32765772
Bn-A06-p18794208	0.991735537	0.991735537	Bn-N6-p20696267	Bn-chrA06-p20132744
Bn-A06-p18807305	0.991735537	0.983471074	Bn-N6-p14561852	Bn-chrA06-p14839232
Bn-A06-p18813274	0.975206612	0.983471074	Bn-N6-p14568050	Bn-chrA06-p14845220
Bn-A06-p18867395	0.975206612	0.966942149	Bn-N6-p14622695	Bn-chrA06-p14896481
Bn-A06-p18872147	1	0.995024876	Bn-N6-p14627458	Bn-chrA06-p14900607
Bn-A06-p18880950	0.933884298	0.925619835	Bn-N6-p14638196	Bn-chrA06-p14909679
Bn-A06-p18882138	0.983471074	0.975206612	Bn-N6-p14639384	Bn-chrA06-p14910867
Bn-A06-p18885366	0.958677686	0.966942149	Bn-N6-p14642612	Bn-chrA06-p14914096
Bn-A06-p18898902	0.983471074	0.966942149	Bn-N6-p14659791	Bn-chrA06-p14928056
Bn-A06-p18998806	0.991735537	0.983471074	Bn-N6-p14747781	Bn-chrA06-p14997621
Bn-A06-p19021973	0.991735537	0.991735537	Bn-N6-p14769905	Bn-chrA06-p15021671
Bn-A06-p19089918	0.975124378	0.975124378	Bn-N6-p14833416	Bn-chrA06-p15093160
Bn-A06-p19102612	0.970149254	0.960199005	Bn-N6-p14840589	Bn-chrC04-p40419964
Bn-A06-p19102725	0.970149254	0.955223881	Bn-N6-p14840702	Bn-chrC04-p40420077

Bn-A06-p19130048	0.985074627	0.985074627	Bn-N6-p14868664	Bn-chrA06-p15132275
Bn-A06-p19151996	0.983388704	0.983388704	Bn-N6-p14891444	Bn-chrA06-p15152616
Bn-A06-p19153385	0.995024876	0.995024876	Bn-N6-p14892834	Bn-chrA06-p15154006
Bn-A06-p19153719	0.985074627	0.985074627	Bn-N6-p14893167	Bn-chrA06-p15154338
Bn-A06-p19163919	0.990033223	0.990033223	Bn-N6-p14903370	Bn-chrA06-p15164541
Bn-A06-p19184868	1	1	Bn-N6-p14925742	Bn-chrA06-p15187882
Bn-A06-p19185585	0.993355482	0.993355482	Bn-N6-p14926457	Bn-chrA06-p15188597
Bn-A06-p19197269	0.983471074	0.983471074	Bn-N6-p14938686	Bn-chrA06-p15200276
Bn-A06-p19226794	0.970149254	0.970149254	Bn-N6-p14966614	Bn-chrA06-p15228203
Bn-A06-p19245025	0.996677741	0.996677741	Bn-N6-p15002038	Bn-chrA06-p15267677
Bn-A06-p19256933	0.950166113	0.950166113	Bn-N6-p15009358	Bn-chrA06-p15274997
Bn-A06-p19263581	0.996677741	0.996677741	Bn-N6-p15021814	Bn-chrA06-p15287450
Bn-A06-p19265815	1	1	Bn-N6-p15024130	Bn-chrA06-p15289767
Bn-A06-p19291105	0.950413223	0.950413223	Bn-N6-p15049571	Bn-chrA06-p15318941
Bn-A06-p19292104	0.917355372	0.917355372	Bn-N6-p15050570	Bn-chrA06-p15319940
Bn-A06-p19296157	0.983471074	0.983471074	Bn-N6-p15054624	Bn-chrA06-p15323994
Bn-A06-p19329736	0.993355482	0.993355482	Bn-N6-p15082549	Bn-chrA06-p15354323
Bn-A06-p19333055	0.991735537	0.991735537	Bn-N6-p15086022	Bn-chrA06-p15357796
Bn-A06-p19333117	0.975206612	0.975206612	Bn-N6-p15086085	Bn-chrA06-p15357859
Bn-A06-p19333349	0.976744186	0.976744186	Bn-N6-p15086316	Bn-chrA06-p15358090
Bn-A06-p19392651	0.991735537	0.991735537	Bn-N6-p15145815	Bn-chrA06-p15410751
Bn-A06-p19395565	0.983471074	0.983471074	Bn-N6-p15149169	Bn-chrA06-p15414105
Bn-A06-p19395890	0.966942149	0.966942149	Bn-N6-p15149536	Bn-chrA06-p15414472
Bn-A06-p19396032	0.950413223	0.950413223	Bn-N6-p15149673	Bn-chrA06-p15414609
Bn-A06-p19404012	0.94214876	0.94214876	Bn-N6-p15152878	Bn-chrA06-p15417774
Bn-A06-p19405765	0.980099502	0.980099502	Bn-N6-p15154641	Bn-chrA06-p15419537
Bn-A06-p19411124	0.950248756	0.950248756	Bn-N6-p15159557	Bn-chrA06-p15424453
Bn-A06-p19416292	1	1	Bn-N6-p15164738	Bn-chrCnn_random-p293165
Bn-A06-p19418423	0.991735537	0.991735537	Bn-N6-p15166878	Bn-chrA06-p15431776
Bn-A06-p19419215	0.966942149	0.966942149	Bn-N6-p15167668	Bn-chrA06-p15432566
Bn-A06-p19434905	0.925619835	0.925619835	Bn-N6-p15184014	Bn-chrA06-p15452021
Bn-A06-p19437625	0.991735537	0.991735537	Bn-N6-p15186773	Bn-chrA06-p15454780
Bn-A06-p19485623	0.925619835	0.925619835	Bn-N6-p15238013	Bn-chrA06_random-p141473
Bn-A06-p19489870	0.993355482	0.993355482	Bn-N6-p15244351	Bn-chrA06-p15502472
Bn-A06-p19501594	0.966777409	0.966777409	Bn-N6-p15251694	Bn-chrA06-p15512066
Bn-A06-p19502324	0.79338843	0.851239669	Bn-N6-p15252402	Bn-chrA06-p15512774
Bn-A06-p19508949	0.378109453	0.378109453	Bn-N13-p40104674	Bn-chrC03-p36103081
Bn-A06-p19533298	0.900826446	0.900826446	Bn-N6-p15275787	Bn-chrA06-p15535021
Bn-A06-p19549093	0.975206612	0.975206612	Bn-N6-p15289677	Bn-chrA06-p15548804
Bn-A06-p19549571	0.818181818	0.818181818	Bn-N6-p15290155	Bn-chrA06-p15549282
Bn-A06-p19555116	0.950413223	0.950413223	Bn-N6-p15297703	Bn-chrA06-p15557174
Bn-A06-p19562187	0.983471074	0.983471074	Bn-N6-p15302019	Bn-chrA06-p15561151
Bn-A06-p19574863	0.986710963	0.986710963	Bn-N6-p15322153	Bn-chrA06-p15582649
Bn-A06-p19579765	0.950413223	0.950413223	Bn-N6-p15323968	Bn-chrA06-p15584464
Bn-A06-p19593266	0.975206612	0.975206612	Bn-N6-p15332394	Bn-chrA06-p15592889
Bn-A06-p19598035	0.859504132	0.859504132	Bn-N13-p39926243	Bn-chrC03-p35920650

Bn-A06-p19602022	0.943521595	0.943521595	Bn-N6-p15339060	Bn-chrA06-p15599313
Bn-A06-p19623637	0.900332226	0.903654485	Bn-N18-p25422137	Bn-chrC08-p22112068
Bn-A06-p19635380	0.975206612	0.975206612	Bn-N6-p15372329	Bn-chrA06-p15634194
Bn-A06-p19646183	0.991735537	0.991735537	Bn-N6-p15386700	Bn-chrA06-p15659492
Bn-A06-p19647126	0.983471074	0.983471074	Bn-N6-p15387647	Bn-chrA06-p15660439
Bn-A06-p19647330	0.983471074	0.983471074	Bn-N6-p15387853	Bn-chrA06-p15660645
Bn-A06-p19647388	0.975206612	0.975206612	Bn-N6-p15387911	Bn-chrA06-p15660703
Bn-A06-p19647543	0.925619835	0.925619835	Bn-N6-p15388177	Bn-chrA06-p15660969
Bn-A06-p19648186	0.975206612	0.975206612	Bn-N6-p15388823	Bn-chrA06-p15661615
Bn-A06-p19648433	0.983471074	0.983471074	Bn-N6-p15389072	Bn-chrA06-p15661864
Bn-A06-p19656363	0.983471074	0.983471074	Bn-N6-p15398477	Bn-chrA06-p15671297
Bn-A06-p19658177	0.970099668	0.970099668	Bn-N6-p15400291	Bn-chrA06-p15673111
Bn-A06-p19680250	0.975206612	0.966942149	Bn-Scaffold00926-p5	Bn-chrAnn_random-p110416
Bn-A06-p19715332	1	1	Bn-Scaffold00926-p2	Bn-chrAnn_random-p110038
Bn-A06-p19739501	0.933554817	0.936877076	Bn-Scaffold00926-p3	Bn-chrAnn_random-p242933
Bn-A06-p19747056	1	0.991735537	Bn-N9-p18219572	Bn-chrA09_random-p213457
Bn-A06-p19757422	0.973421927	0.973421927	Bn-N9-p18229734	Bn-chrA09_random-p212354
Bn-A06-p19759161	0.975206612	0.966942149	Bn-N9-p18231473	Bn-chrA09_random-p212181
Bn-A06-p19788556	0.983471074	0.975206612	Bn-N9-p18268820	Bn-chrA09_random-p208437
Bn-A06-p19792160	0.976744186	0.980066445	Bn-N9-p18272623	Bn-chrA09_random-p208056
Bn-A06-p19794728	0.986710963	0.926910299	Bn-N9-p18275191	Bn-chrC08-p16142045
Bn-A06-p19804872	1	0.991735537	Bn-N9-p18286333	Bn-chrA09_random-p205597
Bn-A06-p19833393	0.970149254	0.975124378	Bn-N9-p18328191	Bn-chrA09_random-p201703
Bn-A06-p19838015	0.995024876	0.995024876	Bn-N13-p46411776	Bn-chrC03-p40955459
Bn-A06-p19885379	0.975124378	0.970149254	Bn-N11-p27032084	Bn-chrC09-p35494961
Bn-A06-p19943194	0.975206612	0.983471074	Bn-N9-p18432032	Bn-chrAnn_random-p151139
Bn-A06-p19968113	0.960199005	0.960199005	Bn-N9-p18462085	Bn-chrA09_random-p192489
Bn-A06-p19971717	0.953488372	0.990033223	Bn-N11-p27031652	Bn-chrA09_random-p192128
Bn-A06-p19972403	0.980099502	0.980099502	Bn-N9-p18466375	Bn-chrA09_random-p192060
Bn-A06-p19976368	0.963414634	0.963414634	Bn-N4-p4143042	Bn-chrAnn_random-p455281
Bn-A06-p20177226	0.935323383	0.955223881	Bn-Scaffold23616-p3	Bn-chrAnn_random-p392814
Bn-A06-p20177791	0.935323383	0.940298507	Bn-Scaffold02656-p1	Bn-chrAnn_random-p149119
Bn-A06-p20211283	0.958677686	0.958677686	Bn-N5-p14768753	Bn-chrA05_random-p145447
Bn-A06-p20320209	0.975206612	0.975206612	Bn-N5-p14653039	Bn-chrA05_random-p127569
Bn-A06-p20320258	0.975206612	0.975206612	Bn-N5-p14652990	Bn-chrA05_random-p127564
Bn-A06-p20476713	1	1	Bn-N5-p14503342	Bn-chrA05-p13321112
Bn-A06-p20494935	0.995024876	0.995024876	Bn-N5-p14482020	Bn-chrA05-p13278278
Bn-A06-p20495127	1	1	Bn-N5-p14481837	Bn-chrA05-p13278095
Bn-A06-p20633479	0.955223881	0.955223881	Bn-N5-p14367193	Bn-chrA05-p13162043
Bn-A06-p20696441	0.965174129	0.965174129	Bn-N5-p14316382	Bn-chrA07-p21495963
Bn-A06-p20696691	0.980099502	0.980099502	Bn-N5-p14316132	Bn-chrA07-p21496213
Bn-A06-p20877321	1	1	Bn-N15-p29457437	Bn-chrC05-p26110548
Bn-A06-p20877670	0.985074627	0.985074627	Bn-N15-p29457089	Bn-chrC05-p26110200
Bn-A06-p20998201	0.995024876	0.995024876	Bn-N5-p1247483	Bn-chrC07-p39822181
Bn-A06-p21031038	1	1	Bn-N15-p29376621	Bn-chrC05-p26018706
Bn-A06-p21041632	1	0.835820896	Bn-N6-p21218604	Bn-chrCnn_random-p747699

Bn-A06-p21045315	0.991735537	1	Bn-N6-p21214921	Bn-chrA06-p20629105
Bn-A06-p21060490	0.940199336	0.990033223	Bn-N17-p34995753	Bn-chrA06-p20611684
Bn-A06-p21062265	0.950413223	0.975206612	Bn-N6-p21197851	Bn-chrA06-p20609909
Bn-A06-p21062492	0.958677686	0.966942149	Bn-N6-p21197627	Bn-chrA06-p20609682
Bn-A06-p21064129	0.991735537	0.983471074	Bn-N6-p21195989	Bn-chrA06-p20608046
Bn-A06-p21064168	0.980099502	0.985074627	Bn-N6-p21195950	Bn-chrA06-p20608007
Bn-A06-p21064290	0.950248756	0.950248756	Bn-N6-p21195828	Bn-chrA06-p20607885
Bn-A06-p21064341	0.94214876	0.94214876	Bn-N17-p35005435	Bn-chrC07-p32216954
Bn-A06-p21065019	0.950413223	0.950413223	Bn-N6-p21195097	Bn-chrA06-p20607154
Bn-A06-p21069394	0.983471074	0.983471074	Bn-N6-p21190723	Bn-chrA06-p20602780
Bn-A06-p21072392	0.983471074	0.983471074	Bn-N6-p21187578	Bn-chrA06-p20598594
Bn-A06-p21080778	0.958677686	0.958677686	Bn-N6-p21179847	Bn-chrA06-p20590863
Bn-A06-p21080915	0.983471074	0.983471074	Bn-N6-p21179482	Bn-chrA06-p20590498
Bn-A06-p21083249	0.983471074	0.983471074	Bn-N6-p21177126	Bn-chrA06-p20588142
Bn-A06-p21083748	0.958677686	0.958677686	Bn-N6-p21176627	Bn-chrA06-p20587643
Bn-A06-p21085532	0.966942149	0.966942149	Bn-N6-p21175337	Bn-chrA06-p20586353
Bn-A06-p21086685	0.628099174	0.628099174	Bn-N6-p21171605	Bn-chrA06_random-p171576
Bn-A06-p21098677	0.970099668	0.970099668	Bn-N6-p21164670	Bn-chrA06-p20581754
Bn-A06-p21101957	0.76119403	0.76119403	Bn-N6-p21161410	Bn-chrA06-p20578496
Bn-A06-p21105437	0.96013289	0.96013289	Bn-N6-p21158483	Bn-chrA06-p20575569
Bn-A06-p21116438	0.966942149	0.966942149	Bn-N6-p21145537	Bn-chrA06-p20562932
Bn-A06-p21126329	0.965174129	0.965174129	Bn-N6-p21137056	Bn-chrA06-p20555399
Bn-A06-p21127138	0.983471074	0.983471074	Bn-N6-p21136444	Bn-chrA06-p20554787
Bn-A06-p21127779	0.990049751	0.990049751	Bn-N6-p21135793	Bn-chrA06-p20554136
Bn-A06-p21127805	0.991735537	0.991735537	Bn-N6-p21135767	Bn-chrA06-p20554110
Bn-A06-p21127973	0.991735537	0.991735537	Bn-N6-p21135599	Bn-chrA06-p20553942
Bn-A06-p21132765	0.966942149	0.966942149	Bn-N6-p21130007	Bn-chrA06-p20548350
Bn-A06-p21134315	0.980066445	0.980066445	Bn-N6-p21128461	Bn-chrA06-p20546804
Bn-A06-p21136673	0.652892562	0.983471074	Bn-N6-p21126373	Bn-chrA06-p20544716
Bn-A06-p21150459	0.995024876	0.995024876	Bn-N6-p21114343	Bn-chrA06-p20536142
Bn-A06-p21150622	0.966942149	0.966942149	Bn-N6-p21114169	Bn-chrA06-p20535968
Bn-A06-p21155152	0.991735537	0.991735537	Bn-N6-p21111251	Bn-chrA06-p20533051
Bn-A06-p21162770	0.965174129	0.965174129	Bn-N6-p21106183	Bn-chrA06-p20527985
Bn-A06-p21163037	0.980099502	0.980099502	Bn-Scaffold74746-p1	Bn-chrA06-p20527716
Bn-A06-p21163950	0.975124378	0.975124378	Bn-N6-p21105000	Bn-chrA06-p20526803
Bn-A06-p21163993	0.975124378	0.975124378	Bn-N6-p21104957	Bn-chrA06-p20526760
Bn-A06-p21164043	0.980099502	0.980099502	Bn-N6-p21104907	Bn-chrA06-p20526710
Bn-A06-p21164284	0.940298507	0.940298507	Bn-N6-p21104666	Bn-chrA06-p20526469
Bn-A06-p21164289	0.970149254	0.970149254	Bn-N6-p21104661	Bn-chrA06-p20526464
Bn-A06-p21183207	0.990033223	0.96345515	Bn-N6-p21085267	Bn-chrA06-p20511932
Bn-A06-p21187999	0.986710963	0.990033223	Bn-N6-p21080416	Bn-chrA06-p20507126
Bn-A06-p21191463	0.966942149	0.975206612	Bn-N6-p21076997	Bn-chrA06-p20503658
Bn-A06-p21194827	0.986710963	0.990033223	Bn-N6-p21068934	Bn-chrA06-p20496458
Bn-A06-p21195115	0.990049751	0.995024876	Bn-N6-p21068646	Bn-chrA06-p20496170
Bn-A06-p21196739	0.985074627	0.980099502	Bn-N6-p21067025	Bn-chrA06-p20494551
Bn-A06-p21221869	0.950413223	0.958677686	Bn-N6-p21039536	Bn-chrA06-p20471116

Bn-A06-p21224575	0.991735537	0.983471074	Bn-N6-p21036830	Bn-chrA06-p20468408
Bn-A06-p21238199	0.991735537	0.983471074	Bn-N6-p21022992	Bn-chrA06-p20458529
Bn-A06-p21247593	0.975206612	0.983471074	Bn-N6-p21013430	Bn-chrA06-p20444159
Bn-A06-p21252883	0.995024876	0.990049751	Bn-N6-p21008140	Bn-chrA06-p20429548
Bn-A06-p21260739	0.991735537	1	Bn-N6-p21000205	Bn-chrA06-p20421943
Bn-A06-p21262030	0.975206612	0.983471074	Bn-N6-p20998914	Bn-chrA06-p20420656
Bn-A06-p21268699	0.965174129	0.965174129	Bn-N17-p35259636	Bn-chrC07-p32478328
Bn-A06-p21268967	0.965174129	0.955223881	Bn-N6-p20991977	Bn-chrA06-p20409097
Bn-A06-p21269830	0.975124378	0.980099502	Bn-N6-p20991114	Bn-chrA06-p20408209
Bn-A06-p21270132	0.970149254	0.930348259	Bn-N6-p20990812	Bn-chrA06-p20407907
Bn-A06-p21273211	0.995024876	0.995024876	Bn-N6-p20987659	Bn-chrA06-p20404601
Bn-A06-p21277332	0.983471074	0.983471074	Bn-N6-p20983251	Bn-chrA06-p20400194
Bn-A06-p21278243	0.94214876	0.94214876	Bn-N6-p20981584	Bn-chrA06-p20399283
Bn-A06-p21280471	0.94214876	0.94214876	Bn-N6-p20979356	Bn-chrA06-p20397055
Bn-A06-p21280942	0.958677686	0.958677686	Bn-N6-p20978885	Bn-chrA06-p20396584
Bn-A06-p21281098	0.958677686	0.958677686	Bn-N6-p20978729	Bn-chrA06-p20396428
Bn-A06-p21293821	0.933884298	0.933884298	Bn-N6-p20966418	Bn-chrA06-p20382468
Bn-A06-p21301745	0.985074627	0.985074627	Bn-N9-p5685405	Bn-chrA08-p13482637
Bn-A06-p21302070	0.985074627	0.985074627	Bn-N9-p5685080	Bn-chrA08-p13482312
Bn-A06-p21308348	0.933554817	0.993355482	Bn-N6-p20957424	Bn-chrA06-p20374356
Bn-A06-p21310030	0.990033223	0.983388704	Bn-N6-p20950951	Bn-chrC07-p32516182
Bn-A06-p21311925	0.983471074	0.983471074	Bn-N6-p20949065	Bn-chrA06-p20370792
Bn-A06-p21315016	0.990049751	0.995024876	Bn-N6-p20945954	Bn-chrA06-p20367718
Bn-A06-p21318925	0.958677686	0.975206612	Bn-N17-p35308010	Bn-chrA06-p20363721
Bn-A06-p21321797	0.933884298	0.933884298	Bn-N6-p20939167	Bn-chrA06-p20360827
Bn-A06-p21324859	0.950413223	0.950413223	Bn-N6-p20930128	Bn-chrA06-p20357750
Bn-A06-p21349507	0.975124378	0.990049751	Bn-N6-p20905511	Bn-chrA06_random-p164409
Bn-A06-p21415201	0.991735537	0.991735537	Bn-N6-p20846412	Bn-chrA06-p20298154
Bn-A06-p21422671	0.985074627	0.990049751	Bn-N6-p20838160	Bn-chrA06-p20294795
Bn-A06-p21426650	1	0.991735537	Bn-Scaffold23928-p5	Bn-chrA06-p20288533
Bn-A06-p21438687	0.955223881	0.960199005	Bn-N6-p20823274	Bn-chrA06-p20268551
Bn-A06-p21445218	0.975206612	0.975206612	Bn-N6-p20816652	Bn-chrA06-p20262006
Bn-A06-p21445439	0.991735537	0.991735537	Bn-N6-p20816431	Bn-chrA06-p20261785
Bn-A06-p21462006	0.990033223	0.983388704	Bn-N6-p20798919	Bn-chrA06-p20244403
Bn-A06-p21463076	0.991735537	0.314049587	Bn-N6-p20797852	Bn-chrA06-p20243347
Bn-A06-p21471436	1	0.991735537	Bn-N6-p20789829	Bn-chrA06_random-p163735
Bn-A06-p21472562	0.990033223	0.970099668	Bn-N6-p20788702	Bn-chrA06_random-p163622
Bn-A06-p21475556	0.993355482	0.990033223	Bn-N6-p20785672	Bn-chrA06_random-p163322
Bn-A06-p21478202	0.975206612	0.975206612	Bn-N17-p35457530	Bn-chrC07-p32671702
Bn-A06-p21490506	0.983471074	0.975206612	Bn-N6-p20769890	Bn-chrA06-p20212148
Bn-A06-p21491454	0.955223881	0.955223881	Bn-N6-p20768943	Bn-chrA06-p20211197
Bn-A06-p21501350	0.990033223	0.993355482	Bn-N6-p20763033	Bn-chrA06-p20200574
Bn-A06-p21509934	0.990033223	0.986710963	Bn-N6-p20752935	Bn-chrA06-p20191321
Bn-A06-p21525576	0.996677741	0.993355482	Bn-N6-p20736799	Bn-chrA06-p20174821
Bn-A06-p21550710	0.996677741	0.993355482	Bn-N6-p20720082	Bn-chrA06-p20161294
Bn-A06-p21572310	0.975206612	0.975206612	Bn-N6-p20702676	Bn-chrA06-p20139150

Bn-A06-p21573640	0.985074627	0.985074627	Bn-N6-p20701073	Bn-chrA06-p20137548
Bn-A06-p21574157	0.965174129	0.965174129	Bn-N6-p20700557	Bn-chrA06-p20137032
Bn-A06-p21589512	0.933884298	0.933884298	Bn-N6-p21229455	Bn-chrA06_random-p169239
Bn-A06-p21591920	0.991735537	1	Bn-N6-p21231863	Bn-chrA06_random-p169001
Bn-A06-p21597170	1	0.991735537	Bn-N6-p21237113	Bn-chrA06_random-p168476
Bn-A06-p21600894	0.975206612	0.966942149	Bn-N6-p21240837	Bn-chrA06_random-p168104
Bn-A06-p21602381	0.983471074	0.991735537	Bn-N6-p21242324	Bn-chrA06_random-p167954
Bn-A06-p21613020	0.966942149	0.958677686	Bn-N6-p21253025	Bn-chrA06_random-p166207
Bn-A06-p21650617	0.983471074	0.991735537	Bn-N6-p21292462	Bn-chrA06-p20668118
Bn-A06-p21653611	0.995024876	0.990049751	Bn-N6-p21295456	Bn-chrA06-p20671111
Bn-A06-p21663690	0.980066445	0.980066445	Bn-N6-p21305280	Bn-chrA06-p20679775
Bn-A06-p21677028	0.990033223	0.986710963	Bn-N6-p21308708	Bn-chrA06-p20689524
Bn-A06-p21796008	0.991735537	1	Bn-N6-p21432182	Bn-chrA06-p20815503
Bn-A06-p21797963	0.94214876	0.950413223	Bn-N6-p21435228	Bn-chrA06-p20817458
Bn-A06-p21800812	0.990033223	0.986710963	Bn-N6-p21438164	Bn-chrA06-p20820870
Bn-A06-p21800927	0.983388704	0.980066445	Bn-N6-p21438279	Bn-chrA06-p20820985
Bn-A06-p21801638	0.983471074	0.991735537	Bn-N6-p21438990	Bn-chrA06-p20821696
Bn-A06-p21804318	0.983471074	0.991735537	Bn-N6-p21441800	Bn-chrA06-p20824360
Bn-A06-p21822204	0.993355482	0.993355482	Bn-N6-p21461446	Bn-chrA06-p20840881
Bn-A06-p21825957	0.983471074	0.983471074	Bn-N6-p21463254	Bn-chrA06-p20842625
Bn-A06-p21826106	0.710743802	0.983471074	Bn-N6-p21483390	Bn-chrA06-p20842774
Bn-A06-p21826609	0.950413223	0.958677686	Bn-N6-p21463844	Bn-chrA06-p20843277
Bn-A06-p21826671	0.958677686	0.950413223	Bn-N6-p21463906	Bn-chrA06-p20843339
Bn-A06-p21826830	0.975206612	0.966942149	Bn-N6-p21475716	Bn-chrA06-p20843498
Bn-A06-p21826979	1	0.991735537	Bn-N6-p21475865	Bn-chrA06-p20843647
Bn-A06-p21827359	0.975206612	0.966942149	Bn-N6-p21476245	Bn-chrA06-p20844027
Bn-A06-p21829647	0.966942149	0.975206612	Bn-N6-p21482563	Bn-chrA06-p20846964
Bn-A06-p21846491	0.955223881	0.960199005	Bn-N6-p21499365	Bn-chrA06-p20863775
Bn-A06-p21848747	0.983471074	0.991735537	Bn-N6-p21501518	Bn-chrA06-p20865944
Bn-A06-p21852261	0.966777409	0.990033223	Bn-N6-p21505032	Bn-chrA06-p20869458
Bn-A06-p21884761	0.900826446	0.975206612	Bn-Scaffold04231-p1	Bn-chrA06-p20902007
Bn-A06-p21888532	0.991735537	0.983471074	Bn-N6-p21546554	Bn-chrA06-p20905778
Bn-A06-p21896359	0.950413223	0.958677686	Bn-N6-p21555491	Bn-chrA06-p20921104
Bn-A06-p21896871	0.983471074	0.991735537	Bn-N6-p21556025	Bn-chrA06-p20922075
Bn-A06-p21899421	0.983471074	0.991735537	Bn-N6-p21558593	Bn-chrA06-p20924642
Bn-A06-p21902421	0.983471074	0.991735537	Bn-N6-p21566527	Bn-chrA06-p20927888
Bn-A06-p21902773	1	0.991735537	Bn-N6-p21566879	Bn-chrA06-p20928240
Bn-A06-p21903683	0.975206612	0.867768595	Bn-N6-p21567789	Bn-chrA06-p20929151
Bn-A06-p21904019	0.983471074	0.991735537	Bn-N6-p21568125	Bn-chrA06-p20930359
Bn-A06-p21904162	0.983471074	0.975206612	Bn-N6-p21568268	Bn-chrA06-p20930500
Bn-A06-p21917612	0.975206612	0.975206612	Bn-N6-p21576432	Bn-chrA06-p20939394
Bn-A06-p21926982	0.965174129	0.960199005	Bn-N6-p21584956	Bn-chrA06-p20949552
Bn-A06-p21930942	0.990033223	0.986710963	Bn-N6-p21589077	Bn-chrA06-p20953512
Bn-A06-p21933122	0.980099502	0.980099502	Bn-N6-p21591265	Bn-chrA06-p20955692
Bn-A06-p21933776	0.975124378	0.975124378	Bn-N6-p21591919	Bn-chrA06-p20956346
Bn-A06-p21933808	0.966292135	0.97752809	Bn-N6-p21591951	Bn-chrA06-p20956378

Bn-A06-p21933870	0.986710963	0.990033223	Bn-N6-p21592013	Bn-chrA06-p20956440
Bn-A06-p21953510	0.990825688	0.990825688	Bn-N6-p21621781	Bn-chrA06-p20985521
Bn-A06-p21954315	0.995024876	0.995024876	Bn-N6-p21622586	Bn-chrA06-p20986326
Bn-A06-p21955208	0.995024876	0.995024876	Bn-N6-p21623479	Bn-chrA06-p20987219
Bn-A06-p21980483	0.970149254	0.970149254	Bn-N6-p21648653	Bn-chrA06-p21014321
Bn-A06-p21980699	0.965174129	0.965174129	Bn-N17-p34313338	Bn-chrC07-p31555914
Bn-A06-p21983885	1	0.991735537	Bn-N6-p21652065	Bn-chrA06-p21017722
Bn-A06-p21986550	0.900826446	0.975206612	Bn-N17-p34303483	Bn-chrA06-p21020661
Bn-A06-p21992536	0.975206612	0.983471074	Bn-N6-p21658465	Bn-chrA06-p21026646
Bn-A06-p21993050	0.79338843	0.991735537	Bn-N6-p21661040	Bn-chrA06-p21027160
Bn-A06-p22001193	0.966942149	0.958677686	Bn-N6-p21663745	Bn-chrA06-p21035269
Bn-A06-p22051862	0.958677686	0.958677686	Bn-N6-p21716689	Bn-chrA06-p21087758
Bn-A06-p22054532	0.94214876	0.958677686	Bn-N6-p21719394	Bn-chrA06-p21090420
Bn-A06-p22056809	0.993355482	0.996677741	Bn-N6-p21721708	Bn-chrA06-p21092688
Bn-A06-p22063657	0.950413223	0.966942149	Bn-N6-p21729844	Bn-chrA06-p21099757
Bn-A06-p22074841	0.975206612	0.975206612	Bn-N6-p21741027	Bn-chrA06-p21111233
Bn-A06-p22077748	0.970149254	0.970149254	Bn-N6-p21743932	Bn-chrA06-p21114138
Bn-A06-p22088550	0.993355482	0.993355482	Bn-N6-p21754214	Bn-chrA06-p21124345
Bn-A06-p22096615	0.940298507	0.940298507	Bn-N6-p21761888	Bn-chrA06-p21132016
Bn-A06-p22105035	0.96345515	0.953488372	Bn-N6-p21770289	Bn-chrA06-p21140417
Bn-A06-p22121666	1	1	Bn-N6-p21788219	Bn-chrA06-p21157047
Bn-A06-p22160452	0.995024876	0.995024876	Bn-N6-p21827996	Bn-chrA06-p21196493
Bn-A06-p22163702	0.995024876	0.995024876	Bn-N6-p21831246	Bn-chrA06-p21199743
Bn-A06-p22173832	1	1	Bn-N6-p21842963	Bn-chrA06_random-p171290
Bn-A06-p22209347	0.991735537	0.991735537	Bn-N6-p21880558	Bn-chrA06-p21245999
Bn-A06-p22231737	0.960199005	0.995024876	Bn-N6-p21903315	Bn-chrA06-p21268721
Bn-A06-p22262511	0.975206612	0.975206612	Bn-N6-p21937803	Bn-chrA06-p21296220
Bn-A06-p22267850	0.995024876	0.995024876	Bn-N6-p21943142	Bn-chrA06-p21301559
Bn-A06-p22268128	0.990049751	0.990049751	Bn-N6-p21943420	Bn-chrA06-p21301837
Bn-A06-p22271048	0.983471074	0.983471074	Bn-N6-p21946340	Bn-chrA06-p21304757
Bn-A06-p22278059	0.720930233	0.720930233	Bn-N6-p21951567	Bn-chrA06-p21312168
Bn-A06-p22278468	0.966777409	0.996677741	Bn-N6-p21951976	Bn-chrA06-p21312577
Bn-A06-p22279513	0.966942149	0.958677686	Bn-N6-p21953030	Bn-chrA06-p21313622
Bn-A06-p22324968	0.983471074	0.966942149	Bn-N6-p21999139	Bn-chrA06-p21356613
Bn-A06-p22326059	0.983471074	0.909090909	Bn-N6-p22000228	Bn-chrA06-p21357195
Bn-A06-p22329026	1	0.996677741	Bn-N6-p22003214	Bn-chrA06-p21362889
Bn-A06-p22329819	0.996677741	1	Bn-N6-p22004007	Bn-chrA06-p21363682
Bn-A06-p22330861	1	1	Bn-N6-p22005049	Bn-chrA06-p21364724
Bn-A06-p22331680	0.995024876	1	Bn-N6-p22005868	Bn-chrA06-p21365691
Bn-A06-p22383556	0.958677686	0.958677686	Bn-N6-p22075229	Bn-chrA06-p21416079
Bn-A06-p22383722	0.966942149	0.966942149	Bn-N6-p22075395	Bn-chrA06-p21416246
Bn-A06-p22391357	0.996677741	0.916943522	Bn-N17-p33472677	Bn-chrC07-p30717183
Bn-A06-p22397061	0.995024876	0.995024876	Bn-N6-p22088890	Bn-chrA06-p21428422
Bn-A06-p22503987	0.985074627	0.985074627	Bn-N6-p22193611	Bn-chrA06-p21526764
Bn-A06-p22505664	0.975206612	0.975206612	Bn-N6-p22195227	Bn-chrA06-p21528698
Bn-A06-p22505810	0.991735537	0.991735537	Bn-N6-p22195373	Bn-chrA06-p21528844

Bn-A06-p22554741	0.983471074	0.983471074	Bn-N6-p22274780	Bn-chrA06-p21597232
Bn-A06-p22555168	0.965174129	0.965174129	Bn-N6-p22275207	Bn-chrA06-p21597660
Bn-A06-p22557239	0.966777409	0.970099668	Bn-N6-p22277277	Bn-chrA06-p21599731
Bn-A06-p22559895	0.966942149	0.975206612	Bn-N6-p22279943	Bn-chrA06-p21602441
Bn-A06-p22577587	0.989583333	1	Bn-N6-p22301881	Bn-chrA06-p21620843
Bn-A06-p22582397	0.965174129	0.990049751	Bn-Scaffold05279-p3	Bn-chrA06-p21623427
Bn-A06-p22588868	0.991735537	1	Bn-N6-p22310361	Bn-chrA06-p21629097
Bn-A06-p22589709	0.993355482	0.996677741	Bn-N6-p22311203	Bn-chrA06-p21629937
Bn-A06-p22594488	0.975206612	0.950413223	Bn-N6-p22319876	Bn-chrC07_random-p169280
Bn-A06-p22595032	0.826446281	0.925619835	Bn-N6-p22320448	Bn-chrA06-p21635677
Bn-A06-p22597668	0.983388704	0.986710963	Bn-N6-p22323689	Bn-chrA06-p21637429
Bn-A06-p22601511	0.976744186	0.980066445	Bn-N6-p22326548	Bn-chrA06-p21640293
Bn-A06-p22601589	0.983471074	0.975206612	Bn-N6-p22326626	Bn-chrA06-p21640371
Bn-A06-p22605217	0.975206612	0.975206612	Bn-N6-p22330266	Bn-chrA06-p21644014
Bn-A06-p22608192	0.993355482	0.993355482	Bn-N6-p22333247	Bn-chrA06-p21646995
Bn-A06-p22644580	0.995024876	0.995024876	Bn-N6-p22375528	Bn-chrA06-p21686790
Bn-A06-p22647955	0.995024876	0.995024876	Bn-N6-p22377809	Bn-chrA06-p21689071
Bn-A06-p22691250	0.980099502	0.980099502	Bn-N6-p22408047	Bn-chrA06-p21718915
Bn-A06-p22709184	1	1	Bn-N17-p32930419	Bn-chrC07-p30219758
Bn-A06-p22709872	0.993355482	0.996677741	Bn-N6-p22430716	Bn-chrA06-p21739928
Bn-A06-p22773574	0.995024876	0.995024876	Bn-N6-p22488281	Bn-chrA06-p21799750
Bn-A06-p22773576	0.990049751	0.990049751	Bn-N6-p22488283	Bn-chrA06-p21799752
Bn-A06-p22781886	0.983471074	0.991735537	Bn-N6-p22495067	Bn-chrA06-p21806706
Bn-A06-p22782335	0.991735537	1	Bn-N6-p22495516	Bn-chrA06-p21807179
Bn-A06-p22784485	0.302325581	0.993355482	Bn-N2-p27875310	Bn-chrA10-p6666723
Bn-A06-p22786948	0.983471074	0.991735537	Bn-N6-p22497187	Bn-chrA06-p21809510
Bn-A06-p22788890	0.966942149	0.975206612	Bn-N6-p22499129	Bn-chrA06-p21811453
Bn-A06-p22796718	0.958677686	0.966942149	Bn-N6-p22507072	Bn-chrA06-p21823370
Bn-A06-p22806976	0.983471074	0.991735537	Bn-N6-p22517233	Bn-chrA06-p21833927
Bn-A06-p22808095	0.993355482	0.993355482	Bn-N6-p22518348	Bn-chrA06-p21835044
Bn-A06-p22809449	0.966942149	0.966942149	Bn-N6-p22519719	Bn-chrA06-p21836415
Bn-A06-p22816078	0.966942149	0.966942149	Bn-N6-p22526298	Bn-chrA06-p21842994
Bn-A06-p22835281	0.933884298	0.966942149	Bn-N6-p22550993	Bn-chrA06-p21858935
Bn-A06-p22847296	0.996677741	0.996677741	Bn-N6-p22564190	Bn-chrA06-p21872123
Bn-A06-p22882207	0.945273632	0.985074627	Bn-N6-p22597496	Bn-chrA06-p21931637
Bn-A06-p22894392	0.953488372	0.983388704	Bn-N17-p32608696	Bn-chrA06-p21943680
Bn-A06-p22910339	0.993355482	0.993355482	Bn-N6-p22625012	Bn-chrA06-p21960156
Bn-A06-p22913862	0.958677686	0.958677686	Bn-N6-p22628561	Bn-chrA06-p21963704
Bn-A06-p22914032	0.966942149	0.966942149	Bn-N6-p22628731	Bn-chrA06-p21963874
Bn-A06-p22914117	0.958677686	0.958677686	Bn-N6-p22628816	Bn-chrA06-p21963959
Bn-A06-p22917369	0.991735537	0.991735537	Bn-N6-p22632278	Bn-chrA06-p21967187
Bn-A06-p22921908	0.980099502	0.980099502	Bn-N6-p22636874	Bn-chrA06-p21971780
Bn-A06-p22922325	0.906976744	0.906976744	Bn-N6-p22637291	Bn-chrA06-p21972197
Bn-A06-p22938934	0.936877076	0.946843854	Bn-N17-p32545000	Bn-chrA06-p21983501
Bn-A06-p22947499	0.991735537	0.983471074	Bn-N6-p22667869	Bn-chrA06-p21994294
Bn-A06-p23041811	0.990049751	0.990049751	Bn-N6-p22769633	Bn-chrA06-p22091906

Bn-A06-p23042397	0.985074627	0.985074627	Bn-N6-p22770219	Bn-chrA06-p22092492
Bn-A06-p23042849	0.960199005	0.960199005	Bn-N6-p22770671	Bn-chrA06-p22092944
Bn-A06-p23043135	0.990049751	0.990049751	Bn-N6-p22770954	Bn-chrA06-p22093227
Bn-A06-p23216260	0.991735537	0.991735537	Bn-N6-p22951881	Bn-chrA06-p22267539
Bn-A06-p23218510	0.958677686	0.958677686	Bn-N6-p22954128	Bn-chrA06-p22269786
Bn-A06-p23219303	0.983471074	0.983471074	Bn-N6-p22954907	Bn-chrA06-p22270565
Bn-A06-p23219336	0.991735537	0.991735537	Bn-N6-p22954942	Bn-chrA06-p22270600
Bn-A06-p23219420	0.991735537	0.991735537	Bn-N6-p22955026	Bn-chrA06-p22270684
Bn-A06-p23226757	1	1	Bn-N6-p22958037	Bn-chrA06-p22273695
Bn-A06-p23226981	0.983471074	0.983471074	Bn-N6-p22958264	Bn-chrA06-p22273922
Bn-A06-p23227091	0.950413223	0.950413223	Bn-N6-p22958374	Bn-chrA06-p22274032
Bn-A06-p23229650	0.991735537	0.983471074	Bn-N6-p22961568	Bn-chrCnn_random-p110187
Bn-A06-p23250542	0.991735537	0.991735537	Bn-N6-p22988707	Bn-chrA06-p22298045
Bn-A06-p23258948	0.752066116	0.752066116	Bn-N6-p22997564	Bn-chrA06-p22306429
Bn-A06-p23259546	0.966942149	0.966942149	Bn-N6-p22998164	Bn-chrA06-p22307029
Bn-A06-p23268021	0.991735537	0.983471074	Bn-N6-p23007190	Bn-chrA06-p22315235
Bn-A06-p23295405	0.925373134	0.930348259	Bn-N6-p23025174	Bn-chrA06-p22333134
Bn-A06-p23341715	0.966942149	0.975206612	Bn-N6-p23118014	Bn-chrA06_random-p178256
Bn-A06-p23347767	0.986710963	0.993355482	Bn-N6-p23125100	Bn-chrA06_random-p178894
Bn-A06-p23350246	0.933884298	0.884297521	Bn-N6-p23129741	Bn-chrA06_random-p179005
Bn-A06-p23406582	1	1	Bn-N6-p23205497	Bn-chrA06-p22381132
Bn-A06-p23442751	0.990049751	0.990049751	Bn-N17-p31512414	Bn-chrC07-p28665096
Bn-A06-p23468803	0.985074627	0.985074627	Bn-N6-p23270696	Bn-chrA06-p22443540
Bn-A06-p23542209	0.851239669	0.983471074	Bn-N6-p23364233	Bn-chrA06-p22518960
Bn-A06-p23585772	0.995024876	0.990049751	Bn-N6-p23400205	Bn-chrA06-p22556366
Bn-A06-p23586019	0.990049751	0.990049751	Bn-N6-p23400452	Bn-chrA06-p22556613
Bn-A06-p23590868	0.983471074	0.991735537	Bn-N6-p23405305	Bn-chrA06-p22561257
Bn-A06-p23591616	0.975206612	0.983471074	Bn-N6-p23406052	Bn-chrA06-p22562004
Bn-A06-p23595655	0.993355482	0.292358804	Bn-N6-p23410265	Unknown
Bn-A06-p23612730	0.983471074	0.991735537	Bn-N6-p23426032	Bn-chrA06-p22588158
Bn-A06-p23621651	0.966942149	0.975206612	Bn-N6-p23435244	Bn-chrA06-p22598628
Bn-A06-p23625426	0.983471074	0.975206612	Bn-N6-p23440104	Bn-chrA06-p22602402
Bn-A06-p23657129	0.936877076	0.933554817	Bn-N6-p23465288	Bn-chrA06-p22640203
Bn-A06-p23660656	0.965174129	0.930348259	Bn-N6-p23467647	Bn-chrA06-p22642755
Bn-A06-p23661679	0.970149254	0.980099502	Bn-N6-p23468682	Bn-chrA06-p22643760
Bn-A06-p23665384	0.991735537	0.768595041	Bn-N6-p23472400	Bn-chrC07-p28232031
Bn-A06-p23665564	0.975206612	0.297520661	Bn-N6-p23472580	Bn-chrC09-p33801225
Bn-A06-p23666378	0.983471074	0.272727273	Bn-N6-p23473395	Bn-chrC02-p17400138
Bn-A06-p23674542	0.950413223	0.958677686	Bn-N6-p23480988	Bn-chrA06-p22658654
Bn-A06-p23677178	0.991735537	0.950413223	Bn-N6-p23483623	Bn-chrA06-p22661330
Bn-A06-p23677200	0.983471074	0.991735537	Bn-N6-p23483645	Bn-chrA06-p22661352
Bn-A06-p23681386	0.983471074	0.991735537	Bn-N6-p23487818	Bn-chrA06-p22666492
Bn-A06-p23684217	1	0.991735537	Bn-N6-p23490648	Bn-chrA06-p22668419
Bn-A06-p23684770	0.966942149	0.975206612	Bn-N6-p23491201	Bn-chrA06-p22668971
Bn-A06-p23687609	0.991735537	0.983471074	Bn-N6-p23494040	Bn-chrA06-p22671823
Bn-A06-p23688372	0.991735537	1	Bn-N6-p23494803	Bn-chrA06-p22672586

Bn-A06-p23695500	0.991735537	1	Bn-N6-p23503112	Bn-chrA06-p22681018
Bn-A06-p23695665	1	0.991735537	Bn-N6-p23503277	Bn-chrA06-p22681183
Bn-A06-p23697182	0.991735537	0.983471074	Bn-N6-p23510478	Bn-chrA06-p22682822
Bn-A06-p23697605	0.94214876	0.925619835	Bn-N6-p23510901	Bn-chrA06-p22683231
Bn-A06-p23699003	0.983471074	0.132231405	Bn-N6-p23512309	Bn-chrC07-p8406688
Bn-A06-p23699420	0.983471074	0.975206612	Bn-N6-p23512722	Bn-chrA06-p22685981
Bn-A06-p23706979	0.983471074	0.991735537	Bn-N6-p23520307	Bn-chrA06-p22694985
Bn-A06-p23710466	0.983471074	0.991735537	Bn-N6-p23523789	Bn-chrA06-p22698328
Bn-A06-p23710506	0.991735537	0.983471074	Bn-N6-p23523829	Bn-chrA06-p22698370
Bn-A06-p23741737	0.995024876	0.995024876	Bn-N6-p23556658	Bn-chrA06-p22728939
Bn-A06-p23744370	0.837209302	0.837209302	Bn-N18-p22925019	Bn-chrCnn_random-p507713
Bn-A06-p23746948	0.532338308	0.830845771	Bn-N6-p23558241	Bn-chrA06-p22727353
Bn-A06-p23748804	0.990049751	0.990049751	Bn-N6-p23560008	Bn-chrA06-p22726963
Bn-A06-p23750653	0.987654321	0.99382716	Bn-N6-p23561852	Bn-chrA06-p22725160
Bn-A06-p23759352	0.983471074	0.975206612	Bn-N6-p23571158	Bn-chrA06-p22715814
Bn-A06-p23768610	0.983471074	0.966942149	Bn-N6-p23581864	Bn-chrA06-p22750650
Bn-A06-p23776151	1	1	Bn-N6-p23591715	Bn-chrA06-p22760586
Bn-A06-p23795599	0.958677686	0.958677686	Bn-N6-p23605693	Bn-chrA06-p22776600
Bn-A06-p23795763	0.950413223	0.966942149	Bn-N6-p23605857	Bn-chrA06-p22776764
Bn-A06-p23796020	0.991735537	0.991735537	Bn-N6-p23606115	Bn-chrA06-p22777022
Bn-A06-p23806343	0.960199005	0.955223881	Bn-N6-p23618000	Bn-chrA06-p22787257
Bn-A06-p23852270	0.983471074	0.983471074	Bn-N6-p23666104	Bn-chrA06-p22839543
Bn-A06-p23857345	0.970149254	0.970149254	Bn-N6-p23672502	Bn-chrA06-p22846229
Bn-A06-p23865356	0.966942149	0.958677686	Bn-N6-p23680500	Bn-chrA06-p22856805
Bn-A06-p23873505	0.983471074	0.991735537	Bn-N6-p23688655	Bn-chrA06-p22863782
Bn-A06-p23892315	0.960199005	0.960199005	Bn-N6-p23703062	Bn-chrA06-p22878151
Bn-A06-p23892404	0.77076412	0.77076412	Bn-N6-p23703151	Bn-chrA06-p22878240
Bn-A06-p23944667	0.975206612	0.983471074	Bn-N6-p23757204	Bn-chrA06-p22930179
Bn-A06-p23968879	0.983471074	0.983471074	Bn-N6-p23786567	Bn-chrAnn_random-p390354
Bn-A06-p23972440	0.876033058	0.876033058	Bn-N6-p23786813	Bn-chrAnn_random-p390351
Bn-A06-p23983648	0.983388704	0.983388704	Bn-N6-p23796915	Bn-chrA06-p22967644
Bn-A06-p23988702	0.950413223	0.950413223	Bn-N6-p23801589	Bn-chrA06-p22975706
Bn-A06-p24000840	0.980099502	0.467661692	Bn-N3-p25075549	Bn-chrA03_random-p569505
Bn-A06-p24005581	0.975206612	0.975206612	Bn-N6-p23818982	Bn-chrA06-p22994275
Bn-A06-p24006085	0.991735537	0.991735537	Bn-Scaffold63681-p1	Bn-chrA06-p22994779
Bn-A06-p24017545	1	1	Bn-N6-p23828776	Bn-chrA06-p23007521
Bn-A06-p24032427	0.966942149	0.966942149	Bn-N6-p23840741	Bn-chrA06-p23030939
Bn-A06-p24040939	0.925619835	0.925619835	Bn-N6-p23849070	Bn-chrA06-p23039388
Bn-A06-p24041870	0.991735537	0.991735537	Bn-N6-p23850001	Bn-chrA06-p23040319
Bn-A06-p24043170	0.983388704	0.983388704	Bn-N6-p23851318	Bn-chrA06-p23041636
Bn-A06-p24045825	0.975206612	0.975206612	Bn-N6-p23855558	Bn-chrA06-p23045876
Bn-A06-p24046488	0.983471074	0.983471074	Bn-N6-p23856221	Bn-chrA06-p23046539
Bn-A06-p24068524	0.884297521	0.859504132	Bn-N6-p23877334	Bn-chrC07_random-p149710
Bn-A06-p24070714	0.958677686	0.958677686	Bn-N6-p23879361	Bn-chrA06-p23070364
Bn-A06-p24075375	0.975206612	0.975206612	Bn-N6-p23883968	Bn-chrA06-p23074971
Bn-A06-p24076443	0.900826446	0.900826446	Bn-N6-p23885034	Bn-chrA06-p23076037

Bn-A06-p24099288	0.983471074	0.975206612	Bn-N6-p23909011	Bn-chrA06-p23095245
Bn-A06-p24132842	0.983471074	0.991735537	Bn-N6-p23944011	Bn-chrA06-p23129284
Bn-A06-p24132956	0.983471074	0.991735537	Bn-N6-p23944125	Bn-chrA06-p23129398
Bn-A06-p24142937	0.958677686	0.94214876	Bn-N6-p23957190	Bn-chrA06-p23133566
Bn-A06-p24145208	0.983471074	0.975206612	Bn-N6-p23959651	Bn-chrA06-p23135452
Bn-A06-p24147820	0.983471074	0.851239669	Bn-N6-p23962263	Bn-chrA06-p23138099
Bn-A06-p24152122	0.965174129	0.965174129	Bn-N6-p23965893	Bn-chrA06-p23141593
Bn-A06-p24156940	0.966942149	0.975206612	Bn-N6-p23973942	Bn-chrA06-p23146420
Bn-A06-p24156988	0.975206612	0.966942149	Bn-N6-p23973990	Bn-chrA06-p23146468
Bn-A06-p24167592	0.991735537	0.991735537	Bn-N6-p23983877	Bn-chrA06-p23155725
Bn-A06-p24168470	0.965174129	0.970149254	Bn-N6-p23984755	Bn-chrA06-p23156603
Bn-A06-p24170361	0.975206612	0.950413223	Bn-N6-p23986647	Bn-chrA06-p23158548
Bn-A06-p24178982	0.966942149	0.966942149	Bn-N6-p23990592	Bn-chrAnn_random-p389847
Bn-A06-p24204030	0.958677686	0.950413223	Bn-N6-p24023698	Bn-chrA06-p23195032
Bn-A06-p24206019	0.94214876	0.933884298	Bn-N6-p24025687	Bn-chrA06-p23197042
Bn-A06-p24206556	0.975206612	0.966942149	Bn-N6-p24026224	Bn-chrA06-p23197587
Bn-A06-p24207308	0.983471074	0.983471074	Bn-N6-p24026977	Bn-chrA06-p23198340
Bn-A06-p24210014	0.983471074	0.975206612	Bn-N6-p24029683	Bn-chrA06-p23201047
Bn-A06-p24218304	0.940199336	0.970099668	Bn-N6-p24038160	Bn-chrA06-p23209373
Bn-A06-p24220121	0.958677686	0.900826446	Bn-N6-p24040134	Bn-chrA06-p23211121
Bn-A06-p24222064	0.983471074	0.975206612	Bn-N6-p24042075	Bn-chrA06-p23213062
Bn-A06-p24236385	0.991735537	0.983471074	Bn-N6-p24056480	Bn-chrA06-p23227411
Bn-A06-p24237899	0.983471074	0.991735537	Bn-N6-p24058038	Bn-chrA06-p23229058
Bn-A06-p24240603	1	0.991735537	Bn-N6-p24060746	Bn-chrA06-p23233343
Bn-A06-p24245615	0.950413223	0.94214876	Bn-N6-p24068112	Bn-chrA06-p23238573
Bn-A06-p24257262	0.950166113	0.96013289	Bn-N6-p24078028	Bn-chrA06-p23254229
Bn-A06-p24267492	0.983388704	0.986710963	Bn-N6-p24088276	Bn-chrA06-p23264491
Bn-A06-p24267499	0.983471074	0.991735537	Bn-N6-p24088283	Bn-chrA06-p23264498
Bn-A06-p24267745	0.975206612	0.975206612	Bn-N6-p24088529	Bn-chrA06-p23264745
Bn-A06-p24270718	0.958677686	0.966942149	Bn-N6-p24091502	Bn-chrA06-p23267850
Bn-A06-p24274213	0.975206612	0.983471074	Bn-N6-p24094998	Bn-chrA06-p23271280
Bn-A06-p24278510	0.991735537	1	Bn-N6-p24099288	Bn-chrA06-p23275564
Bn-A06-p24284014	0.965174129	0.990049751	Bn-N6-p24104837	Bn-chrA06-p23281051
Bn-A06-p24286025	0.966942149	1	Bn-N17-p29727468	Bn-chrA06-p23283297
Bn-A06-p24297216	0.991735537	1	Bn-N6-p24123202	Bn-chrA06-p23296286
Bn-A06-p24307250	0.986710963	0.970099668	Bn-N6-p24135772	Bn-chrA06-p23306063
Bn-A06-p24319329	0.983388704	0.983388704	Bn-N6-p24151274	Bn-chrA06_random-p183763
Bn-A06-p24327317	0.996677741	0.996677741	Bn-N6-p24159659	Bn-chrA06_random-p184555
Bn-A06-p24334015	0.990033223	0.993355482	Bn-N6-p24166589	Bn-chrA06_random-p185280
Bn-A06-p24340685	0.970149254	0.980099502	Bn-N6-p24173273	Bn-chrA06_random-p185955
Bn-A06-p24340830	0.991735537	0.983471074	Bn-N6-p24173419	Bn-chrA06_random-p185969
Bn-A06-p24370009	0.980099502	1	Bn-N6-p24209356	Bn-chrA06_random-p190047
Bn-A06-p24371980	0.991735537	0.983471074	Bn-N6-p24211312	Bn-chrA06_random-p190331
Bn-A06-p24386467	0.975206612	0.958677686	Bn-N6-p24224670	Bn-chrA06-p23320798
Bn-A06-p24395608	0.950413223	0.925619835	Bn-N6-p24233346	Bn-chrA06-p23330529
Bn-A06-p24401543	0.975206612	0.983471074	Bn-N6-p24238112	Bn-chrA06-p23335138

Bn-A06-p24404374	0.983471074	0.983471074	Bn-N6-p24240784	Bn-chrA06-p23337824
Bn-A06-p24404996	0.991735537	0.983471074	Bn-N6-p24241404	Bn-chrA06-p23338446
Bn-A06-p24405065	0.966942149	0.975206612	Bn-N6-p24241473	Bn-chrA06-p23338515
Bn-A06-p24407165	0.958677686	0.958677686	Bn-N6-p24243570	Bn-chrA06-p23340613
Bn-A06-p24407535	0.958677686	0.950413223	Bn-N6-p24243940	Bn-chrA06-p23340987
Bn-A06-p24407609	0.960199005	0.955223881	Bn-N6-p24244014	Bn-chrA06-p23341061
Bn-A06-p24410007	0.966942149	0.966942149	Bn-N6-p24246447	Bn-chrA06-p23343493
Bn-A06-p24428073	0.754152824	0.877076412	Bn-N6-p24262411	Bn-chrA06-p23355973
Bn-A06-p24430448	0.983471074	0.991735537	Bn-N6-p24264284	Bn-chrA06-p23359744
Bn-A06-p24430453	0.991735537	0.983471074	Bn-N6-p24264289	Bn-chrA06-p23359749
Bn-A06-p24431577	0.925619835	0.950413223	Bn-N6-p24265487	Bn-chrA06-p23360856
Bn-A06-p24432451	0.983471074	0.975206612	Bn-N6-p24266787	Bn-chrA06-p23361751
Bn-A06-p24432909	0.867768595	0.859504132	Bn-N6-p24267247	Bn-chrA06-p23362212
Bn-A06-p24433131	0.966942149	0.975206612	Bn-N6-p24267546	Bn-chrA06-p23362511
Bn-A06-p24441882	0.983471074	0.975206612	Bn-N6-p24276952	Bn-chrA06-p23371812
Bn-A06-p24453423	0.966942149	0.983471074	Bn-N17-p29388756	Bn-chrA06-p23394170
Bn-A06-p24465494	0.995024876	1	Bn-N6-p24307735	Bn-chrA06-p23403953
Bn-A06-p24466136	0.966942149	0.958677686	Bn-N6-p24308377	Bn-chrA06-p23404595
Bn-A06-p24467652	0.990033223	0.986710963	Bn-N6-p24309893	Bn-chrA06-p23406115
Bn-A06-p24496051	0.983471074	0.983471074	Bn-N6-p24341316	Bn-chrA06-p23440068
Bn-A06-p24501315	0.991735537	0.983471074	Bn-N6-p24345526	Bn-chrA06-p23444303
Bn-A06-p24501540	0.925619835	0.933884298	Bn-N6-p24345756	Bn-chrA06-p23444534
Bn-A06-p24503033	0.975206612	0.966942149	Bn-N6-p24347205	Bn-chrA06-p23446327
Bn-A06-p24508464	0.966942149	0.966942149	Bn-N6-p24353486	Bn-chrA06-p23452633
Bn-A06-p24536499	1	1	Bn-N6-p24383202	Bn-chrA06-p23486643
Bn-A06-p24544753	0.975206612	0.975206612	Bn-N6-p24393610	Bn-chrA06-p23495918
Bn-A06-p24551529	0.975206612	0.975206612	Bn-N6-p24396660	Bn-chrA06-p23498968
Bn-A06-p24557108	0.986710963	0.986710963	Bn-N6-p24402583	Bn-chrA06-p23504890
Bn-A06-p24557193	0.965174129	0.965174129	Bn-N6-p24402668	Bn-chrA06-p23504975
Bn-A06-p24567939	1	0.991735537	Bn-N6-p24412941	Bn-chrAnn_random-p418174
Bn-A06-p24570179	0.986710963	0.554817276	Bn-N6-p24415191	Bn-chrC07-p26244198
Bn-A06-p24579442	0.966942149	0.958677686	Bn-N6-p24420595	Bn-chrA06_random-p201519
Bn-A06-p24580393	0.975206612	0.966942149	Bn-N6-p24421539	Bn-chrA06_random-p201424
Bn-A06-p24588298	0.975206612	0.983471074	Bn-N6-p24430998	Bn-chrA06_random-p200823
Bn-A06-p24599972	0.958677686	0.966942149	Bn-N6-p24445691	Bn-chrA06_random-p199620
Bn-A06-p24600019	0.950413223	0.958677686	Bn-N6-p24445738	Bn-chrA06_random-p199615
Bn-A06-p24600140	0.991735537	1	Bn-N6-p24445859	Bn-chrA06_random-p199603
Bn-A06-p24602106	0.989583333	1	Bn-N6-p24447825	Bn-chrA06_random-p199411
Bn-A06-p24602719	0.991735537	1	Bn-N6-p24448438	Bn-chrA06_random-p199345
Bn-A06-p24607556	0.975206612	0.966942149	Bn-N6-p24451068	Bn-chrA06_random-p199084
Bn-A06-p24618096	0.966942149	0.958677686	Bn-N6-p24461159	Bn-chrA06_random-p198139
Bn-A06-p24678077	0.970149254	0.970149254	Bn-N6-p24528683	Bn-chrA06_random-p202124
Bn-A06-p24695860	0.990033223	0.973421927	Bn-N6-p24549202	Bn-chrA06-p23595421
Bn-A06-p24705718	0.966942149	0.975206612	Bn-N6-p24557399	Bn-chrA06-p23605060
Bn-A06-p24709822	0.930348259	0.965174129	Bn-N6-p24560947	Bn-chrA06-p23608604
Bn-A06-p24713872	0.950413223	0.94214876	Bn-N6-p24567917	Bn-chrA06-p23617144

Bn-A06-p24717767	0.975206612	0.966942149	Bn-N6-p24571783	Bn-chrA06-p23621029
Bn-A06-p24726203	0.990049751	0.995024876	Bn-N6-p24580308	Bn-chrA06-p23629468
Bn-A06-p24730935	1	1	Bn-N6-p24585353	Bn-chrA06-p23633561
Bn-A06-p24744523	0.985074627	0.980099502	Bn-N6-p24600059	Bn-chrA06-p23647354
Bn-A06-p24746974	0.991735537	1	Bn-N6-p24601888	Bn-chrA06-p23649814
Bn-A06-p24814906	0.983471074	0.933884298	Bn-N6-p24668305	Bn-chrA06-p23719272
Bn-A06-p24822573	0.958677686	0.975206612	Bn-N6-p24675695	Bn-chrA06-p23726920
Bn-A06-p24825884	0.991735537	0.983471074	Bn-N6-p24679001	Bn-chrA06-p23730027
Bn-A06-p24827051	0.996677741	1	Bn-N6-p24680168	Bn-chrA06-p23731195
Bn-A06-p24827783	0.958677686	0.950413223	Bn-N6-p24680900	Bn-chrA06-p23731927
Bn-A06-p24831071	0.950413223	0.958677686	Bn-N6-p24684232	Bn-chrA06-p23739423
Bn-A06-p24834963	0.991735537	1	Bn-N6-p24688430	Bn-chrA06-p23743287
Bn-A06-p24836495	0.976744186	0.976744186	Bn-N17-p27245347	Bn-chrC07-p25471218
Bn-A06-p24849468	0.966942149	0.958677686	Bn-N6-p24703073	Bn-chrA06_random-p196231
Bn-A06-p24886436	0.996677741	1	Bn-N6-p24737560	Bn-chrAnn_random-p424063
Bn-A06-p24890908	0.975206612	0.983471074	Bn-N6-p24742053	Bn-chrA06_random-p191804
Bn-A06-p24913140	0.975206612	0.983471074	Bn-N6-p24747914	Bn-chrAnn_random-p424106
Bn-A06-p24922072	0.900497512	0.925373134	Bn-N6-p24757453	Bn-chrAnn_random-p604901
Bn-A06-p24929938	0.965174129	0.965174129	Bn-N6-p24765269	Bn-chrAnn_random-p604147
Bn-A06-p24934604	0.995024876	0.995024876	Bn-N6-p24772784	Bn-chrAnn_random-p603396
Bn-A06-p24939059	0.991735537	0.991735537	Bn-N6-p24777276	Bn-chrAnn_random-p602975
Bn-A06-p24973824	0.965174129	0.965174129	Bn-N6-p24816725	Bn-chrAnn_random-p598713
Bn-A06-p24976434	0.906976744	0.92358804	Bn-N6-p24819387	Bn-chrAnn_random-p598442
Bn-A06-p25329425	0.966777409	0.986710963	Bn-N6-p25157029	Bn-chrAnn_random-p107767
Bn-A06-p25343454	0.983471074	0.983471074	Bn-N6-p25171248	Bn-chrAnn_random-p107623
Bn-A06-p25345586	0.925619835	0.925619835	Bn-N6-p25174012	Bn-chrAnn_random-p107596
Bn-A06-p25349874	0.983471074	0.983471074	Bn-N6-p25178236	Bn-chrAnn_random-p107553
Bn-A06-p25349932	0.79338843	0.79338843	Bn-N6-p25178294	Bn-chrAnn_random-p107553
Bn-A06-p25350033	0.950413223	0.950413223	Bn-N6-p25178438	Bn-chrAnn_random-p107551
Bn-A06-p25351980	0.983471074	0.983471074	Bn-N6-p25180401	Bn-chrAnn_random-p107532
Bn-A06-p25369364	0.983471074	0.983471074	Bn-N6-p25199191	Bn-chrA06-p23751340
Bn-A06-p25369668	0.966942149	0.966942149	Bn-N6-p25199507	Bn-chrA06-p23751656
Bn-A06-p25369719	0.966942149	0.966942149	Bn-N6-p25199558	Bn-chrA06-p23751707
Bn-A06-p25377321	0.975206612	0.975206612	Bn-N6-p25207097	Bn-chrA06-p23759347
Bn-A06-p25409852	0.990033223	0.990033223	Bn-N6-p25223702	Bn-chrA06-p23780504
Bn-A06-p25471463	0.995024876	1	Bn-N6-p25279787	Bn-chrA06-p23837537
Bn-A06-p25786733	1	1	Bn-N6-p25570516	Bn-chrA06-p24108933
Bn-A06-p25840740	1	1	Bn-N6-p25639058	Bn-chrA06-p24172979
Bn-A06-p25878721	0.790697674	0.790697674	Bn-N6-p25678191	Bn-chrA06-p24211904
Bn-A06-p26011034	0.980099502	0.985074627	Bn-N6-p25807999	Bn-chrA06_random-p214311
Bn-A06-p26023391	0.993355482	0.993355482	Bn-N6-p25820339	Bn-chrA06_random-p215627
Bn-A06-p26039465	0.990049751	0.995024876	Bn-N6-p25832496	Bn-chrA06_random-p216578
Bn-A06-p26043562	0.975206612	0.983471074	Bn-N6-p25837593	Bn-chrA06_random-p216999
Bn-A06-p26045429	0.955223881	0.950248756	Bn-N6-p25839460	Bn-chrA06_random-p217225
Bn-A06-p26058372	0.983471074	0.975206612	Bn-N6-p25852556	Bn-chrA06_random-p218524
Bn-A06-p26058643	0.991735537	0.983471074	Bn-N6-p25852811	Bn-chrA06_random-p218551

Bn-A06-p26066533	0.983388704	0.983388704	Bn-N17-p48616786	Bn-chrC07-p44631326
Bn-A06-p26080915	0.975206612	0.983471074	Bn-N6-p25872261	Bn-chrA06-p24303394
Bn-A06-p26081617	0.913705584	0.974619289	Bn-N6-p25872961	Bn-chrA06-p24304096
Bn-A06-p26083498	0.983471074	0.991735537	Bn-N6-p25873810	Bn-chrA06-p24304953
Bn-A06-p26096667	0.983471074	0.983471074	Bn-N6-p25892133	Bn-chrA06-p24316677
Bn-A06-p26096963	0.975206612	0.975206612	Bn-N6-p25892429	Bn-chrA06-p24316973
Bn-A06-p26097037	0.991735537	0.991735537	Bn-N6-p25892503	Bn-chrA06-p24317047
Bn-A06-p26100486	1	1	Bn-N6-p25895515	Bn-chrA06-p24321284
Bn-A06-p26100652	0.983471074	0.983471074	Bn-N6-p25895681	Bn-chrA06-p24321450
Bn-A06-p26100738	0.975206612	0.975206612	Bn-N6-p25895767	Bn-chrA06-p24321536
Bn-A06-p26100812	1	1	Bn-N6-p25895841	Bn-chrA06-p24321610
Bn-A06-p26101019	0.991735537	0.991735537	Bn-N6-p25896048	Bn-chrA06-p24321817
Bn-A06-p26101080	0.991735537	0.991735537	Bn-N6-p25896109	Bn-chrA06-p24321878
Bn-A06-p26101272	0.991735537	0.991735537	Bn-N6-p25896301	Bn-chrA06-p24322070
Bn-A06-p26108669	0.913621262	0.913621262	Bn-N6-p25904018	Bn-chrA06-p24329786
Bn-A06-p26113448	0.996677741	0.986710963	Bn-N6-p25908744	Bn-chrA06-p24334541
Bn-A06-p26125430	0.990049751	0.990049751	Bn-N6-p18741798	Bn-chrA06-p18266080
Bn-A06-p26135022	0.990049751	0.985074627	Bn-N6-p25921608	Bn-chrA06-p24347416
Bn-A06-p26135038	0.990049751	0.985074627	Bn-N6-p25921624	Bn-chrA06-p24347432
Bn-A06-p26135283	0.993355482	0.993355482	Bn-N6-p25921906	Bn-chrA06-p24347677
Bn-A06-p26135324	0.970149254	0.970149254	Bn-N6-p25921947	Bn-chrA06-p24347718
Bn-A06-p26139717	0.991735537	1	Bn-N6-p25929747	Bn-chrA06-p24352143
Bn-A06-p26144134	0.983471074	0.991735537	Bn-N6-p25934162	Bn-chrA06-p24355163
Bn-A06-p26144417	0.990033223	0.983388704	Bn-N6-p25934445	Bn-chrA06-p24355445
Bn-A06-p26144731	0.985074627	0.995024876	Bn-N6-p25934759	Bn-chrA06-p24355759
Bn-A06-p26152237	0.983471074	0.991735537	Bn-N6-p25942264	Bn-chrA06-p24363263
Bn-A06-p26154413	0.995024876	1	Bn-N6-p25944375	Bn-chrA06-p24365374
Bn-A06-p26155068	0.995024876	1	Bn-N6-p25945030	Bn-chrA06-p24366029
Bn-A06-p26159702	0.991735537	0.983471074	Bn-N6-p25949674	Bn-chrA06-p24370133
Bn-A06-p26173206	0.993355482	0.996677741	Bn-N6-p25964897	Bn-chrA06-p24387670
Bn-A06-p26186229	0.996677741	1	Bn-N6-p25971825	Bn-chrA06_random-p221630
Bn-A06-p26212942	0.985074627	0.990049751	Bn-N6-p26002697	Bn-chrAnn_random-p901139
Bn-A06-p26213014	0.985074627	0.990049751	Bn-N6-p26002769	Bn-chrAnn_random-p901132
Bn-A06-p26213205	0.995024876	0.995024876	Bn-N6-p26002960	Bn-chrAnn_random-p901113
Bn-A06-p26213281	0.996677741	0.996677741	Bn-N6-p26003036	Bn-chrAnn_random-p901105
Bn-A06-p26219274	0.980099502	0.985074627	Bn-N6-p26008063	Bn-chrA06_random-p219076
Bn-A06-p26219617	0.980099502	0.985074627	Bn-N6-p26008403	Bn-chrA06_random-p226498
Bn-A06-p26222718	0.96013289	1	Bn-N6-p26013551	Bn-chrA06_random-p226208
Bn-A06-p26248182	0.970099668	0.970099668	Bn-N6-p26038990	Bn-chrA06_random-p223578
Bn-A07-p53742	1	1	Bn-N7-p47215	Bn-chrA07-p423100
Bn-A07-p73440	0.970099668	0.973421927	Bn-N7-p72884	Bn-chrA07-p396904
Bn-A07-p74011	0.991735537	0.983471074	Bn-N7-p73453	Bn-chrA07-p396335
Bn-A07-p107832	0.987012987	0.993506494	Bn-N7-p122997	Bn-chrA07-p343428
Bn-A07-p108234	0.859504132	0.966942149	Bn-Scaffold37248-p3	Bn-chrA07-p342978
Bn-A07-p116371	0.991735537	1	Bn-N7-p130676	Bn-chrA07-p336154
Bn-A07-p132150	0.935323383	0.955223881	Bn-N7-p144429	Bn-chrA07-p323068

Bn-A07-p144486	0.996677741	0.993355482	Bn-N7-p149125	Bn-chrA07-p312944
Bn-A07-p145272	0.985074627	0.990049751	Bn-Scaffold56364-p2	Bn-chrA07-p312158
Bn-A07-p154378	0.880398671	0.887043189	Bn-N7-p157669	Bn-chrA07-p307100
Bn-A07-p158888	0.976744186	0.980066445	Bn-N7-p172010	Bn-chrA07-p302607
Bn-A07-p160648	0.995024876	0.995024876	Bn-N17-p394283	Bn-chrC07-p609866
Bn-A07-p167614	0.975124378	0.955223881	Bn-N7-p181388	Bn-chrA07-p288197
Bn-A07-p178437	0.893687708	0.900332226	Bn-N7-p195940	Bn-chrA07-p279756
Bn-A07-p184221	0.966942149	0.975206612	Bn-N7-p200286	Bn-chrA07-p273697
Bn-A07-p186971	0.993355482	0.996677741	Bn-N7-p203036	Bn-chrA07-p269131
Bn-A07-p187342	0.876033058	0.884297521	Bn-N7-p203407	Bn-chrA07-p268760
Bn-A07-p187880	0.983471074	0.966942149	Bn-N7-p203667	Bn-chrA07-p268500
Bn-A07-p188720	0.991735537	0.983471074	Bn-N7-p204507	Bn-chrA07-p267662
Bn-A07-p188774	0.966942149	0.975206612	Bn-N7-p204561	Bn-chrA07-p267608
Bn-A07-p189086	0.990033223	0.993355482	Bn-N7-p204873	Bn-chrA07-p267293
Bn-A07-p199473	0.955223881	0.950248756	Bn-N7-p215439	Bn-chrA07-p255458
Bn-A07-p199589	0.966942149	0.966942149	Bn-N7-p221507	Bn-chrA07-p255342
Bn-A07-p211902	0.966942149	0.958677686	Bn-N7-p226830	Bn-chrA07-p244449
Bn-A07-p212488	0.80730897	0.996677741	Bn-N17-p494151	Bn-chrA07-p243867
Bn-A07-p216152	0.991735537	0.991735537	Bn-N7-p231855	Bn-chrA07-p240204
Bn-A07-p241997	0.990049751	0.990049751	Bn-N17-p652619	Bn-chrC07-p877690
Bn-A07-p244051	0.990049751	0.980099502	Bn-N7-p269475	Bn-chrA07-p206928
Bn-A07-p255987	0.991735537	0.991735537	Bn-N7-p284309	Bn-chrA07-p194948
Bn-A07-p267110	1	1	Bn-N7-p296070	Bn-chrA07-p184985
Bn-A07-p275485	0.983471074	0.859504132	Bn-N7-p304493	Bn-chrA07-p176582
Bn-A07-p276584	0.983471074	0.983471074	Bn-N7-p305580	Bn-chrA07-p175504
Bn-A07-p278353	0.900497512	0.900497512	Bn-N17-p700584	Bn-chrC07-p918397
Bn-A07-p286291	0.965174129	0.965174129	Bn-N7-p315372	Bn-chrA07-p165200
Bn-A07-p286993	0.980099502	0.980099502	Bn-N7-p316074	Bn-chrA07-p164498
Bn-A07-p312340	0.990049751	0.990049751	Bn-N7-p340480	Bn-chrA07-p122186
Bn-A07-p315799	0.990049751	0.990049751	Bn-N7-p341722	Bn-chrA07-p120944
Bn-A07-p367569	0.990033223	0.990033223	Bn-N7-p392915	Bn-chrA07-p66485
Bn-A07-p430181	0.975206612	0.983471074	Bn-N1-p26506031	Bn-chrA01-p23046328
Bn-A07-p475640	0.239583333	1	Unknown	Bn-chrA01-p23001304
Bn-A07-p483451	0.198347107	0.991735537	Bn-N3-p2329168	Bn-chrA01-p22994161
Bn-A07-p483535	0.20661157	0.991735537	Unknown	Bn-chrA01-p22994077
Bn-A07-p483541	0.198347107	0.991735537	Bn-Scaffold17351-p6	Bn-chrA01-p22994071
Bn-A07-p541617	0.965116279	0.965116279	Bn-N9-p29000579	Bn-chrC05-p36934828
Bn-A07-p580965	0.975206612	0.983471074	Bn-N1-p26373707	Bn-chrA01_random-p267305
Bn-A07-p607926	0.966942149	0.975206612	Bn-N7-p505550	Bn-chrA07-p503202
Bn-A07-p642484	0.975124378	0.965174129	Bn-N7-p583037	Bn-chrA07-p596159
Bn-A07-p645188	0.983471074	0.975206612	Bn-N7-p585727	Bn-chrA07-p598859
Bn-A07-p653283	0.993355482	0.990033223	Bn-N7-p594197	Bn-chrA07-p606884
Bn-A07-p663772	0.940298507	0.945273632	Bn-N7-p607970	Bn-chrA07-p617360
Bn-A07-p665003	0.933884298	0.958677686	Bn-N17-p1357043	Bn-chrA07-p623675
Bn-A07-p672743	0.910447761	0.487562189	Bn-N7-p615367	Bn-chrC09-p12477416
Bn-A07-p689831	0.985074627	0.985074627	Bn-N7-p633799	Bn-chrA07-p649566

Bn-A07-p689898	0.996677741	0.973421927	Bn-N7-p633866	Bn-chrA07-p649632
Bn-A07-p786330	0.950413223	0.983471074	Bn-N7-p717123	Bn-chrA07-p718209
Bn-A07-p828139	0.983471074	0.975206612	Bn-N7-p751984	Bn-chrA07-p754917
Bn-A07-p829095	1	1	Bn-N7-p752939	Bn-chrA07-p755874
Bn-A07-p831443	0.991735537	1	Bn-N7-p755302	Bn-chrA07-p758169
Bn-A07-p884435	0.975206612	0.975206612	Bn-N7-p867453	Bn-chrA07_random-p44271
Bn-A07-p885219	0.983471074	0.966942149	Bn-N7-p868405	Bn-chrA07_random-p45249
Bn-A07-p897139	0.71641791	0.960199005	Bn-N7-p895965	Bn-chrA07-p19625735
Bn-A07-p897472	0.991735537	0.991735537	Bn-N7-p896298	Bn-chrA07_random-p2967
Bn-A07-p902415	0.996677741	0.996677741	Bn-N7-p902670	Bn-chrA07-p1172007
Bn-A07-p923902	0.985074627	0.985074627	Bn-N7-p926484	Bn-chrA07-p1149162
Bn-A07-p930068	0.991735537	0.983471074	Bn-N7-p929818	Bn-chrA07-p1145826
Bn-A07-p955599	0.975206612	0.958677686	Bn-N7-p970705	Bn-chrA07-p1113459
Bn-A07-p959523	0.991735537	0.991735537	Bn-N7-p974049	Bn-chrA07-p1110150
Bn-A07-p963064	0.993355482	0.990033223	Bn-N7-p977627	Bn-chrA07-p1105731
Bn-A07-p1000144	0.975206612	0.983471074	Bn-N7-p995736	Bn-chrA07_random-p62987
Bn-A07-p1026571	0.970149254	0.980099502	Bn-N7-p1017611	Bn-chrA07_random-p82509
Bn-A07-p1040165	0.975124378	0.980099502	Bn-N18-p5816288	Bn-chrA07_random-p95122
Bn-A07-p1041609	0.970149254	0.975124378	Bn-N18-p5814844	Bn-chrC08-p5573618
Bn-A07-p1093997	0.990033223	0.993355482	Bn-N7-p1112292	Bn-chrA07_random-p144830
Bn-A07-p1096829	0.925619835	0.925619835	Bn-N7-p1115518	Bn-chrA07_random-p147868
Bn-A07-p1098196	0.991735537	0.991735537	Bn-N7-p1119119	Bn-chrA07_random-p151859
Bn-A07-p1118872	0.991735537	0.991735537	Bn-N7-p1152243	Bn-chrA07-p975899
Bn-A07-p1135440	0.966942149	0.958677686	Bn-N7-p1164517	Bn-chrA07-p963624
Bn-A07-p1145134	0.993355482	0.993355482	Bn-N7-p1172665	Bn-chrA07-p954142
Bn-A07-p1158936	1	0.917355372	Bn-N7-p1187686	Bn-chrC07-p3412519
Bn-A07-p1164871	0.603305785	0.47107438	Bn-N7-p1201219	Bn-chrC04-p34284869
Bn-A07-p1177830	0.940298507	0.935323383	Bn-N7-p1213182	Bn-chrA07-p909978
Bn-A07-p1188051	0.991735537	0.983471074	Bn-N7-p1222945	Bn-chrA07-p899565
Bn-A07-p1193071	0.985074627	0.985074627	Bn-N7-p1227963	Bn-chrA07-p894540
Bn-A07-p1193432	0.966942149	0.966942149	Bn-N7-p1228338	Bn-chrA07-p894165
Bn-A07-p1196596	0.983471074	0.975206612	Bn-N7-p1235148	Bn-chrA07-p887591
Bn-A07-p1197450	0.958677686	0.983471074	Bn-N7-p1236669	Bn-chrA07-p886737
Bn-A07-p1197779	0.986710963	0.916943522	Bn-N7-p1237149	Bn-chrA07-p886411
Bn-A07-p1197918	0.990033223	0.92358804	Bn-N7-p1237291	Bn-chrA07-p886269
Bn-A07-p1198037	0.958677686	0.966942149	Bn-N7-p1237410	Bn-chrA07-p886150
Bn-A07-p1198107	0.976744186	0.96013289	Bn-N7-p1237480	Bn-chrA07-p886080
Bn-A07-p1198119	0.980066445	0.96013289	Bn-N7-p1237492	Bn-chrA07-p886068
Bn-A07-p1198281	0.958677686	0.966942149	Bn-N7-p1237654	Bn-chrA07-p885906
Bn-A07-p1210412	0.991735537	0.991735537	Bn-Scaffold06061-p3	Bn-chrA07-p874226
Bn-A07-p1210439	0.983471074	0.983471074	Bn-Scaffold06061-p3	Bn-chrA07-p874199
Bn-A07-p1217535	0.975206612	0.983471074	Bn-N7-p1251465	Bn-chrA07-p871183
Bn-A07-p1217876	1	0.986710963	Bn-N7-p1251806	Bn-chrA07-p870839
Bn-A07-p1227140	0.995024876	0.995024876	Bn-N7-p1262567	Bn-chrA07-p861713
Bn-A07-p1228602	0.975206612	0.958677686	Bn-N7-p1264030	Bn-chrA07-p860252
Bn-A07-p1254564	0.991735537	0.991735537	Bn-N7-p1298977	Bn-chrA07-p1271475

Bn-A07-p1255437	0.950413223	0.79338843	Bn-N7-p1299849	Bn-chrA07-p1270584
Bn-A07-p1292866	1	0.991735537	Bn-N7-p1320908	Bn-chrA07-p1250163
Bn-A07-p1293366	1	1	Bn-N17-p3287749	Bn-chrC07-p3768214
Bn-A07-p1294264	0.809917355	0.909090909	Bn-N7-p1322327	Bn-chrA07-p1248745
Bn-A07-p1300457	0.993355482	0.976744186	Bn-N7-p1329349	Bn-chrA07-p1241723
Bn-A07-p1301127	1	1	Bn-N7-p1330026	Bn-chrA07-p1241046
Bn-A07-p1301402	0.980099502	0.980099502	Bn-N7-p1330301	Bn-chrA07-p1240771
Bn-A07-p1303513	0.983471074	0.983471074	Bn-N7-p1333816	Bn-chrA07-p1237823
Bn-A07-p1303705	0.983471074	0.983471074	Bn-N7-p1334008	Bn-chrA07-p1237631
Bn-A07-p1306286	0.980099502	0.985074627	Bn-N7-p1336584	Bn-chrA07-p1235062
Bn-A07-p1323822	0.970149254	0.975124378	Bn-N7-p1352987	Bn-chrA07-p1218132
Bn-A07-p1324687	0.985074627	0.960199005	Bn-N7-p4743730	Bn-chrA07-p5114301
Bn-A07-p1327427	0.958677686	0.94214876	Bn-N7-p1356597	Bn-chrA07-p1214508
Bn-A07-p1336276	0.983471074	0.966942149	Bn-N7-p1374522	Bn-chrCnn_random-p679782
Bn-A07-p1341355	0.990033223	0.986710963	Bn-N7-p1378886	Bn-chrA07-p1182739
Bn-A07-p1341614	0.975206612	0.975206612	Bn-N7-p1379145	Bn-chrA07-p1182480
Bn-A07-p1341729	0.975206612	0.975206612	Bn-N7-p1379260	Bn-chrA07-p1182365
Bn-A07-p1345521	0.950248756	0.985074627	Bn-N7-p1515424	Bn-chrA07-p1324502
Bn-A07-p1346125	0.975206612	0.983471074	Bn-N17-p3819096	Bn-chrA07-p1323898
Bn-A07-p1349482	0.561983471	1	Bn-N18-p44297707	Bn-chrA07-p1320542
Bn-A07-p1386652	0.933884298	0.983471074	Bn-N17-p3856376	Bn-chrA07_random-p177667
Bn-A07-p1400148	0.975206612	0.983471074	Bn-N7-p1549806	Bn-chrA07_random-p167197
Bn-A07-p1415413	0.913621262	1	Bn-N18-p30622907	Bn-chrAnn_random-p319573
Bn-A07-p1416980	0.876033058	0.950413223	Bn-N18-p30624377	Bn-chrAnn_random-p319588
Bn-A07-p1437386	0.983471074	0.975206612	Bn-N7-p1574595	Bn-chrA07_random-p201838
Bn-A07-p1437777	0.851239669	0.966942149	Bn-N7-p1574926	Bn-chrA07_random-p202229
Bn-A07-p1443503	0.983471074	0.983471074	Bn-N7-p1580667	Bn-chrA07_random-p208497
Bn-A07-p1443824	0.900826446	0.900826446	Bn-N7-p1580988	Bn-chrA07_random-p208818
Bn-A07-p1450730	0.966942149	0.966942149	Bn-N7-p1582176	Bn-chrA07_random-p210004
Bn-A07-p1450961	0.991735537	0.991735537	Bn-N7-p1582412	Bn-chrA07_random-p210238
Bn-A07-p1451303	0.975206612	0.975206612	Bn-N7-p1582753	Bn-chrA07_random-p210579
Bn-A07-p1451559	0.985074627	0.985074627	Bn-N7-p1583009	Bn-chrA07_random-p210835
Bn-A07-p1452210	0.991735537	0.991735537	Bn-N7-p1583660	Bn-chrA07_random-p211486
Bn-A07-p1482858	0.983471074	0.983471074	Bn-N7-p1602681	Bn-chrAnn_random-p379135
Bn-A07-p1501022	0.975206612	0.975206612	Bn-N7-p1621355	Bn-chrA07-p1712262
Bn-A07-p1502636	0.991735537	0.214876033	Bn-N7-p1622968	Unknown
Bn-A07-p1514419	0.958677686	0.958677686	Bn-N17-p4193077	Bn-chrA07-p1689913
Bn-A07-p1516782	0.983471074	0.983471074	Bn-N17-p4195018	Bn-chrA07-p1687972
Bn-A07-p1537589	0.983471074	0.983471074	Bn-N7-p1657598	Bn-chrA07-p1664238
Bn-A07-p1537814	0.991735537	0.991735537	Bn-N7-p1657823	Bn-chrA07-p1664013
Bn-A07-p1557675	0.776859504	0.950413223	Bn-N7-p1675377	Bn-chrA07-p1648134
Bn-A07-p1564369	0.880398671	0.817275748	Bn-N7-p1682483	Bn-chrA07-p1642847
Bn-A07-p1595411	0.993355482	0.996677741	Bn-N7-p1705838	Bn-chrA07-p1620006
Bn-A07-p1602850	0.990033223	0.993355482	Bn-N7-p1713697	Bn-chrA07_random-p189883
Bn-A07-p1619354	1	0.991735537	Bn-N7-p1738710	Bn-chrA07-p1590848
Bn-A07-p1625292	1	0.991735537	Bn-N7-p1748784	Bn-chrA07-p1579244

Bn-A07-p1657613	0.96345515	0.976744186	Bn-N7-p1780390	Bn-chrA07-p1736730
Bn-A07-p1657975	0.989583333	0.989583333	Bn-N7-p1780667	Bn-chrA07-p1737007
Bn-A07-p1658007	0.996677741	0.993355482	Bn-N7-p1780784	Bn-chrA07-p1737124
Bn-A07-p1658486	0.943521595	0.953488372	Bn-N7-p1781263	Bn-chrA07-p1737603
Bn-A07-p1680649	1	0.991735537	Bn-N7-p1789612	Bn-chrA07-p1752216
Bn-A07-p1681059	0.933884298	0.94214876	Bn-N7-p1790020	Bn-chrA07-p1752626
Bn-A07-p1689072	0.973421927	0.903654485	Bn-N7-p1805953	Bn-chrC07-p5101858
Bn-A07-p1701712	0.966942149	0.958677686	Bn-N7-p1818928	Bn-chrA07-p1787533
Bn-A07-p1717993	0.966942149	0.975206612	Bn-N7-p1839170	Bn-chrA07-p1806236
Bn-A07-p1725633	0.966942149	0.975206612	Bn-N7-p1845799	Bn-chrC07_random-p559510
Bn-A07-p1729364	0.990049751	0.990049751	Bn-N7-p1849388	Bn-chrA07-p1812785
Bn-A07-p1734331	0.986710963	0.986710963	Bn-N7-p1855090	Bn-chrA07-p1817767
Bn-A07-p1800556	0.983471074	0.975206612	Bn-N7-p1923077	Bn-chrA07-p1897406
Bn-A07-p1809446	0.94214876	0.933884298	Bn-N7-p1987154	Bn-chrA07-p1969523
Bn-A07-p1809594	0.980099502	0.980099502	Bn-N7-p1987297	Bn-chrA07-p1969667
Bn-A07-p1810459	0.975206612	0.983471074	Bn-N7-p1988166	Bn-chrA07-p1970534
Bn-A07-p1817516	0.983471074	0.975206612	Bn-N7-p2000195	Bn-chrA07-p1977367
Bn-A07-p1822755	0.983471074	0.991735537	Bn-N7-p2003175	Bn-chrA07-p1982996
Bn-A07-p1832580	1	0.991735537	Bn-N7-p2013193	Bn-chrA07-p1994623
Bn-A07-p1835232	0.983471074	0.991735537	Bn-N7-p2015844	Bn-chrA07-p2004494
Bn-A07-p1845182	0.966942149	0.975206612	Bn-Scaffold04025-p2	Bn-chrA07-p2011372
Bn-A07-p1857408	0.737541528	0.737541528	Bn-N13-p31803864	Bn-chrC03-p28317359
Bn-A07-p1861823	0.985074627	0.985074627	Bn-Scaffold02011-p3	Bn-chrCnn_random-p431138
Bn-A07-p1862015	0.980099502	0.980099502	Bn-Scaffold04175-p1	Bn-chrCnn_random-p431226
Bn-A07-p1889752	0.990049751	0.995024876	Bn-Scaffold53518-p2	Bn-chrAnn_random-p228098
Bn-A07-p1962566	0.985074627	0.990049751	Bn-N7-p3790674	Bn-chrA07-p3643691
Bn-A07-p1963551	0.980099502	0.980099502	Bn-N7-p3791659	Bn-chrA07-p3644676
Bn-A07-p1963945	0.985074627	0.985074627	Bn-N7-p3792053	Bn-chrA07-p3645070
Bn-A07-p1963962	0.985074627	0.985074627	Bn-N7-p3792070	Bn-chrA07-p3645087
Bn-A07-p1964708	0.965174129	0.965174129	Bn-N7-p3792814	Bn-chrA07-p3645831
Bn-A07-p1965600	0.985074627	0.985074627	Bn-N7-p3793706	Bn-chrA07-p3646723
Bn-A07-p2264217	1	1	Bn-N7-p4001170	Bn-chrA07-p4006401
Bn-A07-p2278898	0.935323383	0.950248756	Bn-Scaffold01390-p7	Bn-chrAnn_random-p406262
Bn-A07-p2412726	1	1	Bn-N17-p8479168	Bn-chrA07-p3516115
Bn-A07-p2425818	1	1	Bn-N17-p8473452	Bn-chrA07-p3510397
Bn-A07-p2518346	0.993355482	0.993355482	Bn-N7-p4176972	Bn-chrA07-p4504880
Bn-A07-p2524777	0.996677741	1	Bn-N7-p4182170	Bn-chrA07-p4510756
Bn-A07-p2575548	0.980066445	0.993355482	Bn-N7-p4236411	Bn-chrA07-p4563157
Bn-A07-p2578858	0.990049751	0.990049751	Bn-N7-p4242649	Bn-chrA07-p4571739
Bn-A07-p2698690	0.986710963	0.986710963	Bn-N7-p4334144	Bn-chrA07-p4656772
Bn-A07-p2899520	0.996677741	1	Bn-N7-p4513748	Bn-chrA07-p4800872
Bn-A07-p2946329	0.966942149	0.975206612	Bn-Scaffold01112-p3	Bn-chrA07-p4894251
Bn-A07-p2976209	0.983471074	0.991735537	Bn-N7-p4551577	Bn-chrAnn_random-p444773
Bn-A07-p2980414	0.993355482	0.986710963	Bn-N7-p4556230	Bn-chrCnn_random-p114138
Bn-A07-p2990276	0.946843854	0.943521595	Bn-N7-p4568166	Bn-chrA07-p4912091
Bn-A07-p2990468	0.973421927	0.976744186	Bn-N7-p4568360	Bn-chrA07-p4912285

Bn-A07-p2992350	0.986710963	0.993355482	Bn-N7-p4570244	Bn-chrA07-p4914165
Bn-A07-p2992985	0.970149254	0.975124378	Bn-N7-p4570885	Bn-chrA07-p4914807
Bn-A07-p2993312	0.975206612	0.983471074	Bn-N7-p4571219	Bn-chrA07-p4915141
Bn-A07-p2996741	0.601328904	0.734219269	Bn-N19-p45013388	Bn-chrA07-p4922202
Bn-A07-p3013745	0.966942149	0.975206612	Bn-N7-p4599973	Bn-chrA07-p4946165
Bn-A07-p3027332	0.985074627	0.990049751	Bn-N7-p4616996	Bn-chrA07-p4962073
Bn-A07-p3027449	0.993355482	0.996677741	Bn-N7-p4617113	Bn-chrA07-p4962190
Bn-A07-p3028363	0.996677741	0.993355482	Bn-N7-p4617427	Bn-chrA07-p4962504
Bn-A07-p3028546	0.996677741	0.993355482	Bn-N7-p4617610	Bn-chrA07-p4962687
Bn-A07-p3041012	1	0.996677741	Bn-N7-p4632441	Bn-chrA07-p5001002
Bn-A07-p3081602	0.980066445	0.983388704	Bn-N7-p4691361	Bn-chrA07-p5039389
Bn-A07-p3093306	0.991735537	0.983471074	Bn-N7-p4698981	Bn-chrA07-p5046126
Bn-A07-p3102715	0.965174129	0.980099502	Bn-N19-p36899879	Bn-chrA07-p5055535
Bn-A07-p3132316	0.983471074	0.991735537	Bn-N7-p4713420	Bn-chrA07-p5080091
Bn-A07-p3147129	0.990049751	0.990049751	Bn-N18-p38595893	Bn-chrC08-p33251991
Bn-A07-p3161639	0.993355482	0.983388704	Bn-N7-p4742680	Bn-chrA07-p5113262
Bn-A07-p3163343	0.990033223	0.990033223	Bn-N7-p4744409	Bn-chrA07-p5114966
Bn-A07-p3163701	0.993355482	0.990033223	Bn-N7-p4744767	Bn-chrA07-p5115324
Bn-A07-p3164912	0.983471074	0.975206612	Bn-N7-p4746256	Bn-chrA07-p5116512
Bn-A07-p3164991	0.986710963	0.976744186	Bn-N7-p4746335	Bn-chrA07-p5116591
Bn-A07-p3167464	0.995867769	0.991735537	Bn-N7-p4748808	Bn-chrA07-p5119064
Bn-A07-p3183864	0.975124378	0.955223881	Bn-N14-p31648663	Bn-chrCnn_random-p175014
Bn-A07-p3193611	0.993355482	0.986710963	Bn-N7-p4768389	Bn-chrA07-p5137867
Bn-A07-p3202245	0.983471074	0.991735537	Bn-N7-p4780498	Bn-chrA07-p5145367
Bn-A07-p3208245	0.933884298	0.983471074	Bn-N17-p11675532	Bn-chrA07-p5151283
Bn-A07-p3216267	0.983471074	0.983471074	Bn-N7-p4794263	Bn-chrA07-p5160388
Bn-A07-p3252073	0.94214876	0.94214876	Bn-N17-p11731505	Bn-chrC07_random-p302255
Bn-A07-p3262688	0.975206612	0.975206612	Bn-N7-p4845203	Bn-chrA07-p5218239
Bn-A07-p3262752	0.991735537	0.991735537	Bn-N7-p4845267	Bn-chrA07-p5218303
Bn-A07-p3262864	0.983471074	0.983471074	Bn-N7-p4845380	Bn-chrA07-p5218416
Bn-A07-p3275876	0.983471074	0.983471074	Bn-N7-p4866520	Bn-chrA07-p5237792
Bn-A07-p3287486	0.986710963	0.983388704	Bn-N7-p4875256	Bn-chrA07-p5246686
Bn-A07-p3309722	0.996677741	0.996677741	Bn-N7-p4897882	Bn-chrA07-p5268725
Bn-A07-p3323989	0.950413223	0.958677686	Bn-N7-p4911805	Bn-chrA07-p5282644
Bn-A07-p3467400	0.980099502	0.980099502	Bn-N17-p12181043	Bn-chrCnn_random-p162433
Bn-A07-p3474919	0.983471074	0.991735537	Bn-N7-p5112999	Bn-chrA07-p5471075
Bn-A07-p3481474	0.884297521	0.917355372	Bn-N7-p5114373	Bn-chrA07-p5473715
Bn-A07-p3482570	0.966942149	0.958677686	Bn-N7-p5116632	Bn-chrA07-p5474820
Bn-A07-p3482967	0.94214876	0.950413223	Bn-N7-p5117026	Bn-chrA07-p5475200
Bn-A07-p3486022	0.973421927	0.976744186	Bn-N6-p17850899	Bn-chrA06-p17372306
Bn-A07-p3489365	0.983471074	0.909090909	Bn-N7-p5133986	Bn-chrAnn_random-p351259
Bn-A07-p3509187	1	0.991735537	Bn-N7-p5145255	Bn-chrA07-p5495357
Bn-A07-p3517211	0.993355482	0.993355482	Bn-N7-p5153323	Bn-chrA07-p5504139
Bn-A07-p3523290	0.983471074	0.975206612	Bn-N7-p5159374	Bn-chrA07_random-p275030
Bn-A07-p3538601	0.985074627	0.985074627	Bn-N7-p5170034	Bn-chrA07-p5524144
Bn-A07-p3566269	0.980066445	0.980066445	Bn-N7-p5199652	Bn-chrA07-p5552544

Bn-A07-p3568361	0.950413223	0.950413223	Bn-N7-p5201744	Bn-chrA07-p5554636
Bn-A07-p3569093	0.980066445	0.980066445	Bn-N7-p5202476	Bn-chrA07-p5555368
Bn-A07-p3569250	0.983388704	0.983388704	Bn-N7-p5202633	Bn-chrA07-p5555525
Bn-A07-p3571306	0.965174129	0.965174129	Bn-N7-p5204689	Bn-chrA07-p5557581
Bn-A07-p3576219	0.994845361	0.994845361	Bn-Scaffold59091-p1	Bn-chrA07-p5562495
Bn-A07-p3583161	0.983471074	0.983471074	Bn-N7-p5216573	Bn-chrA07-p5569352
Bn-A07-p3584348	0.986710963	0.986710963	Bn-N7-p5217757	Bn-chrA07-p5570536
Bn-A07-p3594657	0.966942149	0.966942149	Bn-N7-p5229386	Bn-chrA07-p5581960
Bn-A07-p3595423	0.966942149	0.966942149	Bn-N7-p5230163	Bn-chrA07-p5582737
Bn-A07-p3595474	0.94214876	0.94214876	Bn-N7-p5230214	Bn-chrA07-p5582788
Bn-A07-p3596286	1	1	Bn-N7-p5231066	Bn-chrA07-p5584152
Bn-A07-p3599603	0.975124378	0.975124378	Bn-N7-p5233222	Bn-chrA07-p5586308
Bn-A07-p3601318	0.966942149	0.966942149	Bn-N7-p5234936	Bn-chrA07-p5588022
Bn-A07-p3609912	0.94214876	0.94214876	Bn-N7-p5235779	Bn-chrA07-p5588865
Bn-A07-p3618045	0.950413223	0.950413223	Bn-N7-p5240893	Bn-chrA07-p5593585
Bn-A07-p3637130	0.950413223	0.950413223	Bn-N5-p6947987	Bn-chrA05-p6186853
Bn-A07-p3646853	0.966942149	0.966942149	Bn-N7-p5260334	Bn-chrA07-p5613758
Bn-A07-p3648246	0.983471074	0.983471074	Bn-N7-p5261722	Bn-chrA07-p5615146
Bn-A07-p3649647	0.975206612	0.975206612	Bn-N7-p5263276	Bn-chrA07-p5616700
Bn-A07-p3659345	0.966942149	0.966942149	Bn-N7-p5279803	Bn-chrA07-p5635533
Bn-A07-p3707777	0.960199005	0.960199005	Bn-N7-p5313502	Bn-chrA07-p5668348
Bn-A07-p3717215	0.980066445	0.980066445	Bn-N7-p5317737	Bn-chrA07-p5672583
Bn-A07-p3718401	0.991735537	0.991735537	Bn-N7-p5318924	Bn-chrA07-p5673770
Bn-A07-p3728535	1	0.958677686	Bn-N7-p5328250	Bn-chrCnn_random-p379870
Bn-A07-p3729992	0.983471074	0.983471074	Bn-N7-p5329729	Bn-chrA07-p5688899
Bn-A07-p3746131	0.983471074	0.975206612	Bn-N7-p5345350	Bn-chrA07-p5706293
Bn-A07-p3747912	0.966942149	0.983471074	Bn-N7-p5347464	Bn-chrA07-p5708072
Bn-A07-p3749471	0.958677686	0.975206612	Bn-N7-p5349029	Bn-chrA07-p5709631
Bn-A07-p3769807	0.768595041	0.983471074	Bn-N7-p5360166	Bn-chrA07-p5729733
Bn-A07-p3771785	0.983471074	0.975206612	Bn-N7-p5361975	Bn-chrA07-p5731711
Bn-A07-p3771821	0.983471074	0.975206612	Bn-N7-p5362011	Bn-chrA07-p5731747
Bn-A07-p3773635	0.950413223	0.966942149	Bn-N7-p5363757	Bn-chrA07-p5733560
Bn-A07-p3793386	0.966942149	0.975206612	Bn-N7-p5378754	Bn-chrA07-p5750839
Bn-A07-p3796068	0.958677686	0.966942149	Bn-N7-p5390231	Bn-chrA07-p5753520
Bn-A07-p3796474	0.986710963	0.973421927	Bn-N7-p5390636	Bn-chrA07-p5753925
Bn-A07-p3796859	0.990049751	0.995024876	Bn-N7-p5391030	Bn-chrA07-p5754310
Bn-A07-p3803780	0.985074627	0.985074627	Bn-N7-p5397899	Bn-chrA07-p5761178
Bn-A07-p3811586	0.980099502	0.995024876	Bn-N7-p5405976	Bn-chrCnn_random-p327766
Bn-A07-p3826249	0.980099502	0.985074627	Bn-N7-p5421931	Bn-chrAnn_random-p358039
Bn-A07-p3856921	0.990033223	0.990033223	Bn-N7-p5441128	Bn-chrA07-p5809630
Bn-A07-p3862192	0.976744186	0.986710963	Bn-N7-p5449697	Bn-chrA07-p5811775
Bn-A07-p3899866	1	1	Bn-N7-p5472422	Bn-chrA07-p5839877
Bn-A07-p3900934	0.975206612	0.958677686	Bn-N7-p5473488	Bn-chrA07-p5840944
Bn-A07-p3915443	0.975206612	0.983471074	Bn-N7-p5477226	Bn-chrA07-p5844663
Bn-A07-p3916567	0.990049751	0.990049751	Bn-N7-p5478132	Bn-chrA07-p5845791
Bn-A07-p3918449	0.890547264	0.880597015	Bn-N7-p5480005	Bn-chrA07-p5847685

Bn-A07-p3921656	0.991735537	0.983471074	Bn-N7-p5485140	Bn-chrA07-p5850789
Bn-A07-p3929927	0.950413223	0.958677686	Bn-N7-p5491433	Bn-chrA07-p5857874
Bn-A07-p3930035	0.933884298	0.925619835	Bn-N7-p5491541	Bn-chrA07-p5857982
Bn-A07-p3930092	0.950413223	0.958677686	Bn-N7-p5491598	Bn-chrA07-p5858047
Bn-A07-p3931338	0.966942149	0.958677686	Bn-N7-p5492846	Bn-chrA07-p5860971
Bn-A07-p3934301	0.876033058	0.876033058	Bn-N7-p5499638	Bn-chrA07-p5863949
Bn-A07-p3957667	0.975124378	0.975124378	Bn-N7-p5523235	Bn-chrA07-p5884688
Bn-A07-p3960247	0.993355482	0.993355482	Bn-N7-p5525814	Bn-chrA07-p5887268
Bn-A07-p3975589	0.976744186	0.996677741	Bn-N7-p5542475	Bn-chrA07-p5906859
Bn-A07-p3976201	0.980099502	0.985074627	Bn-N7-p5543093	Bn-chrA07-p5907473
Bn-A07-p3978709	0.958677686	0.958677686	Bn-N7-p5545742	Bn-chrA07-p5909982
Bn-A07-p3994854	0.805970149	0.805970149	Bn-N7-p5563266	Bn-chrA07-p5927756
Bn-A07-p3995605	0.865671642	0.865671642	Bn-N7-p5563726	Bn-chrA07-p5928216
Bn-A07-p4013256	0.966942149	0.966942149	Bn-N7-p5575771	Bn-chrA07-p5940400
Bn-A07-p4050120	0.993355482	0.993355482	Bn-N7-p5614295	Bn-chrA07-p5977982
Bn-A07-p4050475	0.975124378	0.975124378	Bn-N7-p5614649	Bn-chrA07-p5978336
Bn-A07-p4050743	0.990033223	0.990033223	Bn-N7-p5614917	Bn-chrA07-p5978604
Bn-A07-p4095559	0.996677741	0.993355482	Bn-N7-p5649088	Bn-chrA07-p6012999
Bn-A07-p4097388	0.983471074	0.991735537	Bn-N7-p5650918	Bn-chrA07-p6014828
Bn-A07-p4097690	0.996677741	1	Bn-N7-p5651220	Bn-chrA07-p6015130
Bn-A07-p4109343	0.975206612	0.975206612	Bn-N7-p5661982	Bn-chrA07-p6026999
Bn-A07-p4109459	0.983471074	0.975206612	Bn-N7-p5662098	Bn-chrA07-p6027115
Bn-A07-p4110292	0.925619835	0.917355372	Bn-N7-p5662933	Bn-chrA07-p6027949
Bn-A07-p4129734	0.975206612	0.966942149	Bn-N7-p5677491	Bn-chrA07-p6044995
Bn-A07-p4130487	0.991735537	0.900826446	Bn-N17-p13263351	Bn-chrC07-p13446761
Bn-A07-p4141499	0.925619835	0.975206612	Bn-N7-p5701557	Bn-chrA07-p6059441
Bn-A07-p4142320	0.933884298	0.94214876	Bn-N7-p5705180	Bn-chrA07-p6060262
Bn-A07-p4148934	0.900497512	0.895522388	Bn-N7-p5708942	Bn-chrA07-p6071917
Bn-A07-p4151821	1	0.996677741	Bn-N7-p5711847	Bn-chrA07-p6074800
Bn-A07-p4154271	1	0.996677741	Bn-N7-p5714297	Bn-chrA07-p6077250
Bn-A07-p4155337	1	0.996677741	Bn-N7-p5715363	Bn-chrA07-p6078316
Bn-A07-p4158079	0.980066445	0.983388704	Bn-N7-p5718064	Bn-chrA07-p6081018
Bn-A07-p4161480	0.986710963	0.990033223	Bn-N7-p5721486	Bn-chrA07-p6084438
Bn-A07-p4162513	0.983471074	0.991735537	Bn-N7-p5722520	Bn-chrA07-p6085471
Bn-A07-p4164663	0.990033223	0.993355482	Bn-N7-p5724634	Bn-chrA07-p6087619
Bn-A07-p4173116	0.991735537	0.983471074	Bn-N7-p5732895	Bn-chrA07-p6093107
Bn-A07-p4173583	0.983471074	0.975206612	Bn-N7-p5733362	Bn-chrA07-p6093574
Bn-A07-p4191541	0.996677741	0.993355482	Bn-N7-p5751852	Bn-chrA07-p6111508
Bn-A07-p4191546	0.983471074	0.991735537	Bn-N7-p5751857	Bn-chrA07-p6111513
Bn-A07-p4191832	0.986710963	0.993355482	Bn-N7-p5752143	Bn-chrA07-p6111799
Bn-A07-p4204511	0.983471074	0.975206612	Bn-N7-p5770330	Bn-chrA07-p6125799
Bn-A07-p4204647	0.966942149	0.991735537	Bn-N7-p5770467	Bn-chrA07-p6125935
Bn-A07-p4208528	0.79338843	0.983471074	Bn-N7-p5775000	Bn-chrA07-p6131084
Bn-A07-p4210761	0.991735537	0.983471074	Bn-N7-p5775667	Bn-chrA07-p6133356
Bn-A07-p4289732	0.983471074	0.983471074	Bn-N7-p5861096	Bn-chrA07-p6240941
Bn-A07-p4297035	0.92358804	0.92358804	Bn-N17-p13685676	Bn-chrC07-p11617194

Bn-A07-p4305077	0.950413223	0.950413223	Bn-N7-p5873669	Bn-chrA07-p6253126
Bn-A07-p4305761	0.975206612	0.975206612	Bn-N7-p5874354	Bn-chrA07-p6253811
Bn-A07-p4305829	0.966777409	0.966777409	Bn-N7-p5874422	Bn-chrA07-p6253879
Bn-A07-p4307671	0.983471074	0.983471074	Bn-N7-p5876267	Bn-chrA07-p6255724
Bn-A07-p4309577	1	1	Bn-N7-p5878177	Bn-chrA07-p6257634
Bn-A07-p4324841	0.970149254	0.970149254	Bn-N7-p5893052	Bn-chrA07-p6273728
Bn-A07-p4364075	0.991735537	0.991735537	Bn-N7-p5932176	Bn-chrA07-p6312025
Bn-A07-p4376685	0.993355482	0.993355482	Bn-N7-p5944992	Bn-chrA07-p6324712
Bn-A07-p4380688	0.986710963	0.986710963	Bn-N7-p5949049	Bn-chrA07-p6328755
Bn-A07-p4387388	0.996677741	0.996677741	Bn-N7-p5955748	Bn-chrA07-p6335454
Bn-A07-p4414432	1	1	Bn-N7-p5979312	Bn-chrA07-p6362345
Bn-A07-p4477010	0.975206612	0.975206612	Bn-N7-p6044743	Bn-chrA07-p6430162
Bn-A07-p4480260	0.983388704	0.983388704	Bn-N7-p6046676	Bn-chrA07-p6432212
Bn-A07-p4483935	0.983471074	0.991735537	Bn-N7-p6050372	Bn-chrA07-p6435903
Bn-A07-p4520449	0.983471074	0.991735537	Bn-N7-p6075228	Bn-chrA07-p6462147
Bn-A07-p4522519	0.958677686	0.966942149	Bn-N7-p6077298	Bn-chrA07-p6474301
Bn-A07-p4522573	0.958677686	0.966942149	Bn-N7-p6077352	Bn-chrA07-p6474355
Bn-A07-p4535707	0.950413223	0.975206612	Bn-N7-p6086939	Bn-chrA07-p6479820
Bn-A07-p4536488	0.991735537	0.983471074	Bn-N7-p6087716	Bn-chrA07-p6480601
Bn-A07-p4536608	0.966942149	0.975206612	Bn-N7-p6087836	Bn-chrA07-p6480721
Bn-A07-p4539144	0.950413223	0.966942149	Bn-N7-p6090353	Bn-chrA07-p6483262
Bn-A07-p4554373	0.983471074	0.991735537	Bn-N7-p6110250	Bn-chrA07-p6504425
Bn-A07-p4566975	0.966942149	0.661157025	Bn-N7-p6116331	Bn-chrA07-p6512361
Bn-A07-p4568148	0.950413223	0.975206612	Bn-N7-p6117503	Bn-chrA07-p6513433
Bn-A07-p4578845	0.966942149	0.958677686	Bn-N7-p6129252	Bn-chrA07-p6522553
Bn-A07-p4582463	0.553719008	0.991735537	Bn-N7-p6132922	Bn-chrA07-p6524004
Bn-A07-p4587108	0.73553719	0.958677686	Bn-N17-p14444200	Bn-chrA07-p6528665
Bn-A07-p4607332	0.94214876	0.950413223	Bn-N7-p6164763	Bn-chrA07-p6545846
Bn-A07-p4624421	0.950413223	0.925619835	Bn-N7-p6175718	Bn-chrCnn_random-p338400
Bn-A07-p4639103	0.96	0.965714286	Bn-N18-p6352800	Bn-chrC08-p12549344
Bn-A07-p4644371	0.983471074	0.991735537	Bn-N7-p6189089	Bn-chrAnn_random-p203232
Bn-A07-p4656578	0.94214876	0.950413223	Bn-N7-p6207409	Bn-chrCnn_random-p365643
Bn-A07-p4657440	0.983471074	0.983471074	Bn-N7-p6208272	Bn-chrAnn_random-p30647[illegible]
Bn-A07-p4672563	0.958677686	0.958677686	Bn-N7-p6235419	Bn-chrA07-p6579313
Bn-A07-p4678510	0.983471074	0.983471074	Bn-N7-p6243950	Bn-chrA07-p6585315
Bn-A07-p4684890	0.993355482	0.993355482	Bn-N7-p6250339	Bn-chrA07-p6591848
Bn-A07-p4687740	0.965174129	0.965174129	Bn-N7-p6253189	Bn-chrA07-p6594698
Bn-A07-p4691624	0.985074627	0.985074627	Bn-N7-p6256634	Bn-chrA07-p6598142
Bn-A07-p4693647	1	1	Bn-N7-p6258657	Bn-chrA07-p6600164
Bn-A07-p4704042	0.985074627	0.985074627	Bn-N7-p6267952	Bn-chrA07_random-p422751
Bn-A07-p4708781	1	0.933884298	Bn-N7-p6278815	Bn-chrA07-p6613051
Bn-A07-p4709589	0.990049751	0.985074627	Bn-N7-p6279623	Bn-chrA07-p6613856
Bn-A07-p4745849	0.996677741	0.993355482	Bn-N7-p6311344	Bn-chrA07-p6649572
Bn-A07-p4777275	1	0.991735537	Bn-N7-p6341831	Bn-chrA07-p6666563
Bn-A07-p4802709	0.975206612	0.900826446	Bn-N7-p6366961	Bn-chrA07-p6691862
Bn-A07-p4815239	0.991735537	0.983471074	Bn-N7-p6379719	Bn-chrA07-p6702971

Bn-A07-p4815910	0.983388704	0.983388704	Bn-N7-p6380390	Bn-chrA07-p6703642
Bn-A07-p4817318	0.991735537	0.983471074	Bn-N7-p6381799	Bn-chrA07-p6705049
Bn-A07-p4825210	0.983388704	0.986710963	Bn-N7-p6393903	Bn-chrA07-p6712945
Bn-A07-p4838826	0.966942149	0.975206612	Bn-N7-p6407686	Bn-chrA07-p6725800
Bn-A07-p4851746	0.991735537	0.909090909	Bn-N7-p6420628	Bn-chrA07-p6744499
Bn-A07-p4898790	0.991735537	0.958677686	Bn-N7-p6475131	Bn-chrA07-p6773613
Bn-A07-p4910739	0.980099502	0.975124378	Bn-N7-p6481837	Bn-chrA07-p6781362
Bn-A07-p4911450	0.975206612	0.983471074	Bn-N7-p6482547	Bn-chrA07-p6782073
Bn-A07-p4912091	0.996677741	1	Bn-N7-p6483188	Bn-chrA07-p6782714
Bn-A07-p4912587	0.991735537	1	Bn-N7-p6483683	Bn-chrA07-p6783210
Bn-A07-p4912709	1	0.991735537	Bn-N7-p6483805	Bn-chrA07-p6783332
Bn-A07-p4914608	0.965174129	0.970149254	Bn-N7-p6485705	Bn-chrA07-p6785231
Bn-A07-p4914940	1	0.991735537	Bn-N7-p6486037	Bn-chrA07-p6785563
Bn-A07-p4916203	0.991735537	1	Bn-N7-p6487300	Bn-chrA07-p6786826
Bn-A07-p4916911	0.991735537	0.983471074	Bn-N7-p6488009	Bn-chrA07-p6787534
Bn-A07-p4916995	0.991735537	0.983471074	Bn-N7-p6488093	Bn-chrA07-p6787618
Bn-A07-p4923764	0.975206612	0.966942149	Bn-N7-p6494897	Bn-chrA07-p6794386
Bn-A07-p4923886	0.983471074	0.991735537	Bn-N7-p6495019	Bn-chrA07-p6794508
Bn-A07-p4926294	0.975206612	0.966942149	Bn-N7-p6497490	Bn-chrA07-p6796915
Bn-A07-p4934236	0.975206612	0.983471074	Bn-N7-p6503687	Bn-chrA07-p6805475
Bn-A07-p4937244	0.983471074	0.975206612	Bn-N7-p6506698	Bn-chrA07-p6808482
Bn-A07-p4941605	0.985074627	0.985074627	Bn-N7-p6509274	Bn-chrA07-p6811062
Bn-A07-p4947273	0.945273632	0.945273632	Bn-N7-p6514933	Bn-chrA07-p6816720
Bn-A07-p4954667	0.993355482	0.993355482	Bn-N7-p6520825	Bn-chrA07-p6822420
Bn-A07-p4958794	0.975206612	0.975206612	Bn-N7-p6524952	Bn-chrA07-p6826548
Bn-A07-p4958868	0.970149254	0.970149254	Bn-N7-p6525026	Bn-chrA07-p6826622
Bn-A07-p4959975	0.975206612	0.975206612	Bn-N7-p6526166	Bn-chrA07-p6827762
Bn-A07-p4968765	0.973421927	0.973421927	Bn-N7-p6529612	Bn-chrA07-p6831207
Bn-A07-p4976992	0.965174129	0.965174129	Bn-N7-p6539461	Bn-chrA07-p6840412
Bn-A07-p4997213	0.970149254	0.970149254	Bn-N7-p6558975	Bn-chrA07-p6854122
Bn-A07-p5003344	0.990049751	0.990049751	Bn-N7-p6567828	Bn-chrA07-p6862978
Bn-A07-p5008797	0.983471074	0.983471074	Bn-N7-p6573394	Bn-chrA07-p6868544
Bn-A07-p5016523	0.950413223	0.983471074	Bn-N7-p6580894	Bn-chrA07-p6876098
Bn-A07-p5020495	0.991735537	0.983471074	Bn-N7-p6586715	Bn-chrA07-p6880058
Bn-A07-p5030137	0.966942149	0.975206612	Bn-N7-p6614416	Bn-chrA07-p6894454
Bn-A07-p5039631	0.94214876	0.991735537	Bn-N17-p15223525	Bn-chrA07-p6903535
Bn-A07-p5050160	0.983471074	0.983471074	Bn-N7-p6632110	Bn-chrA07-p6911078
Bn-A07-p5054596	0.980066445	0.980066445	Bn-N7-p6634770	Bn-chrA07-p6913737
Bn-A07-p5072420	0.991735537	0.991735537	Bn-N7-p6659468	Bn-chrA07-p6940676
Bn-A07-p5092272	0.890547264	0.890547264	Bn-N7-p6679874	Bn-chrA07_random-p427173
Bn-A07-p5095657	0.958677686	0.958677686	Bn-N7-p6683612	Bn-chrA07-p6966731
Bn-A07-p5098897	0.983471074	0.983471074	Bn-N7-p6688979	Bn-chrA07-p6974237
Bn-A07-p5100047	0.991735537	0.991735537	Bn-N7-p6690098	Bn-chrA07-p6975355
Bn-A07-p5106046	0.965174129	0.965174129	Bn-N7-p6696116	Bn-chrAnn_random-p412339
Bn-A07-p5110099	0.950413223	0.950413223	Bn-N7-p6699752	Bn-chrA07-p6985387
Bn-A07-p5110555	0.958677686	0.958677686	Bn-N7-p6700208	Bn-chrA07-p6985843

Bn-A07-p5111659	0.991735537	0.991735537	Bn-N7-p6701324	Bn-chrA07-p6986959
Bn-A07-p5112113	0.983471074	0.983471074	Bn-N7-p6701778	Bn-chrA07-p6987413
Bn-A07-p5114487	0.985074627	0.985074627	Bn-N7-p6705645	Bn-chrA07-p6992236
Bn-A07-p5114831	0.958677686	0.958677686	Bn-N7-p6705974	Bn-chrA07-p6992565
Bn-A07-p5114926	0.975206612	0.975206612	Bn-N7-p6706069	Bn-chrA07-p6992660
Bn-A07-p5115445	0.966942149	0.966942149	Bn-N7-p6706589	Bn-chrA07-p6993180
Bn-A07-p5115563	0.975206612	0.975206612	Bn-N7-p6706707	Bn-chrA07-p6993298
Bn-A07-p5118743	0.933884298	0.933884298	Bn-N7-p6710788	Bn-chrA07-p6999113
Bn-A07-p5119149	0.986710963	0.986710963	Bn-N7-p6711189	Bn-chrA07-p6999514
Bn-A07-p5121735	0.966942149	0.966942149	Bn-N7-p6713776	Bn-chrA07-p7002100
Bn-A07-p5126106	0.993355482	0.993355482	Bn-N7-p6718144	Bn-chrA07-p7006468
Bn-A07-p5145011	0.983471074	0.983471074	Bn-N7-p6729961	Bn-chrA07-p7018285
Bn-A07-p5145409	0.990049751	0.990049751	Bn-N7-p6730359	Bn-chrA07-p7018683
Bn-A07-p5150157	0.845771144	0.84079602	Bn-N1-p14708772	Bn-chrA01-p12651212
Bn-A07-p5192258	0.950413223	0.950413223	Bn-N17-p15408165	Bn-chrC07-p13264125
Bn-A07-p5203115	0.966942149	0.966942149	Bn-N7-p6764658	Bn-chrA07-p7052701
Bn-A07-p5211320	0.986710963	0.986710963	Bn-N7-p6774120	Bn-chrA07-p7061895
Bn-A07-p5228632	0.965174129	0.965174129	Bn-N7-p6781198	Bn-chrA07-p7068482
Bn-A07-p5229070	0.94214876	0.94214876	Bn-N7-p6781636	Bn-chrA07-p7068920
Bn-A07-p5234114	0.970149254	0.970149254	Bn-N7-p6798260	Bn-chrA07-p7087959
Bn-A07-p5243525	0.970099668	0.953488372	Bn-N7-p6805566	Bn-chrC07-p13343138
Bn-A07-p5251710	0.925619835	0.925619835	Bn-N7-p6810996	Bn-chrA07-p7101933
Bn-A07-p5255311	0.910299003	0.910299003	Bn-N7-p6822359	Bn-chrA07-p7113392
Bn-A07-p5255375	0.975124378	0.975124378	Bn-N7-p6822423	Bn-chrA07-p7113456
Bn-A07-p5256311	0.976744186	0.976744186	Bn-N7-p6823370	Bn-chrA07-p7114403
Bn-A07-p5259951	0.94214876	0.94214876	Bn-N7-p6826997	Bn-chrA07-p7118032
Bn-A07-p5270463	0.983471074	0.983471074	Bn-N7-p6836706	Bn-chrA07-p7129090
Bn-A07-p5272310	0.970149254	0.970149254	Bn-N7-p6838831	Bn-chrA07-p7131215
Bn-A07-p5288286	0.995024876	0.995024876	Bn-N7-p6852930	Bn-chrA07-p7145344
Bn-A07-p5291077	0.983471074	0.983471074	Bn-N7-p6855593	Bn-chrA07-p7148007
Bn-A07-p5293804	0.991735537	0.991735537	Bn-N7-p6857609	Bn-chrA07-p7150954
Bn-A07-p5295300	1	1	Bn-N7-p6859105	Bn-chrA07-p7152450
Bn-A07-p5304766	0.993355482	0.993355482	Bn-N7-p6866957	Bn-chrA07-p7162155
Bn-A07-p5306358	0.983471074	0.983471074	Bn-N7-p6868549	Bn-chrA07-p7163747
Bn-A07-p5307627	0.996677741	0.996677741	Bn-N7-p6869818	Bn-chrA07-p7165016
Bn-A07-p5310522	0.983471074	0.983471074	Bn-N7-p6872713	Bn-chrA07-p7167911
Bn-A07-p5322075	0.990049751	0.990049751	Bn-N7-p6884009	Bn-chrA07-p7179206
Bn-A07-p5325817	0.990049751	0.990049751	Bn-N7-p6887747	Bn-chrA07-p7182944
Bn-A07-p5325897	0.995024876	0.995024876	Bn-N7-p6887827	Bn-chrA07-p7183024
Bn-A07-p5326470	0.980099502	0.980099502	Bn-N7-p6888399	Bn-chrA07-p7183596
Bn-A07-p5330694	1	1	Bn-N7-p6892622	Bn-chrA07-p7187819
Bn-A07-p5335780	0.985074627	0.985074627	Bn-N7-p6897506	Bn-chrA07-p7192994
Bn-A07-p5340927	0.975206612	0.975206612	Bn-N7-p6902751	Bn-chrA07-p7198237
Bn-A07-p5361286	0.975124378	0.975124378	Bn-N7-p6917189	Bn-chrA07-p7212832
Bn-A07-p5365136	1	1	Bn-N7-p6921020	Bn-chrA07-p7216675
Bn-A07-p5366717	0.993355482	0.993355482	Bn-N7-p6922601	Bn-chrA07-p7218256

Bn-A07-p5367379	0.996677741	0.996677741	Bn-N7-p6923263	Bn-chrA07-p7218918
Bn-A07-p5367982	0.983471074	0.983471074	Bn-N7-p6923866	Bn-chrA07-p7219521
Bn-A07-p5370119	0.995024876	0.995024876	Bn-N7-p6925998	Bn-chrA07-p7221653
Bn-A07-p5385190	0.955223881	0.955223881	Bn-N7-p6935268	Bn-chrA07-p7233141
Bn-A07-p5398751	0.975206612	0.975206612	Bn-N7-p6946488	Bn-chrA07-p7245161
Bn-A07-p5398913	0.983471074	0.983471074	Bn-N7-p6946650	Bn-chrA07-p7245323
Bn-A07-p5400056	0.991735537	0.991735537	Bn-N7-p6947793	Bn-chrA07-p7246466
Bn-A07-p5400130	0.983471074	0.983471074	Bn-N7-p6947867	Bn-chrA07-p7246540
Bn-A07-p5400202	0.983471074	0.983471074	Bn-N7-p6947939	Bn-chrA07-p7246612
Bn-A07-p5401960	0.975206612	0.975206612	Bn-N7-p6949695	Bn-chrA07-p7248366
Bn-A07-p5402372	0.983471074	0.983471074	Bn-N7-p6950841	Bn-chrA07-p7249512
Bn-A07-p5402451	0.991735537	0.991735537	Bn-N7-p6950920	Bn-chrA07-p7249591
Bn-A07-p5402605	0.975206612	0.975206612	Bn-N7-p6951074	Bn-chrA07-p7249745
Bn-A07-p5402835	0.991735537	0.991735537	Bn-N7-p6951305	Bn-chrA07-p7249975
Bn-A07-p5403065	0.966942149	0.966942149	Bn-N7-p6951535	Bn-chrA07-p7250205
Bn-A07-p5407029	0.983471074	0.983471074	Bn-N7-p6955556	Bn-chrA07-p7254224
Bn-A07-p5407143	0.983471074	0.983471074	Bn-N7-p6955670	Bn-chrA07-p7254338
Bn-A07-p5407683	0.996677741	0.996677741	Bn-N7-p6956210	Bn-chrA07-p7254878
Bn-A07-p5408025	0.995024876	0.850746269	Bn-N7-p6963842	Bn-chrAnn_random-p20045[illegible]
Bn-A07-p5412930	0.985074627	0.985074627	Bn-N7-p6968745	Bn-chrA07-p7273076
Bn-A07-p5413164	0.991735537	0.991735537	Bn-N7-p6968978	Bn-chrA07-p7273309
Bn-A07-p5419348	1	1	Bn-N7-p6975366	Bn-chrA07-p7279697
Bn-A07-p5419648	1	1	Bn-N7-p6975666	Bn-chrA07-p7279997
Bn-A07-p5419833	0.995024876	0.995024876	Bn-N7-p6975851	Bn-chrA07-p7280182
Bn-A07-p5427886	0.458471761	0.431893688	Bn-N7-p6982427	Bn-chrA06-p4596998
Bn-A07-p5428283	0.945273632	0.945273632	Bn-N7-p6982935	Bn-chrAnn_random-p345517
Bn-A07-p5431144	0.975206612	0.975206612	Bn-N7-p6985800	Bn-chrAnn_random-p34548[illegible]
Bn-A07-p5436630	1	1	Bn-N7-p6991278	Bn-chrA07-p7295178
Bn-A07-p5438049	0.993355482	0.993355482	Bn-N7-p6992703	Bn-chrA07-p7297472
Bn-A07-p5438333	1	1	Bn-N7-p6992987	Bn-chrA07-p7297756
Bn-A07-p5447322	0.990033223	0.990033223	Bn-N7-p7002121	Bn-chrA07-p7306786
Bn-A07-p5448813	1	1	Bn-N7-p7004361	Bn-chrA07-p7308276
Bn-A07-p5449174	0.991735537	0.991735537	Bn-N7-p7004722	Bn-chrA07-p7308637
Bn-A07-p5455024	0.991735537	0.991735537	Bn-N7-p7010907	Bn-chrA07-p7314823
Bn-A07-p5472218	0.644628099	0.644628099	Bn-N7-p7030133	Bn-chrA07-p7333079
Bn-A07-p5472963	0.990033223	0.990033223	Bn-N7-p7030882	Bn-chrA07-p7333828
Bn-A07-p5482599	0.958677686	0.958677686	Bn-N7-p7047312	Bn-chrA07-p7348913
Bn-A07-p5500390	0.966942149	0.966942149	Bn-N7-p7054812	Bn-chrA07-p7356413
Bn-A07-p5500448	0.950413223	0.950413223	Bn-N7-p7054870	Bn-chrA07-p7356471
Bn-A07-p5516068	0.96345515	0.96345515	Bn-N7-p7072735	Bn-chrA07-p7377104
Bn-A07-p5517100	0.980066445	0.980066445	Bn-N7-p7073770	Bn-chrA07-p7378139
Bn-A07-p5517642	0.96345515	0.96345515	Bn-N7-p7074317	Bn-chrA07-p7378686
Bn-A07-p5518221	0.958677686	0.958677686	Bn-N7-p7074899	Bn-chrA07-p7379268
Bn-A07-p5518604	0.975206612	0.975206612	Bn-N7-p7075281	Bn-chrA07-p7379650
Bn-A07-p5519194	0.966942149	0.966942149	Bn-N7-p7075871	Bn-chrA07-p7380240
Bn-A07-p5519356	0.915422886	0.915422886	Bn-N7-p7076033	Bn-chrA07-p7380402

Bn-A07-p5519439	0.946843854	0.946843854	Bn-N7-p7076116	Bn-chrA07-p7380485
Bn-A07-p5519719	0.524916944	0.521594684	Bn-N3-p15056575	Bn-chrA05-p12048349
Bn-A07-p5539902	0.913621262	0.657807309	Bn-N17-p15997447	Bn-chrC07-p13816694
Bn-A07-p5555349	0.991735537	0.991735537	Bn-N7-p7102527	Bn-chrA07-p7408598
Bn-A07-p5556527	0.991735537	0.991735537	Bn-N7-p7103704	Bn-chrA07-p7411053
Bn-A07-p5557211	0.975206612	0.975206612	Bn-Scaffold09991-p5	Bn-chrA07-p7427832
Bn-A07-p5568504	0.991735537	0.991735537	Bn-N7-p7108520	Bn-chrA07-p7433484
Bn-A07-p5572683	0.993355482	0.993355482	Bn-N7-p7112706	Bn-chrA07-p7437670
Bn-A07-p5574316	0.991735537	0.991735537	Bn-N7-p7114339	Bn-chrA07-p7439303
Bn-A07-p5578410	0.996677741	0.993355482	Bn-N2-p10555970	Bn-chrA02_random-p406285
Bn-A07-p5591930	0.958677686	0.958677686	Bn-N7-p7124905	Bn-chrA07-p7450240
Bn-A07-p5609094	0.970149254	0.955223881	Bn-N15-p33707893	Bn-chrA07-p2272703
Bn-A07-p5609159	0.961538462	0.945054945	Bn-N15-p33707827	Bn-chrA07-p2272637
Bn-A07-p5612740	0.991735537	0.991735537	Bn-N7-p7208352	Bn-chrA07-p7580198
Bn-A07-p5618903	0.975206612	0.975206612	Bn-N7-p7215114	Bn-chrA07-p7586195
Bn-A07-p5619095	0.993355482	0.993355482	Bn-N7-p7215306	Bn-chrA07-p7586387
Bn-A07-p5619285	0.983471074	0.983471074	Bn-N7-p7215496	Bn-chrA07-p7586577
Bn-A07-p5620067	0.966942149	0.966942149	Bn-N7-p7216278	Bn-chrA07-p7587359
Bn-A07-p5633352	0.991735537	0.991735537	Bn-N7-p7229050	Bn-chrA07-p7600131
Bn-A07-p5648697	0.933884298	1	Bn-N7-p7248176	Bn-chrA07-p7618268
Bn-A07-p5660270	0.991735537	0.975206612	Bn-N7-p7266816	Bn-chrC07-p14182743
Bn-A07-p5670936	1	0.991735537	Bn-N7-p7271055	Bn-chrA07-p7648481
Bn-A07-p5684410	0.975206612	0.975206612	Bn-N7-p7292965	Bn-chrA07-p7670385
Bn-A07-p5703663	0.973958333	0.973958333	Bn-N8-p6374413	Bn-chrA08-p6421397
Bn-A07-p5709944	0.975206612	0.983471074	Bn-N7-p7309125	Bn-chrA07-p7686376
Bn-A07-p5710530	0.985074627	0.985074627	Bn-N7-p7309711	Bn-chrA07-p7686961
Bn-A07-p5710766	0.996677741	0.996677741	Bn-N7-p7309947	Bn-chrA07-p7687197
Bn-A07-p5712114	1	0.991735537	Bn-N7-p7311289	Bn-chrA07-p7688651
Bn-A07-p5713057	0.991735537	1	Bn-N7-p7312232	Bn-chrA07-p7689594
Bn-A07-p5733051	0.809917355	0.809917355	Bn-N9-p17510362	Bn-chrA09-p14050688
Bn-A07-p5735606	0.960199005	0.955223881	Bn-N9-p17507822	Bn-chrA09-p14053459
Bn-A07-p5737895	0.917355372	1	Bn-N19-p25902286	Bn-chrA09-p14055748
Bn-A07-p5737991	0.884297521	0.991735537	Bn-N19-p25902382	Bn-chrA09-p14055844
Bn-A07-p5738023	0.752066116	0.983471074	Bn-N19-p25902414	Bn-chrA09-p14055876
Bn-A07-p5757189	0.950413223	0.958677686	Bn-Scaffold75234-p1	Bn-chrA09-p14080027
Bn-A07-p5758215	0.98951049	0.975524476	Bn-N9-p17479230	Bn-chrA09-p14081025
Bn-A07-p5766826	0.818181818	0.32231405	Bn-N7-p7330248	Unknown
Bn-A07-p5768726	0.993355482	0.993355482	Bn-N7-p7332148	Bn-chrA07-p7693670
Bn-A07-p5769927	0.945273632	0.800995025	Bn-N7-p7333349	Bn-chrA07-p7694872
Bn-A07-p5773404	0.983471074	0.991735537	Bn-N7-p7337460	Bn-chrA07-p7698438
Bn-A07-p5786966	1	0.975206612	Bn-N7-p7350875	Bn-chrA07-p7725624
Bn-A07-p5787070	0.991735537	1	Bn-N7-p7350979	Bn-chrA07-p7725728
Bn-A07-p5790807	0.991735537	1	Bn-N7-p7355055	Bn-chrA07-p7729803
Bn-A07-p5793420	0.980099502	0.985074627	Bn-N7-p7357710	Bn-chrA07-p7732445
Bn-A07-p5794504	0.991735537	1	Bn-N7-p7358789	Bn-chrA07-p7733518
Bn-A07-p5810641	0.980099502	0.985074627	Bn-N7-p7375207	Bn-chrA07-p7750341

Bn-A07-p5812342	0.990033223	0.990033223	Bn-N7-p7376908	Bn-chrA07-p7752041
Bn-A07-p5812470	0.993355482	0.986710963	Bn-N7-p7377036	Bn-chrA07-p7752169
Bn-A07-p5813326	0.980099502	0.975124378	Bn-N7-p7377892	Bn-chrA07-p7753025
Bn-A07-p5824940	1	0.991735537	Bn-N7-p7389909	Bn-chrA07-p7765042
Bn-A07-p5826334	0.983471074	0.991735537	Bn-N7-p7391303	Bn-chrA07-p7773242
Bn-A07-p5834691	0.980099502	0.985074627	Bn-N7-p7399582	Bn-chrA07-p7781978
Bn-A07-p5835036	0.975206612	0.983471074	Bn-N7-p7399927	Bn-chrA07-p7782322
Bn-A07-p5835842	0.986710963	0.990033223	Bn-N7-p7400733	Bn-chrA07-p7783128
Bn-A07-p5837375	0.991735537	1	Bn-N7-p7402266	Bn-chrA07-p7784661
Bn-A07-p5849947	0.980066445	0.983388704	Bn-N7-p7413982	Bn-chrA07-p7797774
Bn-A07-p5853302	1	0.991735537	Bn-N7-p7417122	Bn-chrA07-p7800866
Bn-A07-p5859070	0.980099502	0.975124378	Bn-N7-p7423268	Bn-chrA07-p7806853
Bn-A07-p5871275	0.975206612	0.975206612	Bn-N7-p7434664	Bn-chrA07-p7817615
Bn-A07-p5872166	0.966942149	0.966942149	Bn-N7-p7435050	Bn-chrA07-p7818001
Bn-A07-p5877587	0.975206612	0.975206612	Bn-N7-p7441486	Bn-chrA07-p7824442
Bn-A07-p5882920	0.983471074	0.983471074	Bn-N7-p7459607	Bn-chrA07-p7839713
Bn-A07-p5895170	0.991735537	1	Bn-N7-p7471626	Bn-chrA07-p7851552
Bn-A07-p5905223	0.950248756	0.955223881	Bn-N7-p7479495	Bn-chrA07-p7861291
Bn-A07-p5983825	0.995024876	0.990049751	Bn-N7-p7565383	Bn-chrA07-p7918223
Bn-A07-p5995415	0.985074627	0.144278607	Bn-N7-p7574885	Bn-chrA07-p11221386
Bn-A07-p6008398	0.990033223	0.990033223	Bn-N7-p7589923	Bn-chrAnn_random-p423513
Bn-A07-p6012565	0.970149254	0.970149254	Bn-N7-p7593908	Bn-chrA07-p7946436
Bn-A07-p6044939	0.996677741	0.996677741	Bn-N3-p14105117	Bn-chrA03-p12885436
Bn-A07-p6055505	0.96345515	0.986710963	Bn-N3-p7073378	Bn-chrA03-p12874566
Bn-A07-p6068523	0.991735537	1	Bn-N3-p14088911	Bn-chrA03-p12870687
Bn-A07-p6068639	0.975206612	0.983471074	Bn-N3-p14088795	Bn-chrA03-p12870571
Bn-A07-p6071787	0.694214876	0.958677686	Bn-N3-p14083351	Bn-chrA03-p12867434
Bn-A07-p6135654	0.986710963	0.986710963	Bn-N7-p7696387	Bn-chrA07-p8040767
Bn-A07-p6197889	0.993355482	0.990033223	Bn-N7-p7757299	Bn-chrA07-p8101954
Bn-A07-p6206232	0.983388704	0.983388704	Bn-N7-p7765898	Bn-chrA07-p8111919
Bn-A07-p6207835	0.760797342	0.760797342	Bn-N7-p7775654	Bn-chrA07-p8125611
Bn-A07-p6210782	0.966942149	0.975206612	Bn-N7-p7778606	Bn-chrA07-p8128563
Bn-A07-p6215131	0.950413223	0.94214876	Bn-N7-p7782954	Bn-chrA07-p8132910
Bn-A07-p6217329	0.975206612	0.966942149	Bn-N7-p7785152	Bn-chrA07-p8137266
Bn-A07-p6217365	0.983471074	0.975206612	Bn-N7-p7785188	Bn-chrA07-p8137302
Bn-A07-p6238777	0.989583333	0.989583333	Bn-N7-p7808729	Bn-chrA07-p8169465
Bn-A07-p6244683	0.983471074	0.983471074	Bn-N7-p7813114	Bn-chrA07-p8173848
Bn-A07-p6259787	0.986710963	0.986710963	Bn-N7-p7826390	Bn-chrA07-p8188028
Bn-A07-p6262438	0.966942149	0.958677686	Bn-N7-p7829039	Bn-chrA07-p8190679
Bn-A07-p6276115	0.986710963	0.986710963	Bn-N7-p7851921	Bn-chrA07-p8198947
Bn-A07-p6281546	0.890365449	0.890365449	Bn-N7-p7854896	Bn-chrA07-p8201921
Bn-A07-p6282717	0.946843854	0.946843854	Bn-N7-p7856070	Bn-chrA07-p8203095
Bn-A07-p6283126	0.983388704	0.983388704	Bn-N7-p7856478	Bn-chrA07-p8203503
Bn-A07-p6297941	0.986710963	0.986710963	Bn-N7-p7869648	Bn-chrA07-p8218712
Bn-A07-p6305750	0.975124378	0.975124378	Bn-N7-p7873661	Bn-chrA07-p8222755
Bn-A07-p6307545	0.976744186	0.976744186	Bn-N7-p7875459	Bn-chrA07-p8224553

Bn-A07-p6309969	0.986710963	0.986710963	Bn-N7-p7879982	Bn-chrA07-p8229076
Bn-A07-p6327847	0.96013289	0.96013289	Bn-N7-p7902426	Bn-chrA07-p8253105
Bn-A07-p6346472	0.970149254	0.970149254	Bn-N7-p7931431	Bn-chrA07-p8284675
Bn-A07-p6366999	0.970099668	0.970099668	Bn-N7-p7950329	Bn-chrA07-p8311254
Bn-A07-p6369122	0.991735537	0.983471074	Bn-N7-p7952452	Bn-chrA07-p8313386
Bn-A07-p6372584	0.983471074	0.975206612	Bn-N7-p7955899	Bn-chrA07-p8318478
Bn-A07-p6373884	0.983471074	0.991735537	Bn-N7-p7957199	Bn-chrA07-p8322935
Bn-A07-p6382641	0.975206612	0.966942149	Bn-N7-p7965707	Bn-chrA07-p8334083
Bn-A07-p6410362	0.933884298	0.94214876	Bn-N7-p8002314	Bn-chrA07-p8360439
Bn-A07-p6428365	1	0.975206612	Bn-N7-p8019506	Bn-chrA07_random-p485314
Bn-A07-p6432326	0.73553719	0.991735537	Bn-N7-p8023341	Bn-chrA07_random-p481502
Bn-A07-p6432331	0.785123967	0.983471074	Bn-N7-p8023346	Bn-chrA07_random-p481497
Bn-A07-p6437343	0.985074627	0.985074627	Bn-N7-p8025042	Bn-chrA07_random-p479788
Bn-A07-p6439944	0.990033223	0.990033223	Bn-N7-p8026440	Bn-chrA07_random-p478390
Bn-A07-p6443432	0.983471074	0.975206612	Bn-N7-p8029973	Bn-chrA07_random-p474857
Bn-A07-p6444067	0.976744186	0.976744186	Bn-N7-p8030608	Bn-chrA07_random-p474222
Bn-A07-p6444420	0.986710963	0.986710963	Bn-N7-p8030961	Bn-chrA07_random-p473869
Bn-A07-p6445012	0.955223881	0.955223881	Bn-N7-p8031553	Bn-chrA07_random-p473277
Bn-A07-p6460270	0.973421927	0.993355482	Bn-N7-p8037884	Bn-chrA07_random-p464425
Bn-A07-p6494007	0.989583333	1	Bn-N7-p8076082	Bn-chrA07_random-p513924
Bn-A07-p6501207	0.966777409	0.966777409	Bn-N17-p17601417	Bn-chrCnn_random-p437793
Bn-A07-p6509651	0.976744186	0.980066445	Bn-N7-p8088302	Bn-chrA07_random-p501594
Bn-A07-p6516437	0.983388704	0.986710963	Bn-N7-p8094964	Bn-chrA07-p8438861
Bn-A07-p6518480	0.986710963	0.983388704	Bn-N7-p8097006	Bn-chrA07-p8445951
Bn-A07-p6523973	0.960199005	0.990049751	Bn-N17-p17644049	Bn-chrA07-p8460149
Bn-A07-p6524220	0.940298507	0.990049751	Bn-N7-p8102738	Bn-chrA07-p8460396
Bn-A07-p6534557	0.976744186	0.980066445	Bn-N7-p8114866	Bn-chrA07-p8468768
Bn-A07-p6541352	0.980099502	0.975124378	Bn-N7-p8121743	Bn-chrA07-p8475556
Bn-A07-p6542254	0.976744186	0.96345515	Bn-N7-p8122646	Bn-chrA07-p8476356
Bn-A07-p6542812	0.986710963	0.986710963	Bn-N7-p8123197	Bn-chrA07-p8476907
Bn-A07-p6544334	0.991735537	0.991735537	Bn-N7-p8124747	Bn-chrA07-p8478365
Bn-A07-p6544677	0.983471074	0.983471074	Bn-N7-p8125354	Bn-chrA07-p8478712
Bn-A07-p6552161	0.983471074	0.983471074	Bn-N7-p8130886	Bn-chrA07-p8484518
Bn-A07-p6552431	0.966942149	0.966942149	Bn-N7-p8131150	Bn-chrA07-p8484782
Bn-A07-p6553171	0.983471074	0.983471074	Bn-N7-p8131934	Bn-chrA07-p8485566
Bn-A07-p6553231	0.975206612	0.975206612	Bn-N7-p8131997	Bn-chrA07-p8485629
Bn-A07-p6553314	0.991735537	0.991735537	Bn-N7-p8132079	Bn-chrA07-p8485711
Bn-A07-p6553663	0.991735537	0.991735537	Bn-N7-p8132429	Bn-chrA07-p8486061
Bn-A07-p6553774	0.966942149	0.966942149	Bn-N7-p8132540	Bn-chrA07-p8486172
Bn-A07-p6554430	0.973421927	0.973421927	Bn-N7-p8133053	Bn-chrA07-p8486685
Bn-A07-p6554767	0.983471074	0.983471074	Bn-N7-p8133384	Bn-chrA07-p8487016
Bn-A07-p6556246	0.933884298	0.933884298	Bn-N7-p8134382	Bn-chrA07-p8488014
Bn-A07-p6564252	0.950413223	0.950413223	Bn-N7-p8141208	Bn-chrA07-p8497568
Bn-A07-p6564338	0.965174129	0.835820896	Bn-N7-p8141292	Bn-chrA07-p8497652
Bn-A07-p6565672	0.900497512	0.955223881	Bn-N1-p18731406	Bn-chrA07-p8498991
Bn-A07-p6574022	0.991735537	0.47107438	Bn-N7-p8152038	Bn-chrCnn_random-p791664

Bn-A07-p6575320	0.933884298	0.933884298	Bn-N17-p17700584	Bn-chrC07-p15802016
Bn-A07-p6576078	0.975206612	0.975206612	Bn-N7-p8157934	Bn-chrA07-p8518917
Bn-A07-p6576457	0.917355372	0.917355372	Bn-N7-p8158306	Bn-chrA07-p8519289
Bn-A07-p6576689	0.980099502	0.980099502	Bn-N7-p8158538	Bn-chrA07-p8519521
Bn-A07-p6582792	0.980066445	0.983388704	Bn-N5-p25402006	Bn-chrA05-p22585921
Bn-A07-p6606249	0.993355482	0.993355482	Bn-N7-p8187912	Bn-chrA07-p8546733
Bn-A07-p6606278	0.970149254	0.970149254	Bn-N7-p8187941	Bn-chrA07-p8546762
Bn-A07-p6641473	0.996677741	0.996677741	Bn-N7-p8218371	Bn-chrA07-p8574484
Bn-A07-p6644825	0.996677741	0.996677741	Bn-N7-p8221715	Bn-chrA07-p8577828
Bn-A07-p6647096	0.993355482	0.990033223	Bn-N7-p8223986	Bn-chrA07-p8580099
Bn-A07-p6657878	0.985074627	0.985074627	Bn-N7-p8233747	Bn-chrA07-p8589861
Bn-A07-p6660717	1	1	Bn-N7-p8236587	Bn-chrA07-p8592701
Bn-A07-p6711753	0.990033223	0.990033223	Bn-N7-p8292141	Bn-chrA07-p8644097
Bn-A07-p6716678	1	1	Bn-N7-p8297067	Bn-chrA07-p8649023
Bn-A07-p6719583	0.991735537	0.991735537	Bn-N7-p8299963	Bn-chrA07-p8651919
Bn-A07-p6721704	0.970149254	0.975124378	Bn-N7-p8302086	Bn-chrC04-p678795
Bn-A07-p6722244	0.996677741	0.996677741	Bn-N7-p8302625	Bn-chrA07-p8654581
Bn-A07-p6723305	0.996677741	0.996677741	Bn-N7-p8303686	Bn-chrA07-p8655642
Bn-A07-p6735264	0.955223881	0.955223881	Bn-N7-p8323574	Bn-chrA07-p8665783
Bn-A07-p6757359	0.990049751	1	Bn-N7-p8353796	Bn-chrA07-p8687236
Bn-A07-p6759128	0.986710963	0.986710963	Bn-N7-p8355553	Bn-chrA07-p8688995
Bn-A07-p6763846	0.993355482	1	Bn-N7-p8360263	Bn-chrA07-p8693707
Bn-A07-p6765464	0.983388704	0.986710963	Bn-N7-p8361877	Bn-chrA07-p8695325
Bn-A07-p6824682	1	1	Bn-N7-p8399459	Bn-chrA07-p8734224
Bn-A07-p6839228	0.950413223	0.950413223	Bn-N7-p8416169	Bn-chrA07-p8750341
Bn-A07-p6841662	0.975206612	0.991735537	Bn-N17-p18288854	Bn-chrA07-p8752775
Bn-A07-p6850383	0.980099502	0.975124378	Bn-N7-p8426478	Bn-chrA07-p8761753
Bn-A07-p6878338	0.990049751	0.990049751	Bn-N7-p8460839	Bn-chrAnn_random-p45363C
Bn-A07-p6885690	0.990049751	0.990049751	Bn-N7-p8469344	Bn-chrA07_random-p529853
Bn-A07-p6892291	1	1	Bn-N7-p8475994	Bn-chrAnn_random-p475791
Bn-A07-p6894028	1	1	Bn-N7-p8477731	Bn-chrA07_random-p538138
Bn-A07-p6898363	0.985074627	0.985074627	Bn-N7-p8483939	Bn-chrA07_random-p546215
Bn-A07-p6903948	0.950413223	0.950413223	Bn-N7-p8490011	Bn-chrA07_random-p551799
Bn-A07-p6906296	0.435215947	0.986710963	Bn-N13-p53096085	Bn-chrA07_random-p554167
Bn-A07-p6909620	0.965174129	0.970149254	Bn-N7-p8493790	Bn-chrA07_random-p556944
Bn-A07-p6926020	0.996677741	0.983388704	Bn-N7-p8510444	Bn-chrA07_random-p568826
Bn-A07-p6929108	0.646766169	0.646766169	Bn-N12-p12817803	Bn-chrC02-p11844117
Bn-A07-p6942882	0.955223881	0.940298507	Bn-N7-p8523017	Bn-chrC07-p17090011
Bn-A07-p6943725	0.976744186	0.980066445	Bn-N7-p8523858	Bn-chrA07_random-p602835
Bn-A07-p6944436	0.983471074	0.983471074	Bn-N7-p8524569	Bn-chrA07_random-p602124
Bn-A07-p6947953	0.880398671	0.933554817	Bn-N7-p8528691	Bn-chrA07_random-p598002
Bn-A07-p6952910	0.945273632	0.945273632	Bn-N7-p8532856	Bn-chrA07_random-p593837
Bn-A07-p6976405	0.965174129	0.965174129	Bn-N7-p8558397	Bn-chrA07_random-p605657
Bn-A07-p6981435	0.975124378	0.975124378	Bn-N7-p8565060	Bn-chrA07-p8884135
Bn-A07-p6988823	0.983471074	0.983471074	Bn-N7-p8572512	Bn-chrA07-p8891587
Bn-A07-p7009935	0.996677741	0.996677741	Bn-N7-p8592644	Bn-chrA07-p8910847

Bn-A07-p7027400	0.996677741	0.996677741	Bn-N7-p8611224	Bn-chrA07-p8929308
Bn-A07-p7035715	0.760330579	0.760330579	Bn-N7-p8623391	Bn-chrA07-p8939479
Bn-A07-p7039686	0.996677741	0.996677741	Bn-N7-p8624994	Bn-chrA07-p8941082
Bn-A07-p7040554	0.983471074	0.983471074	Bn-N7-p8625862	Bn-chrA07-p8941950
Bn-A07-p7055420	0.995024876	0.995024876	Bn-N7-p8632712	Bn-chrA07-p8948800
Bn-A07-p7069624	0.975206612	0.975206612	Bn-N7-p8643592	Bn-chrA07-p8959654
Bn-A07-p7078464	0.757475083	0.757475083	Bn-N7-p8657424	Bn-chrA07-p8973863
Bn-A07-p7079099	0.986710963	0.986710963	Bn-N7-p8658089	Bn-chrA07-p8974528
Bn-A07-p7082333	0.983471074	0.983471074	Bn-N7-p8661352	Bn-chrA07-p8977790
Bn-A07-p7088617	0.985074627	0.985074627	Bn-N7-p8667801	Bn-chrAnn_random-p401107
Bn-A07-p7092988	0.991735537	0.991735537	Bn-N7-p8672156	Bn-chrA07_random-p712772
Bn-A07-p7099002	0.935323383	0.935323383	Bn-N17-p18732130	Bn-chrC07-p16843494
Bn-A07-p7108284	0.966942149	0.966942149	Bn-N7-p8697388	Bn-chrA07_random-p693512
Bn-A07-p7110257	0.950413223	0.950413223	Bn-N17-p18753704	Bn-chrC07-p16823331
Bn-A07-p7117228	0.975206612	0.975206612	Bn-N7-p8706303	Bn-chrA07_random-p685370
Bn-A07-p7117229	0.975206612	0.975206612	Bn-N7-p8706304	Bn-chrA07_random-p685369
Bn-A07-p7118584	0.995024876	0.995024876	Bn-N7-p8708759	Bn-chrA07_random-p684013
Bn-A07-p7119962	0.996677741	0.993355482	Bn-N7-p8710138	Bn-chrA07_random-p682634
Bn-A07-p7124154	0.993355482	0.993355482	Bn-N7-p8714434	Bn-chrA07_random-p678338
Bn-A07-p7139702	0.975206612	0.975206612	Bn-N7-p8733054	Bn-chrA07_random-p659721
Bn-A07-p7141211	0.966942149	0.966942149	Bn-N7-p8734535	Bn-chrA07_random-p658241
Bn-A07-p7141625	1	1	Bn-N7-p8734949	Bn-chrA07_random-p657827
Bn-A07-p7141806	0.582089552	0.606965174	Bn-N7-p8735130	Bn-chrC01_random-p361825
Bn-A07-p7146249	0.983471074	0.983471074	Bn-N7-p8738366	Bn-chrA07_random-p654187
Bn-A07-p7180352	0.975206612	0.975206612	Bn-N7-p8780388	Bn-chrCnn_random-p126020
Bn-A07-p7184457	0.975206612	0.975206612	Bn-N7-p8783721	Bn-chrAnn_random-p635433
Bn-A07-p7185841	0.991735537	0.991735537	Bn-N7-p8785080	Bn-chrAnn_random-p635297
Bn-A07-p7191306	0.995024876	0.995024876	Bn-N7-p8793041	Bn-chrAnn_random-p634441
Bn-A07-p7194218	0.92039801	0.92039801	Bn-N7-p8798270	Bn-chrA07_random-p616429
Bn-A07-p7196025	0.950413223	0.950413223	Bn-N7-p8800175	Bn-chrAnn_random-p633687
Bn-A07-p7196197	0.983471074	0.983471074	Bn-N7-p8800347	Bn-chrAnn_random-p633670
Bn-A07-p7197488	0.983471074	0.983471074	Bn-N7-p8805645	Bn-chrAnn_random-p633004
Bn-A07-p7197588	0.950413223	0.950413223	Bn-N7-p8805748	Bn-chrAnn_random-p632994
Bn-A07-p7198236	0.980099502	0.980099502	Bn-N7-p8806409	Bn-chrAnn_random-p632928
Bn-A07-p7200963	0.950248756	0.950248756	Bn-N7-p8810529	Bn-chrAnn_random-p632516
Bn-A07-p7211602	0.946843854	0.465116279	Bn-N7-p8821421	Bn-chrA08_random-p132264
Bn-A07-p7215862	0.990033223	0.990033223	Bn-N7-p8826403	Bn-chrAnn_random-p630616
Bn-A07-p7220544	0.975206612	0.966942149	Bn-N7-p8829400	Bn-chrC07-p16650241
Bn-A07-p7230543	0.966942149	0.966942149	Bn-N7-p8834828	Bn-chrAnn_random-p629774
Bn-A07-p7232691	0.983388704	0.983388704	Bn-N7-p8840367	Bn-chrA07_random-p937625
Bn-A07-p7234071	0.966942149	0.966942149	Bn-N7-p8841744	Bn-chrA07_random-p936248
Bn-A07-p7237742	0.965174129	0.965174129	Bn-N7-p8845478	Bn-chrA07_random-p932514
Bn-A07-p7244763	0.983471074	0.983471074	Bn-N7-p8854499	Bn-chrA07_random-p922469
Bn-A07-p7245610	0.993355482	0.993355482	Bn-N7-p8855345	Bn-chrA07_random-p921623
Bn-A07-p7245971	0.983471074	0.983471074	Bn-N7-p8855706	Bn-chrA07_random-p921262
Bn-A07-p7246175	1	1	Bn-N7-p8855910	Bn-chrA07_random-p921058

Bn-A07-p7246452	1	1	Bn-N7-p8856187	Bn-chrA07_random-p920781
Bn-A07-p7252813	0.983471074	0.983471074	Bn-N7-p8856927	Bn-chrA07_random-p920041
Bn-A07-p7255937	0.990049751	0.990049751	Bn-N7-p8859405	Bn-chrA07_random-p917563
Bn-A07-p7261357	0.986710963	0.986710963	Bn-N7-p8864825	Bn-chrA07_random-p912143
Bn-A07-p7261508	0.986710963	0.986710963	Bn-N7-p8864976	Bn-chrA07_random-p911992
Bn-A07-p7268294	0.986710963	0.986710963	Bn-N7-p8874774	Bn-chrAnn_random-p387898
Bn-A07-p7270443	0.990033223	0.990033223	Bn-N7-p10483228	Bn-chrA07_random-p617910
Bn-A07-p7276136	0.996677741	0.996677741	Bn-N7-p8883795	Bn-chrA07_random-p793277
Bn-A07-p7282445	0.975124378	0.975124378	Bn-N7-p8891658	Bn-chrA07_random-p785414
Bn-A07-p7287398	1	1	Bn-N7-p8896085	Bn-chrA07_random-p780987
Bn-A07-p7297758	0.955223881	0.955223881	Bn-N7-p8908837	Bn-chrA07_random-p768859
Bn-A07-p7298176	0.980099502	0.980099502	Bn-N7-p8909255	Bn-chrA07_random-p768441
Bn-A07-p7298603	0.983471074	0.983471074	Bn-N7-p8909681	Bn-chrA07_random-p768015
Bn-A07-p7302034	0.94214876	0.94214876	Bn-N7-p8913071	Bn-chrA07_random-p764625
Bn-A07-p7309287	0.946843854	0.946843854	Bn-N7-p8916937	Bn-chrAnn_random-p476588
Bn-A07-p7310644	0.958677686	0.958677686	Bn-N7-p8918250	Bn-chrAnn_random-p476601
Bn-A07-p7311296	0.975206612	0.975206612	Bn-N7-p8921865	Bn-chrA07_random-p758581
Bn-A07-p7312075	0.890365449	0.890365449	Bn-N7-p8922644	Bn-chrA07_random-p757802
Bn-A07-p7352643	0.966942149	0.966942149	Bn-N7-p8951323	Bn-chrA07_random-p895612
Bn-A07-p7362180	0.966942149	0.966942149	Bn-N7-p8957000	Bn-chrA07_random-p889935
Bn-A07-p7367247	0.975124378	0.975124378	Bn-N7-p8962238	Bn-chrA07_random-p884701
Bn-A07-p7372586	0.991735537	0.991735537	Bn-N7-p8967493	Bn-chrA07_random-p879446
Bn-A07-p7374912	0.990049751	0.990049751	Bn-N7-p8970498	Bn-chrA07_random-p877023
Bn-A07-p7380906	0.975206612	0.975206612	Bn-N7-p8977570	Bn-chrA07_random-p870376
Bn-A07-p7390413	0.966942149	0.966942149	Bn-N7-p8987078	Bn-chrA07_random-p860868
Bn-A07-p7391449	0.970149254	0.970149254	Bn-N7-p8988114	Bn-chrA07_random-p859832
Bn-A07-p7392394	0.980099502	0.980099502	Bn-N7-p8989059	Bn-chrA07_random-p858887
Bn-A07-p7392454	0.975124378	0.975124378	Bn-N7-p8989119	Bn-chrA07_random-p858827
Bn-A07-p7392457	0.975124378	0.975124378	Bn-N7-p8989122	Bn-chrA07_random-p858824
Bn-A07-p7392753	0.950248756	0.950248756	Bn-N7-p8989418	Bn-chrA07_random-p858528
Bn-A07-p7392879	0.955223881	0.955223881	Bn-N7-p8989544	Bn-chrA07_random-p858402
Bn-A07-p7393007	0.975124378	0.975124378	Bn-N7-p8989673	Bn-chrA07_random-p858273
Bn-A07-p7395038	0.983388704	0.983388704	Bn-N7-p8991585	Bn-chrA07_random-p856361
Bn-A07-p7398602	0.966942149	0.966942149	Bn-N7-p8994322	Bn-chrA07_random-p853698
Bn-A07-p7400268	0.958677686	0.958677686	Bn-N7-p8997082	Bn-chrA07_random-p850584
Bn-A07-p7400432	0.983471074	0.983471074	Bn-N7-p8997246	Bn-chrA07_random-p850420
Bn-A07-p7401420	0.983471074	0.983471074	Bn-N7-p8998234	Bn-chrA07_random-p849432
Bn-A07-p7433228	0.986710963	0.986710963	Bn-N7-p9011407	Bn-chrA07_random-p834775
Bn-A07-p7433707	0.990049751	0.990049751	Bn-N7-p9011886	Bn-chrA07_random-p834296
Bn-A07-p7434126	0.991735537	0.991735537	Bn-N7-p9012305	Bn-chrA07_random-p833877
Bn-A07-p7436573	0.935323383	0.935323383	Bn-N7-p9014762	Bn-chrA07_random-p831420
Bn-A07-p7445858	0.893687708	0.893687708	Bn-N7-p9023915	Bn-chrA07_random-p821898
Bn-A07-p7454447	0.950413223	0.950413223	Bn-N7-p9036032	Bn-chrA07_random-p798863
Bn-A07-p7499400	0.991735537	0.991735537	Bn-N7-p9095651	Bn-chrA07-p9281285
Bn-A07-p7509139	0.983471074	0.983471074	Bn-N7-p9105871	Bn-chrA07-p9269921
Bn-A07-p7512738	0.975124378	0.975124378	Bn-N7-p9109470	Bn-chrA07-p9266322

Bn-A07-p7519375	0.966942149	0.966942149	Bn-N7-p9116100	Bn-chrA07-p9259694
Bn-A07-p7528011	0.996677741	0.996677741	Bn-N7-p9125239	Bn-chrA07-p9251073
Bn-A07-p7528095	0.991735537	0.991735537	Bn-N7-p9125323	Bn-chrA07-p9250989
Bn-A07-p7542335	0.986710963	0.986710963	Bn-N7-p9135461	Bn-chrA07-p9240851
Bn-A07-p7551371	0.94214876	0.94214876	Bn-N17-p19834270	Bn-chrC07-p17884737
Bn-A07-p7556616	0.958677686	0.958677686	Bn-N7-p9157495	Bn-chrA07-p9219998
Bn-A07-p7562328	0.986710963	0.986710963	Bn-N7-p9163725	Bn-chrA07-p9213985
Bn-A07-p7578604	0.983471074	0.983471074	Bn-N7-p9180104	Bn-chrA07-p9195273
Bn-A07-p7583086	0.995024876	0.995024876	Bn-N7-p9183288	Bn-chrA07-p9192089
Bn-A07-p7584364	1	1	Bn-N7-p9184566	Bn-chrA07-p9190811
Bn-A07-p7603379	0.966942149	0.966942149	Bn-N7-p9210682	Bn-chrA07-p9164575
Bn-A07-p7603440	0.979166667	0.979166667	Bn-N7-p9210744	Bn-chrA07-p9164444
Bn-A07-p7605204	0.966777409	0.966777409	Bn-N7-p9212198	Bn-chrA07-p9163059
Bn-A07-p7605498	0.983471074	0.983471074	Bn-N7-p9212492	Bn-chrA07-p9162765
Bn-A07-p7605606	0.975206612	0.975206612	Bn-N7-p9212600	Bn-chrA07-p9162657
Bn-A07-p7606040	0.975206612	0.975206612	Bn-N7-p9213040	Bn-chrA07-p9162217
Bn-A07-p7606228	0.983471074	0.983471074	Bn-N7-p9213227	Bn-chrA07-p9162030
Bn-A07-p7606261	0.991735537	0.991735537	Bn-N7-p9213260	Bn-chrA07-p9161997
Bn-A07-p7606760	0.975206612	0.975206612	Bn-N7-p9213749	Bn-chrA07-p9161508
Bn-A07-p7607101	0.966942149	0.966942149	Bn-N7-p9214091	Bn-chrA07-p9161166
Bn-A07-p7607188	0.975206612	0.975206612	Bn-N7-p9214176	Bn-chrA07-p9161081
Bn-A07-p7608035	0.933884298	0.933884298	Bn-N7-p9215081	Bn-chrA07-p9160176
Bn-A07-p7610810	0.991735537	0.991735537	Bn-N7-p9218914	Bn-chrA07-p9156495
Bn-A07-p7611089	0.990033223	0.990033223	Bn-N7-p9219193	Bn-chrA07-p9156216
Bn-A07-p7621496	0.975206612	0.975206612	Bn-N7-p9228674	Bn-chrA07-p9146748
Bn-A07-p7635152	0.936877076	0.936877076	Bn-N7-p9230259	Bn-chrA07-p9145164
Bn-A07-p7637265	0.983471074	0.983471074	Bn-N7-p9232645	Bn-chrA07-p9142789
Bn-A07-p7637621	0.940298507	0.940298507	Bn-N7-p9233000	Bn-chrA07-p9142434
Bn-A07-p7640474	0.930348259	0.930348259	Bn-N2-p15286205	Bn-chrA02_random-p774284
Bn-A07-p7664147	0.958677686	0.958677686	Bn-N7-p9244468	Bn-chrA07-p9131009
Bn-A07-p7673966	0.818181818	0.818181818	Bn-N7-p9249578	Bn-chrA07-p9124272
Bn-A07-p7676201	0.94214876	0.94214876	Bn-N7-p9251048	Bn-chrA07-p9122802
Bn-A07-p7677536	0.991735537	0.991735537	Bn-N7-p9257048	Bn-chrA07-p9116354
Bn-A07-p7677609	0.975206612	0.975206612	Bn-N7-p9257121	Bn-chrA07-p9116281
Bn-A07-p7678236	0.975206612	0.975206612	Bn-N7-p9257749	Bn-chrA07-p9115653
Bn-A07-p7678421	0.975206612	0.975206612	Bn-N7-p9257934	Bn-chrA07-p9115468
Bn-A07-p7678712	0.966942149	0.966942149	Bn-N7-p9258227	Bn-chrA07-p9115175
Bn-A07-p7679469	0.983471074	0.983471074	Bn-N7-p9258984	Bn-chrA07-p9114418
Bn-A07-p7685214	0.958677686	0.958677686	Bn-N7-p9264964	Bn-chrA07-p9109677
Bn-A07-p7686074	0.966942149	0.966942149	Bn-N7-p9265824	Bn-chrA07-p9108817
Bn-A07-p7702326	0.983471074	0.983471074	Bn-N7-p9279689	Bn-chrA07-p9093245
Bn-A07-p7713308	1	1	Bn-N7-p9289403	Bn-chrA07-p9083537
Bn-A07-p7717365	0.983471074	0.983471074	Bn-Scaffold11459-p5	Bn-chrA07-p9074981
Bn-A07-p7723038	0.955223881	0.955223881	Bn-N17-p20195691	Bn-chrA07-p9069184
Bn-A07-p7725873	0.958677686	0.958677686	Bn-N17-p20198699	Bn-chrA07-p9061386
Bn-A07-p7738577	1	1	Bn-N7-p9300146	Bn-chrA07-p9049671

Bn-A07-p7740173	0.950248756	0.950248756	Bn-N7-p9301388	Bn-chrA07-p9048355
Bn-A07-p7773921	0.985074627	0.985074627	Bn-N7-p9336719	Bn-chrA07-p9016768
Bn-A07-p7793608	0.752066116	0.752066116	Bn-N7-p9355544	Bn-chrA07-p8998289
Bn-A07-p7794741	0.985074627	0.985074627	Bn-N7-p9357562	Bn-chrAnn_random-p360163
Bn-A07-p7796025	0.996677741	0.996677741	Bn-N7-p9360955	Bn-chrA07-p8992455
Bn-A07-p7814470	0.991735537	0.991735537	Bn-N7-p9365044	Bn-chrA07-p8988366
Bn-A07-p7872194	0.990049751	0.990049751	Bn-N7-p9420763	Bn-chrA07-p9311944
Bn-A07-p7931519	0.985074627	0.990049751	Bn-N7-p9298824	Bn-chrA07-p9373871
Bn-A07-p8037081	0.975124378	0.975124378	Bn-N7-p9618283	Bn-chrA07-p9496881
Bn-A07-p8046121	0.996677741	0.996677741	Bn-N7-p9627350	Bn-chrA07-p9505949
Bn-A07-p8067557	0.826446281	0.826446281	Bn-N7-p9643189	Bn-chrA07-p9521785
Bn-A07-p8084624	0.990033223	0.990033223	Bn-N7-p9659378	Bn-chrA07-p9537974
Bn-A07-p8096658	0.980066445	0.980066445	Bn-N7-p9667551	Bn-chrA07-p9546112
Bn-A07-p8122640	0.966777409	0.966777409	Bn-N7-p9685775	Bn-chrA07-p9567758
Bn-A07-p8128951	0.991735537	0.991735537	Bn-N7-p9691938	Bn-chrA07-p9573921
Bn-A07-p8131907	0.900826446	0.900826446	Bn-N7-p9694023	Bn-chrA07-p9576159
Bn-A07-p8134950	0.980099502	0.980099502	Bn-N7-p9697098	Bn-chrA07-p9579236
Bn-A07-p8138266	0.975206612	0.975206612	Bn-N7-p9704943	Bn-chrA07-p9587079
Bn-A07-p8143924	0.96345515	0.714285714	Bn-Scaffold41985-p1	Bn-chrA07-p9592718
Bn-A07-p8162493	0.958677686	0.958677686	Bn-N7-p9729237	Bn-chrA07-p9611371
Bn-A07-p8167856	0.993355482	0.993355482	Bn-N7-p9734603	Bn-chrA07-p9616736
Bn-A07-p8175756	0.975206612	0.975206612	Bn-N7-p9742187	Bn-chrA07-p9626537
Bn-A07-p8178839	0.990033223	0.990033223	Bn-N7-p9744672	Bn-chrA07-p9630422
Bn-A07-p8181654	0.983471074	0.983471074	Bn-N7-p9747476	Bn-chrA07-p9633226
Bn-A07-p8183365	0.986710963	0.986710963	Bn-N7-p9750272	Bn-chrA07-p9636022
Bn-A07-p8184393	0.991735537	0.991735537	Bn-N7-p9751304	Bn-chrA07-p9637055
Bn-A07-p8185226	0.966942149	0.966942149	Bn-N7-p9752043	Bn-chrA07-p9637794
Bn-A07-p8186475	0.88372093	0.88372093	Bn-N7-p9753507	Bn-chrA07-p9639265
Bn-A07-p8186509	0.975124378	0.975124378	Bn-N7-p9753541	Bn-chrA07-p9639299
Bn-A07-p8194806	0.925619835	0.925619835	Bn-N17-p20784322	Bn-chrC07-p18806637
Bn-A07-p8197265	0.20661157	0.20661157	Bn-N2-p29112158	Bn-chrC02-p45749618
Bn-A07-p8207791	0.958677686	0.958677686	Bn-N7-p9764669	Bn-chrA07-p9652237
Bn-A07-p8224070	0.970149254	0.970149254	Bn-N7-p9772538	Bn-chrA07-p9660292
Bn-A07-p8224100	0.983471074	0.983471074	Bn-N7-p9772568	Bn-chrA07-p9660322
Bn-A07-p8224244	0.983471074	0.983471074	Bn-N7-p9772712	Bn-chrA07-p9660466
Bn-A07-p8227408	0.975124378	0.975124378	Bn-N7-p9775875	Bn-chrA07-p9663629
Bn-A07-p8227698	0.993355482	0.993355482	Bn-N7-p9776165	Bn-chrA07-p9663919
Bn-A07-p8231877	0.970149254	0.970149254	Bn-N7-p9781891	Bn-chrA07-p9669645
Bn-A07-p8232184	0.993355482	0.993355482	Bn-N7-p9782198	Bn-chrA07-p9669952
Bn-A07-p8243053	0.980066445	0.980066445	Bn-N7-p9794089	Bn-chrA07-p9681842
Bn-A07-p8250951	0.976744186	0.976744186	Bn-N7-p9800102	Bn-chrA07-p9687854
Bn-A07-p8252992	1	1	Bn-N17-p20897295	Bn-chrC07-p18931132
Bn-A07-p8258521	0.990049751	0.990049751	Bn-N7-p9809726	Bn-chrA07-p9698380
Bn-A07-p8259212	0.993355482	0.993355482	Bn-N7-p9810415	Bn-chrA07-p9699069
Bn-A07-p8259218	0.995024876	0.995024876	Bn-N7-p9810421	Bn-chrA07-p9699075
Bn-A07-p8262039	0.985074627	0.985074627	Bn-N7-p9815330	Bn-chrA07-p9704335

Bn-A07-p8265574	0.993355482	0.993355482	Bn-N7-p9818864	Bn-chrA07-p9707869
Bn-A07-p8314613	0.993355482	0.993355482	Bn-N7-p9874342	Bn-chrA07-p9761607
Bn-A07-p8318791	0.940298507	0.940298507	Bn-N7-p9878786	Bn-chrA07-p9765937
Bn-A07-p8323309	0.926910299	0.930232558	Bn-N7-p9881219	Bn-chrA07-p9768370
Bn-A07-p8324017	0.956810631	0.956810631	Bn-N7-p9881938	Bn-chrA07-p9769089
Bn-A07-p8334245	0.993355482	0.993355482	Bn-N7-p9895130	Bn-chrA07-p9781241
Bn-A07-p8348770	1	1	Bn-N7-p9909487	Bn-chrA07-p9795581
Bn-A07-p8359729	0.991735537	0.991735537	Bn-N7-p9921479	Bn-chrA07-p9808086
Bn-A07-p8403118	0.990049751	0.990049751	Bn-N7-p9967606	Bn-chrAnn_random-p441728
Bn-A07-p8434029	0.993355482	0.993355482	Bn-N7-p9982281	Bn-chrA07-p9869111
Bn-A07-p8450971	0.766169154	0.746268657	Bn-N17-p21284274	Bn-chrC07-p19384063
Bn-A07-p8451913	0.991735537	0.991735537	Bn-N7-p10001606	Bn-chrA07-p9888408
Bn-A07-p8498337	0.991735537	0.991735537	Bn-N7-p10042138	Bn-chrA07-p9928765
Bn-A07-p8516100	0.986710963	0.986710963	Bn-N7-p10074233	Bn-chrA07-p9964254
Bn-A07-p8516667	0.983471074	0.983471074	Bn-N7-p10074654	Bn-chrA07-p9964675
Bn-A07-p8520077	0.996677741	0.996677741	Bn-N7-p10078062	Bn-chrA07-p9968083
Bn-A07-p8523568	0.975124378	0.975124378	Bn-N7-p10081261	Bn-chrA07-p9971281
Bn-A07-p8526430	0.950248756	0.950248756	Bn-N7-p10084605	Bn-chrA07-p9976345
Bn-A07-p8530938	0.983471074	0.983471074	Bn-N7-p10088538	Bn-chrA07-p9980278
Bn-A07-p8531068	0.731343284	0.696517413	Bn-N17-p21518315	Bn-chrA07-p9980408
Bn-A07-p8541452	0.995024876	0.995024876	Bn-N7-p10098200	Bn-chrA07-p9989940
Bn-A07-p8545569	1	1	Bn-N7-p10101866	Bn-chrA07-p9993605
Bn-A07-p8558330	0.970099668	0.970099668	Bn-N7-p10114125	Bn-chrA07-p10003444
Bn-A07-p8560948	0.980099502	0.980099502	Bn-N7-p10116766	Bn-chrA07-p10005987
Bn-A07-p8566493	0.970149254	0.970149254	Bn-N7-p10123376	Bn-chrA07-p10012597
Bn-A07-p8567824	0.950413223	0.950413223	Bn-N7-p10124700	Bn-chrA07-p10013921
Bn-A07-p8571463	0.991735537	0.991735537	Bn-N7-p10128380	Bn-chrA07-p10017603
Bn-A07-p8572785	0.990033223	0.990033223	Bn-N7-p10129706	Bn-chrA07-p10018929
Bn-A07-p8584939	0.983471074	0.983471074	Bn-N7-p10139999	Bn-chrA07-p10028753
Bn-A07-p8593730	0.991735537	0.991735537	Bn-N7-p10142914	Bn-chrA07-p10031668
Bn-A07-p8594245	0.950248756	0.950248756	Bn-N7-p10143433	Bn-chrA07-p10032187
Bn-A07-p8598343	0.991735537	0.991735537	Bn-N7-p10161369	Bn-chrA07-p10049867
Bn-A07-p8610828	0.991735537	0.991735537	Bn-N7-p10167179	Bn-chrC07-p19682153
Bn-A07-p8612122	0.985074627	0.985074627	Bn-N7-p10168473	Bn-chrA07-p10056969
Bn-A07-p8615521	0.966942149	0.966942149	Bn-N7-p10171874	Bn-chrA07-p10060370
Bn-A07-p8615961	0.995024876	0.995024876	Bn-N7-p10172313	Bn-chrA07-p10060809
Bn-A07-p8616202	0.996677741	0.996677741	Bn-N7-p10172554	Bn-chrA07-p10061050
Bn-A07-p8617055	1	1	Bn-N7-p10173407	Bn-chrA07-p10061903
Bn-A07-p8617442	1	1	Bn-N7-p10173837	Bn-chrA07-p10062333
Bn-A07-p8618114	1	1	Bn-N7-p10174465	Bn-chrA07-p10062961
Bn-A07-p8619654	0.983471074	0.983471074	Bn-N7-p10176006	Bn-chrA07-p10064502
Bn-A07-p8632859	0.950413223	0.330578512	Bn-N8-p13622446	Unknown
Bn-A07-p8655988	0.983471074	0.983471074	Bn-N7-p10200495	Bn-chrA07-p10078958
Bn-A07-p8656682	0.52892562	0.52892562	Bn-N7-p10201188	Bn-chrA07-p10079651
Bn-A07-p8668003	0.980099502	0.980099502	Bn-N7-p10217619	Bn-chrA07-p10097697
Bn-A07-p8668714	0.983471074	0.983471074	Bn-N7-p10218329	Bn-chrA07-p10098407

Bn-A07-p8669833	0.975206612	0.975206612	Bn-N7-p10219450	Bn-chrA07-p10099528
Bn-A07-p8670132	0.991735537	0.991735537	Bn-N7-p10219757	Bn-chrA07-p10099835
Bn-A07-p8674321	0.768595041	0.768595041	Bn-N7-p10228570	Bn-chrA07-p10108646
Bn-A07-p8674940	0.965174129	0.965174129	Bn-N7-p10229184	Bn-chrA07-p10109260
Bn-A07-p8675164	0.975206612	0.975206612	Bn-N7-p10229408	Bn-chrA07-p10109484
Bn-A07-p8675574	0.970099668	0.970099668	Bn-N7-p10229818	Bn-chrA07-p10109894
Bn-A07-p8696690	0.975206612	0.975206612	Bn-N7-p10241466	Bn-chrA07-p10121491
Bn-A07-p8698580	0.991735537	0.991735537	Bn-N7-p10242428	Bn-chrA07-p10122453
Bn-A07-p8699677	0.983388704	0.983388704	Bn-N7-p10246335	Bn-chrA07-p10128512
Bn-A07-p8701644	0.983471074	0.983471074	Bn-N7-p10248306	Bn-chrA07-p10130483
Bn-A07-p8718607	0.958677686	0.958677686	Bn-N7-p10265373	Bn-chrA07-p10146743
Bn-A07-p8721197	0.985074627	0.985074627	Bn-N7-p10267855	Bn-chrA07-p10149225
Bn-A07-p8724769	0.995024876	0.995024876	Bn-N7-p10270879	Bn-chrA07-p10152249
Bn-A07-p8745024	0.983471074	0.983471074	Bn-N7-p10295054	Bn-chrA07-p10189817
Bn-A07-p8748832	0.993355482	0.993355482	Bn-N7-p10299150	Bn-chrA07-p10193913
Bn-A07-p8752561	0.983388704	0.983388704	Bn-N7-p10302743	Bn-chrA07-p10197608
Bn-A07-p8752606	0.990033223	0.990033223	Bn-N7-p10302788	Bn-chrA07-p10197653
Bn-A07-p8755253	0.975124378	0.975124378	Bn-N7-p10305436	Bn-chrA07-p10202083
Bn-A07-p8758900	0.986710963	0.986710963	Bn-N7-p10308962	Bn-chrA07-p10205609
Bn-A07-p8773140	0.975206612	0.975206612	Bn-N7-p10326697	Bn-chrA07-p10224500
Bn-A07-p8773216	0.958677686	0.958677686	Bn-N7-p10326773	Bn-chrA07-p10224576
Bn-A07-p8775069	0.909090909	0.909090909	Bn-N7-p10328682	Bn-chrA07-p10226485
Bn-A07-p8790021	0.975124378	0.975124378	Bn-N7-p10341860	Bn-chrA07-p10239663
Bn-A07-p8796823	0.88372093	0.88372093	Bn-N7-p10348699	Bn-chrA07-p10246501
Bn-A07-p8824653	0.990049751	0.990049751	Bn-N7-p10397222	Bn-chrA07-p10294321
Bn-A07-p8850191	0.996677741	0.996677741	Bn-N7-p10431293	Bn-chrA07_random-p973533
Bn-A07-p8851644	0.960199005	0.960199005	Bn-N7-p10432761	Bn-chrA07_random-p975001
Bn-A07-p8852716	0.909090909	0.909090909	Bn-N17-p21908599	Bn-chrC07-p20318683
Bn-A07-p8854569	0.996677741	0.996677741	Bn-N7-p10435189	Bn-chrA07_random-p977428
Bn-A07-p8857433	0.958677686	0.958677686	Bn-N7-p10438066	Bn-chrA07_random-p980305
Bn-A07-p8888056	0.916943522	0.916943522	Bn-N17-p42441570	Bn-chrC07-p38867469
Bn-A07-p8895419	0.958677686	0.958677686	Bn-N7-p10474631	Bn-chrA07_random-p102991
Bn-A07-p8896391	0.975206612	0.975206612	Bn-N7-p10475702	Bn-chrA07_random-p102894
Bn-A07-p8898443	0.958677686	0.958677686	Bn-N7-p10477765	Bn-chrA07_random-p102687
Bn-A07-p8903901	0.975206612	0.975206612	Bn-N7-p10502995	Bn-chrA07_random-p102052
Bn-A07-p8911104	0.958677686	0.958677686	Bn-N7-p10511615	Bn-chrA07_random-p101277
Bn-A07-p8929154	0.983471074	0.983471074	Bn-N7-p10527946	Bn-chrA07-p10400980
Bn-A07-p8932587	0.990049751	0.990049751	Bn-N7-p10531124	Bn-chrA07-p10404157
Bn-A07-p8935344	0.983471074	0.983471074	Bn-N7-p10533843	Bn-chrA07-p10406876
Bn-A07-p8941260	0.975206612	0.975206612	Bn-N7-p10539793	Bn-chrA07-p10412830
Bn-A07-p8947431	0.966942149	0.966942149	Bn-N7-p10546669	Bn-chrA07-p10419531
Bn-A07-p8959937	0.985074627	0.985074627	Bn-N7-p10558114	Bn-chrA07-p10430938
Bn-A07-p8962304	0.958677686	0.958677686	Bn-N7-p10560292	Bn-chrA07-p10433116
Bn-A07-p8967890	0.935323383	0.960199005	Bn-N17-p21646039	Bn-chrC07-p20577774
Bn-A07-p8977017	0.930348259	0.930348259	Bn-N7-p10570442	Bn-chrA07-p10442232
Bn-A07-p8977490	0.867768595	0.867768595	Bn-N7-p10570917	Bn-chrA07-p10442707

Bn-A07-p8980166	0.975124378	0.975124378	Bn-N7-p10572009	Bn-chrA07-p10443799
Bn-A07-p8989988	0.975206612	0.975206612	Bn-N7-p10583407	Bn-chrA07-p10455173
Bn-A07-p8997726	0.851239669	0.851239669	Bn-N7-p10591189	Bn-chrA07-p10463092
Bn-A07-p9010472	0.975206612	0.975206612	Bn-N7-p10599216	Bn-chrA07-p10470432
Bn-A07-p9014912	0.990049751	0.990049751	Bn-N7-p10602521	Bn-chrA07-p10473737
Bn-A07-p9020570	0.776859504	0.776859504	Bn-N7-p10604854	Bn-chrA07-p10476070
Bn-A07-p9031421	0.983471074	0.983471074	Bn-N7-p10625124	Bn-chrA07-p10492179
Bn-A07-p9034194	0.910299003	0.910299003	Bn-N17-p22687236	Bn-chrC07-p21025339
Bn-A07-p9041783	0.983471074	0.983471074	Bn-N7-p10635658	Bn-chrA07-p10508233
Bn-A07-p9043136	0.983471074	0.983471074	Bn-N7-p10636903	Bn-chrA07-p10509478
Bn-A07-p9044585	0.157024793	0.198347107	Bn-N13-p41261292	Bn-chrCnn_random-p449876
Bn-A07-p9046330	0.950413223	0.950413223	Bn-N7-p10639867	Bn-chrA07-p10512442
Bn-A07-p9060619	1	1	Bn-N7-p10650407	Bn-chrA07-p10523037
Bn-A07-p9071682	0.991735537	0.991735537	Bn-N7-p10663266	Bn-chrA07-p10534384
Bn-A07-p9079474	0.985074627	0.985074627	Bn-N7-p10670871	Bn-chrA07-p10541988
Bn-A07-p9086600	0.996677741	0.996677741	Bn-N7-p10678215	Bn-chrAnn_random-p274302
Bn-A07-p9089710	0.970149254	0.970149254	Bn-N7-p10681336	Bn-chrA07-p10551849
Bn-A07-p9091839	0.760797342	0.760797342	Bn-N7-p10683458	Bn-chrA07-p10553971
Bn-A07-p9095204	0.990049751	0.990049751	Bn-N7-p10686823	Bn-chrA07-p10557336
Bn-A07-p9095357	0.985074627	0.985074627	Bn-N7-p10686976	Bn-chrA07-p10557489
Bn-A07-p9095557	0.965174129	0.965174129	Bn-N7-p10687176	Bn-chrA07-p10557689
Bn-A07-p9097136	0.731343284	0.686567164	Bn-N7-p10688753	Bn-chrA07-p10559266
Bn-A07-p9111745	0.996677741	0.996677741	Bn-N7-p10697089	Bn-chrA07-p10567589
Bn-A07-p9115370	0.975124378	0.975124378	Bn-N7-p10704581	Bn-chrA07-p10581719
Bn-A07-p9117042	0.983388704	0.983388704	Bn-N7-p10706508	Bn-chrA07-p10583646
Bn-A07-p9120043	0.986710963	0.986710963	Bn-N3-p24974954	Bn-chrUnn_random-p531013
Bn-A07-p9155350	0.940298507	0.940298507	Bn-N7-p10744278	Bn-chrA07-p10619846
Bn-A07-p9180716	0.996677741	0.996677741	Bn-N7-p10777813	Bn-chrA07-p10656778
Bn-A07-p9180953	0.990033223	0.990033223	Bn-N7-p10778048	Bn-chrA07-p10657013
Bn-A07-p9208032	0.965174129	0.965174129	Bn-Scaffold06771-p7	Bn-chrA07-p10676408
Bn-A07-p9216783	0.63681592	0.631840796	Bn-N10-p13213699	Bn-chrC09-p42992868
Bn-A07-p9272000	1	1	Bn-N7-p10832679	Bn-chrAnn_random-p331456
Bn-A07-p9283373	0.996677741	0.996677741	Bn-N7-p10842868	Bn-chrAnn_random-p157411
Bn-A07-p9293372	1	1	Bn-N7-p10852872	Bn-chrAnn_random-p157498
Bn-A07-p9309081	0.965174129	0.965174129	Bn-N6-p21916494	Bn-chrAnn_random-p358639
Bn-A07-p9320476	0.996677741	0.996677741	Bn-N7-p10880110	Bn-chrAnn_random-p157823
Bn-A07-p9321347	0.983388704	0.983388704	Bn-N7-p10880981	Bn-chrAnn_random-p157832
Bn-A07-p9324529	0.945273632	0.945273632	Bn-N7-p10884131	Bn-chrAnn_random-p157863
Bn-A07-p9336662	0.980099502	0.980099502	Bn-N7-p10896201	Bn-chrA07_random-p103842
Bn-A07-p9339366	0.966942149	0.966942149	Bn-N7-p10899898	Bn-chrAnn_random-p247867
Bn-A07-p9344001	0.983471074	0.983471074	Bn-N7-p10904585	Bn-chrAnn_random-p247914
Bn-A07-p9347254	0.965174129	0.965174129	Bn-N7-p10907847	Bn-chrAnn_random-p247947
Bn-A07-p9384306	0.542288557	0.542288557	Bn-N7-p10940482	Bn-chrA07-p10769252
Bn-A07-p9402974	0.983388704	0.983388704	Bn-N7-p10952981	Bn-chrA07-p10781122
Bn-A07-p9429348	0.92358804	0.966777409	Bn-N3-p20309329	Bn-chrA03-p18881165
Bn-A07-p9441022	0.980099502	0.980099502	Bn-N7-p10975141	Bn-chrA07-p10808128

Bn-A07-p9441402	0.975124378	0.975124378	Bn-N7-p10975521	Bn-chrA07-p10808508
Bn-A07-p9441979	0.985074627	0.985074627	Bn-N7-p10976098	Bn-chrA07-p10809085
Bn-A07-p9450863	0.965174129	0.965174129	Bn-N7-p10985289	Bn-chrA07-p10818136
Bn-A07-p9451917	0.909090909	0.909090909	Bn-N7-p10986343	Bn-chrA07-p10819190
Bn-A07-p9462417	0.940298507	0.940298507	Bn-N7-p10990897	Bn-chrA07-p10823504
Bn-A07-p9468382	0.991735537	0.991735537	Bn-N7-p10992576	Bn-chrA07-p10825039
Bn-A07-p9468670	0.983471074	0.983471074	Bn-N7-p10992864	Bn-chrA07-p10825327
Bn-A07-p9480985	0.975124378	0.975124378	Bn-N7-p11006421	Bn-chrA07-p10838446
Bn-A07-p9481452	0.975206612	0.975206612	Bn-N7-p11006889	Bn-chrA07-p10838914
Bn-A07-p9485484	0.993355482	0.993355482	Bn-N7-p11010335	Bn-chrA07-p10842358
Bn-A07-p9486032	0.975206612	0.975206612	Bn-N7-p11010883	Bn-chrA07-p10842906
Bn-A07-p9493282	0.985074627	0.985074627	Bn-N7-p11018459	Bn-chrA07-p10847662
Bn-A07-p9494829	0.975206612	0.975206612	Bn-N7-p11020009	Bn-chrA07-p10849211
Bn-A07-p9509100	0.991735537	0.991735537	Bn-N7-p11033575	Bn-chrA07-p10862930
Bn-A07-p9512042	0.996677741	0.996677741	Bn-N7-p11039410	Bn-chrA07-p10867492
Bn-A07-p9512256	0.996677741	0.996677741	Bn-N7-p11039624	Bn-chrA07-p10867706
Bn-A07-p9516564	0.955223881	0.985074627	Bn-N12-p32764996	Bn-chrA01-p8244451
Bn-A07-p9521819	0.983471074	0.983471074	Bn-N7-p11045846	Bn-chrC07-p21663901
Bn-A07-p9523515	0.983471074	0.983471074	Bn-N7-p11047560	Bn-chrA07-p10875968
Bn-A07-p9525348	0.995024876	0.995024876	Bn-N7-p11049392	Bn-chrA07-p10877800
Bn-A07-p9530382	0.991735537	0.991735537	Bn-N7-p11055877	Bn-chrA07-p10882973
Bn-A07-p9535820	0.966942149	0.966942149	Bn-N9-p23394320	Bn-chrA09-p19578646
Bn-A07-p9564494	0.933884298	0.933884298	Bn-N7-p11071141	Bn-chrA07-p10900603
Bn-A07-p9565657	0.990033223	0.990033223	Bn-N7-p11072298	Bn-chrA07-p10901760
Bn-A07-p9579307	0.94214876	0.94214876	Bn-N7-p11087013	Bn-chrA07-p10917979
Bn-A07-p9583355	0.966942149	0.966942149	Bn-N7-p11089964	Bn-chrA07-p10920929
Bn-A07-p9583457	0.933884298	0.933884298	Bn-N7-p11090066	Bn-chrA07-p10921031
Bn-A07-p9597037	0.985074627	0.985074627	Bn-N7-p11101969	Bn-chrA07-p10932483
Bn-A07-p9611857	0.970149254	0.970149254	Bn-N7-p11109945	Bn-chrA07-p10941135
Bn-A07-p9617534	0.976744186	0.976744186	Bn-N7-p11120243	Bn-chrAnn_random-p335559
Bn-A07-p9632473	0.94214876	0.94214876	Bn-N7-p11134620	Bn-chrA07_random-p104254
Bn-A07-p9637867	0.930348259	0.930348259	Bn-N7-p11138451	Bn-chrA07_random-p104637
Bn-A07-p9639704	0.996677741	0.996677741	Bn-N7-p11140316	Bn-chrA07_random-p104824
Bn-A07-p9641332	0.973421927	0.973421927	Bn-N7-p11141759	Bn-chrA07_random-p104968
Bn-A07-p9661444	0.980099502	0.980099502	Bn-N7-p11159340	Bn-chrAnn_random-p156441
Bn-A07-p9663232	0.993355482	0.993355482	Bn-N7-p11161136	Bn-chrAnn_random-p156459
Bn-A07-p9670298	0.945273632	0.945273632	Bn-N7-p11177134	Bn-chrAnn_random-p156619
Bn-A07-p9684829	0.960199005	0.960199005	Bn-N7-p11191362	Bn-chrAnn_random-p156762
Bn-A07-p9689917	0.986710963	0.986710963	Bn-N7-p11196894	Bn-chrAnn_random-p156817
Bn-A07-p9690172	0.990033223	0.990033223	Bn-N7-p11197149	Bn-chrAnn_random-p156819
Bn-A07-p9690345	0.986710963	0.986710963	Bn-N7-p11197322	Bn-chrAnn_random-p156821
Bn-A07-p9696670	0.970149254	0.970149254	Bn-N7-p11203500	Bn-chrAnn_random-p304995
Bn-A07-p9708357	0.996677741	0.996677741	Bn-N7-p11214875	Bn-chrA07-p10995667
Bn-A07-p9724274	0.940199336	0.940199336	Bn-N17-p24104461	Bn-chrC07-p22015070
Bn-A07-p9760391	0.717607973	0.983388704	Bn-Scaffold72424-p7	Bn-chrAnn_random-p457323
Bn-A07-p9764930	0.740863787	0.740863787	Bn-N7-p11272088	Bn-chrA07-p11058568

Bn-A07-p9791002	0.975206612	0.975206612	Bn-N7-p11291470	Bn-chrA07-p11076585
Bn-A07-p9824132	0.983388704	0.983388704	Bn-N7-p11316552	Bn-chrA07-p11099663
Bn-A07-p9831545	0.990049751	0.990049751	Bn-N7-p11323969	Bn-chrA07-p11107078
Bn-A07-p9841369	0.996677741	0.996677741	Bn-N7-p11333958	Bn-chrA07-p11117064
Bn-A07-p9843341	0.966942149	0.966942149	Bn-N7-p11340642	Bn-chrA07-p11123754
Bn-A07-p9849702	0.950413223	0.950413223	Bn-N7-p11347009	Bn-chrA07-p11131581
Bn-A07-p9867804	0.976744186	0.976744186	Bn-N17-p24349122	Bn-chrC07-p22285461
Bn-A07-p9897363	0.975206612	0.975206612	Bn-N7-p11393929	Bn-chrA07-p11177572
Bn-A07-p9901034	0.970149254	0.970149254	Bn-N7-p11399436	Bn-chrA07-p11183327
Bn-A07-p9903750	0.975206612	0.975206612	Bn-N7-p11401506	Bn-chrA07-p11185397
Bn-A07-p9908315	0.993355482	0.993355482	Bn-N7-p11406142	Bn-chrA07-p11190032
Bn-A07-p9912557	0.975206612	0.975206612	Bn-N7-p11410376	Bn-chrA07-p11194265
Bn-A07-p9912637	0.991735537	0.991735537	Bn-N7-p11410456	Bn-chrA07-p11194345
Bn-A07-p9915554	0.507462687	0.577114428	Bn-N17-p41257075	Bn-chrC07-p37774374
Bn-A07-p9916502	0.975206612	0.975206612	Bn-N7-p11412204	Bn-chrA07-p11196091
Bn-A07-p9916647	0.983388704	0.983388704	Bn-N7-p11412337	Bn-chrA07-p11196224
Bn-A07-p9921856	0.983471074	0.983471074	Bn-N7-p11421553	Bn-chrA07-p11205439
Bn-A07-p9923514	0.647840532	0.647840532	Bn-N7-p11423214	Bn-chrA07-p11207100
Bn-A07-p9924604	0.950413223	0.950413223	Bn-N7-p11424323	Bn-chrA07-p11208121
Bn-A07-p9924929	0.991735537	0.991735537	Bn-N7-p11424648	Bn-chrA07-p11208446
Bn-A07-p9945186	1	1	Bn-N7-p11438033	Bn-chrA07-p11221831
Bn-A07-p9975543	0.950248756	0.950248756	Bn-N7-p11466239	Bn-chrA07-p11250817
Bn-A07-p9985625	0.983388704	0.983388704	Bn-N7-p11476372	Bn-chrA07_random-p105396
Bn-A07-p9987682	0.975206612	0.975206612	Bn-N7-p11478420	Bn-chrA07_random-p105601
Bn-A07-p9988423	0.983471074	0.983471074	Bn-N7-p11479163	Bn-chrA07_random-p105675
Bn-A07-p10003378	0.986710963	0.986710963	Bn-N7-p11491755	Bn-chrA07_random-p106537
Bn-A07-p10004245	0.996677741	0.996677741	Bn-N7-p11492622	Bn-chrA07_random-p106624
Bn-A07-p10010327	0.976744186	0.976744186	Bn-N7-p11498680	Bn-chrA07_random-p107230
Bn-A07-p10022226	0.991735537	0.991735537	Bn-N7-p11502062	Bn-chrA07_random-p107402
Bn-A07-p10033055	0.980066445	0.980066445	Bn-N7-p11508010	Bn-chrA07_random-p107996
Bn-A07-p10037462	0.983388704	0.983388704	Bn-N7-p11512955	Bn-chrA07_random-p108491
Bn-A07-p10092606	0.926910299	0.926910299	Bn-N7-p11576681	Bn-chrA07-p11271232
Bn-A07-p10099786	0.986710963	0.986710963	Bn-N7-p11584293	Bn-chrA07-p11278842
Bn-A07-p10100811	0.956810631	0.956810631	Bn-N7-p11585318	Bn-chrA07-p11279867
Bn-A07-p10101125	0.950413223	0.950413223	Bn-N7-p11585635	Bn-chrA07-p11280184
Bn-A07-p10128190	0.990033223	0.734219269	Bn-N7-p11611316	Bn-chrA07-p11306325
Bn-A07-p10144240	0.983471074	0.983471074	Bn-N7-p11627385	Bn-chrA07-p11321593
Bn-A07-p10169211	0.975206612	0.975206612	Bn-N7-p11645303	Bn-chrA07-p11340785
Bn-A07-p10181071	0.917355372	0.917355372	Bn-N7-p11657842	Bn-chrA07-p11353324
Bn-A07-p10188190	0.890547264	0.895522388	Bn-N9-p6202417	Bn-chrCnn_random-p351750
Bn-A07-p10194155	0.983471074	0.983471074	Bn-N7-p11664233	Bn-chrA07-p11359716
Bn-A07-p10195276	0.966942149	0.966942149	Bn-N7-p11665352	Bn-chrA07-p11360835
Bn-A07-p10200584	0.985074627	0.985074627	Bn-N7-p11670303	Bn-chrA07-p11366373
Bn-A07-p10204148	0.975206612	0.975206612	Bn-N7-p11673828	Bn-chrA07-p11369898
Bn-A07-p10204886	0.958677686	0.958677686	Bn-N7-p11674565	Bn-chrA07-p11370635
Bn-A07-p10207855	0.983471074	0.983471074	Bn-N7-p11677604	Bn-chrA07-p11373483

Bn-A07-p10209077	0.976744186	0.976744186	Bn-N7-p11678835	Bn-chrA07-p11374714
Bn-A07-p10209154	0.950248756	0.950248756	Bn-N7-p11678912	Bn-chrA07-p11374791
Bn-A07-p10220287	0.991735537	0.991735537	Bn-N7-p11690305	Bn-chrA07-p11385431
Bn-A07-p10230704	0.996677741	0.996677741	Bn-N7-p11708200	Bn-chrA07-p11400895
Bn-A07-p10233337	0.983471074	0.983471074	Bn-N7-p11710832	Bn-chrA07-p11403527
Bn-A07-p10236271	0.94214876	0.94214876	Bn-N7-p11713763	Bn-chrA07-p11406459
Bn-A07-p10236552	0.980099502	0.980099502	Bn-N7-p11714044	Bn-chrA07-p11406740
Bn-A07-p10238917	0.983388704	0.983388704	Bn-N7-p11716409	Bn-chrA07-p11409105
Bn-A07-p10253702	0.975206612	0.975206612	Bn-N7-p11734400	Bn-chrA07-p11425961
Bn-A07-p10281077	0.980066445	0.980066445	Bn-N7-p11760233	Bn-chrA07-p11454444
Bn-A07-p10282316	0.983471074	0.983471074	Bn-N7-p11761471	Bn-chrA07-p11455682
Bn-A07-p10282792	0.995024876	0.995024876	Bn-N7-p11761947	Bn-chrA07-p11456158
Bn-A07-p10284031	0.990033223	0.990033223	Bn-N7-p11763083	Bn-chrA07-p11457294
Bn-A07-p10285778	0.965174129	0.965174129	Bn-N7-p11764829	Bn-chrA07-p11459040
Bn-A07-p10289778	0.960199005	0.960199005	Bn-N7-p11768840	Bn-chrA07-p11463051
Bn-A07-p10291035	0.936877076	0.936877076	Bn-N7-p11770094	Bn-chrA07-p11464305
Bn-A07-p10297311	0.966942149	0.966942149	Bn-N7-p11775706	Bn-chrA07-p11469916
Bn-A07-p10299745	0.925619835	0.925619835	Bn-N7-p11778176	Bn-chrA07-p11472386
Bn-A07-p10337326	0.991735537	0.991735537	Bn-N7-p11810475	Bn-chrA07-p11506350
Bn-A07-p10340211	0.966942149	0.975206612	Bn-N7-p11813359	Bn-chrA07-p11509208
Bn-A07-p10347794	0.917355372	0.925619835	Bn-N7-p11826155	Bn-chrA07-p11526727
Bn-A07-p10357533	0.975206612	0.958677686	Bn-N7-p11836386	Bn-chrA07-p11533793
Bn-A07-p10357605	0.966942149	0.950413223	Bn-N7-p11836458	Bn-chrA07-p11533864
Bn-A07-p10357942	0.966942149	0.975206612	Bn-N7-p11836795	Bn-chrA07-p11534201
Bn-A07-p10367676	0.966942149	0.958677686	Bn-N7-p11846968	Bn-chrAnn_random-p453656
Bn-A07-p10368959	0.991735537	1	Bn-N7-p11848410	Bn-chrAnn_random-p453671
Bn-A07-p10370541	0.966942149	0.958677686	Bn-N7-p11849992	Bn-chrA07_random-p113347
Bn-A07-p10379059	0.991735537	0.991735537	Bn-N7-p11858814	Bn-chrA07-p11559981
Bn-A07-p10382757	1	1	Bn-N7-p11862423	Bn-chrA07-p11563554
Bn-A07-p10388044	0.990049751	0.995024876	Bn-N7-p11868057	Bn-chrA07-p11569393
Bn-A07-p10398999	0.983471074	0.975206612	Bn-N7-p11879724	Bn-chrA07-p11582311
Bn-A07-p10399235	0.940298507	0.940298507	Bn-N7-p11879960	Bn-chrA07-p11582547
Bn-A07-p10400825	0.985074627	0.985074627	Bn-N7-p11881550	Bn-chrA07-p11584137
Bn-A07-p10401133	0.992857143	0.992857143	Bn-N7-p11881858	Bn-chrA07-p11584445
Bn-A07-p10409623	0.966942149	0.933884298	Bn-N7-p11890636	Bn-chrA07-p11593150
Bn-A07-p10414624	0.975206612	0.966942149	Bn-N7-p11895271	Bn-chrA07-p11598818
Bn-A07-p10418835	0.935323383	0.935323383	Bn-N7-p11899528	Bn-chrA07-p11603067
Bn-A07-p10421977	0.661157025	0.661157025	Bn-N7-p11910872	Bn-chrA07-p11617091
Bn-A07-p10430301	0.990033223	0.990033223	Bn-N7-p11918244	Bn-chrA07-p11624459
Bn-A07-p10437749	0.945273632	0.945273632	Bn-N7-p11925680	Bn-chrA07-p11632631
Bn-A07-p10437970	0.985074627	0.985074627	Bn-N7-p11925901	Bn-chrA07-p11632852
Bn-A07-p10451411	0.990033223	0.990033223	Bn-N7-p11937147	Bn-chrA07-p11643769
Bn-A07-p10455192	0.975206612	0.975206612	Bn-N7-p11940967	Bn-chrA07-p11647589
Bn-A07-p10466052	0.958677686	0.958677686	Bn-N7-p11944794	Bn-chrA07-p11651416
Bn-A07-p10468928	0.975124378	0.980099502	Bn-N9-p23609949	Bn-chrC04-p14328292
Bn-A07-p10471024	0.976744186	0.976744186	Bn-N7-p11945984	Bn-chrA07-p11652605

Bn-A07-p10473511	0.970099668	0.970099668	Bn-N7-p11948511	Bn-chrA07-p11655130
Bn-A07-p10483339	0.983388704	0.983388704	Bn-N7-p11958317	Bn-chrA07-p11664936
Bn-A07-p10486045	0.980099502	0.980099502	Bn-N7-p11961032	Bn-chrA07-p11667651
Bn-A07-p10494571	0.983471074	0.983471074	Bn-N7-p11969210	Bn-chrA07-p11675829
Bn-A07-p10501097	0.975206612	0.975206612	Bn-N7-p11975561	Bn-chrA07-p11681469
Bn-A07-p10501168	0.975206612	0.975206612	Bn-N7-p11975632	Bn-chrA07-p11681540
Bn-A07-p10532652	0.980099502	0.865671642	Bn-N7-p12010306	Bn-chrC04-p14994520
Bn-A07-p10554944	0.966942149	0.966942149	Bn-N7-p12026837	Bn-chrA07-p11734395
Bn-A07-p10557165	0.950413223	0.950413223	Bn-N7-p12030209	Bn-chrA07-p11737773
Bn-A07-p10557557	0.983471074	0.983471074	Bn-N7-p12030602	Bn-chrA07-p11738166
Bn-A07-p10557559	0.975206612	0.975206612	Bn-N7-p12030604	Bn-chrA07-p11738168
Bn-A07-p10568144	0.925619835	0.925619835	Bn-N7-p12041501	Bn-chrA07-p11748634
Bn-A07-p10573729	0.983471074	0.983471074	Bn-N7-p12047316	Bn-chrA07-p11755300
Bn-A07-p10573931	0.991735537	0.991735537	Bn-N7-p12047513	Bn-chrA07-p11755497
Bn-A07-p10584018	0.991735537	0.991735537	Bn-N7-p12061103	Bn-chrA07-p11769168
Bn-A07-p10635069	0.975206612	0.975206612	Bn-N7-p12108177	Bn-chrA07-p11816844
Bn-A07-p10635266	0.983471074	0.983471074	Bn-N7-p12108376	Bn-chrA07-p11817043
Bn-A07-p10650508	0.983471074	0.983471074	Bn-N7-p12121407	Bn-chrA07-p11830563
Bn-A07-p10651899	0.980099502	0.980099502	Bn-N7-p12122798	Bn-chrA07-p11831954
Bn-A07-p10652292	0.970149254	0.970149254	Bn-N7-p12123191	Bn-chrA07-p11832347
Bn-A07-p10652962	0.980099502	0.980099502	Bn-N7-p12123861	Bn-chrA07-p11833017
Bn-A07-p10653042	0.990049751	0.990049751	Bn-N7-p12123941	Bn-chrA07-p11833097
Bn-A07-p10667459	0.893687708	0.893687708	Bn-N7-p12151327	Bn-chrA07-p11871232
Bn-A07-p10672691	0.845771144	0.985074627	Bn-N7-p12171039	Bn-chrA07-p11888760
Bn-A07-p10673477	0.985074627	0.985074627	Bn-N7-p12171825	Bn-chrA07-p11889546
Bn-A07-p10673700	0.990049751	0.990049751	Bn-N7-p12172048	Bn-chrA07-p11889769
Bn-A07-p10675102	0.975206612	0.975206612	Bn-N7-p12173458	Bn-chrA07-p11891179
Bn-A07-p10725832	0.991735537	0.983471074	Bn-N7-p12215217	Bn-chrA07-p11928474
Bn-A07-p10729654	0.950413223	0.950413223	Bn-N7-p12218493	Bn-chrA07-p11931319
Bn-A07-p10733528	0.983471074	0.983471074	Bn-N7-p12222357	Bn-chrA07-p11935210
Bn-A07-p10750190	1	0.892561983	Bn-N7-p12236021	Bn-chrA07-p11956081
Bn-A07-p10755129	0.983471074	0.983471074	Bn-N7-p12240892	Bn-chrA07-p11961008
Bn-A07-p10775694	0.991735537	1	Bn-N7-p12264998	Bn-chrA07-p11983891
Bn-A07-p10780571	0.975206612	0.975206612	Bn-N7-p12269959	Bn-chrA07-p11988743
Bn-A07-p10798522	0.925619835	0.809917355	Bn-N7-p12284181	Bn-chrC04-p14238191
Bn-A07-p10800383	0.94214876	0.925619835	Bn-N7-p12285347	Bn-chrA07-p12003345
Bn-A07-p10837849	0.975206612	0.975206612	Bn-N7-p12311642	Bn-chrA07-p12038734
Bn-A07-p10847704	0.876033058	0.975206612	Bn-N14-p15981556	Bn-chrA07-p12050115
Bn-A07-p10848104	0.925373134	0.935323383	Bn-N7-p12322177	Bn-chrA07-p12050515
Bn-A07-p10848190	0.910447761	0.92039801	Bn-N7-p12322263	Bn-chrA07-p12050601
Bn-A07-p10849053	0.876033058	0.975206612	Bn-N7-p12323796	Bn-chrA07-p12052490
Bn-A07-p10869578	0.991735537	0.991735537	Bn-N7-p12347990	Bn-chrA07-p12103596
Bn-A07-p10870115	0.989637306	0.989637306	Bn-N7-p12348527	Bn-chrA07-p12104133
Bn-A07-p10873126	0.975206612	0.975206612	Bn-N7-p12351541	Bn-chrA07-p12107128
Bn-A07-p10877038	0.958677686	0.950413223	Bn-N7-p12355448	Bn-chrA07-p12111042
Bn-A07-p10890061	0.970149254	0.970149254	Bn-N7-p12364144	Bn-chrA07-p12121807

Bn-A07-p10891272	0.986710963	0.986710963	Bn-N7-p12365945	Bn-chrA07-p12123608
Bn-A07-p10949782	0.975206612	0.983471074	Bn-N7-p12423923	Bn-chrA07-p12175608
Bn-A07-p10963555	0.966942149	0.966942149	Bn-N7-p12436770	Bn-chrA07-p12188661
Bn-A07-p10965607	0.925619835	0.925619835	Bn-N7-p12438850	Bn-chrA07-p12190774
Bn-A07-p10968184	0.975206612	0.975206612	Bn-N7-p12441442	Bn-chrA07-p12193379
Bn-A07-p10971791	1	0.991735537	Bn-N7-p12445090	Bn-chrA07-p12197008
Bn-A07-p10972081	0.991735537	0.975206612	Bn-N7-p12445379	Bn-chrA07-p12197297
Bn-A07-p10975419	0.983471074	0.975206612	Bn-N7-p12448745	Bn-chrA07-p12200664
Bn-A07-p10979187	1	0.991735537	Bn-N7-p12455553	Bn-chrA07-p12204455
Bn-A07-p10995462	1	0.991735537	Bn-N7-p12475405	Bn-chrA07-p12226231
Bn-A07-p11006909	0.966942149	0.958677686	Bn-N7-p12484986	Bn-chrA07-p12232955
Bn-A07-p11008123	0.983471074	1	Bn-N7-p12486190	Bn-chrA07-p12234169
Bn-A07-p11009397	1	0.991735537	Bn-N7-p12488065	Bn-chrA07-p12235441
Bn-A07-p11010528	0.958677686	0.975206612	Bn-N7-p12488841	Bn-chrA07-p12236738
Bn-A07-p11012468	0.983471074	0.991735537	Bn-N7-p12498446	Bn-chrA07-p12243645
Bn-A07-p11021668	0.834710744	0.917355372	Bn-N7-p12509207	Bn-chrA07-p12252121
Bn-A07-p11041528	0.851239669	0.851239669	Bn-N14-p15554312	Bn-chrC04-p13700644
Bn-A07-p11044717	0.975206612	0.975206612	Bn-N7-p12530729	Bn-chrA07-p12267136
Bn-A07-p11058228	0.933884298	0.933884298	Bn-N7-p12548290	Bn-chrA07-p12296402
Bn-A07-p11059156	0.842975207	0.842975207	Bn-N7-p12549171	Bn-chrA07-p12297283
Bn-A07-p11059209	0.975206612	0.975206612	Bn-N7-p12549224	Bn-chrA07-p12297336
Bn-A07-p11066210	0.950413223	0.950413223	Bn-N7-p12554126	Bn-chrA07-p12312516
Bn-A07-p11068231	0.975206612	0.975206612	Bn-N7-p12556265	Bn-chrA07-p12314655
Bn-A07-p11068735	0.983471074	0.983471074	Bn-N7-p12556769	Bn-chrA07-p12315159
Bn-A07-p11072052	0.884297521	0.884297521	Bn-N7-p12561519	Bn-chrA07-p12319909
Bn-A07-p11074548	0.917355372	0.917355372	Bn-N7-p12562983	Bn-chrA07-p12321373
Bn-A07-p11076587	0.975206612	0.975206612	Bn-N7-p12564995	Bn-chrA07-p12323385
Bn-A07-p11086654	0.983471074	0.983471074	Bn-N7-p12576587	Bn-chrA07-p12333975
Bn-A07-p11103061	0.966942149	0.966942149	Bn-N7-p12590269	Bn-chrA07-p12346577
Bn-A07-p11114150	0.991735537	0.991735537	Bn-N7-p12596579	Bn-chrA07-p12352887
Bn-A07-p11119130	0.925619835	0.925619835	Bn-N7-p12601519	Bn-chrA07-p12357827
Bn-A07-p11138100	0.991735537	0.991735537	Bn-N7-p12620874	Bn-chrA07-p12377532
Bn-A07-p11147817	0.983471074	0.983471074	Bn-N7-p12637135	Bn-chrA07-p12438528
Bn-A07-p11152131	0.991735537	0.991735537	Bn-N7-p12641457	Bn-chrA07-p12442850
Bn-A07-p11156840	0.983471074	0.983471074	Bn-N7-p12646164	Bn-chrA07-p12447556
Bn-A07-p11159936	0.917355372	0.917355372	Bn-N7-p12649312	Bn-chrA07-p12450434
Bn-A07-p11162333	0.975206612	0.975206612	Bn-N7-p12651148	Bn-chrA07-p12452270
Bn-A07-p11163415	0.991735537	0.991735537	Bn-N7-p12652260	Bn-chrA07-p12453382
Bn-A07-p11163481	0.983471074	0.983471074	Bn-N7-p12652326	Bn-chrA07-p12453448
Bn-A07-p11171407	0.991735537	0.991735537	Bn-N7-p12654503	Bn-chrA07-p12455625
Bn-A07-p11175937	0.925619835	0.925619835	Bn-N7-p12660565	Bn-chrA07-p12461687
Bn-A07-p11176924	0.975206612	0.975206612	Bn-N7-p12661562	Bn-chrA07-p12462684
Bn-A07-p11178077	0.933884298	0.933884298	Bn-N7-p12662668	Bn-chrA07-p12463790
Bn-A07-p11178807	0.958677686	0.958677686	Bn-N7-p12663707	Bn-chrA07-p12464829
Bn-A07-p11185122	0.966942149	0.966942149	Bn-N7-p12673921	Bn-chrA07-p12477068
Bn-A07-p11186861	0.950413223	0.950413223	Bn-N7-p12675704	Bn-chrA07-p12478851

Bn-A07-p11191424	0.975206612	0.975206612	Bn-N7-p12679742	Bn-chrA07-p12482889
Bn-A07-p11191776	0.958677686	0.958677686	Bn-N7-p12680094	Bn-chrA07-p12483241
Bn-A07-p11194871	0.79338843	0.79338843	Bn-N14-p15242655	Bn-chrC04_random-p206166
Bn-A07-p11196478	0.984455959	0.984455959	Bn-N7-p12684358	Bn-chrA07-p12487505
Bn-A07-p11196510	0.985074627	0.985074627	Bn-N7-p12684390	Bn-chrA07-p12487537
Bn-A07-p11200338	1	1	Bn-N7-p12688221	Bn-chrA07-p12491367
Bn-A07-p11200772	0.983471074	0.983471074	Bn-N7-p12688656	Bn-chrA07-p12491802
Bn-A07-p11204177	0.983471074	0.983471074	Bn-N7-p12692110	Bn-chrA07-p12495256
Bn-A07-p11208979	0.975206612	0.975206612	Bn-N7-p12696926	Bn-chrA07-p12499917
Bn-A07-p11225243	0.975206612	0.975206612	Bn-N7-p12700881	Bn-chrA07-p12503872
Bn-A07-p11235392	0.991735537	0.991735537	Bn-N7-p12705509	Bn-chrA07-p12508500
Bn-A07-p11245975	0.991735537	0.991735537	Bn-N7-p12716132	Bn-chrA07-p12520307
Bn-A07-p11248281	0.983471074	0.983471074	Bn-N7-p12718432	Bn-chrA07-p12522607
Bn-A07-p11248893	1	1	Bn-N7-p12719042	Bn-chrA07-p12523217
Bn-A07-p11249547	0.991735537	0.991735537	Bn-N7-p12719696	Bn-chrA07-p12523871
Bn-A07-p11255851	0.975206612	0.975206612	Bn-N7-p12728638	Bn-chrA07-p12532067
Bn-A07-p11264644	0.975206612	0.975206612	Bn-N7-p12731005	Bn-chrA07-p12534434
Bn-A07-p11270373	0.983471074	0.983471074	Bn-N7-p12736929	Bn-chrA07-p12540355
Bn-A07-p11276443	0.975206612	0.975206612	Bn-N7-p12741599	Bn-chrA07-p12545024
Bn-A07-p11284412	0.966942149	0.966942149	Bn-N7-p12751000	Bn-chrA07-p12561977
Bn-A07-p11286252	0.975206612	0.975206612	Bn-N7-p12753023	Bn-chrC02-p10081832
Bn-A07-p11287521	0.975206612	0.975206612	Bn-N7-p12754426	Bn-chrAnn_random-p422447
Bn-A07-p11300051	0.983471074	0.983471074	Bn-N7-p12775340	Bn-chrA07-p12716379
Bn-A07-p11304368	0.950413223	0.950413223	Bn-N7-p12783884	Bn-chrA07-p12709112
Bn-A07-p11305778	0.972527473	0.978021978	Bn-N9-p6231801	Bn-chrA09-p3867060
Bn-A07-p11314565	0.768595041	0.768595041	Bn-N7-p12785697	Bn-chrA07-p12707299
Bn-A07-p11317561	0.966942149	0.966942149	Bn-N7-p12788769	Bn-chrA07-p12704227
Bn-A07-p11320753	0.950413223	0.950413223	Bn-N7-p12791997	Bn-chrA07-p12701000
Bn-A07-p11323366	0.996677741	0.996677741	Bn-N7-p12802492	Bn-chrA07-p12689024
Bn-A07-p11324360	0.996677741	0.996677741	Bn-N7-p12803486	Bn-chrA07-p12688030
Bn-A07-p11328250	0.94214876	0.94214876	Bn-N7-p12807401	Bn-chrA07-p12684115
Bn-A07-p11330018	0.985074627	0.985074627	Bn-N7-p12809265	Bn-chrA07-p12682251
Bn-A07-p11331372	0.975124378	0.975124378	Bn-N7-p12810619	Bn-chrA07-p12680897
Bn-A07-p11333494	0.985074627	0.990049751	Bn-N9-p30249970	Bn-chrC08-p32096633
Bn-A07-p11390804	0.935323383	0.935323383	Bn-N5-p9409156	Bn-chrA05-p8407861
Bn-A07-p11403525	0.975124378	0.975124378	Bn-N5-p9420409	Bn-chrA05-p8419114
Bn-A07-p11457443	0.966942149	0.966942149	Bn-N7-p13967611	Bn-chrA07-p13675918
Bn-A07-p11463160	0.990049751	0.990049751	Bn-N7-p13972941	Bn-chrA07-p13681246
Bn-A07-p11472553	0.983388704	0.983388704	Bn-N7-p13984145	Bn-chrA07-p13694490
Bn-A07-p11472623	0.975206612	0.975206612	Bn-N7-p13984215	Bn-chrA07-p13694560
Bn-A07-p11477634	0.958677686	0.958677686	Bn-N7-p13989123	Bn-chrA07-p13699469
Bn-A07-p11492877	0.983471074	0.983471074	Bn-N7-p14004222	Bn-chrA07-p13715904
Bn-A07-p11512981	1	1	Bn-N7-p14023753	Bn-chrAnn_random-p27852C
Bn-A07-p11542386	0.980099502	0.980099502	Bn-N7-p14051166	Bn-chrA07_random-p124807
Bn-A07-p11542638	0.950248756	0.950248756	Bn-N7-p14051418	Bn-chrA07_random-p124782
Bn-A07-p11570097	0.950166113	0.950166113	Bn-N7-p14083332	Bn-chrA07_random-p121591

Bn-A07-p11572985	0.983471074	0.983471074	Bn-N7-p14086176	Bn-chrA07_random-p121307
Bn-A07-p11593493	0.983471074	0.809917355	Bn-N7-p14100576	Bn-chrC06-p17623312
Bn-A07-p11599915	0.991735537	0.900826446	Bn-N7-p14106059	Bn-chrA07_random-p127642
Bn-A07-p11603894	0.870646766	0.975124378	Bn-N7-p14110919	Bn-chrAnn_random-p380319
Bn-A07-p11611255	0.966942149	0.975206612	Bn-N7-p14113132	Bn-chrA07-p13756170
Bn-A07-p11616820	0.973421927	0.966777409	Bn-N7-p14124411	Bn-chrA07-p13762785
Bn-A07-p11627477	0.991735537	0.983471074	Bn-N7-p14141103	Bn-chrA07-p13775857
Bn-A07-p11629852	0.983471074	0.991735537	Bn-N7-p14143467	Bn-chrA07-p13778232
Bn-A07-p11631422	0.950413223	0.958677686	Bn-N7-p14146983	Bn-chrA07-p13779800
Bn-A07-p11631477	0.975206612	0.983471074	Bn-N7-p14147038	Bn-chrA07-p13779855
Bn-A07-p11636619	0.909090909	0.975206612	Bn-N7-p14153762	Bn-chrA07-p13785221
Bn-A07-p11641657	0.995024876	1	Bn-N7-p14169849	Bn-chrA07-p13794256
Bn-A07-p11660810	0.985074627	0.990049751	Bn-N7-p14196781	Bn-chrA07-p13816903
Bn-A07-p11668787	0.991735537	1	Bn-N7-p14201703	Bn-chrA07-p13819516
Bn-A07-p11698185	0.900497512	0.925373134	Bn-N16-p18267063	Bn-chrC06-p17885332
Bn-A07-p11715576	0.989583333	0.989583333	Bn-N7-p14261264	Bn-chrA07-p13878015
Bn-A07-p11724009	0.975124378	0.975124378	Bn-N7-p14269743	Bn-chrA07-p13888622
Bn-A07-p11727700	0.958677686	0.958677686	Bn-N7-p14272015	Bn-chrA07-p13890895
Bn-A07-p11750002	0.990049751	0.985074627	Bn-N7-p14304993	Bn-chrA07-p13903261
Bn-A07-p11764922	1	0.995024876	Bn-N7-p14316847	Bn-chrA07-p13910585
Bn-A07-p11765027	0.950413223	0.950413223	Bn-N7-p14316952	Bn-chrA07-p13910690
Bn-A07-p11765232	0.966942149	0.958677686	Bn-N7-p14317158	Bn-chrA07-p13910894
Bn-A07-p11799578	0.966942149	0.975206612	Bn-N7-p14350560	Bn-chrA07-p13946839
Bn-A07-p11811971	0.975206612	0.966942149	Bn-N7-p14367424	Bn-chrA07-p13964809
Bn-A07-p11813549	0.995024876	0.985074627	Bn-N7-p14370317	Bn-chrA07-p13966389
Bn-A07-p11815679	1	0.909090909	Bn-N7-p14372445	Bn-chrA07-p13968651
Bn-A07-p11815770	0.983471074	0.966942149	Bn-N7-p14372536	Bn-chrA07-p13968742
Bn-A07-p11821917	0.991735537	0.983471074	Bn-N7-p14378988	Bn-chrA07-p13974901
Bn-A07-p11832389	0.993355482	0.996677741	Bn-N7-p14389577	Bn-chrA07-p13987842
Bn-A07-p11838401	0.955223881	0.960199005	Bn-N7-p14394583	Bn-chrA07-p13993892
Bn-A07-p11846645	0.975206612	0.983471074	Bn-N7-p14403862	Bn-chrA07-p14005745
Bn-A07-p11856158	0.930348259	0.930348259	Bn-N7-p14411979	Bn-chrA07-p14013907
Bn-A07-p11861194	0.993355482	0.993355482	Bn-N7-p14417147	Bn-chrA07-p14019073
Bn-A07-p11864371	1	0.996677741	Bn-N7-p14420451	Bn-chrA07-p14021905
Bn-A07-p11870301	0.980066445	0.983388704	Bn-N7-p14429056	Bn-chrA07-p14028255
Bn-A07-p11870468	0.990049751	0.985074627	Bn-N7-p14429223	Bn-chrA07-p14028422
Bn-A07-p11870852	1	0.995024876	Bn-N7-p14429607	Bn-chrA07-p14028806
Bn-A07-p11886650	0.966942149	0.975206612	Bn-N7-p14442513	Bn-chrA07-p14043650
Bn-A07-p11930398	0.983471074	0.975206612	Bn-N7-p14479223	Bn-chrA07_random-p131998
Bn-A07-p11943948	0.980099502	0.985074627	Bn-N7-p14495024	Bn-chrA07_random-p130596
Bn-A07-p11951236	0.985074627	0.995024876	Bn-N7-p14502321	Bn-chrA07_random-p129867
Bn-A07-p11960665	1	0.996677741	Bn-N7-p14511667	Bn-chrA07_random-p128924
Bn-A07-p11966079	0.925619835	0.966942149	Bn-N7-p14518968	Bn-chrA07_random-p128385
Bn-A07-p11979910	0.975206612	0.975206612	Bn-N7-p14533231	Bn-chrA07-p14097364
Bn-A07-p11980798	0.966942149	0.966942149	Bn-N7-p14534121	Bn-chrA07-p14098254
Bn-A07-p11985865	0.995024876	0.995024876	Bn-N7-p14539182	Bn-chrA07-p14103320

Bn-A07-p11987158	0.950413223	0.950413223	Bn-N7-p14540497	Bn-chrA07-p14104255
Bn-A07-p11987538	0.975206612	0.975206612	Bn-N7-p14540877	Bn-chrA07-p14104635
Bn-A07-p11987607	0.975206612	0.975206612	Bn-N7-p14541031	Bn-chrA07-p14104789
Bn-A07-p12003491	0.926910299	0.920265781	Bn-N7-p14555022	Bn-chrA07-p14122821
Bn-A07-p12017702	0.985074627	0.985074627	Bn-N7-p14569170	Bn-chrA07-p14136668
Bn-A07-p12018948	0.983471074	0.983471074	Bn-N7-p14570417	Bn-chrA07-p14137914
Bn-A07-p12050100	0.991735537	1	Bn-N7-p14591244	Bn-chrA07-p14170180
Bn-A07-p12066704	0.990033223	0.990033223	Bn-N7-p14614851	Bn-chrA07-p14216361
Bn-A07-p12075838	0.975206612	0.966942149	Bn-N7-p14631294	Bn-chrA07-p14231905
Bn-A07-p12079803	0.991735537	0.933884298	Bn-N7-p14635537	Bn-chrA07-p14241798
Bn-A07-p12084613	0.990049751	0.985074627	Bn-N7-p14636115	Bn-chrA07-p14242377
Bn-A07-p12086167	1	0.991735537	Bn-N7-p14637728	Bn-chrA07-p14243995
Bn-A07-p12094894	1	0.991735537	Bn-N7-p14645051	Bn-chrA07-p14248647
Bn-A07-p12102140	0.993355482	0.986710963	Bn-N7-p14650210	Bn-chrA07-p14254453
Bn-A07-p12104177	0.991735537	0.983471074	Bn-N7-p14652298	Bn-chrA07-p14256785
Bn-A07-p12113648	0.925619835	0.925619835	Bn-N7-p14661805	Bn-chrA07-p14265986
Bn-A07-p12115911	0.520661157	0.933884298	Bn-N7-p14664185	Bn-chrA07-p14268365
Bn-A07-p12123202	0.965174129	0.965174129	Bn-N7-p14671736	Bn-chrAnn_random-p467334
Bn-A07-p12131198	0.990033223	0.990033223	Bn-N7-p14680023	Bn-chrAnn_random-p326849
Bn-A07-p12171217	0.955223881	0.955223881	Bn-N7-p14706402	Bn-chrA07-p14314065
Bn-A07-p12193031	0.970149254	0.970149254	Bn-N7-p14709044	Bn-chrA07-p14316480
Bn-A07-p12297809	0.925373134	0.92039801	Bn-N7-p14833511	Bn-chrA07-p14423026
Bn-A07-p12318667	0.916943522	0.993355482	Bn-N16-p19985300	Bn-chrA07-p14444588
Bn-A07-p12328863	0.955223881	0.970149254	Bn-N7-p14864972	Bn-chrA07-p14455001
Bn-A07-p12333270	0.980066445	0.980066445	Bn-N7-p14869394	Bn-chrA07-p14459469
Bn-A07-p12337353	0.935323383	0.935323383	Bn-N7-p14873595	Bn-chrA07-p14463553
Bn-A07-p12345399	0.958677686	0.958677686	Bn-N7-p14881658	Bn-chrA07-p14471603
Bn-A07-p12345729	0.965174129	0.970149254	Bn-N7-p14881988	Bn-chrA07-p14471933
Bn-A07-p12348360	0.983471074	0.983471074	Bn-N7-p14884444	Bn-chrA07_random-p135527
Bn-A07-p12362172	0.955223881	0.975124378	Bn-N7-p14888341	Bn-chrA07-p14482344
Bn-A07-p12363575	0.956810631	0.983388704	Bn-N7-p14889664	Bn-chrA07-p14483746
Bn-A07-p12376191	0.995024876	0.995024876	Bn-N7-p14901650	Bn-chrAnn_random-p343749
Bn-A07-p12385490	0.990033223	0.986710963	Bn-N7-p14909637	Bn-chrA07-p14515146
Bn-A07-p12397651	0.983471074	0.933884298	Bn-N7-p14930793	Bn-chrCnn_random-p660973
Bn-A07-p12400157	0.917355372	0.925619835	Bn-N7-p14940049	Bn-chrA07-p14529946
Bn-A07-p12412116	0.996677741	0.976744186	Bn-N7-p14957010	Bn-chrA07-p14546057
Bn-A07-p12415736	0.966942149	0.140495868	Bn-N7-p14967238	Bn-chrA01-p10142491
Bn-A07-p12415996	0.983471074	0.94214876	Bn-N7-p14967498	Bn-chrCnn_random-p655372
Bn-A07-p12418491	1	0.611570248	Bn-N7-p14970009	Bn-chrA07-p14561301
Bn-A07-p12418633	0.991735537	0.785123967	Bn-N7-p14970151	Bn-chrA07-p14566338
Bn-A07-p12419468	0.983471074	0.132231405	Bn-N7-p14970986	Unknown
Bn-A07-p12421128	0.966942149	0.710743802	Bn-N7-p14972660	Bn-chrA07-p14561161
Bn-A07-p12421327	0.975206612	0.595041322	Bn-N7-p14972859	Bn-chrCnn_random-p655561
Bn-A07-p12423315	0.975206612	0.694214876	Bn-N7-p14974827	Bn-chrA06-p16061649
Bn-A07-p12423454	0.991735537	0.173553719	Bn-N7-p14974966	Bn-chrC09-p19559666
Bn-A07-p12423544	0.991735537	0.214876033	Bn-N7-p14975056	Unknown

Bn-A07-p12424387	0.991735537	0.338842975	Bn-N7-p14975958	Bn-chrA07-p14567515
Bn-A07-p12424510	0.991735537	0.20661157	Bn-N7-p14976081	Bn-chrC03-p8527655
Bn-A07-p12424938	1	0.685950413	Bn-N7-p14976509	Bn-chrA07-p14567989
Bn-A07-p12425115	0.991735537	0.223140496	Bn-N7-p14976686	Bn-chrA03-p28675525
Bn-A07-p12425319	0.966942149	0.776859504	Bn-N7-p14976890	Bn-chrA07-p14568358
Bn-A07-p12425566	1	0.305785124	Bn-N7-p14977137	Bn-chrC09-p10407966
Bn-A07-p12426500	0.983471074	0.280991736	Bn-N7-p14978071	Bn-chrAnn_random-p407519
Bn-A07-p12426652	0.991735537	0.181818182	Bn-N7-p14978223	Unknown
Bn-A07-p12426907	0.975206612	0.20661157	Bn-N7-p14978478	Bn-chrC03-p42069240
Bn-A07-p12429159	1	0.380165289	Bn-N7-p14980865	Unknown
Bn-A07-p12430820	0.966942149	0.958677686	Bn-N7-p14982526	Bn-chrA07-p14572647
Bn-A07-p12430965	0.983471074	0.991735537	Bn-N7-p14982671	Bn-chrA07-p14572791
Bn-A07-p12432622	0.983471074	0.826446281	Bn-N7-p14987548	Bn-chrA07-p14573260
Bn-A07-p12432678	0.991735537	0.983471074	Bn-N7-p14987604	Bn-chrA07-p14573316
Bn-A07-p12432832	0.970149254	0.970149254	Bn-N7-p14987758	Bn-chrA07-p14573470
Bn-A07-p12433131	0.983471074	0.983471074	Bn-N7-p14988057	Bn-chrA07-p14573769
Bn-A07-p12443929	0.945273632	0.935323383	Bn-N7-p14998858	Bn-chrA07-p14584383
Bn-A07-p12470367	0.96013289	1	Bn-N16-p20350845	Bn-chrA07-p14621158
Bn-A07-p12486479	0.983471074	0.983471074	Bn-N7-p15039290	Bn-chrA07-p14640151
Bn-A07-p12487549	0.990033223	0.990033223	Bn-N7-p15041071	Bn-chrA07-p14641221
Bn-A07-p12487688	0.975124378	0.975124378	Bn-N7-p15041210	Bn-chrA07-p14641360
Bn-A07-p12487765	0.980099502	0.995024876	Bn-N7-p15041287	Bn-chrA07-p14641437
Bn-A07-p12495414	0.991735537	0.991735537	Bn-N7-p15049714	Bn-chrA07-p14649081
Bn-A07-p12496653	0.983471074	0.983471074	Bn-N7-p15050973	Bn-chrA07-p14650313
Bn-A07-p12502804	0.986710963	0.986710963	Bn-N7-p15057105	Bn-chrA07-p14656444
Bn-A07-p12504230	0.986710963	0.986710963	Bn-N7-p15058527	Bn-chrA07-p14657866
Bn-A07-p12546889	0.950248756	0.950248756	Bn-N7-p15109085	Bn-chrA07_random-p140041
Bn-A07-p12547012	0.990049751	0.990049751	Bn-N7-p15109208	Bn-chrA07_random-p140029
Bn-A07-p12547288	0.990033223	0.990033223	Bn-N7-p15109484	Bn-chrA07_random-p140002
Bn-A07-p12554962	0.993355482	0.993355482	Bn-N7-p15115102	Bn-chrA07_random-p139440
Bn-A07-p12567541	0.986710963	0.986710963	Bn-N7-p15129536	Bn-chrA07_random-p137904
Bn-A07-p12576869	0.975124378	0.975124378	Bn-N7-p15143359	Bn-chrA07_random-p136620
Bn-A07-p12578940	0.975206612	0.975206612	Bn-N7-p15145429	Bn-chrA07_random-p136413
Bn-A07-p12583860	0.980066445	0.980066445	Bn-N7-p15153157	Bn-chrA07_random-p135976
Bn-A07-p12589168	0.953488372	0.953488372	Bn-N7-p15164271	Bn-chrA07_random-p141758
Bn-A07-p12593239	0.958677686	0.958677686	Bn-N7-p15170052	Bn-chrA07-p14694651
Bn-A07-p12594658	0.990033223	0.990033223	Bn-N7-p15171475	Bn-chrA07-p14695791
Bn-A07-p12602102	0.943521595	0.986710963	Bn-N7-p15181717	Bn-chrA07-p14706053
Bn-A07-p12605125	0.950413223	0.950413223	Bn-N7-p15186297	Bn-chrA07-p14709130
Bn-A07-p12609078	0.975124378	0.975124378	Bn-N7-p15189566	Bn-chrA07-p14713091
Bn-A07-p12609202	0.96013289	0.96013289	Bn-N7-p15189690	Bn-chrA07-p14713215
Bn-A07-p12617108	0.960199005	0.960199005	Bn-N16-p20669798	Bn-chrC06-p19379148
Bn-A07-p12617827	0.958677686	0.710743802	Bn-N7-p15198996	Bn-chrC06-p19379958
Bn-A07-p12622860	0.652892562	0.966942149	Bn-N6-p1461701	Bn-chrA07-p14727021
Bn-A07-p12631921	0.983471074	0.991735537	Bn-N16-p20698868	Bn-chrA07-p14739390
Bn-A07-p12679842	1	1	Bn-Scaffold34418-p1	Bn-chrAnn_random-p330753

Bn-A07-p12685266	0.603305785	0.94214876	Bn-N7-p15276004	Bn-chrA07-p14783275
Bn-A07-p12688545	0.990049751	0.945273632	Bn-N7-p15278966	Bn-chrA07-p14789546
Bn-A07-p12693030	0.810631229	0.80730897	Bn-N7-p15282841	Bn-chrA07-p14794619
Bn-A07-p12705404	0.983471074	0.975206612	Bn-N7-p15297868	Bn-chrA07-p14806126
Bn-A07-p12708718	0.913621262	0.913621262	Bn-N7-p15301183	Bn-chrA07-p14809439
Bn-A07-p12710571	0.955223881	0.955223881	Bn-N7-p15303091	Bn-chrA07-p14811347
Bn-A07-p12716261	0.970149254	0.970149254	Bn-N7-p15308770	Bn-chrA07-p14817036
Bn-A07-p12732142	0.975124378	0.965174129	Bn-N7-p15319071	Bn-chrA07-p14828576
Bn-A07-p12734895	0.885572139	0.885572139	Bn-N7-p15321806	Bn-chrA07-p14831307
Bn-A07-p12749234	0.966942149	0.966942149	Bn-N7-p15327587	Bn-chrA07-p14837091
Bn-A07-p12749531	0.966942149	0.966942149	Bn-N7-p15327883	Bn-chrA07-p14837367
Bn-A07-p12751218	0.975206612	0.983471074	Bn-N7-p15329565	Bn-chrA07-p14839056
Bn-A07-p12754144	0.975206612	0.983471074	Bn-N7-p15332485	Bn-chrA07-p14841976
Bn-A07-p12757009	0.960199005	0.960199005	Bn-N7-p15335414	Bn-chrA07-p14844907
Bn-A07-p12787260	0.975206612	0.975206612	Bn-N7-p15363154	Bn-chrA07-p14876988
Bn-A07-p12811691	0.955223881	0.955223881	Bn-N7-p15392729	Bn-chrA07-p14903112
Bn-A07-p12827973	0.975124378	0.980099502	Bn-N7-p15408930	Bn-chrA07-p14919441
Bn-A07-p12828848	0.930348259	0.92039801	Bn-N7-p15409806	Bn-chrA07-p14920317
Bn-A07-p12830017	0.985074627	0.995024876	Bn-N7-p15411027	Bn-chrA07-p14921504
Bn-A07-p12833066	0.975206612	0.991735537	Bn-N7-p15416956	Bn-chrA07-p14930457
Bn-A07-p12833434	0.965174129	0.960199005	Bn-N7-p15417889	Bn-chrA07-p14930824
Bn-A07-p12833496	0.996677741	0.993355482	Bn-N7-p15417951	Bn-chrA07-p14930887
Bn-A07-p12840368	0.96013289	0.976744186	Bn-N16-p21091205	Bn-chrA07-p14937597
Bn-A07-p12841026	0.986710963	0.96345515	Bn-N7-p15425789	Bn-chrA07-p14938253
Bn-A07-p12869509	0.96345515	0.980066445	Bn-N7-p15453526	Bn-chrA07-p14966329
Bn-A07-p12874349	0.983471074	0.950413223	Bn-N7-p15458367	Bn-chrA07-p14971197
Bn-A07-p12876226	0.98540146	0.98540146	Bn-N16-p21154504	Bn-chrC06-p19870231
Bn-A07-p12878986	0.993355482	0.996677741	Bn-N7-p15463003	Bn-chrA07-p14975989
Bn-A07-p12884814	0.983388704	0.996677741	Bn-N7-p15470400	Bn-chrA07-p14981442
Bn-A07-p12904129	0.990049751	0.865671642	Bn-N7-p15489905	Bn-chrCnn_random-p184233
Bn-A07-p12918175	0.975124378	0.7960199	Bn-N7-p15507530	Bn-chrA07-p15018441
Bn-A07-p12921727	1	0.991735537	Bn-N7-p15511082	Bn-chrA07-p15027006
Bn-A07-p12922061	0.991735537	0.983471074	Bn-N7-p15511416	Bn-chrA07-p15027340
Bn-A07-p12926496	0.990049751	0.995024876	Bn-N7-p15515854	Bn-chrA07-p15028961
Bn-A07-p12935086	0.980066445	0.993355482	Bn-N16-p21354499	Bn-chrA07-p15040098
Bn-A07-p12935190	0.995024876	0.995024876	Bn-N16-p21354603	Bn-chrC06-p20078389
Bn-A07-p12947587	0.991735537	0.991735537	Bn-N7-p15540294	Bn-chrA07-p15052697
Bn-A07-p12963622	0.970149254	0.865671642	Bn-N7-p15559143	Bn-chrA07-p15076162
Bn-A07-p12969280	0.975206612	0.975206612	Bn-N7-p15564791	Bn-chrA07-p15081844
Bn-A07-p12992945	0.993355482	1	Bn-N7-p15580229	Bn-chrA07-p15094493
Bn-A07-p13005198	0.933884298	0.933884298	Bn-N7-p15594530	Bn-chrA07-p15108051
Bn-A07-p13007323	0.995024876	0.990049751	Bn-N7-p15598298	Bn-chrA07-p15110148
Bn-A07-p13009858	0.900826446	0.785123967	Bn-N7-p15600741	Bn-chrA07-p15114384
Bn-A07-p13017844	0.995024876	0.970149254	Bn-N7-p15607323	Bn-chrA07-p15121019
Bn-A07-p13037426	0.986710963	0.986710963	Bn-N7-p15631631	Bn-chrAnn_random-p205772
Bn-A07-p13072595	0.995024876	0.995024876	Bn-N7-p15671533	Bn-chrA07-p15145925

Bn-A07-p13090054	0.905472637	0.930348259	Bn-N7-p15689204	Bn-chrA07-p15157288
Bn-A07-p13096286	0.975206612	0.983471074	Bn-N7-p15695444	Bn-chrA07-p15170906
Bn-A07-p13097588	0.983388704	0.986710963	Bn-N7-p15698778	Bn-chrA07-p15172256
Bn-A07-p13097844	1	0.991735537	Bn-N7-p15699034	Bn-chrA07-p15172512
Bn-A07-p13099081	0.950413223	0.94214876	Bn-N7-p15700271	Bn-chrA07-p15173751
Bn-A07-p13101489	0.966942149	0.966942149	Bn-N7-p15702698	Bn-chrA07-p15176797
Bn-A07-p13102686	0.933884298	0.925619835	Bn-N7-p15703877	Bn-chrA09-p27508196
Bn-A07-p13103201	0.966942149	0.975206612	Bn-N7-p15704392	Bn-chrA09-p27508705
Bn-A07-p13110243	0.970149254	0.980099502	Bn-N7-p15711445	Bn-chrA07-p15185406
Bn-A07-p13122160	0.955223881	0.960199005	Bn-N7-p15727166	Bn-chrA07-p15199771
Bn-A07-p13124922	0.983388704	0.993355482	Bn-N7-p15730108	Bn-chrA07-p15205907
Bn-A07-p13125509	0.784053156	0.993355482	Bn-N7-p15730733	Bn-chrA07-p15206494
Bn-A07-p13128413	0.976744186	0.976744186	Bn-N7-p15734269	Bn-chrA07-p15209277
Bn-A07-p13133496	0.950248756	0.970149254	Bn-N7-p15739521	Bn-chrA07-p15214357
Bn-A07-p13137182	0.975206612	0.983471074	Bn-N7-p15743344	Bn-chrA07-p15218750
Bn-A07-p13138010	0.983471074	0.975206612	Bn-N7-p15744343	Bn-chrA07-p15220029
Bn-A07-p13138082	0.983471074	0.975206612	Bn-N7-p15744415	Bn-chrA07-p15220101
Bn-A07-p13138310	0.983471074	0.991735537	Bn-N7-p15744643	Bn-chrA07-p15220329
Bn-A07-p13141694	0.975124378	0.980099502	Bn-N7-p15748242	Bn-chrA07-p15223680
Bn-A07-p13147502	0.996677741	0.993355482	Bn-N7-p15755571	Bn-chrA07-p15227800
Bn-A07-p13147568	0.993355482	0.990033223	Bn-N7-p15755637	Bn-chrA07-p15227866
Bn-A07-p13148032	0.956810631	0.993355482	Bn-N7-p15756101	Bn-chrA07-p15228348
Bn-A07-p13154645	0.955223881	0.955223881	Bn-N7-p15762727	Bn-chrA07-p15234994
Bn-A07-p13156984	0.958677686	0.958677686	Bn-N7-p15765185	Bn-chrA07-p15237452
Bn-A07-p13161975	0.980066445	0.980066445	Bn-N7-p15770750	Bn-chrA07-p15243016
Bn-A07-p13162255	0.990033223	0.990033223	Bn-N7-p15771030	Bn-chrA07-p15243296
Bn-A07-p13162516	0.989583333	0.989583333	Bn-N7-p15771264	Bn-chrA07-p15243530
Bn-A07-p13164874	0.989583333	0.989583333	Bn-N7-p15773710	Bn-chrA07-p15245976
Bn-A07-p13167740	0.991735537	0.991735537	Bn-N7-p15781239	Bn-chrA07-p15251664
Bn-A07-p13167844	0.983471074	0.983471074	Bn-N7-p15781366	Bn-chrA07-p15251791
Bn-A07-p13171850	0.980099502	0.980099502	Bn-N7-p15785405	Bn-chrA07-p15255828
Bn-A07-p13172047	0.956810631	0.956810631	Bn-N7-p15785601	Bn-chrA07-p15256024
Bn-A07-p13184664	0.990033223	0.990033223	Bn-N7-p15800345	Bn-chrA07-p15270767
Bn-A07-p13186090	0.995024876	0.995024876	Bn-N7-p15801770	Bn-chrA07-p15272192
Bn-A07-p13187658	0.993355482	0.993355482	Bn-N7-p15803337	Bn-chrA07-p15273759
Bn-A07-p13188334	0.960199005	0.960199005	Bn-N7-p15804013	Bn-chrA07-p15274435
Bn-A07-p13192299	0.975206612	0.975206612	Bn-N7-p15808423	Bn-chrA07-p15278845
Bn-A07-p13202840	0.975124378	0.975124378	Bn-N7-p15828725	Bn-chrA07_random-p142518
Bn-A07-p13231557	0.975206612	0.975206612	Bn-N7-p15858751	Bn-chrA07_random-p145289
Bn-A07-p13247613	0.983471074	0.958677686	Bn-N7-p15866722	Bn-chrCnn_random-p196523
Bn-A07-p13259825	1	1	Bn-N7-p15876660	Bn-chrA07_random-p147018
Bn-A07-p13260561	0.983471074	0.991735537	Bn-N7-p15877399	Bn-chrA07_random-p147091
Bn-A07-p13273073	0.950248756	0.955223881	Bn-N7-p15888530	Bn-chrA07_random-p153221
Bn-A07-p13278392	0.983471074	0.983471074	Bn-N7-p15893698	Bn-chrA07_random-p152687
Bn-A07-p13283299	0.996677741	0.996677741	Bn-N7-p15899977	Bn-chrA07_random-p152186
Bn-A07-p13292902	0.975206612	0.975206612	Bn-N7-p15906498	Bn-chrA07_random-p151534

Bn-A07-p13305000	0.958677686	0.958677686	Bn-N7-p15914754	Bn-chrA07_random-p150708
Bn-A07-p13320094	0.975206612	0.975206612	Bn-N7-p15934319	Bn-chrA07_random-p149232
Bn-A07-p13320647	0.983388704	0.983388704	Bn-N7-p15934872	Bn-chrA07_random-p149176
Bn-A07-p13323913	0.966942149	0.966942149	Bn-N7-p15943283	Bn-chrA07_random-p148494
Bn-A07-p13324733	0.991735537	0.991735537	Bn-N7-p15945033	Bn-chrA07_random-p148366
Bn-A07-p13334430	0.990033223	0.96345515	Bn-N7-p15960245	Bn-chrC06-p20451781
Bn-A07-p13341778	0.985074627	0.985074627	Bn-N7-p15966140	Bn-chrA07-p15358898
Bn-A07-p13347715	0.940199336	0.943521595	Bn-N7-p15972076	Bn-chrA07-p15364833
Bn-A07-p13350234	0.990049751	0.990049751	Bn-N7-p15974592	Bn-chrA07-p15367349
Bn-A07-p13358954	0.965174129	0.965174129	Bn-N7-p15983477	Bn-chrA07-p15376229
Bn-A07-p13359413	0.983471074	0.983471074	Bn-N7-p15983933	Bn-chrA07-p15376685
Bn-A07-p13382993	0.975206612	0.975206612	Bn-N7-p16005412	Bn-chrA07-p15397961
Bn-A07-p13388181	0.975206612	0.975206612	Bn-N7-p16010414	Bn-chrA07-p15403624
Bn-A07-p13391170	0.991735537	0.991735537	Bn-N7-p16013400	Bn-chrA07-p15406610
Bn-A07-p13391724	0.94214876	0.94214876	Bn-N7-p16013955	Bn-chrA07-p15407165
Bn-A07-p13407262	0.876033058	0.876033058	Bn-N7-p16023329	Bn-chrA07-p15414850
Bn-A07-p13425409	0.995024876	0.995024876	Bn-N7-p16041939	Bn-chrA07-p15433526
Bn-A07-p13481981	0.993355482	0.993355482	Bn-N7-p16102859	Bn-chrA07-p15486725
Bn-A07-p13482160	0.990033223	0.990033223	Bn-N7-p16103038	Bn-chrA07-p15486904
Bn-A07-p13485321	0.980099502	0.980099502	Bn-N7-p16106200	Bn-chrA07-p15490065
Bn-A07-p13485413	0.991735537	0.991735537	Bn-N7-p16106292	Bn-chrA07-p15490157
Bn-A07-p13485722	0.990033223	0.990033223	Bn-N7-p16106600	Bn-chrA07-p15490465
Bn-A07-p13488332	0.980099502	0.980099502	Bn-N7-p16109178	Bn-chrA07-p15493052
Bn-A07-p13488433	0.983471074	0.983471074	Bn-N7-p16109279	Bn-chrA07-p15493153
Bn-A07-p13497516	0.976744186	0.790697674	Bn-N7-p16130688	Bn-chrA07-p15508146
Bn-A07-p13497654	0.980066445	0.744186047	Bn-N7-p16130826	Bn-chrA07-p15508317
Bn-A07-p13498071	0.985074627	0.895522388	Bn-N7-p16131244	Bn-chrA07-p15508684
Bn-A07-p13499840	0.94214876	0.305785124	Bn-N7-p16133012	Bn-chrC08-p31291170
Bn-A07-p13500775	0.958677686	0.553719008	Bn-N7-p16134578	Bn-chrA07-p15513845
Bn-A07-p13512124	0.966942149	0.983471074	Bn-N7-p16149816	Bn-chrA07-p15525833
Bn-A07-p13513308	0.975124378	0.965174129	Bn-N7-p16154029	Bn-chrA07-p15526966
Bn-A07-p13513850	0.986710963	0.980066445	Bn-N7-p16154516	Bn-chrA07-p15527506
Bn-A07-p13516497	0.661157025	0.900826446	Bn-N7-p16157168	Bn-chrA07-p15530186
Bn-A07-p13521038	0.966942149	0.966942149	Bn-N7-p16167784	Bn-chrA07-p15534974
Bn-A07-p13537021	0.980066445	0.980066445	Bn-N7-p16184503	Bn-chrA07-p15551035
Bn-A07-p13543954	0.950413223	0.958677686	Bn-N7-p16191418	Bn-chrA07-p15557946
Bn-A07-p13587753	0.966942149	0.975206612	Bn-N7-p16230522	Bn-chrA07-p15590520
Bn-A07-p13589740	0.980099502	0.980099502	Bn-N7-p16232931	Bn-chrA07-p15592929
Bn-A07-p13599579	0.975206612	0.975206612	Bn-N7-p16236383	Bn-chrA07-p15596380
Bn-A07-p13605650	0.980066445	0.980066445	Bn-N7-p16242457	Bn-chrA07-p15602454
Bn-A07-p13605726	0.970099668	0.970099668	Bn-N7-p16242533	Bn-chrA07-p15602530
Bn-A07-p13613809	0.980099502	0.980099502	Bn-N7-p16248727	Bn-chrA07-p15608724
Bn-A07-p13613886	0.983388704	0.983388704	Bn-N7-p16248804	Bn-chrA07-p15608801
Bn-A07-p13615643	0.995024876	0.995024876	Bn-N7-p16250566	Bn-chrA07-p15610558
Bn-A07-p13639374	1	1	Bn-N7-p16271735	Bn-chrA07-p15631839
Bn-A07-p13650653	0.976744186	0.976744186	Bn-N7-p16283115	Bn-chrA07-p15643197

Bn-A07-p13653958	0.985074627	0.995024876	Bn-N7-p16286422	Bn-chrC06-p21281079
Bn-A07-p13654191	0.996677741	0.77076412	Bn-N7-p16286654	Bn-chrC06-p21281309
Bn-A07-p13657646	0.970149254	0.915422886	Bn-N7-p16290109	Bn-chrA07-p15650183
Bn-A07-p13659939	0.966942149	0.958677686	Bn-N7-p16292466	Bn-chrA07-p15652533
Bn-A07-p13662635	0.993355482	0.990033223	Bn-N7-p16295171	Bn-chrA07-p15655244
Bn-A07-p13665031	0.993355482	0.976744186	Bn-N7-p16297590	Bn-chrA07-p15657669
Bn-A07-p13679221	0.993355482	0.953488372	Bn-N7-p16312495	Bn-chrC06-p21312481
Bn-A07-p13693377	0.966942149	0.966942149	Bn-N7-p16328594	Bn-chrA07-p15689041
Bn-A07-p13705188	0.94214876	0.94214876	Bn-N7-p16339498	Bn-chrA07-p15699545
Bn-A07-p13712520	0.991735537	0.991735537	Bn-N7-p16346921	Bn-chrA07-p15706912
Bn-A07-p13712881	0.970149254	0.975124378	Bn-N7-p16347282	Bn-chrA07-p15707273
Bn-A07-p13715621	0.985074627	0.980099502	Bn-N7-p16350064	Bn-chrA07-p15709982
Bn-A07-p13738704	0.975206612	0.975206612	Bn-N7-p16372392	Bn-chrA07_random-p154957
Bn-A07-p13739429	0.884297521	0.884297521	Bn-N7-p16373144	Bn-chrA07_random-p155032
Bn-A07-p13747722	0.993355482	0.996677741	Bn-N7-p16379790	Bn-chrA07_random-p156120
Bn-A07-p13748498	1	0.995024876	Bn-N7-p16380580	Bn-chrA07_random-p156198
Bn-A07-p13753855	0.983471074	0.975206612	Bn-N7-p16382045	Bn-chrA07_random-p156296
Bn-A07-p13767766	0.970149254	0.975124378	Bn-N7-p16391750	Bn-chrA07_random-p157718
Bn-A07-p13772349	0.975206612	0.975206612	Bn-N7-p16396275	Bn-chrA07_random-p158169
Bn-A07-p13774292	0.990033223	0.986710963	Bn-N7-p16398203	Bn-chrA07_random-p158363
Bn-A07-p13787096	0.865671642	0.990049751	Bn-N7-p16407174	Bn-chrA07_random-p159117
Bn-A07-p13787534	0.991735537	0.983471074	Bn-N7-p16407634	Bn-chrA07_random-p159160
Bn-A07-p13792568	0.966942149	0.975206612	Bn-N7-p16408537	Bn-chrA07_random-p159246
Bn-A07-p13803511	0.909090909	0.909090909	Bn-N16-p23329805	Bn-chrC06-p21487967
Bn-A07-p13828973	0.980099502	0.975124378	Bn-N7-p16440233	Bn-chrA07-p15773663
Bn-A07-p13830862	0.834710744	0.933884298	Bn-N7-p16442121	Bn-chrA07-p15775556
Bn-A07-p13840665	0.996677741	1	Bn-N7-p16451926	Bn-chrA07-p15786850
Bn-A07-p13844810	0.990033223	0.996677741	Bn-N7-p16456074	Bn-chrA07-p15791002
Bn-A07-p13850359	0.956810631	0.986710963	Bn-N7-p16462765	Bn-chrA07-p15799640
Bn-A07-p13855997	0.958677686	0.94214876	Bn-N7-p16469862	Bn-chrA07-p15805906
Bn-A07-p13857064	0.965174129	0.940298507	Bn-N7-p16470929	Bn-chrC06-p21581503
Bn-A07-p13868960	1	0.993355482	Bn-N7-p16480181	Bn-chrA07-p15814482
Bn-A07-p13873942	0.991735537	0.983471074	Bn-N7-p16485186	Bn-chrA07-p15819571
Bn-A07-p13902763	0.88372093	0.893687708	Bn-N7-p16514874	Bn-chrA07-p15840457
Bn-A07-p13926029	0.990049751	0.990049751	Bn-N7-p16533634	Bn-chrA07-p15856116
Bn-A07-p13930308	0.980099502	0.975124378	Bn-N7-p16537885	Bn-chrA07-p15862011
Bn-A07-p13931176	0.983471074	0.991735537	Bn-N7-p16538753	Bn-chrA07-p15862878
Bn-A07-p13936486	0.973421927	0.966777409	Bn-N7-p16543249	Bn-chrA07-p15867344
Bn-A07-p13942362	0.415282392	0.986710963	Unknown	Bn-chrA07-p15872129
Bn-A07-p13943479	0.995024876	0.990049751	Bn-N7-p16548441	Bn-chrA07-p15873199
Bn-A07-p13945924	0.950413223	0.966942149	Bn-N7-p16552692	Bn-chrA07-p15875645
Bn-A07-p13947936	0.990049751	0.990049751	Bn-N7-p16555572	Bn-chrA07-p15876840
Bn-A07-p13947955	0.990049751	0.990049751	Bn-N7-p16555591	Bn-chrA07-p15876859
Bn-A07-p13957160	0.991735537	0.983471074	Bn-N7-p16563873	Bn-chrA07-p15884413
Bn-A07-p13957625	0.983471074	0.975206612	Bn-N7-p16564032	Bn-chrA07-p15884571
Bn-A07-p13963849	0.885572139	0.990049751	Bn-N7-p16570707	Bn-chrA07-p15890559

Bn-A07-p13964923	0.966942149	0.966942149	Bn-N7-p16576302	Bn-chrA07-p15891634
Bn-A07-p13969888	0.966942149	0.876033058	Bn-Scaffold03922-p1	Bn-chrA07-p15897546
Bn-A07-p13971525	0.975124378	0.975124378	Bn-N7-p16587947	Bn-chrA07-p15899182
Bn-A07-p14002586	0.950413223	0.950413223	Bn-N7-p16631479	Bn-chrA07-p15927661
Bn-A07-p14002656	0.986710963	0.983388704	Bn-N7-p16631549	Bn-chrA07-p15927730
Bn-A07-p14005263	0.975206612	0.983471074	Bn-N7-p16634145	Bn-chrA07-p15930325
Bn-A07-p14016405	0.958677686	0.958677686	Bn-N7-p16643860	Bn-chrAnn_random-p370768
Bn-A07-p14024559	0.975206612	0.975206612	Bn-N7-p16652499	Bn-chrA07-p15943506
Bn-A07-p14024677	0.985074627	0.990049751	Bn-N7-p16652617	Bn-chrA07-p15943624
Bn-A07-p14024930	0.995024876	0.985074627	Bn-N7-p16652870	Bn-chrA07-p15943877
Bn-A07-p14026612	0.965174129	0.945273632	Bn-N7-p16654556	Bn-chrA07-p15945563
Bn-A07-p14029820	0.993355482	0.930232558	Bn-N7-p16657716	Bn-chrA07-p15948802
Bn-A07-p14034468	0.975206612	0.983471074	Bn-N7-p16662443	Bn-chrA07-p15954103
Bn-A07-p14045156	0.970099668	0.976744186	Bn-N7-p16672532	Bn-chrA07-p15964218
Bn-A07-p14046607	0.930348259	0.930348259	Bn-N16-p23732072	Bn-chrC06-p21952849
Bn-A07-p14059112	0.996677741	0.996677741	Bn-N7-p16686621	Bn-chrA07-p15977919
Bn-A07-p14062366	0.990049751	0.985074627	Bn-N7-p16694204	Bn-chrA07-p15980704
Bn-A07-p14065231	0.970149254	0.39800995	Bn-N7-p16697045	Bn-chrAnn_random-p407699
Bn-A07-p14071930	0.900826446	0.909090909	Bn-N7-p16703248	Bn-chrA07-p15989056
Bn-A07-p14092905	0.996677741	0.996677741	Bn-N7-p16724230	Bn-chrA07-p16010230
Bn-A07-p14103536	0.966942149	0.958677686	Bn-N7-p16735378	Bn-chrA07-p16020882
Bn-A07-p14110753	0.966942149	0.760330579	Bn-N7-p16742631	Bn-chrA07-p16027847
Bn-A07-p14116283	0.996677741	0.996677741	Bn-N7-p16748138	Bn-chrA07-p16033399
Bn-A07-p14116798	1	0.975206612	Bn-N7-p16748653	Bn-chrA07-p16033927
Bn-A07-p14121891	0.966942149	0.966942149	Bn-N7-p16753893	Bn-chrA07-p16038711
Bn-A07-p14124058	1	0.996677741	Bn-N7-p16758959	Bn-chrA07-p16041295
Bn-A07-p14129550	0.940298507	0.995024876	Bn-N7-p16764574	Bn-chrA07_random-p165373
Bn-A07-p14129566	0.950248756	0.990049751	Bn-N7-p16764589	Bn-chrA07_random-p165372
Bn-A07-p14134237	0.980099502	0.985074627	Bn-N7-p16769121	Bn-chrA07_random-p165944
Bn-A07-p14140428	0.983471074	0.991735537	Bn-N7-p16775433	Bn-chrA07_random-p164121
Bn-A07-p14142383	0.991735537	0.983471074	Bn-N7-p16776505	Bn-chrA07_random-p164014
Bn-A07-p14143419	0.983471074	0.975206612	Bn-N7-p16777541	Bn-chrA07_random-p163907
Bn-A07-p14143422	0.995024876	0.990049751	Bn-N7-p16777544	Bn-chrA07_random-p163907
Bn-A07-p14144205	0.909090909	0.983471074	Bn-N7-p16778327	Bn-chrA07_random-p163829
Bn-A07-p14146406	0.865671642	0.865671642	Bn-N16-p24081844	Bn-chrC06-p22313157
Bn-A07-p14150380	0.958677686	0.966942149	Bn-N7-p16782313	Bn-chrA07_random-p163425
Bn-A07-p14162450	0.986710963	0.986710963	Bn-N7-p16796388	Bn-chrA07_random-p162524
Bn-A07-p14162566	0.945273632	0.940298507	Bn-N7-p16796504	Bn-chrA07_random-p162512
Bn-A07-p14162622	0.983471074	0.991735537	Bn-N7-p16796560	Bn-chrA07_random-p162506
Bn-A07-p14164068	0.990033223	0.880398671	Bn-N7-p16798007	Bn-chrA07_random-p162362
Bn-A07-p14184802	0.996677741	0.993355482	Bn-N7-p16824465	Bn-chrA07_random-p166256
Bn-A07-p14185108	0.950248756	0.970149254	Bn-N7-p16824772	Bn-chrA07_random-p166225
Bn-A07-p14193108	0.990033223	0.983388704	Bn-N7-p16833259	Bn-chrAnn_random-p338842
Bn-A07-p14199690	0.980099502	0.975124378	Bn-N7-p16839482	Bn-chrA07-p16071280
Bn-A07-p14202755	1	0.991735537	Bn-N7-p16843182	Bn-chrA07-p16073173
Bn-A07-p14213371	0.991735537	1	Bn-N7-p16847875	Bn-chrA07-p16076441

Bn-A07-p14225102	1	1	Bn-N7-p16864057	Bn-chrA07-p16092620
Bn-A07-p14225642	0.993355482	0.993355482	Bn-N7-p16864597	Bn-chrA07-p16093160
Bn-A07-p14231567	0.975206612	0.975206612	Bn-N7-p16869831	Bn-chrA07-p16098438
Bn-A07-p14241936	0.983471074	0.983471074	Bn-N7-p16880190	Bn-chrA07-p16109488
Bn-A07-p14263288	0.925619835	0.925619835	Bn-N7-p16901581	Bn-chrA07-p16130767
Bn-A07-p14273852	0.983471074	0.983471074	Bn-N7-p16911986	Bn-chrA07-p16141172
Bn-A07-p14287940	0.983471074	0.983471074	Bn-N7-p16921573	Bn-chrA07-p16155487
Bn-A07-p14288125	0.958677686	0.958677686	Bn-N7-p16921758	Bn-chrA07-p16155672
Bn-A07-p14297369	0.983471074	0.983471074	Bn-N7-p16928874	Bn-chrA07-p16162855
Bn-A07-p14306631	0.818181818	0.818181818	Bn-N7-p16936689	Bn-chrA07-p16170668
Bn-A07-p14325380	1	1	Bn-N7-p16962092	Bn-chrA07-p16196312
Bn-A07-p14328365	1	1	Bn-N7-p16965075	Bn-chrA07-p16199295
Bn-A07-p14329191	0.983471074	0.983471074	Bn-N7-p16965963	Bn-chrA07-p16200182
Bn-A07-p14333680	0.996677741	0.996677741	Bn-N7-p16969944	Bn-chrA07-p16204068
Bn-A07-p14340252	1	1	Bn-N7-p16976492	Bn-chrA07-p16210616
Bn-A07-p14382008	0.996677741	0.996677741	Bn-N7-p17019859	Bn-chrA07-p16256659
Bn-A07-p14390667	0.991735537	0.991735537	Bn-N7-p17028212	Bn-chrA07-p16264839
Bn-A07-p14437313	0.966942149	0.966942149	Bn-N7-p17070776	Bn-chrA07-p16310288
Bn-A07-p14450900	1	0.995024876	Bn-N7-p17088805	Bn-chrA07-p16334853
Bn-A07-p14453848	0.990033223	0.990033223	Bn-N7-p17091758	Bn-chrC06-p23022070
Bn-A07-p14462032	0.966942149	0.966942149	Bn-N7-p17108747	Bn-chrA07-p16348499
Bn-A07-p14462380	0.996677741	0.996677741	Bn-N7-p17109095	Bn-chrA07-p16348847
Bn-A07-p14472064	0.94214876	0.909090909	Bn-N7-p17123463	Bn-chrA07-p16363524
Bn-A07-p14500760	0.950413223	0.94214876	Bn-N7-p17152711	Bn-chrC06-p23208618
Bn-A07-p14511986	0.975206612	0.983471074	Bn-N7-p17155348	Bn-chrA07-p16395075
Bn-A07-p14523096	0.973421927	0.996677741	Bn-N7-p17163479	Bn-chrA07-p16407323
Bn-A07-p14523713	0.955223881	0.940298507	Bn-N7-p17164096	Bn-chrC06-p23238733
Bn-A07-p14525404	0.996677741	0.996677741	Bn-N7-p17165787	Bn-chrA07-p16409629
Bn-A07-p14533453	0.975124378	0.975124378	Bn-N7-p17174266	Bn-chrA07-p16418524
Bn-A07-p14540334	0.980066445	0.973421927	Bn-N7-p17179658	Bn-chrA07-p16423258
Bn-A07-p14562613	0.955223881	0.985074627	Bn-N7-p17201575	Bn-chrA07-p16447645
Bn-A07-p14566670	0.983471074	0.983471074	Bn-N7-p17205609	Bn-chrA07-p16451694
Bn-A07-p14575140	0.995024876	0.995024876	Bn-N7-p17214679	Bn-chrA07-p16459851
Bn-A07-p14585610	0.983471074	0.876033058	Bn-N11-p45063296	Bn-chrC04-p10393832
Bn-A07-p14594344	0.980066445	0.983388704	Bn-N7-p17228169	Bn-chrA07-p16471343
Bn-A07-p14594716	0.983471074	0.975206612	Bn-N7-p17228543	Bn-chrA07-p16471718
Bn-A07-p14596285	0.980066445	0.983388704	Bn-N7-p17230095	Bn-chrA07-p16473279
Bn-A07-p14598430	0.991735537	0.975206612	Bn-N7-p17232223	Bn-chrA07-p16475413
Bn-A07-p14606994	0.990033223	0.986710963	Bn-N7-p17240771	Bn-chrA07-p16492477
Bn-A07-p14611227	0.760330579	0.991735537	Bn-N7-p17247704	Bn-chrA07-p16496706
Bn-A07-p14622638	0.991735537	0.983471074	Bn-N7-p17256364	Bn-chrA07-p16508908
Bn-A07-p14623449	0.983471074	0.975206612	Bn-N7-p17257181	Bn-chrA07-p16509716
Bn-A07-p14627135	0.965174129	0.965174129	Bn-N7-p17260873	Bn-chrA07-p16513397
Bn-A07-p14628930	1	1	Bn-N7-p17262673	Bn-chrA07-p16515197
Bn-A07-p14635438	0.991735537	0.983471074	Bn-N7-p17269083	Bn-chrA07-p16521617
Bn-A07-p14635732	0.983471074	0.975206612	Bn-N7-p17269383	Bn-chrA07-p16521911

Bn-A07-p14635854	0.976744186	0.993355482	Bn-N7-p17269506	Bn-chrA07-p16522033
Bn-A07-p14636039	0.983471074	0.975206612	Bn-N7-p17269691	Bn-chrA07-p16522250
Bn-A07-p14645683	0.980099502	0.975124378	Bn-N7-p17277196	Bn-chrA07-p16540944
Bn-A07-p14646080	0.993355482	0.990033223	Bn-Scaffold23172-p3	Bn-chrA07-p16541342
Bn-A07-p14649790	0.986710963	0.96345515	Bn-N7-p17283432	Bn-chrA07-p16536650
Bn-A07-p14655552	0.975124378	0.980099502	Bn-N7-p17288929	Bn-chrA07-p16549709
Bn-A07-p14664544	0.990033223	0.993355482	Bn-N7-p17292048	Bn-chrA07-p16559113
Bn-A07-p14665161	0.985074627	0.985074627	Bn-N7-p17292666	Bn-chrA07-p16559730
Bn-A07-p14666868	0.913621262	0.913621262	Bn-N7-p17294374	Bn-chrA07-p16561437
Bn-A07-p14682292	0.989583333	1	Bn-N7-p17309322	Bn-chrA07-p16577236
Bn-A07-p14705901	0.966942149	0.958677686	Bn-N7-p17332092	Bn-chrA07-p16596087
Bn-A07-p14735920	0.900332226	0.900332226	Bn-N16-p25343146	Bn-chrC06-p24106262
Bn-A07-p14736696	0.797342193	0.694352159	Bn-N16-p25343920	Bn-chrC06-p34213154
Bn-A07-p14757084	0.980066445	0.980066445	Bn-N7-p17384720	Bn-chrA07-p16641568
Bn-A07-p14759734	0.986710963	0.986710963	Bn-N7-p17387372	Bn-chrA07-p16644226
Bn-A07-p14759792	0.950166113	0.950166113	Bn-N7-p17387430	Bn-chrA07-p16644284
Bn-A07-p14759961	0.900826446	0.909090909	Bn-N7-p17387599	Bn-chrA07-p16644453
Bn-A07-p14759962	0.950166113	0.956810631	Bn-N7-p17387599	Bn-chrA07-p16644453
Bn-A07-p14760232	0.975206612	0.983471074	Bn-N7-p17387872	Bn-chrA07-p16644725
Bn-A07-p14765022	0.958677686	0.958677686	Bn-N7-p17392667	Bn-chrA07-p16649534
Bn-A07-p14766907	0.965174129	0.965174129	Bn-N7-p17394595	Bn-chrA07-p16651461
Bn-A07-p14768265	0.859504132	0.859504132	Bn-N7-p17395698	Bn-chrA07-p16652564
Bn-A07-p14770619	0.975124378	0.975124378	Bn-N7-p17398077	Bn-chrA07-p16654934
Bn-A07-p14780968	0.996677741	0.996677741	Bn-N7-p17406607	Bn-chrAnn_random-p387941
Bn-A07-p14784989	0.955223881	0.955223881	Bn-N7-p17409671	Bn-chrA07-p16664654
Bn-A07-p14792267	0.986710963	0.986710963	Bn-N7-p17416205	Bn-chrA07-p16671171
Bn-A07-p14792772	0.970149254	0.970149254	Bn-N7-p17416711	Bn-chrA07-p16671677
Bn-A07-p14798978	0.990049751	0.990049751	Bn-N7-p17422953	Bn-chrA07-p16678357
Bn-A07-p14804953	0.983471074	0.958677686	Bn-N7-p17428408	Bn-chrC06-p24261735
Bn-A07-p14809393	0.966942149	0.966942149	Bn-N7-p17432993	Bn-chrA07-p16688440
Bn-A07-p14812317	0.970149254	0.970149254	Bn-N7-p17434977	Bn-chrA07_random-p167000
Bn-A07-p14814393	0.980066445	0.990033223	Bn-N7-p17437078	Bn-chrA07_random-p166790
Bn-A07-p14820942	0.975124378	0.975124378	Bn-N7-p17440792	Bn-chrA07-p16693589
Bn-A07-p14823470	0.975206612	0.975206612	Bn-N7-p17443925	Bn-chrA07-p16696722
Bn-A07-p14823761	0.950413223	0.950413223	Bn-N7-p17444215	Bn-chrA07-p16697012
Bn-A07-p14824091	0.995024876	0.995024876	Bn-N7-p17444545	Bn-chrA07-p16697342
Bn-A07-p14836793	0.996677741	0.993355482	Bn-N7-p17454459	Bn-chrA07-p16705306
Bn-A07-p14863875	1	0.950248756	Bn-N7-p17488534	Bn-chrA07-p16731989
Bn-A07-p14865976	0.993355482	0.993355482	Bn-N7-p17490629	Bn-chrA07-p16734023
Bn-A07-p14868938	0.897009967	0.897009967	Bn-N7-p17504580	Bn-chrA07-p16736986
Bn-A07-p14869066	0.980099502	0.985074627	Bn-N7-p17504708	Bn-chrA07-p16737114
Bn-A07-p14870055	0.996677741	1	Bn-N7-p17505696	Bn-chrA07-p16738101
Bn-A07-p14895551	0.983471074	0.975206612	Bn-N7-p17534137	Bn-chrA07-p16764436
Bn-A07-p14895675	0.930348259	0.975124378	Bn-N16-p25671169	Bn-chrA07-p16764560
Bn-A07-p14920018	0.993355482	0.993355482	Bn-N7-p17559082	Bn-chrA07-p16792643
Bn-A07-p14922137	0.980066445	0.986710963	Bn-N7-p17561201	Bn-chrA07-p16794762

Bn-A07-p14934271	0.985074627	0.985074627	Bn-N7-p17572824	Bn-chrA07-p16813772
Bn-A07-p14958606	1	1	Bn-N7-p17595363	Bn-chrA07-p16830450
Bn-A07-p14966705	0.975206612	0.801652893	Bn-N7-p17605318	Bn-chrC06-p24555437
Bn-A07-p14969298	0.926910299	0.926910299	Bn-N16-p25782278	Bn-chrC06-p24561938
Bn-A07-p14970273	0.973421927	0.973421927	Bn-N7-p17609212	Bn-chrA07-p16849089
Bn-A07-p14975152	0.985074627	0.985074627	Bn-N7-p17614051	Bn-chrA07-p16853937
Bn-A07-p15006983	0.993355482	0.993355482	Bn-N7-p17648991	Bn-chrA07-p16889712
Bn-A07-p15007983	0.958677686	0.958677686	Bn-N7-p17649982	Bn-chrA07-p16890703
Bn-A07-p15015434	0.983471074	0.983471074	Bn-N7-p17656934	Bn-chrA07-p16897655
Bn-A07-p15025202	0.966942149	0.966942149	Bn-N7-p17661731	Bn-chrA07-p16902452
Bn-A07-p15033167	0.993355482	0.993355482	Bn-N7-p17670224	Bn-chrA07-p16910731
Bn-A07-p15033648	1	0.995024876	Bn-N7-p17670705	Bn-chrA07-p16911212
Bn-A07-p15035369	0.986710963	0.990033223	Bn-N7-p17672425	Bn-chrA07-p16912933
Bn-A07-p15043209	0.990033223	0.990033223	Bn-N7-p17682010	Bn-chrA07-p16920756
Bn-A07-p15050270	0.983388704	0.946843854	Bn-N7-p17688042	Bn-chrC06-p24725978
Bn-A07-p15054860	0.950248756	0.955223881	Bn-N7-p17692604	Bn-chrA07-p16932495
Bn-A07-p15055528	0.975124378	0.970149254	Bn-N7-p17693272	Bn-chrA07-p16933166
Bn-A07-p15059054	0.976744186	0.976744186	Bn-N7-p17696669	Bn-chrA07-p16936576
Bn-A07-p15064528	0.991735537	0.991735537	Bn-N7-p17703725	Bn-chrA07-p16943856
Bn-A07-p15093743	0.993355482	0.993355482	Bn-N7-p17736927	Bn-chrA07-p16976586
Bn-A07-p15101433	0.991735537	0.991735537	Bn-N7-p17746491	Bn-chrA07-p16987230
Bn-A07-p15107656	1	1	Bn-N7-p17764265	Bn-chrA07-p17007028
Bn-A07-p15120097	0.975206612	0.975206612	Bn-N7-p17781125	Bn-chrA07-p17025623
Bn-A07-p15129531	0.990033223	0.990033223	Bn-N7-p17791210	Bn-chrA07-p17035512
Bn-A07-p15148050	1	1	Bn-N7-p17817928	Bn-chrA07-p17063334
Bn-A07-p15162548	0.925619835	0.925619835	Bn-N7-p17830159	Bn-chrA07-p17075529
Bn-A07-p15162767	0.990033223	0.990033223	Bn-N7-p17830380	Bn-chrA07-p17075750
Bn-A07-p15162837	0.990033223	0.990033223	Bn-N7-p17830450	Bn-chrA07-p17075820
Bn-A07-p15165145	0.995024876	0.995024876	Bn-N7-p17833148	Bn-chrA07-p17078518
Bn-A07-p15175027	0.960199005	0.950248756	Bn-N7-p17842911	Bn-chrA07-p17088242
Bn-A07-p15175141	0.975206612	0.966942149	Bn-N7-p17843024	Bn-chrC06-p25037614
Bn-A07-p15183066	0.973421927	0.990033223	Bn-N16-p26282879	Bn-chrA07-p17092445
Bn-A07-p15220175	0.990033223	0.983388704	Bn-N7-p17895417	Bn-chrA07-p17126282
Bn-A07-p15220891	0.991735537	0.991735537	Bn-N7-p17896133	Bn-chrA07-p17127000
Bn-A07-p15233647	0.966942149	0.958677686	Bn-N7-p17910141	Bn-chrA07-p17140719
Bn-A07-p15236422	0.958677686	0.975206612	Bn-N7-p17912949	Bn-chrA07-p17143530
Bn-A07-p15239170	0.94214876	0.94214876	Bn-N7-p17916052	Bn-chrA07-p17146632
Bn-A07-p15241878	0.991735537	0.991735537	Bn-N7-p17918971	Bn-chrA07-p17149552
Bn-A07-p15242562	0.983471074	0.983471074	Bn-N7-p17919655	Bn-chrA07-p17150236
Bn-A07-p15245952	0.991735537	0.991735537	Bn-N7-p17921873	Bn-chrA07-p17152454
Bn-A07-p15247793	0.983471074	0.983471074	Bn-N7-p17923677	Bn-chrA07-p17154258
Bn-A07-p15248927	0.933884298	0.933884298	Bn-N7-p17924813	Bn-chrA07-p17155394
Bn-A07-p15262851	0.966942149	0.966942149	Bn-N7-p17940636	Bn-chrA07-p17171652
Bn-A07-p15273529	0.983471074	0.983471074	Bn-N7-p17956020	Bn-chrA07-p17189124
Bn-A07-p15276966	0.983471074	0.983471074	Bn-N7-p17960703	Bn-chrA07-p17193808
Bn-A07-p15287374	0.950166113	0.920265781	Bn-N7-p14154863	Bn-chrA03-p13085104

Bn-A07-p15298239	0.983471074	0.983471074	Bn-N7-p17979504	Bn-chrA07-p17214754
Bn-A07-p15298338	0.983471074	0.983471074	Bn-N7-p17979603	Bn-chrA07-p17214853
Bn-A07-p15299046	0.991735537	0.991735537	Bn-N7-p17980311	Bn-chrA07-p17215561
Bn-A07-p15300466	0.970099668	0.970099668	Bn-N7-p17981733	Bn-chrA07-p17216983
Bn-A07-p15303340	0.980066445	0.980066445	Bn-N7-p17984598	Bn-chrA07-p17219848
Bn-A07-p15309387	0.991735537	0.991735537	Bn-N7-p17990900	Bn-chrA07-p17226149
Bn-A07-p15310281	0.983471074	0.983471074	Bn-N7-p17991794	Bn-chrA07-p17227043
Bn-A07-p15321076	0.996677741	0.996677741	Bn-N7-p18003759	Bn-chrA07-p17238471
Bn-A07-p15331892	0.975206612	0.975206612	Bn-N7-p18016065	Bn-chrA07-p17250504
Bn-A07-p15332201	0.780730897	0.780730897	Bn-N7-p18016379	Bn-chrA07-p17250818
Bn-A07-p15352802	1	1	Bn-N7-p18034004	Bn-chrA07-p17269795
Bn-A07-p15354488	0.953488372	0.953488372	Bn-N7-p18037237	Bn-chrA07-p17273028
Bn-A07-p15357890	0.687707641	0.687707641	Bn-N7-p18040641	Bn-chrA07-p17276432
Bn-A07-p15359622	0.996677741	0.996677741	Bn-N7-p18042373	Bn-chrA07-p17278164
Bn-A07-p15364409	0.983471074	0.983471074	Bn-N7-p18048568	Bn-chrA07-p17284203
Bn-A07-p15366978	0.827242525	0.827242525	Bn-N7-p18051146	Bn-chrA07-p17286781
Bn-A07-p15369326	0.990033223	0.990033223	Bn-N7-p18053484	Bn-chrA07-p17289120
Bn-A07-p15372787	0.611295681	0.993355482	Bn-N16-p35692569	Bn-chrA07-p17292583
Bn-A07-p15373643	0.991735537	0.983471074	Bn-N7-p18054702	Bn-chrA07-p17293448
Bn-A07-p15373724	0.991735537	0.983471074	Bn-N7-p18054783	Bn-chrA07-p17293529
Bn-A07-p15381285	0.910299003	0.910299003	Bn-N16-p26797848	Bn-chrC06-p25514431
Bn-A07-p15404895	0.990033223	0.993355482	Bn-N7-p18087198	Bn-chrC06-p25586372
Bn-A07-p15405942	0.953488372	0.983388704	Bn-N7-p18088245	Bn-chrC06-p25587419
Bn-A07-p15410837	0.990033223	0.990033223	Bn-N7-p18093108	Bn-chrA07-p17323892
Bn-A07-p15411766	0.991735537	0.991735537	Bn-N7-p18094003	Bn-chrA07-p17324787
Bn-A07-p15412126	0.975124378	0.975124378	Bn-N10-p7455552	Bn-chrA10-p5516814
Bn-A07-p15415388	0.96013289	0.96013289	Bn-N7-p18097525	Bn-chrA07-p17328308
Bn-A07-p15415913	0.958677686	0.958677686	Bn-N7-p18098049	Bn-chrA07-p17328832
Bn-A07-p15417895	1	1	Bn-N7-p18100041	Bn-chrA07-p17330824
Bn-A07-p15425959	0.983471074	0.983471074	Bn-N7-p18108303	Bn-chrA07-p17339085
Bn-A07-p15426090	0.980099502	0.980099502	Bn-N7-p18108434	Bn-chrA07-p17339216
Bn-A07-p15428458	0.970099668	0.970099668	Bn-N7-p18112046	Bn-chrA07-p17342826
Bn-A07-p15441391	0.983471074	0.983471074	Bn-N7-p18125323	Bn-chrA07_random-p167779
Bn-A07-p15442723	0.966942149	0.966942149	Bn-N7-p18127716	Bn-chrA07-p17351462
Bn-A07-p15444203	0.958677686	0.958677686	Bn-N7-p18132272	Bn-chrA07-p17356018
Bn-A07-p15447440	0.983471074	0.983471074	Bn-N7-p18135869	Bn-chrA07-p17359283
Bn-A07-p15453076	0.993355482	0.996677741	Bn-N7-p18139396	Bn-chrA07-p17362017
Bn-A07-p15459314	0.970149254	0.970149254	Bn-N7-p18149018	Bn-chrA07-p17370464
Bn-A07-p15464739	0.958677686	0.958677686	Bn-N7-p18155765	Bn-chrA07-p17376089
Bn-A07-p15468982	0.950413223	0.842975207	Bn-N7-p18162402	Bn-chrA07_random-p168319
Bn-A07-p15470119	0.991735537	0.991735537	Bn-N7-p18163303	Bn-chrA07_random-p168229
Bn-A07-p15470208	0.991735537	0.991735537	Bn-N7-p18163392	Bn-chrA07_random-p168220
Bn-A07-p15470833	0.991735537	0.991735537	Bn-N7-p18164017	Bn-chrA07_random-p168158
Bn-A07-p15471161	0.991735537	0.991735537	Bn-N7-p18164345	Bn-chrA07_random-p168125
Bn-A07-p15505090	0.966942149	0.966942149	Bn-N7-p18190999	Bn-chrA07-p17408252
Bn-A07-p15524114	0.787375415	0.787375415	Bn-N16-p27213952	Bn-chrC06-p26109570

Bn-A07-p15537891	0.991735537	0.991735537	Bn-N7-p18219144	Bn-chrA07-p17439316
Bn-A07-p15545260	1	0.991735537	Bn-N7-p18227733	Bn-chrC06-p26158375
Bn-A07-p15546893	0.945273632	0.955223881	Bn-N7-p18229666	Bn-chrA07-p17448291
Bn-A07-p15549943	0.996677741	1	Bn-N7-p18232812	Bn-chrA07-p17451383
Bn-A07-p15557239	0.991735537	0.983471074	Bn-N7-p18240107	Bn-chrA07-p17458681
Bn-A07-p15578269	0.980066445	0.980066445	Bn-N7-p18257843	Bn-chrA07-p17475418
Bn-A07-p15587759	0.958677686	0.966942149	Bn-N7-p18263516	Bn-chrA07-p17483901
Bn-A07-p15587835	0.983471074	0.975206612	Bn-N7-p18263592	Bn-chrA07-p17483977
Bn-A07-p15588492	0.975206612	0.983471074	Bn-N7-p18264248	Bn-chrA07-p17484633
Bn-A07-p15588706	0.966942149	0.966942149	Bn-N7-p18264462	Bn-chrA07-p17484847
Bn-A07-p15588927	0.966942149	0.958677686	Bn-N7-p18264684	Bn-chrA07-p17485074
Bn-A07-p15589001	0.983471074	0.958677686	Bn-N7-p18264758	Bn-chrA07-p17485148
Bn-A07-p15591535	0.991735537	0.983471074	Bn-N7-p18267292	Bn-chrA07-p17487704
Bn-A07-p15591699	0.993355482	0.916943522	Bn-N7-p18267456	Bn-chrA07-p17487871
Bn-A07-p15606996	0.991735537	0.983471074	Bn-N7-p18282644	Bn-chrA07-p17504199
Bn-A07-p15612140	0.966942149	0.975206612	Bn-N7-p18287694	Bn-chrA07-p17511344
Bn-A07-p15622870	0.990033223	0.990033223	Bn-N7-p18297859	Bn-chrA07-p17521349
Bn-A07-p15632054	0.965174129	0.965174129	Bn-N7-p18307048	Bn-chrA07-p17530137
Bn-A07-p15644181	0.993355482	0.990033223	Bn-N7-p18317884	Bn-chrA07-p17541687
Bn-A07-p15648620	0.991735537	0.983471074	Bn-N7-p18322939	Bn-chrA07-p17548363
Bn-A07-p15650680	0.966942149	0.966942149	Bn-N7-p18324891	Bn-chrA07_random-p168610
Bn-A07-p15652974	0.991735537	1	Bn-N7-p18326907	Bn-chrA07-p17554927
Bn-A07-p15669200	0.94214876	0.917355372	Bn-N7-p18350619	Bn-chrA07-p17578362
Bn-A07-p15671084	0.950413223	0.892561983	Bn-N7-p18353768	Bn-chrA07-p17580235
Bn-A07-p15674502	0.958677686	0.966942149	Bn-N7-p18354917	Bn-chrA07-p17581390
Bn-A07-p15675658	0.396694215	0.958677686	Unknown	Bn-chrA07-p17582768
Bn-A07-p15675717	0.578512397	0.958677686	Bn-N16-p27523896	Bn-chrA07-p17582826
Bn-A07-p15687112	0.958677686	0.966942149	Bn-N7-p18362200	Bn-chrA07-p17587788
Bn-A07-p15690501	0.950413223	0.958677686	Bn-N7-p18365713	Bn-chrA07-p17594593
Bn-A07-p15691004	0.976744186	0.986710963	Bn-N7-p18366216	Bn-chrA07-p17595097
Bn-A07-p15691716	0.993355482	0.983388704	Bn-N7-p18368781	Bn-chrA07-p17595798
Bn-A07-p15695422	0.94214876	0.975206612	Bn-N7-p18373032	Bn-chrA07-p17600181
Bn-A07-p15698133	0.950413223	0.94214876	Bn-N7-p18375871	Bn-chrA07-p17602484
Bn-A07-p15709440	0.966942149	0.966942149	Bn-N7-p18384800	Bn-chrA07-p17613373
Bn-A07-p15719490	0.993355482	0.993355482	Bn-N7-p18397906	Bn-chrA07-p17624726
Bn-A07-p15728800	0.986710963	0.990033223	Bn-N7-p18407349	Bn-chrA07-p17633960
Bn-A07-p15729378	0.993355482	0.986710963	Bn-N7-p18407916	Bn-chrA07-p17634892
Bn-A07-p15738506	0.966942149	0.975206612	Bn-N7-p18418847	Bn-chrA07-p17647395
Bn-A07-p15756045	0.983471074	0.991735537	Bn-N7-p18437079	Bn-chrA07-p17662711
Bn-A07-p15757524	0.958677686	0.958677686	Bn-N7-p18438689	Bn-chrA07-p17664321
Bn-A07-p15758978	1	0.991735537	Bn-N7-p18440143	Bn-chrA07-p17665775
Bn-A07-p15760227	0.991735537	0.983471074	Bn-N7-p18441392	Bn-chrA07-p17667025
Bn-A07-p15762423	0.983471074	0.991735537	Bn-N7-p18443591	Bn-chrA07-p17668244
Bn-A07-p15768026	1	0.991735537	Bn-N7-p18449193	Bn-chrA07-p17673780
Bn-A07-p15771342	0.958677686	0.950413223	Bn-N7-p18452509	Bn-chrA07-p17677146
Bn-A07-p15773212	0.867768595	0.958677686	Bn-N7-p18454383	Bn-chrA07-p17679028

Bn-A07-p15787164	0.983471074	0.950413223	Bn-N7-p18462244	Bn-chrA07-p17689202
Bn-A07-p15791885	0.996677741	0.996677741	Bn-N7-p18467443	Bn-chrA07-p17699611
Bn-A07-p15797527	0.976744186	0.986710963	Bn-N7-p18472451	Bn-chrA07-p17704623
Bn-A07-p15797607	0.910299003	0.986710963	Bn-N7-p18472531	Bn-chrA07-p17704703
Bn-A07-p15801291	0.975206612	0.983471074	Bn-N7-p18476233	Bn-chrA07-p17708808
Bn-A07-p15802174	0.983471074	0.975206612	Bn-N7-p18477116	Bn-chrA07-p17709685
Bn-A07-p15802229	0.983471074	0.933884298	Bn-N7-p18477171	Bn-chrA07-p17709739
Bn-A07-p15803011	0.966942149	0.950413223	Bn-N7-p18477955	Bn-chrA07-p17710525
Bn-A07-p15809590	1	0.995024876	Bn-N7-p18485768	Bn-chrA07-p17716927
Bn-A07-p15812852	0.990033223	0.986710963	Bn-N7-p18488979	Bn-chrA07-p17720187
Bn-A07-p15814161	0.958677686	0.950413223	Bn-N7-p18490278	Bn-chrA07-p17722226
Bn-A07-p15816246	0.975206612	0.975206612	Bn-N7-p18491537	Bn-chrA07-p17723487
Bn-A07-p15821030	1	1	Bn-N7-p18496325	Bn-chrA07-p17729854
Bn-A07-p15829114	0.958677686	0.958677686	Bn-N7-p18500972	Bn-chrA07-p17734499
Bn-A07-p15829285	0.970149254	0.970149254	Bn-N7-p18501143	Bn-chrA07-p17734670
Bn-A07-p15831043	0.965174129	0.965174129	Bn-N7-p18502907	Bn-chrA07-p17736434
Bn-A07-p15833211	0.94214876	0.94214876	Bn-N7-p18505098	Bn-chrA07-p17738625
Bn-A07-p15856545	0.985074627	0.975124378	Bn-N7-p18531264	Bn-chrA07-p17764264
Bn-A07-p15856978	0.975124378	0.980099502	Bn-N7-p18531697	Bn-chrA07-p17764697
Bn-A07-p15871261	0.933884298	0.925619835	Bn-N7-p18541543	Bn-chrA07-p17777289
Bn-A07-p15872578	0.990033223	0.976744186	Bn-N7-p18542858	Bn-chrAnn_random-p467608
Bn-A07-p15881760	0.950413223	0.950413223	Bn-N7-p18554556	Bn-chrC06-p26250080
Bn-A07-p15885711	0.975206612	0.966942149	Bn-N7-p18559070	Bn-chrA07-p17790472
Bn-A07-p15901533	1	0.976744186	Bn-N7-p18568361	Bn-chrA07-p17803691
Bn-A07-p15903009	1	0.970099668	Bn-N7-p18569547	Bn-chrA07-p17804883
Bn-A07-p15905994	0.983471074	0.983471074	Bn-N7-p18573612	Bn-chrA07-p17807861
Bn-A07-p15913063	0.983471074	0.983471074	Bn-N7-p18575554	Bn-chrA07-p17809471
Bn-A07-p15921259	0.975206612	0.975206612	Bn-N7-p18583594	Bn-chrA07-p17817513
Bn-A07-p15931750	0.986710963	0.996677741	Bn-N7-p18594936	Bn-chrA07-p17829080
Bn-A07-p15938534	0.892561983	0.892561983	Bn-N7-p18601865	Bn-chrA07-p17835860
Bn-A07-p15948472	0.950413223	0.950413223	Bn-N7-p18606472	Bn-chrA07-p17840467
Bn-A07-p15949460	0.991735537	0.991735537	Bn-N7-p18607472	Bn-chrA07-p17841467
Bn-A07-p15957061	0.966942149	0.966942149	Bn-N7-p18631534	Bn-chrA07-p17864883
Bn-A07-p15986673	0.975206612	0.983471074	Bn-N7-p18657326	Bn-chrA07-p17890595
Bn-A07-p15996043	0.975206612	0.975206612	Bn-N7-p18665761	Bn-chrA07-p17899030
Bn-A07-p16005587	0.966942149	0.966942149	Bn-N7-p18685243	Bn-chrA07-p17909671
Bn-A07-p16009799	0.986710963	0.986710963	Bn-N7-p18688438	Bn-chrA07-p17912866
Bn-A07-p16013075	1	1	Bn-N7-p18691714	Bn-chrA07-p17916141
Bn-A07-p16015935	0.991735537	0.991735537	Bn-Scaffold28148-p2	Bn-chrA07-p17919021
Bn-A07-p16020955	0.975206612	0.975206612	Bn-N7-p18696705	Bn-chrC06-p26542914
Bn-A07-p16021617	1	1	Bn-N7-p18697367	Bn-chrA07-p17924399
Bn-A07-p16025294	0.897009967	0.897009967	Bn-N7-p18700670	Bn-chrA07-p17922248
Bn-A07-p16027866	0.966942149	0.966942149	Bn-N7-p18702141	Bn-chrA07-p17929050
Bn-A07-p16028782	0.980066445	0.983388704	Bn-N7-p18703058	Bn-chrA07-p17927283
Bn-A07-p16030550	0.986710963	0.986710963	Bn-N7-p18705419	Bn-chrA07-p17932325
Bn-A07-p16036626	0.983471074	0.983471074	Bn-N7-p18716017	Bn-chrA07-p17951152

Bn-A07-p16039345	0.983471074	0.983471074	Bn-N7-p18719782	Bn-chrA07-p17954918
Bn-A07-p16045526	0.991735537	0.859504132	Bn-N7-p18722837	Bn-chrC06-p26606200
Bn-A07-p16045865	0.966942149	0.966942149	Bn-N7-p18723859	Bn-chrA07_random-p169207
Bn-A07-p16047469	0.975206612	0.975206612	Bn-N7-p18724927	Bn-chrA07_random-p169106
Bn-A07-p16049291	0.983471074	0.983471074	Bn-N7-p18728916	Bn-chrA07-p17963730
Bn-A07-p16049688	0.983471074	0.983471074	Bn-N7-p18729313	Bn-chrA07-p17964127
Bn-A07-p16050076	0.983471074	0.983471074	Bn-N7-p18729717	Bn-chrA07-p17964531
Bn-A07-p16050430	1	1	Bn-N7-p18730071	Bn-chrA07-p17964885
Bn-A07-p16070816	0.970099668	0.970099668	Bn-N7-p18741900	Bn-chrA07-p17973925
Bn-A07-p16074768	0.993355482	0.993355482	Bn-N7-p18746325	Bn-chrA07-p17977596
Bn-A07-p16079602	0.975206612	0.975206612	Bn-N7-p18751294	Bn-chrA07-p17982564
Bn-A07-p16089710	0.958677686	0.958677686	Bn-N7-p18759448	Bn-chrA07-p17988763
Bn-A07-p16090031	0.990049751	0.990049751	Bn-N7-p18759770	Bn-chrA07-p17989085
Bn-A07-p16095589	1	1	Bn-N7-p18765631	Bn-chrA07-p17996553
Bn-A07-p16099690	0.990033223	0.990033223	Bn-N7-p18769734	Bn-chrA07-p18000655
Bn-A07-p16100507	0.983388704	0.983388704	Bn-N7-p18770555	Bn-chrA07-p18001476
Bn-A07-p16101102	0.980066445	0.976744186	Bn-N7-p18771150	Bn-chrA07-p18002071
Bn-A07-p16139853	0.996677741	0.996677741	Bn-N7-p18819466	Bn-chrA07-p18054122
Bn-A07-p16146576	0.950413223	0.950413223	Bn-N7-p18830062	Bn-chrA07-p18067454
Bn-A07-p16146639	0.859504132	0.859504132	Bn-N7-p18830125	Bn-chrA07-p18067517
Bn-A07-p16157761	0.975124378	0.975124378	Bn-N7-p18843538	Bn-chrA07-p18082869
Bn-A07-p16167393	1	0.991735537	Bn-N7-p18852965	Bn-chrAnn_random-p423777
Bn-A07-p16167837	0.990049751	0.945273632	Bn-N7-p18853428	Bn-chrAnn_random-p423773
Bn-A07-p16183869	0.993355482	0.996677741	Bn-N7-p18872267	Bn-chrA07-p18144661
Bn-A07-p16260678	0.970099668	0.993355482	Bn-N7-p18963227	Bn-chrA07-p18224367
Bn-A07-p16261412	1	0.996677741	Bn-N7-p18964562	Bn-chrA07-p18225101
Bn-A07-p16371184	1	1	Bn-N7-p19073905	Bn-chrA07-p18339858
Bn-A07-p16379135	0.760330579	0.760330579	Bn-N7-p19081820	Bn-chrA07-p18348847
Bn-A07-p16383705	0.991735537	0.991735537	Bn-N7-p19085345	Bn-chrA07-p18352372
Bn-A07-p16383888	0.991735537	0.991735537	Bn-N7-p19085528	Bn-chrA07-p18352555
Bn-A07-p16395444	0.983471074	0.983471074	Bn-N7-p19097039	Bn-chrA07-p18364066
Bn-A07-p16399898	1	1	Bn-N7-p19107159	Bn-chrA07-p18374620
Bn-A07-p16404722	0.996677741	0.996677741	Bn-N7-p19114403	Bn-chrA07-p18381863
Bn-A07-p16406072	0.996677741	0.996677741	Bn-N16-p29150061	Bn-chrC06-p27461989
Bn-A07-p16408097	0.989583333	0.989583333	Bn-N7-p19117767	Bn-chrA07-p18385227
Bn-A07-p16423207	0.983388704	0.983388704	Bn-N7-p19132231	Bn-chrA07-p18399690
Bn-A07-p16449661	0.990033223	0.990033223	Bn-N7-p19159713	Bn-chrA07-p18432686
Bn-A07-p16449988	0.956810631	0.96013289	Bn-N7-p19160040	Bn-chrA07-p18433013
Bn-A07-p16461287	0.996677741	0.996677741	Bn-N7-p19171338	Bn-chrA07-p18444310
Bn-A07-p16468333	0.966777409	0.966777409	Bn-N7-p19176714	Bn-chrA07-p18449678
Bn-A07-p16469173	0.975206612	0.975206612	Bn-N7-p19177559	Bn-chrA07-p18450523
Bn-A07-p16491329	0.990049751	0.990049751	Bn-N7-p19204039	Bn-chrA07-p18471602
Bn-A07-p16519850	0.996677741	0.996677741	Bn-N7-p19231704	Bn-chrA07-p18498218
Bn-A07-p16527225	0.966942149	0.966942149	Bn-N7-p19239077	Bn-chrA07-p18507368
Bn-A07-p16553147	0.94214876	0.94214876	Bn-N7-p19263682	Bn-chrA07-p18532017
Bn-A07-p16557893	0.993355482	0.983388704	Bn-N7-p19268240	Bn-chrA07-p18536500

Bn-A07-p16559043	0.983388704	0.973421927	Bn-N7-p19269390	Bn-chrA07-p18537612
Bn-A07-p16566633	0.49833887	0.49833887	Bn-N7-p19277009	Unknown
Bn-A07-p16567234	0.976744186	0.986710963	Bn-N7-p19277609	Bn-chrA07-p18545826
Bn-A07-p16569143	1	0.966777409	Bn-N7-p19279519	Bn-chrA07-p18548227
Bn-A07-p16574221	0.958677686	0.958677686	Bn-N7-p19284610	Bn-chrA07-p18554166
Bn-A07-p16595704	1	0.768595041	Bn-N7-p19308425	Bn-chrA03-p11102000
Bn-A07-p16632905	0.975206612	0.975206612	Bn-N7-p19353739	Bn-chrC06-p27978565
Bn-A07-p16636474	0.995024876	0.995024876	Bn-N7-p19357874	Bn-chrA07_random-p179174
Bn-A07-p16636687	0.970149254	0.970149254	Bn-N7-p19358087	Bn-chrA07_random-p179153
Bn-A07-p16646442	0.983471074	0.330578512	Bn-N7-p19369205	Bn-chrC08-p19388696
Bn-A07-p16659232	0.975124378	0.975124378	Bn-N7-p19383325	Bn-chrA07_random-p176596
Bn-A07-p16659396	0.975206612	0.975206612	Bn-N7-p19383489	Bn-chrA07_random-p176580
Bn-A07-p16668740	0.991735537	0.991735537	Bn-N7-p19392882	Bn-chrA07_random-p175640
Bn-A07-p16677886	0.990033223	0.990033223	Bn-N7-p19401604	Bn-chrA07_random-p174653
Bn-A07-p16680070	0.991735537	0.991735537	Bn-N7-p19403789	Bn-chrA07_random-p174435
Bn-A07-p16683460	0.991735537	0.991735537	Bn-N7-p19407531	Bn-chrA07_random-p174096
Bn-A07-p16687846	0.975206612	0.975206612	Bn-N7-p19411917	Bn-chrA07_random-p173658
Bn-A07-p16694176	0.980066445	0.980066445	Bn-N7-p19418233	Bn-chrAnn_random-p374575
Bn-A07-p16695430	0.986710963	0.986710963	Bn-N7-p19419487	Bn-chrAnn_random-p374588
Bn-A07-p16697491	0.975206612	0.966942149	Bn-N7-p19421634	Bn-chrCnn_random-p780402
Bn-A07-p16709303	0.973421927	0.973421927	Bn-N7-p19433945	Bn-chrA07-p18657421
Bn-A07-p16716464	0.966942149	0.966942149	Bn-N7-p19441212	Bn-chrA07-p18664688
Bn-A07-p16717729	0.966942149	0.876033058	Bn-N7-p19442895	Bn-chrC06-p28233910
Bn-A07-p16721483	0.986710963	0.986710963	Bn-N7-p19450162	Bn-chrA07-p18673521
Bn-A07-p16722346	0.983471074	0.983471074	Bn-N7-p19451034	Bn-chrA07-p18674393
Bn-A07-p16722383	0.933554817	0.933554817	Bn-N7-p19451071	Bn-chrA07-p18674430
Bn-A07-p16722623	0.975206612	0.975206612	Bn-N7-p19451310	Bn-chrA07-p18674669
Bn-A07-p16724761	0.702479339	0.702479339	Bn-N7-p19452597	Bn-chrA07-p18675955
Bn-A07-p16724882	0.94214876	0.94214876	Bn-N7-p19452718	Bn-chrA07-p18676076
Bn-A07-p16726568	0.991735537	0.991735537	Bn-N7-p19453058	Bn-chrA07-p18676416
Bn-A07-p16727616	0.958677686	0.958677686	Bn-N7-p19454364	Bn-chrA07-p18677722
Bn-A07-p16729007	0.970099668	0.970099668	Bn-N7-p19455771	Bn-chrA07-p18679127
Bn-A07-p16729187	0.991735537	0.991735537	Bn-N7-p19455951	Bn-chrA07-p18679307
Bn-A07-p16729233	0.970149254	0.970149254	Bn-N7-p19455998	Bn-chrA07-p18679354
Bn-A07-p16729439	0.975206612	0.975206612	Bn-N7-p19456204	Bn-chrA07-p18679560
Bn-A07-p16729644	0.991735537	0.991735537	Bn-N7-p19456407	Bn-chrA07-p18679763
Bn-A07-p16745833	0.983388704	0.983388704	Bn-N7-p19465227	Bn-chrA07-p18688581
Bn-A07-p16749101	0.975206612	0.975206612	Bn-N7-p19468523	Bn-chrA07-p18691877
Bn-A07-p16755801	0.96345515	0.96345515	Bn-N7-p19475138	Bn-chrA07-p18698475
Bn-A07-p16756509	0.983388704	0.983388704	Bn-N7-p19475846	Bn-chrA07-p18699183
Bn-A07-p16760771	0.950413223	0.950413223	Bn-N7-p19480108	Bn-chrA07-p18703445
Bn-A07-p16765596	0.980066445	0.980066445	Bn-N7-p19484933	Bn-chrA07-p18708270
Bn-A07-p16792991	0.990049751	0.990049751	Bn-N7-p19499588	Bn-chrA07-p18722982
Bn-A07-p16793852	0.986710963	0.986710963	Bn-N7-p19500449	Bn-chrA07-p18723843
Bn-A07-p16806829	0.990033223	0.990033223	Bn-N7-p19518076	Bn-chrA07-p18736503
Bn-A07-p16831414	0.991735537	1	Bn-N7-p19541188	Bn-chrA07-p18760542

Bn-A07-p16833174	1	0.991735537	Bn-N7-p19542578	Bn-chrA07-p18762321
Bn-A07-p16846624	0.975206612	0.975206612	Bn-N7-p19558247	Bn-chrA07-p18775566
Bn-A07-p16871286	0.834710744	0.834710744	Bn-N7-p19574093	Bn-chrA07-p18791466
Bn-A07-p16871349	0.842975207	0.842975207	Bn-N7-p19574144	Bn-chrA07-p18791517
Bn-A07-p16872148	0.943521595	0.940199336	Bn-N7-p19574677	Bn-chrA07-p18792049
Bn-A07-p16895848	0.958677686	0.958677686	Bn-N7-p19595432	Bn-chrA07-p18812151
Bn-A07-p16896370	0.980099502	0.995024876	Bn-N7-p19595976	Bn-chrA07-p18812699
Bn-A07-p16898207	0.983471074	0.983471074	Bn-N7-p19597887	Bn-chrA07-p18814610
Bn-A07-p16904376	0.983471074	0.983471074	Bn-N7-p19604125	Bn-chrA07-p18820750
Bn-A07-p16909798	0.860696517	0.990049751	Bn-N7-p19610807	Bn-chrA07_random-p179998
Bn-A07-p16912963	0.945273632	0.980099502	Bn-N7-p19613978	Bn-chrA07-p18828539
Bn-A07-p16919795	0.910447761	0.960199005	Bn-N7-p19620923	Bn-chrA07-p18835378
Bn-A07-p16919955	0.980066445	0.993355482	Bn-N7-p19621085	Bn-chrA07-p18835538
Bn-A07-p16923741	0.991735537	0.983471074	Bn-N7-p19625350	Bn-chrA07-p18842925
Bn-A07-p16923977	0.985074627	0.930348259	Bn-N7-p19625586	Bn-chrA07-p18843155
Bn-A07-p16924814	0.983388704	0.983388704	Bn-N7-p19626423	Bn-chrA07-p18843992
Bn-A07-p16925201	0.986710963	0.96013289	Bn-N7-p19626810	Bn-chrA07-p18844379
Bn-A07-p16931589	0.956810631	0.953488372	Bn-N7-p19632286	Bn-chrA07-p18852246
Bn-A07-p16934084	0.980099502	0.970149254	Bn-N7-p19635611	Bn-chrA07-p18854733
Bn-A07-p16936529	0.983471074	0.991735537	Bn-N7-p19638052	Bn-chrA07-p18857203
Bn-A07-p16939399	0.993355482	0.993355482	Bn-N7-p19640923	Bn-chrA07-p18860080
Bn-A07-p16946173	0.993355482	0.990033223	Bn-N7-p19647643	Bn-chrA07-p18866806
Bn-A07-p16965943	0.975124378	0.930348259	Bn-N7-p19667355	Bn-chrA07-p18885610
Bn-A07-p16967567	0.980066445	0.986710963	Bn-N7-p19668971	Bn-chrA07-p18887236
Bn-A07-p16970000	0.975124378	0.975124378	Bn-N7-p19671405	Bn-chrA07-p18889670
Bn-A07-p16987468	0.983388704	0.980066445	Bn-N7-p19689583	Bn-chrA07-p18907352
Bn-A07-p16996070	0.993355482	0.993355482	Bn-N7-p19698250	Bn-chrA07-p18916515
Bn-A07-p17001912	0.710743802	0.710743802	Bn-N7-p19705950	Bn-chrA07-p18921183
Bn-A07-p17017277	0.996677741	1	Bn-N7-p19716774	Bn-chrA07-p18931187
Bn-A07-p17026003	0.79338843	0.958677686	Bn-N7-p19728009	Bn-chrA07-p18939821
Bn-A07-p17026027	0.719008264	0.966942149	Bn-N7-p19728033	Bn-chrA07-p18939845
Bn-A07-p17026444	0.94214876	0.94214876	Bn-N7-p19728421	Bn-chrA07-p18940262
Bn-A07-p17047263	0.996677741	0.993355482	Bn-N7-p19743562	Bn-chrA07-p18952770
Bn-A07-p17053061	0.930348259	0.960199005	Bn-N7-p19749483	Bn-chrA07-p18958875
Bn-A07-p17068106	0.950413223	0.950413223	Bn-N7-p19761419	Bn-chrA07-p18970807
Bn-A07-p17074195	0.975206612	0.975206612	Bn-N7-p19768498	Bn-chrA07-p18975177
Bn-A07-p17080877	0.975206612	0.975206612	Bn-N7-p19776570	Bn-chrA07-p18981953
Bn-A07-p17085728	0.707641196	0.707641196	Bn-N7-p19781419	Bn-chrA07-p18986802
Bn-A07-p17087544	0.983471074	0.975206612	Bn-N7-p19783236	Bn-chrA07-p18989097
Bn-A07-p17100649	0.975206612	0.975206612	Bn-N7-p19800272	Bn-chrA07-p19016499
Bn-A07-p17104148	0.975206612	0.975206612	Bn-N7-p19803770	Bn-chrA07-p19019997
Bn-A07-p17123712	0.970149254	0.970149254	Bn-N7-p19825901	Bn-chrA07-p19039999
Bn-A07-p17142058	0.933884298	0.933884298	Bn-N16-p30548052	Bn-chrCnn_random-p167660
Bn-A07-p17148373	0.983471074	0.983471074	Bn-N7-p19873366	Bn-chrA07-p19067845
Bn-A07-p17153938	0.966942149	0.966942149	Bn-N7-p19875521	Bn-chrA07-p19070103
Bn-A07-p17157365	0.991735537	0.991735537	Bn-N7-p19879078	Bn-chrA07-p19074118

Bn-A07-p17160294	0.966942149	0.966942149	Bn-N7-p19880751	Bn-chrA07-p19075791
Bn-A07-p17168790	0.94214876	0.94214876	Bn-N7-p19886150	Bn-chrA07-p19081190
Bn-A07-p17177279	0.933884298	0.94214876	Bn-N7-p19900751	Bn-chrA07-p19094444
Bn-A07-p17186393	0.983471074	0.991735537	Bn-N7-p19913925	Bn-chrC06-p15778507
Bn-A07-p17197152	0.970099668	0.996677741	Bn-N7-p19922466	Bn-chrA07-p19111801
Bn-A07-p17212130	0.983471074	0.983471074	Bn-N7-p19936860	Bn-chrA07-p19126922
Bn-A07-p17212502	1	1	Bn-N7-p19937225	Bn-chrA07-p19127287
Bn-A07-p17214494	0.983471074	0.983471074	Bn-N7-p19939221	Bn-chrA07-p19130320
Bn-A07-p17225853	0.989583333	0.989583333	Bn-N7-p19950600	Bn-chrA07-p19141698
Bn-A07-p17260356	0.979166667	0.979166667	Bn-N16-p30852865	Bn-chrC06-p28949918
Bn-A07-p17298132	0.985074627	0.985074627	Bn-N7-p19989191	Bn-chrA07-p19184691
Bn-A07-p17312272	0.983471074	0.983471074	Bn-N7-p20003104	Bn-chrA07-p19198553
Bn-A07-p17326181	0.980066445	0.980066445	Bn-N7-p20021130	Bn-chrA07-p19217657
Bn-A07-p17339463	0.975206612	0.975206612	Bn-N7-p20035165	Bn-chrA07-p19231822
Bn-A07-p17353806	0.873754153	0.873754153	Bn-N7-p20049427	Bn-chrA07-p19246083
Bn-A07-p17382019	0.983471074	0.983471074	Bn-N7-p20082091	Bn-chrA07-p19282163
Bn-A07-p17382630	0.970099668	0.970099668	Bn-N7-p20082702	Bn-chrA07-p19282774
Bn-A07-p17388536	0.983388704	0.983388704	Bn-N7-p20086829	Bn-chrA07-p19286901
Bn-A07-p17388825	0.993355482	0.993355482	Bn-N7-p20087118	Bn-chrA07-p19287190
Bn-A07-p17400106	0.744186047	0.744186047	Bn-N7-p20100304	Bn-chrA07-p19299385
Bn-A07-p17404116	0.903654485	0.903654485	Bn-N7-p20102443	Bn-chrA07-p19301523
Bn-A07-p17409328	0.991735537	0.991735537	Bn-N7-p20107667	Bn-chrA07-p19306747
Bn-A07-p17411297	0.993355482	0.993355482	Bn-N7-p20109636	Bn-chrA07-p19308716
Bn-A07-p17411599	0.996677741	0.996677741	Bn-N7-p20109938	Bn-chrA07-p19309018
Bn-A07-p17412755	0.990033223	0.990033223	Bn-N7-p20111094	Bn-chrA07-p19310174
Bn-A07-p17418824	0.976744186	0.976744186	Bn-N7-p20117163	Bn-chrA07-p19316243
Bn-A07-p17419179	0.995024876	0.995024876	Bn-N7-p20117518	Bn-chrA07-p19316598
Bn-A07-p17421675	0.956810631	0.956810631	Bn-N7-p20120014	Bn-chrA07-p19319094
Bn-A07-p17434468	0.990033223	0.990033223	Bn-N7-p20132186	Bn-chrA07_random-p180773
Bn-A07-p17435418	0.966942149	0.966942149	Bn-N7-p20133134	Bn-chrA07_random-p180868
Bn-A07-p17439730	0.933554817	0.903654485	Bn-N7-p20137429	Bn-chrA07-p19331222
Bn-A07-p17440585	1	1	Bn-N7-p20138278	Bn-chrA07-p19332073
Bn-A07-p17448936	0.94214876	0.94214876	Bn-N7-p20145873	Bn-chrA07-p19339668
Bn-A07-p17450697	0.993355482	0.993355482	Bn-N7-p20146850	Bn-chrA07-p19340645
Bn-A07-p17465762	0.991735537	0.991735537	Bn-N7-p20160318	Bn-chrA07-p19354083
Bn-A07-p17470966	0.935323383	0.935323383	Bn-N7-p20167404	Bn-chrA07-p19360805
Bn-A07-p17477080	0.991735537	0.991735537	Bn-N7-p20173519	Bn-chrA07-p19366920
Bn-A07-p17483655	0.991735537	0.991735537	Bn-N7-p20180334	Bn-chrA07-p19373485
Bn-A07-p17484397	0.958677686	0.958677686	Bn-N7-p20181076	Bn-chrA07-p19374226
Bn-A07-p17488337	0.983471074	0.983471074	Bn-N7-p20185016	Bn-chrA07-p19378166
Bn-A07-p17488591	0.983471074	0.983471074	Bn-N7-p20185270	Bn-chrA07-p19378420
Bn-A07-p17497283	1	1	Bn-N7-p20193963	Bn-chrA07-p19387277
Bn-A07-p17513051	0.966942149	0.966942149	Bn-N7-p20212462	Bn-chrA07-p19399205
Bn-A07-p17520539	0.990033223	0.990033223	Bn-N7-p20220294	Bn-chrA07-p19406967
Bn-A07-p17523277	0.553719008	0.611570248	Bn-N16-p31427216	Bn-chrC06-p29657277
Bn-A07-p17581698	0.986710963	0.986710963	Bn-N7-p20273866	Bn-chrA07-p19458865

Bn-A07-p17583998	0.993355482	0.993355482	Bn-N7-p20276166	Bn-chrA07-p19461165
Bn-A07-p17587315	0.995024876	0.995024876	Bn-N7-p20279530	Bn-chrA07-p19464529
Bn-A07-p17588031	0.985074627	0.985074627	Bn-N7-p20280260	Bn-chrA07-p19465259
Bn-A07-p17593690	0.975206612	0.975206612	Bn-N7-p20289227	Bn-chrA07-p19475701
Bn-A07-p17593695	0.975206612	0.975206612	Bn-N7-p20289232	Bn-chrA07-p19475706
Bn-A07-p17598687	1	1	Bn-N7-p20294291	Bn-chrA07-p19480764
Bn-A07-p17605668	0.985074627	0.985074627	Bn-N7-p20301104	Bn-chrA07-p19487577
Bn-A07-p17606005	1	1	Bn-N7-p20301441	Bn-chrA07-p19487914
Bn-A07-p17606752	0.975206612	0.975206612	Bn-N7-p20302014	Bn-chrA07-p19488487
Bn-A07-p17607958	0.966942149	0.966942149	Bn-N7-p20305544	Bn-chrA07-p19491505
Bn-A07-p17628920	0.958677686	0.966942149	Bn-N7-p20326009	Bn-chrC06-p30005785
Bn-A07-p17636141	0.970149254	0.970149254	Bn-N7-p20335931	Bn-chrA07-p19519191
Bn-A07-p17639842	0.710743802	0.702479339	Bn-N16-p31739142	Bn-chrA07-p19528120
Bn-A07-p17647906	0.975206612	0.975206612	Bn-N7-p20359160	Bn-chrA07-p19537664
Bn-A07-p17661422	0.893687708	0.887043189	Bn-N7-p6265141	Bn-chrA03-p18581620
Bn-A07-p17679589	0.989583333	0.989583333	Bn-N7-p20396156	Bn-chrAnn_random-p152629
Bn-A07-p17802877	0.958677686	0.958677686	Bn-N7-p20518345	Bn-chrA07-p19645675
Bn-A07-p17804261	0.950413223	0.950413223	Bn-N7-p20518723	Bn-chrA07-p19646053
Bn-A07-p17804272	0.950413223	0.950413223	Bn-N7-p20518734	Bn-chrA07-p19646064
Bn-A07-p17810756	0.975124378	0.975124378	Bn-N7-p20525222	Bn-chrA07-p19652551
Bn-A07-p17814268	0.991735537	0.991735537	Bn-N7-p20528512	Bn-chrA07-p19655841
Bn-A07-p17823544	0.995024876	0.995024876	Bn-N7-p20537658	Bn-chrA07-p19664421
Bn-A07-p17827124	0.945273632	0.945273632	Bn-N7-p20541231	Bn-chrA07-p19667994
Bn-A07-p17839833	0.996677741	0.996677741	Bn-N7-p20556572	Bn-chrA07-p19686123
Bn-A07-p17846018	0.991735537	0.991735537	Bn-N7-p20567913	Bn-chrA07-p19696052
Bn-A07-p17846299	0.986710963	0.946843854	Bn-N7-p20569743	Bn-chrC06-p30379550
Bn-A07-p17860745	0.993355482	0.993355482	Bn-N7-p20582995	Bn-chrA07-p19709751
Bn-A07-p17869552	0.996677741	0.996677741	Bn-N7-p20590722	Bn-chrA07-p19717370
Bn-A07-p17930992	0.990033223	0.990033223	Bn-N7-p20652751	Bn-chrA07-p19778743
Bn-A07-p17959118	0.983471074	0.983471074	Bn-N7-p20681326	Bn-chrA07-p19806736
Bn-A07-p18020732	0.991735537	0.991735537	Bn-N7-p20745840	Bn-chrA07-p19874164
Bn-A07-p18021746	0.842975207	0.842975207	Bn-N7-p20746106	Bn-chrA07-p19874430
Bn-A07-p18028449	0.990049751	0.990049751	Bn-N7-p20752433	Bn-chrA07-p19880757
Bn-A07-p18028951	0.991735537	0.991735537	Bn-N7-p20752942	Bn-chrA07-p19881264
Bn-A07-p18029053	0.983471074	0.983471074	Bn-N7-p20753044	Bn-chrA07-p19881366
Bn-A07-p18035698	0.976744186	0.980066445	Bn-N7-p20760589	Bn-chrA07-p19889045
Bn-A07-p18041441	0.990033223	0.990033223	Bn-N7-p20766228	Bn-chrA07-p19894683
Bn-A07-p18048293	0.983471074	0.983471074	Bn-N7-p20772310	Bn-chrA07-p19900765
Bn-A07-p18049440	0.991735537	0.991735537	Bn-N7-p20773455	Bn-chrA07-p19901910
Bn-A07-p18074241	0.991735537	0.991735537	Bn-N7-p20797217	Bn-chrA07-p19925777
Bn-A07-p18074585	0.99382716	0.99382716	Bn-N7-p20797561	Bn-chrA07-p19926121
Bn-A07-p18076313	0.950413223	0.950413223	Bn-N7-p20798609	Bn-chrA07-p19927169
Bn-A07-p18076429	0.946843854	0.946843854	Bn-N7-p20798725	Bn-chrA07-p19927285
Bn-A07-p18084198	0.975206612	0.975206612	Bn-N7-p20808241	Bn-chrA07-p19936984
Bn-A07-p18088151	0.996677741	0.996677741	Bn-N7-p20812208	Bn-chrA07-p19940951
Bn-A07-p18095670	0.985074627	0.960199005	Bn-N7-p20830966	Bn-chrC06-p30499757

Bn-A07-p18103464	0.985074627	0.985074627	Bn-N7-p20844230	Bn-chrC05-p10277074
Bn-A07-p18103505	0.980099502	0.980099502	Bn-N7-p20844271	Bn-chrC05-p10277033
Bn-A07-p18104828	0.825870647	0.825870647	Bn-N7-p20845604	Bn-chrA07-p19979545
Bn-A07-p18106007	0.995024876	0.995024876	Bn-N7-p20846772	Bn-chrA07-p19980713
Bn-A07-p18106626	0.966942149	0.966942149	Bn-N7-p20847397	Bn-chrA07-p19981338
Bn-A07-p18106750	0.892561983	0.892561983	Bn-N7-p20847520	Bn-chrA07-p19981461
Bn-A07-p18115061	0.651162791	0.983388704	Bn-N7-p20855786	Bn-chrA07-p19989727
Bn-A07-p18115747	0.976744186	0.976744186	Bn-N7-p20856472	Bn-chrA07-p19990413
Bn-A07-p18121365	1	1	Bn-N7-p20862088	Bn-chrA07-p19996028
Bn-A07-p18134446	0.983471074	0.983471074	Bn-N7-p20874908	Bn-chrA07-p20010063
Bn-A07-p18151107	0.975206612	0.975206612	Bn-N7-p20905895	Bn-chrA07-p20041303
Bn-A07-p18157564	0.993355482	0.993355482	Bn-N7-p20910957	Bn-chrA07-p20046365
Bn-A07-p18167424	0.975124378	0.975124378	Bn-N7-p20920433	Bn-chrA07-p20055841
Bn-A07-p18187317	0.900826446	0.909090909	Bn-N16-p32816567	Bn-chrC06-p30720032
Bn-A07-p18196996	0.991735537	0.991735537	Bn-N7-p20961590	Bn-chrA07-p20092385
Bn-A07-p18197951	0.482587065	0.482587065	Unknown	Unknown
Bn-A07-p18201301	0.983471074	0.983471074	Bn-N7-p20963317	Bn-chrA07-p20094112
Bn-A07-p18204966	0.818181818	0.818181818	Bn-N7-p20966934	Bn-chrA07-p20097729
Bn-A07-p18208846	0.983471074	0.983471074	Bn-N7-p20970817	Bn-chrA07-p20101610
Bn-A07-p18210547	0.983471074	0.983471074	Bn-N7-p20972516	Bn-chrA07-p20103309
Bn-A07-p18216943	0.990033223	0.990033223	Bn-N7-p20978354	Bn-chrA07-p20109146
Bn-A07-p18223256	0.991735537	0.991735537	Bn-N7-p20984803	Bn-chrA07-p20115595
Bn-A07-p18226326	0.867768595	0.867768595	Bn-N7-p20987309	Bn-chrA07-p20118101
Bn-A07-p18227328	0.991735537	0.991735537	Bn-N7-p20988316	Bn-chrA07-p20119108
Bn-A07-p18230931	0.966942149	0.966942149	Bn-N7-p20991021	Bn-chrA07-p20121813
Bn-A07-p18231736	0.991735537	0.991735537	Bn-N7-p20991609	Bn-chrA07-p20122401
Bn-A07-p18233390	0.996677741	0.996677741	Bn-N7-p20993262	Bn-chrA07-p20124052
Bn-A07-p18262143	0.973421927	0.973421927	Bn-N7-p21028191	Bn-chrA07-p20156078
Bn-A07-p18265668	0.717607973	0.714285714	Bn-N7-p21031716	Bn-chrA07-p20159602
Bn-A07-p18265772	0.950413223	0.950413223	Bn-N7-p21031821	Bn-chrC06-p31176486
Bn-A07-p18274656	0.990033223	0.990033223	Bn-N7-p21042702	Bn-chrA07-p20170580
Bn-A07-p18282597	0.976744186	0.976744186	Bn-N7-p21050725	Bn-chrA07-p20178603
Bn-A07-p18282722	0.975124378	0.975124378	Bn-N7-p21050850	Bn-chrA07-p20178728
Bn-A07-p18308378	0.991735537	0.991735537	Bn-N7-p21081019	Bn-chrA07-p20209980
Bn-A07-p18319586	0.975206612	0.975206612	Bn-N7-p21092260	Bn-chrA07-p20221220
Bn-A07-p18326762	0.995024876	0.995024876	Bn-N7-p21100078	Bn-chrA07-p20229035
Bn-A07-p18338860	0.958677686	0.925619835	Bn-N16-p33055281	Bn-chrC06-p31294786
Bn-A07-p18346973	0.94214876	0.94214876	Bn-N7-p21120187	Bn-chrA07-p20249998
Bn-A07-p18451074	0.980099502	0.980099502	Bn-Scaffold30091-p2	Bn-chrA07-p20361836
Bn-A07-p18745105	0.996677741	0.996677741	Bn-N7-p21585036	Bn-chrA07-p20708529
Bn-A07-p18812613	0.980099502	0.980099502	Bn-N7-p21658703	Bn-chrA07_random-p186732
Bn-A07-p18822708	0.925619835	0.925619835	Bn-N7-p21671143	Bn-chrA07_random-p187976
Bn-A07-p18832178	0.940298507	0.940298507	Bn-N7-p21681303	Bn-chrA08-p5400815
Bn-A07-p18903215	0.975206612	0.975206612	Bn-N7-p21761161	Bn-chrAnn_random-p245540
Bn-A07-p18914294	0.995024876	0.995024876	Bn-N7-p21785561	Bn-chrA07_random-p188902
Bn-A07-p18919960	0.983471074	0.983471074	Bn-N7-p21791272	Bn-chrA07_random-p188331

Bn-A07-p18922121	0.991735537	0.991735537	Bn-N7-p21793439	Bn-chrA01-p20148728
Bn-A07-p18935257	0.784053156	0.784053156	Bn-N7-p21814012	Bn-chrA07-p20752567
Bn-A07-p18947073	0.980099502	0.980099502	Bn-N7-p21820922	Bn-chrA07-p20759473
Bn-A07-p18954998	0.743801653	0.743801653	Bn-N7-p21828430	Bn-chrA07-p20766981
Bn-A07-p18955989	0.995024876	0.995024876	Bn-N7-p21829421	Bn-chrA07-p20767972
Bn-A07-p18956207	0.950413223	0.950413223	Bn-N7-p21829639	Bn-chrA07-p20768190
Bn-A07-p18957162	0.995024876	0.995024876	Bn-N7-p21830585	Bn-chrA07-p20769136
Bn-A07-p18968214	0.980099502	0.980099502	Bn-N7-p21839242	Bn-chrA07-p20781812
Bn-A07-p18971657	0.991735537	0.991735537	Bn-N7-p21842684	Bn-chrA07-p20785254
Bn-A07-p18973385	0.986710963	0.986710963	Bn-N7-p21844415	Bn-chrA07-p20786985
Bn-A07-p18983802	0.983471074	0.983471074	Bn-N7-p21854973	Bn-chrA07-p20797210
Bn-A07-p18985169	0.920265781	0.920265781	Bn-N7-p21860887	Bn-chrA07-p20803121
Bn-A07-p18990899	0.88372093	0.88372093	Bn-N7-p21861150	Bn-chrA07-p20803384
Bn-A07-p18993118	0.996677741	0.996677741	Bn-N7-p21864012	Bn-chrA07-p20806154
Bn-A07-p19001556	0.970149254	0.970149254	Bn-N7-p21876445	Bn-chrA07-p20818583
Bn-A07-p19001778	0.990049751	0.990049751	Bn-N7-p21876667	Bn-chrA07-p20818805
Bn-A07-p19009397	0.975206612	0.975206612	Bn-N7-p21885980	Bn-chrA07-p20828146
Bn-A07-p19028597	0.966942149	0.966942149	Bn-N7-p21910940	Bn-chrA07_random-p191526
Bn-A07-p19028743	0.973421927	0.973421927	Bn-N7-p21911086	Bn-chrA07_random-p191511
Bn-A07-p19037677	1	1	Bn-N7-p21921197	Bn-chrA07-p20850589
Bn-A07-p19066968	0.973421927	0.973421927	Bn-N7-p21951631	Bn-chrA07-p20879572
Bn-A07-p19090612	0.996677741	0.996677741	Bn-N7-p21968936	Bn-chrA07-p20896681
Bn-A07-p19093712	0.983388704	0.983388704	Bn-N7-p21972098	Bn-chrA07-p20899843
Bn-A07-p19102772	0.983471074	0.983471074	Bn-N7-p21982805	Bn-chrA07-p20909161
Bn-A07-p19109981	0.983471074	0.983471074	Bn-N7-p21990014	Bn-chrA07-p20916370
Bn-A07-p19110333	0.975206612	0.975206612	Bn-N7-p21990366	Bn-chrA07-p20916722
Bn-A07-p19110971	0.983471074	0.983471074	Bn-N7-p21991004	Bn-chrA07-p20917360
Bn-A07-p19111807	0.983471074	0.983471074	Bn-N7-p21991840	Bn-chrA07-p20918196
Bn-A07-p19113444	0.950413223	0.950413223	Bn-N7-p21993479	Bn-chrA07-p20919835
Bn-A07-p19118066	0.983471074	0.966942149	Bn-N7-p21997596	Bn-chrA07-p20923957
Bn-A07-p19129406	0.985074627	0.980099502	Bn-N7-p22009465	Bn-chrA07-p20935320
Bn-A07-p19142808	0.950413223	0.958677686	Bn-N7-p22015948	Bn-chrA07-p20943090
Bn-A07-p19145175	0.867768595	0.611570248	Bn-N7-p22019831	Bn-chrA07-p20944749
Bn-A07-p19150984	0.975206612	0.983471074	Bn-N7-p22021906	Bn-chrA07-p20946811
Bn-A07-p19151274	0.985074627	0.985074627	Bn-N7-p22022196	Bn-chrA07-p20947101
Bn-A07-p19163856	0.986710963	0.983388704	Bn-N7-p22035316	Bn-chrA07-p20959648
Bn-A07-p19166676	0.975206612	0.983471074	Bn-N7-p22038136	Bn-chrA07-p20962471
Bn-A07-p19175358	0.843853821	0.986710963	Bn-N7-p22048526	Bn-chrA07-p20968009
Bn-A07-p19176340	0.930232558	0.993355482	Bn-N16-p34640336	Bn-chrA07-p20968991
Bn-A07-p19186300	0.950413223	0.909090909	Bn-N7-p22059448	Bn-chrA07-p20978981
Bn-A07-p19190510	1	0.996677741	Bn-N7-p22063739	Bn-chrA07-p20983286
Bn-A07-p19192373	0.975206612	0.966942149	Bn-N7-p22065602	Bn-chrA07-p20985155
Bn-A07-p19260238	0.993355482	0.993355482	Bn-N7-p22128099	Bn-chrA07-p21053169
Bn-A07-p19260331	0.993355482	0.993355482	Bn-N7-p22128192	Bn-chrA07-p21053262
Bn-A07-p19311663	0.990049751	0.990049751	Bn-N7-p22177327	Bn-chrA07-p21105713
Bn-A07-p19352007	0.990033223	0.990033223	Bn-N7-p22216779	Bn-chrA07-p21142710

Bn-A07-p19409951	0.993355482	0.993355482	Bn-N7-p22272614	Bn-chrA07-p21200677
Bn-A07-p19423792	0.986710963	0.986710963	Bn-N7-p22291882	Bn-chrAnn_random-p31484C
Bn-A07-p19458370	0.958677686	0.892561983	Bn-N7-p22327509	Bn-chrA07-p21245856
Bn-A07-p19486760	0.986710963	0.993355482	Bn-N7-p22348430	Bn-chrA07-p21263670
Bn-A07-p19487659	0.940298507	0.995024876	Bn-N7-p22349329	Bn-chrA07-p21264568
Bn-A07-p19512603	0.991735537	0.991735537	Bn-N7-p22378556	Bn-chrA07-p21297309
Bn-A07-p19512916	0.983471074	0.983471074	Bn-N7-p22378869	Bn-chrA07-p21297622
Bn-A07-p19515708	1	1	Bn-N7-p22381661	Bn-chrA07-p21300414
Bn-A07-p19563230	1	0.991735537	Bn-N7-p22437065	Bn-chrA07-p21349451
Bn-A07-p19622207	0.990049751	0.990049751	Bn-N7-p22513003	Bn-chrA07-p21425054
Bn-A07-p19622647	0.885572139	1	Bn-N15-p10192518	Bn-chrA07-p21425658
Bn-A07-p19795126	0.965174129	0.965174129	Bn-N7-p22701176	Bn-chrA07-p21608340
Bn-A07-p19824864	0.995024876	0.995024876	Bn-N7-p22725156	Bn-chrA07-p21632032
Bn-A07-p19825063	0.989247312	0.989247312	Bn-N7-p22725356	Bn-chrA07-p21632232
Bn-A07-p19865918	1	1	Bn-N7-p22777396	Bn-chrA07-p21681375
Bn-A07-p19865996	0.991735537	0.991735537	Bn-N7-p22777474	Bn-chrA07-p21681453
Bn-A07-p19871923	0.991735537	0.991735537	Bn-N7-p22783472	Bn-chrA07-p21687450
Bn-A07-p19880756	0.995024876	0.995024876	Bn-N7-p22793838	Bn-chrA07-p21698055
Bn-A07-p19880968	0.958677686	0.958677686	Bn-N7-p22794050	Bn-chrA07-p21698267
Bn-A07-p19900592	0.993355482	0.990033223	Bn-N7-p22818133	Bn-chrA07-p21719776
Bn-A07-p19901175	0.851239669	0.950413223	Bn-N16-p26706551	Bn-chrA07-p21720359
Bn-A07-p19912379	0.983471074	0.991735537	Bn-N7-p22830430	Bn-chrA07-p21731124
Bn-A07-p19915062	0.983388704	0.980066445	Bn-N7-p22833926	Bn-chrA07-p21733726
Bn-A07-p19924753	0.983471074	0.991735537	Bn-N7-p22846933	Bn-chrA07-p21743560
Bn-A07-p19941998	0.993355482	0.993355482	Bn-N7-p22865951	Bn-chrA07-p21760158
Bn-A07-p19957202	1	1	Bn-N7-p22880995	Bn-chrA07-p21777746
Bn-A07-p19961825	0.975124378	0.975124378	Bn-N7-p22885432	Bn-chrA07-p21782148
Bn-A07-p19965401	0.993355482	0.993355482	Bn-N7-p22889167	Bn-chrA07-p21785883
Bn-A07-p19967695	0.985074627	0.985074627	Bn-N7-p22892309	Bn-chrA07-p21789025
Bn-A07-p19975808	0.910447761	0.910447761	Bn-N16-p35914324	Bn-chrC06-p33928181
Bn-A07-p19977445	0.995024876	0.995024876	Bn-N7-p22897765	Bn-chrA07-p21797232
Bn-A07-p19984971	0.996677741	0.996677741	Bn-N7-p22906722	Bn-chrA07-p21806786
Bn-A07-p19987483	0.995024876	0.995024876	Bn-N7-p22914333	Bn-chrA07_random-p193651
Bn-A07-p19987485	0.990049751	0.990049751	Bn-N7-p22914335	Bn-chrA07_random-p193652
Bn-A07-p19995985	0.970149254	0.970149254	Bn-N7-p22923766	Bn-chrA07_random-p194594
Bn-A07-p19999651	0.983388704	0.983388704	Bn-N7-p22927363	Bn-chrA07_random-p194954
Bn-A07-p20000340	0.993355482	0.993355482	Bn-N7-p22928052	Bn-chrA07_random-p195023
Bn-A07-p20003438	0.993355482	0.993355482	Bn-N7-p22931174	Bn-chrA07_random-p195335
Bn-A07-p20005724	0.884297521	0.884297521	Bn-N7-p22933916	Bn-chrA07_random-p195563
Bn-A07-p20006245	0.975206612	0.975206612	Bn-N7-p22934417	Bn-chrA07_random-p195613
Bn-A07-p20029449	0.991735537	0.991735537	Bn-N7-p22957030	Bn-chrA07_random-p197874
Bn-A07-p20044413	0.996677741	0.996677741	Bn-N7-p22973485	Bn-chrA07_random-p192735
Bn-A07-p20090726	0.986710963	0.986710963	Bn-N7-p23020123	Bn-chrA07-p21859060
Bn-A07-p20135392	0.976744186	0.976744186	Bn-N7-p23074790	Bn-chrA07-p21907258
Bn-A07-p20143003	0.96013289	0.96013289	Bn-N7-p23081647	Bn-chrA07-p21913974
Bn-A07-p20143131	0.96013289	0.96013289	Bn-N7-p23081773	Bn-chrA07-p21914100

Bn-A07-p20144687	0.990049751	0.990049751	Bn-N7-p23083318	Bn-chrA07-p21915645
Bn-A07-p20190653	0.996677741	0.983388704	Bn-N7-p23121055	Bn-chrA07-p21951460
Bn-A07-p20192092	0.980066445	0.993355482	Bn-N7-p23122457	Bn-chrA07-p21952862
Bn-A07-p20193221	0.976744186	0.990033223	Bn-N7-p23123586	Bn-chrA07-p21953984
Bn-A07-p20209068	0.990033223	0.990033223	Bn-N7-p23139133	Bn-chrA07-p21969437
Bn-A07-p20221692	0.96013289	0.96013289	Bn-N7-p23146689	Bn-chrA07-p21980362
Bn-A07-p20230189	0.990033223	0.990033223	Bn-N7-p23153248	Bn-chrA07-p21986981
Bn-A07-p20231605	0.970149254	0.970149254	Bn-N7-p23154664	Bn-chrA07-p21988397
Bn-A07-p20248654	0.966942149	0.966942149	Bn-N7-p23164955	Bn-chrA07-p21998687
Bn-A07-p20248822	0.966942149	0.966942149	Bn-N7-p23165123	Bn-chrA07-p21998855
Bn-A07-p20251365	1	0.991735537	Bn-N7-p23167942	Bn-chrA07-p22001674
Bn-A07-p20369599	0.975124378	0.975124378	Bn-N16-p36533958	Bn-chrC06-p34568506
Bn-A07-p20467347	0.983471074	0.991735537	Bn-N7-p23418291	Bn-chrA07-p22239713
Bn-A07-p20469674	0.990049751	0.990049751	Bn-N7-p23420612	Bn-chrA07-p22242397
Bn-A07-p20471091	0.970149254	0.965174129	Bn-N7-p23422028	Bn-chrA07-p22243815
Bn-A07-p20471143	0.975124378	0.970149254	Bn-N7-p23422080	Bn-chrA07-p22243867
Bn-A07-p20477027	0.985074627	0.985074627	Bn-N7-p23430133	Bn-chrA07-p22251919
Bn-A07-p20481682	0.975124378	0.975124378	Bn-N7-p23434792	Bn-chrA07-p22256577
Bn-A07-p20485039	0.960199005	0.960199005	Bn-N7-p23436942	Bn-chrA07-p22258727
Bn-A07-p20511320	0.996677741	0.996677741	Bn-N7-p23459139	Bn-chrA07-p22280456
Bn-A07-p20527962	0.985074627	0.985074627	Bn-N7-p23476183	Bn-chrA07_random-p198254
Bn-A07-p20554194	0.991735537	0.991735537	Bn-N7-p23514296	Bn-chrAnn_random-p970341
Bn-A07-p20558164	0.965174129	0.965174129	Bn-N7-p23518260	Bn-chrAnn_random-p969945
Bn-A07-p20561173	0.993355482	0.993355482	Bn-N7-p23521267	Bn-chrAnn_random-p969644
Bn-A07-p20565057	0.970149254	0.970149254	Bn-N7-p23524919	Bn-chrAnn_random-p969279
Bn-A07-p20565684	0.983471074	0.983471074	Bn-N7-p23525546	Bn-chrAnn_random-p969216
Bn-A07-p20571625	0.990049751	0.990049751	Bn-N7-p23531484	Bn-chrAnn_random-p968623
Bn-A07-p20580512	0.983471074	0.983471074	Bn-N7-p23539797	Bn-chrAnn_random-p967791
Bn-A07-p20589714	0.975124378	0.975124378	Bn-N7-p23549843	Bn-chrAnn_random-p966846
Bn-A07-p20611621	0.975206612	0.975206612	Bn-N7-p23567380	Bn-chrC06_random-p335849
Bn-A07-p20646388	0.983471074	0.983471074	Bn-N7-p23615357	Bn-chrAnn_random-p958834
Bn-A07-p20649978	0.975206612	0.975206612	Bn-N7-p23618921	Bn-chrAnn_random-p958477
Bn-A07-p20658738	0.983471074	0.983471074	Bn-N7-p23619748	Bn-chrAnn_random-p958395
Bn-A07-p20661885	0.970099668	0.970099668	Bn-N7-p23622894	Bn-chrAnn_random-p958080
Bn-A07-p20662200	0.940298507	0.940298507	Bn-N7-p23623208	Bn-chrAnn_random-p958049
Bn-A07-p20671112	0.975206612	0.975206612	Bn-N7-p23632309	Bn-chrAnn_random-p957206
Bn-A07-p20675049	0.823920266	0.823920266	Bn-N7-p23636157	Bn-chrAnn_random-p956821
Bn-A07-p20684260	0.990033223	0.993355482	Bn-N7-p23645045	Bn-chrAnn_random-p955871
Bn-A07-p20688218	0.685950413	0.983471074	Bn-N16-p36915527	Bn-chrAnn_random-p955475
Bn-A07-p20688341	0.867768595	0.975206612	Bn-N16-p36915646	Bn-chrAnn_random-p955463
Bn-A07-p20700508	0.991735537	0.991735537	Bn-N7-p23662679	Bn-chrAnn_random-p375814
Bn-A07-p20732884	1	0.991735537	Bn-N7-p23693757	Bn-chrA07-p22313525
Bn-A07-p20771568	0.996677741	1	Bn-N7-p23721465	Bn-chrA07-p22344000
Bn-A07-p20786814	0.996677741	0.996677741	Bn-N7-p23736962	Bn-chrA07-p22358342
Bn-A07-p20797755	0.991735537	0.983471074	Bn-N7-p23748355	Bn-chrA07-p22371039
Bn-A07-p20801106	0.991735537	1	Bn-N7-p23749440	Bn-chrA07-p22372120

Bn-A07-p20801255	0.983471074	0.991735537	Bn-N7-p23749590	Bn-chrA07-p22372269
Bn-A07-p20801487	0.991735537	0.983471074	Bn-N7-p23749823	Bn-chrA07-p22372501
Bn-A07-p20801586	1	0.991735537	Bn-N7-p23749922	Bn-chrA07-p22372600
Bn-A07-p20801762	0.975206612	0.966942149	Bn-N7-p23750098	Bn-chrA07-p22372776
Bn-A07-p20871163	0.925619835	0.925619835	Bn-N7-p23811865	Bn-chrA07-p22455594
Bn-A07-p20887568	0.953488372	0.953488372	Bn-N7-p23826853	Bn-chrA07-p22470589
Bn-A07-p20894218	0.917355372	0.917355372	Bn-N7-p23833106	Bn-chrA07-p22476713
Bn-A07-p20935217	0.986710963	0.930232558	Bn-N7-p23869260	Bn-chrC06-p35232895
Bn-A07-p20941237	0.985074627	0.791044776	Bn-N16-p37190745	Bn-chrC06-p35236385
Bn-A07-p20941338	0.970149254	0.965174129	Bn-N16-p37190846	Bn-chrC06-p35236486
Bn-A07-p20950895	0.995024876	0.995024876	Bn-Scaffold18867-p3	Bn-chrAnn_random-p452693
Bn-A07-p20951340	0.990049751	0.990049751	Bn-N16-p37224176	Bn-chrC06-p35275477
Bn-A07-p20952117	0.970238095	0.875	Bn-N16-p37226753	Bn-chrC06-p35245199
Bn-A07-p20981410	0.956810631	0.993355482	Bn-N7-p23882909	Bn-chrA07-p22534719
Bn-A07-p20986876	0.993355482	0.993355482	Bn-N7-p23888405	Bn-chrA07-p22540188
Bn-A07-p20999615	0.991735537	0.991735537	Bn-N7-p23898726	Bn-chrA07-p22550509
Bn-A07-p21006643	0.991735537	0.991735537	Bn-N7-p23905791	Bn-chrA07-p22557573
Bn-A07-p21019499	0.996677741	0.996677741	Bn-N7-p23918790	Bn-chrA07-p22569349
Bn-A07-p21078249	0.975124378	0.975124378	Bn-N7-p23974541	Bn-chrA07-p22624670
Bn-A07-p21095697	0.975206612	0.975206612	Bn-N7-p23997641	Bn-chrA07-p22638742
Bn-A07-p21096350	0.983471074	0.983471074	Bn-N7-p23998277	Bn-chrA07-p22639378
Bn-A07-p21096706	0.966942149	0.966942149	Bn-N7-p23998633	Bn-chrA07-p22639734
Bn-A07-p21160078	0.983471074	0.983471074	Bn-N7-p24081454	Bn-chrA07-p22718286
Bn-A07-p21173491	0.983471074	0.958677686	Bn-N7-p24091213	Bn-chrA07-p22731075
Bn-A07-p21173896	0.980066445	0.966777409	Bn-N7-p24091608	Bn-chrA07-p22731478
Bn-A07-p21191683	0.933884298	0.94214876	Bn-N7-p24113156	Bn-chrA07-p22752089
Bn-A07-p21193584	0.537190083	0.958677686	Bn-N7-p24117019	Bn-chrA07-p22754009
Bn-A07-p21195382	0.983471074	0.975206612	Bn-N7-p24122925	Bn-chrA07-p22755821
Bn-A07-p21261518	0.851239669	0.94214876	Bn-N7-p24180943	Bn-chrA07-p22816850
Bn-A07-p21271213	0.975206612	0.983471074	Bn-N7-p24193063	Bn-chrA07-p22827857
Bn-A07-p21280027	0.993355482	0.990033223	Bn-N7-p24201869	Bn-chrA07-p22837720
Bn-A07-p21321699	1	0.990033223	Bn-N7-p24239989	Bn-chrA07-p22875152
Bn-A07-p21328168	1	0.995024876	Bn-N7-p24246510	Bn-chrA07-p22881633
Bn-A07-p21332273	0.983471074	0.702479339	Bn-N7-p24250622	Bn-chrA07-p22885731
Bn-A07-p21337094	0.983471074	0.983471074	Bn-N7-p24254437	Bn-chrA07-p22889570
Bn-A07-p21343543	1	1	Bn-N7-p24260809	Bn-chrA07-p22895947
Bn-A07-p21344843	0.975206612	0.975206612	Bn-N7-p24262085	Bn-chrA07-p22897238
Bn-A07-p21347324	0.958677686	0.966942149	Bn-N7-p24264598	Bn-chrA07-p22899721
Bn-A07-p21354084	0.991735537	0.917355372	Bn-N7-p24271350	Bn-chrC06-p35718425
Bn-A07-p21365616	0.917355372	0.991735537	Bn-Scaffold04407-p9	Bn-chrA07-p22920850
Bn-A07-p21366096	0.752066116	0.983471074	Bn-Scaffold04407-p5	Bn-chrA07-p22921330
Bn-A07-p21366215	0.917355372	0.958677686	Bn-Scaffold04407-p3	Bn-chrA07-p22921449
Bn-A07-p21371577	0.619834711	0.983471074	Bn-Scaffold27418-p2	Bn-chrA07-p22921934
Bn-A07-p21377298	0.396694215	0.966942149	Bn-Scaffold04386-p1	Bn-chrA07-p22927653
Bn-A07-p21395576	0.900826446	0.975206612	Bn-N16-p37733280	Bn-chrA07-p22945981
Bn-A07-p21400508	0.94214876	0.966942149	Bn-N16-p37738493	Bn-chrA07-p22950914

Bn-A07-p21404492	0.827242525	0.993355482	Bn-N16-p37742652	Bn-chrA07-p22954898
Bn-A07-p21406280	0.20661157	1	Bn-N7-p59390	Bn-chrA07-p22956684
Bn-A07-p21410292	0.870646766	0.910447761	Bn-N16-p37750449	Bn-chrA07-p22960604
Bn-A07-p21413042	0.890365449	0.980066445	Bn-N16-p37753254	Bn-chrA07-p22963334
Bn-A07-p21430149	0.624584718	0.734219269	Bn-Scaffold58184-p9	Bn-chrA07-p22979853
Bn-A07-p21434917	0.214876033	0.652892562	Unknown	Bn-chrA02-p8627618
Bn-A07-p21449796	0.774086379	0.953488372	Bn-N16-p37793518	Bn-chrA07-p22993905
Bn-A07-p21463060	0.950413223	0.958677686	Bn-N7-p24313232	Bn-chrA07-p23003258
Bn-A07-p21475300	0.970149254	0.980099502	Bn-N7-p24323648	Bn-chrA07-p23013692
Bn-A07-p21476982	0.991735537	0.991735537	Bn-N7-p24325175	Bn-chrA07-p23015373
Bn-A07-p21478337	0.958677686	0.958677686	Bn-N7-p24327080	Bn-chrA07-p23016728
Bn-A07-p21496070	0.903654485	0.903654485	Bn-N7-p24345207	Bn-chrA07-p23034887
Bn-A07-p21546078	0.884297521	0.884297521	Bn-N16-p37908466	Bn-chrC06-p35958058
Bn-A07-p21555527	0.966942149	0.975206612	Bn-N7-p24411667	Bn-chrA07-p23093446
Bn-A07-p21567127	0.991735537	0.991735537	Bn-N7-p24424123	Bn-chrA07-p23105042
Bn-A07-p21568086	0.58677686	0.991735537	Bn-N7-p24425050	Bn-chrA07-p23105997
Bn-A07-p21569289	0.966942149	0.975206612	Bn-N7-p24426258	Bn-chrA07-p23107202
Bn-A07-p21573107	0.991735537	1	Bn-N7-p24429698	Bn-chrA07-p23110821
Bn-A07-p21577164	0.976744186	0.973421927	Bn-N7-p24433733	Bn-chrA07-p23115057
Bn-A07-p21586613	1	1	Bn-N7-p24443471	Bn-chrA07-p23124464
Bn-A07-p21587819	0.991735537	1	Bn-N7-p24444677	Bn-chrA07-p23132023
Bn-A07-p21600829	0.996677741	0.996677741	Bn-N7-p24457796	Bn-chrA07-p23149328
Bn-A07-p21601293	0.983471074	0.991735537	Bn-N7-p24458260	Bn-chrA07-p23149792
Bn-A07-p21601441	0.966942149	0.94214876	Bn-N7-p24458408	Bn-chrA07-p23149942
Bn-A07-p21601511	0.94214876	0.950413223	Bn-N7-p24458478	Bn-chrA07-p23150013
Bn-A07-p21631043	0.917355372	0.917355372	Bn-N7-p24486028	Bn-chrA07-p23173894
Bn-A07-p21635264	0.975206612	0.975206612	Bn-N7-p24490300	Bn-chrA07-p23178166
Bn-A07-p21636048	0.983471074	0.983471074	Bn-N7-p24491084	Bn-chrA07-p23178950
Bn-A07-p21637923	0.996677741	0.996677741	Bn-N7-p24492964	Bn-chrA07-p23180830
Bn-A07-p21643815	0.983388704	0.983388704	Bn-N7-p24498837	Bn-chrA07-p23186703
Bn-A07-p21668460	0.983471074	0.983471074	Bn-N7-p24524198	Bn-chrA07-p23213705
Bn-A07-p21678440	0.855721393	0.855721393	Bn-N7-p24535423	Bn-chrA07-p23223874
Bn-A07-p21679566	0.996677741	0.996677741	Bn-N7-p24536529	Bn-chrA07-p23224980
Bn-A07-p21680952	0.966777409	0.996677741	Bn-N7-p24537914	Bn-chrA07-p23226365
Bn-A07-p21681332	0.958677686	0.958677686	Bn-N7-p24553714	Bn-chrA07-p23226744
Bn-A07-p21681736	0.991735537	0.991735537	Bn-N7-p24554119	Bn-chrA07-p23227149
Bn-A07-p21699999	0.94214876	0.94214876	Bn-N7-p24556771	Bn-chrA07-p23229605
Bn-A07-p21707007	0.958677686	0.958677686	Bn-N7-p24564042	Bn-chrA07-p23236875
Bn-A07-p21710786	0.966942149	0.966942149	Bn-N7-p24567837	Bn-chrA07-p23240670
Bn-A07-p21790459	0.986710963	0.986710963	Bn-N7-p24572249	Bn-chrA07-p23244500
Bn-A07-p21792030	0.986710963	0.986710963	Bn-N7-p24574746	Bn-chrC06-p36180803
Bn-A07-p21803967	0.980099502	0.980099502	Bn-N7-p24585512	Bn-chrA07-p23258814
Bn-A07-p21808739	0.970149254	0.965174129	Bn-N7-p24590257	Bn-chrA07-p23264738
Bn-A07-p21820686	1	0.990033223	Bn-N7-p24600079	Bn-chrA07-p23276083
Bn-A07-p21835729	0.995024876	0.995024876	Bn-N7-p24613096	Bn-chrA07-p23289070
Bn-A07-p21837742	1	1	Bn-N7-p24619288	Bn-chrA07-p23291083

Bn-A07-p21844673	0.993355482	0.996677741	Bn-N7-p24626217	Bn-chrA07-p23298011
Bn-A07-p21847025	0.991735537	0.991735537	Bn-N7-p24629225	Bn-chrA07-p23301019
Bn-A07-p21852154	0.990033223	0.990033223	Bn-N7-p24635452	Bn-chrA07-p23306482
Bn-A07-p21871055	1	1	Bn-N7-p24657914	Bn-chrA07_random-p205802
Bn-A07-p21875901	0.958677686	0.950413223	Bn-N7-p24662708	Bn-chrC02-p32644352
Bn-A07-p21895875	0.966942149	0.966942149	Bn-N7-p24676089	Bn-chrA07_random-p203288
Bn-A07-p21902068	0.975206612	0.975206612	Bn-N7-p24682266	Bn-chrA07_random-p202670
Bn-A07-p21907519	0.993355482	0.993355482	Bn-N7-p24688508	Bn-chrA07_random-p202101
Bn-A07-p21916201	0.990033223	0.993355482	Bn-N7-p24699466	Bn-chrA07_random-p200843
Bn-A07-p21918901	0.986710963	0.986710963	Bn-N7-p24702165	Bn-chrA07_random-p200573
Bn-A07-p21942650	0.995024876	0.995024876	Bn-N7-p24730179	Bn-chrA09-p15176035
Bn-A07-p21959231	1	1	Bn-N7-p24760074	Bn-chrA07-p23346953
Bn-A07-p21961987	0.966777409	0.966777409	Bn-N7-p24762479	Bn-chrA07-p23349355
Bn-A07-p21962752	0.990049751	0.990049751	Bn-N7-p24768075	Bn-chrA07-p23355328
Bn-A07-p21964088	0.996677741	0.77076412	Bn-N7-p24769411	Bn-chrA07-p23356664
Bn-A07-p21965076	0.986710963	0.986710963	Bn-N7-p24770398	Bn-chrA07-p23357650
Bn-A07-p21970228	1	1	Bn-N7-p24775530	Bn-chrA07-p23362781
Bn-A07-p21979205	0.859504132	0.859504132	Bn-N7-p24784384	Bn-chrA07-p23371817
Bn-A07-p21983885	0.996677741	0.993355482	Bn-N7-p24788202	Bn-chrA07-p23375635
Bn-A07-p21993375	0.983471074	0.991735537	Bn-N7-p24799309	Bn-chrA07-p23385157
Bn-A07-p21994050	0.990033223	0.990033223	Bn-N7-p24800048	Bn-chrA07-p23385896
Bn-A07-p21995054	0.983471074	0.983471074	Bn-N7-p24801058	Bn-chrA07-p23386906
Bn-A07-p22005058	0.980066445	0.966777409	Bn-N7-p24811031	Bn-chrA07-p23396901
Bn-A07-p22005392	0.990033223	0.993355482	Bn-N7-p24811365	Bn-chrA07-p23397235
Bn-A07-p22010671	0.965174129	0.960199005	Bn-N7-p24814522	Bn-chrA07-p23402491
Bn-A07-p22019732	0.983471074	0.20661157	Bn-N7-p24824180	Bn-chrA01-p11352611
Bn-A07-p22034462	0.990033223	0.990033223	Bn-N7-p24839696	Bn-chrA07-p23418664
Bn-A07-p22050904	0.983471074	0.975206612	Bn-N7-p24856389	Bn-chrA07-p23439394
Bn-A07-p22064576	0.990033223	0.990033223	Bn-N7-p24871290	Bn-chrA07-p23453898
Bn-A07-p22077654	0.917355372	0.917355372	Bn-N7-p24881990	Bn-chrA07-p23464598
Bn-A07-p22092610	0.995024876	0.995024876	Bn-N7-p24897405	Bn-chrA07-p23477661
Bn-A07-p22092775	0.995024876	0.995024876	Bn-N7-p24897570	Bn-chrA07-p23477826
Bn-A07-p22095943	0.960199005	0.960199005	Bn-N7-p24900757	Bn-chrA07-p23481012
Bn-A07-p22096180	0.965174129	0.965174129	Bn-N7-p24900987	Bn-chrA07-p23481242
Bn-A07-p22096241	0.990049751	0.990049751	Bn-N7-p24901048	Bn-chrA07-p23481303
Bn-A07-p22103902	0.975206612	0.975206612	Bn-N7-p24910676	Bn-chrA07-p23493169
Bn-A07-p22119502	0.975206612	0.966942149	Bn-N7-p24927237	Bn-chrA07-p23507364
Bn-A07-p22121193	0.986710963	0.990033223	Bn-N7-p24928886	Bn-chrA07-p23509014
Bn-A07-p22121263	0.975206612	0.983471074	Bn-N7-p24928956	Bn-chrA07-p23509084
Bn-A07-p22136602	0.990033223	0.986710963	Bn-N7-p24944312	Bn-chrA07-p23524107
Bn-A07-p22139480	0.990033223	0.990033223	Bn-N7-p24947975	Bn-chrA07-p23526985
Bn-A07-p22140320	0.993355482	0.990033223	Bn-N7-p24948807	Bn-chrA07-p23527825
Bn-A07-p22156609	0.990033223	0.990033223	Bn-N7-p24967569	Bn-chrA07-p23538535
Bn-A07-p22168947	0.985074627	0.980099502	Bn-N7-p24983502	Bn-chrA07-p23555826
Bn-A07-p22169573	0.990033223	0.993355482	Bn-N7-p24984126	Bn-chrA07-p23556452
Bn-A07-p22176569	0.990033223	0.990033223	Bn-N7-p24991322	Bn-chrA07-p23563531

Bn-A07-p22182943	0.983471074	0.983471074	Bn-N7-p25001413	Bn-chrA07-p23573485
Bn-A07-p22198393	0.991735537	0.991735537	Bn-N7-p25015613	Bn-chrA07-p23587684
Bn-A07-p22209952	0.980099502	0.980099502	Bn-N7-p25028678	Bn-chrA07-p23601101
Bn-A07-p22221286	0.958677686	0.958677686	Bn-N7-p25044100	Bn-chrA07-p23619281
Bn-A07-p22231852	0.96013289	0.950166113	Bn-N16-p38701396	Bn-chrA01_random-p38496
Bn-A07-p22237999	0.966942149	0.966942149	Bn-N7-p25062637	Bn-chrA07-p23636506
Bn-A07-p22238668	0.991735537	0.991735537	Bn-N7-p25063313	Bn-chrA07-p23637182
Bn-A07-p22250567	0.996677741	0.996677741	Bn-N7-p25075523	Bn-chrA07-p23649749
Bn-A07-p22251229	0.950248756	0.950248756	Bn-N7-p25076175	Bn-chrA07-p23650401
Bn-A07-p22251906	1	1	Bn-N7-p25076853	Bn-chrA07-p23651079
Bn-A07-p22253687	1	1	Bn-N7-p25078634	Bn-chrA07-p23652860
Bn-A07-p22282923	0.950413223	0.950413223	Bn-N16-p38774659	Bn-chrC06-p36835785
Bn-A07-p22302158	0.79338843	0.79338843	Bn-N7-p25126505	Bn-chrA07-p23698210
Bn-A07-p22306994	0.966942149	0.966942149	Bn-N7-p25131575	Bn-chrA07-p23703280
Bn-A07-p22307021	0.983471074	0.983471074	Bn-N7-p25131602	Bn-chrA07-p23703307
Bn-A07-p22308030	0.935323383	0.935323383	Bn-N7-p25133285	Bn-chrA07-p23705231
Bn-A07-p22309060	1	1	Bn-N7-p25134315	Bn-chrA07-p23706261
Bn-A07-p22309183	0.991735537	0.991735537	Bn-N7-p25134438	Bn-chrA07-p23706384
Bn-A07-p22310107	0.983471074	0.983471074	Bn-N7-p25135147	Bn-chrA07-p23707093
Bn-A07-p22329359	0.991735537	0.991735537	Bn-N7-p25157288	Bn-chrA07-p23730388
Bn-A07-p22362597	0.950166113	0.950166113	Bn-N7-p25187035	Bn-chrA07-p23759752
Bn-A07-p22382675	0.996677741	0.996677741	Bn-N7-p25210618	Bn-chrA07-p23779670
Bn-A07-p22398500	1	0.986710963	Bn-N7-p25229283	Bn-chrA07-p23796382
Bn-A07-p22399700	0.983471074	0.991735537	Bn-N7-p25230685	Bn-chrA07-p23797665
Bn-A07-p22412915	0.976744186	0.993355482	Bn-N7-p25247687	Bn-chrA07-p23811234
Bn-A07-p22440144	1	0.973421927	Bn-N7-p25284734	Bn-chrA07-p23846678
Bn-A07-p22474782	0.991735537	0.991735537	Bn-N7-p25323583	Bn-chrA07-p23885312
Bn-A07-p22476046	0.989189189	0.989189189	Bn-N5-p21597845	Bn-chrA05-p18797100
Bn-A07-p22484069	0.991735537	0.991735537	Bn-Scaffold22667-p4	Bn-chrA07-p23891650
Bn-A07-p22488507	0.996677741	1	Bn-N7-p25334331	Bn-chrA07-p23896065
Bn-A07-p22493972	0.983471074	0.991735537	Bn-N7-p25339799	Bn-chrA07-p23901529
Bn-A07-p22510199	0.983471074	0.983471074	Bn-N7-p25356625	Bn-chrA07-p23918146
Bn-A07-p22510892	0.975206612	0.975206612	Bn-N7-p25357318	Bn-chrA07-p23918839
Bn-A07-p22513026	0.933884298	0.933884298	Bn-N7-p25369605	Bn-chrA07-p23927029
Bn-A07-p22525803	1	0.991735537	Bn-N7-p25392856	Bn-chrA07-p23947641
Bn-A07-p22542288	0.991735537	1	Bn-N7-p25401372	Bn-chrA07-p23956721
Bn-A07-p22575730	0.983471074	0.975206612	Bn-N7-p25437729	Bn-chrA07-p23988307
Bn-A07-p22586713	1	1	Bn-N7-p25457006	Bn-chrA07-p24005201
Bn-A08-p165219	0.950166113	0.950166113	Bn-N8-p295324	Bn-chrA08-p212846
Bn-A08-p177288	0.960199005	0.960199005	Bn-N8-p302806	Bn-chrA08-p220107
Bn-A08-p201236	0.986710963	0.986710963	Bn-N8-p326345	Bn-chrA08-p243516
Bn-A08-p205262	0.996677741	0.996677741	Bn-N8-p330395	Bn-chrA08-p248608
Bn-A08-p211090	0.92039801	0.92039801	Bn-N13-p67517978	Bn-chrC03-p60317095
Bn-A08-p264483	0.996677741	0.993355482	Bn-N8-p398876	Bn-chrA08_random-p177302
Bn-A08-p279186	0.991735537	0.991735537	Bn-N8-p416001	Bn-chrAnn_random-p318303
Bn-A08-p293480	0.980099502	0.980099502	Bn-N13-p67434994	Bn-chrC03-p60268013

Bn-A08-p345270	0.983471074	0.983471074	Bn-N8-p513642	Bn-chrA08-p288777
Bn-A08-p350287	0.996677741	0.996677741	Bn-N8-p518715	Bn-chrA08-p293851
Bn-A08-p355936	0.996677741	0.996677741	Bn-N8-p523067	Bn-chrA08-p298267
Bn-A08-p358866	0.986710963	0.986710963	Bn-N8-p525997	Bn-chrA08-p301197
Bn-A08-p359000	0.986710963	0.986710963	Bn-N8-p526131	Bn-chrA08-p301331
Bn-A08-p359084	0.958677686	0.958677686	Bn-N8-p526215	Bn-chrA08-p301415
Bn-A08-p359138	0.975206612	0.975206612	Bn-N8-p526269	Bn-chrA08-p301469
Bn-A08-p359210	0.975206612	0.975206612	Bn-N8-p526341	Bn-chrA08-p301541
Bn-A08-p359894	0.986710963	0.986710963	Bn-N8-p527025	Bn-chrA08-p302225
Bn-A08-p360373	0.983471074	0.983471074	Bn-N8-p527504	Bn-chrA08-p302704
Bn-A08-p360868	0.983471074	0.983471074	Bn-N8-p527999	Bn-chrA08-p303199
Bn-A08-p360902	0.983471074	0.983471074	Bn-N8-p528033	Bn-chrA08-p303233
Bn-A08-p361063	0.990033223	0.990033223	Bn-N8-p528194	Bn-chrA08-p303394
Bn-A08-p361637	0.983471074	0.983471074	Bn-N8-p528768	Bn-chrA08-p303968
Bn-A08-p361910	0.991735537	0.991735537	Bn-N8-p529041	Bn-chrA08-p304241
Bn-A08-p363107	0.980099502	0.980099502	Bn-N8-p530239	Bn-chrA08-p305439
Bn-A08-p369443	0.991735537	0.991735537	Bn-N8-p535838	Bn-chrA08-p311285
Bn-A08-p372048	0.991735537	0.991735537	Bn-N8-p538443	Bn-chrA08-p313890
Bn-A08-p423689	1	1	Bn-N8-p598765	Bn-chrA08-p375344
Bn-A08-p424009	0.996677741	0.996677741	Bn-N8-p599085	Bn-chrA08-p375664
Bn-A08-p478696	0.950413223	0.950413223	Bn-N8-p655202	Bn-chrAnn_random-p361801
Bn-A08-p482401	0.960199005	0.960199005	Bn-N8-p658897	Bn-chrA08-p414201
Bn-A08-p482505	1	1	Bn-N8-p659001	Bn-chrA08-p414305
Bn-A08-p501373	0.958677686	0.958677686	Bn-N8-p675125	Bn-chrA08-p430892
Bn-A08-p510718	0.993355482	0.993355482	Bn-N8-p684478	Bn-chrA08-p440245
Bn-A08-p523111	0.983471074	0.983471074	Bn-N8-p696829	Bn-chrAnn_random-p437104
Bn-A08-p533195	0.991735537	0.991735537	Bn-N8-p706919	Bn-chrA08-p462653
Bn-A08-p537474	0.958677686	0.958677686	Bn-N8-p711266	Bn-chrA08-p469043
Bn-A08-p543480	0.991735537	0.925619835	Bn-N8-p718156	Bn-chrC03-p59834939
Bn-A08-p570304	0.996677741	0.996677741	Bn-N8-p758575	Bn-chrA08-p518354
Bn-A08-p572531	0.92039801	0.92039801	Bn-N13-p66990904	Bn-chrC03-p59797624
Bn-A08-p575482	0.966942149	0.966942149	Bn-N8-p763823	Bn-chrA08-p523601
Bn-A08-p626649	0.970099668	0.973421927	Bn-N13-p66924325	Bn-chrC03-p59727643
Bn-A08-p657830	0.958677686	0.966942149	Bn-N8-p841969	Bn-chrA08-p598224
Bn-A08-p663199	0.950413223	0.958677686	Bn-N8-p864324	Bn-chrA08-p607207
Bn-A08-p694721	0.983471074	0.983471074	Bn-N8-p900685	Bn-chrA08-p633970
Bn-A08-p697434	0.983471074	0.983471074	Bn-N8-p903392	Bn-chrA08-p636677
Bn-A08-p741480	0.966942149	0.966942149	Bn-N8-p969301	Bn-chrA08_random-p16939
Bn-A08-p742965	0.983471074	0.983471074	Bn-N8-p970786	Bn-chrA08_random-p15454
Bn-A08-p743985	0.991735537	0.991735537	Bn-N8-p971806	Bn-chrA08_random-p14434
Bn-A08-p744725	1	1	Bn-N8-p972494	Bn-chrA08_random-p13746
Bn-A08-p744837	1	1	Bn-N8-p972606	Bn-chrA08_random-p13634
Bn-A08-p745168	0.983471074	0.983471074	Bn-N8-p972937	Bn-chrA08_random-p13303
Bn-A08-p754670	0.955223881	0.955223881	Bn-N8-p982442	Bn-chrA08_random-p3533
Bn-A08-p758590	0.995024876	0.975124378	Bn-N8-p986604	Bn-chrCnn_random-p259004
Bn-A08-p758977	0.993355482	0.817275748	Bn-N8-p986992	Bn-chrCnn_random-p259008

Bn-A08-p782784	0.983388704	0.983388704	Bn-N8-p1011139	Bn-chrA08-p710788
Bn-A08-p792162	0.983471074	0.983471074	Bn-N8-p1020964	Bn-chrA08-p720617
Bn-A08-p797453	0.996677741	0.996677741	Bn-N8-p1026397	Bn-chrA08-p726131
Bn-A08-p798867	1	1	Bn-N8-p1027811	Bn-chrA08-p727545
Bn-A08-p798970	1	1	Bn-N8-p1027914	Bn-chrA08-p727648
Bn-A08-p813316	0.965174129	0.970149254	Bn-N8-p1047794	Bn-chrA08-p741721
Bn-A08-p813510	0.960199005	0.965174129	Bn-N8-p1047988	Bn-chrA08-p741913
Bn-A08-p814234	0.973421927	0.980066445	Bn-N8-p1048712	Bn-chrA08-p742639
Bn-A08-p836396	0.996677741	0.996677741	Bn-N8-p1063418	Bn-chrA08-p759849
Bn-A08-p848067	0.996677741	0.996677741	Bn-N8-p1075920	Bn-chrA08-p771878
Bn-A08-p913985	0.983388704	0.983388704	Bn-N8-p1116507	Bn-chrA08-p826317
Bn-A08-p917004	0.970099668	0.973421927	Bn-N8-p1121008	Bn-chrA08-p830822
Bn-A08-p934417	0.975206612	0.966942149	Bn-N8-p1135564	Bn-chrA08-p851079
Bn-A08-p937299	0.96345515	0.976744186	Bn-N8-p1138446	Bn-chrA08-p853960
Bn-A08-p940619	0.966942149	0.958677686	Bn-N8-p1141887	Bn-chrA08-p857227
Bn-A08-p980768	0.993355482	0.993355482	Bn-N8-p1189412	Bn-chrA08-p898625
Bn-A08-p1002560	0.983471074	0.983471074	Bn-N8-p1212403	Bn-chrA08-p925689
Bn-A08-p1007687	0.975206612	0.975206612	Bn-N8-p1217690	Bn-chrA08-p930811
Bn-A08-p1010548	0.983471074	0.983471074	Bn-N8-p1220551	Bn-chrA08-p933672
Bn-A08-p1022454	0.94214876	0.958677686	Bn-N8-p1229567	Bn-chrA08-p943638
Bn-A08-p1048096	0.966942149	0.958677686	Bn-N8-p1250952	Bn-chrA08-p963670
Bn-A08-p1049012	0.958677686	0.950413223	Bn-N8-p1251878	Bn-chrA08-p964586
Bn-A08-p1059680	0.94214876	0.950413223	Bn-N8-p1259765	Bn-chrA08-p975254
Bn-A08-p1059858	0.983471074	0.975206612	Bn-N8-p1259943	Bn-chrA08-p975432
Bn-A08-p1059966	0.958677686	0.966942149	Bn-N8-p1260051	Bn-chrA08-p975540
Bn-A08-p1068698	0.991735537	0.983471074	Bn-N8-p1270011	Bn-chrA08-p984274
Bn-A08-p1077419	0.980392157	0.986928105	Bn-N8-p1277816	Bn-chrA08-p993141
Bn-A08-p1077573	0.990049751	0.990049751	Bn-N8-p1277970	Bn-chrA08-p993295
Bn-A08-p1084991	0.955223881	0.970149254	Bn-N3-p34880873	Bn-chrC08-p22791979
Bn-A08-p1088499	0.960199005	0.950248756	Bn-N8-p1284504	Bn-chrA08-p999829
Bn-A08-p1088701	0.980099502	0.980099502	Bn-N8-p1284706	Bn-chrA08-p1000028
Bn-A08-p1089123	0.970149254	0.975124378	Bn-N8-p1285128	Bn-chrA08-p1000450
Bn-A08-p1123899	0.993355482	0.990033223	Bn-N8-p1298113	Bn-chrA08-p1012939
Bn-A08-p1136041	0.970149254	0.970149254	Bn-N8-p1301498	Bn-chrA08-p1016504
Bn-A08-p1140651	0.950413223	0.950413223	Bn-N8-p1306069	Bn-chrA08-p1021075
Bn-A08-p1151082	0.966942149	0.966942149	Bn-N8-p1314755	Bn-chrA08-p1030695
Bn-A08-p1166566	0.965174129	0.990049751	Bn-N8-p1331310	Bn-chrA08-p1062877
Bn-A08-p1175573	0.980066445	0.980066445	Bn-N8-p1340230	Bn-chrC03-p58759773
Bn-A08-p1308299	0.966942149	0.966942149	Bn-N5-p9599002	Bn-chrA05-p8611645
Bn-A08-p1313629	0.975124378	0.975124378	Bn-N5-p9593477	Bn-chrA05-p8606121
Bn-A08-p1319548	0.991735537	0.991735537	Bn-N5-p9587568	Bn-chrA05-p8600212
Bn-A08-p1319949	0.983471074	0.983471074	Bn-N5-p9587167	Bn-chrA05-p8599811
Bn-A08-p1324618	1	1	Bn-N5-p9579172	Bn-chrA05-p8589709
Bn-A08-p1325427	1	1	Bn-N5-p9578359	Bn-chrA05-p8588896
Bn-A08-p1332628	0.983471074	0.983471074	Bn-N5-p9571193	Bn-chrA05-p8581731
Bn-A08-p1336421	0.993355482	0.993355482	Bn-N5-p9565508	Bn-chrA05-p8576514

Bn-A08-p1341937	0.966942149	0.966942149	Bn-N5-p9564197	Bn-chrA05-p8575203
Bn-A08-p1342021	0.980099502	0.980099502	Bn-N5-p9564113	Bn-chrA05-p8575119
Bn-A08-p1353718	0.983471074	0.983471074	Bn-N5-p9550657	Bn-chrA05-p8558134
Bn-A08-p1357142	0.983471074	0.983471074	Bn-N5-p9548694	Bn-chrA05-p8556171
Bn-A08-p1364252	0.983471074	0.983471074	Bn-N5-p9541711	Bn-chrA05-p8549188
Bn-A08-p1365508	0.983471074	0.983471074	Bn-N5-p9540466	Bn-chrA05-p8547954
Bn-A08-p1365676	0.983471074	0.983471074	Bn-N5-p9540298	Bn-chrA05-p8547786
Bn-A08-p1369206	0.996677741	0.996677741	Bn-N5-p9527984	Bn-chrAnn_random-p361135
Bn-A08-p1379662	0.983471074	0.983471074	Bn-N5-p9515624	Bn-chrA05-p8524697
Bn-A08-p1379663	0.983471074	0.983471074	Bn-N5-p9515623	Bn-chrA05-p8524696
Bn-A08-p1379996	0.975206612	0.975206612	Bn-N5-p9515292	Bn-chrA05-p8524365
Bn-A08-p1382630	0.975206612	0.975206612	Bn-N5-p9512670	Bn-chrA05-p8521742
Bn-A08-p1400360	1	1	Bn-N5-p9491353	Bn-chrA05-p8494014
Bn-A08-p1404321	0.975124378	0.975124378	Bn-N5-p9487392	Bn-chrA05-p8490053
Bn-A08-p1404439	0.960199005	0.960199005	Bn-N5-p9487275	Bn-chrA05-p8489936
Bn-A08-p1430241	0.955223881	0.995024876	Bn-N5-p9479293	Bn-chrA05-p8481940
Bn-A08-p1506977	0.985074627	0.985074627	Bn-N2-p22485729	Bn-chrA02-p20036272
Bn-A08-p1514861	1	1	Bn-N2-p22497937	Bn-chrA02-p20023549
Bn-A08-p1554055	0.986710963	0.986710963	Bn-N2-p22542153	Bn-chrA02-p19982614
Bn-A08-p1581891	0.993355482	0.993355482	Bn-N2-p22571764	Bn-chrA02-p19949692
Bn-A08-p1584454	0.975206612	0.975206612	Bn-N2-p22574322	Bn-chrA02-p19947136
Bn-A08-p1584455	0.985074627	0.985074627	Bn-N2-p22574322	Bn-chrA02-p19947136
Bn-A08-p1611335	0.985074627	0.985074627	Bn-N2-p22595630	Bn-chrA02-p19925407
Bn-A08-p1645510	1	0.950166113	Bn-N8-p1500819	Bn-chrA08-p1271855
Bn-A08-p1720366	0.975124378	0.980099502	Bn-N8-p1579503	Bn-chrA08-p1360120
Bn-A08-p1786408	0.989583333	0.989583333	Bn-N8-p1659176	Bn-chrA08-p1433573
Bn-A08-p1809204	0.985074627	0.985074627	Bn-N8-p1682130	Bn-chrA08-p1454163
Bn-A08-p1847328	0.990049751	0.990049751	Bn-N8-p1726629	Bn-chrA08-p1497364
Bn-A08-p1880862	0.521594684	0.521594684	Bn-N8-p1772680	Bn-chrA08-p1538262
Bn-A08-p1928132	0.791044776	0.781094527	Bn-N7-p15088627	Bn-chrCnn_random-p116715
Bn-A08-p2184922	0.684385382	0.720930233	Bn-Scaffold78005-p1	Bn-chrA06_random-p167246
Bn-A08-p2274232	0.990049751	0.990049751	Bn-N8-p2133848	Bn-chrA08-p1778992
Bn-A08-p2275410	0.970149254	0.965174129	Bn-N8-p2135016	Bn-chrA08-p1780160
Bn-A08-p2371175	0.991735537	0.991735537	Bn-N8-p2230272	Bn-chrA08_random-p397014
Bn-A08-p2389244	0.950248756	0.950248756	Bn-N8-p2253088	Bn-chrA08-p1839805
Bn-A08-p2394252	0.724252492	0.724252492	Bn-N8-p2255128	Bn-chrA08-p1841846
Bn-A08-p2397885	0.818181818	0.818181818	Bn-N8-p2257095	Bn-chrA08-p1843813
Bn-A08-p2406380	0.991735537	0.991735537	Bn-N8-p2266693	Bn-chrA08-p1852692
Bn-A08-p2415846	0.950413223	0.94214876	Bn-N8-p2281308	Bn-chrA08-p1865603
Bn-A08-p2425057	0.975124378	0.915422886	Bn-N8-p2286125	Bn-chrC08-p2028358
Bn-A08-p2425147	0.990049751	0.965174129	Bn-N8-p2286215	Bn-chrC08-p2028449
Bn-A08-p2425488	0.990049751	0.990049751	Bn-N8-p2286556	Bn-chrC08-p2023563
Bn-A08-p2433393	0.958677686	0.958677686	Bn-N8-p2290308	Bn-chrA08-p1876758
Bn-A08-p2448353	0.991735537	0.991735537	Bn-N8-p2313957	Bn-chrA08-p1901489
Bn-A08-p2451003	0.950413223	0.950413223	Bn-N8-p2316254	Bn-chrA08-p1903764
Bn-A08-p2451084	0.975206612	0.975206612	Bn-N8-p2316334	Bn-chrA08-p1903844

Bn-A08-p2451091	0.958677686	0.958677686	Bn-N8-p2316341	Bn-chrA08-p1903851
Bn-A08-p2461697	0.986710963	0.986710963	Bn-N8-p2321801	Bn-chrA08-p1909311
Bn-A08-p2509113	0.966942149	0.966942149	Bn-N8-p2350131	Bn-chrAnn_random-p287953
Bn-A08-p2511340	0.975206612	0.975206612	Bn-N8-p2353077	Bn-chrA08-p1940818
Bn-A08-p2512020	1	1	Bn-N8-p2353759	Bn-chrA08-p1941499
Bn-A08-p2512477	0.983471074	0.983471074	Bn-N8-p2354217	Bn-chrA08-p1941957
Bn-A08-p2512893	0.990033223	0.990033223	Bn-Scaffold03669-p6	Bn-chrA08-p1942357
Bn-A08-p2517407	0.991735537	0.991735537	Bn-N8-p2359563	Bn-chrA08-p1952315
Bn-A08-p2519736	0.983471074	0.983471074	Bn-N8-p2361866	Bn-chrA08-p1954618
Bn-A08-p2520421	0.991735537	0.991735537	Bn-N8-p2363297	Bn-chrA08-p1955824
Bn-A08-p2531081	0.917355372	0.917355372	Bn-N8-p2373808	Bn-chrA08-p1966333
Bn-A08-p2533244	0.983471074	0.983471074	Bn-N8-p2377256	Bn-chrA08-p1969307
Bn-A08-p2534474	0.945273632	0.945273632	Bn-N8-p2378485	Bn-chrA08-p1970536
Bn-A08-p2536015	0.983388704	0.983388704	Bn-N8-p2380024	Bn-chrA08-p1972075
Bn-A08-p2543882	0.996677741	0.996677741	Bn-N8-p2386707	Bn-chrA08-p1979050
Bn-A08-p2555365	0.983471074	0.983471074	Bn-N8-p2398991	Bn-chrA08-p1992159
Bn-A08-p2561457	0.975124378	0.975124378	Bn-N8-p2405056	Bn-chrA08-p1997675
Bn-A08-p2564131	0.973421927	0.973421927	Bn-N8-p2407710	Bn-chrA08-p2000329
Bn-A08-p2574453	0.983471074	0.983471074	Bn-N8-p2428978	Bn-chrA08-p2012765
Bn-A08-p2576185	0.986710963	0.986710963	Bn-N8-p2430973	Bn-chrA08-p2014693
Bn-A08-p2585031	1	1	Bn-N8-p2437357	Bn-chrA08-p2021230
Bn-A08-p2585836	0.986710963	0.986710963	Bn-N8-p2438159	Bn-chrA08-p2022032
Bn-A08-p2589956	0.990033223	0.990033223	Bn-N8-p2442184	Bn-chrA08-p2026056
Bn-A08-p2590415	0.990033223	0.990033223	Bn-N8-p2442643	Bn-chrA08-p2026515
Bn-A08-p2601211	0.694352159	0.694352159	Bn-N8-p2460266	Bn-chrA08-p2042306
Bn-A08-p2605912	0.993355482	0.993355482	Bn-N8-p2465138	Bn-chrA08-p2046878
Bn-A08-p2618037	0.980099502	0.980099502	Bn-N8-p2477438	Bn-chrA08-p2059177
Bn-A08-p2630666	0.993355482	0.993355482	Bn-N8-p2485755	Bn-chrA08-p2067195
Bn-A08-p2660414	0.975206612	0.975206612	Bn-N8-p2507725	Bn-chrA08-p2088727
Bn-A08-p2675098	0.983471074	0.983471074	Bn-N8-p2517806	Bn-chrA08-p2098808
Bn-A08-p2689572	0.993355482	0.993355482	Bn-N8-p2543342	Bn-chrA08-p2123348
Bn-A08-p2694807	0.986710963	0.986710963	Bn-N8-p2548604	Bn-chrA08-p2128579
Bn-A08-p2696389	0.986710963	0.986710963	Bn-N8-p2550186	Bn-chrA08-p2130161
Bn-A08-p2711497	0.983471074	0.983471074	Bn-N8-p2573766	Bn-chrA08-p2151792
Bn-A08-p2712092	0.983471074	0.983471074	Bn-N8-p2574361	Bn-chrA08-p2152387
Bn-A08-p2715086	0.950413223	0.950413223	Bn-N8-p2577309	Bn-chrA08-p2155338
Bn-A08-p2728670	0.853820598	0.853820598	Bn-N8-p2600395	Bn-chrA08-p2177837
Bn-A08-p2740174	0.991735537	0.991735537	Bn-N8-p2610879	Bn-chrA08-p2187982
Bn-A08-p2741041	0.975206612	0.975206612	Bn-N8-p2611745	Bn-chrC08-p2497899
Bn-A08-p2749328	0.958677686	0.958677686	Bn-N8-p2620476	Bn-chrA08-p2197580
Bn-A08-p2749414	0.966942149	0.966942149	Bn-N8-p2620562	Bn-chrA08-p2197666
Bn-A08-p2757340	0.983471074	0.983471074	Bn-N8-p2628786	Bn-chrA08-p2205791
Bn-A08-p2771143	0.983471074	0.991735537	Bn-N8-p2641528	Bn-chrA08-p2221400
Bn-A08-p2819820	0.975206612	0.983471074	Bn-N8-p2680032	Bn-chrA08-p2266423
Bn-A08-p2822224	0.976744186	0.990033223	Bn-Scaffold04146-p1	Bn-chrA08-p2268802
Bn-A08-p2823223	0.991735537	1	Bn-Scaffold04146-p9	Bn-chrA08-p2269787

Bn-A08-p2841059	0.996677741	1	Bn-N8-p2715964	Bn-chrA08_random-p423550
Bn-A08-p2841643	0.990033223	0.993355482	Bn-N8-p2716550	Bn-chrA08_random-p424135
Bn-A08-p2883203	0.975206612	0.966942149	Bn-N8-p2774613	Bn-chrA08-p2342640
Bn-A08-p2885855	0.991735537	0.983471074	Bn-N8-p2777219	Bn-chrA08-p2344437
Bn-A08-p2897827	0.995024876	0.995024876	Bn-N8-p2784310	Bn-chrA08-p2351520
Bn-A08-p2898446	0.990049751	0.990049751	Bn-N8-p2784929	Bn-chrA08-p2352139
Bn-A08-p2910044	0.991735537	0.991735537	Bn-N8-p2793274	Bn-chrA08-p2365456
Bn-A08-p2910816	0.966942149	0.966942149	Bn-N8-p2794047	Bn-chrA08-p2366229
Bn-A08-p2911101	0.991735537	0.991735537	Bn-N8-p2794332	Bn-chrA08-p2366514
Bn-A08-p2920715	0.966942149	0.966942149	Bn-N8-p2803775	Bn-chrA08-p2375846
Bn-A08-p2922263	0.983471074	0.983471074	Bn-N8-p2805333	Bn-chrA08-p2377403
Bn-A08-p2942782	0.983471074	0.983471074	Bn-N8-p2821958	Bn-chrA08-p2401941
Bn-A08-p2973537	0.990033223	0.993355482	Bn-N8-p2854610	Bn-chrA08-p2445566
Bn-A08-p2989598	1	1	Bn-N8-p2871305	Bn-chrA08-p2473984
Bn-A08-p2995827	0.991735537	0.991735537	Bn-N8-p2874122	Bn-chrA08-p2476800
Bn-A08-p2997093	0.837209302	0.65448505	Bn-N18-p17960198	Bn-chrC06-p18674232
Bn-A08-p3031902	0.958677686	0.958677686	Bn-N8-p2911497	Bn-chrA08-p2513456
Bn-A08-p3064639	0.975206612	0.975206612	Bn-N8-p2941458	Bn-chrA08-p2545051
Bn-A08-p3068539	0.986710963	0.990033223	Bn-N8-p2945356	Bn-chrA08-p2548948
Bn-A08-p3068838	0.966942149	0.966942149	Bn-N8-p2945655	Bn-chrA08-p2549247
Bn-A08-p3079647	0.983471074	0.983471074	Bn-N8-p2959348	Bn-chrA08-p2566406
Bn-A08-p3086727	1	1	Bn-N8-p2963751	Bn-chrA08-p2570810
Bn-A08-p3090858	0.991735537	0.991735537	Bn-N8-p2967886	Bn-chrA08-p2574945
Bn-A08-p3097626	0.990033223	0.990033223	Bn-N8-p2975658	Bn-chrA08-p2582697
Bn-A08-p3104517	0.991735537	0.991735537	Bn-N8-p2983255	Bn-chrA08-p2590294
Bn-A08-p3109145	0.993939394	0.993939394	Bn-N8-p2988031	Bn-chrA08-p2594928
Bn-A08-p3109726	0.990033223	0.990033223	Bn-N8-p2988612	Bn-chrA08-p2595509
Bn-A08-p3120976	0.993355482	0.993355482	Bn-N8-p3000167	Bn-chrA08-p2607542
Bn-A08-p3120986	0.995024876	0.995024876	Bn-N8-p3000176	Bn-chrA08-p2607551
Bn-A08-p3121492	1	1	Bn-N8-p3000683	Bn-chrA08-p2608058
Bn-A08-p3138888	0.990033223	0.990033223	Bn-N8-p3019324	Bn-chrA08-p2619120
Bn-A08-p3161353	0.975206612	0.975206612	Bn-N8-p3040867	Bn-chrA08-p2638396
Bn-A08-p3168874	1	1	Bn-N8-p3048721	Bn-chrA08-p2646273
Bn-A08-p3178977	0.966942149	0.966942149	Bn-N8-p3061223	Bn-chrA08-p2658775
Bn-A08-p3182594	0.975206612	0.975206612	Bn-N8-p3069193	Bn-chrA08-p2666735
Bn-A08-p3182798	0.975206612	0.975206612	Bn-N8-p3069397	Bn-chrA08-p2666939
Bn-A08-p3184233	0.890547264	0.890547264	Bn-N8-p3070767	Bn-chrA08-p2668309
Bn-A08-p3193662	0.975124378	0.975124378	Bn-N8-p3085952	Bn-chrA08-p2683494
Bn-A08-p3195277	0.966942149	0.966942149	Bn-N18-p3962969	Bn-chrC08_random-p955405
Bn-A08-p3221528	0.983471074	0.983471074	Bn-N8-p3100821	Bn-chrA08-p2698798
Bn-A08-p3223002	0.996677741	0.996677741	Bn-N8-p3102300	Bn-chrA08-p2700276
Bn-A08-p3235095	0.966942149	0.966942149	Bn-N8-p3113895	Bn-chrA08-p2712346
Bn-A08-p3237433	0.983471074	0.983471074	Bn-N8-p3116233	Bn-chrA08-p2714684
Bn-A08-p3246905	1	1	Bn-N8-p3125777	Bn-chrA08-p2724399
Bn-A08-p3269074	0.958677686	0.958677686	Bn-N8-p3150393	Bn-chrA08-p2746292
Bn-A08-p3269802	0.975206612	0.975206612	Bn-N8-p3151120	Bn-chrA08-p2747019

Bn-A08-p3270423	0.94214876	0.94214876	Bn-N8-p3151740	Bn-chrA08-p2747639
Bn-A08-p3270600	0.991735537	0.991735537	Bn-N8-p3151917	Bn-chrA08-p2747816
Bn-A08-p3270802	0.966942149	0.966942149	Bn-N8-p3152119	Bn-chrA08-p2748018
Bn-A08-p3284482	0.966942149	0.966942149	Bn-N8-p3166259	Bn-chrA08-p2761828
Bn-A08-p3284666	1	1	Bn-N8-p3166443	Bn-chrA08-p2762012
Bn-A08-p3284817	1	1	Bn-N8-p3166594	Bn-chrA08-p2762163
Bn-A08-p3284955	0.980066445	0.980066445	Bn-N8-p3166732	Bn-chrA08-p2762301
Bn-A08-p3285855	0.933884298	0.933884298	Bn-N8-p3167625	Bn-chrA08-p2763194
Bn-A08-p3298985	0.983388704	0.983388704	Bn-N8-p3183009	Bn-chrA08-p2778155
Bn-A08-p3302700	0.983471074	0.983471074	Bn-N8-p3186724	Bn-chrA08-p2781870
Bn-A08-p3336620	0.991735537	1	Bn-N8-p3220059	Bn-chrA08-p2816817
Bn-A08-p3388906	0.991735537	1	Bn-N8-p3234254	Bn-chrAnn_random-p176756
Bn-A08-p3433222	1	0.993355482	Bn-N8-p3285328	Bn-chrA08-p2845367
Bn-A08-p3433878	0.983471074	0.991735537	Bn-N8-p3285969	Bn-chrA08-p2846024
Bn-A08-p3436359	0.975206612	0.966942149	Bn-N8-p3288453	Bn-chrA08-p2848507
Bn-A08-p3444437	0.970099668	0.96345515	Bn-N8-p3296567	Bn-chrA08-p2856586
Bn-A08-p3464671	0.991735537	1	Bn-N8-p3307360	Bn-chrA08_random-p441390
Bn-A08-p3518817	0.995024876	0.995024876	Bn-N8-p3332340	Bn-chrA08-p2901935
Bn-A08-p3519373	0.983471074	0.983471074	Bn-N8-p3332899	Bn-chrA08-p2902566
Bn-A08-p3523325	0.996677741	0.986710963	Bn-N8-p3337669	Bn-chrA08-p2906735
Bn-A08-p3529278	0.975206612	0.983471074	Bn-N8-p3340392	Bn-chrA08-p2909457
Bn-A08-p3529542	0.991735537	0.983471074	Bn-N8-p3340656	Bn-chrA08-p2909721
Bn-A08-p3530401	0.993355482	0.990033223	Bn-N8-p3341516	Bn-chrA08-p2910580
Bn-A08-p3531112	1	0.996677741	Bn-N8-p3342227	Bn-chrA08-p2911291
Bn-A08-p3536202	0.993355482	0.970099668	Bn-N8-p3347321	Bn-chrA08-p2916374
Bn-A08-p3546013	0.965174129	0.960199005	Bn-N8-p3357184	Bn-chrA08-p2926145
Bn-A08-p3549966	1	1	Bn-N8-p3361108	Bn-chrA08-p2930069
Bn-A08-p3566821	0.975206612	0.966942149	Bn-N8-p3371674	Bn-chrA08-p2940877
Bn-A08-p3573966	0.975206612	0.983471074	Bn-N8-p3384569	Bn-chrA08-p2951513
Bn-A08-p3574400	0.983471074	0.991735537	Bn-N8-p3385002	Bn-chrA08-p2951947
Bn-A08-p3609963	0.980099502	0.985074627	Bn-N8-p3437592	Bn-chrA08-p2989134
Bn-A08-p3614725	0.983471074	0.991735537	Bn-N8-p3442362	Bn-chrAnn_random-p436370
Bn-A08-p3614809	0.991735537	0.983471074	Bn-N8-p3443036	Bn-chrAnn_random-p436369
Bn-A08-p3625318	0.993355482	0.990033223	Bn-N8-p3461825	Bn-chrA08-p3005198
Bn-A08-p3628921	0.990033223	0.900332226	Bn-N8-p3465024	Bn-chrC08-p4688422
Bn-A08-p3737287	0.995024876	1	Bn-N8-p3558110	Bn-chrC08-p5021463
Bn-A08-p3766285	0.990049751	0.990049751	Bn-N8-p3583715	Bn-chrA08-p3126016
Bn-A08-p3814869	0.993355482	0.993355482	Bn-N9-p19734875	Bn-chrAnn_random-p184964
Bn-A08-p3818854	0.986710963	0.986710963	Bn-N9-p19738842	Bn-chrAnn_random-p185359
Bn-A08-p3821120	0.983471074	0.991735537	Bn-Scaffold04750-p4	Bn-chrA08-p3204138
Bn-A08-p3822514	0.991735537	0.991735537	Bn-N8-p3676384	Bn-chrA08-p3205549
Bn-A08-p3840267	0.991735537	1	Bn-N8-p3739128	Bn-chrA08-p3271391
Bn-A08-p3840983	0.900826446	0.892561983	Bn-N8-p3739852	Bn-chrA08-p3272122
Bn-A08-p3846272	0.980099502	0.985074627	Bn-N8-p3749216	Bn-chrA08-p3277674
Bn-A08-p3862608	0.958677686	0.958677686	Bn-N8-p3759596	Bn-chrA08-p3293303
Bn-A08-p3874411	0.958677686	0.991735537	Bn-Scaffold00637-p1	Bn-chrA10-p4337041

Bn-A08-p3875206	0.910447761	0.970149254	Bn-Scaffold00637-p1	Bn-chrA10-p4337830
Bn-A08-p3881061	0.946843854	0.990033223	Bn-Scaffold00637-p1	Bn-chrA10-p4347519
Bn-A08-p3881337	0.900826446	0.975206612	Bn-Scaffold00637-p1	Bn-chrA10-p4347795
Bn-A08-p3881526	0.860465116	0.993355482	Bn-Scaffold00637-p1	Bn-chrA10-p4347984
Bn-A08-p3883962	0.917355372	0.983471074	Bn-N19-p18840612	Bn-chrA10-p4355258
Bn-A08-p3893922	0.94214876	0.983471074	Bn-Scaffold00637-p1	Bn-chrA10-p4358401
Bn-A08-p3894164	0.644628099	0.975206612	Bn-Scaffold00637-p1	Bn-chrA10-p4358643
Bn-A08-p3907214	0.520661157	0.933884298	Bn-N15-p25653509	Bn-chrA10-p4370320
Bn-A08-p3907556	0.791044776	0.955223881	Bn-N17-p9348629	Bn-chrA10-p4370662
Bn-A08-p3919702	0.983471074	0.975206612	Bn-Scaffold00637-p1	Bn-chrA10-p4379392
Bn-A08-p3920722	0.958677686	0.958677686	Bn-Scaffold00637-p1	Bn-chrA10-p4381137
Bn-A08-p3924770	0.991735537	1	Bn-Scaffold00637-p1	Bn-chrA10-p4384264
Bn-A08-p3926038	0.976744186	0.993355482	Bn-Scaffold00637-p1	Bn-chrA10-p4385676
Bn-A08-p3959828	0.991735537	1	Bn-N8-p3818265	Bn-chrA08-p3383007
Bn-A08-p3964610	0.966942149	0.975206612	Bn-N8-p3827464	Bn-chrA08-p3386342
Bn-A08-p3964840	0.966777409	1	Bn-N8-p3827694	Bn-chrA08-p3386572
Bn-A08-p4000353	0.983471074	0.991735537	Bn-N8-p3837598	Bn-chrA08-p3400769
Bn-A08-p4001683	0.966942149	0.958677686	Bn-N8-p3840997	Bn-chrAnn_random-p422612
Bn-A08-p4014130	0.991735537	0.983471074	Bn-N8-p3847813	Bn-chrA08-p3409939
Bn-A08-p4021680	0.917355372	0.892561983	Bn-N8-p3857296	Bn-chrA08-p3419610
Bn-A08-p4022450	0.976744186	0.973421927	Bn-N8-p3858065	Bn-chrA08-p3420381
Bn-A08-p4022688	0.983471074	0.991735537	Bn-N8-p3858303	Bn-chrA08-p3420619
Bn-A08-p4029890	0.958677686	0.950413223	Bn-N8-p3865318	Bn-chrAnn_random-p458008
Bn-A08-p4044726	0.966942149	0.950413223	Bn-N8-p3885493	Bn-chrC08-p5465282
Bn-A08-p4057882	0.989583333	0.989583333	Bn-N8-p3898615	Bn-chrA08-p3464468
Bn-A08-p4060142	0.958677686	0.966942149	Bn-N8-p3900933	Bn-chrA08-p3466785
Bn-A08-p4077507	0.996677741	0.993355482	Bn-N8-p3911207	Bn-chrA08-p3476859
Bn-A08-p4080428	0.893687708	0.877076412	Bn-N8-p3914422	Bn-chrA08-p3481099
Bn-A08-p4096136	0.958677686	0.958677686	Bn-N8-p3927842	Bn-chrA08-p3496509
Bn-A08-p4100297	0.966942149	0.966942149	Bn-N8-p3932489	Bn-chrA08-p3500547
Bn-A08-p4102226	0.983471074	0.983471074	Bn-N8-p3933246	Bn-chrA08-p3501304
Bn-A08-p4107482	0.94214876	0.94214876	Bn-N8-p3944261	Bn-chrA08-p3512368
Bn-A08-p4145883	0.991735537	0.991735537	Bn-N8-p3980652	Bn-chrA08-p3547660
Bn-A08-p4146148	0.976744186	0.976744186	Bn-N8-p3980916	Bn-chrA08-p3547924
Bn-A08-p4146394	0.958677686	0.958677686	Bn-N8-p3981161	Bn-chrA08-p3548169
Bn-A08-p4146828	0.975206612	0.975206612	Bn-N8-p3983021	Bn-chrA08-p3550256
Bn-A08-p4147503	1	1	Bn-N8-p3983696	Bn-chrA08-p3550931
Bn-A08-p4147963	0.983471074	0.983471074	Bn-N8-p3984156	Bn-chrA08-p3551391
Bn-A08-p4148465	1	1	Bn-N8-p3984659	Bn-chrA08-p3551894
Bn-A08-p4148693	0.991735537	0.991735537	Bn-N8-p3984887	Bn-chrA08-p3552122
Bn-A08-p4152083	0.983471074	0.983471074	Bn-N8-p3988274	Bn-chrA08-p3555511
Bn-A08-p4161305	0.966942149	0.966942149	Bn-N8-p4003925	Bn-chrA08-p3571164
Bn-A08-p4164372	0.990033223	0.990033223	Bn-N8-p4006991	Bn-chrA08-p3574230
Bn-A08-p4175442	0.958677686	0.958677686	Bn-N8-p4019235	Bn-chrA08-p3593030
Bn-A08-p4182726	1	1	Bn-N8-p4031637	Bn-chrA08-p3604734
Bn-A08-p4207231	0.975206612	0.975206612	Bn-N8-p4052341	Bn-chrA08-p3626293

Bn-A08-p4209834	0.975206612	0.975206612	Bn-N8-p4054898	Bn-chrA08-p3628850
Bn-A08-p4253915	0.975206612	0.975206612	Bn-N8-p4086978	Bn-chrA08-p3664476
Bn-A08-p4254041	0.989583333	0.989583333	Bn-N8-p4087104	Bn-chrA08-p3664602
Bn-A08-p4255441	0.983471074	0.983471074	Bn-N8-p4092362	Bn-chrA08_random-p468688
Bn-A08-p4255458	0.983471074	0.983471074	Bn-N8-p4092379	Bn-chrA08_random-p468705
Bn-A08-p4267191	0.900826446	0.900826446	Bn-N18-p6297980	Bn-chrC08-p5888132
Bn-A08-p4271010	0.990049751	0.990049751	Bn-N8-p4104241	Bn-chrA08-p3673212
Bn-A08-p4271262	0.855721393	0.855721393	Bn-N8-p4104493	Bn-chrA08-p3673464
Bn-A08-p4273091	0.801652893	0.801652893	Bn-N8-p4106457	Bn-chrA08-p3675428
Bn-A08-p4285785	1	1	Bn-N8-p4118420	Bn-chrA08-p3687154
Bn-A08-p4293226	0.993355482	0.993355482	Bn-N8-p4127375	Bn-chrA08-p3694294
Bn-A08-p4338919	1	1	Bn-N8-p4172538	Bn-chrA08-p3743707
Bn-A08-p4342485	0.989583333	0.989583333	Bn-N8-p4176136	Bn-chrA08-p3747305
Bn-A08-p4347992	1	1	Bn-N8-p4182574	Bn-chrA08-p3753015
Bn-A08-p4350997	0.995024876	0.995024876	Bn-N8-p4185582	Bn-chrA08-p3756023
Bn-A08-p4350998	0.990049751	0.990049751	Bn-N8-p4185583	Bn-chrA08-p3756024
Bn-A08-p4353407	1	1	Bn-N8-p4187992	Bn-chrA08-p3758433
Bn-A08-p4367412	0.991735537	0.991735537	Bn-N8-p4202682	Bn-chrAnn_random-p453127
Bn-A08-p4383325	0.983471074	0.983471074	Bn-N8-p4218244	Bn-chrA08-p3790554
Bn-A08-p4387831	0.991735537	0.94214876	Bn-N8-p4223066	Bn-chrC08_random-p661964
Bn-A08-p4392909	0.991735537	0.991735537	Bn-N8-p4227666	Bn-chrA08-p3800137
Bn-A08-p4394837	0.991735537	0.991735537	Bn-N8-p4229594	Bn-chrA08-p3802065
Bn-A08-p4400287	0.970149254	0.970149254	Bn-N8-p4235153	Bn-chrA08-p3807625
Bn-A08-p4402695	0.983471074	0.983471074	Bn-N8-p4237561	Bn-chrA08-p3810033
Bn-A08-p4404257	1	1	Bn-N8-p4239120	Bn-chrA08-p3811592
Bn-A08-p4407886	0.983471074	0.983471074	Bn-N8-p4242884	Bn-chrA08-p3815149
Bn-A08-p4413411	0.983471074	0.983471074	Bn-N8-p4248758	Bn-chrA08-p3820741
Bn-A08-p4421921	1	1	Bn-N8-p4257117	Bn-chrA08-p3829530
Bn-A08-p4452015	0.975206612	0.975206612	Bn-N8-p4286818	Bn-chrA08-p3864354
Bn-A08-p4452888	1	1	Bn-N8-p4287691	Bn-chrA08-p3865227
Bn-A08-p4453282	0.975206612	0.975206612	Bn-N8-p4288714	Bn-chrA08-p3866386
Bn-A08-p4467910	0.993355482	0.993355482	Bn-N8-p4304291	Bn-chrA08-p3881658
Bn-A08-p4474747	0.990049751	0.990049751	Bn-N8-p4311686	Bn-chrA08-p3888498
Bn-A08-p4475073	0.960199005	0.960199005	Bn-N8-p4312012	Bn-chrA08-p3888824
Bn-A08-p4475542	0.996677741	0.996677741	Bn-N8-p4312481	Bn-chrA08-p3889293
Bn-A08-p4476107	0.980099502	0.980099502	Bn-N8-p4313046	Bn-chrA08-p3889858
Bn-A08-p4476147	0.980099502	0.980099502	Bn-N8-p4313086	Bn-chrA08-p3889898
Bn-A08-p4483232	0.995024876	0.995024876	Bn-N8-p4320169	Bn-chrA08-p3896979
Bn-A08-p4521258	0.991735537	0.991735537	Bn-N8-p4365967	Bn-chrA08_random-p970093
Bn-A08-p4528943	0.996677741	0.996677741	Bn-N8-p4373662	Bn-chrA08-p3947590
Bn-A08-p4537897	0.990049751	0.990049751	Bn-N8-p4382765	Bn-chrA08-p3956547
Bn-A08-p4538107	0.995024876	0.995024876	Bn-N8-p4382975	Bn-chrA08-p3956757
Bn-A08-p4598270	0.966942149	0.966942149	Bn-N8-p4441754	Bn-chrA08-p4018373
Bn-A08-p4602542	0.990033223	0.940199336	Bn-N8-p4444014	Bn-chrCnn_random-p146093
Bn-A08-p4603745	0.983471074	0.983471074	Bn-N8-p4445217	Bn-chrA08-p4021693
Bn-A08-p4624613	0.991735537	0.991735537	Bn-N8-p4466560	Bn-chrA08-p4045680

Bn-A08-p4630998	0.991735537	0.991735537	Bn-N8-p4473089	Bn-chrA06-p8073527
Bn-A08-p4636503	0.991735537	0.991735537	Bn-N8-p4477959	Bn-chrA06-p6191281
Bn-A08-p4645422	0.996677741	0.996677741	Bn-N8-p4486841	Bn-chrAnn_random-p275631
Bn-A08-p4645913	0.993355482	0.993355482	Bn-N8-p4487332	Bn-chrAnn_random-p275627
Bn-A08-p4665843	1	1	Bn-N8-p4504287	Bn-chrA08_random-p876717
Bn-A08-p4666053	0.990033223	0.990033223	Bn-N8-p4504497	Bn-chrA08_random-p876927
Bn-A08-p4668561	0.996677741	0.996677741	Bn-N8-p4507005	Bn-chrA08_random-p879435
Bn-A08-p4670469	0.991735537	0.991735537	Bn-N8-p4508913	Bn-chrA08_random-p881343
Bn-A08-p4687828	0.975206612	0.975206612	Bn-N8-p4525757	Bn-chrAnn_random-p919754
Bn-A08-p4708904	0.983471074	0.983471074	Bn-N8-p4547035	Bn-chrAnn_random-p921847
Bn-A08-p4719687	0.983471074	0.983471074	Bn-N8-p4557678	Bn-chrAnn_random-p924701
Bn-A08-p4737849	0.990033223	0.990033223	Bn-N8-p4573095	Bn-chrAnn_random-p926226
Bn-A08-p4758028	0.983388704	0.983388704	Bn-N8-p4590062	Bn-chrAnn_random-p927835
Bn-A08-p4762095	0.975124378	0.975124378	Bn-N8-p4594148	Bn-chrAnn_random-p928244
Bn-A08-p4765954	0.975206612	0.975206612	Bn-N8-p4601310	Bn-chrAnn_random-p928944
Bn-A08-p4775253	0.980099502	0.980099502	Bn-N8-p4574532	Bn-chrAnn_random-p926370
Bn-A08-p4783045	0.965174129	0.965174129	Bn-N8-p4621136	Bn-chrAnn_random-p930934
Bn-A08-p4837890	1	1	Bn-N8-p4638527	Bn-chrAnn_random-p932673
Bn-A08-p4869575	0.966777409	0.966777409	Bn-N10-p13194150	Bn-chrA10-p12865213
Bn-A08-p4876592	0.990049751	0.980099502	Bn-N10-p13185109	Bn-chrA10-p12859167
Bn-A08-p4881192	0.970149254	0.970149254	Bn-N10-p13179850	Bn-chrA10-p12854475
Bn-A08-p4900525	0.983471074	0.991735537	Bn-N10-p13163477	Bn-chrA10-p12843309
Bn-A08-p4902282	0.950413223	0.958677686	Bn-N10-p13161705	Bn-chrA10-p12841553
Bn-A08-p4931420	0.975206612	0.975206612	Bn-N19-p49643491	Bn-chrC09-p42822208
Bn-A08-p4936436	0.991735537	0.991735537	Bn-N10-p13122698	Bn-chrA10-p12804707
Bn-A08-p4954284	0.985074627	0.985074627	Bn-N10-p13099943	Bn-chrA10-p12779901
Bn-A08-p4993820	0.92039801	0.92039801	Bn-N8-p4783876	Bn-chrA08-p4191988
Bn-A08-p4994186	0.990049751	0.990049751	Bn-N8-p4784242	Bn-chrA08-p4192354
Bn-A08-p4994891	0.980099502	0.980099502	Bn-N8-p4784946	Bn-chrA08-p4193058
Bn-A08-p5006162	0.983471074	0.983471074	Bn-N8-p4796099	Bn-chrA08-p4209443
Bn-A08-p5017136	0.996677741	0.996677741	Bn-N8-p4806558	Bn-chrA08-p4219893
Bn-A08-p5026482	0.976744186	0.976744186	Bn-N8-p4815377	Bn-chrA08-p4228805
Bn-A08-p5031773	0.983471074	0.983471074	Bn-N8-p4820668	Bn-chrA08-p4234098
Bn-A08-p5044814	1	1	Bn-N8-p4831215	Bn-chrA08-p4247662
Bn-A08-p5046769	0.826446281	0.983471074	Bn-N18-p14653842	Bn-chrA08-p4249616
Bn-A08-p5051251	0.986710963	0.986710963	Bn-N8-p4837655	Bn-chrA08-p4254105
Bn-A08-p5054005	1	1	Bn-N8-p4840409	Bn-chrA08-p4256859
Bn-A08-p5054613	1	1	Bn-N8-p4841017	Bn-chrA08-p4257467
Bn-A08-p5056953	0.991735537	0.991735537	Bn-N8-p4843458	Bn-chrA08-p4259908
Bn-A08-p5084300	0.986710963	0.986710963	Bn-N8-p4867184	Bn-chrA08-p4288533
Bn-A08-p5096382	0.996677741	0.993355482	Bn-N8-p4876764	Bn-chrA08-p4300469
Bn-A08-p5104792	0.983471074	0.983471074	Bn-N8-p4885176	Bn-chrA08-p4308879
Bn-A08-p5104945	0.986710963	0.986710963	Bn-N8-p4885324	Bn-chrA08-p4309027
Bn-A08-p5105745	0.983471074	0.983471074	Bn-N8-p4886125	Bn-chrA08-p4309828
Bn-A08-p5109706	0.983471074	0.983471074	Bn-N8-p4890096	Bn-chrA08-p4313797
Bn-A08-p5146710	0.975206612	0.975206612	Bn-N8-p4908806	Bn-chrA08-p4332342

Bn-A08-p5153375	0.94214876	0.94214876	Bn-N8-p4916600	Bn-chrA08-p4340166
Bn-A08-p5155511	0.983471074	0.983471074	Bn-N8-p4924058	Bn-chrA08_random-p497406
Bn-A08-p5174518	0.991735537	0.991735537	Bn-N8-p4925818	Bn-chrA08_random-p499166
Bn-A08-p5174818	0.995024876	0.995024876	Bn-N8-p4926118	Bn-chrA08_random-p499466
Bn-A08-p5212067	0.975124378	0.975124378	Bn-N8-p4965695	Bn-chrA08-p4390842
Bn-A08-p5241894	0.955223881	0.960199005	Bn-N9-p21381610	Bn-chrA09-p17242352
Bn-A08-p5242184	0.960199005	0.965174129	Bn-N9-p21381900	Bn-chrA09-p17242642
Bn-A08-p5249573	0.991735537	0.991735537	Bn-N8-p5021473	Bn-chrA08-p4459465
Bn-A08-p5255647	1	1	Bn-N8-p5025595	Bn-chrA08-p4463444
Bn-A08-p5257044	0.983471074	0.983471074	Bn-N8-p5026993	Bn-chrA08-p4464842
Bn-A08-p5257814	0.991735537	0.991735537	Bn-N8-p5027763	Bn-chrA08-p4465612
Bn-A08-p5263570	0.983471074	0.983471074	Bn-N8-p5033527	Bn-chrA08-p4471376
Bn-A08-p5266776	0.991735537	0.876033058	Bn-N8-p5038061	Bn-chrC09-p23701913
Bn-A08-p5300046	0.983388704	0.983388704	Bn-N8-p5082390	Bn-chrA08-p4522137
Bn-A08-p5300520	0.991735537	0.991735537	Bn-N8-p5082864	Bn-chrA08-p4522611
Bn-A08-p5300648	0.966942149	0.966942149	Bn-N8-p5082992	Bn-chrA08-p4522739
Bn-A08-p5305309	0.975206612	0.975206612	Bn-N8-p5091956	Bn-chrA08-p4538789
Bn-A08-p5333513	0.983471074	0.983471074	Bn-N8-p5113178	Bn-chrA08-p4559806
Bn-A08-p5343622	0.933884298	0.933884298	Bn-N8-p5145014	Bn-chrA08-p4619037
Bn-A08-p5351001	0.975206612	0.975206612	Bn-N8-p5150480	Bn-chrA08-p4624635
Bn-A08-p5361134	0.935323383	0.935323383	Bn-N8-p5161729	Bn-chrA08-p4636053
Bn-A08-p5364653	0.993355482	0.993355482	Bn-N8-p5165248	Bn-chrA08-p4639572
Bn-A08-p5366608	0.993355482	0.990033223	Bn-N8-p5167201	Bn-chrA08-p4641525
Bn-A08-p5420503	0.966942149	0.966942149	Bn-N8-p5216482	Bn-chrA08-p4686498
Bn-A08-p5461032	0.933884298	0.933884298	Bn-N8-p5243086	Bn-chrA08-p4715204
Bn-A08-p5490050	0.993355482	0.993355482	Bn-N8-p5280916	Bn-chrA08-p4748317
Bn-A08-p5491239	1	1	Bn-N8-p5282086	Bn-chrA08-p4749487
Bn-A08-p5539128	0.996677741	0.996677741	Bn-N8-p5326138	Bn-chrA08-p4796609
Bn-A08-p5540892	0.993355482	0.993355482	Bn-N18-p13590482	Bn-chrA08-p4798386
Bn-A08-p5583881	0.991735537	0.991735537	Bn-N8-p5457358	Bn-chrA08-p4837394
Bn-A08-p5591333	0.991735537	0.991735537	Bn-N8-p5464803	Bn-chrA08-p4844837
Bn-A08-p5616343	0.983471074	0.983471074	Bn-N8-p5480169	Bn-chrA08-p4858439
Bn-A08-p5626110	0.991735537	0.991735537	Bn-N8-p5490058	Bn-chrA08-p4868328
Bn-A08-p5626953	0.991735537	0.991735537	Bn-N8-p5490900	Bn-chrA08-p4869170
Bn-A08-p5627117	0.991735537	0.991735537	Bn-N8-p5491064	Bn-chrA08-p4869334
Bn-A08-p5635882	0.996677741	0.996677741	Bn-N8-p5498368	Bn-chrA08-p4876640
Bn-A08-p5636172	0.983388704	0.983388704	Bn-N8-p5498659	Bn-chrA08-p4876931
Bn-A08-p5636755	0.990033223	0.990033223	Bn-N8-p5499242	Bn-chrA08-p4877514
Bn-A08-p5636764	0.975206612	0.975206612	Bn-N8-p5499251	Bn-chrA08-p4877523
Bn-A08-p5638757	0.983471074	0.983471074	Bn-N8-p5501244	Bn-chrA08-p4879516
Bn-A08-p5640946	0.991735537	0.991735537	Bn-N8-p5503440	Bn-chrA08-p4881712
Bn-A08-p5652494	0.990033223	0.990033223	Bn-N8-p5518223	Bn-chrA08-p4895784
Bn-A08-p5664320	0.980066445	0.980066445	Bn-N8-p5534837	Bn-chrAnn_random-p441888
Bn-A08-p5696515	0.975124378	0.975124378	Bn-N8-p5569818	Bn-chrA08-p4946191
Bn-A08-p5697139	0.986710963	0.986710963	Bn-N8-p5570442	Bn-chrA08-p4946815
Bn-A08-p5697224	0.986710963	0.986710963	Bn-N8-p5570527	Bn-chrA08-p4946900

Bn-A08-p5697643	0.990033223	0.990033223	Bn-N8-p5570946	Bn-chrA08-p4947319
Bn-A08-p5697783	0.993355482	0.993355482	Bn-N8-p5571087	Bn-chrA08-p4947460
Bn-A08-p5698262	0.991735537	0.991735537	Bn-N8-p5571566	Bn-chrA08-p4947939
Bn-A08-p5698705	0.980099502	0.980099502	Bn-N8-p5572009	Bn-chrA08-p4948382
Bn-A08-p5699040	0.990049751	0.990049751	Bn-N8-p5572344	Bn-chrA08-p4948717
Bn-A08-p5700152	0.996677741	0.996677741	Bn-N8-p5573456	Bn-chrA08-p4949829
Bn-A08-p5700554	0.991735537	0.991735537	Bn-N8-p5573858	Bn-chrA08-p4950231
Bn-A08-p5702921	1	1	Bn-N8-p5576223	Bn-chrA08-p4952596
Bn-A08-p5705625	1	1	Bn-N8-p5578918	Bn-chrA08-p4955291
Bn-A08-p5721732	1	1	Bn-N8-p5598362	Bn-chrA08-p4971380
Bn-A08-p5729023	0.980066445	0.980066445	Bn-N3-p22879547	Bn-chrAnn_random-p427162
Bn-A08-p5730628	0.991735537	0.991735537	Bn-N8-p5603234	Bn-chrAnn_random-p290912
Bn-A08-p5766271	0.990033223	0.990033223	Bn-N8-p5676908	Bn-chrAnn_random-p428893
Bn-A08-p5806240	1	1	Bn-N8-p5711452	Bn-chrAnn_random-p281382
Bn-A08-p5905574	0.996677741	0.996677741	Bn-N8-p5765973	Bn-chrA08-p4992805
Bn-A08-p5924891	1	1	Bn-N8-p5785368	Bn-chrA08-p5011741
Bn-A08-p5938244	0.995024876	0.995024876	Bn-N8-p5800849	Bn-chrA08-p5031804
Bn-A08-p5975890	0.965174129	0.965174129	Bn-N8-p4759962	Bn-chrA08-p5163423
Bn-A08-p6029041	0.991735537	0.991735537	Bn-N8-p4690333	Bn-chrAnn_random-p404314
Bn-A08-p6066021	0.996677741	0.996677741	Bn-N8-p8377754	Bn-chrAnn_random-p360261
Bn-A08-p6101880	0.975206612	0.975206612	Bn-N8-p8407250	Bn-chrAnn_random-p12218C
Bn-A08-p6104052	0.970149254	0.975124378	Bn-N9-p25655895	Bn-chrAnn_random-p322023
Bn-A08-p6108703	0.966942149	0.94214876	Bn-N8-p8414084	Bn-chrAnn_random-p122301
Bn-A08-p6114046	0.983471074	0.933884298	Bn-N8-p8419492	Bn-chrCnn_random-p103490
Bn-A08-p6133005	0.983471074	0.933884298	Bn-N8-p8423399	Bn-chrAnn_random-p122425
Bn-A08-p6133601	0.991735537	0.190082645	Bn-N8-p8423995	Bn-chrC09-p45348661
Bn-A08-p6148686	0.958677686	0.950413223	Bn-N8-p8428969	Bn-chrC08-p14220763
Bn-A08-p6148996	0.991735537	0.975206612	Bn-N8-p8429279	Bn-chrAnn_random-p122469
Bn-A08-p6157861	0.991735537	0.991735537	Bn-N8-p8439711	Bn-chrA08_random-p123319
Bn-A08-p6159705	0.991735537	0.966942149	Bn-N8-p8441580	Bn-chrAnn_random-p122563
Bn-A08-p6159768	0.983471074	0.991735537	Bn-N8-p8441643	Bn-chrAnn_random-p122563
Bn-A08-p6162616	0.966942149	0.553719008	Bn-N8-p8452246	Bn-chrC08-p14268491
Bn-A08-p6162660	0.985074627	0.731343284	Bn-N8-p8452290	Bn-chrC08-p14268535
Bn-A08-p6164225	0.991735537	0.94214876	Bn-N8-p8453855	Bn-chrA08-p5232301
Bn-A08-p6164968	1	0.950413223	Bn-N8-p8454598	Bn-chrA08-p5233055
Bn-A08-p6165340	0.965174129	0.955223881	Bn-N8-p8454970	Bn-chrA08-p5233422
Bn-A08-p6165665	0.975206612	0.975206612	Bn-N8-p8455295	Bn-chrA08-p5233746
Bn-A08-p6170057	0.985074627	0.915422886	Bn-N8-p8459717	Bn-chrA08-p5238177
Bn-A08-p6185551	0.975206612	0.950413223	Bn-Scaffold09483-p7	Bn-chrA08-p5246505
Bn-A08-p6238237	0.991735537	0.991735537	Bn-N8-p8517296	Bn-chrA08-p5293593
Bn-A08-p6246411	0.980099502	0.960199005	Bn-N8-p8524819	Bn-chrA08-p5307303
Bn-A08-p6248437	0.983471074	0.983471074	Bn-N8-p8526824	Bn-chrA08-p5309306
Bn-A08-p6256690	0.950413223	0.950413223	Bn-N18-p15224594	Bn-chrC08_random-p216232
Bn-A08-p6257665	0.983471074	0.983471074	Bn-N8-p8545244	Bn-chrCnn_random-p806557
Bn-A08-p6268593	0.975124378	0.975124378	Bn-N8-p8553043	Bn-chrA08-p5328103
Bn-A08-p6278699	0.958677686	0.958677686	Bn-N8-p8556054	Bn-chrA08-p5331114

Bn-A08-p6282126	1	1	Bn-N8-p8559481	Bn-chrA08-p5334540
Bn-A08-p6284747	0.970149254	0.970149254	Bn-N8-p8562060	Bn-chrA08-p5337119
Bn-A08-p6297167	0.958677686	0.958677686	Bn-N8-p8569443	Bn-chrA08-p5344266
Bn-A08-p6298340	0.990033223	0.990033223	Bn-N8-p8575382	Bn-chrA08-p5350205
Bn-A08-p6299307	0.958677686	0.958677686	Bn-N8-p8576347	Bn-chrA08-p5351170
Bn-A08-p6299938	0.991735537	0.991735537	Bn-N8-p8576978	Bn-chrA08-p5351801
Bn-A08-p6303585	0.404958678	0.396694215	Bn-N18-p15315854	Bn-chrC08-p14473490
Bn-A08-p6306804	0.437810945	0.437810945	Bn-N16-p20904767	Bn-chrC06-p19612215
Bn-A08-p6319732	0.966942149	0.966942149	Bn-N8-p8587709	Bn-chrA08_random-p131947
Bn-A08-p6319943	0.925619835	0.925619835	Bn-N8-p8587928	Bn-chrA08_random-p131925
Bn-A08-p6320815	0.876033058	0.867768595	Bn-N8-p8588201	Bn-chrA08_random-p131898
Bn-A08-p6332878	0.94214876	0.94214876	Bn-N8-p8593283	Bn-chrA08_random-p131389
Bn-A08-p6343431	0.94214876	0.94214876	Bn-N8-p8604736	Bn-chrA08_random-p130334
Bn-A08-p6366258	0.990033223	0.990033223	Bn-N8-p8626454	Bn-chrA08_random-p128165
Bn-A08-p6369194	0.990049751	0.990049751	Bn-N8-p8629403	Bn-chrA08_random-p127836
Bn-A08-p6371865	0.958677686	0.958677686	Bn-N8-p8632074	Bn-chrA08_random-p127125
Bn-A08-p6392789	0.985074627	0.985074627	Bn-N8-p8650005	Bn-chrA08_random-p125320
Bn-A08-p6394232	0.956810631	0.956810631	Bn-N8-p8651448	Bn-chrA08_random-p284542
Bn-A08-p6400378	0.975206612	0.975206612	Bn-N8-p8656368	Bn-chrA08_random-p279624
Bn-A08-p6488809	0.945273632	0.945273632	Bn-N8-p8740065	Bn-chrA08_random-p190394
Bn-A08-p6523926	0.995024876	0.995024876	Bn-N8-p8769174	Bn-chrAnn_random-p312188
Bn-A08-p6523952	0.990049751	0.990049751	Bn-N8-p8769200	Bn-chrAnn_random-p312188
Bn-A08-p6721530	0.990049751	0.990049751	Bn-N8-p9002151	Bn-chrA08-p5661853
Bn-A08-p6722442	0.985074627	0.995024876	Bn-N8-p9003062	Bn-chrA08-p5662764
Bn-A08-p6744560	0.991735537	0.991735537	Bn-N8-p9035531	Bn-chrA08-p5685034
Bn-A08-p6828854	0.980099502	0.980099502	Bn-N8-p9121537	Bn-chrA08-p5776772
Bn-A08-p6828857	0.985074627	0.985074627	Bn-N8-p9121540	Bn-chrA08-p5776775
Bn-A08-p6829643	0.995024876	0.995024876	Bn-N8-p9122324	Bn-chrA08-p5777559
Bn-A08-p6836766	0.986710963	0.986710963	Bn-N8-p9129554	Bn-chrA08-p5784670
Bn-A08-p6836803	0.995024876	0.995024876	Bn-N8-p9129591	Bn-chrA08-p5784707
Bn-A08-p6887868	0.990033223	0.990033223	Bn-N8-p9180282	Bn-chrC03-p12466596
Bn-A08-p6889067	0.996677741	0.996677741	Bn-N8-p9181481	Bn-chrC03-p12467795
Bn-A08-p6937293	0.976744186	0.956810631	Bn-N8-p9428077	Bn-chrC08_random-p312390
Bn-A08-p6943246	0.980066445	0.966777409	Bn-N8-p9427004	Bn-chrC08_random-p312281
Bn-A08-p6971574	0.945273632	0.945273632	Bn-N8-p9402200	Bn-chrA08-p6022087
Bn-A08-p7018554	0.913621262	0.910299003	Bn-N8-p9362261	Bn-chrA08-p5984401
Bn-A08-p7062593	1	1	Bn-N8-p5917281	Bn-chrA08_random-p547639
Bn-A08-p7063045	0.991735537	0.991735537	Bn-N8-p5917733	Bn-chrA08_random-p547187
Bn-A08-p7064296	0.996677741	0.996677741	Bn-N8-p5918984	Bn-chrA08_random-p545936
Bn-A08-p7066465	0.983471074	0.983471074	Bn-N8-p5920889	Bn-chrA08_random-p544031
Bn-A08-p7066594	0.991735537	0.991735537	Bn-N8-p5921018	Bn-chrA08_random-p543902
Bn-A08-p7067073	0.975206612	0.975206612	Bn-N8-p5921497	Bn-chrA08_random-p543423
Bn-A08-p7071333	0.94214876	0.94214876	Bn-N8-p5927049	Bn-chrA08_random-p538367
Bn-A08-p7075341	0.991735537	0.991735537	Bn-N8-p5931063	Bn-chrA08_random-p534353
Bn-A08-p7078765	0.990033223	0.990033223	Bn-N8-p5952893	Bn-chrA08-p6053787
Bn-A08-p7078920	0.991735537	0.991735537	Bn-N8-p5953048	Bn-chrA08-p6053942

Bn-A08-p7080277	1	1	Bn-N8-p5954405	Bn-chrA08-p6055299
Bn-A08-p7087866	0.993355482	0.993355482	Bn-N8-p5960959	Bn-chrC08-p9028026
Bn-A08-p7087999	0.993355482	0.993355482	Bn-N8-p5961092	Bn-chrC08-p9028159
Bn-A08-p7091133	0.993464052	0.993464052	Bn-N8-p5964228	Bn-chrA08-p6064835
Bn-A08-p7092438	0.993355482	0.993355482	Bn-N8-p5967144	Bn-chrA08-p6067750
Bn-A08-p7099327	1	1	Bn-N8-p5998133	Bn-chrA08-p6097642
Bn-A08-p7110493	0.983471074	0.983471074	Bn-N8-p6013748	Bn-chrA08-p6110308
Bn-A08-p7110561	0.991735537	0.991735537	Bn-N8-p6013816	Bn-chrA08-p6110376
Bn-A08-p7112865	0.975206612	0.975206612	Bn-N8-p6016119	Bn-chrA08-p6112679
Bn-A08-p7120885	0.983471074	0.983471074	Bn-N8-p6021968	Bn-chrA08-p6118527
Bn-A08-p7122254	0.991735537	0.991735537	Bn-N8-p6023345	Bn-chrC02-p39563628
Bn-A08-p7123976	0.991735537	0.991735537	Bn-N8-p6025438	Bn-chrA08-p6121722
Bn-A08-p7139892	0.966942149	0.966942149	Bn-Scaffold31845-p1	Bn-chrA08-p14183556
Bn-A08-p7151447	0.58677686	0.58677686	Bn-N8-p6036123	Bn-chrA08-p6135412
Bn-A08-p7152271	0.933884298	0.933884298	Bn-N8-p6036959	Bn-chrA08-p6136248
Bn-A08-p7162057	0.925619835	0.925619835	Bn-N8-p6043758	Bn-chrA08-p6143047
Bn-A08-p7178765	0.958677686	0.958677686	Bn-N8-p6109564	Bn-chrC08-p9212583
Bn-A08-p7178963	0.983471074	0.983471074	Bn-N8-p6109764	Bn-chrA08-p6218741
Bn-A08-p7202405	0.993355482	0.993355482	Bn-N8-p6148129	Bn-chrA08-p6263721
Bn-A08-p7222585	0.991735537	0.991735537	Bn-Scaffold06770-p1	Bn-chrA08-p6295868
Bn-A08-p7223478	0.859504132	0.859504132	Bn-N18-p8637114	Bn-chrC08-p9328685
Bn-A08-p7243965	0.991735537	0.991735537	Bn-N8-p6168927	Bn-chrA08-p6283773
Bn-A08-p7260514	0.975206612	0.925619835	Bn-N8-p6202597	Bn-chrC08-p9444039
Bn-A08-p7261638	0.991735537	0.801652893	Bn-N8-p6203719	Bn-chrA08-p5345651
Bn-A08-p7261759	0.986710963	0.644518272	Bn-N8-p6203840	Bn-chrUnn_random-p42978[illegible]
Bn-A08-p7263132	0.975206612	0.975206612	Bn-N8-p6212448	Bn-chrA08_random-p477609
Bn-A08-p7263741	0.975206612	0.975206612	Bn-N8-p6213057	Bn-chrA08_random-p478218
Bn-A08-p7264462	0.966942149	0.966942149	Bn-N8-p6213780	Bn-chrA08_random-p478941
Bn-A08-p7264513	0.966942149	0.966942149	Bn-N8-p6213831	Bn-chrA08_random-p478992
Bn-A08-p7265146	0.983471074	0.983471074	Bn-N8-p6214464	Bn-chrA08_random-p479625
Bn-A08-p7265333	0.983471074	0.983471074	Bn-N8-p6214651	Bn-chrA08_random-p479812
Bn-A08-p7265843	0.966942149	0.966942149	Bn-N8-p6215161	Bn-chrA08_random-p480322
Bn-A08-p7265897	0.991735537	0.991735537	Bn-N8-p6215215	Bn-chrA08_random-p480376
Bn-A08-p7271638	0.958677686	0.958677686	Bn-N8-p6220962	Bn-chrA08_random-p486122
Bn-A08-p7273429	0.975206612	0.975206612	Bn-N8-p6223305	Bn-chrA08_random-p488467
Bn-A08-p7276312	0.991735537	0.991735537	Bn-N8-p6227626	Bn-chrAnn_random-p258723
Bn-A08-p7282035	0.983471074	0.983471074	Bn-N8-p6229030	Bn-chrAnn_random-p258737
Bn-A08-p7288153	0.991735537	0.991735537	Bn-N8-p6229608	Bn-chrAnn_random-p258743
Bn-A08-p7303669	0.983471074	0.983471074	Bn-N8-p6298576	Bn-chrA08-p6339520
Bn-A08-p7303995	0.950413223	0.950413223	Bn-N18-p8810165	Bn-chrC08-p9498738
Bn-A08-p7315916	0.685950413	0.685950413	Bn-N8-p6315202	Bn-chrA08-p6356205
Bn-A08-p7318043	0.993355482	0.993355482	Bn-N8-p6317339	Bn-chrA08-p6358342
Bn-A08-p7336214	0.958677686	0.958677686	Bn-N18-p8858481	Bn-chrC08-p9547078
Bn-A08-p7355372	0.966942149	0.966942149	Bn-N18-p8875292	Bn-chrC08-p9556938
Bn-A08-p7366507	1	1	Bn-N8-p6340113	Bn-chrA08-p6384592
Bn-A08-p7374884	0.966942149	0.966942149	Bn-N8-p6348581	Bn-chrA08-p6394092

Bn-A08-p7377024	0.991735537	0.991735537	Bn-N8-p6352925	Bn-chrA08-p6398278
Bn-A08-p7409992	0.996677741	0.996677741	Bn-N8-p6383054	Bn-chrA08-p6430038
Bn-A08-p7410609	0.996677741	0.996677741	Bn-N8-p6383671	Bn-chrA08-p6430655
Bn-A08-p7416849	1	1	Bn-N8-p6390162	Bn-chrA08-p6437146
Bn-A08-p7417420	0.990033223	0.990033223	Bn-N8-p6390733	Bn-chrA08-p6437717
Bn-A08-p7417679	0.996677741	0.996677741	Bn-N8-p6390992	Bn-chrA08-p6437976
Bn-A08-p7417923	0.993355482	0.993355482	Bn-N8-p6391236	Bn-chrA08-p6438220
Bn-A08-p7418001	1	1	Bn-N8-p6391314	Bn-chrA08-p6438298
Bn-A08-p7418132	1	1	Bn-N8-p6391445	Bn-chrA08-p6438429
Bn-A08-p7420214	0.986710963	0.986710963	Bn-N8-p6393526	Bn-chrA08-p6440510
Bn-A08-p7420713	0.983471074	0.983471074	Bn-N8-p6394026	Bn-chrA08-p6441010
Bn-A08-p7425294	0.983471074	0.983471074	Bn-N8-p6398723	Bn-chrA08-p6445707
Bn-A08-p7438002	0.993355482	0.993355482	Bn-N8-p6407282	Bn-chrA08-p6456347
Bn-A08-p7438102	0.993355482	0.993355482	Bn-N8-p6407382	Bn-chrA08-p6456447
Bn-A08-p7439009	0.991735537	0.991735537	Bn-N8-p6408289	Bn-chrA08-p6457354
Bn-A08-p7439711	1	1	Bn-N8-p6409016	Bn-chrA08-p6458081
Bn-A08-p7440367	0.991735537	0.991735537	Bn-N8-p6409672	Bn-chrA08-p6458737
Bn-A08-p7441624	0.975206612	0.975206612	Bn-N8-p6410918	Bn-chrA08-p6459983
Bn-A08-p7443922	0.966942149	0.966942149	Bn-N8-p6413013	Bn-chrA08-p6462078
Bn-A08-p7444336	0.993355482	0.993355482	Bn-N8-p6413426	Bn-chrA08-p6462491
Bn-A08-p7455782	0.975206612	0.975206612	Bn-N8-p6426735	Bn-chrA08-p6477200
Bn-A08-p7458611	1	1	Bn-N8-p6430504	Bn-chrA08-p6480030
Bn-A08-p7496720	0.975124378	0.975124378	Bn-N8-p6452579	Bn-chrA08-p6503838
Bn-A08-p7497342	0.990049751	0.990049751	Bn-N8-p6453201	Bn-chrA08-p6504460
Bn-A08-p7497415	0.975124378	0.975124378	Bn-N8-p6453274	Bn-chrA08-p6504533
Bn-A08-p7497920	0.990049751	0.990049751	Bn-N8-p6453771	Bn-chrA08-p6505030
Bn-A08-p7497925	0.990049751	0.990049751	Bn-N8-p6453776	Bn-chrA08-p6505035
Bn-A08-p7533577	0.980099502	0.980099502	Bn-N8-p6518599	Bn-chrA08-p6547466
Bn-A08-p7563202	0.980099502	0.980099502	Bn-N8-p6550967	Bn-chrA08-p6583204
Bn-A08-p7587591	0.990049751	0.990049751	Bn-N8-p6580521	Bn-chrA08-p6615170
Bn-A08-p7596192	0.985074627	0.985074627	Bn-N18-p9364917	Bn-chrC08-p9962376
Bn-A08-p7611203	1	0.139534884	Bn-N8-p18006234	Unknown
Bn-A08-p7698145	0.996677741	0.996677741	Bn-N8-p6764076	Bn-chrA08_random-p114091
Bn-A08-p7717256	0.996677741	0.996677741	Bn-N8-p6784500	Bn-chrA08_random-p111800
Bn-A08-p7739750	0.991735537	0.991735537	Bn-N8-p6810403	Bn-chrA08_random-p116318
Bn-A08-p7747258	1	1	Bn-N8-p6816991	Bn-chrA08_random-p116977
Bn-A08-p7791369	0.976744186	0.976744186	Bn-N8-p6895245	Bn-chrA08-p6751742
Bn-A08-p7799016	0.975206612	0.975206612	Bn-N8-p6902617	Bn-chrA08-p6759114
Bn-A08-p7803865	0.940199336	0.940199336	Bn-N18-p10209411	Bn-chrC08-p10877552
Bn-A08-p7810122	0.983388704	0.983388704	Bn-Scaffold01327-p8	Bn-chrA08-p6779700
Bn-A08-p7814328	0.975206612	0.975206612	Bn-N8-p6907707	Bn-chrA08-p6786885
Bn-A08-p7814432	0.980066445	0.980066445	Bn-N8-p6907811	Bn-chrA08-p6786989
Bn-A08-p7835995	0.986710963	0.986710963	Bn-N8-p6931659	Bn-chrA08-p6810213
Bn-A08-p7851497	0.950413223	0.950413223	Bn-N8-p6943962	Bn-chrA08-p6825016
Bn-A08-p7856305	0.970149254	0.960199005	Bn-N18-p10249395	Bn-chrC08-p10920324
Bn-A08-p7856849	0.995024876	0.995024876	Bn-N8-p6948450	Bn-chrA08-p6829505

Bn-A08-p7863392	0.991735537	0.991735537	Bn-N8-p6954956	Bn-chrA08-p6836384
Bn-A08-p7881489	0.991735537	1	Bn-N8-p6969834	Bn-chrA08-p6853145
Bn-A08-p7934396	0.970099668	0.990033223	Bn-N5-p5964241	Bn-chrA05-p5445893
Bn-A08-p7947263	0.975206612	0.975206612	Bn-N8-p7067926	Bn-chrA08-p6951592
Bn-A08-p7984584	0.993355482	0.993355482	Bn-N8-p7113378	Bn-chrA08-p6986018
Bn-A08-p7991606	0.983471074	0.983471074	Bn-N8-p7130361	Bn-chrA08-p6998675
Bn-A08-p7998862	0.900826446	0.900826446	Bn-N8-p7147930	Bn-chrA08-p7012135
Bn-A08-p8031289	0.980099502	0.980099502	Bn-N8-p7175077	Bn-chrA08-p7040100
Bn-A08-p8068365	0.909090909	0.909090909	Bn-N8-p7202766	Bn-chrA08-p7066796
Bn-A08-p8073664	0.966942149	0.966942149	Bn-N8-p7207205	Bn-chrA08-p7071236
Bn-A08-p8091811	0.986710963	0.986710963	Bn-N8-p7216514	Bn-chrA08-p7078854
Bn-A08-p8114697	0.884297521	0.884297521	Bn-N8-p7240910	Bn-chrA08-p7103619
Bn-A08-p8126392	0.94214876	0.94214876	Bn-N8-p7242877	Bn-chrA08-p7105587
Bn-A08-p8159163	0.995024876	0.995024876	Bn-N8-p7276065	Bn-chrA08-p7141913
Bn-A08-p8161845	0.991735537	0.991735537	Bn-N8-p7278568	Bn-chrA08-p7144416
Bn-A08-p8162230	0.975206612	0.975206612	Bn-N8-p7278965	Bn-chrA08-p7144813
Bn-A08-p8179629	1	1	Bn-N8-p7643523	Bn-chrA08_random-p116163
Bn-A08-p8180093	0.975206612	0.975206612	Bn-N8-p7643059	Bn-chrA08_random-p116209
Bn-A08-p8194731	1	1	Bn-N8-p7627786	Bn-chrA08_random-p122468
Bn-A08-p8195425	1	1	Bn-N8-p7627092	Bn-chrA08_random-p122398
Bn-A08-p8218376	0.983471074	0.983471074	Bn-N8-p7585015	Bn-chrA08_random-p117955
Bn-A08-p8232026	0.991735537	0.991735537	Bn-N8-p7564088	Bn-chrA08_random-p107945
Bn-A08-p8237891	0.996677741	0.996677741	Bn-N8-p7553881	Bn-chrA08_random-p106895
Bn-A08-p8242214	0.991735537	0.991735537	Bn-N8-p7549400	Bn-chrA08_random-p106446
Bn-A08-p8249952	0.975206612	0.975206612	Bn-N8-p7535491	Bn-chrC01-p37497038
Bn-A08-p8260866	0.975206612	0.975206612	Bn-N8-p7520209	Bn-chrA08_random-p103488
Bn-A08-p8267272	0.892561983	0.892561983	Bn-N8-p7513875	Bn-chrA08_random-p102855
Bn-A08-p8273132	0.975206612	0.975206612	Bn-N8-p7508048	Bn-chrA08_random-p102273
Bn-A08-p8312619	0.966942149	0.917355372	Bn-N8-p7459222	Bn-chrC08_random-p200961
Bn-A08-p8314631	0.975206612	0.975206612	Bn-N8-p7416565	Bn-chrAnn_random-p179548
Bn-A08-p8320380	0.991735537	0.991735537	Bn-N18-p11290982	Bn-chrCnn_random-p339008
Bn-A08-p8334516	0.986710963	0.986710963	Bn-N8-p7392181	Bn-chrAnn_random-p179315
Bn-A08-p8335609	1	1	Bn-N8-p7391080	Bn-chrAnn_random-p179304
Bn-A08-p8351443	0.995024876	0.995024876	Bn-N8-p7375601	Bn-chrA08-p7245557
Bn-A08-p8352143	1	1	Bn-N8-p7374901	Bn-chrA08-p7244857
Bn-A08-p8355054	0.966942149	0.966942149	Bn-N8-p7372220	Bn-chrA08-p7242178
Bn-A08-p8368744	0.983471074	0.983471074	Bn-N8-p7364250	Bn-chrA08-p7233499
Bn-A08-p8372495	0.983471074	0.983471074	Bn-N8-p7363942	Bn-chrA08-p7233191
Bn-A08-p8372634	0.950413223	0.950413223	Bn-N18-p11358848	Bn-chrC08-p11896396
Bn-A08-p8374080	0.991735537	0.991735537	Bn-N8-p7362396	Bn-chrA08-p7231645
Bn-A08-p8378306	0.851239669	0.752066116	Bn-N8-p7358158	Bn-chrCnn_random-p802445
Bn-A08-p8378906	0.991735537	0.991735537	Bn-N8-p7357594	Bn-chrA08-p7228416
Bn-A08-p8385137	0.966942149	0.966942149	Bn-N8-p7351381	Bn-chrA08-p7221120
Bn-A08-p8386874	0.991735537	0.991735537	Bn-N8-p7349644	Bn-chrA08-p7219383
Bn-A08-p8396508	0.990049751	0.990049751	Bn-N8-p7336453	Bn-chrA08-p7205104
Bn-A08-p8397266	0.985074627	0.985074627	Bn-N8-p7335695	Bn-chrA08-p7204346

Bn-A08-p8414586	0.991735537	0.991735537	Bn-N8-p7321405	Bn-chrA08-p7185648
Bn-A08-p8426380	0.950413223	0.950413223	Bn-N18-p11441780	Bn-chrA08-p7167716
Bn-A08-p8432548	0.900826446	0.900826446	Bn-N8-p7654040	Bn-chrA08-p7271108
Bn-A08-p8434044	0.991735537	0.991735537	Bn-N8-p7655528	Bn-chrA08-p7272596
Bn-A08-p8446402	0.991735537	0.991735537	Bn-N8-p7668004	Bn-chrA08-p7285497
Bn-A08-p8447749	0.991735537	0.991735537	Bn-N8-p7669351	Bn-chrA08-p7286844
Bn-A08-p8453264	0.94214876	0.94214876	Bn-N8-p7674868	Bn-chrA08-p7292360
Bn-A08-p8458205	0.993355482	0.990033223	Bn-N8-p7679811	Bn-chrA08-p7297302
Bn-A08-p8470949	0.950413223	0.958677686	Bn-N8-p7689284	Bn-chrA08-p7309118
Bn-A08-p8471051	0.950413223	0.958677686	Bn-N8-p7689384	Bn-chrA08-p7309220
Bn-A08-p8479611	0.986710963	0.986710963	Bn-N8-p7698170	Bn-chrA08-p7329876
Bn-A08-p8479932	0.996677741	0.993355482	Bn-N8-p7698491	Bn-chrA08-p7330197
Bn-A08-p8480536	0.996677741	1	Bn-N8-p7699095	Bn-chrA08-p7330801
Bn-A08-p8480734	0.993355482	0.996677741	Bn-N8-p7699293	Bn-chrA08-p7330999
Bn-A08-p8482679	1	0.995024876	Bn-N8-p7701263	Bn-chrA08-p7333531
Bn-A08-p8493076	0.975124378	0.975124378	Bn-N8-p7710546	Bn-chrA08-p7343067
Bn-A08-p8494172	0.950248756	0.950248756	Bn-N18-p11577952	Bn-chrC08-p12090573
Bn-A08-p8494463	0.966942149	0.966942149	Bn-N8-p7711931	Bn-chrA08-p7344450
Bn-A08-p8495489	0.983471074	0.983471074	Bn-N8-p7712957	Bn-chrA08-p7345476
Bn-A08-p8496013	0.993355482	0.993355482	Bn-N8-p7713481	Bn-chrA08-p7346000
Bn-A08-p8508603	0.975206612	0.983471074	Bn-N8-p7726335	Bn-chrA08-p7358675
Bn-A08-p8520595	0.966942149	0.966942149	Bn-N8-p7738537	Bn-chrA08-p7370630
Bn-A08-p8529251	0.991735537	0.991735537	Bn-N8-p7748382	Bn-chrA08-p7378755
Bn-A08-p8529342	0.930348259	0.930348259	Bn-N8-p7748473	Bn-chrA08-p7378846
Bn-A08-p8533455	0.991735537	0.991735537	Bn-N8-p7752587	Bn-chrA08-p7382960
Bn-A08-p8542502	0.993355482	0.993355482	Bn-N8-p7757996	Bn-chrA08-p7388369
Bn-A08-p8543675	0.965174129	0.965174129	Bn-N8-p7759169	Bn-chrA08-p7389542
Bn-A08-p8543898	0.990033223	0.990033223	Bn-N8-p7759392	Bn-chrA08-p7389765
Bn-A08-p8543911	0.990033223	0.990033223	Bn-N8-p7759405	Bn-chrA08-p7389778
Bn-A08-p8546560	0.996677741	0.996677741	Bn-N8-p7762054	Bn-chrA08-p7392427
Bn-A08-p8567139	0.960199005	0.960199005	Bn-N8-p7799706	Bn-chrA08-p7433018
Bn-A08-p8567867	0.966942149	0.966942149	Bn-N8-p7809353	Bn-chrA08-p7435902
Bn-A08-p8569187	0.958677686	0.958677686	Bn-N8-p7816233	Bn-chrA08-p7442782
Bn-A08-p8570810	0.966942149	0.966942149	Bn-N8-p7792310	Bn-chrA08-p7425622
Bn-A08-p8571258	0.958677686	0.958677686	Bn-N8-p7792759	Bn-chrA08-p7426071
Bn-A08-p8577291	0.818181818	0.818181818	Bn-N8-p7828506	Bn-chrA08-p7455108
Bn-A08-p8577353	0.966942149	0.966942149	Bn-N8-p7828569	Bn-chrA08-p7455171
Bn-A08-p8581123	0.975206612	0.975206612	Bn-N8-p7834013	Bn-chrA08-p7460700
Bn-A08-p8581899	0.980099502	0.980099502	Bn-N8-p7834794	Bn-chrA08-p7461481
Bn-A08-p8582072	0.975206612	0.975206612	Bn-N8-p7834967	Bn-chrA08-p7461654
Bn-A08-p8584951	0.980099502	0.980099502	Bn-N8-p7837852	Bn-chrA08-p7464539
Bn-A08-p8585255	0.966942149	0.966942149	Bn-N8-p7838156	Bn-chrA08-p7464843
Bn-A08-p8586356	0.966942149	0.966942149	Bn-N8-p7839257	Bn-chrA08-p7465944
Bn-A08-p8587056	0.985074627	0.985074627	Bn-N8-p7839957	Bn-chrA08-p7466644
Bn-A08-p8589613	0.980066445	0.983388704	Bn-N8-p7842494	Bn-chrA08-p7469176
Bn-A08-p8601468	0.990049751	0.990049751	Bn-N8-p7859442	Bn-chrA08-p7489479

Bn-A08-p8633780	0.990049751	0.990049751	Bn-N8-p7874940	Bn-chrA08-p7504978
Bn-A08-p8634610	0.990033223	0.990033223	Bn-N8-p7875770	Bn-chrA08-p7505808
Bn-A08-p8644010	0.996677741	0.996677741	Bn-N8-p7884860	Bn-chrA08-p7516073
Bn-A08-p8644734	0.993355482	0.993355482	Bn-N8-p7885583	Bn-chrA08-p7516796
Bn-A08-p8658074	0.993355482	0.993355482	Bn-N8-p7898694	Bn-chrA08-p7529549
Bn-A08-p8672999	0.990033223	0.990033223	Bn-N8-p7915843	Bn-chrA08-p7548444
Bn-A08-p8675006	0.985074627	0.985074627	Bn-N8-p7917936	Bn-chrA08-p7550442
Bn-A08-p8690169	1	1	Bn-N8-p7929559	Bn-chrA08-p7562820
Bn-A08-p8703472	0.955223881	0.940298507	Bn-N8-p7933139	Bn-chrA08-p7565498
Bn-A08-p8710166	0.958677686	0.876033058	Bn-N8-p7940018	Bn-chrC08-p12464832
Bn-A08-p8716833	0.975206612	0.983471074	Bn-N8-p7949374	Bn-chrAnn_random-p331368
Bn-A08-p8721964	1	1	Bn-N8-p7966384	Bn-chrA08-p7580978
Bn-A08-p8727608	0.980066445	0.980066445	Bn-N8-p7972048	Bn-chrA08-p7591188
Bn-A08-p8740002	0.926910299	0.926910299	Bn-N8-p7987878	Bn-chrA08-p7605801
Bn-A08-p8755323	0.996677741	1	Bn-N8-p8005536	Bn-chrA08-p7621329
Bn-A08-p8783951	0.960199005	0.965174129	Bn-N8-p8031999	Bn-chrA08-p7646302
Bn-A08-p8784663	0.996677741	1	Bn-N8-p8032711	Bn-chrA08-p7647014
Bn-A08-p8808046	0.966942149	0.966942149	Bn-N8-p8059826	Bn-chrA08-p7671354
Bn-A08-p8869180	0.966777409	0.966777409	Bn-N8-p8129492	Bn-chrA08-p7738598
Bn-A08-p8872384	0.996677741	1	Bn-N8-p8133241	Bn-chrA08-p7741801
Bn-A08-p8879495	0.986710963	0.976744186	Bn-N8-p8137151	Bn-chrA08-p7745710
Bn-A08-p8909433	0.986710963	0.983388704	Bn-N8-p8162397	Bn-chrA08-p7774655
Bn-A08-p8924822	0.985074627	0.980099502	Bn-N8-p8177958	Bn-chrA08-p7796079
Bn-A08-p8945914	0.996677741	0.976744186	Bn-N8-p8195177	Bn-chrA08-p7812847
Bn-A08-p9133085	0.991735537	1	Bn-N9-p17366777	Bn-chrA09-p14207919
Bn-A08-p9146867	0.996677741	0.993355482	Bn-N9-p17352605	Bn-chrA09-p14222098
Bn-A08-p9172893	0.975206612	0.966942149	Bn-N9-p17329950	Bn-chrA09-p14241872
Bn-A08-p9176343	0.975206612	0.975206612	Bn-N9-p17326481	Bn-chrA09-p14245336
Bn-A08-p9198307	0.958677686	0.966942149	Bn-N9-p17317995	Bn-chrA09-p14265904
Bn-A08-p9199303	0.975206612	0.966942149	Bn-N9-p17317582	Bn-chrA09-p14266900
Bn-A08-p9200154	0.983388704	0.986710963	Bn-N9-p17316731	Bn-chrA09-p14267751
Bn-A08-p9210562	0.990033223	1	Bn-N9-p17292675	Bn-chrA09-p14278251
Bn-A08-p9216064	0.983471074	0.975206612	Bn-N9-p17273441	Bn-chrA09-p14283751
Bn-A08-p9216239	0.976744186	0.980066445	Bn-N9-p17273266	Bn-chrA09-p14283926
Bn-A08-p9216723	0.991735537	0.983471074	Bn-N9-p17272782	Bn-chrA09-p14284410
Bn-A08-p9217601	0.966942149	0.975206612	Bn-N9-p17271892	Bn-chrA09-p14285288
Bn-A08-p9217703	0.983471074	0.975206612	Bn-N9-p17271790	Bn-chrA09-p14285390
Bn-A08-p9218159	0.975206612	0.966942149	Bn-N9-p17271334	Bn-chrA09-p14285846
Bn-A08-p9218449	0.966942149	0.958677686	Bn-N9-p17271044	Bn-chrA09-p14286136
Bn-A08-p9221373	0.859504132	0.933884298	Bn-N9-p17262937	Bn-chrA09-p14289060
Bn-A08-p9226559	0.950413223	0.950413223	Bn-N9-p17252444	Bn-chrA09-p14301049
Bn-A08-p9226756	0.975206612	0.966942149	Bn-N9-p17252246	Bn-chrA09-p14301246
Bn-A08-p9230750	0.975206612	0.983471074	Bn-N9-p17247426	Bn-chrA09-p14305239
Bn-A08-p9232996	0.950413223	0.966942149	Bn-N9-p17245151	Bn-chrA09-p14307520
Bn-A08-p9247445	0.900332226	0.920265781	Bn-N9-p17242160	Bn-chrA09-p14315520
Bn-A08-p9261989	0.983471074	0.975206612	Bn-N9-p17217377	Bn-chrA09-p14330215

Bn-A08-p9265022	0.991735537	0.983471074	Bn-N9-p17214370	Bn-chrA09-p14333248
Bn-A08-p9268915	0.995024876	0.995024876	Bn-N11-p37351569	Bn-chrC01-p32559114
Bn-A08-p9301468	0.983388704	0.96345515	Bn-N9-p17185193	Bn-chrA09-p14372670
Bn-A08-p9308071	0.983471074	0.975206612	Bn-N9-p17179407	Bn-chrA09-p14378514
Bn-A08-p9312404	0.990033223	0.996677741	Bn-N9-p17173515	Bn-chrA09-p14382846
Bn-A08-p9316584	0.991735537	1	Bn-N9-p17160687	Bn-chrA09-p14387026
Bn-A08-p9323955	0.993355482	0.996677741	Bn-N9-p17155996	Bn-chrA09-p14393571
Bn-A08-p9325932	0.993355482	0.996677741	Bn-N9-p17154019	Bn-chrA09-p14395548
Bn-A08-p9326621	0.991735537	0.983471074	Bn-N9-p17153330	Bn-chrA09-p14396237
Bn-A08-p9341774	0.980099502	0.980099502	Bn-N9-p17147048	Bn-chrA09-p14409783
Bn-A08-p9341953	0.985074627	0.985074627	Bn-N9-p17146869	Bn-chrA09-p14409962
Bn-A08-p9343208	0.966777409	1	Bn-N19-p26411957	Bn-chrA09-p14411217
Bn-A08-p9353221	0.884297521	0.966942149	Bn-N19-p26431260	Bn-chrA09-p14422261
Bn-A08-p9355940	0.973421927	0.996677741	Bn-N9-p17130431	Bn-chrA09-p14424988
Bn-A08-p9361440	0.990033223	0.993355482	Bn-N9-p17124929	Bn-chrA09-p14430488
Bn-A08-p9364108	0.993355482	0.990033223	Bn-N9-p17122434	Bn-chrA09-p14432990
Bn-A08-p9367767	0.993355482	0.996677741	Bn-N9-p17118775	Bn-chrA09-p14436647
Bn-A08-p9388258	0.980099502	0.990049751	Bn-N9-p17106253	Bn-chrA09-p14455791
Bn-A08-p9413496	0.950413223	0.983471074	Bn-N9-p17072750	Bn-chrA09-p14482519
Bn-A08-p9429379	0.930232558	0.986710963	Bn-N9-p17049946	Bn-chrA09-p14501550
Bn-A08-p9433046	0.966777409	0.986710963	Bn-N9-p17046292	Bn-chrA09-p14505217
Bn-A08-p9435789	0.873754153	0.857142857	Bn-N9-p17043251	Bn-chrA09-p14507959
Bn-A08-p9436384	0.875621891	0.940298507	Bn-N9-p17042654	Bn-chrA09-p14508554
Bn-A08-p9436578	0.926910299	0.96013289	Bn-N9-p17042471	Bn-chrA09-p14508748
Bn-A08-p9440682	0.970099668	0.956810631	Bn-Scaffold02313-p2	Bn-chrA09-p14512851
Bn-A08-p9450796	0.983471074	0.991735537	Bn-N3-p25865481	Bn-chrA09-p14519701
Bn-A08-p9480351	0.950413223	0.94214876	Bn-N9-p17014714	Bn-chrA09-p14547954
Bn-A08-p9486336	0.702479339	0.983471074	Bn-N9-p17012014	Bn-chrA09-p14554413
Bn-A08-p9486485	0.958677686	0.966942149	Bn-N9-p17011865	Bn-chrA09-p14554562
Bn-A08-p9503231	0.983471074	0.495867769	Bn-N19-p26682267	Bn-chrC09-p22011839
Bn-A08-p9504526	0.983471074	0.991735537	Bn-Scaffold19849-p4	Bn-chrA09-p14572943
Bn-A08-p9505581	0.933884298	0.950413223	Bn-N9-p16991039	Bn-chrA09-p14573998
Bn-A08-p9536971	0.906976744	0.996677741	Bn-N18-p36998192	Bn-chrA07-p5034562
Bn-A08-p9550162	0.970149254	0.985074627	Bn-N2-p13009662	Bn-chrA09-p14617594
Bn-A08-p9550680	0.980099502	0.980099502	Bn-N2-p13010180	Bn-chrA02-p9811985
Bn-A08-p9550767	0.970149254	0.975124378	Bn-N2-p13010267	Bn-chrA09-p14618199
Bn-A08-p9551013	0.975124378	0.980099502	Bn-N2-p13010513	Bn-chrA09-p14618445
Bn-A08-p9551155	0.995024876	0.995024876	Bn-N2-p13010655	Bn-chrA02-p9812460
Bn-A08-p9555028	0.983471074	0.975206612	Bn-N9-p16972814	Bn-chrA09-p14622460
Bn-A08-p9555984	0.966942149	0.958677686	Bn-N9-p16971864	Bn-chrA09-p14623416
Bn-A08-p9567284	0.983471074	0.991735537	Bn-N9-p16949315	Bn-chrA09-p14645907
Bn-A08-p9582412	0.976744186	0.996677741	Bn-N9-p16939667	Bn-chrA09-p14660630
Bn-A08-p9619600	0.933884298	0.933884298	Bn-N9-p16915118	Bn-chrA09-p14688705
Bn-A08-p9656252	1	0.995024876	Bn-N9-p16871598	Bn-chrA09-p14733542
Bn-A08-p9699333	0.965174129	0.985074627	Bn-N9-p16825631	Bn-chrA09-p14774571
Bn-A08-p9705799	0.950413223	0.834710744	Bn-N9-p16824298	Bn-chrA09-p14776335

Bn-A08-p9707204	0.966942149	0.975206612	Bn-N9-p16822886	Bn-chrA09-p14778349
Bn-A08-p9708411	0.933884298	0.94214876	Bn-N9-p16821715	Bn-chrA09-p14779079
Bn-A08-p9713705	0.958677686	0.950413223	Bn-N9-p16812191	Bn-chrA09-p14790460
Bn-A08-p9715574	1	0.991735537	Bn-N9-p16810341	Bn-chrA09-p14797604
Bn-A08-p9718210	0.958677686	0.966942149	Bn-N9-p16807701	Bn-chrAnn_random-p435780
Bn-A08-p9718264	0.966942149	0.966942149	Bn-N9-p16807647	Bn-chrAnn_random-p435780
Bn-A08-p9718553	0.958677686	0.867768595	Bn-N9-p16807358	Bn-chrA09-p14804430
Bn-A08-p9719082	0.983471074	0.991735537	Bn-N9-p16806829	Bn-chrA09-p14804961
Bn-A08-p9720373	0.983471074	0.975206612	Bn-N9-p16805538	Bn-chrA09-p14806233
Bn-A08-p9720439	0.975206612	0.958677686	Bn-N9-p16805472	Bn-chrA09-p14806299
Bn-A08-p9723809	0.743801653	0.975206612	Bn-N19-p27073074	Bn-chrA09-p14809730
Bn-A08-p9729245	0.983471074	0.991735537	Bn-N9-p16796452	Bn-chrA09-p14815388
Bn-A08-p9729282	0.983471074	0.991735537	Bn-N9-p16796415	Bn-chrA09-p14815425
Bn-A08-p9730934	0.983471074	0.975206612	Bn-N9-p16794790	Bn-chrA09-p14821002
Bn-A08-p9739790	0.990033223	0.986710963	Bn-N9-p16777614	Bn-chrA09-p14843969
Bn-A08-p9740508	0.983388704	0.980066445	Bn-N9-p16776889	Bn-chrA09-p14844694
Bn-A08-p9741074	0.983388704	0.976744186	Bn-N9-p16776332	Bn-chrA09-p14845251
Bn-A08-p9749557	0.958677686	0.950413223	Bn-N9-p16766066	Bn-chrA09-p14847574
Bn-A08-p9781356	0.958677686	0.950413223	Bn-N9-p16736839	Bn-chrA09-p14861805
Bn-A08-p9781699	0.983471074	0.917355372	Bn-N9-p16736496	Bn-chrCnn_random-p451470
Bn-A08-p9795033	0.958677686	0.950413223	Bn-N9-p16721191	Bn-chrUnn_random-p481149
Bn-A08-p9795423	0.950248756	0.955223881	Bn-N9-p16720801	Bn-chrA09-p14869360
Bn-A08-p9917420	0.990033223	0.990033223	Bn-N7-p4007819	Bn-chrA07-p4012455
Bn-A08-p9918228	0.980066445	0.980066445	Bn-N7-p4008627	Bn-chrA07-p4013263
Bn-A08-p10026421	0.958677686	0.950413223	Bn-N8-p9593043	Bn-chrA08-p8026233
Bn-A08-p10031059	0.880398671	0.990033223	Bn-N8-p9597287	Bn-chrA08-p8030469
Bn-A08-p10067927	0.975206612	0.975206612	Bn-N8-p9634172	Bn-chrA08-p8069111
Bn-A08-p10068904	0.983471074	0.975206612	Bn-N8-p9637361	Bn-chrA08-p8070063
Bn-A08-p10071208	1	0.991735537	Bn-N8-p9643305	Bn-chrA08-p8077835
Bn-A08-p10098062	0.991735537	0.983471074	Bn-N8-p9676400	Bn-chrA08_random-p133118
Bn-A08-p10101802	1	0.991735537	Bn-N8-p9681559	Bn-chrA08_random-p133631
Bn-A08-p10106723	0.966942149	0.966942149	Bn-N8-p9686586	Bn-chrA08_random-p134391
Bn-A08-p10115835	0.966942149	0.958677686	Bn-N8-p9694008	Bn-chrCnn_random-p173049
Bn-A08-p10118532	0.970149254	0.970149254	Bn-N18-p17777275	Bn-chrCnn_random-p173085
Bn-A08-p10123561	0.876033058	0.94214876	Bn-N8-p9701302	Bn-chrA08_random-p135854
Bn-A08-p10128352	1	0.996677741	Bn-N8-p9705188	Bn-chrA08_random-p136447
Bn-A08-p10128687	0.986710963	0.993355482	Bn-N8-p9705525	Bn-chrA08_random-p136480
Bn-A08-p10128760	0.990033223	0.993355482	Bn-N8-p9705598	Bn-chrA08_random-p136487
Bn-A08-p10136371	0.966942149	0.975206612	Bn-N8-p9724412	Bn-chrA08_random-p137657
Bn-A08-p10147092	0.958677686	0.983471074	Bn-Scaffold01053-p9	Bn-chrA08_random-p138286
Bn-A08-p10180719	0.991735537	0.983471074	Bn-N8-p9759934	Bn-chrAnn_random-p205189
Bn-A08-p10190275	0.983471074	0.975206612	Bn-N8-p9790118	Bn-chrCnn_random-p333095
Bn-A08-p10214814	0.989583333	1	Bn-N8-p9810999	Bn-chrA08-p8162115
Bn-A08-p10215334	0.991735537	0.991735537	Bn-N8-p9811598	Bn-chrA08-p8162714
Bn-A08-p10215623	0.975206612	0.983471074	Bn-N8-p9811890	Bn-chrA08-p8163003
Bn-A08-p10222411	0.933884298	0.975206612	Bn-N8-p9817175	Bn-chrA08-p8166362

Bn-A08-p10270998	0.724252492	0.940199336	Bn-N8-p9938439	Bn-chrA08-p8220484
Bn-A08-p10281337	0.993355482	0.990033223	Bn-N8-p9946893	Bn-chrA08-p8230927
Bn-A08-p10329356	0.996677741	0.996677741	Bn-N8-p9992455	Bn-chrA08-p8285585
Bn-A08-p10329729	0.983471074	0.975206612	Bn-N8-p9992828	Bn-chrA08-p8285958
Bn-A08-p10394354	0.993355482	0.993355482	Bn-N8-p10038893	Bn-chrA08-p8335028
Bn-A08-p10429774	0.592039801	0.980099502	Bn-N7-p11317129	Bn-chrA03-p20854690
Bn-A08-p10439396	0.948275862	0.948275862	Bn-N8-p10085528	Bn-chrA08-p8367791
Bn-A08-p10440699	0.990049751	0.810945274	Bn-N8-p10098600	Bn-chrA08-p8369067
Bn-A08-p10443959	0.991735537	0.983471074	Bn-N8-p10102868	Bn-chrA08-p8372628
Bn-A08-p10452462	0.94214876	0.925619835	Bn-N8-p10109726	Bn-chrC08-p16678522
Bn-A08-p10472777	0.975206612	0.983471074	Bn-N8-p10153445	Bn-chrA08-p8477909
Bn-A08-p10473245	1	0.991735537	Bn-N8-p10153912	Bn-chrA08-p8478419
Bn-A08-p10480848	0.991735537	0.966942149	Bn-N8-p10160106	Bn-chrA08-p8479182
Bn-A08-p10494058	0.991735537	0.983471074	Bn-N8-p10168873	Bn-chrA08-p8490087
Bn-A08-p10509442	0.88372093	0.880398671	Bn-N8-p10184956	Bn-chrA08-p8505225
Bn-A08-p10528898	0.965174129	0.970149254	Bn-N8-p10210186	Bn-chrA08-p8540669
Bn-A08-p10581818	0.900826446	0.900826446	Bn-N8-p10249711	Bn-chrA08-p8586284
Bn-A08-p10593605	0.991735537	0.983471074	Bn-N8-p10277538	Bn-chrA08-p8594558
Bn-A08-p10608550	0.933884298	0.94214876	Bn-N8-p10286217	Bn-chrA08-p8600171
Bn-A08-p10615012	0.985074627	0.985074627	Bn-N8-p10291520	Bn-chrA08-p8612571
Bn-A08-p10626905	0.975206612	0.983471074	Bn-N8-p10306805	Bn-chrA08-p8625305
Bn-A08-p10663544	0.965174129	0.960199005	Bn-N8-p10331185	Bn-chrA08-p8653883
Bn-A08-p10711225	0.990033223	0.973421927	Bn-N8-p10370278	Bn-chrA08_random-p138652
Bn-A08-p10716777	0.996677741	0.96013289	Bn-N8-p10375220	Bn-chrC03-p52144560
Bn-A08-p10733248	1	1	Bn-N8-p9161475	Bn-chrAnn_random-p334454
Bn-A08-p10774582	0.958677686	0.826446281	Bn-Scaffold08042-p4	Bn-chrA08-p8683446
Bn-A08-p10778121	0.975206612	0.966942149	Bn-N8-p10421483	Bn-chrA08-p8686053
Bn-A08-p10789401	0.966942149	0.958677686	Bn-N8-p10435335	Bn-chrC03-p52319011
Bn-A08-p10795996	0.980066445	0.950166113	Bn-N8-p10449072	Bn-chrA08-p8702203
Bn-A08-p10798067	0.986710963	0.990033223	Bn-N8-p10454779	Bn-chrA08-p8704290
Bn-A08-p10809139	0.980066445	0.993355482	Bn-N8-p10465446	Bn-chrA08-p8710654
Bn-A08-p10822662	0.993355482	1	Bn-N8-p10475491	Bn-chrA08-p8724809
Bn-A08-p10824964	1	0.983471074	Bn-N8-p10477792	Bn-chrA08-p8727150
Bn-A08-p10844808	0.996677741	1	Bn-N8-p10496842	Bn-chrA08-p8746227
Bn-A08-p10846901	0.983388704	0.990033223	Bn-N8-p10498935	Bn-chrA08-p8748320
Bn-A08-p10887204	0.933884298	0.933884298	Bn-N8-p10534243	Bn-chrA08-p8782347
Bn-A08-p11212494	0.975206612	0.900826446	Bn-N13-p59513152	Bn-chrC03-p53379280
Bn-A08-p11304602	0.950413223	0.94214876	Bn-N8-p10986366	Bn-chrC03-p53510439
Bn-A08-p11308315	0.876033058	0.983471074	Bn-N13-p59642218	Bn-chrA08-p9169040
Bn-A08-p11310535	0.991735537	0.983471074	Bn-N8-p10992150	Bn-chrA08-p9171259
Bn-A08-p11329864	0.980066445	0.976744186	Bn-N8-p11010771	Bn-chrA08-p9184410
Bn-A08-p11352069	0.975124378	0.975124378	Bn-N8-p11022045	Bn-chrA08-p9197893
Bn-A08-p11380260	0.975124378	0.960199005	Bn-N8-p11049041	Bn-chrA08-p9226978
Bn-A08-p11392487	0.966777409	0.980066445	Bn-N8-p11060899	Bn-chrA08-p9239206
Bn-A08-p11452221	0.94214876	0.950413223	Bn-N8-p11123277	Bn-chrA08-p9295269
Bn-A08-p11458466	0.989583333	0.989583333	Bn-N8-p11129532	Bn-chrA08-p9301524

Bn-A08-p11459293	0.980099502	0.980099502	Bn-N8-p11131297	Bn-chrA08-p9302406
Bn-A08-p11667191	0.991735537	0.991735537	Bn-N8-p11338956	Bn-chrA08-p9509351
Bn-A08-p11679083	0.980066445	0.980066445	Bn-N8-p11352102	Bn-chrA08-p9522791
Bn-A08-p12054745	0.985074627	0.985074627	Bn-N3-p26994721	Bn-chrA03-p25125263
Bn-A08-p12058242	0.990033223	0.990033223	Bn-N3-p26998101	Bn-chrA03-p25128642
Bn-A08-p12064516	0.993355482	0.993355482	Bn-N3-p27004808	Bn-chrA03-p25135300
Bn-A08-p12360518	0.991735537	0.991735537	Bn-N8-p11991228	Bn-chrA08-p10182283
Bn-A08-p12526403	0.905472637	0.905472637	Bn-N8-p12116054	Bn-chrA08-p10304518
Bn-A08-p12555227	0.991735537	0.537190083	Bn-N8-p12138124	Bn-chrCnn_random-p467910
Bn-A08-p12556455	0.970149254	0.975124378	Bn-N8-p12139346	Bn-chrA08-p10335313
Bn-A08-p12559372	0.966942149	0.991735537	Bn-N8-p12142760	Bn-chrA08-p10338218
Bn-A08-p12564228	0.966942149	0.975206612	Bn-N8-p12149002	Bn-chrA08_random-p159385
Bn-A08-p12599446	0.995024876	0.990049751	Bn-N8-p12191131	Bn-chrA08-p10515602
Bn-A08-p12638473	1	0.990033223	Bn-N8-p12235835	Bn-chrA08-p10557348
Bn-A08-p12660208	0.983471074	0.94214876	Bn-N8-p12248695	Bn-chrC03-p56050680
Bn-A08-p12674243	0.980099502	0.975124378	Bn-N8-p12260577	Bn-chrAnn_random-p410058
Bn-A08-p12674577	0.983471074	0.991735537	Bn-N8-p12260911	Bn-chrAnn_random-p410055
Bn-A08-p12698364	0.983471074	0.983471074	Bn-N8-p12279728	Bn-chrA08-p10373237
Bn-A08-p12699181	0.983471074	0.983471074	Bn-N8-p12280545	Bn-chrA08-p10374054
Bn-A08-p12752628	0.983471074	0.991735537	Bn-N8-p12372214	Bn-chrA08-p10449878
Bn-A08-p12761022	0.993355482	0.993355482	Bn-N8-p12380623	Bn-chrA08-p10458232
Bn-A08-p12767403	0.975124378	0.970149254	Bn-N8-p12386789	Bn-chrA08-p10460989
Bn-A08-p12781426	0.958677686	0.966942149	Bn-N8-p12398027	Bn-chrA08-p10473953
Bn-A08-p12794893	0.975206612	0.975206612	Bn-N8-p12401667	Bn-chrA08-p10478871
Bn-A08-p12797532	0.983471074	0.991735537	Bn-N8-p12404325	Bn-chrA08-p10481493
Bn-A08-p12812977	0.995024876	0.810945274	Bn-N8-p12415810	Bn-chrCnn_random-p138513
Bn-A08-p12814556	0.983471074	0.983471074	Bn-N8-p12421629	Bn-chrA08-p10579999
Bn-A08-p12820786	0.67768595	0.983471074	Bn-N8-p12430957	Bn-chrA08-p10587676
Bn-A08-p12828472	0.989583333	0.989583333	Bn-N8-p12438569	Bn-chrA08-p10599319
Bn-A08-p12836248	0.990049751	0.990049751	Bn-N8-p12456939	Bn-chrA08-p10616097
Bn-A08-p12902384	0.958677686	0.966942149	Bn-N8-p12533046	Bn-chrA08-p10682692
Bn-A08-p12904087	0.966942149	0.950413223	Bn-N8-p12534750	Bn-chrA08-p10684389
Bn-A08-p12905848	1	0.892561983	Bn-N8-p12536740	Bn-chrC03-p56466151
Bn-A08-p12913949	0.983471074	0.991735537	Bn-N8-p12546660	Bn-chrA08-p10694561
Bn-A08-p12923799	0.94214876	0.760330579	Bn-N8-p12556531	Bn-chrC03-p56506402
Bn-A08-p12940197	0.983471074	0.975206612	Bn-N8-p12568329	Bn-chrA08-p10716400
Bn-A08-p13009633	0.950413223	0.966942149	Bn-N8-p12643613	Bn-chrA08-p10785647
Bn-A08-p13011710	0.933884298	0.950413223	Bn-N8-p12646066	Bn-chrA08-p10787814
Bn-A08-p13019549	0.990033223	0.990033223	Bn-N13-p63010986	Bn-chrC03-p56642249
Bn-A08-p13030362	0.983471074	0.975206612	Bn-N8-p12708538	Bn-chrA08-p10839191
Bn-A08-p13030439	1	0.991735537	Bn-N8-p12708615	Bn-chrA08-p10839268
Bn-A08-p13036508	0.966942149	0.975206612	Bn-N8-p12716450	Bn-chrA08-p10848585
Bn-A08-p13060591	1	0.995024876	Bn-N8-p12742671	Bn-chrA08-p10872039
Bn-A08-p13066424	0.73553719	0.975206612	Bn-N8-p12754693	Bn-chrA08-p10878219
Bn-A08-p13076304	0.985074627	0.990049751	Bn-N8-p12763413	Bn-chrA08-p10884646
Bn-A08-p13076305	0.950413223	0.958677686	Bn-N8-p12763413	Bn-chrA08-p10884646

Bn-A08-p13091365	0.993355482	0.996677741	Bn-N8-p12779087	Bn-chrAnn_random-p477572
Bn-A08-p13200580	0.985074627	0.980099502	Bn-N8-p12863686	Bn-chrA08_random-p160663
Bn-A08-p13201183	0.995024876	0.990049751	Bn-N8-p12864291	Bn-chrA08_random-p160602
Bn-A08-p13207923	0.995024876	0.980099502	Bn-N13-p63308773	Bn-chrA08-p10953353
Bn-A08-p13214314	1	0.817275748	Bn-N8-p12878798	Bn-chrA08-p10959702
Bn-A08-p13214532	0.985074627	0.915422886	Bn-N8-p12879016	Bn-chrA08-p10959920
Bn-A08-p13221380	0.995024876	0.995024876	Bn-N8-p12891668	Bn-chrA08-p10967903
Bn-A08-p13230287	0.970149254	0.875621891	Bn-N8-p12900897	Bn-chrA08-p10975937
Bn-A08-p13239621	0.990049751	0.990049751	Bn-N8-p12910240	Bn-chrA08-p10991702
Bn-A08-p13239816	0.986710963	0.983388704	Bn-N8-p12910435	Bn-chrA08-p10991897
Bn-A08-p13256383	0.991735537	0.94214876	Bn-N8-p12926416	Bn-chrC03-p57076933
Bn-A08-p13269999	0.985074627	0.985074627	Bn-N2-p25048579	Bn-chrA02-p21311223
Bn-A08-p13270793	0.970149254	0.970149254	Bn-N8-p12940453	Bn-chrA02-p21310429
Bn-A08-p13271519	0.995024876	0.990049751	Bn-N8-p12941278	Bn-chrA08-p11040366
Bn-A08-p13271950	0.985074627	0.990049751	Bn-N8-p12941709	Bn-chrA08-p11040797
Bn-A08-p13284369	0.983471074	0.991735537	Bn-N8-p12952657	Bn-chrA08-p11051828
Bn-A08-p13289070	0.966942149	0.958677686	Bn-N8-p12957539	Bn-chrA08-p11057223
Bn-A08-p13294859	0.991735537	1	Bn-N8-p12961663	Bn-chrA08-p11061347
Bn-A08-p13298788	1	0.364583333	Bn-N8-p12965892	Unknown
Bn-A08-p13303525	1	0.991735537	Bn-N8-p12971477	Bn-chrA08_random-p166492
Bn-A08-p13322654	0.975206612	0.966942149	Bn-N8-p12991220	Bn-chrA08-p11064752
Bn-A08-p13325281	0.983471074	0.983471074	Bn-N8-p12993880	Bn-chrA08-p11067381
Bn-A08-p13327070	0.970149254	0.965174129	Bn-N8-p12999889	Bn-chrA08-p11074344
Bn-A08-p13328135	0.995024876	0.975124378	Bn-N8-p13000954	Bn-chrA08-p11075409
Bn-A08-p13342419	0.990033223	0.990033223	Bn-N8-p13014295	Bn-chrA08-p11088552
Bn-A08-p13352430	0.983471074	0.991735537	Bn-N8-p13023342	Bn-chrA08-p11097577
Bn-A08-p13354792	0.94214876	0.950413223	Bn-N8-p13027358	Bn-chrA08-p11099938
Bn-A08-p13357594	0.94214876	0.950413223	Bn-N8-p13030141	Bn-chrA08-p11102743
Bn-A08-p13357808	0.958677686	0.966942149	Bn-N8-p13030357	Bn-chrA08-p11102958
Bn-A08-p13363830	1	1	Bn-N8-p13038993	Bn-chrA08-p11110869
Bn-A08-p13376746	0.955223881	0.955223881	Bn-N8-p13049362	Bn-chrA08-p11121238
Bn-A08-p13379983	0.975124378	0.726368159	Bn-N8-p13052563	Bn-chrA08-p11124437
Bn-A08-p13382119	0.909090909	0.975206612	Bn-N8-p13054634	Bn-chrA08-p11126579
Bn-A08-p13384059	0.996677741	0.996677741	Bn-N8-p13055365	Bn-chrA08-p11127307
Bn-A08-p13401821	0.94214876	0.950413223	Bn-N8-p13062865	Bn-chrA08-p11146633
Bn-A08-p13409843	0.985074627	0.990049751	Bn-N8-p13070885	Bn-chrA08-p11154553
Bn-A08-p13427247	0.983471074	0.991735537	Bn-N8-p13081679	Bn-chrA08-p11166362
Bn-A08-p13427290	0.966942149	0.991735537	Bn-N8-p13081722	Bn-chrA08-p11166405
Bn-A08-p13428079	0.991735537	1	Bn-N8-p13082114	Bn-chrA08-p11167194
Bn-A08-p13428547	0.991735537	1	Bn-N8-p13082578	Bn-chrA08-p11167662
Bn-A08-p13429015	0.909090909	0.975206612	Bn-N8-p13085109	Bn-chrA08-p11168130
Bn-A08-p13429249	0.966942149	0.975206612	Bn-N8-p13085351	Bn-chrA08-p11168364
Bn-A08-p13431798	0.983471074	0.991735537	Bn-N8-p13089404	Bn-chrA08-p11170913
Bn-A08-p13436029	0.94214876	0.950413223	Bn-N8-p13102695	Bn-chrA08-p11175120
Bn-A08-p13440837	0.991735537	0.983471074	Bn-N8-p13113964	Bn-chrAnn_random-p360729
Bn-A08-p13454167	1	0.991735537	Bn-N8-p13128437	Bn-chrAnn_random-p332075

Bn-A08-p13460499	0.975206612	0.983471074	Bn-N8-p13133724	Bn-chrA08-p11202605
Bn-A08-p13462776	0.958677686	0.950413223	Bn-N8-p13135392	Bn-chrA08-p11212033
Bn-A08-p13465514	0.983471074	0.991735537	Bn-N8-p13142375	Bn-chrA08-p11215882
Bn-A08-p13484039	0.983471074	0.975206612	Bn-N8-p13158827	Bn-chrA08-p11232311
Bn-A08-p13485948	0.983388704	0.976744186	Bn-N8-p13160734	Bn-chrA08-p11234469
Bn-A08-p13486356	0.985074627	0.855721393	Bn-N8-p13161141	Bn-chrA08-p11234854
Bn-A08-p13486650	0.966942149	0.975206612	Bn-N8-p13161435	Bn-chrA08-p11235153
Bn-A08-p13489827	0.975206612	0.966942149	Bn-N8-p13164243	Bn-chrA08-p11236466
Bn-A08-p13504812	0.990033223	0.990033223	Bn-N8-p13183443	Bn-chrA08-p11262922
Bn-A08-p13515130	1	1	Bn-N8-p13195912	Bn-chrA08-p11274768
Bn-A08-p13515686	0.983471074	0.983471074	Bn-N8-p13196478	Bn-chrA08-p11275334
Bn-A08-p13516445	0.975206612	0.975206612	Bn-N8-p13197269	Bn-chrA08-p11276125
Bn-A08-p13518735	0.631229236	0.96013289	Bn-N13-p63947446	Bn-chrAnn_random-p36837C
Bn-A08-p13522752	0.975206612	0.975206612	Bn-N8-p13207024	Bn-chrA08-p11285216
Bn-A08-p13526497	0.991735537	0.991735537	Bn-N8-p13210755	Bn-chrA08-p11288946
Bn-A08-p13527152	0.958677686	0.958677686	Bn-N8-p13211413	Bn-chrA08-p11289604
Bn-A08-p13538895	0.966942149	0.975206612	Bn-N8-p13223769	Bn-chrA08-p11301755
Bn-A08-p13544679	0.958677686	0.884297521	Bn-N8-p13225619	Bn-chrA08-p11303576
Bn-A08-p13559192	0.975206612	0.842975207	Bn-N8-p13239149	Bn-chrA08-p11318524
Bn-A08-p13573114	0.965174129	0.970149254	Bn-N8-p13253873	Bn-chrA08-p11333154
Bn-A08-p13577914	0.96013289	0.96345515	Bn-N8-p13258045	Bn-chrA08-p11336114
Bn-A08-p13580050	0.925619835	0.925619835	Bn-N8-p13260187	Bn-chrA08-p11338232
Bn-A08-p13606285	0.991735537	0.983471074	Bn-N8-p13273836	Bn-chrA08-p11355084
Bn-A08-p13616733	0.995024876	0.990049751	Bn-Scaffold51789-p1	Bn-chrA08-p11367626
Bn-A08-p13626189	0.975206612	0.983471074	Bn-N8-p13290340	Bn-chrA08-p11377260
Bn-A08-p13626982	0.991735537	0.983471074	Bn-N8-p13291138	Bn-chrA08-p11378053
Bn-A08-p13638847	0.975206612	0.983471074	Bn-N8-p13303318	Bn-chrA08-p11390137
Bn-A08-p13670107	0.975206612	0.966942149	Bn-N8-p13329479	Bn-chrA08-p11415638
Bn-A08-p13692951	0.877076412	0.936877076	Bn-N8-p13376528	Bn-chrAnn_random-p379165
Bn-A08-p13707991	0.995633188	0.991266376	Bn-N8-p13387562	Bn-chrA08-p11434427
Bn-A08-p13708497	0.983471074	0.983471074	Bn-N8-p13388037	Bn-chrA08-p11434902
Bn-A08-p13708877	0.950413223	0.950413223	Bn-N8-p13391245	Bn-chrA08-p11437232
Bn-A08-p13709853	0.983471074	0.983471074	Bn-N8-p13392130	Bn-chrA08-p11438117
Bn-A08-p13785392	0.990033223	0.990033223	Bn-N8-p13468253	Bn-chrA08-p11518404
Bn-A08-p13785675	0.976744186	0.976744186	Bn-N8-p13468536	Bn-chrA08-p11518687
Bn-A08-p13803301	0.990033223	0.990033223	Bn-N8-p13486232	Bn-chrA08-p11534091
Bn-A08-p13830207	0.966942149	0.966942149	Bn-N8-p13516299	Bn-chrA08-p11563123
Bn-A08-p13856852	0.975206612	0.958677686	Bn-N8-p13544730	Bn-chrC03_random-p604920
Bn-A08-p13860913	0.991735537	0.991735537	Bn-N8-p13548962	Bn-chrA08-p11589543
Bn-A08-p13864869	1	1	Bn-N8-p13553599	Bn-chrA08-p11593500
Bn-A08-p13876356	0.990033223	0.976744186	Bn-N8-p13565760	Bn-chrA08-p11606302
Bn-A08-p13892926	0.996677741	0.996677741	Bn-N8-p13577434	Bn-chrA08-p11622375
Bn-A08-p13897110	0.986710963	0.980066445	Bn-N8-p13586404	Bn-chrA08-p11628335
Bn-A08-p13901348	0.980099502	0.985074627	Bn-N8-p13590881	Bn-chrA08-p11632694
Bn-A08-p13932634	0.975206612	0.975206612	Bn-N8-p13629386	Bn-chrA08-p11662960
Bn-A08-p13935095	0.966942149	0.958677686	Bn-N8-p13631853	Bn-chrA08-p11663972

Bn-A08-p13938191	0.975206612	0.975206612	Bn-N8-p13635394	Bn-chrA08-p11667067
Bn-A08-p13944679	1	0.991735537	Bn-N8-p13643299	Bn-chrA08-p11673856
Bn-A08-p13946032	1	0.991735537	Bn-N8-p13644648	Bn-chrA08-p11675205
Bn-A08-p13953904	0.991735537	1	Bn-N8-p13652941	Bn-chrA08-p11682796
Bn-A08-p13958465	0.983471074	0.975206612	Bn-N8-p13659060	Bn-chrA08-p11688286
Bn-A08-p13964091	0.986710963	0.797342193	Bn-N8-p13664714	Bn-chrA08-p11693975
Bn-A08-p13966241	0.955223881	0.955223881	Bn-N8-p13668355	Bn-chrA08-p11696159
Bn-A08-p13995190	1	0.991735537	Bn-N8-p13700542	Bn-chrA08-p11722036
Bn-A08-p14002120	0.983471074	0.537190083	Bn-N8-p13705427	Bn-chrAnn_random-p25707C
Bn-A08-p14011080	0.794019934	0.993355482	Bn-N8-p13731971	Bn-chrA08_random-p168914
Bn-A08-p14011808	0.966942149	0.975206612	Bn-N8-p13732712	Bn-chrA08_random-p168841
Bn-A08-p14014365	0.983471074	0.991735537	Bn-N8-p13735227	Bn-chrA08_random-p168583
Bn-A08-p14026432	1	1	Bn-N8-p13745920	Bn-chrA08_random-p167514
Bn-A08-p14028012	0.975206612	0.975206612	Bn-N8-p13747482	Bn-chrA08_random-p167357
Bn-A08-p14045962	0.983471074	0.983471074	Bn-N8-p13767596	Bn-chrA08-p11749179
Bn-A08-p14056618	0.983471074	0.983471074	Bn-N8-p13773599	Bn-chrA08-p11755184
Bn-A08-p14067094	0.990033223	0.980066445	Bn-N8-p13785515	Bn-chrA08-p11767476
Bn-A08-p14067361	0.991735537	1	Bn-N8-p13785782	Bn-chrA08-p11767739
Bn-A08-p14086488	0.975124378	0.975124378	Bn-N8-p13804400	Bn-chrA08-p11777925
Bn-A08-p14086982	0.970149254	0.975124378	Bn-N8-p13804894	Bn-chrA08-p11778419
Bn-A08-p14117791	0.993355482	0.990033223	Bn-N8-p13829513	Bn-chrA08-p11815271
Bn-A08-p14128886	0.991735537	1	Bn-N8-p13838719	Bn-chrA08-p11827449
Bn-A08-p14133917	0.975206612	0.983471074	Bn-N8-p13842510	Bn-chrA08-p11831251
Bn-A08-p14156050	0.983471074	0.991735537	Bn-N8-p13860004	Bn-chrA08-p11851803
Bn-A08-p14163751	0.572139303	0.985074627	Bn-N18-p20069358	Bn-chrA08-p11858903
Bn-A08-p14167389	0.827242525	0.823920266	Bn-N8-p13866389	Bn-chrC08-p18080770
Bn-A08-p14218543	0.995024876	0.995024876	Bn-N8-p13911141	Bn-chrA08-p11905749
Bn-A08-p14218712	0.985074627	0.985074627	Bn-N8-p13910604	Bn-chrA08-p11905918
Bn-A08-p14240368	0.993355482	0.993355482	Bn-N8-p13933961	Bn-chrA08-p11928193
Bn-A08-p14276094	0.933554817	0.933554817	Bn-N8-p13965690	Bn-chrA08-p11962204
Bn-A08-p14327130	0.965174129	0.915422886	Bn-N8-p14035355	Bn-chrC08-p17676991
Bn-A08-p14327570	0.865671642	0.855721393	Bn-N8-p14035077	Bn-chrC08-p17676780
Bn-A08-p14351609	0.973421927	0.973421927	Bn-N8-p14059113	Bn-chrA08-p12051586
Bn-A08-p14351709	0.966777409	0.966777409	Bn-N8-p14059213	Bn-chrA08-p12051686
Bn-A08-p14368762	0.993355482	0.993355482	Bn-N11-p15465238	Bn-chrC01-p13670713
Bn-A08-p14369220	0.990033223	0.990033223	Bn-N8-p14071010	Bn-chrA08-p12061844
Bn-A08-p14375712	0.993355482	0.993355482	Bn-N8-p14071271	Bn-chrA08-p12062105
Bn-A08-p14447288	0.980066445	0.973421927	Bn-N8-p14161558	Bn-chrA08-p12146537
Bn-A08-p14462039	0.986710963	0.986710963	Bn-N8-p14174463	Bn-chrA08-p12159132
Bn-A08-p14493385	0.970099668	0.990033223	Bn-N8-p14218449	Bn-chrA08-p12192500
Bn-A08-p14496135	0.925619835	0.950413223	Bn-N18-p19427490	Bn-chrA08-p12196324
Bn-A08-p14498301	0.92039801	0.910447761	Bn-N8-p14223379	Bn-chrA08-p12198504
Bn-A08-p14500962	0.958677686	0.966942149	Bn-N8-p14225473	Bn-chrA08-p12201972
Bn-A08-p14512862	0.966942149	0.975206612	Bn-N8-p14236375	Bn-chrA08-p12213572
Bn-A08-p14516260	0.910447761	0.915422886	Bn-N8-p14240581	Bn-chrA08-p12216007
Bn-A08-p14538807	0.843853821	0.84717608	Bn-N8-p14264539	Bn-chrA08-p12241951

Bn-A08-p14554907	0.685950413	0.966942149	Bn-N8-p14268064	Bn-chrA08-p12253551
Bn-A08-p14594465	0.960199005	0.825870647	Bn-N8-p14300313	Bn-chrCnn_random-p163435
Bn-A08-p14597378	1	0.55721393	Bn-N8-p14303241	Bn-chrCnn_random-p688368
Bn-A08-p14597858	1	0.84079602	Bn-N8-p14303721	Bn-chrCnn_random-p688364
Bn-A08-p14611553	0.94214876	0.950413223	Bn-N8-p14324042	Bn-chrA08_random-p180035
Bn-A08-p14615989	0.776859504	0.983471074	Bn-N8-p14328606	Bn-chrA08_random-p179442
Bn-A08-p14616048	0.975206612	0.991735537	Bn-N8-p14328665	Bn-chrA08_random-p179436
Bn-A08-p14616742	0.966942149	0.975206612	Bn-N8-p14329641	Bn-chrA08_random-p179367
Bn-A08-p14644041	0.985074627	0.985074627	Bn-N9-p17082767	Bn-chrA01-p10900339
Bn-A08-p14658507	0.966942149	0.958677686	Bn-N8-p14358001	Bn-chrCnn_random-p659335
Bn-A08-p14673671	0.975206612	0.975206612	Bn-N8-p14373101	Bn-chrA08_random-p175400
Bn-A08-p14684717	0.983471074	0.983471074	Bn-N8-p14388297	Bn-chrA08_random-p174465
Bn-A08-p14688219	0.991735537	0.991735537	Bn-N8-p14396900	Bn-chrA08_random-p173623
Bn-A08-p14689466	0.975206612	0.975206612	Bn-N8-p14398163	Bn-chrA08_random-p173497
Bn-A08-p14692156	0.975206612	0.975206612	Bn-N8-p14400848	Bn-chrA08_random-p173229
Bn-A08-p14693257	1	1	Bn-N8-p14401949	Bn-chrA08_random-p173119
Bn-A08-p14693414	0.991735537	0.991735537	Bn-N8-p14402106	Bn-chrA08_random-p173103
Bn-A08-p14694002	1	1	Bn-N8-p14402694	Bn-chrA08_random-p173044
Bn-A08-p14694130	0.991735537	0.991735537	Bn-N8-p14402822	Bn-chrA08_random-p173031
Bn-A08-p14694395	0.996677741	0.996677741	Bn-N8-p14403087	Bn-chrA08_random-p173005
Bn-A08-p14695487	0.975206612	0.975206612	Bn-N8-p14404179	Bn-chrA08_random-p172896
Bn-A08-p14695762	0.966942149	0.966942149	Bn-N8-p14404456	Bn-chrA08_random-p172868
Bn-A08-p14697337	0.991735537	0.991735537	Bn-N8-p14406206	Bn-chrA08-p2954150
Bn-A08-p14698054	0.983471074	0.173553719	Bn-N8-p14406917	Bn-chrA03-p22098848
Bn-A08-p14704782	0.993355482	0.993355482	Bn-N8-p14413106	Bn-chrA08-p12260749
Bn-A08-p14713741	0.983471074	0.983471074	Bn-N8-p14426462	Bn-chrA08-p12274795
Bn-A08-p14715630	0.834710744	0.834710744	Bn-N8-p14428351	Bn-chrA08-p12276684
Bn-A08-p14718347	0.966942149	0.966942149	Bn-N8-p14431283	Bn-chrA08-p12279616
Bn-A08-p14726546	0.975206612	0.975206612	Bn-N8-p14439625	Bn-chrA08-p12291116
Bn-A08-p14729101	0.975206612	0.966942149	Bn-N8-p14442423	Bn-chrA08-p12292664
Bn-A08-p14729648	0.993355482	0.996677741	Bn-N8-p14442970	Bn-chrA08-p12293211
Bn-A08-p14733187	0.966942149	0.958677686	Bn-N8-p14446490	Bn-chrA08-p12296772
Bn-A08-p14734078	0.975206612	0.79338843	Bn-N8-p14447372	Bn-chrA08-p12297645
Bn-A08-p14736390	0.983471074	0.991735537	Bn-N8-p14449686	Bn-chrA08-p12299929
Bn-A08-p14736428	0.991735537	0.983471074	Bn-N8-p14449724	Bn-chrA08-p12299967
Bn-A08-p14737862	0.983471074	0.966942149	Bn-N8-p14451159	Bn-chrA08-p12301318
Bn-A08-p14739774	0.991735537	0.983471074	Bn-N8-p14453064	Bn-chrA08-p12303260
Bn-A08-p14740175	0.991735537	1	Bn-N8-p14453465	Bn-chrA08-p12303661
Bn-A08-p14740662	0.983471074	0.991735537	Bn-N8-p14453952	Bn-chrA08-p12310644
Bn-A08-p14740941	0.983471074	0.991735537	Bn-N8-p14454232	Bn-chrA08-p12310931
Bn-A08-p14749618	0.991735537	0.983471074	Bn-N8-p14469864	Bn-chrA08-p12312967
Bn-A08-p14749890	0.975206612	0.933884298	Bn-N8-p14470136	Bn-chrA08-p12313509
Bn-A08-p14750320	0.983471074	0.991735537	Bn-N8-p14470566	Bn-chrA08-p12313941
Bn-A08-p14750863	0.983471074	0.991735537	Bn-N8-p14471106	Bn-chrA08-p12314445
Bn-A08-p14751184	0.991735537	0.975206612	Bn-N8-p14471427	Bn-chrA08-p12314769
Bn-A08-p14751256	0.983471074	0.958677686	Bn-N8-p14471499	Bn-chrA08-p12314841

Bn-A08-p14751316	0.991735537	0.983471074	Bn-N8-p14471559	Bn-chrA08-p12314901
Bn-A08-p14751458	0.983471074	0.950413223	Bn-N8-p14471701	Bn-chrA08-p12315043
Bn-A08-p14751606	1	0.991735537	Bn-N8-p14471849	Bn-chrA08-p12315190
Bn-A08-p14751701	0.983471074	0.975206612	Bn-N8-p14471944	Bn-chrA08-p12315285
Bn-A08-p14751756	0.975206612	0.975206612	Bn-N8-p14471999	Bn-chrA08-p12315340
Bn-A08-p14752428	0.958677686	0.966942149	Bn-N8-p14472671	Bn-chrA08-p12315986
Bn-A08-p14752486	0.966942149	0.975206612	Bn-N8-p14472729	Bn-chrA08-p12316044
Bn-A08-p14752549	0.975206612	0.983471074	Bn-N8-p14472792	Bn-chrA08-p12316107
Bn-A08-p14752638	1	0.991735537	Bn-N8-p14472881	Bn-chrA08-p12316196
Bn-A08-p14752710	0.983471074	0.991735537	Bn-N8-p14472953	Bn-chrA08-p12316268
Bn-A08-p14759185	0.975206612	0.983471074	Bn-N8-p14479446	Bn-chrA08-p12320997
Bn-A08-p14765750	0.817275748	0.817275748	Bn-N18-p18828841	Bn-chrC08-p17030394
Bn-A08-p14766663	0.950413223	0.950413223	Bn-N8-p14487515	Bn-chrA08-p12328472
Bn-A08-p14784965	0.873754153	0.873754153	Bn-N8-p14506665	Bn-chrA08-p12346434
Bn-A08-p14790412	0.980066445	0.980066445	Bn-N8-p14512675	Bn-chrA08-p12351927
Bn-A08-p14799839	1	1	Bn-N8-p14522893	Bn-chrA08-p12362143
Bn-A08-p14806061	0.993355482	0.986710963	Bn-N8-p14529763	Bn-chrA08-p12369010
Bn-A08-p14813540	0.990049751	0.990049751	Bn-N8-p14537632	Bn-chrA08-p12376879
Bn-A08-p14846467	0.975206612	0.975206612	Bn-N8-p14569947	Bn-chrA08-p12409475
Bn-A08-p14906652	0.996677741	0.996677741	Bn-N8-p14630847	Bn-chrA08-p12464197
Bn-A08-p14955455	0.950413223	0.94214876	Bn-N8-p14684717	Bn-chrAnn_random-p454257
Bn-A08-p14970925	0.970099668	0.970099668	Bn-N8-p14700421	Bn-chrA08_random-p181405
Bn-A08-p14976685	0.991735537	0.991735537	Bn-N8-p14706183	Bn-chrAnn_random-p366546
Bn-A08-p14977157	0.958677686	0.966942149	Bn-N8-p14706655	Bn-chrA08-p12509902
Bn-A08-p14980805	0.996677741	0.993355482	Bn-N8-p14710303	Bn-chrA08-p12513565
Bn-A08-p14988862	0.983471074	0.975206612	Bn-N8-p14720218	Bn-chrA08-p12516168
Bn-A08-p15027690	0.975206612	0.958677686	Bn-N8-p1516253	Bn-chrA08-p1309918
Bn-A08-p15031334	0.966942149	0.958677686	Bn-N8-p14762593	Bn-chrA08-p12564032
Bn-A08-p15031416	1	0.991735537	Bn-N8-p14762675	Bn-chrA08-p12564114
Bn-A08-p15031530	0.966942149	0.975206612	Bn-N8-p14762789	Bn-chrA08-p12564228
Bn-A08-p15031658	0.818181818	0.991735537	Bn-N8-p14762917	Bn-chrA08-p12564356
Bn-A08-p15033008	0.983471074	0.975206612	Bn-N8-p14764084	Bn-chrA08-p12565706
Bn-A08-p15034231	0.966942149	0.958677686	Bn-N8-p14765362	Bn-chrA08-p12566929
Bn-A08-p15034494	0.776859504	0.975206612	Bn-N8-p14766244	Bn-chrA08-p12567190
Bn-A08-p15035231	0.991735537	0.983471074	Bn-N8-p14768560	Bn-chrA08-p12567927
Bn-A08-p15041593	0.983471074	0.975206612	Bn-N8-p14771356	Bn-chrA08-p12569611
Bn-A08-p15042741	0.933884298	0.702479339	Bn-N8-p14772503	Bn-chrCnn_random-p633060
Bn-A08-p15043392	1	0.991735537	Bn-N8-p14773149	Bn-chrA08-p12570973
Bn-A08-p15053710	0.975206612	0.966942149	Bn-N8-p14784176	Bn-chrAnn_random-p375314
Bn-A08-p15067659	0.983388704	0.986710963	Bn-N8-p14800175	Bn-chrA08-p12610509
Bn-A08-p15067721	0.980066445	0.983388704	Bn-N8-p14800237	Bn-chrA08-p12610571
Bn-A08-p15075119	0.991735537	0.983471074	Bn-Scaffold59374-p1	Bn-chrA08-p12616808
Bn-A08-p15095047	0.976744186	0.983388704	Bn-N8-p14827328	Bn-chrA08-p12634445
Bn-A08-p15122707	0.893687708	0.740863787	Bn-N8-p14852936	Bn-chrA08-p12664222
Bn-A08-p15127926	0.976744186	0.92358804	Bn-N8-p14858153	Bn-chrC03-p51217077
Bn-A08-p15177299	0.970149254	0.970149254	Bn-N8-p14908178	Bn-chrA08-p12735302

Bn-A08-p15213340	0.870431894	0.993355482	Bn-N13-p57224861	Bn-chrA08-p12767479
Bn-A08-p15223029	0.983471074	0.983471074	Bn-N8-p14952957	Bn-chrA08-p12777088
Bn-A08-p15231480	0.983471074	0.975206612	Bn-N8-p14967895	Bn-chrA08-p12789601
Bn-A08-p15239295	0.980099502	0.980099502	Bn-N8-p14975889	Bn-chrA08-p12798060
Bn-A08-p15239790	0.990049751	0.990049751	Bn-N8-p14976383	Bn-chrA08-p12798554
Bn-A08-p15243115	0.983471074	0.975206612	Bn-N8-p14979752	Bn-chrA08-p12801878
Bn-A08-p15258493	1	0.996677741	Bn-N8-p14993857	Bn-chrA08-p12815796
Bn-A08-p15259781	1	0.991735537	Bn-N8-p14995145	Bn-chrA08-p12817391
Bn-A08-p15265602	0.991735537	1	Bn-N8-p15001020	Bn-chrA08-p12823267
Bn-A08-p15265969	0.917355372	0.925619835	Bn-N8-p15001382	Bn-chrA08-p12823628
Bn-A08-p15268077	1	0.991735537	Bn-N8-p15003490	Bn-chrA08-p12825732
Bn-A08-p15273100	0.986710963	0.986710963	Bn-N8-p15008502	Bn-chrA08-p12830853
Bn-A08-p15281365	1	1	Bn-N8-p15021155	Bn-chrA08-p12843502
Bn-A08-p15283513	0.975206612	0.925619835	Bn-N8-p15023302	Bn-chrA08-p12845649
Bn-A08-p15301389	0.993355482	0.993355482	Bn-N8-p15047950	Bn-chrA08-p12868946
Bn-A08-p15305183	0.764119601	0.970099668	Bn-N8-p15051789	Bn-chrA08-p12872783
Bn-A08-p15322011	0.983471074	0.983471074	Bn-N8-p15068724	Bn-chrA08-p12889669
Bn-A08-p15326882	0.973421927	0.970099668	Bn-N13-p56974805	Bn-chrA08-p12894574
Bn-A08-p15341166	0.983388704	0.983388704	Bn-N8-p15080346	Bn-chrA08-p12901288
Bn-A08-p15342008	0.980066445	0.980066445	Bn-N8-p15081192	Bn-chrA08-p12902134
Bn-A08-p15353881	0.983471074	0.983471074	Bn-N8-p15090232	Bn-chrA08-p12910226
Bn-A08-p15362382	0.996677741	0.996677741	Bn-N8-p15099462	Bn-chrA08-p12918737
Bn-A08-p15367242	0.787375415	0.787375415	Bn-N8-p15104608	Bn-chrA08-p12923598
Bn-A08-p15437367	0.975124378	0.985074627	Bn-Scaffold43944-p2	Bn-chrC03-p50525702
Bn-A08-p15448484	0.996677741	1	Bn-N8-p15181502	Bn-chrA08-p13018992
Bn-A08-p15466459	0.996677741	0.996677741	Bn-N8-p15200793	Bn-chrA08-p13039120
Bn-A08-p15471679	0.996677741	0.996677741	Bn-N8-p15207492	Bn-chrA08-p13044416
Bn-A08-p15484420	0.993355482	0.993355482	Bn-N8-p15219950	Bn-chrA08-p13056870
Bn-A08-p15489686	0.958677686	0.958677686	Bn-N8-p15224551	Bn-chrA08-p13061471
Bn-A08-p15498461	0.993355482	0.993355482	Bn-N8-p15240411	Bn-chrA08-p13081161
Bn-A08-p15502867	0.991735537	0.991735537	Bn-N8-p15244335	Bn-chrA08-p13085085
Bn-A08-p15507426	0.983471074	0.983471074	Bn-N8-p15250232	Bn-chrA08-p13089647
Bn-A08-p15594956	1	1	Bn-N8-p15335573	Bn-chrA08-p13116538
Bn-A08-p15614498	0.966942149	0.966942149	Bn-N13-p56349992	Bn-chrC03-p50177193
Bn-A08-p15615914	0.985074627	0.985074627	Bn-N8-p15354868	Bn-chrA08-p13146464
Bn-A08-p15616595	0.990033223	0.990033223	Bn-N8-p15355549	Bn-chrA08-p13147145
Bn-A08-p15619709	0.990049751	0.990049751	Bn-N8-p15358663	Bn-chrA08-p13150259
Bn-A08-p15620062	0.990049751	0.990049751	Bn-N8-p15359016	Bn-chrA08-p13150612
Bn-A08-p15620471	0.985074627	0.985074627	Bn-N8-p15359431	Bn-chrA08-p13151027
Bn-A08-p15620702	0.995024876	0.995024876	Bn-N8-p15359662	Bn-chrA08-p13151258
Bn-A08-p15700195	0.980099502	0.980099502	Bn-N8-p15416748	Bn-chrA08-p13211797
Bn-A08-p15701695	0.991735537	1	Bn-N8-p15416987	Bn-chrA08-p13212036
Bn-A08-p15710460	0.983471074	0.983471074	Bn-N8-p15426114	Bn-chrA08-p13216601
Bn-A08-p15710698	0.983471074	0.983471074	Bn-N8-p15426352	Bn-chrA08-p13216839
Bn-A08-p15723645	1	0.950413223	Bn-N8-p15440582	Bn-chrA07-p3012054
Bn-A08-p15736750	0.983471074	0.94214876	Bn-N8-p15451568	Bn-chrA08-p13238714

Bn-A08-p15737285	1	0.991735537	Bn-N8-p15452103	Bn-chrA08-p13239246
Bn-A08-p15740524	0.996677741	0.996677741	Bn-N8-p15455358	Bn-chrA08-p13242508
Bn-A08-p15767590	0.990033223	0.990033223	Bn-N8-p15492485	Bn-chrAnn_random-p320314
Bn-A08-p15782077	0.960199005	0.960199005	Bn-N8-p15503840	Bn-chrA08-p13277167
Bn-A08-p15782130	0.955223881	0.955223881	Bn-N8-p15503893	Bn-chrA08-p13277220
Bn-A08-p15782229	0.970149254	0.970149254	Bn-N8-p15503992	Bn-chrA08-p13277319
Bn-A08-p15782241	0.975124378	0.975124378	Bn-N8-p15504004	Bn-chrA08-p13277331
Bn-A08-p15782307	0.960199005	0.960199005	Bn-N8-p15504070	Bn-chrA08-p13277397
Bn-A08-p15790373	0.993355482	0.993355482	Bn-N8-p15512424	Bn-chrA08-p13286035
Bn-A08-p15792069	0.985074627	0.985074627	Bn-N8-p15514120	Bn-chrA08-p13287731
Bn-A08-p15792369	0.990049751	0.990049751	Bn-N8-p15514420	Bn-chrA08-p13288031
Bn-A08-p15792684	0.975124378	0.975124378	Bn-N8-p15514735	Bn-chrA08-p13288346
Bn-A08-p15792942	0.970149254	0.970149254	Bn-N8-p15514993	Bn-chrA08-p13288604
Bn-A08-p15802943	0.925619835	0.925619835	Bn-N8-p15526236	Bn-chrA08-p13299987
Bn-A08-p15804301	0.975206612	0.975206612	Bn-N8-p15527596	Bn-chrA08-p13301345
Bn-A08-p15819039	0.990033223	0.990033223	Bn-N8-p15532995	Bn-chrA08-p13306747
Bn-A08-p15819293	0.996677741	0.996677741	Bn-N8-p15533249	Bn-chrA08-p13307001
Bn-A08-p15872628	0.991735537	0.983471074	Bn-N8-p15573047	Bn-chrA08-p13340515
Bn-A08-p15888048	0.983471074	0.991735537	Bn-N8-p15591070	Bn-chrA08-p13355956
Bn-A08-p15906423	0.950413223	0.958677686	Bn-N8-p15608508	Bn-chrA08-p13375269
Bn-A08-p15914038	0.983471074	0.975206612	Bn-N8-p15614950	Bn-chrA08-p13386256
Bn-A08-p15945454	1	1	Bn-N8-p15640322	Bn-chrA08-p13407212
Bn-A08-p15952558	0.975206612	0.983471074	Bn-N8-p15649647	Bn-chrA08-p13415860
Bn-A08-p15954904	0.983471074	0.983471074	Bn-N8-p15652276	Bn-chrA08-p13419263
Bn-A08-p15961655	0.990033223	0.990033223	Bn-N19-p54773025	Bn-chrC09-p47480062
Bn-A08-p15967287	0.983471074	0.933884298	Bn-N8-p15659901	Bn-chrC03-p49701222
Bn-A08-p15972135	0.983471074	0.966942149	Bn-N8-p15666870	Bn-chrA08-p13429614
Bn-A08-p15991441	0.996677741	0.857142857	Bn-N8-p15686563	Bn-chrA08-p13450528
Bn-A08-p15994149	0.990033223	0.980066445	Bn-N8-p15687942	Bn-chrA08-p13452346
Bn-A08-p16028375	0.94214876	0.950413223	Bn-N8-p15714872	Bn-chrA08-p13488659
Bn-A08-p16030898	0.958677686	0.958677686	Bn-N8-p15717300	Bn-chrA08-p13491098
Bn-A08-p16039443	0.991735537	0.991735537	Bn-N8-p15726713	Bn-chrA08-p13499255
Bn-A08-p16039755	0.983471074	0.983471074	Bn-N8-p15727025	Bn-chrA08-p13499567
Bn-A08-p16052172	0.950166113	0.96345515	Bn-N8-p15738676	Bn-chrA08-p13511208
Bn-A08-p16061843	0.975124378	0.975124378	Bn-N8-p15748397	Bn-chrA08-p13520926
Bn-A08-p16077598	0.966942149	0.966942149	Bn-N8-p15763493	Bn-chrA08-p13536078
Bn-A08-p16111871	0.975206612	0.966942149	Bn-N8-p15798281	Bn-chrA08-p13571425
Bn-A08-p16115851	0.893687708	0.661129568	Bn-N8-p15804412	Bn-chrA08-p13575244
Bn-A08-p16125811	1	1	Bn-N8-p15814496	Bn-chrA08-p13582160
Bn-A08-p16132462	0.975124378	0.980099502	Bn-N8-p15829618	Bn-chrA08-p13593711
Bn-A08-p16141425	0.985074627	0.980099502	Bn-N8-p15834199	Bn-chrA08-p13598304
Bn-A08-p16149197	0.996677741	0.933554817	Bn-N8-p15844918	Bn-chrC03-p49347437
Bn-A08-p16171460	0.966942149	0.933884298	Bn-N8-p15863075	Bn-chrC03-p49301410
Bn-A08-p16183708	0.991735537	0.983471074	Bn-N8-p15876364	Bn-chrA08-p13645128
Bn-A08-p16201029	0.950166113	0.946843854	Bn-N8-p15898446	Bn-chrA08-p13664327
Bn-A08-p16208029	0.983471074	0.983471074	Bn-N8-p15905574	Bn-chrA08-p13670662

Bn-A08-p16210589	0.983471074	0.983471074	Bn-N8-p15907885	Bn-chrA08-p13672973
Bn-A08-p16211013	0.980066445	0.993355482	Bn-N8-p15908307	Bn-chrA08-p13673395
Bn-A08-p16216893	0.973421927	0.970099668	Bn-N8-p15914242	Bn-chrA08-p13680115
Bn-A08-p16232132	0.926910299	0.926910299	Bn-N8-p15928868	Bn-chrA08-p13695472
Bn-A08-p16248227	1	0.991735537	Bn-N8-p15952855	Bn-chrA08-p13722699
Bn-A08-p16248633	0.880398671	0.990033223	Bn-N8-p15953256	Bn-chrA08-p13723105
Bn-A08-p16253366	0.983471074	0.330578512	Bn-N5-p7058169	Unknown
Bn-A08-p16253891	0.991735537	0.876033058	Bn-N5-p7057644	Bn-chrC09-p40808693
Bn-A08-p16259976	0.966942149	0.859504132	Bn-N8-p15960786	Bn-chrC03_random-p472905
Bn-A08-p16260215	0.993355482	0.823920266	Bn-N8-p15961025	Bn-chrC03_random-p472929
Bn-A08-p16267662	0.986710963	0.986710963	Bn-N8-p15969400	Bn-chrA08-p13739038
Bn-A08-p16286008	0.980066445	0.980066445	Bn-N8-p15985095	Bn-chrA08-p13754930
Bn-A08-p16286082	0.965174129	0.965174129	Bn-N8-p15985169	Bn-chrA08-p13755004
Bn-A08-p16293275	0.993355482	0.973421927	Bn-N8-p15992471	Bn-chrA08-p13762139
Bn-A08-p16299351	1	0.995024876	Bn-N8-p15998537	Bn-chrA08-p13768161
Bn-A08-p16306425	0.966942149	0.975206612	Bn-N8-p16006346	Bn-chrA08-p13775800
Bn-A08-p16313016	0.995024876	0.990049751	Bn-N8-p16012975	Bn-chrA08-p13782792
Bn-A08-p16314818	0.983471074	0.975206612	Bn-N8-p16014776	Bn-chrA08-p13784496
Bn-A08-p16317873	0.983471074	0.421487603	Bn-N8-p16017715	Bn-chrC07-p14105854
Bn-A08-p16318709	0.990033223	0.913621262	Bn-N8-p16018551	Bn-chrC03-p49009017
Bn-A08-p16318890	0.993355482	0.910299003	Bn-N8-p16018732	Bn-chrC03-p49008836
Bn-A08-p16325858	0.950413223	0.950413223	Bn-N8-p16025918	Bn-chrA08-p13791056
Bn-A08-p16328281	0.966942149	0.966942149	Bn-N8-p16028989	Bn-chrA08-p13793502
Bn-A08-p16344396	0.842975207	0.842975207	Bn-N8-p16042964	Bn-chrA08-p13807406
Bn-A08-p16349875	0.926910299	0.926910299	Bn-N8-p16048457	Bn-chrA08-p13812899
Bn-A08-p16353006	0.966942149	0.966942149	Bn-N8-p16051119	Bn-chrA08-p13815560
Bn-A08-p16366766	0.960199005	0.960199005	Bn-N8-p16068959	Bn-chrA08-p13830656
Bn-A08-p16382380	0.991735537	0.983471074	Bn-N8-p16085763	Bn-chrA08-p13850663
Bn-A08-p16382662	0.893687708	0.983388704	Bn-N8-p16086043	Bn-chrA08-p13850945
Bn-A08-p16392743	0.983471074	0.983471074	Bn-N8-p16096353	Bn-chrA08-p13861247
Bn-A08-p16392957	0.965174129	0.965174129	Bn-N8-p16096567	Bn-chrA08-p13861461
Bn-A08-p16403550	0.993355482	0.993355482	Bn-N8-p16111116	Bn-chrA08-p13870471
Bn-A08-p16412952	0.958677686	0.958677686	Bn-N8-p16117503	Bn-chrA08-p13876464
Bn-A08-p16416932	0.986710963	0.986710963	Bn-N8-p16121325	Bn-chrA08-p13880272
Bn-A08-p16418684	0.970149254	0.970149254	Bn-N8-p16123081	Bn-chrAnn_random-p387202
Bn-A08-p16454339	0.966942149	0.966942149	Bn-N8-p16159609	Bn-chrA08-p13925605
Bn-A08-p16457656	0.980099502	0.84079602	Bn-N8-p16162924	Bn-chrA08-p13928964
Bn-A08-p16459840	0.975206612	0.983471074	Bn-N8-p16165865	Bn-chrA08-p13931148
Bn-A08-p16474855	0.991735537	0.983471074	Bn-N8-p16180982	Bn-chrA08-p13946231
Bn-A08-p16477837	0.983471074	0.991735537	Bn-N8-p16185287	Bn-chrA08-p13949213
Bn-A08-p16477916	0.983471074	0.983471074	Bn-N8-p16185365	Bn-chrA08-p13949292
Bn-A08-p16477986	0.991735537	0.983471074	Bn-N8-p16185435	Bn-chrA08-p13949362
Bn-A08-p16514789	0.975206612	0.727272727	Bn-N8-p16220256	Bn-chrCnn_random-p417763
Bn-A08-p16521179	0.991735537	0.991735537	Bn-N8-p16232261	Bn-chrA08-p13985520
Bn-A08-p16523108	0.966942149	0.933884298	Bn-N8-p16234300	Bn-chrC03-p48834362
Bn-A08-p16549391	0.983471074	0.983471074	Bn-N8-p16259892	Bn-chrA08-p14018574

Bn-A08-p16551334	0.859504132	0.719008264	Bn-N8-p16261831	Bn-chrA08-p14020510
Bn-A08-p16559652	0.975206612	0.966942149	Bn-N8-p16267240	Bn-chrA08-p14028501
Bn-A08-p16562035	1	0.991735537	Bn-N8-p16270995	Bn-chrA08-p14030897
Bn-A08-p16572658	0.991735537	0.991735537	Bn-N8-p16282351	Bn-chrA08-p14042723
Bn-A08-p16575548	0.955223881	0.940298507	Bn-N8-p16284975	Bn-chrA08-p14045348
Bn-A08-p16575796	0.958677686	0.966942149	Bn-N8-p16285223	Bn-chrA08-p14045596
Bn-A08-p16582191	0.966942149	0.958677686	Bn-N8-p16291688	Bn-chrA08-p14052062
Bn-A08-p16621345	0.94214876	0.917355372	Bn-N8-p16330542	Bn-chrA08-p14078745
Bn-A08-p16631459	0.958677686	0.94214876	Bn-N8-p16340598	Bn-chrA08-p14088818
Bn-A08-p16632230	0.950413223	0.958677686	Bn-N8-p16341369	Bn-chrA08-p14089576
Bn-A08-p16638835	0.975206612	0.966942149	Bn-N8-p16347723	Bn-chrA08-p14094786
Bn-A08-p16640519	0.975206612	0.157024793	Bn-N8-p16349555	Unknown
Bn-A08-p16640580	0.991735537	0.198347107	Bn-N8-p16349616	Unknown
Bn-A08-p16640710	0.975206612	0.983471074	Bn-N8-p16349746	Bn-chrA08-p14095944
Bn-A08-p16644247	0.983471074	0.991735537	Bn-N8-p16353988	Bn-chrA08-p14101308
Bn-A08-p16644519	0.983471074	0.991735537	Bn-N8-p16354260	Bn-chrA08-p14101580
Bn-A08-p16653908	0.983471074	0.975206612	Bn-N8-p16363326	Bn-chrA08-p14117137
Bn-A08-p16655249	0.983471074	0.991735537	Bn-N8-p16364668	Bn-chrA08-p14118474
Bn-A08-p16672855	0.996677741	0.996677741	Bn-N8-p16381367	Bn-chrA08-p14135238
Bn-A08-p16673732	0.985074627	0.985074627	Bn-N8-p16382244	Bn-chrA08-p14136115
Bn-A08-p16676967	0.983471074	0.983471074	Bn-N8-p16385477	Bn-chrA08-p14139341
Bn-A08-p16704644	1	0.991735537	Bn-Scaffold11609-p2	Bn-chrA08-p14162185
Bn-A08-p16714223	0.966942149	0.958677686	Bn-N8-p16423300	Bn-chrA08-p14173458
Bn-A08-p16716010	0.975206612	0.79338843	Bn-N8-p16425097	Bn-chrA08-p14175007
Bn-A08-p16728548	0.975206612	0.958677686	Bn-N8-p16442873	Bn-chrC03_random-p467957
Bn-A08-p16739744	0.983471074	0.975206612	Bn-N8-p16454651	Bn-chrA08-p14211954
Bn-A08-p16742428	0.983471074	0.958677686	Bn-N8-p16457335	Bn-chrA08-p14214607
Bn-A08-p16744972	1	0.79338843	Bn-N8-p16459885	Bn-chrA08-p14215421
Bn-A08-p16770669	0.975206612	0.975206612	Bn-N8-p16480922	Bn-chrA08-p14238151
Bn-A08-p16770923	0.966942149	0.975206612	Bn-N8-p16481176	Bn-chrA08-p14238405
Bn-A08-p16771030	0.983471074	0.991735537	Bn-N8-p16481283	Bn-chrA08-p14238512
Bn-A08-p16771161	0.996677741	0.990033223	Bn-N8-p16481414	Bn-chrA08-p14238643
Bn-A08-p16793918	0.970149254	0.975124378	Bn-N8-p16503845	Bn-chrA08-p14265059
Bn-A08-p16799210	0.950413223	0.958677686	Bn-N8-p16509119	Bn-chrA08-p14272143
Bn-A08-p16802197	0.94214876	0.925619835	Bn-N8-p16512118	Bn-chrA08-p14275366
Bn-A08-p16809551	0.958677686	0.966942149	Bn-N8-p16517927	Bn-chrA08-p14282906
Bn-A08-p16829757	0.84717608	0.837209302	Bn-N8-p16534122	Bn-chrA08-p14305282
Bn-A08-p16842687	0.991735537	0.983471074	Bn-N8-p16547530	Bn-chrA08-p14316985
Bn-A08-p16842690	0.975206612	0.983471074	Bn-N8-p16547533	Bn-chrA08-p14316988
Bn-A08-p16846184	0.991735537	0.719008264	Bn-N8-p16551503	Bn-chrA08-p14320518
Bn-A08-p16846907	0.975206612	0.975206612	Bn-N8-p16552226	Bn-chrA08-p14321245
Bn-A08-p16853718	0.983471074	0.975206612	Bn-N8-p16556673	Bn-chrA08-p14327144
Bn-A08-p16859028	0.975206612	0.983471074	Bn-N8-p16560650	Bn-chrA08-p14337112
Bn-A08-p16859321	0.966942149	0.958677686	Bn-N8-p16560959	Bn-chrA08-p14337680
Bn-A08-p16870021	0.976744186	0.973421927	Bn-N13-p53640332	Bn-chrA08-p14349298
Bn-A08-p16894122	0.983471074	0.983471074	Bn-N8-p16596516	Bn-chrA08-p14373600

Bn-A08-p16894466	0.966942149	0.966942149	Bn-N8-p16596861	Bn-chrA08-p14373945
Bn-A08-p16895440	0.983471074	0.983471074	Bn-N8-p16599347	Bn-chrA08-p14376431
Bn-A08-p16913215	0.975206612	0.975206612	Bn-N8-p16615084	Bn-chrA08-p14393035
Bn-A08-p16938935	1	1	Bn-N8-p16637946	Bn-chrA08-p14411526
Bn-A08-p16943139	0.990033223	0.990033223	Bn-N8-p16642216	Bn-chrA08-p14415779
Bn-A08-p16943263	0.996677741	0.996677741	Bn-N8-p16642340	Bn-chrA08-p14415903
Bn-A08-p16956285	0.993355482	0.996677741	Bn-N5-p25200682	Bn-chrA05_random-p291102
Bn-A08-p16958537	0.983471074	0.991735537	Bn-N8-p16663666	Bn-chrA08-p14431455
Bn-A08-p16965932	0.985074627	0.985074627	Bn-N8-p16671094	Bn-chrA08-p14438883
Bn-A08-p16966023	0.980099502	0.980099502	Bn-N8-p16671185	Bn-chrA08-p14438974
Bn-A08-p16980526	0.993355482	0.993355482	Bn-N8-p16685917	Bn-chrC08-p6869540
Bn-A08-p16990347	0.965174129	0.965174129	Bn-N8-p16695728	Bn-chrA08-p14465747
Bn-A08-p16992690	0.991735537	0.991735537	Bn-N8-p16698073	Bn-chrA08-p14468091
Bn-A08-p16998644	0.966942149	0.966942149	Bn-N8-p16708644	Bn-chrA08-p14476890
Bn-A08-p17000507	0.973421927	0.973421927	Bn-N8-p16710385	Bn-chrA08-p14478630
Bn-A08-p17005826	0.975206612	0.975206612	Bn-N8-p16715506	Bn-chrA08-p14483750
Bn-A08-p17050758	0.880398671	0.880398671	Bn-N8-p16750357	Bn-chrA08-p14524027
Bn-A08-p17061248	0.991735537	0.991735537	Bn-N8-p16759199	Bn-chrA08-p14532868
Bn-A08-p17062689	0.983471074	0.975206612	Bn-N8-p16760643	Bn-chrA08-p14534308
Bn-A08-p17067481	0.986710963	0.990033223	Bn-N8-p16765433	Bn-chrA08-p14539085
Bn-A08-p17069605	0.975206612	0.983471074	Bn-N8-p16767555	Bn-chrA08-p14541240
Bn-A08-p17070496	0.983471074	0.975206612	Bn-N8-p16768413	Bn-chrA08-p14542091
Bn-A08-p17078349	0.983471074	0.983471074	Bn-N8-p16777245	Bn-chrA08-p14549936
Bn-A08-p17088758	0.945273632	0.945273632	Bn-N8-p16786044	Bn-chrA08-p14562376
Bn-A08-p17094421	0.975206612	0.983471074	Bn-N8-p16791557	Bn-chrA08-p14568038
Bn-A08-p17105262	0.976744186	0.976744186	Bn-N8-p16802291	Bn-chrA08-p14578815
Bn-A08-p17108442	0.996677741	0.996677741	Bn-N8-p16811409	Bn-chrA08-p14587173
Bn-A08-p17111194	0.986710963	0.986710963	Bn-N8-p16814168	Bn-chrA08-p14589932
Bn-A08-p17124630	1	1	Bn-N8-p16829820	Bn-chrA08-p14609856
Bn-A08-p17125271	0.996677741	0.996677741	Bn-N8-p16830461	Bn-chrA08-p14610497
Bn-A08-p17130151	0.986710963	0.986710963	Bn-N8-p16834676	Bn-chrA08-p14614711
Bn-A08-p17144811	0.966942149	0.975206612	Bn-N8-p16853191	Bn-chrA08-p14631236
Bn-A08-p17152372	0.975206612	0.950413223	Bn-N8-p16858120	Bn-chrA08-p14636989
Bn-A08-p17158655	1	0.983388704	Bn-N8-p16867251	Bn-chrA08-p14643274
Bn-A08-p17159464	1	0.991735537	Bn-N8-p16868060	Bn-chrA08-p14644083
Bn-A08-p17161182	0.983471074	0.975206612	Bn-N8-p16869534	Bn-chrA08-p14645518
Bn-A08-p17162841	0.867768595	0.933884298	Bn-N8-p16870493	Bn-chrA08-p14647879
Bn-A08-p17186790	0.996677741	0.996677741	Bn-N8-p16894646	Bn-chrA08-p14672402
Bn-A08-p17187064	0.980099502	0.980099502	Bn-N8-p16894920	Bn-chrA08-p14672676
Bn-A08-p17190098	0.975124378	0.975124378	Bn-N8-p16897922	Bn-chrA08-p14675678
Bn-A08-p17191196	0.990049751	0.990049751	Bn-N13-p52891907	Bn-chrC03-p47240439
Bn-A08-p17191740	0.990049751	0.990049751	Bn-N8-p16899564	Bn-chrA08-p14677320
Bn-A08-p17192841	0.995024876	0.995024876	Bn-N8-p16900662	Bn-chrA08-p14678418
Bn-A08-p17193797	0.980099502	0.980099502	Bn-N8-p16901618	Bn-chrA08-p14679374
Bn-A08-p17195176	1	1	Bn-N8-p16902997	Bn-chrA08-p14680753
Bn-A08-p17202184	0.958677686	0.958677686	Bn-N8-p16911036	Bn-chrA08-p14688784

Bn-A08-p17202527	0.958677686	0.958677686	Bn-N8-p16911379	Bn-chrA08-p14689127
Bn-A08-p17202584	0.991735537	0.991735537	Bn-N8-p16911436	Bn-chrA08-p14689184
Bn-A08-p17202745	0.975206612	0.975206612	Bn-N8-p16911597	Bn-chrA08-p14689345
Bn-A08-p17209652	0.983471074	0.983471074	Bn-N8-p16919093	Bn-chrA08-p14696841
Bn-A08-p17217296	0.990049751	0.990049751	Bn-N8-p16923386	Bn-chrA08-p14701134
Bn-A08-p17221534	0.958677686	0.958677686	Bn-N8-p16927699	Bn-chrA08-p14705447
Bn-A08-p17235030	0.996677741	0.996677741	Bn-N8-p16945126	Bn-chrAnn_random-p289498
Bn-A08-p17236071	1	1	Bn-N8-p16946167	Bn-chrAnn_random-p289508
Bn-A08-p17238995	0.915422886	0.915422886	Bn-N13-p52787768	Bn-chrC03-p46895083
Bn-A08-p17249190	0.975206612	0.975206612	Bn-N8-p16959265	Bn-chrAnn_random-p241658
Bn-A08-p17254975	0.993355482	0.993355482	Bn-N8-p16967474	Bn-chrAnn_random-p241716
Bn-A08-p17255997	0.996677741	0.996677741	Bn-N8-p16968469	Bn-chrAnn_random-p241726
Bn-A08-p17256848	0.990049751	0.985074627	Bn-N8-p16969322	Bn-chrAnn_random-p241734
Bn-A08-p17291598	0.973421927	0.980066445	Bn-N8-p17004603	Bn-chrA08-p14720670
Bn-A08-p17291679	0.976744186	0.976744186	Bn-N8-p17004684	Bn-chrA08-p14720751
Bn-A08-p17293594	0.897009967	0.88372093	Bn-N8-p17006572	Bn-chrA08-p14722637
Bn-A08-p17297704	0.993355482	0.976744186	Bn-N8-p17010670	Bn-chrA08-p14726724
Bn-A08-p17297854	0.983471074	0.975206612	Bn-N8-p17010820	Bn-chrA08-p14726874
Bn-A08-p17301557	0.958677686	0.966942149	Bn-N8-p17014513	Bn-chrA08-p14730267
Bn-A08-p17306802	0.970149254	0.975124378	Bn-N8-p17019485	Bn-chrA08-p14735481
Bn-A08-p17307247	0.980099502	0.980099502	Bn-N8-p17019930	Bn-chrA08-p14735926
Bn-A08-p17315378	0.983471074	0.975206612	Bn-N8-p17036250	Bn-chrA08-p14753749
Bn-A08-p17327135	0.991735537	0.983471074	Bn-N8-p17047112	Bn-chrA08-p14773171
Bn-A08-p17330668	0.975206612	0.983471074	Bn-N8-p17059779	Bn-chrA08-p14783439
Bn-A08-p17331714	0.975206612	0.983471074	Bn-N8-p17061695	Bn-chrA08-p14784723
Bn-A08-p17345947	0.958677686	0.966942149	Bn-N8-p17070454	Bn-chrA08-p14792248
Bn-A08-p17351964	0.983471074	0.966942149	Bn-N8-p17076675	Bn-chrA08-p14797308
Bn-A08-p17353404	0.991735537	0.67768595	Bn-N8-p17078115	Bn-chrA08-p14798773
Bn-A08-p17353995	0.991735537	0.983471074	Bn-N8-p17078706	Bn-chrA08_random-p186394
Bn-A08-p17354167	0.975206612	0.983471074	Bn-N8-p17078878	Bn-chrA08_random-p186377
Bn-A08-p17355609	0.993355482	0.996677741	Bn-N8-p17081155	Bn-chrA08_random-p186233
Bn-A08-p17355672	0.985074627	0.980099502	Bn-N8-p17081218	Bn-chrA08_random-p186227
Bn-A08-p17363588	0.970149254	0.965174129	Bn-N8-p17094950	Bn-chrA08-p14819137
Bn-A08-p17366299	0.986710963	0.897009967	Bn-N8-p17097654	Bn-chrA08-p14821822
Bn-A08-p17381323	0.966942149	0.760330579	Bn-N13-p52431923	Bn-chrA08-p14836624
Bn-A08-p17382135	0.991735537	0.991735537	Bn-N8-p17115644	Bn-chrA08-p14837454
Bn-A08-p17382231	0.983471074	0.983471074	Bn-N8-p17115740	Bn-chrA08-p14837550
Bn-A08-p17386548	0.975206612	0.975206612	Bn-N8-p17120056	Bn-chrA08-p14842805
Bn-A08-p17387138	0.958677686	0.958677686	Bn-N8-p17120646	Bn-chrA08-p14843395
Bn-A08-p17393018	0.975206612	0.983471074	Bn-N8-p17126529	Bn-chrA08-p14852060
Bn-A08-p17393046	0.983471074	0.975206612	Bn-N8-p17126557	Bn-chrA08-p14852088
Bn-A08-p17400256	0.975206612	0.991735537	Bn-N8-p17135076	Bn-chrA08-p14865757
Bn-A08-p17402165	0.975124378	0.970149254	Bn-N8-p17136992	Bn-chrA08-p14867667
Bn-A08-p17403283	0.936877076	0.950166113	Bn-N8-p17138121	Bn-chrA08-p14868795
Bn-A08-p17434041	0.958677686	0.884297521	Bn-N8-p17157955	Bn-chrA08-p14897716
Bn-A08-p17435209	0.958677686	0.933884298	Bn-N8-p17159126	Bn-chrA08-p14898784

Bn-A08-p17435316	0.917355372	0.950413223	Bn-N8-p17159233	Bn-chrA08-p14898888
Bn-A08-p17437898	0.990049751	0.995024876	Bn-N8-p17161812	Bn-chrA08-p14901378
Bn-A08-p17446253	0.966942149	0.966942149	Bn-N8-p17163957	Bn-chrA08-p14903514
Bn-A08-p17449031	0.990049751	0.990049751	Bn-N3-p31715748	Bn-chrA03-p28972500
Bn-A08-p17449089	0.970149254	0.970149254	Bn-N3-p31715690	Bn-chrA03-p28972442
Bn-A08-p17449196	0.960199005	0.960199005	Bn-N8-p17166986	Bn-chrAnn_random-p402377
Bn-A08-p17449410	0.995024876	0.995024876	Bn-N8-p17167199	Bn-chrAnn_random-p40238C
Bn-A08-p17461463	0.993355482	0.993355482	Bn-N8-p17179409	Bn-chrA08-p14922079
Bn-A08-p17462017	0.990033223	0.990033223	Bn-N8-p17179963	Bn-chrA08-p14922633
Bn-A08-p17468541	0.975206612	0.975206612	Bn-N8-p17186481	Bn-chrA08-p14929150
Bn-A08-p17470755	0.983471074	0.975206612	Bn-N8-p17188807	Bn-chrA08-p14931406
Bn-A08-p17472208	0.983471074	0.991735537	Bn-N8-p17190258	Bn-chrA08-p14932854
Bn-A08-p17507343	0.975206612	0.983471074	Bn-N8-p17222025	Bn-chrA08-p14963506
Bn-A08-p17513789	0.897009967	0.897009967	Bn-N8-p17227705	Bn-chrA08-p14969703
Bn-A08-p17530580	0.863787375	0.910299003	Bn-N8-p17245366	Bn-chrA08-p14979843
Bn-A08-p17530617	0.744186047	0.794019934	Bn-N8-p17245403	Bn-chrA08-p14979880
Bn-A08-p17534858	0.976744186	0.986710963	Bn-N8-p17249326	Bn-chrA08-p14984073
Bn-A08-p17535851	1	0.991735537	Bn-N8-p17250317	Bn-chrA08-p14985066
Bn-A08-p17547975	0.991735537	1	Bn-N8-p17255122	Bn-chrA08-p14996566
Bn-A08-p17559236	0.991735537	0.983471074	Bn-N8-p17270027	Bn-chrA08-p15013335
Bn-A08-p17565873	0.96345515	0.966777409	Bn-N8-p17275506	Bn-chrA08-p15018416
Bn-A08-p17570589	0.958677686	0.975206612	Bn-N8-p17282600	Bn-chrA08-p15023117
Bn-A08-p17578990	0.996677741	0.993355482	Bn-N8-p17290924	Bn-chrA08-p15031430
Bn-A08-p17586676	0.996677741	0.993355482	Bn-N8-p17307028	Bn-chrA08-p15039054
Bn-A08-p17612777	0.96345515	0.980066445	Bn-N8-p17333121	Bn-chrA08-p15071970
Bn-A08-p17612865	0.991735537	1	Bn-N8-p17333209	Bn-chrA08-p15072058
Bn-A08-p17613494	1	0.991735537	Bn-N8-p17333838	Bn-chrA08-p15072687
Bn-A08-p17613870	0.991735537	1	Bn-N8-p17334214	Bn-chrA08-p15073063
Bn-A08-p17618201	0.993355482	0.790697674	Bn-N8-p17338547	Bn-chrA08-p15077395
Bn-A08-p17639419	0.993355482	0.986710963	Bn-N8-p17373422	Bn-chrC03_random-p456880
Bn-A08-p17647291	0.884297521	0.975206612	Bn-N8-p17383066	Bn-chrA08-p15094064
Bn-A08-p17647346	0.94214876	0.958677686	Bn-N8-p17383122	Bn-chrA08-p15094119
Bn-A08-p17648611	0.983471074	0.991735537	Bn-N8-p17386226	Bn-chrA08-p15095384
Bn-A08-p17649841	0.991735537	1	Bn-N8-p17387381	Bn-chrA08-p15096962
Bn-A08-p17654313	0.94214876	0.958677686	Bn-N8-p17391218	Bn-chrA08-p15101547
Bn-A08-p17654367	0.94214876	0.958677686	Bn-N8-p17391268	Bn-chrA08-p15101601
Bn-A08-p17654459	0.94214876	0.950413223	Bn-N8-p17391360	Bn-chrA08-p15101693
Bn-A08-p17655953	0.983471074	0.975206612	Bn-N8-p17392856	Bn-chrA08-p15103203
Bn-A08-p17663954	0.983471074	0.892561983	Bn-N8-p17400111	Bn-chrC03-p46781208
Bn-A08-p17680057	0.991735537	0.991735537	Bn-N8-p17413065	Bn-chrA08-p15125384
Bn-A08-p17681802	0.975206612	0.975206612	Bn-N8-p17414476	Bn-chrA08-p15126794
Bn-A08-p17686366	0.860465116	0.983388704	Bn-N8-p17418926	Bn-chrA08-p15131401
Bn-A08-p17686371	0.700996678	0.990033223	Bn-N8-p17418931	Bn-chrA08-p15131406
Bn-A08-p17686730	0.975206612	0.983471074	Bn-N8-p17419292	Bn-chrA08-p15131714
Bn-A08-p17687210	0.975206612	0.41322314	Bn-N8-p17419776	Bn-chrA05-p3563509
Bn-A08-p17687648	0.991735537	0.983471074	Bn-N8-p17420251	Bn-chrA08-p15132571

Bn-A08-p17696796	0.966942149	0.975206612	Bn-N8-p17425347	Bn-chrA08-p15137219
Bn-A08-p17698912	0.985074627	0.990049751	Bn-N8-p17427151	Bn-chrA08-p15144318
Bn-A08-p17702727	0.983471074	0.991735537	Bn-N8-p17436342	Bn-chrA08-p15148135
Bn-A08-p17709729	0.966942149	0.966942149	Bn-N8-p17443568	Bn-chrA08-p15153652
Bn-A08-p17710867	0.993355482	0.996677741	Bn-N8-p17444751	Bn-chrA08-p15154790
Bn-A08-p17721455	0.983471074	0.966942149	Bn-N8-p17457023	Bn-chrA08-p15164285
Bn-A08-p17723283	0.966942149	0.966942149	Bn-N8-p17458818	Bn-chrA08-p15166088
Bn-A08-p17724619	0.975206612	0.975206612	Bn-N8-p17459974	Bn-chrA08-p15167242
Bn-A08-p17735673	0.991735537	0.991735537	Bn-N8-p17469786	Bn-chrA08-p15175664
Bn-A08-p17740796	0.966942149	0.966942149	Bn-N8-p17474950	Bn-chrA08-p15182798
Bn-A08-p17748884	0.983388704	0.986710963	Bn-N8-p17488093	Bn-chrA08-p15190894
Bn-A08-p17772887	0.991735537	0.983471074	Bn-N8-p17511887	Bn-chrA08-p15222296
Bn-A08-p17813465	0.983471074	0.966942149	Bn-N8-p17543400	Bn-chrA08-p15261330
Bn-A08-p17816612	0.958677686	0.991735537	Bn-N8-p17545263	Bn-chrA08-p15264476
Bn-A08-p17823277	0.966942149	0.975206612	Bn-N8-p17555865	Bn-chrA08-p15271140
Bn-A08-p17824285	1	0.991735537	Bn-N8-p17556900	Bn-chrA08-p15272148
Bn-A08-p17830772	0.950413223	0.94214876	Bn-N8-p17565821	Bn-chrA08-p15279326
Bn-A08-p17848907	0.92039801	0.92039801	Bn-N18-p27148653	Bn-chrAnn_random-p19254[illegible]
Bn-A08-p17849621	0.880398671	0.916943522	Bn-N8-p17573103	Bn-chrA08-p15302837
Bn-A08-p17851106	0.970099668	0.970099668	Bn-N8-p17574579	Bn-chrA08-p15304302
Bn-A08-p17857870	0.993355482	0.980066445	Bn-N8-p17579698	Bn-chrA08-p15310499
Bn-A08-p17859330	1	0.996677741	Bn-N8-p17581149	Bn-chrA08-p15312006
Bn-A08-p17863903	0.983471074	0.975206612	Bn-N8-p17592686	Bn-chrA08-p15316581
Bn-A08-p17887796	0.975124378	0.975124378	Bn-N8-p17612784	Bn-chrA07-p2230559
Bn-A08-p17932590	0.991735537	0.983471074	Bn-N8-p17671040	Bn-chrA08-p15372463
Bn-A08-p17938347	0.986710963	0.986710963	Bn-N5-p24244068	Bn-chrA01-p15761782
Bn-A08-p17981717	0.96345515	0.96345515	Bn-N8-p17711290	Bn-chrA08-p15427992
Bn-A08-p18000876	0.975124378	0.975124378	Bn-N8-p17732398	Bn-chrA08-p15449250
Bn-A08-p18045254	0.983388704	0.983388704	Bn-N8-p17778288	Bn-chrA08-p15495543
Bn-A08-p18069035	0.996677741	0.996677741	Bn-N8-p17795827	Bn-chrA08-p15513079
Bn-A08-p18070928	0.996677741	0.996677741	Bn-N8-p17798612	Bn-chrA08-p15515864
Bn-A08-p18078817	0.993355482	0.993355482	Bn-N8-p17803801	Bn-chrA08-p15521052
Bn-A08-p18107648	0.983471074	0.950413223	Bn-N8-p17836140	Bn-chrA08-p15552056
Bn-A08-p18112619	0.993355482	0.993355482	Bn-N18-p26552559	Bn-chrA08-p15559693
Bn-A08-p18144819	0.983388704	0.943521595	Bn-N8-p17878011	Bn-chrA08-p15590782
Bn-A08-p18153504	0.983388704	0.983388704	Bn-N8-p17884807	Bn-chrA08-p15597635
Bn-A08-p18200896	0.986710963	0.986710963	Bn-N8-p17928683	Bn-chrA08-p15642416
Bn-A08-p18234758	0.980066445	0.790697674	Bn-N8-p17964906	Bn-chrA08-p15675850
Bn-A08-p18248601	0.991735537	0.991735537	Bn-N8-p17974255	Bn-chrA08-p15683273
Bn-A08-p18286374	0.970149254	0.970149254	Bn-N8-p18021601	Bn-chrA08-p15736567
Bn-A08-p18290748	0.996677741	0.996677741	Bn-N8-p18030952	Bn-chrA08-p15742984
Bn-A08-p18291268	0.986710963	0.986710963	Bn-N8-p18031474	Bn-chrA08-p15743506
Bn-A08-p18310412	1	1	Bn-N8-p18048620	Bn-chrA08-p15760442
Bn-A08-p18321643	0.993355482	0.970099668	Bn-N8-p18067482	Bn-chrC08_random-p336682[illegible]
Bn-A08-p18331012	0.996677741	0.996677741	Bn-N8-p18076240	Bn-chrA08-p15783248
Bn-A08-p18353208	0.990033223	0.990033223	Bn-N8-p18098656	Bn-chrA08-p15805817

Bn-A08-p18356257	0.986710963	0.983388704	Bn-N8-p18101702	Bn-chrA08-p15808881
Bn-A08-p18375745	0.996677741	0.996677741	Bn-N8-p18113675	Bn-chrA08-p15825017
Bn-A08-p18409864	0.970149254	0.472636816	Bn-N8-p18146353	Bn-chrC08-p22595326
Bn-A08-p18432711	0.983388704	0.973421927	Bn-N8-p18182156	Bn-chrA08-p15873934
Bn-A08-p18432800	0.983388704	0.966777409	Bn-N8-p18182245	Bn-chrA08-p15874023
Bn-A08-p18432919	0.993355482	0.980066445	Bn-N8-p18182364	Bn-chrA08-p15874142
Bn-A08-p18433093	0.993355482	0.990033223	Bn-N8-p18182538	Bn-chrA08-p15874316
Bn-A08-p18433672	0.993355482	0.993355482	Bn-N8-p18183114	Bn-chrA08-p15874892
Bn-A08-p18462668	0.993355482	0.973421927	Bn-N8-p18208682	Bn-chrA08-p15896507
Bn-A08-p18463273	0.996677741	0.966777409	Bn-N8-p18209287	Bn-chrA08-p15897113
Bn-A08-p18505527	0.996677741	0.996677741	Bn-N8-p18249329	Bn-chrA08-p15926788
Bn-A08-p18524830	0.996677741	0.996677741	Bn-N8-p18274413	Bn-chrA08-p15949224
Bn-A08-p18526873	0.983388704	0.986710963	Bn-N8-p18279878	Bn-chrA08-p15951274
Bn-A08-p18538166	1	1	Bn-N8-p18290523	Bn-chrA08-p15961919
Bn-A08-p18543984	0.990049751	0.990049751	Bn-N8-p18304756	Bn-chrA08-p15967144
Bn-A08-p18592850	0.993355482	0.970099668	Bn-N8-p18351042	Bn-chrA08-p16010278
Bn-A08-p18597209	1	1	Bn-N8-p18355625	Bn-chrA08-p16015918
Bn-A08-p18605816	0.975206612	0.975206612	Bn-N8-p18359822	Bn-chrA08-p16019785
Bn-A08-p18630178	0.817275748	0.687707641	Bn-N18-p25494078	Bn-chrA08-p16035635
Bn-A08-p18631047	0.584717608	0.734219269	Bn-N18-p25490266	Bn-chrCnn_random-p252530
Bn-A08-p18680503	0.973421927	0.973421927	Bn-N8-p18436703	Bn-chrCnn_random-p695310
Bn-A08-p18704383	0.986710963	0.986710963	Bn-N8-p18453392	Bn-chrA08_random-p192191
Bn-A08-p18720560	1	0.983471074	Bn-N8-p18468951	Bn-chrA08_random-p191393
Bn-A08-p18726458	0.975206612	0.983471074	Bn-N8-p18479671	Bn-chrA08_random-p190530
Bn-A08-p18733383	1	1	Bn-N8-p18489511	Bn-chrAnn_random-p350195
Bn-A08-p18750116	0.985074627	0.985074627	Bn-N8-p18503671	Bn-chrA08-p16102429
Bn-A08-p18750253	0.985074627	0.985074627	Bn-N8-p18503808	Bn-chrA08-p16102566
Bn-A08-p18754717	0.930232558	0.930232558	Bn-N8-p18507294	Bn-chrA08-p16106052
Bn-A08-p18781667	0.953488372	0.953488372	Bn-N8-p18537299	Bn-chrA08-p16135777
Bn-A08-p18784592	0.983388704	0.983388704	Bn-Scaffold02717-p1	Bn-chrA08-p16138727
Bn-A08-p18785448	0.916943522	0.916943522	Bn-N8-p18541103	Bn-chrA08-p16139581
Bn-A08-p18789560	0.993355482	0.993355482	Bn-N8-p18545244	Bn-chrA08-p16143722
Bn-A08-p18798063	0.950166113	0.950166113	Bn-N8-p18564132	Bn-chrA08-p16160904
Bn-A08-p18820218	0.993355482	0.993355482	Bn-N8-p18585891	Bn-chrA08-p16173558
Bn-A08-p18827698	0.983388704	0.983388704	Bn-N8-p18593363	Bn-chrA08-p16181030
Bn-A08-p18953939	0.990033223	0.990033223	Bn-N8-p18721579	Bn-chrA08-p16308452
Bn-A08-p18954204	0.993355482	0.993355482	Bn-N8-p18721844	Bn-chrA08-p16308717
Bn-A08-p18966828	0.993355482	0.993355482	Bn-N8-p18734465	Bn-chrA08_random-p194009
Bn-A08-p18980694	0.993355482	0.993355482	Bn-N8-p18748850	Bn-chrA08-p16324163
Bn-A08-p18989194	0.996677741	0.973421927	Bn-N8-p18757420	Bn-chrA08-p16331510
Bn-A08-p18989271	0.996677741	0.976744186	Bn-N8-p18757497	Bn-chrA08-p16331587
Bn-A08-p19005550	0.976744186	0.980066445	Bn-N8-p18773926	Bn-chrA08-p16347914
Bn-A08-p19007302	0.985074627	0.980099502	Bn-N8-p18775647	Bn-chrA08-p16350939
Bn-A08-p19008575	0.983388704	0.953488372	Bn-N8-p18776922	Bn-chrA08-p16352212
Bn-A08-p19008671	0.970099668	0.913621262	Bn-N8-p18777018	Bn-chrC08-p21450859
Bn-A08-p19009254	0.965174129	0.970149254	Bn-N8-p18777760	Bn-chrA08-p16352894

Bn-A08-p19017689	0.943521595	0.940199336	Bn-N8-p18786566	Bn-chrA08-p16360983
Bn-A08-p19020094	0.990049751	0.990049751	Bn-N8-p18788063	Bn-chrA08-p16363386
Bn-A08-p19035945	0.975124378	0.76119403	Bn-N8-p18799600	Bn-chrA08-p16374128
Bn-A08-p19052340	0.990049751	0.990049751	Bn-N8-p18819257	Bn-chrA08-p16389356
Bn-A08-p19052519	0.985074627	0.975124378	Bn-N8-p18819436	Bn-chrA08-p16389535
Bn-A08-p19056219	0.991735537	0.991735537	Bn-N8-p18823136	Bn-chrA08-p16393234
Bn-A08-p19056545	0.983388704	0.983388704	Bn-N8-p18823461	Bn-chrA08-p16393559
Bn-A08-p19069388	0.965174129	0.965174129	Bn-N8-p18833258	Bn-chrA08-p16403356
Bn-A08-p19084386	0.989583333	0.989583333	Bn-N8-p18848906	Bn-chrA08-p16421190
Bn-A08-p19122274	1	1	Bn-N8-p18888323	Bn-chrA08-p16458414
Bn-A08-p19173201	0.983471074	0.983471074	Bn-N8-p18941616	Bn-chrA08-p16511598
Bn-A08-p19176979	0.960199005	0.960199005	Bn-N8-p18945362	Bn-chrA08-p16515343
Bn-A08-p19191183	1	1	Bn-N8-p18960253	Bn-chrA08-p16530093
Bn-A08-p19191695	0.507462687	0.497512438	Bn-N6-p6366249	Unknown
Bn-A08-p19197746	0.966942149	0.966942149	Bn-N8-p18966855	Bn-chrA08-p16536694
Bn-A08-p19198891	0.935323383	0.935323383	Bn-N8-p18968019	Bn-chrA08-p16537857
Bn-A08-p19199087	0.980099502	0.980099502	Bn-N8-p18968220	Bn-chrA08-p16538058
Bn-A08-p19199175	0.973421927	0.973421927	Bn-N8-p18968308	Bn-chrA08-p16538146
Bn-A08-p19217869	0.990049751	0.990049751	Bn-N8-p18989146	Bn-chrA08-p16557154
Bn-A08-p19243127	0.996677741	0.996677741	Bn-N8-p19014257	Bn-chrA08-p16583267
Bn-A08-p19244116	0.996677741	0.996677741	Bn-N8-p19015246	Bn-chrA08-p16584256
Bn-A08-p19261458	0.960199005	0.960199005	Bn-N8-p19031077	Bn-chrA08-p16600087
Bn-A08-p19284258	0.993355482	0.993355482	Bn-N8-p19053908	Bn-chrA08-p16623848
Bn-A08-p19287037	0.990033223	0.990033223	Bn-N8-p19056687	Bn-chrA08-p16626627
Bn-A08-p19316413	0.930232558	0.986710963	Bn-N17-p19292488	Bn-chrA09_random-p143104
Bn-A08-p19326795	0.973421927	0.973421927	Bn-N8-p19108900	Bn-chrA08-p16679455
Bn-A08-p19327655	0.933884298	0.933884298	Bn-N18-p24128500	Bn-chrC08-p20839667
Bn-A08-p19346186	0.863787375	0.910299003	Bn-N18-p24079296	Bn-chrC08-p20791290
Bn-A08-p19346369	0.970149254	0.970149254	Bn-N8-p19133093	Bn-chrA08-p16703150
Bn-A08-p19363491	0.996677741	0.996677741	Bn-N8-p19150840	Bn-chrA08-p16720696
Bn-A08-p19365594	0.996677741	0.996677741	Bn-N8-p19152943	Bn-chrA08-p16722799
Bn-A08-p19407950	0.983388704	0.96345515	Bn-N8-p19208221	Bn-chrA08-p16773869
Bn-A08-p19410224	0.983471074	0.975206612	Bn-N8-p19210493	Bn-chrA08-p16776098
Bn-A08-p19428204	0.958677686	0.933884298	Bn-N8-p19229698	Bn-chrA08-p16803539
Bn-A08-p19435224	0.958677686	0.950413223	Bn-N8-p19236615	Bn-chrA08-p16810375
Bn-A08-p19440125	0.983471074	0.950413223	Bn-N8-p19241486	Bn-chrA08-p16815244
Bn-A08-p19443099	1	0.983471074	Bn-N8-p19243590	Bn-chrA08-p16817228
Bn-A08-p19449546	0.966942149	0.975206612	Bn-N8-p19246156	Bn-chrA08-p16819482
Bn-A08-p19452520	0.900826446	0.884297521	Bn-N8-p19249147	Bn-chrA08-p16822372
Bn-A08-p19453024	0.983471074	0.975206612	Bn-N8-p19249640	Bn-chrA08-p16822847
Bn-A08-p19454534	0.975206612	0.983471074	Bn-N8-p19251282	Bn-chrA08-p16825039
Bn-A08-p19454590	0.958677686	0.966942149	Bn-N8-p19251339	Bn-chrA08-p16825096
Bn-A08-p19460659	0.980066445	0.983388704	Bn-N8-p19258338	Bn-chrA08-p16831561
Bn-A08-p19483726	0.830845771	0.830845771	Bn-N18-p23810373	Bn-chrC08-p20543329
Bn-A08-p19487830	0.986710963	0.986710963	Bn-N8-p19289995	Bn-chrA08-p16861867
Bn-A08-p19504101	0.966942149	0.966942149	Bn-N8-p19303607	Bn-chrA08_random-p203660

Bn-A08-p19530023	1	1	Bn-N8-p19333368	Bn-chrA08_random-p197608
Bn-A08-p19538361	0.973421927	0.973421927	Bn-N8-p19341873	Bn-chrA08_random-p198459
Bn-A08-p19541990	0.966942149	0.966942149	Bn-N8-p19345416	Bn-chrA08_random-p198813
Bn-A08-p19553756	0.983471074	0.991735537	Bn-N8-p19357480	Bn-chrA08_random-p200183
Bn-A08-p19573947	0.966942149	0.975206612	Bn-N8-p19378150	Bn-chrA08_random-p202631
Bn-A08-p19574453	0.966942149	0.991735537	Bn-N8-p19378654	Bn-chrA08_random-p202682
Bn-A08-p19575209	0.991735537	1	Bn-N8-p19379598	Bn-chrA08_random-p202757
Bn-A08-p19578180	0.933554817	0.986710963	Bn-N18-p23545795	Bn-chrA08_random-p203061
Bn-A08-p19599590	0.945273632	0.945273632	Bn-N8-p19399357	Bn-chrA08-p16920654
Bn-A08-p19608779	0.976744186	0.983388704	Bn-N8-p19409129	Bn-chrA08-p16930426
Bn-A08-p19619504	0.906976744	0.990033223	Bn-N8-p19420061	Bn-chrA08-p16940789
Bn-A08-p19626269	0.973421927	0.973421927	Bn-N8-p19426783	Bn-chrA08-p16947575
Bn-A08-p19642040	0.991735537	0.991735537	Bn-N8-p19441441	Bn-chrA08-p16962231
Bn-A08-p19646345	1	1	Bn-N8-p19448615	Bn-chrA08-p16969405
Bn-A08-p19659002	0.983471074	0.991735537	Bn-N8-p19461301	Bn-chrA08-p16982083
Bn-A08-p19675091	0.993355482	0.993355482	Bn-N8-p19477234	Bn-chrA08-p17005547
Bn-A08-p19676486	0.975124378	0.970149254	Bn-N8-p19476198	Bn-chrA08-p17006942
Bn-A08-p19676498	0.980099502	0.975124378	Bn-N8-p19476210	Bn-chrA08-p17006954
Bn-A08-p19676653	0.955223881	0.985074627	Bn-N18-p23318644	Bn-chrA08-p17007109
Bn-A08-p19676751	0.950248756	0.970149254	Bn-N18-p23318546	Bn-chrA08-p17007207
Bn-A08-p19683899	0.495867769	0.991735537	Bn-N11-p34110475	Bn-chrA08_random-p204106
Bn-A08-p19686197	1	0.495867769	Bn-N8-p19486014	Unknown
Bn-A08-p19690298	0.991735537	0.94214876	Bn-N8-p19490115	Bn-chrA08-p17012925
Bn-A08-p19690393	0.991735537	1	Bn-N8-p19490210	Bn-chrA08-p17013021
Bn-A08-p19699523	0.991735537	0.983471074	Bn-N8-p19499341	Bn-chrA08-p17026238
Bn-A08-p19715503	0.966942149	0.975206612	Bn-N8-p19517411	Bn-chrA08-p17040124
Bn-A08-p19719692	0.975206612	0.983471074	Bn-N8-p19520626	Bn-chrA08-p17044527
Bn-A08-p19736692	0.991735537	1	Bn-N8-p19543607	Bn-chrA08-p17061912
Bn-A08-p19736774	0.950413223	0.991735537	Bn-N18-p23160340	Bn-chrA08-p17061994
Bn-A08-p19757192	0.940199336	0.946843854	Bn-N8-p19559029	Bn-chrA08-p17079491
Bn-A08-p19782306	0.975124378	0.975124378	Bn-N8-p19586029	Bn-chrA08-p17103088
Bn-A08-p19782433	0.980099502	0.980099502	Bn-N8-p19586156	Bn-chrA08-p17103215
Bn-A08-p19792067	0.996677741	0.996677741	Bn-N8-p19595799	Bn-chrA08-p17112854
Bn-A08-p19799335	0.991735537	1	Bn-N8-p19603144	Bn-chrA08-p17120104
Bn-A08-p19799789	0.991735537	1	Bn-N8-p19603599	Bn-chrA08-p17120558
Bn-A08-p19800532	0.975206612	0.983471074	Bn-N8-p19604368	Bn-chrA08-p17121303
Bn-A08-p19802597	0.950413223	0.94214876	Bn-N8-p19617708	Bn-chrA08-p17123369
Bn-A08-p19821458	0.991735537	0.991735537	Bn-N8-p19638198	Bn-chrA08-p17144027
Bn-A08-p19834705	0.991735537	0.991735537	Bn-N8-p19651757	Bn-chrA08-p17156901
Bn-A08-p19871877	0.980099502	0.980099502	Bn-N8-p19687672	Bn-chrA08-p17178051
Bn-A08-p19879173	0.995024876	0.995024876	Bn-N8-p19696516	Bn-chrA08-p17185347
Bn-A08-p19879285	0.980099502	0.980099502	Bn-N8-p19696628	Bn-chrA08-p17185459
Bn-A08-p19879343	0.990049751	0.990049751	Bn-N8-p19696686	Bn-chrA08-p17185517
Bn-A08-p19879488	0.985074627	0.985074627	Bn-N8-p19696831	Bn-chrA08-p17185662
Bn-A08-p19879499	0.985074627	0.985074627	Bn-N8-p19696842	Bn-chrA08-p17185673
Bn-A08-p19888431	0.986710963	0.986710963	Bn-N8-p19707140	Bn-chrA08-p17195234

Bn-A08-p19903284	0.994923858	0.994923858	Bn-N8-p19719543	Bn-chrA08-p17206406
Bn-A08-p19905748	0.975206612	0.966942149	Bn-N8-p19722055	Bn-chrA08-p17208865
Bn-A08-p19907560	0.996677741	0.996677741	Bn-N8-p19723958	Bn-chrA08-p17210673
Bn-A08-p19908763	0.986710963	0.986710963	Bn-N8-p19725161	Bn-chrA08-p17211876
Bn-A08-p19919658	0.92358804	0.92358804	Bn-N8-p19737576	Bn-chrA08-p17222422
Bn-A08-p19919901	0.946843854	0.946843854	Bn-N8-p19737820	Bn-chrA08-p17222666
Bn-A08-p19921292	0.973421927	0.973421927	Bn-N8-p19739247	Bn-chrA08-p17224093
Bn-A08-p19924214	0.966777409	0.966777409	Bn-N8-p19741701	Bn-chrA08-p17226544
Bn-A08-p19960214	0.975206612	0.975206612	Bn-N8-p19762815	Bn-chrA08-p17249929
Bn-A08-p19982640	0.991735537	0.991735537	Bn-N8-p19783814	Bn-chrA08-p17268843
Bn-A08-p19983972	0.983388704	0.983388704	Bn-N8-p19785184	Bn-chrA08-p17270213
Bn-A08-p19998345	0.975206612	0.975206612	Bn-N8-p19796826	Bn-chrA08-p17281373
Bn-A08-p20002292	0.980099502	0.985074627	Bn-N8-p19801314	Bn-chrA08-p17285863
Bn-A08-p20014208	0.993355482	0.993355482	Bn-N8-p19815193	Bn-chrA08-p17298816
Bn-A08-p20020511	0.990049751	0.990049751	Bn-N8-p19822201	Bn-chrA08-p17306277
Bn-A08-p20025646	0.986710963	0.986710963	Bn-N8-p19824393	Bn-chrA08-p17308469
Bn-A08-p20037426	0.976744186	0.976744186	Bn-N8-p19835979	Bn-chrA08-p17319677
Bn-A08-p20042299	0.980099502	0.980099502	Bn-N8-p19837252	Bn-chrA08-p17320691
Bn-A08-p20068678	0.993355482	0.993355482	Bn-N8-p19858500	Bn-chrA08-p17341793
Bn-A08-p20068977	0.990033223	0.990033223	Bn-N8-p19858799	Bn-chrA08-p17342092
Bn-A08-p20069265	0.996677741	0.996677741	Bn-N8-p19859087	Bn-chrA08-p17342380
Bn-A08-p20070433	0.996677741	0.996677741	Bn-N8-p19860255	Bn-chrA08-p17343548
Bn-A08-p20072457	0.966942149	0.966942149	Bn-N8-p19862815	Bn-chrA08-p17346108
Bn-A08-p20072731	0.985074627	0.985074627	Bn-N8-p19863089	Bn-chrA08-p17346382
Bn-A08-p20075062	0.996677741	0.996677741	Bn-N8-p19865386	Bn-chrA08-p17348679
Bn-A08-p20085504	1	0.893687708	Bn-N8-p19875990	Bn-chrC08-p19659450
Bn-A08-p20091818	0.996677741	0.996677741	Bn-N8-p19882294	Bn-chrA08-p17365627
Bn-A08-p20092115	0.990033223	0.990033223	Bn-N8-p19882591	Bn-chrA08-p17365924
Bn-A08-p20096558	1	1	Bn-N8-p19886966	Bn-chrA08-p17370298
Bn-A08-p20104274	0.986710963	0.986710963	Bn-N8-p19894223	Bn-chrA08-p17377531
Bn-A08-p20106099	0.917355372	0.917355372	Bn-N8-p19896084	Bn-chrA08-p17379392
Bn-A08-p20106529	0.935323383	0.935323383	Bn-N8-p19896514	Bn-chrA08-p17379822
Bn-A08-p20114959	1	1	Bn-N8-p19905288	Bn-chrA08_random-p204919
Bn-A08-p20134420	1	1	Bn-N8-p19915403	Bn-chrA08_random-p207499
Bn-A08-p20169768	0.983471074	0.983471074	Bn-N8-p19933880	Bn-chrA08_random-p205651
Bn-A08-p20170453	0.975206612	0.975206612	Bn-N8-p19934563	Bn-chrA08_random-p205583
Bn-A08-p20173109	0.958677686	0.966942149	Bn-N8-p19937163	Bn-chrA08_random-p205323
Bn-A08-p20189027	0.980099502	0.980099502	Bn-N8-p19957910	Bn-chrA08-p17402892
Bn-A08-p20216535	0.993355482	0.993355482	Bn-Scaffold02041-p1	Bn-chrA08-p17422209
Bn-A08-p20216905	0.993355482	0.993355482	Bn-Scaffold02041-p1	Bn-chrA08-p17422579
Bn-A08-p20226034	0.995024876	0.995024876	Bn-N8-p19983571	Bn-chrA08-p17431707
Bn-A08-p20238985	0.993355482	0.993355482	Bn-N8-p19996576	Bn-chrA08-p17444713
Bn-A08-p20244218	0.950413223	0.950413223	Bn-N8-p20001777	Bn-chrA08-p17449914
Bn-A08-p20245485	0.975206612	0.975206612	Bn-N8-p20003418	Bn-chrA08-p17451554
Bn-A08-p20262207	0.990033223	0.990033223	Bn-N8-p20017482	Bn-chrA08-p17465653
Bn-A08-p20277144	0.983388704	0.983388704	Bn-N8-p20048541	Bn-chrA08-p17498639

Bn-A08-p20286379	0.985074627	0.985074627	Bn-N8-p20051389	Bn-chrA08-p17501486
Bn-A08-p20294693	0.976744186	0.976744186	Bn-N8-p20061467	Bn-chrA08-p17510123
Bn-A08-p20305036	0.983471074	0.983471074	Bn-N8-p20348461	Bn-chrA08-p17775586
Bn-A08-p20306059	0.983471074	0.983471074	Bn-N8-p20351252	Bn-chrA08-p17777716
Bn-A08-p20306463	0.983388704	0.983388704	Bn-N8-p20351656	Bn-chrA08-p17778120
Bn-A08-p20306567	0.983471074	0.983471074	Bn-N8-p20351760	Bn-chrA08-p17778224
Bn-A08-p20314411	0.983471074	0.983471074	Bn-N8-p20356688	Bn-chrA08-p17783150
Bn-A08-p20319696	0.960199005	0.955223881	Bn-N8-p20361964	Bn-chrA08-p17788429
Bn-A08-p20319815	0.993355482	0.993355482	Bn-N8-p20362083	Bn-chrA08-p17788548
Bn-A08-p20321558	0.976744186	0.976744186	Bn-N8-p20363828	Bn-chrA08-p17790293
Bn-A08-p20324326	0.916943522	0.916943522	Bn-N8-p20366583	Bn-chrA08-p17793048
Bn-A08-p20336178	0.995024876	0.995024876	Bn-N8-p20379990	Bn-chrA08-p17804632
Bn-A08-p20341221	0.198347107	0.975206612	Bn-N12-p25172344	Bn-chrA08-p17809528
Bn-A08-p20343735	0.851239669	0.94214876	Bn-N18-p21457522	Bn-chrA08-p17811886
Bn-A08-p20347040	0.909090909	0.884297521	Bn-N8-p20392010	Bn-chrA08-p17815236
Bn-A08-p20354125	0.396694215	0.983471074	Unknown	Bn-chrA08-p17821926
Bn-A08-p20354609	0.900826446	0.909090909	Bn-N8-p20398894	Bn-chrA08-p17822432
Bn-A08-p20355213	0.991735537	0.694214876	Bn-N8-p20399499	Bn-chrA08-p17823039
Bn-A08-p20356745	0.94214876	0.94214876	Bn-N8-p20401030	Bn-chrA08-p17824616
Bn-A08-p20360306	0.936507937	0.952380952	Bn-N8-p20404594	Bn-chrA08-p17828216
Bn-A08-p20373317	0.990049751	1	Bn-N8-p20410459	Bn-chrA08-p17834206
Bn-A08-p20373400	0.960199005	0.910447761	Bn-N8-p20410542	Bn-chrC08-p18814963
Bn-A08-p20394042	0.966942149	0.966942149	Bn-N8-p20432212	Bn-chrA08-p17871535
Bn-A08-p20395388	1	1	Bn-N8-p20433558	Bn-chrA08-p17872881
Bn-A08-p20416050	0.985074627	0.985074627	Bn-N8-p20463692	Bn-chrA08-p17901518
Bn-A08-p20441889	0.943521595	0.943521595	Bn-N8-p20495674	Bn-chrA08-p17924717
Bn-A08-p20474660	0.996677741	1	Bn-N8-p20534215	Bn-chrA08-p17956052
Bn-A08-p20476359	1	0.991735537	Bn-N8-p20535921	Bn-chrA08-p17957758
Bn-A08-p20480348	0.980099502	0.985074627	Bn-N8-p20539877	Bn-chrA08-p17961724
Bn-A08-p20485374	0.989583333	0.989583333	Bn-N8-p20547159	Bn-chrA08-p17966722
Bn-A08-p20490539	0.933884298	0.933884298	Bn-N8-p20552338	Bn-chrA08-p17971901
Bn-A08-p20490691	0.980066445	0.980066445	Bn-N8-p20552490	Bn-chrA08-p17972053
Bn-A08-p20529396	0.993355482	0.976744186	Bn-N8-p20593300	Bn-chrA08-p18008746
Bn-A08-p20530487	0.991735537	0.983471074	Bn-N8-p20594384	Bn-chrA08-p18009832
Bn-A08-p20537757	0.661157025	0.950413223	Bn-N18-p21006384	Bn-chrA08-p18015014
Bn-A08-p20538517	0.966942149	0.975206612	Bn-N8-p20600222	Bn-chrA08-p18015778
Bn-A08-p20546003	0.975206612	0.983471074	Bn-N8-p20606125	Bn-chrA08-p18026358
Bn-A08-p20546044	0.983471074	0.975206612	Bn-N8-p20606166	Bn-chrA08-p18026399
Bn-A08-p20546110	0.983471074	0.991735537	Bn-N8-p20606232	Bn-chrA08-p18026465
Bn-A08-p20547091	0.900332226	0.990033223	Bn-N8-p20606688	Bn-chrA08-p18026924
Bn-A08-p20548890	0.983471074	0.975206612	Bn-N8-p20608466	Bn-chrA08-p18028721
Bn-A08-p20551301	0.993355482	0.993355482	Bn-N8-p20610895	Bn-chrA08-p18031138
Bn-A08-p20554540	0.990049751	0.985074627	Bn-N8-p20614128	Bn-chrA08-p18034374
Bn-A08-p20555629	0.980099502	0.985074627	Bn-N8-p20615247	Bn-chrA08-p18035463
Bn-A08-p20577960	0.991735537	0.983471074	Bn-N8-p20631070	Bn-chrA08_random-p208491
Bn-A08-p20587556	1	0.989583333	Bn-N8-p20641784	Bn-chrA08-p18061887

Bn-A08-p20594698	0.975206612	0.983471074	Bn-N8-p20649161	Bn-chrA08-p18068968
Bn-A08-p20594785	0.958677686	0.975206612	Bn-N8-p20649248	Bn-chrA08-p18069055
Bn-A08-p20608675	0.966942149	0.991735537	Bn-N8-p20662249	Bn-chrA08-p18081290
Bn-A08-p20679328	0.983388704	0.863787375	Bn-N8-p20744873	Bn-chrA08-p18169942
Bn-A08-p20692045	0.986710963	0.990033223	Bn-N8-p20760472	Bn-chrAnn_random-p457895
Bn-A08-p20717142	0.946843854	0.946843854	Bn-N18-p2025883	Bn-chrC08-p1769127
Bn-A08-p20778097	0.996677741	0.996677741	Bn-N8-p20847011	Bn-chrA08-p18934509
Bn-A08-p20801363	0.993355482	0.993355482	Bn-N8-p20869624	Bn-chrA08-p18912430
Bn-A08-p20803469	0.990033223	0.990033223	Bn-N8-p20871753	Bn-chrA08-p18910301
Bn-A08-p20820768	0.996677741	0.996677741	Bn-N8-p20879154	Bn-chrA08-p18902660
Bn-A08-p20838949	0.986710963	0.986710963	Bn-N8-p20900858	Bn-chrA08-p18878158
Bn-A08-p20839304	0.986710963	0.986710963	Bn-N8-p20901235	Bn-chrA08-p18877781
Bn-A08-p20846182	0.993355482	0.993355482	Bn-N8-p20908110	Bn-chrA08-p18870906
Bn-A08-p20855631	0.497512438	0.497512438	Unknown	Bn-chrA08-p18861277
Bn-A08-p20865005	0.996677741	0.996677741	Bn-N8-p20927494	Bn-chrA08-p18854100
Bn-A08-p20874645	0.996677741	0.996677741	Bn-N8-p20937151	Bn-chrA08-p18844443
Bn-A08-p20904948	0.980099502	0.980099502	Bn-N8-p20968196	Bn-chrA08-p18815494
Bn-A08-p20913891	1	0.993355482	Bn-N8-p20977267	Bn-chrA08-p18806436
Bn-A08-p20927339	0.794019934	0.990033223	Bn-N8-p20989516	Bn-chrA08-p18794177
Bn-A08-p20931438	0.975206612	0.983471074	Bn-N8-p20993661	Bn-chrA08-p18787714
Bn-A08-p20931690	0.996677741	0.996677741	Bn-N8-p20994132	Bn-chrA08-p18787462
Bn-A08-p20934184	0.996677741	0.983388704	Bn-N8-p20996625	Bn-chrA08-p18784968
Bn-A08-p20945277	0.970149254	0.960199005	Bn-N8-p21005755	Bn-chrA08-p18767829
Bn-A08-p20950199	0.996677741	0.986710963	Bn-N8-p21010676	Bn-chrA08-p18762900
Bn-A08-p20965113	0.996677741	0.936877076	Bn-N8-p21028995	Bn-chrA08-p18739906
Bn-A08-p20967165	0.970099668	0.96345515	Bn-N8-p21031047	Bn-chrA08-p18737854
Bn-A08-p20968239	0.970149254	0.970149254	Bn-N18-p1551819	Bn-chrC08-p1284899
Bn-A08-p20971092	0.955223881	0.955223881	Bn-N8-p21035086	Bn-chrA08-p18733636
Bn-A08-p20993982	0.996677741	0.996677741	Bn-N8-p21055202	Bn-chrA08-p18709824
Bn-A08-p21036778	0.993355482	0.986710963	Bn-N8-p21100372	Bn-chrAnn_random-p293696
Bn-A08-p21062076	0.990033223	0.990033223	Bn-N18-p1305030	Bn-chrA08-p18657105
Bn-A08-p21070052	0.917355372	0.917355372	Bn-N8-p21131090	Bn-chrA08-p18649127
Bn-A08-p21076939	0.991735537	0.950413223	Bn-N8-p21140255	Bn-chrA08-p18640126
Bn-A08-p21076987	0.94214876	0.900826446	Bn-N8-p21140303	Bn-chrA08-p18640078
Bn-A08-p21077284	0.79338843	0.991735537	Bn-N8-p21140570	Bn-chrA08-p18638573
Bn-A08-p21132027	0.800995025	0.855721393	Bn-N18-p1122222	Bn-chrC08_random-p368595
Bn-A08-p21149542	0.993355482	0.993355482	Bn-N8-p21210689	Bn-chrA08-p18567341
Bn-A08-p21168203	0.983388704	0.983388704	Bn-N8-p21226954	Bn-chrA08-p18551076
Bn-A08-p21189645	1	1	Bn-N8-p21264787	Bn-chrA08-p18530138
Bn-A08-p21199124	0.990033223	0.990033223	Bn-N8-p21274010	Bn-chrA08-p18520916
Bn-A08-p21227076	0.965174129	0.965174129	Bn-N8-p21302699	Bn-chrA08-p18491194
Bn-A08-p21228678	0.990033223	0.990033223	Bn-N8-p21304221	Bn-chrA08-p18489672
Bn-A08-p21241586	0.990049751	0.990049751	Bn-N8-p21319986	Bn-chrAnn_random-p424164
Bn-A08-p21282852	0.976744186	0.976744186	Bn-N8-p21361253	Bn-chrA08-p18442078
Bn-A08-p21283107	0.943521595	0.943521595	Bn-N18-p774166	Bn-chrC08-p476779
Bn-A08-p21283562	0.976744186	0.976744186	Bn-N8-p21361961	Bn-chrA08-p18441370

Bn-A08-p21319486	0.980099502	0.980099502	Bn-N8-p21399260	Bn-chrA08-p18406752
Bn-A08-p21330967	0.996677741	0.996677741	Bn-N8-p21408616	Bn-chrA08-p18395587
Bn-A08-p21334007	0.990033223	0.990033223	Bn-N8-p21411623	Bn-chrA08-p18392580
Bn-A08-p21348310	0.617940199	0.617940199	Bn-N8-p21425973	Bn-chrA08-p18381527
Bn-A08-p21352716	0.970099668	0.970099668	Bn-N8-p21430377	Bn-chrA08-p18377123
Bn-A08-p21383689	0.973421927	0.973421927	Bn-N8-p21458623	Bn-chrA08-p18350928
Bn-A08-p21385759	0.996677741	0.996677741	Bn-N8-p21460702	Bn-chrA08-p18349485
Bn-A08-p21401348	0.993355482	0.993355482	Bn-N8-p21478311	Bn-chrA08-p18334855
Bn-A08-p21417592	0.950166113	0.950166113	Bn-N8-p21495935	Bn-chrA08-p18320317
Bn-A08-p21434747	0.993355482	0.993355482	Bn-N8-p21511255	Bn-chrA08-p18304328
Bn-A08-p21463036	0.940199336	0.940199336	Bn-N8-p21536587	Bn-chrA08-p18278869
Bn-A08-p21557215	0.991735537	0.991735537	Bn-N4-p14606227	Bn-chrA04-p13258687
Bn-A08-p21557880	0.983471074	0.991735537	Bn-N4-p14606853	Bn-chrA10-p11749601
Bn-A08-p21557975	0.983471074	0.983471074	Bn-N4-p14606948	Bn-chrA04-p13259408
Bn-A08-p21591824	0.993355482	0.993355482	Bn-N8-p21680666	Bn-chrAnn_random-p188494
Bn-A09-p5992	0.983471074	0.975206612	Bn-N9-p903369	Bn-chrA09-p677779
Bn-A09-p15262	0.991735537	0.983471074	Bn-N9-p890009	Bn-chrA09-p668181
Bn-A09-p24404	0.975206612	0.983471074	Bn-N9-p881001	Bn-chrA09-p659170
Bn-A09-p33280	0.991735537	0.909090909	Bn-N9-p872947	Bn-chrA09-p650991
Bn-A09-p34830	0.980099502	0.990049751	Bn-N9-p871366	Bn-chrA09-p649450
Bn-A09-p42845	0.975206612	0.966942149	Bn-N9-p838203	Bn-chrA09-p638061
Bn-A09-p43101	0.983471074	0.975206612	Bn-N9-p837947	Bn-chrA09-p637805
Bn-A09-p86293	0.936877076	0.936877076	Bn-N9-p796829	Bn-chrA09-p599166
Bn-A09-p94205	0.995024876	0.985074627	Bn-N9-p787778	Bn-chrC01-p28860538
Bn-A09-p100554	0.985074627	0.945273632	Bn-N9-p780587	Bn-chrC09-p68250
Bn-A09-p100631	0.975124378	0.970149254	Bn-N9-p780510	Bn-chrA09-p575338
Bn-A09-p100643	0.970149254	0.975124378	Bn-N9-p780498	Bn-chrA09-p575326
Bn-A09-p109917	0.940298507	0.940298507	Bn-N19-p1200873	Bn-chrC09-p57733
Bn-A09-p112102	0.980099502	0.975124378	Bn-N9-p760351	Bn-chrA09-p564264
Bn-A09-p135892	0.983388704	0.990033223	Bn-N9-p737667	Bn-chrA09-p539846
Bn-A09-p146640	0.990049751	0.995024876	Bn-N9-p726908	Bn-chrA09-p528295
Bn-A09-p149032	0.965174129	0.965174129	Bn-N19-p1132101	Bn-chrCnn_random-p178229
Bn-A09-p149317	0.995024876	1	Bn-N9-p722153	Bn-chrA09-p525720
Bn-A09-p149667	0.975206612	0.983471074	Bn-N9-p721803	Bn-chrA09-p525370
Bn-A09-p156691	0.966942149	0.975206612	Bn-N9-p714555	Bn-chrA09-p519524
Bn-A09-p160775	0.966942149	0.958677686	Bn-N9-p709677	Bn-chrA09-p515411
Bn-A09-p174327	0.991735537	0.966942149	Bn-N9-p697821	Bn-chrCnn_random-p178589
Bn-A09-p175303	0.935323383	0.766169154	Bn-N9-p691433	Bn-chrA09-p504959
Bn-A09-p184319	1	0.983388704	Bn-N9-p678827	Bn-chrA09-p499980
Bn-A09-p219538	0.94214876	0.950413223	Bn-N9-p658759	Bn-chrA09-p479995
Bn-A09-p222701	0.975206612	0.983471074	Bn-N9-p655576	Bn-chrA09-p476810
Bn-A09-p234493	0.993355482	0.990033223	Bn-N19-p55978520	Bn-chrAnn_random-p229494
Bn-A09-p239851	0.996677741	0.993355482	Bn-N19-p55973158	Bn-chrAnn_random-p229548
Bn-A09-p260819	1	1	Bn-N19-p55962413	Bn-chrUnn_random-p494718
Bn-A09-p264743	0.990033223	0.966777409	Bn-N19-p55958615	Bn-chrA09-p429423
Bn-A09-p264855	0.983471074	0.900826446	Bn-N19-p55958503	Bn-chrA09-p429311

Bn-A09-p281844	0.983471074	0.991735537	Bn-N19-p55940966	Bn-chrA09-p408228
Bn-A09-p285148	0.966942149	0.975206612	Bn-N19-p55937663	Bn-chrA09-p404925
Bn-A09-p297029	0.970149254	0.960199005	Bn-N19-p55922079	Bn-chrCnn_random-p218284
Bn-A09-p301754	0.983471074	0.991735537	Bn-N19-p55917195	Bn-chrC02-p26368454
Bn-A09-p301830	0.996677741	0.990033223	Bn-N19-p55917119	Bn-chrA09-p389323
Bn-A09-p302062	0.955223881	0.985074627	Bn-N19-p965061	Bn-chrA09-p389091
Bn-A09-p321430	0.776859504	0.933884298	Bn-N19-p1101880	Bn-chrA09-p371063
Bn-A09-p329151	0.702479339	0.710743802	Bn-N19-p55893528	Bn-chrA09-p369504
Bn-A09-p362846	0.991735537	0.983471074	Bn-N19-p55841194	Bn-chrAnn_random-p274144
Bn-A09-p381059	0.190082645	0.983471074	Bn-N15-p486904	Bn-chrAnn_random-p310619
Bn-A09-p381724	0.859504132	0.991735537	Bn-N8-p11911764	Bn-chrAnn_random-p310612
Bn-A09-p384043	0.752066116	0.983471074	Bn-N16-p29859813	Bn-chrA09-p9336182
Bn-A09-p388989	0.985074627	0.338308458	Bn-N19-p55820334	Unknown
Bn-A09-p403519	0.925619835	0.925619835	Bn-N19-p849968	Bn-chrCnn_random-p133834
Bn-A09-p408412	0.955223881	0.955223881	Bn-N19-p55797571	Bn-chrA09-p245679
Bn-A09-p417110	1	0.991735537	Bn-N19-p55792619	Bn-chrA09-p240186
Bn-A09-p427855	0.966942149	0.950413223	Bn-N19-p55785038	Bn-chrA09-p222862
Bn-A09-p433176	0.991735537	1	Bn-N19-p55781367	Bn-chrA09-p219209
Bn-A09-p469758	0.986710963	0.986710963	Bn-N19-p55748855	Bn-chrA09-p185273
Bn-A09-p487360	0.975124378	0.975124378	Bn-N19-p55735477	Bn-chrA09-p171845
Bn-A09-p487432	0.756218905	0.756218905	Bn-N19-p55735405	Bn-chrA09-p171773
Bn-A09-p574205	0.958677686	0.950413223	Bn-N19-p55641089	Bn-chrA09_random-p308423
Bn-A09-p584756	1	0.991735537	Bn-N19-p55632004	Bn-chrA09_random-p10928
Bn-A09-p586357	0.966942149	0.958677686	Bn-N19-p55630399	Bn-chrA09_random-p12533
Bn-A09-p594794	0.983471074	0.975206612	Bn-N19-p55621802	Bn-chrA09_random-p21430
Bn-A09-p596014	0.950413223	0.975206612	Bn-N19-p55620559	Bn-chrA09_random-p22672
Bn-A09-p599316	0.991735537	1	Bn-N19-p55612694	Bn-chrA09_random-p27433
Bn-A09-p603278	0.991735537	1	Bn-N19-p55607436	Bn-chrA09_random-p31184
Bn-A09-p614715	0.966942149	0.958677686	Bn-N9-p641361	Bn-chrA09-p1638078
Bn-A09-p616919	0.975206612	0.983471074	Bn-N9-p640406	Bn-chrA09-p1637130
Bn-A09-p620595	0.975206612	0.966942149	Bn-N9-p637593	Bn-chrA09-p1634318
Bn-A09-p631411	0.966942149	0.966942149	Bn-N9-p626805	Bn-chrA09_random-p4669
Bn-A09-p632449	0.991735537	0.983471074	Bn-N9-p626353	Bn-chrCnn_random-p167676
Bn-A09-p642046	0.966942149	0.991735537	Bn-N19-p496077	Bn-chrAnn_random-p312248
Bn-A09-p643947	0.991735537	0.983471074	Bn-N9-p614870	Bn-chrAnn_random-p312267
Bn-A09-p652580	0.991735537	1	Bn-N9-p604052	Bn-chrA09_random-p188696
Bn-A09-p660753	0.991735537	0.983471074	Bn-N9-p595878	Bn-chrA09_random-p196873
Bn-A09-p660775	0.991735537	0.983471074	Bn-N9-p595856	Bn-chrA09_random-p196895
Bn-A09-p664845	0.983471074	0.966942149	Bn-N9-p590754	Bn-chrA09_random-p200866
Bn-A09-p675589	0.991735537	0.966942149	Bn-N9-p583670	Bn-chrCnn_random-p173255
Bn-A09-p678387	0.996677741	0.976744186	Bn-N9-p580796	Bn-chrA09_random-p208870
Bn-A09-p679276	0.917355372	0.958677686	Bn-N9-p579908	Bn-chrA09_random-p209759
Bn-A09-p688895	0.983471074	0.975206612	Bn-N9-p572906	Bn-chrCnn_random-p174939
Bn-A09-p694190	0.985074627	0.985074627	Bn-N19-p417038	Bn-chrCnn_random-p175445
Bn-A09-p696636	0.925619835	0.94214876	Bn-N19-p413458	Bn-chrA09_random-p227483
Bn-A09-p702173	0.860696517	0.990049751	Bn-N9-p562015	Bn-chrA09_random-p231786

Bn-A09-p704028	0.983471074	0.991735537	Bn-N9-p560125	Bn-chrA09_random-p233682
Bn-A09-p706265	0.991735537	0.991735537	Bn-N9-p557891	Bn-chrA09_random-p235909
Bn-A09-p708660	1	0.991735537	Bn-N9-p555610	Bn-chrA09_random-p238152
Bn-A09-p718444	0.991735537	0.983471074	Bn-N9-p545897	Bn-chrA09_random-p256694
Bn-A09-p731402	0.983471074	0.991735537	Bn-N9-p530413	Bn-chrA09-p136838
Bn-A09-p733947	0.906976744	0.973421927	Bn-N9-p527767	Bn-chrA09-p134192
Bn-A09-p739088	0.966942149	0.975206612	Bn-N9-p525116	Bn-chrA09-p131540
Bn-A09-p750455	0.953488372	0.993355482	Bn-N19-p320463	Bn-chrA09-p121716
Bn-A09-p751817	0.950413223	0.958677686	Bn-N9-p513856	Bn-chrA09-p120354
Bn-A09-p757653	0.970099668	0.983388704	Bn-N9-p511108	Bn-chrA09-p117508
Bn-A09-p763204	0.818181818	0.801652893	Bn-N9-p504875	Bn-chrA09-p106558
Bn-A09-p765293	0.991735537	0.991735537	Bn-N9-p503975	Bn-chrA09-p105581
Bn-A09-p768355	0.983471074	0.851239669	Bn-N9-p500911	Bn-chrCnn_random-p186951
Bn-A09-p775340	0.958677686	0.991735537	Bn-N19-p292554	Bn-chrA09-p94649
Bn-A09-p788109	0.983471074	0.991735537	Bn-N9-p479492	Bn-chrA09-p82113
Bn-A09-p788595	0.986710963	0.990033223	Bn-N9-p479006	Bn-chrA09-p81627
Bn-A09-p854992	0.966942149	0.925619835	Bn-N9-p445585	Bn-chrA09-p41573
Bn-A09-p855112	0.991735537	0.983471074	Bn-N9-p445465	Bn-chrA09-p41453
Bn-A09-p865058	0.909090909	0.917355372	Bn-N9-p441889	Bn-chrA09-p38592
Bn-A09-p865922	0.94214876	0.966942149	Bn-N9-p440784	Bn-chrA09-p37730
Bn-A09-p867036	0.958677686	0.884297521	Bn-N9-p439804	Bn-chrA09-p36744
Bn-A09-p868589	0.983471074	0.991735537	Bn-N9-p438953	Bn-chrA09-p35224
Bn-A09-p869372	0.958677686	0.975206612	Bn-N9-p438171	Bn-chrA09-p34442
Bn-A09-p941202	0.917355372	0.925619835	Bn-N9-p358440	Bn-chrA09_random-p112232
Bn-A09-p942734	0.917355372	0.917355372	Bn-N9-p356896	Bn-chrA09_random-p113784
Bn-A09-p951202	0.991735537	0.983471074	Bn-N9-p348382	Bn-chrA09_random-p122268
Bn-A09-p957959	0.975206612	0.966942149	Bn-N9-p342104	Bn-chrA09_random-p126378
Bn-A09-p962254	0.983471074	0.983471074	Bn-N9-p338221	Bn-chrA09_random-p130265
Bn-A09-p972043	0.991735537	0.991735537	Bn-N9-p328484	Bn-chrA09_random-p141481
Bn-A09-p984186	0.975206612	0.983471074	Bn-N9-p312970	Bn-chrA09_random-p155347
Bn-A09-p985671	1	0.966942149	Bn-N9-p311492	Bn-chrCnn_random-p208666
Bn-A09-p996577	0.983471074	0.991735537	Bn-N9-p301255	Bn-chrA09_random-p166729
Bn-A09-p999098	0.991735537	1	Bn-N9-p297475	Bn-chrA09_random-p169262
Bn-A09-p1011107	0.892561983	0.950413223	Bn-N9-p279051	Bn-chrA09_random-p56709
Bn-A09-p1018965	0.983471074	0.991735537	Bn-N9-p271223	Bn-chrA09_random-p64533
Bn-A09-p1032996	0.975206612	0.983471074	Bn-N9-p258264	Bn-chrAnn_random-p330902
Bn-A09-p1165212	0.958677686	0.966942149	Bn-N9-p1277924	Bn-chrA09-p940331
Bn-A09-p1188091	0.909090909	0.917355372	Bn-N9-p1297967	Bn-chrA09-p963280
Bn-A09-p1227639	1	0.991735537	Bn-N9-p1349039	Bn-chrA09-p1004078
Bn-A09-p1236359	0.990049751	0.990049751	Bn-N9-p1354789	Bn-chrA09-p1007423
Bn-A09-p1243032	1	1	Bn-N9-p1360264	Bn-chrA09-p1017494
Bn-A09-p1252014	0.980099502	0.980099502	Bn-N9-p1369261	Bn-chrA09-p1026490
Bn-A09-p1252150	0.975124378	0.975124378	Bn-N9-p1369397	Bn-chrA09-p1026626
Bn-A09-p1272298	0.991735537	1	Bn-N9-p1392583	Bn-chrA09-p1053245
Bn-A09-p1404415	0.985074627	0.985074627	Bn-N9-p1517886	Bn-chrAnn_random-p476968
Bn-A09-p1405207	0.990049751	0.990049751	Bn-N9-p1518678	Bn-chrAnn_random-p47696[illegible]

Bn-A09-p1541696	0.990033223	0.990033223	Bn-N9-p2840404	Bn-chrA09-p2385155
Bn-A09-p1550543	0.958677686	0.958677686	Bn-N9-p2830317	Bn-chrA09-p2375544
Bn-A09-p1552993	0.950413223	0.950413223	Bn-N9-p2829986	Bn-chrA09-p2375213
Bn-A09-p1558529	0.983388704	0.983388704	Bn-N9-p2825810	Bn-chrA09-p2371037
Bn-A09-p1567071	0.970149254	0.970149254	Bn-N9-p2813202	Bn-chrA09-p2362537
Bn-A09-p1570558	0.991735537	0.991735537	Bn-N9-p2809617	Bn-chrA09-p2358949
Bn-A09-p1588624	0.991735537	0.991735537	Bn-N9-p2793812	Bn-chrA09-p2343400
Bn-A09-p1603105	0.991735537	0.991735537	Bn-N9-p2776241	Bn-chrA09-p2324670
Bn-A09-p1603211	0.983471074	0.983471074	Bn-N9-p2776135	Bn-chrA09-p2324564
Bn-A09-p1603989	0.933884298	0.933884298	Bn-N9-p2775357	Bn-chrA09-p2323786
Bn-A09-p1608051	0.785123967	0.983471074	Bn-N9-p2769892	Bn-chrA09-p2318314
Bn-A09-p1610631	0.980066445	0.976744186	Bn-N9-p2767268	Bn-chrA09-p2315761
Bn-A09-p1617368	0.986710963	0.996677741	Bn-N9-p2759695	Bn-chrA09-p2309279
Bn-A09-p1623725	0.983471074	0.983471074	Bn-N9-p2753341	Bn-chrA09-p2302907
Bn-A09-p1630301	1	1	Bn-N9-p2746790	Bn-chrA09-p2296382
Bn-A09-p1631944	0.993355482	0.993355482	Bn-N9-p2745147	Bn-chrA09-p2294741
Bn-A09-p1634229	0.996677741	0.993355482	Bn-N9-p2742902	Bn-chrA09-p2292497
Bn-A09-p1634504	0.958677686	0.958677686	Bn-N9-p2742627	Bn-chrA09-p2292222
Bn-A09-p1634652	0.983471074	0.991735537	Bn-N9-p2742479	Bn-chrA09-p2292074
Bn-A09-p1657554	0.958677686	0.966942149	Bn-N19-p3710582	Bn-chrA09-p2274332
Bn-A09-p1670138	0.975206612	0.983471074	Bn-N9-p2711221	Bn-chrA09-p2263576
Bn-A09-p1670189	0.950413223	0.966942149	Bn-N9-p2711170	Bn-chrA09-p2263525
Bn-A09-p1670665	0.966942149	0.355371901	Bn-N9-p2710696	Unknown
Bn-A09-p1673884	0.917355372	0.925619835	Bn-N9-p2710090	Bn-chrA09-p2262645
Bn-A09-p1674552	0.958677686	0.975206612	Bn-N9-p2709417	Bn-chrA09-p2261833
Bn-A09-p1674796	0.892561983	0.991735537	Bn-N9-p2709176	Bn-chrA09-p2261544
Bn-A09-p1680594	0.991735537	0.917355372	Bn-N9-p2697938	Bn-chrA09-p2250227
Bn-A09-p1681240	0.991735537	0.925619835	Bn-N9-p2697292	Bn-chrC09-p2264418
Bn-A09-p1690633	0.975206612	0.983471074	Bn-N9-p2677352	Bn-chrA09-p2233585
Bn-A09-p1691589	0.917355372	0.917355372	Bn-N9-p2676396	Bn-chrA09-p2232704
Bn-A09-p1698812	0.991735537	0.983471074	Bn-N9-p2668659	Bn-chrA09-p2227131
Bn-A09-p1699080	0.966942149	0.958677686	Bn-N9-p2668389	Bn-chrA09-p2226863
Bn-A09-p1699131	0.958677686	0.966942149	Bn-N9-p2668338	Bn-chrA09-p2226812
Bn-A09-p1700881	0.991735537	0.983471074	Bn-N9-p2663496	Bn-chrA09-p2225063
Bn-A09-p1702352	0.983471074	0.991735537	Bn-N9-p2662024	Bn-chrA09-p2223592
Bn-A09-p1702403	0.975206612	0.983471074	Bn-N9-p2661973	Bn-chrA09-p2223541
Bn-A09-p1711423	1	0.991735537	Bn-N9-p2653738	Bn-chrAnn_random-p38615[illegible]
Bn-A09-p1715714	0.983471074	0.991735537	Bn-N9-p2650517	Bn-chrAnn_random-p325294
Bn-A09-p1716772	0.983471074	0.983471074	Bn-N9-p2649372	Bn-chrAnn_random-p32528[illegible]
Bn-A09-p1719501	0.925619835	0.710743802	Bn-N9-p2641425	Bn-chrA09-p2215713
Bn-A09-p1719614	0.917355372	0.917355372	Bn-N9-p2641312	Bn-chrA09-p2215600
Bn-A09-p1721492	0.970149254	0.975124378	Bn-N9-p2638139	Bn-chrA09-p2213722
Bn-A09-p1721507	0.970149254	0.970149254	Bn-N9-p2638124	Bn-chrA09-p2213707
Bn-A09-p1727484	0.247933884	0.983471074	Unknown	Bn-chrA09-p2207100
Bn-A09-p1727915	0.694214876	0.975206612	Bn-N19-p3573201	Bn-chrA09-p2206661
Bn-A09-p1727971	0.404958678	0.975206612	Bn-N19-p3572693	Bn-chrA09-p2206605

Bn-A09-p1746285	0.975124378	0.970149254	Bn-N9-p2611432	Bn-chrA09-p2188167
Bn-A09-p1746688	0.985074627	0.990049751	Bn-N9-p2611029	Bn-chrA09-p2187760
Bn-A09-p1746892	0.995024876	1	Bn-N9-p2610825	Bn-chrA09-p2187556
Bn-A09-p1756759	0.983471074	0.983471074	Bn-N9-p2596904	Bn-chrA09-p2180010
Bn-A09-p1760983	0.933884298	0.933884298	Bn-N9-p2592969	Bn-chrA09-p2176199
Bn-A09-p1761036	0.966942149	0.966942149	Bn-N9-p2592916	Bn-chrA09-p2176146
Bn-A09-p1765907	0.991735537	0.991735537	Bn-N9-p2588046	Bn-chrA09-p2171498
Bn-A09-p1766799	0.983471074	0.876033058	Bn-N9-p2586941	Bn-chrC09-p2167390
Bn-A09-p1780211	0.966942149	0.983471074	Bn-N9-p2570397	Bn-chrA06-p9501666
Bn-A09-p1785717	0.966942149	0.966942149	Bn-N9-p2566120	Bn-chrA09-p2141350
Bn-A09-p1785718	0.966942149	0.966942149	Bn-N9-p2566119	Bn-chrA09-p2141349
Bn-A09-p1786726	0.975124378	0.980099502	Bn-N9-p2565112	Bn-chrA09-p2140342
Bn-A09-p1796337	0.985074627	0.980099502	Bn-N9-p2559897	Bn-chrA09-p2135409
Bn-A09-p1821136	0.993355482	0.877076412	Bn-N9-p2528409	Bn-chrA09-p2115521
Bn-A09-p1829952	1	0.945273632	Bn-N9-p2520215	Bn-chrA09-p2104338
Bn-A09-p1832760	0.975206612	0.975206612	Bn-N9-p2517306	Bn-chrA09-p2101519
Bn-A09-p1841381	1	1	Bn-N9-p2507237	Bn-chrA09-p2094647
Bn-A09-p1846673	0.950413223	0.958677686	Bn-N9-p2498569	Bn-chrA09-p2089368
Bn-A09-p1856828	0.983471074	0.983471074	Bn-N9-p2488499	Bn-chrA09-p2079503
Bn-A09-p1858563	0.983471074	0.975206612	Bn-N9-p2487164	Bn-chrA09-p2078018
Bn-A09-p1858591	0.975206612	0.983471074	Bn-N9-p2487136	Bn-chrA09-p2077990
Bn-A09-p1858913	0.950413223	0.958677686	Bn-N9-p2486814	Bn-chrA09-p2077668
Bn-A09-p1860063	0.983471074	0.975206612	Bn-N9-p2485176	Bn-chrA09-p2076518
Bn-A09-p1926105	0.975206612	0.975206612	Bn-N9-p2422534	Bn-chrA09-p2029650
Bn-A09-p1929245	0.991735537	0.991735537	Bn-N9-p2419388	Bn-chrA09-p2026507
Bn-A09-p1931318	1	0.991735537	Bn-N9-p2417293	Bn-chrA09-p2024422
Bn-A09-p1956137	0.933884298	0.933884298	Bn-N9-p2389532	Bn-chrA09-p1999319
Bn-A09-p1973528	0.958677686	0.975206612	Bn-N9-p2371422	Bn-chrA09-p1981616
Bn-A09-p1989556	0.991735537	0.991735537	Bn-N9-p2340141	Bn-chrA09-p1964559
Bn-A09-p2009904	0.983471074	0.966942149	Bn-N9-p2324723	Bn-chrC09-p1882297
Bn-A09-p2010675	0.983471074	0.859504132	Bn-N9-p2323952	Bn-chrC09-p1881649
Bn-A09-p2033221	0.991735537	0.991735537	Bn-N9-p2301751	Bn-chrA09-p1928551
Bn-A09-p2033767	0.985074627	0.990049751	Bn-N9-p2301205	Bn-chrA09-p1928005
Bn-A09-p2044817	0.983388704	0.996677741	Bn-N9-p2293605	Bn-chrA09-p1920224
Bn-A09-p2065608	0.983471074	0.983471074	Bn-N9-p2275621	Bn-chrA09-p1901569
Bn-A09-p2076591	0.26446281	0.975206612	Bn-N18-p12013192	Bn-chrA09-p1890153
Bn-A09-p2076856	0.247933884	0.975206612	Unknown	Bn-chrA09-p1887194
Bn-A09-p2081648	0.985074627	0.990049751	Bn-N9-p2266736	Bn-chrA09-p1883773
Bn-A09-p2082298	0.965174129	1	Bn-N9-p2266088	Bn-chrA09-p1883108
Bn-A09-p2083933	0.958677686	0.958677686	Bn-N9-p2264448	Bn-chrA09-p1881472
Bn-A09-p2116947	1	0.991735537	Bn-N9-p1613782	Bn-chrA09-p1287776
Bn-A09-p2126525	0.867768595	0.876033058	Bn-N9-p1625877	Bn-chrA09-p1298185
Bn-A09-p2131637	0.933884298	0.958677686	Bn-N19-p2220394	Bn-chrA09-p1300713
Bn-A09-p2131851	0.950413223	0.94214876	Bn-N9-p1635553	Bn-chrA09-p1300927
Bn-A09-p2157775	0.991735537	0.983471074	Bn-N9-p1655795	Bn-chrA09-p1332204
Bn-A09-p2160040	0.991735537	0.983471074	Bn-N9-p1659079	Bn-chrA09-p1337134

Bn-A09-p2208929	0.990049751	0.995024876	Bn-N9-p1703140	Bn-chrA09-p1382821
Bn-A09-p2209142	0.995024876	0.990049751	Bn-N9-p1703353	Bn-chrA09-p1383032
Bn-A09-p2232416	1	0.190082645	Bn-N9-p1725120	Bn-chrC09-p1224999
Bn-A09-p2241625	0.991735537	0.983471074	Bn-N9-p1733463	Bn-chrA09_random-p354724
Bn-A09-p2280336	0.915422886	0.935323383	Bn-N9-p1783351	Bn-chrA09-p1440112
Bn-A09-p2298880	0.958677686	0.991735537	Bn-N9-p1802613	Bn-chrA09-p1458923
Bn-A09-p2323366	0.976744186	0.956810631	Bn-N9-p1865629	Bn-chrA09-p1519270
Bn-A09-p2343357	0.991735537	0.983471074	Bn-N9-p1889552	Bn-chrA09-p1539811
Bn-A09-p2343613	1	0.991735537	Bn-N9-p1889809	Bn-chrA09-p1540068
Bn-A09-p2346796	0.958677686	0.966942149	Bn-N9-p1894272	Bn-chrA09-p1544289
Bn-A09-p2374845	0.975206612	0.223140496	Bn-N9-p1922972	Bn-chrC02-p39879102
Bn-A09-p2415458	0.975206612	0.983471074	Bn-N9-p1955218	Bn-chrA09-p1603382
Bn-A09-p2416564	0.970149254	0.975124378	Bn-N9-p1956314	Bn-chrA09-p1604488
Bn-A09-p2419287	0.989583333	0.989583333	Bn-N9-p1957998	Bn-chrA09-p1606177
Bn-A09-p2429402	0.975206612	0.966942149	Bn-N9-p1969293	Bn-chrA09-p1618380
Bn-A09-p2462013	0.910299003	0.299003322	Bn-N17-p42275257	Unknown
Bn-A09-p2538450	0.983471074	1	Bn-N9-p2071219	Bn-chrA09-p1733591
Bn-A09-p2548356	0.991735537	0.991735537	Bn-N9-p2081207	Bn-chrA09-p1742205
Bn-A09-p2548974	0.975206612	0.975206612	Bn-N9-p2081825	Bn-chrA09-p1742822
Bn-A09-p2555630	0.960451977	0.988700565	Bn-N9-p2091834	Bn-chrA09-p1747587
Bn-A09-p2557243	0.990049751	0.2039801	Bn-N9-p2093451	Bn-chrA07-p17810540
Bn-A09-p2570919	0.903654485	0.900332226	Bn-N9-p2120112	Bn-chrA09-p1766498
Bn-A09-p2609043	0.983471074	0.975206612	Bn-N9-p2150419	Bn-chrA09-p1796434
Bn-A09-p2710152	1	0.991735537	Bn-N9-p2242314	Bn-chrA09-p1859732
Bn-A09-p2711315	0.94214876	0.950413223	Bn-N9-p2242831	Bn-chrAnn_random-p350614
Bn-A09-p2730673	0.985074627	0.980099502	Bn-N9-p3154514	Bn-chrA09-p2674966
Bn-A09-p2733282	0.983471074	0.983471074	Bn-N9-p3157123	Bn-chrA09-p2677575
Bn-A09-p2963981	0.991735537	0.991735537	Bn-N9-p3362431	Bn-chrA09-p2872740
Bn-A09-p3024539	0.991735537	0.991735537	Bn-N9-p3420487	Bn-chrA09-p2944565
Bn-A09-p3029767	0.991735537	0.991735537	Bn-N9-p3425767	Bn-chrA09-p2949845
Bn-A09-p3047090	0.991735537	0.991735537	Bn-N9-p3442536	Bn-chrA09-p2966603
Bn-A09-p3051349	0.991735537	0.991735537	Bn-N9-p3446970	Bn-chrA09-p2971335
Bn-A09-p3053532	0.975206612	0.975206612	Bn-N9-p3449149	Bn-chrA09-p2973512
Bn-A09-p3053860	0.991735537	0.991735537	Bn-N9-p3449476	Bn-chrA09-p2973839
Bn-A09-p3064935	0.975206612	0.975206612	Bn-N9-p3472083	Bn-chrA09-p2999148
Bn-A09-p3097420	0.991735537	0.991735537	Bn-N9-p3504500	Bn-chrA09-p3031274
Bn-A09-p3116738	1	1	Bn-N9-p3524348	Bn-chrA09-p3051910
Bn-A09-p3173488	0.995024876	0.995024876	Bn-N19-p4919686	Bn-chrC09_random-p46673
Bn-A09-p3225300	0.983471074	0.975206612	Bn-N9-p3609648	Bn-chrA09-p3124127
Bn-A09-p3233005	0.985074627	0.985074627	Bn-N19-p5017304	Bn-chrC09-p3600506
Bn-A09-p3233129	0.990049751	0.990049751	Bn-N19-p5017427	Bn-chrC09-p3600629
Bn-A09-p3234323	0.970149254	1	Bn-N9-p3618744	Bn-chrA09-p3135041
Bn-A09-p3237193	0.991735537	1	Bn-N9-p3626169	Bn-chrA09-p3137907
Bn-A09-p3247915	0.975206612	0.966942149	Bn-N9-p3630504	Bn-chrA09-p3140801
Bn-A09-p3251545	0.958677686	0.867768595	Bn-N9-p3633743	Bn-chrCnn_random-p147198
Bn-A09-p3252267	0.966942149	0.966942149	Bn-N9-p3636650	Bn-chrA09-p3144939

Bn-A09-p3257371	0.966101695	0.97740113	Bn-N9-p3640940	Bn-chrA09-p3154626
Bn-A09-p3275736	0.975206612	0.991735537	Bn-N9-p3663349	Bn-chrA09-p3167406
Bn-A09-p3347911	0.983471074	0.983471074	Bn-N9-p3734435	Bn-chrA09-p3233706
Bn-A09-p3387327	0.966942149	0.966942149	Bn-N9-p3780524	Bn-chrA09_random-p382430
Bn-A09-p3452203	0.940298507	0.940298507	Bn-N9-p3844406	Bn-chrA09-p3280516
Bn-A09-p3453706	0.950413223	0.950413223	Bn-N9-p3846144	Bn-chrA09-p3282254
Bn-A09-p3546619	0.990033223	0.990033223	Bn-N14-p1519904	Bn-chrC04-p1322838
Bn-A09-p3908310	0.980066445	0.667774086	Bn-N9-p4796113	Bn-chrA09-p4094922
Bn-A09-p3940412	0.900826446	0.900826446	Bn-N9-p4763249	Bn-chrA09-p4058383
Bn-A09-p3940779	0.983471074	0.983471074	Bn-N9-p4762870	Bn-chrA09-p4058004
Bn-A09-p3955243	0.995024876	0.995024876	Bn-N9-p4748422	Bn-chrA09-p4038248
Bn-A09-p3994064	0.925619835	0.925619835	Bn-N9-p4710049	Bn-chrA09-p3999692
Bn-A09-p3994437	0.991735537	0.991735537	Bn-N9-p4709675	Bn-chrA09-p3999318
Bn-A09-p4010053	0.975206612	0.975206612	Bn-N9-p4692834	Bn-chrA09_random-p558867
Bn-A09-p4011631	0.991735537	0.991735537	Bn-N9-p4691264	Bn-chrC04-p14713327
Bn-A09-p4012239	0.991735537	0.991735537	Bn-N9-p4690656	Bn-chrC04-p14713935
Bn-A09-p4021115	0.991735537	0.991735537	Bn-N9-p4684959	Bn-chrA09-p3982306
Bn-A09-p4024494	0.991735537	0.991735537	Bn-N9-p4681963	Bn-chrA09-p3979310
Bn-A09-p4024534	0.983471074	0.983471074	Bn-N9-p4681923	Bn-chrA09-p3979270
Bn-A09-p4038403	0.975206612	0.975206612	Bn-N9-p4670569	Bn-chrA09-p3967914
Bn-A09-p4044084	0.909090909	0.909090909	Bn-N19-p6866842	Bn-chrCnn_random-p805809
Bn-A09-p4096474	0.975206612	0.975206612	Bn-N9-p4609099	Bn-chrA09-p3906694
Bn-A09-p4163474	0.958677686	0.966942149	Bn-N9-p4535903	Bn-chrA09-p3835016
Bn-A09-p4163989	0.970149254	0.970149254	Bn-N9-p4535388	Bn-chrA09-p3834501
Bn-A09-p4164576	0.970149254	0.975124378	Bn-N9-p4534801	Bn-chrA09-p3833914
Bn-A09-p4168039	0.985074627	0.975124378	Bn-N9-p4531182	Bn-chrA09-p3831397
Bn-A09-p4206179	0.991735537	0.983471074	Bn-N9-p4487566	Bn-chrA09-p3791683
Bn-A09-p4207679	0.990033223	0.990033223	Bn-N9-p4486066	Bn-chrA09-p3790183
Bn-A09-p4241780	0.966942149	0.966942149	Bn-N9-p4449979	Bn-chrA09_random-p530798
Bn-A09-p4262547	0.980099502	0.980099502	Bn-N9-p4426894	Bn-chrA09_random-p510554
Bn-A09-p4275713	0.993355482	0.993355482	Bn-N9-p4414546	Bn-chrA09_random-p498282
Bn-A09-p4282686	0.970149254	0.970149254	Bn-N9-p4407270	Bn-chrA09_random-p490069
Bn-A09-p4286317	1	1	Bn-N9-p4403689	Bn-chrA09-p3776220
Bn-A09-p4288843	0.991735537	0.991735537	Bn-N9-p4401156	Bn-chrA09-p3773687
Bn-A09-p4291965	0.892561983	0.892561983	Bn-N9-p4396387	Bn-chrA09-p3769152
Bn-A09-p4300103	0.986710963	0.986710963	Bn-N9-p4392624	Bn-chrA09-p3765390
Bn-A09-p4347835	0.995024876	0.995024876	Bn-N9-p4352083	Bn-chrA09-p3722709
Bn-A09-p4351470	0.983471074	0.983471074	Bn-N9-p4351793	Bn-chrA09-p3722419
Bn-A09-p4356331	0.983471074	0.983471074	Bn-N9-p4348335	Bn-chrA09-p3718961
Bn-A09-p4384911	0.94214876	0.94214876	Bn-N9-p4323622	Bn-chrA09-p3695622
Bn-A09-p4416731	0.950248756	0.935323383	Bn-N9-p4288025	Bn-chrA09-p3669694
Bn-A09-p4433127	0.960199005	0.960199005	Bn-N9-p5083824	Bn-chrA09-p4344766
Bn-A09-p4433252	0.985074627	0.985074627	Bn-N9-p5083949	Bn-chrA09-p4344891
Bn-A09-p4436808	1	1	Bn-N15-p42514834	Bn-chrCnn_random-p269741
Bn-A09-p4447029	0.983471074	0.983471074	Bn-N9-p5098146	Bn-chrA09-p4358812
Bn-A09-p4459348	0.950413223	0.950413223	Bn-N9-p5109900	Bn-chrA09-p4370354

Bn-A09-p4463675	0.966942149	0.966942149	Bn-N9-p5116009	Bn-chrA09-p4376902
Bn-A09-p4465163	0.975206612	0.975206612	Bn-N9-p5117226	Bn-chrA09-p4378118
Bn-A09-p4475636	0.966942149	0.966942149	Bn-N9-p5130279	Bn-chrA09-p4388002
Bn-A09-p4509627	0.983471074	0.983471074	Bn-N9-p5162393	Bn-chrA09-p4419828
Bn-A09-p4542819	0.956810631	0.990033223	Bn-N1-p14839416	Bn-chrA06_random-p225317
Bn-A09-p4616864	0.970149254	0.92039801	Bn-N9-p5271971	Bn-chrA09-p4528743
Bn-A09-p4652268	0.7960199	0.985074627	Bn-N19-p7859662	Bn-chrA09-p4571935
Bn-A09-p4655322	0.955223881	0.975124378	Bn-N9-p5312617	Bn-chrA09-p4574941
Bn-A09-p4671467	0.92358804	0.900332226	Bn-N9-p5330263	Bn-chrA09-p4592049
Bn-A09-p4681749	1	0.996677741	Bn-N9-p5334119	Bn-chrA09-p4595909
Bn-A09-p4683160	0.993355482	0.986710963	Bn-N9-p5335527	Bn-chrA09-p4597320
Bn-A09-p4683384	0.975124378	0.985074627	Bn-N9-p5335753	Bn-chrA09-p4597544
Bn-A09-p4690204	0.975206612	0.966942149	Bn-N9-p5337979	Bn-chrA09-p4600598
Bn-A09-p4735640	0.958677686	0.958677686	Bn-N9-p5370922	Bn-chrA09-p4635583
Bn-A09-p4776093	0.966942149	0.966942149	Bn-N9-p5408553	Bn-chrAnn_random-p40617S
Bn-A09-p4777728	0.933884298	0.933884298	Bn-N9-p5410548	Bn-chrA09-p4677312
Bn-A09-p4824263	0.966942149	0.966942149	Bn-N9-p5465764	Bn-chrA09-p4732728
Bn-A09-p4831706	0.983471074	0.983471074	Bn-N9-p5473395	Bn-chrA09-p4740361
Bn-A09-p4835780	1	1	Bn-N9-p5477470	Bn-chrA09-p4744435
Bn-A09-p4838767	0.945273632	0.925373134	Bn-N19-p8115030	Bn-chrA09-p4747389
Bn-A09-p4848610	0.966942149	0.966942149	Bn-N9-p5489993	Bn-chrA09-p4756513
Bn-A09-p4849057	0.776859504	0.776859504	Bn-N9-p5490132	Bn-chrA09-p4756652
Bn-A09-p4850116	0.980099502	0.980099502	Bn-N9-p5491191	Bn-chrA09-p4757711
Bn-A09-p4850180	0.950248756	0.950248756	Bn-N19-p8127893	Bn-chrC09-p6292658
Bn-A09-p4862871	0.950413223	0.950413223	Bn-N9-p5504192	Bn-chrA09-p4769933
Bn-A09-p4863472	0.995024876	0.990049751	Bn-N9-p5504797	Bn-chrA09-p4770538
Bn-A09-p4895204	0.983471074	0.975206612	Bn-N9-p5549040	Bn-chrA09-p4802810
Bn-A09-p4895637	0.950413223	0.958677686	Bn-N9-p5549473	Bn-chrA09-p4803243
Bn-A09-p4905706	0.986710963	0.990033223	Bn-N9-p5572690	Bn-chrA09-p4813515
Bn-A09-p4905766	0.995024876	0.990049751	Bn-N9-p5572750	Bn-chrA09-p4813575
Bn-A09-p4913177	0.966942149	0.94214876	Bn-N9-p5585362	Bn-chrA09_random-p679073
Bn-A09-p4913432	0.991735537	1	Bn-N9-p5585617	Bn-chrA09_random-p679327
Bn-A09-p4916858	0.877076412	0.624584718	Bn-N9-p5591815	Bn-chrA09_random-p739540
Bn-A09-p4927893	0.990049751	0.965174129	Bn-N9-p5608180	Bn-chrA09_random-p693654
Bn-A09-p4932188	0.966942149	0.958677686	Bn-N9-p5613255	Bn-chrA09_random-p698357
Bn-A09-p4939799	0.975124378	0.985074627	Bn-N9-p5620591	Bn-chrA09_random-p703819
Bn-A09-p4963985	0.983471074	0.983471074	Bn-N9-p5644963	Bn-chrA09_random-p663016
Bn-A09-p4965726	0.975206612	0.975206612	Bn-N9-p5646681	Bn-chrA09_random-p664734
Bn-A09-p4976736	0.980099502	0.980099502	Bn-N9-p34842590	Bn-chrA09-p29555599
Bn-A09-p4990811	0.996677741	0.996677741	Bn-N9-p34857648	Bn-chrA09-p29570841
Bn-A09-p4996710	0.986710963	0.986710963	Bn-N9-p34863516	Bn-chrA09-p29576708
Bn-A09-p4997670	0.983388704	0.983388704	Bn-N9-p34864471	Bn-chrA09-p29577663
Bn-A09-p5006353	0.930348259	0.930348259	Bn-N9-p34878046	Bn-chrA09-p29591092
Bn-A09-p5013589	0.973421927	0.973421927	Bn-N9-p34884858	Bn-chrA09-p29598719
Bn-A09-p5031583	0.996677741	0.996677741	Bn-N9-p34904763	Bn-chrA09-p29617337
Bn-A09-p5033479	0.983471074	0.157024793	Bn-N9-p34906777	Bn-chrA01-p2682928

Bn-A09-p5034747	0.983471074	0.983471074	Bn-N9-p34909042	Bn-chrA09-p29631060
Bn-A09-p5037318	0.983471074	0.983471074	Bn-N9-p34911377	Bn-chrA09-p29633395
Bn-A09-p5039439	0.834710744	0.991735537	Bn-N18-p38382574	Bn-chrA09-p29637110
Bn-A09-p5040112	0.983471074	0.975206612	Bn-N9-p34913853	Bn-chrA09-p29637783
Bn-A09-p5040525	0.975206612	0.966942149	Bn-N9-p34914407	Bn-chrA09-p29638196
Bn-A09-p5054103	0.956810631	0.996677741	Bn-N18-p38400259	Bn-chrA09-p29652021
Bn-A09-p5061646	0.991735537	0.983471074	Bn-N9-p34937224	Bn-chrA09-p29660839
Bn-A09-p5062101	0.980099502	0.990049751	Bn-N9-p34937678	Bn-chrA09-p29661294
Bn-A09-p5118197	0.983471074	0.983471074	Bn-N9-p34980596	Bn-chrA09-p29701179
Bn-A09-p5132918	0.991735537	0.983471074	Bn-N9-p34992197	Bn-chrA09-p29713336
Bn-A09-p5144107	1	0.991735537	Bn-N9-p34994095	Bn-chrA09-p29715213
Bn-A09-p5149149	0.867109635	0.996677741	Bn-N9-p35000993	Bn-chrA09-p29720742
Bn-A09-p5157169	0.940298507	0.955223881	Bn-N9-p35009210	Bn-chrA09-p29734048
Bn-A09-p5168276	0.975124378	0.975124378	Bn-N9-p35019518	Bn-chrA09-p29744561
Bn-A09-p5189883	0.991735537	0.991735537	Bn-N9-p5679339	Bn-chrA09-p4861884
Bn-A09-p5190180	0.991735537	0.991735537	Bn-N9-p5679640	Bn-chrA09-p4862185
Bn-A09-p5190641	0.975206612	0.975206612	Bn-N9-p5680093	Bn-chrA09-p4862908
Bn-A09-p5200256	0.996677741	0.996677741	Bn-N9-p5696393	Bn-chrA09-p4873875
Bn-A09-p5215814	0.985074627	0.990049751	Bn-N9-p5709910	Bn-chrA09-p4887456
Bn-A09-p5219527	0.983471074	0.991735537	Bn-N9-p5713613	Bn-chrA09-p4891166
Bn-A09-p5225650	1	0.991735537	Bn-N9-p5719792	Bn-chrA09-p4897381
Bn-A09-p5232967	0.966942149	0.966942149	Bn-N9-p5727103	Bn-chrA09-p4910036
Bn-A09-p5244764	0.983471074	0.991735537	Bn-N9-p5733624	Bn-chrA09-p4917431
Bn-A09-p5246171	0.975206612	0.983471074	Bn-N9-p5735034	Bn-chrA09-p4918834
Bn-A09-p5246229	0.966942149	0.958677686	Bn-N9-p5735092	Bn-chrA09-p4918892
Bn-A09-p5251134	0.966942149	0.958677686	Bn-N9-p5742657	Bn-chrA09-p4926711
Bn-A09-p5255753	0.975206612	0.983471074	Bn-N9-p5747944	Bn-chrA09-p4931333
Bn-A09-p5258401	0.975206612	0.950413223	Bn-N9-p5752550	Bn-chrA09-p4933982
Bn-A09-p5267535	0.966942149	0.603305785	Bn-N9-p5766413	Bn-chrA10-p15656938
Bn-A09-p5274560	0.966942149	0.975206612	Bn-N7-p15084779	Bn-chrA07-p14683833
Bn-A09-p5285034	0.991735537	1	Bn-N9-p5777736	Bn-chrA09-p4955240
Bn-A09-p5286216	0.983471074	0.991735537	Bn-N9-p5778911	Bn-chrA09-p4956422
Bn-A09-p5298381	0.986710963	0.976744186	Bn-N9-p5791053	Bn-chrAnn_random-p395326
Bn-A09-p5298521	0.975206612	0.983471074	Bn-N9-p5791193	Bn-chrAnn_random-p395324
Bn-A09-p5322252	0.857142857	0.857142857	Bn-N9-p5811564	Bn-chrCnn_random-p438561
Bn-A09-p5327731	0.991735537	0.991735537	Bn-N9-p5816719	Bn-chrA09-p5008448
Bn-A09-p5329868	0.966942149	0.975206612	Bn-N9-p5818815	Bn-chrAnn_random-p341472
Bn-A09-p5331841	0.975206612	0.983471074	Bn-N9-p5820542	Bn-chrA09-p5012312
Bn-A09-p5332906	0.991735537	0.198347107	Bn-N9-p5821751	Bn-chrC01_random-p156011
Bn-A09-p5334820	0.956810631	0.96013289	Bn-N9-p5824269	Bn-chrAnn_random-p376634
Bn-A09-p5337372	0.966942149	0.958677686	Bn-N9-p5826817	Bn-chrC09-p6445695
Bn-A09-p5343993	0.975206612	0.983471074	Bn-N9-p5833415	Bn-chrC09-p6451189
Bn-A09-p5348717	0.983471074	0.983471074	Bn-N9-p5838737	Bn-chrC09-p6459045
Bn-A09-p5382769	0.975206612	0.991735537	Bn-N9-p5857929	Bn-chrA09_random-p724908
Bn-A09-p5406597	0.975124378	0.975124378	Bn-N9-p5883070	Bn-chrCnn_random-p583027
Bn-A09-p5406959	0.995024876	0.995024876	Bn-N9-p5883432	Bn-chrCnn_random-p583031

Bn-A09-p5416801	0.940298507	0.945273632	Bn-N9-p5892568	Bn-chrAnn_random-p271673
Bn-A09-p5418207	1	1	Bn-N9-p5893980	Bn-chrAnn_random-p271687
Bn-A09-p5418342	0.990049751	0.990049751	Bn-N9-p5894115	Bn-chrAnn_random-p271689
Bn-A09-p5431352	0.933884298	0.619834711	Bn-N9-p5907084	Bn-chrC07-p2485038
Bn-A09-p5432697	0.966942149	0.975206612	Bn-N9-p5908425	Bn-chrA09_random-p747708
Bn-A09-p5442608	0.966942149	0.975206612	Bn-N9-p5915429	Bn-chrA09_random-p755472
Bn-A09-p5444807	0.975206612	0.950413223	Bn-N9-p5918197	Bn-chrUnn_random-p165656
Bn-A09-p5454067	0.933884298	0.958677686	Bn-N9-p5929470	Bn-chrA09-p5013687
Bn-A09-p5455608	0.933884298	0.933884298	Bn-N9-p5931012	Bn-chrA09-p5015235
Bn-A09-p5470888	0.991735537	0.983471074	Bn-N9-p5950456	Bn-chrA09-p5036357
Bn-A09-p5476558	0.975206612	0.983471074	Bn-N9-p5951635	Bn-chrA09-p5037525
Bn-A09-p5601358	0.983471074	0.983471074	Bn-N9-p6071385	Bn-chrA09-p5173513
Bn-A09-p5603209	0.991735537	0.975206612	Bn-N9-p6073226	Bn-chrC09-p6893401
Bn-A09-p5631441	0.983471074	0.983471074	Bn-N9-p6107811	Bn-chrA09-p5213793
Bn-A09-p5678953	0.983471074	0.983471074	Bn-N9-p6148597	Bn-chrA09-p5238666
Bn-A09-p5680812	0.985074627	0.985074627	Bn-N9-p6150457	Bn-chrC09-p7035649
Bn-A09-p5781490	0.995024876	0.995024876	Bn-N9-p6266106	Bn-chrA09-p5341112
Bn-A09-p5958009	0.995024876	0.995024876	Bn-N9-p6441022	Bn-chrA09_random-p793769
Bn-A09-p5958764	0.980099502	0.980099502	Bn-N9-p6441777	Bn-chrCnn_random-p508860
Bn-A09-p5966342	0.960199005	0.960199005	Bn-N1-p1112754	Bn-chrA01-p667438
Bn-A09-p5966858	0.990049751	0.990049751	Bn-N1-p1112176	Bn-chrA01-p666860
Bn-A09-p5975583	0.925619835	0.958677686	Bn-N18-p38563178	Bn-chrA09-p29769050
Bn-A09-p5977032	0.990049751	0.985074627	Bn-N9-p35045978	Bn-chrA09-p29770499
Bn-A09-p6010593	0.851239669	0.851239669	Bn-N9-p7796053	Bn-chrA09-p6668848
Bn-A09-p6021939	0.975206612	0.975206612	Bn-N9-p7788237	Bn-chrA09-p6661032
Bn-A09-p6057858	0.979166667	0.979166667	Bn-N19-p12032376	Bn-chrCnn_random-p421672
Bn-A09-p6090256	0.986710963	0.986710963	Bn-N4-p19097673	Bn-chrA04_random-p132707
Bn-A09-p6133524	0.989583333	0.989583333	Bn-N9-p7648675	Bn-chrA09-p6512725
Bn-A09-p6140805	0.993355482	0.993355482	Bn-N9-p7641319	Bn-chrA09-p6505369
Bn-A09-p6295669	0.768595041	0.768595041	Bn-Scaffold01618-p2	Bn-chrC02-p19861735
Bn-A09-p6357468	0.900332226	0.943521595	Bn-N16-p13386879	Bn-chrA05-p16888985
Bn-A09-p6445844	0.985074627	0.985074627	Bn-N9-p7397656	Bn-chrA09-p6249155
Bn-A09-p6459935	0.983471074	0.983471074	Bn-N9-p7378715	Bn-chrA09-p6280469
Bn-A09-p6546149	0.996677741	0.996677741	Bn-N9-p7288994	Bn-chrA09-p6455963
Bn-A09-p6562874	0.995024876	0.995024876	Bn-N9-p7273082	Bn-chrA09-p6440229
Bn-A09-p6574470	0.996677741	0.996677741	Bn-N9-p7264636	Bn-chrA09-p6430156
Bn-A09-p6615722	1	1	Bn-N19-p11198841	Bn-chrC09-p8519141
Bn-A09-p6616042	0.993355482	0.993355482	Bn-N9-p7199737	Bn-chrA09-p6369463
Bn-A09-p6616405	0.92039801	0.92039801	Bn-N9-p7199373	Bn-chrA09-p6369099
Bn-A09-p6630867	0.990033223	0.990033223	Bn-N9-p7187127	Bn-chrA09-p6356065
Bn-A09-p6632506	1	1	Bn-N19-p11176935	Bn-chrC09-p8498474
Bn-A09-p6674663	0.933884298	0.933884298	Bn-N9-p33605370	Bn-chrA09-p28477295
Bn-A09-p6783843	0.993355482	0.990033223	Bn-N9-p7072330	Bn-chrA09_random-p837197
Bn-A09-p6817144	0.983471074	0.991735537	Bn-N9-p7042231	Bn-chrAnn_random-p215668
Bn-A09-p6829125	0.991735537	1	Bn-N9-p7029693	Bn-chrAnn_random-p215546
Bn-A09-p6852464	0.950413223	0.94214876	Bn-N9-p7007642	Bn-chrA09-p5992180

Bn-A09-p6866176	0.975206612	0.966942149	Bn-N9-p6995894	Bn-chrA09-p5969526
Bn-A09-p6879008	0.975206612	0.983471074	Bn-N9-p6977042	Bn-chrA09-p5957165
Bn-A09-p6922467	0.910447761	0.910447761	Bn-N9-p6924911	Bn-chrA09-p5918716
Bn-A09-p6950255	0.958677686	0.950413223	Bn-N9-p6894750	Bn-chrA09-p5891505
Bn-A09-p6970158	0.966942149	0.94214876	Bn-N9-p6878342	Bn-chrC09-p8284283
Bn-A09-p7047022	0.624584718	0.950166113	Bn-N9-p6806606	Bn-chrC09-p8121161
Bn-A09-p7071738	0.991735537	0.991735537	Bn-N9-p6779439	Bn-chrA09-p5789403
Bn-A09-p7086863	0.985074627	0.985074627	Bn-N9-p6738311	Bn-chrA09-p5748886
Bn-A09-p7087772	0.985074627	0.985074627	Bn-N9-p6737402	Bn-chrA09-p5747977
Bn-A09-p7096041	0.990033223	0.990033223	Bn-N9-p6728798	Bn-chrA09-p5739373
Bn-A09-p7101179	0.870431894	0.870431894	Bn-N9-p6723665	Bn-chrA09-p5731227
Bn-A09-p7174944	0.825870647	0.805970149	Bn-N19-p10270316	Bn-chrC09-p7886445
Bn-A09-p7176234	0.983388704	0.983388704	Bn-N9-p6651242	Bn-chrA09-p5655884
Bn-A09-p7191362	0.945273632	0.945273632	Bn-N9-p6639323	Bn-chrA09-p5643968
Bn-A09-p7191369	0.989583333	0.989583333	Bn-N9-p6639316	Bn-chrA09-p5643961
Bn-A09-p7199203	0.989583333	0.989583333	Bn-N9-p6631275	Bn-chrA09-p5628970
Bn-A09-p7287912	0.975124378	0.975124378	Bn-N9-p6549979	Bn-chrA09-p5572503
Bn-A09-p7317278	1	1	Bn-N9-p6531524	Bn-chrA09-p5553806
Bn-A09-p7318713	0.993355482	0.993355482	Bn-N9-p6530089	Bn-chrA09-p5552371
Bn-A09-p7327691	0.983471074	0.983471074	Bn-N9-p6520804	Bn-chrA09-p5544661
Bn-A09-p7329993	0.975206612	0.975206612	Bn-N9-p6518504	Bn-chrA09-p5542361
Bn-A09-p7331034	0.983471074	0.983471074	Bn-N9-p6517474	Bn-chrA09-p5541331
Bn-A09-p7331063	0.991735537	0.991735537	Bn-N9-p6517445	Bn-chrA09-p5541302
Bn-A09-p7332680	0.966942149	0.966942149	Bn-N9-p6515827	Bn-chrA09-p5539684
Bn-A09-p7397116	0.96013289	0.96013289	Bn-N9-p6457254	Bn-chrA09-p5484453
Bn-A09-p7454442	0.991735537	0.991735537	Bn-N9-p7886394	Bn-chrA09-p6949348
Bn-A09-p7472401	0.993355482	0.993355482	Bn-N9-p7909361	Bn-chrA09-p6974166
Bn-A09-p7490528	0.975206612	0.975206612	Bn-N9-p7926307	Bn-chrA09-p6991433
Bn-A09-p7490626	0.990049751	0.990049751	Bn-N9-p7926405	Bn-chrA09-p6991531
Bn-A09-p7491672	0.980099502	0.980099502	Bn-N9-p7927451	Bn-chrA09-p6992577
Bn-A09-p7494889	0.720930233	0.950166113	Bn-N9-p7931501	Bn-chrA09-p6996627
Bn-A09-p7499345	0.983471074	0.983471074	Bn-N9-p7944875	Bn-chrA09-p7009860
Bn-A09-p7503009	0.975124378	0.975124378	Bn-N9-p7947558	Bn-chrA09-p7012542
Bn-A09-p7510345	0.900826446	0.900826446	Bn-N9-p7954063	Bn-chrA09-p7019047
Bn-A09-p7517353	1	1	Bn-N9-p7966391	Bn-chrA09-p7029936
Bn-A09-p7560188	0.983471074	0.983471074	Bn-N9-p8009411	Bn-chrA09-p7077529
Bn-A09-p7663062	0.956810631	0.956810631	Bn-N8-p10523379	Bn-chrC07-p15319884
Bn-A09-p7741917	0.980099502	0.980099502	Bn-N9-p8204076	Bn-chrA09-p6875368
Bn-A09-p7892057	0.975124378	0.975124378	Bn-N7-p9784621	Bn-chrA07-p9672375
Bn-A09-p8044910	1	1	Bn-N9-p8533234	Bn-chrA09-p7500510
Bn-A09-p8239411	0.970149254	0.970149254	Bn-N9-p8711466	Bn-chrA09-p7690582
Bn-A09-p8291726	0.975124378	0.975124378	Bn-N9-p8781510	Bn-chrA09-p7770339
Bn-A09-p8591730	0.975206612	0.975206612	Bn-N9-p9040963	Bn-chrA09-p8020165
Bn-A09-p8591746	0.983471074	0.983471074	Bn-N9-p9040980	Bn-chrA09-p8020182
Bn-A09-p8598652	0.945273632	0.945273632	Bn-N9-p9047569	Bn-chrA09-p8026807
Bn-A09-p8611536	0.970149254	0.970149254	Bn-N9-p9063319	Bn-chrA09-p8043494

Bn-A09-p8634971	0.985074627	0.985074627	Bn-N9-p9082262	Bn-chrA09-p8061969
Bn-A09-p8635608	0.975124378	0.975124378	Bn-N9-p9082898	Bn-chrA09-p8062605
Bn-A09-p8635777	0.975124378	0.975124378	Bn-N19-p14542553	Bn-chrC09-p11082801
Bn-A09-p8679695	0.818181818	0.809917355	Bn-N9-p9158552	Bn-chrA09_random-p101600
Bn-A09-p8713449	0.985074627	0.985074627	Bn-N9-p26243348	Bn-chrA09-p22153897
Bn-A09-p8770590	0.983471074	0.983471074	Bn-N9-p9235144	Bn-chrA09-p7176904
Bn-A09-p8773859	0.950413223	0.950413223	Bn-N9-p9237807	Bn-chrA09-p7180213
Bn-A09-p8783204	0.985074627	0.985074627	Bn-N8-p16807091	Bn-chrA08-p14582855
Bn-A09-p8784896	0.995024876	0.995024876	Bn-N8-p16809123	Bn-chrA08-p14584887
Bn-A09-p8787239	0.991735537	0.991735537	Bn-N9-p9239465	Bn-chrA09-p7182503
Bn-A09-p8787573	0.685950413	0.685950413	Bn-N9-p9239763	Bn-chrA09-p7182801
Bn-A09-p8788225	0.859504132	0.859504132	Bn-N9-p9239826	Bn-chrA09-p7182864
Bn-A09-p8791527	0.966942149	0.966942149	Bn-N9-p9242003	Bn-chrA09-p7185041
Bn-A09-p8793873	0.958677686	0.958677686	Bn-N9-p9244418	Bn-chrA09-p7187419
Bn-A09-p8798692	0.925373134	0.925373134	Bn-N9-p9249565	Bn-chrA09-p7192237
Bn-A09-p8798760	0.920529801	0.920529801	Bn-N9-p9249633	Bn-chrA09-p7192305
Bn-A09-p8800446	0.983471074	0.983471074	Bn-N9-p9251306	Bn-chrA09-p7193978
Bn-A09-p8810292	0.958677686	0.958677686	Bn-N9-p9271391	Bn-chrA09-p8067544
Bn-A09-p8823608	0.966942149	0.966942149	Bn-N9-p9297879	Bn-chrA09-p8090164
Bn-A09-p8823667	0.809917355	0.809917355	Bn-N9-p9297938	Bn-chrA09-p8090223
Bn-A09-p8828707	0.94214876	0.94214876	Bn-N9-p9302972	Bn-chrA09-p8095257
Bn-A09-p8828806	0.950413223	0.950413223	Bn-N9-p9303069	Bn-chrA09-p8095354
Bn-A09-p8877444	0.991735537	0.991735537	Bn-N9-p9356085	Bn-chrA09-p8144897
Bn-A09-p8891015	1	1	Bn-N9-p9359933	Bn-chrA09-p8148746
Bn-A09-p8891277	0.991735537	0.991735537	Bn-N9-p9360195	Bn-chrA09-p8149008
Bn-A09-p8911821	0.917355372	0.917355372	Bn-N9-p9391946	Bn-chrA09-p8181543
Bn-A09-p8942646	0.990049751	0.990049751	Bn-N9-p9413894	Bn-chrA09_random-p105089
Bn-A09-p8943559	0.990049751	0.990049751	Bn-N9-p9414806	Bn-chrA09_random-p104998
Bn-A09-p8957856	0.958677686	0.958677686	Bn-N9-p9429440	Bn-chrA09_random-p103139
Bn-A09-p9030563	0.995024876	0.995024876	Bn-N9-p9511675	Bn-chrA09-p8204948
Bn-A09-p9040117	0.983471074	0.950413223	Bn-N9-p9521664	Bn-chrC09-p11435454
Bn-A09-p9063447	0.991735537	0.991735537	Bn-N9-p9549530	Bn-chrA09-p8247154
Bn-A09-p9083090	0.895522388	0.895522388	Bn-N9-p9563274	Bn-chrA09-p8260898
Bn-A09-p9083366	0.980099502	0.980099502	Bn-N9-p9563498	Bn-chrA09-p8261122
Bn-A09-p9083445	0.721393035	0.721393035	Bn-N9-p9563578	Bn-chrA09-p8261202
Bn-A09-p9097901	0.983471074	0.983471074	Bn-N9-p9582793	Bn-chrA09-p8279439
Bn-A09-p9098267	0.991735537	0.991735537	Bn-N9-p9583159	Bn-chrA09-p8279805
Bn-A09-p9100536	0.851239669	0.851239669	Bn-N9-p9583624	Bn-chrA09-p8280270
Bn-A09-p9100736	0.983471074	0.983471074	Bn-N9-p9583824	Bn-chrA09-p8280470
Bn-A09-p9101925	0.991735537	0.991735537	Bn-N9-p9584861	Bn-chrA09-p8281507
Bn-A09-p9103739	0.975206612	0.975206612	Bn-N9-p9586678	Bn-chrA09-p8283324
Bn-A09-p9135388	0.975206612	0.975206612	Bn-N9-p9626225	Bn-chrA09-p8316936
Bn-A09-p9206874	1	1	Bn-N9-p9708058	Bn-chrA09-p8402909
Bn-A09-p9315814	0.983471074	0.983471074	Bn-N9-p9829575	Bn-chrAnn_random-p227357
Bn-A09-p9315824	0.975206612	0.975206612	Bn-N9-p9829585	Bn-chrAnn_random-p227357
Bn-A09-p9317714	1	1	Bn-N9-p9831471	Bn-chrAnn_random-p227338

Bn-A09-p9325852	0.958677686	0.958677686	Bn-N9-p9852768	Bn-chrA09-p8414656
Bn-A09-p9325966	0.983471074	0.983471074	Bn-N9-p9852882	Bn-chrA09-p8414770
Bn-A09-p9354510	0.970099668	0.970099668	Bn-N9-p9880509	Bn-chrA09-p8442294
Bn-A09-p9371106	0.943521595	0.943521595	Bn-N19-p15847047	Bn-chrC09_random-p990810
Bn-A09-p9379347	0.966942149	0.966942149	Bn-N9-p9899071	Bn-chrA09-p8460857
Bn-A09-p9421306	0.96345515	0.96345515	Bn-N9-p9941353	Bn-chrA09-p8503632
Bn-A09-p9423176	1	1	Bn-N9-p9943223	Bn-chrA09-p8505502
Bn-A09-p9443127	0.966942149	0.966942149	Bn-N9-p9963358	Bn-chrA09-p8525835
Bn-A09-p9444998	0.991735537	0.991735537	Bn-N9-p9965229	Bn-chrA09-p8527706
Bn-A09-p9445474	0.970099668	0.970099668	Bn-N9-p9965705	Bn-chrA09-p8528182
Bn-A09-p9446903	0.990033223	0.990033223	Bn-N9-p9967134	Bn-chrA09-p8529611
Bn-A09-p9450845	0.991735537	0.991735537	Bn-N9-p9971086	Bn-chrA09-p8533557
Bn-A09-p9450916	0.991735537	0.991735537	Bn-N9-p9971157	Bn-chrA09-p8533628
Bn-A09-p9452891	0.993355482	0.993355482	Bn-N9-p9973131	Bn-chrA09-p8535602
Bn-A09-p9463840	0.975206612	0.975206612	Bn-N9-p9984527	Bn-chrA09_random-p112672
Bn-A09-p9464949	0.991735537	0.991735537	Bn-N9-p9985636	Bn-chrA09_random-p112561
Bn-A09-p9480770	0.991735537	0.975206612	Bn-N9-p10002632	Bn-chrC09-p12141790
Bn-A09-p9497384	0.975206612	0.975206612	Bn-N9-p10019520	Bn-chrA09-p8571800
Bn-A09-p9512316	0.986710963	0.986710963	Bn-N9-p10035291	Bn-chrA09-p8587229
Bn-A09-p9558015	0.993355482	0.993355482	Bn-N9-p10074811	Bn-chrA09-p8626921
Bn-A09-p9558692	0.985074627	1	Bn-N9-p10075488	Bn-chrA09-p8627598
Bn-A09-p9558834	0.983388704	0.983388704	Bn-N9-p10076529	Bn-chrA09-p8627740
Bn-A09-p9559997	0.975124378	0.995024876	Bn-N19-p16199574	Bn-chrA09-p8628903
Bn-A09-p9563670	0.965174129	0.965174129	Bn-N9-p10082549	Bn-chrA09-p8632478
Bn-A09-p9563948	0.960199005	0.960199005	Bn-N9-p10082810	Bn-chrA09-p8632739
Bn-A09-p9613588	0.975206612	0.975206612	Bn-N9-p10141608	Bn-chrA09-p8682890
Bn-A09-p9624934	0.965174129	0.980099502	Bn-N19-p16287034	Bn-chrA09-p8694234
Bn-A09-p9627969	1	1	Bn-Scaffold21522-p2	Bn-chrA09-p8697289
Bn-A09-p9655424	0.958677686	0.958677686	Bn-N9-p10205795	Bn-chrA09-p8725873
Bn-A09-p9656145	0.991735537	0.991735537	Bn-N9-p10206510	Bn-chrA09-p8726588
Bn-A09-p9667631	0.983471074	0.983471074	Bn-N9-p10214694	Bn-chrC09-p12539813
Bn-A09-p9669847	0.991735537	0.991735537	Bn-Scaffold69548-p1	Bn-chrC09-p12542018
Bn-A09-p9671359	0.960199005	0.955223881	Bn-N9-p10218400	Bn-chrA09-p8740005
Bn-A09-p9691088	0.989583333	0.989583333	Bn-N9-p10235813	Bn-chrA09_random-p111901
Bn-A09-p9702226	0.933884298	0.958677686	Bn-N13-p39819389	Bn-chrA10-p15717446
Bn-A09-p9724274	0.986710963	0.990033223	Bn-N9-p10267456	Bn-chrA09-p8782103
Bn-A09-p9728715	0.990049751	0.985074627	Bn-N3-p19023544	Bn-chrA03-p17662776
Bn-A09-p9747402	0.990033223	0.990033223	Bn-N9-p10287002	Bn-chrA09-p8808982
Bn-A09-p9765092	0.945273632	0.940298507	Bn-N9-p10316084	Bn-chrA09-p8818903
Bn-A09-p9771068	1	0.614583333	Bn-N9-p10316902	Bn-chrAnn_random-p913622
Bn-A09-p9774483	0.970149254	0.975124378	Bn-N9-p10322050	Bn-chrA09-p8832233
Bn-A09-p9774806	0.383084577	0.467661692	Unknown	Unknown
Bn-A09-p9788623	0.983471074	0.983471074	Bn-N9-p10332343	Bn-chrA09-p8844199
Bn-A09-p9789590	0.983471074	0.983471074	Bn-N9-p10333704	Bn-chrC09-p12707888
Bn-A09-p9789898	0.950248756	0.965174129	Bn-N9-p10334013	Bn-chrA09-p8845474
Bn-A09-p9789943	0.975206612	0.975206612	Bn-N9-p10334058	Bn-chrA09-p8845519

Bn-A09-p9845028	0.990033223	0.986710963	Bn-N9-p10418352	Bn-chrA09-p8876364
Bn-A09-p9854305	0.991735537	0.991735537	Bn-N9-p10425303	Bn-chrA09-p8882624
Bn-A09-p9862616	0.993355482	0.993355482	Bn-N9-p10435565	Bn-chrA09-p8891285
Bn-A09-p9863993	0.983471074	0.991735537	Bn-N9-p10436941	Bn-chrA09-p8892662
Bn-A09-p9865556	0.917355372	0.933884298	Bn-N9-p10441284	Bn-chrA09-p8894225
Bn-A09-p9877560	1	0.991735537	Bn-N9-p10446505	Bn-chrA09-p8899405
Bn-A09-p9878665	0.983471074	0.991735537	Bn-N9-p10447610	Bn-chrA09-p8900509
Bn-A09-p9980036	0.991735537	0.991735537	Bn-N9-p10504374	Bn-chrA09-p8962563
Bn-A09-p9987709	0.986710963	0.966777409	Bn-N9-p10512040	Bn-chrC09_random-p965920
Bn-A09-p10019827	0.991735537	0.983471074	Bn-N9-p10531605	Bn-chrA09-p9005708
Bn-A09-p10023881	0.970149254	0.975124378	Bn-N9-p10533975	Bn-chrA09-p9008110
Bn-A09-p10024669	0.991735537	0.983471074	Bn-N9-p10534763	Bn-chrA09-p9008904
Bn-A09-p10046047	0.958677686	0.975206612	Bn-N9-p10552438	Bn-chrA09-p9028295
Bn-A09-p10064024	0.92039801	0.910447761	Bn-N9-p10576518	Bn-chrA09-p9050799
Bn-A09-p10064773	0.975206612	0.966942149	Bn-N9-p10577267	Bn-chrA09-p9051547
Bn-A09-p10092753	0.990049751	0.985074627	Bn-N9-p10609821	Bn-chrA09-p9086363
Bn-A09-p10109790	0.970099668	0.970099668	Bn-N9-p10616686	Bn-chrA09-p9093202
Bn-A09-p10119818	0.975124378	0.975124378	Bn-N9-p10628606	Bn-chrA09-p9103340
Bn-A09-p10172534	1	0.991735537	Bn-N9-p10681875	Bn-chrA09-p9249593
Bn-A09-p10176936	0.983471074	0.991735537	Bn-N9-p10686284	Bn-chrA09-p9254004
Bn-A09-p10192314	0.975206612	0.966942149	Bn-N9-p10704712	Bn-chrAnn_random-p272049
Bn-A09-p10194119	0.966942149	0.975206612	Bn-N9-p10706644	Bn-chrAnn_random-p272032
Bn-A09-p10212596	0.983471074	0.909090909	Bn-N9-p10722058	Bn-chrA09-p9293672
Bn-A09-p10212674	0.975206612	0.801652893	Bn-N9-p10722136	Bn-chrA09-p9293742
Bn-A09-p10212726	0.966942149	0.933884298	Bn-N9-p10722188	Bn-chrA09-p9293794
Bn-A09-p10247512	0.975206612	0.900826446	Bn-N9-p10753227	Bn-chrA09-p9327141
Bn-A09-p10296510	0.983471074	0.991735537	Bn-N9-p10803696	Bn-chrA09-p9517587
Bn-A09-p10297982	0.991735537	1	Bn-N9-p10805806	Bn-chrA09-p9519057
Bn-A09-p10306216	1	0.995024876	Bn-N9-p10813951	Bn-chrA09-p9527219
Bn-A09-p10318807	0.975206612	0.966942149	Bn-N9-p10830558	Bn-chrA09-p9550579
Bn-A09-p10325493	0.867768595	0.958677686	Bn-N9-p10840516	Bn-chrA09-p9571283
Bn-A09-p10329846	0.958677686	0.950413223	Bn-N9-p10844888	Bn-chrA09-p9575678
Bn-A09-p10336331	0.991735537	0.983471074	Bn-N9-p10851555	Bn-chrA09-p9582216
Bn-A09-p10343752	1	0.990049751	Bn-N9-p10859008	Bn-chrA09-p9589816
Bn-A09-p10493965	0.991735537	1	Bn-N9-p18121358	Bn-chrAnn_random-p485112
Bn-A09-p10495974	0.933884298	0.917355372	Bn-N9-p18119333	Bn-chrAnn_random-p484905
Bn-A09-p10504507	0.996677741	0.996677741	Bn-N9-p18110773	Bn-chrAnn_random-p483966
Bn-A09-p10531672	0.950413223	0.94214876	Bn-N9-p18081166	Bn-chrAnn_random-p480433
Bn-A09-p10536531	0.970149254	0.980099502	Bn-N9-p18076111	Bn-chrAnn_random-p479953
Bn-A09-p10536718	0.983471074	0.991735537	Bn-N9-p18075927	Bn-chrAnn_random-p479935
Bn-A09-p10544697	0.991735537	0.867768595	Bn-N9-p18067653	Bn-chrC09-p24735639
Bn-A09-p10549276	0.9375	0.9375	Bn-N9-p18060090	Bn-chrAnn_random-p279432
Bn-A09-p10558501	0.945273632	0.945273632	Bn-Scaffold04658-p9	Bn-chrA06-p13778617
Bn-A09-p10577283	0.990033223	0.677740864	Bn-N8-p14299611	Bn-chrA02-p24468610
Bn-A09-p10580560	0.993355482	0.990033223	Bn-N9-p18007159	Bn-chrAnn_random-p474556
Bn-A09-p10602037	1	1	Bn-N9-p17995366	Bn-chrAnn_random-p473350

Bn-A09-p10628904	0.980099502	0.985074627	Bn-N9-p15428410	Bn-chrAnn_random-p470501
Bn-A09-p10663133	0.966942149	0.975206612	Bn-N9-p15462335	Bn-chrAnn_random-p467529
Bn-A09-p10712987	0.990033223	0.990033223	Bn-N9-p15511903	Bn-chrA09_random-p163199
Bn-A09-p10713953	1	1	Bn-N9-p15512869	Bn-chrA09_random-p163103
Bn-A09-p10714432	0.990049751	0.995024876	Bn-N9-p15513349	Bn-chrA09_random-p163055
Bn-A09-p10756737	0.975124378	0.975124378	Bn-N9-p15566288	Bn-chrA09_random-p158455
Bn-A09-p10757309	0.96345515	0.96345515	Bn-N9-p15566860	Bn-chrAnn_random-p373673
Bn-A09-p10759604	0.990049751	0.990049751	Bn-N9-p15569138	Bn-chrAnn_random-p373696
Bn-A09-p10761012	0.965174129	0.970149254	Bn-N9-p15574843	Bn-chrAnn_random-p428280
Bn-A09-p10771119	0.975206612	0.966942149	Bn-N9-p15621159	Bn-chrAnn_random-p143168
Bn-A09-p10836683	0.958677686	0.958677686	Bn-N9-p15685303	Bn-chrAnn_random-p119155
Bn-A09-p10841953	0.975206612	0.966942149	Bn-N9-p15690644	Bn-chrAnn_random-p118979
Bn-A09-p10842108	0.991735537	1	Bn-N9-p15690799	Bn-chrAnn_random-p118977
Bn-A09-p10842287	1	0.991735537	Bn-N9-p15690978	Bn-chrAnn_random-p118976
Bn-A09-p10850022	0.975206612	0.966942149	Bn-N9-p15698668	Bn-chrAnn_random-p118913
Bn-A09-p10859693	0.983471074	0.859504132	Bn-N9-p15712145	Bn-chrAnn_random-p118852
Bn-A09-p10873462	0.983471074	0.975206612	Bn-N9-p15720591	Bn-chrAnn_random-p118768
Bn-A09-p10881353	0.975206612	0.983471074	Bn-N9-p15728652	Bn-chrAnn_random-p118687
Bn-A09-p10893080	0.983471074	0.991735537	Bn-N9-p15740763	Bn-chrAnn_random-p118569
Bn-A09-p10900375	0.983471074	0.975206612	Bn-N9-p15748420	Bn-chrAnn_random-p118443
Bn-A09-p10901534	0.975206612	0.966942149	Bn-N9-p15749576	Bn-chrAnn_random-p118427
Bn-A09-p10903227	0.991735537	1	Bn-N9-p15751268	Bn-chrAnn_random-p118410
Bn-A09-p10904572	1	0.991735537	Bn-N9-p15752614	Bn-chrAnn_random-p118397
Bn-A09-p10912706	0.975206612	0.975206612	Bn-N9-p15761049	Bn-chrCnn_random-p163486
Bn-A09-p10946429	0.991735537	0.983471074	Bn-N9-p15796403	Bn-chrCnn_random-p164066
Bn-A09-p10947108	0.991735537	1	Bn-N9-p15797086	Bn-chrCnn_random-p164072
Bn-A09-p10971822	0.991735537	1	Bn-N9-p15821901	Bn-chrCnn_random-p164387
Bn-A09-p11006522	1	0.876033058	Bn-N9-p15858031	Bn-chrA09-p15565663
Bn-A09-p11039999	0.983471074	0.991735537	Bn-N9-p15888727	Bn-chrA09-p15598955
Bn-A09-p11110730	0.975124378	0.980099502	Bn-N9-p15963440	Bn-chrA09-p15663349
Bn-A09-p11114990	1	0.970149254	Bn-N8-p9070360	Bn-chrC09-p5968310
Bn-A09-p11126016	0.958677686	0.966942149	Bn-N9-p15970210	Bn-chrA09-p15672350
Bn-A09-p11126583	0.983471074	0.991735537	Bn-N9-p15970794	Bn-chrA09-p15672841
Bn-A09-p11135440	0.995024876	0.975124378	Bn-N9-p15983128	Bn-chrA09-p15676987
Bn-A09-p11139874	0.958677686	0.950413223	Bn-N9-p15987880	Bn-chrA09-p15685531
Bn-A09-p11141656	0.986710963	0.910299003	Bn-N9-p15989661	Bn-chrA09-p15687290
Bn-A09-p11141987	0.993355482	0.986710963	Bn-N9-p15990007	Bn-chrA09-p15687636
Bn-A09-p11149722	0.983471074	0.991735537	Bn-N9-p15997865	Bn-chrA09-p15692182
Bn-A09-p11152092	0.917355372	0.925619835	Bn-N9-p16000235	Bn-chrA09-p15694548
Bn-A09-p11164079	0.925619835	0.892561983	Bn-N9-p37035630	Bn-chrC01-p38675458
Bn-A09-p11180241	0.983471074	0.991735537	Bn-N9-p16018975	Bn-chrA09-p15433479
Bn-A09-p11181581	0.975206612	0.983471074	Bn-N9-p16020312	Bn-chrA09-p15434831
Bn-A09-p11190460	0.94214876	0.933884298	Bn-N9-p16028839	Bn-chrA09-p15443193
Bn-A09-p11210109	0.980099502	0.960199005	Bn-N9-p16054816	Bn-chrA09-p15447679
Bn-A09-p11239885	0.985074627	0.990049751	Bn-N9-p16068452	Bn-chrA09-p15465224
Bn-A09-p11242530	0.991735537	0.983471074	Bn-N9-p16071098	Bn-chrA09-p15467859

Bn-A09-p11243089	0.983471074	0.991735537	Bn-N9-p16071655	Bn-chrA09-p15468417
Bn-A09-p11251794	0.945273632	0.940298507	Bn-N6-p10702686	Bn-chrAnn_random-p261353
Bn-A09-p11252662	0.960199005	0.960199005	Bn-N6-p10703554	Bn-chrAnn_random-p276302
Bn-A09-p11260023	0.990033223	0.986710963	Bn-N9-p16095518	Bn-chrA09-p15534227
Bn-A09-p11267401	0.980066445	0.983388704	Bn-N9-p16102961	Bn-chrA09-p15563859
Bn-A09-p11278998	0.966942149	0.975206612	Bn-N9-p16110199	Bn-chrA09-p15424017
Bn-A09-p11279200	0.801652893	0.79338843	Bn-N9-p16110401	Bn-chrA09-p15423812
Bn-A09-p11284877	0.975206612	0.983471074	Bn-N9-p16112246	Bn-chrA09-p15421992
Bn-A09-p11284928	0.958677686	0.966942149	Bn-N9-p16112297	Bn-chrA09-p15421941
Bn-A09-p11297498	0.991735537	0.958677686	Bn-N9-p16116311	Bn-chrA09-p15416323
Bn-A09-p11297772	0.958677686	0.966942149	Bn-N9-p16116586	Bn-chrA09-p15416054
Bn-A09-p11393003	0.975206612	0.198347107	Bn-N9-p16245534	Bn-chrCnn_random-p233083
Bn-A09-p11400911	0.991735537	0.983471074	Bn-N9-p16253701	Bn-chrC09-p22909785
Bn-A09-p11461510	0.950413223	0.950413223	Bn-N9-p16299854	Bn-chrA09-p15263371
Bn-A09-p11476056	0.983471074	0.991735537	Bn-N9-p16328394	Bn-chrA09-p15233112
Bn-A09-p11477205	0.950413223	0.958677686	Bn-N9-p16329543	Bn-chrA09-p15232060
Bn-A09-p11487820	0.950413223	0.917355372	Bn-N9-p16334881	Bn-chrC09-p22810667
Bn-A09-p11491871	0.603305785	0.834710744	Bn-N19-p27886982	Bn-chrC09-p22792144
Bn-A09-p11498983	1	0.991735537	Bn-N9-p16350840	Bn-chrA09-p15186713
Bn-A09-p11505022	0.983471074	0.975206612	Bn-N9-p16363388	Bn-chrA09-p15180348
Bn-A09-p11512637	0.966942149	0.958677686	Bn-N9-p16369220	Bn-chrA09-p15169858
Bn-A09-p11530621	0.958677686	0.966942149	Bn-N9-p16398040	Bn-chrA09-p15146101
Bn-A09-p11539075	1	0.991735537	Bn-N9-p16419871	Bn-chrA09-p15136507
Bn-A09-p11542176	0.991735537	0.983471074	Bn-N9-p16422997	Bn-chrA09-p15128320
Bn-A09-p11544785	0.975206612	0.966942149	Bn-N9-p16424577	Bn-chrA09-p15126743
Bn-A09-p11558689	0.983471074	0.991735537	Bn-N9-p16427701	Bn-chrA09-p15123648
Bn-A09-p11589729	0.991735537	1	Bn-N9-p16459418	Bn-chrA09-p15090252
Bn-A09-p11595635	0.983471074	0.975206612	Bn-N9-p16465322	Bn-chrA09-p15084349
Bn-A09-p11614521	0.975206612	0.950413223	Bn-N9-p16467427	Bn-chrA09-p15082825
Bn-A09-p11617195	0.983471074	0.975206612	Bn-N9-p16470089	Bn-chrA09-p15080152
Bn-A09-p11630515	0.983471074	0.991735537	Bn-N9-p16483090	Bn-chrA09-p15067165
Bn-A09-p11689854	0.950413223	0.958677686	Bn-N9-p16545437	Bn-chrA03_random-p275698
Bn-A09-p11690216	0.983471074	0.991735537	Bn-N9-p16545799	Bn-chrA03_random-p275734
Bn-A09-p11692396	0.983471074	0.991735537	Bn-N9-p16547109	Bn-chrAnn_random-p392549
Bn-A09-p11727553	0.991735537	0.991735537	Bn-N9-p16601487	Bn-chrA09-p14981688
Bn-A09-p12080295	0.990033223	0.990033223	Bn-N6-p16660113	Bn-chrA06-p16273934
Bn-A09-p12327781	0.989583333	0.989583333	Bn-N9-p24723362	Bn-chrA09-p20707891
Bn-A09-p12466272	0.976744186	0.96345515	Bn-N10-p10526881	Bn-chrA02-p13117253
Bn-A09-p12557952	1	1	Bn-N9-p24945547	Bn-chrA09-p20919348
Bn-A09-p12577050	0.996677741	0.996677741	Bn-N9-p24964622	Bn-chrA09-p20938334
Bn-A09-p12737707	0.965174129	0.965174129	Bn-N15-p12200281	Bn-chrUnn_random-p405829
Bn-A09-p12913174	0.890547264	0.890547264	Bn-N9-p12188021	Bn-chrA09-p10773958
Bn-A09-p12929078	0.983471074	0.983471074	Bn-N9-p12175652	Bn-chrA09-p10761537
Bn-A09-p13021095	0.985074627	0.985074627	Bn-N16-p2227302	Bn-chrC06-p1864701
Bn-A09-p13151129	0.960199005	0.950248756	Bn-N19-p19870862	Bn-chrA09-p10553703
Bn-A09-p13251498	0.965174129	0.960199005	Bn-N9-p11902359	Bn-chrAnn_random-p384609

Bn-A09-p13253434	0.983388704	0.980066445	Bn-N19-p19760365	Bn-chrAnn_random-p38459C
Bn-A09-p13278092	0.839041096	0.924657534	Bn-Scaffold02693-p2	Bn-chrA09-p10434882
Bn-A09-p13384525	0.995024876	1	Bn-N19-p18863999	Bn-chrC09-p15180543
Bn-A09-p13482934	0.980099502	0.990049751	Bn-N19-p18685776	Bn-chrCnn_random-p349841
Bn-A09-p13513734	0.958677686	0.958677686	Bn-N9-p11717361	Bn-chrA09-p10258084
Bn-A09-p13515300	0.991735537	0.991735537	Bn-N9-p11716429	Bn-chrA09-p10257152
Bn-A09-p13515849	0.983471074	0.983471074	Bn-N9-p11710975	Bn-chrA09-p10251698
Bn-A09-p13525202	0.991735537	0.991735537	Bn-N9-p11703399	Bn-chrA09-p10244124
Bn-A09-p13525353	0.73553719	0.73553719	Bn-N9-p11703248	Bn-chrA09-p10243973
Bn-A09-p13564702	0.983471074	0.983471074	Bn-N9-p11655190	Bn-chrA09-p10187157
Bn-A09-p13565633	0.983471074	0.983471074	Bn-N9-p11654259	Bn-chrA09-p10186226
Bn-A09-p13581136	0.975124378	0.975124378	Bn-N9-p11637805	Bn-chrA09-p10171021
Bn-A09-p13582084	0.966942149	0.966942149	Bn-N9-p11636851	Bn-chrA09-p10170067
Bn-A09-p13584189	0.966942149	0.966942149	Bn-N9-p11634724	Bn-chrA09-p10167940
Bn-A09-p13585262	0.983471074	0.983471074	Bn-N9-p11633658	Bn-chrA09-p10166874
Bn-A09-p13587429	0.975206612	0.975206612	Bn-N9-p11631306	Bn-chrA09-p10163461
Bn-A09-p13590924	0.950413223	0.950413223	Bn-N9-p11627733	Bn-chrA09-p10159889
Bn-A09-p13599590	0.884297521	0.884297521	Bn-N9-p11617475	Bn-chrA09-p10151170
Bn-A09-p13600151	0.975206612	0.975206612	Bn-N9-p11616918	Bn-chrA09-p10150613
Bn-A09-p13614414	0.933884298	0.933884298	Bn-N9-p11601998	Bn-chrA09-p10135694
Bn-A09-p13614625	0.975206612	0.975206612	Bn-N9-p11601789	Bn-chrA09-p10135485
Bn-A09-p13616262	0.983471074	0.975206612	Bn-N19-p18424309	Bn-chrC09-p14509034
Bn-A09-p13625959	0.975206612	0.975206612	Bn-N9-p11592321	Bn-chrA09-p10124977
Bn-A09-p13626678	0.991735537	0.991735537	Bn-N9-p11591260	Bn-chrA09-p10123916
Bn-A09-p13628315	0.975206612	0.975206612	Bn-N9-p11589614	Bn-chrA09-p10122270
Bn-A09-p13628485	0.966942149	0.966942149	Bn-N9-p11589443	Bn-chrA09-p10122099
Bn-A09-p13641153	0.966942149	0.966942149	Bn-N9-p11580280	Bn-chrA09-p10113230
Bn-A09-p13654188	0.611570248	0.611570248	Bn-N9-p11569753	Bn-chrA09-p10103104
Bn-A09-p13666209	0.966942149	0.966942149	Bn-N9-p11568358	Bn-chrA09-p10101972
Bn-A09-p13667207	0.909090909	0.909090909	Bn-N9-p11567366	Bn-chrA09-p10100980
Bn-A09-p13667337	0.983471074	0.983471074	Bn-N9-p11567236	Bn-chrA09-p10100850
Bn-A09-p13682690	0.975206612	0.975206612	Bn-N9-p11558427	Bn-chrA09-p10091897
Bn-A09-p13692953	0.859504132	0.884297521	Bn-N9-p11548008	Bn-chrA09-p10068860
Bn-A09-p13693010	0.900826446	0.975206612	Bn-N9-p11547952	Bn-chrA09-p10068803
Bn-A09-p13693499	0.94214876	0.892561983	Bn-N9-p11547474	Bn-chrC09_random-p106524
Bn-A09-p13696043	0.917355372	0.991735537	Bn-N19-p18326124	Bn-chrA09-p10065809
Bn-A09-p13703014	0.975206612	0.966942149	Bn-N9-p11537892	Bn-chrA09-p10058626
Bn-A09-p13718575	0.973421927	0.926910299	Bn-N9-p11533710	Bn-chrA09-p10054446
Bn-A09-p13723999	0.991735537	0.983471074	Bn-N9-p11528578	Bn-chrA09-p10049290
Bn-A09-p13725417	0.975206612	0.966942149	Bn-N9-p11527161	Bn-chrA09-p10043720
Bn-A09-p13727740	0.983471074	0.991735537	Bn-N9-p11523018	Bn-chrA09-p10041407
Bn-A09-p13727794	0.975206612	0.983471074	Bn-N9-p11522964	Bn-chrA09-p10041353
Bn-A09-p13729060	0.966942149	0.958677686	Bn-N9-p11521698	Bn-chrA09-p10040092
Bn-A09-p13729175	0.975206612	0.966942149	Bn-N9-p11521583	Bn-chrA09-p10039977
Bn-A09-p13729226	0.958677686	0.966942149	Bn-N9-p11521532	Bn-chrA09-p10039926
Bn-A09-p13730527	0.975206612	0.983471074	Bn-N9-p11520485	Bn-chrA09-p10038891

Bn-A09-p13730999	0.933884298	0.900826446	Bn-N9-p11520013	Bn-chrA09-p10038419
Bn-A09-p13731601	0.991735537	0.983471074	Bn-N9-p11519411	Bn-chrA09-p10037809
Bn-A09-p13731617	0.975206612	0.983471074	Bn-N9-p11519395	Bn-chrA09-p10037793
Bn-A09-p13731672	0.966942149	0.975206612	Bn-N9-p11519340	Bn-chrA09-p10037738
Bn-A09-p13736860	0.920265781	0.980066445	Bn-N9-p11514133	Bn-chrA09-p10027424
Bn-A09-p13737711	0.976744186	0.933554817	Bn-N9-p11513284	Bn-chrCnn_random-p185211
Bn-A09-p13740488	0.900826446	0.991735537	Bn-N19-p18282073	Bn-chrA09-p10023894
Bn-A09-p13759470	0.990033223	0.986710963	Bn-N9-p11496848	Bn-chrA09-p10007994
Bn-A09-p13776245	0.995024876	0.995024876	Bn-N9-p11480426	Bn-chrA09-p9990299
Bn-A09-p13781551	0.975206612	0.776859504	Bn-N9-p11472502	Bn-chrAnn_random-p390416
Bn-A09-p13794246	0.975206612	0.933884298	Bn-N9-p11448948	Bn-chrCnn_random-p184488
Bn-A09-p13824652	0.990049751	0.990049751	Bn-N9-p11418426	Bn-chrA09-p9950391
Bn-A09-p13827265	0.958677686	0.958677686	Bn-N9-p11414820	Bn-chrA09-p9946784
Bn-A09-p13827481	0.983471074	0.983471074	Bn-N9-p11414604	Bn-chrA09-p9946568
Bn-A09-p13827572	0.991735537	0.991735537	Bn-N9-p11414513	Bn-chrA09-p9946477
Bn-A09-p13827633	0.983471074	0.983471074	Bn-N9-p11414452	Bn-chrA09-p9946416
Bn-A09-p13836909	0.975206612	0.975206612	Bn-N9-p11412132	Bn-chrA09-p9944096
Bn-A09-p13883964	0.966942149	0.966942149	Bn-N9-p11390755	Bn-chrA09-p9905888
Bn-A09-p13905294	0.958677686	0.958677686	Bn-N9-p11375369	Bn-chrA09-p9889260
Bn-A09-p13905850	0.975206612	0.975206612	Bn-N9-p11374815	Bn-chrC09-p14277489
Bn-A09-p13952461	0.990049751	0.990049751	Bn-N19-p17992612	Bn-chrC09-p14265471
Bn-A09-p13957074	0.94214876	0.94214876	Bn-N19-p17988021	Bn-chrC09-p14225458
Bn-A09-p13969906	0.983471074	0.983471074	Bn-N9-p11269877	Bn-chrA09-p9771822
Bn-A09-p13970641	0.975206612	0.975206612	Bn-N9-p11269142	Bn-chrA09-p9771087
Bn-A09-p13983057	0.958677686	0.950413223	Bn-N9-p11256724	Bn-chrA09-p9757615
Bn-A09-p13983626	0.966942149	0.975206612	Bn-N9-p11256156	Bn-chrA09-p9757053
Bn-A09-p14016577	0.975206612	0.975206612	Bn-N9-p11223068	Bn-chrA09-p9730641
Bn-A09-p14017321	0.966942149	0.611570248	Bn-N9-p11222324	Bn-chrC09-p14174801
Bn-A09-p14024256	0.976744186	0.980066445	Bn-N9-p11212160	Bn-chrA09-p9723302
Bn-A09-p14024436	0.986710963	0.990033223	Bn-N9-p11211980	Bn-chrA09-p9723122
Bn-A09-p14031358	0.980099502	0.980099502	Bn-N9-p11204941	Bn-chrA09-p9717275
Bn-A09-p14052967	0.983471074	0.983471074	Bn-N9-p11182344	Bn-chrA09-p9693898
Bn-A09-p14081496	0.92358804	0.990033223	Bn-N9-p11148216	Bn-chrA09_random-p122093
Bn-A09-p14105923	0.990049751	0.990049751	Bn-N19-p17836522	Bn-chrCnn_random-p724460
Bn-A09-p14106312	1	1	Bn-N9-p11126377	Bn-chrA09-p9643591
Bn-A09-p14115991	0.966942149	0.991735537	Bn-N9-p11111353	Bn-chrA09-p9627958
Bn-A09-p14130668	0.826446281	0.834710744	Bn-N15-p22218432	Bn-chrC07-p34838051
Bn-A09-p14132945	0.776859504	0.983471074	Bn-Scaffold03270-p2	Bn-chrA09-p9228082
Bn-A09-p14133028	0.925619835	0.975206612	Bn-N9-p11081141	Bn-chrA09-p9227999
Bn-A09-p14137381	1	1	Bn-N9-p11076747	Bn-chrA09-p9224656
Bn-A09-p14137599	0.925619835	0.925619835	Bn-N9-p11076529	Bn-chrA09-p9224438
Bn-A09-p14137607	0.917355372	0.917355372	Bn-N9-p11076521	Bn-chrA09-p9224430
Bn-A09-p14138079	0.983471074	0.975206612	Bn-N9-p11076061	Bn-chrA09-p9223970
Bn-A09-p14147641	0.983471074	0.67768595	Bn-N9-p11065518	Bn-chrA09-p1978474
Bn-A09-p14147772	0.958677686	0.32231405	Bn-N9-p11065387	Bn-chrCnn_random-p539255
Bn-A09-p14147959	0.966942149	0.958677686	Bn-N9-p11065199	Bn-chrA09-p9216946

Bn-A09-p14149101	0.966942149	0.958677686	Bn-N9-p11060175	Bn-chrA09-p9215806
Bn-A09-p14161665	0.933884298	0.94214876	Bn-N9-p11047301	Bn-chrA09-p9192251
Bn-A09-p14166446	0.986710963	0.980066445	Bn-N9-p11027623	Bn-chrA09-p9189423
Bn-A09-p14167293	0.990049751	0.990049751	Bn-N19-p17754029	Bn-chrC09-p13994729
Bn-A09-p14179631	0.965174129	0.771144279	Bn-Scaffold02250-p7	Bn-chrC04-p20826159
Bn-A09-p14182601	0.910299003	0.890365449	Bn-N9-p11012988	Bn-chrA09-p9175809
Bn-A09-p14199197	0.993355482	0.993355482	Bn-N9-p10996357	Bn-chrA09-p9159166
Bn-A09-p14204447	0.975206612	0.983471074	Bn-N9-p10989283	Bn-chrA09-p9151651
Bn-A09-p14204721	0.842975207	0.652892562	Bn-N9-p10989009	Bn-chrA09-p9151379
Bn-A09-p14205256	0.983471074	0.991735537	Bn-N9-p10988848	Bn-chrA09-p9151211
Bn-A09-p14205358	0.991735537	0.991735537	Bn-N9-p10988746	Bn-chrA09-p9151109
Bn-A09-p14217456	0.993355482	0.561461794	Bn-N9-p10974729	Bn-chrC02-p37333978
Bn-A09-p14218230	0.990033223	0.335548173	Bn-N9-p10973955	Bn-chrCnn_random-p474606
Bn-A09-p14223034	0.993355482	0.966777409	Bn-N9-p10969018	Bn-chrA09-p9616944
Bn-A09-p14246053	0.991735537	1	Bn-N9-p10941036	Bn-chrA09_random-p113956
Bn-A09-p14266174	0.980099502	0.975124378	Bn-N9-p10912967	Bn-chrCnn_random-p384766
Bn-A09-p14267034	0.990049751	0.985074627	Bn-N9-p10912114	Bn-chrAnn_random-p466858
Bn-A09-p14282683	0.943521595	0.970099668	Bn-N9-p10891172	Bn-chrA09_random-p117140
Bn-A09-p14283410	0.991735537	0.983471074	Bn-N9-p10890447	Bn-chrA09_random-p117271
Bn-A09-p14284094	0.702479339	0.991735537	Bn-N9-p10889753	Bn-chrA09_random-p117340
Bn-A09-p14285217	1	0.926910299	Bn-N9-p10888630	Bn-chrA09_random-p117440
Bn-A09-p14287005	1	1	Bn-N19-p17533617	Bn-chrCnn_random-p408663
Bn-A09-p14287061	0.845771144	0.850746269	Bn-N9-p10886785	Bn-chrA09_random-p117762
Bn-A09-p14296015	0.991735537	1	Bn-N9-p10877826	Bn-chrA09_random-p118657
Bn-A09-p14302455	1	0.991735537	Bn-N9-p10871387	Bn-chrA09_random-p119301
Bn-A09-p14302563	0.983471074	0.983471074	Bn-N9-p10871280	Bn-chrA09_random-p119312
Bn-A09-p14330510	0.983471074	0.975206612	Bn-N9-p10869354	Bn-chrAnn_random-p384164
Bn-A09-p14330786	0.983471074	0.991735537	Bn-N9-p10869078	Bn-chrAnn_random-p384173
Bn-A09-p14330816	0.991735537	0.983471074	Bn-N9-p10869048	Bn-chrAnn_random-p384174
Bn-A09-p14376353	0.990049751	0.980099502	Bn-N9-p14376189	Bn-chrA09-p12714926
Bn-A09-p14432426	0.92358804	0.920265781	Bn-N9-p14339612	Bn-chrA09-p12675514
Bn-A09-p14452314	0.966777409	0.966777409	Bn-Scaffold02042-p1	Bn-chrA09-p12655434
Bn-A09-p14468231	0.985074627	0.990049751	Bn-N9-p14306878	Bn-chrA09-p12641161
Bn-A09-p14501539	1	0.989583333	Bn-N9-p14269523	Bn-chrA09-p12609042
Bn-A09-p14535292	0.970149254	0.975124378	Bn-N9-p14240731	Bn-chrA09_random-p155441
Bn-A09-p14585104	0.964705882	0.958823529	Bn-N4-p5280160	Bn-chrA01-p2607948
Bn-A09-p14630762	0.950413223	0.958677686	Bn-N9-p14125939	Bn-chrAnn_random-p149572
Bn-A09-p14631585	0.991735537	0.991735537	Bn-N9-p14125115	Bn-chrAnn_random-p149561
Bn-A09-p14647697	0.965174129	0.975124378	Bn-N9-p27415413	Bn-chrAnn_random-p475653
Bn-A09-p14653854	0.980066445	0.980066445	Bn-N1-p26062353	Bn-chrAnn_random-p383962
Bn-A09-p14702686	0.975206612	0.983471074	Bn-N9-p14079175	Bn-chrA09_random-p127952
Bn-A09-p14751809	0.993355482	0.930232558	Bn-N9-p14047667	Bn-chrA09-p12385669
Bn-A09-p14783306	0.94214876	0.958677686	Bn-Scaffold05113-p1	Bn-chrA09-p12355017
Bn-A09-p14867417	0.947916667	1	Bn-N9-p13932511	Bn-chrA09-p12251268
Bn-A09-p14986234	0.965174129	0.970149254	Bn-N9-p13916818	Bn-chrA09-p12145053
Bn-A09-p14986845	0.800995025	0.731343284	Bn-N9-p13916238	Bn-chrA09-p12144442

Bn-A09-p14987165	0.990049751	1	Bn-N9-p13915917	Bn-chrA09-p12144123
Bn-A09-p15005673	0.910447761	0.945273632	Bn-Scaffold45658-p2	Bn-chrA09_random-p146214
Bn-A09-p15042985	0.996677741	0.993355482	Bn-N9-p13871495	Bn-chrA09_random-p148679
Bn-A09-p15090180	0.975124378	0.970149254	Bn-N9-p13830137	Bn-chrA09-p12023106
Bn-A09-p15105347	0.983471074	0.991735537	Bn-N9-p13813037	Bn-chrA09-p12008779
Bn-A09-p15112850	0.990033223	0.976744186	Bn-N5-p13759318	Bn-chrA05-p13780594
Bn-A09-p15134898	0.980099502	0.985074627	Bn-N9-p13777668	Bn-chrA09-p11974607
Bn-A09-p15192631	0.990049751	0.985074627	Bn-N9-p13706969	Bn-chrA09-p11896107
Bn-A09-p15202528	0.985074627	0.975124378	Bn-N5-p17318324	Bn-chrA09-p6716322
Bn-A09-p15203129	0.960199005	0.980099502	Bn-N5-p17318924	Bn-chrA09-p6716922
Bn-A09-p15203190	0.960199005	0.980099502	Bn-N5-p17318985	Bn-chrA09-p6716983
Bn-A09-p15203308	0.895522388	0.930348259	Bn-N5-p17319103	Bn-chrA09-p6717101
Bn-A09-p15203468	0.92039801	0.955223881	Bn-N5-p17319263	Bn-chrA09-p6717261
Bn-A09-p15270033	0.893687708	0.893687708	Bn-N9-p13607675	Bn-chrA09-p11786805
Bn-A09-p15353026	0.991735537	0.991735537	Bn-N9-p13523937	Bn-chrA09-p11697422
Bn-A09-p15362032	0.833887043	0.833887043	Bn-N9-p13517032	Bn-chrA09-p11690519
Bn-A09-p15416895	0.859504132	0.975206612	Bn-N9-p8687712	Bn-chrC09-p10631816
Bn-A09-p15420800	0.996677741	0.996677741	Bn-N9-p13467557	Bn-chrA09-p11637714
Bn-A09-p15497972	0.933554817	0.933554817	Bn-N9-p13402087	Bn-chrA09-p11571040
Bn-A09-p15556325	0.965174129	1	Bn-N19-p7430775	Bn-chrAnn_random-p327774
Bn-A09-p15648941	0.965174129	0.965174129	Bn-N9-p13291249	Bn-chrA09-p11460220
Bn-A09-p15665552	0.985074627	0.985074627	Bn-N9-p13274560	Bn-chrA09-p11441632
Bn-A09-p15826536	0.975124378	0.975124378	Bn-N9-p13106070	Bn-chrAnn_random-p697280
Bn-A09-p15852129	0.986710963	0.986710963	Bn-N19-p21305223	Bn-chrC09_random-p145296
Bn-A09-p15935674	0.983471074	0.983471074	Bn-N9-p12989680	Bn-chrAnn_random-p442343
Bn-A09-p15935917	0.991735537	0.933884298	Bn-N9-p12989436	Bn-chrC06-p16056291
Bn-A09-p15952662	0.966942149	0.966942149	Bn-N9-p12958162	Bn-chrAnn_random-p713059
Bn-A09-p15959411	0.975206612	0.975206612	Bn-N9-p12950239	Bn-chrAnn_random-p713934
Bn-A09-p15960787	0.975206612	0.975206612	Bn-N9-p12948863	Bn-chrAnn_random-p714071
Bn-A09-p15960945	0.983471074	0.983471074	Bn-N9-p12948705	Bn-chrAnn_random-p714087
Bn-A09-p15975138	0.983471074	0.983471074	Bn-N9-p12935518	Bn-chrAnn_random-p715584
Bn-A09-p15981176	0.975206612	0.975206612	Bn-N9-p12919896	Bn-chrAnn_random-p717251
Bn-A09-p15982584	0.983471074	0.983471074	Bn-N9-p12918548	Bn-chrAnn_random-p717386
Bn-A09-p15984968	1	1	Bn-N9-p12916177	Bn-chrAnn_random-p717623
Bn-A09-p15986311	0.991735537	0.991735537	Bn-N9-p12914836	Bn-chrAnn_random-p717757
Bn-A09-p16009460	0.975206612	0.305785124	Bn-N10-p2572095	Unknown
Bn-A09-p16090255	0.975206612	0.975206612	Bn-N9-p12790459	Bn-chrA09-p11293885
Bn-A09-p16094451	0.966942149	0.966942149	Bn-N9-p12784463	Bn-chrAnn_random-p308003
Bn-A09-p16157117	0.966942149	0.966942149	Bn-N9-p12710331	Bn-chrA09-p11193249
Bn-A09-p16172780	1	1	Bn-N9-p12699309	Bn-chrA09-p11183058
Bn-A09-p16184825	0.94214876	0.94214876	Bn-N9-p12682794	Bn-chrA09-p11166547
Bn-A09-p16187931	0.983471074	0.983471074	Bn-N9-p12677112	Bn-chrA09-p11160085
Bn-A09-p16195258	0.991735537	0.991735537	Bn-N9-p12670251	Bn-chrAnn_random-p414997
Bn-A09-p16322417	0.504132231	0.495867769	Bn-N16-p13319458	Bn-chrC06-p13188650
Bn-A09-p16479468	0.995024876	0.985074627	Bn-N1-p9572063	Bn-chrCnn_random-p489655
Bn-A09-p16479915	0.995024876	0.995024876	Bn-Scaffold00929-p4	Bn-chrCnn_random-p489659

Bn-A09-p16519073	0.990033223	0.993355482	Bn-N9-p12592845	Bn-chrA09-p11054102
Bn-A09-p16540542	0.983388704	0.986710963	Bn-N9-p12563631	Bn-chrA09-p11039718
Bn-A09-p16569660	0.930232558	0.916943522	Bn-N9-p12528584	Bn-chrA09-p11024277
Bn-A09-p16600344	0.925373134	0.92039801	Bn-N9-p12505036	Bn-chrA09-p10994074
Bn-A09-p16620672	0.933554817	0.933554817	Bn-N9-p12491512	Bn-chrA09-p10978543
Bn-A09-p16639867	0.990033223	0.993355482	Bn-N9-p12472837	Bn-chrA09-p10946807
Bn-A09-p16640011	0.990033223	0.983388704	Bn-N9-p12472693	Bn-chrA09-p10946663
Bn-A09-p16655896	0.90625	1	Bn-N9-p12447851	Bn-chrA09-p10927374
Bn-A09-p16690775	0.983388704	0.983388704	Bn-N9-p12403575	Bn-chrAnn_random-p12311[illegible]
Bn-A09-p16702435	0.996677741	1	Bn-N9-p12398328	Bn-chrAnn_random-p12317[illegible]
Bn-A09-p16718038	0.986710963	0.986710963	Bn-N9-p12383443	Bn-chrAnn_random-p12338[illegible]
Bn-A09-p16722238	0.960199005	0.955223881	Bn-N9-p12379294	Bn-chrAnn_random-p12342[illegible]
Bn-A09-p16722618	0.993355482	0.990033223	Bn-N9-p12378918	Bn-chrAnn_random-p12342[illegible]
Bn-A09-p16722814	0.993355482	0.980066445	Bn-N9-p12378717	Bn-chrAnn_random-p12342[illegible]
Bn-A09-p16727301	1	0.936877076	Bn-N9-p12374059	Bn-chrC09-p15335586
Bn-A09-p16728084	0.926910299	0.996677741	Bn-N19-p19250298	Bn-chrAnn_random-p12343[illegible]
Bn-A09-p16766275	0.990033223	0.986710963	Bn-N9-p12339754	Bn-chrAnn_random-p18006[illegible]
Bn-A09-p16771727	1	0.995024876	Bn-N9-p12335993	Bn-chrA09-p10920066
Bn-A09-p16781669	1	0.991735537	Bn-N9-p12323611	Bn-chrA09-p10910403
Bn-A09-p16805527	1	0.991735537	Bn-N9-p12298129	Bn-chrA09-p10886480
Bn-A09-p16818644	0.991735537	1	Bn-N9-p12284734	Bn-chrA09-p10873130
Bn-A09-p16833397	0.993355482	0.986710963	Bn-N9-p12269675	Bn-chrA09-p10853406
Bn-A09-p16835122	0.960199005	0.950248756	Bn-N9-p12267932	Bn-chrA09-p10851681
Bn-A09-p16846786	0.986710963	0.986710963	Bn-N9-p12257181	Bn-chrA09-p10841676
Bn-A09-p16967653	0.993355482	0.986710963	Bn-N9-p14472424	Bn-chrA09-p12803156
Bn-A09-p16982554	0.983471074	0.991735537	Bn-N9-p14491141	Bn-chrA09-p12817787
Bn-A09-p16998291	0.993355482	0.990033223	Bn-N9-p14504409	Bn-chrA09-p12833142
Bn-A09-p17035876	0.986710963	0.986710963	Bn-N9-p14543140	Bn-chrA09-p12876168
Bn-A09-p17162263	0.991735537	0.991735537	Bn-N9-p14682425	Bn-chrA09-p12990822
Bn-A09-p17332686	0.975206612	0.950413223	Bn-N9-p14808789	Bn-chrA09-p13114773
Bn-A09-p17342092	0.991735537	0.983471074	Bn-N9-p14818185	Bn-chrA09-p13123382
Bn-A09-p17354112	1	0.991735537	Bn-N9-p14830054	Bn-chrA09-p13134913
Bn-A09-p17415894	0.960199005	0.975124378	Bn-N9-p14892095	Bn-chrA09-p13189265
Bn-A09-p17415909	0.825870647	0.945273632	Bn-N19-p24370712	Bn-chrA09-p13189280
Bn-A09-p17416038	0.965174129	0.975124378	Bn-N9-p14892239	Bn-chrA09-p13189409
Bn-A09-p17433463	0.991735537	0.991735537	Bn-N9-p14921077	Bn-chrA09-p13211756
Bn-A09-p17440923	0.975206612	0.975206612	Bn-N9-p14921872	Bn-chrA09-p13212551
Bn-A09-p17474059	0.990049751	0.970149254	Bn-N2-p7814288	Bn-chrC04-p42440466
Bn-A09-p17483853	0.985074627	0.985074627	Bn-N9-p14960945	Bn-chrA09-p13249946
Bn-A09-p17483960	0.955223881	0.955223881	Bn-N9-p14961052	Bn-chrA09-p13250053
Bn-A09-p17484188	0.965174129	0.965174129	Bn-N9-p14961293	Bn-chrA09-p13250294
Bn-A09-p17509906	0.985074627	0.980099502	Bn-N9-p14992403	Bn-chrA09-p13269950
Bn-A09-p17509910	0.985074627	0.980099502	Bn-N9-p14992407	Bn-chrA09-p13269954
Bn-A09-p17511656	0.975206612	0.983471074	Bn-N9-p14994154	Bn-chrA09-p13271701
Bn-A09-p17538848	0.983471074	0.991735537	Bn-N9-p15014693	Bn-chrA09-p13296992
Bn-A09-p17582590	0.993355482	0.993355482	Bn-N9-p15058440	Bn-chrA09-p13323793

Bn-A09-p17591228	1	0.996677741	Bn-N9-p15063619	Bn-chrA09-p13328973
Bn-A09-p17600384	0.980099502	0.980099502	Bn-N13-p41406354	Bn-chrC03-p37261484
Bn-A09-p17600725	0.985074627	0.985074627	Bn-N13-p41406016	Bn-chrC03-p37261146
Bn-A09-p17609859	0.990033223	0.970099668	Bn-N9-p15084076	Bn-chrA09-p13343803
Bn-A09-p17624347	0.966942149	0.966942149	Bn-N9-p15104855	Bn-chrA09-p13368132
Bn-A09-p17657506	0.950413223	0.950413223	Bn-N9-p15156045	Bn-chrA09-p13414334
Bn-A09-p17657662	0.94214876	0.94214876	Bn-N9-p15156199	Bn-chrA09-p13414488
Bn-A09-p17669440	0.983388704	0.787375415	Bn-N9-p15169042	Bn-chrA09-p13428122
Bn-A09-p17672066	0.94214876	0.950413223	Bn-N9-p15171674	Bn-chrA09-p13430723
Bn-A09-p17672507	0.950413223	0.917355372	Bn-N9-p15172124	Bn-chrA09-p13431175
Bn-A09-p17672835	0.966942149	0.975206612	Bn-N9-p15172451	Bn-chrA09-p13431502
Bn-A09-p17674977	0.966942149	0.958677686	Bn-N9-p15174595	Bn-chrA09-p13443136
Bn-A09-p17686638	0.910299003	0.900332226	Bn-N9-p15185916	Bn-chrA09-p13456735
Bn-A09-p17690155	0.958677686	0.966942149	Bn-N9-p15191750	Bn-chrA09-p13460265
Bn-A09-p17698726	1	0.991735537	Bn-N9-p15197185	Bn-chrA09-p13469669
Bn-A09-p17700863	0.986710963	0.986710963	Bn-N9-p15199292	Bn-chrA09-p13471812
Bn-A09-p17723940	0.958677686	0.966942149	Bn-N9-p15234056	Bn-chrA09-p13494956
Bn-A09-p17735771	0.950413223	0.991735537	Bn-N9-p15240895	Bn-chrA09-p13500438
Bn-A09-p17739465	0.983471074	0.991735537	Bn-N9-p15243816	Bn-chrA09-p13504674
Bn-A09-p17765321	0.991735537	1	Bn-N9-p15256372	Bn-chrA09-p13532859
Bn-A09-p17834144	0.958677686	0.950413223	Bn-Scaffold01090-p7	Bn-chrA09-p13578422
Bn-A09-p17846566	0.840531561	0.827242525	Bn-N9-p17975283	Bn-chrA09-p13585752
Bn-A09-p17869345	0.983471074	0.975206612	Bn-N9-p17952775	Bn-chrA09-p13622907
Bn-A09-p17915424	0.867768595	0.876033058	Bn-N9-p17890231	Bn-chrA09-p13686155
Bn-A09-p17938318	0.975206612	0.966942149	Bn-N9-p17876301	Bn-chrA09_random-p229242
Bn-A09-p17938471	0.983471074	0.991735537	Bn-N9-p17876148	Bn-chrA09_random-p229258
Bn-A09-p17954328	0.975206612	0.966942149	Bn-N9-p17854902	Bn-chrA09-p13715842
Bn-A09-p17955698	1	0.927083333	Bn-N9-p17848232	Bn-chrA09-p13717163
Bn-A09-p18049051	0.983471074	0	Bn-N9-p17749989	-
Bn-A09-p18092741	0.958677686	0.958677686	Bn-N9-p17701932	Bn-chrA09-p13860740
Bn-A09-p18132317	0.980099502	0.980099502	Bn-N9-p17685023	Bn-chrA09-p13877648
Bn-A09-p18156994	0.975124378	0.965174129	Bn-N9-p17853801	Bn-chrA09-p13896850
Bn-A09-p18180174	0.983471074	0.983471074	Bn-Scaffold65434-p1	Bn-chrA09-p13953363
Bn-A09-p18258259	1	1	Bn-N19-p25877131	Bn-chrA09-p14026503
Bn-A09-p18258443	0.995024876	0.995024876	Bn-N9-p17531358	Bn-chrA09-p14026687
Bn-A09-p18269045	0.958677686	0.966942149	Bn-N7-p1507700	Bn-chrA07-p1340609
Bn-A09-p18273980	0.986710963	0.986710963	Bn-N7-p1502640	Bn-chrA07-p1345543
Bn-A09-p18274708	0.975206612	0.983471074	Bn-N7-p1501912	Bn-chrA07-p1346272
Bn-A09-p18274781	0.980066445	0.983388704	Bn-N7-p1501839	Bn-chrA07-p1346345
Bn-A09-p18274919	0.966942149	0.975206612	Bn-N7-p1501701	Bn-chrA07-p1346483
Bn-A09-p18275501	0.983471074	0.983471074	Bn-N7-p1501119	Bn-chrA07-p1347065
Bn-A09-p18275618	0.993355482	0.993355482	Bn-N7-p1501002	Bn-chrA07-p1347182
Bn-A09-p18275862	0.983471074	0.966942149	Bn-N7-p1500758	Bn-chrA07-p1347426
Bn-A09-p18283186	0.996677741	1	Bn-N7-p1493424	Bn-chrA07-p1357541
Bn-A09-p18289551	0.970149254	0.970149254	Bn-N7-p1486473	Bn-chrA07-p1364902
Bn-A09-p18297719	1	0.983471074	Bn-N7-p1478167	Bn-chrC07-p21857199

Bn-A09-p18306551	0.983471074	0.991735537	Bn-N7-p1468976	Bn-chrA07-p1380194
Bn-A09-p18306845	0.991735537	0.983471074	Bn-N7-p1468682	Bn-chrA07-p1380488
Bn-A09-p18316084	0.991735537	0.983471074	Bn-N7-p1458970	Bn-chrA07-p1403299
Bn-A09-p18324038	0.966942149	0.958677686	Bn-N7-p1450968	Bn-chrA07-p1431141
Bn-A09-p18352122	0.985074627	0.960199005	Bn-N7-p1428003	Bn-chrA07-p1487627
Bn-A09-p18355295	0.975124378	0.985074627	Bn-N7-p1425432	Bn-chrA07-p1499368
Bn-A09-p18403803	0.980099502	0.980099502	Bn-N14-p19608855	Bn-chrUnn_random-p17652[illegible]
Bn-A09-p18588652	0.960199005	0.960199005	Bn-N6-p19142761	Bn-chrA06-p18667390
Bn-A09-p18670679	0.990033223	0.993355482	Bn-N9-p19400231	Bn-chrAnn_random-p14914[illegible]
Bn-A09-p18713965	0.983471074	0.991735537	Bn-N9-p19587628	Bn-chrAnn_random-p16926[illegible]
Bn-A09-p18745571	0.980066445	0.976744186	Bn-N9-p19644679	Bn-chrAnn_random-p17608[illegible]
Bn-A09-p18753596	0.975206612	0.983471074	Bn-N9-p19654882	Bn-chrAnn_random-p17711[illegible]
Bn-A09-p18764246	0.958677686	0.950413223	Bn-N9-p19670077	Bn-chrAnn_random-p17816[illegible]
Bn-A09-p18765584	0.990033223	0.990033223	Bn-N9-p19671414	Bn-chrAnn_random-p17829[illegible]
Bn-A09-p18765663	0.990033223	0.990033223	Bn-N9-p19671493	Bn-chrAnn_random-p17830[illegible]
Bn-A09-p18789989	0.990033223	0.990033223	Bn-N9-p19691926	Bn-chrAnn_random-p17986[illegible]
Bn-A09-p18803128	0.955223881	0.955223881	Bn-N9-p19697558	Bn-chrAnn_random-p18042[illegible]
Bn-A09-p18803850	0.813953488	0.813953488	Bn-N9-p19698280	Bn-chrAnn_random-p18051[illegible]
Bn-A09-p18898633	0.983388704	0.980066445	Bn-N9-p19783482	Bn-chrAnn_random-p18952[illegible]
Bn-A09-p18922925	1	0.991735537	Bn-N9-p19796827	Bn-chrAnn_random-p19042[illegible]
Bn-A09-p18923026	0.991735537	0.991735537	Bn-N9-p19796928	Bn-chrAnn_random-p19043[illegible]
Bn-A09-p18979474	0.975206612	0.983471074	Bn-N9-p19835205	Bn-chrAnn_random-p19379[illegible]
Bn-A09-p18982971	0.970099668	0.970099668	Bn-N9-p19836436	Bn-chrAnn_random-p19391[illegible]
Bn-A09-p18986611	0.983471074	0.983471074	Bn-N9-p19840053	Bn-chrAnn_random-p19428[illegible]
Bn-A09-p19040325	0.986710963	0.478405316	Bn-N9-p19890789	Bn-chrA09-p15727609
Bn-A09-p19043442	0.993355482	0.990033223	Bn-N9-p19893906	Bn-chrA09-p15730745
Bn-A09-p19055902	0.900826446	0.966942149	Bn-N9-p19901593	Bn-chrA09-p15744432
Bn-A09-p19057312	0.917355372	0.966942149	Bn-N9-p19902266	Bn-chrA09-p15745353
Bn-A09-p19068517	0.983471074	0.975206612	Bn-N9-p19925308	Bn-chrA09-p15756037
Bn-A09-p19069970	0.94214876	0.950413223	Bn-N9-p19925877	Bn-chrA09-p15757543
Bn-A09-p19087834	0.94214876	0.950413223	Bn-N9-p19939466	Bn-chrA09-p15768310
Bn-A09-p19089743	0.950413223	0.958677686	Bn-N9-p19943964	Bn-chrA09-p15770214
Bn-A09-p19106825	0.859504132	0.892561983	Bn-N16-p11116943	Bn-chrA09-p15788936
Bn-A09-p19107481	0.975206612	0.966942149	Bn-N9-p19976883	Bn-chrA09-p15792986
Bn-A09-p19112065	0.986710963	0.970099668	Bn-N9-p19981786	Bn-chrA09-p15803541
Bn-A09-p19113008	0.991735537	1	Bn-N9-p19982729	Bn-chrA09-p15804486
Bn-A09-p19145862	0.993355482	0.990033223	Bn-N9-p20009585	Bn-chrA09-p15859344
Bn-A09-p19152331	0.990033223	0.986710963	Bn-N9-p20013522	Bn-chrA09-p15862132
Bn-A09-p19165649	0.975206612	0.851239669	Bn-N9-p20026151	Bn-chrA09-p15853268
Bn-A09-p19168927	0.993355482	0.996677741	Bn-N9-p20029444	Bn-chrA09-p15882406
Bn-A09-p19169299	0.950413223	0.94214876	Bn-N9-p20029843	Bn-chrA09-p15882805
Bn-A09-p19171202	0.958677686	0.950413223	Bn-N9-p20031744	Bn-chrA09-p15884707
Bn-A09-p19172018	0.991735537	0.983471074	Bn-N9-p20032571	Bn-chrA09-p15885534
Bn-A09-p19181075	0.983388704	0.980066445	Bn-N9-p20036621	Bn-chrA09-p15893612
Bn-A09-p19187527	0.983471074	0.975206612	Bn-N9-p20043345	Bn-chrA09-p15903878
Bn-A09-p19187628	0.975206612	0.983471074	Bn-N9-p20043446	Bn-chrA09-p15903979

Bn-A09-p19188090	0.975124378	0.975124378	Bn-N9-p20043908	Bn-chrA09-p15904438
Bn-A09-p19198146	0.983471074	0.991735537	Bn-N9-p20051749	Bn-chrA09-p15969289
Bn-A09-p19198948	0.960199005	0.990049751	Bn-N9-p20052453	Bn-chrA09-p15970002
Bn-A09-p19208887	0.975206612	0.983471074	Bn-N9-p20065491	Bn-chrA09-p15982638
Bn-A09-p19209425	0.966942149	0.958677686	Bn-N9-p20066030	Bn-chrA09-p15983177
Bn-A09-p19209609	0.975206612	0.983471074	Bn-N9-p20066214	Bn-chrA09-p15983361
Bn-A09-p19209695	0.991735537	0.983471074	Bn-N9-p20066300	Bn-chrA09-p15983447
Bn-A09-p19225304	0.975124378	0.990049751	Bn-N9-p20081662	Bn-chrA09-p15985678
Bn-A09-p19225424	0.958677686	0.966942149	Bn-N9-p20081781	Bn-chrA09-p15985798
Bn-A09-p19227824	1	0.991735537	Bn-N9-p20083067	Bn-chrA09-p15986534
Bn-A09-p19242388	0.94214876	0.933884298	Bn-N9-p20105976	Bn-chrA09-p16001575
Bn-A09-p19244125	0.966942149	0.958677686	Bn-N9-p20107711	Bn-chrA09-p16004897
Bn-A09-p19244566	0.983471074	0.975206612	Bn-N9-p20108152	Bn-chrA09-p16005338
Bn-A09-p19267307	0.983471074	0.851239669	Bn-N9-p20115017	Bn-chrA09-p16012118
Bn-A09-p19268137	0.915422886	0.985074627	Bn-N9-p20115869	Bn-chrA09-p16012982
Bn-A09-p19268663	0.980099502	0.990049751	Bn-N9-p20116395	Bn-chrA09-p16013509
Bn-A09-p19272074	0.239669421	0.94214876	Bn-Scaffold13432-p7	Bn-chrA09-p16022292
Bn-A09-p19272189	0.314049587	0.966942149	Bn-N9-p10197711	Bn-chrA09-p16022407
Bn-A09-p19272475	0.256198347	0.966942149	Unknown	Bn-chrA09-p16022692
Bn-A09-p19282217	0.958677686	0.966942149	Bn-N9-p20134956	Bn-chrA09-p16029384
Bn-A09-p19283001	0.983471074	0.983471074	Bn-N9-p20135740	Bn-chrA09-p16030165
Bn-A09-p19297523	0.980099502	0.975124378	Bn-N9-p20149962	Bn-chrA09-p16052604
Bn-A09-p19316704	0.990033223	0.986710963	Bn-N9-p20163072	Bn-chrA09-p16064783
Bn-A09-p19320500	0.991735537	0.867768595	Bn-N9-p20169759	Bn-chrA09-p16068068
Bn-A09-p19320658	0.26446281	0.991735537	Unknown	Bn-chrA09-p16068226
Bn-A09-p19320740	0.181818182	0.950413223	Unknown	Bn-chrA09-p16068308
Bn-A09-p19320810	0.256198347	0.950413223	Bn-Scaffold01100-p3	Bn-chrA09-p16068373
Bn-A09-p19321164	0.966942149	0.966942149	Bn-N9-p20170019	Bn-chrA09-p16068727
Bn-A09-p19347448	0.966942149	0.983471074	Bn-N16-p11601742	Bn-chrA09-p16098739
Bn-A09-p19348447	0.958677686	0.966942149	Bn-N9-p20213558	Bn-chrA09-p16099714
Bn-A09-p19364150	0.983471074	0.94214876	Bn-N9-p20235031	Bn-chrA09-p16115331
Bn-A09-p19370807	0.991735537	1	Bn-N9-p20241662	Bn-chrA09-p16126243
Bn-A09-p19370880	1	0.991735537	Bn-N9-p20241735	Bn-chrA09-p16126316
Bn-A09-p19379073	0.955223881	0.960199005	Bn-N9-p20242017	Bn-chrA09-p16126603
Bn-A09-p19379912	0.991735537	0.983471074	Bn-N9-p20242854	Bn-chrA09-p16127447
Bn-A09-p19392577	0.991735537	0.983471074	Bn-N9-p20255746	Bn-chrA09-p16141228
Bn-A09-p19400561	0.32231405	1	Bn-N6-p17651976	Bn-chrA09-p16149218
Bn-A09-p19400882	0.223140496	0.991735537	Bn-N14-p55112990	Bn-chrA09-p16149539
Bn-A09-p19406640	0.935323383	0.995024876	Bn-Scaffold06439-p7	Bn-chrA09-p16155476
Bn-A09-p19412946	0.355371901	0.785123967	Bn-N11-p24651711	Bn-chrA09-p16161243
Bn-A09-p19413084	0.20661157	0.991735537	Bn-N15-p17860803	Bn-chrA09-p16161381
Bn-A09-p19415047	0.917355372	0.975206612	Bn-N9-p20267604	Bn-chrA09-p16163341
Bn-A09-p19417282	0.495867769	1	Bn-N19-p16182056	Bn-chrA09-p16165578
Bn-A09-p19418268	0.950413223	0.975206612	Bn-N9-p20270961	Bn-chrA09-p16166564
Bn-A09-p19427814	0.79338843	0.966942149	Bn-N16-p11764018	Bn-chrA09-p16176132
Bn-A09-p19438042	0.966777409	0.993355482	Bn-N9-p20283011	Bn-chrA09-p16178988

Bn-A09-p19444777	0.595041322	1	Bn-N15-p18597677	Bn-chrA09-p16185306
Bn-A09-p19447656	0.917355372	0.975206612	Bn-N15-p50281238	Bn-chrA09-p16188195
Bn-A09-p19451773	0.297520661	0.983471074	Bn-N15-p50279502	Bn-chrA09-p16192129
Bn-A09-p19452055	0.867768595	0.958677686	Bn-N15-p50279298	Bn-chrA09-p16192411
Bn-A09-p19452153	0.859504132	0.983471074	Bn-N15-p50279196	Bn-chrA09-p16192508
Bn-A09-p19452209	0.801652893	0.983471074	Bn-N15-p50279106	Bn-chrA09-p16192564
Bn-A09-p19452462	0.661157025	0.991735537	Bn-N9-p20304223	Bn-chrA09-p16192817
Bn-A09-p19452677	0.355371901	0.983471074	Bn-N16-p27719926	Bn-chrA09-p16193032
Bn-A09-p19452740	0.173553719	1	Unknown	Bn-chrA09-p16193095
Bn-A09-p19452814	0.239669421	1	Bn-N13-p43962529	Bn-chrA09-p16193169
Bn-A09-p19453248	0.603305785	0.975206612	Bn-N9-p20310275	Bn-chrA09-p16193603
Bn-A09-p19454867	0.950413223	0.991735537	Bn-N15-p50267769	Bn-chrA09-p16196011
Bn-A09-p19462041	0.986710963	0.980066445	Bn-N9-p20321729	Bn-chrA09-p16202626
Bn-A09-p19489065	0.975206612	0.966942149	Bn-N9-p20344006	Bn-chrA09-p16237534
Bn-A09-p19494485	0.993355482	0.993355482	Bn-N9-p20348114	Bn-chrA09-p16241649
Bn-A09-p19507076	0.983471074	0.966942149	Bn-N9-p20368724	Bn-chrA09-p16261593
Bn-A09-p19513927	0.991735537	0.983471074	Bn-N9-p20377953	Bn-chrA09-p16267438
Bn-A09-p19514024	0.991735537	0.983471074	Bn-N9-p20378050	Bn-chrA09-p16267535
Bn-A09-p19514405	0.991735537	0.983471074	Bn-N9-p20378431	Bn-chrA09-p16267912
Bn-A09-p19514416	0.983471074	0.991735537	Bn-N9-p20378442	Bn-chrA09-p16267923
Bn-A09-p19514576	0.983471074	0.975206612	Bn-N9-p20378602	Bn-chrA09-p16268083
Bn-A09-p19515165	0.983471074	0.94214876	Bn-N9-p20379191	Bn-chrA09-p16269514
Bn-A09-p19515287	0.958677686	0.966942149	Bn-N9-p20379315	Bn-chrA09-p16269642
Bn-A09-p19520460	0.966942149	0.975206612	Bn-N9-p20384494	Bn-chrA09-p16274680
Bn-A09-p19524439	0.985074627	0.955223881	Bn-N9-p20394121	Bn-chrA09-p16277440
Bn-A09-p19533134	0.991735537	0.950413223	Bn-N9-p20402837	Bn-chrA09-p16283813
Bn-A09-p19537178	0.975206612	0.983471074	Bn-N9-p20408559	Bn-chrA09-p16288181
Bn-A09-p19539215	0.966942149	0.760330579	Bn-N9-p20410595	Bn-chrA09-p16290779
Bn-A09-p19542797	1	0.991735537	Bn-N9-p20412733	Bn-chrA09-p16293461
Bn-A09-p19545956	0.991735537	0.983471074	Bn-N9-p20416550	Bn-chrA09-p16296661
Bn-A09-p19548419	0.965174129	0.940298507	Bn-N9-p20419049	Bn-chrA09-p16299119
Bn-A09-p19551395	0.958677686	0.966942149	Bn-N9-p20422010	Bn-chrA09-p16301919
Bn-A09-p19551714	0.966942149	0.958677686	Bn-N9-p20422330	Bn-chrA09-p16302238
Bn-A09-p19551816	0.94214876	0.933884298	Bn-N9-p20422432	Bn-chrA09-p16302340
Bn-A09-p19552388	0.975206612	0.966942149	Bn-N9-p20422993	Bn-chrA09-p16302912
Bn-A09-p19552538	0.993355482	0.990033223	Bn-N9-p20423143	Bn-chrA09-p16303062
Bn-A09-p19566744	0.975206612	0.991735537	Bn-N9-p20439249	Bn-chrCnn_random-p526899
Bn-A09-p19571341	0.983471074	0.991735537	Bn-N9-p20443615	Bn-chrA09-p16321742
Bn-A09-p19571521	0.966942149	0.975206612	Bn-N9-p20443795	Bn-chrA09-p16321922
Bn-A09-p19571970	0.958677686	0.950413223	Bn-N9-p20444245	Bn-chrA09-p16322371
Bn-A09-p19572122	0.966942149	0.958677686	Bn-N9-p20444397	Bn-chrA09-p16322523
Bn-A09-p19578073	0.975206612	0.983471074	Bn-N9-p20466259	Bn-chrA09-p16328474
Bn-A09-p19578479	0.983471074	0.975206612	Bn-N9-p20466667	Bn-chrA09-p16328880
Bn-A09-p19579332	0.966942149	0.975206612	Bn-N9-p20467520	Bn-chrA09-p16329733
Bn-A09-p19581060	0.983471074	0.975206612	Bn-N9-p20469267	Bn-chrA09-p16331643
Bn-A09-p19583295	0.991735537	0.983471074	Bn-N9-p20471265	Bn-chrA09-p16333878

Bn-A09-p19589751	0.993355482	0.96345515	Bn-N9-p20474138	Bn-chrA09-p16339265
Bn-A09-p19591679	0.975206612	0.966942149	Bn-N9-p20476072	Bn-chrA09-p16341192
Bn-A09-p19592030	0.975206612	0.966942149	Bn-N9-p20476423	Bn-chrA09-p16341543
Bn-A09-p19592609	0.993355482	0.966777409	Bn-N9-p20477002	Bn-chrA09-p16342122
Bn-A09-p19596070	0.991735537	0.983471074	Bn-N9-p20480508	Bn-chrA09-p16345583
Bn-A09-p19596462	0.966942149	0.702479339	Bn-N9-p20480892	Bn-chrA09-p16345975
Bn-A09-p19596886	0.996677741	0.803986711	Bn-N9-p20481351	Bn-chrCnn_random-p529288
Bn-A09-p19614389	0.966942149	0.958677686	Bn-N9-p20504819	Bn-chrA09-p16363388
Bn-A09-p19616778	0.975206612	0.983471074	Bn-N9-p20507902	Bn-chrA09-p16365776
Bn-A09-p19619009	0.990033223	0.990033223	Bn-N9-p20510163	Bn-chrA09-p16368007
Bn-A09-p19625255	0.446280992	0.975206612	Bn-N11-p39213512	Bn-chrA09-p16374209
Bn-A09-p19641357	0.950413223	0.958677686	Bn-N9-p20515947	Bn-chrA09-p16389470
Bn-A09-p19649335	0.996677741	0.92358804	Bn-N9-p20530509	Bn-chrA09-p16397444
Bn-A09-p19649635	0.983388704	0.986710963	Bn-N9-p20530828	Bn-chrA09-p16397744
Bn-A09-p19649882	0.986710963	0.983388704	Bn-N9-p20531075	Bn-chrA09-p16397991
Bn-A09-p19650716	0.983471074	0.975206612	Bn-N9-p20531910	Bn-chrA09-p16398825
Bn-A09-p19651041	0.991735537	1	Bn-N9-p20532235	Bn-chrA09-p16399150
Bn-A09-p19651425	0.975206612	0.966942149	Bn-N9-p20532619	Bn-chrA09-p16399534
Bn-A09-p19651913	0.996677741	0.986710963	Bn-N9-p20533108	Bn-chrA09-p16400022
Bn-A09-p19652139	0.975206612	0.966942149	Bn-N9-p20533333	Bn-chrA09-p16400248
Bn-A09-p19653446	0.996677741	0.986710963	Bn-Scaffold01251-p3	Bn-chrA09-p16401555
Bn-A09-p19653782	0.966942149	0.958677686	Bn-Scaffold01251-p3	Bn-chrA09-p16401891
Bn-A09-p19654219	0.991735537	0.983471074	Bn-Scaffold01251-p4	Bn-chrA09-p16402328
Bn-A09-p19658011	0.930232558	0.920265781	Bn-Scaffold01251-p8	Bn-chrA09-p16406085
Bn-A09-p19666970	0.983471074	0.991735537	Bn-N9-p20540420	Bn-chrA09-p16415005
Bn-A09-p19667399	0.983471074	0.975206612	Bn-N9-p20540848	Bn-chrA09-p16415434
Bn-A09-p19667648	0.986710963	0.980066445	Bn-N9-p20541097	Bn-chrA09-p16415683
Bn-A09-p19667700	0.983471074	0.975206612	Bn-N9-p20541149	Bn-chrA09-p16415735
Bn-A09-p19668151	0.991735537	0.983471074	Bn-N9-p20541600	Bn-chrA09-p16416186
Bn-A09-p19668698	0.966942149	0.958677686	Bn-N9-p20542149	Bn-chrA09-p16416733
Bn-A09-p19668818	0.983471074	0.991735537	Bn-N9-p20542269	Bn-chrA09-p16416853
Bn-A09-p19668996	0.975206612	0.983471074	Bn-N9-p20542447	Bn-chrA09-p16417031
Bn-A09-p19669270	0.958677686	0.966942149	Bn-N9-p20542730	Bn-chrA09-p16417305
Bn-A09-p19670121	0.960199005	0.950248756	Bn-N9-p20543581	Bn-chrA09-p16418156
Bn-A09-p19670340	0.966942149	0.975206612	Bn-N9-p20543799	Bn-chrA09-p16418375
Bn-A09-p19671062	0.983471074	0.975206612	Bn-N9-p20544519	Bn-chrA09-p16419097
Bn-A09-p19671192	0.991735537	0.983471074	Bn-N9-p20544649	Bn-chrA09-p16419227
Bn-A09-p19671394	0.986710963	0.973421927	Bn-N9-p20544851	Bn-chrA09-p16419429
Bn-A09-p19671542	0.991735537	0.983471074	Bn-N9-p20544999	Bn-chrA09-p16419577
Bn-A09-p19675265	0.975206612	0.983471074	Bn-N9-p20548719	Bn-chrA09-p16423300
Bn-A09-p19688476	0.983471074	0.975206612	Bn-N9-p20754163	Bn-chrAnn_random-p284955
Bn-A09-p19689884	0.975206612	0.966942149	Bn-N9-p20755441	Bn-chrAnn_random-p284969
Bn-A09-p19690311	0.975206612	0.966942149	Bn-N9-p20755926	Bn-chrA09-p16679682
Bn-A09-p19690392	0.966942149	0.975206612	Bn-N9-p20756007	Bn-chrA09-p16679763
Bn-A09-p19690521	0.975206612	0.983471074	Bn-N9-p20756136	Bn-chrA09-p16679892
Bn-A09-p19690574	0.983471074	0.975206612	Bn-N9-p20756189	Bn-chrA09-p16679945

Bn-A09-p19690777	0.975206612	0.983471074	Bn-N9-p20756395	Bn-chrA09-p16680148
Bn-A09-p19692416	0.975206612	0.983471074	Bn-N9-p20759076	Bn-chrA09-p16681787
Bn-A09-p19718543	0.809917355	0.966942149	Bn-N9-p20759500	Bn-chrAnn_random-p400171
Bn-A09-p19718581	0.975206612	0.983471074	Bn-N9-p20759538	Bn-chrAnn_random-p400172
Bn-A09-p19718831	0.958677686	0.950413223	Bn-N9-p20760301	Bn-chrAnn_random-p400174
Bn-A09-p19725386	0.983471074	0.975206612	Bn-N9-p20761278	Bn-chrAnn_random-p400184
Bn-A09-p19743333	0.975206612	0.966942149	Bn-N9-p20775891	Bn-chrA09-p16723804
Bn-A09-p19743818	0.983471074	0.975206612	Bn-N9-p20776377	Bn-chrA09-p16724289
Bn-A09-p19749530	0.975206612	0.983471074	Bn-N9-p20799064	Bn-chrA09-p16730613
Bn-A09-p19753460	1	0.991735537	Bn-N9-p20813980	Bn-chrA09-p16736419
Bn-A09-p19754463	0.834710744	0.975206612	Bn-N9-p20814798	Bn-chrA09-p16737422
Bn-A09-p19766078	0.570247934	0.991735537	Bn-N9-p20815172	Bn-chrA09-p16749817
Bn-A09-p19766741	0.983471074	0.991735537	Bn-N9-p20815835	Bn-chrA09-p16750480
Bn-A09-p19766911	0.991735537	0.983471074	Bn-N9-p20816011	Bn-chrA09-p16750650
Bn-A09-p19770493	0.983471074	0.975206612	Bn-N9-p20821285	Bn-chrA09-p16754362
Bn-A09-p19770545	0.975206612	0.983471074	Bn-N9-p20821337	Bn-chrA09-p16754414
Bn-A09-p19770719	0.983471074	0.975206612	Bn-N9-p20821511	Bn-chrA09-p16754588
Bn-A09-p19771174	0.983471074	0.975206612	Bn-N9-p20821966	Bn-chrA09-p16755043
Bn-A09-p19774967	0.966942149	0.975206612	Bn-N9-p20826808	Bn-chrA09-p16757879
Bn-A09-p19775158	0.991735537	1	Bn-N9-p20826999	Bn-chrA09-p16758070
Bn-A09-p19775290	0.983471074	0.991735537	Bn-N9-p20827131	Bn-chrA09-p16758202
Bn-A09-p19779966	0.975206612	0.983471074	Bn-N9-p20830877	Bn-chrA09-p16762877
Bn-A09-p19783822	0.983471074	0.975206612	Bn-N9-p20834722	Bn-chrA09-p16766724
Bn-A09-p19796502	0.983471074	0.975206612	Bn-N9-p20842970	Bn-chrA09-p16778338
Bn-A09-p19796581	0.983471074	0.975206612	Bn-N9-p20843049	Bn-chrA09-p16778417
Bn-A09-p19796626	0.975206612	0.983471074	Bn-N9-p20843094	Bn-chrA09-p16778462
Bn-A09-p19796916	0.991735537	0.983471074	Bn-N9-p20843384	Bn-chrA09-p16778752
Bn-A09-p19799940	1	0.990033223	Bn-N9-p20846787	Bn-chrA09-p16781776
Bn-A09-p19803046	0.79338843	0.983471074	Bn-N8-p3933839	Bn-chrA09-p16784882
Bn-A09-p19803535	0.991735537	0.983471074	Bn-N9-p20850384	Bn-chrA09-p16785370
Bn-A09-p19805361	0.991735537	0.983471074	Bn-N9-p20852212	Bn-chrA09-p16787196
Bn-A09-p19835714	0.945273632	0.975124378	Bn-N9-p20882154	Bn-chrA09-p16823174
Bn-A09-p19851523	0.950413223	0.958677686	Bn-N9-p20901243	Bn-chrA09-p16836714
Bn-A09-p19865476	0.958677686	0.966942149	Bn-N9-p20921309	Bn-chrA09-p16848871
Bn-A09-p19872952	0.980099502	0.970149254	Bn-N9-p20930698	Bn-chrA09-p16857092
Bn-A09-p19873361	0.975206612	0.966942149	Bn-N9-p20931108	Bn-chrA09-p16857501
Bn-A09-p19939967	0.985074627	0.985074627	Bn-N9-p20995741	Bn-chrA09-p16912117
Bn-A09-p19941634	0.991735537	0.983471074	Bn-N9-p20998101	Bn-chrA09-p16914495
Bn-A09-p19948856	0.966942149	0.975206612	Bn-N9-p21004835	Bn-chrA09-p16921253
Bn-A09-p20006096	0.945273632	0.960199005	Bn-N9-p21088934	Bn-chrA09-p16996654
Bn-A09-p20006533	0.965174129	0.975124378	Bn-N9-p21089371	Bn-chrA09-p16997091
Bn-A09-p20051040	0.950413223	0.958677686	Bn-N9-p21133492	Bn-chrA09-p17032718
Bn-A09-p20052851	0.52892562	0.991735537	Bn-N9-p21134661	Bn-chrA09-p17033808
Bn-A09-p20053737	0.760330579	0.983471074	Bn-N11-p41990267	Bn-chrA09-p17034711
Bn-A09-p20059101	0.925619835	0.991735537	Bn-N9-p21147399	Bn-chrA09-p17039858
Bn-A09-p20061287	0.983471074	0.983471074	Bn-N9-p21152940	Bn-chrA09-p17042052

Bn-A09-p20074633	0.996677741	0.993355482	Bn-N9-p17204046	Bn-chrA09-p14344730
Bn-A09-p20081587	0.933884298	0.876033058	Bn-N9-p21169587	Bn-chrA09-p17057545
Bn-A09-p20099237	0.991735537	0.983471074	Bn-N9-p21186381	Bn-chrC05-p19056412
Bn-A09-p20113369	0.966942149	0.909090909	Bn-N9-p21199737	Bn-chrC05-p19036243
Bn-A09-p20115072	0.983471074	0.991735537	Bn-N9-p21201409	Bn-chrA09-p17092645
Bn-A09-p20122747	0.975124378	0.970149254	Bn-N9-p21227224	Bn-chrA09-p17103977
Bn-A09-p20124340	0.975206612	0.975206612	Bn-N9-p21228811	Bn-chrA09-p17105561
Bn-A09-p20127421	0.983471074	0.983471074	Bn-N9-p21231037	Bn-chrA09-p17107775
Bn-A09-p20129620	0.983471074	0.983471074	Bn-N9-p21233232	Bn-chrA09-p17114069
Bn-A09-p20133806	0.966942149	0.966942149	Bn-N9-p21237430	Bn-chrA09-p17118268
Bn-A09-p20140028	0.983471074	0.983471074	Bn-N9-p21260266	Bn-chrA09-p17138648
Bn-A09-p20140775	0.958677686	0.958677686	Bn-N9-p21261015	Bn-chrA09-p17139397
Bn-A09-p20140928	0.950413223	0.950413223	Bn-N9-p21261169	Bn-chrA09-p17139551
Bn-A09-p20145310	0.966942149	0.966942149	Bn-N9-p21265547	Bn-chrA09-p17143929
Bn-A09-p20149594	0.991735537	0.991735537	Bn-N9-p21269761	Bn-chrA09-p17148142
Bn-A09-p20155229	0.991735537	0.991735537	Bn-N9-p21275441	Bn-chrA09-p17153822
Bn-A09-p20156214	0.983471074	0.983471074	Bn-N9-p21276426	Bn-chrA09-p17154807
Bn-A09-p20156448	0.983471074	0.983471074	Bn-N9-p21276660	Bn-chrA09-p17155041
Bn-A09-p20156587	1	1	Bn-N9-p21276799	Bn-chrA09-p17155180
Bn-A09-p20156878	0.983471074	0.983471074	Bn-N9-p21277129	Bn-chrA09-p17155510
Bn-A09-p20157191	0.983471074	0.983471074	Bn-N9-p21277444	Bn-chrA09-p17155825
Bn-A09-p20157367	0.975206612	0.975206612	Bn-N9-p21277620	Bn-chrA09-p17156001
Bn-A09-p20163233	0.991735537	0.859504132	Bn-N9-p21279713	Bn-chrA09-p17157027
Bn-A09-p20163796	0.991735537	0.991735537	Bn-N9-p21280276	Bn-chrA09-p17157590
Bn-A09-p20165725	0.983471074	0.983471074	Bn-N9-p21282919	Bn-chrA09-p17160233
Bn-A09-p20168622	1	1	Bn-N9-p21285817	Bn-chrA09-p17163131
Bn-A09-p20169504	0.991735537	0.991735537	Bn-N9-p21286699	Bn-chrA09-p17164013
Bn-A09-p20173294	0.991735537	0.991735537	Bn-N9-p21291564	Bn-chrA09-p17169955
Bn-A09-p20176250	0.975206612	0.975206612	Bn-N9-p21294516	Bn-chrA09-p17172905
Bn-A09-p20185990	0.721393035	0.721393035	Bn-N9-p21302696	Bn-chrA09-p17181085
Bn-A09-p20188481	0.991735537	0.991735537	Bn-N9-p21303875	Bn-chrA09-p17182265
Bn-A09-p20198503	0.991735537	0.991735537	Bn-N9-p21316856	Bn-chrA09-p17193842
Bn-A09-p20198816	0.991735537	0.991735537	Bn-N9-p21317170	Bn-chrA09-p17194156
Bn-A09-p20198871	0.991735537	0.991735537	Bn-N9-p21317225	Bn-chrA09-p17194211
Bn-A09-p20208747	0.983471074	0.933884298	Bn-N9-p21328668	Bn-chrC05-p18762247
Bn-A09-p20209419	0.983471074	0.94214876	Bn-N9-p21329340	Bn-chrC05-p18758340
Bn-A09-p20212765	0.991735537	0.991735537	Bn-N9-p21332519	Bn-chrA09_random-p234902
Bn-A09-p20214059	0.991735537	0.991735537	Bn-N9-p21333811	Bn-chrA09_random-p235031
Bn-A09-p20215345	1	1	Bn-N9-p21335130	Bn-chrAnn_random-p369171
Bn-A09-p20263302	0.995024876	0.995024876	Bn-N9-p21411328	Bn-chrA09-p17272801
Bn-A09-p20327880	0.991735537	0.991735537	Bn-N9-p21469757	Bn-chrA09-p17344589
Bn-A09-p20336623	0.989583333	0.989583333	Bn-N9-p21486612	Bn-chrA09-p17362025
Bn-A09-p20429966	0.970149254	0.661691542	Bn-N3-p26958637	Bn-chrC05-p30290282
Bn-A09-p20512896	0.991735537	0.991735537	Bn-N9-p21642695	Bn-chrA09-p17460505
Bn-A09-p20516599	0.991735537	0.991735537	Bn-N9-p21646806	Bn-chrA09-p17468220
Bn-A09-p20516744	0.983471074	0.983471074	Bn-N9-p21646951	Bn-chrA09-p17468365

Bn-A09-p20517062	0.991735537	0.991735537	Bn-N9-p21647269	Bn-chrA09-p17468683
Bn-A09-p20517413	0.975206612	0.975206612	Bn-N9-p21647621	Bn-chrA09-p17469035
Bn-A09-p20517911	1	1	Bn-N9-p21648116	Bn-chrA09-p17469530
Bn-A09-p20523068	0.991735537	0.991735537	Bn-N9-p21653354	Bn-chrCnn_random-p611878
Bn-A09-p20526508	0.975206612	0.975206612	Bn-N9-p21656791	Bn-chrA09-p17475767
Bn-A09-p20554631	0.859504132	0.859504132	Bn-N9-p21680116	Bn-chrA09-p17498551
Bn-A09-p20566614	0.958677686	0.958677686	Bn-N9-p21684500	Bn-chrA09-p17502935
Bn-A09-p20569783	0.990049751	0.990049751	Bn-N9-p21687646	Bn-chrA09-p17506081
Bn-A09-p20569958	0.966942149	0.966942149	Bn-N9-p21687819	Bn-chrA09-p17506254
Bn-A09-p20649745	0.909090909	0.909090909	Bn-N9-p21750677	Bn-chrA09-p17580753
Bn-A09-p20652846	0.975206612	0.975206612	Bn-N9-p21753724	Bn-chrA09-p17583800
Bn-A09-p20672617	0.983471074	0.983471074	Bn-N9-p21774140	Bn-chrA09-p17607040
Bn-A09-p20678910	0.966942149	0.966942149	Bn-N9-p21784493	Bn-chrA09-p17616651
Bn-A09-p20684557	0.966942149	0.966942149	Bn-N9-p21795303	Bn-chrA09-p17627834
Bn-A09-p20694373	0.94214876	0.94214876	Bn-N9-p21807254	Bn-chrA09-p17640187
Bn-A09-p20720084	0.995024876	0.995024876	Bn-N9-p21834523	Bn-chrA09-p18076140
Bn-A09-p20856553	0.983388704	0.993355482	Bn-N6-p15504305	Bn-chrA06_random-p140718
Bn-A09-p20863459	0.990033223	0.986710963	Bn-N10-p7091939	Bn-chrA10_random-p536639
Bn-A09-p21224256	0.990049751	0.990049751	Bn-N9-p22389886	Bn-chrA09-p18562827
Bn-A09-p21352125	0.990049751	0.990049751	Bn-N7-p2503302	Bn-chrA09-p14858134
Bn-A09-p21795879	1	1	Bn-N9-p22994345	Bn-chrA09-p19190708
Bn-A09-p21821777	0.059800664	0.986710963	Bn-N6-p23840296	Bn-chrC01-p31701751
Bn-A09-p21821779	0.059800664	0.986710963	Bn-N6-p23840294	Bn-chrC01-p31701753
Bn-A09-p21865966	0.993355482	0.993355482	Bn-N9-p23057667	Bn-chrA09-p19253716
Bn-A09-p21914887	0.986710963	0.986710963	Bn-N9-p23111780	Bn-chrA09-p19304555
Bn-A09-p21921236	0.985074627	0.985074627	Bn-N9-p23118175	Bn-chrA09-p19310897
Bn-A09-p21921500	0.985074627	0.985074627	Bn-N9-p23118439	Bn-chrA09-p19311161
Bn-A09-p21922178	0.996677741	0.996677741	Bn-N9-p23119117	Bn-chrA09-p19311839
Bn-A09-p21922383	1	1	Bn-N9-p23119322	Bn-chrA09-p19312044
Bn-A09-p21923295	0.996677741	0.996677741	Bn-N9-p23120233	Bn-chrA09-p19312955
Bn-A09-p21924011	0.986710963	0.986710963	Bn-N9-p23120949	Bn-chrA09-p19313671
Bn-A09-p21931377	0.996677741	0.996677741	Bn-N9-p23128427	Bn-chrA09-p19321149
Bn-A09-p21932600	0.996677741	0.996677741	Bn-N9-p23129657	Bn-chrA09-p19322379
Bn-A09-p21935602	1	1	Bn-N9-p23132665	Bn-chrA09-p19325387
Bn-A09-p21939406	1	1	Bn-N9-p23137502	Bn-chrA09-p19330163
Bn-A09-p21969014	0.976744186	0.976744186	Bn-N9-p23158205	Bn-chrA09-p19353266
Bn-A09-p22003936	0.926910299	0.926910299	Bn-N9-p23206074	Bn-chrA09-p19386196
Bn-A09-p22021632	0.995024876	0.995024876	Bn-N9-p23224373	Bn-chrA09-p19399010
Bn-A09-p22070817	0.991735537	0.991735537	Bn-N9-p23280812	Bn-chrA09-p19462063
Bn-A09-p22070848	0.983471074	0.983471074	Bn-N9-p23280843	Bn-chrA09-p19462094
Bn-A09-p22071523	0.94214876	0.94214876	Bn-N9-p23281519	Bn-chrA09-p19462770
Bn-A09-p22166884	0.983388704	0.983388704	Bn-N9-p23367443	Bn-chrA09-p19550695
Bn-A09-p22170209	0.970149254	0.970149254	Bn-N9-p23370768	Bn-chrA09-p19554020
Bn-A09-p22197988	0.950248756	0.950248756	Bn-N9-p23401115	Bn-chrA09-p19585441
Bn-A09-p22212378	0.980066445	0.980066445	Bn-N15-p18968009	Bn-chrCnn_random-p371454
Bn-A09-p22344523	0.960199005	0.960199005	Bn-N9-p23586076	Bn-chrA09-p19761317

Bn-A09-p22345800	0.990049751	0.990049751	Bn-N9-p23587353	Bn-chrA09-p19762594
Bn-A09-p22462771	1	1	Bn-N9-p23693324	Bn-chrA09-p19863055
Bn-A09-p22495700	1	1	Bn-N9-p23728533	Bn-chrA09-p19896878
Bn-A09-p22612980	0.976744186	0.976744186	Bn-N6-p23714983	Bn-chrA06-p22891982
Bn-A09-p22656762	0.983471074	0.975206612	Bn-N10-p531120	Bn-chrA10-p496940
Bn-A09-p22657080	0.991735537	0.983471074	Bn-N10-p529148	Bn-chrA10-p497258
Bn-A09-p22873685	1	1	Bn-N9-p25335523	Bn-chrA09-p21270411
Bn-A09-p22953858	1	1	Bn-N9-p25382332	Bn-chrA09-p21326254
Bn-A09-p22965277	0.966942149	0.966942149	Bn-N9-p25400496	Bn-chrA09-p21343356
Bn-A09-p23089160	0.995024876	0.995024876	Bn-N9-p25535207	Bn-chrA09-p21452668
Bn-A09-p23089510	0.995	0.99	Bn-Scaffold48718-p2	Bn-chrA09-p21453018
Bn-A09-p23107846	0.990049751	0.990049751	Bn-N9-p25553145	Bn-chrA09-p21471416
Bn-A09-p23107847	0.995024876	0.990049751	Bn-N9-p25553145	Bn-chrA09-p21471416
Bn-A09-p23144404	0.990049751	0.985074627	Bn-N9-p25588982	Bn-chrA09-p21511895
Bn-A09-p23144931	1	0.991735537	Bn-N9-p25589509	Bn-chrA09-p21512422
Bn-A09-p23189250	0.955223881	0.960199005	Bn-N9-p25621035	Bn-chrA09-p21553072
Bn-A09-p23200280	0.996677741	0.993355482	Bn-N9-p25624388	Bn-chrA09-p21559186
Bn-A09-p23221438	0.975206612	0.966942149	Bn-Scaffold05099-p5	Bn-chrA09-p21588834
Bn-A09-p23250129	0.983471074	0.966942149	Bn-N9-p25679946	Bn-chrA09-p21623034
Bn-A09-p23254203	0.950413223	0.958677686	Bn-N9-p25683497	Bn-chrA09-p21626606
Bn-A09-p23268918	0.975206612	0.983471074	Bn-N9-p25696717	Bn-chrA09-p21632778
Bn-A09-p23269680	0.986710963	0.993355482	Bn-N9-p25697479	Bn-chrA09-p21633541
Bn-A09-p23325795	0.965174129	0.990049751	Bn-N9-p25772129	Bn-chrA09-p21714064
Bn-A09-p23329474	0.990049751	0.990049751	Bn-N3-p21891747	Bn-chrA03-p20410775
Bn-A09-p23330048	0.983471074	0.20661157	Bn-N9-p25776480	Bn-chrC02-p23716597
Bn-A09-p23330054	0.991735537	0.148760331	Bn-N9-p25776486	Unknown
Bn-A09-p23331059	0.975206612	0.983471074	Bn-N9-p25777492	Bn-chrA09-p21725107
Bn-A09-p23331135	0.983471074	0.991735537	Bn-N9-p25777568	Bn-chrA09-p21725183
Bn-A09-p23331742	1	0.991735537	Bn-N9-p25778175	Bn-chrA09-p21725790
Bn-A09-p23334889	0.990033223	0.993355482	Bn-N9-p25781439	Bn-chrA09-p21729739
Bn-A09-p23345719	0.993355482	0.976744186	Bn-N9-p25790598	Bn-chrA09-p21738895
Bn-A09-p23354310	0.991735537	0.975206612	Bn-N9-p25797589	Bn-chrA09-p21747322
Bn-A09-p23354810	0.983471074	0.991735537	Bn-N9-p25798089	Bn-chrA09-p21747821
Bn-A09-p23354848	0.990033223	0.996677741	Bn-N9-p25798127	Bn-chrA09-p21747859
Bn-A09-p23356793	0.983471074	0.991735537	Bn-N9-p25800680	Bn-chrA09-p21761502
Bn-A09-p23356947	0.991735537	0.991735537	Bn-N9-p25800834	Bn-chrA09-p21761655
Bn-A09-p23379304	0.966942149	0.834710744	Bn-N9-p25821951	Bn-chrA09-p21778318
Bn-A09-p23379933	0.892561983	0.94214876	Bn-N9-p25831122	Bn-chrA09-p21783425
Bn-A09-p23382659	0.930232558	0.933554817	Bn-N9-p25833805	Bn-chrA09-p21786130
Bn-A09-p23410187	0.983471074	0.991735537	Bn-N9-p25861303	Bn-chrA09-p21819393
Bn-A09-p23416800	0.973421927	0.996677741	Bn-N9-p25867929	Bn-chrA09-p21826013
Bn-A09-p23419710	0.958677686	0.950413223	Bn-N9-p25871519	Bn-chrC05-p13671510
Bn-A09-p23436500	0.973421927	0.933554817	Bn-N9-p25890318	Bn-chrA09-p21843123
Bn-A09-p23438753	0.966942149	0.966942149	Bn-N9-p25892571	Bn-chrA09-p21845192
Bn-A09-p23445424	0.991735537	0.983471074	Bn-N9-p25898872	Bn-chrA09-p21849699
Bn-A09-p23461886	0.991735537	0.826446281	Bn-N9-p25909719	Bn-chrC05-p13592118

Bn-A09-p23489920	0.991735537	0.983471074	Bn-N9-p25944749	Bn-chrA09-p21880350
Bn-A09-p23494647	1	0.983471074	Bn-N9-p25949479	Bn-chrA09-p21886223
Bn-A09-p23515910	0.991735537	0.991735537	Bn-N9-p25967343	Bn-chrA09-p21894367
Bn-A09-p23516882	0.991735537	0.991735537	Bn-N9-p25968315	Bn-chrA09-p21895338
Bn-A09-p23519665	0.991735537	0.991735537	Bn-N9-p25971131	Bn-chrA09-p21898156
Bn-A09-p23525690	0.975206612	0.975206612	Bn-N9-p25975943	Bn-chrA09-p21902968
Bn-A09-p23540416	0.996677741	0.996677741	Bn-N9-p25990686	Bn-chrA09-p21919166
Bn-A09-p23541526	0.989583333	0.989583333	Bn-N9-p25991796	Bn-chrA09-p21920276
Bn-A09-p23545925	0.990033223	0.990033223	Bn-N9-p25996118	Bn-chrA09-p21924702
Bn-A09-p23546242	0.996677741	0.996677741	Bn-N9-p25996435	Bn-chrA09-p21925019
Bn-A09-p23552075	1	0.991735537	Bn-N9-p26001801	Bn-chrA09-p21931328
Bn-A09-p23552530	0.965174129	0.980099502	Bn-N9-p26002256	Bn-chrA09-p21931781
Bn-A09-p23553955	0.950413223	0.859504132	Bn-N9-p26003681	Bn-chrA09-p21933211
Bn-A09-p23554212	0.975206612	0.975206612	Bn-N9-p26003936	Bn-chrA09-p21933466
Bn-A09-p23554351	0.983471074	0.983471074	Bn-N9-p26004075	Bn-chrA09-p21933605
Bn-A09-p23554402	0.983388704	0.983388704	Bn-N9-p26004126	Bn-chrA09-p21933656
Bn-A09-p23649871	0.991735537	1	Bn-N9-p26094288	Bn-chrA09-p22022668
Bn-A09-p23693948	0.975206612	0.966942149	Bn-N9-p26149263	Bn-chrA09-p22065803
Bn-A09-p23707462	0.991735537	0.983471074	Bn-N9-p26158727	Bn-chrA09-p22081699
Bn-A09-p23708692	0.983471074	0.991735537	Bn-N9-p26159948	Bn-chrA09-p22082925
Bn-A09-p23713670	0.993355482	0.933554817	Bn-N9-p26164927	Bn-chrA09-p22090076
Bn-A09-p23717291	0.996677741	0.990033223	Bn-N9-p26168547	Bn-chrA09-p22093865
Bn-A09-p23722611	0.983471074	0.991735537	Bn-N9-p26173888	Bn-chrA09-p22099180
Bn-A09-p23724102	0.975206612	0.983471074	Bn-N9-p26175353	Bn-chrA09-p22100671
Bn-A09-p23724105	0.975206612	0.983471074	Bn-N9-p26175356	Bn-chrA09-p22100674
Bn-A09-p23724303	0.94214876	0.958677686	Bn-N9-p26175551	Bn-chrA09-p22100872
Bn-A09-p23756115	0.975206612	0.966942149	Bn-N9-p26198959	Bn-chrA09-p22128629
Bn-A09-p23773295	0.966942149	0.958677686	Bn-N9-p26221834	Bn-chrA09-p22140895
Bn-A09-p23778678	0.975206612	0.983471074	Bn-N9-p26227392	Bn-chrA09-p22142527
Bn-A09-p23780921	0.991735537	0.983471074	Bn-N9-p26229635	Bn-chrA09-p22144773
Bn-A09-p23790907	0.990049751	0.990049751	Bn-N6-p2562452	Bn-chrA09-p22154333
Bn-A09-p23802586	0.976744186	0.984496124	Bn-N9-p26255772	Bn-chrA09-p22166007
Bn-A09-p23802640	0.972826087	0.97826087	Bn-N9-p26255827	Bn-chrA09-p22166062
Bn-A09-p23802762	0.980099502	0.975124378	Bn-N9-p26255949	Bn-chrA09-p22166184
Bn-A09-p23802813	0.985074627	0.980099502	Bn-N9-p26256000	Bn-chrA09-p22166235
Bn-A09-p23808067	0.935323383	0.970149254	Bn-N15-p14289776	Bn-chrA09-p22170499
Bn-A09-p23838725	0.991735537	1	Bn-N9-p26291038	Bn-chrA09-p22206616
Bn-A09-p23852034	0.950413223	0.958677686	Bn-N9-p26302301	Bn-chrA09-p22217935
Bn-A09-p23861045	0.993355482	0.990033223	Bn-N9-p26314030	Bn-chrA09-p22226914
Bn-A09-p23865794	0.94214876	0.933884298	Bn-N9-p26318746	Bn-chrA09-p22231633
Bn-A09-p23868070	0.990033223	0.966777409	Bn-N9-p26321051	Bn-chrA09-p22233930
Bn-A09-p23904657	0.966942149	0.975206612	Bn-N9-p26354871	Bn-chrA09-p22263937
Bn-A09-p23904905	0.975206612	0.966942149	Bn-N9-p26355119	Bn-chrA09-p22264185
Bn-A09-p23904964	0.991735537	0.983471074	Bn-N9-p26355178	Bn-chrA09-p22264244
Bn-A09-p23913406	0.990033223	0.993355482	Bn-N9-p26364529	Bn-chrA09-p22272660
Bn-A09-p23918548	0.980066445	0.970099668	Bn-N9-p26371099	Bn-chrA09-p22290731

Bn-A09-p23921036	0.976744186	0.970099668	Bn-N9-p26379411	Bn-chrA09-p22293217
Bn-A09-p23928124	0.983471074	0.983471074	Bn-N9-p26389403	Bn-chrA09-p22308493
Bn-A09-p23928417	0.966942149	0.975206612	Bn-N9-p26389700	Bn-chrA09-p22308789
Bn-A09-p23928674	0.983471074	0.991735537	Bn-N9-p26391719	Bn-chrA09_random-p272415
Bn-A09-p23940132	1	0.991735537	Bn-N9-p26395463	Bn-chrA09_random-p271906
Bn-A09-p23948738	0.94214876	0.94214876	Bn-N9-p26404078	Bn-chrA09_random-p270428
Bn-A09-p23986523	0.980099502	0.975124378	Bn-N9-p26438351	Bn-chrA09_random-p269982
Bn-A09-p23993823	0.94214876	0.950413223	Bn-N9-p26445648	Bn-chrA09_random-p269252
Bn-A09-p23994818	0.975206612	0.983471074	Bn-N9-p26446630	Bn-chrA09_random-p269153
Bn-A09-p23995326	0.73553719	0.743801653	Bn-N9-p26447131	Bn-chrA09_random-p269101
Bn-A09-p24003401	0.973421927	0.976744186	Bn-N9-p26454668	Bn-chrA09-p22313439
Bn-A09-p24003969	0.983471074	0.983471074	Bn-N9-p26455236	Bn-chrA09-p22314006
Bn-A09-p24014257	0.619834711	0.983471074	Bn-N15-p13815516	Bn-chrA09-p22323588
Bn-A09-p24086990	0.983471074	0.975206612	Bn-N9-p26530164	Bn-chrA09-p23048282
Bn-A09-p24088098	0.975206612	0.975206612	Bn-N9-p26531272	Bn-chrA09-p23049394
Bn-A09-p24104666	0.980099502	0.985074627	Bn-N9-p26566800	Bn-chrA09-p23062383
Bn-A09-p24105838	0.991735537	1	Bn-N9-p26567975	Bn-chrA09-p23063555
Bn-A09-p24106965	0.966942149	0.975206612	Bn-N9-p26569102	Bn-chrA09-p23064653
Bn-A09-p24113289	0.925373134	0.960199005	Bn-N15-p13633484	Bn-chrA09-p23069753
Bn-A09-p24113399	0.945273632	0.985074627	Bn-N15-p13633374	Bn-chrA09-p23069863
Bn-A09-p24131450	0.965174129	0.960199005	Bn-Scaffold49903-p2	Bn-chrA06-p10385815
Bn-A09-p24216715	0.990049751	0.990049751	Bn-N9-p26668010	Bn-chrA09-p23163166
Bn-A09-p24400007	0.975124378	0.975124378	Bn-N9-p26844877	Bn-chrA09-p22443783
Bn-A09-p24506395	0.993355482	0.993355482	Bn-N9-p26961826	Bn-chrA09-p22566411
Bn-A09-p24520612	0.966942149	0.966942149	Bn-N5-p17195093	Bn-chrA05-p15546430
Bn-A09-p24556003	0.892561983	0.892561983	Bn-N15-p12912611	Bn-chrC05-p11746563
Bn-A09-p24564546	0.950413223	0.950413223	Bn-N9-p27024443	Bn-chrAnn_random-p309666
Bn-A09-p24597678	0.993355482	0.993355482	Bn-N9-p27057726	Bn-chrA09-p22656475
Bn-A09-p24598452	0.990033223	0.993355482	Bn-N9-p27058500	Bn-chrA09-p22657249
Bn-A09-p24718991	0.983388704	0.953488372	Bn-N9-p27526304	Bn-chrC05-p10855859
Bn-A09-p24720890	0.996677741	0.993355482	Bn-N9-p27527541	Bn-chrAnn_random-p412392
Bn-A09-p24724042	0.990033223	0.980066445	Bn-N9-p27530683	Bn-chrAnn_random-p412077
Bn-A09-p24724971	0.996677741	1	Bn-N9-p27531612	Bn-chrAnn_random-p411984
Bn-A09-p24737500	0.976744186	0.983388704	Bn-N9-p27537731	Bn-chrAnn_random-p410758
Bn-A09-p24738552	0.950413223	0.975206612	Bn-N9-p27538778	Bn-chrAnn_random-p410653
Bn-A09-p24791667	0.88372093	0.996677741	Bn-N9-p27568900	Bn-chrAnn_random-p404139
Bn-A09-p24854950	0.966942149	0.950413223	Bn-N9-p27639806	Bn-chrAnn_random-p396841
Bn-A09-p24855012	0.975206612	0.966942149	Bn-N9-p27639868	Bn-chrAnn_random-p396835
Bn-A09-p24867641	0.990033223	0.956810631	Bn-N9-p27643720	Bn-chrAnn_random-p396464
Bn-A09-p24907634	0.950413223	0.958677686	Bn-N9-p27663688	Bn-chrAnn_random-p393643
Bn-A09-p24909461	0.983471074	0.975206612	Bn-N9-p27665633	Bn-chrAnn_random-p393563
Bn-A09-p24921401	0.966942149	0.73553719	Bn-N9-p27674282	Bn-chrAnn_random-p393065
Bn-A09-p24932536	0.975206612	0.983471074	Bn-N9-p27688885	Bn-chrAnn_random-p391983
Bn-A09-p24938815	0.991735537	0.983471074	Bn-N9-p27694903	Bn-chrAnn_random-p391376
Bn-A09-p24938986	0.983471074	0.983471074	Bn-N9-p27695074	Bn-chrAnn_random-p391359
Bn-A09-p24942194	0.983471074	0.991735537	Bn-N9-p27698114	Bn-chrAnn_random-p391055

Bn-A09-p24943615	0.975206612	0.966942149	Bn-N9-p27699535	Bn-chrAnn_random-p390913
Bn-A09-p24943679	0.983471074	0.991735537	Bn-N9-p27699599	Bn-chrAnn_random-p390906
Bn-A09-p24973274	0.940298507	0.945273632	Bn-Scaffold11833-p4	Bn-chrAnn_random-p184632
Bn-A09-p24976222	0.989583333	0.989583333	Bn-N9-p27726792	Bn-chrAnn_random-p388126
Bn-A09-p24979982	0.983471074	0.975206612	Bn-N9-p27730512	Bn-chrAnn_random-p387561
Bn-A09-p24980725	0.983471074	0.991735537	Bn-N9-p27731254	Bn-chrAnn_random-p387487
Bn-A09-p24984795	0.966942149	0.958677686	Bn-N9-p27735332	Bn-chrAnn_random-p387041
Bn-A09-p24986160	0.94214876	0.950413223	Bn-N9-p27736696	Bn-chrAnn_random-p386844
Bn-A09-p24987467	0.975206612	0.950413223	Bn-N9-p27738166	Bn-chrAnn_random-p386714
Bn-A09-p24987744	0.991735537	1	Bn-N9-p27738443	Bn-chrAnn_random-p386687
Bn-A09-p25004061	0.993355482	0.990033223	Bn-Scaffold06475-p6	Bn-chrAnn_random-p385987
Bn-A09-p25018422	0.966942149	0.983471074	Bn-N9-p27749557	Bn-chrAnn_random-p384807
Bn-A09-p25031535	1	0.993355482	Bn-Scaffold01613-p3	Bn-chrAnn_random-p382607
Bn-A09-p25042008	0.983471074	0.983471074	Bn-N9-p27768923	Bn-chrAnn_random-p382248
Bn-A09-p25042124	0.995024876	0.990049751	Bn-N9-p27769039	Bn-chrAnn_random-p382233
Bn-A09-p25045741	0.887043189	0.880398671	Bn-N9-p27770538	Bn-chrAnn_random-p382014
Bn-A09-p25046127	0.958677686	0.950413223	Bn-N9-p27770924	Bn-chrAnn_random-p381976
Bn-A09-p25046263	0.983471074	0.975206612	Bn-N9-p27771060	Bn-chrAnn_random-p381962
Bn-A09-p25050662	0.975206612	0.966942149	Bn-N9-p27776915	Bn-chrAnn_random-p381520
Bn-A09-p25054325	0.966942149	0.983471074	Bn-N9-p27780592	Bn-chrAnn_random-p381162
Bn-A09-p25076813	0.991735537	0.983471074	Bn-N9-p27802369	Bn-chrAnn_random-p379049
Bn-A09-p25090295	1	0.996677741	Bn-N9-p27827308	Bn-chrAnn_random-p377480
Bn-A09-p25113239	0.966942149	0.958677686	Bn-N9-p27852465	Bn-chrAnn_random-p376167
Bn-A09-p25113520	0.94214876	0.933884298	Bn-N9-p27852746	Bn-chrAnn_random-p376139
Bn-A09-p25128018	0.958677686	0.917355372	Bn-N9-p27866476	Bn-chrAnn_random-p374839
Bn-A09-p25133968	0.975206612	0.983471074	Bn-N9-p27870905	Bn-chrAnn_random-p374377
Bn-A09-p25139418	0.892561983	0.991735537	Bn-N18-p29525655	Bn-chrAnn_random-p372834
Bn-A09-p25139597	0.867768595	0.909090909	Bn-N9-p27874482	Bn-chrAnn_random-p372816
Bn-A09-p25141537	0.876033058	0.975206612	Bn-N9-p27868798	Bn-chrAnn_random-p372623
Bn-A09-p25155338	0.975124378	0.980099502	Bn-N9-p27889728	Bn-chrAnn_random-p127584
Bn-A09-p25156399	0.950413223	0.884297521	Bn-N9-p27890797	Bn-chrAnn_random-p127574
Bn-A09-p25166951	0.983471074	0.991735537	Bn-N9-p27896050	Bn-chrAnn_random-p413661
Bn-A09-p25174630	0.983471074	0.983471074	Bn-N9-p27913724	Bn-chrAnn_random-p127433
Bn-A09-p25187073	0.950413223	0.958677686	Bn-N9-p27923341	Bn-chrUnn_random-p364954
Bn-A09-p25212730	0.991735537	0.702479339	Bn-N9-p27943335	Bn-chrC08_random-p364955
Bn-A09-p25224568	0.983471074	0.991735537	Bn-N9-p27953897	Bn-chrAnn_random-p126893
Bn-A09-p25236454	0.996677741	0.993355482	Bn-N9-p27969318	Bn-chrA09-p23258876
Bn-A09-p25236781	0.995024876	0.995024876	Bn-N9-p27969646	Bn-chrA09-p23259203
Bn-A09-p25237569	0.985074627	0.980099502	Bn-N9-p27970434	Bn-chrA09-p23259991
Bn-A09-p25238294	0.966942149	0.975206612	Bn-N9-p27971158	Bn-chrA09-p23260715
Bn-A09-p25243519	0.995024876	1	Bn-N9-p27976405	Bn-chrA09-p23265945
Bn-A09-p25253827	0.958677686	0.966942149	Bn-N9-p27990229	Bn-chrA09-p23276125
Bn-A09-p25269364	0.958677686	0.966942149	Bn-N9-p28005744	Bn-chrA09-p23287069
Bn-A09-p25280192	0.975206612	0.966942149	Bn-N9-p28016542	Bn-chrA09-p23297962
Bn-A09-p25291094	0.983471074	0.975206612	Bn-N9-p28030287	Bn-chrA09-p23317947
Bn-A09-p25309174	0.950413223	0.958677686	Bn-N9-p28042769	Bn-chrA09-p23345004

Bn-A09-p25309514	0.975206612	0.983471074	Bn-N9-p28043109	Bn-chrA09-p23345365
Bn-A09-p25316014	0.983471074	0.991735537	Bn-N9-p28048755	Bn-chrA09-p23360705
Bn-A09-p25316922	0.867768595	0.876033058	Bn-N9-p28049663	Bn-chrA09-p23361615
Bn-A09-p25319349	0.975206612	0.966942149	Bn-N9-p28052666	Bn-chrA09-p23363209
Bn-A09-p25319672	0.986710963	0.980066445	Bn-N9-p28052989	Bn-chrA09-p23363532
Bn-A09-p25320119	0.991735537	0.983471074	Bn-N9-p28053436	Bn-chrA09-p23363964
Bn-A09-p25320259	0.991735537	1	Bn-N9-p28053576	Bn-chrA09-p23364104
Bn-A09-p25320373	0.983471074	0.975206612	Bn-N9-p28053690	Bn-chrA09-p23364218
Bn-A09-p25320436	0.991735537	1	Bn-N9-p28053753	Bn-chrA09-p23365238
Bn-A09-p25324332	0.876033058	0.900826446	Bn-N9-p28065524	Bn-chrA09-p23369168
Bn-A09-p25336380	0.983471074	0.991735537	Bn-N9-p28084381	Bn-chrA09-p23469075
Bn-A09-p25336452	0.950413223	0.925619835	Bn-N9-p28084453	Bn-chrA09-p23469148
Bn-A09-p25372544	0.966942149	0.966942149	Bn-N9-p28125783	Bn-chrA09-p23517032
Bn-A09-p25376723	0.991735537	1	Bn-N9-p28129971	Bn-chrA09-p23521213
Bn-A09-p25440365	0.980066445	0.980066445	Bn-N9-p28189974	Bn-chrA09-p23589414
Bn-A09-p25443253	0.958677686	0.958677686	Bn-N9-p28191613	Bn-chrA09-p23591055
Bn-A09-p25443581	0.950413223	0.950413223	Bn-N9-p28192052	Bn-chrA09-p23591493
Bn-A09-p25455888	0.966942149	0.966942149	Bn-N9-p28204510	Bn-chrA09-p23603952
Bn-A09-p25491749	1	1	Bn-N9-p28240118	Bn-chrA09-p23639690
Bn-A09-p25492079	0.991735537	0.991735537	Bn-N9-p28243130	Bn-chrA09-p23642702
Bn-A09-p25492986	0.991735537	0.94214876	Bn-N9-p28245118	Bn-chrC08_random-p341483
Bn-A09-p25493173	0.991735537	0.851239669	Bn-N9-p28245307	Bn-chrC08_random-p341755
Bn-A09-p25508290	0.975206612	0.975206612	Bn-N9-p28272802	Bn-chrA09_random-p274197
Bn-A09-p25523186	0.958677686	0.958677686	Bn-N9-p28280800	Bn-chrA09-p23646774
Bn-A09-p25532977	0.990033223	0.990033223	Bn-N9-p28290861	Bn-chrA09-p23656835
Bn-A09-p25536905	0.991735537	0.991735537	Bn-N9-p28294732	Bn-chrA09-p23660706
Bn-A09-p25543730	0.966942149	0.966942149	Bn-N9-p28296749	Bn-chrA09-p23662722
Bn-A09-p25558236	0.983471074	0.983471074	Bn-N9-p28312002	Bn-chrA09-p23678179
Bn-A09-p25575397	0.991735537	1	Bn-N9-p28333542	Bn-chrA09-p23692880
Bn-A09-p25618420	0.980066445	0.993355482	Bn-N9-p28356167	Bn-chrA09-p23733993
Bn-A09-p25633407	0.975206612	0.966942149	Bn-N9-p28368440	Bn-chrA09-p23747002
Bn-A09-p25635243	0.926910299	0.946843854	Bn-N9-p28370262	Bn-chrA09-p23751069
Bn-A09-p25636375	0.991735537	1	Bn-N9-p28371394	Bn-chrA09-p23752197
Bn-A09-p25637924	0.983471074	0.983471074	Bn-N9-p28372911	Bn-chrA09-p23754375
Bn-A09-p25638072	0.966942149	0.975206612	Bn-N9-p28373061	Bn-chrA09-p23754525
Bn-A09-p25638579	0.958677686	0.966942149	Bn-N9-p28373572	Bn-chrA09-p23755035
Bn-A09-p25652958	0.960199005	0.965174129	Bn-N9-p28393923	Bn-chrA09_random-p275878
Bn-A09-p25652968	0.966942149	0.958677686	Bn-N9-p28393932	Bn-chrA09_random-p275877
Bn-A09-p25653162	0.965174129	0.965174129	Bn-N9-p28394127	Bn-chrA09_random-p275857
Bn-A09-p25654791	0.990049751	0.985074627	Bn-N9-p28395783	Bn-chrA09_random-p275694
Bn-A09-p25656794	0.983471074	0.983471074	Bn-N9-p28398684	Bn-chrA09_random-p276013
Bn-A09-p25660797	0.983471074	0.983471074	Bn-N9-p28402264	Bn-chrA09_random-p276384
Bn-A09-p25662730	0.985074627	0.985074627	Bn-N9-p28405313	Bn-chrAnn_random-p348574
Bn-A09-p25670603	0.966942149	0.966942149	Bn-N9-p28416914	Bn-chrAnn_random-p262841
Bn-A09-p25680334	0.776859504	0.776859504	Bn-N4-p2791426	Bn-chrA04-p2579716
Bn-A09-p25695942	0.991735537	0.991735537	Bn-N9-p28429762	Bn-chrAnn_random-p204928

Bn-A09-p25735963	0.991735537	0.991735537	Bn-N9-p28473102	Bn-chrA09-p23820972
Bn-A09-p25736089	0.991735537	1	Bn-N9-p28473228	Bn-chrA09-p23821098
Bn-A09-p25736905	1	0.991735537	Bn-N9-p28474044	Bn-chrA09-p23821914
Bn-A09-p25738255	0.991735537	0.983471074	Bn-N9-p28475394	Bn-chrA09-p23823264
Bn-A09-p25738298	0.991735537	0.983471074	Bn-N9-p28475437	Bn-chrA09-p23823307
Bn-A09-p25739064	1	0.996677741	Bn-N9-p28476031	Bn-chrA09-p23823902
Bn-A09-p25740281	0.975206612	0.966942149	Bn-N9-p28477281	Bn-chrA09-p23825142
Bn-A09-p25747213	0.983471074	0.983471074	Bn-N9-p28484581	Bn-chrA09-p23840458
Bn-A09-p25747305	0.991735537	0.983471074	Bn-N9-p28484673	Bn-chrA09-p23840550
Bn-A09-p25749386	0.983471074	0.975206612	Bn-N9-p28486752	Bn-chrA09-p23842630
Bn-A09-p25750328	0.991735537	1	Bn-N9-p28487694	Bn-chrA09-p23843572
Bn-A09-p25777776	1	0.913621262	Bn-N9-p28522183	Bn-chrC08-p25373425
Bn-A09-p25779397	0.993355482	0.970099668	Bn-N9-p28524521	Bn-chrA09-p23873063
Bn-A09-p25793645	0.986710963	0.986710963	Bn-N9-p28547385	Bn-chrA09-p23889266
Bn-A09-p25804956	0.880398671	0.880398671	Bn-N9-p28553180	Bn-chrA09-p23894193
Bn-A09-p25821544	0.950413223	0.958677686	Bn-N9-p28569905	Bn-chrA09-p23910901
Bn-A09-p25823689	0.996677741	0.993355482	Bn-N9-p28573901	Bn-chrA09-p23913044
Bn-A09-p25832607	1	1	Bn-N9-p28578976	Bn-chrA09-p23921964
Bn-A09-p25852108	0.960199005	0.965174129	Bn-N9-p28603223	Bn-chrA09-p23942047
Bn-A09-p25872184	0.983388704	0.980066445	Bn-N9-p28622282	Bn-chrA09-p23963297
Bn-A09-p25884785	0.980099502	0.975124378	Bn-Scaffold02294-p2	Bn-chrAnn_random-p381467
Bn-A09-p25886596	0.986710963	0.986710963	Bn-N9-p28652697	Bn-chrA09-p23975465
Bn-A09-p25894427	0.966942149	0.975206612	Bn-N9-p33286114	Bn-chrA09-p28206667
Bn-A09-p25899239	0.983471074	0.991735537	Bn-N9-p33289819	Bn-chrA09-p28210372
Bn-A09-p25899875	0.991735537	0.983471074	Bn-N9-p33290456	Bn-chrA09-p28211039
Bn-A09-p25904062	0.983471074	0.966942149	Bn-N9-p33294913	Bn-chrA09-p28216809
Bn-A09-p25923686	0.983388704	0.983388704	Bn-N9-p28689657	Bn-chrA09-p24009613
Bn-A09-p25942252	0.975206612	0.975206612	Bn-N9-p28706548	Bn-chrA09-p24026651
Bn-A09-p25960397	0.996677741	0.996677741	Bn-N9-p28720457	Bn-chrA09-p24040761
Bn-A09-p25980030	0.986710963	0.986710963	Bn-N9-p28739667	Bn-chrA09-p24059225
Bn-A09-p25980096	0.986710963	0.986710963	Bn-N9-p28739733	Bn-chrA09-p24059291
Bn-A09-p25980581	0.986710963	0.986710963	Bn-N9-p28740217	Bn-chrA09-p24059775
Bn-A09-p26008548	0.996677741	0.996677741	Bn-N9-p28765285	Bn-chrA09-p24085619
Bn-A09-p26008706	1	1	Bn-N9-p28765443	Bn-chrA09-p24085777
Bn-A09-p26054532	0.990033223	0.990033223	Bn-N9-p28812517	Bn-chrA09-p24130754
Bn-A09-p26062484	0.986710963	0.986710963	Bn-N9-p28820417	Bn-chrA09-p24138651
Bn-A09-p26081899	0.925373134	0.990049751	Bn-Scaffold02355-p1	Bn-chrA09-p24151571
Bn-A09-p26101323	0.925619835	0.925619835	Bn-N9-p28841041	Bn-chrA09-p24170242
Bn-A09-p26133745	0.990033223	0.990033223	Bn-N9-p28867305	Bn-chrA09-p24197951
Bn-A09-p26136023	0.966777409	0.966777409	Bn-N18-p30854660	Bn-chrC08-p25733332
Bn-A09-p26138669	0.955223881	0.955223881	Bn-N9-p28872231	Bn-chrA09-p24202877
Bn-A09-p26141650	0.834710744	0.834710744	Bn-N9-p28875220	Bn-chrA09-p24205866
Bn-A09-p26142571	0.96013289	0.96013289	Bn-N9-p28878040	Bn-chrA09-p24209584
Bn-A09-p26144798	0.973421927	0.973421927	Bn-N9-p28879070	Bn-chrA09-p24210614
Bn-A09-p26158627	0.966942149	0.966942149	Bn-N9-p28892885	Bn-chrA09-p24221433
Bn-A09-p26158694	0.94214876	0.94214876	Bn-N9-p28892951	Bn-chrA09-p24221499

Bn-A09-p26186084	0.950413223	0.950413223	Bn-N9-p28939752	Bn-chrA09-p24301957
Bn-A09-p26244223	0.958677686	0.958677686	Bn-N9-p29011190	Bn-chrA09-p24365321
Bn-A09-p26246760	0.794019934	0.681063123	Bn-N18-p31030092	Bn-chrC09-p33211770
Bn-A09-p26294118	0.776859504	0.776859504	Bn-N9-p29045126	Bn-chrA09-p24383798
Bn-A09-p26301343	0.983471074	0.983471074	Bn-N9-p29047761	Bn-chrA09-p24386433
Bn-A09-p26304509	1	1	Bn-N9-p29048975	Bn-chrA09-p24387646
Bn-A09-p26307738	0.950413223	0.950413223	Bn-N9-p29051051	Bn-chrA09-p24389722
Bn-A09-p26323563	0.996677741	0.996677741	Bn-N9-p29067546	Bn-chrA09-p24406410
Bn-A09-p26346701	0.867768595	0.867768595	Bn-N18-p31142179	Bn-chrC08-p26026846
Bn-A09-p26347498	0.975206612	0.975206612	Bn-N9-p29084069	Bn-chrA09-p24422382
Bn-A09-p26347709	0.966942149	0.966942149	Bn-N9-p29084285	Bn-chrA09-p24422598
Bn-A09-p26349839	1	1	Bn-N9-p29086413	Bn-chrA09-p24424726
Bn-A09-p26353266	0.975206612	0.975206612	Bn-N9-p29089836	Bn-chrA09-p24428149
Bn-A09-p26355161	0.993355482	0.993355482	Bn-N9-p29091725	Bn-chrC09-p19111647
Bn-A09-p26394411	0.991735537	0.991735537	Bn-N9-p29130258	Bn-chrA09-p24468548
Bn-A09-p26397574	0.991735537	0.991735537	Bn-N9-p29134808	Bn-chrA09-p24473408
Bn-A09-p26400930	0.983471074	0.983471074	Bn-N9-p29138543	Bn-chrA09-p24477143
Bn-A09-p26451184	0.991735537	0.991735537	Bn-N9-p29176439	Bn-chrA09-p24513278
Bn-A09-p26456605	0.955223881	0.955223881	Bn-N12-p20595735	Bn-chrA09-p24525936
Bn-A09-p26461272	0.996677741	0.996677741	Bn-N9-p29186842	Bn-chrA09-p24530606
Bn-A09-p26469418	0.995024876	0.995024876	Bn-N18-p29024228	Bn-chrC08-p24079515
Bn-A09-p26477673	0.983388704	0.983388704	Bn-N9-p29201424	Bn-chrA09-p24545088
Bn-A09-p26481517	0.991735537	0.991735537	Bn-N9-p29205268	Bn-chrA09-p24548932
Bn-A09-p26492309	0.714285714	0.647840532	Bn-N19-p39309662	Bn-chrA09_random-p760180
Bn-A09-p26517614	0.985074627	0.985074627	Bn-N9-p29226221	Bn-chrA09-p24569621
Bn-A09-p26521987	0.975206612	0.975206612	Bn-N9-p29233451	Bn-chrA09-p24576865
Bn-A09-p26522069	0.983471074	0.983471074	Bn-N9-p29233546	Bn-chrA09-p24576960
Bn-A09-p26524436	0.966942149	0.966942149	Bn-N9-p29234836	Bn-chrA09-p24578250
Bn-A09-p26530290	0.809917355	0.809917355	Bn-N9-p29237576	Bn-chrA09-p24580990
Bn-A09-p26532740	0.991735537	0.991735537	Bn-N9-p29240043	Bn-chrA09-p24583457
Bn-A09-p26532777	0.975206612	0.975206612	Bn-N9-p29240080	Bn-chrA09-p24583494
Bn-A09-p26543723	0.970099668	0.970099668	Bn-N9-p29245542	Bn-chrA09-p24591804
Bn-A09-p26547937	0.990049751	1	Bn-N12-p26462670	Bn-chrCnn_random-p775377
Bn-A09-p26570681	0.842975207	0.842975207	Bn-N9-p29274861	Bn-chrA09-p24621767
Bn-A09-p26577478	0.991735537	0.991735537	Bn-N9-p29281657	Bn-chrA09-p24628559
Bn-A09-p26578444	0.991735537	0.991735537	Bn-N9-p29282623	Bn-chrA09-p24629525
Bn-A09-p26578867	0.925373134	0.925373134	Bn-N9-p29283046	Bn-chrA09-p24629948
Bn-A09-p26606418	0.993355482	0.993355482	Bn-N9-p29321581	Bn-chrA09-p24663232
Bn-A09-p26620030	0.993355482	0.993355482	Bn-N9-p29334455	Bn-chrA09-p24676230
Bn-A09-p26620447	0.980066445	0.990033223	Bn-N9-p29334872	Bn-chrA09-p24676646
Bn-A09-p26625665	0.993355482	0.993355482	Bn-N9-p29339916	Bn-chrA09-p24685305
Bn-A09-p26637002	0.983471074	0.983471074	Bn-N9-p29352829	Bn-chrA09-p24696780
Bn-A09-p26662535	0.996677741	0.996677741	Bn-N9-p29378149	Bn-chrA09-p24735000
Bn-A09-p26721456	0.965174129	0.965174129	Bn-N9-p29426266	Bn-chrA09-p24783116
Bn-A09-p26721683	0.986710963	0.986710963	Bn-N9-p29426493	Bn-chrA09-p24783343
Bn-A09-p26721815	0.983388704	0.983388704	Bn-N9-p29426625	Bn-chrA09-p24783475

Bn-A09-p26727566	0.976744186	0.976744186	Bn-N18-p31610682	Bn-chrCnn_random-p294235
Bn-A09-p26761130	1	1	Bn-N9-p29468639	Bn-chrA09-p24822161
Bn-A09-p26765098	0.975206612	0.975206612	Bn-N9-p29472606	Bn-chrA09-p24826711
Bn-A09-p26809036	0.910447761	0.910447761	Bn-N9-p29525166	Bn-chrA09-p24885811
Bn-A09-p26812073	0.958677686	0.958677686	Bn-N9-p29530710	Bn-chrC08-p26593796
Bn-A09-p26825500	0.940199336	0.940199336	Bn-N9-p29536449	Bn-chrA09-p24897094
Bn-A09-p26831710	0.980099502	0.980099502	Bn-N9-p29542578	Bn-chrA09-p24903223
Bn-A09-p26864664	0.975206612	0.975206612	Bn-N9-p29569315	Bn-chrA09-p24929959
Bn-A09-p26873627	0.942222222	0.942222222	Bn-N9-p29572979	Bn-chrA09-p24933622
Bn-A09-p26874249	0.975124378	0.975124378	Bn-N9-p29573677	Bn-chrA09-p24934320
Bn-A09-p26874996	0.983471074	0.983471074	Bn-N9-p29574431	Bn-chrA09-p24935074
Bn-A09-p26875060	0.975206612	0.975206612	Bn-N9-p29574495	Bn-chrA09-p24935138
Bn-A09-p26877444	0.950413223	0.950413223	Bn-N9-p29577031	Bn-chrA09-p24937414
Bn-A09-p26881902	0.996677741	0.996677741	Bn-N9-p29593584	Bn-chrA09-p24952473
Bn-A09-p27036273	0.990049751	0.990049751	Bn-N9-p29785710	Bn-chrA09-p25035037
Bn-A09-p27037200	0.990049751	0.990049751	Bn-N9-p29786637	Bn-chrA09-p25035964
Bn-A09-p27103683	0.986710963	0.986710963	Bn-N9-p29855688	Bn-chrA09-p25104079
Bn-A09-p27108615	0.993355482	0.993355482	Bn-N9-p29859398	Bn-chrA09-p25108813
Bn-A09-p27109839	0.986710963	0.986710963	Bn-N9-p29860633	Bn-chrA09-p25110048
Bn-A09-p27351028	0.774086379	0.774086379	Bn-N9-p30139703	Bn-chrA09-p25392041
Bn-A09-p27351047	0.840531561	0.840531561	Bn-N9-p30139722	Bn-chrA09-p25392060
Bn-A09-p27370873	0.950166113	0.950166113	Bn-N9-p30157942	Bn-chrA09-p25409627
Bn-A09-p27436536	0.946843854	0.946843854	Bn-N9-p30231342	Bn-chrA09_random-p299445
Bn-A09-p27456915	0.990033223	0.990033223	Bn-N9-p30251910	Bn-chrA09_random-p297317
Bn-A09-p27529804	0.993355482	0.993355482	Bn-N9-p30304134	Bn-chrA09-p25452627
Bn-A09-p27531548	0.980066445	0.980066445	Bn-N9-p30306145	Bn-chrA09-p25454638
Bn-A09-p27579758	0.970099668	0.913621262	Bn-N9-p30342302	Bn-chrC08-p27575103
Bn-A09-p27580497	0.985074627	0.985074627	Bn-N9-p30348738	Bn-chrA09-p25498625
Bn-A09-p27607559	0.966942149	0.966942149	Bn-N9-p30375851	Bn-chrA09-p25526951
Bn-A09-p27610064	0.983471074	0.983471074	Bn-N9-p30378356	Bn-chrA09-p25529456
Bn-A09-p27611114	0.958677686	0.958677686	Bn-N9-p30379405	Bn-chrA09-p25530505
Bn-A09-p27614316	0.983471074	0.983471074	Bn-N9-p30382608	Bn-chrA09-p25533708
Bn-A09-p27628825	0.991735537	0.991735537	Bn-N9-p30393396	Bn-chrA09-p25547139
Bn-A09-p27637967	0.996677741	0.996677741	Bn-N9-p30405003	Bn-chrA09-p25559195
Bn-A09-p27642687	0.818181818	0.818181818	Bn-N9-p30409551	Bn-chrA09-p25563743
Bn-A09-p27642788	0.851239669	0.851239669	Bn-N9-p30409635	Bn-chrA09-p25563827
Bn-A09-p27642842	0.966942149	0.966942149	Bn-N9-p30409688	Bn-chrA09-p25563880
Bn-A09-p27652538	0.991735537	0.991735537	Bn-N9-p30430661	Bn-chrA09-p25575823
Bn-A09-p27656599	0.900826446	0.900826446	Bn-N18-p32885433	Bn-chrC08-p27734596
Bn-A09-p27658438	1	1	Bn-N9-p30442138	Bn-chrA09-p25585683
Bn-A09-p27666329	0.996677741	0.996677741	Bn-N9-p30447396	Bn-chrA09-p25590941
Bn-A09-p27673543	0.983471074	0.983471074	Bn-N9-p30452736	Bn-chrA09-p25596369
Bn-A09-p27678823	0.850498339	0.850498339	Bn-N9-p30458166	Bn-chrA09-p25601799
Bn-A09-p27688300	0.975124378	0.975124378	Bn-N9-p30469243	Bn-chrA09-p25613051
Bn-A09-p27772299	0.975206612	0.975206612	Bn-N9-p30542775	Bn-chrA09-p25687742
Bn-A09-p27775326	0.975124378	0.975124378	Bn-N9-p30545802	Bn-chrA09-p25690769

Bn-A09-p27777480	0.966942149	0.975206612	Bn-N9-p30548397	Bn-chrAnn_random-p453356
Bn-A09-p27787013	0.669421488	0.520661157	Bn-N14-p30724961	Bn-chrA09-p25714758
Bn-A09-p27830289	0.995024876	0.995024876	Bn-N9-p30606147	Bn-chrA09-p25756535
Bn-A09-p27864717	0.995024876	0.995024876	Bn-N9-p30651397	Bn-chrA09-p25796467
Bn-A09-p27867800	0.975206612	0.975206612	Bn-N9-p30655752	Bn-chrA09-p25800659
Bn-A09-p27869414	0.990033223	0.990033223	Bn-N9-p30657437	Bn-chrA09-p25802344
Bn-A09-p27873343	0.966942149	0.966942149	Bn-N9-p30662430	Bn-chrA09-p25807336
Bn-A09-p27886551	0.975206612	0.975206612	Bn-N9-p30670574	Bn-chrA09-p25815479
Bn-A09-p27887024	0.933884298	0.933884298	Bn-N9-p30671055	Bn-chrA09-p25815960
Bn-A09-p27908768	0.975124378	0.975124378	Bn-N9-p30694169	Bn-chrA09-p25839997
Bn-A09-p27918462	0.975124378	0.975124378	Bn-N9-p30700772	Bn-chrA09-p25847306
Bn-A09-p27939579	1	1	Bn-N9-p30719026	Bn-chrA09-p25867596
Bn-A09-p27943991	0.958333333	0.958333333	Bn-N9-p30723387	Bn-chrA09-p25871957
Bn-A09-p27944522	0.995024876	0.995024876	Bn-N9-p30723969	Bn-chrA09-p25872539
Bn-A09-p27958031	0.991735537	0.991735537	Bn-N9-p30739360	Bn-chrA09-p25889557
Bn-A09-p27965397	0.950413223	0.950413223	Bn-N9-p30743842	Bn-chrAnn_random-p417698
Bn-A09-p27968747	0.990049751	0.885572139	Bn-N9-p30746404	Bn-chrC08-p28116498
Bn-A09-p27969744	0.975206612	0.975206612	Bn-N9-p30747409	Bn-chrA09-p25899202
Bn-A09-p27974695	0.876033058	0.876033058	Bn-N9-p30772878	Bn-chrA05-p20265628
Bn-A09-p27975967	0.975206612	0.975206612	Bn-N9-p30773181	Bn-chrA05-p20265931
Bn-A09-p27976341	0.983471074	0.983471074	Bn-N9-p30773561	Bn-chrA05-p20266311
Bn-A09-p27982460	0.995024876	0.995024876	Bn-N9-p30779698	Bn-chrA05-p20272448
Bn-A09-p27982567	1	1	Bn-N9-p30779805	Bn-chrA05-p20272555
Bn-A09-p27983743	0.966942149	0.966942149	Bn-N9-p30780983	Bn-chrA05-p20273733
Bn-A09-p27984417	0.884297521	0.884297521	Bn-N9-p30782147	Bn-chrC08-p28132337
Bn-A09-p27992030	0.958677686	0.958677686	Bn-N9-p30787119	Bn-chrA05-p20279440
Bn-A09-p28003463	0.990033223	0.990033223	Bn-N9-p30801320	Bn-chrA09_random-p304305
Bn-A09-p28017415	0.991735537	0.991735537	Bn-N9-p30823116	Bn-chrA09-p25950687
Bn-A09-p28025169	0.975206612	0.966942149	Bn-N9-p30826677	Bn-chrA09-p25954248
Bn-A09-p28026997	0.983471074	0.983471074	Bn-N9-p30828505	Bn-chrAnn_random-p404517
Bn-A09-p28027675	0.991735537	0.991735537	Bn-N9-p30829188	Bn-chrAnn_random-p404524
Bn-A09-p28027779	0.903654485	0.903654485	Bn-N9-p30829292	Bn-chrAnn_random-p404525
Bn-A09-p28041600	0.966942149	0.966942149	Bn-N7-p11656567	Bn-chrA07-p11352049
Bn-A09-p28043424	0.960199005	0.960199005	Bn-N9-p30856696	Bn-chrA09-p25977219
Bn-A09-p28044674	0.876033058	0.876033058	Bn-N18-p33429704	Bn-chrC08-p28202675
Bn-A09-p28060237	0.975206612	0.975206612	Bn-N9-p30874268	Bn-chrA09-p25994485
Bn-A09-p28070330	0.996677741	0.993355482	Bn-N9-p30881386	Bn-chrA09-p26001334
Bn-A09-p28074719	0.990049751	0.990049751	Bn-N9-p30885788	Bn-chrA09-p26005735
Bn-A09-p28076316	1	1	Bn-N9-p30887376	Bn-chrA09-p26007323
Bn-A09-p28082973	0.925619835	0.545454545	Bn-N9-p30892261	Bn-chrA09-p26014847
Bn-A09-p28083029	0.975206612	0.842975207	Bn-N9-p30892317	Bn-chrA09-p26014903
Bn-A09-p28127211	0.880597015	0.980099502	Bn-N18-p33520528	Bn-chrA09-p26047485
Bn-A09-p28137068	0.996677741	0.996677741	Bn-N9-p30949594	Bn-chrA09-p26058498
Bn-A09-p28142154	0.983471074	0.983471074	Bn-N9-p30954699	Bn-chrA09-p26063602
Bn-A09-p28142685	0.983471074	0.983471074	Bn-N9-p30955230	Bn-chrA09-p26064133
Bn-A09-p28149545	1	1	Bn-N9-p30962086	Bn-chrA09-p26070989

Bn-A09-p28161041	1	1	Bn-N9-p30973560	Bn-chrA09-p26082481
Bn-A09-p28161429	0.993355482	0.993355482	Bn-N9-p30973952	Bn-chrA09-p26082873
Bn-A09-p28191601	0.975206612	0.975206612	Bn-N9-p31005702	Bn-chrA09-p26114332
Bn-A09-p28202906	1	1	Bn-N9-p31017020	Bn-chrA09-p26125650
Bn-A09-p28203756	0.955223881	0.955223881	Bn-N9-p31020917	Bn-chrA09-p26129548
Bn-A09-p28209274	0.986710963	0.966777409	Bn-Scaffold12533-p5	Bn-chrA09-p26135201
Bn-A09-p28212233	0.983471074	0.975206612	Bn-N9-p31029615	Bn-chrA09-p26138145
Bn-A09-p28219155	0.991735537	0.991735537	Bn-N9-p31036560	Bn-chrA09-p26148421
Bn-A09-p28225347	0.966942149	0.966942149	Bn-N9-p31042574	Bn-chrA09-p26154348
Bn-A09-p28237378	0.940298507	0.965174129	Bn-N9-p31048763	Bn-chrAnn_random-p352991
Bn-A09-p28249213	0.975206612	0.975206612	Bn-N9-p31069164	Bn-chrAnn_random-p155496
Bn-A09-p28249307	0.983471074	0.983471074	Bn-N9-p31069258	Bn-chrAnn_random-p155495
Bn-A09-p28249925	0.900826446	0.958677686	Bn-N9-p31069920	Bn-chrAnn_random-p155489
Bn-A09-p28259332	0.993355482	0.993355482	Bn-N9-p31078829	Bn-chrAnn_random-p155400
Bn-A09-p28265844	0.628099174	0.628099174	Bn-N9-p31085389	Bn-chrAnn_random-p155334
Bn-A09-p28266331	0.995024876	0.995024876	Bn-N9-p31085656	Bn-chrAnn_random-p155331
Bn-A09-p28269530	0.785123967	0.950413223	Bn-N9-p31088605	Bn-chrAnn_random-p155302
Bn-A09-p28282617	0.975206612	0.975206612	Bn-N9-p31103721	Bn-chrA09-p26185354
Bn-A09-p28290848	1	1	Bn-N9-p31116025	Bn-chrA09-p26193329
Bn-A09-p28291555	0.991735537	0.991735537	Bn-N9-p31116735	Bn-chrA09-p26194038
Bn-A09-p28302787	0.975206612	0.975206612	Bn-N9-p31131767	Bn-chrA09-p26211831
Bn-A09-p28308685	0.989583333	0.989583333	Bn-N9-p31137285	Bn-chrA09-p26217348
Bn-A09-p28322032	0.980099502	0.980099502	Bn-N9-p31159129	Bn-chrA09-p26234685
Bn-A09-p28322383	0.965174129	0.965174129	Bn-N9-p31159480	Bn-chrA09-p26235036
Bn-A09-p28365530	1	1	Bn-N9-p31192632	Bn-chrA09-p26271620
Bn-A09-p28387669	0.950413223	0.950413223	Bn-N9-p31216757	Bn-chrA09-p26295939
Bn-A09-p28435678	0.94214876	0.94214876	Bn-N9-p31268166	Bn-chrA09-p26340695
Bn-A09-p28463402	0.975206612	0.975206612	Bn-N9-p31293195	Bn-chrA09-p26361367
Bn-A09-p28466750	0.975206612	0.975206612	Bn-N9-p31296497	Bn-chrA09-p26364669
Bn-A09-p28500123	0.975124378	0.975124378	Bn-N9-p31319402	Bn-chrA09-p26387619
Bn-A09-p28510409	0.933554817	0.933554817	Bn-N9-p31335274	Bn-chrA09-p26403680
Bn-A09-p28511369	0.897009967	0.897009967	Bn-N9-p31336234	Bn-chrA09-p26404640
Bn-A09-p28518619	0.986710963	0.986710963	Bn-N9-p31343479	Bn-chrA09-p26411885
Bn-A09-p28565181	0.820598007	0.860465116	Bn-N9-p31390320	Bn-chrC08-p28970577
Bn-A09-p28565331	0.920265781	0.920265781	Bn-N9-p31390472	Bn-chrA09-p26462419
Bn-A09-p28566621	0.980066445	0.980066445	Bn-N9-p31391762	Bn-chrA09-p26463709
Bn-A09-p28580489	0.996677741	0.996677741	Bn-N9-p31408736	Bn-chrA09-p26480682
Bn-A09-p28589299	0.993355482	0.993355482	Bn-N9-p31418962	Bn-chrA09-p26494717
Bn-A09-p28602135	0.986710963	0.986710963	Bn-N9-p31431455	Bn-chrA09-p26507209
Bn-A09-p28603202	0.990033223	0.990033223	Bn-N9-p31432522	Bn-chrA09-p26508276
Bn-A09-p28603388	0.995024876	0.995024876	Bn-N9-p31432706	Bn-chrA09-p26508460
Bn-A09-p28628739	0.975206612	0.975206612	Bn-N9-p31460265	Bn-chrAnn_random-p463785
Bn-A09-p28654058	0.900826446	0.900826446	Bn-N9-p31485819	Bn-chrA09-p26580614
Bn-A09-p28670849	0.991735537	0.991735537	Bn-N9-p31504176	Bn-chrA09-p26599069
Bn-A09-p28681656	0.991735537	0.991735537	Bn-N9-p31518695	Bn-chrA09-p26617385
Bn-A09-p28695042	0.993355482	0.993355482	Bn-N9-p31531960	Bn-chrA09-p26632132

Bn-A09-p28699275	0.986710963	0.986710963	Bn-N9-p31535973	Bn-chrA09-p26636145
Bn-A09-p28705871	1	0.996677741	Bn-N9-p31541144	Bn-chrA09-p26642733
Bn-A09-p28712265	0.943521595	0.96345515	Bn-N9-p31547775	Bn-chrA09-p26648963
Bn-A09-p28717690	0.834710744	0.834710744	Bn-N9-p31557538	Bn-chrA09-p26655621
Bn-A09-p28721875	0.917355372	0.876033058	Bn-N18-p34373489	Bn-chrC08-p29222246
Bn-A09-p28722989	0.975206612	0.958677686	Bn-N9-p31562739	Bn-chrA09-p26660856
Bn-A09-p28723813	0.975206612	0.983471074	Bn-N9-p31563563	Bn-chrA09-p26661672
Bn-A09-p28726474	0.970149254	0.975124378	Bn-N9-p31566224	Bn-chrC08-p29226831
Bn-A09-p28737145	0.996677741	0.996677741	Bn-N9-p31576342	Bn-chrA09-p26675793
Bn-A09-p28754133	0.993355482	0.986710963	Bn-N9-p31588842	Bn-chrA09-p26690641
Bn-A09-p28758854	0.975206612	0.983471074	Bn-N9-p31594681	Bn-chrAnn_random-p253552
Bn-A09-p28759144	0.950413223	0.950413223	Bn-N9-p31594971	Bn-chrAnn_random-p253555
Bn-A09-p28759763	0.983471074	0.975206612	Bn-N9-p31595590	Bn-chrAnn_random-p253562
Bn-A09-p28765063	0.966942149	0.32231405	Bn-N9-p31600889	Bn-chrC08-p25789761
Bn-A09-p28779232	0.989473684	0.989473684	Bn-N2-p9384090	Bn-chrA09-p26711160
Bn-A09-p28797379	0.903654485	0.900332226	Bn-N9-p31636279	Bn-chrA09-p26729954
Bn-A09-p28802268	0.958677686	0.958677686	Bn-N9-p31641206	Bn-chrA09-p26734204
Bn-A09-p28822639	0.809917355	0.809917355	Bn-N9-p31661184	Bn-chrAnn_random-p390144
Bn-A09-p28865744	0.993355482	0.96013289	Bn-N9-p31689507	Bn-chrA09-p26782974
Bn-A09-p28881091	0.983388704	0.983388704	Bn-N9-p31705116	Bn-chrA09-p26807401
Bn-A09-p28885939	0.980066445	0.976744186	Bn-N9-p31708492	Bn-chrA09-p26810813
Bn-A09-p28887563	0.995024876	0.990049751	Bn-N9-p31710112	Bn-chrA09-p26812433
Bn-A09-p28888373	0.965174129	0.955223881	Bn-N9-p31713110	Bn-chrA09-p26813844
Bn-A09-p28888465	0.980099502	0.980099502	Bn-N9-p31713202	Bn-chrA09-p26813936
Bn-A09-p28910059	0.993355482	0.986710963	Bn-N9-p31735651	Bn-chrA09-p26840515
Bn-A09-p28910407	0.996677741	0.993355482	Bn-N9-p31735999	Bn-chrA09-p26840863
Bn-A09-p28914189	1	0.995024876	Bn-N9-p31740922	Bn-chrA09-p26843581
Bn-A09-p28918272	0.991735537	1	Bn-N9-p31745010	Bn-chrA09-p26847662
Bn-A09-p28919016	0.983471074	0.975206612	Bn-N9-p31745774	Bn-chrA09-p26848422
Bn-A09-p28919262	0.966942149	0.975206612	Bn-N9-p31746019	Bn-chrA09-p26848668
Bn-A09-p28921828	1	0.991735537	Bn-N9-p31748280	Bn-chrA09-p26855319
Bn-A09-p28925363	0.983471074	0.975206612	Bn-N9-p31752023	Bn-chrA09-p26858796
Bn-A09-p28951443	0.996677741	0.993355482	Bn-N9-p31774310	Bn-chrA09-p26880504
Bn-A09-p28966938	0.92039801	0.92039801	Bn-N18-p34658354	Bn-chrC08-p29497587
Bn-A09-p28975119	0.970149254	0.960199005	Bn-N9-p31798647	Bn-chrA09-p26898516
Bn-A09-p28985128	0.995024876	0.995024876	Bn-N18-p34672855	Bn-chrC08-p29514542
Bn-A09-p28987525	0.975124378	0.975124378	Bn-N18-p34675526	Bn-chrC08-p29522080
Bn-A09-p28988322	0.980099502	0.980099502	Bn-N9-p31811100	Bn-chrA09-p26913936
Bn-A09-p28988381	0.975206612	0.975206612	Bn-N9-p31811168	Bn-chrA09-p26914004
Bn-A09-p28990334	0.991735537	0.67768595	Bn-N9-p31813084	Bn-chrA09-p26916059
Bn-A09-p28990432	0.975206612	0.578512397	Bn-N9-p31813182	Bn-chrA09-p26916156
Bn-A09-p28990648	0.94214876	0.851239669	Bn-N9-p31813620	Bn-chrC08-p29541054
Bn-A09-p28996871	0.983471074	0.975206612	Bn-N9-p31819799	Bn-chrA09-p26922619
Bn-A09-p29000054	0.925619835	0.925619835	Bn-N9-p31822992	Bn-chrA09-p26925801
Bn-A09-p29012402	0.975206612	0.752066116	Bn-N9-p31836170	Bn-chrA09_random-p305024
Bn-A09-p29012798	0.986710963	0.96013289	Bn-N9-p31836564	Bn-chrA09_random-p305064

Bn-A09-p29019543	0.983471074	0.983471074	Bn-N9-p31843357	Bn-chrA09_random-p305743
Bn-A09-p29035265	0.980066445	0.980066445	Bn-N9-p31860303	Bn-chrA09_random-p307426
Bn-A09-p29048790	0.991735537	0.991735537	Bn-N9-p31866772	Bn-chrA09_random-p308072
Bn-A09-p29052515	0.983471074	0.983471074	Bn-N9-p31870504	Bn-chrA09_random-p308446
Bn-A09-p29052616	0.966942149	0.966942149	Bn-N9-p31870605	Bn-chrA09_random-p308456
Bn-A09-p29070287	0.991735537	0.991735537	Bn-N9-p31894085	Bn-chrA09_random-p310827
Bn-A09-p29084281	0.983471074	0.983471074	Bn-N9-p31910359	Bn-chrA09_random-p312382
Bn-A09-p29086743	0.794019934	0.916943522	Bn-N18-p34816481	Bn-chrA09_random-p312624
Bn-A09-p29087590	0.975124378	0.975124378	Bn-N9-p31913654	Bn-chrA09_random-p312711
Bn-A09-p29092594	0.975206612	0.975206612	Bn-N9-p31918651	Bn-chrA09_random-p313211
Bn-A09-p29101305	0.966942149	0.966942149	Bn-N9-p31925728	Bn-chrA09_random-p313951
Bn-A09-p29101339	0.970099668	0.970099668	Bn-N9-p31925762	Bn-chrA09_random-p313955
Bn-A09-p29106108	0.892561983	0.892561983	Bn-N9-p31932518	Bn-chrA09_random-p314563
Bn-A09-p29134207	0.995024876	1	Bn-Scaffold09268-p8	Bn-chrA09_random-p316487
Bn-A09-p29134633	0.945273632	0.945273632	Bn-N9-p31964176	Bn-chrA09_random-p316530
Bn-A09-p29146030	0.975206612	0.975206612	Bn-N9-p31976046	Bn-chrAnn_random-p410408
Bn-A09-p29146468	0.991735537	0.983471074	Bn-N9-p31976483	Bn-chrAnn_random-p410404
Bn-A09-p29150680	0.991735537	0.983471074	Bn-N9-p31979327	Bn-chrA09_random-p317614
Bn-A09-p29151179	0.975206612	0.884297521	Bn-N9-p31979825	Bn-chrA09-p27032068
Bn-A09-p29163307	0.976744186	0.946843854	Bn-N9-p31985627	Bn-chrA09-p27041084
Bn-A09-p29171797	0.983471074	0.991735537	Bn-N9-p31992479	Bn-chrA09-p27047244
Bn-A09-p29172005	1	0.991735537	Bn-N9-p31992687	Bn-chrA09-p27047452
Bn-A09-p29186255	0.983471074	0.991735537	Bn-N9-p32008041	Bn-chrA09-p27063736
Bn-A09-p29196000	1	0.363636364	Bn-N9-p32017704	Bn-chrC08-p24784280
Bn-A09-p29196648	0.958677686	0.363636364	Bn-N9-p32018352	Bn-chrC01_random-p299637
Bn-A09-p29198562	0.970099668	0.993355482	Bn-N9-p32020396	Bn-chrA09-p27087932
Bn-A09-p29201922	0.983471074	0.983471074	Bn-N9-p32023756	Bn-chrA09-p27090718
Bn-A09-p29215200	0.985074627	0.985074627	Bn-N7-p12774188	Bn-chrA07-p12717531
Bn-A09-p29229941	0.933884298	0.900826446	Bn-N9-p32051469	Bn-chrA09-p27112968
Bn-A09-p29238214	0.990049751	0.985074627	Bn-N4-p5035820	Bn-chrA04-p4701465
Bn-A09-p29241904	0.983471074	0.975206612	Bn-N9-p32058156	Bn-chrA09-p27121056
Bn-A09-p29245415	0.950248756	0.935323383	Bn-N9-p32065164	Bn-chrC08_random-p382076
Bn-A09-p29249462	0.958677686	0.958677686	Bn-N9-p32068978	Bn-chrA09-p27128569
Bn-A09-p29250072	0.958677686	0.545454545	Bn-N9-p32069575	Bn-chrA09-p27129164
Bn-A09-p29250220	0.983471074	0.975206612	Bn-N9-p32069723	Bn-chrA09-p27129312
Bn-A09-p29250304	0.986710963	0.986710963	Bn-N9-p32069808	Bn-chrA09-p27129396
Bn-A09-p29250607	0.970149254	0.970149254	Bn-N9-p32070075	Bn-chrA09-p27129663
Bn-A09-p29265453	0.993355482	0.990033223	Bn-N9-p32096014	Bn-chrA09-p27155456
Bn-A09-p29272014	0.991735537	0.991735537	Bn-N9-p32102552	Bn-chrA09-p27162009
Bn-A09-p29275947	0.991735537	0.966942149	Bn-N9-p32106534	Bn-chrA09-p27166013
Bn-A09-p29282189	0.980066445	0.970099668	Bn-N9-p32111185	Bn-chrA09-p27171639
Bn-A09-p29310462	0.983388704	0.993355482	Bn-N9-p32142609	Bn-chrA09-p27195067
Bn-A09-p29321510	0.983471074	0.983471074	Bn-N9-p32155024	Bn-chrA09-p27205229
Bn-A09-p29322628	0.983471074	0.983471074	Bn-N9-p32157265	Bn-chrA09-p27206364
Bn-A09-p29331470	0.980099502	0.975124378	Bn-N9-p32161397	Bn-chrC08-p29966621
Bn-A09-p29346712	0.970149254	0.970149254	Bn-N9-p32202430	Bn-chrAnn_random-p386993

Bn-A09-p29348596	0.933554817	0.933554817	Bn-N9-p32203903	Bn-chrAnn_random-p387008
Bn-A09-p29368287	0.958677686	0.958677686	Bn-N9-p32221491	Bn-chrAnn_random-p305898
Bn-A09-p29378096	0.983388704	0.983388704	Bn-N9-p32231258	Bn-chrA09-p27243323
Bn-A09-p29379607	0.985074627	0.985074627	Bn-N9-p32232490	Bn-chrA09-p27244553
Bn-A09-p29388908	1	1	Bn-N9-p32241927	Bn-chrA09-p27253776
Bn-A09-p29399896	1	1	Bn-N9-p32254540	Bn-chrA09-p27265327
Bn-A09-p29400976	1	1	Bn-N9-p32255620	Bn-chrA09-p27266407
Bn-A09-p29416708	0.975206612	0.975206612	Bn-N9-p32271265	Bn-chrA09-p27282049
Bn-A09-p29420099	0.991735537	0.991735537	Bn-N9-p32274681	Bn-chrA09-p27285464
Bn-A09-p29444457	0.958677686	0.958677686	Bn-N9-p32292944	Bn-chrA09-p27306447
Bn-A09-p29446045	0.652892562	0.975206612	Bn-N13-p21307672	Bn-chrA03-p21359327
Bn-A09-p29449180	0.975206612	0.975206612	Bn-N9-p32300548	Bn-chrA09-p27314053
Bn-A09-p29454619	0.986710963	0.986710963	Bn-N9-p32301340	Bn-chrA09-p27314845
Bn-A09-p29457801	0.950166113	0.950166113	Bn-N15-p37646368	Bn-chrC05-p33233802
Bn-A09-p29480022	0.950413223	0.950413223	Bn-N9-p32317450	Bn-chrA09-p27330955
Bn-A09-p29493614	0.917355372	0.917355372	Bn-N9-p32326702	Bn-chrA09-p27339782
Bn-A09-p29494065	0.96345515	0.96345515	Bn-N9-p32327157	Bn-chrA09-p27340237
Bn-A09-p29496129	0.996677741	0.996677741	Bn-N9-p32329210	Bn-chrA09-p27342290
Bn-A09-p29496571	0.983471074	0.983471074	Bn-N9-p32329653	Bn-chrA09-p27342733
Bn-A09-p29496588	0.966942149	0.966942149	Bn-N9-p32329670	Bn-chrA09-p27342750
Bn-A09-p29500238	0.970149254	0.962686567	Bn-N1-p1385015	Bn-chrA10-p9720358
Bn-A09-p29500640	0.975124378	0.950248756	Bn-N2-p26894781	Bn-chrAnn_random-p346113
Bn-A09-p29500865	0.960199005	0.960199005	Bn-N2-p26895006	Bn-chrA10-p9719805
Bn-A09-p29507416	0.983471074	0.983471074	Bn-N9-p32336610	Bn-chrA09-p27349693
Bn-A09-p29508036	0.990049751	0.990049751	Bn-N9-p32337224	Bn-chrA09-p27350307
Bn-A09-p29519072	0.985074627	0.985074627	Bn-N9-p32350553	Bn-chrAnn_random-p337363
Bn-A09-p29617756	0.990049751	0.990049751	Bn-N9-p32470393	Bn-chrA09-p27436059
Bn-A09-p29617813	0.970149254	0.970149254	Bn-N9-p32470450	Bn-chrA09-p27436116
Bn-A09-p29628819	0.989130435	0.989130435	Bn-N9-p32481633	Bn-chrA09-p27447512
Bn-A09-p29629155	0.990049751	0.990049751	Bn-N9-p32481969	Bn-chrA09-p27447848
Bn-A09-p29630906	0.995024876	0.995024876	Bn-N9-p32483720	Bn-chrA09-p27449599
Bn-A09-p29631122	0.995024876	0.995024876	Bn-N9-p32483936	Bn-chrA09-p27449815
Bn-A09-p29676939	0.976744186	0.976744186	Bn-N9-p32522340	Bn-chrAnn_random-p408595
Bn-A09-p29699253	0.983388704	0.983388704	Bn-N9-p32550436	Bn-chrA09-p27524302
Bn-A09-p29747854	0.996677741	0.996677741	Bn-N9-p32596386	Bn-chrA09-p27569181
Bn-A09-p29756068	0.966942149	0.760330579	Bn-N9-p32604634	Bn-chrA09-p27577699
Bn-A09-p29756845	0.966942149	0.975206612	Bn-N9-p32605456	Bn-chrA09-p27578175
Bn-A09-p29763444	0.990049751	0.985074627	Bn-N9-p32612149	Bn-chrC04-p27902063
Bn-A09-p29785600	1	0.989583333	Bn-N9-p32641466	Bn-chrA09-p27620549
Bn-A09-p29797321	0.542288557	0.497512438	Bn-N18-p14431261	Bn-chrC05-p30714464
Bn-A09-p29859019	0.996677741	0.996677741	Bn-N9-p32696994	Bn-chrA09-p27656205
Bn-A09-p29882032	0.989583333	0.989583333	Bn-N9-p32722574	Bn-chrA09-p27637683
Bn-A09-p29890020	0.995024876	0.995024876	Bn-N9-p32746935	Bn-chrA09_random-p326189
Bn-A09-p29944855	0.973421927	0.973421927	Bn-N9-p32794553	Bn-chrA09-p27756531
Bn-A09-p29951785	0.995024876	0.995024876	Bn-N18-p35851104	Bn-chrC08-p30727434
Bn-A09-p29953651	0.963636364	0.963636364	Bn-N9-p32804994	Bn-chrA09-p27766971

Bn-A09-p29968210	0.991735537	0.991735537	Bn-N9-p32817223	Bn-chrA09-p27779056
Bn-A09-p29968302	1	1	Bn-N9-p32817315	Bn-chrA09-p27779148
Bn-A09-p29990966	0.970149254	0.970149254	Bn-N9-p32847349	Bn-chrA09-p27815143
Bn-A09-p29991084	0.975124378	0.975124378	Bn-N9-p32847467	Bn-chrA09-p27815261
Bn-A09-p29991443	0.925925926	0.925925926	Unknown	Unknown
Bn-A09-p30002747	0.965174129	0.965174129	Bn-N9-p32857331	Bn-chrA09-p27828064
Bn-A09-p30007298	0.980099502	0.980099502	Bn-N9-p32861877	Bn-chrC08_random-p390805
Bn-A09-p30010889	0.993355482	0.993355482	Bn-N9-p32865468	Bn-chrA09-p27836192
Bn-A09-p30016810	0.388059701	0.980099502	Bn-N15-p26658362	Bn-chrA04-p14148774
Bn-A09-p30032125	0.986710963	0.986710963	Bn-N9-p32881718	Bn-chrA09-p27850169
Bn-A09-p30058110	1	0.991735537	Bn-N9-p32904919	Bn-chrA09-p27873120
Bn-A09-p30058247	0.975124378	0.975124378	Bn-N9-p32905058	Bn-chrA09-p27873259
Bn-A09-p30073403	0.975206612	0.975206612	Bn-N9-p32921836	Bn-chrA09-p27887992
Bn-A09-p30075198	1	0.991735537	Bn-N9-p32923631	Bn-chrA09-p27889785
Bn-A09-p30102296	0.986710963	0.986710963	Bn-N9-p32949596	Bn-chrA09-p27916700
Bn-A09-p30105255	0.983471074	0.983471074	Bn-N9-p32952552	Bn-chrA09-p27920128
Bn-A09-p30105467	0.966942149	0.966942149	Bn-N9-p32952992	Bn-chrA09-p27920568
Bn-A09-p30108245	0.983388704	0.983388704	Bn-N9-p32955823	Bn-chrA09-p27923669
Bn-A09-p30108430	0.975206612	0.975206612	Bn-N9-p32956008	Bn-chrA09-p27923854
Bn-A09-p30112316	0.991735537	0.991735537	Bn-N9-p32962302	Bn-chrA09-p27929459
Bn-A09-p30115693	0.958677686	0.958677686	Bn-N9-p32965678	Bn-chrA09-p27932908
Bn-A09-p30116087	0.876033058	0.876033058	Bn-N9-p32966071	Bn-chrA09-p27933300
Bn-A09-p30117412	0.975206612	0.983471074	Bn-N9-p32969627	Bn-chrA09-p27935312
Bn-A09-p30117469	0.975206612	0.983471074	Bn-N9-p32969683	Bn-chrA09-p27935369
Bn-A09-p30119978	0.975206612	0.966942149	Bn-N9-p32972182	Bn-chrA09-p27937960
Bn-A09-p30121269	0.947089947	0.947089947	Bn-N9-p32972363	Bn-chrA09-p27938141
Bn-A09-p30129045	0.975206612	0.983471074	Bn-N9-p32976209	Bn-chrA09-p27941839
Bn-A09-p30141773	0.966942149	0.909090909	Bn-N9-p32984137	Bn-chrC08-p30933363
Bn-A09-p30145025	0.975206612	0.966942149	Bn-N9-p32987165	Bn-chrA09-p27948009
Bn-A09-p30147263	0.917355372	0.909090909	Bn-N9-p32989401	Bn-chrA09-p27950254
Bn-A09-p30171993	0.991735537	1	Bn-N9-p33029660	Bn-chrA09_random-p329727
Bn-A09-p30191789	1	0.991735537	Bn-N9-p33048056	Bn-chrA09_random-p327875
Bn-A09-p30206959	0.983471074	0.991735537	Bn-N9-p33063221	Bn-chrA09_random-p326306
Bn-A09-p30233844	0.991735537	0.983471074	Bn-N9-p33089970	Bn-chrA09-p28002503
Bn-A09-p30257404	0.991735537	0.983471074	Bn-N9-p33114966	Bn-chrA09-p28026215
Bn-A09-p30260475	0.975206612	0.966942149	Bn-N9-p33118038	Bn-chrA09-p28037461
Bn-A09-p30260757	0.985074627	0.970149254	Bn-N9-p33118320	Bn-chrA09-p28037743
Bn-A09-p30264461	0.991735537	0.727272727	Bn-N9-p33122024	Bn-chrC08-p31165524
Bn-A09-p30264538	1	0.983471074	Bn-N9-p33122101	Bn-chrA09-p28041400
Bn-A09-p30265013	0.983471074	0.991735537	Bn-N9-p33122578	Bn-chrA09-p28041878
Bn-A09-p30269740	0.975206612	0.983471074	Bn-N9-p33127388	Bn-chrA09-p28047798
Bn-A09-p30270747	0.991735537	1	Bn-N9-p33131332	Bn-chrA09-p28049361
Bn-A09-p30278622	0.975206612	0.958677686	Bn-N9-p33139365	Bn-chrA09-p28055705
Bn-A09-p30280052	0.983471074	0.975206612	Bn-N9-p33140796	Bn-chrA09-p28059070
Bn-A09-p30280973	0.991735537	0.983471074	Bn-N9-p33141717	Bn-chrA09-p28059989
Bn-A09-p30282007	0.993355482	0.996677741	Bn-N9-p33142751	Bn-chrA09-p28061015

Bn-A09-p30284632	0.983471074	0.966942149	Bn-N9-p33145376	Bn-chrA09-p28063610
Bn-A09-p30284813	0.990033223	0.996677741	Bn-N9-p33145557	Bn-chrA09-p28063791
Bn-A09-p30292279	0.990033223	0.990033223	Bn-N9-p33151933	Bn-chrA09-p28070215
Bn-A09-p30329420	0.983388704	0.990033223	Bn-N9-p33199716	Bn-chrA09-p28112032
Bn-A09-p30329663	0.983471074	0.983471074	Bn-N9-p33199959	Bn-chrA09-p28112275
Bn-A09-p30339735	0.943521595	0.970099668	Bn-N9-p33210275	Bn-chrA09-p28121888
Bn-A09-p30344978	0.980066445	0.996677741	Bn-N9-p33215764	Bn-chrA09-p28127638
Bn-A09-p30348281	0.960199005	0.975124378	Bn-N9-p33219048	Bn-chrA09-p28130930
Bn-A09-p30358198	0.970149254	0.975124378	Bn-N9-p33228943	Bn-chrA09-p28142634
Bn-A09-p30371768	0.983388704	0.983388704	Bn-N9-p33245743	Bn-chrA09-p28155813
Bn-A09-p30373180	0.975206612	0.966942149	Bn-N9-p33247228	Bn-chrA09-p28157301
Bn-A09-p30387343	0.96013289	0.96013289	Bn-N6-p7674500	Bn-chrA06-p7621130
Bn-A09-p30391674	0.975206612	0.983471074	Bn-N9-p33258675	Bn-chrA09-p28174153
Bn-A09-p30395018	0.966942149	0.975206612	Bn-N9-p33261440	Bn-chrA09-p28180323
Bn-A09-p30404228	0.990033223	0.986710963	Bn-N9-p33269311	Bn-chrA09-p28188769
Bn-A09-p30406985	0.965174129	0.965174129	Bn-N9-p33272059	Bn-chrA09-p28191526
Bn-A09-p30417650	0.983471074	0.975206612	Bn-N9-p33299829	Bn-chrA09-p28221468
Bn-A09-p30422813	1	0.983471074	Bn-N9-p33300106	Bn-chrA09-p28221743
Bn-A09-p30422888	1	0.991735537	Bn-N9-p33300181	Bn-chrA09-p28221823
Bn-A09-p30424208	0.975206612	0.94214876	Bn-N9-p33301488	Bn-chrA09-p28223147
Bn-A09-p30424373	0.983471074	0.975206612	Bn-N9-p33301653	Bn-chrA09-p28223646
Bn-A09-p30431025	0.986710963	0.986710963	Bn-N9-p33307742	Bn-chrA09-p28229776
Bn-A09-p30437673	0.986710963	0.993355482	Bn-N9-p33314575	Bn-chrA09-p28236600
Bn-A09-p30439085	0.991735537	0.983471074	Bn-N9-p33316006	Bn-chrA09-p28238031
Bn-A09-p30463234	0.991735537	1	Bn-N9-p33350256	Bn-chrA09-p28257927
Bn-A09-p30464712	0.702479339	0.950413223	Bn-N18-p36549521	Bn-chrA09-p28259407
Bn-A09-p30466030	0.983471074	0.983471074	Bn-N9-p33353187	Bn-chrA09-p28260725
Bn-A09-p30488258	0.975124378	0.985074627	Bn-N18-p36861419	Bn-chrC08-p31676740
Bn-A09-p30494224	0.996677741	0.996677741	Bn-N9-p33380487	Bn-chrA09-p28296365
Bn-A09-p30495797	1	0.996677741	Bn-N9-p33382060	Bn-chrA09-p28297938
Bn-A09-p30506895	0.930232558	0.933554817	Bn-N9-p33396040	Bn-chrA09-p28310845
Bn-A09-p30507984	0.541528239	0.906976744	Bn-N9-p33397140	Bn-chrA09-p28311934
Bn-A09-p30523149	0.990033223	0.986710963	Bn-N9-p33408206	Bn-chrA09-p28326350
Bn-A09-p30535727	0.980099502	0.606965174	Bn-N9-p33418187	Bn-chrA09-p28336207
Bn-A09-p30559416	0.993355482	0.993355482	Bn-N9-p33439588	Bn-chrA09_random-p332585
Bn-A09-p30561532	0.991735537	0.991735537	Bn-N9-p33441701	Bn-chrA09_random-p335362
Bn-A09-p30570466	1	0.983471074	Bn-N9-p33448292	Bn-chrCnn_random-p423668
Bn-A09-p30584271	0.983471074	0.619834711	Bn-N9-p33458092	Bn-chrA09_random-p101557
Bn-A09-p30587291	0.785123967	0.785123967	Bn-N9-p33461107	Bn-chrCnn_random-p423834
Bn-A09-p30592138	1	1	Bn-N9-p33464335	Bn-chrA09_random-p334737
Bn-A09-p30601385	0.884297521	0.892561983	Bn-N9-p33474135	Bn-chrA09_random-p340988
Bn-A09-p30611158	0.975206612	0.975206612	Bn-N9-p33478617	Bn-chrA09_random-p340539
Bn-A09-p30611162	0.975206612	0.975206612	Bn-N9-p33478621	Bn-chrA09_random-p340539
Bn-A09-p30612667	0.857142857	0.857142857	Bn-N9-p33479503	Bn-chrA09_random-p340451
Bn-A09-p30627540	0.94214876	0.94214876	Bn-N9-p33495047	Bn-chrC08-p31545263
Bn-A09-p30651428	1	1	Bn-N9-p33518923	Bn-chrA09_random-p336805

Bn-A09-p30654305	0.975124378	0.975124378	Bn-N9-p33520847	Bn-chrA09_random-p336612
Bn-A09-p30674713	0.989583333	0.989583333	Bn-N9-p33545748	Bn-chrA09-p28423701
Bn-A09-p30678275	0.983471074	1	Bn-N9-p33549263	Bn-chrA09-p28427228
Bn-A09-p30681640	0.991735537	0.983471074	Bn-N9-p33552629	Bn-chrA09-p28430592
Bn-A09-p30687979	0.996677741	0.996677741	Bn-N9-p33567036	Bn-chrA09-p28438430
Bn-A09-p30697212	0.995024876	0.995024876	Bn-N18-p36808723	Bn-chrC08-p31625335
Bn-A09-p30707823	0.966942149	0.975206612	Bn-N9-p33586753	Bn-chrA09-p28458799
Bn-A09-p30707848	0.975206612	0.983471074	Bn-N9-p33586778	Bn-chrA09-p28458824
Bn-A09-p30709081	0.991735537	0.966942149	Bn-N9-p33588010	Bn-chrA09-p28460052
Bn-A09-p30710881	0.983471074	0.975206612	Bn-N9-p33589806	Bn-chrA09-p28461846
Bn-A09-p30711004	0.966942149	0.958677686	Bn-N9-p33589929	Bn-chrA09-p28461969
Bn-A09-p30718480	0.877076412	0.990033223	Bn-N9-p33597407	Bn-chrA09-p28469372
Bn-A09-p30724789	0.986710963	0.986710963	Bn-N9-p33603704	Bn-chrA09-p28475632
Bn-A09-p30725020	0.985074627	0.990049751	Bn-N9-p33603935	Bn-chrA09-p28475863
Bn-A09-p30729719	0.993355482	0.993355482	Bn-N9-p33609108	Bn-chrA09-p28479204
Bn-A09-p30733739	0.983388704	0.983388704	Bn-N9-p33613606	Bn-chrA09-p28483543
Bn-A09-p30740855	0.990033223	0.990033223	Bn-N9-p33620834	Bn-chrA09-p28494010
Bn-A09-p30755882	0.958677686	0.983471074	Bn-N9-p33645118	Bn-chrA09-p28514081
Bn-A09-p30763709	0.996677741	0.990033223	Bn-N2-p12964585	Bn-chrA09-p28516856
Bn-A09-p30771886	0.966942149	0.975206612	Bn-N9-p33660113	Bn-chrA09-p28528192
Bn-A09-p30780421	0.965174129	0.985074627	Bn-N9-p33672788	Bn-chrA09-p28536896
Bn-A09-p30780679	0.960199005	0.970149254	Bn-N9-p33673061	Bn-chrC08-p31717071
Bn-A09-p30780890	0.985074627	0.985074627	Bn-N9-p33673272	Bn-chrA09-p28537365
Bn-A09-p30788157	0.976744186	0.976744186	Bn-N9-p33676446	Bn-chrA09-p28540539
Bn-A09-p30800136	0.966942149	0.975206612	Bn-N9-p33685511	Bn-chrA09-p28552451
Bn-A09-p30805314	0.966942149	0.925619835	Bn-N9-p33690679	Bn-chrA09-p28557636
Bn-A09-p30805387	0.991735537	0.991735537	Bn-N9-p33690752	Bn-chrA09-p28557709
Bn-A09-p30807188	0.966942149	0.652892562	Bn-N9-p33692553	Bn-chrC08-p31741398
Bn-A09-p30830947	0.571428571	0.96345515	Bn-N9-p33732394	Bn-chrC08-p31774886
Bn-A09-p30835062	0.94214876	0.966942149	Bn-N9-p33736043	Bn-chrA09-p28582712
Bn-A09-p30846003	0.996677741	1	Bn-N9-p33751179	Bn-chrA09-p28591878
Bn-A09-p30847721	0.979166667	0.989583333	Bn-N9-p33752814	Bn-chrA09-p28593511
Bn-A09-p30856402	0.983471074	0.991735537	Bn-N9-p33765205	Bn-chrA09-p28604662
Bn-A09-p30869609	0.983388704	0.956810631	Bn-N9-p33775584	Bn-chrA09-p28614011
Bn-A09-p30876251	0.991735537	1	Bn-N9-p33782289	Bn-chrA09-p28621846
Bn-A09-p30877022	0.993355482	0.990033223	Bn-N9-p33783060	Bn-chrA09-p28622617
Bn-A09-p30877241	0.935323383	0.910447761	Bn-N9-p33783279	Bn-chrA09-p28622836
Bn-A09-p30877372	0.975124378	0.980099502	Bn-N9-p33783410	Bn-chrA09-p28622967
Bn-A09-p30887157	1	0.991735537	Bn-N9-p33788816	Bn-chrA09-p28628531
Bn-A09-p30890768	0.983471074	0.991735537	Bn-N9-p33799097	Bn-chrA09-p28632144
Bn-A09-p30892887	0.985074627	0.990049751	Bn-N9-p33801213	Bn-chrA09-p28638676
Bn-A09-p30897594	0.995024876	0.995024876	Bn-N9-p33805923	Bn-chrA09-p28643381
Bn-A09-p30900431	0.990033223	0.986710963	Bn-N9-p33810425	Bn-chrA09-p28646218
Bn-A09-p30901490	0.945273632	0.985074627	Bn-N18-p37070442	Bn-chrA09-p28647278
Bn-A09-p30908648	0.995024876	0.990049751	Bn-N9-p33818148	Bn-chrA09-p28653928
Bn-A09-p30909393	0.993355482	0.986710963	Bn-N9-p33818892	Bn-chrA09-p28655613

Bn-A09-p30917919	0.983471074	0.983471074	Bn-N9-p33824170	Bn-chrA09-p28661945
Bn-A09-p30918224	0.966942149	0.892561983	Bn-N9-p33824534	Bn-chrA09-p28662308
Bn-A09-p30921980	0.966942149	0.966942149	Bn-N9-p33826250	Bn-chrA09-p28664405
Bn-A09-p30925903	0.94214876	0.933884298	Bn-N9-p33830199	Bn-chrA09-p28668257
Bn-A09-p30943916	0.851239669	0.975206612	Bn-N9-p33850117	Bn-chrA09-p28683382
Bn-A09-p30954374	0.983388704	0.96013289	Bn-N9-p33859639	Bn-chrC08_random-p398960
Bn-A09-p30956202	0.958677686	0.966942149	Bn-N9-p33861474	Bn-chrA09-p28695684
Bn-A09-p30965242	0.983471074	0.983471074	Bn-N9-p33866174	Bn-chrA09-p28702844
Bn-A09-p30971760	0.975206612	0.958677686	Bn-N9-p33872670	Bn-chrA09-p28709319
Bn-A09-p30971818	0.966942149	0.950413223	Bn-N9-p33872728	Bn-chrA09-p28709377
Bn-A09-p31013896	0.991735537	0.991735537	Bn-N9-p33924543	Bn-chrA09_random-p342875
Bn-A09-p31036504	0.991735537	0.991735537	Bn-N9-p33947421	Bn-chrA09_random-p345404
Bn-A09-p31040590	0.958677686	0.958677686	Bn-N9-p33951505	Bn-chrA09_random-p345813
Bn-A09-p31041328	0.950413223	0.950413223	Bn-N9-p33952242	Bn-chrA09_random-p345887
Bn-A09-p31049155	0.996677741	0.996677741	Bn-N9-p33957338	Bn-chrA09_random-p346411
Bn-A09-p31050548	0.983471074	0.983471074	Bn-N9-p33958685	Bn-chrA09_random-p346546
Bn-A09-p31070303	0.975206612	0.975206612	Bn-N9-p33982194	Bn-chrA09-p28744408
Bn-A09-p31071610	0.990033223	0.990033223	Bn-N9-p33990448	Bn-chrA09-p28754004
Bn-A09-p31118981	0.958677686	0.958677686	Bn-N9-p34034550	Bn-chrA09-p28809121
Bn-A09-p31152287	0.970149254	0.970149254	Bn-N9-p34064022	Bn-chrA09-p28839399
Bn-A09-p31204477	0.970149254	0.970149254	Bn-N9-p34109805	Bn-chrAnn_random-p326726
Bn-A09-p31204602	0.955223881	0.955223881	Bn-N9-p34109930	Bn-chrAnn_random-p326725
Bn-A09-p31231616	0.975124378	0.975124378	Bn-N9-p34136236	Bn-chrA09-p28912707
Bn-A09-p31270539	0.995024876	0.995024876	Bn-N9-p34170667	Bn-chrA09-p28947134
Bn-A09-p31377029	0.991735537	0.983471074	Bn-N9-p34308381	Bn-chrA09-p29072653
Bn-A09-p31388253	0.955223881	0.965174129	Bn-N9-p34319221	Bn-chrA09-p29083330
Bn-A09-p31393136	0.966942149	0.950413223	Bn-N9-p34324408	Bn-chrA09-p29088987
Bn-A09-p31393314	0.94214876	0.950413223	Bn-N9-p34324586	Bn-chrA09-p29089176
Bn-A09-p31395071	0.991735537	0.983471074	Bn-N9-p34326342	Bn-chrA09-p29089739
Bn-A09-p31402193	1	0.996677741	Bn-N9-p34333751	Bn-chrA09-p29098459
Bn-A09-p31412669	0.92039801	0.935323383	Bn-N18-p37714208	Bn-chrCnn_random-p465210
Bn-A09-p31413712	0.975206612	0.983471074	Bn-N9-p34344730	Bn-chrA09-p29114753
Bn-A09-p31416695	0.991735537	0.975206612	Bn-N9-p34347725	Bn-chrA09-p29117734
Bn-A09-p31427478	1	0.991735537	Bn-N9-p34356769	Bn-chrA09-p29125594
Bn-A09-p31453659	0.876033058	0.867768595	Bn-N9-p34385444	Bn-chrA09-p29149932
Bn-A09-p31492693	1	0.991735537	Bn-N9-p34419806	Bn-chrA09-p29184324
Bn-A09-p31493152	0.884297521	0.950413223	Bn-N18-p37805730	Bn-chrA09-p29184783
Bn-A09-p31494058	0.991735537	0.983471074	Bn-N9-p34420779	Bn-chrA09-p29185689
Bn-A09-p31505811	0.975206612	0.966942149	Bn-N9-p34433697	Bn-chrA09-p29203529
Bn-A09-p31549008	0.991735537	0.991735537	Bn-N9-p34475965	Bn-chrA09-p29244197
Bn-A09-p31549208	1	0.975206612	Bn-N9-p34476165	Bn-chrA09-p29244378
Bn-A09-p31549492	0.975206612	0.950413223	Bn-N9-p34482324	Bn-chrA09-p29244639
Bn-A09-p31549655	1	0.710743802	Bn-N9-p34482487	Bn-chrA09-p29244802
Bn-A09-p31554401	0.950413223	0.958677686	Bn-N9-p34484216	Bn-chrA09-p29246531
Bn-A09-p31554524	0.975206612	0.983471074	Bn-N9-p34484339	Bn-chrA09-p29246654
Bn-A09-p31576965	0.980066445	0.976744186	Bn-N9-p34509014	Bn-chrA09-p29267540

Bn-A09-p31578167	0.983471074	0.975206612	Bn-N9-p34510216	Bn-chrA09-p29268741
Bn-A09-p31580606	1	0.991735537	Bn-N9-p34512655	Bn-chrA09-p29271604
Bn-A09-p31582088	0.933884298	0.991735537	Bn-N9-p34514127	Bn-chrA09-p29273078
Bn-A09-p31587667	0.986710963	0.571428571	Bn-N9-p34523424	Bn-chrC01-p805856
Bn-A09-p31597547	0.986710963	0.986710963	Bn-N9-p34531203	Bn-chrA09-p29284793
Bn-A09-p31610860	0.933884298	0.876033058	Bn-N9-p34548555	Bn-chrA09-p29298488
Bn-A09-p31611193	0.983471074	0.991735537	Bn-N9-p34548887	Bn-chrA09-p29298819
Bn-A09-p31611279	0.983471074	0.991735537	Bn-N9-p34548973	Bn-chrA09-p29298905
Bn-A09-p31611436	0.983471074	0.991735537	Bn-N9-p34549130	Bn-chrA09-p29299062
Bn-A09-p31611521	0.983471074	0.975206612	Bn-N9-p34549215	Bn-chrA09-p29299147
Bn-A09-p31611638	0.958677686	0.966942149	Bn-N9-p34549332	Bn-chrA09-p29299264
Bn-A09-p31612154	0.975206612	0.975206612	Bn-N9-p34549848	Bn-chrA09-p29299788
Bn-A09-p31613718	0.983471074	0.983471074	Bn-N9-p34552105	Bn-chrA09-p29301394
Bn-A09-p31614055	0.975206612	0.983471074	Bn-N9-p34552442	Bn-chrA09-p29301729
Bn-A09-p31614130	0.975206612	0.983471074	Bn-N9-p34552517	Bn-chrA09-p29301804
Bn-A09-p31614376	0.991735537	0.983471074	Bn-N9-p34552763	Bn-chrA09-p29302052
Bn-A09-p31615419	0.990033223	0.980066445	Bn-N9-p34553913	Bn-chrA09-p29302565
Bn-A09-p31629427	0.96345515	0.996677741	Bn-N9-p34567489	Bn-chrAnn_random-p447619
Bn-A09-p31635684	0.965174129	0.990049751	Bn-N9-p34575261	Bn-chrA09-p29326800
Bn-A09-p31651915	1	0.996677741	Bn-N9-p34590897	Bn-chrA09-p29341563
Bn-A09-p31658403	0.966942149	0.975206612	Bn-N9-p34630115	Bn-chrA09-p29345051
Bn-A09-p31660206	0.965174129	0.980099502	Bn-N9-p34632030	Bn-chrA09-p29346854
Bn-A09-p31663954	1	0.991735537	Bn-N9-p34634217	Bn-chrA09-p29350796
Bn-A09-p31678038	0.909090909	0.966942149	Bn-N9-p34647945	Bn-chrA09-p29364278
Bn-A09-p31704785	0.985074627	0.980099502	Bn-N9-p34674821	Bn-chrA09-p29394048
Bn-A09-p31706338	0.991735537	1	Bn-N9-p34676377	Bn-chrA09-p29395601
Bn-A09-p31706416	0.983471074	0.975206612	Bn-N9-p34676455	Bn-chrA09-p29395679
Bn-A09-p31706472	0.975206612	0.983471074	Bn-N9-p34676511	Bn-chrA09-p29395737
Bn-A09-p31709548	0.983471074	0.975206612	Bn-N9-p34679107	Bn-chrA09-p29400518
Bn-A09-p31716654	0.996677741	0.990033223	Bn-N9-p34692151	Bn-chrA09-p29406530
Bn-A09-p31725940	0.991735537	0.991735537	Bn-N18-p38087406	Bn-chrC08-p32793923
Bn-A09-p31726007	0.991735537	0.991735537	Bn-N18-p38087473	Bn-chrC08-p32793990
Bn-A09-p31726014	0.983471074	0.983471074	Bn-N18-p38087480	Bn-chrC08-p32793997
Bn-A09-p31730182	0.975206612	0.975206612	Bn-N9-p34704348	Bn-chrA09-p29417595
Bn-A09-p31740290	0.933884298	0.950413223	Bn-N9-p34710910	Bn-chrA09-p29426359
Bn-A09-p31741551	0.983388704	0.983388704	Bn-N9-p34712133	Bn-chrA09-p29427591
Bn-A09-p31781629	1	1	Bn-N9-p34738467	Bn-chrA09-p29453276
Bn-A09-p31792154	0.993355482	0.966777409	Bn-N9-p34750082	Bn-chrC08_random-p403504
Bn-A09-p31810193	1	1	Bn-N9-p34770315	Bn-chrA09_random-p351690
Bn-A09-p31821192	0.991735537	0.991735537	Bn-N9-p34793920	Bn-chrA09-p29503872
Bn-A09-p31870750	1	1	Bn-N9-p34840747	Bn-chrA09-p29553712
Bn-A09-p31884693	0.991735537	1	Bn-N9-p35071970	Bn-chrA09-p29782892
Bn-A09-p31888714	0.966942149	0.966942149	Bn-N9-p35075971	Bn-chrA09-p29786920
Bn-A09-p31890957	0.983471074	0.991735537	Bn-N9-p35079343	Bn-chrA09-p29790102
Bn-A09-p31891306	0.603305785	0.983471074	Bn-N13-p59578622	Bn-chrA09-p29790451
Bn-A09-p31905309	0.975206612	0.983471074	Bn-N9-p35106448	Bn-chrA09_random-p351059

Bn-A09-p31941551	0.976744186	0.973421927	Bn-N4-p19472911	Bn-chrA04-p17626033
Bn-A09-p31949837	0.990049751	0.995024876	Bn-N9-p35141266	Bn-chrA09_random-p348928
Bn-A09-p31952879	0.975206612	0.983471074	Bn-N9-p35144313	Bn-chrA09_random-p349232
Bn-A09-p31953653	0.966942149	0.975206612	Bn-N9-p35145350	Bn-chrA09_random-p349310
Bn-A09-p31957772	0.975206612	0.983471074	Bn-N9-p35149981	Bn-chrA09_random-p349720
Bn-A09-p31968693	1	0.859504132	Bn-N9-p35163703	Bn-chrC08-p33333815
Bn-A09-p31969888	0.975206612	0.94214876	Bn-N9-p35164898	Bn-chrC08-p33335419
Bn-A09-p31979689	0.834710744	0.834710744	Bn-N18-p38701686	Bn-chrC08_random-p412254
Bn-A09-p31982487	0.975206612	0.991735537	Bn-N9-p35179771	Bn-chrA09-p29882803
Bn-A09-p31987382	0.975206612	0.983471074	Bn-N9-p35185360	Bn-chrA09-p29887631
Bn-A09-p31987655	0.991735537	0.966942149	Bn-N9-p35185640	Bn-chrA09-p29887904
Bn-A09-p32025164	0.975206612	0.983471074	Bn-N9-p35228335	Bn-chrA09-p29917466
Bn-A09-p32027483	1	0.991735537	Bn-N9-p35235452	Bn-chrA09-p29919785
Bn-A09-p32066349	0.980099502	0.975124378	Bn-N9-p35273375	Bn-chrA09-p29956410
Bn-A09-p32104929	0.950248756	0.955223881	Bn-N9-p35316363	Bn-chrA09-p29995462
Bn-A09-p32104974	0.965174129	0.965174129	Bn-N9-p35316407	Bn-chrA09-p29995506
Bn-A09-p32105188	0.985074627	0.980099502	Bn-N9-p35316622	Bn-chrA09-p29995721
Bn-A09-p32161150	0.933884298	0.975206612	Bn-N9-p35370518	Bn-chrA09-p30024927
Bn-A09-p32165581	0.995024876	0.995024876	Bn-Scaffold06905-p1	Bn-chrA08-p15762659
Bn-A09-p32167322	0.975206612	0.983471074	Bn-N9-p35372167	Bn-chrA09-p30026113
Bn-A09-p32172515	1	0.995024876	Bn-N9-p35375186	Bn-chrA09-p30032192
Bn-A09-p32223480	0.991735537	0.991735537	Bn-N9-p35410636	Bn-chrA09-p30076451
Bn-A09-p32225646	0.809917355	0.983471074	Bn-N9-p35412775	Bn-chrA09-p30078611
Bn-A09-p32254190	0.966942149	0.958677686	Bn-N9-p35432640	Bn-chrA09-p30101931
Bn-A09-p32257776	1	0.991735537	Bn-N9-p35438207	Bn-chrA09-p30105516
Bn-A09-p32258181	1	0.991735537	Bn-N9-p35438612	Bn-chrA09-p30105921
Bn-A09-p32269283	0.996677741	0.996677741	Bn-N9-p35447010	Bn-chrA09-p30114935
Bn-A09-p32270596	0.996677741	0.996677741	Bn-N9-p35448377	Bn-chrA09-p30116302
Bn-A09-p32275168	0.989583333	0.989583333	Bn-N9-p35451183	Bn-chrA09-p30119108
Bn-A09-p32321534	0.966942149	0.975206612	Bn-N9-p35482679	Bn-chrA09-p30146204
Bn-A09-p32344408	0.950413223	0.950413223	Bn-N9-p35504808	Bn-chrA09-p30167232
Bn-A09-p32441297	1	1	Bn-N9-p35602251	Bn-chrA09-p30259721
Bn-A09-p32553111	0.995024876	0.995024876	Bn-N9-p35716954	Bn-chrA09-p30361588
Bn-A09-p32590303	1	1	Bn-N9-p35741674	Bn-chrA09-p30386496
Bn-A09-p32624472	0.993355482	0.993355482	Bn-N8-p5130068	Bn-chrA08-p4604055
Bn-A09-p32656218	0.950248756	0.995024876	Bn-N18-p13436786	Bn-chrAnn_random-p292999
Bn-A09-p32665710	0.983471074	0.983471074	Bn-N9-p35804452	Bn-chrA09-p30447461
Bn-A09-p32666778	1	1	Bn-N9-p35805520	Bn-chrA09-p30448529
Bn-A09-p32669666	0.983471074	0.983471074	Bn-N9-p35808409	Bn-chrA09-p30451418
Bn-A09-p32713083	0.966942149	0.975206612	Bn-N9-p35854469	Bn-chrA09-p30496141
Bn-A09-p32717048	0.67768595	0.685950413	Bn-N9-p35858419	Bn-chrA09-p30500500
Bn-A09-p32787589	0.991735537	0.983471074	Bn-N9-p35916330	Bn-chrAnn_random-p253033
Bn-A09-p32811261	0.980066445	0.983388704	Bn-N9-p35936226	Bn-chrA09_random-p358542
Bn-A09-p32822165	0.685950413	0.628099174	Bn-N9-p35948073	Bn-chrC08-p34427034
Bn-A09-p32826561	0.991735537	0.983471074	Bn-N9-p35952538	Bn-chrA09_random-p357003
Bn-A09-p32841826	0.965174129	0.965174129	Bn-N9-p35964800	Bn-chrCnn_random-p545271

Bn-A09-p32849664	0.933554817	0.996677741	Bn-N18-p39671307	Bn-chrUnn_random-p264606
Bn-A09-p32862905	0.983471074	0.975206612	Bn-N9-p35986275	Bn-chrA09-p30615218
Bn-A09-p32864411	0.986710963	0.986710963	Bn-N9-p35987797	Bn-chrA09-p30616740
Bn-A09-p32884256	0.985074627	0.985074627	Bn-N9-p36008248	Bn-chrA09-p30637434
Bn-A09-p32903518	0.996677741	0.996677741	Bn-N9-p36028024	Bn-chrA09-p30656895
Bn-A09-p32923447	0.991735537	0.991735537	Bn-N9-p36039181	Bn-chrA09-p30668240
Bn-A09-p32923478	0.983471074	0.983471074	Bn-N9-p36039212	Bn-chrA09-p30668271
Bn-A09-p32937856	0.966942149	0.958677686	Bn-N9-p36054518	Bn-chrA09-p30681505
Bn-A09-p32944206	0.950248756	0.935323383	Bn-N9-p36060868	Bn-chrA09-p30687779
Bn-A09-p32945202	0.966942149	0.958677686	Bn-N9-p36061864	Bn-chrC08-p34238965
Bn-A09-p32954471	0.950248756	0.995024876	Bn-N9-p36071116	Bn-chrA09-p30697654
Bn-A09-p32959480	0.975206612	0.983471074	Bn-N9-p36076241	Bn-chrA09-p30702732
Bn-A09-p32974956	0.994594595	0.989189189	Bn-N9-p36091644	Bn-chrA09-p30726020
Bn-A09-p32978334	0.983388704	0.983388704	Bn-N9-p36095294	Bn-chrA09-p30729395
Bn-A09-p32980100	0.826446281	0.991735537	Bn-N9-p36096719	Bn-chrA09-p30730820
Bn-A09-p32980153	0.826446281	0.975206612	Bn-N9-p36096753	Bn-chrA09-p30730873
Bn-A09-p32993230	0.96013289	0.990033223	Bn-N9-p36116723	Bn-chrA09-p30752819
Bn-A09-p32995843	0.983471074	0.975206612	Bn-N9-p36120868	Bn-chrA09-p30755422
Bn-A09-p33006858	0.993355482	0.996677741	Bn-N9-p36131162	Bn-chrA09-p30768955
Bn-A09-p33009761	0.975124378	0.975124378	Bn-N9-p36133036	Bn-chrA09-p30770843
Bn-A09-p33013128	0.976744186	0.990033223	Bn-N9-p36137660	Bn-chrA09-p30774190
Bn-A09-p33015862	0.980099502	1	Bn-N5-p25188826	Bn-chrA07-p17505815
Bn-A09-p33025970	0.975206612	0.975206612	Bn-N9-p36147880	Bn-chrA09-p30781455
Bn-A09-p33029336	0.975206612	0.983471074	Bn-N9-p36156613	Bn-chrA09-p30784818
Bn-A09-p33037403	0.995024876	0.995024876	Bn-N9-p36166069	Bn-chrA09-p30792887
Bn-A09-p33040441	0.993355482	0.996677741	Bn-N9-p36169883	Bn-chrA09-p30795923
Bn-A09-p33058738	0.900332226	1	Bn-N9-p36175971	Bn-chrA09-p30810487
Bn-A09-p33061472	0.996677741	0.996677741	Bn-N9-p36178644	Bn-chrA09-p30813160
Bn-A09-p33073037	0.991735537	0.991735537	Bn-N9-p36190273	Bn-chrA09-p30824789
Bn-A09-p33073469	0.993355482	0.993355482	Bn-N9-p36190705	Bn-chrA09-p30825221
Bn-A09-p33100320	0.990049751	0.990049751	Bn-N9-p36219008	Bn-chrAnn_random-p200704
Bn-A09-p33105121	0.990049751	0.990049751	Bn-N9-p36229150	Bn-chrAnn_random-p200827
Bn-A09-p33105204	0.985074627	0.985074627	Bn-N9-p36229233	Bn-chrAnn_random-p200828
Bn-A09-p33179406	0.966942149	0.966942149	Bn-N9-p36291870	Bn-chrA09-p30892974
Bn-A09-p33193573	0.991735537	0.991735537	Bn-N9-p36308603	Bn-chrA09-p30909707
Bn-A09-p33256856	0.993355482	0.993355482	Bn-N9-p36353297	Bn-chrA09-p30953917
Bn-A09-p33271433	1	0.564784053	Bn-N9-p36365521	Bn-chrAnn_random-p137408
Bn-A09-p33278240	0.897009967	0.877076412	Bn-N9-p36372253	Bn-chrAnn_random-p140250
Bn-A09-p33344815	0.966942149	0.975206612	Bn-N9-p36426233	Bn-chrA09_random-p364169
Bn-A09-p33347853	0.983471074	0.983471074	Bn-N9-p36429268	Bn-chrA09_random-p364474
Bn-A09-p33347915	0.991735537	0.983471074	Bn-N9-p36429330	Bn-chrA09_random-p364480
Bn-A09-p33348200	0.983471074	0.975206612	Bn-N9-p36429642	Bn-chrA09_random-p364514
Bn-A09-p33348598	0.966942149	0.975206612	Bn-N9-p36430038	Bn-chrA09_random-p364554
Bn-A09-p33349373	0.983471074	0.991735537	Bn-N9-p36432086	Bn-chrA09_random-p364631
Bn-A09-p33349948	0.94214876	0.950413223	Bn-N9-p36432665	Bn-chrA09_random-p364689
Bn-A09-p33358168	0.905472637	0.975124378	Bn-N9-p36445006	Bn-chrA09_random-p365574

Bn-A09-p33358298	0.945273632	0.955223881	Bn-N9-p36445136	Bn-chrA09_random-p365587
Bn-A09-p33358356	0.935323383	0.955223881	Bn-N9-p36445193	Bn-chrA09_random-p365593
Bn-A09-p33369348	0.975206612	0.958677686	Bn-N9-p36456929	Bn-chrA09_random-p366797
Bn-A09-p33372131	0.990049751	0.985074627	Bn-N9-p36462585	Bn-chrA09_random-p367225
Bn-A09-p33372204	0.980099502	0.985074627	Bn-N9-p36462658	Bn-chrA09_random-p367232
Bn-A09-p33372571	0.990033223	0.983388704	Bn-N9-p36463025	Bn-chrA09_random-p367269
Bn-A09-p33372831	0.966942149	0.958677686	Bn-N9-p36463525	Bn-chrA09_random-p367361
Bn-A09-p33372891	0.966942149	0.975206612	Bn-N9-p36463585	Bn-chrA09_random-p367367
Bn-A09-p33372925	0.955223881	0.960199005	Bn-N9-p36463619	Bn-chrA09_random-p367371
Bn-A09-p33374339	0.990049751	0.990049751	Bn-N9-p36464997	Bn-chrA09_random-p367520
Bn-A09-p33382398	0.975206612	0.966942149	Bn-N9-p36472028	Bn-chrA09_random-p368019
Bn-A09-p33391361	1	0.991735537	Bn-N9-p36477539	Bn-chrA09_random-p368569
Bn-A09-p33398481	0.993355482	0.993355482	Bn-N9-p36484837	Bn-chrA09_random-p369611
Bn-A09-p33399849	0.983471074	0.975206612	Bn-N9-p36486132	Bn-chrA09_random-p369741
Bn-A09-p33400463	0.970149254	0.995024876	Bn-N9-p36486746	Bn-chrA09_random-p369803
Bn-A09-p33400597	0.958677686	0.950413223	Bn-N9-p36486878	Bn-chrA09_random-p369816
Bn-A09-p33406111	0.975206612	0.983471074	Bn-N9-p36491914	Bn-chrA09_random-p370372
Bn-A09-p33406350	0.990049751	0.985074627	Bn-N9-p36492153	Bn-chrA09_random-p370396
Bn-A09-p33406899	1	0.995024876	Bn-N9-p36492702	Bn-chrA09_random-p370451
Bn-A09-p33415187	0.983471074	0.975206612	Bn-N9-p36501303	Bn-chrA09_random-p370948
Bn-A09-p33419515	0.983471074	0.975206612	Bn-N9-p36505650	Bn-chrA09_random-p371380
Bn-A09-p33427256	0.966942149	0.975206612	Bn-N9-p36523886	Bn-chrA09_random-p372151
Bn-A09-p33428597	0.966942149	0.966942149	Bn-N9-p36525236	Bn-chrA09_random-p372285
Bn-A09-p33431499	0.925619835	0.933884298	Bn-N9-p36528797	Bn-chrA09_random-p372578
Bn-A09-p33434100	0.975124378	0.980099502	Bn-N9-p36531397	Bn-chrA09_random-p372838
Bn-A09-p33434187	0.975124378	0.980099502	Bn-N9-p36531483	Bn-chrA09_random-p372846
Bn-A09-p33450690	0.94214876	0.950413223	Bn-N9-p36559690	Bn-chrA09_random-p374549
Bn-A09-p33456655	0.993355482	0.996677741	Bn-N9-p36565327	Bn-chrA09_random-p375140
Bn-A09-p33458611	0.983471074	0.983471074	Bn-N9-p36567352	Bn-chrA09_random-p375351
Bn-A09-p33459299	0.966942149	0.958677686	Bn-N9-p36568041	Bn-chrA09_random-p375419
Bn-A09-p33464355	0.975206612	0.983471074	Bn-N9-p36573079	Bn-chrA09_random-p375904
Bn-A09-p33473254	0.975206612	0.966942149	Bn-N9-p36582096	Bn-chrA09_random-p376613
Bn-A09-p33475518	0.983471074	0.975206612	Bn-N9-p36583438	Bn-chrA09_random-p376810
Bn-A09-p33485784	0.958677686	0.950413223	Bn-N9-p36584923	Bn-chrA09_random-p376963
Bn-A09-p33487356	1	0.991735537	Bn-N9-p36590161	Bn-chrA09_random-p377120
Bn-A09-p33491501	0.996677741	0.996677741	Bn-N9-p36594305	Bn-chrA09_random-p377534
Bn-A09-p33496868	0.975206612	0.983471074	Bn-N9-p36599650	Bn-chrA09_random-p378071
Bn-A09-p33497174	0.991735537	0.983471074	Bn-N9-p36599957	Bn-chrA09_random-p378102
Bn-A09-p33499505	1	0.991735537	Bn-N9-p36603036	Bn-chrA09_random-p378335
Bn-A09-p33499917	0.991735537	0.983471074	Bn-N9-p36603451	Bn-chrA09_random-p378376
Bn-A09-p33500473	0.983471074	0.256198347	Bn-N9-p36604316	Bn-chrA06-p23274740
Bn-A09-p33512900	0.985074627	0.990049751	Bn-N9-p36606030	Bn-chrA09_random-p385791
Bn-A09-p33518379	0.94214876	0.975206612	Bn-N18-p40423128	Bn-chrA09_random-p385243
Bn-A09-p33521094	0.983471074	0.991735537	Bn-N9-p36614056	Bn-chrA09_random-p384971
Bn-A09-p33535361	0.995024876	1	Bn-N9-p36626698	Bn-chrA09_random-p383574
Bn-A09-p33539118	0.950413223	0.966942149	Bn-N9-p36630445	Bn-chrA09_random-p383199

Bn-A09-p33542334	0.983471074	0.975206612	Bn-N9-p36634833	Bn-chrA09_random-p382878
Bn-A09-p33544191	0.990049751	0.990049751	Bn-N9-p36638933	Bn-chrA09_random-p382687
Bn-A09-p33568547	0.983471074	0.966942149	Bn-N9-p36658566	Bn-chrC08-p34886654
Bn-A09-p33574107	0.991735537	1	Bn-N9-p36663023	Bn-chrA09_random-p379527
Bn-A09-p33582715	0.991735537	0.983471074	Bn-N9-p36673067	Bn-chrA09_random-p378667
Bn-A09-p33587432	0.983471074	0.991735537	Bn-N9-p36677429	Bn-chrA09_random-p404416
Bn-A09-p33595011	0.990033223	0.983388704	Bn-N9-p36690132	Bn-chrA09_random-p391715
Bn-A09-p33595174	0.958677686	0.950413223	Bn-N9-p36690293	Bn-chrA09_random-p391699
Bn-A09-p33641390	0.976744186	0.976744186	Bn-N9-p36740648	Bn-chrA09_random-p386620
Bn-A09-p33650317	0.990049751	0.990049751	Bn-N9-p36752556	Bn-chrAnn_random-p426557
Bn-A09-p33660289	1	1	Bn-N9-p36764678	Bn-chrAnn_random-p272651
Bn-A09-p33694154	0.990049751	0.990049751	Bn-N9-p36805010	Bn-chrA09-p30983145
Bn-A09-p33694194	0.990049751	0.990049751	Bn-N9-p36805050	Bn-chrA09-p30983185
Bn-A09-p33706030	0.965174129	0.965174129	Bn-N9-p36808386	Bn-chrA09-p30986521
Bn-A09-p33716233	0.975206612	0.958677686	Bn-N9-p36819201	Bn-chrC08-p35005324
Bn-A09-p33722405	0.983471074	0.975206612	Bn-N9-p36828993	Bn-chrC08-p35012244
Bn-A09-p33723162	1	0.818181818	Bn-N9-p36829751	Bn-chrC08-p35013454
Bn-A09-p33723456	0.991735537	0.983471074	Bn-N9-p36830045	Bn-chrC08-p35013714
Bn-A09-p33724136	0.975206612	0.975206612	Bn-N9-p36830723	Bn-chrC08-p35015346
Bn-A09-p33724178	0.983471074	0.975206612	Bn-N9-p36830765	Bn-chrC08-p35015388
Bn-A09-p33726811	0.958677686	0.966942149	Bn-N9-p36832994	Bn-chrC08-p35017630
Bn-A09-p33730723	0.991735537	1	Bn-N9-p36835540	Bn-chrC08-p35020346
Bn-A09-p33731681	0.950413223	0.958677686	Bn-N9-p36836802	Bn-chrC08-p35021305
Bn-A09-p33744089	0.990033223	0.990033223	Bn-N9-p36849654	Bn-chrA09-p31020404
Bn-A09-p33777546	0.975206612	0.925619835	Bn-N9-p36868438	Bn-chrA09-p31044722
Bn-A09-p33777987	1	0.991735537	Bn-N9-p36868879	Bn-chrA09-p31045661
Bn-A09-p33783680	1	0.991735537	Bn-N9-p36871178	Bn-chrA09-p31047950
Bn-A09-p33792068	0.991735537	0.983471074	Bn-N9-p36881124	Bn-chrA09-p31055253
Bn-A09-p33803933	0.983471074	0.991735537	Bn-N9-p36888071	Bn-chrA09-p31060860
Bn-A09-p33815163	0.983471074	0.991735537	Bn-N9-p36900705	Bn-chrA09-p31074689
Bn-A09-p33817337	1	0.991735537	Bn-N9-p36905931	Bn-chrA09-p31076849
Bn-A09-p33821269	0.975206612	0.966942149	Bn-N9-p36908320	Bn-chrA09-p31082881
Bn-A09-p33822952	1	0.991735537	Bn-N9-p36909912	Bn-chrA09-p31084553
Bn-A09-p33830942	0.983471074	0.636363636	Bn-N9-p36917760	Bn-chrC08-p35127439
Bn-A09-p33841506	0.975206612	0.966942149	Bn-N9-p36936101	Bn-chrA09_random-p395154
Bn-A09-p33853347	0.975206612	0.983471074	Bn-N9-p36947964	Bn-chrA09_random-p393836
Bn-A09-p33853604	0.966942149	0.975206612	Bn-N9-p36948221	Bn-chrA09_random-p393811
Bn-A09-p33855795	0.980099502	0.880597015	Bn-N9-p36951692	Bn-chrA09_random-p393578
Bn-A09-p33867909	0.991735537	0.983471074	Bn-N9-p36962068	Bn-chrA09_random-p396993
Bn-A09-p33875531	0.975206612	0.966942149	Bn-N9-p36971733	Bn-chrA09_random-p397751
Bn-A09-p33875784	0.985074627	0.955223881	Bn-N9-p36971983	Bn-chrA09_random-p397776
Bn-A09-p33892344	0.991735537	0.983471074	Bn-N9-p36996507	Bn-chrA09_random-p400244
Bn-A09-p33894393	0.960199005	0.955223881	Bn-N18-p40907881	Bn-chrC08-p35217854
Bn-A09-p33915936	0.966942149	0.958677686	Bn-N9-p37023133	Bn-chrA09_random-p403261
Bn-A09-p33925635	0.983471074	0.975206612	Bn-N9-p37045447	Bn-chrA09-p31175871
Bn-A09-p33926185	0.983471074	0.991735537	Bn-N9-p37045997	Bn-chrA09-p31176420

Bn-A09-p33927099	0.991735537	0.983471074	Bn-N9-p37046903	Bn-chrA09-p31177335
Bn-A09-p33942716	0.991735537	0.983471074	Bn-N9-p37062740	Bn-chrA09-p31193171
Bn-A09-p33948825	0.991735537	1	Bn-N9-p37068849	Bn-chrC08-p35305919
Bn-A09-p33971771	0.983471074	0.991735537	Bn-N9-p37090730	Bn-chrA09-p31205667
Bn-A09-p33972258	0.983471074	0.842975207	Bn-N9-p37091217	Bn-chrA09-p31206155
Bn-A09-p33973275	0.991735537	0.983471074	Bn-N9-p37092568	Bn-chrA09-p31207202
Bn-A09-p33975396	0.966942149	0.975206612	Bn-N9-p37095133	Bn-chrA09-p31209285
Bn-A09-p33975959	0.983471074	0.958677686	Bn-N9-p37098326	Bn-chrA09-p31209844
Bn-A09-p33976066	0.991735537	1	Bn-N9-p37098433	Bn-chrA09-p31209952
Bn-A09-p33976347	0.975206612	0.966942149	Bn-N9-p37098714	Bn-chrA09-p31210233
Bn-A09-p33976464	0.958677686	0.966942149	Bn-N9-p37098831	Bn-chrA09-p31210585
Bn-A09-p33976515	0.966942149	0.975206612	Bn-N9-p37098882	Bn-chrA09-p31210636
Bn-A09-p33981445	0.933884298	0.94214876	Bn-N9-p37102108	Bn-chrA09-p31213065
Bn-A09-p33983999	0.973421927	0.983388704	Bn-N18-p41042789	Bn-chrA09-p31215545
Bn-A09-p34005292	0.94214876	0.983471074	Bn-Scaffold04485-p1	Bn-chrA09-p31245032
Bn-A09-p34018790	0.983471074	0.966942149	Bn-N9-p37138842	Bn-chrA09-p31256312
Bn-A09-p34022183	0.970149254	0.965174129	Bn-N9-p37142220	Bn-chrA09-p31262998
Bn-A09-p34028225	0.991735537	0.975206612	Bn-N9-p37148234	Bn-chrAnn_random-p426041
Bn-A09-p34029934	0.950413223	0.958677686	Bn-N9-p37149943	Bn-chrAnn_random-p426058
Bn-A09-p34037290	0.900826446	0.909090909	Bn-N9-p37157379	Bn-chrA09-p31289575
Bn-A09-p34039082	0.958677686	0.925619835	Bn-N9-p37159171	Bn-chrA09-p31291367
Bn-A09-p34044914	0.96345515	0.986710963	Bn-N9-p37165019	Bn-chrA09-p31297241
Bn-A09-p34045999	0.975206612	0.917355372	Bn-N9-p37166104	Bn-chrA09-p31298321
Bn-A09-p34046789	0.991735537	0.983471074	Bn-N9-p37166894	Bn-chrA09-p31299111
Bn-A09-p34053851	1	1	Bn-N9-p37173971	Bn-chrA09-p31306339
Bn-A09-p34058517	0.966942149	0.966942149	Bn-N9-p37180008	Bn-chrA09-p31311059
Bn-A09-p34063724	0.966942149	0.958677686	Bn-N9-p37185217	Bn-chrA09-p31316772
Bn-A09-p34067269	1	0.991735537	Bn-N9-p37188753	Bn-chrA09-p31320312
Bn-A09-p34247590	0.917355372	0.809917355	Bn-N18-p41334426	Bn-chrC08-p35303459
Bn-A09-p34308105	1	1	Bn-N9-p37439196	Bn-chrA09-p31557576
Bn-A09-p34331338	0.983471074	0.983471074	Bn-N18-p41429158	Bn-chrC08-p35709662
Bn-A09-p34373261	0.989583333	0.989583333	Bn-N9-p37494557	Bn-chrA09-p31612581
Bn-A09-p34390255	0.995024876	0.995024876	Bn-N9-p37513186	Bn-chrA09-p31629368
Bn-A09-p34393068	0.993355482	0.993355482	Bn-N9-p37516308	Bn-chrA09-p31632488
Bn-A09-p34437367	0.995024876	0.995024876	Bn-N9-p37559980	Bn-chrC08-p35852166
Bn-A09-p34469671	0.989583333	0.989583333	Bn-N9-p37594587	Bn-chrA09-p31707835
Bn-A09-p34477765	0.975206612	1	Bn-N18-p41616716	Bn-chrA09-p31716073
Bn-A09-p34482075	0.970149254	0.970149254	Bn-N9-p37607705	Bn-chrC08-p35917738
Bn-A09-p34591600	0.965174129	0.970149254	Bn-N18-p41737490	Bn-chrC08-p36034672
Bn-A09-p34779068	0.980099502	0.980099502	Bn-N9-p37895290	Bn-chrA09-p32025074
Bn-A09-p34794738	0.996677741	0.996677741	Bn-N9-p37921048	Bn-chrA09-p32045531
Bn-A09-p34995048	0.986710963	0.986710963	Bn-N9-p38145589	Bn-chrA09-p32205577
Bn-A09-p34995922	0.991735537	0.991735537	Bn-N9-p38146463	Bn-chrA09-p32206451
Bn-A09-p35162641	0.966942149	0.867768595	Bn-N9-p38325101	Bn-chrA09-p32375998
Bn-A09-p35221507	0.991735537	0.991735537	Bn-N9-p38388156	Bn-chrA09_random-p407590
Bn-A09-p35228442	0.990033223	0.986710963	Bn-N9-p38395580	Bn-chrA09_random-p406773

Bn-A09-p35262679	0.980066445	0.980066445	Bn-N9-p38427349	Bn-chrA09-p32395536
Bn-A09-p35278018	0.946843854	1	Bn-N18-p42699168	Bn-chrA09-p32408555
Bn-A09-p35279845	1	1	Bn-N9-p38443531	Bn-chrA09-p32410398
Bn-A09-p35292945	0.983388704	0.976744186	Bn-N9-p38458444	Bn-chrA09-p32424986
Bn-A09-p35299111	0.983388704	0.980066445	Bn-N9-p38466601	Bn-chrA09-p32431188
Bn-A09-p35303838	0.960199005	0.960199005	Bn-N9-p38476156	Bn-chrA09-p32435926
Bn-A09-p35306248	0.960199005	0.955223881	Bn-N9-p38478570	Bn-chrA09-p32438352
Bn-A09-p35321301	0.943521595	0.936877076	Bn-N9-p38495271	Bn-chrA09-p32456183
Bn-A09-p35368467	0.990033223	0.986710963	Bn-N9-p38544569	Bn-chrA09-p32514369
Bn-A09-p35372226	0.943521595	0.983388704	Bn-N9-p38548418	Bn-chrA09-p32518453
Bn-A09-p35389045	0.936877076	0.996677741	Bn-N9-p38571470	Bn-chrA09-p32536940
Bn-A09-p35404670	0.983388704	0.983388704	Bn-N9-p38588486	Bn-chrA09-p32550722
Bn-A09-p35425963	0.990033223	0.990033223	Bn-N9-p38607905	Bn-chrA09-p32570440
Bn-A09-p35426888	1	1	Bn-N9-p38608830	Bn-chrA09-p32571365
Bn-A09-p35437924	0.996677741	0.996677741	Bn-N9-p38619816	Bn-chrA09-p32582352
Bn-A09-p35466517	0.930232558	0.930232558	Bn-N9-p38651614	Bn-chrA09-p32608739
Bn-A09-p35472661	0.983471074	0.983471074	Bn-N9-p38658292	Bn-chrA09-p32615418
Bn-A09-p35476619	0.948387097	0.948387097	Bn-N9-p38662262	Bn-chrA09-p32619387
Bn-A09-p35477936	0.980099502	0.980099502	Bn-N9-p38663579	Bn-chrA09-p32620704
Bn-A09-p35479723	0.993355482	0.993355482	Bn-N9-p38665367	Bn-chrA09-p32622492
Bn-A09-p35480425	0.975206612	0.975206612	Bn-N9-p38666096	Bn-chrA09-p32623221
Bn-A09-p35480484	0.975206612	0.975206612	Bn-N9-p38666155	Bn-chrA09-p32623280
Bn-A09-p35481880	0.980099502	0.980099502	Bn-N9-p38667551	Bn-chrA09-p32624676
Bn-A09-p35483201	1	1	Bn-N9-p38668868	Bn-chrA09-p32625993
Bn-A09-p35484843	0.955223881	0.955223881	Bn-N9-p38670515	Bn-chrA09-p32627640
Bn-A09-p35485049	0.985074627	0.985074627	Bn-N9-p38670721	Bn-chrA09-p32627846
Bn-A09-p35485458	0.930348259	0.930348259	Bn-N9-p38671130	Bn-chrA09-p32628255
Bn-A09-p35489554	0.975206612	0.975206612	Bn-N9-p38675403	Bn-chrAnn_random-p304108
Bn-A09-p35492133	0.989583333	0.822916667	Bn-N9-p38677942	Bn-chrAnn_random-p304082
Bn-A09-p35498001	0.996677741	0.996677741	Bn-N9-p38682231	Bn-chrA09-p32642973
Bn-A09-p35505057	0.983471074	0.983471074	Bn-N9-p38689050	Bn-chrA09-p32651645
Bn-A09-p35505120	0.950413223	0.950413223	Bn-N9-p38689113	Bn-chrA09-p32651708
Bn-A09-p35508720	0.966942149	0.966942149	Bn-N9-p38692821	Bn-chrA09-p32655415
Bn-A09-p35508955	0.975206612	0.975206612	Bn-N9-p38693055	Bn-chrA09-p32655649
Bn-A09-p35509044	0.986710963	0.986710963	Bn-N9-p38693144	Bn-chrA09-p32655738
Bn-A09-p35510262	0.825870647	0.825870647	Bn-N9-p38694350	Bn-chrA09-p32656944
Bn-A09-p35510324	0.900497512	0.900497512	Bn-N9-p38694412	Bn-chrA09-p32657006
Bn-A09-p35512602	0.966777409	0.817275748	Bn-Scaffold04901-p9	Bn-chrC03-p9738085
Bn-A09-p35519543	0.986710963	0.986710963	Bn-N9-p38697778	Bn-chrA09-p32660234
Bn-A09-p35520884	0.975206612	0.975206612	Bn-N9-p38699119	Bn-chrA09-p32661575
Bn-A09-p35520944	0.986710963	0.986710963	Bn-N9-p38699179	Bn-chrA09-p32661635
Bn-A09-p35529865	0.945273632	0.945273632	Bn-N9-p38708446	Bn-chrA09-p32670736
Bn-A09-p35531421	0.965174129	0.965174129	Bn-N9-p38710002	Bn-chrA09-p32672292
Bn-A09-p35534652	0.983388704	0.983388704	Bn-N9-p38713689	Bn-chrA09_random-p413139
Bn-A09-p35559972	0.975206612	0.975206612	Bn-N9-p38738924	Bn-chrAnn_random-p486509
Bn-A09-p35563022	0.976744186	0.976744186	Bn-N9-p38745937	Bn-chrA09-p32699117

Bn-A09-p35571119	0.995024876	0.995024876	Bn-N9-p38754020	Bn-chrA09-p32707199
Bn-A09-p35594338	0.950413223	0.950413223	Bn-N9-p38771901	Bn-chrA09-p32724480
Bn-A09-p35598320	0.834710744	0.834710744	Bn-N18-p43084500	Bn-chrC08-p37129790
Bn-A09-p35604358	0.983471074	0.983471074	Bn-N9-p38781367	Bn-chrA09-p32733945
Bn-A09-p35609789	0.970149254	0.970149254	Bn-N9-p38788175	Bn-chrA09-p32738600
Bn-A09-p35611451	0.996677741	0.996677741	Bn-N9-p38789837	Bn-chrA09_random-p405456
Bn-A09-p35624679	0.925619835	0.925619835	Bn-N9-p38804159	Bn-chrA09-p32761825
Bn-A09-p35624767	0.991735537	0.991735537	Bn-N9-p38804252	Bn-chrA09-p32761918
Bn-A09-p35647477	0.993355482	0.993355482	Bn-N9-p38824701	Bn-chrA09-p32778940
Bn-A09-p35656352	0.993355482	0.993355482	Bn-N9-p38833762	Bn-chrA09-p32788001
Bn-A09-p35661872	0.966777409	0.976744186	Bn-N9-p38840970	Bn-chrA09-p32793509
Bn-A09-p35672030	0.940298507	0.950248756	Bn-Scaffold01321-p9	Bn-chrAnn_random-p312774
Bn-A09-p35681738	0.958677686	0.966942149	Bn-N9-p38854907	Bn-chrA09-p32820066
Bn-A09-p35691478	0.94214876	0.950413223	Bn-N9-p38864737	Bn-chrA09-p32829725
Bn-A09-p35703402	0.986710963	0.986710963	Bn-N9-p38874891	Bn-chrA09-p32840874
Bn-A09-p35707505	0.479338843	0.991735537	Bn-N18-p43252029	Bn-chrA09-p32845031
Bn-A09-p35724973	0.958677686	0.958677686	Bn-N9-p38894139	Bn-chrA09-p32874049
Bn-A09-p35728172	0.991735537	0.991735537	Bn-N9-p38897339	Bn-chrA09-p32877249
Bn-A09-p35753486	0.991735537	0.991735537	Bn-N9-p38920621	Bn-chrA09-p32900829
Bn-A09-p35790555	0.996677741	0.996677741	Bn-N9-p38950008	Bn-chrA09-p32932211
Bn-A09-p35797282	0.990033223	0.990033223	Bn-N9-p38956667	Bn-chrA09-p32938870
Bn-A09-p35797678	0.983388704	0.983388704	Bn-N9-p38957063	Bn-chrA09-p32939266
Bn-A09-p35848630	0.985074627	0.985074627	Bn-N18-p43381931	Bn-chrC08-p37976149
Bn-A09-p35866195	0.995024876	0.995024876	Bn-N18-p43399598	Bn-chrC08-p37959234
Bn-A09-p35867904	0.900497512	0.940298507	Bn-N18-p43401280	Bn-chrAnn_random-p167344
Bn-A09-p35872518	0.975206612	0.975206612	Bn-N9-p39014446	Bn-chrAnn_random-p167293
Bn-A09-p35872842	0.958677686	0.958677686	Bn-N9-p39014767	Bn-chrAnn_random-p167290
Bn-A09-p35872885	0.991735537	0.991735537	Bn-N9-p39014810	Bn-chrAnn_random-p167290
Bn-A09-p35879759	1	0.991735537	Bn-N9-p39021453	Bn-chrAnn_random-p167226
Bn-A09-p35890304	0.983471074	0.975206612	Bn-N9-p39030093	Bn-chrA09-p32971629
Bn-A09-p35901593	0.990033223	0.976744186	Bn-N9-p39043844	Bn-chrA09-p32983885
Bn-A09-p35907846	0.966942149	0.958677686	Bn-N9-p39049709	Bn-chrA09-p32989727
Bn-A09-p35908013	0.966942149	0.958677686	Bn-N9-p39049875	Bn-chrA09-p32989894
Bn-A09-p35914702	0.983388704	0.983388704	Bn-N9-p39056889	Bn-chrA09-p33029777
Bn-A09-p35935166	0.983388704	0.983388704	Bn-N9-p39076233	Bn-chrA09-p33050798
Bn-A09-p35964883	0.983388704	0.986710963	Bn-N9-p39103864	Bn-chrA09-p33088190
Bn-A09-p35979119	0.986710963	0.986710963	Bn-N9-p39118362	Bn-chrC08-p37842484
Bn-A09-p35981493	0.990049751	0.990049751	Bn-N9-p39120736	Bn-chrA09-p33104863
Bn-A09-p35981539	0.995024876	0.995024876	Bn-N9-p39120782	Bn-chrA09-p33104909
Bn-A09-p36019854	0.873754153	0.873754153	Bn-N9-p39140028	Bn-chrA09-p33124214
Bn-A09-p36033827	0.993355482	0.993355482	Bn-N9-p39160240	Bn-chrA09-p33144413
Bn-A09-p36062760	0.960199005	0.960199005	Bn-N9-p39188012	Bn-chrA09-p33176628
Bn-A09-p36062825	0.950248756	0.950248756	Bn-N9-p39188077	Bn-chrA09-p33176693
Bn-A09-p36112515	0.996677741	0.996677741	Bn-N9-p39245304	Bn-chrA09-p33233969
Bn-A09-p36120469	0.933554817	0.933554817	Bn-N9-p39265479	Bn-chrC08-p37691955
Bn-A09-p36136583	0.989583333	0.989583333	Bn-N9-p39281186	Bn-chrA09-p33257617

Bn-A09-p36172635	0.966777409	0.966777409	Bn-N18-p43731092	Bn-chrC08-p37631294
Bn-A09-p36173579	0.973421927	0.973421927	Bn-N9-p39319479	Bn-chrA09-p33295299
Bn-A09-p36200543	0.983471074	0.991735537	Bn-N9-p39351742	Bn-chrAnn_random-p172632
Bn-A09-p36201113	0.996677741	1	Bn-N9-p39352315	Bn-chrAnn_random-p172638
Bn-A09-p36204736	0.991735537	0.983471074	Bn-N9-p39355938	Bn-chrAnn_random-p172674
Bn-A09-p36206114	1	0.983471074	Bn-N9-p39357316	Bn-chrAnn_random-p172688
Bn-A09-p36210166	0.983471074	0.991735537	Bn-N9-p39361424	Bn-chrAnn_random-p172729
Bn-A09-p36210247	0.960199005	0.960199005	Bn-N9-p39361505	Bn-chrAnn_random-p172729
Bn-A09-p36211880	0.983471074	0.991735537	Bn-N9-p39363652	Bn-chrAnn_random-p172746
Bn-A09-p36221958	0.950413223	0.94214876	Bn-N9-p39380185	Bn-chrAnn_random-p172854
Bn-A09-p36248653	0.983471074	0.966942149	Bn-N9-p39406857	Bn-chrA09-p33336393
Bn-A09-p36254037	1	0.986710963	Bn-N9-p39412270	Bn-chrA09-p33341775
Bn-A09-p36259207	0.983471074	0.991735537	Bn-N9-p39415213	Bn-chrA09-p33344515
Bn-A09-p36268830	0.933884298	0.925619835	Bn-N18-p43850890	Bn-chrA09-p33361478
Bn-A09-p36290340	0.985074627	0.985074627	Bn-N9-p39449185	Bn-chrA09-p33381022
Bn-A09-p36298053	0.983388704	0.990033223	Bn-N9-p39457788	Bn-chrA09-p33389446
Bn-A09-p36327491	0.983388704	0.983388704	Bn-N9-p39493609	Bn-chrAnn_random-p132194
Bn-A09-p36330477	0.937172775	0.921465969	Bn-N18-p43997860	Bn-chrAnn_random-p132172
Bn-A09-p36340882	0.993355482	0.993355482	Bn-N9-p39504442	Bn-chrAnn_random-p132093
Bn-A09-p36363739	0.996677741	0.996677741	Bn-N9-p39526986	Bn-chrAnn_random-p131866
Bn-A09-p36385502	0.993355482	0.993355482	Bn-N9-p39549647	Bn-chrAnn_random-p131631
Bn-A09-p36397959	0.983471074	0.983471074	Bn-N9-p39555642	Bn-chrAnn_random-p131571
Bn-A09-p36402398	0.92358804	0.92358804	Bn-N9-p39560080	Bn-chrAnn_random-p131526
Bn-A09-p36402581	0.991735537	0.991735537	Bn-N9-p39560263	Bn-chrAnn_random-p131524
Bn-A09-p36403465	0.975206612	0.975206612	Bn-N9-p39566523	Bn-chrA09-p33415100
Bn-A09-p36410312	0.966942149	0.966942149	Bn-N9-p39573277	Bn-chrA09-p33421857
Bn-A09-p36410528	0.917355372	0.917355372	Bn-N9-p39573489	Bn-chrA09-p33422069
Bn-A09-p36429976	0.958677686	0.958677686	Bn-N9-p39594876	Bn-chrA09-p33437818
Bn-A09-p36434436	0.996677741	0.996677741	Bn-N9-p39599314	Bn-chrA09-p33442256
Bn-A09-p36455112	0.975206612	0.983471074	Bn-N9-p39620061	Bn-chrA09-p33463015
Bn-A09-p36456115	0.983471074	0.975206612	Bn-N9-p39621064	Bn-chrA09-p33464016
Bn-A09-p36456505	0.970099668	0.993355482	Bn-N9-p39621454	Bn-chrA09-p33464422
Bn-A09-p36462450	0.991735537	0.983471074	Bn-N9-p39627068	Bn-chrA09-p33470049
Bn-A09-p36467520	0.996677741	0.956810631	Bn-N9-p39632142	Bn-chrA09-p33475135
Bn-A09-p36490411	0.991735537	1	Bn-N9-p39652543	Bn-chrA09-p33495612
Bn-A09-p36491715	0.958677686	0.966942149	Bn-N9-p39658156	Bn-chrA09-p33496929
Bn-A09-p36550750	0.966942149	0.966942149	Bn-N9-p39722802	Bn-chrA09-p33561270
Bn-A09-p36551108	0.983471074	0.983471074	Bn-N9-p39723385	Bn-chrA09-p33561852
Bn-A09-p36558536	0.950413223	0.950413223	Bn-N9-p39730749	Bn-chrA09-p33569215
Bn-A09-p36619537	1	1	Bn-N9-p39792978	Bn-chrA09-p33627983
Bn-A09-p36727719	0.96013289	0.96013289	Bn-N18-p44485147	Bn-chrC08-p38339634
Bn-A09-p36803825	0.352159468	0.996677741	Bn-N13-p55061222	Bn-chrA09-p33753773
Bn-A09-p36805327	0.252491694	0.993355482	Bn-N12-p3110619	Bn-chrA09-p33755275
Bn-A09-p36807928	0.551495017	1	Bn-N19-p37045962	Bn-chrA09-p33757865
Bn-A09-p36812148	0.915422886	1	Bn-N9-p40040706	Bn-chrA09-p33762084
Bn-A09-p36815767	0.947916667	1	Bn-N9-p39935452	Bn-chrA09-p33765762

Bn-A09-p36842526	0.995024876	0.995024876	Bn-N1-p7271038	Bn-chrA10-p6316563
Bn-A09-p36884091	0.917355372	0.958677686	Bn-N9-p40058346	Bn-chrA09-p33828578
Bn-A09-p36886022	0.685950413	0.958677686	Bn-N9-p40060791	Bn-chrA09-p33832953
Bn-A09-p36891089	0.917355372	0.917355372	Bn-N9-p40067761	Bn-chrCnn_random-p803799
Bn-A09-p36891502	0.651741294	0.985074627	Bn-N9-p40068467	Bn-chrA09-p33839248
Bn-A09-p36906070	0.511627907	0.980066445	Bn-N9-p40085805	Bn-chrA09-p33861419
Bn-A09-p36915816	0.956810631	0.986710963	Bn-Scaffold06015-p6	Bn-chrAnn_random-p298487
Bn-A09-p36939006	0.975206612	0.975206612	Bn-N9-p40118295	Bn-chrCnn_random-p809115
Bn-A09-p36939814	0.970099668	0.893687708	Bn-N9-p40120303	Bn-chrCnn_random-p809316
Bn-A09-p36969978	0.893687708	0.475083056	Bn-Scaffold03369-p1	Bn-chrCnn_random-p812370
Bn-A09-p36970657	0.860465116	0.275747508	Bn-Scaffold03369-p2	Bn-chrCnn_random-p812435
Bn-A09-p36975005	0.380165289	0.52892562	Bn-N3-p6574617	Bn-chrA10-p6197370
Bn-A09-p36991758	0.925619835	0.834710744	Bn-N9-p40174955	Bn-chrCnn_random-p814873
Bn-A09-p37001475	0.876033058	0.239669421	Bn-N9-p40187847	Bn-chrC05-p18917490
Bn-A09-p37010341	0.446280992	0.462809917	Bn-N11-p11529222	Bn-chrC02-p2474009
Bn-A09-p37019231	0.933884298	0.892561983	Bn-N9-p40209530	Bn-chrC07-p37969657
Bn-A09-p37024383	0.960199005	0.860696517	Bn-N18-p44587258	Bn-chrA10-p117675
Bn-A09-p37029924	0.826446281	0.859504132	Bn-N9-p40255542	Bn-chrCnn_random-p822685
Bn-A09-p37031129	0.950413223	0.776859504	Bn-N9-p40257902	Bn-chrCnn_random-p822804
Bn-A09-p37034993	0.73089701	0.681063123	Bn-N18-p44622559	Bn-chrCnn_random-p823186
Bn-A09-p37048611	0.953488372	0.953488372	Bn-N18-p44637869	Bn-chrCnn_random-p824663
Bn-A09-p37056545	0.677740864	0.983388704	Bn-Scaffold39641-p5	Bn-chrCnn_random-p825450
Bn-A09-p37070158	0.785123967	0.94214876	Bn-Scaffold25174-p5	Bn-chrCnn_random-p827060
Bn-A09-p37075737	0.991735537	0.983471074	Bn-N18-p44677507	Bn-chrCnn_random-p828983
Bn-A09-p37082082	0.975206612	0.966942149	Bn-N18-p44683502	Bn-chrCnn_random-p829039
Bn-A09-p37097229	0.685950413	0.611570248	Bn-N18-p44691660	Bn-chrC08-p31627311
Bn-A09-p37112947	0.214876033	0.214876033	Bn-N4-p15620062	Bn-chrC09-p30668855
Bn-A10-p862	0.965174129	0.990049751	Bn-Scaffold00637-p9	Bn-chrAnn_random-p223677
Bn-A10-p2282	0.990049751	0.975124378	Bn-Scaffold00637-p9	Bn-chrAnn_random-p223663
Bn-A10-p8941	0.966942149	0.983471074	Bn-Scaffold00637-p8	Bn-chrA10-p4433136
Bn-A10-p9026	1	0.991735537	Bn-Scaffold00637-p8	Bn-chrA10-p4433221
Bn-A10-p11422	1	0.991735537	Bn-Scaffold00637-p8	Bn-chrA10-p4434401
Bn-A10-p37005	1	1	Bn-Scaffold00637-p7	Bn-chrA10-p4443943
Bn-A10-p46022	0.983471074	0.983471074	Bn-Scaffold00637-p6	Bn-chrA10-p4452227
Bn-A10-p46331	0.991735537	0.991735537	Bn-Scaffold00637-p6	Bn-chrA10-p4452535
Bn-A10-p58713	0.917355372	0.983471074	Bn-N19-p32815092	Bn-chrA10-p4528000
Bn-A10-p58959	0.495867769	0.991735537	Bn-N19-p32814862	Bn-chrA10-p4528247
Bn-A10-p62077	0.917355372	0.983471074	Bn-N12-p32993630	Bn-chrA10-p4531612
Bn-A10-p64365	0.925619835	0.991735537	Bn-N12-p32991271	Bn-chrA10-p4533898
Bn-A10-p65275	0.352159468	0.993355482	Bn-N7-p4182888	Bn-chrA10-p4534808
Bn-A10-p68823	0.925619835	0.991735537	Bn-N6-p16240705	Bn-chrA10-p4538587
Bn-A10-p73025	0.910447761	0.985074627	Bn-N15-p26030363	Bn-chrA10-p4542801
Bn-A10-p73255	0.930348259	0.980099502	Bn-N5-p12497761	Bn-chrA10-p4543031
Bn-A10-p78356	0.950166113	0.980066445	Bn-N6-p16291304	Bn-chrA10-p4548430
Bn-A10-p84343	0.429752066	0.429752066	Bn-N10-p6891775	Bn-chrA10_random-p874915
Bn-A10-p89338	0.520661157	0.991735537	Bn-N12-p32998662	Bn-chrA10-p4567458

Bn-A10-p90069	0.917355372	0.991735537	Bn-Scaffold00637-p2	Bn-chrA10-p4568189
Bn-A10-p95651	0.983471074	0.983471074	Bn-Scaffold00637-p1	Bn-chrA10-p4573724
Bn-A10-p110116	0.991735537	1	Bn-Scaffold00637-p1	Bn-chrA10-p4588049
Bn-A10-p112405	0.991735537	0.991735537	Bn-Scaffold00637-p5	Bn-chrA10-p4588944
Bn-A10-p112610	1	1	Bn-Scaffold00637-p3	Bn-chrA10-p4589149
Bn-A10-p112852	1	0.991735537	Bn-Scaffold00637-p6	Bn-chrA10-p4589392
Bn-A10-p113985	0.975206612	0.975206612	Bn-N10-p4482689	Bn-chrA10-p4593199
Bn-A10-p114515	0.975206612	0.975206612	Bn-N10-p4482159	Bn-chrA10-p4593729
Bn-A10-p119498	0.975206612	0.966942149	Bn-N10-p4477227	Bn-chrA10-p4598662
Bn-A10-p119690	0.876033058	0.966942149	Bn-N10-p4477053	Bn-chrA10-p4598854
Bn-A10-p126180	0.950166113	1	Bn-N19-p32582381	Bn-chrA10-p4604401
Bn-A10-p135462	1	1	Bn-N10-p4452891	Bn-chrA10-p4613763
Bn-A10-p157450	0.958677686	0.958677686	Bn-N10-p4390384	Bn-chrAnn_random-p313066
Bn-A10-p158082	0.966942149	0.966942149	Bn-N10-p4389759	Bn-chrAnn_random-p313060
Bn-A10-p173508	0.958677686	0.958677686	Bn-N10-p4373431	Bn-chrA10-p3552781
Bn-A10-p173989	0.975206612	0.966942149	Bn-N10-p4372949	Bn-chrA10-p3552300
Bn-A10-p174519	1	0.991735537	Bn-N10-p4372416	Bn-chrA10-p3551770
Bn-A10-p175613	0.991735537	0.983471074	Bn-N10-p4371322	Bn-chrA10-p3550676
Bn-A10-p175661	0.983471074	0.975206612	Bn-N10-p4371274	Bn-chrA10-p3550628
Bn-A10-p180934	0.991735537	0.991735537	Bn-N10-p4362516	Bn-chrA10-p3545358
Bn-A10-p182060	0.996677741	1	Bn-N10-p4361353	Bn-chrA10-p3544200
Bn-A10-p189638	1	1	Bn-N10-p4353644	Bn-chrA10-p3536485
Bn-A10-p189778	1	1	Bn-N10-p4353504	Bn-chrA10-p3536345
Bn-A10-p190381	0.983471074	0.975206612	Bn-N10-p4352901	Bn-chrA10-p3535742
Bn-A10-p191051	0.983471074	0.983471074	Bn-N10-p4352235	Bn-chrA10-p3535072
Bn-A10-p191555	0.989583333	1	Bn-N10-p4351731	Bn-chrA10-p3534568
Bn-A10-p191700	0.989583333	1	Bn-N10-p4351611	Bn-chrA10-p3534448
Bn-A10-p206167	0.976744186	0.980066445	Bn-N10-p4337361	Bn-chrA10-p3520080
Bn-A10-p212837	0.983471074	0.983471074	Bn-N10-p4330533	Bn-chrA10-p3513252
Bn-A10-p213607	0.966942149	0.966942149	Bn-N10-p4329678	Bn-chrA10-p3512398
Bn-A10-p226207	0.980099502	0.985074627	Bn-N10-p4316969	Bn-chrA10-p3499604
Bn-A10-p234189	0.92358804	0.990033223	Bn-N10-p4309006	Bn-chrA10-p3491617
Bn-A10-p235238	0.975206612	0.975206612	Bn-N10-p4307956	Bn-chrA10-p3490568
Bn-A10-p237443	0.983471074	0.983471074	Bn-N10-p4305751	Bn-chrA10-p3488364
Bn-A10-p246700	0.950413223	0.950413223	Bn-N10-p4296319	Bn-chrA10-p3479030
Bn-A10-p284701	0.975206612	0.983471074	Bn-N10-p4264922	Bn-chrA10-p3443090
Bn-A10-p284815	0.980066445	0.986710963	Bn-N10-p4264808	Bn-chrA10-p3442976
Bn-A10-p285080	0.991735537	0.991735537	Bn-N10-p4264542	Bn-chrA10-p3442710
Bn-A10-p285203	0.991735537	0.991735537	Bn-N10-p4264419	Bn-chrA10-p3442587
Bn-A10-p285279	0.991735537	0.983471074	Bn-N10-p4264343	Bn-chrA10-p3442511
Bn-A10-p285533	0.975206612	0.983471074	Bn-N10-p4264089	Bn-chrA10-p3442257
Bn-A10-p285601	0.983471074	0.983471074	Bn-N10-p4264021	Bn-chrA10-p3442189
Bn-A10-p286279	0.975206612	0.983471074	Bn-N10-p4263343	Bn-chrA10-p3441511
Bn-A10-p286335	0.983471074	0.975206612	Bn-N10-p4263287	Bn-chrA10-p3441455
Bn-A10-p292846	1	0.996677741	Bn-N10-p4256807	Bn-chrA10-p3429070
Bn-A10-p302099	0.975206612	0.966942149	Bn-N10-p4247550	Bn-chrA10-p3418854

Bn-A10-p303664	0.983471074	0.983471074	Bn-N10-p4245985	Bn-chrA10-p3417296
Bn-A10-p306851	0.983471074	0.991735537	Bn-N10-p4242768	Bn-chrA10-p3413923
Bn-A10-p309723	0.685950413	0.702479339	Bn-N10-p4240741	Bn-chrA10-p3412790
Bn-A10-p320201	0.867768595	0.983471074	Bn-N19-p31827576	Bn-chrA10-p3410770
Bn-A10-p320209	0.842975207	0.983471074	Bn-N10-p4233581	Bn-chrA10-p3410762
Bn-A10-p326170	0.958677686	0.958677686	Bn-N10-p4227604	Bn-chrA10-p3404809
Bn-A10-p327056	0.950413223	0.950413223	Bn-N10-p4226591	Bn-chrA10-p3403796
Bn-A10-p348441	0.991735537	0.991735537	Bn-N10-p4224497	Bn-chrA10-p3401703
Bn-A10-p349032	0.702479339	0.702479339	Bn-N10-p4223912	Bn-chrA10-p3401118
Bn-A10-p349436	0.950413223	0.950413223	Bn-N10-p4223165	Bn-chrA10-p3400371
Bn-A10-p353725	0.975206612	0.975206612	Bn-N10-p4218844	Bn-chrA10-p3396052
Bn-A10-p366002	0.975206612	0.975206612	Bn-N10-p4211524	Bn-chrA10-p3388732
Bn-A10-p378724	0.983471074	0.983471074	Bn-N10-p4195288	Bn-chrAnn_random-p247023
Bn-A10-p382954	0.983471074	0.983471074	Bn-N10-p4192233	Bn-chrAnn_random-p465394
Bn-A10-p395236	0.851239669	0.851239669	Bn-N10-p4189695	Bn-chrCnn_random-p492289
Bn-A10-p408589	0.983471074	0.983471074	Bn-Scaffold01165-p1	Bn-chrAnn_random-p265853
Bn-A10-p466849	0.993355482	0.990033223	Bn-N10-p4150990	Bn-chrAnn_random-p232255
Bn-A10-p467507	1	1	Bn-Scaffold41279-p1	Bn-chrAnn_random-p232249
Bn-A10-p496266	0.305785124	0.966942149	Unknown	Bn-chrA06-p17793705
Bn-A10-p502169	0.980099502	0.980099502	Bn-N10-p6508713	Bn-chrA10-p3000523
Bn-A10-p510846	0.983471074	0.983471074	Bn-N10-p6506271	Bn-chrA10-p2998076
Bn-A10-p515442	0.917355372	0.975206612	Bn-N10-p6489493	Bn-chrA10-p2969301
Bn-A10-p529306	0.995024876	0.995024876	Bn-Scaffold46515-p1	Bn-chrC05-p1517299
Bn-A10-p585176	0.986710963	0.980066445	Bn-N10-p3413422	Bn-chrA10_random-p104070
Bn-A10-p592763	0.983471074	0.975206612	Bn-N10-p3404650	Bn-chrC06-p57811
Bn-A10-p600021	1	0.995024876	Bn-N10-p3397365	Bn-chrA10-p2824438
Bn-A10-p634822	0.996677741	0.405315615	Bn-N10-p3363254	Bn-chrC01-p13549085
Bn-A10-p688412	0.983471074	0.991735537	Bn-N10-p3316169	Bn-chrA10_random-p103665
Bn-A10-p693390	0.950413223	0.950413223	Bn-N10-p3309566	Bn-chrA10_random-p133108
Bn-A10-p700896	0.652892562	0.652892562	Bn-N10-p3308467	Bn-chrA10_random-p133219
Bn-A10-p708566	0.966942149	0.975206612	Bn-N10-p3294734	Bn-chrA10-p2738174
Bn-A10-p709526	0.991735537	1	Bn-N10-p3293777	Bn-chrA10-p2737220
Bn-A10-p709917	0.975206612	0.975206612	Bn-N10-p3293386	Bn-chrA10-p2736830
Bn-A10-p718048	0.936877076	0.936877076	Bn-N10-p3290241	Bn-chrA10-p2732348
Bn-A10-p749870	0.995024876	0.980099502	Bn-N10-p3250971	Bn-chrA10-p2696512
Bn-A10-p750464	0.983471074	0.975206612	Bn-N10-p3250354	Bn-chrA10-p2695895
Bn-A10-p750738	0.966942149	0.446280992	Bn-N10-p3250050	Bn-chrAnn_random-p465358
Bn-A10-p754316	0.975206612	0.975206612	Bn-N10-p3240611	Bn-chrA10-p2691584
Bn-A10-p754475	0.94214876	0.925619835	Bn-N10-p3240452	Bn-chrA10-p2691425
Bn-A10-p758707	0.986710963	0.983388704	Bn-N10-p3233916	Bn-chrA10-p2683711
Bn-A10-p788337	0.970149254	0.930348259	Bn-N10-p3206951	Bn-chrC05-p2569823
Bn-A10-p902245	1	0.991735537	Bn-N10-p3094856	Bn-chrA10-p2597792
Bn-A10-p922511	0.974874372	0.974874372	Bn-N11-p36978921	Bn-chrC01-p32149812
Bn-A10-p923788	0.970149254	0.970149254	Bn-N7-p20013217	Bn-chrA07-p19211704
Bn-A10-p926733	0.960199005	0.950248756	Bn-N10-p3083156	Bn-chrA10-p2578557
Bn-A10-p971793	0.975206612	0.975206612	Bn-N10-p3037089	Bn-chrA10-p2542629

Bn-A10-p973131	0.975206612	0.975206612	Bn-N10-p3032751	Bn-chrA10-p2541324
Bn-A10-p983013	0.966942149	0.966942149	Bn-N10-p3021668	Bn-chrA10-p2533892
Bn-A10-p991199	0.991735537	0.991735537	Bn-N10-p3017724	Bn-chrA10-p2531178
Bn-A10-p991279	0.983471074	0.983471074	Bn-N10-p3017644	Bn-chrA10-p2531098
Bn-A10-p992281	0.933884298	0.933884298	Bn-N10-p3016644	Bn-chrA10-p2530098
Bn-A10-p1001670	0.991735537	0.983471074	Bn-N10-p3007255	Bn-chrA10-p2520710
Bn-A10-p1002178	0.991735537	0.983471074	Bn-N10-p3006747	Bn-chrA10-p2520202
Bn-A10-p1002706	0.983471074	0.991735537	Bn-N10-p3006219	Bn-chrA10-p2519674
Bn-A10-p1003667	0.993355482	0.990033223	Bn-N10-p3005258	Bn-chrA10-p2518713
Bn-A10-p1010963	0.955223881	0.955223881	Bn-N10-p2994980	Bn-chrA10-p2506052
Bn-A10-p1023294	0.980099502	0.975124378	Bn-N10-p2981798	Bn-chrCnn_random-p549983
Bn-A10-p1040224	0.986710963	0.986710963	Bn-N10-p2958901	Bn-chrA10_random-p176964
Bn-A10-p1041199	0.983471074	0.991735537	Bn-N10-p2957926	Bn-chrA10_random-p177939
Bn-A10-p1108975	0.983388704	0.980066445	Bn-N10-p2886382	Bn-chrA10-p2432736
Bn-A10-p1123189	0.991735537	0.983471074	Bn-N10-p2868701	Bn-chrA10-p2418430
Bn-A10-p1125100	0.991735537	0.983471074	Bn-N10-p2866782	Bn-chrA10-p2416805
Bn-A10-p1125156	0.983471074	0.975206612	Bn-N10-p2866726	Bn-chrA10-p2416749
Bn-A10-p1131424	0.993355482	0.996677741	Bn-N10-p2860391	Bn-chrA10-p2410258
Bn-A10-p1149411	0.993355482	0.993355482	Bn-N10-p2837553	Bn-chrA10-p2386964
Bn-A10-p1153553	0.983471074	0.975206612	Bn-N10-p2833210	Bn-chrC05-p2359514
Bn-A10-p1159591	0.985074627	0.990049751	Bn-N17-p38304797	Bn-chrCnn_random-p757626
Bn-A10-p1161115	0.991735537	1	Bn-N10-p2820042	Bn-chrA10_random-p271436
Bn-A10-p1162422	0.545454545	0.661157025	Bn-N10-p2819852	Bn-chrA10_random-p270951
Bn-A10-p1162937	0.760330579	0.958677686	Bn-N10-p2819368	Bn-chrA10_random-p270624
Bn-A10-p1166564	0.834710744	0.785123967	Bn-N10-p2813063	Bn-chrC05-p2354470
Bn-A10-p1172344	0.983471074	0.975206612	Bn-N10-p2811830	Bn-chrA10_random-p264963
Bn-A10-p1187541	0.996677741	0.993355482	Bn-N10-p2795757	Bn-chrA10_random-p248894
Bn-A10-p1193336	0.975206612	0.983471074	Bn-N10-p2790114	Bn-chrA10_random-p242849
Bn-A10-p1194688	0.94214876	0.94214876	Bn-N10-p2788769	Bn-chrA10_random-p241501
Bn-A10-p1196851	0.991735537	0.991735537	Bn-N10-p2786588	Bn-chrA10_random-p239321
Bn-A10-p1215039	0.975206612	0.966942149	Bn-N10-p2759191	Bn-chrA10_random-p216771
Bn-A10-p1215054	0.975206612	0.983471074	Bn-N10-p2759176	Bn-chrA10_random-p216756
Bn-A10-p1264986	0.950248756	0.990049751	Bn-N15-p2523137	Bn-chrA10-p2307972
Bn-A10-p1268549	0.966942149	0.958677686	Bn-N10-p2691862	Bn-chrA10-p2304472
Bn-A10-p1269116	0.976744186	0.976744186	Bn-N15-p2518877	Bn-chrC05-p2265786
Bn-A10-p1273491	0.983471074	0.983471074	Bn-N10-p2686920	Bn-chrA10-p2299545
Bn-A10-p1303093	0.966942149	0.966942149	Bn-N10-p2679239	Bn-chrA10-p2290506
Bn-A10-p1313220	0.993355482	0.990033223	Bn-N10-p2672199	Bn-chrA10-p2281299
Bn-A10-p1318385	0.966942149	0.958677686	Bn-N10-p2667034	Bn-chrC05-p2235671
Bn-A10-p1345255	0.990049751	0.985074627	Bn-N10-p2631797	Bn-chrA10-p2252291
Bn-A10-p1354965	0.876033058	0.975206612	Bn-N15-p2451181	Bn-chrA10-p2246372
Bn-A10-p1359047	0.995024876	0.995024876	Bn-N15-p2448948	Bn-chrC05-p2183521
Bn-A10-p1370599	0.966942149	0.975206612	Bn-N10-p2610745	Bn-chrA10-p2230972
Bn-A10-p1406327	0.958677686	0.975206612	Bn-N15-p2409529	Bn-chrA10_random-p317627
Bn-A10-p1414990	0.983471074	0.983471074	Bn-N10-p2569210	Bn-chrA10_random-p314082
Bn-A10-p1416938	0.983471074	0.983471074	Bn-N10-p2567262	Bn-chrA10_random-p312126

Bn-A10-p1458849	0.990049751	0.995024876	Bn-N10-p2525099	Bn-chrA10_random-p596103
Bn-A10-p1459953	0.990049751	0.985074627	Bn-N10-p2523995	Bn-chrA10_random-p594960
Bn-A10-p1461575	0.970149254	0.975124378	Bn-N10-p2521304	Bn-chrC05_random-p17071
Bn-A10-p1461642	0.970149254	0.975124378	Bn-N10-p2521237	Bn-chrC05_random-p17138
Bn-A10-p1461745	0.970149254	0.970149254	Bn-N10-p2521134	Bn-chrA10_random-p285993
Bn-A10-p1468837	0.991735537	0.47107438	Bn-N10-p2509513	Bn-chrC03_random-p501818
Bn-A10-p1474859	0.88372093	0.88372093	Bn-N7-p1893662	Bn-chrA07-p1859183
Bn-A10-p1484979	0.993355482	0.990033223	Bn-N10-p2484816	Bn-chrA10_random-p279294
Bn-A10-p1484980	0.991735537	0.983471074	Bn-N10-p2484815	Bn-chrA10_random-p279293
Bn-A10-p1486296	0.975206612	0.975206612	Bn-N10-p2483504	Bn-chrA10_random-p277995
Bn-A10-p1513127	0.643835616	0.996575342	Bn-N10-p2450672	Bn-chrA10-p2209130
Bn-A10-p1580627	0.975206612	0.983471074	Bn-N10-p2372591	Bn-chrA10-p2123183
Bn-A10-p1582619	0.983471074	0.991735537	Bn-N10-p2371138	Bn-chrA10-p2121730
Bn-A10-p1582783	0.991735537	1	Bn-N10-p2370974	Bn-chrA10-p2121566
Bn-A10-p1590088	0.991735537	0.867768595	Bn-N10-p2360121	Bn-chrC05-p1999532
Bn-A10-p1592490	0.991735537	0.983471074	Bn-N10-p2357719	Bn-chrA10-p2111865
Bn-A10-p1595235	0.983471074	0.991735537	Bn-N10-p2354972	Bn-chrA10-p2109113
Bn-A10-p1596948	0.990033223	0.986710963	Bn-N10-p2353258	Bn-chrA10-p2107400
Bn-A10-p1606279	0.995024876	0.995024876	Bn-N10-p2343996	Bn-chrA10-p2098135
Bn-A10-p1611838	0.990049751	0.990049751	Bn-N10-p2336662	Bn-chrA10-p2093309
Bn-A10-p1611973	0.990049751	0.990049751	Bn-N10-p2336527	Bn-chrA10-p2093174
Bn-A10-p1629545	0.975124378	0.965174129	Bn-N3-p7151642	Bn-chrA03-p6379577
Bn-A10-p1655784	0.990033223	0.990033223	Bn-N10-p2288176	Bn-chrA10-p1857796
Bn-A10-p1698746	1	1	Bn-N10-p2222171	Bn-chrA10-p1795993
Bn-A10-p1746455	0.975206612	0.983471074	Bn-N10-p2160505	Bn-chrA10-p2018501
Bn-A10-p1782825	0.925619835	0.966942149	Bn-N10-p2132362	Bn-chrA10-p1989571
Bn-A10-p1787348	0.94214876	0.958677686	Bn-N15-p2061201	Bn-chrA10-p1985037
Bn-A10-p1791974	0.991735537	1	Bn-N10-p2123204	Bn-chrA10-p1980375
Bn-A10-p1800811	0.975124378	0.975124378	Bn-N10-p2116614	Bn-chrA10-p1973838
Bn-A10-p1881790	0.958677686	0.917355372	Bn-N10-p1998840	Bn-chrC04-p10310040
Bn-A10-p1900932	0.945273632	0.940298507	Bn-N10-p1978938	Bn-chrAnn_random-p211687
Bn-A10-p1919293	0.996677741	0.993355482	Bn-N10-p1969996	Bn-chrA10-p1780461
Bn-A10-p1920466	0.993355482	0.996677741	Bn-N10-p1968860	Bn-chrA10-p1779296
Bn-A10-p1939668	0.94214876	0.933884298	Bn-N10-p1954589	Bn-chrA10-p1770364
Bn-A10-p1939809	0.986710963	0.983388704	Bn-N10-p1954448	Bn-chrA10-p1770223
Bn-A10-p1956075	0.950413223	0.94214876	Bn-N10-p1935600	Bn-chrA10-p1750365
Bn-A10-p1956491	0.983471074	0.975206612	Bn-N10-p1935184	Bn-chrA10-p1749949
Bn-A10-p1957591	0.990033223	0.983388704	Bn-N10-p1933228	Bn-chrA10-p1748850
Bn-A10-p1962854	0.983471074	0.975206612	Bn-N10-p1920427	Bn-chrA10-p1743200
Bn-A10-p2008903	1	0.983388704	Bn-N10-p1865632	Bn-chrA10-p1708254
Bn-A10-p2010205	0.983471074	0.983471074	Bn-N10-p1860093	Bn-chrA10-p1706958
Bn-A10-p2012635	0.396694215	0.975206612	Bn-N8-p748003	Bn-chrA10-p1704363
Bn-A10-p2022381	0.991735537	1	Bn-N10-p1848892	Bn-chrA10-p1691303
Bn-A10-p2034005	0.986710963	0.986710963	Bn-N10-p1844715	Bn-chrA10-p1685918
Bn-A10-p2034006	0.958677686	0.966942149	Bn-N10-p1844714	Bn-chrA10-p1685917
Bn-A10-p2035810	0.991735537	0.983471074	Bn-N15-p1843710	Bn-chrA10-p1684109

Bn-A10-p2042679	0.991735537	0.983471074	Bn-N10-p1832985	Bn-chrA10-p1676950
Bn-A10-p2042772	0.983471074	0.991735537	Bn-N10-p1832892	Bn-chrA10-p1676857
Bn-A10-p2044339	0.996677741	0.976744186	Bn-N10-p1831325	Bn-chrA10-p1675290
Bn-A10-p2048753	0.986710963	0.973421927	Bn-N10-p1827250	Bn-chrA10-p1671209
Bn-A10-p2048923	0.983471074	0.975206612	Bn-N10-p1827080	Bn-chrA10-p1671039
Bn-A10-p2056053	0.991735537	0.983471074	Bn-N10-p1819797	Bn-chrA10-p1656737
Bn-A10-p2059382	1	0.991735537	Bn-N10-p1816479	Bn-chrA10-p1653398
Bn-A10-p2062185	0.966942149	0.958677686	Bn-N10-p1813677	Bn-chrA10-p1650585
Bn-A10-p2065185	0.983471074	0.991735537	Bn-N10-p1810479	Bn-chrA10-p1649404
Bn-A10-p2065818	0.991735537	0.983471074	Bn-N10-p1806302	Bn-chrA10-p1648771
Bn-A10-p2065917	0.991735537	1	Bn-N10-p1806203	Bn-chrA10-p1648672
Bn-A10-p2074000	0.983471074	0.975206612	Bn-N10-p1798135	Bn-chrA10-p1640663
Bn-A10-p2084182	0.986710963	0.983388704	Bn-N10-p1789509	Bn-chrC05-p1548612
Bn-A10-p2086836	1	0.996677741	Bn-N10-p1786872	Bn-chrC05-p1545959
Bn-A10-p2092612	0.983471074	0.983471074	Bn-N10-p1786559	Bn-chrC05-p1545646
Bn-A10-p2096179	0.980099502	0.980099502	Bn-N10-p1783003	Bn-chrA10-p1624464
Bn-A10-p2096482	0.983471074	0.983471074	Bn-N10-p1782700	Bn-chrA10-p1624161
Bn-A10-p2104477	0.975206612	0.975206612	Bn-N10-p1773677	Bn-chrA10-p1615139
Bn-A10-p2106781	1	1	Bn-N10-p1771367	Bn-chrA10-p1612829
Bn-A10-p2107649	0.983471074	0.983471074	Bn-N10-p1770499	Bn-chrA10-p1611961
Bn-A10-p2107673	0.983471074	0.983471074	Bn-N10-p1770475	Bn-chrA10-p1611937
Bn-A10-p2113222	0.955223881	0.955223881	Bn-N10-p1764249	Bn-chrA10-p1606174
Bn-A10-p2153847	0.983388704	0.983388704	Bn-N10-p1723691	Bn-chrAnn_random-p133913
Bn-A10-p2156484	0.975206612	0.975206612	Bn-N10-p1721079	Bn-chrAnn_random-p133939
Bn-A10-p2166959	0.950413223	0.950413223	Bn-N10-p1708569	Bn-chrAnn_random-p134063
Bn-A10-p2171166	0.993355482	0.993355482	Bn-N10-p1704397	Bn-chrAnn_random-p134124
Bn-A10-p2174043	1	1	Bn-N10-p1701520	Bn-chrAnn_random-p134153
Bn-A10-p2174162	1	1	Bn-N10-p1701401	Bn-chrAnn_random-p134154
Bn-A10-p2174249	0.983471074	0.983471074	Bn-N10-p1701314	Bn-chrAnn_random-p134155
Bn-A10-p2176252	0.983388704	0.983388704	Bn-N10-p1699311	Bn-chrAnn_random-p134175
Bn-A10-p2196623	0.983388704	0.757475083	Bn-N10-p1674904	Bn-chrC05_random-p331705
Bn-A10-p2208158	0.991735537	0.73553719	Bn-N10-p1653257	Bn-chrAnn_random-p134473
Bn-A10-p2240635	0.991735537	0.991735537	Bn-N10-p1629949	Bn-chrA10-p1592086
Bn-A10-p2246688	0.965174129	0.965174129	Bn-N10-p1626379	Bn-chrA10-p1588517
Bn-A10-p2247699	0.991735537	0.991735537	Bn-N10-p1625365	Bn-chrA10-p1587503
Bn-A10-p2260170	0.966942149	0.966942149	Bn-N15-p1632525	Bn-chrC05-p1409266
Bn-A10-p2262873	0.983471074	0.983471074	Bn-N10-p1612682	Bn-chrA10-p1574821
Bn-A10-p2266029	0.933884298	0.933884298	Bn-N10-p1610466	Bn-chrA10-p1572605
Bn-A10-p2268467	0.975206612	0.975206612	Bn-N10-p1608071	Bn-chrA10-p1570210
Bn-A10-p2268881	0.991735537	0.991735537	Bn-N10-p1607657	Bn-chrA10-p1569796
Bn-A10-p2279962	0.990033223	0.990033223	Bn-N10-p1603334	Bn-chrA10-p1565470
Bn-A10-p2292633	0.983471074	0.983471074	Bn-N10-p1597356	Bn-chrA10-p1559419
Bn-A10-p2294849	0.958677686	0.958677686	Bn-N10-p1594492	Bn-chrA10-p1556890
Bn-A10-p2295027	0.990049751	0.990049751	Bn-N10-p1594314	Bn-chrA10-p1556712
Bn-A10-p2309307	0.975206612	0.975206612	Bn-N10-p1580565	Bn-chrA10-p1542965
Bn-A10-p2309584	0.986710963	0.986710963	Bn-N10-p1580288	Bn-chrA10-p1542688

Bn-A10-p2311797	0.958677686	0.958677686	Bn-N10-p1578075	Bn-chrA10-p1540475
Bn-A10-p2312209	0.757475083	0.757475083	Bn-N10-p1577663	Bn-chrA10-p1540063
Bn-A10-p2315491	1	1	Bn-N10-p5685437	Bn-chrA10_random-p132550
Bn-A10-p2315612	0.985074627	0.990049751	Bn-N10-p5685558	Bn-chrA10_random-p132538
Bn-A10-p2337558	0.986710963	0.986710963	Bn-N10-p1554391	Bn-chrA10-p1519222
Bn-A10-p2356781	1	0.916943522	Bn-N10-p1536384	Bn-chrA10-p1501970
Bn-A10-p2357401	0.965174129	0.970149254	Bn-N10-p1535766	Bn-chrA10-p1501354
Bn-A10-p2367479	0.909090909	0.94214876	Bn-N10-p1533523	Bn-chrA10-p1495747
Bn-A10-p2369024	0.970149254	0.960199005	Bn-N10-p1532307	Bn-chrA10-p1494481
Bn-A10-p2369622	0.950413223	0.94214876	Bn-N10-p1531709	Bn-chrA10-p1493884
Bn-A10-p2374648	0.958677686	0.884297521	Bn-N10-p1526705	Bn-chrC05-p1348182
Bn-A10-p2385591	0.983471074	0.983471074	Bn-N10-p1515178	Bn-chrA10-p1477375
Bn-A10-p2403813	0.983471074	0.991735537	Bn-N10-p1507795	Bn-chrA10-p1469352
Bn-A10-p2415609	0.975206612	0.975206612	Bn-N10-p1497520	Bn-chrA10-p1459479
Bn-A10-p2416648	0.975206612	0.975206612	Bn-N10-p1496521	Bn-chrA10-p1458480
Bn-A10-p2425437	0.603305785	0.603305785	Bn-N15-p1517237	Bn-chrC05_random-p32530
Bn-A10-p2431843	0.975206612	0.975206612	Bn-N10-p1479552	Bn-chrA10-p1441434
Bn-A10-p2443900	0.685950413	0.685950413	Bn-N10-p1471073	Bn-chrA10-p1432955
Bn-A10-p2444751	0.983471074	0.983471074	Bn-N10-p1470228	Bn-chrA10-p1432110
Bn-A10-p2444761	0.983471074	0.983471074	Bn-N10-p1470218	Bn-chrA10-p1432100
Bn-A10-p2449120	0.785123967	0.785123967	Bn-N10-p1467040	Bn-chrA10-p1428922
Bn-A10-p2461822	0.991735537	0.991735537	Bn-N10-p1454204	Bn-chrA10-p1416087
Bn-A10-p2465908	0.975206612	0.975206612	Bn-N10-p1450152	Bn-chrA10-p1412035
Bn-A10-p2468308	0.991735537	0.991735537	Bn-N10-p1447753	Bn-chrA10-p1409636
Bn-A10-p2468805	0.909090909	0.909090909	Bn-N10-p1447254	Bn-chrA10-p1409137
Bn-A10-p2489044	0.983471074	0.983471074	Bn-N10-p1432789	Bn-chrA10-p1394709
Bn-A10-p2495533	0.975206612	0.975206612	Bn-N10-p1425461	Bn-chrA10-p1387118
Bn-A10-p2495652	0.975206612	0.975206612	Bn-N10-p1425342	Bn-chrA10-p1386999
Bn-A10-p2500393	0.975206612	0.975206612	Bn-N10-p1420348	Bn-chrA10-p1382003
Bn-A10-p2500523	0.983471074	0.743801653	Bn-N10-p1419901	Bn-chrA10-p1381556
Bn-A10-p2523110	1	1	Bn-N10-p1398053	Bn-chrA10-p1361140
Bn-A10-p2523239	0.94214876	0.94214876	Bn-N10-p1397924	Bn-chrA10-p1361011
Bn-A10-p2529144	0.966942149	0.966942149	Bn-N10-p1388373	Bn-chrA10_random-p5195
Bn-A10-p2532668	0.983471074	0.983471074	Bn-N10-p1384825	Bn-chrA10_random-p8741
Bn-A10-p2535072	0.958677686	0.950413223	Bn-N10-p1379545	Bn-chrA10_random-p13530
Bn-A10-p2536196	0.983471074	0.983471074	Bn-N10-p1373823	Bn-chrA10_random-p19252
Bn-A10-p2545412	1	1	Bn-N10-p1367113	Bn-chrA10_random-p26186
Bn-A10-p2553059	0.958677686	0.958677686	Bn-N17-p36624160	Bn-chrC07-p33687399
Bn-A10-p2554535	0.991735537	0.991735537	Bn-N10-p1364049	Bn-chrA10_random-p29250
Bn-A10-p2557960	0.950413223	0.950413223	Bn-N10-p1361370	Bn-chrA10_random-p31429
Bn-A10-p2580811	0.996677741	0.996677741	Bn-N10-p1337003	Bn-chrA10-p1326306
Bn-A10-p2589024	0.996677741	0.996677741	Bn-N10-p1328701	Bn-chrA10-p1318117
Bn-A10-p2687844	0.965174129	0.965174129	Bn-N10-p1233095	Bn-chrA10-p1222282
Bn-A10-p2710961	0.975206612	0.975206612	Bn-N10-p1213968	Bn-chrA10-p1208915
Bn-A10-p2713478	0.966942149	0.966942149	Bn-N10-p1211711	Bn-chrA10-p1206658
Bn-A10-p2728481	0.993355482	0.993355482	Bn-N10-p1200010	Bn-chrA10-p1194856

Bn-A10-p2742493	1	1	Bn-N15-p1214934	Bn-chrC05-p1048823
Bn-A10-p2747164	0.958677686	0.958677686	Bn-N10-p1181109	Bn-chrA10-p1177516
Bn-A10-p2762385	0.958677686	0.958677686	Bn-N10-p1170949	Bn-chrA10-p1167496
Bn-A10-p2764779	0.990049751	0.990049751	Bn-N10-p1168468	Bn-chrA10-p1165110
Bn-A10-p2765290	0.991735537	0.991735537	Bn-N10-p1167957	Bn-chrA10-p1164599
Bn-A10-p2776972	0.986710963	0.986710963	Bn-N10-p1141207	Bn-chrA10-p1155156
Bn-A10-p2777921	0.996677741	0.996677741	Bn-N10-p1140260	Bn-chrA10-p1154209
Bn-A10-p2804340	0.991735537	0.991735537	Bn-N10-p1126191	Bn-chrA10-p1140141
Bn-A10-p2805505	0.985074627	0.985074627	Bn-N10-p1125026	Bn-chrC05-p1002544
Bn-A10-p2809123	0.975206612	0.975206612	Bn-N10-p1122803	Bn-chrA10-p1136753
Bn-A10-p2819947	0.966942149	0.966942149	Bn-N10-p1108499	Bn-chrA10-p1123045
Bn-A10-p2833336	0.983471074	0.983471074	Bn-N10-p1093225	Bn-chrA10-p1106751
Bn-A10-p2849300	0.995024876	0.995024876	Bn-N10-p1085667	Bn-chrA10-p1083359
Bn-A10-p2852183	0.983471074	0.983471074	Bn-N10-p1082760	Bn-chrA10-p1080441
Bn-A10-p2890008	0.990033223	0.990033223	Bn-N7-p3299226	Bn-chrAnn_random-p339987
Bn-A10-p2899850	0.993355482	0.990033223	Bn-N7-p3305498	Bn-chrC07-p8445221
Bn-A10-p2937911	0.986710963	0.990033223	Bn-N7-p3325254	Bn-chrA07_random-p299696
Bn-A10-p2939364	0.993355482	0.990033223	Bn-N7-p3326707	Bn-chrA07_random-p301149
Bn-A10-p2941216	0.970149254	0.990049751	Bn-N7-p3328567	Bn-chrA07_random-p303001
Bn-A10-p2947958	0.985074627	0.985074627	Bn-N7-p3337702	Bn-chrAnn_random-p467141
Bn-A10-p2970564	0.955223881	0.955223881	Bn-N7-p3357261	Bn-chrA07_random-p324220
Bn-A10-p2972293	0.986710963	0.986710963	Bn-N7-p3358990	Bn-chrA07_random-p325948
Bn-A10-p2976330	0.956810631	0.96345515	Bn-N12-p44495879	Bn-chrC09-p48076822
Bn-A10-p2977092	0.976744186	0.976744186	Bn-N12-p44496640	Bn-chrC02-p45390482
Bn-A10-p2992421	0.985074627	0.985074627	Bn-N7-p3366747	Bn-chrA07_random-p332617
Bn-A10-p2992463	0.985074627	0.985074627	Bn-N7-p3366789	Bn-chrA07_random-p332659
Bn-A10-p2993544	0.975206612	0.975206612	Bn-N7-p3367870	Bn-chrA07_random-p333741
Bn-A10-p2993567	0.983388704	0.980066445	Bn-N7-p3367893	Bn-chrA07_random-p333764
Bn-A10-p2997098	0.916943522	0.92358804	Bn-N7-p3371486	Bn-chrA07_random-p339043
Bn-A10-p2997237	0.950413223	0.950413223	Bn-N7-p3371625	Bn-chrA07_random-p339182
Bn-A10-p3042880	0.990033223	0.993355482	Bn-N7-p3415813	Bn-chrA07-p4121682
Bn-A10-p3044426	0.887043189	0.956810631	Bn-N7-p3417386	Bn-chrA07-p4125193
Bn-A10-p3053568	1	1	Bn-N7-p3426705	Bn-chrA07-p4129822
Bn-A10-p3063220	1	1	Bn-N7-p3441726	Bn-chrA07-p4149628
Bn-A10-p3078181	0.993355482	0.993355482	Bn-N7-p3453733	Bn-chrA07-p4162142
Bn-A10-p3100309	0.980099502	0.980099502	Bn-N7-p3470447	Bn-chrA07-p4180167
Bn-A10-p3116203	0.88372093	0.88372093	Bn-N7-p3494433	Bn-chrA07-p4207522
Bn-A10-p3118613	0.993355482	0.993355482	Bn-N7-p3496829	Bn-chrA07-p4209918
Bn-A10-p3182217	1	1	Bn-N7-p3577936	Bn-chrA07-p4283656
Bn-A10-p3229296	0.945273632	0.990049751	Bn-Scaffold36417-p2	Bn-chrC02-p20624904
Bn-A10-p3235646	0.996677741	0.993355482	Bn-N7-p3620878	Bn-chrA07-p4345441
Bn-A10-p3243369	0.996677741	0.996677741	Bn-N7-p3630026	Bn-chrA07-p4353958
Bn-A10-p3266570	0.991735537	0.983471074	Bn-N7-p3668540	Bn-chrA07-p4388133
Bn-A10-p3329131	0.980099502	0.975124378	Bn-N7-p3706531	Bn-chrA07-p4431904
Bn-A10-p3512464	0.915422886	0.985074627	Bn-N12-p29232701	Bn-chrA10-p6872876
Bn-A10-p3587974	0.965174129	0.985074627	Bn-N6-p5042673	Bn-chrA10-p6799275

Bn-A10-p3605582	0.965174129	0.970149254	Bn-N10-p4791358	Bn-chrA10-p6783122
Bn-A10-p3605726	0.985074627	0.980099502	Bn-N10-p4791502	Bn-chrA10-p6782978
Bn-A10-p3605865	0.993355482	0.996677741	Bn-N10-p4791641	Bn-chrA10-p6782839
Bn-A10-p3648582	1	0.991735537	Bn-N10-p4837668	Bn-chrA10-p6741899
Bn-A10-p3649288	0.990033223	0.993355482	Bn-N10-p4838374	Bn-chrA10-p6741193
Bn-A10-p3649424	0.980066445	0.980066445	Bn-N10-p4838510	Bn-chrA10-p6741057
Bn-A10-p3649434	0.980066445	0.980066445	Bn-N10-p4838520	Bn-chrA10-p6741047
Bn-A10-p3649554	0.983388704	0.983388704	Bn-N10-p4838640	Bn-chrA10-p6740927
Bn-A10-p3651562	0.990033223	0.986710963	Bn-N10-p4840648	Bn-chrA10-p6738919
Bn-A10-p3652309	0.990033223	0.993355482	Bn-N10-p4841394	Bn-chrA10-p6738172
Bn-A10-p3652425	1	0.991735537	Bn-N10-p4841510	Bn-chrA10-p6738056
Bn-A10-p3669813	0.986710963	0.986710963	Bn-N10-p4857374	Bn-chrA10-p6723869
Bn-A10-p3677315	0.993355482	0.996677741	Bn-N10-p4864871	Bn-chrA10-p6716373
Bn-A10-p3677867	0.993355482	0.990033223	Bn-N10-p4865423	Bn-chrA10-p6715821
Bn-A10-p3700919	0.935323383	0.960199005	Bn-N10-p4887900	Bn-chrA10-p6695112
Bn-A10-p3710328	0.991735537	1	Bn-N10-p4900264	Bn-chrA10-p6686573
Bn-A10-p3711074	0.993355482	0.996677741	Bn-N10-p4901009	Bn-chrA10-p6685827
Bn-A10-p3725533	0.966777409	0.980066445	Bn-N10-p4931132	Bn-chrA10-p6647374
Bn-A10-p3728041	0.983471074	0.975206612	Bn-N10-p4933619	Bn-chrA10-p6642741
Bn-A10-p3744140	0.975206612	0.966942149	Bn-N10-p4945826	Bn-chrA10-p6630310
Bn-A10-p3745157	0.995024876	0.995024876	Bn-N10-p4946843	Bn-chrA10-p6629294
Bn-A10-p3745691	0.996677741	1	Bn-N10-p4947377	Bn-chrA10-p6628760
Bn-A10-p3745973	0.990049751	0.995024876	Bn-N10-p4947658	Bn-chrA10-p6628479
Bn-A10-p3747583	0.996677741	0.993355482	Bn-N10-p4949266	Bn-chrA10-p6626871
Bn-A10-p3782582	0.980099502	0.915422886	Bn-N10-p1041846	Bn-chrC05-p992399
Bn-A10-p3782643	0.970149254	0.925373134	Bn-N10-p1041785	Bn-chrC05-p992460
Bn-A10-p3806889	0.960199005	0.960199005	Bn-N10-p1018570	Bn-chrA10-p1013426
Bn-A10-p3809773	0.950413223	0.950413223	Bn-N10-p1015618	Bn-chrA10-p1010539
Bn-A10-p3825331	0.960199005	0.960199005	Bn-N10-p1004835	Bn-chrA10-p999757
Bn-A10-p3845546	0.986710963	0.986710963	Bn-N10-p984144	Bn-chrAnn_random-p363692
Bn-A10-p3856673	0.983388704	0.983388704	Bn-N10-p976840	Bn-chrA10-p975073
Bn-A10-p3894990	0.965174129	0.965174129	Bn-Scaffold03504-p1	Bn-chrA10-p934083
Bn-A10-p3903845	0.983471074	0.983471074	Bn-N10-p932923	Bn-chrA10-p918887
Bn-A10-p3905100	1	1	Bn-N10-p931657	Bn-chrA10-p917621
Bn-A10-p3905295	0.958677686	0.958677686	Bn-N10-p931462	Bn-chrA10-p917426
Bn-A10-p3906905	1	1	Bn-N10-p930130	Bn-chrA10-p916094
Bn-A10-p3909275	0.990033223	0.990033223	Bn-N10-p927565	Bn-chrA10-p913570
Bn-A10-p3921433	0.851239669	0.851239669	Bn-N15-p1027942	Bn-chrC05-p925127
Bn-A10-p3944803	0.973421927	0.970099668	Bn-Scaffold03150-p9	Bn-chrC03-p53671067
Bn-A10-p3955901	0.973421927	0.973421927	Bn-N10-p912738	Bn-chrA10-p898749
Bn-A10-p3966740	0.991735537	0.991735537	Bn-N10-p898703	Bn-chrA10-p885133
Bn-A10-p3970485	0.993355482	0.993355482	Bn-N10-p894962	Bn-chrA10-p881392
Bn-A10-p3978242	0.94214876	0.94214876	Bn-N10-p886752	Bn-chrA10-p873182
Bn-A10-p3980498	0.983471074	0.983471074	Bn-N10-p884502	Bn-chrA10-p870935
Bn-A10-p3981548	0.900826446	0.900826446	Bn-N10-p877220	Bn-chrAnn_random-p441578
Bn-A10-p3988046	0.980066445	0.980066445	Bn-N10-p866437	Bn-chrA10-p855892

Bn-A10-p3991393	0.933884298	0.933884298	Bn-N10-p860471	Bn-chrA10-p849955
Bn-A10-p3991855	0.983471074	0.983471074	Bn-N10-p859998	Bn-chrA10-p849482
Bn-A10-p4038884	0.863787375	0.863787375	Bn-N10-p831013	Bn-chrA10-p821148
Bn-A10-p4040298	0.970099668	0.970099668	Bn-N10-p829577	Bn-chrA10-p819712
Bn-A10-p4076884	0.983471074	0.983471074	Bn-N10-p795050	Bn-chrA10-p784758
Bn-A10-p4078158	0.958677686	0.958677686	Bn-N10-p793644	Bn-chrA10-p783352
Bn-A10-p4079529	0.991735537	0.991735537	Bn-N10-p792273	Bn-chrA10-p781981
Bn-A10-p4080011	0.991735537	0.991735537	Bn-N10-p791792	Bn-chrA10-p781500
Bn-A10-p4088438	0.976744186	0.976744186	Bn-N10-p782670	Bn-chrA10-p772418
Bn-A10-p4089670	0.996677741	0.996677741	Bn-N10-p781432	Bn-chrA10-p771180
Bn-A10-p4101984	0.966777409	0.966777409	Bn-N10-p772347	Bn-chrA10-p762097
Bn-A10-p4104015	0.983471074	0.983471074	Bn-N10-p770327	Bn-chrA10-p760077
Bn-A10-p4108968	0.991735537	0.991735537	Bn-N10-p766357	Bn-chrA10-p756108
Bn-A10-p4112636	0.925619835	0.925619835	Bn-N10-p763440	Bn-chrA10-p753190
Bn-A10-p4114397	1	1	Bn-N10-p761887	Bn-chrA10-p751637
Bn-A10-p4114477	0.991735537	0.991735537	Bn-N10-p761807	Bn-chrA10-p751557
Bn-A10-p4115062	0.991735537	0.991735537	Bn-N10-p761222	Bn-chrA10-p750972
Bn-A10-p4118433	0.975206612	0.975206612	Bn-N10-p757971	Bn-chrA10-p747721
Bn-A10-p4120782	0.991735537	0.991735537	Bn-N10-p756176	Bn-chrA10-p745926
Bn-A10-p4127274	0.983471074	0.983471074	Bn-N10-p749727	Bn-chrA10-p738650
Bn-A10-p4134125	0.96345515	0.96345515	Bn-N15-p791259	Bn-chrC05-p737896
Bn-A10-p4138782	0.975206612	0.975206612	Bn-N10-p738423	Bn-chrA10-p727348
Bn-A10-p4138801	0.975206612	0.975206612	Bn-N10-p738404	Bn-chrA10-p727329
Bn-A10-p4151991	0.983471074	0.983471074	Bn-N10-p723263	Bn-chrA10-p713005
Bn-A10-p4154238	0.995024876	0.995024876	Bn-N10-p719711	Bn-chrA10-p709454
Bn-A10-p4197906	0.966942149	0.966942149	Bn-N10-p665064	Bn-chrA10-p638064
Bn-A10-p4211081	0.983471074	0.983471074	Bn-Scaffold68868-p2	Bn-chrA10-p625725
Bn-A10-p4212056	0.950413223	0.950413223	Bn-N10-p650685	Bn-chrA10-p623732
Bn-A10-p4214836	0.993355482	0.993355482	Bn-N10-p647918	Bn-chrA10-p620965
Bn-A10-p4268080	0.996677741	0.996677741	Bn-N10-p587140	Bn-chrA10-p560431
Bn-A10-p4272881	1	0.991735537	Bn-N1-p854381	Bn-chrA06-p5337119
Bn-A10-p4287815	0.975124378	0.975124378	Bn-N10-p575055	Bn-chrA10-p546615
Bn-A10-p4296387	0.989583333	0.989583333	Bn-N10-p566482	Bn-chrA10-p538043
Bn-A10-p4304707	0.859504132	0.859504132	Bn-N12-p16947465	Bn-chrC02-p16421709
Bn-A10-p4305337	0.504132231	0.743801653	Bn-N4-p18648945	Bn-chrCnn_random-p831293
Bn-A10-p4311291	0.966942149	0.966942149	Bn-N10-p556861	Bn-chrA10-p528348
Bn-A10-p4332413	0.905472637	0.975124378	Bn-N10-p10148935	Bn-chrA10_random-p154644
Bn-A10-p4339494	0.958677686	0.958677686	Bn-N10-p532284	Bn-chrA10-p498104
Bn-A10-p4347862	0.983388704	0.983388704	Bn-N10-p518121	Bn-chrA10-p486258
Bn-A10-p4355593	0.975124378	0.975124378	Bn-N10-p508692	Bn-chrA10-p475698
Bn-A10-p4355648	0.975206612	0.975206612	Bn-N10-p508637	Bn-chrA10-p475643
Bn-A10-p4355682	0.989583333	0.989583333	Bn-N10-p508603	Bn-chrA10-p475609
Bn-A10-p4355750	1	1	Bn-N10-p508535	Bn-chrA10-p475541
Bn-A10-p4355840	0.966942149	0.966942149	Bn-N10-p508445	Bn-chrA10-p475451
Bn-A10-p4356450	0.991735537	0.991735537	Bn-N10-p507835	Bn-chrA10-p474841
Bn-A10-p4381139	0.969957082	0.974248927	Bn-N15-p615074	Bn-chrC05-p555749

Bn-A10-p4418919	1	1	Bn-N10-p467845	Bn-chrA10-p435215
Bn-A10-p4425066	0.980099502	0.980099502	Bn-N10-p457441	Bn-chrA10-p424957
Bn-A10-p4425312	0.970149254	0.970149254	Bn-N10-p457194	Bn-chrA10-p424710
Bn-A10-p4437644	0.986710963	0.986710963	Bn-N10-p446346	Bn-chrA10-p412132
Bn-A10-p4460099	1	1	Bn-N10-p411194	Bn-chrA10-p375372
Bn-A10-p4466886	0.991735537	0.991735537	Bn-N10-p403891	Bn-chrA10-p369938
Bn-A10-p4467083	0.980099502	0.980099502	Bn-N10-p403693	Bn-chrA10-p369740
Bn-A10-p4469775	0.983471074	0.983471074	Bn-N10-p401915	Bn-chrA10-p367962
Bn-A10-p4477499	0.966942149	0.966942149	Bn-N10-p400233	Bn-chrA10-p366280
Bn-A10-p4477601	0.983471074	0.983471074	Bn-N10-p400131	Bn-chrA10-p366178
Bn-A10-p4477643	0.991735537	0.991735537	Bn-N10-p400089	Bn-chrA10-p366136
Bn-A10-p4478046	0.991735537	0.991735537	Bn-N10-p399683	Bn-chrA10-p365730
Bn-A10-p4478343	0.983471074	0.983471074	Bn-N10-p399384	Bn-chrA10-p365431
Bn-A10-p4478469	0.991735537	0.991735537	Bn-N10-p399258	Bn-chrA10-p365305
Bn-A10-p4478517	0.991735537	0.991735537	Bn-N10-p399210	Bn-chrA10-p365257
Bn-A10-p4480010	0.966942149	0.966942149	Bn-N10-p397772	Bn-chrA10-p363819
Bn-A10-p4504517	0.966942149	0.975206612	Bn-N10-p380557	Bn-chrA10-p346227
Bn-A10-p4504641	0.983471074	0.983471074	Bn-N10-p380433	Bn-chrA10-p346103
Bn-A10-p4506059	0.950413223	0.950413223	Bn-N10-p379016	Bn-chrA10-p344708
Bn-A10-p4506119	0.966942149	0.966942149	Bn-N10-p378956	Bn-chrA10-p344648
Bn-A10-p4512626	0.983471074	0.991735537	Bn-N10-p375499	Bn-chrA10-p341154
Bn-A10-p4553957	0.993355482	0.973421927	Bn-N10-p341281	Bn-chrC05-p369884
Bn-A10-p4555527	0.993355482	0.993355482	Bn-N10-p338135	Bn-chrA10-p305469
Bn-A10-p4558688	0.965174129	0.965174129	Bn-N10-p334970	Bn-chrA10-p302306
Bn-A10-p4563194	0.975206612	0.975206612	Bn-N10-p330463	Bn-chrA10-p297800
Bn-A10-p4566109	0.996677741	0.996677741	Bn-N4-p7895252	Bn-chrA09-p31278328
Bn-A10-p4574423	0.980066445	0.980066445	Bn-N10-p322875	Bn-chrA10-p290847
Bn-A10-p4575458	0.980099502	0.980099502	Bn-N10-p321834	Bn-chrA10-p289806
Bn-A10-p4577455	0.983471074	0.983471074	Bn-N10-p319837	Bn-chrA10-p287809
Bn-A10-p4605721	0.96345515	0.956810631	Bn-N10-p298208	Bn-chrA10-p264884
Bn-A10-p4606815	0.991735537	0.991735537	Bn-N10-p297188	Bn-chrA07-p3129213
Bn-A10-p4613784	0.991735537	0.983471074	Bn-N10-p290219	Bn-chrA10-p256324
Bn-A10-p4613978	0.991735537	0.983471074	Bn-N10-p290025	Bn-chrA10-p256130
Bn-A10-p4619653	0.975206612	0.983471074	Bn-N10-p285898	Bn-chrA10-p252528
Bn-A10-p4619719	0.991735537	0.983471074	Bn-N10-p285832	Bn-chrA10-p252462
Bn-A10-p4621384	0.950413223	0.925619835	Bn-N10-p284169	Bn-chrA10-p250090
Bn-A10-p4621842	0.996677741	0.953488372	Bn-N10-p283711	Bn-chrA10-p249635
Bn-A10-p4622176	0.983471074	0.991735537	Bn-N10-p283377	Bn-chrA10-p249309
Bn-A10-p4622209	0.991735537	0.983471074	Bn-N10-p283344	Bn-chrA10-p249276
Bn-A10-p4622333	0.991735537	0.966942149	Bn-N10-p283220	Bn-chrA10-p249152
Bn-A10-p4622728	0.991735537	1	Bn-N10-p282813	Bn-chrA10-p248713
Bn-A10-p4622812	0.991735537	1	Bn-N10-p282729	Bn-chrA10-p248629
Bn-A10-p4623073	0.983471074	0.991735537	Bn-N10-p282468	Bn-chrA10-p248366
Bn-A10-p4623186	0.983471074	0.975206612	Bn-N10-p282355	Bn-chrA10-p248253
Bn-A10-p4623492	0.991735537	0.603305785	Bn-N10-p282049	Bn-chrC05-p307568
Bn-A10-p4624712	0.983471074	0.991735537	Bn-N10-p280829	Bn-chrA10-p246719

Bn-A10-p4625350	0.983471074	0.991735537	Bn-N10-p280191	Bn-chrA10-p246113
Bn-A10-p4627479	0.983471074	0.975206612	Bn-N10-p275292	Bn-chrA10-p243983
Bn-A10-p4639202	0.983388704	0.983388704	Bn-N10-p260628	Bn-chrA10-p233866
Bn-A10-p4642637	0.986710963	0.986710963	Bn-N10-p257232	Bn-chrA10-p227346
Bn-A10-p4653595	0.966942149	0.94214876	Bn-N10-p249578	Bn-chrC05-p285497
Bn-A10-p4663138	0.995024876	0.990049751	Bn-N10-p240236	Bn-chrA10-p209876
Bn-A10-p4695520	0.991735537	0.991735537	Bn-N10-p205455	Bn-chrA10-p184983
Bn-A10-p4701756	0.644628099	0.94214876	Bn-N10-p195559	Bn-chrA10-p178576
Bn-A10-p4706425	0.950413223	0.867768595	Bn-N10-p190611	Bn-chrC05-p196563
Bn-A10-p4725713	0.990033223	0.993355482	Bn-N10-p176298	Bn-chrA10-p161850
Bn-A10-p4727374	0.986710963	0.986710963	Bn-N10-p174643	Bn-chrA10-p160196
Bn-A10-p4731968	0.993355482	0.993355482	Bn-N10-p172505	Bn-chrA10-p158058
Bn-A10-p4742940	0.990033223	0.986710963	Bn-N10-p165000	Bn-chrA10-p150860
Bn-A10-p4755899	0.975124378	0.975124378	Bn-N10-p146549	Bn-chrA10-p141392
Bn-A10-p4757850	0.991735537	0.991735537	Bn-N10-p144599	Bn-chrA10-p139442
Bn-A10-p4786596	0.990033223	0.990033223	Bn-N10-p109572	Bn-chrA10-p95476
Bn-A10-p4792793	0.986710963	0.986710963	Bn-N10-p100701	Bn-chrA10-p89330
Bn-A10-p4798449	0.933333333	0.923809524	Bn-N4-p13097371	Bn-chrA06-p2062191
Bn-A10-p4802341	0.985074627	0.985074627	Bn-N10-p88239	Bn-chrA10-p82442
Bn-A10-p4813171	0.990033223	0.996677741	Bn-N10-p64218	Bn-chrCnn_random-p586879
Bn-A10-p4821527	0.983388704	0.983388704	Bn-N10-p56284	Bn-chrA10-p56676
Bn-A10-p4829546	1	1	Bn-N10-p53102	Bn-chrA10-p53496
Bn-A10-p4840892	0.986710963	0.983388704	Bn-N10-p41211	Bn-chrA10-p41912
Bn-A10-p4859385	0.983388704	0.983388704	Bn-N10-p9994	Bn-chrAnn_random-p412931
Bn-A10-p4871099	0.990033223	0.993355482	Bn-N10-p6693573	Bn-chrA10-p3185007
Bn-A10-p4897556	1	0.991735537	Bn-N10-p6662625	Bn-chrA10-p3155918
Bn-A10-p4922362	0.990049751	0.995024876	Bn-N10-p6637421	Bn-chrA10-p3131493
Bn-A10-p4935553	0.991735537	1	Bn-N10-p5719885	Bn-chrA10_random-p638340
Bn-A10-p4950011	0.986710963	0.986710963	Bn-N10-p5727893	Bn-chrA10_random-p646388
Bn-A10-p4962508	0.991735537	1	Bn-N10-p5740874	Bn-chrA10_random-p665537
Bn-A10-p4966927	0.983471074	0.991735537	Bn-N10-p5745295	Bn-chrA10_random-p669957
Bn-A10-p4968720	0.991735537	0.983471074	Bn-N10-p5747087	Bn-chrA10_random-p671750
Bn-A10-p4968727	0.983471074	0.991735537	Bn-N10-p5747094	Bn-chrA10_random-p671757
Bn-A10-p4968918	0.991735537	0.983471074	Bn-N10-p5747285	Bn-chrA10_random-p671948
Bn-A10-p4969803	1	0.991735537	Bn-N10-p5748170	Bn-chrA10_random-p672834
Bn-A10-p4972228	0.991735537	1	Bn-N10-p5750611	Bn-chrA10_random-p675298
Bn-A10-p4972604	0.991735537	0.983471074	Bn-N10-p5750987	Bn-chrA10_random-p675674
Bn-A10-p4972839	0.991735537	0.983471074	Bn-N10-p5751222	Bn-chrA10_random-p675909
Bn-A10-p4972939	0.990033223	0.993355482	Bn-N10-p5751322	Bn-chrA10_random-p676009
Bn-A10-p4983427	0.975206612	0.983471074	Bn-N10-p5761745	Bn-chrA10_random-p688830
Bn-A10-p4985950	1	0.991735537	Bn-N10-p5764268	Bn-chrA10_random-p691351
Bn-A10-p4986275	0.855721393	0.860696517	Bn-N10-p5764593	Bn-chrA10_random-p691676
Bn-A10-p4995181	0.975206612	0.859504132	Bn-N10-p5773994	Bn-chrA10_random-p704227
Bn-A10-p5004521	1	0.991735537	Bn-N10-p5781764	Bn-chrA10_random-p711791
Bn-A10-p5005845	0.983471074	0.991735537	Bn-N10-p5783069	Bn-chrA10_random-p713075
Bn-A10-p5007924	0.990033223	0.993355482	Bn-N10-p5785115	Bn-chrA10_random-p715125

Bn-A10-p5035362	0.975206612	0.966942149	Bn-N10-p5812384	Bn-chrA10_random-p744095
Bn-A10-p5039340	0.991735537	0.983471074	Bn-N10-p5816362	Bn-chrA10_random-p748077
Bn-A10-p5040430	0.983471074	0.991735537	Bn-N10-p5817452	Bn-chrA10_random-p749165
Bn-A10-p5047195	0.983471074	0.983471074	Bn-N10-p5824214	Bn-chrA10_random-p755927
Bn-A10-p5050557	0.991735537	1	Bn-N10-p5833000	Bn-chrA10_random-p759266
Bn-A10-p5052658	0.983471074	0.991735537	Bn-N10-p5835379	Bn-chrA10_random-p759839
Bn-A10-p5066911	0.975206612	0.983471074	Bn-N10-p5856884	Bn-chrA10_random-p770651
Bn-A10-p5070820	0.991735537	0.983471074	Bn-N10-p5860812	Bn-chrA10_random-p774545
Bn-A10-p5127309	0.859504132	0.975206612	Bn-N10-p5934599	Bn-chrA10-p4685786
Bn-A10-p5128067	0.955223881	0.960199005	Bn-N19-p36422057	Bn-chrA10-p4686544
Bn-A10-p5132669	0.991735537	1	Bn-N10-p5940004	Bn-chrA10-p4693476
Bn-A10-p5135356	1	0.991735537	Bn-N10-p5942691	Bn-chrA10-p4696163
Bn-A10-p5143067	1	0.995024876	Bn-N10-p5949663	Bn-chrA10-p4733256
Bn-A10-p5143614	0.990049751	0.995024876	Bn-N10-p5950210	Bn-chrA10-p4733803
Bn-A10-p5151975	0.983471074	0.991735537	Bn-N10-p5958597	Bn-chrA10-p4743257
Bn-A10-p5178249	0.991735537	0.991735537	Bn-N10-p5989733	Bn-chrA10-p4765965
Bn-A10-p5183795	0.991735537	1	Bn-N10-p5995748	Bn-chrA10-p4769301
Bn-A10-p5183890	1	0.991735537	Bn-N10-p5995843	Bn-chrA10-p4769396
Bn-A10-p5185255	1	0.991735537	Bn-N10-p5997210	Bn-chrA10-p4770762
Bn-A10-p5202031	0.966942149	0.909090909	Bn-N10-p6018661	Bn-chrC09-p31643005
Bn-A10-p5209915	0.983471074	0.975206612	Bn-N10-p6030433	Bn-chrA10-p4798429
Bn-A10-p5210009	0.983471074	0.991735537	Bn-N10-p6030527	Bn-chrA10-p4798523
Bn-A10-p5210045	0.983471074	0.991735537	Bn-N10-p6030563	Bn-chrA10-p4798559
Bn-A10-p5210204	0.991735537	0.983471074	Bn-N10-p6030722	Bn-chrA10-p4798718
Bn-A10-p5210238	0.983471074	0.991735537	Bn-N10-p6030756	Bn-chrA10-p4798752
Bn-A10-p5210317	0.983471074	0.991735537	Bn-N10-p6030835	Bn-chrA10-p4798831
Bn-A10-p5210325	0.983471074	0.991735537	Bn-N10-p6030843	Bn-chrA10-p4798839
Bn-A10-p5210388	0.966942149	0.958677686	Bn-N10-p6030906	Bn-chrA10-p4798902
Bn-A10-p5212114	0.966942149	0.94214876	Bn-N10-p6032633	Bn-chrA10-p4801763
Bn-A10-p5213281	0.966942149	0.958677686	Bn-N10-p6034014	Bn-chrA10-p4802921
Bn-A10-p5218803	0.975206612	0.966942149	Bn-N10-p6039535	Bn-chrA10-p4806086
Bn-A10-p5219035	0.975206612	0.966942149	Bn-N10-p6039767	Bn-chrA10-p4806318
Bn-A10-p5219089	0.975206612	0.983471074	Bn-N10-p6039821	Bn-chrA10-p4806372
Bn-A10-p5224680	0.958677686	0.892561983	Bn-N10-p6045274	Bn-chrA10-p4813756
Bn-A10-p5225089	0.991735537	0.975206612	Bn-N10-p6045683	Bn-chrA10-p4814163
Bn-A10-p5225094	0.983471074	0.983471074	Bn-N10-p6045688	Bn-chrA10-p4814168
Bn-A10-p5234281	0.975206612	0.966942149	Bn-N10-p6055135	Bn-chrA10-p4821347
Bn-A10-p5236071	0.991735537	1	Bn-N10-p6056925	Bn-chrA10-p4822721
Bn-A10-p5239454	0.966942149	0.975206612	Bn-N10-p6057930	Bn-chrA10-p4823723
Bn-A10-p5239634	0.983471074	0.991735537	Bn-N10-p6058110	Bn-chrA10-p4823903
Bn-A10-p5239938	0.991735537	0.983471074	Bn-N10-p6058414	Bn-chrA10-p4824207
Bn-A10-p5240072	1	0.991735537	Bn-N10-p6058548	Bn-chrA10-p4824341
Bn-A10-p5240139	0.966942149	0.958677686	Bn-N10-p6058615	Bn-chrA10-p4824408
Bn-A10-p5240656	0.991735537	0.983471074	Bn-N10-p6059132	Bn-chrA10-p4824925
Bn-A10-p5242221	0.991735537	1	Bn-N10-p6060610	Bn-chrA10-p4826528
Bn-A10-p5244792	0.991735537	0.975206612	Bn-N10-p6063181	Bn-chrA10-p4829848

Bn-A10-p5250586	0.975206612	0.991735537	Bn-N10-p6068974	Bn-chrA10-p4840318
Bn-A10-p5267588	0.991735537	1	Bn-N10-p6088040	Bn-chrA10-p4854320
Bn-A10-p5299784	1	0.991735537	Bn-N10-p6114631	Bn-chrA10-p4878039
Bn-A10-p5302094	1	0.991735537	Bn-N10-p6116941	Bn-chrA10-p4880349
Bn-A10-p5317804	0.983471074	0.975206612	Bn-N10-p6132376	Bn-chrA10-p4895932
Bn-A10-p5330675	0.865671642	0.865671642	Bn-N5-p13909044	Bn-chrA05-p13916957
Bn-A10-p5341726	1	0.991735537	Bn-N10-p6144711	Bn-chrA10-p4920744
Bn-A10-p5342414	0.958677686	0.966942149	Bn-N10-p6145399	Bn-chrA10-p4921117
Bn-A10-p5342465	0.950413223	0.94214876	Bn-N10-p6145450	Bn-chrA10-p4921168
Bn-A10-p5525609	0.966942149	0.975206612	Bn-N10-p6371122	Bn-chrA05-p11793247
Bn-A10-p5532832	0.94214876	0.710743802	Bn-N10-p6372119	Bn-chrA10-p5167370
Bn-A10-p5604463	0.975124378	0.975124378	Bn-N10-p7152115	Bn-chrA10-p5244977
Bn-A10-p5606181	0.980099502	0.980099502	Bn-N10-p7153825	Bn-chrA10-p5246687
Bn-A10-p5635816	0.983471074	0.933884298	Bn-N10-p7187678	Bn-chrA10-p5271673
Bn-A10-p5662796	0.983471074	0.983471074	Bn-N10-p7217768	Bn-chrA10-p5304433
Bn-A10-p5666711	0.966942149	0.958677686	Bn-N10-p7221687	Bn-chrA10-p5311140
Bn-A10-p5669630	0.975206612	0.966942149	Bn-N10-p7224607	Bn-chrA10-p5314064
Bn-A10-p5670945	0.983471074	0.991735537	Bn-N10-p7225922	Bn-chrA10-p5315379
Bn-A10-p5673195	0.983471074	0.991735537	Bn-N10-p7228172	Bn-chrA10-p5317647
Bn-A10-p5673588	1	0.958677686	Bn-N10-p7228565	Bn-chrA10-p5318035
Bn-A10-p5675953	0.966942149	0.958677686	Bn-N10-p7230929	Bn-chrA10-p5320399
Bn-A10-p5679914	0.966942149	0.975206612	Bn-N10-p7235080	Bn-chrA10-p5324731
Bn-A10-p5680678	0.958677686	0.867768595	Bn-N10-p7235844	Bn-chrA10-p5325490
Bn-A10-p5684074	0.983471074	0.975206612	Bn-N10-p7239240	Bn-chrA10-p5328883
Bn-A10-p5712509	0.975206612	0.892561983	Bn-N10-p7265104	Bn-chrA10-p5338364
Bn-A10-p5721147	0.966942149	0.975206612	Bn-N10-p7276667	Bn-chrAnn_random-p386627
Bn-A10-p5727780	0.983471074	0.991735537	Bn-N10-p7285331	Bn-chrA10-p5358238
Bn-A10-p5730775	0.983471074	0.975206612	Bn-N10-p7287797	Bn-chrA10-p5365326
Bn-A10-p5746168	0.958677686	0.958677686	Bn-N10-p7303335	Bn-chrA10-p5380555
Bn-A10-p5762378	0.983471074	0.983471074	Bn-N10-p7330505	Bn-chrA10-p5402907
Bn-A10-p5774002	0.983471074	0.983471074	Bn-N10-p7336747	Bn-chrA10-p5410684
Bn-A10-p5778609	0.983471074	0.983471074	Bn-N10-p7341628	Bn-chrA10-p5414874
Bn-A10-p5781113	0.975206612	0.975206612	Bn-N10-p7344842	Bn-chrA10-p5417394
Bn-A10-p5799347	0.980099502	0.985074627	Bn-N10-p7364123	Bn-chrA10-p5436592
Bn-A10-p5819027	0.966942149	0.975206612	Bn-N10-p7382826	Bn-chrA10-p5451195
Bn-A10-p5831782	0.975206612	0.983471074	Bn-N10-p7396078	Bn-chrA10-p5464373
Bn-A10-p5832298	0.958677686	0.950413223	Bn-N10-p7396594	Bn-chrA10-p5464888
Bn-A10-p5832645	0.950413223	0.991735537	Bn-N10-p7396938	Bn-chrA10-p5465235
Bn-A10-p5834005	1	0.991735537	Bn-N10-p7399371	Bn-chrA10-p5466595
Bn-A10-p5845294	0.983471074	0.983471074	Bn-N10-p7406294	Bn-chrA10-p5477103
Bn-A10-p5860913	0.983471074	0.991735537	Bn-N10-p7422645	Bn-chrA10-p5490838
Bn-A10-p5869175	0.933884298	0.975206612	Bn-N10-p7430860	Bn-chrA10-p5499100
Bn-A10-p5873429	0.958677686	0.966942149	Bn-N10-p7435086	Bn-chrA10-p5503354
Bn-A10-p5888302	0.900826446	0.892561983	Bn-N10-p7453775	Bn-chrA10-p5515045
Bn-A10-p5892580	0.995024876	0.990049751	Bn-N10-p7455880	Bn-chrA10-p5517142
Bn-A10-p5996125	0.958677686	0.933884298	Bn-N10-p7589626	Bn-chrAnn_random-p296742

Bn-A10-p6012827	0.97311828	0.97311828	Bn-N19-p38254946	Bn-chrC09-p33299679
Bn-A10-p6055143	0.996677741	0.996677741	Bn-N10-p7631082	Bn-chrA10-p5676993
Bn-A10-p6120400	0.983388704	0.983388704	Bn-N10-p7704661	Bn-chrA10-p5741892
Bn-A10-p6126590	0.966942149	0.975206612	Bn-N10-p7711290	Bn-chrA10-p5748118
Bn-A10-p6137434	0.975124378	0.975124378	Bn-N10-p7723073	Bn-chrA10-p5758808
Bn-A10-p6148523	0.975206612	0.983471074	Bn-N10-p7751010	Bn-chrA10-p5770010
Bn-A10-p6159693	0.980066445	0.980066445	Bn-N10-p7762914	Bn-chrA10-p5779900
Bn-A10-p6160352	0.970149254	0.970149254	Bn-N10-p7763571	Bn-chrA10-p5780557
Bn-A10-p6162838	0.990033223	0.990033223	Bn-N10-p7765879	Bn-chrA10-p5783106
Bn-A10-p6166631	0.890547264	0.925373134	Bn-N10-p7769600	Bn-chrA10-p5785301
Bn-A10-p6176593	0.991735537	0.983471074	Bn-N10-p7779320	Bn-chrA10-p5794103
Bn-A10-p6177431	0.991735537	0.983471074	Bn-N10-p7780378	Bn-chrA10-p5794550
Bn-A10-p6180693	0.976744186	0.976744186	Bn-N10-p7783581	Bn-chrA10_random-p119484
Bn-A10-p6192971	0.94214876	0.94214876	Bn-N10-p7796722	Bn-chrCnn_random-p425066
Bn-A10-p6195984	0.867768595	0.876033058	Bn-N10-p7799739	Bn-chrA10_random-p117858
Bn-A10-p6198988	0.990033223	0.996677741	Bn-N10-p7802731	Bn-chrA10_random-p117667
Bn-A10-p6204788	0.975124378	0.985074627	Bn-N10-p7808581	Bn-chrA10_random-p117081
Bn-A10-p6228003	0.966942149	0.975206612	Bn-N10-p7833539	Bn-chrA10-p7053442
Bn-A10-p6233556	0.966942149	0.966942149	Bn-N10-p7839095	Bn-chrA10-p7058974
Bn-A10-p6240637	0.958677686	0.958677686	Bn-N10-p7846042	Bn-chrA10-p7065862
Bn-A10-p6247519	0.975206612	0.983471074	Bn-N10-p7856544	Bn-chrA10-p7075242
Bn-A10-p6270494	0.975206612	0.966942149	Bn-N10-p7880012	Bn-chrA10-p7079634
Bn-A10-p6271467	0.991735537	1	Bn-N10-p7880985	Bn-chrA10-p7080546
Bn-A10-p6271554	0.958677686	0.966942149	Bn-N10-p7881072	Bn-chrA10-p7080633
Bn-A10-p6271776	0.94214876	0.958677686	Bn-N10-p7881288	Bn-chrA10-p7080855
Bn-A10-p6278596	1	0.991735537	Bn-N10-p7888819	Bn-chrA10-p7088670
Bn-A10-p6280436	0.975206612	0.966942149	Bn-N10-p7890668	Bn-chrA10-p7090507
Bn-A10-p6290949	0.983471074	0.975206612	Bn-N10-p7901247	Bn-chrA10-p7108308
Bn-A10-p6299839	0.958677686	0.950413223	Bn-N10-p7905901	Bn-chrA10-p7112954
Bn-A10-p6309983	0.975206612	0.975206612	Bn-N10-p7916258	Bn-chrA10-p7121941
Bn-A10-p6322788	0.975206612	0.983471074	Bn-N10-p7929103	Bn-chrA10-p7135999
Bn-A10-p6323527	0.966942149	0.975206612	Bn-N10-p7929842	Bn-chrA10-p7136739
Bn-A10-p6324850	0.975206612	0.983471074	Bn-N10-p7931165	Bn-chrA10-p7138064
Bn-A10-p6332974	0.966942149	0.975206612	Bn-N19-p38934920	Bn-chrA10-p7163408
Bn-A10-p6344414	0.991735537	0.983471074	Bn-N10-p7956059	Bn-chrA10-p7151899
Bn-A10-p6364237	0.910447761	0.990049751	Bn-N10-p7982887	Bn-chrA10-p7187933
Bn-A10-p6371190	0.990033223	0.996677741	Bn-N10-p7989875	Bn-chrA10-p7194726
Bn-A10-p6405472	0.966942149	0.958677686	Bn-N10-p8026286	Bn-chrA10_random-p133576
Bn-A10-p6412022	0.958677686	0.950413223	Bn-N10-p8033876	Bn-chrA10-p7242620
Bn-A10-p6418601	0.991735537	0.983471074	Bn-N10-p8040443	Bn-chrA10-p7249551
Bn-A10-p6457220	0.900826446	0.909090909	Bn-N10-p8076350	Bn-chrA10-p7258624
Bn-A10-p6459258	0.983471074	0.966942149	Bn-N10-p8078561	Bn-chrA10-p7259032
Bn-A10-p6459782	0.94214876	0.776859504	Bn-N10-p8079085	Bn-chrA10-p7259562
Bn-A10-p6460164	0.834710744	0.958677686	Bn-N10-p8079448	Bn-chrA10-p7259896
Bn-A10-p6464869	0.975206612	0.975206612	Bn-N10-p8085336	Bn-chrA10-p7273108
Bn-A10-p6468609	0.917355372	0.644628099	Bn-N10-p8089061	Bn-chrA10-p7276846

Bn-A10-p6537045	0.966942149	0.975206612	Bn-N10-p8126020	Bn-chrA10-p8124311
Bn-A10-p6539423	0.991735537	0.975206612	Bn-N10-p8128397	Bn-chrC09-p34336178
Bn-A10-p6540548	0.950413223	0.958677686	Bn-N10-p8129526	Bn-chrA10-p8140388
Bn-A10-p6541418	0.958677686	0.950413223	Bn-N10-p8130396	Bn-chrA10-p8141257
Bn-A10-p6555288	0.983471074	0.884297521	Bn-N10-p8149171	Bn-chrC09-p34369194
Bn-A10-p6558564	0.991735537	0.983471074	Bn-N10-p8152524	Bn-chrA10-p8170419
Bn-A10-p6561569	0.958677686	0.958677686	Bn-N19-p39467239	Bn-chrC09-p34377755
Bn-A10-p6582015	0.975206612	0.950413223	Bn-N10-p8179837	Bn-chrA10-p8198841
Bn-A10-p6600373	0.990049751	0.990049751	Bn-N10-p8197048	Bn-chrA10-p8216123
Bn-A10-p6600844	0.983471074	0.983471074	Bn-N10-p8197519	Bn-chrA10-p8216594
Bn-A10-p6604928	0.975206612	0.743801653	Bn-N10-p8201602	Bn-chrC09-p34481867
Bn-A10-p6609801	0.966942149	0.966942149	Bn-N10-p8206554	Bn-chrAnn_random-p274352
Bn-A10-p6624071	0.991735537	0.991735537	Bn-N10-p8220366	Bn-chrA10-p8228879
Bn-A10-p6632002	0.975206612	0.975206612	Bn-N10-p8228294	Bn-chrA10-p8238827
Bn-A10-p6638864	0.991735537	0.991735537	Bn-N10-p8235727	Bn-chrA10-p8245839
Bn-A10-p6639652	0.958677686	0.958677686	Bn-N10-p8236514	Bn-chrA10-p8246626
Bn-A10-p6643953	0.983471074	0.983471074	Bn-N10-p8242779	Bn-chrA10-p8252112
Bn-A10-p6671842	0.975206612	0.975206612	Bn-N10-p8274092	Bn-chrA10-p8279975
Bn-A10-p6678779	0.966942149	0.966942149	Bn-N10-p8282150	Bn-chrA10-p8286760
Bn-A10-p6724918	0.809917355	0.710743802	Bn-N10-p8317394	Bn-chrA10-p8318132
Bn-A10-p6730588	0.970149254	0.970149254	Bn-N10-p8322916	Bn-chrA10-p8323654
Bn-A10-p6743896	0.990049751	0.995024876	Bn-N10-p8345249	Bn-chrA10-p8352376
Bn-A10-p6760255	0.990049751	0.990049751	Bn-N10-p8361662	Bn-chrA10-p8367666
Bn-A10-p6760664	0.980099502	0.980099502	Bn-N10-p8362071	Bn-chrA10-p8368075
Bn-A10-p6760904	0.975124378	0.975124378	Bn-N10-p8362311	Bn-chrA10-p8368315
Bn-A10-p6761330	0.980099502	0.980099502	Bn-N10-p8362736	Bn-chrA10-p8368740
Bn-A10-p6763376	0.958677686	0.958677686	Bn-N10-p8365468	Bn-chrA10-p8370783
Bn-A10-p6765523	0.975206612	0.966942149	Bn-N10-p8367620	Bn-chrA10-p8372929
Bn-A10-p6770590	0.652892562	0.652892562	Bn-N12-p30401317	Bn-chrC02-p32636509
Bn-A10-p6773336	0.809917355	0.966942149	Bn-N17-p25486115	Bn-chrA10-p8447440
Bn-A10-p6779480	0.950413223	0.991735537	Bn-N19-p39864173	Bn-chrA10-p8441249
Bn-A10-p6792798	0.966942149	0.975206612	Bn-N19-p39887885	Bn-chrA10-p8429118
Bn-A10-p6796120	0.975206612	0.983471074	Bn-N10-p8395757	Bn-chrA10-p8425539
Bn-A10-p6838885	0.975124378	0.955223881	Bn-N10-p8410308	Bn-chrA10-p8409934
Bn-A10-p6851091	0.983471074	0.966942149	Bn-N10-p8427566	Bn-chrC09-p35000959
Bn-A10-p6851375	0.980099502	0.990049751	Bn-N10-p8427848	Bn-chrA10-p8403752
Bn-A10-p6864839	0.958677686	0.983471074	Bn-N10-p8447579	Bn-chrA10-p8389803
Bn-A10-p6880184	0.975124378	0.970149254	Bn-N10-p8458434	Bn-chrA10-p8460576
Bn-A10-p6881287	0.983471074	0.983471074	Bn-N10-p8459537	Bn-chrA10-p8461679
Bn-A10-p6896063	0.950413223	0.958677686	Bn-N10-p8472459	Bn-chrA10-p8474298
Bn-A10-p6915231	0.958677686	0.958677686	Bn-N10-p8499914	Bn-chrA10-p8501855
Bn-A10-p6967288	0.966942149	0.983471074	Bn-N19-p40237283	Bn-chrA10-p8555160
Bn-A10-p6968402	0.933884298	0.94214876	Bn-N10-p8549974	Bn-chrA10-p8559688
Bn-A10-p7065016	0.945273632	0.975124378	Bn-N10-p8633594	Bn-chrA10-p8647552
Bn-A10-p7069659	0.996677741	0.996677741	Bn-N10-p8638254	Bn-chrA10-p8652209
Bn-A10-p7076810	0.976744186	0.976744186	Bn-N10-p8648033	Bn-chrA10-p8665825

Bn-A10-p7078693	0.983388704	0.983388704	Bn-N10-p8649923	Bn-chrA10-p8667715
Bn-A10-p7078767	0.990033223	0.990033223	Bn-N10-p8649997	Bn-chrA10-p8667789
Bn-A10-p7109137	0.995024876	0.995024876	Bn-N10-p8674036	Bn-chrA10-p8694317
Bn-A10-p7118112	0.985074627	0.985074627	Bn-N10-p8683108	Bn-chrA10-p8703409
Bn-A10-p7182541	0.991735537	0.991735537	Bn-N10-p8753600	Bn-chrA10-p8781237
Bn-A10-p7186465	0.975206612	0.975206612	Bn-N19-p40656394	Bn-chrA10-p8786764
Bn-A10-p7231726	1	1	Bn-N10-p8794663	Bn-chrA10-p8868748
Bn-A10-p7247322	0.900826446	0.892561983	Bn-N10-p8810546	Bn-chrA10-p8883802
Bn-A10-p7250703	0.96013289	0.96013289	Bn-N10-p8818594	Bn-chrA10-p8893827
Bn-A10-p7252424	1	0.991735537	Bn-N10-p8820235	Bn-chrA10-p8895466
Bn-A10-p7260660	0.925619835	0.933884298	Bn-N10-p8832756	Bn-chrA10-p8903549
Bn-A10-p7262868	0.983471074	0.991735537	Bn-N10-p8834966	Bn-chrA10-p8905758
Bn-A10-p7278102	0.975206612	0.966942149	Bn-N10-p8840505	Bn-chrA10-p8920313
Bn-A10-p7278188	0.990033223	0.986710963	Bn-N10-p8840591	Bn-chrA10-p8920399
Bn-A10-p7283605	1	0.996677741	Bn-N10-p8846180	Bn-chrA10-p8925994
Bn-A10-p7309029	0.94214876	0.950413223	Bn-N10-p8869981	Bn-chrA10-p8968405
Bn-A10-p7326339	0.980099502	0.980099502	Bn-N10-p8881369	Bn-chrA10_random-p153725
Bn-A10-p7341107	0.985074627	0.925373134	Bn-Scaffold17729-p3	Bn-chrC09_random-p353582
Bn-A10-p7346569	0.996677741	0.990033223	Bn-N10-p8919875	Bn-chrA10-p8998128
Bn-A10-p7347530	0.966942149	0.950413223	Bn-N10-p8920828	Bn-chrA10-p8999081
Bn-A10-p7349925	0.990049751	0.995024876	Bn-N10-p8924295	Bn-chrA10-p9011582
Bn-A10-p7357100	0.996677741	0.966777409	Bn-N10-p8938488	Bn-chrA10-p9019949
Bn-A10-p7357442	0.958677686	0.925619835	Bn-N10-p8938830	Bn-chrA10-p9020292
Bn-A10-p7357555	0.980099502	0.935323383	Bn-N10-p8938943	Bn-chrA10-p9020402
Bn-A10-p7360229	0.79338843	0.909090909	Bn-N10-p8940805	Bn-chrA10-p9030142
Bn-A10-p7365704	0.933884298	0.966942149	Bn-N19-p41150470	Bn-chrA10-p9030869
Bn-A10-p7376910	0.975206612	0.966942149	Bn-N10-p8951417	Bn-chrA10-p9053011
Bn-A10-p7388008	0.950413223	0.958677686	Bn-N19-p41235285	Bn-chrA10-p9060857
Bn-A10-p7393471	0.990033223	0.986710963	Bn-N10-p8969857	Bn-chrA10-p9066114
Bn-A10-p7393632	0.983471074	0.975206612	Bn-N10-p8970018	Bn-chrA10-p9066275
Bn-A10-p7394180	0.953488372	0.986710963	Bn-N10-p8970618	Bn-chrA10-p9066823
Bn-A10-p7394578	0.958677686	0.966942149	Bn-N10-p8971040	Bn-chrA10-p9067231
Bn-A10-p7410271	0.986710963	0.990033223	Bn-N10-p8986806	Bn-chrA10-p9082803
Bn-A10-p7444203	0.983471074	0.983471074	Bn-N10-p9020878	Bn-chrA09-p5779828
Bn-A10-p7453695	0.752066116	0.983471074	Bn-N19-p41394658	Bn-chrA05-p14455512
Bn-A10-p7454278	0.983471074	0.975206612	Bn-N10-p9023298	Bn-chrA05-p14454929
Bn-A10-p7454604	0.917355372	1	Bn-N10-p9023632	Bn-chrA05-p14454603
Bn-A10-p7480192	0.958677686	0.958677686	Bn-N10-p9035369	Bn-chrAnn_random-p217544
Bn-A10-p7480918	0.810631229	0.837209302	Bn-N19-p41478141	Bn-chrAnn_random-p217551
Bn-A10-p7482568	0.990033223	0.983388704	Bn-N19-p41479811	Bn-chrC09-p36380938
Bn-A10-p7492957	0.737541528	0.996677741	Bn-N10-p9054105	Bn-chrA10-p9182745
Bn-A10-p7500176	0.975206612	0.94214876	Bn-N10-p9061322	Bn-chrC09-p36413349
Bn-A10-p7502551	0.950413223	0.94214876	Bn-N10-p9063787	Bn-chrA10-p9194707
Bn-A10-p7502935	0.933884298	0.950413223	Bn-N10-p9064197	Bn-chrA10-p9195091
Bn-A10-p7504317	0.976744186	0.976744186	Bn-N10-p9065708	Bn-chrA10-p9196483
Bn-A10-p7510542	0.973421927	0.973421927	Bn-N10-p9071873	Bn-chrA10-p9202710

Bn-A10-p7513409	0.986710963	0.986710963	Bn-N10-p9074740	Bn-chrA10-p9205581
Bn-A10-p7554677	0.970149254	0.970149254	Bn-N10-p9122739	Bn-chrA10-p9251499
Bn-A10-p7560857	0.990033223	0.990033223	Bn-N10-p9130002	Bn-chrA10-p9257906
Bn-A10-p7578202	0.965174129	0.965174129	Bn-N10-p9147320	Bn-chrA10-p9275204
Bn-A10-p7586742	0.975206612	0.983471074	Bn-N10-p9155142	Bn-chrA10-p9283019
Bn-A10-p7587166	0.958677686	0.950413223	Bn-N10-p9155596	Bn-chrA10-p9283455
Bn-A10-p7588274	0.975206612	0.966942149	Bn-N10-p9156704	Bn-chrA10-p9285417
Bn-A10-p7599250	0.966942149	0.975206612	Bn-N10-p9167144	Bn-chrA10-p9290430
Bn-A10-p7600250	1	0.991735537	Bn-N10-p9168144	Bn-chrA10-p9291431
Bn-A10-p7611277	0.94214876	0.950413223	Bn-N10-p9178214	Bn-chrA10-p9309646
Bn-A10-p7611361	0.983471074	0.975206612	Bn-N10-p9178298	Bn-chrA10-p9309730
Bn-A10-p7628181	0.993355482	0.993355482	Bn-N10-p9200324	Bn-chrA10_random-p148723
Bn-A10-p7636136	0.966942149	0.966942149	Bn-N10-p9210764	Bn-chrA10_random-p147623
Bn-A10-p7644362	0.966942149	0.966942149	Bn-N10-p9221793	Bn-chrA10_random-p146797
Bn-A10-p7647619	0.983471074	0.983471074	Bn-N19-p41876207	Bn-chrCnn_random-p787562
Bn-A10-p7650629	0.958677686	0.958677686	Bn-N10-p9228035	Bn-chrA10_random-p146172
Bn-A10-p7650750	0.983471074	0.983471074	Bn-N10-p9228156	Bn-chrA10_random-p146160
Bn-A10-p7650844	0.990033223	0.990033223	Bn-N10-p9228250	Bn-chrA10_random-p146150
Bn-A10-p7652354	0.995024876	0.995024876	Bn-N10-p9229760	Bn-chrA10_random-p145999
Bn-A10-p7652705	1	1	Bn-N10-p9230111	Bn-chrA10_random-p145964
Bn-A10-p7657767	0.983471074	0.983471074	Bn-N10-p9235166	Bn-chrA10_random-p145458
Bn-A10-p7669513	1	1	Bn-N10-p9245890	Bn-chrA10_random-p144293
[truncated: 813,528 more chars]
