# Supplementary material for: A high-density SNP genotyping array for Brassica napus and its ancestral diploid species based on optimised selection of single-locus markers in the allotetraploid genome
Source: Theor Appl Genet. 2016 Jun 30;129(10):1887–99. doi: 10.1007/s00122-016-2746-7 (PMC5025514; doi:10.1007/s00122-016-2746-7)
Supplement: Supplementary file 6 — Complete scoring matrix for genetic map for Brassica napus based on SG DH population (DH12075 x PSA12). (PDF 26859 kb) [file 122_2016_2746_MOESM6_ESM.pdf]

Table 4: Complete genetic map for Brassica napus based on SG population

Order	DH12075v4_ID	New_name_rapa	locus_name	LG	Bin	Distance	SG_152
1	Bn-N1-p148713	Bn-A01-p137814	Bn-A01-p137814	A1	1	0	B
2	Bn-N1-p148768	Bn-A01-p137869	Bn-A01-p137869	A1	1	0	B
3	Bn-N1-p189666	Bn-A01-p178709	Bn-A01-p178709	A1	1	0	B
4	Bn-N1-p208162	Bn-A01-p195253	Bn-A01-p195253	A1	1	0	B
5	Bn-N1-p240997	Bn-A01-p234898	Bn-A01-p234898	A1	1	0	B
6	Bn-N1-p241218	Bn-A01-p235115	Bn-A01-p235115	A1	1	0	B
7	Bn-N1-p241562	Bn-A01-p235484	Bn-A01-p235484	A1	1	0	B
8	Bn-N1-p241855	Bn-A01-p235781	Bn-A01-p235781	A1	1	0	B
9	Bn-N1-p251681	Bn-A01-p250759	Bn-A01-p250759	A1	1	0	B
10	Bn-N1-p325704	Bn-A01-p324562	Bn-A01-p324562	A1	2	1.9	B
11	Bn-N1-p405672	Bn-C1-p529102	Bn-scaff_19244_1-p44173(	A1	2	1.9	B
12	Bn-N1-p442085	Bn-A01-p442492	Bn-A01-p442492	A1	2	1.9	B
13	Bn-N1-p455627	Bn-A01-p460072	Bn-A01-p460072	A1	2	1.9	B
14	Bn-N1-p463330	Bn-A01-p467816	Bn-A01-p467816	A1	2	1.9	B
15	Bn-N1-p504157	Bn-A01-p515600	Bn-A01-p515600	A1	2	1.9	B
16	Bn-N1-p595502	Bn-A01-p595651	Bn-A01-p595651	A1	2	1.9	B
17	Bn-N1-p602504	Bn-A01-p598242	Bn-A01-p598242	A1	2	1.9	B
18	Bn-N1-p604190	Bn-A01-p599910	Bn-A01-p599910	A1	2	1.9	B
19	Bn-N1-p613419	Bn-A01-p609857	Bn-A01-p609857	A1	2	1.9	B
20	Bn-N1-p615539	Bn-A01-p611977	Bn-A01-p611977	A1	2	1.9	B
21	Bn-N1-p627350	Bn-A01-p622550	Bn-A01-p622550	A1	2	1.9	B
22	Bn-N1-p648675	Bn-A01-p644410	Bn-A01-p644410	A1	2	1.9	B
23	Bn-N1-p650216	Bn-A01-p645951	Bn-A01-p645951	A1	2	1.9	B
24	Bn-N1-p652446	Bn-A01-p648196	Bn-A01-p648196	A1	2	1.9	B
25	Bn-N1-p654018	Bn-A01-p649805	Bn-A01-p649805	A1	2	1.9	B
26	Bn-N1-p663269	Bn-A01-p663088	Bn-A01-p663088	A1	2	1.9	B
27	Bn-N1-p687818	Bn-A01-p688355	Bn-A01-p688355	A1	2	1.9	B
28	Bn-N1-p704843	Bn-A01-p704525	Bn-A01-p704525	A1	2	1.9	B
29	Bn-N1-p791511	Bn-A01-p777123	Bn-A01-p777123	A1	3	1.9	B
30	Bn-N1-p792427	Bn-A01-p778048	Bn-A01-p778048	A1	3	1.9	B
31	Bn-N1-p819843	Bn-C2-p12033716	Bn-scaff_20461_1-p16932(	A1	3	1.9	B
32	Bn-N1-p839949	Bn-A01-p820317	Bn-A01-p820317	A1	3	1.9	B
33	Bn-N11-p2427698	Bn-A01-p975213	Bn-A01-p975213	A1	4	3	B
34	Bn-N11-p2713205	Bn-A01-p1188928	Bn-A01-p1188928	A1	4	3	B
35	Bn-N1-p1001647	Bn-A01-p974934	Bn-A01-p974934	A1	4	3	B
36	Bn-N1-p1005541	Bn-A01-p978860	Bn-A01-p978860	A1	4	3	B
37	Bn-N1-p1030519	Bn-A01-p1000115	Bn-A01-p1000115	A1	4	3	B
38	Bn-N1-p1056422	Bn-A01-p1029558	Bn-A01-p1029558	A1	4	3	B
39	Bn-N1-p1056532	Bn-A01-p1029668	Bn-A01-p1029668	A1	4	3	B
40	Bn-N1-p1069090	Bn-A01-p1041947	Bn-A01-p1041947	A1	4	3	B
41	Bn-N1-p1099188	Bn-A01-p1067371	Bn-A01-p1067371	A1	4	3	B
42	Bn-N1-p1169679	Bn-A01-p1132885	Bn-A01-p1132885	A1	4	3	B
43	Bn-N1-p1196607	Bn-A01-p1148760	Bn-A01-p1148760	A1	4	3	B
44	Bn-N1-p1197545	Bn-A01-p1149698	Bn-A01-p1149698	A1	4	3	B
45	Bn-N1-p1198704	Bn-A01-p1150857	Bn-A01-p1150857	A1	4	3	B

46	Bn-N1-p1199814	Bn-A01-p1151961	Bn-A01-p1151961	A1	4	3	B
47	Bn-N1-p1200224	Bn-A01-p1152369	Bn-A01-p1152369	A1	4	3	0
48	Bn-N1-p1238133	Bn-A01-p1188004	Bn-A01-p1188004	A1	4	3	B
49	Bn-N1-p891435	Bn-A01-p866260	Bn-A01-p866260	A1	4	3	B
50	Bn-N1-p894187	Bn-C1-p1241973	Bn-scaff_15838_1-p51787[illegible]	A1	4	3	B
51	Bn-N1-p900297	Bn-A01-p878256	Bn-A01-p878256	A1	4	3	B
52	Bn-N1-p902872	Bn-A01-p879755	Bn-A01-p879755	A1	4	3	B
53	Bn-N1-p932870	Bn-A01-p912298	Bn-A01-p912298	A1	4	3	B
54	Bn-N1-p936377	Bn-A01-p916774	Bn-A01-p916774	A1	4	3	B
55	Bn-N1-p958851	Bn-A01-p938887	Bn-A01-p938887	A1	4	3	B
56	Bn-N1-p959173	Bn-A01-p939202	Bn-A01-p939202	A1	4	3	B
57	Bn-N1-p996151	Bn-A01-p968465	Bn-A01-p968465	A1	4	3	B
58	Bn-N1-p1281775	Bn-A01-p1236927	Bn-A01-p1236927	A1	5	4.1	B
59	Bn-N1-p1290014	Bn-A01-p1247899	Bn-A01-p1247899	A1	5	4.1	B
60	Bn-N1-p1292716	Bn-A01-p1250595	Bn-A01-p1250595	A1	5	4.1	B
61	Bn-N1-p1304643	Bn-A01-p1261017	Bn-A01-p1261017	A1	5	4.1	B
62	Bn-N1-p1319351	Bn-A01-p1276486	Bn-A01-p1276486	A1	5	4.1	B
63	Bn-N1-p1319369	Bn-A01-p1276504	Bn-A01-p1276504	A1	5	4.1	B
64	Bn-N1-p1355558	Bn-A01-p1314440	Bn-A01-p1314440	A1	5	4.1	B
65	Bn-N1-p1418752	Bn-A01-p1372430	Bn-A01-p1372430	A1	6	6.3	B
66	Bn-N1-p1464686	Bn-A01-p1409723	Bn-A01-p1409723	A1	6	6.3	B
67	Bn-N1-p1477507	Bn-A01-p1423163	Bn-A01-p1423163	A1	6	6.3	B
68	Bn-N1-p1495817	Bn-A01-p1440009	Bn-A01-p1440009	A1	6	6.3	B
69	Bn-N1-p1509145	Bn-A01-p1453156	Bn-A01-p1453156	A1	6	6.3	B
70	Bn-N1-p1509590	Bn-A01-p1453560	Bn-A01-p1453560	A1	6	6.3	B
71	Bn-N1-p1512420	Bn-A01-p1456256	Bn-A01-p1456256	A1	6	6.3	B
72	Bn-N1-p1530325	Bn-A01-p1473188	Bn-A01-p1473188	A1	6	6.3	B
73	Bn-N11-p3326058	Bn-A01-p1678541	Bn-A01-p1678541	A1	7	8.5	B
74	Bn-N11-p3334929	Bn-A01-p1684485	Bn-A01-p1684485	A1	7	8.5	B
75	Bn-N1-p1683410	Bn-C1-p2176739	Bn-scaff_15838_1-p14526[illegible]	A1	7	8.5	B
76	Bn-N1-p1703757	Bn-A01-p1661180	Bn-A01-p1661180	A1	7	8.5	B
77	Bn-N1-p1709347	Bn-A01-p1666732	Bn-A01-p1666732	A1	7	8.5	B
78	Bn-N1-p1722929	Bn-A01-p14559770	Bn-A01-p14559770	A1	7	8.5	B
79	Bn-N1-p1806273	Bn-A01-p1742846	Bn-A01-p1742846	A1	8	9.6	B
80	Bn-N1-p1807914	Bn-A01-p1745099	Bn-A01-p1745099	A1	8	9.6	B
81	Bn-N1-p1812677	Bn-A01-p1746226	Bn-A01-p1746226	A1	8	9.6	B
82	Bn-N1-p1840607	Bn-A01-p1770291	Bn-A01-p1770291	A1	9	10.7	B
83	Bn-N1-p1840799	Bn-A01-p1770482	Bn-A01-p1770482	A1	9	10.7	B
84	Bn-N1-p1841352	Bn-A01-p1772621	Bn-A01-p1772621	A1	9	10.7	B
85	Bn-N1-p1842065	Bn-A01-p1773289	Bn-A01-p1773289	A1	9	10.7	B
86	Bn-N1-p1864630	Bn-A01-p1793928	Bn-A01-p1793928	A1	9	10.7	B
87	Bn-N1-p1897288	Bn-A01-p1823705	Bn-A01-p1823705	A1	9	10.7	B
88	Bn-N1-p1905152	Bn-A01-p1831507	Bn-A01-p1831507	A1	9	10.7	B
89	Bn-N1-p1911681	Bn-A01-p1838960	Bn-A01-p1838960	A1	9	10.7	B
90	Bn-N1-p1931677	Bn-A01-p1855903	Bn-A01-p1855903	A1	9	10.7	B
91	Bn-N1-p1934009	Bn-A01-p1858279	Bn-A01-p1858279	A1	9	10.7	B
92	Bn-N1-p1960659	Bn-A01-p1880406	Bn-A01-p1880406	A1	9	10.7	B
93	Bn-N1-p1969454	Bn-A01-p1890102	Bn-A01-p1890102	A1	9	10.7	B
94	Bn-N1-p2040832	Bn-A01-p1990755	Bn-A01-p1990755	A1	10	11.8	B

95	Bn-N1-p2044259	Bn-A01-p1993302	Bn-A01-p1993302	A1	10	11.8	B
96	Bn-N1-p2055534	Bn-A01-p2004336	Bn-A01-p2004336	A1	10	11.8	B
97	Bn-N1-p2093225	Bn-A01-p2042209	Bn-A01-p2042209	A1	10	11.8	0
98	Bn-N1-p2104938	Bn-A01-p2053320	Bn-A01-p2053320	A1	10	11.8	B
99	Bn-N1-p2120803	Bn-A01-p2069488	Bn-A01-p2069488	A1	10	11.8	B
100	Bn-N11-p3917104	Bn-A01-p2121224	Bn-A01-p2121224	A1	11	12.9	B
101	Bn-N1-p2145056	Bn-A01-p2094793	Bn-A01-p2094793	A1	11	12.9	B
102	Bn-N1-p2151261	Bn-A01-p2100996	Bn-A01-p2100996	A1	11	12.9	B
103	Bn-N11-p3967814	Bn-A01-p2177864	Bn-A01-p2177864	A1	12	14	B
104	Bn-N1-p2211868	Bn-A01-p2171217	Bn-A01-p2171217	A1	12	14	B
105	Bn-N11-p4104265	Bn-C1-p2985151	Bn-scaff_15838_1-p22610!	A1	13	16.2	0
106	Bn-N1-p2284563	Bn-A01-p2242890	Bn-A01-p2242890	A1	13	16.2	B
107	Bn-N1-p2290291	Bn-A01-p2244327	Bn-A01-p2244327	A1	13	16.2	B
108	Bn-N1-p2308181	Bn-A01-p2260859	Bn-A01-p2260859	A1	13	16.2	B
109	Bn-N1-p2309230	Bn-A01-p2261903	Bn-A01-p2261903	A1	13	16.2	B
110	Bn-N1-p2349273	Bn-A01-p2311015	Bn-A01-p2311015	A1	13	16.2	B
111	Bn-N1-p2361058	Bn-A01-p2318972	Bn-A01-p2318972	A1	13	16.2	B
112	Bn-N1-p2457821	Bn-A01-p2421445	Bn-A01-p2421445	A1	13	16.2	B
113	Bn-N1-p2550026	Bn-A01-p2504370	Bn-A01-p2504370	A1	13	16.2	B
114	Bn-N1-p2552952	Bn-A01-p2501423	Bn-A01-p2501423	A1	13	16.2	B
115	Bn-N1-p2577241	Bn-A01-p2478054	Bn-A01-p2478054	A1	13	16.2	B
116	Bn-N1-p2595584	Bn-A01-p2457750	Bn-A01-p2457750	A1	13	16.2	B
117	Bn-N1-p2600258	Bn-A01-p2448162	Bn-A01-p2448162	A1	13	16.2	B
118	Bn-N1-p2609944	Bn-A01-p2569303	Bn-A01-p2569303	A1	13	16.2	B
119	Bn-N1-p2637711	Bn-A01-p2587709	Bn-A01-p2587709	A1	13	16.2	B
120	Bn-N1-p2657233	Bn-A01-p2608660	Bn-A01-p2608660	A1	14	19	B
121	Bn-N1-p2671811	Bn-A01-p2624222	Bn-A01-p2624222	A1	14	19	B
122	Bn-N1-p2683740	Bn-A01-p2634563	Bn-A01-p2634563	A1	14	19	B
123	Bn-N1-p2720419	Bn-A01-p2667471	Bn-A01-p2667471	A1	14	19	B
124	Bn-N1-p2720635	Bn-A01-p2667691	Bn-A01-p2667691	A1	14	19	B
125	Bn-N1-p2720711	Bn-A01-p2667767	Bn-A01-p2667767	A1	14	19	B
126	Bn-N1-p2770797	Bn-A01-p2713528	Bn-A01-p2713528	A1	14	19	B
127	Bn-N1-p2770852	Bn-A01-p2713583	Bn-A01-p2713583	A1	14	19	B
128	Bn-N1-p2775946	Bn-A01-p2718126	Bn-A01-p2718126	A1	14	19	B
129	Bn-N8-p12837754	Bn-A01-p2713439	Bn-A01-p2713439	A1	14	19	B
130	Bn-N1-p2879286	Bn-A01-p2825565	Bn-A01-p2825565	A1	15	20.4	B
131	Bn-N1-p2921846	Bn-A01-p2867284	Bn-A01-p2867284	A1	16	21.8	B
132	Bn-N1-p2927409	Bn-A01-p2873007	Bn-A01-p2873007	A1	16	21.8	B
133	Bn-N1-p2941286	Bn-A01-p2884934	Bn-A01-p2884934	A1	16	21.8	B
134	Bn-N1-p2981735	Bn-A01-p2915231	Bn-A01-p2915231	A1	16	21.8	B
135	Bn-N1-p2982319	Bn-A01-p2915821	Bn-A01-p2915821	A1	16	21.8	B
136	Bn-N1-p2997150	Bn-A01-p2928824	Bn-A01-p2928824	A1	16	21.8	B
137	Bn-N1-p3051730	Bn-A01-p2990104	Bn-A01-p2990104	A1	16	21.8	B
138	Bn-N1-p3065971	Bn-A01-p3005310	Bn-A01-p3005310	A1	16	21.8	B
139	Bn-N1-p3066454	Bn-A01-p3005793	Bn-A01-p3005793	A1	16	21.8	B
140	Bn-N1-p3104967	Bn-A01-p3045214	Bn-A01-p3045214	A1	16	21.8	B
141	Bn-N1-p3105729	Bn-A01-p3045984	Bn-A01-p3045984	A1	16	21.8	B
142	Bn-N1-p3110102	Bn-A01-p3050613	Bn-A01-p3050613	A1	16	21.8	B

143	Bn-N1-p3119720	Bn-A01-p3061223	Bn-A01-p3061223	A1	16	21.8	B
144	Bn-N1-p3129154	Bn-A01-p3070657	Bn-A01-p3070657	A1	16	21.8	B
145	Bn-N1-p3134899	Bn-A01-p3076531	Bn-A01-p3076531	A1	16	21.8	B
146	Bn-N1-p3135680	Bn-A01-p3077309	Bn-A01-p3077309	A1	16	21.8	B
147	Bn-N1-p3199609	Bn-A01-p3136468	Bn-A01-p3136468	A1	16	21.8	B
148	Bn-N1-p3238299	Bn-A01-p3169855	Bn-A01-p3169855	A1	16	21.8	B
149	Bn-Scaffold03534-p130	Bn-A01-p3173607	Bn-A01-p3173607	A1	16	21.8	B
150	Bn-Scaffold03534-p2004	Bn-A01-p3175614	Bn-A01-p3175614	A1	16	21.8	B
151	Bn-N1-p3247018	Bn-A01-p3183888	Bn-A01-p3183888	A1	17	23.2	B
152	Bn-N1-p3356185	Bn-A01-p3306526	Bn-A01-p3306526	A1	18	24.4	B
153	Bn-N1-p3356763	Bn-A01-p3307173	Bn-A01-p3307173	A1	18	24.4	B
154	Bn-N1-p3363215	Bn-A01-p3323476	Bn-A01-p3323476	A1	18	24.4	B
155	Bn-N1-p3522836	Bn-A01-p3487320	Bn-A01-p3487320	A1	18	24.4	B
156	Bn-N1-p3534473	Bn-A01-p3495476	Bn-A01-p3495476	A1	18	24.4	B
157	Bn-N1-p3612730	Bn-A01-p3567127	Bn-A01-p3567127	A1	18	24.4	B
158	Bn-N1-p3618633	Bn-A01-p3573045	Bn-A01-p3573045	A1	18	24.4	B
159	Bn-N1-p3621665	Bn-A01-p3576068	Bn-A01-p3576068	A1	18	24.4	B
160	Bn-N1-p3626398	Bn-A01-p3579887	Bn-A01-p3579887	A1	18	24.4	B
161	Bn-N1-p3643891	Bn-A01-p3586744	Bn-A01-p3586744	A1	18	24.4	B
162	Bn-N1-p3655215	Bn-A01-p3598097	Bn-A01-p3598097	A1	18	24.4	B
163	Bn-N1-p3665703	Bn-A01-p3610530	Bn-A01-p3610530	A1	18	24.4	B
164	Bn-N1-p3679483	Bn-A01-p3626821	Bn-A01-p3626821	A1	18	24.4	B
165	Bn-N1-p3690151	Bn-A01-p3631990	Bn-A01-p3631990	A1	18	24.4	B
166	Bn-N1-p3700085	Bn-A01-p3643352	Bn-A01-p3643352	A1	18	24.4	B
167	Bn-N1-p3701452	Bn-A01-p3644770	Bn-A01-p3644770	A1	18	24.4	B
168	Bn-N11-p6991883	Bn-C1-p5809315	Bn-scaff_19193_1-p65805(	A1	19	26.7	B
169	Bn-N11-p6991936	Bn-C1-p5809368	Bn-scaff_19193_1-p657997	A1	19	26.7	B
170	Bn-N1-p3952570	Bn-A01-p3904495	Bn-A01-p3904495	A1	19	26.7	B
171	Bn-N1-p3955040	Bn-A01-p3906965	Bn-A01-p3906965	A1	19	26.7	B
172	Bn-N1-p3987645	Bn-A01-p3939508	Bn-A01-p3939508	A1	19	26.7	B
173	Bn-N1-p4036442	Bn-A01-p3984534	Bn-A01-p3984534	A1	19	26.7	B
174	Bn-N1-p4084437	Bn-A01-p4027402	Bn-A01-p4027402	A1	19	26.7	B
175	Bn-N1-p4129839	Bn-A01-p4070059	Bn-A01-p4070059	A1	19	26.7	B
176	Bn-N1-p4171006	Bn-A01-p4116110	Bn-A01-p4116110	A1	19	26.7	B
177	Bn-N1-p4274490	Bn-A01-p4227703	Bn-A01-p4227703	A1	19	26.7	B
178	Bn-N1-p4293684	Bn-A01-p4242269	Bn-A01-p4242269	A1	19	26.7	B
179	Bn-N1-p4297042	Bn-A01-p4246586	Bn-A01-p4246586	A1	19	26.7	B
180	Bn-N1-p4354411	Bn-A01-p4311134	Bn-A01-p4311134	A1	19	26.7	B
181	Bn-N1-p4367773	Bn-A01-p4322340	Bn-A01-p4322340	A1	19	26.7	B
182	Bn-N1-p4400568	Bn-A01-p4354753	Bn-A01-p4354753	A1	19	26.7	B
183	Bn-N1-p4421854	Bn-A01-p4377760	Bn-A01-p4377760	A1	19	26.7	B
184	Bn-N1-p4486639	Bn-A01-p26064479	Bn-A01-p26064479	A1	19	26.7	B
185	Bn-N1-p4488299	Bn-A01-p4452779	Bn-A01-p4452779	A1	19	26.7	B
186	Bn-N1-p4614510	Bn-A01-p4580719	Bn-A01-p4580719	A1	19	26.7	B
187	Bn-N1-p4642962	Bn-A01-p4600975	Bn-A01-p4600975	A1	19	26.7	B
188	Bn-N1-p4651489	Bn-A01-p4612881	Bn-A01-p4612881	A1	19	26.7	B
189	Bn-N1-p4057880	Bn-A01-p4003991	Bn-A01-p4003991	A1	20	26.7	B
190	Bn-N1-p4747599	Bn-C1-p6623334	Bn-scaff_17731_1-p15587(	A1	20	26.7	B
191	Bn-N1-p4757019	Bn-A01-p4747874	Bn-A01-p4747874	A1	20	26.7	B

192	Bn-N1-p4759874	Bn-A01-p4750734	Bn-A01-p4750734	A1	20	26.7	B
193	Bn-N1-p4785853	Bn-A01-p4773772	Bn-A01-p4773772	A1	20	26.7	B
194	Bn-N1-p4786146	Bn-A01-p4774065	Bn-A01-p4774065	A1	20	26.7	B
195	Bn-N1-p4802753	Bn-A01-p4786754	Bn-A01-p4786754	A1	20	26.7	B
196	Bn-N1-p4802786	Bn-A01-p4786787	Bn-A01-p4786787	A1	20	26.7	B
197	Bn-N1-p4803559	Bn-A01-p4787515	Bn-A01-p4787515	A1	20	26.7	B
198	Bn-N1-p4819255	Bn-A01-p4803773	Bn-A01-p4803773	A1	20	26.7	B
199	Bn-N1-p4828077	Bn-A01-p4812269	Bn-A01-p4812269	A1	20	26.7	B
200	Bn-N1-p4830975	Bn-A01-p4820187	Bn-A01-p4820187	A1	20	26.7	B
201	Bn-N1-p4839663	Bn-A01-p4832074	Bn-A01-p4832074	A1	20	26.7	B
202	Bn-N1-p4840575	Bn-A01-p4833020	Bn-A01-p4833020	A1	20	26.7	B
203	Bn-N1-p4844234	Bn-A01-p4836338	Bn-A01-p4836338	A1	20	26.7	B
204	Bn-N1-p4847934	Bn-A01-p4840973	Bn-A01-p4840973	A1	20	26.7	B
205	Bn-N1-p4881303	Bn-A01-p4874972	Bn-A01-p4874972	A1	20	26.7	B
206	Bn-N1-p4892640	Bn-A01-p4883188	Bn-A01-p4883188	A1	20	26.7	B
207	Bn-N1-p4918604	Bn-A01-p4908924	Bn-A01-p4908924	A1	20	26.7	B
208	Bn-N1-p4918744	Bn-A01-p4909065	Bn-A01-p4909065	A1	20	26.7	B
209	Bn-N1-p4922079	Bn-A01-p4920622	Bn-A01-p4920622	A1	20	26.7	B
210	Bn-N1-p4924007	Bn-A01-p4922557	Bn-A01-p4922557	A1	20	26.7	B
211	Bn-N1-p4926465	Bn-A01-p4925004	Bn-A01-p4925004	A1	20	26.7	B
212	Bn-N1-p4952041	Bn-A01-p4947577	Bn-A01-p4947577	A1	20	26.7	B
213	Bn-N1-p4983719	Bn-A01-p4985329	Bn-A01-p4985329	A1	20	26.7	B
214	Bn-N1-p5034368	Bn-A01-p5057753	Bn-A01-p5057753	A1	20	26.7	B
215	Bn-N1-p5078759	Bn-A01-p5106042	Bn-A01-p5106042	A1	21	27.6	B
216	Bn-N1-p5157847	Bn-A01-p5193639	Bn-A01-p5193639	A1	22	28.5	B
217	Bn-N11-p8814470	Bn-A01-p5385928	Bn-A01-p5385928	A1	23	29.5	B
218	Bn-N11-p9349312	Bn-A01-p5718049	Bn-A01-p5718049	A1	23	29.5	B
219	Bn-N1-p5202234	Bn-A01-p5238564	Bn-A01-p5238564	A1	23	29.5	B
220	Bn-N1-p5206638	Bn-A01-p5242967	Bn-A01-p5242967	A1	23	29.5	B
221	Bn-N1-p5206700	Bn-A01-p5243029	Bn-A01-p5243029	A1	23	29.5	B
222	Bn-N1-p5206707	Bn-A01-p5243036	Bn-A01-p5243036	A1	23	29.5	B
223	Bn-N1-p5206852	Bn-A01-p5243181	Bn-A01-p5243181	A1	23	29.5	B
224	Bn-N1-p5236162	Bn-A01-p5265598	Bn-A01-p5265598	A1	23	29.5	B
225	Bn-N1-p5270686	Bn-A01-p5296564	Bn-A01-p5296564	A1	23	29.5	B
226	Bn-N1-p5289158	Bn-A01-p5318059	Bn-A01-p5318059	A1	23	29.5	B
227	Bn-N1-p5294832	Bn-A01-p5323726	Bn-A01-p5323726	A1	23	29.5	B
228	Bn-N1-p5295505	Bn-A01-p5324399	Bn-A01-p5324399	A1	23	29.5	B
229	Bn-N1-p5295578	Bn-A01-p5324472	Bn-A01-p5324472	A1	23	29.5	B
230	Bn-N1-p5300332	Bn-A01-p5328220	Bn-A01-p5328220	A1	23	29.5	B
231	Bn-N1-p5302294	Bn-A01-p5330192	Bn-A01-p5330192	A1	23	29.5	B
232	Bn-N1-p5305947	Bn-A01-p5332773	Bn-A01-p5332773	A1	23	29.5	B
233	Bn-N1-p5307651	Bn-A01-p5334474	Bn-A01-p5334474	A1	23	29.5	B
234	Bn-N1-p5309927	Bn-A01-p5335218	Bn-A01-p5335218	A1	23	29.5	B
235	Bn-N1-p5310032	Bn-A01-p5335323	Bn-A01-p5335323	A1	23	29.5	B
236	Bn-N1-p5310082	Bn-A01-p5335372	Bn-A01-p5335372	A1	23	29.5	B
237	Bn-N1-p5370787	Bn-A01-p5391551	Bn-A01-p5391551	A1	23	29.5	B
238	Bn-N1-p5397830	Bn-A01-p5418396	Bn-A01-p5418396	A1	23	29.5	B
239	Bn-N1-p5442893	Bn-A01-p5469727	Bn-A01-p5469727	A1	23	29.5	B
240	Bn-N1-p5452118	Bn-A01-p5481004	Bn-A01-p5481004	A1	23	29.5	B

241	Bn-N1-p5456726	Bn-A01-p5485980	Bn-A01-p5485980	A1	23	29.5	B
242	Bn-N1-p5456850	Bn-A01-p5486104	Bn-A01-p5486104	A1	23	29.5	B
243	Bn-N1-p5499937	Bn-A01-p5522403	Bn-A01-p5522403	A1	23	29.5	B
244	Bn-N1-p5510839	Bn-A01-p5536695	Bn-A01-p5536695	A1	23	29.5	B
245	Bn-N1-p5539534	Bn-A01-p5550226	Bn-A01-p5550226	A1	23	29.5	B
246	Bn-N1-p5556653	Bn-A01-p5567077	Bn-A01-p5567077	A1	23	29.5	B
247	Bn-N1-p5557170	Bn-A01-p5567840	Bn-A01-p5567840	A1	23	29.5	B
248	Bn-N1-p5562764	Bn-A01-p5570110	Bn-A01-p5570110	A1	23	29.5	B
249	Bn-N1-p5562878	Bn-A01-p5570221	Bn-A01-p5570221	A1	23	29.5	B
250	Bn-N1-p5583095	Bn-A01-p5585933	Bn-A01-p5585933	A1	23	29.5	B
251	Bn-N1-p5584119	Bn-A01-p5586671	Bn-A01-p5586671	A1	23	29.5	B
252	Bn-N1-p5584302	Bn-A01-p5586854	Bn-A01-p5586854	A1	23	29.5	B
253	Bn-N1-p5585331	Bn-A01-p5587840	Bn-A01-p5587840	A1	23	29.5	B
254	Bn-N1-p5588331	Bn-A01-p5590998	Bn-A01-p5590998	A1	23	29.5	B
255	Bn-N1-p5592645	Bn-A01-p5595319	Bn-A01-p5595319	A1	23	29.5	B
256	Bn-N1-p5596422	Bn-A01-p5601232	Bn-A01-p5601232	A1	23	29.5	B
257	Bn-N1-p5613276	Bn-A01-p5621392	Bn-A01-p5621392	A1	23	29.5	B
258	Bn-N1-p5644004	Bn-A01-p5659083	Bn-A01-p5659083	A1	23	29.5	B
259	Bn-N1-p5687622	Bn-A01-p5697706	Bn-A01-p5697706	A1	23	29.5	B
260	Bn-N1-p5700098	Bn-A01-p5711920	Bn-A01-p5711920	A1	23	29.5	B
261	Bn-N1-p5719044	Bn-A01-p5730640	Bn-A01-p5730640	A1	23	29.5	B
262	Bn-N1-p5719833	Bn-A01-p5731429	Bn-A01-p5731429	A1	23	29.5	B
263	Bn-N1-p5720630	Bn-A01-p5732226	Bn-A01-p5732226	A1	23	29.5	B
264	Bn-N1-p5728610	Bn-A01-p5737901	Bn-A01-p5737901	A1	23	29.5	B
265	Bn-N1-p5759503	Bn-A01-p5762190	Bn-A01-p5762190	A1	23	29.5	B
266	Bn-N1-p5766660	Bn-A01-p5775331	Bn-A01-p5775331	A1	23	29.5	B
267	Bn-N1-p5768493	Bn-A01-p5777117	Bn-A01-p5777117	A1	23	29.5	B
268	Bn-N1-p5797482	Bn-A01-p5797296	Bn-A01-p5797296	A1	23	29.5	B
269	Bn-N1-p5806986	Bn-A01-p5806635	Bn-A01-p5806635	A1	23	29.5	B
270	Bn-N1-p5822192	Bn-A01-p5821651	Bn-A01-p5821651	A1	23	29.5	B
271	Bn-N1-p5822556	Bn-A01-p5822015	Bn-A01-p5822015	A1	23	29.5	B
272	Bn-N1-p5878881	Bn-A01-p5868598	Bn-A01-p5868598	A1	23	29.5	B
273	Bn-N1-p5883788	Bn-A01-p5873504	Bn-A01-p5873504	A1	23	29.5	B
274	Bn-N1-p5888445	Bn-A01-p5878485	Bn-A01-p5878485	A1	23	29.5	B
275	Bn-N1-p5914797	Bn-A01-p5899092	Bn-A01-p5899092	A1	23	29.5	B
276	Bn-N1-p5959680	Bn-A01-p5949582	Bn-A01-p5949582	A1	23	29.5	B
277	Bn-N1-p5959763	Bn-A01-p5949665	Bn-A01-p5949665	A1	23	29.5	B
278	Bn-N1-p5963407	Bn-A01-p5959700	Bn-A01-p5959700	A1	23	29.5	B
279	Bn-N1-p5992032	Bn-A01-p5977820	Bn-A01-p5977820	A1	23	29.5	B
280	Bn-N1-p6006584	Bn-A01-p5990203	Bn-A01-p5990203	A1	23	29.5	B
281	Bn-N1-p6011242	Bn-A01-p5994840	Bn-A01-p5994840	A1	23	29.5	B
282	Bn-N1-p6011382	Bn-A01-p5994980	Bn-A01-p5994980	A1	23	29.5	B
283	Bn-N1-p6059919	Bn-A01-p6038249	Bn-A01-p6038249	A1	23	29.5	B
284	Bn-N1-p6134186	Bn-A01-p6111016	Bn-A01-p6111016	A1	24	30.4	B
285	Bn-N1-p6192158	Bn-A01-p6168163	Bn-A01-p6168163	A1	24	30.4	B
286	Bn-N1-p6206809	Bn-A01-p6182710	Bn-A01-p6182710	A1	24	30.4	B
287	Bn-N1-p6212733	Bn-A01-p6188347	Bn-A01-p6188347	A1	25	32.3	B
288	Bn-N1-p6240730	Bn-A01-p6217011	Bn-A01-p6217011	A1	25	32.3	B
289	Bn-N1-p6244388	Bn-A01-p6220667	Bn-A01-p6220667	A1	25	32.3	B

290	Bn-N1-p6255985	Bn-A01-p6232061	Bn-A01-p6232061	A1	25	32.3	B
291	Bn-N1-p6284809	Bn-A01-p6272642	Bn-A01-p6272642	A1	25	32.3	B
292	Bn-N1-p6286302	Bn-A01-p6274135	Bn-A01-p6274135	A1	25	32.3	B
293	Bn-N1-p6287952	Bn-A01-p6275784	Bn-A01-p6275784	A1	25	32.3	B
294	Bn-N1-p6331851	Bn-A01-p6333810	Bn-A01-p6333810	A1	25	32.3	B
295	Bn-N1-p6331950	Bn-A01-p6333910	Bn-A01-p6333910	A1	25	32.3	B
296	Bn-N1-p6348505	Bn-A01-p6349933	Bn-A01-p6349933	A1	25	32.3	B
297	Bn-N1-p6482538	Bn-A01-p6482543	Bn-A01-p6482543	A1	25	32.3	B
298	Bn-N1-p6556551	Bn-A01-p6560270	Bn-A01-p6560270	A1	25	32.3	B
299	Bn-N1-p6660632	Bn-A01-p6678914	Bn-A01-p6678914	A1	25	32.3	B
300	Bn-N1-p6671771	Bn-A01-p6687707	Bn-A01-p6687707	A1	26	34.2	B
301	Bn-N1-p6671903	Bn-A01-p6687839	Bn-A01-p6687839	A1	26	34.2	B
302	Bn-N1-p6683633	Bn-C1-p9736868	Bn-scaff_20638_1-p11630	A1	26	34.2	B
303	Bn-N1-p6691596	Bn-A01-p6708539	Bn-A01-p6708539	A1	26	34.2	B
304	Bn-N1-p6723020	Bn-A01-p6747706	Bn-A01-p6747706	A1	26	34.2	B
305	Bn-N1-p6755980	Bn-A01-p6776634	Bn-A01-p6776634	A1	26	34.2	B
306	Bn-N1-p6810101	Bn-A01-p6825673	Bn-A01-p6825673	A1	26	34.2	B
307	Bn-N1-p6810755	Bn-A01-p6826327	Bn-A01-p6826327	A1	26	34.2	B
308	Bn-N1-p6828366	Bn-A01-p6838738	Bn-A01-p6838738	A1	26	34.2	B
309	Bn-N1-p6894209	Bn-A01-p6898291	Bn-A01-p6898291	A1	27	35.1	B
310	Bn-N1-p6946745	Bn-A01-p6958863	Bn-A01-p6958863	A1	27	35.1	B
311	Bn-N1-p6947123	Bn-A01-p6959248	Bn-A01-p6959248	A1	27	35.1	B
312	Bn-N1-p6952513	Bn-A01-p6965221	Bn-A01-p6965221	A1	27	35.1	B
313	Bn-N1-p6955983	Bn-A01-p6968751	Bn-A01-p6968751	A1	27	35.1	B
314	Bn-N1-p6956429	Bn-A01-p6969197	Bn-A01-p6969197	A1	27	35.1	B
315	Bn-N1-p6956516	Bn-A01-p6969284	Bn-A01-p6969284	A1	27	35.1	B
316	Bn-N1-p6956590	Bn-A01-p6969358	Bn-A01-p6969358	A1	27	35.1	B
317	Bn-N1-p6961785	Bn-A01-p6972779	Bn-A01-p6972779	A1	27	35.1	B
318	Bn-N1-p7146539	Bn-A01-p7132729	Bn-A01-p7132729	A1	27	35.1	B
319	Bn-N11-p12469663	Bn-C1-p11167303	Bn-scaff_17592_1-p36152	A1	28	36	B
320	Bn-N1-p7166601	Bn-A01-p7160702	Bn-A01-p7160702	A1	28	36	B
321	Bn-N1-p7373856	Bn-A01-p7363480	Bn-A01-p7363480	A1	28	36	B
322	Bn-N1-p7523127	Bn-A01-p7487861	Bn-A01-p7487861	A1	28	36	B
323	Bn-N1-p7683662	Bn-C1-p11409530	Bn-scaff_17592_1-p60375	A1	28	36	B
324	Bn-N1-p7686806	Bn-A01-p7648929	Bn-A01-p7648929	A1	28	36	B
325	Unknown	Bn-A01-p7646601	Bn-A01-p7646601	A1	28	36	B
326	Bn-N1-p7886156	Bn-A01-p7825044	Bn-A01-p7825044	A1	29	37.9	B
327	Bn-N1-p7911511	Bn-A01-p7852440	Bn-A01-p7852440	A1	29	37.9	B
328	Bn-N1-p7920296	Bn-A01-p7861283	Bn-A01-p7861283	A1	29	37.9	B
329	Bn-N1-p7947519	Bn-A01-p7889385	Bn-A01-p7889385	A1	29	37.9	B
330	Bn-N1-p7948308	Bn-A01-p7890174	Bn-A01-p7890174	A1	29	37.9	B
331	Bn-N1-p7973148	Bn-A01-p7926287	Bn-A01-p7926287	A1	29	37.9	B
332	Bn-N1-p7988862	Bn-A01-p7942548	Bn-A01-p7942548	A1	29	37.9	B
333	Bn-N1-p7998583	Bn-A01-p7953001	Bn-A01-p7953001	A1	29	37.9	B
334	Bn-N1-p8008195	Bn-A01-p7961620	Bn-A01-p7961620	A1	30	38.8	B
335	Bn-N1-p8033912	Bn-A01-p7979458	Bn-A01-p7979458	A1	30	38.8	B
336	Bn-N11-p13267055	Bn-A01-p7982220	Bn-A01-p7982220	A1	31	39.7	B
337	Bn-N11-p13398541	Bn-C1-p12012770	Bn-scaff_17369_1-p84137	A1	31	39.7	B
338	Bn-N1-p8037687	Bn-A01-p7983241	Bn-A01-p7983241	A1	31	39.7	B

339	Bn-N1-p8044316	Bn-A01-p7987687	Bn-A01-p7987687	A1	31	39.7	B
340	Bn-N1-p8048407	Bn-C1-p11900248	Bn-scaff_17369_1-p95389	A1	31	39.7	B
341	Bn-N1-p8071288	Bn-A01-p8016433	Bn-A01-p8016433	A1	31	39.7	B
342	Bn-N1-p8072235	Bn-A01-p8017380	Bn-A01-p8017380	A1	31	39.7	B
343	Bn-N1-p8095898	Bn-A01-p8045760	Bn-A01-p8045760	A1	31	39.7	B
344	Bn-N1-p8096280	Bn-A01-p8046142	Bn-A01-p8046142	A1	31	39.7	B
345	Bn-N1-p8106618	Bn-A01-p8051804	Bn-A01-p8051804	A1	31	39.7	B
346	Bn-N1-p8113120	Bn-A01-p8058255	Bn-A01-p8058255	A1	31	39.7	B
347	Bn-N1-p8113339	Bn-A01-p8058475	Bn-A01-p8058475	A1	31	39.7	B
348	Bn-N1-p8117459	Bn-A01-p8063652	Bn-A01-p8063652	A1	31	39.7	B
349	Bn-N11-p13444522	Bn-C1-p12059994	Bn-scaff_17369_1-p79414	A1	32	40.6	B
350	Bn-N11-p15137831	Bn-C1-p13626442	Bn-scaff_19614_1-p29327	A1	32	40.6	B
351	Bn-N11-p15138260	Bn-C1-p13626872	Bn-scaff_19614_1-p29284	A1	32	40.6	B
352	Bn-N11-p15138321	Bn-C1-p13626933	Bn-scaff_19614_1-p29278	A1	32	40.6	B
353	Bn-N12-p24679216	Bn-C2-p30460921	Bn-scaff_18507_1-p40822	A1	32	40.6	B
354	Bn-N1-p8129897	Bn-A01-p8076080	Bn-A01-p8076080	A1	32	40.6	B
355	Bn-N1-p8150073	Bn-A01-p8108178	Bn-A01-p8108178	A1	32	40.6	B
356	Bn-N1-p8153093	Bn-A01-p8111196	Bn-A01-p8111196	A1	32	40.6	B
357	Bn-N1-p8154622	Bn-A01-p8115849	Bn-A01-p8115849	A1	32	40.6	B
358	Bn-N1-p8155064	Bn-A01-p8116314	Bn-A01-p8116314	A1	32	40.6	B
359	Bn-N1-p8256146	Bn-A01-p8240363	Bn-A01-p8240363	A1	32	40.6	B
360	Bn-N1-p8273622	Bn-A01-p8246641	Bn-A01-p8246641	A1	32	40.6	B
361	Bn-N1-p8446285	Bn-A01-p8402568	Bn-A01-p8402568	A1	32	40.6	B
362	Bn-N1-p8461229	Bn-A01-p8418102	Bn-A01-p8418102	A1	32	40.6	B
363	Bn-N1-p8518161	Bn-A01-p8470314	Bn-A01-p8470314	A1	32	40.6	B
364	Bn-N1-p8549776	Bn-A01-p8504134	Bn-A01-p8504134	A1	32	40.6	B
365	Bn-N1-p8562814	Bn-A01-p8515129	Bn-A01-p8515129	A1	32	40.6	B
366	Bn-N1-p8581598	Bn-A01-p8543512	Bn-A01-p8543512	A1	32	40.6	B
367	Bn-N1-p8600760	Bn-A01-p8564149	Bn-A01-p8564149	A1	32	40.6	B
368	Bn-N1-p8687484	Bn-A01-p8647625	Bn-A01-p8647625	A1	32	40.6	B
369	Bn-N1-p8721483	Bn-A01-p8675601	Bn-A01-p8675601	A1	32	40.6	B
370	Bn-N1-p8721906	Bn-A01-p8676024	Bn-A01-p8676024	A1	32	40.6	B
371	Bn-N1-p8722026	Bn-A01-p8676144	Bn-A01-p8676144	A1	32	40.6	B
372	Bn-N1-p8725314	Bn-A01-p8681309	Bn-A01-p8681309	A1	32	40.6	B
373	Bn-N1-p8725561	Bn-A01-p8681556	Bn-A01-p8681556	A1	32	40.6	B
374	Bn-N1-p8725762	Bn-A01-p8681757	Bn-A01-p8681757	A1	32	40.6	B
375	Bn-N1-p8751591	Bn-A01-p9180340	Bn-A01-p9180340	A1	32	40.6	B
376	Bn-N1-p8752082	Bn-A01-p9180828	Bn-A01-p9180828	A1	32	40.6	B
377	Bn-N1-p8807849	Bn-A01-p9254925	Bn-A01-p9254925	A1	32	40.6	B
378	Bn-N1-p8808969	Bn-A01-p9256011	Bn-A01-p9256011	A1	32	40.6	B
379	Bn-N1-p8811102	Bn-A01-p9258144	Bn-A01-p9258144	A1	32	40.6	B
380	Bn-N1-p8812569	Bn-A01-p9259611	Bn-A01-p9259611	A1	32	40.6	B
381	Bn-N1-p8813031	Bn-A01-p9260073	Bn-A01-p9260073	A1	32	40.6	B
382	Bn-N1-p8822872	Bn-A01-p9264081	Bn-A01-p9264081	A1	32	40.6	B
383	Bn-N1-p8884533	Bn-A01-p9331463	Bn-A01-p9331463	A1	32	40.6	B
384	Bn-N1-p8886114	Bn-A01-p9333956	Bn-A01-p9333956	A1	32	40.6	B
385	Bn-N1-p8887264	Bn-A01-p9337252	Bn-A01-p9337252	A1	32	40.6	B
386	Bn-N1-p8904052	Bn-A01-p9360043	Bn-A01-p9360043	A1	32	40.6	B
387	Bn-N1-p8948927	Bn-C1-p13401609	Bn-scaff_18636_1-p17733	A1	32	40.6	B

388	Bn-N1-p8995481	Bn-A01-p9442471	Bn-A01-p9442471	A1	32	40.6	B
389	Bn-N1-p8997612	Bn-A01-p9445754	Bn-A01-p9445754	A1	32	40.6	B
390	Bn-N1-p9013686	Bn-A01-p9476520	Bn-A01-p9476520	A1	32	40.6	B
391	Bn-N1-p9029564	Bn-A01-p9499100	Bn-A01-p9499100	A1	32	40.6	B
392	Bn-N1-p9047680	Bn-A01-p9519478	Bn-A01-p9519478	A1	32	40.6	B
393	Bn-N1-p9080805	Bn-A01-p9565360	Bn-A01-p9565360	A1	32	40.6	B
394	Bn-N1-p9084007	Bn-A01-p9568888	Bn-A01-p9568888	A1	32	40.6	B
395	Bn-N1-p9123417	Bn-A01-p9614730	Bn-A01-p9614730	A1	32	40.6	B
396	Bn-N1-p9213589	Bn-A01-p9699152	Bn-A01-p9699152	A1	33	41.5	B
397	Bn-N1-p9221164	Bn-A01-p9711809	Bn-A01-p9711809	A1	33	41.5	B
398	Bn-N1-p9221963	Bn-A01-p9712591	Bn-A01-p9712591	A1	33	41.5	B
399	Bn-N1-p9378768	Bn-A01-p9866468	Bn-A01-p9866468	A1	33	41.5	B
400	Bn-N1-p9398357	Bn-A01-p9884449	Bn-A01-p9884449	A1	33	41.5	B
401	Bn-N1-p9438573	Bn-A01-p9922741	Bn-A01-p9922741	A1	33	41.5	B
402	Bn-N1-p9529910	Bn-A01-p9989240	Bn-A01-p9989240	A1	34	42.4	B
403	Bn-N1-p9540400	Bn-A01-p9996240	Bn-A01-p9996240	A1	34	42.4	B
404	Bn-N1-p9540618	Bn-A01-p9996458	Bn-A01-p9996458	A1	34	42.4	B
405	Bn-N1-p9782678	Bn-A01-p10231780	Bn-A01-p10231780	A1	35	43.3	B
406	Bn-N1-p9793020	Bn-A01-p10242217	Bn-A01-p10242217	A1	35	43.3	B
407	Bn-N1-p9793104	Bn-A01-p10242298	Bn-A01-p10242298	A1	35	43.3	B
408	Bn-N1-p9797752	Bn-A01-p10249225	Bn-A01-p10249225	A1	35	43.3	B
409	Bn-N1-p9799309	Bn-A01-p10251052	Bn-A01-p10251052	A1	35	43.3	B
410	Bn-N1-p9805260	Bn-A01-p10257919	Bn-A01-p10257919	A1	35	43.3	B
411	Bn-N1-p9819446	Bn-A01-p10268662	Bn-A01-p10268662	A1	35	43.3	B
412	Bn-N1-p9830448	Bn-A01-p10278752	Bn-A01-p10278752	A1	35	43.3	B
413	Bn-N1-p9839493	Bn-A01-p10285074	Bn-A01-p10285074	A1	35	43.3	B
414	Bn-N1-p9847473	Bn-A01-p10297732	Bn-A01-p10297732	A1	35	43.3	B
415	Bn-N1-p9848920	Bn-A01-p10299187	Bn-A01-p10299187	A1	35	43.3	B
416	Bn-N1-p9915747	Bn-A01-p10343401	Bn-A01-p10343401	A1	35	43.3	B
417	Bn-N1-p9916844	Bn-A01-p10344498	Bn-A01-p10344498	A1	35	43.3	B
418	Bn-N1-p9958899	Bn-A01-p10398937	Bn-A01-p10398937	A1	35	43.3	B
419	Bn-Scaffold18942-p478	Bn-A01-p10250255	Bn-A01-p10250255	A1	35	43.3	B
420	Bn-N11-p16439822	Bn-C1-p14940042	Bn-scaff_15747_1-p77520	A1	36	44.2	B
421	Bn-N11-p16440735	Bn-C1-p14940954	Bn-scaff_15747_1-p77611	A1	36	44.2	B
422	Bn-N11-p27777155	Bn-A01-p16223356	Bn-A01-p16223356	A1	36	44.2	B
423	Bn-N1-p10024494	Bn-C4-p24903660	Bn-scaff_16217_1-p14334	A1	36	44.2	B
424	Bn-N1-p10129316	Bn-A01-p10590357	Bn-A01-p10590357	A1	36	44.2	B
425	Bn-N1-p10175252	Bn-A01-p10636637	Bn-A01-p10636637	A1	36	44.2	B
426	Bn-N1-p10608511	Bn-A01-p11091663	Bn-A01-p11091663	A1	36	44.2	B
427	Bn-N1-p14619802	Bn-A01-p15090383	Bn-A01-p15090383	A1	36	44.2	B
428	Bn-N1-p18826458	Bn-A01-p18178802	Bn-A01-p18178802	A1	36	44.2	B
429	Bn-N11-p22634236	Bn-C1-p20869430	Bn-scaff_17959_1-p28421	A1	37	45.1	B
430	Bn-N11-p22659762	Bn-C1-p20893873	Bn-scaff_17959_1-p25977	A1	37	45.1	B
431	Bn-N11-p22659823	Bn-C1-p20893934	Bn-scaff_17959_1-p25971	A1	37	45.1	B
432	Bn-N11-p28029104	Bn-C1-p26243221	Bn-scaff_21820_1-p17449	A1	37	45.1	B
433	Bn-N11-p28029782	Bn-C1-p26243898	Bn-scaff_21820_1-p17381	A1	37	45.1	B
434	Bn-N11-p28033664	Bn-C1-p26247775	Bn-scaff_21820_1-p16993	A1	37	45.1	B
435	Bn-N11-p28075665	Bn-A01-p16103425	Bn-A01-p16103425	A1	37	45.1	B
436	Bn-N11-p30524343	Bn-A01-p17137863	Bn-A01-p17137863	A1	37	45.1	B

437	Bn-N11-p32273256	Bn-A01-p18121827	Bn-A01-p18121827	A1	37	45.1	0
438	Bn-N12-p42320001	Bn-A01-p17787446	Bn-A01-p17787446	A1	37	45.1	B
439	Bn-N13-p26682708	Bn-A01-p17136539	Bn-A01-p17136539	A1	37	45.1	B
440	Bn-N13-p39160420	Bn-A01-p16093558	Bn-A01-p16093558	A1	37	45.1	B
441	Bn-N13-p67705972	Bn-A01-p16222479	Bn-A01-p16222479	A1	37	45.1	B
442	Bn-N14-p31433813	Bn-A01-p16093901	Bn-A01-p16093901	A1	37	45.1	B
443	Bn-N14-p49614979	Bn-A01-p14181463	Bn-A01-p14181463	A1	37	45.1	B
444	Bn-N14-p49615167	Bn-A01-p14181651	Bn-A01-p14181651	A1	37	45.1	B
445	Bn-N14-p49615345	Bn-A01-p14181829	Bn-A01-p14181829	A1	37	45.1	B
446	Bn-N17-p45120061	Bn-A01-p16221475	Bn-A01-p16221475	A1	37	45.1	B
447	Bn-N1-p10489679	Bn-A01-p10934377	Bn-A01-p10934377	A1	37	45.1	B
448	Bn-N1-p10503300	Bn-A01-p10938883	Bn-A01-p10938883	A1	37	45.1	B
449	Bn-N1-p10503591	Bn-A01-p10939144	Bn-A01-p10939144	A1	37	45.1	B
450	Bn-N1-p10507360	Bn-A01-p10945930	Bn-A01-p10945930	A1	37	45.1	B
451	Bn-N1-p10544311	Bn-A01-p10991782	Bn-A01-p10991782	A1	37	45.1	B
452	Bn-N1-p10564116	Bn-A01-p11011196	Bn-A01-p11011196	A1	37	45.1	B
453	Bn-N1-p10597648	Bn-A01-p11054015	Bn-A01-p11054015	A1	37	45.1	B
454	Bn-N1-p10604872	Bn-A01-p11077627	Bn-A01-p11077627	A1	37	45.1	B
455	Bn-N1-p10628794	Bn-A01-p11120493	Bn-A01-p11120493	A1	37	45.1	B
456	Bn-N1-p10656374	Bn-A01-p11142752	Bn-A01-p11142752	A1	37	45.1	B
457	Bn-N1-p10742834	Bn-A01-p11233207	Bn-A01-p11233207	A1	37	45.1	B
458	Bn-N1-p10860453	Bn-A01-p11330661	Bn-A01-p11330661	A1	37	45.1	B
459	Bn-N1-p10936827	Bn-A01-p11407167	Bn-A01-p11407167	A1	37	45.1	B
460	Bn-N1-p11000718	Bn-A01-p11469992	Bn-A01-p11469992	A1	37	45.1	B
461	Bn-N1-p11002226	Bn-A01-p11471489	Bn-A01-p11471489	A1	37	45.1	B
462	Bn-N1-p11009759	Bn-A01-p11479565	Bn-A01-p11479565	A1	37	45.1	B
463	Bn-N1-p11101231	Bn-A01-p11543126	Bn-A01-p11543126	A1	37	45.1	B
464	Bn-N1-p11101339	Bn-A01-p11543191	Bn-A01-p11543191	A1	37	45.1	B
465	Bn-N1-p11187449	Bn-A01-p11702957	Bn-A01-p11702957	A1	37	45.1	B
466	Bn-N1-p11228267	Bn-A01-p11744233	Bn-A01-p11744233	A1	37	45.1	B
467	Bn-N1-p11294556	Bn-A01-p11807124	Bn-A01-p11807124	A1	37	45.1	B
468	Bn-N1-p11295584	Bn-A01-p11808121	Bn-A01-p11808121	A1	37	45.1	B
469	Bn-N1-p11305925	Bn-A01-p11817698	Bn-A01-p11817698	A1	37	45.1	B
470	Bn-N1-p11307133	Bn-A01-p11818901	Bn-A01-p11818901	A1	37	45.1	B
471	Bn-N1-p11321978	Bn-A01-p11825880	Bn-A01-p11825880	A1	37	45.1	B
472	Bn-N1-p11424272	Bn-Scaffold000444-p3	Bn-Scaffold000444-p344	A1	37	45.1	B
473	Bn-N1-p11456056	Bn-A01-p11947255	Bn-A01-p11947255	A1	37	45.1	B
474	Bn-N1-p11460402	Bn-A01-p11951605	Bn-A01-p11951605	A1	37	45.1	B
475	Bn-N1-p11464433	Bn-A01-p11955645	Bn-A01-p11955645	A1	37	45.1	B
476	Bn-N1-p11508229	Bn-A01-p12077110	Bn-A01-p12077110	A1	37	45.1	B
477	Bn-N1-p11606752	Bn-A01-p12178646	Bn-A01-p12178646	A1	37	45.1	B
478	Bn-N1-p11607855	Bn-A01-p12179784	Bn-A01-p12179784	A1	37	45.1	B
479	Bn-N1-p11635701	Bn-A01-p12233209	Bn-A01-p12233209	A1	37	45.1	B
480	Bn-N1-p11698424	Bn-A01-p12302708	Bn-A01-p12302708	A1	37	45.1	B
481	Bn-N1-p11702332	Bn-A01-p12302861	Bn-A01-p12302861	A1	37	45.1	B
482	Bn-N1-p11703847	Bn-A01-p12305025	Bn-A01-p12305025	A1	37	45.1	B
483	Bn-N1-p11704650	Bn-A01-p12305828	Bn-A01-p12305828	A1	37	45.1	B
484	Bn-N1-p11705015	Bn-A01-p12306195	Bn-A01-p12306195	A1	37	45.1	B
485	Bn-N1-p11707740	Bn-A01-p12308897	Bn-A01-p12308897	A1	37	45.1	B

486	Bn-N1-p11708590	Bn-A01-p12309767	Bn-A01-p12309767	A1	37	45.1	B
487	Bn-N1-p11714916	Bn-A01-p12314813	Bn-A01-p12314813	A1	37	45.1	B
488	Bn-N1-p11862661	Bn-A01-p12454306	Bn-A01-p12454306	A1	37	45.1	B
489	Bn-N1-p11866943	Bn-A01-p12456089	Bn-A01-p12456089	A1	37	45.1	B
490	Bn-N1-p11891352	Bn-A01-p12516944	Bn-A01-p12516944	A1	37	45.1	B
491	Bn-N1-p11892376	Bn-A01-p12484368	Bn-A01-p12484368	A1	37	45.1	B
492	Bn-N1-p11922690	Bn-A01-p12510328	Bn-A01-p12510328	A1	37	45.1	B
493	Bn-N1-p11922928	Bn-A01-p12510566	Bn-A01-p12510566	A1	37	45.1	B
494	Bn-N1-p11939222	Bn-A01-p12522369	Bn-A01-p12522369	A1	37	45.1	B
495	Bn-N1-p11942054	Bn-Scaffold003507-p1	Bn-Scaffold003507-p142	A1	37	45.1	B
496	Bn-N1-p11942661	Bn-A01-p12533991	Bn-A01-p12533991	A1	37	45.1	B
497	Bn-N1-p11944135	Bn-A01-p12535269	Bn-A01-p12535269	A1	37	45.1	B
498	Bn-N1-p11944482	Bn-A01-p12535612	Bn-A01-p12535612	A1	37	45.1	B
499	Bn-N1-p11949187	Bn-A01-p12539655	Bn-A01-p12539655	A1	37	45.1	B
500	Bn-N1-p11949787	Bn-A01-p12539953	Bn-A01-p12539953	A1	37	45.1	B
501	Bn-N1-p11953827	Bn-A01-p12543678	Bn-A01-p12543678	A1	37	45.1	B
502	Bn-N1-p11954216	Bn-A01-p12544054	Bn-A01-p12544054	A1	37	45.1	B
503	Bn-N1-p11966937	Bn-A01-p12560952	Bn-A01-p12560952	A1	37	45.1	B
504	Bn-N1-p12015522	Bn-A01-p12593173	Bn-A01-p12593173	A1	37	45.1	B
505	Bn-N1-p12046264	Bn-Scaffold000300-p4	Bn-Scaffold000300-p4564	A1	37	45.1	B
506	Bn-N1-p12057480	Bn-Scaffold000300-p1	Bn-Scaffold000300-p15526	A1	37	45.1	B
507	Bn-N1-p12058978	Bn-Scaffold000300-p1	Bn-Scaffold000300-p17011	A1	37	45.1	B
508	Bn-N1-p12062192	Bn-Scaffold000300-p2	Bn-Scaffold000300-p20118	A1	37	45.1	B
509	Bn-N1-p12095406	Bn-A01-p12618357	Bn-A01-p12618357	A1	37	45.1	B
510	Bn-N1-p12099404	Bn-A01-p12622123	Bn-A01-p12622123	A1	37	45.1	B
511	Bn-N1-p12143466	Bn-A01-p12665045	Bn-A01-p12665045	A1	37	45.1	B
512	Bn-N1-p12143671	Bn-A01-p12665250	Bn-A01-p12665250	A1	37	45.1	B
513	Bn-N1-p12154035	Bn-A01-p12672899	Bn-A01-p12672899	A1	37	45.1	B
514	Bn-N1-p12210563	Bn-A01-p12734805	Bn-A01-p12734805	A1	37	45.1	B
515	Bn-N1-p12541186	Bn-A01-p15865197	Bn-A01-p15865197	A1	37	45.1	B
516	Bn-N1-p13554075	Bn-A01-p14001039	Bn-A01-p14001039	A1	37	45.1	B
517	Bn-N1-p13653384	Bn-A01-p14074268	Bn-A01-p14074268	A1	37	45.1	B
518	Bn-N1-p13683365	Bn-A01-p14102222	Bn-A01-p14102222	A1	37	45.1	B
519	Bn-N1-p13715421	Bn-A01-p14134232	Bn-A01-p14134232	A1	37	45.1	B
520	Bn-N1-p13717849	Bn-A01-p14138072	Bn-A01-p14138072	A1	37	45.1	B
521	Bn-N1-p13727990	Bn-A01-p14148071	Bn-A01-p14148071	A1	37	45.1	B
522	Bn-N1-p13766078	Bn-A01-p14197545	Bn-A01-p14197545	A1	37	45.1	B
523	Bn-N1-p13769494	Bn-A01-p14200787	Bn-A01-p14200787	A1	37	45.1	B
524	Bn-N1-p13779649	Bn-A01-p14210994	Bn-A01-p14210994	A1	37	45.1	B
525	Bn-N1-p13839796	Bn-A01-p14238948	Bn-A01-p14238948	A1	37	45.1	B
526	Bn-N1-p13929322	Bn-A01-p14328823	Bn-A01-p14328823	A1	37	45.1	B
527	Bn-N1-p14402784	Bn-A01-p14905298	Bn-A01-p14905298	A1	37	45.1	B
528	Bn-N1-p14404126	Bn-A01-p14906403	Bn-A01-p14906403	A1	37	45.1	B
529	Bn-N1-p14404217	Bn-A01-p14906494	Bn-A01-p14906494	A1	37	45.1	B
530	Bn-N1-p14415406	Bn-A01-p14917697	Bn-A01-p14917697	A1	37	45.1	B
531	Bn-N1-p14433010	Bn-A01-p14934844	Bn-A01-p14934844	A1	37	45.1	B
532	Bn-N1-p14445402	Bn-A01-p14947053	Bn-A01-p14947053	A1	37	45.1	B
533	Bn-N1-p14616211	Bn-A01-p15086787	Bn-A01-p15086787	A1	37	45.1	B
534	Bn-N1-p14616469	Bn-A01-p15087045	Bn-A01-p15087045	A1	37	45.1	B

535	Bn-N1-p14616595	Bn-A01-p15087171	Bn-A01-p15087171	A1	37	45.1	B
536	Bn-N1-p14624637	Bn-A01-p15095213	Bn-A01-p15095213	A1	37	45.1	B
537	Bn-N1-p14624738	Bn-A01-p15095314	Bn-A01-p15095314	A1	37	45.1	B
538	Bn-N1-p14624914	Bn-A01-p15095524	Bn-A01-p15095524	A1	37	45.1	B
539	Bn-N1-p14920683	Bn-A01-p15401259	Bn-A01-p15401259	A1	37	45.1	B
540	Bn-N1-p14921746	Bn-A01-p15402291	Bn-A01-p15402291	A1	37	45.1	B
541	Bn-N1-p14928214	Bn-A01-p15408721	Bn-A01-p15408721	A1	37	45.1	B
542	Bn-N1-p14933346	Bn-A01-p15413589	Bn-A01-p15413589	A1	37	45.1	B
543	Bn-N1-p14971773	Bn-A01-p15441407	Bn-A01-p15441407	A1	37	45.1	B
544	Bn-N1-p14978764	Bn-A01-p15446864	Bn-A01-p15446864	A1	37	45.1	B
545	Bn-N1-p15012196	Bn-A01-p15474986	Bn-A01-p15474986	A1	37	45.1	0
546	Bn-N1-p15012249	Bn-A01-p15475038	Bn-A01-p15475038	A1	37	45.1	B
547	Bn-N1-p15013837	Bn-A01-p15476640	Bn-A01-p15476640	A1	37	45.1	B
548	Bn-N1-p15020118	Bn-A01-p15481986	Bn-A01-p15481986	A1	37	45.1	B
549	Bn-N1-p15024042	Bn-A01-p15487080	Bn-A01-p15487080	A1	37	45.1	B
550	Bn-N1-p15030987	Bn-A01-p15489266	Bn-A01-p15489266	A1	37	45.1	B
551	Bn-N1-p15038338	Bn-A01-p15494260	Bn-A01-p15494260	A1	37	45.1	B
552	Bn-N1-p15040901	Bn-A01-p15496639	Bn-A01-p15496639	A1	37	45.1	B
553	Bn-N1-p15041035	Bn-A01-p15496772	Bn-A01-p15496772	A1	37	45.1	B
554	Bn-N1-p15041452	Bn-A01-p15497190	Bn-A01-p15497190	A1	37	45.1	B
555	Bn-N1-p15042884	Bn-A01-p15502589	Bn-A01-p15502589	A1	37	45.1	B
556	Bn-N1-p15057423	Bn-A01-p15517260	Bn-A01-p15517260	A1	37	45.1	B
557	Bn-N1-p15059254	Bn-A01-p15519090	Bn-A01-p15519090	A1	37	45.1	B
558	Bn-N1-p15097995	Bn-A01-p15568028	Bn-A01-p15568028	A1	37	45.1	B
559	Bn-N1-p16512681	Bn-A01-p15957543	Bn-A01-p15957543	A1	37	45.1	B
560	Bn-N1-p16669166	Bn-A01-p16086381	Bn-A01-p16086381	A1	37	45.1	B
561	Bn-N1-p16697490	Bn-A01-p16106314	Bn-A01-p16106314	A1	37	45.1	B
562	Bn-N1-p16697548	Bn-A01-p16106372	Bn-A01-p16106372	A1	37	45.1	B
563	Bn-N1-p16723243	Bn-A01-p16123649	Bn-A01-p16123649	A1	37	45.1	B
564	Bn-N1-p16749259	Bn-A01-p16150568	Bn-A01-p16150568	A1	37	45.1	B
565	Bn-N1-p16764051	Bn-A01-p16173770	Bn-A01-p16173770	A1	37	45.1	B
566	Bn-N1-p16766349	Bn-A01-p16175909	Bn-A01-p16175909	A1	37	45.1	B
567	Bn-N1-p16785995	Bn-A01-p16200217	Bn-A01-p16200217	A1	37	45.1	B
568	Bn-N1-p16804153	Bn-A01-p16217917	Bn-A01-p16217917	A1	37	45.1	B
569	Bn-N1-p16805316	Bn-A01-p16222086	Bn-A01-p16222086	A1	37	45.1	B
570	Bn-N1-p16805397	Bn-A01-p16222167	Bn-A01-p16222167	A1	37	45.1	B
571	Bn-N1-p16830009	Bn-A01-p16257043	Bn-A01-p16257043	A1	37	45.1	B
572	Bn-N1-p16849435	Bn-A01-p16276961	Bn-A01-p16276961	A1	37	45.1	B
573	Bn-N1-p16898020	Bn-A01-p16324010	Bn-A01-p16324010	A1	37	45.1	B
574	Bn-N1-p16976972	Bn-A01-p16389291	Bn-A01-p16389291	A1	37	45.1	B
575	Bn-N1-p17016754	Bn-A01-p16418499	Bn-A01-p16418499	A1	37	45.1	B
576	Bn-N1-p17035841	Bn-A01-p16432946	Bn-A01-p16432946	A1	37	45.1	B
577	Bn-N1-p17074427	Bn-A01-p16474128	Bn-A01-p16474128	A1	37	45.1	B
578	Bn-N1-p17110858	Bn-A01-p16512818	Bn-A01-p16512818	A1	37	45.1	B
579	Bn-N1-p17136099	Bn-A01-p16538323	Bn-A01-p16538323	A1	37	45.1	B
580	Bn-N1-p17136363	Bn-A01-p16538587	Bn-A01-p16538587	A1	37	45.1	B
581	Bn-N1-p17137026	Bn-A01-p16539250	Bn-A01-p16539250	A1	37	45.1	B
582	Bn-N1-p17154159	Bn-A01-p16557636	Bn-A01-p16557636	A1	37	45.1	B
583	Bn-N1-p17229848	Bn-A01-p16662544	Bn-A01-p16662544	A1	37	45.1	B

584	Bn-N1-p17302028	Bn-A01-p16715637	Bn-A01-p16715637	A1	37	45.1	B
585	Bn-N1-p17303927	Bn-C1-p27154552	Bn-scaff_20452_1-p20150	A1	37	45.1	B
586	Bn-N1-p17482527	Bn-A01-p16893578	Bn-A01-p16893578	A1	37	45.1	B
587	Bn-N1-p17592603	Bn-A01-p16994459	Bn-A01-p16994459	A1	37	45.1	B
588	Bn-N1-p17625591	Bn-A01-p17015383	Bn-A01-p17015383	A1	37	45.1	B
589	Bn-N1-p17653794	Bn-A01-p17036109	Bn-A01-p17036109	A1	37	45.1	B
590	Bn-N1-p17668847	Bn-A01-p17040457	Bn-A01-p17040457	A1	37	45.1	B
591	Bn-N1-p17677345	Bn-A01-p17049439	Bn-A01-p17049439	A1	37	45.1	B
592	Bn-N1-p17678314	Bn-A01-p17050461	Bn-A01-p17050461	A1	37	45.1	B
593	Bn-N1-p17714097	Bn-A01-p17081120	Bn-A01-p17081120	A1	37	45.1	B
594	Bn-N1-p17734691	Bn-A01-p17105194	Bn-A01-p17105194	A1	37	45.1	B
595	Bn-N1-p17734869	Bn-A01-p17105372	Bn-A01-p17105372	A1	37	45.1	B
596	Bn-N1-p17735169	Bn-A01-p17105672	Bn-A01-p17105672	A1	37	45.1	B
597	Bn-N1-p17764703	Bn-A01-p17150604	Bn-A01-p17150604	A1	37	45.1	B
598	Bn-N1-p17764974	Bn-A01-p17150875	Bn-A01-p17150875	A1	37	45.1	B
599	Bn-N1-p17772446	Bn-A01-p17156534	Bn-A01-p17156534	A1	37	45.1	B
600	Bn-N1-p17776308	Bn-A01-p17160355	Bn-A01-p17160355	A1	37	45.1	B
601	Bn-N1-p17781589	Bn-A01-p17165052	Bn-A01-p17165052	A1	37	45.1	B
602	Bn-N1-p17781871	Bn-A01-p17165303	Bn-A01-p17165303	A1	37	45.1	B
603	Bn-N1-p17820486	Bn-A01-p17187327	Bn-A01-p17187327	A1	37	45.1	B
604	Bn-N1-p17831692	Bn-C1-p27977077	Bn-scaff_22770_1-p33433	A1	37	45.1	B
605	Bn-N1-p17910900	Bn-A01-p17258931	Bn-A01-p17258931	A1	37	45.1	B
606	Bn-N1-p17936190	Bn-A01-p17283208	Bn-A01-p17283208	A1	37	45.1	B
607	Bn-N1-p17985376	Bn-A01-p17326645	Bn-A01-p17326645	A1	37	45.1	B
608	Bn-N1-p18163081	Bn-A01-p17506817	Bn-A01-p17506817	A1	37	45.1	B
609	Bn-N1-p18166634	Bn-A01-p17518104	Bn-A01-p17518104	A1	37	45.1	B
610	Bn-N1-p18166843	Bn-A01-p17518305	Bn-A01-p17518305	A1	37	45.1	B
611	Bn-N1-p18167006	Bn-A01-p17518468	Bn-A01-p17518468	A1	37	45.1	B
612	Bn-N1-p18176351	Bn-A01-p17527782	Bn-A01-p17527782	A1	37	45.1	B
613	Bn-N1-p18183475	Bn-C1-p28692725	Bn-scaff_16929_1-p88751	A1	37	45.1	B
614	Bn-N1-p18222096	Bn-A01-p17574483	Bn-A01-p17574483	A1	37	45.1	B
615	Bn-N1-p18225517	Bn-A01-p17577903	Bn-A01-p17577903	A1	37	45.1	B
616	Bn-N1-p18323834	Bn-A01-p17649002	Bn-A01-p17649002	A1	37	45.1	B
617	Bn-N1-p18356262	Bn-A01-p17677926	Bn-A01-p17677926	A1	37	45.1	B
618	Bn-N1-p18375745	Bn-A01-p17691799	Bn-A01-p17691799	A1	37	45.1	B
619	Bn-N1-p18377386	Bn-A01-p17693439	Bn-A01-p17693439	A1	37	45.1	B
620	Bn-N1-p18396319	Bn-A01-p17714021	Bn-A01-p17714021	A1	37	45.1	B
621	Bn-N1-p18402890	Bn-A01-p17716773	Bn-A01-p17716773	A1	37	45.1	B
622	Bn-N1-p18414830	Bn-A01-p17728772	Bn-A01-p17728772	A1	37	45.1	B
623	Bn-N1-p18434525	Bn-A01-p17751647	Bn-A01-p17751647	A1	37	45.1	B
624	Bn-N1-p18441884	Bn-A01-p17769742	Bn-A01-p17769742	A1	37	45.1	B
625	Bn-N1-p18443557	Bn-A01-p17771411	Bn-A01-p17771411	A1	37	45.1	B
626	Bn-N1-p18497212	Bn-C1-p29201819	Bn-scaff_16929_1-p37842	A1	37	45.1	B
627	Bn-N1-p18535795	Bn-A01-p17885385	Bn-A01-p17885385	A1	37	45.1	B
628	Bn-N1-p18536222	Bn-A01-p17885812	Bn-A01-p17885812	A1	37	45.1	B
629	Bn-N1-p18537005	Bn-A01-p17886595	Bn-A01-p17886595	A1	37	45.1	B
630	Bn-N1-p18537545	Bn-A01-p17887135	Bn-A01-p17887135	A1	37	45.1	B
631	Bn-N1-p18537624	Bn-A01-p17887214	Bn-A01-p17887214	A1	37	45.1	B
632	Bn-N1-p18538345	Bn-A01-p17887935	Bn-A01-p17887935	A1	37	45.1	B

633	Bn-N1-p18539315	Bn-A01-p17888905	Bn-A01-p17888905	A1	37	45.1	B
634	Bn-N1-p18542985	Bn-A01-p17892299	Bn-A01-p17892299	A1	37	45.1	B
635	Bn-N1-p18550465	Bn-A01-p17899781	Bn-A01-p17899781	A1	37	45.1	B
636	Bn-N1-p18559022	Bn-A01-p17905950	Bn-A01-p17905950	A1	37	45.1	B
637	Bn-N1-p18559394	Bn-A01-p17906322	Bn-A01-p17906322	A1	37	45.1	B
638	Bn-N1-p18559471	Bn-A01-p17906399	Bn-A01-p17906399	A1	37	45.1	B
639	Bn-N1-p18559837	Bn-A01-p17906765	Bn-A01-p17906765	A1	37	45.1	B
640	Bn-N1-p18560131	Bn-A01-p17907059	Bn-A01-p17907059	A1	37	45.1	B
641	Bn-N1-p18560635	Bn-A01-p17907563	Bn-A01-p17907563	A1	37	45.1	B
642	Bn-N1-p18563874	Bn-A01-p17910798	Bn-A01-p17910798	A1	37	45.1	B
643	Bn-N1-p18564275	Bn-A01-p17911199	Bn-A01-p17911199	A1	37	45.1	B
644	Bn-N1-p18564997	Bn-A01-p17911921	Bn-A01-p17911921	A1	37	45.1	B
645	Bn-N1-p18569082	Bn-A01-p17916004	Bn-A01-p17916004	A1	37	45.1	B
646	Bn-N1-p18577929	Bn-A01-p17930058	Bn-A01-p17930058	A1	37	45.1	B
647	Bn-N1-p18578999	Bn-A01-p17931128	Bn-A01-p17931128	A1	37	45.1	B
648	Bn-N1-p18784280	Bn-A01-p18124795	Bn-A01-p18124795	A1	37	45.1	B
649	Bn-N1-p18852932	Bn-A01-p18200921	Bn-A01-p18200921	A1	37	45.1	B
650	Bn-N1-p18860025	Bn-A01-p18205455	Bn-A01-p18205455	A1	37	45.1	B
651	Bn-N1-p18907894	Bn-A01-p18266537	Bn-A01-p18266537	A1	37	45.1	B
652	Bn-N1-p19330175	Bn-A01-p19340658	Bn-A01-p19340658	A1	37	45.1	B
653	Bn-N1-p19443005	Bn-A01-p19447794	Bn-A01-p19447794	A1	37	45.1	B
654	Bn-N1-p19492902	Bn-A01-p19498222	Bn-A01-p19498222	A1	37	45.1	B
655	Bn-N1-p19551786	Bn-A01-p19579243	Bn-A01-p19579243	A1	37	45.1	B
656	Bn-N1-p19589887	Bn-A01-p19617963	Bn-A01-p19617963	A1	37	45.1	B
657	Bn-N1-p19594827	Bn-A01-p19623880	Bn-A01-p19623880	A1	37	45.1	B
658	Bn-N1-p19594932	Bn-A01-p19623985	Bn-A01-p19623985	A1	37	45.1	B
659	Bn-N1-p19595070	Bn-A01-p19624123	Bn-A01-p19624123	A1	37	45.1	B
660	Bn-N1-p19595163	Bn-A01-p19624216	Bn-A01-p19624216	A1	37	45.1	B
661	Bn-N1-p19597950	Bn-A01-p19633815	Bn-A01-p19633815	A1	37	45.1	B
662	Bn-N1-p19605856	Bn-A01-p19650666	Bn-A01-p19650666	A1	37	45.1	B
663	Bn-N1-p19644864	Bn-A01-p19700004	Bn-A01-p19700004	A1	37	45.1	B
664	Bn-N1-p19655672	Bn-A01-p19722138	Bn-A01-p19722138	A1	37	45.1	B
665	Bn-N1-p19662300	Bn-A01-p19731678	Bn-A01-p19731678	A1	37	45.1	B
666	Bn-N1-p2018972	Bn-A01-p18160311	Bn-A01-p18160311	A1	37	45.1	B
667	Bn-N5-p15435464	Bn-A01-p11238778	Bn-A01-p11238778	A1	37	45.1	B
668	Bn-Scaffold04366-p1376	Bn-A01-p16262292	Bn-A01-p16262292	A1	37	45.1	B
669	Bn-Scaffold04366-p383	Bn-A01-p16261297	Bn-A01-p16261297	A1	37	45.1	B
670	Bn-Scaffold05442-p451	Bn-A01-p16088721	Bn-A01-p16088721	A1	37	45.1	B
671	Bn-Scaffold07305-p531	Bn-A01-p16260285	Bn-A01-p16260285	A1	37	45.1	B
672	Bn-Scaffold20063-p580	Bn-A01-p11492050	Bn-A01-p11492050	A1	37	45.1	B
673	Unknown	Bn-A01-p16094037	Bn-A01-p16094037	A1	37	45.1	B
674	Unknown	Bn-A01-p16221732	Bn-A01-p16221732	A1	37	45.1	B
675	Unknown	Bn-A01-p16222098	Bn-A01-p16222098	A1	37	45.1	B
676	Unknown	Bn-A01-p17783808	Bn-A01-p17783808	A1	37	45.1	B
677	Unknown	Bn-A01-p17783900	Bn-A01-p17783900	A1	37	45.1	B
678	Bn-N1-p19905997	Bn-A01-p19983739	Bn-A01-p19983739	A1	38	46	B
679	Bn-N1-p19915857	Bn-A01-p19993388	Bn-A01-p19993388	A1	38	46	B
680	Bn-N1-p20044897	Bn-A01-p20139530	Bn-A01-p20139530	A1	38	46	B
681	Bn-N1-p20058240	Bn-A01-p20144346	Bn-A01-p20144346	A1	38	46	B

682	Bn-N1-p20074024	Bn-Scaffold014697-p8	Bn-Scaffold014697-p80	A1	38	46	B
683	Bn-N1-p20076099	Bn-A01-p20159960	Bn-A01-p20159960	A1	38	46	B
684	Bn-N1-p20284986	Bn-A01-p20397807	Bn-A01-p20397807	A1	38	46	B
685	Bn-N1-p20324743	Bn-A01-p20430407	Bn-A01-p20430407	A1	38	46	B
686	Bn-N1-p20327047	Bn-Scaffold001599-p5	Bn-Scaffold001599-p514	A1	38	46	B
687	Bn-N1-p20330193	Bn-A01-p20431228	Bn-A01-p20431228	A1	38	46	B
688	Bn-N1-p20338159	Bn-A04-p2881354	Bn-A04-p2881354	A1	38	46	B
689	Bn-N1-p20341020	Bn-A01-p20442457	Bn-A01-p20442457	A1	38	46	B
690	Bn-N1-p20355727	Bn-A01-p20471502	Bn-A01-p20471502	A1	38	46	B
691	Bn-N1-p20367448	Bn-A01-p20482459	Bn-A01-p20482459	A1	38	46	B
692	Bn-N1-p20368960	Bn-A01-p20483938	Bn-A01-p20483938	A1	38	46	B
693	Bn-N1-p20664815	Bn-A01-p20753932	Bn-A01-p20753932	A1	38	46	B
694	Bn-N1-p20714527	Bn-A01-p20805506	Bn-A01-p20805506	A1	38	46	B
695	Bn-N1-p20714634	Bn-A01-p20805613	Bn-A01-p20805613	A1	38	46	B
696	Bn-N1-p20744534	Bn-A01-p20836664	Bn-A01-p20836664	A1	38	46	B
697	Bn-N1-p20768390	Bn-A01-p20866022	Bn-A01-p20866022	A1	38	46	B
698	Bn-N1-p20768885	Bn-A01-p20866518	Bn-A01-p20866518	A1	38	46	B
699	Bn-N1-p20777150	Bn-C1-p33121580	Bn-scaff_16534_2-p30796	A1	38	46	0
700	Bn-N1-p20809964	Bn-A01-p20909816	Bn-A01-p20909816	A1	38	46	B
701	Bn-N1-p20874310	Bn-A01-p20990218	Bn-A01-p20990218	A1	38	46	B
702	Bn-Scaffold07211-p725	Bn-A01-p20933189	Bn-A01-p20933189	A1	38	46	B
703	Bn-N11-p35064282	Bn-A01-p20433111	Bn-A01-p20433111	A1	39	46.9	0
704	Bn-N11-p36342172	Bn-C1-p33540030	Bn-scaff_15879_1-p40391	A1	39	46.9	B
705	Bn-N17-p21262009	Bn-C2-p44217500	Bn-scaff_15712_2-p14588	A1	39	46.9	B
706	Bn-N1-p20906257	Bn-A01-p21035670	Bn-A01-p21035670	A1	39	46.9	0
707	Bn-N1-p20913951	Bn-A01-p21038677	Bn-A01-p21038677	A1	39	46.9	B
708	Bn-N1-p20954233	Bn-A01-p21079129	Bn-A01-p21079129	A1	39	46.9	B
709	Bn-N1-p20954885	Bn-A01-p21079774	Bn-A01-p21079774	A1	39	46.9	B
710	Bn-N1-p20955020	Bn-A01-p21079909	Bn-A01-p21079909	A1	39	46.9	B
711	Bn-N1-p21381690	Bn-A01-p21393260	Bn-A01-p21393260	A1	39	46.9	B
712	Bn-N1-p21383096	Bn-A01-p21394483	Bn-A01-p21394483	A1	39	46.9	B
713	Bn-N1-p21434496	Bn-A01-p21444041	Bn-A01-p21444041	A1	39	46.9	B
714	Bn-N1-p21471174	Bn-A01-p21473161	Bn-A01-p21473161	A1	39	46.9	0
715	Bn-N1-p21508826	Bn-A01-p21500509	Bn-A01-p21500509	A1	39	46.9	B
716	Bn-N1-p21514264	Bn-A01-p21519780	Bn-A01-p21519780	A1	39	46.9	B
717	Bn-N1-p21519435	Bn-A01-p21524150	Bn-A01-p21524150	A1	39	46.9	B
718	Bn-N1-p21522465	Bn-A01-p21527187	Bn-A01-p21527187	A1	39	46.9	B
719	Bn-N1-p21526893	Bn-A01-p21532512	Bn-A01-p21532512	A1	39	46.9	B
720	Bn-N1-p21548844	Bn-A01-p21556957	Bn-A01-p21556957	A1	39	46.9	B
721	Bn-Scaffold36130-p289	Bn-A01-p21224647	Bn-A01-p21224647	A1	39	46.9	0
722	Bn-N1-p21636899	Bn-A01-p21651835	Bn-A01-p21651835	A1	40	47.7	B
723	Bn-N1-p21649843	Bn-A01-p21663900	Bn-A01-p21663900	A1	40	47.7	B
724	Bn-N1-p21689831	Bn-A01-p21709916	Bn-A01-p21709916	A1	40	47.7	B
725	Bn-N1-p21695724	Bn-A01-p21715162	Bn-A01-p21715162	A1	40	47.7	B
726	Bn-N1-p21706353	Bn-C1-p34708753	Bn-scaff_15794_2-p25123	A1	41	48.5	0
727	Bn-N1-p21832963	Bn-A01-p21855770	Bn-A01-p21855770	A1	41	48.5	B
728	Bn-N1-p21900463	Bn-A01-p21923141	Bn-A01-p21923141	A1	42	49.3	0
729	Bn-N1-p21901887	Bn-A01-p21926431	Bn-A01-p21926431	A1	42	49.3	B
730	Bn-N1-p21903951	Bn-A01-p21928497	Bn-A01-p21928497	A1	42	49.3	B

731	Bn-N1-p21903985	Bn-A01-p21928531	Bn-A01-p21928531	A1	42	49.3	B
732	Bn-N1-p21904381	Bn-A01-p21928927	Bn-A01-p21928927	A1	42	49.3	B
733	Bn-N1-p21906045	Bn-A01-p21930611	Bn-A01-p21930611	A1	42	49.3	B
734	Bn-N1-p21906172	Bn-A01-p21930738	Bn-A01-p21930738	A1	42	49.3	B
735	Bn-N1-p21906662	Bn-A01-p21931228	Bn-A01-p21931228	A1	42	49.3	B
736	Bn-N1-p21906932	Bn-A01-p21931498	Bn-A01-p21931498	A1	42	49.3	B
737	Bn-N1-p21907287	Bn-A01-p21931853	Bn-A01-p21931853	A1	42	49.3	0
738	Bn-N1-p21914912	Bn-A01-p21937985	Bn-A01-p21937985	A1	42	49.3	B
739	Bn-N1-p21915672	Bn-A01-p21938751	Bn-A01-p21938751	A1	42	49.3	0
740	Bn-N1-p21959787	Bn-A01-p21980567	Bn-A01-p21980567	A1	42	49.3	B
741	Bn-N1-p21978487	Bn-A01-p21997686	Bn-A01-p21997686	A1	42	49.3	B
742	Bn-N1-p22001815	Bn-A01-p22014179	Bn-A01-p22014179	A1	42	49.3	B
743	Bn-N1-p22003987	Bn-A01-p22016353	Bn-A01-p22016353	A1	42	49.3	B
744	Bn-N1-p22010652	Bn-A01-p22018616	Bn-A01-p22018616	A1	42	49.3	0
745	Bn-N1-p22014059	Bn-A01-p22022023	Bn-A01-p22022023	A1	42	49.3	B
746	Bn-N1-p22039892	Bn-A01-p22051510	Bn-A01-p22051510	A1	42	49.3	B
747	Bn-N11-p38162747	Bn-C1-p35315258	Bn-scaff_15844_1-p11921(	A1	43	50.1	B
748	Bn-N1-p22090041	Bn-A01-p22087629	Bn-A01-p22087629	A1	43	50.1	B
749	Bn-N1-p22154564	Bn-C1-p35360034	Bn-scaff_15844_1-p16399:	A1	43	50.1	B
750	Bn-N1-p22242804	Bn-A01-p22681496	Bn-A01-p22681496	A1	44	50.9	B
751	Bn-N1-p22245609	Bn-A01-p22684301	Bn-A01-p22684301	A1	44	50.9	B
752	Bn-N1-p22311096	Bn-A01-p22768523	Bn-A01-p22768523	A1	44	50.9	B
753	Bn-N1-p22378076	Bn-A01-p22841314	Bn-A01-p22841314	A1	44	50.9	B
754	Bn-N1-p22522332	Bn-A01-p23020726	Bn-A01-p23020726	A1	44	50.9	B
755	Bn-N1-p22597454	Bn-A01-p23095567	Bn-A01-p23095567	A1	44	50.9	B
756	Bn-N1-p22598034	Bn-A01-p23096147	Bn-A01-p23096147	A1	44	50.9	B
757	Bn-N1-p22599580	Bn-A01-p23097693	Bn-A01-p23097693	A1	44	50.9	B
758	Bn-N1-p22603336	Bn-A01-p23103120	Bn-A01-p23103120	A1	44	50.9	B
759	Bn-N1-p22607387	Bn-A01-p23111844	Bn-A01-p23111844	A1	44	50.9	0
760	Bn-N1-p22651446	Bn-A01-p23175111	Bn-A01-p23175111	A1	44	50.9	B
761	Bn-N1-p22658800	Bn-A01-p23181293	Bn-A01-p23181293	A1	44	50.9	B
762	Bn-N1-p22672895	Bn-A01-p23196022	Bn-A01-p23196022	A1	44	50.9	B
763	Bn-N1-p22729837	Bn-A01-p23253678	Bn-A01-p23253678	A1	44	50.9	B
764	Bn-N1-p22735485	Bn-A01-p23259034	Bn-A01-p23259034	A1	44	50.9	B
765	Bn-N1-p22736190	Bn-A01-p23259741	Bn-A01-p23259741	A1	44	50.9	B
766	Bn-N1-p22813949	Bn-A01-p23321507	Bn-A01-p23321507	A1	44	50.9	B
767	Bn-N1-p22814213	Bn-A01-p23321771	Bn-A01-p23321771	A1	44	50.9	B
768	Bn-N1-p22814819	Bn-A01-p23322377	Bn-A01-p23322377	A1	44	50.9	B
769	Bn-N1-p22814880	Bn-A01-p23322438	Bn-A01-p23322438	A1	44	50.9	B
770	Bn-N1-p22868765	Bn-A01-p23370802	Bn-A01-p23370802	A1	44	50.9	B
771	Bn-Scaffold01970-p1093	Bn-A01-p23279247	Bn-A01-p23279247	A1	44	50.9	B
772	Bn-Scaffold01970-p3716	Bn-A01-p23281878	Bn-A01-p23281878	A1	44	50.9	B
773	Bn-Scaffold01970-p3839	Bn-A01-p23282001	Bn-A01-p23282001	A1	44	50.9	B
774	Bn-N1-p22954653	Bn-A01-p23448661	Bn-A01-p23448661	A1	45	55.2	A
775	Bn-N1-p22981209	Bn-A01-p23472646	Bn-A01-p23472646	A1	45	55.2	A
776	Bn-N1-p22982035	Bn-A01-p23473514	Bn-A01-p23473514	A1	45	55.2	A
777	Bn-N1-p23001301	Bn-A01-p23493920	Bn-A01-p23493920	A1	45	55.2	A
778	Bn-N1-p23039208	Bn-A01-p23538856	Bn-A01-p23538856	A1	45	55.2	A
779	Bn-N1-p23039656	Bn-A01-p23539305	Bn-A01-p23539305	A1	45	55.2	A

780	Bn-N1-p23066223	Bn-A01-p23560747	Bn-A01-p23560747	A1	45	55.2	A
781	Bn-N1-p23075763	Bn-A01-p23568963	Bn-A01-p23568963	A1	45	55.2	A
782	Bn-N1-p23082579	Bn-A06-p9371495	Bn-A06-p9371495	A1	45	55.2	A
783	Bn-N1-p23129730	Bn-C1-p37030858	Bn-scaff_17515_1-p51302	A1	46	56	A
784	Bn-N1-p23132864	Bn-A01-p23614494	Bn-A01-p23614494	A1	47	56.8	A
785	Bn-N1-p23133257	Bn-A01-p23614887	Bn-A01-p23614887	A1	47	56.8	A
786	Bn-N1-p23135594	Bn-A01-p23616839	Bn-A01-p23616839	A1	47	56.8	A
787	Bn-N1-p23155057	Bn-A01-p23634753	Bn-A01-p23634753	A1	47	56.8	A
788	Bn-N1-p23159907	Bn-A01-p23638107	Bn-A01-p23638107	A1	47	56.8	A
789	Bn-N11-p40097101	Bn-A01-p23718726	Bn-A01-p23718726	A1	48	57.6	A
790	Bn-N1-p23199810	Bn-A01-p23685978	Bn-A01-p23685978	A1	48	57.6	A
791	Bn-N1-p23209338	Bn-A01-p23694782	Bn-A01-p23694782	A1	48	57.6	A
792	Bn-Scaffold20519-p138	Bn-A01-p23763782	Bn-A01-p23763782	A1	48	57.6	0
793	Bn-N11-p40660108	Bn-C1-p37970950	Bn-scaff_22115_1-p86172	A1	49	59.3	A
794	Bn-N1-p23361899	Bn-A01-p23842865	Bn-A01-p23842865	A1	49	59.3	A
795	Bn-N1-p23362064	Bn-A01-p23843030	Bn-A01-p23843030	A1	49	59.3	A
796	Bn-N1-p23460955	Bn-A01-p23950559	Bn-A01-p23950559	A1	49	59.3	A
797	Bn-N1-p23522034	Bn-A01-p24006789	Bn-A01-p24006789	A1	49	59.3	A
798	Bn-N1-p23526404	Bn-A01-p24020373	Bn-A01-p24020373	A1	49	59.3	A
799	Bn-N1-p23626240	Bn-A01-p24390991	Bn-A01-p24390991	A1	49	59.3	0
800	Bn-N1-p23631798	Bn-A01-p24396906	Bn-A01-p24396906	A1	49	59.3	A
801	Bn-N1-p23676567	Bn-A01-p24431478	Bn-A01-p24431478	A1	49	59.3	A
802	Bn-N1-p23680494	Bn-A01-p24435452	Bn-A01-p24435452	A1	49	59.3	A
803	Bn-N1-p23728227	Bn-A01-p24487852	Bn-A01-p24487852	A1	49	59.3	A
804	Bn-N1-p23749741	Bn-A01-p24504495	Bn-A01-p24504495	A1	49	59.3	A
805	Bn-N1-p23749821	Bn-A01-p24504575	Bn-A01-p24504575	A1	49	59.3	A
806	Bn-N1-p23752586	Bn-A01-p24507466	Bn-A01-p24507466	A1	49	59.3	0
807	Bn-N1-p23755791	Bn-A01-p24510656	Bn-A01-p24510656	A1	49	59.3	A
808	Bn-N1-p23767382	Bn-A01-p24520244	Bn-A01-p24520244	A1	49	59.3	A
809	Bn-N1-p23769250	Bn-C1-p38392764	Bn-scaff_27278_1-p26361	A1	49	59.3	A
810	Bn-N1-p23774769	Bn-A01-p24526982	Bn-A01-p24526982	A1	49	59.3	A
811	Bn-N3-p4535279	Bn-A01-p24319792	Bn-A01-p24319792	A1	49	59.3	A
812	Unknown	Bn-A01-p24320794	Bn-A01-p24320794	A1	49	59.3	A
813	Bn-N1-p23799383	Bn-A01-p24556230	Bn-A01-p24556230	A1	50	61	A
814	Bn-N1-p23806529	Bn-A01-p24565084	Bn-A01-p24565084	A1	50	61	A
815	Bn-N1-p23890376	Bn-A01-p24646334	Bn-A01-p24646334	A1	50	61	A
816	Bn-N1-p23899338	Bn-A01-p24655110	Bn-A01-p24655110	A1	50	61	A
817	Bn-N1-p23941762	Bn-C4-p5742743	Bn-scaff_16534_1-p24195	A1	50	61	A
818	Bn-N1-p23943419	Bn-A01-p24696367	Bn-A01-p24696367	A1	50	61	A
819	Bn-N1-p23944238	Bn-A01-p24697185	Bn-A01-p24697185	A1	50	61	A
820	Bn-N1-p23949100	Bn-A01-p24701885	Bn-A01-p24701885	A1	51	62.7	A
821	Bn-N1-p23950352	Bn-A01-p24703150	Bn-A01-p24703150	A1	51	62.7	A
822	Bn-N1-p24028871	Bn-A01-p24811222	Bn-A01-p24811222	A1	51	62.7	A
823	Bn-N1-p24029810	Bn-A01-p24813515	Bn-A01-p24813515	A1	51	62.7	A
824	Bn-N1-p24056843	Bn-A01-p24848629	Bn-A01-p24848629	A1	51	62.7	0
825	Bn-N1-p24141141	Bn-A01-p24920662	Bn-A01-p24920662	A1	51	62.7	A
826	Bn-N1-p24141641	Bn-A01-p24921162	Bn-A01-p24921162	A1	51	62.7	A
827	Bn-N1-p24141834	Bn-A01-p24921355	Bn-A01-p24921355	A1	51	62.7	A
828	Bn-N1-p24223765	Bn-Scaffold000164-p2	Bn-Scaffold000164-p24772	A1	51	62.7	A

829	Bn-N1-p24226735	Bn-Scaffold000164-p2	Bn-Scaffold000164-p24475	A1	51	62.7	0
830	Bn-N1-p24226858	Bn-Scaffold000164-p2	Bn-Scaffold000164-p24463	A1	51	62.7	A
831	Bn-N1-p24277294	Bn-Scaffold000164-p1	Bn-Scaffold000164-p19629	A1	51	62.7	A
832	Bn-N1-p24277298	Bn-Scaffold000164-p1	Bn-Scaffold000164-p19628	A1	51	62.7	A
833	Bn-N1-p24334938	Bn-Scaffold000164-p1	Bn-Scaffold000164-p13392	A1	51	62.7	A
834	Bn-Scaffold01007-p26798	Bn-Scaffold000164-p2	Bn-Scaffold000164-p28395	A1	51	62.7	A
835	Bn-Scaffold01007-p3201	Bn-Scaffold000164-p3	Bn-Scaffold000164-p31054	A1	51	62.7	A
836	Bn-Scaffold01007-p45996	Bn-Scaffold000164-p2	Bn-Scaffold000164-p26751	A1	51	62.7	A
837	Bn-Scaffold01007-p50626	Bn-Scaffold000164-p2	Bn-Scaffold000164-p26288	A1	51	62.7	A
838	Bn-Scaffold04620-p169	Bn-Scaffold000164-p3	Bn-Scaffold000164-p30083	A1	51	62.7	A
839	Bn-Scaffold04620-p768	Bn-Scaffold000164-p3	Bn-Scaffold000164-p30024	A1	51	62.7	A
840	Bn-Scaffold04620-p829	Bn-Scaffold000164-p3	Bn-Scaffold000164-p30018	A1	51	62.7	A
841	Unknown	Bn-A01-p24705790	Bn-A01-p24705790	A1	51	62.7	A
842	Bn-N11-p42649504	Bn-A01-p26369426	Bn-A01-p26369426	A1	52	65.2	A
843	Bn-N11-p42743962	Bn-A01-p26426004	Bn-A01-p26426004	A1	52	65.2	A
844	Bn-N1-p24408926	Bn-Scaffold000164-p5	Bn-Scaffold000164-p55747	A1	52	65.2	A
845	Bn-N1-p24418714	Bn-Scaffold000164-p4	Bn-Scaffold000164-p46170	A1	52	65.2	A
846	Bn-N1-p24428525	Bn-Scaffold000164-p3	Bn-Scaffold000164-p37834	A1	52	65.2	A
847	Bn-N1-p24541048	Bn-A01-p26423587	Bn-A01-p26423587	A1	52	65.2	A
848	Bn-N1-p24543088	Bn-A01-p26425810	Bn-A01-p26425810	A1	52	65.2	A
849	Bn-N1-p24724459	Bn-A01-p26594603	Bn-A01-p26594603	A1	52	65.2	A
850	Bn-N1-p24735170	Bn-A01-p26604864	Bn-A01-p26604864	A1	52	65.2	A
851	Bn-N1-p24744652	Bn-A01-p26614232	Bn-A01-p26614232	A1	52	65.2	A
852	Bn-N5-p22509511	Bn-A01-p26571596	Bn-A01-p26571596	A1	52	65.2	A
853	Bn-Scaffold02085-p3765	Bn-A04-p4601980	Bn-A04-p4601980	A1	52	65.2	A
854	Bn-Scaffold02085-p4479	Bn-A04-p4602694	Bn-A04-p4602694	A1	52	65.2	A
855	Bn-Scaffold02085-p523	Bn-A04-p4598738	Bn-A04-p4598738	A1	52	65.2	A
856	Bn-N1-p24829825	Bn-A01-p26688333	Bn-A01-p26688333	A1	53	66	A
857	Bn-N1-p24855634	Bn-A01-p26716060	Bn-A01-p26716060	A1	53	66	A
858	Bn-N1-p24860523	Bn-A01-p26722319	Bn-A01-p26722319	A1	53	66	A
859	Bn-N1-p24868250	Bn-Scaffold000386-p1	Bn-Scaffold000386-p1325	A1	53	66	A
860	Bn-N1-p24873219	Bn-Scaffold000386-p4	Bn-Scaffold000386-p4150	A1	53	66	A
861	Bn-N11-p43448595	Bn-C1-p40468734	Bn-scaff_16691_1-p135001	A1	54	66.8	A
862	Bn-N11-p43736360	Bn-A01-p27301264	Bn-A01-p27301264	A1	54	66.8	A
863	Bn-N1-p24989268	Bn-A01-p27119250	Bn-A01-p27119250	A1	54	66.8	A
864	Bn-N1-p25086811	Bn-C1-p40521005	Bn-scaff_16691_1-p14022	A1	54	66.8	A
865	Bn-N1-p25216255	Bn-A01-p27331467	Bn-A01-p27331467	A1	54	66.8	A
866	Bn-N1-p25235735	Bn-A01-p27360503	Bn-A01-p27360503	A1	54	66.8	A
867	Bn-N1-p25308592	Bn-A01-p27417163	Bn-A01-p27417163	A1	54	66.8	A
868	Bn-N1-p25347145	Bn-A01-p27463135	Bn-A01-p27463135	A1	54	66.8	A
869	Bn-Scaffold03168-p766	Bn-A01-p27392534	Bn-A01-p27392534	A1	54	66.8	A
870	Bn-N1-p25466344	Bn-A01-p27580681	Bn-A01-p27580681	A1	55	70.2	A
871	Bn-N1-p25466719	Bn-A01-p27581056	Bn-A01-p27581056	A1	55	70.2	A
872	Bn-N1-p25475203	Bn-A01-p27589456	Bn-A01-p27589456	A1	55	70.2	A
873	Bn-N1-p25801701	Bn-A01-p27866419	Bn-A01-p27866419	A1	56	71	A
874	Bn-N11-p45297311	Bn-A01-p28261145	Bn-A01-p28261145	A1	57	71.8	A
875	Bn-N11-p45319575	Bn-A01-p28278192	Bn-A01-p28278192	A1	57	71.8	A
876	Bn-N11-p45415669	Bn-C1-p42279571	Bn-scaff_16342_1-p66673	A1	57	71.8	A
877	Bn-N11-p45451389	Bn-Scaffold000215-p6	Bn-Scaffold000215-p67874	A1	57	71.8	A

878	Bn-N11-p45461247	Bn-Scaffold000215-p7	Bn-Scaffold000215-p78165	A1	57	71.8	A
879	Bn-N1-p25965645	Bn-A01-p27988223	Bn-A01-p27988223	A1	57	71.8	A
880	Bn-N1-p26094193	Bn-A01-p28102080	Bn-A01-p28102080	A1	57	71.8	A
881	Bn-N1-p26143514	Bn-C1-p41933500	Bn-scaff_22321_1-p13103	A1	57	71.8	A
882	Bn-N1-p26161144	Bn-C1-p41972176	Bn-scaff_17972_1-p11306€	A1	57	71.8	A
883	Bn-Scaffold01107-p12950	Bn-A01-p28217427	Bn-A01-p28217427	A1	57	71.8	A
884	Bn-Scaffold01107-p19733	Bn-A01-p28207450	Bn-A01-p28207450	A1	57	71.8	A
885	Bn-Scaffold01107-p8092	Bn-A01-p28222170	Bn-A01-p28222170	A1	57	71.8	A
886	Bn-Scaffold01107-p9803	Bn-A01-p28220430	Bn-A01-p28220430	A1	57	71.8	A
887	Bn-Scaffold01504-p6253	Bn-A01-p28186318	Bn-A01-p28186318	A1	57	71.8	A
888	Bn-N1-p26290806	Bn-A01-p28462071	Bn-A01-p28462071	A1	58	74.3	A
889	Bn-N1-p26332052	Bn-A01-p28497362	Bn-A01-p28497362	A1	58	74.3	A
890	Bn-N1-p26332590	Bn-A01-p28497900	Bn-A01-p28497900	A1	58	74.3	A
891	Bn-N1-p26335066	Bn-A01-p28500378	Bn-A01-p28500378	A1	58	74.3	A
892	Bn-N4-p15324215	Bn-C13824437-p146	Bn-C13824437-p146	A1	58	74.3	A
893	Bn-N1-p26506031	Bn-A07-p430181	Bn-A07-p430181	A1	59	75.1	A
894	Bn-N1-p26638166	Bn-A01-p25577348	Bn-A01-p25577348	A1	59	75.1	A
895	Bn-N1-p26698258	Bn-A01-p25517197	Bn-A01-p25517197	A1	59	75.1	A
896	Bn-N1-p26702479	Bn-A01-p25512934	Bn-A01-p25512934	A1	59	75.1	A
897	Bn-N1-p26770166	Bn-A01-p25453874	Bn-A01-p25453874	A1	59	75.1	A
898	Bn-N1-p26804360	Bn-A01-p25326722	Bn-A01-p25326722	A1	59	75.1	A
899	Bn-N1-p26840028	Bn-A01-p25279161	Bn-A01-p25279161	A1	59	75.1	A
900	Bn-N1-p26854811	Bn-A01-p25267377	Bn-A01-p25267377	A1	59	75.1	A
901	Bn-N3-p2329168	Bn-A07-p483451	Bn-A07-p483451	A1	59	75.1	A
902	Bn-N4-p12310391	Bn-A04-p10288476	Bn-A04-p10288476	A1	59	75.1	A
903	Bn-Scaffold17351-p645	Bn-A07-p483541	Bn-A07-p483541	A1	59	75.1	A
904	Unknown	Bn-A07-p475640	Bn-A07-p475640	A1	59	75.1	A
905	Unknown	Bn-A07-p483535	Bn-A07-p483535	A1	59	75.1	A
906	Bn-N1-p26927669	Bn-C5-p46445047	Bn-scaff_15856_1-p50968	A1	60	75.9	A
907	Bn-N1-p26985780	Bn-A01-p25072800	Bn-A01-p25072800	A1	60	75.9	A
908	Bn-N1-p27009705	Bn-Scaffold000656-p3	Bn-Scaffold000656-p3142	A1	60	75.9	A
909	Bn-N1-p27020484	Bn-A01-p25040577	Bn-A01-p25040577	A1	60	75.9	A
910	Bn-N1-p27064751	Bn-Scaffold000237-p1	Bn-Scaffold000237-p12082	A1	60	75.9	A
911	Bn-Scaffold00891-p81308	Bn-A01-p28577026	Bn-A01-p28577026	A1	60	75.9	A
912	Bn-Scaffold00891-p82305	Bn-A01-p28578023	Bn-A01-p28578023	A1	60	75.9	A
913	Bn-Scaffold01260-p4889	Bn-A01-p25032772	Bn-A01-p25032772	A1	60	75.9	A
914	Bn-N2-p142072	Bn-A02-p1485915	Bn-A02-p1485915	A2	1	0	A
915	Bn-N2-p15550	Bn-A02-p1396892	Bn-A02-p1396892	A2	1	0	A
916	Bn-N2-p181357	Bn-A01-p13622238	Bn-A01-p13622238	A2	1	0	A
917	Bn-N2-p230194	Bn-A02-p1552305	Bn-A02-p1552305	A2	1	0	A
918	Bn-N2-p249451	Bn-A02-p1576350	Bn-A02-p1576350	A2	1	0	A
919	Bn-N2-p34829	Bn-A02-p1411508	Bn-A02-p1411508	A2	1	0	A
920	Bn-N2-p372010	Bn-A02-p1687398	Bn-A02-p1687398	A2	1	0	A
921	Bn-N2-p379523	Bn-A02-p1698015	Bn-A02-p1698015	A2	1	0	A
922	Bn-N2-p392255	Bn-A02-p1707420	Bn-A02-p1707420	A2	1	0	A
923	Bn-N2-p461838	Bn-C2-p577703	Bn-scaff_18321_1-p29016	A2	1	0	A
924	Bn-N2-p828865	Bn-A02-p2128775	Bn-A02-p2128775	A2	1	0	A
925	Bn-N2-p834279	Bn-A02-p2134112	Bn-A02-p2134112	A2	1	0	A
926	Bn-N2-p835204	Bn-A02-p2135053	Bn-A02-p2135053	A2	1	0	A

927	Bn-N2-p836188	Bn-A02-p2136039	Bn-A02-p2136039	A2	1	0	A
928	Bn-N2-p839451	Bn-A02-p2138408	Bn-A02-p2138408	A2	1	0	A
929	Bn-N2-p881955	Bn-A02-p2182742	Bn-A02-p2182742	A2	1	0	A
930	Bn-N2-p952606	Bn-A02-p2258714	Bn-A02-p2258714	A2	1	0	A
931	Bn-N2-p991484	Bn-A02-p2287712	Bn-A02-p2287712	A2	1	0	A
932	Bn-N2-p991976	Bn-A02-p2288207	Bn-A02-p2288207	A2	1	0	A
933	Bn-N2-p991985	Bn-A02-p2288216	Bn-A02-p2288216	A2	1	0	A
934	Bn-N2-p1000576	Bn-A02-p2292871	Bn-A02-p2292871	A2	2	1.8	A
935	Bn-N2-p1068047	Bn-A02-p2364069	Bn-A02-p2364069	A2	3	5.3	A
936	Bn-N2-p1226262	Bn-A02-p2527082	Bn-A02-p2527082	A2	3	5.3	A
937	Bn-N2-p1244624	Bn-A02-p2542065	Bn-A02-p2542065	A2	3	5.3	A
938	Bn-N2-p1274020	Bn-A02-p2572625	Bn-A02-p2572625	A2	4	6.2	A
939	Bn-N2-p1284147	Bn-A02-p2582999	Bn-A02-p2582999	A2	4	6.2	A
940	Bn-N2-p1314947	Bn-A02-p2617701	Bn-A02-p2617701	A2	5	7.1	A
941	Bn-N2-p1315027	Bn-A02-p2617781	Bn-A02-p2617781	A2	5	7.1	A
942	Bn-N2-p1321568	Bn-A02-p2624375	Bn-A02-p2624375	A2	5	7.1	A
943	Bn-N2-p1336947	Bn-A02-p2638031	Bn-A02-p2638031	A2	5	7.1	A
944	Bn-N2-p1341829	Bn-A02-p2642904	Bn-A02-p2642904	A2	5	7.1	A
945	Bn-N2-p1341995	Bn-A02-p2643078	Bn-A02-p2643078	A2	5	7.1	0
946	Bn-N2-p1394706	Bn-A02-p2695870	Bn-A02-p2695870	A2	5	7.1	A
947	Bn-N2-p1397046	Bn-A02-p2698217	Bn-A02-p2698217	A2	5	7.1	A
948	Bn-N2-p1408616	Bn-A02-p2708161	Bn-A02-p2708161	A2	5	7.1	A
949	Unknown	Bn-A02-p2707979	Bn-A02-p2707979	A2	5	7.1	A
950	Bn-N2-p1453265	Bn-A02-p2739888	Bn-A02-p2739888	A2	6	8	A
951	Bn-N2-p1465935	Bn-A02-p2753403	Bn-A02-p2753403	A2	6	8	A
952	Bn-N2-p1466504	Bn-A02-p2753972	Bn-A02-p2753972	A2	6	8	A
953	Bn-N2-p1537227	Bn-A02-p1341101	Bn-A02-p1341101	A2	6	8	A
954	Bn-N2-p1612584	Bn-A02-p1264906	Bn-A02-p1264906	A2	6	8	A
955	Bn-N2-p1619108	Bn-A02-p1259929	Bn-A02-p1259929	A2	6	8	A
956	Bn-N2-p1659106	Bn-A02-p1220275	Bn-A02-p1220275	A2	6	8	A
957	Bn-N2-p1786578	Bn-A02-p1096082	Bn-A02-p1096082	A2	7	9.8	A
958	Bn-N2-p1790534	Bn-A02-p1092146	Bn-A02-p1092146	A2	8	10.6	A
959	Bn-N2-p1812897	Bn-A02-p1068542	Bn-A02-p1068542	A2	9	11.4	A
960	Bn-N2-p1874242	Bn-A02-p2799146	Bn-A02-p2799146	A2	9	11.4	A
961	Bn-N2-p1875323	Bn-A02-p2800227	Bn-A02-p2800227	A2	9	11.4	A
962	Bn-N2-p1875675	Bn-A02-p2800579	Bn-A02-p2800579	A2	9	11.4	A
963	Bn-N2-p1920922	Bn-A02-p2851231	Bn-A02-p2851231	A2	9	11.4	A
964	Bn-N2-p1952858	Bn-A02-p2877222	Bn-A02-p2877222	A2	9	11.4	A
965	Bn-N2-p1983351	Bn-A02-p2923889	Bn-A02-p2923889	A2	10	12.2	A
966	Bn-N2-p1987002	Bn-A02-p2927357	Bn-A02-p2927357	A2	10	12.2	A
967	Bn-N2-p1991401	Bn-A02-p2931725	Bn-A02-p2931725	A2	10	12.2	A
968	Bn-N2-p2069982	Bn-A02-p3011103	Bn-A02-p3011103	A2	11	17.1	A
969	Bn-N2-p2091947	Bn-A02-p3033620	Bn-A02-p3033620	A2	11	17.1	A
970	Bn-N2-p2151215	Bn-A02-p3074016	Bn-A02-p3074016	A2	11	17.1	A
971	Bn-N2-p2196718	Bn-A02-p3121742	Bn-A02-p3121742	A2	12	17.9	A
972	Bn-N2-p2223558	Bn-A02-p3145923	Bn-A02-p3145923	A2	12	17.9	A
973	Bn-N2-p2228465	Bn-A02-p3150860	Bn-A02-p3150860	A2	12	17.9	A
974	Bn-N2-p2238075	Bn-A02-p3160461	Bn-A02-p3160461	A2	13	18.7	A
975	Bn-N2-p2256921	Bn-A02-p3175610	Bn-A02-p3175610	A2	13	18.7	A

976	Bn-N2-p2261789	Bn-A02-p3180162	Bn-A02-p3180162	A2	13	18.7	A
977	Bn-N2-p2263070	Bn-A02-p3181443	Bn-A02-p3181443	A2	13	18.7	A
978	Bn-N2-p2269507	Bn-A02-p3190650	Bn-A02-p3190650	A2	13	18.7	A
979	Bn-N2-p2278952	Bn-A02-p3207085	Bn-A02-p3207085	A2	13	18.7	A
980	Bn-N2-p2280143	Bn-A02-p3208275	Bn-A02-p3208275	A2	13	18.7	A
981	Bn-N2-p2286772	Bn-A02-p3214903	Bn-A02-p3214903	A2	13	18.7	A
982	Bn-N2-p2304775	Bn-A02-p3233816	Bn-A02-p3233816	A2	14	19.5	A
983	Bn-N2-p2345136	Bn-A02-p3260536	Bn-A02-p3260536	A2	14	19.5	A
984	Bn-N2-p2380926	Bn-A02-p3294682	Bn-A02-p3294682	A2	15	21.9	A
985	Bn-N2-p2383804	Bn-A02-p3297592	Bn-A02-p3297592	A2	16	22.7	A
986	Bn-N2-p2387091	Bn-A02-p3300731	Bn-A02-p3300731	A2	16	22.7	A
987	Bn-N2-p2389079	Bn-A02-p3302725	Bn-A02-p3302725	A2	16	22.7	A
988	Bn-N2-p2434012	Bn-A02-p3344176	Bn-A02-p3344176	A2	16	22.7	A
989	Bn-N2-p2438776	Bn-A02-p3349026	Bn-A02-p3349026	A2	16	22.7	A
990	Bn-N2-p2442666	Bn-A02-p3352863	Bn-A02-p3352863	A2	16	22.7	A
991	Bn-N2-p2458886	Bn-A02-p3374678	Bn-A02-p3374678	A2	17	23.5	A
992	Bn-N2-p2482742	Bn-A02-p3398506	Bn-A02-p3398506	A2	18	24.3	A
993	Bn-N2-p2482946	Bn-A02-p3398710	Bn-A02-p3398710	A2	18	24.3	A
994	Bn-N2-p2524901	Bn-A02-p3427640	Bn-A02-p3427640	A2	18	24.3	A
995	Bn-N2-p2566292	Bn-A02-p3488996	Bn-A02-p3488996	A2	18	24.3	A
996	Bn-N2-p2567267	Bn-A02-p3489974	Bn-A02-p3489974	A2	18	24.3	A
997	Bn-N2-p2616924	Bn-A02-p3542024	Bn-A02-p3542024	A2	19	25.1	A
998	Bn-N2-p2708860	Bn-A02-p3633790	Bn-A02-p3633790	A2	19	25.1	A
999	Bn-N2-p2787089	Bn-A02-p3716898	Bn-A02-p3716898	A2	19	25.1	A
1000	Bn-N2-p2788609	Bn-A02-p3718340	Bn-A02-p3718340	A2	19	25.1	A
1001	Bn-N2-p2868097	Bn-A02-p3803477	Bn-A02-p3803477	A2	19	25.1	A
1002	Bn-N2-p2891505	Bn-C2-p3780809	Bn-scaff_15714_1-p977841	A2	19	25.1	A
1003	Bn-N2-p2892084	Bn-A02-p3822985	Bn-A02-p3822985	A2	19	25.1	A
1004	Bn-N2-p2924938	Bn-A02-p3859399	Bn-A02-p3859399	A2	19	25.1	A
1005	Bn-N2-p2975056	Bn-A02-p3914269	Bn-A02-p3914269	A2	19	25.1	A
1006	Bn-N2-p2975407	Bn-C7-p32456571	Bn-scaff_16130_1-p22592	A2	19	25.1	A
1007	Bn-N2-p3016075	Bn-A02-p3950581	Bn-A02-p3950581	A2	19	25.1	A
1008	Bn-Scaffold03677-p1641	Bn-A02-p3934729	Bn-A02-p3934729	A2	19	25.1	A
1009	Bn-N2-p3119297	Bn-A02-p4035808	Bn-A02-p4035808	A2	20	25.9	A
1010	Bn-N2-p3120499	Bn-A02-p4037028	Bn-A02-p4037028	A2	20	25.9	A
1011	Bn-N2-p3121692	Bn-A02-p4038224	Bn-A02-p4038224	A2	20	25.9	A
1012	Bn-N2-p3125736	Bn-A02-p4044933	Bn-A02-p4044933	A2	20	25.9	A
1013	Bn-N2-p3172570	Bn-A02-p4103543	Bn-A02-p4103543	A2	20	25.9	A
1014	Bn-N2-p3174149	Bn-C2-p4142672	Bn-scaff_15714_1-p61597	A2	20	25.9	A
1015	Bn-N2-p3222603	Bn-A02-p4151292	Bn-A02-p4151292	A2	20	25.9	A
1016	Bn-N2-p3249801	Bn-A02-p4183553	Bn-A02-p4183553	A2	20	25.9	A
1017	Bn-N2-p3261807	Bn-A02-p4199792	Bn-A02-p4199792	A2	20	25.9	A
1018	Bn-N2-p3263201	Bn-A02-p4201122	Bn-A02-p4201122	A2	20	25.9	A
1019	Bn-N2-p3314254	Bn-A02-p4241100	Bn-A02-p4241100	A2	20	25.9	A
1020	Bn-N2-p3355557	Bn-A02-p4277649	Bn-A02-p4277649	A2	21	26.7	A
1021	Bn-N2-p3380125	Bn-A02-p4298976	Bn-A02-p4298976	A2	21	26.7	A
1022	Bn-N2-p3380234	Bn-A02-p4299085	Bn-A02-p4299085	A2	21	26.7	A
1023	Bn-N2-p3380557	Bn-A02-p4299411	Bn-A02-p4299411	A2	21	26.7	A
1024	Bn-N2-p3387309	Bn-A02-p4306196	Bn-A02-p4306196	A2	21	26.7	A

1025	Bn-N2-p3433468	Bn-A02-p4352396	Bn-A02-p4352396	A2	21	26.7	A
1026	Bn-N12-p5156932	Bn-C2-p5371476	Bn-scaff_22527_1-p18586	A2	22	28.3	A
1027	Bn-N2-p3649128	Bn-A02-p4568023	Bn-A02-p4568023	A2	22	28.3	A
1028	Bn-N2-p3778378	Bn-A02-p4698141	Bn-A02-p4698141	A2	22	28.3	A
1029	Bn-N2-p3865572	Bn-A02-p4773307	Bn-A02-p4773307	A2	22	28.3	A
1030	Bn-N2-p3866360	Bn-A02-p4774095	Bn-A02-p4774095	A2	22	28.3	A
1031	Bn-N2-p3866363	Bn-A02-p4774099	Bn-A02-p4774099	A2	22	28.3	A
1032	Bn-N2-p4461220	Bn-A02-p5326222	Bn-A02-p5326222	A2	23	32.3	A
1033	Bn-N12-p6060989	Bn-A02-p5394958	Bn-A02-p5394958	A2	24	33.1	A
1034	Bn-N2-p4567982	Bn-A02-p5429003	Bn-A02-p5429003	A2	25	37.1	B
1035	Bn-N2-p4585093	Bn-C2-p6336063	Bn-scaff_17522_1-p14692	A2	25	37.1	B
1036	Bn-N12-p6289690	Bn-A02-p5517878	Bn-A02-p5517878	A2	26	37.9	B
1037	Bn-N2-p4660762	Bn-A02-p5507193	Bn-A02-p5507193	A2	26	37.9	B
1038	Bn-N2-p4661101	Bn-A02-p5507530	Bn-A02-p5507530	A2	26	37.9	B
1039	Bn-N2-p4668207	Bn-A02-p5516551	Bn-A02-p5516551	A2	26	37.9	B
1040	Bn-N2-p4674659	Bn-A02-p5519388	Bn-A02-p5519388	A2	26	37.9	B
1041	Bn-N2-p4720861	Bn-A02-p5570144	Bn-A02-p5570144	A2	26	37.9	B
1042	Bn-N2-p4725469	Bn-A02-p5574727	Bn-A02-p5574727	A2	26	37.9	B
1043	Bn-N2-p4733516	Bn-A02-p5582861	Bn-A02-p5582861	A2	26	37.9	B
1044	Bn-N2-p4769587	Bn-A02-p5601991	Bn-A02-p5601991	A2	27	38.7	B
1045	Bn-N2-p4769867	Bn-A02-p5602271	Bn-A02-p5602271	A2	27	38.7	B
1046	Bn-N2-p4837452	Bn-A02-p5660188	Bn-A02-p5660188	A2	27	38.7	B
1047	Bn-N2-p4938229	Bn-A02-p5743951	Bn-A02-p5743951	A2	27	38.7	B
1048	Bn-N2-p4979108	Bn-A02-p5799113	Bn-A02-p5799113	A2	27	38.7	B
1049	Bn-N2-p4984177	Bn-A02-p5803164	Bn-A02-p5803164	A2	27	38.7	B
1050	Bn-N2-p4989347	Bn-A02-p5812506	Bn-A02-p5812506	A2	27	38.7	B
1051	Bn-N2-p5035622	Bn-A02-p5859447	Bn-A02-p5859447	A2	27	38.7	B
1052	Bn-N2-p5101425	Bn-A02-p5907701	Bn-A02-p5907701	A2	28	40.3	B
1053	Bn-N2-p5179695	Bn-A02-p5985314	Bn-A02-p5985314	A2	28	40.3	B
1054	Bn-N2-p5180066	Bn-A02-p5985692	Bn-A02-p5985692	A2	28	40.3	B
1055	Bn-N12-p7467056	Bn-A02-p6166584	Bn-A02-p6166584	A2	29	42.7	B
1056	Bn-N19-p25826330	Bn-C9-p28906494	Bn-scaff_17339_1-p16096	A2	29	42.7	B
1057	Bn-N2-p5291239	Bn-A02-p6084757	Bn-A02-p6084757	A2	29	42.7	B
1058	Bn-N2-p5369382	Bn-A02-p6171263	Bn-A02-p6171263	A2	29	42.7	B
1059	Bn-N2-p5401114	Bn-A02-p6202881	Bn-A02-p6202881	A2	29	42.7	B
1060	Bn-N2-p5401292	Bn-A02-p6203059	Bn-A02-p6203059	A2	29	42.7	B
1061	Bn-N2-p5404859	Bn-A02-p6206533	Bn-A02-p6206533	A2	29	42.7	B
1062	Bn-N2-p5405111	Bn-A02-p6206785	Bn-A02-p6206785	A2	29	42.7	B
1063	Bn-N2-p5408093	Bn-A02-p6209563	Bn-A02-p6209563	A2	29	42.7	B
1064	Bn-N2-p5444958	Bn-A02-p6237474	Bn-A02-p6237474	A2	29	42.7	B
1065	Bn-N2-p5474711	Bn-A02-p6256499	Bn-A02-p6256499	A2	29	42.7	B
1066	Bn-N2-p5483616	Bn-A02-p6265274	Bn-A02-p6265274	A2	29	42.7	B
1067	Bn-N2-p5485530	Bn-A02-p6267188	Bn-A02-p6267188	A2	29	42.7	B
1068	Bn-N2-p5485642	Bn-A02-p6267300	Bn-A02-p6267300	A2	29	42.7	B
1069	Bn-N2-p5492381	Bn-A02-p6274305	Bn-A02-p6274305	A2	29	42.7	B
1070	Bn-N2-p5496117	Bn-A02-p6277812	Bn-A02-p6277812	A2	29	42.7	B
1071	Bn-N2-p5498804	Bn-A02-p6280517	Bn-A02-p6280517	A2	29	42.7	B
1072	Bn-N2-p5531394	Bn-A02-p6315978	Bn-A02-p6315978	A2	29	42.7	B
1073	Bn-N2-p5568974	Bn-C2-p8376781	Bn-scaff_16269_1-p53463	A2	29	42.7	B

1074	Bn-N2-p5570408	Bn-A02-p6360867	Bn-A02-p6360867	A2	29	42.7	B
1075	Bn-N2-p5578109	Bn-A02-p6366262	Bn-A02-p6366262	A2	29	42.7	B
1076	Bn-N2-p5617114	Bn-A02-p6390043	Bn-A02-p6390043	A2	29	42.7	B
1077	Bn-N2-p5619470	Bn-A02-p6392450	Bn-A02-p6392450	A2	29	42.7	B
1078	Bn-N2-p5627536	Bn-A02-p6399820	Bn-A02-p6399820	A2	29	42.7	B
1079	Bn-N2-p5627603	Bn-C2-p8426580	Bn-scaff_16269_1-p48484(	A2	29	42.7	B
1080	Bn-N2-p5699607	Bn-C2-p8581973	Bn-scaff_16269_1-p329447	A2	30	43.5	B
1081	Bn-N2-p5707926	Bn-A02-p6473898	Bn-A02-p6473898	A2	30	43.5	B
1082	Bn-N2-p5708131	Bn-A02-p6474102	Bn-A02-p6474102	A2	30	43.5	B
1083	Bn-N2-p5711970	Bn-A02-p6478309	Bn-A02-p6478309	A2	30	43.5	B
1084	Bn-N12-p8313110	Bn-A02-p6587201	Bn-A02-p6587201	A2	31	44.3	B
1085	Bn-N2-p5831138	Bn-A02-p6577065	Bn-A02-p6577065	A2	31	44.3	B
1086	Bn-N2-p5832950	Bn-A02-p6578877	Bn-A02-p6578877	A2	31	44.3	B
1087	Bn-N2-p5838571	Bn-A02-p6584506	Bn-A02-p6584506	A2	31	44.3	B
1088	Bn-N2-p5870990	Bn-A02-p6615821	Bn-A02-p6615821	A2	31	44.3	B
1089	Bn-N2-p5914507	Bn-A02-p6653554	Bn-A02-p6653554	A2	31	44.3	B
1090	Bn-N2-p5919336	Bn-A02-p6658392	Bn-A02-p6658392	A2	31	44.3	B
1091	Bn-N2-p5970711	Bn-A02-p6711900	Bn-A02-p6711900	A2	31	44.3	B
1092	Bn-N12-p10169815	Bn-A02-p7784914	Bn-A02-p7784914	A2	32	45.1	B
1093	Bn-N12-p10418828	Bn-A02-p7885731	Bn-A02-p7885731	A2	32	45.1	B
1094	Bn-N12-p10646815	Bn-C2-p11813441	Bn-scaff_21224_1-p69022	A2	32	45.1	B
1095	Bn-N12-p11602434	Bn-A02-p8549867	Bn-A02-p8549867	A2	32	45.1	B
1096	Bn-N12-p8810941	Bn-C2-p9758520	Bn-scaff_16804_1-p63598(	A2	32	45.1	B
1097	Bn-N12-p9988211	Bn-A02-p7675505	Bn-A02-p7675505	A2	32	45.1	B
1098	Bn-N17-p44448751	Bn-A02-p7756308	Bn-A02-p7756308	A2	32	45.1	B
1099	Bn-N18-p2292250	Bn-Scaffold010197-p1	Bn-Scaffold010197-p119	A2	32	45.1	B
1100	Bn-N1-p20941934	Bn-Scaffold005540-p5	Bn-Scaffold005540-p56	A2	32	45.1	B
1101	Bn-N2-p6071971	Bn-Scaffold009013-p2	Bn-Scaffold009013-p209	A2	32	45.1	B
1102	Bn-N2-p6114654	Bn-A02-p6859259	Bn-A02-p6859259	A2	32	45.1	B
1103	Bn-N2-p6118405	Bn-A02-p6862619	Bn-A02-p6862619	A2	32	45.1	B
1104	Bn-N2-p6234189	Bn-C2-p9505806	Bn-scaff_16804_1-p38327!	A2	32	45.1	B
1105	Bn-N2-p6316820	Bn-A02-p7043311	Bn-A02-p7043311	A2	32	45.1	B
1106	Bn-N2-p6326082	Bn-A02-p7056194	Bn-A02-p7056194	A2	32	45.1	B
1107	Bn-N2-p6327490	Bn-A02-p7057571	Bn-A02-p7057571	A2	32	45.1	B
1108	Bn-N2-p6336359	Bn-A02-p7067463	Bn-A02-p7067463	A2	32	45.1	B
1109	Bn-N2-p6511735	Bn-A02-p7236427	Bn-A02-p7236427	A2	32	45.1	B
1110	Bn-N2-p6522023	Bn-A02-p7245763	Bn-A02-p7245763	A2	32	45.1	B
1111	Bn-N2-p6526880	Bn-A02-p7250607	Bn-A02-p7250607	A2	32	45.1	B
1112	Bn-N2-p6537031	Bn-A02-p7260577	Bn-A02-p7260577	A2	32	45.1	B
1113	Bn-N2-p6537400	Bn-A02-p7260946	Bn-A02-p7260946	A2	32	45.1	B
1114	Bn-N2-p6552902	Bn-A02-p7278213	Bn-A02-p7278213	A2	32	45.1	B
1115	Bn-N2-p6556606	Bn-A02-p7281627	Bn-A02-p7281627	A2	32	45.1	B
1116	Bn-N2-p6557094	Bn-A02-p7282115	Bn-A02-p7282115	A2	32	45.1	B
1117	Bn-N2-p6595573	Bn-A02-p7319800	Bn-A02-p7319800	A2	32	45.1	B
1118	Bn-N2-p6624910	Bn-A02-p7349063	Bn-A02-p7349063	A2	32	45.1	B
1119	Bn-N2-p6625937	Bn-A02-p7350064	Bn-A02-p7350064	A2	32	45.1	B
1120	Bn-N2-p6626072	Bn-A02-p7350199	Bn-A02-p7350199	A2	32	45.1	B
1121	Bn-N2-p6626975	Bn-A02-p7351109	Bn-A02-p7351109	A2	32	45.1	B
1122	Bn-N2-p6627478	Bn-A02-p7351619	Bn-A02-p7351619	A2	32	45.1	B

1123	Bn-N2-p6627657	Bn-A02-p7351746	Bn-A02-p7351746	A2	32	45.1	B
1124	Bn-N2-p6641973	Bn-A02-p7361744	Bn-A02-p7361744	A2	32	45.1	B
1125	Bn-N2-p6678856	Bn-A02-p7396082	Bn-A02-p7396082	A2	32	45.1	B
1126	Bn-N2-p6746526	Bn-A02-p7453258	Bn-A02-p7453258	A2	32	45.1	B
1127	Bn-N2-p6762423	Bn-A02-p7463965	Bn-A02-p7463965	A2	32	45.1	B
1128	Bn-N2-p6763298	Bn-A02-p7464840	Bn-A02-p7464840	A2	32	45.1	B
1129	Bn-N2-p6763455	Bn-A02-p7464997	Bn-A02-p7464997	A2	32	45.1	B
1130	Bn-N2-p6780617	Bn-C2-p10634974	Bn-scaff_27180_1-p44567	A2	32	45.1	B
1131	Bn-N2-p6796960	Bn-A02-p7498192	Bn-A02-p7498192	A2	32	45.1	B
1132	Bn-N2-p6805120	Bn-A02-p7510882	Bn-A02-p7510882	A2	32	45.1	B
1133	Bn-N2-p6811103	Bn-A02-p7516732	Bn-A02-p7516732	A2	32	45.1	B
1134	Bn-N2-p6825182	Bn-A02-p7531907	Bn-A02-p7531907	A2	32	45.1	B
1135	Bn-N2-p6865368	Bn-A02-p7575736	Bn-A02-p7575736	A2	32	45.1	B
1136	Bn-N2-p6885350	Bn-A02-p7595911	Bn-A02-p7595911	A2	32	45.1	B
1137	Bn-N2-p6886805	Bn-A02-p7597314	Bn-A02-p7597314	A2	32	45.1	B
1138	Bn-N2-p6934143	Bn-A02-p7647776	Bn-A02-p7647776	A2	32	45.1	B
1139	Bn-N2-p7010305	Bn-A02-p7712926	Bn-A02-p7712926	A2	32	45.1	B
1140	Bn-N2-p7040922	Bn-A02-p7750368	Bn-A02-p7750368	A2	32	45.1	B
1141	Bn-N2-p7044394	Bn-A02-p7753579	Bn-A02-p7753579	A2	32	45.1	B
1142	Bn-N2-p7104651	Bn-A02-p7801111	Bn-A02-p7801111	A2	32	45.1	B
1143	Bn-N2-p7107216	Bn-A02-p7804291	Bn-A02-p7804291	A2	32	45.1	B
1144	Bn-N2-p7156963	Bn-A02-p7848143	Bn-A02-p7848143	A2	32	45.1	B
1145	Bn-N2-p7161904	Bn-A02-p7853194	Bn-A02-p7853194	A2	32	45.1	B
1146	Bn-N2-p7167173	Bn-A02-p7862412	Bn-A02-p7862412	A2	32	45.1	B
1147	Bn-N2-p7188525	Bn-A02-p7882027	Bn-A02-p7882027	A2	32	45.1	B
1148	Bn-N2-p7198690	Bn-A02-p7892272	Bn-A02-p7892272	A2	32	45.1	B
1149	Bn-N2-p7198972	Bn-A02-p7892561	Bn-A02-p7892561	A2	32	45.1	B
1150	Bn-N2-p7203033	Bn-A02-p7896923	Bn-A02-p7896923	A2	32	45.1	B
1151	Bn-N2-p7275411	Bn-A02-p7976278	Bn-A02-p7976278	A2	32	45.1	B
1152	Bn-N2-p7277351	Bn-A02-p7978230	Bn-A02-p7978230	A2	32	45.1	B
1153	Bn-N2-p7355591	Bn-A02-p17938666	Bn-A02-p17938666	A2	32	45.1	B
1154	Bn-N2-p7418858	Bn-A02-p8090196	Bn-A02-p8090196	A2	32	45.1	B
1155	Bn-N2-p7437059	Bn-A02-p8111245	Bn-A02-p8111245	A2	32	45.1	B
1156	Bn-N2-p7467376	Bn-A02-p8140997	Bn-A02-p8140997	A2	32	45.1	B
1157	Bn-N2-p7468288	Bn-A02-p8141909	Bn-A02-p8141909	A2	32	45.1	B
1158	Bn-N2-p7472055	Bn-A02-p8145676	Bn-A02-p8145676	A2	32	45.1	B
1159	Bn-N2-p7510164	Bn-A02-p8190375	Bn-A02-p8190375	A2	32	45.1	B
1160	Bn-N2-p7510888	Bn-A02-p8191099	Bn-A02-p8191099	A2	32	45.1	B
1161	Bn-N2-p7559860	Bn-A02-p8234333	Bn-A02-p8234333	A2	32	45.1	B
1162	Bn-N2-p7560012	Bn-A02-p8234485	Bn-A02-p8234485	A2	32	45.1	B
1163	Bn-N2-p7560389	Bn-A02-p8234978	Bn-A02-p8234978	A2	32	45.1	B
1164	Bn-N2-p7569475	Bn-A02-p8243789	Bn-A02-p8243789	A2	32	45.1	B
1165	Bn-N2-p7576288	Bn-A02-p8250856	Bn-A02-p8250856	A2	32	45.1	B
1166	Bn-N2-p7576690	Bn-A02-p8251258	Bn-A02-p8251258	A2	32	45.1	B
1167	Bn-N2-p7636096	Bn-A02-p8323616	Bn-A02-p8323616	A2	32	45.1	B
1168	Bn-N2-p7654564	Bn-A02-p8335312	Bn-A02-p8335312	A2	32	45.1	B
1169	Bn-N2-p7662364	Bn-A02-p8336294	Bn-A02-p8336294	A2	32	45.1	B
1170	Bn-N2-p7776012	Bn-C2-p12708356	Bn-scaff_17725_1-p50521[illegible]	A2	32	45.1	B
1171	Bn-N2-p7880377	Bn-A02-p8517135	Bn-A02-p8517135	A2	32	45.1	B

1172	Bn-N2-p7894091	Bn-A02-p8527757	Bn-A02-p8527757	A2	32	45.1	B
1173	Bn-N2-p7896474	Bn-A02-p8530142	Bn-A02-p8530142	A2	32	45.1	B
1174	Bn-N2-p7899040	Bn-A02-p8532901	Bn-A02-p8532901	A2	32	45.1	B
1175	Bn-N2-p7921645	Bn-A02-p8549373	Bn-A02-p8549373	A2	32	45.1	B
1176	Bn-N2-p7927221	Bn-A02-p8554870	Bn-A02-p8554870	A2	32	45.1	B
1177	Bn-N3-p35398375	Bn-A02-p7738248	Bn-A02-p7738248	A2	32	45.1	B
1178	Bn-N6-p10498898	Bn-Scaffold010196-p8	Bn-Scaffold010196-p86	A2	32	45.1	B
1179	Bn-Scaffold39323-p240	Bn-A02-p7975165	Bn-A02-p7975165	A2	32	45.1	B
1180	Bn-Scaffold50752-p245	Bn-A02-p7264855	Bn-A02-p7264855	A2	32	45.1	B
1181	Unknown	Bn-A02-p7675384	Bn-A02-p7675384	A2	32	45.1	B
1182	Unknown	Bn-A02-p7736604	Bn-A02-p7736604	A2	32	45.1	B
1183	Unknown	Bn-A02-p20055831	Bn-A02-p20055831	A2	32	45.1	B
1184	Unknown	Bn-Scaffold010197-p2	Bn-Scaffold010197-p200	A2	32	45.1	B
1185	Bn-N2-p7984224	Bn-A02-p8625851	Bn-A02-p8625851	A2	33	46.7	B
1186	Bn-N4-p12410028	Bn-A10-p15849160	Bn-A10-p15849160	A2	33	46.7	B
1187	Bn-N2-p7995626	Bn-A02-p8637915	Bn-A02-p8637915	A2	34	47.5	B
1188	Bn-N2-p7996421	Bn-C2-p13221043	Bn-scaff_20942_1-p69551	A2	34	47.5	B
1189	Bn-N2-p8013649	Bn-C2-p13260878	Bn-scaff_20942_1-p65567	A2	34	47.5	B
1190	Bn-N2-p8016026	Bn-A02-p8660234	Bn-A02-p8660234	A2	34	47.5	B
1191	Bn-N2-p8017864	Bn-A02-p8663297	Bn-A02-p8663297	A2	34	47.5	B
1192	Bn-N2-p8036705	Bn-A02-p8682290	Bn-A02-p8682290	A2	34	47.5	B
1193	Bn-N2-p8129832	Bn-A02-p8767145	Bn-A02-p8767145	A2	34	47.5	B
1194	Bn-N2-p8138197	Bn-A02-p8777846	Bn-A02-p8777846	A2	34	47.5	B
1195	Bn-N2-p8215157	Bn-A02-p8865764	Bn-A02-p8865764	A2	35	48.3	B
1196	Bn-N2-p8216900	Bn-A02-p8867507	Bn-A02-p8867507	A2	35	48.3	B
1197	Bn-N2-p8217055	Bn-A02-p8867662	Bn-A02-p8867662	A2	35	48.3	B
1198	Bn-N2-p8221887	Bn-A02-p8872271	Bn-A02-p8872271	A2	35	48.3	B
1199	Bn-N2-p8223245	Bn-A02-p8873630	Bn-A02-p8873630	A2	35	48.3	B
1200	Bn-N2-p8223479	Bn-A02-p8873864	Bn-A02-p8873864	A2	35	48.3	B
1201	Bn-N2-p8223608	Bn-A02-p8873993	Bn-A02-p8873993	A2	35	48.3	B
1202	Bn-N2-p8223631	Bn-A02-p8874016	Bn-A02-p8874016	A2	35	48.3	B
1203	Bn-N2-p8223701	Bn-A02-p8874086	Bn-A02-p8874086	A2	35	48.3	B
1204	Bn-N2-p8223914	Bn-A02-p8874299	Bn-A02-p8874299	A2	35	48.3	B
1205	Bn-N2-p8223995	Bn-A02-p8874380	Bn-A02-p8874380	A2	35	48.3	B
1206	Bn-N2-p8226678	Bn-A02-p8877059	Bn-A02-p8877059	A2	35	48.3	B
1207	Bn-N2-p8227206	Bn-A02-p8877587	Bn-A02-p8877587	A2	35	48.3	B
1208	Bn-N2-p8227406	Bn-A02-p8877788	Bn-A02-p8877788	A2	35	48.3	B
1209	Bn-N2-p8227944	Bn-A02-p8878326	Bn-A02-p8878326	A2	35	48.3	B
1210	Bn-N2-p8229849	Bn-A02-p8880230	Bn-A02-p8880230	A2	35	48.3	B
1211	Bn-N2-p8230083	Bn-A02-p8880464	Bn-A02-p8880464	A2	35	48.3	B
1212	Bn-N2-p8230689	Bn-A02-p8881070	Bn-A02-p8881070	A2	35	48.3	B
1213	Bn-N2-p8230870	Bn-A02-p8881251	Bn-A02-p8881251	A2	35	48.3	B
1214	Bn-N2-p8231867	Bn-A02-p8883792	Bn-A02-p8883792	A2	35	48.3	B
1215	Bn-N2-p8232317	Bn-A02-p8884242	Bn-A02-p8884242	A2	35	48.3	B
1216	Bn-N2-p8232787	Bn-A02-p8884712	Bn-A02-p8884712	A2	35	48.3	B
1217	Bn-N2-p8233079	Bn-A02-p8885004	Bn-A02-p8885004	A2	35	48.3	B
1218	Bn-N2-p8233284	Bn-A02-p8885209	Bn-A02-p8885209	A2	35	48.3	B
1219	Bn-N2-p8233983	Bn-A02-p8885908	Bn-A02-p8885908	A2	35	48.3	B
1220	Bn-N2-p8234313	Bn-A02-p8886238	Bn-A02-p8886238	A2	35	48.3	B

1221	Bn-N2-p8235574	Bn-A02-p8891703	Bn-A02-p8891703	A2	35	48.3	B
1222	Bn-N2-p8239496	Bn-A02-p8895388	Bn-A02-p8895388	A2	35	48.3	B
1223	Bn-N2-p8243784	Bn-A02-p8899305	Bn-A02-p8899305	A2	35	48.3	B
1224	Bn-N12-p12568136	Bn-A02-p9161498	Bn-A02-p9161498	A2	36	49.1	B
1225	Bn-N2-p8400457	Bn-C2-p14030661	Bn-scaff_23813_1-p22281	A2	36	49.1	B
1226	Bn-N2-p8418126	Bn-A02-p9064726	Bn-A02-p9064726	A2	36	49.1	B
1227	Bn-N2-p8438742	Bn-A02-p9080423	Bn-A02-p9080423	A2	36	49.1	B
1228	Bn-N2-p8539183	Bn-A02-p9187768	Bn-A02-p9187768	A2	36	49.1	B
1229	Bn-N2-p8575828	Bn-A02-p9225798	Bn-A02-p9225798	A2	36	49.1	B
1230	Bn-N2-p8604770	Bn-A02-p9259325	Bn-A02-p9259325	A2	36	49.1	B
1231	Bn-N2-p8656021	Bn-A02-p9308233	Bn-A02-p9308233	A2	36	49.1	B
1232	Bn-N2-p8688243	Bn-A02-p9339288	Bn-A02-p9339288	A2	36	49.1	B
1233	Bn-N2-p8688469	Bn-A02-p9339514	Bn-A02-p9339514	A2	36	49.1	B
1234	Bn-N2-p8688957	Bn-A02-p9340002	Bn-A02-p9340002	A2	36	49.1	B
1235	Bn-N2-p8709878	Bn-A02-p9361967	Bn-A02-p9361967	A2	36	49.1	B
1236	Bn-N2-p8769849	Bn-A02-p9408651	Bn-A02-p9408651	A2	36	49.1	B
1237	Bn-N2-p8770998	Bn-A02-p9409799	Bn-A02-p9409799	A2	36	49.1	B
1238	Bn-N2-p8777642	Bn-A02-p9415380	Bn-A02-p9415380	A2	36	49.1	B
1239	Bn-N2-p8777898	Bn-A02-p9415636	Bn-A02-p9415636	A2	36	49.1	B
1240	Bn-N2-p8778163	Bn-A02-p9415901	Bn-A02-p9415901	A2	36	49.1	B
1241	Bn-N2-p8778357	Bn-A02-p9416095	Bn-A02-p9416095	A2	36	49.1	B
1242	Bn-N2-p8785290	Bn-A02-p9420837	Bn-A02-p9420837	A2	36	49.1	B
1243	Bn-N2-p8786433	Bn-A02-p9422112	Bn-A02-p9422112	A2	36	49.1	B
1244	Bn-N2-p8867444	Bn-A02-p9513250	Bn-A02-p9513250	A2	36	49.1	B
1245	Bn-N2-p8969710	Bn-A02-p9635795	Bn-A02-p9635795	A2	36	49.1	B
1246	Bn-N2-p8969868	Bn-A02-p9635953	Bn-A02-p9635953	A2	36	49.1	B
1247	Bn-N2-p8982954	Bn-A02-p9643734	Bn-A02-p9643734	A2	36	49.1	B
1248	Bn-N2-p8985287	Bn-A02-p9646068	Bn-A02-p9646068	A2	36	49.1	B
1249	Bn-N2-p8987694	Bn-A02-p9648484	Bn-A02-p9648484	A2	36	49.1	B
1250	Bn-N2-p9002188	Bn-A02-p9664226	Bn-A02-p9664226	A2	36	49.1	B
1251	Bn-N2-p9008665	Bn-C3-p59330474	Bn-scaff_15794_3-p62430	A2	36	49.1	B
1252	Bn-N2-p9038746	Bn-A02-p9703128	Bn-A02-p9703128	A2	36	49.1	B
1253	Bn-N2-p9043636	Bn-A02-p9706613	Bn-A02-p9706613	A2	36	49.1	B
1254	Bn-N2-p9044332	Bn-A02-p9707309	Bn-A02-p9707309	A2	36	49.1	B
1255	Bn-N2-p9059358	Bn-A02-p9722276	Bn-A02-p9722276	A2	36	49.1	B
1256	Bn-N2-p9062262	Bn-A02-p9726742	Bn-A02-p9726742	A2	36	49.1	B
1257	Bn-N3-p27918313	Bn-A02-p9497598	Bn-A02-p9497598	A2	36	49.1	B
1258	Bn-N12-p13750534	Bn-C8-p24221710	Bn-scaff_17807_1-p98331	A2	37	49.9	B
1259	Bn-N12-p14167805	Bn-A02-p10228189	Bn-A02-p10228189	A2	37	49.9	B
1260	Bn-N12-p14438175	Bn-A02-p10404084	Bn-A02-p10404084	A2	37	49.9	B
1261	Bn-N2-p10002325	Bn-A02-p10568947	Bn-A02-p10568947	A2	37	49.9	B
1262	Bn-N2-p10010255	Bn-A02-p10574526	Bn-A02-p10574526	A2	37	49.9	B
1263	Bn-N2-p10020924	Bn-A02-p10587170	Bn-A02-p10587170	A2	37	49.9	B
1264	Bn-N2-p10020986	Bn-A02-p10587232	Bn-A02-p10587232	A2	37	49.9	B
1265	Bn-N2-p10054527	Bn-A02-p10614602	Bn-A02-p10614602	A2	37	49.9	B
1266	Bn-N2-p10056794	Bn-A02-p10616871	Bn-A02-p10616871	A2	37	49.9	B
1267	Bn-N2-p10064457	Bn-A02-p10624373	Bn-A02-p10624373	A2	37	49.9	B
1268	Bn-N2-p10065821	Bn-A02-p10625720	Bn-A02-p10625720	A2	37	49.9	B
1269	Bn-N2-p10156665	Bn-A02-p10742809	Bn-A02-p10742809	A2	37	49.9	B

1270	Bn-N2-p10159573	Bn-A02-p10745711	Bn-A02-p10745711	A2	37	49.9	B
1271	Bn-N2-p10160429	Bn-A02-p10746556	Bn-A02-p10746556	A2	37	49.9	B
1272	Bn-N2-p10166906	Bn-A02-p10753552	Bn-A02-p10753552	A2	37	49.9	B
1273	Bn-N2-p10167349	Bn-C2-p17262530	Bn-scaff_27039_1-p48366	A2	37	49.9	B
1274	Bn-N2-p10175805	Bn-A02-p10763116	Bn-A02-p10763116	A2	37	49.9	B
1275	Bn-N2-p10282963	Bn-C2-p17290586	Bn-scaff_27039_1-p51172	A2	37	49.9	B
1276	Bn-N2-p10290173	Bn-A02-p10781906	Bn-A02-p10781906	A2	37	49.9	B
1277	Bn-N2-p10294498	Bn-A02-p10786230	Bn-A02-p10786230	A2	37	49.9	B
1278	Bn-N2-p10295372	Bn-A02-p10787104	Bn-A02-p10787104	A2	37	49.9	B
1279	Bn-N2-p10295648	Bn-A02-p10787380	Bn-A02-p10787380	A2	37	49.9	B
1280	Bn-N2-p10297213	Bn-A02-p10788285	Bn-A02-p10788285	A2	37	49.9	B
1281	Bn-N2-p10323540	Bn-A02-p10815211	Bn-A02-p10815211	A2	37	49.9	B
1282	Bn-N2-p10323900	Bn-A02-p10815571	Bn-A02-p10815571	A2	37	49.9	B
1283	Bn-N2-p10324651	Bn-A02-p10815969	Bn-A02-p10815969	A2	37	49.9	B
1284	Bn-N2-p10342502	Bn-A02-p10839131	Bn-A02-p10839131	A2	37	49.9	B
1285	Bn-N2-p10387849	Bn-A02-p10869120	Bn-A02-p10869120	A2	37	49.9	B
1286	Bn-N2-p10390357	Bn-A02-p10879119	Bn-A02-p10879119	A2	37	49.9	B
1287	Bn-N2-p10435256	Bn-A02-p10930249	Bn-A02-p10930249	A2	37	49.9	B
1288	Bn-N2-p10444355	Bn-A01-p16630613	Bn-A01-p16630613	A2	37	49.9	B
1289	Bn-N2-p10453749	Bn-A01-p16627043	Bn-A01-p16627043	A2	37	49.9	B
1290	Bn-N2-p10473667	Bn-A02-p10949322	Bn-A02-p10949322	A2	37	49.9	B
1291	Bn-N2-p10505168	Bn-A02-p10983922	Bn-A02-p10983922	A2	37	49.9	B
1292	Bn-N2-p10519407	Bn-A02-p10996151	Bn-A02-p10996151	A2	37	49.9	B
1293	Bn-N2-p10555970	Bn-A07-p5578410	Bn-A07-p5578410	A2	37	49.9	B
1294	Bn-N2-p10576393	Bn-A02-p11038401	Bn-A02-p11038401	A2	37	49.9	B
1295	Bn-N2-p10579677	Bn-A02-p11041726	Bn-A02-p11041726	A2	37	49.9	B
1296	Bn-N2-p10602102	Bn-A02-p11060975	Bn-A02-p11060975	A2	37	49.9	B
1297	Bn-N2-p10604255	Bn-A02-p11062457	Bn-A02-p11062457	A2	37	49.9	B
1298	Bn-N2-p10608743	Bn-A02-p11068431	Bn-A02-p11068431	A2	37	49.9	B
1299	Bn-N2-p10613271	Bn-A02-p11073162	Bn-A02-p11073162	A2	37	49.9	B
1300	Bn-N2-p10619539	Bn-A02-p11080706	Bn-A02-p11080706	A2	37	49.9	B
1301	Bn-N2-p10631489	Bn-A02-p11096221	Bn-A02-p11096221	A2	37	49.9	B
1302	Bn-N2-p10684688	Bn-A02-p11153317	Bn-A02-p11153317	A2	37	49.9	B
1303	Bn-N2-p10716672	Bn-C2-p18174658	Bn-scaff_15838_3-p61315	A2	37	49.9	B
1304	Bn-N2-p10736093	Bn-A02-p11187572	Bn-A02-p11187572	A2	37	49.9	B
1305	Bn-N2-p10738485	Bn-A02-p11192451	Bn-A02-p11192451	A2	37	49.9	B
1306	Bn-N2-p10747278	Bn-A02-p11200305	Bn-A02-p11200305	A2	37	49.9	B
1307	Bn-N2-p10800937	Bn-A02-p11248642	Bn-A02-p11248642	A2	37	49.9	B
1308	Bn-N2-p10810899	Bn-A02-p11259297	Bn-A02-p11259297	A2	37	49.9	B
1309	Bn-N2-p10841741	Bn-A02-p11278529	Bn-A02-p11278529	A2	37	49.9	B
1310	Bn-N2-p10858464	Bn-A02-p11293986	Bn-A02-p11293986	A2	37	49.9	B
1311	Bn-N2-p10883664	Bn-C2-p18567464	Bn-scaff_15838_3-p22034	A2	37	49.9	B
1312	Bn-N2-p10903083	Bn-A02-p11342711	Bn-A02-p11342711	A2	37	49.9	B
1313	Bn-N2-p10906282	Bn-A02-p11345557	Bn-A02-p11345557	A2	37	49.9	B
1314	Bn-N2-p9203173	Bn-A02-p9829719	Bn-A02-p9829719	A2	37	49.9	B
1315	Bn-N2-p9219826	Bn-A02-p9846691	Bn-A02-p9846691	A2	37	49.9	B
1316	Bn-N2-p9220034	Bn-A02-p9846899	Bn-A02-p9846899	A2	37	49.9	B
1317	Bn-N2-p9285739	Bn-A06-p4838544	Bn-A06-p4838544	A2	37	49.9	B
1318	Bn-N2-p9300950	Bn-A02-p9888208	Bn-A02-p9888208	A2	37	49.9	B

1319	Bn-N2-p9304477	Bn-A02-p9891733	Bn-A02-p9891733	A2	37	49.9	B
1320	Bn-N2-p9330806	Bn-C2-p15386517	Bn-scaff_21453_1-p79485	A2	37	49.9	B
1321	Bn-N2-p9336590	Bn-A02-p9927808	Bn-A02-p9927808	A2	37	49.9	B
1322	Bn-N2-p9406491	Bn-A02-p9990613	Bn-A02-p9990613	A2	37	49.9	B
1323	Bn-N2-p9413235	Bn-A02-p9996703	Bn-A02-p9996703	A2	37	49.9	B
1324	Bn-N2-p9430743	Bn-A02-p10019636	Bn-A02-p10019636	A2	37	49.9	B
1325	Bn-N2-p9431338	Bn-A02-p10020231	Bn-A02-p10020231	A2	37	49.9	B
1326	Bn-N2-p9467968	Bn-A02-p10067386	Bn-A02-p10067386	A2	37	49.9	B
1327	Bn-N2-p9479375	Bn-A02-p10078171	Bn-A02-p10078171	A2	37	49.9	B
1328	Bn-N2-p9495098	Bn-A02-p10092191	Bn-A02-p10092191	A2	37	49.9	B
1329	Bn-N2-p9499093	Bn-A02-p10096185	Bn-A02-p10096185	A2	37	49.9	B
1330	Bn-N2-p9525819	Bn-C2-p15801750	Bn-scaff_16565_1-p97701!	A2	37	49.9	B
1331	Bn-N2-p9546173	Bn-A02-p10126530	Bn-A02-p10126530	A2	37	49.9	B
1332	Bn-N2-p9642839	Bn-A02-p10218812	Bn-A02-p10218812	A2	37	49.9	B
1333	Bn-N2-p9652004	Bn-A02-p10227986	Bn-A02-p10227986	A2	37	49.9	B
1334	Bn-N2-p9814560	Bn-A02-p10396907	Bn-A02-p10396907	A2	37	49.9	B
1335	Bn-N2-p9854027	Bn-A02-p10418131	Bn-A02-p10418131	A2	37	49.9	B
1336	Bn-N2-p9876477	Bn-Scaffold000172-p:	Bn-Scaffold000172-p22100	A2	37	49.9	B
1337	Bn-N2-p9900258	Bn-A02-p10477549	Bn-A02-p10477549	A2	37	49.9	B
1338	Bn-N2-p9908983	Bn-C2-p16557685	Bn-scaff_16565_1-p22108(	A2	37	49.9	B
1339	Bn-N2-p9908997	Bn-A02-p10485644	Bn-A02-p10485644	A2	37	49.9	B
1340	Bn-N2-p9930276	Bn-A02-p10507801	Bn-A02-p10507801	A2	37	49.9	B
1341	Bn-N2-p9930339	Bn-A02-p10507864	Bn-A02-p10507864	A2	37	49.9	B
1342	Bn-N2-p9930550	Bn-A02-p10508075	Bn-A02-p10508075	A2	37	49.9	B
1343	Bn-N2-p9937231	Bn-A02-p10510996	Bn-A02-p10510996	A2	37	49.9	B
1344	Bn-N2-p9961771	Bn-A02-p10524842	Bn-A02-p10524842	A2	37	49.9	B
1345	Bn-N2-p9964003	Bn-A02-p10527075	Bn-A02-p10527075	A2	37	49.9	B
1346	Bn-N2-p9965542	Bn-A02-p10528586	Bn-A02-p10528586	A2	37	49.9	B
1347	Bn-N2-p9968278	Bn-A02-p10530978	Bn-A02-p10530978	A2	37	49.9	B
1348	Bn-N2-p9971336	Bn-A02-p10533300	Bn-A02-p10533300	A2	37	49.9	B
1349	Bn-N2-p9980461	Bn-A02-p10540052	Bn-A02-p10540052	A2	37	49.9	B
1350	Bn-N2-p9980557	Bn-A02-p10540148	Bn-A02-p10540148	A2	37	49.9	B
1351	Bn-N2-p9991356	Bn-A02-p10556758	Bn-A02-p10556758	A2	37	49.9	B
1352	Bn-Scaffold04901-p978	Bn-A09-p35512602	Bn-A09-p35512602	A2	37	49.9	B
1353	Bn-Scaffold23806-p188	Bn-A02-p10323998	Bn-A02-p10323998	A2	37	49.9	B
1354	Bn-Scaffold44522-p270	Bn-C2-p15325235	Bn-scaff_21453_1-p18203	A2	37	49.9	B
1355	Unknown	Bn-A02-p11195341	Bn-A02-p11195341	A2	37	49.9	B
1356	Bn-N2-p10907135	Bn-A02-p11346389	Bn-A02-p11346389	A2	38	50.7	B
1357	Bn-N2-p10917650	Bn-A02-p11356944	Bn-A02-p11356944	A2	38	50.7	B
1358	Bn-N2-p10926253	Bn-A02-p11365167	Bn-A02-p11365167	A2	38	50.7	B
1359	Bn-N2-p11118270	Bn-A02-p11415015	Bn-A02-p11415015	A2	38	50.7	B
1360	Bn-N2-p11144428	Bn-A02-p11439590	Bn-A02-p11439590	A2	38	50.7	B
1361	Bn-N2-p11145734	Bn-A02-p11440895	Bn-A02-p11440895	A2	38	50.7	B
1362	Bn-N2-p11146551	Bn-A02-p11441711	Bn-A02-p11441711	A2	38	50.7	B
1363	Bn-N2-p11155737	Bn-A02-p11449348	Bn-A02-p11449348	A2	38	50.7	B
1364	Bn-N2-p11159233	Bn-A02-p11451035	Bn-A02-p11451035	A2	38	50.7	B
1365	Bn-N2-p11166930	Bn-A02-p11454573	Bn-A02-p11454573	A2	38	50.7	B
1366	Bn-N2-p11167023	Bn-A02-p11454658	Bn-A02-p11454658	A2	38	50.7	B
1367	Bn-N2-p11167111	Bn-A02-p11454746	Bn-A02-p11454746	A2	38	50.7	B

1368	Bn-N2-p11167436	Bn-A02-p11455068	Bn-A02-p11455068	A2	38	50.7	B
1369	Bn-N2-p11167750	Bn-A02-p11455369	Bn-A02-p11455369	A2	38	50.7	B
1370	Bn-N2-p11168040	Bn-A02-p11455659	Bn-A02-p11455659	A2	38	50.7	B
1371	Bn-N2-p11171997	Bn-A02-p11457727	Bn-A02-p11457727	A2	38	50.7	B
1372	Bn-N2-p11194498	Bn-A02-p11479169	Bn-A02-p11479169	A2	38	50.7	B
1373	Bn-N2-p11195020	Bn-A02-p11479678	Bn-A02-p11479678	A2	38	50.7	B
1374	Bn-N2-p11226065	Bn-A02-p11513420	Bn-A02-p11513420	A2	38	50.7	B
1375	Bn-N2-p11388027	Bn-A02-p11660867	Bn-A02-p11660867	A2	38	50.7	B
1376	Bn-N2-p11393857	Bn-A02-p11666688	Bn-A02-p11666688	A2	38	50.7	B
1377	Bn-N2-p11406038	Bn-A02-p11673455	Bn-A02-p11673455	A2	38	50.7	B
1378	Bn-N2-p11429219	Bn-A02-p11703005	Bn-A02-p11703005	A2	38	50.7	B
1379	Bn-N2-p11442395	Bn-A02-p11713499	Bn-A02-p11713499	A2	38	50.7	B
1380	Bn-N2-p11453116	Bn-A02-p11723698	Bn-A02-p11723698	A2	38	50.7	B
1381	Bn-N2-p11467352	Bn-A02-p11737945	Bn-A02-p11737945	A2	38	50.7	B
1382	Bn-N2-p11468303	Bn-A02-p11738896	Bn-A02-p11738896	A2	38	50.7	B
1383	Bn-N2-p11476302	Bn-A02-p11746891	Bn-A02-p11746891	A2	38	50.7	B
1384	Bn-N2-p11478014	Bn-A02-p11748602	Bn-A02-p11748602	A2	38	50.7	B
1385	Bn-N2-p11480152	Bn-A02-p11750740	Bn-A02-p11750740	A2	38	50.7	B
1386	Bn-N2-p11485420	Bn-A02-p11759281	Bn-A02-p11759281	A2	38	50.7	0
1387	Bn-N2-p11526101	Bn-A02-p11793499	Bn-A02-p11793499	A2	38	50.7	B
1388	Bn-N2-p11560901	Bn-A02-p11816790	Bn-A02-p11816790	A2	39	51.5	B
1389	Bn-N2-p11561943	Bn-A02-p11817742	Bn-A02-p11817742	A2	39	51.5	B
1390	Bn-N2-p11591140	Bn-A02-p11847986	Bn-A02-p11847986	A2	40	53.1	B
1391	Bn-N2-p11592565	Bn-A02-p11849409	Bn-A02-p11849409	A2	40	53.1	B
1392	Bn-N2-p11592727	Bn-A02-p11849571	Bn-A02-p11849571	A2	40	53.1	B
1393	Bn-N2-p11607484	Bn-A02-p11863349	Bn-A02-p11863349	A2	40	53.1	B
1394	Bn-N2-p11607816	Bn-A02-p11863681	Bn-A02-p11863681	A2	40	53.1	B
1395	Bn-N2-p11614129	Bn-A02-p11870177	Bn-A02-p11870177	A2	40	53.1	B
1396	Bn-N2-p11638890	Bn-A02-p11896538	Bn-A02-p11896538	A2	40	53.1	B
1397	Bn-N2-p11649060	Bn-A02-p11906704	Bn-A02-p11906704	A2	40	53.1	B
1398	Bn-N2-p11673849	Bn-A02-p11929538	Bn-A02-p11929538	A2	40	53.1	B
1399	Bn-N2-p11679355	Bn-A02-p11935002	Bn-A02-p11935002	A2	40	53.1	B
1400	Bn-N2-p11681573	Bn-A02-p11937221	Bn-A02-p11937221	A2	40	53.1	B
1401	Bn-N2-p11707244	Bn-A02-p11960741	Bn-A02-p11960741	A2	40	53.1	B
1402	Bn-N2-p11708084	Bn-A02-p11961581	Bn-A02-p11961581	A2	40	53.1	B
1403	Bn-N2-p11748445	Bn-A02-p11994999	Bn-A02-p11994999	A2	40	53.1	B
1404	Bn-N2-p11749120	Bn-A02-p11995673	Bn-A02-p11995673	A2	40	53.1	B
1405	Bn-N2-p11757214	Bn-A02-p12003125	Bn-A02-p12003125	A2	40	53.1	B
1406	Bn-N2-p11757306	Bn-A02-p12003217	Bn-A02-p12003217	A2	40	53.1	B
1407	Bn-N2-p11905764	Bn-A02-p12145607	Bn-A02-p12145607	A2	40	53.1	B
1408	Bn-N12-p17242648	Bn-A02-p12287615	Bn-A02-p12287615	A2	41	53.9	B
1409	Bn-N12-p17699543	Bn-A02-p12627047	Bn-A02-p12627047	A2	41	53.9	B
1410	Bn-N12-p17987100	Bn-A02-p12819772	Bn-A02-p12819772	A2	41	53.9	B
1411	Bn-N12-p17989621	Bn-A02-p12822311	Bn-A02-p12822311	A2	41	53.9	B
1412	Bn-N1-p22788352	Bn-A02-p13252137	Bn-A02-p13252137	A2	41	53.9	B
1413	Bn-N2-p12031940	Bn-A02-p12278577	Bn-A02-p12278577	A2	41	53.9	B
1414	Bn-N2-p12037386	Bn-A02-p12284078	Bn-A02-p12284078	A2	41	53.9	B
1415	Bn-N2-p12038192	Bn-A02-p12284882	Bn-A02-p12284882	A2	41	53.9	B
1416	Bn-N2-p12045225	Bn-A02-p12292026	Bn-A02-p12292026	A2	41	53.9	B

1417	Bn-N2-p12045967	Bn-A02-p12292768	Bn-A02-p12292768	A2	41	53.9	B
1418	Bn-N2-p12046587	Bn-A02-p12293388	Bn-A02-p12293388	A2	41	53.9	B
1419	Bn-N2-p12048480	Bn-A02-p12295281	Bn-A02-p12295281	A2	41	53.9	B
1420	Bn-N2-p12048881	Bn-A02-p12295682	Bn-A02-p12295682	A2	41	53.9	B
1421	Bn-N2-p12055344	Bn-A02-p12297473	Bn-A02-p12297473	A2	41	53.9	B
1422	Bn-N2-p12065913	Bn-A02-p12314542	Bn-A02-p12314542	A2	41	53.9	B
1423	Bn-N2-p12094985	Bn-A02-p12336037	Bn-A02-p12336037	A2	41	53.9	B
1424	Bn-N2-p12104783	Bn-A02-p12342256	Bn-A02-p12342256	A2	41	53.9	B
1425	Bn-N2-p12123041	Bn-A02-p12363140	Bn-A02-p12363140	A2	41	53.9	B
1426	Bn-N2-p12223211	Bn-A02-p12393535	Bn-A02-p12393535	A2	41	53.9	B
1427	Bn-N2-p12247905	Bn-A02-p12415095	Bn-A02-p12415095	A2	41	53.9	B
1428	Bn-N2-p12297288	Bn-A02-p12460628	Bn-A02-p12460628	A2	41	53.9	B
1429	Bn-N2-p12351103	Bn-A02-p12512328	Bn-A02-p12512328	A2	41	53.9	B
1430	Bn-N2-p12351308	Bn-A02-p12512533	Bn-A02-p12512533	A2	41	53.9	B
1431	Bn-N2-p12352956	Bn-A02-p12514179	Bn-A02-p12514179	A2	41	53.9	B
1432	Bn-N2-p12357618	Bn-A02-p12516859	Bn-A02-p12516859	A2	41	53.9	B
1433	Bn-N2-p12365464	Bn-A02-p12521545	Bn-A02-p12521545	A2	41	53.9	B
1434	Bn-N2-p12368772	Bn-A02-p12524852	Bn-A02-p12524852	A2	41	53.9	B
1435	Bn-N2-p12398641	Bn-A02-p12554865	Bn-A02-p12554865	A2	41	53.9	B
1436	Bn-N2-p12403756	Bn-A02-p12559982	Bn-A02-p12559982	A2	41	53.9	B
1437	Bn-N2-p12451189	Bn-A02-p12595278	Bn-A02-p12595278	A2	41	53.9	B
1438	Bn-N2-p12498200	Bn-A02-p12628141	Bn-A02-p12628141	A2	41	53.9	B
1439	Bn-N2-p12501085	Bn-A02-p12632166	Bn-A02-p12632166	A2	41	53.9	B
1440	Bn-N2-p12506479	Bn-A02-p12637974	Bn-A02-p12637974	A2	41	53.9	B
1441	Bn-N2-p12507345	Bn-A02-p12642708	Bn-A02-p12642708	A2	41	53.9	B
1442	Bn-N2-p12560760	Bn-A02-p12686417	Bn-A02-p12686417	A2	41	53.9	B
1443	Bn-N2-p12569885	Bn-A02-p12693516	Bn-A02-p12693516	A2	41	53.9	B
1444	Bn-N2-p12597648	Bn-A02-p12739678	Bn-A02-p12739678	A2	41	53.9	B
1445	Bn-N2-p12636981	Bn-A02-p12752915	Bn-A02-p12752915	A2	41	53.9	B
1446	Bn-N2-p12640956	Bn-A02-p12756887	Bn-A02-p12756887	A2	41	53.9	B
1447	Bn-N2-p12702925	Bn-C2-p21802267	Bn-scaff_20735_1-p10055	A2	41	53.9	B
1448	Bn-N2-p12709133	Bn-A02-p12815817	Bn-A02-p12815817	A2	41	53.9	B
1449	Bn-N2-p12761827	Bn-Scaffold010908-p[illegible]	Bn-Scaffold010908-p102	A2	41	53.9	B
1450	Bn-N2-p12766232	Bn-A02-p12848961	Bn-A02-p12848961	A2	41	53.9	B
1451	Bn-N2-p12769090	Bn-A02-p12851818	Bn-A02-p12851818	A2	41	53.9	B
1452	Bn-N2-p12784484	Bn-A03-p31160337	Bn-A03-p31160337	A2	41	53.9	B
1453	Bn-N2-p12805548	Bn-A02-p12943042	Bn-A02-p12943042	A2	41	53.9	B
1454	Bn-N2-p12812391	Bn-A02-p12939509	Bn-A02-p12939509	A2	41	53.9	B
1455	Bn-N2-p12832242	Bn-A02-p12972954	Bn-A02-p12972954	A2	41	53.9	B
1456	Bn-N2-p12836031	Bn-A02-p12976547	Bn-A02-p12976547	A2	41	53.9	B
1457	Bn-N2-p12861986	Bn-A02-p13000494	Bn-A02-p13000494	A2	41	53.9	B
1458	Bn-N2-p12877301	Bn-A02-p13019236	Bn-A02-p13019236	A2	41	53.9	B
1459	Bn-N2-p12981704	Bn-A02-p13118529	Bn-A02-p13118529	A2	41	53.9	B
1460	Bn-N2-p12992463	Bn-A02-p13130726	Bn-A02-p13130726	A2	41	53.9	B
1461	Bn-N2-p13095850	Bn-A02-p13197154	Bn-A02-p13197154	A2	41	53.9	B
1462	Bn-N2-p13103660	Bn-A02-p13212386	Bn-A02-p13212386	A2	41	53.9	B
1463	Bn-N2-p13305246	Bn-A02-p13411303	Bn-A02-p13411303	A2	41	53.9	B
1464	Bn-N2-p13359983	Bn-A02-p13570742	Bn-A02-p13570742	A2	41	53.9	B
1465	Bn-N2-p13390510	Bn-A02-p13598238	Bn-A02-p13598238	A2	41	53.9	B

1466	Bn-N2-p13425886	Bn-A02-p13626431	Bn-A02-p13626431	A2	41	53.9	B
1467	Bn-N2-p13429147	Bn-C2-p23160294	Bn-scaff_23108_1-p36293	A2	41	53.9	B
1468	Bn-N2-p13470531	Bn-A02-p13662225	Bn-A02-p13662225	A2	41	53.9	B
1469	Bn-N2-p13585870	Bn-A02-p13751737	Bn-A02-p13751737	A2	41	53.9	B
1470	Bn-N2-p13613868	Bn-A02-p13782884	Bn-A02-p13782884	A2	41	53.9	B
1471	Bn-N2-p13632476	Bn-A02-p13794668	Bn-A02-p13794668	A2	41	53.9	B
1472	Bn-N2-p13634711	Bn-A02-p13798655	Bn-A02-p13798655	A2	41	53.9	B
1473	Bn-N2-p13640071	Bn-A02-p13803383	Bn-A02-p13803383	A2	41	53.9	B
1474	Bn-N2-p13640123	Bn-A02-p13803435	Bn-A02-p13803435	A2	41	53.9	B
1475	Bn-N2-p13676706	Bn-A02-p13822105	Bn-A02-p13822105	A2	41	53.9	B
1476	Bn-N2-p13723961	Bn-A02-p13874627	Bn-A02-p13874627	A2	41	53.9	B
1477	Bn-N2-p13724898	Bn-A02-p13875459	Bn-A02-p13875459	A2	41	53.9	B
1478	Bn-N2-p13805885	Bn-A02-p13972998	Bn-A02-p13972998	A2	41	53.9	B
1479	Bn-N2-p13832071	Bn-A02-p13999424	Bn-A02-p13999424	A2	41	53.9	B
1480	Bn-N2-p13865264	Bn-A02-p14025825	Bn-A02-p14025825	A2	41	53.9	B
1481	Bn-N2-p13866011	Bn-A02-p14026570	Bn-A02-p14026570	A2	41	53.9	B
1482	Bn-N2-p13866808	Bn-A02-p14028000	Bn-A02-p14028000	A2	41	53.9	B
1483	Bn-N2-p13867560	Bn-A02-p14028770	Bn-A02-p14028770	A2	41	53.9	B
1484	Bn-N2-p13879570	Bn-A02-p14040220	Bn-A02-p14040220	A2	41	53.9	B
1485	Bn-N9-p22402618	Bn-C9-p26742887	Bn-scaff_19661_1-p17454	A2	41	53.9	B
1486	Bn-N9-p22403153	Bn-A02-p13498319	Bn-A02-p13498319	A2	41	53.9	0
1487	Bn-Scaffold00856b-p44156	Bn-A02-p13556392	Bn-A02-p13556392	A2	41	53.9	B
1488	Bn-Scaffold00856b-p44262	Bn-A02-p13556497	Bn-A02-p13556497	A2	41	53.9	B
1489	Bn-N2-p13922267	Bn-A02-p14078497	Bn-A02-p14078497	A2	42	54.7	B
1490	Bn-N2-p13969128	Bn-A02-p14127042	Bn-A02-p14127042	A2	42	54.7	B
1491	Bn-N2-p13985787	Bn-A02-p14145586	Bn-A02-p14145586	A2	42	54.7	B
1492	Bn-N2-p13988837	Bn-A02-p14148076	Bn-A02-p14148076	A2	42	54.7	B
1493	Bn-N2-p13993986	Bn-A02-p14151274	Bn-A02-p14151274	A2	42	54.7	B
1494	Bn-N2-p14003361	Bn-A02-p14167623	Bn-A02-p14167623	A2	42	54.7	B
1495	Bn-N2-p14003528	Bn-C2-p24353082	Bn-scaff_16903_1-p41297	A2	42	54.7	B
1496	Bn-N2-p14009194	Bn-A02-p14170476	Bn-A02-p14170476	A2	42	54.7	B
1497	Bn-N2-p14075845	Bn-A02-p14227678	Bn-A02-p14227678	A2	43	55.5	B
1498	Bn-N2-p14078284	Bn-A02-p14229894	Bn-A02-p14229894	A2	43	55.5	B
1499	Bn-N2-p14133848	Bn-A02-p14287428	Bn-A02-p14287428	A2	43	55.5	B
1500	Bn-N2-p14137752	Bn-A02-p14293315	Bn-A02-p14293315	A2	43	55.5	B
1501	Bn-N2-p14160230	Bn-A02-p14328410	Bn-A02-p14328410	A2	43	55.5	B
1502	Bn-Scaffold21546-p444	Bn-A02-p14215327	Bn-A02-p14215327	A2	43	55.5	B
1503	Bn-N2-p14254515	Bn-Scaffold000262-p	Bn-Scaffold000262-p25395	A2	44	56.3	B
1504	Bn-N2-p14271524	Bn-Scaffold000262-p	Bn-Scaffold000262-p33927	A2	44	56.3	B
1505	Bn-N2-p14408299	Bn-A02-p14526876	Bn-A02-p14526876	A2	44	56.3	B
1506	Bn-N2-p14408413	Bn-A02-p14526990	Bn-A02-p14526990	A2	44	56.3	B
1507	Bn-N2-p14409203	Bn-A02-p14527781	Bn-A02-p14527781	A2	44	56.3	B
1508	Bn-N2-p14411210	Bn-A02-p14529778	Bn-A02-p14529778	A2	44	56.3	B
1509	Bn-N2-p14412131	Bn-A02-p14530693	Bn-A02-p14530693	A2	44	56.3	B
1510	Bn-N2-p14437752	Bn-A02-p14564880	Bn-A02-p14564880	A2	44	56.3	B
1511	Bn-N2-p14443818	Bn-A02-p14576479	Bn-A02-p14576479	A2	44	56.3	B
1512	Bn-N2-p14448487	Bn-A02-p14579685	Bn-A02-p14579685	A2	44	56.3	B
1513	Bn-N2-p14469002	Bn-A02-p14608076	Bn-A02-p14608076	A2	44	56.3	B
1514	Bn-N2-p14524253	Bn-A02-p14650284	Bn-A02-p14650284	A2	44	56.3	B

1515	Bn-N2-p14535027	Bn-A02-p14653729	Bn-A02-p14653729	A2	44	56.3	B
1516	Bn-N2-p14550513	Bn-C2-p25131998	Bn-scaff_18675_1-p95593(	A2	44	56.3	B
1517	Bn-N2-p14576541	Bn-A02-p14695760	Bn-A02-p14695760	A2	44	56.3	B
1518	Bn-N2-p14666326	Bn-A02-p14775699	Bn-A02-p14775699	A2	44	56.3	B
1519	Bn-N2-p14671951	Bn-A02-p14781371	Bn-A02-p14781371	A2	44	56.3	B
1520	Bn-N2-p14838349	Bn-A02-p14926973	Bn-A02-p14926973	A2	44	56.3	B
1521	Bn-N2-p14875554	Bn-A02-p14956864	Bn-A02-p14956864	A2	44	56.3	B
1522	Bn-N2-p14911092	Bn-A02-p14992706	Bn-A02-p14992706	A2	44	56.3	B
1523	Bn-N2-p14911167	Bn-A02-p14992781	Bn-A02-p14992781	A2	44	56.3	B
1524	Bn-N2-p14918578	Bn-A02-p14996107	Bn-A02-p14996107	A2	44	56.3	B
1525	Bn-N2-p15064754	Bn-A02-p15184172	Bn-A02-p15184172	A2	44	56.3	B
1526	Bn-N2-p15525546	Bn-A02-p15637932	Bn-A02-p15637932	A2	45	57.9	B
1527	Bn-N2-p15585036	Bn-A02-p15693192	Bn-A02-p15693192	A2	45	57.9	B
1528	Bn-N2-p15605193	Bn-A02-p15717143	Bn-A02-p15717143	A2	45	57.9	B
1529	Bn-N2-p15740491	Bn-A02-p15870327	Bn-A02-p15870327	A2	45	57.9	B
1530	Bn-N2-p15752161	Bn-A02-p15884717	Bn-A02-p15884717	A2	45	57.9	B
1531	Bn-N2-p15783884	Bn-A02-p15912199	Bn-A02-p15912199	A2	45	57.9	B
1532	Bn-N12-p23403089	Bn-C2-p28864983	Bn-scaff_22749_1-p31852:	A2	46	58.7	B
1533	Bn-N12-p23403111	Bn-C2-p28865005	Bn-scaff_22749_1-p31854	A2	46	58.7	B
1534	Bn-N12-p24329965	Bn-C2-p30108085	Bn-scaff_18507_1-p55389	A2	46	58.7	B
1535	Bn-N12-p24331530	Bn-C2-p30109650	Bn-scaff_18507_1-p56954	A2	46	58.7	B
1536	Bn-N12-p24565810	Bn-C2-p30352290	Bn-scaff_18507_1-p29959	A2	46	58.7	B
1537	Bn-N12-p24988057	Bn-C2-p30763003	Bn-scaff_18507_1-p71030	A2	46	58.7	B
1538	Bn-N19-p28239158	Bn-Scaffold01798-p1	Bn-scaff_21016_1-p1898	A2	46	58.7	B
1539	Bn-N2-p16160231	Bn-A02-p16259084	Bn-A02-p16259084	A2	46	58.7	B
1540	Bn-N2-p16160445	Bn-A02-p16259298	Bn-A02-p16259298	A2	46	58.7	B
1541	Bn-N2-p16333048	Bn-A02-p16428766	Bn-A02-p16428766	A2	46	58.7	B
1542	Bn-N2-p16432374	Bn-A02-p16520874	Bn-A02-p16520874	A2	46	58.7	B
1543	Bn-N2-p16440230	Bn-A02-p16528486	Bn-A02-p16528486	A2	46	58.7	B
1544	Bn-N2-p16589487	Bn-A05-p9225459	Bn-A05-p9225459	A2	46	58.7	B
1545	Bn-N2-p16701742	Bn-A05-p9118240	Bn-A05-p9118240	A2	46	58.7	B
1546	Bn-N2-p16739682	Bn-A05-p9080340	Bn-A05-p9080340	A2	46	58.7	B
1547	Bn-N2-p16740260	Bn-A05-p9079762	Bn-A05-p9079762	A2	46	58.7	B
1548	Bn-N2-p16740896	Bn-A05-p9079126	Bn-A05-p9079126	A2	46	58.7	B
1549	Bn-N2-p16741239	Bn-A05-p9078783	Bn-A05-p9078783	A2	46	58.7	B
1550	Bn-N2-p16741709	Bn-A05-p9078313	Bn-A05-p9078313	A2	46	58.7	B
1551	Bn-N2-p16741741	Bn-A05-p9078281	Bn-A05-p9078281	A2	46	58.7	B
1552	Bn-N2-p16742963	Bn-A05-p9077059	Bn-A05-p9077059	A2	46	58.7	B
1553	Bn-N2-p16743318	Bn-A05-p9076704	Bn-A05-p9076704	A2	46	58.7	B
1554	Bn-N2-p16743869	Bn-A05-p9076153	Bn-A05-p9076153	A2	46	58.7	B
1555	Bn-N2-p16762042	Bn-A05-p9063726	Bn-A05-p9063726	A2	46	58.7	B
1556	Bn-N2-p16771429	Bn-A05-p9058906	Bn-A05-p9058906	A2	46	58.7	B
1557	Bn-N2-p16777070	Bn-C2-p29104725	Bn-scaff_22749_1-p55826	A2	46	58.7	B
1558	Bn-N2-p16891506	Bn-A05-p8943009	Bn-A05-p8943009	A2	46	58.7	B
1559	Bn-N2-p16950420	Bn-A05-p8892295	Bn-A05-p8892295	A2	46	58.7	B
1560	Bn-N2-p17049997	Bn-A05-p8786891	Bn-A05-p8786891	A2	46	58.7	B
1561	Bn-N2-p17367373	Bn-C2-p30940449	Bn-scaff_18507_1-p88775:	A2	46	58.7	B
1562	Bn-N2-p18281250	Bn-A02-p17381818	Bn-A02-p17381818	A2	47	59.5	B
1563	Bn-Scaffold00810-p12320	Bn-Scaffold000206-p	Bn-Scaffold000206-p4837	A2	47	59.5	B

1564	Bn-N2-p20034994	Bn-Scaffold000167-p9	Bn-Scaffold000167-p98940	A2	48	62.7	B
1565	Bn-N2-p20510858	Bn-A02-p17910994	Bn-A02-p17910994	A2	48	62.7	B
1566	Bn-N2-p20662672	Bn-A02-p18101171	Bn-A02-p18101171	A2	48	62.7	B
1567	Bn-N2-p20982980	Bn-A02-p18438691	Bn-A02-p18438691	A2	48	62.7	B
1568	Bn-N2-p21124875	Bn-A02-p18599005	Bn-A02-p18599005	A2	48	62.7	B
1569	Bn-N2-p21457111	Bn-A02-p18917772	Bn-A02-p18917772	A2	48	62.7	B
1570	Bn-N17-p18413201	Bn-A02-p19602796	Bn-A02-p19602796	A2	49	63.5	B
1571	Bn-N2-p21506168	Bn-A02-p18974322	Bn-A02-p18974322	A2	49	63.5	B
1572	Bn-N2-p21513905	Bn-A02-p18979982	Bn-A02-p18979982	A2	49	63.5	B
1573	Bn-N2-p21516778	Bn-A02-p18985754	Bn-A02-p18985754	A2	49	63.5	B
1574	Bn-N2-p21519375	Bn-C2-p40691248	Bn-scaff_17289_1-p78006	A2	49	63.5	B
1575	Bn-N2-p21524415	Bn-A02-p18994312	Bn-A02-p18994312	A2	49	63.5	B
1576	Bn-N2-p21524575	Bn-A02-p18994472	Bn-A02-p18994472	A2	49	63.5	B
1577	Bn-N2-p21528916	Bn-A02-p18998627	Bn-A02-p18998627	A2	49	63.5	B
1578	Bn-N2-p21534227	Bn-A02-p19003944	Bn-A02-p19003944	A2	49	63.5	B
1579	Bn-N2-p21584042	Bn-A02-p19045730	Bn-A02-p19045730	A2	49	63.5	B
1580	Bn-N2-p21597671	Bn-C2-p40844107	Bn-scaff_17289_1-p62720	A2	49	63.5	B
1581	Bn-N2-p21697642	Bn-A02-p19125023	Bn-A02-p19125023	A2	49	63.5	B
1582	Bn-N2-p21739691	Bn-A02-p19170689	Bn-A02-p19170689	A2	49	63.5	B
1583	Bn-N2-p21750802	Bn-A02-p19190669	Bn-A02-p19190669	A2	49	63.5	B
1584	Bn-N2-p21760905	Bn-A02-p19207431	Bn-A02-p19207431	A2	49	63.5	B
1585	Bn-N2-p21763435	Bn-A02-p19209964	Bn-A02-p19209964	A2	49	63.5	B
1586	Bn-N2-p21773179	Bn-A02-p19212766	Bn-A02-p19212766	A2	49	63.5	B
1587	Bn-N2-p21778766	Bn-A02-p19218108	Bn-A02-p19218108	A2	49	63.5	B
1588	Bn-N2-p21805259	Bn-A02-p19244695	Bn-A02-p19244695	A2	49	63.5	B
1589	Bn-N2-p21821497	Bn-A02-p19260818	Bn-A02-p19260818	A2	49	63.5	B
1590	Bn-N2-p21822532	Bn-A02-p19261853	Bn-A02-p19261853	A2	49	63.5	B
1591	Bn-N2-p21834040	Bn-A02-p19285211	Bn-A02-p19285211	A2	49	63.5	B
1592	Bn-N2-p21838446	Bn-A02-p19294461	Bn-A02-p19294461	A2	49	63.5	B
1593	Bn-N2-p21843849	Bn-A02-p19299222	Bn-A02-p19299222	A2	49	63.5	B
1594	Bn-N2-p21844083	Bn-A02-p19299455	Bn-A02-p19299455	A2	49	63.5	B
1595	Bn-N2-p21856271	Bn-A02-p19316657	Bn-A02-p19316657	A2	49	63.5	B
1596	Bn-N2-p21857257	Bn-A02-p19317643	Bn-A02-p19317643	A2	49	63.5	B
1597	Bn-N2-p21897001	Bn-A02-p19348348	Bn-A02-p19348348	A2	49	63.5	B
1598	Bn-N2-p21907037	Bn-A02-p19373757	Bn-A02-p19373757	A2	49	63.5	B
1599	Bn-N2-p21907105	Bn-A02-p19373825	Bn-A02-p19373825	A2	49	63.5	B
1600	Bn-N2-p21908063	Bn-A02-p19374782	Bn-A02-p19374782	A2	49	63.5	B
1601	Bn-N2-p21909141	Bn-A02-p19375860	Bn-A02-p19375860	A2	49	63.5	B
1602	Bn-N2-p21910271	Bn-A02-p19385896	Bn-A02-p19385896	A2	49	63.5	B
1603	Bn-N2-p21910361	Bn-A02-p19385986	Bn-A02-p19385986	A2	49	63.5	B
1604	Bn-N2-p21920826	Bn-A02-p19395795	Bn-A02-p19395795	A2	49	63.5	B
1605	Bn-N2-p21926877	Bn-A02-p19401844	Bn-A02-p19401844	A2	49	63.5	B
1606	Bn-N2-p21927122	Bn-A02-p19402089	Bn-A02-p19402089	A2	49	63.5	B
1607	Bn-N2-p21937682	Bn-A02-p19410459	Bn-A02-p19410459	A2	49	63.5	B
1608	Bn-N2-p21979947	Bn-A02-p19450017	Bn-A02-p19450017	A2	49	63.5	B
1609	Bn-N2-p21980348	Bn-A02-p19450418	Bn-A02-p19450418	A2	49	63.5	B
1610	Bn-N2-p21981310	Bn-A02-p19451380	Bn-A02-p19451380	A2	49	63.5	B
1611	Bn-N2-p21987946	Bn-A02-p19460299	Bn-A02-p19460299	A2	49	63.5	B
1612	Bn-N2-p22199661	Bn-C4-p43278255	Bn-scaff_21956_1-p25590(	A2	49	63.5	B

1613	Bn-N2-p22234936	Bn-A02-p19521910	Bn-A02-p19521910	A2	49	63.5	B
1614	Bn-N2-p22245724	Bn-A02-p19534217	Bn-A02-p19534217	A2	49	63.5	B
1615	Bn-N2-p22266105	Bn-A02-p19549587	Bn-A02-p19549587	A2	49	63.5	B
1616	Bn-N2-p22285981	Bn-A02-p19573867	Bn-A02-p19573867	A2	49	63.5	B
1617	Bn-N2-p22287665	Bn-A02-p19575587	Bn-A02-p19575587	A2	49	63.5	B
1618	Bn-N2-p22291612	Bn-A02-p19587474	Bn-A02-p19587474	A2	49	63.5	B
1619	Bn-N2-p22331062	Bn-A02-p19645773	Bn-A02-p19645773	A2	49	63.5	B
1620	Bn-N2-p22331923	Bn-C9-p20718703	Bn-scaff_15650_1-p70761[illegible]	A2	49	63.5	0
1621	Bn-N2-p22355660	Bn-A02-p19662482	Bn-A02-p19662482	A2	49	63.5	B
1622	Bn-N2-p22368745	Bn-A02-p19674831	Bn-A02-p19674831	A2	49	63.5	B
1623	Bn-N2-p22376878	Bn-A02-p19682984	Bn-A02-p19682984	A2	49	63.5	B
1624	Bn-N2-p22414997	Bn-A02-p19702955	Bn-A02-p19702955	A2	49	63.5	B
1625	Bn-N2-p22416723	Bn-A02-p19704677	Bn-A02-p19704677	A2	49	63.5	B
1626	Bn-N2-p22485729	Bn-A08-p1506977	Bn-A08-p1506977	A2	49	63.5	B
1627	Bn-N2-p22497937	Bn-A08-p1514861	Bn-A08-p1514861	A2	49	63.5	B
1628	Bn-N2-p22542153	Bn-A08-p1554055	Bn-A08-p1554055	A2	49	63.5	B
1629	Bn-N2-p22571764	Bn-A08-p1581891	Bn-A08-p1581891	A2	49	63.5	B
1630	Bn-N2-p22574322	Bn-A08-p1584454	Bn-A08-p1584454	A2	49	63.5	B
1631	Bn-N2-p22574322	Bn-A08-p1584455	Bn-A08-p1584455	A2	49	63.5	B
1632	Bn-N2-p22695143	Bn-A02-p21211198	Bn-A02-p21211198	A2	49	63.5	B
1633	Unknown	Bn-A02-p19589605	Bn-A02-p19589605	A2	49	63.5	B
1634	Bn-N12-p34848776	Bn-A02-p21009288	Bn-A02-p21009288	A2	50	64.3	B
1635	Bn-N12-p36336452	Bn-C2-p43669503	Bn-scaff_15712_6-p83645[illegible]	A2	50	64.3	B
1636	Bn-N2-p22881947	Bn-A02-p21061002	Bn-A02-p21061002	A2	50	64.3	B
1637	Bn-N2-p22913975	Bn-A02-p21032956	Bn-A02-p21032956	A2	50	64.3	B
1638	Bn-N2-p22923264	Bn-A02-p21023727	Bn-A02-p21023727	A2	50	64.3	B
1639	Bn-N2-p23031682	Bn-C2-p42386973	Bn-scaff_18360_1-p91556[illegible]	A2	50	64.3	B
1640	Bn-N2-p23092699	Bn-A02-p20882153	Bn-A02-p20882153	A2	50	64.3	B
1641	Bn-N2-p23134950	Bn-A02-p20845905	Bn-A02-p20845905	A2	50	64.3	B
1642	Bn-N2-p23202407	Bn-C2-p42767682	Bn-scaff_18360_1-p12962[illegible]	A2	50	64.3	B
1643	Bn-N2-p23202762	Bn-A02-p20776266	Bn-A02-p20776266	A2	50	64.3	B
1644	Bn-N2-p23249831	Bn-A02-p20718651	Bn-A02-p20718651	A2	50	64.3	0
1645	Bn-N2-p23249911	Bn-A02-p20718571	Bn-A02-p20718571	A2	50	64.3	B
1646	Bn-N2-p23400250	Bn-A02-p20571280	Bn-A02-p20571280	A2	50	64.3	B
1647	Bn-N2-p23402078	Bn-A02-p20568585	Bn-A02-p20568585	A2	50	64.3	B
1648	Bn-N2-p23428442	Bn-A02-p20536885	Bn-A02-p20536885	A2	50	64.3	B
1649	Bn-N2-p23437887	Bn-A02-p20526431	Bn-A02-p20526431	A2	50	64.3	B
1650	Bn-N2-p23498494	Bn-A02-p20462487	Bn-A02-p20462487	A2	50	64.3	B
1651	Bn-N2-p23499487	Bn-A02-p20461477	Bn-A02-p20461477	A2	50	64.3	B
1652	Bn-N2-p23510709	Bn-C2-p43303515	Bn-scaff_15712_6-p47046[illegible]	A2	50	64.3	B
1653	Bn-N2-p23598377	Bn-A02-p20363776	Bn-A02-p20363776	A2	50	64.3	B
1654	Bn-N2-p23716622	Bn-A02-p20207870	Bn-A02-p20207870	A2	50	64.3	B
1655	Bn-N2-p23761268	Bn-A02-p20166067	Bn-A02-p20166067	A2	50	64.3	B
1656	Bn-N2-p23788971	Bn-C2-p43862435	Bn-scaff_15712_6-p10293[illegible]	A2	50	64.3	B
1657	Bn-N5-p14316382	Bn-A06-p20696441	Bn-A06-p20696441	A2	50	64.3	B
1658	Bn-N13-p4512976	Bn-C4-p32237957	Bn-scaff_18776_1-p861	A2	51	65.1	B
1659	Bn-N13-p65531037	Bn-C4-p32243844	Bn-scaff_18776_1-p6748	A2	51	65.1	B
1660	Bn-N2-p24122314	Bn-A02-p19756499	Bn-A02-p19756499	A2	51	65.1	B
1661	Bn-N2-p24218337	Bn-A02-p22847472	Bn-A02-p22847472	A2	51	65.1	B

1662	Bn-N2-p24221591	Bn-A02-p22843446	Bn-A02-p22843446	A2	52	67.5	B
1663	Bn-N2-p24273663	Bn-A02-p22798107	Bn-A02-p22798107	A2	53	68.3	B
1664	Bn-N2-p24276200	Bn-A02-p22787085	Bn-A02-p22787085	A2	53	68.3	B
1665	Bn-N2-p24299028	Bn-A02-p22757902	Bn-A02-p22757902	A2	53	68.3	B
1666	Bn-N2-p24333529	Bn-Scaffold000270-p3	Bn-Scaffold000270-p33166	A2	54	69.1	B
1667	Bn-N2-p24355954	Bn-Scaffold000270-p1	Bn-Scaffold000270-p11524	A2	54	69.1	B
1668	Bn-N2-p24416123	Bn-A02-p22443535	Bn-A02-p22443535	A2	54	69.1	B
1669	Bn-N2-p24452906	Bn-A02-p22409779	Bn-A02-p22409779	A2	54	69.1	B
1670	Bn-N2-p24462745	Bn-A02-p22395060	Bn-A02-p22395060	A2	54	69.1	B
1671	Bn-N2-p24463173	Bn-A02-p22394632	Bn-A02-p22394632	A2	54	69.1	B
1672	Bn-N2-p24492412	Bn-A02-p22371956	Bn-A02-p22371956	A2	54	69.1	B
1673	Bn-N2-p24508335	Bn-A02-p22352518	Bn-A02-p22352518	A2	54	69.1	B
1674	Bn-N2-p24931155	Bn-A02-p21930156	Bn-A02-p21930156	A2	54	69.1	B
1675	Bn-Scaffold05113-p1075	Bn-A09-p14783306	Bn-A09-p14783306	A2	54	69.1	B
1676	Bn-Scaffold05113-p161	Bn-A02-p22365337	Bn-A02-p22365337	A2	54	69.1	B
1677	Bn-Scaffold05962-p1105	Bn-A02-p22460758	Bn-A02-p22460758	A2	54	69.1	B
1678	Bn-N12-p39636693	Bn-C2-p46403354	Bn-scaff_17109_4-p97410	A2	55	70.7	B
1679	Bn-N12-p39637714	Bn-C2-p46404374	Bn-scaff_17109_4-p96390	A2	55	70.7	B
1680	Bn-N2-p25226448	Bn-A02-p21639869	Bn-A02-p21639869	A2	55	70.7	B
1681	Bn-N2-p25304341	Bn-A02-p21563911	Bn-A02-p21563911	A2	55	70.7	B
1682	Bn-N2-p25780097	Bn-A02-p23362842	Bn-A02-p23362842	A2	55	70.7	B
1683	Bn-N2-p25829195	Bn-A02-p23411852	Bn-A02-p23411852	A2	55	70.7	B
1684	Bn-N2-p25834471	Bn-A02-p23414614	Bn-A02-p23414614	A2	55	70.7	B
1685	Bn-N2-p25841676	Bn-A02-p23423377	Bn-A02-p23423377	A2	55	70.7	B
1686	Bn-N2-p25843437	Bn-A02-p23425517	Bn-A02-p23425517	A2	55	70.7	B
1687	Bn-N2-p25846391	Bn-A02-p23428461	Bn-A02-p23428461	A2	55	70.7	B
1688	Bn-N2-p25852350	Bn-A02-p23432823	Bn-A02-p23432823	A2	55	70.7	B
1689	Bn-N2-p25856614	Bn-A02-p23436829	Bn-A02-p23436829	A2	55	70.7	B
1690	Bn-N2-p25867943	Bn-A02-p23448271	Bn-A02-p23448271	A2	55	70.7	B
1691	Bn-N2-p25873549	Bn-A02-p23456103	Bn-A02-p23456103	A2	55	70.7	B
1692	Bn-N2-p25888075	Bn-A02-p23472944	Bn-A02-p23472944	A2	55	70.7	B
1693	Bn-N2-p25888938	Bn-A02-p23473807	Bn-A02-p23473807	A2	55	70.7	B
1694	Bn-N2-p25889440	Bn-A02-p23474309	Bn-A02-p23474309	A2	55	70.7	B
1695	Bn-N2-p25895492	Bn-A02-p23477774	Bn-A02-p23477774	A2	55	70.7	B
1696	Bn-N2-p25895693	Bn-A02-p23477968	Bn-A02-p23477968	A2	55	70.7	B
1697	Bn-N2-p25930169	Bn-A02-p23509986	Bn-A02-p23509986	A2	55	70.7	B
1698	Bn-N2-p25955982	Bn-Scaffold016439-p4	Bn-Scaffold016439-p48	A2	56	71.5	B
1699	Bn-N10-p9972797	Bn-A02-p23708516	Bn-A02-p23708516	A2	57	73.1	B
1700	Bn-N12-p40444818	Bn-A02-p23700858	Bn-A02-p23700858	A2	57	73.1	B
1701	Bn-N12-p40512125	Bn-A02-p23726200	Bn-A02-p23726200	A2	57	73.1	B
1702	Bn-N12-p40524642	Bn-C2-p47312418	Bn-scaff_17109_1-p71578	A2	57	73.1	B
1703	Bn-N12-p40526823	Bn-A02-p23730904	Bn-A02-p23730904	A2	57	73.1	B
1704	Bn-N17-p35241902	Bn-A02-p23708299	Bn-A02-p23708299	A2	57	73.1	B
1705	Bn-N2-p26087020	Bn-A02-p23668607	Bn-A02-p23668607	A2	57	73.1	B
1706	Bn-N2-p26088247	Bn-A02-p23669898	Bn-A02-p23669898	A2	57	73.1	B
1707	Bn-N2-p26100891	Bn-A02-p23686560	Bn-A02-p23686560	A2	57	73.1	B
1708	Unknown	Bn-A02-p23708117	Bn-A02-p23708117	A2	57	73.1	B
1709	Unknown	Bn-A02-p23708678	Bn-A02-p23708678	A2	57	73.1	B
1710	Bn-N12-p40545058	Bn-C2-p47336929	Bn-scaff_17109_1-p69127	A2	58	74.2	B

1711	Bn-N12-p40545513	Bn-C2-p47337384	Bn-scaff_17109_1-p690819	A2	58	74.2	B
1712	Bn-N2-p26176003	Bn-A02-p23788301	Bn-A02-p23788301	A2	59	76.4	B
1713	Bn-N2-p26180452	Bn-A02-p23792703	Bn-A02-p23792703	A2	59	76.4	B
1714	Bn-N2-p26207834	Bn-A02-p23819701	Bn-A02-p23819701	A2	60	77.2	B
1715	Bn-N2-p26207929	Bn-A02-p23819797	Bn-A02-p23819797	A2	60	77.2	B
1716	Bn-N2-p26212825	Bn-C2-p47458745	Bn-scaff_17109_1-p569458	A2	60	77.2	B
1717	Bn-N2-p26213639	Bn-A02-p23824343	Bn-A02-p23824343	A2	60	77.2	B
1718	Bn-N2-p26215628	Bn-A02-p23826324	Bn-A02-p23826324	A2	60	77.2	B
1719	Bn-N2-p26220424	Bn-A02-p23832547	Bn-A02-p23832547	A2	60	77.2	B
1720	Bn-N2-p26223574	Bn-A02-p23835683	Bn-A02-p23835683	A2	60	77.2	B
1721	Bn-N2-p26224099	Bn-A02-p23836232	Bn-A02-p23836232	A2	60	77.2	B
1722	Bn-N2-p26229880	Bn-A02-p23842103	Bn-A02-p23842103	A2	60	77.2	B
1723	Bn-N2-p26249411	Bn-A02-p23856500	Bn-A02-p23856500	A2	60	77.2	B
1724	Bn-N2-p26250302	Bn-A02-p23857384	Bn-A02-p23857384	A2	60	77.2	B
1725	Bn-N2-p26268225	Bn-A02-p23875682	Bn-A02-p23875682	A2	60	77.2	B
1726	Bn-N2-p26279361	Bn-A02-p23889188	Bn-A02-p23889188	A2	60	77.2	B
1727	Bn-N2-p26287727	Bn-A02-p23897014	Bn-A02-p23897014	A2	60	77.2	B
1728	Bn-N2-p26386995	Bn-A02-p24015556	Bn-A02-p24015556	A2	60	77.2	B
1729	Bn-N2-p26534947	Bn-A02-p24178094	Bn-A02-p24178094	A2	60	77.2	B
1730	Bn-N12-p41622271	Bn-A02-p24401267	Bn-A02-p24401267	A2	61	78.8	B
1731	Bn-N2-p26676979	Bn-A02-p24316498	Bn-A02-p24316498	A2	61	78.8	B
1732	Bn-N2-p26678351	Bn-A02-p24317870	Bn-A02-p24317870	A2	61	78.8	B
1733	Bn-N2-p26695494	Bn-A02-p24345550	Bn-A02-p24345550	A2	61	78.8	B
1734	Bn-N2-p26695782	Bn-A02-p24345838	Bn-A02-p24345838	A2	61	78.8	B
1735	Bn-N2-p26698268	Bn-A02-p24351316	Bn-A02-p24351316	A2	61	78.8	B
1736	Bn-N2-p26701541	Bn-A02-p24353955	Bn-A02-p24353955	A2	61	78.8	B
1737	Bn-N2-p26701583	Bn-A02-p24353997	Bn-A02-p24353997	A2	61	78.8	B
1738	Bn-N2-p26702462	Bn-A02-p24354876	Bn-A02-p24354876	A2	61	78.8	B
1739	Bn-N2-p26707970	Bn-A02-p24359370	Bn-A02-p24359370	A2	61	78.8	B
1740	Bn-N2-p26731687	Bn-A02-p24378297	Bn-A02-p24378297	A2	61	78.8	B
1741	Bn-N2-p26740390	Bn-A02-p24386848	Bn-A02-p24386848	A2	61	78.8	B
1742	Bn-N2-p26752657	Bn-A02-p24399308	Bn-A02-p24399308	A2	61	78.8	B
1743	Bn-N2-p26755405	Bn-A02-p24402133	Bn-A02-p24402133	A2	61	78.8	B
1744	Bn-N2-p26761096	Bn-A02-p24415454	Bn-A02-p24415454	A2	61	78.8	B
1745	Bn-N2-p26799071	Bn-A02-p24449690	Bn-A02-p24449690	A2	62	79.6	B
1746	Bn-N2-p26882288	Bn-A02-p24522983	Bn-A02-p24522983	A2	63	80.4	B
1747	Bn-N2-p26899397	Bn-A02-p24532892	Bn-A02-p24532892	A2	63	80.4	B
1748	Bn-N2-p26907160	Bn-A02-p24533751	Bn-A02-p24533751	A2	63	80.4	B
1749	Bn-N2-p26913794	Bn-A02-p24543172	Bn-A02-p24543172	A2	63	80.4	B
1750	Bn-N2-p26923562	Bn-A02-p24552203	Bn-A02-p24552203	A2	63	80.4	0
1751	Bn-N2-p26923836	Bn-A02-p24552476	Bn-A02-p24552476	A2	63	80.4	B
1752	Bn-N2-p26923902	Bn-A02-p24552542	Bn-A02-p24552542	A2	63	80.4	B
1753	Bn-N2-p26924001	Bn-A02-p24552639	Bn-A02-p24552639	A2	63	80.4	B
1754	Bn-N2-p26924084	Bn-A02-p24552721	Bn-A02-p24552721	A2	63	80.4	B
1755	Bn-N2-p26924199	Bn-A02-p24552840	Bn-A02-p24552840	A2	63	80.4	B
1756	Bn-N2-p26924202	Bn-A02-p24552843	Bn-A02-p24552843	A2	63	80.4	B
1757	Bn-N2-p26931265	Bn-A02-p24559792	Bn-A02-p24559792	A2	63	80.4	B
1758	Bn-N2-p26947248	Bn-C2-p48678069	Bn-scaff_17623_1-p47244(	A2	63	80.4	B
1759	Bn-Scaffold13473-p314	Bn-A02-p24718110	Bn-A02-p24718110	A2	63	80.4	B

1760	Bn-N2-p27068181	Bn-C2-p49029761	Bn-scaff_17623_1-p120748	A2	64	81.2	B
1761	Bn-N2-p27068249	Bn-C2-p49029828	Bn-scaff_17623_1-p120681	A2	64	81.2	B
1762	Bn-N12-p42592318	Bn-C2-p49443681	Bn-scaff_17721_1-p644860	A2	65	83.6	B
1763	Bn-N2-p27283251	Bn-A02-p24928311	Bn-A02-p24928311	A2	65	83.6	B
1764	Bn-N2-p27306231	Bn-A02-p24945785	Bn-A02-p24945785	A2	65	83.6	B
1765	Bn-N2-p27309412	Bn-A02-p24948938	Bn-A02-p24948938	A2	65	83.6	B
1766	Bn-N2-p27323063	Bn-C2-p49439958	Bn-scaff_17721_1-p648583	A2	65	83.6	B
1767	Bn-N2-p27330481	Bn-A02-p24971607	Bn-A02-p24971607	A2	65	83.6	B
1768	Bn-N2-p27435756	Bn-A02-p25100451	Bn-A02-p25100451	A2	66	84.4	B
1769	Bn-N3-p20735120	Bn-A02-p25118816	Bn-A02-p25118816	A2	66	84.4	B
1770	Bn-N2-p27515009	Bn-A02-p25180996	Bn-A02-p25180996	A2	67	85.2	B
1771	Bn-N2-p27515640	Bn-A02-p25181474	Bn-A02-p25181474	A2	67	85.2	B
1772	Bn-N2-p27518281	Bn-A02-p25188041	Bn-A02-p25188041	A2	67	85.2	B
1773	Bn-N2-p27602638	Bn-A02-p25260066	Bn-A02-p25260066	A2	67	85.2	B
1774	Bn-N2-p27670381	Bn-A02-p25375825	Bn-A02-p25375825	A2	67	85.2	B
1775	Bn-N2-p27681881	Bn-A02-p25389902	Bn-A02-p25389902	A2	68	86	B
1776	Bn-N2-p27686740	Bn-A02-p25400515	Bn-A02-p25400515	A2	68	86	B
1777	Bn-N2-p27686816	Bn-A02-p25400591	Bn-A02-p25400591	A2	68	86	B
1778	Bn-N2-p27687764	Bn-A02-p25402286	Bn-A02-p25402286	A2	68	86	B
1779	Bn-N2-p27704681	Bn-A02-p25419536	Bn-A02-p25419536	A2	68	86	B
1780	Bn-N2-p27705618	Bn-A02-p25429624	Bn-A02-p25429624	A2	68	86	B
1781	Bn-N2-p27714267	Bn-A02-p25436510	Bn-A02-p25436510	A2	68	86	B
1782	Bn-N2-p27715822	Bn-A02-p25438684	Bn-A02-p25438684	A2	68	86	B
1783	Bn-N2-p27722794	Bn-A02-p25445157	Bn-A02-p25445157	A2	68	86	B
1784	Bn-N2-p27724158	Bn-A02-p25446521	Bn-A02-p25446521	A2	68	86	B
1785	Bn-N2-p27725728	Bn-A02-p25448092	Bn-A02-p25448092	A2	68	86	B
1786	Bn-N2-p27730776	Bn-A02-p25453070	Bn-A02-p25453070	A2	68	86	B
1787	Bn-N2-p27731154	Bn-A02-p25453447	Bn-A02-p25453447	A2	68	86	B
1788	Bn-N2-p27732531	Bn-A02-p25454242	Bn-A02-p25454242	A2	68	86	B
1789	Bn-N2-p27733184	Bn-A02-p25454882	Bn-A02-p25454882	A2	68	86	B
1790	Bn-N2-p27735438	Bn-A02-p25456985	Bn-A02-p25456985	A2	68	86	B
1791	Bn-N2-p27735498	Bn-A02-p25457045	Bn-A02-p25457045	A2	68	86	B
1792	Bn-N2-p27735941	Bn-A02-p25457843	Bn-A02-p25457843	A2	68	86	B
1793	Bn-N2-p27740104	Bn-A02-p25463582	Bn-A02-p25463582	A2	68	86	B
1794	Bn-N2-p27741906	Bn-A02-p25465508	Bn-A02-p25465508	A2	68	86	B
1795	Bn-N2-p27741985	Bn-A02-p25465589	Bn-A02-p25465589	A2	68	86	B
1796	Bn-N2-p27760434	Bn-A02-p25479893	Bn-A02-p25479893	A2	68	86	B
1797	Bn-N2-p27765188	Bn-A02-p25484876	Bn-A02-p25484876	A2	68	86	B
1798	Bn-N2-p27765939	Bn-A02-p25485627	Bn-A02-p25485627	A2	68	86	B
1799	Bn-N2-p27786711	Bn-A02-p25530111	Bn-A02-p25530111	A2	68	86	B
1800	Bn-N2-p27787594	Bn-A02-p25530994	Bn-A02-p25530994	A2	68	86	B
1801	Bn-N2-p27810682	Bn-A02-p25554878	Bn-A02-p25554878	A2	69	87.6	B
1802	Bn-N2-p27824731	Bn-A02-p25572877	Bn-A02-p25572877	A2	69	87.6	B
1803	Bn-N2-p27883015	Bn-A02-p25615285	Bn-A02-p25615285	A2	69	87.6	B
1804	Bn-N2-p27892772	Bn-A02-p25625124	Bn-A02-p25625124	A2	69	87.6	B
1805	Bn-N2-p27894166	Bn-A02-p25626500	Bn-A02-p25626500	A2	69	87.6	B
1806	Bn-N2-p27898241	Bn-A02-p25630745	Bn-A02-p25630745	A2	69	87.6	B
1807	Bn-N2-p27898927	Bn-A02-p25631431	Bn-A02-p25631431	A2	69	87.6	B
1808	Bn-N2-p27900178	Bn-A02-p25632681	Bn-A02-p25632681	A2	69	87.6	B

1809	Bn-N2-p27903643	Bn-A02-p25636038	Bn-A02-p25636038	A2	69	87.6	B
1810	Bn-N2-p27903741	Bn-A02-p25636136	Bn-A02-p25636136	A2	69	87.6	B
1811	Bn-N2-p27921272	Bn-A02-p25655528	Bn-A02-p25655528	A2	69	87.6	B
1812	Bn-N2-p27937138	Bn-A02-p25670193	Bn-A02-p25670193	A2	69	87.6	B
1813	Bn-N2-p27945749	Bn-A02-p25678882	Bn-A02-p25678882	A2	69	87.6	B
1814	Bn-N2-p27948371	Bn-A02-p25681480	Bn-A02-p25681480	A2	69	87.6	B
1815	Bn-N2-p27956264	Bn-A02-p25687470	Bn-A02-p25687470	A2	69	87.6	B
1816	Bn-N2-p28047144	Bn-A02-p25762081	Bn-A02-p25762081	A2	69	87.6	B
1817	Bn-N2-p28058120	Bn-A02-p25795588	Bn-A02-p25795588	A2	69	87.6	B
1818	Bn-N12-p43888045	Bn-A02-p25814719	Bn-A02-p25814719	A2	70	89.2	B
1819	Bn-N2-p28086130	Bn-A02-p25820450	Bn-A02-p25820450	A2	70	89.2	B
1820	Bn-N2-p28092398	Bn-A02-p25826690	Bn-A02-p25826690	A2	70	89.2	B
1821	Bn-N2-p28101502	Bn-A02-p25841130	Bn-A02-p25841130	A2	71	90	B
1822	Bn-N2-p28101630	Bn-A02-p25841252	Bn-A02-p25841252	A2	71	90	B
1823	Bn-N2-p28101760	Bn-A02-p25841382	Bn-A02-p25841382	A2	71	90	B
1824	Bn-N2-p28102100	Bn-A02-p25841722	Bn-A02-p25841722	A2	71	90	B
1825	Bn-N2-p28103234	Bn-A02-p25842875	Bn-A02-p25842875	A2	71	90	B
1826	Bn-N2-p28107422	Bn-A02-p25844300	Bn-A02-p25844300	A2	71	90	B
1827	Bn-N2-p28108452	Bn-A02-p25845339	Bn-A02-p25845339	A2	71	90	B
1828	Bn-N2-p28124925	Bn-A02-p25864853	Bn-A02-p25864853	A2	72	90.8	B
1829	Bn-N2-p28125432	Bn-A02-p25865360	Bn-A02-p25865360	A2	72	90.8	B
1830	Bn-N12-p44049052	Bn-A02-p26345558	Bn-A02-p26345558	A2	73	97.4	B
1831	Bn-N2-p28398142	Bn-A02-p26193572	Bn-A02-p26193572	A2	73	97.4	B
1832	Bn-N2-p28494437	Bn-A02-p26304354	Bn-A02-p26304354	A2	73	97.4	B
1833	Bn-N2-p28512399	Bn-A02-p26322916	Bn-A02-p26322916	A2	73	97.4	B
1834	Bn-N2-p28575801	Bn-A02-p26380384	Bn-A02-p26380384	A2	73	97.4	B
1835	Bn-N2-p28771949	Bn-A02-p26982864	Bn-A02-p26982864	A2	73	97.4	B
1836	Bn-N12-p44751216	Bn-A02-p27215745	Bn-A02-p27215745	A2	74	98.2	B
1837	Bn-N2-p28804745	Bn-A02-p27022909	Bn-A02-p27022909	A2	74	98.2	B
1838	Bn-N2-p28841236	Bn-A02-p27055844	Bn-A02-p27055844	A2	74	98.2	B
1839	Bn-N2-p28841264	Bn-A02-p27055872	Bn-A02-p27055872	A2	74	98.2	B
1840	Bn-N2-p28957063	Bn-A02-p27179350	Bn-A02-p27179350	A2	74	98.2	B
1841	Bn-N2-p28957777	Bn-A02-p27180049	Bn-A02-p27180049	A2	74	98.2	B
1842	Bn-N2-p28990408	Bn-A02-p27203061	Bn-A02-p27203061	A2	74	98.2	B
1843	Bn-N2-p29014558	Bn-A02-p27226579	Bn-A02-p27226579	A2	74	98.2	B
1844	Bn-N2-p29032604	Bn-A02-p27245861	Bn-A02-p27245861	A2	74	98.2	B
1845	Bn-N2-p29061532	Bn-A02-p27262588	Bn-A02-p27262588	A2	74	98.2	B
1846	Bn-N2-p29080099	Bn-A02-p27286923	Bn-A02-p27286923	A2	74	98.2	B
1847	Bn-N2-p29084819	Bn-A02-p27288011	Bn-A02-p27288011	A2	74	98.2	B
1848	Bn-N2-p29084852	Bn-A02-p27288044	Bn-A02-p27288044	A2	74	98.2	B
1849	Bn-N2-p29084929	Bn-A02-p27288121	Bn-A02-p27288121	A2	74	98.2	B
1850	Bn-N2-p29090784	Bn-A02-p27304105	Bn-A02-p27304105	A2	74	98.2	B
1851	Bn-N2-p29103428	Bn-A02-p27327804	Bn-A02-p27327804	A2	74	98.2	0
1852	Bn-N2-p29115133	Bn-A02-p27336483	Bn-A02-p27336483	A2	74	98.2	B
1853	Bn-N2-p29138890	Bn-A02-p27358734	Bn-A02-p27358734	A2	74	98.2	B
1854	Bn-N2-p29279740	Bn-A02-p27490396	Bn-A02-p27490396	A2	74	98.2	B
1855	Bn-N2-p29416753	Bn-A02-p27645672	Bn-A02-p27645672	A2	74	98.2	B
1856	Bn-N2-p29533207	Bn-A02-p27743910	Bn-A02-p27743910	A2	74	98.2	B
1857	Bn-N2-p29544132	Bn-A02-p27755843	Bn-A02-p27755843	A2	74	98.2	B

1858	Bn-N2-p29609872	Bn-A02-p27831174	Bn-A02-p27831174	A2	74	98.2	B
1859	Unknown	Bn-A02-p27122935	Bn-A02-p27122935	A2	74	98.2	B
1860	Bn-N3-p330552	Bn-A03-p312762	Bn-A03-p312762	A3	1	0	B
1861	Bn-N3-p417823	Bn-A03-p403559	Bn-A03-p403559	A3	1	0	B
1862	Bn-N3-p439036	Bn-A03-p373134	Bn-A03-p373134	A3	1	0	B
1863	Bn-N3-p494001	Bn-A03-p498320	Bn-A03-p498320	A3	1	0	B
1864	Bn-N3-p662130	Bn-A03-p657593	Bn-A03-p657593	A3	1	0	B
1865	Bn-N3-p671635	Bn-A03-p671279	Bn-A03-p671279	A3	1	0	B
1866	Bn-N3-p102637	Bn-A03-p90436	Bn-A03-p90436	A3	2	0.8	B
1867	Bn-N3-p1810	Bn-A03-p12026	Bn-A03-p12026	A3	2	0.8	B
1868	Bn-N3-p198287	Bn-A03-p187017	Bn-A03-p187017	A3	2	0.8	B
1869	Bn-N3-p210139	Bn-A03-p196636	Bn-A03-p196636	A3	2	0.8	B
1870	Bn-N3-p211867	Bn-A03-p198822	Bn-A03-p198822	A3	2	0.8	B
1871	Bn-N3-p215656	Bn-A03-p203000	Bn-A03-p203000	A3	2	0.8	B
1872	Bn-N3-p253211	Bn-A03-p235039	Bn-A03-p235039	A3	2	0.8	B
1873	Bn-N3-p712599	Bn-A03-p708787	Bn-A03-p708787	A3	2	0.8	B
1874	Bn-N3-p764664	Bn-A03-p757592	Bn-A03-p757592	A3	2	0.8	B
1875	Bn-N3-p772129	Bn-A03-p765077	Bn-A03-p765077	A3	2	0.8	B
1876	Bn-N3-p839004	Bn-A03-p831433	Bn-A03-p831433	A3	2	0.8	B
1877	Bn-N3-p910599	Bn-A03-p898067	Bn-A03-p898067	A3	2	0.8	B
1878	Bn-N3-p960953	Bn-A03-p943531	Bn-A03-p943531	A3	2	0.8	B
1879	Bn-N3-p1010731	Bn-A03-p991949	Bn-A03-p991949	A3	3	1.6	B
1880	Unknown	Bn-A03-p974894	Bn-A03-p974894	A3	3	1.6	B
1881	Bn-N3-p1025736	Bn-A03-p1008445	Bn-A03-p1008445	A3	4	3.2	B
1882	Bn-N3-p1080752	Bn-A03-p1050437	Bn-A03-p1050437	A3	4	3.2	B
1883	Bn-N3-p1081207	Bn-A03-p1050893	Bn-A03-p1050893	A3	4	3.2	B
1884	Bn-N3-p1092881	Bn-A03-p1065055	Bn-A03-p1065055	A3	4	3.2	B
1885	Bn-N3-p1098950	Bn-A03-p1070680	Bn-A03-p1070680	A3	4	3.2	B
1886	Bn-N3-p1104084	Bn-A03-p1075837	Bn-A03-p1075837	A3	4	3.2	B
1887	Bn-N3-p1107969	Bn-A03-p1084819	Bn-A03-p1084819	A3	4	3.2	B
1888	Bn-N3-p1114121	Bn-A03-p1089875	Bn-A03-p1089875	A3	4	3.2	B
1889	Bn-N3-p1250119	Bn-A03-p1238329	Bn-A03-p1238329	A3	4	3.2	B
1890	Bn-N3-p1322771	Bn-A03-p1308820	Bn-A03-p1308820	A3	4	3.2	B
1891	Bn-N3-p1362960	Bn-A03-p1352996	Bn-A03-p1352996	A3	4	3.2	B
1892	Bn-N3-p1363269	Bn-A03-p1353309	Bn-A03-p1353309	A3	4	3.2	B
1893	Bn-N3-p1364248	Bn-A03-p1354206	Bn-A03-p1354206	A3	4	3.2	B
1894	Bn-N3-p1364418	Bn-A03-p1354375	Bn-A03-p1354375	A3	4	3.2	B
1895	Bn-Scaffold03750-p1288	Bn-A03-p1068165	Bn-A03-p1068165	A3	4	3.2	B
1896	Bn-Scaffold03750-p438	Bn-A03-p1067313	Bn-A03-p1067313	A3	4	3.2	B
1897	Bn-N3-p1475985	Bn-A03-p1461633	Bn-A03-p1461633	A3	5	4.8	B
1898	Bn-N3-p1499294	Bn-A03-p1488306	Bn-A03-p1488306	A3	5	4.8	B
1899	Bn-N3-p1513506	Bn-A03-p1503462	Bn-A03-p1503462	A3	5	4.8	B
1900	Bn-N3-p1517990	Bn-A03-p1507769	Bn-A03-p1507769	A3	5	4.8	B
1901	Bn-N3-p1524562	Bn-A03-p1514927	Bn-A03-p1514927	A3	5	4.8	B
1902	Bn-N3-p1543888	Bn-A03-p1532114	Bn-A03-p1532114	A3	5	4.8	B
1903	Bn-N3-p1568936	Bn-A03-p1559742	Bn-A03-p1559742	A3	5	4.8	B
1904	Bn-N3-p1571317	Bn-A03-p1562119	Bn-A03-p1562119	A3	5	4.8	B
1905	Bn-N3-p1572813	Bn-A03-p1563613	Bn-A03-p1563613	A3	5	4.8	B
1906	Bn-N3-p1607870	Bn-A03-p1596091	Bn-A03-p1596091	A3	5	4.8	B

1907	Bn-N3-p1740688	Bn-A03-p1698016	Bn-A03-p1698016	A3	5	4.8	B
1908	Bn-N3-p1755477	Bn-A03-p1711856	Bn-A03-p1711856	A3	6	5.6	B
1909	Bn-N3-p1759871	Bn-A03-p1716217	Bn-A03-p1716217	A3	6	5.6	B
1910	Bn-N3-p1761406	Bn-A03-p1719791	Bn-A03-p1719791	A3	6	5.6	B
1911	Bn-N3-p1795652	Bn-Scaffold000906-p:	Bn-Scaffold000906-p1518	A3	6	5.6	B
1912	Bn-N3-p1797273	Bn-A03-p1755651	Bn-A03-p1755651	A3	6	5.6	B
1913	Bn-N3-p1873254	Bn-A03-p1817556	Bn-A03-p1817556	A3	6	5.6	B
1914	Bn-N3-p1980524	Bn-A03-p1894119	Bn-A03-p1894119	A3	6	5.6	B
1915	Bn-N3-p2016565	Bn-A03-p1922712	Bn-A03-p1922712	A3	7	7.2	B
1916	Bn-N3-p2019723	Bn-A03-p1926809	Bn-A03-p1926809	A3	7	7.2	B
1917	Bn-N13-p2605446	Bn-A03-p1990974	Bn-A03-p1990974	A3	8	8.8	B
1918	Bn-N13-p3023239	Bn-A03-p2344066	Bn-A03-p2344066	A3	8	8.8	B
1919	Bn-N3-p2055094	Bn-A03-p1954639	Bn-A03-p1954639	A3	8	8.8	B
1920	Bn-N3-p2060235	Bn-A03-p1959964	Bn-A03-p1959964	A3	8	8.8	B
1921	Bn-N3-p2060586	Bn-A03-p1960313	Bn-A03-p1960313	A3	8	8.8	B
1922	Bn-N3-p2060923	Bn-A03-p1960694	Bn-A03-p1960694	A3	8	8.8	B
1923	Bn-N3-p2177223	Bn-A03-p2074890	Bn-A03-p2074890	A3	8	8.8	B
1924	Bn-N3-p2233968	Bn-A03-p2140542	Bn-A03-p2140542	A3	8	8.8	B
1925	Bn-N3-p2278303	Bn-A03-p2190638	Bn-A03-p2190638	A3	8	8.8	B
1926	Bn-N3-p2339537	Bn-A03-p2271873	Bn-A03-p2271873	A3	8	8.8	B
1927	Bn-N3-p2387210	Bn-A03-p2311607	Bn-A03-p2311607	A3	8	8.8	B
1928	Bn-N3-p2512255	Bn-C3-p3015428	Bn-scaff_18936_1-p17769	A3	8	8.8	B
1929	Bn-N3-p2525430	Bn-A03-p2431404	Bn-A03-p2431404	A3	8	8.8	B
1930	Bn-N3-p2541663	Bn-A03-p2449290	Bn-A03-p2449290	A3	8	8.8	B
1931	Bn-N3-p2579332	Bn-A03-p2479213	Bn-A03-p2479213	A3	8	8.8	B
1932	Bn-N3-p2632049	Bn-A03-p2520008	Bn-A03-p2520008	A3	8	8.8	B
1933	Bn-N3-p2633204	Bn-A03-p2521128	Bn-A03-p2521128	A3	8	8.8	B
1934	Bn-N3-p2645979	Bn-A03-p2532699	Bn-A03-p2532699	A3	8	8.8	B
1935	Bn-N3-p2657992	Bn-A03-p2548187	Bn-A03-p2548187	A3	9	9.6	B
1936	Bn-N3-p2679025	Bn-A03-p2579866	Bn-A03-p2579866	A3	9	9.6	B
1937	Bn-N3-p2682880	Bn-C3-p3196551	Bn-scaff_18936_1-p35882	A3	9	9.6	B
1938	Bn-N3-p2695450	Bn-A03-p2596410	Bn-A03-p2596410	A3	9	9.6	B
1939	Bn-N3-p2724321	Bn-A03-p2627552	Bn-A03-p2627552	A3	10	10.4	B
1940	Bn-N3-p2739794	Bn-A03-p2643134	Bn-A03-p2643134	A3	10	10.4	B
1941	Bn-N3-p2742051	Bn-A03-p2645391	Bn-A03-p2645391	A3	10	10.4	B
1942	Bn-N3-p2765209	Bn-A03-p2665731	Bn-A03-p2665731	A3	11	11.2	B
1943	Bn-N3-p2767202	Bn-A03-p2667726	Bn-A03-p2667726	A3	11	11.2	B
1944	Bn-N3-p2793803	Bn-A03-p2695311	Bn-A03-p2695311	A3	11	11.2	B
1945	Bn-N3-p2807568	Bn-A03-p2704795	Bn-A03-p2704795	A3	11	11.2	B
1946	Bn-N3-p2816954	Bn-A03-p2714251	Bn-A03-p2714251	A3	11	11.2	B
1947	Bn-N3-p2832338	Bn-A03-p2727877	Bn-A03-p2727877	A3	11	11.2	B
1948	Bn-N3-p2839591	Bn-A03-p2737338	Bn-A03-p2737338	A3	11	11.2	B
1949	Bn-N3-p2852260	Bn-C3-p3420579	Bn-scaff_18936_1-p58285(	A3	11	11.2	B
1950	Bn-N3-p2892489	Bn-A03-p2793288	Bn-A03-p2793288	A3	11	11.2	B
1951	Bn-N3-p2940916	Bn-A03-p2858882	Bn-A03-p2858882	A3	11	11.2	B
1952	Bn-N3-p2942605	Bn-A03-p2860489	Bn-A03-p2860489	A3	11	11.2	B
1953	Bn-N3-p2945138	Bn-A03-p2862687	Bn-A03-p2862687	A3	11	11.2	B
1954	Bn-N3-p2948138	Bn-A03-p2872671	Bn-A03-p2872671	A3	11	11.2	B
1955	Bn-N3-p2954702	Bn-A03-p2879773	Bn-A03-p2879773	A3	11	11.2	B

1956	Bn-N3-p2966126	Bn-A03-p2891056	Bn-A03-p2891056	A3	11	11.2	B
1957	Bn-N3-p2966999	Bn-A03-p2891934	Bn-A03-p2891934	A3	11	11.2	B
1958	Bn-N3-p2987792	Bn-A03-p2914046	Bn-A03-p2914046	A3	11	11.2	B
1959	Bn-N3-p3055256	Bn-A03-p2978588	Bn-A03-p2978588	A3	11	11.2	B
1960	Bn-N3-p3110843	Bn-A03-p3036102	Bn-A03-p3036102	A3	11	11.2	B
1961	Bn-N3-p3074368	Bn-A03-p2997270	Bn-A03-p2997270	A3	12	13.6	B
1962	Bn-N3-p3078563	Bn-A03-p3001467	Bn-A03-p3001467	A3	12	13.6	B
1963	Bn-N3-p3157400	Bn-A03-p3074927	Bn-A03-p3074927	A3	12	13.6	B
1964	Bn-N3-p3157903	Bn-A03-p3075427	Bn-A03-p3075427	A3	12	13.6	B
1965	Bn-N3-p3167245	Bn-A03-p3084593	Bn-A03-p3084593	A3	12	13.6	B
1966	Bn-N3-p3167518	Bn-A03-p3084866	Bn-A03-p3084866	A3	12	13.6	B
1967	Bn-N3-p3182643	Bn-A03-p3098813	Bn-A03-p3098813	A3	13	14.4	B
1968	Bn-N3-p3183725	Bn-A03-p3099901	Bn-A03-p3099901	A3	13	14.4	B
1969	Bn-N13-p4106390	Bn-A03-p3156872	Bn-A03-p3156872	A3	14	15.2	B
1970	Bn-N13-p4250640	Bn-C3-p4098946	Bn-scaff_15877_1-p18481[illegible]	A3	14	15.2	B
1971	Bn-N13-p4250691	Bn-C3-p4098997	Bn-scaff_15877_1-p18486[illegible]	A3	14	15.2	B
1972	Bn-N13-p4496684	Bn-A03-p3476410	Bn-A03-p3476410	A3	14	15.2	B
1973	Bn-N13-p4510999	Bn-C3-p4390165	Bn-scaff_15877_1-p47603[illegible]	A3	14	15.2	0
1974	Bn-N3-p3317486	Bn-A03-p3219484	Bn-A03-p3219484	A3	14	15.2	B
1975	Bn-N3-p3406926	Bn-A03-p3319357	Bn-A03-p3319357	A3	14	15.2	B
1976	Bn-N3-p3477614	Bn-A03-p3392103	Bn-A03-p3392103	A3	14	15.2	B
1977	Bn-N13-p5303096	Bn-A03-p3674265	Bn-A03-p3674265	A3	15	16.8	B
1978	Bn-N3-p3752600	Bn-C3-p4629817	Bn-scaff_15877_1-p71568[illegible]	A3	15	16.8	B
1979	Bn-N3-p3772359	Bn-A03-p3692048	Bn-A03-p3692048	A3	15	16.8	B
1980	Bn-N3-p3885826	Bn-A03-p3795299	Bn-A03-p3795299	A3	16	17.6	B
1981	Bn-N3-p3886050	Bn-A03-p3795523	Bn-A03-p3795523	A3	16	17.6	B
1982	Bn-N3-p3892311	Bn-A03-p3801626	Bn-A03-p3801626	A3	16	17.6	B
1983	Bn-N3-p3910025	Bn-A03-p3819549	Bn-A03-p3819549	A3	16	17.6	B
1984	Bn-N3-p3913548	Bn-A03-p3823071	Bn-A03-p3823071	A3	16	17.6	B
1985	Bn-N3-p3948397	Bn-A03-p3857752	Bn-A03-p3857752	A3	16	17.6	B
1986	Bn-N3-p3952637	Bn-A03-p3861991	Bn-A03-p3861991	A3	16	17.6	B
1987	Bn-N3-p3952950	Bn-A03-p3862302	Bn-A03-p3862302	A3	16	17.6	B
1988	Bn-N3-p4065533	Bn-A03-p3969278	Bn-A03-p3969278	A3	16	17.6	B
1989	Bn-N3-p4184014	Bn-A03-p4096019	Bn-A03-p4096019	A3	16	17.6	B
1990	Bn-N3-p4210706	Bn-A03-p4116886	Bn-A03-p4116886	A3	16	17.6	B
1991	Bn-N3-p4314795	Bn-A03-p4193022	Bn-A03-p4193022	A3	16	17.6	B
1992	Bn-N3-p4315303	Bn-A03-p4196989	Bn-A03-p4196989	A3	16	17.6	B
1993	Bn-N3-p4365825	Bn-A03-p4244641	Bn-A03-p4244641	A3	16	17.6	B
1994	Bn-N3-p4390447	Bn-A03-p4279693	Bn-A03-p4279693	A3	16	17.6	B
1995	Bn-N3-p4421100	Bn-A03-p4309562	Bn-A03-p4309562	A3	16	17.6	B
1996	Bn-N3-p4448837	Bn-A03-p4339301	Bn-A03-p4339301	A3	16	17.6	B
1997	Bn-N3-p4451981	Bn-A03-p4342338	Bn-A03-p4342338	A3	16	17.6	B
1998	Bn-N3-p4458513	Bn-A03-p4348997	Bn-A03-p4348997	A3	16	17.6	B
1999	Bn-N3-p4500151	Bn-A03-p4399285	Bn-A03-p4399285	A3	16	17.6	B
2000	Bn-N3-p4518862	Bn-A03-p4416817	Bn-A03-p4416817	A3	16	17.6	B
2001	Bn-N3-p4529861	Bn-A03-p4423604	Bn-A03-p4423604	A3	16	17.6	B
2002	Bn-N3-p4577573	Bn-A03-p4473205	Bn-A03-p4473205	A3	16	17.6	B
2003	Bn-N3-p4585484	Bn-A03-p4488639	Bn-A03-p4488639	A3	16	17.6	B
2004	Bn-N3-p4593459	Bn-A03-p4498766	Bn-A03-p4498766	A3	16	17.6	B

2005	Bn-N3-p4604576	Bn-A03-p4509102	Bn-A03-p4509102	A3	16	17.6	B
2006	Bn-Scaffold01563-p6986	Bn-A03-p3808441	Bn-A03-p3808441	A3	16	17.6	B
2007	Bn-Scaffold20404-p489	Bn-A03-p4548924	Bn-A03-p4548924	A3	16	17.6	B
2008	Bn-N3-p4675147	Bn-A03-p4572110	Bn-A03-p4572110	A3	17	18.4	B
2009	Bn-N3-p4759424	Bn-A03-p4673503	Bn-A03-p4673503	A3	17	18.4	0
2010	Bn-N3-p4759429	Bn-A03-p4673508	Bn-A03-p4673508	A3	17	18.4	B
2011	Bn-N3-p4817421	Bn-A03-p4769653	Bn-A03-p4769653	A3	17	18.4	B
2012	Bn-N3-p4831677	Bn-A03-p4783485	Bn-A03-p4783485	A3	17	18.4	B
2013	Bn-N3-p4851767	Bn-A03-p4799142	Bn-A03-p4799142	A3	17	18.4	B
2014	Bn-N3-p4919913	Bn-A03-p4871103	Bn-A03-p4871103	A3	18	19.2	B
2015	Bn-N3-p4928778	Bn-A03-p4879812	Bn-A03-p4879812	A3	18	19.2	B
2016	Bn-N3-p4958930	Bn-A03-p4909233	Bn-A03-p4909233	A3	18	19.2	B
2017	Bn-N3-p4964782	Bn-A03-p4916224	Bn-A03-p4916224	A3	18	19.2	B
2018	Bn-N13-p7326743	Bn-C5-p26606684	Bn-scaff_22195_1-p31290(	A3	19	20.8	B
2019	Bn-N3-p5059196	Bn-A03-p5025156	Bn-A03-p5025156	A3	19	20.8	B
2020	Bn-N3-p5078686	Bn-A03-p5045095	Bn-A03-p5045095	A3	19	20.8	B
2021	Bn-N3-p5106348	Bn-A03-p5072729	Bn-A03-p5072729	A3	19	20.8	B
2022	Bn-N3-p5111964	Bn-A03-p5078310	Bn-A03-p5078310	A3	19	20.8	B
2023	Bn-N3-p5115640	Bn-A03-p5083198	Bn-A03-p5083198	A3	19	20.8	B
2024	Bn-N3-p5115828	Bn-A03-p5083386	Bn-A03-p5083386	A3	19	20.8	B
2025	Bn-N3-p5115872	Bn-A03-p5083430	Bn-A03-p5083430	A3	19	20.8	B
2026	Bn-N3-p5149867	Bn-A03-p5111446	Bn-A03-p5111446	A3	19	20.8	B
2027	Bn-N3-p5185451	Bn-A03-p5153735	Bn-A03-p5153735	A3	19	20.8	B
2028	Bn-N3-p5191753	Bn-A03-p5159930	Bn-A03-p5159930	A3	19	20.8	B
2029	Bn-N3-p5191911	Bn-A03-p5160088	Bn-A03-p5160088	A3	19	20.8	B
2030	Bn-N3-p5194879	Bn-A03-p5163056	Bn-A03-p5163056	A3	19	20.8	B
2031	Bn-N3-p5202612	Bn-A03-p5170706	Bn-A03-p5170706	A3	19	20.8	B
2032	Bn-N3-p5211714	Bn-A03-p5177830	Bn-A03-p5177830	A3	19	20.8	B
2033	Bn-N3-p5214539	Bn-A03-p5180655	Bn-A03-p5180655	A3	19	20.8	B
2034	Bn-Scaffold08851-p752	Bn-A03-p5052289	Bn-A03-p5052289	A3	19	20.8	B
2035	Bn-N13-p7615820	Bn-A03-p5408678	Bn-A03-p5408678	A3	20	24.9	A
2036	Bn-N3-p5300453	Bn-A03-p5268272	Bn-A03-p5268272	A3	20	24.9	A
2037	Bn-N3-p5347595	Bn-Scaffold01280-p4:	Bn-scaff_18523_1-p438	A3	20	24.9	A
2038	Bn-N3-p5352536	Bn-A03-p5333699	Bn-A03-p5333699	A3	20	24.9	A
2039	Bn-N3-p5394308	Bn-A03-p5374417	Bn-A03-p5374417	A3	20	24.9	A
2040	Bn-N3-p5400514	Bn-A03-p5380857	Bn-A03-p5380857	A3	20	24.9	A
2041	Bn-N3-p5434725	Bn-A03-p5414946	Bn-A03-p5414946	A3	20	24.9	A
2042	Bn-N3-p5455764	Bn-C3-p6902159	Bn-scaff_18322_1-p27310!	A3	20	24.9	A
2043	Bn-N3-p5456465	Bn-A03-p5434265	Bn-A03-p5434265	A3	20	24.9	A
2044	Bn-N3-p5475261	Bn-A03-p5454100	Bn-A03-p5454100	A3	20	24.9	A
2045	Bn-N3-p5480987	Bn-A03-p5462418	Bn-A03-p5462418	A3	20	24.9	A
2046	Bn-N3-p5490652	Bn-A03-p5468447	Bn-A03-p5468447	A3	20	24.9	A
2047	Bn-N3-p5493961	Bn-A03-p5471743	Bn-A03-p5471743	A3	20	24.9	A
2048	Bn-N3-p5535586	Bn-A03-p5502308	Bn-A03-p5502308	A3	20	24.9	A
2049	Bn-N3-p5537135	Bn-A03-p5503857	Bn-A03-p5503857	A3	20	24.9	A
2050	Bn-N3-p5567045	Bn-A03-p5532043	Bn-A03-p5532043	A3	20	24.9	A
2051	Bn-N3-p5586379	Bn-A03-p5551018	Bn-A03-p5551018	A3	20	24.9	A
2052	Bn-N3-p5599272	Bn-A03-p5564849	Bn-A03-p5564849	A3	20	24.9	A
2053	Bn-N3-p5602300	Bn-C6-p34381571	Bn-scaff_27421_1-p7441	A3	20	24.9	A

2054	Bn-N3-p5607743	Bn-A03-p5578777	Bn-A03-p5578777	A3	20	24.9	A
2055	Bn-N3-p5639957	Bn-A03-p5608927	Bn-A03-p5608927	A3	20	24.9	A
2056	Bn-N3-p5668974	Bn-A03-p5626575	Bn-A03-p5626575	A3	20	24.9	A
2057	Bn-N3-p5669472	Bn-A03-p5627074	Bn-A03-p5627074	A3	20	24.9	A
2058	Bn-N3-p5677021	Bn-A03-p5643775	Bn-A03-p5643775	A3	20	24.9	A
2059	Bn-N3-p5682437	Bn-A03-p5649178	Bn-A03-p5649178	A3	20	24.9	A
2060	Bn-N3-p5710737	Bn-A03-p5682911	Bn-A03-p5682911	A3	21	25.7	0
2061	Bn-N3-p5723046	Bn-A03-p5694401	Bn-A03-p5694401	A3	21	25.7	A
2062	Bn-N3-p5775835	Bn-A03-p5741340	Bn-A03-p5741340	A3	21	25.7	A
2063	Bn-Scaffold01911-p2656	Bn-A03-p5721171	Bn-A03-p5721171	A3	21	25.7	A
2064	Bn-Scaffold01911-p2716	Bn-A03-p5721231	Bn-A03-p5721231	A3	21	25.7	A
2065	Bn-Scaffold01911-p2928	Bn-A03-p5721443	Bn-A03-p5721443	A3	21	25.7	A
2066	Bn-Scaffold01911-p3102	Bn-A03-p5721617	Bn-A03-p5721617	A3	21	25.7	A
2067	Bn-Scaffold01911-p4065	Bn-A03-p5722579	Bn-A03-p5722579	A3	21	25.7	0
2068	Bn-N3-p5849472	Bn-A03-p5809786	Bn-A03-p5809786	A3	22	26.5	A
2069	Bn-N3-p5849546	Bn-A03-p5809860	Bn-A03-p5809860	A3	22	26.5	A
2070	Bn-N3-p5854130	Bn-A03-p5814447	Bn-A03-p5814447	A3	22	26.5	A
2071	Bn-N3-p6077412	Bn-A03-p6036521	Bn-A03-p6036521	A3	22	26.5	A
2072	Bn-N3-p6079930	Bn-A03-p6039029	Bn-A03-p6039029	A3	22	26.5	A
2073	Bn-N3-p6183478	Bn-A03-p6136484	Bn-A03-p6136484	A3	23	27.3	A
2074	Bn-N3-p6196755	Bn-A03-p6149485	Bn-A03-p6149485	A3	23	27.3	A
2075	Bn-N3-p6205135	Bn-A03-p6157864	Bn-A03-p6157864	A3	23	27.3	A
2076	Bn-N3-p6205483	Bn-A03-p6158211	Bn-A03-p6158211	A3	23	27.3	A
2077	Bn-N3-p6308534	Bn-A03-p6274828	Bn-A03-p6274828	A3	23	27.3	A
2078	Bn-N3-p6310134	Bn-A03-p6276385	Bn-A03-p6276385	A3	23	27.3	A
2079	Bn-N3-p6356236	Bn-A03-p6317002	Bn-A03-p6317002	A3	23	27.3	A
2080	Bn-N3-p6374184	Bn-A03-p6332797	Bn-A03-p6332797	A3	23	27.3	A
2081	Bn-N3-p6376681	Bn-A03-p6335597	Bn-A03-p6335597	A3	23	27.3	A
2082	Bn-N3-p6389292	Bn-C3-p8116956	Bn-scaff_18322_1-p151625	A3	23	27.3	A
2083	Bn-N3-p6445278	Bn-A03-p6385958	Bn-A03-p6385958	A3	23	27.3	A
2084	Bn-N3-p6568754	Bn-A03-p6528914	Bn-A03-p6528914	A3	23	27.3	A
2085	Bn-Scaffold67092-p107	Bn-A03-p6456584	Bn-A03-p6456584	A3	23	27.3	A
2086	Bn-N3-p6629360	Bn-A03-p6585645	Bn-A03-p6585645	A3	24	28.9	A
2087	Bn-N3-p6630151	Bn-A03-p6586436	Bn-A03-p6586436	A3	24	28.9	A
2088	Bn-N3-p6633766	Bn-A03-p6590051	Bn-A03-p6590051	A3	24	28.9	A
2089	Bn-N3-p6650892	Bn-A03-p6602851	Bn-A03-p6602851	A3	24	28.9	A
2090	Bn-N3-p6704528	Bn-A03-p6668259	Bn-A03-p6668259	A3	25	32.1	A
2091	Bn-N3-p6710985	Bn-A03-p6677819	Bn-A03-p6677819	A3	25	32.1	A
2092	Bn-N3-p6770990	Bn-A03-p6744344	Bn-A03-p6744344	A3	25	32.1	A
2093	Bn-N3-p6795796	Bn-A03-p6765024	Bn-A03-p6765024	A3	25	32.1	A
2094	Bn-N3-p6815796	Bn-A03-p6789784	Bn-A03-p6789784	A3	25	32.1	A
2095	Bn-N3-p6817864	Bn-A03-p6791851	Bn-A03-p6791851	A3	25	32.1	A
2096	Bn-N18-p17540552	Bn-A03-p6898220	Bn-A03-p6898220	A3	26	32.9	A
2097	Bn-N3-p7039005	Bn-A03-p7007376	Bn-A03-p7007376	A3	26	32.9	A
2098	Bn-N3-p7039006	Bn-A03-p7007377	Bn-A03-p7007377	A3	26	32.9	A
2099	Bn-N3-p7049530	Bn-A03-p7015536	Bn-A03-p7015536	A3	26	32.9	A
2100	Bn-N3-p7118530	Bn-A03-p7067563	Bn-A03-p7067563	A3	27	33.8	A
2101	Bn-N3-p7216160	Bn-A03-p7159916	Bn-A03-p7159916	A3	27	33.8	A
2102	Bn-N3-p7234314	Bn-A03-p7177504	Bn-A03-p7177504	A3	27	33.8	A

2103	Bn-N3-p7234546	Bn-A03-p7177776	Bn-A03-p7177776	A3	27	33.8	A
2104	Bn-N3-p7235355	Bn-A03-p7178551	Bn-A03-p7178551	A3	27	33.8	A
2105	Bn-N3-p7235454	Bn-A03-p7178650	Bn-A03-p7178650	A3	27	33.8	A
2106	Bn-N3-p7235721	Bn-A03-p7178917	Bn-A03-p7178917	A3	27	33.8	A
2107	Bn-N3-p7237090	Bn-A03-p7180288	Bn-A03-p7180288	A3	27	33.8	A
2108	Bn-N3-p7240873	Bn-C3-p9312773	Bn-scaff_18322_1-p32043	A3	27	33.8	A
2109	Bn-N3-p7242704	Bn-A03-p7186958	Bn-A03-p7186958	A3	27	33.8	A
2110	Bn-N3-p7435856	Bn-A03-p7366783	Bn-A03-p7366783	A3	28	35.5	A
2111	Bn-N3-p7478253	Bn-A03-p7411648	Bn-A03-p7411648	A3	28	35.5	A
2112	Bn-N13-p10704969	Bn-A03-p7629219	Bn-A03-p7629219	A3	29	36.4	A
2113	Bn-N13-p10706102	Bn-A03-p7630376	Bn-A03-p7630376	A3	29	36.4	A
2114	Bn-N3-p7487460	Bn-A03-p7420835	Bn-A03-p7420835	A3	29	36.4	A
2115	Bn-N3-p7487804	Bn-A03-p7421178	Bn-A03-p7421178	A3	29	36.4	A
2116	Bn-N3-p7523983	Bn-A03-p7458972	Bn-A03-p7458972	A3	29	36.4	A
2117	Bn-N3-p7525249	Bn-A03-p7460244	Bn-A03-p7460244	A3	29	36.4	A
2118	Bn-N3-p7541615	Bn-A03-p7478438	Bn-A03-p7478438	A3	29	36.4	A
2119	Bn-N3-p7559200	Bn-A03-p7500107	Bn-A03-p7500107	A3	29	36.4	A
2120	Bn-N3-p7612737	Bn-A03-p7555264	Bn-A03-p7555264	A3	29	36.4	A
2121	Bn-N3-p7620724	Bn-C1-p930986	Bn-scaff_19244_1-p84361	A3	29	36.4	A
2122	Bn-N3-p7630005	Bn-A03-p7571364	Bn-A03-p7571364	A3	29	36.4	A
2123	Bn-N3-p7630748	Bn-A03-p7572128	Bn-A03-p7572128	A3	29	36.4	A
2124	Bn-N3-p7641496	Bn-A03-p7582235	Bn-A03-p7582235	A3	29	36.4	A
2125	Bn-N3-p7669799	Bn-A03-p7613494	Bn-A03-p7613494	A3	29	36.4	A
2126	Bn-N3-p7687041	Bn-A03-p7630628	Bn-A03-p7630628	A3	29	36.4	A
2127	Bn-N3-p7698799	Bn-A03-p7642692	Bn-A03-p7642692	A3	29	36.4	A
2128	Bn-N3-p7699902	Bn-A03-p7643795	Bn-A03-p7643795	A3	29	36.4	A
2129	Bn-N3-p7728895	Bn-A03-p7672403	Bn-A03-p7672403	A3	29	36.4	A
2130	Bn-N3-p7743233	Bn-A03-p7685279	Bn-A03-p7685279	A3	29	36.4	A
2131	Bn-N3-p7781093	Bn-A03-p7738300	Bn-A03-p7738300	A3	29	36.4	A
2132	Bn-N3-p7994167	Bn-A03-p7939679	Bn-A03-p7939679	A3	30	37.3	A
2133	Bn-N3-p8049152	Bn-A03-p7988522	Bn-A03-p7988522	A3	30	37.3	A
2134	Bn-N3-p8049297	Bn-A03-p7988667	Bn-A03-p7988667	A3	30	37.3	0
2135	Bn-N3-p8049450	Bn-A03-p7988820	Bn-A03-p7988820	A3	30	37.3	A
2136	Bn-N13-p11968682	Bn-A03-p8420805	Bn-A03-p8420805	A3	31	39	A
2137	Bn-N13-p41521362	Bn-A03-p8360894	Bn-A03-p8360894	A3	31	39	A
2138	Bn-N3-p8103434	Bn-A03-p8039401	Bn-A03-p8039401	A3	31	39	A
2139	Bn-N3-p8178415	Bn-A03-p8122222	Bn-A03-p8122222	A3	31	39	A
2140	Bn-N3-p8190537	Bn-A03-p8133096	Bn-A03-p8133096	A3	31	39	A
2141	Bn-N3-p8195522	Bn-A03-p8152842	Bn-A03-p8152842	A3	31	39	A
2142	Bn-N3-p8212799	Bn-A03-p8169179	Bn-A03-p8169179	A3	31	39	A
2143	Bn-N3-p8214576	Bn-A03-p8170734	Bn-A03-p8170734	A3	31	39	A
2144	Bn-N3-p8215053	Bn-A03-p8171211	Bn-A03-p8171211	A3	31	39	A
2145	Bn-N3-p8226524	Bn-A03-p8181187	Bn-A03-p8181187	A3	31	39	A
2146	Bn-N3-p8237866	Bn-A03-p8190847	Bn-A03-p8190847	A3	31	39	A
2147	Bn-N3-p8246931	Bn-A03-p8204479	Bn-A03-p8204479	A3	31	39	A
2148	Bn-N3-p8260182	Bn-A03-p8217276	Bn-A03-p8217276	A3	31	39	A
2149	Bn-N3-p8260263	Bn-A03-p8217358	Bn-A03-p8217358	A3	31	39	A
2150	Bn-N3-p8261863	Bn-A03-p8218946	Bn-A03-p8218946	A3	31	39	A
2151	Bn-N3-p8324319	Bn-A03-p8279849	Bn-A03-p8279849	A3	31	39	A

2152	Bn-N3-p8327365	Bn-A03-p8282545	Bn-A03-p8282545	A3	31	39	A
2153	Bn-N3-p8330205	Bn-A03-p8285385	Bn-A03-p8285385	A3	31	39	A
2154	Bn-N3-p8332322	Bn-A03-p8288895	Bn-A03-p8288895	A3	31	39	A
2155	Bn-N3-p8341560	Bn-A03-p8298067	Bn-A03-p8298067	A3	31	39	A
2156	Bn-N3-p8383999	Bn-A03-p8333536	Bn-A03-p8333536	A3	31	39	A
2157	Bn-N3-p8387612	Bn-A03-p8337135	Bn-A03-p8337135	A3	31	39	A
2158	Bn-N3-p8432862	Bn-A03-p8381012	Bn-A03-p8381012	A3	31	39	0
2159	Bn-N3-p8453706	Bn-A03-p8395621	Bn-A03-p8395621	A3	31	39	A
2160	Bn-N3-p8455128	Bn-C4-p7833219	Bn-scaff_19248_1-p523097	A3	31	39	A
2161	Bn-N3-p8464679	Bn-A03-p8407999	Bn-A03-p8407999	A3	31	39	A
2162	Bn-N3-p8465382	Bn-C4-p7667313	Bn-scaff_19248_1-p357191	A3	31	39	A
2163	Bn-N3-p8465479	Bn-A03-p8408815	Bn-A03-p8408815	A3	31	39	A
2164	Bn-N3-p8561479	Bn-C3-p11357442	Bn-scaff_19111_1-p140517	A3	31	39	A
2165	Bn-N3-p8564933	Bn-A03-p8480669	Bn-A03-p8480669	A3	31	39	A
2166	Bn-N3-p8567970	Bn-A03-p8483706	Bn-A03-p8483706	A3	31	39	A
2167	Bn-N3-p8582113	Bn-A03-p8497543	Bn-A03-p8497543	A3	31	39	A
2168	Bn-N3-p8707588	Bn-C3-p11620848	Bn-scaff_23954_1-p111112	A3	31	39	A
2169	Bn-N3-p8718378	Bn-A03-p8637914	Bn-A03-p8637914	A3	31	39	A
2170	Bn-N3-p8718723	Bn-A03-p8638217	Bn-A03-p8638217	A3	31	39	A
2171	Bn-N3-p8728661	Bn-A03-p8645654	Bn-A03-p8645654	A3	31	39	A
2172	Bn-Scaffold17470-p139	Bn-A03-p8260551	Bn-A03-p8260551	A3	31	39	A
2173	Bn-Scaffold50563-p132	Bn-C3-p11631735	Bn-scaff_23954_1-p110023	A3	31	39	A
2174	Bn-N13-p13328692	Bn-C3-p12590927	Bn-scaff_23954_1-p141044	A3	32	41.6	A
2175	Bn-N13-p13418695	Bn-C3-p12684608	Bn-scaff_23954_1-p47363	A3	32	41.6	A
2176	Bn-N18-p36207298	Bn-A03-p9303850	Bn-A03-p9303850	A3	32	41.6	A
2177	Bn-N3-p8747803	Bn-A03-p8663803	Bn-A03-p8663803	A3	32	41.6	A
2178	Bn-N3-p8755325	Bn-A03-p8671468	Bn-A03-p8671468	A3	32	41.6	A
2179	Bn-N3-p8755556	Bn-A03-p8671697	Bn-A03-p8671697	A3	32	41.6	A
2180	Bn-N3-p8832993	Bn-A03-p8735039	Bn-A03-p8735039	A3	32	41.6	A
2181	Bn-N3-p8842895	Bn-A03-p8741978	Bn-A03-p8741978	A3	32	41.6	A
2182	Bn-N3-p8842913	Bn-A03-p8741996	Bn-A03-p8741996	A3	32	41.6	A
2183	Bn-N3-p8872133	Bn-A03-p8764251	Bn-A03-p8764251	A3	32	41.6	A
2184	Bn-N3-p8872358	Bn-A03-p8764481	Bn-A03-p8764481	A3	32	41.6	A
2185	Bn-N3-p8929217	Bn-A03-p8802360	Bn-A03-p8802360	A3	32	41.6	A
2186	Bn-N3-p8949754	Bn-A03-p8822734	Bn-A03-p8822734	A3	32	41.6	A
2187	Bn-N3-p8949789	Bn-C3-p11914392	Bn-scaff_23954_1-p817579	A3	32	41.6	A
2188	Bn-N3-p8961102	Bn-A03-p8833016	Bn-A03-p8833016	A3	32	41.6	A
2189	Bn-N3-p8980740	Bn-A03-p8850377	Bn-A03-p8850377	A3	32	41.6	A
2190	Bn-N3-p8980899	Bn-A03-p8851043	Bn-A03-p8851043	A3	32	41.6	A
2191	Bn-N3-p9029427	Bn-A03-p8897362	Bn-A03-p8897362	A3	32	41.6	A
2192	Bn-N3-p9057017	Bn-A03-p8924852	Bn-A03-p8924852	A3	32	41.6	A
2193	Bn-N3-p9069361	Bn-C3-p12109334	Bn-scaff_23954_1-p622637	A3	32	41.6	A
2194	Bn-N3-p9081014	Bn-A03-p8952053	Bn-A03-p8952053	A3	32	41.6	A
2195	Bn-N3-p9090488	Bn-A03-p8960332	Bn-A03-p8960332	A3	32	41.6	A
2196	Bn-N3-p9183454	Bn-A03-p9047380	Bn-A03-p9047380	A3	32	41.6	A
2197	Bn-N3-p9247475	Bn-A03-p9115020	Bn-A03-p9115020	A3	32	41.6	A
2198	Bn-N3-p9272497	Bn-C3-p12477037	Bn-scaff_23954_1-p254934	A3	32	41.6	A
2199	Bn-N3-p9356784	Bn-A03-p9203389	Bn-A03-p9203389	A3	32	41.6	A
2200	Bn-N3-p9356933	Bn-A03-p9203536	Bn-A03-p9203536	A3	32	41.6	A

2201	Bn-N3-p9357231	Bn-A03-p9203834	Bn-A03-p9203834	A3	32	41.6	A
2202	Bn-N3-p9357389	Bn-A03-p9203992	Bn-A03-p9203992	A3	32	41.6	A
2203	Bn-N3-p9357396	Bn-A03-p9203999	Bn-A03-p9203999	A3	32	41.6	A
2204	Bn-N3-p9362802	Bn-A03-p9208735	Bn-A03-p9208735	A3	32	41.6	A
2205	Bn-N3-p9386412	Bn-A03-p9233645	Bn-A03-p9233645	A3	32	41.6	A
2206	Bn-N3-p9397787	Bn-A03-p9243701	Bn-A03-p9243701	A3	32	41.6	A
2207	Bn-N3-p9398282	Bn-A03-p9244195	Bn-A03-p9244195	A3	32	41.6	A
2208	Bn-N3-p9400025	Bn-A03-p9245937	Bn-A03-p9245937	A3	32	41.6	A
2209	Bn-N3-p9400309	Bn-A03-p9246222	Bn-A03-p9246222	A3	32	41.6	A
2210	Bn-N3-p9401808	Bn-A03-p9247798	Bn-A03-p9247798	A3	32	41.6	A
2211	Bn-N3-p9405991	Bn-A03-p9250832	Bn-A03-p9250832	A3	32	41.6	A
2212	Bn-N3-p9422013	Bn-A03-p9265554	Bn-A03-p9265554	A3	32	41.6	A
2213	Bn-N3-p9422787	Bn-A03-p9266328	Bn-A03-p9266328	A3	32	41.6	A
2214	Bn-N3-p9461892	Bn-A03-p9300575	Bn-A03-p9300575	A3	32	41.6	A
2215	Bn-N3-p9464474	Bn-A03-p9301541	Bn-A03-p9301541	A3	32	41.6	A
2216	Bn-N3-p9466679	Bn-A03-p9304563	Bn-A03-p9304563	A3	32	41.6	A
2217	Bn-N3-p9548800	Bn-A03-p9382281	Bn-A03-p9382281	A3	32	41.6	A
2218	Bn-N3-p9610692	Bn-A03-p9429437	Bn-A03-p9429437	A3	32	41.6	A
2219	Bn-N3-p9621591	Bn-A03-p9439354	Bn-A03-p9439354	A3	32	41.6	A
2220	Bn-N3-p9622130	Bn-A03-p9439893	Bn-A03-p9439893	A3	32	41.6	A
2221	Bn-N3-p9631681	Bn-A03-p9449316	Bn-A03-p9449316	A3	32	41.6	A
2222	Bn-N3-p9650408	Bn-A03-p9468025	Bn-A03-p9468025	A3	32	41.6	A
2223	Bn-N3-p9679562	Bn-A03-p9497697	Bn-A03-p9497697	A3	33	42.5	0
2224	Bn-N3-p9706667	Bn-A03-p9537155	Bn-A03-p9537155	A3	33	42.5	A
2225	Bn-N3-p9735568	Bn-A03-p9567767	Bn-A03-p9567767	A3	33	42.5	A
2226	Bn-N3-p9768372	Bn-A03-p9601581	Bn-A03-p9601581	A3	33	42.5	A
2227	Unknown	Bn-A03-p9602074	Bn-A03-p9602074	A3	33	42.5	A
2228	Bn-N13-p14663572	Bn-A03-p10008905	Bn-A03-p10008905	A3	34	43.4	A
2229	Bn-N3-p10018687	Bn-A03-p9823618	Bn-A03-p9823618	A3	34	43.4	A
2230	Bn-N3-p10020110	Bn-A03-p9825032	Bn-A03-p9825032	A3	34	43.4	A
2231	Bn-N3-p10026056	Bn-A03-p9833648	Bn-A03-p9833648	A3	34	43.4	A
2232	Bn-N3-p10040187	Bn-A03-p9847680	Bn-A03-p9847680	A3	34	43.4	A
2233	Bn-N3-p10048973	Bn-A03-p9856465	Bn-A03-p9856465	A3	34	43.4	A
2234	Bn-N3-p10056625	Bn-A03-p9863138	Bn-A03-p9863138	A3	34	43.4	A
2235	Bn-N3-p10057039	Bn-A03-p9863553	Bn-A03-p9863553	A3	34	43.4	A
2236	Bn-N3-p10057086	Bn-A03-p9863600	Bn-A03-p9863600	A3	34	43.4	A
2237	Bn-N3-p10063572	Bn-A03-p9869156	Bn-A03-p9869156	A3	34	43.4	A
2238	Bn-N3-p10066296	Bn-A03-p9873327	Bn-A03-p9873327	A3	34	43.4	A
2239	Bn-N3-p10066353	Bn-A03-p9873384	Bn-A03-p9873384	A3	34	43.4	A
2240	Bn-N3-p10069357	Bn-A03-p9876378	Bn-A03-p9876378	A3	34	43.4	A
2241	Bn-N3-p10076547	Bn-A03-p9883544	Bn-A03-p9883544	A3	34	43.4	A
2242	Bn-N3-p10086814	Bn-A03-p9899417	Bn-A03-p9899417	A3	34	43.4	A
2243	Bn-N3-p10122165	Bn-A03-p9939018	Bn-A03-p9939018	A3	34	43.4	A
2244	Bn-N3-p10215077	Bn-A03-p10027020	Bn-A03-p10027020	A3	34	43.4	A
2245	Bn-N3-p9835434	Bn-A03-p9657864	Bn-A03-p9657864	A3	34	43.4	A
2246	Bn-N3-p9838370	Bn-A03-p9660869	Bn-A03-p9660869	A3	34	43.4	A
2247	Bn-N3-p9845555	Bn-A03-p9677969	Bn-A03-p9677969	A3	34	43.4	A
2248	Bn-N3-p9846554	Bn-A03-p9678981	Bn-A03-p9678981	A3	34	43.4	A
2249	Bn-N3-p9847047	Bn-A03-p9680882	Bn-A03-p9680882	A3	34	43.4	A

2250	Bn-N3-p9870689	Bn-A03-p9705481	Bn-A03-p9705481	A3	34	43.4	A
2251	Bn-N3-p9882221	Bn-C3-p13312667	Bn-scaff_16002_1-p199281	A3	34	43.4	A
2252	Bn-N13-p14770283	Bn-A03-p10086496	Bn-A03-p10086496	A3	35	44.3	A
2253	Bn-N3-p10278812	Bn-A03-p10084501	Bn-A03-p10084501	A3	35	44.3	A
2254	Bn-N3-p10518229	Bn-A03-p10288202	Bn-A03-p10288202	A3	35	44.3	A
2255	Bn-N3-p10525409	Bn-A03-p10296969	Bn-A03-p10296969	A3	35	44.3	A
2256	Bn-N3-p10556684	Bn-C3-p14306821	Bn-scaff_16002_1-p99865[illegible]	A3	35	44.3	A
2257	Bn-N3-p10611530	Bn-A03-p10376862	Bn-A03-p10376862	A3	35	44.3	A
2258	Bn-N3-p10653298	Bn-A03-p10414176	Bn-A03-p10414176	A3	35	44.3	A
2259	Bn-N3-p10704304	Bn-A03-p10464551	Bn-A03-p10464551	A3	36	45.2	A
2260	Bn-N3-p10708311	Bn-A03-p10458923	Bn-A03-p10458923	A3	36	45.2	A
2261	Bn-N3-p10724531	Bn-A03-p10487754	Bn-A03-p10487754	A3	36	45.2	A
2262	Bn-N3-p10757283	Bn-A03-p10517056	Bn-A03-p10517056	A3	36	45.2	A
2263	Bn-N3-p10812473	Bn-A03-p10562274	Bn-A03-p10562274	A3	36	45.2	A
2264	Bn-N3-p10836704	Bn-A03-p10583411	Bn-A03-p10583411	A3	36	45.2	A
2265	Bn-N3-p10837322	Bn-A03-p10584028	Bn-A03-p10584028	A3	36	45.2	A
2266	Bn-N3-p10840760	Bn-A03-p10587547	Bn-A03-p10587547	A3	36	45.2	A
2267	Bn-N3-p10850182	Bn-A03-p10600103	Bn-A03-p10600103	A3	36	45.2	A
2268	Bn-N3-p10850644	Bn-A03-p10600567	Bn-A03-p10600567	A3	36	45.2	A
2269	Bn-N3-p10866539	Bn-A03-p10608519	Bn-A03-p10608519	A3	36	45.2	A
2270	Bn-N3-p10913353	Bn-A03-p10649410	Bn-A03-p10649410	A3	36	45.2	A
2271	Bn-N3-p10915390	Bn-A03-p10651420	Bn-A03-p10651420	A3	36	45.2	A
2272	Bn-N13-p16498143	Bn-A03-p11138137	Bn-A03-p11138137	A3	37	46.1	A
2273	Bn-N18-p1332696	Bn-C5-p46639345	Bn-scaff_20809_1-p16431[illegible]	A3	37	46.1	A
2274	Bn-N3-p10980676	Bn-A03-p10714724	Bn-A03-p10714724	A3	37	46.1	A
2275	Bn-N3-p10999696	Bn-A03-p10732934	Bn-A03-p10732934	A3	37	46.1	A
2276	Bn-N3-p11028842	Bn-A03-p10757525	Bn-A03-p10757525	A3	37	46.1	A
2277	Bn-N3-p11054307	Bn-A03-p10773321	Bn-A03-p10773321	A3	37	46.1	A
2278	Bn-N3-p11054325	Bn-A03-p10773339	Bn-A03-p10773339	A3	37	46.1	A
2279	Bn-N3-p11072359	Bn-A03-p10794513	Bn-A03-p10794513	A3	37	46.1	A
2280	Bn-N3-p11075281	Bn-A03-p10797405	Bn-A03-p10797405	A3	37	46.1	A
2281	Bn-N3-p11082099	Bn-A03-p10803927	Bn-A03-p10803927	A3	37	46.1	A
2282	Bn-N3-p11092942	Bn-A03-p10814591	Bn-A03-p10814591	A3	37	46.1	A
2283	Bn-N3-p11103919	Bn-A03-p10823720	Bn-A03-p10823720	A3	37	46.1	A
2284	Bn-N3-p11109054	Bn-A03-p10830360	Bn-A03-p10830360	A3	37	46.1	A
2285	Bn-N3-p11156706	Bn-A03-p10873434	Bn-A03-p10873434	A3	37	46.1	A
2286	Bn-N3-p11171589	Bn-A03-p10883930	Bn-A03-p10883930	A3	37	46.1	A
2287	Bn-N3-p11176825	Bn-A03-p10889554	Bn-A03-p10889554	A3	37	46.1	A
2288	Bn-N3-p11183158	Bn-A03-p10897572	Bn-A03-p10897572	A3	37	46.1	A
2289	Bn-N3-p11183810	Bn-A03-p10898289	Bn-A03-p10898289	A3	37	46.1	A
2290	Bn-N3-p11186329	Bn-A03-p10900813	Bn-A03-p10900813	A3	37	46.1	A
2291	Bn-N3-p11206326	Bn-A03-p10928669	Bn-A03-p10928669	A3	37	46.1	A
2292	Bn-N3-p11208153	Bn-A03-p10930541	Bn-A03-p10930541	A3	37	46.1	A
2293	Bn-N3-p11240899	Bn-A03-p10959387	Bn-A03-p10959387	A3	37	46.1	A
2294	Bn-N3-p11240951	Bn-A03-p10959439	Bn-A03-p10959439	A3	37	46.1	A
2295	Bn-N3-p11242269	Bn-A03-p10962307	Bn-A03-p10962307	A3	37	46.1	A
2296	Bn-N3-p11242606	Bn-A03-p10962645	Bn-A03-p10962645	A3	37	46.1	A
2297	Bn-N3-p11242930	Bn-A03-p10962969	Bn-A03-p10962969	A3	37	46.1	A
2298	Bn-N3-p11244349	Bn-A03-p10964394	Bn-A03-p10964394	A3	37	46.1	A

2299	Bn-N3-p11244639	Bn-A03-p10964682	Bn-A03-p10964682	A3	37	46.1	A
2300	Bn-N3-p11249111	Bn-A03-p10969109	Bn-A03-p10969109	A3	37	46.1	A
2301	Bn-N3-p11276586	Bn-A03-p10993904	Bn-A03-p10993904	A3	37	46.1	A
2302	Bn-N3-p11348345	Bn-A03-p11053857	Bn-A03-p11053857	A3	37	46.1	A
2303	Bn-N3-p11366690	Bn-A03-p11070958	Bn-A03-p11070958	A3	37	46.1	A
2304	Bn-N3-p11413127	Bn-A03-p11133743	Bn-A03-p11133743	A3	37	46.1	A
2305	Bn-N3-p11425938	Bn-A03-p11143696	Bn-A03-p11143696	A3	37	46.1	A
2306	Bn-N3-p11447194	Bn-C3-p15923925	Bn-scaff_22466_1-p61834	A3	37	46.1	A
2307	Bn-N3-p11450935	Bn-Scaffold001128-p	Bn-Scaffold001128-p1186	A3	37	46.1	A
2308	Bn-N3-p11458348	Bn-A03-p11177892	Bn-A03-p11177892	A3	37	46.1	A
2309	Bn-N3-p11468882	Bn-A03-p11188500	Bn-A03-p11188500	A3	37	46.1	A
2310	Bn-N3-p11472341	Bn-A03-p11191805	Bn-A03-p11191805	A3	37	46.1	A
2311	Bn-N3-p11485348	Bn-A03-p11202719	Bn-A03-p11202719	A3	37	46.1	A
2312	Bn-N3-p11501278	Bn-A03-p11218468	Bn-A03-p11218468	A3	37	46.1	A
2313	Bn-N3-p11515431	Bn-A03-p11232586	Bn-A03-p11232586	A3	37	46.1	A
2314	Bn-N3-p11346446	Bn-A03-p11051970	Bn-A03-p11051970	A3	38	47	A
2315	Bn-N3-p11530996	Bn-C3-p16053891	Bn-scaff_22466_1-p74831	A3	38	47	A
2316	Bn-N3-p11538775	Bn-A03-p11256138	Bn-A03-p11256138	A3	38	47	A
2317	Bn-N3-p11539029	Bn-A03-p11256392	Bn-A03-p11256392	A3	38	47	A
2318	Bn-N3-p11539306	Bn-A03-p11256664	Bn-A03-p11256664	A3	38	47	A
2319	Bn-N3-p11540371	Bn-A03-p11257708	Bn-A03-p11257708	A3	38	47	A
2320	Bn-N3-p11581195	Bn-A03-p11294370	Bn-A03-p11294370	A3	38	47	A
2321	Bn-N3-p11599250	Bn-A03-p11319722	Bn-A03-p11319722	A3	38	47	A
2322	Bn-N3-p11599429	Bn-A03-p11319901	Bn-A03-p11319901	A3	38	47	A
2323	Bn-N3-p11640091	Bn-A03-p11364656	Bn-A03-p11364656	A3	38	47	A
2324	Bn-N3-p11653289	Bn-A03-p11381125	Bn-A03-p11381125	A3	38	47	A
2325	Bn-N3-p11658215	Bn-A03-p11383138	Bn-A03-p11383138	A3	38	47	A
2326	Bn-N3-p11666230	Bn-C3-p16401873	Bn-scaff_22466_1-p10962	A3	38	47	A
2327	Bn-N3-p11685041	Bn-A03-p11399915	Bn-A03-p11399915	A3	38	47	A
2328	Bn-N3-p11699513	Bn-A03-p11418849	Bn-A03-p11418849	A3	38	47	A
2329	Bn-Scaffold43347-p83	Bn-A01-p22937843	Bn-A01-p22937843	A3	38	47	A
2330	Bn-N13-p17554706	Bn-Scaffold00966-p2	Bn-scaff_17658_1-p25183	A3	39	47.8	A
2331	Bn-N3-p11784223	Bn-A03-p11499523	Bn-A03-p11499523	A3	39	47.8	A
2332	Bn-N3-p11789875	Bn-A03-p11505658	Bn-A03-p11505658	A3	39	47.8	A
2333	Bn-N3-p11803507	Bn-A03-p11517597	Bn-A03-p11517597	A3	39	47.8	A
2334	Bn-N3-p11804914	Bn-A03-p11519012	Bn-A03-p11519012	A3	39	47.8	A
2335	Bn-N3-p11805166	Bn-A03-p11519264	Bn-A03-p11519264	A3	39	47.8	A
2336	Bn-N3-p11806708	Bn-A03-p11520156	Bn-A03-p11520156	A3	39	47.8	A
2337	Bn-N3-p11825806	Bn-A03-p11534030	Bn-A03-p11534030	A3	39	47.8	A
2338	Bn-N3-p11884861	Bn-C3-p16819499	Bn-scaff_16352_1-p17155	A3	39	47.8	A
2339	Bn-N3-p11892910	Bn-A03-p11590315	Bn-A03-p11590315	A3	39	47.8	A
2340	Bn-N3-p11919294	Bn-A03-p11605644	Bn-A03-p11605644	A3	39	47.8	A
2341	Bn-N3-p11937837	Bn-A03-p11621375	Bn-A03-p11621375	A3	39	47.8	A
2342	Bn-N3-p11941042	Bn-A03-p11624578	Bn-A03-p11624578	A3	39	47.8	A
2343	Bn-N3-p11942749	Bn-A03-p11626279	Bn-A03-p11626279	A3	39	47.8	A
2344	Bn-N3-p11957456	Bn-A03-p11640168	Bn-A03-p11640168	A3	39	47.8	A
2345	Bn-N3-p11973191	Bn-A03-p11655161	Bn-A03-p11655161	A3	39	47.8	A
2346	Bn-N3-p11973725	Bn-A03-p11655433	Bn-A03-p11655433	A3	39	47.8	A
2347	Bn-N3-p11974044	Bn-A03-p11655749	Bn-A03-p11655749	A3	39	47.8	A

2348	Bn-N3-p11974609	Bn-A03-p11656333	Bn-A03-p11656333	A3	39	47.8	A
2349	Bn-N3-p12015363	Bn-A03-p11704711	Bn-A03-p11704711	A3	39	47.8	A
2350	Bn-N3-p12061079	Bn-A03-p11744082	Bn-A03-p11744082	A3	39	47.8	A
2351	Bn-N3-p12075423	Bn-A03-p11751694	Bn-A03-p11751694	A3	39	47.8	A
2352	Bn-N3-p12122804	Bn-A03-p11792328	Bn-A03-p11792328	A3	39	47.8	A
2353	Bn-N3-p12129486	Bn-A03-p11798309	Bn-A03-p11798309	A3	39	47.8	A
2354	Bn-N3-p12165148	Bn-A03-p11831815	Bn-A03-p11831815	A3	39	47.8	A
2355	Bn-N3-p12169254	Bn-C3-p17299260	Bn-scaff_16352_1-p49691(	A3	39	47.8	A
2356	Bn-N3-p12215088	Bn-A03-p11883572	Bn-A03-p11883572	A3	39	47.8	A
2357	Bn-N13-p18473637	Bn-C3-p17640797	Bn-scaff_16352_1-p83845:	A3	40	49.4	A
2358	Bn-N3-p12426160	Bn-A03-p12076661	Bn-A03-p12076661	A3	40	49.4	A
2359	Bn-N3-p12537961	Bn-A03-p12186958	Bn-A03-p12186958	A3	40	49.4	A
2360	Bn-N3-p12553115	Bn-A03-p12201775	Bn-A03-p12201775	A3	40	49.4	A
2361	Bn-N3-p12560738	Bn-A03-p12213267	Bn-A03-p12213267	A3	40	49.4	A
2362	Bn-N3-p12612221	Bn-A03-p12250401	Bn-A03-p12250401	A3	40	49.4	A
2363	Bn-N3-p12670776	Bn-A03-p12322277	Bn-A03-p12322277	A3	40	49.4	A
2364	Bn-N3-p12695564	Bn-A03-p12342812	Bn-A03-p12342812	A3	40	49.4	A
2365	Bn-N3-p12696451	Bn-A03-p12343742	Bn-A03-p12343742	A3	40	49.4	A
2366	Bn-N3-p12710315	Bn-A03-p12353218	Bn-A03-p12353218	A3	40	49.4	A
2367	Bn-N3-p12710471	Bn-A03-p12353370	Bn-A03-p12353370	A3	40	49.4	A
2368	Bn-N3-p12716589	Bn-A03-p12359936	Bn-A03-p12359936	A3	40	49.4	A
2369	Bn-N3-p12750656	Bn-A03-p12393626	Bn-A03-p12393626	A3	40	49.4	A
2370	Bn-N3-p12750742	Bn-A03-p12393711	Bn-A03-p12393711	A3	40	49.4	A
2371	Bn-N3-p12802514	Bn-A03-p12426461	Bn-A03-p12426461	A3	40	49.4	A
2372	Bn-N3-p12805622	Bn-A03-p12429205	Bn-A03-p12429205	A3	40	49.4	A
2373	Bn-N3-p12807491	Bn-A03-p12431376	Bn-A03-p12431376	A3	40	49.4	A
2374	Bn-N3-p12830096	Bn-A03-p12453877	Bn-A03-p12453877	A3	40	49.4	A
2375	Bn-N3-p12856222	Bn-A03-p12477334	Bn-A03-p12477334	A3	40	49.4	A
2376	Bn-N3-p12920844	Bn-A03-p12541037	Bn-A03-p12541037	A3	41	51	A
2377	Bn-N3-p12921014	Bn-A03-p12541207	Bn-A03-p12541207	A3	41	51	A
2378	Bn-N3-p12921144	Bn-A03-p12541337	Bn-A03-p12541337	A3	41	51	A
2379	Bn-N3-p13028750	Bn-A03-p12639200	Bn-A03-p12639200	A3	41	51	A
2380	Bn-N3-p13028869	Bn-A03-p12639319	Bn-A03-p12639319	A3	41	51	A
2381	Bn-N3-p13217701	Bn-A03-p12820905	Bn-A03-p12820905	A3	41	51	A
2382	Bn-N3-p13323285	Bn-A03-p12932399	Bn-A03-p12932399	A3	41	51	A
2383	Bn-N3-p13323965	Bn-A03-p12933099	Bn-A03-p12933099	A3	41	51	A
2384	Bn-N3-p13385371	Bn-A03-p12990610	Bn-A03-p12990610	A3	42	51.8	A
2385	Bn-N3-p13435771	Bn-A03-p13039285	Bn-A03-p13039285	A3	42	51.8	A
2386	Bn-N3-p13439822	Bn-A03-p13044950	Bn-A03-p13044950	A3	42	51.8	A
2387	Bn-N3-p13443875	Bn-C3-p19369252	Bn-scaff_20646_1-p60086	A3	42	51.8	A
2388	Bn-N3-p13458036	Bn-A03-p13061709	Bn-A03-p13061709	A3	42	51.8	A
2389	Bn-N3-p13480093	Bn-A03-p13094043	Bn-A03-p13094043	A3	42	51.8	A
2390	Bn-Scaffold01261-p10675	Bn-A03-p12973462	Bn-A03-p12973462	A3	42	51.8	A
2391	Bn-N13-p20803975	Bn-A03-p13236840	Bn-A03-p13236840	A3	43	52.6	A
2392	Bn-N13-p20804689	Bn-C3-p19813954	Bn-scaff_27677_1-p10529(	A3	43	52.6	A
2393	Bn-N13-p20831776	Bn-A03-p13254564	Bn-A03-p13254564	A3	43	52.6	A
2394	Bn-N13-p20897815	Bn-A03-p13302882	Bn-A03-p13302882	A3	43	52.6	A
2395	Bn-N13-p20967497	Bn-A03-p13335946	Bn-A03-p13335946	A3	43	52.6	A
2396	Bn-N13-p21100276	Bn-C3-p20054801	Bn-scaff_27870_1-p20867:	A3	43	52.6	A

2397	Bn-N13-p21701987	Bn-A03-p13653603	Bn-A03-p13653603	A3	43	52.6	A
2398	Bn-N13-p21713728	Bn-C3-p20702735	Bn-scaff_19523_1-p36620:	A3	43	52.6	A
2399	Bn-N13-p21999599	Bn-C3-p20958865	Bn-scaff_19523_1-p62233:	A3	43	52.6	A
2400	Bn-N18-p40161473	Bn-A03-p13325522	Bn-A03-p13325522	A3	43	52.6	A
2401	Bn-N3-p13485759	Bn-A03-p13099476	Bn-A03-p13099476	A3	43	52.6	A
2402	Bn-N3-p13489967	Bn-A03-p13104283	Bn-A03-p13104283	A3	43	52.6	A
2403	Bn-N3-p13503467	Bn-A03-p13109267	Bn-A03-p13109267	A3	43	52.6	A
2404	Bn-N3-p13512412	Bn-A03-p13131453	Bn-A03-p13131453	A3	43	52.6	A
2405	Bn-N3-p13547494	Bn-A03-p13160528	Bn-A03-p13160528	A3	43	52.6	A
2406	Bn-N3-p13645159	Bn-A03-p13286574	Bn-A03-p13286574	A3	43	52.6	A
2407	Bn-N3-p13646257	Bn-A03-p13287670	Bn-A03-p13287670	A3	43	52.6	A
2408	Bn-N3-p13650548	Bn-A03-p13291935	Bn-A03-p13291935	A3	43	52.6	A
2409	Bn-N3-p13654777	Bn-A03-p13298734	Bn-A03-p13298734	A3	43	52.6	A
2410	Bn-N3-p13663950	Bn-A03-p13306819	Bn-A03-p13306819	A3	43	52.6	A
2411	Bn-N3-p13687875	Bn-A03-p13335275	Bn-A03-p13335275	A3	43	52.6	A
2412	Bn-N3-p13744415	Bn-A03-p13421039	Bn-A03-p13421039	A3	43	52.6	A
2413	Bn-N3-p13762395	Bn-A03-p13440035	Bn-A03-p13440035	A3	43	52.6	A
2414	Bn-N3-p13787184	Bn-A03-p13465062	Bn-A03-p13465062	A3	43	52.6	A
2415	Bn-N3-p13800648	Bn-C3-p20284114	Bn-scaff_20741_1-p20543	A3	43	52.6	A
2416	Bn-N3-p13801088	Bn-C3-p20284553	Bn-scaff_20741_1-p20982	A3	43	52.6	A
2417	Bn-N3-p13834517	Bn-A03-p13502336	Bn-A03-p13502336	A3	43	52.6	A
2418	Bn-N3-p13835018	Bn-A03-p13502830	Bn-A03-p13502830	A3	43	52.6	A
2419	Bn-N3-p13865572	Bn-A03-p13533904	Bn-A03-p13533904	A3	43	52.6	A
2420	Bn-N3-p13940936	Bn-A03-p13585261	Bn-A03-p13585261	A3	43	52.6	A
2421	Bn-N3-p13961114	Bn-A03-p13605344	Bn-A03-p13605344	A3	43	52.6	A
2422	Bn-N3-p13966548	Bn-A03-p13610858	Bn-A03-p13610858	A3	43	52.6	A
2423	Bn-N3-p13985853	Bn-A03-p13624310	Bn-A03-p13624310	A3	43	52.6	A
2424	Bn-N3-p13989606	Bn-Scaffold01031-p2:	Bn-scaff_28509_1-p22130	A3	43	52.6	A
2425	Bn-N3-p13991377	Bn-A03-p13630048	Bn-A03-p13630048	A3	43	52.6	A
2426	Bn-N3-p14017333	Bn-A03-p13658460	Bn-A03-p13658460	A3	43	52.6	A
2427	Bn-N3-p14023187	Bn-A03-p13664657	Bn-A03-p13664657	A3	43	52.6	A
2428	Bn-N3-p14037826	Bn-A03-p13679854	Bn-A03-p13679854	A3	43	52.6	A
2429	Bn-N3-p14064161	Bn-C3-p20762378	Bn-scaff_19523_1-p42584(	A3	43	52.6	A
2430	Bn-N3-p14077688	Bn-A03-p13746713	Bn-A03-p13746713	A3	43	52.6	A
2431	Bn-N3-p14078069	Bn-A03-p13747074	Bn-A03-p13747074	A3	43	52.6	A
2432	Bn-N3-p14105117	Bn-A07-p6044939	Bn-A07-p6044939	A3	43	52.6	A
2433	Bn-N3-p14134241	Bn-A03-p13777203	Bn-A03-p13777203	A3	43	52.6	A
2434	Bn-N3-p14140217	Bn-A03-p13783263	Bn-A03-p13783263	A3	43	52.6	A
2435	Bn-N3-p14145741	Bn-A03-p13802162	Bn-A03-p13802162	A3	43	52.6	A
2436	Bn-N3-p14158600	Bn-C3-p20946177	Bn-scaff_19523_1-p60964!	A3	43	52.6	A
2437	Bn-N3-p14159620	Bn-A03-p13815665	Bn-A03-p13815665	A3	43	52.6	A
2438	Bn-N3-p14175996	Bn-A03-p13853558	Bn-A03-p13853558	A3	43	52.6	A
2439	Bn-N3-p14182950	Bn-A03-p13861679	Bn-A03-p13861679	A3	43	52.6	A
2440	Bn-N3-p14183025	Bn-A03-p13861749	Bn-A03-p13861749	A3	43	52.6	A
2441	Bn-N3-p14210407	Bn-A03-p13883754	Bn-A03-p13883754	A3	43	52.6	A
2442	Bn-N3-p14232208	Bn-A03-p13906492	Bn-A03-p13906492	A3	43	52.6	A
2443	Bn-N3-p14234295	Bn-A03-p13908787	Bn-A03-p13908787	A3	43	52.6	A
2444	Bn-N3-p14236228	Bn-A03-p13912476	Bn-A03-p13912476	A3	43	52.6	A
2445	Bn-N3-p14248744	Bn-A03-p13922405	Bn-A03-p13922405	A3	43	52.6	A

2446	Bn-N3-p14253184	Bn-A03-p13925731	Bn-A03-p13925731	A3	43	52.6	A
2447	Bn-N3-p14273849	Bn-A03-p13943992	Bn-A03-p13943992	A3	43	52.6	A
2448	Bn-N3-p14294755	Bn-C3-p21205872	Bn-scaff_19523_1-p86934(	A3	43	52.6	A
2449	Bn-N3-p14303293	Bn-A03-p13968921	Bn-A03-p13968921	A3	43	52.6	A
2450	Bn-N3-p14304481	Bn-A03-p13970114	Bn-A03-p13970114	A3	43	52.6	A
2451	Bn-N3-p14309064	Bn-A03-p13974725	Bn-A03-p13974725	A3	43	52.6	A
2452	Bn-N3-p14311970	Bn-A03-p13977740	Bn-A03-p13977740	A3	43	52.6	A
2453	Bn-N3-p14331357	Bn-A03-p14003701	Bn-A03-p14003701	A3	43	52.6	A
2454	Bn-N3-p14341517	Bn-A03-p14015779	Bn-A03-p14015779	A3	43	52.6	A
2455	Bn-N3-p14348717	Bn-A03-p14023392	Bn-A03-p14023392	A3	43	52.6	A
2456	Bn-N3-p7073378	Bn-A07-p6055505	Bn-A07-p6055505	A3	43	52.6	A
2457	Bn-Scaffold20951-p171	Bn-A03-p13266036	Bn-A03-p13266036	A3	43	52.6	A
2458	Bn-Scaffold29317-p155	Bn-A03-p13616280	Bn-A03-p13616280	A3	43	52.6	A
2459	Unknown	Bn-A03-p13324647	Bn-A03-p13324647	A3	43	52.6	A
2460	Bn-N3-p14438774	Bn-A03-p14112910	Bn-A03-p14112910	A3	44	56.7	A
2461	Bn-N3-p14439353	Bn-A03-p14113489	Bn-A03-p14113489	A3	44	56.7	A
2462	Bn-N3-p14453580	Bn-A03-p14127649	Bn-A03-p14127649	A3	44	56.7	A
2463	Bn-N3-p14508983	Bn-A03-p14175904	Bn-A03-p14175904	A3	45	60.7	A
2464	Bn-N3-p14509547	Bn-A03-p14176469	Bn-A03-p14176469	A3	45	60.7	A
2465	Bn-N3-p14531428	Bn-A03-p14197151	Bn-A03-p14197151	A3	45	60.7	A
2466	Bn-N3-p14535159	Bn-A03-p14201146	Bn-A03-p14201146	A3	45	60.7	A
2467	Bn-N3-p14543573	Bn-A03-p14209971	Bn-A03-p14209971	A3	45	60.7	A
2468	Bn-N3-p14611514	Bn-A03-p14276328	Bn-A03-p14276328	A3	45	60.7	A
2469	Bn-N3-p14617650	Bn-A03-p14282460	Bn-A03-p14282460	A3	45	60.7	A
2470	Bn-N3-p14627320	Bn-A03-p14291952	Bn-A03-p14291952	A3	45	60.7	A
2471	Bn-N3-p14630797	Bn-A03-p14295396	Bn-A03-p14295396	A3	45	60.7	A
2472	Bn-N13-p22939644	Bn-A03-p14418556	Bn-A03-p14418556	A3	46	62.3	A
2473	Bn-N3-p14641501	Bn-A03-p14306440	Bn-A03-p14306440	A3	46	62.3	A
2474	Bn-N3-p14644864	Bn-A03-p14309803	Bn-A03-p14309803	A3	46	62.3	A
2475	Bn-N3-p14645446	Bn-A03-p14310386	Bn-A03-p14310386	A3	46	62.3	A
2476	Bn-N3-p14645865	Bn-A03-p14310805	Bn-A03-p14310805	A3	46	62.3	A
2477	Bn-N3-p14665052	Bn-C3-p21735313	Bn-scaff_18482_1-p44703(	A3	46	62.3	A
2478	Bn-N3-p14687684	Bn-A03-p14353080	Bn-A03-p14353080	A3	46	62.3	A
2479	Bn-N3-p14688286	Bn-A03-p14355646	Bn-A03-p14355646	A3	46	62.3	A
2480	Bn-N3-p14693565	Bn-A03-p14369287	Bn-A03-p14369287	A3	46	62.3	A
2481	Bn-N3-p14728197	Bn-A03-p14398744	Bn-A03-p14398744	A3	46	62.3	A
2482	Bn-N3-p14729444	Bn-A03-p14399972	Bn-A03-p14399972	A3	46	62.3	A
2483	Bn-N3-p14729492	Bn-A03-p14400020	Bn-A03-p14400020	A3	46	62.3	A
2484	Bn-N3-p14738917	Bn-A03-p14413451	Bn-A03-p14413451	A3	46	62.3	A
2485	Bn-N3-p14747648	Bn-C3-p21860069	Bn-scaff_18482_1-p32228(	A3	46	62.3	A
2486	Bn-N3-p14752111	Bn-A03-p14423477	Bn-A03-p14423477	A3	46	62.3	A
2487	Bn-N3-p14764950	Bn-A03-p14434665	Bn-A03-p14434665	A3	46	62.3	A
2488	Bn-N11-p45457579	Bn-C3-p22684406	Bn-scaff_22067_1-p22595(	A3	47	63.1	A
2489	Bn-N3-p14777622	Bn-A03-p14447238	Bn-A03-p14447238	A3	47	63.1	A
2490	Bn-N3-p14793905	Bn-A03-p14462148	Bn-A03-p14462148	A3	47	63.1	A
2491	Bn-N3-p14805303	Bn-A03-p14473296	Bn-A03-p14473296	A3	47	63.1	A
2492	Bn-N3-p14806463	Bn-A03-p14474478	Bn-A03-p14474478	A3	47	63.1	A
2493	Bn-N3-p14845698	Bn-A03-p14511431	Bn-A03-p14511431	A3	47	63.1	A
2494	Bn-N3-p14890862	Bn-A03-p14552086	Bn-A03-p14552086	A3	47	63.1	A

2495	Bn-N3-p14930339	Bn-A03-p14585799	Bn-A03-p14585799	A3	47	63.1	A
2496	Bn-N3-p14940380	Bn-A03-p14598476	Bn-A03-p14598476	A3	47	63.1	A
2497	Bn-N3-p15067937	Bn-A03-p14692579	Bn-A03-p14692579	A3	47	63.1	A
2498	Bn-N3-p15133628	Bn-A03-p14784764	Bn-A03-p14784764	A3	47	63.1	A
2499	Bn-N3-p15168702	Bn-A03-p14811653	Bn-A03-p14811653	A3	47	63.1	A
2500	Bn-N3-p15285911	Bn-A03-p14927037	Bn-A03-p14927037	A3	47	63.1	A
2501	Bn-N3-p15323141	Bn-A03-p14962288	Bn-A03-p14962288	A3	47	63.1	A
2502	Bn-N3-p15359664	Bn-A03-p15002477	Bn-A03-p15002477	A3	47	63.1	A
2503	Bn-N3-p15361204	Bn-A03-p15004059	Bn-A03-p15004059	A3	47	63.1	A
2504	Bn-N3-p15371131	Bn-C3-p22697798	Bn-scaff_22067_1-p23935	A3	47	63.1	A
2505	Bn-N3-p15373470	Bn-A03-p15015706	Bn-A03-p15015706	A3	47	63.1	A
2506	Bn-N3-p15430341	Bn-A03-p15077006	Bn-A03-p15077006	A3	47	63.1	A
2507	Bn-N3-p15445011	Bn-C3-p22815348	Bn-scaff_17521_1-p14002	A3	47	63.1	A
2508	Bn-N3-p15474549	Bn-A03-p15120410	Bn-A03-p15120410	A3	47	63.1	A
2509	Bn-N3-p15495724	Bn-A03-p15141821	Bn-A03-p15141821	A3	47	63.1	A
2510	Bn-N3-p15508378	Bn-A03-p15156393	Bn-A03-p15156393	A3	47	63.1	A
2511	Bn-N3-p15559804	Bn-A03-p15200901	Bn-A03-p15200901	A3	47	63.1	A
2512	Bn-N3-p15570064	Bn-A03-p15207594	Bn-A03-p15207594	A3	47	63.1	A
2513	Bn-N3-p15595799	Bn-A03-p15237693	Bn-A03-p15237693	A3	47	63.1	A
2514	Bn-N3-p15623742	Bn-A03-p15265791	Bn-A03-p15265791	A3	47	63.1	A
2515	Bn-N3-p15677762	Bn-A03-p15326805	Bn-A03-p15326805	A3	47	63.1	A
2516	Bn-N3-p15688638	Bn-A03-p15337088	Bn-A03-p15337088	A3	47	63.1	A
2517	Bn-N3-p15694477	Bn-A03-p15347967	Bn-A03-p15347967	A3	47	63.1	A
2518	Bn-N3-p15696427	Bn-A03-p15349925	Bn-A03-p15349925	A3	47	63.1	A
2519	Bn-N3-p15698667	Bn-A03-p15351982	Bn-A03-p15351982	A3	47	63.1	A
2520	Bn-Scaffold01107-p16315	Bn-A03-p15245600	Bn-A03-p15245600	A3	47	63.1	A
2521	Bn-N3-p15710902	Bn-A03-p15359867	Bn-A03-p15359867	A3	48	64.7	A
2522	Bn-N3-p15718306	Bn-A03-p15365259	Bn-A03-p15365259	A3	48	64.7	A
2523	Bn-N3-p15720729	Bn-C3-p23161298	Bn-scaff_17521_1-p10542	A3	48	64.7	A
2524	Bn-N3-p15724491	Bn-C3-p23165081	Bn-scaff_17521_1-p10505	A3	48	64.7	A
2525	Bn-N3-p15727046	Bn-A03-p15368328	Bn-A03-p15368328	A3	48	64.7	A
2526	Bn-N3-p15727175	Bn-C3-p23190299	Bn-scaff_17521_1-p10252	A3	48	64.7	A
2527	Bn-N3-p15754783	Bn-A03-p15404736	Bn-A03-p15404736	A3	48	64.7	A
2528	Bn-N3-p15758953	Bn-A03-p15405498	Bn-A03-p15405498	A3	48	64.7	A
2529	Bn-N3-p15779216	Bn-A03-p15424536	Bn-A03-p15424536	A3	48	64.7	A
2530	Bn-N3-p15791833	Bn-A03-p15435114	Bn-A03-p15435114	A3	48	64.7	A
2531	Bn-N3-p15791893	Bn-A03-p15435174	Bn-A03-p15435174	A3	48	64.7	A
2532	Bn-N3-p15792170	Bn-A03-p15435442	Bn-A03-p15435442	A3	48	64.7	A
2533	Bn-N3-p15918295	Bn-A03-p15546590	Bn-A03-p15546590	A3	48	64.7	A
2534	Bn-N3-p15921837	Bn-A03-p15548506	Bn-A03-p15548506	A3	48	64.7	A
2535	Bn-N3-p15957400	Bn-A03-p15577849	Bn-A03-p15577849	A3	49	65.5	A
2536	Bn-N3-p15980808	Bn-A03-p15605355	Bn-A03-p15605355	A3	49	65.5	A
2537	Bn-N3-p16057209	Bn-A03-p15708192	Bn-A03-p15708192	A3	49	65.5	A
2538	Bn-N3-p16057306	Bn-A03-p15708289	Bn-A03-p15708289	A3	49	65.5	A
2539	Bn-N3-p16075709	Bn-A03-p15725925	Bn-A03-p15725925	A3	49	65.5	A
2540	Bn-N3-p16090079	Bn-A03-p15739646	Bn-A03-p15739646	A3	49	65.5	A
2541	Bn-N3-p16118711	Bn-A03-p15766386	Bn-A03-p15766386	A3	49	65.5	A
2542	Bn-N3-p16124912	Bn-C3-p23750735	Bn-scaff_17521_1-p46484	A3	49	65.5	A
2543	Bn-N3-p16129355	Bn-A03-p15776756	Bn-A03-p15776756	A3	49	65.5	A

2544	Bn-N3-p16156278	Bn-A03-p15801208	Bn-A03-p15801208	A3	49	65.5	A
2545	Bn-N3-p16176575	Bn-A03-p15818896	Bn-A03-p15818896	A3	49	65.5	A
2546	Bn-N3-p16183335	Bn-A03-p15824796	Bn-A03-p15824796	A3	49	65.5	A
2547	Bn-N3-p16186372	Bn-A03-p15828397	Bn-A03-p15828397	A3	49	65.5	A
2548	Bn-N3-p16242827	Bn-A03-p15892296	Bn-A03-p15892296	A3	49	65.5	A
2549	Bn-N3-p16309095	Bn-A03-p15954297	Bn-A03-p15954297	A3	50	67.1	A
2550	Bn-N3-p16312745	Bn-A03-p15957657	Bn-A03-p15957657	A3	50	67.1	A
2551	Bn-N3-p16313138	Bn-A03-p15958035	Bn-A03-p15958035	A3	50	67.1	A
2552	Bn-N3-p16314717	Bn-A03-p15960001	Bn-A03-p15960001	A3	50	67.1	A
2553	Bn-N3-p16328200	Bn-A03-p15973005	Bn-A03-p15973005	A3	50	67.1	A
2554	Bn-N3-p16417695	Bn-A03-p16051661	Bn-A03-p16051661	A3	50	67.1	A
2555	Bn-N3-p16447196	Bn-A03-p16081434	Bn-A03-p16081434	A3	50	67.1	A
2556	Bn-N3-p16469347	Bn-A03-p16103891	Bn-A03-p16103891	A3	50	67.1	A
2557	Bn-N3-p16525656	Bn-A03-p16158248	Bn-A03-p16158248	A3	50	67.1	A
2558	Bn-N3-p16558996	Bn-A03-p16185537	Bn-A03-p16185537	A3	50	67.1	A
2559	Bn-N3-p16583768	Bn-A03-p16211692	Bn-A03-p16211692	A3	50	67.1	A
2560	Bn-N3-p16606777	Bn-A03-p16229294	Bn-A03-p16229294	A3	50	67.1	A
2561	Bn-N3-p16653377	Bn-A03-p16278251	Bn-A03-p16278251	A3	50	67.1	A
2562	Bn-N3-p16745660	Bn-A03-p16371467	Bn-A03-p16371467	A3	50	67.1	A
2563	Bn-N3-p16755208	Bn-A03-p16380304	Bn-A03-p16380304	A3	50	67.1	A
2564	Bn-N3-p16755441	Bn-C3-p24879581	Bn-scaff_17298_1-p66390(	A3	50	67.1	0
2565	Bn-N3-p16777694	Bn-C3-p24902744	Bn-scaff_17298_1-p68706	A3	50	67.1	0
2566	Bn-N3-p16781840	Bn-A03-p16409353	Bn-A03-p16409353	A3	50	67.1	A
2567	Bn-N3-p16801093	Bn-A03-p16431100	Bn-A03-p16431100	A3	50	67.1	A
2568	Bn-N3-p16961243	Bn-A03-p16607714	Bn-A03-p16607714	A3	51	68.7	A
2569	Bn-N3-p16964899	Bn-A03-p16611349	Bn-A03-p16611349	A3	51	68.7	A
2570	Bn-N3-p16966877	Bn-C3-p25144091	Bn-scaff_17298_1-p92841(	A3	51	68.7	A
2571	Bn-N3-p17029747	Bn-A05-p6918694	Bn-A05-p6918694	A3	51	68.7	A
2572	Bn-N3-p17112362	Bn-A03-p16742011	Bn-A03-p16742011	A3	51	68.7	A
2573	Bn-N3-p17132728	Bn-C3-p25389699	Bn-scaff_17298_1-p117401	A3	52	69.5	A
2574	Bn-N3-p17184568	Bn-A03-p16811562	Bn-A03-p16811562	A3	52	69.5	A
2575	Bn-N3-p17185957	Bn-A03-p16812953	Bn-A03-p16812953	A3	52	69.5	A
2576	Bn-N13-p26848688	Bn-C3-p25537230	Bn-scaff_17298_1-p13215	A3	53	70.3	A
2577	Bn-N3-p17230620	Bn-A03-p16852209	Bn-A03-p16852209	A3	53	70.3	A
2578	Bn-N3-p17289286	Bn-A03-p16901041	Bn-A03-p16901041	A3	53	70.3	A
2579	Bn-N3-p17304550	Bn-A03-p16915877	Bn-A03-p16915877	A3	53	70.3	0
2580	Bn-N3-p17352306	Bn-A03-p16955732	Bn-A03-p16955732	A3	53	70.3	A
2581	Bn-N3-p17416648	Bn-A03-p17019251	Bn-A03-p17019251	A3	53	70.3	A
2582	Bn-N3-p17438283	Bn-A03-p17038064	Bn-A03-p17038064	A3	53	70.3	A
2583	Bn-N3-p17439104	Bn-A03-p17038887	Bn-A03-p17038887	A3	53	70.3	A
2584	Bn-N3-p17439181	Bn-A03-p17038965	Bn-A03-p17038965	A3	53	70.3	A
2585	Bn-N3-p17455648	Bn-A03-p17056395	Bn-A03-p17056395	A3	53	70.3	A
2586	Bn-N3-p17471016	Bn-C3-p25960459	Bn-scaff_17298_1-p17447	A3	53	70.3	A
2587	Bn-N3-p17542662	Bn-A03-p17139931	Bn-A03-p17139931	A3	53	70.3	A
2588	Bn-N3-p17584688	Bn-A03-p17182494	Bn-A03-p17182494	A3	54	71.1	A
2589	Bn-N3-p17618294	Bn-A03-p17217528	Bn-A03-p17217528	A3	54	71.1	A
2590	Bn-N3-p17683591	Bn-A03-p17281617	Bn-A03-p17281617	A3	54	71.1	A
2591	Bn-N3-p18120292	Bn-A03-p17782292	Bn-A03-p17782292	A3	55	71.9	A
2592	Bn-N3-p18370833	Bn-A03-p18019822	Bn-A03-p18019822	A3	55	71.9	A

2593	Bn-N3-p18376463	Bn-A03-p18025565	Bn-A03-p18025565	A3	55	71.9	A
2594	Bn-N3-p18376651	Bn-A03-p18025753	Bn-A03-p18025753	A3	55	71.9	A
2595	Bn-N3-p18475063	Bn-A03-p18120938	Bn-A03-p18120938	A3	56	72.7	A
2596	Bn-N3-p18538515	Bn-A03-p18183550	Bn-A03-p18183550	A3	56	72.7	A
2597	Bn-N3-p18559964	Bn-A03-p18198495	Bn-A03-p18198495	A3	56	72.7	A
2598	Bn-N13-p30250915	Bn-C3-p28979805	Bn-scaff_17395_1-p20236	A3	57	73.5	A
2599	Bn-N3-p18780518	Bn-A03-p18414643	Bn-A03-p18414643	A3	57	73.5	A
2600	Bn-N3-p18817217	Bn-A03-p18455020	Bn-A03-p18455020	A3	57	73.5	A
2601	Bn-N3-p18828574	Bn-A03-p18461597	Bn-A03-p18461597	A3	57	73.5	A
2602	Bn-N3-p18828755	Bn-A03-p18461778	Bn-A03-p18461778	A3	57	73.5	A
2603	Bn-N3-p18829258	Bn-A03-p18462261	Bn-A03-p18462261	A3	57	73.5	A
2604	Bn-N3-p18839043	Bn-A03-p18471820	Bn-A03-p18471820	A3	57	73.5	A
2605	Bn-N3-p18897762	Bn-C9-p50683166	Bn-scaff_17109_2-p31590	A3	57	73.5	A
2606	Bn-N3-p18949264	Bn-A03-p18570046	Bn-A03-p18570046	A3	57	73.5	A
2607	Bn-N3-p18977923	Bn-A03-p18593371	Bn-A03-p18593371	A3	57	73.5	A
2608	Bn-N3-p19001295	Bn-A03-p18618990	Bn-A03-p18618990	A3	57	73.5	A
2609	Bn-N3-p19147826	Bn-A03-p18775919	Bn-A03-p18775919	A3	57	73.5	A
2610	Bn-N3-p19147979	Bn-A03-p18776071	Bn-A03-p18776071	A3	57	73.5	A
2611	Bn-N3-p19154466	Bn-A03-p18782352	Bn-A03-p18782352	A3	57	73.5	A
2612	Bn-N3-p19154530	Bn-A03-p18782416	Bn-A03-p18782416	A3	57	73.5	A
2613	Bn-N3-p19161041	Bn-A03-p18788929	Bn-A03-p18788929	A3	57	73.5	A
2614	Bn-N3-p19188356	Bn-A03-p18811487	Bn-A03-p18811487	A3	57	73.5	A
2615	Bn-N3-p19198936	Bn-A03-p18824580	Bn-A03-p18824580	A3	57	73.5	A
2616	Bn-N3-p19200841	Bn-A03-p18826478	Bn-A03-p18826478	A3	57	73.5	A
2617	Bn-N3-p19203009	Bn-A03-p18828645	Bn-A03-p18828645	A3	57	73.5	A
2618	Bn-N3-p19216285	Bn-A03-p18836509	Bn-A03-p18836509	A3	57	73.5	A
2619	Bn-N3-p19222659	Bn-A03-p18846268	Bn-A03-p18846268	A3	58	74.3	A
2620	Bn-N13-p31123378	Bn-A03-p19094554	Bn-A03-p19094554	A3	59	75.1	A
2621	Bn-N13-p32798196	Bn-A03-p19751864	Bn-A03-p19751864	A3	59	75.1	A
2622	Bn-N3-p19334061	Bn-A03-p19068030	Bn-A03-p19068030	A3	59	75.1	A
2623	Bn-N3-p19349248	Bn-A03-p19078721	Bn-A03-p19078721	A3	59	75.1	A
2624	Bn-N3-p19405475	Bn-A03-p19132431	Bn-A03-p19132431	A3	59	75.1	A
2625	Bn-N3-p19431402	Bn-A03-p19147644	Bn-A03-p19147644	A3	59	75.1	A
2626	Bn-N3-p19480829	Bn-A03-p19189483	Bn-A03-p19189483	A3	59	75.1	A
2627	Bn-N3-p19490620	Bn-A03-p19198939	Bn-A03-p19198939	A3	59	75.1	A
2628	Bn-N3-p19493925	Bn-A03-p19202899	Bn-A03-p19202899	A3	59	75.1	A
2629	Bn-N3-p19494521	Bn-A03-p19203494	Bn-A03-p19203494	A3	59	75.1	A
2630	Bn-N3-p19495568	Bn-A03-p19204483	Bn-A03-p19204483	A3	59	75.1	A
2631	Bn-N3-p19501669	Bn-A03-p19210113	Bn-A03-p19210113	A3	59	75.1	A
2632	Bn-N3-p19532856	Bn-A03-p19243266	Bn-A03-p19243266	A3	59	75.1	A
2633	Bn-N3-p19541020	Bn-A03-p19252196	Bn-A03-p19252196	A3	59	75.1	A
2634	Bn-N3-p19545827	Bn-A03-p19254792	Bn-A03-p19254792	A3	59	75.1	A
2635	Bn-N3-p19566361	Bn-A03-p19274997	Bn-A03-p19274997	A3	59	75.1	A
2636	Bn-N3-p19638274	Bn-A03-p19344007	Bn-A03-p19344007	A3	59	75.1	A
2637	Bn-N3-p19653242	Bn-A03-p19359327	Bn-A03-p19359327	A3	59	75.1	A
2638	Bn-N3-p19669593	Bn-A03-p19375820	Bn-A03-p19375820	A3	59	75.1	A
2639	Bn-N3-p19733694	Bn-A03-p19438796	Bn-A03-p19438796	A3	59	75.1	A
2640	Bn-N3-p19734381	Bn-C3-p30526558	Bn-scaff_16092_1-p86691	A3	59	75.1	A
2641	Bn-N3-p19736425	Bn-A03-p19441544	Bn-A03-p19441544	A3	59	75.1	A

2642	Bn-N3-p19752040	Bn-A03-p19462036	Bn-A03-p19462036	A3	59	75.1	A
2643	Bn-N3-p19795423	Bn-A03-p19501529	Bn-A03-p19501529	A3	59	75.1	A
2644	Bn-N3-p19814265	Bn-A03-p19510486	Bn-A03-p19510486	A3	59	75.1	A
2645	Bn-N3-p19817951	Bn-A03-p19512402	Bn-A03-p19512402	A3	59	75.1	A
2646	Bn-N3-p19820402	Bn-A03-p19519674	Bn-A03-p19519674	A3	59	75.1	A
2647	Bn-N3-p19870417	Bn-A03-p19573902	Bn-A03-p19573902	A3	59	75.1	A
2648	Bn-N3-p19877863	Bn-A03-p19582066	Bn-A03-p19582066	A3	59	75.1	A
2649	Bn-N3-p20078266	Bn-A03-p19772056	Bn-A03-p19772056	A3	59	75.1	A
2650	Bn-N3-p20104982	Bn-A03-p19806018	Bn-A03-p19806018	A3	59	75.1	A
2651	Bn-N3-p20105153	Bn-C3-p31329470	Bn-scaff_18917_1-p28690	A3	59	75.1	A
2652	Bn-N3-p20133234	Bn-A03-p19832980	Bn-A03-p19832980	A3	59	75.1	A
2653	Bn-N3-p20151796	Bn-A03-p19844661	Bn-A03-p19844661	A3	59	75.1	A
2654	Bn-N3-p20178063	Bn-A03-p19868485	Bn-A03-p19868485	A3	59	75.1	A
2655	Bn-N3-p20192016	Bn-A03-p19880918	Bn-A03-p19880918	A3	59	75.1	A
2656	Bn-N13-p33240254	Bn-A03-p19896843	Bn-A03-p19896843	A3	60	75.9	A
2657	Bn-N3-p20214153	Bn-A03-p19897251	Bn-A03-p19897251	A3	60	75.9	A
2658	Bn-N3-p20219485	Bn-A03-p19902796	Bn-A03-p19902796	A3	60	75.9	A
2659	Bn-N3-p20231441	Bn-A03-p19916449	Bn-A03-p19916449	A3	60	75.9	A
2660	Bn-N3-p20240617	Bn-A03-p19925593	Bn-A03-p19925593	A3	60	75.9	A
2661	Bn-N3-p20262720	Bn-A03-p19956358	Bn-A03-p19956358	A3	60	75.9	A
2662	Bn-N3-p20283079	Bn-A03-p19973423	Bn-A03-p19973423	A3	60	75.9	A
2663	Bn-N3-p20284442	Bn-A03-p19974784	Bn-A03-p19974784	A3	60	75.9	A
2664	Bn-N3-p20285197	Bn-A03-p19975539	Bn-A03-p19975539	A3	60	75.9	A
2665	Bn-N3-p20301366	Bn-A03-p20000930	Bn-A03-p20000930	A3	60	75.9	A
2666	Bn-N3-p20304899	Bn-A03-p20005216	Bn-A03-p20005216	A3	60	75.9	A
2667	Bn-N3-p20306827	Bn-A03-p20007146	Bn-A03-p20007146	A3	60	75.9	A
2668	Bn-N3-p20309329	Bn-A07-p9429348	Bn-A07-p9429348	A3	60	75.9	A
2669	Bn-N3-p20329049	Bn-A03-p20033408	Bn-A03-p20033408	A3	60	75.9	A
2670	Bn-N3-p20371571	Bn-A03-p20079452	Bn-A03-p20079452	A3	61	76.7	A
2671	Bn-N3-p20408835	Bn-A03-p20114910	Bn-A03-p20114910	A3	62	77.5	A
2672	Bn-N3-p20417490	Bn-A03-p20122616	Bn-A03-p20122616	A3	62	77.5	A
2673	Bn-N3-p20420513	Bn-A03-p20125361	Bn-A03-p20125361	A3	62	77.5	A
2674	Bn-N3-p20445824	Bn-A03-p20151244	Bn-A03-p20151244	A3	62	77.5	A
2675	Bn-N3-p20469807	Bn-A03-p20180463	Bn-A03-p20180463	A3	63	79.1	A
2676	Bn-N4-p3960452	Bn-A03-p20221017	Bn-A03-p20221017	A3	63	79.1	A
2677	Bn-N3-p20532008	Bn-A03-p20237681	Bn-A03-p20237681	A3	64	79.9	A
2678	Bn-N3-p20584427	Bn-A03-p20286113	Bn-A03-p20286113	A3	64	79.9	A
2679	Bn-N3-p20624118	Bn-A03-p20318892	Bn-A03-p20318892	A3	64	79.9	A
2680	Bn-N3-p20624573	Bn-A03-p20319347	Bn-A03-p20319347	A3	64	79.9	A
2681	Bn-N3-p20630056	Bn-A03-p20324951	Bn-A03-p20324951	A3	64	79.9	A
2682	Bn-N3-p20670177	Bn-A03-p20358050	Bn-A03-p20358050	A3	64	79.9	A
2683	Bn-N3-p20684551	Bn-A03-p20369417	Bn-A03-p20369417	A3	64	79.9	A
2684	Bn-N3-p20684656	Bn-A03-p20369521	Bn-A03-p20369521	A3	64	79.9	A
2685	Bn-N3-p20702073	Bn-A03-p20383979	Bn-A03-p20383979	A3	64	79.9	A
2686	Bn-N3-p20706806	Bn-A03-p20395531	Bn-A03-p20395531	A3	64	79.9	A
2687	Bn-N3-p20706859	Bn-A03-p20395584	Bn-A03-p20395584	A3	64	79.9	A
2688	Bn-N3-p20710100	Bn-A03-p20398340	Bn-A03-p20398340	A3	64	79.9	A
2689	Bn-N3-p20710471	Bn-A03-p20398711	Bn-A03-p20398711	A3	64	79.9	A
2690	Bn-N3-p20717493	Bn-A03-p20409233	Bn-A03-p20409233	A3	64	79.9	A

2691	Bn-N3-p20805694	Bn-A03-p20495253	Bn-A03-p20495253	A3	64	79.9	A
2692	Bn-N3-p20806631	Bn-C3-p32680543	Bn-scaff_15695_1-p39296[illegible]	A3	64	79.9	A
2693	Bn-N19-p51507834	Bn-A01-p13825941	Bn-A01-p13825941	A3	65	81.5	A
2694	Bn-N19-p51509406	Bn-A01-p13827513	Bn-A01-p13827513	A3	65	81.5	0
2695	Bn-N19-p51509910	Bn-A01-p13828017	Bn-A01-p13828017	A3	65	81.5	0
2696	Bn-N19-p51510207	Bn-A01-p13828314	Bn-A01-p13828314	A3	65	81.5	0
2697	Bn-N3-p20829184	Bn-A03-p20518761	Bn-A03-p20518761	A3	65	81.5	A
2698	Bn-N3-p20834180	Bn-A03-p20523585	Bn-A03-p20523585	A3	65	81.5	A
2699	Bn-N3-p20836118	Bn-A03-p20525474	Bn-A03-p20525474	A3	65	81.5	A
2700	Bn-N3-p20836235	Bn-A03-p20525591	Bn-A03-p20525591	A3	65	81.5	A
2701	Bn-N3-p20885422	Bn-A03-p20560569	Bn-A03-p20560569	A3	65	81.5	A
2702	Bn-N3-p20893626	Bn-A03-p20568764	Bn-A03-p20568764	A3	65	81.5	A
2703	Bn-N3-p20899843	Bn-A03-p20584351	Bn-A03-p20584351	A3	65	81.5	A
2704	Bn-N3-p20913968	Bn-A03-p20604949	Bn-A03-p20604949	A3	65	81.5	A
2705	Bn-N3-p20919598	Bn-A03-p20609910	Bn-A03-p20609910	A3	65	81.5	A
2706	Bn-N3-p20943128	Bn-A03-p20656717	Bn-A03-p20656717	A3	65	81.5	A
2707	Bn-N3-p20950682	Bn-A03-p20662048	Bn-A03-p20662048	A3	65	81.5	A
2708	Bn-N3-p20956058	Bn-C3-p33131567	Bn-scaff_18855_1-p83104[illegible]	A3	65	81.5	A
2709	Bn-N3-p20988449	Bn-A03-p20701991	Bn-A03-p20701991	A3	65	81.5	A
2710	Bn-N3-p21005324	Bn-A03-p20727133	Bn-A03-p20727133	A3	65	81.5	A
2711	Bn-N17-p38456629	Bn-A03-p21076805	Bn-A03-p21076805	A3	66	83.9	A
2712	Bn-N3-p21159284	Bn-A03-p20909567	Bn-A03-p20909567	A3	66	83.9	A
2713	Bn-N3-p21187651	Bn-A03-p20937268	Bn-A03-p20937268	A3	66	83.9	A
2714	Bn-N3-p21231831	Bn-A03-p20983297	Bn-A03-p20983297	A3	66	83.9	A
2715	Bn-N3-p21231932	Bn-A03-p20983398	Bn-A03-p20983398	A3	66	83.9	A
2716	Bn-N3-p21310390	Bn-A03-p21075664	Bn-A03-p21075664	A3	66	83.9	A
2717	Bn-N3-p21500288	Bn-A03-p21238466	Bn-A03-p21238466	A3	67	84.7	A
2718	Bn-N3-p21524087	Bn-A03-p21252366	Bn-A03-p21252366	A3	67	84.7	A
2719	Bn-N3-p21543364	Bn-A03-p21271941	Bn-A03-p21271941	A3	67	84.7	A
2720	Bn-N3-p21561811	Bn-A03-p21283570	Bn-A03-p21283570	A3	67	84.7	A
2721	Bn-N3-p21568955	Bn-A03-p21292773	Bn-A03-p21292773	A3	67	84.7	A
2722	Bn-N3-p21572010	Bn-A03-p21294836	Bn-A03-p21294836	A3	67	84.7	A
2723	Bn-N3-p21616626	Bn-A03-p21329715	Bn-A03-p21329715	A3	67	84.7	A
2724	Bn-N3-p21637085	Bn-A03-p21344477	Bn-A03-p21344477	A3	67	84.7	A
2725	Bn-N3-p21667099	Bn-A03-p21375969	Bn-A03-p21375969	A3	68	85.5	A
2726	Bn-N3-p21713680	Bn-A03-p21422696	Bn-A03-p21422696	A3	68	85.5	A
2727	Bn-N13-p55033728	Bn-A03-p21503102	Bn-A03-p21503102	A3	69	86.3	A
2728	Bn-N17-p39038160	Bn-A03-p21504871	Bn-A03-p21504871	A3	69	86.3	A
2729	Bn-N3-p21778969	Bn-A03-p21505530	Bn-A03-p21505530	A3	69	86.3	A
2730	Bn-N3-p21779059	Bn-A03-p21505440	Bn-A03-p21505440	A3	69	86.3	A
2731	Bn-N3-p21779068	Bn-A03-p21505431	Bn-A03-p21505431	A3	69	86.3	A
2732	Bn-N3-p21779136	Bn-A03-p21505363	Bn-A03-p21505363	A3	69	86.3	A
2733	Bn-N3-p21900335	Bn-A03-p21632587	Bn-A03-p21632587	A3	69	86.3	A
2734	Bn-N3-p21947903	Bn-A03-p21685480	Bn-A03-p21685480	A3	70	87.1	A
2735	Bn-N3-p22019268	Bn-A03-p21744993	Bn-A03-p21744993	A3	70	87.1	A
2736	Bn-N3-p22024535	Bn-A03-p21750559	Bn-A03-p21750559	A3	70	87.1	A
2737	Bn-N3-p22041349	Bn-A03-p21782249	Bn-A03-p21782249	A3	70	87.1	A
2738	Bn-N3-p22042117	Bn-A03-p21783015	Bn-A03-p21783015	A3	70	87.1	A
2739	Bn-N3-p22045211	Bn-A03-p21786943	Bn-A03-p21786943	A3	70	87.1	A

2740	Bn-N3-p22047478	Bn-A03-p21787407	Bn-A03-p21787407	A3	70	87.1	A
2741	Bn-N3-p22047905	Bn-A03-p21787813	Bn-A03-p21787813	A3	70	87.1	A
2742	Bn-N3-p22053427	Bn-A03-p21798303	Bn-A03-p21798303	A3	70	87.1	A
2743	Bn-N3-p22063994	Bn-A03-p21813117	Bn-A03-p21813117	A3	70	87.1	A
2744	Bn-N3-p22082635	Bn-A03-p21830330	Bn-A03-p21830330	A3	70	87.1	A
2745	Bn-N3-p22091569	Bn-A03-p21839200	Bn-A03-p21839200	A3	70	87.1	A
2746	Bn-N3-p22104485	Bn-A03-p21846413	Bn-A03-p21846413	A3	70	87.1	A
2747	Bn-N3-p22121774	Bn-A03-p21864294	Bn-A03-p21864294	A3	70	87.1	A
2748	Bn-Scaffold07531-p476	Bn-A03-p21752426	Bn-A03-p21752426	A3	70	87.1	A
2749	Bn-Scaffold07531-p786	Bn-A03-p21752115	Bn-A03-p21752115	A3	70	87.1	A
2750	Bn-N13-p21307672	Bn-A09-p29446045	Bn-A09-p29446045	A3	71	87.9	A
2751	Bn-N3-p22166287	Bn-A03-p21904842	Bn-A03-p21904842	A3	71	87.9	A
2752	Bn-N3-p22170891	Bn-A03-p21907151	Bn-A03-p21907151	A3	71	87.9	A
2753	Bn-N3-p22173162	Bn-A03-p21909917	Bn-A03-p21909917	A3	71	87.9	A
2754	Bn-N3-p22190031	Bn-A03-p21921653	Bn-A03-p21921653	A3	71	87.9	A
2755	Bn-N3-p22258835	Bn-A03-p21979477	Bn-A03-p21979477	A3	71	87.9	A
2756	Bn-N3-p22259691	Bn-A03-p21980336	Bn-A03-p21980336	A3	71	87.9	A
2757	Bn-N3-p22287140	Bn-A03-p22003165	Bn-A03-p22003165	A3	71	87.9	A
2758	Bn-N3-p22296381	Bn-A03-p22012070	Bn-A03-p22012070	A3	71	87.9	A
2759	Bn-N3-p22308052	Bn-A03-p22028778	Bn-A03-p22028778	A3	71	87.9	A
2760	Bn-N3-p22363065	Bn-A03-p22090414	Bn-A03-p22090414	A3	71	87.9	A
2761	Bn-N3-p22371208	Bn-A03-p22098429	Bn-A03-p22098429	A3	71	87.9	A
2762	Bn-N3-p22393687	Bn-A03-p22121980	Bn-A03-p22121980	A3	71	87.9	A
2763	Bn-N3-p22410574	Bn-A03-p22138059	Bn-A03-p22138059	A3	71	87.9	A
2764	Bn-N3-p22417163	Bn-A03-p22145945	Bn-A03-p22145945	A3	71	87.9	A
2765	Bn-N3-p22420219	Bn-A03-p22149000	Bn-A03-p22149000	A3	71	87.9	A
2766	Bn-N3-p22469046	Bn-A03-p22183905	Bn-A03-p22183905	A3	71	87.9	A
2767	Bn-N3-p22561791	Bn-A03-p22285105	Bn-A03-p22285105	A3	71	87.9	A
2768	Bn-N3-p22576660	Bn-A03-p22299476	Bn-A03-p22299476	A3	71	87.9	A
2769	Bn-N3-p22580370	Bn-A03-p22304555	Bn-A03-p22304555	A3	71	87.9	A
2770	Bn-N3-p22582859	Bn-A03-p22306212	Bn-A03-p22306212	A3	71	87.9	A
2771	Bn-N3-p22583395	Bn-A03-p22306743	Bn-A03-p22306743	A3	71	87.9	A
2772	Bn-N3-p22591655	Bn-A03-p22313737	Bn-A03-p22313737	A3	71	87.9	A
2773	Bn-N3-p22664130	Bn-A03-p22378361	Bn-A03-p22378361	A3	71	87.9	A
2774	Bn-N3-p22691366	Bn-A03-p22398141	Bn-A03-p22398141	A3	71	87.9	A
2775	Bn-N3-p22848336	Bn-A03-p22556722	Bn-A03-p22556722	A3	71	87.9	A
2776	Bn-N3-p22880397	Bn-A03-p22582673	Bn-A03-p22582673	A3	71	87.9	A
2777	Bn-N3-p22880586	Bn-A03-p22582862	Bn-A03-p22582862	A3	71	87.9	A
2778	Bn-N3-p22886076	Bn-A03-p22588041	Bn-A03-p22588041	A3	71	87.9	A
2779	Bn-N3-p22888749	Bn-A03-p22590442	Bn-A03-p22590442	A3	71	87.9	A
2780	Bn-N3-p22893279	Bn-A03-p22595213	Bn-A03-p22595213	A3	71	87.9	A
2781	Bn-N3-p22902373	Bn-A03-p22610222	Bn-A03-p22610222	A3	71	87.9	A
2782	Bn-N3-p22908167	Bn-A03-p22619997	Bn-A03-p22619997	A3	71	87.9	A
2783	Bn-N3-p22916771	Bn-C7-p40194832	Bn-scaff_16069_1-p48489[illegible]	A3	71	87.9	A
2784	Bn-N3-p22918251	Bn-A03-p22626240	Bn-A03-p22626240	A3	71	87.9	A
2785	Bn-N3-p22922739	Bn-A03-p22630782	Bn-A03-p22630782	A3	71	87.9	A
2786	Bn-N3-p22925476	Bn-A03-p22633445	Bn-A03-p22633445	A3	71	87.9	A
2787	Bn-N3-p22927684	Bn-A03-p22635426	Bn-A03-p22635426	A3	71	87.9	A
2788	Bn-N3-p22936404	Bn-A03-p22643433	Bn-A03-p22643433	A3	71	87.9	A

2789	Bn-N3-p22938039	Bn-A03-p22651625	Bn-A03-p22651625	A3	71	87.9	A
2790	Bn-N3-p22944155	Bn-A03-p22655575	Bn-A03-p22655575	A3	71	87.9	A
2791	Bn-N3-p23049133	Bn-A03-p22739253	Bn-A03-p22739253	A3	71	87.9	A
2792	Bn-N3-p23049409	Bn-A03-p22739529	Bn-A03-p22739529	A3	71	87.9	A
2793	Bn-N3-p23054682	Bn-A03-p22744766	Bn-A03-p22744766	A3	71	87.9	A
2794	Bn-N3-p23059076	Bn-A03-p22750972	Bn-A03-p22750972	A3	71	87.9	A
2795	Bn-N3-p23060917	Bn-A03-p22752812	Bn-A03-p22752812	A3	71	87.9	A
2796	Bn-N3-p23060976	Bn-A03-p22752871	Bn-A03-p22752871	A3	71	87.9	A
2797	Bn-N3-p23087925	Bn-A03-p22779259	Bn-A03-p22779259	A3	71	87.9	A
2798	Bn-Scaffold61537-p153	Bn-A03-p22582234	Bn-A03-p22582234	A3	71	87.9	A
2799	Bn-N17-p17253028	Bn-A03-p22816854	Bn-A03-p22816854	A3	72	88.7	A
2800	Bn-N3-p23119593	Bn-A03-p22820867	Bn-A03-p22820867	A3	72	88.7	0
2801	Bn-N3-p23119669	Bn-A03-p22820943	Bn-A03-p22820943	A3	72	88.7	A
2802	Bn-N3-p23119722	Bn-A03-p22820996	Bn-A03-p22820996	A3	72	88.7	A
2803	Bn-N3-p23140811	Bn-A03-p22838495	Bn-A03-p22838495	A3	72	88.7	A
2804	Unknown	Bn-A03-p22816593	Bn-A03-p22816593	A3	72	88.7	A
2805	Unknown	Bn-A03-p22816713	Bn-A03-p22816713	A3	72	88.7	A
2806	Unknown	Bn-A03-p22816724	Bn-A03-p22816724	A3	72	88.7	A
2807	Bn-N3-p23181434	Bn-A03-p22882585	Bn-A03-p22882585	A3	73	89.5	A
2808	Bn-N17-p41072072	Bn-A03-p23077236	Bn-A03-p23077236	A3	74	90.3	A
2809	Bn-N17-p41083571	Bn-A03-p23086442	Bn-A03-p23086442	A3	74	90.3	A
2810	Bn-N17-p41248233	Bn-C7-p40912764	Bn-scaff_16069_1-p12028	A3	74	90.3	A
2811	Bn-N3-p23342059	Bn-A03-p23017354	Bn-A03-p23017354	A3	74	90.3	A
2812	Bn-N3-p23365036	Bn-A03-p23043608	Bn-A03-p23043608	A3	74	90.3	A
2813	Bn-N3-p23370355	Bn-A03-p23047749	Bn-A03-p23047749	A3	74	90.3	A
2814	Bn-N3-p23411579	Bn-A03-p23088716	Bn-A03-p23088716	A3	74	90.3	A
2815	Bn-N3-p23429793	Bn-A03-p23123128	Bn-A03-p23123128	A3	74	90.3	A
2816	Bn-N3-p23435010	Bn-A03-p23127658	Bn-A03-p23127658	A3	74	90.3	A
2817	Bn-N3-p23506348	Bn-A03-p23205791	Bn-A03-p23205791	A3	74	90.3	A
2818	Bn-N3-p23667615	Bn-A03-p29571938	Bn-A03-p29571938	A3	75	92.7	A
2819	Bn-N3-p24031518	Bn-A03-p23735715	Bn-A03-p23735715	A3	76	93.5	A
2820	Bn-N3-p24031897	Bn-A03-p23736096	Bn-A03-p23736096	A3	76	93.5	A
2821	Bn-N14-p5281224	Bn-C3-p36400180	Bn-scaff_18356_1-p18168	A3	77	95.1	0
2822	Bn-N3-p24101110	Bn-A03-p23810014	Bn-A03-p23810014	A3	77	95.1	A
2823	Bn-N3-p24105409	Bn-A03-p23822093	Bn-A03-p23822093	A3	77	95.1	A
2824	Bn-N3-p24126497	Bn-A03-p23860825	Bn-A03-p23860825	A3	77	95.1	A
2825	Bn-N3-p24127148	Bn-A03-p23861481	Bn-A03-p23861481	A3	77	95.1	A
2826	Bn-N3-p24127685	Bn-A03-p23862018	Bn-A03-p23862018	A3	77	95.1	A
2827	Bn-N3-p24128350	Bn-A03-p23862683	Bn-A03-p23862683	A3	77	95.1	A
2828	Bn-N3-p24133071	Bn-A03-p23868030	Bn-A03-p23868030	A3	77	95.1	A
2829	Bn-N3-p24237586	Bn-A03-p23973475	Bn-A03-p23973475	A3	77	95.1	A
2830	Bn-N3-p24283710	Bn-A03-p24016551	Bn-A03-p24016551	A3	77	95.1	A
2831	Bn-N3-p24284469	Bn-A03-p24017311	Bn-A03-p24017311	A3	77	95.1	A
2832	Bn-N3-p24289836	Bn-A03-p24022648	Bn-A03-p24022648	A3	77	95.1	A
2833	Bn-N3-p24315585	Bn-A03-p24032564	Bn-A03-p24032564	A3	77	95.1	A
2834	Bn-N3-p24358747	Bn-A03-p24090020	Bn-A03-p24090020	A3	77	95.1	A
2835	Bn-N3-p24362517	Bn-A03-p24092986	Bn-A03-p24092986	A3	77	95.1	A
2836	Bn-N3-p24431801	Bn-A03-p24176332	Bn-A03-p24176332	A3	78	95.9	A
2837	Bn-N3-p24446072	Bn-A03-p24179750	Bn-A03-p24179750	A3	78	95.9	A

2838	Bn-N3-p24447158	Bn-A03-p24181569	Bn-A03-p24181569	A3	78	95.9	A
2839	Bn-N3-p24521572	Bn-A03-p24255868	Bn-A03-p24255868	A3	78	95.9	A
2840	Bn-N11-p31790301	Bn-A03-p24606883	Bn-A03-p24606883	A3	79	98.3	A
2841	Bn-N12-p13991406	Bn-A03-p24897111	Bn-A03-p24897111	A3	79	98.3	A
2842	Bn-N13-p52649385	Bn-A03-p18886205	Bn-A03-p18886205	A3	79	98.3	A
2843	Bn-N17-p43120499	Bn-A03-p24998976	Bn-A03-p24998976	A3	79	98.3	A
2844	Bn-N17-p43120844	Bn-A03-p24999716	Bn-A03-p24999716	A3	79	98.3	A
2845	Bn-N17-p43143511	Bn-A03-p25032014	Bn-A03-p25032014	A3	79	98.3	A
2846	Bn-N17-p43145031	Bn-A03-p25033468	Bn-A03-p25033468	A3	79	98.3	A
2847	Bn-N19-p52550305	Bn-A03-p24902152	Bn-A03-p24902152	A3	79	98.3	A
2848	Bn-N3-p11952122	Bn-A03-p24902631	Bn-A03-p24902631	A3	79	98.3	A
2849	Bn-N3-p24553962	Bn-A03-p24294571	Bn-A03-p24294571	A3	79	98.3	A
2850	Bn-N3-p24559224	Bn-A03-p24298999	Bn-A03-p24298999	A3	79	98.3	A
2851	Bn-N3-p24599088	Bn-A03-p24345237	Bn-A03-p24345237	A3	79	98.3	A
2852	Bn-N3-p24614035	Bn-A03-p24355827	Bn-A03-p24355827	A3	79	98.3	A
2853	Bn-N3-p24614086	Bn-A03-p24355878	Bn-A03-p24355878	A3	79	98.3	A
2854	Bn-N3-p24621501	Bn-A03-p24363260	Bn-A03-p24363260	A3	79	98.3	A
2855	Bn-N3-p24622052	Bn-A03-p24363811	Bn-A03-p24363811	A3	79	98.3	A
2856	Bn-N3-p24623582	Bn-A03-p24365383	Bn-A03-p24365383	A3	79	98.3	A
2857	Bn-N3-p24630045	Bn-A03-p24392068	Bn-A03-p24392068	A3	79	98.3	A
2858	Bn-N3-p24631719	Bn-A03-p24394796	Bn-A03-p24394796	A3	79	98.3	A
2859	Bn-N3-p24632590	Bn-A03-p24395668	Bn-A03-p24395668	A3	79	98.3	A
2860	Bn-N3-p24632888	Bn-A03-p24396004	Bn-A03-p24396004	A3	79	98.3	A
2861	Bn-N3-p24634841	Bn-A03-p24397912	Bn-A03-p24397912	A3	79	98.3	A
2862	Bn-N3-p24634845	Bn-A03-p24397916	Bn-A03-p24397916	A3	79	98.3	A
2863	Bn-N3-p24635348	Bn-A03-p24398419	Bn-A03-p24398419	A3	79	98.3	A
2864	Bn-N3-p24635504	Bn-A03-p24400338	Bn-A03-p24400338	A3	79	98.3	A
2865	Bn-N3-p24639573	Bn-A03-p24411222	Bn-A03-p24411222	A3	79	98.3	A
2866	Bn-N3-p24641971	Bn-A03-p24415246	Bn-A03-p24415246	A3	79	98.3	A
2867	Bn-N3-p24643877	Bn-A03-p24417118	Bn-A03-p24417118	A3	79	98.3	A
2868	Bn-N3-p24646980	Bn-A03-p24419229	Bn-A03-p24419229	A3	79	98.3	A
2869	Bn-N3-p24650940	Bn-A03-p24420093	Bn-A03-p24420093	A3	79	98.3	A
2870	Bn-N3-p24651687	Bn-A03-p24420842	Bn-A03-p24420842	A3	79	98.3	A
2871	Bn-N3-p24653094	Bn-A03-p24423536	Bn-A03-p24423536	A3	79	98.3	A
2872	Bn-N3-p24653317	Bn-A03-p24423762	Bn-A03-p24423762	A3	79	98.3	A
2873	Bn-N3-p24653352	Bn-A03-p24423797	Bn-A03-p24423797	A3	79	98.3	A
2874	Bn-N3-p24653729	Bn-A03-p24424174	Bn-A03-p24424174	A3	79	98.3	A
2875	Bn-N3-p24653786	Bn-A03-p24424231	Bn-A03-p24424231	A3	79	98.3	A
2876	Bn-N3-p24653841	Bn-A03-p24424286	Bn-A03-p24424286	A3	79	98.3	A
2877	Bn-N3-p24654359	Bn-A03-p24424807	Bn-A03-p24424807	A3	79	98.3	A
2878	Bn-N3-p24655654	Bn-A03-p24426088	Bn-A03-p24426088	A3	79	98.3	A
2879	Bn-N3-p24678709	Bn-A03-p24445966	Bn-A03-p24445966	A3	79	98.3	A
2880	Bn-N3-p24721128	Bn-A03-p24482649	Bn-A03-p24482649	A3	79	98.3	A
2881	Bn-N3-p24724768	Bn-A03-p24486881	Bn-A03-p24486881	A3	79	98.3	A
2882	Bn-N3-p24728771	Bn-A03-p24494026	Bn-A03-p24494026	A3	79	98.3	A
2883	Bn-N3-p24728955	Bn-A03-p24494224	Bn-A03-p24494224	A3	79	98.3	A
2884	Bn-N3-p24751925	Bn-A03-p24512946	Bn-A03-p24512946	A3	79	98.3	A
2885	Bn-N3-p24771219	Bn-A03-p24527313	Bn-A03-p24527313	A3	79	98.3	A
2886	Bn-N3-p24787485	Bn-C5-p14124591	Bn-scaff_18338_1-p92045[illegible]	A3	79	98.3	A

2887	Bn-N3-p24794707	Bn-A03-p24565421	Bn-A03-p24565421	A3	79	98.3	A
2888	Bn-N3-p24806668	Bn-A03-p24576670	Bn-A03-p24576670	A3	79	98.3	A
2889	Bn-N3-p24810883	Bn-A03-p24581655	Bn-A03-p24581655	A3	79	98.3	A
2890	Bn-N3-p24832519	Bn-C7-p42387916	Bn-scaff_16069_1-p267797	A3	79	98.3	A
2891	Bn-N3-p24843059	Bn-A03-p24613172	Bn-A03-p24613172	A3	79	98.3	A
2892	Bn-N3-p24847663	Bn-A03-p24627047	Bn-A03-p24627047	A3	79	98.3	A
2893	Bn-N3-p24866051	Bn-A03-p24650359	Bn-A03-p24650359	A3	79	98.3	A
2894	Bn-N3-p24910174	Bn-A03-p24688180	Bn-A03-p24688180	A3	79	98.3	A
2895	Bn-N3-p24912822	Bn-A03-p24690789	Bn-A03-p24690789	A3	79	98.3	A
2896	Bn-N3-p24956620	Bn-A03-p24745951	Bn-A03-p24745951	A3	79	98.3	A
2897	Bn-N3-p24956676	Bn-A03-p24746007	Bn-A03-p24746007	A3	79	98.3	A
2898	Bn-N3-p24957877	Bn-A03-p24748004	Bn-A03-p24748004	A3	79	98.3	A
2899	Bn-N3-p24963556	Bn-A03-p24753667	Bn-A03-p24753667	A3	79	98.3	A
2900	Bn-N3-p24963718	Bn-A03-p24753829	Bn-A03-p24753829	A3	79	98.3	A
2901	Bn-N3-p25046301	Bn-A03-p24824357	Bn-A03-p24824357	A3	79	98.3	A
2902	Bn-N3-p25048900	Bn-A03-p24827646	Bn-A03-p24827646	A3	79	98.3	A
2903	Bn-N3-p25066947	Bn-A03-p24842021	Bn-A03-p24842021	A3	79	98.3	A
2904	Bn-N3-p25075549	Bn-A06-p24000840	Bn-A06-p24000840	A3	79	98.3	0
2905	Bn-N3-p25095312	Bn-A03-p24864494	Bn-A03-p24864494	A3	79	98.3	A
2906	Bn-N3-p25124773	Bn-A03-p24906226	Bn-A03-p24906226	A3	79	98.3	A
2907	Bn-N3-p25183358	Bn-A03-p24971161	Bn-A03-p24971161	A3	79	98.3	A
2908	Bn-N3-p25183557	Bn-A03-p24971360	Bn-A03-p24971360	A3	79	98.3	A
2909	Bn-N3-p25201832	Bn-A03-p24990059	Bn-A03-p24990059	A3	79	98.3	A
2910	Bn-N3-p25207170	Bn-A03-p25009736	Bn-A03-p25009736	A3	79	98.3	A
2911	Bn-N3-p25207353	Bn-A03-p25009919	Bn-A03-p25009919	A3	79	98.3	A
2912	Bn-N3-p25207359	Bn-A03-p25009925	Bn-A03-p25009925	A3	79	98.3	A
2913	Bn-N3-p25214769	Bn-A03-p25024647	Bn-A03-p25024647	A3	79	98.3	A
2914	Bn-N3-p25214871	Bn-A03-p25024749	Bn-A03-p25024749	A3	79	98.3	A
2915	Bn-N3-p25215047	Bn-A03-p25024928	Bn-A03-p25024928	A3	79	98.3	A
2916	Bn-N3-p25215488	Bn-A03-p25025360	Bn-A03-p25025360	A3	79	98.3	A
2917	Bn-N3-p25222063	Bn-A03-p25037423	Bn-A03-p25037423	A3	79	98.3	A
2918	Bn-N3-p25232380	Bn-A03-p25047750	Bn-A03-p25047750	A3	79	98.3	A
2919	Bn-N3-p25256225	Bn-A03-p25071259	Bn-A03-p25071259	A3	79	98.3	A
2920	Bn-N3-p25258404	Bn-A03-p25073363	Bn-A03-p25073363	A3	79	98.3	A
2921	Bn-N3-p25499702	Bn-A03-p25314490	Bn-A03-p25314490	A3	79	98.3	A
2922	Bn-N3-p25499782	Bn-A03-p25314570	Bn-A03-p25314570	A3	79	98.3	A
2923	Bn-N3-p25531054	Bn-A03-p25343812	Bn-A03-p25343812	A3	79	98.3	A
2924	Bn-N3-p25545269	Bn-A03-p25361950	Bn-A03-p25361950	A3	79	98.3	A
2925	Bn-N3-p25545643	Bn-A03-p25362364	Bn-A03-p25362364	A3	79	98.3	A
2926	Bn-N3-p25594093	Bn-A03-p25393494	Bn-A03-p25393494	A3	79	98.3	A
2927	Bn-N4-p19627187	Bn-A03-p24422801	Bn-A03-p24422801	A3	79	98.3	A
2928	Bn-N8-p8225339	Bn-A03-p24896477	Bn-A03-p24896477	A3	79	98.3	A
2929	Bn-Scaffold01419-p7069	Bn-A03-p25008908	Bn-A03-p25008908	A3	79	98.3	A
2930	Unknown	Bn-A03-p24669270	Bn-A03-p24669270	A3	79	98.3	0
2931	Unknown	Bn-A03-p24896531	Bn-A03-p24896531	A3	79	98.3	A
2932	Unknown	Bn-A03-p24896769	Bn-A03-p24896769	A3	79	98.3	A
2933	Unknown	Bn-A03-p24896884	Bn-A03-p24896884	A3	79	98.3	A
2934	Unknown	Bn-A03-p24897118	Bn-A03-p24897118	A3	79	98.3	A
2935	Unknown	Bn-A03-p24897286	Bn-A03-p24897286	A3	79	98.3	A

2936	Unknown	Bn-A03-p24900647	Bn-A03-p24900647	A3	79	98.3	A
2937	Bn-N3-p25600454	Bn-A03-p25402301	Bn-A03-p25402301	A3	80	99.1	A
2938	Bn-N3-p25600870	Bn-A03-p25402717	Bn-A03-p25402717	A3	80	99.1	A
2939	Bn-N3-p25601946	Bn-A03-p25403786	Bn-A03-p25403786	A3	80	99.1	A
2940	Bn-N3-p25605061	Bn-A03-p25408779	Bn-A03-p25408779	A3	80	99.1	A
2941	Bn-N3-p25624074	Bn-A03-p25423612	Bn-A03-p25423612	A3	80	99.1	A
2942	Bn-N3-p25693625	Bn-A03-p25494539	Bn-A03-p25494539	A3	80	99.1	A
2943	Bn-N3-p25724701	Bn-A03-p25525612	Bn-A03-p25525612	A3	81	99.9	A
2944	Bn-N3-p25804790	Bn-A03-p25591818	Bn-A03-p25591818	A3	82	100.7	A
2945	Bn-N3-p25828301	Bn-A03-p25614406	Bn-A03-p25614406	A3	82	100.7	A
2946	Bn-N3-p25845246	Bn-A03-p25631316	Bn-A03-p25631316	A3	83	101.5	A
2947	Bn-N3-p25849712	Bn-A03-p25636300	Bn-A03-p25636300	A3	83	101.5	A
2948	Bn-N3-p25850691	Bn-A03-p25637279	Bn-A03-p25637279	A3	83	101.5	A
2949	Bn-N3-p25855762	Bn-A03-p25640844	Bn-A03-p25640844	A3	83	101.5	A
2950	Bn-N3-p25904465	Bn-A03-p25679775	Bn-A03-p25679775	A3	83	101.5	A
2951	Bn-N3-p25982788	Bn-A03-p25729227	Bn-A03-p25729227	A3	83	101.5	A
2952	Bn-N3-p25982866	Bn-A03-p25729305	Bn-A03-p25729305	A3	83	101.5	A
2953	Bn-N3-p25988524	Bn-A03-p25734907	Bn-A03-p25734907	A3	83	101.5	A
2954	Bn-N3-p26001616	Bn-A03-p25751759	Bn-A03-p25751759	A3	83	101.5	A
2955	Bn-N3-p26003524	Bn-A03-p25756310	Bn-A03-p25756310	A3	83	101.5	A
2956	Bn-N3-p26026651	Bn-A03-p25785875	Bn-A03-p25785875	A3	83	101.5	A
2957	Bn-N3-p26119211	Bn-A03-p25879876	Bn-A03-p25879876	A3	83	101.5	A
2958	Bn-N3-p26180052	Bn-A03-p25949532	Bn-A03-p25949532	A3	84	102.3	A
2959	Bn-N3-p26180196	Bn-A03-p25949676	Bn-A03-p25949676	A3	84	102.3	A
2960	Bn-N3-p26181711	Bn-A03-p25951191	Bn-A03-p25951191	A3	84	102.3	A
2961	Bn-N3-p26183591	Bn-A03-p25952725	Bn-A03-p25952725	A3	84	102.3	A
2962	Bn-N3-p26188816	Bn-A03-p25957465	Bn-A03-p25957465	A3	84	102.3	A
2963	Bn-N3-p26231379	Bn-A03-p25993601	Bn-A03-p25993601	A3	84	102.3	A
2964	Bn-N17-p44067603	Bn-A03-p26013651	Bn-A03-p26013651	A3	85	103.1	A
2965	Bn-N3-p26244407	Bn-A03-p26004171	Bn-A03-p26004171	A3	85	103.1	A
2966	Bn-N3-p26255287	Bn-A03-p26021585	Bn-A03-p26021585	A3	85	103.1	A
2967	Bn-N3-p26287335	Bn-A03-p26066930	Bn-A03-p26066930	A3	85	103.1	A
2968	Bn-N3-p26500219	Bn-Scaffold000379-p1	Bn-Scaffold000379-p15742	A3	86	103.9	A
2969	Bn-N3-p26505748	Bn-A03-p26272560	Bn-A03-p26272560	A3	86	103.9	A
2970	Bn-N3-p26508632	Bn-A03-p26275444	Bn-A03-p26275444	A3	86	103.9	A
2971	Bn-N3-p26508803	Bn-A03-p26275615	Bn-A03-p26275615	A3	86	103.9	A
2972	Bn-N3-p26509537	Bn-A03-p26276349	Bn-A03-p26276349	A3	86	103.9	A
2973	Bn-N3-p26517534	Bn-A03-p26284882	Bn-A03-p26284882	A3	86	103.9	A
2974	Bn-N3-p26521343	Bn-A03-p26288769	Bn-A03-p26288769	A3	87	104.7	A
2975	Bn-N3-p26551674	Bn-A03-p26309336	Bn-A03-p26309336	A3	87	104.7	A
2976	Bn-N3-p26551888	Bn-A03-p26309550	Bn-A03-p26309550	A3	87	104.7	A
2977	Bn-N3-p26552691	Bn-A03-p26310351	Bn-A03-p26310351	A3	87	104.7	A
2978	Bn-N3-p26570714	Bn-A03-p26311193	Bn-A03-p26311193	A3	87	104.7	A
2979	Bn-N3-p26621508	Bn-A03-p26359851	Bn-A03-p26359851	A3	87	104.7	A
2980	Bn-N3-p26663953	Bn-A03-p26405367	Bn-A03-p26405367	A3	87	104.7	A
2981	Bn-N3-p26664961	Bn-A03-p26406372	Bn-A03-p26406372	A3	87	104.7	A
2982	Bn-N3-p26685356	Bn-C7-p44189196	Bn-scaff_16069_1-p44792!	A3	87	104.7	A
2983	Bn-N3-p26687748	Bn-A03-p26433672	Bn-A03-p26433672	A3	87	104.7	A
2984	Bn-N3-p26687923	Bn-A03-p26433849	Bn-A03-p26433849	A3	87	104.7	A

2985	Bn-N3-p26687986	Bn-A03-p26433912	Bn-A03-p26433912	A3	87	104.7	A
2986	Bn-N3-p26692066	Bn-A03-p26437565	Bn-A03-p26437565	A3	87	104.7	A
2987	Bn-N3-p26702481	Bn-A03-p26444464	Bn-A03-p26444464	A3	87	104.7	A
2988	Bn-N3-p26702962	Bn-A03-p26444945	Bn-A03-p26444945	A3	87	104.7	A
2989	Bn-N3-p26703045	Bn-A03-p26445028	Bn-A03-p26445028	A3	87	104.7	A
2990	Bn-N3-p26736040	Bn-A03-p26477533	Bn-A03-p26477533	A3	88	105.5	A
2991	Bn-N3-p26796689	Bn-A03-p26528958	Bn-A03-p26528958	A3	88	105.5	A
2992	Bn-N3-p26828341	Bn-A03-p26560194	Bn-A03-p26560194	A3	88	105.5	A
2993	Bn-N3-p26835139	Bn-A03-p26567111	Bn-A03-p26567111	A3	88	105.5	A
2994	Bn-N3-p26840399	Bn-A03-p26571083	Bn-A03-p26571083	A3	88	105.5	A
2995	Bn-N3-p26873097	Bn-A03-p26603077	Bn-A03-p26603077	A3	88	105.5	A
2996	Bn-N3-p26874117	Bn-A03-p26604097	Bn-A03-p26604097	A3	88	105.5	A
2997	Bn-N3-p26874599	Bn-A03-p26604578	Bn-A03-p26604578	A3	88	105.5	A
2998	Bn-N3-p26878086	Bn-A03-p26607921	Bn-A03-p26607921	A3	88	105.5	A
2999	Bn-N3-p26994721	Bn-A08-p12054745	Bn-A08-p12054745	A3	88	105.5	A
3000	Bn-N3-p27058874	Bn-A03-p26771482	Bn-A03-p26771482	A3	89	109.5	A
3001	Bn-N3-p27060036	Bn-A03-p26775994	Bn-A03-p26775994	A3	89	109.5	A
3002	Bn-N3-p27070755	Bn-A03-p26786549	Bn-A03-p26786549	A3	89	109.5	A
3003	Bn-N3-p27073110	Bn-A03-p26788875	Bn-A03-p26788875	A3	89	109.5	A
3004	Bn-N3-p27073988	Bn-A03-p26789755	Bn-A03-p26789755	A3	89	109.5	A
3005	Bn-N3-p27083185	Bn-A03-p26798652	Bn-A03-p26798652	A3	89	109.5	A
3006	Bn-N3-p27085064	Bn-A03-p26800384	Bn-A03-p26800384	A3	89	109.5	A
3007	Bn-N3-p27095429	Bn-A03-p26809706	Bn-A03-p26809706	A3	89	109.5	A
3008	Bn-N3-p27096750	Bn-A03-p26811025	Bn-A03-p26811025	A3	89	109.5	A
3009	Bn-N3-p27114764	Bn-A03-p26815896	Bn-A03-p26815896	A3	89	109.5	A
3010	Bn-N3-p27137550	Bn-A03-p26833555	Bn-A03-p26833555	A3	89	109.5	A
3011	Bn-N3-p27367099	Bn-A03-p27066000	Bn-A03-p27066000	A3	90	110.3	A
3012	Bn-N3-p27371835	Bn-A03-p27070872	Bn-A03-p27070872	A3	90	110.3	A
3013	Bn-N3-p27410908	Bn-A03-p27111541	Bn-A03-p27111541	A3	90	110.3	A
3014	Bn-N3-p27412155	Bn-A03-p27112789	Bn-A03-p27112789	A3	90	110.3	A
3015	Bn-N3-p27503944	Bn-A03-p27156983	Bn-A03-p27156983	A3	90	110.3	A
3016	Bn-N3-p27508522	Bn-A03-p27164178	Bn-A03-p27164178	A3	90	110.3	A
3017	Bn-N3-p27512703	Bn-A03-p27168311	Bn-A03-p27168311	A3	90	110.3	A
3018	Bn-N3-p27522943	Bn-A03-p27179255	Bn-A03-p27179255	A3	90	110.3	A
3019	Bn-N3-p27602495	Bn-A03-p27253035	Bn-A03-p27253035	A3	90	110.3	A
3020	Bn-N3-p27603138	Bn-A03-p27253678	Bn-A03-p27253678	A3	90	110.3	A
3021	Bn-N3-p27605270	Bn-A03-p27255790	Bn-A03-p27255790	A3	90	110.3	A
3022	Bn-N3-p27605832	Bn-A03-p27256355	Bn-A03-p27256355	A3	90	110.3	A
3023	Bn-N3-p27649606	Bn-A03-p27288521	Bn-A03-p27288521	A3	90	110.3	A
3024	Bn-N3-p27728417	Bn-A03-p27360321	Bn-A03-p27360321	A3	90	110.3	A
3025	Bn-N3-p27744103	Bn-A03-p27372281	Bn-A03-p27372281	A3	90	110.3	A
3026	Bn-N3-p27796507	Bn-A03-p27423379	Bn-A03-p27423379	A3	90	110.3	A
3027	Bn-N3-p27810899	Bn-A03-p27438081	Bn-A03-p27438081	A3	90	110.3	A
3028	Bn-N3-p27818670	Bn-A03-p27445819	Bn-A03-p27445819	A3	90	110.3	A
3029	Bn-N3-p27857151	Bn-A03-p27484868	Bn-A03-p27484868	A3	90	110.3	A
3030	Bn-N3-p27861973	Bn-A03-p27489686	Bn-A03-p27489686	A3	90	110.3	A
3031	Bn-N3-p27914811	Bn-A03-p27541115	Bn-A03-p27541115	A3	90	110.3	A
3032	Bn-N3-p27923490	Bn-A03-p27546971	Bn-A03-p27546971	A3	90	110.3	A
3033	Bn-N3-p27923776	Bn-A03-p27547270	Bn-A03-p27547270	A3	90	110.3	A

3034	Bn-N3-p27937067	Bn-C7-p45273449	Bn-scaff_16110_1-p30543(	A3	90	110.3	A
3035	Bn-N3-p27940257	Bn-A03-p27564692	Bn-A03-p27564692	A3	90	110.3	A
3036	Bn-N3-p27946080	Bn-A03-p27568225	Bn-A03-p27568225	A3	90	110.3	A
3037	Bn-N3-p27947509	Bn-A03-p27568580	Bn-A03-p27568580	A3	90	110.3	A
3038	Bn-N3-p27957944	Bn-A03-p27581773	Bn-A03-p27581773	A3	90	110.3	A
3039	Bn-N3-p27988913	Bn-A03-p27596068	Bn-A03-p27596068	A3	90	110.3	A
3040	Bn-N3-p27993022	Bn-A03-p27600547	Bn-A03-p27600547	A3	90	110.3	A
3041	Bn-N4-p15251270	Bn-A04-p13173919	Bn-A04-p13173919	A3	90	110.3	A
3042	Bn-N17-p46115361	Bn-A03-p28154970	Bn-A03-p28154970	A3	91	111.1	A
3043	Bn-N3-p28005407	Bn-A03-p27612791	Bn-A03-p27612791	A3	91	111.1	A
3044	Bn-N3-p28008656	Bn-A03-p27616297	Bn-A03-p27616297	A3	91	111.1	A
3045	Bn-N3-p28035188	Bn-A03-p27650553	Bn-A03-p27650553	A3	91	111.1	A
3046	Bn-N3-p28044630	Bn-A03-p27663888	Bn-A03-p27663888	A3	91	111.1	A
3047	Bn-N3-p28068549	Bn-A03-p27683723	Bn-A03-p27683723	A3	91	111.1	A
3048	Bn-N3-p28068732	Bn-A03-p27683903	Bn-A03-p27683903	A3	91	111.1	A
3049	Bn-N3-p28069814	Bn-A03-p27685078	Bn-A03-p27685078	A3	91	111.1	A
3050	Bn-N3-p28071097	Bn-A03-p27686257	Bn-A03-p27686257	A3	91	111.1	A
3051	Bn-N3-p28071325	Bn-A03-p27686487	Bn-A03-p27686487	A3	91	111.1	A
3052	Bn-N3-p28091486	Bn-A03-p27686879	Bn-A03-p27686879	A3	91	111.1	A
3053	Bn-N3-p28135958	Bn-A03-p27712521	Bn-A03-p27712521	A3	91	111.1	A
3054	Bn-N3-p28136742	Bn-A03-p27713307	Bn-A03-p27713307	A3	91	111.1	A
3055	Bn-N3-p28137408	Bn-A03-p27713975	Bn-A03-p27713975	A3	91	111.1	A
3056	Bn-N3-p28139016	Bn-A03-p27715593	Bn-A03-p27715593	A3	91	111.1	A
3057	Bn-N3-p28143273	Bn-A03-p27719757	Bn-A03-p27719757	A3	91	111.1	A
3058	Bn-N3-p28143561	Bn-A03-p27720045	Bn-A03-p27720045	A3	91	111.1	A
3059	Bn-N3-p28157816	Bn-A03-p27741110	Bn-A03-p27741110	A3	91	111.1	A
3060	Bn-N3-p28159664	Bn-A03-p27742950	Bn-A03-p27742950	A3	91	111.1	A
3061	Bn-N3-p28163911	Bn-A03-p27746955	Bn-A03-p27746955	A3	91	111.1	A
3062	Bn-N3-p28164136	Bn-A03-p27747178	Bn-A03-p27747178	A3	91	111.1	A
3063	Bn-N3-p28247743	Bn-A03-p27838177	Bn-A03-p27838177	A3	91	111.1	A
3064	Bn-N3-p28262385	Bn-A03-p27853312	Bn-A03-p27853312	A3	91	111.1	A
3065	Bn-N3-p28341690	Bn-A03-p27906760	Bn-A03-p27906760	A3	91	111.1	A
3066	Bn-N3-p28376955	Bn-C7-p45606248	Bn-scaff_16110_1-p27215(	A3	91	111.1	A
3067	Bn-N3-p28380203	Bn-A03-p27951979	Bn-A03-p27951979	A3	91	111.1	A
3068	Bn-N3-p28469456	Bn-A03-p28039749	Bn-A03-p28039749	A3	91	111.1	A
3069	Bn-N3-p28478610	Bn-A03-p28043216	Bn-A03-p28043216	A3	91	111.1	A
3070	Bn-N3-p28480905	Bn-A03-p28046101	Bn-A03-p28046101	A3	91	111.1	A
3071	Bn-N3-p28530811	Bn-A03-p28091909	Bn-A03-p28091909	A3	91	111.1	A
3072	Bn-N3-p28549660	Bn-A03-p28109103	Bn-A03-p28109103	A3	91	111.1	A
3073	Bn-N3-p28558388	Bn-A03-p28117836	Bn-A03-p28117836	A3	91	111.1	A
3074	Bn-N3-p28574150	Bn-A03-p28153736	Bn-A03-p28153736	A3	91	111.1	A
3075	Bn-N3-p28574211	Bn-A03-p28153797	Bn-A03-p28153797	A3	91	111.1	A
3076	Bn-N3-p28574467	Bn-A03-p28154053	Bn-A03-p28154053	A3	91	111.1	A
3077	Bn-N3-p28575244	Bn-A03-p28154830	Bn-A03-p28154830	A3	91	111.1	A
3078	Bn-N3-p28609414	Bn-A03-p28184972	Bn-A03-p28184972	A3	91	111.1	A
3079	Bn-N3-p28636899	Bn-A03-p28207594	Bn-A03-p28207594	A3	91	111.1	A
3080	Bn-N3-p28656504	Bn-A03-p28227026	Bn-A03-p28227026	A3	91	111.1	A
3081	Bn-N3-p28658838	Bn-A03-p28229360	Bn-A03-p28229360	A3	91	111.1	A
3082	Bn-N3-p28662679	Bn-A03-p28233201	Bn-A03-p28233201	A3	91	111.1	A

3083	Bn-N3-p28663179	Bn-A03-p28233701	Bn-A03-p28233701	A3	91	111.1	A
3084	Bn-N3-p28663645	Bn-A03-p28234166	Bn-A03-p28234166	A3	91	111.1	A
3085	Bn-N3-p28692893	Bn-A03-p28256169	Bn-A03-p28256169	A3	91	111.1	A
3086	Bn-N3-p28724592	Bn-A03-p28279796	Bn-A03-p28279796	A3	91	111.1	A
3087	Bn-N17-p46855285	Bn-C7-p46436238	Bn-scaff_16110_1-p18915[illegible]	A3	92	111.9	A
3088	Bn-N3-p28916350	Bn-A03-p28491294	Bn-A03-p28491294	A3	92	111.9	A
3089	Bn-N3-p28971341	Bn-A03-p28535638	Bn-A03-p28535638	A3	92	111.9	A
3090	Bn-N3-p28984296	Bn-A03-p28541411	Bn-A03-p28541411	A3	92	111.9	A
3091	Bn-N3-p29254604	Bn-A03-p28808705	Bn-A03-p28808705	A3	92	111.9	A
3092	Bn-N3-p29315535	Bn-A03-p28878698	Bn-A03-p28878698	A3	92	111.9	A
3093	Bn-N3-p29315540	Bn-A03-p28878703	Bn-A03-p28878703	A3	92	111.9	A
3094	Bn-N3-p29316444	Bn-A03-p28879610	Bn-A03-p28879610	A3	92	111.9	A
3095	Bn-N3-p29404545	Bn-A03-p28977058	Bn-A03-p28977058	A3	92	111.9	A
3096	Bn-N3-p29408925	Bn-A03-p28981296	Bn-A03-p28981296	A3	92	111.9	A
3097	Bn-N3-p29438003	Bn-A03-p29002618	Bn-A03-p29002618	A3	92	111.9	A
3098	Bn-N3-p29446118	Bn-A03-p29009401	Bn-A03-p29009401	A3	92	111.9	A
3099	Bn-N3-p29475909	Bn-A03-p29046284	Bn-A03-p29046284	A3	92	111.9	A
3100	Bn-N3-p29679770	Bn-A03-p29198982	Bn-A03-p29198982	A3	92	111.9	A
3101	Bn-N3-p29696617	Bn-A03-p29216506	Bn-A03-p29216506	A3	92	111.9	A
3102	Bn-N3-p29696722	Bn-A03-p29216612	Bn-A03-p29216612	A3	92	111.9	A
3103	Bn-N3-p29715620	Bn-A03-p29264946	Bn-A03-p29264946	A3	92	111.9	A
3104	Bn-N3-p29720055	Bn-A03-p29269385	Bn-A03-p29269385	A3	92	111.9	A
3105	Bn-N3-p29751828	Bn-A03-p29234677	Bn-A03-p29234677	A3	92	111.9	A
3106	Bn-N3-p29796029	Bn-A03-p29335920	Bn-A03-p29335920	A3	92	111.9	A
3107	Bn-N3-p29824338	Bn-A03-p29364596	Bn-A03-p29364596	A3	92	111.9	A
3108	Bn-N3-p30604879	Bn-A03-p30183760	Bn-A03-p30183760	A3	92	111.9	A
3109	Bn-N3-p31683892	Bn-A03-p31049094	Bn-A03-p31049094	A3	92	111.9	A
3110	Bn-Scaffold12474-p782	Bn-A03-p28910430	Bn-A03-p28910430	A3	92	111.9	A
3111	Bn-N14-p20375333	Bn-Scaffold00842-p3[illegible]	Bn-scaff_28703_1-p34884	A3	93	112.7	A
3112	Bn-N17-p47469311	Bn-C7-p46998691	Bn-scaff_16110_1-p13291[illegible]	A3	93	112.7	A
3113	Bn-N3-p30007309	Bn-A03-p29564970	Bn-A03-p29564970	A3	93	112.7	A
3114	Bn-N3-p30007426	Bn-A03-p29565087	Bn-A03-p29565087	A3	93	112.7	A
3115	Bn-N3-p30016471	Bn-A03-p29573204	Bn-A03-p29573204	A3	93	112.7	A
3116	Bn-N3-p30022628	Bn-A03-p29583640	Bn-A03-p29583640	A3	93	112.7	A
3117	Bn-N3-p30026567	Bn-A03-p29587388	Bn-A03-p29587388	A3	93	112.7	A
3118	Bn-N3-p30027124	Bn-A03-p29587945	Bn-A03-p29587945	A3	93	112.7	A
3119	Bn-N3-p30028438	Bn-A03-p29590349	Bn-A03-p29590349	A3	93	112.7	A
3120	Bn-N3-p30118965	Bn-A03-p29656801	Bn-A03-p29656801	A3	93	112.7	A
3121	Bn-N3-p30123893	Bn-A03-p29661431	Bn-A03-p29661431	A3	93	112.7	A
3122	Bn-N3-p30486960	Bn-A03-p30052524	Bn-A03-p30052524	A3	93	112.7	A
3123	Bn-N3-p30585268	Bn-A03-p30170124	Bn-A03-p30170124	A3	93	112.7	A
3124	Bn-N3-p30605056	Bn-A03-p30183937	Bn-A03-p30183937	A3	93	112.7	A
3125	Bn-N3-p30665384	Bn-A03-p30243799	Bn-A03-p30243799	A3	93	112.7	A
3126	Bn-N3-p30731942	Bn-A03-p30291719	Bn-A03-p30291719	A3	93	112.7	A
3127	Bn-N3-p31074167	Bn-A03-p30564852	Bn-A03-p30564852	A3	93	112.7	A
3128	Bn-N3-p31198615	Bn-A03-p30658785	Bn-A03-p30658785	A3	93	112.7	A
3129	Bn-N3-p31207684	Bn-A03-p30667694	Bn-A03-p30667694	A3	93	112.7	A
3130	Bn-N3-p31246596	Bn-A03-p30693412	Bn-A03-p30693412	A3	93	112.7	A
3131	Bn-N3-p31375211	Bn-A03-p30831188	Bn-A03-p30831188	A3	93	112.7	A

3132	Bn-N3-p31433263	Bn-A03-p30876909	Bn-A03-p30876909	A3	93	112.7	A
3133	Bn-N3-p31436223	Bn-A03-p30879004	Bn-A03-p30879004	A3	93	112.7	A
3134	Bn-N3-p31437667	Bn-A03-p30879164	Bn-A03-p30879164	A3	93	112.7	A
3135	Bn-N3-p31446102	Bn-A03-p30888263	Bn-A03-p30888263	A3	93	112.7	A
3136	Bn-N3-p31523807	Bn-A03-p30951542	Bn-A03-p30951542	A3	93	112.7	A
3137	Bn-N3-p31643527	Bn-C1-p8024658	Bn-scaff_17827_1-p49625	A3	93	112.7	A
3138	Bn-N3-p31661304	Bn-A03-p31030338	Bn-A03-p31030338	A3	93	112.7	A
3139	Bn-N3-p31714734	Bn-A03-p31077034	Bn-A03-p31077034	A3	93	112.7	A
3140	Bn-N3-p31728620	Bn-A03-p31091148	Bn-A03-p31091148	A3	93	112.7	A
3141	Bn-N3-p31733173	Bn-A03-p31095221	Bn-A03-p31095221	A3	93	112.7	A
3142	Bn-N3-p31751578	Bn-A03-p31113968	Bn-A03-p31113968	A3	93	112.7	A
3143	Bn-N3-p32159871	Bn-A03-p31579593	Bn-A03-p31579593	A3	93	112.7	A
3144	Bn-N3-p32213844	Bn-A03-p31147953	Bn-A03-p31147953	A3	93	112.7	A
3145	Bn-N3-p32823407	Bn-Scaffold000157-p	Bn-Scaffold000157-p91759	A3	93	112.7	A
3146	Bn-N3-p33233203	Bn-Scaffold000162-p	Bn-Scaffold000162-p32052	A3	93	112.7	A
3147	Bn-N3-p33241744	Bn-Scaffold000162-p	Bn-Scaffold000162-p32093	A3	93	112.7	A
3148	Bn-N3-p33255811	Bn-Scaffold000096-p	Bn-Scaffold000096-p92871	A3	93	112.7	A
3149	Bn-N3-p33346228	Bn-Scaffold000096-p	Bn-Scaffold000096-p87840	A3	93	112.7	A
3150	Bn-N3-p33349256	Bn-Scaffold000096-p	Bn-Scaffold000096-p85899	A3	93	112.7	A
3151	Bn-N3-p33357021	Bn-Scaffold000096-p	Bn-Scaffold000096-p85721	A3	93	112.7	0
3152	Bn-N3-p33411210	Bn-C6-p13224543	Bn-scaff_16064_1-p14684	A3	93	112.7	A
3153	Bn-N3-p33597563	Bn-Scaffold000096-p	Bn-Scaffold000096-p76330	A3	93	112.7	A
3154	Bn-N3-p33597953	Bn-Scaffold000096-p	Bn-Scaffold000096-p76291	A3	93	112.7	A
3155	Bn-N3-p33598434	Bn-Scaffold000096-p	Bn-Scaffold000096-p76242	A3	93	112.7	A
3156	Bn-N3-p33635049	Bn-Scaffold000096-p	Bn-Scaffold000096-p71836	A3	93	112.7	A
3157	Bn-N3-p33636493	Bn-Scaffold000096-p	Bn-Scaffold000096-p71691	A3	93	112.7	A
3158	Bn-N3-p33636820	Bn-Scaffold000096-p	Bn-Scaffold000096-p71659	A3	93	112.7	A
3159	Bn-N3-p33639132	Bn-Scaffold000096-p	Bn-Scaffold000096-p70674	A3	93	112.7	A
3160	Bn-N3-p33639500	Bn-Scaffold000096-p	Bn-Scaffold000096-p70637	A3	93	112.7	A
3161	Bn-N3-p33712271	Bn-Scaffold000096-p	Bn-Scaffold000096-p63128	A3	93	112.7	A
3162	Bn-N3-p33731872	Bn-Scaffold000096-p	Bn-Scaffold000096-p58813	A3	93	112.7	A
3163	Bn-N3-p33742588	Bn-Scaffold000096-p	Bn-Scaffold000096-p57756	A3	93	112.7	A
3164	Bn-N3-p33747273	Bn-Scaffold000096-p	Bn-Scaffold000096-p56186	A3	93	112.7	A
3165	Bn-N3-p33748455	Bn-Scaffold000096-p	Bn-Scaffold000096-p56068	A3	93	112.7	A
3166	Bn-N3-p33760051	Bn-Scaffold000096-p	Bn-Scaffold000096-p54070	A3	93	112.7	A
3167	Bn-N3-p33761597	Bn-Scaffold000096-p	Bn-Scaffold000096-p53930	A3	93	112.7	A
3168	Bn-N3-p33767428	Bn-Scaffold000096-p	Bn-Scaffold000096-p53338	A3	93	112.7	A
3169	Bn-N3-p33767642	Bn-Scaffold000096-p	Bn-Scaffold000096-p53317	A3	93	112.7	A
3170	Bn-N3-p33767785	Bn-Scaffold000096-p	Bn-Scaffold000096-p53303	A3	93	112.7	A
3171	Bn-N3-p33772313	Bn-Scaffold000096-p	Bn-Scaffold000096-p51880	A3	93	112.7	A
3172	Bn-N3-p33775293	Bn-Scaffold000096-p	Bn-Scaffold000096-p51525	A3	93	112.7	A
3173	Bn-N3-p33779936	Bn-Scaffold000096-p	Bn-Scaffold000096-p51060	A3	93	112.7	A
3174	Bn-N3-p33787798	Bn-Scaffold000096-p	Bn-Scaffold000096-p50329	A3	93	112.7	A
3175	Bn-N3-p33810490	Bn-C4-p22743714	Bn-scaff_15911_1-p53948	A3	93	112.7	A
3176	Bn-N3-p34205085	Bn-Scaffold000096-p	Bn-Scaffold000096-p89560	A3	93	112.7	A
3177	Bn-N3-p34262739	Bn-Scaffold000096-p	Bn-Scaffold000096-p23421	A3	93	112.7	A
3178	Bn-N3-p34720786	Bn-A01-p22265175	Bn-A01-p22265175	A3	93	112.7	A
3179	Bn-N3-p34977114	Bn-Scaffold000149-p	Bn-Scaffold000149-p32290	A3	93	112.7	A
3180	Bn-N3-p35058408	Bn-Scaffold000149-p	Bn-Scaffold000149-p26978	A3	93	112.7	A

3181	Bn-N3-p35200352	Bn-C13743529-p205	Bn-C13743529-p205	A3	93	112.7	A
3182	Bn-N3-p35267553	Bn-Scaffold000149-p1	Bn-Scaffold000149-p13107	A3	93	112.7	A
3183	Bn-N3-p35449707	Bn-Scaffold000162-p1	Bn-Scaffold000162-p16932	A3	93	112.7	A
3184	Bn-N3-p35550017	Bn-Scaffold000209-p6	Bn-Scaffold000209-p62055	A3	93	112.7	A
3185	Bn-N3-p35607330	Bn-Scaffold000220-p2	Bn-Scaffold000220-p29775	A3	93	112.7	A
3186	Bn-Scaffold02077-p2679	Bn-A03-p31088264	Bn-A03-p31088264	A3	93	112.7	A
3187	Bn-Scaffold02077-p2883	Bn-A03-p31088060	Bn-A03-p31088060	A3	93	112.7	A
3188	Bn-Scaffold02570-p2976	Bn-C4-p38713160	Bn-scaff_17525_1-p302132	A3	93	112.7	A
3189	Bn-N3-p28566839	Bn-A03-p28126287	Bn-A03-p28126287	A3	94	114.3	A
3190	Bn-N4-p193375	Bn-A04-p122280	Bn-A04-p122280	A4	1	0	A
3191	Bn-N4-p204073	Bn-A04-p113641	Bn-A04-p113641	A4	1	0	A
3192	Bn-N4-p239356	Bn-A04-p233588	Bn-A04-p233588	A4	1	0	A
3193	Bn-N4-p240436	Bn-A04-p234664	Bn-A04-p234664	A4	1	0	A
3194	Bn-N4-p249828	Bn-A04-p248884	Bn-A04-p248884	A4	1	0	A
3195	Bn-N4-p252312	Bn-A04-p251383	Bn-A04-p251383	A4	1	0	A
3196	Bn-N4-p263953	Bn-A04-p261340	Bn-A04-p261340	A4	1	0	A
3197	Bn-N14-p25636843	Bn-C4-p24627268	Bn-scaff_16217_1-p419736	A4	2	0.8	A
3198	Bn-N14-p26057912	Bn-A04-p534430	Bn-A04-p534430	A4	2	0.8	A
3199	Bn-N14-p26252095	Bn-C4-p25437626	Bn-scaff_16714_1-p252822	A4	2	0.8	A
3200	Bn-N4-p269934	Bn-A04-p267294	Bn-A04-p267294	A4	2	0.8	A
3201	Bn-N4-p298174	Bn-A04-p288906	Bn-A04-p288906	A4	2	0.8	A
3202	Bn-N4-p299261	Bn-A04-p289974	Bn-A04-p289974	A4	2	0.8	A
3203	Bn-N4-p327652	Bn-A04-p312324	Bn-A04-p312324	A4	2	0.8	A
3204	Bn-N4-p328419	Bn-A04-p313091	Bn-A04-p313091	A4	2	0.8	A
3205	Bn-N4-p328738	Bn-A04-p313410	Bn-A04-p313410	A4	2	0.8	A
3206	Bn-N4-p329768	Bn-A04-p314440	Bn-A04-p314440	A4	2	0.8	A
3207	Bn-N4-p334926	Bn-A04-p320972	Bn-A04-p320972	A4	2	0.8	A
3208	Bn-N4-p336773	Bn-A04-p322820	Bn-A04-p322820	A4	2	0.8	A
3209	Bn-N4-p338158	Bn-A04-p324144	Bn-A04-p324144	A4	2	0.8	A
3210	Bn-N4-p339717	Bn-A04-p326007	Bn-A04-p326007	A4	2	0.8	A
3211	Bn-N4-p367121	Bn-A04-p354688	Bn-A04-p354688	A4	2	0.8	A
3212	Bn-N4-p369005	Bn-A04-p356570	Bn-A04-p356570	A4	2	0.8	A
3213	Bn-N4-p369707	Bn-A04-p357272	Bn-A04-p357272	A4	2	0.8	A
3214	Bn-N4-p398455	Bn-A04-p398807	Bn-A04-p398807	A4	2	0.8	A
3215	Bn-N4-p404783	Bn-A04-p405060	Bn-A04-p405060	A4	2	0.8	A
3216	Bn-N4-p534732	Bn-A04-p534845	Bn-A04-p534845	A4	2	0.8	A
3217	Bn-N4-p537891	Bn-A04-p541336	Bn-A04-p541336	A4	2	0.8	A
3218	Bn-N4-p575467	Bn-A04-p582626	Bn-A04-p582626	A4	2	0.8	A
3219	Bn-N4-p575660	Bn-A04-p582979	Bn-A04-p582979	A4	2	0.8	A
3220	Bn-N4-p575717	Bn-A04-p583036	Bn-A04-p583036	A4	2	0.8	A
3221	Bn-N4-p575923	Bn-A04-p583242	Bn-A04-p583242	A4	2	0.8	A
3222	Bn-N4-p575935	Bn-A04-p583254	Bn-A04-p583254	A4	2	0.8	A
3223	Bn-N4-p608275	Bn-A04-p597296	Bn-A04-p597296	A4	2	0.8	A
3224	Bn-N4-p610824	Bn-A04-p599845	Bn-A04-p599845	A4	2	0.8	A
3225	Bn-N4-p611202	Bn-A04-p600223	Bn-A04-p600223	A4	2	0.8	A
3226	Bn-N4-p618611	Bn-C4-p25346911	Bn-scaff_16714_1-p343537	A4	2	0.8	A
3227	Bn-N4-p627886	Bn-A04-p619001	Bn-A04-p619001	A4	2	0.8	A
3228	Bn-N4-p653373	Bn-A04-p641998	Bn-A04-p641998	A4	2	0.8	A
3229	Bn-N4-p655223	Bn-A04-p643876	Bn-A04-p643876	A4	2	0.8	A

3230	Bn-N4-p657044	Bn-A04-p645702	Bn-A04-p645702	A4	2	0.8	A
3231	Bn-N4-p671810	Bn-A04-p665932	Bn-A04-p665932	A4	2	0.8	A
3232	Bn-N4-p674606	Bn-A04-p668378	Bn-A04-p668378	A4	2	0.8	A
3233	Bn-N4-p730447	Bn-A04-p726356	Bn-A04-p726356	A4	2	0.8	A
3234	Bn-N14-p26646935	Bn-C4-p25744913	Bn-scaff_18656_1-p54366	A4	3	3.2	A
3235	Bn-N4-p1078166	Bn-A04-p1145007	Bn-A04-p1145007	A4	3	3.2	A
3236	Bn-N4-p1083852	Bn-A04-p1156390	Bn-A04-p1156390	A4	3	3.2	A
3237	Bn-N4-p1095696	Bn-A04-p1161563	Bn-A04-p1161563	A4	3	3.2	A
3238	Bn-N4-p1110879	Bn-A04-p1176453	Bn-A04-p1176453	A4	3	3.2	A
3239	Bn-N4-p748522	Bn-A04-p745024	Bn-A04-p745024	A4	3	3.2	A
3240	Bn-N4-p756721	Bn-A04-p757668	Bn-A04-p757668	A4	3	3.2	A
3241	Bn-N4-p759440	Bn-A04-p761383	Bn-A04-p761383	A4	3	3.2	A
3242	Bn-N4-p781449	Bn-A04-p783339	Bn-A04-p783339	A4	3	3.2	A
3243	Bn-N4-p782818	Bn-A04-p784708	Bn-A04-p784708	A4	3	3.2	A
3244	Bn-N4-p783164	Bn-C4-p25743594	Bn-scaff_18656_1-p53047	A4	3	3.2	A
3245	Bn-N4-p783742	Bn-A04-p785628	Bn-A04-p785628	A4	3	3.2	A
3246	Bn-N4-p784701	Bn-A04-p786588	Bn-A04-p786588	A4	3	3.2	A
3247	Bn-N4-p790541	Bn-A04-p792399	Bn-A04-p792399	A4	3	3.2	A
3248	Bn-N4-p801225	Bn-A04-p803315	Bn-A04-p803315	A4	3	3.2	A
3249	Bn-N4-p806747	Bn-A04-p808824	Bn-A04-p808824	A4	3	3.2	A
3250	Bn-N4-p806757	Bn-A04-p808834	Bn-A04-p808834	A4	3	3.2	A
3251	Bn-N4-p810348	Bn-A04-p809984	Bn-A04-p809984	A4	3	3.2	A
3252	Bn-N4-p810403	Bn-A04-p810039	Bn-A04-p810039	A4	3	3.2	A
3253	Bn-N4-p810530	Bn-A04-p810166	Bn-A04-p810166	A4	3	3.2	A
3254	Bn-N4-p811294	Bn-A04-p810935	Bn-A04-p810935	A4	3	3.2	A
3255	Bn-N4-p813484	Bn-A04-p813173	Bn-A04-p813173	A4	3	3.2	A
3256	Bn-N4-p814343	Bn-A04-p814033	Bn-A04-p814033	A4	3	3.2	A
3257	Bn-N4-p830188	Bn-A04-p831099	Bn-A04-p831099	A4	3	3.2	A
3258	Bn-N4-p855702	Bn-A04-p860212	Bn-A04-p860212	A4	3	3.2	A
3259	Bn-N4-p859905	Bn-A04-p865058	Bn-A04-p865058	A4	3	3.2	A
3260	Bn-N4-p864354	Bn-A04-p869491	Bn-A04-p869491	A4	3	3.2	A
3261	Bn-N4-p865228	Bn-A04-p870365	Bn-A04-p870365	A4	3	3.2	A
3262	Bn-N4-p869997	Bn-A04-p874008	Bn-A04-p874008	A4	3	3.2	A
3263	Bn-N4-p915890	Bn-A04-p926916	Bn-A04-p926916	A4	3	3.2	A
3264	Bn-N4-p923006	Bn-A04-p934150	Bn-A04-p934150	A4	3	3.2	A
3265	Bn-N4-p965926	Bn-A04-p1032336	Bn-A04-p1032336	A4	3	3.2	A
3266	Bn-N4-p975963	Bn-A04-p1042273	Bn-A04-p1042273	A4	3	3.2	A
3267	Bn-N4-p976801	Bn-A04-p1043111	Bn-A04-p1043111	A4	3	3.2	A
3268	Bn-N4-p982842	Bn-A04-p1048288	Bn-A04-p1048288	A4	3	3.2	A
3269	Bn-N4-p1148070	Bn-A04-p1213196	Bn-A04-p1213196	A4	4	4	A
3270	Bn-N4-p1164595	Bn-A04-p1231047	Bn-A04-p1231047	A4	4	4	A
3271	Bn-N4-p1164675	Bn-A04-p1231127	Bn-A04-p1231127	A4	4	4	A
3272	Bn-N4-p1171078	Bn-A04-p1240196	Bn-A04-p1240196	A4	4	4	A
3273	Bn-N4-p1214158	Bn-A04-p1274596	Bn-A04-p1274596	A4	4	4	A
3274	Bn-N4-p1215871	Bn-A04-p1276308	Bn-A04-p1276308	A4	4	4	A
3275	Bn-N4-p1223168	Bn-A04-p1282528	Bn-A04-p1282528	A4	4	4	A
3276	Bn-N4-p1237518	Bn-Scaffold000993-p1	Bn-Scaffold000993-p187	A4	4	4	A
3277	Bn-N4-p1252454	Bn-A04-p1317446	Bn-A04-p1317446	A4	5	4.8	A
3278	Bn-N4-p1253627	Bn-A04-p1318613	Bn-A04-p1318613	A4	5	4.8	A

3279	Bn-N4-p1274801	Bn-A04-p1346738	Bn-A04-p1346738	A4	5	4.8	A
3280	Bn-N4-p1277334	Bn-A04-p1349255	Bn-A04-p1349255	A4	5	4.8	A
3281	Bn-N4-p1279432	Bn-A04-p1351337	Bn-A04-p1351337	A4	5	4.8	A
3282	Bn-N4-p1305593	Bn-A04-p1377226	Bn-A04-p1377226	A4	6	5.6	A
3283	Bn-N4-p1324713	Bn-A04-p1390449	Bn-A04-p1390449	A4	6	5.6	A
3284	Bn-N4-p1354082	Bn-A04-p1416287	Bn-A04-p1416287	A4	6	5.6	A
3285	Bn-N4-p1355252	Bn-A04-p1417481	Bn-A04-p1417481	A4	6	5.6	A
3286	Bn-N4-p1370880	Bn-A04-p1429850	Bn-A04-p1429850	A4	6	5.6	A
3287	Bn-N4-p1392198	Bn-A04-p1454434	Bn-A04-p1454434	A4	7	6.4	A
3288	Bn-N4-p1567651	Bn-A04-p1623151	Bn-A04-p1623151	A4	7	6.4	A
3289	Bn-N4-p1589334	Bn-A04-p1655416	Bn-A04-p1655416	A4	8	7.2	A
3290	Bn-N4-p1607740	Bn-A04-p1680506	Bn-A04-p1680506	A4	9	8.8	A
3291	Bn-N4-p1686812	Bn-A04-p1745832	Bn-A04-p1745832	A4	10	9.6	A
3292	Bn-N4-p1700455	Bn-A04-p1759257	Bn-A04-p1759257	A4	10	9.6	A
3293	Bn-N4-p1706479	Bn-A04-p1764916	Bn-A04-p1764916	A4	10	9.6	A
3294	Bn-N4-p1722642	Bn-A04-p1774031	Bn-A04-p1774031	A4	10	9.6	A
3295	Bn-N4-p1723549	Bn-A04-p1774926	Bn-A04-p1774926	A4	10	9.6	A
3296	Bn-N4-p1723774	Bn-A04-p1775155	Bn-A04-p1775155	A4	10	9.6	A
3297	Bn-N4-p1753561	Bn-A04-p1815404	Bn-A04-p1815404	A4	10	9.6	A
3298	Bn-N4-p1777273	Bn-A04-p1827230	Bn-A04-p1827230	A4	10	9.6	A
3299	Bn-N4-p1787089	Bn-A04-p1837535	Bn-A04-p1837535	A4	10	9.6	A
3300	Bn-N4-p1807140	Bn-A04-p1867423	Bn-A04-p1867423	A4	10	9.6	A
3301	Bn-N4-p1821292	Bn-A04-p1869508	Bn-A04-p1869508	A4	10	9.6	A
3302	Bn-N4-p1836032	Bn-A04-p1880654	Bn-A04-p1880654	A4	10	9.6	A
3303	Bn-N4-p1837609	Bn-A04-p1882240	Bn-A04-p1882240	A4	10	9.6	A
3304	Bn-N14-p29487619	Bn-A04-p1950544	Bn-A04-p1950544	A4	11	10.4	A
3305	Bn-N14-p29795870	Bn-C4-p28706375	Bn-scaff_26391_1-p192467	A4	11	10.4	A
3306	Bn-N14-p30466686	Bn-C4-p29261678	Bn-scaff_17180_1-p153332	A4	11	10.4	A
3307	Bn-N14-p31134719	Bn-A04-p2841305	Bn-A04-p2841305	A4	11	10.4	A
3308	Bn-N14-p31538783	Bn-C4-p30448934	Bn-scaff_16128_1-p115117	A4	11	10.4	A
3309	Bn-N4-p1847854	Bn-A04-p1893968	Bn-A04-p1893968	A4	11	10.4	A
3310	Bn-N4-p1852249	Bn-A04-p1897541	Bn-A04-p1897541	A4	11	10.4	A
3311	Bn-N4-p1855279	Bn-A04-p1900422	Bn-A04-p1900422	A4	11	10.4	A
3312	Bn-N4-p1868652	Bn-A04-p1913537	Bn-A04-p1913537	A4	11	10.4	A
3313	Bn-N4-p1871339	Bn-A04-p1931502	Bn-A04-p1931502	A4	11	10.4	A
3314	Bn-N4-p1882542	Bn-A04-p1927138	Bn-A04-p1927138	A4	11	10.4	A
3315	Bn-N4-p1883711	Bn-A04-p1928290	Bn-A04-p1928290	A4	11	10.4	A
3316	Bn-N4-p1892856	Bn-A04-p2330084	Bn-A04-p2330084	A4	11	10.4	A
3317	Bn-N4-p2016910	Bn-A04-p2191612	Bn-A04-p2191612	A4	11	10.4	A
3318	Bn-N4-p2030254	Bn-A04-p2178146	Bn-A04-p2178146	A4	11	10.4	A
3319	Bn-N4-p2053034	Bn-A04-p2152528	Bn-A04-p2152528	A4	11	10.4	A
3320	Bn-N4-p2056282	Bn-A04-p2149214	Bn-A04-p2149214	A4	11	10.4	A
3321	Bn-N4-p2059248	Bn-A04-p2146459	Bn-A04-p2146459	A4	11	10.4	A
3322	Bn-N4-p2063013	Bn-A04-p2137317	Bn-A04-p2137317	A4	11	10.4	A
3323	Bn-N4-p2065538	Bn-A04-p2135204	Bn-A04-p2135204	A4	11	10.4	A
3324	Bn-N4-p2068901	Bn-A04-p2126294	Bn-A04-p2126294	A4	11	10.4	A
3325	Bn-N4-p2068964	Bn-A04-p2126232	Bn-A04-p2126232	A4	11	10.4	A
3326	Bn-N4-p2103753	Bn-A04-p2094818	Bn-A04-p2094818	A4	11	10.4	A
3327	Bn-N4-p2135748	Bn-C4-p28606425	Bn-scaff_26391_1-p292417	A4	11	10.4	A

3328	Bn-N4-p2149677	Bn-A04-p2048587	Bn-A04-p2048587	A4	11	10.4	A
3329	Bn-N4-p2172423	Bn-A04-p2029030	Bn-A04-p2029030	A4	11	10.4	A
3330	Bn-N4-p2187255	Bn-A04-p2010971	Bn-A04-p2010971	A4	11	10.4	A
3331	Bn-N4-p2188750	Bn-A04-p2009476	Bn-A04-p2009476	A4	11	10.4	A
3332	Bn-N4-p2198222	Bn-A04-p2000268	Bn-A04-p2000268	A4	11	10.4	A
3333	Bn-N4-p2205665	Bn-A04-p1993598	Bn-A04-p1993598	A4	11	10.4	A
3334	Bn-N4-p2207545	Bn-A04-p1991843	Bn-A04-p1991843	A4	11	10.4	A
3335	Bn-N4-p2224803	Bn-A04-p1971842	Bn-A04-p1971842	A4	11	10.4	A
3336	Bn-N4-p2230820	Bn-A04-p1962888	Bn-A04-p1962888	A4	11	10.4	A
3337	Bn-N4-p2246771	Bn-A04-p1948034	Bn-A04-p1948034	A4	11	10.4	A
3338	Bn-N4-p2251056	Bn-A04-p1943749	Bn-A04-p1943749	A4	11	10.4	A
3339	Bn-N4-p2253675	Bn-A04-p1941188	Bn-A04-p1941188	A4	11	10.4	A
3340	Bn-N4-p2256529	Bn-A04-p1938332	Bn-A04-p1938332	A4	11	10.4	A
3341	Bn-N4-p2259968	Bn-A04-p1935147	Bn-A04-p1935147	A4	11	10.4	A
3342	Bn-N4-p2262439	Bn-A04-p1932352	Bn-A04-p1932352	A4	11	10.4	A
3343	Bn-N4-p2262491	Bn-A04-p1932300	Bn-A04-p1932300	A4	11	10.4	A
3344	Bn-N4-p2294513	Bn-A04-p2367374	Bn-A04-p2367374	A4	11	10.4	A
3345	Bn-N4-p2332536	Bn-A04-p2429853	Bn-A04-p2429853	A4	11	10.4	A
3346	Bn-N4-p2334217	Bn-A04-p2434674	Bn-A04-p2434674	A4	11	10.4	A
3347	Bn-N4-p2340231	Bn-A04-p2439163	Bn-A04-p2439163	A4	11	10.4	A
3348	Bn-N4-p2347721	Bn-A04-p2448580	Bn-A04-p2448580	A4	11	10.4	A
3349	Bn-N4-p2373181	Bn-A04-p2475793	Bn-A04-p2475793	A4	11	10.4	A
3350	Bn-N4-p2415593	Bn-A04-p2498254	Bn-A04-p2498254	A4	11	10.4	A
3351	Bn-N4-p2460369	Bn-A04-p2537756	Bn-A04-p2537756	A4	11	10.4	A
3352	Bn-N4-p2464595	Bn-A04-p2542000	Bn-A04-p2542000	A4	11	10.4	A
3353	Bn-N4-p2479855	Bn-A04-p2562005	Bn-A04-p2562005	A4	11	10.4	A
3354	Bn-N4-p2482817	Bn-A04-p2566546	Bn-A04-p2566546	A4	11	10.4	A
3355	Bn-N4-p2484663	Bn-A04-p2568394	Bn-A04-p2568394	A4	11	10.4	A
3356	Bn-N4-p2505134	Bn-A04-p2592215	Bn-A04-p2592215	A4	11	10.4	A
3357	Bn-N4-p2506550	Bn-A04-p2593658	Bn-A04-p2593658	A4	11	10.4	A
3358	Bn-N4-p2528793	Bn-A04-p2616582	Bn-A04-p2616582	A4	11	10.4	A
3359	Bn-N4-p2562922	Bn-A04-p2648823	Bn-A04-p2648823	A4	11	10.4	A
3360	Bn-N4-p2564526	Bn-A04-p2650427	Bn-A04-p2650427	A4	11	10.4	A
3361	Bn-N4-p2565270	Bn-A04-p2651171	Bn-A04-p2651171	A4	11	10.4	A
3362	Bn-N4-p2571679	Bn-A04-p2658023	Bn-A04-p2658023	A4	11	10.4	A
3363	Bn-N4-p2574324	Bn-A04-p2660465	Bn-A04-p2660465	A4	11	10.4	A
3364	Bn-N4-p2577983	Bn-A04-p2664124	Bn-A04-p2664124	A4	11	10.4	A
3365	Bn-N4-p2579461	Bn-A04-p2665421	Bn-A04-p2665421	A4	11	10.4	A
3366	Bn-N4-p2582248	Bn-A04-p2668053	Bn-A04-p2668053	A4	11	10.4	A
3367	Bn-N4-p2593065	Bn-A04-p2680732	Bn-A04-p2680732	A4	11	10.4	A
3368	Bn-N4-p2604056	Bn-A04-p2689124	Bn-A04-p2689124	A4	11	10.4	A
3369	Bn-N4-p2623471	Bn-A04-p2713517	Bn-A04-p2713517	A4	11	10.4	A
3370	Bn-N4-p2641392	Bn-A04-p2730690	Bn-A04-p2730690	A4	11	10.4	A
3371	Bn-N4-p2658421	Bn-A04-p2750401	Bn-A04-p2750401	A4	11	10.4	A
3372	Bn-N4-p2659118	Bn-A04-p2751097	Bn-A04-p2751097	A4	11	10.4	A
3373	Bn-N4-p2661812	Bn-A04-p2753796	Bn-A04-p2753796	A4	11	10.4	A
3374	Bn-N4-p2666235	Bn-A04-p2760477	Bn-A04-p2760477	A4	11	10.4	A
3375	Bn-N4-p2666824	Bn-A04-p2761067	Bn-A04-p2761067	A4	11	10.4	A
3376	Bn-N4-p2668661	Bn-A04-p2762891	Bn-A04-p2762891	A4	11	10.4	A

3377	Bn-N4-p2669192	Bn-A04-p2763423	Bn-A04-p2763423	A4	11	10.4	A
3378	Bn-N4-p2671264	Bn-A04-p2765547	Bn-A04-p2765547	A4	11	10.4	A
3379	Bn-N4-p2687264	Bn-A04-p2780974	Bn-A04-p2780974	A4	11	10.4	A
3380	Bn-N4-p2688141	Bn-A04-p2781853	Bn-A04-p2781853	A4	11	10.4	A
3381	Bn-N4-p2688543	Bn-A04-p2782258	Bn-A04-p2782258	A4	11	10.4	A
3382	Bn-N4-p2689459	Bn-A04-p2783207	Bn-A04-p2783207	A4	11	10.4	A
3383	Bn-N4-p2692610	Bn-A04-p2789250	Bn-A04-p2789250	A4	11	10.4	A
3384	Bn-N4-p2696256	Bn-A04-p2793807	Bn-A04-p2793807	A4	11	10.4	A
3385	Bn-N4-p2705775	Bn-A04-p2802411	Bn-A04-p2802411	A4	11	10.4	A
3386	Bn-N4-p2719497	Bn-A04-p2817722	Bn-A04-p2817722	A4	11	10.4	A
3387	Bn-N4-p2719870	Bn-A04-p2818095	Bn-A04-p2818095	A4	11	10.4	A
3388	Bn-N4-p2721433	Bn-A04-p2825294	Bn-A04-p2825294	A4	11	10.4	A
3389	Bn-N4-p2721501	Bn-A04-p2825362	Bn-A04-p2825362	A4	11	10.4	A
3390	Bn-N4-p2747567	Bn-A04-p2847914	Bn-A04-p2847914	A4	11	10.4	A
3391	Bn-N4-p2767110	Bn-A04-p2889239	Bn-A04-p2889239	A4	11	10.4	A
3392	Bn-N4-p2770374	Bn-A04-p2892557	Bn-A04-p2892557	A4	11	10.4	A
3393	Bn-N4-p2770540	Bn-A04-p2892722	Bn-A04-p2892722	A4	11	10.4	A
3394	Bn-N4-p2770803	Bn-A04-p2892985	Bn-A04-p2892985	A4	11	10.4	A
3395	Bn-N4-p2770871	Bn-A04-p2893053	Bn-A04-p2893053	A4	11	10.4	A
3396	Bn-N4-p2778620	Bn-A04-p2903530	Bn-A04-p2903530	A4	11	10.4	A
3397	Bn-N4-p2783770	Bn-A04-p2913939	Bn-A04-p2913939	A4	11	10.4	A
3398	Bn-N4-p2784240	Bn-A04-p2914409	Bn-A04-p2914409	A4	11	10.4	A
3399	Bn-N4-p2787268	Bn-A04-p2921313	Bn-A04-p2921313	A4	11	10.4	A
3400	Bn-N4-p2787637	Bn-A04-p2921682	Bn-A04-p2921682	A4	11	10.4	A
3401	Bn-N4-p2787787	Bn-A04-p2921832	Bn-A04-p2921832	A4	11	10.4	A
3402	Bn-N4-p2789585	Bn-A04-p2923396	Bn-A04-p2923396	A4	11	10.4	A
3403	Bn-N4-p2791426	Bn-A09-p25680334	Bn-A09-p25680334	A4	11	10.4	A
3404	Bn-N4-p2797228	Bn-A04-p2925273	Bn-A04-p2925273	A4	11	10.4	A
3405	Bn-N4-p2803713	Bn-A04-p2932086	Bn-A04-p2932086	A4	11	10.4	A
3406	Bn-N4-p2813079	Bn-A04-p2941201	Bn-A04-p2941201	A4	11	10.4	A
3407	Bn-N4-p2816602	Bn-A04-p2944723	Bn-A04-p2944723	A4	11	10.4	A
3408	Bn-N4-p2819871	Bn-A04-p2947993	Bn-A04-p2947993	A4	11	10.4	A
3409	Bn-N4-p2821197	Bn-A04-p2948936	Bn-A04-p2948936	A4	11	10.4	A
3410	Bn-N4-p2825854	Bn-A04-p2954791	Bn-A04-p2954791	A4	11	10.4	A
3411	Bn-N4-p2825887	Bn-A04-p2954824	Bn-A04-p2954824	A4	11	10.4	A
3412	Bn-N4-p2851926	Bn-C4-p30176665	Bn-scaff_16263_2-p55262(	A4	11	10.4	A
3413	Bn-N4-p2873842	Bn-A04-p2963387	Bn-A04-p2963387	A4	11	10.4	A
3414	Bn-N4-p2887116	Bn-A04-p2983187	Bn-A04-p2983187	A4	11	10.4	A
3415	Bn-N4-p2897148	Bn-A04-p2999394	Bn-A04-p2999394	A4	11	10.4	A
3416	Bn-N4-p2906851	Bn-A04-p3004068	Bn-A04-p3004068	A4	11	10.4	A
3417	Bn-N4-p2913345	Bn-A04-p3016121	Bn-A04-p3016121	A4	11	10.4	A
3418	Bn-N4-p2915145	Bn-A04-p3022681	Bn-A04-p3022681	A4	11	10.4	A
3419	Bn-N4-p2990898	Bn-A04-p3095652	Bn-A04-p3095652	A4	11	10.4	A
3420	Bn-N4-p3021601	Bn-A04-p3126855	Bn-A04-p3126855	A4	11	10.4	A
3421	Bn-N4-p3045094	Bn-A04-p3144839	Bn-A04-p3144839	A4	11	10.4	A
3422	Bn-N4-p3052809	Bn-A04-p3152494	Bn-A04-p3152494	A4	11	10.4	A
3423	Bn-N4-p3052869	Bn-A04-p3152554	Bn-A04-p3152554	A4	11	10.4	A
3424	Bn-N4-p3075892	Bn-A04-p3172854	Bn-A04-p3172854	A4	11	10.4	A
3425	Bn-N4-p3088237	Bn-A04-p3181336	Bn-A04-p3181336	A4	11	10.4	A

3426	Bn-N4-p3089856	Bn-A04-p3182956	Bn-A04-p3182956	A4	11	10.4	A
3427	Bn-Scaffold01376-p6621	Bn-A05-p18147040	Bn-A05-p18147040	A4	11	10.4	A
3428	Bn-Scaffold07981-p895	Bn-A04-p2692131	Bn-A04-p2692131	A4	11	10.4	A
3429	Unknown	Bn-A04-p1976999	Bn-A04-p1976999	A4	11	10.4	A
3430	Bn-N4-p3096077	Bn-A04-p3190463	Bn-A04-p3190463	A4	12	12	A
3431	Bn-N4-p3129508	Bn-A04-p3225119	Bn-A04-p3225119	A4	12	12	A
3432	Bn-N4-p3160241	Bn-A04-p3254177	Bn-A04-p3254177	A4	12	12	A
3433	Bn-N4-p3161205	Bn-A04-p3255140	Bn-A04-p3255140	A4	12	12	A
3434	Bn-N4-p3161237	Bn-A04-p3255172	Bn-A04-p3255172	A4	12	12	0
3435	Bn-N4-p3170115	Bn-A04-p3262432	Bn-A04-p3262432	A4	12	12	A
3436	Bn-N4-p3175665	Bn-A04-p3267803	Bn-A04-p3267803	A4	12	12	A
3437	Bn-N4-p3187032	Bn-A04-p3272740	Bn-A04-p3272740	A4	12	12	A
3438	Bn-N4-p3190776	Bn-A04-p3283282	Bn-A04-p3283282	A4	12	12	A
3439	Bn-N4-p3207023	Bn-C4-p30948032	Bn-scaff_18839_1-p34332	A4	12	12	A
3440	Bn-N4-p3272981	Bn-A04-p3357700	Bn-A04-p3357700	A4	12	12	A
3441	Bn-N4-p3299665	Bn-A04-p3379410	Bn-A04-p3379410	A4	12	12	A
3442	Bn-N4-p3310984	Bn-A04-p3390331	Bn-A04-p3390331	A4	12	12	A
3443	Bn-N4-p3317226	Bn-A04-p3395103	Bn-A04-p3395103	A4	12	12	A
3444	Bn-N4-p3332635	Bn-A04-p3414140	Bn-A04-p3414140	A4	12	12	A
3445	Bn-N4-p3332727	Bn-A04-p3414232	Bn-A04-p3414232	A4	12	12	A
3446	Bn-N4-p3332942	Bn-A04-p3414449	Bn-A04-p3414449	A4	12	12	A
3447	Bn-N4-p3334529	Bn-A04-p3420532	Bn-A04-p3420532	A4	12	12	A
3448	Bn-N4-p3335114	Bn-A04-p3421117	Bn-A04-p3421117	A4	12	12	A
3449	Bn-N4-p3370906	Bn-A04-p3457753	Bn-A04-p3457753	A4	12	12	A
3450	Bn-N4-p3376272	Bn-A04-p3462758	Bn-A04-p3462758	A4	12	12	A
3451	Bn-N4-p3386454	Bn-A04-p3471699	Bn-A04-p3471699	A4	12	12	A
3452	Bn-N4-p3388770	Bn-A04-p3473990	Bn-A04-p3473990	A4	12	12	A
3453	Bn-N4-p3392990	Bn-A04-p3483780	Bn-A04-p3483780	A4	12	12	A
3454	Bn-N4-p3498786	Bn-A04-p3583216	Bn-A04-p3583216	A4	12	12	A
3455	Bn-N14-p32588755	Bn-A04-p3690435	Bn-A04-p3690435	A4	13	13.6	A
3456	Bn-N4-p3548333	Bn-A04-p3632226	Bn-A04-p3632226	A4	13	13.6	A
3457	Bn-N4-p3583041	Bn-A04-p3668627	Bn-A04-p3668627	A4	13	13.6	A
3458	Bn-N4-p3621830	Bn-A04-p3719381	Bn-A04-p3719381	A4	13	13.6	A
3459	Bn-N4-p3642885	Bn-A04-p3743549	Bn-A04-p3743549	A4	14	14.4	A
3460	Bn-N4-p3653112	Bn-A04-p3750975	Bn-A04-p3750975	A4	14	14.4	A
3462	Bn-N17-p9292883	Bn-C7-p8708127	Bn-scaff_16975_1-p12236	A4	15	16.8	A
3463	Bn-N4-p4088273	Bn-A06-p5285976	Bn-A06-p5285976	A4	15	16.8	A
3464	Bn-N4-p4090036	Bn-A06-p5284260	Bn-A06-p5284260	A4	15	16.8	A
3465	Bn-N4-p4094226	Bn-A06-p5280225	Bn-A06-p5280225	A4	15	16.8	A
3466	Bn-N4-p5614929	Bn-A04-p5183306	Bn-A04-p5183306	A4	15	16.8	A
3467	Bn-N4-p5827825	Bn-A04-p5378408	Bn-A04-p5378408	A4	15	16.8	A
3468	Bn-N4-p5946136	Bn-A04-p5398352	Bn-A04-p5398352	A4	15	16.8	A
3469	Bn-N4-p6128312	Bn-A04-p2411039	Bn-A04-p2411039	A4	15	16.8	A
3470	Bn-N4-p6130608	Bn-A04-p5576673	Bn-A04-p5576673	A4	15	16.8	A
3471	Bn-N4-p6139904	Bn-C4-p34314904	Bn-scaff_20814_1-p25186	A4	15	16.8	A
3472	Bn-N4-p6269396	Bn-A04-p5715780	Bn-A04-p5715780	A4	15	16.8	A
3473	Bn-N4-p6452475	Bn-Scaffold000256-p	Bn-Scaffold000256-p23238	A4	15	16.8	A
3474	Bn-N4-p7102629	Bn-Scaffold000104-p	Bn-Scaffold000104-p20201	A4	15	16.8	A
3475	Bn-N4-p7105150	Bn-Scaffold000104-p	Bn-Scaffold000104-p19912	A4	15	16.8	A

3476	Bn-N4-p7105171	Bn-Scaffold000104-p1	Bn-Scaffold000104-p19907	A4	15	16.8	A
3477	Bn-N4-p7110281	Bn-Scaffold000104-p1	Bn-Scaffold000104-p19396	A4	15	16.8	A
3478	Bn-N4-p7134450	Bn-Scaffold000104-p1	Bn-Scaffold000104-p17679	A4	15	16.8	0
3479	Bn-N4-p7136195	Bn-Scaffold000104-p1	Bn-Scaffold000104-p17505	A4	15	16.8	A
3480	Bn-N4-p7136302	Bn-Scaffold000104-p1	Bn-Scaffold000104-p17494	A4	15	16.8	A
3481	Bn-N4-p7136984	Bn-Scaffold000104-p1	Bn-Scaffold000104-p17428	A4	15	16.8	A
3482	Bn-N4-p7137306	Bn-Scaffold000104-p1	Bn-Scaffold000104-p17396	A4	15	16.8	A
3483	Bn-N4-p7137515	Bn-Scaffold000104-p1	Bn-Scaffold000104-p17375	A4	15	16.8	A
3484	Bn-N4-p7143821	Bn-Scaffold000104-p1	Bn-Scaffold000104-p16733	A4	15	16.8	A
3485	Bn-N4-p7200335	Bn-Scaffold000104-p9	Bn-Scaffold000104-p98567	A4	15	16.8	A
3486	Bn-N4-p7271031	Bn-Scaffold000104-p3	Bn-Scaffold000104-p33765	A4	15	16.8	A
3487	Bn-N4-p7427323	Bn-A04-p5809264	Bn-A04-p5809264	A4	15	16.8	A
3488	Bn-N4-p7449869	Bn-A04-p5827901	Bn-A04-p5827901	A4	15	16.8	A
3489	Bn-N4-p7460179	Bn-A04-p5852413	Bn-A04-p5852413	A4	15	16.8	A
3490	Bn-N4-p7489397	Bn-A04-p5880992	Bn-A04-p5880992	A4	15	16.8	A
3491	Bn-N4-p7560106	Bn-Scaffold001080-p1	Bn-Scaffold001080-p1208	A4	15	16.8	A
3492	Bn-N4-p7608030	Bn-A04-p5959753	Bn-A04-p5959753	A4	15	16.8	A
3493	Bn-N4-p7612487	Bn-A04-p5964175	Bn-A04-p5964175	A4	15	16.8	A
3494	Bn-N4-p7613904	Bn-A04-p5965590	Bn-A04-p5965590	A4	15	16.8	A
3495	Bn-N4-p7621193	Bn-A04-p5967828	Bn-A04-p5967828	A4	15	16.8	A
3496	Bn-N4-p7629728	Bn-A04-p5971620	Bn-A04-p5971620	A4	15	16.8	A
3497	Bn-N4-p7631623	Bn-A04-p5973348	Bn-A04-p5973348	A4	15	16.8	A
3498	Bn-N4-p7632194	Bn-A04-p5973919	Bn-A04-p5973919	A4	15	16.8	A
3499	Bn-N4-p7632470	Bn-A04-p5974195	Bn-A04-p5974195	A4	15	16.8	A
3500	Bn-N4-p7637415	Bn-A04-p5979118	Bn-A04-p5979118	A4	15	16.8	A
3501	Bn-N4-p7640512	Bn-A04-p5982123	Bn-A04-p5982123	A4	15	16.8	A
3502	Bn-N4-p7641981	Bn-A04-p5983577	Bn-A04-p5983577	A4	15	16.8	A
3503	Bn-N4-p7686443	Bn-A04-p6028097	Bn-A04-p6028097	A4	15	16.8	A
3504	Bn-N4-p7686677	Bn-A04-p6028334	Bn-A04-p6028334	A4	15	16.8	A
3505	Bn-N4-p7688234	Bn-A04-p6029797	Bn-A04-p6029797	A4	15	16.8	A
3506	Bn-N4-p7704798	Bn-A04-p6047600	Bn-A04-p6047600	A4	15	16.8	A
3507	Bn-N4-p7707855	Bn-A04-p6050616	Bn-A04-p6050616	A4	15	16.8	A
3508	Bn-N4-p7751442	Bn-A04-p6100884	Bn-A04-p6100884	A4	15	16.8	A
3509	Bn-N4-p7830973	Bn-A04-p6165743	Bn-A04-p6165743	A4	15	16.8	A
3510	Bn-N12-p16947465	Bn-A10-p4304707	Bn-A10-p4304707	A4	16	17.6	A
3511	Bn-N14-p32977648	Bn-A04-p4002585	Bn-A04-p4002585	A4	16	17.6	A
3512	Bn-N14-p33298973	Bn-A06-p5217195	Bn-A06-p5217195	A4	16	17.6	A
3513	Bn-N14-p34631291	Bn-A04-p4881807	Bn-A04-p4881807	A4	16	17.6	A
3514	Bn-N14-p37220173	Bn-A04-p6228211	Bn-A04-p6228211	A4	16	17.6	A
3515	Bn-N14-p37652911	Bn-A04-p6533593	Bn-A04-p6533593	A4	16	17.6	A
3516	Bn-N14-p38133852	Bn-A04-p7044446	Bn-A04-p7044446	A4	16	17.6	A
3517	Bn-N14-p38166443	Bn-A04-p7066595	Bn-A04-p7066595	A4	16	17.6	A
3518	Bn-N14-p38367131	Bn-Scaffold00578-p3	Bn-scaff_18817_1-p31981	A4	16	17.6	A
3519	Bn-N18-p21724826	Bn-Scaffold000469-p2	Bn-Scaffold000469-p2820	A4	16	17.6	A
3520	Bn-N19-p1337021	Bn-A01-p25050109	Bn-A01-p25050109	A4	16	17.6	A
3521	Bn-N4-p18648945	Bn-A10-p4305337	Bn-A10-p4305337	A4	16	17.6	A
3522	Bn-N4-p3700659	Bn-A04-p3820381	Bn-A04-p3820381	A4	16	17.6	A
3523	Bn-N4-p3744805	Bn-A04-p3875034	Bn-A04-p3875034	A4	16	17.6	A
3524	Bn-N4-p3839135	Bn-A04-p3990611	Bn-A04-p3990611	A4	16	17.6	A

3525	Bn-N4-p3839352	Bn-A04-p3990828	Bn-A04-p3990828	A4	16	17.6	A
3526	Bn-N4-p3922972	Bn-A06-p5441991	Bn-A06-p5441991	A4	16	17.6	A
3527	Bn-N4-p3927266	Bn-A06-p5436800	Bn-A06-p5436800	A4	16	17.6	A
3528	Bn-N4-p3947979	Bn-A06-p5427889	Bn-A06-p5427889	A4	16	17.6	A
3529	Bn-N4-p3990078	Bn-A06-p5390277	Bn-A06-p5390277	A4	16	17.6	A
3530	Bn-N4-p3990597	Bn-A06-p5389758	Bn-A06-p5389758	A4	16	17.6	A
3531	Bn-N4-p3993216	Bn-A06-p5386579	Bn-A06-p5386579	A4	16	17.6	A
3532	Bn-N4-p3995321	Bn-A06-p5384725	Bn-A06-p5384725	A4	16	17.6	A
3533	Bn-N4-p4000303	Bn-A06-p5372653	Bn-A06-p5372653	A4	16	17.6	A
3534	Bn-N4-p4033677	Bn-A06-p5339433	Bn-A06-p5339433	A4	16	17.6	A
3535	Bn-N4-p4038127	Bn-A06-p5335605	Bn-A06-p5335605	A4	16	17.6	A
3536	Bn-N4-p4040503	Bn-A06-p5333246	Bn-A06-p5333246	A4	16	17.6	A
3537	Bn-N4-p4094961	Bn-A06-p5279490	Bn-A06-p5279490	A4	16	17.6	A
3538	Bn-N4-p4100424	Bn-A06-p5274160	Bn-A06-p5274160	A4	16	17.6	A
3539	Bn-N4-p4178354	Bn-A06-p5236016	Bn-A06-p5236016	A4	16	17.6	A
3540	Bn-N4-p4181313	Bn-A06-p5232462	Bn-A06-p5232462	A4	16	17.6	A
3541	Bn-N4-p4219149	Bn-A06-p5197898	Bn-A06-p5197898	A4	16	17.6	A
3542	Bn-N4-p4243391	Bn-A06-p5182164	Bn-A06-p5182164	A4	16	17.6	A
3543	Bn-N4-p4294633	Bn-A04-p4056517	Bn-A04-p4056517	A4	16	17.6	A
3544	Bn-N4-p4302052	Bn-A04-p4060806	Bn-A04-p4060806	A4	16	17.6	A
3545	Bn-N4-p4315492	Bn-A04-p4074166	Bn-A04-p4074166	A4	16	17.6	A
3546	Bn-N4-p4357591	Bn-A04-p4112460	Bn-A04-p4112460	A4	16	17.6	A
3547	Bn-N4-p4452107	Bn-A04-p4203564	Bn-A04-p4203564	A4	16	17.6	A
3548	Bn-N4-p4454024	Bn-A04-p4204119	Bn-A04-p4204119	A4	16	17.6	A
3549	Bn-N4-p4454683	Bn-A04-p4204775	Bn-A04-p4204775	A4	16	17.6	A
3550	Bn-N4-p4457320	Bn-A04-p4207198	Bn-A04-p4207198	A4	16	17.6	A
3551	Bn-N4-p4491938	Bn-A04-p4221603	Bn-A04-p4221603	A4	16	17.6	A
3552	Bn-N4-p4494197	Bn-A04-p4223859	Bn-A04-p4223859	A4	16	17.6	A
3553	Bn-N4-p4494988	Bn-A04-p4224646	Bn-A04-p4224646	A4	16	17.6	A
3554	Bn-N4-p4495047	Bn-A04-p4224705	Bn-A04-p4224705	A4	16	17.6	A
3555	Bn-N4-p4523660	Bn-A04-p4249132	Bn-A04-p4249132	A4	16	17.6	A
3556	Bn-N4-p4532621	Bn-A04-p4265606	Bn-A04-p4265606	A4	16	17.6	A
3557	Bn-N4-p4547759	Bn-A04-p4276334	Bn-A04-p4276334	A4	16	17.6	A
3558	Bn-N4-p4577694	Bn-A04-p4286343	Bn-A04-p4286343	A4	16	17.6	A
3559	Bn-N4-p4625457	Bn-A04-p4330419	Bn-A04-p4330419	A4	16	17.6	A
3560	Bn-N4-p4641714	Bn-A04-p4346492	Bn-A04-p4346492	A4	16	17.6	A
3561	Bn-N4-p4644935	Bn-A04-p4349715	Bn-A04-p4349715	A4	16	17.6	A
3562	Bn-N4-p4649324	Bn-A04-p4350372	Bn-A04-p4350372	A4	16	17.6	A
3563	Bn-N4-p4649459	Bn-A04-p4350555	Bn-A04-p4350555	A4	16	17.6	A
3564	Bn-N4-p4689011	Bn-A04-p4392113	Bn-A04-p4392113	A4	16	17.6	A
3565	Bn-N4-p4706883	Bn-A04-p4409348	Bn-A04-p4409348	A4	16	17.6	A
3566	Bn-N4-p4707005	Bn-A04-p4409463	Bn-A04-p4409463	A4	16	17.6	A
3567	Bn-N4-p4707284	Bn-A04-p4409752	Bn-A04-p4409752	A4	16	17.6	A
3568	Bn-N4-p4709874	Bn-A04-p4410113	Bn-A04-p4410113	A4	16	17.6	A
3569	Bn-N4-p4709905	Bn-A04-p4410144	Bn-A04-p4410144	A4	16	17.6	A
3570	Bn-N4-p4709957	Bn-A04-p4410196	Bn-A04-p4410196	A4	16	17.6	A
3571	Bn-N4-p4719841	Bn-A04-p4418670	Bn-A04-p4418670	A4	16	17.6	A
3572	Bn-N4-p4719886	Bn-A04-p4418715	Bn-A04-p4418715	A4	16	17.6	A
3573	Bn-N4-p4806174	Bn-A04-p4529360	Bn-A04-p4529360	A4	16	17.6	A

3574	Bn-N4-p4891177	Bn-A01-p11653461	Bn-A01-p11653461	A4	16	17.6	A
3575	Bn-N4-p4891849	Bn-A01-p11652789	Bn-A01-p11652789	A4	16	17.6	A
3576	Bn-N4-p4901993	Bn-A01-p11642772	Bn-A01-p11642772	A4	16	17.6	A
3577	Bn-N4-p4902089	Bn-A01-p11642677	Bn-A01-p11642677	A4	16	17.6	A
3578	Bn-N4-p4902101	Bn-A01-p11642664	Bn-A01-p11642664	A4	16	17.6	A
3579	Bn-N4-p5035820	Bn-A09-p29238214	Bn-A09-p29238214	A4	16	17.6	A
3580	Bn-N4-p5062253	Bn-A04-p4730302	Bn-A04-p4730302	A4	16	17.6	A
3581	Bn-N4-p5091187	Bn-A04-p4742377	Bn-A04-p4742377	A4	16	17.6	A
3582	Bn-N4-p5091242	Bn-A04-p4742432	Bn-A04-p4742432	A4	16	17.6	A
3583	Bn-N4-p5188763	Bn-A04-p4825669	Bn-A04-p4825669	A4	16	17.6	A
3584	Bn-N4-p5205440	Bn-A04-p4834943	Bn-A04-p4834943	A4	16	17.6	A
3585	Bn-N4-p5205593	Bn-A04-p4835096	Bn-A04-p4835096	A4	16	17.6	A
3586	Bn-N4-p5205743	Bn-A04-p4835244	Bn-A04-p4835244	A4	16	17.6	A
3587	Bn-N4-p5206381	Bn-A04-p4835883	Bn-A04-p4835883	A4	16	17.6	A
3588	Bn-N4-p5206944	Bn-A04-p4836499	Bn-A04-p4836499	A4	16	17.6	A
3589	Bn-N4-p5214354	Bn-A04-p4848743	Bn-A04-p4848743	A4	16	17.6	A
3590	Bn-N4-p5219184	Bn-A04-p4864900	Bn-A04-p4864900	A4	16	17.6	A
3591	Bn-N4-p5221092	Bn-A04-p4866786	Bn-A04-p4866786	A4	16	17.6	A
3592	Bn-N4-p5229923	Bn-A04-p4872836	Bn-A04-p4872836	A4	16	17.6	A
3593	Bn-N4-p5231353	Bn-A04-p4874261	Bn-A04-p4874261	A4	16	17.6	A
3594	Bn-N4-p5232333	Bn-A04-p4874840	Bn-A04-p4874840	A4	16	17.6	A
3595	Bn-N4-p5236813	Bn-A04-p4879262	Bn-A04-p4879262	A4	16	17.6	A
3596	Bn-N4-p5238319	Bn-A04-p4880759	Bn-A04-p4880759	A4	16	17.6	A
3597	Bn-N4-p5238741	Bn-A04-p4881168	Bn-A04-p4881168	A4	16	17.6	A
3598	Bn-N4-p5310615	Bn-A04-p4891169	Bn-A04-p4891169	A4	16	17.6	A
3599	Bn-N4-p5326434	Bn-A04-p4909402	Bn-A04-p4909402	A4	16	17.6	A
3600	Bn-N4-p5327114	Bn-A04-p4910033	Bn-A04-p4910033	A4	16	17.6	A
3601	Bn-N4-p5345059	Bn-A04-p4932716	Bn-A04-p4932716	A4	16	17.6	A
3602	Bn-N4-p5363976	Bn-C4-p33496025	Bn-scaff_20191_1-p14334	A4	16	17.6	A
3603	Bn-N4-p5380466	Bn-A04-p4976932	Bn-A04-p4976932	A4	16	17.6	A
3604	Bn-N4-p5381022	Bn-A04-p4977488	Bn-A04-p4977488	A4	16	17.6	A
3605	Bn-N4-p5382012	Bn-A04-p4978478	Bn-A04-p4978478	A4	16	17.6	A
3606	Bn-N4-p5382026	Bn-A04-p4978492	Bn-A04-p4978492	A4	16	17.6	A
3607	Bn-N4-p5382314	Bn-A04-p4978780	Bn-A04-p4978780	A4	16	17.6	A
3608	Bn-N4-p5564369	Bn-C4-p33708474	Bn-scaff_26139_1-p31357	A4	16	17.6	A
3609	Bn-N4-p5565820	Bn-A04-p5133653	Bn-A04-p5133653	A4	16	17.6	A
3610	Bn-N4-p5569488	Bn-A04-p5137321	Bn-A04-p5137321	A4	16	17.6	A
3611	Bn-N4-p7917200	Bn-A04-p6240855	Bn-A04-p6240855	A4	16	17.6	A
3612	Bn-N4-p7926047	Bn-A04-p6255997	Bn-A04-p6255997	A4	16	17.6	A
3613	Bn-N4-p7931828	Bn-A04-p6263702	Bn-A04-p6263702	A4	16	17.6	A
3614	Bn-N4-p7932174	Bn-A04-p6264043	Bn-A04-p6264043	A4	16	17.6	A
3615	Bn-N4-p7952695	Bn-C4-p35925431	Bn-scaff_16394_1-p11549	A4	16	17.6	A
3616	Bn-N4-p7970235	Bn-A04-p6277634	Bn-A04-p6277634	A4	16	17.6	A
3617	Bn-N4-p7992709	Bn-A04-p6296526	Bn-A04-p6296526	A4	16	17.6	A
3618	Bn-N4-p8242241	Bn-A04-p6550369	Bn-A04-p6550369	A4	16	17.6	A
3461	Bn-N4-p8242628	Bn-A04-p6550757	Bn-A04-p6550757	A4	16	17.6	0
3619	Bn-N4-p8247861	Bn-A04-p6555913	Bn-A04-p6555913	A4	16	17.6	A
3620	Bn-N4-p8248353	Bn-A04-p6556405	Bn-A04-p6556405	A4	16	17.6	A
3621	Bn-N4-p8296575	Bn-A04-p6588935	Bn-A04-p6588935	A4	16	17.6	A

3622	Bn-N4-p8348683	Bn-A04-p6632126	Bn-A04-p6632126	A4	16	17.6	A
3623	Bn-N4-p8357278	Bn-A04-p6637458	Bn-A04-p6637458	A4	16	17.6	A
3624	Bn-N4-p8373347	Bn-A04-p6653508	Bn-A04-p6653508	A4	16	17.6	A
3625	Bn-N4-p8377182	Bn-A04-p6657342	Bn-A04-p6657342	A4	16	17.6	A
3626	Bn-N4-p8391311	Bn-A04-p6673120	Bn-A04-p6673120	A4	16	17.6	A
3627	Bn-N4-p8406762	Bn-A04-p6685371	Bn-A04-p6685371	A4	16	17.6	A
3628	Bn-N4-p8437700	Bn-A04-p6718464	Bn-A04-p6718464	A4	16	17.6	A
3629	Bn-N4-p8456270	Bn-A04-p3229572	Bn-A04-p3229572	A4	16	17.6	0
3630	Bn-N4-p8481482	Bn-A04-p6747807	Bn-A04-p6747807	A4	16	17.6	A
3631	Bn-N4-p8482498	Bn-A04-p6748822	Bn-A04-p6748822	A4	16	17.6	A
3632	Bn-N4-p8482749	Bn-A04-p6749073	Bn-A04-p6749073	A4	16	17.6	A
3633	Bn-N4-p8482790	Bn-A04-p6749114	Bn-A04-p6749114	A4	16	17.6	A
3634	Bn-N4-p8483047	Bn-A04-p6749322	Bn-A04-p6749322	A4	16	17.6	A
3635	Bn-N4-p8486515	Bn-A04-p6752299	Bn-A04-p6752299	A4	16	17.6	A
3636	Bn-N4-p8487195	Bn-A04-p6752979	Bn-A04-p6752979	A4	16	17.6	A
3637	Bn-N4-p8494359	Bn-A04-p6760294	Bn-A04-p6760294	A4	16	17.6	A
3638	Bn-N4-p8500149	Bn-A04-p6764233	Bn-A04-p6764233	A4	16	17.6	A
3639	Bn-N4-p8500961	Bn-A04-p6765045	Bn-A04-p6765045	A4	16	17.6	A
3640	Bn-N4-p8507147	Bn-A04-p6771264	Bn-A04-p6771264	A4	16	17.6	A
3641	Bn-N4-p8509867	Bn-A04-p6773984	Bn-A04-p6773984	A4	16	17.6	A
3642	Bn-N4-p8510915	Bn-A04-p6775008	Bn-A04-p6775008	A4	16	17.6	A
3643	Bn-N4-p8512504	Bn-A04-p6776597	Bn-A04-p6776597	A4	16	17.6	A
3644	Bn-N4-p8512693	Bn-A04-p6776786	Bn-A04-p6776786	A4	16	17.6	A
3645	Bn-N4-p8519231	Bn-A04-p6783717	Bn-A04-p6783717	A4	16	17.6	A
3646	Bn-N4-p8525888	Bn-A04-p6790373	Bn-A04-p6790373	A4	16	17.6	A
3647	Bn-N4-p8719557	Bn-A04-p6966154	Bn-A04-p6966154	A4	16	17.6	A
3648	Bn-N4-p8724710	Bn-A04-p6968899	Bn-A04-p6968899	A4	16	17.6	A
3649	Bn-N4-p8729888	Bn-A04-p6973605	Bn-A04-p6973605	A4	16	17.6	A
3650	Bn-N4-p8729995	Bn-A04-p6973712	Bn-A04-p6973712	A4	16	17.6	A
3651	Bn-N4-p8730489	Bn-A04-p6974206	Bn-A04-p6974206	A4	16	17.6	A
3652	Bn-N4-p8731938	Bn-A04-p6975646	Bn-A04-p6975646	A4	16	17.6	A
3653	Bn-N4-p8734411	Bn-A04-p6978108	Bn-A04-p6978108	A4	16	17.6	A
3654	Bn-N4-p8734466	Bn-A04-p6978163	Bn-A04-p6978163	A4	16	17.6	A
3655	Bn-N4-p8735429	Bn-A04-p6979129	Bn-A04-p6979129	A4	16	17.6	A
3656	Bn-N4-p8736901	Bn-A04-p6980459	Bn-A04-p6980459	A4	16	17.6	A
3657	Bn-N4-p8794886	Bn-A04-p7066181	Bn-A04-p7066181	A4	16	17.6	A
3658	Bn-N4-p8797594	Bn-A04-p7068079	Bn-A04-p7068079	A4	16	17.6	A
3659	Bn-N4-p8819355	Bn-A04-p7088336	Bn-A04-p7088336	A4	16	17.6	A
3660	Bn-N4-p8825886	Bn-Scaffold00578-p2(	Bn-scaff_18817_1-p20803(	A4	16	17.6	A
3661	Bn-N4-p8849472	Bn-A04-p7107858	Bn-A04-p7107858	A4	16	17.6	A
3662	Bn-N4-p8850965	Bn-A04-p7109346	Bn-A04-p7109346	A4	16	17.6	A
3663	Bn-N4-p8851142	Bn-A04-p7109625	Bn-A04-p7109625	A4	16	17.6	A
3664	Bn-N4-p8853018	Bn-A04-p7112803	Bn-A04-p7112803	A4	16	17.6	A
3665	Bn-N4-p8853259	Bn-A04-p7113045	Bn-A04-p7113045	A4	16	17.6	A
3666	Bn-N4-p8853369	Bn-A04-p7113156	Bn-A04-p7113156	A4	16	17.6	A
3667	Bn-N4-p8853496	Bn-A04-p7113281	Bn-A04-p7113281	A4	16	17.6	A
3668	Bn-N4-p8853714	Bn-A04-p7113498	Bn-A04-p7113498	A4	16	17.6	A
3669	Bn-N4-p8856051	Bn-A04-p7133952	Bn-A04-p7133952	A4	16	17.6	A
3670	Bn-N4-p8856121	Bn-A04-p7134022	Bn-A04-p7134022	A4	16	17.6	A

3671	Bn-N4-p8862101	Bn-A04-p7139140	Bn-A04-p7139140	A4	16	17.6	A
3672	Bn-N4-p8866013	Bn-A04-p7141171	Bn-A04-p7141171	A4	16	17.6	A
3673	Bn-N4-p8867424	Bn-A04-p7142597	Bn-A04-p7142597	A4	16	17.6	A
3674	Bn-N4-p8868696	Bn-A04-p7143891	Bn-A04-p7143891	A4	16	17.6	A
3675	Bn-N4-p8873308	Bn-A04-p7149396	Bn-A04-p7149396	A4	16	17.6	A
3676	Bn-N4-p8873974	Bn-A04-p7150064	Bn-A04-p7150064	A4	16	17.6	A
3677	Bn-N4-p8874014	Bn-A04-p7150104	Bn-A04-p7150104	A4	16	17.6	A
3678	Bn-N4-p8874239	Bn-A04-p7150329	Bn-A04-p7150329	A4	16	17.6	A
3679	Bn-N4-p8886866	Bn-Scaffold000355-p1	Bn-Scaffold000355-p19729	A4	16	17.6	A
3680	Bn-N4-p8897087	Bn-A04-p7161326	Bn-A04-p7161326	A4	16	17.6	A
3681	Bn-N4-p8898451	Bn-A04-p7162690	Bn-A04-p7162690	A4	16	17.6	A
3682	Bn-N4-p8940673	Bn-A04-p7212436	Bn-A04-p7212436	A4	16	17.6	A
3683	Bn-N4-p8999628	Bn-A04-p7251871	Bn-A04-p7251871	A4	16	17.6	A
3684	Bn-N4-p9024078	Bn-A04-p7275318	Bn-A04-p7275318	A4	16	17.6	A
3685	Bn-N4-p9084663	Bn-A04-p7323275	Bn-A04-p7323275	A4	16	17.6	A
3686	Bn-N4-p9101667	Bn-Scaffold000281-p4	Bn-Scaffold000281-p4606	A4	16	17.6	A
3687	Bn-N4-p9127651	Bn-Scaffold000281-p2	Bn-Scaffold000281-p25625	A4	16	17.6	A
3688	Bn-N4-p9162577	Bn-A04-p7350870	Bn-A04-p7350870	A4	16	17.6	A
3689	Bn-N4-p9196667	Bn-C4-p37018768	Bn-scaff_16876_1-p124048	A4	16	17.6	A
3690	Bn-N4-p9203787	Bn-A04-p7389149	Bn-A04-p7389149	A4	16	17.6	A
3691	Bn-N4-p9228932	Bn-A04-p7407657	Bn-A04-p7407657	A4	16	17.6	A
3692	Bn-N4-p9232963	Bn-A04-p7410699	Bn-A04-p7410699	A4	16	17.6	A
3693	Bn-N4-p9245171	Bn-A04-p7422354	Bn-A04-p7422354	A4	16	17.6	A
3694	Bn-N4-p9247238	Bn-A04-p7425359	Bn-A04-p7425359	A4	16	17.6	A
3695	Bn-N4-p9247576	Bn-A04-p7425697	Bn-A04-p7425697	A4	16	17.6	A
3696	Bn-N4-p9248406	Bn-A04-p7426527	Bn-A04-p7426527	A4	16	17.6	A
3697	Bn-N4-p9252792	Bn-A04-p7431245	Bn-A04-p7431245	A4	16	17.6	A
3698	Bn-N4-p9253344	Bn-A04-p7431816	Bn-A04-p7431816	A4	16	17.6	A
3699	Bn-N4-p9256354	Bn-A04-p7441018	Bn-A04-p7441018	A4	16	17.6	A
3700	Bn-N4-p9256990	Bn-A04-p7441659	Bn-A04-p7441659	A4	16	17.6	A
3701	Bn-N4-p9257693	Bn-A04-p7442353	Bn-A04-p7442353	A4	16	17.6	A
3702	Bn-N4-p9258226	Bn-A04-p7442886	Bn-A04-p7442886	A4	16	17.6	A
3703	Bn-N4-p9258735	Bn-A04-p7443395	Bn-A04-p7443395	A4	16	17.6	A
3704	Bn-N4-p9273747	Bn-A04-p7450678	Bn-A04-p7450678	A4	16	17.6	A
3705	Bn-N4-p9291667	Bn-A05-p20676409	Bn-A05-p20676409	A4	16	17.6	A
3706	Bn-N4-p9348797	Bn-A04-p7488675	Bn-A04-p7488675	A4	16	17.6	A
3707	Bn-N4-p9354869	Bn-A04-p7493966	Bn-A04-p7493966	A4	16	17.6	A
3708	Bn-N4-p9451011	Bn-A04-p7582986	Bn-A04-p7582986	A4	16	17.6	A
3709	Bn-N4-p9452812	Bn-A04-p7584788	Bn-A04-p7584788	A4	16	17.6	A
3710	Bn-N4-p9472834	Bn-A04-p7601638	Bn-A04-p7601638	A4	16	17.6	A
3711	Bn-N4-p9488385	Bn-A04-p7616574	Bn-A04-p7616574	A4	16	17.6	A
3712	Bn-N4-p9489640	Bn-A04-p7617794	Bn-A04-p7617794	A4	16	17.6	A
3713	Bn-N4-p9498896	Bn-A04-p7625503	Bn-A04-p7625503	A4	16	17.6	A
3714	Bn-N4-p9510178	Bn-A04-p7635112	Bn-A04-p7635112	A4	16	17.6	A
3715	Bn-N4-p9540723	Bn-A04-p7666812	Bn-A04-p7666812	A4	16	17.6	A
3716	Bn-N4-p9540892	Bn-A04-p7666981	Bn-A04-p7666981	A4	16	17.6	A
3717	Bn-N4-p9544207	Bn-A04-p7670170	Bn-A04-p7670170	A4	16	17.6	A
3718	Bn-N4-p9545821	Bn-A04-p7671731	Bn-A04-p7671731	A4	16	17.6	A
3719	Bn-N4-p9546315	Bn-A04-p7672240	Bn-A04-p7672240	A4	16	17.6	A

3720	Bn-N4-p9549843	Bn-A04-p7681107	Bn-A04-p7681107	A4	16	17.6	A
3721	Bn-N4-p9550299	Bn-A04-p7681563	Bn-A04-p7681563	A4	16	17.6	A
3722	Bn-N4-p9552405	Bn-A04-p7683677	Bn-A04-p7683677	A4	16	17.6	A
3723	Bn-N4-p9554937	Bn-A04-p7685115	Bn-A04-p7685115	A4	16	17.6	A
3724	Bn-N4-p9562834	Bn-A04-p7694288	Bn-A04-p7694288	A4	16	17.6	A
3725	Bn-N4-p9573185	Bn-Scaffold035543-p3	Bn-Scaffold035543-p33	A4	16	17.6	A
3726	Bn-N4-p9647741	Bn-A04-p7760488	Bn-A04-p7760488	A4	16	17.6	A
3727	Bn-N4-p9659964	Bn-A04-p7769990	Bn-A04-p7769990	A4	16	17.6	A
3728	Bn-N4-p9660892	Bn-A04-p7770904	Bn-A04-p7770904	A4	16	17.6	A
3729	Bn-N4-p9666609	Bn-A04-p7776319	Bn-A04-p7776319	A4	16	17.6	A
3730	Bn-N4-p9689667	Bn-A04-p7797300	Bn-A04-p7797300	A4	16	17.6	A
3731	Bn-N4-p9701242	Bn-A04-p7808804	Bn-A04-p7808804	A4	16	17.6	A
3732	Bn-N4-p9706103	Bn-A04-p7813672	Bn-A04-p7813672	A4	16	17.6	A
3733	Bn-N4-p9710410	Bn-A04-p7819525	Bn-A04-p7819525	A4	16	17.6	A
3734	Bn-N4-p9713393	Bn-A04-p7822519	Bn-A04-p7822519	A4	16	17.6	A
3735	Bn-N4-p9713459	Bn-A04-p7822585	Bn-A04-p7822585	A4	16	17.6	A
3736	Bn-N4-p9775987	Bn-A04-p7883120	Bn-A04-p7883120	A4	16	17.6	A
3737	Bn-Scaffold07321-p1160	Bn-Scaffold000962-p2	Bn-Scaffold000962-p273	A4	16	17.6	A
3738	Bn-Scaffold27301-p352	Bn-A04-p7297317	Bn-A04-p7297317	A4	16	17.6	A
3739	Bn-Scaffold35790-p367	Bn-Scaffold005956-p2	Bn-Scaffold005956-p293	A4	16	17.6	A
3740	Bn-N14-p39410658	Bn-C4-p37792292	Bn-scaff_16876_1-p46695	A4	17	18.4	A
3741	Bn-N14-p39410726	Bn-C4-p37792360	Bn-scaff_16876_1-p46688	A4	17	18.4	A
3742	Bn-N14-p39410859	Bn-C4-p37792493	Bn-scaff_16876_1-p46675	A4	17	18.4	A
3743	Bn-N14-p39411649	Bn-C4-p37793283	Bn-scaff_16876_1-p46596	A4	17	18.4	A
3744	Bn-N14-p39411758	Bn-C4-p37793392	Bn-scaff_16876_1-p46585	A4	17	18.4	A
3745	Bn-N14-p39461741	Bn-C4-p37844978	Bn-scaff_16876_1-p41427	A4	17	18.4	A
3746	Bn-N14-p39841509	Bn-C4-p38251889	Bn-scaff_16876_1-p7359	A4	17	18.4	A
3747	Bn-N14-p39943784	Bn-C4-p38348792	Bn-scaff_17525_1-p66650	A4	17	18.4	A
3748	Bn-N14-p39943851	Bn-C4-p38348859	Bn-scaff_17525_1-p66643	A4	17	18.4	A
3749	Bn-N4-p10061305	Bn-A04-p8178997	Bn-A04-p8178997	A4	17	18.4	A
3750	Bn-N4-p10069080	Bn-A04-p8186888	Bn-A04-p8186888	A4	17	18.4	A
3751	Bn-N4-p10084261	Bn-A04-p8208799	Bn-A04-p8208799	A4	17	18.4	A
3752	Bn-N4-p10186151	Bn-A04-p8287776	Bn-A04-p8287776	A4	17	18.4	A
3753	Bn-N4-p10186282	Bn-A04-p8287906	Bn-A04-p8287906	A4	17	18.4	A
3754	Bn-N4-p10191316	Bn-A04-p8292499	Bn-A04-p8292499	A4	17	18.4	A
3755	Bn-N4-p10197873	Bn-A04-p8299391	Bn-A04-p8299391	A4	17	18.4	A
3756	Bn-N4-p10209082	Bn-A04-p8310242	Bn-A04-p8310242	A4	17	18.4	A
3757	Bn-N4-p10209166	Bn-A04-p8310328	Bn-A04-p8310328	A4	17	18.4	A
3758	Bn-N4-p10213376	Bn-A04-p8328216	Bn-A04-p8328216	A4	17	18.4	A
3759	Bn-N4-p10214706	Bn-A04-p8329549	Bn-A04-p8329549	A4	17	18.4	A
3760	Bn-N4-p10228066	Bn-A04-p8344866	Bn-A04-p8344866	A4	17	18.4	A
3761	Bn-N4-p10230334	Bn-A04-p8345666	Bn-A04-p8345666	A4	17	18.4	A
3762	Bn-N4-p10238186	Bn-A04-p8348145	Bn-A04-p8348145	A4	17	18.4	A
3763	Bn-N4-p10238804	Bn-A04-p8348750	Bn-A04-p8348750	A4	17	18.4	A
3764	Bn-N4-p10238860	Bn-A04-p8348806	Bn-A04-p8348806	A4	17	18.4	A
3765	Bn-N4-p10325716	Bn-A04-p8403728	Bn-A04-p8403728	A4	17	18.4	A
3766	Bn-N4-p10327890	Bn-A04-p8407945	Bn-A04-p8407945	A4	17	18.4	A
3767	Bn-N4-p10393619	Bn-A04-p8473768	Bn-A04-p8473768	A4	17	18.4	A
3768	Bn-N4-p10401209	Bn-A04-p8481222	Bn-A04-p8481222	A4	17	18.4	A

3769	Bn-N4-p10459058	Bn-C4-p38742748	Bn-scaff_17525_1-p27254	A4	17	18.4	A
3770	Bn-N4-p10461522	Bn-A04-p8559580	Bn-A04-p8559580	A4	17	18.4	A
3771	Bn-N4-p10466045	Bn-A04-p8564072	Bn-A04-p8564072	A4	17	18.4	A
3772	Bn-N4-p10474725	Bn-A04-p8575908	Bn-A04-p8575908	A4	17	18.4	A
3773	Bn-N4-p10484006	Bn-A04-p8587019	Bn-A04-p8587019	A4	17	18.4	A
3774	Bn-N4-p9747560	Bn-A04-p7857679	Bn-A04-p7857679	A4	17	18.4	A
3775	Bn-N4-p9747603	Bn-A04-p7857722	Bn-A04-p7857722	A4	17	18.4	A
3776	Bn-N4-p9761069	Bn-A04-p7870001	Bn-A04-p7870001	A4	17	18.4	A
3777	Bn-N4-p9778175	Bn-A04-p7886817	Bn-A04-p7886817	A4	17	18.4	A
3778	Bn-N4-p9778920	Bn-A04-p7887561	Bn-A04-p7887561	A4	17	18.4	A
3779	Bn-N4-p9781792	Bn-A04-p7888986	Bn-A04-p7888986	A4	17	18.4	A
3780	Bn-N4-p9782736	Bn-A04-p7889763	Bn-A04-p7889763	A4	17	18.4	A
3781	Bn-N4-p9784019	Bn-A04-p7897465	Bn-A04-p7897465	A4	17	18.4	A
3782	Bn-N4-p9822880	Bn-A04-p7931854	Bn-A04-p7931854	A4	17	18.4	A
3783	Bn-N4-p9855100	Bn-A04-p7955059	Bn-A04-p7955059	A4	17	18.4	A
3784	Bn-N4-p9982801	Bn-A04-p8114074	Bn-A04-p8114074	A4	17	18.4	A
3785	Bn-N4-p9982806	Bn-A04-p8114079	Bn-A04-p8114079	A4	17	18.4	A
3786	Bn-N4-p9986143	Bn-A04-p8117244	Bn-A04-p8117244	A4	17	18.4	A
3787	Bn-Scaffold78125-p176	Bn-A04-p8312347	Bn-A04-p8312347	A4	17	18.4	A
3788	Bn-N14-p40467836	Bn-A04-p8609799	Bn-A04-p8609799	A4	18	19.2	A
3789	Bn-N4-p10512694	Bn-A04-p8612311	Bn-A04-p8612311	A4	18	19.2	A
3790	Bn-N4-p10588664	Bn-A04-p8686116	Bn-A04-p8686116	A4	18	19.2	A
3791	Bn-N4-p10589490	Bn-A04-p8686934	Bn-A04-p8686934	A4	18	19.2	A
3792	Bn-N4-p10721042	Bn-A04-p8788445	Bn-A04-p8788445	A4	18	19.2	A
3793	Bn-N4-p10722959	Bn-A04-p8790105	Bn-A04-p8790105	A4	18	19.2	A
3794	Bn-N4-p10723288	Bn-A04-p8790433	Bn-A04-p8790433	A4	18	19.2	A
3795	Bn-N4-p10741775	Bn-A04-p8796688	Bn-A04-p8796688	A4	18	19.2	A
3796	Bn-N4-p10741793	Bn-A04-p8796706	Bn-A04-p8796706	A4	18	19.2	A
3797	Bn-N4-p10744229	Bn-A04-p8803630	Bn-A04-p8803630	A4	18	19.2	A
3798	Bn-N4-p10747215	Bn-A04-p8807708	Bn-A04-p8807708	A4	18	19.2	A
3799	Bn-N4-p10751522	Bn-A04-p8811740	Bn-A04-p8811740	A4	18	19.2	A
3800	Bn-N4-p10752832	Bn-A04-p8813038	Bn-A04-p8813038	A4	18	19.2	A
3801	Bn-N4-p10760090	Bn-A04-p8817780	Bn-A04-p8817780	A4	18	19.2	A
3802	Bn-N4-p10776079	Bn-A04-p8824695	Bn-A04-p8824695	A4	18	19.2	A
3803	Bn-N4-p10779088	Bn-A04-p8828462	Bn-A04-p8828462	A4	18	19.2	A
3804	Bn-N4-p10781189	Bn-A04-p8830454	Bn-A04-p8830454	A4	18	19.2	A
3805	Bn-N4-p10784937	Bn-A04-p8834077	Bn-A04-p8834077	A4	18	19.2	A
3806	Bn-N4-p10785120	Bn-A04-p8834259	Bn-A04-p8834259	A4	18	19.2	A
3807	Bn-N4-p10791067	Bn-A04-p8840146	Bn-A04-p8840146	A4	18	19.2	A
3808	Bn-N4-p10799340	Bn-A04-p8847485	Bn-A04-p8847485	A4	18	19.2	A
3809	Bn-N4-p10802012	Bn-A04-p8850036	Bn-A04-p8850036	A4	18	19.2	A
3810	Bn-N4-p10804298	Bn-C4-p39348941	Bn-scaff_16694_1-p22548	A4	18	19.2	A
3811	Bn-N4-p10804490	Bn-C4-p39355679	Bn-scaff_16694_1-p15810	A4	18	19.2	A
3812	Bn-N4-p10818166	Bn-A04-p8865894	Bn-A04-p8865894	A4	18	19.2	A
3813	Bn-N4-p10843822	Bn-A04-p8889253	Bn-A04-p8889253	A4	18	19.2	A
3814	Bn-N4-p10846981	Bn-A04-p8892416	Bn-A04-p8892416	A4	18	19.2	A
3815	Bn-N4-p10861544	Bn-A04-p8906594	Bn-A04-p8906594	A4	18	19.2	A
3816	Bn-N4-p10868856	Bn-C4-p39430832	Bn-scaff_19208_1-p59244	A4	18	19.2	A
3817	Bn-N14-p41597682	Bn-A04-p9296465	Bn-A04-p9296465	A4	19	20	A

3818	Bn-N14-p41670941	Bn-C4-p39997449	Bn-scaff_25870_1-p81302	A4	19	20	A
3819	Bn-N14-p44320717	Bn-C4-p42399426	Bn-scaff_15852_1-p23456	A4	19	20	A
3820	Bn-N14-p45329352	Bn-C4-p43176727	Bn-scaff_21956_1-p15437	A4	19	20	A
3821	Bn-N14-p45433812	Bn-C4-p43279394	Bn-scaff_21956_1-p25703	A4	19	20	A
3822	Bn-N14-p45448306	Bn-A04-p11595428	Bn-A04-p11595428	A4	19	20	A
3823	Bn-N14-p45664344	Bn-Scaffold01551-p2	Bn-scaff_25139_1-p2069	A4	19	20	A
3824	Bn-N15-p11244670	Bn-A06-p11368090	Bn-A06-p11368090	A4	19	20	A
3825	Bn-N4-p11036134	Bn-A04-p9086923	Bn-A04-p9086923	A4	19	20	A
3826	Bn-N4-p11037233	Bn-A04-p9088043	Bn-A04-p9088043	A4	19	20	A
3827	Bn-N4-p11037947	Bn-A04-p9088760	Bn-A04-p9088760	A4	19	20	A
3828	Bn-N4-p11071258	Bn-A04-p9119079	Bn-A04-p9119079	A4	19	20	A
3829	Bn-N4-p11071321	Bn-A04-p9119142	Bn-A04-p9119142	A4	19	20	A
3830	Bn-N4-p11072046	Bn-A04-p9119867	Bn-A04-p9119867	A4	19	20	A
3831	Bn-N4-p11169034	Bn-A04-p9232024	Bn-A04-p9232024	A4	19	20	A
3832	Bn-N4-p11235730	Bn-A04-p9296314	Bn-A04-p9296314	A4	19	20	A
3833	Bn-N4-p11252760	Bn-A04-p9309213	Bn-A04-p9309213	A4	19	20	A
3834	Bn-N4-p11260262	Bn-A04-p9317798	Bn-A04-p9317798	A4	19	20	A
3835	Bn-N4-p11264561	Bn-A04-p9322160	Bn-A04-p9322160	A4	19	20	A
3836	Bn-N4-p11302129	Bn-A04-p9363550	Bn-A04-p9363550	A4	19	20	A
3837	Bn-N4-p11330582	Bn-A04-p9379937	Bn-A04-p9379937	A4	19	20	A
3838	Bn-N4-p11349375	Bn-A04-p9392577	Bn-A04-p9392577	A4	19	20	A
3839	Bn-N4-p11364987	Bn-A04-p9400393	Bn-A04-p9400393	A4	19	20	A
3840	Bn-N4-p11398821	Bn-A04-p9422027	Bn-A04-p9422027	A4	19	20	A
3841	Bn-N4-p11403256	Bn-A04-p9426523	Bn-A04-p9426523	A4	19	20	A
3842	Bn-N4-p11472813	Bn-A04-p9484747	Bn-A04-p9484747	A4	19	20	A
3843	Bn-N4-p11490001	Bn-A04-p9509486	Bn-A04-p9509486	A4	19	20	A
3844	Bn-N4-p11490738	Bn-A04-p9510223	Bn-A04-p9510223	A4	19	20	A
3845	Bn-N4-p11491266	Bn-A04-p9510750	Bn-A04-p9510750	A4	19	20	A
3846	Bn-N4-p11491280	Bn-A04-p9510764	Bn-A04-p9510764	A4	19	20	A
3847	Bn-N4-p11491921	Bn-A04-p9511386	Bn-A04-p9511386	A4	19	20	A
3848	Bn-N4-p11513641	Bn-A04-p9524800	Bn-A04-p9524800	A4	19	20	A
3849	Bn-N4-p11612168	Bn-A04-p9623505	Bn-A04-p9623505	A4	19	20	A
3850	Bn-N4-p11625156	Bn-A04-p9632786	Bn-A04-p9632786	A4	19	20	A
3851	Bn-N4-p11656499	Bn-A04-p9658559	Bn-A04-p9658559	A4	19	20	A
3852	Bn-N4-p11667162	Bn-A04-p9673337	Bn-A04-p9673337	A4	19	20	A
3853	Bn-N4-p11669634	Bn-A04-p9675790	Bn-A04-p9675790	A4	19	20	A
3854	Bn-N4-p11790336	Bn-A04-p9808875	Bn-A04-p9808875	A4	19	20	A
3855	Bn-N4-p11801084	Bn-A04-p9819724	Bn-A04-p9819724	A4	19	20	A
3856	Bn-N4-p11801243	Bn-A04-p9819885	Bn-A04-p9819885	A4	19	20	A
3857	Bn-N4-p11823878	Bn-A04-p9841094	Bn-A04-p9841094	A4	19	20	A
3858	Bn-N4-p11826893	Bn-A04-p9844020	Bn-A04-p9844020	A4	19	20	A
3859	Bn-N4-p11831435	Bn-A04-p9848849	Bn-A04-p9848849	A4	19	20	A
3860	Bn-N4-p11843496	Bn-A04-p9861037	Bn-A04-p9861037	A4	19	20	A
3861	Bn-N4-p11845785	Bn-A04-p9863329	Bn-A04-p9863329	A4	19	20	A
3862	Bn-N4-p11852935	Bn-A04-p9864983	Bn-A04-p9864983	A4	19	20	A
3863	Bn-N4-p11853578	Bn-A04-p9865629	Bn-A04-p9865629	A4	19	20	A
3864	Bn-N4-p11853659	Bn-A04-p9865712	Bn-A04-p9865712	A4	19	20	A
3865	Bn-N4-p11856220	Bn-A04-p9868258	Bn-A04-p9868258	A4	19	20	A
3866	Bn-N4-p11858133	Bn-A04-p9870167	Bn-A04-p9870167	A4	19	20	A

3867	Bn-N4-p11859341	Bn-A04-p9870879	Bn-A04-p9870879	A4	19	20	A
3868	Bn-N4-p11866661	Bn-A04-p9878192	Bn-A04-p9878192	A4	19	20	A
3869	Bn-N4-p11866946	Bn-A04-p9878444	Bn-A04-p9878444	A4	19	20	A
3870	Bn-N4-p11910916	Bn-A04-p9920827	Bn-A04-p9920827	A4	19	20	A
3871	Bn-N4-p11910962	Bn-A04-p9920873	Bn-A04-p9920873	A4	19	20	A
3872	Bn-N4-p11911966	Bn-A04-p9921876	Bn-A04-p9921876	A4	19	20	A
3873	Bn-N4-p11920065	Bn-A04-p9930371	Bn-A04-p9930371	A4	19	20	A
3874	Bn-N4-p11922280	Bn-A04-p9932606	Bn-A04-p9932606	A4	19	20	A
3875	Bn-N4-p11965185	Bn-A04-p9983165	Bn-A04-p9983165	A4	19	20	A
3876	Bn-N4-p11983093	Bn-A04-p9991606	Bn-A04-p9991606	A4	19	20	A
3877	Bn-N4-p12005681	Bn-A04-p10016854	Bn-A04-p10016854	A4	19	20	A
3878	Bn-N4-p12128700	Bn-A04-p10113831	Bn-A04-p10113831	A4	19	20	A
3879	Bn-N4-p12128838	Bn-A04-p10113969	Bn-A04-p10113969	A4	19	20	A
3880	Bn-N4-p12141458	Bn-A04-p10129538	Bn-A04-p10129538	A4	19	20	A
3881	Bn-N4-p12164658	Bn-A04-p10154400	Bn-A04-p10154400	A4	19	20	A
3882	Bn-N4-p12166165	Bn-A04-p10155907	Bn-A04-p10155907	A4	19	20	A
3883	Bn-N4-p12196262	Bn-A04-p10176387	Bn-A04-p10176387	A4	19	20	A
3884	Bn-N4-p12202408	Bn-A04-p10182453	Bn-A04-p10182453	A4	19	20	A
3885	Bn-N4-p12207503	Bn-A04-p10188827	Bn-A04-p10188827	A4	19	20	A
3886	Bn-N4-p12216719	Bn-C4-p41227593	Bn-scaff_15798_1-p41905	A4	19	20	A
3887	Bn-N4-p12637478	Bn-A04-p10383434	Bn-A04-p10383434	A4	19	20	A
3888	Bn-N4-p12641858	Bn-A04-p10387804	Bn-A04-p10387804	A4	19	20	A
3889	Bn-N4-p12647481	Bn-A04-p10393460	Bn-A04-p10393460	A4	19	20	A
3890	Bn-N4-p12728711	Bn-A04-p10464942	Bn-A04-p10464942	A4	19	20	A
3891	Bn-N4-p12731491	Bn-A04-p10458946	Bn-A04-p10458946	A4	19	20	A
3892	Bn-N4-p12830886	Bn-A04-p10555408	Bn-A04-p10555408	A4	19	20	A
3893	Bn-N4-p12835744	Bn-A04-p10559481	Bn-A04-p10559481	A4	19	20	A
3894	Bn-N4-p12837177	Bn-A04-p10560896	Bn-A04-p10560896	A4	19	20	A
3895	Bn-N4-p12842304	Bn-A04-p10566018	Bn-A04-p10566018	A4	19	20	A
3896	Bn-N4-p12872786	Bn-A04-p10600455	Bn-A04-p10600455	A4	19	20	A
3897	Bn-N4-p12882182	Bn-A04-p10608772	Bn-A04-p10608772	A4	19	20	A
3898	Bn-N4-p12893052	Bn-A04-p10628977	Bn-A04-p10628977	A4	19	20	A
3899	Bn-N4-p12928183	Bn-A04-p10661890	Bn-A04-p10661890	A4	19	20	A
3900	Bn-N4-p12928224	Bn-A04-p10661931	Bn-A04-p10661931	A4	19	20	A
3901	Bn-N4-p12928696	Bn-A04-p10662404	Bn-A04-p10662404	A4	19	20	A
3902	Bn-N4-p12936968	Bn-A04-p10671164	Bn-A04-p10671164	A4	19	20	A
3903	Bn-N4-p12937505	Bn-A04-p10671721	Bn-A04-p10671721	A4	19	20	A
3904	Bn-N4-p12960006	Bn-A04-p10688504	Bn-A04-p10688504	A4	19	20	A
3905	Bn-N4-p13003736	Bn-A04-p10744003	Bn-A04-p10744003	A4	19	20	A
3906	Bn-N4-p13009208	Bn-A04-p10748559	Bn-A04-p10748559	A4	19	20	A
3907	Bn-N4-p13027215	Bn-A04-p10769811	Bn-A04-p10769811	A4	19	20	A
3908	Bn-N4-p13032331	Bn-A04-p10774641	Bn-A04-p10774641	A4	19	20	A
3909	Bn-N4-p13035554	Bn-A04-p10786704	Bn-A04-p10786704	A4	19	20	A
3910	Bn-N4-p13041412	Bn-A04-p10792201	Bn-A04-p10792201	A4	19	20	A
3911	Bn-N4-p13064299	Bn-A04-p10808508	Bn-A04-p10808508	A4	19	20	A
3912	Bn-N4-p13064596	Bn-A04-p10808805	Bn-A04-p10808805	A4	19	20	A
3913	Bn-N4-p13065377	Bn-C4-p42198893	Bn-scaff_15852_1-p43510	A4	19	20	A
3914	Bn-N4-p13065428	Bn-A04-p10809637	Bn-A04-p10809637	A4	19	20	A
3915	Bn-N4-p13110030	Bn-A04-p10833713	Bn-A04-p10833713	A4	19	20	A

3916	Bn-N4-p13148883	Bn-A04-p10861826	Bn-A04-p10861826	A4	19	20	A
3917	Bn-N4-p13153794	Bn-A04-p10867512	Bn-A04-p10867512	A4	19	20	A
3918	Bn-N4-p13172647	Bn-A04-p10881502	Bn-A04-p10881502	A4	19	20	A
3919	Bn-N4-p13200712	Bn-A04-p10914108	Bn-A04-p10914108	A4	19	20	A
3920	Bn-N4-p13291720	Bn-A04-p10980342	Bn-A04-p10980342	A4	19	20	A
3921	Bn-N4-p13295084	Bn-A04-p10983805	Bn-A04-p10983805	A4	19	20	A
3922	Bn-N4-p13331211	Bn-A04-p11020444	Bn-A04-p11020444	A4	19	20	A
3923	Bn-N4-p13340907	Bn-A04-p11026664	Bn-A04-p11026664	A4	19	20	A
3924	Bn-N4-p13372031	Bn-A04-p11058870	Bn-A04-p11058870	A4	19	20	A
3925	Bn-N4-p13385133	Bn-C4-p42640094	Bn-scaff_16700_1-p6000	A4	19	20	A
3926	Bn-N4-p13412903	Bn-A04-p11119216	Bn-A04-p11119216	A4	19	20	A
3927	Bn-N4-p13413271	Bn-A04-p11119582	Bn-A04-p11119582	A4	19	20	A
3928	Bn-N4-p13482331	Bn-A04-p11191754	Bn-A04-p11191754	A4	19	20	A
3929	Bn-N4-p13483986	Bn-A04-p11192862	Bn-A04-p11192862	A4	19	20	A
3930	Bn-N4-p13491897	Bn-A04-p11197346	Bn-A04-p11197346	A4	19	20	A
3931	Bn-N4-p13492258	Bn-A04-p11197707	Bn-A04-p11197707	A4	19	20	A
3932	Bn-N4-p13515177	Bn-A04-p11209663	Bn-A04-p11209663	A4	19	20	A
3933	Bn-N4-p13515340	Bn-A04-p11209827	Bn-A04-p11209827	A4	19	20	A
3934	Bn-N4-p13518558	Bn-A04-p11213108	Bn-A04-p11213108	A4	19	20	A
3935	Bn-N4-p13553512	Bn-A04-p11243983	Bn-A04-p11243983	A4	19	20	A
3936	Bn-N4-p13553523	Bn-A04-p11243994	Bn-A04-p11243994	A4	19	20	A
3937	Bn-N4-p13554052	Bn-C4-p42859914	Bn-scaff_24979_1-p40845	A4	19	20	A
3938	Bn-N4-p13556851	Bn-A04-p11247206	Bn-A04-p11247206	A4	19	20	A
3939	Bn-N4-p13559742	Bn-A04-p11250035	Bn-A04-p11250035	A4	19	20	A
3940	Bn-N4-p13559848	Bn-A04-p11250141	Bn-A04-p11250141	A4	19	20	A
3941	Bn-N4-p13584873	Bn-A04-p11291596	Bn-A04-p11291596	A4	19	20	A
3942	Bn-N4-p13621425	Bn-A04-p11319703	Bn-A04-p11319703	A4	19	20	A
3943	Bn-N4-p13621735	Bn-A04-p11320045	Bn-A04-p11320045	A4	19	20	A
3944	Bn-N4-p13625199	Bn-A04-p11325008	Bn-A04-p11325008	A4	19	20	A
3945	Bn-N4-p13636377	Bn-A04-p11329756	Bn-A04-p11329756	A4	19	20	A
3946	Bn-N4-p13667364	Bn-A04-p11359006	Bn-A04-p11359006	A4	19	20	A
3947	Bn-N4-p13679025	Bn-A04-p11364790	Bn-A04-p11364790	A4	19	20	A
3948	Bn-N4-p13688766	Bn-A04-p11376488	Bn-A04-p11376488	A4	19	20	A
3949	Bn-N4-p13690012	Bn-A04-p11377702	Bn-A04-p11377702	A4	19	20	A
3950	Bn-N4-p13690084	Bn-A04-p11377775	Bn-A04-p11377775	A4	19	20	A
3951	Bn-N4-p13701647	Bn-A04-p11386737	Bn-A04-p11386737	A4	19	20	A
3952	Bn-N4-p13716359	Bn-A04-p11402649	Bn-A04-p11402649	A4	19	20	A
3953	Bn-N4-p13749037	Bn-A04-p11425304	Bn-A04-p11425304	A4	19	20	A
3954	Bn-N4-p13751811	Bn-A04-p11428085	Bn-A04-p11428085	A4	19	20	A
3955	Bn-N4-p13776401	Bn-A04-p11450339	Bn-A04-p11450339	A4	19	20	A
3956	Bn-N4-p13778611	Bn-A04-p11452549	Bn-A04-p11452549	A4	19	20	A
3957	Bn-N4-p13781466	Bn-C4-p43117488	Bn-scaff_21956_1-p95133	A4	19	20	A
3958	Bn-N4-p13789033	Bn-A04-p11465588	Bn-A04-p11465588	A4	19	20	A
3959	Bn-N4-p13836054	Bn-A04-p11510797	Bn-A04-p11510797	A4	19	20	A
3960	Bn-N4-p13837635	Bn-A04-p11512379	Bn-A04-p11512379	A4	19	20	A
3961	Bn-N4-p13838707	Bn-A04-p11513352	Bn-A04-p11513352	A4	19	20	A
3962	Bn-N4-p13975906	Bn-C4-p43351397	Bn-scaff_21198_1-p16130	A4	19	20	A
3963	Bn-N4-p14044443	Bn-A04-p11734372	Bn-A04-p11734372	A4	19	20	A
3964	Bn-N4-p14069590	Bn-A04-p11765380	Bn-A04-p11765380	A4	19	20	A

3965	Bn-N4-p14083506	Bn-A04-p11791366	Bn-A04-p11791366	A4	19	20	A
3966	Bn-N4-p14088376	Bn-A04-p11795779	Bn-A04-p11795779	A4	19	20	A
3967	Bn-N4-p14114079	Bn-A04-p11818064	Bn-A04-p11818064	A4	19	20	A
3968	Bn-N4-p14162714	Bn-C4-p43594891	Bn-scaff_22278_1-p23832¦	A4	19	20	A
3969	Bn-N4-p14170993	Bn-A04-p11865854	Bn-A04-p11865854	A4	19	20	A
3970	Bn-N4-p14210018	Bn-C4-p43695465	Bn-scaff_22278_1-p13775	A4	19	20	A
3971	Bn-N4-p14213799	Bn-A04-p11890416	Bn-A04-p11890416	A4	19	20	A
3972	Bn-N4-p14244731	Bn-A04-p11922910	Bn-A04-p11922910	A4	19	20	A
3973	Bn-N4-p14245131	Bn-A04-p11923310	Bn-A04-p11923310	A4	19	20	A
3974	Bn-N4-p14245332	Bn-A04-p11923511	Bn-A04-p11923511	A4	19	20	A
3975	Bn-N4-p14246038	Bn-C4-p43748590	Bn-scaff_22278_1-p84630	A4	19	20	A
3976	Bn-N9-p33089970	Bn-A09-p30233844	Bn-A09-p30233844	A4	19	20	A
3977	Bn-N4-p14285273	Bn-A04-p11957518	Bn-A04-p11957518	A4	20	21.6	A
3978	Bn-N4-p14285802	Bn-A04-p11958047	Bn-A04-p11958047	A4	20	21.6	A
3979	Bn-N4-p14293805	Bn-A04-p11961175	Bn-A04-p11961175	A4	20	21.6	A
3980	Bn-N4-p14342001	Bn-A04-p12010066	Bn-A04-p12010066	A4	20	21.6	A
3981	Bn-N4-p14343231	Bn-A04-p12011281	Bn-A04-p12011281	A4	20	21.6	A
3982	Bn-N4-p14449321	Bn-A04-p12112148	Bn-A04-p12112148	A4	20	21.6	A
3983	Bn-N4-p14466779	Bn-Scaffold004542-p4	Bn-Scaffold004542-p45	A4	20	21.6	A
3984	Bn-N4-p14483050	Bn-A04-p12151317	Bn-A04-p12151317	A4	20	21.6	A
3985	Bn-N4-p14573838	Bn-A04-p12236240	Bn-A04-p12236240	A4	21	23.2	A
3986	Bn-N4-p14580040	Bn-A04-p12243311	Bn-A04-p12243311	A4	21	23.2	A
3987	Bn-N4-p14596496	Bn-A04-p12259499	Bn-A04-p12259499	A4	21	23.2	A
3988	Bn-N14-p21187050	Bn-C4-p20399045	Bn-scaff_19575_1-p66188	A4	22	24.8	A
3989	Bn-N14-p46551005	Bn-A04-p12651819	Bn-A04-p12651819	A4	22	24.8	A
3990	Bn-N14-p47548179	Bn-A04-p13246454	Bn-A04-p13246454	A4	22	24.8	A
3991	Bn-N4-p14621921	Bn-A04-p12286484	Bn-A04-p12286484	A4	22	24.8	A
3992	Bn-N4-p14624049	Bn-A04-p12288635	Bn-A04-p12288635	A4	22	24.8	A
3993	Bn-N4-p14660450	Bn-A04-p12333381	Bn-A04-p12333381	A4	22	24.8	A
3994	Bn-N4-p14671293	Bn-A04-p12344157	Bn-A04-p12344157	A4	22	24.8	A
3995	Bn-N4-p14671478	Bn-A04-p12344342	Bn-A04-p12344342	A4	22	24.8	A
3996	Bn-N4-p14681626	Bn-A04-p12348826	Bn-A04-p12348826	A4	22	24.8	A
3997	Bn-N4-p14683376	Bn-A04-p12350578	Bn-A04-p12350578	A4	22	24.8	A
3998	Bn-N4-p14697921	Bn-A04-p12360869	Bn-A04-p12360869	A4	22	24.8	A
3999	Bn-N4-p14711545	Bn-A04-p12372810	Bn-A04-p12372810	A4	22	24.8	A
4000	Bn-N4-p14722671	Bn-A04-p12655669	Bn-A04-p12655669	A4	22	24.8	A
4001	Bn-N4-p14726255	Bn-A04-p12659254	Bn-A04-p12659254	A4	22	24.8	A
4002	Bn-N4-p14726403	Bn-A04-p12659402	Bn-A04-p12659402	A4	22	24.8	A
4003	Bn-N4-p14745871	Bn-A04-p12670129	Bn-A04-p12670129	A4	22	24.8	A
4004	Bn-N4-p14753696	Bn-A04-p12677961	Bn-A04-p12677961	A4	22	24.8	A
4005	Bn-N4-p14763421	Bn-A04-p12687510	Bn-A04-p12687510	A4	22	24.8	A
4006	Bn-N4-p14766672	Bn-A04-p12690759	Bn-A04-p12690759	A4	22	24.8	0
4007	Bn-N4-p14767153	Bn-A04-p12691245	Bn-A04-p12691245	A4	22	24.8	A
4008	Bn-N4-p14778835	Bn-A04-p12712772	Bn-A04-p12712772	A4	22	24.8	A
4009	Bn-N4-p14781922	Bn-A04-p12715859	Bn-A04-p12715859	A4	22	24.8	A
4010	Bn-N4-p14785933	Bn-A04-p12719682	Bn-A04-p12719682	A4	22	24.8	A
4011	Bn-N4-p14794411	Bn-A04-p12724637	Bn-A04-p12724637	A4	22	24.8	A
4012	Bn-N4-p14795370	Bn-A04-p12725596	Bn-A04-p12725596	A4	22	24.8	A
4013	Bn-N4-p14795841	Bn-A04-p12726067	Bn-A04-p12726067	A4	22	24.8	A

4014	Bn-N4-p14798136	Bn-A04-p12728362	Bn-A04-p12728362	A4	22	24.8	A
4015	Bn-N4-p14804042	Bn-A04-p12734346	Bn-A04-p12734346	A4	22	24.8	A
4016	Bn-N4-p14808644	Bn-A04-p12735594	Bn-A04-p12735594	A4	22	24.8	A
4017	Bn-N4-p14815362	Bn-A04-p12749707	Bn-A04-p12749707	A4	22	24.8	A
4018	Bn-N4-p14817423	Bn-A04-p12751774	Bn-A04-p12751774	A4	22	24.8	A
4019	Bn-N4-p14822272	Bn-A04-p12756666	Bn-A04-p12756666	A4	22	24.8	A
4020	Bn-N4-p14825038	Bn-A04-p12759219	Bn-A04-p12759219	A4	22	24.8	A
4021	Bn-N4-p14829864	Bn-A04-p12764046	Bn-A04-p12764046	A4	22	24.8	A
4022	Bn-N4-p14835031	Bn-A04-p12769407	Bn-A04-p12769407	A4	22	24.8	A
4023	Bn-N4-p14837837	Bn-A04-p12772506	Bn-A04-p12772506	A4	22	24.8	A
4024	Bn-N4-p14859732	Bn-A04-p12802914	Bn-A04-p12802914	A4	22	24.8	A
4025	Bn-N4-p14986900	Bn-A04-p12942400	Bn-A04-p12942400	A4	22	24.8	A
4026	Bn-N4-p15005514	Bn-A04-p12959668	Bn-A04-p12959668	A4	22	24.8	A
4027	Bn-N4-p15093867	Bn-A04-p13052013	Bn-A04-p13052013	A4	22	24.8	A
4028	Bn-N4-p15102956	Bn-A04-p13056382	Bn-A04-p13056382	A4	22	24.8	A
4029	Bn-N4-p15106045	Bn-A04-p13059485	Bn-A04-p13059485	A4	22	24.8	A
4030	Bn-N4-p15107691	Bn-A04-p13061139	Bn-A04-p13061139	A4	22	24.8	A
4031	Bn-N4-p15110146	Bn-A04-p13062081	Bn-A04-p13062081	A4	22	24.8	A
4032	Bn-N4-p15111227	Bn-A04-p13063145	Bn-A04-p13063145	A4	22	24.8	A
4033	Bn-N4-p15114834	Bn-A04-p13068545	Bn-A04-p13068545	A4	22	24.8	A
4034	Bn-N4-p15122228	Bn-A04-p13078078	Bn-A04-p13078078	A4	22	24.8	A
4035	Bn-N4-p15151940	Bn-Scaffold000251-p3	Bn-Scaffold000251-p34051	A4	22	24.8	A
4036	Bn-N4-p15316208	Bn-A04-p13235708	Bn-A04-p13235708	A4	22	24.8	A
4037	Bn-N4-p15322956	Bn-A04-p13241959	Bn-A04-p13241959	A4	22	24.8	A
4038	Bn-N4-p15328477	Bn-A04-p13245778	Bn-A04-p13245778	A4	22	24.8	A
4039	Bn-N4-p15329791	Bn-A04-p13247108	Bn-A04-p13247108	A4	22	24.8	A
4040	Bn-N4-p15332454	Bn-A04-p13249011	Bn-A04-p13249011	A4	22	24.8	A
4041	Bn-N4-p15337468	Bn-A04-p13269135	Bn-A04-p13269135	A4	22	24.8	A
4042	Bn-N4-p15349049	Bn-A04-p13286494	Bn-A04-p13286494	A4	22	24.8	A
4043	Bn-N4-p15352692	Bn-A04-p13293545	Bn-A04-p13293545	A4	22	24.8	A
4044	Bn-N4-p15352787	Bn-A04-p13293640	Bn-A04-p13293640	A4	22	24.8	A
4045	Bn-N4-p15353469	Bn-A04-p13294322	Bn-A04-p13294322	A4	22	24.8	A
4046	Bn-N4-p15370445	Bn-A04-p13302511	Bn-A04-p13302511	A4	22	24.8	A
4047	Bn-N4-p15377894	Bn-A04-p13306369	Bn-A04-p13306369	A4	22	24.8	A
4048	Bn-N4-p15435930	Bn-A04-p13368998	Bn-A04-p13368998	A4	22	24.8	A
4049	Bn-N4-p15439797	Bn-A04-p13372886	Bn-A04-p13372886	A4	22	24.8	A
4050	Bn-N4-p15444121	Bn-A04-p13384606	Bn-A04-p13384606	A4	22	24.8	A
4051	Bn-N4-p15477342	Bn-A04-p13418563	Bn-A04-p13418563	A4	22	24.8	A
4052	Bn-N4-p15486644	Bn-A04-p13428243	Bn-A04-p13428243	A4	22	24.8	A
4053	Bn-N4-p15486725	Bn-A04-p13428324	Bn-A04-p13428324	A4	22	24.8	A
4054	Bn-N4-p15487928	Bn-A04-p13429557	Bn-A04-p13429557	A4	22	24.8	A
4055	Bn-N4-p15488520	Bn-A04-p13430155	Bn-A04-p13430155	A4	22	24.8	A
4056	Bn-N4-p15489372	Bn-A04-p13431005	Bn-A04-p13431005	A4	22	24.8	A
4057	Bn-N4-p15527167	Bn-A04-p13497608	Bn-A04-p13497608	A4	22	24.8	A
4058	Bn-N4-p15531244	Bn-A04-p13501943	Bn-A04-p13501943	A4	22	24.8	A
4059	Bn-N4-p15533186	Bn-A04-p13504670	Bn-A04-p13504670	A4	22	24.8	A
4060	Bn-N4-p15533527	Bn-A04-p13505017	Bn-A04-p13505017	A4	22	24.8	A
4061	Bn-N4-p15535912	Bn-A04-p13510301	Bn-A04-p13510301	A4	22	24.8	A
4062	Bn-N4-p15540324	Bn-A04-p13516223	Bn-A04-p13516223	A4	22	24.8	A

4063	Bn-N4-p15540386	Bn-A04-p13516285	Bn-A04-p13516285	A4	22	24.8	A
4064	Bn-N4-p15546886	Bn-A04-p13516411	Bn-A04-p13516411	A4	22	24.8	A
4065	Bn-N4-p15547601	Bn-A04-p13517158	Bn-A04-p13517158	A4	22	24.8	A
4066	Bn-N4-p15547838	Bn-A04-p13520736	Bn-A04-p13520736	A4	22	24.8	A
4067	Bn-N4-p15548809	Bn-A04-p13521376	Bn-A04-p13521376	A4	22	24.8	A
4068	Bn-N4-p15548838	Bn-A04-p13521405	Bn-A04-p13521405	A4	22	24.8	A
4069	Bn-N4-p15595126	Bn-C4-p45796054	Bn-scaff_16517_1-p91597	A4	22	24.8	A
4070	Bn-N4-p15595532	Bn-A04-p13572341	Bn-A04-p13572341	A4	22	24.8	A
4071	Bn-N4-p15605130	Bn-A04-p13583325	Bn-A04-p13583325	A4	22	24.8	A
4072	Bn-N14-p48360333	Bn-C4-p46265970	Bn-scaff_16517_1-p56151	A4	23	25.6	A
4073	Bn-N4-p15622445	Bn-A04-p13600415	Bn-A04-p13600415	A4	23	25.6	A
4074	Bn-N4-p15674600	Bn-A04-p13655958	Bn-A04-p13655958	A4	23	25.6	A
4075	Bn-N4-p15678260	Bn-A04-p13659690	Bn-A04-p13659690	A4	23	25.6	A
4076	Bn-N4-p15688616	Bn-A04-p13670045	Bn-A04-p13670045	A4	23	25.6	A
4077	Bn-N4-p15701910	Bn-A04-p13685561	Bn-A04-p13685561	A4	23	25.6	A
4078	Bn-N4-p15707430	Bn-A04-p13689816	Bn-A04-p13689816	A4	23	25.6	A
4079	Bn-N4-p15714656	Bn-A04-p13705410	Bn-A04-p13705410	A4	23	25.6	A
4080	Bn-N4-p15714794	Bn-A04-p13705548	Bn-A04-p13705548	A4	23	25.6	A
4081	Bn-N4-p15714880	Bn-A04-p13705636	Bn-A04-p13705636	A4	23	25.6	A
4082	Bn-N4-p15715005	Bn-A04-p13705760	Bn-A04-p13705760	A4	23	25.6	A
4083	Bn-N4-p15771314	Bn-A04-p13761897	Bn-A04-p13761897	A4	23	25.6	A
4084	Bn-N4-p15777042	Bn-A04-p13767868	Bn-A04-p13767868	A4	23	25.6	A
4085	Bn-N4-p15894606	Bn-A04-p13866861	Bn-A04-p13866861	A4	23	25.6	A
4086	Bn-N4-p16108021	Bn-C4-p46736296	Bn-scaff_16804_2-p15792	A4	24	26.4	A
4087	Bn-N14-p48976254	Bn-A04-p14156506	Bn-A04-p14156506	A4	25	28	A
4088	Bn-N4-p16162014	Bn-C4-p46830608	Bn-scaff_16804_2-p63611	A4	25	28	A
4089	Bn-N4-p16255878	Bn-A04-p14255771	Bn-A04-p14255771	A4	26	29.6	A
4090	Bn-N4-p16258547	Bn-A04-p14271110	Bn-A04-p14271110	A4	26	29.6	A
4091	Bn-N4-p16258598	Bn-A04-p14271161	Bn-A04-p14271161	A4	26	29.6	A
4092	Bn-N4-p16322475	Bn-A04-p14333001	Bn-A04-p14333001	A4	26	29.6	A
4093	Bn-N16-p29869414	Bn-A04-p14756394	Bn-A04-p14756394	A4	27	32.8	A
4094	Bn-N4-p16378096	Bn-A04-p14380140	Bn-A04-p14380140	A4	27	32.8	A
4095	Bn-N4-p16401198	Bn-A04-p14397957	Bn-A04-p14397957	A4	27	32.8	A
4096	Bn-N4-p16401576	Bn-A04-p14398335	Bn-A04-p14398335	A4	27	32.8	A
4097	Bn-N4-p16454593	Bn-A04-p14483727	Bn-A04-p14483727	A4	27	32.8	A
4098	Bn-N4-p16518169	Bn-A04-p14559897	Bn-A04-p14559897	A4	27	32.8	A
4099	Bn-N4-p16523294	Bn-A04-p14565138	Bn-A04-p14565138	A4	27	32.8	A
4100	Bn-N4-p16524041	Bn-A04-p14565883	Bn-A04-p14565883	A4	27	32.8	A
4101	Bn-N4-p16711540	Bn-A04-p14756001	Bn-A04-p14756001	A4	27	32.8	A
4102	Bn-N14-p50385352	Bn-A04-p14986920	Bn-A04-p14986920	A4	28	36.8	B
4103	Bn-N4-p16884111	Bn-A04-p14917114	Bn-A04-p14917114	A4	28	36.8	B
4104	Bn-N4-p16889546	Bn-A04-p14922562	Bn-A04-p14922562	A4	28	36.8	B
4105	Bn-N4-p16911000	Bn-A04-p14944336	Bn-A04-p14944336	A4	28	36.8	B
4106	Bn-N4-p16924672	Bn-A04-p14961814	Bn-A04-p14961814	A4	28	36.8	B
4107	Bn-N4-p16929784	Bn-A04-p14965926	Bn-A04-p14965926	A4	28	36.8	B
4108	Bn-N4-p16963218	Bn-A04-p14999714	Bn-A04-p14999714	A4	28	36.8	B
4109	Bn-N4-p16963400	Bn-A04-p15001710	Bn-A04-p15001710	A4	28	36.8	B
4110	Bn-N4-p16965424	Bn-A04-p15007146	Bn-A04-p15007146	A4	28	36.8	B
4111	Bn-N4-p16967420	Bn-A04-p15009639	Bn-A04-p15009639	A4	28	36.8	B

4112	Bn-N4-p16968929	Bn-A04-p15011193	Bn-A04-p15011193	A4	28	36.8	B
4113	Bn-N4-p17082281	Bn-A04-p15152016	Bn-A04-p15152016	A4	29	37.6	B
4114	Bn-N4-p17271998	Bn-A04-p15298740	Bn-A04-p15298740	A4	29	37.6	B
4115	Bn-Scaffold03862-p1699	Bn-A04-p15168889	Bn-A04-p15168889	A4	29	37.6	B
4116	Bn-N14-p50489503	Bn-C4-p48717057	Bn-scaff_15585_1-p10605[illegible]	A4	30	38.4	B
4117	Bn-N4-p17297494	Bn-A04-p15315594	Bn-A04-p15315594	A4	30	38.4	B
4118	Bn-N4-p17369430	Bn-A04-p15404568	Bn-A04-p15404568	A4	30	38.4	B
4119	Bn-N4-p17378738	Bn-A04-p15409836	Bn-A04-p15409836	A4	30	38.4	B
4120	Bn-N4-p17402396	Bn-A04-p15436139	Bn-A04-p15436139	A4	30	38.4	B
4121	Bn-N4-p17447676	Bn-A04-p15471964	Bn-A04-p15471964	A4	30	38.4	B
4122	Bn-N4-p17450818	Bn-A04-p15475759	Bn-A04-p15475759	A4	30	38.4	B
4123	Bn-N4-p17465788	Bn-A04-p15492182	Bn-A04-p15492182	A4	30	38.4	B
4124	Bn-N4-p17478812	Bn-A04-p15500804	Bn-A04-p15500804	A4	30	38.4	B
4125	Bn-N5-p5223997	Bn-C4-p48635290	Bn-scaff_15585_1-p97878[illegible]	A4	30	38.4	B
4126	Bn-N4-p17499224	Bn-A04-p15514869	Bn-A04-p15514869	A4	31	39.2	B
4127	Bn-N4-p17504210	Bn-A04-p15519877	Bn-A04-p15519877	A4	31	39.2	B
4128	Bn-N4-p17513547	Bn-A04-p15530059	Bn-A04-p15530059	A4	31	39.2	B
4129	Bn-N4-p17518121	Bn-A04-p15534626	Bn-A04-p15534626	A4	31	39.2	B
4130	Bn-N4-p17518498	Bn-A04-p15535011	Bn-A04-p15535011	A4	31	39.2	B
4131	Bn-N4-p17547137	Bn-A04-p15555443	Bn-A04-p15555443	A4	31	39.2	B
4132	Bn-N4-p17593159	Bn-A04-p15600337	Bn-A04-p15600337	A4	31	39.2	B
4133	Bn-N4-p17648503	Bn-A04-p15655213	Bn-A04-p15655213	A4	31	39.2	B
4134	Bn-N4-p17703045	Bn-A04-p15707649	Bn-A04-p15707649	A4	31	39.2	B
4135	Bn-N4-p17722846	Bn-A04-p15727549	Bn-A04-p15727549	A4	31	39.2	B
4136	Bn-Scaffold31010-p309	Bn-A04-p15835310	Bn-A04-p15835310	A4	32	40	B
4137	Bn-N7-p15002311	Bn-A04-p15895339	Bn-A04-p15895339	A4	33	41.6	B
4138	Bn-Scaffold00305b-p73895	Bn-A04-p15885427	Bn-A04-p15885427	A4	33	41.6	B
4139	Bn-Scaffold00305b-p82022	Bn-A04-p15877051	Bn-A04-p15877051	A4	33	41.6	B
4140	Bn-Scaffold01341-p370	Bn-A04-p15870508	Bn-A04-p15870508	A4	33	41.6	B
4141	Unknown	Bn-A04-p15895735	Bn-A04-p15895735	A4	33	41.6	B
4142	Bn-N4-p17983696	Bn-A04-p16056186	Bn-A04-p16056186	A4	34	42.4	B
4143	Bn-N4-p18198567	Bn-A04-p16233024	Bn-A04-p16233024	A4	35	43.4	B
4144	Bn-N4-p18198920	Bn-A04-p16233377	Bn-A04-p16233377	A4	35	43.4	B
4145	Bn-N4-p18235505	Bn-A04-p16264494	Bn-A04-p16264494	A4	35	43.4	B
4146	Bn-N4-p18239986	Bn-A04-p16268792	Bn-A04-p16268792	A4	35	43.4	B
4147	Bn-N4-p18246343	Bn-A04-p16275472	Bn-A04-p16275472	A4	35	43.4	B
4148	Bn-N4-p18248210	Bn-A04-p16277340	Bn-A04-p16277340	A4	35	43.4	B
4149	Bn-N4-p18251300	Bn-A04-p16280279	Bn-A04-p16280279	A4	35	43.4	B
4150	Bn-N4-p18253060	Bn-A04-p16282039	Bn-A04-p16282039	A4	35	43.4	B
4151	Bn-N4-p18258871	Bn-A04-p16287850	Bn-A04-p16287850	A4	35	43.4	B
4152	Bn-N4-p18295066	Bn-C4-p50000919	Bn-scaff_16888_1-p10235[illegible]	A4	35	43.4	B
4153	Bn-N4-p18302479	Bn-C4-p50008719	Bn-scaff_16888_1-p10313[illegible]	A4	35	43.4	B
4154	Bn-N4-p18308270	Bn-A04-p16336301	Bn-A04-p16336301	A4	35	43.4	B
4155	Bn-N4-p18420558	Bn-A04-p16445225	Bn-A04-p16445225	A4	36	44.4	B
4156	Bn-N4-p18420961	Bn-A04-p16445602	Bn-A04-p16445602	A4	36	44.4	B
4157	Bn-N4-p18422343	Bn-A04-p16446976	Bn-A04-p16446976	A4	36	44.4	B
4158	Bn-N4-p18422584	Bn-A04-p16447217	Bn-A04-p16447217	A4	36	44.4	B
4159	Bn-N4-p18427467	Bn-A04-p16452098	Bn-A04-p16452098	A4	36	44.4	B
4160	Bn-N4-p18442566	Bn-A04-p16477853	Bn-A04-p16477853	A4	36	44.4	B

4161	Bn-N4-p18443986	Bn-A04-p16479236	Bn-A04-p16479236	A4	36	44.4	B
4162	Bn-N4-p18447576	Bn-A04-p16486340	Bn-A04-p16486340	A4	36	44.4	B
4163	Bn-N4-p18460684	Bn-A04-p16503326	Bn-A04-p16503326	A4	36	44.4	B
4164	Bn-N4-p18475690	Bn-C4-p50251051	Bn-scaff_16888_1-p127363	A4	36	44.4	B
4165	Bn-N4-p18478004	Bn-A04-p16523116	Bn-A04-p16523116	A4	36	44.4	B
4166	Bn-N4-p18538501	Bn-A04-p16598514	Bn-A04-p16598514	A4	37	45.4	B
4167	Bn-N4-p18548589	Bn-A04-p16608584	Bn-A04-p16608584	A4	37	45.4	B
4168	Bn-N4-p18620726	Bn-A04-p16663552	Bn-A04-p16663552	A4	37	45.4	B
4169	Bn-N4-p18646102	Bn-A04-p16684288	Bn-A04-p16684288	A4	37	45.4	B
4170	Bn-N14-p52198548	Bn-C4-p50844987	Bn-scaff_16888_1-p186757	A4	38	46.4	B
4171	Bn-N4-p18737197	Bn-A04-p17058367	Bn-A04-p17058367	A4	38	46.4	B
4172	Bn-N4-p18739727	Bn-C4-p50908452	Bn-scaff_16888_1-p193103	A4	38	46.4	B
4173	Bn-N4-p18743497	Bn-A04-p17052071	Bn-A04-p17052071	A4	38	46.4	B
4174	Bn-N4-p18749074	Bn-A04-p17046250	Bn-A04-p17046250	A4	38	46.4	B
4175	Bn-N4-p18751707	Bn-A04-p17045348	Bn-A04-p17045348	A4	38	46.4	B
4176	Bn-N4-p18752848	Bn-A04-p17044228	Bn-A04-p17044228	A4	38	46.4	B
4177	Bn-N4-p18775521	Bn-A04-p17019529	Bn-A04-p17019529	A4	38	46.4	B
4178	Bn-N4-p18788355	Bn-A04-p17003395	Bn-A04-p17003395	A4	38	46.4	B
4179	Bn-N4-p18791320	Bn-A04-p16998111	Bn-A04-p16998111	A4	38	46.4	B
4180	Bn-N4-p18808278	Bn-A04-p16980928	Bn-A04-p16980928	A4	38	46.4	B
4181	Bn-N4-p18859626	Bn-A04-p16919672	Bn-A04-p16919672	A4	38	46.4	B
4182	Bn-N4-p18859650	Bn-A04-p16919648	Bn-A04-p16919648	A4	38	46.4	B
4183	Bn-N4-p18888967	Bn-A04-p16892915	Bn-A04-p16892915	A4	38	46.4	B
4184	Bn-N4-p18935470	Bn-A04-p16841462	Bn-A04-p16841462	A4	38	46.4	B
4185	Bn-N4-p18943797	Bn-A04-p16832545	Bn-A04-p16832545	A4	38	46.4	B
4186	Bn-N4-p18957489	Bn-A04-p16819445	Bn-A04-p16819445	A4	38	46.4	B
4187	Bn-N4-p18963932	Bn-C4-p50565299	Bn-scaff_16888_1-p158788	A4	38	46.4	B
4188	Bn-N4-p18969850	Bn-A04-p16805391	Bn-A04-p16805391	A4	38	46.4	B
4189	Bn-N4-p18973231	Bn-A04-p16798852	Bn-A04-p16798852	A4	38	46.4	B
4190	Bn-N4-p18980996	Bn-A04-p16791082	Bn-A04-p16791082	A4	38	46.4	B
4191	Bn-N4-p19021064	Bn-A04-p16751677	Bn-A04-p16751677	A4	38	46.4	B
4192	Bn-N4-p19024648	Bn-A04-p16748162	Bn-A04-p16748162	A4	38	46.4	B
4193	Bn-N4-p19027130	Bn-A04-p16745356	Bn-A04-p16745356	A4	38	46.4	B
4194	Bn-N4-p19028469	Bn-A04-p16744035	Bn-A04-p16744035	A4	38	46.4	B
4195	Bn-N4-p19028528	Bn-A04-p16743976	Bn-A04-p16743976	A4	38	46.4	B
4196	Bn-N4-p19106540	Bn-A04-p17170974	Bn-A04-p17170974	A4	38	46.4	B
4197	Bn-N4-p19106930	Bn-A04-p17171349	Bn-A04-p17171349	A4	38	46.4	B
4198	Bn-N4-p19107894	Bn-A04-p17172246	Bn-A04-p17172246	A4	38	46.4	B
4199	Bn-N4-p19115278	Bn-A04-p17179870	Bn-A04-p17179870	A4	38	46.4	B
4200	Bn-N14-p52521816	Bn-C4-p51194144	Bn-scaff_20270_1-p210772	A4	39	47.2	B
4201	Bn-N4-p19251899	Bn-C4-p51208243	Bn-scaff_20270_1-p224871	A4	39	47.2	B
4202	Bn-N4-p19254592	Bn-A04-p17312041	Bn-A04-p17312041	A4	39	47.2	B
4203	Bn-N4-p19262174	Bn-A04-p17322241	Bn-A04-p17322241	A4	39	47.2	B
4204	Bn-N4-p19271681	Bn-A04-p17331915	Bn-A04-p17331915	A4	39	47.2	B
4205	Bn-N4-p19332706	Bn-A04-p17383739	Bn-A04-p17383739	A4	39	47.2	B
4206	Bn-N4-p19342177	Bn-A04-p17391802	Bn-A04-p17391802	A4	39	47.2	B
4207	Bn-N4-p19346776	Bn-A04-p17394552	Bn-A04-p17394552	A4	39	47.2	B
4208	Bn-N4-p19346960	Bn-A04-p17394736	Bn-A04-p17394736	A4	39	47.2	B
4209	Bn-N4-p19364932	Bn-A04-p17412823	Bn-A04-p17412823	A4	39	47.2	B

4210	Bn-N4-p19374651	Bn-A04-p17422800	Bn-A04-p17422800	A4	39	47.2	B
4211	Bn-N4-p19440153	Bn-A04-p17490425	Bn-A04-p17490425	A4	39	47.2	B
4212	Bn-N4-p19446545	Bn-A04-p17496899	Bn-A04-p17496899	A4	39	47.2	B
4213	Bn-N4-p19490724	Bn-A04-p17535104	Bn-A04-p17535104	A4	39	47.2	B
4214	Bn-N4-p19542103	Bn-A04-p17574162	Bn-A04-p17574162	A4	39	47.2	B
4215	Bn-N4-p19545572	Bn-A04-p17577700	Bn-A04-p17577700	A4	39	47.2	B
4216	Bn-N4-p19546312	Bn-A04-p17578444	Bn-A04-p17578444	A4	39	47.2	B
4217	Bn-N4-p19548845	Bn-A04-p17580804	Bn-A04-p17580804	A4	39	47.2	B
4218	Bn-N4-p19649505	Bn-A04-p17686228	Bn-A04-p17686228	A4	40	48	B
4219	Bn-N4-p19655215	Bn-A04-p17692138	Bn-A04-p17692138	A4	40	48	B
4220	Bn-N4-p19662384	Bn-A04-p17698780	Bn-A04-p17698780	A4	40	48	B
4221	Bn-N4-p19668024	Bn-A04-p17704458	Bn-A04-p17704458	A4	40	48	B
4222	Bn-N4-p19669966	Bn-A04-p17706333	Bn-A04-p17706333	A4	40	48	B
4223	Bn-N4-p19675646	Bn-A04-p17712184	Bn-A04-p17712184	A4	40	48	B
4224	Bn-N4-p19705522	Bn-A04-p17740663	Bn-A04-p17740663	A4	41	48.8	B
4225	Bn-N4-p19720981	Bn-A04-p17756059	Bn-A04-p17756059	A4	41	48.8	B
4226	Bn-N4-p19725124	Bn-A04-p17760280	Bn-A04-p17760280	A4	41	48.8	B
4227	Bn-N4-p19725713	Bn-A04-p17760869	Bn-A04-p17760869	A4	41	48.8	B
4228	Bn-N4-p19775221	Bn-A04-p17811740	Bn-A04-p17811740	A4	41	48.8	B
4229	Bn-N4-p19780571	Bn-A04-p17816969	Bn-A04-p17816969	A4	41	48.8	0
4230	Bn-N4-p19780403	Bn-A04-p17816796	Bn-A04-p17816796	A4	42	49.6	B
4231	Bn-N4-p19827880	Bn-A04-p17864572	Bn-A04-p17864572	A4	43	50.4	B
4232	Bn-N4-p19832166	Bn-A04-p17870056	Bn-A04-p17870056	A4	43	50.4	B
4233	Bn-N4-p19834650	Bn-A04-p17871505	Bn-A04-p17871505	A4	43	50.4	B
4234	Bn-N4-p19932331	Bn-A04-p17969838	Bn-A04-p17969838	A4	43	50.4	B
4235	Bn-N4-p20005862	Bn-A04-p18031439	Bn-A04-p18031439	A4	43	50.4	0
4236	Bn-N4-p20009256	Bn-C4-p52239303	Bn-scaff_18903_1-p82359	A4	43	50.4	B
4237	Bn-N4-p20065336	Bn-A04-p18085734	Bn-A04-p18085734	A4	43	50.4	B
4238	Bn-N4-p20065825	Bn-A04-p18086224	Bn-A04-p18086224	A4	43	50.4	B
4239	Bn-N4-p20067340	Bn-A04-p18087738	Bn-A04-p18087738	A4	43	50.4	B
4240	Unknown	Bn-A04-p17940767	Bn-A04-p17940767	A4	43	50.4	B
4241	Bn-N4-p20125262	Bn-A04-p18136301	Bn-A04-p18136301	A4	44	51.2	B
4242	Bn-N14-p53703397	Bn-A04-p18168733	Bn-A04-p18168733	A4	45	52	B
4243	Bn-N4-p20169038	Bn-A04-p18180777	Bn-A04-p18180777	A4	45	52	B
4244	Bn-N4-p20260734	Bn-A04-p18273048	Bn-A04-p18273048	A4	46	52.8	B
4245	Bn-N4-p20357803	Bn-A04-p18377757	Bn-A04-p18377757	A4	47	55.2	B
4246	Bn-N4-p20360648	Bn-C4-p52700090	Bn-scaff_18903_1-p54314(	A4	47	55.2	B
4247	Bn-N4-p20419859	Bn-A04-p18429573	Bn-A04-p18429573	A4	47	55.2	B
4248	Bn-N4-p20483625	Bn-A04-p18487715	Bn-A04-p18487715	A4	48	56	B
4249	Bn-N4-p20498001	Bn-A04-p18502854	Bn-A04-p18502854	A4	48	56	B
4250	Bn-N4-p20520720	Bn-A04-p18522691	Bn-A04-p18522691	A4	48	56	B
4251	Bn-N4-p20574835	Bn-A04-p18572841	Bn-A04-p18572841	A4	48	56	B
4252	Bn-N4-p20579460	Bn-A04-p18577466	Bn-A04-p18577466	A4	49	56.8	B
4253	Bn-N4-p20602010	Bn-A04-p18595858	Bn-A04-p18595858	A4	49	56.8	B
4254	Bn-N4-p20610287	Bn-C4-p53013307	Bn-scaff_18903_1-p85636:	A4	49	56.8	B
4255	Bn-N4-p20612582	Bn-A04-p18606660	Bn-A04-p18606660	A4	49	56.8	B
4256	Bn-N4-p20621135	Bn-A04-p18613344	Bn-A04-p18613344	A4	49	56.8	B
4257	Bn-N4-p20625106	Bn-A04-p18617343	Bn-A04-p18617343	A4	49	56.8	B
4258	Bn-N4-p20626993	Bn-A04-p18620226	Bn-A04-p18620226	A4	49	56.8	B

4259	Bn-N4-p20641939	Bn-A04-p18639840	Bn-A04-p18639840	A4	49	56.8	B
4260	Bn-N4-p20731935	Bn-A04-p18731541	Bn-A04-p18731541	A4	49	56.8	B
4261	Bn-N4-p20732350	Bn-C4-p53151093	Bn-scaff_18903_1-p99414	A4	49	56.8	B
4262	Bn-N4-p20778423	Bn-A04-p18763435	Bn-A04-p18763435	A4	49	56.8	B
4263	Bn-N9-p34604389	Bn-A01-p25429100	Bn-A01-p25429100	A4	49	56.8	B
4264	Bn-N9-p34605210	Bn-A01-p25428278	Bn-A01-p25428278	A4	49	56.8	B
4265	Bn-N4-p21036845	Bn-A01-p25199395	Bn-A01-p25199395	A4	50	57.6	B
4266	Bn-N4-p21054418	Bn-A01-p25183707	Bn-A01-p25183707	A4	50	57.6	B
4267	Bn-N4-p21062470	Bn-A01-p25175675	Bn-A01-p25175675	A4	50	57.6	B
4268	Bn-N4-p21066278	Bn-A01-p25171870	Bn-A01-p25171870	A4	50	57.6	B
4269	Bn-Scaffold00895-p35448	Bn-A01-p25350397	Bn-A01-p25350397	A4	50	57.6	B
4270	Bn-Scaffold00895-p36729	Bn-A01-p25351684	Bn-A01-p25351684	A4	50	57.6	B
4271	Bn-Scaffold00895-p52753	Bn-A01-p25363566	Bn-A01-p25363566	A4	50	57.6	B
4272	Bn-Scaffold00895-p62181	Bn-A01-p25377739	Bn-A01-p25377739	A4	50	57.6	B
4273	Bn-Scaffold00895-p76429	Bn-A01-p25391997	Bn-A01-p25391997	A4	50	57.6	B
4274	Bn-Scaffold00895-p89635	Bn-A01-p25405698	Bn-A01-p25405698	A4	50	57.6	B
4275	Bn-N11-p1777591	Bn-A05-p541565	Bn-A05-p541565	A5	1	0	A
4276	Bn-N5-p272544	Bn-A05-p244051	Bn-A05-p244051	A5	1	0	A
4277	Bn-N5-p384683	Bn-A05-p346822	Bn-A05-p346822	A5	1	0	A
4278	Bn-N5-p407617	Bn-Scaffold000403-p1	Bn-Scaffold000403-p12455	A5	1	0	A
4279	Bn-N5-p407860	Bn-Scaffold000403-p1	Bn-Scaffold000403-p12697	A5	1	0	A
4280	Bn-N5-p410256	Bn-A05-p355200	Bn-A05-p355200	A5	1	0	A
4281	Bn-N5-p524531	Bn-A05-p449086	Bn-A05-p449086	A5	1	0	A
4282	Bn-N5-p604085	Bn-A05-p529716	Bn-A05-p529716	A5	1	0	A
4283	Bn-N5-p622657	Bn-A05-p547582	Bn-A05-p547582	A5	1	0	0
4284	Bn-N5-p625889	Bn-A05-p550807	Bn-A05-p550807	A5	1	0	A
4285	Bn-N5-p637306	Bn-A05-p559597	Bn-A05-p559597	A5	1	0	A
4286	Bn-N5-p654857	Bn-A05-p575873	Bn-A05-p575873	A5	1	0	0
4287	Bn-N5-p663919	Bn-A05-p579882	Bn-A05-p579882	A5	1	0	A
4288	Bn-N5-p666924	Bn-A05-p583972	Bn-A05-p583972	A5	1	0	0
4289	Bn-N5-p673281	Bn-A05-p589658	Bn-A05-p589658	A5	1	0	A
4290	Bn-N5-p684459	Bn-A05-p600196	Bn-A05-p600196	A5	1	0	0
4291	Bn-N5-p701378	Bn-A05-p619336	Bn-A05-p619336	A5	1	0	A
4292	Bn-N5-p725761	Bn-C4-p944414	Bn-scaff_23946_1-p74217	A5	1	0	A
4293	Bn-N5-p738760	Bn-A05-p653606	Bn-A05-p653606	A5	1	0	A
4294	Bn-N5-p749162	Bn-A05-p673050	Bn-A05-p673050	A5	1	0	A
4295	Bn-N5-p759307	Bn-A05-p679803	Bn-A05-p679803	A5	2	0.8	A
4296	Bn-N5-p766534	Bn-A05-p686912	Bn-A05-p686912	A5	2	0.8	A
4297	Bn-N5-p767412	Bn-A05-p687790	Bn-A05-p687790	A5	2	0.8	A
4298	Bn-N5-p773928	Bn-A05-p701389	Bn-A05-p701389	A5	2	0.8	A
4299	Bn-N5-p802595	Bn-A05-p726481	Bn-A05-p726481	A5	2	0.8	A
4300	Bn-N5-p827007	Bn-A05-p757624	Bn-A05-p757624	A5	2	0.8	0
4301	Bn-N5-p831536	Bn-A05-p761283	Bn-A05-p761283	A5	2	0.8	0
4302	Bn-N5-p880252	Bn-A05-p780844	Bn-A05-p780844	A5	2	0.8	A
4303	Bn-N5-p894417	Bn-A05-p794760	Bn-A05-p794760	A5	2	0.8	A
4304	Bn-N5-p902155	Bn-A05-p800515	Bn-A05-p800515	A5	2	0.8	A
4305	Bn-N5-p902189	Bn-A05-p800549	Bn-A05-p800549	A5	2	0.8	A
4306	Bn-N5-p916272	Bn-A05-p815322	Bn-A05-p815322	A5	2	0.8	A
4307	Bn-N5-p916761	Bn-A05-p816963	Bn-A05-p816963	A5	2	0.8	A

4308	Bn-N5-p916834	Bn-A05-p817036	Bn-A05-p817036	A5	2	0.8	A
4309	Bn-N5-p923666	Bn-A05-p822597	Bn-A05-p822597	A5	2	0.8	A
4310	Bn-N5-p928178	Bn-A05-p825915	Bn-A05-p825915	A5	2	0.8	A
4311	Bn-N5-p945099	Bn-A05-p837013	Bn-A05-p837013	A5	2	0.8	0
4312	Bn-N5-p951999	Bn-A05-p849712	Bn-A05-p849712	A5	2	0.8	A
4313	Bn-N5-p955303	Bn-A05-p853364	Bn-A05-p853364	A5	2	0.8	0
4314	Bn-N5-p1000999	Bn-A05-p892520	Bn-A05-p892520	A5	3	2.4	A
4315	Bn-N5-p1012825	Bn-A05-p904751	Bn-A05-p904751	A5	3	2.4	A
4316	Bn-N5-p1015308	Bn-A05-p907258	Bn-A05-p907258	A5	3	2.4	A
4317	Bn-N5-p1030016	Bn-A05-p922813	Bn-A05-p922813	A5	3	2.4	A
4318	Bn-N5-p1033381	Bn-A05-p926361	Bn-A05-p926361	A5	3	2.4	0
4319	Bn-N5-p1046398	Bn-A05-p933536	Bn-A05-p933536	A5	3	2.4	A
4320	Bn-N5-p1068268	Bn-A05-p958740	Bn-A05-p958740	A5	3	2.4	A
4321	Bn-N5-p1245455	Bn-A05-p1134104	Bn-A05-p1134104	A5	3	2.4	A
4322	Bn-N14-p1292630	Bn-A05-p910039	Bn-A05-p910039	A5	4	2.4	A
4323	Bn-N5-p1077255	Bn-A05-p967575	Bn-A05-p967575	A5	4	2.4	A
4324	Bn-N5-p1080492	Bn-A05-p970816	Bn-A05-p970816	A5	4	2.4	A
4325	Bn-N5-p1174032	Bn-A05-p1071371	Bn-A05-p1071371	A5	5	2.4	A
4326	Bn-N5-p1182966	Bn-A05-p1074864	Bn-A05-p1074864	A5	5	2.4	A
4327	Bn-N5-p1362258	Bn-A05-p1259869	Bn-A05-p1259869	A5	6	4.8	A
4328	Bn-N5-p1371434	Bn-A05-p1267683	Bn-A05-p1267683	A5	6	4.8	0
4329	Bn-N5-p1383086	Bn-A05-p1279304	Bn-A05-p1279304	A5	6	4.8	A
4330	Bn-N5-p1384962	Bn-A05-p1281138	Bn-A05-p1281138	A5	6	4.8	A
4331	Bn-N5-p1410753	Bn-A05-p1308471	Bn-A05-p1308471	A5	6	4.8	A
4332	Bn-Scaffold03131-p1061	Bn-A05-p1310329	Bn-A05-p1310329	A5	6	4.8	A
4333	Bn-N5-p1436231	Bn-A05-p1335771	Bn-A05-p1335771	A5	7	6.4	A
4334	Bn-N5-p1451685	Bn-A05-p1347246	Bn-A05-p1347246	A5	8	7.2	A
4335	Bn-N5-p1453955	Bn-A05-p1349520	Bn-A05-p1349520	A5	8	7.2	A
4336	Bn-N5-p1455247	Bn-A05-p1350976	Bn-A05-p1350976	A5	8	7.2	A
4337	Bn-N5-p1459257	Bn-A05-p1356378	Bn-A05-p1356378	A5	8	7.2	0
4338	Bn-N5-p1480737	Bn-A05-p1372057	Bn-A05-p1372057	A5	8	7.2	0
4339	Bn-N5-p1493971	Bn-A05-p1384677	Bn-A05-p1384677	A5	8	7.2	A
4340	Bn-N5-p1494482	Bn-A05-p1385188	Bn-A05-p1385188	A5	8	7.2	A
4341	Bn-N5-p1505543	Bn-A05-p1396162	Bn-A05-p1396162	A5	8	7.2	A
4342	Bn-N5-p1505947	Bn-A05-p1396567	Bn-A05-p1396567	A5	8	7.2	A
4343	Bn-N5-p1565456	Bn-A05-p1434959	Bn-A05-p1434959	A5	8	7.2	A
4344	Bn-N5-p1568006	Bn-A05-p1436011	Bn-A05-p1436011	A5	8	7.2	A
4345	Bn-N5-p1583214	Bn-A05-p1457058	Bn-A05-p1457058	A5	8	7.2	A
4346	Bn-N5-p1602173	Bn-A05-p1475369	Bn-A05-p1475369	A5	8	7.2	A
4347	Bn-N5-p1605466	Bn-A05-p1479325	Bn-A05-p1479325	A5	8	7.2	A
4348	Bn-N5-p1619320	Bn-A05-p1492327	Bn-A05-p1492327	A5	8	7.2	A
4349	Bn-N13-p66045454	Bn-C3-p21156760	Bn-scaff_19523_1-p82022	A5	9	10.4	0
4350	Bn-N5-p1722333	Bn-A05-p1554943	Bn-A05-p1554943	A5	9	10.4	A
4351	Bn-N5-p1753871	Bn-A05-p1578074	Bn-A05-p1578074	A5	9	10.4	A
4352	Bn-N5-p1879670	Bn-A05-p1676024	Bn-A05-p1676024	A5	9	10.4	A
4353	Bn-N5-p1891553	Bn-A05-p1690598	Bn-A05-p1690598	A5	9	10.4	A
4354	Bn-N5-p1893365	Bn-A05-p1692254	Bn-A05-p1692254	A5	9	10.4	A
4355	Bn-N5-p1893801	Bn-A05-p1692692	Bn-A05-p1692692	A5	9	10.4	A
4356	Bn-N5-p1894784	Bn-A05-p1695394	Bn-A05-p1695394	A5	9	10.4	A

4357	Bn-N5-p1895717	Bn-A05-p1696330	Bn-A05-p1696330	A5	9	10.4	A
4358	Bn-N5-p1896002	Bn-A05-p1696613	Bn-A05-p1696613	A5	9	10.4	A
4359	Bn-N5-p1896407	Bn-A05-p1697018	Bn-A05-p1697018	A5	9	10.4	A
4360	Bn-N5-p1901300	Bn-A05-p1701030	Bn-A05-p1701030	A5	9	10.4	A
4361	Bn-N5-p1949635	Bn-A05-p1761473	Bn-A05-p1761473	A5	10	11.2	A
4362	Bn-N5-p2039192	Bn-A05-p1857946	Bn-A05-p1857946	A5	11	12	A
4363	Bn-N5-p2075840	Bn-A05-p1898273	Bn-A05-p1898273	A5	12	14.4	A
4364	Bn-N5-p2097213	Bn-A05-p1919060	Bn-A05-p1919060	A5	12	14.4	A
4365	Bn-N5-p2214915	Bn-A05-p2024975	Bn-A05-p2024975	A5	12	14.4	A
4366	Bn-N5-p2220726	Bn-A05-p2030789	Bn-A05-p2030789	A5	12	14.4	0
4367	Bn-N5-p2230561	Bn-A05-p2039690	Bn-A05-p2039690	A5	12	14.4	A
4368	Bn-N5-p2252597	Bn-A05-p2062917	Bn-A05-p2062917	A5	12	14.4	A
4369	Bn-N5-p2257407	Bn-A05-p2067722	Bn-A05-p2067722	A5	12	14.4	A
4370	Bn-N14-p3739605	Bn-A05-p2262914	Bn-A05-p2262914	A5	13	16.8	0
4371	Bn-N5-p2298341	Bn-A05-p2106680	Bn-A05-p2106680	A5	13	16.8	A
4372	Bn-N5-p2299339	Bn-A05-p2107808	Bn-A05-p2107808	A5	13	16.8	A
4373	Bn-N5-p2300244	Bn-A05-p2108713	Bn-A05-p2108713	A5	13	16.8	A
4374	Bn-N5-p2303825	Bn-A05-p2111165	Bn-A05-p2111165	A5	13	16.8	A
4375	Bn-N5-p2340764	Bn-A05-p2143241	Bn-A05-p2143241	A5	13	16.8	A
4376	Bn-N5-p2353516	Bn-A05-p2151202	Bn-A05-p2151202	A5	13	16.8	A
4377	Bn-N5-p2357631	Bn-A05-p2155466	Bn-A05-p2155466	A5	13	16.8	0
4378	Bn-N5-p2369335	Bn-A05-p2167188	Bn-A05-p2167188	A5	13	16.8	A
4379	Bn-N5-p2369416	Bn-A05-p2167269	Bn-A05-p2167269	A5	13	16.8	A
4380	Bn-N5-p2407963	Bn-A05-p2208960	Bn-A05-p2208960	A5	13	16.8	A
4381	Bn-N5-p2440678	Bn-A05-p2246038	Bn-A05-p2246038	A5	13	16.8	A
4382	Bn-N5-p2483134	Bn-A05-p2287815	Bn-A05-p2287815	A5	13	16.8	A
4383	Bn-N5-p2490392	Bn-A05-p2293577	Bn-A05-p2293577	A5	13	16.8	A
4384	Bn-N5-p2493077	Bn-A05-p2296307	Bn-A05-p2296307	A5	13	16.8	A
4385	Bn-N5-p2509507	Bn-A05-p2303112	Bn-A05-p2303112	A5	14	17.6	A
4386	Bn-N14-p3918239	Bn-A05-p2345233	Bn-A05-p2345233	A5	15	20	A
4387	Bn-N5-p2562704	Bn-A05-p2367876	Bn-A05-p2367876	A5	16	22.4	A
4388	Bn-N5-p2594467	Bn-C4-p3824098	Bn-scaff_16534_1-p50090(	A5	16	22.4	0
4389	Bn-N5-p2649995	Bn-A05-p2460244	Bn-A05-p2460244	A5	16	22.4	A
4390	Bn-N5-p2658699	Bn-A05-p2469338	Bn-A05-p2469338	A5	16	22.4	A
4391	Bn-N5-p2680108	Bn-A05-p2495697	Bn-A05-p2495697	A5	16	22.4	A
4392	Bn-N5-p2673519	Bn-C4-p3971988	Bn-scaff_16534_1-p64879(	A5	17	23.2	A
4393	Bn-N5-p2675450	Bn-A05-p2489101	Bn-A05-p2489101	A5	17	23.2	A
4394	Bn-N5-p2675497	Bn-C4-p3974036	Bn-scaff_16534_1-p65083[illegible]	A5	17	23.2	0
4395	Bn-N5-p2676866	Bn-A05-p2492451	Bn-A05-p2492451	A5	17	23.2	A
4396	Bn-N5-p2679473	Bn-A05-p2495066	Bn-A05-p2495066	A5	17	23.2	A
4397	Bn-N5-p2679827	Bn-A05-p2495419	Bn-A05-p2495419	A5	17	23.2	0
4398	Bn-N5-p2686383	Bn-A05-p2501988	Bn-A05-p2501988	A5	17	23.2	A
4399	Bn-N5-p2698863	Bn-A05-p2523179	Bn-A05-p2523179	A5	17	23.2	A
4400	Bn-N5-p2778500	Bn-A05-p2592847	Bn-A05-p2592847	A5	17	23.2	A
4401	Bn-N5-p2791865	Bn-A05-p2600118	Bn-A05-p2600118	A5	17	23.2	A
4402	Bn-N5-p2795396	Bn-A05-p2604957	Bn-A05-p2604957	A5	17	23.2	A
4403	Bn-N5-p2797098	Bn-A05-p2606660	Bn-A05-p2606660	A5	17	23.2	A
4404	Bn-N5-p2816986	Bn-A05-p2610006	Bn-A05-p2610006	A5	17	23.2	A
4405	Bn-N5-p2836831	Bn-A05-p2641800	Bn-A05-p2641800	A5	18	24	A

4406	Bn-N5-p2848406	Bn-A05-p2653139	Bn-A05-p2653139	A5	18	24	0
4407	Bn-N5-p2851329	Bn-A05-p2656042	Bn-A05-p2656042	A5	18	24	0
4408	Bn-N5-p2870666	Bn-A05-p2671921	Bn-A05-p2671921	A5	19	26.4	A
4409	Bn-N5-p2893352	Bn-A05-p2688101	Bn-A05-p2688101	A5	19	26.4	0
4410	Bn-N5-p2902787	Bn-A05-p2711007	Bn-A05-p2711007	A5	19	26.4	A
4411	Bn-N5-p2902988	Bn-A05-p2711208	Bn-A05-p2711208	A5	19	26.4	A
4412	Bn-N5-p2920992	Bn-A05-p2726301	Bn-A05-p2726301	A5	19	26.4	A
4413	Bn-N5-p2975363	Bn-A05-p2778577	Bn-A05-p2778577	A5	19	26.4	A
4414	Bn-N5-p2981394	Bn-A05-p2791353	Bn-A05-p2791353	A5	19	26.4	A
4415	Bn-N5-p2992931	Bn-A05-p2804703	Bn-A05-p2804703	A5	19	26.4	A
4416	Bn-N5-p3028806	Bn-A05-p2839985	Bn-A05-p2839985	A5	19	26.4	A
4417	Bn-N5-p3032379	Bn-A05-p2843347	Bn-A05-p2843347	A5	19	26.4	A
4418	Bn-N5-p3033090	Bn-A05-p2844059	Bn-A05-p2844059	A5	19	26.4	A
4419	Bn-N5-p3043378	Bn-A05-p2852544	Bn-A05-p2852544	A5	19	26.4	A
4420	Bn-N5-p3053056	Bn-A05-p2860757	Bn-A05-p2860757	A5	19	26.4	A
4421	Bn-N5-p3055382	Bn-A05-p2863301	Bn-A05-p2863301	A5	19	26.4	A
4422	Bn-N5-p3056016	Bn-A05-p2863674	Bn-A05-p2863674	A5	19	26.4	A
4423	Bn-N5-p3056777	Bn-A05-p2864686	Bn-A05-p2864686	A5	19	26.4	A
4424	Bn-N5-p3057390	Bn-A05-p2865380	Bn-A05-p2865380	A5	19	26.4	A
4425	Bn-N5-p3057889	Bn-A05-p2865634	Bn-A05-p2865634	A5	19	26.4	A
4426	Bn-N5-p3057960	Bn-A05-p2865716	Bn-A05-p2865716	A5	19	26.4	A
4427	Bn-N5-p3142611	Bn-A05-p2924872	Bn-A05-p2924872	A5	19	26.4	A
4428	Bn-N5-p3144077	Bn-A05-p2925195	Bn-A05-p2925195	A5	19	26.4	A
4429	Bn-N5-p3152646	Bn-A05-p2929698	Bn-A05-p2929698	A5	19	26.4	A
4430	Bn-N14-p5324758	Bn-A05-p3188714	Bn-A05-p3188714	A5	20	27.2	A
4431	Bn-N5-p3236393	Bn-A05-p3005109	Bn-A05-p3005109	A5	20	27.2	A
4432	Bn-N5-p3249549	Bn-A05-p3019980	Bn-A05-p3019980	A5	20	27.2	A
4433	Bn-N5-p3251235	Bn-A05-p3021668	Bn-A05-p3021668	A5	20	27.2	A
4434	Bn-N5-p3274081	Bn-A05-p3042172	Bn-A05-p3042172	A5	20	27.2	A
4435	Bn-N5-p3274082	Bn-A05-p3042173	Bn-A05-p3042173	A5	20	27.2	A
4436	Bn-N5-p3275694	Bn-A05-p3043365	Bn-A05-p3043365	A5	20	27.2	A
4437	Bn-N5-p3278028	Bn-A05-p3045689	Bn-A05-p3045689	A5	20	27.2	A
4438	Bn-N5-p3305012	Bn-A05-p3071868	Bn-A05-p3071868	A5	20	27.2	A
4439	Bn-N5-p3361502	Bn-A05-p3117520	Bn-A05-p3117520	A5	20	27.2	A
4440	Bn-N5-p3436050	Bn-A05-p3188172	Bn-A05-p3188172	A5	20	27.2	A
4441	Bn-N5-p3439087	Bn-A05-p3191390	Bn-A05-p3191390	A5	20	27.2	A
4442	Bn-N5-p3492083	Bn-A05-p3242299	Bn-A05-p3242299	A5	20	27.2	A
4443	Bn-N5-p3498024	Bn-C4-p5223786	Bn-scaff_16534_1-p19005	A5	20	27.2	A
4444	Bn-N5-p3515043	Bn-A05-p3264129	Bn-A05-p3264129	A5	20	27.2	A
4445	Bn-N5-p3520163	Bn-A05-p3275289	Bn-A05-p3275289	A5	20	27.2	A
4446	Bn-N5-p3523738	Bn-A05-p3278514	Bn-A05-p3278514	A5	20	27.2	A
4447	Bn-N5-p3525724	Bn-A05-p3280592	Bn-A05-p3280592	A5	20	27.2	A
4448	Bn-N5-p3526947	Bn-A05-p3282089	Bn-A05-p3282089	A5	20	27.2	A
4449	Bn-N14-p5843790	Bn-C4-p5748084	Bn-scaff_16534_1-p24248	A5	21	28.8	A
4450	Bn-N5-p3545250	Bn-A05-p3300112	Bn-A05-p3300112	A5	21	28.8	A
4451	Bn-N5-p3553066	Bn-A05-p3307736	Bn-A05-p3307736	A5	21	28.8	A
4452	Bn-N5-p3557125	Bn-A05-p3311786	Bn-A05-p3311786	A5	21	28.8	A
4453	Bn-N5-p3558019	Bn-A05-p3312679	Bn-A05-p3312679	A5	21	28.8	A
4454	Bn-N5-p3631412	Bn-A05-p3394311	Bn-A05-p3394311	A5	21	28.8	A

4455	Bn-N5-p3666804	Bn-A05-p3421628	Bn-A05-p3421628	A5	21	28.8	A
4456	Bn-N5-p3671044	Bn-A05-p3427883	Bn-A05-p3427883	A5	21	28.8	A
4457	Bn-N5-p3671154	Bn-A05-p3427993	Bn-A05-p3427993	A5	21	28.8	A
4458	Bn-N5-p3707907	Bn-A05-p3467036	Bn-A05-p3467036	A5	21	28.8	A
4459	Bn-N5-p3707931	Bn-A05-p3467060	Bn-A05-p3467060	A5	21	28.8	A
4460	Bn-N5-p3708559	Bn-A05-p3467688	Bn-A05-p3467688	A5	21	28.8	A
4461	Bn-N5-p3709000	Bn-A05-p3468129	Bn-A05-p3468129	A5	21	28.8	A
4462	Bn-N5-p3709075	Bn-A05-p3468204	Bn-A05-p3468204	A5	21	28.8	A
4463	Bn-N5-p3710345	Bn-A05-p3469996	Bn-A05-p3469996	A5	21	28.8	A
4464	Bn-N5-p3749456	Bn-A05-p3507831	Bn-A05-p3507831	A5	22	29.6	A
4465	Bn-N5-p3787183	Bn-A05-p3550203	Bn-A05-p3550203	A5	22	29.6	A
4466	Bn-N5-p3872235	Bn-A05-p3617320	Bn-A05-p3617320	A5	23	31.2	A
4467	Bn-N5-p3873260	Bn-A05-p3618356	Bn-A05-p3618356	A5	23	31.2	A
4468	Bn-N5-p3920087	Bn-A05-p3649985	Bn-A05-p3649985	A5	23	31.2	A
4469	Bn-N5-p3920168	Bn-A05-p3650066	Bn-A05-p3650066	A5	23	31.2	A
4470	Bn-N5-p3963432	Bn-A05-p3684668	Bn-A05-p3684668	A5	23	31.2	A
4471	Bn-N5-p3970509	Bn-A05-p3703643	Bn-A05-p3703643	A5	23	31.2	A
4472	Bn-N5-p4104667	Bn-A05-p3834445	Bn-A05-p3834445	A5	23	31.2	A
4473	Bn-N5-p4104862	Bn-A05-p3834639	Bn-A05-p3834639	A5	23	31.2	A
4474	Bn-N5-p4115183	Bn-A05-p3846005	Bn-A05-p3846005	A5	23	31.2	A
4475	Bn-N14-p6804962	Bn-C4-p6693136	Bn-scaff_15908_1-p77093	A5	24	32	A
4476	Bn-N5-p4182501	Bn-A05-p3923692	Bn-A05-p3923692	A5	24	32	A
4477	Bn-N5-p4182623	Bn-A05-p3923814	Bn-A05-p3923814	A5	24	32	A
4478	Bn-N5-p4230168	Bn-A05-p3966848	Bn-A05-p3966848	A5	25	32.8	A
4479	Bn-N5-p4249850	Bn-A05-p3988279	Bn-A05-p3988279	A5	25	32.8	A
4480	Bn-N5-p4250529	Bn-A05-p3988958	Bn-A05-p3988958	A5	26	33.6	A
4481	Bn-N5-p4268889	Bn-A05-p4006770	Bn-A05-p4006770	A5	26	33.6	A
4482	Bn-N5-p4285459	Bn-A05-p4023464	Bn-A05-p4023464	A5	26	33.6	A
4483	Bn-N5-p4323014	Bn-A05-p4059534	Bn-A05-p4059534	A5	26	33.6	A
4484	Bn-N5-p4325214	Bn-A05-p4061730	Bn-A05-p4061730	A5	26	33.6	A
4485	Bn-N5-p4325462	Bn-A05-p4061978	Bn-A05-p4061978	A5	26	33.6	A
4486	Bn-N15-p15895383	Bn-C5-p14702534	Bn-scaff_18338_1-p34251	A5	27	34.4	A
4487	Bn-N5-p4333154	Bn-A05-p4073230	Bn-A05-p4073230	A5	27	34.4	A
4488	Bn-N5-p4333921	Bn-A05-p4073997	Bn-A05-p4073997	A5	27	34.4	A
4489	Bn-N5-p4338418	Bn-A05-p4078496	Bn-A05-p4078496	A5	27	34.4	A
4490	Bn-N5-p4365753	Bn-A05-p4102906	Bn-A05-p4102906	A5	27	34.4	A
4491	Bn-N5-p4366930	Bn-A05-p4104070	Bn-A05-p4104070	A5	27	34.4	A
4492	Bn-N5-p4372981	Bn-A05-p4108818	Bn-A05-p4108818	A5	27	34.4	A
4493	Bn-N5-p4374186	Bn-A05-p4110015	Bn-A05-p4110015	A5	27	34.4	A
4494	Bn-N5-p4386273	Bn-A05-p4121158	Bn-A05-p4121158	A5	27	34.4	A
4495	Bn-N5-p4463509	Bn-A05-p4212213	Bn-A05-p4212213	A5	27	34.4	A
4496	Bn-N14-p7638046	Bn-C4-p7476463	Bn-scaff_19248_1-p16634	A5	28	38.4	A
4497	Bn-N5-p4565661	Bn-A05-p4316642	Bn-A05-p4316642	A5	28	38.4	A
4498	Bn-N5-p4593820	Bn-A05-p4333409	Bn-A05-p4333409	A5	28	38.4	A
4499	Bn-N5-p4597112	Bn-A05-p4336667	Bn-A05-p4336667	A5	28	38.4	A
4500	Bn-N5-p4607613	Bn-C4-p7303440	Bn-scaff_25040_1-p24261	A5	28	38.4	A
4501	Bn-N5-p4607614	Bn-A05-p4353454	Bn-A05-p4353454	A5	28	38.4	A
4502	Bn-N5-p4608601	Bn-A05-p4354442	Bn-A05-p4354442	A5	28	38.4	A
4503	Bn-N5-p4625600	Bn-A05-p4376029	Bn-A05-p4376029	A5	28	38.4	A

4504	Bn-N5-p4720302	Bn-A05-p4498290	Bn-A05-p4498290	A5	28	38.4	A
4505	Bn-N5-p4720410	Bn-A05-p4498398	Bn-A05-p4498398	A5	28	38.4	A
4506	Bn-N5-p4775030	Bn-A05-p4516526	Bn-A05-p4516526	A5	28	38.4	A
4507	Bn-N5-p4812406	Bn-A05-p4549959	Bn-A05-p4549959	A5	28	38.4	A
4508	Bn-N5-p4812912	Bn-A05-p4550465	Bn-A05-p4550465	A5	28	38.4	A
4509	Bn-N5-p4831145	Bn-A05-p4569670	Bn-A05-p4569670	A5	28	38.4	A
4510	Bn-N5-p4848123	Bn-A05-p4593001	Bn-A05-p4593001	A5	28	38.4	A
4511	Bn-N5-p4863246	Bn-A05-p4612911	Bn-A05-p4612911	A5	28	38.4	A
4512	Bn-N5-p4866381	Bn-A05-p4616151	Bn-A05-p4616151	A5	28	38.4	A
4513	Bn-N5-p4868223	Bn-A05-p4618158	Bn-A05-p4618158	A5	28	38.4	A
4514	Bn-N5-p4869368	Bn-A05-p4619303	Bn-A05-p4619303	A5	28	38.4	A
4515	Bn-N5-p4871145	Bn-A05-p4621087	Bn-A05-p4621087	A5	28	38.4	A
4516	Bn-N5-p4872017	Bn-A05-p4621949	Bn-A05-p4621949	A5	28	38.4	A
4517	Bn-N5-p4875635	Bn-A05-p4625019	Bn-A05-p4625019	A5	28	38.4	A
4518	Bn-N5-p4885817	Bn-A05-p4632407	Bn-A05-p4632407	A5	28	38.4	A
4519	Bn-N5-p4902267	Bn-A05-p4644984	Bn-A05-p4644984	A5	28	38.4	A
4520	Bn-N5-p4932628	Bn-A05-p4665099	Bn-A05-p4665099	A5	28	38.4	A
4521	Bn-N5-p4945932	Bn-A05-p4679663	Bn-A05-p4679663	A5	28	38.4	A
4522	Bn-N5-p4959227	Bn-A05-p4696360	Bn-A05-p4696360	A5	29	39.2	A
4523	Bn-N5-p5063788	Bn-C4-p7980371	Bn-scaff_18440_1-p13233[illegible]	A5	29	39.2	A
4524	Bn-N5-p5070399	Bn-A05-p4792988	Bn-A05-p4792988	A5	29	39.2	A
4525	Bn-N5-p5071552	Bn-A05-p4794141	Bn-A05-p4794141	A5	29	39.2	A
4526	Bn-N5-p5164920	Bn-A05-p4904405	Bn-A05-p4904405	A5	29	39.2	A
4527	Bn-N5-p5171809	Bn-A05-p4915516	Bn-A05-p4915516	A5	29	39.2	A
4528	Bn-N5-p5176166	Bn-A05-p4919899	Bn-A05-p4919899	A5	29	39.2	A
4529	Bn-N14-p7418066	Bn-C4-p7265427	Bn-scaff_17983_1-p71628	A5	30	40	A
4530	Bn-N5-p5194031	Bn-A05-p4940676	Bn-A05-p4940676	A5	30	40	A
4531	Bn-N5-p5194068	Bn-A05-p4940713	Bn-A05-p4940713	A5	30	40	A
4532	Bn-N5-p5194134	Bn-A05-p4940779	Bn-A05-p4940779	A5	30	40	A
4533	Bn-N5-p5194586	Bn-A05-p4941234	Bn-A05-p4941234	A5	30	40	A
4534	Bn-N5-p5206299	Bn-A05-p4959282	Bn-A05-p4959282	A5	30	40	A
4535	Bn-N5-p5276623	Bn-A05-p5020058	Bn-A05-p5020058	A5	30	40	A
4536	Bn-N5-p5284473	Bn-A05-p5026139	Bn-A05-p5026139	A5	30	40	A
4537	Bn-N14-p9128434	Bn-A05-p5283304	Bn-A05-p5283304	A5	31	41.6	A
4538	Bn-N5-p5361751	Bn-A05-p5093548	Bn-A05-p5093548	A5	31	41.6	A
4539	Bn-N5-p5370597	Bn-A05-p5102120	Bn-A05-p5102120	A5	31	41.6	A
4540	Bn-N5-p5376501	Bn-A05-p5104821	Bn-A05-p5104821	A5	31	41.6	A
4541	Bn-N5-p5403580	Bn-A05-p5135426	Bn-A05-p5135426	A5	31	41.6	A
4542	Bn-N5-p5552874	Bn-A05-p5279229	Bn-A05-p5279229	A5	31	41.6	A
4543	Bn-N5-p5553661	Bn-A05-p5280016	Bn-A05-p5280016	A5	31	41.6	A
4544	Bn-N5-p5555132	Bn-A05-p5281478	Bn-A05-p5281478	A5	31	41.6	A
4545	Bn-N5-p5602569	Bn-A05-p5328959	Bn-A05-p5328959	A5	31	41.6	A
4546	Bn-N5-p5611393	Bn-A05-p5337620	Bn-A05-p5337620	A5	31	41.6	A
4547	Bn-N5-p5612892	Bn-A05-p5344859	Bn-A05-p5344859	A5	31	41.6	A
4548	Bn-N5-p5617991	Bn-A05-p5349944	Bn-A05-p5349944	A5	31	41.6	A
4549	Bn-N5-p5618612	Bn-A05-p5350567	Bn-A05-p5350567	A5	31	41.6	A
4550	Bn-N5-p5618923	Bn-A05-p5350892	Bn-A05-p5350892	A5	31	41.6	A
4551	Bn-N5-p5368766	Bn-C4-p8573479	Bn-scaff_23907_1-p49739[illegible]	A5	32	43.2	A
4552	Bn-N5-p5652046	Bn-A05-p5372015	Bn-A05-p5372015	A5	32	43.2	A

4553	Bn-N5-p5654806	Bn-A05-p5374000	Bn-A05-p5374000	A5	32	43.2	A
4554	Bn-N5-p5657971	Bn-C4-p9031482	Bn-scaff_23907_1-p955394	A5	32	43.2	A
4555	Bn-N5-p5672976	Bn-A05-p5391937	Bn-A05-p5391937	A5	32	43.2	A
4556	Bn-N14-p9686458	Bn-C4-p9255611	Bn-scaff_18439_1-p106249	A5	33	44	A
4557	Bn-N5-p5746359	Bn-A05-p5475992	Bn-A05-p5475992	A5	33	44	A
4558	Bn-N5-p5788080	Bn-A05-p5519729	Bn-A05-p5519729	A5	33	44	A
4559	Bn-N5-p5789631	Bn-A05-p5521242	Bn-A05-p5521242	A5	33	44	A
4560	Bn-N5-p5790600	Bn-A05-p5522210	Bn-A05-p5522210	A5	33	44	A
4561	Bn-N5-p5799452	Bn-A05-p5533987	Bn-A05-p5533987	A5	33	44	A
4562	Bn-N5-p5806607	Bn-A05-p5541232	Bn-A05-p5541232	A5	33	44	A
4563	Bn-N5-p5807936	Bn-A05-p5542434	Bn-A05-p5542434	A5	33	44	A
4564	Bn-N5-p5817607	Bn-A05-p5552246	Bn-A05-p5552246	A5	33	44	A
4565	Bn-N5-p5831310	Bn-A05-p5565945	Bn-A05-p5565945	A5	33	44	A
4566	Bn-N5-p5831558	Bn-A05-p5566193	Bn-A05-p5566193	A5	33	44	A
4567	Bn-N5-p5841821	Bn-A05-p5575695	Bn-A05-p5575695	A5	33	44	A
4568	Bn-N5-p5852889	Bn-A05-p5586839	Bn-A05-p5586839	A5	33	44	A
4569	Bn-N5-p5854323	Bn-A05-p5588271	Bn-A05-p5588271	A5	33	44	A
4570	Bn-N5-p5885217	Bn-A05-p5614651	Bn-A05-p5614651	A5	33	44	A
4571	Bn-N5-p5916356	Bn-A05-p5644210	Bn-A05-p5644210	A5	33	44	A
4572	Bn-N5-p5917146	Bn-A05-p5645000	Bn-A05-p5645000	A5	33	44	A
4573	Bn-N5-p5929818	Bn-A05-p5657708	Bn-A05-p5657708	A5	33	44	A
4574	Bn-N5-p5937171	Bn-A05-p5664658	Bn-A05-p5664658	A5	33	44	A
4575	Bn-N5-p5941716	Bn-A05-p5670181	Bn-A05-p5670181	A5	33	44	A
4576	Bn-N5-p5962932	Bn-A05-p5700216	Bn-A05-p5700216	A5	33	44	A
4577	Bn-N5-p5963607	Bn-A05-p5700891	Bn-A05-p5700891	A5	33	44	A
4578	Bn-N5-p5978793	Bn-A05-p5714702	Bn-A05-p5714702	A5	33	44	A
4579	Bn-N5-p5978847	Bn-A05-p5714756	Bn-A05-p5714756	A5	33	44	A
4580	Bn-N5-p5978927	Bn-A05-p5714836	Bn-A05-p5714836	A5	33	44	A
4581	Bn-N5-p6077945	Bn-A05-p5805445	Bn-A05-p5805445	A5	34	44.8	A
4582	Bn-N5-p6081172	Bn-A05-p5808654	Bn-A05-p5808654	A5	34	44.8	A
4583	Bn-N5-p6081413	Bn-A05-p5808895	Bn-A05-p5808895	A5	34	44.8	A
4584	Bn-N5-p6106417	Bn-A05-p5838524	Bn-A05-p5838524	A5	34	44.8	A
4585	Bn-N5-p6109754	Bn-A05-p5839222	Bn-A05-p5839222	A5	34	44.8	A
4586	Bn-N5-p6109970	Bn-A05-p5839438	Bn-A05-p5839438	A5	34	44.8	A
4587	Bn-N5-p6113510	Bn-A05-p5842980	Bn-A05-p5842980	A5	34	44.8	A
4588	Bn-N5-p6148874	Bn-A05-p5878329	Bn-A05-p5878329	A5	34	44.8	A
4589	Bn-N5-p6167768	Bn-A05-p5897513	Bn-A05-p5897513	A5	34	44.8	A
4590	Bn-N5-p6195750	Bn-A05-p5924319	Bn-A05-p5924319	A5	35	45.6	A
4591	Bn-N5-p6198075	Bn-A05-p5926631	Bn-A05-p5926631	A5	35	45.6	A
4592	Bn-N5-p6250576	Bn-A05-p5970916	Bn-A05-p5970916	A5	35	45.6	A
4593	Bn-N5-p6282683	Bn-A05-p5992612	Bn-A05-p5992612	A5	35	45.6	A
4594	Bn-N5-p6434195	Bn-A05-p6154871	Bn-A05-p6154871	A5	36	47.2	A
4595	Bn-N5-p6435225	Bn-A05-p6155901	Bn-A05-p6155901	A5	36	47.2	A
4596	Bn-N5-p6457548	Bn-A05-p6176943	Bn-A05-p6176943	A5	36	47.2	A
4597	Bn-N5-p6514458	Bn-A05-p6236807	Bn-A05-p6236807	A5	36	47.2	A
4598	Bn-N5-p6540668	Bn-A05-p6261796	Bn-A05-p6261796	A5	36	47.2	A
4599	Bn-N5-p6599347	Bn-A05-p6308471	Bn-A05-p6308471	A5	36	47.2	A
4600	Bn-N5-p6599419	Bn-A05-p6308543	Bn-A05-p6308543	A5	36	47.2	A
4601	Bn-N5-p6622776	Bn-A05-p6335652	Bn-A05-p6335652	A5	36	47.2	A

4602	Bn-N5-p6686533	Bn-A05-p6402777	Bn-A05-p6402777	A5	36	47.2	A
4603	Bn-N5-p6721334	Bn-A05-p6441813	Bn-A05-p6441813	A5	36	47.2	A
4604	Bn-N5-p6767318	Bn-A05-p6473296	Bn-A05-p6473296	A5	36	47.2	A
4605	Bn-N5-p6773964	Bn-A05-p6480168	Bn-A05-p6480168	A5	36	47.2	A
4606	Bn-N5-p6774703	Bn-A05-p6480907	Bn-A05-p6480907	A5	36	47.2	A
4607	Bn-N5-p6774777	Bn-A05-p6480981	Bn-A05-p6480981	A5	36	47.2	A
4608	Bn-N5-p6774794	Bn-C4-p10890203	Bn-scaff_17869_1-p70914	A5	36	47.2	A
4609	Bn-N5-p6784054	Bn-A05-p6494313	Bn-A05-p6494313	A5	36	47.2	A
4610	Bn-N5-p6830606	Bn-A05-p6534544	Bn-A05-p6534544	A5	36	47.2	A
4611	Bn-N5-p6835694	Bn-A05-p6654419	Bn-A05-p6654419	A5	36	47.2	A
4612	Bn-N5-p6835828	Bn-A05-p6654285	Bn-A05-p6654285	A5	36	47.2	A
4613	Bn-N5-p6932079	Bn-A05-p6571330	Bn-A05-p6571330	A5	36	47.2	A
4614	Bn-N5-p6932082	Bn-A05-p6571327	Bn-A05-p6571327	A5	36	47.2	A
4615	Bn-N5-p6966372	Bn-A05-p6542040	Bn-A05-p6542040	A5	36	47.2	A
4616	Bn-N5-p6966977	Bn-A05-p6541429	Bn-A05-p6541429	A5	36	47.2	A
4617	Bn-N5-p6968830	Bn-A05-p6539730	Bn-A05-p6539730	A5	36	47.2	A
4618	Bn-N5-p6991428	Bn-A05-p6688695	Bn-A05-p6688695	A5	36	47.2	A
4619	Bn-N14-p12313445	Bn-A05-p6898696	Bn-A05-p6898696	A5	37	48	A
4620	Bn-N14-p13209662	Bn-Scaffold01145-p1	Bn-scaff_17759_1-p12468	A5	37	48	A
4621	Bn-N16-p13514935	Bn-A05-p9849941	Bn-A05-p9849941	A5	37	48	A
4622	Bn-N5-p10023837	Bn-C6-p32970334	Bn-scaff_17088_3-p11728	A5	37	48	A
4623	Bn-N5-p10023951	Bn-A05-p10423564	Bn-A05-p10423564	A5	37	48	A
4624	Bn-N5-p10026726	Bn-A05-p10426347	Bn-A05-p10426347	A5	37	48	A
4625	Bn-N5-p10038696	Bn-A05-p10437937	Bn-A05-p10437937	A5	37	48	A
4626	Bn-N5-p10057051	Bn-A05-p10452570	Bn-A05-p10452570	A5	37	48	A
4627	Bn-N5-p10251993	Bn-A05-p10622526	Bn-A05-p10622526	A5	37	48	A
4628	Bn-N5-p10254457	Bn-A05-p10625034	Bn-A05-p10625034	A5	37	48	A
4629	Bn-N5-p10254659	Bn-A05-p10625236	Bn-A05-p10625236	A5	37	48	A
4630	Bn-N5-p10321363	Bn-A05-p10697862	Bn-A05-p10697862	A5	37	48	A
4631	Bn-N5-p10361634	Bn-A05-p10719967	Bn-A05-p10719967	A5	37	48	A
4632	Bn-N5-p7081718	Bn-A05-p6786862	Bn-A05-p6786862	A5	37	48	A
4633	Bn-N5-p7082020	Bn-A05-p6787164	Bn-A05-p6787164	A5	37	48	A
4634	Bn-N5-p7082349	Bn-C4-p8105773	Bn-scaff_23907_1-p29685	A5	37	48	A
4635	Bn-N5-p7163028	Bn-A05-p6867531	Bn-A05-p6867531	A5	37	48	A
4636	Bn-N5-p7172426	Bn-A05-p6878538	Bn-A05-p6878538	A5	37	48	A
4637	Bn-N5-p7188085	Bn-A05-p6897871	Bn-A05-p6897871	A5	37	48	A
4638	Bn-N5-p7201619	Bn-A05-p6910402	Bn-A05-p6910402	A5	37	48	A
4639	Bn-N5-p7239361	Bn-A05-p6951707	Bn-A05-p6951707	A5	37	48	A
4640	Bn-N5-p7245977	Bn-A05-p6957948	Bn-A05-p6957948	A5	37	48	A
4641	Bn-N5-p7276887	Bn-A05-p6984362	Bn-A05-p6984362	A5	37	48	A
4642	Bn-N5-p7292023	Bn-A05-p6995896	Bn-A05-p6995896	A5	37	48	A
4643	Bn-N5-p7304200	Bn-A05-p7011916	Bn-A05-p7011916	A5	37	48	A
4644	Bn-N5-p7357542	Bn-Scaffold000397-p	Bn-Scaffold000397-p7197	A5	37	48	A
4645	Bn-N5-p7378912	Bn-A05-p7062784	Bn-A05-p7062784	A5	37	48	A
4646	Bn-N5-p7398390	Bn-A05-p7076442	Bn-A05-p7076442	A5	37	48	A
4647	Bn-N5-p7485167	Bn-A05-p7187425	Bn-A05-p7187425	A5	37	48	A
4648	Bn-N5-p7509876	Bn-A05-p7222795	Bn-A05-p7222795	A5	37	48	A
4649	Bn-N5-p7521211	Bn-A05-p7227877	Bn-A05-p7227877	A5	37	48	A
4650	Bn-N5-p7525358	Bn-A05-p7231369	Bn-A05-p7231369	A5	37	48	A

4651	Bn-N5-p7631525	Bn-A05-p7346478	Bn-A05-p7346478	A5	37	48	A
4652	Bn-N5-p7650848	Bn-A05-p7370869	Bn-A05-p7370869	A5	37	48	A
4653	Bn-N5-p7971834	Bn-A05-p7613069	Bn-A05-p7613069	A5	37	48	A
4654	Bn-N5-p8005891	Bn-A05-p7651411	Bn-A05-p7651411	A5	37	48	A
4655	Bn-N5-p8083716	Bn-A05-p7729367	Bn-A05-p7729367	A5	37	48	A
4656	Bn-N5-p8128096	Bn-A05-p7778186	Bn-A05-p7778186	A5	37	48	A
4657	Bn-N5-p8153993	Bn-A05-p7791550	Bn-A05-p7791550	A5	37	48	A
4658	Bn-N5-p8179626	Bn-A05-p7811409	Bn-A05-p7811409	A5	37	48	A
4659	Bn-N5-p8207484	Bn-A05-p7844876	Bn-A05-p7844876	A5	37	48	A
4660	Bn-N5-p8218313	Bn-A05-p7855766	Bn-A05-p7855766	A5	37	48	A
4661	Bn-N5-p8230878	Bn-A05-p7874994	Bn-A05-p7874994	A5	37	48	A
4662	Bn-N5-p8260953	Bn-A05-p7911054	Bn-A05-p7911054	A5	37	48	A
4663	Bn-N5-p8494946	Bn-A05-p9555189	Bn-A05-p9555189	A5	37	48	A
4664	Bn-N5-p8636774	Bn-A05-p9389251	Bn-A05-p9389251	A5	37	48	A
4665	Bn-N5-p8644809	Bn-A05-p9385617	Bn-A05-p9385617	A5	37	48	A
4666	Bn-N5-p8645321	Bn-A05-p9384270	Bn-A05-p9384270	A5	37	48	A
4667	Bn-N5-p8652776	Bn-A05-p9376879	Bn-A05-p9376879	A5	37	48	A
4668	Bn-N5-p8702877	Bn-A05-p9319851	Bn-A05-p9319851	A5	37	48	A
4669	Bn-N5-p8778514	Bn-A05-p9602710	Bn-A05-p9602710	A5	37	48	A
4670	Bn-N5-p8778701	Bn-A05-p9602897	Bn-A05-p9602897	A5	37	48	A
4671	Bn-N5-p8804262	Bn-A05-p9638089	Bn-A05-p9638089	A5	37	48	A
4672	Bn-N5-p8807023	Bn-A05-p9638802	Bn-A05-p9638802	A5	37	48	A
4673	Bn-N5-p8813919	Bn-A05-p9646716	Bn-A05-p9646716	A5	37	48	A
4674	Bn-N5-p8832703	Bn-A05-p9660152	Bn-A05-p9660152	A5	37	48	A
4675	Bn-N5-p8856168	Bn-A05-p9668222	Bn-A05-p9668222	A5	37	48	A
4676	Bn-N5-p8936796	Bn-A05-p9758517	Bn-A05-p9758517	A5	37	48	A
4677	Bn-N5-p8944491	Bn-A05-p9765872	Bn-A05-p9765872	A5	37	48	A
4678	Bn-N5-p8944953	Bn-A05-p9766332	Bn-A05-p9766332	A5	37	48	A
4679	Bn-N5-p8945238	Bn-A05-p9766628	Bn-A05-p9766628	A5	37	48	A
4680	Bn-N5-p8951234	Bn-A05-p9773183	Bn-A05-p9773183	A5	37	48	A
4681	Bn-N5-p8962481	Bn-A05-p9777250	Bn-A05-p9777250	A5	37	48	A
4682	Bn-N5-p9030784	Bn-A05-p9849049	Bn-A05-p9849049	A5	37	48	A
4683	Bn-N5-p9031562	Bn-A05-p9849822	Bn-A05-p9849822	A5	37	48	A
4684	Bn-N5-p9084379	Bn-A01-p11996408	Bn-A01-p11996408	A5	37	48	A
4685	Bn-N5-p9085684	Bn-A01-p11993589	Bn-A01-p11993589	A5	37	48	A
4686	Bn-N5-p9086994	Bn-A01-p11992281	Bn-A01-p11992281	A5	37	48	A
4687	Bn-N5-p9087685	Bn-A01-p11991521	Bn-A01-p11991521	A5	37	48	A
4688	Bn-N5-p9103213	Bn-A05-p9890807	Bn-A05-p9890807	A5	37	48	A
4689	Bn-N5-p9112819	Bn-A05-p9900987	Bn-A05-p9900987	A5	37	48	A
4690	Bn-N5-p9213866	Bn-A05-p10009197	Bn-A05-p10009197	A5	37	48	A
4691	Bn-N5-p9214153	Bn-A05-p10009485	Bn-A05-p10009485	A5	37	48	A
4692	Bn-N5-p9420409	Bn-A07-p11403525	Bn-A07-p11403525	A5	37	48	A
4693	Bn-N5-p9479293	Bn-A08-p1430241	Bn-A08-p1430241	A5	37	48	A
4694	Bn-N5-p9487275	Bn-A08-p1404439	Bn-A08-p1404439	A5	37	48	A
4695	Bn-N5-p9487392	Bn-A08-p1404321	Bn-A08-p1404321	A5	37	48	A
4696	Bn-N5-p9491353	Bn-A08-p1400360	Bn-A08-p1400360	A5	37	48	A
4697	Bn-N5-p9512670	Bn-A08-p1382630	Bn-A08-p1382630	A5	37	48	A
4698	Bn-N5-p9527984	Bn-A08-p1369206	Bn-A08-p1369206	A5	37	48	A
4699	Bn-N5-p9540298	Bn-A08-p1365676	Bn-A08-p1365676	A5	37	48	A

4700	Bn-N5-p9540466	Bn-A08-p1365508	Bn-A08-p1365508	A5	37	48	A
4701	Bn-N5-p9541711	Bn-A08-p1364252	Bn-A08-p1364252	A5	37	48	A
4702	Bn-N5-p9548694	Bn-A08-p1357142	Bn-A08-p1357142	A5	37	48	A
4703	Bn-N5-p9550657	Bn-A08-p1353718	Bn-A08-p1353718	A5	37	48	A
4704	Bn-N5-p9564113	Bn-A08-p1342021	Bn-A08-p1342021	A5	37	48	A
4705	Bn-N5-p9564197	Bn-A08-p1341937	Bn-A08-p1341937	A5	37	48	A
4706	Bn-N5-p9565240	Bn-C6-p25895547	Bn-scaff_18439_1-p49815[illegible]	A5	37	48	A
4707	Bn-N5-p9565508	Bn-A08-p1336421	Bn-A08-p1336421	A5	37	48	A
4708	Bn-N5-p9571193	Bn-A08-p1332628	Bn-A08-p1332628	A5	37	48	A
4709	Bn-N5-p9578359	Bn-A08-p1325427	Bn-A08-p1325427	A5	37	48	A
4710	Bn-N5-p9579172	Bn-A08-p1324618	Bn-A08-p1324618	A5	37	48	A
4711	Bn-N5-p9587167	Bn-A08-p1319949	Bn-A08-p1319949	A5	37	48	A
4712	Bn-N5-p9587568	Bn-A08-p1319548	Bn-A08-p1319548	A5	37	48	A
4713	Bn-N5-p9593477	Bn-A08-p1313629	Bn-A08-p1313629	A5	37	48	A
4714	Bn-N5-p9599002	Bn-A08-p1308299	Bn-A08-p1308299	A5	37	48	A
4715	Bn-N5-p9615398	Bn-Scaffold000218-p[illegible]	Bn-Scaffold000218-p16790	A5	37	48	A
4716	Bn-N5-p9619586	Bn-Scaffold000218-p[illegible]	Bn-Scaffold000218-p18252	A5	37	48	A
4717	Bn-N5-p9626470	Bn-Scaffold000218-p[illegible]	Bn-Scaffold000218-p27195	A5	37	48	A
4718	Bn-N5-p9830494	Bn-A05-p10269046	Bn-A05-p10269046	A5	37	48	A
4719	Bn-N5-p9842754	Bn-A05-p10278245	Bn-A05-p10278245	A5	37	48	A
4720	Bn-N5-p9845857	Bn-A05-p10281337	Bn-A05-p10281337	A5	37	48	A
4721	Bn-N5-p9880048	Bn-A05-p10305352	Bn-A05-p10305352	A5	37	48	A
4722	Bn-N5-p9895872	Bn-A05-p10320143	Bn-A05-p10320143	A5	37	48	A
4723	Bn-N5-p9896230	Bn-A05-p10320501	Bn-A05-p10320501	A5	37	48	A
4724	Bn-N5-p9897309	Bn-A05-p10321580	Bn-A05-p10321580	A5	37	48	A
4725	Bn-N5-p9924616	Bn-A05-p10349551	Bn-A05-p10349551	A5	37	48	A
4726	Bn-N5-p9942033	Bn-A05-p10364013	Bn-A05-p10364013	A5	37	48	A
4727	Bn-N5-p9948397	Bn-A05-p10367279	Bn-A05-p10367279	A5	37	48	A
4728	Bn-N11-p22136508	Bn-A05-p14960328	Bn-A05-p14960328	A5	38	49.6	A
4729	Bn-N12-p4812469	Bn-A05-p12891987	Bn-A05-p12891987	A5	38	49.6	A
4730	Bn-N12-p4813205	Bn-A05-p12891251	Bn-A05-p12891251	A5	38	49.6	A
4731	Bn-N13-p36788938	Bn-A05-p13778123	Bn-A05-p13778123	A5	38	49.6	A
4732	Bn-N14-p50588041	Bn-C6-p10997513	Bn-scaff_15892_1-p20678	A5	38	49.6	A
4733	Bn-N15-p25498290	Bn-A05-p11687380	Bn-A05-p11687380	A5	38	49.6	A
4734	Bn-N15-p25890644	Bn-A05-p11593933	Bn-A05-p11593933	A5	38	49.6	A
4735	Bn-N15-p28476724	Bn-A05-p13760191	Bn-A05-p13760191	A5	38	49.6	A
4736	Bn-N15-p28604572	Bn-A05-p13811322	Bn-A05-p13811322	A5	38	49.6	A
4737	Bn-N15-p29457089	Bn-A06-p20877670	Bn-A06-p20877670	A5	38	49.6	A
4738	Bn-N15-p29458176	Bn-C5-p28190173	Bn-scaff_15705_2-p83267[illegible]	A5	38	49.6	A
4739	Bn-N15-p29501516	Bn-C5-p28224210	Bn-scaff_15705_2-p79863[illegible]	A5	38	49.6	A
4740	Bn-N15-p29502817	Bn-C5-p28225511	Bn-scaff_15705_2-p79733[illegible]	A5	38	49.6	A
4741	Bn-N15-p29534218	Bn-C5-p28256284	Bn-scaff_15705_2-p76656[illegible]	A5	38	49.6	A
4742	Bn-N15-p31120570	Bn-A05-p14537104	Bn-A05-p14537104	A5	38	49.6	A
4743	Bn-N15-p31198686	Bn-A05-p14555358	Bn-A05-p14555358	A5	38	49.6	A
4744	Bn-N15-p31200429	Bn-A05-p14557992	Bn-A05-p14557992	A5	38	49.6	A
4745	Bn-N15-p31200528	Bn-A05-p14558091	Bn-A05-p14558091	A5	38	49.6	A
4746	Bn-N15-p37409471	Bn-A05-p14959506	Bn-A05-p14959506	A5	38	49.6	A
4747	Bn-N16-p9311278	Bn-A05-p10778859	Bn-A05-p10778859	A5	38	49.6	A
4748	Bn-N17-p6935980	Bn-A05-p13778538	Bn-A05-p13778538	A5	38	49.6	A

4749	Bn-N17-p872796	Bn-A05-p14533489	Bn-A05-p14533489	A5	38	49.6	A
4750	Bn-N1-p2911278	Bn-A05-p14525918	Bn-A05-p14525918	A5	38	49.6	A
4751	Bn-N5-p10393746	Bn-A05-p10752346	Bn-A05-p10752346	A5	38	49.6	A
4752	Bn-N5-p10401975	Bn-A05-p10760570	Bn-A05-p10760570	A5	38	49.6	A
4753	Bn-N5-p10416785	Bn-A05-p10780480	Bn-A05-p10780480	A5	38	49.6	A
4754	Bn-N5-p10419340	Bn-A05-p10783057	Bn-A05-p10783057	A5	38	49.6	A
4755	Bn-N5-p10524003	Bn-A05-p10868044	Bn-A05-p10868044	A5	38	49.6	A
4756	Bn-N5-p10577272	Bn-A05-p10922835	Bn-A05-p10922835	A5	38	49.6	A
4757	Bn-N5-p10578539	Bn-A05-p10924105	Bn-A05-p10924105	A5	38	49.6	A
4758	Bn-N5-p10599417	Bn-A05-p10939686	Bn-A05-p10939686	A5	38	49.6	A
4759	Bn-N5-p10600132	Bn-A05-p10940401	Bn-A05-p10940401	A5	38	49.6	A
4760	Bn-N5-p10607584	Bn-A05-p10947783	Bn-A05-p10947783	A5	38	49.6	A
4761	Bn-N5-p10608734	Bn-A05-p10948934	Bn-A05-p10948934	A5	38	49.6	A
4762	Bn-N5-p10627144	Bn-A05-p10964994	Bn-A05-p10964994	A5	38	49.6	A
4763	Bn-N5-p10631100	Bn-A05-p10966950	Bn-A05-p10966950	A5	38	49.6	A
4764	Bn-N5-p10648004	Bn-A05-p10981427	Bn-A05-p10981427	A5	38	49.6	A
4765	Bn-N5-p10656438	Bn-A05-p10999977	Bn-A05-p10999977	A5	38	49.6	A
4766	Bn-N5-p10656497	Bn-A05-p11000036	Bn-A05-p11000036	A5	38	49.6	A
4767	Bn-N5-p10656719	Bn-A05-p11000261	Bn-A05-p11000261	A5	38	49.6	A
4768	Bn-N5-p10663727	Bn-A05-p11013933	Bn-A05-p11013933	A5	38	49.6	A
4769	Bn-N5-p10699281	Bn-A05-p11068866	Bn-A05-p11068866	A5	38	49.6	A
4770	Bn-N5-p10708377	Bn-A05-p11084469	Bn-A05-p11084469	A5	38	49.6	A
4771	Bn-N5-p10712086	Bn-A05-p11088814	Bn-A05-p11088814	A5	38	49.6	A
4772	Bn-N5-p10755775	Bn-A05-p11120195	Bn-A05-p11120195	A5	38	49.6	A
4773	Bn-N5-p10755854	Bn-A05-p11120274	Bn-A05-p11120274	A5	38	49.6	A
4774	Bn-N5-p10776513	Bn-A05-p11143078	Bn-A05-p11143078	A5	38	49.6	A
4775	Bn-N5-p10779764	Bn-A05-p11146315	Bn-A05-p11146315	A5	38	49.6	A
4776	Bn-N5-p10780019	Bn-A05-p11146571	Bn-A05-p11146571	A5	38	49.6	A
4777	Bn-N5-p10788978	Bn-A05-p11151246	Bn-A05-p11151246	A5	38	49.6	A
4778	Bn-N5-p10801969	Bn-A05-p11168272	Bn-A05-p11168272	A5	38	49.6	A
4779	Bn-N5-p10802109	Bn-A05-p11168423	Bn-A05-p11168423	A5	38	49.6	A
4780	Bn-N5-p11229549	Bn-A05-p11607344	Bn-A05-p11607344	A5	38	49.6	A
4781	Bn-N5-p11297083	Bn-A05-p11686214	Bn-A05-p11686214	A5	38	49.6	A
4782	Bn-N5-p11300721	Bn-A05-p11689852	Bn-A05-p11689852	A5	38	49.6	A
4783	Bn-N5-p11350515	Bn-A05-p11737404	Bn-A05-p11737404	A5	38	49.6	A
4784	Bn-N5-p11353647	Bn-A05-p11740536	Bn-A05-p11740536	A5	38	49.6	A
4785	Bn-N5-p11364647	Bn-A05-p11751401	Bn-A05-p11751401	A5	38	49.6	A
4786	Bn-N5-p11385605	Bn-A05-p11776888	Bn-A05-p11776888	A5	38	49.6	A
4787	Bn-N5-p11395386	Bn-A05-p11786537	Bn-A05-p11786537	A5	38	49.6	A
4788	Bn-N5-p11423120	Bn-A05-p11812265	Bn-A05-p11812265	A5	38	49.6	A
4789	Bn-N5-p11445225	Bn-A05-p11840240	Bn-A05-p11840240	A5	38	49.6	A
4790	Bn-N5-p11517673	Bn-Scaffold000172-p9	Bn-Scaffold000172-p99636	A5	38	49.6	A
4791	Bn-N5-p11530724	Bn-Scaffold000172-p1	Bn-Scaffold000172-p11084	A5	38	49.6	A
4792	Bn-N5-p11536006	Bn-Scaffold000172-p1	Bn-Scaffold000172-p11612	A5	38	49.6	A
4793	Bn-N5-p11547071	Bn-Scaffold000172-p1	Bn-Scaffold000172-p13258	A5	38	49.6	A
4794	Bn-N5-p11553008	Bn-Scaffold000172-p1	Bn-Scaffold000172-p13538	A5	38	49.6	A
4795	Bn-N5-p11683983	Bn-Scaffold000172-p2	Bn-Scaffold000172-p24309	A5	38	49.6	A
4796	Bn-N5-p11716231	Bn-Scaffold000172-p2	Bn-Scaffold000172-p25436	A5	38	49.6	A
4797	Bn-N5-p11753241	Bn-Scaffold000172-p2	Bn-Scaffold000172-p27675	A5	38	49.6	A

4798	Bn-N5-p11762470	Bn-A05-p13184792	Bn-A05-p13184792	A5	38	49.6	A
4799	Bn-N5-p11768537	Bn-A05-p13179258	Bn-A05-p13179258	A5	38	49.6	A
4800	Bn-N5-p11768840	Bn-A05-p13178955	Bn-A05-p13178955	A5	38	49.6	A
4801	Bn-N5-p11770044	Bn-A05-p13175224	Bn-A05-p13175224	A5	38	49.6	A
4802	Bn-N5-p11780191	Bn-A05-p13168139	Bn-A05-p13168139	A5	38	49.6	A
4803	Bn-N5-p11787351	Bn-A05-p13165406	Bn-A05-p13165406	A5	38	49.6	A
4804	Bn-N5-p11825012	Bn-Scaffold000324-p3	Bn-Scaffold000324-p3687	A5	38	49.6	A
4805	Bn-N5-p11825268	Bn-Scaffold000324-p3	Bn-Scaffold000324-p3944	A5	38	49.6	A
4806	Bn-N5-p11833451	Bn-Scaffold000324-p1	Bn-Scaffold000324-p10085	A5	38	49.6	A
4807	Bn-N5-p11834989	Bn-Scaffold000324-p1	Bn-Scaffold000324-p11634	A5	38	49.6	A
4808	Bn-N5-p11835261	Bn-Scaffold000324-p1	Bn-Scaffold000324-p11905	A5	38	49.6	A
4809	Bn-N5-p11876624	Bn-C5-p24904514	Bn-scaff_17092_1-p42620	A5	38	49.6	A
4810	Bn-N5-p11897495	Bn-A05-p11961924	Bn-A05-p11961924	A5	38	49.6	A
4811	Bn-N5-p11942848	Bn-A05-p12002033	Bn-A05-p12002033	A5	38	49.6	A
4812	Bn-N5-p11960281	Bn-A05-p12018989	Bn-A05-p12018989	A5	38	49.6	A
4813	Bn-N5-p11961000	Bn-A05-p12019708	Bn-A05-p12019708	A5	38	49.6	A
4814	Bn-N5-p11963021	Bn-A05-p12021730	Bn-A05-p12021730	A5	38	49.6	A
4815	Bn-N5-p11963267	Bn-A05-p12021976	Bn-A05-p12021976	A5	38	49.6	A
4816	Bn-N5-p11963831	Bn-A05-p12022540	Bn-A05-p12022540	A5	38	49.6	A
4817	Bn-N5-p11967408	Bn-A05-p12026117	Bn-A05-p12026117	A5	38	49.6	A
4818	Bn-N5-p11974229	Bn-A05-p12032938	Bn-A05-p12032938	A5	38	49.6	A
4819	Bn-N5-p11984567	Bn-A05-p12046650	Bn-A05-p12046650	A5	38	49.6	A
4820	Bn-N5-p11997358	Bn-A05-p12059250	Bn-A05-p12059250	A5	38	49.6	A
4821	Bn-N5-p12005202	Bn-A05-p12067093	Bn-A05-p12067093	A5	38	49.6	A
4822	Bn-N5-p12042428	Bn-A05-p12102885	Bn-A05-p12102885	A5	38	49.6	A
4823	Bn-N5-p12042975	Bn-A05-p12103432	Bn-A05-p12103432	A5	38	49.6	A
4824	Bn-N5-p12044483	Bn-A05-p12104940	Bn-A05-p12104940	A5	38	49.6	A
4825	Bn-N5-p12064364	Bn-A05-p12115583	Bn-A05-p12115583	A5	38	49.6	A
4826	Bn-N5-p12068040	Bn-A05-p12119364	Bn-A05-p12119364	A5	38	49.6	A
4827	Bn-N5-p12079902	Bn-A05-p12131090	Bn-A05-p12131090	A5	38	49.6	A
4828	Bn-N5-p12138543	Bn-A05-p12193915	Bn-A05-p12193915	A5	38	49.6	A
4829	Bn-N5-p12144089	Bn-A05-p12199879	Bn-A05-p12199879	A5	38	49.6	A
4830	Bn-N5-p12144860	Bn-C5-p24024140	Bn-scaff_16454_1-p56466	A5	38	49.6	A
4831	Bn-N5-p12153049	Bn-A05-p12208840	Bn-A05-p12208840	A5	38	49.6	A
4832	Bn-N5-p12153338	Bn-A05-p12209129	Bn-A05-p12209129	A5	38	49.6	A
4833	Bn-N5-p12160934	Bn-A05-p12216620	Bn-A05-p12216620	A5	38	49.6	A
4834	Bn-N5-p12243956	Bn-A05-p12298408	Bn-A05-p12298408	A5	38	49.6	A
4835	Bn-N5-p12311564	Bn-A05-p12362411	Bn-A05-p12362411	A5	38	49.6	A
4836	Bn-N5-p12329634	Bn-A05-p12378382	Bn-A05-p12378382	A5	38	49.6	A
4837	Bn-N5-p12372532	Bn-A05-p12418828	Bn-A05-p12418828	A5	38	49.6	A
4838	Bn-N5-p12392526	Bn-A05-p12450816	Bn-A05-p12450816	A5	38	49.6	A
4839	Bn-N5-p12394037	Bn-A05-p12453362	Bn-A05-p12453362	A5	38	49.6	A
4840	Bn-N5-p12426980	Bn-A05-p12497800	Bn-A05-p12497800	A5	38	49.6	A
4841	Bn-N5-p12436378	Bn-A05-p12507206	Bn-A05-p12507206	A5	38	49.6	A
4842	Bn-N5-p12442262	Bn-A05-p12511706	Bn-A05-p12511706	A5	38	49.6	A
4843	Bn-N5-p12521345	Bn-A05-p12597194	Bn-A05-p12597194	A5	38	49.6	A
4844	Bn-N5-p12558838	Bn-A05-p12643056	Bn-A05-p12643056	A5	38	49.6	A
4845	Bn-N5-p12597351	Bn-A05-p12684736	Bn-A05-p12684736	A5	38	49.6	A
4846	Bn-N5-p12647061	Bn-A05-p12734993	Bn-A05-p12734993	A5	38	49.6	A

4847	Bn-N5-p12867251	Bn-A05-p12930251	Bn-A05-p12930251	A5	38	49.6	A
4848	Bn-N5-p13343674	Bn-A05-p13688426	Bn-A05-p13688426	A5	38	49.6	A
4849	Bn-N5-p13344230	Bn-A05-p13688982	Bn-A05-p13688982	A5	38	49.6	A
4850	Bn-N5-p13344625	Bn-C5-p27396772	Bn-scaff_15648_1-p53856{	A5	38	49.6	A
4851	Bn-N5-p13415953	Bn-A05-p13751450	Bn-A05-p13751450	A5	38	49.6	A
4852	Bn-N5-p13423511	Bn-A05-p13760281	Bn-A05-p13760281	A5	38	49.6	A
4853	Bn-N5-p13488373	Bn-A05-p13811525	Bn-A05-p13811525	A5	38	49.6	A
4854	Bn-N5-p13489952	Bn-A05-p13813146	Bn-A05-p13813146	A5	38	49.6	A
4855	Bn-N5-p13493842	Bn-A05-p13816756	Bn-A05-p13816756	A5	38	49.6	A
4856	Bn-N5-p13493912	Bn-A05-p13816826	Bn-A05-p13816826	A5	38	49.6	A
4857	Bn-N5-p13493928	Bn-A05-p13816842	Bn-A05-p13816842	A5	38	49.6	A
4858	Bn-N5-p13494239	Bn-A05-p13817153	Bn-A05-p13817153	A5	38	49.6	A
4859	Bn-N5-p13499507	Bn-A05-p13822414	Bn-A05-p13822414	A5	38	49.6	A
4860	Bn-N5-p13499709	Bn-A05-p13822616	Bn-A05-p13822616	A5	38	49.6	A
4861	Bn-N5-p13502511	Bn-A05-p13825402	Bn-A05-p13825402	A5	38	49.6	A
4862	Bn-N5-p13503120	Bn-A05-p13826013	Bn-A05-p13826013	A5	38	49.6	A
4863	Bn-N5-p13514127	Bn-A05-p13845255	Bn-A05-p13845255	A5	38	49.6	A
4864	Bn-N5-p13551129	Bn-A05-p13863624	Bn-A05-p13863624	A5	38	49.6	A
4865	Bn-N5-p13551178	Bn-A05-p13863673	Bn-A05-p13863673	A5	38	49.6	A
4866	Bn-N5-p13551480	Bn-A05-p13863975	Bn-A05-p13863975	A5	38	49.6	A
4867	Bn-N5-p13551569	Bn-A05-p13864064	Bn-A05-p13864064	A5	38	49.6	A
4868	Bn-N5-p13551638	Bn-A05-p13864133	Bn-A05-p13864133	A5	38	49.6	A
4869	Bn-N5-p13551973	Bn-A05-p13864467	Bn-A05-p13864467	A5	38	49.6	A
4870	Bn-N5-p13558528	Bn-A05-p13876782	Bn-A05-p13876782	A5	38	49.6	A
4871	Bn-N5-p13562138	Bn-A05-p13880377	Bn-A05-p13880377	A5	38	49.6	A
4872	Bn-N5-p13564460	Bn-A05-p13882697	Bn-A05-p13882697	A5	38	49.6	A
4873	Bn-N5-p13568722	Bn-A05-p13886960	Bn-A05-p13886960	A5	38	49.6	A
4874	Bn-N5-p13576841	Bn-A05-p13895100	Bn-A05-p13895100	A5	38	49.6	A
4875	Bn-N5-p13576894	Bn-A05-p13895153	Bn-A05-p13895153	A5	38	49.6	A
4876	Bn-N5-p13578663	Bn-A05-p13896924	Bn-A05-p13896924	A5	38	49.6	A
4877	Bn-N5-p13579644	Bn-A05-p13897888	Bn-A05-p13897888	A5	38	49.6	A
4878	Bn-N5-p13593274	Bn-A05-p13907424	Bn-A05-p13907424	A5	38	49.6	A
4879	Bn-N5-p13593939	Bn-A05-p13908499	Bn-A05-p13908499	A5	38	49.6	A
4880	Bn-N5-p13683744	Bn-A05-p13965323	Bn-A05-p13965323	A5	38	49.6	A
4881	Bn-N5-p13690810	Bn-A05-p13973628	Bn-A05-p13973628	A5	38	49.6	A
4882	Bn-N5-p13759318	Bn-A09-p15112850	Bn-A09-p15112850	A5	38	49.6	A
4883	Bn-N5-p13829896	Bn-A05-p14106384	Bn-A05-p14106384	A5	38	49.6	A
4884	Bn-N5-p13864056	Bn-A05-p14121401	Bn-A05-p14121401	A5	38	49.6	A
4885	Bn-N5-p13890086	Bn-A05-p14144406	Bn-A05-p14144406	A5	38	49.6	A
4886	Bn-N5-p13891987	Bn-A05-p14146305	Bn-A05-p14146305	A5	38	49.6	A
4887	Bn-N5-p13959974	Bn-A05-p14198626	Bn-A05-p14198626	A5	38	49.6	A
4888	Bn-N5-p14031409	Bn-A05-p14256951	Bn-A05-p14256951	A5	38	49.6	A
4889	Bn-N5-p14652990	Bn-A06-p20320258	Bn-A06-p20320258	A5	38	49.6	A
4890	Bn-N5-p14653039	Bn-A06-p20320209	Bn-A06-p20320209	A5	38	49.6	A
4891	Bn-N5-p14768753	Bn-A06-p20211283	Bn-A06-p20211283	A5	38	49.6	A
4892	Bn-N5-p14881123	Bn-A05-p14347603	Bn-A05-p14347603	A5	38	49.6	A
4893	Bn-N5-p14909937	Bn-A05-p14372759	Bn-A05-p14372759	A5	38	49.6	A
4894	Bn-N5-p14911377	Bn-A05-p14374199	Bn-A05-p14374199	A5	38	49.6	A
4895	Bn-N5-p14911806	Bn-A05-p14374632	Bn-A05-p14374632	A5	38	49.6	A

4896	Bn-N5-p14919338	Bn-A05-p14380049	Bn-A05-p14380049	A5	38	49.6	A
4897	Bn-N5-p14932487	Bn-A05-p14394895	Bn-A05-p14394895	A5	38	49.6	A
4898	Bn-N5-p14938829	Bn-A05-p14402155	Bn-A05-p14402155	A5	38	49.6	A
4899	Bn-N5-p14984918	Bn-A05-p14464476	Bn-A05-p14464476	A5	38	49.6	A
4900	Bn-N5-p14992271	Bn-A05-p14477462	Bn-A05-p14477462	A5	38	49.6	A
4901	Bn-N5-p15012795	Bn-A05-p14483099	Bn-A05-p14483099	A5	38	49.6	A
4902	Bn-N5-p15021872	Bn-A05-p14496771	Bn-A05-p14496771	A5	38	49.6	A
4903	Bn-N5-p15023658	Bn-Scaffold008008-p1	Bn-Scaffold008008-p158	A5	38	49.6	A
4904	Bn-N5-p15026050	Bn-A05-p14505946	Bn-A05-p14505946	A5	38	49.6	A
4905	Bn-N5-p15036982	Bn-A05-p14521315	Bn-A05-p14521315	A5	38	49.6	A
4906	Bn-N5-p15044093	Bn-A05-p14529716	Bn-A05-p14529716	A5	38	49.6	A
4907	Bn-N5-p15088228	Bn-A05-p14588431	Bn-A05-p14588431	A5	38	49.6	A
4908	Bn-N5-p15101132	Bn-C5-p29764083	Bn-scaff_16228_1-p17171	A5	38	49.6	A
4909	Bn-N5-p15110184	Bn-A05-p14602709	Bn-A05-p14602709	A5	38	49.6	A
4910	Bn-N5-p15110344	Bn-A05-p14602869	Bn-A05-p14602869	A5	38	49.6	A
4911	Bn-N5-p15115218	Bn-A05-p14612446	Bn-A05-p14612446	A5	38	49.6	A
4912	Bn-N5-p15127575	Bn-A05-p14624727	Bn-A05-p14624727	A5	38	49.6	A
4913	Bn-N5-p15127632	Bn-A05-p14624784	Bn-A05-p14624784	A5	38	49.6	A
4914	Bn-N5-p15139750	Bn-A05-p14636717	Bn-A05-p14636717	A5	38	49.6	A
4915	Bn-N5-p15216588	Bn-A05-p14709014	Bn-A05-p14709014	A5	38	49.6	A
4916	Bn-N5-p15216944	Bn-A05-p14709370	Bn-A05-p14709370	A5	38	49.6	A
4917	Bn-N5-p15218846	Bn-A05-p14712935	Bn-A05-p14712935	A5	38	49.6	A
4918	Bn-N5-p15221014	Bn-A05-p14715097	Bn-A05-p14715097	A5	38	49.6	A
4919	Bn-N5-p15229344	Bn-A05-p14723523	Bn-A05-p14723523	A5	38	49.6	A
4920	Bn-N5-p15238883	Bn-A05-p14726545	Bn-A05-p14726545	A5	38	49.6	A
4921	Bn-N5-p15239028	Bn-A05-p14726690	Bn-A05-p14726690	A5	38	49.6	A
4922	Bn-N5-p15247403	Bn-A05-p14734937	Bn-A05-p14734937	A5	38	49.6	A
4923	Bn-N5-p15251706	Bn-A05-p14739008	Bn-A05-p14739008	A5	38	49.6	A
4924	Bn-N5-p15251778	Bn-A05-p14739080	Bn-A05-p14739080	A5	38	49.6	A
4925	Bn-N5-p15253702	Bn-A05-p14740992	Bn-A05-p14740992	A5	38	49.6	A
4926	Bn-N5-p15255426	Bn-A05-p14742715	Bn-A05-p14742715	A5	38	49.6	A
4927	Bn-N5-p15255738	Bn-A05-p14743027	Bn-A05-p14743027	A5	38	49.6	A
4928	Bn-N5-p15260421	Bn-A05-p14747774	Bn-A05-p14747774	A5	38	49.6	A
4929	Bn-N5-p15272447	Bn-A05-p14755242	Bn-A05-p14755242	A5	38	49.6	A
4930	Bn-N5-p15415387	Bn-A05-p14882151	Bn-A05-p14882151	A5	38	49.6	A
4931	Bn-N5-p15560869	Bn-A05-p15013585	Bn-A05-p15013585	A5	38	49.6	A
4932	Bn-N5-p15563350	Bn-A05-p15016066	Bn-A05-p15016066	A5	38	49.6	A
4933	Bn-N5-p15577934	Bn-A05-p15035058	Bn-A05-p15035058	A5	38	49.6	A
4934	Bn-N5-p15752905	Bn-A05-p15236669	Bn-A05-p15236669	A5	38	49.6	A
4935	Bn-N5-p15761117	Bn-A05-p15250834	Bn-A05-p15250834	A5	38	49.6	A
4936	Bn-N5-p15770182	Bn-A05-p15281306	Bn-A05-p15281306	A5	38	49.6	A
4937	Bn-N5-p15770643	Bn-A05-p15281766	Bn-A05-p15281766	A5	38	49.6	A
4938	Bn-N5-p15771033	Bn-A05-p15282156	Bn-A05-p15282156	A5	38	49.6	A
4939	Bn-N5-p15778804	Bn-A05-p15290524	Bn-A05-p15290524	A5	38	49.6	A
4940	Bn-N5-p15782664	Bn-A05-p15297460	Bn-A05-p15297460	A5	38	49.6	A
4941	Bn-N5-p15801621	Bn-A05-p15320768	Bn-A05-p15320768	A5	38	49.6	A
4942	Bn-N5-p15803039	Bn-A05-p15322236	Bn-A05-p15322236	A5	38	49.6	A
4943	Bn-N5-p15821977	Bn-Scaffold000464-p5	Bn-Scaffold000464-p5975	A5	38	49.6	A
4944	Bn-N5-p15832847	Bn-A05-p15351341	Bn-A05-p15351341	A5	38	49.6	A

4945	Bn-N5-p15862879	Bn-A05-p15383133	Bn-A05-p15383133	A5	38	49.6	A
4946	Bn-N5-p15877196	Bn-A05-p15397425	Bn-A05-p15397425	A5	38	49.6	A
4947	Bn-N5-p15911825	Bn-A05-p15426487	Bn-A05-p15426487	A5	38	49.6	A
4948	Bn-N5-p16112613	Bn-A05-p15894915	Bn-A05-p15894915	A5	38	49.6	A
4949	Bn-N5-p17698898	Bn-A05-p13372155	Bn-A05-p13372155	A5	38	49.6	A
4950	Bn-Scaffold01037-p11884	Bn-Scaffold000254-p3	Bn-Scaffold000254-p39427	A5	38	49.6	A
4951	Bn-Scaffold03512-p1023	Bn-C5-p27393720	Bn-scaff_15648_1-p54162(	A5	38	49.6	A
4952	Bn-Scaffold03512-p1309	Bn-C5-p27394006	Bn-scaff_15648_1-p541334	A5	38	49.6	A
4953	Bn-Scaffold03512-p2287	Bn-A05-p13687507	Bn-A05-p13687507	A5	38	49.6	A
4954	Bn-Scaffold03828-p2404	Bn-A05-p13766174	Bn-A05-p13766174	A5	38	49.6	A
4955	Bn-Scaffold04206-p1459	Bn-Scaffold000555-p3	Bn-Scaffold000555-p3863	A5	38	49.6	A
4956	Unknown	Bn-A05-p13778371	Bn-A05-p13778371	A5	38	49.6	A
4957	Unknown	Bn-A05-p14547107	Bn-A05-p14547107	A5	38	49.6	A
4958	Bn-N11-p17875360	Bn-A05-p17330707	Bn-A05-p17330707	A5	39	50.4	A
4959	Bn-N12-p10310867	Bn-C2-p11475163	Bn-scaff_15712_5-p269157	A5	39	50.4	A
4960	Bn-N12-p10312888	Bn-C2-p11477184	Bn-scaff_15712_5-p26713(	A5	39	50.4	A
4961	Bn-N12-p10313053	Bn-C2-p11477349	Bn-scaff_15712_5-p266971	A5	39	50.4	A
4962	Bn-N12-p10314008	Bn-C2-p11478304	Bn-scaff_15712_5-p26601(	A5	39	50.4	A
4963	Bn-N12-p10314103	Bn-C2-p11478399	Bn-scaff_15712_5-p265921	A5	39	50.4	A
4964	Bn-N12-p10315049	Bn-C2-p11479345	Bn-scaff_15712_5-p264975	A5	39	50.4	A
4965	Bn-N15-p36517662	Bn-A05-p17417422	Bn-A05-p17417422	A5	39	50.4	A
4966	Bn-N15-p37531187	Bn-A05-p17913760	Bn-A05-p17913760	A5	39	50.4	A
4967	Bn-N5-p16370464	Bn-A05-p16092993	Bn-A05-p16092993	A5	39	50.4	A
4968	Bn-N5-p16377651	Bn-A05-p16099943	Bn-A05-p16099943	A5	39	50.4	A
4969	Bn-N5-p16377653	Bn-A05-p16099945	Bn-A05-p16099945	A5	39	50.4	A
4970	Bn-N5-p16389529	Bn-A05-p16106702	Bn-A05-p16106702	A5	39	50.4	A
4971	Bn-N5-p16389794	Bn-A05-p16106977	Bn-A05-p16106977	A5	39	50.4	A
4972	Bn-N5-p16392905	Bn-A05-p16109920	Bn-A05-p16109920	A5	39	50.4	A
4973	Bn-N5-p16450714	Bn-A05-p16167725	Bn-A05-p16167725	A5	39	50.4	A
4974	Bn-N5-p16465968	Bn-A05-p16183886	Bn-A05-p16183886	A5	39	50.4	A
4975	Bn-N5-p16472430	Bn-A05-p16186321	Bn-A05-p16186321	A5	39	50.4	A
4976	Bn-N5-p16479473	Bn-A05-p16195531	Bn-A05-p16195531	A5	39	50.4	A
4977	Bn-N5-p16480057	Bn-A05-p16196113	Bn-A05-p16196113	A5	39	50.4	A
4978	Bn-N5-p16494801	Bn-A05-p16209763	Bn-A05-p16209763	A5	39	50.4	A
4979	Bn-N5-p16512265	Bn-A05-p16224115	Bn-A05-p16224115	A5	39	50.4	A
4980	Bn-N5-p16512423	Bn-A05-p16224273	Bn-A05-p16224273	A5	39	50.4	A
4981	Bn-N5-p16513246	Bn-A05-p16225090	Bn-A05-p16225090	A5	39	50.4	A
4982	Bn-N5-p16521414	Bn-A05-p16234543	Bn-A05-p16234543	A5	39	50.4	A
4983	Bn-N5-p16535070	Bn-A05-p16245038	Bn-A05-p16245038	A5	39	50.4	A
4984	Bn-N5-p16557571	Bn-A05-p16274076	Bn-A05-p16274076	A5	39	50.4	A
4985	Bn-N5-p16690718	Bn-A05-p16392699	Bn-A05-p16392699	A5	39	50.4	A
4986	Bn-N5-p16931011	Bn-C4-p30743218	Bn-scaff_18839_1-p138513	A5	39	50.4	0
4987	Bn-N5-p16965358	Bn-A05-p16637476	Bn-A05-p16637476	A5	39	50.4	A
4988	Bn-N5-p16982177	Bn-A05-p16651858	Bn-A05-p16651858	A5	39	50.4	A
4989	Bn-N5-p16995647	Bn-A05-p16652401	Bn-A05-p16652401	A5	39	50.4	A
4990	Bn-N5-p17012472	Bn-A05-p16671522	Bn-A05-p16671522	A5	39	50.4	A
4991	Bn-N5-p17084123	Bn-A05-p16747797	Bn-A05-p16747797	A5	39	50.4	A
4992	Bn-N5-p17096806	Bn-A05-p16754780	Bn-A05-p16754780	A5	39	50.4	A
4993	Bn-N5-p17098424	Bn-A05-p16755625	Bn-A05-p16755625	A5	39	50.4	A

4994	Bn-N5-p17126736	Bn-A05-p16789633	Bn-A05-p16789633	A5	39	50.4	A
4995	Bn-N5-p17126911	Bn-A05-p16789808	Bn-A05-p16789808	A5	39	50.4	A
4996	Bn-N5-p17172322	Bn-A05-p16834136	Bn-A05-p16834136	A5	39	50.4	A
4997	Bn-N5-p17173754	Bn-A05-p16835570	Bn-A05-p16835570	A5	39	50.4	A
4998	Bn-N5-p17174702	Bn-A05-p16836518	Bn-A05-p16836518	A5	39	50.4	A
4999	Bn-N5-p17181358	Bn-A05-p16842656	Bn-A05-p16842656	A5	39	50.4	A
5000	Bn-N5-p17182905	Bn-A05-p16844203	Bn-A05-p16844203	A5	39	50.4	A
5001	Bn-N5-p17183206	Bn-A05-p16844504	Bn-A05-p16844504	A5	39	50.4	A
5002	Bn-N5-p17185581	Bn-A05-p16846880	Bn-A05-p16846880	A5	39	50.4	A
5003	Bn-N5-p17187456	Bn-A05-p16848755	Bn-A05-p16848755	A5	39	50.4	A
5004	Bn-N5-p17213370	Bn-A05-p16862109	Bn-A05-p16862109	A5	39	50.4	A
5005	Bn-N5-p17215616	Bn-A05-p16863502	Bn-A05-p16863502	A5	39	50.4	A
5006	Bn-N5-p17219560	Bn-A05-p16867347	Bn-A05-p16867347	A5	39	50.4	A
5007	Bn-N5-p17224711	Bn-A05-p16892759	Bn-A05-p16892759	A5	39	50.4	A
5008	Bn-N5-p17229653	Bn-A05-p16897600	Bn-A05-p16897600	A5	39	50.4	A
5009	Bn-N5-p17230322	Bn-A05-p16898305	Bn-A05-p16898305	A5	39	50.4	A
5010	Bn-N5-p17242220	Bn-A05-p16909798	Bn-A05-p16909798	A5	39	50.4	A
5011	Bn-N5-p17242378	Bn-A05-p16909956	Bn-A05-p16909956	A5	39	50.4	A
5012	Bn-N5-p17263146	Bn-A05-p16938263	Bn-A05-p16938263	A5	39	50.4	A
5013	Bn-N5-p18111176	Bn-A05-p17203695	Bn-A05-p17203695	A5	39	50.4	A
5014	Bn-N5-p18137448	Bn-A05-p17215153	Bn-A05-p17215153	A5	39	50.4	A
5015	Bn-N5-p18167468	Bn-A05-p17243214	Bn-A05-p17243214	A5	39	50.4	A
5016	Bn-N5-p18232665	Bn-A05-p17292224	Bn-A05-p17292224	A5	39	50.4	A
5017	Bn-N5-p18314195	Bn-A05-p17392998	Bn-A05-p17392998	A5	39	50.4	A
5018	Bn-N5-p18337436	Bn-A05-p17419591	Bn-A05-p17419591	A5	39	50.4	A
5019	Bn-N5-p18338329	Bn-A05-p17420484	Bn-A05-p17420484	A5	39	50.4	A
5020	Bn-N5-p18338458	Bn-A05-p17420613	Bn-A05-p17420613	A5	39	50.4	A
5021	Bn-N5-p18571997	Bn-A05-p17564038	Bn-A05-p17564038	A5	39	50.4	A
5022	Bn-N5-p18574806	Bn-A05-p17566846	Bn-A05-p17566846	A5	39	50.4	A
5023	Bn-N5-p18575139	Bn-A05-p17567179	Bn-A05-p17567179	A5	39	50.4	A
5024	Bn-N5-p18589546	Bn-C5-p34714713	Bn-scaff_16263_1-p44609[illegible]	A5	39	50.4	A
5025	Bn-N5-p18660925	Bn-A05-p17675829	Bn-A05-p17675829	A5	39	50.4	A
5026	Bn-N5-p18759377	Bn-A05-p17739455	Bn-A05-p17739455	A5	39	50.4	A
5027	Bn-N5-p18765736	Bn-A05-p17750022	Bn-A05-p17750022	A5	39	50.4	A
5028	Bn-N5-p18779379	Bn-A05-p17758084	Bn-A05-p17758084	A5	39	50.4	A
5029	Bn-N5-p18787203	Bn-A05-p17761769	Bn-A05-p17761769	A5	39	50.4	A
5030	Bn-N5-p18803472	Bn-A05-p17774694	Bn-A05-p17774694	A5	39	50.4	A
5031	Bn-N5-p18886242	Bn-A05-p17880874	Bn-A05-p17880874	A5	39	50.4	A
5032	Bn-N5-p18888688	Bn-C5-p35301945	Bn-scaff_16166_1-p42174[illegible]	A5	39	50.4	A
5033	Bn-N5-p18900369	Bn-A05-p17894045	Bn-A05-p17894045	A5	39	50.4	A
5034	Bn-N5-p18901492	Bn-C5-p35316283	Bn-scaff_16166_1-p40741[illegible]	A5	39	50.4	A
5035	Bn-N5-p18901501	Bn-A05-p17895197	Bn-A05-p17895197	A5	39	50.4	A
5036	Bn-N5-p18936830	Bn-A05-p17931505	Bn-A05-p17931505	A5	39	50.4	0
5037	Bn-N5-p19501941	Bn-A05-p18547040	Bn-A05-p18547040	A5	39	50.4	A
5038	Bn-N15-p39132084	Bn-C5-p37041078	Bn-scaff_15690_1-p37228[illegible]	A5	40	51.2	A
5039	Bn-N15-p39168145	Bn-C5-p37075162	Bn-scaff_15690_1-p40637[illegible]	A5	40	51.2	A
5040	Bn-N5-p19205661	Bn-A05-p18229790	Bn-A05-p18229790	A5	40	51.2	A
5041	Bn-N5-p19261411	Bn-A05-p18306160	Bn-A05-p18306160	A5	40	51.2	A
5042	Bn-N5-p19266967	Bn-A05-p18309576	Bn-A05-p18309576	A5	40	51.2	A

5043	Bn-N5-p19268047	Bn-A05-p18310658	Bn-A05-p18310658	A5	40	51.2	A
5044	Bn-N5-p19268227	Bn-A05-p18310838	Bn-A05-p18310838	A5	40	51.2	A
5045	Bn-N5-p19350950	Bn-A05-p18391581	Bn-A05-p18391581	A5	40	51.2	A
5046	Bn-N5-p19376008	Bn-C5-p36220918	Bn-scaff_21015_1-p89425	A5	40	51.2	A
5047	Bn-N5-p19412722	Bn-A05-p18452462	Bn-A05-p18452462	A5	40	51.2	A
5048	Bn-N5-p19412824	Bn-A05-p18452563	Bn-A05-p18452563	A5	40	51.2	A
5049	Bn-N5-p19413401	Bn-A05-p18453140	Bn-A05-p18453140	A5	40	51.2	A
5050	Bn-N5-p19422118	Bn-A05-p18458181	Bn-A05-p18458181	A5	40	51.2	A
5051	Bn-N5-p19492197	Bn-A05-p18528112	Bn-A05-p18528112	A5	40	51.2	A
5052	Bn-N5-p19498971	Bn-A05-p18543933	Bn-A05-p18543933	A5	40	51.2	A
5053	Bn-N5-p19546408	Bn-A05-p18598001	Bn-A05-p18598001	A5	40	51.2	A
5054	Bn-N5-p19635519	Bn-A05-p18682729	Bn-A05-p18682729	A5	40	51.2	A
5055	Bn-N5-p19639821	Bn-A05-p18687031	Bn-A05-p18687031	A5	40	51.2	A
5056	Bn-N5-p19673681	Bn-A05-p18730286	Bn-A05-p18730286	A5	40	51.2	A
5057	Bn-N5-p19704288	Bn-A05-p18744312	Bn-A05-p18744312	A5	40	51.2	A
5058	Bn-N5-p19708503	Bn-A05-p18753257	Bn-A05-p18753257	A5	40	51.2	A
5059	Bn-N5-p19720494	Bn-A05-p18761878	Bn-A05-p18761878	A5	40	51.2	A
5060	Bn-N5-p19772031	Bn-A05-p18807426	Bn-A05-p18807426	A5	40	51.2	A
5061	Bn-Scaffold00820-p96864	Bn-A05-p18627784	Bn-A05-p18627784	A5	40	51.2	A
5062	Bn-N5-p19863530	Bn-A05-p18905070	Bn-A05-p18905070	A5	41	52	A
5063	Bn-N5-p19908496	Bn-A05-p18958059	Bn-A05-p18958059	A5	42	54.5	A
5064	Bn-N5-p19946962	Bn-A05-p19001293	Bn-A05-p19001293	A5	42	54.5	A
5065	Bn-N5-p19975569	Bn-A05-p19031269	Bn-A05-p19031269	A5	42	54.5	A
5066	Bn-N5-p19976489	Bn-A05-p19032247	Bn-A05-p19032247	A5	42	54.5	A
5067	Bn-N5-p19986389	Bn-A05-p19041695	Bn-A05-p19041695	A5	42	54.5	A
5068	Bn-N5-p19993850	Bn-A05-p19048941	Bn-A05-p19048941	A5	42	54.5	A
5069	Bn-N5-p20005163	Bn-A05-p19064234	Bn-A05-p19064234	A5	42	54.5	A
5070	Bn-N5-p20015866	Bn-A05-p19070214	Bn-A05-p19070214	A5	42	54.5	A
5071	Bn-N5-p20015936	Bn-A05-p19070284	Bn-A05-p19070284	A5	42	54.5	A
5072	Bn-N5-p20060451	Bn-A05-p19116891	Bn-A05-p19116891	A5	43	55.3	A
5073	Bn-N5-p20127318	Bn-A05-p19178139	Bn-A05-p19178139	A5	43	55.3	A
5074	Bn-N5-p20142943	Bn-A05-p19195181	Bn-A05-p19195181	A5	43	55.3	A
5075	Bn-N5-p20146553	Bn-scaffold5213-p158	Bn-scaffold5213-p158	A5	43	55.3	A
5076	Bn-N5-p20153412	Bn-A05-p19207735	Bn-A05-p19207735	A5	43	55.3	A
5077	Bn-N5-p20155541	Bn-A05-p19209866	Bn-A05-p19209866	A5	43	55.3	A
5078	Bn-N5-p20191672	Bn-A05-p19238078	Bn-A05-p19238078	A5	43	55.3	A
5079	Bn-N5-p20191728	Bn-A05-p19238134	Bn-A05-p19238134	A5	43	55.3	A
5080	Bn-N5-p20209716	Bn-A05-p19267100	Bn-A05-p19267100	A5	43	55.3	A
5081	Bn-N5-p20209769	Bn-A05-p19267153	Bn-A05-p19267153	A5	43	55.3	A
5082	Bn-N5-p20211837	Bn-A05-p19268141	Bn-A05-p19268141	A5	43	55.3	A
5083	Bn-N5-p20230377	Bn-A05-p19278249	Bn-A05-p19278249	A5	43	55.3	A
5084	Bn-N5-p20230566	Bn-A05-p19278438	Bn-A05-p19278438	A5	43	55.3	A
5085	Bn-N5-p20308057	Bn-A05-p19359433	Bn-A05-p19359433	A5	43	55.3	A
5086	Bn-N5-p20308067	Bn-A05-p19359443	Bn-A05-p19359443	A5	43	55.3	A
5087	Bn-N5-p20349142	Bn-C5-p38444024	Bn-scaff_16516_1-p57960	A5	43	55.3	A
5088	Bn-N5-p20449834	Bn-C5-p38710444	Bn-scaff_18826_1-p10469	A5	43	55.3	A
5089	Bn-N5-p20467794	Bn-A05-p19514459	Bn-A05-p19514459	A5	43	55.3	A
5090	Bn-N5-p20486481	Bn-A05-p19532660	Bn-A05-p19532660	A5	43	55.3	A
5091	Bn-N5-p20494828	Bn-A05-p19539167	Bn-A05-p19539167	A5	43	55.3	A

5092	Bn-N5-p20494896	Bn-A05-p19539235	Bn-A05-p19539235	A5	43	55.3	A
5093	Bn-N5-p20502885	Bn-A05-p19547378	Bn-A05-p19547378	A5	43	55.3	A
5094	Bn-N5-p20509798	Bn-A05-p19554281	Bn-A05-p19554281	A5	43	55.3	A
5095	Bn-N5-p20537173	Bn-A05-p19575123	Bn-A05-p19575123	A5	43	55.3	A
5096	Bn-N5-p20543965	Bn-A05-p19583714	Bn-A05-p19583714	A5	43	55.3	A
5097	Bn-N5-p20565652	Bn-A05-p19599809	Bn-A05-p19599809	A5	43	55.3	A
5098	Bn-N5-p20590239	Bn-A05-p19622826	Bn-A05-p19622826	A5	43	55.3	A
5099	Bn-Scaffold09660-p256	Bn-A05-p19599390	Bn-A05-p19599390	A5	43	55.3	A
5100	Bn-N15-p40940767	Bn-A05-p19728614	Bn-A05-p19728614	A5	44	56.1	A
5101	Bn-N5-p20653226	Bn-A05-p19650278	Bn-A05-p19650278	A5	44	56.1	A
5102	Bn-N5-p20653946	Bn-A05-p19650965	Bn-A05-p19650965	A5	44	56.1	A
5103	Bn-N5-p20656318	Bn-A05-p19653084	Bn-A05-p19653084	A5	44	56.1	A
5104	Bn-N5-p20663333	Bn-A05-p19660820	Bn-A05-p19660820	A5	44	56.1	A
5105	Bn-N5-p20668684	Bn-A05-p19666434	Bn-A05-p19666434	A5	44	56.1	A
5106	Bn-N5-p20670585	Bn-A05-p19668317	Bn-A05-p19668317	A5	44	56.1	A
5107	Bn-N5-p20670900	Bn-A05-p19668632	Bn-A05-p19668632	A5	44	56.1	A
5108	Bn-N5-p20746277	Bn-A05-p19745674	Bn-A05-p19745674	A5	44	56.1	A
5109	Bn-N5-p20765698	Bn-C5-p39124525	Bn-scaff_18826_1-p63291[illegible]	A5	44	56.1	A
5110	Bn-N5-p20796236	Bn-A05-p19802577	Bn-A05-p19802577	A5	45	59.4	A
5111	Bn-N15-p41361882	Bn-C5-p39430565	Bn-scaff_18826_1-p32687[illegible]	A5	46	60.2	A
5112	Bn-N5-p20921026	Bn-A05-p19919111	Bn-A05-p19919111	A5	46	60.2	A
5113	Bn-N5-p20921094	Bn-A05-p19919179	Bn-A05-p19919179	A5	46	60.2	A
5114	Bn-N5-p20933033	Bn-A05-p19930910	Bn-A05-p19930910	A5	46	60.2	A
5115	Bn-N5-p20945128	Bn-C3-p26329901	Bn-scaff_17298_1-p21142[illegible]	A5	46	60.2	A
5116	Bn-N5-p20945637	Bn-C5-p39400259	Bn-scaff_18826_1-p35718[illegible]	A5	46	60.2	A
5117	Bn-N5-p20971492	Bn-A05-p19978706	Bn-A05-p19978706	A5	46	60.2	A
5118	Bn-N5-p20975583	Bn-A05-p19990020	Bn-A05-p19990020	A5	46	60.2	A
5119	Bn-N5-p20990105	Bn-A05-p20009925	Bn-A05-p20009925	A5	46	60.2	A
5120	Bn-N5-p20999054	Bn-A05-p20019130	Bn-A05-p20019130	A5	46	60.2	A
5121	Bn-N5-p21075694	Bn-A05-p20105944	Bn-A05-p20105944	A5	46	60.2	A
5122	Bn-N5-p21081970	Bn-A05-p20109948	Bn-A05-p20109948	A5	46	60.2	A
5123	Bn-N5-p21087722	Bn-A05-p20113630	Bn-A05-p20113630	A5	46	60.2	A
5124	Bn-N5-p21103930	Bn-A05-p20124425	Bn-A05-p20124425	A5	46	60.2	A
5125	Bn-N5-p21107745	Bn-A05-p20125478	Bn-A05-p20125478	A5	46	60.2	A
5126	Bn-N5-p21108248	Bn-A05-p20125982	Bn-A05-p20125982	A5	46	60.2	A
5127	Bn-N5-p21108337	Bn-A05-p20126071	Bn-A05-p20126071	A5	46	60.2	A
5128	Bn-N5-p21109638	Bn-A05-p20127293	Bn-A05-p20127293	A5	46	60.2	A
5129	Bn-N5-p21109891	Bn-A05-p20127547	Bn-A05-p20127547	A5	46	60.2	A
5130	Bn-N5-p21111137	Bn-A05-p20128796	Bn-A05-p20128796	A5	46	60.2	A
5131	Bn-N5-p21112649	Bn-A05-p20129266	Bn-A05-p20129266	A5	46	60.2	A
5132	Bn-N5-p21122742	Bn-A05-p20138451	Bn-A05-p20138451	A5	46	60.2	A
5133	Bn-N5-p21124163	Bn-A05-p20139872	Bn-A05-p20139872	A5	46	60.2	A
5134	Bn-N5-p21126172	Bn-A05-p20141883	Bn-A05-p20141883	A5	46	60.2	A
5135	Bn-N5-p21127093	Bn-A05-p20142804	Bn-A05-p20142804	A5	46	60.2	A
5136	Bn-N5-p21148095	Bn-A05-p20161210	Bn-A05-p20161210	A5	46	60.2	A
5137	Bn-N5-p21150003	Bn-A05-p20163118	Bn-A05-p20163118	A5	46	60.2	0
5138	Bn-N5-p21154067	Bn-A05-p20167176	Bn-A05-p20167176	A5	46	60.2	A
5139	Bn-N5-p21155697	Bn-A05-p20168806	Bn-A05-p20168806	A5	46	60.2	A
5140	Bn-N5-p21155799	Bn-A05-p20168909	Bn-A05-p20168909	A5	46	60.2	A

5141	Bn-N5-p21156090	Bn-C5-p39660753	Bn-scaff_18826_1-p96686	A5	46	60.2	A
5142	Bn-N5-p21632033	Bn-A05-p20623573	Bn-A05-p20623573	A5	46	60.2	A
5143	Bn-Scaffold16449-p328	Bn-A05-p20020286	Bn-A05-p20020286	A5	46	60.2	A
5144	Bn-N5-p21252649	Bn-A05-p20254809	Bn-A05-p20254809	A5	47	61	A
5145	Bn-N5-p21310204	Bn-Scaffold002092-p!	Bn-Scaffold002092-p583	A5	47	61	A
5146	Bn-N5-p21317537	Bn-A05-p20316923	Bn-A05-p20316923	A5	47	61	A
5147	Bn-N5-p21320240	Bn-A05-p20319622	Bn-A05-p20319622	A5	47	61	A
5148	Bn-N5-p21383427	Bn-A05-p20377770	Bn-A05-p20377770	A5	47	61	A
5149	Bn-N5-p21387104	Bn-A05-p20379970	Bn-A05-p20379970	A5	47	61	A
5150	Bn-N5-p21387764	Bn-A05-p20380629	Bn-A05-p20380629	A5	47	61	A
5151	Bn-N5-p21394148	Bn-A05-p20386700	Bn-A05-p20386700	A5	47	61	A
5152	Bn-N5-p21397579	Bn-A05-p20390129	Bn-A05-p20390129	A5	47	61	A
5153	Bn-N5-p21409222	Bn-A05-p20399452	Bn-A05-p20399452	A5	47	61	A
5154	Bn-N5-p21413614	Bn-A05-p20404099	Bn-A05-p20404099	A5	47	61	A
5155	Bn-N5-p21430848	Bn-A05-p20425648	Bn-A05-p20425648	A5	47	61	A
5156	Bn-N5-p21434617	Bn-A05-p20429403	Bn-A05-p20429403	A5	47	61	A
5157	Bn-N5-p21445934	Bn-A05-p20443393	Bn-A05-p20443393	A5	47	61	A
5158	Bn-N5-p21446601	Bn-A05-p20449058	Bn-A05-p20449058	A5	47	61	A
5159	Bn-N5-p21454386	Bn-A05-p20458590	Bn-A05-p20458590	A5	47	61	A
5160	Bn-N5-p21490939	Bn-A05-p20492525	Bn-A05-p20492525	A5	48	62.7	A
5161	Bn-N5-p21551551	Bn-A05-p20549826	Bn-A05-p20549826	A5	48	62.7	A
5162	Bn-N5-p21556284	Bn-A05-p20554567	Bn-A05-p20554567	A5	48	62.7	A
5163	Bn-N5-p21557718	Bn-A05-p20555996	Bn-A05-p20555996	A5	48	62.7	A
5164	Bn-N5-p21566392	Bn-A05-p20564729	Bn-A05-p20564729	A5	48	62.7	A
5165	Bn-N5-p21589324	Bn-A05-p20591895	Bn-A05-p20591895	A5	48	62.7	A
5166	Bn-N5-p21589393	Bn-A05-p20591964	Bn-A05-p20591964	A5	48	62.7	A
5167	Bn-N5-p21593116	Bn-A05-p20594196	Bn-A05-p20594196	A5	48	62.7	A
5168	Bn-N5-p21596300	Bn-A05-p20597839	Bn-A05-p20597839	A5	48	62.7	A
5169	Bn-N5-p21640251	Bn-A05-p20632153	Bn-A05-p20632153	A5	48	62.7	A
5170	Bn-N5-p21686601	Bn-A05-p20673293	Bn-A05-p20673293	A5	48	62.7	A
5171	Bn-N5-p21694862	Bn-A05-p20681871	Bn-A05-p20681871	A5	48	62.7	A
5172	Bn-N5-p21700221	Bn-A05-p20690058	Bn-A05-p20690058	A5	48	62.7	A
5173	Bn-N5-p21702875	Bn-A05-p20692714	Bn-A05-p20692714	A5	48	62.7	A
5174	Bn-N5-p21705718	Bn-A05-p20697145	Bn-A05-p20697145	A5	48	62.7	A
5175	Bn-N5-p21705983	Bn-A05-p20697410	Bn-A05-p20697410	A5	48	62.7	A
5176	Bn-N5-p21706043	Bn-A05-p20697473	Bn-A05-p20697473	A5	48	62.7	A
5177	Bn-N5-p21706401	Bn-A05-p20697824	Bn-A05-p20697824	A5	48	62.7	A
5178	Bn-N5-p21706477	Bn-A05-p20697908	Bn-A05-p20697908	A5	48	62.7	A
5179	Bn-N5-p21707895	Bn-A05-p20699671	Bn-A05-p20699671	A5	48	62.7	A
5180	Bn-N5-p21721917	Bn-A05-p20712388	Bn-A05-p20712388	A5	48	62.7	A
5181	Bn-N5-p21723709	Bn-A05-p20714207	Bn-A05-p20714207	A5	48	62.7	A
5182	Bn-N5-p21723804	Bn-A05-p20714302	Bn-A05-p20714302	A5	48	62.7	A
5183	Bn-N5-p21724083	Bn-A05-p20714581	Bn-A05-p20714581	A5	48	62.7	A
5184	Bn-N5-p21726006	Bn-A05-p20716500	Bn-A05-p20716500	A5	48	62.7	A
5185	Bn-N5-p21728375	Bn-A05-p20718776	Bn-A05-p20718776	A5	48	62.7	A
5186	Bn-N5-p21728928	Bn-A05-p20719343	Bn-A05-p20719343	A5	48	62.7	A
5187	Bn-N5-p21737810	Bn-A05-p20728232	Bn-A05-p20728232	A5	48	62.7	A
5188	Bn-N5-p21762829	Bn-A05-p20747913	Bn-A05-p20747913	A5	48	62.7	A
5189	Bn-N5-p21843939	Bn-A05-p20824936	Bn-A05-p20824936	A5	48	62.7	A

5190	Bn-N5-p21844288	Bn-A05-p20825280	Bn-A05-p20825280	A5	48	62.7	A
5191	Bn-N5-p21848115	Bn-A05-p20828693	Bn-A05-p20828693	A5	48	62.7	A
5192	Bn-N5-p21858583	Bn-A05-p20839044	Bn-A05-p20839044	A5	48	62.7	A
5193	Bn-N5-p21861878	Bn-A05-p20844496	Bn-A05-p20844496	A5	48	62.7	A
5194	Bn-N5-p21869120	Bn-A05-p20852240	Bn-A05-p20852240	A5	48	62.7	A
5195	Bn-N5-p21955512	Bn-A05-p20940971	Bn-A05-p20940971	A5	49	65.2	A
5196	Bn-N5-p21968005	Bn-A05-p20955184	Bn-A05-p20955184	A5	49	65.2	A
5197	Bn-N5-p21986809	Bn-A05-p20972649	Bn-A05-p20972649	A5	49	65.2	A
5198	Bn-N5-p21995827	Bn-A05-p20974054	Bn-A05-p20974054	A5	49	65.2	A
5199	Bn-N5-p22021124	Bn-A05-p21000008	Bn-A05-p21000008	A5	49	65.2	A
5200	Bn-N5-p22021619	Bn-A05-p21000614	Bn-A05-p21000614	A5	49	65.2	A
5201	Bn-N5-p22049826	Bn-A05-p21028051	Bn-A05-p21028051	A5	50	66	A
5202	Bn-N5-p22051769	Bn-A05-p21029850	Bn-A05-p21029850	A5	50	66	A
5203	Bn-N5-p22051829	Bn-A05-p21029910	Bn-A05-p21029910	A5	50	66	A
5204	Bn-N5-p22075109	Bn-A05-p21052552	Bn-A05-p21052552	A5	50	66	A
5205	Bn-N5-p22080486	Bn-A05-p21057936	Bn-A05-p21057936	A5	50	66	A
5206	Bn-N5-p22080734	Bn-A05-p21058156	Bn-A05-p21058156	A5	50	66	A
5207	Bn-N5-p22081012	Bn-A05-p21058435	Bn-A05-p21058435	A5	50	66	A
5208	Bn-N5-p22087896	Bn-A05-p21065087	Bn-A05-p21065087	A5	50	66	A
5209	Bn-N5-p22120041	Bn-C5-p41398106	Bn-scaff_21369_1-p381222	A5	50	66	A
5210	Bn-N5-p22126612	Bn-A05-p21107181	Bn-A05-p21107181	A5	50	66	A
5211	Bn-N5-p22222880	Bn-A05-p21188442	Bn-A05-p21188442	A5	51	66.8	A
5212	Bn-N5-p22236093	Bn-A05-p21201908	Bn-A05-p21201908	A5	51	66.8	A
5213	Bn-N5-p22236141	Bn-A05-p21201956	Bn-A05-p21201956	A5	51	66.8	A
5214	Bn-N15-p43379026	Bn-C5-p41574636	Bn-scaff_21369_1-p204692	A5	52	67.6	A
5215	Bn-N5-p22242588	Bn-A05-p21208394	Bn-A05-p21208394	A5	52	67.6	A
5216	Bn-N5-p22244271	Bn-A05-p21210078	Bn-A05-p21210078	A5	52	67.6	A
5217	Bn-N5-p22260441	Bn-A05-p21221296	Bn-A05-p21221296	A5	52	67.6	A
5218	Bn-N5-p22267180	Bn-A05-p21233666	Bn-A05-p21233666	A5	52	67.6	A
5219	Bn-N5-p22281026	Bn-A05-p21240761	Bn-A05-p21240761	A5	52	67.6	A
5220	Bn-N5-p22284184	Bn-A05-p21243931	Bn-A05-p21243931	A5	52	67.6	A
5221	Bn-N5-p22288664	Bn-A05-p21248382	Bn-A05-p21248382	A5	52	67.6	A
5222	Bn-N5-p22365600	Bn-A05-p21326091	Bn-A05-p21326091	A5	53	69.3	A
5223	Bn-N5-p22392693	Bn-A05-p21347821	Bn-A05-p21347821	A5	54	70.1	A
5224	Bn-N5-p22475280	Bn-A05-p21370435	Bn-A05-p21370435	A5	54	70.1	A
5225	Bn-N5-p22475463	Bn-A05-p21370618	Bn-A05-p21370618	A5	54	70.1	A
5226	Bn-N5-p22483019	Bn-A05-p21381782	Bn-A05-p21381782	A5	54	70.1	A
5227	Bn-N5-p22489174	Bn-C5-p41848809	Bn-scaff_18901_1-p197391	A5	54	70.1	A
5228	Bn-N5-p22571580	Bn-A05-p21470614	Bn-A05-p21470614	A5	55	73.4	A
5229	Bn-N5-p22584410	Bn-A05-p21484113	Bn-A05-p21484113	A5	55	73.4	A
5230	Bn-N5-p22592447	Bn-A05-p21491115	Bn-A05-p21491115	A5	55	73.4	A
5231	Bn-N5-p22595155	Bn-A05-p21493851	Bn-A05-p21493851	A5	55	73.4	A
5232	Bn-N5-p22822682	Bn-C5-p42395326	Bn-scaff_17441_1-p349027	A5	55	73.4	A
5233	Bn-N15-p44190242	Bn-A05-p21698558	Bn-A05-p21698558	A5	56	74.2	A
5234	Bn-N5-p22581296	Bn-A05-p21480994	Bn-A05-p21480994	A5	56	74.2	A
5235	Bn-N5-p22635384	Bn-A05-p21532839	Bn-A05-p21532839	A5	56	74.2	A
5236	Bn-N5-p22695386	Bn-A05-p21587317	Bn-A05-p21587317	A5	56	74.2	A
5237	Bn-N5-p22707327	Bn-A05-p21600954	Bn-A05-p21600954	A5	56	74.2	A
5238	Bn-N5-p22736616	Bn-A05-p21631586	Bn-A05-p21631586	A5	56	74.2	A

5239	Bn-N5-p22746726	Bn-A05-p21641604	Bn-A05-p21641604	A5	56	74.2	A
5240	Bn-N5-p22746738	Bn-A05-p21641616	Bn-A05-p21641616	A5	56	74.2	A
5241	Bn-N5-p22751748	Bn-A05-p21646684	Bn-A05-p21646684	A5	56	74.2	A
5242	Bn-N5-p22762138	Bn-A05-p21660030	Bn-A05-p21660030	A5	56	74.2	A
5243	Bn-N5-p22762196	Bn-A05-p21660088	Bn-A05-p21660088	A5	56	74.2	A
5244	Bn-N5-p22762393	Bn-A05-p21661113	Bn-A05-p21661113	A5	56	74.2	A
5245	Bn-N5-p22798882	Bn-A05-p21699314	Bn-A05-p21699314	A5	56	74.2	A
5246	Bn-N15-p44269414	Bn-C5-p42434733	Bn-scaff_17441_1-p38843	A5	57	75	A
5247	Bn-N5-p22895992	Bn-A05-p21806633	Bn-A05-p21806633	A5	57	75	A
5248	Bn-N5-p22896160	Bn-A05-p21806801	Bn-A05-p21806801	A5	57	75	A
5249	Bn-N5-p22899077	Bn-A05-p21809718	Bn-A05-p21809718	A5	57	75	A
5250	Bn-N5-p22909386	Bn-A05-p21852563	Bn-A05-p21852563	A5	57	75	A
5251	Bn-N5-p22951199	Bn-A05-p21889289	Bn-A05-p21889289	A5	58	79.2	A
5252	Bn-N5-p22994971	Bn-A05-p21930978	Bn-A05-p21930978	A5	59	80	A
5253	Bn-N5-p23203724	Bn-A05-p22152482	Bn-A05-p22152482	A5	60	83.3	A
5254	Bn-N5-p23033256	Bn-A05-p21973951	Bn-A05-p21973951	A5	61	83.3	A
5255	Bn-N5-p23079653	Bn-A05-p22015412	Bn-A05-p22015412	A5	61	83.3	A
5256	Bn-N5-p23135358	Bn-A05-p22089650	Bn-A05-p22089650	A5	61	83.3	A
5257	Bn-N5-p23176773	Bn-A05-p22141046	Bn-A05-p22141046	A5	61	83.3	A
5258	Bn-N5-p23280486	Bn-A05-p22218776	Bn-A05-p22218776	A5	62	85	A
5259	Bn-N5-p23311561	Bn-A05-p22249401	Bn-A05-p22249401	A5	62	85	A
5260	Bn-N15-p45058666	Bn-A05-p22276626	Bn-A05-p22276626	A5	63	85.8	A
5261	Bn-N5-p23328990	Bn-A05-p22266340	Bn-A05-p22266340	A5	63	85.8	A
5262	Bn-N5-p23329583	Bn-A05-p22266933	Bn-A05-p22266933	A5	63	85.8	A
5263	Bn-N5-p23332175	Bn-A05-p22269511	Bn-A05-p22269511	A5	63	85.8	A
5264	Bn-N5-p23348012	Bn-A05-p22282750	Bn-A05-p22282750	A5	64	86.6	A
5265	Bn-N5-p23348130	Bn-A05-p22282814	Bn-A05-p22282814	A5	64	86.6	A
5266	Bn-N5-p23349971	Bn-A05-p22284501	Bn-A05-p22284501	A5	64	86.6	A
5267	Bn-N5-p23387315	Bn-A05-p22324761	Bn-A05-p22324761	A5	64	86.6	A
5268	Bn-N5-p23503111	Bn-A05-p22427412	Bn-A05-p22427412	A5	64	86.6	A
5269	Bn-N5-p23529568	Bn-A05-p22454211	Bn-A05-p22454211	A5	64	86.6	A
5270	Bn-N5-p23529914	Bn-A05-p22454557	Bn-A05-p22454557	A5	64	86.6	A
5271	Bn-N5-p23534788	Bn-A05-p22459438	Bn-A05-p22459438	A5	64	86.6	A
5272	Bn-N5-p23562937	Bn-A05-p22488454	Bn-A05-p22488454	A5	65	87.4	A
5273	Bn-N5-p23571097	Bn-A05-p22498838	Bn-A05-p22498838	A5	65	87.4	A
5274	Bn-N5-p23578251	Bn-A05-p22506006	Bn-A05-p22506006	A5	65	87.4	A
5275	Bn-N5-p23586216	Bn-A05-p22511813	Bn-A05-p22511813	A5	65	87.4	A
5276	Bn-N5-p23632498	Bn-A05-p22563719	Bn-A05-p22563719	A5	66	88.2	A
5277	Bn-N5-p23645999	Bn-A05-p22585118	Bn-A05-p22585118	A5	66	88.2	A
5278	Bn-N5-p23651326	Bn-A05-p22589874	Bn-A05-p22589874	A5	66	88.2	A
5279	Bn-N5-p23652525	Bn-A05-p22591072	Bn-A05-p22591072	A5	66	88.2	A
5280	Bn-N5-p23653076	Bn-A05-p22591626	Bn-A05-p22591626	A5	66	88.2	A
5281	Bn-N5-p23662711	Bn-A05-p22599241	Bn-A05-p22599241	A5	66	88.2	A
5282	Bn-N5-p23676910	Bn-A05-p22613926	Bn-A05-p22613926	A5	67	89	A
5283	Bn-N5-p23716430	Bn-A05-p22650582	Bn-A05-p22650582	A5	67	89	A
5284	Bn-N5-p23716993	Bn-A05-p22651145	Bn-A05-p22651145	A5	67	89	A
5285	Bn-N5-p23719329	Bn-A05-p22653151	Bn-A05-p22653151	A5	67	89	A
5286	Bn-N5-p23723629	Bn-A05-p22657528	Bn-A05-p22657528	A5	67	89	A
5287	Bn-N5-p23724605	Bn-A05-p22658512	Bn-A05-p22658512	A5	67	89	A

5288	Bn-N5-p23759203	Bn-A05-p22691469	Bn-A05-p22691469	A5	68	89.8	A
5289	Bn-N5-p23775665	Bn-A05-p22712783	Bn-A05-p22712783	A5	68	89.8	A
5290	Bn-N5-p23835201	Bn-A05-p22767106	Bn-A05-p22767106	A5	69	90.6	A
5291	Bn-N5-p23850516	Bn-A05-p22780670	Bn-A05-p22780670	A5	69	90.6	A
5292	Bn-N5-p23856136	Bn-A05-p22786339	Bn-A05-p22786339	A5	69	90.6	A
5293	Bn-N5-p23859137	Bn-A05-p22789344	Bn-A05-p22789344	A5	70	91.4	A
5294	Bn-N5-p23859503	Bn-A05-p22789711	Bn-A05-p22789711	A5	70	91.4	A
5295	Bn-N5-p23859766	Bn-A05-p22789974	Bn-A05-p22789974	A5	70	91.4	A
5296	Bn-N5-p23926491	Bn-A05-p22863641	Bn-A05-p22863641	A5	71	92.2	A
5297	Bn-N5-p23932304	Bn-A05-p22868682	Bn-A05-p22868682	A5	71	92.2	A
5298	Bn-N5-p23989852	Bn-A05-p22889788	Bn-A05-p22889788	A5	71	92.2	A
5299	Bn-N5-p23997708	Bn-A05-p22899595	Bn-A05-p22899595	A5	71	92.2	A
5300	Bn-N5-p24009871	Bn-A05-p22911906	Bn-A05-p22911906	A5	71	92.2	A
5301	Bn-N5-p24009932	Bn-C5-p44033542	Bn-scaff_20219_1-p30832(	A5	71	92.2	A
5302	Bn-N5-p24010509	Bn-A05-p22912544	Bn-A05-p22912544	A5	71	92.2	A
5303	Bn-N5-p24015388	Bn-A05-p22917436	Bn-A05-p22917436	A5	71	92.2	A
5304	Bn-N15-p46318493	Bn-A02-p23297789	Bn-A02-p23297789	A5	72	93	A
5305	Bn-N15-p46318587	Bn-A02-p23297690	Bn-A02-p23297690	A5	72	93	A
5306	Bn-N5-p24043719	Bn-A05-p22948751	Bn-A05-p22948751	A5	72	93	A
5307	Bn-N5-p24063948	Bn-A05-p22973324	Bn-A05-p22973324	A5	72	93	A
5308	Bn-N5-p24074498	Bn-A05-p22981266	Bn-A05-p22981266	A5	72	93	A
5309	Bn-N5-p24106491	Bn-C5-p44218272	Bn-scaff_23186_1-p432591	A5	72	93	A
5310	Bn-N5-p24114691	Bn-A05-p23019620	Bn-A05-p23019620	A5	72	93	A
5311	Bn-N5-p24115095	Bn-A05-p23020834	Bn-A05-p23020834	A5	72	93	A
5312	Bn-N5-p24122493	Bn-A05-p23027881	Bn-A05-p23027881	A5	72	93	A
5313	Bn-N5-p24126846	Bn-A05-p23030143	Bn-A05-p23030143	A5	72	93	A
5314	Bn-N5-p24129511	Bn-A05-p23032803	Bn-A05-p23032803	A5	72	93	A
5315	Bn-N5-p24153291	Bn-A05-p23044521	Bn-A05-p23044521	A5	72	93	A
5316	Bn-N5-p24227096	Bn-A05-p23126108	Bn-A05-p23126108	A5	72	93	A
5317	Bn-N5-p24252879	Bn-A05-p23141080	Bn-A05-p23141080	A5	72	93	A
5318	Bn-N5-p24276239	Bn-C5-p44425675	Bn-scaff_23186_1-p22518[illegible]	A5	72	93	A
5319	Bn-N5-p24277022	Bn-A05-p23165568	Bn-A05-p23165568	A5	72	93	A
5320	Bn-N5-p24278778	Bn-A05-p23166612	Bn-A05-p23166612	A5	72	93	A
5321	Bn-N5-p24278874	Bn-A05-p23166708	Bn-A05-p23166708	A5	72	93	A
5322	Bn-N5-p24279379	Bn-A05-p23167201	Bn-A05-p23167201	A5	72	93	A
5323	Bn-N5-p24285127	Bn-A05-p23172786	Bn-A05-p23172786	A5	72	93	A
5324	Bn-N5-p24290658	Bn-A05-p23179980	Bn-A05-p23179980	A5	72	93	A
5325	Bn-N5-p24291874	Bn-C5-p44450488	Bn-scaff_23186_1-p20037[illegible]	A5	72	93	A
5326	Bn-N5-p24299145	Bn-A05-p23188259	Bn-A05-p23188259	A5	72	93	A
5327	Bn-N5-p24299292	Bn-A05-p23188406	Bn-A05-p23188406	A5	72	93	A
5328	Bn-N5-p24302511	Bn-A05-p23196656	Bn-A05-p23196656	A5	72	93	A
5329	Bn-N5-p24312531	Bn-A02-p23309610	Bn-A02-p23309610	A5	72	93	A
5330	Bn-N5-p24327677	Bn-A02-p23295254	Bn-A02-p23295254	A5	72	93	A
5331	Bn-N5-p24337534	Bn-A02-p23280869	Bn-A02-p23280869	A5	72	93	A
5332	Bn-N5-p24337931	Bn-A02-p23280472	Bn-A02-p23280472	A5	72	93	A
5333	Bn-N15-p46394060	Bn-C5-p44598197	Bn-scaff_23186_1-p52666	A5	73	93.8	A
5334	Bn-N5-p24461823	Bn-A02-p23163100	Bn-A02-p23163100	A5	73	93.8	A
5335	Bn-N5-p24463861	Bn-A02-p23161914	Bn-A02-p23161914	A5	73	93.8	A
5336	Bn-N5-p24499063	Bn-A02-p23128571	Bn-A02-p23128571	A5	73	93.8	A

5337	Bn-N5-p24518497	Bn-A02-p23108517	Bn-A02-p23108517	A5	73	93.8	A
5338	Bn-N5-p24533762	Bn-A02-p23095572	Bn-A02-p23095572	A5	73	93.8	A
5339	Bn-N5-p24537998	Bn-A02-p23092476	Bn-A02-p23092476	A5	73	93.8	A
5340	Bn-N5-p24618267	Bn-A05-p23253826	Bn-A05-p23253826	A5	74	94.6	A
5341	Bn-N5-p24638929	Bn-A05-p23276053	Bn-A05-p23276053	A5	74	94.6	A
5342	Bn-N5-p24655514	Bn-A05-p23285926	Bn-A05-p23285926	A5	74	94.6	A
5343	Bn-N5-p24657940	Bn-A05-p23288913	Bn-A05-p23288913	A5	74	94.6	A
5344	Bn-N5-p24738956	Bn-A05-p23377419	Bn-A05-p23377419	A5	74	94.6	A
5345	Bn-N5-p24761238	Bn-A05-p23397606	Bn-A05-p23397606	A5	74	94.6	A
5346	Bn-N5-p24768396	Bn-A05-p23405117	Bn-A05-p23405117	A5	74	94.6	A
5347	Bn-N5-p24772897	Bn-A05-p23415646	Bn-A05-p23415646	A5	74	94.6	A
5348	Bn-N5-p24842502	Bn-A05-p23478913	Bn-A05-p23478913	A5	74	94.6	A
5349	Bn-N5-p24861878	Bn-A05-p23498540	Bn-A05-p23498540	A5	74	94.6	A
5350	Bn-N5-p24865653	Bn-A05-p23502993	Bn-A05-p23502993	A5	74	94.6	A
5351	Bn-N5-p24875589	Bn-A05-p23512931	Bn-A05-p23512931	A5	74	94.6	A
5352	Bn-N5-p24929824	Bn-Scaffold000203-p!	Bn-Scaffold000203-p57343	A5	74	94.6	A
5353	Bn-N5-p24935182	Bn-Scaffold000203-p!	Bn-Scaffold000203-p50466	A5	75	96.3	A
5354	Bn-N5-p24955605	Bn-Scaffold000203-p:	Bn-Scaffold000203-p29938	A5	75	96.3	A
5355	Bn-N5-p24969277	Bn-Scaffold000203-p!	Bn-Scaffold000203-p9544	A5	75	96.3	A
5356	Bn-N5-p24969336	Bn-Scaffold000203-p!	Bn-Scaffold000203-p9485	A5	75	96.3	A
5357	Bn-N5-p25031207	Bn-Scaffold000191-p!	Bn-Scaffold000191-p53301	A5	75	96.3	A
5358	Bn-N5-p25180238	Bn-A02-p26901243	Bn-A02-p26901243	A5	76	98	A
5359	Bn-N5-p25180545	Bn-A02-p26900937	Bn-A02-p26900937	A5	76	98	A
5360	Bn-N5-p25199646	Bn-A02-p26883269	Bn-A02-p26883269	A5	76	98	A
5361	Bn-N5-p25200682	Bn-A08-p16956285	Bn-A08-p16956285	A5	76	98	A
5362	Bn-Scaffold00893-p13729	Bn-A02-p26857230	Bn-A02-p26857230	A5	76	98	A
5363	Bn-Scaffold00893-p20358	Bn-A02-p26854933	Bn-A02-p26854933	A5	76	98	A
5364	Bn-Scaffold00893-p20411	Bn-A02-p26854880	Bn-A02-p26854880	A5	76	98	A
5365	Bn-Scaffold00893-p49364	Bn-A02-p26832454	Bn-A02-p26832454	A5	76	98	A
5366	Bn-Scaffold00893-p56997	Bn-A02-p26824823	Bn-A02-p26824823	A5	76	98	A
5367	Bn-Scaffold00893-p57520	Bn-A02-p26824300	Bn-A02-p26824300	A5	76	98	A
5368	Bn-Scaffold00893-p57581	Bn-A02-p26824239	Bn-A02-p26824239	A5	76	98	A
5369	Bn-Scaffold00893-p62131	Bn-A02-p26819608	Bn-A02-p26819608	A5	76	98	A
5370	Bn-Scaffold00893-p62609	Bn-A02-p26819130	Bn-A02-p26819130	A5	76	98	A
5371	Bn-Scaffold00893-p65233	Bn-A02-p26816506	Bn-A02-p26816506	A5	76	98	A
5372	Bn-Scaffold00893-p65633	Bn-A02-p26816101	Bn-A02-p26816101	A5	76	98	A
5373	Bn-N5-p25279974	Bn-A02-p26713316	Bn-A02-p26713316	A5	77	98.8	A
5374	Bn-N5-p25304193	Bn-A02-p26689319	Bn-A02-p26689319	A5	77	98.8	A
5375	Bn-N5-p25336681	Bn-A02-p26665282	Bn-A02-p26665282	A5	77	98.8	A
5376	Bn-N5-p25348367	Bn-A02-p26653626	Bn-A02-p26653626	A5	77	98.8	A
5377	Bn-N5-p25355861	Bn-A02-p26646040	Bn-A02-p26646040	A5	77	98.8	A
5378	Bn-N5-p25376687	Bn-A02-p26625696	Bn-A02-p26625696	A5	77	98.8	A
5379	Bn-N5-p25377236	Bn-A02-p26625153	Bn-A02-p26625153	A5	77	98.8	A
5380	Bn-N5-p25381220	Bn-A02-p26621160	Bn-A02-p26621160	A5	77	98.8	A
5381	Bn-N5-p25421902	Bn-A05-p23553029	Bn-A05-p23553029	A5	77	98.8	A
5382	Bn-Scaffold00893-p77746	Bn-A02-p26804091	Bn-A02-p26804091	A5	77	98.8	A
5383	Bn-Scaffold00893-p77934	Bn-A02-p26803902	Bn-A02-p26803902	A5	77	98.8	A
5384	Bn-N5-p25471487	Bn-A05-p23598510	Bn-A05-p23598510	A5	78	99.6	A
5385	Bn-N5-p25550142	Bn-A05-p23680925	Bn-A05-p23680925	A5	78	99.6	A

5386	Bn-N5-p25561134	Bn-A05-p23691939	Bn-A05-p23691939	A5	78		99.6	A
5387	Bn-N5-p25561438	Bn-A05-p23692249	Bn-A05-p23692249	A5	78		99.6	A
5388	Bn-N5-p25562767	Bn-A05-p23693572	Bn-A05-p23693572	A5	78		99.6	A
5389	Bn-N5-p25659292	Bn-A05-p23776343	Bn-A05-p23776343	A5	78		99.6	A
5390	Bn-N5-p25665315	Bn-A05-p23782123	Bn-A05-p23782123	A5	78		99.6	A
5391	Bn-N5-p25665496	Bn-A05-p23782304	Bn-A05-p23782304	A5	78		99.6	A
5392	Bn-Scaffold01069-p18397	Bn-A05-p23848037	Bn-A05-p23848037	A5	78		99.6	A
5393	Bn-Scaffold01069-p22210	Bn-A05-p23844225	Bn-A05-p23844225	A5	78		99.6	A
5394	Bn-Scaffold01069-p22378	Bn-A05-p23844016	Bn-A05-p23844016	A5	78		99.6	A
5395	Bn-Scaffold67398-p81	Bn-A05-p23876386	Bn-A05-p23876386	A5	78		99.6	0
5396	Bn-N16-p6542105	Bn-A06-p908271	Bn-A06-p908271	A6		1	0	B
5397	Bn-N5-p25729964	Bn-A06-p18783	Bn-A06-p18783	A6		1	0	B
5398	Bn-N5-p25750407	Bn-A06-p49287	Bn-A06-p49287	A6		1	0	B
5399	Bn-N5-p25751506	Bn-A06-p50386	Bn-A06-p50386	A6		1	0	B
5400	Bn-N5-p25754637	Bn-A06-p53517	Bn-A06-p53517	A6		1	0	B
5401	Bn-N5-p25755799	Bn-A06-p54687	Bn-A06-p54687	A6		1	0	B
5402	Bn-N5-p25781557	Bn-A06-p87536	Bn-A06-p87536	A6		1	0	B
5403	Bn-N6-p108297	Bn-A06-p195449	Bn-A06-p195449	A6		1	0	B
5404	Bn-N6-p110450	Bn-A06-p197604	Bn-A06-p197604	A6		1	0	B
5405	Bn-N6-p117217	Bn-A06-p205050	Bn-A06-p205050	A6		1	0	0
5406	Bn-N6-p171195	Bn-A06-p267765	Bn-A06-p267765	A6		1	0	B
5407	Bn-N6-p179121	Bn-A06-p275949	Bn-A06-p275949	A6		1	0	B
5408	Bn-N6-p184034	Bn-A06-p279045	Bn-A06-p279045	A6		1	0	B
5409	Bn-N6-p190792	Bn-A06-p280351	Bn-A06-p280351	A6		1	0	B
5410	Bn-N6-p192087	Bn-A06-p281559	Bn-A06-p281559	A6		1	0	0
5411	Bn-N6-p192402	Bn-A06-p281874	Bn-A06-p281874	A6		1	0	B
5412	Bn-N6-p193379	Bn-A06-p282853	Bn-A06-p282853	A6		1	0	B
5413	Bn-N6-p193951	Bn-A06-p283425	Bn-A06-p283425	A6		1	0	0
5414	Bn-N6-p19617	Bn-A06-p112339	Bn-A06-p112339	A6		1	0	B
5415	Bn-N6-p198857	Bn-A06-p283640	Bn-A06-p283640	A6		1	0	B
5416	Bn-N6-p199623	Bn-A06-p284408	Bn-A06-p284408	A6		1	0	B
5417	Bn-N6-p200730	Bn-A06-p285515	Bn-A06-p285515	A6		1	0	B
5418	Bn-N6-p206062	Bn-A06-p290355	Bn-A06-p290355	A6		1	0	B
5419	Bn-N6-p21178	Bn-A06-p113900	Bn-A06-p113900	A6		1	0	B
5420	Bn-N6-p249355	Bn-A06-p329217	Bn-A06-p329217	A6		1	0	B
5421	Bn-N6-p26608	Bn-A06-p115948	Bn-A06-p115948	A6		1	0	B
5422	Bn-N6-p294802	Bn-A06-p362402	Bn-A06-p362402	A6		1	0	B
5423	Bn-N6-p327818	Bn-A06-p393328	Bn-A06-p393328	A6		1	0	B
5424	Bn-N6-p345216	Bn-A06-p410058	Bn-A06-p410058	A6		1	0	B
5425	Bn-N6-p444427	Bn-A06-p506366	Bn-A06-p506366	A6		1	0	B
5426	Bn-N6-p530605	Bn-A06-p569582	Bn-A06-p569582	A6		1	0	B
5427	Bn-N6-p552786	Bn-A06-p600029	Bn-A06-p600029	A6		1	0	B
5428	Bn-N6-p561236	Bn-A06-p608554	Bn-A06-p608554	A6		1	0	B
5429	Bn-N6-p561252	Bn-A06-p608570	Bn-A06-p608570	A6		1	0	B
5430	Bn-N6-p564271	Bn-A06-p611589	Bn-A06-p611589	A6		1	0	B
5431	Bn-N6-p566321	Bn-A06-p621695	Bn-A06-p621695	A6		1	0	B
5432	Bn-N6-p566471	Bn-A06-p613789	Bn-A06-p613789	A6		1	0	B
5433	Bn-N6-p577726	Bn-C6-p32177150	Bn-scaff_18936_1-p14035	A6		1	0	B
5434	Bn-N6-p63276	Bn-A06-p149145	Bn-A06-p149145	A6		1	0	B

5435	Bn-N6-p653650	Bn-A06-p690460	Bn-A06-p690460	A6	1	0	B
5436	Bn-N6-p668466	Bn-A06-p709547	Bn-A06-p709547	A6	1	0	B
5437	Bn-N6-p727954	Bn-A06-p781674	Bn-A06-p781674	A6	1	0	B
5438	Bn-N6-p757450	Bn-A06-p798821	Bn-A06-p798821	A6	1	0	B
5439	Bn-N6-p784591	Bn-A06-p825900	Bn-A06-p825900	A6	1	0	B
5440	Bn-N6-p821079	Bn-A06-p860631	Bn-A06-p860631	A6	1	0	B
5441	Bn-N6-p85190	Bn-A06-p167382	Bn-A06-p167382	A6	1	0	B
5442	Bn-N6-p863299	Bn-A06-p899811	Bn-A06-p899811	A6	1	0	B
5443	Bn-N6-p89072	Bn-A06-p172731	Bn-A06-p172731	A6	1	0	B
5444	Bn-N6-p95728	Bn-A06-p183811	Bn-A06-p183811	A6	1	0	B
5445	Bn-N8-p10329715	Bn-A06-p556859	Bn-A06-p556859	A6	1	0	B
5446	Bn-N16-p5825826	Bn-A06-p1135789	Bn-A06-p1135789	A6	2	0.8	B
5447	Bn-N6-p1011787	Bn-A06-p1054104	Bn-A06-p1054104	A6	2	0.8	B
5448	Bn-N6-p1128866	Bn-A06-p1176062	Bn-A06-p1176062	A6	2	0.8	B
5449	Bn-N6-p1132418	Bn-C6-p33894306	Bn-scaff_20773_1-p15517[illegible]	A6	2	0.8	B
5450	Bn-N6-p1158451	Bn-A06-p1217543	Bn-A06-p1217543	A6	2	0.8	B
5451	Bn-N6-p1172718	Bn-A06-p1232267	Bn-A06-p1232267	A6	2	0.8	B
5452	Bn-N6-p884932	Bn-A06-p918871	Bn-A06-p918871	A6	2	0.8	B
5453	Bn-N6-p904664	Bn-A06-p947740	Bn-A06-p947740	A6	2	0.8	B
5454	Bn-N6-p961197	Bn-A06-p999044	Bn-A06-p999044	A6	2	0.8	B
5455	Bn-N6-p976643	Bn-A06-p1016417	Bn-A06-p1016417	A6	2	0.8	B
5456	Bn-N6-p990954	Bn-C6-p33574116	Bn-scaff_16647_1-p39842[illegible]	A6	2	0.8	B
5457	Bn-N8-p1704686	Bn-A06-p1142557	Bn-A06-p1142557	A6	2	0.8	B
5458	Bn-N6-p1256849	Bn-A06-p1322724	Bn-A06-p1322724	A6	3	1.6	B
5459	Bn-N6-p1264649	Bn-A06-p1330567	Bn-A06-p1330567	A6	3	1.6	B
5460	Bn-N6-p1279393	Bn-A06-p1345642	Bn-A06-p1345642	A6	3	1.6	B
5461	Bn-N6-p1282105	Bn-A06-p1348404	Bn-A06-p1348404	A6	3	1.6	B
5462	Bn-N6-p1284288	Bn-A06-p1350786	Bn-A06-p1350786	A6	3	1.6	B
5463	Bn-N6-p1292497	Bn-A06-p1358955	Bn-A06-p1358955	A6	3	1.6	B
5464	Bn-N6-p1299195	Bn-A06-p1366713	Bn-A06-p1366713	A6	3	1.6	B
5465	Bn-N6-p1311866	Bn-A06-p1378913	Bn-A06-p1378913	A6	3	1.6	B
5466	Bn-N6-p1337715	Bn-A06-p1404859	Bn-A06-p1404859	A6	4	2.4	B
5467	Bn-N6-p1343345	Bn-A06-p1408273	Bn-A06-p1408273	A6	4	2.4	B
5468	Bn-N6-p1367548	Bn-A06-p1427206	Bn-A06-p1427206	A6	4	2.4	B
5469	Bn-N6-p1372772	Bn-A06-p1432446	Bn-A06-p1432446	A6	4	2.4	B
5470	Bn-N6-p1373346	Bn-A06-p1433021	Bn-A06-p1433021	A6	4	2.4	B
5471	Bn-N6-p1378530	Bn-C6-p34676013	Bn-scaff_16677_1-p10271[illegible]	A6	4	2.4	B
5472	Bn-N6-p1379601	Bn-C6-p34674939	Bn-scaff_16677_1-p10164[illegible]	A6	4	2.4	B
5473	Bn-N6-p1384557	Bn-A06-p1443684	Bn-A06-p1443684	A6	4	2.4	B
5474	Bn-N6-p1400741	Bn-A06-p1450741	Bn-A06-p1450741	A6	4	2.4	B
5475	Bn-N6-p1406092	Bn-A06-p1455751	Bn-A06-p1455751	A6	4	2.4	B
5476	Bn-N6-p1406122	Bn-A06-p1455781	Bn-A06-p1455781	A6	4	2.4	B
5477	Bn-N6-p1406862	Bn-A06-p1456521	Bn-A06-p1456521	A6	4	2.4	B
5478	Bn-N6-p1410403	Bn-A06-p1460039	Bn-A06-p1460039	A6	4	2.4	B
5479	Bn-N16-p4752677	Bn-A06-p1507197	Bn-A06-p1507197	A6	5	3.2	B
5480	Bn-N6-p1524549	Bn-C6-p34973904	Bn-scaff_18344_1-p39887[illegible]	A6	6	4	B
5481	Bn-N6-p1614708	Bn-A06-p1658095	Bn-A06-p1658095	A6	6	4	B
5482	Bn-N6-p1700511	Bn-A06-p1743446	Bn-A06-p1743446	A6	6	4	B
5483	Bn-N6-p1726972	Bn-A06-p1768254	Bn-A06-p1768254	A6	7	4.8	B

5484	Bn-N6-p1727635	Bn-A06-p1768918	Bn-A06-p1768918	A6	7	4.8	B
5485	Bn-N6-p1750370	Bn-A06-p1793586	Bn-A06-p1793586	A6	7	4.8	B
5486	Bn-N6-p1758485	Bn-A06-p1802123	Bn-A06-p1802123	A6	7	4.8	B
5487	Bn-N6-p1826429	Bn-C6-p35753652	Bn-scaff_16485_1-p59260{	A6	7	4.8	B
5488	Bn-N6-p1837548	Bn-A06-p1872484	Bn-A06-p1872484	A6	7	4.8	B
5489	Bn-N6-p1837661	Bn-A06-p1872597	Bn-A06-p1872597	A6	7	4.8	B
5490	Bn-N6-p1847127	Bn-A06-p1892397	Bn-A06-p1892397	A6	7	4.8	B
5491	Bn-N9-p9366899	Bn-A01-p23728095	Bn-A01-p23728095	A6	7	4.8	B
5492	Bn-N6-p1911301	Bn-A06-p1947247	Bn-A06-p1947247	A6	8	5.6	B
5493	Bn-N6-p2052031	Bn-A06-p2065936	Bn-A06-p2065936	A6	8	5.6	B
5494	Bn-N6-p2062066	Bn-A06-p2077171	Bn-A06-p2077171	A6	8	5.6	B
5495	Bn-N6-p2144614	Bn-A06-p2165351	Bn-A06-p2165351	A6	9	6.4	B
5496	Bn-N6-p2166386	Bn-A06-p2185099	Bn-A06-p2185099	A6	9	6.4	B
5497	Bn-N6-p2205250	Bn-A06-p2218956	Bn-A06-p2218956	A6	9	6.4	B
5498	Bn-N6-p2229916	Bn-A06-p2234696	Bn-A06-p2234696	A6	9	6.4	B
5499	Bn-N6-p2229938	Bn-A06-p2234718	Bn-A06-p2234718	A6	9	6.4	B
5500	Bn-N16-p3154580	Bn-C6-p36818201	Bn-scaff_16984_1-p35351	A6	10	7.2	B
5501	Bn-N6-p2243184	Bn-A06-p2251910	Bn-A06-p2251910	A6	10	7.2	B
5502	Bn-N16-p3090573	Bn-A06-p2275754	Bn-A06-p2275754	A6	11	11.2	B
5503	Bn-N16-p3092008	Bn-C6-p36930361	Bn-scaff_16553_1-p33772	A6	11	11.2	B
5504	Bn-N16-p3092416	Bn-C6-p36929954	Bn-scaff_16553_1-p34179	A6	11	11.2	B
5505	Bn-N6-p2261524	Bn-A06-p2268393	Bn-A06-p2268393	A6	11	11.2	B
5506	Bn-N6-p2267445	Bn-A06-p2274318	Bn-A06-p2274318	A6	11	11.2	B
5507	Bn-N6-p11658526	Bn-A06-p2390609	Bn-A06-p2390609	A6	12	14.4	B
5508	Bn-N6-p2286818	Bn-A06-p2293069	Bn-A06-p2293069	A6	12	14.4	B
5509	Bn-N6-p2307066	Bn-A06-p2307291	Bn-A06-p2307291	A6	12	14.4	B
5510	Bn-N6-p2342384	Bn-A06-p2341154	Bn-A06-p2341154	A6	12	14.4	B
5511	Bn-N6-p2346938	Bn-A06-p2345966	Bn-A06-p2345966	A6	12	14.4	B
5512	Bn-N6-p2348726	Bn-A06-p2347754	Bn-A06-p2347754	A6	12	14.4	B
5513	Bn-N6-p2349397	Bn-A06-p2348428	Bn-A06-p2348428	A6	12	14.4	B
5514	Bn-N6-p2358735	Bn-A06-p2357647	Bn-A06-p2357647	A6	12	14.4	B
5515	Bn-N6-p2407250	Bn-A06-p2407080	Bn-A06-p2407080	A6	12	14.4	B
5516	Bn-N6-p2468768	Bn-A06-p2514723	Bn-A06-p2514723	A6	13	16	B
5517	Bn-N6-p2492906	Bn-A06-p2542722	Bn-A06-p2542722	A6	14	16.8	B
5518	Bn-N6-p2513183	Bn-A06-p2563025	Bn-A06-p2563025	A6	14	16.8	B
5519	Bn-N6-p2539378	Bn-A06-p2582613	Bn-A06-p2582613	A6	14	16.8	B
5520	Bn-N6-p2568453	Bn-A06-p2617079	Bn-A06-p2617079	A6	15	18.4	B
5521	Bn-N6-p2570466	Bn-A06-p2619089	Bn-A06-p2619089	A6	15	18.4	B
5522	Bn-N6-p2590382	Bn-A06-p2633224	Bn-A06-p2633224	A6	15	18.4	B
5523	Bn-N6-p2590921	Bn-A06-p2633764	Bn-A06-p2633764	A6	15	18.4	B
5524	Bn-N6-p2601945	Bn-A06-p2646572	Bn-A06-p2646572	A6	15	18.4	0
5525	Bn-N6-p2605783	Bn-A06-p2650442	Bn-A06-p2650442	A6	15	18.4	B
5526	Bn-N15-p2981931	Bn-C5-p2936192	Bn-scaff_20901_1-p12932	A6	16	19.2	B
5527	Bn-N15-p2990291	Bn-A06-p2682929	Bn-A06-p2682929	A6	16	19.2	B
5528	Bn-N6-p2616610	Bn-A06-p2676314	Bn-A06-p2676314	A6	16	19.2	B
5529	Bn-N15-p3135500	Bn-Scaffold01028-p2	Bn-scaff_28179_1-p21528	A6	17	21.6	B
5530	Bn-N6-p2660431	Bn-A06-p2717967	Bn-A06-p2717967	A6	17	21.6	B
5531	Bn-N6-p2661302	Bn-A06-p2718852	Bn-A06-p2718852	A6	17	21.6	B
5532	Bn-N6-p2671625	Bn-A06-p2729577	Bn-A06-p2729577	A6	17	21.6	B

5533	Bn-N6-p2687616	Bn-A06-p2756247	Bn-A06-p2756247	A6	17	21.6	B
5534	Bn-N6-p2689864	Bn-A06-p2758495	Bn-A06-p2758495	A6	17	21.6	B
5535	Bn-N6-p2705931	Bn-A06-p2778072	Bn-A06-p2778072	A6	17	21.6	B
5536	Bn-N6-p2706688	Bn-A06-p2778829	Bn-A06-p2778829	A6	17	21.6	B
5537	Bn-N6-p2706696	Bn-A06-p2778837	Bn-A06-p2778837	A6	17	21.6	B
5538	Bn-N6-p2707101	Bn-A06-p2779243	Bn-A06-p2779243	A6	17	21.6	B
5539	Bn-N6-p2716511	Bn-A06-p2788585	Bn-A06-p2788585	A6	17	21.6	B
5540	Bn-N6-p2729429	Bn-A06-p2801792	Bn-A06-p2801792	A6	17	21.6	B
5541	Bn-N6-p2749101	Bn-A06-p2826630	Bn-A06-p2826630	A6	17	21.6	B
5542	Bn-N6-p2761621	Bn-A06-p2842757	Bn-A06-p2842757	A6	17	21.6	B
5543	Bn-N6-p2785638	Bn-A06-p2870413	Bn-A06-p2870413	A6	17	21.6	B
5544	Bn-N6-p2789474	Bn-A06-p2874216	Bn-A06-p2874216	A6	17	21.6	B
5545	Bn-N6-p2816336	Bn-A06-p2891175	Bn-A06-p2891175	A6	17	21.6	B
5546	Bn-N6-p2816451	Bn-A06-p2891290	Bn-A06-p2891290	A6	17	21.6	B
5547	Bn-N6-p2854863	Bn-A06-p2929852	Bn-A06-p2929852	A6	17	21.6	B
5548	Bn-N6-p2855167	Bn-A06-p2930156	Bn-A06-p2930156	A6	17	21.6	B
5549	Bn-N6-p2861174	Bn-A06-p2936438	Bn-A06-p2936438	A6	18	22.4	B
5550	Bn-N15-p3364850	Bn-A06-p2984184	Bn-A06-p2984184	A6	19	23.2	B
5551	Bn-N6-p2876401	Bn-A06-p2954752	Bn-A06-p2954752	A6	19	23.2	B
5552	Bn-N6-p2879696	Bn-A06-p2958095	Bn-A06-p2958095	A6	19	23.2	B
5553	Bn-N6-p2885692	Bn-A06-p2966556	Bn-A06-p2966556	A6	19	23.2	B
5554	Bn-N6-p2901029	Bn-C5-p3278100	Bn-scaff_20901_1-p951365	A6	19	23.2	B
5555	Bn-N6-p2908047	Bn-A06-p2987202	Bn-A06-p2987202	A6	19	23.2	B
5556	Bn-N6-p2930003	Bn-A06-p3012876	Bn-A06-p3012876	A6	19	23.2	B
5557	Bn-N6-p2933241	Bn-A06-p3016186	Bn-A06-p3016186	A6	19	23.2	B
5558	Bn-N6-p2933412	Bn-A06-p3016356	Bn-A06-p3016356	A6	19	23.2	B
5559	Bn-N6-p2939511	Bn-C5-p3344588	Bn-scaff_20901_1-p884877	A6	19	23.2	0
5560	Bn-N6-p2968512	Bn-A06-p3050747	Bn-A06-p3050747	A6	20	24.8	B
5561	Bn-N6-p2969695	Bn-A06-p3051928	Bn-A06-p3051928	A6	20	24.8	B
5562	Bn-N6-p2984592	Bn-A06-p3066038	Bn-A06-p3066038	A6	20	24.8	B
5563	Bn-N6-p2991285	Bn-A06-p3072724	Bn-A06-p3072724	A6	20	24.8	B
5564	Bn-N6-p3020965	Bn-A06-p3093741	Bn-A06-p3093741	A6	20	24.8	B
5565	Bn-N6-p3024061	Bn-A06-p3096813	Bn-A06-p3096813	A6	20	24.8	B
5566	Bn-N6-p3040061	Bn-A06-p3114818	Bn-A06-p3114818	A6	20	24.8	B
5567	Bn-N6-p3081425	Bn-A06-p3146367	Bn-A06-p3146367	A6	20	24.8	B
5568	Bn-N6-p3081783	Bn-A06-p3146720	Bn-A06-p3146720	A6	20	24.8	B
5569	Bn-N6-p3115162	Bn-A06-p3184395	Bn-A06-p3184395	A6	20	24.8	B
5570	Bn-N6-p3119656	Bn-A06-p3188819	Bn-A06-p3188819	A6	20	24.8	B
5571	Bn-N6-p3120329	Bn-A06-p3189490	Bn-A06-p3189490	A6	20	24.8	B
5572	Bn-N6-p3133254	Bn-A06-p3202098	Bn-A06-p3202098	A6	20	24.8	B
5573	Bn-N6-p3133744	Bn-A06-p3202615	Bn-A06-p3202615	A6	20	24.8	B
5574	Bn-N6-p3135944	Bn-Scaffold000283-p2	Bn-Scaffold000283-p24053	A6	20	24.8	B
5575	Bn-N6-p3138340	Bn-A06-p3206017	Bn-A06-p3206017	A6	20	24.8	0
5576	Bn-N6-p3139949	Bn-A06-p3207623	Bn-A06-p3207623	A6	20	24.8	B
5577	Bn-N6-p3144086	Bn-A06-p3210791	Bn-A06-p3210791	A6	20	24.8	B
5578	Bn-N6-p3150293	Bn-A06-p3226693	Bn-A06-p3226693	A6	20	24.8	B
5579	Bn-N6-p3178901	Bn-A06-p3269320	Bn-A06-p3269320	A6	20	24.8	B
5580	Bn-N6-p3212001	Bn-A06-p3302657	Bn-A06-p3302657	A6	20	24.8	B
5581	Bn-N6-p3227453	Bn-A06-p3316040	Bn-A06-p3316040	A6	20	24.8	B

5582	Bn-N6-p3227622	Bn-A06-p3316207	Bn-A06-p3316207	A6	20	24.8	B
5583	Bn-N15-p3913044	Bn-A06-p3339435	Bn-A06-p3339435	A6	21	25.6	B
5584	Bn-N15-p4041812	Bn-A06-p3434498	Bn-A06-p3434498	A6	21	25.6	B
5585	Bn-N6-p3236858	Bn-A06-p3325502	Bn-A06-p3325502	A6	21	25.6	B
5586	Bn-N6-p3236922	Bn-A06-p3325566	Bn-A06-p3325566	A6	21	25.6	B
5587	Bn-N6-p3274422	Bn-A06-p3361377	Bn-A06-p3361377	A6	21	25.6	B
5588	Bn-N6-p3276226	Bn-A06-p3363188	Bn-A06-p3363188	A6	21	25.6	B
5589	Bn-N6-p3294131	Bn-A06-p3380495	Bn-A06-p3380495	A6	21	25.6	B
5590	Bn-N6-p3312066	Bn-A06-p3399869	Bn-A06-p3399869	A6	21	25.6	B
5591	Bn-N6-p3312170	Bn-A06-p3399973	Bn-A06-p3399973	A6	21	25.6	B
5592	Bn-N6-p3318240	Bn-A06-p3405779	Bn-A06-p3405779	A6	21	25.6	B
5593	Bn-N6-p3318501	Bn-A06-p3406041	Bn-A06-p3406041	A6	21	25.6	B
5594	Bn-N6-p3326604	Bn-A06-p3414128	Bn-A06-p3414128	A6	21	25.6	B
5595	Bn-N6-p3329194	Bn-A06-p3416803	Bn-A06-p3416803	A6	21	25.6	B
5596	Bn-N6-p3343630	Bn-A06-p3428214	Bn-A06-p3428214	A6	21	25.6	B
5597	Bn-N6-p3352894	Bn-A06-p3437409	Bn-A06-p3437409	A6	21	25.6	B
5598	Bn-N11-p43809110	Bn-A06-p3708256	Bn-A06-p3708256	A6	22	26.4	B
5599	Bn-N15-p4594195	Bn-A06-p3818421	Bn-A06-p3818421	A6	22	26.4	B
5600	Bn-N15-p4594266	Bn-A06-p3818484	Bn-A06-p3818484	A6	22	26.4	B
5601	Bn-N6-p3411122	Bn-A06-p3491241	Bn-A06-p3491241	A6	22	26.4	B
5602	Bn-N6-p3412183	Bn-A06-p3492295	Bn-A06-p3492295	A6	22	26.4	B
5603	Bn-N6-p3600793	Bn-A06-p3691343	Bn-A06-p3691343	A6	22	26.4	B
5604	Bn-N6-p3608896	Bn-A06-p3703307	Bn-A06-p3703307	A6	22	26.4	B
5605	Bn-N6-p3809412	Bn-A06-p3900404	Bn-A06-p3900404	A6	23	28	B
5606	Bn-N6-p3871081	Bn-A06-p3953661	Bn-A06-p3953661	A6	23	28	B
5607	Bn-N6-p3893142	Bn-A06-p3970972	Bn-A06-p3970972	A6	23	28	B
5608	Bn-N6-p3921192	Bn-A06-p3992289	Bn-A06-p3992289	A6	24	28.8	B
5609	Bn-N6-p3926060	Bn-A06-p3997307	Bn-A06-p3997307	A6	24	28.8	B
5610	Bn-N6-p3927346	Bn-A06-p3998593	Bn-A06-p3998593	A6	24	28.8	B
5611	Bn-N6-p3928739	Bn-A06-p3999986	Bn-A06-p3999986	A6	24	28.8	B
5612	Bn-N6-p3958445	Bn-A06-p4019827	Bn-A06-p4019827	A6	24	28.8	B
5613	Bn-N6-p3961778	Bn-A06-p4021930	Bn-A06-p4021930	A6	25	29.6	B
5614	Bn-N6-p3965563	Bn-A06-p4025613	Bn-A06-p4025613	A6	25	29.6	B
5615	Bn-N6-p3966708	Bn-A06-p4026453	Bn-A06-p4026453	A6	25	29.6	B
5616	Bn-N6-p3967285	Bn-A06-p4027051	Bn-A06-p4027051	A6	25	29.6	B
5617	Bn-N6-p3985083	Bn-A06-p4039206	Bn-A06-p4039206	A6	25	29.6	B
5618	Bn-N6-p4046119	Bn-A06-p4098532	Bn-A06-p4098532	A6	26	30.4	B
5619	Bn-N6-p4046387	Bn-A06-p4098800	Bn-A06-p4098800	A6	26	30.4	B
5620	Bn-N6-p4111353	Bn-A06-p4179318	Bn-A06-p4179318	A6	27	31.2	B
5621	Bn-N6-p4118639	Bn-A06-p4196008	Bn-A06-p4196008	A6	27	31.2	B
5622	Bn-N6-p4149689	Bn-A06-p4218801	Bn-A06-p4218801	A6	27	31.2	B
5623	Bn-N6-p4167424	Bn-A06-p4229919	Bn-A06-p4229919	A6	27	31.2	B
5624	Bn-N6-p4169661	Bn-C5-p5034235	Bn-scaff_21496_1-p38238(	A6	27	31.2	B
5625	Bn-N15-p5380511	Bn-A06-p4435926	Bn-A06-p4435926	A6	28	32.8	B
5626	Bn-N6-p4253412	Bn-A06-p4315934	Bn-A06-p4315934	A6	28	32.8	B
5627	Bn-N6-p4304145	Bn-A06-p4371627	Bn-A06-p4371627	A6	28	32.8	B
5628	Bn-N6-p4310063	Bn-Scaffold01390-p9ٰ	Bn-scaff_17482_1-p9837	A6	28	32.8	B
5629	Bn-N6-p4341969	Bn-A06-p4418231	Bn-A06-p4418231	A6	28	32.8	B
5630	Bn-N6-p4343325	Bn-A06-p4419571	Bn-A06-p4419571	A6	28	32.8	B

5631	Bn-N6-p4344015	Bn-A06-p4420263	Bn-A06-p4420263	A6	28	32.8	B
5632	Bn-N6-p4344970	Bn-A06-p4421214	Bn-A06-p4421214	A6	28	32.8	B
5633	Bn-N6-p4361910	Bn-A06-p4436168	Bn-A06-p4436168	A6	28	32.8	B
5634	Bn-N6-p4362910	Bn-A06-p4437120	Bn-A06-p4437120	A6	28	32.8	B
5635	Bn-N6-p4376374	Bn-A06-p4452778	Bn-A06-p4452778	A6	28	32.8	B
5636	Bn-N6-p4385362	Bn-A06-p4461241	Bn-A06-p4461241	A6	28	32.8	B
5637	Bn-N6-p4418843	Bn-A06-p4508774	Bn-A06-p4508774	A6	29	33.6	B
5638	Bn-N6-p4511003	Bn-A06-p4597746	Bn-A06-p4597746	A6	29	33.6	B
5639	Bn-N6-p4524219	Bn-A06-p4619013	Bn-A06-p4619013	A6	29	33.6	B
5640	Bn-N6-p4528589	Bn-A06-p4623401	Bn-A06-p4623401	A6	29	33.6	B
5641	Bn-N6-p4574042	Bn-A06-p4703366	Bn-A06-p4703366	A6	29	33.6	B
5642	Bn-N6-p4606368	Bn-A06-p4729500	Bn-A06-p4729500	A6	29	33.6	B
5643	Bn-N6-p4613334	Bn-A06-p4731996	Bn-A06-p4731996	A6	29	33.6	B
5644	Bn-N6-p4752756	Bn-Scaffold000240-p3	Bn-Scaffold000240-p33373	A6	30	34.4	B
5645	Bn-N6-p4795418	Bn-A06-p4874556	Bn-A06-p4874556	A6	30	34.4	B
5646	Bn-N15-p6144435	Bn-A06-p4927344	Bn-A06-p4927344	A6	31	35.2	B
5647	Bn-N15-p6327444	Bn-C5-p6620772	Bn-scaff_18181_1-p16031	A6	31	35.2	B
5648	Bn-N6-p4802388	Bn-A06-p4881785	Bn-A06-p4881785	A6	31	35.2	B
5649	Bn-N6-p4832997	Bn-A06-p4896084	Bn-A06-p4896084	A6	31	35.2	B
5650	Bn-N6-p4837924	Bn-A06-p4901091	Bn-A06-p4901091	A6	31	35.2	B
5651	Bn-N6-p4838282	Bn-A06-p4901457	Bn-A06-p4901457	A6	31	35.2	B
5652	Bn-N6-p4874165	Bn-A06-p4929018	Bn-A06-p4929018	A6	31	35.2	B
5653	Bn-N6-p4879063	Bn-A06-p4933932	Bn-A06-p4933932	A6	31	35.2	B
5654	Bn-N6-p4999092	Bn-C5-p6425990	Bn-scaff_18181_1-p17979	A6	31	35.2	B
5655	Bn-N6-p5005346	Bn-A04-p9339599	Bn-A04-p9339599	A6	31	35.2	B
5656	Bn-N6-p5017241	Bn-A06-p5091252	Bn-A06-p5091252	A6	31	35.2	B
5657	Bn-N6-p5041218	Bn-A06-p5114770	Bn-A06-p5114770	A6	31	35.2	B
5658	Bn-N6-p5081581	Bn-A06-p5151254	Bn-A06-p5151254	A6	31	35.2	B
5659	Bn-N6-p5127629	Bn-A06-p5535537	Bn-A06-p5535537	A6	31	35.2	B
5660	Bn-N6-p5130414	Bn-A06-p5540327	Bn-A06-p5540327	A6	31	35.2	B
5661	Bn-N6-p5136130	Bn-A06-p5546411	Bn-A06-p5546411	A6	31	35.2	B
5662	Bn-N6-p5200409	Bn-A06-p5616164	Bn-A06-p5616164	A6	32	36.8	B
5663	Bn-N6-p5202546	Bn-A06-p5618302	Bn-A06-p5618302	A6	32	36.8	B
5664	Bn-N6-p5228100	Bn-A06-p5641863	Bn-A06-p5641863	A6	32	36.8	B
5665	Bn-N15-p7101313	Bn-C5-p7073072	Bn-scaff_18181_1-p11508	A6	33	37.6	B
5666	Bn-N19-p41466660	Bn-A06-p5826493	Bn-A06-p5826493	A6	33	37.6	B
5667	Bn-N6-p5293277	Bn-C5-p6881457	Bn-scaff_18181_1-p13425	A6	33	37.6	B
5668	Bn-N6-p5325363	Bn-A06-p5739682	Bn-A06-p5739682	A6	33	37.6	B
5669	Bn-N6-p5330191	Bn-A06-p5741557	Bn-A06-p5741557	A6	33	37.6	B
5670	Bn-N6-p5341945	Bn-A06-p5747694	Bn-A06-p5747694	A6	33	37.6	B
5671	Bn-N6-p5362304	Bn-A06-p5767097	Bn-A06-p5767097	A6	33	37.6	B
5672	Bn-N6-p5378080	Bn-A06-p5780982	Bn-A06-p5780982	A6	33	37.6	B
5673	Bn-N6-p5412407	Bn-A06-p5693735	Bn-A06-p5693735	A6	33	37.6	B
5674	Bn-N6-p5414690	Bn-A06-p5824546	Bn-A06-p5824546	A6	33	37.6	B
5675	Bn-N6-p5420524	Bn-A06-p5830298	Bn-A06-p5830298	A6	33	37.6	B
5676	Bn-N6-p5428823	Bn-A06-p5844403	Bn-A06-p5844403	A6	33	37.6	0
5677	Bn-N6-p5429962	Bn-A06-p5845542	Bn-A06-p5845542	A6	33	37.6	B
5678	Bn-N6-p5455035	Bn-A06-p5868273	Bn-A06-p5868273	A6	33	37.6	B
5679	Bn-N6-p5467747	Bn-A06-p5880980	Bn-A06-p5880980	A6	33	37.6	B

5680	Bn-N6-p5475639	Bn-A06-p5888779	Bn-A06-p5888779	A6	33	37.6	B
5681	Bn-N6-p5495718	Bn-A06-p5904190	Bn-A06-p5904190	A6	33	37.6	B
5682	Bn-N6-p5499009	Bn-A06-p5907449	Bn-A06-p5907449	A6	33	37.6	B
5683	Bn-N6-p5547966	Bn-A06-p5947781	Bn-A06-p5947781	A6	34	38.4	B
5684	Bn-N6-p5565642	Bn-A06-p5956274	Bn-A06-p5956274	A6	35	39.2	B
5685	Bn-N6-p5613149	Bn-A06-p5995778	Bn-A06-p5995778	A6	35	39.2	B
5686	Bn-N6-p5614218	Bn-A06-p5996847	Bn-A06-p5996847	A6	35	39.2	B
5687	Bn-N6-p5616830	Bn-A06-p6006425	Bn-A06-p6006425	A6	35	39.2	B
5688	Bn-N6-p5656244	Bn-A06-p6047002	Bn-A06-p6047002	A6	35	39.2	B
5689	Bn-N6-p5661359	Bn-A06-p6056844	Bn-A06-p6056844	A6	35	39.2	B
5690	Bn-N6-p5672077	Bn-A06-p6066073	Bn-A06-p6066073	A6	35	39.2	B
5691	Bn-N6-p5701636	Bn-A06-p6091453	Bn-A06-p6091453	A6	35	39.2	B
5692	Bn-N6-p5707075	Bn-A06-p6103844	Bn-A06-p6103844	A6	36	40	B
5693	Bn-N6-p5713810	Bn-Scaffold012485-p5	Bn-Scaffold012485-p58	A6	36	40	B
5694	Bn-N6-p5736788	Bn-C5-p7593891	Bn-scaff_18181_1-p630071	A6	36	40	B
5695	Bn-N6-p5760603	Bn-A06-p6182046	Bn-A06-p6182046	A6	37	40.8	B
5696	Bn-N6-p5761153	Bn-A06-p6182593	Bn-A06-p6182593	A6	37	40.8	B
5697	Bn-N6-p5762420	Bn-A06-p6183859	Bn-A06-p6183859	A6	37	40.8	B
5698	Bn-N6-p5763016	Bn-A06-p6184455	Bn-A06-p6184455	A6	37	40.8	B
5699	Bn-N6-p5780087	Bn-A06-p6196005	Bn-A06-p6196005	A6	37	40.8	B
5700	Bn-N6-p5782654	Bn-A06-p6198128	Bn-A06-p6198128	A6	37	40.8	B
5701	Bn-N6-p5898614	Bn-A06-p6311231	Bn-A06-p6311231	A6	38	41.6	B
5702	Bn-N6-p5898672	Bn-A06-p6311289	Bn-A06-p6311289	A6	38	41.6	B
5703	Bn-N15-p8111890	Bn-A06-p6408922	Bn-A06-p6408922	A6	39	42.4	B
5704	Bn-N15-p8359521	Bn-A06-p6569468	Bn-A06-p6569468	A6	39	42.4	B
5705	Bn-N6-p5934735	Bn-A06-p6343931	Bn-A06-p6343931	A6	39	42.4	B
5706	Bn-N6-p5946940	Bn-A06-p6356463	Bn-A06-p6356463	A6	39	42.4	B
5707	Bn-N6-p5975051	Bn-A06-p6382773	Bn-A06-p6382773	A6	39	42.4	B
5708	Bn-N6-p6005927	Bn-C5-p8006158	Bn-scaff_18181_1-p217804	A6	39	42.4	B
5709	Bn-N6-p6020344	Bn-A06-p6432467	Bn-A06-p6432467	A6	39	42.4	B
5710	Bn-N6-p6024972	Bn-A06-p6437068	Bn-A06-p6437068	A6	39	42.4	B
5711	Bn-N6-p6027275	Bn-A06-p6439396	Bn-A06-p6439396	A6	39	42.4	B
5712	Bn-N6-p6040067	Bn-A06-p6451055	Bn-A06-p6451055	A6	39	42.4	B
5713	Bn-N6-p6041172	Bn-A06-p6452160	Bn-A06-p6452160	A6	39	42.4	B
5714	Bn-N6-p6047598	Bn-A06-p6458266	Bn-A06-p6458266	A6	39	42.4	B
5715	Bn-N6-p6067046	Bn-A06-p6477943	Bn-A06-p6477943	A6	39	42.4	B
5716	Bn-N6-p6099643	Bn-A06-p6517823	Bn-A06-p6517823	A6	39	42.4	B
5717	Bn-N6-p6118014	Bn-A06-p6532740	Bn-A06-p6532740	A6	39	42.4	B
5718	Bn-N6-p6151640	Bn-A06-p6555456	Bn-A06-p6555456	A6	39	42.4	B
5719	Bn-N6-p6226240	Bn-A06-p6624071	Bn-A06-p6624071	A6	40	43.2	B
5720	Bn-N6-p6239300	Bn-A06-p6636202	Bn-A06-p6636202	A6	40	43.2	B
5721	Bn-N6-p6245632	Bn-A06-p6646249	Bn-A06-p6646249	A6	40	43.2	B
5722	Bn-N6-p6246719	Bn-A06-p6647323	Bn-A06-p6647323	A6	41	44	B
5723	Bn-N6-p6296354	Bn-A06-p6692851	Bn-A06-p6692851	A6	41	44	B
5724	Bn-N6-p6306290	Bn-A06-p6705911	Bn-A06-p6705911	A6	41	44	B
5725	Bn-N6-p6306446	Bn-A06-p6706067	Bn-A06-p6706067	A6	41	44	B
5726	Bn-N6-p6319916	Bn-A06-p6723804	Bn-A06-p6723804	A6	41	44	B
5727	Bn-N6-p6340136	Bn-A06-p6744737	Bn-A06-p6744737	A6	41	44	B
5728	Bn-N6-p6342582	Bn-A06-p6747460	Bn-A06-p6747460	A6	41	44	B

5729	Bn-N14-p7108144	Bn-C5-p8939233	Bn-scaff_16082_1-p278297	A6	42	44.8	B
5730	Bn-N15-p9456872	Bn-A06-p7384367	Bn-A06-p7384367	A6	42	44.8	B
5731	Bn-N15-p9841752	Bn-C5-p9592634	Bn-scaff_22183_1-p422126	A6	42	44.8	B
5732	Bn-N1-p25931195	Bn-A02-p23910066	Bn-A02-p23910066	A6	42	44.8	B
5733	Bn-N6-p6349603	Bn-A06-p6761232	Bn-A06-p6761232	A6	42	44.8	B
5734	Bn-N6-p6349612	Bn-A06-p6761241	Bn-A06-p6761241	A6	42	44.8	B
5735	Bn-N6-p6349702	Bn-A06-p6761331	Bn-A06-p6761331	A6	42	44.8	B
5736	Bn-N6-p6367200	Bn-A06-p6782162	Bn-A06-p6782162	A6	42	44.8	B
5737	Bn-N6-p6368018	Bn-A06-p6782980	Bn-A06-p6782980	A6	42	44.8	B
5738	Bn-N6-p6443334	Bn-A06-p6864079	Bn-A06-p6864079	A6	42	44.8	B
5739	Bn-N6-p6553172	Bn-A06-p6919765	Bn-A06-p6919765	A6	42	44.8	B
5740	Bn-N6-p6583392	Bn-A06-p6951503	Bn-A06-p6951503	A6	42	44.8	B
5741	Bn-N6-p6590676	Bn-A06-p6959460	Bn-A06-p6959460	A6	42	44.8	B
5742	Bn-N6-p6592285	Bn-A06-p6961069	Bn-A06-p6961069	A6	42	44.8	B
5743	Bn-N6-p6601765	Bn-A06-p6970539	Bn-A06-p6970539	A6	42	44.8	B
5744	Bn-N6-p6602868	Bn-A06-p6971737	Bn-A06-p6971737	A6	42	44.8	B
5745	Bn-N6-p6603126	Bn-A06-p6971900	Bn-A06-p6971900	A6	42	44.8	B
5746	Bn-N6-p6603567	Bn-A06-p6972345	Bn-A06-p6972345	A6	42	44.8	B
5747	Bn-N6-p6604777	Bn-C5-p8673583	Bn-scaff_16082_1-p543947	A6	42	44.8	B
5748	Bn-N6-p6621007	Bn-A06-p6985717	Bn-A06-p6985717	A6	42	44.8	B
5749	Bn-N6-p6625781	Bn-A06-p6990496	Bn-A06-p6990496	A6	42	44.8	B
5750	Bn-N6-p6629147	Bn-A06-p6993862	Bn-A06-p6993862	A6	42	44.8	B
5751	Bn-N6-p6630777	Bn-A06-p6995492	Bn-A06-p6995492	A6	42	44.8	B
5752	Bn-N6-p6638576	Bn-A06-p7003598	Bn-A06-p7003598	A6	42	44.8	B
5753	Bn-N6-p6659814	Bn-A06-p7023598	Bn-A06-p7023598	A6	42	44.8	B
5754	Bn-N6-p6660020	Bn-A06-p7023804	Bn-A06-p7023804	A6	42	44.8	B
5755	Bn-N6-p6660034	Bn-A06-p7023818	Bn-A06-p7023818	A6	42	44.8	B
5756	Bn-N6-p6660140	Bn-A06-p7023924	Bn-A06-p7023924	A6	42	44.8	B
5757	Bn-N6-p6711796	Bn-A06-p7083415	Bn-A06-p7083415	A6	42	44.8	B
5758	Bn-N6-p6753013	Bn-A06-p7119339	Bn-A06-p7119339	A6	42	44.8	0
5759	Bn-N6-p6779221	Bn-A06-p7134425	Bn-A06-p7134425	A6	42	44.8	B
5760	Bn-N6-p6912944	Bn-A06-p7247810	Bn-A06-p7247810	A6	42	44.8	B
5761	Bn-N6-p6967901	Bn-A06-p7288881	Bn-A06-p7288881	A6	42	44.8	B
5762	Bn-N6-p6968408	Bn-A06-p7289388	Bn-A06-p7289388	A6	42	44.8	B
5763	Bn-N6-p6969531	Bn-A06-p7290511	Bn-A06-p7290511	A6	42	44.8	B
5764	Bn-N6-p6969852	Bn-A06-p7290893	Bn-A06-p7290893	A6	42	44.8	B
5765	Bn-N6-p6969910	Bn-A06-p7290973	Bn-A06-p7290973	A6	42	44.8	B
5766	Bn-N6-p6977846	Bn-A06-p7302651	Bn-A06-p7302651	A6	42	44.8	B
5767	Bn-N6-p6988346	Bn-A06-p7313589	Bn-A06-p7313589	A6	42	44.8	B
5768	Bn-N6-p6989144	Bn-A06-p7314403	Bn-A06-p7314403	A6	42	44.8	B
5769	Bn-N6-p7111117	Bn-A06-p7420466	Bn-A06-p7420466	A6	42	44.8	B
5770	Bn-N6-p7138783	Bn-A06-p7444483	Bn-A06-p7444483	A6	42	44.8	B
5771	Bn-N6-p7263744	Bn-A06-p7560098	Bn-A06-p7560098	A6	42	44.8	B
5772	Bn-N6-p7290539	Bn-A06-p7583628	Bn-A06-p7583628	A6	42	44.8	B
5773	Bn-N15-p9848110	Bn-A06-p7589883	Bn-A06-p7589883	A6	43	45.6	B
5774	Bn-N6-p7324192	Bn-A06-p7616430	Bn-A06-p7616430	A6	43	45.6	B
5775	Bn-N6-p7332959	Bn-A06-p7624783	Bn-A06-p7624783	A6	43	45.6	B
5776	Bn-N6-p7345346	Bn-A06-p7636623	Bn-A06-p7636623	A6	43	45.6	B
5777	Bn-N6-p7345452	Bn-A06-p7636729	Bn-A06-p7636729	A6	43	45.6	B

5778	Bn-N6-p7350279	Bn-A06-p7641474	Bn-A06-p7641474	A6	43	45.6	B
5779	Bn-N6-p7381857	Bn-A06-p7670615	Bn-A06-p7670615	A6	43	45.6	B
5780	Bn-N6-p7381938	Bn-A06-p7670696	Bn-A06-p7670696	A6	43	45.6	B
5781	Bn-N6-p7413359	Bn-A06-p7701069	Bn-A06-p7701069	A6	44	47.2	B
5782	Bn-N6-p7415532	Bn-A06-p7703242	Bn-A06-p7703242	A6	44	47.2	B
5783	Bn-N6-p7419989	Bn-A06-p7707699	Bn-A06-p7707699	A6	44	47.2	B
5784	Bn-N6-p7639231	Bn-A06-p7927767	Bn-A06-p7927767	A6	44	47.2	B
5785	Bn-N6-p7644220	Bn-A06-p7931177	Bn-A06-p7931177	A6	44	47.2	B
5786	Bn-N6-p7658347	Bn-A06-p7949147	Bn-A06-p7949147	A6	44	47.2	B
5787	Bn-N6-p7670105	Bn-A06-p7959476	Bn-A06-p7959476	A6	44	47.2	B
5788	Bn-N6-p7677089	Bn-A06-p7962905	Bn-A06-p7962905	A6	44	47.2	B
5789	Bn-N6-p7679228	Bn-A06-p7965038	Bn-A06-p7965038	A6	44	47.2	B
5790	Bn-N6-p7680265	Bn-A06-p7965415	Bn-A06-p7965415	A6	44	47.2	B
5791	Bn-N6-p7683289	Bn-A06-p7973826	Bn-A06-p7973826	A6	44	47.2	B
5792	Bn-N6-p7684039	Bn-A06-p7974986	Bn-A06-p7974986	A6	44	47.2	B
5793	Bn-N6-p7699586	Bn-A06-p7993829	Bn-A06-p7993829	A6	44	47.2	B
5794	Bn-N6-p7719603	Bn-A06-p8021875	Bn-A06-p8021875	A6	44	47.2	B
5795	Bn-N6-p7719785	Bn-A06-p8022058	Bn-A06-p8022058	A6	44	47.2	B
5796	Bn-N6-p7728912	Bn-A06-p8033701	Bn-A06-p8033701	A6	44	47.2	B
5797	Bn-N6-p7888552	Bn-A06-p8201495	Bn-A06-p8201495	A6	44	47.2	B
5798	Bn-N6-p7888638	Bn-A06-p8201581	Bn-A06-p8201581	A6	44	47.2	B
5799	Bn-N1-p12676284	Bn-Scaffold013153-p1	Bn-Scaffold013153-p127	A6	45	48	B
5800	Bn-N6-p7956675	Bn-A06-p8263325	Bn-A06-p8263325	A6	45	48	B
5801	Bn-N6-p7961563	Bn-A06-p8277116	Bn-A06-p8277116	A6	45	48	B
5802	Bn-N6-p8082983	Bn-A06-p8402978	Bn-A06-p8402978	A6	45	48	B
5803	Bn-N6-p8109487	Bn-A06-p8426071	Bn-A06-p8426071	A6	45	48	B
5804	Bn-N6-p8112214	Bn-A06-p8430642	Bn-A06-p8430642	A6	45	48	B
5805	Bn-N6-p8115655	Bn-A06-p8434088	Bn-A06-p8434088	A6	45	48	B
5806	Bn-N18-p28491976	Bn-A06-p9399854	Bn-A06-p9399854	A6	46	51.2	B
5807	Bn-N6-p8125820	Bn-A06-p8446816	Bn-A06-p8446816	A6	46	51.2	B
5808	Bn-N6-p8152434	Bn-A06-p8475485	Bn-A06-p8475485	A6	46	51.2	B
5809	Bn-N6-p8197103	Bn-A06-p8536023	Bn-A06-p8536023	A6	46	51.2	B
5810	Bn-N6-p8206489	Bn-A06-p8546149	Bn-A06-p8546149	A6	46	51.2	B
5811	Bn-N6-p8207038	Bn-A06-p8546698	Bn-A06-p8546698	A6	46	51.2	B
5812	Bn-N6-p8293012	Bn-A06-p8614872	Bn-A06-p8614872	A6	46	51.2	B
5813	Bn-N6-p8293955	Bn-A06-p8615815	Bn-A06-p8615815	A6	46	51.2	B
5814	Bn-N6-p8316471	Bn-A06-p8633786	Bn-A06-p8633786	A6	46	51.2	B
5815	Bn-N6-p8316851	Bn-A06-p8634166	Bn-A06-p8634166	A6	46	51.2	B
5816	Bn-N6-p8317333	Bn-A06-p8634648	Bn-A06-p8634648	A6	46	51.2	B
5817	Bn-N6-p8429433	Bn-A06-p8766088	Bn-A06-p8766088	A6	46	51.2	B
5818	Bn-N6-p8462651	Bn-A06-p8784719	Bn-A06-p8784719	A6	46	51.2	B
5819	Bn-N6-p8496421	Bn-A06-p8810900	Bn-A06-p8810900	A6	46	51.2	B
5820	Bn-N6-p8523572	Bn-A06-p8837290	Bn-A06-p8837290	A6	46	51.2	B
5821	Bn-N6-p8634880	Bn-A06-p8948420	Bn-A06-p8948420	A6	46	51.2	B
5822	Bn-N6-p8652804	Bn-A06-p8968750	Bn-A06-p8968750	A6	46	51.2	B
5823	Bn-N6-p8680322	Bn-A06-p8992364	Bn-A06-p8992364	A6	46	51.2	B
5824	Bn-N6-p8686530	Bn-A06-p8998572	Bn-A06-p8998572	A6	46	51.2	B
5825	Bn-N6-p8695592	Bn-A06-p9009861	Bn-A06-p9009861	A6	46	51.2	B
5826	Bn-N6-p8696705	Bn-A06-p9010952	Bn-A06-p9010952	A6	46	51.2	B

5827	Bn-N6-p8739420	Bn-A06-p9074653	Bn-A06-p9074653	A6	46	51.2	B
5828	Bn-N6-p8757728	Bn-A06-p9090817	Bn-A06-p9090817	A6	46	51.2	B
5829	Bn-N6-p8762986	Bn-A06-p9094772	Bn-A06-p9094772	A6	46	51.2	B
5830	Bn-N6-p8774702	Bn-A02-p14938941	Bn-A02-p14938941	A6	46	51.2	B
5831	Bn-N6-p8953040	Bn-A06-p9287101	Bn-A06-p9287101	A6	46	51.2	B
5832	Bn-N6-p8955235	Bn-A06-p9289355	Bn-A06-p9289355	A6	46	51.2	B
5833	Bn-N6-p8956427	Bn-A06-p9291582	Bn-A06-p9291582	A6	46	51.2	B
5834	Bn-N6-p8957102	Bn-A06-p9292257	Bn-A06-p9292257	A6	46	51.2	B
5835	Bn-N6-p9147293	Bn-A06-p9452981	Bn-A06-p9452981	A6	46	51.2	B
5836	Bn-N6-p9159585	Bn-A06-p9465709	Bn-A06-p9465709	A6	46	51.2	B
5837	Bn-N6-p9170777	Bn-A06-p9475900	Bn-A06-p9475900	A6	46	51.2	B
5838	Bn-N6-p9200968	Bn-A06-p9517147	Bn-A06-p9517147	A6	46	51.2	B
5839	Bn-N6-p9265150	Bn-A06-p9562200	Bn-A06-p9562200	A6	46	51.2	B
5840	Bn-N6-p9286025	Bn-A06-p9593892	Bn-A06-p9593892	A6	46	51.2	B
5841	Bn-N6-p9350124	Bn-A06-p9665402	Bn-A06-p9665402	A6	46	51.2	B
5842	Bn-N6-p9366293	Bn-A06-p9684583	Bn-A06-p9684583	A6	46	51.2	B
5843	Bn-N6-p9595488	Bn-A06-p9936409	Bn-A06-p9936409	A6	46	51.2	B
5844	Bn-N6-p9692002	Bn-A06-p10018992	Bn-A06-p10018992	A6	46	51.2	B
5845	Bn-N6-p9692591	Bn-A06-p10019580	Bn-A06-p10019580	A6	46	51.2	B
5846	Bn-N6-p9694318	Bn-A06-p10021272	Bn-A06-p10021272	A6	46	51.2	B
5847	Bn-N6-p9694385	Bn-A06-p10021339	Bn-A06-p10021339	A6	46	51.2	B
5848	Bn-N6-p9694439	Bn-A06-p10021393	Bn-A06-p10021393	A6	46	51.2	B
5849	Bn-N6-p9695291	Bn-A06-p10022213	Bn-A06-p10022213	A6	46	51.2	B
5850	Bn-N6-p9695423	Bn-A06-p10022347	Bn-A06-p10022347	A6	46	51.2	B
5851	Bn-N6-p9695826	Bn-A06-p10022751	Bn-A06-p10022751	A6	46	51.2	B
5852	Bn-N6-p9696647	Bn-A06-p10023556	Bn-A06-p10023556	A6	46	51.2	B
5853	Bn-N6-p9709277	Bn-A06-p10031660	Bn-A06-p10031660	A6	46	51.2	B
5854	Bn-N6-p9710715	Bn-A06-p10032901	Bn-A06-p10032901	A6	46	51.2	B
5855	Bn-N6-p9711212	Bn-A06-p10033398	Bn-A06-p10033398	A6	46	51.2	B
5856	Bn-N6-p9711532	Bn-A06-p10033718	Bn-A06-p10033718	A6	46	51.2	B
5857	Bn-N6-p9711717	Bn-A06-p10033903	Bn-A06-p10033903	A6	46	51.2	B
5858	Bn-N6-p9730325	Bn-A06-p10053057	Bn-A06-p10053057	A6	46	51.2	B
5859	Bn-N6-p9731068	Bn-A06-p10053801	Bn-A06-p10053801	A6	46	51.2	B
5860	Bn-N6-p9743287	Bn-A06-p10064955	Bn-A06-p10064955	A6	46	51.2	B
5861	Bn-N6-p9747331	Bn-A06-p10068703	Bn-A06-p10068703	A6	46	51.2	B
5862	Bn-N6-p9747779	Bn-A06-p10069150	Bn-A06-p10069150	A6	46	51.2	B
5863	Bn-N6-p9768805	Bn-A06-p10085385	Bn-A06-p10085385	A6	46	51.2	B
5864	Bn-N6-p9770089	Bn-A06-p10086667	Bn-A06-p10086667	A6	46	51.2	B
5865	Bn-N13-p38258630	Bn-C3-p36269883	Bn-scaff_18356_1-p311981	A6	47	52.8	B
5866	Bn-N13-p40104674	Bn-A06-p19508949	Bn-A06-p19508949	A6	47	52.8	B
5867	Bn-N13-p47843647	Bn-A06-p13726921	Bn-A06-p13726921	A6	47	52.8	B
5868	Bn-N13-p48108178	Bn-Scaffold00874-p5	Bn-scaff_18013_1-p194773	A6	47	52.8	B
5869	Bn-N19-p40743796	Bn-C4-p44226463	Bn-scaff_20079_1-p517025	A6	47	52.8	B
5870	Bn-N2-p20810162	Bn-A05-p17835705	Bn-A05-p17835705	A6	47	52.8	B
5871	Bn-N6-p10000718	Bn-A06-p10330066	Bn-A06-p10330066	A6	47	52.8	B
5872	Bn-N6-p10020752	Bn-A06-p10349427	Bn-A06-p10349427	A6	47	52.8	B
5873	Bn-N6-p10022930	Bn-A06-p10351604	Bn-A06-p10351604	A6	47	52.8	B
5874	Bn-N6-p10059495	Bn-A06-p10391346	Bn-A06-p10391346	A6	47	52.8	B
5875	Bn-N6-p10160456	Bn-A06-p10480404	Bn-A06-p10480404	A6	47	52.8	B

5876	Bn-N6-p10259845	Bn-A06-p10574739	Bn-A06-p10574739	A6	47	52.8	B
5877	Bn-N6-p10264860	Bn-A06-p10578969	Bn-A06-p10578969	A6	47	52.8	B
5878	Bn-N6-p10265506	Bn-A06-p10579615	Bn-A06-p10579615	A6	47	52.8	B
5879	Bn-N6-p10282763	Bn-A06-p10594170	Bn-A06-p10594170	A6	47	52.8	B
5880	Bn-N6-p10298354	Bn-A06-p10609574	Bn-A06-p10609574	A6	47	52.8	B
5881	Bn-N6-p10326476	Bn-A06-p10642761	Bn-A06-p10642761	A6	47	52.8	0
5882	Bn-N6-p10470960	Bn-A06-p10781335	Bn-A06-p10781335	A6	47	52.8	B
5883	Bn-N6-p10486461	Bn-A06-p10795156	Bn-A06-p10795156	A6	47	52.8	B
5884	Bn-N6-p10517430	Bn-C3-p48811508	Bn-scaff_21867_1-p80938	A6	47	52.8	B
5885	Bn-N6-p10519745	Bn-A06-p10832056	Bn-A06-p10832056	A6	47	52.8	B
5886	Bn-N6-p10521827	Bn-A06-p10833219	Bn-A06-p10833219	A6	47	52.8	B
5887	Bn-N6-p10543512	Bn-A06-p10859915	Bn-A06-p10859915	A6	47	52.8	B
5888	Bn-N6-p10547446	Bn-A06-p10863673	Bn-A06-p10863673	A6	47	52.8	B
5889	Bn-N6-p11060957	Bn-A06-p11344945	Bn-A06-p11344945	A6	47	52.8	B
5890	Bn-N6-p11123038	Bn-A06-p11415584	Bn-A06-p11415584	A6	47	52.8	B
5891	Bn-N6-p11161601	Bn-A06-p11455914	Bn-A06-p11455914	A6	47	52.8	B
5892	Bn-N6-p11224509	Bn-A06-p11500765	Bn-A06-p11500765	A6	47	52.8	0
5893	Bn-N6-p11506638	Bn-A06-p13958812	Bn-A06-p13958812	A6	47	52.8	B
5894	Bn-N6-p11558487	Bn-C3-p43173157	Bn-scaff_17004_1-p57157	A6	47	52.8	B
5895	Bn-N6-p11573284	Bn-A06-p13872191	Bn-A06-p13872191	A6	47	52.8	B
5896	Bn-N6-p11588034	Bn-A06-p13859891	Bn-A06-p13859891	A6	47	52.8	B
5897	Bn-N6-p11750499	Bn-A06-p13732668	Bn-A06-p13732668	A6	47	52.8	B
5898	Bn-N6-p11975677	Bn-Scaffold000796-p	Bn-Scaffold000796-p1119	A6	47	52.8	B
5899	Bn-N6-p12075465	Bn-Scaffold000100-p	Bn-Scaffold000100-p62402	A6	47	52.8	B
5900	Bn-N6-p12296394	Bn-C3-p46480810	Bn-scaff_19186_1-p46645	A6	47	52.8	B
5901	Bn-N6-p12416764	Bn-Scaffold000100-p	Bn-Scaffold000100-p20920	A6	47	52.8	B
5902	Bn-N6-p12433138	Bn-Scaffold000100-p	Bn-Scaffold000100-p19348	A6	47	52.8	B
5903	Bn-N6-p12435496	Bn-Scaffold000100-p	Bn-Scaffold000100-p19121	A6	47	52.8	B
5904	Bn-N6-p12454313	Bn-Scaffold000100-p	Bn-Scaffold000100-p17147	A6	47	52.8	B
5905	Bn-N6-p12463399	Bn-Scaffold000100-p	Bn-Scaffold000100-p16189	A6	47	52.8	B
5906	Bn-N6-p12517626	Bn-Scaffold000100-p	Bn-Scaffold000100-p67842	A6	47	52.8	B
5907	Bn-N6-p12653871	Bn-A06-p13610210	Bn-A06-p13610210	A6	47	52.8	B
5908	Bn-N6-p13048689	Bn-A06-p13190352	Bn-A06-p13190352	A6	47	52.8	B
5909	Bn-N6-p13073203	Bn-A06-p13144922	Bn-A06-p13144922	A6	47	52.8	B
5910	Bn-N6-p13415001	Bn-A06-p12775802	Bn-A06-p12775802	A6	47	52.8	B
5911	Bn-N6-p13525814	Bn-A06-p12632207	Bn-A06-p12632207	A6	47	52.8	B
5912	Bn-N6-p13693924	Bn-A06-p12438791	Bn-A06-p12438791	A6	47	52.8	B
5913	Bn-N6-p13723822	Bn-A06-p12404848	Bn-A06-p12404848	A6	47	52.8	B
5914	Bn-N6-p14037351	Bn-A06-p12135104	Bn-A06-p12135104	A6	47	52.8	B
5915	Bn-N6-p14081442	Bn-A06-p12093192	Bn-A06-p12093192	A6	47	52.8	B
5916	Bn-N6-p14278810	Bn-A06-p11919396	Bn-A06-p11919396	A6	47	52.8	B
5917	Bn-N6-p14360023	Bn-A06-p11848767	Bn-A06-p11848767	A6	47	52.8	B
5918	Bn-N6-p14360023	Bn-A06-p11848770	Bn-A06-p11848770	A6	47	52.8	B
5919	Bn-N6-p14360239	Bn-A06-p11848553	Bn-A06-p11848553	A6	47	52.8	B
5920	Bn-N6-p14372265	Bn-A06-p11840908	Bn-A06-p11840908	A6	47	52.8	B
5921	Bn-N6-p14390646	Bn-A06-p11819954	Bn-A06-p11819954	A6	47	52.8	B
5922	Bn-N6-p14395105	Bn-A06-p11819912	Bn-A06-p11819912	A6	47	52.8	B
5923	Bn-N6-p14522882	Bn-A06-p11708819	Bn-A06-p11708819	A6	47	52.8	B
5924	Bn-N6-p14526584	Bn-A06-p11704401	Bn-A06-p11704401	A6	47	52.8	B

5925	Bn-N6-p14568050	Bn-A06-p18813274	Bn-A06-p18813274	A6	47	52.8	B
5926	Bn-N6-p14769905	Bn-A06-p19021973	Bn-A06-p19021973	A6	47	52.8	B
5927	Bn-N6-p14891444	Bn-A06-p19151996	Bn-A06-p19151996	A6	47	52.8	B
5928	Bn-N6-p14903370	Bn-A06-p19163919	Bn-A06-p19163919	A6	47	52.8	B
5929	Bn-N6-p14925742	Bn-A06-p19184868	Bn-A06-p19184868	A6	47	52.8	B
5930	Bn-N6-p14938686	Bn-A06-p19197269	Bn-A06-p19197269	A6	47	52.8	B
5931	Bn-N6-p15024130	Bn-A06-p19265815	Bn-A06-p19265815	A6	47	52.8	B
5932	Bn-N6-p15050570	Bn-A06-p19292104	Bn-A06-p19292104	A6	47	52.8	B
5933	Bn-N6-p15054624	Bn-A06-p19296157	Bn-A06-p19296157	A6	47	52.8	B
5934	Bn-N6-p15082549	Bn-A06-p19329736	Bn-A06-p19329736	A6	47	52.8	B
5935	Bn-N6-p15086022	Bn-A06-p19333055	Bn-A06-p19333055	A6	47	52.8	B
5936	Bn-N6-p15086316	Bn-A06-p19333349	Bn-A06-p19333349	A6	47	52.8	B
5937	Bn-N6-p15145815	Bn-A06-p19392651	Bn-A06-p19392651	A6	47	52.8	B
5938	Bn-N6-p15149169	Bn-A06-p19395565	Bn-A06-p19395565	A6	47	52.8	B
5939	Bn-N6-p15149536	Bn-A06-p19395890	Bn-A06-p19395890	A6	47	52.8	B
5940	Bn-N6-p15149673	Bn-A06-p19396032	Bn-A06-p19396032	A6	47	52.8	B
5941	Bn-N6-p15152878	Bn-A06-p19404012	Bn-A06-p19404012	A6	47	52.8	B
5942	Bn-N6-p15164738	Bn-A06-p19416292	Bn-A06-p19416292	A6	47	52.8	B
5943	Bn-N6-p15166878	Bn-A06-p19418423	Bn-A06-p19418423	A6	47	52.8	B
5944	Bn-N6-p15167668	Bn-A06-p19419215	Bn-A06-p19419215	A6	47	52.8	B
5945	Bn-N6-p15184014	Bn-A06-p19434905	Bn-A06-p19434905	A6	47	52.8	B
5946	Bn-N6-p15186773	Bn-A06-p19437625	Bn-A06-p19437625	A6	47	52.8	B
5947	Bn-N6-p15238013	Bn-A06-p19485623	Bn-A06-p19485623	A6	47	52.8	B
5948	Bn-N6-p15251694	Bn-A06-p19501594	Bn-A06-p19501594	A6	47	52.8	B
5949	Bn-N6-p15252402	Bn-A06-p19502324	Bn-A06-p19502324	A6	47	52.8	B
5950	Bn-N6-p15275787	Bn-A06-p19533298	Bn-A06-p19533298	A6	47	52.8	B
5951	Bn-N6-p15323968	Bn-A06-p19579765	Bn-A06-p19579765	A6	47	52.8	B
5952	Bn-N6-p15332394	Bn-A06-p19593266	Bn-A06-p19593266	A6	47	52.8	B
5953	Bn-N6-p15332652	Bn-C3-p2674942	Bn-scaff_27198_1-p44558?	A6	47	52.8	B
5954	Bn-N6-p15339060	Bn-A06-p19602022	Bn-A06-p19602022	A6	47	52.8	B
5955	Bn-N6-p15372329	Bn-A06-p19635380	Bn-A06-p19635380	A6	47	52.8	B
5956	Bn-N6-p15386700	Bn-A06-p19646183	Bn-A06-p19646183	A6	47	52.8	B
5957	Bn-N6-p15387647	Bn-A06-p19647126	Bn-A06-p19647126	A6	47	52.8	B
5958	Bn-N6-p15387853	Bn-A06-p19647330	Bn-A06-p19647330	A6	47	52.8	B
5959	Bn-N6-p15389072	Bn-A06-p19648433	Bn-A06-p19648433	A6	47	52.8	B
5960	Bn-N6-p15462382	Bn-A06-p14121814	Bn-A06-p14121814	A6	47	52.8	B
5961	Bn-N6-p15501716	Bn-A06-p14163715	Bn-A06-p14163715	A6	47	52.8	B
5962	Bn-N6-p15516387	Bn-A06-p14179943	Bn-A06-p14179943	A6	47	52.8	B
5963	Bn-N6-p15533259	Bn-A06-p14189236	Bn-A06-p14189236	A6	47	52.8	0
5964	Bn-N6-p15542913	Bn-A06-p14198240	Bn-A06-p14198240	A6	47	52.8	B
5965	Bn-N6-p15550976	Bn-A06-p14207547	Bn-A06-p14207547	A6	47	52.8	B
5966	Bn-N6-p15551621	Bn-A06-p14212186	Bn-A06-p14212186	A6	47	52.8	B
5967	Bn-N6-p15554070	Bn-A06-p14214930	Bn-A06-p14214930	A6	47	52.8	B
5968	Bn-N6-p15573174	Bn-A06-p14241294	Bn-A06-p14241294	A6	47	52.8	B
5969	Bn-N6-p15606489	Bn-A06-p14281185	Bn-A06-p14281185	A6	47	52.8	B
5970	Bn-N6-p15610450	Bn-A06-p14285203	Bn-A06-p14285203	A6	47	52.8	B
5971	Bn-N6-p15623085	Bn-A06-p14300543	Bn-A06-p14300543	A6	47	52.8	B
5972	Bn-N6-p15623184	Bn-A06-p14300642	Bn-A06-p14300642	A6	47	52.8	B
5973	Bn-N6-p15660129	Bn-A06-p14327501	Bn-A06-p14327501	A6	47	52.8	B

5974	Bn-N6-p15718977	Bn-A06-p14373935	Bn-A06-p14373935	A6	47	52.8	B
5975	Bn-N6-p15729660	Bn-A06-p14389679	Bn-A06-p14389679	A6	47	52.8	B
5976	Bn-N6-p15731885	Bn-A06-p14391905	Bn-A06-p14391905	A6	47	52.8	B
5977	Bn-N6-p15741968	Bn-A06-p14399524	Bn-A06-p14399524	A6	47	52.8	B
5978	Bn-N6-p15750171	Bn-A06-p14402043	Bn-A06-p14402043	A6	47	52.8	B
5979	Bn-N6-p15782151	Bn-C3-p36829294	Bn-scaff_19310_1-p24733[illegible]	A6	47	52.8	B
5980	Bn-N6-p15782391	Bn-A06-p14428186	Bn-A06-p14428186	A6	47	52.8	B
5981	Bn-N6-p15782498	Bn-A06-p14428293	Bn-A06-p14428293	A6	47	52.8	B
5982	Bn-N6-p15797519	Bn-A06-p14439178	Bn-A06-p14439178	A6	47	52.8	B
5983	Bn-N6-p15797741	Bn-A06-p14439400	Bn-A06-p14439400	A6	47	52.8	B
5984	Bn-N6-p15852302	Bn-A06-p14495889	Bn-A06-p14495889	A6	47	52.8	B
5985	Bn-N6-p15921749	Bn-A06-p14578177	Bn-A06-p14578177	A6	47	52.8	B
5986	Bn-N6-p15921875	Bn-A06-p14578303	Bn-A06-p14578303	A6	47	52.8	B
5987	Bn-N6-p15953299	Bn-Scaffold000141-p[illegible]	Bn-Scaffold000141-p19856	A6	47	52.8	B
5988	Bn-N6-p16068499	Bn-Scaffold000141-p[illegible]	Bn-Scaffold000141-p13250	A6	47	52.8	B
5989	Bn-N6-p16297067	Bn-Scaffold000141-p[illegible]	Bn-Scaffold000141-p39713	A6	47	52.8	B
5990	Bn-N6-p16372986	Bn-A01-p24057724	Bn-A01-p24057724	A6	47	52.8	B
5991	Bn-N6-p16384260	Bn-Scaffold013287-p[illegible]	Bn-Scaffold013287-p77	A6	47	52.8	B
5992	Bn-N6-p16384895	Bn-A01-p24070704	Bn-A01-p24070704	A6	47	52.8	B
5993	Bn-N6-p16565866	Bn-A06-p14616522	Bn-A06-p14616522	A6	47	52.8	B
5994	Bn-N6-p16607904	Bn-A06-p14664213	Bn-A06-p14664213	A6	47	52.8	B
5995	Bn-N6-p16746780	Bn-A06-p14813811	Bn-A06-p14813811	A6	47	52.8	B
5996	Bn-N6-p16749183	Bn-A06-p14816143	Bn-A06-p14816143	A6	47	52.8	B
5997	Bn-N6-p16757684	Bn-A06-p14829925	Bn-A06-p14829925	A6	47	52.8	B
5998	Bn-N6-p16760737	Bn-A06-p14834424	Bn-A06-p14834424	A6	47	52.8	B
5999	Bn-N6-p16762388	Bn-A06-p14836066	Bn-A06-p14836066	A6	47	52.8	B
6000	Bn-N6-p16762802	Bn-A06-p14836486	Bn-A06-p14836486	A6	47	52.8	B
6001	Bn-N6-p16776339	Bn-A06-p14848662	Bn-A06-p14848662	A6	47	52.8	B
6002	Bn-N6-p16780381	Bn-A06-p14852091	Bn-A06-p14852091	A6	47	52.8	B
6003	Bn-N6-p16780447	Bn-A06-p14852157	Bn-A06-p14852157	A6	47	52.8	B
6004	Bn-N6-p16959442	Bn-A06-p15037349	Bn-A06-p15037349	A6	47	52.8	B
6005	Bn-N6-p16980240	Bn-A06-p15059166	Bn-A06-p15059166	A6	47	52.8	B
6006	Bn-N6-p17044210	Bn-A06-p15101156	Bn-A06-p15101156	A6	47	52.8	B
6007	Bn-N6-p17142669	Bn-A06-p15210596	Bn-A06-p15210596	A6	47	52.8	B
6008	Bn-N6-p3986853	Bn-A06-p4040976	Bn-A06-p4040976	A6	47	52.8	B
6009	Bn-N6-p9805692	Bn-A06-p10124135	Bn-A06-p10124135	A6	47	52.8	B
6010	Bn-N6-p9866941	Bn-A06-p10182944	Bn-A06-p10182944	A6	47	52.8	B
6011	Bn-N6-p9870439	Bn-A06-p10200920	Bn-A06-p10200920	A6	47	52.8	B
6012	Bn-N6-p9871396	Bn-A06-p10202557	Bn-A06-p10202557	A6	47	52.8	B
6013	Bn-N6-p9884626	Bn-A06-p10210368	Bn-A06-p10210368	A6	47	52.8	B
6014	Bn-N6-p9919737	Bn-A06-p10238741	Bn-A06-p10238741	A6	47	52.8	B
6015	Bn-N6-p9919979	Bn-A06-p10238985	Bn-A06-p10238985	A6	47	52.8	B
6016	Bn-N6-p9921350	Bn-A06-p10240329	Bn-A06-p10240329	A6	47	52.8	B
6017	Bn-N6-p9961478	Bn-A06-p10297153	Bn-A06-p10297153	A6	47	52.8	B
6018	Bn-N6-p9971045	Bn-A06-p10300664	Bn-A06-p10300664	A6	47	52.8	B
6019	Bn-N6-p9981241	Bn-A06-p10308910	Bn-A06-p10308910	A6	47	52.8	B
6020	Bn-N6-p9997457	Bn-C3-p49746338	Bn-scaff_23407_1-p12533[illegible]	A6	47	52.8	B
6021	Bn-N6-p9998566	Bn-A06-p10327909	Bn-A06-p10327909	A6	47	52.8	B
6022	Bn-Scaffold00776-p6668	Bn-A06-p14232703	Bn-A06-p14232703	A6	47	52.8	B

6023	Bn-Scaffold00776-p6669	Bn-A06-p14232702	Bn-A06-p14232702	A6	47	52.8	B
6024	Bn-Scaffold01110-p24274	Bn-Scaffold000416-p5	Bn-Scaffold000416-p5550	A6	47	52.8	B
6025	Bn-Scaffold01415-p4730	Bn-Scaffold002005-p3	Bn-Scaffold002005-p385	A6	47	52.8	B
6026	Bn-Scaffold01415-p7340	Bn-Scaffold002229-p3	Bn-Scaffold002229-p373	A6	47	52.8	B
6027	Bn-Scaffold17123-p307	Bn-Scaffold001630-p4	Bn-Scaffold001630-p45	A6	47	52.8	B
6028	Bn-Scaffold26936-p472	Bn-Scaffold000873-p1	Bn-Scaffold000873-p1368	A6	47	52.8	B
6029	Bn-Scaffold48758-p176	Bn-A06-p11025660	Bn-A06-p11025660	A6	47	52.8	B
6030	Bn-N13-p35942732	Bn-A06-p16262957	Bn-A06-p16262957	A6	48	53.6	B
6031	Bn-N6-p17186112	Bn-A06-p15258692	Bn-A06-p15258692	A6	48	53.6	B
6032	Bn-N6-p17342922	Bn-A06-p15420581	Bn-A06-p15420581	A6	48	53.6	B
6033	Bn-N6-p17343361	Bn-A06-p15421020	Bn-A06-p15421020	A6	48	53.6	B
6034	Bn-N6-p17365262	Bn-A06-p15432171	Bn-A06-p15432171	A6	48	53.6	B
6035	Bn-N6-p17369634	Bn-A06-p15434445	Bn-A06-p15434445	A6	48	53.6	B
6036	Bn-N6-p17417175	Bn-A06-p15489475	Bn-A06-p15489475	A6	48	53.6	B
6037	Bn-N6-p17437593	Bn-A06-p15513288	Bn-A06-p15513288	A6	48	53.6	B
6038	Bn-N6-p17439650	Bn-A06-p15515345	Bn-A06-p15515345	A6	48	53.6	B
6039	Bn-N6-p17553824	Bn-A06-p15632434	Bn-A06-p15632434	A6	48	53.6	B
6040	Bn-N6-p17574012	Bn-A06-p15649917	Bn-A06-p15649917	A6	48	53.6	B
6041	Bn-N6-p17585697	Bn-A06-p15661345	Bn-A06-p15661345	A6	48	53.6	B
6042	Bn-N6-p17590888	Bn-A06-p15662331	Bn-A06-p15662331	A6	48	53.6	B
6043	Bn-N6-p17626600	Bn-A06-p15691701	Bn-A06-p15691701	A6	48	53.6	B
6044	Bn-N6-p17628829	Bn-A06-p15700525	Bn-A06-p15700525	A6	48	53.6	B
6045	Bn-N6-p17629388	Bn-A06-p15701094	Bn-A06-p15701094	A6	48	53.6	B
6046	Bn-N6-p17630548	Bn-A06-p15703535	Bn-A06-p15703535	A6	48	53.6	B
6047	Bn-N6-p17639162	Bn-A06-p15709579	Bn-A06-p15709579	A6	48	53.6	B
6048	Bn-N6-p17692316	Bn-A06-p15760165	Bn-A06-p15760165	A6	48	53.6	B
6049	Bn-N6-p17707009	Bn-A06-p15775625	Bn-A06-p15775625	A6	48	53.6	B
6050	Bn-N6-p17724325	Bn-A06-p15791244	Bn-A06-p15791244	A6	48	53.6	0
6051	Bn-N6-p17733531	Bn-A06-p15801373	Bn-A06-p15801373	A6	48	53.6	B
6052	Bn-N6-p17832537	Bn-A06-p15894910	Bn-A06-p15894910	A6	48	53.6	B
6053	Bn-N6-p17910735	Bn-A06-p15966650	Bn-A06-p15966650	A6	48	53.6	B
6054	Bn-N6-p17911611	Bn-A06-p15967529	Bn-A06-p15967529	A6	48	53.6	B
6055	Bn-N6-p17911764	Bn-A06-p15967682	Bn-A06-p15967682	A6	48	53.6	B
6056	Bn-N6-p17929879	Bn-A06-p15988623	Bn-A06-p15988623	A6	48	53.6	B
6057	Bn-N6-p17931561	Bn-A06-p15990313	Bn-A06-p15990313	A6	48	53.6	B
6058	Bn-N6-p17960475	Bn-A06-p16014870	Bn-A06-p16014870	A6	48	53.6	B
6059	Bn-N6-p17961288	Bn-A06-p16015727	Bn-A06-p16015727	A6	48	53.6	B
6060	Bn-N6-p17961356	Bn-A06-p16015795	Bn-A06-p16015795	A6	48	53.6	B
6061	Bn-N6-p17980477	Bn-A06-p16032028	Bn-A06-p16032028	A6	48	53.6	B
6062	Bn-N6-p18007169	Bn-A06-p16064239	Bn-A06-p16064239	A6	48	53.6	B
6063	Bn-N6-p18017612	Bn-A06-p16071214	Bn-A06-p16071214	A6	48	53.6	B
6064	Bn-N6-p18042370	Bn-A06-p16102726	Bn-A06-p16102726	A6	48	53.6	B
6065	Bn-N6-p18073029	Bn-A06-p16133328	Bn-A06-p16133328	A6	48	53.6	B
6066	Bn-N6-p18088613	Bn-A06-p16148736	Bn-A06-p16148736	A6	48	53.6	B
6067	Bn-N6-p18090242	Bn-A06-p16150365	Bn-A06-p16150365	A6	48	53.6	B
6068	Bn-N6-p18111085	Bn-A06-p16166965	Bn-A06-p16166965	A6	48	53.6	B
6069	Bn-N6-p18152112	Bn-A06-p16181242	Bn-A06-p16181242	A6	48	53.6	B
6070	Bn-N6-p18220641	Bn-A06-p16256906	Bn-A06-p16256906	A6	48	53.6	B
6071	Bn-N6-p18248307	Bn-A06-p16303805	Bn-A06-p16303805	A6	48	53.6	B

6072	Bn-N6-p18248362	Bn-A06-p16303860	Bn-A06-p16303860	A6	48	53.6	B
6073	Bn-N6-p18253330	Bn-A06-p16308544	Bn-A06-p16308544	A6	48	53.6	B
6074	Bn-N6-p18320935	Bn-A06-p16362555	Bn-A06-p16362555	A6	48	53.6	B
6075	Bn-N6-p18340438	Bn-A06-p16381299	Bn-A06-p16381299	A6	48	53.6	B
6076	Bn-N6-p18419519	Bn-A06-p16449642	Bn-A06-p16449642	A6	48	53.6	B
6077	Bn-N6-p18513041	Bn-A06-p16554297	Bn-A06-p16554297	A6	48	53.6	B
6078	Bn-N6-p18549378	Bn-A06-p16588120	Bn-A06-p16588120	A6	48	53.6	B
6079	Bn-N6-p18575447	Bn-A06-p16613538	Bn-A06-p16613538	A6	48	53.6	0
6080	Bn-N6-p18582061	Bn-A06-p16620858	Bn-A06-p16620858	A6	48	53.6	B
6081	Bn-N6-p18582187	Bn-A06-p16620984	Bn-A06-p16620984	A6	48	53.6	B
6082	Bn-N6-p18585322	Bn-A06-p16624574	Bn-A06-p16624574	A6	48	53.6	B
6083	Bn-N6-p18590300	Bn-A06-p16629547	Bn-A06-p16629547	A6	48	53.6	B
6084	Bn-N6-p18624752	Bn-A06-p16657894	Bn-A06-p16657894	A6	49	54.4	B
6085	Bn-N6-p18653034	Bn-A06-p16689717	Bn-A06-p16689717	A6	49	54.4	B
6086	Bn-N6-p18661738	Bn-A06-p16701773	Bn-A06-p16701773	A6	49	54.4	B
6087	Bn-N6-p18681628	Bn-A06-p16718752	Bn-A06-p16718752	A6	49	54.4	B
6088	Bn-N6-p18685741	Bn-A06-p16728885	Bn-A06-p16728885	A6	49	54.4	B
6089	Bn-N6-p18716297	Bn-A06-p16840167	Bn-A06-p16840167	A6	49	54.4	B
6090	Bn-N17-p37815441	Bn-C7-p37619722	Bn-scaff_15705_1-p14330!	A6	50	55.2	B
6091	Bn-N6-p18861927	Bn-A06-p16985788	Bn-A06-p16985788	A6	50	55.2	B
6092	Bn-N6-p18874537	Bn-A06-p16993923	Bn-A06-p16993923	A6	50	55.2	B
6093	Bn-N6-p18884318	Bn-A06-p17003685	Bn-A06-p17003685	A6	50	55.2	B
6094	Bn-N6-p18887432	Bn-A06-p17006799	Bn-A06-p17006799	A6	50	55.2	B
6095	Bn-N6-p18888408	Bn-A06-p17007775	Bn-A06-p17007775	A6	50	55.2	B
6096	Bn-N6-p18901434	Bn-A06-p17021956	Bn-A06-p17021956	A6	50	55.2	B
6097	Bn-N6-p18903462	Bn-A06-p17024179	Bn-A06-p17024179	A6	50	55.2	B
6098	Bn-N6-p18905561	Bn-A06-p17026294	Bn-A06-p17026294	A6	50	55.2	B
6099	Bn-N6-p18911850	Bn-A06-p17032583	Bn-A06-p17032583	A6	50	55.2	B
6100	Bn-N6-p18912491	Bn-A06-p17033313	Bn-A06-p17033313	A6	50	55.2	B
6101	Bn-N6-p18917241	Bn-A06-p17037912	Bn-A06-p17037912	A6	50	55.2	B
6102	Bn-N6-p18969189	Bn-A06-p17093738	Bn-A06-p17093738	A6	50	55.2	B
6103	Bn-N6-p18976375	Bn-A06-p17099451	Bn-A06-p17099451	A6	50	55.2	B
6104	Bn-N6-p18987149	Bn-A06-p16933880	Bn-A06-p16933880	A6	50	55.2	B
6105	Bn-N6-p19011860	Bn-A06-p17143376	Bn-A06-p17143376	A6	51	56	B
6106	Bn-N6-p19058882	Bn-A06-p17173552	Bn-A06-p17173552	A6	51	56	B
6107	Bn-N6-p19061389	Bn-A06-p17176086	Bn-A06-p17176086	A6	51	56	B
6108	Bn-N6-p19092598	Bn-A06-p17208027	Bn-A06-p17208027	A6	51	56	B
6109	Bn-N6-p19105195	Bn-A06-p17220153	Bn-A06-p17220153	A6	51	56	B
6110	Bn-N6-p19142761	Bn-A09-p18588652	Bn-A09-p18588652	A6	51	56	B
6111	Bn-N6-p19153944	Bn-A06-p17289947	Bn-A06-p17289947	A6	51	56	B
6112	Bn-N6-p19157485	Bn-A06-p17291868	Bn-A06-p17291868	A6	51	56	B
6113	Bn-N6-p19167067	Bn-A06-p17301456	Bn-A06-p17301456	A6	51	56	B
6114	Bn-N6-p19178905	Bn-A06-p17313345	Bn-A06-p17313345	A6	51	56	B
6115	Bn-N6-p19187566	Bn-A06-p17321987	Bn-A06-p17321987	A6	51	56	B
6116	Bn-N6-p19196368	Bn-A06-p17331074	Bn-A06-p17331074	A6	51	56	B
6117	Bn-N6-p19203092	Bn-A06-p17337842	Bn-A06-p17337842	A6	51	56	B
6118	Bn-N6-p19241908	Bn-A06-p17375407	Bn-A06-p17375407	A6	51	56	B
6119	Bn-N6-p19242383	Bn-A06-p17375882	Bn-A06-p17375882	A6	51	56	B
6120	Bn-Scaffold08133-p758	Bn-A06-p17175388	Bn-A06-p17175388	A6	51	56	B

6121	Bn-N6-p19419365	Bn-A06-p17548258	Bn-A06-p17548258	A6	52	56.8	B
6122	Bn-N6-p19419678	Bn-C7-p37027858	Bn-scaff_15705_1-p841235	A6	52	56.8	B
6123	Bn-N6-p19420003	Bn-A06-p17548894	Bn-A06-p17548894	A6	52	56.8	B
6124	Bn-N6-p19420456	Bn-A06-p17549347	Bn-A06-p17549347	A6	52	56.8	B
6125	Bn-N6-p19420713	Bn-A06-p17549603	Bn-A06-p17549603	A6	52	56.8	B
6126	Bn-N6-p19470789	Bn-C7-p36966851	Bn-scaff_15705_1-p780228	A6	52	56.8	B
6127	Bn-N17-p36730215	Bn-C7-p36515796	Bn-scaff_15705_1-p329173	A6	53	57.6	B
6128	Bn-N17-p36776915	Bn-C7-p36560811	Bn-scaff_15705_1-p374188	A6	53	57.6	B
6129	Bn-N6-p19553577	Bn-A06-p17659127	Bn-A06-p17659127	A6	53	57.6	B
6130	Bn-N6-p19565919	Bn-A06-p17671185	Bn-A06-p17671185	A6	53	57.6	B
6131	Bn-N6-p19641317	Bn-A06-p17741868	Bn-A06-p17741868	A6	53	57.6	B
6132	Bn-N6-p19677313	Bn-A06-p17778079	Bn-A06-p17778079	A6	53	57.6	B
6133	Bn-N6-p19677443	Bn-A06-p17778210	Bn-A06-p17778210	A6	53	57.6	B
6134	Bn-N6-p19684269	Bn-A06-p17785331	Bn-A06-p17785331	A6	53	57.6	B
6135	Bn-N6-p19689270	Bn-A06-p17790290	Bn-A06-p17790290	A6	53	57.6	B
6136	Bn-N6-p19690501	Bn-A06-p17791496	Bn-A06-p17791496	A6	53	57.6	B
6137	Bn-N6-p19690751	Bn-A06-p17791746	Bn-A06-p17791746	A6	53	57.6	B
6138	Bn-N6-p19706005	Bn-C7-p36682638	Bn-scaff_15705_1-p496015	A6	53	57.6	B
6139	Bn-N6-p19706174	Bn-A06-p17823115	Bn-A06-p17823115	A6	53	57.6	B
6140	Bn-N6-p19760321	Bn-A06-p17887060	Bn-A06-p17887060	A6	53	57.6	B
6141	Bn-N6-p19762621	Bn-A06-p17889311	Bn-A06-p17889311	A6	53	57.6	B
6142	Bn-N6-p19763799	Bn-C7-p36618244	Bn-scaff_15705_1-p431621	A6	53	57.6	0
6143	Bn-N6-p19783532	Bn-A06-p17908957	Bn-A06-p17908957	A6	53	57.6	B
6144	Bn-N6-p19795122	Bn-A06-p17920968	Bn-A06-p17920968	A6	53	57.6	B
6145	Bn-N6-p19805639	Bn-A06-p17932959	Bn-A06-p17932959	A6	53	57.6	B
6146	Bn-N6-p19809927	Bn-A06-p17938497	Bn-A06-p17938497	A6	53	57.6	B
6147	Bn-N6-p19816609	Bn-A06-p17939632	Bn-A06-p17939632	A6	53	57.6	B
6148	Bn-N6-p19850690	Bn-A06-p17967505	Bn-A06-p17967505	A6	53	57.6	B
6149	Bn-N6-p19901068	Bn-A06-p18009980	Bn-A06-p18009980	A6	54	58.4	B
6150	Bn-N6-p19920755	Bn-A06-p18022188	Bn-A06-p18022188	A6	54	58.4	B
6151	Bn-N6-p19927994	Bn-A06-p18029021	Bn-A06-p18029021	A6	54	58.4	B
6152	Bn-N6-p19928590	Bn-A06-p18029621	Bn-A06-p18029621	A6	54	58.4	B
6153	Bn-N6-p19929931	Bn-A06-p18033006	Bn-A06-p18033006	A6	54	58.4	B
6154	Bn-N6-p19937370	Bn-A06-p18040423	Bn-A06-p18040423	A6	54	58.4	B
6155	Bn-N6-p19951816	Bn-A06-p18059678	Bn-A06-p18059678	A6	54	58.4	B
6156	Bn-N6-p20013669	Bn-A06-p18102164	Bn-A06-p18102164	A6	54	58.4	B
6157	Bn-N6-p20025477	Bn-A06-p18113924	Bn-A06-p18113924	A6	54	58.4	B
6158	Bn-N6-p20027175	Bn-A06-p18115817	Bn-A06-p18115817	A6	54	58.4	B
6159	Bn-N6-p20047298	Bn-A06-p18129979	Bn-A06-p18129979	A6	54	58.4	B
6160	Bn-N6-p20052081	Bn-A06-p18131525	Bn-A06-p18131525	A6	54	58.4	B
6161	Bn-N6-p20058827	Bn-A06-p18136235	Bn-A06-p18136235	A6	54	58.4	B
6162	Bn-N6-p20063792	Bn-A06-p18141167	Bn-A06-p18141167	A6	54	58.4	B
6163	Bn-N6-p20066752	Bn-A06-p18144127	Bn-A06-p18144127	A6	54	58.4	B
6164	Bn-N6-p20075480	Bn-A06-p18152497	Bn-A06-p18152497	A6	54	58.4	B
6165	Bn-N6-p20136498	Bn-A06-p18219362	Bn-A06-p18219362	A6	55	59.2	B
6166	Bn-N6-p20137469	Bn-A06-p18220327	Bn-A06-p18220327	A6	55	59.2	B
6167	Bn-N6-p20156394	Bn-A06-p18235310	Bn-A06-p18235310	A6	55	59.2	B
6168	Bn-N6-p20158797	Bn-A06-p18237512	Bn-A06-p18237512	A6	55	59.2	B
6169	Bn-N6-p20165039	Bn-A06-p18247490	Bn-A06-p18247490	A6	55	59.2	B

6170	Bn-N6-p20166468	Bn-A06-p18248917	Bn-A06-p18248917	A6	55	59.2	B
6171	Bn-N6-p20166605	Bn-A06-p18249054	Bn-A06-p18249054	A6	55	59.2	B
6172	Bn-N6-p20170308	Bn-A06-p18252711	Bn-A06-p18252711	A6	55	59.2	B
6173	Bn-N6-p20172474	Bn-A06-p18254877	Bn-A06-p18254877	A6	55	59.2	B
6174	Bn-N6-p20193140	Bn-A06-p18277724	Bn-A06-p18277724	A6	55	59.2	B
6175	Bn-N6-p20193287	Bn-A06-p18277802	Bn-A06-p18277802	A6	55	59.2	B
6176	Bn-N6-p20277325	Bn-A06-p18364834	Bn-A06-p18364834	A6	56	60	B
6177	Bn-N6-p20281720	Bn-A06-p18369013	Bn-A06-p18369013	A6	56	60	B
6178	Bn-N6-p20293170	Bn-A06-p18380196	Bn-A06-p18380196	A6	56	60	B
6179	Bn-N6-p20301015	Bn-A06-p18388041	Bn-A06-p18388041	A6	56	60	B
6180	Bn-N6-p20301809	Bn-A06-p18388778	Bn-A06-p18388778	A6	56	60	B
6181	Bn-N6-p20303918	Bn-A06-p18390889	Bn-A06-p18390889	A6	56	60	B
6182	Bn-N6-p20305492	Bn-A06-p18392463	Bn-A06-p18392463	A6	56	60	B
6183	Bn-N6-p20310652	Bn-A06-p18397592	Bn-A06-p18397592	A6	56	60	B
6184	Bn-N6-p20315760	Bn-A06-p18402713	Bn-A06-p18402713	A6	57	61.6	B
6185	Bn-N6-p20355116	Bn-A06-p18445250	Bn-A06-p18445250	A6	57	61.6	0
6186	Bn-N6-p20389510	Bn-C7-p35880911	Bn-scaff_17291_1-p305613	A6	57	61.6	B
6187	Bn-N17-p35812600	Bn-A06-p18614110	Bn-A06-p18614110	A6	58	62.4	B
6188	Bn-N6-p20405390	Bn-A06-p18500596	Bn-A06-p18500596	A6	58	62.4	B
6189	Bn-N6-p20407149	Bn-A06-p18502399	Bn-A06-p18502399	A6	58	62.4	B
6190	Bn-N6-p20476134	Bn-A06-p18573338	Bn-A06-p18573338	A6	58	62.4	B
6191	Bn-N6-p20504508	Bn-A06-p18600460	Bn-A06-p18600460	A6	58	62.4	B
6192	Bn-N6-p20533644	Bn-A06-p18624741	Bn-A06-p18624741	A6	58	62.4	B
6193	Bn-N6-p20543306	Bn-A06-p18634396	Bn-A06-p18634396	A6	59	63.2	B
6194	Bn-N6-p20550993	Bn-A06-p18642085	Bn-A06-p18642085	A6	59	63.2	B
6195	Bn-N6-p20608453	Bn-A06-p18709596	Bn-A06-p18709596	A6	59	63.2	B
6196	Bn-N6-p20616335	Bn-A06-p18717323	Bn-A06-p18717323	A6	59	63.2	B
6197	Bn-N6-p20617678	Bn-A06-p18718829	Bn-A06-p18718829	A6	59	63.2	B
6198	Bn-N6-p20630481	Bn-A06-p18729660	Bn-A06-p18729660	A6	59	63.2	B
6199	Bn-N6-p20651519	Bn-A06-p18755859	Bn-A06-p18755859	A6	59	63.2	B
6200	Bn-N6-p20652953	Bn-A06-p18757339	Bn-A06-p18757339	A6	59	63.2	B
6201	Bn-N6-p20720082	Bn-A06-p21550710	Bn-A06-p21550710	A6	59	63.2	B
6202	Bn-N6-p20736799	Bn-A06-p21525576	Bn-A06-p21525576	A6	59	63.2	B
6203	Bn-N6-p20752935	Bn-A06-p21509934	Bn-A06-p21509934	A6	59	63.2	B
6204	Bn-N6-p20763033	Bn-A06-p21501350	Bn-A06-p21501350	A6	59	63.2	B
6205	Bn-N6-p20769890	Bn-A06-p21490506	Bn-A06-p21490506	A6	59	63.2	B
6206	Bn-Scaffold23928-p505	Bn-A06-p21426650	Bn-A06-p21426650	A6	59	63.2	B
6207	Bn-N17-p35259821	Bn-C7-p34577497	Bn-scaff_18520_1-p621993	A6	60	64	B
6208	Bn-N6-p20905511	Bn-A06-p21349507	Bn-A06-p21349507	A6	60	64	B
6209	Bn-N6-p20978729	Bn-A06-p21281098	Bn-A06-p21281098	A6	60	64	B
6210	Bn-N6-p20978885	Bn-A06-p21280942	Bn-A06-p21280942	A6	60	64	B
6211	Bn-N6-p20987659	Bn-A06-p21273211	Bn-A06-p21273211	A6	60	64	B
6212	Bn-N6-p20990812	Bn-A06-p21270132	Bn-A06-p21270132	A6	60	64	B
6213	Bn-N6-p20991114	Bn-A06-p21269830	Bn-A06-p21269830	A6	60	64	B
6214	Bn-N6-p20991977	Bn-A06-p21268967	Bn-A06-p21268967	A6	60	64	B
6215	Bn-N6-p21008140	Bn-A06-p21252883	Bn-A06-p21252883	A6	60	64	B
6216	Bn-N6-p21039536	Bn-A06-p21221869	Bn-A06-p21221869	A6	61	64.8	B
6217	Bn-N6-p21067025	Bn-A06-p21196739	Bn-A06-p21196739	A6	61	64.8	B
6218	Bn-N6-p21068646	Bn-A06-p21195115	Bn-A06-p21195115	A6	61	64.8	B

6219	Bn-N6-p21080416	Bn-A06-p21187999	Bn-A06-p21187999	A6	61	64.8	B
6220	Bn-N6-p21085267	Bn-A06-p21183207	Bn-A06-p21183207	A6	61	64.8	B
6221	Bn-N6-p21104957	Bn-A06-p21163993	Bn-A06-p21163993	A6	61	64.8	B
6222	Bn-Scaffold74746-p122	Bn-A06-p21163037	Bn-A06-p21163037	A6	62	65.6	B
6223	Bn-N6-p21106756	Bn-C7-p34444146	Bn-scaff_18520_1-p48864	A6	63	66.4	B
6224	Bn-N6-p21128461	Bn-A06-p21134315	Bn-A06-p21134315	A6	64	67.2	B
6225	Bn-N6-p21136444	Bn-A06-p21127138	Bn-A06-p21127138	A6	64	67.2	B
6226	Bn-N6-p21130007	Bn-A06-p21132765	Bn-A06-p21132765	A6	65	68	B
6227	Bn-N6-p21161410	Bn-A06-p21101957	Bn-A06-p21101957	A6	65	68	B
6228	Bn-N6-p21164670	Bn-A06-p21098677	Bn-A06-p21098677	A6	65	68	B
6229	Bn-N6-p21171605	Bn-A06-p21086685	Bn-A06-p21086685	A6	65	68	B
6230	Bn-N6-p21175337	Bn-A06-p21085532	Bn-A06-p21085532	A6	65	68	B
6231	Bn-N6-p21176627	Bn-A06-p21083748	Bn-A06-p21083748	A6	65	68	B
6232	Bn-N6-p21177126	Bn-A06-p21083249	Bn-A06-p21083249	A6	65	68	B
6233	Bn-N6-p21179482	Bn-A06-p21080915	Bn-A06-p21080915	A6	65	68	B
6234	Bn-N6-p21179847	Bn-A06-p21080778	Bn-A06-p21080778	A6	65	68	B
6235	Bn-N17-p34995753	Bn-A06-p21060490	Bn-A06-p21060490	A6	66	68.8	0
6236	Bn-N17-p35005435	Bn-A06-p21064341	Bn-A06-p21064341	A6	66	68.8	B
6237	Bn-N17-p35005654	Bn-C7-p34317893	Bn-scaff_18520_1-p36238	A6	66	68.8	B
6238	Bn-N17-p35005681	Bn-C7-p34317920	Bn-scaff_18520_1-p36241	A6	66	68.8	B
6239	Bn-N6-p21187578	Bn-A06-p21072392	Bn-A06-p21072392	A6	66	68.8	B
6240	Bn-N6-p21190723	Bn-A06-p21069394	Bn-A06-p21069394	A6	66	68.8	B
6241	Bn-N6-p21195828	Bn-A06-p21064290	Bn-A06-p21064290	A6	66	68.8	B
6242	Bn-N6-p21195950	Bn-A06-p21064168	Bn-A06-p21064168	A6	66	68.8	B
6243	Bn-N6-p21195989	Bn-A06-p21064129	Bn-A06-p21064129	A6	66	68.8	B
6244	Bn-N6-p21197627	Bn-A06-p21062492	Bn-A06-p21062492	A6	66	68.8	B
6245	Bn-N6-p21197851	Bn-A06-p21062265	Bn-A06-p21062265	A6	66	68.8	B
6246	Bn-N6-p21214921	Bn-A06-p21045315	Bn-A06-p21045315	A6	66	68.8	B
6247	Bn-N6-p21218604	Bn-A06-p21041632	Bn-A06-p21041632	A6	66	68.8	B
6248	Bn-N6-p21231863	Bn-A06-p21591920	Bn-A06-p21591920	A6	66	68.8	B
6249	Bn-N6-p21237113	Bn-A06-p21597170	Bn-A06-p21597170	A6	66	68.8	B
6250	Bn-N6-p21240837	Bn-A06-p21600894	Bn-A06-p21600894	A6	66	68.8	B
6251	Bn-N6-p21242324	Bn-A06-p21602381	Bn-A06-p21602381	A6	66	68.8	B
6252	Bn-N6-p21292462	Bn-A06-p21650617	Bn-A06-p21650617	A6	66	68.8	B
6253	Bn-N6-p21305280	Bn-A06-p21663690	Bn-A06-p21663690	A6	66	68.8	B
6254	Bn-N6-p21308708	Bn-A06-p21677028	Bn-A06-p21677028	A6	66	68.8	B
6255	Bn-N6-p21432182	Bn-A06-p21796008	Bn-A06-p21796008	A6	67	70.4	B
6256	Bn-N6-p21438164	Bn-A06-p21800812	Bn-A06-p21800812	A6	68	71.2	B
6257	Bn-N6-p21438279	Bn-A06-p21800927	Bn-A06-p21800927	A6	68	71.2	B
6258	Bn-N6-p21438990	Bn-A06-p21801638	Bn-A06-p21801638	A6	68	71.2	B
6259	Bn-N6-p21441800	Bn-A06-p21804318	Bn-A06-p21804318	A6	68	71.2	B
6260	Bn-N6-p21463254	Bn-A06-p21825957	Bn-A06-p21825957	A6	69	72	B
6261	Bn-N6-p21463844	Bn-A06-p21826609	Bn-A06-p21826609	A6	69	72	B
6262	Bn-N6-p21463906	Bn-A06-p21826671	Bn-A06-p21826671	A6	69	72	B
6263	Bn-N6-p21475716	Bn-A06-p21826830	Bn-A06-p21826830	A6	69	72	B
6264	Bn-N6-p21475865	Bn-A06-p21826979	Bn-A06-p21826979	A6	69	72	B
6265	Bn-N6-p21476245	Bn-A06-p21827359	Bn-A06-p21827359	A6	69	72	B
6266	Bn-N6-p21482563	Bn-A06-p21829647	Bn-A06-p21829647	A6	69	72	B
6267	Bn-N17-p34303483	Bn-A06-p21986550	Bn-A06-p21986550	A6	70	73.6	B

6268	Bn-N6-p21499365	Bn-A06-p21846491	Bn-A06-p21846491	A6	70	73.6	B
6269	Bn-N6-p21501518	Bn-A06-p21848747	Bn-A06-p21848747	A6	70	73.6	B
6270	Bn-N6-p21514334	Bn-C7-p33879552	Bn-scaff_21711_1-p75853	A6	70	73.6	B
6271	Bn-N6-p21555491	Bn-A06-p21896359	Bn-A06-p21896359	A6	70	73.6	B
6272	Bn-N6-p21576432	Bn-A06-p21917612	Bn-A06-p21917612	A6	70	73.6	B
6273	Bn-N6-p21584956	Bn-A06-p21926982	Bn-A06-p21926982	A6	70	73.6	B
6274	Bn-N6-p21589077	Bn-A06-p21930942	Bn-A06-p21930942	A6	70	73.6	B
6275	Bn-N6-p21591919	Bn-A06-p21933776	Bn-A06-p21933776	A6	70	73.6	B
6276	Bn-N6-p21591951	Bn-A06-p21933808	Bn-A06-p21933808	A6	70	73.6	0
6277	Bn-N6-p21592013	Bn-A06-p21933870	Bn-A06-p21933870	A6	70	73.6	B
6278	Bn-N6-p21652065	Bn-A06-p21983885	Bn-A06-p21983885	A6	70	73.6	B
6279	Bn-N6-p21658465	Bn-A06-p21992536	Bn-A06-p21992536	A6	70	73.6	B
6280	Bn-N6-p21663745	Bn-A06-p22001193	Bn-A06-p22001193	A6	70	73.6	B
6281	Bn-N6-p21770289	Bn-A06-p22105035	Bn-A06-p22105035	A6	70	73.6	B
6282	Bn-N6-p21788219	Bn-A06-p22121666	Bn-A06-p22121666	A6	70	73.6	B
6283	Bn-Scaffold04231-p1614	Bn-A06-p21884761	Bn-A06-p21884761	A6	70	73.6	B
6284	Bn-N17-p33716246	Bn-C7-p34820496	Bn-scaff_17972_1-p61014(	A6	71	74.4	B
6285	Bn-N6-p21827996	Bn-A06-p22160452	Bn-A06-p22160452	A6	71	74.4	B
6286	Bn-N6-p21880558	Bn-A06-p22209347	Bn-A06-p22209347	A6	71	74.4	B
6287	Bn-N6-p21903315	Bn-A06-p22231737	Bn-A06-p22231737	A6	71	74.4	B
6288	Bn-N6-p21937803	Bn-A06-p22262511	Bn-A06-p22262511	A6	71	74.4	B
6289	Bn-N6-p21943420	Bn-A06-p22268128	Bn-A06-p22268128	A6	71	74.4	B
6290	Bn-N6-p21946340	Bn-A06-p22271048	Bn-A06-p22271048	A6	71	74.4	B
6291	Bn-N6-p21999139	Bn-A06-p22324968	Bn-A06-p22324968	A6	71	74.4	B
6292	Bn-N6-p22000228	Bn-A06-p22326059	Bn-A06-p22326059	A6	71	74.4	B
6293	Bn-N6-p22075229	Bn-A06-p22383556	Bn-A06-p22383556	A6	71	74.4	B
6294	Bn-N6-p22075395	Bn-A06-p22383722	Bn-A06-p22383722	A6	71	74.4	B
6295	Bn-N6-p22430716	Bn-A06-p22709872	Bn-A06-p22709872	A6	72	76	B
6296	Bn-N6-p22667869	Bn-A06-p22947499	Bn-A06-p22947499	A6	73	76.8	B
6297	Bn-N6-p22770954	Bn-A06-p23043135	Bn-A06-p23043135	A6	73	76.8	B
6298	Bn-N6-p22951881	Bn-A06-p23216260	Bn-A06-p23216260	A6	74	78.4	B
6299	Bn-N6-p22954128	Bn-A06-p23218510	Bn-A06-p23218510	A6	74	78.4	B
6300	Bn-N6-p22954907	Bn-A06-p23219303	Bn-A06-p23219303	A6	74	78.4	B
6301	Bn-N6-p22954942	Bn-A06-p23219336	Bn-A06-p23219336	A6	74	78.4	B
6302	Bn-N6-p22955026	Bn-A06-p23219420	Bn-A06-p23219420	A6	74	78.4	B
6303	Bn-N6-p22958037	Bn-A06-p23226757	Bn-A06-p23226757	A6	74	78.4	B
6304	Bn-N6-p22958264	Bn-A06-p23226981	Bn-A06-p23226981	A6	74	78.4	B
6305	Bn-N6-p22958374	Bn-A06-p23227091	Bn-A06-p23227091	A6	74	78.4	B
6306	Bn-N6-p22961568	Bn-A06-p23229650	Bn-A06-p23229650	A6	74	78.4	B
6307	Bn-N6-p22988707	Bn-A06-p23250542	Bn-A06-p23250542	A6	74	78.4	B
6308	Bn-N6-p22998164	Bn-A06-p23259546	Bn-A06-p23259546	A6	74	78.4	B
6309	Bn-N6-p23025174	Bn-A06-p23295405	Bn-A06-p23295405	A6	74	78.4	B
6310	Bn-N6-p23125100	Bn-A06-p23347767	Bn-A06-p23347767	A6	74	78.4	B
6311	Bn-N6-p23129741	Bn-A06-p23350246	Bn-A06-p23350246	A6	74	78.4	B
6312	Bn-N6-p23270696	Bn-A06-p23468803	Bn-A06-p23468803	A6	74	78.4	B
6313	Bn-N6-p23406052	Bn-A06-p23591616	Bn-A06-p23591616	A6	75	79.2	B
6314	Bn-N6-p23426032	Bn-A06-p23612730	Bn-A06-p23612730	A6	75	79.2	B
6315	Bn-N6-p23435244	Bn-A06-p23621651	Bn-A06-p23621651	A6	75	79.2	B
6316	Bn-N6-p23440104	Bn-A06-p23625426	Bn-A06-p23625426	A6	75	79.2	B

6317	Bn-N6-p23472400	Bn-A06-p23665384	Bn-A06-p23665384	A6	76	80.8	B
6318	Bn-N6-p23472580	Bn-A06-p23665564	Bn-A06-p23665564	A6	76	80.8	B
6319	Bn-N6-p23473395	Bn-A06-p23666378	Bn-A06-p23666378	A6	76	80.8	B
6320	Bn-N6-p23512722	Bn-A06-p23699420	Bn-A06-p23699420	A6	76	80.8	B
6321	Bn-N6-p23523789	Bn-A06-p23710466	Bn-A06-p23710466	A6	76	80.8	B
6322	Bn-N6-p23556658	Bn-A06-p23741737	Bn-A06-p23741737	A6	76	80.8	B
6323	Bn-N6-p23560008	Bn-A06-p23748804	Bn-A06-p23748804	A6	76	80.8	B
6324	Bn-N6-p23561852	Bn-A06-p23750653	Bn-A06-p23750653	A6	76	80.8	B
6325	Bn-N6-p23571158	Bn-A06-p23759352	Bn-A06-p23759352	A6	76	80.8	B
6326	Bn-N6-p23591715	Bn-A06-p23776151	Bn-A06-p23776151	A6	76	80.8	B
6327	Bn-N6-p23605693	Bn-A06-p23795599	Bn-A06-p23795599	A6	76	80.8	B
6328	Bn-N6-p23605857	Bn-A06-p23795763	Bn-A06-p23795763	A6	76	80.8	B
6329	Bn-N6-p23606115	Bn-A06-p23796020	Bn-A06-p23796020	A6	76	80.8	B
6330	Bn-N6-p23666104	Bn-A06-p23852270	Bn-A06-p23852270	A6	77	81.6	B
6331	Bn-N6-p23801589	Bn-A06-p23988702	Bn-A06-p23988702	A6	78	83.2	B
6332	Bn-N6-p23818982	Bn-A06-p24005581	Bn-A06-p24005581	A6	78	83.2	B
6333	Bn-N6-p23840741	Bn-A06-p24032427	Bn-A06-p24032427	A6	78	83.2	B
6334	Bn-N6-p23879361	Bn-A06-p24070714	Bn-A06-p24070714	A6	78	83.2	B
6335	Bn-N6-p23883968	Bn-A06-p24075375	Bn-A06-p24075375	A6	78	83.2	B
6336	Bn-N6-p23885034	Bn-A06-p24076443	Bn-A06-p24076443	A6	78	83.2	B
6337	Bn-N17-p29727468	Bn-A06-p24286025	Bn-A06-p24286025	A6	79	84	B
6338	Bn-N6-p23909011	Bn-A06-p24099288	Bn-A06-p24099288	A6	79	84	B
6339	Bn-N6-p23944011	Bn-A06-p24132842	Bn-A06-p24132842	A6	79	84	B
6340	Bn-N6-p23944125	Bn-A06-p24132956	Bn-A06-p24132956	A6	79	84	B
6341	Bn-N6-p23973942	Bn-A06-p24156940	Bn-A06-p24156940	A6	79	84	B
6342	Bn-N6-p24023698	Bn-A06-p24204030	Bn-A06-p24204030	A6	79	84	B
6343	Bn-N6-p24026224	Bn-A06-p24206556	Bn-A06-p24206556	A6	79	84	B
6344	Bn-N6-p24040134	Bn-A06-p24220121	Bn-A06-p24220121	A6	79	84	B
6345	Bn-N6-p24056480	Bn-A06-p24236385	Bn-A06-p24236385	A6	79	84	B
6346	Bn-N6-p24058038	Bn-A06-p24237899	Bn-A06-p24237899	A6	79	84	B
6347	Bn-N6-p24078028	Bn-A06-p24257262	Bn-A06-p24257262	A6	79	84	B
6348	Bn-N6-p24104837	Bn-A06-p24284014	Bn-A06-p24284014	A6	79	84	B
6349	Bn-N6-p24159659	Bn-A06-p24327317	Bn-A06-p24327317	A6	79	84	B
6350	Bn-N6-p24166589	Bn-A06-p24334015	Bn-A06-p24334015	A6	79	84	B
6351	Bn-N6-p24173273	Bn-A06-p24340685	Bn-A06-p24340685	A6	79	84	B
6352	Bn-Scaffold05907-p297	Bn-C7-p29229779	Bn-scaff_15754_1-p496098	A6	79	84	B
6353	Bn-N6-p24209356	Bn-A06-p24370009	Bn-A06-p24370009	A6	80	84.8	B
6354	Bn-N6-p24224670	Bn-A06-p24386467	Bn-A06-p24386467	A6	80	84.8	B
6355	Bn-N17-p29388756	Bn-A06-p24453423	Bn-A06-p24453423	A6	81	85.6	B
6356	Bn-N6-p24239358	Bn-C7-p29028654	Bn-scaff_15754_1-p29497	A6	81	85.6	B
6357	Bn-N6-p24240784	Bn-A06-p24404374	Bn-A06-p24404374	A6	81	85.6	B
6358	Bn-N6-p24241404	Bn-A06-p24404996	Bn-A06-p24404996	A6	81	85.6	B
6359	Bn-N6-p24241473	Bn-A06-p24405065	Bn-A06-p24405065	A6	81	85.6	B
6360	Bn-N6-p24246447	Bn-A06-p24410007	Bn-A06-p24410007	A6	81	85.6	B
6361	Bn-N6-p24276952	Bn-A06-p24441882	Bn-A06-p24441882	A6	81	85.6	B
6362	Bn-N6-p24307735	Bn-A06-p24465494	Bn-A06-p24465494	A6	81	85.6	0
6363	Bn-N6-p24308377	Bn-A06-p24466136	Bn-A06-p24466136	A6	81	85.6	B
6364	Bn-N6-p24309893	Bn-A06-p24467652	Bn-A06-p24467652	A6	81	85.6	B
6365	Bn-N6-p24341316	Bn-A06-p24496051	Bn-A06-p24496051	A6	82	87.2	B

6366	Bn-N6-p24345526	Bn-A06-p24501315	Bn-A06-p24501315	A6	82	87.2	B
6367	Bn-N6-p24347205	Bn-A06-p24503033	Bn-A06-p24503033	A6	83	88	B
6368	Bn-N6-p24353486	Bn-A06-p24508464	Bn-A06-p24508464	A6	83	88	B
6369	Bn-N6-p24393610	Bn-A06-p24544753	Bn-A06-p24544753	A6	83	88	B
6370	Bn-N6-p24396660	Bn-A06-p24551529	Bn-A06-p24551529	A6	83	88	B
6371	Bn-N6-p24412941	Bn-A06-p24567939	Bn-A06-p24567939	A6	83	88	B
6372	Bn-N6-p24415191	Bn-A06-p24570179	Bn-A06-p24570179	A6	83	88	B
6373	Bn-N6-p24445738	Bn-A06-p24600019	Bn-A06-p24600019	A6	83	88	B
6374	Bn-N6-p24445859	Bn-A06-p24600140	Bn-A06-p24600140	A6	83	88	B
6375	Bn-N6-p24448438	Bn-A06-p24602719	Bn-A06-p24602719	A6	83	88	B
6376	Bn-N17-p27245347	Bn-A06-p24836495	Bn-A06-p24836495	A6	84	90.4	B
6377	Bn-N17-p27277424	Bn-C7-p27801000	Bn-scaff_15715_1-p20294(	A6	84	90.4	B
6378	Bn-N6-p24553714	Bn-C7-p28265764	Bn-scaff_19724_1-p467818	A6	84	90.4	B
6379	Bn-N6-p24557399	Bn-A06-p24705718	Bn-A06-p24705718	A6	84	90.4	B
6380	Bn-N6-p24567917	Bn-A06-p24713872	Bn-A06-p24713872	A6	84	90.4	B
6381	Bn-N6-p24571783	Bn-A06-p24717767	Bn-A06-p24717767	A6	84	90.4	B
6382	Bn-N6-p24580308	Bn-A06-p24726203	Bn-A06-p24726203	A6	84	90.4	B
6383	Bn-N6-p24601888	Bn-A06-p24746974	Bn-A06-p24746974	A6	84	90.4	B
6384	Bn-N6-p24679001	Bn-A06-p24825884	Bn-A06-p24825884	A6	84	90.4	B
6385	Bn-N6-p24680168	Bn-A06-p24827051	Bn-A06-p24827051	A6	84	90.4	B
6386	Bn-N6-p24680900	Bn-A06-p24827783	Bn-A06-p24827783	A6	84	90.4	B
6387	Bn-N6-p24703073	Bn-A06-p24849468	Bn-A06-p24849468	A6	84	90.4	B
6388	Bn-N6-p24705727	Bn-C7-p27802408	Bn-scaff_15715_1-p204348	A6	84	90.4	B
6389	Bn-N6-p24742053	Bn-A06-p24890908	Bn-A06-p24890908	A6	84	90.4	B
6390	Bn-N6-p24757453	Bn-A06-p24922072	Bn-A06-p24922072	A6	84	90.4	B
6391	Bn-N6-p24777276	Bn-A06-p24939059	Bn-A06-p24939059	A6	84	90.4	B
6392	Bn-N6-p24806475	Bn-C7-p27578386	Bn-scaff_21268_1-p193486	A6	85	91.2	B
6393	Bn-N6-p25085426	Bn-C7-p26650096	Bn-scaff_15712_1-p113701	A6	86	97.7	A
6394	Bn-N6-p25171248	Bn-A06-p25343454	Bn-A06-p25343454	A6	87	98.5	A
6395	Bn-N6-p25174012	Bn-A06-p25345586	Bn-A06-p25345586	A6	87	98.5	A
6396	Bn-N6-p25178236	Bn-A06-p25349874	Bn-A06-p25349874	A6	87	98.5	A
6397	Bn-N6-p25178294	Bn-A06-p25349932	Bn-A06-p25349932	A6	87	98.5	A
6398	Bn-N6-p25178438	Bn-A06-p25350033	Bn-A06-p25350033	A6	87	98.5	A
6399	Bn-N6-p25199191	Bn-A06-p25369364	Bn-A06-p25369364	A6	87	98.5	A
6400	Bn-N6-p25199507	Bn-A06-p25369668	Bn-A06-p25369668	A6	87	98.5	A
6401	Bn-N6-p25570516	Bn-A06-p25786733	Bn-A06-p25786733	A6	88	99.3	A
6402	Bn-N6-p25807999	Bn-A06-p26011034	Bn-A06-p26011034	A6	89	100.1	A
6403	Bn-N6-p25872261	Bn-A06-p26080915	Bn-A06-p26080915	A6	89	100.1	A
6404	Bn-N6-p25873810	Bn-A06-p26083498	Bn-A06-p26083498	A6	89	100.1	A
6405	Bn-N6-p25895515	Bn-A06-p26100486	Bn-A06-p26100486	A6	89	100.1	A
6406	Bn-N6-p25895681	Bn-A06-p26100652	Bn-A06-p26100652	A6	89	100.1	A
6407	Bn-N6-p25895767	Bn-A06-p26100738	Bn-A06-p26100738	A6	89	100.1	A
6408	Bn-N6-p25895841	Bn-A06-p26100812	Bn-A06-p26100812	A6	89	100.1	A
6409	Bn-N6-p25896048	Bn-A06-p26101019	Bn-A06-p26101019	A6	89	100.1	A
6410	Bn-N6-p25896109	Bn-A06-p26101080	Bn-A06-p26101080	A6	89	100.1	A
6411	Bn-N6-p25896301	Bn-A06-p26101272	Bn-A06-p26101272	A6	89	100.1	A
6412	Bn-N6-p25934445	Bn-A06-p26144417	Bn-A06-p26144417	A6	89	100.1	A
6413	Bn-N6-p25934759	Bn-A06-p26144731	Bn-A06-p26144731	A6	89	100.1	A
6414	Bn-N6-p25971825	Bn-A06-p26186229	Bn-A06-p26186229	A6	89	100.1	A

6481	Bn-N17-p1357043	Bn-A07-p665003	Bn-A07-p665003	A7	1	0	B
6482	Bn-N17-p3856376	Bn-A07-p1386652	Bn-A07-p1386652	A7	1	0	B
6483	Bn-N17-p4639605	Bn-C7-p4158666	Bn-scaff_27815_1-p35807	A7	1	0	B
6484	Bn-N18-p44297707	Bn-A07-p1349482	Bn-A07-p1349482	A7	1	0	B
6485	Bn-N7-p1087645	Bn-Scaffold02893-p7(	Bn-scaff_27955_1-p76	A7	1	0	B
6486	Bn-N7-p1119119	Bn-A07-p1098196	Bn-A07-p1098196	A7	1	0	B
6487	Bn-N7-p1152243	Bn-A07-p1118872	Bn-A07-p1118872	A7	1	0	B
6488	Bn-N7-p1164517	Bn-A07-p1135440	Bn-A07-p1135440	A7	1	0	B
6489	Bn-N7-p1227963	Bn-A07-p1193071	Bn-A07-p1193071	A7	1	0	B
6490	Bn-N7-p1228338	Bn-A07-p1193432	Bn-A07-p1193432	A7	1	0	B
6491	Bn-N7-p122997	Bn-A07-p107832	Bn-A07-p107832	A7	1	0	B
6492	Bn-N7-p1237149	Bn-A07-p1197779	Bn-A07-p1197779	A7	1	0	B
6493	Bn-N7-p1237291	Bn-A07-p1197918	Bn-A07-p1197918	A7	1	0	B
6494	Bn-N7-p1237492	Bn-A07-p1198119	Bn-A07-p1198119	A7	1	0	B
6495	Bn-N7-p1262567	Bn-A07-p1227140	Bn-A07-p1227140	A7	1	0	B
6496	Bn-N7-p1268169	Bn-C3-p37401129	Bn-scaff_25323_1-p7608	A7	1	0	B
6497	Bn-N7-p1299849	Bn-A07-p1255437	Bn-A07-p1255437	A7	1	0	B
6498	Bn-N7-p1320908	Bn-A07-p1292866	Bn-A07-p1292866	A7	1	0	B
6499	Bn-N7-p1333816	Bn-A07-p1303513	Bn-A07-p1303513	A7	1	0	B
6500	Bn-N7-p1334008	Bn-A07-p1303705	Bn-A07-p1303705	A7	1	0	B
6501	Bn-N7-p1374522	Bn-A07-p1336276	Bn-A07-p1336276	A7	1	0	B
6502	Bn-N7-p1380806	Bn-C7-p2066710	Bn-scaff_15762_1-p11327:	A7	1	0	B
6503	Bn-N7-p1450968	Bn-A09-p18324038	Bn-A09-p18324038	A7	1	0	B
6504	Bn-N7-p1458970	Bn-A09-p18316084	Bn-A09-p18316084	A7	1	0	B
6505	Bn-N7-p1468682	Bn-A09-p18306845	Bn-A09-p18306845	A7	1	0	B
6506	Bn-N7-p1468976	Bn-A09-p18306551	Bn-A09-p18306551	A7	1	0	B
6507	Bn-N7-p1486473	Bn-A09-p18289551	Bn-A09-p18289551	A7	1	0	B
6508	Bn-N7-p1493424	Bn-A09-p18283186	Bn-A09-p18283186	A7	1	0	B
6509	Bn-N7-p1501002	Bn-A09-p18275618	Bn-A09-p18275618	A7	1	0	B
6510	Bn-N7-p1501701	Bn-A09-p18274919	Bn-A09-p18274919	A7	1	0	B
6511	Bn-N7-p1501839	Bn-A09-p18274781	Bn-A09-p18274781	A7	1	0	B
6512	Bn-N7-p1501912	Bn-A09-p18274708	Bn-A09-p18274708	A7	1	0	B
6513	Bn-N7-p1502640	Bn-A09-p18273980	Bn-A09-p18273980	A7	1	0	B
6514	Bn-N7-p1507700	Bn-A09-p18269045	Bn-A09-p18269045	A7	1	0	B
6515	Bn-N7-p1515424	Bn-A07-p1345521	Bn-A07-p1345521	A7	1	0	B
6516	Bn-N7-p1549806	Bn-A07-p1400148	Bn-A07-p1400148	A7	1	0	B
6517	Bn-N7-p1574595	Bn-A07-p1437386	Bn-A07-p1437386	A7	1	0	B
6518	Bn-N7-p1574926	Bn-A07-p1437777	Bn-A07-p1437777	A7	1	0	B
6519	Bn-N7-p1582176	Bn-A07-p1450730	Bn-A07-p1450730	A7	1	0	B
6520	Bn-N7-p1582412	Bn-A07-p1450961	Bn-A07-p1450961	A7	1	0	B
6521	Bn-N7-p1582753	Bn-A07-p1451303	Bn-A07-p1451303	A7	1	0	B
6522	Bn-N7-p1583660	Bn-A07-p1452210	Bn-A07-p1452210	A7	1	0	B
6523	Bn-N7-p1602681	Bn-A07-p1482858	Bn-A07-p1482858	A7	1	0	B
6524	Bn-N7-p1675377	Bn-A07-p1557675	Bn-A07-p1557675	A7	1	0	B
6525	Bn-N7-p1700315	Bn-C7-p3827070	Bn-scaff_27815_1-p36740:	A7	1	0	B
6526	Bn-N7-p1705838	Bn-A07-p1595411	Bn-A07-p1595411	A7	1	0	B
6527	Bn-N7-p172010	Bn-A07-p158888	Bn-A07-p158888	A7	1	0	B
6528	Bn-N7-p1748784	Bn-A07-p1625292	Bn-A07-p1625292	A7	1	0	B
6529	Bn-N7-p1780390	Bn-A07-p1657613	Bn-A07-p1657613	A7	1	0	B

6530	Bn-N7-p1780667	Bn-A07-p1657975	Bn-A07-p1657975	A7	1	0	B
6531	Bn-N7-p1781263	Bn-A07-p1658486	Bn-A07-p1658486	A7	1	0	B
6532	Bn-N7-p1789612	Bn-A07-p1680649	Bn-A07-p1680649	A7	1	0	B
6533	Bn-N7-p181388	Bn-A07-p167614	Bn-A07-p167614	A7	1	0	B
6534	Bn-N7-p226830	Bn-A07-p211902	Bn-A07-p211902	A7	1	0	B
6535	Bn-N7-p304493	Bn-A07-p275485	Bn-A07-p275485	A7	1	0	B
6536	Bn-N7-p502106	Bn-Scaffold003472-p5	Bn-Scaffold003472-p57	A7	1	0	B
6537	Bn-N7-p555405	Bn-Scaffold001988-p6	Bn-Scaffold001988-p613	A7	1	0	B
6538	Bn-N7-p571421	Bn-Scaffold001356-p3	Bn-Scaffold001356-p370	A7	1	0	B
6539	Bn-N7-p578418	Bn-Scaffold002856-p5	Bn-Scaffold002856-p544	A7	1	0	B
6540	Bn-N7-p583037	Bn-A07-p642484	Bn-A07-p642484	A7	1	0	B
6541	Bn-N7-p717123	Bn-A07-p786330	Bn-A07-p786330	A7	1	0	B
6542	Bn-N7-p752939	Bn-A07-p829095	Bn-A07-p829095	A7	1	0	B
6543	Bn-N7-p795379	Bn-Scaffold000232-p5	Bn-Scaffold000232-p57461	A7	1	0	B
6544	Bn-N7-p867453	Bn-A07-p884435	Bn-A07-p884435	A7	1	0	B
6545	Bn-N7-p868405	Bn-A07-p885219	Bn-A07-p885219	A7	1	0	B
6546	Bn-N7-p955943	Bn-C7-p2030608	Bn-scaff_15762_1-p10966	A7	1	0	B
6547	Bn-N7-p974049	Bn-A07-p959523	Bn-A07-p959523	A7	1	0	B
6548	Bn-N8-p4313086	Bn-A08-p4476147	Bn-A08-p4476147	A7	1	0	B
6476	Bn-N17-p5270230	Bn-C7-p4742582	Bn-scaff_17461_1-p17771	A7	2	0.8	B
6477	Bn-N17-p5589574	Bn-A10-p11783750	Bn-A10-p11783750	A7	2	0.8	B
6478	Bn-N17-p5598959	Bn-A10-p11789263	Bn-A10-p11789263	A7	2	0.8	B
6549	Bn-N7-p1818928	Bn-A07-p1701712	Bn-A07-p1701712	A7	2	0.8	B
6550	Bn-N7-p1839170	Bn-A07-p1717993	Bn-A07-p1717993	A7	2	0.8	B
6551	Bn-N7-p1849388	Bn-A07-p1729364	Bn-A07-p1729364	A7	2	0.8	B
6552	Bn-N7-p2066071	Bn-A10-p11494631	Bn-A10-p11494631	A7	2	0.8	B
6553	Bn-N7-p2093868	Bn-A10-p11527601	Bn-A10-p11527601	A7	2	0.8	B
6554	Bn-N7-p2111403	Bn-A10-p11541544	Bn-A10-p11541544	A7	2	0.8	B
6555	Bn-N7-p2180386	Bn-A10-p11601681	Bn-A10-p11601681	A7	2	0.8	B
6556	Bn-N7-p2186117	Bn-A10-p11607526	Bn-A10-p11607526	A7	2	0.8	B
6557	Bn-N7-p2187711	Bn-A10-p11608926	Bn-A10-p11608926	A7	2	0.8	B
6558	Bn-N7-p2187754	Bn-A10-p11608969	Bn-A10-p11608969	A7	2	0.8	B
6559	Bn-N7-p2188112	Bn-A10-p11609327	Bn-A10-p11609327	A7	2	0.8	B
6560	Bn-N7-p2192314	Bn-A10-p11613529	Bn-A10-p11613529	A7	2	0.8	B
6561	Bn-N7-p2213671	Bn-A10-p11637678	Bn-A10-p11637678	A7	2	0.8	B
6562	Bn-N7-p2215189	Bn-A10-p11639214	Bn-A10-p11639214	A7	2	0.8	B
6563	Bn-N7-p2294109	Bn-A10-p11723249	Bn-A10-p11723249	A7	2	0.8	B
6564	Bn-N7-p2294742	Bn-A10-p11723882	Bn-A10-p11723882	A7	2	0.8	B
6479	Bn-N7-p2306631	Bn-A10-p11744746	Bn-A10-p11744746	A7	2	0.8	B
6565	Bn-N7-p2307111	Bn-A10-p11745226	Bn-A10-p11745226	A7	2	0.8	B
6566	Bn-N7-p2312607	Bn-A10-p11749802	Bn-A10-p11749802	A7	2	0.8	B
6567	Bn-N7-p2312792	Bn-A10-p11749987	Bn-A10-p11749987	A7	2	0.8	B
6568	Bn-N7-p2351058	Bn-A10-p11822315	Bn-A10-p11822315	A7	2	0.8	B
6569	Bn-N7-p2361466	Bn-A10-p11831091	Bn-A10-p11831091	A7	2	0.8	B
6570	Bn-N7-p2370973	Bn-A10-p11839709	Bn-A10-p11839709	A7	2	0.8	B
6571	Bn-N7-p2374936	Bn-A10-p11843630	Bn-A10-p11843630	A7	2	0.8	B
6572	Bn-N7-p2382505	Bn-A10-p11850386	Bn-A10-p11850386	A7	2	0.8	B
6573	Bn-N7-p2382949	Bn-A10-p11850829	Bn-A10-p11850829	A7	2	0.8	B
6574	Bn-N7-p2387397	Bn-A10-p11858346	Bn-A10-p11858346	A7	2	0.8	B

6575	Bn-N7-p2421665	Bn-A10-p11908544	Bn-A10-p11908544	A7	2	0.8	B
6480	Bn-Scaffold00599-p267529	Bn-A10-p11585663	Bn-A10-p11585663	A7	2	0.8	B
6576	Bn-N6-p15192686	Bn-A10-p12201411	Bn-A10-p12201411	A7	3	1.6	B
6577	Bn-N7-p2429224	Bn-A10-p11914509	Bn-A10-p11914509	A7	3	1.6	B
6578	Bn-N7-p2433118	Bn-A10-p11918400	Bn-A10-p11918400	A7	3	1.6	B
6579	Bn-N7-p2492673	Bn-A10-p12014652	Bn-A10-p12014652	A7	3	1.6	B
6580	Bn-N7-p2562923	Bn-Scaffold000632-p2	Bn-Scaffold000632-p2930	A7	3	1.6	B
6581	Bn-N7-p2586869	Bn-A10-p12065852	Bn-A10-p12065852	A7	3	1.6	B
6582	Bn-N7-p2588156	Bn-A10-p12066688	Bn-A10-p12066688	A7	3	1.6	B
6583	Bn-N7-p2588390	Bn-A10-p12066927	Bn-A10-p12066927	A7	3	1.6	B
6584	Bn-N7-p2588483	Bn-A10-p12067020	Bn-A10-p12067020	A7	3	1.6	B
6585	Bn-N7-p2588668	Bn-A10-p12067206	Bn-A10-p12067206	A7	3	1.6	B
6586	Bn-N7-p2588880	Bn-A10-p12067415	Bn-A10-p12067415	A7	3	1.6	B
6587	Bn-N7-p2588939	Bn-A10-p12067474	Bn-A10-p12067474	A7	3	1.6	B
6588	Bn-N7-p2592632	Bn-A10-p12072376	Bn-A10-p12072376	A7	3	1.6	B
6589	Bn-N7-p2592882	Bn-A10-p12072631	Bn-A10-p12072631	A7	3	1.6	B
6590	Bn-N7-p2592909	Bn-A10-p12072657	Bn-A10-p12072657	A7	3	1.6	B
6591	Bn-N7-p2619398	Bn-A10-p12099007	Bn-A10-p12099007	A7	3	1.6	B
6592	Bn-N7-p2619450	Bn-A10-p12099059	Bn-A10-p12099059	A7	3	1.6	B
6593	Bn-N7-p2619693	Bn-A10-p12099302	Bn-A10-p12099302	A7	3	1.6	B
6594	Bn-N7-p2731925	Bn-A10-p12225681	Bn-A10-p12225681	A7	3	1.6	B
6595	Bn-N7-p2737927	Bn-A10-p12231523	Bn-A10-p12231523	A7	3	1.6	B
6596	Bn-N17-p10799935	Bn-C7-p10230099	Bn-scaff_15766_1-p10372	A7	4	2.4	B
6597	Bn-N17-p10799956	Bn-C7-p10230119	Bn-scaff_15766_1-p10374	A7	4	2.4	B
6598	Bn-N17-p10800064	Bn-C7-p10230228	Bn-scaff_15766_1-p10385	A7	4	2.4	B
6599	Bn-N17-p13263351	Bn-A07-p4130487	Bn-A07-p4130487	A7	4	2.4	B
6600	Bn-N17-p6734291	Bn-A10-p12317050	Bn-A10-p12317050	A7	4	2.4	B
6601	Bn-N17-p7049566	Bn-C7-p6555070	Bn-scaff_17882_1-p91047	A7	4	2.4	B
6602	Bn-N17-p7352346	Bn-A10-p12603365	Bn-A10-p12603365	A7	4	2.4	0
6603	Bn-N17-p7707586	Bn-A10-p12774471	Bn-A10-p12774471	A7	4	2.4	B
6604	Bn-N18-p36998192	Bn-A08-p9536971	Bn-A08-p9536971	A7	4	2.4	B
6605	Bn-N7-p10326773	Bn-A07-p8773216	Bn-A07-p8773216	A7	4	2.4	B
6606	Bn-N7-p2809465	Bn-A10-p12307115	Bn-A10-p12307115	A7	4	2.4	B
6607	Bn-N7-p2816873	Bn-A10-p12311753	Bn-A10-p12311753	A7	4	2.4	B
6608	Bn-N7-p2816937	Bn-A10-p12311817	Bn-A10-p12311817	A7	4	2.4	B
6609	Bn-N7-p2818500	Bn-A10-p12313235	Bn-A10-p12313235	A7	4	2.4	B
6610	Bn-N7-p2818878	Bn-A10-p12313613	Bn-A10-p12313613	A7	4	2.4	B
6611	Bn-N7-p2821118	Bn-A10-p12315853	Bn-A10-p12315853	A7	4	2.4	B
6612	Bn-N7-p2821213	Bn-A10-p12315948	Bn-A10-p12315948	A7	4	2.4	B
6613	Bn-N7-p2821655	Bn-A10-p12316390	Bn-A10-p12316390	A7	4	2.4	B
6614	Bn-N7-p2821685	Bn-A10-p12316420	Bn-A10-p12316420	A7	4	2.4	B
6615	Bn-N7-p2821752	Bn-A10-p12316487	Bn-A10-p12316487	A7	4	2.4	B
6616	Bn-N7-p2822165	Bn-A10-p12316900	Bn-A10-p12316900	A7	4	2.4	B
6617	Bn-N7-p2822528	Bn-A10-p12317263	Bn-A10-p12317263	A7	4	2.4	B
6618	Bn-N7-p2829327	Bn-A10-p12324365	Bn-A10-p12324365	A7	4	2.4	B
6619	Bn-N7-p2838826	Bn-A10-p12331294	Bn-A10-p12331294	A7	4	2.4	B
6620	Bn-N7-p2838968	Bn-A10-p12331790	Bn-A10-p12331790	A7	4	2.4	B
6621	Bn-N7-p2854426	Bn-A10-p12353464	Bn-A10-p12353464	A7	4	2.4	B
6622	Bn-N7-p2864667	Bn-A10-p12368127	Bn-A10-p12368127	A7	4	2.4	B

6623	Bn-N7-p2887965	Bn-A10-p12382686	Bn-A10-p12382686	A7	4	2.4	B
6624	Bn-N7-p2970827	Bn-A10-p12463322	Bn-A10-p12463322	A7	4	2.4	B
6625	Bn-N7-p2973434	Bn-A10-p12465530	Bn-A10-p12465530	A7	4	2.4	B
6626	Bn-N7-p3069431	Bn-A10-p12574115	Bn-A10-p12574115	A7	4	2.4	B
6627	Bn-N7-p3080446	Bn-A10-p12597952	Bn-A10-p12597952	A7	4	2.4	B
6628	Bn-N7-p3086279	Bn-A10-p12604580	Bn-A10-p12604580	A7	4	2.4	B
6629	Bn-N7-p3093734	Bn-A10-p12611994	Bn-A10-p12611994	A7	4	2.4	B
6630	Bn-N7-p3114500	Bn-A10-p12627247	Bn-A10-p12627247	A7	4	2.4	B
6631	Bn-N7-p3118881	Bn-A10-p12631480	Bn-A10-p12631480	A7	4	2.4	B
6632	Bn-N7-p3119107	Bn-A10-p12631706	Bn-A10-p12631706	A7	4	2.4	B
6633	Bn-N7-p3119690	Bn-A10-p12632289	Bn-A10-p12632289	A7	4	2.4	B
6634	Bn-N7-p3120753	Bn-A10-p12633352	Bn-A10-p12633352	A7	4	2.4	B
6635	Bn-N7-p3120870	Bn-A10-p12633469	Bn-A10-p12633469	A7	4	2.4	B
6636	Bn-N7-p3121064	Bn-A10-p12633663	Bn-A10-p12633663	A7	4	2.4	B
6637	Bn-N7-p3126983	Bn-A10-p12639538	Bn-A10-p12639538	A7	4	2.4	B
6638	Bn-N7-p3170849	Bn-A10-p12680158	Bn-A10-p12680158	A7	4	2.4	B
6639	Bn-N7-p3171157	Bn-A10-p12680466	Bn-A10-p12680466	A7	4	2.4	B
6640	Bn-N7-p3178480	Bn-A10-p12690502	Bn-A10-p12690502	A7	4	2.4	B
6641	Bn-N7-p3204966	Bn-A10-p12714738	Bn-A10-p12714738	A7	4	2.4	B
6642	Bn-N7-p3212343	Bn-A10-p12718710	Bn-A10-p12718710	A7	4	2.4	B
6643	Bn-N7-p3299226	Bn-A10-p2890008	Bn-A10-p2890008	A7	4	2.4	B
6644	Bn-N7-p3328567	Bn-A10-p2941216	Bn-A10-p2941216	A7	4	2.4	B
6645	Bn-N7-p3337702	Bn-A10-p2947958	Bn-A10-p2947958	A7	4	2.4	B
6646	Bn-N7-p3341585	Bn-C7-p7328292	Bn-scaff_22310_1-p81763[illegible]	A7	4	2.4	B
6647	Bn-N7-p3341782	Bn-C7-p7328485	Bn-scaff_22310_1-p81744[illegible]	A7	4	2.4	B
6648	Bn-N7-p3357261	Bn-A10-p2970564	Bn-A10-p2970564	A7	4	2.4	B
6649	Bn-N7-p3366789	Bn-A10-p2992463	Bn-A10-p2992463	A7	4	2.4	B
6650	Bn-N7-p3367870	Bn-A10-p2993544	Bn-A10-p2993544	A7	4	2.4	B
6651	Bn-N7-p3367893	Bn-A10-p2993567	Bn-A10-p2993567	A7	4	2.4	B
6652	Bn-N7-p3371486	Bn-A10-p2997098	Bn-A10-p2997098	A7	4	2.4	B
6653	Bn-N7-p3371625	Bn-A10-p2997237	Bn-A10-p2997237	A7	4	2.4	B
6654	Bn-N7-p3417386	Bn-A10-p3044426	Bn-A10-p3044426	A7	4	2.4	B
6655	Bn-N7-p3426705	Bn-A10-p3053568	Bn-A10-p3053568	A7	4	2.4	B
6656	Bn-N7-p3441726	Bn-A10-p3063220	Bn-A10-p3063220	A7	4	2.4	B
6657	Bn-N7-p3453733	Bn-A10-p3078181	Bn-A10-p3078181	A7	4	2.4	B
6658	Bn-N7-p3556955	Bn-C7-p7588561	Bn-scaff_22310_1-p55736[illegible]	A7	4	2.4	B
6659	Bn-N7-p3620878	Bn-A10-p3235646	Bn-A10-p3235646	A7	4	2.4	B
6660	Bn-N7-p4182170	Bn-A07-p2524777	Bn-A07-p2524777	A7	4	2.4	B
6661	Bn-N7-p4513748	Bn-A07-p2899520	Bn-A07-p2899520	A7	4	2.4	B
6662	Bn-N7-p4568166	Bn-A07-p2990276	Bn-A07-p2990276	A7	4	2.4	B
6663	Bn-N7-p4570885	Bn-A07-p2992985	Bn-A07-p2992985	A7	4	2.4	B
6664	Bn-N7-p4571219	Bn-A07-p2993312	Bn-A07-p2993312	A7	4	2.4	B
6665	Bn-N7-p4597398	Bn-C4-p51413650	Bn-scaff_20270_1-p43027[illegible]	A7	4	2.4	B
6666	Bn-N7-p4599973	Bn-A07-p3013745	Bn-A07-p3013745	A7	4	2.4	B
6667	Bn-N7-p4616996	Bn-A07-p3027332	Bn-A07-p3027332	A7	4	2.4	B
6668	Bn-N7-p4617427	Bn-A07-p3028363	Bn-A07-p3028363	A7	4	2.4	B
6669	Bn-N7-p4617610	Bn-A07-p3028546	Bn-A07-p3028546	A7	4	2.4	B
6670	Bn-N7-p4622165	Bn-C3-p63352774	Bn-scaff_23098_1-p12058[illegible]	A7	4	2.4	B
6671	Bn-N7-p4632441	Bn-A07-p3041012	Bn-A07-p3041012	A7	4	2.4	B

6672	Bn-N7-p4691361	Bn-A07-p3081602	Bn-A07-p3081602	A7	4	2.4	B
6673	Bn-N7-p4742680	Bn-A07-p3161639	Bn-A07-p3161639	A7	4	2.4	B
6674	Bn-N7-p4744409	Bn-A07-p3163343	Bn-A07-p3163343	A7	4	2.4	B
6675	Bn-N7-p4744767	Bn-A07-p3163701	Bn-A07-p3163701	A7	4	2.4	B
6676	Bn-N7-p4746335	Bn-A07-p3164991	Bn-A07-p3164991	A7	4	2.4	B
6677	Bn-N7-p4780498	Bn-A07-p3202245	Bn-A07-p3202245	A7	4	2.4	B
6678	Bn-N7-p4794263	Bn-A07-p3216267	Bn-A07-p3216267	A7	4	2.4	B
6679	Bn-N7-p4911805	Bn-A07-p3323989	Bn-A07-p3323989	A7	4	2.4	B
6680	Bn-N7-p5117026	Bn-A07-p3482967	Bn-A07-p3482967	A7	4	2.4	B
6681	Bn-N7-p5117478	Bn-C7-p12006278	Bn-scaff_16721_1-p15512	A7	4	2.4	B
6682	Bn-N7-p5145255	Bn-A07-p3509187	Bn-A07-p3509187	A7	4	2.4	B
6683	Bn-N7-p5159374	Bn-A07-p3523290	Bn-A07-p3523290	A7	4	2.4	B
6684	Bn-N7-p5170034	Bn-A07-p3538601	Bn-A07-p3538601	A7	4	2.4	B
6685	Bn-N7-p5186510	Bn-C4-p21838013	Bn-scaff_19170_1-p11053	A7	4	2.4	B
6686	Bn-N7-p5199652	Bn-A07-p3566269	Bn-A07-p3566269	A7	4	2.4	B
6687	Bn-N7-p5201744	Bn-A07-p3568361	Bn-A07-p3568361	A7	4	2.4	B
6688	Bn-N7-p5202476	Bn-A07-p3569093	Bn-A07-p3569093	A7	4	2.4	B
6689	Bn-N7-p5202633	Bn-A07-p3569250	Bn-A07-p3569250	A7	4	2.4	B
6690	Bn-N7-p5204689	Bn-A07-p3571306	Bn-A07-p3571306	A7	4	2.4	B
6691	Bn-N7-p5208939	Bn-C7-p12260974	Bn-scaff_16721_1-p18059	A7	4	2.4	B
6692	Bn-N7-p5345350	Bn-A07-p3746131	Bn-A07-p3746131	A7	4	2.4	B
6693	Bn-N7-p5347464	Bn-A07-p3747912	Bn-A07-p3747912	A7	4	2.4	B
6694	Bn-N7-p5349029	Bn-A07-p3749471	Bn-A07-p3749471	A7	4	2.4	B
6695	Bn-N7-p5361975	Bn-A07-p3771785	Bn-A07-p3771785	A7	4	2.4	B
6696	Bn-N7-p5362011	Bn-A07-p3771821	Bn-A07-p3771821	A7	4	2.4	B
6697	Bn-N7-p5363757	Bn-A07-p3773635	Bn-A07-p3773635	A7	4	2.4	B
6698	Bn-N7-p5378754	Bn-A07-p3793386	Bn-A07-p3793386	A7	4	2.4	B
6699	Bn-N7-p5390231	Bn-A07-p3796068	Bn-A07-p3796068	A7	4	2.4	B
6700	Bn-N7-p5390636	Bn-A07-p3796474	Bn-A07-p3796474	A7	4	2.4	B
6701	Bn-N7-p5391030	Bn-A07-p3796859	Bn-A07-p3796859	A7	4	2.4	B
6702	Bn-N7-p5441128	Bn-A07-p3856921	Bn-A07-p3856921	A7	4	2.4	B
6703	Bn-N7-p5449697	Bn-A07-p3862192	Bn-A07-p3862192	A7	4	2.4	B
6704	Bn-N7-p5472422	Bn-A07-p3899866	Bn-A07-p3899866	A7	4	2.4	B
6705	Bn-N7-p5477226	Bn-A07-p3915443	Bn-A07-p3915443	A7	4	2.4	B
6706	Bn-N7-p5491433	Bn-A07-p3929927	Bn-A07-p3929927	A7	4	2.4	B
6707	Bn-N7-p5491541	Bn-A07-p3930035	Bn-A07-p3930035	A7	4	2.4	B
6708	Bn-N7-p5491598	Bn-A07-p3930092	Bn-A07-p3930092	A7	4	2.4	B
6709	Bn-N7-p5492846	Bn-A07-p3931338	Bn-A07-p3931338	A7	4	2.4	B
6710	Bn-N7-p5542475	Bn-A07-p3975589	Bn-A07-p3975589	A7	4	2.4	B
6711	Bn-N7-p5543093	Bn-A07-p3976201	Bn-A07-p3976201	A7	4	2.4	B
6712	Bn-N7-p5545742	Bn-A07-p3978709	Bn-A07-p3978709	A7	4	2.4	B
6713	Bn-N7-p5563266	Bn-A07-p3994854	Bn-A07-p3994854	A7	4	2.4	B
6714	Bn-N7-p5563726	Bn-A07-p3995605	Bn-A07-p3995605	A7	4	2.4	B
6715	Bn-N7-p5575771	Bn-A07-p4013256	Bn-A07-p4013256	A7	4	2.4	B
6716	Bn-N7-p5614295	Bn-A07-p4050120	Bn-A07-p4050120	A7	4	2.4	B
6717	Bn-N7-p5614649	Bn-A07-p4050475	Bn-A07-p4050475	A7	4	2.4	B
6718	Bn-N7-p5614917	Bn-A07-p4050743	Bn-A07-p4050743	A7	4	2.4	B
6719	Bn-N7-p5649088	Bn-A07-p4095559	Bn-A07-p4095559	A7	4	2.4	B
6720	Bn-N7-p5650918	Bn-A07-p4097388	Bn-A07-p4097388	A7	4	2.4	B

6721	Bn-N7-p5651220	Bn-A07-p4097690	Bn-A07-p4097690	A7	4	2.4	B
6722	Bn-N7-p5661982	Bn-A07-p4109343	Bn-A07-p4109343	A7	4	2.4	B
6723	Bn-N7-p5662098	Bn-A07-p4109459	Bn-A07-p4109459	A7	4	2.4	B
6724	Bn-N7-p5662933	Bn-A07-p4110292	Bn-A07-p4110292	A7	4	2.4	B
6725	Bn-N7-p5677491	Bn-A07-p4129734	Bn-A07-p4129734	A7	4	2.4	B
6726	Bn-N7-p5701557	Bn-A07-p4141499	Bn-A07-p4141499	A7	4	2.4	B
6727	Bn-N7-p5705180	Bn-A07-p4142320	Bn-A07-p4142320	A7	4	2.4	B
6728	Bn-N7-p5708942	Bn-A07-p4148934	Bn-A07-p4148934	A7	4	2.4	B
6729	Bn-N7-p5711847	Bn-A07-p4151821	Bn-A07-p4151821	A7	4	2.4	B
6730	Bn-N7-p5715363	Bn-A07-p4155337	Bn-A07-p4155337	A7	4	2.4	B
6731	Bn-N7-p5718064	Bn-A07-p4158079	Bn-A07-p4158079	A7	4	2.4	B
6732	Bn-N7-p5721486	Bn-A07-p4161480	Bn-A07-p4161480	A7	4	2.4	B
6733	Bn-N7-p5722520	Bn-A07-p4162513	Bn-A07-p4162513	A7	4	2.4	B
6734	Bn-N7-p5732895	Bn-A07-p4173116	Bn-A07-p4173116	A7	4	2.4	B
6735	Bn-N7-p5733362	Bn-A07-p4173583	Bn-A07-p4173583	A7	4	2.4	B
6736	Bn-N7-p5751857	Bn-A07-p4191546	Bn-A07-p4191546	A7	4	2.4	B
6737	Bn-N7-p5770330	Bn-A07-p4204511	Bn-A07-p4204511	A7	4	2.4	B
6738	Bn-N7-p5770467	Bn-A07-p4204647	Bn-A07-p4204647	A7	4	2.4	B
6739	Bn-N7-p5775000	Bn-A07-p4208528	Bn-A07-p4208528	A7	4	2.4	B
6740	Bn-N7-p5775667	Bn-A07-p4210761	Bn-A07-p4210761	A7	4	2.4	B
6741	Bn-N7-p5777093	Bn-C7-p13238296	Bn-scaff_16371_1-p110691	A7	4	2.4	B
6742	Bn-N7-p5861096	Bn-A07-p4289732	Bn-A07-p4289732	A7	4	2.4	B
6743	Bn-N7-p5874422	Bn-A07-p4305829	Bn-A07-p4305829	A7	4	2.4	B
6744	Bn-N7-p5878177	Bn-A07-p4309577	Bn-A07-p4309577	A7	4	2.4	B
6745	Bn-N7-p5932176	Bn-A07-p4364075	Bn-A07-p4364075	A7	4	2.4	B
6746	Bn-N7-p6044743	Bn-A07-p4477010	Bn-A07-p4477010	A7	4	2.4	B
6747	Bn-N7-p6050372	Bn-A07-p4483935	Bn-A07-p4483935	A7	4	2.4	B
6748	Bn-N7-p6077298	Bn-A07-p4522519	Bn-A07-p4522519	A7	4	2.4	B
6749	Bn-N7-p6077352	Bn-A07-p4522573	Bn-A07-p4522573	A7	4	2.4	B
6750	Bn-N7-p6086939	Bn-A07-p4535707	Bn-A07-p4535707	A7	4	2.4	B
6751	Bn-N7-p6087716	Bn-A07-p4536488	Bn-A07-p4536488	A7	4	2.4	B
6752	Bn-N7-p6087836	Bn-A07-p4536608	Bn-A07-p4536608	A7	4	2.4	B
6753	Bn-N7-p6090353	Bn-A07-p4539144	Bn-A07-p4539144	A7	4	2.4	B
6754	Bn-N7-p6110250	Bn-A07-p4554373	Bn-A07-p4554373	A7	4	2.4	B
6755	Bn-N7-p6117503	Bn-A07-p4568148	Bn-A07-p4568148	A7	4	2.4	B
6756	Bn-N7-p6129252	Bn-A07-p4578845	Bn-A07-p4578845	A7	4	2.4	B
6757	Bn-N7-p6137560	Bn-A10-p10877107	Bn-A10-p10877107	A7	4	2.4	B
6758	Bn-N7-p6137618	Bn-A10-p10877049	Bn-A10-p10877049	A7	4	2.4	B
6759	Bn-N7-p6138258	Bn-A10-p10876460	Bn-A10-p10876460	A7	4	2.4	B
6760	Bn-N7-p6164763	Bn-A07-p4607332	Bn-A07-p4607332	A7	4	2.4	B
6761	Bn-N7-p6175718	Bn-A07-p4624421	Bn-A07-p4624421	A7	4	2.4	B
6762	Bn-N7-p6203463	Bn-C7-p14321962	Bn-scaff_17584_1-p223738	A7	4	2.4	B
6763	Bn-N7-p6243950	Bn-A07-p4678510	Bn-A07-p4678510	A7	4	2.4	B
6764	Bn-N7-p6250339	Bn-A07-p4684890	Bn-A07-p4684890	A7	4	2.4	B
6765	Bn-N7-p6278815	Bn-A07-p4708781	Bn-A07-p4708781	A7	4	2.4	B
6766	Bn-N7-p6279623	Bn-A07-p4709589	Bn-A07-p4709589	A7	4	2.4	B
6767	Bn-N7-p6379719	Bn-A07-p4815239	Bn-A07-p4815239	A7	4	2.4	B
6768	Bn-N7-p6380390	Bn-A07-p4815910	Bn-A07-p4815910	A7	4	2.4	B
6769	Bn-Scaffold01112-p30312	Bn-A07-p2946329	Bn-A07-p2946329	A7	4	2.4	B

6770	Bn-Scaffold33778-p211	Bn-C7-p33560805	Bn-scaff_16514_1-p257216	A7	4	2.4	B
6771	Bn-Scaffold59091-p190	Bn-A07-p3576219	Bn-A07-p3576219	A7	4	2.4	B
6429	Bn-N17-p15223525	Bn-A07-p5039631	Bn-A07-p5039631	A7	5	3.2	B
6430	Bn-N1-p14708772	Bn-A07-p5150157	Bn-A07-p5150157	A7	5	3.2	B
6431	Bn-N7-p505550	Bn-A07-p607926	Bn-A07-p607926	A7	5	3.2	0
6432	Bn-N7-p6487300	Bn-A07-p4916203	Bn-A07-p4916203	A7	5	3.2	B
6433	Bn-N7-p6506698	Bn-A07-p4937244	Bn-A07-p4937244	A7	5	3.2	B
6434	Bn-N7-p6509274	Bn-A07-p4941605	Bn-A07-p4941605	A7	5	3.2	B
6435	Bn-N7-p6514933	Bn-A07-p4947273	Bn-A07-p4947273	A7	5	3.2	B
6436	Bn-N7-p6524952	Bn-A07-p4958794	Bn-A07-p4958794	A7	5	3.2	B
6437	Bn-N7-p6525026	Bn-A07-p4958868	Bn-A07-p4958868	A7	5	3.2	B
6438	Bn-N7-p6526166	Bn-A07-p4959975	Bn-A07-p4959975	A7	5	3.2	B
6439	Bn-N7-p6529612	Bn-A07-p4968765	Bn-A07-p4968765	A7	5	3.2	B
6440	Bn-N7-p6539461	Bn-A07-p4976992	Bn-A07-p4976992	A7	5	3.2	B
6441	Bn-N7-p6552257	Bn-C1-p14552454	Bn-scaff_15747_1-p387612	A7	5	3.2	B
6442	Bn-N7-p6558975	Bn-A07-p4997213	Bn-A07-p4997213	A7	5	3.2	B
6443	Bn-N7-p6567828	Bn-A07-p5003344	Bn-A07-p5003344	A7	5	3.2	B
6444	Bn-N7-p6573394	Bn-A07-p5008797	Bn-A07-p5008797	A7	5	3.2	B
6445	Bn-N7-p6632110	Bn-A07-p5050160	Bn-A07-p5050160	A7	5	3.2	B
6446	Bn-N7-p6659468	Bn-A07-p5072420	Bn-A07-p5072420	A7	5	3.2	B
6447	Bn-N7-p6672692	Bn-C7-p15049139	Bn-scaff_15626_1-p711237	A7	5	3.2	B
6448	Bn-N7-p6679874	Bn-A07-p5092272	Bn-A07-p5092272	A7	5	3.2	0
6449	Bn-N7-p6683612	Bn-A07-p5095657	Bn-A07-p5095657	A7	5	3.2	B
6450	Bn-N7-p6688979	Bn-A07-p5098897	Bn-A07-p5098897	A7	5	3.2	B
6451	Bn-N7-p6696116	Bn-A07-p5106046	Bn-A07-p5106046	A7	5	3.2	B
6452	Bn-N7-p6699752	Bn-A07-p5110099	Bn-A07-p5110099	A7	5	3.2	B
6453	Bn-N7-p6700208	Bn-A07-p5110555	Bn-A07-p5110555	A7	5	3.2	B
6454	Bn-N7-p6701324	Bn-A07-p5111659	Bn-A07-p5111659	A7	5	3.2	B
6455	Bn-N7-p6701778	Bn-A07-p5112113	Bn-A07-p5112113	A7	5	3.2	B
6456	Bn-N7-p6705645	Bn-A07-p5114487	Bn-A07-p5114487	A7	5	3.2	B
6457	Bn-N7-p6705974	Bn-A07-p5114831	Bn-A07-p5114831	A7	5	3.2	B
6458	Bn-N7-p6706069	Bn-A07-p5114926	Bn-A07-p5114926	A7	5	3.2	B
6459	Bn-N7-p6706589	Bn-A07-p5115445	Bn-A07-p5115445	A7	5	3.2	B
6460	Bn-N7-p6706707	Bn-A07-p5115563	Bn-A07-p5115563	A7	5	3.2	B
6461	Bn-N7-p6710788	Bn-A07-p5118743	Bn-A07-p5118743	A7	5	3.2	B
6462	Bn-N7-p6713776	Bn-A07-p5121735	Bn-A07-p5121735	A7	5	3.2	B
6463	Bn-N7-p6718144	Bn-A07-p5126106	Bn-A07-p5126106	A7	5	3.2	B
6464	Bn-N7-p6730359	Bn-A07-p5145409	Bn-A07-p5145409	A7	5	3.2	B
6465	Bn-N7-p6781198	Bn-A07-p5228632	Bn-A07-p5228632	A7	5	3.2	B
6466	Bn-N7-p6805566	Bn-A07-p5243525	Bn-A07-p5243525	A7	5	3.2	B
6467	Bn-N7-p6810996	Bn-A07-p5251710	Bn-A07-p5251710	A7	5	3.2	B
6468	Bn-N7-p6822359	Bn-A07-p5255311	Bn-A07-p5255311	A7	5	3.2	B
6469	Bn-N7-p6822423	Bn-A07-p5255375	Bn-A07-p5255375	A7	5	3.2	B
6470	Bn-N7-p6834202	Bn-Scaffold001110-p4	Bn-Scaffold001110-p441	A7	5	3.2	B
6471	Bn-N7-p6836706	Bn-A07-p5270463	Bn-A07-p5270463	A7	5	3.2	B
6472	Bn-N7-p6838831	Bn-A07-p5272310	Bn-A07-p5272310	A7	5	3.2	B
6473	Bn-N7-p6869818	Bn-A07-p5307627	Bn-A07-p5307627	A7	5	3.2	B
6474	Bn-N7-p6921020	Bn-A07-p5365136	Bn-A07-p5365136	A7	5	3.2	B
6475	Bn-N7-p6923866	Bn-A07-p5367982	Bn-A07-p5367982	A7	5	3.2	B

6415	Bn-N3-p15056575	Bn-A07-p5519719	Bn-A07-p5519719	A7	6	4.1	B
6416	Bn-N7-p6956210	Bn-A07-p5407683	Bn-A07-p5407683	A7	6	4.1	B
6417	Bn-N7-p6968745	Bn-A07-p5412930	Bn-A07-p5412930	A7	6	4.1	B
6418	Bn-N7-p7002121	Bn-A07-p5447322	Bn-A07-p5447322	A7	6	4.1	B
6419	Bn-N7-p7018282	Bn-C8-p21285243	Bn-scaff_16231_1-p13434(	A7	6	4.1	B
6420	Bn-N7-p7054812	Bn-A07-p5500390	Bn-A07-p5500390	A7	6	4.1	B
6421	Bn-N7-p7054870	Bn-A07-p5500448	Bn-A07-p5500448	A7	6	4.1	B
6422	Bn-N7-p7074317	Bn-A07-p5517642	Bn-A07-p5517642	A7	6	4.1	B
6423	Bn-N7-p7075871	Bn-A07-p5519194	Bn-A07-p5519194	A7	6	4.1	B
6424	Bn-N7-p7076033	Bn-A07-p5519356	Bn-A07-p5519356	A7	6	4.1	B
6425	Bn-N7-p7103704	Bn-A07-p5556527	Bn-A07-p5556527	A7	6	4.1	B
6426	Bn-N7-p7112706	Bn-A07-p5572683	Bn-A07-p5572683	A7	6	4.1	B
6427	Bn-N7-p7117612	Bn-C7-p15787754	Bn-scaff_23140_1-p68699	A7	6	4.1	B
6428	Bn-N7-p8970498	Bn-A07-p7374912	Bn-A07-p7374912	A7	6	4.1	B
6772	Bn-N13-p41261292	Bn-A07-p9044585	Bn-A07-p9044585	A7	7	5	B
6773	Bn-N14-p26638654	Bn-Scaffold20259-p2(	Bn-scaff_20074_1-p266	A7	7	5	B
6774	Bn-N15-p17550245	Bn-C5-p18052529	Bn-scaff_16062_1-p19174{	A7	7	5	B
6775	Bn-N15-p17550854	Bn-C5-p18053138	Bn-scaff_16062_1-p19235;	A7	7	5	B
6776	Bn-N17-p17601417	Bn-A07-p6501207	Bn-A07-p6501207	A7	7	5	B
6777	Bn-N17-p17700584	Bn-A07-p6575320	Bn-A07-p6575320	A7	7	5	B
6778	Bn-N17-p18753704	Bn-A07-p7110257	Bn-A07-p7110257	A7	7	5	B
6779	Bn-N17-p19834270	Bn-A07-p7551371	Bn-A07-p7551371	A7	7	5	B
6780	Bn-N17-p20198699	Bn-A07-p7725873	Bn-A07-p7725873	A7	7	5	B
6781	Bn-N17-p20784322	Bn-A07-p8194806	Bn-A07-p8194806	A7	7	5	B
6782	Bn-N17-p21646039	Bn-A07-p8967890	Bn-A07-p8967890	A7	7	5	0
6783	Bn-N17-p22589667	Bn-C7-p21469580	Bn-scaff_23400_1-p65305	A7	7	5	B
6784	Bn-N2-p29112158	Bn-A07-p8197265	Bn-A07-p8197265	A7	7	5	B
6785	Bn-N7-p10001606	Bn-A07-p8451913	Bn-A07-p8451913	A7	7	5	B
6786	Bn-N7-p10042138	Bn-A07-p8498337	Bn-A07-p8498337	A7	7	5	B
6787	Bn-N7-p10074654	Bn-A07-p8516667	Bn-A07-p8516667	A7	7	5	B
6788	Bn-N7-p10078062	Bn-A07-p8520077	Bn-A07-p8520077	A7	7	5	B
6789	Bn-N7-p10088538	Bn-A07-p8530938	Bn-A07-p8530938	A7	7	5	B
6790	Bn-N7-p10139999	Bn-A07-p8584939	Bn-A07-p8584939	A7	7	5	B
6791	Bn-N7-p10142914	Bn-A07-p8593730	Bn-A07-p8593730	A7	7	5	B
6792	Bn-N7-p10143433	Bn-A07-p8594245	Bn-A07-p8594245	A7	7	5	B
6793	Bn-N7-p10168473	Bn-A07-p8612122	Bn-A07-p8612122	A7	7	5	B
6794	Bn-N7-p10171874	Bn-A07-p8615521	Bn-A07-p8615521	A7	7	5	B
6795	Bn-N7-p10173407	Bn-A07-p8617055	Bn-A07-p8617055	A7	7	5	B
6796	Bn-N7-p10173837	Bn-A07-p8617442	Bn-A07-p8617442	A7	7	5	B
6797	Bn-N7-p10204519	Bn-Scaffold007063-p2	Bn-Scaffold007063-p202	A7	7	5	B
6798	Bn-N7-p10218329	Bn-A07-p8668714	Bn-A07-p8668714	A7	7	5	B
6799	Bn-N7-p10219450	Bn-A07-p8669833	Bn-A07-p8669833	A7	7	5	B
6800	Bn-N7-p10219757	Bn-A07-p8670132	Bn-A07-p8670132	A7	7	5	B
6801	Bn-N7-p10228570	Bn-A07-p8674321	Bn-A07-p8674321	A7	7	5	B
6802	Bn-N7-p10229184	Bn-A07-p8674940	Bn-A07-p8674940	A7	7	5	B
6803	Bn-N7-p10229408	Bn-A07-p8675164	Bn-A07-p8675164	A7	7	5	B
6804	Bn-N7-p10241466	Bn-A07-p8696690	Bn-A07-p8696690	A7	7	5	B
6805	Bn-N7-p10265373	Bn-A07-p8718607	Bn-A07-p8718607	A7	7	5	B
6806	Bn-N7-p10295054	Bn-A07-p8745024	Bn-A07-p8745024	A7	7	5	B

6807	Bn-N7-p10302788	Bn-A07-p8752606	Bn-A07-p8752606	A7	7	5	B
6808	Bn-N7-p10326697	Bn-A07-p8773140	Bn-A07-p8773140	A7	7	5	B
6809	Bn-N7-p10341860	Bn-A07-p8790021	Bn-A07-p8790021	A7	7	5	B
6810	Bn-N7-p10348699	Bn-A07-p8796823	Bn-A07-p8796823	A7	7	5	B
6811	Bn-N7-p10397222	Bn-A07-p8824653	Bn-A07-p8824653	A7	7	5	B
6812	Bn-N7-p10475702	Bn-A07-p8896391	Bn-A07-p8896391	A7	7	5	B
6813	Bn-N7-p10477765	Bn-A07-p8898443	Bn-A07-p8898443	A7	7	5	B
6814	Bn-N7-p10483228	Bn-A07-p7270443	Bn-A07-p7270443	A7	7	5	B
6815	Bn-N7-p10502995	Bn-A07-p8903901	Bn-A07-p8903901	A7	7	5	B
6816	Bn-N7-p10539793	Bn-A07-p8941260	Bn-A07-p8941260	A7	7	5	B
6817	Bn-N7-p10558114	Bn-A07-p8959937	Bn-A07-p8959937	A7	7	5	B
6818	Bn-N7-p10560292	Bn-A07-p8962304	Bn-A07-p8962304	A7	7	5	B
6819	Bn-N7-p10570917	Bn-A07-p8977490	Bn-A07-p8977490	A7	7	5	B
6820	Bn-N7-p10572009	Bn-A07-p8980166	Bn-A07-p8980166	A7	7	5	B
6821	Bn-N7-p10591189	Bn-A07-p8997726	Bn-A07-p8997726	A7	7	5	B
6822	Bn-N7-p10599216	Bn-A07-p9010472	Bn-A07-p9010472	A7	7	5	B
6823	Bn-N7-p10635658	Bn-A07-p9041783	Bn-A07-p9041783	A7	7	5	B
6824	Bn-N7-p10636903	Bn-A07-p9043136	Bn-A07-p9043136	A7	7	5	B
6825	Bn-N7-p10670871	Bn-A07-p9079474	Bn-A07-p9079474	A7	7	5	B
6826	Bn-N7-p10678215	Bn-A07-p9086600	Bn-A07-p9086600	A7	7	5	B
6827	Bn-N7-p10686823	Bn-A07-p9095204	Bn-A07-p9095204	A7	7	5	B
6828	Bn-N7-p10687176	Bn-A07-p9095557	Bn-A07-p9095557	A7	7	5	B
6829	Bn-N7-p10688753	Bn-A07-p9097136	Bn-A07-p9097136	A7	7	5	B
6830	Bn-N7-p10697089	Bn-A07-p9111745	Bn-A07-p9111745	A7	7	5	B
6831	Bn-N7-p10706508	Bn-A07-p9117042	Bn-A07-p9117042	A7	7	5	B
6832	Bn-N7-p7136845	Bn-A01-p10513529	Bn-A01-p10513529	A7	7	5	B
6833	Bn-N7-p7142992	Bn-A01-p10518110	Bn-A01-p10518110	A7	7	5	B
6834	Bn-N7-p7148322	Bn-A01-p10523194	Bn-A01-p10523194	A7	7	5	B
6835	Bn-N7-p7148961	Bn-A01-p10523833	Bn-A01-p10523833	A7	7	5	B
6836	Bn-N7-p7153279	Bn-A01-p10525357	Bn-A01-p10525357	A7	7	5	B
6837	Bn-N7-p7153728	Bn-A01-p10525805	Bn-A01-p10525805	A7	7	5	B
6838	Bn-N7-p7153977	Bn-A01-p10526054	Bn-A01-p10526054	A7	7	5	B
6839	Bn-N7-p7154482	Bn-A01-p10526561	Bn-A01-p10526561	A7	7	5	B
6840	Bn-N7-p7173907	Bn-A01-p10550486	Bn-A01-p10550486	A7	7	5	B
6841	Bn-N7-p7248176	Bn-A07-p5648697	Bn-A07-p5648697	A7	7	5	B
6842	Bn-N7-p7266816	Bn-A07-p5660270	Bn-A07-p5660270	A7	7	5	B
6843	Bn-N7-p7271055	Bn-A07-p5670936	Bn-A07-p5670936	A7	7	5	B
6844	Bn-N7-p7292965	Bn-A07-p5684410	Bn-A07-p5684410	A7	7	5	B
6845	Bn-N7-p7400733	Bn-A07-p5835842	Bn-A07-p5835842	A7	7	5	B
6846	Bn-N7-p7413982	Bn-A07-p5849947	Bn-A07-p5849947	A7	7	5	B
6847	Bn-N7-p7434664	Bn-A07-p5871275	Bn-A07-p5871275	A7	7	5	B
6848	Bn-N7-p7441486	Bn-A07-p5877587	Bn-A07-p5877587	A7	7	5	B
6849	Bn-N7-p7459607	Bn-A07-p5882920	Bn-A07-p5882920	A7	7	5	B
6850	Bn-N7-p7574885	Bn-A07-p5995415	Bn-A07-p5995415	A7	7	5	B
6851	Bn-N7-p7757299	Bn-A07-p6197889	Bn-A07-p6197889	A7	7	5	B
6852	Bn-N7-p7765898	Bn-A07-p6206232	Bn-A07-p6206232	A7	7	5	B
6853	Bn-N7-p7775654	Bn-A07-p6207835	Bn-A07-p6207835	A7	7	5	B
6854	Bn-N7-p7808729	Bn-A07-p6238777	Bn-A07-p6238777	A7	7	5	B
6855	Bn-N7-p7851921	Bn-A07-p6276115	Bn-A07-p6276115	A7	7	5	B

6856	Bn-N7-p7856070	Bn-A07-p6282717	Bn-A07-p6282717	A7	7	5	B
6857	Bn-N7-p7856478	Bn-A07-p6283126	Bn-A07-p6283126	A7	7	5	B
6858	Bn-N7-p7869648	Bn-A07-p6297941	Bn-A07-p6297941	A7	7	5	B
6859	Bn-N7-p7873661	Bn-A07-p6305750	Bn-A07-p6305750	A7	7	5	B
6860	Bn-N7-p7875459	Bn-A07-p6307545	Bn-A07-p6307545	A7	7	5	B
6861	Bn-N7-p7879982	Bn-A07-p6309969	Bn-A07-p6309969	A7	7	5	B
6862	Bn-N7-p7902426	Bn-A07-p6327847	Bn-A07-p6327847	A7	7	5	B
6863	Bn-N7-p8023341	Bn-A07-p6432326	Bn-A07-p6432326	A7	7	5	B
6864	Bn-N7-p8025042	Bn-A07-p6437343	Bn-A07-p6437343	A7	7	5	B
6865	Bn-N7-p8026440	Bn-A07-p6439944	Bn-A07-p6439944	A7	7	5	B
6866	Bn-N7-p8030608	Bn-A07-p6444067	Bn-A07-p6444067	A7	7	5	B
6867	Bn-N7-p8030961	Bn-A07-p6444420	Bn-A07-p6444420	A7	7	5	B
6868	Bn-N7-p8031553	Bn-A07-p6445012	Bn-A07-p6445012	A7	7	5	B
6869	Bn-N7-p8076082	Bn-A07-p6494007	Bn-A07-p6494007	A7	7	5	B
6870	Bn-N7-p8121743	Bn-A07-p6541352	Bn-A07-p6541352	A7	7	5	B
6871	Bn-N7-p8124747	Bn-A07-p6544334	Bn-A07-p6544334	A7	7	5	B
6872	Bn-N7-p8125354	Bn-A07-p6544677	Bn-A07-p6544677	A7	7	5	B
6873	Bn-N7-p8130886	Bn-A07-p6552161	Bn-A07-p6552161	A7	7	5	B
6874	Bn-N7-p8131150	Bn-A07-p6552431	Bn-A07-p6552431	A7	7	5	B
6875	Bn-N7-p8131934	Bn-A07-p6553171	Bn-A07-p6553171	A7	7	5	B
6876	Bn-N7-p8131997	Bn-A07-p6553231	Bn-A07-p6553231	A7	7	5	B
6877	Bn-N7-p8132079	Bn-A07-p6553314	Bn-A07-p6553314	A7	7	5	B
6878	Bn-N7-p8132429	Bn-A07-p6553663	Bn-A07-p6553663	A7	7	5	B
6879	Bn-N7-p8132540	Bn-A07-p6553774	Bn-A07-p6553774	A7	7	5	B
6880	Bn-N7-p8133053	Bn-A07-p6554430	Bn-A07-p6554430	A7	7	5	B
6881	Bn-N7-p8133384	Bn-A07-p6554767	Bn-A07-p6554767	A7	7	5	B
6882	Bn-N7-p8134382	Bn-A07-p6556246	Bn-A07-p6556246	A7	7	5	B
6883	Bn-N7-p8141292	Bn-A07-p6564338	Bn-A07-p6564338	A7	7	5	B
6884	Bn-N7-p8152038	Bn-A07-p6574022	Bn-A07-p6574022	A7	7	5	B
6885	Bn-N7-p8157934	Bn-A07-p6576078	Bn-A07-p6576078	A7	7	5	B
6886	Bn-N7-p8158306	Bn-A07-p6576457	Bn-A07-p6576457	A7	7	5	B
6887	Bn-N7-p8158538	Bn-A07-p6576689	Bn-A07-p6576689	A7	7	5	B
6888	Bn-N7-p8187912	Bn-A07-p6606249	Bn-A07-p6606249	A7	7	5	B
6889	Bn-N7-p8221715	Bn-A07-p6644825	Bn-A07-p6644825	A7	7	5	B
6890	Bn-N7-p8233747	Bn-A07-p6657878	Bn-A07-p6657878	A7	7	5	B
6891	Bn-N7-p8236587	Bn-A07-p6660717	Bn-A07-p6660717	A7	7	5	B
6892	Bn-N7-p8297067	Bn-A07-p6716678	Bn-A07-p6716678	A7	7	5	B
6893	Bn-N7-p8299963	Bn-A07-p6719583	Bn-A07-p6719583	A7	7	5	B
6894	Bn-N7-p8302625	Bn-A07-p6722244	Bn-A07-p6722244	A7	7	5	B
6895	Bn-N7-p8360263	Bn-A07-p6763846	Bn-A07-p6763846	A7	7	5	B
6896	Bn-N7-p8361877	Bn-A07-p6765464	Bn-A07-p6765464	A7	7	5	B
6897	Bn-N7-p8365999	Bn-C7-p17884086	Bn-scaff_28429_1-p32571(	A7	7	5	B
6898	Bn-N7-p8399459	Bn-A07-p6824682	Bn-A07-p6824682	A7	7	5	B
6899	Bn-N7-p8460839	Bn-A07-p6878338	Bn-A07-p6878338	A7	7	5	B
6900	Bn-N7-p8469344	Bn-A07-p6885690	Bn-A07-p6885690	A7	7	5	B
6901	Bn-N7-p8483939	Bn-A07-p6898363	Bn-A07-p6898363	A7	7	5	B
6902	Bn-N7-p8523858	Bn-A07-p6943725	Bn-A07-p6943725	A7	7	5	B
6903	Bn-N7-p8528691	Bn-A07-p6947953	Bn-A07-p6947953	A7	7	5	B
6904	Bn-N7-p8532856	Bn-A07-p6952910	Bn-A07-p6952910	A7	7	5	B

6905	Bn-N7-p8538942	Bn-C5-p28607594	Bn-scaff_15705_2-p41525[illegible]	A7	7	5	B
6906	Bn-N7-p8542634	Bn-Scaffold002961-p[illegible]	Bn-Scaffold002961-p365	A7	7	5	B
6907	Bn-N7-p8558397	Bn-A07-p6976405	Bn-A07-p6976405	A7	7	5	B
6908	Bn-N7-p8565060	Bn-A07-p6981435	Bn-A07-p6981435	A7	7	5	B
6909	Bn-N7-p8611224	Bn-A07-p7027400	Bn-A07-p7027400	A7	7	5	B
6910	Bn-N7-p8632712	Bn-A07-p7055420	Bn-A07-p7055420	A7	7	5	B
6911	Bn-N7-p8657424	Bn-A07-p7078464	Bn-A07-p7078464	A7	7	5	B
6912	Bn-N7-p8667801	Bn-A07-p7088617	Bn-A07-p7088617	A7	7	5	B
6913	Bn-N7-p8672156	Bn-A07-p7092988	Bn-A07-p7092988	A7	7	5	B
6914	Bn-N7-p8697388	Bn-A07-p7108284	Bn-A07-p7108284	A7	7	5	B
6915	Bn-N7-p8706303	Bn-A07-p7117228	Bn-A07-p7117228	A7	7	5	B
6916	Bn-N7-p8706304	Bn-A07-p7117229	Bn-A07-p7117229	A7	7	5	B
6917	Bn-N7-p8710138	Bn-A07-p7119962	Bn-A07-p7119962	A7	7	5	B
6918	Bn-N7-p8714434	Bn-A07-p7124154	Bn-A07-p7124154	A7	7	5	B
6919	Bn-N7-p8733054	Bn-A07-p7139702	Bn-A07-p7139702	A7	7	5	B
6920	Bn-N7-p8734535	Bn-A07-p7141211	Bn-A07-p7141211	A7	7	5	B
6921	Bn-N7-p8734949	Bn-A07-p7141625	Bn-A07-p7141625	A7	7	5	B
6922	Bn-N7-p8735130	Bn-A07-p7141806	Bn-A07-p7141806	A7	7	5	B
6923	Bn-N7-p8738366	Bn-A07-p7146249	Bn-A07-p7146249	A7	7	5	B
6924	Bn-N7-p8780388	Bn-A07-p7180352	Bn-A07-p7180352	A7	7	5	B
6925	Bn-N7-p8785080	Bn-A07-p7185841	Bn-A07-p7185841	A7	7	5	B
6926	Bn-N7-p8793041	Bn-A07-p7191306	Bn-A07-p7191306	A7	7	5	B
6927	Bn-N7-p8798270	Bn-A07-p7194218	Bn-A07-p7194218	A7	7	5	B
6928	Bn-N7-p8800175	Bn-A07-p7196025	Bn-A07-p7196025	A7	7	5	B
6929	Bn-N7-p8800347	Bn-A07-p7196197	Bn-A07-p7196197	A7	7	5	B
6930	Bn-N7-p8805645	Bn-A07-p7197488	Bn-A07-p7197488	A7	7	5	B
6931	Bn-N7-p8805748	Bn-A07-p7197588	Bn-A07-p7197588	A7	7	5	B
6932	Bn-N7-p8821421	Bn-A07-p7211602	Bn-A07-p7211602	A7	7	5	B
6933	Bn-N7-p8826403	Bn-A07-p7215862	Bn-A07-p7215862	A7	7	5	B
6934	Bn-N7-p8854499	Bn-A07-p7244763	Bn-A07-p7244763	A7	7	5	B
6935	Bn-N7-p8855706	Bn-A07-p7245971	Bn-A07-p7245971	A7	7	5	B
6936	Bn-N7-p8855910	Bn-A07-p7246175	Bn-A07-p7246175	A7	7	5	B
6937	Bn-N7-p8856187	Bn-A07-p7246452	Bn-A07-p7246452	A7	7	5	B
6938	Bn-N7-p8856927	Bn-A07-p7252813	Bn-A07-p7252813	A7	7	5	B
6939	Bn-N7-p8864825	Bn-A07-p7261357	Bn-A07-p7261357	A7	7	5	B
6940	Bn-N7-p8864976	Bn-A07-p7261508	Bn-A07-p7261508	A7	7	5	B
6941	Bn-N7-p8874774	Bn-A07-p7268294	Bn-A07-p7268294	A7	7	5	B
6942	Bn-N7-p8883795	Bn-A07-p7276136	Bn-A07-p7276136	A7	7	5	B
6943	Bn-N7-p8891658	Bn-A07-p7282445	Bn-A07-p7282445	A7	7	5	B
6944	Bn-N7-p8896085	Bn-A07-p7287398	Bn-A07-p7287398	A7	7	5	B
6945	Bn-N7-p8909255	Bn-A07-p7298176	Bn-A07-p7298176	A7	7	5	B
6946	Bn-N7-p8913071	Bn-A07-p7302034	Bn-A07-p7302034	A7	7	5	B
6947	Bn-N7-p8916937	Bn-A07-p7309287	Bn-A07-p7309287	A7	7	5	B
6948	Bn-N7-p8918250	Bn-A07-p7310644	Bn-A07-p7310644	A7	7	5	B
6949	Bn-N7-p8957000	Bn-A07-p7362180	Bn-A07-p7362180	A7	7	5	B
6950	Bn-N7-p8962238	Bn-A07-p7367247	Bn-A07-p7367247	A7	7	5	B
6951	Bn-N7-p8987078	Bn-A07-p7390413	Bn-A07-p7390413	A7	7	5	B
6952	Bn-N7-p9023915	Bn-A07-p7445858	Bn-A07-p7445858	A7	7	5	B
6953	Bn-N7-p9036032	Bn-A07-p7454447	Bn-A07-p7454447	A7	7	5	B

6954	Bn-N7-p9105871	Bn-A07-p7509139	Bn-A07-p7509139	A7	7	5	B
6955	Bn-N7-p9157495	Bn-A07-p7556616	Bn-A07-p7556616	A7	7	5	B
6956	Bn-N7-p9180104	Bn-A07-p7578604	Bn-A07-p7578604	A7	7	5	B
6957	Bn-N7-p9210682	Bn-A07-p7603379	Bn-A07-p7603379	A7	7	5	B
6958	Bn-N7-p9210744	Bn-A07-p7603440	Bn-A07-p7603440	A7	7	5	B
6959	Bn-N7-p9212492	Bn-A07-p7605498	Bn-A07-p7605498	A7	7	5	B
6960	Bn-N7-p9212600	Bn-A07-p7605606	Bn-A07-p7605606	A7	7	5	B
6961	Bn-N7-p9213040	Bn-A07-p7606040	Bn-A07-p7606040	A7	7	5	B
6962	Bn-N7-p9213227	Bn-A07-p7606228	Bn-A07-p7606228	A7	7	5	B
6963	Bn-N7-p9213260	Bn-A07-p7606261	Bn-A07-p7606261	A7	7	5	B
6964	Bn-N7-p9213749	Bn-A07-p7606760	Bn-A07-p7606760	A7	7	5	B
6965	Bn-N7-p9214091	Bn-A07-p7607101	Bn-A07-p7607101	A7	7	5	B
6966	Bn-N7-p9214176	Bn-A07-p7607188	Bn-A07-p7607188	A7	7	5	B
6967	Bn-N7-p9215081	Bn-A07-p7608035	Bn-A07-p7608035	A7	7	5	B
6968	Bn-N7-p9218914	Bn-A07-p7610810	Bn-A07-p7610810	A7	7	5	B
6969	Bn-N7-p9228674	Bn-A07-p7621496	Bn-A07-p7621496	A7	7	5	B
6970	Bn-N7-p9230259	Bn-A07-p7635152	Bn-A07-p7635152	A7	7	5	B
6971	Bn-N7-p9232645	Bn-A07-p7637265	Bn-A07-p7637265	A7	7	5	B
6972	Bn-N7-p9244468	Bn-A07-p7664147	Bn-A07-p7664147	A7	7	5	B
6973	Bn-N7-p9249578	Bn-A07-p7673966	Bn-A07-p7673966	A7	7	5	B
6974	Bn-N7-p9251048	Bn-A07-p7676201	Bn-A07-p7676201	A7	7	5	B
6975	Bn-N7-p9257048	Bn-A07-p7677536	Bn-A07-p7677536	A7	7	5	B
6976	Bn-N7-p9257121	Bn-A07-p7677609	Bn-A07-p7677609	A7	7	5	B
6977	Bn-N7-p9257749	Bn-A07-p7678236	Bn-A07-p7678236	A7	7	5	B
6978	Bn-N7-p9257934	Bn-A07-p7678421	Bn-A07-p7678421	A7	7	5	B
6979	Bn-N7-p9258227	Bn-A07-p7678712	Bn-A07-p7678712	A7	7	5	B
6980	Bn-N7-p9258984	Bn-A07-p7679469	Bn-A07-p7679469	A7	7	5	B
6981	Bn-N7-p9264964	Bn-A07-p7685214	Bn-A07-p7685214	A7	7	5	B
6982	Bn-N7-p9265824	Bn-A07-p7686074	Bn-A07-p7686074	A7	7	5	B
6983	Bn-N7-p9300146	Bn-A07-p7738577	Bn-A07-p7738577	A7	7	5	B
6984	Bn-N7-p9301388	Bn-A07-p7740173	Bn-A07-p7740173	A7	7	5	B
6985	Bn-N7-p9336719	Bn-A07-p7773921	Bn-A07-p7773921	A7	7	5	B
6986	Bn-N7-p9691938	Bn-A07-p8128951	Bn-A07-p8128951	A7	7	5	B
6987	Bn-N7-p9694023	Bn-A07-p8131907	Bn-A07-p8131907	A7	7	5	B
6988	Bn-N7-p9697098	Bn-A07-p8134950	Bn-A07-p8134950	A7	7	5	B
6989	Bn-N7-p9704943	Bn-A07-p8138266	Bn-A07-p8138266	A7	7	5	B
6990	Bn-N7-p9721973	Bn-C7-p20485897	Bn-scaff_19106_1-p42662[illegible]	A7	7	5	B
6991	Bn-N7-p9729237	Bn-A07-p8162493	Bn-A07-p8162493	A7	7	5	B
6992	Bn-N7-p9747476	Bn-A07-p8181654	Bn-A07-p8181654	A7	7	5	B
6993	Bn-N7-p9750272	Bn-A07-p8183365	Bn-A07-p8183365	A7	7	5	B
6994	Bn-N7-p9751304	Bn-A07-p8184393	Bn-A07-p8184393	A7	7	5	B
6995	Bn-N7-p9752043	Bn-A07-p8185226	Bn-A07-p8185226	A7	7	5	B
6996	Bn-N7-p9753541	Bn-A07-p8186509	Bn-A07-p8186509	A7	7	5	B
6997	Bn-N7-p9878786	Bn-A07-p8318791	Bn-A07-p8318791	A7	7	5	B
6998	Bn-N7-p9881219	Bn-A07-p8323309	Bn-A07-p8323309	A7	7	5	B
6999	Bn-N7-p9881938	Bn-A07-p8324017	Bn-A07-p8324017	A7	7	5	B
7000	Bn-N7-p9895130	Bn-A07-p8334245	Bn-A07-p8334245	A7	7	5	B
7001	Bn-N7-p9909487	Bn-A07-p8348770	Bn-A07-p8348770	A7	7	5	B
7002	Bn-N7-p9921479	Bn-A07-p8359729	Bn-A07-p8359729	A7	7	5	B

7003	Bn-Scaffold41985-p191	Bn-A07-p8143924	Bn-A07-p8143924	A7	7	5	B
7004	Bn-N7-p10744278	Bn-A07-p9155350	Bn-A07-p9155350	A7	8	6.6	B
7005	Bn-N7-p10832679	Bn-A07-p9272000	Bn-A07-p9272000	A7	8	6.6	B
7006	Bn-N7-p10842868	Bn-A07-p9283373	Bn-A07-p9283373	A7	8	6.6	B
7007	Bn-N7-p10852872	Bn-A07-p9293372	Bn-A07-p9293372	A7	8	6.6	B
7008	Bn-N7-p10880110	Bn-A07-p9320476	Bn-A07-p9320476	A7	8	6.6	B
7009	Bn-N7-p10880981	Bn-A07-p9321347	Bn-A07-p9321347	A7	8	6.6	B
7010	Bn-N7-p10890045	Bn-C7-p22906675	Bn-scaff_18501_1-p47151	A7	8	6.6	B
7011	Bn-N7-p10896201	Bn-A07-p9336662	Bn-A07-p9336662	A7	8	6.6	B
7012	Bn-N7-p10899898	Bn-A07-p9339366	Bn-A07-p9339366	A7	8	6.6	B
7013	Bn-N7-p10904585	Bn-A07-p9344001	Bn-A07-p9344001	A7	8	6.6	B
7014	Bn-N7-p10907847	Bn-A07-p9347254	Bn-A07-p9347254	A7	8	6.6	B
7015	Bn-N7-p10952981	Bn-A07-p9402974	Bn-A07-p9402974	A7	8	6.6	B
7016	Bn-N7-p11010883	Bn-A07-p9486032	Bn-A07-p9486032	A7	8	6.6	B
7017	Bn-N7-p11033575	Bn-A07-p9509100	Bn-A07-p9509100	A7	8	6.6	B
7018	Bn-N7-p11045846	Bn-A07-p9521819	Bn-A07-p9521819	A7	8	6.6	B
7019	Bn-N7-p11049392	Bn-A07-p9525348	Bn-A07-p9525348	A7	8	6.6	B
7020	Bn-N7-p11071141	Bn-A07-p9564494	Bn-A07-p9564494	A7	8	6.6	B
7021	Bn-N7-p11134620	Bn-A07-p9632473	Bn-A07-p9632473	A7	8	6.6	B
7022	Bn-Scaffold06771-p798	Bn-A07-p9208032	Bn-A07-p9208032	A7	8	6.6	B
7023	Bn-N7-p11159340	Bn-A07-p9661444	Bn-A07-p9661444	A7	9	8.3	B
7024	Bn-N7-p11161136	Bn-A07-p9663232	Bn-A07-p9663232	A7	9	8.3	B
7025	Bn-N7-p11177134	Bn-A07-p9670298	Bn-A07-p9670298	A7	9	8.3	B
7026	Bn-N7-p11272088	Bn-A07-p9764930	Bn-A07-p9764930	A7	9	8.3	B
7027	Bn-N17-p41257075	Bn-A07-p9915554	Bn-A07-p9915554	A7	10	9.1	B
7028	Bn-N7-p11291470	Bn-A07-p9791002	Bn-A07-p9791002	A7	10	9.1	B
7029	Bn-N7-p11316552	Bn-A07-p9824132	Bn-A07-p9824132	A7	10	9.1	B
7030	Bn-N7-p11333958	Bn-A07-p9841369	Bn-A07-p9841369	A7	10	9.1	B
7031	Bn-N7-p11410376	Bn-A07-p9912557	Bn-A07-p9912557	A7	10	9.1	B
7032	Bn-N7-p11410456	Bn-A07-p9912637	Bn-A07-p9912637	A7	10	9.1	B
7033	Bn-N7-p11412204	Bn-A07-p9916502	Bn-A07-p9916502	A7	10	9.1	B
7034	Bn-N7-p11412337	Bn-A07-p9916647	Bn-A07-p9916647	A7	10	9.1	B
7035	Bn-N7-p11421553	Bn-A07-p9921856	Bn-A07-p9921856	A7	10	9.1	B
7036	Bn-N7-p11478420	Bn-A07-p9987682	Bn-A07-p9987682	A7	10	9.1	B
7037	Bn-N7-p11479163	Bn-A07-p9988423	Bn-A07-p9988423	A7	10	9.1	B
7038	Bn-N7-p11491755	Bn-A07-p10003378	Bn-A07-p10003378	A7	10	9.1	B
7039	Bn-N7-p11492622	Bn-A07-p10004245	Bn-A07-p10004245	A7	10	9.1	B
7040	Bn-N7-p11545666	Bn-C7-p24352991	Bn-scaff_18202_1-p11599	A7	10	9.1	B
7041	Bn-N7-p11576681	Bn-A07-p10092606	Bn-A07-p10092606	A7	10	9.1	0
7042	Bn-N7-p11584293	Bn-A07-p10099786	Bn-A07-p10099786	A7	10	9.1	B
7043	Bn-N7-p11585635	Bn-A07-p10101125	Bn-A07-p10101125	A7	10	9.1	B
7044	Bn-N7-p11645303	Bn-A07-p10169211	Bn-A07-p10169211	A7	10	9.1	B
7045	Bn-N7-p11656567	Bn-A09-p28041600	Bn-A09-p28041600	A7	10	9.1	B
7046	Bn-N7-p11657842	Bn-A07-p10181071	Bn-A07-p10181071	A7	10	9.1	B
7047	Bn-N7-p11664233	Bn-A07-p10194155	Bn-A07-p10194155	A7	10	9.1	B
7048	Bn-N7-p11665352	Bn-A07-p10195276	Bn-A07-p10195276	A7	10	9.1	B
7049	Bn-N7-p11670303	Bn-A07-p10200584	Bn-A07-p10200584	A7	10	9.1	B
7050	Bn-N7-p11673828	Bn-A07-p10204148	Bn-A07-p10204148	A7	10	9.1	B
7051	Bn-N7-p11674565	Bn-A07-p10204886	Bn-A07-p10204886	A7	10	9.1	B

7052	Bn-N7-p11690305	Bn-A07-p10220287	Bn-A07-p10220287	A7	10	9.1	B
7053	Bn-N7-p11697120	Bn-Scaffold000684-p2	Bn-Scaffold000684-p2321	A7	10	9.1	B
7054	Bn-N7-p11708200	Bn-A07-p10230704	Bn-A07-p10230704	A7	10	9.1	B
7055	Bn-N7-p11710832	Bn-A07-p10233337	Bn-A07-p10233337	A7	10	9.1	B
7056	Bn-N7-p11713763	Bn-A07-p10236271	Bn-A07-p10236271	A7	10	9.1	B
7057	Bn-N7-p11716409	Bn-A07-p10238917	Bn-A07-p10238917	A7	10	9.1	B
7058	Bn-N7-p11734400	Bn-A07-p10253702	Bn-A07-p10253702	A7	10	9.1	B
7059	Bn-N7-p11826155	Bn-A07-p10347794	Bn-A07-p10347794	A7	10	9.1	B
7060	Bn-N7-p11836386	Bn-A07-p10357533	Bn-A07-p10357533	A7	10	9.1	B
7061	Bn-N7-p11836458	Bn-A07-p10357605	Bn-A07-p10357605	A7	10	9.1	B
7062	Bn-N7-p11836795	Bn-A07-p10357942	Bn-A07-p10357942	A7	10	9.1	B
7063	Bn-N7-p11846968	Bn-A07-p10367676	Bn-A07-p10367676	A7	10	9.1	B
7064	Bn-N7-p11848410	Bn-A07-p10368959	Bn-A07-p10368959	A7	10	9.1	B
7065	Bn-N7-p11849992	Bn-A07-p10370541	Bn-A07-p10370541	A7	10	9.1	B
7066	Bn-N7-p11881550	Bn-A07-p10400825	Bn-A07-p10400825	A7	10	9.1	B
7067	Bn-N7-p11881858	Bn-A07-p10401133	Bn-A07-p10401133	A7	10	9.1	B
7068	Bn-N7-p11890636	Bn-A07-p10409623	Bn-A07-p10409623	A7	10	9.1	B
7069	Bn-N7-p11895271	Bn-A07-p10414624	Bn-A07-p10414624	A7	10	9.1	B
7070	Bn-N7-p11895733	Bn-C4-p16662101	Bn-scaff_19253_1-p25672	A7	10	9.1	B
7071	Bn-N7-p11899528	Bn-A07-p10418835	Bn-A07-p10418835	A7	10	9.1	B
7072	Bn-N7-p11940967	Bn-A07-p10455192	Bn-A07-p10455192	A7	10	9.1	B
7073	Bn-N7-p11944794	Bn-A07-p10466052	Bn-A07-p10466052	A7	10	9.1	B
7074	Bn-N7-p11945984	Bn-A07-p10471024	Bn-A07-p10471024	A7	10	9.1	B
7075	Bn-N7-p11958317	Bn-A07-p10483339	Bn-A07-p10483339	A7	10	9.1	B
7076	Bn-N7-p12010306	Bn-A07-p10532652	Bn-A07-p10532652	A7	10	9.1	B
7077	Bn-N7-p12041501	Bn-A07-p10568144	Bn-A07-p10568144	A7	10	9.1	B
7078	Bn-N7-p12122798	Bn-A07-p10651899	Bn-A07-p10651899	A7	10	9.1	B
7079	Bn-N7-p12123191	Bn-A07-p10652292	Bn-A07-p10652292	A7	10	9.1	B
7080	Bn-N7-p12123861	Bn-A07-p10652962	Bn-A07-p10652962	A7	10	9.1	B
7081	Bn-N7-p12123941	Bn-A07-p10653042	Bn-A07-p10653042	A7	10	9.1	B
7082	Bn-N7-p12171039	Bn-A07-p10672691	Bn-A07-p10672691	A7	10	9.1	B
7083	Bn-N7-p12171622	Bn-C4-p15827144	Bn-scaff_18505_1-p25457	A7	10	9.1	B
7084	Bn-N7-p12173458	Bn-A07-p10675102	Bn-A07-p10675102	A7	10	9.1	B
7085	Bn-N7-p12652326	Bn-A07-p11163481	Bn-A07-p11163481	A7	10	9.1	0
7086	Bn-N14-p15981556	Bn-A07-p10847704	Bn-A07-p10847704	A7	11	10	B
7087	Bn-N14-p22781634	Bn-A02-p187256	Bn-A02-p187256	A7	11	10	B
7088	Bn-N16-p16301190	Bn-A02-p647706	Bn-A02-p647706	A7	11	10	B
7089	Bn-N19-p32747479	Bn-A02-p185917	Bn-A02-p185917	A7	11	10	B
7090	Bn-N7-p12218493	Bn-A07-p10729654	Bn-A07-p10729654	A7	11	10	B
7091	Bn-N7-p12236021	Bn-A07-p10750190	Bn-A07-p10750190	A7	11	10	B
7092	Bn-N7-p12269959	Bn-A07-p10780571	Bn-A07-p10780571	A7	11	10	B
7093	Bn-N7-p12347990	Bn-A07-p10869578	Bn-A07-p10869578	A7	11	10	B
7094	Bn-N7-p12355448	Bn-A07-p10877038	Bn-A07-p10877038	A7	11	10	B
7095	Bn-N7-p12364284	Bn-C4-p15358286	Bn-scaff_18712_1-p32669	A7	11	10	B
7096	Bn-N7-p12365945	Bn-A07-p10891272	Bn-A07-p10891272	A7	11	10	B
7097	Bn-N7-p12441442	Bn-A07-p10968184	Bn-A07-p10968184	A7	11	10	B
7098	Bn-N7-p12471476	Bn-C4-p15126972	Bn-scaff_18712_1-p95379	A7	11	10	B
7099	Bn-N7-p12475405	Bn-A07-p10995462	Bn-A07-p10995462	A7	11	10	B
7100	Bn-N7-p12488841	Bn-A07-p11010528	Bn-A07-p11010528	A7	11	10	B

7101	Bn-N7-p12509207	Bn-A07-p11021668	Bn-A07-p11021668	A7	11	10	B
7102	Bn-N7-p12530729	Bn-A07-p11044717	Bn-A07-p11044717	A7	11	10	B
7103	Bn-N7-p12601519	Bn-A07-p11119130	Bn-A07-p11119130	A7	11	10	B
7104	Bn-N7-p12620874	Bn-A07-p11138100	Bn-A07-p11138100	A7	11	10	B
7105	Bn-N7-p12637135	Bn-A07-p11147817	Bn-A07-p11147817	A7	11	10	B
7106	Bn-N7-p12646164	Bn-A07-p11156840	Bn-A07-p11156840	A7	11	10	B
7107	Bn-N7-p12649312	Bn-A07-p11159936	Bn-A07-p11159936	A7	11	10	B
7108	Bn-N7-p12651148	Bn-A07-p11162333	Bn-A07-p11162333	A7	11	10	B
7109	Bn-N7-p12652260	Bn-A07-p11163415	Bn-A07-p11163415	A7	11	10	B
7110	Bn-N7-p12654503	Bn-A07-p11171407	Bn-A07-p11171407	A7	11	10	B
7111	Bn-N7-p12660565	Bn-A07-p11175937	Bn-A07-p11175937	A7	11	10	B
7112	Bn-N7-p12661562	Bn-A07-p11176924	Bn-A07-p11176924	A7	11	10	B
7113	Bn-N7-p12662668	Bn-A07-p11178077	Bn-A07-p11178077	A7	11	10	B
7114	Bn-N7-p12663707	Bn-A07-p11178807	Bn-A07-p11178807	A7	11	10	B
7115	Bn-N7-p12673921	Bn-A07-p11185122	Bn-A07-p11185122	A7	11	10	B
7116	Bn-N7-p12675704	Bn-A07-p11186861	Bn-A07-p11186861	A7	11	10	B
7117	Bn-N7-p12679742	Bn-A07-p11191424	Bn-A07-p11191424	A7	11	10	0
7118	Bn-N7-p12680094	Bn-A07-p11191776	Bn-A07-p11191776	A7	11	10	B
7119	Bn-N7-p12688221	Bn-A07-p11200338	Bn-A07-p11200338	A7	11	10	B
7120	Bn-N7-p12688656	Bn-A07-p11200772	Bn-A07-p11200772	A7	11	10	B
7121	Bn-N7-p12692110	Bn-A07-p11204177	Bn-A07-p11204177	A7	11	10	B
7122	Bn-N7-p12696926	Bn-A07-p11208979	Bn-A07-p11208979	A7	11	10	B
7123	Bn-N7-p12700881	Bn-A07-p11225243	Bn-A07-p11225243	A7	11	10	B
7124	Bn-N7-p12705509	Bn-A07-p11235392	Bn-A07-p11235392	A7	11	10	B
7125	Bn-N7-p12719042	Bn-A07-p11248893	Bn-A07-p11248893	A7	11	10	B
7126	Bn-N7-p12719696	Bn-A07-p11249547	Bn-A07-p11249547	A7	11	10	B
7127	Bn-N7-p12721004	Bn-A02-p188454	Bn-A02-p188454	A7	11	10	B
7128	Bn-N7-p12736929	Bn-A07-p11270373	Bn-A07-p11270373	A7	11	10	B
7129	Bn-N7-p12741599	Bn-A07-p11276443	Bn-A07-p11276443	A7	11	10	B
7130	Bn-N7-p12751000	Bn-A07-p11284412	Bn-A07-p11284412	A7	11	10	B
7131	Bn-N7-p12754426	Bn-A07-p11287521	Bn-A07-p11287521	A7	11	10	B
7132	Bn-N7-p12775340	Bn-A07-p11300051	Bn-A07-p11300051	A7	11	10	B
7133	Bn-N7-p12803486	Bn-A07-p11324360	Bn-A07-p11324360	A7	11	10	B
7134	Bn-N7-p12805127	Bn-C4-p14443871	Bn-scaff_16402_1-p16404	A7	11	10	B
7135	Bn-N7-p12807401	Bn-A07-p11328250	Bn-A07-p11328250	A7	11	10	B
7136	Bn-N7-p12810619	Bn-A07-p11331372	Bn-A07-p11331372	A7	11	10	B
7137	Bn-N7-p12857776	Bn-Scaffold000317-p2	Bn-Scaffold000317-p23246	A7	11	10	B
7138	Bn-N7-p12867764	Bn-A02-p1198	Bn-A02-p1198	A7	11	10	B
7139	Bn-N7-p12869742	Bn-A02-p3174	Bn-A02-p3174	A7	11	10	B
7140	Bn-N7-p12962144	Bn-A02-p85725	Bn-A02-p85725	A7	11	10	B
7141	Bn-N7-p13043612	Bn-A02-p174249	Bn-A02-p174249	A7	11	10	B
7142	Bn-N7-p13044968	Bn-A02-p175605	Bn-A02-p175605	A7	11	10	B
7143	Bn-N7-p13049089	Bn-A02-p179557	Bn-A02-p179557	A7	11	10	B
7144	Bn-N7-p13052148	Bn-A02-p182618	Bn-A02-p182618	A7	11	10	B
7145	Bn-N7-p13052996	Bn-A02-p183438	Bn-A02-p183438	A7	11	10	B
7146	Bn-N7-p13053144	Bn-A02-p183586	Bn-A02-p183586	A7	11	10	B
7147	Bn-N7-p13053398	Bn-A02-p183840	Bn-A02-p183840	A7	11	10	B
7148	Bn-N7-p13062919	Bn-A02-p202316	Bn-A02-p202316	A7	11	10	B
7149	Bn-N7-p13063095	Bn-A02-p202493	Bn-A02-p202493	A7	11	10	B

7150	Bn-N7-p13063423	Bn-A02-p202819	Bn-A02-p202819	A7	11	10	B
7151	Bn-N7-p13069652	Bn-A02-p209474	Bn-A02-p209474	A7	11	10	B
7152	Bn-N7-p13072225	Bn-A02-p212959	Bn-A02-p212959	A7	11	10	0
7153	Bn-N7-p13133534	Bn-A02-p266070	Bn-A02-p266070	A7	11	10	B
7154	Bn-N7-p13133586	Bn-A02-p266121	Bn-A02-p266121	A7	11	10	B
7155	Bn-N7-p13166456	Bn-A02-p294984	Bn-A02-p294984	A7	11	10	B
7156	Bn-N7-p13184963	Bn-A02-p300727	Bn-A02-p300727	A7	11	10	B
7157	Bn-N7-p13186813	Bn-A02-p302582	Bn-A02-p302582	A7	11	10	B
7158	Bn-N7-p13187310	Bn-A02-p303085	Bn-A02-p303085	A7	11	10	B
7159	Bn-N7-p13187468	Bn-A02-p303243	Bn-A02-p303243	A7	11	10	B
7160	Bn-N7-p13187560	Bn-A02-p303330	Bn-A02-p303330	A7	11	10	B
7161	Bn-N7-p13187605	Bn-A02-p303375	Bn-A02-p303375	A7	11	10	B
7162	Bn-N7-p13187745	Bn-A02-p303515	Bn-A02-p303515	A7	11	10	B
7163	Bn-N7-p13188692	Bn-A02-p304056	Bn-A02-p304056	A7	11	10	B
7164	Bn-N7-p13189642	Bn-A02-p305007	Bn-A02-p305007	A7	11	10	B
7165	Bn-N7-p13196779	Bn-A02-p307657	Bn-A02-p307657	A7	11	10	B
7166	Bn-N7-p13197088	Bn-A02-p308370	Bn-A02-p308370	A7	11	10	B
7167	Bn-N7-p13214023	Bn-A02-p325065	Bn-A02-p325065	A7	11	10	B
7168	Bn-N7-p13214746	Bn-A02-p325788	Bn-A02-p325788	A7	11	10	B
7169	Bn-N7-p13282682	Bn-A02-p393636	Bn-A02-p393636	A7	11	10	B
7170	Bn-N7-p13296926	Bn-A02-p416231	Bn-A02-p416231	A7	11	10	B
7171	Bn-N7-p13316291	Bn-A02-p441751	Bn-A02-p441751	A7	11	10	B
7172	Bn-N7-p13371113	Bn-A02-p488160	Bn-A02-p488160	A7	11	10	B
7173	Bn-N7-p13383260	Bn-A02-p500418	Bn-A02-p500418	A7	11	10	B
7174	Bn-N7-p13383887	Bn-C6-p22849690	Bn-scaff_15818_1-p49304	A7	11	10	B
7175	Bn-N7-p13389583	Bn-A02-p501869	Bn-A02-p501869	A7	11	10	B
7176	Bn-N7-p13412718	Bn-A02-p510727	Bn-A02-p510727	A7	11	10	B
7177	Bn-N7-p13423790	Bn-A02-p518958	Bn-A02-p518958	A7	11	10	0
7178	Bn-N7-p13444606	Bn-A02-p545783	Bn-A02-p545783	A7	11	10	B
7179	Bn-N7-p13456681	Bn-A02-p557523	Bn-A02-p557523	A7	11	10	B
7180	Bn-N7-p13493436	Bn-A02-p588093	Bn-A02-p588093	A7	11	10	B
7181	Bn-N7-p13499429	Bn-A02-p594109	Bn-A02-p594109	A7	11	10	B
7182	Bn-N7-p13511104	Bn-C6-p22510266	Bn-scaff_15818_1-p83246	A7	11	10	B
7183	Bn-N7-p13513951	Bn-A02-p616247	Bn-A02-p616247	A7	11	10	B
7184	Bn-N7-p13523326	Bn-C6-p22483936	Bn-scaff_15818_1-p85879	A7	11	10	B
7185	Bn-N7-p13548076	Bn-A02-p647024	Bn-A02-p647024	A7	11	10	B
7186	Bn-N7-p13551729	Bn-A02-p652623	Bn-A02-p652623	A7	11	10	B
7187	Bn-N7-p13556789	Bn-A02-p655765	Bn-A02-p655765	A7	11	10	B
7188	Bn-N7-p13984215	Bn-A07-p11472623	Bn-A07-p11472623	A7	11	10	B
7189	Bn-Scaffold12647-p249	Bn-A02-p276572	Bn-A02-p276572	A7	11	10	B
7190	Unknown	Bn-A02-p188268	Bn-A02-p188268	A7	11	10	B
7191	Bn-N16-p16873742	Bn-A02-p761524	Bn-A02-p761524	A7	12	10.8	B
7192	Bn-N7-p13572791	Bn-A02-p677501	Bn-A02-p677501	A7	12	10.8	B
7193	Bn-N7-p13632028	Bn-A02-p725112	Bn-A02-p725112	A7	12	10.8	B
7194	Bn-N7-p13676055	Bn-A02-p771313	Bn-A02-p771313	A7	12	10.8	B
7195	Bn-N7-p13676693	Bn-A02-p771951	Bn-A02-p771951	A7	12	10.8	B
7196	Bn-N7-p13676973	Bn-A04-p8595035	Bn-A04-p8595035	A7	12	10.8	B
7197	Bn-N7-p13707097	Bn-A02-p799930	Bn-A02-p799930	A7	12	10.8	B
7198	Bn-N7-p13712824	Bn-A02-p806025	Bn-A02-p806025	A7	12	10.8	B

7199	Bn-N7-p13715521	Bn-A02-p808711	Bn-A02-p808711	A7	12	10.8	B
7200	Bn-N7-p13718122	Bn-A02-p809627	Bn-A02-p809627	A7	12	10.8	B
7201	Bn-N7-p13740475	Bn-A02-p823586	Bn-A02-p823586	A7	12	10.8	B
7202	Bn-N7-p13768733	Bn-A02-p847638	Bn-A02-p847638	A7	12	10.8	B
7203	Bn-N7-p13795116	Bn-A02-p870494	Bn-A02-p870494	A7	12	10.8	B
7204	Bn-N7-p13796203	Bn-A02-p873656	Bn-A02-p873656	A7	12	10.8	B
7205	Bn-N7-p13796317	Bn-A02-p873770	Bn-A02-p873770	A7	12	10.8	B
7206	Bn-N7-p13806454	Bn-C6-p21590465	Bn-scaff_15818_2-p48516(	A7	12	10.8	B
7207	Bn-N7-p13808846	Bn-A02-p892640	Bn-A02-p892640	A7	12	10.8	B
7208	Bn-N7-p13833514	Bn-A02-p918384	Bn-A02-p918384	A7	12	10.8	B
7209	Bn-N7-p13838353	Bn-A02-p925207	Bn-A02-p925207	A7	12	10.8	B
7210	Bn-N7-p13902795	Bn-A02-p980390	Bn-A02-p980390	A7	12	10.8	B
7211	Bn-N7-p13984145	Bn-A07-p11472553	Bn-A07-p11472553	A7	12	10.8	B
7212	Bn-N7-p14004222	Bn-A07-p11492877	Bn-A07-p11492877	A7	12	10.8	B
7213	Bn-N7-p14023753	Bn-A07-p11512981	Bn-A07-p11512981	A7	12	10.8	B
7214	Bn-N7-p14051166	Bn-A07-p11542386	Bn-A07-p11542386	A7	13	11.6	B
7215	Bn-N7-p14083332	Bn-A07-p11570097	Bn-A07-p11570097	A7	13	11.6	B
7216	Bn-N7-p14143467	Bn-A07-p11629852	Bn-A07-p11629852	A7	13	11.6	B
7217	Bn-N7-p14146983	Bn-A07-p11631422	Bn-A07-p11631422	A7	13	11.6	B
7218	Bn-N7-p14147038	Bn-A07-p11631477	Bn-A07-p11631477	A7	13	11.6	B
7219	Bn-N7-p14201703	Bn-A07-p11668787	Bn-A07-p11668787	A7	13	11.6	B
7220	Bn-N16-p18267063	Bn-A07-p11698185	Bn-A07-p11698185	A7	14	12.2	B
7221	Bn-N16-p19985300	Bn-A07-p12318667	Bn-A07-p12318667	A7	14	12.2	B
7222	Bn-N16-p20669798	Bn-A07-p12617108	Bn-A07-p12617108	A7	14	12.2	B
7223	Bn-N6-p1461701	Bn-A07-p12622860	Bn-A07-p12622860	A7	14	12.2	B
7224	Bn-N7-p14316847	Bn-A07-p11764922	Bn-A07-p11764922	A7	14	12.2	B
7225	Bn-N7-p14378988	Bn-A07-p11821917	Bn-A07-p11821917	A7	14	12.2	B
7226	Bn-N7-p14394583	Bn-A07-p11838401	Bn-A07-p11838401	A7	14	12.2	B
7227	Bn-N7-p14403862	Bn-A07-p11846645	Bn-A07-p11846645	A7	14	12.2	B
7228	Bn-N7-p14417147	Bn-A07-p11861194	Bn-A07-p11861194	A7	14	12.2	B
7229	Bn-N7-p14429056	Bn-A07-p11870301	Bn-A07-p11870301	A7	14	12.2	B
7230	Bn-N7-p14429223	Bn-A07-p11870468	Bn-A07-p11870468	A7	14	12.2	B
7231	Bn-N7-p14429607	Bn-A07-p11870852	Bn-A07-p11870852	A7	14	12.2	B
7232	Bn-N7-p14449439	Bn-Scaffold000164-p1	Bn-Scaffold000164-p12357	A7	14	12.2	B
7233	Bn-N7-p14555022	Bn-A07-p12003491	Bn-A07-p12003491	A7	14	12.2	B
7234	Bn-N7-p14591244	Bn-A07-p12050100	Bn-A07-p12050100	A7	14	12.2	B
7235	Bn-N7-p14622816	Bn-C6-p19450685	Bn-scaff_18206_2-p32123	A7	14	12.2	B
7236	Bn-N7-p14671736	Bn-A07-p12123202	Bn-A07-p12123202	A7	14	12.2	B
7237	Bn-N7-p14706402	Bn-A07-p12171217	Bn-A07-p12171217	A7	14	12.2	B
7238	Bn-N7-p14709044	Bn-A07-p12193031	Bn-A07-p12193031	A7	14	12.2	B
7239	Bn-N7-p14869730	Bn-C6-p18813759	Bn-scaff_18206_3-p53695(	A7	14	12.2	B
7240	Bn-N7-p14881658	Bn-A07-p12345399	Bn-A07-p12345399	A7	14	12.2	B
7241	Bn-N7-p14881988	Bn-A07-p12345729	Bn-A07-p12345729	A7	14	12.2	B
7242	Bn-N7-p14889664	Bn-A07-p12363575	Bn-A07-p12363575	A7	14	12.2	B
7243	Bn-N7-p14909637	Bn-A07-p12385490	Bn-A07-p12385490	A7	14	12.2	B
7244	Bn-N7-p14957010	Bn-A07-p12412116	Bn-A07-p12412116	A7	14	12.2	B
7245	Bn-N7-p14967238	Bn-A07-p12415736	Bn-A07-p12415736	A7	14	12.2	B
7246	Bn-N7-p14967498	Bn-A07-p12415996	Bn-A07-p12415996	A7	14	12.2	B
7247	Bn-N7-p14970009	Bn-A07-p12418491	Bn-A07-p12418491	A7	14	12.2	B

7248	Bn-N7-p14970151	Bn-A07-p12418633	Bn-A07-p12418633	A7	14	12.2	B
7249	Bn-N7-p14970986	Bn-A07-p12419468	Bn-A07-p12419468	A7	14	12.2	B
7250	Bn-N7-p14972660	Bn-A07-p12421128	Bn-A07-p12421128	A7	14	12.2	B
7251	Bn-N7-p14974827	Bn-A07-p12423315	Bn-A07-p12423315	A7	14	12.2	B
7252	Bn-N7-p14974966	Bn-A07-p12423454	Bn-A07-p12423454	A7	14	12.2	B
7253	Bn-N7-p14975056	Bn-A07-p12423544	Bn-A07-p12423544	A7	14	12.2	B
7254	Bn-N7-p14975958	Bn-A07-p12424387	Bn-A07-p12424387	A7	14	12.2	B
7255	Bn-N7-p14976081	Bn-A07-p12424510	Bn-A07-p12424510	A7	14	12.2	B
7256	Bn-N7-p14976509	Bn-A07-p12424938	Bn-A07-p12424938	A7	14	12.2	B
7257	Bn-N7-p14976686	Bn-A07-p12425115	Bn-A07-p12425115	A7	14	12.2	B
7258	Bn-N7-p14976890	Bn-A07-p12425319	Bn-A07-p12425319	A7	14	12.2	B
7259	Bn-N7-p14977137	Bn-A07-p12425566	Bn-A07-p12425566	A7	14	12.2	B
7260	Bn-N7-p14978223	Bn-A07-p12426652	Bn-A07-p12426652	A7	14	12.2	B
7261	Bn-N7-p14978478	Bn-A07-p12426907	Bn-A07-p12426907	A7	14	12.2	B
7262	Bn-N7-p14980865	Bn-A07-p12429159	Bn-A07-p12429159	A7	14	12.2	B
7263	Bn-N7-p14982671	Bn-A07-p12430965	Bn-A07-p12430965	A7	14	12.2	B
7264	Bn-N7-p14987604	Bn-A07-p12432678	Bn-A07-p12432678	A7	14	12.2	B
7265	Bn-N7-p14998858	Bn-A07-p12443929	Bn-A07-p12443929	A7	14	12.2	B
7266	Bn-N7-p15039290	Bn-A07-p12486479	Bn-A07-p12486479	A7	14	12.2	B
7267	Bn-N7-p15041071	Bn-A07-p12487549	Bn-A07-p12487549	A7	14	12.2	B
7268	Bn-N7-p15041210	Bn-A07-p12487688	Bn-A07-p12487688	A7	14	12.2	B
7269	Bn-N7-p15041287	Bn-A07-p12487765	Bn-A07-p12487765	A7	14	12.2	B
7270	Bn-N7-p15049714	Bn-A07-p12495414	Bn-A07-p12495414	A7	14	12.2	B
7271	Bn-N7-p15057105	Bn-A07-p12502804	Bn-A07-p12502804	A7	14	12.2	B
7272	Bn-N7-p15084779	Bn-A09-p5274560	Bn-A09-p5274560	A7	14	12.2	B
7273	Bn-N7-p15153157	Bn-A07-p12583860	Bn-A07-p12583860	A7	14	12.2	B
7274	Bn-N7-p15164271	Bn-A07-p12589168	Bn-A07-p12589168	A7	14	12.2	B
7275	Bn-N7-p15181717	Bn-A07-p12602102	Bn-A07-p12602102	A7	14	12.2	B
7276	Bn-N7-p15189690	Bn-A07-p12609202	Bn-A07-p12609202	A7	14	12.2	B
7277	Bn-N7-p15198996	Bn-A07-p12617827	Bn-A07-p12617827	A7	14	12.2	B
7278	Bn-N7-p15297868	Bn-A07-p12705404	Bn-A07-p12705404	A7	14	12.2	B
7279	Bn-N7-p15301183	Bn-A07-p12708718	Bn-A07-p12708718	A7	14	12.2	B
7280	Bn-N7-p15319071	Bn-A07-p12732142	Bn-A07-p12732142	A7	14	12.2	B
7281	Bn-N7-p14888341	Bn-A07-p12362172	Bn-A07-p12362172	A7	15	13.1	B
7282	Bn-N7-p15335414	Bn-A07-p12757009	Bn-A07-p12757009	A7	16	14	B
7283	Bn-N16-p21091205	Bn-A07-p12840368	Bn-A07-p12840368	A7	17	17.5	B
7284	Bn-N16-p21354499	Bn-A07-p12935086	Bn-A07-p12935086	A7	17	17.5	B
7285	Bn-N16-p21354754	Bn-C6-p17389549	Bn-scaff_15763_1-p50613€	A7	17	17.5	B
7286	Bn-N7-p15416956	Bn-A07-p12833066	Bn-A07-p12833066	A7	17	17.5	B
7287	Bn-N7-p15417889	Bn-A07-p12833434	Bn-A07-p12833434	A7	17	17.5	B
7288	Bn-N7-p15417951	Bn-A07-p12833496	Bn-A07-p12833496	A7	17	17.5	B
7289	Bn-N7-p15458367	Bn-A07-p12874349	Bn-A07-p12874349	A7	17	17.5	B
7290	Bn-N7-p15463003	Bn-A07-p12878986	Bn-A07-p12878986	A7	17	17.5	B
7291	Bn-N7-p15507530	Bn-A07-p12918175	Bn-A07-p12918175	A7	17	17.5	B
7292	Bn-N7-p15511082	Bn-A07-p12921727	Bn-A07-p12921727	A7	17	17.5	B
7293	Bn-N7-p15515854	Bn-A07-p12926496	Bn-A07-p12926496	A7	17	17.5	B
7294	Bn-N7-p15540294	Bn-A07-p12947587	Bn-A07-p12947587	A7	17	17.5	B
7295	Bn-N7-p15580229	Bn-A07-p12992945	Bn-A07-p12992945	A7	17	17.5	B
7296	Bn-N7-p15585204	Bn-C6-p17258189	Bn-scaff_15763_1-p63749€	A7	17	17.5	B

7297	Bn-N7-p15671533	Bn-A07-p13072595	Bn-A07-p13072595	A7	18	19.2	B
7298	Bn-N7-p15711445	Bn-A07-p13110243	Bn-A07-p13110243	A7	18	19.2	B
7299	Bn-N7-p15727166	Bn-A07-p13122160	Bn-A07-p13122160	A7	18	19.2	B
7300	Bn-N7-p15730108	Bn-A07-p13124922	Bn-A07-p13124922	A7	18	19.2	B
7301	Bn-N7-p15734269	Bn-A07-p13128413	Bn-A07-p13128413	A7	18	19.2	B
7302	Bn-N7-p15739521	Bn-A07-p13133496	Bn-A07-p13133496	A7	18	19.2	B
7303	Bn-N7-p15755571	Bn-A07-p13147502	Bn-A07-p13147502	A7	18	19.2	B
7304	Bn-N7-p15755637	Bn-A07-p13147568	Bn-A07-p13147568	A7	18	19.2	B
7305	Bn-N7-p15756101	Bn-A07-p13148032	Bn-A07-p13148032	A7	18	19.2	B
7306	Bn-N7-p15764803	Bn-C6-p16866125	Bn-scaff_15763_1-p10295(	A7	18	19.2	B
7307	Bn-N7-p15765185	Bn-A07-p13156984	Bn-A07-p13156984	A7	18	19.2	B
7308	Bn-N7-p15773710	Bn-A07-p13164874	Bn-A07-p13164874	A7	18	19.2	B
7309	Bn-N7-p15785405	Bn-A07-p13171850	Bn-A07-p13171850	A7	18	19.2	B
7310	Bn-N7-p15785601	Bn-A07-p13172047	Bn-A07-p13172047	A7	18	19.2	B
7311	Bn-N7-p15800345	Bn-A07-p13184664	Bn-A07-p13184664	A7	18	19.2	B
7312	Bn-N7-p15800681	Bn-C6-p16805710	Bn-scaff_15763_1-p10899/	A7	18	19.2	0
7313	Bn-N7-p15808423	Bn-A07-p13192299	Bn-A07-p13192299	A7	18	19.2	B
7314	Bn-N7-p15858751	Bn-A07-p13231557	Bn-A07-p13231557	A7	18	19.2	B
7315	Bn-N7-p15876337	Bn-Scaffold014949-p:	Bn-Scaffold014949-p111	A7	18	19.2	B
7316	Bn-N7-p15893698	Bn-A07-p13278392	Bn-A07-p13278392	A7	18	19.2	B
7317	Bn-N7-p15899977	Bn-A07-p13283299	Bn-A07-p13283299	A7	18	19.2	B
7318	Bn-N7-p15914754	Bn-A07-p13305000	Bn-A07-p13305000	A7	18	19.2	B
7319	Bn-N7-p15934319	Bn-A07-p13320094	Bn-A07-p13320094	A7	18	19.2	B
7320	Bn-N7-p15943283	Bn-A07-p13323913	Bn-A07-p13323913	A7	18	19.2	B
7321	Bn-N7-p15945033	Bn-A07-p13324733	Bn-A07-p13324733	A7	18	19.2	B
7322	Bn-N7-p15960245	Bn-A07-p13334430	Bn-A07-p13334430	A7	18	19.2	B
7323	Bn-N7-p16010414	Bn-A07-p13388181	Bn-A07-p13388181	A7	18	19.2	B
7324	Bn-N7-p16106600	Bn-A07-p13485722	Bn-A07-p13485722	A7	18	19.2	B
7325	Bn-N7-p16130688	Bn-A07-p13497516	Bn-A07-p13497516	A7	18	19.2	B
7326	Bn-N7-p16130826	Bn-A07-p13497654	Bn-A07-p13497654	A7	18	19.2	B
7327	Bn-N7-p16146078	Bn-C6-p16007343	Bn-scaff_15818_1-p15038(	A7	18	19.2	B
7328	Bn-N7-p16190523	Bn-C6-p15931286	Bn-scaff_15818_1-p15799!	A7	19	20.1	B
7329	Bn-N7-p16230522	Bn-A07-p13587753	Bn-A07-p13587753	A7	19	20.1	B
7330	Bn-N7-p16236383	Bn-A07-p13599579	Bn-A07-p13599579	A7	19	20.1	B
7331	Bn-N7-p16271735	Bn-A07-p13639374	Bn-A07-p13639374	A7	20	21	B
7332	Bn-N7-p16292466	Bn-A07-p13659939	Bn-A07-p13659939	A7	20	21	B
7333	Bn-N7-p16308315	Bn-C6-p15639241	Bn-scaff_15818_1-p18719(	A7	20	21	B
7334	Bn-N7-p16312495	Bn-A07-p13679221	Bn-A07-p13679221	A7	20	21	B
7335	Bn-N7-p16250566	Bn-A07-p13615643	Bn-A07-p13615643	A7	21	21.9	B
7336	Bn-N7-p16350064	Bn-A07-p13715621	Bn-A07-p13715621	A7	21	21.9	B
7337	Bn-N7-p16372392	Bn-A07-p13738704	Bn-A07-p13738704	A7	21	21.9	B
7338	Bn-N7-p16373144	Bn-A07-p13739429	Bn-A07-p13739429	A7	21	21.9	B
7339	Bn-N16-p23732072	Bn-A07-p14046607	Bn-A07-p14046607	A7	22	22.8	B
7340	Bn-N16-p24064734	Bn-C6-p14634455	Bn-scaff_15818_1-p28767{	A7	22	22.8	B
7341	Bn-N7-p16379790	Bn-A07-p13747722	Bn-A07-p13747722	A7	22	22.8	B
7342	Bn-N7-p16380580	Bn-A07-p13748498	Bn-A07-p13748498	A7	22	22.8	B
7343	Bn-N7-p16382045	Bn-A07-p13753855	Bn-A07-p13753855	A7	22	22.8	B
7344	Bn-N7-p16396275	Bn-A07-p13772349	Bn-A07-p13772349	A7	22	22.8	B
7345	Bn-N7-p16398203	Bn-A07-p13774292	Bn-A07-p13774292	A7	22	22.8	B

7346	Bn-N7-p16407634	Bn-A07-p13787534	Bn-A07-p13787534	A7	22	22.8	B
7347	Bn-N7-p16408537	Bn-A07-p13792568	Bn-A07-p13792568	A7	22	22.8	B
7348	Bn-N7-p16440233	Bn-A07-p13828973	Bn-A07-p13828973	A7	22	22.8	B
7349	Bn-N7-p16451926	Bn-A07-p13840665	Bn-A07-p13840665	A7	22	22.8	B
7350	Bn-N7-p16456074	Bn-A07-p13844810	Bn-A07-p13844810	A7	22	22.8	B
7351	Bn-N7-p16469862	Bn-A07-p13855997	Bn-A07-p13855997	A7	22	22.8	B
7352	Bn-N7-p16470929	Bn-A07-p13857064	Bn-A07-p13857064	A7	22	22.8	B
7353	Bn-N7-p16485186	Bn-A07-p13873942	Bn-A07-p13873942	A7	22	22.8	B
7354	Bn-N7-p16548441	Bn-A07-p13943479	Bn-A07-p13943479	A7	22	22.8	B
7355	Bn-N7-p16555572	Bn-A07-p13947936	Bn-A07-p13947936	A7	22	22.8	B
7356	Bn-N7-p16555591	Bn-A07-p13947955	Bn-A07-p13947955	A7	22	22.8	B
7357	Bn-N7-p16555660	Bn-C6-p15145831	Bn-scaff_15818_1-p23654(	A7	22	22.8	B
7358	Bn-N7-p16555776	Bn-C6-p15145715	Bn-scaff_15818_1-p23655Z	A7	22	22.8	B
7359	Bn-N7-p16563873	Bn-A07-p13957160	Bn-A07-p13957160	A7	22	22.8	B
7360	Bn-N7-p16564032	Bn-A07-p13957625	Bn-A07-p13957625	A7	22	22.8	B
7361	Bn-N7-p16631479	Bn-A07-p14002586	Bn-A07-p14002586	A7	22	22.8	B
7362	Bn-N7-p16631549	Bn-A07-p14002656	Bn-A07-p14002656	A7	22	22.8	B
7363	Bn-N7-p16634145	Bn-A07-p14005263	Bn-A07-p14005263	A7	22	22.8	B
7364	Bn-N7-p16654556	Bn-A07-p14026612	Bn-A07-p14026612	A7	22	22.8	B
7365	Bn-N7-p16672343	Bn-C6-p14968020	Bn-scaff_15818_1-p254321	A7	22	22.8	B
7366	Bn-N7-p16724230	Bn-A07-p14092905	Bn-A07-p14092905	A7	22	22.8	B
7367	Bn-N7-p16735378	Bn-A07-p14103536	Bn-A07-p14103536	A7	22	22.8	B
7368	Bn-N7-p16748653	Bn-A07-p14116798	Bn-A07-p14116798	A7	22	22.8	B
7369	Bn-N7-p16753893	Bn-A07-p14121891	Bn-A07-p14121891	A7	22	22.8	B
7370	Bn-N7-p16764574	Bn-A07-p14129550	Bn-A07-p14129550	A7	22	22.8	B
7371	Bn-N7-p16764589	Bn-A07-p14129566	Bn-A07-p14129566	A7	22	22.8	B
7372	Bn-N7-p16796504	Bn-A07-p14162566	Bn-A07-p14162566	A7	22	22.8	B
7373	Bn-N7-p16796560	Bn-A07-p14162622	Bn-A07-p14162622	A7	22	22.8	B
7374	Bn-N7-p16798007	Bn-A07-p14164068	Bn-A07-p14164068	A7	22	22.8	B
7375	Bn-N7-p16864057	Bn-A07-p14225102	Bn-A07-p14225102	A7	22	22.8	B
7376	Bn-N7-p16864597	Bn-A07-p14225642	Bn-A07-p14225642	A7	22	22.8	B
7377	Bn-N7-p16880190	Bn-A07-p14241936	Bn-A07-p14241936	A7	22	22.8	B
7378	Bn-N7-p16921758	Bn-A07-p14288125	Bn-A07-p14288125	A7	22	22.8	B
7379	Bn-N7-p16936689	Bn-A07-p14306631	Bn-A07-p14306631	A7	22	22.8	B
7380	Bn-N7-p16960659	Bn-C6-p14203057	Bn-scaff_15818_1-p33081{	A7	22	22.8	B
7381	Bn-N7-p16976492	Bn-A07-p14340252	Bn-A07-p14340252	A7	22	22.8	B
7382	Bn-N7-p16979304	Bn-C6-p14172347	Bn-scaff_15818_1-p33388(	A7	22	22.8	B
7383	Bn-N7-p17028212	Bn-A07-p14390667	Bn-A07-p14390667	A7	22	22.8	B
7384	Bn-Scaffold03922-p1695	Bn-A07-p13969888	Bn-A07-p13969888	A7	22	22.8	B
7385	Bn-N7-p17091758	Bn-A07-p14453848	Bn-A07-p14453848	A7	23	23.7	B
7386	Bn-N7-p17109095	Bn-A07-p14462380	Bn-A07-p14462380	A7	23	23.7	B
7387	Bn-N7-p17123463	Bn-A07-p14472064	Bn-A07-p14472064	A7	23	23.7	B
7388	Bn-N16-p24914804	Bn-C6-p13716781	Bn-scaff_15746_1-p210487	A7	24	24.7	B
7389	Bn-N16-p24980162	Bn-C6-p13660220	Bn-scaff_15746_1-p15392(	A7	24	24.7	B
7390	Bn-N7-p17152711	Bn-A07-p14500760	Bn-A07-p14500760	A7	24	24.7	B
7391	Bn-N7-p17155348	Bn-A07-p14511986	Bn-A07-p14511986	A7	24	24.7	B
7392	Bn-N7-p17163479	Bn-A07-p14523096	Bn-A07-p14523096	A7	24	24.7	B
7393	Bn-N7-p17174266	Bn-A07-p14533453	Bn-A07-p14533453	A7	24	24.7	B
7394	Bn-N7-p17179658	Bn-A07-p14540334	Bn-A07-p14540334	A7	24	24.7	B

7395	Bn-N7-p17232223	Bn-A07-p14598430	Bn-A07-p14598430	A7	24	24.7	B
7396	Bn-N7-p17233798	Bn-C6-p13563731	Bn-scaff_15746_1-p57437	A7	24	24.7	B
7397	Bn-N7-p17256364	Bn-A07-p14622638	Bn-A07-p14622638	A7	24	24.7	B
7398	Bn-N7-p17257181	Bn-A07-p14623449	Bn-A07-p14623449	A7	24	24.7	B
7399	Bn-N7-p17262673	Bn-A07-p14628930	Bn-A07-p14628930	A7	24	24.7	B
7400	Bn-N7-p17270757	Bn-C6-p13507593	Bn-scaff_15746_1-p1299	A7	24	24.7	B
7401	Bn-N7-p17277196	Bn-A07-p14645683	Bn-A07-p14645683	A7	24	24.7	B
7402	Bn-N7-p17283432	Bn-A07-p14649790	Bn-A07-p14649790	A7	24	24.7	B
7403	Bn-N7-p17288929	Bn-A07-p14655552	Bn-A07-p14655552	A7	24	24.7	B
7404	Bn-N7-p17292048	Bn-A07-p14664544	Bn-A07-p14664544	A7	24	24.7	B
7405	Bn-N7-p17292666	Bn-A07-p14665161	Bn-A07-p14665161	A7	24	24.7	B
7406	Bn-N7-p17295637	Bn-C6-p13426768	Bn-scaff_18439_1-p79427	A7	24	24.7	B
7407	Bn-N7-p17309322	Bn-A07-p14682292	Bn-A07-p14682292	A7	24	24.7	B
7408	Bn-N7-p17332092	Bn-A07-p14705901	Bn-A07-p14705901	A7	24	24.7	B
7409	Bn-N16-p25671169	Bn-A07-p14895675	Bn-A07-p14895675	A7	25	25.6	B
7410	Bn-N7-p17387599	Bn-A07-p14759961	Bn-A07-p14759961	A7	25	25.6	B
7411	Bn-N7-p17387599	Bn-A07-p14759962	Bn-A07-p14759962	A7	25	25.6	B
7412	Bn-N7-p17387872	Bn-A07-p14760232	Bn-A07-p14760232	A7	25	25.6	B
7413	Bn-N7-p17428408	Bn-A07-p14804953	Bn-A07-p14804953	A7	25	25.6	B
7414	Bn-N7-p17434977	Bn-A07-p14812317	Bn-A07-p14812317	A7	25	25.6	B
7415	Bn-N7-p17440792	Bn-A07-p14820942	Bn-A07-p14820942	A7	25	25.6	B
7416	Bn-N7-p17443925	Bn-A07-p14823470	Bn-A07-p14823470	A7	25	25.6	B
7417	Bn-N7-p17444215	Bn-A07-p14823761	Bn-A07-p14823761	A7	25	25.6	B
7418	Bn-N7-p17454459	Bn-A07-p14836793	Bn-A07-p14836793	A7	25	25.6	B
7419	Bn-N7-p17488534	Bn-A07-p14863875	Bn-A07-p14863875	A7	25	25.6	B
7420	Bn-N7-p17504708	Bn-A07-p14869066	Bn-A07-p14869066	A7	25	25.6	B
7421	Bn-N7-p17505696	Bn-A07-p14870055	Bn-A07-p14870055	A7	25	25.6	B
7422	Bn-N7-p17534137	Bn-A07-p14895551	Bn-A07-p14895551	A7	25	25.6	B
7423	Bn-N7-p17595363	Bn-A07-p14958606	Bn-A07-p14958606	A7	25	25.6	B
7424	Bn-N7-p17649982	Bn-A07-p15007983	Bn-A07-p15007983	A7	25	25.6	B
7425	Bn-N7-p17656934	Bn-A07-p15015434	Bn-A07-p15015434	A7	25	25.6	B
7426	Bn-N7-p17661731	Bn-A07-p15025202	Bn-A07-p15025202	A7	25	25.6	B
7427	Bn-N7-p17670705	Bn-A07-p15033648	Bn-A07-p15033648	A7	25	25.6	B
7428	Bn-N7-p17692604	Bn-A07-p15054860	Bn-A07-p15054860	A7	25	25.6	B
7429	Bn-N7-p17693272	Bn-A07-p15055528	Bn-A07-p15055528	A7	25	25.6	B
7430	Bn-N7-p17696669	Bn-A07-p15059054	Bn-A07-p15059054	A7	25	25.6	B
7431	Bn-N7-p17703725	Bn-A07-p15064528	Bn-A07-p15064528	A7	25	25.6	B
7432	Bn-N7-p17830159	Bn-A07-p15162548	Bn-A07-p15162548	A7	25	25.6	B
7433	Bn-N7-p17830380	Bn-A07-p15162767	Bn-A07-p15162767	A7	25	25.6	B
7434	Bn-N7-p17830450	Bn-A07-p15162837	Bn-A07-p15162837	A7	25	25.6	B
7435	Bn-N7-p17833148	Bn-A07-p15165145	Bn-A07-p15165145	A7	25	25.6	B
7436	Bn-N7-p17842911	Bn-A07-p15175027	Bn-A07-p15175027	A7	25	25.6	B
7437	Bn-N7-p17910141	Bn-A07-p15233647	Bn-A07-p15233647	A7	25	25.6	0
7438	Bn-N7-p17916052	Bn-A07-p15239170	Bn-A07-p15239170	A7	25	25.6	B
7439	Bn-N7-p17918971	Bn-A07-p15241878	Bn-A07-p15241878	A7	25	25.6	B
7440	Bn-N7-p17919655	Bn-A07-p15242562	Bn-A07-p15242562	A7	25	25.6	B
7441	Bn-N7-p17923677	Bn-A07-p15247793	Bn-A07-p15247793	A7	25	25.6	B
7442	Bn-N7-p17960703	Bn-A07-p15276966	Bn-A07-p15276966	A7	25	25.6	B
7443	Bn-N7-p17979504	Bn-A07-p15298239	Bn-A07-p15298239	A7	25	25.6	B

7444	Bn-N7-p17979603	Bn-A07-p15298338	Bn-A07-p15298338	A7	25	25.6	B
7445	Bn-N7-p17980311	Bn-A07-p15299046	Bn-A07-p15299046	A7	25	25.6	B
7446	Bn-N7-p17990900	Bn-A07-p15309387	Bn-A07-p15309387	A7	25	25.6	B
7447	Bn-N7-p17991794	Bn-A07-p15310281	Bn-A07-p15310281	A7	25	25.6	B
7448	Bn-N16-p35692569	Bn-A07-p15372787	Bn-A07-p15372787	A7	26	26.5	B
7449	Bn-N7-p18003759	Bn-A07-p15321076	Bn-A07-p15321076	A7	26	26.5	B
7450	Bn-N7-p18016065	Bn-A07-p15331892	Bn-A07-p15331892	A7	26	26.5	B
7451	Bn-N7-p18034004	Bn-A07-p15352802	Bn-A07-p15352802	A7	26	26.5	B
7452	Bn-N7-p18037237	Bn-A07-p15354488	Bn-A07-p15354488	A7	26	26.5	B
7453	Bn-N7-p18040641	Bn-A07-p15357890	Bn-A07-p15357890	A7	26	26.5	B
7454	Bn-N7-p18042373	Bn-A07-p15359622	Bn-A07-p15359622	A7	26	26.5	B
7455	Bn-N7-p18051146	Bn-A07-p15366978	Bn-A07-p15366978	A7	26	26.5	B
7456	Bn-N7-p18054702	Bn-A07-p15373643	Bn-A07-p15373643	A7	26	26.5	B
7457	Bn-N7-p18054783	Bn-A07-p15373724	Bn-A07-p15373724	A7	26	26.5	B
7458	Bn-N7-p18087198	Bn-A07-p15404895	Bn-A07-p15404895	A7	26	26.5	B
7459	Bn-N7-p18094003	Bn-A07-p15411766	Bn-A07-p15411766	A7	26	26.5	B
7460	Bn-N7-p18097525	Bn-A07-p15415388	Bn-A07-p15415388	A7	26	26.5	B
7461	Bn-N7-p18098049	Bn-A07-p15415913	Bn-A07-p15415913	A7	26	26.5	B
7462	Bn-N7-p18108303	Bn-A07-p15425959	Bn-A07-p15425959	A7	26	26.5	B
7463	Bn-N7-p18127716	Bn-A07-p15442723	Bn-A07-p15442723	A7	26	26.5	B
7464	Bn-N7-p18132272	Bn-A07-p15444203	Bn-A07-p15444203	A7	26	26.5	B
7465	Bn-N7-p18135869	Bn-A07-p15447440	Bn-A07-p15447440	A7	26	26.5	B
7466	Bn-N7-p18155765	Bn-A07-p15464739	Bn-A07-p15464739	A7	26	26.5	B
7467	Bn-N7-p18162402	Bn-A07-p15468982	Bn-A07-p15468982	A7	26	26.5	B
7468	Bn-N7-p18163303	Bn-A07-p15470119	Bn-A07-p15470119	A7	26	26.5	B
7469	Bn-N7-p18163392	Bn-A07-p15470208	Bn-A07-p15470208	A7	26	26.5	B
7470	Bn-N7-p18164017	Bn-A07-p15470833	Bn-A07-p15470833	A7	26	26.5	B
7471	Bn-N7-p18164345	Bn-A07-p15471161	Bn-A07-p15471161	A7	26	26.5	B
7472	Bn-N7-p18188389	Bn-C6-p11520051	Bn-scaff_20807_1-p10454	A7	26	26.5	B
7473	Bn-N7-p18229666	Bn-A07-p15546893	Bn-A07-p15546893	A7	26	26.5	B
7474	Bn-N7-p18232812	Bn-A07-p15549943	Bn-A07-p15549943	A7	26	26.5	B
7475	Bn-N7-p18257843	Bn-A07-p15578269	Bn-A07-p15578269	A7	27	27.4	B
7476	Bn-N7-p18267292	Bn-A07-p15591535	Bn-A07-p15591535	A7	27	27.4	B
7477	Bn-N7-p18287694	Bn-A07-p15612140	Bn-A07-p15612140	A7	27	27.4	B
7478	Bn-N7-p18297859	Bn-A07-p15622870	Bn-A07-p15622870	A7	27	27.4	B
7479	Bn-N7-p18322939	Bn-A07-p15648620	Bn-A07-p15648620	A7	27	27.4	B
7480	Bn-N7-p18324891	Bn-A07-p15650680	Bn-A07-p15650680	A7	27	27.4	B
7481	Bn-N7-p18326907	Bn-A07-p15652974	Bn-A07-p15652974	A7	27	27.4	B
7482	Bn-N7-p18368781	Bn-A07-p15691716	Bn-A07-p15691716	A7	27	27.4	B
7483	Bn-N7-p18407349	Bn-A07-p15728800	Bn-A07-p15728800	A7	27	27.4	B
7484	Bn-N7-p18407916	Bn-A07-p15729378	Bn-A07-p15729378	A7	27	27.4	B
7485	Bn-N7-p18418847	Bn-A07-p15738506	Bn-A07-p15738506	A7	27	27.4	B
7486	Bn-N7-p18449193	Bn-A07-p15768026	Bn-A07-p15768026	A7	27	27.4	B
7487	Bn-N7-p18467443	Bn-A07-p15791885	Bn-A07-p15791885	A7	27	27.4	B
7488	Bn-N7-p18472531	Bn-A07-p15797607	Bn-A07-p15797607	A7	27	27.4	B
7489	Bn-N7-p18476233	Bn-A07-p15801291	Bn-A07-p15801291	A7	27	27.4	B
7490	Bn-N7-p18477171	Bn-A07-p15802229	Bn-A07-p15802229	A7	27	27.4	B
7491	Bn-N7-p18477955	Bn-A07-p15803011	Bn-A07-p15803011	A7	27	27.4	B
7492	Bn-N7-p18485768	Bn-A07-p15809590	Bn-A07-p15809590	A7	27	27.4	B

7493	Bn-N7-p18488979	Bn-A07-p15812852	Bn-A07-p15812852	A7	27	27.4	B
7494	Bn-N7-p18500972	Bn-A07-p15829114	Bn-A07-p15829114	A7	27	27.4	B
7495	Bn-N7-p18501143	Bn-A07-p15829285	Bn-A07-p15829285	A7	27	27.4	B
7496	Bn-N7-p18502907	Bn-A07-p15831043	Bn-A07-p15831043	A7	27	27.4	B
7497	Bn-N7-p18583594	Bn-A07-p15921259	Bn-A07-p15921259	A7	27	27.4	B
7498	Bn-N7-p18607472	Bn-A07-p15949460	Bn-A07-p15949460	A7	28	29.2	B
7499	Bn-N7-p18657326	Bn-A07-p15986673	Bn-A07-p15986673	A7	28	29.2	B
7500	Bn-N7-p18685243	Bn-A07-p16005587	Bn-A07-p16005587	A7	28	29.2	B
7501	Bn-N7-p18688438	Bn-A07-p16009799	Bn-A07-p16009799	A7	28	29.2	B
7502	Bn-N7-p18691714	Bn-A07-p16013075	Bn-A07-p16013075	A7	28	29.2	B
7503	Bn-N7-p18696705	Bn-A07-p16020955	Bn-A07-p16020955	A7	28	29.2	B
7504	Bn-N7-p18722837	Bn-A07-p16045526	Bn-A07-p16045526	A7	28	29.2	B
7505	Bn-N7-p18723859	Bn-A07-p16045865	Bn-A07-p16045865	A7	28	29.2	B
7506	Bn-N7-p18724927	Bn-A07-p16047469	Bn-A07-p16047469	A7	28	29.2	B
7507	Bn-N7-p18730071	Bn-A07-p16050430	Bn-A07-p16050430	A7	28	29.2	B
7508	Bn-Scaffold28148-p207	Bn-A07-p16015935	Bn-A07-p16015935	A7	28	29.2	B
7509	Bn-N7-p18746325	Bn-A07-p16074768	Bn-A07-p16074768	A7	29	30.1	B
7510	Bn-N7-p18830062	Bn-A07-p16146576	Bn-A07-p16146576	A7	29	30.1	B
7511	Bn-N16-p29001909	Bn-C6-p9536177	Bn-scaff_15892_1-p14820	A7	30	31	B
7512	Bn-N7-p18872267	Bn-A07-p16183869	Bn-A07-p16183869	A7	30	31	B
7513	Bn-N7-p18963227	Bn-A07-p16260678	Bn-A07-p16260678	A7	30	31	B
7514	Bn-N7-p18964562	Bn-A07-p16261412	Bn-A07-p16261412	A7	30	31	B
7515	Bn-N7-p19073905	Bn-A07-p16371184	Bn-A07-p16371184	A7	30	31	B
7516	Bn-N7-p19081820	Bn-A07-p16379135	Bn-A07-p16379135	A7	30	31	B
7517	Bn-N7-p19085345	Bn-A07-p16383705	Bn-A07-p16383705	A7	30	31	B
7518	Bn-N7-p19085528	Bn-A07-p16383888	Bn-A07-p16383888	A7	30	31	B
7519	Bn-N7-p19160040	Bn-A07-p16449988	Bn-A07-p16449988	A7	31	31.9	B
7520	Bn-N7-p19176714	Bn-A07-p16468333	Bn-A07-p16468333	A7	31	31.9	B
7521	Bn-N7-p19177559	Bn-A07-p16469173	Bn-A07-p16469173	A7	31	31.9	B
7522	Bn-N7-p19231704	Bn-A07-p16519850	Bn-A07-p16519850	A7	31	31.9	B
7523	Bn-N7-p19239077	Bn-A07-p16527225	Bn-A07-p16527225	A7	31	31.9	B
7524	Bn-N7-p19268240	Bn-A07-p16557893	Bn-A07-p16557893	A7	31	31.9	B
7525	Bn-N7-p19277609	Bn-A07-p16567234	Bn-A07-p16567234	A7	31	31.9	B
7526	Bn-N7-p19357874	Bn-A07-p16636474	Bn-A07-p16636474	A7	31	31.9	B
7527	Bn-N7-p19383325	Bn-A07-p16659232	Bn-A07-p16659232	A7	31	31.9	B
7528	Bn-N7-p19383489	Bn-A07-p16659396	Bn-A07-p16659396	A7	31	31.9	B
7529	Bn-N7-p19392882	Bn-A07-p16668740	Bn-A07-p16668740	A7	31	31.9	B
7530	Bn-N7-p19441212	Bn-A07-p16716464	Bn-A07-p16716464	A7	31	31.9	B
7531	Bn-N7-p19442895	Bn-A07-p16717729	Bn-A07-p16717729	A7	31	31.9	B
7532	Bn-N7-p19451034	Bn-A07-p16722346	Bn-A07-p16722346	A7	31	31.9	B
7533	Bn-N7-p19451071	Bn-A07-p16722383	Bn-A07-p16722383	A7	31	31.9	B
7534	Bn-N7-p19452718	Bn-A07-p16724882	Bn-A07-p16724882	A7	31	31.9	B
7535	Bn-N7-p19453058	Bn-A07-p16726568	Bn-A07-p16726568	A7	31	31.9	B
7536	Bn-N7-p19454364	Bn-A07-p16727616	Bn-A07-p16727616	A7	31	31.9	B
7537	Bn-N7-p19455771	Bn-A07-p16729007	Bn-A07-p16729007	A7	31	31.9	B
7538	Bn-N7-p19455951	Bn-A07-p16729187	Bn-A07-p16729187	A7	31	31.9	B
7539	Bn-N7-p19455998	Bn-A07-p16729233	Bn-A07-p16729233	A7	31	31.9	B
7540	Bn-N7-p19456204	Bn-A07-p16729439	Bn-A07-p16729439	A7	31	31.9	B
7541	Bn-N7-p19456407	Bn-A07-p16729644	Bn-A07-p16729644	A7	31	31.9	B

7542	Bn-N7-p19468523	Bn-A07-p16749101	Bn-A07-p16749101	A7	31	31.9	B
7543	Bn-N7-p19468658	Bn-C6-p8587890	Bn-scaff_16116_1-p367607	A7	31	31.9	B
7544	Bn-N7-p19474459	Bn-C6-p8581646	Bn-scaff_16116_1-p373851	A7	31	31.9	B
7545	Bn-N7-p19475846	Bn-A07-p16756509	Bn-A07-p16756509	A7	31	31.9	B
7546	Bn-N7-p19480108	Bn-A07-p16760771	Bn-A07-p16760771	A7	31	31.9	B
7547	Bn-N7-p19484933	Bn-A07-p16765596	Bn-A07-p16765596	A7	31	31.9	B
7548	Bn-N7-p19541188	Bn-A07-p16831414	Bn-A07-p16831414	A7	31	31.9	B
7549	Bn-N7-p19542578	Bn-A07-p16833174	Bn-A07-p16833174	A7	31	31.9	B
7550	Bn-N7-p19574144	Bn-A07-p16871349	Bn-A07-p16871349	A7	31	31.9	B
7551	Bn-N7-p19574677	Bn-A07-p16872148	Bn-A07-p16872148	A7	31	31.9	B
7552	Bn-N7-p19595976	Bn-A07-p16896370	Bn-A07-p16896370	A7	31	31.9	B
7553	Bn-N7-p19613978	Bn-A07-p16912963	Bn-A07-p16912963	A7	31	31.9	B
7554	Bn-N7-p19626810	Bn-A07-p16925201	Bn-A07-p16925201	A7	31	31.9	B
7555	Bn-N7-p19635611	Bn-A07-p16934084	Bn-A07-p16934084	A7	31	31.9	B
7556	Bn-N7-p19638052	Bn-A07-p16936529	Bn-A07-p16936529	A7	31	31.9	B
7557	Bn-N7-p19640923	Bn-A07-p16939399	Bn-A07-p16939399	A7	31	31.9	B
7558	Bn-N7-p19647643	Bn-A07-p16946173	Bn-A07-p16946173	A7	31	31.9	B
7559	Bn-N7-p19671405	Bn-A07-p16970000	Bn-A07-p16970000	A7	31	31.9	B
7560	Bn-N7-p19705950	Bn-A07-p17001912	Bn-A07-p17001912	A7	31	31.9	B
7561	Bn-N7-p19728033	Bn-A07-p17026027	Bn-A07-p17026027	A7	31	31.9	B
7562	Bn-N7-p19783236	Bn-A07-p17087544	Bn-A07-p17087544	A7	31	31.9	B
7563	Bn-N7-p19825901	Bn-A07-p17123712	Bn-A07-p17123712	A7	31	31.9	B
7564	Bn-N7-p19936860	Bn-A07-p17212130	Bn-A07-p17212130	A7	32	32.8	B
7565	Bn-N7-p19939221	Bn-A07-p17214494	Bn-A07-p17214494	A7	32	32.8	B
7566	Bn-N7-p19950600	Bn-A07-p17225853	Bn-A07-p17225853	A7	32	32.8	B
7567	Bn-N7-p19990324	Bn-C6-p7678381	Bn-scaff_17436_1-p76050	A7	32	32.8	B
7568	Bn-N7-p20003104	Bn-A07-p17312272	Bn-A07-p17312272	A7	32	32.8	B
7569	Bn-N7-p20021130	Bn-A07-p17326181	Bn-A07-p17326181	A7	32	32.8	B
7570	Bn-N7-p20049427	Bn-A07-p17353806	Bn-A07-p17353806	A7	32	32.8	B
7571	Bn-N7-p20082702	Bn-A07-p17382630	Bn-A07-p17382630	A7	32	32.8	B
7572	Bn-N7-p20100304	Bn-A07-p17400106	Bn-A07-p17400106	A7	32	32.8	B
7573	Bn-N7-p20107667	Bn-A07-p17409328	Bn-A07-p17409328	A7	32	32.8	B
7574	Bn-N7-p20109938	Bn-A07-p17411599	Bn-A07-p17411599	A7	32	32.8	B
7575	Bn-N7-p20111094	Bn-A07-p17412755	Bn-A07-p17412755	A7	32	32.8	B
7576	Bn-N7-p20117518	Bn-A07-p17419179	Bn-A07-p17419179	A7	32	32.8	B
7577	Bn-N7-p20120014	Bn-A07-p17421675	Bn-A07-p17421675	A7	32	32.8	B
7578	Bn-N7-p20137429	Bn-A07-p17439730	Bn-A07-p17439730	A7	32	32.8	B
7579	Bn-N7-p20237616	Bn-C8-p33902403	Bn-scaff_16197_1-p249902	A7	32	32.8	B
7580	Bn-N7-p20289227	Bn-A07-p17593690	Bn-A07-p17593690	A7	32	32.8	B
7581	Bn-N7-p20289232	Bn-A07-p17593695	Bn-A07-p17593695	A7	32	32.8	B
7582	Bn-N7-p20301104	Bn-A07-p17605668	Bn-A07-p17605668	A7	32	32.8	B
7583	Bn-N7-p20301441	Bn-A07-p17606005	Bn-A07-p17606005	A7	32	32.8	B
7584	Bn-N7-p20302014	Bn-A07-p17606752	Bn-A07-p17606752	A7	32	32.8	B
7585	Bn-N7-p20396156	Bn-A07-p17679589	Bn-A07-p17679589	A7	32	32.8	B
7586	Bn-N7-p20518723	Bn-A07-p17804261	Bn-A07-p17804261	A7	33	33.7	B
7587	Bn-N7-p20518734	Bn-A07-p17804272	Bn-A07-p17804272	A7	33	33.7	B
7588	Bn-N7-p20556572	Bn-A07-p17839833	Bn-A07-p17839833	A7	33	33.7	B
7589	Bn-N7-p20567913	Bn-A07-p17846018	Bn-A07-p17846018	A7	33	33.7	B
7590	Bn-N7-p20569743	Bn-A07-p17846299	Bn-A07-p17846299	A7	33	33.7	B

7591	Bn-N16-p32785030	Bn-C6-p6022107	Bn-scaff_23957_1-p17504	A7	34	34.6	B
7592	Bn-N7-p20652751	Bn-A07-p17930992	Bn-A07-p17930992	A7	34	34.6	B
7593	Bn-N7-p20725214	Bn-C6-p6352391	Bn-scaff_18807_1-p15514	A7	34	34.6	B
7594	Bn-N7-p20745840	Bn-A07-p18020732	Bn-A07-p18020732	A7	34	34.6	B
7595	Bn-N7-p20746106	Bn-A07-p18021746	Bn-A07-p18021746	A7	34	34.6	B
7596	Bn-N7-p20752942	Bn-A07-p18028951	Bn-A07-p18028951	A7	34	34.6	B
7597	Bn-N7-p20753044	Bn-A07-p18029053	Bn-A07-p18029053	A7	34	34.6	B
7598	Bn-N7-p20772310	Bn-A07-p18048293	Bn-A07-p18048293	A7	34	34.6	B
7599	Bn-N7-p20773455	Bn-A07-p18049440	Bn-A07-p18049440	A7	34	34.6	B
7600	Bn-N7-p20798609	Bn-A07-p18076313	Bn-A07-p18076313	A7	34	34.6	B
7601	Bn-N7-p20798725	Bn-A07-p18076429	Bn-A07-p18076429	A7	34	34.6	B
7602	Bn-N7-p20808241	Bn-A07-p18084198	Bn-A07-p18084198	A7	34	34.6	B
7603	Bn-N7-p20844230	Bn-A07-p18103464	Bn-A07-p18103464	A7	34	34.6	B
7604	Bn-N7-p20844271	Bn-A07-p18103505	Bn-A07-p18103505	A7	34	34.6	B
7605	Bn-N7-p20846772	Bn-A07-p18106007	Bn-A07-p18106007	A7	34	34.6	B
7606	Bn-N7-p20846821	Bn-C6-p6159516	Bn-scaff_23957_1-p37633	A7	34	34.6	B
7607	Bn-N7-p20847397	Bn-A07-p18106626	Bn-A07-p18106626	A7	34	34.6	B
7608	Bn-N7-p20847520	Bn-A07-p18106750	Bn-A07-p18106750	A7	34	34.6	B
7609	Bn-N7-p20850674	Bn-C6-p6150659	Bn-scaff_23957_1-p46490	A7	34	34.6	B
7610	Bn-N7-p20868387	Bn-C6-p6105816	Bn-scaff_23957_1-p91333	A7	34	34.6	B
7611	Bn-N7-p20905895	Bn-A07-p18151107	Bn-A07-p18151107	A7	34	34.6	B
7612	Bn-N7-p20910957	Bn-A07-p18157564	Bn-A07-p18157564	A7	34	34.6	B
7613	Bn-N7-p20961590	Bn-A07-p18196996	Bn-A07-p18196996	A7	34	34.6	B
7614	Bn-N7-p20963317	Bn-A07-p18201301	Bn-A07-p18201301	A7	34	34.6	B
7615	Bn-N7-p20966934	Bn-A07-p18204966	Bn-A07-p18204966	A7	34	34.6	B
7616	Bn-N7-p20984803	Bn-A07-p18223256	Bn-A07-p18223256	A7	34	34.6	B
7617	Bn-N7-p20988316	Bn-A07-p18227328	Bn-A07-p18227328	A7	34	34.6	B
7618	Bn-N7-p20991021	Bn-A07-p18230931	Bn-A07-p18230931	A7	34	34.6	B
7619	Bn-N7-p21031716	Bn-A07-p18265668	Bn-A07-p18265668	A7	34	34.6	B
7620	Bn-N7-p21031821	Bn-A07-p18265772	Bn-A07-p18265772	A7	34	34.6	B
7621	Bn-N7-p21050850	Bn-A07-p18282722	Bn-A07-p18282722	A7	34	34.6	B
7622	Bn-N7-p21081019	Bn-A07-p18308378	Bn-A07-p18308378	A7	34	34.6	B
7623	Bn-N7-p21092260	Bn-A07-p18319586	Bn-A07-p18319586	A7	34	34.6	B
7624	Bn-N7-p21100078	Bn-A07-p18326762	Bn-A07-p18326762	A7	34	34.6	B
7625	Bn-N7-p21120187	Bn-A07-p18346973	Bn-A07-p18346973	A7	34	34.6	B
7626	Unknown	Bn-A07-p18197951	Bn-A07-p18197951	A7	34	34.6	B
7627	Bn-N7-p21671143	Bn-A07-p18822708	Bn-A07-p18822708	A7	35	35.5	B
7628	Bn-N7-p21681303	Bn-A07-p18832178	Bn-A07-p18832178	A7	35	35.5	B
7629	Bn-N7-p21761161	Bn-A07-p18903215	Bn-A07-p18903215	A7	35	35.5	B
7630	Bn-N7-p21793439	Bn-A07-p18922121	Bn-A07-p18922121	A7	35	35.5	B
7631	Bn-N7-p21814012	Bn-A07-p18935257	Bn-A07-p18935257	A7	35	35.5	B
7632	Bn-N7-p21829421	Bn-A07-p18955989	Bn-A07-p18955989	A7	35	35.5	B
7633	Bn-N7-p21830585	Bn-A07-p18957162	Bn-A07-p18957162	A7	35	35.5	B
7634	Bn-N7-p21839242	Bn-A07-p18968214	Bn-A07-p18968214	A7	35	35.5	B
7635	Bn-N7-p21854973	Bn-A07-p18983802	Bn-A07-p18983802	A7	35	35.5	B
7636	Bn-N7-p21876445	Bn-A07-p19001556	Bn-A07-p19001556	A7	35	35.5	B
7637	Bn-N7-p21876667	Bn-A07-p19001778	Bn-A07-p19001778	A7	35	35.5	B
7638	Bn-N7-p21885980	Bn-A07-p19009397	Bn-A07-p19009397	A7	35	35.5	B
7639	Bn-N7-p21911086	Bn-A07-p19028743	Bn-A07-p19028743	A7	35	35.5	B

7640	Bn-N7-p21991004	Bn-A07-p19110971	Bn-A07-p19110971	A7	35	35.5	B
7641	Bn-N7-p22009465	Bn-A07-p19129406	Bn-A07-p19129406	A7	35	35.5	B
7642	Bn-N7-p22063739	Bn-A07-p19190510	Bn-A07-p19190510	A7	36	38.2	B
7643	Bn-N7-p22065602	Bn-A07-p19192373	Bn-A07-p19192373	A7	36	38.2	B
7644	Bn-N7-p22327509	Bn-A07-p19458370	Bn-A07-p19458370	A7	37	39.1	B
7645	Bn-N7-p22378869	Bn-A07-p19512916	Bn-A07-p19512916	A7	37	39.1	B
7646	Bn-N7-p22429034	Bn-Scaffold00722-p2(	Bn-scaff_19937_1-p20028	A7	37	39.1	B
7647	Bn-N7-p22513003	Bn-A07-p19622207	Bn-A07-p19622207	A7	37	39.1	B
7648	Bn-N16-p35639331	Bn-C6-p3437667	Bn-scaff_20294_1-p25068	A7	38	40	B
7649	Bn-N16-p35639500	Bn-C6-p3437498	Bn-scaff_20294_1-p25051	A7	38	40	B
7650	Bn-N7-p22701176	Bn-A07-p19795126	Bn-A07-p19795126	A7	38	40	B
7651	Bn-N7-p22777474	Bn-A07-p19865996	Bn-A07-p19865996	A7	38	40	B
7652	Bn-N7-p22833926	Bn-A07-p19915062	Bn-A07-p19915062	A7	38	40	B
7653	Bn-N16-p35914324	Bn-A07-p19975808	Bn-A07-p19975808	A7	39	42.5	B
7654	Bn-N2-p15286205	Bn-A07-p7640474	Bn-A07-p7640474	A7	39	42.5	B
7655	Bn-N7-p22880995	Bn-A07-p19957202	Bn-A07-p19957202	A7	39	42.5	B
7656	Bn-N7-p22885432	Bn-A07-p19961825	Bn-A07-p19961825	A7	39	42.5	B
7657	Bn-N7-p22889167	Bn-A07-p19965401	Bn-A07-p19965401	A7	39	42.5	B
7658	Bn-N7-p22923766	Bn-A07-p19995985	Bn-A07-p19995985	A7	39	42.5	B
7659	Bn-N7-p23083318	Bn-A07-p20144687	Bn-A07-p20144687	A7	40	43.6	B
7660	Bn-N7-p23122457	Bn-A07-p20192092	Bn-A07-p20192092	A7	40	43.6	B
7661	Bn-N7-p23146689	Bn-A07-p20221692	Bn-A07-p20221692	A7	40	43.6	B
7662	Bn-N7-p23153248	Bn-A07-p20230189	Bn-A07-p20230189	A7	40	43.6	B
7663	Bn-N7-p23154664	Bn-A07-p20231605	Bn-A07-p20231605	A7	40	43.6	B
7664	Bn-N7-p23164955	Bn-A07-p20248654	Bn-A07-p20248654	A7	40	43.6	B
7665	Bn-N7-p23165123	Bn-A07-p20248822	Bn-A07-p20248822	A7	40	43.6	B
7666	Bn-N7-p23167942	Bn-A07-p20251365	Bn-A07-p20251365	A7	40	43.6	B
7667	Bn-N7-p23418291	Bn-A07-p20467347	Bn-A07-p20467347	A7	41	44.5	B
7668	Bn-N7-p23434792	Bn-A07-p20481682	Bn-A07-p20481682	A7	41	44.5	B
7669	Bn-N7-p23436942	Bn-A07-p20485039	Bn-A07-p20485039	A7	41	44.5	B
7670	Bn-N16-p36915527	Bn-A07-p20688218	Bn-A07-p20688218	A7	42	47.7	B
7671	Bn-N16-p36915646	Bn-A07-p20688341	Bn-A07-p20688341	A7	42	47.7	B
7672	Bn-N7-p23514296	Bn-A07-p20554194	Bn-A07-p20554194	A7	42	47.7	B
7673	Bn-N7-p23518260	Bn-A07-p20558164	Bn-A07-p20558164	A7	42	47.7	B
7674	Bn-N7-p23524919	Bn-A07-p20565057	Bn-A07-p20565057	A7	42	47.7	B
7675	Bn-N7-p23525546	Bn-A07-p20565684	Bn-A07-p20565684	A7	42	47.7	B
7676	Bn-N7-p23539797	Bn-A07-p20580512	Bn-A07-p20580512	A7	42	47.7	B
7677	Bn-N7-p23549843	Bn-A07-p20589714	Bn-A07-p20589714	A7	42	47.7	B
7678	Bn-N7-p23615357	Bn-A07-p20646388	Bn-A07-p20646388	A7	42	47.7	B
7679	Bn-N7-p23618921	Bn-A07-p20649978	Bn-A07-p20649978	A7	42	47.7	B
7680	Bn-N7-p23619748	Bn-A07-p20658738	Bn-A07-p20658738	A7	42	47.7	B
7681	Bn-N7-p23623208	Bn-A07-p20662200	Bn-A07-p20662200	A7	42	47.7	B
7682	Bn-N7-p23721465	Bn-A07-p20771568	Bn-A07-p20771568	A7	42	47.7	B
7683	Bn-N7-p23749440	Bn-A07-p20801106	Bn-A07-p20801106	A7	42	47.7	B
7684	Bn-N7-p23749590	Bn-A07-p20801255	Bn-A07-p20801255	A7	42	47.7	B
7685	Bn-N7-p23749823	Bn-A07-p20801487	Bn-A07-p20801487	A7	42	47.7	B
7686	Bn-N7-p23749922	Bn-A07-p20801586	Bn-A07-p20801586	A7	42	47.7	B
7687	Bn-N7-p23750098	Bn-A07-p20801762	Bn-A07-p20801762	A7	42	47.7	B
7688	Bn-N7-p23918790	Bn-A07-p21019499	Bn-A07-p21019499	A7	43	55	B

7689	Bn-N7-p24106556	Bn-C6-p1629681	Bn-scaff_17799_1-p10534!	A7	43	55	B
7690	Bn-N7-p24113156	Bn-A07-p21191683	Bn-A07-p21191683	A7	43	55	B
7691	Bn-N7-p23833106	Bn-A07-p20894218	Bn-A07-p20894218	A7	44	57.7	B
7692	Bn-N7-p23882909	Bn-A07-p20981410	Bn-A07-p20981410	A7	44	57.7	B
7693	Bn-N7-p23888405	Bn-A07-p20986876	Bn-A07-p20986876	A7	44	57.7	B
7694	Bn-N7-p23905791	Bn-A07-p21006643	Bn-A07-p21006643	A7	44	57.7	B
7695	Bn-N7-p23974541	Bn-A07-p21078249	Bn-A07-p21078249	A7	44	57.7	B
7696	Bn-N7-p24117019	Bn-A07-p21193584	Bn-A07-p21193584	A7	44	57.7	B
7697	Bn-N7-p24180943	Bn-A07-p21261518	Bn-A07-p21261518	A7	44	57.7	B
7698	Bn-N16-p37750449	Bn-A07-p21410292	Bn-A07-p21410292	A7	45	58.7	B
7699	Bn-N16-p37793518	Bn-A07-p21449796	Bn-A07-p21449796	A7	45	58.7	B
7700	Bn-N7-p24325175	Bn-A07-p21476982	Bn-A07-p21476982	A7	45	58.7	B
7701	Bn-Scaffold04386-p1414	Bn-A07-p21377298	Bn-A07-p21377298	A7	45	58.7	B
7702	Bn-Scaffold04407-p991	Bn-A07-p21365616	Bn-A07-p21365616	A7	45	58.7	B
7703	Bn-Scaffold27418-p265	Bn-A07-p21371577	Bn-A07-p21371577	A7	45	58.7	B
7704	Bn-Scaffold58184-p90	Bn-A07-p21430149	Bn-A07-p21430149	A7	45	58.7	B
7705	Bn-N16-p37719630	Bn-C6-p1416583	Bn-scaff_17799_1-p84035	A7	45	58.7	B
7706	Bn-N7-p24239989	Bn-A07-p21321699	Bn-A07-p21321699	A7	45	58.7	B
7707	Bn-N7-p24327080	Bn-A07-p21478337	Bn-A07-p21478337	A7	45	58.7	B
7747	Bn-N16-p37908466	Bn-A07-p21546078	Bn-A07-p21546078	A7	46	60	B
7708	Bn-N16-p38623586	Bn-C6-p553028	Bn-scaff_24104_1-p38670	A7	46	60	B
7709	Bn-N16-p38701396	Bn-A07-p22231852	Bn-A07-p22231852	A7	46	60	B
7746	Bn-N7-p24426258	Bn-A07-p21569289	Bn-A07-p21569289	A7	46	60	B
7710	Bn-N7-p24429698	Bn-A07-p21573107	Bn-A07-p21573107	A7	46	60	B
7711	Bn-N7-p24433733	Bn-A07-p21577164	Bn-A07-p21577164	A7	46	60	B
7712	Bn-N7-p24444677	Bn-A07-p21587819	Bn-A07-p21587819	A7	46	60	B
7713	Bn-N7-p24519134	Bn-C6-p1084072	Bn-scaff_17799_1-p50784	A7	46	60	B
7714	Bn-N7-p24535423	Bn-A07-p21678440	Bn-A07-p21678440	A7	46	60	B
7715	Bn-N7-p24537914	Bn-A07-p21680952	Bn-A07-p21680952	A7	46	60	B
7716	Bn-N7-p24553714	Bn-A07-p21681332	Bn-A07-p21681332	A7	46	60	B
7717	Bn-N7-p24554119	Bn-A07-p21681736	Bn-A07-p21681736	A7	46	60	B
7718	Bn-N7-p24626217	Bn-A07-p21844673	Bn-A07-p21844673	A7	46	60	B
7719	Bn-N7-p24662708	Bn-A07-p21875901	Bn-A07-p21875901	A7	46	60	B
7720	Bn-N7-p24760074	Bn-A07-p21959231	Bn-A07-p21959231	A7	46	60	B
7721	Bn-N7-p24775530	Bn-A07-p21970228	Bn-A07-p21970228	A7	46	60	B
7722	Bn-N7-p24799309	Bn-A07-p21993375	Bn-A07-p21993375	A7	46	60	B
7723	Bn-N7-p24801058	Bn-A07-p21995054	Bn-A07-p21995054	A7	46	60	B
7724	Bn-N7-p24811031	Bn-A07-p22005058	Bn-A07-p22005058	A7	46	60	B
7725	Bn-N7-p24811365	Bn-A07-p22005392	Bn-A07-p22005392	A7	46	60	B
7726	Bn-N7-p24814522	Bn-A07-p22010671	Bn-A07-p22010671	A7	46	60	B
7727	Bn-N7-p24824180	Bn-A07-p22019732	Bn-A07-p22019732	A7	46	60	B
7728	Bn-N7-p24856683	Bn-C6-p726719	Bn-scaff_17799_1-p15048	A7	46	60	B
7729	Bn-N7-p24901048	Bn-A07-p22096241	Bn-A07-p22096241	A7	46	60	B
7730	Bn-N7-p24927237	Bn-A07-p22119502	Bn-A07-p22119502	A7	46	60	B
7731	Bn-N7-p24928956	Bn-A07-p22121263	Bn-A07-p22121263	A7	46	60	B
7732	Bn-N7-p24948807	Bn-A07-p22140320	Bn-A07-p22140320	A7	46	60	B
7733	Bn-N7-p25028678	Bn-A07-p22209952	Bn-A07-p22209952	A7	46	60	B
7734	Bn-N7-p25062637	Bn-A07-p22237999	Bn-A07-p22237999	A7	46	60	B
7735	Bn-N7-p25063313	Bn-A07-p22238668	Bn-A07-p22238668	A7	46	60	B

7736	Bn-N7-p25075523	Bn-A07-p22250567	Bn-A07-p22250567	A7	46	60	B
7737	Bn-N7-p25078634	Bn-A07-p22253687	Bn-A07-p22253687	A7	46	60	B
7738	Bn-N7-p25126505	Bn-A07-p22302158	Bn-A07-p22302158	A7	46	60	B
7739	Bn-N7-p25131575	Bn-A07-p22306994	Bn-A07-p22306994	A7	46	60	B
7740	Bn-N7-p25131602	Bn-A07-p22307021	Bn-A07-p22307021	A7	46	60	B
7741	Bn-N7-p25133285	Bn-A07-p22308030	Bn-A07-p22308030	A7	46	60	B
7742	Bn-N7-p25134315	Bn-A07-p22309060	Bn-A07-p22309060	A7	46	60	B
7743	Bn-N7-p25134438	Bn-A07-p22309183	Bn-A07-p22309183	A7	46	60	B
7744	Bn-N7-p25135147	Bn-A07-p22310107	Bn-A07-p22310107	A7	46	60	B
7745	Bn-N7-p25157288	Bn-A07-p22329359	Bn-A07-p22329359	A7	46	60	B
7748	Bn-N13-p64939357	Bn-C3-p62191846	Bn-scaff_18559_1-p77834	A8	1	0	B
7749	Bn-N13-p66171597	Bn-C3-p63382423	Bn-scaff_23098_1-p90934	A8	1	0	B
7750	Bn-N13-p66924325	Bn-A08-p626649	Bn-A08-p626649	A8	1	0	B
7751	Bn-N13-p66990904	Bn-A08-p572531	Bn-A08-p572531	A8	1	0	B
7752	Bn-N13-p67434994	Bn-A08-p293480	Bn-A08-p293480	A8	1	0	B
7753	Bn-N13-p67517978	Bn-A08-p211090	Bn-A08-p211090	A8	1	0	B
7754	Bn-N8-p1020964	Bn-A08-p792162	Bn-A08-p792162	A8	1	0	B
7755	Bn-N8-p1047794	Bn-A08-p813316	Bn-A08-p813316	A8	1	0	B
7756	Bn-N8-p1075920	Bn-A08-p848067	Bn-A08-p848067	A8	1	0	B
7757	Bn-N8-p1138446	Bn-A08-p937299	Bn-A08-p937299	A8	1	0	B
7758	Bn-N8-p1141887	Bn-A08-p940619	Bn-A08-p940619	A8	1	0	B
7759	Bn-N8-p1189412	Bn-A08-p980768	Bn-A08-p980768	A8	1	0	B
7760	Bn-N8-p1217690	Bn-A08-p1007687	Bn-A08-p1007687	A8	1	0	B
7761	Bn-N8-p1220551	Bn-A08-p1010548	Bn-A08-p1010548	A8	1	0	B
7762	Bn-N8-p1229567	Bn-A08-p1022454	Bn-A08-p1022454	A8	1	0	B
7763	Bn-N8-p1251878	Bn-A08-p1049012	Bn-A08-p1049012	A8	1	0	B
7764	Bn-N8-p1259765	Bn-A08-p1059680	Bn-A08-p1059680	A8	1	0	B
7765	Bn-N8-p1259943	Bn-A08-p1059858	Bn-A08-p1059858	A8	1	0	B
7766	Bn-N8-p1260051	Bn-A08-p1059966	Bn-A08-p1059966	A8	1	0	B
7767	Bn-N8-p1277970	Bn-A08-p1077573	Bn-A08-p1077573	A8	1	0	B
7768	Bn-N8-p1284504	Bn-A08-p1088499	Bn-A08-p1088499	A8	1	0	0
7769	Bn-N8-p1284706	Bn-A08-p1088701	Bn-A08-p1088701	A8	1	0	B
7770	Bn-N8-p1331310	Bn-A08-p1166566	Bn-A08-p1166566	A8	1	0	B
7771	Bn-N8-p1387662	Bn-C3-p63153426	Bn-scaff_23098_1-p31993	A8	1	0	B
7772	Bn-N8-p1516253	Bn-A08-p15027690	Bn-A08-p15027690	A8	1	0	B
7773	Bn-N8-p1558964	Bn-C3-p62785421	Bn-scaff_16148_1-p12132	A8	1	0	B
7774	Bn-N8-p1579503	Bn-A08-p1720366	Bn-A08-p1720366	A8	1	0	B
7775	Bn-N8-p1603580	Bn-Scaffold000477-p	Bn-Scaffold000477-p7116	A8	1	0	B
7776	Bn-N8-p1682130	Bn-A08-p1809204	Bn-A08-p1809204	A8	1	0	B
7777	Bn-N8-p2137845	Bn-C3-p62192308	Bn-scaff_18559_1-p77372	A8	1	0	B
7778	Bn-N8-p2255128	Bn-A08-p2394252	Bn-A08-p2394252	A8	1	0	B
7779	Bn-N8-p2257095	Bn-A08-p2397885	Bn-A08-p2397885	A8	1	0	B
7780	Bn-N8-p2266693	Bn-A08-p2406380	Bn-A08-p2406380	A8	1	0	B
7781	Bn-N8-p2286125	Bn-A08-p2425057	Bn-A08-p2425057	A8	1	0	B
7782	Bn-N8-p2286215	Bn-A08-p2425147	Bn-A08-p2425147	A8	1	0	B
7783	Bn-N8-p2290308	Bn-A08-p2433393	Bn-A08-p2433393	A8	1	0	B
7784	Bn-N8-p2321801	Bn-A08-p2461697	Bn-A08-p2461697	A8	1	0	B
7785	Bn-N8-p2350131	Bn-A08-p2509113	Bn-A08-p2509113	A8	1	0	B
7786	Bn-N8-p2353077	Bn-A08-p2511340	Bn-A08-p2511340	A8	1	0	B

7787	Bn-N8-p2354217	Bn-A08-p2512477	Bn-A08-p2512477	A8	1	0	B
7788	Bn-N8-p2386707	Bn-A08-p2543882	Bn-A08-p2543882	A8	1	0	B
7789	Bn-N8-p2430973	Bn-A08-p2576185	Bn-A08-p2576185	A8	1	0	B
7790	Bn-N8-p2438159	Bn-A08-p2585836	Bn-A08-p2585836	A8	1	0	B
7791	Bn-N8-p2442184	Bn-A08-p2589956	Bn-A08-p2589956	A8	1	0	B
7792	Bn-N8-p2460266	Bn-A08-p2601211	Bn-A08-p2601211	A8	1	0	B
7793	Bn-N8-p2465138	Bn-A08-p2605912	Bn-A08-p2605912	A8	1	0	B
7794	Bn-N8-p2477438	Bn-A08-p2618037	Bn-A08-p2618037	A8	1	0	B
7795	Bn-N8-p2505061	Bn-C8-p2371103	Bn-scaff_16022_1-p14361	A8	1	0	B
7796	Bn-N8-p2507725	Bn-A08-p2660414	Bn-A08-p2660414	A8	1	0	B
7797	Bn-N8-p2517806	Bn-A08-p2675098	Bn-A08-p2675098	A8	1	0	B
7798	Bn-N8-p2680032	Bn-A08-p2819820	Bn-A08-p2819820	A8	1	0	B
7799	Bn-N8-p2694844	Bn-Scaffold00685-p8	Bn-scaff_27765_1-p33897	A8	1	0	B
7800	Bn-N8-p2706828	Bn-Scaffold00685-p8	Bn-scaff_27765_1-p33220	A8	1	0	B
7801	Bn-N8-p2715964	Bn-A08-p2841059	Bn-A08-p2841059	A8	1	0	B
7802	Bn-N8-p2716550	Bn-A08-p2841643	Bn-A08-p2841643	A8	1	0	B
7803	Bn-N8-p2774613	Bn-A08-p2883203	Bn-A08-p2883203	A8	1	0	B
7804	Bn-N8-p2777219	Bn-A08-p2885855	Bn-A08-p2885855	A8	1	0	B
7805	Bn-N8-p2784929	Bn-A08-p2898446	Bn-A08-p2898446	A8	1	0	B
7806	Bn-N8-p2793274	Bn-A08-p2910044	Bn-A08-p2910044	A8	1	0	B
7807	Bn-N8-p2794047	Bn-A08-p2910816	Bn-A08-p2910816	A8	1	0	B
7808	Bn-N8-p2794332	Bn-A08-p2911101	Bn-A08-p2911101	A8	1	0	B
7809	Bn-N8-p2803775	Bn-A08-p2920715	Bn-A08-p2920715	A8	1	0	B
7810	Bn-N8-p295324	Bn-A08-p165219	Bn-A08-p165219	A8	1	0	B
7811	Bn-N8-p302806	Bn-A08-p177288	Bn-A08-p177288	A8	1	0	B
7812	Bn-N8-p4619176	Bn-C5-p34051129	Bn-scaff_22002_1-p78733	A8	1	0	0
7813	Bn-N8-p599085	Bn-A08-p424009	Bn-A08-p424009	A8	1	0	B
7814	Bn-N8-p658897	Bn-A08-p482401	Bn-A08-p482401	A8	1	0	B
7815	Bn-N8-p711266	Bn-A08-p537474	Bn-A08-p537474	A8	1	0	B
7816	Bn-N8-p718156	Bn-A08-p543480	Bn-A08-p543480	A8	1	0	B
7817	Bn-N8-p758575	Bn-A08-p570304	Bn-A08-p570304	A8	1	0	B
7830	Bn-N8-p972937	Bn-A08-p745168	Bn-A08-p745168	A8	1	0	B
7831	Bn-Scaffold02025-p4211	Bn-Scaffold040234-p	Bn-Scaffold040234-p71	A8	1	0	0
7832	Bn-Scaffold03669-p603	Bn-A08-p2512893	Bn-A08-p2512893	A8	1	0	B
7833	Bn-Scaffold04146-p966	Bn-A08-p2823223	Bn-A08-p2823223	A8	1	0	B
7835	Bn-N13-p12708164	Bn-C2-p23559226	Bn-scaff_15636_1-p29470	A8	2	0.8	B
7836	Bn-N18-p10209411	Bn-A08-p7803865	Bn-A08-p7803865	A8	2	0.8	B
7837	Bn-N18-p11290982	Bn-A08-p8320380	Bn-A08-p8320380	A8	2	0.8	B
7838	Bn-N18-p11358848	Bn-A08-p8372634	Bn-A08-p8372634	A8	2	0.8	B
7839	Bn-N18-p13590482	Bn-A08-p5540892	Bn-A08-p5540892	A8	2	0.8	B
7840	Bn-N18-p14653842	Bn-A08-p5046769	Bn-A08-p5046769	A8	2	0.8	B
7842	Bn-N18-p3962969	Bn-A08-p3195277	Bn-A08-p3195277	A8	2	0.8	B
7843	Bn-N18-p4698077	Bn-C8-p3555347	Bn-scaff_21003_1-p45712	A8	2	0.8	B
7844	Bn-N18-p6297980	Bn-A08-p4267191	Bn-A08-p4267191	A8	2	0.8	B
7845	Bn-N18-p8810165	Bn-A08-p7303995	Bn-A08-p7303995	A8	2	0.8	B
7846	Bn-N18-p8858481	Bn-A08-p7336214	Bn-A08-p7336214	A8	2	0.8	B
7847	Bn-N6-p16074386	Bn-Scaffold000180-p	Bn-Scaffold000180-p11220	A8	2	0.8	B
7848	Bn-N8-p24575	Bn-Scaffold000180-p	Bn-Scaffold000180-p12803	A8	2	0.8	B
7849	Bn-N8-p2854610	Bn-A08-p2973537	Bn-A08-p2973537	A8	2	0.8	B

7850	Bn-N8-p2871305	Bn-A08-p2989598	Bn-A08-p2989598	A8	2	0.8	B
7851	Bn-N8-p2874122	Bn-A08-p2995827	Bn-A08-p2995827	A8	2	0.8	B
7852	Bn-N8-p2911497	Bn-A08-p3031902	Bn-A08-p3031902	A8	2	0.8	B
7853	Bn-N8-p2941458	Bn-A08-p3064639	Bn-A08-p3064639	A8	2	0.8	B
7854	Bn-N8-p2945655	Bn-A08-p3068838	Bn-A08-p3068838	A8	2	0.8	B
7855	Bn-N8-p3000167	Bn-A08-p3120976	Bn-A08-p3120976	A8	2	0.8	B
7856	Bn-N8-p3019324	Bn-A08-p3138888	Bn-A08-p3138888	A8	2	0.8	B
7857	Bn-N8-p3048721	Bn-A08-p3168874	Bn-A08-p3168874	A8	2	0.8	B
7858	Bn-N8-p3061223	Bn-A08-p3178977	Bn-A08-p3178977	A8	2	0.8	B
7859	Bn-N8-p3069193	Bn-A08-p3182594	Bn-A08-p3182594	A8	2	0.8	B
7860	Bn-N8-p3069397	Bn-A08-p3182798	Bn-A08-p3182798	A8	2	0.8	B
7861	Bn-N8-p3070767	Bn-A08-p3184233	Bn-A08-p3184233	A8	2	0.8	B
7862	Bn-N8-p3100821	Bn-A08-p3221528	Bn-A08-p3221528	A8	2	0.8	B
7863	Bn-N8-p3116233	Bn-A08-p3237433	Bn-A08-p3237433	A8	2	0.8	B
7864	Bn-N8-p3125777	Bn-A08-p3246905	Bn-A08-p3246905	A8	2	0.8	B
7865	Bn-N8-p3166259	Bn-A08-p3284482	Bn-A08-p3284482	A8	2	0.8	B
7866	Bn-N8-p3166443	Bn-A08-p3284666	Bn-A08-p3284666	A8	2	0.8	B
7867	Bn-N8-p3166594	Bn-A08-p3284817	Bn-A08-p3284817	A8	2	0.8	B
7868	Bn-N8-p3166732	Bn-A08-p3284955	Bn-A08-p3284955	A8	2	0.8	B
7869	Bn-N8-p3167625	Bn-A08-p3285855	Bn-A08-p3285855	A8	2	0.8	B
7870	Bn-N8-p3186724	Bn-A08-p3302700	Bn-A08-p3302700	A8	2	0.8	B
7871	Bn-N8-p3285328	Bn-A08-p3433222	Bn-A08-p3433222	A8	2	0.8	B
7872	Bn-N8-p3285969	Bn-A08-p3433878	Bn-A08-p3433878	A8	2	0.8	B
7873	Bn-N8-p3288453	Bn-A08-p3436359	Bn-A08-p3436359	A8	2	0.8	B
7874	Bn-N8-p3307360	Bn-A08-p3464671	Bn-A08-p3464671	A8	2	0.8	B
7875	Bn-N8-p3332899	Bn-A08-p3519373	Bn-A08-p3519373	A8	2	0.8	B
7876	Bn-N8-p3340656	Bn-A08-p3529542	Bn-A08-p3529542	A8	2	0.8	B
7877	Bn-N8-p3357184	Bn-A08-p3546013	Bn-A08-p3546013	A8	2	0.8	B
7878	Bn-N8-p3371674	Bn-A08-p3566821	Bn-A08-p3566821	A8	2	0.8	B
7879	Bn-N8-p3384569	Bn-A08-p3573966	Bn-A08-p3573966	A8	2	0.8	B
7880	Bn-N8-p3385002	Bn-A08-p3574400	Bn-A08-p3574400	A8	2	0.8	B
7881	Bn-N8-p3443036	Bn-A08-p3614809	Bn-A08-p3614809	A8	2	0.8	B
7882	Bn-N8-p3465024	Bn-A08-p3628921	Bn-A08-p3628921	A8	2	0.8	B
7883	Bn-N8-p36538	Bn-Scaffold000180-p[illegible]	Bn-Scaffold000180-p13522	A8	2	0.8	B
7884	Bn-N8-p3739852	Bn-A08-p3840983	Bn-A08-p3840983	A8	2	0.8	B
7885	Bn-N8-p37449	Bn-Scaffold000180-p[illegible]	Bn-Scaffold000180-p13613	A8	2	0.8	B
7886	Bn-N8-p3749216	Bn-A08-p3846272	Bn-A08-p3846272	A8	2	0.8	B
7887	Bn-N8-p3759596	Bn-A08-p3862608	Bn-A08-p3862608	A8	2	0.8	B
7888	Bn-N8-p3788226	Bn-Scaffold000259-p[illegible]	Bn-Scaffold000259-p29271	A8	2	0.8	B
7889	Bn-N8-p3789729	Bn-Scaffold000259-p[illegible]	Bn-Scaffold000259-p30688	A8	2	0.8	B
7890	Bn-N8-p3796116	Bn-Scaffold000259-p[illegible]	Bn-Scaffold000259-p35623	A8	2	0.8	B
7891	Bn-N8-p3797614	Bn-Scaffold000259-p[illegible]	Bn-Scaffold000259-p37148	A8	2	0.8	B
7892	Bn-N8-p3798406	Bn-Scaffold000259-p[illegible]	Bn-Scaffold000259-p37845	A8	2	0.8	B
7893	Bn-N8-p3827464	Bn-A08-p3964610	Bn-A08-p3964610	A8	2	0.8	B
7894	Bn-N8-p3827694	Bn-A08-p3964840	Bn-A08-p3964840	A8	2	0.8	B
7895	Bn-N8-p3837598	Bn-A08-p4000353	Bn-A08-p4000353	A8	2	0.8	B
7896	Bn-N8-p3840997	Bn-A08-p4001683	Bn-A08-p4001683	A8	2	0.8	B
7897	Bn-N8-p3847813	Bn-A08-p4014130	Bn-A08-p4014130	A8	2	0.8	B
7898	Bn-N8-p3857296	Bn-A08-p4021680	Bn-A08-p4021680	A8	2	0.8	B

7899	Bn-N8-p3858065	Bn-A08-p4022450	Bn-A08-p4022450	A8	2	0.8	B
7900	Bn-N8-p3858303	Bn-A08-p4022688	Bn-A08-p4022688	A8	2	0.8	B
7901	Bn-N8-p3865318	Bn-A08-p4029890	Bn-A08-p4029890	A8	2	0.8	B
7902	Bn-N8-p3869304	Bn-Scaffold000654-p	Bn-Scaffold000654-p721	A8	2	0.8	B
7903	Bn-N8-p3870953	Bn-Scaffold000654-p	Bn-Scaffold000654-p2388	A8	2	0.8	B
7904	Bn-N8-p3988274	Bn-A08-p4152083	Bn-A08-p4152083	A8	2	0.8	B
7905	Bn-N8-p4031637	Bn-A08-p4182726	Bn-A08-p4182726	A8	2	0.8	0
7906	Bn-N8-p4086978	Bn-A08-p4253915	Bn-A08-p4253915	A8	2	0.8	B
7907	Bn-N8-p4087104	Bn-A08-p4254041	Bn-A08-p4254041	A8	2	0.8	B
7908	Bn-N8-p4104241	Bn-A08-p4271010	Bn-A08-p4271010	A8	2	0.8	B
7909	Bn-N8-p4104493	Bn-A08-p4271262	Bn-A08-p4271262	A8	2	0.8	B
7910	Bn-N8-p4106457	Bn-A08-p4273091	Bn-A08-p4273091	A8	2	0.8	B
7911	Bn-N8-p41514	Bn-Scaffold000180-p	Bn-Scaffold000180-p14020	A8	2	0.8	B
7912	Bn-N8-p4227666	Bn-A08-p4392909	Bn-A08-p4392909	A8	2	0.8	B
7913	Bn-N8-p42472	Bn-Scaffold000180-p	Bn-Scaffold000180-p14116	A8	2	0.8	B
7914	Bn-N8-p42643	Bn-Scaffold000180-p	Bn-Scaffold000180-p14133	A8	2	0.8	B
7915	Bn-N8-p42872	Bn-Scaffold000180-p	Bn-Scaffold000180-p14156	A8	2	0.8	B
7916	Bn-N8-p4288714	Bn-A08-p4453282	Bn-A08-p4453282	A8	2	0.8	B
7917	Bn-N8-p4304291	Bn-A08-p4467910	Bn-A08-p4467910	A8	2	0.8	B
7918	Bn-N8-p43821	Bn-Scaffold000180-p	Bn-Scaffold000180-p14251	A8	2	0.8	B
7919	Bn-N8-p4441754	Bn-A08-p4598270	Bn-A08-p4598270	A8	2	0.8	B
7920	Bn-N8-p4444014	Bn-A08-p4602542	Bn-A08-p4602542	A8	2	0.8	B
7921	Bn-N8-p4445217	Bn-A08-p4603745	Bn-A08-p4603745	A8	2	0.8	B
7922	Bn-N8-p4477959	Bn-A08-p4636503	Bn-A08-p4636503	A8	2	0.8	B
7923	Bn-N8-p4486841	Bn-A08-p4645422	Bn-A08-p4645422	A8	2	0.8	B
7924	Bn-N8-p4487332	Bn-A08-p4645913	Bn-A08-p4645913	A8	2	0.8	B
7925	Bn-N8-p4504497	Bn-A08-p4666053	Bn-A08-p4666053	A8	2	0.8	B
7926	Bn-N8-p4507005	Bn-A08-p4668561	Bn-A08-p4668561	A8	2	0.8	B
7927	Bn-N8-p4508913	Bn-A08-p4670469	Bn-A08-p4670469	A8	2	0.8	B
7928	Bn-N8-p4525757	Bn-A08-p4687828	Bn-A08-p4687828	A8	2	0.8	B
7929	Bn-N8-p4547035	Bn-A08-p4708904	Bn-A08-p4708904	A8	2	0.8	B
7930	Bn-N8-p4557678	Bn-A08-p4719687	Bn-A08-p4719687	A8	2	0.8	B
7931	Bn-N8-p4601310	Bn-A08-p4765954	Bn-A08-p4765954	A8	2	0.8	B
7932	Bn-N8-p4690333	Bn-A08-p6029041	Bn-A08-p6029041	A8	2	0.8	B
7933	Bn-N8-p4759962	Bn-A08-p5975890	Bn-A08-p5975890	A8	2	0.8	B
7934	Bn-N8-p4783876	Bn-A08-p4993820	Bn-A08-p4993820	A8	2	0.8	B
7935	Bn-N8-p4784242	Bn-A08-p4994186	Bn-A08-p4994186	A8	2	0.8	B
7936	Bn-N8-p4796099	Bn-A08-p5006162	Bn-A08-p5006162	A8	2	0.8	B
7937	Bn-N8-p4806558	Bn-A08-p5017136	Bn-A08-p5017136	A8	2	0.8	B
7938	Bn-N8-p4820668	Bn-A08-p5031773	Bn-A08-p5031773	A8	2	0.8	B
7939	Bn-N8-p4831215	Bn-A08-p5044814	Bn-A08-p5044814	A8	2	0.8	B
7940	Bn-N8-p4840409	Bn-A08-p5054005	Bn-A08-p5054005	A8	2	0.8	B
7941	Bn-N8-p4841017	Bn-A08-p5054613	Bn-A08-p5054613	A8	2	0.8	B
7942	Bn-N8-p4843458	Bn-A08-p5056953	Bn-A08-p5056953	A8	2	0.8	B
7943	Bn-N8-p4867184	Bn-A08-p5084300	Bn-A08-p5084300	A8	2	0.8	B
7944	Bn-N8-p4876764	Bn-A08-p5096382	Bn-A08-p5096382	A8	2	0.8	B
7945	Bn-N8-p4885324	Bn-A08-p5104945	Bn-A08-p5104945	A8	2	0.8	B
7946	Bn-N8-p4886125	Bn-A08-p5105745	Bn-A08-p5105745	A8	2	0.8	B
7947	Bn-N8-p4890096	Bn-A08-p5109706	Bn-A08-p5109706	A8	2	0.8	B

7948	Bn-N8-p4908806	Bn-A08-p5146710	Bn-A08-p5146710	A8	2	0.8	B
7949	Bn-N8-p4916600	Bn-A08-p5153375	Bn-A08-p5153375	A8	2	0.8	B
7950	Bn-N8-p4924058	Bn-A08-p5155511	Bn-A08-p5155511	A8	2	0.8	B
7951	Bn-N8-p4925818	Bn-A08-p5174518	Bn-A08-p5174518	A8	2	0.8	B
7952	Bn-N8-p4926118	Bn-A08-p5174818	Bn-A08-p5174818	A8	2	0.8	B
7953	Bn-N8-p4965695	Bn-A08-p5212067	Bn-A08-p5212067	A8	2	0.8	B
7954	Bn-N8-p5021473	Bn-A08-p5249573	Bn-A08-p5249573	A8	2	0.8	B
7955	Bn-N8-p5025595	Bn-A08-p5255647	Bn-A08-p5255647	A8	2	0.8	B
7956	Bn-N8-p5026993	Bn-A08-p5257044	Bn-A08-p5257044	A8	2	0.8	B
7957	Bn-N8-p5027763	Bn-A08-p5257814	Bn-A08-p5257814	A8	2	0.8	B
7958	Bn-N8-p50317	Bn-Scaffold000180-p1	Bn-Scaffold000180-p14904	A8	2	0.8	B
7959	Bn-N8-p5033527	Bn-A08-p5263570	Bn-A08-p5263570	A8	2	0.8	B
7960	Bn-N8-p5038061	Bn-A08-p5266776	Bn-A08-p5266776	A8	2	0.8	B
7961	Bn-N8-p5082390	Bn-A08-p5300046	Bn-A08-p5300046	A8	2	0.8	B
7962	Bn-N8-p5082864	Bn-A08-p5300520	Bn-A08-p5300520	A8	2	0.8	B
7963	Bn-N8-p5082992	Bn-A08-p5300648	Bn-A08-p5300648	A8	2	0.8	B
7964	Bn-N8-p5091956	Bn-A08-p5305309	Bn-A08-p5305309	A8	2	0.8	B
7965	Bn-N8-p5113178	Bn-A08-p5333513	Bn-A08-p5333513	A8	2	0.8	B
7966	Bn-N8-p5130068	Bn-A09-p32624472	Bn-A09-p32624472	A8	2	0.8	B
7967	Bn-N8-p5145014	Bn-A08-p5343622	Bn-A08-p5343622	A8	2	0.8	B
7968	Bn-N8-p5150480	Bn-A08-p5351001	Bn-A08-p5351001	A8	2	0.8	B
7969	Bn-N8-p5161729	Bn-A08-p5361134	Bn-A08-p5361134	A8	2	0.8	B
7970	Bn-N8-p5165248	Bn-A08-p5364653	Bn-A08-p5364653	A8	2	0.8	B
7971	Bn-N8-p5201219	Bn-C1-p11901149	Bn-scaff_17369_1-p952992	A8	2	0.8	B
7972	Bn-N8-p5216482	Bn-A08-p5420503	Bn-A08-p5420503	A8	2	0.8	B
7973	Bn-N8-p5243086	Bn-A08-p5461032	Bn-A08-p5461032	A8	2	0.8	B
7974	Bn-N8-p5280916	Bn-A08-p5490050	Bn-A08-p5490050	A8	2	0.8	B
7975	Bn-N8-p5282086	Bn-A08-p5491239	Bn-A08-p5491239	A8	2	0.8	B
7976	Bn-N8-p5326138	Bn-A08-p5539128	Bn-A08-p5539128	A8	2	0.8	B
7977	Bn-N8-p5457358	Bn-A08-p5583881	Bn-A08-p5583881	A8	2	0.8	B
7978	Bn-N8-p5464803	Bn-A08-p5591333	Bn-A08-p5591333	A8	2	0.8	B
7979	Bn-N8-p5490058	Bn-A08-p5626110	Bn-A08-p5626110	A8	2	0.8	B
7980	Bn-N8-p5490900	Bn-A08-p5626953	Bn-A08-p5626953	A8	2	0.8	B
7981	Bn-N8-p5491064	Bn-A08-p5627117	Bn-A08-p5627117	A8	2	0.8	B
7982	Bn-N8-p5498368	Bn-A08-p5635882	Bn-A08-p5635882	A8	2	0.8	B
7983	Bn-N8-p5498659	Bn-A08-p5636172	Bn-A08-p5636172	A8	2	0.8	B
7984	Bn-N8-p5499242	Bn-A08-p5636755	Bn-A08-p5636755	A8	2	0.8	B
7985	Bn-N8-p5499251	Bn-A08-p5636764	Bn-A08-p5636764	A8	2	0.8	B
7986	Bn-N8-p5501244	Bn-A08-p5638757	Bn-A08-p5638757	A8	2	0.8	B
7987	Bn-N8-p5503440	Bn-A08-p5640946	Bn-A08-p5640946	A8	2	0.8	B
7988	Bn-N8-p5518223	Bn-A08-p5652494	Bn-A08-p5652494	A8	2	0.8	B
7989	Bn-N8-p5534837	Bn-A08-p5664320	Bn-A08-p5664320	A8	2	0.8	B
7990	Bn-N8-p5569818	Bn-A08-p5696515	Bn-A08-p5696515	A8	2	0.8	B
7991	Bn-N8-p5570442	Bn-A08-p5697139	Bn-A08-p5697139	A8	2	0.8	B
7992	Bn-N8-p5570527	Bn-A08-p5697224	Bn-A08-p5697224	A8	2	0.8	B
7993	Bn-N8-p5570946	Bn-A08-p5697643	Bn-A08-p5697643	A8	2	0.8	B
7994	Bn-N8-p5571566	Bn-A08-p5698262	Bn-A08-p5698262	A8	2	0.8	B
7995	Bn-N8-p5572009	Bn-A08-p5698705	Bn-A08-p5698705	A8	2	0.8	B
7996	Bn-N8-p5572344	Bn-A08-p5699040	Bn-A08-p5699040	A8	2	0.8	B

7997	Bn-N8-p5573456	Bn-A08-p5700152	Bn-A08-p5700152	A8	2	0.8	B
7998	Bn-N8-p5573858	Bn-A08-p5700554	Bn-A08-p5700554	A8	2	0.8	B
7999	Bn-N8-p5576223	Bn-A08-p5702921	Bn-A08-p5702921	A8	2	0.8	B
8000	Bn-N8-p5598362	Bn-A08-p5721732	Bn-A08-p5721732	A8	2	0.8	B
8001	Bn-N8-p5603234	Bn-A08-p5730628	Bn-A08-p5730628	A8	2	0.8	B
8002	Bn-N8-p5650374	Bn-C3-p58591565	Bn-scaff_24108_1-p41173(	A8	2	0.8	B
8003	Bn-N8-p5676908	Bn-A08-p5766271	Bn-A08-p5766271	A8	2	0.8	B
8004	Bn-N8-p5765973	Bn-A08-p5905574	Bn-A08-p5905574	A8	2	0.8	B
8005	Bn-N8-p5785368	Bn-A08-p5924891	Bn-A08-p5924891	A8	2	0.8	B
8006	Bn-N8-p5917281	Bn-A08-p7062593	Bn-A08-p7062593	A8	2	0.8	B
8007	Bn-N8-p5917733	Bn-A08-p7063045	Bn-A08-p7063045	A8	2	0.8	B
8008	Bn-N8-p5918984	Bn-A08-p7064296	Bn-A08-p7064296	A8	2	0.8	B
8009	Bn-N8-p5920889	Bn-A08-p7066465	Bn-A08-p7066465	A8	2	0.8	B
8010	Bn-N8-p5921018	Bn-A08-p7066594	Bn-A08-p7066594	A8	2	0.8	B
8011	Bn-N8-p5921497	Bn-A08-p7067073	Bn-A08-p7067073	A8	2	0.8	B
8012	Bn-N8-p5927049	Bn-A08-p7071333	Bn-A08-p7071333	A8	2	0.8	B
8013	Bn-N8-p5931063	Bn-A08-p7075341	Bn-A08-p7075341	A8	2	0.8	B
8014	Bn-N8-p5952893	Bn-A08-p7078765	Bn-A08-p7078765	A8	2	0.8	B
8015	Bn-N8-p5953048	Bn-A08-p7078920	Bn-A08-p7078920	A8	2	0.8	B
8016	Bn-N8-p5954405	Bn-A08-p7080277	Bn-A08-p7080277	A8	2	0.8	B
8017	Bn-N8-p5960959	Bn-A08-p7087866	Bn-A08-p7087866	A8	2	0.8	B
8018	Bn-N8-p5961092	Bn-A08-p7087999	Bn-A08-p7087999	A8	2	0.8	B
8019	Bn-N8-p5967144	Bn-A08-p7092438	Bn-A08-p7092438	A8	2	0.8	B
8020	Bn-N8-p5980385	Bn-Scaffold000360-p8	Bn-Scaffold000360-p8925	A8	2	0.8	B
8021	Bn-N8-p5998133	Bn-A08-p7099327	Bn-A08-p7099327	A8	2	0.8	B
8022	Bn-N8-p6003831	Bn-C8-p7086339	Bn-scaff_16273_1-p29128:	A8	2	0.8	B
8023	Bn-N8-p6013748	Bn-A08-p7110493	Bn-A08-p7110493	A8	2	0.8	B
8024	Bn-N8-p6013816	Bn-A08-p7110561	Bn-A08-p7110561	A8	2	0.8	B
8025	Bn-N8-p6016119	Bn-A08-p7112865	Bn-A08-p7112865	A8	2	0.8	B
8026	Bn-N8-p6021968	Bn-A08-p7120885	Bn-A08-p7120885	A8	2	0.8	B
8027	Bn-N8-p6023345	Bn-A08-p7122254	Bn-A08-p7122254	A8	2	0.8	B
8028	Bn-N8-p6025438	Bn-A08-p7123976	Bn-A08-p7123976	A8	2	0.8	B
8029	Bn-N8-p6036959	Bn-A08-p7152271	Bn-A08-p7152271	A8	2	0.8	B
8030	Bn-N8-p6043758	Bn-A08-p7162057	Bn-A08-p7162057	A8	2	0.8	B
8031	Bn-N8-p6109564	Bn-A08-p7178765	Bn-A08-p7178765	A8	2	0.8	B
8032	Bn-N8-p6109764	Bn-A08-p7178963	Bn-A08-p7178963	A8	2	0.8	B
8033	Bn-N8-p6113808	Bn-C8-p7229018	Bn-scaff_16273_1-p43396:	A8	2	0.8	B
8034	Bn-N8-p6119605	Bn-Scaffold07641-p2:	Bn-scaff_28908_1-p210	A8	2	0.8	B
8035	Bn-N8-p6125714	Bn-C9-p10014122	Bn-scaff_17487_1-p14903:	A8	2	0.8	B
8036	Bn-N8-p6168927	Bn-A08-p7243965	Bn-A08-p7243965	A8	2	0.8	B
8037	Bn-N8-p6202597	Bn-A08-p7260514	Bn-A08-p7260514	A8	2	0.8	B
8038	Bn-N8-p6203719	Bn-A08-p7261638	Bn-A08-p7261638	A8	2	0.8	B
8039	Bn-N8-p6203840	Bn-A08-p7261759	Bn-A08-p7261759	A8	2	0.8	B
8040	Bn-N8-p6212448	Bn-A08-p7263132	Bn-A08-p7263132	A8	2	0.8	B
8041	Bn-N8-p6213057	Bn-A08-p7263741	Bn-A08-p7263741	A8	2	0.8	B
8042	Bn-N8-p6213780	Bn-A08-p7264462	Bn-A08-p7264462	A8	2	0.8	B
8043	Bn-N8-p6213831	Bn-A08-p7264513	Bn-A08-p7264513	A8	2	0.8	B
8044	Bn-N8-p6214464	Bn-A08-p7265146	Bn-A08-p7265146	A8	2	0.8	B
8045	Bn-N8-p6214651	Bn-A08-p7265333	Bn-A08-p7265333	A8	2	0.8	B

8046	Bn-N8-p6215161	Bn-A08-p7265843	Bn-A08-p7265843	A8	2	0.8	B
8047	Bn-N8-p6215215	Bn-A08-p7265897	Bn-A08-p7265897	A8	2	0.8	B
8048	Bn-N8-p6220962	Bn-A08-p7271638	Bn-A08-p7271638	A8	2	0.8	B
8049	Bn-N8-p6223305	Bn-A08-p7273429	Bn-A08-p7273429	A8	2	0.8	B
8050	Bn-N8-p6227626	Bn-A08-p7276312	Bn-A08-p7276312	A8	2	0.8	B
8051	Bn-N8-p6229030	Bn-A08-p7282035	Bn-A08-p7282035	A8	2	0.8	B
8052	Bn-N8-p6229608	Bn-A08-p7288153	Bn-A08-p7288153	A8	2	0.8	B
8053	Bn-N8-p6298576	Bn-A08-p7303669	Bn-A08-p7303669	A8	2	0.8	B
8054	Bn-N8-p6315202	Bn-A08-p7315916	Bn-A08-p7315916	A8	2	0.8	B
8055	Bn-N8-p6317339	Bn-A08-p7318043	Bn-A08-p7318043	A8	2	0.8	B
8056	Bn-N8-p6331205	Bn-C8-p7573323	Bn-scaff_16766_1-p28912	A8	2	0.8	B
8057	Bn-N8-p6340113	Bn-A08-p7366507	Bn-A08-p7366507	A8	2	0.8	B
8058	Bn-N8-p6348581	Bn-A08-p7374884	Bn-A08-p7374884	A8	2	0.8	B
8059	Bn-N8-p6352925	Bn-A08-p7377024	Bn-A08-p7377024	A8	2	0.8	B
8060	Bn-N8-p6374413	Bn-A07-p5703663	Bn-A07-p5703663	A8	2	0.8	B
8061	Bn-N8-p6383054	Bn-A08-p7409992	Bn-A08-p7409992	A8	2	0.8	B
8062	Bn-N8-p6383671	Bn-A08-p7410609	Bn-A08-p7410609	A8	2	0.8	B
8063	Bn-N8-p6390162	Bn-A08-p7416849	Bn-A08-p7416849	A8	2	0.8	B
8064	Bn-N8-p6390733	Bn-A08-p7417420	Bn-A08-p7417420	A8	2	0.8	B
8065	Bn-N8-p6390992	Bn-A08-p7417679	Bn-A08-p7417679	A8	2	0.8	B
8066	Bn-N8-p6391236	Bn-A08-p7417923	Bn-A08-p7417923	A8	2	0.8	B
8067	Bn-N8-p6391314	Bn-A08-p7418001	Bn-A08-p7418001	A8	2	0.8	B
8068	Bn-N8-p6391445	Bn-A08-p7418132	Bn-A08-p7418132	A8	2	0.8	B
8069	Bn-N8-p6393526	Bn-A08-p7420214	Bn-A08-p7420214	A8	2	0.8	B
8070	Bn-N8-p6394026	Bn-A08-p7420713	Bn-A08-p7420713	A8	2	0.8	B
8071	Bn-N8-p6398723	Bn-A08-p7425294	Bn-A08-p7425294	A8	2	0.8	B
8072	Bn-N8-p6407282	Bn-A08-p7438002	Bn-A08-p7438002	A8	2	0.8	B
8073	Bn-N8-p6407382	Bn-A08-p7438102	Bn-A08-p7438102	A8	2	0.8	B
8074	Bn-N8-p6408289	Bn-A08-p7439009	Bn-A08-p7439009	A8	2	0.8	B
8075	Bn-N8-p6409016	Bn-A08-p7439711	Bn-A08-p7439711	A8	2	0.8	B
8076	Bn-N8-p6409672	Bn-A08-p7440367	Bn-A08-p7440367	A8	2	0.8	B
8077	Bn-N8-p6410918	Bn-A08-p7441624	Bn-A08-p7441624	A8	2	0.8	B
8078	Bn-N8-p6413013	Bn-A08-p7443922	Bn-A08-p7443922	A8	2	0.8	B
8079	Bn-N8-p6413426	Bn-A08-p7444336	Bn-A08-p7444336	A8	2	0.8	B
8080	Bn-N8-p6426735	Bn-A08-p7455782	Bn-A08-p7455782	A8	2	0.8	B
8081	Bn-N8-p6430504	Bn-A08-p7458611	Bn-A08-p7458611	A8	2	0.8	B
8082	Bn-N8-p66238	Bn-Scaffold000180-p	Bn-Scaffold000180-p16334	A8	2	0.8	B
8083	Bn-N8-p6784500	Bn-A08-p7717256	Bn-A08-p7717256	A8	2	0.8	B
8084	Bn-N8-p6810403	Bn-A08-p7739750	Bn-A08-p7739750	A8	2	0.8	B
8085	Bn-N8-p6816991	Bn-A08-p7747258	Bn-A08-p7747258	A8	2	0.8	B
8086	Bn-N8-p68778	Bn-Scaffold000180-p	Bn-Scaffold000180-p16595	A8	2	0.8	B
8087	Bn-N8-p6895245	Bn-A08-p7791369	Bn-A08-p7791369	A8	2	0.8	B
8088	Bn-N8-p6902617	Bn-A08-p7799016	Bn-A08-p7799016	A8	2	0.8	B
8089	Bn-N8-p6907707	Bn-A08-p7814328	Bn-A08-p7814328	A8	2	0.8	B
8090	Bn-N8-p6907811	Bn-A08-p7814432	Bn-A08-p7814432	A8	2	0.8	B
8091	Bn-N8-p6931659	Bn-A08-p7835995	Bn-A08-p7835995	A8	2	0.8	B
8092	Bn-N8-p6943962	Bn-A08-p7851497	Bn-A08-p7851497	A8	2	0.8	B
8093	Bn-N8-p6954956	Bn-A08-p7863392	Bn-A08-p7863392	A8	2	0.8	B
8094	Bn-N8-p6969834	Bn-A08-p7881489	Bn-A08-p7881489	A8	2	0.8	B

8095	Bn-N8-p7067926	Bn-A08-p7947263	Bn-A08-p7947263	A8	2	0.8	B
8096	Bn-N8-p7130361	Bn-A08-p7991606	Bn-A08-p7991606	A8	2	0.8	B
8097	Bn-N8-p7147930	Bn-A08-p7998862	Bn-A08-p7998862	A8	2	0.8	B
8098	Bn-N8-p7207205	Bn-A08-p8073664	Bn-A08-p8073664	A8	2	0.8	B
8099	Bn-N8-p7216514	Bn-A08-p8091811	Bn-A08-p8091811	A8	2	0.8	B
8100	Bn-N8-p7240910	Bn-A08-p8114697	Bn-A08-p8114697	A8	2	0.8	B
8101	Bn-N8-p7276065	Bn-A08-p8159163	Bn-A08-p8159163	A8	2	0.8	B
8102	Bn-N8-p7278568	Bn-A08-p8161845	Bn-A08-p8161845	A8	2	0.8	B
8103	Bn-N8-p7278965	Bn-A08-p8162230	Bn-A08-p8162230	A8	2	0.8	B
8104	Bn-N8-p7321405	Bn-A08-p8414586	Bn-A08-p8414586	A8	2	0.8	B
8105	Bn-N8-p7335695	Bn-A08-p8397266	Bn-A08-p8397266	A8	2	0.8	B
8106	Bn-N8-p7336453	Bn-A08-p8396508	Bn-A08-p8396508	A8	2	0.8	B
8107	Bn-N8-p7357594	Bn-A08-p8378906	Bn-A08-p8378906	A8	2	0.8	B
8108	Bn-N8-p7358158	Bn-A08-p8378306	Bn-A08-p8378306	A8	2	0.8	B
8109	Bn-N8-p7362396	Bn-A08-p8374080	Bn-A08-p8374080	A8	2	0.8	B
8110	Bn-N8-p7363942	Bn-A08-p8372495	Bn-A08-p8372495	A8	2	0.8	B
8111	Bn-N8-p7364250	Bn-A08-p8368744	Bn-A08-p8368744	A8	2	0.8	B
8112	Bn-N8-p7372220	Bn-A08-p8355054	Bn-A08-p8355054	A8	2	0.8	B
8113	Bn-N8-p7374438	Bn-C8-p9809099	Bn-scaff_16142_1-p41148	A8	2	0.8	B
8114	Bn-N8-p7374901	Bn-A08-p8352143	Bn-A08-p8352143	A8	2	0.8	0
8115	Bn-N8-p7375601	Bn-A08-p8351443	Bn-A08-p8351443	A8	2	0.8	B
8116	Bn-N8-p7391080	Bn-A08-p8335609	Bn-A08-p8335609	A8	2	0.8	B
8117	Bn-N8-p7406442	Bn-Scaffold000519-p	Bn-Scaffold000519-p5715	A8	2	0.8	B
8118	Bn-N8-p7416565	Bn-A08-p8314631	Bn-A08-p8314631	A8	2	0.8	B
8119	Bn-N8-p7459222	Bn-A08-p8312619	Bn-A08-p8312619	A8	2	0.8	B
8120	Bn-N8-p7508048	Bn-A08-p8273132	Bn-A08-p8273132	A8	2	0.8	B
8121	Bn-N8-p7513875	Bn-A08-p8267272	Bn-A08-p8267272	A8	2	0.8	B
8122	Bn-N8-p7520209	Bn-A08-p8260866	Bn-A08-p8260866	A8	2	0.8	B
8123	Bn-N8-p7535491	Bn-A08-p8249952	Bn-A08-p8249952	A8	2	0.8	B
8124	Bn-N8-p7549400	Bn-A08-p8242214	Bn-A08-p8242214	A8	2	0.8	B
8125	Bn-N8-p7553881	Bn-A08-p8237891	Bn-A08-p8237891	A8	2	0.8	B
8126	Bn-N8-p7564088	Bn-A08-p8232026	Bn-A08-p8232026	A8	2	0.8	B
8127	Bn-N8-p7585015	Bn-A08-p8218376	Bn-A08-p8218376	A8	2	0.8	B
8128	Bn-N8-p7627092	Bn-A08-p8195425	Bn-A08-p8195425	A8	2	0.8	B
8129	Bn-N8-p7627786	Bn-A08-p8194731	Bn-A08-p8194731	A8	2	0.8	B
8130	Bn-N8-p7643059	Bn-A08-p8180093	Bn-A08-p8180093	A8	2	0.8	B
8131	Bn-N8-p7643523	Bn-A08-p8179629	Bn-A08-p8179629	A8	2	0.8	B
8132	Bn-N8-p7654040	Bn-A08-p8432548	Bn-A08-p8432548	A8	2	0.8	B
8133	Bn-N8-p7669351	Bn-A08-p8447749	Bn-A08-p8447749	A8	2	0.8	B
8134	Bn-N8-p7674868	Bn-A08-p8453264	Bn-A08-p8453264	A8	2	0.8	B
8135	Bn-N8-p7679811	Bn-A08-p8458205	Bn-A08-p8458205	A8	2	0.8	B
8136	Bn-N8-p7698170	Bn-A08-p8479611	Bn-A08-p8479611	A8	2	0.8	B
8137	Bn-N8-p7698491	Bn-A08-p8479932	Bn-A08-p8479932	A8	2	0.8	B
8138	Bn-N8-p7699293	Bn-A08-p8480734	Bn-A08-p8480734	A8	2	0.8	B
8139	Bn-N8-p7710546	Bn-A08-p8493076	Bn-A08-p8493076	A8	2	0.8	B
8140	Bn-N8-p7711931	Bn-A08-p8494463	Bn-A08-p8494463	A8	2	0.8	B
8141	Bn-N8-p7712957	Bn-A08-p8495489	Bn-A08-p8495489	A8	2	0.8	B
8142	Bn-N8-p7726335	Bn-A08-p8508603	Bn-A08-p8508603	A8	2	0.8	B
8143	Bn-N8-p7738537	Bn-A08-p8520595	Bn-A08-p8520595	A8	2	0.8	B

8144	Bn-N8-p7743305	Bn-C8-p10122780	Bn-scaff_16142_1-p97803	A8	2	0.8	B
8145	Bn-N8-p7748382	Bn-A08-p8529251	Bn-A08-p8529251	A8	2	0.8	B
8146	Bn-N8-p7748473	Bn-A08-p8529342	Bn-A08-p8529342	A8	2	0.8	B
8147	Bn-N8-p7752587	Bn-A08-p8533455	Bn-A08-p8533455	A8	2	0.8	B
8148	Bn-N8-p7757996	Bn-A08-p8542502	Bn-A08-p8542502	A8	2	0.8	B
8149	Bn-N8-p7759169	Bn-A08-p8543675	Bn-A08-p8543675	A8	2	0.8	B
8150	Bn-N8-p7759392	Bn-A08-p8543898	Bn-A08-p8543898	A8	2	0.8	B
8151	Bn-N8-p7759405	Bn-A08-p8543911	Bn-A08-p8543911	A8	2	0.8	B
8152	Bn-N8-p7762054	Bn-A08-p8546560	Bn-A08-p8546560	A8	2	0.8	B
8212	Bn-Scaffold01327-p858	Bn-A08-p7810122	Bn-A08-p7810122	A8	2	0.8	B
8213	Bn-Scaffold05379-p1204	Bn-Scaffold001041-p9	Bn-Scaffold001041-p911	A8	2	0.8	B
8214	Bn-Scaffold05379-p1504	Bn-Scaffold001041-p1	Bn-Scaffold001041-p1211	A8	2	0.8	B
8215	Bn-Scaffold06770-p1113	Bn-A08-p7222585	Bn-A08-p7222585	A8	2	0.8	B
8216	Bn-Scaffold19869-p426	Bn-A06-p17681092	Bn-A06-p17681092	A8	2	0.8	B
8217	Bn-Scaffold31845-p178	Bn-A08-p7139892	Bn-A08-p7139892	A8	2	0.8	B
7818	Bn-N8-p7792310	Bn-A08-p8570810	Bn-A08-p8570810	A8	3	1.6	B
7819	Bn-N8-p7792759	Bn-A08-p8571258	Bn-A08-p8571258	A8	3	1.6	B
7820	Bn-N8-p7809353	Bn-A08-p8567867	Bn-A08-p8567867	A8	3	1.6	B
7821	Bn-N8-p7816233	Bn-A08-p8569187	Bn-A08-p8569187	A8	3	1.6	B
7822	Bn-N8-p7828506	Bn-A08-p8577291	Bn-A08-p8577291	A8	3	1.6	B
7823	Bn-N8-p7828569	Bn-A08-p8577353	Bn-A08-p8577353	A8	3	1.6	B
7824	Bn-N8-p7834013	Bn-A08-p8581123	Bn-A08-p8581123	A8	3	1.6	B
7825	Bn-N8-p7834794	Bn-A08-p8581899	Bn-A08-p8581899	A8	3	1.6	B
7826	Bn-N8-p7834967	Bn-A08-p8582072	Bn-A08-p8582072	A8	3	1.6	B
7827	Bn-N8-p7837852	Bn-A08-p8584951	Bn-A08-p8584951	A8	3	1.6	B
7828	Bn-N8-p7838156	Bn-A08-p8585255	Bn-A08-p8585255	A8	3	1.6	B
7829	Bn-N8-p7839257	Bn-A08-p8586356	Bn-A08-p8586356	A8	3	1.6	B
7834	Bn-N11-p17297622	Bn-A03-p17336041	Bn-A03-p17336041	A8	4	4	B
7841	Bn-N18-p15303076	Bn-Scaffold000664-p1	Bn-Scaffold000664-p1838	A8	4	4	B
8153	Bn-N8-p7859442	Bn-A08-p8601468	Bn-A08-p8601468	A8	4	4	B
8154	Bn-N8-p7874940	Bn-A08-p8633780	Bn-A08-p8633780	A8	4	4	B
8155	Bn-N8-p7875770	Bn-A08-p8634610	Bn-A08-p8634610	A8	4	4	B
8156	Bn-N8-p7898694	Bn-A08-p8658074	Bn-A08-p8658074	A8	4	4	B
8157	Bn-N8-p7915843	Bn-A08-p8672999	Bn-A08-p8672999	A8	4	4	B
8158	Bn-N8-p7929559	Bn-A08-p8690169	Bn-A08-p8690169	A8	4	4	B
8159	Bn-N8-p7940018	Bn-A08-p8710166	Bn-A08-p8710166	A8	4	4	B
8160	Bn-N8-p7949374	Bn-A08-p8716833	Bn-A08-p8716833	A8	4	4	B
8161	Bn-N8-p7966384	Bn-A08-p8721964	Bn-A08-p8721964	A8	4	4	B
8162	Bn-N8-p7987878	Bn-A08-p8740002	Bn-A08-p8740002	A8	4	4	B
8163	Bn-N8-p8005536	Bn-A08-p8755323	Bn-A08-p8755323	A8	4	4	B
8164	Bn-N8-p8032711	Bn-A08-p8784663	Bn-A08-p8784663	A8	4	4	B
8165	Bn-N8-p8059826	Bn-A08-p8808046	Bn-A08-p8808046	A8	4	4	B
8166	Bn-N8-p8129492	Bn-A08-p8869180	Bn-A08-p8869180	A8	4	4	B
8167	Bn-N8-p8133241	Bn-A08-p8872384	Bn-A08-p8872384	A8	4	4	B
8168	Bn-N8-p8134585	Bn-C8-p10616569	Bn-scaff_16287_1-p395887	A8	4	4	B
8169	Bn-N8-p8137151	Bn-A08-p8879495	Bn-A08-p8879495	A8	4	4	B
8170	Bn-N8-p8423995	Bn-A08-p6133601	Bn-A08-p6133601	A8	4	4	B
8171	Bn-N8-p8428969	Bn-A08-p6148686	Bn-A08-p6148686	A8	4	4	B
8172	Bn-N8-p8429279	Bn-A08-p6148996	Bn-A08-p6148996	A8	4	4	B

8173	Bn-N8-p8439711	Bn-A08-p6157861	Bn-A08-p6157861	A8	4	4	B
8174	Bn-N8-p8441580	Bn-A08-p6159705	Bn-A08-p6159705	A8	4	4	B
8175	Bn-N8-p8441643	Bn-A08-p6159768	Bn-A08-p6159768	A8	4	4	B
8176	Bn-N8-p8452246	Bn-A08-p6162616	Bn-A08-p6162616	A8	4	4	B
8177	Bn-N8-p8452290	Bn-A08-p6162660	Bn-A08-p6162660	A8	4	4	B
8178	Bn-N8-p8453855	Bn-A08-p6164225	Bn-A08-p6164225	A8	4	4	B
8179	Bn-N8-p8454598	Bn-A08-p6164968	Bn-A08-p6164968	A8	4	4	B
8180	Bn-N8-p8454970	Bn-A08-p6165340	Bn-A08-p6165340	A8	4	4	B
8181	Bn-N8-p8455295	Bn-A08-p6165665	Bn-A08-p6165665	A8	4	4	B
8182	Bn-N8-p8459717	Bn-A08-p6170057	Bn-A08-p6170057	A8	4	4	B
8183	Bn-N8-p8524819	Bn-A08-p6246411	Bn-A08-p6246411	A8	4	4	B
8184	Bn-N8-p8556054	Bn-A08-p6278699	Bn-A08-p6278699	A8	4	4	B
8185	Bn-N8-p8559481	Bn-A08-p6282126	Bn-A08-p6282126	A8	4	4	B
8186	Bn-N8-p8562060	Bn-A08-p6284747	Bn-A08-p6284747	A8	4	4	0
8187	Bn-N8-p8575382	Bn-A08-p6298340	Bn-A08-p6298340	A8	4	4	B
8188	Bn-N8-p8576347	Bn-A08-p6299307	Bn-A08-p6299307	A8	4	4	B
8189	Bn-N8-p8593283	Bn-A08-p6332878	Bn-A08-p6332878	A8	4	4	B
8190	Bn-N8-p8604736	Bn-A08-p6343431	Bn-A08-p6343431	A8	4	4	B
8191	Bn-N8-p8618284	Bn-C8-p13445569	Bn-scaff_21250_1-p41073	A8	4	4	B
8192	Bn-N8-p8626454	Bn-A08-p6366258	Bn-A08-p6366258	A8	4	4	B
8193	Bn-N8-p8629403	Bn-A08-p6369194	Bn-A08-p6369194	A8	4	4	B
8194	Bn-N8-p8632074	Bn-A08-p6371865	Bn-A08-p6371865	A8	4	4	B
8195	Bn-N8-p8651448	Bn-A08-p6394232	Bn-A08-p6394232	A8	4	4	B
8196	Bn-N8-p8740065	Bn-A08-p6488809	Bn-A08-p6488809	A8	4	4	B
8197	Bn-N8-p8769174	Bn-A08-p6523926	Bn-A08-p6523926	A8	4	4	B
8198	Bn-N8-p8769200	Bn-A08-p6523952	Bn-A08-p6523952	A8	4	4	B
8199	Bn-N8-p9002151	Bn-A08-p6721530	Bn-A08-p6721530	A8	4	4	B
8200	Bn-N8-p9003062	Bn-A08-p6722442	Bn-A08-p6722442	A8	4	4	B
8201	Bn-N8-p9035531	Bn-A08-p6744560	Bn-A08-p6744560	A8	4	4	B
8202	Bn-N8-p9121537	Bn-A08-p6828854	Bn-A08-p6828854	A8	4	4	B
8203	Bn-N8-p9121540	Bn-A08-p6828857	Bn-A08-p6828857	A8	4	4	B
8204	Bn-N8-p9121603	Bn-C8-p14583492	Bn-scaff_17257_1-p97933	A8	4	4	B
8205	Bn-N8-p9122324	Bn-A08-p6829643	Bn-A08-p6829643	A8	4	4	B
8206	Bn-N8-p9240429	Bn-Scaffold000219-p	Bn-Scaffold000219-p66499	A8	4	4	B
8207	Bn-N8-p9299555	Bn-Scaffold000219-p	Bn-Scaffold000219-p12098	A8	4	4	B
8208	Bn-N8-p9362261	Bn-A08-p7018554	Bn-A08-p7018554	A8	4	4	B
8209	Bn-N8-p9428077	Bn-A08-p6937293	Bn-A08-p6937293	A8	4	4	B
8210	Bn-N8-p9437820	Bn-C8-p15150502	Bn-scaff_20497_1-p50935	A8	4	4	B
8211	Bn-N8-p9505869	Bn-Scaffold000247-p	Bn-Scaffold000247-p34082	A8	4	4	B
8218	Bn-N8-p9613284	Bn-C8-p15465315	Bn-scaff_18275_1-p12858	A8	5	4.8	B
8219	Bn-N8-p9634172	Bn-A08-p10067927	Bn-A08-p10067927	A8	5	4.8	B
8220	Bn-N8-p9705188	Bn-A08-p10128352	Bn-A08-p10128352	A8	5	4.8	B
8221	Bn-N8-p9705525	Bn-A08-p10128687	Bn-A08-p10128687	A8	5	4.8	B
8222	Bn-N8-p9811598	Bn-A08-p10215334	Bn-A08-p10215334	A8	5	4.8	B
8223	Bn-Scaffold01053-p9109	Bn-A08-p10147092	Bn-A08-p10147092	A8	5	4.8	B
8224	Bn-N8-p9946893	Bn-A08-p10281337	Bn-A08-p10281337	A8	6	5.6	B
8225	Bn-N13-p58561976	Bn-C3-p56635713	Bn-scaff_15703_1-p18631	A8	7	6.4	B
8226	Bn-N8-p10085528	Bn-A08-p10439396	Bn-A08-p10439396	A8	7	6.4	B
8227	Bn-N8-p10098600	Bn-A08-p10440699	Bn-A08-p10440699	A8	7	6.4	B

8228	Bn-N8-p10109726	Bn-A08-p10452462	Bn-A08-p10452462	A8	7	6.4	B
8229	Bn-N8-p10121013	Bn-Scaffold000316-p1	Bn-Scaffold000316-p15795	A8	7	6.4	B
8230	Bn-N8-p10184956	Bn-A08-p10509442	Bn-A08-p10509442	A8	7	6.4	B
8231	Bn-N8-p10277538	Bn-A08-p10593605	Bn-A08-p10593605	A8	7	6.4	B
8232	Bn-N8-p10291520	Bn-A08-p10615012	Bn-A08-p10615012	A8	7	6.4	B
8233	Bn-N8-p10331185	Bn-A08-p10663544	Bn-A08-p10663544	A8	7	6.4	B
8234	Bn-N8-p10435335	Bn-A08-p10789401	Bn-A08-p10789401	A8	7	6.4	B
8235	Bn-N8-p10496842	Bn-A08-p10844808	Bn-A08-p10844808	A8	7	6.4	B
8236	Bn-N8-p10498935	Bn-A08-p10846901	Bn-A08-p10846901	A8	7	6.4	B
8237	Bn-N8-p10545891	Bn-C3-p56635826	Bn-scaff_15703_1-p18642	A8	7	6.4	B
8238	Bn-N7-p15088627	Bn-A08-p1928132	Bn-A08-p1928132	A8	8	7.2	B
8239	Bn-N8-p10979512	Bn-C3-p57295296	Bn-scaff_17042_1-p35973	A8	8	7.2	B
8240	Bn-N8-p10979559	Bn-C3-p57295343	Bn-scaff_17042_1-p35977	A8	8	7.2	B
8241	Bn-N8-p11123277	Bn-A08-p11452221	Bn-A08-p11452221	A8	8	7.2	B
8242	Bn-N8-p11129532	Bn-A08-p11458466	Bn-A08-p11458466	A8	8	7.2	B
8243	Bn-N8-p11131297	Bn-A08-p11459293	Bn-A08-p11459293	A8	8	7.2	B
8244	Bn-N8-p11338956	Bn-A08-p11667191	Bn-A08-p11667191	A8	8	7.2	B
8245	Bn-N10-p5812384	Bn-A10-p5035362	Bn-A10-p5035362	A8	9	9.6	B
8246	Bn-N13-p62236423	Bn-C3-p59723852	Bn-scaff_15794_1-p23672	A8	9	9.6	B
8247	Bn-N13-p62236523	Bn-C3-p59723951	Bn-scaff_15794_1-p23662	A8	9	9.6	B
8248	Bn-N8-p12138124	Bn-A08-p12555227	Bn-A08-p12555227	A8	9	9.6	B
8249	Bn-N8-p12139346	Bn-A08-p12556455	Bn-A08-p12556455	A8	9	9.6	B
8250	Bn-N8-p12142760	Bn-A08-p12559372	Bn-A08-p12559372	A8	9	9.6	B
8251	Bn-N8-p12149002	Bn-A08-p12564228	Bn-A08-p12564228	A8	9	9.6	B
8252	Bn-N8-p12191131	Bn-A08-p12599446	Bn-A08-p12599446	A8	9	9.6	B
8253	Bn-N8-p12235835	Bn-A08-p12638473	Bn-A08-p12638473	A8	9	9.6	B
8254	Bn-N8-p12248695	Bn-A08-p12660208	Bn-A08-p12660208	A8	9	9.6	B
8255	Bn-N8-p12260577	Bn-A08-p12674243	Bn-A08-p12674243	A8	9	9.6	B
8256	Bn-N8-p12260911	Bn-A08-p12674577	Bn-A08-p12674577	A8	9	9.6	B
8257	Bn-N8-p12279728	Bn-A08-p12698364	Bn-A08-p12698364	A8	9	9.6	B
8258	Bn-N8-p12280545	Bn-A08-p12699181	Bn-A08-p12699181	A8	9	9.6	B
8259	Bn-N8-p12331547	Bn-C5-p6374716	Bn-scaff_18181_1-p18492	A8	9	9.6	B
8260	Bn-N8-p12372214	Bn-A08-p12752628	Bn-A08-p12752628	A8	9	9.6	B
8261	Bn-N8-p12380623	Bn-A08-p12761022	Bn-A08-p12761022	A8	9	9.6	B
8262	Bn-N8-p12386789	Bn-A08-p12767403	Bn-A08-p12767403	A8	9	9.6	B
8263	Bn-N8-p12398027	Bn-A08-p12781426	Bn-A08-p12781426	A8	9	9.6	B
8264	Bn-N8-p12401667	Bn-A08-p12794893	Bn-A08-p12794893	A8	9	9.6	B
8265	Bn-N8-p12404325	Bn-A08-p12797532	Bn-A08-p12797532	A8	9	9.6	B
8266	Bn-N8-p12415213	Bn-C3-p60164882	Bn-scaff_16996_1-p33514	A8	9	9.6	B
8267	Bn-N8-p12415810	Bn-A08-p12812977	Bn-A08-p12812977	A8	9	9.6	B
8268	Bn-N8-p12421629	Bn-A08-p12814556	Bn-A08-p12814556	A8	9	9.6	B
8269	Bn-N8-p12430957	Bn-A08-p12820786	Bn-A08-p12820786	A8	9	9.6	B
8270	Bn-N8-p12438569	Bn-A08-p12828472	Bn-A08-p12828472	A8	9	9.6	B
8271	Bn-N8-p12533046	Bn-A08-p12902384	Bn-A08-p12902384	A8	9	9.6	B
8272	Bn-N8-p12534750	Bn-A08-p12904087	Bn-A08-p12904087	A8	9	9.6	B
8273	Bn-N8-p12536740	Bn-A08-p12905848	Bn-A08-p12905848	A8	9	9.6	B
8274	Bn-N8-p12546660	Bn-A08-p12913949	Bn-A08-p12913949	A8	9	9.6	B
8275	Bn-N8-p12556531	Bn-A08-p12923799	Bn-A08-p12923799	A8	9	9.6	B
8276	Bn-N8-p12643613	Bn-A08-p13009633	Bn-A08-p13009633	A8	9	9.6	B

8277	Bn-N8-p12646066	Bn-A08-p13011710	Bn-A08-p13011710	A8	9	9.6	B
8278	Bn-N8-p12686182	Bn-Scaffold000320-p1	Bn-Scaffold000320-p11480	A8	9	9.6	B
8279	Bn-N8-p12708538	Bn-A08-p13030362	Bn-A08-p13030362	A8	9	9.6	B
8280	Bn-N8-p12716450	Bn-A08-p13036508	Bn-A08-p13036508	A8	9	9.6	B
8281	Bn-N8-p12762599	Bn-C3-p60619434	Bn-scaff_17821_1-p11931(	A8	9	9.6	B
8282	Bn-N8-p12763413	Bn-A08-p13076304	Bn-A08-p13076304	A8	9	9.6	B
8283	Bn-N8-p12763413	Bn-A08-p13076305	Bn-A08-p13076305	A8	9	9.6	B
8284	Bn-N8-p12864291	Bn-A08-p13201183	Bn-A08-p13201183	A8	9	9.6	B
8285	Bn-N8-p12877637	Bn-C3-p60750810	Bn-scaff_17119_1-p85292	A8	9	9.6	B
8286	Bn-N8-p12878798	Bn-A08-p13214314	Bn-A08-p13214314	A8	9	9.6	B
8287	Bn-N8-p12879016	Bn-A08-p13214532	Bn-A08-p13214532	A8	9	9.6	B
8288	Bn-N8-p12891309	Bn-C3-p60779962	Bn-scaff_17119_1-p11444	A8	9	9.6	B
8289	Bn-N8-p12891668	Bn-A08-p13221380	Bn-A08-p13221380	A8	9	9.6	B
8290	Bn-N8-p12900897	Bn-A08-p13230287	Bn-A08-p13230287	A8	9	9.6	B
8291	Bn-N8-p12971477	Bn-A08-p13303525	Bn-A08-p13303525	A8	9	9.6	B
8292	Bn-N8-p12991220	Bn-A08-p13322654	Bn-A08-p13322654	A8	9	9.6	B
8293	Bn-N8-p12999889	Bn-A08-p13327070	Bn-A08-p13327070	A8	9	9.6	B
8294	Bn-N8-p13014295	Bn-A08-p13342419	Bn-A08-p13342419	A8	9	9.6	B
8295	Bn-N8-p13023342	Bn-A08-p13352430	Bn-A08-p13352430	A8	10	10.4	B
8296	Bn-N8-p13027358	Bn-A08-p13354792	Bn-A08-p13354792	A8	10	10.4	0
8297	Bn-N8-p13030141	Bn-A08-p13357594	Bn-A08-p13357594	A8	10	10.4	B
8298	Bn-N8-p13030357	Bn-A08-p13357808	Bn-A08-p13357808	A8	10	10.4	B
8299	Bn-N8-p13038993	Bn-A08-p13363830	Bn-A08-p13363830	A8	11	12.8	B
8300	Bn-N8-p13049362	Bn-A08-p13376746	Bn-A08-p13376746	A8	11	12.8	B
8301	Bn-N8-p13052563	Bn-A08-p13379983	Bn-A08-p13379983	A8	11	12.8	B
8302	Bn-N8-p13054634	Bn-A08-p13382119	Bn-A08-p13382119	A8	11	12.8	B
8303	Bn-N8-p13055365	Bn-A08-p13384059	Bn-A08-p13384059	A8	11	12.8	B
8304	Bn-N8-p13062865	Bn-A08-p13401821	Bn-A08-p13401821	A8	11	12.8	B
8305	Bn-N8-p13070885	Bn-A08-p13409843	Bn-A08-p13409843	A8	11	12.8	B
8306	Bn-N8-p13074265	Bn-C3-p61195260	Bn-scaff_23761_1-p35445	A8	11	12.8	B
8307	Bn-N13-p63947446	Bn-A08-p13518735	Bn-A08-p13518735	A8	12	13.6	B
8308	Bn-N8-p13128437	Bn-A08-p13454167	Bn-A08-p13454167	A8	12	13.6	B
8309	Bn-N8-p13133724	Bn-A08-p13460499	Bn-A08-p13460499	A8	12	13.6	B
8310	Bn-N8-p13135392	Bn-A08-p13462776	Bn-A08-p13462776	A8	12	13.6	B
8311	Bn-N8-p13161435	Bn-A08-p13486650	Bn-A08-p13486650	A8	12	13.6	B
8312	Bn-N8-p13195912	Bn-A08-p13515130	Bn-A08-p13515130	A8	12	13.6	B
8313	Bn-N8-p13196478	Bn-A08-p13515686	Bn-A08-p13515686	A8	12	13.6	B
8314	Bn-N8-p13197269	Bn-A08-p13516445	Bn-A08-p13516445	A8	12	13.6	B
8315	Bn-N8-p13207024	Bn-A08-p13522752	Bn-A08-p13522752	A8	12	13.6	B
8316	Bn-N8-p13210755	Bn-A08-p13526497	Bn-A08-p13526497	A8	12	13.6	B
8317	Bn-N8-p13211413	Bn-A08-p13527152	Bn-A08-p13527152	A8	12	13.6	B
8318	Bn-N8-p13223769	Bn-A08-p13538895	Bn-A08-p13538895	A8	12	13.6	B
8319	Bn-N8-p13225619	Bn-A08-p13544679	Bn-A08-p13544679	A8	12	13.6	B
8320	Bn-N8-p13239149	Bn-A08-p13559192	Bn-A08-p13559192	A8	12	13.6	B
8321	Bn-N8-p13258045	Bn-A08-p13577914	Bn-A08-p13577914	A8	12	13.6	B
8322	Bn-N8-p13290340	Bn-A08-p13626189	Bn-A08-p13626189	A8	13	16.8	B
8323	Bn-N8-p13303318	Bn-A08-p13638847	Bn-A08-p13638847	A8	13	16.8	B
8324	Bn-N8-p13329479	Bn-A08-p13670107	Bn-A08-p13670107	A8	13	16.8	B
8325	Bn-N8-p13348171	Bn-C3-p61585003	Bn-scaff_23761_1-p42518	A8	13	16.8	B

8326	Bn-N8-p13376528	Bn-A08-p13692951	Bn-A08-p13692951	A8	13	16.8	B
8327	Bn-N8-p13391245	Bn-A08-p13708877	Bn-A08-p13708877	A8	14	17.6	B
8328	Bn-N8-p13534677	Bn-C3-p61864079	Bn-scaff_23761_1-p70426	A8	14	17.6	B
8329	Bn-N8-p13544730	Bn-A08-p13856852	Bn-A08-p13856852	A8	14	17.6	B
8330	Bn-N8-p13548962	Bn-A08-p13860913	Bn-A08-p13860913	A8	14	17.6	B
8331	Bn-N8-p13553599	Bn-A08-p13864869	Bn-A08-p13864869	A8	14	17.6	B
8332	Bn-N8-p13565760	Bn-A08-p13876356	Bn-A08-p13876356	A8	14	17.6	B
8333	Bn-N8-p13590881	Bn-A08-p13901348	Bn-A08-p13901348	A8	14	17.6	B
8334	Bn-N8-p13622446	Bn-A07-p8632859	Bn-A07-p8632859	A8	14	17.6	B
8335	Bn-N8-p13629386	Bn-A08-p13932634	Bn-A08-p13932634	A8	14	17.6	B
8336	Bn-N8-p13631853	Bn-A08-p13935095	Bn-A08-p13935095	A8	14	17.6	B
8337	Bn-N8-p13635394	Bn-A08-p13938191	Bn-A08-p13938191	A8	14	17.6	B
8338	Bn-N8-p13643299	Bn-A08-p13944679	Bn-A08-p13944679	A8	14	17.6	B
8339	Bn-N8-p13644648	Bn-A08-p13946032	Bn-A08-p13946032	A8	14	17.6	B
8340	Bn-N8-p13652941	Bn-A08-p13953904	Bn-A08-p13953904	A8	14	17.6	B
8341	Bn-N8-p13659060	Bn-A08-p13958465	Bn-A08-p13958465	A8	14	17.6	B
8342	Bn-N8-p13664714	Bn-A08-p13964091	Bn-A08-p13964091	A8	14	17.6	B
8343	Bn-N8-p13700542	Bn-A08-p13995190	Bn-A08-p13995190	A8	14	17.6	B
8344	Bn-N8-p13705427	Bn-A08-p14002120	Bn-A08-p14002120	A8	14	17.6	B
8345	Bn-N8-p13732712	Bn-A08-p14011808	Bn-A08-p14011808	A8	14	17.6	B
8346	Bn-N8-p13735227	Bn-A08-p14014365	Bn-A08-p14014365	A8	14	17.6	B
8347	Bn-N8-p13745920	Bn-A08-p14026432	Bn-A08-p14026432	A8	14	17.6	B
8348	Bn-N8-p13747482	Bn-A08-p14028012	Bn-A08-p14028012	A8	14	17.6	B
8349	Bn-N8-p13767596	Bn-A08-p14045962	Bn-A08-p14045962	A8	14	17.6	B
8350	Bn-N8-p13773599	Bn-A08-p14056618	Bn-A08-p14056618	A8	14	17.6	B
8351	Bn-N8-p13779459	Bn-Scaffold002031-p	Bn-Scaffold002031-p167	A8	14	17.6	B
8352	Bn-N8-p13785515	Bn-A08-p14067094	Bn-A08-p14067094	A8	14	17.6	B
8353	Bn-N8-p13785782	Bn-A08-p14067361	Bn-A08-p14067361	A8	14	17.6	B
8354	Bn-N8-p13804400	Bn-A08-p14086488	Bn-A08-p14086488	A8	14	17.6	B
8355	Bn-N8-p13829513	Bn-A08-p14117791	Bn-A08-p14117791	A8	14	17.6	B
8356	Bn-N8-p13838719	Bn-A08-p14128886	Bn-A08-p14128886	A8	14	17.6	B
8357	Bn-N8-p13842510	Bn-A08-p14133917	Bn-A08-p14133917	A8	14	17.6	B
8358	Bn-N8-p13911141	Bn-A08-p14218543	Bn-A08-p14218543	A8	14	17.6	B
8359	Bn-N8-p13965690	Bn-A08-p14276094	Bn-A08-p14276094	A8	14	17.6	B
8360	Bn-N8-p14059213	Bn-A08-p14351709	Bn-A08-p14351709	A8	14	17.6	B
8361	Bn-N8-p14071010	Bn-A08-p14369220	Bn-A08-p14369220	A8	14	17.6	B
8362	Bn-N8-p14071271	Bn-A08-p14375712	Bn-A08-p14375712	A8	14	17.6	B
8363	Bn-N8-p14223379	Bn-A08-p14498301	Bn-A08-p14498301	A8	15	18.4	B
8364	Bn-N8-p14268064	Bn-A08-p14554907	Bn-A08-p14554907	A8	15	18.4	B
8365	Bn-N18-p18828841	Bn-A08-p14765750	Bn-A08-p14765750	A8	16	19.2	B
8366	Bn-N8-p14328665	Bn-A08-p14616048	Bn-A08-p14616048	A8	16	19.2	B
8367	Bn-N8-p14329641	Bn-A08-p14616742	Bn-A08-p14616742	A8	16	19.2	B
8368	Bn-N8-p14398163	Bn-A08-p14689466	Bn-A08-p14689466	A8	16	19.2	B
8369	Bn-N8-p14426462	Bn-A08-p14713741	Bn-A08-p14713741	A8	16	19.2	B
8370	Bn-N8-p14428351	Bn-A08-p14715630	Bn-A08-p14715630	A8	16	19.2	B
8371	Bn-N8-p14431283	Bn-A08-p14718347	Bn-A08-p14718347	A8	16	19.2	B
8372	Bn-N8-p14439625	Bn-A08-p14726546	Bn-A08-p14726546	A8	16	19.2	B
8373	Bn-N8-p14442423	Bn-A08-p14729101	Bn-A08-p14729101	A8	16	19.2	B
8374	Bn-N8-p14442970	Bn-A08-p14729648	Bn-A08-p14729648	A8	16	19.2	B

8375	Bn-N8-p14447372	Bn-A08-p14734078	Bn-A08-p14734078	A8	16	19.2	B
8376	Bn-N8-p14487515	Bn-A08-p14766663	Bn-A08-p14766663	A8	16	19.2	B
8377	Bn-N8-p14506665	Bn-A08-p14784965	Bn-A08-p14784965	A8	16	19.2	B
8378	Bn-N8-p14512675	Bn-A08-p14790412	Bn-A08-p14790412	A8	16	19.2	B
8379	Bn-N8-p14537632	Bn-A08-p14813540	Bn-A08-p14813540	A8	17	20	B
8380	Bn-N8-p14670055	Bn-C3-p55722563	Bn-scaff_18602_1-p244845	A8	18	20.8	B
8381	Bn-N8-p14670111	Bn-C3-p55722507	Bn-scaff_18602_1-p244789	A8	18	20.8	B
8382	Bn-N8-p14700421	Bn-A08-p14970925	Bn-A08-p14970925	A8	18	20.8	0
8383	Bn-N8-p14706183	Bn-A08-p14976685	Bn-A08-p14976685	A8	18	20.8	B
8384	Bn-N8-p14706655	Bn-A08-p14977157	Bn-A08-p14977157	A8	18	20.8	B
8385	Bn-N8-p14710303	Bn-A08-p14980805	Bn-A08-p14980805	A8	18	20.8	B
8386	Bn-N8-p14720218	Bn-A08-p14988862	Bn-A08-p14988862	A8	18	20.8	B
8387	Bn-N8-p14766244	Bn-A08-p15034494	Bn-A08-p15034494	A8	18	20.8	B
8388	Bn-N8-p14784176	Bn-A08-p15053710	Bn-A08-p15053710	A8	18	20.8	B
8389	Bn-N8-p14800175	Bn-A08-p15067659	Bn-A08-p15067659	A8	18	20.8	B
8390	Bn-N8-p14852936	Bn-A08-p15122707	Bn-A08-p15122707	A8	18	20.8	B
8391	Bn-N8-p14896931	Bn-C3-p55303106	Bn-scaff_16394_2-p174513	A8	18	20.8	B
8392	Bn-N8-p14908178	Bn-A08-p15177299	Bn-A08-p15177299	A8	18	20.8	B
8393	Bn-N8-p14967895	Bn-A08-p15231480	Bn-A08-p15231480	A8	18	20.8	B
8394	Bn-N8-p15001020	Bn-A08-p15265602	Bn-A08-p15265602	A8	18	20.8	B
8395	Bn-N8-p15001382	Bn-A08-p15265969	Bn-A08-p15265969	A8	18	20.8	B
8396	Bn-N8-p15003490	Bn-A08-p15268077	Bn-A08-p15268077	A8	18	20.8	B
8397	Bn-N8-p15068724	Bn-A08-p15322011	Bn-A08-p15322011	A8	18	20.8	B
8398	Bn-Scaffold59374-p127	Bn-A08-p15075119	Bn-A08-p15075119	A8	18	20.8	B
8399	Bn-N8-p15090232	Bn-A08-p15353881	Bn-A08-p15353881	A8	19	21.6	B
8400	Bn-N8-p15207492	Bn-A08-p15471679	Bn-A08-p15471679	A8	19	21.6	B
8401	Bn-N8-p15224551	Bn-A08-p15489686	Bn-A08-p15489686	A8	19	21.6	B
8402	Bn-N8-p15244335	Bn-A08-p15502867	Bn-A08-p15502867	A8	19	21.6	B
8403	Bn-N13-p56349992	Bn-A08-p15614498	Bn-A08-p15614498	A8	20	22.4	B
8404	Bn-N8-p15416987	Bn-A08-p15701695	Bn-A08-p15701695	A8	20	22.4	B
8405	Bn-N8-p15440582	Bn-A08-p15723645	Bn-A08-p15723645	A8	20	22.4	B
8406	Bn-N8-p15503840	Bn-A08-p15782077	Bn-A08-p15782077	A8	20	22.4	B
8407	Bn-N8-p15503893	Bn-A08-p15782130	Bn-A08-p15782130	A8	20	22.4	B
8408	Bn-N8-p15503992	Bn-A08-p15782229	Bn-A08-p15782229	A8	20	22.4	B
8409	Bn-N8-p15504004	Bn-A08-p15782241	Bn-A08-p15782241	A8	20	22.4	B
8410	Bn-N8-p15504070	Bn-A08-p15782307	Bn-A08-p15782307	A8	20	22.4	B
8411	Bn-N8-p15512424	Bn-A08-p15790373	Bn-A08-p15790373	A8	20	22.4	B
8412	Bn-N8-p15514120	Bn-A08-p15792069	Bn-A08-p15792069	A8	20	22.4	B
8413	Bn-N8-p15514420	Bn-A08-p15792369	Bn-A08-p15792369	A8	20	22.4	B
8414	Bn-N8-p15514735	Bn-A08-p15792684	Bn-A08-p15792684	A8	20	22.4	B
8415	Bn-N8-p15514993	Bn-A08-p15792942	Bn-A08-p15792942	A8	20	22.4	B
8416	Bn-N8-p15573047	Bn-A08-p15872628	Bn-A08-p15872628	A8	20	22.4	B
8417	Bn-N8-p15591070	Bn-A08-p15888048	Bn-A08-p15888048	A8	20	22.4	B
8418	Bn-N8-p15608508	Bn-A08-p15906423	Bn-A08-p15906423	A8	20	22.4	B
8419	Bn-N8-p15614950	Bn-A08-p15914038	Bn-A08-p15914038	A8	20	22.4	B
8420	Bn-N8-p15652276	Bn-A08-p15954904	Bn-A08-p15954904	A8	20	22.4	B
8421	Bn-N8-p15666870	Bn-A08-p15972135	Bn-A08-p15972135	A8	20	22.4	B
8422	Bn-N8-p15686563	Bn-A08-p15991441	Bn-A08-p15991441	A8	21	23.2	B
8423	Bn-N8-p15696130	Bn-C3-p53579689	Bn-scaff_24631_1-p745795	A8	21	23.2	B

8424	Bn-N8-p15720124	Bn-C3-p53635749	Bn-scaff_24631_1-p801855	A8	21	23.2	B
8425	Bn-N8-p15738676	Bn-A08-p16052172	Bn-A08-p16052172	A8	21	23.2	B
8426	Bn-N8-p15748397	Bn-A08-p16061843	Bn-A08-p16061843	A8	21	23.2	B
8427	Bn-N8-p15798281	Bn-A08-p16111871	Bn-A08-p16111871	A8	21	23.2	B
8428	Bn-N8-p15804412	Bn-A08-p16115851	Bn-A08-p16115851	A8	21	23.2	B
8429	Bn-N8-p15829618	Bn-A08-p16132462	Bn-A08-p16132462	A8	21	23.2	B
8430	Bn-N8-p15844918	Bn-A08-p16149197	Bn-A08-p16149197	A8	21	23.2	B
8431	Bn-N8-p15863075	Bn-A08-p16171460	Bn-A08-p16171460	A8	21	23.2	B
8432	Bn-N8-p15875587	Bn-C3-p53229192	Bn-scaff_24631_1-p395298	A8	22	24	B
8433	Bn-N8-p15876364	Bn-A08-p16183708	Bn-A08-p16183708	A8	22	24	B
8434	Bn-N8-p15884213	Bn-C3-p53217448	Bn-scaff_24631_1-p383554	A8	22	24	B
8435	Bn-N8-p15885084	Bn-C3-p53217234	Bn-scaff_24631_1-p383340	A8	22	24	B
8436	Bn-N8-p15914242	Bn-A08-p16216893	Bn-A08-p16216893	A8	23	24.8	B
8437	Bn-N8-p15952855	Bn-A08-p16248227	Bn-A08-p16248227	A8	24	25.6	B
8438	Bn-N8-p15953256	Bn-A08-p16248633	Bn-A08-p16248633	A8	24	25.6	B
8439	Bn-N8-p15960786	Bn-A08-p16259976	Bn-A08-p16259976	A8	24	25.6	B
8440	Bn-N8-p15971865	Bn-C3-p53049850	Bn-scaff_24631_1-p215956	A8	24	25.6	B
8441	Bn-N8-p15985169	Bn-A08-p16286082	Bn-A08-p16286082	A8	25	26.4	B
8442	Bn-N8-p16006346	Bn-A08-p16306425	Bn-A08-p16306425	A8	25	26.4	B
8443	Bn-N8-p16017715	Bn-A08-p16317873	Bn-A08-p16317873	A8	25	26.4	B
8444	Bn-N8-p16018551	Bn-A08-p16318709	Bn-A08-p16318709	A8	25	26.4	B
8445	Bn-N8-p16042964	Bn-A08-p16344396	Bn-A08-p16344396	A8	25	26.4	B
8446	Bn-N8-p16048457	Bn-A08-p16349875	Bn-A08-p16349875	A8	25	26.4	B
8447	Bn-N8-p16051119	Bn-A08-p16353006	Bn-A08-p16353006	A8	25	26.4	B
8448	Bn-N8-p16068959	Bn-A08-p16366766	Bn-A08-p16366766	A8	25	26.4	B
8449	Bn-N8-p16085763	Bn-A08-p16382380	Bn-A08-p16382380	A8	25	26.4	B
8450	Bn-N8-p16096353	Bn-A08-p16392743	Bn-A08-p16392743	A8	25	26.4	B
8451	Bn-N8-p16096567	Bn-A08-p16392957	Bn-A08-p16392957	A8	25	26.4	B
8452	Bn-N8-p16121325	Bn-A08-p16416932	Bn-A08-p16416932	A8	25	26.4	B
8453	Bn-N8-p16159609	Bn-A08-p16454339	Bn-A08-p16454339	A8	25	26.4	B
8454	Bn-N8-p16162924	Bn-A08-p16457656	Bn-A08-p16457656	A8	25	26.4	B
8455	Bn-N8-p16165865	Bn-A08-p16459840	Bn-A08-p16459840	A8	25	26.4	B
8456	Bn-N8-p16203810	Bn-Scaffold000827-p4	Bn-Scaffold000827-p421	A8	25	26.4	B
8457	Bn-N8-p16220256	Bn-A08-p16514789	Bn-A08-p16514789	A8	25	26.4	B
8458	Bn-N8-p16232261	Bn-A08-p16521179	Bn-A08-p16521179	A8	25	26.4	B
8459	Bn-N8-p16234300	Bn-A08-p16523108	Bn-A08-p16523108	A8	25	26.4	B
8460	Bn-N8-p16267240	Bn-A08-p16559652	Bn-A08-p16559652	A8	25	26.4	B
8461	Bn-N8-p16270995	Bn-A08-p16562035	Bn-A08-p16562035	A8	25	26.4	B
8462	Bn-N8-p16284975	Bn-A08-p16575548	Bn-A08-p16575548	A8	25	26.4	B
8463	Bn-N8-p16285223	Bn-A08-p16575796	Bn-A08-p16575796	A8	25	26.4	B
8464	Bn-N8-p16340598	Bn-A08-p16631459	Bn-A08-p16631459	A8	25	26.4	B
8465	Bn-N8-p16347723	Bn-A08-p16638835	Bn-A08-p16638835	A8	25	26.4	B
8466	Bn-N8-p16349555	Bn-A08-p16640519	Bn-A08-p16640519	A8	25	26.4	B
8467	Bn-N8-p16349616	Bn-A08-p16640580	Bn-A08-p16640580	A8	25	26.4	B
8468	Bn-N8-p16349746	Bn-A08-p16640710	Bn-A08-p16640710	A8	25	26.4	B
8469	Bn-N8-p16353988	Bn-A08-p16644247	Bn-A08-p16644247	A8	25	26.4	B
8470	Bn-N8-p16354260	Bn-A08-p16644519	Bn-A08-p16644519	A8	25	26.4	B
8471	Bn-N8-p16442873	Bn-A08-p16728548	Bn-A08-p16728548	A8	25	26.4	B
8472	Bn-N8-p16481176	Bn-A08-p16770923	Bn-A08-p16770923	A8	25	26.4	B

8473	Bn-N8-p16481283	Bn-A08-p16771030	Bn-A08-p16771030	A8	25	26.4	B
8474	Bn-N8-p16481414	Bn-A08-p16771161	Bn-A08-p16771161	A8	25	26.4	B
8475	Bn-N8-p16517927	Bn-A08-p16809551	Bn-A08-p16809551	A8	25	26.4	B
8476	Bn-N8-p16547530	Bn-A08-p16842687	Bn-A08-p16842687	A8	25	26.4	B
8477	Bn-N8-p16547533	Bn-A08-p16842690	Bn-A08-p16842690	A8	25	26.4	B
8478	Bn-Scaffold11609-p264	Bn-A08-p16704644	Bn-A08-p16704644	A8	25	26.4	B
8479	Bn-N8-p16552226	Bn-A08-p16846907	Bn-A08-p16846907	A8	26	27.2	B
8480	Bn-N8-p16560650	Bn-A08-p16859028	Bn-A08-p16859028	A8	26	27.2	B
8481	Bn-N8-p16560959	Bn-A08-p16859321	Bn-A08-p16859321	A8	26	27.2	B
8482	Bn-N8-p16596516	Bn-A08-p16894122	Bn-A08-p16894122	A8	26	27.2	B
8483	Bn-N8-p16615084	Bn-A08-p16913215	Bn-A08-p16913215	A8	27	28	B
8484	Bn-N8-p16663666	Bn-A08-p16958537	Bn-A08-p16958537	A8	27	28	B
8485	Bn-N8-p16685917	Bn-A08-p16980526	Bn-A08-p16980526	A8	28	28.8	B
8486	Bn-N8-p16698073	Bn-A08-p16992690	Bn-A08-p16992690	A8	28	28.8	B
8487	Bn-N8-p16767555	Bn-A08-p17069605	Bn-A08-p17069605	A8	28	28.8	B
8488	Bn-N8-p16777245	Bn-A08-p17078349	Bn-A08-p17078349	A8	28	28.8	B
8489	Bn-N8-p16786044	Bn-A08-p17088758	Bn-A08-p17088758	A8	28	28.8	B
8490	Bn-N8-p16802291	Bn-A08-p17105262	Bn-A08-p17105262	A8	28	28.8	B
8491	Bn-N8-p16807091	Bn-A09-p8783204	Bn-A09-p8783204	A8	28	28.8	B
8492	Bn-N8-p16814168	Bn-A08-p17111194	Bn-A08-p17111194	A8	28	28.8	B
8493	Bn-N8-p16829820	Bn-A08-p17124630	Bn-A08-p17124630	A8	28	28.8	B
8494	Bn-N8-p16834676	Bn-A08-p17130151	Bn-A08-p17130151	A8	28	28.8	B
8495	Bn-N8-p16853191	Bn-A08-p17144811	Bn-A08-p17144811	A8	28	28.8	B
8496	Bn-N8-p16858120	Bn-A08-p17152372	Bn-A08-p17152372	A8	28	28.8	B
8497	Bn-N8-p16867251	Bn-A08-p17158655	Bn-A08-p17158655	A8	28	28.8	B
8498	Bn-N8-p16868060	Bn-A08-p17159464	Bn-A08-p17159464	A8	28	28.8	B
8499	Bn-N8-p16869534	Bn-A08-p17161182	Bn-A08-p17161182	A8	28	28.8	B
8500	Bn-N8-p16870493	Bn-A08-p17162841	Bn-A08-p17162841	A8	28	28.8	B
8501	Bn-N13-p52787768	Bn-A08-p17238995	Bn-A08-p17238995	A8	29	31.2	B
8502	Bn-N8-p16894920	Bn-A08-p17187064	Bn-A08-p17187064	A8	29	31.2	B
8503	Bn-N8-p16911036	Bn-A08-p17202184	Bn-A08-p17202184	A8	29	31.2	B
8504	Bn-N8-p16911379	Bn-A08-p17202527	Bn-A08-p17202527	A8	29	31.2	B
8505	Bn-N8-p16911436	Bn-A08-p17202584	Bn-A08-p17202584	A8	29	31.2	B
8506	Bn-N8-p16911597	Bn-A08-p17202745	Bn-A08-p17202745	A8	29	31.2	B
8507	Bn-N8-p16927699	Bn-A08-p17221534	Bn-A08-p17221534	A8	29	31.2	B
8508	Bn-N8-p16959265	Bn-A08-p17249190	Bn-A08-p17249190	A8	29	31.2	B
8509	Bn-N8-p16968469	Bn-A08-p17255997	Bn-A08-p17255997	A8	29	31.2	B
8510	Bn-N8-p17004603	Bn-A08-p17291598	Bn-A08-p17291598	A8	30	32	B
8511	Bn-N8-p17004684	Bn-A08-p17291679	Bn-A08-p17291679	A8	30	32	B
8512	Bn-N8-p17006572	Bn-A08-p17293594	Bn-A08-p17293594	A8	30	32	B
8513	Bn-N13-p52207197	Bn-C3-p50371274	Bn-scaff_16135_1-p31546	A8	31	32.8	B
8514	Bn-N13-p52431923	Bn-A08-p17381323	Bn-A08-p17381323	A8	31	32.8	B
8515	Bn-N8-p17014513	Bn-A08-p17301557	Bn-A08-p17301557	A8	31	32.8	B
8516	Bn-N8-p17036250	Bn-A08-p17315378	Bn-A08-p17315378	A8	31	32.8	B
8517	Bn-N8-p17047112	Bn-A08-p17327135	Bn-A08-p17327135	A8	31	32.8	B
8518	Bn-N8-p17059779	Bn-A08-p17330668	Bn-A08-p17330668	A8	31	32.8	B
8519	Bn-N8-p17070454	Bn-A08-p17345947	Bn-A08-p17345947	A8	31	32.8	B
8520	Bn-N8-p17078706	Bn-A08-p17353995	Bn-A08-p17353995	A8	31	32.8	B
8521	Bn-N8-p17078878	Bn-A08-p17354167	Bn-A08-p17354167	A8	31	32.8	B

8522	Bn-N8-p17081155	Bn-A08-p17355609	Bn-A08-p17355609	A8	31	32.8	B
8523	Bn-N8-p17081218	Bn-A08-p17355672	Bn-A08-p17355672	A8	31	32.8	B
8524	Bn-N8-p17097654	Bn-A08-p17366299	Bn-A08-p17366299	A8	31	32.8	B
8525	Bn-N8-p17098025	Bn-C3-p50615411	Bn-scaff_20790_1-p264415	A8	31	32.8	B
8526	Bn-N8-p17115740	Bn-A08-p17382231	Bn-A08-p17382231	A8	31	32.8	B
8527	Bn-N8-p17136992	Bn-A08-p17402165	Bn-A08-p17402165	A8	31	32.8	B
8528	Bn-N8-p17159126	Bn-A08-p17435209	Bn-A08-p17435209	A8	31	32.8	B
8529	Bn-N8-p17186481	Bn-A08-p17468541	Bn-A08-p17468541	A8	31	32.8	B
8530	Bn-N8-p17222025	Bn-A08-p17507343	Bn-A08-p17507343	A8	31	32.8	B
8531	Bn-N8-p17227705	Bn-A08-p17513789	Bn-A08-p17513789	A8	32	35.2	B
8532	Bn-N8-p17245403	Bn-A08-p17530617	Bn-A08-p17530617	A8	33	39.2	B
8533	Bn-N8-p17249326	Bn-A08-p17534858	Bn-A08-p17534858	A8	33	39.2	B
8534	Bn-N8-p17255122	Bn-A08-p17547975	Bn-A08-p17547975	A8	33	39.2	B
8535	Bn-N8-p17270027	Bn-A08-p17559236	Bn-A08-p17559236	A8	33	39.2	B
8536	Bn-N8-p17275506	Bn-A08-p17565873	Bn-A08-p17565873	A8	33	39.2	B
8537	Bn-N8-p17307028	Bn-A08-p17586676	Bn-A08-p17586676	A8	33	39.2	B
8538	Bn-N8-p17333121	Bn-A08-p17612777	Bn-A08-p17612777	A8	33	39.2	B
8539	Bn-N8-p17333209	Bn-A08-p17612865	Bn-A08-p17612865	A8	33	39.2	B
8540	Bn-N8-p17333838	Bn-A08-p17613494	Bn-A08-p17613494	A8	33	39.2	B
8541	Bn-N8-p17334214	Bn-A08-p17613870	Bn-A08-p17613870	A8	33	39.2	B
8542	Bn-N8-p17338547	Bn-A08-p17618201	Bn-A08-p17618201	A8	33	39.2	B
8543	Bn-N8-p17373422	Bn-A08-p17639419	Bn-A08-p17639419	A8	33	39.2	B
8544	Bn-N8-p17418926	Bn-A08-p17686366	Bn-A08-p17686366	A8	33	39.2	B
8545	Bn-N8-p17418931	Bn-A08-p17686371	Bn-A08-p17686371	A8	33	39.2	B
8546	Bn-N8-p17419292	Bn-A08-p17686730	Bn-A08-p17686730	A8	33	39.2	B
8547	Bn-N8-p17419776	Bn-A08-p17687210	Bn-A08-p17687210	A8	33	39.2	B
8548	Bn-N8-p17420251	Bn-A08-p17687648	Bn-A08-p17687648	A8	33	39.2	B
8549	Bn-N8-p17427151	Bn-A08-p17698912	Bn-A08-p17698912	A8	33	39.2	B
8550	Bn-N8-p17436342	Bn-A08-p17702727	Bn-A08-p17702727	A8	33	39.2	B
8551	Bn-N8-p17443568	Bn-A08-p17709729	Bn-A08-p17709729	A8	33	39.2	B
8552	Bn-N8-p17457023	Bn-A08-p17721455	Bn-A08-p17721455	A8	33	39.2	B
8553	Bn-N8-p17458818	Bn-A08-p17723283	Bn-A08-p17723283	A8	33	39.2	B
8554	Bn-N8-p17459974	Bn-A08-p17724619	Bn-A08-p17724619	A8	33	39.2	B
8555	Bn-N8-p17469786	Bn-A08-p17735673	Bn-A08-p17735673	A8	33	39.2	B
8556	Bn-N8-p17488093	Bn-A08-p17748884	Bn-A08-p17748884	A8	34	40	B
8557	Bn-N8-p17543400	Bn-A08-p17813465	Bn-A08-p17813465	A8	34	40	B
8558	Bn-N8-p17545263	Bn-A08-p17816612	Bn-A08-p17816612	A8	34	40	0
8559	Bn-N8-p17555865	Bn-A08-p17823277	Bn-A08-p17823277	A8	34	40	B
8560	Bn-N8-p17556900	Bn-A08-p17824285	Bn-A08-p17824285	A8	34	40	B
8561	Bn-N8-p17565821	Bn-A08-p17830772	Bn-A08-p17830772	A8	34	40	B
8562	Bn-N8-p17573103	Bn-A08-p17849621	Bn-A08-p17849621	A8	34	40	B
8563	Bn-N8-p17574579	Bn-A08-p17851106	Bn-A08-p17851106	A8	34	40	B
8564	Bn-N8-p17592686	Bn-A08-p17863903	Bn-A08-p17863903	A8	35	41.6	B
8565	Bn-N8-p17612784	Bn-A08-p17887796	Bn-A08-p17887796	A8	35	41.6	B
8566	Bn-N8-p17732398	Bn-A08-p18000876	Bn-A08-p18000876	A8	36	43.2	B
8567	Bn-N18-p26552559	Bn-A08-p18112619	Bn-A08-p18112619	A8	37	44	B
8568	Bn-N8-p17778288	Bn-A08-p18045254	Bn-A08-p18045254	A8	37	44	B
8569	Bn-N8-p17798612	Bn-A08-p18070928	Bn-A08-p18070928	A8	37	44	B
8570	Bn-N8-p17803801	Bn-A08-p18078817	Bn-A08-p18078817	A8	37	44	B

8571	Bn-N8-p17836140	Bn-A08-p18107648	Bn-A08-p18107648	A8	37	44	B
8572	Bn-N8-p17928683	Bn-A08-p18200896	Bn-A08-p18200896	A8	37	44	B
8573	Bn-N8-p18006234	Bn-A08-p7611203	Bn-A08-p7611203	A8	38	49.7	B
8574	Bn-N8-p18030952	Bn-A08-p18290748	Bn-A08-p18290748	A8	38	49.7	B
8575	Bn-N8-p18031474	Bn-A08-p18291268	Bn-A08-p18291268	A8	38	49.7	B
8576	Bn-N8-p18042252	Bn-A05-p6672638	Bn-A05-p6672638	A8	38	49.7	B
8577	Bn-N8-p18048620	Bn-A08-p18310412	Bn-A08-p18310412	A8	38	49.7	B
8578	Bn-N8-p18067482	Bn-A08-p18321643	Bn-A08-p18321643	A8	38	49.7	B
8579	Bn-N8-p18304756	Bn-A08-p18543984	Bn-A08-p18543984	A8	39	52.9	B
8580	Bn-N8-p18351042	Bn-A08-p18592850	Bn-A08-p18592850	A8	39	52.9	B
8581	Bn-N8-p18359822	Bn-A08-p18605816	Bn-A08-p18605816	A8	40	53.7	B
8582	Bn-N8-p18393020	Bn-C8-p22823813	Bn-scaff_15923_1-p10961	A8	40	53.7	B
8583	Bn-N8-p18436703	Bn-A08-p18680503	Bn-A08-p18680503	A8	40	53.7	B
8584	Bn-N18-p25076370	Bn-C8-p22424775	Bn-scaff_16231_1-p20387	A8	41	54.5	B
8585	Bn-N8-p18468951	Bn-A08-p18720560	Bn-A08-p18720560	A8	41	54.5	B
8586	Bn-N8-p18503671	Bn-A08-p18750116	Bn-A08-p18750116	A8	41	54.5	B
8587	Bn-N8-p18507294	Bn-A08-p18754717	Bn-A08-p18754717	A8	41	54.5	B
8588	Bn-N8-p18564132	Bn-A08-p18798063	Bn-A08-p18798063	A8	41	54.5	B
8589	Bn-N8-p18585891	Bn-A08-p18820218	Bn-A08-p18820218	A8	41	54.5	B
8590	Bn-N8-p18593363	Bn-A08-p18827698	Bn-A08-p18827698	A8	41	54.5	B
8591	Bn-Scaffold25034-p422	Bn-C8-p22650548	Bn-scaff_15923_1-p12693	A8	41	54.5	B
8592	Bn-N18-p24679255	Bn-C8-p22051543	Bn-scaff_16231_1-p57710	A8	42	57.7	0
8593	Bn-N8-p18775647	Bn-A08-p19007302	Bn-A08-p19007302	A8	42	57.7	B
8594	Bn-N8-p18776922	Bn-A08-p19008575	Bn-A08-p19008575	A8	42	57.7	B
8595	Bn-N8-p18777018	Bn-A08-p19008671	Bn-A08-p19008671	A8	42	57.7	B
8596	Bn-N8-p18786566	Bn-A08-p19017689	Bn-A08-p19017689	A8	42	57.7	B
8597	Bn-N8-p18823461	Bn-A08-p19056545	Bn-A08-p19056545	A8	42	57.7	B
8598	Bn-N8-p18848906	Bn-A08-p19084386	Bn-A08-p19084386	A8	42	57.7	B
8599	Bn-N8-p18888323	Bn-A08-p19122274	Bn-A08-p19122274	A8	42	57.7	B
8600	Bn-N8-p18961142	Bn-C8-p21827288	Bn-scaff_16231_1-p80135	A8	42	57.7	B
8601	Bn-N8-p18966855	Bn-A08-p19197746	Bn-A08-p19197746	A8	42	57.7	B
8602	Bn-N8-p18968019	Bn-A08-p19198891	Bn-A08-p19198891	A8	42	57.7	B
8603	Bn-N8-p19031077	Bn-A08-p19261458	Bn-A08-p19261458	A8	42	57.7	B
8604	Bn-N8-p19062530	Bn-C8-p21592037	Bn-scaff_16231_1-p103661	A8	43	60.1	B
8605	Bn-N18-p23810373	Bn-A08-p19483726	Bn-A08-p19483726	A8	44	62.5	B
8606	Bn-N8-p19229698	Bn-A08-p19428204	Bn-A08-p19428204	A8	44	62.5	B
8607	Bn-N8-p19236615	Bn-A08-p19435224	Bn-A08-p19435224	A8	44	62.5	B
8608	Bn-N8-p19289995	Bn-A08-p19487830	Bn-A08-p19487830	A8	44	62.5	B
8609	Bn-N8-p19303607	Bn-A08-p19504101	Bn-A08-p19504101	A8	44	62.5	B
8610	Bn-N18-p23545795	Bn-A08-p19578180	Bn-A08-p19578180	A8	45	64.1	0
8611	Bn-N18-p23614287	Bn-C8-p21143733	Bn-scaff_16231_1-p148491	A8	45	64.1	B
8612	Bn-N8-p19333368	Bn-A08-p19530023	Bn-A08-p19530023	A8	45	64.1	B
8613	Bn-N8-p19345416	Bn-A08-p19541990	Bn-A08-p19541990	A8	45	64.1	B
8614	Bn-N8-p19357480	Bn-A08-p19553756	Bn-A08-p19553756	A8	45	64.1	B
8615	Bn-N8-p19378150	Bn-A08-p19573947	Bn-A08-p19573947	A8	45	64.1	B
8616	Bn-N8-p19379598	Bn-A08-p19575209	Bn-A08-p19575209	A8	45	64.1	B
8617	Bn-N8-p19399357	Bn-A08-p19599590	Bn-A08-p19599590	A8	45	64.1	B
8618	Bn-N8-p19409129	Bn-A08-p19608779	Bn-A08-p19608779	A8	45	64.1	B
8619	Bn-N8-p19441441	Bn-A08-p19642040	Bn-A08-p19642040	A8	45	64.1	B

8620	Bn-N8-p19448615	Bn-A08-p19646345	Bn-A08-p19646345	A8	46	64.9	B
8621	Bn-N11-p34110475	Bn-A08-p19683899	Bn-A08-p19683899	A8	47	68.1	B
8622	Bn-N18-p23318546	Bn-A08-p19676751	Bn-A08-p19676751	A8	47	68.1	B
8623	Bn-N18-p23318644	Bn-A08-p19676653	Bn-A08-p19676653	A8	47	68.1	B
8624	Bn-N8-p19476198	Bn-A08-p19676486	Bn-A08-p19676486	A8	47	68.1	B
8625	Bn-N8-p19476210	Bn-A08-p19676498	Bn-A08-p19676498	A8	47	68.1	B
8626	Bn-N8-p19488518	Bn-C8-p20787189	Bn-scaff_16231_1-p18414[illegible]	A8	47	68.1	B
8627	Bn-N8-p19490115	Bn-A08-p19690298	Bn-A08-p19690298	A8	47	68.1	B
8628	Bn-N8-p19490210	Bn-A08-p19690393	Bn-A08-p19690393	A8	47	68.1	B
8629	Bn-N8-p19543607	Bn-A08-p19736692	Bn-A08-p19736692	A8	47	68.1	B
8630	Bn-N8-p19586029	Bn-A08-p19782306	Bn-A08-p19782306	A8	47	68.1	B
8631	Bn-N8-p19651757	Bn-A08-p19834705	Bn-A08-p19834705	A8	48	73	B
8632	Bn-N8-p19707140	Bn-A08-p19888431	Bn-A08-p19888431	A8	48	73	B
8633	Bn-N8-p19725161	Bn-A08-p19908763	Bn-A08-p19908763	A8	48	73	B
8634	Bn-N8-p19762815	Bn-A08-p19960214	Bn-A08-p19960214	A8	49	73.9	B
8635	Bn-N8-p19783814	Bn-A08-p19982640	Bn-A08-p19982640	A8	49	73.9	B
8636	Bn-N8-p19858500	Bn-A08-p20068678	Bn-A08-p20068678	A8	50	76.2	B
8637	Bn-N8-p19862815	Bn-A08-p20072457	Bn-A08-p20072457	A8	50	76.2	B
8638	Bn-N8-p19882294	Bn-A08-p20091818	Bn-A08-p20091818	A8	50	76.2	B
8639	Bn-N8-p19882591	Bn-A08-p20092115	Bn-A08-p20092115	A8	50	76.2	B
8640	Bn-N8-p19886966	Bn-A08-p20096558	Bn-A08-p20096558	A8	50	76.2	B
8641	Bn-N8-p19896084	Bn-A08-p20106099	Bn-A08-p20106099	A8	50	76.2	B
8642	Bn-N8-p19896514	Bn-A08-p20106529	Bn-A08-p20106529	A8	50	76.2	B
8643	Bn-N8-p19915403	Bn-A08-p20134420	Bn-A08-p20134420	A8	50	76.2	B
8644	Bn-N8-p19928029	Bn-C8-p19994689	Bn-scaff_21786_1-p52917	A8	50	76.2	B
8645	Bn-N8-p19928494	Bn-C8-p19994218	Bn-scaff_21786_1-p52446	A8	50	76.2	B
8646	Bn-N8-p19937163	Bn-A08-p20173109	Bn-A08-p20173109	A8	50	76.2	B
8647	Bn-N8-p19957910	Bn-A08-p20189027	Bn-A08-p20189027	A8	50	76.2	B
8648	Bn-N8-p19983571	Bn-A08-p20226034	Bn-A08-p20226034	A8	50	76.2	B
8649	Bn-N8-p20003418	Bn-A08-p20245485	Bn-A08-p20245485	A8	50	76.2	B
8650	Bn-N8-p20048541	Bn-A08-p20277144	Bn-A08-p20277144	A8	50	76.2	B
8651	Bn-N8-p20051389	Bn-A08-p20286379	Bn-A08-p20286379	A8	50	76.2	B
8652	Bn-N18-p21801715	Bn-A05-p8210067	Bn-A05-p8210067	A8	51	77	B
8653	Bn-N8-p20113458	Bn-C8-p19607543	Bn-scaff_17227_1-p11012[illegible]	A8	51	77	B
8654	Bn-N8-p20277216	Bn-A05-p8297352	Bn-A05-p8297352	A8	51	77	B
8655	Bn-N8-p20299320	Bn-A05-p8318433	Bn-A05-p8318433	A8	51	77	B
8656	Bn-N12-p25172344	Bn-A08-p20341221	Bn-A08-p20341221	A8	52	77.8	B
8657	Bn-N18-p21048503	Bn-C8-p18660653	Bn-scaff_17227_1-p15434[illegible]	A8	52	77.8	B
8658	Bn-N18-p21457522	Bn-A08-p20343735	Bn-A08-p20343735	A8	52	77.8	B
8659	Bn-N8-p20348461	Bn-A08-p20305036	Bn-A08-p20305036	A8	52	77.8	B
8660	Bn-N8-p20351252	Bn-A08-p20306059	Bn-A08-p20306059	A8	52	77.8	B
8661	Bn-N8-p20351760	Bn-A08-p20306567	Bn-A08-p20306567	A8	52	77.8	B
8662	Bn-N8-p20356688	Bn-A08-p20314411	Bn-A08-p20314411	A8	52	77.8	B
8663	Bn-N8-p20362083	Bn-A08-p20319815	Bn-A08-p20319815	A8	52	77.8	B
8664	Bn-N8-p20392010	Bn-A08-p20347040	Bn-A08-p20347040	A8	52	77.8	B
8665	Bn-N8-p20404594	Bn-A08-p20360306	Bn-A08-p20360306	A8	52	77.8	B
8666	Bn-N8-p20432212	Bn-A08-p20394042	Bn-A08-p20394042	A8	52	77.8	B
8667	Bn-N8-p20433558	Bn-A08-p20395388	Bn-A08-p20395388	A8	52	77.8	B
8668	Bn-N8-p20495674	Bn-A08-p20441889	Bn-A08-p20441889	A8	52	77.8	B

8669	Bn-N8-p20535921	Bn-A08-p20476359	Bn-A08-p20476359	A8	52	77.8	B
8670	Bn-N8-p20552338	Bn-A08-p20490539	Bn-A08-p20490539	A8	52	77.8	B
8671	Bn-N8-p20552490	Bn-A08-p20490691	Bn-A08-p20490691	A8	52	77.8	B
8672	Bn-N8-p20600222	Bn-A08-p20538517	Bn-A08-p20538517	A8	52	77.8	B
8673	Bn-N8-p20606125	Bn-A08-p20546003	Bn-A08-p20546003	A8	52	77.8	B
8674	Bn-N8-p20606166	Bn-A08-p20546044	Bn-A08-p20546044	A8	52	77.8	B
8675	Bn-N8-p20606232	Bn-A08-p20546110	Bn-A08-p20546110	A8	52	77.8	B
8676	Bn-N8-p20608466	Bn-A08-p20548890	Bn-A08-p20548890	A8	52	77.8	B
8677	Bn-N8-p20614128	Bn-A08-p20554540	Bn-A08-p20554540	A8	52	77.8	B
8678	Bn-N8-p20615247	Bn-A08-p20555629	Bn-A08-p20555629	A8	52	77.8	B
8679	Bn-N8-p20631070	Bn-A08-p20577960	Bn-A08-p20577960	A8	52	77.8	B
8680	Bn-N8-p20641784	Bn-A08-p20587556	Bn-A08-p20587556	A8	52	77.8	B
8681	Bn-N8-p20649161	Bn-A08-p20594698	Bn-A08-p20594698	A8	52	77.8	B
8682	Bn-N8-p20649248	Bn-A08-p20594785	Bn-A08-p20594785	A8	52	77.8	B
8683	Bn-N8-p20662249	Bn-A08-p20608675	Bn-A08-p20608675	A8	52	77.8	B
8684	Bn-N8-p20879154	Bn-A08-p20820768	Bn-A08-p20820768	A8	53	81.8	B
8685	Bn-N8-p20900858	Bn-A08-p20838949	Bn-A08-p20838949	A8	53	81.8	B
8686	Bn-N8-p20968196	Bn-A08-p20904948	Bn-A08-p20904948	A8	53	81.8	B
8687	Bn-N8-p20989516	Bn-A08-p20927339	Bn-A08-p20927339	A8	53	81.8	0
8688	Unknown	Bn-A08-p20855631	Bn-A08-p20855631	A8	53	81.8	B
8689	Bn-N18-p1551819	Bn-A08-p20968239	Bn-A08-p20968239	A8	54	84.2	B
8690	Bn-N8-p21005755	Bn-A08-p20945277	Bn-A08-p20945277	A8	54	84.2	B
8691	Bn-N8-p21010352	Bn-C8-p1367788	Bn-scaff_26293_1-p15848(	A8	54	84.2	B
8692	Bn-N8-p21010676	Bn-A08-p20950199	Bn-A08-p20950199	A8	54	84.2	B
8693	Bn-N8-p21028995	Bn-A08-p20965113	Bn-A08-p20965113	A8	54	84.2	B
8694	Bn-N8-p21131090	Bn-A08-p21070052	Bn-A08-p21070052	A8	54	84.2	0
8695	Bn-N8-p21274010	Bn-A08-p21199124	Bn-A08-p21199124	A8	55	85	B
8696	Bn-N8-p21361961	Bn-A08-p21283562	Bn-A08-p21283562	A8	55	85	B
8697	Bn-N8-p21425973	Bn-A08-p21348310	Bn-A08-p21348310	A8	55	85	B
8698	Bn-N8-p21430377	Bn-A08-p21352716	Bn-A08-p21352716	A8	55	85	B
8699	Bn-N8-p21478311	Bn-A08-p21401348	Bn-A08-p21401348	A8	55	85	B
8700	Bn-N8-p21495935	Bn-A08-p21417592	Bn-A08-p21417592	A8	55	85	B
8701	Bn-N9-p342104	Bn-A09-p957959	Bn-A09-p957959	A9	1	0	A
8702	Bn-N9-p348382	Bn-A09-p951202	Bn-A09-p951202	A9	1	0	A
8703	Bn-N9-p358440	Bn-A09-p941202	Bn-A09-p941202	A9	1	0	A
8704	Bn-N9-p438953	Bn-A09-p868589	Bn-A09-p868589	A9	1	0	A
8705	Bn-N19-p320463	Bn-A09-p750455	Bn-A09-p750455	A9	2	0.8	A
8706	Bn-N19-p413458	Bn-A09-p696636	Bn-A09-p696636	A9	2	0.8	A
8707	Bn-N19-p55620559	Bn-A09-p596014	Bn-A09-p596014	A9	2	0.8	A
8708	Bn-N19-p55621802	Bn-A09-p594794	Bn-A09-p594794	A9	2	0.8	A
8709	Bn-N19-p55630399	Bn-A09-p586357	Bn-A09-p586357	A9	2	0.8	A
8710	Bn-N9-p504875	Bn-A09-p763204	Bn-A09-p763204	A9	2	0.8	A
8711	Bn-N9-p560125	Bn-A09-p704028	Bn-A09-p704028	A9	2	0.8	A
8712	Bn-N9-p640406	Bn-A09-p616919	Bn-A09-p616919	A9	2	0.8	A
8713	Bn-N9-p641361	Bn-A09-p614715	Bn-A09-p614715	A9	2	0.8	A
8714	Bn-N9-p780943	Bn-C9-p1254655	Bn-scaff_15783_1-p26293(	A9	3	2.1	A
8715	Bn-N9-p796829	Bn-A09-p86293	Bn-A09-p86293	A9	3	2.1	A
8716	Bn-N9-p1014810	Bn-A01-p26969210	Bn-A01-p26969210	A9	4	3.4	0
8717	Bn-N9-p1015065	Bn-A01-p26968952	Bn-A01-p26968952	A9	4	3.4	A

8718	Bn-N9-p1017642	Bn-A01-p26966407	Bn-A01-p26966407	A9	4	3.4	A
8719	Bn-N9-p1023417	Bn-A01-p26956455	Bn-A01-p26956455	A9	4	3.4	A
8720	Bn-N9-p1082272	Bn-A01-p26921219	Bn-A01-p26921219	A9	4	3.4	A
8721	Bn-N9-p1090077	Bn-A01-p26914512	Bn-A01-p26914512	A9	4	3.4	A
8722	Bn-N9-p911561	Bn-A01-p27061075	Bn-A01-p27061075	A9	4	3.4	A
8723	Bn-N9-p912273	Bn-A01-p27060364	Bn-A01-p27060364	A9	4	3.4	A
8724	Bn-N9-p934832	Bn-A01-p27037472	Bn-A01-p27037472	A9	4	3.4	A
8725	Bn-N9-p943089	Bn-A01-p27029254	Bn-A01-p27029254	A9	4	3.4	A
8726	Bn-N9-p943236	Bn-A01-p27029107	Bn-A01-p27029107	A9	4	3.4	A
8727	Bn-N9-p952448	Bn-A01-p27019745	Bn-A01-p27019745	A9	4	3.4	A
8728	Bn-N9-p980814	Bn-A01-p26998962	Bn-A01-p26998962	A9	4	3.4	A
8729	Bn-N9-p992054	Bn-A01-p26990131	Bn-A01-p26990131	A9	4	3.4	A
8730	Bn-N9-p1150435	Bn-A01-p26837207	Bn-A01-p26837207	A9	5	4.3	A
8731	Bn-N9-p1277924	Bn-A09-p1165212	Bn-A09-p1165212	A9	6	5.2	A
8732	Bn-N9-p1297967	Bn-A09-p1188091	Bn-A09-p1188091	A9	6	5.2	A
8733	Bn-N9-p1392583	Bn-A09-p1272298	Bn-A09-p1272298	A9	7	7.9	A
8734	Bn-N9-p1613782	Bn-A09-p2116947	Bn-A09-p2116947	A9	7	7.9	A
8735	Bn-N18-p12013192	Bn-A09-p2076591	Bn-A09-p2076591	A9	8	8.8	A
8736	Bn-N9-p1655795	Bn-A09-p2157775	Bn-A09-p2157775	A9	8	8.8	A
8737	Bn-N9-p1703140	Bn-A09-p2208929	Bn-A09-p2208929	A9	8	8.8	A
8738	Bn-N9-p1725120	Bn-A09-p2232416	Bn-A09-p2232416	A9	8	8.8	A
8739	Bn-N9-p1733463	Bn-A09-p2241625	Bn-A09-p2241625	A9	8	8.8	A
8740	Bn-N9-p1802613	Bn-A09-p2298880	Bn-A09-p2298880	A9	8	8.8	A
8741	Bn-N9-p1922972	Bn-A09-p2374845	Bn-A09-p2374845	A9	8	8.8	A
8742	Bn-N9-p2071219	Bn-A09-p2538450	Bn-A09-p2538450	A9	8	8.8	A
8743	Bn-N9-p2150419	Bn-A09-p2609043	Bn-A09-p2609043	A9	8	8.8	A
8744	Bn-N9-p2242314	Bn-A09-p2710152	Bn-A09-p2710152	A9	8	8.8	A
8745	Bn-N9-p2264448	Bn-A09-p2083933	Bn-A09-p2083933	A9	8	8.8	A
8746	Bn-N9-p2293605	Bn-A09-p2044817	Bn-A09-p2044817	A9	8	8.8	A
8747	Bn-N9-p2301751	Bn-A09-p2033221	Bn-A09-p2033221	A9	8	8.8	A
8748	Bn-N9-p2323952	Bn-A09-p2010675	Bn-A09-p2010675	A9	8	8.8	A
8749	Bn-N9-p2340141	Bn-A09-p1989556	Bn-A09-p1989556	A9	8	8.8	A
8750	Bn-N9-p2371422	Bn-A09-p1973528	Bn-A09-p1973528	A9	8	8.8	A
8751	Bn-N9-p2389532	Bn-A09-p1956137	Bn-A09-p1956137	A9	8	8.8	A
8752	Unknown	Bn-A09-p2076856	Bn-A09-p2076856	A9	8	8.8	A
8753	Bn-N9-p2419388	Bn-A09-p1929245	Bn-A09-p1929245	A9	9	9.7	A
8754	Bn-N19-p3573201	Bn-A09-p1727915	Bn-A09-p1727915	A9	10	10.6	A
8755	Bn-N9-p2488499	Bn-A09-p1856828	Bn-A09-p1856828	A9	10	10.6	A
8756	Bn-N9-p2565112	Bn-A09-p1786726	Bn-A09-p1786726	A9	10	10.6	A
8757	Bn-N9-p2566119	Bn-A09-p1785718	Bn-A09-p1785718	A9	10	10.6	A
8758	Bn-N9-p2566120	Bn-A09-p1785717	Bn-A09-p1785717	A9	10	10.6	A
8759	Bn-N9-p2588046	Bn-A09-p1765907	Bn-A09-p1765907	A9	10	10.6	A
8760	Bn-N9-p2592916	Bn-A09-p1761036	Bn-A09-p1761036	A9	10	10.6	A
8761	Bn-N9-p2592969	Bn-A09-p1760983	Bn-A09-p1760983	A9	10	10.6	A
8762	Bn-N9-p2634083	Bn-C9-p3558946	Bn-scaff_17526_1-p22081[illegible]	A9	10	10.6	A
8763	Bn-N9-p2641312	Bn-A09-p1719614	Bn-A09-p1719614	A9	10	10.6	A
8764	Bn-N9-p2742479	Bn-A09-p1634652	Bn-A09-p1634652	A9	10	10.6	A
8765	Bn-N9-p2742627	Bn-A09-p1634504	Bn-A09-p1634504	A9	10	10.6	A
8766	Bn-N9-p2742902	Bn-A09-p1634229	Bn-A09-p1634229	A9	10	10.6	A

8767	Bn-N9-p2745147	Bn-A09-p1631944	Bn-A09-p1631944	A9	10	10.6	A
8768	Bn-N9-p2746790	Bn-A09-p1630301	Bn-A09-p1630301	A9	10	10.6	A
8769	Bn-N9-p2759695	Bn-A09-p1617368	Bn-A09-p1617368	A9	10	10.6	A
8770	Bn-N9-p2767268	Bn-A09-p1610631	Bn-A09-p1610631	A9	10	10.6	A
8771	Bn-N9-p2769892	Bn-A09-p1608051	Bn-A09-p1608051	A9	10	10.6	A
8772	Bn-N9-p2775357	Bn-A09-p1603989	Bn-A09-p1603989	A9	10	10.6	A
8773	Bn-N9-p2776241	Bn-A09-p1603105	Bn-A09-p1603105	A9	10	10.6	A
8774	Bn-N9-p2793812	Bn-A09-p1588624	Bn-A09-p1588624	A9	10	10.6	A
8775	Bn-N9-p2809617	Bn-A09-p1570558	Bn-A09-p1570558	A9	11	12.3	A
8776	Bn-N9-p2829986	Bn-A09-p1552993	Bn-A09-p1552993	A9	12	13.1	A
8777	Bn-N9-p4609099	Bn-A09-p4096474	Bn-A09-p4096474	A9	13	24.6	A
8778	Bn-N9-p4681923	Bn-A09-p4024534	Bn-A09-p4024534	A9	13	24.6	A
8779	Bn-N9-p4681963	Bn-A09-p4024494	Bn-A09-p4024494	A9	13	24.6	A
8780	Bn-N9-p4684959	Bn-A09-p4021115	Bn-A09-p4021115	A9	13	24.6	A
8781	Bn-N9-p4690656	Bn-A09-p4012239	Bn-A09-p4012239	A9	13	24.6	A
8782	Bn-N9-p4709675	Bn-A09-p3994437	Bn-A09-p3994437	A9	13	24.6	A
8783	Bn-N9-p4710049	Bn-A09-p3994064	Bn-A09-p3994064	A9	13	24.6	A
8784	Bn-N9-p4762870	Bn-A09-p3940779	Bn-A09-p3940779	A9	13	24.6	A
8785	Bn-N9-p4763249	Bn-A09-p3940412	Bn-A09-p3940412	A9	13	24.6	A
8786	Bn-N9-p4855859	Bn-Scaffold000178-p3	Bn-Scaffold000178-p33587	A9	14	25.4	A
8787	Bn-N9-p4861842	Bn-C9-p7147395	Bn-scaff_17423_1-p76038(	A9	14	25.4	A
8788	Bn-N9-p4868714	Bn-Scaffold000178-p4	Bn-Scaffold000178-p47905	A9	14	25.4	A
8789	Bn-N9-p5116009	Bn-A09-p4463675	Bn-A09-p4463675	A9	15	26.2	A
8790	Bn-N9-p5117226	Bn-A09-p4465163	Bn-A09-p4465163	A9	15	26.2	A
8791	Bn-N9-p5130279	Bn-A09-p4475636	Bn-A09-p4475636	A9	15	26.2	A
8792	Bn-N9-p5162393	Bn-A09-p4509627	Bn-A09-p4509627	A9	16	27	A
8793	Bn-N19-p7859662	Bn-A09-p4652268	Bn-A09-p4652268	A9	17	28.6	A
8794	Bn-N9-p5312617	Bn-A09-p4655322	Bn-A09-p4655322	A9	17	28.6	A
8795	Bn-N9-p5334119	Bn-A09-p4681749	Bn-A09-p4681749	A9	17	28.6	A
8796	Bn-N9-p5335527	Bn-A09-p4683160	Bn-A09-p4683160	A9	17	28.6	A
8797	Bn-N9-p5335753	Bn-A09-p4683384	Bn-A09-p4683384	A9	17	28.6	A
8798	Bn-N9-p5337979	Bn-A09-p4690204	Bn-A09-p4690204	A9	17	28.6	A
8799	Bn-N9-p5370922	Bn-A09-p4735640	Bn-A09-p4735640	A9	17	28.6	A
8800	Bn-N9-p5473395	Bn-A09-p4831706	Bn-A09-p4831706	A9	17	28.6	A
8801	Bn-N9-p5489993	Bn-A09-p4848610	Bn-A09-p4848610	A9	17	28.6	A
8802	Bn-N9-p5504192	Bn-A09-p4862871	Bn-A09-p4862871	A9	17	28.6	A
8803	Bn-N9-p5504797	Bn-A09-p4863472	Bn-A09-p4863472	A9	17	28.6	A
8804	Bn-N9-p5549473	Bn-A09-p4895637	Bn-A09-p4895637	A9	17	28.6	A
8805	Bn-N9-p5585362	Bn-A09-p4913177	Bn-A09-p4913177	A9	17	28.6	A
8806	Bn-N9-p5585617	Bn-A09-p4913432	Bn-A09-p4913432	A9	17	28.6	A
8807	Bn-N9-p5608180	Bn-A09-p4927893	Bn-A09-p4927893	A9	17	28.6	A
8808	Bn-N9-p5613255	Bn-A09-p4932188	Bn-A09-p4932188	A9	17	28.6	A
8809	Bn-N9-p5644963	Bn-A09-p4963985	Bn-A09-p4963985	A9	17	28.6	A
8810	Bn-N9-p5646681	Bn-A09-p4965726	Bn-A09-p4965726	A9	17	28.6	A
8811	Bn-N9-p5709910	Bn-A09-p5215814	Bn-A09-p5215814	A9	17	28.6	A
8812	Bn-N9-p5713613	Bn-A09-p5219527	Bn-A09-p5219527	A9	17	28.6	A
8813	Bn-N9-p5719792	Bn-A09-p5225650	Bn-A09-p5225650	A9	17	28.6	A
8814	Bn-N9-p5727103	Bn-A09-p5232967	Bn-A09-p5232967	A9	17	28.6	A
8815	Bn-N9-p5735034	Bn-A09-p5246171	Bn-A09-p5246171	A9	17	28.6	A

8816	Bn-N9-p5735092	Bn-A09-p5246229	Bn-A09-p5246229	A9	17	28.6	A
8817	Bn-N9-p5742657	Bn-A09-p5251134	Bn-A09-p5251134	A9	17	28.6	A
8818	Bn-N9-p5747944	Bn-A09-p5255753	Bn-A09-p5255753	A9	17	28.6	A
8819	Bn-N9-p5766413	Bn-A09-p5267535	Bn-A09-p5267535	A9	17	28.6	A
8820	Bn-N9-p5777736	Bn-A09-p5285034	Bn-A09-p5285034	A9	17	28.6	A
8821	Bn-N9-p5778911	Bn-A09-p5286216	Bn-A09-p5286216	A9	17	28.6	A
8822	Bn-N9-p5791053	Bn-A09-p5298381	Bn-A09-p5298381	A9	17	28.6	A
8823	Bn-N9-p5791193	Bn-A09-p5298521	Bn-A09-p5298521	A9	17	28.6	A
8824	Bn-N9-p5816719	Bn-A09-p5327731	Bn-A09-p5327731	A9	17	28.6	A
8825	Bn-N9-p5818815	Bn-A09-p5329868	Bn-A09-p5329868	A9	17	28.6	A
8826	Bn-N9-p5820542	Bn-A09-p5331841	Bn-A09-p5331841	A9	17	28.6	A
8827	Bn-N9-p5821751	Bn-A09-p5332906	Bn-A09-p5332906	A9	17	28.6	A
8828	Bn-N9-p5826817	Bn-A09-p5337372	Bn-A09-p5337372	A9	17	28.6	A
8829	Bn-N9-p5833415	Bn-A09-p5343993	Bn-A09-p5343993	A9	17	28.6	A
8830	Bn-N9-p5838737	Bn-A09-p5348717	Bn-A09-p5348717	A9	17	28.6	A
8831	Bn-N9-p5892568	Bn-A09-p5416801	Bn-A09-p5416801	A9	17	28.6	A
8832	Bn-N9-p5908425	Bn-A09-p5432697	Bn-A09-p5432697	A9	17	28.6	A
8833	Bn-N9-p5915429	Bn-A09-p5442608	Bn-A09-p5442608	A9	17	28.6	A
8834	Bn-N9-p5931012	Bn-A09-p5455608	Bn-A09-p5455608	A9	18	29.4	A
8835	Bn-N9-p5950456	Bn-A09-p5470888	Bn-A09-p5470888	A9	18	29.4	A
8836	Bn-N9-p5951635	Bn-A09-p5476558	Bn-A09-p5476558	A9	18	29.4	A
8837	Bn-N9-p6148597	Bn-A09-p5678953	Bn-A09-p5678953	A9	18	29.4	A
8838	Bn-N9-p6202417	Bn-A07-p10188190	Bn-A07-p10188190	A9	19	30.2	A
8839	Bn-N9-p6457254	Bn-A09-p7397116	Bn-A09-p7397116	A9	19	30.2	A
8840	Bn-N9-p6517445	Bn-A09-p7331063	Bn-A09-p7331063	A9	19	30.2	A
8841	Bn-N9-p6517474	Bn-A09-p7331034	Bn-A09-p7331034	A9	19	30.2	A
8842	Bn-N9-p6518504	Bn-A09-p7329993	Bn-A09-p7329993	A9	19	30.2	A
8843	Bn-N9-p6531524	Bn-A09-p7317278	Bn-A09-p7317278	A9	19	30.2	A
8844	Bn-N19-p10270316	Bn-A09-p7174944	Bn-A09-p7174944	A9	20	31	A
8845	Bn-N9-p6631275	Bn-A09-p7199203	Bn-A09-p7199203	A9	20	31	A
8846	Bn-N9-p6639316	Bn-A09-p7191369	Bn-A09-p7191369	A9	20	31	A
8847	Bn-N9-p6639323	Bn-A09-p7191362	Bn-A09-p7191362	A9	20	31	A
8848	Bn-N9-p6806606	Bn-A09-p7047022	Bn-A09-p7047022	A9	21	34.2	A
8849	Bn-N9-p6924911	Bn-A09-p6922467	Bn-A09-p6922467	A9	22	36.6	A
8850	Bn-N9-p7210196	Bn-C9-p11083912	Bn-scaff_17683_1-p28029	A9	22	36.6	A
8851	Bn-N9-p7210255	Bn-C9-p11083971	Bn-scaff_17683_1-p28023	A9	22	36.6	A
8852	Bn-N9-p6995894	Bn-A09-p6866176	Bn-A09-p6866176	A9	23	38.2	A
8853	Bn-N9-p7052384	Bn-C9-p10793169	Bn-scaff_20428_1-p13308	A9	23	38.2	A
8854	Bn-N9-p7199373	Bn-A09-p6616405	Bn-A09-p6616405	A9	23	38.2	A
8855	Bn-N19-p11693818	Bn-C9-p11618288	Bn-scaff_21656_1-p10938	A9	24	39	A
8856	Bn-N19-p14915925	Bn-C9-p14453533	Bn-scaff_22835_1-p31916	A9	24	39	A
8857	Bn-N1-p10414413	Bn-Scaffold01946-p2	Bn-scaff_25535_1-p2682	A9	24	39	A
8858	Bn-N9-p7648675	Bn-A09-p6133524	Bn-A09-p6133524	A9	24	39	A
8859	Bn-N9-p7796053	Bn-A09-p6010593	Bn-A09-p6010593	A9	24	39	A
8860	Bn-N9-p8115406	Bn-C9-p12378314	Bn-scaff_15701_1-p73786	A9	24	39	A
8861	Bn-N9-p8204076	Bn-A09-p7741917	Bn-A09-p7741917	A9	24	39	A
8862	Bn-N9-p8533234	Bn-A09-p8044910	Bn-A09-p8044910	A9	24	39	A
8863	Bn-N9-p8687712	Bn-A09-p15416895	Bn-A09-p15416895	A9	24	39	A
8864	Bn-N9-p8781510	Bn-A09-p8291726	Bn-A09-p8291726	A9	24	39	A

8865	Bn-N9-p9040963	Bn-A09-p8591730	Bn-A09-p8591730	A9	24	39	A
8866	Bn-N9-p9040980	Bn-A09-p8591746	Bn-A09-p8591746	A9	24	39	A
8867	Bn-N9-p9062851	Bn-C3-p64468889	Bn-scaff_16755_1-p515807	A9	24	39	A
8868	Bn-N9-p9158552	Bn-A09-p8679695	Bn-A09-p8679695	A9	24	39	A
8869	Bn-N9-p9235144	Bn-A09-p8770590	Bn-A09-p8770590	A9	24	39	A
8870	Bn-N9-p9237807	Bn-A09-p8773859	Bn-A09-p8773859	A9	24	39	A
8871	Bn-N9-p9239465	Bn-A09-p8787239	Bn-A09-p8787239	A9	24	39	A
8872	Bn-N9-p9239763	Bn-A09-p8787573	Bn-A09-p8787573	A9	24	39	A
8873	Bn-N9-p9239826	Bn-A09-p8788225	Bn-A09-p8788225	A9	24	39	A
8874	Bn-N9-p9242003	Bn-A09-p8791527	Bn-A09-p8791527	A9	24	39	A
8875	Bn-N9-p9244418	Bn-A09-p8793873	Bn-A09-p8793873	A9	24	39	A
8876	Bn-N9-p9271391	Bn-A09-p8810292	Bn-A09-p8810292	A9	24	39	A
8877	Bn-N9-p9359933	Bn-A09-p8891015	Bn-A09-p8891015	A9	24	39	A
8878	Bn-N9-p9360195	Bn-A09-p8891277	Bn-A09-p8891277	A9	24	39	A
8879	Bn-N9-p9391946	Bn-A09-p8911821	Bn-A09-p8911821	A9	24	39	A
8880	Bn-N9-p9413894	Bn-A09-p8942646	Bn-A09-p8942646	A9	24	39	A
8881	Bn-N9-p9429440	Bn-A09-p8957856	Bn-A09-p8957856	A9	24	39	A
8882	Bn-N9-p9563274	Bn-A09-p9083090	Bn-A09-p9083090	A9	24	39	A
8883	Bn-N9-p9563498	Bn-A09-p9083366	Bn-A09-p9083366	A9	24	39	A
8884	Bn-N9-p9563578	Bn-A09-p9083445	Bn-A09-p9083445	A9	24	39	A
8885	Bn-N9-p9583159	Bn-A09-p9098267	Bn-A09-p9098267	A9	24	39	A
8886	Bn-N9-p9584861	Bn-A09-p9101925	Bn-A09-p9101925	A9	24	39	A
8887	Bn-N9-p9626225	Bn-A09-p9135388	Bn-A09-p9135388	A9	24	39	A
8888	Bn-N9-p9708058	Bn-A09-p9206874	Bn-A09-p9206874	A9	24	39	A
8889	Bn-N9-p9829575	Bn-A09-p9315814	Bn-A09-p9315814	A9	24	39	A
8890	Bn-N9-p9829585	Bn-A09-p9315824	Bn-A09-p9315824	A9	24	39	A
8891	Bn-N9-p9831471	Bn-A09-p9317714	Bn-A09-p9317714	A9	24	39	A
8892	Bn-N9-p9852882	Bn-A09-p9325966	Bn-A09-p9325966	A9	24	39	A
8893	Bn-N9-p10002632	Bn-A09-p9480770	Bn-A09-p9480770	A9	25	39.8	A
8894	Bn-N9-p9899071	Bn-A09-p9379347	Bn-A09-p9379347	A9	25	39.8	A
8895	Bn-N9-p9941353	Bn-A09-p9421306	Bn-A09-p9421306	A9	25	39.8	A
8896	Bn-N9-p9963358	Bn-A09-p9443127	Bn-A09-p9443127	A9	25	39.8	A
8897	Bn-N9-p9965229	Bn-A09-p9444998	Bn-A09-p9444998	A9	25	39.8	A
8898	Bn-N9-p9965705	Bn-A09-p9445474	Bn-A09-p9445474	A9	25	39.8	A
8899	Bn-N9-p9967134	Bn-A09-p9446903	Bn-A09-p9446903	A9	25	39.8	A
8900	Bn-N9-p9971086	Bn-A09-p9450845	Bn-A09-p9450845	A9	25	39.8	A
8901	Bn-N9-p9971157	Bn-A09-p9450916	Bn-A09-p9450916	A9	25	39.8	A
8902	Bn-N9-p9973131	Bn-A09-p9452891	Bn-A09-p9452891	A9	25	39.8	A
8903	Bn-N9-p9984527	Bn-A09-p9463840	Bn-A09-p9463840	A9	25	39.8	A
8904	Bn-N9-p10035291	Bn-A09-p9512316	Bn-A09-p9512316	A9	26	40.6	A
8905	Bn-N9-p10082549	Bn-A09-p9563670	Bn-A09-p9563670	A9	26	40.6	A
8906	Bn-N9-p10082810	Bn-A09-p9563948	Bn-A09-p9563948	A9	26	40.6	A
8907	Bn-N9-p10218400	Bn-A09-p9671359	Bn-A09-p9671359	A9	26	40.6	A
8908	Bn-N9-p10235813	Bn-A09-p9691088	Bn-A09-p9691088	A9	26	40.6	A
8909	Bn-Scaffold69548-p178	Bn-A09-p9669847	Bn-A09-p9669847	A9	26	40.6	A
8910	Bn-N19-p17529037	Bn-C9-p16936646	Bn-scaff_18100_1-p137189	A9	27	41.4	A
8911	Bn-N9-p10418352	Bn-A09-p9845028	Bn-A09-p9845028	A9	27	41.4	A
8912	Bn-N9-p10441284	Bn-A09-p9865556	Bn-A09-p9865556	A9	27	41.4	A
8913	Bn-N9-p10446505	Bn-A09-p9877560	Bn-A09-p9877560	A9	27	41.4	A

8914	Bn-N9-p10447610	Bn-A09-p9878665	Bn-A09-p9878665	A9	27	41.4	A
8915	Bn-N9-p10531605	Bn-A09-p10019827	Bn-A09-p10019827	A9	27	41.4	A
8916	Bn-N9-p10533975	Bn-A09-p10023881	Bn-A09-p10023881	A9	27	41.4	A
8917	Bn-N9-p10534763	Bn-A09-p10024669	Bn-A09-p10024669	A9	27	41.4	A
8918	Bn-N9-p10576518	Bn-A09-p10064024	Bn-A09-p10064024	A9	27	41.4	A
8919	Bn-N9-p10577267	Bn-A09-p10064773	Bn-A09-p10064773	A9	27	41.4	A
8920	Bn-N9-p10609821	Bn-A09-p10092753	Bn-A09-p10092753	A9	27	41.4	A
8921	Bn-N9-p10616686	Bn-A09-p10109790	Bn-A09-p10109790	A9	27	41.4	A
8922	Bn-N9-p10681875	Bn-A09-p10172534	Bn-A09-p10172534	A9	27	41.4	A
8923	Bn-N9-p10686284	Bn-A09-p10176936	Bn-A09-p10176936	A9	27	41.4	A
8924	Bn-N9-p10704712	Bn-A09-p10192314	Bn-A09-p10192314	A9	27	41.4	A
8925	Bn-N9-p10706644	Bn-A09-p10194119	Bn-A09-p10194119	A9	27	41.4	A
8926	Bn-N9-p10805806	Bn-A09-p10297982	Bn-A09-p10297982	A9	27	41.4	A
8927	Bn-N9-p10859008	Bn-A09-p10343752	Bn-A09-p10343752	A9	27	41.4	A
8928	Bn-N9-p10869048	Bn-A09-p14330816	Bn-A09-p14330816	A9	27	41.4	A
8929	Bn-N9-p10869078	Bn-A09-p14330786	Bn-A09-p14330786	A9	27	41.4	A
8930	Bn-N9-p10869354	Bn-A09-p14330510	Bn-A09-p14330510	A9	27	41.4	A
8931	Bn-N9-p10871280	Bn-A09-p14302563	Bn-A09-p14302563	A9	27	41.4	A
8932	Bn-N9-p10877826	Bn-A09-p14296015	Bn-A09-p14296015	A9	27	41.4	A
8933	Bn-Scaffold42719-p83	Bn-C9-p16383820	Bn-scaff_17174_1-p38864	A9	27	41.4	A
8934	Bn-N18-p23083283	Bn-A02-p15457681	Bn-A02-p15457681	A9	28	43	A
8935	Bn-N19-p18326124	Bn-A09-p13696043	Bn-A09-p13696043	A9	28	43	A
8936	Bn-N9-p10912114	Bn-A09-p14267034	Bn-A09-p14267034	A9	28	43	A
8937	Bn-N9-p10912967	Bn-A09-p14266174	Bn-A09-p14266174	A9	28	43	A
8938	Bn-N9-p10941036	Bn-A09-p14246053	Bn-A09-p14246053	A9	28	43	A
8939	Bn-N9-p11027623	Bn-A09-p14166446	Bn-A09-p14166446	A9	28	43	A
8940	Bn-N9-p11076521	Bn-A09-p14137607	Bn-A09-p14137607	A9	28	43	A
8941	Bn-N9-p11076529	Bn-A09-p14137599	Bn-A09-p14137599	A9	28	43	A
8942	Bn-N9-p11076747	Bn-A09-p14137381	Bn-A09-p14137381	A9	28	43	A
8943	Bn-N9-p11256724	Bn-A09-p13983057	Bn-A09-p13983057	A9	28	43	A
8944	Bn-N9-p11411101	Bn-C9-p17562545	Bn-scaff_18100_1-p76308	A9	28	43	A
8945	Bn-N9-p11412132	Bn-A09-p13836909	Bn-A09-p13836909	A9	28	43	A
8946	Bn-N9-p11414452	Bn-A09-p13827633	Bn-A09-p13827633	A9	28	43	A
8947	Bn-N9-p11418426	Bn-A09-p13824652	Bn-A09-p13824652	A9	28	43	A
8948	Bn-N9-p11533710	Bn-A09-p13718575	Bn-A09-p13718575	A9	28	43	A
8949	Bn-N9-p11547474	Bn-A09-p13693499	Bn-A09-p13693499	A9	28	43	A
8950	Bn-N9-p11558427	Bn-A09-p13682690	Bn-A09-p13682690	A9	28	43	A
8951	Bn-N9-p11567366	Bn-A09-p13667207	Bn-A09-p13667207	A9	28	43	A
8952	Bn-N9-p11568358	Bn-A09-p13666209	Bn-A09-p13666209	A9	28	43	A
8953	Bn-N9-p11569753	Bn-A09-p13654188	Bn-A09-p13654188	A9	28	43	A
8954	Bn-N9-p11589443	Bn-A09-p13628485	Bn-A09-p13628485	A9	28	43	A
8955	Bn-N9-p11591260	Bn-A09-p13626678	Bn-A09-p13626678	A9	28	43	A
8956	Bn-N9-p11592321	Bn-A09-p13625959	Bn-A09-p13625959	A9	28	43	A
8957	Bn-N9-p11601789	Bn-A09-p13614625	Bn-A09-p13614625	A9	28	43	A
8958	Bn-N9-p11601998	Bn-A09-p13614414	Bn-A09-p13614414	A9	28	43	A
8959	Bn-N9-p11617475	Bn-A09-p13599590	Bn-A09-p13599590	A9	28	43	A
8960	Bn-N9-p11627733	Bn-A09-p13590924	Bn-A09-p13590924	A9	28	43	A
8961	Bn-N9-p11631306	Bn-A09-p13587429	Bn-A09-p13587429	A9	28	43	A
8962	Bn-N9-p11633658	Bn-A09-p13585262	Bn-A09-p13585262	A9	28	43	A

8963	Bn-N9-p11636851	Bn-A09-p13582084	Bn-A09-p13582084	A9	28	43	A
8964	Bn-N9-p11637805	Bn-A09-p13581136	Bn-A09-p13581136	A9	28	43	A
8965	Bn-N9-p11638371	Bn-C9-p17924346	Bn-scaff_18100_1-p11248	A9	28	43	A
8966	Bn-N9-p11654259	Bn-A09-p13565633	Bn-A09-p13565633	A9	28	43	A
8967	Bn-N9-p11655190	Bn-A09-p13564702	Bn-A09-p13564702	A9	28	43	A
8968	Bn-N9-p11703248	Bn-A09-p13525353	Bn-A09-p13525353	A9	28	43	A
8969	Bn-N9-p11703399	Bn-A09-p13525202	Bn-A09-p13525202	A9	28	43	A
8970	Bn-N9-p11710975	Bn-A09-p13515849	Bn-A09-p13515849	A9	28	43	A
8971	Bn-N9-p11716429	Bn-A09-p13515300	Bn-A09-p13515300	A9	28	43	A
8972	Bn-N9-p11717361	Bn-A09-p13513734	Bn-A09-p13513734	A9	28	43	A
8973	Bn-N9-p12175652	Bn-A09-p12929078	Bn-A09-p12929078	A9	28	43	A
8974	Bn-N9-p12188021	Bn-A09-p12913174	Bn-A09-p12913174	A9	28	43	A
8975	Bn-N9-p12258463	Bn-C9-p18749332	Bn-scaff_17888_1-p43530	A9	28	43	A
8976	Bn-N9-p12323611	Bn-A09-p16781669	Bn-A09-p16781669	A9	28	43	A
8977	Bn-Scaffold02250-p718	Bn-A09-p14179631	Bn-A09-p14179631	A9	28	43	A
8978	Bn-N9-p12472837	Bn-A09-p16639867	Bn-A09-p16639867	A9	29	43.8	A
8979	Bn-N9-p12677112	Bn-A09-p16187931	Bn-A09-p16187931	A9	29	43.8	A
8980	Bn-N9-p12682794	Bn-A09-p16184825	Bn-A09-p16184825	A9	29	43.8	A
8981	Bn-N9-p12784463	Bn-A09-p16094451	Bn-A09-p16094451	A9	29	43.8	A
8982	Bn-N9-p12790459	Bn-A09-p16090255	Bn-A09-p16090255	A9	29	43.8	A
8983	Bn-N9-p12914836	Bn-A09-p15986311	Bn-A09-p15986311	A9	29	43.8	A
8984	Bn-N9-p12916177	Bn-A09-p15984968	Bn-A09-p15984968	A9	29	43.8	A
8985	Bn-N9-p12918548	Bn-A09-p15982584	Bn-A09-p15982584	A9	29	43.8	A
8986	Bn-N9-p12919896	Bn-A09-p15981176	Bn-A09-p15981176	A9	29	43.8	A
8987	Bn-N9-p12948705	Bn-A09-p15960945	Bn-A09-p15960945	A9	29	43.8	A
8988	Bn-N9-p12948863	Bn-A09-p15960787	Bn-A09-p15960787	A9	29	43.8	A
8989	Bn-N9-p12950239	Bn-A09-p15959411	Bn-A09-p15959411	A9	29	43.8	A
8990	Bn-N9-p12958162	Bn-A09-p15952662	Bn-A09-p15952662	A9	29	43.8	A
8991	Bn-N9-p12989436	Bn-A09-p15935917	Bn-A09-p15935917	A9	29	43.8	A
8992	Bn-N9-p12989680	Bn-A09-p15935674	Bn-A09-p15935674	A9	29	43.8	0
8993	Bn-N9-p13013602	Bn-C9-p20154608	Bn-scaff_20903_1-p39889	A9	29	43.8	A
8994	Bn-N9-p13640142	Bn-C9-p21533473	Bn-scaff_23293_1-p23919	A9	30	45.4	A
8995	Bn-N9-p14240731	Bn-A09-p14535292	Bn-A09-p14535292	A9	31	47	A
8996	Bn-N9-p14269523	Bn-A09-p14501539	Bn-A09-p14501539	A9	31	47	A
8997	Bn-N9-p14339612	Bn-A09-p14432426	Bn-A09-p14432426	A9	31	47	A
8998	Bn-N9-p14491622	Bn-C9-p23060065	Bn-scaff_18424_1-p41119	A9	31	47	A
8999	Bn-N11-p16279385	Bn-C1-p14792518	Bn-scaff_15747_1-p62767	A9	32	47.8	A
9000	Bn-N19-p25877131	Bn-A09-p18258259	Bn-A09-p18258259	A9	32	47.8	A
9001	Bn-N19-p25902286	Bn-A07-p5737895	Bn-A07-p5737895	A9	32	47.8	A
9002	Bn-N19-p25902382	Bn-A07-p5737991	Bn-A07-p5737991	A9	32	47.8	A
9003	Bn-N19-p25902414	Bn-A07-p5738023	Bn-A07-p5738023	A9	32	47.8	A
9004	Bn-N9-p14682425	Bn-A09-p17162263	Bn-A09-p17162263	A9	32	47.8	A
9005	Bn-N9-p14892095	Bn-A09-p17415894	Bn-A09-p17415894	A9	32	47.8	A
9006	Bn-N9-p14921077	Bn-A09-p17433463	Bn-A09-p17433463	A9	32	47.8	A
9007	Bn-N9-p14921872	Bn-A09-p17440923	Bn-A09-p17440923	A9	32	47.8	A
9008	Bn-N9-p14960945	Bn-A09-p17483853	Bn-A09-p17483853	A9	32	47.8	A
9009	Bn-N9-p14961052	Bn-A09-p17483960	Bn-A09-p17483960	A9	32	47.8	A
9010	Bn-N9-p14992403	Bn-A09-p17509906	Bn-A09-p17509906	A9	32	47.8	A
9011	Bn-N9-p15073173	Bn-C9-p23795818	Bn-scaff_18944_1-p39426	A9	32	47.8	A

9012	Bn-N9-p15169042	Bn-A09-p17669440	Bn-A09-p17669440	A9	32	47.8	A
9013	Bn-N9-p15171674	Bn-A09-p17672066	Bn-A09-p17672066	A9	32	47.8	A
9014	Bn-N9-p15172124	Bn-A09-p17672507	Bn-A09-p17672507	A9	32	47.8	A
9015	Bn-N9-p15172451	Bn-A09-p17672835	Bn-A09-p17672835	A9	32	47.8	A
9016	Bn-N9-p15174595	Bn-A09-p17674977	Bn-A09-p17674977	A9	32	47.8	A
9017	Bn-N9-p15191750	Bn-A09-p17690155	Bn-A09-p17690155	A9	32	47.8	A
9018	Bn-N9-p15197185	Bn-A09-p17698726	Bn-A09-p17698726	A9	32	47.8	A
9019	Bn-N9-p15199292	Bn-A09-p17700863	Bn-A09-p17700863	A9	32	47.8	A
9020	Bn-N9-p17466235	Bn-Scaffold000362-p1	Bn-Scaffold000362-p15191	A9	32	47.8	A
9021	Bn-N9-p17479230	Bn-A07-p5758215	Bn-A07-p5758215	A9	32	47.8	A
9022	Bn-N9-p17531358	Bn-A09-p18258443	Bn-A09-p18258443	A9	32	47.8	A
9023	Bn-N9-p17685023	Bn-A09-p18132317	Bn-A09-p18132317	A9	32	47.8	A
9024	Bn-N9-p17853801	Bn-A09-p18156994	Bn-A09-p18156994	A9	32	47.8	A
9025	Bn-N9-p17854902	Bn-A09-p17954328	Bn-A09-p17954328	A9	32	47.8	A
9026	Bn-N9-p17876148	Bn-A09-p17938471	Bn-A09-p17938471	A9	32	47.8	A
9027	Bn-N9-p17876301	Bn-A09-p17938318	Bn-A09-p17938318	A9	32	47.8	A
9028	Bn-N9-p17914357	Bn-C9-p24233069	Bn-scaff_19691_1-p20446	A9	32	47.8	A
9029	Bn-N9-p17952775	Bn-A09-p17869345	Bn-A09-p17869345	A9	32	47.8	A
9030	Bn-N10-p4468256	Bn-A01-p8782649	Bn-A01-p8782649	A9	33	49.4	A
9031	Bn-N11-p24651711	Bn-A09-p19412946	Bn-A09-p19412946	A9	33	49.4	A
9032	Bn-N11-p41990267	Bn-A09-p20053737	Bn-A09-p20053737	A9	33	49.4	A
9033	Bn-N12-p32482899	Bn-C2-p39825959	Bn-scaff_16449_1-p92326	A9	33	49.4	A
9034	Bn-N13-p43962529	Bn-A09-p19452814	Bn-A09-p19452814	A9	33	49.4	A
9035	Bn-N14-p55112990	Bn-A09-p19400882	Bn-A09-p19400882	A9	33	49.4	A
9036	Bn-N15-p17860803	Bn-A09-p19413084	Bn-A09-p19413084	A9	33	49.4	A
9037	Bn-N15-p18597677	Bn-A09-p19444777	Bn-A09-p19444777	A9	33	49.4	A
9038	Bn-N15-p50267769	Bn-A09-p19454867	Bn-A09-p19454867	A9	33	49.4	A
9039	Bn-N15-p50279106	Bn-A09-p19452209	Bn-A09-p19452209	A9	33	49.4	A
9040	Bn-N15-p50279196	Bn-A09-p19452153	Bn-A09-p19452153	A9	33	49.4	A
9041	Bn-N15-p50279298	Bn-A09-p19452055	Bn-A09-p19452055	A9	33	49.4	A
9042	Bn-N15-p50279502	Bn-A09-p19451773	Bn-A09-p19451773	A9	33	49.4	A
9043	Bn-N15-p50281238	Bn-A09-p19447656	Bn-A09-p19447656	A9	33	49.4	A
9044	Bn-N16-p10614826	Bn-Scaffold01163-p1	Bn-scaff_27218_1-p19546	A9	33	49.4	A
9045	Bn-N16-p10862059	Bn-C6-p28028765	Bn-scaff_17984_1-p31787	A9	33	49.4	A
9046	Bn-N16-p11601742	Bn-A09-p19347448	Bn-A09-p19347448	A9	33	49.4	A
9047	Bn-N16-p11764018	Bn-A09-p19427814	Bn-A09-p19427814	A9	33	49.4	A
9048	Bn-N16-p20798263	Bn-Scaffold000185-p1	Bn-Scaffold000185-p10665	A9	33	49.4	A
9049	Bn-N16-p27719926	Bn-A09-p19452677	Bn-A09-p19452677	A9	33	49.4	A
9050	Bn-N16-p9070780	Bn-C6-p30271121	Bn-scaff_18002_1-p94815	A9	33	49.4	A
9051	Bn-N19-p16182056	Bn-A09-p19417282	Bn-A09-p19417282	A9	33	49.4	A
9052	Bn-N19-p26431260	Bn-A08-p9353221	Bn-A08-p9353221	A9	33	49.4	A
9053	Bn-N19-p26573216	Bn-C9-p28266859	Bn-scaff_16569_1-p59372	A9	33	49.4	A
9054	Bn-N19-p26682267	Bn-A08-p9503231	Bn-A08-p9503231	A9	33	49.4	A
9055	Bn-N19-p27073074	Bn-A08-p9723809	Bn-A08-p9723809	A9	33	49.4	A
9056	Bn-N1-p17194917	Bn-A01-p16597123	Bn-A01-p16597123	A9	33	49.4	A
9057	Bn-N6-p16509218	Bn-A01-p24229645	Bn-A01-p24229645	A9	33	49.4	A
9058	Bn-N8-p3933839	Bn-A09-p19803046	Bn-A09-p19803046	A9	33	49.4	A
9059	Bn-N9-p10197711	Bn-A09-p19272189	Bn-A09-p19272189	A9	33	49.4	A
9060	Bn-N9-p15658113	Bn-C1-p38213167	Bn-scaff_19168_1-p13064	A9	33	49.4	A

9061	Bn-N9-p15690644	Bn-A09-p10841953	Bn-A09-p10841953	A9	33	49.4	A
9062	Bn-N9-p15690799	Bn-A09-p10842108	Bn-A09-p10842108	A9	33	49.4	A
9063	Bn-N9-p15690978	Bn-A09-p10842287	Bn-A09-p10842287	A9	33	49.4	A
9064	Bn-N9-p15698668	Bn-A09-p10850022	Bn-A09-p10850022	A9	33	49.4	A
9065	Bn-N9-p15712145	Bn-A09-p10859693	Bn-A09-p10859693	A9	33	49.4	A
9066	Bn-N9-p15728652	Bn-A09-p10881353	Bn-A09-p10881353	A9	33	49.4	A
9067	Bn-N9-p15740763	Bn-A09-p10893080	Bn-A09-p10893080	A9	33	49.4	A
9068	Bn-N9-p15749576	Bn-A09-p10901534	Bn-A09-p10901534	A9	33	49.4	A
9069	Bn-N9-p15751268	Bn-A09-p10903227	Bn-A09-p10903227	A9	33	49.4	A
9070	Bn-N9-p15761049	Bn-A09-p10912706	Bn-A09-p10912706	A9	33	49.4	A
9071	Bn-N9-p15796403	Bn-A09-p10946429	Bn-A09-p10946429	A9	33	49.4	A
9072	Bn-N9-p15797086	Bn-A09-p10947108	Bn-A09-p10947108	A9	33	49.4	A
9073	Bn-N9-p15821901	Bn-A09-p10971822	Bn-A09-p10971822	A9	33	49.4	A
9074	Bn-N9-p15858031	Bn-A09-p11006522	Bn-A09-p11006522	A9	33	49.4	A
9075	Bn-N9-p15970794	Bn-A09-p11126583	Bn-A09-p11126583	A9	33	49.4	A
9076	Bn-N9-p15989661	Bn-A09-p11141656	Bn-A09-p11141656	A9	33	49.4	A
9077	Bn-N9-p15990007	Bn-A09-p11141987	Bn-A09-p11141987	A9	33	49.4	A
9078	Bn-N9-p15997865	Bn-A09-p11149722	Bn-A09-p11149722	A9	33	49.4	A
9079	Bn-N9-p16000235	Bn-A09-p11152092	Bn-A09-p11152092	A9	33	49.4	A
9080	Bn-N9-p16018975	Bn-A09-p11180241	Bn-A09-p11180241	A9	33	49.4	A
9081	Bn-N9-p16071098	Bn-A09-p11242530	Bn-A09-p11242530	A9	33	49.4	A
9082	Bn-N9-p16071655	Bn-A09-p11243089	Bn-A09-p11243089	A9	33	49.4	A
9083	Bn-N9-p16079452	Bn-Scaffold000398-p8	Bn-Scaffold000398-p8508	A9	33	49.4	A
9084	Bn-N9-p16102961	Bn-A09-p11267401	Bn-A09-p11267401	A9	33	49.4	A
9085	Bn-N9-p16110401	Bn-A09-p11279200	Bn-A09-p11279200	A9	33	49.4	A
9086	Bn-N9-p16116311	Bn-A09-p11297498	Bn-A09-p11297498	A9	33	49.4	A
9087	Bn-N9-p16245534	Bn-A09-p11393003	Bn-A09-p11393003	A9	33	49.4	A
9088	Bn-N9-p16299854	Bn-A09-p11461510	Bn-A09-p11461510	A9	33	49.4	A
9089	Bn-N9-p16328394	Bn-A09-p11476056	Bn-A09-p11476056	A9	33	49.4	A
9090	Bn-N9-p16329543	Bn-A09-p11477205	Bn-A09-p11477205	A9	33	49.4	A
9091	Bn-N9-p16424577	Bn-A09-p11544785	Bn-A09-p11544785	A9	33	49.4	A
9092	Bn-N9-p16547109	Bn-A09-p11692396	Bn-A09-p11692396	A9	33	49.4	A
9093	Bn-N9-p16720801	Bn-A08-p9795423	Bn-A08-p9795423	A9	33	49.4	A
9094	Bn-N9-p16721191	Bn-A08-p9795033	Bn-A08-p9795033	A9	33	49.4	A
9095	Bn-N9-p16736496	Bn-A08-p9781699	Bn-A08-p9781699	A9	33	49.4	A
9096	Bn-N9-p16736839	Bn-A08-p9781356	Bn-A08-p9781356	A9	33	49.4	A
9097	Bn-N9-p16766066	Bn-A08-p9749557	Bn-A08-p9749557	A9	33	49.4	A
9098	Bn-N9-p16776332	Bn-A08-p9741074	Bn-A08-p9741074	A9	33	49.4	A
9099	Bn-N9-p16776889	Bn-A08-p9740508	Bn-A08-p9740508	A9	33	49.4	A
9100	Bn-N9-p16777614	Bn-A08-p9739790	Bn-A08-p9739790	A9	33	49.4	A
9101	Bn-N9-p16794790	Bn-A08-p9730934	Bn-A08-p9730934	A9	33	49.4	A
9102	Bn-N9-p16796415	Bn-A08-p9729282	Bn-A08-p9729282	A9	33	49.4	A
9103	Bn-N9-p16796452	Bn-A08-p9729245	Bn-A08-p9729245	A9	33	49.4	A
9104	Bn-N9-p16805472	Bn-A08-p9720439	Bn-A08-p9720439	A9	33	49.4	A
9105	Bn-N9-p16805538	Bn-A08-p9720373	Bn-A08-p9720373	A9	33	49.4	A
9106	Bn-N9-p16806829	Bn-A08-p9719082	Bn-A08-p9719082	A9	33	49.4	A
9107	Bn-N9-p16807358	Bn-A08-p9718553	Bn-A08-p9718553	A9	33	49.4	A
9108	Bn-N9-p16807647	Bn-A08-p9718264	Bn-A08-p9718264	A9	33	49.4	A
9109	Bn-N9-p16807701	Bn-A08-p9718210	Bn-A08-p9718210	A9	33	49.4	A

9110	Bn-N9-p16810341	Bn-A08-p9715574	Bn-A08-p9715574	A9	33	49.4	A
9111	Bn-N9-p16812191	Bn-A08-p9713705	Bn-A08-p9713705	A9	33	49.4	A
9112	Bn-N9-p16822886	Bn-A08-p9707204	Bn-A08-p9707204	A9	33	49.4	A
9113	Bn-N9-p16825631	Bn-A08-p9699333	Bn-A08-p9699333	A9	33	49.4	A
9114	Bn-N9-p16871598	Bn-A08-p9656252	Bn-A08-p9656252	A9	33	49.4	A
9115	Bn-N9-p16939667	Bn-A08-p9582412	Bn-A08-p9582412	A9	33	49.4	A
9116	Bn-N9-p16949315	Bn-A08-p9567284	Bn-A08-p9567284	A9	33	49.4	A
9117	Bn-N9-p16971864	Bn-A08-p9555984	Bn-A08-p9555984	A9	33	49.4	A
9118	Bn-N9-p16972814	Bn-A08-p9555028	Bn-A08-p9555028	A9	33	49.4	A
9119	Bn-N9-p16991039	Bn-A08-p9505581	Bn-A08-p9505581	A9	33	49.4	A
9120	Bn-N9-p17011865	Bn-A08-p9486485	Bn-A08-p9486485	A9	33	49.4	A
9121	Bn-N9-p17012014	Bn-A08-p9486336	Bn-A08-p9486336	A9	33	49.4	A
9122	Bn-N9-p17014714	Bn-A08-p9480351	Bn-A08-p9480351	A9	33	49.4	A
9123	Bn-N9-p17042471	Bn-A08-p9436578	Bn-A08-p9436578	A9	33	49.4	A
9124	Bn-N9-p17043251	Bn-A08-p9435789	Bn-A08-p9435789	A9	33	49.4	A
9125	Bn-N9-p17046292	Bn-A08-p9433046	Bn-A08-p9433046	A9	33	49.4	A
9126	Bn-N9-p17049946	Bn-A08-p9429379	Bn-A08-p9429379	A9	33	49.4	A
9127	Bn-N9-p17057302	Bn-C9-p28240913	Bn-scaff_16569_1-p567777	A9	33	49.4	A
9128	Bn-N9-p17072750	Bn-A08-p9413496	Bn-A08-p9413496	A9	33	49.4	A
9129	Bn-N9-p17106253	Bn-A08-p9388258	Bn-A08-p9388258	A9	33	49.4	A
9130	Bn-N9-p17118775	Bn-A08-p9367767	Bn-A08-p9367767	A9	33	49.4	A
9131	Bn-N9-p17122434	Bn-A08-p9364108	Bn-A08-p9364108	A9	33	49.4	A
9132	Bn-N9-p17130431	Bn-A08-p9355940	Bn-A08-p9355940	A9	33	49.4	A
9133	Bn-N9-p17153330	Bn-A08-p9326621	Bn-A08-p9326621	A9	33	49.4	A
9134	Bn-N9-p17154019	Bn-A08-p9325932	Bn-A08-p9325932	A9	33	49.4	A
9135	Bn-N9-p17155996	Bn-A08-p9323955	Bn-A08-p9323955	A9	33	49.4	A
9136	Bn-N9-p17160687	Bn-A08-p9316584	Bn-A08-p9316584	A9	33	49.4	A
9137	Bn-N9-p17173515	Bn-A08-p9312404	Bn-A08-p9312404	A9	33	49.4	A
9138	Bn-N9-p17179407	Bn-A08-p9308071	Bn-A08-p9308071	A9	33	49.4	A
9139	Bn-N9-p17185193	Bn-A08-p9301468	Bn-A08-p9301468	A9	33	49.4	A
9140	Bn-N9-p17217377	Bn-A08-p9261989	Bn-A08-p9261989	A9	33	49.4	A
9141	Bn-N9-p17219821	Bn-A10-p17125825	Bn-A10-p17125825	A9	33	49.4	A
9142	Bn-N9-p17242160	Bn-A08-p9247445	Bn-A08-p9247445	A9	33	49.4	A
9143	Bn-N9-p17245151	Bn-A08-p9232996	Bn-A08-p9232996	A9	33	49.4	A
9144	Bn-N9-p17247426	Bn-A08-p9230750	Bn-A08-p9230750	A9	33	49.4	A
9145	Bn-N9-p17262937	Bn-A08-p9221373	Bn-A08-p9221373	A9	33	49.4	A
9146	Bn-N9-p17271334	Bn-A08-p9218159	Bn-A08-p9218159	A9	33	49.4	A
9147	Bn-N9-p17316731	Bn-A08-p9200154	Bn-A08-p9200154	A9	33	49.4	A
9148	Bn-N9-p17317582	Bn-A08-p9199303	Bn-A08-p9199303	A9	33	49.4	A
9149	Bn-N9-p17317995	Bn-A08-p9198307	Bn-A08-p9198307	A9	33	49.4	A
9150	Bn-N9-p18067653	Bn-A09-p10544697	Bn-A09-p10544697	A9	33	49.4	A
9151	Bn-N9-p18075927	Bn-A09-p10536718	Bn-A09-p10536718	A9	33	49.4	A
9152	Bn-N9-p18081166	Bn-A09-p10531672	Bn-A09-p10531672	A9	33	49.4	A
9153	Bn-N9-p18121358	Bn-A09-p10493965	Bn-A09-p10493965	A9	33	49.4	A
9154	Bn-N9-p18209130	Bn-Scaffold000428-p(	Bn-Scaffold000428-p6186	A9	33	49.4	A
9155	Bn-N9-p18219572	Bn-A06-p19747056	Bn-A06-p19747056	A9	33	49.4	A
9156	Bn-N9-p18229734	Bn-A06-p19757422	Bn-A06-p19757422	A9	33	49.4	A
9157	Bn-N9-p18231473	Bn-A06-p19759161	Bn-A06-p19759161	A9	33	49.4	A
9158	Bn-N9-p18268820	Bn-A06-p19788556	Bn-A06-p19788556	A9	33	49.4	A

9159	Bn-N9-p18328191	Bn-A06-p19833393	Bn-A06-p19833393	A9	33	49.4	A
9160	Bn-N9-p18588951	Bn-A01-p8788567	Bn-A01-p8788567	A9	33	49.4	A
9161	Bn-N9-p18605492	Bn-A01-p8805177	Bn-A01-p8805177	A9	33	49.4	A
9162	Bn-N9-p18606839	Bn-A01-p8806524	Bn-A01-p8806524	A9	33	49.4	A
9163	Bn-N9-p18625383	Bn-A01-p8821722	Bn-A01-p8821722	A9	33	49.4	A
9164	Bn-N9-p18661213	Bn-A01-p8859217	Bn-A01-p8859217	A9	33	49.4	A
9165	Bn-N9-p18681639	Bn-A01-p8874473	Bn-A01-p8874473	A9	33	49.4	A
9166	Bn-N9-p18728866	Bn-A01-p8901327	Bn-A01-p8901327	A9	33	49.4	A
9167	Bn-N9-p18729029	Bn-A01-p8901490	Bn-A01-p8901490	A9	33	49.4	A
9168	Bn-N9-p18745608	Bn-A01-p8914737	Bn-A01-p8914737	A9	33	49.4	A
9169	Bn-N9-p19200012	Bn-C8-p10921094	Bn-scaff_18310_1-p11206[illegible]	A9	33	49.4	A
9170	Bn-N9-p19452871	Bn-C3-p59237784	Bn-scaff_15794_3-p15512[illegible]	A9	33	49.4	A
9171	Bn-N9-p19644679	Bn-A09-p18745571	Bn-A09-p18745571	A9	33	49.4	A
9172	Bn-N9-p19654882	Bn-A09-p18753596	Bn-A09-p18753596	A9	33	49.4	A
9173	Bn-N9-p19691926	Bn-A09-p18789989	Bn-A09-p18789989	A9	33	49.4	A
9174	Bn-N9-p19734875	Bn-A08-p3814869	Bn-A08-p3814869	A9	33	49.4	A
9175	Bn-N9-p19738842	Bn-A08-p3818854	Bn-A08-p3818854	A9	33	49.4	A
9176	Bn-N9-p19796928	Bn-A09-p18923026	Bn-A09-p18923026	A9	33	49.4	A
9177	Bn-N9-p19808183	Bn-C6-p28028853	Bn-scaff_17984_1-p31796[illegible]	A9	33	49.4	A
9178	Bn-N9-p19836436	Bn-A09-p18982971	Bn-A09-p18982971	A9	33	49.4	A
9179	Bn-N9-p19840053	Bn-A09-p18986611	Bn-A09-p18986611	A9	33	49.4	A
9180	Bn-N9-p19890789	Bn-A09-p19040325	Bn-A09-p19040325	A9	33	49.4	A
9181	Bn-N9-p19893906	Bn-A09-p19043442	Bn-A09-p19043442	A9	33	49.4	A
9182	Bn-N9-p19939466	Bn-A09-p19087834	Bn-A09-p19087834	A9	33	49.4	A
9183	Bn-N9-p19943964	Bn-A09-p19089743	Bn-A09-p19089743	A9	33	49.4	A
9184	Bn-N9-p19981786	Bn-A09-p19112065	Bn-A09-p19112065	A9	33	49.4	A
9185	Bn-N9-p20020859	Bn-Scaffold002662-p[illegible]	Bn-Scaffold002662-p387	A9	33	49.4	A
9186	Bn-N9-p20029444	Bn-A09-p19168927	Bn-A09-p19168927	A9	33	49.4	A
9187	Bn-N9-p20036621	Bn-A09-p19181075	Bn-A09-p19181075	A9	33	49.4	A
9188	Bn-N9-p20043345	Bn-A09-p19187527	Bn-A09-p19187527	A9	33	49.4	A
9189	Bn-N9-p20043446	Bn-A09-p19187628	Bn-A09-p19187628	A9	33	49.4	A
9190	Bn-N9-p20043908	Bn-A09-p19188090	Bn-A09-p19188090	A9	33	49.4	A
9191	Bn-N9-p20051749	Bn-A09-p19198146	Bn-A09-p19198146	A9	33	49.4	A
9192	Bn-N9-p20052453	Bn-A09-p19198948	Bn-A09-p19198948	A9	33	49.4	A
9193	Bn-N9-p20065491	Bn-A09-p19208887	Bn-A09-p19208887	A9	33	49.4	A
9194	Bn-N9-p20066030	Bn-A09-p19209425	Bn-A09-p19209425	A9	33	49.4	A
9195	Bn-N9-p20066214	Bn-A09-p19209609	Bn-A09-p19209609	A9	33	49.4	A
9196	Bn-N9-p20066300	Bn-A09-p19209695	Bn-A09-p19209695	A9	33	49.4	A
9197	Bn-N9-p20081662	Bn-A09-p19225304	Bn-A09-p19225304	A9	33	49.4	A
9198	Bn-N9-p20081781	Bn-A09-p19225424	Bn-A09-p19225424	A9	33	49.4	A
9199	Bn-N9-p20115869	Bn-A09-p19268137	Bn-A09-p19268137	A9	33	49.4	A
9200	Bn-N9-p20116008	Bn-C5-p28060501	Bn-scaff_15705_2-p96234[illegible]	A9	33	49.4	A
9201	Bn-N9-p20116395	Bn-A09-p19268663	Bn-A09-p19268663	A9	33	49.4	A
9202	Bn-N9-p20135740	Bn-A09-p19283001	Bn-A09-p19283001	A9	33	49.4	A
9203	Bn-N9-p20169759	Bn-A09-p19320500	Bn-A09-p19320500	A9	33	49.4	A
9204	Bn-N9-p20170019	Bn-A09-p19321164	Bn-A09-p19321164	A9	33	49.4	A
9205	Bn-N9-p20213558	Bn-A09-p19348447	Bn-A09-p19348447	A9	33	49.4	A
9206	Bn-N9-p20235031	Bn-A09-p19364150	Bn-A09-p19364150	A9	33	49.4	A
9207	Bn-N9-p20241662	Bn-A09-p19370807	Bn-A09-p19370807	A9	33	49.4	A

9208	Bn-N9-p20241735	Bn-A09-p19370880	Bn-A09-p19370880	A9	33	49.4	A
9209	Bn-N9-p20242017	Bn-A09-p19379073	Bn-A09-p19379073	A9	33	49.4	A
9210	Bn-N9-p20242854	Bn-A09-p19379912	Bn-A09-p19379912	A9	33	49.4	A
9211	Bn-N9-p20255746	Bn-A09-p19392577	Bn-A09-p19392577	A9	33	49.4	A
9212	Bn-N9-p20270961	Bn-A09-p19418268	Bn-A09-p19418268	A9	33	49.4	A
9213	Bn-N9-p20304223	Bn-A09-p19452462	Bn-A09-p19452462	A9	33	49.4	A
9214	Bn-N9-p20310275	Bn-A09-p19453248	Bn-A09-p19453248	A9	33	49.4	A
9215	Bn-N9-p20321729	Bn-A09-p19462041	Bn-A09-p19462041	A9	33	49.4	A
9216	Bn-N9-p20344006	Bn-A09-p19489065	Bn-A09-p19489065	A9	33	49.4	A
9217	Bn-N9-p20366760	Bn-Scaffold000584-p4	Bn-Scaffold000584-p4211	A9	33	49.4	A
9218	Bn-N9-p20377953	Bn-A09-p19513927	Bn-A09-p19513927	A9	33	49.4	A
9219	Bn-N9-p20378050	Bn-A09-p19514024	Bn-A09-p19514024	A9	33	49.4	A
9220	Bn-N9-p20378431	Bn-A09-p19514405	Bn-A09-p19514405	A9	33	49.4	A
9221	Bn-N9-p20378442	Bn-A09-p19514416	Bn-A09-p19514416	A9	33	49.4	A
9222	Bn-N9-p20378602	Bn-A09-p19514576	Bn-A09-p19514576	A9	33	49.4	A
9223	Bn-N9-p20379191	Bn-A09-p19515165	Bn-A09-p19515165	A9	33	49.4	A
9224	Bn-N9-p20379315	Bn-A09-p19515287	Bn-A09-p19515287	A9	33	49.4	A
9225	Bn-N9-p20384494	Bn-A09-p19520460	Bn-A09-p19520460	A9	33	49.4	A
9226	Bn-N9-p20394121	Bn-A09-p19524439	Bn-A09-p19524439	A9	33	49.4	A
9227	Bn-N9-p20402837	Bn-A09-p19533134	Bn-A09-p19533134	A9	33	49.4	A
9228	Bn-N9-p20408559	Bn-A09-p19537178	Bn-A09-p19537178	A9	33	49.4	A
9229	Bn-N9-p20412376	Bn-C6-p26758003	Bn-scaff_17044_1-p31088	A9	33	49.4	A
9230	Bn-N9-p20412733	Bn-A09-p19542797	Bn-A09-p19542797	A9	33	49.4	A
9231	Bn-N9-p20416550	Bn-A09-p19545956	Bn-A09-p19545956	A9	33	49.4	A
9232	Bn-N9-p20419049	Bn-A09-p19548419	Bn-A09-p19548419	A9	33	49.4	A
9233	Bn-N9-p20422010	Bn-A09-p19551395	Bn-A09-p19551395	A9	33	49.4	A
9234	Bn-N9-p20422330	Bn-A09-p19551714	Bn-A09-p19551714	A9	33	49.4	A
9235	Bn-N9-p20422432	Bn-A09-p19551816	Bn-A09-p19551816	A9	33	49.4	A
9236	Bn-N9-p20422993	Bn-A09-p19552388	Bn-A09-p19552388	A9	33	49.4	A
9237	Bn-N9-p20423143	Bn-A09-p19552538	Bn-A09-p19552538	A9	33	49.4	A
9238	Bn-N9-p20439249	Bn-A09-p19566744	Bn-A09-p19566744	A9	33	49.4	A
9239	Bn-N9-p20443615	Bn-A09-p19571341	Bn-A09-p19571341	A9	33	49.4	A
9240	Bn-N9-p20443795	Bn-A09-p19571521	Bn-A09-p19571521	A9	33	49.4	A
9241	Bn-N9-p20444245	Bn-A09-p19571970	Bn-A09-p19571970	A9	33	49.4	A
9242	Bn-N9-p20444397	Bn-A09-p19572122	Bn-A09-p19572122	A9	33	49.4	A
9243	Bn-N9-p20466259	Bn-A09-p19578073	Bn-A09-p19578073	A9	33	49.4	A
9244	Bn-N9-p20466667	Bn-A09-p19578479	Bn-A09-p19578479	A9	33	49.4	A
9245	Bn-N9-p20467520	Bn-A09-p19579332	Bn-A09-p19579332	A9	33	49.4	A
9246	Bn-N9-p20469267	Bn-A09-p19581060	Bn-A09-p19581060	A9	33	49.4	A
9247	Bn-N9-p20471265	Bn-A09-p19583295	Bn-A09-p19583295	A9	33	49.4	A
9248	Bn-N9-p20476072	Bn-A09-p19591679	Bn-A09-p19591679	A9	33	49.4	A
9249	Bn-N9-p20476423	Bn-A09-p19592030	Bn-A09-p19592030	A9	33	49.4	A
9250	Bn-N9-p20480508	Bn-A09-p19596070	Bn-A09-p19596070	A9	33	49.4	A
9251	Bn-N9-p20480892	Bn-A09-p19596462	Bn-A09-p19596462	A9	33	49.4	A
9252	Bn-N9-p20504819	Bn-A09-p19614389	Bn-A09-p19614389	A9	33	49.4	A
9253	Bn-N9-p20507902	Bn-A09-p19616778	Bn-A09-p19616778	A9	33	49.4	A
9254	Bn-N9-p20515947	Bn-A09-p19641357	Bn-A09-p19641357	A9	33	49.4	A
9255	Bn-N9-p20530509	Bn-A09-p19649335	Bn-A09-p19649335	A9	33	49.4	A
9256	Bn-N9-p20530828	Bn-A09-p19649635	Bn-A09-p19649635	A9	33	49.4	A

9257	Bn-N9-p20531075	Bn-A09-p19649882	Bn-A09-p19649882	A9	33	49.4	A
9258	Bn-N9-p20531910	Bn-A09-p19650716	Bn-A09-p19650716	A9	33	49.4	A
9259	Bn-N9-p20532235	Bn-A09-p19651041	Bn-A09-p19651041	A9	33	49.4	A
9260	Bn-N9-p20532619	Bn-A09-p19651425	Bn-A09-p19651425	A9	33	49.4	A
9261	Bn-N9-p20533108	Bn-A09-p19651913	Bn-A09-p19651913	A9	33	49.4	A
9262	Bn-N9-p20533333	Bn-A09-p19652139	Bn-A09-p19652139	A9	33	49.4	A
9263	Bn-N9-p20540420	Bn-A09-p19666970	Bn-A09-p19666970	A9	33	49.4	A
9264	Bn-N9-p20540848	Bn-A09-p19667399	Bn-A09-p19667399	A9	33	49.4	A
9265	Bn-N9-p20541149	Bn-A09-p19667700	Bn-A09-p19667700	A9	33	49.4	A
9266	Bn-N9-p20541600	Bn-A09-p19668151	Bn-A09-p19668151	A9	33	49.4	A
9267	Bn-N9-p20542149	Bn-A09-p19668698	Bn-A09-p19668698	A9	33	49.4	A
9268	Bn-N9-p20542269	Bn-A09-p19668818	Bn-A09-p19668818	A9	33	49.4	A
9269	Bn-N9-p20542447	Bn-A09-p19668996	Bn-A09-p19668996	A9	33	49.4	A
9270	Bn-N9-p20542730	Bn-A09-p19669270	Bn-A09-p19669270	A9	33	49.4	A
9271	Bn-N9-p20543799	Bn-A09-p19670340	Bn-A09-p19670340	A9	33	49.4	A
9272	Bn-N9-p20544519	Bn-A09-p19671062	Bn-A09-p19671062	A9	33	49.4	A
9273	Bn-N9-p20544649	Bn-A09-p19671192	Bn-A09-p19671192	A9	33	49.4	A
9274	Bn-N9-p20544851	Bn-A09-p19671394	Bn-A09-p19671394	A9	33	49.4	A
9275	Bn-N9-p20544999	Bn-A09-p19671542	Bn-A09-p19671542	A9	33	49.4	A
9276	Bn-N9-p20548719	Bn-A09-p19675265	Bn-A09-p19675265	A9	33	49.4	A
9277	Bn-N9-p20559636	Bn-Scaffold000499-p	Bn-Scaffold000499-p5529	A9	33	49.4	A
9278	Bn-N9-p20586669	Bn-Scaffold000185-p	Bn-Scaffold000185-p20375	A9	33	49.4	A
9279	Bn-N9-p20605512	Bn-Scaffold000185-p	Bn-Scaffold000185-p18572	A9	33	49.4	A
9280	Bn-N9-p20610117	Bn-Scaffold000185-p	Bn-Scaffold000185-p15887	A9	33	49.4	A
9281	Bn-N9-p20614160	Bn-Scaffold000185-p	Bn-Scaffold000185-p15578	A9	33	49.4	A
9282	Bn-N9-p20618131	Bn-Scaffold000185-p	Bn-Scaffold000185-p15309	A9	33	49.4	A
9283	Bn-N9-p20641263	Bn-Scaffold000185-p	Bn-Scaffold000185-p13195	A9	33	49.4	A
9284	Bn-N9-p20641441	Bn-Scaffold000185-p	Bn-Scaffold000185-p13177	A9	33	49.4	A
9285	Bn-N9-p20642767	Bn-Scaffold000185-p	Bn-Scaffold000185-p13037	A9	33	49.4	A
9286	Bn-N9-p20650410	Bn-Scaffold000185-p	Bn-Scaffold000185-p12855	A9	33	49.4	A
9287	Bn-N9-p20653505	Bn-Scaffold000367-p	Bn-Scaffold000367-p2871	A9	33	49.4	A
9288	Bn-N9-p20655457	Bn-Scaffold000367-p	Bn-Scaffold000367-p4795	A9	33	49.4	A
9289	Bn-N9-p20655694	Bn-Scaffold000367-p	Bn-Scaffold000367-p5046	A9	33	49.4	A
9290	Bn-N9-p20656898	Bn-Scaffold000367-p	Bn-Scaffold000367-p6435	A9	33	49.4	A
9291	Bn-N9-p20657334	Bn-Scaffold000367-p	Bn-Scaffold000367-p6861	A9	33	49.4	A
9292	Bn-N9-p20660820	Bn-Scaffold000185-p	Bn-Scaffold000185-p8472	A9	33	49.4	A
9293	Bn-N9-p20667230	Bn-Scaffold000185-p	Bn-Scaffold000185-p15635	A9	33	49.4	A
9294	Bn-N9-p20667583	Bn-Scaffold000185-p	Bn-Scaffold000185-p15983	A9	33	49.4	A
9295	Bn-N9-p20668462	Bn-Scaffold000185-p	Bn-Scaffold000185-p16862	A9	33	49.4	A
9296	Bn-N9-p20670637	Bn-Scaffold000185-p	Bn-Scaffold000185-p18794	A9	33	49.4	A
9297	Bn-N9-p20677801	Bn-Scaffold000185-p	Bn-Scaffold000185-p26330	A9	33	49.4	A
9298	Bn-N9-p20694463	Bn-Scaffold000185-p	Bn-Scaffold000185-p54518	A9	33	49.4	A
9299	Bn-N9-p20694643	Bn-Scaffold000185-p	Bn-Scaffold000185-p54702	A9	33	49.4	A
9300	Bn-N9-p20694930	Bn-Scaffold000185-p	Bn-Scaffold000185-p54995	A9	33	49.4	A
9301	Bn-N9-p20695310	Bn-Scaffold000185-p	Bn-Scaffold000185-p55372	A9	33	49.4	A
9302	Bn-N9-p20695449	Bn-Scaffold000185-p	Bn-Scaffold000185-p55511	A9	33	49.4	A
9303	Bn-N9-p20696140	Bn-Scaffold000185-p	Bn-Scaffold000185-p56198	A9	33	49.4	A
9304	Bn-N9-p20696180	Bn-Scaffold000185-p	Bn-Scaffold000185-p56238	A9	33	49.4	A
9305	Bn-N9-p20697723	Bn-Scaffold000185-p	Bn-Scaffold000185-p57756	A9	33	49.4	A

9306	Bn-N9-p20697785	Bn-Scaffold000185-p5	Bn-Scaffold000185-p57818	A9	33	49.4	A
9307	Bn-N9-p20718340	Bn-Scaffold000185-p8	Bn-Scaffold000185-p82192	A9	33	49.4	A
9308	Bn-N9-p20719466	Bn-Scaffold000185-p8	Bn-Scaffold000185-p83366	A9	33	49.4	A
9309	Bn-N9-p20741809	Bn-Scaffold000185-p1	Bn-Scaffold000185-p10409	A9	33	49.4	A
9310	Bn-N9-p20743560	Bn-Scaffold000185-p1	Bn-Scaffold000185-p10585	A9	33	49.4	A
9311	Bn-N9-p20745030	Bn-Scaffold000185-p1	Bn-Scaffold000185-p11655	A9	33	49.4	A
9312	Bn-N9-p20745032	Bn-Scaffold000185-p1	Bn-Scaffold000185-p11655	A9	33	49.4	A
9313	Bn-N9-p20745327	Bn-A06-p11432316	Bn-A06-p11432316	A9	33	49.4	A
9314	Bn-N9-p20754163	Bn-A09-p19688476	Bn-A09-p19688476	A9	33	49.4	A
9315	Bn-N9-p20755441	Bn-A09-p19689884	Bn-A09-p19689884	A9	33	49.4	A
9316	Bn-N9-p20755926	Bn-A09-p19690311	Bn-A09-p19690311	A9	33	49.4	A
9317	Bn-N9-p20756007	Bn-A09-p19690392	Bn-A09-p19690392	A9	33	49.4	A
9318	Bn-N9-p20756136	Bn-A09-p19690521	Bn-A09-p19690521	A9	33	49.4	A
9319	Bn-N9-p20756189	Bn-A09-p19690574	Bn-A09-p19690574	A9	33	49.4	A
9320	Bn-N9-p20756395	Bn-A09-p19690777	Bn-A09-p19690777	A9	33	49.4	A
9321	Bn-N9-p20759076	Bn-A09-p19692416	Bn-A09-p19692416	A9	33	49.4	A
9322	Bn-N9-p20759500	Bn-A09-p19718543	Bn-A09-p19718543	A9	33	49.4	A
9323	Bn-N9-p20759538	Bn-A09-p19718581	Bn-A09-p19718581	A9	33	49.4	A
9324	Bn-N9-p20760301	Bn-A09-p19718831	Bn-A09-p19718831	A9	33	49.4	A
9325	Bn-N9-p20761278	Bn-A09-p19725386	Bn-A09-p19725386	A9	33	49.4	A
9326	Bn-N9-p20775891	Bn-A09-p19743333	Bn-A09-p19743333	A9	33	49.4	A
9327	Bn-N9-p20776377	Bn-A09-p19743818	Bn-A09-p19743818	A9	33	49.4	A
9328	Bn-N9-p20799064	Bn-A09-p19749530	Bn-A09-p19749530	A9	33	49.4	A
9329	Bn-N9-p20813980	Bn-A09-p19753460	Bn-A09-p19753460	A9	33	49.4	A
9330	Bn-N9-p20814798	Bn-A09-p19754463	Bn-A09-p19754463	A9	33	49.4	A
9331	Bn-N9-p20815172	Bn-A09-p19766078	Bn-A09-p19766078	A9	33	49.4	A
9332	Bn-N9-p20815835	Bn-A09-p19766741	Bn-A09-p19766741	A9	33	49.4	A
9333	Bn-N9-p20816011	Bn-A09-p19766911	Bn-A09-p19766911	A9	33	49.4	A
9334	Bn-N9-p20821285	Bn-A09-p19770493	Bn-A09-p19770493	A9	33	49.4	A
9335	Bn-N9-p20821337	Bn-A09-p19770545	Bn-A09-p19770545	A9	33	49.4	A
9336	Bn-N9-p20821511	Bn-A09-p19770719	Bn-A09-p19770719	A9	33	49.4	A
9337	Bn-N9-p20821966	Bn-A09-p19771174	Bn-A09-p19771174	A9	33	49.4	A
9338	Bn-N9-p20826808	Bn-A09-p19774967	Bn-A09-p19774967	A9	33	49.4	A
9339	Bn-N9-p20826999	Bn-A09-p19775158	Bn-A09-p19775158	A9	33	49.4	A
9340	Bn-N9-p20827131	Bn-A09-p19775290	Bn-A09-p19775290	A9	33	49.4	A
9341	Bn-N9-p20830877	Bn-A09-p19779966	Bn-A09-p19779966	A9	33	49.4	A
9342	Bn-N9-p20834722	Bn-A09-p19783822	Bn-A09-p19783822	A9	33	49.4	A
9343	Bn-N9-p20842970	Bn-A09-p19796502	Bn-A09-p19796502	A9	33	49.4	A
9344	Bn-N9-p20843049	Bn-A09-p19796581	Bn-A09-p19796581	A9	33	49.4	A
9345	Bn-N9-p20843094	Bn-A09-p19796626	Bn-A09-p19796626	A9	33	49.4	A
9346	Bn-N9-p20843384	Bn-A09-p19796916	Bn-A09-p19796916	A9	33	49.4	A
9347	Bn-N9-p20850384	Bn-A09-p19803535	Bn-A09-p19803535	A9	33	49.4	A
9348	Bn-N9-p20852212	Bn-A09-p19805361	Bn-A09-p19805361	A9	33	49.4	A
9349	Bn-N9-p20882154	Bn-A09-p19835714	Bn-A09-p19835714	A9	33	49.4	A
9350	Bn-N9-p21133492	Bn-A09-p20051040	Bn-A09-p20051040	A9	33	49.4	A
9351	Bn-N9-p21134661	Bn-A09-p20052851	Bn-A09-p20052851	A9	33	49.4	A
9352	Bn-N9-p21147399	Bn-A09-p20059101	Bn-A09-p20059101	A9	33	49.4	A
9353	Bn-N9-p21169587	Bn-A09-p20081587	Bn-A09-p20081587	A9	33	49.4	A
9354	Bn-N9-p21186381	Bn-A09-p20099237	Bn-A09-p20099237	A9	33	49.4	A

9355	Bn-N9-p21199737	Bn-A09-p20113369	Bn-A09-p20113369	A9	33	49.4	A
9356	Bn-N9-p21201409	Bn-A09-p20115072	Bn-A09-p20115072	A9	33	49.4	A
9357	Bn-N9-p21227224	Bn-A09-p20122747	Bn-A09-p20122747	A9	33	49.4	A
9358	Bn-N9-p21411328	Bn-A09-p20263302	Bn-A09-p20263302	A9	33	49.4	A
9359	Bn-N9-p21415673	Bn-C5-p15983985	Bn-scaff_15959_1-p92102(	A9	33	49.4	A
9360	Bn-N9-p21469757	Bn-A09-p20327880	Bn-A09-p20327880	A9	33	49.4	A
9361	Bn-N9-p21486612	Bn-A09-p20336623	Bn-A09-p20336623	A9	33	49.4	A
9362	Bn-N9-p21642695	Bn-A09-p20512896	Bn-A09-p20512896	A9	33	49.4	A
9363	Bn-N9-p21646806	Bn-A09-p20516599	Bn-A09-p20516599	A9	33	49.4	A
9364	Bn-N9-p21646951	Bn-A09-p20516744	Bn-A09-p20516744	A9	33	49.4	A
9365	Bn-N9-p21647269	Bn-A09-p20517062	Bn-A09-p20517062	A9	33	49.4	A
9366	Bn-N9-p21647621	Bn-A09-p20517413	Bn-A09-p20517413	A9	33	49.4	A
9367	Bn-N9-p21648116	Bn-A09-p20517911	Bn-A09-p20517911	A9	33	49.4	A
9368	Bn-N9-p21656791	Bn-A09-p20526508	Bn-A09-p20526508	A9	33	49.4	A
9369	Bn-N9-p21680116	Bn-A09-p20554631	Bn-A09-p20554631	A9	33	49.4	A
9370	Bn-N9-p21684500	Bn-A09-p20566614	Bn-A09-p20566614	A9	33	49.4	A
9371	Bn-N9-p21687819	Bn-A09-p20569958	Bn-A09-p20569958	A9	33	49.4	A
9372	Bn-N9-p21750677	Bn-A09-p20649745	Bn-A09-p20649745	A9	33	49.4	A
9373	Bn-N9-p21753724	Bn-A09-p20652846	Bn-A09-p20652846	A9	33	49.4	A
9374	Bn-N9-p21774140	Bn-A09-p20672617	Bn-A09-p20672617	A9	33	49.4	A
9375	Bn-N9-p21784493	Bn-A09-p20678910	Bn-A09-p20678910	A9	33	49.4	A
9376	Bn-N9-p21807254	Bn-A09-p20694373	Bn-A09-p20694373	A9	33	49.4	A
9377	Bn-N9-p22538152	Bn-C8-p7462469	Bn-scaff_16766_1-p17827(	A9	33	49.4	A
9378	Bn-N9-p23111780	Bn-A09-p21914887	Bn-A09-p21914887	A9	33	49.4	A
9379	Bn-N9-p23119322	Bn-A09-p21922383	Bn-A09-p21922383	A9	33	49.4	A
9380	Bn-N9-p23120233	Bn-A09-p21923295	Bn-A09-p21923295	A9	33	49.4	A
9381	Bn-N9-p23120949	Bn-A09-p21924011	Bn-A09-p21924011	A9	33	49.4	A
9382	Bn-N9-p23128427	Bn-A09-p21931377	Bn-A09-p21931377	A9	33	49.4	A
9383	Bn-N9-p23158205	Bn-A09-p21969014	Bn-A09-p21969014	A9	33	49.4	A
9384	Bn-N9-p23206074	Bn-A09-p22003936	Bn-A09-p22003936	A9	33	49.4	A
9385	Bn-N9-p23280812	Bn-A09-p22070817	Bn-A09-p22070817	A9	33	49.4	A
9386	Bn-N9-p23280843	Bn-A09-p22070848	Bn-A09-p22070848	A9	33	49.4	A
9387	Bn-N9-p23281519	Bn-A09-p22071523	Bn-A09-p22071523	A9	33	49.4	A
9388	Bn-N9-p23286990	Bn-C9-p18034405	Bn-scaff_18100_1-p12349	A9	33	49.4	A
9389	Bn-N9-p23307296	Bn-C5-p19644068	Bn-scaff_16478_1-p52885	A9	33	49.4	A
9390	Bn-Scaffold00926-p52964	Bn-A06-p19680250	Bn-A06-p19680250	A9	33	49.4	A
9391	Bn-Scaffold01251-p3740	Bn-A09-p19653782	Bn-A09-p19653782	A9	33	49.4	A
9392	Bn-Scaffold01251-p4171	Bn-A09-p19654219	Bn-A09-p19654219	A9	33	49.4	A
9393	Bn-Scaffold13347-p102	Bn-Scaffold000096-p	Bn-Scaffold000096-p70937	A9	33	49.4	A
9394	Bn-Scaffold13432-p799	Bn-A09-p19272074	Bn-A09-p19272074	A9	33	49.4	A
9395	Bn-Scaffold19849-p435	Bn-A08-p9504526	Bn-A08-p9504526	A9	33	49.4	A
9396	Unknown	Bn-A09-p19272475	Bn-A09-p19272475	A9	33	49.4	A
9397	Unknown	Bn-A09-p19452740	Bn-A09-p19452740	A9	33	49.4	A
9398	Bn-N9-p23728533	Bn-A09-p22495700	Bn-A09-p22495700	A9	34	53.4	B
9399	Bn-N13-p16057699	Bn-C3-p15381003	Bn-scaff_22466_1-p75425	A9	35	54.2	B
9400	Bn-N15-p16737228	Bn-C5-p17470620	Bn-scaff_17869_1-p12727	A9	35	54.2	B
9401	Bn-N15-p16144070	Bn-C5-p14929455	Bn-scaff_18338_1-p11559	A9	36	55	B
9402	Bn-N9-p25335523	Bn-A09-p22873685	Bn-A09-p22873685	A9	36	55	B
9403	Bn-N9-p25382332	Bn-A09-p22953858	Bn-A09-p22953858	A9	36	55	B

9404	Bn-N9-p25400496	Bn-A09-p22965277	Bn-A09-p22965277	A9	36	55	B
9405	Bn-N9-p25553145	Bn-A09-p23107846	Bn-A09-p23107846	A9	36	55	B
9406	Bn-N9-p25588982	Bn-A09-p23144404	Bn-A09-p23144404	A9	37	55.8	B
9407	Bn-N9-p25621035	Bn-A09-p23189250	Bn-A09-p23189250	A9	37	55.8	B
9408	Bn-N9-p25624388	Bn-A09-p23200280	Bn-A09-p23200280	A9	37	55.8	B
9409	Bn-N15-p15311138	Bn-C5-p14133297	Bn-scaff_18338_1-p91175:	A9	38	56.6	B
9410	Bn-N9-p25679946	Bn-A09-p23250129	Bn-A09-p23250129	A9	38	56.6	B
9411	Bn-N9-p25696717	Bn-A09-p23268918	Bn-A09-p23268918	A9	38	56.6	B
9412	Bn-N9-p25772129	Bn-A09-p23325795	Bn-A09-p23325795	A9	38	56.6	B
9413	Bn-N9-p25790598	Bn-A09-p23345719	Bn-A09-p23345719	A9	38	56.6	B
9414	Bn-N9-p25797589	Bn-A09-p23354310	Bn-A09-p23354310	A9	39	57.4	B
9415	Bn-N9-p25798089	Bn-A09-p23354810	Bn-A09-p23354810	A9	39	57.4	B
9416	Bn-N9-p25798127	Bn-A09-p23354848	Bn-A09-p23354848	A9	39	57.4	B
9417	Bn-N9-p25800680	Bn-A09-p23356793	Bn-A09-p23356793	A9	39	57.4	B
9418	Bn-N9-p25800834	Bn-A09-p23356947	Bn-A09-p23356947	A9	39	57.4	B
9419	Bn-N9-p25821951	Bn-A09-p23379304	Bn-A09-p23379304	A9	39	57.4	B
9420	Bn-N9-p25831122	Bn-A09-p23379933	Bn-A09-p23379933	A9	39	57.4	B
9421	Bn-N9-p25867929	Bn-A09-p23416800	Bn-A09-p23416800	A9	40	59	B
9422	Bn-N9-p25892571	Bn-A09-p23438753	Bn-A09-p23438753	A9	40	59	B
9423	Bn-N9-p25898872	Bn-A09-p23445424	Bn-A09-p23445424	A9	40	59	B
9424	Bn-N9-p25944749	Bn-A09-p23489920	Bn-A09-p23489920	A9	40	59	B
9425	Bn-N9-p25949479	Bn-A09-p23494647	Bn-A09-p23494647	A9	40	59	B
9426	Bn-N9-p26001801	Bn-A09-p23552075	Bn-A09-p23552075	A9	41	59.8	B
9427	Bn-N9-p26173888	Bn-A09-p23722611	Bn-A09-p23722611	A9	41	59.8	B
9428	Bn-N9-p26175353	Bn-A09-p23724102	Bn-A09-p23724102	A9	41	59.8	B
9429	Bn-N9-p26175356	Bn-A09-p23724105	Bn-A09-p23724105	A9	41	59.8	B
9430	Bn-N9-p26175551	Bn-A09-p23724303	Bn-A09-p23724303	A9	41	59.8	B
9431	Bn-N6-p2562452	Bn-A09-p23790907	Bn-A09-p23790907	A9	42	61.4	B
9432	Bn-N9-p26198959	Bn-A09-p23756115	Bn-A09-p23756115	A9	42	61.4	B
9433	Bn-N9-p26221834	Bn-A09-p23773295	Bn-A09-p23773295	A9	42	61.4	B
9434	Bn-N9-p26227392	Bn-A09-p23778678	Bn-A09-p23778678	A9	42	61.4	B
9435	Bn-N9-p26229635	Bn-A09-p23780921	Bn-A09-p23780921	A9	42	61.4	B
9436	Bn-N9-p26355119	Bn-A09-p23904905	Bn-A09-p23904905	A9	43	64.6	B
9437	Bn-N9-p26364529	Bn-A09-p23913406	Bn-A09-p23913406	A9	43	64.6	B
9438	Bn-N9-p26379411	Bn-A09-p23921036	Bn-A09-p23921036	A9	43	64.6	B
9439	Bn-N9-p26395463	Bn-A09-p23940132	Bn-A09-p23940132	A9	43	64.6	B
9440	Bn-N9-p26404078	Bn-A09-p23948738	Bn-A09-p23948738	A9	43	64.6	B
9441	Bn-N9-p26454668	Bn-A09-p24003401	Bn-A09-p24003401	A9	43	64.6	B
9442	Bn-N9-p26455236	Bn-A09-p24003969	Bn-A09-p24003969	A9	43	64.6	B
9443	Bn-N9-p26530164	Bn-A09-p24086990	Bn-A09-p24086990	A9	44	65.4	B
9444	Bn-N9-p26566800	Bn-A09-p24104666	Bn-A09-p24104666	A9	44	65.4	B
9445	Bn-N9-p26567912	Bn-C5-p12998755	Bn-scaff_21338_1-p97205!	A9	44	65.4	B
9446	Bn-N9-p26567975	Bn-A09-p24105838	Bn-A09-p24105838	A9	44	65.4	B
9447	Bn-N9-p26569102	Bn-A09-p24106965	Bn-A09-p24106965	A9	44	65.4	B
9448	Bn-N9-p26823882	Bn-Scaffold003668-p2	Bn-Scaffold003668-p282	A9	45	67	B
9449	Bn-N9-p26997825	Bn-C5-p12386090	Bn-scaff_21338_1-p35939	A9	46	69.4	B
9450	Bn-N9-p27024443	Bn-A09-p24564546	Bn-A09-p24564546	A9	47	70.2	B
9451	Bn-N9-p27165724	Bn-Scaffold000282-p(	Bn-Scaffold000282-p6257	A9	47	70.2	B
9452	Bn-N9-p27491742	Bn-A05-p15802321	Bn-A05-p15802321	A9	48	71	B

9453	Bn-N9-p27495421	Bn-A05-p15805806	Bn-A05-p15805806	A9	49	71.8	B
9454	Bn-N9-p27504187	Bn-A05-p15808942	Bn-A05-p15808942	A9	49	71.8	B
9455	Bn-N9-p27510150	Bn-A05-p15812933	Bn-A05-p15812933	A9	49	71.8	B
9456	Bn-N9-p27665633	Bn-A09-p24909461	Bn-A09-p24909461	A9	49	71.8	B
9457	Bn-N9-p27674282	Bn-A09-p24921401	Bn-A09-p24921401	A9	49	71.8	B
9458	Bn-N9-p27695074	Bn-A09-p24938986	Bn-A09-p24938986	A9	49	71.8	B
9459	Bn-N9-p27698114	Bn-A09-p24942194	Bn-A09-p24942194	A9	49	71.8	B
9460	Bn-N9-p27699599	Bn-A09-p24943679	Bn-A09-p24943679	A9	49	71.8	B
9461	Bn-N9-p27769039	Bn-A09-p25042124	Bn-A09-p25042124	A9	49	71.8	B
9462	Bn-N9-p27770538	Bn-A09-p25045741	Bn-A09-p25045741	A9	49	71.8	B
9463	Bn-N9-p27770924	Bn-A09-p25046127	Bn-A09-p25046127	A9	49	71.8	B
9464	Bn-N9-p27771060	Bn-A09-p25046263	Bn-A09-p25046263	A9	49	71.8	B
9465	Bn-N9-p27802369	Bn-A09-p25076813	Bn-A09-p25076813	A9	49	71.8	B
9466	Bn-N9-p27827308	Bn-A09-p25090295	Bn-A09-p25090295	A9	49	71.8	B
9467	Bn-N9-p27969318	Bn-A09-p25236454	Bn-A09-p25236454	A9	49	71.8	B
9468	Bn-N9-p27970434	Bn-A09-p25237569	Bn-A09-p25237569	A9	49	71.8	B
9469	Bn-N9-p27971158	Bn-A09-p25238294	Bn-A09-p25238294	A9	49	71.8	B
9470	Bn-N9-p28005744	Bn-A09-p25269364	Bn-A09-p25269364	A9	49	71.8	B
9471	Bn-N9-p28042769	Bn-A09-p25309174	Bn-A09-p25309174	A9	49	71.8	B
9472	Bn-N9-p28043109	Bn-A09-p25309514	Bn-A09-p25309514	A9	49	71.8	B
9473	Bn-N9-p28049663	Bn-A09-p25316922	Bn-A09-p25316922	A9	49	71.8	B
9474	Bn-N9-p28053436	Bn-A09-p25320119	Bn-A09-p25320119	A9	49	71.8	B
9475	Bn-N9-p28125783	Bn-A09-p25372544	Bn-A09-p25372544	A9	49	71.8	B
9476	Bn-N9-p28129971	Bn-A09-p25376723	Bn-A09-p25376723	A9	49	71.8	B
9477	Bn-N9-p28204510	Bn-A09-p25455888	Bn-A09-p25455888	A9	50	72.6	B
9478	Bn-N9-p28240118	Bn-A09-p25491749	Bn-A09-p25491749	A9	50	72.6	B
9479	Bn-N9-p28243130	Bn-A09-p25492079	Bn-A09-p25492079	A9	50	72.6	B
9480	Bn-N9-p28245118	Bn-A09-p25492986	Bn-A09-p25492986	A9	50	72.6	B
9481	Bn-N9-p28245307	Bn-A09-p25493173	Bn-A09-p25493173	A9	50	72.6	B
9482	Bn-N9-p28260162	Bn-C8-p27930921	Bn-scaff_16770_1-p31421[illegible]	A9	50	72.6	B
9483	Bn-N9-p28272802	Bn-A09-p25508290	Bn-A09-p25508290	A9	50	72.6	B
9484	Bn-N9-p28280800	Bn-A09-p25523186	Bn-A09-p25523186	A9	50	72.6	B
9485	Bn-N9-p28290861	Bn-A09-p25532977	Bn-A09-p25532977	A9	50	72.6	B
9486	Bn-N9-p28294732	Bn-A09-p25536905	Bn-A09-p25536905	A9	50	72.6	B
9487	Bn-N9-p28296749	Bn-A09-p25543730	Bn-A09-p25543730	A9	50	72.6	B
9488	Bn-N9-p28333542	Bn-A09-p25575397	Bn-A09-p25575397	A9	50	72.6	B
9489	Bn-N9-p28351905	Bn-C8-p27841046	Bn-scaff_16770_1-p30522[illegible]	A9	50	72.6	B
9490	Bn-N9-p28372911	Bn-A09-p25637924	Bn-A09-p25637924	A9	50	72.6	B
9491	Bn-N9-p28402264	Bn-A09-p25660797	Bn-A09-p25660797	A9	50	72.6	B
9492	Bn-N9-p28416914	Bn-A09-p25670603	Bn-A09-p25670603	A9	50	72.6	B
9493	Bn-N9-p28429762	Bn-A09-p25695942	Bn-A09-p25695942	A9	50	72.6	B
9494	Bn-N9-p28473228	Bn-A09-p25736089	Bn-A09-p25736089	A9	50	72.6	B
9495	Bn-N9-p28475437	Bn-A09-p25738298	Bn-A09-p25738298	A9	50	72.6	B
9496	Bn-N9-p28476031	Bn-A09-p25739064	Bn-A09-p25739064	A9	50	72.6	B
9497	Bn-N9-p28484581	Bn-A09-p25747213	Bn-A09-p25747213	A9	50	72.6	B
9498	Bn-N9-p28484673	Bn-A09-p25747305	Bn-A09-p25747305	A9	50	72.6	B
9499	Bn-N9-p28487694	Bn-A09-p25750328	Bn-A09-p25750328	A9	50	72.6	B
9500	Bn-N9-p28522183	Bn-A09-p25777776	Bn-A09-p25777776	A9	50	72.6	B
9501	Bn-N9-p28524521	Bn-A09-p25779397	Bn-A09-p25779397	A9	50	72.6	B

9502	Bn-N9-p28547385	Bn-A09-p25793645	Bn-A09-p25793645	A9	50	72.6	B
9503	Bn-N9-p28553180	Bn-A09-p25804956	Bn-A09-p25804956	A9	50	72.6	B
9504	Bn-N9-p28569905	Bn-A09-p25821544	Bn-A09-p25821544	A9	50	72.6	0
9505	Bn-N9-p28573901	Bn-A09-p25823689	Bn-A09-p25823689	A9	50	72.6	B
9506	Bn-N9-p28603223	Bn-A09-p25852108	Bn-A09-p25852108	A9	50	72.6	B
9507	Bn-N9-p28622282	Bn-A09-p25872184	Bn-A09-p25872184	A9	50	72.6	B
9508	Bn-N9-p28652697	Bn-A09-p25886596	Bn-A09-p25886596	A9	50	72.6	B
9509	Bn-N9-p28706548	Bn-A09-p25942252	Bn-A09-p25942252	A9	50	72.6	B
9510	Bn-N9-p28765285	Bn-A09-p26008548	Bn-A09-p26008548	A9	50	72.6	B
9511	Bn-N9-p28765443	Bn-A09-p26008706	Bn-A09-p26008706	A9	50	72.6	B
9512	Bn-N9-p28883745	Bn-C8-p27366728	Bn-scaff_16770_1-p25779	A9	50	72.6	0
9513	Bn-Scaffold02294-p2503	Bn-A09-p25884785	Bn-A09-p25884785	A9	50	72.6	B
9514	Bn-N18-p30854660	Bn-A09-p26136023	Bn-A09-p26136023	A9	51	73.4	B
9515	Bn-N18-p31030092	Bn-A09-p26246760	Bn-A09-p26246760	A9	51	73.4	B
9516	Bn-N9-p28841041	Bn-A09-p26101323	Bn-A09-p26101323	A9	51	73.4	B
9517	Bn-N9-p28872231	Bn-A09-p26138669	Bn-A09-p26138669	A9	51	73.4	B
9518	Bn-N9-p28875220	Bn-A09-p26141650	Bn-A09-p26141650	A9	51	73.4	B
9519	Bn-N9-p28878040	Bn-A09-p26142571	Bn-A09-p26142571	A9	51	73.4	B
9520	Bn-N9-p28879070	Bn-A09-p26144798	Bn-A09-p26144798	A9	51	73.4	B
9521	Bn-N9-p28892885	Bn-A09-p26158627	Bn-A09-p26158627	A9	51	73.4	B
9522	Bn-N9-p28892951	Bn-A09-p26158694	Bn-A09-p26158694	A9	51	73.4	B
9523	Bn-N9-p28939752	Bn-A09-p26186084	Bn-A09-p26186084	A9	51	73.4	B
9524	Bn-N9-p29030766	Bn-C8-p28033991	Bn-scaff_16770_1-p32452	A9	51	73.4	B
9525	Bn-N9-p29045126	Bn-A09-p26294118	Bn-A09-p26294118	A9	51	73.4	B
9526	Bn-N9-p29047761	Bn-A09-p26301343	Bn-A09-p26301343	A9	51	73.4	B
9527	Bn-N9-p29051051	Bn-A09-p26307738	Bn-A09-p26307738	A9	51	73.4	B
9528	Bn-N9-p29084285	Bn-A09-p26347709	Bn-A09-p26347709	A9	51	73.4	B
9529	Bn-N9-p29176439	Bn-A09-p26451184	Bn-A09-p26451184	A9	51	73.4	B
9530	Bn-N9-p29186842	Bn-A09-p26461272	Bn-A09-p26461272	A9	51	73.4	B
9531	Bn-N9-p29237576	Bn-A09-p26530290	Bn-A09-p26530290	A9	51	73.4	B
9532	Bn-N9-p29283046	Bn-A09-p26578867	Bn-A09-p26578867	A9	52	74.2	B
9533	Bn-N9-p29321581	Bn-A09-p26606418	Bn-A09-p26606418	A9	52	74.2	B
9534	Bn-N9-p29339916	Bn-A09-p26625665	Bn-A09-p26625665	A9	52	74.2	B
9535	Bn-N9-p29468639	Bn-A09-p26761130	Bn-A09-p26761130	A9	52	74.2	B
9536	Bn-N9-p29472606	Bn-A09-p26765098	Bn-A09-p26765098	A9	52	74.2	B
9537	Bn-N9-p29569315	Bn-A09-p26864664	Bn-A09-p26864664	A9	52	74.2	B
9538	Bn-N9-p29593584	Bn-A09-p26881902	Bn-A09-p26881902	A9	52	74.2	B
9539	Bn-N18-p32012310	Bn-C8-p29008548	Bn-scaff_16770_1-p42197	A9	53	75.8	B
9540	Bn-N18-p32016663	Bn-C8-p29012185	Bn-scaff_16770_1-p42234	A9	53	75.8	B
9541	Bn-N9-p29855688	Bn-A09-p27103683	Bn-A09-p27103683	A9	53	75.8	B
9542	Bn-N9-p29859398	Bn-A09-p27108615	Bn-A09-p27108615	A9	53	75.8	B
9543	Bn-N9-p29860633	Bn-A09-p27109839	Bn-A09-p27109839	A9	53	75.8	B
9544	Bn-N18-p32742430	Bn-C8-p29720065	Bn-scaff_16361_1-p84144	A9	54	76.6	B
9545	Bn-N18-p32885433	Bn-A09-p27656599	Bn-A09-p27656599	A9	54	76.6	B
9546	Bn-N9-p30306145	Bn-A09-p27531548	Bn-A09-p27531548	A9	54	76.6	B
9547	Bn-N9-p30348738	Bn-A09-p27580497	Bn-A09-p27580497	A9	54	76.6	B
9548	Bn-N9-p30393396	Bn-A09-p27628825	Bn-A09-p27628825	A9	54	76.6	B
9549	Bn-N9-p30405003	Bn-A09-p27637967	Bn-A09-p27637967	A9	54	76.6	B
9550	Bn-N9-p30447396	Bn-A09-p27666329	Bn-A09-p27666329	A9	54	76.6	B

9551	Bn-N9-p30458166	Bn-A09-p27678823	Bn-A09-p27678823	A9	54	76.6	B
9552	Bn-N9-p30469243	Bn-A09-p27688300	Bn-A09-p27688300	A9	54	76.6	B
9553	Bn-N9-p30545802	Bn-A09-p27775326	Bn-A09-p27775326	A9	54	76.6	B
9554	Bn-N9-p30655752	Bn-A09-p27867800	Bn-A09-p27867800	A9	54	76.6	B
9555	Bn-N9-p30657437	Bn-A09-p27869414	Bn-A09-p27869414	A9	54	76.6	B
9556	Bn-N9-p30662430	Bn-A09-p27873343	Bn-A09-p27873343	A9	54	76.6	B
9557	Bn-N9-p30670574	Bn-A09-p27886551	Bn-A09-p27886551	A9	54	76.6	B
9558	Bn-N9-p30672935	Bn-C8-p30199706	Bn-scaff_16361_1-p56378[illegible]	A9	54	76.6	B
9559	Bn-N9-p30694169	Bn-A09-p27908768	Bn-A09-p27908768	A9	54	76.6	B
9560	Bn-N9-p30700772	Bn-A09-p27918462	Bn-A09-p27918462	A9	54	76.6	B
9561	Bn-N9-p30719026	Bn-A09-p27939579	Bn-A09-p27939579	A9	54	76.6	B
9562	Bn-N9-p30746404	Bn-A09-p27968747	Bn-A09-p27968747	A9	54	76.6	B
9563	Bn-N9-p30747409	Bn-A09-p27969744	Bn-A09-p27969744	A9	54	76.6	B
9564	Bn-N9-p30772878	Bn-A09-p27974695	Bn-A09-p27974695	A9	54	76.6	B
9565	Bn-N9-p30773181	Bn-A09-p27975967	Bn-A09-p27975967	A9	54	76.6	B
9566	Bn-N9-p30773561	Bn-A09-p27976341	Bn-A09-p27976341	A9	54	76.6	B
9567	Bn-N9-p30779805	Bn-A09-p27982567	Bn-A09-p27982567	A9	54	76.6	B
9568	Bn-N9-p30823116	Bn-A09-p28017415	Bn-A09-p28017415	A9	54	76.6	B
9569	Bn-N9-p30826677	Bn-A09-p28025169	Bn-A09-p28025169	A9	54	76.6	B
9570	Bn-N9-p30828505	Bn-A09-p28026997	Bn-A09-p28026997	A9	54	76.6	B
9571	Bn-N9-p30829188	Bn-A09-p28027675	Bn-A09-p28027675	A9	54	76.6	B
9572	Bn-N9-p30829292	Bn-A09-p28027779	Bn-A09-p28027779	A9	54	76.6	B
9573	Bn-N9-p30892261	Bn-A09-p28082973	Bn-A09-p28082973	A9	54	76.6	B
9574	Bn-N9-p30949594	Bn-A09-p28137068	Bn-A09-p28137068	A9	54	76.6	B
9575	Bn-N9-p30954699	Bn-A09-p28142154	Bn-A09-p28142154	A9	54	76.6	B
9576	Bn-N9-p30955230	Bn-A09-p28142685	Bn-A09-p28142685	A9	54	76.6	B
9577	Bn-N9-p30973560	Bn-A09-p28161041	Bn-A09-p28161041	A9	54	76.6	B
9578	Bn-N9-p30973952	Bn-A09-p28161429	Bn-A09-p28161429	A9	54	76.6	B
9579	Bn-N9-p31020917	Bn-A09-p28203756	Bn-A09-p28203756	A9	54	76.6	B
9580	Bn-N9-p31029615	Bn-A09-p28212233	Bn-A09-p28212233	A9	54	76.6	B
9581	Bn-N9-p31641206	Bn-A09-p28802268	Bn-A09-p28802268	A9	54	76.6	B
9582	Bn-Scaffold12533-p573	Bn-A09-p28209274	Bn-A09-p28209274	A9	54	76.6	B
9583	Bn-N9-p31042574	Bn-A09-p28225347	Bn-A09-p28225347	A9	55	77.4	B
9584	Bn-N9-p31054476	Bn-C8-p30616202	Bn-scaff_16361_1-p98028[illegible]	A9	55	77.4	B
9585	Bn-N9-p31069164	Bn-A09-p28249213	Bn-A09-p28249213	A9	55	77.4	B
9586	Bn-N9-p31069258	Bn-A09-p28249307	Bn-A09-p28249307	A9	55	77.4	B
9587	Bn-N9-p31069920	Bn-A09-p28249925	Bn-A09-p28249925	A9	55	77.4	B
9588	Bn-N9-p31088605	Bn-A09-p28269530	Bn-A09-p28269530	A9	55	77.4	B
9589	Bn-N9-p31103721	Bn-A09-p28282617	Bn-A09-p28282617	A9	55	77.4	B
9590	Bn-N9-p31116735	Bn-A09-p28291555	Bn-A09-p28291555	A9	55	77.4	B
9591	Bn-N9-p31131767	Bn-A09-p28302787	Bn-A09-p28302787	A9	55	77.4	B
9592	Bn-N9-p31268166	Bn-A09-p28435678	Bn-A09-p28435678	A9	55	77.4	B
9593	Bn-N9-p31541144	Bn-A09-p28705871	Bn-A09-p28705871	A9	56	80.6	B
9594	Bn-N9-p31547775	Bn-A09-p28712265	Bn-A09-p28712265	A9	56	80.6	B
9595	Bn-N9-p31557538	Bn-A09-p28717690	Bn-A09-p28717690	A9	56	80.6	B
9596	Bn-N9-p31562739	Bn-A09-p28722989	Bn-A09-p28722989	A9	56	80.6	B
9597	Bn-N9-p31563563	Bn-A09-p28723813	Bn-A09-p28723813	A9	56	80.6	B
9598	Bn-N9-p31594681	Bn-A09-p28758854	Bn-A09-p28758854	A9	56	80.6	B
9599	Bn-N9-p31594971	Bn-A09-p28759144	Bn-A09-p28759144	A9	56	80.6	B

9600	Bn-N9-p31595590	Bn-A09-p28759763	Bn-A09-p28759763	A9	56	80.6	B
9601	Bn-N9-p31600889	Bn-A09-p28765063	Bn-A09-p28765063	A9	56	80.6	B
9602	Bn-N9-p31811168	Bn-A09-p28988381	Bn-A09-p28988381	A9	56	80.6	B
9603	Bn-N9-p31813084	Bn-A09-p28990334	Bn-A09-p28990334	A9	56	80.6	B
9604	Bn-N9-p31813182	Bn-A09-p28990432	Bn-A09-p28990432	A9	56	80.6	B
9605	Bn-N9-p31813620	Bn-A09-p28990648	Bn-A09-p28990648	A9	56	80.6	B
9606	Bn-N9-p31822992	Bn-A09-p29000054	Bn-A09-p29000054	A9	56	80.6	B
9607	Bn-N9-p31836170	Bn-A09-p29012402	Bn-A09-p29012402	A9	56	80.6	B
9608	Bn-N9-p31843357	Bn-A09-p29019543	Bn-A09-p29019543	A9	56	80.6	B
9609	Bn-N9-p31866772	Bn-A09-p29048790	Bn-A09-p29048790	A9	56	80.6	B
9610	Bn-N9-p31870504	Bn-A09-p29052515	Bn-A09-p29052515	A9	56	80.6	B
9611	Bn-N9-p31870605	Bn-A09-p29052616	Bn-A09-p29052616	A9	56	80.6	B
9612	Bn-N9-p31913654	Bn-A09-p29087590	Bn-A09-p29087590	A9	56	80.6	B
9613	Bn-N9-p31932518	Bn-A09-p29106108	Bn-A09-p29106108	A9	56	80.6	B
9614	Bn-N9-p31976046	Bn-A09-p29146030	Bn-A09-p29146030	A9	56	80.6	B
9615	Bn-N9-p31976483	Bn-A09-p29146468	Bn-A09-p29146468	A9	56	80.6	B
9616	Bn-N9-p31979327	Bn-A09-p29150680	Bn-A09-p29150680	A9	56	80.6	B
9617	Bn-N9-p31979825	Bn-A09-p29151179	Bn-A09-p29151179	A9	56	80.6	B
9618	Bn-N9-p31985627	Bn-A09-p29163307	Bn-A09-p29163307	A9	56	80.6	B
9619	Bn-Scaffold09268-p853	Bn-A09-p29134207	Bn-A09-p29134207	A9	56	80.6	B
9620	Bn-N9-p32008041	Bn-A09-p29186255	Bn-A09-p29186255	A9	57	82.2	B
9621	Bn-N9-p32017704	Bn-A09-p29196000	Bn-A09-p29196000	A9	57	82.2	B
9622	Bn-N9-p32065164	Bn-A09-p29245415	Bn-A09-p29245415	A9	58	83.8	B
9623	Bn-N9-p32069808	Bn-A09-p29250304	Bn-A09-p29250304	A9	58	83.8	0
9624	Bn-N9-p32102552	Bn-A09-p29272014	Bn-A09-p29272014	A9	58	83.8	B
9625	Bn-N9-p32106534	Bn-A09-p29275947	Bn-A09-p29275947	A9	58	83.8	B
9626	Bn-N9-p32155024	Bn-A09-p29321510	Bn-A09-p29321510	A9	59	84.6	B
9627	Bn-N9-p32157265	Bn-A09-p29322628	Bn-A09-p29322628	A9	59	84.6	B
9628	Bn-N9-p32161397	Bn-A09-p29331470	Bn-A09-p29331470	A9	59	84.6	B
9629	Bn-N9-p32202430	Bn-A09-p29346712	Bn-A09-p29346712	A9	59	84.6	B
9630	Bn-N9-p32203903	Bn-A09-p29348596	Bn-A09-p29348596	A9	59	84.6	B
9631	Bn-N9-p32221491	Bn-A09-p29368287	Bn-A09-p29368287	A9	59	84.6	B
9632	Bn-N9-p32231258	Bn-A09-p29378096	Bn-A09-p29378096	A9	59	84.6	B
9633	Bn-N9-p32241927	Bn-A09-p29388908	Bn-A09-p29388908	A9	59	84.6	B
9634	Bn-N9-p32271265	Bn-A09-p29416708	Bn-A09-p29416708	A9	59	84.6	B
9635	Bn-N9-p32292944	Bn-A09-p29444457	Bn-A09-p29444457	A9	59	84.6	B
9636	Bn-N9-p32301340	Bn-A09-p29454619	Bn-A09-p29454619	A9	59	84.6	B
9637	Bn-N9-p32317450	Bn-A09-p29480022	Bn-A09-p29480022	A9	60	85.4	B
9638	Bn-N9-p32326702	Bn-A09-p29493614	Bn-A09-p29493614	A9	60	85.4	B
9639	Bn-N9-p32327157	Bn-A09-p29494065	Bn-A09-p29494065	A9	60	85.4	B
9640	Bn-N9-p32329210	Bn-A09-p29496129	Bn-A09-p29496129	A9	60	85.4	B
9641	Bn-N9-p32329653	Bn-A09-p29496571	Bn-A09-p29496571	A9	60	85.4	B
9642	Bn-N9-p32329670	Bn-A09-p29496588	Bn-A09-p29496588	A9	60	85.4	B
9643	Bn-N9-p32336610	Bn-A09-p29507416	Bn-A09-p29507416	A9	60	85.4	B
9644	Bn-N9-p32337224	Bn-A09-p29508036	Bn-A09-p29508036	A9	60	85.4	B
9645	Bn-N18-p35442628	Bn-C8-p32395391	Bn-scaff_16361_1-p27938[illegible]	A9	61	86.2	B
9646	Bn-N9-p32470450	Bn-A09-p29617813	Bn-A09-p29617813	A9	61	86.2	B
9647	Bn-N9-p32483720	Bn-A09-p29630906	Bn-A09-p29630906	A9	61	86.2	0
9648	Bn-N18-p14431261	Bn-A09-p29797321	Bn-A09-p29797321	A9	62	87	B

9649	Bn-N9-p32550436	Bn-A09-p29699253	Bn-A09-p29699253	A9	62	87	B
9650	Bn-N9-p32596386	Bn-A09-p29747854	Bn-A09-p29747854	A9	62	87	B
9651	Bn-N9-p32605456	Bn-A09-p29756845	Bn-A09-p29756845	A9	62	87	B
9652	Bn-N9-p32612149	Bn-A09-p29763444	Bn-A09-p29763444	A9	62	87	B
9653	Bn-N9-p32662229	Bn-C8-p32695741	Bn-scaff_16361_1-p309417	A9	62	87	B
9654	Bn-N9-p32696994	Bn-A09-p29859019	Bn-A09-p29859019	A9	62	87	B
9655	Bn-N9-p32722574	Bn-A09-p29882032	Bn-A09-p29882032	A9	62	87	B
9656	Bn-N9-p32746935	Bn-A09-p29890020	Bn-A09-p29890020	A9	62	87	B
9657	Bn-N9-p32794553	Bn-A09-p29944855	Bn-A09-p29944855	A9	62	87	B
9658	Bn-N18-p36314513	Bn-C8-p33289073	Bn-scaff_16197_1-p311235	A9	63	87.8	B
9659	Bn-N18-p36314585	Bn-C8-p33289146	Bn-scaff_16197_1-p311228	A9	63	87.8	B
9660	Bn-N9-p32817315	Bn-A09-p29968302	Bn-A09-p29968302	A9	63	87.8	B
9661	Bn-N9-p32857331	Bn-A09-p30002747	Bn-A09-p30002747	A9	63	87.8	B
9662	Bn-N9-p32865468	Bn-A09-p30010889	Bn-A09-p30010889	A9	63	87.8	B
9663	Bn-N9-p32905058	Bn-A09-p30058247	Bn-A09-p30058247	A9	63	87.8	B
9664	Bn-N9-p32923631	Bn-A09-p30075198	Bn-A09-p30075198	A9	63	87.8	B
9665	Bn-N9-p32949596	Bn-A09-p30102296	Bn-A09-p30102296	A9	63	87.8	B
9666	Bn-N9-p32952552	Bn-A09-p30105255	Bn-A09-p30105255	A9	63	87.8	B
9667	Bn-N9-p32952992	Bn-A09-p30105467	Bn-A09-p30105467	A9	63	87.8	B
9668	Bn-N9-p32955823	Bn-A09-p30108245	Bn-A09-p30108245	A9	63	87.8	B
9669	Bn-N9-p32956008	Bn-A09-p30108430	Bn-A09-p30108430	A9	63	87.8	B
9670	Bn-N9-p32962302	Bn-A09-p30112316	Bn-A09-p30112316	A9	63	87.8	B
9671	Bn-N9-p32969627	Bn-A09-p30117412	Bn-A09-p30117412	A9	63	87.8	B
9672	Bn-N9-p32969683	Bn-A09-p30117469	Bn-A09-p30117469	A9	63	87.8	B
9673	Bn-N9-p32976209	Bn-A09-p30129045	Bn-A09-p30129045	A9	63	87.8	B
9674	Bn-N9-p32984137	Bn-A09-p30141773	Bn-A09-p30141773	A9	63	87.8	B
9675	Bn-N9-p33029660	Bn-A09-p30171993	Bn-A09-p30171993	A9	63	87.8	B
9676	Bn-N9-p33114966	Bn-A09-p30257404	Bn-A09-p30257404	A9	63	87.8	B
9677	Bn-N9-p33118038	Bn-A09-p30260475	Bn-A09-p30260475	A9	63	87.8	B
9678	Bn-N9-p33118320	Bn-A09-p30260757	Bn-A09-p30260757	A9	63	87.8	B
9679	Bn-N9-p33122024	Bn-A09-p30264461	Bn-A09-p30264461	A9	63	87.8	B
9680	Bn-N9-p33122101	Bn-A09-p30264538	Bn-A09-p30264538	A9	63	87.8	B
9681	Bn-N9-p33122578	Bn-A09-p30265013	Bn-A09-p30265013	A9	63	87.8	B
9682	Bn-N9-p33127388	Bn-A09-p30269740	Bn-A09-p30269740	A9	63	87.8	B
9683	Bn-N9-p33131332	Bn-A09-p30270747	Bn-A09-p30270747	A9	63	87.8	B
9684	Bn-N9-p33139365	Bn-A09-p30278622	Bn-A09-p30278622	A9	63	87.8	B
9685	Bn-N9-p33140796	Bn-A09-p30280052	Bn-A09-p30280052	A9	63	87.8	B
9686	Bn-N9-p33141717	Bn-A09-p30280973	Bn-A09-p30280973	A9	63	87.8	B
9687	Bn-N9-p33142751	Bn-A09-p30282007	Bn-A09-p30282007	A9	63	87.8	B
9688	Bn-N9-p33145376	Bn-A09-p30284632	Bn-A09-p30284632	A9	63	87.8	B
9689	Bn-N9-p33145557	Bn-A09-p30284813	Bn-A09-p30284813	A9	63	87.8	B
9690	Bn-N18-p36549521	Bn-A09-p30464712	Bn-A09-p30464712	A9	64	90.2	B
9691	Bn-N9-p33307742	Bn-A09-p30431025	Bn-A09-p30431025	A9	64	90.2	B
9692	Bn-N9-p33350256	Bn-A09-p30463234	Bn-A09-p30463234	A9	64	90.2	B
9693	Bn-N9-p33353187	Bn-A09-p30466030	Bn-A09-p30466030	A9	64	90.2	B
9694	Bn-N9-p33382060	Bn-A09-p30495797	Bn-A09-p30495797	A9	64	90.2	B
9695	Bn-N9-p33397140	Bn-A09-p30507984	Bn-A09-p30507984	A9	64	90.2	B
9696	Bn-N9-p33408206	Bn-A09-p30523149	Bn-A09-p30523149	A9	64	90.2	B
9697	Bn-N9-p33458092	Bn-A09-p30584271	Bn-A09-p30584271	A9	64	90.2	B

9698	Bn-N9-p33461107	Bn-A09-p30587291	Bn-A09-p30587291	A9	64	90.2	B
9699	Bn-N9-p33464335	Bn-A09-p30592138	Bn-A09-p30592138	A9	64	90.2	B
9700	Bn-N9-p33474135	Bn-A09-p30601385	Bn-A09-p30601385	A9	64	90.2	B
9701	Bn-N9-p33495047	Bn-A09-p30627540	Bn-A09-p30627540	A9	64	90.2	B
9702	Bn-N9-p33518923	Bn-A09-p30651428	Bn-A09-p30651428	A9	64	90.2	B
9703	Bn-N9-p33520847	Bn-A09-p30654305	Bn-A09-p30654305	A9	64	90.2	B
9704	Bn-N9-p33545748	Bn-A09-p30674713	Bn-A09-p30674713	A9	64	90.2	B
9705	Bn-N9-p33552629	Bn-A09-p30681640	Bn-A09-p30681640	A9	64	90.2	B
9706	Bn-N9-p33589806	Bn-A09-p30710881	Bn-A09-p30710881	A9	65	91	B
9707	Bn-N9-p33597407	Bn-A09-p30718480	Bn-A09-p30718480	A9	65	91	B
9708	Bn-N9-p33603704	Bn-A09-p30724789	Bn-A09-p30724789	A9	65	91	B
9709	Bn-N9-p33603935	Bn-A09-p30725020	Bn-A09-p30725020	A9	65	91	B
9710	Bn-N9-p33613606	Bn-A09-p30733739	Bn-A09-p30733739	A9	65	91	B
9711	Bn-N9-p33685511	Bn-A09-p30800136	Bn-A09-p30800136	A9	65	91	B
9712	Bn-N9-p33751179	Bn-A09-p30846003	Bn-A09-p30846003	A9	65	91	B
9713	Bn-N9-p33752814	Bn-A09-p30847721	Bn-A09-p30847721	A9	65	91	B
9714	Bn-N9-p33788816	Bn-A09-p30887157	Bn-A09-p30887157	A9	65	91	B
9715	Bn-N9-p33799097	Bn-A09-p30890768	Bn-A09-p30890768	A9	65	91	B
9716	Bn-N9-p33810425	Bn-A09-p30900431	Bn-A09-p30900431	A9	65	91	B
9717	Bn-N9-p33866174	Bn-A09-p30965242	Bn-A09-p30965242	A9	65	91	B
9718	Bn-N9-p33872670	Bn-A09-p30971760	Bn-A09-p30971760	A9	65	91	B
9719	Bn-N9-p33872728	Bn-A09-p30971818	Bn-A09-p30971818	A9	65	91	B
9720	Bn-N9-p33924543	Bn-A09-p31013896	Bn-A09-p31013896	A9	65	91	B
9721	Bn-N9-p34308381	Bn-A09-p31377029	Bn-A09-p31377029	A9	65	91	B
9722	Bn-N9-p34319221	Bn-A09-p31388253	Bn-A09-p31388253	A9	66	91.8	B
9723	Bn-N9-p34324408	Bn-A09-p31393136	Bn-A09-p31393136	A9	66	91.8	B
9724	Bn-N9-p34324586	Bn-A09-p31393314	Bn-A09-p31393314	A9	66	91.8	B
9725	Bn-N9-p34326342	Bn-A09-p31395071	Bn-A09-p31395071	A9	66	91.8	B
9726	Bn-N9-p34333751	Bn-A09-p31402193	Bn-A09-p31402193	A9	66	91.8	B
9727	Bn-N9-p34344730	Bn-A09-p31413712	Bn-A09-p31413712	A9	66	91.8	B
9728	Bn-N9-p34347725	Bn-A09-p31416695	Bn-A09-p31416695	A9	66	91.8	B
9729	Bn-N18-p38236074	Bn-C8-p35208546	Bn-scaff_16197_1-p11928[illegible]	A9	67	92.6	B
9730	Bn-N18-p38275203	Bn-C8-p35245087	Bn-scaff_16197_1-p11563[illegible]	A9	67	92.6	B
9731	Bn-N18-p38279055	Bn-C8-p35252252	Bn-scaff_16197_1-p11491[illegible]	A9	67	92.6	B
9732	Bn-N18-p38400259	Bn-A09-p5054103	Bn-A09-p5054103	A9	67	92.6	B
9733	Bn-N18-p38644759	Bn-C8-p35613978	Bn-scaff_16197_1-p78745[illegible]	A9	67	92.6	B
9734	Bn-N18-p38644812	Bn-C8-p35614031	Bn-scaff_16197_1-p78740[illegible]	A9	67	92.6	B
9735	Bn-N18-p38649089	Bn-C8-p35615162	Bn-scaff_16197_1-p78627[illegible]	A9	67	92.6	0
9736	Bn-N18-p38836466	Bn-C8-p35771333	Bn-scaff_16197_1-p63009[illegible]	A9	67	92.6	B
9737	Bn-N9-p34419806	Bn-A09-p31492693	Bn-A09-p31492693	A9	67	92.6	B
9738	Bn-N9-p34420779	Bn-A09-p31494058	Bn-A09-p31494058	A9	67	92.6	B
9739	Bn-N9-p34433697	Bn-A09-p31505811	Bn-A09-p31505811	A9	67	92.6	B
9740	Bn-N9-p34476165	Bn-A09-p31549208	Bn-A09-p31549208	A9	67	92.6	B
9741	Bn-N9-p34482324	Bn-A09-p31549492	Bn-A09-p31549492	A9	67	92.6	B
9742	Bn-N9-p34482487	Bn-A09-p31549655	Bn-A09-p31549655	A9	67	92.6	B
9743	Bn-N9-p34484216	Bn-A09-p31554401	Bn-A09-p31554401	A9	67	92.6	B
9744	Bn-N9-p34484339	Bn-A09-p31554524	Bn-A09-p31554524	A9	67	92.6	B
9745	Bn-N9-p34510216	Bn-A09-p31578167	Bn-A09-p31578167	A9	67	92.6	B
9746	Bn-N9-p34512655	Bn-A09-p31580606	Bn-A09-p31580606	A9	67	92.6	B

9747	Bn-N9-p34514127	Bn-A09-p31582088	Bn-A09-p31582088	A9	67	92.6	B
9748	Bn-N9-p34548887	Bn-A09-p31611193	Bn-A09-p31611193	A9	67	92.6	B
9749	Bn-N9-p34548973	Bn-A09-p31611279	Bn-A09-p31611279	A9	67	92.6	B
9750	Bn-N9-p34549130	Bn-A09-p31611436	Bn-A09-p31611436	A9	67	92.6	B
9751	Bn-N9-p34549215	Bn-A09-p31611521	Bn-A09-p31611521	A9	67	92.6	B
9752	Bn-N9-p34549332	Bn-A09-p31611638	Bn-A09-p31611638	A9	67	92.6	B
9753	Bn-N9-p34549848	Bn-A09-p31612154	Bn-A09-p31612154	A9	67	92.6	B
9754	Bn-N9-p34552105	Bn-A09-p31613718	Bn-A09-p31613718	A9	67	92.6	B
9755	Bn-N9-p34552442	Bn-A09-p31614055	Bn-A09-p31614055	A9	67	92.6	B
9756	Bn-N9-p34552517	Bn-A09-p31614130	Bn-A09-p31614130	A9	67	92.6	B
9757	Bn-N9-p34552763	Bn-A09-p31614376	Bn-A09-p31614376	A9	67	92.6	B
9758	Bn-N9-p34567489	Bn-A09-p31629427	Bn-A09-p31629427	A9	67	92.6	B
9759	Bn-N9-p34575261	Bn-A09-p31635684	Bn-A09-p31635684	A9	67	92.6	B
9760	Bn-N9-p34590897	Bn-A09-p31651915	Bn-A09-p31651915	A9	67	92.6	B
9761	Bn-N9-p34630115	Bn-A09-p31658403	Bn-A09-p31658403	A9	67	92.6	B
9762	Bn-N9-p34632030	Bn-A09-p31660206	Bn-A09-p31660206	A9	67	92.6	B
9763	Bn-N9-p34635021	Bn-C8-p35013219	Bn-scaff_16197_1-p138821	A9	67	92.6	B
9764	Bn-N9-p34647945	Bn-A09-p31678038	Bn-A09-p31678038	A9	67	92.6	B
9765	Bn-N9-p34674821	Bn-A09-p31704785	Bn-A09-p31704785	A9	67	92.6	B
9766	Bn-N9-p34676377	Bn-A09-p31706338	Bn-A09-p31706338	A9	67	92.6	B
9767	Bn-N9-p34676455	Bn-A09-p31706416	Bn-A09-p31706416	A9	67	92.6	B
9768	Bn-N9-p34676511	Bn-A09-p31706472	Bn-A09-p31706472	A9	67	92.6	0
9769	Bn-N9-p34692151	Bn-A09-p31716654	Bn-A09-p31716654	A9	67	92.6	B
9770	Bn-N9-p34704348	Bn-A09-p31730182	Bn-A09-p31730182	A9	67	92.6	B
9771	Bn-N9-p34710910	Bn-A09-p31740290	Bn-A09-p31740290	A9	67	92.6	B
9772	Bn-N9-p34738467	Bn-A09-p31781629	Bn-A09-p31781629	A9	67	92.6	B
9773	Bn-N9-p34746044	Bn-C8-p35143701	Bn-scaff_16197_1-p125773	A9	67	92.6	B
9774	Bn-N9-p34770315	Bn-A09-p31810193	Bn-A09-p31810193	A9	67	92.6	B
9775	Bn-N9-p34840747	Bn-A09-p31870750	Bn-A09-p31870750	A9	67	92.6	B
9776	Bn-N9-p34842590	Bn-A09-p4976736	Bn-A09-p4976736	A9	67	92.6	B
9777	Bn-N9-p34863516	Bn-A09-p4996710	Bn-A09-p4996710	A9	67	92.6	B
9778	Bn-N9-p34864471	Bn-A09-p4997670	Bn-A09-p4997670	A9	67	92.6	B
9779	Bn-N9-p34884858	Bn-A09-p5013589	Bn-A09-p5013589	A9	67	92.6	B
9780	Bn-N9-p34906777	Bn-A09-p5033479	Bn-A09-p5033479	A9	67	92.6	B
9781	Bn-N9-p34909042	Bn-A09-p5034747	Bn-A09-p5034747	A9	67	92.6	B
9782	Bn-N9-p34911377	Bn-A09-p5037318	Bn-A09-p5037318	A9	67	92.6	B
9783	Bn-N9-p34913853	Bn-A09-p5040112	Bn-A09-p5040112	A9	67	92.6	B
9784	Bn-N9-p34914407	Bn-A09-p5040525	Bn-A09-p5040525	A9	67	92.6	B
9785	Bn-N9-p34937224	Bn-A09-p5061646	Bn-A09-p5061646	A9	67	92.6	B
9786	Bn-N18-p38701686	Bn-A09-p31979689	Bn-A09-p31979689	A9	68	93.4	B
9787	Bn-N9-p34992197	Bn-A09-p5132918	Bn-A09-p5132918	A9	68	93.4	B
9788	Bn-N9-p35000993	Bn-A09-p5149149	Bn-A09-p5149149	A9	68	93.4	B
9789	Bn-N9-p35019518	Bn-A09-p5168276	Bn-A09-p5168276	A9	68	93.4	B
9790	Bn-N9-p35045978	Bn-A09-p5977032	Bn-A09-p5977032	A9	68	93.4	0
9791	Bn-N9-p35046023	Bn-C8-p35531039	Bn-scaff_16197_1-p870393	A9	68	93.4	B
9792	Bn-N9-p35079343	Bn-A09-p31890957	Bn-A09-p31890957	A9	68	93.4	B
9793	Bn-N9-p35106448	Bn-A09-p31905309	Bn-A09-p31905309	A9	68	93.4	B
9794	Bn-N9-p35141266	Bn-A09-p31949837	Bn-A09-p31949837	A9	68	93.4	B
9795	Bn-N9-p35144313	Bn-A09-p31952879	Bn-A09-p31952879	A9	68	93.4	B

9796	Bn-N9-p35149981	Bn-A09-p31957772	Bn-A09-p31957772	A9	68	93.4	B
9797	Bn-N9-p35163703	Bn-A09-p31968693	Bn-A09-p31968693	A9	68	93.4	B
9798	Bn-N9-p35164898	Bn-A09-p31969888	Bn-A09-p31969888	A9	68	93.4	0
9799	Bn-N9-p35185360	Bn-A09-p31987382	Bn-A09-p31987382	A9	68	93.4	B
9800	Bn-N9-p35228335	Bn-A09-p32025164	Bn-A09-p32025164	A9	68	93.4	B
9801	Bn-N9-p35370518	Bn-A09-p32161150	Bn-A09-p32161150	A9	68	93.4	B
9802	Bn-N9-p35372167	Bn-A09-p32167322	Bn-A09-p32167322	A9	68	93.4	B
9803	Bn-N9-p35404050	Bn-C5-p33482752	Bn-scaff_15695_2-p42386	A9	68	93.4	B
9804	Bn-N9-p35410636	Bn-A09-p32223480	Bn-A09-p32223480	A9	68	93.4	B
9805	Bn-N9-p35412775	Bn-A09-p32225646	Bn-A09-p32225646	A9	68	93.4	B
9806	Bn-N9-p35418407	Bn-Scaffold011301-p	Bn-Scaffold011301-p120	A9	68	93.4	B
9807	Bn-N9-p35432640	Bn-A09-p32254190	Bn-A09-p32254190	A9	68	93.4	B
9808	Bn-N9-p35438207	Bn-A09-p32257776	Bn-A09-p32257776	A9	68	93.4	B
9809	Bn-N9-p35448377	Bn-A09-p32270596	Bn-A09-p32270596	A9	68	93.4	B
9810	Bn-N9-p35482679	Bn-A09-p32321534	Bn-A09-p32321534	A9	68	93.4	B
9811	Bn-N9-p35504808	Bn-A09-p32344408	Bn-A09-p32344408	A9	68	93.4	B
9812	Bn-N18-p39279119	Bn-C8-p36223426	Bn-scaff_16197_1-p17800	A9	69	95.8	A
9813	Bn-N18-p39576040	Bn-C8-p36478180	Bn-scaff_16445_1-p28653	A9	69	95.8	A
9814	Bn-N6-p15587040	Bn-A06-p2732731	Bn-A06-p2732731	A9	69	95.8	A
9815	Bn-N9-p35716954	Bn-A09-p32553111	Bn-A09-p32553111	A9	69	95.8	B
9816	Bn-N9-p35741674	Bn-A09-p32590303	Bn-A09-p32590303	A9	69	95.8	B
9817	Bn-N9-p35805520	Bn-A09-p32666778	Bn-A09-p32666778	A9	69	95.8	B
9818	Bn-N9-p35808409	Bn-A09-p32669666	Bn-A09-p32669666	A9	69	95.8	B
9819	Bn-N9-p35854469	Bn-A09-p32713083	Bn-A09-p32713083	A9	69	95.8	B
9820	Bn-N9-p35881998	Bn-C8-p36387444	Bn-scaff_16197_1-p13988	A9	69	95.8	A
9821	Bn-N9-p35916330	Bn-A09-p32787589	Bn-A09-p32787589	A9	69	95.8	0
9822	Bn-N9-p35948073	Bn-A09-p32822165	Bn-A09-p32822165	A9	69	95.8	A
9823	Bn-N9-p35952538	Bn-A09-p32826561	Bn-A09-p32826561	A9	69	95.8	0
9824	Bn-N9-p35964800	Bn-A09-p32841826	Bn-A09-p32841826	A9	69	95.8	0
9825	Bn-N9-p36039181	Bn-A09-p32923447	Bn-A09-p32923447	A9	69	95.8	0
9826	Bn-N9-p36039212	Bn-A09-p32923478	Bn-A09-p32923478	A9	69	95.8	0
9827	Bn-Scaffold04520-p171	Bn-A04-p3851969	Bn-A04-p3851969	A9	69	95.8	A
9828	Bn-N9-p36096719	Bn-A09-p32980100	Bn-A09-p32980100	A9	70	96.6	B
9829	Bn-N9-p36120868	Bn-A09-p32995843	Bn-A09-p32995843	A9	70	96.6	B
9830	Bn-N9-p36131162	Bn-A09-p33006858	Bn-A09-p33006858	A9	70	96.6	B
9831	Bn-N9-p36137660	Bn-A09-p33013128	Bn-A09-p33013128	A9	71	97.4	B
9832	Bn-N9-p36169883	Bn-A09-p33040441	Bn-A09-p33040441	A9	71	97.4	B
9833	Bn-N9-p36175971	Bn-A09-p33058738	Bn-A09-p33058738	A9	71	97.4	B
9834	Bn-N9-p36190705	Bn-A09-p33073469	Bn-A09-p33073469	A9	71	97.4	B
9835	Bn-N9-p36116723	Bn-A09-p32993230	Bn-A09-p32993230	A9	71	97.4	B
9836	Bn-N9-p36190273	Bn-A09-p33073037	Bn-A09-p33073037	A9	71	97.4	B
9837	Bn-N9-p36445006	Bn-A09-p33358168	Bn-A09-p33358168	A9	72	99	B
9838	Bn-N9-p36445136	Bn-A09-p33358298	Bn-A09-p33358298	A9	72	99	B
9839	Bn-N9-p36445193	Bn-A09-p33358356	Bn-A09-p33358356	A9	72	99	B
9840	Bn-N9-p36456929	Bn-A09-p33369348	Bn-A09-p33369348	A9	72	99	B
9841	Bn-N9-p36462585	Bn-A09-p33372131	Bn-A09-p33372131	A9	72	99	B
9842	Bn-N9-p36462658	Bn-A09-p33372204	Bn-A09-p33372204	A9	72	99	B
9843	Bn-N9-p36463025	Bn-A09-p33372571	Bn-A09-p33372571	A9	72	99	B
9844	Bn-N9-p36463525	Bn-A09-p33372831	Bn-A09-p33372831	A9	72	99	B

9845	Bn-N9-p36463585	Bn-A09-p33372891	Bn-A09-p33372891	A9	72	99	B
9846	Bn-N9-p36463619	Bn-A09-p33372925	Bn-A09-p33372925	A9	72	99	B
9847	Bn-N9-p36472028	Bn-A09-p33382398	Bn-A09-p33382398	A9	72	99	B
9848	Bn-N9-p36477539	Bn-A09-p33391361	Bn-A09-p33391361	A9	72	99	B
9849	Bn-N9-p36484837	Bn-A09-p33398481	Bn-A09-p33398481	A9	72	99	B
9850	Bn-N9-p36486746	Bn-A09-p33400463	Bn-A09-p33400463	A9	72	99	B
9851	Bn-N9-p36486878	Bn-A09-p33400597	Bn-A09-p33400597	A9	72	99	B
9852	Bn-N9-p36492153	Bn-A09-p33406350	Bn-A09-p33406350	A9	72	99	B
9853	Bn-N9-p36505650	Bn-A09-p33419515	Bn-A09-p33419515	A9	72	99	B
9854	Bn-N9-p36525236	Bn-A09-p33428597	Bn-A09-p33428597	A9	72	99	B
9855	Bn-N9-p36573079	Bn-A09-p33464355	Bn-A09-p33464355	A9	73	99.8	B
9856	Bn-N9-p36583438	Bn-A09-p33475518	Bn-A09-p33475518	A9	73	99.8	B
9857	Bn-N18-p40419966	Bn-C8-p37330048	Bn-scaff_16445_1-p20134[illegible]	A9	74	100.6	B
9858	Bn-N9-p36590161	Bn-A09-p33487356	Bn-A09-p33487356	A9	74	100.6	B
9859	Bn-N9-p36599650	Bn-A09-p33496868	Bn-A09-p33496868	A9	74	100.6	B
9860	Bn-N9-p36599957	Bn-A09-p33497174	Bn-A09-p33497174	A9	74	100.6	B
9861	Bn-N9-p36603036	Bn-A09-p33499505	Bn-A09-p33499505	A9	74	100.6	B
9862	Bn-N9-p36603451	Bn-A09-p33499917	Bn-A09-p33499917	A9	74	100.6	B
9863	Bn-N9-p36604316	Bn-A09-p33500473	Bn-A09-p33500473	A9	74	100.6	B
9864	Bn-N9-p36606030	Bn-A09-p33512900	Bn-A09-p33512900	A9	74	100.6	B
9865	Bn-N9-p36614056	Bn-A09-p33521094	Bn-A09-p33521094	A9	74	100.6	B
9866	Bn-N9-p36663023	Bn-A09-p33574107	Bn-A09-p33574107	A9	74	100.6	B
9867	Bn-N9-p36673067	Bn-A09-p33582715	Bn-A09-p33582715	A9	74	100.6	B
9868	Bn-N9-p36677429	Bn-A09-p33587432	Bn-A09-p33587432	A9	74	100.6	B
9869	Bn-N9-p36690132	Bn-A09-p33595011	Bn-A09-p33595011	A9	74	100.6	B
9870	Bn-N9-p36690293	Bn-A09-p33595174	Bn-A09-p33595174	A9	74	100.6	B
9871	Bn-N9-p36758903	Bn-C8-p37503537	Bn-scaff_16445_1-p18399[illegible]	A9	74	100.6	B
9872	Bn-N9-p36805010	Bn-A09-p33694154	Bn-A09-p33694154	A9	74	100.6	B
9873	Bn-N9-p36819201	Bn-A09-p33716233	Bn-A09-p33716233	A9	74	100.6	B
9874	Bn-N9-p36828993	Bn-A09-p33722405	Bn-A09-p33722405	A9	74	100.6	B
9875	Bn-N9-p36829751	Bn-A09-p33723162	Bn-A09-p33723162	A9	74	100.6	B
9876	Bn-N9-p36830723	Bn-A09-p33724136	Bn-A09-p33724136	A9	74	100.6	B
9877	Bn-N9-p36830765	Bn-A09-p33724178	Bn-A09-p33724178	A9	74	100.6	B
9878	Bn-N9-p36835540	Bn-A09-p33730723	Bn-A09-p33730723	A9	74	100.6	B
9879	Bn-N9-p36836802	Bn-A09-p33731681	Bn-A09-p33731681	A9	74	100.6	B
9880	Bn-N9-p36868438	Bn-A09-p33777546	Bn-A09-p33777546	A9	74	100.6	B
9881	Bn-N9-p36868879	Bn-A09-p33777987	Bn-A09-p33777987	A9	74	100.6	B
9882	Bn-N9-p36888071	Bn-A09-p33803933	Bn-A09-p33803933	A9	74	100.6	B
9883	Bn-N9-p36905931	Bn-A09-p33817337	Bn-A09-p33817337	A9	74	100.6	B
9884	Bn-N9-p36917760	Bn-A09-p33830942	Bn-A09-p33830942	A9	74	100.6	B
9885	Bn-N18-p41042789	Bn-A09-p33983999	Bn-A09-p33983999	A9	75	101.4	B
9886	Bn-N9-p37142220	Bn-A09-p34022183	Bn-A09-p34022183	A9	75	101.4	B
9887	Bn-N9-p37148234	Bn-A09-p34028225	Bn-A09-p34028225	A9	75	101.4	0
9888	Bn-N9-p37149943	Bn-A09-p34029934	Bn-A09-p34029934	A9	75	101.4	B
9889	Bn-N9-p37159171	Bn-A09-p34039082	Bn-A09-p34039082	A9	75	101.4	B
9890	Bn-N9-p37166894	Bn-A09-p34046789	Bn-A09-p34046789	A9	75	101.4	B
9891	Bn-N9-p37185217	Bn-A09-p34063724	Bn-A09-p34063724	A9	75	101.4	B
9892	Bn-N9-p37188753	Bn-A09-p34067269	Bn-A09-p34067269	A9	75	101.4	B
9893	Bn-N18-p41757106	Bn-C8-p38599944	Bn-scaff_16445_1-p74355[illegible]	A9	76	103.8	0

9894	Bn-N9-p37607705	Bn-A09-p34482075	Bn-A09-p34482075	A9	76	103.8	B
9895	Bn-N9-p37895290	Bn-A09-p34779068	Bn-A09-p34779068	A9	77	105.4	B
9896	Bn-N9-p38143693	Bn-C8-p39030057	Bn-scaff_16445_1-p31344(	A9	78	106.2	B
9897	Bn-N9-p38145589	Bn-A09-p34995048	Bn-A09-p34995048	A9	78	106.2	B
9898	Bn-N9-p38146463	Bn-A09-p34995922	Bn-A09-p34995922	A9	78	106.2	B
9899	Bn-N9-p38325101	Bn-A09-p35162641	Bn-A09-p35162641	A9	79	107.8	B
9900	Bn-N9-p38388156	Bn-A09-p35221507	Bn-A09-p35221507	A9	79	107.8	B
9901	Bn-N18-p42699168	Bn-A09-p35278018	Bn-A09-p35278018	A9	80	108.6	B
9902	Bn-N9-p38427349	Bn-A09-p35262679	Bn-A09-p35262679	A9	80	108.6	B
9903	Bn-N9-p38466601	Bn-A09-p35299111	Bn-A09-p35299111	A9	80	108.6	B
9904	Bn-N9-p38495271	Bn-A09-p35321301	Bn-A09-p35321301	A9	80	108.6	B
9905	Bn-Scaffold01568-p1838	Bn-A05-p3796206	Bn-A05-p3796206	A9	80	108.6	B
9906	Bn-N9-p38544569	Bn-A09-p35368467	Bn-A09-p35368467	A9	81	109.4	B
9907	Bn-N9-p38548418	Bn-A09-p35372226	Bn-A09-p35372226	A9	81	109.4	B
9908	Bn-N9-p38571470	Bn-A09-p35389045	Bn-A09-p35389045	A9	81	109.4	B
9909	Bn-N9-p38608830	Bn-A09-p35426888	Bn-A09-p35426888	A9	81	109.4	B
9910	Bn-N18-p43084500	Bn-A09-p35598320	Bn-A09-p35598320	A9	82	110.2	B
9911	Bn-N9-p38661089	Bn-C8-p39803460	Bn-scaff_21269_1-p11894(	A9	82	110.2	B
9912	Bn-N9-p38663579	Bn-A09-p35477936	Bn-A09-p35477936	A9	82	110.2	B
9913	Bn-N9-p38665367	Bn-A09-p35479723	Bn-A09-p35479723	A9	82	110.2	B
9914	Bn-N9-p38670721	Bn-A09-p35485049	Bn-A09-p35485049	A9	82	110.2	B
9915	Bn-N9-p38671130	Bn-A09-p35485458	Bn-A09-p35485458	A9	82	110.2	B
9916	Bn-N9-p38675403	Bn-A09-p35489554	Bn-A09-p35489554	A9	82	110.2	B
9917	Bn-N9-p38677942	Bn-A09-p35492133	Bn-A09-p35492133	A9	82	110.2	B
9918	Bn-N9-p38689050	Bn-A09-p35505057	Bn-A09-p35505057	A9	82	110.2	B
9919	Bn-N9-p38689113	Bn-A09-p35505120	Bn-A09-p35505120	A9	82	110.2	B
9920	Bn-N9-p38692821	Bn-A09-p35508720	Bn-A09-p35508720	A9	82	110.2	B
9921	Bn-N9-p38693055	Bn-A09-p35508955	Bn-A09-p35508955	A9	82	110.2	B
9922	Bn-N9-p38693144	Bn-A09-p35509044	Bn-A09-p35509044	A9	82	110.2	B
9923	Bn-N9-p38699119	Bn-A09-p35520884	Bn-A09-p35520884	A9	82	110.2	B
9924	Bn-N9-p38777005	Bn-C8-p39970021	Bn-scaff_21269_1-p28551(	A9	82	110.2	B
9925	Bn-N9-p39160240	Bn-A09-p36033827	Bn-A09-p36033827	A9	82	110.2	0
9926	Bn-N18-p43252029	Bn-A09-p35707505	Bn-A09-p35707505	A9	83	111	B
9927	Bn-N9-p38804159	Bn-A09-p35624679	Bn-A09-p35624679	A9	83	111	B
9928	Bn-N9-p38804252	Bn-A09-p35624767	Bn-A09-p35624767	A9	83	111	B
9929	Bn-N9-p38833762	Bn-A09-p35656352	Bn-A09-p35656352	A9	83	111	B
9930	Bn-N9-p38883377	Bn-C8-p40040359	Bn-scaff_21269_1-p35584(	A9	83	111	B
9931	Bn-N9-p38894139	Bn-A09-p35724973	Bn-A09-p35724973	A9	83	111	0
9932	Bn-N9-p38920621	Bn-A09-p35753486	Bn-A09-p35753486	A9	83	111	B
9933	Bn-N9-p38950008	Bn-A09-p35790555	Bn-A09-p35790555	A9	83	111	B
9934	Bn-N18-p43463516	Bn-C8-p40312788	Bn-scaff_16389_1-p21560(	A9	84	112.6	B
9935	Bn-N9-p39021453	Bn-A09-p35879759	Bn-A09-p35879759	A9	84	112.6	B
9936	Bn-N9-p39049709	Bn-A09-p35907846	Bn-A09-p35907846	A9	84	112.6	B
9937	Bn-N9-p39049875	Bn-A09-p35908013	Bn-A09-p35908013	A9	84	112.6	B
9938	Bn-N9-p39056889	Bn-A09-p35914702	Bn-A09-p35914702	A9	84	112.6	B
9939	Bn-N9-p39103864	Bn-A09-p35964883	Bn-A09-p35964883	A9	84	112.6	B
9940	Bn-N14-p49714674	Bn-A06-p8545758	Bn-A06-p8545758	A9	85	113.4	B
9941	Bn-N18-p43731092	Bn-A09-p36172635	Bn-A09-p36172635	A9	85	113.4	B
9942	Bn-N9-p39188012	Bn-A09-p36062760	Bn-A09-p36062760	A9	85	113.4	B

9943	Bn-N9-p39188077	Bn-A09-p36062825	Bn-A09-p36062825	A9	85	113.4	B
9944	Bn-N9-p39245304	Bn-A09-p36112515	Bn-A09-p36112515	A9	85	113.4	B
9945	Bn-N9-p39319479	Bn-A09-p36173579	Bn-A09-p36173579	A9	85	113.4	B
9946	Bn-N9-p39351742	Bn-A09-p36200543	Bn-A09-p36200543	A9	86	117.5	A
9947	Bn-N9-p39352315	Bn-A09-p36201113	Bn-A09-p36201113	A9	86	117.5	A
9948	Bn-N9-p39355938	Bn-A09-p36204736	Bn-A09-p36204736	A9	86	117.5	A
9949	Bn-N9-p39357316	Bn-A09-p36206114	Bn-A09-p36206114	A9	86	117.5	A
9950	Bn-N9-p39361505	Bn-A09-p36210247	Bn-A09-p36210247	A9	86	117.5	A
9951	Bn-N9-p39363652	Bn-A09-p36211880	Bn-A09-p36211880	A9	86	117.5	A
9952	Bn-N9-p39380185	Bn-A09-p36221958	Bn-A09-p36221958	A9	86	117.5	A
9953	Bn-N9-p39406857	Bn-A09-p36248653	Bn-A09-p36248653	A9	86	117.5	A
9954	Bn-N9-p39412270	Bn-A09-p36254037	Bn-A09-p36254037	A9	86	117.5	A
9955	Bn-N9-p39415213	Bn-A09-p36259207	Bn-A09-p36259207	A9	86	117.5	A
9956	Bn-N9-p39560080	Bn-A09-p36402398	Bn-A09-p36402398	A9	87	118.3	A
9957	Bn-N9-p39560263	Bn-A09-p36402581	Bn-A09-p36402581	A9	87	118.3	A
9958	Bn-N9-p39566523	Bn-A09-p36403465	Bn-A09-p36403465	A9	87	118.3	A
9959	Bn-N9-p39573277	Bn-A09-p36410312	Bn-A09-p36410312	A9	87	118.3	A
9960	Bn-N9-p39573489	Bn-A09-p36410528	Bn-A09-p36410528	A9	87	118.3	A
9961	Bn-N9-p39599314	Bn-A09-p36434436	Bn-A09-p36434436	A9	87	118.3	A
9962	Bn-N9-p39621064	Bn-A09-p36456115	Bn-A09-p36456115	A9	87	118.3	A
9963	Bn-N9-p39652543	Bn-A09-p36490411	Bn-A09-p36490411	A9	87	118.3	A
9964	Bn-N9-p39658156	Bn-A09-p36491715	Bn-A09-p36491715	A9	87	118.3	A
9965	Bn-N11-p11529222	Bn-A09-p37010341	Bn-A09-p37010341	A9	88	125	A
9966	Bn-N12-p3110619	Bn-A09-p36805327	Bn-A09-p36805327	A9	88	125	A
9967	Bn-N13-p55061222	Bn-A09-p36803825	Bn-A09-p36803825	A9	88	125	A
9968	Bn-N18-p44691660	Bn-A09-p37097229	Bn-A09-p37097229	A9	88	125	A
9969	Bn-N19-p37045962	Bn-A09-p36807928	Bn-A09-p36807928	A9	88	125	A
9970	Bn-N4-p15620062	Bn-A09-p37112947	Bn-A09-p37112947	A9	88	125	A
9971	Bn-N9-p39935452	Bn-A09-p36815767	Bn-A09-p36815767	A9	88	125	A
9972	Bn-N9-p40060791	Bn-A09-p36886022	Bn-A09-p36886022	A9	88	125	A
9973	Bn-N9-p40068467	Bn-A09-p36891502	Bn-A09-p36891502	A9	88	125	A
9974	Bn-N9-p40255542	Bn-A09-p37029924	Bn-A09-p37029924	A9	88	125	A
9975	Bn-Scaffold03369-p1628	Bn-A09-p36969978	Bn-A09-p36969978	A9	88	125	A
9976	Bn-N18-p44587485	Bn-C8-p41591217	Bn-scaff_16021_1-p16746[illegible]	A9	89	127.5	A
9977	Bn-N18-p44635485	Bn-C8-p41643256	Bn-scaff_16021_1-p11543[illegible]	A9	89	127.5	A
9978	Bn-N9-p40208874	Bn-C8-p41583107	Bn-scaff_16021_1-p17557[illegible]	A9	89	127.5	A
9979	Bn-Scaffold20020-p366	Bn-C8-p41696816	Bn-scaff_16021_1-p61870	A9	89	127.5	A
9980	Bn-N10-p144599	Bn-A10-p4757850	Bn-A10-p4757850	A10	1	0	A
9981	Bn-N10-p146549	Bn-A10-p4755899	Bn-A10-p4755899	A10	1	0	A
9982	Bn-N10-p165000	Bn-A10-p4742940	Bn-A10-p4742940	A10	1	0	A
9983	Bn-N10-p174643	Bn-A10-p4727374	Bn-A10-p4727374	A10	1	0	A
9984	Bn-N10-p176298	Bn-A10-p4725713	Bn-A10-p4725713	A10	1	0	A
9985	Bn-N10-p190611	Bn-A10-p4706425	Bn-A10-p4706425	A10	1	0	A
9986	Bn-N10-p205455	Bn-A10-p4695520	Bn-A10-p4695520	A10	1	0	A
9987	Bn-N10-p249578	Bn-A10-p4653595	Bn-A10-p4653595	A10	1	0	A
9988	Bn-N10-p41211	Bn-A10-p4840892	Bn-A10-p4840892	A10	1	0	0
9989	Bn-N10-p53102	Bn-A10-p4829546	Bn-A10-p4829546	A10	1	0	A
9990	Bn-N10-p330463	Bn-A10-p4563194	Bn-A10-p4563194	A10	2	0.8	0
9991	Bn-N10-p334540	Bn-C5-p335866	Bn-scaff_16414_1-p16798[illegible]	A10	2	0.8	A

9992	Bn-N10-p338135	Bn-A10-p4555527	Bn-A10-p4555527	A10	2	0.8	A
9993	Bn-N10-p297188	Bn-A10-p4606815	Bn-A10-p4606815	A10	3	0.8	A
9994	Bn-N10-p341281	Bn-A10-p4553957	Bn-A10-p4553957	A10	3	0.8	A
9995	Bn-N10-p380433	Bn-A10-p4504641	Bn-A10-p4504641	A10	3	0.8	A
9996	Bn-N10-p457441	Bn-A10-p4425066	Bn-A10-p4425066	A10	3	0.8	A
9997	Bn-N10-p566482	Bn-A10-p4296387	Bn-A10-p4296387	A10	3	0.8	A
9998	Bn-N10-p587140	Bn-A10-p4268080	Bn-A10-p4268080	A10	3	0.8	A
9999	Bn-N10-p650685	Bn-A10-p4212056	Bn-A10-p4212056	A10	3	0.8	A
10000	Bn-N10-p665064	Bn-A10-p4197906	Bn-A10-p4197906	A10	3	0.8	A
10001	Bn-N10-p738404	Bn-A10-p4138801	Bn-A10-p4138801	A10	3	0.8	0
10002	Bn-N10-p738423	Bn-A10-p4138782	Bn-A10-p4138782	A10	3	0.8	A
10003	Bn-N10-p757971	Bn-A10-p4118433	Bn-A10-p4118433	A10	3	0.8	A
10004	Bn-N10-p761807	Bn-A10-p4114477	Bn-A10-p4114477	A10	3	0.8	A
10005	Bn-N10-p761887	Bn-A10-p4114397	Bn-A10-p4114397	A10	3	0.8	A
10006	Bn-N10-p763440	Bn-A10-p4112636	Bn-A10-p4112636	A10	3	0.8	A
10007	Bn-N10-p770327	Bn-A10-p4104015	Bn-A10-p4104015	A10	3	0.8	A
10008	Bn-N10-p781432	Bn-A10-p4089670	Bn-A10-p4089670	A10	3	0.8	A
10009	Bn-N10-p782670	Bn-A10-p4088438	Bn-A10-p4088438	A10	3	0.8	A
10010	Bn-N10-p791792	Bn-A10-p4080011	Bn-A10-p4080011	A10	3	0.8	A
10011	Bn-N10-p792273	Bn-A10-p4079529	Bn-A10-p4079529	A10	3	0.8	A
10012	Bn-N10-p793644	Bn-A10-p4078158	Bn-A10-p4078158	A10	3	0.8	A
10013	Bn-N10-p795050	Bn-A10-p4076884	Bn-A10-p4076884	A10	3	0.8	A
10014	Bn-N10-p829577	Bn-A10-p4040298	Bn-A10-p4040298	A10	3	0.8	A
10015	Bn-N10-p831013	Bn-A10-p4038884	Bn-A10-p4038884	A10	3	0.8	A
10016	Bn-N10-p884502	Bn-A10-p3980498	Bn-A10-p3980498	A10	3	0.8	A
10017	Bn-N10-p886752	Bn-A10-p3978242	Bn-A10-p3978242	A10	3	0.8	A
10018	Bn-N10-p898703	Bn-A10-p3966740	Bn-A10-p3966740	A10	3	0.8	A
10019	Bn-N10-p912738	Bn-A10-p3955901	Bn-A10-p3955901	A10	4	1.6	A
10020	Bn-N15-p1027942	Bn-A10-p3921433	Bn-A10-p3921433	A10	4	1.6	A
10021	Bn-Scaffold03150-p963	Bn-A10-p3944803	Bn-A10-p3944803	A10	4	1.6	A
10022	Bn-N10-p1004835	Bn-A10-p3825331	Bn-A10-p3825331	A10	5	2.4	A
10023	Bn-N10-p1014379	Bn-C5-p983949	Bn-scaff_16414_1-p103171	A10	5	2.4	A
10024	Bn-N10-p1015618	Bn-A10-p3809773	Bn-A10-p3809773	A10	5	2.4	A
10025	Bn-N10-p1018570	Bn-A10-p3806889	Bn-A10-p3806889	A10	5	2.4	A
10026	Bn-N10-p1082760	Bn-A10-p2852183	Bn-A10-p2852183	A10	5	2.4	A
10027	Bn-N10-p932923	Bn-A10-p3903845	Bn-A10-p3903845	A10	5	2.4	A
10028	Bn-N10-p984144	Bn-A10-p3845546	Bn-A10-p3845546	A10	5	2.4	A
10029	Bn-Scaffold03504-p1128	Bn-A10-p3894990	Bn-A10-p3894990	A10	5	2.4	A
10030	Bn-N10-p1108499	Bn-A10-p2819947	Bn-A10-p2819947	A10	6	3.2	A
10031	Bn-N10-p1125026	Bn-A10-p2805505	Bn-A10-p2805505	A10	6	3.2	A
10032	Bn-N10-p1126191	Bn-A10-p2804340	Bn-A10-p2804340	A10	6	3.2	A
10033	Bn-N10-p1213968	Bn-A10-p2710961	Bn-A10-p2710961	A10	6	3.2	A
10034	Bn-N10-p1361370	Bn-A10-p2557960	Bn-A10-p2557960	A10	7	4	A
10035	Bn-N10-p1364049	Bn-A10-p2554535	Bn-A10-p2554535	A10	7	4	A
10036	Bn-N10-p1367113	Bn-A10-p2545412	Bn-A10-p2545412	A10	7	4	A
10037	Bn-N10-p1373823	Bn-A10-p2536196	Bn-A10-p2536196	A10	7	4	A
10038	Bn-N10-p1379545	Bn-A10-p2535072	Bn-A10-p2535072	A10	7	4	A
10039	Bn-N10-p1388373	Bn-A10-p2529144	Bn-A10-p2529144	A10	7	4	A
10040	Bn-N10-p1384825	Bn-A10-p2532668	Bn-A10-p2532668	A10	8	4.8	A

10041	Bn-N10-p1398053	Bn-A10-p2523110	Bn-A10-p2523110	A10	8	4.8	A
10042	Bn-N10-p1419901	Bn-A10-p2500523	Bn-A10-p2500523	A10	8	4.8	A
10043	Bn-N10-p1420348	Bn-A10-p2500393	Bn-A10-p2500393	A10	8	4.8	A
10044	Bn-N10-p1425342	Bn-A10-p2495652	Bn-A10-p2495652	A10	8	4.8	A
10045	Bn-N10-p1425461	Bn-A10-p2495533	Bn-A10-p2495533	A10	8	4.8	A
10046	Bn-N10-p1432789	Bn-A10-p2489044	Bn-A10-p2489044	A10	8	4.8	A
10047	Bn-N10-p1447254	Bn-A10-p2468805	Bn-A10-p2468805	A10	8	4.8	A
10048	Bn-N10-p1447753	Bn-A10-p2468308	Bn-A10-p2468308	A10	8	4.8	A
10049	Bn-N10-p1450152	Bn-A10-p2465908	Bn-A10-p2465908	A10	8	4.8	A
10050	Bn-N10-p1467040	Bn-A10-p2449120	Bn-A10-p2449120	A10	8	4.8	A
10051	Bn-N10-p1470218	Bn-A10-p2444761	Bn-A10-p2444761	A10	8	4.8	A
10052	Bn-N10-p1470228	Bn-A10-p2444751	Bn-A10-p2444751	A10	8	4.8	A
10053	Bn-N10-p1471073	Bn-A10-p2443900	Bn-A10-p2443900	A10	8	4.8	A
10054	Bn-N10-p1479552	Bn-A10-p2431843	Bn-A10-p2431843	A10	8	4.8	A
10055	Bn-N10-p1496521	Bn-A10-p2416648	Bn-A10-p2416648	A10	8	4.8	A
10056	Bn-N10-p1497520	Bn-A10-p2415609	Bn-A10-p2415609	A10	8	4.8	A
10057	Bn-N10-p1515178	Bn-A10-p2385591	Bn-A10-p2385591	A10	8	4.8	A
10058	Bn-N10-p1526705	Bn-A10-p2374648	Bn-A10-p2374648	A10	8	4.8	A
10059	Bn-N10-p1531709	Bn-A10-p2369622	Bn-A10-p2369622	A10	8	4.8	A
10060	Bn-N10-p1532307	Bn-A10-p2369024	Bn-A10-p2369024	A10	8	4.8	A
10061	Bn-N10-p1535766	Bn-A10-p2357401	Bn-A10-p2357401	A10	8	4.8	A
10062	Bn-N10-p1578075	Bn-A10-p2311797	Bn-A10-p2311797	A10	8	4.8	A
10063	Bn-N10-p1580565	Bn-A10-p2309307	Bn-A10-p2309307	A10	8	4.8	A
10064	Bn-N10-p1594314	Bn-A10-p2295027	Bn-A10-p2295027	A10	8	4.8	A
10065	Bn-N10-p1594492	Bn-A10-p2294849	Bn-A10-p2294849	A10	8	4.8	A
10066	Bn-N10-p1597356	Bn-A10-p2292633	Bn-A10-p2292633	A10	8	4.8	A
10067	Bn-N10-p1607657	Bn-A10-p2268881	Bn-A10-p2268881	A10	8	4.8	A
10068	Bn-N10-p1608071	Bn-A10-p2268467	Bn-A10-p2268467	A10	8	4.8	A
10069	Bn-N10-p1626379	Bn-A10-p2246688	Bn-A10-p2246688	A10	8	4.8	A
10070	Bn-N10-p1644361	Bn-C5-p1587557	Bn-scaff_16414_1-p428111	A10	8	4.8	A
10071	Bn-N10-p1653257	Bn-A10-p2208158	Bn-A10-p2208158	A10	8	4.8	A
10072	Bn-N10-p1674904	Bn-A10-p2196623	Bn-A10-p2196623	A10	8	4.8	A
10073	Bn-N10-p1699311	Bn-A10-p2176252	Bn-A10-p2176252	A10	8	4.8	A
10074	Bn-N10-p1701314	Bn-A10-p2174249	Bn-A10-p2174249	A10	8	4.8	A
10075	Bn-N10-p1701520	Bn-A10-p2174043	Bn-A10-p2174043	A10	8	4.8	A
10076	Bn-N10-p1708569	Bn-A10-p2166959	Bn-A10-p2166959	A10	8	4.8	A
10077	Bn-N10-p1721079	Bn-A10-p2156484	Bn-A10-p2156484	A10	8	4.8	A
10078	Bn-N10-p1723691	Bn-A10-p2153847	Bn-A10-p2153847	A10	8	4.8	A
10079	Bn-N10-p1770475	Bn-A10-p2107673	Bn-A10-p2107673	A10	8	4.8	A
10080	Bn-N10-p1770499	Bn-A10-p2107649	Bn-A10-p2107649	A10	8	4.8	A
10081	Bn-N10-p1786872	Bn-A10-p2086836	Bn-A10-p2086836	A10	8	4.8	A
10082	Bn-N10-p1789509	Bn-A10-p2084182	Bn-A10-p2084182	A10	8	4.8	A
10083	Bn-N10-p1798135	Bn-A10-p2074000	Bn-A10-p2074000	A10	8	4.8	A
10084	Bn-N10-p1827080	Bn-A10-p2048923	Bn-A10-p2048923	A10	8	4.8	A
10085	Bn-N10-p1827250	Bn-A10-p2048753	Bn-A10-p2048753	A10	8	4.8	A
10086	Bn-N10-p1831325	Bn-A10-p2044339	Bn-A10-p2044339	A10	8	4.8	A
10087	Bn-N10-p1968860	Bn-A10-p1920466	Bn-A10-p1920466	A10	8	4.8	0
10088	Bn-N10-p1969996	Bn-A10-p1919293	Bn-A10-p1919293	A10	8	4.8	A
10089	Bn-N10-p1978938	Bn-A10-p1900932	Bn-A10-p1900932	A10	8	4.8	A

10090	Bn-N10-p2123204	Bn-A10-p1791974	Bn-A10-p1791974	A10	9	5.6	A
10091	Bn-N10-p2222171	Bn-A10-p1698746	Bn-A10-p1698746	A10	10	6.4	A
10092	Bn-N10-p2336527	Bn-A10-p1611973	Bn-A10-p1611973	A10	10	6.4	A
10093	Bn-N10-p2370974	Bn-A10-p1582783	Bn-A10-p1582783	A10	10	6.4	A
10094	Bn-N10-p2371138	Bn-A10-p1582619	Bn-A10-p1582619	A10	10	6.4	A
10095	Bn-N10-p2521330	Bn-C5-p2336950	Bn-scaff_20901_1-p18925	A10	11	7.2	A
10096	Bn-N10-p2525099	Bn-A10-p1458849	Bn-A10-p1458849	A10	11	7.2	A
10097	Bn-N10-p2567262	Bn-A10-p1416938	Bn-A10-p1416938	A10	11	7.2	A
10098	Bn-N10-p2569210	Bn-A10-p1414990	Bn-A10-p1414990	A10	11	7.2	A
10099	Bn-N15-p2384036	Bn-Scaffold032460-p	Bn-Scaffold032460-p85	A10	11	7.2	A
10100	Bn-N10-p2450672	Bn-A10-p1513127	Bn-A10-p1513127	A10	12	8	A
10101	Bn-N10-p2631797	Bn-A10-p1345255	Bn-A10-p1345255	A10	12	8	A
10102	Bn-N10-p2667034	Bn-A10-p1318385	Bn-A10-p1318385	A10	12	8	A
10103	Bn-N10-p2672199	Bn-A10-p1313220	Bn-A10-p1313220	A10	12	8	A
10104	Bn-N10-p2686920	Bn-A10-p1273491	Bn-A10-p1273491	A10	12	8	A
10105	Bn-N10-p2759176	Bn-A10-p1215054	Bn-A10-p1215054	A10	12	8	A
10106	Bn-N10-p2759191	Bn-A10-p1215039	Bn-A10-p1215039	A10	12	8	A
10107	Bn-N10-p2786588	Bn-A10-p1196851	Bn-A10-p1196851	A10	12	8	A
10108	Bn-N10-p2788769	Bn-A10-p1194688	Bn-A10-p1194688	A10	12	8	A
10109	Bn-N10-p2795757	Bn-A10-p1187541	Bn-A10-p1187541	A10	12	8	A
10110	Bn-N10-p2811830	Bn-A10-p1172344	Bn-A10-p1172344	A10	12	8	A
10111	Bn-N10-p2819368	Bn-A10-p1162937	Bn-A10-p1162937	A10	12	8	A
10112	Bn-N10-p2819852	Bn-A10-p1162422	Bn-A10-p1162422	A10	12	8	A
10113	Bn-N10-p2820042	Bn-A10-p1161115	Bn-A10-p1161115	A10	12	8	A
10114	Bn-N10-p2833210	Bn-A10-p1153553	Bn-A10-p1153553	A10	12	8	A
10115	Bn-N10-p2837553	Bn-A10-p1149411	Bn-A10-p1149411	A10	12	8	A
10116	Bn-N10-p2957926	Bn-A10-p1041199	Bn-A10-p1041199	A10	12	8	A
10117	Bn-N10-p2958901	Bn-A10-p1040224	Bn-A10-p1040224	A10	12	8	A
10118	Bn-N10-p2994980	Bn-A10-p1010963	Bn-A10-p1010963	A10	12	8	A
10119	Bn-N10-p2995296	Bn-C5-p2652150	Bn-scaff_20901_1-p15773	A10	12	8	A
10120	Bn-N10-p3006747	Bn-A10-p1002178	Bn-A10-p1002178	A10	12	8	A
10121	Bn-N15-p2522639	Bn-C5-p2485950	Bn-scaff_20901_1-p17435	A10	12	8	A
10122	Bn-N10-p3233916	Bn-A10-p758707	Bn-A10-p758707	A10	13	8.8	A
10123	Bn-N10-p3240452	Bn-A10-p754475	Bn-A10-p754475	A10	13	8.8	A
10124	Bn-N10-p3240611	Bn-A10-p754316	Bn-A10-p754316	A10	13	8.8	A
10125	Bn-N10-p3250050	Bn-A10-p750738	Bn-A10-p750738	A10	13	8.8	A
10126	Bn-N10-p3250971	Bn-A10-p749870	Bn-A10-p749870	A10	13	8.8	A
10127	Bn-N10-p3251139	Bn-C6-p37675978	Bn-scaff_18140_1-p53632	A10	13	8.8	A
10128	Bn-N10-p3316169	Bn-A10-p688412	Bn-A10-p688412	A10	13	8.8	A
10129	Bn-N10-p4082185	Bn-Scaffold000123-p	Bn-Scaffold000123-p12263	A10	13	8.8	A
10130	Bn-N10-p4189695	Bn-A10-p395236	Bn-A10-p395236	A10	13	8.8	A
10131	Bn-N10-p4233581	Bn-A10-p320209	Bn-A10-p320209	A10	13	8.8	A
10132	Bn-N10-p4242768	Bn-A10-p306851	Bn-A10-p306851	A10	13	8.8	A
10133	Bn-N10-p4247550	Bn-A10-p302099	Bn-A10-p302099	A10	13	8.8	A
10134	Bn-N10-p4256807	Bn-A10-p292846	Bn-A10-p292846	A10	13	8.8	A
10135	Bn-N10-p4263287	Bn-A10-p286335	Bn-A10-p286335	A10	13	8.8	A
10136	Bn-N10-p4263343	Bn-A10-p286279	Bn-A10-p286279	A10	13	8.8	A
10137	Bn-N10-p4264089	Bn-A10-p285533	Bn-A10-p285533	A10	13	8.8	A
10138	Bn-N10-p4264343	Bn-A10-p285279	Bn-A10-p285279	A10	13	8.8	A

10139	Bn-N10-p4264808	Bn-A10-p284815	Bn-A10-p284815	A10	13	8.8	A
10140	Bn-N10-p4264922	Bn-A10-p284701	Bn-A10-p284701	A10	13	8.8	A
10141	Bn-N10-p4351611	Bn-A10-p191700	Bn-A10-p191700	A10	13	8.8	A
10142	Bn-N10-p4351731	Bn-A10-p191555	Bn-A10-p191555	A10	13	8.8	A
10143	Bn-N10-p4352901	Bn-A10-p190381	Bn-A10-p190381	A10	13	8.8	A
10144	Bn-N10-p4371274	Bn-A10-p175661	Bn-A10-p175661	A10	13	8.8	A
10145	Bn-N10-p4371322	Bn-A10-p175613	Bn-A10-p175613	A10	13	8.8	A
10146	Bn-N10-p4372416	Bn-A10-p174519	Bn-A10-p174519	A10	13	8.8	A
10147	Bn-N10-p4372949	Bn-A10-p173989	Bn-A10-p173989	A10	13	8.8	A
10148	Bn-N10-p4477053	Bn-A10-p119690	Bn-A10-p119690	A10	13	8.8	A
10149	Bn-N10-p4477227	Bn-A10-p119498	Bn-A10-p119498	A10	13	8.8	A
10150	Bn-N10-p4504548	Bn-Scaffold000211-p	Bn-Scaffold000211-p80994	A10	13	8.8	A
10151	Bn-N10-p4506013	Bn-Scaffold000211-p	Bn-Scaffold000211-p79530	A10	13	8.8	A
10152	Bn-N10-p4514941	Bn-Scaffold000211-p	Bn-Scaffold000211-p65635	A10	13	8.8	A
10153	Bn-N10-p4526640	Bn-Scaffold000211-p	Bn-Scaffold000211-p32515	A10	13	8.8	A
10154	Bn-N10-p4572601	Bn-A04-p12639105	Bn-A04-p12639105	A10	13	8.8	A
10155	Bn-N10-p4575735	Bn-A04-p12635986	Bn-A04-p12635986	A10	13	8.8	A
10156	Bn-N10-p4576615	Bn-A04-p12635106	Bn-A04-p12635106	A10	13	8.8	A
10157	Bn-N10-p4576865	Bn-A04-p12634856	Bn-A04-p12634856	A10	13	8.8	A
10158	Bn-N10-p4577391	Bn-A04-p12634330	Bn-A04-p12634330	A10	13	8.8	A
10159	Bn-N10-p4580182	Bn-A04-p12631523	Bn-A04-p12631523	A10	13	8.8	A
10160	Bn-N10-p4607805	Bn-A04-p12600635	Bn-A04-p12600635	A10	13	8.8	A
10161	Bn-N10-p4608596	Bn-A04-p12599843	Bn-A04-p12599843	A10	13	8.8	A
10162	Bn-N10-p4608812	Bn-A04-p12599627	Bn-A04-p12599627	A10	13	8.8	A
10163	Bn-N10-p4625760	Bn-A04-p12575115	Bn-A04-p12575115	A10	13	8.8	A
10164	Bn-N10-p4627837	Bn-A04-p12572164	Bn-A04-p12572164	A10	13	8.8	A
10165	Bn-N10-p4716216	Bn-A04-p12526741	Bn-A04-p12526741	A10	13	8.8	A
10166	Bn-N10-p4838510	Bn-A10-p3649424	Bn-A10-p3649424	A10	13	8.8	A
10167	Bn-N10-p4838640	Bn-A10-p3649554	Bn-A10-p3649554	A10	13	8.8	A
10168	Bn-N10-p4901009	Bn-A10-p3711074	Bn-A10-p3711074	A10	13	8.8	A
10169	Bn-N10-p4931132	Bn-A10-p3725533	Bn-A10-p3725533	A10	13	8.8	A
10170	Bn-N10-p4945826	Bn-A10-p3744140	Bn-A10-p3744140	A10	13	8.8	A
10171	Bn-N10-p4947377	Bn-A10-p3745691	Bn-A10-p3745691	A10	13	8.8	A
10172	Bn-N10-p4947658	Bn-A10-p3745973	Bn-A10-p3745973	A10	13	8.8	A
10173	Bn-N10-p4949266	Bn-A10-p3747583	Bn-A10-p3747583	A10	13	8.8	A
10174	Bn-N10-p5105446	Bn-A01-p19174115	Bn-A01-p19174115	A10	13	8.8	A
10175	Bn-N10-p5108815	Bn-A01-p19170760	Bn-A01-p19170760	A10	13	8.8	A
10176	Bn-N10-p5115427	Bn-A01-p19163275	Bn-A01-p19163275	A10	13	8.8	A
10177	Bn-N10-p5115709	Bn-A01-p19162993	Bn-A01-p19162993	A10	13	8.8	A
10178	Bn-N10-p5120351	Bn-A01-p19158362	Bn-A01-p19158362	A10	13	8.8	A
10179	Bn-N10-p5120442	Bn-A01-p19158271	Bn-A01-p19158271	A10	13	8.8	A
10180	Bn-N10-p5122536	Bn-A01-p19156177	Bn-A01-p19156177	A10	13	8.8	A
10181	Bn-N10-p5164097	Bn-A01-p19125668	Bn-A01-p19125668	A10	13	8.8	A
10182	Bn-N10-p5167876	Bn-A01-p19122048	Bn-A01-p19122048	A10	13	8.8	A
10183	Bn-N10-p5173194	Bn-A01-p19116819	Bn-A01-p19116819	A10	13	8.8	A
10184	Bn-N10-p5174007	Bn-A01-p19116006	Bn-A01-p19116006	A10	13	8.8	A
10185	Bn-N10-p5174141	Bn-A01-p19115872	Bn-A01-p19115872	A10	13	8.8	A
10186	Bn-N10-p5174483	Bn-A01-p19115530	Bn-A01-p19115530	A10	13	8.8	A
10187	Bn-N10-p5174992	Bn-A01-p19115021	Bn-A01-p19115021	A10	13	8.8	A

10188	Bn-N10-p5175044	Bn-A01-p19114969	Bn-A01-p19114969	A10	13	8.8	A
10189	Bn-N10-p5178700	Bn-A01-p19111305	Bn-A01-p19111305	A10	13	8.8	A
10190	Bn-N10-p5178759	Bn-A01-p19111246	Bn-A01-p19111246	A10	13	8.8	A
10191	Bn-N10-p5179497	Bn-A01-p19110508	Bn-A01-p19110508	A10	13	8.8	A
10192	Bn-N10-p5179621	Bn-A01-p19110384	Bn-A01-p19110384	A10	13	8.8	A
10193	Bn-N10-p5181156	Bn-A01-p19108849	Bn-A01-p19108849	A10	13	8.8	A
10194	Bn-N10-p5181262	Bn-A01-p19108743	Bn-A01-p19108743	A10	13	8.8	A
10195	Bn-N10-p5181566	Bn-A01-p19108439	Bn-A01-p19108439	A10	13	8.8	A
10196	Bn-N10-p5182232	Bn-A01-p19107773	Bn-A01-p19107773	A10	13	8.8	A
10197	Bn-N10-p5182806	Bn-A01-p19107198	Bn-A01-p19107198	A10	13	8.8	A
10198	Bn-N10-p5183002	Bn-A01-p19107003	Bn-A01-p19107003	A10	13	8.8	A
10199	Bn-N10-p5191096	Bn-A01-p19098776	Bn-A01-p19098776	A10	13	8.8	A
10200	Bn-N10-p5191658	Bn-A01-p19098195	Bn-A01-p19098195	A10	13	8.8	A
10201	Bn-N10-p5405133	Bn-A01-p18902391	Bn-A01-p18902391	A10	13	8.8	A
10202	Bn-N10-p5408243	Bn-A01-p18896245	Bn-A01-p18896245	A10	13	8.8	A
10203	Bn-N10-p5409861	Bn-A01-p18894627	Bn-A01-p18894627	A10	13	8.8	A
10204	Bn-N10-p5414239	Bn-A01-p18890238	Bn-A01-p18890238	A10	13	8.8	A
10205	Bn-N10-p5422236	Bn-A01-p18882626	Bn-A01-p18882626	A10	13	8.8	A
10206	Bn-N10-p5423154	Bn-A01-p18881723	Bn-A01-p18881723	A10	13	8.8	A
10207	Bn-N10-p5429018	Bn-A01-p18876940	Bn-A01-p18876940	A10	13	8.8	A
10208	Bn-N10-p5435069	Bn-A01-p18870174	Bn-A01-p18870174	A10	13	8.8	A
10209	Bn-N10-p5455380	Bn-A01-p18850969	Bn-A01-p18850969	A10	13	8.8	A
10210	Bn-N10-p5566002	Bn-A01-p18739675	Bn-A01-p18739675	A10	13	8.8	A
10211	Bn-N10-p5603551	Bn-A01-p18711867	Bn-A01-p18711867	A10	13	8.8	A
10212	Bn-N10-p5610557	Bn-A01-p18704834	Bn-A01-p18704834	A10	13	8.8	A
10213	Bn-N10-p5611186	Bn-A01-p18704205	Bn-A01-p18704205	A10	13	8.8	A
10214	Bn-N10-p5612208	Bn-A01-p18703183	Bn-A01-p18703183	A10	13	8.8	A
10215	Bn-N10-p5621947	Bn-A01-p18692686	Bn-A01-p18692686	A10	13	8.8	A
10216	Bn-N10-p5677577	Bn-A01-p18636804	Bn-A01-p18636804	A10	13	8.8	A
10217	Bn-N10-p5719885	Bn-A10-p4935553	Bn-A10-p4935553	A10	13	8.8	A
10218	Bn-N10-p5727893	Bn-A10-p4950011	Bn-A10-p4950011	A10	13	8.8	A
10219	Bn-N10-p5745295	Bn-A10-p4966927	Bn-A10-p4966927	A10	13	8.8	A
10220	Bn-N10-p5817452	Bn-A10-p5040430	Bn-A10-p5040430	A10	13	8.8	A
10221	Bn-N10-p6006657	Bn-C9-p34982708	Bn-scaff_20619_1-p21059	A10	13	8.8	A
10222	Bn-N10-p6693573	Bn-A10-p4871099	Bn-A10-p4871099	A10	13	8.8	A
10223	Bn-N10-p6891775	Bn-A10-p84343	Bn-A10-p84343	A10	13	8.8	A
10224	Bn-N10-p7320434	Bn-Scaffold000296-p	Bn-Scaffold000296-p13776	A10	13	8.8	A
10225	Bn-N10-p7322138	Bn-Scaffold000296-p	Bn-Scaffold000296-p12138	A10	13	8.8	A
10226	Bn-N10-p7322764	Bn-Scaffold000296-p	Bn-Scaffold000296-p11526	A10	13	8.8	A
10227	Bn-N10-p8427848	Bn-A10-p6851375	Bn-A10-p6851375	A10	13	8.8	A
10228	Bn-N11-p21945402	Bn-Scaffold000123-p	Bn-Scaffold000123-p13487	A10	13	8.8	A
10229	Bn-N12-p32991271	Bn-A10-p64365	Bn-A10-p64365	A10	13	8.8	A
10230	Bn-N12-p32993630	Bn-A10-p62077	Bn-A10-p62077	A10	13	8.8	A
10231	Bn-N12-p32998662	Bn-A10-p89338	Bn-A10-p89338	A10	13	8.8	A
10232	Bn-N14-p16012148	Bn-A06-p12078648	Bn-A06-p12078648	A10	13	8.8	A
10233	Bn-N14-p31864314	Bn-Scaffold000296-p	Bn-Scaffold000296-p9362	A10	13	8.8	A
10234	Bn-N15-p25653509	Bn-A08-p3907214	Bn-A08-p3907214	A10	13	8.8	A
10235	Bn-N15-p26030363	Bn-A10-p73025	Bn-A10-p73025	A10	13	8.8	A
10236	Bn-N15-p27775038	Bn-Scaffold000286-p	Bn-Scaffold000286-p27367	A10	13	8.8	A

10237	Bn-N15-p36079405	Bn-Scaffold000286-p1	Bn-Scaffold000286-p19036	A10	13	8.8	A
10238	Bn-N16-p934994	Bn-Scaffold000296-p7	Bn-Scaffold000296-p7640	A10	13	8.8	A
10239	Bn-N17-p45148395	Bn-Scaffold01079-p1	Bn-scaff_26033_1-p11857	A10	13	8.8	A
10240	Bn-N18-p5758358	Bn-Scaffold000286-p1	Bn-Scaffold000286-p17553	A10	13	8.8	A
10241	Bn-N19-p18840612	Bn-A08-p3883962	Bn-A08-p3883962	A10	13	8.8	A
10242	Bn-N19-p2815272	Bn-Scaffold000286-p1	Bn-Scaffold000286-p19675	A10	13	8.8	A
10243	Bn-N19-p31827576	Bn-A10-p320201	Bn-A10-p320201	A10	13	8.8	A
10244	Bn-N19-p32814862	Bn-A10-p58959	Bn-A10-p58959	A10	13	8.8	A
10245	Bn-N19-p32815092	Bn-A10-p58713	Bn-A10-p58713	A10	13	8.8	A
10246	Bn-N19-p33702564	Bn-Scaffold000465-p5	Bn-Scaffold000465-p5464	A10	13	8.8	A
10247	Bn-N19-p33702727	Bn-Scaffold000465-p5	Bn-Scaffold000465-p5300	A10	13	8.8	A
10248	Bn-N19-p34974217	Bn-C9-p33193100	Bn-scaff_16619_1-p22907	A10	13	8.8	A
10249	Bn-N2-p27875310	Bn-A06-p22784485	Bn-A06-p22784485	A10	13	8.8	A
10250	Bn-N2-p7913103	Bn-Scaffold000286-p1	Bn-Scaffold000286-p17668	A10	13	8.8	A
10251	Bn-N2-p7913198	Bn-Scaffold000286-p1	Bn-Scaffold000286-p17572	A10	13	8.8	A
10252	Bn-N3-p18799870	Bn-Scaffold000286-p3	Bn-Scaffold000286-p31161	A10	13	8.8	A
10253	Bn-N3-p32358473	Bn-Scaffold000296-p7	Bn-Scaffold000296-p7135	A10	13	8.8	A
10254	Bn-N5-p12497761	Bn-A10-p73255	Bn-A10-p73255	A10	13	8.8	A
10255	Bn-N6-p16240705	Bn-A10-p68823	Bn-A10-p68823	A10	13	8.8	A
10256	Bn-N7-p3097784	Bn-Scaffold000286-p2	Bn-Scaffold000286-p20113	A10	13	8.8	A
10257	Bn-N7-p4182888	Bn-A10-p65275	Bn-A10-p65275	A10	13	8.8	A
10258	Bn-N8-p12940453	Bn-A08-p13270793	Bn-A08-p13270793	A10	13	8.8	A
10259	Bn-N9-p27720056	Bn-Scaffold000123-p1	Bn-Scaffold000123-p13334	A10	13	8.8	A
10260	Bn-N9-p28303189	Bn-Scaffold000286-p3	Bn-Scaffold000286-p31072	A10	13	8.8	A
10261	Bn-Scaffold00637-p105943	Bn-Scaffold000560-p4	Bn-Scaffold000560-p4391	A10	13	8.8	A
10262	Bn-Scaffold00637-p106177	Bn-Scaffold000560-p4	Bn-Scaffold000560-p4157	A10	13	8.8	A
10263	Bn-Scaffold00637-p106336	Bn-Scaffold000560-p4	Bn-Scaffold000560-p4001	A10	13	8.8	A
10264	Bn-Scaffold00637-p106660	Bn-Scaffold000560-p3	Bn-Scaffold000560-p3677	A10	13	8.8	A
10265	Bn-Scaffold00637-p108286	Bn-Scaffold000560-p2	Bn-Scaffold000560-p2232	A10	13	8.8	A
10266	Bn-Scaffold00637-p108401	Bn-Scaffold000560-p2	Bn-Scaffold000560-p2117	A10	13	8.8	A
10267	Bn-Scaffold00637-p108504	Bn-Scaffold000560-p2	Bn-Scaffold000560-p2014	A10	13	8.8	A
10268	Bn-Scaffold00637-p132659	Bn-A08-p3926038	Bn-A08-p3926038	A10	13	8.8	A
10269	Bn-Scaffold00637-p133912	Bn-A08-p3924770	Bn-A08-p3924770	A10	13	8.8	A
10270	Bn-Scaffold00637-p138781	Bn-A08-p3919702	Bn-A08-p3919702	A10	13	8.8	A
10271	Bn-Scaffold00637-p1399	Bn-A10-p110116	Bn-A10-p110116	A10	13	8.8	A
10272	Bn-Scaffold00637-p157321	Bn-A08-p3894164	Bn-A08-p3894164	A10	13	8.8	A
10273	Bn-Scaffold00637-p161366	Bn-A08-p3881526	Bn-A08-p3881526	A10	13	8.8	A
10274	Bn-Scaffold00637-p161554	Bn-A08-p3881337	Bn-A08-p3881337	A10	13	8.8	A
10275	Bn-Scaffold00637-p161830	Bn-A08-p3881061	Bn-A08-p3881061	A10	13	8.8	A
10276	Bn-Scaffold00637-p209964	Bn-Scaffold000465-p8	Bn-Scaffold000465-p8854	A10	13	8.8	A
10277	Bn-Scaffold00637-p61	Bn-A10-p112852	Bn-A10-p112852	A10	13	8.8	A
10278	Bn-Scaffold00637-p87295	Bn-A10-p11422	Bn-A10-p11422	A10	13	8.8	A
10279	Bn-Scaffold00637-p88558	Bn-A10-p8941	Bn-A10-p8941	A10	13	8.8	A
10280	Bn-Scaffold01163-p22177	Bn-Scaffold000286-p2	Bn-Scaffold000286-p20880	A10	13	8.8	A
10281	Bn-Scaffold01179-p21888	Bn-Scaffold000296-p1	Bn-Scaffold000296-p16696	A10	13	8.8	A
10282	Bn-Scaffold02078-p3897	Bn-A01-p18715909	Bn-A01-p18715909	A10	13	8.8	A
10283	Bn-Scaffold04847-p1225	Bn-Scaffold000296-p2	Bn-Scaffold000296-p20578	A10	13	8.8	A
10284	Bn-Scaffold28109-p102	Bn-Scaffold000286-p2	Bn-Scaffold000286-p20329	A10	13	8.8	A
10285	Bn-Scaffold28109-p69	Bn-Scaffold000286-p2	Bn-Scaffold000286-p20362	A10	13	8.8	A

10286	Unknown	Bn-Scaffold000123-p1	Bn-Scaffold000123-p13371	A10	13	8.8	A
10287	Unknown	Bn-Scaffold000123-p1	Bn-Scaffold000123-p13498	A10	13	8.8	A
10288	Bn-N10-p6132376	Bn-A10-p5317804	Bn-A10-p5317804	A10	14	9.6	A
10289	Bn-N10-p6371122	Bn-A10-p5525609	Bn-A10-p5525609	A10	14	9.6	A
10290	Bn-N10-p6372119	Bn-A10-p5532832	Bn-A10-p5532832	A10	14	9.6	A
10291	Bn-N10-p7152115	Bn-A10-p5604463	Bn-A10-p5604463	A10	14	9.6	A
10292	Bn-N10-p7153825	Bn-A10-p5606181	Bn-A10-p5606181	A10	14	9.6	A
10293	Bn-N10-p7217768	Bn-A10-p5662796	Bn-A10-p5662796	A10	14	9.6	A
10294	Bn-N10-p7221687	Bn-A10-p5666711	Bn-A10-p5666711	A10	14	9.6	A
10295	Bn-N10-p7225922	Bn-A10-p5670945	Bn-A10-p5670945	A10	14	9.6	A
10296	Bn-N10-p7228172	Bn-A10-p5673195	Bn-A10-p5673195	A10	14	9.6	A
10297	Bn-N10-p7228565	Bn-A10-p5673588	Bn-A10-p5673588	A10	14	9.6	A
10298	Bn-N10-p7235080	Bn-A10-p5679914	Bn-A10-p5679914	A10	14	9.6	A
10299	Bn-N10-p7235844	Bn-A10-p5680678	Bn-A10-p5680678	A10	14	9.6	A
10300	Bn-N10-p7239240	Bn-A10-p5684074	Bn-A10-p5684074	A10	14	9.6	A
10301	Bn-N10-p7341628	Bn-A10-p5778609	Bn-A10-p5778609	A10	14	9.6	A
10302	Bn-N10-p7344842	Bn-A10-p5781113	Bn-A10-p5781113	A10	14	9.6	A
10303	Bn-N10-p7364351	Bn-C9-p36256859	Bn-scaff_17367_1-p86610	A10	14	9.6	A
10304	Bn-N10-p7382826	Bn-A10-p5819027	Bn-A10-p5819027	A10	14	9.6	A
10305	Bn-N10-p7396078	Bn-A10-p5831782	Bn-A10-p5831782	A10	14	9.6	A
10306	Bn-N10-p7396938	Bn-A10-p5832645	Bn-A10-p5832645	A10	14	9.6	A
10307	Bn-N10-p7430860	Bn-A10-p5869175	Bn-A10-p5869175	A10	14	9.6	A
10308	Bn-N10-p7435086	Bn-A10-p5873429	Bn-A10-p5873429	A10	14	9.6	0
10309	Bn-N10-p7453775	Bn-A10-p5888302	Bn-A10-p5888302	A10	14	9.6	A
10310	Bn-N10-p7769600	Bn-A10-p6166631	Bn-A10-p6166631	A10	14	9.6	A
10311	Bn-N10-p7779320	Bn-A10-p6176593	Bn-A10-p6176593	A10	14	9.6	A
10312	Bn-N10-p7780378	Bn-A10-p6177431	Bn-A10-p6177431	A10	14	9.6	A
10313	Bn-N10-p7783581	Bn-A10-p6180693	Bn-A10-p6180693	A10	14	9.6	A
10314	Bn-N10-p7796722	Bn-A10-p6192971	Bn-A10-p6192971	A10	14	9.6	A
10315	Bn-N10-p7802731	Bn-A10-p6198988	Bn-A10-p6198988	A10	14	9.6	A
10316	Bn-N10-p7808581	Bn-A10-p6204788	Bn-A10-p6204788	A10	14	9.6	A
10317	Bn-N10-p7880985	Bn-A10-p6271467	Bn-A10-p6271467	A10	14	9.6	A
10318	Bn-N10-p7881072	Bn-A10-p6271554	Bn-A10-p6271554	A10	14	9.6	A
10319	Bn-N10-p7881288	Bn-A10-p6271776	Bn-A10-p6271776	A10	14	9.6	A
10320	Bn-N10-p7929103	Bn-A10-p6322788	Bn-A10-p6322788	A10	14	9.6	A
10321	Bn-N10-p7929842	Bn-A10-p6323527	Bn-A10-p6323527	A10	14	9.6	A
10322	Bn-N10-p7931165	Bn-A10-p6324850	Bn-A10-p6324850	A10	14	9.6	A
10323	Bn-N10-p7956059	Bn-A10-p6344414	Bn-A10-p6344414	A10	14	9.6	A
10324	Bn-N10-p7982887	Bn-A10-p6364237	Bn-A10-p6364237	A10	14	9.6	A
10325	Bn-N10-p7989875	Bn-A10-p6371190	Bn-A10-p6371190	A10	14	9.6	A
10326	Bn-N10-p8026286	Bn-A10-p6405472	Bn-A10-p6405472	A10	14	9.6	A
10327	Bn-N10-p8033876	Bn-A10-p6412022	Bn-A10-p6412022	A10	14	9.6	A
10328	Bn-N10-p8089061	Bn-A10-p6468609	Bn-A10-p6468609	A10	14	9.6	A
10329	Bn-N10-p8129526	Bn-A10-p6540548	Bn-A10-p6540548	A10	14	9.6	A
10330	Bn-N10-p8130396	Bn-A10-p6541418	Bn-A10-p6541418	A10	14	9.6	A
10331	Bn-N10-p8149171	Bn-A10-p6555288	Bn-A10-p6555288	A10	14	9.6	A
10332	Bn-N10-p8152524	Bn-A10-p6558564	Bn-A10-p6558564	A10	14	9.6	A
10333	Bn-N10-p8274092	Bn-A10-p6671842	Bn-A10-p6671842	A10	14	9.6	A
10334	Bn-N10-p8361662	Bn-A10-p6760255	Bn-A10-p6760255	A10	14	9.6	A

10335	Bn-N10-p8362311	Bn-A10-p6760904	Bn-A10-p6760904	A10	14	9.6	A
10336	Bn-N10-p8367620	Bn-A10-p6765523	Bn-A10-p6765523	A10	14	9.6	A
10337	Bn-N10-p8395757	Bn-A10-p6796120	Bn-A10-p6796120	A10	14	9.6	A
10338	Bn-N10-p8410308	Bn-A10-p6838885	Bn-A10-p6838885	A10	14	9.6	A
10339	Bn-N10-p8410591	Bn-C9-p38256586	Bn-scaff_16456_1-p57033	A10	14	9.6	A
10340	Bn-N10-p8472459	Bn-A10-p6896063	Bn-A10-p6896063	A10	14	9.6	A
10341	Bn-N10-p8633594	Bn-A10-p7065016	Bn-A10-p7065016	A10	14	9.6	A
10342	Bn-N10-p8638254	Bn-A10-p7069659	Bn-A10-p7069659	A10	14	9.6	A
10343	Bn-N10-p8674036	Bn-A10-p7109137	Bn-A10-p7109137	A10	14	9.6	A
10344	Bn-N10-p8753600	Bn-A10-p7182541	Bn-A10-p7182541	A10	14	9.6	A
10345	Bn-N10-p8756392	Bn-C9-p39048669	Bn-scaff_16042_1-p98299	A10	14	9.6	A
10346	Bn-N10-p8794663	Bn-A10-p7231726	Bn-A10-p7231726	A10	14	9.6	A
10347	Bn-N10-p8810546	Bn-A10-p7247322	Bn-A10-p7247322	A10	14	9.6	A
10348	Bn-N10-p8818594	Bn-A10-p7250703	Bn-A10-p7250703	A10	14	9.6	A
10349	Bn-N10-p8820235	Bn-A10-p7252424	Bn-A10-p7252424	A10	14	9.6	A
10350	Bn-N10-p8870416	Bn-C9-p39281687	Bn-scaff_16042_1-p33131	A10	14	9.6	A
10351	Bn-N10-p8881369	Bn-A10-p7326339	Bn-A10-p7326339	A10	14	9.6	A
10352	Bn-N10-p8924295	Bn-A10-p7349925	Bn-A10-p7349925	A10	14	9.6	A
10353	Bn-N10-p8940805	Bn-A10-p7360229	Bn-A10-p7360229	A10	14	9.6	A
10354	Bn-N10-p8951417	Bn-A10-p7376910	Bn-A10-p7376910	A10	14	9.6	A
10355	Bn-N10-p8969857	Bn-A10-p7393471	Bn-A10-p7393471	A10	14	9.6	A
10356	Bn-N10-p8970018	Bn-A10-p7393632	Bn-A10-p7393632	A10	14	9.6	A
10357	Bn-N10-p8970618	Bn-A10-p7394180	Bn-A10-p7394180	A10	14	9.6	A
10358	Bn-N10-p8971040	Bn-A10-p7394578	Bn-A10-p7394578	A10	14	9.6	A
10359	Bn-N10-p8986806	Bn-A10-p7410271	Bn-A10-p7410271	A10	14	9.6	A
10360	Bn-N10-p8989526	Bn-Scaffold000516-p	Bn-Scaffold000516-p949	A10	14	9.6	A
10361	Bn-N10-p9054105	Bn-A10-p7492957	Bn-A10-p7492957	A10	14	9.6	A
10362	Bn-N10-p9061322	Bn-A10-p7500176	Bn-A10-p7500176	A10	14	9.6	A
10363	Bn-N10-p9063787	Bn-A10-p7502551	Bn-A10-p7502551	A10	14	9.6	A
10364	Bn-N10-p9064197	Bn-A10-p7502935	Bn-A10-p7502935	A10	14	9.6	A
10365	Bn-N10-p9071873	Bn-A10-p7510542	Bn-A10-p7510542	A10	14	9.6	A
10366	Bn-N10-p9074740	Bn-A10-p7513409	Bn-A10-p7513409	A10	14	9.6	A
10367	Bn-N10-p9122739	Bn-A10-p7554677	Bn-A10-p7554677	A10	14	9.6	A
10368	Bn-N10-p9130002	Bn-A10-p7560857	Bn-A10-p7560857	A10	14	9.6	A
10369	Bn-N10-p9178214	Bn-A10-p7611277	Bn-A10-p7611277	A10	14	9.6	A
10370	Bn-N10-p9281717	Bn-A10-p7706894	Bn-A10-p7706894	A10	14	9.6	A
10371	Bn-N10-p9299579	Bn-A10-p7720931	Bn-A10-p7720931	A10	14	9.6	A
10372	Bn-N10-p9341147	Bn-A10-p7757976	Bn-A10-p7757976	A10	14	9.6	A
10373	Bn-N10-p9343697	Bn-A10-p7760481	Bn-A10-p7760481	A10	14	9.6	A
10374	Bn-N10-p9352782	Bn-A10-p7767853	Bn-A10-p7767853	A10	14	9.6	A
10375	Bn-N10-p9354689	Bn-A10-p7769785	Bn-A10-p7769785	A10	14	9.6	A
10376	Bn-N10-p9358717	Bn-A10-p7772340	Bn-A10-p7772340	A10	14	9.6	A
10377	Bn-N10-p9367093	Bn-A10-p7774373	Bn-A10-p7774373	A10	14	9.6	A
10378	Bn-N10-p9373679	Bn-A10-p7790953	Bn-A10-p7790953	A10	14	9.6	A
10379	Bn-N10-p9374265	Bn-A10-p7791536	Bn-A10-p7791536	A10	14	9.6	A
10380	Bn-N10-p9375798	Bn-A10-p7793015	Bn-A10-p7793015	A10	14	9.6	A
10381	Bn-N10-p9377313	Bn-A10-p7795745	Bn-A10-p7795745	A10	14	9.6	A
10382	Bn-N10-p9377475	Bn-A10-p7795907	Bn-A10-p7795907	A10	14	9.6	A
10383	Bn-N10-p9396668	Bn-A10-p7802218	Bn-A10-p7802218	A10	14	9.6	A

10384	Bn-N10-p9403733	Bn-A10-p7809041	Bn-A10-p7809041	A10	14	9.6	A
10385	Bn-N10-p9409040	Bn-A10-p7814196	Bn-A10-p7814196	A10	14	9.6	A
10386	Bn-N10-p9409116	Bn-A10-p7814272	Bn-A10-p7814272	A10	14	9.6	A
10387	Bn-N10-p9420518	Bn-A10-p7824378	Bn-A10-p7824378	A10	14	9.6	A
10388	Bn-N10-p9431923	Bn-A10-p7835472	Bn-A10-p7835472	A10	14	9.6	A
10389	Bn-N10-p9465140	Bn-A10-p7867506	Bn-A10-p7867506	A10	14	9.6	A
10390	Bn-N12-p30401317	Bn-A10-p6770590	Bn-A10-p6770590	A10	14	9.6	A
10391	Bn-N19-p37510536	Bn-C9-p35951476	Bn-scaff_17367_1-p56072(	A10	14	9.6	A
10392	Bn-N19-p38934920	Bn-A10-p6332974	Bn-A10-p6332974	A10	14	9.6	A
10393	Bn-N19-p39805148	Bn-C9-p37879111	Bn-scaff_17919_1-p12901	A10	14	9.6	A
10394	Bn-N19-p42197153	Bn-A10-p7805981	Bn-A10-p7805981	A10	14	9.6	A
10395	Bn-N4-p1379161	Bn-A10-p7787682	Bn-A10-p7787682	A10	14	9.6	A
10396	Bn-N10-p9510368	Bn-A10-p7904737	Bn-A10-p7904737	A10	15	10.4	A
10397	Bn-N10-p9510690	Bn-A10-p7905059	Bn-A10-p7905059	A10	15	10.4	0
10398	Bn-N10-p9511912	Bn-A10-p7906281	Bn-A10-p7906281	A10	15	10.4	A
10399	Bn-N10-p9518968	Bn-A10-p7913022	Bn-A10-p7913022	A10	15	10.4	A
10400	Bn-N10-p9555515	Bn-A10-p7946574	Bn-A10-p7946574	A10	15	10.4	A
10401	Bn-N10-p9579052	Bn-A10-p7976115	Bn-A10-p7976115	A10	15	10.4	A
10402	Bn-N10-p9665194	Bn-A10-p8036516	Bn-A10-p8036516	A10	15	10.4	A
10403	Bn-N10-p9665336	Bn-A10-p8036658	Bn-A10-p8036658	A10	15	10.4	A
10404	Bn-N10-p9679248	Bn-A10-p8044126	Bn-A10-p8044126	A10	15	10.4	A
10405	Bn-N10-p9679310	Bn-A10-p8044188	Bn-A10-p8044188	A10	15	10.4	A
10406	Bn-N10-p9687334	Bn-A10-p8049073	Bn-A10-p8049073	A10	15	10.4	A
10407	Bn-N10-p9697812	Bn-A10-p8063505	Bn-A10-p8063505	A10	15	10.4	A
10408	Bn-N10-p9703670	Bn-A10-p8070229	Bn-A10-p8070229	A10	15	10.4	A
10409	Bn-N10-p9718949	Bn-A10-p8083623	Bn-A10-p8083623	A10	15	10.4	A
10410	Bn-N10-p9720896	Bn-A10-p8087617	Bn-A10-p8087617	A10	15	10.4	A
10411	Bn-N10-p9728515	Bn-A10-p8093150	Bn-A10-p8093150	A10	15	10.4	A
10412	Bn-N10-p9735208	Bn-A10-p8104368	Bn-A10-p8104368	A10	15	10.4	A
10413	Bn-N10-p9735319	Bn-A10-p8104479	Bn-A10-p8104479	A10	15	10.4	A
10414	Bn-N10-p9738343	Bn-A10-p8106826	Bn-A10-p8106826	A10	15	10.4	A
10415	Bn-N10-p9740322	Bn-A10-p8108242	Bn-A10-p8108242	A10	15	10.4	A
10416	Bn-N10-p9748204	Bn-A10-p8125136	Bn-A10-p8125136	A10	15	10.4	A
10417	Bn-N10-p9752948	Bn-A10-p8129578	Bn-A10-p8129578	A10	15	10.4	A
10418	Bn-N19-p42508285	Bn-A10-p7975206	Bn-A10-p7975206	A10	15	10.4	A
10419	Bn-N10-p10020007	Bn-A10-p8419569	Bn-A10-p8419569	A10	16	11.2	A
10420	Bn-N10-p10024667	Bn-C9-p41482416	Bn-scaff_15808_1-p33708‹	A10	16	11.2	A
10421	Bn-N10-p10055397	Bn-A10-p8441474	Bn-A10-p8441474	A10	16	11.2	A
10422	Bn-N10-p10057518	Bn-A10-p8443505	Bn-A10-p8443505	A10	16	11.2	A
10423	Bn-N10-p10066242	Bn-A10-p8452135	Bn-A10-p8452135	A10	16	11.2	A
10424	Bn-N10-p10070492	Bn-A10-p8456410	Bn-A10-p8456410	A10	16	11.2	A
10425	Bn-N10-p10079641	Bn-A10-p8465363	Bn-A10-p8465363	A10	16	11.2	A
10426	Bn-N10-p10080669	Bn-A10-p8466391	Bn-A10-p8466391	A10	16	11.2	A
10427	Bn-N10-p10080685	Bn-A10-p8466407	Bn-A10-p8466407	A10	16	11.2	A
10428	Bn-N10-p10089831	Bn-A10-p8475601	Bn-A10-p8475601	A10	16	11.2	A
10429	Bn-N10-p10121072	Bn-C9-p41664555	Bn-scaff_15808_1-p51922:	A10	16	11.2	A
10430	Bn-N10-p10121257	Bn-A10-p8503752	Bn-A10-p8503752	A10	16	11.2	A
10431	Bn-N10-p10131155	Bn-A10-p8518310	Bn-A10-p8518310	A10	16	11.2	0
10432	Bn-N10-p10148935	Bn-A10-p4332413	Bn-A10-p4332413	A10	16	11.2	A

10433	Bn-N10-p10181576	Bn-A10-p8553366	Bn-A10-p8553366	A10	16	11.2	A
10434	Bn-N10-p10187618	Bn-A10-p8559413	Bn-A10-p8559413	A10	16	11.2	A
10435	Bn-N10-p10192915	Bn-A10-p8564698	Bn-A10-p8564698	A10	16	11.2	A
10436	Bn-N10-p10219233	Bn-A10-p8583543	Bn-A10-p8583543	A10	16	11.2	A
10437	Bn-N10-p10220535	Bn-A10-p8584829	Bn-A10-p8584829	A10	16	11.2	A
10438	Bn-N10-p10222721	Bn-A10-p8587028	Bn-A10-p8587028	A10	16	11.2	A
10439	Bn-N10-p10224379	Bn-A10-p8589353	Bn-A10-p8589353	A10	16	11.2	A
10440	Bn-N10-p10236420	Bn-A10-p8608729	Bn-A10-p8608729	A10	16	11.2	A
10441	Bn-N10-p10246201	Bn-A10-p8620702	Bn-A10-p8620702	A10	16	11.2	A
10442	Bn-N10-p10247735	Bn-A10-p8622237	Bn-A10-p8622237	A10	16	11.2	A
10443	Bn-N10-p9814537	Bn-A10-p8202766	Bn-A10-p8202766	A10	16	11.2	A
10444	Bn-N10-p9857551	Bn-A10-p8257748	Bn-A10-p8257748	A10	16	11.2	A
10445	Bn-N10-p9880744	Bn-A10-p8290065	Bn-A10-p8290065	A10	16	11.2	A
10446	Bn-N10-p9881123	Bn-A10-p8290448	Bn-A10-p8290448	A10	16	11.2	A
10447	Bn-N10-p9901618	Bn-A10-p8308769	Bn-A10-p8308769	A10	16	11.2	A
10448	Bn-N10-p9925071	Bn-A10-p8331925	Bn-A10-p8331925	A10	16	11.2	A
10449	Bn-N10-p9934261	Bn-A10-p8340529	Bn-A10-p8340529	A10	16	11.2	A
10450	Bn-N10-p9952651	Bn-A10-p8359538	Bn-A10-p8359538	A10	16	11.2	A
10451	Bn-N10-p9952788	Bn-A10-p8359675	Bn-A10-p8359675	A10	16	11.2	A
10452	Bn-N10-p9953103	Bn-A10-p8359990	Bn-A10-p8359990	A10	16	11.2	A
10453	Bn-N10-p9992962	Bn-A10-p8398873	Bn-A10-p8398873	A10	16	11.2	A
10454	Bn-N14-p47135656	Bn-C4-p44950306	Bn-scaff_16804_1-p72250(	A10	16	11.2	A
10455	Bn-N14-p47136893	Bn-C4-p44951543	Bn-scaff_16804_1-p72373	A10	16	11.2	A
10456	Bn-N19-p43117189	Bn-A10-p8285049	Bn-A10-p8285049	A10	16	11.2	A
10457	Bn-N19-p43212085	Bn-A10-p8309983	Bn-A10-p8309983	A10	16	11.2	A
10458	Bn-N19-p43213003	Bn-C9-p41195651	Bn-scaff_15808_1-p50319	A10	16	11.2	A
10459	Bn-N19-p43251724	Bn-C9-p41237723	Bn-scaff_15808_1-p92391	A10	16	11.2	A
10460	Bn-N19-p43496389	Bn-C9-p41482784	Bn-scaff_15808_1-p33745	A10	16	11.2	A
10461	Bn-N19-p43560319	Bn-A10-p8444450	Bn-A10-p8444450	A10	16	11.2	A
10462	Bn-Scaffold04795-p231	Bn-A10-p8544988	Bn-A10-p8544988	A10	16	11.2	0
10463	Bn-Scaffold30630-p351	Bn-C9-p41176590	Bn-scaff_15808_1-p31258	A10	16	11.2	A
10464	Bn-N10-p10343369	Bn-A10-p8722713	Bn-A10-p8722713	A10	17	12	A
10465	Bn-N10-p10343794	Bn-A10-p8723171	Bn-A10-p8723171	A10	17	12	A
10466	Bn-N10-p10361952	Bn-A10-p8733734	Bn-A10-p8733734	A10	17	12	A
10467	Bn-N10-p10371085	Bn-A10-p8741886	Bn-A10-p8741886	A10	17	12	A
10468	Bn-N10-p10374851	Bn-A10-p8743143	Bn-A10-p8743143	A10	17	12	0
10469	Bn-N10-p10375902	Bn-A10-p8744180	Bn-A10-p8744180	A10	17	12	A
10470	Bn-N10-p10377573	Bn-A10-p8745850	Bn-A10-p8745850	A10	17	12	A
10471	Bn-N10-p10378785	Bn-A10-p8747062	Bn-A10-p8747062	A10	17	12	A
10472	Bn-N10-p10493335	Bn-C9-p42422620	Bn-scaff_21924_1-p88938	A10	17	12	A
10473	Bn-N10-p10502521	Bn-A10-p8867175	Bn-A10-p8867175	A10	18	13.6	A
10474	Bn-N10-p10544389	Bn-A10-p8904827	Bn-A10-p8904827	A10	18	13.6	A
10475	Bn-N10-p10553880	Bn-A10-p8917543	Bn-A10-p8917543	A10	18	13.6	A
10476	Bn-N10-p10709112	Bn-A10-p9084421	Bn-A10-p9084421	A10	19	14.4	A
10477	Bn-N10-p10730133	Bn-A10-p9099489	Bn-A10-p9099489	A10	19	14.4	A
10478	Bn-N10-p10748774	Bn-A10-p9117984	Bn-A10-p9117984	A10	19	14.4	A
10479	Bn-N10-p10752076	Bn-A10-p9121306	Bn-A10-p9121306	A10	19	14.4	A
10480	Bn-N10-p10755322	Bn-A10-p9124552	Bn-A10-p9124552	A10	19	14.4	A
10481	Bn-N10-p10771171	Bn-A10-p9150150	Bn-A10-p9150150	A10	19	14.4	A

10482	Bn-N10-p10771380	Bn-A10-p9150359	Bn-A10-p9150359	A10	19	14.4	A
10483	Bn-N10-p10790201	Bn-A10-p9166440	Bn-A10-p9166440	A10	19	14.4	A
10484	Bn-N10-p10790265	Bn-A10-p9166504	Bn-A10-p9166504	A10	19	14.4	A
10485	Bn-N10-p10810668	Bn-A10-p9181902	Bn-A10-p9181902	A10	19	14.4	A
10486	Bn-N10-p10841185	Bn-A10-p9209872	Bn-A10-p9209872	A10	19	14.4	A
10487	Bn-N12-p21757913	Bn-A10-p8961320	Bn-A10-p8961320	A10	19	14.4	A
10488	Bn-N14-p38133147	Bn-A10-p8948483	Bn-A10-p8948483	A10	19	14.4	A
10489	Bn-N14-p5208637	Bn-A10-p8961465	Bn-A10-p8961465	A10	19	14.4	A
10490	Bn-N15-p42456444	Bn-A10-p8960651	Bn-A10-p8960651	A10	19	14.4	A
10491	Bn-N19-p44732365	Bn-A10-p8957186	Bn-A10-p8957186	A10	19	14.4	A
10492	Bn-N19-p44763440	Bn-A10-p8965800	Bn-A10-p8965800	A10	19	14.4	A
10493	Bn-N3-p22188456	Bn-A10-p8964484	Bn-A10-p8964484	A10	19	14.4	A
10494	Unknown	Bn-A10-p8960805	Bn-A10-p8960805	A10	19	14.4	A
10495	Bn-N10-p10868858	Bn-A10-p9245966	Bn-A10-p9245966	A10	20	15.2	A
10496	Bn-N10-p10875719	Bn-A10-p9255041	Bn-A10-p9255041	A10	20	15.2	A
10497	Bn-N10-p10948364	Bn-A10-p9318940	Bn-A10-p9318940	A10	20	15.2	A
10498	Bn-N10-p10963914	Bn-A10-p9329804	Bn-A10-p9329804	A10	20	15.2	A
10499	Bn-N10-p11000973	Bn-A10-p9370046	Bn-A10-p9370046	A10	21	16	A
10500	Bn-N10-p11022928	Bn-A10-p9396364	Bn-A10-p9396364	A10	22	17.6	A
10501	Bn-N10-p11091769	Bn-A10-p9459037	Bn-A10-p9459037	A10	22	17.6	A
10502	Bn-N10-p11098941	Bn-A10-p9470646	Bn-A10-p9470646	A10	22	17.6	A
10503	Bn-N10-p11108265	Bn-A10-p9473804	Bn-A10-p9473804	A10	22	17.6	A
10504	Bn-N10-p11149986	Bn-A10-p9514802	Bn-A10-p9514802	A10	22	17.6	A
10505	Bn-N10-p11164906	Bn-A10-p9528776	Bn-A10-p9528776	A10	22	17.6	A
10506	Bn-N10-p11212698	Bn-A10-p9561249	Bn-A10-p9561249	A10	22	17.6	A
10507	Bn-N10-p11215353	Bn-A10-p9558311	Bn-A10-p9558311	A10	22	17.6	A
10508	Bn-N10-p11215987	Bn-A10-p9579123	Bn-A10-p9579123	A10	22	17.6	A
10509	Bn-N10-p11226589	Bn-A10-p9581812	Bn-A10-p9581812	A10	22	17.6	A
10510	Bn-N19-p45741636	Bn-A10-p9436205	Bn-A10-p9436205	A10	22	17.6	A
10511	Bn-N10-p11235601	Bn-A10-p9594548	Bn-A10-p9594548	A10	23	18.4	A
10512	Bn-N10-p11255699	Bn-A10-p9607076	Bn-A10-p9607076	A10	23	18.4	A
10513	Bn-N10-p11302212	Bn-A10-p9648940	Bn-A10-p9648940	A10	23	18.4	A
10514	Bn-N10-p11368340	Bn-A10-p9732666	Bn-A10-p9732666	A10	23	18.4	A
10515	Bn-N10-p11378086	Bn-A10-p9740870	Bn-A10-p9740870	A10	23	18.4	A
10516	Bn-N10-p11382854	Bn-A10-p9747217	Bn-A10-p9747217	A10	23	18.4	A
10517	Bn-N10-p11383186	Bn-A10-p9747551	Bn-A10-p9747551	A10	23	18.4	A
10518	Bn-N19-p46194919	Bn-A10-p9694236	Bn-A10-p9694236	A10	23	18.4	A
10519	Bn-N19-p46198937	Bn-A10-p9695953	Bn-A10-p9695953	A10	23	18.4	A
10520	Bn-N19-p46199326	Bn-A10-p9696331	Bn-A10-p9696331	A10	23	18.4	A
10521	Bn-N7-p13661104	Bn-A10-p9618292	Bn-A10-p9618292	A10	23	18.4	A
10522	Bn-Scaffold16324-p428	Bn-A10-p9592692	Bn-A10-p9592692	A10	23	18.4	A
10523	Bn-N10-p11495739	Bn-A10-p9868333	Bn-A10-p9868333	A10	24	19.2	A
10524	Bn-N10-p11590458	Bn-A10-p9966843	Bn-A10-p9966843	A10	24	19.2	A
10525	Bn-N10-p11604706	Bn-A10-p9973634	Bn-A10-p9973634	A10	24	19.2	A
10526	Bn-N10-p11604883	Bn-A10-p9973811	Bn-A10-p9973811	A10	24	19.2	A
10527	Bn-N10-p11657804	Bn-A10-p10020060	Bn-A10-p10020060	A10	24	19.2	A
10528	Bn-N10-p11689867	Bn-A10-p10070473	Bn-A10-p10070473	A10	24	19.2	A
10529	Bn-N10-p11696979	Bn-A10-p10082237	Bn-A10-p10082237	A10	24	19.2	A
10530	Bn-N19-p46930573	Bn-A10-p10026077	Bn-A10-p10026077	A10	24	19.2	A

10531	Bn-Scaffold03327-p124	Bn-A10-p9888229	Bn-A10-p9888229	A10	24	19.2	A
10532	Bn-N10-p11718757	Bn-C9-p44593285	Bn-scaff_17088_1-p35376!	A10	25	20.8	A
10533	Bn-N10-p11769661	Bn-A10-p10141249	Bn-A10-p10141249	A10	25	20.8	A
10534	Bn-N10-p11818254	Bn-A10-p10186330	Bn-A10-p10186330	A10	25	20.8	A
10535	Bn-N10-p11868078	Bn-A10-p10244709	Bn-A10-p10244709	A10	25	20.8	A
10536	Bn-N10-p11875373	Bn-A10-p10252741	Bn-A10-p10252741	A10	25	20.8	A
10537	Bn-N10-p11912249	Bn-A10-p10298013	Bn-A10-p10298013	A10	25	20.8	A
10538	Bn-N10-p11964992	Bn-A10-p10361031	Bn-A10-p10361031	A10	25	20.8	A
10539	Bn-N10-p12009044	Bn-A10-p10395990	Bn-A10-p10395990	A10	25	20.8	A
10540	Bn-N10-p12039050	Bn-A10-p10423500	Bn-A10-p10423500	A10	25	20.8	A
10541	Bn-N10-p12078376	Bn-A10-p10464159	Bn-A10-p10464159	A10	26	21.6	A
10542	Bn-Scaffold01263-p707	Bn-A10-p10613361	Bn-A10-p10613361	A10	26	21.6	A
10543	Bn-N10-p12243716	Bn-A10-p10647252	Bn-A10-p10647252	A10	27	26.5	B
10544	Bn-N10-p12249467	Bn-A10-p10652964	Bn-A10-p10652964	A10	27	26.5	B
10545	Bn-N10-p12250552	Bn-A10-p10654061	Bn-A10-p10654061	A10	27	26.5	B
10546	Bn-N10-p12265600	Bn-A10-p10668111	Bn-A10-p10668111	A10	27	26.5	B
10547	Bn-N10-p12266804	Bn-A10-p10669303	Bn-A10-p10669303	A10	27	26.5	B
10548	Bn-N10-p12274787	Bn-A10-p10677237	Bn-A10-p10677237	A10	27	26.5	B
10549	Bn-N10-p12279547	Bn-A10-p10680827	Bn-A10-p10680827	A10	27	26.5	B
10550	Bn-N10-p12327683	Bn-A10-p10726590	Bn-A10-p10726590	A10	27	26.5	B
10551	Bn-N10-p12328932	Bn-A10-p10728078	Bn-A10-p10728078	A10	27	26.5	B
10552	Bn-N10-p12352772	Bn-A10-p10755216	Bn-A10-p10755216	A10	27	26.5	B
10553	Bn-N10-p12364300	Bn-C9-p45711255	Bn-scaff_15576_1-p15959	A10	27	26.5	B
10554	Bn-N10-p12365473	Bn-A10-p10764928	Bn-A10-p10764928	A10	27	26.5	B
10555	Bn-N10-p12385408	Bn-A10-p10777142	Bn-A10-p10777142	A10	27	26.5	B
10556	Bn-N10-p12419347	Bn-A10-p10814517	Bn-A10-p10814517	A10	27	26.5	B
10557	Bn-N10-p12461167	Bn-A10-p10866946	Bn-A10-p10866946	A10	27	26.5	B
10558	Bn-N10-p12474103	Bn-C9-p45925736	Bn-scaff_15576_1-p37407	A10	27	26.5	B
10559	Bn-N19-p36899078	Bn-Scaffold000104-p	Bn-Scaffold000104-p40497	A10	27	26.5	B
10560	Bn-N10-p12513083	Bn-A10-p10914713	Bn-A10-p10914713	A10	28	27.3	B
10561	Bn-N10-p12513860	Bn-A10-p10918889	Bn-A10-p10918889	A10	28	27.3	B
10562	Bn-N10-p12516680	Bn-A10-p10921712	Bn-A10-p10921712	A10	28	27.3	B
10563	Bn-N10-p12526796	Bn-A10-p10927965	Bn-A10-p10927965	A10	28	27.3	B
10564	Bn-N10-p12537825	Bn-A10-p10936559	Bn-A10-p10936559	A10	28	27.3	B
10565	Bn-N10-p12557367	Bn-A10-p10949218	Bn-A10-p10949218	A10	28	27.3	B
10566	Bn-N10-p12659756	Bn-A10-p11059267	Bn-A10-p11059267	A10	28	27.3	B
10567	Bn-N10-p12675560	Bn-A10-p11075240	Bn-A10-p11075240	A10	28	27.3	B
10568	Bn-N10-p12689085	Bn-A10-p11082862	Bn-A10-p11082862	A10	28	27.3	B
10569	Bn-N10-p12717224	Bn-A10-p11105998	Bn-A10-p11105998	A10	29	28.1	B
10570	Bn-N10-p12755352	Bn-A10-p11138874	Bn-A10-p11138874	A10	29	28.1	B
10571	Bn-N10-p12783717	Bn-A10-p11183840	Bn-A10-p11183840	A10	30	29.7	B
10572	Bn-N10-p12845954	Bn-C9-p46799291	Bn-scaff_27705_1-p16118	A10	30	29.7	B
10573	Bn-N10-p12846026	Bn-A10-p11230418	Bn-A10-p11230418	A10	30	29.7	B
10574	Bn-N10-p12880595	Bn-A10-p11272184	Bn-A10-p11272184	A10	30	29.7	B
10575	Bn-N10-p12917622	Bn-A10-p11302032	Bn-A10-p11302032	A10	30	29.7	B
10576	Bn-N10-p12923291	Bn-Scaffold01471-p7	Bn-scaff_20835_1-p7749	A10	30	29.7	B
10577	Bn-N10-p12928294	Bn-A10-p11312705	Bn-A10-p11312705	A10	30	29.7	B
10578	Bn-N10-p13024539	Bn-A10-p11396195	Bn-A10-p11396195	A10	31	30.5	B
10579	Bn-N10-p13063530	Bn-A10-p11427179	Bn-A10-p11427179	A10	32	32.1	B

10580	Bn-N10-p13076227	Bn-A10-p11451545	Bn-A10-p11451545	A10	32	32.1	B
10581	Bn-N10-p13122698	Bn-A08-p4936436	Bn-A08-p4936436	A10	32	32.1	B
10582	Bn-N19-p49643491	Bn-A08-p4931420	Bn-A08-p4931420	A10	32	32.1	B
10583	Bn-N19-p49762022	Bn-C9-p47439482	Bn-scaff_17028_1-p44445	A10	33	32.9	B
10584	Bn-N10-p13241309	Bn-A10-p12842513	Bn-A10-p12842513	A10	34	34.5	B
10585	Bn-N10-p13261881	Bn-A10-p12857310	Bn-A10-p12857310	A10	34	34.5	B
10586	Bn-N10-p13268179	Bn-A10-p12863402	Bn-A10-p12863402	A10	34	34.5	B
10587	Bn-N10-p13269012	Bn-A10-p12864230	Bn-A10-p12864230	A10	34	34.5	B
10588	Bn-N10-p13286395	Bn-A10-p12885978	Bn-A10-p12885978	A10	34	34.5	B
10589	Bn-N10-p13307673	Bn-A10-p12900258	Bn-A10-p12900258	A10	34	34.5	B
10590	Bn-N10-p13341433	Bn-A10-p12933288	Bn-A10-p12933288	A10	35	35.3	B
10591	Bn-N10-p13389062	Bn-A10-p12996989	Bn-A10-p12996989	A10	35	35.3	B
10592	Bn-N10-p13396013	Bn-C9-p47754143	Bn-scaff_21841_1-p29725	A10	35	35.3	B
10593	Bn-N10-p13404021	Bn-A10-p13009957	Bn-A10-p13009957	A10	35	35.3	B
10594	Bn-N10-p13429661	Bn-A10-p13031723	Bn-A10-p13031723	A10	35	35.3	B
10595	Bn-N10-p13449164	Bn-A10-p13051361	Bn-A10-p13051361	A10	35	35.3	B
10596	Bn-N10-p13530217	Bn-A10-p13130002	Bn-A10-p13130002	A10	36	36.1	B
10597	Bn-N10-p13560204	Bn-A10-p13138769	Bn-A10-p13138769	A10	36	36.1	B
10598	Bn-N10-p13561070	Bn-A10-p13139635	Bn-A10-p13139635	A10	36	36.1	B
10599	Bn-N10-p13563545	Bn-A10-p13142215	Bn-A10-p13142215	A10	36	36.1	B
10600	Bn-N10-p13584582	Bn-A10-p13162021	Bn-A10-p13162021	A10	36	36.1	B
10601	Bn-N10-p13586951	Bn-A10-p13164379	Bn-A10-p13164379	A10	36	36.1	B
10602	Bn-N10-p13587108	Bn-A10-p13164536	Bn-A10-p13164536	A10	36	36.1	B
10603	Bn-N10-p13591796	Bn-A10-p13169213	Bn-A10-p13169213	A10	36	36.1	B
10604	Bn-N10-p13625784	Bn-A10-p13192787	Bn-A10-p13192787	A10	36	36.1	B
10605	Bn-N10-p13636114	Bn-A10-p13207985	Bn-A10-p13207985	A10	37	36.9	B
10606	Bn-N10-p13642700	Bn-A10-p13218309	Bn-A10-p13218309	A10	37	36.9	B
10607	Bn-N10-p13642816	Bn-A10-p13218425	Bn-A10-p13218425	A10	37	36.9	B
10608	Bn-N10-p13643620	Bn-A10-p13219229	Bn-A10-p13219229	A10	37	36.9	B
10609	Bn-N10-p13667961	Bn-A10-p13243690	Bn-A10-p13243690	A10	37	36.9	B
10610	Bn-N10-p13696841	Bn-A10-p13263227	Bn-A10-p13263227	A10	38	37.7	B
10611	Bn-N10-p13699784	Bn-A10-p13266157	Bn-A10-p13266157	A10	38	37.7	B
10612	Bn-N10-p13729447	Bn-A10-p13289368	Bn-A10-p13289368	A10	38	37.7	B
10613	Bn-N10-p13739986	Bn-A10-p13299250	Bn-A10-p13299250	A10	38	37.7	B
10614	Bn-N10-p13775604	Bn-A10-p13327024	Bn-A10-p13327024	A10	38	37.7	B
10615	Bn-N10-p13792515	Bn-A10-p13343454	Bn-A10-p13343454	A10	39	38.5	B
10616	Bn-N10-p13806192	Bn-A10-p13358504	Bn-A10-p13358504	A10	39	38.5	B
10617	Bn-N10-p13841241	Bn-A10-p13393956	Bn-A10-p13393956	A10	39	38.5	B
10618	Bn-N10-p13843438	Bn-A10-p13396422	Bn-A10-p13396422	A10	39	38.5	B
10619	Bn-N10-p13860320	Bn-A10-p13410287	Bn-A10-p13410287	A10	40	39.3	B
10620	Bn-N10-p13875193	Bn-A10-p13423663	Bn-A10-p13423663	A10	40	39.3	B
10621	Bn-N10-p13888278	Bn-A10-p13433604	Bn-A10-p13433604	A10	40	39.3	B
10622	Bn-N10-p13900036	Bn-A10-p13446086	Bn-A10-p13446086	A10	40	39.3	B
10623	Bn-N10-p13901846	Bn-A10-p13447899	Bn-A10-p13447899	A10	40	39.3	B
10624	Bn-N10-p13901905	Bn-A10-p13447958	Bn-A10-p13447958	A10	40	39.3	B
10625	Bn-N10-p13929187	Bn-A10-p13475821	Bn-A10-p13475821	A10	40	39.3	B
10626	Bn-N10-p13931599	Bn-A10-p13479456	Bn-A10-p13479456	A10	40	39.3	B
10627	Bn-N10-p13936578	Bn-A10-p13489723	Bn-A10-p13489723	A10	40	39.3	B
10628	Bn-N10-p13938984	Bn-A10-p13495547	Bn-A10-p13495547	A10	40	39.3	B

10629	Bn-N10-p13941662	Bn-A10-p13498211	Bn-A10-p13498211	A10	40	39.3	B
10630	Bn-N10-p14028553	Bn-A10-p13577876	Bn-A10-p13577876	A10	40	39.3	B
10631	Bn-N10-p14100175	Bn-A10-p13638489	Bn-A10-p13638489	A10	41	40.1	B
10632	Bn-N10-p14102032	Bn-A10-p13640346	Bn-A10-p13640346	A10	41	40.1	B
10633	Bn-N10-p14128485	Bn-A10-p13669875	Bn-A10-p13669875	A10	42	41.7	B
10634	Bn-N10-p14140344	Bn-A10-p13677412	Bn-A10-p13677412	A10	42	41.7	B
10635	Bn-N10-p14144802	Bn-A10-p13680808	Bn-A10-p13680808	A10	42	41.7	B
10636	Bn-N10-p14145095	Bn-A10-p13681096	Bn-A10-p13681096	A10	42	41.7	B
10637	Bn-N10-p14189516	Bn-A10-p13723140	Bn-A10-p13723140	A10	42	41.7	B
10638	Bn-N10-p14223512	Bn-A10-p13758377	Bn-A10-p13758377	A10	42	41.7	B
10639	Bn-N10-p14243763	Bn-A10-p13779014	Bn-A10-p13779014	A10	42	41.7	B
10640	Bn-N10-p14253316	Bn-A10-p13788629	Bn-A10-p13788629	A10	42	41.7	B
10641	Bn-N10-p14254267	Bn-A10-p13789579	Bn-A10-p13789579	A10	42	41.7	B
10642	Bn-N10-p14259392	Bn-C9-p49154154	Bn-scaff_22082_1-p36768[illegible]	A10	42	41.7	B
10643	Bn-N10-p14260941	Bn-A10-p13796417	Bn-A10-p13796417	A10	42	41.7	B
10644	Bn-N10-p14263300	Bn-A10-p13798602	Bn-A10-p13798602	A10	42	41.7	B
10645	Bn-N10-p14280519	Bn-A10-p13815263	Bn-A10-p13815263	A10	43	42.5	B
10646	Bn-N10-p14283825	Bn-A10-p13818569	Bn-A10-p13818569	A10	43	42.5	B
10647	Bn-N10-p14307564	Bn-A10-p13837373	Bn-A10-p13837373	A10	43	42.5	B
10648	Bn-N10-p14396084	Bn-A10-p13938504	Bn-A10-p13938504	A10	43	42.5	B
10649	Bn-N10-p14454462	Bn-A10-p13995725	Bn-A10-p13995725	A10	43	42.5	B
10650	Bn-N10-p14456091	Bn-A10-p13997359	Bn-A10-p13997359	A10	43	42.5	B
10651	Bn-N10-p14461495	Bn-A10-p14012013	Bn-A10-p14012013	A10	43	42.5	B
10652	Bn-N10-p14505713	Bn-A10-p14058257	Bn-A10-p14058257	A10	43	42.5	B
10653	Bn-N10-p14515412	Bn-C9-p49559953	Bn-scaff_19757_1-p22787	A10	43	42.5	B
10654	Bn-N10-p14549323	Bn-A10-p14097282	Bn-A10-p14097282	A10	43	42.5	B
10655	Bn-N10-p14568248	Bn-A10-p14119080	Bn-A10-p14119080	A10	43	42.5	B
10656	Bn-N10-p14568268	Bn-A10-p14119100	Bn-A10-p14119100	A10	43	42.5	B
10657	Bn-N10-p14568674	Bn-A10-p14119507	Bn-A10-p14119507	A10	43	42.5	B
10658	Bn-N10-p14582760	Bn-A10-p14131328	Bn-A10-p14131328	A10	43	42.5	B
10659	Bn-N10-p14595313	Bn-A10-p14143770	Bn-A10-p14143770	A10	43	42.5	B
10660	Bn-N10-p14612957	Bn-A10-p14160561	Bn-A10-p14160561	A10	43	42.5	B
10661	Bn-N10-p14628365	Bn-C9-p49710217	Bn-scaff_17109_1-p21307[illegible]	A10	43	42.5	B
10662	Bn-N10-p14795786	Bn-A10-p14340467	Bn-A10-p14340467	A10	43	42.5	B
10663	Bn-N10-p14983813	Bn-A10-p14528560	Bn-A10-p14528560	A10	43	42.5	B
10664	Bn-N10-p14715060	Bn-A10-p14254487	Bn-A10-p14254487	A10	44	43.3	B
10665	Bn-N10-p14755806	Bn-A10-p14299800	Bn-A10-p14299800	A10	44	43.3	B
10666	Bn-N10-p14778504	Bn-C1-p12052503	Bn-scaff_17369_1-p80163[illegible]	A10	44	43.3	B
10667	Bn-N10-p14810503	Bn-A10-p14353770	Bn-A10-p14353770	A10	44	43.3	B
10668	Bn-N10-p14860657	Bn-A10-p14402509	Bn-A10-p14402509	A10	44	43.3	B
10669	Bn-N10-p14870789	Bn-A10-p14412790	Bn-A10-p14412790	A10	44	43.3	B
10670	Bn-N10-p14879359	Bn-A10-p14420633	Bn-A10-p14420633	A10	44	43.3	B
10671	Bn-N10-p14891913	Bn-A10-p14433226	Bn-A10-p14433226	A10	44	43.3	B
10672	Bn-N10-p14919806	Bn-A10-p14472446	Bn-A10-p14472446	A10	44	43.3	B
10673	Bn-N10-p14935901	Bn-A10-p14488358	Bn-A10-p14488358	A10	44	43.3	B
10674	Bn-N10-p14980347	Bn-A10-p14524788	Bn-A10-p14524788	A10	44	43.3	B
10675	Bn-N10-p14980622	Bn-A10-p14525063	Bn-A10-p14525063	A10	44	43.3	B
10676	Bn-N10-p14981788	Bn-A10-p14526228	Bn-A10-p14526228	A10	44	43.3	B
10677	Bn-N10-p14981849	Bn-A10-p14526289	Bn-A10-p14526289	A10	44	43.3	B

10678	Bn-N17-p32319280	Bn-C7-p32203508	Bn-scaff_16130_1-p20062	A10	44	43.3	B
10679	Bn-N17-p32320599	Bn-C7-p32204827	Bn-scaff_16130_1-p20075	A10	44	43.3	B
10680	Bn-N10-p15065815	Bn-A10-p14623802	Bn-A10-p14623802	A10	45	44.9	B
10681	Bn-N10-p15083378	Bn-A10-p14638980	Bn-A10-p14638980	A10	45	44.9	B
10682	Bn-N10-p15090804	Bn-A10-p14646222	Bn-A10-p14646222	A10	45	44.9	B
10683	Bn-N10-p15091407	Bn-A10-p14646825	Bn-A10-p14646825	A10	45	44.9	B
10684	Bn-N10-p15103352	Bn-A10-p14658742	Bn-A10-p14658742	A10	45	44.9	B
10685	Bn-N10-p15109709	Bn-A10-p14664967	Bn-A10-p14664967	A10	45	44.9	B
10686	Bn-N10-p15109806	Bn-A10-p14665064	Bn-A10-p14665064	A10	45	44.9	B
10687	Bn-N10-p15137512	Bn-C9-p50482480	Bn-scaff_17109_2-p51658	A10	45	44.9	B
10688	Bn-N10-p15151684	Bn-A10-p14697526	Bn-A10-p14697526	A10	45	44.9	B
10689	Bn-N10-p15166733	Bn-A10-p14711221	Bn-A10-p14711221	A10	45	44.9	B
10690	Bn-N10-p15275238	Bn-A10-p14809234	Bn-A10-p14809234	A10	45	44.9	B
10691	Bn-N10-p15275509	Bn-A10-p14809502	Bn-A10-p14809502	A10	45	44.9	B
10692	Bn-N10-p15286509	Bn-A10-p14820463	Bn-A10-p14820463	A10	45	44.9	B
10693	Bn-N10-p15287902	Bn-A10-p14821861	Bn-A10-p14821861	A10	45	44.9	B
10694	Bn-N10-p15289945	Bn-A10-p14823906	Bn-A10-p14823906	A10	45	44.9	B
10695	Bn-N10-p15307795	Bn-A10-p14834518	Bn-A10-p14834518	A10	45	44.9	B
10696	Bn-N10-p15322850	Bn-A10-p14844217	Bn-A10-p14844217	A10	45	44.9	B
10697	Bn-N10-p15347259	Bn-A10-p14872849	Bn-A10-p14872849	A10	45	44.9	B
10698	Bn-N10-p15347883	Bn-A10-p14873471	Bn-A10-p14873471	A10	45	44.9	B
10699	Bn-N10-p15359194	Bn-A10-p14883536	Bn-A10-p14883536	A10	45	44.9	B
10700	Bn-N10-p15382906	Bn-A10-p14897192	Bn-A10-p14897192	A10	45	44.9	B
10701	Bn-N10-p15400027	Bn-C9-p50792064	Bn-scaff_17109_2-p20700	A10	45	44.9	B
10702	Bn-N10-p15404656	Bn-A10-p14918537	Bn-A10-p14918537	A10	45	44.9	B
10703	Bn-N10-p15438914	Bn-A10-p14950115	Bn-A10-p14950115	A10	45	44.9	B
10704	Bn-N10-p15449313	Bn-A10-p14962307	Bn-A10-p14962307	A10	45	44.9	B
10705	Bn-N10-p15496671	Bn-A10-p15006053	Bn-A10-p15006053	A10	45	44.9	B
10706	Bn-N10-p15581153	Bn-A10-p15086931	Bn-A10-p15086931	A10	46	45.8	B
10707	Bn-N10-p15639341	Bn-A10-p15156277	Bn-A10-p15156277	A10	46	45.8	B
10708	Bn-N10-p15707679	Bn-A10-p16451834	Bn-A10-p16451834	A10	46	45.8	B
10709	Bn-N10-p15708239	Bn-C9-p51324542	Bn-scaff_17750_1-p18718	A10	46	45.8	B
10710	Bn-N10-p15709291	Bn-A10-p16450218	Bn-A10-p16450218	A10	46	45.8	B
10711	Bn-N10-p15711490	Bn-A10-p16448121	Bn-A10-p16448121	A10	46	45.8	B
10712	Bn-N10-p15717099	Bn-A10-p16442531	Bn-A10-p16442531	A10	46	45.8	B
10713	Bn-N10-p15720562	Bn-A10-p16439150	Bn-A10-p16439150	A10	46	45.8	B
10714	Bn-N10-p15722580	Bn-A10-p16437131	Bn-A10-p16437131	A10	46	45.8	B
10715	Bn-N10-p15732177	Bn-C9-p51347961	Bn-scaff_17750_1-p18484	A10	47	46.7	B
10716	Bn-N10-p15733314	Bn-A10-p16425155	Bn-A10-p16425155	A10	47	46.7	B
10717	Bn-N10-p15738014	Bn-A10-p16421672	Bn-A10-p16421672	A10	47	46.7	B
10718	Bn-N10-p15743728	Bn-A10-p16415851	Bn-A10-p16415851	A10	47	46.7	B
10719	Bn-N10-p15753717	Bn-A10-p16405686	Bn-A10-p16405686	A10	47	46.7	B
10720	Bn-N10-p15756132	Bn-A10-p16404507	Bn-A10-p16404507	A10	47	46.7	B
10721	Bn-N10-p15761632	Bn-A10-p16401982	Bn-A10-p16401982	A10	47	46.7	B
10722	Bn-N10-p15761738	Bn-A10-p16401876	Bn-A10-p16401876	A10	47	46.7	B
10723	Bn-N10-p15784965	Bn-A10-p16377145	Bn-A10-p16377145	A10	47	46.7	B
10724	Bn-N10-p15813657	Bn-A10-p16354622	Bn-A10-p16354622	A10	48	51.8	B
10725	Bn-N10-p15825188	Bn-A10-p16344917	Bn-A10-p16344917	A10	48	51.8	B
10726	Bn-N10-p15830303	Bn-A10-p16336708	Bn-A10-p16336708	A10	48	51.8	B

10727	Bn-N10-p15839226	Bn-A10-p16326058	Bn-A10-p16326058	A10	48	51.8	B
10728	Bn-N10-p15847683	Bn-A10-p16317798	Bn-A10-p16317798	A10	48	51.8	B
10729	Bn-N10-p15878279	Bn-A10-p16285282	Bn-A10-p16285282	A10	49	52.7	B
10730	Bn-N10-p15918848	Bn-A10-p16249484	Bn-A10-p16249484	A10	49	52.7	B
10731	Bn-N10-p15931040	Bn-A10-p16237178	Bn-A10-p16237178	A10	49	52.7	B
10732	Bn-N10-p15939293	Bn-A10-p16224024	Bn-A10-p16224024	A10	49	52.7	B
10733	Bn-N10-p15949852	Bn-A10-p16215351	Bn-A10-p16215351	A10	49	52.7	B
10734	Bn-N10-p15950038	Bn-A10-p16215165	Bn-A10-p16215165	A10	49	52.7	B
10735	Bn-N10-p15955037	Bn-A10-p16210702	Bn-A10-p16210702	A10	49	52.7	B
10736	Bn-N10-p15957847	Bn-A10-p16207908	Bn-A10-p16207908	A10	49	52.7	B
10737	Bn-N19-p54057799	Bn-C9-p51503921	Bn-scaff_17750_1-p16924{	A10	49	52.7	B
10738	Bn-N10-p16068992	Bn-A10-p16087066	Bn-A10-p16087066	A10	50	53.6	B
10739	Bn-N10-p16083357	Bn-A10-p16074706	Bn-A10-p16074706	A10	51	54.5	B
10740	Bn-N10-p16090488	Bn-A10-p16067573	Bn-A10-p16067573	A10	51	54.5	B
10741	Bn-N10-p16101812	Bn-A10-p16061207	Bn-A10-p16061207	A10	51	54.5	B
10742	Bn-N10-p16296245	Bn-A10-p15863143	Bn-A10-p15863143	A10	51	54.5	B
10743	Bn-N10-p16295331	Bn-A10-p15864056	Bn-A10-p15864056	A10	52	56.2	B
10744	Bn-N10-p16340492	Bn-A10-p15817344	Bn-A10-p15817344	A10	52	56.2	B
10745	Bn-N10-p16366292	Bn-A10-p15793623	Bn-A10-p15793623	A10	52	56.2	B
10746	Bn-N10-p16382105	Bn-A10-p15774860	Bn-A10-p15774860	A10	52	56.2	B
10747	Bn-N10-p16423703	Bn-A10-p15742689	Bn-A10-p15742689	A10	52	56.2	B
10748	Bn-N10-p16424105	Bn-A10-p15742287	Bn-A10-p15742287	A10	52	56.2	B
10749	Bn-N10-p16424265	Bn-A10-p15742125	Bn-A10-p15742125	A10	52	56.2	B
10750	Bn-N10-p16424568	Bn-A10-p15741819	Bn-A10-p15741819	A10	52	56.2	B
10751	Bn-N10-p16425491	Bn-A10-p15740896	Bn-A10-p15740896	A10	52	56.2	B
10752	Bn-N10-p16427579	Bn-A10-p15738808	Bn-A10-p15738808	A10	52	56.2	B
10753	Bn-N10-p16429012	Bn-A10-p15737423	Bn-A10-p15737423	A10	52	56.2	B
10754	Bn-N10-p16433190	Bn-A10-p15734598	Bn-A10-p15734598	A10	52	56.2	B
10755	Bn-N10-p16434436	Bn-A10-p15731773	Bn-A10-p15731773	A10	52	56.2	B
10756	Bn-N10-p16441651	Bn-A10-p15727608	Bn-A10-p15727608	A10	52	56.2	B
10757	Bn-N10-p16455179	Bn-C5-p10336544	Bn-scaff_26075_1-p20662	A10	52	56.2	B
10758	Bn-N10-p16457656	Bn-A10-p15711469	Bn-A10-p15711469	A10	52	56.2	B
10759	Bn-N10-p16497369	Bn-A10-p15670267	Bn-A10-p15670267	A10	52	56.2	B
10760	Bn-N10-p16510169	Bn-A10-p15657263	Bn-A10-p15657263	A10	52	56.2	B
10761	Bn-N10-p16515942	Bn-A10-p15651383	Bn-A10-p15651383	A10	52	56.2	B
10762	Bn-N10-p16533851	Bn-A10-p15633321	Bn-A10-p15633321	A10	52	56.2	B
10763	Bn-N19-p54662336	Bn-A10-p15824883	Bn-A10-p15824883	A10	52	56.2	B
10764	Bn-N4-p8343331	Bn-A10-p15736726	Bn-A10-p15736726	A10	52	56.2	B
10765	Bn-N4-p8344080	Bn-A10-p15735290	Bn-A10-p15735290	A10	52	56.2	B
10766	Bn-N10-p16559905	Bn-A10-p15607468	Bn-A10-p15607468	A10	53	57.1	B
10767	Bn-N10-p16561832	Bn-A10-p15605556	Bn-A10-p15605556	A10	53	57.1	B
10768	Bn-N10-p16566578	Bn-A10-p15601027	Bn-A10-p15601027	A10	53	57.1	B
10769	Bn-N10-p16570303	Bn-A10-p15597364	Bn-A10-p15597364	A10	54	58	B
10770	Bn-N10-p16576269	Bn-A10-p15591443	Bn-A10-p15591443	A10	54	58	B
10771	Bn-N10-p16681932	Bn-A10-p15507350	Bn-A10-p15507350	A10	54	58	B
10772	Bn-N10-p16691768	Bn-A10-p15497556	Bn-A10-p15497556	A10	54	58	B
10773	Bn-N10-p16692507	Bn-A10-p15496817	Bn-A10-p15496817	A10	54	58	B
10774	Bn-N10-p16703595	Bn-A10-p15485139	Bn-A10-p15485139	A10	54	58	B
10775	Bn-N10-p16711667	Bn-A10-p15476300	Bn-A10-p15476300	A10	54	58	B

10776	Bn-N10-p16732070	Bn-A10-p15458720	Bn-A10-p15458720	A10	54	58	B
10777	Bn-N10-p16745200	Bn-A10-p15447032	Bn-A10-p15447032	A10	54	58	B
10778	Bn-N10-p16746202	Bn-A10-p15446038	Bn-A10-p15446038	A10	54	58	B
10779	Bn-N10-p16748021	Bn-A10-p15444223	Bn-A10-p15444223	A10	54	58	B
10780	Bn-N10-p16754844	Bn-A10-p15437444	Bn-A10-p15437444	A10	54	58	B
10781	Bn-N10-p16794775	Bn-A10-p15401999	Bn-A10-p15401999	A10	54	58	B
10782	Bn-N10-p16791314	Bn-A10-p15405149	Bn-A10-p15405149	A10	55	58.9	B
10783	Bn-N10-p16798686	Bn-A10-p15398137	Bn-A10-p15398137	A10	55	58.9	B
10784	Bn-N19-p55179397	Bn-A10-p15433413	Bn-A10-p15433413	A10	55	58.9	B
10785	Bn-N19-p55220975	Bn-A10-p15408188	Bn-A10-p15408188	A10	55	58.9	B
10786	Bn-N10-p16811029	Bn-A10-p15387894	Bn-A10-p15387894	A10	56	59.8	B
10787	Bn-N10-p16811262	Bn-A10-p15387634	Bn-A10-p15387634	A10	56	59.8	B
10788	Bn-N10-p16832459	Bn-A10-p15367240	Bn-A10-p15367240	A10	57	60.7	B
10789	Bn-N10-p16838219	Bn-A10-p15361519	Bn-A10-p15361519	A10	57	60.7	B
10790	Bn-Scaffold05692-p530	Bn-A10-p15378551	Bn-A10-p15378551	A10	57	60.7	B
10791	Bn-N10-p16874115	Bn-C9-p52786547	Bn-scaff_17750_1-p40985[illegible]	A10	58	61.6	B
10792	Bn-N10-p16875078	Bn-A10-p15330596	Bn-A10-p15330596	A10	58	61.6	B
10793	Bn-N10-p16875146	Bn-A10-p15330528	Bn-A10-p15330528	A10	58	61.6	B
10794	Bn-N10-p16889689	Bn-A10-p15312311	Bn-A10-p15312311	A10	58	61.6	B
10795	Bn-N10-p16901840	Bn-A10-p15299272	Bn-A10-p15299272	A10	58	61.6	B
10796	Bn-N10-p16945504	Bn-A10-p15265102	Bn-A10-p15265102	A10	59	62.5	B
10797	Bn-N10-p16973256	Bn-A10-p15237975	Bn-A10-p15237975	A10	59	62.5	B
10798	Bn-N10-p16973367	Bn-A10-p15237864	Bn-A10-p15237864	A10	59	62.5	B
10799	Bn-N10-p17075724	Bn-A10-p16522658	Bn-A10-p16522658	A10	59	62.5	B
10800	Bn-N19-p55393806	Bn-A10-p15276444	Bn-A10-p15276444	A10	59	62.5	B
10801	Bn-N19-p55444882	Bn-A10-p15238153	Bn-A10-p15238153	A10	59	62.5	B
10802	Bn-N10-p17174060	Bn-A10-p16620627	Bn-A10-p16620627	A10	60	63.4	B
10803	Bn-N10-p17179621	Bn-A10-p16626615	Bn-A10-p16626615	A10	60	63.4	B
10804	Bn-N14-p1859330	Bn-A10-p16862809	Bn-A10-p16862809	A10	61	64.2	B
10805	Bn-N12-p421642	Bn-A10-p16935603	Bn-A10-p16935603	A10	62	73.2	B
10806	Bn-N10-p17829335	Bn-A10-p17262954	Bn-A10-p17262954	A10	63	75.6	B
10807	Bn-N10-p17850418	Bn-A10-p17279612	Bn-A10-p17279612	A10	63	75.6	B
10808	Bn-N10-p17867565	Bn-A10-p17302908	Bn-A10-p17302908	A10	63	75.6	B
10809	Bn-N18-p44745193	Bn-A10-p17568393	Bn-A10-p17568393	A10	63	75.6	B
10810	Bn-N18-p44750629	Bn-A10-p17566833	Bn-A10-p17566833	A10	63	75.6	B
10811	Bn-N18-p44762126	Bn-A10-p17561217	Bn-A10-p17561217	A10	63	75.6	B
10812	Bn-N18-p44798703	Bn-A10-p17555032	Bn-A10-p17555032	A10	63	75.6	B
10813	Bn-N18-p44817535	Bn-A10-p17541781	Bn-A10-p17541781	A10	63	75.6	B
10814	Bn-N18-p44933930	Bn-A10-p17434951	Bn-A10-p17434951	A10	63	75.6	B
10815	Bn-N18-p44961280	Bn-A10-p17421322	Bn-A10-p17421322	A10	63	75.6	B
10816	Bn-N18-p45005347	Bn-C9-p54361811	Bn-scaff_22481_1-p23102[illegible]	A10	63	75.6	B
10817	Bn-N7-p9323096	Bn-A10-p17321665	Bn-A10-p17321665	A10	63	75.6	B
10818	Bn-Scaffold01176-p12149	Bn-A10-p17414621	Bn-A10-p17414621	A10	63	75.6	B
10819	Bn-Scaffold06346-p696	Bn-C9-p54507412	Bn-scaff_22481_1-p85423	A10	63	75.6	0
10820	Bn-Scaffold06346-p855	Bn-C9-p54507570	Bn-scaff_22481_1-p85265	A10	63	75.6	0
10821	Bn-N11-p1183319	Bn-C1-p3840	Bn-scaff_20320_1-p83433	C1	1	0	A
10822	Bn-N11-p1234353	Bn-C1-p62516	Bn-scaff_20320_1-p24757	C1	1	0	A
10823	Bn-N11-p1310425	Bn-C1-p140531	Bn-scaff_19244_1-p53159	C1	1	0	A
10824	Bn-N11-p1380612	Bn-C1-p210515	Bn-scaff_19244_1-p12314[illegible]	C1	1	0	A

10825	Bn-N11-p1459224	Bn-C1-p284003	Bn-scaff_19244_1-p19663	C1	1	0	0
10826	Bn-N11-p1469527	Bn-C1-p294457	Bn-scaff_19244_1-p20708	C1	1	0	A
10827	Bn-N11-p1471141	Bn-C1-p296096	Bn-scaff_19244_1-p20872	C1	1	0	A
10828	Bn-N11-p2038166	Bn-C1-p903714	Bn-scaff_19244_1-p81634	C1	2	4.3	B
10829	Bn-N11-p2335230	Bn-A01-p907721	Bn-A01-p907721	C1	3	8.6	B
10830	Bn-N11-p2374032	Bn-C1-p1310534	Bn-scaff_15838_1-p58643	C1	3	8.6	B
10831	Bn-N11-p2529209	Bn-C1-p1447998	Bn-scaff_15838_1-p72390	C1	3	8.6	0
10832	Bn-N11-p2546553	Bn-C1-p1464979	Bn-scaff_15838_1-p74088	C1	3	8.6	B
10833	Bn-N11-p2546757	Bn-C1-p1465169	Bn-scaff_15838_1-p74107	C1	3	8.6	B
10834	Bn-N11-p2579966	Bn-C1-p1491848	Bn-scaff_15838_1-p76775	C1	3	8.6	B
10835	Bn-N11-p2591521	Bn-C1-p1499664	Bn-scaff_15838_1-p77556	C1	3	8.6	0
10836	Bn-N11-p2603155	Bn-C1-p1511282	Bn-scaff_15838_1-p78718	C1	3	8.6	0
10837	Bn-N11-p2665753	Bn-C1-p1573439	Bn-scaff_15838_1-p84934	C1	3	8.6	B
10838	Bn-N11-p2972339	Bn-C1-p1914447	Bn-scaff_15838_1-p11903	C1	4	10.3	B
10839	Bn-N11-p3006442	Bn-C1-p1942263	Bn-scaff_15838_1-p12181	C1	4	10.3	0
10840	Bn-N11-p3379560	Bn-C1-p2298052	Bn-scaff_15838_1-p15739	C1	5	14	B
10841	Bn-N1-p1769679	Bn-C1-p2296948	Bn-scaff_15838_1-p15728	C1	5	14	B
10842	Bn-N11-p3868546	Bn-C1-p2753186	Bn-scaff_15838_1-p20290	C1	6	19.6	0
10843	Bn-N11-p3873556	Bn-C1-p2760078	Bn-scaff_15838_1-p20359	C1	6	19.6	B
10844	Bn-N11-p3877237	Bn-C1-p2763705	Bn-scaff_15838_1-p20396	C1	6	19.6	0
10845	Bn-N11-p3938379	Bn-C1-p2828337	Bn-scaff_15838_1-p21042	C1	6	19.6	B
10846	Bn-N11-p3976230	Bn-C1-p2869084	Bn-scaff_15838_1-p21449	C1	6	19.6	0
10847	Bn-N11-p4046663	Bn-C1-p2937023	Bn-scaff_15838_1-p22129	C1	6	19.6	0
10848	Bn-N11-p4453494	Bn-C1-p3320931	Bn-scaff_15838_5-p15173	C1	7	21.3	B
10849	Bn-N11-p5126350	Bn-C1-p3948263	Bn-scaff_15838_5-p77907	C1	8	25.6	0
10850	Bn-N11-p5429066	Bn-C1-p4212311	Bn-scaff_15838_5-p10431	C1	8	25.6	B
10851	Bn-N11-p5551650	Bn-C1-p4327378	Bn-scaff_15838_5-p11581	C1	9	27.6	0
10852	Bn-N11-p5555812	Bn-C1-p4331531	Bn-scaff_15838_5-p11623	C1	9	27.6	0
10853	Bn-N11-p5567645	Bn-C1-p4342864	Bn-scaff_15838_5-p11736	C1	9	27.6	0
10854	Bn-N11-p5574849	Bn-C1-p4357587	Bn-scaff_15838_5-p11883	C1	9	27.6	0
10855	Bn-N11-p5575143	Bn-C1-p4357881	Bn-scaff_15838_5-p11886	C1	9	27.6	0
10856	Bn-N11-p5576647	Bn-C1-p4359368	Bn-scaff_15838_5-p11901	C1	9	27.6	0
10857	Bn-N11-p5583239	Bn-C1-p4377879	Bn-scaff_15838_5-p12086	C1	9	27.6	0
10858	Bn-N11-p5583312	Bn-C1-p4377952	Bn-scaff_15838_5-p12087	C1	9	27.6	0
10859	Bn-N11-p5583445	Bn-C1-p4378085	Bn-scaff_15838_5-p12088	C1	9	27.6	0
10860	Bn-N11-p5589950	Bn-C1-p4379150	Bn-scaff_15838_5-p12099	C1	9	27.6	0
10861	Bn-N11-p5590431	Bn-C1-p4379637	Bn-scaff_15838_5-p12104	C1	9	27.6	0
10862	Bn-N11-p5590968	Bn-C1-p4380172	Bn-scaff_15838_5-p12109	C1	9	27.6	0
10863	Bn-N11-p5591637	Bn-C1-p4380841	Bn-scaff_15838_5-p12116	C1	9	27.6	0
10864	Bn-N11-p5595317	Bn-C1-p4384890	Bn-scaff_15838_5-p12156	C1	9	27.6	0
10865	Bn-N11-p5601673	Bn-C1-p4390487	Bn-scaff_15838_5-p12212	C1	9	27.6	0
10866	Bn-N11-p5602517	Bn-C1-p4396925	Bn-scaff_15838_5-p12277	C1	9	27.6	0
10867	Bn-N11-p5731470	Bn-C1-p4463284	Bn-scaff_25149_1-p15397	C1	9	27.6	0
10868	Bn-N11-p5731811	Bn-C1-p4463622	Bn-scaff_25149_1-p15735	C1	9	27.6	0
10869	Bn-N11-p5733639	Bn-C1-p4465448	Bn-scaff_25149_1-p17561	C1	9	27.6	0
10870	Bn-N11-p5755692	Bn-C1-p4493595	Bn-scaff_25149_1-p45708	C1	9	27.6	0
10871	Bn-N11-p5759633	Bn-C1-p4502891	Bn-scaff_25149_1-p55004	C1	9	27.6	0
10872	Bn-N11-p5759846	Bn-C1-p4503105	Bn-scaff_25149_1-p55218	C1	9	27.6	0
10873	Bn-N11-p5765240	Bn-C1-p4524264	Bn-scaff_25149_1-p76377	C1	9	27.6	0

10874	Bn-N11-p5776901	Bn-C1-p4538432	Bn-scaff_25149_1-p90545	C1	9	27.6 B
10875	Bn-N11-p5813219	Bn-C1-p4583026	Bn-scaff_17978_1-p22148	C1	9	27.6 B
10876	Bn-N11-p5828086	Bn-C1-p4584486	Bn-scaff_17978_1-p23608	C1	9	27.6 B
10877	Bn-N11-p5851752	Bn-C1-p4608470	Bn-scaff_17978_1-p47592	C1	9	27.6 0
10878	Bn-N11-p5856566	Bn-C1-p4613291	Bn-scaff_17978_1-p52413	C1	9	27.6 0
10879	Bn-N11-p5857009	Bn-C1-p4613734	Bn-scaff_17978_1-p52856	C1	9	27.6 0
10880	Bn-N11-p6024852	Bn-C1-p4786011	Bn-scaff_17978_1-p22513	C1	9	27.6 0
10881	Bn-N11-p6344269	Bn-C1-p5147865	Bn-scaff_19193_1-p13195	C1	9	27.6 0
10882	Bn-N11-p6369546	Bn-C1-p5176883	Bn-scaff_19193_1-p12904	C1	9	27.6 0
10883	Bn-N11-p6371431	Bn-C1-p5178771	Bn-scaff_19193_1-p12885	C1	9	27.6 0
10884	Bn-N11-p6413595	Bn-C1-p5214383	Bn-scaff_19193_1-p12529	C1	9	27.6 B
10885	Bn-N11-p6452056	Bn-C1-p5252062	Bn-scaff_19193_1-p12153	C1	9	27.6 B
10886	Bn-N11-p6480326	Bn-C1-p5275436	Bn-scaff_19193_1-p11919	C1	9	27.6 0
10887	Bn-N11-p6482703	Bn-C1-p5277886	Bn-scaff_19193_1-p11894	C1	9	27.6 0
10888	Bn-N11-p6682469	Bn-C1-p5461248	Bn-scaff_19193_1-p10061	C1	9	27.6 0
10889	Bn-N11-p6742595	Bn-C1-p5539398	Bn-scaff_19193_1-p92796	C1	9	27.6 B
10890	Bn-N11-p6749296	Bn-C1-p5550647	Bn-scaff_19193_1-p91671	C1	9	27.6 0
10891	Bn-N11-p6916358	Bn-C1-p5755438	Bn-scaff_19193_1-p71192	C1	9	27.6 0
10892	Bn-N11-p7044918	Bn-C1-p5864232	Bn-scaff_19193_1-p60313	C1	9	27.6 B
10893	Bn-N11-p7086451	Bn-A01-p4220987	Bn-A01-p4220987	C1	9	27.6 B
10894	Bn-N11-p7114660	Bn-Scaffold10778-p4	Bn-scaff_28371_1-p431	C1	9	27.6 B
10895	Bn-N11-p7117379	Bn-Scaffold02163-p2	Bn-scaff_21313_1-p2300	C1	9	27.6 B
10896	Bn-N11-p7117911	Bn-Scaffold02362-p1	Bn-scaff_27215_1-p1538	C1	9	27.6 B
10897	Bn-N11-p7118051	Bn-Scaffold02362-p1	Bn-scaff_27215_1-p1398	C1	9	27.6 B
10898	Bn-N11-p7120745	Bn-C1-p5939768	Bn-scaff_19193_1-p52759	C1	9	27.6 B
10899	Bn-N11-p7158601	Bn-C1-p5979233	Bn-scaff_19193_1-p48813	C1	9	27.6 B
10900	Bn-N11-p7300337	Bn-C1-p6110842	Bn-scaff_19193_1-p35652	C1	9	27.6 B
10901	Bn-N11-p7308199	Bn-C1-p6121681	Bn-scaff_19193_1-p34568	C1	9	27.6 B
10902	Bn-N11-p7462236	Bn-C1-p6238327	Bn-scaff_19193_1-p22903	C1	9	27.6 B
10903	Bn-N11-p7486885	Bn-C1-p6268898	Bn-scaff_19193_1-p19846	C1	9	27.6 B
10904	Bn-N11-p7633637	Bn-C1-p6380938	Bn-scaff_19193_1-p86427	C1	9	27.6 B
10905	Bn-N11-p7717213	Bn-C1-p6453216	Bn-scaff_19193_1-p14149	C1	9	27.6 B
10906	Bn-N11-p7718188	Bn-C1-p6454004	Bn-scaff_19193_1-p13361	C1	9	27.6 B
10907	Bn-N11-p7724724	Bn-C1-p6460576	Bn-scaff_19193_1-p6789	C1	9	27.6 B
10908	Bn-N11-p7806758	Bn-C1-p6921597	Bn-scaff_17731_1-p45413	C1	9	27.6 B
10909	Bn-N11-p7924588	Bn-C1-p6699273	Bn-scaff_17731_1-p23180	C1	9	27.6 B
10910	Bn-N11-p7975313	Bn-C1-p6753304	Bn-scaff_17731_1-p28584	C1	9	27.6 B
10911	Bn-N11-p7975400	Bn-C1-p6753391	Bn-scaff_17731_1-p28592	C1	9	27.6 B
10912	Bn-N11-p8009289	Bn-C1-p6776408	Bn-scaff_17731_1-p30894	C1	9	27.6 B
10913	Bn-N11-p8063778	Bn-C1-p6826183	Bn-scaff_17731_1-p35871	C1	9	27.6 B
10914	Bn-N11-p8154312	Bn-C1-p6897124	Bn-scaff_17731_1-p42966	C1	9	27.6 B
10915	Bn-N11-p8156072	Bn-C1-p6898773	Bn-scaff_17731_1-p43130	C1	9	27.6 B
10916	Bn-N11-p8237562	Bn-C1-p7004066	Bn-scaff_17731_1-p53660	C1	9	27.6 B
10917	Bn-N11-p8237694	Bn-C1-p7004197	Bn-scaff_17731_1-p53673	C1	9	27.6 B
10918	Bn-N11-p8237748	Bn-C1-p7004251	Bn-scaff_17731_1-p53678	C1	9	27.6 B
10919	Bn-N11-p8237978	Bn-C1-p7004482	Bn-scaff_17731_1-p53701	C1	9	27.6 B
10920	Bn-N11-p8238061	Bn-C1-p7004578	Bn-scaff_17731_1-p53711	C1	9	27.6 B
10921	Bn-N11-p8238787	Bn-C1-p7005354	Bn-scaff_17731_1-p53789	C1	9	27.6 B
10922	Bn-N11-p8239767	Bn-C1-p7006081	Bn-scaff_17731_1-p53861	C1	9	27.6 B

10923	Bn-N11-p8464708	Bn-C1-p7215880	Bn-scaff_17731_1-p74841	C1	9	27.6	B
10924	Bn-N11-p8464813	Bn-C1-p7215982	Bn-scaff_17731_1-p74851	C1	9	27.6	B
10925	Bn-N11-p8464817	Bn-C1-p7215986	Bn-scaff_17731_1-p74852	C1	9	27.6	B
10926	Bn-N11-p8465055	Bn-C1-p7216224	Bn-scaff_17731_1-p74876	C1	9	27.6	B
10927	Bn-N11-p8465521	Bn-C1-p7216718	Bn-scaff_17731_1-p74925	C1	9	27.6	B
10928	Bn-N11-p8465667	Bn-C1-p7216921	Bn-scaff_17731_1-p74945	C1	9	27.6	B
10929	Bn-N11-p8465720	Bn-C1-p7216974	Bn-scaff_17731_1-p74951	C1	9	27.6	B
10930	Bn-N11-p8466133	Bn-C1-p7217383	Bn-scaff_17731_1-p74991	C1	9	27.6	B
10931	Bn-N11-p8466317	Bn-C1-p7222892	Bn-scaff_17731_1-p75542	C1	9	27.6	B
10932	Bn-N11-p8466325	Bn-C1-p7222900	Bn-scaff_17731_1-p75543	C1	9	27.6	B
10933	Bn-N11-p8467815	Bn-C1-p7224413	Bn-scaff_17731_1-p75694	C1	9	27.6	B
10934	Bn-N11-p8468439	Bn-C1-p7225124	Bn-scaff_17731_1-p75766	C1	9	27.6	B
10935	Bn-N11-p8535692	Bn-C1-p7294038	Bn-scaff_17731_1-p82657	C1	9	27.6	B
10936	Bn-N11-p8648715	Bn-C1-p7392509	Bn-scaff_17731_1-p92504	C1	9	27.6	B
10937	Bn-N11-p8649272	Bn-C1-p7392944	Bn-scaff_17731_1-p92548	C1	9	27.6	B
10938	Bn-N14-p24100586	Bn-scaffold9512-p788	Bn-scaffold9512-p788	C1	9	27.6	B
10939	Bn-Scaffold04132-p1972	Bn-C13838053-p225	Bn-C13838053-p225	C1	9	27.6	0
10940	Bn-N11-p8709150	Bn-C1-p7446976	Bn-scaff_17731_1-p97951	C1	10	28.6	B
10941	Bn-N11-p8729492	Bn-C1-p7469700	Bn-scaff_17731_1-p10022	C1	10	28.6	B
10942	Bn-N11-p8730195	Bn-C1-p7470348	Bn-scaff_17731_1-p10028	C1	10	28.6	B
10943	Bn-N11-p8731195	Bn-C1-p7470872	Bn-scaff_17731_1-p10034	C1	10	28.6	B
10944	Bn-N11-p8732454	Bn-C1-p7475347	Bn-scaff_17731_1-p10078	C1	10	28.6	B
10945	Bn-N11-p8733053	Bn-C1-p7475947	Bn-scaff_17731_1-p10084	C1	10	28.6	B
10946	Bn-N11-p8733313	Bn-C1-p7476207	Bn-scaff_17731_1-p10087	C1	10	28.6	B
10947	Bn-N11-p8743000	Bn-C1-p7484703	Bn-scaff_17731_1-p10172	C1	10	28.6	B
10948	Bn-N11-p8758607	Bn-C1-p7521519	Bn-scaff_17731_1-p10540	C1	10	28.6	B
10949	Bn-N11-p8770158	Bn-C1-p7530885	Bn-scaff_17827_1-p2478	C1	10	28.6	B
10950	Bn-N11-p8788639	Bn-C1-p7548620	Bn-scaff_17827_1-p20213	C1	10	28.6	B
10951	Bn-N11-p8834382	Bn-C1-p7598025	Bn-scaff_17827_1-p69618	C1	10	28.6	B
10952	Bn-N11-p8872360	Bn-C1-p7639508	Bn-scaff_17827_1-p111101	C1	10	28.6	B
10953	Bn-N11-p8872467	Bn-C1-p7639615	Bn-scaff_17827_1-p11120	C1	10	28.6	B
10954	Bn-N11-p8873169	Bn-C1-p7640327	Bn-scaff_17827_1-p11192	C1	10	28.6	B
10955	Bn-N11-p8995749	Bn-C1-p7800379	Bn-scaff_17827_1-p27197	C1	10	28.6	B
10956	Bn-N11-p9291289	Bn-C1-p8141825	Bn-scaff_17827_1-p61341	C1	11	29.6	B
10957	Bn-N11-p9291345	Bn-C1-p8141881	Bn-scaff_17827_1-p61347	C1	11	29.6	B
10958	Bn-N11-p9303916	Bn-C1-p8150383	Bn-scaff_17827_1-p62197	C1	12	30.6	B
10959	Bn-N11-p9304089	Bn-C1-p8150557	Bn-scaff_17827_1-p62215	C1	12	30.6	B
10960	Bn-N11-p9304146	Bn-C1-p8150614	Bn-scaff_17827_1-p62220	C1	12	30.6	B
10961	Bn-N11-p9304502	Bn-C1-p8150970	Bn-scaff_17827_1-p62256	C1	12	30.6	B
10962	Bn-N11-p9305288	Bn-C1-p8151751	Bn-scaff_17827_1-p62334	C1	12	30.6	B
10963	Bn-N11-p9305320	Bn-C1-p8151783	Bn-scaff_17827_1-p62337	C1	12	30.6	B
10964	Bn-N11-p9310649	Bn-C1-p8157116	Bn-scaff_17827_1-p62870	C1	12	30.6	B
10965	Bn-N11-p9311021	Bn-C1-p8157488	Bn-scaff_17827_1-p629081	C1	12	30.6	B
10966	Bn-N11-p9437941	Bn-C1-p8288604	Bn-scaff_17827_1-p76019	C1	12	30.6	B
10967	Bn-N11-p9450477	Bn-C1-p8300752	Bn-scaff_17827_1-p77234	C1	12	30.6	B
10968	Bn-N11-p9462817	Bn-C1-p8311299	Bn-scaff_17827_1-p78289	C1	12	30.6	B
10969	Bn-N11-p9514839	Bn-C1-p8365332	Bn-scaff_17827_1-p83692	C1	12	30.6	B
10970	Bn-N11-p9525813	Bn-C1-p8378210	Bn-scaff_17827_1-p84980	C1	12	30.6	B
10971	Bn-N11-p9624669	Bn-C1-p8464929	Bn-scaff_17827_1-p93652	C1	12	30.6	B

10972	Bn-N11-p9661445	Bn-C1-p8491995	Bn-scaff_17827_1-p963588	C1	12	30.6	B
10973	Bn-N11-p9669126	Bn-C1-p8501083	Bn-scaff_17827_1-p972676	C1	12	30.6	B
10974	Bn-N11-p9701887	Bn-C1-p8539408	Bn-scaff_17827_1-p101100	C1	12	30.6	B
10975	Bn-N11-p9714385	Bn-C1-p8553868	Bn-scaff_17827_1-p102546	C1	12	30.6	B
10976	Bn-N11-p9716628	Bn-C1-p8555977	Bn-scaff_17827_1-p102757	C1	12	30.6	B
10977	Bn-N11-p9717508	Bn-C1-p8556859	Bn-scaff_17827_1-p102845	C1	12	30.6	B
10978	Bn-N11-p9750208	Bn-Scaffold01143-p1	Bn-scaff_21305_1-p13647	C1	12	30.6	B
10979	Bn-N11-p9835133	Bn-C1-p8635674	Bn-scaff_20210_1-p449263	C1	12	30.6	B
10980	Bn-N11-p9835704	Bn-C1-p8636259	Bn-scaff_20210_1-p448678	C1	12	30.6	B
10981	Bn-N11-p9840034	Bn-C1-p8640056	Bn-scaff_20210_1-p444881	C1	12	30.6	B
10982	Bn-N14-p36294897	Bn-C1-p8549157	Bn-scaff_17827_1-p102075	C1	12	30.6	B
10983	Bn-N11-p10017244	Bn-C1-p8817529	Bn-scaff_20210_1-p267408	C1	13	32.1	B
10984	Bn-N11-p10097199	Bn-C1-p8907461	Bn-scaff_20210_1-p177476	C1	13	32.1	B
10985	Bn-N11-p10112638	Bn-C1-p8922961	Bn-scaff_20210_1-p161976	C1	13	32.1	B
10986	Bn-N11-p10120705	Bn-C1-p8930419	Bn-scaff_20210_1-p154518	C1	13	32.1	B
10987	Bn-N11-p10673559	Bn-C1-p9496207	Bn-scaff_16128_2-p180117	C1	13	32.1	B
10988	Bn-N11-p10703519	Bn-C1-p9525413	Bn-scaff_16128_2-p150911	C1	13	32.1	B
10989	Bn-N11-p10704840	Bn-C1-p9526722	Bn-scaff_16128_2-p149602	C1	13	32.1	B
10990	Bn-N11-p10726850	Bn-C1-p9544440	Bn-scaff_16128_2-p131884	C1	13	32.1	B
10991	Bn-N11-p10727340	Bn-C1-p9544962	Bn-scaff_16128_2-p131362	C1	13	32.1	B
10992	Bn-N11-p10733555	Bn-C1-p9552045	Bn-scaff_16128_2-p124279	C1	13	32.1	B
10993	Bn-N11-p10734730	Bn-C1-p9553219	Bn-scaff_16128_2-p123105	C1	13	32.1	B
10994	Bn-N11-p10747419	Bn-C1-p9562914	Bn-scaff_16128_2-p113410	C1	13	32.1	B
10995	Bn-N11-p10747666	Bn-C1-p9563161	Bn-scaff_16128_2-p113163	C1	13	32.1	B
10996	Bn-N11-p10760565	Bn-C1-p9570074	Bn-scaff_16128_2-p106250	C1	13	32.1	B
10997	Bn-N11-p10852512	Bn-C1-p9647037	Bn-scaff_16128_2-p29287	C1	14	33.6	B
10998	Bn-N11-p10908712	Bn-C1-p9702414	Bn-scaff_20638_1-p150757	C1	14	33.6	B
10999	Bn-N11-p10997090	Bn-C1-p9770761	Bn-scaff_20638_1-p82410	C1	14	33.6	B
11000	Bn-N11-p11114850	Bn-C1-p9854551	Bn-scaff_28666_1-p32984	C1	14	33.6	B
11001	Bn-N11-p11566204	Bn-C1-p10298103	Bn-scaff_24869_1-p152736	C1	15	34.6	B
11002	Bn-N11-p11569442	Bn-C1-p10303140	Bn-scaff_24869_1-p147699	C1	15	34.6	B
11003	Bn-N11-p12604644	Bn-C1-p11291634	Bn-scaff_17592_1-p485859	C1	15	34.6	B
11004	Bn-N11-p12624910	Bn-C1-p11312193	Bn-scaff_17592_1-p506418	C1	15	34.6	B
11005	Bn-N11-p12644159	Bn-C1-p11336483	Bn-scaff_17592_1-p530708	C1	15	34.6	B
11006	Bn-N11-p12644631	Bn-C1-p11336955	Bn-scaff_17592_1-p531180	C1	15	34.6	B
11007	Bn-N17-p21549995	Bn-C7-p21425341	Bn-scaff_23400_1-p109544	C1	15	34.6	B
11008	Bn-N11-p12953772	Bn-C1-p11604573	Bn-scaff_17592_1-p798798	C1	16	35.6	B
11009	Bn-N11-p12954035	Bn-C1-p11604836	Bn-scaff_17592_1-p799061	C1	16	35.6	B
11010	Bn-N11-p12958722	Bn-C1-p11609365	Bn-scaff_17369_1-p124477	C1	16	35.6	B
11011	Bn-N11-p12958795	Bn-C1-p11609438	Bn-scaff_17369_1-p124470	C1	16	35.6	B
11012	Bn-N11-p13063113	Bn-C1-p11703836	Bn-scaff_17369_1-p115030	C1	16	35.6	B
11013	Bn-N11-p13063126	Bn-C1-p11703849	Bn-scaff_17369_1-p115029	C1	16	35.6	B
11014	Bn-N11-p13063241	Bn-C1-p11703964	Bn-scaff_17369_1-p115017	C1	16	35.6	B
11015	Bn-N11-p13100683	Bn-C1-p11741511	Bn-scaff_17369_1-p111263	C1	17	36.6	B
11016	Bn-N11-p13101589	Bn-C1-p11742417	Bn-scaff_17369_1-p111172	C1	17	36.6	B
11017	Bn-N11-p13114617	Bn-C1-p11754972	Bn-scaff_17369_1-p109916	C1	17	36.6	B
11018	Bn-N11-p13132361	Bn-C1-p11765514	Bn-scaff_17369_1-p108862	C1	17	36.6	B
11019	Bn-N11-p13157952	Bn-C1-p11793383	Bn-scaff_17369_1-p106075	C1	17	36.6	B
11020	Bn-N11-p13201905	Bn-C1-p11837138	Bn-scaff_17369_1-p101700	C1	17	36.6	B

11021	Bn-N11-p13229265	Bn-C1-p11861892	Bn-scaff_17369_1-p992249	C1	18	37.6	B
11022	Bn-N11-p13242643	Bn-C1-p11872982	Bn-scaff_17369_1-p981159	C1	18	37.6	B
11023	Bn-N11-p13278652	Bn-C1-p11899755	Bn-scaff_17369_1-p954386	C1	18	37.6	B
11024	Bn-N11-p13383971	Bn-C1-p11998398	Bn-scaff_17369_1-p855743	C1	18	37.6	B
11025	Bn-N11-p13392417	Bn-C1-p12006398	Bn-scaff_17369_1-p847743	C1	18	37.6	B
11026	Bn-N11-p13392517	Bn-C1-p12006498	Bn-scaff_17369_1-p847643	C1	18	37.6	B
11027	Bn-N11-p13396823	Bn-C1-p12011052	Bn-scaff_17369_1-p843089	C1	19	39.6	B
11028	Bn-N11-p13409174	Bn-C1-p12024696	Bn-scaff_17369_1-p829445	C1	19	39.6	B
11029	Bn-N11-p13428669	Bn-C1-p12044103	Bn-scaff_17369_1-p810038	C1	19	39.6	B
11030	Bn-N11-p13434430	Bn-C1-p12049859	Bn-scaff_17369_1-p804282	C1	19	39.6	B
11031	Bn-N11-p13436458	Bn-C1-p12051887	Bn-scaff_17369_1-p802254	C1	19	39.6	B
11032	Bn-N11-p13472293	Bn-C1-p12092818	Bn-scaff_17369_1-p761323	C1	19	39.6	B
11033	Bn-N11-p13493897	Bn-C1-p12113583	Bn-scaff_17369_1-p740558	C1	19	39.6	B
11034	Bn-N11-p13551026	Bn-C1-p12174838	Bn-scaff_17369_1-p679303	C1	19	39.6	B
11035	Bn-N11-p13553258	Bn-C1-p12177076	Bn-scaff_17369_1-p677065	C1	19	39.6	B
11036	Bn-N11-p13604090	Bn-C1-p12228961	Bn-scaff_17369_1-p625180	C1	20	40.6	B
11037	Bn-N11-p13608098	Bn-C1-p12232992	Bn-scaff_17369_1-p621149	C1	20	40.6	B
11038	Bn-N11-p13616822	Bn-C1-p12247264	Bn-scaff_17369_1-p606877	C1	20	40.6	B
11039	Bn-N11-p13686160	Bn-C1-p12294431	Bn-scaff_17369_1-p559710	C1	20	40.6	B
11040	Bn-N11-p13834154	Bn-C1-p12467566	Bn-scaff_17369_1-p386575	C1	20	40.6	B
11041	Bn-N11-p13928723	Bn-C1-p12582975	Bn-scaff_17369_1-p271166	C1	21	41.6	B
11042	Bn-N11-p14109221	Bn-C1-p12738046	Bn-scaff_17369_1-p116095	C1	22	43.6	B
11043	Bn-N11-p14151844	Bn-C1-p12770524	Bn-scaff_17369_1-p83617	C1	22	43.6	B
11044	Bn-N11-p14229035	Bn-C1-p12821338	Bn-scaff_17369_1-p32803	C1	22	43.6	B
11045	Bn-N11-p14259285	Bn-C1-p12850747	Bn-scaff_17369_1-p3394	C1	22	43.6	B
11046	Bn-N11-p14307087	Bn-C1-p12903498	Bn-scaff_24947_1-p112023	C1	22	43.6	B
11047	Bn-N11-p14312299	Bn-C1-p12908708	Bn-scaff_24947_1-p106813	C1	22	43.6	B
11048	Bn-N11-p14315450	Bn-C1-p12911864	Bn-scaff_24947_1-p103657	C1	22	43.6	B
11049	Bn-N11-p14320251	Bn-C1-p12916669	Bn-scaff_24947_1-p98852	C1	22	43.6	B
11050	Bn-N11-p14327744	Bn-C1-p12925636	Bn-scaff_24947_1-p89885	C1	22	43.6	B
11051	Bn-N11-p14327891	Bn-C1-p12925783	Bn-scaff_24947_1-p89738	C1	22	43.6	B
11052	Bn-N11-p14327948	Bn-C1-p12925840	Bn-scaff_24947_1-p89681	C1	22	43.6	B
11053	Bn-N11-p14328440	Bn-C1-p12926333	Bn-scaff_24947_1-p89188	C1	22	43.6	B
11054	Bn-N11-p14329807	Bn-C1-p12927700	Bn-scaff_24947_1-p87821	C1	22	43.6	B
11055	Bn-N11-p14330213	Bn-C1-p12928106	Bn-scaff_24947_1-p87415	C1	22	43.6	B
11056	Bn-N11-p14330790	Bn-C1-p12928683	Bn-scaff_24947_1-p86838	C1	22	43.6	B
11057	Bn-N11-p14333785	Bn-C1-p12931470	Bn-scaff_24947_1-p84051	C1	22	43.6	B
11058	Bn-N11-p14333988	Bn-C1-p12931673	Bn-scaff_24947_1-p83848	C1	22	43.6	B
11059	Bn-N11-p14338670	Bn-C1-p12936353	Bn-scaff_24947_1-p79168	C1	22	43.6	B
11060	Bn-N11-p14377169	Bn-C1-p12966748	Bn-scaff_24947_1-p48773	C1	22	43.6	B
11061	Bn-N11-p14378924	Bn-C1-p12968509	Bn-scaff_24947_1-p47012	C1	22	43.6	B
11062	Bn-N11-p14379712	Bn-C1-p12969297	Bn-scaff_24947_1-p46224	C1	22	43.6	B
11063	Bn-N11-p14379852	Bn-C1-p12969437	Bn-scaff_24947_1-p46084	C1	22	43.6	B
11064	Bn-N11-p14382144	Bn-C1-p12971717	Bn-scaff_24947_1-p43804	C1	22	43.6	B
11065	Bn-N11-p14382333	Bn-C1-p12971906	Bn-scaff_24947_1-p43615	C1	22	43.6	0
11066	Bn-N11-p14383922	Bn-C1-p12976646	Bn-scaff_24947_1-p38875	C1	22	43.6	B
11067	Bn-N11-p14397369	Bn-C1-p12988110	Bn-scaff_24947_1-p27411	C1	22	43.6	B
11068	Bn-N11-p14402159	Bn-C1-p12992873	Bn-scaff_24947_1-p22648	C1	22	43.6	B
11069	Bn-N11-p14412175	Bn-C1-p13002376	Bn-scaff_24947_1-p13145	C1	22	43.6	B

11070	Bn-N11-p14415569	Bn-C1-p13005765	Bn-scaff_24947_1-p9756	C1	22	43.6	B
11071	Bn-N11-p14418144	Bn-C1-p13008330	Bn-scaff_24947_1-p7191	C1	22	43.6	B
11072	Bn-N11-p15104442	Bn-C1-p13594012	Bn-scaff_19614_1-p325704	C1	23	46.6	B
11073	Bn-N11-p15124720	Bn-C1-p13614132	Bn-scaff_19614_1-p305584	C1	23	46.6	B
11074	Bn-N11-p15168215	Bn-C1-p13661237	Bn-scaff_19614_1-p258479	C1	23	46.6	B
11075	Bn-N11-p15348140	Bn-C1-p13866232	Bn-scaff_19614_1-p53484	C1	24	48.6	B
11076	Bn-N11-p15353887	Bn-C1-p13883693	Bn-scaff_19614_1-p36023	C1	24	48.6	B
11077	Bn-N11-p15364111	Bn-C1-p13892721	Bn-scaff_19614_1-p26995	C1	24	48.6	B
11078	Bn-N11-p15447324	Bn-C1-p13989834	Bn-scaff_27129_1-p70019	C1	24	48.6	B
11079	Bn-N11-p15472735	Bn-C1-p14018166	Bn-scaff_27129_1-p98351	C1	24	48.6	B
11080	Bn-N11-p15477919	Bn-C1-p14023031	Bn-scaff_27129_1-p103216	C1	24	48.6	B
11081	Bn-N11-p15479954	Bn-C1-p14025065	Bn-scaff_27129_1-p105250	C1	24	48.6	B
11082	Bn-N11-p15492774	Bn-C1-p14030679	Bn-scaff_27129_1-p110864	C1	24	48.6	B
11083	Bn-N11-p15498551	Bn-C1-p14032066	Bn-scaff_27129_1-p112251	C1	24	48.6	B
11084	Bn-N12-p10245784	Bn-C2-p11411592	Bn-scaff_15712_5-p332728	C1	24	48.6	B
11085	Bn-N1-p9440919	Bn-C1-p14039763	Bn-scaff_27129_1-p119948	C1	24	48.6	B
11086	Bn-N11-p15748495	Bn-C1-p14268880	Bn-scaff_15747_1-p104038	C1	25	49.6	B
11087	Bn-N11-p15750118	Bn-C1-p14270475	Bn-scaff_15747_1-p105633	C1	25	49.6	0
11088	Bn-N11-p15753797	Bn-C1-p14273019	Bn-scaff_15747_1-p108177	C1	25	49.6	0
11089	Bn-N11-p15812621	Bn-C1-p14332796	Bn-scaff_15747_1-p167954	C1	26	50.5	B
11090	Bn-N4-p19097673	Bn-A09-p6090256	Bn-A09-p6090256	C1	26	50.5	B
11091	Bn-N11-p16052156	Bn-C1-p14565806	Bn-scaff_15747_1-p400964	C1	27	52.4	B
11092	Bn-N11-p16053796	Bn-C1-p14566338	Bn-scaff_15747_1-p401496	C1	27	52.4	B
11093	Bn-N11-p16054429	Bn-C1-p14566970	Bn-scaff_15747_1-p402128	C1	27	52.4	B
11094	Bn-N11-p16093672	Bn-C1-p14610861	Bn-scaff_15747_1-p446019	C1	27	52.4	B
11095	Bn-N11-p16226717	Bn-C1-p14756484	Bn-scaff_15747_1-p591642	C1	27	52.4	B
11096	Bn-N11-p16293667	Bn-C1-p14808184	Bn-scaff_15747_1-p643342	C1	27	52.4	B
11097	Bn-N11-p16766478	Bn-C1-p15251711	Bn-scaff_15803_1-p574757	C1	27	52.4	B
11098	Bn-N11-p16767481	Bn-C1-p15252712	Bn-scaff_15803_1-p573756	C1	27	52.4	B
11099	Bn-N11-p16859764	Bn-C1-p15343509	Bn-scaff_15803_1-p482959	C1	27	52.4	B
11100	Bn-N11-p16877789	Bn-C1-p15360189	Bn-scaff_15803_1-p466279	C1	27	52.4	B
11101	Bn-N11-p16889137	Bn-C1-p15370409	Bn-scaff_15803_1-p456059	C1	27	52.4	B
11102	Bn-N11-p16890850	Bn-C1-p15372120	Bn-scaff_15803_1-p454348	C1	27	52.4	B
11103	Bn-N11-p16890857	Bn-C1-p15372127	Bn-scaff_15803_1-p454341	C1	27	52.4	B
11104	Bn-N11-p17494544	Bn-C1-p15889924	Bn-scaff_17203_1-p551127	C1	27	52.4	B
11105	Bn-N11-p17501513	Bn-C1-p15896569	Bn-scaff_17203_1-p544482	C1	27	52.4	B
11106	Bn-N11-p17723407	Bn-C1-p16122777	Bn-scaff_17203_1-p318274	C1	27	52.4	B
11107	Bn-N11-p17726397	Bn-C1-p16125761	Bn-scaff_17203_1-p315290	C1	27	52.4	B
11108	Bn-N11-p18316438	Bn-C1-p16621131	Bn-scaff_22790_1-p179981	C1	27	52.4	B
11109	Bn-N11-p18318665	Bn-C1-p16623358	Bn-scaff_22790_1-p182208	C1	27	52.4	B
11110	Bn-N11-p18327293	Bn-C1-p16628246	Bn-scaff_22790_1-p187096	C1	27	52.4	B
11111	Bn-N11-p18479753	Bn-C1-p16812030	Bn-scaff_22790_1-p370880	C1	27	52.4	B
11112	Bn-N11-p18486935	Bn-C1-p16819210	Bn-scaff_22790_1-p378060	C1	27	52.4	B
11113	Bn-N11-p18564588	Bn-C1-p16895151	Bn-scaff_22790_1-p454001	C1	27	52.4	B
11114	Bn-N11-p18568478	Bn-C1-p16898667	Bn-scaff_22790_1-p457517	C1	27	52.4	B
11115	Bn-N11-p18569526	Bn-C1-p16899714	Bn-scaff_22790_1-p458564	C1	27	52.4	B
11116	Bn-N11-p18589218	Bn-C1-p16916869	Bn-scaff_22790_1-p475719	C1	27	52.4	B
11117	Bn-N11-p18799884	Bn-C1-p17113803	Bn-scaff_22790_1-p672653	C1	27	52.4	B
11118	Bn-N11-p18837092	Bn-C1-p17170280	Bn-scaff_22790_1-p729130	C1	27	52.4	B

11119	Bn-N1-p9970782	Bn-A01-p10410665	Bn-A01-p10410665	C1	27	52.4	B
11120	Bn-N11-p18914484	Bn-C1-p17234515	Bn-scaff_22790_1-p79336	C1	28	54.3	B
11121	Bn-N11-p18932545	Bn-C1-p17250519	Bn-scaff_22790_1-p80936	C1	28	54.3	B
11122	Bn-N11-p18946156	Bn-C1-p17264222	Bn-scaff_22790_1-p82307	C1	28	54.3	B
11123	Bn-N11-p20941887	Bn-scaffold7263-p39	Bn-scaffold7263-p395	C1	28	54.3	B
11124	Bn-N11-p21189148	Bn-scaffold9702-p25	Bn-scaffold9702-p250	C1	28	54.3	B
11125	Bn-N11-p24674648	Bn-scaffold9986-p66	Bn-scaffold9986-p660	C1	28	54.3	B
11126	Bn-N11-p24674717	Bn-scaffold9986-p59	Bn-scaffold9986-p592	C1	28	54.3	B
11127	Bn-N11-p24724911	Bn-C14045238-p669	Bn-C14045238-p669	C1	28	54.3	B
11128	Bn-N11-p24725779	Bn-scaffold17178-p2	Bn-scaffold17178-p245	C1	28	54.3	B
11129	Bn-N11-p25098816	Bn-A01-p15000034	Bn-A01-p15000034	C1	28	54.3	B
11130	Bn-N11-p25172656	Bn-A01-p15043967	Bn-A01-p15043967	C1	28	54.3	B
11131	Bn-N11-p25656722	Bn-A01-p15295874	Bn-A01-p15295874	C1	28	54.3	0
11132	Bn-N11-p27186372	Bn-Scaffold022794-p	Bn-Scaffold022794-p55	C1	28	54.3	B
11133	Bn-N11-p31369543	Bn-A01-p17549137	Bn-A01-p17549137	C1	28	54.3	B
11134	Bn-N1-p13748387	Bn-A01-p14169412	Bn-A01-p14169412	C1	28	54.3	B
11135	Bn-N1-p18190500	Bn-A01-p17550125	Bn-A01-p17550125	C1	28	54.3	B
11136	Bn-Scaffold01971-p4450	Bn-C14045238-p1241	Bn-C14045238-p1241	C1	28	54.3	B
11137	Bn-N11-p19034482	Bn-C1-p17361225	Bn-scaff_22790_1-p92007	C1	29	54.3	B
11138	Bn-N11-p19066405	Bn-C1-p17393542	Bn-scaff_22790_1-p95239	C1	29	54.3	B
11139	Bn-N11-p19121843	Bn-C1-p17452130	Bn-scaff_22790_1-p10109	C1	29	54.3	B
11140	Bn-N11-p19123140	Bn-C1-p17453424	Bn-scaff_22790_1-p10122	C1	29	54.3	B
11141	Bn-N11-p19123140	Bn-C1-p17453425	Bn-scaff_22790_1-p10122	C1	29	54.3	B
11142	Bn-N11-p19123934	Bn-C1-p17454221	Bn-scaff_22790_1-p10130	C1	29	54.3	B
11143	Bn-N11-p19132449	Bn-C1-p17462658	Bn-scaff_22790_1-p10215	C1	29	54.3	B
11144	Bn-N11-p19135762	Bn-C1-p17465971	Bn-scaff_22790_1-p10248	C1	29	54.3	B
11145	Bn-N11-p19137363	Bn-C1-p17467572	Bn-scaff_22790_1-p10264	C1	29	54.3	B
11146	Bn-N11-p19155470	Bn-C1-p17484930	Bn-scaff_22790_1-p10437	C1	29	54.3	B
11147	Bn-N11-p19184274	Bn-C1-p17512501	Bn-scaff_22790_1-p10713	C1	29	54.3	B
11148	Bn-N11-p19234940	Bn-C1-p17566140	Bn-scaff_22790_1-p11249	C1	29	54.3	B
11149	Bn-N11-p19239636	Bn-C1-p17570835	Bn-scaff_22790_1-p11296	C1	29	54.3	B
11150	Bn-N11-p19247459	Bn-C1-p17583214	Bn-scaff_22790_1-p11420	C1	29	54.3	B
11151	Bn-N11-p19260809	Bn-C1-p17596483	Bn-scaff_22790_1-p11553	C1	29	54.3	B
11152	Bn-N11-p19273183	Bn-C1-p17610688	Bn-scaff_22790_1-p11695	C1	29	54.3	B
11153	Bn-N11-p19275553	Bn-C1-p17612827	Bn-scaff_22790_1-p11716	C1	29	54.3	B
11154	Bn-N11-p19333774	Bn-C1-p17673967	Bn-scaff_22790_1-p12328	C1	29	54.3	B
11155	Bn-N11-p19334750	Bn-C1-p17674943	Bn-scaff_22790_1-p12337	C1	29	54.3	B
11156	Bn-N11-p19357910	Bn-C1-p17706136	Bn-scaff_22790_1-p12649	C1	29	54.3	B
11157	Bn-N11-p19361291	Bn-C1-p17709517	Bn-scaff_22790_1-p12683	C1	29	54.3	B
11158	Bn-N11-p19364095	Bn-C1-p17712320	Bn-scaff_22790_1-p12711	C1	29	54.3	B
11159	Bn-N11-p19366342	Bn-C1-p17714510	Bn-scaff_22790_1-p12733	C1	29	54.3	B
11160	Bn-N11-p19367008	Bn-C1-p17715176	Bn-scaff_22790_1-p12740	C1	29	54.3	B
11161	Bn-N11-p19425351	Bn-C1-p17776261	Bn-scaff_22790_1-p13351	C1	29	54.3	B
11162	Bn-N11-p19431092	Bn-C1-p17781987	Bn-scaff_22790_1-p13408	C1	29	54.3	B
11163	Bn-N11-p19512320	Bn-C1-p17865111	Bn-scaff_20250_1-p82478	C1	29	54.3	B
11164	Bn-N11-p19525137	Bn-C1-p17878102	Bn-scaff_20250_1-p95469	C1	29	54.3	B
11165	Bn-N11-p19593226	Bn-C1-p17947612	Bn-scaff_20250_1-p16497	C1	29	54.3	B
11166	Bn-N11-p19616684	Bn-C1-p17973590	Bn-scaff_20250_1-p19095	C1	29	54.3	B
11167	Bn-N11-p19645414	Bn-C1-p17996003	Bn-scaff_20250_1-p21337	C1	29	54.3	B

11168	Bn-N11-p19672238	Bn-C1-p18022645	Bn-scaff_20250_1-p24001	C1	29	54.3	B
11169	Bn-N11-p19684767	Bn-C1-p18035150	Bn-scaff_20250_1-p25251	C1	29	54.3	B
11170	Bn-N11-p19684941	Bn-C1-p18035324	Bn-scaff_20250_1-p25269	C1	29	54.3	B
11171	Bn-N11-p19744590	Bn-C1-p18093055	Bn-scaff_20250_1-p31042	C1	29	54.3	B
11172	Bn-N11-p19754782	Bn-C1-p18103249	Bn-scaff_20250_1-p32061	C1	29	54.3	0
11173	Bn-N11-p19823085	Bn-C1-p18169638	Bn-scaff_20250_1-p38700	C1	29	54.3	B
11174	Bn-N11-p19830322	Bn-C1-p18171157	Bn-scaff_20250_1-p38852	C1	29	54.3	B
11175	Bn-N11-p19835449	Bn-C1-p18176635	Bn-scaff_20250_1-p39400	C1	29	54.3	B
11176	Bn-N11-p19836710	Bn-C1-p18177893	Bn-scaff_20250_1-p39526	C1	29	54.3	B
11177	Bn-N11-p19840199	Bn-C1-p18184735	Bn-scaff_20250_1-p40210	C1	29	54.3	B
11178	Bn-N11-p19840254	Bn-C1-p18184790	Bn-scaff_20250_1-p40215	C1	29	54.3	B
11179	Bn-N11-p19840589	Bn-C1-p18185124	Bn-scaff_20250_1-p40249	C1	29	54.3	B
11180	Bn-N11-p19889874	Bn-C1-p18212417	Bn-scaff_20250_1-p42978	C1	29	54.3	B
11181	Bn-N11-p19896508	Bn-C1-p18217739	Bn-scaff_20250_1-p43510	C1	29	54.3	B
11182	Bn-N11-p19897691	Bn-C1-p18218922	Bn-scaff_20250_1-p43628	C1	29	54.3	B
11183	Bn-N11-p19903416	Bn-C1-p18224647	Bn-scaff_20250_1-p44201	C1	29	54.3	B
11184	Bn-N11-p19905733	Bn-C1-p18226964	Bn-scaff_20250_1-p44433	C1	29	54.3	B
11185	Bn-N11-p19905777	Bn-C1-p18227008	Bn-scaff_20250_1-p44437	C1	29	54.3	B
11186	Bn-N11-p20019737	Bn-C1-p18341736	Bn-scaff_20250_2-p25576	C1	29	54.3	B
11187	Bn-N11-p20020192	Bn-C1-p18342192	Bn-scaff_20250_2-p25530	C1	29	54.3	B
11188	Bn-N11-p20037658	Bn-C1-p18356367	Bn-scaff_20250_2-p24112	C1	29	54.3	B
11189	Bn-N11-p20071364	Bn-C1-p18389077	Bn-scaff_20250_2-p20841	C1	29	54.3	B
11190	Bn-N11-p20071508	Bn-C1-p18389221	Bn-scaff_20250_2-p20827	C1	29	54.3	B
11191	Bn-N11-p20073020	Bn-C1-p18390733	Bn-scaff_20250_2-p20676	C1	29	54.3	B
11192	Bn-N11-p20077295	Bn-C1-p18395008	Bn-scaff_20250_2-p20248	C1	29	54.3	B
11193	Bn-N11-p20121226	Bn-C1-p18435404	Bn-scaff_20250_2-p16209	C1	29	54.3	B
11194	Bn-N11-p20134343	Bn-C1-p18449936	Bn-scaff_20250_2-p14756	C1	29	54.3	B
11195	Bn-N11-p20143905	Bn-C1-p18468075	Bn-scaff_20250_2-p12942	C1	29	54.3	B
11196	Bn-N11-p20188774	Bn-C1-p18515001	Bn-scaff_20250_2-p82495	C1	29	54.3	B
11197	Bn-N11-p20198536	Bn-C1-p18526263	Bn-scaff_20250_2-p71233	C1	29	54.3	B
11198	Bn-N11-p20198686	Bn-C1-p18526413	Bn-scaff_20250_2-p71083	C1	29	54.3	B
11199	Bn-N11-p20199049	Bn-C1-p18526776	Bn-scaff_20250_2-p70720	C1	29	54.3	B
11200	Bn-N11-p20199265	Bn-C1-p18526992	Bn-scaff_20250_2-p70504	C1	29	54.3	B
11201	Bn-N11-p20199776	Bn-C1-p18527503	Bn-scaff_20250_2-p69993	C1	29	54.3	B
11202	Bn-N11-p20199933	Bn-C1-p18527660	Bn-scaff_20250_2-p69836	C1	29	54.3	B
11203	Bn-N11-p20200905	Bn-C1-p18528632	Bn-scaff_20250_2-p68864	C1	29	54.3	B
11204	Bn-N11-p20200966	Bn-C1-p18528693	Bn-scaff_20250_2-p68803	C1	29	54.3	B
11205	Bn-N11-p20202197	Bn-C1-p18530032	Bn-scaff_20250_2-p67464	C1	29	54.3	B
11206	Bn-N11-p20217620	Bn-C1-p18543008	Bn-scaff_20250_2-p54488	C1	29	54.3	B
11207	Bn-N11-p20218343	Bn-C1-p18543710	Bn-scaff_20250_2-p53786	C1	29	54.3	B
11208	Bn-N11-p20250688	Bn-C1-p18574208	Bn-scaff_20250_2-p23288	C1	29	54.3	B
11209	Bn-N11-p20260538	Bn-C1-p18582781	Bn-scaff_20250_2-p14715	C1	29	54.3	B
11210	Bn-N11-p20262459	Bn-C1-p18584702	Bn-scaff_20250_2-p12794	C1	29	54.3	B
11211	Bn-N11-p20262697	Bn-C1-p18584940	Bn-scaff_20250_2-p12556	C1	29	54.3	B
11212	Bn-N11-p20262783	Bn-C1-p18585026	Bn-scaff_20250_2-p12470	C1	29	54.3	B
11213	Bn-N11-p20263188	Bn-C1-p18585431	Bn-scaff_20250_2-p12065	C1	29	54.3	B
11214	Bn-N11-p20263243	Bn-C1-p18585486	Bn-scaff_20250_2-p12010	C1	29	54.3	B
11215	Bn-N11-p20277044	Bn-C1-p18609938	Bn-scaff_16056_1-p12343	C1	29	54.3	B
11216	Bn-N11-p20280169	Bn-C1-p18613063	Bn-scaff_16056_1-p15468	C1	29	54.3	B

11217	Bn-N11-p20280257	Bn-C1-p18613151	Bn-scaff_16056_1-p15556	C1	29	54.3	B
11218	Bn-N11-p20281023	Bn-C1-p18613917	Bn-scaff_16056_1-p16322	C1	29	54.3	B
11219	Bn-N11-p20288123	Bn-C1-p18621022	Bn-scaff_16056_1-p23427	C1	29	54.3	B
11220	Bn-N11-p20288182	Bn-C1-p18621081	Bn-scaff_16056_1-p23486	C1	29	54.3	B
11221	Bn-N11-p20290072	Bn-C1-p18622971	Bn-scaff_16056_1-p25376	C1	29	54.3	B
11222	Bn-N11-p20310738	Bn-C1-p18643148	Bn-scaff_16627_1-p107038	C1	29	54.3	B
11223	Bn-N11-p20312370	Bn-C1-p18644780	Bn-scaff_16627_1-p105406	C1	29	54.3	B
11224	Bn-N11-p20332736	Bn-C1-p18683029	Bn-scaff_16627_1-p67157	C1	29	54.3	B
11225	Bn-N11-p20332793	Bn-C1-p18683086	Bn-scaff_16627_1-p67100	C1	29	54.3	B
11226	Bn-N11-p20333697	Bn-C1-p18683990	Bn-scaff_16627_1-p66196	C1	29	54.3	B
11227	Bn-N11-p20346378	Bn-C1-p18697884	Bn-scaff_16627_1-p52302	C1	29	54.3	B
11228	Bn-N11-p20391616	Bn-C1-p18742426	Bn-scaff_16627_1-p7760	C1	29	54.3	B
11229	Bn-N11-p20627780	Bn-C1-p18861393	Bn-scaff_19364_1-p650716	C1	29	54.3	B
11230	Bn-N11-p20676204	Bn-C1-p18911448	Bn-scaff_19364_1-p600661	C1	29	54.3	0
11231	Bn-N11-p20684883	Bn-C1-p18934303	Bn-scaff_19364_1-p577806	C1	29	54.3	B
11232	Bn-N11-p20687404	Bn-C1-p18936831	Bn-scaff_19364_1-p575278	C1	29	54.3	B
11233	Bn-N11-p20693166	Bn-C1-p18942451	Bn-scaff_19364_1-p569658	C1	29	54.3	B
11234	Bn-N11-p20700809	Bn-C1-p18950129	Bn-scaff_19364_1-p561980	C1	29	54.3	B
11235	Bn-N11-p20703590	Bn-C1-p18953166	Bn-scaff_19364_1-p558943	C1	29	54.3	B
11236	Bn-N11-p20726621	Bn-C1-p18975007	Bn-scaff_19364_1-p537102	C1	29	54.3	B
11237	Bn-N11-p20737582	Bn-C1-p18985585	Bn-scaff_19364_1-p526524	C1	29	54.3	B
11238	Bn-N11-p20737737	Bn-C1-p18985740	Bn-scaff_19364_1-p526369	C1	29	54.3	B
11239	Bn-N11-p20737851	Bn-C1-p18985854	Bn-scaff_19364_1-p526255	C1	29	54.3	B
11240	Bn-N11-p20744021	Bn-C1-p18987414	Bn-scaff_19364_1-p524695	C1	29	54.3	B
11241	Bn-N11-p20744306	Bn-C1-p18987699	Bn-scaff_19364_1-p524410	C1	29	54.3	B
11242	Bn-N11-p20744652	Bn-C1-p18988045	Bn-scaff_19364_1-p524064	C1	29	54.3	B
11243	Bn-N11-p20756421	Bn-C1-p19002783	Bn-scaff_19364_1-p509326	C1	29	54.3	B
11244	Bn-N11-p20765316	Bn-C1-p19042549	Bn-scaff_19364_1-p469560	C1	29	54.3	B
11245	Bn-N11-p20766233	Bn-C1-p19043465	Bn-scaff_19364_1-p468644	C1	29	54.3	B
11246	Bn-N11-p20766689	Bn-C1-p19043921	Bn-scaff_19364_1-p468188	C1	29	54.3	B
11247	Bn-N11-p20767629	Bn-C1-p19044883	Bn-scaff_19364_1-p467226	C1	29	54.3	B
11248	Bn-N11-p20767717	Bn-C1-p19044971	Bn-scaff_19364_1-p467138	C1	29	54.3	B
11249	Bn-N11-p20767842	Bn-C1-p19045096	Bn-scaff_19364_1-p467013	C1	29	54.3	B
11250	Bn-N11-p20768113	Bn-C1-p19045373	Bn-scaff_19364_1-p466736	C1	29	54.3	B
11251	Bn-N11-p20768206	Bn-C1-p19045466	Bn-scaff_19364_1-p466643	C1	29	54.3	B
11252	Bn-N11-p20768644	Bn-C1-p19045904	Bn-scaff_19364_1-p466205	C1	29	54.3	B
11253	Bn-N11-p20769021	Bn-C1-p19046280	Bn-scaff_19364_1-p465829	C1	29	54.3	B
11254	Bn-N11-p20771519	Bn-C1-p19048840	Bn-scaff_19364_1-p463269	C1	29	54.3	B
11255	Bn-N11-p20772976	Bn-C1-p19049481	Bn-scaff_19364_1-p462628	C1	29	54.3	B
11256	Bn-N11-p20773409	Bn-C1-p19049910	Bn-scaff_19364_1-p462199	C1	29	54.3	B
11257	Bn-N11-p20773461	Bn-C1-p19049962	Bn-scaff_19364_1-p462147	C1	29	54.3	B
11258	Bn-N11-p20773470	Bn-C1-p19049971	Bn-scaff_19364_1-p462138	C1	29	54.3	B
11259	Bn-N11-p20774309	Bn-C1-p19050822	Bn-scaff_19364_1-p461287	C1	29	54.3	B
11260	Bn-N11-p20774567	Bn-C1-p19051080	Bn-scaff_19364_1-p461029	C1	29	54.3	B
11261	Bn-N11-p20775617	Bn-C1-p19052142	Bn-scaff_19364_1-p459967	C1	29	54.3	B
11262	Bn-N11-p20788085	Bn-C1-p19056223	Bn-scaff_19364_1-p455886	C1	29	54.3	B
11263	Bn-N11-p20788446	Bn-C1-p19056584	Bn-scaff_19364_1-p455525	C1	29	54.3	B
11264	Bn-N11-p20789527	Bn-C1-p19058694	Bn-scaff_19364_1-p453415	C1	29	54.3	B
11265	Bn-N11-p20802561	Bn-C1-p19067867	Bn-scaff_19364_1-p444242	C1	29	54.3	B

11266	Bn-N11-p20803036	Bn-C1-p19068343	Bn-scaff_19364_1-p44376	C1	29	54.3	B
11267	Bn-N11-p20803656	Bn-C1-p19068966	Bn-scaff_19364_1-p44314	C1	29	54.3	B
11268	Bn-N11-p20826482	Bn-C1-p19088498	Bn-scaff_19364_1-p42361	C1	29	54.3	B
11269	Bn-N11-p20858391	Bn-C1-p19122298	Bn-scaff_19364_1-p38981	C1	29	54.3	B
11270	Bn-N11-p20858550	Bn-C1-p19122457	Bn-scaff_19364_1-p38965	C1	29	54.3	B
11271	Bn-N11-p20858750	Bn-C1-p19122657	Bn-scaff_19364_1-p38945	C1	29	54.3	B
11272	Bn-N11-p20858886	Bn-C1-p19122793	Bn-scaff_19364_1-p38931	C1	29	54.3	B
11273	Bn-N11-p20859135	Bn-C1-p19123042	Bn-scaff_19364_1-p38906	C1	29	54.3	B
11274	Bn-N11-p20859192	Bn-C1-p19123099	Bn-scaff_19364_1-p38901	C1	29	54.3	B
11275	Bn-N11-p20859411	Bn-C1-p19123313	Bn-scaff_19364_1-p38879	C1	29	54.3	B
11276	Bn-N11-p20859627	Bn-C1-p19123529	Bn-scaff_19364_1-p38858	C1	29	54.3	B
11277	Bn-N11-p20859647	Bn-C1-p19123549	Bn-scaff_19364_1-p38856	C1	29	54.3	B
11278	Bn-N11-p20861366	Bn-C1-p19130589	Bn-scaff_19364_1-p38152	C1	29	54.3	B
11279	Bn-N11-p20861973	Bn-C1-p19131196	Bn-scaff_19364_1-p38091	C1	29	54.3	B
11280	Bn-N11-p20862245	Bn-C1-p19131468	Bn-scaff_19364_1-p38064	C1	29	54.3	B
11281	Bn-N11-p20863960	Bn-C1-p19133179	Bn-scaff_19364_1-p37893	C1	29	54.3	B
11282	Bn-N11-p20866688	Bn-C1-p19135798	Bn-scaff_19364_1-p37631	C1	29	54.3	B
11283	Bn-N11-p20866952	Bn-C1-p19136062	Bn-scaff_19364_1-p37604	C1	29	54.3	B
11284	Bn-N11-p20878380	Bn-C1-p19148230	Bn-scaff_19364_1-p36387	C1	29	54.3	B
11285	Bn-N11-p20878901	Bn-C1-p19148763	Bn-scaff_19364_1-p36334	C1	29	54.3	B
11286	Bn-N11-p20880062	Bn-C1-p19149924	Bn-scaff_19364_1-p36218	C1	29	54.3	B
11287	Bn-N11-p20880181	Bn-C1-p19150043	Bn-scaff_19364_1-p36206	C1	29	54.3	B
11288	Bn-N11-p20880198	Bn-C1-p19150060	Bn-scaff_19364_1-p36204	C1	29	54.3	B
11289	Bn-N11-p20880413	Bn-C1-p19150275	Bn-scaff_19364_1-p36183	C1	29	54.3	B
11290	Bn-N11-p20880807	Bn-C1-p19150678	Bn-scaff_19364_1-p36143	C1	29	54.3	B
11291	Bn-N11-p20887431	Bn-C1-p19155057	Bn-scaff_19364_1-p35705	C1	29	54.3	B
11292	Bn-N11-p20905804	Bn-C1-p19170807	Bn-scaff_19364_1-p34130	C1	29	54.3	B
11293	Bn-N11-p20907683	Bn-C1-p19172689	Bn-scaff_19364_1-p33942	C1	29	54.3	B
11294	Bn-N11-p20908414	Bn-C1-p19173423	Bn-scaff_19364_1-p33868	C1	29	54.3	B
11295	Bn-N11-p20908618	Bn-C1-p19173627	Bn-scaff_19364_1-p33848	C1	29	54.3	B
11296	Bn-N11-p20933321	Bn-C1-p19199445	Bn-scaff_19364_1-p31266	C1	29	54.3	B
11297	Bn-N11-p20934859	Bn-C1-p19200594	Bn-scaff_19364_1-p31151	C1	29	54.3	B
11298	Bn-N11-p20939036	Bn-C1-p19204758	Bn-scaff_19364_1-p30735	C1	29	54.3	B
11299	Bn-N11-p20941664	Bn-C1-p19207471	Bn-scaff_19364_1-p30463	C1	29	54.3	B
11300	Bn-N11-p20945271	Bn-C1-p19211008	Bn-scaff_19364_1-p30110	C1	29	54.3	B
11301	Bn-N11-p20945564	Bn-C1-p19211301	Bn-scaff_19364_1-p30080	C1	29	54.3	B
11302	Bn-N11-p20946406	Bn-C1-p19212143	Bn-scaff_19364_1-p29996	C1	29	54.3	B
11303	Bn-N11-p20952017	Bn-C1-p19219288	Bn-scaff_19364_1-p29282	C1	29	54.3	B
11304	Bn-N11-p20952152	Bn-C1-p19219423	Bn-scaff_19364_1-p29268	C1	29	54.3	B
11305	Bn-N11-p20975440	Bn-C1-p19242773	Bn-scaff_19364_1-p26933	C1	29	54.3	B
11306	Bn-N11-p20975448	Bn-C1-p19242781	Bn-scaff_19364_1-p26932	C1	29	54.3	B
11307	Bn-N11-p21010087	Bn-C1-p19281387	Bn-scaff_19364_1-p23072	C1	29	54.3	B
11308	Bn-N11-p21012465	Bn-C1-p19283797	Bn-scaff_19364_1-p22831	C1	29	54.3	B
11309	Bn-N11-p21013982	Bn-C1-p19285313	Bn-scaff_19364_1-p22679	C1	29	54.3	B
11310	Bn-N11-p21014533	Bn-C1-p19285866	Bn-scaff_19364_1-p22624	C1	29	54.3	B
11311	Bn-N11-p21023307	Bn-C1-p19287657	Bn-scaff_19364_1-p22445	C1	29	54.3	B
11312	Bn-N11-p21023404	Bn-C1-p19287754	Bn-scaff_19364_1-p22435	C1	29	54.3	B
11313	Bn-N11-p21023486	Bn-C1-p19287836	Bn-scaff_19364_1-p22427	C1	29	54.3	B
11314	Bn-N11-p21023695	Bn-C1-p19288045	Bn-scaff_19364_1-p22406	C1	29	54.3	B

11315	Bn-N11-p21023699	Bn-C1-p19288049	Bn-scaff_19364_1-p22406(	C1	29	54.3	B
11316	Bn-N11-p21025581	Bn-C1-p19289931	Bn-scaff_19364_1-p22217{	C1	29	54.3	B
11317	Bn-N11-p21025799	Bn-C1-p19290149	Bn-scaff_19364_1-p22196(	C1	29	54.3	B
11318	Bn-N11-p21025944	Bn-C1-p19290294	Bn-scaff_19364_1-p22181!	C1	29	54.3	B
11319	Bn-N11-p21031806	Bn-C1-p19299356	Bn-scaff_19364_1-p21275:	C1	29	54.3	B
11320	Bn-N11-p21032668	Bn-C1-p19300222	Bn-scaff_19364_1-p21188	C1	29	54.3	B
11321	Bn-N11-p21032905	Bn-C1-p19300459	Bn-scaff_19364_1-p21165(	C1	29	54.3	B
11322	Bn-N11-p21042250	Bn-C1-p19327682	Bn-scaff_19364_1-p18442	C1	29	54.3	B
11323	Bn-N11-p21050787	Bn-C1-p19336198	Bn-scaff_19364_1-p17591:	C1	29	54.3	B
11324	Bn-N11-p21050926	Bn-C1-p19336338	Bn-scaff_19364_1-p17577:	C1	29	54.3	B
11325	Bn-N11-p21058791	Bn-C1-p19344525	Bn-scaff_19364_1-p16758	C1	29	54.3	B
11326	Bn-N11-p21066531	Bn-C1-p19359750	Bn-scaff_19364_1-p15235!	C1	29	54.3	B
11327	Bn-N11-p21066650	Bn-C1-p19359869	Bn-scaff_19364_1-p15224(	C1	29	54.3	B
11328	Bn-N11-p21066853	Bn-C1-p19360072	Bn-scaff_19364_1-p15203	C1	29	54.3	B
11329	Bn-N11-p21067168	Bn-C1-p19360386	Bn-scaff_19364_1-p15172:	C1	29	54.3	B
11330	Bn-N11-p21067275	Bn-C1-p19360493	Bn-scaff_19364_1-p15161(	C1	29	54.3	B
11331	Bn-N11-p21067453	Bn-C1-p19360670	Bn-scaff_19364_1-p15143!	C1	29	54.3	B
11332	Bn-N11-p21067473	Bn-C1-p19360690	Bn-scaff_19364_1-p15141!	C1	29	54.3	B
11333	Bn-N11-p21068022	Bn-C1-p19361221	Bn-scaff_19364_1-p15088{	C1	29	54.3	B
11334	Bn-N11-p21097297	Bn-C1-p19428609	Bn-scaff_19364_1-p83500	C1	29	54.3	B
11335	Bn-N11-p21107510	Bn-C1-p19434934	Bn-scaff_19364_1-p77175	C1	29	54.3	B
11336	Bn-N11-p21108691	Bn-C1-p19436116	Bn-scaff_19364_1-p75993	C1	29	54.3	B
11337	Bn-N11-p21177661	Bn-C1-p19486805	Bn-scaff_19364_1-p25304	C1	29	54.3	B
11338	Bn-N11-p21177741	Bn-C1-p19486884	Bn-scaff_19364_1-p25225	C1	29	54.3	B
11339	Bn-N11-p21177863	Bn-C1-p19487008	Bn-scaff_19364_1-p25101	C1	29	54.3	B
11340	Bn-N11-p21177894	Bn-C1-p19487039	Bn-scaff_19364_1-p25070	C1	29	54.3	B
11341	Bn-N11-p21181154	Bn-C1-p19490370	Bn-scaff_19364_1-p21739	C1	29	54.3	B
11342	Bn-N11-p21186364	Bn-C1-p19495535	Bn-scaff_19364_1-p16574	C1	29	54.3	B
11343	Bn-N11-p21188658	Bn-C1-p19497830	Bn-scaff_19364_1-p14279	C1	29	54.3	B
11344	Bn-N11-p21195783	Bn-C1-p19501569	Bn-scaff_19364_1-p10540	C1	29	54.3	B
11345	Bn-N11-p21245681	Bn-Scaffold00751-p8:	Bn-scaff_16809_1-p82365	C1	29	54.3	B
11346	Bn-N11-p21258772	Bn-Scaffold00751-p7(	Bn-scaff_16809_1-p70319	C1	29	54.3	B
11347	Bn-N11-p21259157	Bn-Scaffold00751-p6!	Bn-scaff_16809_1-p69934	C1	29	54.3	B
11348	Bn-N11-p21259600	Bn-Scaffold00751-p6!	Bn-scaff_16809_1-p69491	C1	29	54.3	B
11349	Bn-N11-p21259608	Bn-Scaffold00751-p6!	Bn-scaff_16809_1-p69483	C1	29	54.3	B
11350	Bn-N11-p21259688	Bn-Scaffold00751-p6!	Bn-scaff_16809_1-p69403	C1	29	54.3	B
11351	Bn-N11-p21261958	Bn-Scaffold00751-p6(	Bn-scaff_16809_1-p66728	C1	29	54.3	B
11352	Bn-N11-p21261990	Bn-Scaffold00751-p6(	Bn-scaff_16809_1-p66696	C1	29	54.3	B
11353	Bn-N11-p21267286	Bn-Scaffold00751-p6(	Bn-scaff_16809_1-p60560	C1	29	54.3	B
11354	Bn-N11-p21271925	Bn-Scaffold00751-p5(	Bn-scaff_16809_1-p56352	C1	29	54.3	B
11355	Bn-N11-p21272497	Bn-Scaffold00751-p5!	Bn-scaff_16809_1-p55782	C1	29	54.3	B
11356	Bn-N11-p21272882	Bn-Scaffold00751-p5!	Bn-scaff_16809_1-p55357	C1	29	54.3	B
11357	Bn-N11-p21281556	Bn-Scaffold00751-p5	Bn-scaff_16809_1-p54602	C1	29	54.3	B
11358	Bn-N11-p21301412	Bn-Scaffold00751-p4(	Bn-scaff_16809_1-p46229	C1	29	54.3	B
11359	Bn-N11-p21544900	Bn-C1-p19772195	Bn-scaff_24721_1-p48736	C1	29	54.3	B
11360	Bn-N11-p21560509	Bn-C1-p19788229	Bn-scaff_24721_1-p32702	C1	29	54.3	B
11361	Bn-N11-p21573276	Bn-C1-p19804388	Bn-scaff_24721_1-p16543	C1	29	54.3	B
11362	Bn-N11-p21679538	Bn-C1-p19866469	Bn-scaff_18559_2-p29727(	C1	29	54.3	B
11363	Bn-N11-p21744150	Bn-C1-p19921490	Bn-scaff_18559_2-p24224!	C1	29	54.3	B

11364	Bn-N11-p21761366	Bn-C1-p19937586	Bn-scaff_18559_2-p22615[illegible]	C1	29	54.3	B
11365	Bn-N11-p21809457	Bn-C1-p19990416	Bn-scaff_18559_2-p17332[illegible]	C1	29	54.3	B
11366	Bn-N11-p21849460	Bn-C1-p20034728	Bn-scaff_18559_2-p12901[illegible]	C1	29	54.3	B
11367	Bn-N11-p21873570	Bn-C1-p20057930	Bn-scaff_18559_2-p10580[illegible]	C1	29	54.3	B
11368	Bn-N11-p21878268	Bn-C1-p20062883	Bn-scaff_18559_2-p10085[illegible]	C1	29	54.3	B
11369	Bn-N11-p21915062	Bn-C1-p20104080	Bn-scaff_18559_2-p59659	C1	29	54.3	B
11370	Bn-N11-p21915524	Bn-C1-p20104547	Bn-scaff_18559_2-p59192	C1	29	54.3	B
11371	Bn-N11-p21940110	Bn-C1-p20128562	Bn-scaff_18559_2-p35177	C1	29	54.3	B
11372	Bn-N11-p22010654	Bn-C1-p20188020	Bn-scaff_17685_1-p24182	C1	29	54.3	B
11373	Bn-N11-p22046673	Bn-C1-p20221725	Bn-scaff_17685_1-p57887	C1	29	54.3	B
11374	Bn-N11-p22050569	Bn-C1-p20225036	Bn-scaff_17685_1-p61198	C1	29	54.3	B
11375	Bn-N11-p22096816	Bn-C1-p20281981	Bn-scaff_17685_1-p11814[illegible]	C1	29	54.3	B
11376	Bn-N11-p22136003	Bn-C1-p20323026	Bn-scaff_17685_1-p15918[illegible]	C1	29	54.3	B
11377	Bn-N11-p22158161	Bn-C1-p20340374	Bn-scaff_17685_1-p17653[illegible]	C1	29	54.3	B
11378	Bn-N11-p22218776	Bn-C1-p20393555	Bn-scaff_17685_1-p22971[illegible]	C1	29	54.3	B
11379	Bn-N11-p22265651	Bn-C1-p20499885	Bn-scaff_17959_1-p65375[illegible]	C1	29	54.3	B
11380	Bn-N11-p22407100	Bn-C1-p20636675	Bn-scaff_17959_1-p51696[illegible]	C1	29	54.3	B
11381	Bn-N11-p22413706	Bn-C1-p20643315	Bn-scaff_17959_1-p51032[illegible]	C1	29	54.3	B
11382	Bn-N11-p22586639	Bn-C1-p20823827	Bn-scaff_17959_1-p32981[illegible]	C1	29	54.3	B
11383	Bn-N11-p22835851	Bn-C1-p21076541	Bn-scaff_17959_1-p77103	C1	29	54.3	B
11384	Bn-N11-p22836365	Bn-C1-p21077055	Bn-scaff_17959_1-p76589	C1	29	54.3	B
11385	Bn-N11-p22866520	Bn-C1-p21106185	Bn-scaff_17959_1-p47459	C1	29	54.3	B
11386	Bn-N11-p22868351	Bn-C1-p21108016	Bn-scaff_17959_1-p45628	C1	29	54.3	B
11387	Bn-N11-p22958867	Bn-C1-p21177309	Bn-scaff_24236_1-p23566	C1	29	54.3	B
11388	Bn-N11-p22959058	Bn-C1-p21177501	Bn-scaff_24236_1-p23758	C1	29	54.3	B
11389	Bn-N11-p22959543	Bn-C1-p21177982	Bn-scaff_24236_1-p24239	C1	29	54.3	B
11390	Bn-N11-p22959871	Bn-C1-p21178313	Bn-scaff_24236_1-p24570	C1	29	54.3	B
11391	Bn-N11-p22961470	Bn-C1-p21179853	Bn-scaff_24236_1-p26110	C1	29	54.3	B
11392	Bn-N11-p22961933	Bn-C1-p21180317	Bn-scaff_24236_1-p26574	C1	29	54.3	B
11393	Bn-N11-p22972879	Bn-C1-p21189586	Bn-scaff_24236_1-p35843	C1	29	54.3	B
11394	Bn-N11-p22974466	Bn-C1-p21191172	Bn-scaff_24236_1-p37429	C1	29	54.3	B
11395	Bn-N11-p22974501	Bn-C1-p21191207	Bn-scaff_24236_1-p37464	C1	29	54.3	B
11396	Bn-N11-p22974552	Bn-C1-p21191258	Bn-scaff_24236_1-p37515	C1	29	54.3	B
11397	Bn-N11-p22979123	Bn-C1-p21195752	Bn-scaff_24236_1-p42009	C1	29	54.3	B
11398	Bn-N11-p22995540	Bn-C1-p21209839	Bn-scaff_24236_1-p56096	C1	29	54.3	B
11399	Bn-N11-p22996085	Bn-C1-p21210382	Bn-scaff_24236_1-p56639	C1	29	54.3	B
11400	Bn-N11-p22996188	Bn-C1-p21210485	Bn-scaff_24236_1-p56742	C1	29	54.3	B
11401	Bn-N11-p23022045	Bn-C1-p21236215	Bn-scaff_24236_1-p82472	C1	29	54.3	B
11402	Bn-N11-p23022756	Bn-C1-p21236927	Bn-scaff_24236_1-p83184	C1	29	54.3	B
11403	Bn-N11-p23022817	Bn-C1-p21236988	Bn-scaff_24236_1-p83245	C1	29	54.3	B
11404	Bn-N11-p23029694	Bn-C1-p21243824	Bn-scaff_24236_1-p90081	C1	29	54.3	B
11405	Bn-N11-p23030506	Bn-C1-p21244627	Bn-scaff_24236_1-p90884	C1	29	54.3	B
11406	Bn-N11-p23030863	Bn-C1-p21244984	Bn-scaff_24236_1-p91241	C1	29	54.3	B
11407	Bn-N11-p23031142	Bn-C1-p21245263	Bn-scaff_24236_1-p91520	C1	29	54.3	B
11408	Bn-N11-p23076654	Bn-C1-p21261120	Bn-scaff_24236_1-p10737[illegible]	C1	29	54.3	B
11409	Bn-N11-p23086417	Bn-C1-p21267872	Bn-scaff_24236_1-p11412[illegible]	C1	29	54.3	B
11410	Bn-N11-p23191903	Bn-C1-p21353523	Bn-scaff_24236_1-p19978[illegible]	C1	29	54.3	B
11411	Bn-N11-p23201068	Bn-C1-p21354569	Bn-scaff_24236_1-p20082[illegible]	C1	29	54.3	B
11412	Bn-N11-p23240027	Bn-C1-p21397353	Bn-scaff_24236_1-p24361[illegible]	C1	29	54.3	B

11413	Bn-N11-p23241491	Bn-C1-p21398817	Bn-scaff_24236_1-p24507[illegible]	C1	29	54.3	B
11414	Bn-N11-p23250294	Bn-C1-p21412275	Bn-scaff_24236_1-p25853[illegible]	C1	29	54.3	B
11415	Bn-N11-p23268401	Bn-C1-p21430100	Bn-scaff_24236_1-p27635[illegible]	C1	29	54.3	B
11416	Bn-N11-p23269686	Bn-C1-p21431386	Bn-scaff_24236_1-p27764[illegible]	C1	29	54.3	B
11417	Bn-N11-p23269797	Bn-C1-p21431497	Bn-scaff_24236_1-p27775[illegible]	C1	29	54.3	B
11418	Bn-N11-p23274904	Bn-C1-p21436540	Bn-scaff_24236_1-p28279[illegible]	C1	29	54.3	B
11419	Bn-N11-p23275112	Bn-C1-p21436737	Bn-scaff_24236_1-p28299[illegible]	C1	29	54.3	B
11420	Bn-N11-p23279220	Bn-C1-p21440789	Bn-scaff_24236_1-p28704[illegible]	C1	29	54.3	B
11421	Bn-N11-p23281353	Bn-C1-p21442923	Bn-scaff_24236_1-p28918[illegible]	C1	29	54.3	B
11422	Bn-N11-p23296756	Bn-C1-p21447640	Bn-scaff_24236_1-p29389[illegible]	C1	29	54.3	B
11423	Bn-N11-p23296809	Bn-C1-p21447692	Bn-scaff_24236_1-p29394[illegible]	C1	29	54.3	B
11424	Bn-N11-p23300500	Bn-C1-p21451378	Bn-scaff_24236_1-p29763[illegible]	C1	29	54.3	B
11425	Bn-N11-p23308319	Bn-C1-p21460561	Bn-scaff_24236_1-p30681[illegible]	C1	29	54.3	B
11426	Bn-N11-p23309426	Bn-C1-p21461667	Bn-scaff_24236_1-p30792[illegible]	C1	29	54.3	B
11427	Bn-N11-p23317640	Bn-C1-p21470536	Bn-scaff_24236_1-p31679[illegible]	C1	29	54.3	B
11428	Bn-N11-p23328600	Bn-C1-p21490266	Bn-scaff_24236_1-p33652[illegible]	C1	29	54.3	B
11429	Bn-N11-p23329188	Bn-C1-p21490854	Bn-scaff_24236_1-p33711[illegible]	C1	29	54.3	B
11430	Bn-N11-p23329250	Bn-C1-p21490916	Bn-scaff_24236_1-p33717[illegible]	C1	29	54.3	B
11431	Bn-N11-p23335829	Bn-C1-p21497508	Bn-scaff_24236_1-p34376[illegible]	C1	29	54.3	B
11432	Bn-N11-p23335999	Bn-C1-p21497678	Bn-scaff_24236_1-p34393[illegible]	C1	29	54.3	B
11433	Bn-N11-p23338993	Bn-C1-p21500658	Bn-scaff_24236_1-p34691[illegible]	C1	29	54.3	B
11434	Bn-N11-p23340200	Bn-C1-p21501866	Bn-scaff_24236_1-p34812[illegible]	C1	29	54.3	B
11435	Bn-N11-p23343151	Bn-C1-p21504647	Bn-scaff_24236_1-p35090[illegible]	C1	29	54.3	B
11436	Bn-N11-p23356394	Bn-C1-p21520442	Bn-scaff_24236_1-p36669[illegible]	C1	29	54.3	B
11437	Bn-N11-p23359820	Bn-C1-p21523817	Bn-scaff_24236_1-p37007[illegible]	C1	29	54.3	B
11438	Bn-N11-p23362050	Bn-C1-p21526045	Bn-scaff_24236_1-p37230[illegible]	C1	29	54.3	B
11439	Bn-N11-p23362570	Bn-C1-p21526564	Bn-scaff_24236_1-p37282[illegible]	C1	29	54.3	B
11440	Bn-N11-p23379974	Bn-C1-p21545632	Bn-scaff_24236_1-p39188[illegible]	C1	29	54.3	B
11441	Bn-N11-p23380139	Bn-C1-p21545797	Bn-scaff_24236_1-p39205[illegible]	C1	29	54.3	B
11442	Bn-N11-p23381475	Bn-C1-p21547101	Bn-scaff_24236_1-p39335[illegible]	C1	29	54.3	B
11443	Bn-N11-p23388875	Bn-C1-p21553575	Bn-scaff_24236_1-p39983[illegible]	C1	29	54.3	B
11444	Bn-N11-p23415883	Bn-C1-p21563746	Bn-scaff_24236_1-p41000[illegible]	C1	29	54.3	B
11445	Bn-N11-p23429529	Bn-C1-p21577388	Bn-scaff_24236_1-p42364[illegible]	C1	29	54.3	B
11446	Bn-N11-p23429654	Bn-C1-p21577513	Bn-scaff_24236_1-p42377[illegible]	C1	29	54.3	B
11447	Bn-N11-p23429845	Bn-C1-p21577693	Bn-scaff_24236_1-p42395[illegible]	C1	29	54.3	B
11448	Bn-N11-p23429889	Bn-C1-p21577737	Bn-scaff_24236_1-p42399[illegible]	C1	29	54.3	B
11449	Bn-N11-p23432037	Bn-C1-p21579448	Bn-scaff_24236_1-p42570[illegible]	C1	29	54.3	B
11450	Bn-N11-p23432369	Bn-C1-p21579777	Bn-scaff_24236_1-p42603[illegible]	C1	29	54.3	B
11451	Bn-N11-p23433210	Bn-C1-p21584458	Bn-scaff_24236_1-p43071[illegible]	C1	29	54.3	B
11452	Bn-N11-p23433323	Bn-C1-p21584571	Bn-scaff_24236_1-p43082[illegible]	C1	29	54.3	B
11453	Bn-N11-p23434171	Bn-C1-p21585418	Bn-scaff_24236_1-p43167[illegible]	C1	29	54.3	B
11454	Bn-N11-p23434545	Bn-C1-p21585792	Bn-scaff_24236_1-p43204[illegible]	C1	29	54.3	B
11455	Bn-N11-p23451923	Bn-C1-p21600671	Bn-scaff_24236_1-p44692[illegible]	C1	29	54.3	B
11456	Bn-N11-p23451997	Bn-C1-p21600745	Bn-scaff_24236_1-p44700[illegible]	C1	29	54.3	B
11457	Bn-N11-p23452142	Bn-C1-p21600890	Bn-scaff_24236_1-p44714[illegible]	C1	29	54.3	B
11458	Bn-N11-p23453285	Bn-C1-p21602032	Bn-scaff_24236_1-p44828[illegible]	C1	29	54.3	B
11459	Bn-N11-p23453779	Bn-C1-p21602526	Bn-scaff_24236_1-p44878[illegible]	C1	29	54.3	B
11460	Bn-N11-p23453945	Bn-C1-p21602692	Bn-scaff_24236_1-p44894[illegible]	C1	29	54.3	B
11461	Bn-N11-p23454446	Bn-C1-p21603206	Bn-scaff_24236_1-p44946[illegible]	C1	29	54.3	B

11462	Bn-N11-p23454462	Bn-C1-p21603222	Bn-scaff_24236_1-p449479	C1	29	54.3	B
11463	Bn-N11-p23457269	Bn-C1-p21606073	Bn-scaff_24236_1-p452330	C1	29	54.3	B
11464	Bn-N11-p23457758	Bn-C1-p21606579	Bn-scaff_24236_1-p452836	C1	29	54.3	B
11465	Bn-N11-p23457896	Bn-C1-p21606717	Bn-scaff_24236_1-p452974	C1	29	54.3	B
11466	Bn-N11-p23458472	Bn-C1-p21607294	Bn-scaff_24236_1-p453551	C1	29	54.3	B
11467	Bn-N11-p23459603	Bn-C1-p21608423	Bn-scaff_24236_1-p454680	C1	29	54.3	B
11468	Bn-N11-p23460012	Bn-C1-p21608832	Bn-scaff_24236_1-p455089	C1	29	54.3	B
11469	Bn-N11-p23460103	Bn-C1-p21608923	Bn-scaff_24236_1-p455180	C1	29	54.3	B
11470	Bn-N11-p23460304	Bn-C1-p21609124	Bn-scaff_24236_1-p455381	C1	29	54.3	B
11471	Bn-N11-p23460369	Bn-C1-p21609189	Bn-scaff_24236_1-p455446	C1	29	54.3	B
11472	Bn-N11-p23460886	Bn-C1-p21609706	Bn-scaff_24236_1-p455963	C1	29	54.3	B
11473	Bn-N11-p23461941	Bn-C1-p21610787	Bn-scaff_24236_1-p457044	C1	29	54.3	B
11474	Bn-N11-p23462261	Bn-C1-p21611108	Bn-scaff_24236_1-p457365	C1	29	54.3	B
11475	Bn-N11-p23462560	Bn-C1-p21611407	Bn-scaff_24236_1-p457664	C1	29	54.3	B
11476	Bn-N11-p23463872	Bn-C1-p21612692	Bn-scaff_24236_1-p458949	C1	29	54.3	B
11477	Bn-N11-p23464126	Bn-C1-p21612946	Bn-scaff_24236_1-p459203	C1	29	54.3	B
11478	Bn-N11-p23464265	Bn-C1-p21613085	Bn-scaff_24236_1-p459342	C1	29	54.3	B
11479	Bn-N11-p23464365	Bn-C1-p21613185	Bn-scaff_24236_1-p459442	C1	29	54.3	B
11480	Bn-N11-p23464637	Bn-C1-p21613457	Bn-scaff_24236_1-p459714	C1	29	54.3	B
11481	Bn-N11-p23473376	Bn-C1-p21624900	Bn-scaff_24236_1-p471157	C1	29	54.3	B
11482	Bn-N11-p23473725	Bn-C1-p21625244	Bn-scaff_24236_1-p471501	C1	29	54.3	B
11483	Bn-N11-p23475740	Bn-C1-p21626941	Bn-scaff_24236_1-p473198	C1	29	54.3	B
11484	Bn-N11-p23476159	Bn-C1-p21627401	Bn-scaff_24236_1-p473658	C1	29	54.3	B
11485	Bn-N11-p23476279	Bn-C1-p21627512	Bn-scaff_24236_1-p473769	C1	29	54.3	B
11486	Bn-N11-p23484779	Bn-C1-p21631778	Bn-scaff_24236_1-p478035	C1	29	54.3	B
11487	Bn-N11-p23485217	Bn-C1-p21632216	Bn-scaff_24236_1-p478473	C1	29	54.3	B
11488	Bn-N11-p23486429	Bn-C1-p21633427	Bn-scaff_24236_1-p479684	C1	29	54.3	B
11489	Bn-N11-p23492367	Bn-C1-p21639702	Bn-scaff_24236_1-p485959	C1	29	54.3	B
11490	Bn-N11-p23493582	Bn-C1-p22053730	Bn-scaff_19183_1-p478485	C1	29	54.3	B
11491	Bn-N11-p23499567	Bn-C1-p22062613	Bn-scaff_19183_1-p469602	C1	29	54.3	B
11492	Bn-N11-p23504196	Bn-C1-p22067112	Bn-scaff_19183_1-p465103	C1	29	54.3	B
11493	Bn-N11-p23514733	Bn-C1-p22077390	Bn-scaff_19183_1-p454825	C1	29	54.3	B
11494	Bn-N11-p23514945	Bn-C1-p22077601	Bn-scaff_19183_1-p454614	C1	29	54.3	B
11495	Bn-N11-p23516435	Bn-C1-p22079255	Bn-scaff_19183_1-p452960	C1	29	54.3	B
11496	Bn-N11-p23521900	Bn-C1-p22080935	Bn-scaff_19183_1-p451280	C1	29	54.3	B
11497	Bn-N11-p23521951	Bn-C1-p22080986	Bn-scaff_19183_1-p451229	C1	29	54.3	B
11498	Bn-N11-p23548839	Bn-C1-p22107009	Bn-scaff_19183_1-p425206	C1	29	54.3	B
11499	Bn-N11-p23550184	Bn-C1-p22108293	Bn-scaff_19183_1-p423922	C1	29	54.3	B
11500	Bn-N11-p23550723	Bn-C1-p22108831	Bn-scaff_19183_1-p423384	C1	29	54.3	B
11501	Bn-N11-p23550983	Bn-C1-p22109091	Bn-scaff_19183_1-p423124	C1	29	54.3	B
11502	Bn-N11-p23551999	Bn-C1-p22110105	Bn-scaff_19183_1-p422110	C1	29	54.3	B
11503	Bn-N11-p23552082	Bn-C1-p22110188	Bn-scaff_19183_1-p422027	C1	29	54.3	B
11504	Bn-N11-p23552194	Bn-C1-p22110300	Bn-scaff_19183_1-p421915	C1	29	54.3	B
11505	Bn-N11-p23552873	Bn-C1-p22110979	Bn-scaff_19183_1-p421236	C1	29	54.3	B
11506	Bn-N11-p23553453	Bn-C1-p22111559	Bn-scaff_19183_1-p420656	C1	29	54.3	B
11507	Bn-N11-p23553952	Bn-C1-p22112056	Bn-scaff_19183_1-p420159	C1	29	54.3	B
11508	Bn-N11-p23562448	Bn-C1-p22119761	Bn-scaff_19183_1-p412454	C1	29	54.3	B
11509	Bn-N11-p23566952	Bn-C1-p22124177	Bn-scaff_19183_1-p408038	C1	29	54.3	B
11510	Bn-N11-p23567196	Bn-C1-p22124421	Bn-scaff_19183_1-p407794	C1	29	54.3	B

11511	Bn-N11-p23569290	Bn-C1-p22130290	Bn-scaff_19183_1-p401925	C1	29	54.3	B
11512	Bn-N11-p23586172	Bn-C1-p22134371	Bn-scaff_19183_1-p397844	C1	29	54.3	B
11513	Bn-N11-p23597947	Bn-C1-p22140623	Bn-scaff_19183_1-p391592	C1	29	54.3	B
11514	Bn-N11-p23609303	Bn-C1-p21648607	Bn-scaff_22563_1-p143668	C1	29	54.3	B
11515	Bn-N11-p23609540	Bn-C1-p21648846	Bn-scaff_22563_1-p143429	C1	29	54.3	B
11516	Bn-N11-p23611659	Bn-C1-p21650996	Bn-scaff_22563_1-p141279	C1	29	54.3	0
11517	Bn-N11-p23612153	Bn-C1-p21651490	Bn-scaff_22563_1-p140785	C1	29	54.3	B
11518	Bn-N11-p23632859	Bn-C1-p21677785	Bn-scaff_22563_1-p114490	C1	29	54.3	B
11519	Bn-N11-p23634188	Bn-C1-p21679089	Bn-scaff_22563_1-p113186	C1	29	54.3	B
11520	Bn-N11-p23634397	Bn-C1-p21679298	Bn-scaff_22563_1-p112977	C1	29	54.3	B
11521	Bn-N11-p23635095	Bn-C1-p21679996	Bn-scaff_22563_1-p112279	C1	29	54.3	B
11522	Bn-N11-p23635125	Bn-C1-p21680026	Bn-scaff_22563_1-p112249	C1	29	54.3	B
11523	Bn-N11-p23635453	Bn-C1-p21680354	Bn-scaff_22563_1-p111921	C1	29	54.3	0
11524	Bn-N11-p23635532	Bn-C1-p21680433	Bn-scaff_22563_1-p111842	C1	29	54.3	B
11525	Bn-N11-p23648451	Bn-C1-p21692228	Bn-scaff_22563_1-p100047	C1	29	54.3	B
11526	Bn-N11-p23648759	Bn-C1-p21692536	Bn-scaff_22563_1-p99739	C1	29	54.3	B
11527	Bn-N11-p23649079	Bn-C1-p21692860	Bn-scaff_22563_1-p99415	C1	29	54.3	B
11528	Bn-N11-p23655092	Bn-C1-p21698875	Bn-scaff_22563_1-p93400	C1	29	54.3	B
11529	Bn-N11-p23668308	Bn-C1-p21705608	Bn-scaff_22563_1-p86667	C1	29	54.3	B
11530	Bn-N11-p23668438	Bn-C1-p21705737	Bn-scaff_22563_1-p86538	C1	29	54.3	B
11531	Bn-N11-p23669124	Bn-C1-p21706249	Bn-scaff_22563_1-p86026	C1	29	54.3	B
11532	Bn-N11-p23669359	Bn-C1-p21706484	Bn-scaff_22563_1-p85791	C1	29	54.3	B
11533	Bn-N11-p23677840	Bn-C1-p21715277	Bn-scaff_22563_1-p76998	C1	29	54.3	B
11534	Bn-N11-p23677891	Bn-C1-p21715328	Bn-scaff_22563_1-p76947	C1	29	54.3	B
11535	Bn-N11-p23678031	Bn-C1-p21715468	Bn-scaff_22563_1-p76807	C1	29	54.3	B
11536	Bn-N11-p23678681	Bn-C1-p21716097	Bn-scaff_22563_1-p76178	C1	29	54.3	B
11537	Bn-N11-p23679642	Bn-C1-p21717058	Bn-scaff_22563_1-p75217	C1	29	54.3	B
11538	Bn-N11-p23681380	Bn-C1-p21718797	Bn-scaff_22563_1-p73478	C1	29	54.3	B
11539	Bn-N11-p23681462	Bn-C1-p21718880	Bn-scaff_22563_1-p73395	C1	29	54.3	B
11540	Bn-N11-p23681841	Bn-C1-p21719259	Bn-scaff_22563_1-p73016	C1	29	54.3	B
11541	Bn-N11-p23682758	Bn-C1-p21720175	Bn-scaff_22563_1-p72100	C1	29	54.3	0
11542	Bn-N11-p23698390	Bn-C1-p21745511	Bn-scaff_22563_1-p46764	C1	29	54.3	B
11543	Bn-N11-p23698453	Bn-C1-p21745574	Bn-scaff_22563_1-p46701	C1	29	54.3	B
11544	Bn-N11-p23699721	Bn-C1-p21746843	Bn-scaff_22563_1-p45432	C1	29	54.3	B
11545	Bn-N11-p23700166	Bn-C1-p21747288	Bn-scaff_22563_1-p44987	C1	29	54.3	B
11546	Bn-N11-p23700330	Bn-C1-p21747452	Bn-scaff_22563_1-p44823	C1	29	54.3	B
11547	Bn-N11-p23700778	Bn-C1-p21747900	Bn-scaff_22563_1-p44375	C1	29	54.3	B
11548	Bn-N11-p23702433	Bn-C1-p21749551	Bn-scaff_22563_1-p42724	C1	29	54.3	B
11549	Bn-N11-p23727912	Bn-C1-p21766042	Bn-scaff_22563_1-p26233	C1	29	54.3	B
11550	Bn-N11-p23728006	Bn-C1-p21766136	Bn-scaff_22563_1-p26139	C1	29	54.3	B
11551	Bn-N11-p23728918	Bn-C1-p21767049	Bn-scaff_22563_1-p25226	C1	29	54.3	B
11552	Bn-N11-p23729382	Bn-C1-p21767523	Bn-scaff_22563_1-p24752	C1	29	54.3	B
11553	Bn-N11-p23729840	Bn-C1-p21767980	Bn-scaff_22563_1-p24295	C1	29	54.3	B
11554	Bn-N11-p23730962	Bn-C1-p21769142	Bn-scaff_22563_1-p23133	C1	29	54.3	B
11555	Bn-N11-p23734125	Bn-C1-p21776207	Bn-scaff_22563_1-p16068	C1	29	54.3	B
11556	Bn-N11-p23755251	Bn-C1-p21829823	Bn-scaff_23948_1-p37449	C1	29	54.3	B
11557	Bn-N11-p23756069	Bn-C1-p21829007	Bn-scaff_23948_1-p36633	C1	29	54.3	B
11558	Bn-N11-p23767521	Bn-C1-p21818225	Bn-scaff_23948_1-p25851	C1	29	54.3	B
11559	Bn-N11-p23768658	Bn-C1-p21817087	Bn-scaff_23948_1-p24713	C1	29	54.3	B

11560	Bn-N11-p23904928	Bn-C1-p21895086	Bn-scaff_22795_1-p147897	C1	29	54.3	B
11561	Bn-N11-p23981922	Bn-C1-p21940422	Bn-scaff_22795_1-p102561	C1	29	54.3	B
11562	Bn-N11-p23982329	Bn-C1-p21940835	Bn-scaff_22795_1-p102148	C1	29	54.3	B
11563	Bn-N11-p23991554	Bn-C1-p21951640	Bn-scaff_22795_1-p91343	C1	29	54.3	B
11564	Bn-N11-p23991747	Bn-C1-p21951833	Bn-scaff_22795_1-p91150	C1	29	54.3	B
11565	Bn-N11-p23991938	Bn-C1-p21952025	Bn-scaff_22795_1-p90958	C1	29	54.3	B
11566	Bn-N11-p23992003	Bn-C1-p21952090	Bn-scaff_22795_1-p90893	C1	29	54.3	B
11567	Bn-N11-p23992738	Bn-C1-p21952837	Bn-scaff_22795_1-p90146	C1	29	54.3	B
11568	Bn-N11-p23994632	Bn-C1-p21954731	Bn-scaff_22795_1-p88252	C1	29	54.3	B
11569	Bn-N11-p23994760	Bn-C1-p21954859	Bn-scaff_22795_1-p88124	C1	29	54.3	B
11570	Bn-N11-p23995025	Bn-C1-p21955124	Bn-scaff_22795_1-p87859	C1	29	54.3	B
11571	Bn-N11-p24037637	Bn-C1-p21995413	Bn-scaff_22795_1-p47570	C1	29	54.3	B
11572	Bn-N11-p24037754	Bn-C1-p21995530	Bn-scaff_22795_1-p47453	C1	29	54.3	B
11573	Bn-N11-p24037968	Bn-C1-p21995744	Bn-scaff_22795_1-p47239	C1	29	54.3	B
11574	Bn-N11-p24038247	Bn-C1-p21996023	Bn-scaff_22795_1-p46960	C1	29	54.3	B
11575	Bn-N11-p24038411	Bn-C1-p21996187	Bn-scaff_22795_1-p46796	C1	29	54.3	B
11576	Bn-N11-p24038712	Bn-C1-p21996489	Bn-scaff_22795_1-p46494	C1	29	54.3	B
11577	Bn-N11-p24044157	Bn-C1-p22001945	Bn-scaff_22795_1-p41038	C1	29	54.3	B
11578	Bn-N11-p24044427	Bn-C1-p22002215	Bn-scaff_22795_1-p40768	C1	29	54.3	B
11579	Bn-N11-p24044430	Bn-C1-p22002218	Bn-scaff_22795_1-p40765	C1	29	54.3	B
11580	Bn-N11-p24050714	Bn-C1-p22008311	Bn-scaff_22795_1-p34672	C1	29	54.3	B
11581	Bn-N11-p24051020	Bn-C1-p22008617	Bn-scaff_22795_1-p34366	C1	29	54.3	B
11582	Bn-N11-p24051556	Bn-C1-p22009153	Bn-scaff_22795_1-p33830	C1	29	54.3	B
11583	Bn-N11-p24052272	Bn-C1-p22015267	Bn-scaff_22795_1-p27716	C1	29	54.3	B
11584	Bn-N11-p24074205	Bn-C1-p22033068	Bn-scaff_22795_1-p9915	C1	29	54.3	B
11585	Bn-N11-p24097304	Bn-C1-p22520456	Bn-scaff_19183_1-p11759	C1	29	54.3	B
11586	Bn-N11-p24097497	Bn-C1-p22520263	Bn-scaff_19183_1-p11952	C1	29	54.3	B
11587	Bn-N11-p24098559	Bn-C1-p22519202	Bn-scaff_19183_1-p13013	C1	29	54.3	B
11588	Bn-N11-p24105042	Bn-C1-p22513109	Bn-scaff_19183_1-p19106	C1	29	54.3	B
11589	Bn-N11-p24118404	Bn-C1-p22506925	Bn-scaff_19183_1-p25290	C1	29	54.3	B
11590	Bn-N11-p24118487	Bn-C1-p22506842	Bn-scaff_19183_1-p25373	C1	29	54.3	B
11591	Bn-N11-p24118574	Bn-C1-p22506755	Bn-scaff_19183_1-p25460	C1	29	54.3	B
11592	Bn-N11-p24119619	Bn-C1-p22505719	Bn-scaff_19183_1-p26496	C1	29	54.3	B
11593	Bn-N11-p24119777	Bn-C1-p22505561	Bn-scaff_19183_1-p26654	C1	29	54.3	B
11594	Bn-N11-p24120038	Bn-C1-p22505300	Bn-scaff_19183_1-p26915	C1	29	54.3	B
11595	Bn-N11-p24121013	Bn-C1-p22504735	Bn-scaff_19183_1-p27480	C1	29	54.3	B
11596	Bn-N11-p24121172	Bn-C1-p22504576	Bn-scaff_19183_1-p27639	C1	29	54.3	B
11597	Bn-N11-p24121621	Bn-C1-p22504131	Bn-scaff_19183_1-p28084	C1	29	54.3	B
11598	Bn-N11-p24122082	Bn-C1-p22503670	Bn-scaff_19183_1-p28545	C1	29	54.3	B
11599	Bn-N11-p24122187	Bn-C1-p22503565	Bn-scaff_19183_1-p28650	C1	29	54.3	B
11600	Bn-N11-p24122461	Bn-C1-p22503291	Bn-scaff_19183_1-p28924	C1	29	54.3	B
11601	Bn-N11-p24122882	Bn-C1-p22502870	Bn-scaff_19183_1-p29345	C1	29	54.3	B
11602	Bn-N11-p24129880	Bn-C1-p22495818	Bn-scaff_19183_1-p36397	C1	29	54.3	B
11603	Bn-N11-p24145982	Bn-C1-p22479276	Bn-scaff_19183_1-p52939	C1	29	54.3	B
11604	Bn-N11-p24147836	Bn-C1-p22477430	Bn-scaff_19183_1-p54785	C1	29	54.3	B
11605	Bn-N11-p24155103	Bn-C1-p22470893	Bn-scaff_19183_1-p61322	C1	29	54.3	B
11606	Bn-N11-p24172833	Bn-C1-p22462530	Bn-scaff_19183_1-p69685	C1	29	54.3	B
11607	Bn-N11-p24181473	Bn-C1-p22453886	Bn-scaff_19183_1-p78329	C1	29	54.3	B
11608	Bn-N11-p24184876	Bn-C1-p22450482	Bn-scaff_19183_1-p81733	C1	29	54.3	B

11609	Bn-N11-p24205557	Bn-C1-p22431721	Bn-scaff_19183_1-p100494	C1	29	54.3	B
11610	Bn-N11-p24209173	Bn-C1-p22427906	Bn-scaff_19183_1-p104309	C1	29	54.3	B
11611	Bn-N11-p24225499	Bn-C1-p22412762	Bn-scaff_19183_1-p119453	C1	29	54.3	B
11612	Bn-N11-p24225959	Bn-C1-p22412305	Bn-scaff_19183_1-p119910	C1	29	54.3	B
11613	Bn-N11-p24227535	Bn-C1-p22410729	Bn-scaff_19183_1-p121486	C1	29	54.3	B
11614	Bn-N11-p24229075	Bn-C1-p22409190	Bn-scaff_19183_1-p123025	C1	29	54.3	B
11615	Bn-N11-p24242456	Bn-C1-p22401339	Bn-scaff_19183_1-p130876	C1	29	54.3	B
11616	Bn-N11-p24251385	Bn-C1-p22392383	Bn-scaff_19183_1-p139832	C1	29	54.3	B
11617	Bn-N11-p24251779	Bn-C1-p22391988	Bn-scaff_19183_1-p140227	C1	29	54.3	B
11618	Bn-N11-p24251882	Bn-C1-p22391885	Bn-scaff_19183_1-p140330	C1	29	54.3	B
11619	Bn-N11-p24252090	Bn-C1-p22391677	Bn-scaff_19183_1-p140538	C1	29	54.3	B
11620	Bn-N11-p24252302	Bn-C1-p22391465	Bn-scaff_19183_1-p140750	C1	29	54.3	B
11621	Bn-N11-p24253537	Bn-C1-p22390237	Bn-scaff_19183_1-p141978	C1	29	54.3	B
11622	Bn-N11-p24256753	Bn-C1-p22386971	Bn-scaff_19183_1-p145244	C1	29	54.3	B
11623	Bn-N11-p24256894	Bn-C1-p22386830	Bn-scaff_19183_1-p145385	C1	29	54.3	B
11624	Bn-N11-p24257027	Bn-C1-p22386697	Bn-scaff_19183_1-p145518	C1	29	54.3	B
11625	Bn-N11-p24257186	Bn-C1-p22386538	Bn-scaff_19183_1-p145677	C1	29	54.3	B
11626	Bn-N11-p24257502	Bn-C1-p22380411	Bn-scaff_19183_1-p151804	C1	29	54.3	B
11627	Bn-N11-p24257727	Bn-C1-p22380186	Bn-scaff_19183_1-p152029	C1	29	54.3	B
11628	Bn-N11-p24260008	Bn-C1-p22377207	Bn-scaff_19183_1-p155008	C1	29	54.3	B
11629	Bn-N11-p24260341	Bn-C1-p22376874	Bn-scaff_19183_1-p155341	C1	29	54.3	B
11630	Bn-N11-p24260928	Bn-C1-p22376287	Bn-scaff_19183_1-p155928	C1	29	54.3	B
11631	Bn-N11-p24261554	Bn-C1-p22375653	Bn-scaff_19183_1-p156562	C1	29	54.3	B
11632	Bn-N11-p24265340	Bn-C1-p22369224	Bn-scaff_19183_1-p162991	C1	29	54.3	B
11633	Bn-N11-p24265553	Bn-C1-p22369010	Bn-scaff_19183_1-p163205	C1	29	54.3	B
11634	Bn-N11-p24265939	Bn-C1-p22368590	Bn-scaff_19183_1-p163625	C1	29	54.3	B
11635	Bn-N11-p24265994	Bn-C1-p22368535	Bn-scaff_19183_1-p163680	C1	29	54.3	B
11636	Bn-N11-p24266142	Bn-C1-p22368387	Bn-scaff_19183_1-p163828	C1	29	54.3	B
11637	Bn-N11-p24266458	Bn-C1-p22368071	Bn-scaff_19183_1-p164144	C1	29	54.3	B
11638	Bn-N11-p24266687	Bn-C1-p22367842	Bn-scaff_19183_1-p164373	C1	29	54.3	B
11639	Bn-N11-p24266799	Bn-C1-p22367730	Bn-scaff_19183_1-p164485	C1	29	54.3	B
11640	Bn-N11-p24294177	Bn-C1-p22357306	Bn-scaff_19183_1-p174909	C1	29	54.3	B
11641	Bn-N11-p24297820	Bn-C1-p22352573	Bn-scaff_19183_1-p179642	C1	29	54.3	B
11642	Bn-N11-p24298171	Bn-C1-p22352222	Bn-scaff_19183_1-p179993	C1	29	54.3	B
11643	Bn-N11-p24298306	Bn-C1-p22352086	Bn-scaff_19183_1-p180129	C1	29	54.3	B
11644	Bn-N11-p24298380	Bn-C1-p22352016	Bn-scaff_19183_1-p180199	C1	29	54.3	B
11645	Bn-N11-p24298726	Bn-C1-p22351671	Bn-scaff_19183_1-p180544	C1	29	54.3	B
11646	Bn-N11-p24299036	Bn-C1-p22351361	Bn-scaff_19183_1-p180854	C1	29	54.3	B
11647	Bn-N11-p24299675	Bn-C1-p22350722	Bn-scaff_19183_1-p181493	C1	29	54.3	B
11648	Bn-N11-p24301165	Bn-C1-p22349235	Bn-scaff_19183_1-p182980	C1	29	54.3	B
11649	Bn-N11-p24301793	Bn-C1-p22348607	Bn-scaff_19183_1-p183608	C1	29	54.3	B
11650	Bn-N11-p24301886	Bn-C1-p22348515	Bn-scaff_19183_1-p183700	C1	29	54.3	B
11651	Bn-N11-p24302982	Bn-C1-p22347382	Bn-scaff_19183_1-p184833	C1	29	54.3	B
11652	Bn-N11-p24303283	Bn-C1-p22347081	Bn-scaff_19183_1-p185134	C1	29	54.3	B
11653	Bn-N11-p24303701	Bn-C1-p22346663	Bn-scaff_19183_1-p185552	C1	29	54.3	B
11654	Bn-N11-p24303845	Bn-C1-p22346519	Bn-scaff_19183_1-p185696	C1	29	54.3	B
11655	Bn-N11-p24303935	Bn-C1-p22346429	Bn-scaff_19183_1-p185786	C1	29	54.3	B
11656	Bn-N11-p24305509	Bn-C1-p22344855	Bn-scaff_19183_1-p187360	C1	29	54.3	B
11657	Bn-N11-p24305684	Bn-C1-p22344680	Bn-scaff_19183_1-p187535	C1	29	54.3	B

11658	Bn-N11-p24306478	Bn-C1-p22343886	Bn-scaff_19183_1-p188329	C1	29	54.3	B
11659	Bn-N11-p24315976	Bn-C1-p22334491	Bn-scaff_19183_1-p197724	C1	29	54.3	B
11660	Bn-N11-p24317045	Bn-C1-p22333572	Bn-scaff_19183_1-p198643	C1	29	54.3	B
11661	Bn-N11-p24329908	Bn-C1-p22322345	Bn-scaff_19183_1-p209870	C1	29	54.3	B
11662	Bn-N11-p24329964	Bn-C1-p22322288	Bn-scaff_19183_1-p209927	C1	29	54.3	B
11663	Bn-N11-p24343187	Bn-C1-p22303074	Bn-scaff_19183_1-p229141	C1	29	54.3	B
11664	Bn-N11-p24343245	Bn-C1-p22303016	Bn-scaff_19183_1-p229199	C1	29	54.3	B
11665	Bn-N11-p24343310	Bn-C1-p22302951	Bn-scaff_19183_1-p229264	C1	29	54.3	B
11666	Bn-N11-p24352249	Bn-C1-p22289703	Bn-scaff_19183_1-p242512	C1	29	54.3	B
11667	Bn-N11-p24353365	Bn-C1-p22288587	Bn-scaff_19183_1-p243628	C1	29	54.3	B
11668	Bn-N11-p24354290	Bn-C1-p22287638	Bn-scaff_19183_1-p244577	C1	29	54.3	B
11669	Bn-N11-p24359155	Bn-C1-p22282805	Bn-scaff_19183_1-p249410	C1	29	54.3	B
11670	Bn-N11-p24364341	Bn-C1-p22277605	Bn-scaff_19183_1-p254610	C1	29	54.3	B
11671	Bn-N11-p24364971	Bn-C1-p22277013	Bn-scaff_19183_1-p255202	C1	29	54.3	B
11672	Bn-N11-p24365096	Bn-C1-p22276888	Bn-scaff_19183_1-p255327	C1	29	54.3	B
11673	Bn-N11-p24376638	Bn-C1-p22266188	Bn-scaff_19183_1-p266027	C1	29	54.3	B
11674	Bn-N11-p24380531	Bn-C1-p22262257	Bn-scaff_19183_1-p269958	C1	29	54.3	B
11675	Bn-N11-p24384272	Bn-C1-p22258522	Bn-scaff_19183_1-p273693	C1	29	54.3	B
11676	Bn-N11-p24420704	Bn-C1-p22221807	Bn-scaff_19183_1-p310408	C1	29	54.3	B
11677	Bn-N11-p24421557	Bn-C1-p22220952	Bn-scaff_19183_1-p311263	C1	29	54.3	B
11678	Bn-N11-p24421816	Bn-C1-p22220693	Bn-scaff_19183_1-p311522	C1	29	54.3	B
11679	Bn-N11-p24421884	Bn-C1-p22220624	Bn-scaff_19183_1-p311591	C1	29	54.3	B
11680	Bn-N11-p24422054	Bn-C1-p22220454	Bn-scaff_19183_1-p311761	C1	29	54.3	B
11681	Bn-N11-p24422150	Bn-C1-p22220358	Bn-scaff_19183_1-p311857	C1	29	54.3	B
11682	Bn-N11-p24424881	Bn-C1-p22217612	Bn-scaff_19183_1-p314603	C1	29	54.3	B
11683	Bn-N11-p24425896	Bn-C1-p22216582	Bn-scaff_19183_1-p315633	C1	29	54.3	B
11684	Bn-N11-p24426322	Bn-C1-p22216156	Bn-scaff_19183_1-p316059	C1	29	54.3	B
11685	Bn-N11-p24426406	Bn-C1-p22216072	Bn-scaff_19183_1-p316143	C1	29	54.3	B
11686	Bn-N11-p24426939	Bn-C1-p22215535	Bn-scaff_19183_1-p316680	C1	29	54.3	B
11687	Bn-N11-p24427291	Bn-C1-p22215183	Bn-scaff_19183_1-p317032	C1	29	54.3	B
11688	Bn-N11-p24427360	Bn-C1-p22215114	Bn-scaff_19183_1-p317101	C1	29	54.3	B
11689	Bn-N11-p24427361	Bn-C1-p22215113	Bn-scaff_19183_1-p317102	C1	29	54.3	B
11690	Bn-N11-p24427514	Bn-C1-p22214960	Bn-scaff_19183_1-p317255	C1	29	54.3	B
11691	Bn-N11-p24428267	Bn-C1-p22214207	Bn-scaff_19183_1-p318008	C1	29	54.3	B
11692	Bn-N11-p24428588	Bn-C1-p22213886	Bn-scaff_19183_1-p318329	C1	29	54.3	B
11693	Bn-N11-p24431802	Bn-C1-p22202803	Bn-scaff_19183_1-p329412	C1	29	54.3	B
11694	Bn-N11-p24432340	Bn-C1-p22202265	Bn-scaff_19183_1-p329950	C1	29	54.3	B
11695	Bn-N11-p24432854	Bn-C1-p22201752	Bn-scaff_19183_1-p330463	C1	29	54.3	B
11696	Bn-N11-p24456635	Bn-C1-p22178917	Bn-scaff_19183_1-p353298	C1	29	54.3	B
11697	Bn-N11-p24458601	Bn-C1-p22176957	Bn-scaff_19183_1-p355258	C1	29	54.3	B
11698	Bn-N11-p24459682	Bn-C1-p22175869	Bn-scaff_19183_1-p356346	C1	29	54.3	B
11699	Bn-N11-p24462528	Bn-C1-p22175329	Bn-scaff_19183_1-p356886	C1	29	54.3	B
11700	Bn-N11-p24465114	Bn-C1-p22172741	Bn-scaff_19183_1-p359474	C1	29	54.3	B
11701	Bn-N11-p24480410	Bn-C1-p22164371	Bn-scaff_19183_1-p367844	C1	29	54.3	B
11702	Bn-N11-p24482026	Bn-C1-p22162728	Bn-scaff_19183_1-p369487	C1	29	54.3	B
11703	Bn-N11-p24482130	Bn-C1-p22162624	Bn-scaff_19183_1-p369591	C1	29	54.3	B
11704	Bn-N11-p24482188	Bn-C1-p22162566	Bn-scaff_19183_1-p369649	C1	29	54.3	B
11705	Bn-N11-p24482605	Bn-C1-p22162149	Bn-scaff_19183_1-p370066	C1	29	54.3	B
11706	Bn-N11-p24482973	Bn-C1-p22161785	Bn-scaff_19183_1-p370430	C1	29	54.3	B

11707	Bn-N11-p24483205	Bn-C1-p22161553	Bn-scaff_19183_1-p370662	C1	29	54.3	B
11708	Bn-N11-p24483935	Bn-C1-p22160823	Bn-scaff_19183_1-p371392	C1	29	54.3	B
11709	Bn-N11-p24490904	Bn-C1-p22157485	Bn-scaff_19183_1-p374730	C1	29	54.3	B
11710	Bn-N11-p24491785	Bn-C1-p22156646	Bn-scaff_19183_1-p375569	C1	29	54.3	B
11711	Bn-N11-p24515280	Bn-C1-p22534883	Bn-scaff_16691_1-p110736	C1	29	54.3	B
11712	Bn-N11-p24519756	Bn-C1-p22539351	Bn-scaff_16691_1-p110289	C1	29	54.3	B
11713	Bn-N11-p24609299	Bn-C1-p22626361	Bn-scaff_16691_1-p101588	C1	29	54.3	B
11714	Bn-N11-p24609813	Bn-C1-p22626890	Bn-scaff_16691_1-p101535	C1	29	54.3	B
11715	Bn-N11-p24610152	Bn-C1-p22627231	Bn-scaff_16691_1-p101501	C1	29	54.3	B
11716	Bn-N11-p24610223	Bn-C1-p22627302	Bn-scaff_16691_1-p101494	C1	29	54.3	B
11717	Bn-N11-p24610542	Bn-C1-p22627621	Bn-scaff_16691_1-p101462	C1	29	54.3	B
11718	Bn-N11-p24616214	Bn-C1-p22633387	Bn-scaff_16691_1-p100885	C1	29	54.3	B
11719	Bn-N11-p24657805	Bn-C1-p22671691	Bn-scaff_16691_1-p970553	C1	29	54.3	B
11720	Bn-N11-p24658803	Bn-C1-p22672688	Bn-scaff_16691_1-p969556	C1	29	54.3	B
11721	Bn-N11-p24659868	Bn-C1-p22673737	Bn-scaff_16691_1-p968507	C1	29	54.3	B
11722	Bn-N11-p24660103	Bn-C1-p22673972	Bn-scaff_16691_1-p968272	C1	29	54.3	B
11723	Bn-N11-p24660166	Bn-C1-p22674035	Bn-scaff_16691_1-p968209	C1	29	54.3	B
11724	Bn-N11-p24660687	Bn-C1-p22674556	Bn-scaff_16691_1-p967688	C1	29	54.3	B
11725	Bn-N11-p24660806	Bn-C1-p22674676	Bn-scaff_16691_1-p967568	C1	29	54.3	B
11726	Bn-N11-p24661098	Bn-C1-p22674968	Bn-scaff_16691_1-p967276	C1	29	54.3	B
11727	Bn-N11-p24661247	Bn-C1-p22675117	Bn-scaff_16691_1-p967127	C1	29	54.3	B
11728	Bn-N11-p24698121	Bn-C1-p22723702	Bn-scaff_16691_1-p918542	C1	29	54.3	B
11729	Bn-N11-p24698229	Bn-C1-p22723810	Bn-scaff_16691_1-p918434	C1	29	54.3	B
11730	Bn-N11-p24712695	Bn-C1-p22728209	Bn-scaff_16691_1-p914035	C1	29	54.3	B
11731	Bn-N11-p24712788	Bn-C1-p22728302	Bn-scaff_16691_1-p913942	C1	29	54.3	B
11732	Bn-N11-p24716701	Bn-C1-p22678649	Bn-scaff_16691_1-p963595	C1	29	54.3	B
11733	Bn-N11-p24720453	Bn-C1-p22687905	Bn-scaff_16691_1-p954339	C1	29	54.3	B
11734	Bn-N11-p24760424	Bn-C1-p22761151	Bn-scaff_16691_1-p881093	C1	29	54.3	0
11735	Bn-N11-p24764420	Bn-C1-p22764343	Bn-scaff_16691_1-p877901	C1	29	54.3	B
11736	Bn-N11-p24766134	Bn-C1-p22766065	Bn-scaff_16691_1-p876179	C1	29	54.3	B
11737	Bn-N11-p24766238	Bn-C1-p22766169	Bn-scaff_16691_1-p876075	C1	29	54.3	B
11738	Bn-N11-p24766495	Bn-C1-p22766426	Bn-scaff_16691_1-p875818	C1	29	54.3	B
11739	Bn-N11-p24766498	Bn-C1-p22766429	Bn-scaff_16691_1-p875815	C1	29	54.3	B
11740	Bn-N11-p24766893	Bn-C1-p22766824	Bn-scaff_16691_1-p875420	C1	29	54.3	B
11741	Bn-N11-p24767536	Bn-C1-p22767468	Bn-scaff_16691_1-p874776	C1	29	54.3	B
11742	Bn-N11-p24767748	Bn-C1-p22767659	Bn-scaff_16691_1-p874585	C1	29	54.3	B
11743	Bn-N11-p24767915	Bn-C1-p22767826	Bn-scaff_16691_1-p874418	C1	29	54.3	0
11744	Bn-N11-p24768221	Bn-C1-p22768132	Bn-scaff_16691_1-p874112	C1	29	54.3	B
11745	Bn-N11-p24768333	Bn-C1-p22768244	Bn-scaff_16691_1-p874000	C1	29	54.3	B
11746	Bn-N11-p24768933	Bn-C1-p22768854	Bn-scaff_16691_1-p873390	C1	29	54.3	B
11747	Bn-N11-p24769263	Bn-C1-p22769184	Bn-scaff_16691_1-p873060	C1	29	54.3	B
11748	Bn-N11-p24775916	Bn-C1-p22775672	Bn-scaff_16691_1-p866572	C1	29	54.3	B
11749	Bn-N11-p24775925	Bn-C1-p22775681	Bn-scaff_16691_1-p866563	C1	29	54.3	B
11750	Bn-N11-p24776184	Bn-C1-p22776114	Bn-scaff_16691_1-p866130	C1	29	54.3	B
11751	Bn-N11-p24777432	Bn-C1-p22777363	Bn-scaff_16691_1-p864881	C1	29	54.3	B
11752	Bn-N11-p24784558	Bn-C1-p22784383	Bn-scaff_16691_1-p857861	C1	29	54.3	B
11753	Bn-N11-p24785421	Bn-C1-p22785245	Bn-scaff_16691_1-p856999	C1	29	54.3	B
11754	Bn-N11-p24785584	Bn-C1-p22785408	Bn-scaff_16691_1-p856836	C1	29	54.3	B
11755	Bn-N11-p24785777	Bn-C1-p22785601	Bn-scaff_16691_1-p856643	C1	29	54.3	B

11756	Bn-N11-p24786002	Bn-C1-p22785826	Bn-scaff_16691_1-p856418	C1	29	54.3	B
11757	Bn-N11-p24786230	Bn-C1-p22786054	Bn-scaff_16691_1-p856190	C1	29	54.3	B
11758	Bn-N11-p24787984	Bn-C1-p22787846	Bn-scaff_16691_1-p854398	C1	29	54.3	B
11759	Bn-N11-p24788078	Bn-C1-p22787940	Bn-scaff_16691_1-p854304	C1	29	54.3	B
11760	Bn-N11-p24788199	Bn-C1-p22788061	Bn-scaff_16691_1-p854183	C1	29	54.3	B
11761	Bn-N11-p24788554	Bn-C1-p22788437	Bn-scaff_16691_1-p853807	C1	29	54.3	B
11762	Bn-N11-p24788647	Bn-C1-p22788530	Bn-scaff_16691_1-p853714	C1	29	54.3	B
11763	Bn-N11-p24789457	Bn-C1-p22789455	Bn-scaff_16691_1-p852789	C1	29	54.3	B
11764	Bn-N11-p24797950	Bn-C1-p22790649	Bn-scaff_16691_1-p851595	C1	29	54.3	B
11765	Bn-N11-p24803175	Bn-C1-p22795869	Bn-scaff_16691_1-p846375	C1	29	54.3	B
11766	Bn-N11-p24804037	Bn-C1-p22796732	Bn-scaff_16691_1-p845512	C1	29	54.3	B
11767	Bn-N11-p24808713	Bn-C1-p22802185	Bn-scaff_16691_1-p840059	C1	29	54.3	B
11768	Bn-N11-p24808839	Bn-C1-p22802311	Bn-scaff_16691_1-p839933	C1	29	54.3	B
11769	Bn-N11-p24816112	Bn-C1-p22818118	Bn-scaff_16691_1-p824126	C1	29	54.3	B
11770	Bn-N11-p24816167	Bn-C1-p22818173	Bn-scaff_16691_1-p824071	C1	29	54.3	B
11771	Bn-N11-p24816301	Bn-C1-p22818307	Bn-scaff_16691_1-p823937	C1	29	54.3	B
11772	Bn-N11-p24817179	Bn-C1-p22819190	Bn-scaff_16691_1-p823054	C1	29	54.3	B
11773	Bn-N11-p24824003	Bn-C1-p22823729	Bn-scaff_16691_1-p818515	C1	29	54.3	B
11774	Bn-N11-p24824126	Bn-C1-p22823852	Bn-scaff_16691_1-p818392	C1	29	54.3	B
11775	Bn-N11-p24826198	Bn-C1-p22826130	Bn-scaff_16691_1-p816114	C1	29	54.3	B
11776	Bn-N11-p24826487	Bn-C1-p22826419	Bn-scaff_16691_1-p815825	C1	29	54.3	B
11777	Bn-N11-p24826747	Bn-C1-p22826681	Bn-scaff_16691_1-p815563	C1	29	54.3	B
11778	Bn-N11-p24826899	Bn-C1-p22826833	Bn-scaff_16691_1-p815411	C1	29	54.3	B
11779	Bn-N11-p24841174	Bn-C1-p22837522	Bn-scaff_16691_1-p804722	C1	29	54.3	B
11780	Bn-N11-p24841595	Bn-C1-p22837943	Bn-scaff_16691_1-p804301	C1	29	54.3	B
11781	Bn-N11-p24843969	Bn-C1-p22840307	Bn-scaff_16691_1-p801937	C1	29	54.3	B
11782	Bn-N11-p24845010	Bn-C1-p22841348	Bn-scaff_16691_1-p800896	C1	29	54.3	B
11783	Bn-N11-p24845947	Bn-C1-p22842286	Bn-scaff_16691_1-p799958	C1	29	54.3	B
11784	Bn-N11-p24846345	Bn-C1-p22842684	Bn-scaff_16691_1-p799560	C1	29	54.3	B
11785	Bn-N11-p24848288	Bn-C1-p22848889	Bn-scaff_16691_1-p793355	C1	29	54.3	B
11786	Bn-N11-p24852051	Bn-C1-p22849166	Bn-scaff_16691_1-p793078	C1	29	54.3	B
11787	Bn-N11-p24852150	Bn-C1-p22849265	Bn-scaff_16691_1-p792979	C1	29	54.3	B
11788	Bn-N11-p24852408	Bn-C1-p22849523	Bn-scaff_16691_1-p792721	C1	29	54.3	B
11789	Bn-N11-p24852722	Bn-C1-p22849836	Bn-scaff_16691_1-p792408	C1	29	54.3	B
11790	Bn-N11-p24852911	Bn-C1-p22850025	Bn-scaff_16691_1-p792219	C1	29	54.3	B
11791	Bn-N11-p24853074	Bn-C1-p22850188	Bn-scaff_16691_1-p792056	C1	29	54.3	B
11792	Bn-N11-p24854019	Bn-C1-p22851128	Bn-scaff_16691_1-p791116	C1	29	54.3	B
11793	Bn-N11-p24854475	Bn-C1-p22851586	Bn-scaff_16691_1-p790658	C1	29	54.3	B
11794	Bn-N11-p24855920	Bn-C1-p22853030	Bn-scaff_16691_1-p789214	C1	29	54.3	B
11795	Bn-N11-p24858869	Bn-C1-p22856497	Bn-scaff_16691_1-p785747	C1	29	54.3	B
11796	Bn-N11-p24862496	Bn-C1-p22860133	Bn-scaff_16691_1-p782111	C1	29	54.3	B
11797	Bn-N11-p24862598	Bn-C1-p22860235	Bn-scaff_16691_1-p782009	C1	29	54.3	B
11798	Bn-N11-p24862674	Bn-C1-p22860310	Bn-scaff_16691_1-p781934	C1	29	54.3	B
11799	Bn-N11-p24863000	Bn-C1-p22860636	Bn-scaff_16691_1-p781608	C1	29	54.3	B
11800	Bn-N11-p24868774	Bn-C1-p22865821	Bn-scaff_16691_1-p776423	C1	29	54.3	B
11801	Bn-N11-p24870622	Bn-C1-p22867651	Bn-scaff_16691_1-p774593	C1	29	54.3	B
11802	Bn-N11-p24872179	Bn-C1-p22869181	Bn-scaff_16691_1-p773063	C1	29	54.3	B
11803	Bn-N11-p24876074	Bn-C1-p22873075	Bn-scaff_16691_1-p769169	C1	29	54.3	B
11804	Bn-N11-p24878438	Bn-C1-p22875431	Bn-scaff_16691_1-p766813	C1	29	54.3	B

11805	Bn-N11-p24883126	Bn-C1-p22876823	Bn-scaff_16691_1-p765421	C1	29	54.3	B
11806	Bn-N11-p24883455	Bn-C1-p22877152	Bn-scaff_16691_1-p765092	C1	29	54.3	B
11807	Bn-N11-p24883845	Bn-C1-p22877542	Bn-scaff_16691_1-p764702	C1	29	54.3	B
11808	Bn-N11-p24883987	Bn-C1-p22877685	Bn-scaff_16691_1-p764559	C1	29	54.3	B
11809	Bn-N11-p24884497	Bn-C1-p22878196	Bn-scaff_16691_1-p764048	C1	29	54.3	B
11810	Bn-N11-p24888781	Bn-C1-p22882395	Bn-scaff_16691_1-p759849	C1	29	54.3	B
11811	Bn-N11-p24888844	Bn-C1-p22882458	Bn-scaff_16691_1-p759786	C1	29	54.3	B
11812	Bn-N11-p24889105	Bn-C1-p22882718	Bn-scaff_16691_1-p759526	C1	29	54.3	B
11813	Bn-N11-p24889392	Bn-C1-p22883005	Bn-scaff_16691_1-p759239	C1	29	54.3	B
11814	Bn-N11-p24890088	Bn-C1-p22883699	Bn-scaff_16691_1-p758545	C1	29	54.3	B
11815	Bn-N11-p24891629	Bn-C1-p22885240	Bn-scaff_16691_1-p757004	C1	29	54.3	B
11816	Bn-N11-p24891961	Bn-C1-p22885571	Bn-scaff_16691_1-p756673	C1	29	54.3	B
11817	Bn-N11-p24892608	Bn-C1-p22886198	Bn-scaff_16691_1-p756046	C1	29	54.3	B
11818	Bn-N11-p24895612	Bn-C1-p22889201	Bn-scaff_16691_1-p753043	C1	29	54.3	B
11819	Bn-N11-p24918130	Bn-C1-p22910878	Bn-scaff_16691_1-p731366	C1	29	54.3	B
11820	Bn-N11-p24918454	Bn-C1-p22911197	Bn-scaff_16691_1-p731047	C1	29	54.3	B
11821	Bn-N11-p24950057	Bn-C1-p22945136	Bn-scaff_16691_1-p697108	C1	29	54.3	B
11822	Bn-N11-p24967680	Bn-C1-p22963843	Bn-scaff_16691_1-p678401	C1	29	54.3	B
11823	Bn-N11-p24974314	Bn-C1-p22970670	Bn-scaff_16691_1-p671574	C1	29	54.3	B
11824	Bn-N11-p24974328	Bn-C1-p22970684	Bn-scaff_16691_1-p671560	C1	29	54.3	B
11825	Bn-N11-p24974381	Bn-C1-p22970737	Bn-scaff_16691_1-p671507	C1	29	54.3	B
11826	Bn-N11-p24988335	Bn-C1-p22978720	Bn-scaff_16691_1-p663524	C1	29	54.3	B
11827	Bn-N11-p24988397	Bn-C1-p22978782	Bn-scaff_16691_1-p663462	C1	29	54.3	B
11828	Bn-N11-p24991759	Bn-C1-p22982173	Bn-scaff_16691_1-p660071	C1	29	54.3	B
11829	Bn-N11-p24992183	Bn-C1-p22982597	Bn-scaff_16691_1-p659647	C1	29	54.3	B
11830	Bn-N11-p24992236	Bn-C1-p22982650	Bn-scaff_16691_1-p659594	C1	29	54.3	B
11831	Bn-N11-p24992913	Bn-C1-p22983327	Bn-scaff_16691_1-p658917	C1	29	54.3	B
11832	Bn-N11-p24993165	Bn-C1-p22983579	Bn-scaff_16691_1-p658665	C1	29	54.3	B
11833	Bn-N11-p24993388	Bn-C1-p22983802	Bn-scaff_16691_1-p658442	C1	29	54.3	B
11834	Bn-N11-p24994602	Bn-C1-p22985020	Bn-scaff_16691_1-p657224	C1	29	54.3	B
11835	Bn-N11-p24995712	Bn-C1-p22986149	Bn-scaff_16691_1-p656095	C1	29	54.3	B
11836	Bn-N11-p25000889	Bn-C1-p22991375	Bn-scaff_16691_1-p650869	C1	29	54.3	B
11837	Bn-N11-p25007147	Bn-C1-p22997476	Bn-scaff_16691_1-p644768	C1	29	54.3	B
11838	Bn-N11-p25008159	Bn-C1-p22998487	Bn-scaff_16691_1-p643757	C1	29	54.3	B
11839	Bn-N11-p25008302	Bn-C1-p22998635	Bn-scaff_16691_1-p643609	C1	29	54.3	B
11840	Bn-N11-p25008426	Bn-C1-p22998759	Bn-scaff_16691_1-p643485	C1	29	54.3	B
11841	Bn-N11-p25008525	Bn-C1-p22998858	Bn-scaff_16691_1-p643386	C1	29	54.3	B
11842	Bn-N11-p25008989	Bn-C1-p22999322	Bn-scaff_16691_1-p642922	C1	29	54.3	B
11843	Bn-N11-p25009191	Bn-C1-p22999524	Bn-scaff_16691_1-p642720	C1	29	54.3	B
11844	Bn-N11-p25009430	Bn-C1-p22999763	Bn-scaff_16691_1-p642481	C1	29	54.3	B
11845	Bn-N11-p25009550	Bn-C1-p22999883	Bn-scaff_16691_1-p642361	C1	29	54.3	B
11846	Bn-N11-p25010862	Bn-C1-p23001214	Bn-scaff_16691_1-p641030	C1	29	54.3	B
11847	Bn-N11-p25011498	Bn-C1-p23001836	Bn-scaff_16691_1-p640408	C1	29	54.3	B
11848	Bn-N11-p25012671	Bn-C1-p23003017	Bn-scaff_16691_1-p639227	C1	29	54.3	B
11849	Bn-N11-p25012776	Bn-C1-p23003122	Bn-scaff_16691_1-p639122	C1	29	54.3	B
11850	Bn-N11-p25014199	Bn-C1-p23004429	Bn-scaff_16691_1-p637815	C1	29	54.3	B
11851	Bn-N11-p25015860	Bn-C1-p23006079	Bn-scaff_16691_1-p636165	C1	29	54.3	B
11852	Bn-N11-p25015908	Bn-C1-p23006127	Bn-scaff_16691_1-p636117	C1	29	54.3	B
11853	Bn-N11-p25016116	Bn-C1-p23006335	Bn-scaff_16691_1-p635909	C1	29	54.3	B

11854	Bn-N11-p25016409	Bn-C1-p23006628	Bn-scaff_16691_1-p63561[illegible]	C1	29	54.3	B
11855	Bn-N11-p25016710	Bn-C1-p23006929	Bn-scaff_16691_1-p63531[illegible]	C1	29	54.3	B
11856	Bn-N11-p25016726	Bn-C1-p23006945	Bn-scaff_16691_1-p63529[illegible]	C1	29	54.3	B
11857	Bn-N11-p25016941	Bn-C1-p23007160	Bn-scaff_16691_1-p63508[illegible]	C1	29	54.3	B
11858	Bn-N11-p25057283	Bn-C1-p23040936	Bn-scaff_16691_1-p60130[illegible]	C1	29	54.3	B
11859	Bn-N11-p25080265	Bn-C1-p23065968	Bn-scaff_16691_1-p57627[illegible]	C1	29	54.3	B
11860	Bn-N11-p25080523	Bn-C1-p23066227	Bn-scaff_16691_1-p57601[illegible]	C1	29	54.3	B
11861	Bn-N11-p25083666	Bn-C1-p23069374	Bn-scaff_16691_1-p57287[illegible]	C1	29	54.3	0
11862	Bn-N11-p25083754	Bn-C1-p23069462	Bn-scaff_16691_1-p57278[illegible]	C1	29	54.3	B
11863	Bn-N11-p25083838	Bn-C1-p23069546	Bn-scaff_16691_1-p57269[illegible]	C1	29	54.3	B
11864	Bn-N11-p25096155	Bn-C1-p23076916	Bn-scaff_16691_1-p56532[illegible]	C1	29	54.3	B
11865	Bn-N11-p25126753	Bn-C1-p23098028	Bn-scaff_16691_1-p54421[illegible]	C1	29	54.3	B
11866	Bn-N11-p25157991	Bn-C1-p23126696	Bn-scaff_16691_1-p51554[illegible]	C1	29	54.3	B
11867	Bn-N11-p25185591	Bn-C1-p23159148	Bn-scaff_16691_1-p48309[illegible]	C1	29	54.3	B
11868	Bn-N11-p25413831	Bn-C1-p23396526	Bn-scaff_16691_1-p24571[illegible]	C1	29	54.3	B
11869	Bn-N11-p25541239	Bn-C1-p23497529	Bn-scaff_16691_1-p14471[illegible]	C1	29	54.3	B
11870	Bn-N11-p25614872	Bn-C1-p23575152	Bn-scaff_16691_1-p67092	C1	29	54.3	B
11871	Bn-N11-p25619160	Bn-C1-p23579966	Bn-scaff_16691_1-p62278	C1	29	54.3	B
11872	Bn-N11-p25619981	Bn-C1-p23580781	Bn-scaff_16691_1-p61463	C1	29	54.3	B
11873	Bn-N11-p25899810	Bn-C1-p23840786	Bn-scaff_20589_1-p19844[illegible]	C1	29	54.3	B
11874	Bn-N11-p26607893	Bn-C1-p24542652	Bn-scaff_20478_1-p14543[illegible]	C1	29	54.3	B
11875	Bn-N11-p26609111	Bn-C1-p24543870	Bn-scaff_20478_1-p14421[illegible]	C1	29	54.3	B
11876	Bn-N11-p26609114	Bn-C1-p24543873	Bn-scaff_20478_1-p14420[illegible]	C1	29	54.3	B
11877	Bn-N11-p26609397	Bn-C1-p24544156	Bn-scaff_20478_1-p14392[illegible]	C1	29	54.3	B
11878	Bn-N11-p26609622	Bn-C1-p24544381	Bn-scaff_20478_1-p14370[illegible]	C1	29	54.3	B
11879	Bn-N11-p26610123	Bn-C1-p24544882	Bn-scaff_20478_1-p14320[illegible]	C1	29	54.3	B
11880	Bn-N11-p26617163	Bn-C1-p24552973	Bn-scaff_20478_1-p13510[illegible]	C1	29	54.3	B
11881	Bn-N11-p26627721	Bn-C1-p24564778	Bn-scaff_20478_1-p12330[illegible]	C1	29	54.3	B
11882	Bn-N11-p26647996	Bn-C1-p24584623	Bn-scaff_20478_1-p10345[illegible]	C1	29	54.3	B
11883	Bn-N11-p26688064	Bn-C1-p24626718	Bn-scaff_20478_1-p61364	C1	29	54.3	B
11884	Bn-N11-p26695119	Bn-C1-p24633650	Bn-scaff_20478_1-p54432	C1	29	54.3	B
11885	Bn-N11-p26714402	Bn-C1-p24651319	Bn-scaff_20478_1-p36763	C1	29	54.3	B
11886	Bn-N11-p26714787	Bn-C1-p24651704	Bn-scaff_20478_1-p36378	C1	29	54.3	B
11887	Bn-N11-p26715194	Bn-C1-p24652111	Bn-scaff_20478_1-p35971	C1	29	54.3	B
11888	Bn-N11-p26777094	Bn-C1-p24884230	Bn-scaff_15906_1-p45864[illegible]	C1	29	54.3	B
11889	Bn-N11-p26777326	Bn-C1-p24884002	Bn-scaff_15906_1-p45887[illegible]	C1	29	54.3	B
11890	Bn-N11-p26800526	Bn-C1-p24861651	Bn-scaff_15906_1-p48122[illegible]	C1	29	54.3	B
11891	Bn-N11-p26800800	Bn-C1-p24861377	Bn-scaff_15906_1-p48150[illegible]	C1	29	54.3	B
11892	Bn-N11-p26832424	Bn-C1-p24828497	Bn-scaff_15906_1-p51438[illegible]	C1	29	54.3	B
11893	Bn-N11-p26866207	Bn-C1-p24794605	Bn-scaff_15906_1-p54827[illegible]	C1	29	54.3	B
11894	Bn-N11-p26894685	Bn-C1-p24765856	Bn-scaff_15906_1-p57702[illegible]	C1	29	54.3	B
11895	Bn-N11-p26894798	Bn-C1-p24765743	Bn-scaff_15906_1-p57713[illegible]	C1	29	54.3	B
11896	Bn-N11-p26896807	Bn-C1-p24763723	Bn-scaff_15906_1-p57915[illegible]	C1	29	54.3	B
11897	Bn-N11-p26901019	Bn-C1-p24751419	Bn-scaff_15906_1-p59145[illegible]	C1	29	54.3	B
11898	Bn-N11-p26902329	Bn-C1-p24750107	Bn-scaff_15906_1-p59277[illegible]	C1	29	54.3	B
11899	Bn-N11-p26902843	Bn-C1-p24749595	Bn-scaff_15906_1-p59328[illegible]	C1	29	54.3	B
11900	Bn-N11-p27063832	Bn-C1-p24988320	Bn-scaff_15906_1-p35455[illegible]	C1	29	54.3	B
11901	Bn-N11-p27064013	Bn-C1-p24988501	Bn-scaff_15906_1-p35437[illegible]	C1	29	54.3	B
11902	Bn-N11-p27096595	Bn-C1-p25023329	Bn-scaff_15906_1-p31954[illegible]	C1	29	54.3	B

11903	Bn-N11-p27108777	Bn-C1-p25035468	Bn-scaff_15906_1-p307409	C1	29	54.3	B
11904	Bn-N11-p27110122	Bn-C1-p25036813	Bn-scaff_15906_1-p306064	C1	29	54.3	B
11905	Bn-N11-p27110294	Bn-C1-p25036985	Bn-scaff_15906_1-p305892	C1	29	54.3	B
11906	Bn-N11-p27110539	Bn-C1-p25037230	Bn-scaff_15906_1-p305647	C1	29	54.3	B
11907	Bn-N11-p27116292	Bn-C1-p25042920	Bn-scaff_15906_1-p299957	C1	29	54.3	B
11908	Bn-N11-p27121223	Bn-C1-p25047874	Bn-scaff_15906_1-p295003	C1	29	54.3	B
11909	Bn-N11-p27125347	Bn-C1-p25051989	Bn-scaff_15906_1-p290888	C1	29	54.3	B
11910	Bn-N11-p27126632	Bn-C1-p25053289	Bn-scaff_15906_1-p289588	C1	29	54.3	B
11911	Bn-N11-p27127546	Bn-C1-p25054189	Bn-scaff_15906_1-p288688	C1	29	54.3	B
11912	Bn-N11-p27128980	Bn-C1-p25055623	Bn-scaff_15906_1-p287254	C1	29	54.3	B
11913	Bn-N11-p27129061	Bn-C1-p25055705	Bn-scaff_15906_1-p287172	C1	29	54.3	B
11914	Bn-N11-p27129081	Bn-C1-p25055725	Bn-scaff_15906_1-p287152	C1	29	54.3	B
11915	Bn-N11-p27131083	Bn-C1-p25057724	Bn-scaff_15906_1-p285153	C1	29	54.3	B
11916	Bn-N11-p27132349	Bn-C1-p25058992	Bn-scaff_15906_1-p283885	C1	29	54.3	B
11917	Bn-N11-p27142023	Bn-C1-p25066805	Bn-scaff_15906_1-p276072	C1	29	54.3	B
11918	Bn-N11-p27192712	Bn-C1-p25121217	Bn-scaff_15906_1-p221660	C1	29	54.3	0
11919	Bn-N11-p27254011	Bn-C1-p25152009	Bn-scaff_15906_1-p190868	C1	29	54.3	B
11920	Bn-N11-p27254017	Bn-C1-p25152015	Bn-scaff_15906_1-p190862	C1	29	54.3	B
11921	Bn-N11-p27254606	Bn-C1-p25152608	Bn-scaff_15906_1-p190269	C1	29	54.3	B
11922	Bn-N11-p27254790	Bn-C1-p25152792	Bn-scaff_15906_1-p190085	C1	29	54.3	B
11923	Bn-N11-p27255336	Bn-C1-p25153338	Bn-scaff_15906_1-p189539	C1	29	54.3	B
11924	Bn-N11-p27274250	Bn-C1-p25167285	Bn-scaff_15906_1-p175592	C1	29	54.3	B
11925	Bn-N11-p27292216	Bn-C1-p25185224	Bn-scaff_15906_1-p157653	C1	29	54.3	B
11926	Bn-N11-p27309702	Bn-C1-p25197579	Bn-scaff_15906_1-p145298	C1	29	54.3	B
11927	Bn-N11-p27318920	Bn-C1-p25206728	Bn-scaff_15906_1-p136149	C1	29	54.3	B
11928	Bn-N11-p27319247	Bn-C1-p25207056	Bn-scaff_15906_1-p135821	C1	29	54.3	B
11929	Bn-N11-p27319819	Bn-C1-p25207627	Bn-scaff_15906_1-p135250	C1	29	54.3	B
11930	Bn-N11-p27327054	Bn-C1-p25214399	Bn-scaff_15906_1-p128478	C1	29	54.3	B
11931	Bn-N11-p27328577	Bn-C1-p25215931	Bn-scaff_15906_1-p126946	C1	29	54.3	B
11932	Bn-N11-p27329647	Bn-C1-p25217003	Bn-scaff_15906_1-p125874	C1	29	54.3	B
11933	Bn-N11-p27329699	Bn-C1-p25217055	Bn-scaff_15906_1-p125822	C1	29	54.3	B
11934	Bn-N11-p27331374	Bn-C1-p25218730	Bn-scaff_15906_1-p124147	C1	29	54.3	B
11935	Bn-N11-p27334446	Bn-C1-p25221807	Bn-scaff_15906_1-p121070	C1	29	54.3	B
11936	Bn-N11-p27334661	Bn-C1-p25222022	Bn-scaff_15906_1-p120855	C1	29	54.3	B
11937	Bn-N11-p27334680	Bn-C1-p25222041	Bn-scaff_15906_1-p120836	C1	29	54.3	B
11938	Bn-N11-p27337390	Bn-C1-p25224751	Bn-scaff_15906_1-p118126	C1	29	54.3	B
11939	Bn-N11-p27337467	Bn-C1-p25224828	Bn-scaff_15906_1-p118049	C1	29	54.3	B
11940	Bn-N11-p27338787	Bn-C1-p25226154	Bn-scaff_15906_1-p116723	C1	29	54.3	B
11941	Bn-N11-p27338946	Bn-C1-p25226313	Bn-scaff_15906_1-p116564	C1	29	54.3	B
11942	Bn-N11-p27340030	Bn-C1-p25227396	Bn-scaff_15906_1-p115481	C1	29	54.3	B
11943	Bn-N11-p27340703	Bn-C1-p25228069	Bn-scaff_15906_1-p114808	C1	29	54.3	B
11944	Bn-N11-p27342527	Bn-C1-p25229893	Bn-scaff_15906_1-p112984	C1	29	54.3	B
11945	Bn-N11-p27344090	Bn-C1-p25231456	Bn-scaff_15906_1-p111421	C1	29	54.3	B
11946	Bn-N11-p27362878	Bn-C1-p25251322	Bn-scaff_15906_1-p91555	C1	29	54.3	B
11947	Bn-N11-p27364511	Bn-C1-p25252970	Bn-scaff_15906_1-p89907	C1	29	54.3	B
11948	Bn-N11-p27379748	Bn-C1-p25268035	Bn-scaff_15906_1-p74842	C1	29	54.3	B
11949	Bn-N11-p27382535	Bn-C1-p25270818	Bn-scaff_15906_1-p72059	C1	29	54.3	B
11950	Bn-N11-p27384583	Bn-C1-p25272540	Bn-scaff_15906_1-p70337	C1	29	54.3	B
11951	Bn-N11-p27388478	Bn-C1-p25275867	Bn-scaff_15906_1-p67010	C1	29	54.3	B

11952	Bn-N11-p27389042	Bn-C1-p25280597	Bn-scaff_15906_1-p62280	C1	29	54.3	B
11953	Bn-N11-p27389421	Bn-C1-p25280976	Bn-scaff_15906_1-p61901	C1	29	54.3	B
11954	Bn-N11-p27396890	Bn-C1-p25288647	Bn-scaff_15906_1-p54230	C1	29	54.3	B
11955	Bn-N11-p27397064	Bn-C1-p25288821	Bn-scaff_15906_1-p54056	C1	29	54.3	B
11956	Bn-N11-p27406149	Bn-C1-p25292761	Bn-scaff_15906_1-p50116	C1	29	54.3	B
11957	Bn-N11-p27410762	Bn-C1-p25297660	Bn-scaff_15906_1-p45217	C1	29	54.3	B
11958	Bn-N11-p27414464	Bn-C1-p25301347	Bn-scaff_15906_1-p41530	C1	29	54.3	B
11959	Bn-N11-p27419963	Bn-C1-p25306993	Bn-scaff_15906_1-p35884	C1	29	54.3	B
11960	Bn-N11-p27420595	Bn-C1-p25307631	Bn-scaff_15906_1-p35246	C1	29	54.3	B
11961	Bn-N11-p27420658	Bn-C1-p25307694	Bn-scaff_15906_1-p35183	C1	29	54.3	B
11962	Bn-N11-p27424652	Bn-C1-p25311739	Bn-scaff_15906_1-p31138	C1	29	54.3	B
11963	Bn-N11-p27425071	Bn-C1-p25312158	Bn-scaff_15906_1-p30719	C1	29	54.3	B
11964	Bn-N11-p27427663	Bn-C1-p25314737	Bn-scaff_15906_1-p28140	C1	29	54.3	B
11965	Bn-N11-p27431782	Bn-C1-p25318865	Bn-scaff_15906_1-p24012	C1	29	54.3	B
11966	Bn-N11-p27432084	Bn-C1-p25319166	Bn-scaff_15906_1-p23711	C1	29	54.3	B
11967	Bn-N11-p27763190	Bn-C1-p25343748	Bn-scaff_21820_1-p10739(	C1	29	54.3	B
11968	Bn-N11-p27763502	Bn-C1-p25345640	Bn-scaff_21820_1-p10720	C1	29	54.3	B
11969	Bn-N12-p24341211	Bn-C1-p23330977	Bn-scaff_16691_1-p31126	C1	29	54.3	B
11970	Bn-N13-p37272369	Bn-C1-p23125737	Bn-scaff_16691_1-p51650	C1	29	54.3	B
11971	Bn-N14-p22718473	Bn-C1-p18944315	Bn-scaff_19364_1-p56779	C1	29	54.3	B
11972	Bn-N15-p22429840	Bn-C1-p24944055	Bn-scaff_15906_1-p39882	C1	29	54.3	B
11973	Bn-N1-p12057902	Bn-C1-p17996301	Bn-scaff_20250_1-p21366	C1	29	54.3	B
11974	Bn-N1-p14032392	Bn-C1-p21847362	Bn-scaff_22795_1-p19562	C1	29	54.3	B
11975	Bn-N1-p14800251	Bn-C1-p23591665	Bn-scaff_16691_1-p50579	C1	29	54.3	B
11976	Bn-N1-p15221054	Bn-C1-p25073862	Bn-scaff_15906_1-p26901	C1	29	54.3	B
11977	Bn-Scaffold06268-p1146	Bn-C1-p22366531	Bn-scaff_19183_1-p16568	C1	29	54.3	B
11978	Bn-Scaffold07400-p469	Bn-C1-p22676257	Bn-scaff_16691_1-p96598	C1	29	54.3	B
11979	Bn-Scaffold07400-p857	Bn-C1-p22675869	Bn-scaff_16691_1-p96637	C1	29	54.3	B
11980	Bn-Scaffold07400-p881	Bn-C1-p22675845	Bn-scaff_16691_1-p96639	C1	29	54.3	B
11981	Bn-Scaffold09318-p699	Bn-C1-p24538147	Bn-scaff_20478_1-p14993	C1	29	54.3	B
11982	Bn-Scaffold59414-p177	Bn-Scaffold00751-p4	Bn-scaff_16809_1-p43861	C1	29	54.3	B
11983	Bn-N11-p16242179	Bn-C3-p40219319	Bn-scaff_19702_1-p61645	C1	30	54.3	B
11984	Bn-N11-p17608345	Bn-C6-p35484852	Bn-scaff_19569_1-p2814	C1	30	54.3	B
11985	Bn-N11-p19400845	Bn-Scaffold11992-p4	Bn-scaff_20126_1-p457	C1	30	54.3	B
11986	Bn-N11-p21610886	Bn-Scaffold00976-p2	Bn-scaff_25750_1-p26267	C1	30	54.3	B
11987	Bn-N11-p24506360	Bn-Scaffold01557-p7	Bn-scaff_25373_1-p711	C1	30	54.3	B
11988	Bn-N11-p24506672	Bn-Scaffold01557-p3	Bn-scaff_25373_1-p399	C1	30	54.3	B
11989	Bn-N11-p27067162	Bn-Scaffold00919-p2	Bn-scaff_22976_1-p23202	C1	30	54.3	B
11990	Bn-N11-p27077496	Bn-C2-p40151081	Bn-scaff_21705_1-p43509	C1	30	54.3	B
11991	Bn-N11-p27470078	Bn-Scaffold01107-p8	Bn-scaff_19458_1-p8019	C1	30	54.3	B
11992	Bn-N11-p27514854	Bn-C7-p8532856	Bn-scaff_22441_1-p43103	C1	30	54.3	B
11993	Bn-N11-p27514957	Bn-C7-p8532750	Bn-scaff_22441_1-p43209	C1	30	54.3	B
11994	Bn-N11-p27515544	Bn-C7-p8532163	Bn-scaff_22441_1-p43796	C1	30	54.3	B
11995	Bn-N11-p27516739	Bn-C7-p8530971	Bn-scaff_22441_1-p44988	C1	30	54.3	B
11996	Bn-N11-p27517770	Bn-C7-p8529941	Bn-scaff_22441_1-p46018	C1	30	54.3	B
11997	Bn-N11-p27520985	Bn-Scaffold00581-p2	Bn-scaff_15791_1-p21050	C1	30	54.3	B
11998	Bn-N11-p27521729	Bn-Scaffold00581-p2	Bn-scaff_15791_1-p20975	C1	30	54.3	B
11999	Bn-N11-p27522681	Bn-Scaffold00581-p2	Bn-scaff_15791_1-p20877	C1	30	54.3	B
12000	Bn-N11-p27535011	Bn-Scaffold00581-p1	Bn-scaff_15791_1-p19628	C1	30	54.3	B

12001	Bn-N11-p27535776	Bn-Scaffold00581-p19	Bn-scaff_15791_1-p195580	C1	30	54.3	B
12002	Bn-N11-p27588269	Bn-Scaffold00581-p15	Bn-scaff_15791_1-p159632	C1	30	54.3	B
12003	Bn-N11-p27588655	Bn-Scaffold00581-p15	Bn-scaff_15791_1-p159264	C1	30	54.3	B
12004	Bn-N11-p27607036	Bn-Scaffold00581-p13	Bn-scaff_15791_1-p139192	C1	30	54.3	B
12005	Bn-N11-p27607140	Bn-Scaffold00581-p13	Bn-scaff_15791_1-p139088	C1	30	54.3	B
12006	Bn-N11-p27607183	Bn-Scaffold00581-p13	Bn-scaff_15791_1-p139045	C1	30	54.3	B
12007	Bn-N11-p27608880	Bn-Scaffold00581-p13	Bn-scaff_15791_1-p137348	C1	30	54.3	B
12008	Bn-N11-p27609529	Bn-Scaffold00581-p13	Bn-scaff_15791_1-p136765	C1	30	54.3	B
12009	Bn-N11-p27610335	Bn-Scaffold00581-p13	Bn-scaff_15791_1-p135959	C1	30	54.3	B
12010	Bn-N11-p27618060	Bn-Scaffold00581-p12	Bn-scaff_15791_1-p128271	C1	30	54.3	B
12011	Bn-N11-p27629921	Bn-Scaffold00581-p11	Bn-scaff_15791_1-p117382	C1	30	54.3	B
12012	Bn-N11-p27629949	Bn-Scaffold00581-p11	Bn-scaff_15791_1-p117355	C1	30	54.3	B
12013	Bn-N11-p27630030	Bn-Scaffold00581-p11	Bn-scaff_15791_1-p117274	C1	30	54.3	B
12014	Bn-N11-p27630102	Bn-Scaffold00581-p11	Bn-scaff_15791_1-p117202	C1	30	54.3	B
12015	Bn-N11-p27630615	Bn-Scaffold00581-p11	Bn-scaff_15791_1-p116689	C1	30	54.3	B
12016	Bn-N11-p27630806	Bn-Scaffold00581-p11	Bn-scaff_15791_1-p116498	C1	30	54.3	B
12017	Bn-N11-p27631911	Bn-Scaffold00581-p11	Bn-scaff_15791_1-p115392	C1	30	54.3	B
12018	Bn-N11-p27631969	Bn-Scaffold00581-p11	Bn-scaff_15791_1-p115334	C1	30	54.3	B
12019	Bn-N11-p27634395	Bn-Scaffold00581-p11	Bn-scaff_15791_1-p112906	C1	30	54.3	B
12020	Bn-N11-p27645371	Bn-Scaffold00581-p10	Bn-scaff_15791_1-p105333	C1	30	54.3	B
12021	Bn-N11-p27665016	Bn-Scaffold00581-p94	Bn-scaff_15791_1-p94980	C1	30	54.3	B
12022	Bn-N11-p27665346	Bn-Scaffold00581-p94	Bn-scaff_15791_1-p94650	C1	30	54.3	B
12023	Bn-N11-p27666191	Bn-Scaffold00581-p93	Bn-scaff_15791_1-p93803	C1	30	54.3	B
12024	Bn-N11-p27667558	Bn-Scaffold00581-p92	Bn-scaff_15791_1-p92475	C1	30	54.3	B
12025	Bn-N11-p27668650	Bn-Scaffold00581-p91	Bn-scaff_15791_1-p91375	C1	30	54.3	B
12026	Bn-N11-p27669644	Bn-Scaffold00581-p90	Bn-scaff_15791_1-p90377	C1	30	54.3	B
12027	Bn-N11-p27670233	Bn-Scaffold00581-p89	Bn-scaff_15791_1-p89788	C1	30	54.3	B
12028	Bn-N11-p27670718	Bn-Scaffold00581-p89	Bn-scaff_15791_1-p89304	C1	30	54.3	B
12029	Bn-N11-p27681247	Bn-Scaffold00581-p75	Bn-scaff_15791_1-p75723	C1	30	54.3	B
12030	Bn-N11-p27689022	Bn-Scaffold00581-p67	Bn-scaff_15791_1-p67268	C1	30	54.3	B
12031	Bn-N11-p27711838	Bn-Scaffold00581-p43	Bn-scaff_15791_1-p43592	C1	30	54.3	B
12032	Bn-N11-p27718899	Bn-Scaffold00581-p36	Bn-scaff_15791_1-p36503	C1	30	54.3	B
12033	Bn-N11-p27722735	Bn-Scaffold00581-p32	Bn-scaff_15791_1-p32569	C1	30	54.3	B
12034	Bn-N11-p27744463	Bn-Scaffold00581-p17	Bn-scaff_15791_1-p17957	C1	30	54.3	B
12035	Bn-N11-p27744807	Bn-Scaffold00581-p17	Bn-scaff_15791_1-p17613	C1	30	54.3	B
12036	Bn-N11-p27749353	Bn-Scaffold00581-p13	Bn-scaff_15791_1-p13102	C1	30	54.3	B
12037	Bn-N11-p27750826	Bn-Scaffold00581-p11	Bn-scaff_15791_1-p11657	C1	30	54.3	B
12038	Bn-N11-p27752115	Bn-Scaffold00581-p10	Bn-scaff_15791_1-p10371	C1	30	54.3	B
12039	Bn-N11-p27758566	Bn-Scaffold00581-p39	Bn-scaff_15791_1-p3927	C1	30	54.3	B
12040	Bn-N11-p27764259	Bn-C1-p25346396	Bn-scaff_21820_1-p107131	C1	30	54.3	B
12041	Bn-N11-p27765786	Bn-C1-p25347893	Bn-scaff_21820_1-p106981	C1	30	54.3	B
12042	Bn-N11-p27793533	Bn-C1-p26498241	Bn-scaff_15749_1-p448039	C1	30	54.3	B
12043	Bn-N11-p27793842	Bn-C1-p26497932	Bn-scaff_15749_1-p448348	C1	30	54.3	B
12044	Bn-N11-p27794499	Bn-C1-p26497274	Bn-scaff_15749_1-p449006	C1	30	54.3	B
12045	Bn-N11-p27794712	Bn-C1-p26497057	Bn-scaff_15749_1-p449223	C1	30	54.3	0
12046	Bn-N11-p27795192	Bn-C1-p26496577	Bn-scaff_15749_1-p449703	C1	30	54.3	B
12047	Bn-N11-p27832862	Bn-C1-p26468504	Bn-scaff_15749_1-p477776	C1	30	54.3	B
12048	Bn-N11-p27844219	Bn-C1-p26421834	Bn-scaff_16532_1-p8308	C1	30	54.3	B
12049	Bn-N11-p27858924	Bn-C1-p26461320	Bn-scaff_15749_1-p484960	C1	30	54.3	B

12050	Bn-N11-p27863123	Bn-C1-p26075796	Bn-scaff_21820_1-p341916	C1	30	54.3	B
12051	Bn-N11-p27864665	Bn-C1-p26077337	Bn-scaff_21820_1-p340375	C1	30	54.3	B
12052	Bn-N11-p27866349	Bn-C1-p26078941	Bn-scaff_21820_1-p338771	C1	30	54.3	B
12053	Bn-N11-p27868467	Bn-C1-p26081063	Bn-scaff_21820_1-p336649	C1	30	54.3	B
12054	Bn-N11-p27870801	Bn-C1-p26083072	Bn-scaff_21820_1-p334640	C1	30	54.3	B
12055	Bn-N11-p27872008	Bn-C1-p26084278	Bn-scaff_21820_1-p333434	C1	30	54.3	B
12056	Bn-N11-p27876463	Bn-C1-p26091421	Bn-scaff_21820_1-p326291	C1	30	54.3	B
12057	Bn-N11-p27877128	Bn-C1-p26092086	Bn-scaff_21820_1-p325626	C1	30	54.3	B
12058	Bn-N11-p27877773	Bn-C1-p26092729	Bn-scaff_21820_1-p324983	C1	30	54.3	B
12059	Bn-N11-p27877950	Bn-C1-p26092906	Bn-scaff_21820_1-p324806	C1	30	54.3	B
12060	Bn-N11-p27915289	Bn-C1-p26122249	Bn-scaff_21820_1-p295463	C1	30	54.3	B
12061	Bn-N11-p27916510	Bn-C1-p26123417	Bn-scaff_21820_1-p294295	C1	30	54.3	B
12062	Bn-N11-p27926463	Bn-C1-p26136933	Bn-scaff_21820_1-p280779	C1	30	54.3	B
12063	Bn-N11-p27927350	Bn-C1-p26137818	Bn-scaff_21820_1-p279894	C1	30	54.3	B
12064	Bn-N11-p27943415	Bn-C1-p26157415	Bn-scaff_21820_1-p260297	C1	30	54.3	B
12065	Bn-N11-p27953985	Bn-C1-p26165970	Bn-scaff_21820_1-p251742	C1	30	54.3	B
12066	Bn-N11-p27955085	Bn-C1-p26167046	Bn-scaff_21820_1-p250666	C1	30	54.3	B
12067	Bn-N11-p27957168	Bn-C1-p26168809	Bn-scaff_21820_1-p248903	C1	30	54.3	B
12068	Bn-N11-p27957473	Bn-C1-p26169113	Bn-scaff_21820_1-p248599	C1	30	54.3	B
12069	Bn-N11-p27957861	Bn-C1-p26169501	Bn-scaff_21820_1-p248211	C1	30	54.3	B
12070	Bn-N11-p27958002	Bn-C1-p26169642	Bn-scaff_21820_1-p248070	C1	30	54.3	B
12071	Bn-N11-p27958479	Bn-C1-p26170133	Bn-scaff_21820_1-p247579	C1	30	54.3	B
12072	Bn-N11-p27959270	Bn-C1-p26170925	Bn-scaff_21820_1-p246787	C1	30	54.3	B
12073	Bn-N11-p27959420	Bn-C1-p26171075	Bn-scaff_21820_1-p246637	C1	30	54.3	B
12074	Bn-N11-p27973296	Bn-C1-p26184972	Bn-scaff_21820_1-p232740	C1	30	54.3	B
12075	Bn-N11-p27973574	Bn-C1-p26185251	Bn-scaff_21820_1-p232461	C1	30	54.3	B
12076	Bn-N11-p27976987	Bn-C1-p26189000	Bn-scaff_21820_1-p228712	C1	30	54.3	B
12077	Bn-N11-p27979934	Bn-C1-p26191951	Bn-scaff_21820_1-p225761	C1	30	54.3	B
12078	Bn-N11-p27980611	Bn-C1-p26192775	Bn-scaff_21820_1-p224937	C1	30	54.3	B
12079	Bn-N11-p27981503	Bn-C1-p26193670	Bn-scaff_21820_1-p224042	C1	30	54.3	B
12080	Bn-N11-p27981658	Bn-C1-p26193825	Bn-scaff_21820_1-p223887	C1	30	54.3	B
12081	Bn-N11-p27982070	Bn-C1-p26194239	Bn-scaff_21820_1-p223473	C1	30	54.3	B
12082	Bn-N11-p27982397	Bn-C1-p26194567	Bn-scaff_21820_1-p223145	C1	30	54.3	B
12083	Bn-N11-p27982731	Bn-C1-p26194902	Bn-scaff_21820_1-p222810	C1	30	54.3	B
12084	Bn-N11-p28000654	Bn-C1-p26206809	Bn-scaff_21820_1-p210903	C1	30	54.3	B
12085	Bn-N11-p28000668	Bn-C1-p26206823	Bn-scaff_21820_1-p210889	C1	30	54.3	0
12086	Bn-N11-p28000719	Bn-C1-p26206874	Bn-scaff_21820_1-p210838	C1	30	54.3	B
12087	Bn-N11-p28002007	Bn-C1-p26208121	Bn-scaff_21820_1-p209591	C1	30	54.3	B
12088	Bn-N11-p28002448	Bn-C1-p26208557	Bn-scaff_21820_1-p209155	C1	30	54.3	B
12089	Bn-N11-p28013441	Bn-C1-p26220641	Bn-scaff_21820_1-p197071	C1	30	54.3	B
12090	Bn-N11-p28013999	Bn-C1-p26221200	Bn-scaff_21820_1-p196512	C1	30	54.3	B
12091	Bn-N11-p28022892	Bn-C1-p26238076	Bn-scaff_21820_1-p179636	C1	30	54.3	B
12092	Bn-N11-p28061794	Bn-C1-p26272916	Bn-scaff_21820_1-p144796	C1	30	54.3	B
12093	Bn-N11-p28068210	Bn-C1-p26279491	Bn-scaff_21820_1-p138221	C1	30	54.3	B
12094	Bn-N11-p28069398	Bn-C1-p26280679	Bn-scaff_21820_1-p137033	C1	30	54.3	B
12095	Bn-N11-p28069429	Bn-C1-p26280710	Bn-scaff_21820_1-p137002	C1	30	54.3	B
12096	Bn-N11-p28070273	Bn-C1-p26281546	Bn-scaff_21820_1-p136166	C1	30	54.3	B
12097	Bn-N11-p28070987	Bn-C1-p26282262	Bn-scaff_21820_1-p135450	C1	30	54.3	B
12098	Bn-N11-p28072189	Bn-C1-p26283464	Bn-scaff_21820_1-p134248	C1	30	54.3	B

12099	Bn-N11-p28073271	Bn-C1-p26284554	Bn-scaff_21820_1-p133158	C1	30	54.3	B
12100	Bn-N11-p28073541	Bn-C1-p26284815	Bn-scaff_21820_1-p132897	C1	30	54.3	B
12101	Bn-N11-p28073606	Bn-C1-p26284880	Bn-scaff_21820_1-p132832	C1	30	54.3	B
12102	Bn-N11-p28075512	Bn-C1-p26286808	Bn-scaff_21820_1-p130904	C1	30	54.3	B
12103	Bn-N11-p28077423	Bn-C1-p26288559	Bn-scaff_21820_1-p129153	C1	30	54.3	B
12104	Bn-N11-p28096802	Bn-C1-p26308460	Bn-scaff_21820_1-p109252	C1	30	54.3	B
12105	Bn-N11-p28096950	Bn-C1-p26308601	Bn-scaff_21820_1-p109111	C1	30	54.3	B
12106	Bn-N11-p28106784	Bn-C1-p26318495	Bn-scaff_21820_1-p99217	C1	30	54.3	B
12107	Bn-N11-p28107882	Bn-C1-p26319600	Bn-scaff_21820_1-p98112	C1	30	54.3	B
12108	Bn-N11-p28111587	Bn-C1-p26324554	Bn-scaff_21820_1-p93158	C1	30	54.3	B
12109	Bn-N11-p28137119	Bn-C1-p26360967	Bn-scaff_21820_1-p56745	C1	30	54.3	B
12110	Bn-N11-p28140621	Bn-C1-p26374966	Bn-scaff_21820_1-p42746	C1	30	54.3	B
12111	Bn-N11-p28140743	Bn-C1-p26375088	Bn-scaff_21820_1-p42624	C1	30	54.3	B
12112	Bn-N11-p28141051	Bn-C1-p26375395	Bn-scaff_21820_1-p42317	C1	30	54.3	B
12113	Bn-N11-p28141244	Bn-C1-p26375608	Bn-scaff_21820_1-p42104	C1	30	54.3	B
12114	Bn-N11-p28141314	Bn-C1-p26375679	Bn-scaff_21820_1-p42033	C1	30	54.3	B
12115	Bn-N11-p28142147	Bn-C1-p26376501	Bn-scaff_21820_1-p41211	C1	30	54.3	B
12116	Bn-N11-p28142503	Bn-C1-p26376857	Bn-scaff_21820_1-p40855	C1	30	54.3	B
12117	Bn-N11-p28213199	Bn-C1-p26394131	Bn-scaff_21820_1-p23581	C1	30	54.3	B
12118	Bn-N11-p28214843	Bn-C1-p26395410	Bn-scaff_21820_1-p22302	C1	30	54.3	B
12119	Bn-N11-p28216723	Bn-C1-p26397479	Bn-scaff_21820_1-p20233	C1	30	54.3	B
12120	Bn-N11-p28216908	Bn-C1-p26397665	Bn-scaff_21820_1-p20047	C1	30	54.3	B
12121	Bn-N11-p28220842	Bn-C1-p26401594	Bn-scaff_21820_1-p16118	C1	30	54.3	B
12122	Bn-N11-p28226406	Bn-C1-p26402345	Bn-scaff_21820_1-p15367	C1	30	54.3	B
12123	Bn-N11-p28227910	Bn-C1-p26404816	Bn-scaff_21820_1-p12896	C1	30	54.3	B
12124	Bn-N11-p28228225	Bn-C1-p26405131	Bn-scaff_21820_1-p12581	C1	30	54.3	B
12125	Bn-N11-p28228405	Bn-C1-p26405317	Bn-scaff_21820_1-p12395	C1	30	54.3	B
12126	Bn-N11-p28237398	Bn-Scaffold00686-p14	Bn-scaff_26101_1-p147422	C1	30	54.3	B
12127	Bn-N11-p28237433	Bn-Scaffold00686-p14	Bn-scaff_26101_1-p147387	C1	30	54.3	B
12128	Bn-N11-p28237579	Bn-Scaffold00686-p14	Bn-scaff_26101_1-p147241	C1	30	54.3	B
12129	Bn-N11-p28246253	Bn-Scaffold00686-p14	Bn-scaff_26101_1-p146982	C1	30	54.3	B
12130	Bn-N11-p28246320	Bn-Scaffold00686-p14	Bn-scaff_26101_1-p146915	C1	30	54.3	B
12131	Bn-N11-p28246828	Bn-Scaffold00686-p14	Bn-scaff_26101_1-p146408	C1	30	54.3	B
12132	Bn-N11-p28249435	Bn-Scaffold00686-p13	Bn-scaff_26101_1-p139236	C1	30	54.3	B
12133	Bn-N11-p28249523	Bn-Scaffold00686-p13	Bn-scaff_26101_1-p139146	C1	30	54.3	B
12134	Bn-N11-p28249578	Bn-Scaffold00686-p13	Bn-scaff_26101_1-p139091	C1	30	54.3	B
12135	Bn-N11-p28249601	Bn-Scaffold00686-p13	Bn-scaff_26101_1-p139068	C1	30	54.3	B
12136	Bn-N11-p28249673	Bn-Scaffold00686-p13	Bn-scaff_26101_1-p138996	C1	30	54.3	B
12137	Bn-N11-p28251132	Bn-Scaffold00686-p13	Bn-scaff_26101_1-p137481	C1	30	54.3	B
12138	Bn-N11-p28252137	Bn-Scaffold00686-p13	Bn-scaff_26101_1-p136501	C1	30	54.3	B
12139	Bn-N11-p28252414	Bn-Scaffold00686-p13	Bn-scaff_26101_1-p136221	C1	30	54.3	B
12140	Bn-N11-p28252617	Bn-Scaffold00686-p13	Bn-scaff_26101_1-p136001	C1	30	54.3	B
12141	Bn-N11-p28257046	Bn-Scaffold00686-p13	Bn-scaff_26101_1-p134349	C1	30	54.3	B
12142	Bn-N11-p28262949	Bn-Scaffold00686-p12	Bn-scaff_26101_1-p120558	C1	30	54.3	B
12143	Bn-N11-p28263433	Bn-Scaffold00686-p12	Bn-scaff_26101_1-p120080	C1	30	54.3	B
12144	Bn-N11-p28263549	Bn-Scaffold00686-p11	Bn-scaff_26101_1-p119963	C1	30	54.3	B
12145	Bn-N11-p28263644	Bn-Scaffold00686-p11	Bn-scaff_26101_1-p119868	C1	30	54.3	B
12146	Bn-N11-p28263802	Bn-Scaffold00686-p11	Bn-scaff_26101_1-p119709	C1	30	54.3	B
12147	Bn-N11-p28263858	Bn-Scaffold00686-p11	Bn-scaff_26101_1-p119653	C1	30	54.3	B

12148	Bn-N11-p28263925	Bn-Scaffold00686-p1	Bn-scaff_26101_1-p119586	C1	30	54.3	B
12149	Bn-N11-p28264388	Bn-Scaffold00686-p1	Bn-scaff_26101_1-p119122	C1	30	54.3	B
12150	Bn-N11-p28264579	Bn-Scaffold00686-p1	Bn-scaff_26101_1-p118931	C1	30	54.3	B
12151	Bn-N11-p28265074	Bn-Scaffold00686-p1	Bn-scaff_26101_1-p118436	C1	30	54.3	B
12152	Bn-N11-p28265326	Bn-Scaffold00686-p1	Bn-scaff_26101_1-p118180	C1	30	54.3	B
12153	Bn-N11-p28266093	Bn-Scaffold00686-p1	Bn-scaff_26101_1-p117587	C1	30	54.3	B
12154	Bn-N11-p28266647	Bn-Scaffold00686-p1	Bn-scaff_26101_1-p117027	C1	30	54.3	B
12155	Bn-N11-p28267666	Bn-Scaffold00686-p1	Bn-scaff_26101_1-p113978	C1	30	54.3	B
12156	Bn-N11-p28269078	Bn-Scaffold00686-p1	Bn-scaff_26101_1-p112869	C1	30	54.3	B
12157	Bn-N11-p28269158	Bn-Scaffold00686-p1	Bn-scaff_26101_1-p112789	C1	30	54.3	B
12158	Bn-N11-p28336637	Bn-Scaffold00686-p2	Bn-scaff_26101_1-p25897	C1	30	54.3	B
12159	Bn-N11-p28437576	Bn-C1-p26049570	Bn-scaff_21820_1-p368142	C1	30	54.3	B
12160	Bn-N11-p28440254	Bn-C1-p26046897	Bn-scaff_21820_1-p370815	C1	30	54.3	B
12161	Bn-N11-p28440610	Bn-C1-p26046536	Bn-scaff_21820_1-p371176	C1	30	54.3	B
12162	Bn-N11-p28441089	Bn-C1-p26046057	Bn-scaff_21820_1-p371655	C1	30	54.3	B
12163	Bn-N11-p28446131	Bn-C1-p26039652	Bn-scaff_21820_1-p378060	C1	30	54.3	B
12164	Bn-N11-p28446272	Bn-C1-p26039511	Bn-scaff_21820_1-p378201	C1	30	54.3	B
12165	Bn-N11-p28484289	Bn-C1-p25995469	Bn-scaff_21820_1-p422243	C1	30	54.3	B
12166	Bn-N11-p28491042	Bn-C1-p25991531	Bn-scaff_21820_1-p426181	C1	30	54.3	B
12167	Bn-N11-p28521571	Bn-C1-p25953312	Bn-scaff_21820_1-p464400	C1	30	54.3	B
12168	Bn-N11-p28521635	Bn-C1-p25953248	Bn-scaff_21820_1-p464464	C1	30	54.3	B
12169	Bn-N11-p28521754	Bn-C1-p25953127	Bn-scaff_21820_1-p464585	C1	30	54.3	B
12170	Bn-N11-p28521788	Bn-C1-p25953093	Bn-scaff_21820_1-p464619	C1	30	54.3	B
12171	Bn-N11-p28532581	Bn-C1-p25941565	Bn-scaff_21820_1-p476147	C1	30	54.3	B
12172	Bn-N11-p28533240	Bn-C1-p25940902	Bn-scaff_21820_1-p476810	C1	30	54.3	B
12173	Bn-N11-p28533375	Bn-C1-p25940767	Bn-scaff_21820_1-p476945	C1	30	54.3	B
12174	Bn-N11-p28533569	Bn-C1-p25940573	Bn-scaff_21820_1-p477139	C1	30	54.3	B
12175	Bn-N11-p28533626	Bn-C1-p25940516	Bn-scaff_21820_1-p477196	C1	30	54.3	B
12176	Bn-N11-p28544341	Bn-C1-p25927212	Bn-scaff_21820_1-p490500	C1	30	54.3	B
12177	Bn-N11-p28551418	Bn-C1-p25918680	Bn-scaff_21820_1-p499032	C1	30	54.3	B
12178	Bn-N11-p28551881	Bn-C1-p25918218	Bn-scaff_21820_1-p499494	C1	30	54.3	B
12179	Bn-N11-p28553813	Bn-C1-p25916759	Bn-scaff_21820_1-p500953	C1	30	54.3	B
12180	Bn-N11-p28556122	Bn-C1-p25913915	Bn-scaff_21820_1-p503797	C1	30	54.3	B
12181	Bn-N11-p28557756	Bn-C1-p25908776	Bn-scaff_21820_1-p508936	C1	30	54.3	B
12182	Bn-N11-p28558235	Bn-C1-p25908341	Bn-scaff_21820_1-p509371	C1	30	54.3	B
12183	Bn-N11-p28558724	Bn-C1-p25907712	Bn-scaff_21820_1-p510000	C1	30	54.3	B
12184	Bn-N11-p28559564	Bn-C1-p25906837	Bn-scaff_21820_1-p510875	C1	30	54.3	B
12185	Bn-N11-p28585012	Bn-C1-p25886755	Bn-scaff_21820_1-p530957	C1	30	54.3	B
12186	Bn-N11-p28587396	Bn-C1-p25884371	Bn-scaff_21820_1-p533341	C1	30	54.3	B
12187	Bn-N11-p28590617	Bn-C1-p25881026	Bn-scaff_21820_1-p536686	C1	30	54.3	B
12188	Bn-N11-p28591477	Bn-C1-p25880409	Bn-scaff_21820_1-p537303	C1	30	54.3	0
12189	Bn-N11-p28604763	Bn-C1-p25873938	Bn-scaff_21820_1-p543774	C1	30	54.3	B
12190	Bn-N11-p28606416	Bn-C1-p25872297	Bn-scaff_21820_1-p545415	C1	30	54.3	B
12191	Bn-N11-p28608163	Bn-C1-p25870551	Bn-scaff_21820_1-p547161	C1	30	54.3	B
12192	Bn-N11-p28608194	Bn-C1-p25870520	Bn-scaff_21820_1-p547192	C1	30	54.3	B
12193	Bn-N11-p28609000	Bn-C1-p25864515	Bn-scaff_21820_1-p553197	C1	30	54.3	B
12194	Bn-N11-p28609707	Bn-C1-p25863809	Bn-scaff_21820_1-p553903	C1	30	54.3	B
12195	Bn-N11-p28609789	Bn-C1-p25863727	Bn-scaff_21820_1-p553985	C1	30	54.3	B
12196	Bn-N11-p28622962	Bn-C1-p25850675	Bn-scaff_21820_1-p567037	C1	30	54.3	B

12197	Bn-N11-p28623127	Bn-C1-p25850510	Bn-scaff_21820_1-p56720[illegible]	C1	30	54.3	B
12198	Bn-N11-p28623218	Bn-C1-p25850419	Bn-scaff_21820_1-p56729[illegible]	C1	30	54.3	B
12199	Bn-N11-p28624286	Bn-C1-p25849351	Bn-scaff_21820_1-p56836[illegible]	C1	30	54.3	B
12200	Bn-N11-p28624527	Bn-C1-p25849110	Bn-scaff_21820_1-p56860[illegible]	C1	30	54.3	B
12201	Bn-N11-p28625088	Bn-C1-p25848549	Bn-scaff_21820_1-p56916[illegible]	C1	30	54.3	B
12202	Bn-N11-p28625283	Bn-C1-p25848354	Bn-scaff_21820_1-p56935[illegible]	C1	30	54.3	B
12203	Bn-N11-p28626138	Bn-C1-p25847497	Bn-scaff_21820_1-p57021[illegible]	C1	30	54.3	B
12204	Bn-N11-p28629080	Bn-C1-p25844566	Bn-scaff_21820_1-p57314[illegible]	C1	30	54.3	B
12205	Bn-N11-p28630172	Bn-C1-p25843469	Bn-scaff_21820_1-p57424[illegible]	C1	30	54.3	B
12206	Bn-N11-p28630778	Bn-C1-p25842865	Bn-scaff_21820_1-p57484[illegible]	C1	30	54.3	B
12207	Bn-N11-p28631025	Bn-C1-p25842617	Bn-scaff_21820_1-p57509[illegible]	C1	30	54.3	B
12208	Bn-N11-p28631973	Bn-C1-p25841668	Bn-scaff_21820_1-p57604[illegible]	C1	30	54.3	B
12209	Bn-N11-p28632177	Bn-C1-p25841464	Bn-scaff_21820_1-p57624[illegible]	C1	30	54.3	B
12210	Bn-N11-p28632264	Bn-C1-p25841377	Bn-scaff_21820_1-p57633[illegible]	C1	30	54.3	B
12211	Bn-N11-p28632458	Bn-C1-p25841183	Bn-scaff_21820_1-p57652[illegible]	C1	30	54.3	B
12212	Bn-N11-p28632815	Bn-C1-p25840827	Bn-scaff_21820_1-p57688[illegible]	C1	30	54.3	B
12213	Bn-N11-p28633214	Bn-C1-p25840433	Bn-scaff_21820_1-p57727[illegible]	C1	30	54.3	B
12214	Bn-N11-p28633472	Bn-C1-p25840175	Bn-scaff_21820_1-p57753[illegible]	C1	30	54.3	B
12215	Bn-N11-p28633761	Bn-C1-p25839886	Bn-scaff_21820_1-p57782[illegible]	C1	30	54.3	B
12216	Bn-N11-p28634019	Bn-C1-p25839628	Bn-scaff_21820_1-p57808[illegible]	C1	30	54.3	B
12217	Bn-N11-p28634299	Bn-C1-p25839348	Bn-scaff_21820_1-p57836[illegible]	C1	30	54.3	B
12218	Bn-N11-p28634964	Bn-C1-p25838684	Bn-scaff_21820_1-p57902[illegible]	C1	30	54.3	B
12219	Bn-N11-p28635196	Bn-C1-p25838452	Bn-scaff_21820_1-p57926[illegible]	C1	30	54.3	B
12220	Bn-N11-p28635629	Bn-C1-p25838066	Bn-scaff_21820_1-p57964[illegible]	C1	30	54.3	B
12221	Bn-N11-p28637275	Bn-C1-p25836423	Bn-scaff_21820_1-p58128[illegible]	C1	30	54.3	B
12222	Bn-N11-p28639873	Bn-C1-p25835867	Bn-scaff_21820_1-p58184[illegible]	C1	30	54.3	B
12223	Bn-N11-p28640234	Bn-C1-p25835506	Bn-scaff_21820_1-p58220[illegible]	C1	30	54.3	B
12224	Bn-N11-p28640561	Bn-C1-p25835180	Bn-scaff_21820_1-p58253[illegible]	C1	30	54.3	B
12225	Bn-N11-p28640781	Bn-C1-p25834960	Bn-scaff_21820_1-p58275[illegible]	C1	30	54.3	B
12226	Bn-N11-p28641056	Bn-C1-p25834685	Bn-scaff_21820_1-p58302[illegible]	C1	30	54.3	B
12227	Bn-N11-p28641903	Bn-C1-p25833838	Bn-scaff_21820_1-p58387[illegible]	C1	30	54.3	B
12228	Bn-N11-p28643455	Bn-C1-p25832286	Bn-scaff_21820_1-p58542[illegible]	C1	30	54.3	B
12229	Bn-N11-p28644716	Bn-C1-p25831071	Bn-scaff_21820_1-p58664[illegible]	C1	30	54.3	B
12230	Bn-N11-p28645158	Bn-C1-p25830629	Bn-scaff_21820_1-p58708[illegible]	C1	30	54.3	B
12231	Bn-N11-p28645656	Bn-C1-p25830176	Bn-scaff_21820_1-p58753[illegible]	C1	30	54.3	B
12232	Bn-N11-p28645720	Bn-C1-p25830112	Bn-scaff_21820_1-p58760[illegible]	C1	30	54.3	B
12233	Bn-N11-p28646840	Bn-C1-p25828993	Bn-scaff_21820_1-p58871[illegible]	C1	30	54.3	B
12234	Bn-N11-p28647989	Bn-C1-p25827845	Bn-scaff_21820_1-p58986[illegible]	C1	30	54.3	B
12235	Bn-N11-p28649421	Bn-C1-p25826415	Bn-scaff_21820_1-p59129[illegible]	C1	30	54.3	B
12236	Bn-N11-p28667119	Bn-C1-p25816994	Bn-scaff_21820_1-p60071[illegible]	C1	30	54.3	B
12237	Bn-N11-p28723412	Bn-C1-p25653290	Bn-scaff_21820_1-p76442[illegible]	C1	30	54.3	B
12238	Bn-N11-p28724338	Bn-C1-p25652364	Bn-scaff_21820_1-p76534[illegible]	C1	30	54.3	B
12239	Bn-N11-p28792068	Bn-C1-p25585409	Bn-scaff_21820_1-p83230[illegible]	C1	30	54.3	B
12240	Bn-N11-p28842440	Bn-C1-p25548301	Bn-scaff_21820_1-p86941[illegible]	C1	30	54.3	B
12241	Bn-N11-p28842883	Bn-C1-p25547858	Bn-scaff_21820_1-p86985[illegible]	C1	30	54.3	B
12242	Bn-N11-p28913652	Bn-C1-p25469937	Bn-scaff_21820_1-p94777[illegible]	C1	30	54.3	B
12243	Bn-N11-p28923213	Bn-C1-p25468150	Bn-scaff_21820_1-p94956[illegible]	C1	30	54.3	B
12244	Bn-N11-p28932192	Bn-C1-p25461543	Bn-scaff_21820_1-p95616[illegible]	C1	30	54.3	B
12245	Bn-N11-p28932972	Bn-C1-p25460763	Bn-scaff_21820_1-p95694[illegible]	C1	30	54.3	B

12246	Bn-N11-p28945480	Bn-C1-p25452227	Bn-scaff_21820_1-p965485	C1	30	54.3	B
12247	Bn-N11-p28958150	Bn-C1-p25441180	Bn-scaff_21820_1-p976532	C1	30	54.3	B
12248	Bn-N11-p28968507	Bn-C1-p25431313	Bn-scaff_21820_1-p986399	C1	30	54.3	B
12249	Bn-N11-p28968844	Bn-C1-p25430975	Bn-scaff_21820_1-p986737	C1	30	54.3	B
12250	Bn-N11-p28970560	Bn-C1-p25429239	Bn-scaff_21820_1-p988473	C1	30	54.3	B
12251	Bn-N11-p28970635	Bn-C1-p25429164	Bn-scaff_21820_1-p988548	C1	30	54.3	B
12252	Bn-N11-p28981860	Bn-C1-p25424568	Bn-scaff_21820_1-p993144	C1	30	54.3	B
12253	Bn-N11-p28987394	Bn-C1-p25419006	Bn-scaff_21820_1-p998706	C1	30	54.3	B
12254	Bn-N11-p28994866	Bn-C1-p25411352	Bn-scaff_21820_1-p100636	C1	30	54.3	B
12255	Bn-N11-p28996572	Bn-C1-p25406539	Bn-scaff_21820_1-p101117	C1	30	54.3	B
12256	Bn-N11-p28997195	Bn-C1-p25405915	Bn-scaff_21820_1-p101179	C1	30	54.3	B
12257	Bn-N11-p29002003	Bn-C1-p25400734	Bn-scaff_21820_1-p101697	C1	30	54.3	B
12258	Bn-N11-p29002112	Bn-C1-p25400625	Bn-scaff_21820_1-p101708	C1	30	54.3	B
12259	Bn-N11-p29002681	Bn-C1-p25400057	Bn-scaff_21820_1-p101765	C1	30	54.3	B
12260	Bn-N11-p29003630	Bn-C1-p25399107	Bn-scaff_21820_1-p101860	C1	30	54.3	B
12261	Bn-N11-p29005040	Bn-C1-p25397699	Bn-scaff_21820_1-p102001	C1	30	54.3	B
12262	Bn-N11-p29006047	Bn-C1-p25396692	Bn-scaff_21820_1-p102102	C1	30	54.3	B
12263	Bn-N11-p29006310	Bn-C1-p25396429	Bn-scaff_21820_1-p102128	C1	30	54.3	B
12264	Bn-N11-p29006463	Bn-C1-p25396276	Bn-scaff_21820_1-p102143	C1	30	54.3	B
12265	Bn-N11-p29012731	Bn-C1-p25389337	Bn-scaff_21820_1-p102837	C1	30	54.3	B
12266	Bn-N11-p29013909	Bn-C1-p25388157	Bn-scaff_21820_1-p102955	C1	30	54.3	B
12267	Bn-N11-p29014272	Bn-C1-p25387794	Bn-scaff_21820_1-p102991	C1	30	54.3	B
12268	Bn-N11-p29014829	Bn-C1-p25387239	Bn-scaff_21820_1-p103047	C1	30	54.3	B
12269	Bn-N11-p29015557	Bn-C1-p25386511	Bn-scaff_21820_1-p103120	C1	30	54.3	B
12270	Bn-N11-p29062116	Bn-C1-p26512639	Bn-scaff_15749_1-p433641	C1	30	54.3	B
12271	Bn-N11-p29062198	Bn-C1-p26512721	Bn-scaff_15749_1-p433559	C1	30	54.3	B
12272	Bn-N11-p29083561	Bn-C1-p26536803	Bn-scaff_15749_1-p409477	C1	30	54.3	B
12273	Bn-N11-p29083652	Bn-C1-p26536894	Bn-scaff_15749_1-p409386	C1	30	54.3	B
12274	Bn-N11-p29083799	Bn-C1-p26537019	Bn-scaff_15749_1-p409261	C1	30	54.3	B
12275	Bn-N11-p29086683	Bn-C1-p26539904	Bn-scaff_15749_1-p406376	C1	30	54.3	B
12276	Bn-N11-p29086791	Bn-C1-p26540012	Bn-scaff_15749_1-p406268	C1	30	54.3	B
12277	Bn-N11-p29087019	Bn-C1-p26540244	Bn-scaff_15749_1-p406036	C1	30	54.3	B
12278	Bn-N11-p29087702	Bn-C1-p26540927	Bn-scaff_15749_1-p405353	C1	30	54.3	B
12279	Bn-N11-p29088139	Bn-C1-p26541363	Bn-scaff_15749_1-p404917	C1	30	54.3	B
12280	Bn-N11-p29091933	Bn-C1-p26545160	Bn-scaff_15749_1-p401120	C1	30	54.3	B
12281	Bn-N11-p29107180	Bn-C1-p26572463	Bn-scaff_15749_1-p373817	C1	30	54.3	B
12282	Bn-N11-p29116849	Bn-C1-p26581833	Bn-scaff_15749_1-p364447	C1	30	54.3	B
12283	Bn-N11-p29151626	Bn-C1-p26606377	Bn-scaff_15749_1-p339903	C1	30	54.3	B
12284	Bn-N11-p29152032	Bn-C1-p26606783	Bn-scaff_15749_1-p339497	C1	30	54.3	B
12285	Bn-N11-p29152101	Bn-C1-p26606839	Bn-scaff_15749_1-p339441	C1	30	54.3	B
12286	Bn-N11-p29152397	Bn-C1-p26607135	Bn-scaff_15749_1-p339145	C1	30	54.3	B
12287	Bn-N11-p29152506	Bn-C1-p26607244	Bn-scaff_15749_1-p339036	C1	30	54.3	B
12288	Bn-N11-p29152552	Bn-C1-p26607290	Bn-scaff_15749_1-p338990	C1	30	54.3	B
12289	Bn-N11-p29152699	Bn-C1-p26607437	Bn-scaff_15749_1-p338843	C1	30	54.3	B
12290	Bn-N11-p29152829	Bn-C1-p26607567	Bn-scaff_15749_1-p338713	C1	30	54.3	B
12291	Bn-N11-p29153163	Bn-C1-p26607898	Bn-scaff_15749_1-p338382	C1	30	54.3	B
12292	Bn-N11-p29153190	Bn-C1-p26607925	Bn-scaff_15749_1-p338355	C1	30	54.3	B
12293	Bn-N11-p29153459	Bn-C1-p26608197	Bn-scaff_15749_1-p338083	C1	30	54.3	B
12294	Bn-N11-p29153695	Bn-C1-p26608433	Bn-scaff_15749_1-p337847	C1	30	54.3	B

12295	Bn-N11-p29154795	Bn-C1-p26609543	Bn-scaff_15749_1-p336737	C1	30	54.3	B
12296	Bn-N11-p29154982	Bn-C1-p26610691	Bn-scaff_15749_1-p335589	C1	30	54.3	B
12297	Bn-N11-p29157392	Bn-C1-p26613100	Bn-scaff_15749_1-p333180	C1	30	54.3	B
12298	Bn-N11-p29157783	Bn-C1-p26613497	Bn-scaff_15749_1-p332783	C1	30	54.3	B
12299	Bn-N11-p29169805	Bn-C1-p26624868	Bn-scaff_15749_1-p321412	C1	30	54.3	B
12300	Bn-N11-p29182525	Bn-C1-p26634486	Bn-scaff_15749_1-p311794	C1	30	54.3	B
12301	Bn-N11-p29182606	Bn-C1-p26634567	Bn-scaff_15749_1-p311713	C1	30	54.3	B
12302	Bn-N11-p29183105	Bn-C1-p26635072	Bn-scaff_15749_1-p311208	C1	30	54.3	B
12303	Bn-N11-p29183241	Bn-C1-p26635208	Bn-scaff_15749_1-p311072	C1	30	54.3	B
12304	Bn-N11-p29183322	Bn-C1-p26635289	Bn-scaff_15749_1-p310991	C1	30	54.3	B
12305	Bn-N11-p29183407	Bn-C1-p26635374	Bn-scaff_15749_1-p310906	C1	30	54.3	B
12306	Bn-N11-p29183772	Bn-C1-p26635765	Bn-scaff_15749_1-p310515	C1	30	54.3	B
12307	Bn-N11-p29183857	Bn-C1-p26635850	Bn-scaff_15749_1-p310430	C1	30	54.3	B
12308	Bn-N11-p29183921	Bn-C1-p26635914	Bn-scaff_15749_1-p310366	C1	30	54.3	B
12309	Bn-N11-p29184702	Bn-C1-p26636707	Bn-scaff_15749_1-p309573	C1	30	54.3	B
12310	Bn-N11-p29184759	Bn-C1-p26636764	Bn-scaff_15749_1-p309516	C1	30	54.3	B
12311	Bn-N11-p29184856	Bn-C1-p26636861	Bn-scaff_15749_1-p309419	C1	30	54.3	B
12312	Bn-N11-p29185056	Bn-C1-p26637061	Bn-scaff_15749_1-p309219	C1	30	54.3	B
12313	Bn-N11-p29196927	Bn-C1-p26637917	Bn-scaff_15749_1-p308363	C1	30	54.3	B
12314	Bn-N11-p29199701	Bn-C1-p26645483	Bn-scaff_15749_1-p300797	C1	30	54.3	B
12315	Bn-N11-p29242172	Bn-C1-p26689776	Bn-scaff_15749_1-p256504	C1	30	54.3	B
12316	Bn-N11-p29242499	Bn-C1-p26690141	Bn-scaff_15749_1-p256139	C1	30	54.3	B
12317	Bn-N11-p29285484	Bn-C1-p26723719	Bn-scaff_15749_1-p222561	C1	30	54.3	B
12318	Bn-N11-p29285985	Bn-C1-p26724225	Bn-scaff_15749_1-p222055	C1	30	54.3	B
12319	Bn-N11-p29286175	Bn-C1-p26724415	Bn-scaff_15749_1-p221865	C1	30	54.3	B
12320	Bn-N11-p29286486	Bn-C1-p26729693	Bn-scaff_15749_1-p216587	C1	30	54.3	B
12321	Bn-N11-p29287391	Bn-C1-p26730602	Bn-scaff_15749_1-p215678	C1	30	54.3	B
12322	Bn-N11-p29287739	Bn-C1-p26730950	Bn-scaff_15749_1-p215330	C1	30	54.3	B
12323	Bn-N11-p29287946	Bn-C1-p26731157	Bn-scaff_15749_1-p215123	C1	30	54.3	B
12324	Bn-N11-p29324700	Bn-C1-p26773812	Bn-scaff_15749_1-p172468	C1	30	54.3	B
12325	Bn-N11-p29324894	Bn-C1-p26774006	Bn-scaff_15749_1-p172274	C1	30	54.3	B
12326	Bn-N11-p29385870	Bn-C1-p26827042	Bn-scaff_15749_1-p119238	C1	30	54.3	B
12327	Bn-N11-p29401774	Bn-C1-p26842101	Bn-scaff_15749_1-p104179	C1	30	54.3	B
12328	Bn-N11-p29449605	Bn-C1-p26890907	Bn-scaff_15749_1-p55373	C1	30	54.3	B
12329	Bn-N11-p29452227	Bn-C1-p26893532	Bn-scaff_15749_1-p52748	C1	30	54.3	B
12330	Bn-N11-p29505644	Bn-C1-p26951882	Bn-scaff_20452_1-p404172	C1	30	54.3	B
12331	Bn-N11-p29604622	Bn-C1-p27048135	Bn-scaff_20452_1-p307919	C1	30	54.3	B
12332	Bn-N11-p29792340	Bn-C1-p27209597	Bn-scaff_20452_1-p146457	C1	30	54.3	B
12333	Bn-N11-p29825339	Bn-C1-p27241169	Bn-scaff_20452_1-p114885	C1	30	54.3	B
12334	Bn-N11-p29841394	Bn-C1-p27269204	Bn-scaff_20452_1-p86850	C1	30	54.3	B
12335	Bn-N11-p29846146	Bn-C1-p27273938	Bn-scaff_20452_1-p82116	C1	30	54.3	B
12336	Bn-N11-p29876222	Bn-C1-p27313865	Bn-scaff_20452_1-p42189	C1	30	54.3	B
12337	Bn-N11-p29879195	Bn-C1-p27317317	Bn-scaff_20452_1-p38737	C1	30	54.3	B
12338	Bn-N11-p29890733	Bn-C1-p27327914	Bn-scaff_20452_1-p28140	C1	30	54.3	B
12339	Bn-N11-p29891274	Bn-C1-p27328442	Bn-scaff_20452_1-p27612	C1	30	54.3	B
12340	Bn-N11-p29901914	Bn-C1-p27332422	Bn-scaff_20452_1-p23632	C1	30	54.3	B
12341	Bn-N11-p29902861	Bn-C1-p27333347	Bn-scaff_20452_1-p22707	C1	30	54.3	B
12342	Bn-N11-p29937298	Bn-C1-p27369576	Bn-scaff_24608_1-p13423	C1	30	54.3	B
12343	Bn-N11-p29938793	Bn-C1-p27371070	Bn-scaff_24608_1-p14917	C1	30	54.3	B

12344	Bn-N11-p29941193	Bn-C1-p27372534	Bn-scaff_24608_1-p16381	C1	30	54.3	B
12345	Bn-N11-p29941350	Bn-C1-p27372691	Bn-scaff_24608_1-p16538	C1	30	54.3	B
12346	Bn-N11-p29964521	Bn-C1-p27392994	Bn-scaff_24608_1-p36841	C1	30	54.3	B
12347	Bn-N11-p29964699	Bn-C1-p27393172	Bn-scaff_24608_1-p37019	C1	30	54.3	B
12348	Bn-N11-p30131857	Bn-C1-p27511171	Bn-scaff_24608_1-p15501{	C1	30	54.3	B
12349	Bn-N17-p28902950	Bn-C3-p49956905	Bn-scaff_19740_1-p19210(	C1	30	54.3	B
12350	Bn-N19-p33321646	Bn-Scaffold05273-p1	Bn-scaff_16391_1-p142	C1	30	54.3	B
12351	Bn-N1-p16639456	Bn-C1-p26248464	Bn-scaff_21820_1-p16924	C1	30	54.3	B
12352	Bn-N1-p16685045	Bn-C1-p26273252	Bn-scaff_21820_1-p14446(	C1	30	54.3	B
12353	Bn-N1-p16690463	Bn-C1-p26273564	Bn-scaff_21820_1-p14414	C1	30	54.3	B
12354	Bn-N1-p16957107	Bn-C1-p26695131	Bn-scaff_15749_1-p25114	C1	30	54.3	B
12355	Bn-N1-p17527182	Bn-C1-p27451324	Bn-scaff_24608_1-p95171	C1	30	54.3	B
12356	Bn-N3-p2316917	Bn-C1-p26477023	Bn-scaff_15749_1-p46925	C1	30	54.3	B
12357	Bn-Scaffold01095-p61	Bn-C1-p25348315	Bn-scaff_21820_1-p10693	C1	30	54.3	B
12358	Bn-Scaffold10058-p797	Bn-Scaffold03608-p2	Bn-scaff_19803_1-p221	C1	30	54.3	B
12359	Bn-N11-p15942695	Bn-C1-p31718451	Bn-scaff_23795_1-p82795	C1	31	55.2	B
12360	Bn-N11-p31293589	Bn-C1-p28633025	Bn-scaff_16929_1-p94721	C1	31	55.2	B
12361	Bn-N11-p31358345	Bn-C1-p28693838	Bn-scaff_16929_1-p88640	C1	31	55.2	B
12362	Bn-N11-p31358488	Bn-C1-p28693981	Bn-scaff_16929_1-p88626	C1	31	55.2	B
12363	Bn-N11-p31429521	Bn-C1-p28769732	Bn-scaff_16929_1-p81051	C1	31	55.2	B
12364	Bn-N11-p31454315	Bn-C1-p28783151	Bn-scaff_16929_1-p79709	C1	31	55.2	B
12365	Bn-N11-p31539226	Bn-C1-p28854671	Bn-scaff_16929_1-p72557	C1	31	55.2	B
12366	Bn-N11-p31550758	Bn-C1-p28860161	Bn-scaff_16929_1-p72008	C1	31	55.2	B
12367	Bn-N11-p31551362	Bn-C1-p28860761	Bn-scaff_16929_1-p71948	C1	31	55.2	B
12368	Bn-N11-p31551556	Bn-C1-p28860955	Bn-scaff_16929_1-p71928	C1	31	55.2	B
12369	Bn-N11-p31564568	Bn-C1-p28898488	Bn-scaff_16929_1-p68175	C1	31	55.2	B
12370	Bn-N11-p31564655	Bn-C1-p28898575	Bn-scaff_16929_1-p68166	C1	31	55.2	B
12371	Bn-N11-p31564694	Bn-C1-p28898614	Bn-scaff_16929_1-p68162	C1	31	55.2	B
12372	Bn-N11-p31613450	Bn-C1-p28954046	Bn-scaff_16929_1-p62619	C1	31	55.2	B
12373	Bn-N11-p31641734	Bn-C1-p29001994	Bn-scaff_16929_1-p57824	C1	31	55.2	B
12374	Bn-N11-p31651903	Bn-C1-p29003610	Bn-scaff_16929_1-p57663	C1	31	55.2	B
12375	Bn-N11-p31653069	Bn-C1-p29011860	Bn-scaff_16929_1-p56838	C1	31	55.2	B
12376	Bn-N11-p31656775	Bn-C1-p29015489	Bn-scaff_16929_1-p56475	C1	31	55.2	B
12377	Bn-N11-p31657941	Bn-C1-p29019075	Bn-scaff_16929_1-p56116	C1	31	55.2	B
12378	Bn-N11-p31658116	Bn-C1-p29019249	Bn-scaff_16929_1-p56099	C1	31	55.2	B
12379	Bn-N11-p31666478	Bn-C1-p29026823	Bn-scaff_16929_1-p55342(	C1	31	55.2	B
12380	Bn-N11-p31720559	Bn-C1-p29058145	Bn-scaff_16929_1-p52209	C1	31	55.2	B
12381	Bn-N11-p31765906	Bn-C1-p29100992	Bn-scaff_16929_1-p47925	C1	31	55.2	B
12382	Bn-N11-p31898289	Bn-C1-p29263612	Bn-scaff_16929_1-p31663	C1	31	55.2	B
12383	Bn-N11-p31904805	Bn-C1-p29270103	Bn-scaff_16929_1-p31014(	C1	31	55.2	B
12384	Bn-N11-p31918291	Bn-C1-p29285534	Bn-scaff_16929_1-p29470	C1	31	55.2	B
12385	Bn-N11-p31941918	Bn-C1-p29290911	Bn-scaff_16929_1-p28933	C1	31	55.2	B
12386	Bn-N11-p31943696	Bn-C1-p29292684	Bn-scaff_16929_1-p28755	C1	31	55.2	B
12387	Bn-N11-p31943831	Bn-C1-p29292819	Bn-scaff_16929_1-p28742	C1	31	55.2	B
12388	Bn-N11-p31944259	Bn-C1-p29293247	Bn-scaff_16929_1-p28699	C1	31	55.2	B
12389	Bn-N11-p31946894	Bn-C1-p29295800	Bn-scaff_16929_1-p28444	C1	31	55.2	B
12390	Bn-N11-p31947766	Bn-C1-p29301749	Bn-scaff_16929_1-p27849	C1	31	55.2	B
12391	Bn-N11-p31954822	Bn-C1-p29308124	Bn-scaff_16929_1-p27211	C1	31	55.2	B
12392	Bn-N11-p31956512	Bn-C1-p29309865	Bn-scaff_16929_1-p27037	C1	31	55.2	B

12393	Bn-N11-p32002353	Bn-C1-p29385004	Bn-scaff_16929_1-p19523	C1	31	55.2	B
12394	Bn-N11-p32019665	Bn-C1-p29404254	Bn-scaff_16929_1-p17598	C1	31	55.2	B
12395	Bn-N11-p32044246	Bn-C1-p29427800	Bn-scaff_16929_1-p15244	C1	31	55.2	B
12396	Bn-N11-p32491906	Bn-C1-p29867401	Bn-scaff_16055_1-p11982	C1	31	55.2	B
12397	Bn-N11-p32648494	Bn-C1-p30008318	Bn-scaff_16055_1-p26073	C1	31	55.2	B
12398	Bn-N11-p32780728	Bn-C1-p30146793	Bn-scaff_16055_1-p39921	C1	31	55.2	B
12399	Bn-N11-p32797548	Bn-C1-p30157680	Bn-scaff_16055_1-p41010	C1	31	55.2	B
12400	Bn-N11-p32798142	Bn-C1-p30158262	Bn-scaff_16055_1-p41068	C1	31	55.2	B
12401	Bn-N11-p32856998	Bn-C1-p30196547	Bn-scaff_16055_1-p44896	C1	31	55.2	B
12402	Bn-N11-p32857049	Bn-C1-p30196598	Bn-scaff_16055_1-p44901	C1	31	55.2	B
12403	Bn-N11-p32859459	Bn-C1-p30199007	Bn-scaff_16055_1-p45142	C1	31	55.2	B
12404	Bn-N11-p32880692	Bn-C1-p30222999	Bn-scaff_16055_1-p47542	C1	31	55.2	B
12405	Bn-N11-p32911419	Bn-C1-p30250927	Bn-scaff_16055_1-p50334	C1	31	55.2	B
12406	Bn-N11-p32915070	Bn-C1-p30255445	Bn-scaff_16055_1-p50786	C1	31	55.2	B
12407	Bn-N11-p32924218	Bn-C1-p30263592	Bn-scaff_16055_1-p51601	C1	31	55.2	B
12408	Bn-N11-p32924291	Bn-C1-p30263665	Bn-scaff_16055_1-p51608	C1	31	55.2	B
12409	Bn-N11-p32924501	Bn-C1-p30263875	Bn-scaff_16055_1-p51629	C1	31	55.2	B
12410	Bn-N11-p33153100	Bn-C1-p30489837	Bn-scaff_16055_1-p74225	C1	31	55.2	B
12411	Bn-N11-p33178831	Bn-C1-p30521237	Bn-scaff_16055_1-p77365	C1	31	55.2	B
12412	Bn-N11-p33181320	Bn-C1-p30523679	Bn-scaff_16055_1-p77610	C1	31	55.2	B
12413	Bn-N11-p33183128	Bn-C1-p30525487	Bn-scaff_16055_1-p77790	C1	31	55.2	B
12414	Bn-N11-p33240890	Bn-C1-p30594314	Bn-scaff_16055_1-p84673	C1	31	55.2	B
12415	Bn-N11-p33313331	Bn-C1-p30670307	Bn-scaff_16055_1-p92272	C1	31	55.2	B
12416	Bn-N11-p33378452	Bn-Scaffold01215-p1	Bn-scaff_17744_1-p10005	C1	31	55.2	B
12417	Bn-N11-p33783802	Bn-C1-p31211352	Bn-scaff_16055_1-p14637	C1	31	55.2	B
12418	Bn-N11-p33865097	Bn-C1-p31291231	Bn-scaff_16055_1-p15436	C1	31	55.2	B
12419	Bn-N11-p33886125	Bn-C1-p31310347	Bn-scaff_16055_1-p15627	C1	31	55.2	B
12420	Bn-N11-p33934911	Bn-C1-p31329981	Bn-scaff_23795_1-p47126	C1	31	55.2	B
12421	Bn-N11-p33961423	Bn-C1-p31352820	Bn-scaff_23795_1-p44842	C1	31	55.2	B
12422	Bn-N11-p33974428	Bn-C1-p31363060	Bn-scaff_23795_1-p43818	C1	31	55.2	B
12423	Bn-N11-p33992362	Bn-C1-p31388583	Bn-scaff_23795_1-p41266	C1	31	55.2	B
12424	Bn-N11-p34463730	Bn-Scaffold01066-p9	Bn-scaff_15919_1-p9462	C1	31	55.2	B
12425	Bn-N11-p34550763	Bn-C1-p31879708	Bn-scaff_17517_1-p78363	C1	31	55.2	B
12426	Bn-N11-p46156258	Bn-C1-p31397802	Bn-scaff_23795_1-p40344	C1	31	55.2	B
12427	Bn-N12-p28007755	Bn-C2-p34886562	Bn-scaff_18245_1-p21935	C1	31	55.2	B
12428	Bn-N17-p17635966	Bn-C2-p1343191	Bn-scaff_22970_1-p43312	C1	31	55.2	B
12429	Bn-N18-p17073148	Bn-C1-p31517467	Bn-scaff_23795_1-p28377	C1	31	55.2	B
12430	Bn-N9-p21545946	Bn-C9-p15854065	Bn-scaff_16912_1-p83219	C1	31	55.2	B
12431	Bn-N11-p35043881	Bn-Scaffold01540-p4	Bn-scaff_21163_1-p4255	C1	32	56.1	B
12432	Bn-N11-p35046298	Bn-Scaffold01540-p1	Bn-scaff_21163_1-p1838	C1	32	56.1	B
12433	Bn-N11-p35051624	Bn-C1-p32288671	Bn-scaff_17517_1-p48732	C1	32	56.1	B
12434	Bn-N11-p35057959	Bn-C1-p32294875	Bn-scaff_17517_1-p49353	C1	32	56.1	B
12435	Bn-N11-p35059430	Bn-C1-p32296346	Bn-scaff_17517_1-p49500	C1	32	56.1	B
12436	Bn-N11-p35060358	Bn-C1-p32297274	Bn-scaff_17517_1-p49592	C1	32	56.1	B
12437	Bn-N11-p35066672	Bn-C1-p32303586	Bn-scaff_17517_1-p50224	C1	32	56.1	B
12438	Bn-N11-p35098504	Bn-C1-p32340536	Bn-scaff_17517_1-p53919	C1	32	56.1	B
12439	Bn-N11-p35580061	Bn-C1-p32782283	Bn-scaff_17517_1-p98093	C1	32	56.1	B
12440	Bn-N11-p35669804	Bn-C1-p32885617	Bn-scaff_16534_2-p72001	C1	32	56.1	B
12441	Bn-N11-p35675700	Bn-C1-p32892047	Bn-scaff_16534_2-p78431	C1	32	56.1	B

12442	Bn-N11-p35676328	Bn-C1-p32892675	Bn-scaff_16534_2-p79059	C1	32	56.1	B
12443	Bn-N11-p35676652	Bn-C1-p32892999	Bn-scaff_16534_2-p79383	C1	32	56.1	B
12444	Bn-N11-p35677529	Bn-C1-p32893876	Bn-scaff_16534_2-p80260	C1	32	56.1	B
12445	Bn-N11-p35677781	Bn-C1-p32894128	Bn-scaff_16534_2-p80512	C1	32	56.1	B
12446	Bn-N11-p35678583	Bn-C1-p32894904	Bn-scaff_16534_2-p81288	C1	32	56.1	B
12447	Bn-N11-p35678999	Bn-C1-p32895320	Bn-scaff_16534_2-p81704	C1	32	56.1	B
12448	Bn-N11-p35679075	Bn-C1-p32895396	Bn-scaff_16534_2-p81780	C1	32	56.1	B
12449	Bn-N11-p35679319	Bn-C1-p32895641	Bn-scaff_16534_2-p82025	C1	32	56.1	B
12450	Bn-N11-p35684155	Bn-C1-p32900572	Bn-scaff_16534_2-p86956	C1	32	56.1	B
12451	Bn-N11-p35685490	Bn-C1-p32901908	Bn-scaff_16534_2-p88292	C1	32	56.1	B
12452	Bn-N11-p35686331	Bn-C1-p32903084	Bn-scaff_16534_2-p89468	C1	32	56.1	B
12453	Bn-N11-p35724778	Bn-C1-p32938807	Bn-scaff_16534_2-p125191	C1	32	56.1	B
12454	Bn-N11-p35726072	Bn-C1-p32940096	Bn-scaff_16534_2-p126480	C1	32	56.1	B
12455	Bn-N11-p35726151	Bn-C1-p32940175	Bn-scaff_16534_2-p126559	C1	32	56.1	B
12456	Bn-N11-p35731496	Bn-C1-p32942993	Bn-scaff_16534_2-p129377	C1	32	56.1	0
12457	Bn-N11-p35745434	Bn-C1-p32958111	Bn-scaff_16534_2-p144495	C1	32	56.1	B
12458	Bn-N11-p35813955	Bn-C1-p33027962	Bn-scaff_16534_2-p214346	C1	32	56.1	B
12459	Bn-N11-p35828586	Bn-C1-p33049033	Bn-scaff_16534_2-p235417	C1	32	56.1	B
12460	Bn-N11-p35862158	Bn-C1-p33057651	Bn-scaff_16534_2-p244035	C1	32	56.1	B
12461	Bn-N11-p35862572	Bn-C1-p33058061	Bn-scaff_16534_2-p244445	C1	32	56.1	B
12462	Bn-N11-p35863018	Bn-C1-p33058507	Bn-scaff_16534_2-p244891	C1	32	56.1	B
12463	Bn-N11-p35866652	Bn-C1-p33062193	Bn-scaff_16534_2-p248577	C1	32	56.1	B
12464	Bn-N11-p35906181	Bn-C1-p33089422	Bn-scaff_16534_2-p275806	C1	32	56.1	B
12465	Bn-N11-p35990591	Bn-C1-p33157135	Bn-scaff_15879_1-p21018	C1	33	57	B
12466	Bn-N11-p35998696	Bn-C1-p33165246	Bn-scaff_15879_1-p29129	C1	33	57	B
12467	Bn-N11-p35998945	Bn-C1-p33165496	Bn-scaff_15879_1-p29379	C1	33	57	B
12468	Bn-N11-p36001553	Bn-C1-p33168082	Bn-scaff_15879_1-p31965	C1	33	57	B
12469	Bn-N11-p36050362	Bn-C1-p33212057	Bn-scaff_15879_1-p75940	C1	33	57	B
12470	Bn-N11-p36054138	Bn-C1-p33215849	Bn-scaff_15879_1-p79732	C1	33	57	B
12471	Bn-N11-p36327188	Bn-C1-p33522942	Bn-scaff_15879_1-p386825	C1	33	57	B
12472	Bn-N11-p36627296	Bn-C1-p33826648	Bn-scaff_15879_1-p690531	C1	33	57	B
12473	Bn-N11-p36652805	Bn-C1-p33851501	Bn-scaff_15879_1-p715384	C1	33	57	B
12474	Bn-N11-p36781364	Bn-A01-p21277841	Bn-A01-p21277841	C1	33	57	B
12475	Bn-N1-p20809797	Bn-A01-p20909649	Bn-A01-p20909649	C1	33	57	B
12476	Bn-N11-p36788702	Bn-Scaffold01385-p62	Bn-scaff_27823_1-p6273	C1	34	57.9	B
12477	Bn-N11-p36813497	Bn-C1-p34021154	Bn-scaff_21225_1-p144434	C1	34	57.9	B
12478	Bn-N11-p36840053	Bn-C1-p34029393	Bn-scaff_21225_1-p136195	C1	34	57.9	B
12479	Bn-N11-p36868804	Bn-C1-p34054969	Bn-scaff_21225_1-p110619	C1	34	57.9	B
12480	Bn-N11-p36875306	Bn-C1-p34060778	Bn-scaff_21225_1-p104810	C1	34	57.9	B
12481	Bn-N11-p36904309	Bn-C1-p34090627	Bn-scaff_21225_1-p74961	C1	35	59.7	B
12482	Bn-N11-p37051326	Bn-C1-p34212547	Bn-scaff_17036_1-p180029	C1	35	59.7	B
12483	Bn-N11-p37051579	Bn-C1-p34212800	Bn-scaff_17036_1-p179776	C1	35	59.7	B
12484	Bn-N11-p37059462	Bn-C1-p34221184	Bn-scaff_17036_1-p171392	C1	35	59.7	B
12485	Bn-N11-p37128229	Bn-C1-p34300850	Bn-scaff_17036_1-p91726	C1	35	59.7	B
12486	Bn-N11-p37198739	Bn-C1-p34370806	Bn-scaff_17036_1-p21770	C1	35	59.7	B
12487	Bn-N11-p37201307	Bn-C1-p34373374	Bn-scaff_17036_1-p19202	C1	35	59.7	B
12488	Bn-N11-p37202622	Bn-C1-p34374690	Bn-scaff_17036_1-p17886	C1	35	59.7	B
12489	Bn-N11-p37204245	Bn-C1-p34376310	Bn-scaff_17036_1-p16266	C1	35	59.7	B
12490	Bn-N11-p37215620	Bn-C1-p34387623	Bn-scaff_17036_1-p4953	C1	35	59.7	B

12491	Bn-N11-p37217678	Bn-C1-p34389671	Bn-scaff_17036_1-p2905	C1	35	59.7	B
12492	Bn-N11-p37351767	Bn-C5-p36166279	Bn-scaff_21015_1-p34786	C1	35	59.7	B
12493	Bn-N11-p37352034	Bn-Scaffold000587-p3	Bn-Scaffold000587-p3379	C1	35	59.7	B
12494	Bn-N11-p37514523	Bn-C1-p34672404	Bn-scaff_15794_2-p214881	C1	35	59.7	B
12495	Bn-N11-p37518141	Bn-C1-p34676006	Bn-scaff_15794_2-p218483	C1	35	59.7	B
12496	Bn-N11-p37523915	Bn-C1-p34686873	Bn-scaff_15794_2-p229350	C1	35	59.7	B
12497	Bn-N11-p37702579	Bn-C1-p34853248	Bn-scaff_18039_1-p103923	C1	35	59.7	B
12498	Bn-N11-p37758563	Bn-C1-p34916132	Bn-scaff_18039_1-p166807	C1	35	59.7	B
12499	Bn-N11-p37774954	Bn-C1-p34932550	Bn-scaff_18039_1-p183225	C1	35	59.7	B
12500	Bn-N11-p37774962	Bn-C1-p34932558	Bn-scaff_18039_1-p183233	C1	35	59.7	B
12501	Bn-N11-p37782132	Bn-C1-p34939688	Bn-scaff_18039_1-p190363	C1	35	59.7	B
12502	Bn-N11-p37782623	Bn-C1-p34940179	Bn-scaff_18039_1-p190854	C1	35	59.7	B
12503	Bn-N11-p37790852	Bn-C1-p34950204	Bn-scaff_18039_1-p200879	C1	35	59.7	B
12504	Bn-N11-p37796044	Bn-C1-p34955367	Bn-scaff_18039_1-p206042	C1	35	59.7	B
12505	Bn-N11-p37796140	Bn-C1-p34955463	Bn-scaff_18039_1-p206138	C1	35	59.7	B
12506	Bn-N11-p37796604	Bn-C1-p34955922	Bn-scaff_18039_1-p206597	C1	35	59.7	B
12507	Bn-N11-p37809238	Bn-C1-p34968554	Bn-scaff_18039_1-p219229	C1	35	59.7	B
12508	Bn-N11-p37810107	Bn-C1-p34969392	Bn-scaff_18039_1-p220067	C1	35	59.7	B
12509	Bn-N11-p37810191	Bn-C1-p34969478	Bn-scaff_18039_1-p220153	C1	35	59.7	B
12510	Bn-N11-p37813724	Bn-C1-p34973189	Bn-scaff_18039_1-p223864	C1	35	59.7	B
12511	Bn-N11-p37825995	Bn-C1-p34986635	Bn-scaff_18039_1-p237310	C1	35	59.7	B
12512	Bn-N11-p37828725	Bn-C1-p34989362	Bn-scaff_18039_1-p240037	C1	35	59.7	B
12513	Bn-N11-p37835326	Bn-C1-p34996026	Bn-scaff_18039_1-p246701	C1	35	59.7	B
12514	Bn-N11-p37859731	Bn-C1-p35015104	Bn-scaff_18039_1-p265779	C1	35	59.7	B
12515	Bn-Scaffold01915-p2700	Bn-Scaffold01349-p2	Bn-scaff_20764_1-p2534	C1	35	59.7	B
12516	Bn-N11-p38505222	Bn-C3-p21365850	Bn-scaff_18482_1-p816502	C1	36	60.6	B
12517	Bn-N11-p38532120	Bn-C1-p35696048	Bn-scaff_21857_1-p231820	C1	36	60.6	B
12518	Bn-N11-p39029778	Bn-Scaffold00763-p5	Bn-scaff_16962_1-p655721	C1	37	61.5	B
12519	Bn-N11-p39063578	Bn-A01-p23120368	Bn-A01-p23120368	C1	37	61.5	B
12520	Bn-N11-p39077884	Bn-Scaffold01680-p4	Bn-scaff_16226_1-p4282	C1	37	61.5	B
12521	Bn-N11-p39097945	Bn-C1-p36199428	Bn-scaff_24466_1-p16327	C1	37	61.5	B
12522	Bn-N11-p39103282	Bn-C1-p36204772	Bn-scaff_24466_1-p21671	C1	37	61.5	B
12523	Bn-N11-p39104201	Bn-C1-p36205716	Bn-scaff_24466_1-p22615	C1	37	61.5	B
12524	Bn-N11-p39122963	Bn-C1-p36222644	Bn-scaff_24466_1-p39543	C1	37	61.5	B
12525	Bn-N11-p39123031	Bn-C1-p36222712	Bn-scaff_24466_1-p39611	C1	37	61.5	B
12526	Bn-N11-p39134216	Bn-C1-p36236636	Bn-scaff_24466_1-p53535	C1	37	61.5	B
12527	Bn-N11-p39145122	Bn-C1-p36245355	Bn-scaff_24466_1-p62254	C1	37	61.5	B
12528	Bn-N11-p39145569	Bn-C1-p36245802	Bn-scaff_24466_1-p62701	C1	37	61.5	B
12529	Bn-N11-p39146398	Bn-C1-p36246631	Bn-scaff_24466_1-p63530	C1	37	61.5	B
12530	Bn-N11-p39159719	Bn-C1-p36259916	Bn-scaff_24466_1-p76815	C1	37	61.5	B
12531	Bn-N11-p39231104	Bn-C1-p36332494	Bn-scaff_17515_1-p749666	C1	37	61.5	B
12532	Bn-N11-p39246443	Bn-C1-p36339246	Bn-scaff_17515_1-p742914	C1	37	61.5	B
12533	Bn-N11-p39368319	Bn-C1-p36457731	Bn-scaff_17515_1-p624429	C1	37	61.5	B
12534	Bn-N11-p39404075	Bn-C1-p36493265	Bn-scaff_17515_1-p588895	C1	37	61.5	B
12535	Bn-N11-p34926527	Bn-C1-p32179274	Bn-scaff_17517_1-p377929	C1	38	62.4	B
12536	Bn-N11-p39429215	Bn-C1-p36518681	Bn-scaff_17515_1-p563479	C1	38	62.4	B
12537	Bn-N11-p39431367	Bn-C1-p36521576	Bn-scaff_17515_1-p560584	C1	38	62.4	B
12538	Bn-N11-p39434331	Bn-C1-p36524540	Bn-scaff_17515_1-p557620	C1	38	62.4	B
12539	Bn-N11-p39456097	Bn-C1-p36547633	Bn-scaff_17515_1-p534527	C1	38	62.4	B

12540	Bn-N11-p39735207	Bn-C1-p36815442	Bn-scaff_17515_1-p266718	C1	38	62.4	B
12541	Bn-N11-p39740882	Bn-C1-p36820996	Bn-scaff_17515_1-p261164	C1	38	62.4	B
12542	Bn-N11-p39750305	Bn-C1-p36830419	Bn-scaff_17515_1-p251741	C1	38	62.4	B
12543	Bn-N11-p39752007	Bn-C1-p36832121	Bn-scaff_17515_1-p250039	C1	38	62.4	B
12544	Bn-N11-p39756533	Bn-C1-p36836647	Bn-scaff_17515_1-p245513	C1	38	62.4	B
12545	Bn-N11-p39756985	Bn-C1-p36837099	Bn-scaff_17515_1-p245061	C1	38	62.4	B
12546	Bn-N11-p39757095	Bn-C1-p36837209	Bn-scaff_17515_1-p244951	C1	38	62.4	B
12547	Bn-N11-p39757239	Bn-C1-p36837353	Bn-scaff_17515_1-p244807	C1	38	62.4	B
12548	Bn-N11-p39757331	Bn-C1-p36837445	Bn-scaff_17515_1-p244715	C1	38	62.4	B
12549	Bn-N11-p39757542	Bn-C1-p36837656	Bn-scaff_17515_1-p244504	C1	38	62.4	B
12550	Bn-N11-p39758177	Bn-C1-p36838291	Bn-scaff_17515_1-p243869	C1	38	62.4	B
12551	Bn-N11-p39758268	Bn-C1-p36838382	Bn-scaff_17515_1-p243778	C1	38	62.4	B
12552	Bn-N11-p39758361	Bn-C1-p36838475	Bn-scaff_17515_1-p243685	C1	38	62.4	B
12553	Bn-N11-p39784930	Bn-C1-p36864947	Bn-scaff_17515_1-p217213	C1	38	62.4	B
12554	Bn-N11-p39797629	Bn-C1-p36878905	Bn-scaff_17515_1-p203255	C1	38	62.4	B
12555	Bn-N11-p39801964	Bn-C1-p36883242	Bn-scaff_17515_1-p198918	C1	38	62.4	B
12556	Bn-N11-p39802797	Bn-C1-p36884075	Bn-scaff_17515_1-p198085	C1	38	62.4	B
12557	Bn-N11-p39803234	Bn-C1-p36884512	Bn-scaff_17515_1-p197648	C1	38	62.4	B
12558	Bn-N11-p39805741	Bn-C1-p36887019	Bn-scaff_17515_1-p195141	C1	38	62.4	B
12559	Bn-N11-p39805820	Bn-C1-p36887098	Bn-scaff_17515_1-p195062	C1	38	62.4	B
12560	Bn-N11-p39805923	Bn-C1-p36887201	Bn-scaff_17515_1-p194959	C1	38	62.4	B
12561	Bn-N11-p39805925	Bn-C1-p36887203	Bn-scaff_17515_1-p194957	C1	38	62.4	B
12562	Bn-N11-p39806669	Bn-C1-p36887947	Bn-scaff_17515_1-p194213	C1	38	62.4	B
12563	Bn-N11-p39811240	Bn-C1-p36892484	Bn-scaff_17515_1-p189676	C1	38	62.4	B
12564	Bn-N11-p39818568	Bn-C1-p36912121	Bn-scaff_17515_1-p170039	C1	38	62.4	B
12565	Bn-N11-p39859061	Bn-C1-p36953645	Bn-scaff_17515_1-p128515	C1	38	62.4	B
12566	Bn-N11-p39872657	Bn-C1-p36966875	Bn-scaff_17515_1-p115285	C1	38	62.4	B
12567	Bn-N11-p39873857	Bn-C1-p36968075	Bn-scaff_17515_1-p114085	C1	38	62.4	B
12568	Bn-N11-p39875951	Bn-C1-p36970335	Bn-scaff_17515_1-p111825	C1	38	62.4	B
12569	Bn-N11-p39876649	Bn-C1-p36971033	Bn-scaff_17515_1-p111127	C1	38	62.4	B
12570	Bn-N11-p39878530	Bn-C1-p36972914	Bn-scaff_17515_1-p109246	C1	38	62.4	B
12571	Bn-N11-p39879452	Bn-C1-p36973836	Bn-scaff_17515_1-p108324	C1	38	62.4	B
12572	Bn-N11-p39881352	Bn-C1-p36975738	Bn-scaff_17515_1-p106422	C1	38	62.4	B
12573	Bn-N11-p39967820	Bn-A01-p23642494	Bn-A01-p23642494	C1	38	62.4	B
12574	Bn-N11-p40119754	Bn-C1-p37223041	Bn-scaff_18672_1-p140782	C1	38	62.4	B
12575	Bn-N11-p40125378	Bn-C1-p37251006	Bn-scaff_18672_1-p168747	C1	38	62.4	B
12576	Bn-N11-p40126660	Bn-C1-p37252249	Bn-scaff_18672_1-p169990	C1	38	62.4	B
12577	Bn-N11-p40168272	Bn-C1-p37305057	Bn-scaff_18672_1-p222798	C1	38	62.4	B
12578	Bn-N11-p40203549	Bn-C1-p37336127	Bn-scaff_23799_1-p9079	C1	38	62.4	B
12579	Bn-N11-p40218956	Bn-C1-p37348319	Bn-scaff_23799_1-p21271	C1	38	62.4	B
12580	Bn-N11-p40262413	Bn-C1-p37378466	Bn-scaff_23799_1-p51418	C1	38	62.4	B
12581	Bn-N11-p40272279	Bn-C1-p37389124	Bn-scaff_23799_1-p62076	C1	38	62.4	B
12582	Bn-N11-p40453048	Bn-C1-p37792029	Bn-scaff_23347_1-p92650	C1	38	62.4	B
12583	Bn-N11-p40453259	Bn-C1-p37792240	Bn-scaff_23347_1-p92439	C1	38	62.4	B
12584	Bn-N11-p40463237	Bn-C1-p37800334	Bn-scaff_23347_1-p84345	C1	38	62.4	B
12585	Bn-N11-p40463436	Bn-C1-p37800533	Bn-scaff_23347_1-p84146	C1	38	62.4	B
12586	Bn-N11-p40464221	Bn-C1-p37801308	Bn-scaff_23347_1-p83371	C1	38	62.4	B
12587	Bn-N11-p40464360	Bn-C1-p37801447	Bn-scaff_23347_1-p83232	C1	38	62.4	B
12588	Bn-N11-p40464590	Bn-C1-p37801670	Bn-scaff_23347_1-p83009	C1	38	62.4	B

12589	Bn-N11-p40495954	Bn-C1-p37826306	Bn-scaff_23347_1-p58373	C1	38	62.4	B
12590	Bn-N11-p40496430	Bn-C1-p37826769	Bn-scaff_23347_1-p57910	C1	38	62.4	B
12591	Bn-N11-p40694758	Bn-C1-p38005397	Bn-scaff_22115_1-p120619	C1	38	62.4	B
12592	Bn-N11-p40841834	Bn-C1-p38125259	Bn-scaff_22115_1-p240481	C1	38	62.4	B
12593	Bn-N11-p40842651	Bn-C1-p38126081	Bn-scaff_22115_1-p241303	C1	38	62.4	B
12594	Bn-N1-p23599350	Bn-A01-p24369239	Bn-A01-p24369239	C1	38	62.4	0
12595	Bn-N11-p40934166	Bn-C1-p38234084	Bn-scaff_19168_1-p109723	C1	39	63.3	B
12596	Bn-N11-p41026310	Bn-C1-p38333894	Bn-scaff_19168_1-p9913	C1	39	63.3	B
12597	Bn-N11-p41026603	Bn-C1-p38334187	Bn-scaff_19168_1-p9620	C1	39	63.3	B
12598	Bn-N11-p41028010	Bn-C1-p38335594	Bn-scaff_19168_1-p8213	C1	39	63.3	B
12599	Bn-N11-p41029557	Bn-C1-p38337212	Bn-scaff_19168_1-p6595	C1	39	63.3	B
12600	Bn-N11-p41029727	Bn-C1-p38337382	Bn-scaff_19168_1-p6425	C1	39	63.3	B
12601	Bn-N11-p41069302	Bn-C1-p38351503	Bn-scaff_18096_1-p30025	C1	39	63.3	B
12602	Bn-N11-p41096963	Bn-C3-p25774151	Bn-scaff_17298_1-p155847	C1	39	63.3	B
12603	Bn-N11-p41109555	Bn-C1-p38399555	Bn-scaff_27278_1-p19570	C1	39	63.3	B
12604	Bn-N11-p41426008	Bn-C1-p38664551	Bn-scaff_15936_1-p245327	C1	39	63.3	B
12605	Bn-N11-p41451524	Bn-C1-p38690139	Bn-scaff_15936_1-p270915	C1	39	63.3	B
12606	Bn-N11-p41530620	Bn-C1-p38774747	Bn-scaff_15936_1-p355523	C1	39	63.3	B
12607	Bn-N11-p41532779	Bn-C1-p38776889	Bn-scaff_15936_1-p357665	C1	39	63.3	B
12608	Bn-N11-p41533914	Bn-C1-p38778024	Bn-scaff_15936_1-p358800	C1	39	63.3	B
12609	Bn-N11-p41634652	Bn-C1-p38864217	Bn-scaff_15936_1-p444993	C1	39	63.3	B
12610	Bn-N11-p41718129	Bn-C1-p38946816	Bn-scaff_15936_1-p527592	C1	39	63.3	B
12611	Bn-N11-p41922487	Bn-C1-p39154016	Bn-scaff_16567_1-p98742	C1	40	64.2	B
12612	Bn-N11-p42143293	Bn-C1-p39343227	Bn-scaff_21884_1-p154748	C1	40	64.2	B
12613	Bn-N11-p42177018	Bn-C1-p39370506	Bn-scaff_21884_1-p182027	C1	40	64.2	B
12614	Bn-N11-p42180964	Bn-C1-p39374207	Bn-scaff_21884_1-p185728	C1	40	64.2	B
12615	Bn-N11-p42181026	Bn-C1-p39374269	Bn-scaff_21884_1-p185790	C1	40	64.2	B
12616	Bn-N11-p42211669	Bn-C1-p39408342	Bn-scaff_21884_1-p219863	C1	40	64.2	B
12617	Bn-N11-p42215454	Bn-C1-p39412099	Bn-scaff_21884_1-p223620	C1	40	64.2	B
12618	Bn-N11-p42219363	Bn-C1-p39416008	Bn-scaff_21884_1-p227529	C1	40	64.2	B
12619	Bn-N11-p42248116	Bn-C1-p39444161	Bn-scaff_21884_1-p255682	C1	40	64.2	B
12620	Bn-N1-p24449515	Bn-Scaffold000164-p1	Bn-Scaffold000164-p15854	C1	40	64.2	B
12621	Bn-Scaffold09830-p788	Bn-C1-p39423755	Bn-scaff_21884_1-p235276	C1	40	64.2	B
12622	Bn-N11-p42748068	Bn-C1-p39827382	Bn-scaff_21884_1-p638903	C1	41	65.1	B
12623	Bn-N11-p42748431	Bn-C1-p39827768	Bn-scaff_21884_1-p639289	C1	41	65.1	B
12624	Bn-N11-p42751086	Bn-C1-p39830722	Bn-scaff_21884_1-p642243	C1	41	65.1	B
12625	Bn-N11-p42751440	Bn-C1-p39831076	Bn-scaff_21884_1-p642597	C1	41	65.1	B
12626	Bn-N11-p42751845	Bn-C1-p39831480	Bn-scaff_21884_1-p643001	C1	41	65.1	B
12627	Bn-N11-p42752020	Bn-C1-p39831652	Bn-scaff_21884_1-p643173	C1	41	65.1	B
12628	Bn-N11-p42752134	Bn-C1-p39832665	Bn-scaff_21884_1-p644186	C1	41	65.1	B
12629	Bn-N11-p42805570	Bn-C1-p39901740	Bn-scaff_21884_1-p713261	C1	41	65.1	B
12630	Bn-N11-p42915401	Bn-C1-p39998825	Bn-scaff_21884_1-p810346	C1	41	65.1	B
12631	Bn-N11-p42950789	Bn-C1-p40008203	Bn-scaff_21884_1-p819724	C1	41	65.1	B
12632	Bn-N11-p42951023	Bn-C1-p40008437	Bn-scaff_21884_1-p819958	C1	41	65.1	B
12633	Bn-N11-p42951037	Bn-C1-p40008451	Bn-scaff_21884_1-p819972	C1	41	65.1	B
12634	Bn-N11-p42984402	Bn-C1-p40032402	Bn-scaff_21884_1-p843923	C1	41	65.1	B
12635	Bn-Scaffold13510-p168	Bn-C1-p40011006	Bn-scaff_21884_1-p822527	C1	41	65.1	B
12636	Bn-Scaffold13510-p356	Bn-C1-p40010819	Bn-scaff_21884_1-p822340	C1	41	65.1	B
12637	Bn-Scaffold67684-p169	Bn-A01-p26518304	Bn-A01-p26518304	C1	41	65.1	B

12638	Bn-N11-p43521596	Bn-A01-p27225628	Bn-A01-p27225628	C1	42	67.7	B
12639	Bn-N11-p43536774	Bn-C1-p40553550	Bn-scaff_16691_1-p143482	C1	42	67.7	B
12640	Bn-N11-p43775094	Bn-A01-p27147782	Bn-A01-p27147782	C1	42	67.7	B
12641	Bn-N11-p43819864	Bn-A01-p27125649	Bn-A01-p27125649	C1	42	67.7	0
12642	Bn-N1-p24795592	Bn-C1-p40154533	Bn-scaff_21884_1-p96605	C1	42	67.7	B
12643	Bn-N11-p43652872	Bn-C1-p40710950	Bn-scaff_15712_3-p80420	C1	43	68.6	B
12644	Bn-N11-p43677917	Bn-C1-p40729829	Bn-scaff_15712_3-p78532	C1	43	68.6	B
12645	Bn-N11-p43732014	Bn-C1-p40642222	Bn-scaff_22987_1-p12324	C1	43	68.6	B
12646	Bn-N11-p43916598	Bn-C1-p40811862	Bn-scaff_15712_3-p70329	C1	43	68.6	B
12647	Bn-N11-p43970726	Bn-C1-p40861632	Bn-scaff_15712_3-p65352	C1	44	69.5	B
12648	Bn-N11-p43984419	Bn-C1-p40875666	Bn-scaff_15712_3-p63948	C1	45	70.4	B
12649	Bn-N11-p43984512	Bn-C1-p40875759	Bn-scaff_15712_3-p63939	C1	45	70.4	B
12650	Bn-N11-p43984525	Bn-C1-p40875772	Bn-scaff_15712_3-p63938	C1	45	70.4	B
12651	Bn-N11-p43984578	Bn-C1-p40875825	Bn-scaff_15712_3-p63933	C1	45	70.4	B
12652	Bn-N11-p43984756	Bn-C1-p40876002	Bn-scaff_15712_3-p63915	C1	45	70.4	B
12653	Bn-N11-p43984768	Bn-C1-p40876014	Bn-scaff_15712_3-p63914	C1	45	70.4	B
12654	Bn-N11-p43985539	Bn-C1-p40876782	Bn-scaff_15712_3-p63837	C1	45	70.4	B
12655	Bn-N11-p43985775	Bn-C1-p40877022	Bn-scaff_15712_3-p63813	C1	45	70.4	B
12656	Bn-N11-p43985932	Bn-C1-p40877180	Bn-scaff_15712_3-p63797	C1	45	70.4	B
12657	Bn-N11-p43986178	Bn-C1-p40878134	Bn-scaff_15712_3-p63702	C1	45	70.4	B
12658	Bn-N11-p43986238	Bn-C1-p40878194	Bn-scaff_15712_3-p63696	C1	45	70.4	B
12659	Bn-N11-p43986347	Bn-C1-p40878294	Bn-scaff_15712_3-p63686	C1	45	70.4	B
12660	Bn-N11-p43988507	Bn-C1-p40878460	Bn-scaff_15712_3-p63669	C1	45	70.4	B
12661	Bn-N11-p43997466	Bn-C1-p40887219	Bn-scaff_15712_3-p62793	C1	45	70.4	B
12662	Bn-N11-p43997931	Bn-C1-p40887694	Bn-scaff_15712_3-p62746	C1	45	70.4	B
12663	Bn-N11-p43998196	Bn-C1-p40887963	Bn-scaff_15712_3-p62719	C1	45	70.4	B
12664	Bn-N11-p44005319	Bn-C1-p40890605	Bn-scaff_15712_3-p62455	C1	45	70.4	B
12665	Bn-N11-p44005382	Bn-C1-p40890668	Bn-scaff_15712_3-p62448	C1	45	70.4	B
12666	Bn-N11-p44038218	Bn-C1-p40924091	Bn-scaff_15712_3-p59106	C1	45	70.4	B
12667	Bn-N11-p44038400	Bn-C1-p40924274	Bn-scaff_15712_3-p59088	C1	45	70.4	B
12668	Bn-N11-p44038622	Bn-C1-p40924496	Bn-scaff_15712_3-p59065	C1	45	70.4	B
12669	Bn-N11-p44049532	Bn-C1-p40927511	Bn-scaff_15712_3-p58764	C1	45	70.4	B
12670	Bn-N11-p44050058	Bn-C1-p40928066	Bn-scaff_15712_3-p58708	C1	45	70.4	B
12671	Bn-N11-p44053532	Bn-C1-p40928420	Bn-scaff_15712_3-p58673	C1	45	70.4	0
12672	Bn-N11-p44053651	Bn-C1-p40928540	Bn-scaff_15712_3-p58661	C1	45	70.4	B
12673	Bn-N11-p44053817	Bn-C1-p40928707	Bn-scaff_15712_3-p58644	C1	45	70.4	B
12674	Bn-N11-p44068605	Bn-C1-p40972031	Bn-scaff_15712_3-p54312	C1	45	70.4	B
12675	Bn-N11-p44075765	Bn-C1-p40978774	Bn-scaff_15712_3-p53638	C1	45	70.4	B
12676	Bn-N11-p44082387	Bn-C1-p40989099	Bn-scaff_15712_3-p52605	C1	45	70.4	B
12677	Bn-N11-p44092162	Bn-C1-p40996946	Bn-scaff_15712_3-p51820	C1	45	70.4	B
12678	Bn-N11-p44094037	Bn-C1-p40998767	Bn-scaff_15712_3-p51638	C1	45	70.4	B
12679	Bn-N11-p44096289	Bn-C1-p41005283	Bn-scaff_15712_3-p50987	C1	45	70.4	B
12680	Bn-N11-p44101308	Bn-C1-p41010292	Bn-scaff_15712_3-p50486	C1	45	70.4	B
12681	Bn-N11-p44111285	Bn-C1-p41021785	Bn-scaff_15712_3-p49337	C1	45	70.4	B
12682	Bn-N11-p44124243	Bn-C1-p41025416	Bn-scaff_15712_3-p48973	C1	45	70.4	B
12683	Bn-N11-p44131158	Bn-C1-p41032535	Bn-scaff_15712_3-p48262	C1	45	70.4	B
12684	Bn-N11-p44132771	Bn-C1-p41034146	Bn-scaff_15712_3-p48100	C1	45	70.4	B
12685	Bn-N11-p44133050	Bn-C1-p41034424	Bn-scaff_15712_3-p48073	C1	45	70.4	B
12686	Bn-N11-p44133692	Bn-C1-p41035066	Bn-scaff_15712_3-p48008	C1	45	70.4	B

12687	Bn-N11-p44219422	Bn-C1-p41124995	Bn-scaff_15712_3-p39016(	C1	45	70.4	B
12688	Bn-N8-p4052341	Bn-A08-p4207231	Bn-A08-p4207231	C1	45	70.4	A
12689	Bn-Scaffold01128-p11149	Bn-C1-p40966402	Bn-scaff_15712_3-p54875:	C1	45	70.4	B
12690	Bn-Scaffold01128-p11161	Bn-C1-p40966414	Bn-scaff_15712_3-p54874:	C1	45	70.4	B
12691	Bn-Scaffold01128-p11324	Bn-C1-p40966574	Bn-scaff_15712_3-p54858:	C1	45	70.4	B
12692	Bn-Scaffold01128-p11465	Bn-C1-p40966715	Bn-scaff_15712_3-p54844(	C1	45	70.4	B
12693	Bn-Scaffold01128-p3952	Bn-C1-p40952930	Bn-scaff_15712_3-p56222!	C1	45	70.4	B
12694	Bn-Scaffold01128-p4228	Bn-C1-p40953246	Bn-scaff_15712_3-p56190!	C1	45	70.4	B
12695	Bn-Scaffold01128-p4315	Bn-C1-p40953333	Bn-scaff_15712_3-p56182:	C1	45	70.4	B
12696	Bn-Scaffold01128-p4664	Bn-C1-p40953681	Bn-scaff_15712_3-p56147-	C1	45	70.4	B
12697	Bn-Scaffold01128-p4794	Bn-C1-p40953811	Bn-scaff_15712_3-p56134-	C1	45	70.4	B
12698	Bn-Scaffold01128-p5264	Bn-C1-p40954285	Bn-scaff_15712_3-p56087(	C1	45	70.4	B
12699	Bn-Scaffold01128-p61	Bn-C1-p40949044	Bn-scaff_15712_3-p56611:	C1	45	70.4	B
12700	Bn-N11-p44370484	Bn-C1-p41263976	Bn-scaff_15712_3-p25117!	C1	46	71.3	B
12701	Bn-N11-p44413050	Bn-C1-p41306370	Bn-scaff_15712_3-p20878!	C1	46	71.3	B
12702	Bn-N11-p44592876	Bn-C1-p41468412	Bn-scaff_15712_3-p46743	C1	46	71.3	B
12703	Bn-N12-p283366	Bn-C2-p671385	Bn-scaff_18321_1-p12269{	C2	1	0	A
12704	Bn-N12-p296762	Bn-C2-p677863	Bn-scaff_18321_1-p12917(	C2	1	0	A
12705	Bn-N12-p368232	Bn-C2-p781626	Bn-scaff_17752_1-p12834:	C2	1	0	A
12706	Bn-N12-p392347	Bn-C2-p798196	Bn-scaff_17752_1-p11177:	C2	1	0	A
12707	Bn-N12-p456664	Bn-C2-p764300	Bn-scaff_18321_1-p21561:	C2	1	0	A
12708	Bn-N12-p461371	Bn-C2-p833469	Bn-scaff_17752_1-p76499	C2	1	0	A
12709	Bn-N12-p468235	Bn-C2-p836134	Bn-scaff_17752_1-p73834	C2	1	0	A
12710	Bn-N12-p593242	Bn-C2-p942438	Bn-scaff_22970_1-p32371	C2	1	0	A
12711	Bn-N12-p593831	Bn-C2-p943030	Bn-scaff_22970_1-p32963	C2	1	0	A
12712	Bn-N12-p601873	Bn-C2-p951762	Bn-scaff_22970_1-p41695	C2	1	0	A
12713	Bn-N12-p629050	Bn-C2-p982343	Bn-scaff_22970_1-p72276	C2	1	0	A
12714	Bn-N12-p706413	Bn-C2-p1034829	Bn-scaff_22970_1-p12476:	C2	1	0	A
12715	Bn-N12-p716178	Bn-C2-p1046159	Bn-scaff_22970_1-p13609:	C2	1	0	A
12716	Bn-N12-p716293	Bn-C2-p1046270	Bn-scaff_22970_1-p13620:	C2	1	0	A
12717	Bn-N12-p716760	Bn-C2-p1046727	Bn-scaff_22970_1-p13666(	C2	1	0	A
12718	Bn-N12-p717105	Bn-C2-p1047072	Bn-scaff_22970_1-p13700!	C2	1	0	A
12719	Bn-N12-p717358	Bn-C2-p1047323	Bn-scaff_22970_1-p13725(	C2	1	0	A
12720	Bn-N12-p717774	Bn-C2-p1047768	Bn-scaff_22970_1-p13770:	C2	1	0	A
12721	Bn-N12-p718219	Bn-C2-p1048212	Bn-scaff_22970_1-p13814!	C2	1	0	A
12722	Bn-N12-p718353	Bn-C2-p1048344	Bn-scaff_22970_1-p13827:	C2	1	0	A
12723	Bn-N12-p774769	Bn-C2-p1123874	Bn-scaff_22970_1-p21380:	C2	1	0	A
12724	Bn-N12-p779310	Bn-C2-p1128622	Bn-scaff_22970_1-p21855!	C2	1	0	A
12725	Bn-N12-p826479	Bn-C2-p1174085	Bn-scaff_22970_1-p26401{	C2	1	0	A
12726	Bn-N12-p840214	Bn-C2-p1187367	Bn-scaff_22970_1-p27730(	C2	1	0	A
12727	Bn-N12-p875999	Bn-C2-p1231545	Bn-scaff_22970_1-p32147{	C2	1	0	A
12728	Bn-N2-p337117	Bn-A02-p1671689	Bn-A02-p1671689	C2	1	0	A
12729	Bn-N2-p378026	Bn-C2-p489300	Bn-scaff_23167_1-p59288	C2	1	0	A
12730	Bn-N2-p991502	Bn-C2-p1294641	Bn-scaff_22970_1-p38457-	C2	1	0	A
12731	Bn-N9-p35082571	Bn-C2-p1265030	Bn-scaff_22970_1-p35496:	C2	1	0	A
12732	Bn-N12-p1012301	Bn-C2-p1388138	Bn-scaff_22970_1-p47807:	C2	2	1.7	A
12733	Bn-N12-p1033747	Bn-C2-p1409897	Bn-scaff_22970_1-p49983(	C2	2	1.7	A
12734	Bn-N12-p1346764	Bn-C2-p1763304	Bn-scaff_15714_1-p29953-	C2	2	1.7	A
12735	Bn-N12-p936581	Bn-C2-p1296749	Bn-scaff_22970_1-p38668:	C2	2	1.7	A

12736	Bn-N2-p1225989	Bn-C2-p1620056	Bn-scaff_15714_1-p313859	C2	2	1.7	A
12737	Bn-N2-p1508906	Bn-A02-p1370581	Bn-A02-p1370581	C2	2	1.7	A
12738	Bn-N12-p1352422	Bn-C2-p1768713	Bn-scaff_15714_1-p298993	C2	3	2.5	A
12739	Bn-N12-p1363645	Bn-C2-p1780579	Bn-scaff_15714_1-p297807	C2	3	2.5	A
12740	Bn-N12-p1445265	Bn-C2-p1863621	Bn-scaff_15714_1-p289502	C2	3	2.5	A
12741	Bn-N12-p1445332	Bn-C2-p1863688	Bn-scaff_15714_1-p289496	C2	3	2.5	A
12742	Bn-N12-p1537049	Bn-A02-p1344870	Bn-A02-p1344870	C2	3	2.5	A
12743	Bn-N12-p1640332	Bn-C2-p2054626	Bn-scaff_15714_1-p270402	C2	4	6.7	A
12744	Bn-N2-p1714788	Bn-A02-p1157373	Bn-A02-p1157373	C2	4	6.7	A
12745	Bn-N2-p1716440	Bn-C2-p2191462	Bn-scaff_15714_1-p256718	C2	4	6.7	A
12746	Bn-N2-p1849700	Bn-A02-p2776634	Bn-A02-p2776634	C2	4	6.7	A
12747	Bn-N12-p1886204	Bn-C2-p2277308	Bn-scaff_15714_1-p248134	C2	5	7.5	A
12748	Bn-N12-p1967083	Bn-C2-p2374956	Bn-scaff_15714_1-p238369	C2	5	7.5	A
12749	Bn-N12-p2037536	Bn-C2-p2427796	Bn-scaff_15714_1-p233085	C2	5	7.5	A
12750	Bn-N12-p2037830	Bn-C2-p2428090	Bn-scaff_15714_1-p233056	C2	5	7.5	A
12751	Bn-N12-p2055236	Bn-C2-p2459365	Bn-scaff_15714_1-p229928	C2	5	7.5	A
12752	Bn-N12-p2067130	Bn-C2-p2460452	Bn-scaff_15714_1-p229819	C2	5	7.5	A
12753	Bn-N2-p1840707	Bn-C2-p2366337	Bn-scaff_15714_1-p239231	C2	5	7.5	A
12754	Bn-N12-p2402343	Bn-C2-p2775008	Bn-scaff_15714_1-p198364	C2	6	9.6	A
12755	Bn-N12-p2411136	Bn-C2-p2791893	Bn-scaff_15714_1-p196675	C2	6	9.6	A
12756	Bn-N12-p2429835	Bn-C2-p2809270	Bn-scaff_15714_1-p194938	C2	6	9.6	A
12757	Bn-N2-p2157365	Bn-A02-p3080150	Bn-A02-p3080150	C2	6	9.6	A
12758	Bn-N12-p2498010	Bn-C2-p2865732	Bn-scaff_15714_1-p189291	C2	7	11.7	A
12759	Bn-N12-p2499977	Bn-C2-p2867700	Bn-scaff_15714_1-p189095	C2	7	11.7	A
12760	Bn-N12-p2500003	Bn-C2-p2867726	Bn-scaff_15714_1-p189092	C2	7	11.7	A
12761	Bn-N12-p2500715	Bn-C2-p2868435	Bn-scaff_15714_1-p189021	C2	7	11.7	A
12762	Bn-N12-p2526933	Bn-C2-p2890787	Bn-scaff_15714_1-p186786	C2	7	11.7	A
12763	Bn-N12-p2530571	Bn-C2-p2894380	Bn-scaff_15714_1-p186427	C2	7	11.7	A
12764	Bn-N12-p2959247	Bn-A02-p3507057	Bn-A02-p3507057	C2	8	13.4	A
12765	Bn-N2-p2570456	Bn-A02-p3493190	Bn-A02-p3493190	C2	8	13.4	A
12766	Bn-N12-p3440999	Bn-C2-p3792621	Bn-scaff_15714_1-p966029	C2	9	14.2	A
12767	Bn-N12-p3571369	Bn-C2-p3877560	Bn-scaff_15714_1-p881090	C2	9	14.2	A
12768	Bn-N12-p3587102	Bn-C2-p3893052	Bn-scaff_15714_1-p865598	C2	9	14.2	A
12769	Bn-N12-p3621253	Bn-C2-p3923191	Bn-scaff_15714_1-p835459	C2	9	14.2	A
12770	Bn-N12-p3622358	Bn-C2-p3924331	Bn-scaff_15714_1-p834319	C2	9	14.2	A
12771	Bn-N12-p3622726	Bn-C2-p3924740	Bn-scaff_15714_1-p833910	C2	9	14.2	A
12772	Bn-N12-p3628062	Bn-C2-p3925687	Bn-scaff_15714_1-p832963	C2	9	14.2	A
12773	Bn-N12-p3628123	Bn-C2-p3925748	Bn-scaff_15714_1-p832902	C2	9	14.2	A
12774	Bn-N12-p3657735	Bn-C2-p3955018	Bn-scaff_15714_1-p803632	C2	9	14.2	A
12775	Bn-N12-p3660577	Bn-C2-p3957242	Bn-scaff_15714_1-p801408	C2	9	14.2	A
12776	Bn-N12-p3660631	Bn-C2-p3957296	Bn-scaff_15714_1-p801354	C2	9	14.2	A
12777	Bn-N12-p3671764	Bn-C2-p3967508	Bn-scaff_15714_1-p791142	C2	9	14.2	A
12778	Bn-N12-p3672556	Bn-C2-p3968301	Bn-scaff_15714_1-p790349	C2	9	14.2	A
12779	Bn-N12-p3672638	Bn-C2-p3968382	Bn-scaff_15714_1-p790268	C2	9	14.2	A
12780	Bn-N12-p3675333	Bn-C2-p3972920	Bn-scaff_15714_1-p785730	C2	9	14.2	A
12781	Bn-N12-p3675538	Bn-C2-p3973125	Bn-scaff_15714_1-p785525	C2	9	14.2	A
12782	Bn-N12-p3679894	Bn-C2-p3978097	Bn-scaff_15714_1-p780553	C2	9	14.2	A
12783	Bn-N12-p3686258	Bn-C2-p3984147	Bn-scaff_15714_1-p774503	C2	9	14.2	A
12784	Bn-N12-p3687622	Bn-C2-p3985195	Bn-scaff_15714_1-p773455	C2	9	14.2	A

12785	Bn-N12-p3688718	Bn-C2-p3986192	Bn-scaff_15714_1-p772458	C2	9	14.2	0
12786	Bn-N12-p3688769	Bn-C2-p3986243	Bn-scaff_15714_1-p772407	C2	9	14.2	0
12787	Bn-N12-p3715066	Bn-C2-p4013448	Bn-scaff_15714_1-p745202	C2	9	14.2	A
12788	Bn-N12-p3725056	Bn-C2-p4017515	Bn-scaff_15714_1-p741135	C2	9	14.2	A
12789	Bn-N12-p3731814	Bn-C2-p4022192	Bn-scaff_15714_1-p736458	C2	9	14.2	A
12790	Bn-N12-p3733880	Bn-C2-p4023888	Bn-scaff_15714_1-p734762	C2	9	14.2	A
12791	Bn-N12-p3735401	Bn-C2-p4025392	Bn-scaff_15714_1-p733258	C2	9	14.2	A
12792	Bn-N12-p3740745	Bn-C2-p4030736	Bn-scaff_15714_1-p727914	C2	9	14.2	A
12793	Bn-N12-p3744326	Bn-C2-p4033516	Bn-scaff_15714_1-p725134	C2	9	14.2	A
12794	Bn-N12-p3746591	Bn-C2-p4035169	Bn-scaff_15714_1-p723481	C2	9	14.2	A
12795	Bn-N12-p3763252	Bn-C2-p4050029	Bn-scaff_15714_1-p708621	C2	9	14.2	A
12796	Bn-N2-p2877737	Bn-C2-p3767921	Bn-scaff_15714_1-p990729	C2	9	14.2	A
12797	Bn-N12-p3912844	Bn-C2-p4204945	Bn-scaff_15714_1-p553705	C2	10	15	A
12798	Bn-N12-p3965150	Bn-C2-p4241123	Bn-scaff_15714_1-p517527	C2	10	15	A
12799	Bn-N12-p4034840	Bn-C2-p4337757	Bn-scaff_15714_1-p420893	C2	10	15	A
12800	Bn-N2-p3309912	Bn-A02-p4237047	Bn-A02-p4237047	C2	10	15	A
12801	Bn-N12-p4118896	Bn-C2-p4380637	Bn-scaff_15714_1-p378013	C2	11	15.8	A
12802	Bn-N12-p4121985	Bn-C2-p4390372	Bn-scaff_15714_1-p368278	C2	11	15.8	A
12803	Bn-N12-p4133708	Bn-C2-p4401054	Bn-scaff_15714_1-p357596	C2	11	15.8	A
12804	Bn-N12-p4142029	Bn-C2-p4412359	Bn-scaff_15714_1-p346291	C2	11	15.8	A
12805	Bn-N12-p4142123	Bn-C2-p4412452	Bn-scaff_15714_1-p346198	C2	11	15.8	A
12806	Bn-N12-p4155019	Bn-C2-p4429894	Bn-scaff_15714_1-p328756	C2	11	15.8	A
12807	Bn-N12-p4180680	Bn-C2-p4448067	Bn-scaff_15714_1-p310583	C2	11	15.8	A
12808	Bn-N12-p4251431	Bn-C2-p4548163	Bn-scaff_15714_1-p210487	C2	11	15.8	A
12809	Bn-N2-p3349263	Bn-A02-p4262796	Bn-A02-p4262796	C2	11	15.8	A
12810	Bn-N2-p3349374	Bn-A02-p4262907	Bn-A02-p4262907	C2	11	15.8	A
12811	Bn-N12-p4336290	Bn-C2-p4623747	Bn-scaff_15714_1-p134903	C2	12	18.3	A
12812	Bn-N12-p4350638	Bn-C2-p4640139	Bn-scaff_15714_1-p118511	C2	12	18.3	A
12813	Bn-N12-p4390494	Bn-C2-p4789633	Bn-scaff_29061_1-p30884	C2	12	18.3	A
12814	Bn-N12-p4455936	Bn-C2-p4867778	Bn-scaff_16825_1-p56159	C2	12	18.3	A
12815	Bn-N12-p4478930	Bn-C2-p4874994	Bn-scaff_16825_1-p63375	C2	12	18.3	A
12816	Bn-N12-p4530254	Bn-C2-p4905989	Bn-scaff_16825_1-p94370	C2	12	18.3	A
12817	Bn-N12-p4541916	Bn-C2-p4915017	Bn-scaff_16825_1-p103398	C2	12	18.3	A
12818	Bn-N12-p4542148	Bn-C2-p4915249	Bn-scaff_16825_1-p103630	C2	12	18.3	A
12819	Bn-N12-p4542988	Bn-C2-p4916390	Bn-scaff_16825_1-p104771	C2	12	18.3	A
12820	Bn-N12-p4543118	Bn-C2-p4916520	Bn-scaff_16825_1-p104901	C2	12	18.3	A
12821	Bn-N12-p4604754	Bn-Scaffold00629-p1	Bn-scaff_27670_1-p359444	C2	12	18.3	A
12822	Bn-N12-p5062255	Bn-C2-p5280785	Bn-scaff_22527_1-p276556	C2	12	18.3	A
12823	Bn-N12-p5181761	Bn-C2-p5401218	Bn-scaff_22527_1-p156123	C2	12	18.3	A
12824	Bn-N12-p5182359	Bn-C2-p5401816	Bn-scaff_22527_1-p155525	C2	12	18.3	A
12825	Bn-N12-p5182425	Bn-C2-p5401881	Bn-scaff_22527_1-p155460	C2	12	18.3	A
12826	Bn-N12-p5246337	Bn-C2-p5469228	Bn-scaff_22527_1-p88113	C2	12	18.3	A
12827	Bn-N12-p5452068	Bn-C2-p5685990	Bn-scaff_23176_1-p55098	C2	12	18.3	A
12828	Bn-N12-p5477324	Bn-C2-p5710544	Bn-scaff_23176_1-p79652	C2	12	18.3	A
12829	Bn-N12-p5920409	Bn-C2-p6103224	Bn-scaff_17522_1-p170209	C2	12	18.3	A
12830	Bn-N12-p5972039	Bn-C2-p6156765	Bn-scaff_17522_1-p164855	C2	12	18.3	A
12831	Bn-N17-p36240263	Bn-C7-p36085186	Bn-scaff_17291_1-p101338	C2	12	18.3	A
12832	Bn-Scaffold05721-p543	Bn-C2-p6214343	Bn-scaff_17522_1-p159097	C2	12	18.3	A
12833	Bn-N12-p6117636	Bn-C2-p6321978	Bn-scaff_17522_1-p148333	C2	13	19.1	A

12834	Bn-N12-p6668685	Bn-C2-p6897111	Bn-scaff_17522_1-p90820	C2	13	19.1	A
12835	Bn-N12-p6790271	Bn-C2-p7028110	Bn-scaff_17522_1-p77720	C2	13	19.1	A
12836	Bn-N12-p6790451	Bn-C2-p7028290	Bn-scaff_17522_1-p77702	C2	13	19.1	A
12837	Bn-N12-p6790701	Bn-C2-p7028540	Bn-scaff_17522_1-p77677	C2	13	19.1	A
12838	Bn-N12-p6813864	Bn-C2-p7059190	Bn-scaff_17522_1-p74612	C2	13	19.1	A
12839	Bn-N12-p6846412	Bn-C2-p7100417	Bn-scaff_17522_1-p70489	C2	13	19.1	A
12840	Bn-N12-p6882910	Bn-C2-p7150986	Bn-scaff_17522_1-p65432	C2	13	19.1	A
12841	Bn-N12-p6883698	Bn-C2-p7151774	Bn-scaff_17522_1-p65354	C2	13	19.1	A
12842	Bn-N12-p6883912	Bn-C2-p7151988	Bn-scaff_17522_1-p65332	C2	13	19.1	A
12843	Bn-N12-p6884849	Bn-C2-p7152926	Bn-scaff_17522_1-p65238	C2	13	19.1	A
12844	Bn-N12-p6885723	Bn-C2-p7153800	Bn-scaff_17522_1-p65151	C2	13	19.1	A
12845	Bn-N12-p6885929	Bn-C2-p7154006	Bn-scaff_17522_1-p65130	C2	13	19.1	A
12846	Bn-N12-p6886001	Bn-C2-p7154078	Bn-scaff_17522_1-p65123	C2	13	19.1	A
12847	Bn-N12-p6913455	Bn-C2-p7180337	Bn-scaff_17522_1-p62497	C2	13	19.1	A
12848	Bn-N12-p6924407	Bn-C2-p7191290	Bn-scaff_17522_1-p61402	C2	13	19.1	A
12849	Bn-N12-p6961348	Bn-C2-p7228787	Bn-scaff_17522_1-p57652	C2	13	19.1	A
12850	Bn-N12-p6993629	Bn-C2-p7263844	Bn-scaff_17522_1-p54147	C2	13	19.1	A
12851	Bn-N12-p7014156	Bn-C2-p7287813	Bn-scaff_17522_1-p51750	C2	13	19.1	A
12852	Bn-N12-p7026719	Bn-C2-p7299983	Bn-scaff_17522_1-p50533	C2	13	19.1	A
12853	Bn-N12-p7054227	Bn-C2-p7324237	Bn-scaff_17522_1-p48107	C2	13	19.1	A
12854	Bn-N12-p7063826	Bn-C2-p7334504	Bn-scaff_17522_1-p47081	C2	13	19.1	A
12855	Bn-N12-p7073691	Bn-C2-p7349024	Bn-scaff_17522_1-p45629	C2	13	19.1	A
12856	Bn-N12-p7097604	Bn-C2-p7377765	Bn-scaff_17522_1-p42755	C2	13	19.1	A
12857	Bn-N12-p7126688	Bn-C2-p7407639	Bn-scaff_17522_1-p39767	C2	13	19.1	A
12858	Bn-N12-p7246803	Bn-C2-p7546669	Bn-scaff_17522_1-p25864	C2	13	19.1	A
12859	Bn-N12-p7325403	Bn-C2-p7674352	Bn-scaff_17522_1-p13096	C2	13	19.1	A
12860	Bn-N12-p7334512	Bn-C2-p7680554	Bn-scaff_17522_1-p12476	C2	13	19.1	A
12861	Bn-N12-p7397916	Bn-C2-p7744653	Bn-scaff_17522_1-p60662	C2	13	19.1	A
12862	Bn-N12-p7444881	Bn-C2-p7818335	Bn-scaff_16934_1-p42198	C2	13	19.1	A
12863	Bn-N12-p7511633	Bn-C2-p7893661	Bn-scaff_16934_1-p34665	C2	13	19.1	A
12864	Bn-N12-p7644666	Bn-C2-p8027888	Bn-scaff_16934_1-p21242	C2	13	19.1	A
12865	Bn-N12-p7677424	Bn-C2-p8073268	Bn-scaff_16934_1-p16704	C2	13	19.1	A
12866	Bn-N12-p7696665	Bn-C2-p8091986	Bn-scaff_16934_1-p14833	C2	13	19.1	A
12867	Bn-N12-p7696665	Bn-C2-p8091988	Bn-scaff_16934_1-p14832	C2	13	19.1	A
12868	Bn-N12-p7698472	Bn-C2-p8093795	Bn-scaff_16934_1-p14652	C2	13	19.1	A
12869	Bn-N12-p7699011	Bn-C2-p8094318	Bn-scaff_16934_1-p14599	C2	13	19.1	A
12870	Bn-N12-p7700939	Bn-C2-p8096246	Bn-scaff_16934_1-p14407	C2	13	19.1	A
12871	Bn-N12-p7777183	Bn-C2-p8247302	Bn-scaff_16269_1-p66411	C2	13	19.1	A
12872	Bn-N12-p7780451	Bn-C2-p8250570	Bn-scaff_16269_1-p66085	C2	13	19.1	A
12873	Bn-N12-p7827768	Bn-C2-p8301802	Bn-scaff_16269_1-p60961	C2	13	19.1	A
12874	Bn-N12-p8138186	Bn-C2-p8603157	Bn-scaff_16269_1-p30826	C2	13	19.1	A
12875	Bn-N12-p8144274	Bn-C2-p8609649	Bn-scaff_16269_1-p30177	C2	13	19.1	A
12876	Bn-N14-p54904923	Bn-C2-p8804148	Bn-scaff_16269_1-p10727	C2	13	19.1	A
12877	Bn-N16-p21721715	Bn-C6-p39285533	Bn-scaff_18549_1-p36469	C2	13	19.1	A
12878	Bn-N2-p4807150	Bn-A02-p5628094	Bn-A02-p5628094	C2	13	19.1	A
12879	Bn-N2-p4807529	Bn-A02-p5628473	Bn-A02-p5628473	C2	13	19.1	A
12880	Bn-N2-p5732602	Bn-C2-p8615159	Bn-scaff_16269_1-p29626	C2	13	19.1	A
12881	Bn-Scaffold01300-p5097	Bn-C2-p8146434	Bn-scaff_16934_1-p93882	C2	13	19.1	A
12882	Bn-Scaffold01300-p8957	Bn-C2-p8142209	Bn-scaff_16934_1-p98107	C2	13	19.1	A

12883	Bn-Scaffold03748-p1431	Bn-C2-p7930407	Bn-scaff_16934_1-p30990	C2	13	19.1	A
12884	Bn-Scaffold03762-p1145	Bn-C2-p7887624	Bn-scaff_16934_1-p35269	C2	13	19.1	A
12885	Bn-Scaffold03762-p527	Bn-C2-p7888242	Bn-scaff_16934_1-p35207	C2	13	19.1	A
12886	Bn-N12-p10142081	Bn-C2-p11288793	Bn-scaff_15712_5-p45552	C2	14	20.8	A
12887	Bn-N12-p10151393	Bn-C2-p11298722	Bn-scaff_15712_5-p44559	C2	14	20.8	A
12888	Bn-N12-p10154081	Bn-C2-p11301303	Bn-scaff_15712_5-p44301	C2	14	20.8	A
12889	Bn-N12-p10154306	Bn-C2-p11301528	Bn-scaff_15712_5-p44279	C2	14	20.8	A
12890	Bn-N12-p10154312	Bn-C2-p11301534	Bn-scaff_15712_5-p44278	C2	14	20.8	A
12891	Bn-N12-p10171164	Bn-C2-p11318197	Bn-scaff_15712_5-p42612	C2	14	20.8	A
12892	Bn-N12-p10487548	Bn-C2-p11655272	Bn-scaff_15712_5-p89048	C2	14	20.8	A
12893	Bn-N12-p10699569	Bn-C2-p11859174	Bn-scaff_21224_1-p11475	C2	14	20.8	A
12894	Bn-N12-p10721184	Bn-C2-p11880582	Bn-scaff_20461_1-p32246	C2	14	20.8	A
12895	Bn-N12-p10721865	Bn-C2-p11881284	Bn-scaff_20461_1-p32176	C2	14	20.8	A
12896	Bn-N12-p10729580	Bn-C2-p11888846	Bn-scaff_20461_1-p31419	C2	14	20.8	A
12897	Bn-N12-p10758177	Bn-C2-p11926342	Bn-scaff_20461_1-p27670	C2	14	20.8	A
12898	Bn-N12-p10789608	Bn-C2-p11953072	Bn-scaff_20461_1-p24997	C2	14	20.8	A
12899	Bn-N12-p10794789	Bn-C2-p11964834	Bn-scaff_20461_1-p23821	C2	14	20.8	A
12900	Bn-N12-p10800292	Bn-C2-p11970354	Bn-scaff_20461_1-p23269	C2	14	20.8	A
12901	Bn-N12-p10810117	Bn-C2-p11981218	Bn-scaff_20461_1-p22182	C2	14	20.8	A
12902	Bn-N12-p10811744	Bn-C2-p11982838	Bn-scaff_20461_1-p22020	C2	14	20.8	A
12903	Bn-N12-p10823326	Bn-C2-p11994400	Bn-scaff_20461_1-p20864	C2	14	20.8	A
12904	Bn-N12-p10833926	Bn-C2-p12003389	Bn-scaff_20461_1-p19965	C2	14	20.8	A
12905	Bn-N12-p10849524	Bn-C2-p12024624	Bn-scaff_20461_1-p17842	C2	14	20.8	A
12906	Bn-N12-p10857215	Bn-C2-p12033015	Bn-scaff_20461_1-p17003	C2	14	20.8	A
12907	Bn-N12-p10859339	Bn-C2-p12035149	Bn-scaff_20461_1-p16789	C2	14	20.8	A
12908	Bn-N12-p10891919	Bn-C2-p12081462	Bn-scaff_20461_1-p12158	C2	14	20.8	A
12909	Bn-N12-p10894035	Bn-C2-p12083227	Bn-scaff_20461_1-p11981	C2	14	20.8	A
12910	Bn-N12-p10894269	Bn-C2-p12083459	Bn-scaff_20461_1-p11958	C2	14	20.8	A
12911	Bn-N12-p10896364	Bn-C2-p12086551	Bn-scaff_20461_1-p11649	C2	14	20.8	A
12912	Bn-N12-p10934499	Bn-C2-p12152512	Bn-scaff_20461_1-p50533	C2	14	20.8	A
12913	Bn-N12-p11010886	Bn-C2-p9326050	Bn-scaff_16804_1-p20351	C2	14	20.8	A
12914	Bn-N12-p11395982	Bn-C2-p12717701	Bn-scaff_17725_1-p51455	C2	14	20.8	A
12915	Bn-N12-p11769252	Bn-C2-p13181723	Bn-scaff_20942_1-p73483	C2	14	20.8	A
12916	Bn-N12-p11778989	Bn-C2-p13197655	Bn-scaff_20942_1-p71890	C2	14	20.8	A
12917	Bn-N12-p11797223	Bn-C2-p13222020	Bn-scaff_20942_1-p69453	C2	14	20.8	A
12918	Bn-N12-p11801756	Bn-C2-p13227059	Bn-scaff_20942_1-p68949	C2	14	20.8	A
12919	Bn-N12-p11802373	Bn-C2-p13227674	Bn-scaff_20942_1-p68888	C2	14	20.8	A
12920	Bn-N12-p11802677	Bn-C2-p13227986	Bn-scaff_20942_1-p68856	C2	14	20.8	A
12921	Bn-N12-p11844944	Bn-C2-p13279329	Bn-scaff_20942_1-p63722	C2	14	20.8	A
12922	Bn-N12-p11845052	Bn-C2-p13279437	Bn-scaff_20942_1-p63711	C2	14	20.8	A
12923	Bn-N12-p11846053	Bn-C2-p13280410	Bn-scaff_20942_1-p63614	C2	14	20.8	A
12924	Bn-N12-p11851337	Bn-C2-p13289785	Bn-scaff_20942_1-p62677	C2	14	20.8	A
12925	Bn-N12-p11910254	Bn-C2-p13424589	Bn-scaff_20942_1-p49196	C2	14	20.8	A
12926	Bn-N12-p11927731	Bn-C2-p13401551	Bn-scaff_20942_1-p51500	C2	14	20.8	A
12927	Bn-N12-p11969252	Bn-C2-p13476449	Bn-scaff_20942_1-p44010	C2	14	20.8	A
12928	Bn-N12-p11969742	Bn-C2-p13476939	Bn-scaff_20942_1-p43961	C2	14	20.8	A
12929	Bn-N12-p11969830	Bn-C2-p13477027	Bn-scaff_20942_1-p43952	C2	14	20.8	A
12930	Bn-N12-p8508779	Bn-C2-p9300673	Bn-scaff_16804_1-p17814	C2	14	20.8	A
12931	Bn-N12-p8512903	Bn-C2-p9304554	Bn-scaff_16804_1-p18202	C2	14	20.8	A

12932	Bn-N12-p8582218	Bn-C2-p9377358	Bn-scaff_16804_1-p254827	C2	14	20.8	A
12933	Bn-N12-p8857836	Bn-Scaffold01089-p23	Bn-scaff_18374_1-p23965	C2	14	20.8	A
12934	Bn-N12-p8895997	Bn-C2-p9892816	Bn-scaff_16804_4-p107537	C2	14	20.8	A
12935	Bn-N12-p8900166	Bn-C2-p9896967	Bn-scaff_16804_4-p111688	C2	14	20.8	A
12936	Bn-N12-p8900611	Bn-C2-p9897415	Bn-scaff_16804_4-p112136	C2	14	20.8	A
12937	Bn-N12-p9029594	Bn-C2-p10052040	Bn-scaff_16804_4-p266761	C2	14	20.8	A
12938	Bn-N12-p9126635	Bn-C2-p10182914	Bn-scaff_18514_1-p77519	C2	14	20.8	A
12939	Bn-N12-p9129825	Bn-C2-p10186106	Bn-scaff_18514_1-p74327	C2	14	20.8	A
12940	Bn-N12-p9166358	Bn-C2-p10220345	Bn-scaff_18514_1-p40088	C2	14	20.8	A
12941	Bn-N12-p9174657	Bn-C2-p10232432	Bn-scaff_18514_1-p28001	C2	14	20.8	A
12942	Bn-N12-p9175400	Bn-C2-p10233176	Bn-scaff_18514_1-p27257	C2	14	20.8	A
12943	Bn-N12-p9432295	Bn-C2-p10430606	Bn-scaff_22670_1-p170074	C2	14	20.8	A
12944	Bn-N12-p9516320	Bn-C2-p10518566	Bn-scaff_15712_13-p33062	C2	14	20.8	A
12945	Bn-N12-p9521145	Bn-C2-p10523642	Bn-scaff_15712_13-p38138	C2	14	20.8	A
12946	Bn-N12-p9526815	Bn-C2-p10528672	Bn-scaff_15712_13-p43168	C2	14	20.8	A
12947	Bn-N12-p9549136	Bn-C2-p10546871	Bn-scaff_15712_13-p61367	C2	14	20.8	A
12948	Bn-N12-p9694321	Bn-C2-p10723215	Bn-scaff_15712_5-p102110	C2	14	20.8	A
12949	Bn-N12-p9742807	Bn-C2-p10802760	Bn-scaff_15712_5-p941560	C2	14	20.8	A
12950	Bn-N2-p6964999	Bn-C2-p11077703	Bn-scaff_15712_5-p666617	C2	14	20.8	A
12951	Bn-N2-p7521421	Bn-A02-p8199891	Bn-A02-p8199891	C2	14	20.8	A
12952	Bn-N2-p8007110	Bn-C2-p13243720	Bn-scaff_20942_1-p672835	C2	14	20.8	A
12953	Bn-N2-p8034681	Bn-C2-p13309156	Bn-scaff_20942_1-p607399	C2	14	20.8	A
12954	Bn-N9-p19570497	Bn-C2-p11963491	Bn-scaff_20461_1-p239554	C2	14	20.8	A
12955	Bn-Scaffold01396-p6634	Bn-C2-p12823138	Bn-scaff_20942_1-p109341	C2	14	20.8	A
12956	Bn-Scaffold01917-p4110	Bn-C2-p10548828	Bn-scaff_15712_13-p63324	C2	14	20.8	A
12957	Bn-Scaffold03999-p1898	Bn-C2-p13450609	Bn-scaff_20942_1-p465946	C2	14	20.8	A
12958	Bn-Scaffold05217-p1132	Bn-C2-p12103399	Bn-scaff_20461_1-p99646	C2	14	20.8	A
12959	Bn-Scaffold06823-p795	Bn-C2-p12254470	Bn-scaff_17725_1-p51326	C2	14	20.8	A
12960	Bn-Scaffold11550-p843	Bn-C2-p11939639	Bn-scaff_20461_1-p263406	C2	14	20.8	A
12961	Bn-N12-p12139142	Bn-C2-p13653557	Bn-scaff_20942_1-p262998	C2	15	22.5	A
12962	Bn-N12-p12152182	Bn-C2-p13664030	Bn-scaff_20942_1-p252525	C2	15	22.5	A
12963	Bn-N12-p12155460	Bn-C2-p13668830	Bn-scaff_20942_1-p247725	C2	15	22.5	A
12964	Bn-N12-p12156251	Bn-C2-p13669641	Bn-scaff_20942_1-p246914	C2	15	22.5	A
12965	Bn-N12-p12156587	Bn-C2-p13669977	Bn-scaff_20942_1-p246578	C2	15	22.5	A
12966	Bn-N12-p12157513	Bn-C2-p13670901	Bn-scaff_20942_1-p245654	C2	15	22.5	A
12967	Bn-N12-p12157747	Bn-C2-p13671137	Bn-scaff_20942_1-p245418	C2	15	22.5	A
12968	Bn-N12-p12158331	Bn-C2-p13671752	Bn-scaff_20942_1-p244803	C2	15	22.5	A
12969	Bn-N12-p12158438	Bn-C2-p13671859	Bn-scaff_20942_1-p244696	C2	15	22.5	A
12970	Bn-N12-p12159368	Bn-C2-p13672790	Bn-scaff_20942_1-p243765	C2	15	22.5	A
12971	Bn-N12-p12168361	Bn-C2-p13681680	Bn-scaff_20942_1-p234875	C2	15	22.5	A
12972	Bn-N12-p12168857	Bn-C2-p13682186	Bn-scaff_20942_1-p234369	C2	15	22.5	A
12973	Bn-N12-p12168944	Bn-C2-p13682273	Bn-scaff_20942_1-p234282	C2	15	22.5	A
12974	Bn-N12-p12177614	Bn-C2-p13689615	Bn-scaff_20942_1-p226940	C2	15	22.5	0
12975	Bn-N12-p12177665	Bn-C2-p13689666	Bn-scaff_20942_1-p226889	C2	15	22.5	A
12976	Bn-N12-p12178876	Bn-Scaffold00998-p48	Bn-scaff_25704_1-p4837	C2	15	22.5	A
12977	Bn-N12-p12178932	Bn-Scaffold00998-p48	Bn-scaff_25704_1-p4893	C2	15	22.5	A
12978	Bn-N12-p12180846	Bn-Scaffold00998-p91	Bn-scaff_25704_1-p9158	C2	15	22.5	A
12979	Bn-N12-p12185379	Bn-Scaffold00998-p13	Bn-scaff_25704_1-p13849	C2	15	22.5	A
12980	Bn-N12-p12185494	Bn-Scaffold00998-p13	Bn-scaff_25704_1-p13964	C2	15	22.5	A

12981	Bn-N12-p12188141	Bn-Scaffold00998-p15	Bn-scaff_25704_1-p15568	C2	15	22.5	A
12982	Bn-N12-p12188154	Bn-Scaffold00998-p15	Bn-scaff_25704_1-p15581	C2	15	22.5	A
12983	Bn-N12-p12189410	Bn-Scaffold00998-p16	Bn-scaff_25704_1-p16835	C2	15	22.5	A
12984	Bn-N12-p12192191	Bn-Scaffold00998-p19	Bn-scaff_25704_1-p19623	C2	15	22.5	A
12985	Bn-N12-p12193156	Bn-Scaffold00998-p20	Bn-scaff_25704_1-p20587	C2	15	22.5	A
12986	Bn-N12-p12208822	Bn-Scaffold00998-p34	Bn-scaff_25704_1-p34917	C2	15	22.5	A
12987	Bn-N12-p12214761	Bn-C2-p13731942	Bn-scaff_20942_1-p184613	C2	15	22.5	A
12988	Bn-N12-p12218298	Bn-C2-p13735536	Bn-scaff_20942_1-p181019	C2	15	22.5	A
12989	Bn-N12-p12218351	Bn-C2-p13735589	Bn-scaff_20942_1-p180966	C2	15	22.5	A
12990	Bn-N12-p12218443	Bn-C2-p13735681	Bn-scaff_20942_1-p180874	C2	15	22.5	A
12991	Bn-N12-p12218938	Bn-C2-p13736166	Bn-scaff_20942_1-p180389	C2	15	22.5	A
12992	Bn-N12-p12219054	Bn-C2-p13736281	Bn-scaff_20942_1-p180274	C2	15	22.5	A
12993	Bn-N12-p12220021	Bn-C2-p13737249	Bn-scaff_20942_1-p179306	C2	15	22.5	A
12994	Bn-N12-p12233419	Bn-C2-p13751007	Bn-scaff_20942_1-p165548	C2	15	22.5	A
12995	Bn-N12-p12241429	Bn-C2-p13762643	Bn-scaff_20942_1-p153912	C2	15	22.5	A
12996	Bn-N12-p12241518	Bn-C2-p13762732	Bn-scaff_20942_1-p153823	C2	15	22.5	A
12997	Bn-N12-p12243091	Bn-C2-p13764309	Bn-scaff_20942_1-p152246	C2	15	22.5	A
12998	Bn-N2-p8301981	Bn-A02-p8955992	Bn-A02-p8955992	C2	15	22.5	A
12999	Bn-N2-p8579132	Bn-A02-p9229113	Bn-A02-p9229113	C2	15	22.5	A
13000	Bn-Scaffold01535-p5046	Bn-C2-p13794620	Bn-scaff_20942_1-p121935	C2	15	22.5	A
13001	Bn-Scaffold29307-p182	Bn-C2-p13847064	Bn-scaff_20942_1-p69491	C2	15	22.5	A
13002	Bn-N12-p12889447	Bn-C2-p14539885	Bn-scaff_23813_1-p531505	C2	16	23.3	A
13003	Bn-N12-p13756282	Bn-A02-p9973927	Bn-A02-p9973927	C2	16	23.3	A
13004	Bn-N12-p13847011	Bn-C2-p15609445	Bn-scaff_16565_1-p116932	C2	16	23.3	A
13005	Bn-N12-p13852227	Bn-C2-p15613492	Bn-scaff_16565_1-p116527	C2	16	23.3	A
13006	Bn-N12-p13935070	Bn-C2-p15709508	Bn-scaff_16565_1-p106925	C2	16	23.3	A
13007	Bn-N12-p14039214	Bn-C2-p15862858	Bn-scaff_16565_1-p915907	C2	16	23.3	A
13008	Bn-N12-p14078054	Bn-C2-p15895718	Bn-scaff_16565_1-p883047	C2	16	23.3	A
13009	Bn-N12-p14087783	Bn-C2-p15918743	Bn-scaff_16565_1-p860022	C2	16	23.3	A
13010	Bn-N12-p14131896	Bn-C2-p15974945	Bn-scaff_16565_1-p803820	C2	16	23.3	A
13011	Bn-N12-p14157397	Bn-C2-p16000360	Bn-scaff_16565_1-p778405	C2	16	23.3	A
13012	Bn-N12-p14157401	Bn-C2-p16000364	Bn-scaff_16565_1-p778401	C2	16	23.3	A
13013	Bn-N12-p14157567	Bn-C2-p16000530	Bn-scaff_16565_1-p778235	C2	16	23.3	A
13014	Bn-N12-p14164098	Bn-C2-p16006955	Bn-scaff_16565_1-p771810	C2	16	23.3	A
13015	Bn-N12-p14168049	Bn-C2-p16010913	Bn-scaff_16565_1-p767852	C2	16	23.3	A
13016	Bn-N12-p14174464	Bn-C2-p16017330	Bn-scaff_16565_1-p761435	C2	16	23.3	A
13017	Bn-N12-p14305902	Bn-C2-p16274758	Bn-scaff_16565_1-p504007	C2	16	23.3	A
13018	Bn-N12-p14804742	Bn-C2-p17090347	Bn-scaff_27039_1-p311483	C2	16	23.3	A
13019	Bn-N12-p14837782	Bn-C9-p39271293	Bn-scaff_16042_1-p320923	C2	16	23.3	A
13020	Bn-N12-p14838229	Bn-C2-p17116322	Bn-scaff_27039_1-p337458	C2	16	23.3	A
13021	Bn-N12-p14838558	Bn-C2-p17116641	Bn-scaff_27039_1-p337777	C2	16	23.3	A
13022	Bn-N12-p14838796	Bn-C9-p39270281	Bn-scaff_16042_1-p319911	C2	16	23.3	A
13023	Bn-N12-p14880222	Bn-A02-p10775283	Bn-A02-p10775283	C2	16	23.3	A
13024	Bn-N12-p14907463	Bn-C2-p17317508	Bn-scaff_27039_1-p538644	C2	16	23.3	A
13025	Bn-N12-p14915242	Bn-C2-p17324647	Bn-scaff_27039_1-p545783	C2	16	23.3	A
13026	Bn-N12-p14978007	Bn-C2-p17356580	Bn-scaff_27039_1-p577716	C2	16	23.3	A
13027	Bn-N12-p15004559	Bn-C2-p17464137	Bn-scaff_27039_1-p685273	C2	16	23.3	A
13028	Bn-N12-p15009058	Bn-C2-p17468651	Bn-scaff_27039_1-p689787	C2	16	23.3	A
13029	Bn-N12-p15009128	Bn-C2-p17468721	Bn-scaff_27039_1-p689857	C2	16	23.3	A

13030	Bn-N12-p15101795	Bn-C2-p17561360	Bn-scaff_21607_1-p66467	C2	16	23.3	A
13031	Bn-N12-p15107221	Bn-C2-p17567073	Bn-scaff_21607_1-p72180	C2	16	23.3	A
13032	Bn-N12-p15255100	Bn-C2-p17723686	Bn-scaff_23291_1-p85817	C2	16	23.3	A
13033	Bn-N12-p15260676	Bn-C2-p17728700	Bn-scaff_23291_1-p90831	C2	16	23.3	A
13034	Bn-N12-p16154321	Bn-C2-p18921952	Bn-scaff_20979_1-p498237	C2	16	23.3	A
13035	Bn-N12-p16183314	Bn-C2-p18963757	Bn-scaff_20979_1-p456432	C2	16	23.3	A
13036	Bn-N12-p16323493	Bn-C2-p19266963	Bn-scaff_20979_1-p153226	C2	16	23.3	A
13037	Bn-N12-p16388017	Bn-C2-p19361122	Bn-scaff_20979_1-p59067	C2	16	23.3	A
13038	Bn-N18-p25195523	Bn-C2-p17178800	Bn-scaff_27039_1-p399936	C2	16	23.3	A
13039	Bn-N18-p25196353	Bn-C2-p17179627	Bn-scaff_27039_1-p400763	C2	16	23.3	A
13040	Bn-N19-p40902786	Bn-C9-p39270473	Bn-scaff_16042_1-p320103	C2	16	23.3	A
13041	Bn-N2-p10848744	Bn-A02-p11284285	Bn-A02-p11284285	C2	16	23.3	A
13042	Bn-N2-p10881716	Bn-A02-p11320789	Bn-A02-p11320789	C2	16	23.3	A
13043	Bn-N2-p9575002	Bn-A02-p10153359	Bn-A02-p10153359	C2	16	23.3	A
13044	Bn-N2-p9746334	Bn-A02-p10332863	Bn-A02-p10332863	C2	16	23.3	A
13045	Bn-Scaffold00980-p19074	Bn-C2-p17390536	Bn-scaff_27039_1-p611672	C2	16	23.3	A
13046	Bn-Scaffold01196-p14616	Bn-C2-p17198764	Bn-scaff_27039_1-p419900	C2	16	23.3	A
13047	Bn-Scaffold01196-p1467	Bn-C2-p17208467	Bn-scaff_27039_1-p429603	C2	16	23.3	A
13048	Bn-Scaffold01196-p19707	Bn-C2-p17193451	Bn-scaff_27039_1-p414587	C2	16	23.3	A
13049	Bn-Scaffold01196-p22574	Bn-C2-p17184829	Bn-scaff_27039_1-p405965	C2	16	23.3	A
13050	Bn-Scaffold01386-p3579	Bn-C2-p16908432	Bn-scaff_27039_1-p129568	C2	16	23.3	A
13051	Bn-Scaffold01435-p3659	Bn-C2-p16850627	Bn-scaff_27039_1-p71763	C2	16	23.3	A
13052	Bn-Scaffold01508-p3893	Bn-C2-p16919486	Bn-scaff_27039_1-p140622	C2	16	23.3	A
13053	Bn-Scaffold01513-p4655	Bn-C2-p16878578	Bn-scaff_27039_1-p99714	C2	16	23.3	A
13054	Bn-Scaffold01803-p3337	Bn-C2-p19108688	Bn-scaff_20979_1-p311501	C2	16	23.3	A
13055	Bn-Scaffold02199-p1417	Bn-C2-p17264561	Bn-scaff_27039_1-p485697	C2	16	23.3	A
13056	Bn-Scaffold02199-p3036	Bn-C2-p17262663	Bn-scaff_27039_1-p483799	C2	16	23.3	A
13057	Bn-Scaffold02664-p1493	Bn-C2-p15878926	Bn-scaff_16565_1-p899839	C2	16	23.3	A
13058	Bn-Scaffold02815-p131	Bn-C2-p17791220	Bn-scaff_23291_1-p153351	C2	16	23.3	A
13059	Bn-Scaffold04993-p1265	Bn-C2-p16867389	Bn-scaff_27039_1-p88525	C2	16	23.3	A
13060	Bn-Scaffold04993-p1379	Bn-C2-p16867503	Bn-scaff_27039_1-p88639	C2	16	23.3	A
13061	Bn-Scaffold45154-p203	Bn-C2-p17392447	Bn-scaff_27039_1-p613583	C2	16	23.3	A
13062	Bn-Scaffold84019-p106	Bn-C2-p16852297	Bn-scaff_27039_1-p73433	C2	16	23.3	A
13063	Bn-N12-p16538763	Bn-C2-p19642551	Bn-scaff_15839_1-p183517	C2	17	24.1	A
13064	Bn-N12-p16545765	Bn-C2-p19649557	Bn-scaff_15839_1-p190523	C2	17	24.1	A
13065	Bn-N12-p16549909	Bn-C2-p19652456	Bn-scaff_15839_1-p193422	C2	17	24.1	A
13066	Bn-N12-p16601159	Bn-C2-p19720967	Bn-scaff_18702_1-p61195	C2	17	24.1	A
13067	Bn-N12-p16654539	Bn-A02-p11772983	Bn-A02-p11772983	C2	17	24.1	A
13068	Bn-N12-p16686185	Bn-C2-p19817943	Bn-scaff_18702_1-p158171	C2	17	24.1	A
13069	Bn-N12-p16705932	Bn-C2-p19840955	Bn-scaff_18702_1-p181183	C2	17	24.1	A
13070	Bn-N12-p16729238	Bn-C2-p19861280	Bn-scaff_18702_1-p201508	C2	18	24.9	A
13071	Bn-N12-p16773174	Bn-C2-p19891309	Bn-scaff_18702_1-p231537	C2	18	24.9	A
13072	Bn-N12-p16832045	Bn-C2-p19949397	Bn-scaff_18702_1-p289625	C2	18	24.9	A
13073	Bn-N12-p16833531	Bn-C2-p19950883	Bn-scaff_18702_1-p291111	C2	18	24.9	A
13074	Bn-N12-p16843014	Bn-C2-p19976623	Bn-scaff_18702_1-p316851	C2	18	24.9	A
13075	Bn-N12-p16851567	Bn-C2-p19989096	Bn-scaff_18702_1-p329324	C2	18	24.9	A
13076	Bn-N12-p16879377	Bn-C2-p20018099	Bn-scaff_18702_1-p358327	C2	18	24.9	A
13077	Bn-N12-p16880099	Bn-C2-p20018821	Bn-scaff_18702_1-p359049	C2	18	24.9	A
13078	Bn-N12-p16886230	Bn-C2-p20024952	Bn-scaff_18702_1-p365180	C2	18	24.9	A

13079	Bn-N12-p16888587	Bn-C2-p20027309	Bn-scaff_18702_1-p367537	C2	18	24.9	A
13080	Bn-N12-p16889036	Bn-C2-p20027758	Bn-scaff_18702_1-p367986	C2	18	24.9	A
13081	Bn-N12-p16902429	Bn-C2-p20044211	Bn-scaff_18702_1-p384439	C2	18	24.9	A
13082	Bn-N12-p16974309	Bn-C2-p20122635	Bn-scaff_18702_1-p462863	C2	18	24.9	A
13083	Bn-N12-p17010755	Bn-C2-p20157033	Bn-scaff_18702_1-p497261	C2	18	24.9	A
13084	Bn-N12-p17214604	Bn-C2-p20430663	Bn-scaff_17566_1-p140421	C2	18	24.9	A
13085	Bn-N12-p17215404	Bn-C2-p20431515	Bn-scaff_17566_1-p141273	C2	18	24.9	A
13086	Bn-N12-p17328481	Bn-C2-p20971245	Bn-scaff_17077_1-p149538	C2	18	24.9	A
13087	Bn-N12-p17480815	Bn-C2-p21140588	Bn-scaff_17077_1-p318881	C2	18	24.9	A
13088	Bn-N12-p17619195	Bn-C2-p21363282	Bn-scaff_17077_1-p541575	C2	18	24.9	A
13089	Bn-N12-p17636309	Bn-C2-p21379988	Bn-scaff_17077_1-p558281	C2	18	24.9	A
13090	Bn-N12-p17804964	Bn-Scaffold01144-p7	Bn-scaff_27485_1-p7330	C2	18	24.9	A
13091	Bn-N12-p17842746	Bn-C2-p21691691	Bn-scaff_16437_1-p63154	C2	18	24.9	A
13092	Bn-N12-p17872979	Bn-C2-p21718732	Bn-scaff_16437_1-p36113	C2	18	24.9	A
13093	Bn-N12-p17896241	Bn-C2-p21735536	Bn-scaff_16437_1-p19309	C2	18	24.9	A
13094	Bn-N12-p17903328	Bn-C2-p21742550	Bn-scaff_16437_1-p12295	C2	18	24.9	A
13095	Bn-N12-p17969762	Bn-C2-p21818854	Bn-scaff_16369_1-p302817	C2	18	24.9	A
13096	Bn-N12-p17970248	Bn-C2-p21819340	Bn-scaff_16369_1-p302331	C2	18	24.9	A
13097	Bn-N13-p41406354	Bn-A09-p17600384	Bn-A09-p17600384	C2	18	24.9	A
13098	Bn-N15-p12418473	Bn-C2-p47082298	Bn-scaff_17109_1-p945905	C2	18	24.9	A
13099	Bn-N2-p12022544	Bn-C2-p20617726	Bn-scaff_17079_1-p144459	C2	18	24.9	A
13100	Bn-N2-p12022558	Bn-C2-p20617712	Bn-scaff_17079_1-p144473	C2	18	24.9	A
13101	Bn-N2-p12022895	Bn-C2-p20617435	Bn-scaff_17079_1-p144750	C2	18	24.9	A
13102	Bn-N2-p12023062	Bn-C2-p20617266	Bn-scaff_17079_1-p144919	C2	18	24.9	A
13103	Bn-N2-p12182955	Bn-C2-p20927460	Bn-scaff_17077_1-p105753	C2	18	24.9	A
13104	Bn-N2-p12198451	Bn-C2-p20947329	Bn-scaff_17077_1-p125622	C2	18	24.9	A
13105	Bn-N2-p12363988	Bn-C2-p21151673	Bn-scaff_17077_1-p329966	C2	18	24.9	A
13106	Bn-N4-p12338448	Bn-C2-p20610730	Bn-scaff_21188_1-p1514	C2	18	24.9	A
13107	Bn-N4-p12338818	Bn-C2-p20611102	Bn-scaff_21188_1-p1142	C2	18	24.9	A
13108	Bn-N4-p12339241	Bn-C2-p20611525	Bn-scaff_21188_1-p719	C2	18	24.9	A
13109	Bn-N4-p12340040	Bn-C2-p20616142	Bn-scaff_17079_1-p146043	C2	18	24.9	A
13110	Bn-N4-p12455274	Bn-C2-p20682522	Bn-scaff_17079_1-p79663	C2	18	24.9	A
13111	Bn-N4-p12510262	Bn-C7-p30886761	Bn-scaff_16130_1-p689480	C2	18	24.9	A
13112	Bn-N4-p12510420	Bn-C7-p30886919	Bn-scaff_16130_1-p689638	C2	18	24.9	A
13113	Bn-N4-p12541782	Bn-C7-p30930932	Bn-scaff_16130_1-p733651	C2	18	24.9	A
13114	Bn-N9-p23741429	Bn-C2-p20620982	Bn-scaff_17079_1-p141203	C2	18	24.9	A
13115	Bn-Scaffold01330-p8167	Bn-C2-p20460572	Bn-scaff_21861_1-p17903	C2	18	24.9	A
13116	Bn-Scaffold01376-p3913	Bn-C2-p20476496	Bn-scaff_21861_1-p33827	C2	18	24.9	A
13117	Bn-Scaffold01376-p3978	Bn-C2-p20476561	Bn-scaff_21861_1-p33892	C2	18	24.9	A
13118	Bn-Scaffold01376-p7358	Bn-C2-p20479941	Bn-scaff_21861_1-p37272	C2	18	24.9	A
13119	Bn-Scaffold01674-p4177	Bn-C2-p20722788	Bn-scaff_17079_1-p39397	C2	18	24.9	A
13120	Bn-Scaffold02261-p3865	Bn-C2-p21768270	Bn-scaff_20735_1-p44052	C2	18	24.9	A
13121	Bn-Scaffold02261-p3914	Bn-C2-p21768320	Bn-scaff_20735_1-p44002	C2	18	24.9	A
13122	Bn-Scaffold05569-p723	Bn-C2-p20603041	Bn-scaff_21188_1-p9203	C2	18	24.9	A
13123	Bn-Scaffold09727-p953	Bn-C2-p19983140	Bn-scaff_18702_1-p323368	C2	18	24.9	A
13124	Bn-Scaffold10161-p928	Bn-C2-p20100655	Bn-scaff_18702_1-p440883	C2	18	24.9	A
13125	Bn-Scaffold17798-p366	Bn-C4-p22049694	Bn-scaff_23432_1-p155986	C2	18	24.9	A
13126	Bn-N12-p18195253	Bn-C2-p22031369	Bn-scaff_16369_1-p90302	C2	19	25.7	A
13127	Bn-N12-p18203639	Bn-C2-p22039277	Bn-scaff_16369_1-p82394	C2	19	25.7	A

13128	Bn-N12-p18483884	Bn-C2-p22353101	Bn-scaff_16704_1-p503958	C2	19	25.7	A
13129	Bn-N12-p18524753	Bn-C2-p22394649	Bn-scaff_16704_1-p462410	C2	19	25.7	A
13130	Bn-N12-p18526047	Bn-C2-p22395945	Bn-scaff_16704_1-p461114	C2	19	25.7	A
13131	Bn-N12-p18526159	Bn-C2-p22396057	Bn-scaff_16704_1-p461002	C2	19	25.7	A
13132	Bn-N12-p18526434	Bn-C2-p22396332	Bn-scaff_16704_1-p460727	C2	19	25.7	A
13133	Bn-N12-p18527482	Bn-C2-p22397408	Bn-scaff_16704_1-p459651	C2	19	25.7	A
13134	Bn-N12-p18532897	Bn-A02-p13167989	Bn-A02-p13167989	C2	19	25.7	A
13135	Bn-N12-p18572532	Bn-C2-p22448670	Bn-scaff_16704_1-p408389	C2	19	25.7	A
13136	Bn-N12-p18582490	Bn-C2-p22464524	Bn-scaff_16704_1-p392535	C2	19	25.7	A
13137	Bn-N12-p18584669	Bn-C2-p22466687	Bn-scaff_16704_1-p390372	C2	19	25.7	A
13138	Bn-N12-p18607361	Bn-C2-p22481832	Bn-scaff_16704_1-p375227	C2	19	25.7	A
13139	Bn-N12-p18623592	Bn-C2-p22503526	Bn-scaff_16704_1-p353533	C2	19	25.7	A
13140	Bn-N12-p18626646	Bn-C2-p22508003	Bn-scaff_16704_1-p349056	C2	19	25.7	A
13141	Bn-N12-p18666056	Bn-C2-p22584388	Bn-scaff_16704_1-p272671	C2	19	25.7	A
13142	Bn-N12-p18666116	Bn-C2-p22584448	Bn-scaff_16704_1-p272611	C2	19	25.7	A
13143	Bn-N12-p18668891	Bn-C2-p22587309	Bn-scaff_16704_1-p269750	C2	19	25.7	A
13144	Bn-N12-p18678167	Bn-C2-p22588787	Bn-scaff_16704_1-p268272	C2	19	25.7	A
13145	Bn-N12-p18678277	Bn-C2-p22588899	Bn-scaff_16704_1-p268160	C2	19	25.7	A
13146	Bn-N12-p18678390	Bn-C2-p22589012	Bn-scaff_16704_1-p268047	C2	19	25.7	A
13147	Bn-N12-p18683683	Bn-C2-p22594239	Bn-scaff_16704_1-p262820	C2	19	25.7	A
13148	Bn-N12-p18716143	Bn-C2-p22638585	Bn-scaff_16704_1-p218474	C2	19	25.7	A
13149	Bn-N12-p18723893	Bn-C2-p22639919	Bn-scaff_16704_1-p217140	C2	19	25.7	A
13150	Bn-N12-p18745084	Bn-C2-p22682789	Bn-scaff_16704_1-p174270	C2	19	25.7	A
13151	Bn-N12-p18745570	Bn-C2-p22683270	Bn-scaff_16704_1-p173789	C2	19	25.7	A
13152	Bn-N12-p18770988	Bn-C2-p22706853	Bn-scaff_16704_1-p150206	C2	19	25.7	A
13153	Bn-N12-p18771117	Bn-C2-p22706982	Bn-scaff_16704_1-p150077	C2	19	25.7	A
13154	Bn-N12-p18817966	Bn-C2-p22736506	Bn-scaff_16704_1-p120553	C2	19	25.7	A
13155	Bn-N12-p18818639	Bn-C2-p22737175	Bn-scaff_16704_1-p119884	C2	19	25.7	A
13156	Bn-N12-p18819447	Bn-C2-p22737985	Bn-scaff_16704_1-p119074	C2	19	25.7	A
13157	Bn-N12-p18895780	Bn-C2-p23082339	Bn-scaff_23108_1-p284977	C2	19	25.7	A
13158	Bn-N12-p19479890	Bn-C2-p23898427	Bn-scaff_22093_1-p169534	C2	19	25.7	A
13159	Bn-N12-p19527774	Bn-C2-p23964162	Bn-scaff_22093_1-p103799	C2	19	25.7	A
13160	Bn-N12-p19527848	Bn-C2-p23964236	Bn-scaff_22093_1-p103725	C2	19	25.7	A
13161	Bn-N12-p19528479	Bn-C2-p23964868	Bn-scaff_22093_1-p103093	C2	19	25.7	A
13162	Bn-N12-p19529660	Bn-C2-p23966049	Bn-scaff_22093_1-p101912	C2	19	25.7	A
13163	Bn-N12-p19537059	Bn-C2-p23970795	Bn-scaff_22093_1-p97166	C2	19	25.7	A
13164	Bn-N12-p19614672	Bn-C2-p24164288	Bn-scaff_25262_1-p114443	C2	19	25.7	A
13165	Bn-N12-p19643524	Bn-C2-p24082830	Bn-scaff_21034_1-p41433	C2	19	25.7	A
13166	Bn-N12-p20248197	Bn-C2-p25019477	Bn-scaff_18675_1-p106845	C2	19	25.7	A
13167	Bn-N13-p52976797	Bn-Scaffold01073-p4	Bn-scaff_27747_1-p4819	C2	19	25.7	A
13168	Bn-N13-p64772470	Bn-C2-p23120937	Bn-scaff_23108_1-p323575	C2	19	25.7	A
13169	Bn-N2-p13797789	Bn-C2-p23974542	Bn-scaff_22093_1-p93419	C2	19	25.7	A
13170	Bn-Scaffold01388-p10940	Bn-C2-p22508661	Bn-scaff_16704_1-p348398	C2	19	25.7	A
13171	Bn-Scaffold04349-p1465	Bn-C2-p22471533	Bn-scaff_16704_1-p385526	C2	19	25.7	A
13172	Bn-Scaffold04571-p920	Bn-C2-p22528804	Bn-scaff_16704_1-p328255	C2	19	25.7	A
13173	Bn-Scaffold04826-p698	Bn-C2-p22807447	Bn-scaff_23108_1-p10085	C2	19	25.7	A
13174	Unknown	Bn-C2-p24113566	Bn-scaff_21034_1-p10697	C2	19	25.7	A
13175	Bn-N12-p20416446	Bn-C2-p25275891	Bn-scaff_18675_1-p812043	C2	20	29	A
13176	Bn-N12-p20496877	Bn-C2-p25538602	Bn-scaff_18675_1-p549332	C2	20	29	A

13177	Bn-N12-p20505324	Bn-C2-p25560076	Bn-scaff_18675_1-p527858	C2	20	29	A
13178	Bn-N12-p20532674	Bn-C2-p25594987	Bn-scaff_18675_1-p492947	C2	20	29	A
13179	Bn-N12-p20563773	Bn-C2-p25619057	Bn-scaff_18675_1-p468877	C2	20	29	A
13180	Bn-N12-p20566309	Bn-C2-p25621669	Bn-scaff_18675_1-p466265	C2	20	29	A
13181	Bn-N12-p20600290	Bn-C2-p25661100	Bn-scaff_18675_1-p426834	C2	20	29	A
13182	Bn-N12-p20603376	Bn-C2-p25664751	Bn-scaff_18675_1-p423183	C2	20	29	A
13183	Bn-N12-p20604574	Bn-C2-p25665962	Bn-scaff_18675_1-p421972	C2	20	29	A
13184	Bn-N12-p20604625	Bn-C2-p25666013	Bn-scaff_18675_1-p421921	C2	20	29	A
13185	Bn-N12-p20605315	Bn-C2-p25666703	Bn-scaff_18675_1-p421231	C2	20	29	A
13186	Bn-N12-p20614752	Bn-C2-p25672368	Bn-scaff_18675_1-p415566	C2	20	29	A
13187	Bn-N12-p20618150	Bn-C2-p25676918	Bn-scaff_18675_1-p411016	C2	20	29	A
13188	Bn-N12-p20636681	Bn-C2-p25691296	Bn-scaff_18675_1-p396638	C2	20	29	A
13189	Bn-N12-p20637717	Bn-C2-p25692332	Bn-scaff_18675_1-p395602	C2	20	29	A
13190	Bn-N12-p20638533	Bn-C2-p25693150	Bn-scaff_18675_1-p394784	C2	20	29	A
13191	Bn-N12-p20640133	Bn-C2-p25694750	Bn-scaff_18675_1-p393184	C2	20	29	A
13192	Bn-N12-p20643003	Bn-C2-p25700628	Bn-scaff_18675_1-p387306	C2	20	29	A
13193	Bn-N12-p20660455	Bn-C2-p25713736	Bn-scaff_18675_1-p374198	C2	20	29	A
13194	Bn-N12-p20685737	Bn-C2-p25740147	Bn-scaff_18675_1-p347787	C2	20	29	A
13195	Bn-N12-p20740837	Bn-C2-p25823908	Bn-scaff_18675_1-p264026	C2	20	29	A
13196	Bn-N12-p20777885	Bn-C2-p25857217	Bn-scaff_18675_1-p230717	C2	20	29	A
13197	Bn-N12-p20784843	Bn-C2-p25867009	Bn-scaff_18675_1-p220925	C2	20	29	A
13198	Bn-N12-p20821578	Bn-C2-p25913678	Bn-scaff_18675_1-p174256	C2	20	29	A
13199	Bn-N12-p20850466	Bn-C2-p25992303	Bn-scaff_18675_1-p95631	C2	20	29	A
13200	Bn-N12-p20865032	Bn-C2-p26002689	Bn-scaff_18675_1-p85245	C2	20	29	A
13201	Bn-N12-p20866909	Bn-C2-p26005806	Bn-scaff_18675_1-p82128	C2	20	29	A
13202	Bn-N12-p20937803	Bn-C2-p26079625	Bn-scaff_18675_1-p8309	C2	20	29	A
13203	Bn-N12-p20997148	Bn-C2-p26141780	Bn-scaff_16300_1-p126630	C2	20	29	A
13204	Bn-N12-p20997156	Bn-C2-p26141788	Bn-scaff_16300_1-p126629	C2	20	29	A
13205	Bn-N12-p20997951	Bn-C2-p26142585	Bn-scaff_16300_1-p126549	C2	20	29	A
13206	Bn-N12-p21000745	Bn-C2-p26145424	Bn-scaff_16300_1-p126265	C2	20	29	A
13207	Bn-N12-p21002465	Bn-C2-p26147167	Bn-scaff_16300_1-p126091	C2	20	29	A
13208	Bn-N12-p21002512	Bn-C2-p26147214	Bn-scaff_16300_1-p126086	C2	20	29	A
13209	Bn-N12-p21002572	Bn-C2-p26147274	Bn-scaff_16300_1-p126080	C2	20	29	A
13210	Bn-N12-p21008746	Bn-C2-p26153522	Bn-scaff_16300_1-p125455	C2	20	29	A
13211	Bn-N12-p21016076	Bn-C2-p26159348	Bn-scaff_16300_1-p124873	C2	20	29	A
13212	Bn-N12-p21025279	Bn-C2-p26173469	Bn-scaff_16300_1-p123461	C2	20	29	A
13213	Bn-N12-p21025981	Bn-C2-p26174171	Bn-scaff_16300_1-p123391	C2	20	29	A
13214	Bn-N12-p21031049	Bn-C2-p26179116	Bn-scaff_16300_1-p122896	C2	20	29	A
13215	Bn-N12-p21036612	Bn-C2-p26184816	Bn-scaff_16300_1-p122326	C2	20	29	A
13216	Bn-N12-p21038950	Bn-C2-p26187145	Bn-scaff_16300_1-p122093	C2	20	29	A
13217	Bn-N12-p21060139	Bn-C2-p26207733	Bn-scaff_16300_1-p120034	C2	20	29	A
13218	Bn-N12-p21111292	Bn-C2-p26302023	Bn-scaff_16300_1-p110605	C2	20	29	A
13219	Bn-N12-p21114228	Bn-C2-p26298279	Bn-scaff_16300_1-p110980	C2	20	29	A
13220	Bn-N12-p21207373	Bn-C2-p26437190	Bn-scaff_16300_1-p970891	C2	20	29	A
13221	Bn-N12-p21208880	Bn-C2-p26438732	Bn-scaff_16300_1-p969349	C2	20	29	A
13222	Bn-N12-p21209800	Bn-C2-p26439652	Bn-scaff_16300_1-p968429	C2	20	29	A
13223	Bn-N12-p21218284	Bn-C2-p26444417	Bn-scaff_16300_1-p963664	C2	20	29	A
13224	Bn-N12-p21224511	Bn-C2-p26451038	Bn-scaff_16300_1-p957043	C2	20	29	A
13225	Bn-N12-p21237357	Bn-C2-p26466140	Bn-scaff_16300_1-p941941	C2	20	29	A

13226	Bn-N12-p21252345	Bn-C2-p26475057	Bn-scaff_16300_1-p933024	C2	20	29	A
13227	Bn-N12-p21295987	Bn-C2-p26517836	Bn-scaff_16300_1-p890245	C2	20	29	A
13228	Bn-N12-p21303464	Bn-C2-p26525119	Bn-scaff_16300_1-p882962	C2	20	29	A
13229	Bn-N12-p21303507	Bn-C2-p26525162	Bn-scaff_16300_1-p882919	C2	20	29	A
13230	Bn-N12-p21303939	Bn-C2-p26525594	Bn-scaff_16300_1-p882487	C2	20	29	A
13231	Bn-Scaffold00998-p11382	Bn-C2-p25492969	Bn-scaff_18675_1-p594965	C2	20	29	A
13232	Bn-Scaffold00998-p14494	Bn-C2-p25496078	Bn-scaff_18675_1-p591856	C2	20	29	A
13233	Bn-Scaffold00998-p14666	Bn-C2-p25496250	Bn-scaff_18675_1-p591684	C2	20	29	A
13234	Bn-Scaffold00998-p14684	Bn-C2-p25496268	Bn-scaff_18675_1-p591666	C2	20	29	A
13235	Bn-Scaffold00998-p14931	Bn-C2-p25496520	Bn-scaff_18675_1-p591414	C2	20	29	A
13236	Bn-Scaffold00998-p15194	Bn-C2-p25496821	Bn-scaff_18675_1-p591113	C2	20	29	A
13237	Bn-Scaffold00998-p30545	Bn-C2-p25509529	Bn-scaff_18675_1-p578405	C2	20	29	A
13238	Bn-Scaffold00998-p30672	Bn-C2-p25509655	Bn-scaff_18675_1-p578279	C2	20	29	A
13239	Bn-Scaffold00998-p34029	Bn-C2-p25511914	Bn-scaff_18675_1-p576020	C2	20	29	A
13240	Bn-Scaffold00998-p46648	Bn-C2-p25526366	Bn-scaff_18675_1-p561568	C2	20	29	A
13241	Bn-Scaffold01103-p2595	Bn-C2-p25486683	Bn-scaff_18675_1-p601251	C2	20	29	A
13242	Bn-Scaffold01103-p27034	Bn-C2-p25464725	Bn-scaff_18675_1-p623209	C2	20	29	A
13243	Bn-Scaffold01103-p27436	Bn-C2-p25464323	Bn-scaff_18675_1-p623611	C2	20	29	A
13244	Bn-Scaffold01103-p3236	Bn-C2-p25482586	Bn-scaff_18675_1-p605348	C2	20	29	A
13245	Bn-Scaffold01103-p8364	Bn-C2-p25478670	Bn-scaff_18675_1-p609264	C2	20	29	A
13246	Bn-Scaffold01103-p8529	Bn-C2-p25478505	Bn-scaff_18675_1-p609429	C2	20	29	A
13247	Bn-Scaffold01103-p9100	Bn-C2-p25478158	Bn-scaff_18675_1-p609776	C2	20	29	A
13248	Bn-Scaffold01103-p9327	Bn-C2-p25477931	Bn-scaff_18675_1-p610003	C2	20	29	A
13249	Bn-Scaffold01552-p4436	Bn-C2-p26397251	Bn-scaff_16300_1-p101083	C2	20	29	A
13250	Bn-Scaffold02740-p2519	Bn-C2-p25695496	Bn-scaff_18675_1-p392438	C2	20	29	A
13251	Bn-Scaffold02890-p428	Bn-C2-p25412576	Bn-scaff_18675_1-p675358	C2	20	29	A
13252	Bn-Scaffold07693-p537	Bn-C2-p25415082	Bn-scaff_18675_1-p672852	C2	20	29	A
13253	Bn-Scaffold26874-p274	Bn-C2-p25656807	Bn-scaff_18675_1-p431127	C2	20	29	A
13254	Unknown	Bn-C2-p25774621	Bn-scaff_18675_1-p313313	C2	20	29	A
13255	Bn-N11-p57044	Bn-C2-p32814943	Bn-scaff_16328_1-p751831	C2	21	29.8	A
13256	Bn-N11-p60993	Bn-C2-p32818889	Bn-scaff_16328_1-p755777	C2	21	29.8	A
13257	Bn-N12-p21583340	Bn-C2-p26782405	Bn-scaff_16300_1-p625676	C2	21	29.8	A
13258	Bn-N12-p21707052	Bn-C2-p26931578	Bn-scaff_16300_1-p476503	C2	21	29.8	A
13259	Bn-N12-p21755870	Bn-C2-p26987666	Bn-scaff_16300_1-p420415	C2	21	29.8	A
13260	Bn-N12-p21911938	Bn-C2-p27158670	Bn-scaff_16300_1-p249411	C2	21	29.8	A
13261	Bn-N12-p21928198	Bn-C2-p27177053	Bn-scaff_16300_1-p231028	C2	21	29.8	A
13262	Bn-N12-p21934986	Bn-C2-p27185224	Bn-scaff_16300_1-p222857	C2	21	29.8	A
13263	Bn-N12-p21935234	Bn-C2-p27185472	Bn-scaff_16300_1-p222609	C2	21	29.8	A
13264	Bn-N12-p21940431	Bn-C2-p27190670	Bn-scaff_16300_1-p217411	C2	21	29.8	A
13265	Bn-N12-p21941303	Bn-C2-p27191542	Bn-scaff_16300_1-p216539	C2	21	29.8	A
13266	Bn-N12-p21942987	Bn-C2-p27193225	Bn-scaff_16300_1-p214856	C2	21	29.8	A
13267	Bn-N12-p21943840	Bn-C2-p27194078	Bn-scaff_16300_1-p214003	C2	21	29.8	A
13268	Bn-N12-p21944222	Bn-C2-p27194460	Bn-scaff_16300_1-p213621	C2	21	29.8	A
13269	Bn-N12-p21944505	Bn-C2-p27194743	Bn-scaff_16300_1-p213338	C2	21	29.8	A
13270	Bn-N12-p21956254	Bn-C2-p27211344	Bn-scaff_16300_1-p196737	C2	21	29.8	A
13271	Bn-N12-p22036052	Bn-C2-p27259420	Bn-scaff_16300_1-p148661	C2	21	29.8	A
13272	Bn-N12-p22036981	Bn-C2-p27260349	Bn-scaff_16300_1-p147732	C2	21	29.8	A
13273	Bn-N12-p22141431	Bn-C2-p27374104	Bn-scaff_16300_1-p33977	C2	21	29.8	A
13274	Bn-N12-p22142478	Bn-C2-p27376782	Bn-scaff_16300_1-p31299	C2	21	29.8	A

13275	Bn-N12-p22284032	Bn-C2-p27601989	Bn-scaff_22451_1-p193809	C2	21	29.8	A
13276	Bn-N12-p22301662	Bn-C2-p27610826	Bn-scaff_22451_1-p202646	C2	21	29.8	A
13277	Bn-N12-p22307741	Bn-C2-p27616342	Bn-scaff_22451_1-p208162	C2	21	29.8	A
13278	Bn-N12-p22391675	Bn-C2-p27716342	Bn-scaff_22451_1-p308162	C2	21	29.8	A
13279	Bn-N12-p22643316	Bn-C2-p27952515	Bn-scaff_22451_2-p87375	C2	21	29.8	A
13280	Bn-N12-p22647981	Bn-C2-p27957292	Bn-scaff_22451_2-p92152	C2	21	29.8	A
13281	Bn-N12-p22714171	Bn-C2-p28038658	Bn-scaff_22451_2-p173518	C2	21	29.8	A
13282	Bn-N12-p22840493	Bn-C2-p28188458	Bn-scaff_22451_2-p323318	C2	21	29.8	A
13283	Bn-N12-p22995928	Bn-C2-p28390998	Bn-scaff_22451_2-p525858	C2	21	29.8	A
13284	Bn-N12-p23272710	Bn-C2-p28690664	Bn-scaff_22749_1-p144203	C2	21	29.8	A
13285	Bn-N12-p23284104	Bn-C2-p28701223	Bn-scaff_22749_1-p154762	C2	21	29.8	A
13286	Bn-N12-p23351949	Bn-C2-p28801849	Bn-scaff_22749_1-p255388	C2	21	29.8	A
13287	Bn-N12-p23359867	Bn-C2-p28806917	Bn-scaff_22749_1-p260456	C2	21	29.8	A
13288	Bn-N12-p23439030	Bn-C2-p28939196	Bn-scaff_22749_1-p392735	C2	21	29.8	A
13289	Bn-N12-p23447983	Bn-C2-p28947119	Bn-scaff_22749_1-p400658	C2	21	29.8	A
13290	Bn-N12-p23461091	Bn-C2-p29261512	Bn-scaff_18199_1-p448318	C2	21	29.8	A
13291	Bn-N12-p23707718	Bn-C2-p29035093	Bn-scaff_22749_1-p488632	C2	21	29.8	A
13292	Bn-N12-p23721731	Bn-C2-p29021284	Bn-scaff_22749_1-p474823	C2	21	29.8	A
13293	Bn-N12-p23727213	Bn-C2-p29010316	Bn-scaff_22749_1-p463855	C2	21	29.8	A
13294	Bn-N12-p23757234	Bn-C2-p28987718	Bn-scaff_22749_1-p441257	C2	21	29.8	A
13295	Bn-N12-p23788446	Bn-C2-p29274749	Bn-scaff_18199_1-p435081	C2	21	29.8	A
13296	Bn-N12-p24208091	Bn-C2-p29977523	Bn-scaff_20221_1-p75074	C2	21	29.8	A
13297	Bn-N12-p24214468	Bn-C2-p29984166	Bn-scaff_20221_1-p68431	C2	21	29.8	A
13298	Bn-N12-p24214961	Bn-C2-p29984659	Bn-scaff_20221_1-p67938	C2	21	29.8	A
13299	Bn-N12-p24217822	Bn-C2-p29987520	Bn-scaff_20221_1-p65077	C2	21	29.8	A
13300	Bn-N12-p24217918	Bn-C2-p29987616	Bn-scaff_20221_1-p64981	C2	21	29.8	A
13301	Bn-N12-p24238424	Bn-C2-p30007561	Bn-scaff_20221_1-p45036	C2	21	29.8	A
13302	Bn-N12-p24264140	Bn-C2-p30033412	Bn-scaff_20221_1-p19185	C2	21	29.8	A
13303	Bn-N12-p24281208	Bn-C2-p30059666	Bn-scaff_18507_1-p6970	C2	21	29.8	A
13304	Bn-N12-p24284782	Bn-C2-p30063240	Bn-scaff_18507_1-p10544	C2	21	29.8	A
13305	Bn-N12-p24285151	Bn-C2-p30063609	Bn-scaff_18507_1-p10913	C2	21	29.8	A
13306	Bn-N12-p24285849	Bn-C2-p30064307	Bn-scaff_18507_1-p11611	C2	21	29.8	A
13307	Bn-N12-p24286400	Bn-C2-p30064858	Bn-scaff_18507_1-p12162	C2	21	29.8	A
13308	Bn-N12-p24286744	Bn-C2-p30065202	Bn-scaff_18507_1-p12506	C2	21	29.8	A
13309	Bn-N12-p24286804	Bn-C2-p30065262	Bn-scaff_18507_1-p12566	C2	21	29.8	A
13310	Bn-N12-p24287202	Bn-C2-p30065660	Bn-scaff_18507_1-p12964	C2	21	29.8	A
13311	Bn-N12-p24290175	Bn-C2-p30069309	Bn-scaff_18507_1-p16613	C2	21	29.8	A
13312	Bn-N12-p24291338	Bn-C2-p30070472	Bn-scaff_18507_1-p17776	C2	21	29.8	A
13313	Bn-N12-p24291393	Bn-C2-p30070527	Bn-scaff_18507_1-p17831	C2	21	29.8	A
13314	Bn-N12-p24291559	Bn-C2-p30070693	Bn-scaff_18507_1-p17997	C2	21	29.8	A
13315	Bn-N12-p24291967	Bn-C2-p30071101	Bn-scaff_18507_1-p18405	C2	21	29.8	A
13316	Bn-N12-p24292237	Bn-C2-p30071371	Bn-scaff_18507_1-p18675	C2	21	29.8	A
13317	Bn-N12-p24292497	Bn-C2-p30071631	Bn-scaff_18507_1-p18935	C2	21	29.8	A
13318	Bn-N12-p24293251	Bn-C2-p30072385	Bn-scaff_18507_1-p19689	C2	21	29.8	A
13319	Bn-N12-p24293759	Bn-C2-p30072893	Bn-scaff_18507_1-p20197	C2	21	29.8	A
13320	Bn-N12-p24294026	Bn-C2-p30073160	Bn-scaff_18507_1-p20464	C2	21	29.8	A
13321	Bn-N12-p24303623	Bn-C2-p30082463	Bn-scaff_18507_1-p29767	C2	21	29.8	A
13322	Bn-N12-p24303683	Bn-C2-p30082523	Bn-scaff_18507_1-p29827	C2	21	29.8	A
13323	Bn-N12-p24334195	Bn-C2-p30112037	Bn-scaff_18507_1-p59341	C2	21	29.8	A

13324	Bn-N12-p24343030	Bn-C2-p30120723	Bn-scaff_18507_1-p68027	C2	21	29.8	A
13325	Bn-N12-p24370521	Bn-C2-p30154357	Bn-scaff_18507_1-p10166	C2	21	29.8	A
13326	Bn-N12-p24370803	Bn-C2-p30154639	Bn-scaff_18507_1-p10194	C2	21	29.8	A
13327	Bn-N12-p24457135	Bn-C2-p30244132	Bn-scaff_18507_1-p19143	C2	21	29.8	A
13328	Bn-N12-p24469702	Bn-C2-p30256598	Bn-scaff_18507_1-p20390	C2	21	29.8	A
13329	Bn-N12-p24554755	Bn-C2-p30350599	Bn-scaff_18507_1-p29790	C2	21	29.8	A
13330	Bn-N12-p24622961	Bn-C2-p30404862	Bn-scaff_18507_1-p35216	C2	21	29.8	A
13331	Bn-N12-p24624848	Bn-C2-p30406749	Bn-scaff_18507_1-p35405	C2	21	29.8	A
13332	Bn-N12-p24625320	Bn-C2-p30407221	Bn-scaff_18507_1-p35452	C2	21	29.8	A
13333	Bn-N12-p24625761	Bn-C2-p30407662	Bn-scaff_18507_1-p35496	C2	21	29.8	A
13334	Bn-N12-p24625875	Bn-C2-p30407776	Bn-scaff_18507_1-p35508	C2	21	29.8	A
13335	Bn-N12-p24641592	Bn-C2-p30423492	Bn-scaff_18507_1-p37079	C2	21	29.8	A
13336	Bn-N12-p24667933	Bn-C2-p30449578	Bn-scaff_18507_1-p39688	C2	21	29.8	A
13337	Bn-N12-p24682629	Bn-C2-p30464334	Bn-scaff_18507_1-p41163	C2	21	29.8	A
13338	Bn-N12-p24682793	Bn-C2-p30464498	Bn-scaff_18507_1-p41180	C2	21	29.8	A
13339	Bn-N12-p25013237	Bn-C2-p30788372	Bn-scaff_18507_1-p73567	C2	21	29.8	A
13340	Bn-N12-p25038650	Bn-C2-p30811292	Bn-scaff_18507_1-p75859	C2	21	29.8	A
13341	Bn-N12-p25038780	Bn-C2-p30811422	Bn-scaff_18507_1-p75872	C2	21	29.8	A
13342	Bn-N12-p25039908	Bn-C2-p30812550	Bn-scaff_18507_1-p75985	C2	21	29.8	A
13343	Bn-N12-p25047114	Bn-C2-p30820110	Bn-scaff_18507_1-p76741	C2	21	29.8	A
13344	Bn-N12-p25047696	Bn-C2-p30820692	Bn-scaff_18507_1-p76799	C2	21	29.8	A
13345	Bn-N12-p25049959	Bn-C2-p30822955	Bn-scaff_18507_1-p77025	C2	21	29.8	A
13346	Bn-N12-p25054404	Bn-C2-p30827402	Bn-scaff_18507_1-p77470	C2	21	29.8	A
13347	Bn-N12-p25058189	Bn-C2-p30830838	Bn-scaff_18507_1-p77814	C2	21	29.8	A
13348	Bn-N12-p25060802	Bn-C2-p30833451	Bn-scaff_18507_1-p78075	C2	21	29.8	A
13349	Bn-N12-p25060875	Bn-C2-p30833524	Bn-scaff_18507_1-p78082	C2	21	29.8	A
13350	Bn-N12-p25073328	Bn-C2-p30845965	Bn-scaff_18507_1-p79326	C2	21	29.8	A
13351	Bn-N12-p25094900	Bn-Scaffold01010-p2	Bn-scaff_19372_1-p26232	C2	21	29.8	A
13352	Bn-N12-p25129395	Bn-C2-p30899655	Bn-scaff_18507_1-p84695	C2	21	29.8	A
13353	Bn-N12-p25131387	Bn-C2-p30901647	Bn-scaff_18507_1-p84895	C2	21	29.8	A
13354	Bn-N12-p25133023	Bn-C2-p30902832	Bn-scaff_18507_1-p85013	C2	21	29.8	A
13355	Bn-N12-p25143699	Bn-C2-p30913497	Bn-scaff_18507_1-p86080	C2	21	29.8	A
13356	Bn-N12-p25144151	Bn-C2-p30913949	Bn-scaff_18507_1-p86125	C2	21	29.8	A
13357	Bn-N12-p25149027	Bn-C2-p30918477	Bn-scaff_18507_1-p86578	C2	21	29.8	A
13358	Bn-N12-p25150025	Bn-C2-p30919475	Bn-scaff_18507_1-p86677	C2	21	29.8	A
13359	Bn-N12-p25150755	Bn-C2-p30920205	Bn-scaff_18507_1-p86750	C2	21	29.8	A
13360	Bn-N12-p25150919	Bn-C2-p30920369	Bn-scaff_18507_1-p86767	C2	21	29.8	A
13361	Bn-N12-p25174257	Bn-C2-p30942623	Bn-scaff_18507_1-p88992	C2	21	29.8	A
13362	Bn-N12-p25207946	Bn-C2-p30975849	Bn-scaff_18507_1-p92315	C2	21	29.8	A
13363	Bn-N12-p25208606	Bn-C2-p30976509	Bn-scaff_18507_1-p92381	C2	21	29.8	A
13364	Bn-N12-p25208944	Bn-C2-p30976847	Bn-scaff_18507_1-p92415	C2	21	29.8	A
13365	Bn-N12-p25209171	Bn-C2-p30977074	Bn-scaff_18507_1-p92437	C2	21	29.8	A
13366	Bn-N12-p25227650	Bn-C2-p31000586	Bn-scaff_18507_1-p94789	C2	21	29.8	A
13367	Bn-N12-p25229826	Bn-C2-p31002762	Bn-scaff_18507_1-p95006	C2	21	29.8	A
13368	Bn-N12-p25231124	Bn-C2-p31004060	Bn-scaff_18507_1-p95136	C2	21	29.8	A
13369	Bn-N12-p25266780	Bn-C2-p31038857	Bn-scaff_18507_1-p98616	C2	21	29.8	A
13370	Bn-N12-p25324673	Bn-C2-p31098592	Bn-scaff_18507_1-p10458	C2	21	29.8	A
13371	Bn-N12-p25327751	Bn-C2-p31101667	Bn-scaff_18507_1-p10489	C2	21	29.8	A
13372	Bn-N12-p25341577	Bn-C2-p31116561	Bn-scaff_18507_1-p10638	C2	21	29.8	A

13373	Bn-N12-p25402026	Bn-C2-p31194792	Bn-scaff_18507_1-p114209	C2	21	29.8	A
13374	Bn-N12-p25435541	Bn-C2-p31227002	Bn-scaff_18507_1-p117430	C2	21	29.8	A
13375	Bn-N12-p25439616	Bn-C2-p31230778	Bn-scaff_18507_1-p117808	C2	21	29.8	A
13376	Bn-N12-p25442130	Bn-C2-p31233292	Bn-scaff_18507_1-p118059	C2	21	29.8	A
13377	Bn-N12-p25442502	Bn-C2-p31233664	Bn-scaff_18507_1-p118096	C2	21	29.8	A
13378	Bn-N12-p25442627	Bn-C2-p31233789	Bn-scaff_18507_1-p118109	C2	21	29.8	A
13379	Bn-N12-p25450848	Bn-C2-p31242274	Bn-scaff_18507_1-p118957	C2	21	29.8	A
13380	Bn-N12-p25459918	Bn-C2-p31251345	Bn-scaff_18507_1-p119864	C2	21	29.8	A
13381	Bn-N12-p25465935	Bn-C2-p31257363	Bn-scaff_18507_1-p120466	C2	21	29.8	A
13382	Bn-N12-p25467278	Bn-C2-p31258706	Bn-scaff_18507_1-p120601	C2	21	29.8	A
13383	Bn-N12-p25467701	Bn-C2-p31259129	Bn-scaff_18507_1-p120643	C2	21	29.8	A
13384	Bn-N12-p25468626	Bn-C2-p31260054	Bn-scaff_18507_1-p120735	C2	21	29.8	A
13385	Bn-N12-p25469431	Bn-C2-p31260859	Bn-scaff_18507_1-p120816	C2	21	29.8	A
13386	Bn-N12-p25469795	Bn-C2-p31261223	Bn-scaff_18507_1-p120852	C2	21	29.8	A
13387	Bn-N12-p25469962	Bn-C2-p31261390	Bn-scaff_18507_1-p120869	C2	21	29.8	A
13388	Bn-N12-p25470250	Bn-C2-p31261678	Bn-scaff_18507_1-p120898	C2	21	29.8	A
13389	Bn-N12-p25471085	Bn-C2-p31262513	Bn-scaff_18507_1-p120981	C2	21	29.8	A
13390	Bn-N12-p25471256	Bn-C2-p31262684	Bn-scaff_18507_1-p120998	C2	21	29.8	A
13391	Bn-N12-p25472736	Bn-C2-p31264307	Bn-scaff_18507_1-p121161	C2	21	29.8	A
13392	Bn-N12-p25491107	Bn-C2-p31277021	Bn-scaff_18507_1-p122432	C2	21	29.8	A
13393	Bn-N12-p25497627	Bn-C2-p31283541	Bn-scaff_18507_1-p123084	C2	21	29.8	A
13394	Bn-N12-p25503659	Bn-C2-p31289889	Bn-scaff_18507_1-p123719	C2	21	29.8	A
13395	Bn-N12-p25508201	Bn-C2-p31294431	Bn-scaff_18507_1-p124173	C2	21	29.8	A
13396	Bn-N12-p25509996	Bn-C2-p31296226	Bn-scaff_18507_1-p124353	C2	21	29.8	A
13397	Bn-N12-p25510344	Bn-C2-p31296574	Bn-scaff_18507_1-p124387	C2	21	29.8	A
13398	Bn-N12-p25544893	Bn-C2-p31333229	Bn-scaff_18507_1-p128053	C2	21	29.8	A
13399	Bn-N12-p25562760	Bn-C2-p31351976	Bn-scaff_18507_1-p129928	C2	21	29.8	A
13400	Bn-N12-p25652738	Bn-C2-p31446544	Bn-scaff_18507_1-p139384	C2	21	29.8	A
13401	Bn-N12-p25686195	Bn-C2-p31474785	Bn-scaff_18507_1-p142208	C2	21	29.8	A
13402	Bn-N12-p25689084	Bn-C2-p31477674	Bn-scaff_18507_1-p142497	C2	21	29.8	A
13403	Bn-N12-p25693884	Bn-C2-p31482505	Bn-scaff_18507_1-p142980	C2	21	29.8	A
13404	Bn-N12-p25704471	Bn-C2-p31501310	Bn-scaff_26317_1-p113632	C2	21	29.8	A
13405	Bn-N12-p25704638	Bn-C2-p31501477	Bn-scaff_26317_1-p113465	C2	21	29.8	A
13406	Bn-N12-p25704738	Bn-C2-p31501577	Bn-scaff_26317_1-p113365	C2	21	29.8	A
13407	Bn-N12-p25704857	Bn-C2-p31501696	Bn-scaff_26317_1-p113246	C2	21	29.8	A
13408	Bn-N12-p25705198	Bn-C2-p31502037	Bn-scaff_26317_1-p112905	C2	21	29.8	A
13409	Bn-N12-p25705552	Bn-C2-p31502391	Bn-scaff_26317_1-p112551	C2	21	29.8	A
13410	Bn-N12-p25705663	Bn-C2-p31502502	Bn-scaff_26317_1-p112440	C2	21	29.8	A
13411	Bn-N12-p25705798	Bn-C2-p31502637	Bn-scaff_26317_1-p112305	C2	21	29.8	A
13412	Bn-N12-p25705892	Bn-C2-p31502731	Bn-scaff_26317_1-p112211	C2	21	29.8	A
13413	Bn-N12-p25708534	Bn-C2-p31505374	Bn-scaff_26317_1-p109568	C2	21	29.8	A
13414	Bn-N12-p25708711	Bn-C2-p31505551	Bn-scaff_26317_1-p109391	C2	21	29.8	A
13415	Bn-N12-p25708854	Bn-C2-p31505694	Bn-scaff_26317_1-p109248	C2	21	29.8	A
13416	Bn-N12-p25709190	Bn-C2-p31506030	Bn-scaff_26317_1-p108912	C2	21	29.8	A
13417	Bn-N12-p25709198	Bn-C2-p31506038	Bn-scaff_26317_1-p108904	C2	21	29.8	A
13418	Bn-N12-p25709277	Bn-C2-p31506117	Bn-scaff_26317_1-p108825	C2	21	29.8	A
13419	Bn-N12-p25713410	Bn-C2-p31510186	Bn-scaff_26317_1-p104756	C2	21	29.8	A
13420	Bn-N12-p25750020	Bn-C2-p31547004	Bn-scaff_26317_1-p67938	C2	21	29.8	A
13421	Bn-N12-p25850530	Bn-C2-p31628261	Bn-scaff_23401_1-p13220	C2	21	29.8	A

13422	Bn-N12-p25850942	Bn-C2-p31628673	Bn-scaff_23401_1-p13632	C2	21	29.8	A
13423	Bn-N12-p25851028	Bn-C2-p31628759	Bn-scaff_23401_1-p13718	C2	21	29.8	A
13424	Bn-N12-p25851194	Bn-C2-p31628925	Bn-scaff_23401_1-p13884	C2	21	29.8	A
13425	Bn-N12-p25851344	Bn-C2-p31629075	Bn-scaff_23401_1-p14034	C2	21	29.8	A
13426	Bn-N12-p25851685	Bn-C2-p31629416	Bn-scaff_23401_1-p14375	C2	21	29.8	A
13427	Bn-N12-p25852533	Bn-C2-p31630264	Bn-scaff_23401_1-p15223	C2	21	29.8	A
13428	Bn-N12-p25896981	Bn-C2-p31687664	Bn-scaff_23401_1-p72623	C2	21	29.8	A
13429	Bn-N12-p25897164	Bn-C2-p31687847	Bn-scaff_23401_1-p72806	C2	21	29.8	A
13430	Bn-N12-p25897906	Bn-C2-p31688589	Bn-scaff_23401_1-p73548	C2	21	29.8	A
13431	Bn-N12-p25902011	Bn-C2-p31693426	Bn-scaff_23401_1-p78385	C2	21	29.8	A
13432	Bn-N12-p25902027	Bn-C2-p31693442	Bn-scaff_23401_1-p78401	C2	21	29.8	A
13433	Bn-N12-p25907820	Bn-C2-p31699033	Bn-scaff_23401_1-p83992	C2	21	29.8	A
13434	Bn-N12-p25908331	Bn-C2-p31699544	Bn-scaff_23401_1-p84503	C2	21	29.8	A
13435	Bn-N12-p25909507	Bn-C2-p31700722	Bn-scaff_23401_1-p85681	C2	21	29.8	A
13436	Bn-N12-p25910268	Bn-C2-p31701483	Bn-scaff_23401_1-p86442	C2	21	29.8	A
13437	Bn-N12-p25911413	Bn-C2-p31702628	Bn-scaff_23401_1-p87587	C2	21	29.8	A
13438	Bn-N12-p26016599	Bn-C2-p31819805	Bn-scaff_23401_1-p204764	C2	21	29.8	A
13439	Bn-N12-p26116413	Bn-C2-p31931463	Bn-scaff_23401_1-p316422	C2	21	29.8	A
13440	Bn-N12-p26133662	Bn-C2-p31948044	Bn-scaff_23401_1-p333003	C2	21	29.8	A
13441	Bn-N12-p26149924	Bn-C2-p31964459	Bn-scaff_23401_1-p349418	C2	21	29.8	A
13442	Bn-N12-p26150604	Bn-C2-p31965138	Bn-scaff_23401_1-p350097	C2	21	29.8	A
13443	Bn-N12-p26162618	Bn-C2-p31976520	Bn-scaff_23401_1-p361479	C2	21	29.8	A
13444	Bn-N12-p26170529	Bn-C2-p31984409	Bn-scaff_23401_1-p369368	C2	21	29.8	A
13445	Bn-N12-p26171423	Bn-C2-p31985303	Bn-scaff_23401_1-p370262	C2	21	29.8	A
13446	Bn-N12-p26171499	Bn-C2-p31985379	Bn-scaff_23401_1-p370338	C2	21	29.8	A
13447	Bn-N12-p26186441	Bn-C2-p32001052	Bn-scaff_23401_1-p386011	C2	21	29.8	A
13448	Bn-N12-p26187301	Bn-C2-p32001912	Bn-scaff_23401_1-p386871	C2	21	29.8	A
13449	Bn-N12-p26187599	Bn-C2-p32002210	Bn-scaff_23401_1-p387169	C2	21	29.8	A
13450	Bn-N12-p26187845	Bn-C2-p32002456	Bn-scaff_23401_1-p387415	C2	21	29.8	A
13451	Bn-N12-p26187901	Bn-C2-p32002512	Bn-scaff_23401_1-p387471	C2	21	29.8	A
13452	Bn-N12-p26188807	Bn-C2-p32003418	Bn-scaff_23401_1-p388377	C2	21	29.8	A
13453	Bn-N12-p26188951	Bn-C2-p32003562	Bn-scaff_23401_1-p388521	C2	21	29.8	A
13454	Bn-N12-p26189162	Bn-C2-p32003773	Bn-scaff_23401_1-p388732	C2	21	29.8	A
13455	Bn-N12-p26189328	Bn-C2-p32003939	Bn-scaff_23401_1-p388898	C2	21	29.8	A
13456	Bn-N12-p26189648	Bn-C2-p32004259	Bn-scaff_23401_1-p389218	C2	21	29.8	A
13457	Bn-N12-p26189649	Bn-C2-p32004260	Bn-scaff_23401_1-p389219	C2	21	29.8	A
13458	Bn-N12-p26189724	Bn-C2-p32004335	Bn-scaff_23401_1-p389294	C2	21	29.8	A
13459	Bn-N12-p26189811	Bn-C2-p32004422	Bn-scaff_23401_1-p389381	C2	21	29.8	A
13460	Bn-N12-p26190099	Bn-C2-p32004710	Bn-scaff_23401_1-p389669	C2	21	29.8	A
13461	Bn-N12-p26190259	Bn-C2-p32004870	Bn-scaff_23401_1-p389829	C2	21	29.8	A
13462	Bn-N12-p26190279	Bn-C2-p32004890	Bn-scaff_23401_1-p389849	C2	21	29.8	A
13463	Bn-N12-p26190335	Bn-C2-p32004946	Bn-scaff_23401_1-p389905	C2	21	29.8	A
13464	Bn-N12-p26190483	Bn-C2-p32005094	Bn-scaff_23401_1-p390053	C2	21	29.8	A
13465	Bn-N12-p26190698	Bn-C2-p32005309	Bn-scaff_23401_1-p390268	C2	21	29.8	A
13466	Bn-N12-p26192237	Bn-C2-p32006848	Bn-scaff_23401_1-p391807	C2	21	29.8	A
13467	Bn-N12-p26192594	Bn-C2-p32007205	Bn-scaff_23401_1-p392164	C2	21	29.8	A
13468	Bn-N12-p26194012	Bn-C2-p32008623	Bn-scaff_23401_1-p393582	C2	21	29.8	A
13469	Bn-N12-p26194316	Bn-C2-p32008927	Bn-scaff_23401_1-p393886	C2	21	29.8	A
13470	Bn-N12-p26194846	Bn-C2-p32009457	Bn-scaff_23401_1-p394416	C2	21	29.8	A

13471	Bn-N12-p26195303	Bn-C2-p32009914	Bn-scaff_23401_1-p394873	C2	21	29.8	A
13472	Bn-N12-p26196058	Bn-C2-p32010553	Bn-scaff_23401_1-p395512	C2	21	29.8	A
13473	Bn-N12-p26196317	Bn-C2-p32010812	Bn-scaff_23401_1-p395771	C2	21	29.8	A
13474	Bn-N12-p26197030	Bn-C2-p32011525	Bn-scaff_23401_1-p396484	C2	21	29.8	A
13475	Bn-N12-p26197150	Bn-C2-p32011645	Bn-scaff_23401_1-p396604	C2	21	29.8	A
13476	Bn-N12-p26199050	Bn-C2-p32013545	Bn-scaff_23401_1-p398504	C2	21	29.8	A
13477	Bn-N12-p26199418	Bn-C2-p32013913	Bn-scaff_23401_1-p398872	C2	21	29.8	A
13478	Bn-N12-p26199557	Bn-C2-p32014052	Bn-scaff_23401_1-p399011	C2	21	29.8	A
13479	Bn-N12-p26200853	Bn-C2-p32015348	Bn-scaff_23401_1-p400307	C2	21	29.8	A
13480	Bn-N12-p26200951	Bn-C2-p32015446	Bn-scaff_23401_1-p400405	C2	21	29.8	A
13481	Bn-N12-p26201458	Bn-C2-p32015953	Bn-scaff_23401_1-p400912	C2	21	29.8	A
13482	Bn-N12-p26204408	Bn-C2-p32018575	Bn-scaff_23401_1-p403534	C2	21	29.8	A
13483	Bn-N12-p26206078	Bn-C2-p32020245	Bn-scaff_23401_1-p405204	C2	21	29.8	A
13484	Bn-N12-p26228851	Bn-C2-p32041974	Bn-scaff_23401_1-p426933	C2	21	29.8	A
13485	Bn-N12-p26230142	Bn-C2-p32043266	Bn-scaff_23401_1-p428225	C2	21	29.8	A
13486	Bn-N12-p26258685	Bn-C2-p32085041	Bn-scaff_16328_1-p21929	C2	21	29.8	A
13487	Bn-N12-p26262416	Bn-C2-p32088662	Bn-scaff_16328_1-p25550	C2	21	29.8	A
13488	Bn-N12-p26265323	Bn-C2-p32091566	Bn-scaff_16328_1-p28454	C2	21	29.8	A
13489	Bn-N12-p26268029	Bn-C2-p32094272	Bn-scaff_16328_1-p31160	C2	21	29.8	A
13490	Bn-N12-p26268996	Bn-C2-p32095239	Bn-scaff_16328_1-p32127	C2	21	29.8	A
13491	Bn-N12-p26269069	Bn-C2-p32095312	Bn-scaff_16328_1-p32200	C2	21	29.8	A
13492	Bn-N12-p26269820	Bn-C2-p32096063	Bn-scaff_16328_1-p32951	C2	21	29.8	A
13493	Bn-N12-p26269986	Bn-C2-p32096229	Bn-scaff_16328_1-p33117	C2	21	29.8	A
13494	Bn-N12-p26270544	Bn-C2-p32096787	Bn-scaff_16328_1-p33675	C2	21	29.8	A
13495	Bn-N12-p26270644	Bn-C2-p32096887	Bn-scaff_16328_1-p33775	C2	21	29.8	A
13496	Bn-N12-p26270980	Bn-C2-p32097223	Bn-scaff_16328_1-p34111	C2	21	29.8	A
13497	Bn-N12-p26280539	Bn-C2-p32106587	Bn-scaff_16328_1-p43475	C2	21	29.8	A
13498	Bn-N12-p26293540	Bn-C2-p32062074	Bn-scaff_23401_1-p447033	C2	21	29.8	A
13499	Bn-N12-p26294137	Bn-C2-p32062671	Bn-scaff_23401_1-p447630	C2	21	29.8	A
13500	Bn-N12-p26294261	Bn-C2-p32062795	Bn-scaff_23401_1-p447754	C2	21	29.8	A
13501	Bn-N12-p26295757	Bn-C14160250-p885	Bn-C14160250-p885	C2	21	29.8	A
13502	Bn-N12-p26300175	Bn-C2-p32113003	Bn-scaff_16328_1-p49891	C2	21	29.8	A
13503	Bn-N12-p26300230	Bn-C2-p32113058	Bn-scaff_16328_1-p49946	C2	21	29.8	A
13504	Bn-N12-p26305695	Bn-C2-p32118545	Bn-scaff_16328_1-p55433	C2	21	29.8	A
13505	Bn-N12-p26305993	Bn-C2-p32118843	Bn-scaff_16328_1-p55731	C2	21	29.8	A
13506	Bn-N12-p26306140	Bn-C2-p32118990	Bn-scaff_16328_1-p55878	C2	21	29.8	A
13507	Bn-N12-p26306412	Bn-C2-p32119263	Bn-scaff_16328_1-p56151	C2	21	29.8	A
13508	Bn-N12-p26306550	Bn-C2-p32119400	Bn-scaff_16328_1-p56288	C2	21	29.8	A
13509	Bn-N12-p26306604	Bn-C2-p32119454	Bn-scaff_16328_1-p56342	C2	21	29.8	A
13510	Bn-N12-p26306903	Bn-C2-p32119753	Bn-scaff_16328_1-p56641	C2	21	29.8	A
13511	Bn-N12-p26307126	Bn-C2-p32119976	Bn-scaff_16328_1-p56864	C2	21	29.8	A
13512	Bn-N12-p26307886	Bn-C2-p32120736	Bn-scaff_16328_1-p57624	C2	21	29.8	A
13513	Bn-N12-p26307888	Bn-C2-p32120738	Bn-scaff_16328_1-p57626	C2	21	29.8	A
13514	Bn-N12-p26307987	Bn-C2-p32120837	Bn-scaff_16328_1-p57725	C2	21	29.8	A
13515	Bn-N12-p26308193	Bn-C2-p32121043	Bn-scaff_16328_1-p57931	C2	21	29.8	A
13516	Bn-N12-p26309140	Bn-C2-p32121990	Bn-scaff_16328_1-p58878	C2	21	29.8	A
13517	Bn-N12-p26331470	Bn-C2-p32134967	Bn-scaff_16328_1-p71855	C2	21	29.8	A
13518	Bn-N12-p26332190	Bn-C2-p32135687	Bn-scaff_16328_1-p72575	C2	21	29.8	A
13519	Bn-N12-p26333574	Bn-C2-p32142472	Bn-scaff_16328_1-p79360	C2	21	29.8	A

13520	Bn-N12-p26335848	Bn-C2-p32144746	Bn-scaff_16328_1-p81634	C2	21	29.8	A
13521	Bn-N12-p26338822	Bn-C2-p32147720	Bn-scaff_16328_1-p84608	C2	21	29.8	A
13522	Bn-N12-p26339002	Bn-C2-p32147900	Bn-scaff_16328_1-p84788	C2	21	29.8	A
13523	Bn-N12-p26339154	Bn-C2-p32148052	Bn-scaff_16328_1-p84940	C2	21	29.8	A
13524	Bn-N12-p26340048	Bn-C2-p32148946	Bn-scaff_16328_1-p85834	C2	21	29.8	A
13525	Bn-N12-p26345904	Bn-C2-p32154830	Bn-scaff_16328_1-p91718	C2	21	29.8	A
13526	Bn-N12-p26346727	Bn-C2-p32155653	Bn-scaff_16328_1-p92541	C2	21	29.8	A
13527	Bn-N12-p26347342	Bn-C2-p32156268	Bn-scaff_16328_1-p93156	C2	21	29.8	A
13528	Bn-N12-p26348174	Bn-C2-p32157100	Bn-scaff_16328_1-p93988	C2	21	29.8	A
13529	Bn-N12-p26349642	Bn-C2-p32158568	Bn-scaff_16328_1-p95456	C2	21	29.8	A
13530	Bn-N12-p26359770	Bn-C2-p32169505	Bn-scaff_16328_1-p106393	C2	21	29.8	A
13531	Bn-N12-p26361725	Bn-C2-p32171723	Bn-scaff_16328_1-p108611	C2	21	29.8	A
13532	Bn-N12-p26362290	Bn-C2-p32172288	Bn-scaff_16328_1-p109176	C2	21	29.8	A
13533	Bn-N12-p26367078	Bn-C2-p32177074	Bn-scaff_16328_1-p113962	C2	21	29.8	A
13534	Bn-N12-p26367803	Bn-C2-p32177799	Bn-scaff_16328_1-p114687	C2	21	29.8	A
13535	Bn-N12-p26368420	Bn-C2-p32178416	Bn-scaff_16328_1-p115304	C2	21	29.8	A
13536	Bn-N12-p26413199	Bn-C2-p32220313	Bn-scaff_16328_1-p157201	C2	21	29.8	A
13537	Bn-N12-p26415138	Bn-C2-p32222252	Bn-scaff_16328_1-p159140	C2	21	29.8	A
13538	Bn-N12-p26417776	Bn-C4-p29539702	Bn-scaff_17180_1-p431356	C2	21	29.8	A
13539	Bn-N12-p26440391	Bn-C2-p32247277	Bn-scaff_16328_1-p184165	C2	21	29.8	A
13540	Bn-N12-p26448661	Bn-C2-p32256924	Bn-scaff_16328_1-p193812	C2	21	29.8	A
13541	Bn-N12-p26451965	Bn-C2-p32259299	Bn-scaff_16328_1-p196187	C2	21	29.8	A
13542	Bn-N12-p26456968	Bn-C2-p32261354	Bn-scaff_16328_1-p198242	C2	21	29.8	A
13543	Bn-N12-p26458551	Bn-C2-p32263506	Bn-scaff_16328_1-p200394	C2	21	29.8	A
13544	Bn-N12-p26460146	Bn-C2-p32272978	Bn-scaff_16328_1-p209866	C2	21	29.8	A
13545	Bn-N12-p26464736	Bn-C2-p32277769	Bn-scaff_16328_1-p214657	C2	21	29.8	A
13546	Bn-N12-p26567921	Bn-C2-p32388663	Bn-scaff_16328_1-p325551	C2	21	29.8	A
13547	Bn-N12-p26576187	Bn-C2-p32395435	Bn-scaff_16328_1-p332323	C2	21	29.8	A
13548	Bn-N12-p26578889	Bn-C2-p32398008	Bn-scaff_16328_1-p334896	C2	21	29.8	A
13549	Bn-N12-p26650208	Bn-C2-p32468174	Bn-scaff_16328_1-p405062	C2	21	29.8	A
13550	Bn-N12-p26714576	Bn-C2-p32544453	Bn-scaff_16328_1-p481341	C2	21	29.8	A
13551	Bn-N12-p26722593	Bn-C2-p32552438	Bn-scaff_16328_1-p489326	C2	21	29.8	A
13552	Bn-N12-p26724390	Bn-C2-p32554226	Bn-scaff_16328_1-p491114	C2	21	29.8	A
13553	Bn-N12-p26726239	Bn-C2-p32556073	Bn-scaff_16328_1-p492961	C2	21	29.8	A
13554	Bn-N12-p26727961	Bn-C2-p32560064	Bn-scaff_16328_1-p496952	C2	21	29.8	A
13555	Bn-N12-p26728086	Bn-C2-p32560191	Bn-scaff_16328_1-p497079	C2	21	29.8	A
13556	Bn-N12-p26737696	Bn-C2-p32569515	Bn-scaff_16328_1-p506403	C2	21	29.8	A
13557	Bn-N12-p26752013	Bn-C2-p32588191	Bn-scaff_16328_1-p525079	C2	21	29.8	A
13558	Bn-N12-p26764791	Bn-C2-p32600790	Bn-scaff_16328_1-p537678	C2	21	29.8	A
13559	Bn-N12-p26772873	Bn-C2-p32611096	Bn-scaff_16328_1-p547984	C2	21	29.8	A
13560	Bn-N12-p26773769	Bn-C2-p32611992	Bn-scaff_16328_1-p548880	C2	21	29.8	A
13561	Bn-N12-p26773937	Bn-C2-p32612160	Bn-scaff_16328_1-p549048	C2	21	29.8	A
13562	Bn-N12-p26774277	Bn-C2-p32612500	Bn-scaff_16328_1-p549388	C2	21	29.8	A
13563	Bn-N12-p26805159	Bn-C2-p32634952	Bn-scaff_16328_1-p571840	C2	21	29.8	A
13564	Bn-N12-p26810022	Bn-C2-p32635329	Bn-scaff_16328_1-p572217	C2	21	29.8	A
13565	Bn-N12-p26818623	Bn-C2-p32643944	Bn-scaff_16328_1-p580832	C2	21	29.8	A
13566	Bn-N12-p26818891	Bn-C2-p32644212	Bn-scaff_16328_1-p581100	C2	21	29.8	A
13567	Bn-N12-p26820230	Bn-C2-p32645551	Bn-scaff_16328_1-p582439	C2	21	29.8	A
13568	Bn-N12-p26820495	Bn-C2-p32645838	Bn-scaff_16328_1-p582726	C2	21	29.8	A

13569	Bn-N12-p26820759	Bn-C2-p32646102	Bn-scaff_16328_1-p58299	C2	21	29.8	A
13570	Bn-N12-p26820952	Bn-C2-p32646295	Bn-scaff_16328_1-p58318	C2	21	29.8	A
13571	Bn-N12-p26828142	Bn-C2-p32659448	Bn-scaff_16328_1-p59633	C2	21	29.8	A
13572	Bn-N12-p26831488	Bn-C2-p32662810	Bn-scaff_16328_1-p59969	C2	21	29.8	A
13573	Bn-N12-p26852673	Bn-C2-p32685664	Bn-scaff_16328_1-p62255	C2	21	29.8	A
13574	Bn-N12-p26853599	Bn-C2-p32686630	Bn-scaff_16328_1-p62351	C2	21	29.8	A
13575	Bn-N12-p26855626	Bn-C2-p32688680	Bn-scaff_16328_1-p62556	C2	21	29.8	A
13576	Bn-N12-p26868420	Bn-C2-p32699898	Bn-scaff_16328_1-p63678	C2	21	29.8	A
13577	Bn-N12-p26910673	Bn-C2-p32746793	Bn-scaff_16328_1-p683681	C2	21	29.8	A
13578	Bn-N12-p27971946	Bn-C2-p34851309	Bn-scaff_18245_1-p18409	C2	21	29.8	A
13579	Bn-N12-p29951312	Bn-C2-p37181623	Bn-scaff_16545_1-p85558	C2	21	29.8	A
13580	Bn-N12-p29973913	Bn-C2-p37206785	Bn-scaff_16545_1-p83042	C2	21	29.8	A
13581	Bn-N12-p29973914	Bn-C2-p37206786	Bn-scaff_16545_1-p83042	C2	21	29.8	A
13582	Bn-N12-p29977734	Bn-C2-p37210603	Bn-scaff_16545_1-p82660	C2	21	29.8	A
13583	Bn-N12-p29981591	Bn-C2-p37214460	Bn-scaff_16545_1-p82275	C2	21	29.8	A
13584	Bn-N12-p29987011	Bn-C2-p37219880	Bn-scaff_16545_1-p81733	C2	21	29.8	A
13585	Bn-N12-p30022357	Bn-C2-p37254117	Bn-scaff_16545_1-p78309	C2	21	29.8	A
13586	Bn-N12-p30056571	Bn-C2-p37285344	Bn-scaff_16545_1-p75186	C2	21	29.8	A
13587	Bn-N12-p30065224	Bn-C2-p37293777	Bn-scaff_16545_1-p74343	C2	21	29.8	A
13588	Bn-N12-p30065938	Bn-C2-p37294492	Bn-scaff_16545_1-p74272	C2	21	29.8	A
13589	Bn-N12-p30067100	Bn-C2-p37295654	Bn-scaff_16545_1-p74155	C2	21	29.8	A
13590	Bn-N12-p30067891	Bn-C2-p37296445	Bn-scaff_16545_1-p74076	C2	21	29.8	A
13591	Bn-N12-p30081737	Bn-C2-p37310063	Bn-scaff_16545_1-p72714	C2	21	29.8	A
13592	Bn-N12-p30096572	Bn-C2-p37324899	Bn-scaff_16545_1-p71231	C2	21	29.8	A
13593	Bn-N12-p30164809	Bn-C2-p37385186	Bn-scaff_16545_1-p65202	C2	21	29.8	A
13594	Bn-N12-p30172925	Bn-C2-p37395605	Bn-scaff_16545_1-p64160	C2	21	29.8	A
13595	Bn-N12-p30274348	Bn-C2-p37494143	Bn-scaff_16545_1-p54306	C2	21	29.8	A
13596	Bn-N12-p30274896	Bn-C2-p37494691	Bn-scaff_16545_1-p542521	C2	21	29.8	A
13597	Bn-N12-p30281541	Bn-C2-p37501332	Bn-scaff_16545_1-p53588	C2	21	29.8	A
13598	Bn-N12-p30319163	Bn-C2-p37540869	Bn-scaff_16545_1-p49634	C2	21	29.8	A
13599	Bn-N12-p30344870	Bn-C2-p37563587	Bn-scaff_16545_1-p47362	C2	21	29.8	A
13600	Bn-N12-p30345832	Bn-C2-p37564557	Bn-scaff_16545_1-p47265	C2	21	29.8	A
13601	Bn-N12-p30347202	Bn-C2-p37565927	Bn-scaff_16545_1-p47128	C2	21	29.8	A
13602	Bn-N12-p30368435	Bn-C2-p37588034	Bn-scaff_16545_1-p44917	C2	21	29.8	A
13603	Bn-N12-p30373479	Bn-C2-p37593029	Bn-scaff_16545_1-p44418	C2	21	29.8	A
13604	Bn-N12-p30399644	Bn-C2-p37619453	Bn-scaff_16545_1-p41775	C2	21	29.8	A
13605	Bn-N12-p30416037	Bn-Scaffold01184-p1	Bn-scaff_21552_1-p17328	C2	21	29.8	A
13606	Bn-N12-p30508651	Bn-C2-p37680440	Bn-scaff_16545_1-p35677	C2	21	29.8	A
13607	Bn-N12-p30510455	Bn-C2-p37682243	Bn-scaff_16545_1-p35496	C2	21	29.8	A
13608	Bn-N12-p30515913	Bn-C2-p37687824	Bn-scaff_16545_1-p34938	C2	21	29.8	A
13609	Bn-N12-p30667874	Bn-C2-p38147066	Bn-scaff_26023_1-p10596	C2	21	29.8	A
13610	Bn-N12-p30711719	Bn-C2-p38197716	Bn-scaff_23104_1-p40868	C2	21	29.8	A
13611	Bn-N12-p30865553	Bn-C2-p38345381	Bn-scaff_23104_1-p26102	C2	21	29.8	A
13612	Bn-N12-p30865710	Bn-C2-p38345538	Bn-scaff_23104_1-p26086	C2	21	29.8	A
13613	Bn-N12-p30894939	Bn-C2-p38374264	Bn-scaff_23104_1-p23213	C2	21	29.8	A
13614	Bn-N12-p30935964	Bn-C2-p38415038	Bn-scaff_23104_1-p19136	C2	21	29.8	A
13615	Bn-N12-p31068687	Bn-C2-p38564188	Bn-scaff_23104_1-p42215	C2	21	29.8	A
13616	Bn-N12-p31209137	Bn-Scaffold00418-p1	Bn-scaff_21131_1-p16768	C2	21	29.8	A
13617	Bn-N12-p31488960	Bn-C2-p38901825	Bn-scaff_16449_1-p10164	C2	21	29.8	A

13618	Bn-N12-p31515527	Bn-C2-p38928501	Bn-scaff_16449_1-p98978	C2	21	29.8	A
13619	Bn-N12-p31596925	Bn-C2-p39005818	Bn-scaff_16449_1-p91246	C2	21	29.8	A
13620	Bn-N12-p31623395	Bn-C2-p39042310	Bn-scaff_16449_1-p87597	C2	21	29.8	A
13621	Bn-N12-p31638130	Bn-C2-p39067758	Bn-scaff_16449_1-p85052	C2	21	29.8	A
13622	Bn-N12-p31790175	Bn-C2-p39206630	Bn-scaff_16449_1-p71165	C2	21	29.8	A
13623	Bn-N12-p31871586	Bn-C2-p39248088	Bn-scaff_16449_1-p67019	C2	21	29.8	A
13624	Bn-N15-p28103924	Bn-C5-p27002289	Bn-scaff_22099_1-p25174	C2	21	29.8	A
13625	Bn-N15-p4440761	Bn-C2-p29967725	Bn-scaff_20221_1-p84872	C2	21	29.8	A
13626	Bn-N16-p39272178	Bn-C2-p38764329	Bn-scaff_16298_1-p15782	C2	21	29.8	A
13627	Bn-N16-p39272458	Bn-C2-p38764609	Bn-scaff_16298_1-p15810	C2	21	29.8	A
13628	Bn-N16-p39332033	Bn-C2-p38821738	Bn-scaff_16298_1-p21523	C2	21	29.8	A
13629	Bn-N16-p39394124	Bn-C2-p38873910	Bn-scaff_16298_1-p26740	C2	21	29.8	A
13630	Bn-N2-p17657106	Bn-C2-p31918787	Bn-scaff_23401_1-p30374	C2	21	29.8	A
13631	Bn-Scaffold01075-p24883	Bn-C2-p38652864	Bn-scaff_16298_1-p46362	C2	21	29.8	A
13632	Bn-Scaffold02442-p3912	Bn-C2-p37510836	Bn-scaff_16545_1-p52637	C2	21	29.8	A
13633	Bn-Scaffold03421-p2519	Bn-Scaffold01010-p3	Bn-scaff_19372_1-p3915	C2	21	29.8	A
13634	Bn-Scaffold24311-p390	Bn-C2-p38175317	Bn-scaff_23104_1-p43108	C2	21	29.8	A
13635	Unknown	Bn-C2-p38682009	Bn-scaff_16298_1-p75507	C2	21	29.8	A
13636	Bn-N12-p20973295	Bn-C9-p8598640	Bn-scaff_17487_1-p74837	C2	22	29.8	A
13637	Bn-N12-p32980114	Bn-C2-p40361876	Bn-scaff_21705_1-p22429	C2	22	29.8	A
13638	Bn-N12-p32987804	Bn-C2-p40369565	Bn-scaff_21705_1-p21660	C2	22	29.8	A
13639	Bn-N12-p32997064	Bn-C2-p40378827	Bn-scaff_21705_1-p20734	C2	22	29.8	A
13640	Bn-N12-p32997611	Bn-C2-p40379374	Bn-scaff_21705_1-p20679	C2	22	29.8	A
13641	Bn-N12-p32998290	Bn-C2-p40380053	Bn-scaff_21705_1-p20611	C2	22	29.8	A
13642	Bn-N12-p33000454	Bn-C2-p40382217	Bn-scaff_21705_1-p20395	C2	22	29.8	A
13643	Bn-N12-p33023859	Bn-C2-p40411162	Bn-scaff_21705_1-p17501	C2	22	29.8	A
13644	Bn-N12-p33025778	Bn-C2-p40413081	Bn-scaff_21705_1-p17309	C2	22	29.8	A
13645	Bn-N12-p33051140	Bn-C2-p40436800	Bn-scaff_21705_1-p14937	C2	22	29.8	A
13646	Bn-N12-p33081680	Bn-C2-p40466509	Bn-scaff_21705_1-p11966	C2	22	29.8	A
13647	Bn-N12-p33082045	Bn-C2-p40466875	Bn-scaff_21705_1-p11929	C2	22	29.8	A
13648	Bn-N12-p33141342	Bn-C2-p40534309	Bn-scaff_21705_1-p51863	C2	22	29.8	A
13649	Bn-N12-p33411370	Bn-C2-p40796369	Bn-scaff_17289_1-p67494	C2	22	29.8	A
13650	Bn-N12-p33441790	Bn-C2-p40815609	Bn-scaff_17289_1-p65570	C2	22	29.8	A
13651	Bn-N12-p33635052	Bn-C2-p41012763	Bn-scaff_17289_1-p45854	C2	22	29.8	A
13652	Bn-N2-p14848453	Bn-Scaffold000564-p	Bn-Scaffold000564-p4733	C2	22	29.8	A
13653	Bn-N2-p21591340	Bn-A02-p19052873	Bn-A02-p19052873	C2	22	29.8	A
13654	Bn-N2-p21760848	Bn-C2-p41009774	Bn-scaff_17289_1-p46153	C2	22	29.8	A
13655	Bn-N12-p30962630	Bn-C2-p38441136	Bn-scaff_23104_1-p16526	C2	23	30.6	A
13656	Bn-N12-p33993923	Bn-C2-p41452655	Bn-scaff_17289_1-p18657	C2	23	30.6	A
13657	Bn-N12-p34013662	Bn-C2-p41475606	Bn-scaff_18360_1-p4195	C2	23	30.6	A
13658	Bn-N12-p34143722	Bn-A02-p19717843	Bn-A02-p19717843	C2	23	30.6	A
13659	Bn-N12-p34155889	Bn-C2-p41623284	Bn-scaff_18360_1-p15187	C2	23	30.6	A
13660	Bn-N12-p34253460	Bn-C2-p41669140	Bn-scaff_18360_1-p19772	C2	23	30.6	A
13661	Bn-N12-p34390482	Bn-C2-p41835524	Bn-scaff_18360_1-p36411	C2	23	30.6	A
13662	Bn-N12-p34458282	Bn-C3-p24936161	Bn-scaff_17298_1-p72048	C2	23	30.6	A
13663	Bn-N12-p34666411	Bn-C2-p42102165	Bn-scaff_18360_1-p63075	C2	23	30.6	A
13664	Bn-N12-p34954671	Bn-C2-p42341949	Bn-scaff_18360_1-p87053	C2	23	30.6	A
13665	Bn-N12-p35577944	Bn-C2-p42960500	Bn-scaff_15712_6-p12744	C2	23	30.6	A
13666	Bn-N12-p35598457	Bn-C2-p42990299	Bn-scaff_15712_6-p15724	C2	23	30.6	A

13667	Bn-N12-p35616226	Bn-C2-p43017775	Bn-scaff_15712_6-p184722	C2	23	30.6	A
13668	Bn-N12-p35622484	Bn-C2-p43019379	Bn-scaff_15712_6-p186326	C2	23	30.6	A
13669	Bn-N12-p35629888	Bn-C2-p43023296	Bn-scaff_15712_6-p190243	C2	23	30.6	A
13670	Bn-N12-p35630008	Bn-C2-p43023416	Bn-scaff_15712_6-p190363	C2	23	30.6	A
13671	Bn-N12-p35642727	Bn-C2-p43040258	Bn-scaff_15712_6-p207205	C2	23	30.6	A
13672	Bn-N12-p35649637	Bn-C2-p43047282	Bn-scaff_15712_6-p214229	C2	23	30.6	A
13673	Bn-N12-p35675873	Bn-C2-p43073844	Bn-scaff_15712_6-p240791	C2	23	30.6	A
13674	Bn-N12-p35676097	Bn-C2-p43074069	Bn-scaff_15712_6-p241016	C2	23	30.6	A
13675	Bn-N12-p35691261	Bn-C2-p43094842	Bn-scaff_15712_6-p261789	C2	23	30.6	A
13676	Bn-N12-p35699434	Bn-C2-p43107004	Bn-scaff_15712_6-p273951	C2	23	30.6	A
13677	Bn-N12-p35699448	Bn-C2-p43107018	Bn-scaff_15712_6-p273965	C2	23	30.6	A
13678	Bn-N12-p35708916	Bn-C2-p43115664	Bn-scaff_15712_6-p282611	C2	23	30.6	A
13679	Bn-N12-p35713815	Bn-C2-p43125524	Bn-scaff_15712_6-p292471	C2	23	30.6	A
13680	Bn-N12-p35753179	Bn-C2-p43165142	Bn-scaff_15712_6-p332089	C2	23	30.6	A
13681	Bn-N12-p35764942	Bn-C2-p43179017	Bn-scaff_15712_6-p345964	C2	23	30.6	A
13682	Bn-N12-p35765047	Bn-C2-p43179122	Bn-scaff_15712_6-p346069	C2	23	30.6	A
13683	Bn-N12-p35784219	Bn-C2-p43190977	Bn-scaff_15712_6-p357924	C2	23	30.6	A
13684	Bn-N12-p35847923	Bn-Scaffold01367-p1(	Bn-scaff_17122_1-p1008	C2	23	30.6	A
13685	Bn-N12-p35864821	Bn-C13496975-p159	Bn-C13496975-p159	C2	23	30.6	A
13686	Bn-N12-p36031075	Bn-C2-p43394551	Bn-scaff_15712_6-p561498	C2	23	30.6	A
13687	Bn-N12-p36041273	Bn-C2-p43404775	Bn-scaff_15712_6-p571722	C2	23	30.6	A
13688	Bn-N12-p36042483	Bn-C2-p43405979	Bn-scaff_15712_6-p572926	C2	23	30.6	A
13689	Bn-N12-p36044282	Bn-C2-p43407780	Bn-scaff_15712_6-p574727	C2	23	30.6	A
13690	Bn-N12-p36044388	Bn-C2-p43407887	Bn-scaff_15712_6-p574834	C2	23	30.6	A
13691	Bn-N12-p36052747	Bn-C2-p43411714	Bn-scaff_15712_6-p578661	C2	23	30.6	A
13692	Bn-N12-p36058732	Bn-C2-p43417723	Bn-scaff_15712_6-p584670	C2	23	30.6	A
13693	Bn-N12-p36059458	Bn-C2-p43418448	Bn-scaff_15712_6-p585395	C2	23	30.6	A
13694	Bn-N12-p36077001	Bn-C2-p43432608	Bn-scaff_15712_6-p599555	C2	23	30.6	A
13695	Bn-N12-p36077513	Bn-C2-p43433120	Bn-scaff_15712_6-p600067	C2	23	30.6	A
13696	Bn-N12-p36077707	Bn-C2-p43433314	Bn-scaff_15712_6-p600261	C2	23	30.6	A
13697	Bn-N12-p36077903	Bn-C2-p43433510	Bn-scaff_15712_6-p600457	C2	23	30.6	A
13698	Bn-N12-p36077984	Bn-C2-p43433591	Bn-scaff_15712_6-p600538	C2	23	30.6	A
13699	Bn-N12-p36078578	Bn-C2-p43434194	Bn-scaff_15712_6-p601141	C2	23	30.6	A
13700	Bn-N12-p36078645	Bn-C2-p43434261	Bn-scaff_15712_6-p601208	C2	23	30.6	A
13701	Bn-N12-p36079693	Bn-C2-p43435309	Bn-scaff_15712_6-p602256	C2	23	30.6	A
13702	Bn-N12-p36124076	Bn-C2-p43482022	Bn-scaff_15712_6-p648969	C2	23	30.6	A
13703	Bn-N12-p36141571	Bn-C2-p43495160	Bn-scaff_15712_6-p662107	C2	23	30.6	A
13704	Bn-N12-p36249744	Bn-C2-p43608836	Bn-scaff_15712_6-p775783	C2	23	30.6	A
13705	Bn-N12-p36277091	Bn-C2-p43635023	Bn-scaff_15712_6-p801970	C2	23	30.6	A
13706	Bn-N12-p36277830	Bn-C2-p43635761	Bn-scaff_15712_6-p802708	C2	23	30.6	A
13707	Bn-N12-p36278296	Bn-C2-p43636228	Bn-scaff_15712_6-p803175	C2	23	30.6	A
13708	Bn-N12-p36278502	Bn-C2-p43636434	Bn-scaff_15712_6-p803381	C2	23	30.6	A
13709	Bn-N12-p36310024	Bn-C2-p43644794	Bn-scaff_15712_6-p811741	C2	23	30.6	A
13710	Bn-N12-p36314937	Bn-C2-p43645857	Bn-scaff_15712_6-p812804	C2	23	30.6	A
13711	Bn-N12-p36315209	Bn-C2-p43646129	Bn-scaff_15712_6-p813076	C2	23	30.6	A
13712	Bn-N12-p36316821	Bn-C2-p43650093	Bn-scaff_15712_6-p817040	C2	23	30.6	A
13713	Bn-N12-p36334266	Bn-C2-p43667571	Bn-scaff_15712_6-p834518	C2	23	30.6	A
13714	Bn-N12-p36334323	Bn-C2-p43667628	Bn-scaff_15712_6-p834575	C2	23	30.6	A
13715	Bn-N12-p36334325	Bn-C2-p43667630	Bn-scaff_15712_6-p834577	C2	23	30.6	A

13716	Bn-N12-p36346769	Bn-C2-p43685794	Bn-scaff_15712_6-p852741	C2	23	30.6	A
13717	Bn-N12-p36441719	Bn-C2-p43765283	Bn-scaff_15712_6-p93223	C2	23	30.6	A
13718	Bn-N12-p36447814	Bn-C2-p43767465	Bn-scaff_15712_6-p93441	C2	23	30.6	A
13719	Bn-N12-p36497080	Bn-C2-p43838092	Bn-scaff_15712_6-p10050	C2	23	30.6	A
13720	Bn-N12-p36566038	Bn-C2-p43892155	Bn-scaff_15712_6-p10591	C2	23	30.6	A
13721	Bn-N12-p36698228	Bn-C2-p44042485	Bn-scaff_15712_6-p12094	C2	23	30.6	A
13722	Bn-N12-p36714945	Bn-C2-p44074294	Bn-scaff_15712_6-p12412	C2	23	30.6	A
13723	Bn-N12-p36894742	Bn-C2-p44221762	Bn-scaff_15712_2-p18850	C2	23	30.6	A
13724	Bn-N12-p36895178	Bn-C2-p44222188	Bn-scaff_15712_2-p19276	C2	23	30.6	A
13725	Bn-N12-p36938036	Bn-C2-p44276943	Bn-scaff_15712_2-p74031	C2	23	30.6	A
13726	Bn-N12-p36965541	Bn-C2-p44307534	Bn-scaff_15712_2-p10462	C2	23	30.6	A
13727	Bn-N12-p37264603	Bn-C2-p44568521	Bn-scaff_15712_2-p36560	C2	23	30.6	A
13728	Bn-N12-p37434976	Bn-C2-p44715308	Bn-scaff_15712_2-p51239	C2	23	30.6	A
13729	Bn-N15-p28702121	Bn-C5-p27664100	Bn-scaff_15648_1-p27124	C2	23	30.6	A
13730	Bn-N2-p23510539	Bn-C2-p43303345	Bn-scaff_15712_6-p47029	C2	23	30.6	A
13731	Bn-N12-p23949867	Bn-C2-p29443551	Bn-scaff_18199_1-p26627	C2	24	33.1	A
13732	Bn-N12-p37700534	Bn-C2-p44970383	Bn-scaff_15712_2-p767471	C2	24	33.1	A
13733	Bn-N12-p37704366	Bn-C2-p44974215	Bn-scaff_15712_2-p77130	C2	24	33.1	A
13734	Bn-N12-p38135032	Bn-Scaffold00285-p2	Bn-scaff_15712_9-p55269	C2	24	33.1	A
13735	Bn-N12-p38201135	Bn-Scaffold00285-p3	Bn-scaff_15712_9-p62111	C2	24	33.1	A
13736	Bn-N12-p38214040	Bn-Scaffold00285-p3	Bn-scaff_15712_9-p63476	C2	24	33.1	A
13737	Bn-N12-p38392821	Bn-Scaffold00285-p5	Bn-scaff_15712_9-p80146	C2	24	33.1	A
13738	Bn-N12-p38412000	Bn-Scaffold00285-p5	Bn-scaff_15712_9-p822331	C2	24	33.1	A
13739	Bn-N12-p38594202	Bn-C2-p45299880	Bn-scaff_22144_1-p20784	C2	24	33.1	A
13740	Bn-N12-p38611586	Bn-C2-p45315597	Bn-scaff_22144_1-p22356	C2	24	33.1	A
13741	Bn-N12-p38631428	Bn-C9-p44243832	Bn-scaff_17088_1-p4312	C2	24	33.1	A
13742	Bn-N12-p38931227	Bn-C2-p45581279	Bn-scaff_16209_1-p12895	C2	24	33.1	A
13743	Bn-N12-p38940207	Bn-C2-p45593219	Bn-scaff_16209_1-p11701	C2	24	33.1	A
13744	Bn-N12-p39217401	Bn-A02-p21713818	Bn-A02-p21713818	C2	24	33.1	A
13745	Bn-N12-p39217463	Bn-A02-p21713756	Bn-A02-p21713756	C2	24	33.1	A
13746	Bn-N12-p39235226	Bn-C2-p45916432	Bn-scaff_17831_1-p206101	C2	24	33.1	A
13747	Bn-N12-p39239883	Bn-C2-p45921458	Bn-scaff_17831_1-p21112	C2	24	33.1	A
13748	Bn-N12-p39499870	Bn-Scaffold00992-p2	Bn-scaff_26712_1-p24644	C2	25	33.9	A
13749	Bn-N12-p39500318	Bn-Scaffold00992-p2	Bn-scaff_26712_1-p25092	C2	25	33.9	A
13750	Bn-N12-p39500508	Bn-Scaffold00992-p2	Bn-scaff_26712_1-p25284	C2	25	33.9	A
13751	Bn-N12-p39501053	Bn-Scaffold00992-p2	Bn-scaff_26712_1-p25842	C2	25	33.9	A
13752	Bn-N12-p39501250	Bn-Scaffold00992-p2	Bn-scaff_26712_1-p26029	C2	25	33.9	A
13753	Bn-N12-p39531765	Bn-C2-p46193494	Bn-scaff_17109_4-p30727	C2	25	33.9	A
13754	Bn-N12-p39531860	Bn-C2-p46193590	Bn-scaff_17109_4-p30717	C2	25	33.9	A
13755	Bn-N12-p39537468	Bn-C2-p46198466	Bn-scaff_17109_4-p30229	C2	25	33.9	A
13756	Bn-N12-p39537627	Bn-C2-p46198624	Bn-scaff_17109_4-p30214	C2	25	33.9	A
13757	Bn-N12-p39539197	Bn-C2-p46200447	Bn-scaff_17109_4-p30031	C2	25	33.9	A
13758	Bn-N12-p39539847	Bn-C2-p46201931	Bn-scaff_17109_4-p29883	C2	25	33.9	A
13759	Bn-N12-p39544144	Bn-C2-p46217719	Bn-scaff_17109_4-p28304	C2	25	33.9	A
13760	Bn-N12-p39583257	Bn-C2-p46341547	Bn-scaff_17109_4-p15921	C2	25	33.9	A
13761	Bn-N12-p39591477	Bn-C2-p46349806	Bn-scaff_17109_4-p15095	C2	25	33.9	A
13762	Bn-N12-p39622498	Bn-C2-p46388838	Bn-scaff_17109_4-p11192	C2	25	33.9	A
13763	Bn-N2-p25339339	Bn-Scaffold00992-p2	Bn-scaff_26712_1-p26624	C2	25	33.9	A
13764	Bn-N2-p25392820	Bn-A02-p21489620	Bn-A02-p21489620	C2	25	33.9	A

13765	Bn-N12-p40164030	Bn-C2-p46969947	Bn-scaff_17109_1-p105825	C2	26	35.5	A
13766	Bn-N12-p40357683	Bn-C2-p47138435	Bn-scaff_17109_1-p889768	C2	27	37.1	A
13767	Bn-N2-p26054274	Bn-C2-p47159535	Bn-scaff_17109_1-p868668	C2	27	37.1	A
13768	Bn-N12-p40391461	Bn-C2-p47170554	Bn-scaff_17109_1-p857649	C2	28	37.9	A
13769	Bn-N12-p40412523	Bn-C2-p47190527	Bn-scaff_17109_1-p837676	C2	28	37.9	A
13770	Bn-N12-p40417447	Bn-C2-p47195429	Bn-scaff_17109_1-p832774	C2	28	37.9	A
13771	Bn-N12-p40422883	Bn-C2-p47200887	Bn-scaff_17109_1-p827316	C2	28	37.9	A
13772	Bn-N12-p40544490	Bn-C2-p47336361	Bn-scaff_17109_1-p691842	C2	29	38.7	A
13773	Bn-N12-p40621403	Bn-C2-p47401630	Bn-scaff_17109_1-p626573	C2	29	38.7	A
13774	Bn-N12-p40828857	Bn-C9-p21776536	Bn-scaff_17109_1-p428616	C2	30	39.5	A
13775	Bn-N12-p40838707	Bn-Scaffold00847-p5	Bn-scaff_15712_12-p5675	C2	30	39.5	A
13776	Bn-N12-p40838951	Bn-Scaffold00847-p5	Bn-scaff_15712_12-p5921	C2	30	39.5	A
13777	Bn-N12-p40851833	Bn-Scaffold00847-p2	Bn-scaff_15712_12-p21418	C2	30	39.5	A
13778	Bn-N12-p40050744	Bn-C2-p46860272	Bn-scaff_17109_1-p116793	C2	31	41.1	A
13779	Bn-N12-p40971090	Bn-C2-p47678596	Bn-scaff_15918_1-p82985	C2	31	41.1	A
13780	Bn-N12-p40971890	Bn-C2-p47679396	Bn-scaff_15918_1-p83785	C2	31	41.1	A
13781	Bn-N12-p41038481	Bn-C2-p47792315	Bn-scaff_15918_1-p196704	C2	31	41.1	A
13782	Bn-N12-p41048342	Bn-C2-p47756727	Bn-scaff_15918_1-p161116	C2	31	41.1	A
13783	Bn-N12-p41056747	Bn-C2-p47823363	Bn-scaff_15918_1-p227752	C2	31	41.1	A
13784	Bn-N12-p41058738	Bn-C2-p47825283	Bn-scaff_15918_1-p229672	C2	31	41.1	A
13785	Bn-N12-p41059001	Bn-C2-p47825546	Bn-scaff_15918_1-p229935	C2	31	41.1	A
13786	Bn-N12-p41059053	Bn-C2-p47825598	Bn-scaff_15918_1-p229987	C2	31	41.1	A
13787	Bn-N12-p41062332	Bn-C2-p47826217	Bn-scaff_15918_1-p230606	C2	31	41.1	A
13788	Bn-N12-p41062427	Bn-C2-p47826312	Bn-scaff_15918_1-p230701	C2	31	41.1	A
13789	Bn-N12-p41133522	Bn-C2-p47898905	Bn-scaff_15918_1-p303294	C2	31	41.1	A
13790	Bn-N12-p41134045	Bn-C2-p47899427	Bn-scaff_15918_1-p303816	C2	31	41.1	A
13791	Bn-N12-p41140847	Bn-C2-p47906246	Bn-scaff_15918_1-p310635	C2	31	41.1	A
13792	Bn-N12-p41142342	Bn-C2-p47907751	Bn-scaff_15918_1-p312140	C2	31	41.1	A
13793	Bn-N12-p41142936	Bn-C2-p47908344	Bn-scaff_15918_1-p312733	C2	31	41.1	A
13794	Bn-N12-p41143701	Bn-C2-p47913804	Bn-scaff_15918_1-p318193	C2	31	41.1	A
13795	Bn-N12-p41143975	Bn-C2-p47914076	Bn-scaff_15918_1-p318465	C2	31	41.1	A
13796	Bn-N12-p41334437	Bn-C2-p48091803	Bn-scaff_27946_1-p1047	C2	32	42.7	A
13797	Bn-N12-p41775492	Bn-C2-p48551446	Bn-scaff_17623_1-p599063	C2	33	45.2	A
13798	Bn-N12-p41815038	Bn-C2-p48603850	Bn-scaff_17623_1-p546659	C2	34	50.2	A
13799	Bn-N11-p10640405	Bn-C3-p49912971	Bn-scaff_19740_1-p236040	C2	35	51	A
13800	Bn-N12-p42045007	Bn-C2-p48893518	Bn-scaff_17623_1-p256991	C2	36	51.8	A
13801	Bn-N12-p42045401	Bn-C2-p48893912	Bn-scaff_17623_1-p256597	C2	36	51.8	A
13802	Bn-N12-p42046279	Bn-C2-p48894790	Bn-scaff_17623_1-p255719	C2	36	51.8	A
13803	Bn-N12-p42046853	Bn-C2-p48895364	Bn-scaff_17623_1-p255145	C2	36	51.8	A
13804	Bn-N12-p42047233	Bn-C2-p48895744	Bn-scaff_17623_1-p254765	C2	36	51.8	A
13805	Bn-N12-p42047304	Bn-C2-p48895815	Bn-scaff_17623_1-p254694	C2	36	51.8	A
13806	Bn-N12-p42047374	Bn-C2-p48895885	Bn-scaff_17623_1-p254624	C2	36	51.8	A
13807	Bn-N12-p42047885	Bn-C2-p48896396	Bn-scaff_17623_1-p254113	C2	36	51.8	A
13808	Bn-N12-p42049544	Bn-C2-p48898055	Bn-scaff_17623_1-p252454	C2	36	51.8	A
13809	Bn-N12-p42052725	Bn-C2-p48901234	Bn-scaff_17623_1-p249275	C2	36	51.8	A
13810	Bn-N12-p42052891	Bn-C2-p48901400	Bn-scaff_17623_1-p249109	C2	36	51.8	A
13811	Bn-N12-p42058172	Bn-C2-p48907328	Bn-scaff_17623_1-p243181	C2	36	51.8	A
13812	Bn-N12-p42059710	Bn-C2-p48910401	Bn-scaff_17623_1-p240108	C2	36	51.8	A
13813	Bn-N12-p42070700	Bn-C2-p48916250	Bn-scaff_17623_1-p234259	C2	36	51.8	A

13814	Bn-N12-p42077971	Bn-C2-p48922806	Bn-scaff_17623_1-p22770	C2	36	51.8	A
13815	Bn-N12-p42089903	Bn-C2-p48933499	Bn-scaff_17623_1-p21701	C2	36	51.8	A
13816	Bn-N12-p42092655	Bn-C2-p48936266	Bn-scaff_17623_1-p21424	C2	36	51.8	A
13817	Bn-N12-p42093138	Bn-C2-p48936754	Bn-scaff_17623_1-p21375	C2	36	51.8	A
13818	Bn-N12-p42104665	Bn-C2-p48950413	Bn-scaff_17623_1-p20009	C2	36	51.8	A
13819	Bn-N12-p42151714	Bn-C2-p49008788	Bn-scaff_17623_1-p14172	C2	36	51.8	A
13820	Bn-N12-p42233163	Bn-C2-p49079464	Bn-scaff_17623_1-p71045	C2	37	53.4	A
13821	Bn-N12-p42288646	Bn-Scaffold00934-p1	Bn-scaff_25071_1-p14638	C2	37	53.4	A
13822	Bn-N12-p42323785	Bn-C2-p49178771	Bn-scaff_17721_1-p90977	C2	37	53.4	A
13823	Bn-N12-p42326091	Bn-C2-p49182188	Bn-scaff_17721_1-p90635	C2	37	53.4	A
13824	Bn-N12-p42372330	Bn-C2-p49239289	Bn-scaff_17721_1-p84925	C2	37	53.4	A
13825	Bn-N12-p42382054	Bn-C2-p49254335	Bn-scaff_17721_1-p83420	C2	37	53.4	A
13826	Bn-N12-p42458740	Bn-C2-p49294923	Bn-scaff_17721_1-p79361	C2	37	53.4	A
13827	Bn-N12-p42598517	Bn-C2-p49449909	Bn-scaff_17721_1-p63863	C2	38	54.2	A
13828	Bn-N12-p42608319	Bn-C2-p49459660	Bn-scaff_17721_1-p62888	C2	38	54.2	A
13829	Bn-N12-p42614610	Bn-C2-p49465257	Bn-scaff_17721_1-p62328	C2	38	54.2	A
13830	Bn-N12-p42673050	Bn-C2-p49556733	Bn-scaff_17721_1-p53180	C2	38	54.2	A
13831	Bn-N12-p42698621	Bn-A02-p25027576	Bn-A02-p25027576	C2	38	54.2	A
13832	Bn-N12-p42861044	Bn-C2-p49736299	Bn-scaff_17721_1-p35224	C2	38	54.2	A
13833	Bn-N12-p42864036	Bn-C2-p49740986	Bn-scaff_17721_1-p34755	C2	38	54.2	A
13834	Bn-N12-p42906812	Bn-C2-p49751205	Bn-scaff_17721_1-p33733	C2	38	54.2	A
13835	Bn-N12-p42909964	Bn-C2-p49755867	Bn-scaff_17721_1-p33267	C2	38	54.2	A
13836	Bn-N12-p42911382	Bn-C2-p49758059	Bn-scaff_17721_1-p33048	C2	38	54.2	A
13837	Bn-N12-p42911456	Bn-C2-p49758133	Bn-scaff_17721_1-p33040	C2	38	54.2	A
13838	Bn-N12-p42919829	Bn-C2-p49779404	Bn-scaff_17721_1-p30913	C2	38	54.2	A
13839	Bn-N12-p42961905	Bn-C2-p49814777	Bn-scaff_17721_1-p27376	C2	38	54.2	A
13840	Bn-N12-p42963432	Bn-C2-p49816293	Bn-scaff_17721_1-p27224	C2	38	54.2	A
13841	Bn-N12-p42965315	Bn-C2-p49817102	Bn-scaff_17721_1-p27143	C2	38	54.2	A
13842	Bn-N12-p42972781	Bn-C2-p49824583	Bn-scaff_17721_1-p26395	C2	38	54.2	A
13843	Bn-N12-p43004107	Bn-C2-p49853877	Bn-scaff_17721_1-p23466	C2	38	54.2	A
13844	Bn-N12-p43006455	Bn-C2-p49856264	Bn-scaff_17721_1-p23227	C2	38	54.2	A
13845	Bn-N12-p43006603	Bn-C2-p49856412	Bn-scaff_17721_1-p23212	C2	38	54.2	A
13846	Bn-N12-p43017370	Bn-C2-p49866993	Bn-scaff_17721_1-p22154	C2	38	54.2	A
13847	Bn-N12-p43035107	Bn-C2-p49884745	Bn-scaff_17721_1-p20379	C2	38	54.2	A
13848	Bn-N12-p43035991	Bn-C2-p49885723	Bn-scaff_17721_1-p20281	C2	38	54.2	A
13849	Bn-N12-p43047068	Bn-C2-p49895935	Bn-scaff_17721_1-p19260	C2	38	54.2	A
13850	Bn-N12-p43047245	Bn-C2-p49896112	Bn-scaff_17721_1-p19242	C2	38	54.2	A
13851	Bn-N12-p43049742	Bn-C2-p49898609	Bn-scaff_17721_1-p18993	C2	38	54.2	A
13852	Bn-N12-p43049835	Bn-C2-p49898703	Bn-scaff_17721_1-p18983	C2	38	54.2	A
13853	Bn-N12-p43051312	Bn-C2-p49900179	Bn-scaff_17721_1-p18836	C2	38	54.2	A
13854	Bn-N12-p43051666	Bn-C2-p49900533	Bn-scaff_17721_1-p18800	C2	38	54.2	A
13855	Bn-N12-p43053223	Bn-C2-p29094764	Bn-scaff_22749_1-p54830	C2	38	54.2	A
13856	Bn-N12-p43064211	Bn-C2-p49913078	Bn-scaff_17721_1-p17546	C2	38	54.2	A
13857	Bn-N12-p43148129	Bn-A02-p25367886	Bn-A02-p25367886	C2	38	54.2	A
13858	Bn-N12-p43151336	Bn-C2-p50028137	Bn-scaff_17721_1-p60404	C2	38	54.2	A
13859	Bn-N12-p43153206	Bn-C2-p50030402	Bn-scaff_17721_1-p58139	C2	38	54.2	A
13860	Bn-N12-p43162207	Bn-C2-p50037587	Bn-scaff_17721_1-p50954	C2	38	54.2	A
13861	Bn-N12-p43267542	Bn-C2-p50135404	Bn-scaff_17177_1-p95457	C2	38	54.2	A
13862	Bn-N12-p43270382	Bn-C2-p50138244	Bn-scaff_17177_1-p95173	C2	38	54.2	A

13863	Bn-N12-p43270617	Bn-C2-p50138479	Bn-scaff_17177_1-p951499	C2	38	54.2	A
13864	Bn-N12-p43275051	Bn-C2-p50142907	Bn-scaff_17177_1-p947071	C2	38	54.2	A
13865	Bn-N12-p43286229	Bn-C2-p50154086	Bn-scaff_17177_1-p935892	C2	38	54.2	A
13866	Bn-N12-p43385608	Bn-C2-p50253849	Bn-scaff_17177_1-p836129	C2	38	54.2	A
13867	Bn-N12-p43385700	Bn-C2-p50253941	Bn-scaff_17177_1-p836037	C2	38	54.2	A
13868	Bn-N12-p43444195	Bn-C2-p50314263	Bn-scaff_17177_1-p775715	C2	38	54.2	A
13869	Bn-N12-p43445982	Bn-C2-p50316050	Bn-scaff_17177_1-p773928	C2	38	54.2	A
13870	Bn-N12-p43447821	Bn-C2-p50317869	Bn-scaff_17177_1-p772109	C2	38	54.2	A
13871	Bn-N2-p27515892	Bn-A02-p25181726	Bn-A02-p25181726	C2	38	54.2	A
13872	Bn-Scaffold17689-p352	Bn-C2-p50038337	Bn-scaff_17721_1-p50204	C2	38	54.2	A
13873	Bn-Scaffold17689-p566	Bn-C2-p50038554	Bn-scaff_17721_1-p49987	C2	38	54.2	A
13874	Bn-N12-p43682190	Bn-C2-p50543794	Bn-scaff_17177_1-p546184	C2	39	56.7	A
13875	Bn-N12-p43724232	Bn-C2-p50557457	Bn-scaff_17177_1-p532521	C2	39	56.7	A
13876	Bn-N12-p43747760	Bn-C2-p50581246	Bn-scaff_17177_1-p508732	C2	39	56.7	A
13877	Bn-N12-p44011551	Bn-C2-p51343986	Bn-scaff_26086_1-p11779	C2	40	59.2	A
13878	Bn-N12-p44382055	Bn-C2-p51836264	Bn-scaff_16139_1-p129512	C2	41	64.2	A
13879	Bn-N2-p28735992	Bn-C2-p51794167	Bn-scaff_16139_1-p133722	C2	41	64.2	A
13880	Bn-N12-p44386210	Bn-C2-p51840262	Bn-scaff_16139_1-p129112	C2	42	65	A
13881	Bn-N12-p44392234	Bn-C2-p51846290	Bn-scaff_16139_1-p128509	C2	42	65	A
13882	Bn-N12-p44394465	Bn-C2-p51848531	Bn-scaff_16139_1-p128285	C2	42	65	A
13883	Bn-N12-p44395621	Bn-C2-p51849689	Bn-scaff_16139_1-p128169	C2	42	65	A
13884	Bn-N12-p44396399	Bn-C2-p51850467	Bn-scaff_16139_1-p128092	C2	42	65	A
13885	Bn-N12-p44397732	Bn-C2-p51851800	Bn-scaff_16139_1-p127958	C2	42	65	A
13886	Bn-N12-p44399515	Bn-C2-p51853582	Bn-scaff_16139_1-p127780	C2	42	65	A
13887	Bn-N12-p44405940	Bn-C2-p51854718	Bn-scaff_16139_1-p127667	C2	42	65	A
13888	Bn-N12-p44406832	Bn-C2-p51855424	Bn-scaff_16139_1-p127596	C2	42	65	A
13889	Bn-N12-p44407411	Bn-C2-p51856003	Bn-scaff_16139_1-p127538	C2	42	65	A
13890	Bn-N12-p44412997	Bn-C2-p51859664	Bn-scaff_16139_1-p127172	C2	42	65	A
13891	Bn-N12-p44413028	Bn-C2-p51859695	Bn-scaff_16139_1-p127169	C2	42	65	A
13892	Bn-N12-p44417891	Bn-C2-p51863893	Bn-scaff_16139_1-p126749	C2	42	65	A
13893	Bn-N12-p44418063	Bn-C2-p51864071	Bn-scaff_16139_1-p126731	C2	42	65	A
13894	Bn-N12-p44549746	Bn-C2-p51996636	Bn-scaff_16139_1-p113475	C2	42	65	A
13895	Bn-N12-p44588081	Bn-C2-p52028173	Bn-scaff_16139_1-p110321	C2	42	65	A
13896	Bn-N12-p44588219	Bn-C2-p52028311	Bn-scaff_16139_1-p110307	C2	42	65	A
13897	Bn-N12-p44588448	Bn-C2-p52028541	Bn-scaff_16139_1-p110284	C2	42	65	A
13898	Bn-N12-p44612979	Bn-C2-p52059322	Bn-scaff_16139_1-p107206	C2	43	65.8	A
13899	Bn-N12-p44629331	Bn-C2-p52067557	Bn-scaff_16139_1-p106383	C2	43	65.8	A
13900	Bn-N12-p44640689	Bn-C2-p52080311	Bn-scaff_16139_1-p105107	C2	43	65.8	A
13901	Bn-N12-p44662380	Bn-C2-p52104030	Bn-scaff_16139_1-p102735	C2	43	65.8	A
13902	Bn-N12-p44703940	Bn-C2-p52153018	Bn-scaff_16139_1-p978370	C2	43	65.8	A
13903	Bn-N12-p44722917	Bn-C2-p52172015	Bn-scaff_16139_1-p959373	C2	44	66.6	A
13904	Bn-N12-p44732203	Bn-C2-p52179807	Bn-scaff_16139_1-p951581	C2	44	66.6	A
13905	Bn-N12-p44754248	Bn-C2-p52202717	Bn-scaff_16139_1-p928671	C2	44	66.6	A
13906	Bn-N12-p44755147	Bn-C2-p52203619	Bn-scaff_16139_1-p927769	C2	44	66.6	A
13907	Bn-N12-p44759550	Bn-C2-p52207948	Bn-scaff_16139_1-p923440	C2	44	66.6	A
13908	Bn-N12-p44763209	Bn-C2-p52209830	Bn-scaff_16139_1-p921558	C2	44	66.6	A
13909	Bn-N12-p44763559	Bn-C2-p52210180	Bn-scaff_16139_1-p921208	C2	44	66.6	A
13910	Bn-N12-p44954857	Bn-Scaffold00615-p1[illegible]	Bn-scaff_16139_1-p720716	C2	45	67.4	A
13911	Bn-N12-p44999360	Bn-Scaffold00615-p8[illegible]	Bn-scaff_16139_1-p670451	C2	45	67.4	A

13912	Bn-N12-p45006999	Bn-Scaffold00615-p74	Bn-scaff_16139_1-p662939	C2	45	67.4	A
13913	Bn-N12-p45012799	Bn-Scaffold00615-p71	Bn-scaff_16139_1-p660500	C2	45	67.4	A
13914	Bn-N12-p45015845	Bn-Scaffold00615-p60	Bn-scaff_16139_1-p655249	C2	45	67.4	A
13915	Bn-N12-p45017517	Bn-Scaffold00615-p64	Bn-scaff_16139_1-p653514	C2	45	67.4	A
13916	Bn-N12-p45032138	Bn-Scaffold00615-p50	Bn-scaff_16139_1-p645644	C2	45	67.4	A
13917	Bn-N12-p45034016	Bn-Scaffold00615-p54	Bn-scaff_16139_1-p643335	C2	45	67.4	A
13918	Bn-N12-p45034055	Bn-Scaffold00615-p54	Bn-scaff_16139_1-p643296	C2	45	67.4	A
13919	Bn-N12-p45035574	Bn-Scaffold00615-p52	Bn-scaff_16139_1-p641045	C2	45	67.4	A
13920	Bn-N12-p45533153	Bn-C2-p52762110	Bn-scaff_16139_1-p462655	C2	45	67.4	A
13921	Bn-N12-p45533177	Bn-C2-p52762134	Bn-scaff_16139_1-p462679	C2	45	67.4	A
13922	Bn-N12-p45453768	Bn-C2-p52680379	Bn-scaff_16139_1-p380924	C2	46	68.2	A
13923	Bn-N12-p45096888	Bn-C2-p52299943	Bn-scaff_16139_1-p488	C2	47	69.8	A
13924	Bn-N12-p45138655	Bn-C2-p52340672	Bn-scaff_16139_1-p41217	C2	47	69.8	A
13925	Bn-N12-p45198056	Bn-C2-p52425623	Bn-scaff_16139_1-p126168	C2	47	69.8	A
13926	Bn-N12-p45198890	Bn-C2-p52426456	Bn-scaff_16139_1-p127001	C2	47	69.8	A
13927	Bn-N12-p45199819	Bn-C2-p52427414	Bn-scaff_16139_1-p127959	C2	47	69.8	A
13928	Bn-N12-p45208239	Bn-C2-p52438267	Bn-scaff_16139_1-p138812	C2	47	69.8	A
13929	Bn-N12-p45242214	Bn-C2-p52460028	Bn-scaff_16139_1-p160573	C2	47	69.8	A
13930	Bn-N12-p45253473	Bn-C2-p52489550	Bn-scaff_16139_1-p190095	C2	47	69.8	A
13931	Bn-N12-p45253706	Bn-C2-p52489783	Bn-scaff_16139_1-p190328	C2	47	69.8	A
13932	Bn-N12-p45262173	Bn-C2-p52497994	Bn-scaff_16139_1-p198539	C2	47	69.8	A
13933	Bn-N12-p45272049	Bn-C2-p52508117	Bn-scaff_16139_1-p208662	C2	47	69.8	A
13934	Bn-N12-p45291918	Bn-C2-p52536631	Bn-scaff_16139_1-p237176	C2	47	69.8	A
13935	Bn-N12-p45293778	Bn-C2-p52538491	Bn-scaff_16139_1-p239036	C2	47	69.8	A
13936	Bn-N12-p45311604	Bn-C2-p52558744	Bn-scaff_16139_1-p259289	C2	47	69.8	A
13937	Bn-N12-p45312136	Bn-C2-p52559269	Bn-scaff_16139_1-p259814	C2	47	69.8	A
13938	Bn-N12-p45313252	Bn-C2-p52559793	Bn-scaff_16139_1-p260338	C2	47	69.8	A
13939	Bn-N12-p45313388	Bn-C2-p52559929	Bn-scaff_16139_1-p260474	C2	47	69.8	A
13940	Bn-N12-p45313402	Bn-C2-p52559943	Bn-scaff_16139_1-p260488	C2	47	69.8	A
13941	Bn-N12-p45356549	Bn-C2-p52588501	Bn-scaff_16139_1-p289046	C2	47	69.8	A
13942	Bn-N2-p29468717	Bn-A02-p27682762	Bn-A02-p27682762	C2	47	69.8	0
13943	Bn-N2-p29527207	Bn-C2-p52459977	Bn-scaff_16139_1-p160522	C2	47	69.8	A
13944	Bn-N13-p2992378	Bn-C3-p2915302	Bn-scaff_18936_1-p77573	C3	1	0	A
13945	Bn-N13-p3013440	Bn-C3-p2935373	Bn-scaff_18936_1-p97644	C3	1	0	A
13946	Bn-N13-p3018551	Bn-C3-p2940484	Bn-scaff_18936_1-p102755	C3	1	0	A
13947	Bn-N13-p3375183	Bn-C3-p3310082	Bn-scaff_18936_1-p472353	C3	2	4	A
13948	Bn-N13-p3557440	Bn-C3-p3481719	Bn-scaff_18936_1-p643990	C3	3	4.8	A
13949	Bn-N3-p2839443	Bn-A03-p3571622	Bn-A03-p2737190	C3	3	4.8	A
13950	Bn-N13-p3762152	Bn-C3-p3699157	Bn-scaff_18936_1-p861428	C3	4	6.5	A
13951	Bn-N13-p4017874	Bn-C3-p3903737	Bn-scaff_18936_1-p1066000	C3	5	7.3	0
13952	Bn-N13-p4085409	Bn-C3-p3953647	Bn-scaff_15877_1-p39516	C3	5	7.3	A
13953	Bn-N3-p3122409	Bn-C3-p3818785	Bn-scaff_18936_1-p981056	C3	5	7.3	A
13954	Bn-N13-p4178380	Bn-C3-p4037974	Bn-scaff_15877_1-p123843	C3	6	8.1	A
13955	Bn-N13-p4252360	Bn-C3-p4100659	Bn-scaff_15877_1-p186528	C3	6	8.1	A
13956	Bn-N13-p4633364	Bn-A03-p4682207	Bn-A03-p3571622	C3	7	12.3	A
13957	Bn-N13-p5299943	Bn-C3-p4632221	Bn-scaff_15877_1-p718090	C3	7	12.3	A
13958	Bn-N13-p5938734	Bn-C3-p5164120	Bn-scaff_21778_1-p163428	C3	8	15.7	A
13959	Bn-N3-p4145513	Bn-C3-p5068957	Bn-scaff_21778_1-p68265	C3	8	15.7	A
13960	Bn-N13-p5986297	Bn-C3-p5218330	Bn-scaff_21778_1-p217638	C3	9	16.5	A

13961	Bn-N13-p6035825	Bn-C3-p5262525	Bn-scaff_21778_1-p261833	C3	9	16.5	A
13962	Bn-N13-p6193914	Bn-C3-p5434023	Bn-scaff_22728_1-p144738	C3	10	19	A
13963	Bn-N13-p6237613	Bn-C3-p5475554	Bn-scaff_22728_1-p140584	C3	10	19	A
13964	Bn-N13-p6242022	Bn-C3-p5479816	Bn-scaff_22728_1-p140158	C3	10	19	A
13965	Bn-N13-p6244288	Bn-C3-p5482090	Bn-scaff_22728_1-p139931	C3	10	19	A
13966	Bn-N13-p6244396	Bn-C3-p5482198	Bn-scaff_22728_1-p139920	C3	10	19	A
13967	Bn-N13-p6244888	Bn-C3-p5482690	Bn-scaff_22728_1-p139871	C3	10	19	A
13968	Bn-N13-p6245154	Bn-C3-p5482956	Bn-scaff_22728_1-p139844	C3	10	19	A
13969	Bn-N13-p6245599	Bn-C3-p5483401	Bn-scaff_22728_1-p139800	C3	10	19	A
13970	Bn-N13-p6246061	Bn-C3-p5483864	Bn-scaff_22728_1-p139753	C3	10	19	A
13971	Bn-N13-p6246142	Bn-C3-p5483945	Bn-scaff_22728_1-p139745	C3	10	19	A
13972	Bn-N13-p6246158	Bn-C3-p5483961	Bn-scaff_22728_1-p139744	C3	10	19	A
13973	Bn-N13-p6246329	Bn-C3-p5484132	Bn-scaff_22728_1-p139727	C3	10	19	A
13974	Bn-N13-p6246453	Bn-C3-p5484256	Bn-scaff_22728_1-p139714	C3	10	19	A
13975	Bn-N13-p6246507	Bn-C3-p5484310	Bn-scaff_22728_1-p139709	C3	10	19	A
13976	Bn-N13-p6246511	Bn-C3-p5484314	Bn-scaff_22728_1-p139708	C3	10	19	A
13977	Bn-N3-p19263737	Bn-C3-p5479342	Bn-scaff_22728_1-p140206	C3	10	19	A
13978	Bn-N3-p19263883	Bn-C3-p5479196	Bn-scaff_22728_1-p140220	C3	10	19	A
13979	Bn-N13-p6395291	Bn-C3-p5622237	Bn-scaff_22728_1-p125916	C3	11	19.8	A
13980	Bn-N13-p6465866	Bn-C3-p5694509	Bn-scaff_22728_1-p118689	C3	11	19.8	A
13981	Bn-N13-p6471383	Bn-C3-p5699682	Bn-scaff_22728_1-p118172	C3	11	19.8	0
13982	Bn-N13-p6593204	Bn-C3-p5800427	Bn-scaff_22728_1-p108097	C3	12	21.5	A
13983	Bn-N13-p6618287	Bn-C3-p5829124	Bn-scaff_22728_1-p105227	C3	12	21.5	A
13984	Bn-N13-p6630129	Bn-A03-p8112016	Bn-A03-p4682207	C3	12	21.5	A
13985	Bn-N13-p6634029	Bn-C3-p5856599	Bn-scaff_22728_1-p102480	C3	12	21.5	A
13986	Bn-N13-p7464782	Bn-C4-p42681345	Bn-scaff_16700_1-p47251	C3	13	24	A
13987	Bn-N13-p8015497	Bn-C3-p7275851	Bn-scaff_18322_1-p235736	C3	14	24.8	A
13988	Bn-N13-p8243761	Bn-C3-p7484139	Bn-scaff_18322_1-p214907	C3	15	25.6	A
13989	Bn-N13-p8245744	Bn-C3-p7486013	Bn-scaff_18322_1-p214719	C3	15	25.6	A
13990	Bn-N13-p8377292	Bn-C3-p7617283	Bn-scaff_18322_1-p201592	C3	15	25.6	A
13991	Bn-N13-p8898820	Bn-C3-p8162553	Bn-scaff_18322_1-p147065	C3	15	25.6	A
13992	Bn-N13-p8366065	Bn-C3-p7604728	Bn-scaff_18322_1-p202848	C3	16	26.4	A
13993	Bn-N13-p8425349	Bn-C2-p31814630	Bn-scaff_23401_1-p199589	C3	16	26.4	A
13994	Bn-N3-p6086296	Bn-C3-p7722899	Bn-scaff_18322_1-p191031	C3	16	26.4	A
13995	Bn-N13-p8747939	Bn-C3-p8000487	Bn-scaff_18322_1-p163272	C3	17	28.1	A
13996	Bn-N13-p8761234	Bn-C3-p8017908	Bn-scaff_18322_1-p161530	C3	17	28.1	A
13997	Bn-N13-p8809720	Bn-C3-p8066876	Bn-scaff_18322_1-p156633	C3	17	28.1	A
13998	Bn-N13-p8860985	Bn-C3-p8122864	Bn-scaff_18322_1-p151034	C3	17	28.1	A
13999	Bn-N13-p8864977	Bn-C3-p8123383	Bn-scaff_18322_1-p150982	C3	17	28.1	A
14000	Bn-N13-p8865105	Bn-C3-p8123511	Bn-scaff_18322_1-p150970	C3	17	28.1	A
14001	Bn-N13-p8865143	Bn-C3-p8123549	Bn-scaff_18322_1-p150966	C3	17	28.1	A
14002	Bn-N13-p8978895	Bn-C3-p8242424	Bn-scaff_18322_1-p139078	C3	17	28.1	A
14003	Bn-N13-p9055329	Bn-C3-p8317665	Bn-scaff_18322_1-p131554	C3	17	28.1	A
14004	Bn-N3-p6464189	Bn-C3-p8268101	Bn-scaff_18322_1-p136511	C3	17	28.1	0
14005	Bn-N13-p9110162	Bn-C3-p8403185	Bn-scaff_18322_1-p123002	C3	18	28.9	A
14006	Bn-N13-p9416709	Bn-C3-p8714497	Bn-scaff_18322_1-p918715	C3	18	28.9	A
14007	Bn-N13-p9531073	Bn-C3-p8829675	Bn-scaff_18322_1-p803537	C3	18	28.9	A
14008	Bn-N13-p9886904	Bn-C3-p9135177	Bn-scaff_18322_1-p498035	C3	19	29.7	A
14009	Bn-N13-p9901359	Bn-C3-p9149378	Bn-scaff_18322_1-p483834	C3	19	29.7	A

14010	Bn-N13-p9919516	Bn-C3-p9158172	Bn-scaff_18322_1-p47504	C3	19	29.7	A
14011	Bn-N13-p11190096	Bn-C3-p10380565	Bn-scaff_21312_1-p69450	C3	20	30.5	A
14012	Bn-N13-p11373962	Bn-A03-p8359092	Bn-A03-p8112016	C3	20	30.5	A
14013	Bn-N13-p10664194	Bn-C3-p9868408	Bn-scaff_21312_1-p12066	C3	21	32.9	A
14014	Bn-N13-p11865252	Bn-C3-p11172822	Bn-scaff_19111_1-p32513	C3	21	32.9	A
14015	Bn-N14-p21282996	Bn-C4-p20491244	Bn-scaff_19575_1-p75408	C3	21	32.9	A
14016	Bn-N3-p8414225	Bn-A03-p8475614	Bn-A03-p8359092	C3	21	32.9	A
14017	Bn-N3-p8559885	Bn-A03-p8479803	Bn-A03-p8475614	C3	21	32.9	A
14018	Bn-N3-p8564064	Bn-A06-p10609641	Bn-A03-p8479803	C3	21	32.9	A
14019	Bn-N3-p9034675	Bn-C3-p12070773	Bn-scaff_23954_1-p66119	C3	22	34.5	A
14020	Bn-N13-p13544313	Bn-C3-p12808223	Bn-scaff_16002_1-p24972	C3	23	35.3	A
14021	Bn-N13-p13845543	Bn-C3-p13083725	Bn-scaff_16002_1-p22217	C3	23	35.3	A
14022	Bn-N13-p13849646	Bn-C3-p13088118	Bn-scaff_16002_1-p22173	C3	23	35.3	A
14023	Bn-N13-p13897846	Bn-C3-p13121745	Bn-scaff_16002_1-p21837	C3	23	35.3	A
14024	Bn-N13-p13932388	Bn-C3-p13152240	Bn-scaff_16002_1-p21532	C3	23	35.3	A
14025	Bn-N3-p9413372	Bn-C3-p12683751	Bn-scaff_23954_1-p48220	C3	23	35.3	A
14026	Bn-N13-p14031815	Bn-Scaffold00930-p1	Bn-scaff_26231_1-p10230	C3	24	37	A
14027	Bn-N13-p14033002	Bn-C3-p13252556	Bn-scaff_16002_1-p20529	C3	24	37	A
14028	Bn-N13-p14066106	Bn-C3-p13279392	Bn-scaff_16002_1-p20260	C3	24	37	A
14029	Bn-N13-p14258453	Bn-C3-p13478875	Bn-scaff_16002_1-p18266	C3	24	37	A
14030	Bn-N13-p14346911	Bn-C3-p13574082	Bn-scaff_16002_1-p17313	C3	24	37	A
14031	Bn-N13-p14421104	Bn-C3-p13629037	Bn-scaff_16002_1-p16764	C3	24	37	A
14032	Bn-N13-p14477862	Bn-C3-p13677270	Bn-scaff_16002_1-p16282	C3	25	37.8	A
14033	Bn-N13-p14477937	Bn-C3-p13677345	Bn-scaff_16002_1-p16281	C3	25	37.8	A
14034	Bn-N13-p14759341	Bn-C3-p13950647	Bn-scaff_16002_1-p13548	C3	25	37.8	A
14035	Bn-N13-p14810260	Bn-C3-p14005974	Bn-scaff_16002_1-p12995	C3	25	37.8	A
14036	Bn-N13-p15596827	Bn-C3-p14822737	Bn-scaff_16002_1-p48274	C3	26	39.5	A
14037	Bn-N13-p15627092	Bn-C3-p14859675	Bn-scaff_16002_1-p44580	C3	26	39.5	A
14038	Bn-N13-p15627095	Bn-C3-p14859678	Bn-scaff_16002_1-p44580	C3	26	39.5	A
14039	Bn-N13-p15639876	Bn-C3-p14869270	Bn-scaff_16002_1-p43620	C3	26	39.5	A
14040	Bn-N13-p15641108	Bn-C3-p14870502	Bn-scaff_16002_1-p43497	C3	26	39.5	A
14041	Bn-N13-p15641195	Bn-C3-p14870589	Bn-scaff_16002_1-p43489	C3	26	39.5	A
14042	Bn-N13-p15654878	Bn-C3-p14885146	Bn-scaff_16002_1-p42033	C3	26	39.5	A
14043	Bn-N13-p15751427	Bn-C3-p14964071	Bn-scaff_16002_1-p34140	C3	26	39.5	A
14044	Bn-N13-p15795667	Bn-C3-p15006696	Bn-scaff_16002_1-p29878	C3	26	39.5	A
14045	Bn-N13-p16059949	Bn-C3-p15383253	Bn-scaff_22466_1-p77675	C3	26	39.5	A
14046	Bn-N3-p10944658	Bn-C3-p14917165	Bn-scaff_16002_1-p38831	C3	26	39.5	A
14047	Bn-N3-p10967854	Bn-A03-p11156293	Bn-A03-p10703085	C3	26	39.5	A
14048	Bn-Scaffold04110-p1500	Bn-C3-p14794214	Bn-scaff_16002_1-p51126	C3	26	39.5	A
14049	Bn-N11-p35047619	Bn-scaffold5052-p14	Bn-scaffold5052-p146	C3	27	41.3	A
14050	Bn-N13-p16262071	Bn-C3-p15591671	Bn-scaff_22466_1-p28609	C3	27	41.3	A
14051	Bn-N13-p16484575	Bn-C3-p15798466	Bn-scaff_22466_1-p49288	C3	27	41.3	A
14052	Bn-N13-p16651680	Bn-C3-p15960783	Bn-scaff_22466_1-p65520	C3	27	41.3	A
14053	Bn-N13-p16713045	Bn-C3-p16012661	Bn-scaff_22466_1-p70708	C3	27	41.3	A
14054	Bn-N13-p16748300	Bn-C3-p16039243	Bn-scaff_22466_1-p73366	C3	27	41.3	A
14055	Bn-N13-p16923327	Bn-C3-p16224017	Bn-scaff_22466_1-p91843	C3	27	41.3	A
14056	Bn-N13-p16926791	Bn-C3-p16227518	Bn-scaff_22466_1-p92194	C3	27	41.3	A
14057	Bn-N13-p16962715	Bn-C3-p16248023	Bn-scaff_22466_1-p94244	C3	27	41.3	A
14058	Bn-N13-p16965535	Bn-C3-p16251222	Bn-scaff_22466_1-p94564	C3	27	41.3	A

14059	Bn-N13-p16965654	Bn-C3-p16251341	Bn-scaff_22466_1-p94576[illegible]	C3	27	41.3	A
14060	Bn-N13-p16965815	Bn-C3-p16251503	Bn-scaff_22466_1-p94592[illegible]	C3	27	41.3	A
14061	Bn-N13-p16966499	Bn-C3-p16252187	Bn-scaff_22466_1-p94660[illegible]	C3	27	41.3	A
14062	Bn-N13-p16966870	Bn-C3-p16252558	Bn-scaff_22466_1-p94698[illegible]	C3	27	41.3	A
14063	Bn-N13-p16968923	Bn-C3-p16260263	Bn-scaff_22466_1-p95468[illegible]	C3	27	41.3	A
14064	Bn-N13-p16969341	Bn-C3-p16260681	Bn-scaff_22466_1-p95510[illegible]	C3	27	41.3	A
14065	Bn-N13-p16970003	Bn-C3-p16261718	Bn-scaff_22466_1-p95614[illegible]	C3	27	41.3	A
14066	Bn-N13-p16974720	Bn-C3-p16266456	Bn-scaff_22466_1-p96087[illegible]	C3	27	41.3	A
14067	Bn-N13-p17003166	Bn-C3-p16292466	Bn-scaff_22466_1-p98688[illegible]	C3	27	41.3	A
14068	Bn-N13-p17003799	Bn-C3-p16293099	Bn-scaff_22466_1-p98752[illegible]	C3	27	41.3	A
14069	Bn-N13-p17004461	Bn-C3-p16293761	Bn-scaff_22466_1-p98818[illegible]	C3	27	41.3	A
14070	Bn-N13-p17009898	Bn-C3-p16299197	Bn-scaff_22466_1-p99361[illegible]	C3	27	41.3	A
14071	Bn-N13-p17010556	Bn-C3-p16299854	Bn-scaff_22466_1-p99427[illegible]	C3	27	41.3	A
14072	Bn-N13-p17010633	Bn-C3-p16299931	Bn-scaff_22466_1-p99435[illegible]	C3	27	41.3	A
14073	Bn-N13-p17010803	Bn-C3-p16300101	Bn-scaff_22466_1-p99452[illegible]	C3	27	41.3	A
14074	Bn-N13-p17011013	Bn-C3-p16300311	Bn-scaff_22466_1-p99473[illegible]	C3	27	41.3	A
14075	Bn-N13-p17011843	Bn-C3-p16301141	Bn-scaff_22466_1-p99556[illegible]	C3	27	41.3	A
14076	Bn-N13-p17011925	Bn-C3-p16301223	Bn-scaff_22466_1-p99564[illegible]	C3	27	41.3	A
14077	Bn-N13-p17012182	Bn-C3-p16301480	Bn-scaff_22466_1-p99590[illegible]	C3	27	41.3	A
14078	Bn-N13-p17012396	Bn-C3-p16301694	Bn-scaff_22466_1-p99611[illegible]	C3	27	41.3	A
14079	Bn-N13-p17012689	Bn-C3-p16301987	Bn-scaff_22466_1-p99640[illegible]	C3	27	41.3	A
14080	Bn-N13-p17013757	Bn-C3-p16303055	Bn-scaff_22466_1-p99747[illegible]	C3	27	41.3	A
14081	Bn-N13-p17606958	Bn-C3-p16846335	Bn-scaff_16352_1-p43991	C3	27	41.3	A
14082	Bn-N13-p17611839	Bn-C3-p16851194	Bn-scaff_16352_1-p48850	C3	27	41.3	A
14083	Bn-N13-p17626384	Bn-C3-p16864987	Bn-scaff_16352_1-p62643	C3	27	41.3	A
14084	Bn-N13-p17627653	Bn-C3-p16866931	Bn-scaff_16352_1-p64587	C3	27	41.3	A
14085	Bn-N13-p17646137	Bn-C3-p16884735	Bn-scaff_16352_1-p82391	C3	27	41.3	A
14086	Bn-N13-p17646915	Bn-C3-p16885513	Bn-scaff_16352_1-p83169	C3	27	41.3	A
14087	Bn-N13-p17648371	Bn-C3-p16887064	Bn-scaff_16352_1-p84720	C3	27	41.3	A
14088	Bn-N13-p17687993	Bn-C3-p16920096	Bn-scaff_16352_1-p11775[illegible]	C3	27	41.3	A
14089	Bn-N13-p17691227	Bn-C3-p16922721	Bn-scaff_16352_1-p12037[illegible]	C3	27	41.3	A
14090	Bn-N13-p17758573	Bn-C3-p16992467	Bn-scaff_16352_1-p19012[illegible]	C3	27	41.3	A
14091	Bn-N13-p17759330	Bn-C3-p16993247	Bn-scaff_16352_1-p19090[illegible]	C3	27	41.3	A
14092	Bn-N13-p17760058	Bn-C3-p16993975	Bn-scaff_16352_1-p19163[illegible]	C3	27	41.3	A
14093	Bn-N13-p17760444	Bn-C3-p16994361	Bn-scaff_16352_1-p19201[illegible]	C3	27	41.3	A
14094	Bn-N13-p17761710	Bn-C3-p16995627	Bn-scaff_16352_1-p19328[illegible]	C3	27	41.3	A
14095	Bn-N13-p17767517	Bn-C3-p17002239	Bn-scaff_16352_1-p19989[illegible]	C3	27	41.3	A
14096	Bn-N13-p17838976	Bn-C3-p17058301	Bn-scaff_16352_1-p25595[illegible]	C3	27	41.3	A
14097	Bn-N13-p17860422	Bn-C3-p17076741	Bn-scaff_16352_1-p27439[illegible]	C3	27	41.3	A
14098	Bn-N13-p17860526	Bn-C3-p17076845	Bn-scaff_16352_1-p27450[illegible]	C3	27	41.3	A
14099	Bn-N13-p17861520	Bn-C3-p17077838	Bn-scaff_16352_1-p27549[illegible]	C3	27	41.3	A
14100	Bn-N13-p17865835	Bn-C3-p17082319	Bn-scaff_16352_1-p27997[illegible]	C3	27	41.3	A
14101	Bn-N13-p17866172	Bn-C3-p17082657	Bn-scaff_16352_1-p28031[illegible]	C3	27	41.3	A
14102	Bn-N13-p17873946	Bn-C3-p17090390	Bn-scaff_16352_1-p28804[illegible]	C3	27	41.3	A
14103	Bn-N13-p17898341	Bn-C3-p17110907	Bn-scaff_16352_1-p30856[illegible]	C3	27	41.3	A
14104	Bn-N13-p17922312	Bn-C3-p17128064	Bn-scaff_16352_1-p32572[illegible]	C3	27	41.3	A
14105	Bn-N13-p17926506	Bn-C3-p17131855	Bn-scaff_16352_1-p32951[illegible]	C3	27	41.3	A
14106	Bn-N13-p17930482	Bn-C3-p17135991	Bn-scaff_16352_1-p33364[illegible]	C3	27	41.3	A
14107	Bn-N13-p17932153	Bn-C3-p17137634	Bn-scaff_16352_1-p33529[illegible]	C3	27	41.3	A

14108	Bn-N13-p17934840	Bn-C3-p17140411	Bn-scaff_16352_1-p33806	C3	27	41.3	A
14109	Bn-N13-p17935675	Bn-C3-p17141244	Bn-scaff_16352_1-p33890	C3	27	41.3	A
14110	Bn-N13-p17961634	Bn-C3-p17165032	Bn-scaff_16352_1-p36268	C3	27	41.3	A
14111	Bn-N13-p18290029	Bn-C3-p17441539	Bn-scaff_16352_1-p63919	C3	27	41.3	A
14112	Bn-N13-p18296442	Bn-C3-p17449576	Bn-scaff_16352_1-p64723	C3	27	41.3	A
14113	Bn-N13-p18371385	Bn-C3-p17522963	Bn-scaff_16352_1-p72061	C3	27	41.3	A
14114	Bn-N13-p18396411	Bn-C3-p17546711	Bn-scaff_16352_1-p74436	C3	27	41.3	A
14115	Bn-N13-p18407242	Bn-C3-p17565032	Bn-scaff_16352_1-p76268	C3	27	41.3	A
14116	Bn-N13-p18815692	Bn-C3-p18015345	Bn-scaff_16352_1-p12130	C3	27	41.3	A
14117	Bn-N13-p18896653	Bn-C3-p18090194	Bn-scaff_16352_1-p12878	C3	27	41.3	A
14118	Bn-N13-p18932437	Bn-C3-p18117405	Bn-scaff_16352_1-p13150	C3	27	41.3	A
14119	Bn-N13-p18937276	Bn-C3-p18122267	Bn-scaff_16352_1-p13199	C3	27	41.3	A
14120	Bn-N13-p18970196	Bn-C3-p18166036	Bn-scaff_16352_1-p13636	C3	27	41.3	A
14121	Bn-N13-p18970958	Bn-C3-p18166819	Bn-scaff_16352_1-p13644	C3	27	41.3	A
14122	Bn-N13-p18970964	Bn-C3-p18166825	Bn-scaff_16352_1-p13644	C3	27	41.3	A
14123	Bn-N13-p19011195	Bn-C3-p19684218	Bn-scaff_15782_1-p25478	C3	27	41.3	A
14124	Bn-N13-p19019578	Bn-C3-p19675912	Bn-scaff_15782_1-p24647	C3	27	41.3	0
14125	Bn-N13-p19052362	Bn-C3-p19621717	Bn-scaff_15782_1-p19228	C3	27	41.3	A
14126	Bn-N13-p19076536	Bn-C3-p19601906	Bn-scaff_15782_1-p17246	C3	27	41.3	A
14127	Bn-N13-p19149929	Bn-C3-p19567138	Bn-scaff_15782_1-p13770	C3	27	41.3	A
14128	Bn-N13-p19151048	Bn-C3-p19564907	Bn-scaff_15782_1-p13547	C3	27	41.3	A
14129	Bn-N13-p19160947	Bn-C3-p19560091	Bn-scaff_15782_1-p13065	C3	27	41.3	A
14130	Bn-N13-p19170302	Bn-C3-p19559717	Bn-scaff_15782_1-p13028	C3	27	41.3	A
14131	Bn-N13-p19170562	Bn-C3-p19559457	Bn-scaff_15782_1-p13002	C3	27	41.3	A
14132	Bn-N13-p19170644	Bn-C3-p19559375	Bn-scaff_15782_1-p12993	C3	27	41.3	A
14133	Bn-N13-p19170788	Bn-C3-p19559232	Bn-scaff_15782_1-p12979	C3	27	41.3	A
14134	Bn-N13-p19171485	Bn-C3-p19558575	Bn-scaff_15782_1-p12913	C3	27	41.3	A
14135	Bn-N13-p19171548	Bn-C3-p19558512	Bn-scaff_15782_1-p12907	C3	27	41.3	A
14136	Bn-N13-p19185267	Bn-C3-p19547070	Bn-scaff_15782_1-p11763	C3	27	41.3	A
14137	Bn-N13-p19185461	Bn-C3-p19546872	Bn-scaff_15782_1-p11743	C3	27	41.3	A
14138	Bn-N13-p19185570	Bn-C3-p19546763	Bn-scaff_15782_1-p11732	C3	27	41.3	A
14139	Bn-N13-p19186969	Bn-C3-p19545365	Bn-scaff_15782_1-p11592	C3	27	41.3	A
14140	Bn-N13-p19187283	Bn-C3-p19545051	Bn-scaff_15782_1-p11561	C3	27	41.3	A
14141	Bn-N13-p19187453	Bn-C3-p19544881	Bn-scaff_15782_1-p11544	C3	27	41.3	A
14142	Bn-N13-p19187604	Bn-C3-p19544730	Bn-scaff_15782_1-p11529	C3	27	41.3	A
14143	Bn-N13-p19190063	Bn-C3-p19540655	Bn-scaff_15782_1-p11121	C3	27	41.3	A
14144	Bn-N13-p19190208	Bn-C3-p19540513	Bn-scaff_15782_1-p11107	C3	27	41.3	A
14145	Bn-N13-p19193718	Bn-C3-p19536954	Bn-scaff_15782_1-p10751	C3	27	41.3	A
14146	Bn-N13-p19193771	Bn-C3-p19536901	Bn-scaff_15782_1-p10746	C3	27	41.3	A
14147	Bn-N13-p19214993	Bn-C3-p19500500	Bn-scaff_15782_1-p71063	C3	27	41.3	A
14148	Bn-N13-p19230341	Bn-C3-p19486405	Bn-scaff_15782_1-p56968	C3	27	41.3	A
14149	Bn-N13-p19249747	Bn-C3-p19467497	Bn-scaff_15782_1-p38060	C3	27	41.3	A
14150	Bn-N13-p19249797	Bn-C3-p19467447	Bn-scaff_15782_1-p38010	C3	27	41.3	A
14151	Bn-N13-p19249996	Bn-C3-p19467248	Bn-scaff_15782_1-p37811	C3	27	41.3	A
14152	Bn-N13-p41428166	Bn-C3-p19665281	Bn-scaff_15782_1-p23584	C3	27	41.3	A
14153	Bn-N3-p11438941	Bn-A03-p13572495	Bn-A03-p11156293	C3	27	41.3	A
14154	Bn-N3-p11515723	Bn-C3-p16026291	Bn-scaff_22466_1-p72071	C3	27	41.3	A
14155	Bn-N3-p12663515	Bn-C3-p19649477	Bn-scaff_15782_1-p22004	C3	27	41.3	A
14156	Bn-Scaffold38786-p370	Bn-C3-p17090967	Bn-scaff_16352_1-p28862	C3	27	41.3	A

14157	Bn-N13-p19497975	Bn-C3-p18351712	Bn-scaff_20646_1-p10776[illegible]	C3	28	43.9	A
14158	Bn-N13-p19500387	Bn-C3-p18354125	Bn-scaff_20646_1-p10752[illegible]	C3	28	43.9	A
14159	Bn-N13-p19519392	Bn-C3-p18363418	Bn-scaff_20646_1-p10659[illegible]	C3	28	43.9	A
14160	Bn-N13-p19662752	Bn-C3-p18508931	Bn-scaff_20646_1-p92040[illegible]	C3	28	43.9	A
14161	Bn-N13-p19519007	Bn-C3-p18363033	Bn-scaff_20646_1-p10663[illegible]	C3	29	44.8	A
14162	Bn-N13-p19751255	Bn-C3-p18596511	Bn-scaff_20646_1-p83282[illegible]	C3	29	44.8	A
14163	Bn-N13-p19775095	Bn-C3-p18618581	Bn-scaff_20646_1-p81075[illegible]	C3	29	44.8	A
14164	Bn-N13-p19780641	Bn-C3-p18624006	Bn-scaff_20646_1-p80533[illegible]	C3	29	44.8	A
14165	Bn-N13-p19784425	Bn-C3-p18627767	Bn-scaff_20646_1-p80157[illegible]	C3	29	44.8	A
14166	Bn-N13-p19845918	Bn-C3-p18694001	Bn-scaff_20646_1-p73533[illegible]	C3	29	44.8	A
14167	Bn-N13-p19900033	Bn-C3-p18753832	Bn-scaff_20646_1-p67550[illegible]	C3	30	45.7	A
14168	Bn-N13-p19900213	Bn-C3-p18754012	Bn-scaff_20646_1-p67532[illegible]	C3	30	45.7	A
14169	Bn-N13-p19901744	Bn-C3-p18755542	Bn-scaff_20646_1-p67379[illegible]	C3	30	45.7	A
14170	Bn-N13-p19903819	Bn-C3-p18758934	Bn-scaff_20646_1-p67040[illegible]	C3	30	45.7	A
14171	Bn-N13-p19922796	Bn-C3-p18777763	Bn-scaff_20646_1-p65157[illegible]	C3	30	45.7	A
14172	Bn-N13-p19935910	Bn-C3-p18789478	Bn-scaff_20646_1-p63986[illegible]	C3	30	45.7	A
14173	Bn-N13-p19935997	Bn-C3-p18789565	Bn-scaff_20646_1-p63977[illegible]	C3	30	45.7	A
14174	Bn-N13-p19936061	Bn-C3-p18789629	Bn-scaff_20646_1-p63970[illegible]	C3	30	45.7	A
14175	Bn-N13-p19941198	Bn-C3-p18794925	Bn-scaff_20646_1-p63441[illegible]	C3	30	45.7	A
14176	Bn-N13-p19941294	Bn-C3-p18795021	Bn-scaff_20646_1-p63431[illegible]	C3	30	45.7	A
14177	Bn-N13-p19941602	Bn-C3-p18795329	Bn-scaff_20646_1-p63400[illegible]	C3	30	45.7	A
14178	Bn-N13-p20120863	Bn-C3-p19007194	Bn-scaff_20646_1-p42214[illegible]	C3	30	45.7	A
14179	Bn-N13-p20156123	Bn-C3-p19031998	Bn-scaff_20646_1-p39734[illegible]	C3	30	45.7	A
14180	Bn-N13-p20159801	Bn-C3-p19036839	Bn-scaff_20646_1-p39249[illegible]	C3	30	45.7	A
14181	Bn-N13-p20171050	Bn-C3-p19050634	Bn-scaff_20646_1-p37870[illegible]	C3	30	45.7	A
14182	Bn-N13-p20172306	Bn-C3-p19051888	Bn-scaff_20646_1-p37745[illegible]	C3	30	45.7	A
14183	Bn-N13-p20259790	Bn-C3-p19070782	Bn-scaff_20646_1-p35855[illegible]	C3	30	45.7	A
14184	Bn-N13-p20314070	Bn-C3-p19129254	Bn-scaff_20646_1-p30008[illegible]	C3	30	45.7	A
14185	Bn-N13-p20314379	Bn-C3-p19129563	Bn-scaff_20646_1-p29977[illegible]	C3	30	45.7	A
14186	Bn-N13-p20327603	Bn-C3-p19138762	Bn-scaff_20646_1-p29057[illegible]	C3	30	45.7	A
14187	Bn-N13-p20341907	Bn-C3-p19152333	Bn-scaff_20646_1-p27700[illegible]	C3	30	45.7	A
14188	Bn-N13-p20343814	Bn-C3-p19154284	Bn-scaff_20646_1-p27505[illegible]	C3	30	45.7	A
14189	Bn-N13-p20369415	Bn-C3-p19181268	Bn-scaff_20646_1-p24807[illegible]	C3	30	45.7	A
14190	Bn-N13-p20369631	Bn-C3-p19181484	Bn-scaff_20646_1-p24785[illegible]	C3	30	45.7	A
14191	Bn-N13-p20377373	Bn-C3-p19191376	Bn-scaff_20646_1-p23796[illegible]	C3	30	45.7	A
14192	Bn-N13-p20388907	Bn-C3-p19202481	Bn-scaff_20646_1-p22685[illegible]	C3	30	45.7	A
14193	Bn-N13-p20409055	Bn-C3-p19221247	Bn-scaff_20646_1-p20809[illegible]	C3	30	45.7	A
14194	Bn-N13-p20409850	Bn-C3-p19222041	Bn-scaff_20646_1-p20729[illegible]	C3	30	45.7	A
14195	Bn-N13-p20434338	Bn-C3-p19246652	Bn-scaff_20646_1-p18268[illegible]	C3	30	45.7	A
14196	Bn-N13-p20482329	Bn-C3-p19296509	Bn-scaff_20646_1-p13282[illegible]	C3	30	45.7	A
14197	Bn-N11-p34573051	Bn-C3-p20167522	Bn-scaff_27870_1-p95950	C3	31	51	A
14198	Bn-N13-p20649361	Bn-C3-p19981581	Bn-scaff_27677_1-p27292[illegible]	C3	31	51	A
14199	Bn-N13-p20672417	Bn-C3-p19959296	Bn-scaff_27677_1-p25063[illegible]	C3	31	51	A
14200	Bn-N13-p20682700	Bn-C3-p19945144	Bn-scaff_27677_1-p23648[illegible]	C3	31	51	A
14201	Bn-N13-p20775060	Bn-C3-p19844047	Bn-scaff_27677_1-p13538[illegible]	C3	31	51	A
14202	Bn-N3-p13528428	Bn-C3-p19956238	Bn-scaff_27677_1-p24758[illegible]	C3	31	51	A
14203	Bn-N3-p13529439	Bn-C3-p19955130	Bn-scaff_27677_1-p24647[illegible]	C3	31	51	A
14204	Bn-Scaffold01310-p11254	Bn-C3-p19995197	Bn-scaff_27677_1-p28653[illegible]	C3	31	51	A
14205	Bn-N13-p21545093	Bn-C3-p20525188	Bn-scaff_19523_1-p18865[illegible]	C3	32	51.9	A

14206	Bn-N13-p21573788	Bn-C3-p20568555	Bn-scaff_19523_1-p232023	C3	32	51.9	A
14207	Bn-N3-p13926123	Bn-A03-p14735831	Bn-A03-p13572495	C3	32	51.9	A
14208	Bn-N13-p22495983	Bn-C3-p21443157	Bn-scaff_18482_1-p739195	C3	33	58.6	B
14209	Bn-N13-p22573010	Bn-C3-p21509796	Bn-scaff_18482_1-p672556	C3	33	58.6	B
14210	Bn-N13-p22836602	Bn-C3-p21737145	Bn-scaff_18482_1-p445207	C3	33	58.6	B
14211	Bn-N13-p22836878	Bn-C3-p21737421	Bn-scaff_18482_1-p444931	C3	33	58.6	B
14212	Bn-N13-p22959916	Bn-C3-p21881587	Bn-scaff_18482_1-p300765	C3	33	58.6	B
14213	Bn-N13-p23025187	Bn-C3-p21935411	Bn-scaff_18482_1-p246941	C3	33	58.6	B
14214	Bn-N13-p23025757	Bn-C3-p21935944	Bn-scaff_18482_1-p246408	C3	33	58.6	B
14215	Bn-N13-p23299576	Bn-C3-p22202335	Bn-scaff_22751_1-p19884	C3	34	59.4	B
14216	Bn-N3-p14967476	Bn-C3-p22221956	Bn-scaff_22751_1-p39505	C3	34	59.4	B
14217	Bn-N3-p15096208	Bn-A03-p15417676	Bn-A03-p14735831	C3	34	59.4	B
14218	Bn-N13-p23741532	Bn-C3-p22562109	Bn-scaff_22067_1-p103662	C3	35	61.1	B
14219	Bn-N4-p15980627	Bn-C3-p22468917	Bn-scaff_22067_1-p10470	C3	35	61.1	B
14220	Bn-N13-p23772660	Bn-C3-p22600584	Bn-scaff_22067_1-p142137	C3	36	61.9	B
14221	Bn-N13-p23778608	Bn-C3-p22606381	Bn-scaff_22067_1-p147934	C3	36	61.9	B
14222	Bn-Scaffold33763-p112	Bn-C3-p22599648	Bn-scaff_22067_1-p141201	C3	36	61.9	B
14223	Bn-N13-p24102290	Bn-C3-p22945691	Bn-scaff_17521_1-p126989	C3	37	66.8	B
14224	Bn-N13-p24436448	Bn-A03-p15983019	Bn-A03-p15417676	C3	38	67.6	B
14225	Bn-N13-p24554201	Bn-C3-p23372614	Bn-scaff_17521_1-p842968	C3	38	67.6	B
14226	Bn-N13-p24562269	Bn-C3-p23377786	Bn-scaff_17521_1-p837796	C3	38	67.6	B
14227	Bn-N13-p24596917	Bn-C3-p23411252	Bn-scaff_17521_1-p804330	C3	38	67.6	B
14228	Bn-N13-p24612465	Bn-C3-p23424506	Bn-scaff_17521_1-p791076	C3	38	67.6	B
14229	Bn-N13-p24692113	Bn-C3-p23499957	Bn-scaff_17521_1-p715625	C3	38	67.6	B
14230	Bn-N13-p24692790	Bn-C3-p23500456	Bn-scaff_17521_1-p715126	C3	38	67.6	B
14231	Bn-N13-p24752673	Bn-C3-p23558552	Bn-scaff_17521_1-p657030	C3	38	67.6	B
14232	Bn-N13-p24839164	Bn-C3-p23635748	Bn-scaff_17521_1-p579834	C3	38	67.6	B
14233	Bn-N13-p24927465	Bn-C3-p23704359	Bn-scaff_17521_1-p511223	C3	39	68.4	B
14234	Bn-N13-p24927635	Bn-C3-p23704528	Bn-scaff_17521_1-p511054	C3	39	68.4	B
14235	Bn-N13-p24942235	Bn-C3-p23709531	Bn-scaff_17521_1-p506051	C3	39	68.4	B
14236	Bn-N13-p24942542	Bn-C3-p23709838	Bn-scaff_17521_1-p505744	C3	39	68.4	B
14237	Bn-N13-p24961890	Bn-C3-p23733649	Bn-scaff_17521_1-p481933	C3	39	68.4	B
14238	Bn-N13-p25135770	Bn-C3-p23913111	Bn-scaff_17521_1-p302471	C3	39	68.4	B
14239	Bn-N13-p25138690	Bn-C3-p23916065	Bn-scaff_17521_1-p299517	C3	39	68.4	B
14240	Bn-N13-p25138955	Bn-C3-p23916330	Bn-scaff_17521_1-p299252	C3	39	68.4	B
14241	Bn-N13-p25148813	Bn-C3-p23926412	Bn-scaff_17521_1-p289170	C3	39	68.4	B
14242	Bn-N13-p25148893	Bn-C3-p23926492	Bn-scaff_17521_1-p289090	C3	39	68.4	B
14243	Bn-N13-p25151830	Bn-C3-p23929476	Bn-scaff_17521_1-p286106	C3	39	68.4	B
14244	Bn-N13-p25148157	Bn-C3-p23925519	Bn-scaff_17521_1-p290063	C3	40	70	B
14245	Bn-N13-p25244880	Bn-C3-p24021907	Bn-scaff_17521_1-p193675	C3	40	70	B
14246	Bn-N13-p25279346	Bn-C3-p24078563	Bn-scaff_17521_1-p137019	C3	40	70	B
14247	Bn-N13-p25295898	Bn-C3-p24090407	Bn-scaff_17521_1-p125175	C3	40	70	B
14248	Bn-N13-p25431348	Bn-C3-p24214181	Bn-scaff_17521_1-p1401	C3	40	70	B
14249	Bn-N13-p25431360	Bn-C3-p24214192	Bn-scaff_17521_1-p1390	C3	40	70	B
14250	Bn-N13-p25431415	Bn-C3-p24214247	Bn-scaff_17521_1-p1335	C3	40	70	B
14251	Bn-N3-p16338309	Bn-A03-p16990947	Bn-A03-p15983019	C3	40	70	B
14252	Bn-N13-p25461966	Bn-Scaffold01023-p1	Bn-scaff_28562_1-p11758	C3	41	70.8	B
14253	Bn-N13-p25462781	Bn-Scaffold01023-p1(	Bn-scaff_28562_1-p10940	C3	41	70.8	B
14254	Bn-N13-p25492661	Bn-C3-p24229563	Bn-scaff_17298_1-p13882	C3	41	70.8	B

14255	Bn-N13-p25497074	Bn-C3-p24233954	Bn-scaff_17298_1-p18273	C3	41	70.8	B
14256	Bn-N13-p25497265	Bn-C3-p24234212	Bn-scaff_17298_1-p18531	C3	41	70.8	B
14257	Bn-N13-p25513727	Bn-C3-p24250851	Bn-scaff_17298_1-p35170	C3	41	70.8	B
14258	Bn-N13-p25533723	Bn-C3-p24268101	Bn-scaff_17298_1-p52420	C3	41	70.8	B
14259	Bn-N13-p25534029	Bn-C3-p24268405	Bn-scaff_17298_1-p52724	C3	41	70.8	B
14260	Bn-N13-p25534099	Bn-C3-p24268475	Bn-scaff_17298_1-p52794	C3	41	70.8	B
14261	Bn-N13-p25537535	Bn-C3-p24268722	Bn-scaff_17298_1-p53041	C3	41	70.8	B
14262	Bn-N13-p25537569	Bn-C3-p24268755	Bn-scaff_17298_1-p53074	C3	41	70.8	B
14263	Bn-N13-p25538220	Bn-C3-p24269246	Bn-scaff_17298_1-p53565	C3	41	70.8	B
14264	Bn-N13-p25538360	Bn-C3-p24269386	Bn-scaff_17298_1-p53705	C3	41	70.8	B
14265	Bn-N13-p25538813	Bn-C3-p24269843	Bn-scaff_17298_1-p54162	C3	41	70.8	B
14266	Bn-N13-p25538911	Bn-C3-p24269941	Bn-scaff_17298_1-p54260	C3	41	70.8	B
14267	Bn-N13-p25554878	Bn-C3-p24276849	Bn-scaff_17298_1-p61168	C3	41	70.8	B
14268	Bn-N13-p25554948	Bn-C3-p24276919	Bn-scaff_17298_1-p61238	C3	41	70.8	B
14269	Bn-N13-p25556095	Bn-C3-p24278059	Bn-scaff_17298_1-p62378	C3	41	70.8	B
14270	Bn-N13-p25557972	Bn-C3-p24279702	Bn-scaff_17298_1-p64021	C3	41	70.8	B
14271	Bn-N13-p25558134	Bn-C3-p24279864	Bn-scaff_17298_1-p64183	C3	41	70.8	B
14272	Bn-N13-p25558234	Bn-C3-p24279964	Bn-scaff_17298_1-p64283	C3	41	70.8	B
14273	Bn-N13-p25558599	Bn-C3-p24280306	Bn-scaff_17298_1-p64625	C3	41	70.8	B
14274	Bn-N13-p25558765	Bn-C3-p24280472	Bn-scaff_17298_1-p64791	C3	41	70.8	B
14275	Bn-N13-p25560586	Bn-C3-p24291539	Bn-scaff_17298_1-p75858	C3	41	70.8	B
14276	Bn-N13-p25560751	Bn-C3-p24291704	Bn-scaff_17298_1-p76023	C3	41	70.8	B
14277	Bn-N13-p25561023	Bn-C3-p24291976	Bn-scaff_17298_1-p76295	C3	41	70.8	B
14278	Bn-N13-p25561228	Bn-C3-p24292181	Bn-scaff_17298_1-p76500	C3	41	70.8	B
14279	Bn-N13-p25561406	Bn-C3-p24292359	Bn-scaff_17298_1-p76678	C3	41	70.8	B
14280	Bn-N13-p25561632	Bn-C3-p24292585	Bn-scaff_17298_1-p76904	C3	41	70.8	B
14281	Bn-N13-p25561952	Bn-C3-p24292905	Bn-scaff_17298_1-p77224	C3	41	70.8	B
14282	Bn-N13-p25570135	Bn-C3-p24299756	Bn-scaff_17298_1-p84075	C3	41	70.8	B
14283	Bn-N13-p25570904	Bn-C3-p24300542	Bn-scaff_17298_1-p84861	C3	41	70.8	B
14284	Bn-N13-p25571368	Bn-C3-p24301058	Bn-scaff_17298_1-p85377	C3	41	70.8	B
14285	Bn-N13-p25573631	Bn-C3-p24303319	Bn-scaff_17298_1-p87638	C3	41	70.8	B
14286	Bn-N13-p25574365	Bn-C3-p24304073	Bn-scaff_17298_1-p88392	C3	41	70.8	B
14287	Bn-N13-p25574624	Bn-C3-p24304335	Bn-scaff_17298_1-p88654	C3	41	70.8	B
14288	Bn-N13-p25575081	Bn-C3-p24304792	Bn-scaff_17298_1-p89111	C3	41	70.8	B
14289	Bn-N13-p25580237	Bn-C3-p24316657	Bn-scaff_17298_1-p10097(	C3	41	70.8	B
14290	Bn-N13-p25581282	Bn-C3-p24317700	Bn-scaff_17298_1-p10201(	C3	41	70.8	B
14291	Bn-N13-p25585841	Bn-C3-p24324458	Bn-scaff_17298_1-p10877(	C3	41	70.8	B
14292	Bn-N13-p25609110	Bn-C3-p24354456	Bn-scaff_17298_1-p13877(	C3	42	71.6	B
14293	Bn-N13-p25609494	Bn-C3-p24354840	Bn-scaff_17298_1-p13915(	C3	42	71.6	B
14294	Bn-N13-p25611164	Bn-C3-p24356528	Bn-scaff_17298_1-p14084(	C3	42	71.6	B
14295	Bn-N13-p25614607	Bn-C3-p24359976	Bn-scaff_17298_1-p14429(	C3	42	71.6	B
14296	Bn-N13-p25672924	Bn-C3-p24418455	Bn-scaff_17298_1-p20277(	C3	42	71.6	B
14297	Bn-N13-p25689730	Bn-Scaffold01626-p6(	Bn-scaff_21894_1-p6693	C3	42	71.6	B
14298	Bn-N13-p26100579	Bn-C3-p24842847	Bn-scaff_17298_1-p62716(	C3	43	72.4	B
14299	Bn-N13-p26157491	Bn-C3-p24895511	Bn-scaff_17298_1-p67983(	C3	44	73.2	B
14300	Bn-N13-p26177116	Bn-C3-p24915174	Bn-scaff_17298_1-p69949(	C3	44	73.2	0
14301	Bn-N13-p26320612	Bn-C3-p25026440	Bn-scaff_17298_1-p81075(	C3	44	73.2	B
14302	Bn-N13-p26321115	Bn-C3-p25026943	Bn-scaff_17298_1-p81126(	C3	44	73.2	B
14303	Bn-N13-p26321738	Bn-C3-p25027566	Bn-scaff_17298_1-p81188(	C3	44	73.2	B

14304	Bn-N13-p26338990	Bn-C3-p25043116	Bn-scaff_17298_1-p827435	C3	44	73.2	B
14305	Bn-N13-p26339470	Bn-C3-p25043560	Bn-scaff_17298_1-p827879	C3	44	73.2	B
14306	Bn-N13-p26440961	Bn-C3-p25124784	Bn-scaff_17298_1-p909103	C3	44	73.2	B
14307	Bn-N13-p26462604	Bn-C3-p25150419	Bn-scaff_17298_1-p934738	C3	44	73.2	B
14308	Bn-N13-p26464329	Bn-C3-p25152026	Bn-scaff_17298_1-p936345	C3	44	73.2	B
14309	Bn-N13-p26575324	Bn-C3-p25270752	Bn-scaff_17298_1-p105507	C3	45	75.6	B
14310	Bn-N13-p26584639	Bn-C3-p25280042	Bn-scaff_17298_1-p106436	C3	45	75.6	B
14311	Bn-N13-p26688062	Bn-C3-p25374138	Bn-scaff_17298_1-p115845	C3	45	75.6	B
14312	Bn-N13-p26688968	Bn-C3-p25375072	Bn-scaff_17298_1-p115939	C3	45	75.6	B
14313	Bn-N13-p26701563	Bn-C3-p25390018	Bn-scaff_17298_1-p117433	C3	45	75.6	B
14314	Bn-N13-p26708939	Bn-C3-p25397175	Bn-scaff_17298_1-p118149	C3	45	75.6	B
14315	Bn-N13-p26783811	Bn-C3-p25465034	Bn-scaff_17298_1-p124935	C3	45	75.6	B
14316	Bn-N13-p26784330	Bn-C3-p25465520	Bn-scaff_17298_1-p124983	C3	45	75.6	B
14317	Bn-N13-p26802824	Bn-C3-p25486459	Bn-scaff_17298_1-p127077	C3	45	75.6	B
14318	Bn-N13-p26834187	Bn-C3-p25518677	Bn-scaff_17298_1-p130299	C3	46	77.2	B
14319	Bn-N13-p26844738	Bn-C3-p25533201	Bn-scaff_17298_1-p131752	C3	46	77.2	B
14320	Bn-N13-p26848042	Bn-C3-p25536567	Bn-scaff_17298_1-p132088	C3	46	77.2	B
14321	Bn-N11-p28886236	Bn-C3-p26267183	Bn-scaff_17298_1-p205150	C3	47	78	B
14322	Bn-N13-p26945357	Bn-C3-p25627940	Bn-scaff_17298_1-p141225	C3	47	78	B
14323	Bn-N13-p26946388	Bn-C3-p25628971	Bn-scaff_17298_1-p141329	C3	47	78	A
14324	Bn-N13-p26998039	Bn-C3-p25687563	Bn-scaff_17298_1-p147188	C3	47	78	B
14325	Bn-N13-p27488482	Bn-C3-p26179931	Bn-scaff_17298_1-p196425	C3	47	78	B
14326	Bn-N13-p27568024	Bn-C3-p26311974	Bn-scaff_17298_1-p209629	C3	47	78	B
14327	Bn-N13-p27569145	Bn-C3-p26313117	Bn-scaff_17298_1-p209743	C3	47	78	B
14328	Bn-N13-p27706047	Bn-C3-p26447612	Bn-scaff_23350_1-p16694	C3	47	78	B
14329	Bn-N13-p27861994	Bn-C3-p26636990	Bn-scaff_23350_1-p206072	C3	47	78	B
14330	Bn-N13-p28238895	Bn-C3-p27025096	Bn-scaff_23350_1-p594178	C3	47	78	B
14331	Bn-N13-p28249182	Bn-C3-p27035456	Bn-scaff_23350_1-p604538	C3	47	78	B
14332	Bn-N13-p29643841	Bn-C3-p28322482	Bn-scaff_16310_1-p33031	C3	47	78	0
14333	Bn-N3-p17390270	Bn-A03-p19071304	Bn-A03-p16990947	C3	47	78	B
14334	Bn-N5-p20306526	Bn-C3-p26188160	Bn-scaff_17298_1-p197247	C3	47	78	B
14335	Bn-N13-p28304014	Bn-C3-p27074199	Bn-scaff_23350_1-p643281	C3	48	78.8	B
14336	Bn-N13-p28393850	Bn-C3-p27196443	Bn-scaff_23350_1-p765525	C3	48	78.8	B
14337	Bn-N13-p28646335	Bn-C6-p6993764	Bn-scaff_23821_1-p94162	C3	48	78.8	B
14338	Bn-N13-p28938029	Bn-C3-p27797465	Bn-scaff_20103_1-p309490	C3	48	78.8	B
14339	Bn-N13-p28955083	Bn-C3-p27786014	Bn-scaff_20103_1-p298039	C3	48	78.8	B
14340	Bn-N13-p29179097	Bn-C3-p27577747	Bn-scaff_20103_1-p89772	C3	48	78.8	B
14341	Bn-N13-p29186520	Bn-C3-p27570263	Bn-scaff_20103_1-p82288	C3	48	78.8	B
14342	Bn-N13-p29206377	Bn-C3-p27530996	Bn-scaff_20103_1-p43021	C3	48	78.8	B
14343	Bn-N13-p29287418	Bn-C3-p27462443	Bn-scaff_28640_1-p25433	C3	48	78.8	B
14344	Bn-N13-p29291252	Bn-C3-p27458727	Bn-scaff_28640_1-p29149	C3	48	78.8	B
14345	Bn-N13-p29383446	Bn-C3-p28076430	Bn-scaff_16310_1-p279083	C3	48	78.8	B
14346	Bn-N13-p29521316	Bn-C3-p28269677	Bn-scaff_16310_1-p85836	C3	48	78.8	B
14347	Bn-N13-p29523456	Bn-C3-p28271800	Bn-scaff_16310_1-p83713	C3	48	78.8	B
14348	Bn-N13-p29547671	Bn-C3-p28288885	Bn-scaff_16310_1-p66628	C3	48	78.8	B
14349	Bn-N13-p29629902	Bn-C3-p28309062	Bn-scaff_16310_1-p46451	C3	48	78.8	B
14350	Bn-N13-p29630336	Bn-C3-p28309496	Bn-scaff_16310_1-p46017	C3	48	78.8	B
14351	Bn-N13-p29630619	Bn-C3-p28309780	Bn-scaff_16310_1-p45733	C3	48	78.8	B
14352	Bn-N13-p29631267	Bn-C3-p28310416	Bn-scaff_16310_1-p45097	C3	48	78.8	B

14353	Bn-N13-p29632914	Bn-C3-p28310642	Bn-scaff_16310_1-p44871	C3	48	78.8	B
14354	Bn-N13-p29768150	Bn-C3-p28448163	Bn-scaff_16925_1-p349728	C3	48	78.8	B
14355	Bn-N13-p29785923	Bn-C3-p28454343	Bn-scaff_16925_1-p343548	C3	48	78.8	B
14356	Bn-N13-p29786643	Bn-C3-p28455063	Bn-scaff_16925_1-p342828	C3	48	78.8	B
14357	Bn-N13-p29848682	Bn-C3-p28536620	Bn-scaff_16925_1-p261271	C3	48	78.8	B
14358	Bn-N13-p29860056	Bn-C3-p28547802	Bn-scaff_16925_1-p250089	C3	48	78.8	B
14359	Bn-N13-p29861310	Bn-C3-p28547949	Bn-scaff_16925_1-p249942	C3	48	78.8	B
14360	Bn-N13-p29861420	Bn-C3-p28548059	Bn-scaff_16925_1-p249832	C3	48	78.8	B
14361	Bn-N13-p29864198	Bn-C3-p28550804	Bn-scaff_16925_1-p247087	C3	48	78.8	B
14362	Bn-N13-p29876160	Bn-C3-p28562290	Bn-scaff_16925_1-p235601	C3	48	78.8	B
14363	Bn-N13-p29880296	Bn-C3-p28566788	Bn-scaff_16925_1-p231103	C3	48	78.8	B
14364	Bn-N13-p29884060	Bn-C3-p28570341	Bn-scaff_16925_1-p227550	C3	48	78.8	B
14365	Bn-N13-p29903419	Bn-C3-p28581852	Bn-scaff_16925_1-p216039	C3	48	78.8	B
14366	Bn-N13-p29934621	Bn-C3-p28614770	Bn-scaff_16925_1-p183121	C3	48	78.8	B
14367	Bn-N13-p29935256	Bn-C3-p28615645	Bn-scaff_16925_1-p182246	C3	48	78.8	B
14368	Bn-N13-p29943421	Bn-C3-p28614266	Bn-scaff_16925_1-p183625	C3	48	78.8	B
14369	Bn-N13-p29990553	Bn-C3-p28668762	Bn-scaff_16925_1-p129129	C3	48	78.8	B
14370	Bn-N13-p30000316	Bn-C3-p28682270	Bn-scaff_16925_1-p115621	C3	48	78.8	B
14371	Bn-N13-p30000610	Bn-C3-p28682860	Bn-scaff_16925_1-p115031	C3	48	78.8	B
14372	Bn-N13-p30000912	Bn-C3-p28683163	Bn-scaff_16925_1-p114728	C3	48	78.8	B
14373	Bn-N13-p30026926	Bn-C3-p28692761	Bn-scaff_16925_1-p105130	C3	48	78.8	B
14374	Bn-N13-p30029256	Bn-C3-p28695706	Bn-scaff_16925_1-p102185	C3	48	78.8	B
14375	Bn-N13-p30039532	Bn-C3-p28701990	Bn-scaff_16925_1-p95901	C3	48	78.8	B
14376	Bn-N13-p30046234	Bn-C3-p28710859	Bn-scaff_16925_1-p87032	C3	48	78.8	B
14377	Bn-N3-p18369291	Bn-C3-p27796098	Bn-scaff_20103_1-p308123	C3	48	78.8	B
14378	Bn-N7-p12809265	Bn-A08-p11212494	Bn-A07-p11330018	C3	48	78.8	B
14379	Bn-Scaffold59840-p86	Bn-C3-p28272074	Bn-scaff_16310_1-p83439	C3	48	78.8	B
14380	Bn-N13-p30096565	Bn-C3-p28808057	Bn-scaff_17395_1-p191984	C3	49	79.6	B
14381	Bn-N13-p30222940	Bn-C3-p28952864	Bn-scaff_17395_1-p47177	C3	49	79.6	B
14382	Bn-N13-p30230884	Bn-C3-p28956782	Bn-scaff_17395_1-p43259	C3	49	79.6	B
14383	Bn-N13-p30419774	Bn-C3-p29136800	Bn-scaff_19026_1-p316166	C3	49	79.6	B
14384	Bn-N13-p30423059	Bn-C3-p29140061	Bn-scaff_19026_1-p312905	C3	49	79.6	B
14385	Bn-N13-p30430335	Bn-C3-p29148886	Bn-scaff_19026_1-p304080	C3	49	79.6	B
14386	Bn-N13-p30441403	Bn-C3-p29160609	Bn-scaff_19026_1-p292357	C3	49	79.6	B
14387	Bn-N13-p30446027	Bn-C3-p29170208	Bn-scaff_19026_1-p282758	C3	49	79.6	B
14388	Bn-N13-p30451951	Bn-C3-p29175347	Bn-scaff_19026_1-p277619	C3	49	79.6	B
14389	Bn-N13-p30473444	Bn-C3-p29196028	Bn-scaff_19026_1-p256938	C3	49	79.6	B
14390	Bn-N13-p30475713	Bn-C3-p29198913	Bn-scaff_19026_1-p254053	C3	49	79.6	B
14391	Bn-N13-p30481045	Bn-C3-p29203539	Bn-scaff_19026_1-p249427	C3	49	79.6	B
14392	Bn-N13-p30499129	Bn-C3-p29207169	Bn-scaff_19026_1-p245797	C3	49	79.6	B
14393	Bn-N13-p30515717	Bn-C3-p29220702	Bn-scaff_19026_1-p232264	C3	49	79.6	B
14394	Bn-N13-p30519747	Bn-C3-p29224749	Bn-scaff_19026_1-p228217	C3	49	79.6	B
14395	Bn-N13-p30521310	Bn-C3-p29225851	Bn-scaff_19026_1-p227115	C3	49	79.6	B
14396	Bn-N13-p30521892	Bn-C3-p29226388	Bn-scaff_19026_1-p226578	C3	49	79.6	B
14397	Bn-N13-p30523025	Bn-C3-p29227552	Bn-scaff_19026_1-p225414	C3	49	79.6	B
14398	Bn-N13-p30523369	Bn-C3-p29233609	Bn-scaff_19026_1-p219357	C3	49	79.6	B
14399	Bn-N13-p30523458	Bn-C3-p29233698	Bn-scaff_19026_1-p219268	C3	49	79.6	B
14400	Bn-N13-p30523486	Bn-C3-p29233726	Bn-scaff_19026_1-p219240	C3	49	79.6	B
14401	Bn-N13-p30524277	Bn-C3-p29237017	Bn-scaff_19026_1-p215949	C3	49	79.6	B

14402	Bn-N13-p30524428	Bn-C3-p29237168	Bn-scaff_19026_1-p215798	C3	49	79.6	B
14403	Bn-N13-p30526520	Bn-C3-p29239262	Bn-scaff_19026_1-p213704	C3	49	79.6	B
14404	Bn-N13-p30527124	Bn-C3-p29239863	Bn-scaff_19026_1-p213103	C3	49	79.6	B
14405	Bn-N13-p30527315	Bn-C3-p29240054	Bn-scaff_19026_1-p212912	C3	49	79.6	B
14406	Bn-N13-p30534607	Bn-C3-p29247322	Bn-scaff_19026_1-p205644	C3	49	79.6	B
14407	Bn-N13-p30537190	Bn-C3-p29249868	Bn-scaff_19026_1-p203098	C3	49	79.6	B
14408	Bn-N13-p30537513	Bn-C3-p29250190	Bn-scaff_19026_1-p202776	C3	49	79.6	B
14409	Bn-N13-p30537611	Bn-C3-p29250288	Bn-scaff_19026_1-p202678	C3	49	79.6	B
14410	Bn-N13-p30537940	Bn-C3-p29250615	Bn-scaff_19026_1-p202351	C3	49	79.6	B
14411	Bn-N13-p30538281	Bn-C3-p29250956	Bn-scaff_19026_1-p202010	C3	49	79.6	B
14412	Bn-N13-p30538565	Bn-C3-p29251240	Bn-scaff_19026_1-p201726	C3	49	79.6	B
14413	Bn-N13-p30539366	Bn-C3-p29252069	Bn-scaff_19026_1-p200897	C3	49	79.6	B
14414	Bn-N13-p30546802	Bn-C3-p29259510	Bn-scaff_19026_1-p193456	C3	49	79.6	B
14415	Bn-N13-p30547866	Bn-C3-p29260550	Bn-scaff_19026_1-p192416	C3	49	79.6	B
14416	Bn-N13-p30547931	Bn-C3-p29260615	Bn-scaff_19026_1-p192351	C3	49	79.6	B
14417	Bn-N13-p30548002	Bn-C3-p29260686	Bn-scaff_19026_1-p192280	C3	49	79.6	B
14418	Bn-N13-p30548752	Bn-C3-p29261430	Bn-scaff_19026_1-p191536	C3	49	79.6	B
14419	Bn-N13-p30548960	Bn-C3-p29261641	Bn-scaff_19026_1-p191325	C3	49	79.6	B
14420	Bn-N13-p30549475	Bn-C3-p29262157	Bn-scaff_19026_1-p190809	C3	49	79.6	B
14421	Bn-N13-p30550397	Bn-C3-p29263080	Bn-scaff_19026_1-p189886	C3	49	79.6	B
14422	Bn-N13-p30550491	Bn-C3-p29263174	Bn-scaff_19026_1-p189792	C3	49	79.6	B
14423	Bn-N13-p30550614	Bn-C3-p29263297	Bn-scaff_19026_1-p189669	C3	49	79.6	B
14424	Bn-N13-p30550635	Bn-C3-p29263318	Bn-scaff_19026_1-p189648	C3	49	79.6	B
14425	Bn-N13-p30550826	Bn-C3-p29263509	Bn-scaff_19026_1-p189457	C3	49	79.6	B
14426	Bn-N13-p30550917	Bn-C3-p29263628	Bn-scaff_19026_1-p189338	C3	49	79.6	B
14427	Bn-N13-p30551546	Bn-C3-p29264625	Bn-scaff_19026_1-p188341	C3	49	79.6	B
14428	Bn-N13-p30551986	Bn-C3-p29265045	Bn-scaff_19026_1-p187921	C3	49	79.6	B
14429	Bn-N13-p30552079	Bn-C3-p29265138	Bn-scaff_19026_1-p187828	C3	49	79.6	B
14430	Bn-N13-p30555341	Bn-C3-p29268124	Bn-scaff_19026_1-p184842	C3	49	79.6	B
14431	Bn-N13-p30605049	Bn-C3-p29315183	Bn-scaff_19026_1-p137783	C3	49	79.6	B
14432	Bn-N13-p30634252	Bn-C3-p29347171	Bn-scaff_19026_1-p105795	C3	49	79.6	B
14433	Bn-N13-p30636549	Bn-C3-p29349404	Bn-scaff_19026_1-p103562	C3	49	79.6	B
14434	Bn-N13-p30637387	Bn-C3-p29350284	Bn-scaff_19026_1-p102682	C3	49	79.6	B
14435	Bn-N13-p30659786	Bn-C3-p29368575	Bn-scaff_19026_1-p84391	C3	49	79.6	B
14436	Bn-N13-p30697394	Bn-C3-p29397253	Bn-scaff_19026_1-p55713	C3	49	79.6	B
14437	Bn-N13-p30864833	Bn-C3-p29520104	Bn-scaff_16822_1-p139438	C3	49	79.6	B
14438	Bn-N13-p30886079	Bn-C3-p29549322	Bn-scaff_16822_1-p110220	C3	49	79.6	B
14439	Bn-N13-p30887941	Bn-C3-p29551281	Bn-scaff_16822_1-p108261	C3	49	79.6	B
14440	Bn-N13-p30888036	Bn-C3-p29551376	Bn-scaff_16822_1-p108166	C3	49	79.6	B
14441	Bn-N13-p30900486	Bn-C3-p29562681	Bn-scaff_16822_1-p96861	C3	49	79.6	B
14442	Bn-N13-p30900756	Bn-C3-p29562951	Bn-scaff_16822_1-p96591	C3	49	79.6	B
14443	Bn-N13-p30901285	Bn-C3-p29563485	Bn-scaff_16822_1-p96057	C3	49	79.6	B
14444	Bn-N13-p30901539	Bn-C3-p29563742	Bn-scaff_16822_1-p95800	C3	49	79.6	B
14445	Bn-N13-p30904800	Bn-C3-p29566670	Bn-scaff_16822_1-p92872	C3	49	79.6	B
14446	Bn-N13-p30904877	Bn-C3-p29566746	Bn-scaff_16822_1-p92796	C3	49	79.6	B
14447	Bn-N13-p31113875	Bn-C3-p29683155	Bn-scaff_16092_1-p23514	C3	49	79.6	B
14448	Bn-N13-p31149196	Bn-C3-p29729445	Bn-scaff_16092_1-p69804	C3	49	79.6	B
14449	Bn-N13-p31303280	Bn-C3-p29813047	Bn-scaff_16092_1-p153406	C3	49	79.6	B
14450	Bn-N13-p31303374	Bn-C3-p29813141	Bn-scaff_16092_1-p153500	C3	49	79.6	B

14451	Bn-N13-p31628419	Bn-C3-p30122421	Bn-scaff_16092_1-p46278	C3	49	79.6	B
14452	Bn-N13-p31632697	Bn-C3-p30126626	Bn-scaff_16092_1-p46698	C3	49	79.6	B
14453	Bn-N13-p31632960	Bn-C3-p30126846	Bn-scaff_16092_1-p46720	C3	49	79.6	B
14454	Bn-N13-p31633695	Bn-C3-p30127624	Bn-scaff_16092_1-p46798	C3	49	79.6	B
14455	Bn-N13-p31642393	Bn-C3-p30136191	Bn-scaff_16092_1-p47655	C3	49	79.6	B
14456	Bn-N13-p31678280	Bn-C3-p30162482	Bn-scaff_16092_1-p50284	C3	49	79.6	B
14457	Bn-N13-p31681963	Bn-C3-p30167452	Bn-scaff_16092_1-p50781	C3	49	79.6	B
14458	Bn-N13-p31712456	Bn-C3-p30200812	Bn-scaff_16092_1-p54117	C3	49	79.6	B
14459	Bn-N13-p31719517	Bn-C3-p30208050	Bn-scaff_16092_1-p54840	C3	49	79.6	B
14460	Bn-N13-p31745550	Bn-C3-p30230800	Bn-scaff_16092_1-p57115	C3	49	79.6	B
14461	Bn-N13-p31788253	Bn-C3-p30284550	Bn-scaff_16092_1-p62490	C3	49	79.6	B
14462	Bn-N13-p31791492	Bn-C3-p30287300	Bn-scaff_16092_1-p62765	C3	49	79.6	B
14463	Bn-N13-p31811247	Bn-C3-p30305877	Bn-scaff_16092_1-p64623	C3	49	79.6	B
14464	Bn-N13-p31812292	Bn-C3-p30306922	Bn-scaff_16092_1-p64728	C3	49	79.6	B
14465	Bn-N13-p31828686	Bn-C3-p30322532	Bn-scaff_16092_1-p66289	C3	49	79.6	B
14466	Bn-N13-p31842414	Bn-C3-p30336084	Bn-scaff_16092_1-p67644	C3	49	79.6	B
14467	Bn-N13-p31842588	Bn-C3-p30336258	Bn-scaff_16092_1-p67661	C3	49	79.6	B
14468	Bn-N13-p31842599	Bn-C3-p30336269	Bn-scaff_16092_1-p67662	C3	49	79.6	B
14469	Bn-N13-p31854535	Bn-C3-p30349060	Bn-scaff_16092_1-p68941	C3	49	79.6	B
14470	Bn-N13-p31855096	Bn-C3-p30349618	Bn-scaff_16092_1-p68997	C3	49	79.6	B
14471	Bn-N13-p31867027	Bn-C3-p30362886	Bn-scaff_16092_1-p70324	C3	49	79.6	B
14472	Bn-N13-p31867090	Bn-C3-p30362948	Bn-scaff_16092_1-p70330	C3	49	79.6	B
14473	Bn-N13-p31867440	Bn-C3-p30363298	Bn-scaff_16092_1-p70365	C3	49	79.6	B
14474	Bn-N13-p31868680	Bn-C3-p30364537	Bn-scaff_16092_1-p70489	C3	49	79.6	B
14475	Bn-N13-p31917295	Bn-C3-p30413083	Bn-scaff_16092_1-p75344	C3	49	79.6	B
14476	Bn-N13-p31987138	Bn-C3-p30479250	Bn-scaff_16092_1-p81960	C3	49	79.6	B
14477	Bn-N13-p32002561	Bn-C3-p30494568	Bn-scaff_16092_1-p83492	C3	49	79.6	B
14478	Bn-N13-p32033287	Bn-C3-p30524777	Bn-scaff_16092_1-p86513	C3	49	79.6	B
14479	Bn-N13-p32038310	Bn-C3-p30529798	Bn-scaff_16092_1-p87015	C3	49	79.6	B
14480	Bn-N13-p32047176	Bn-C3-p30538566	Bn-scaff_16092_1-p87892	C3	49	79.6	B
14481	Bn-N13-p32088412	Bn-C3-p30583421	Bn-scaff_16092_1-p92378	C3	49	79.6	B
14482	Bn-N13-p32089397	Bn-C3-p30584407	Bn-scaff_16092_1-p92476	C3	49	79.6	B
14483	Bn-N13-p32108816	Bn-Scaffold01004-p1	Bn-scaff_25970_1-p12375	C3	49	79.6	B
14484	Bn-N13-p32188354	Bn-C3-p30639437	Bn-scaff_18917_1-p97693	C3	49	79.6	B
14485	Bn-N13-p32255473	Bn-C3-p30670300	Bn-scaff_18917_1-p94607	C3	49	79.6	B
14486	Bn-N13-p32257752	Bn-C3-p30674099	Bn-scaff_18917_1-p94227	C3	49	79.6	B
14487	Bn-N13-p32298597	Bn-C3-p30707550	Bn-scaff_18917_1-p90882	C3	49	79.6	B
14488	Bn-N13-p32312348	Bn-C3-p30719552	Bn-scaff_18917_1-p89681	C3	49	79.6	B
14489	Bn-N13-p32314780	Bn-C3-p30722089	Bn-scaff_18917_1-p89428	C3	49	79.6	B
14490	Bn-N13-p32332629	Bn-C3-p30741138	Bn-scaff_18917_1-p87523	C3	49	79.6	B
14491	Bn-N3-p19142981	Bn-C3-p29258384	Bn-scaff_19026_1-p19458	C3	49	79.6	B
14492	Bn-N3-p19337910	Bn-A03-p20375108	Bn-A03-p19071304	C3	49	79.6	B
14493	Bn-N8-p5615925	Bn-C3-p29351747	Bn-scaff_19026_1-p10121	C3	49	79.6	B
14494	Bn-N13-p32339346	Bn-C3-p30751017	Bn-scaff_18917_1-p86535	C3	50	81.2	B
14495	Bn-N13-p32344580	Bn-C3-p30759235	Bn-scaff_18917_1-p85713	C3	50	81.2	B
14496	Bn-N13-p32366878	Bn-C3-p30777255	Bn-scaff_18917_1-p83911	C3	50	81.2	B
14497	Bn-N13-p32381614	Bn-C3-p30799116	Bn-scaff_18917_1-p81725	C3	50	81.2	B
14498	Bn-N13-p32444822	Bn-C3-p30872016	Bn-scaff_18917_1-p74435	C3	51	82	B
14499	Bn-N13-p32448833	Bn-C3-p30873178	Bn-scaff_18917_1-p74319	C3	51	82	B

14500	Bn-N13-p32451972	Bn-C3-p30874934	Bn-scaff_18917_1-p741437	C3	51	82	B
14501	Bn-N13-p32469555	Bn-C3-p30896342	Bn-scaff_18917_1-p720029	C3	52	82.8	B
14502	Bn-N13-p32501068	Bn-C3-p30926648	Bn-scaff_18917_1-p689723	C3	52	82.8	B
14503	Bn-N13-p32501325	Bn-C3-p30926907	Bn-scaff_18917_1-p689464	C3	52	82.8	B
14504	Bn-N13-p32520072	Bn-C3-p30949166	Bn-scaff_18917_1-p667205	C3	53	84.4	B
14505	Bn-N13-p32525760	Bn-C3-p30957993	Bn-scaff_18917_1-p658378	C3	53	84.4	B
14506	Bn-N13-p32526185	Bn-C3-p30958419	Bn-scaff_18917_1-p657952	C3	53	84.4	B
14507	Bn-N13-p32527256	Bn-C3-p30958820	Bn-scaff_18917_1-p657551	C3	53	84.4	B
14508	Bn-N13-p32529700	Bn-C3-p30961268	Bn-scaff_18917_1-p655103	C3	53	84.4	B
14509	Bn-N13-p32535433	Bn-C3-p30962374	Bn-scaff_18917_1-p653997	C3	53	84.4	B
14510	Bn-N13-p32536074	Bn-C3-p30963016	Bn-scaff_18917_1-p653355	C3	53	84.4	B
14511	Bn-N13-p32536680	Bn-C3-p30963623	Bn-scaff_18917_1-p652748	C3	53	84.4	B
14512	Bn-N13-p32547080	Bn-C3-p30993624	Bn-scaff_18917_1-p622747	C3	53	84.4	B
14513	Bn-N13-p32547386	Bn-C3-p30993930	Bn-scaff_18917_1-p622441	C3	53	84.4	B
14514	Bn-N13-p32560628	Bn-C3-p30997395	Bn-scaff_18917_1-p618976	C3	53	84.4	B
14515	Bn-N13-p32575203	Bn-C3-p31012578	Bn-scaff_18917_1-p603793	C3	53	84.4	B
14516	Bn-N13-p32575299	Bn-C3-p31012673	Bn-scaff_18917_1-p603698	C3	53	84.4	B
14517	Bn-N13-p32601491	Bn-C3-p31021199	Bn-scaff_18917_1-p595172	C3	53	84.4	B
14518	Bn-N13-p32620186	Bn-C3-p31064730	Bn-scaff_18917_1-p551641	C3	53	84.4	B
14519	Bn-N13-p32623241	Bn-C3-p31067873	Bn-scaff_18917_1-p548498	C3	53	84.4	B
14520	Bn-N13-p32627973	Bn-C3-p31074164	Bn-scaff_18917_1-p542207	C3	53	84.4	B
14521	Bn-N13-p32632922	Bn-C3-p31079660	Bn-scaff_18917_1-p536711	C3	53	84.4	B
14522	Bn-N13-p32633049	Bn-C3-p31079785	Bn-scaff_18917_1-p536586	C3	53	84.4	B
14523	Bn-N13-p32636143	Bn-C3-p31082872	Bn-scaff_18917_1-p533499	C3	53	84.4	B
14524	Bn-N13-p32640752	Bn-C3-p31090831	Bn-scaff_18917_1-p525540	C3	53	84.4	B
14525	Bn-N13-p32647170	Bn-C3-p31101837	Bn-scaff_18917_1-p514534	C3	54	85.2	B
14526	Bn-N13-p32647656	Bn-C3-p31102315	Bn-scaff_18917_1-p514056	C3	54	85.2	B
14527	Bn-N13-p32657324	Bn-C3-p31123697	Bn-scaff_18917_1-p492674	C3	54	85.2	B
14528	Bn-N13-p32684911	Bn-C3-p31143746	Bn-scaff_18917_1-p472625	C3	54	85.2	B
14529	Bn-N13-p32697270	Bn-C3-p31151783	Bn-scaff_18917_1-p464588	C3	54	85.2	B
14530	Bn-N13-p32697303	Bn-C3-p31151816	Bn-scaff_18917_1-p464555	C3	54	85.2	B
14531	Bn-N13-p32721670	Bn-C3-p31172022	Bn-scaff_18917_1-p444349	C3	54	85.2	B
14532	Bn-N13-p32732195	Bn-C3-p31174419	Bn-scaff_18917_1-p441952	C3	54	85.2	B
14533	Bn-N13-p32748227	Bn-C3-p31182800	Bn-scaff_18917_1-p433571	C3	54	85.2	B
14534	Bn-N13-p32873836	Bn-C3-p31296084	Bn-scaff_18917_1-p320287	C3	54	85.2	B
14535	Bn-N13-p32892410	Bn-C3-p31333707	Bn-scaff_18917_1-p282664	C3	54	85.2	B
14536	Bn-N13-p32911267	Bn-C3-p31348970	Bn-scaff_18917_1-p267401	C3	54	85.2	B
14537	Bn-N13-p32965462	Bn-C3-p31384015	Bn-scaff_18917_1-p232356	C3	54	85.2	B
14538	Bn-N13-p33112798	Bn-C3-p31516965	Bn-scaff_18917_1-p99406	C3	54	85.2	B
14539	Bn-N13-p33233104	Bn-C3-p31525660	Bn-scaff_18917_1-p90711	C3	54	85.2	B
14540	Bn-N13-p33485836	Bn-C3-p31832053	Bn-scaff_16130_5-p10410	C3	54	85.2	B
14541	Bn-N13-p33513301	Bn-C7-p31396736	Bn-scaff_16130_1-p119945	C3	54	85.2	B
14542	Bn-N13-p33513441	Bn-C3-p31842689	Bn-scaff_16130_2-p801510	C3	54	85.2	B
14543	Bn-N13-p33513490	Bn-C3-p31842739	Bn-scaff_16130_2-p801460	C3	54	85.2	B
14544	Bn-N13-p33781131	Bn-C3-p32119982	Bn-scaff_16130_2-p524217	C3	54	85.2	B
14545	Bn-N15-p43061512	Bn-C5-p41257114	Bn-scaff_21369_1-p522214	C3	54	85.2	B
14546	Bn-N13-p33816297	Bn-C3-p32159218	Bn-scaff_16130_2-p484981	C3	55	86	B
14547	Bn-N13-p33816414	Bn-C3-p32159335	Bn-scaff_16130_2-p484864	C3	55	86	B
14548	Bn-N13-p33830390	Bn-C3-p32171936	Bn-scaff_16130_2-p472263	C3	55	86	B

14549	Bn-N13-p33831861	Bn-C3-p32173406	Bn-scaff_16130_2-p47079[illegible]	C3	55	86	B
14550	Bn-N13-p33895652	Bn-C3-p32224822	Bn-scaff_16130_2-p41937[illegible]	C3	55	86	B
14551	Bn-N13-p33896499	Bn-C3-p32225669	Bn-scaff_16130_2-p41853[illegible]	C3	55	86	B
14552	Bn-N13-p33896562	Bn-C3-p32225732	Bn-scaff_16130_2-p41846[illegible]	C3	55	86	B
14553	Bn-N13-p33896873	Bn-C3-p32226043	Bn-scaff_16130_2-p41815[illegible]	C3	55	86	B
14554	Bn-N13-p33897228	Bn-C3-p32226398	Bn-scaff_16130_2-p41780[illegible]	C3	55	86	B
14555	Bn-N13-p33897270	Bn-C3-p32226440	Bn-scaff_16130_2-p41775[illegible]	C3	55	86	B
14556	Bn-N13-p33898970	Bn-C3-p32228140	Bn-scaff_16130_2-p41605[illegible]	C3	55	86	B
14557	Bn-N13-p33899178	Bn-C3-p32228348	Bn-scaff_16130_2-p41585[illegible]	C3	55	86	B
14558	Bn-N13-p33900929	Bn-C3-p32229835	Bn-scaff_16130_2-p41436[illegible]	C3	55	86	B
14559	Bn-N13-p33901032	Bn-C3-p32229938	Bn-scaff_16130_2-p41426[illegible]	C3	55	86	B
14560	Bn-N13-p33901106	Bn-C3-p32230012	Bn-scaff_16130_2-p41418[illegible]	C3	55	86	B
14561	Bn-N13-p33901312	Bn-C3-p32230218	Bn-scaff_16130_2-p41398[illegible]	C3	55	86	B
14562	Bn-N13-p33901391	Bn-C3-p32230297	Bn-scaff_16130_2-p41390[illegible]	C3	55	86	B
14563	Bn-N13-p33901560	Bn-C3-p32230466	Bn-scaff_16130_2-p41373[illegible]	C3	55	86	B
14564	Bn-N13-p33901767	Bn-C3-p32230673	Bn-scaff_16130_2-p41352[illegible]	C3	55	86	B
14565	Bn-N13-p33903503	Bn-C3-p32232409	Bn-scaff_16130_2-p41179[illegible]	C3	55	86	B
14566	Bn-N13-p33912030	Bn-C3-p32240804	Bn-scaff_16130_2-p40339[illegible]	C3	55	86	B
14567	Bn-N13-p33928148	Bn-C3-p32265829	Bn-scaff_16130_2-p37837[illegible]	C3	55	86	B
14568	Bn-N13-p33968181	Bn-Scaffold01374-p5[illegible]	Bn-scaff_24459_1-p5391	C3	55	86	B
14569	Bn-N13-p33981091	Bn-C3-p32295639	Bn-scaff_15695_1-p77786[illegible]	C3	55	86	B
14570	Bn-N13-p33982581	Bn-C3-p32297126	Bn-scaff_15695_1-p77637[illegible]	C3	55	86	B
14571	Bn-N13-p33983156	Bn-C3-p32297703	Bn-scaff_15695_1-p77580[illegible]	C3	55	86	B
14572	Bn-N13-p34174138	Bn-A03-p2737190	Bn-A03-p20375108	C3	55	86	B
14573	Bn-N13-p34174231	Bn-C3-p32459744	Bn-scaff_15695_1-p61376[illegible]	C3	55	86	B
14574	Bn-N13-p34176483	Bn-C3-p32461996	Bn-scaff_15695_1-p61150[illegible]	C3	55	86	B
14575	Bn-N13-p34233201	Bn-C3-p32508698	Bn-scaff_15695_1-p56480[illegible]	C3	55	86	B
14576	Bn-N13-p34322177	Bn-C3-p32602753	Bn-scaff_15695_1-p47075[illegible]	C3	55	86	B
14577	Bn-N13-p34416114	Bn-C3-p32690493	Bn-scaff_15695_1-p38301[illegible]	C3	55	86	B
14578	Bn-N13-p34416175	Bn-C3-p32690554	Bn-scaff_15695_1-p38295[illegible]	C3	55	86	B
14579	Bn-N13-p34418492	Bn-C3-p32692870	Bn-scaff_15695_1-p38063[illegible]	C3	55	86	B
14580	Bn-N13-p34418646	Bn-C3-p32693020	Bn-scaff_15695_1-p38048[illegible]	C3	55	86	B
14581	Bn-N13-p34439334	Bn-C3-p32719798	Bn-scaff_26320_1-p29859[illegible]	C3	55	86	B
14582	Bn-N13-p34439630	Bn-C3-p32720094	Bn-scaff_26320_1-p29829[illegible]	C3	55	86	B
14583	Bn-N13-p34440251	Bn-C3-p32720714	Bn-scaff_26320_1-p29767[illegible]	C3	55	86	B
14584	Bn-N13-p34452249	Bn-C3-p32736042	Bn-scaff_26320_1-p28234[illegible]	C3	55	86	B
14585	Bn-N13-p34452352	Bn-C3-p32736145	Bn-scaff_26320_1-p28224[illegible]	C3	55	86	B
14586	Bn-N13-p34458934	Bn-C3-p32747139	Bn-scaff_26320_1-p27124[illegible]	C3	55	86	B
14587	Bn-N13-p34464756	Bn-C3-p32747917	Bn-scaff_26320_1-p27047[illegible]	C3	55	86	B
14588	Bn-N13-p34464835	Bn-C3-p32747996	Bn-scaff_26320_1-p27039[illegible]	C3	55	86	B
14589	Bn-N13-p34465164	Bn-C3-p32748325	Bn-scaff_26320_1-p27006[illegible]	C3	55	86	B
14590	Bn-N13-p34465777	Bn-C3-p32748938	Bn-scaff_26320_1-p26945[illegible]	C3	55	86	B
14591	Bn-N13-p34477558	Bn-C3-p32759599	Bn-scaff_26320_1-p25878[illegible]	C3	55	86	0
14592	Bn-N13-p34478583	Bn-C3-p32760624	Bn-scaff_26320_1-p25776[illegible]	C3	55	86	B
14593	Bn-N13-p34479990	Bn-C3-p32762014	Bn-scaff_26320_1-p25637[illegible]	C3	55	86	B
14594	Bn-N13-p34499745	Bn-C3-p32781687	Bn-scaff_26320_1-p23670[illegible]	C3	55	86	B
14595	Bn-N13-p34500911	Bn-C3-p32782853	Bn-scaff_26320_1-p23553[illegible]	C3	55	86	B
14596	Bn-N13-p34514243	Bn-C3-p32797926	Bn-scaff_26320_1-p22046[illegible]	C3	55	86	B
14597	Bn-N13-p34514266	Bn-C3-p32797963	Bn-scaff_26320_1-p22042[illegible]	C3	55	86	B

14598	Bn-N13-p34883782	Bn-C3-p33167179	Bn-scaff_18855_1-p79543	C3	55	86	B
14599	Bn-N13-p35432096	Bn-C3-p33715848	Bn-scaff_18855_1-p24676	C3	55	86	B
14600	Bn-N13-p35521866	Bn-C3-p33819147	Bn-scaff_18855_1-p14346	C3	55	86	B
14601	Bn-N13-p35652489	Bn-C3-p33936981	Bn-scaff_18855_1-p25630	C3	55	86	B
14602	Bn-N13-p35652722	Bn-C3-p33937214	Bn-scaff_18855_1-p25397	C3	55	86	B
14603	Bn-N13-p35664026	Bn-C3-p33946015	Bn-scaff_18855_1-p16596	C3	55	86	B
14604	Bn-N13-p35665701	Bn-C3-p33947690	Bn-scaff_18855_1-p14921	C3	55	86	B
14605	Bn-N13-p35666759	Bn-C3-p33948754	Bn-scaff_18855_1-p13857	C3	55	86	B
14606	Bn-N13-p35678142	Bn-C3-p33951411	Bn-scaff_18855_1-p11200	C3	55	86	B
14607	Bn-N13-p35715570	Bn-C3-p33971853	Bn-scaff_25094_1-p9143	C3	55	86	B
14608	Bn-N13-p35717299	Bn-C3-p33975839	Bn-scaff_25094_1-p13129	C3	55	86	B
14609	Bn-N13-p35717355	Bn-C3-p33975894	Bn-scaff_25094_1-p13184	C3	55	86	B
14610	Bn-N13-p35724962	Bn-C3-p33988770	Bn-scaff_25094_1-p26060	C3	55	86	B
14611	Bn-N13-p35725238	Bn-C3-p33989052	Bn-scaff_25094_1-p26342	C3	55	86	B
14612	Bn-N13-p35726272	Bn-C3-p33989915	Bn-scaff_25094_1-p27205	C3	55	86	B
14613	Bn-N13-p35827137	Bn-C3-p34058698	Bn-scaff_25094_1-p95988	C3	55	86	B
14614	Bn-N13-p35827652	Bn-C3-p34059213	Bn-scaff_25094_1-p96503	C3	55	86	B
14615	Bn-N13-p35852017	Bn-Scaffold01027-p3	Bn-scaff_19109_1-p33401	C3	55	86	B
14616	Bn-N13-p35852112	Bn-Scaffold01027-p3	Bn-scaff_19109_1-p33306	C3	55	86	B
14617	Bn-N13-p35853192	Bn-Scaffold01027-p3	Bn-scaff_19109_1-p30884	C3	55	86	B
14618	Bn-N13-p35859216	Bn-Scaffold01027-p2	Bn-scaff_19109_1-p24726	C3	55	86	B
14619	Bn-N13-p35861653	Bn-Scaffold01027-p2	Bn-scaff_19109_1-p22283	C3	55	86	B
14620	Bn-N13-p35886546	Bn-C3-p34082692	Bn-scaff_25094_1-p11998	C3	55	86	B
14621	Bn-N13-p35901567	Bn-C3-p34087722	Bn-scaff_25094_1-p12501	C3	55	86	B
14622	Bn-N13-p35903137	Bn-C3-p34089291	Bn-scaff_25094_1-p12658	C3	55	86	B
14623	Bn-N5-p12207672	Bn-C3-p33817586	Bn-scaff_18855_1-p14502	C3	55	86	B
14624	Bn-N8-p15558094	Bn-C3-p32775588	Bn-scaff_26320_1-p24280	C3	55	86	B
14625	Bn-Scaffold01107-p12231	Bn-C3-p33817643	Bn-scaff_18855_1-p14496	C3	55	86	B
14626	Bn-Scaffold01107-p12252	Bn-C3-p33817664	Bn-scaff_18855_1-p14494	C3	55	86	B
14627	Bn-N13-p36646684	Bn-C3-p34846496	Bn-scaff_17044_1-p54533	C3	56	86.8	B
14628	Bn-N13-p36660268	Bn-C3-p34860207	Bn-scaff_17044_1-p53162	C3	56	86.8	B
14629	Bn-N13-p36662124	Bn-C3-p34862294	Bn-scaff_17044_1-p52953	C3	56	86.8	B
14630	Bn-N13-p36665403	Bn-C3-p34865613	Bn-scaff_17044_1-p52621	C3	56	86.8	B
14631	Bn-N13-p36665940	Bn-C3-p34866111	Bn-scaff_17044_1-p52572	C3	56	86.8	B
14632	Bn-N13-p36668857	Bn-C3-p34869029	Bn-scaff_17044_1-p52280	C3	56	86.8	B
14633	Bn-N13-p37011996	Bn-C3-p35168382	Bn-scaff_15747_2-p12730	C3	57	89.2	B
14634	Bn-N13-p37129127	Bn-C3-p35228355	Bn-scaff_15747_2-p67333	C3	57	89.2	B
14635	Bn-N14-p9266093	Bn-C3-p29600126	Bn-scaff_16822_1-p59416	C3	57	89.2	B
14636	Bn-N13-p38217511	Bn-C3-p36232123	Bn-scaff_18356_1-p34974	C3	58	92.4	B
14637	Bn-N13-p38299095	Bn-C3-p36288903	Bn-scaff_18356_1-p29296	C3	58	92.4	B
14638	Bn-N13-p38345444	Bn-C3-p36350685	Bn-scaff_18356_1-p23117	C3	58	92.4	B
14639	Bn-N13-p38363334	Bn-C3-p36361548	Bn-scaff_18356_1-p22031	C3	58	92.4	B
14640	Bn-N6-p16737937	Bn-C3-p36309663	Bn-scaff_18356_1-p27220	C3	58	92.4	B
14641	Bn-N13-p38520334	Bn-C3-p36538236	Bn-scaff_18356_1-p43628	C3	59	93.2	B
14642	Bn-N13-p38520690	Bn-C3-p36538592	Bn-scaff_18356_1-p43272	C3	59	93.2	B
14643	Bn-N13-p38524463	Bn-C3-p36542401	Bn-scaff_18356_1-p39463	C3	59	93.2	B
14644	Bn-N6-p16544357	Bn-A06-p19885379	Bn-A06-p14594965	C3	59	93.2	B
14645	Bn-N13-p35494025	Bn-C3-p33783470	Bn-scaff_18855_1-p17914	C3	60	94.8	B
14646	Bn-N13-p35494152	Bn-C3-p33778273	Bn-scaff_18855_1-p18433	C3	60	94.8	B

14647	Bn-N13-p38631850	Bn-C3-p36648793	Bn-scaff_19310_1-p66830	C3	60	94.8	B
14648	Bn-N13-p38638636	Bn-C3-p36655589	Bn-scaff_19310_1-p73626	C3	60	94.8	B
14649	Bn-N13-p38640224	Bn-C3-p36657177	Bn-scaff_19310_1-p75214	C3	60	94.8	B
14650	Bn-N13-p38641798	Bn-C3-p36658108	Bn-scaff_19310_1-p76145	C3	60	94.8	B
14651	Bn-N13-p38644912	Bn-C3-p36661221	Bn-scaff_19310_1-p79258	C3	60	94.8	B
14652	Bn-N13-p38648039	Bn-C3-p36668904	Bn-scaff_19310_1-p86941	C3	60	94.8	B
14653	Bn-N13-p38670958	Bn-C3-p36698517	Bn-scaff_19310_1-p116554	C3	60	94.8	B
14654	Bn-N13-p38675075	Bn-C3-p36706321	Bn-scaff_19310_1-p124358	C3	60	94.8	B
14655	Bn-N13-p38749837	Bn-C3-p36784398	Bn-scaff_19310_1-p202435	C3	60	94.8	B
14656	Bn-N13-p38756837	Bn-C3-p36791160	Bn-scaff_19310_1-p209197	C3	60	94.8	B
14657	Bn-N13-p38788490	Bn-C3-p36836062	Bn-scaff_19310_1-p254099	C3	60	94.8	B
14658	Bn-N13-p38811779	Bn-C3-p36858011	Bn-scaff_19310_1-p276048	C3	60	94.8	B
14659	Bn-N13-p38812341	Bn-C3-p36858504	Bn-scaff_19310_1-p276541	C3	60	94.8	B
14660	Bn-N13-p38815640	Bn-C3-p36861824	Bn-scaff_19310_1-p279861	C3	60	94.8	B
14661	Bn-N13-p38870659	Bn-C3-p36933151	Bn-scaff_19310_1-p351188	C3	60	94.8	B
14662	Bn-N13-p38871641	Bn-C3-p36934467	Bn-scaff_19310_1-p352504	C3	60	94.8	B
14663	Bn-N13-p38878449	Bn-C3-p36943908	Bn-scaff_19310_1-p361945	C3	60	94.8	B
14664	Bn-N13-p38891019	Bn-C3-p36958710	Bn-scaff_19310_1-p376747	C3	60	94.8	B
14665	Bn-N13-p38945132	Bn-C3-p37014678	Bn-scaff_19310_1-p432715	C3	60	94.8	B
14666	Bn-N13-p38945187	Bn-C3-p37014733	Bn-scaff_19310_1-p432770	C3	60	94.8	B
14667	Bn-N13-p38945189	Bn-C3-p37014735	Bn-scaff_19310_1-p432772	C3	60	94.8	B
14668	Bn-N13-p38945934	Bn-C3-p37015479	Bn-scaff_19310_1-p433516	C3	60	94.8	B
14669	Bn-N13-p39436608	Bn-C3-p37083937	Bn-scaff_19310_1-p501974	C3	60	94.8	B
14670	Bn-N13-p39436858	Bn-C3-p37084189	Bn-scaff_19310_1-p502226	C3	60	94.8	B
14671	Bn-N13-p39436947	Bn-C3-p37084278	Bn-scaff_19310_1-p502315	C3	60	94.8	B
14672	Bn-N13-p39442480	Bn-C3-p37091616	Bn-scaff_19310_1-p509653	C3	60	94.8	B
14673	Bn-N13-p39444599	Bn-C3-p37093750	Bn-scaff_19310_1-p511787	C3	60	94.8	B
14674	Bn-N13-p39463755	Bn-C3-p37113154	Bn-scaff_19310_1-p531191	C3	60	94.8	B
14675	Bn-N13-p39486947	Bn-C3-p37144070	Bn-scaff_19310_1-p562107	C3	60	94.8	B
14676	Bn-N13-p39487486	Bn-C3-p37144609	Bn-scaff_19310_1-p562646	C3	60	94.8	B
14677	Bn-N13-p39487557	Bn-C3-p37144680	Bn-scaff_19310_1-p562717	C3	60	94.8	B
14678	Bn-N13-p39488905	Bn-C3-p37146027	Bn-scaff_19310_1-p564064	C3	60	94.8	B
14679	Bn-N13-p39569343	Bn-C3-p37210210	Bn-scaff_19310_1-p628247	C3	60	94.8	B
14680	Bn-N13-p39572716	Bn-C3-p37213591	Bn-scaff_19310_1-p631628	C3	60	94.8	B
14681	Bn-N13-p39579082	Bn-C3-p37226553	Bn-scaff_19310_1-p644590	C3	60	94.8	B
14682	Bn-N13-p39579176	Bn-C3-p37226647	Bn-scaff_19310_1-p644684	C3	60	94.8	B
14683	Bn-N13-p39702172	Bn-C3-p37366742	Bn-scaff_27076_1-p26680	C3	60	94.8	B
14684	Bn-N13-p39789050	Bn-C3-p37437762	Bn-scaff_17249_1-p132072	C3	60	94.8	B
14685	Bn-N13-p39818348	Bn-C3-p37462532	Bn-scaff_17249_1-p129595	C3	60	94.8	B
14686	Bn-N13-p40369251	Bn-C3-p38009199	Bn-scaff_17249_1-p749288	C3	60	94.8	B
14687	Bn-N13-p40472912	Bn-C3-p38126042	Bn-scaff_17249_1-p632445	C3	60	94.8	B
14688	Bn-N13-p40552674	Bn-C3-p38193605	Bn-scaff_17249_1-p564882	C3	60	94.8	B
14689	Bn-N13-p40786318	Bn-C3-p38458079	Bn-scaff_17249_1-p300408	C3	60	94.8	B
14690	Bn-N13-p41187292	Bn-C3-p38853970	Bn-scaff_17573_1-p95384	C3	60	94.8	B
14691	Bn-N13-p41601991	Bn-C3-p39352732	Bn-scaff_16350_1-p661907	C3	60	94.8	B
14692	Bn-N13-p41971769	Bn-C3-p39841895	Bn-scaff_16350_1-p172744	C3	60	94.8	B
14693	Bn-N13-p41977354	Bn-C3-p39836534	Bn-scaff_16350_1-p178105	C3	60	94.8	B
14694	Bn-N13-p42001439	Bn-C3-p39809422	Bn-scaff_16350_1-p205217	C3	60	94.8	B
14695	Bn-N13-p43203897	Bn-C3-p44218506	Bn-scaff_15758_3-p559128	C3	60	94.8	B

14696	Bn-N13-p43387442	Bn-C3-p44391298	Bn-scaff_15758_3-p73192(	C3	60	94.8	B
14697	Bn-N13-p44035034	Bn-C3-p40540813	Bn-scaff_19702_1-p29496(	C3	60	94.8	0
14698	Bn-N13-p44120126	Bn-Scaffold01131-p3(	Bn-scaff_17636_1-p3673	C3	60	94.8	B
14699	Bn-N13-p44314137	Bn-C3-p40630897	Bn-scaff_19702_1-p20487(	C3	60	94.8	B
14700	Bn-N13-p44489742	Bn-C3-p40908336	Bn-scaff_19965_1-p72464	C3	60	94.8	B
14701	Bn-N13-p44639592	Bn-C3-p41060885	Bn-scaff_19965_1-p22501:	C3	60	94.8	B
14702	Bn-N13-p45008685	Bn-C3-p41808850	Bn-scaff_15758_1-p36244	C3	60	94.8	B
14703	Bn-N13-p45953324	Bn-C3-p45357569	Bn-scaff_21851_1-p36812(	C3	60	94.8	B
14704	Bn-N13-p46410417	Bn-C3-p45722717	Bn-scaff_21330_1-p35297!	C3	60	94.8	B
14705	Bn-N13-p46473155	Bn-C3-p45783876	Bn-scaff_21330_1-p29181(	C3	60	94.8	B
14706	Bn-N13-p46500968	Bn-C3-p45816380	Bn-scaff_21330_1-p25931:	C3	60	94.8	B
14707	Bn-N13-p46502754	Bn-C3-p45818157	Bn-scaff_21330_1-p25753!	C3	60	94.8	B
14708	Bn-N13-p46727768	Bn-C3-p46100031	Bn-scaff_19186_1-p84723!	C3	60	94.8	B
14709	Bn-N13-p46744387	Bn-C3-p46122333	Bn-scaff_19186_1-p82493:	C3	60	94.8	B
14710	Bn-N13-p49629240	Bn-C3-p47772261	Bn-scaff_15611_1-p69883!	C3	60	94.8	B
14711	Bn-N13-p50048707	Bn-C3-p48085859	Bn-scaff_20881_1-p19298	C3	60	94.8	B
14712	Bn-N13-p50449853	Bn-C3-p48551593	Bn-scaff_17977_1-p14691:	C3	60	94.8	B
14713	Bn-N13-p50788583	Bn-C3-p48893599	Bn-scaff_21867_1-p72729:	C3	60	94.8	B
14714	Bn-N13-p50818978	Bn-C3-p48934557	Bn-scaff_21867_1-p68633!	C3	60	94.8	B
14715	Bn-N13-p52438725	Bn-C3-p50595928	Bn-scaff_20790_1-p28389{	C3	60	94.8	B
14716	Bn-N14-p28544333	Bn-C3-p37066011	Bn-scaff_19310_1-p48404{	C3	60	94.8	B
14717	Bn-N6-p10298421	Bn-A06-p14576278	Bn-A06-p10609641	C3	60	94.8	B
14718	Bn-N6-p11810477	Bn-scaffold19029-p3:	Bn-Scaffold000100-p87356	C3	60	94.8	B
14719	Bn-N6-p15587768	Bn-C3-p37118435	Bn-scaff_19310_1-p53647:	C3	60	94.8	B
14720	Bn-N6-p15919859	Bn-A06-p14594965	Bn-A06-p14576278	C3	60	94.8	B
14721	Bn-Scaffold02177-p3359	Bn-C3-p41809796	Bn-scaff_15758_1-p37190	C3	60	94.8	B
14722	Bn-Scaffold13992-p275	Bn-C3-p37083170	Bn-scaff_19310_1-p50120?	C3	60	94.8	B
14723	Bn-N13-p51208823	Bn-C3-p49533128	Bn-scaff_21867_1-p87764	C3	61	95.6	B
14724	Bn-N13-p51259014	Bn-C3-p49570914	Bn-scaff_21867_1-p49978	C3	61	95.6	B
14725	Bn-N13-p51263530	Bn-C3-p49581184	Bn-scaff_21867_1-p39708	C3	61	95.6	B
14726	Bn-N13-p51273270	Bn-C3-p49590360	Bn-scaff_21867_1-p30532	C3	61	95.6	B
14727	Bn-N13-p51450003	Bn-C3-p49743619	Bn-scaff_23407_1-p12805?	C3	62	96.4	B
14728	Bn-N13-p51457114	Bn-C3-p49751093	Bn-scaff_23407_1-p12058:	C3	62	96.4	B
14729	Bn-N13-p51493654	Bn-C3-p49784531	Bn-scaff_23407_1-p87145	C3	62	96.4	B
14730	Bn-N13-p51493679	Bn-C3-p49784556	Bn-scaff_23407_1-p87120	C3	62	96.4	B
14731	Bn-N13-p51493972	Bn-C3-p49784832	Bn-scaff_23407_1-p86844	C3	62	96.4	B
14732	Bn-N13-p51495468	Bn-C3-p49786328	Bn-scaff_23407_1-p85348	C3	62	96.4	B
14733	Bn-N13-p51495500	Bn-C3-p49786360	Bn-scaff_23407_1-p85316	C3	62	96.4	B
14734	Bn-N13-p51495715	Bn-C3-p49786575	Bn-scaff_23407_1-p85101	C3	62	96.4	B
14735	Bn-N13-p51496071	Bn-C3-p49786930	Bn-scaff_23407_1-p84746	C3	62	96.4	B
14736	Bn-N13-p51496961	Bn-C3-p49787820	Bn-scaff_23407_1-p83856	C3	62	96.4	B
14737	Bn-N13-p51497319	Bn-C3-p49788178	Bn-scaff_23407_1-p83498	C3	62	96.4	B
14738	Bn-N13-p51497394	Bn-C3-p49788253	Bn-scaff_23407_1-p83423	C3	62	96.4	B
14739	Bn-N13-p51497493	Bn-C3-p49788352	Bn-scaff_23407_1-p83324	C3	62	96.4	B
14740	Bn-N13-p51497666	Bn-C3-p49788525	Bn-scaff_23407_1-p83151	C3	62	96.4	B
14741	Bn-N13-p51519082	Bn-C3-p49810151	Bn-scaff_23407_1-p61525	C3	62	96.4	B
14742	Bn-N13-p51519304	Bn-C3-p49810373	Bn-scaff_23407_1-p61303	C3	62	96.4	B
14743	Bn-N13-p51544919	Bn-C3-p49835464	Bn-scaff_23407_1-p36212	C3	62	96.4	B
14744	Bn-N13-p51545525	Bn-C3-p49836070	Bn-scaff_23407_1-p35606	C3	62	96.4	B

14745	Bn-N13-p51545680	Bn-C3-p49836225	Bn-scaff_23407_1-p35451	C3	62	96.4	B
14746	Bn-N13-p51553878	Bn-C3-p49844242	Bn-scaff_23407_1-p27434	C3	62	96.4	B
14747	Bn-N13-p51554299	Bn-C3-p49844663	Bn-scaff_23407_1-p27013	C3	62	96.4	B
14748	Bn-N13-p51612439	Bn-C3-p49848601	Bn-scaff_23407_1-p23075	C3	62	96.4	B
14749	Bn-N13-p51781470	Bn-C3-p50052688	Bn-scaff_19740_1-p96323	C3	62	96.4	B
14750	Bn-N13-p51787936	Bn-C3-p50057758	Bn-scaff_19740_1-p91253	C3	62	96.4	B
14751	Bn-N13-p51812788	Bn-C3-p50081209	Bn-scaff_19740_1-p67802	C3	62	96.4	B
14752	Bn-N13-p51813749	Bn-C3-p50082170	Bn-scaff_19740_1-p66841	C3	62	96.4	B
14753	Bn-N13-p51835215	Bn-C3-p50103458	Bn-scaff_19740_1-p45553	C3	62	96.4	B
14754	Bn-N13-p51868312	Bn-C3-p50139811	Bn-scaff_19740_1-p9200	C3	62	96.4	B
14755	Bn-N13-p51895110	Bn-C13868303-p376	Bn-C13679819-p142	C3	62	96.4	B
14756	Bn-N13-p52072295	Bn-C3-p50225807	Bn-scaff_16135_1-p17000	C3	62	96.4	B
14757	Bn-N13-p52085695	Bn-C3-p50240085	Bn-scaff_16135_1-p18428	C3	62	96.4	B
14758	Bn-N13-p52098514	Bn-C3-p50251780	Bn-scaff_16135_1-p19597	C3	62	96.4	B
14759	Bn-N13-p52124627	Bn-C3-p50286221	Bn-scaff_16135_1-p23041	C3	62	96.4	0
14760	Bn-N13-p52152019	Bn-C3-p50313134	Bn-scaff_16135_1-p25732	C3	62	96.4	B
14761	Bn-N13-p52195586	Bn-C3-p50365382	Bn-scaff_16135_1-p30957	C3	62	96.4	B
14762	Bn-N13-p52197718	Bn-C3-p50367512	Bn-scaff_16135_1-p31170	C3	62	96.4	B
14763	Bn-N6-p9942897	Bn-C3-p49845275	Bn-scaff_23407_1-p26401	C3	62	96.4	B
14764	Bn-N13-p52659189	Bn-C3-p50876390	Bn-scaff_20790_1-p3436	C3	63	98	B
14765	Bn-N13-p52684406	Bn-C3-p50892430	Bn-scaff_18849_1-p35049	C3	63	98	B
14766	Bn-N11-p15574205	Bn-C3-p51036492	Bn-scaff_18849_1-p20643	C3	64	102	B
14767	Bn-N11-p27032084	Bn-A07-p11330018	Bn-A06-p19885379	C3	64	102	B
14768	Bn-N13-p52808696	Bn-C3-p51012387	Bn-scaff_18849_1-p23054	C3	64	102	B
14769	Bn-N13-p52808715	Bn-C3-p51012407	Bn-scaff_18849_1-p23052	C3	64	102	B
14770	Bn-N13-p52809088	Bn-C3-p51012792	Bn-scaff_18849_1-p23013	C3	64	102	B
14771	Bn-N13-p52840897	Bn-C3-p51048812	Bn-scaff_18849_1-p19411	C3	64	102	B
14772	Bn-N13-p52841334	Bn-C3-p51049226	Bn-scaff_18849_1-p19370	C3	64	102	B
14773	Bn-N13-p52841472	Bn-C3-p51049364	Bn-scaff_18849_1-p19356	C3	64	102	B
14774	Bn-N13-p52842794	Bn-C3-p51050684	Bn-scaff_18849_1-p19224	C3	64	102	B
14775	Bn-N13-p52843234	Bn-C3-p51051124	Bn-scaff_18849_1-p19180	C3	64	102	B
14776	Bn-N13-p52843875	Bn-C3-p51051765	Bn-scaff_18849_1-p19116	C3	64	102	B
14777	Bn-N13-p52845515	Bn-C3-p51053396	Bn-scaff_18849_1-p18953	C3	64	102	B
14778	Bn-N13-p52845624	Bn-C3-p51053505	Bn-scaff_18849_1-p18942	C3	64	102	B
14779	Bn-N13-p52845694	Bn-C3-p51053575	Bn-scaff_18849_1-p18935	C3	64	102	B
14780	Bn-N13-p52845885	Bn-C3-p51053767	Bn-scaff_18849_1-p18916	C3	64	102	B
14781	Bn-N13-p52904401	Bn-C3-p51106133	Bn-scaff_18849_1-p13679	C3	64	102	B
14782	Bn-N13-p52924645	Bn-C3-p51136494	Bn-scaff_18849_1-p10643	C3	64	102	B
14783	Bn-N15-p21427152	Bn-C4-p42011416	Bn-scaff_15852_1-p62257	C3	64	102	B
14784	Bn-N2-p14552801	Bn-C3-p51033808	Bn-scaff_18849_1-p20912	C3	64	102	B
14785	Bn-N8-p16897915	Bn-C3-p51097191	Bn-scaff_18849_1-p14573	C3	64	102	B
14786	Bn-N8-p16897922	Bn-A08-p8334516	Bn-A08-p17190098	C3	64	102	B
14787	Bn-N8-p16918207	Bn-C3-p51012844	Bn-scaff_18849_1-p23008	C3	64	102	B
14788	Bn-Scaffold46029-p89	Bn-C3-p51033230	Bn-scaff_18849_1-p20969	C3	64	102	B
14789	Bn-N13-p53059228	Bn-C3-p51220360	Bn-scaff_18849_1-p22568	C3	65	103.6	B
14790	Bn-N13-p53103572	Bn-C3-p51270477	Bn-scaff_17440_1-p93854	C3	65	103.6	B
14791	Bn-N13-p53103642	Bn-C3-p51270547	Bn-scaff_17440_1-p93847	C3	65	103.6	B
14792	Bn-N13-p53103969	Bn-C3-p51270935	Bn-scaff_17440_1-p93808	C3	65	103.6	B
14793	Bn-N13-p53167783	Bn-C3-p51341012	Bn-scaff_17440_1-p86800	C3	65	103.6	B

14794	Bn-N13-p53200707	Bn-C3-p51376361	Bn-scaff_17440_1-p832657	C3	65	103.6	B
14795	Bn-N13-p53204129	Bn-C3-p51379725	Bn-scaff_17440_1-p829293	C3	65	103.6	B
14796	Bn-N13-p53374845	Bn-C3-p51549327	Bn-scaff_17440_1-p659691	C3	65	103.6	B
14797	Bn-N13-p53442652	Bn-C3-p51615442	Bn-scaff_17440_1-p593576	C3	65	103.6	B
14798	Bn-N13-p53443386	Bn-C3-p51616176	Bn-scaff_17440_1-p592842	C3	65	103.6	B
14799	Bn-N13-p53445599	Bn-C3-p51618353	Bn-scaff_17440_1-p590665	C3	66	104.4	B
14800	Bn-N13-p53499955	Bn-C3-p51656147	Bn-scaff_17440_1-p552871	C3	66	104.4	B
14801	Bn-N13-p53527538	Bn-C3-p51685151	Bn-scaff_17440_1-p523867	C3	66	104.4	B
14802	Bn-N13-p53546844	Bn-C3-p51704544	Bn-scaff_17440_1-p504474	C3	66	104.4	B
14803	Bn-N13-p53574023	Bn-C3-p51736449	Bn-scaff_17440_1-p472569	C3	66	104.4	B
14804	Bn-N13-p53651160	Bn-C3-p51797405	Bn-scaff_17440_1-p411613	C3	66	104.4	B
14805	Bn-N13-p53692374	Bn-C3-p51836130	Bn-scaff_17440_1-p372888	C3	66	104.4	B
14806	Bn-N13-p53775106	Bn-C3-p51892437	Bn-scaff_17440_1-p316581	C3	67	105.2	B
14807	Bn-N13-p53780464	Bn-C3-p51897790	Bn-scaff_17440_1-p311228	C3	67	105.2	B
14808	Bn-N13-p53785208	Bn-C3-p51902479	Bn-scaff_17440_1-p306539	C3	67	105.2	B
14809	Bn-N13-p53789724	Bn-C3-p51909947	Bn-scaff_17440_1-p299071	C3	67	105.2	B
14810	Bn-N13-p53820816	Bn-C3-p51940041	Bn-scaff_17440_1-p268977	C3	67	105.2	B
14811	Bn-N13-p53834225	Bn-C3-p51947104	Bn-scaff_17440_1-p261914	C3	67	105.2	B
14812	Bn-N13-p53837698	Bn-C3-p51950882	Bn-scaff_17440_1-p258136	C3	67	105.2	B
14813	Bn-N13-p53850474	Bn-C3-p51964975	Bn-scaff_17440_1-p244043	C3	67	105.2	B
14814	Bn-N13-p53894484	Bn-C3-p52016973	Bn-scaff_17440_1-p192045	C3	67	105.2	B
14815	Bn-N13-p53904438	Bn-C3-p52031480	Bn-scaff_17440_1-p177538	C3	67	105.2	B
14816	Bn-N13-p53984842	Bn-C3-p52101988	Bn-scaff_17440_1-p107030	C3	67	105.2	B
14817	Bn-N13-p54026938	Bn-C3-p52140962	Bn-scaff_17440_1-p68056	C3	67	105.2	B
14818	Bn-N13-p54051447	Bn-C3-p52163606	Bn-scaff_17440_1-p45412	C3	67	105.2	B
14819	Bn-N13-p54063279	Bn-C3-p52183164	Bn-scaff_17440_1-p25854	C3	67	105.2	B
14820	Bn-N13-p54069581	Bn-C3-p52188423	Bn-scaff_17440_1-p20595	C3	67	105.2	B
14821	Bn-N13-p54080578	Bn-C3-p52198679	Bn-scaff_17440_1-p10339	C3	67	105.2	B
14822	Bn-N13-p54099150	Bn-C3-p52222301	Bn-scaff_16372_1-p13184	C3	67	105.2	B
14823	Bn-N13-p54173879	Bn-C3-p52298120	Bn-scaff_16372_1-p89003	C3	67	105.2	B
14824	Bn-N13-p54247904	Bn-C3-p52389904	Bn-scaff_24726_1-p90002	C3	67	105.2	B
14825	Bn-N13-p54249545	Bn-C3-p52391546	Bn-scaff_24726_1-p88360	C3	67	105.2	B
14826	Bn-Scaffold01549-p3827	Bn-C3-p52150246	Bn-scaff_17440_1-p58772	C3	67	105.2	B
14827	Bn-Scaffold01549-p4600	Bn-C3-p52151020	Bn-scaff_17440_1-p57998	C3	67	105.2	B
14828	Bn-N13-p54320108	Bn-C3-p52463498	Bn-scaff_24726_1-p16408	C3	68	106	B
14829	Bn-N13-p54326105	Bn-C13931552-p379	Bn-C13868303-p376	C3	68	106	B
14830	Bn-N13-p54333399	Bn-Scaffold01946-p4	Bn-scaff_25535_1-p416	C3	68	106	B
14831	Bn-N13-p54348188	Bn-C3-p52500055	Bn-scaff_16665_1-p20050	C3	68	106	B
14832	Bn-N13-p54375312	Bn-C3-p52528545	Bn-scaff_16665_1-p48540	C3	68	106	B
14833	Bn-N13-p54375363	Bn-C3-p52528596	Bn-scaff_16665_1-p48591	C3	68	106	B
14834	Bn-N13-p54375434	Bn-C3-p52528667	Bn-scaff_16665_1-p48662	C3	68	106	B
14835	Bn-N13-p54376554	Bn-C3-p52529798	Bn-scaff_16665_1-p49793	C3	68	106	B
14836	Bn-N13-p54376745	Bn-C3-p52530027	Bn-scaff_16665_1-p50022	C3	68	106	B
14837	Bn-N13-p54385536	Bn-C3-p52534642	Bn-scaff_16665_1-p54637	C3	68	106	B
14838	Bn-N13-p54393200	Bn-C3-p52544882	Bn-scaff_16665_1-p64877	C3	68	106	B
14839	Bn-N13-p54400116	Bn-C3-p52548805	Bn-scaff_16665_1-p68800	C3	68	106	B
14840	Bn-N13-p54401340	Bn-C3-p52550144	Bn-scaff_16665_1-p70139	C3	68	106	B
14841	Bn-N13-p54450120	Bn-C3-p52577134	Bn-scaff_16665_1-p97129	C3	68	106	B
14842	Bn-N13-p54455052	Bn-C3-p52578769	Bn-scaff_16665_1-p98764	C3	68	106	B

14843	Bn-N13-p54456158	Bn-C3-p52579879	Bn-scaff_16665_1-p99874	C3	68	106	B
14844	Bn-N13-p54498507	Bn-C3-p52638341	Bn-scaff_16665_1-p15833	C3	68	106	B
14845	Bn-N13-p54498633	Bn-C3-p52638467	Bn-scaff_16665_1-p15846	C3	68	106	B
14846	Bn-N13-p54499644	Bn-C3-p52649067	Bn-scaff_16665_1-p16906	C3	68	106	B
14847	Bn-N13-p54525534	Bn-C3-p52668609	Bn-scaff_16665_1-p18860	C3	68	106	B
14848	Bn-N13-p54531614	Bn-C3-p52671210	Bn-scaff_16665_1-p19120	C3	68	106	B
14849	Bn-N13-p54554829	Bn-C3-p52678240	Bn-scaff_16665_1-p19823	C3	68	106	B
14850	Bn-N13-p54554899	Bn-C3-p52678309	Bn-scaff_16665_1-p19830	C3	68	106	B
14851	Bn-N13-p54554959	Bn-C3-p52678369	Bn-scaff_16665_1-p19836	C3	68	106	B
14852	Bn-N13-p54554978	Bn-C3-p52678388	Bn-scaff_16665_1-p19838	C3	68	106	B
14853	Bn-N13-p54555726	Bn-C3-p52679137	Bn-scaff_16665_1-p19913	C3	68	106	B
14854	Bn-N13-p54555784	Bn-C3-p52679195	Bn-scaff_16665_1-p19919	C3	68	106	B
14855	Bn-N13-p54555897	Bn-C3-p52679308	Bn-scaff_16665_1-p19930	C3	68	106	B
14856	Bn-N13-p54556007	Bn-C3-p52679425	Bn-scaff_16665_1-p19942	C3	68	106	B
14857	Bn-N13-p54559689	Bn-C3-p52680269	Bn-scaff_16665_1-p20026	C3	68	106	B
14858	Bn-N13-p54767699	Bn-C3-p52897428	Bn-scaff_24631_1-p63534	C3	68	106	B
14859	Bn-N13-p54890638	Bn-C3-p53018880	Bn-scaff_24631_1-p18498	C3	69	106.8	B
14860	Bn-N13-p54892951	Bn-C3-p53021205	Bn-scaff_24631_1-p18731	C3	69	106.8	B
14861	Bn-N13-p55004318	Bn-C3-p53102126	Bn-scaff_24631_1-p26823	C3	70	108.4	B
14862	Bn-N13-p55008263	Bn-C3-p53105434	Bn-scaff_24631_1-p27154	C3	70	108.4	B
14863	Bn-N13-p55013233	Bn-C3-p53110410	Bn-scaff_24631_1-p27651	C3	70	108.4	B
14864	Bn-N13-p55032494	Bn-C3-p53130345	Bn-scaff_24631_1-p29645	C3	70	108.4	B
14865	Bn-N13-p55034552	Bn-C3-p53131198	Bn-scaff_24631_1-p29730	C3	70	108.4	B
14866	Bn-N13-p55036817	Bn-C3-p53133169	Bn-scaff_24631_1-p29927	C3	70	108.4	B
14867	Bn-N13-p55036932	Bn-C3-p53133325	Bn-scaff_24631_1-p29943	C3	70	108.4	B
14868	Bn-N11-p4172101	Bn-C3-p53182462	Bn-scaff_24631_1-p34856	C3	71	109.2	B
14869	Bn-N13-p55065650	Bn-C3-p53172050	Bn-scaff_24631_1-p33815	C3	71	109.2	B
14870	Bn-N13-p55109430	Bn-C3-p53206876	Bn-scaff_24631_1-p37298	C3	71	109.2	B
14871	Bn-N13-p55120897	Bn-C3-p53207846	Bn-scaff_24631_1-p37395	C3	71	109.2	B
14872	Bn-N13-p55140852	Bn-C3-p53225796	Bn-scaff_24631_1-p39190	C3	71	109.2	B
14873	Bn-N2-p21093121	Bn-C3-p53238536	Bn-scaff_24631_1-p40464	C3	71	109.2	B
14874	Bn-N13-p55167270	Bn-C3-p53249881	Bn-scaff_24631_1-p41598	C3	72	110.8	B
14875	Bn-N13-p55173407	Bn-C3-p53256125	Bn-scaff_24631_1-p42223	C3	72	110.8	B
14876	Bn-N13-p55173554	Bn-C3-p53256271	Bn-scaff_24631_1-p42237	C3	72	110.8	B
14877	Bn-N13-p55490354	Bn-C3-p53619703	Bn-scaff_24631_1-p78580	C3	72	110.8	B
14878	Bn-N13-p55490361	Bn-C3-p53619696	Bn-scaff_24631_1-p78580	C3	72	110.8	B
14879	Bn-N13-p55535153	Bn-C3-p53573401	Bn-scaff_24631_1-p73950	C3	72	110.8	B
14880	Bn-N13-p55535171	Bn-C3-p53573383	Bn-scaff_24631_1-p73948	C3	72	110.8	B
14881	Bn-N13-p55535987	Bn-C3-p53572597	Bn-scaff_24631_1-p73870	C3	72	110.8	B
14882	Bn-N13-p55880297	Bn-C3-p53979059	Bn-scaff_16394_2-p14985	C3	73	111.6	B
14883	Bn-N13-p55884043	Bn-C3-p53982861	Bn-scaff_16394_2-p14947	C3	73	111.6	B
14884	Bn-N13-p55920691	Bn-C3-p54016664	Bn-scaff_16394_2-p14609	C3	73	111.6	B
14885	Bn-N13-p55943902	Bn-C3-p54031554	Bn-scaff_16394_2-p14460	C3	73	111.6	B
14886	Bn-N13-p56066278	Bn-C3-p54100388	Bn-scaff_16394_2-p13772	C3	74	112.4	B
14887	Bn-N13-p56069547	Bn-C3-p54102673	Bn-scaff_16394_2-p13749	C3	74	112.4	B
14888	Bn-N13-p56070424	Bn-C3-p54103303	Bn-scaff_16394_2-p13743	C3	74	112.4	B
14889	Bn-N13-p56070738	Bn-C3-p54103693	Bn-scaff_16394_2-p13739	C3	74	112.4	B
14890	Bn-N13-p56163948	Bn-C3-p54212650	Bn-scaff_16394_2-p12649	C3	74	112.4	B
14891	Bn-N13-p56245794	Bn-C3-p54299542	Bn-scaff_16394_2-p11780	C3	74	112.4	B

14892	Bn-N13-p56315266	Bn-C3-p54369460	Bn-scaff_16394_2-p110815	C3	74	112.4 B
14893	Bn-N13-p56317556	Bn-C3-p54371712	Bn-scaff_16394_2-p110590	C3	74	112.4 B
14894	Bn-N13-p56338358	Bn-C3-p54390989	Bn-scaff_16394_2-p108663	C3	74	112.4 B
14895	Bn-N13-p56483433	Bn-C3-p54484753	Bn-scaff_16394_2-p992866	C3	75	113.2 B
14896	Bn-N13-p56688554	Bn-C3-p54690736	Bn-scaff_16394_2-p786883	C3	75	113.2 B
14897	Bn-N13-p56688986	Bn-C3-p54691168	Bn-scaff_16394_2-p786451	C3	75	113.2 B
14898	Bn-N13-p56690037	Bn-C3-p54692219	Bn-scaff_16394_2-p785400	C3	75	113.2 B
14899	Bn-N13-p56690360	Bn-C3-p54692542	Bn-scaff_16394_2-p785077	C3	75	113.2 B
14900	Bn-N13-p56690389	Bn-C3-p54692571	Bn-scaff_16394_2-p785048	C3	75	113.2 B
14901	Bn-N13-p56690853	Bn-C3-p54693035	Bn-scaff_16394_2-p784584	C3	75	113.2 B
14902	Bn-N13-p56710012	Bn-scaffold19029-p39	Bn-scaffold19029-p329	C3	75	113.2 B
14903	Bn-N13-p56710075	Bn-scaffold5052-p146	Bn-scaffold19029-p392	C3	75	113.2 B
14904	Bn-N13-p56714612	Bn-Scaffold01531-p24	Bn-scaff_28554_1-p2486	C3	75	113.2 B
14905	Bn-N13-p56715271	Bn-Scaffold01531-p18	Bn-scaff_28554_1-p1828	C3	75	113.2 B
14906	Bn-N13-p56720979	Bn-C3-p54750916	Bn-scaff_16394_2-p726703	C3	75	113.2 B
14907	Bn-N13-p56722294	Bn-C3-p54751954	Bn-scaff_16394_2-p725665	C3	75	113.2 B
14908	Bn-N13-p56722582	Bn-C3-p54752242	Bn-scaff_16394_2-p725377	C3	75	113.2 B
14909	Bn-N13-p56722992	Bn-C3-p54752652	Bn-scaff_16394_2-p724967	C3	75	113.2 B
14910	Bn-N13-p56764124	Bn-C3-p54782101	Bn-scaff_16394_2-p695518	C3	75	113.2 B
14911	Bn-N13-p56764339	Bn-C3-p54782308	Bn-scaff_16394_2-p695311	C3	75	113.2 B
14912	Bn-N13-p56764619	Bn-C3-p54782588	Bn-scaff_16394_2-p695031	C3	75	113.2 B
14913	Bn-N13-p56776197	Bn-C3-p54800300	Bn-scaff_16394_2-p677319	C3	75	113.2 B
14914	Bn-N13-p56779756	Bn-C3-p54803795	Bn-scaff_16394_2-p673824	C3	75	113.2 B
14915	Bn-N13-p56780911	Bn-C3-p54804929	Bn-scaff_16394_2-p672690	C3	75	113.2 B
14916	Bn-N13-p56780988	Bn-C3-p54805006	Bn-scaff_16394_2-p672613	C3	75	113.2 B
14917	Bn-N13-p56783980	Bn-C3-p54807116	Bn-scaff_16394_2-p670503	C3	75	113.2 B
14918	Bn-N13-p56805193	Bn-C3-p54835654	Bn-scaff_16394_2-p641965	C3	75	113.2 B
14919	Bn-N13-p56809186	Bn-C3-p54839644	Bn-scaff_16394_2-p637975	C3	75	113.2 B
14920	Bn-N13-p56812165	Bn-C3-p54842405	Bn-scaff_16394_2-p635214	C3	75	113.2 B
14921	Bn-N13-p56816909	Bn-C3-p54847202	Bn-scaff_16394_2-p630417	C3	75	113.2 B
14922	Bn-N13-p56851744	Bn-C3-p54912441	Bn-scaff_16394_2-p565178	C3	75	113.2 B
14923	Bn-N13-p56852428	Bn-C3-p54913125	Bn-scaff_16394_2-p564494	C3	75	113.2 B
14924	Bn-N13-p56858064	Bn-C3-p54918817	Bn-scaff_16394_2-p558802	C3	75	113.2 B
14925	Bn-N13-p56859087	Bn-C3-p54919166	Bn-scaff_16394_2-p558453	C3	75	113.2 B
14926	Bn-N13-p56859229	Bn-C3-p54919308	Bn-scaff_16394_2-p558311	C3	75	113.2 B
14927	Bn-N13-p56859321	Bn-C3-p54919421	Bn-scaff_16394_2-p558198	C3	75	113.2 B
14928	Bn-N13-p56862865	Bn-C3-p54922892	Bn-scaff_16394_2-p554727	C3	75	113.2 B
14929	Bn-N13-p56863778	Bn-C3-p54923804	Bn-scaff_16394_2-p553815	C3	75	113.2 B
14930	Bn-N13-p56863804	Bn-C3-p54923830	Bn-scaff_16394_2-p553789	C3	75	113.2 B
14931	Bn-N13-p56871176	Bn-C3-p54925889	Bn-scaff_16394_2-p551730	C3	75	113.2 B
14932	Bn-N13-p56871585	Bn-C3-p54926298	Bn-scaff_16394_2-p551321	C3	75	113.2 B
14933	Bn-N13-p56871904	Bn-C3-p54926617	Bn-scaff_16394_2-p551002	C3	75	113.2 B
14934	Bn-N13-p56886005	Bn-C3-p54935079	Bn-scaff_16394_2-p542540	C3	75	113.2 B
14935	Bn-N13-p56894043	Bn-C3-p54947089	Bn-scaff_16394_2-p530530	C3	75	113.2 B
14936	Bn-N13-p56896419	Bn-C3-p54949675	Bn-scaff_16394_2-p527944	C3	75	113.2 B
14937	Bn-N13-p56896673	Bn-C3-p54949930	Bn-scaff_16394_2-p527689	C3	75	113.2 B
14938	Bn-N13-p56899119	Bn-C3-p54952450	Bn-scaff_16394_2-p525169	C3	75	113.2 B
14939	Bn-N13-p56899215	Bn-C3-p54952546	Bn-scaff_16394_2-p525073	C3	75	113.2 B
14940	Bn-N13-p56909381	Bn-C3-p54962511	Bn-scaff_16394_2-p515108	C3	75	113.2 B

14941	Bn-N13-p56912831	Bn-C3-p54965961	Bn-scaff_16394_2-p511658	C3	75	113.2	B
14942	Bn-N13-p56913833	Bn-C3-p54967557	Bn-scaff_16394_2-p510062	C3	75	113.2	B
14943	Bn-Scaffold00990-p24835	Bn-Scaffold01007-p6(	Bn-scaff_28265_1-p6630	C3	75	113.2	B
14944	Bn-Scaffold00990-p25636	Bn-Scaffold01007-p7	Bn-scaff_28265_1-p7428	C3	75	113.2	B
14945	Bn-Scaffold00990-p38024	Bn-Scaffold01007-p1(	Bn-scaff_28265_1-p16036	C3	75	113.2	B
14946	Bn-Scaffold02437-p857	Bn-C3-p52472009	Bn-scaff_24726_1-p7897	C3	75	113.2	B
14947	Bn-Scaffold03561-p2560	Bn-Scaffold01007-p2(	Bn-scaff_28265_1-p20757	C3	75	113.2	B
14948	Bn-Scaffold03561-p860	Bn-Scaffold01007-p1	Bn-scaff_28265_1-p19042	C3	75	113.2	B
14949	Bn-N13-p56956424	Bn-C3-p55005964	Bn-scaff_16394_2-p471655	C3	76	117.2	A
14950	Bn-N13-p56958818	Bn-C3-p55008225	Bn-scaff_16394_2-p469394	C3	76	117.2	A
14951	Bn-N13-p56981601	Bn-C3-p55029418	Bn-scaff_16394_2-p448201	C3	76	117.2	A
14952	Bn-N13-p57080292	Bn-C3-p55129421	Bn-scaff_16394_2-p348198	C3	77	118	A
14953	Bn-N13-p57080525	Bn-C3-p55129659	Bn-scaff_16394_2-p347960	C3	77	118	A
14954	Bn-N13-p57107393	Bn-C3-p55146962	Bn-scaff_16394_2-p330657	C3	77	118	A
14955	Bn-N13-p57107623	Bn-C3-p55147192	Bn-scaff_16394_2-p330427	C3	77	118	A
14956	Bn-N13-p57399905	Bn-C3-p55406195	Bn-scaff_16394_2-p71424	C3	78	118.8	A
14957	Bn-N13-p57400045	Bn-C3-p55406334	Bn-scaff_16394_2-p71285	C3	78	118.8	A
14958	Bn-N13-p57407005	Bn-C3-p55420852	Bn-scaff_16394_2-p56767	C3	78	118.8	A
14959	Bn-N13-p57407151	Bn-C3-p55421002	Bn-scaff_16394_2-p56617	C3	78	118.8	A
14960	Bn-N13-p57408001	Bn-C3-p55421691	Bn-scaff_16394_2-p55928	C3	78	118.8	A
14961	Bn-N13-p57408206	Bn-C3-p55421896	Bn-scaff_16394_2-p55723	C3	78	118.8	A
14962	Bn-N13-p57409117	Bn-C3-p55422785	Bn-scaff_16394_2-p54834	C3	78	118.8	A
14963	Bn-N13-p57414491	Bn-C3-p55424016	Bn-scaff_16394_2-p53603	C3	78	118.8	A
14964	Bn-N13-p57415179	Bn-C3-p55424707	Bn-scaff_16394_2-p52912	C3	78	118.8	A
14965	Bn-N13-p57416061	Bn-C3-p55426747	Bn-scaff_16394_2-p50872	C3	78	118.8	A
14966	Bn-N13-p57416115	Bn-C3-p55426801	Bn-scaff_16394_2-p50818	C3	78	118.8	A
14967	Bn-N13-p57421892	Bn-C3-p55427186	Bn-scaff_16394_2-p50433	C3	78	118.8	A
14968	Bn-N13-p57436782	Bn-C3-p55437048	Bn-scaff_16394_2-p40571	C3	78	118.8	A
14969	Bn-N13-p57439589	Bn-C3-p55452998	Bn-scaff_16394_2-p24621	C3	78	118.8	A
14970	Bn-N13-p57470149	Bn-C3-p55491656	Bn-scaff_18602_1-p13938	C3	78	118.8	A
14971	Bn-N13-p57487802	Bn-C3-p55498311	Bn-scaff_18602_1-p20593	C3	78	118.8	A
14972	Bn-N13-p57488604	Bn-C3-p55499116	Bn-scaff_18602_1-p21398	C3	78	118.8	0
14973	Bn-N13-p57491318	Bn-C3-p55501722	Bn-scaff_18602_1-p24004	C3	78	118.8	A
14974	Bn-N13-p57491436	Bn-C3-p55501840	Bn-scaff_18602_1-p24122	C3	78	118.8	A
14975	Bn-N13-p57491719	Bn-C3-p55502123	Bn-scaff_18602_1-p24405	C3	78	118.8	A
14976	Bn-N13-p57491725	Bn-C3-p55502128	Bn-scaff_18602_1-p24410	C3	78	118.8	A
14977	Bn-N13-p57494719	Bn-C3-p55523801	Bn-scaff_18602_1-p46083	C3	79	119.6	A
14978	Bn-N13-p57540903	Bn-C3-p55566792	Bn-scaff_18602_1-p89074	C3	80	120.4	A
14979	Bn-N13-p57545175	Bn-C3-p55573223	Bn-scaff_18602_1-p95505	C3	80	120.4	A
14980	Bn-N13-p57567953	Bn-C3-p55590808	Bn-scaff_18602_1-p113090	C3	80	120.4	A
14981	Bn-N13-p57582753	Bn-C3-p55616845	Bn-scaff_18602_1-p139127	C3	80	120.4	A
14982	Bn-N13-p57589082	Bn-C3-p55620332	Bn-scaff_18602_1-p142614	C3	80	120.4	A
14983	Bn-N13-p57697131	Bn-C3-p55735911	Bn-scaff_18602_1-p258193	C3	80	120.4	A
14984	Bn-N13-p57700845	Bn-C3-p55739552	Bn-scaff_18602_1-p261834	C3	80	120.4	A
14985	Bn-N13-p57701017	Bn-C3-p55739724	Bn-scaff_18602_1-p262006	C3	80	120.4	A
14986	Bn-N13-p57701192	Bn-C3-p55739899	Bn-scaff_18602_1-p262181	C3	80	120.4	A
14987	Bn-N13-p57701931	Bn-C3-p55740638	Bn-scaff_18602_1-p262920	C3	80	120.4	A
14988	Bn-N13-p57702220	Bn-C3-p55740925	Bn-scaff_18602_1-p263207	C3	80	120.4	A
14989	Bn-Scaffold17674-p322	Bn-C3-p55739020	Bn-scaff_18602_1-p261302	C3	80	120.4	A

14990	Bn-N13-p57793747	Bn-C3-p55817169	Bn-scaff_16182_1-p632134	C3	81	121.2	A
14991	Bn-N13-p57826468	Bn-C3-p55903363	Bn-scaff_16182_1-p545940	C3	81	121.2	A
14992	Bn-N13-p57828272	Bn-C3-p55906940	Bn-scaff_16182_1-p542363	C3	81	121.2	A
14993	Bn-N13-p57852769	Bn-C3-p55923809	Bn-scaff_16182_1-p525494	C3	81	121.2	A
14994	Bn-N13-p57852952	Bn-C3-p55923990	Bn-scaff_16182_1-p525313	C3	81	121.2	A
14995	Bn-N13-p57853190	Bn-C3-p55924228	Bn-scaff_16182_1-p525075	C3	81	121.2	A
14996	Bn-N13-p57853423	Bn-C3-p55924461	Bn-scaff_16182_1-p524842	C3	81	121.2	A
14997	Bn-N13-p57858491	Bn-C3-p55926453	Bn-scaff_16182_1-p522850	C3	81	121.2	A
14998	Bn-N13-p57922320	Bn-C3-p55997644	Bn-scaff_16182_1-p451659	C3	81	121.2	A
14999	Bn-N13-p58054641	Bn-C3-p56130180	Bn-scaff_16182_1-p319123	C3	81	121.2	A
15000	Bn-N13-p58077787	Bn-C3-p56152632	Bn-scaff_16182_1-p296671	C3	81	121.2	A
15001	Bn-N13-p58446455	Bn-C3-p56493426	Bn-scaff_15703_1-p44024	C3	81	121.2	A
15002	Bn-N13-p58451156	Bn-C3-p56502711	Bn-scaff_15703_1-p53309	C3	81	121.2	A
15003	Bn-N13-p58483638	Bn-C3-p56545732	Bn-scaff_15703_1-p96330	C3	81	121.2	A
15004	Bn-N8-p7392181	Bn-C13679819-p142	Bn-A08-p8334516	C3	81	121.2	A
15005	Bn-Scaffold01185-p13173	Bn-C3-p55867417	Bn-scaff_16182_1-p581886	C3	81	121.2	A
15006	Bn-Scaffold01185-p13552	Bn-C3-p55867792	Bn-scaff_16182_1-p581511	C3	81	121.2	A
15007	Bn-Scaffold01185-p14243	Bn-C3-p55888567	Bn-scaff_16182_1-p560736	C3	81	121.2	A
15008	Bn-Scaffold01185-p21672	Bn-C3-p55881230	Bn-scaff_16182_1-p568073	C3	81	121.2	A
15009	Bn-N13-p58686520	Bn-Scaffold00889-p2	Bn-scaff_26846_1-p26261	C3	82	122	A
15010	Bn-N13-p58695204	Bn-Scaffold00889-p3	Bn-scaff_26846_1-p30953	C3	82	122	A
15011	Bn-N13-p58699520	Bn-Scaffold00889-p3	Bn-scaff_26846_1-p38699	C3	82	122	A
15012	Bn-N13-p58766071	Bn-Scaffold00887-p2	Bn-scaff_18206_1-p510833	C3	82	122	A
15013	Bn-N13-p58768236	Bn-Scaffold00887-p2	Bn-scaff_18206_1-p508712	C3	82	122	A
15014	Bn-N13-p58771645	Bn-Scaffold00887-p1	Bn-scaff_18206_1-p505259	C3	82	122	A
15015	Bn-N13-p58771856	Bn-Scaffold00887-p1	Bn-scaff_18206_1-p505048	C3	82	122	A
15016	Bn-N13-p58772282	Bn-Scaffold00887-p1	Bn-scaff_18206_1-p504621	C3	82	122	A
15017	Bn-N13-p58796102	Bn-Scaffold01128-p2	Bn-scaff_18206_1-p484563	C3	82	122	A
15018	Bn-N13-p58796758	Bn-Scaffold01128-p2	Bn-scaff_18206_1-p483957	C3	82	122	A
15019	Bn-N13-p58808365	Bn-Scaffold01128-p1	Bn-scaff_18206_1-p479271	C3	82	122	A
15020	Bn-N13-p58812745	Bn-Scaffold01128-p1	Bn-scaff_18206_1-p475382	C3	82	122	A
15021	Bn-N13-p58812805	Bn-Scaffold01128-p1	Bn-scaff_18206_1-p475430	C3	82	122	A
15022	Bn-N13-p58813295	Bn-Scaffold01128-p1	Bn-scaff_18206_1-p475918	C3	82	122	A
15023	Bn-N13-p58813453	Bn-Scaffold01128-p1	Bn-scaff_18206_1-p476077	C3	82	122	A
15024	Bn-N13-p58813808	Bn-Scaffold01128-p1	Bn-scaff_18206_1-p476439	C3	82	122	A
15025	Bn-N13-p58813868	Bn-Scaffold01128-p1	Bn-scaff_18206_1-p476499	C3	82	122	A
15026	Bn-N13-p58889203	Bn-Scaffold00883-p3	Bn-scaff_21386_1-p39609	C3	82	122	A
15027	Bn-N13-p58889429	Bn-Scaffold00883-p3	Bn-scaff_21386_1-p39383	C3	82	122	A
15028	Bn-N13-p58891853	Bn-Scaffold00883-p3	Bn-scaff_21386_1-p37139	C3	82	122	A
15029	Bn-N13-p58892102	Bn-Scaffold00883-p3	Bn-scaff_21386_1-p36891	C3	82	122	A
15030	Bn-N13-p58899509	Bn-Scaffold00883-p3	Bn-scaff_21386_1-p36155	C3	82	122	A
15031	Bn-N13-p58899746	Bn-Scaffold00883-p3	Bn-scaff_21386_1-p35918	C3	82	122	0
15032	Bn-N13-p58899963	Bn-Scaffold00883-p3	Bn-scaff_21386_1-p35701	C3	82	122	A
15033	Bn-N13-p58900027	Bn-Scaffold00883-p3	Bn-scaff_21386_1-p35636	C3	82	122	A
15034	Bn-N13-p58900103	Bn-Scaffold00883-p3	Bn-scaff_21386_1-p35560	C3	82	122	A
15035	Bn-N13-p58900165	Bn-Scaffold00883-p3	Bn-scaff_21386_1-p35498	C3	82	122	A
15036	Bn-N13-p58910766	Bn-Scaffold00883-p3	Bn-scaff_21386_1-p30267	C3	82	122	A
15037	Bn-N13-p58911922	Bn-Scaffold00883-p2	Bn-scaff_21386_1-p29359	C3	82	122	A
15038	Bn-N13-p58912738	Bn-Scaffold00883-p2	Bn-scaff_21386_1-p26868	C3	82	122	A

15039	Bn-N13-p58917243	Bn-Scaffold00883-p1	Bn-scaff_21386_1-p17474	C3	82	122	A
15040	Bn-N13-p58917426	Bn-Scaffold00883-p1	Bn-scaff_21386_1-p17291	C3	82	122	A
15041	Bn-N13-p58941120	Bn-Scaffold01468-p5	Bn-scaff_24457_1-p5761	C3	82	122	A
15042	Bn-N13-p58941326	Bn-Scaffold01468-p5	Bn-scaff_24457_1-p5555	C3	82	122	A
15043	Bn-N13-p58941397	Bn-Scaffold01468-p5	Bn-scaff_24457_1-p5483	C3	82	122	A
15044	Bn-N13-p58941539	Bn-Scaffold01468-p5	Bn-scaff_24457_1-p5341	C3	82	122	A
15045	Bn-N13-p58942124	Bn-Scaffold01468-p4	Bn-scaff_24457_1-p4791	C3	82	122	A
15046	Bn-N13-p58943357	Bn-Scaffold01468-p4	Bn-scaff_24457_1-p4062	C3	82	122	A
15047	Bn-N13-p58943493	Bn-Scaffold01468-p3	Bn-scaff_24457_1-p3926	C3	82	122	A
15048	Bn-N13-p58943767	Bn-Scaffold01468-p3	Bn-scaff_24457_1-p3651	C3	82	122	A
15049	Bn-N13-p58944067	Bn-Scaffold01468-p3	Bn-scaff_24457_1-p3350	C3	82	122	A
15050	Bn-N13-p58944702	Bn-Scaffold01468-p2	Bn-scaff_24457_1-p2690	C3	82	122	A
15051	Bn-N13-p58946328	Bn-Scaffold01468-p1	Bn-scaff_24457_1-p1741	C3	82	122	A
15052	Bn-N13-p58947211	Bn-Scaffold01468-p8	Bn-scaff_24457_1-p839	C3	82	122	A
15053	Bn-N13-p58950093	Bn-Scaffold01468-p3	Bn-scaff_24457_1-p325	C3	82	122	A
15054	Bn-N13-p58950145	Bn-Scaffold01468-p2	Bn-scaff_24457_1-p273	C3	82	122	A
15055	Bn-N13-p58950918	Bn-C3-p56909471	Bn-scaff_18669_1-p16438	C3	82	122	A
15056	Bn-N13-p58952245	Bn-C3-p56910426	Bn-scaff_18669_1-p17393	C3	82	122	A
15057	Bn-N13-p58970442	Bn-C3-p56924769	Bn-scaff_18669_1-p31736	C3	82	122	A
15058	Bn-N13-p58972692	Bn-C3-p56927004	Bn-scaff_18669_1-p33971	C3	82	122	A
15059	Bn-N13-p58984125	Bn-C3-p56934819	Bn-scaff_18669_1-p41786	C3	82	122	A
15060	Bn-N13-p59028157	Bn-C3-p56744658	Bn-scaff_26642_1-p5287	C3	82	122	A
15061	Bn-N13-p59028427	Bn-C3-p56744887	Bn-scaff_26642_1-p5516	C3	82	122	A
15062	Bn-N13-p59028608	Bn-C3-p56751945	Bn-scaff_26642_1-p12574	C3	82	122	A
15063	Bn-N13-p59029570	Bn-C3-p56752788	Bn-scaff_26642_1-p13417	C3	82	122	A
15064	Bn-N13-p59029803	Bn-C3-p56753021	Bn-scaff_26642_1-p13650	C3	82	122	A
15065	Bn-N13-p59030085	Bn-C3-p56753302	Bn-scaff_26642_1-p13931	C3	82	122	A
15066	Bn-N13-p59030125	Bn-C3-p56753342	Bn-scaff_26642_1-p13971	C3	82	122	A
15067	Bn-N13-p59030194	Bn-C3-p56753411	Bn-scaff_26642_1-p14040	C3	82	122	A
15068	Bn-N13-p59030649	Bn-C3-p56753859	Bn-scaff_26642_1-p14488	C3	82	122	A
15069	Bn-N13-p59035029	Bn-C3-p56758530	Bn-scaff_26642_1-p19159	C3	82	122	A
15070	Bn-N13-p59035753	Bn-C3-p56759595	Bn-scaff_26642_1-p20224	C3	82	122	A
15071	Bn-N13-p59035904	Bn-C3-p56759746	Bn-scaff_26642_1-p20375	C3	82	122	A
15072	Bn-N13-p59036134	Bn-C3-p56759976	Bn-scaff_26642_1-p20605	C3	82	122	A
15073	Bn-N13-p59036528	Bn-C3-p56760487	Bn-scaff_26642_1-p21116	C3	82	122	A
15074	Bn-N13-p59036613	Bn-C3-p56760572	Bn-scaff_26642_1-p21201	C3	82	122	A
15075	Bn-N13-p59040713	Bn-C3-p56764078	Bn-scaff_26642_1-p24707	C3	82	122	A
15076	Bn-N13-p59040979	Bn-C3-p56764646	Bn-scaff_26642_1-p25275	C3	82	122	A
15077	Bn-N13-p59041416	Bn-C3-p56767867	Bn-scaff_26642_1-p28496	C3	82	122	A
15078	Bn-N13-p59041521	Bn-C3-p56767972	Bn-scaff_26642_1-p28601	C3	82	122	A
15079	Bn-N13-p59055198	Bn-C3-p56776186	Bn-scaff_26642_1-p36815	C3	82	122	A
15080	Bn-N13-p59057608	Bn-C3-p56783755	Bn-scaff_26642_1-p44384	C3	82	122	A
15081	Bn-N13-p59061860	Bn-C3-p56786468	Bn-scaff_26642_1-p47097	C3	82	122	A
15082	Bn-N13-p59069525	Bn-C3-p56790297	Bn-scaff_26642_1-p50926	C3	82	122	A
15083	Bn-N13-p59070008	Bn-C3-p56790787	Bn-scaff_26642_1-p51416	C3	82	122	A
15084	Bn-N13-p59071429	Bn-C3-p56792205	Bn-scaff_26642_1-p52834	C3	82	122	A
15085	Bn-N13-p59071522	Bn-C3-p56792300	Bn-scaff_26642_1-p52929	C3	82	122	A
15086	Bn-N13-p59073007	Bn-C3-p56792945	Bn-scaff_26642_1-p53574	C3	82	122	A
15087	Bn-N13-p59073110	Bn-C3-p56793048	Bn-scaff_26642_1-p53677	C3	82	122	A

15088	Bn-N13-p59074644	Bn-C3-p56794784	Bn-scaff_26642_1-p55413	C3	82	122	A
15089	Bn-N13-p59074735	Bn-C3-p56794875	Bn-scaff_26642_1-p55504	C3	82	122	A
15090	Bn-N13-p59074844	Bn-C3-p56794984	Bn-scaff_26642_1-p55613	C3	82	122	A
15091	Bn-N13-p59074958	Bn-C3-p56795096	Bn-scaff_26642_1-p55725	C3	82	122	A
15092	Bn-N13-p59110705	Bn-C3-p56817028	Bn-scaff_26642_1-p77657	C3	82	122	A
15093	Bn-N13-p59127987	Bn-C3-p56843811	Bn-scaff_26642_1-p10444	C3	82	122	A
15094	Bn-N13-p59130047	Bn-C3-p56847933	Bn-scaff_26642_1-p10856	C3	82	122	A
15095	Bn-N13-p59130374	Bn-C3-p56848262	Bn-scaff_26642_1-p10889	C3	82	122	A
15096	Bn-N13-p59130502	Bn-C3-p56848390	Bn-scaff_26642_1-p10901	C3	82	122	A
15097	Bn-N13-p59130621	Bn-C3-p56848510	Bn-scaff_26642_1-p10913	C3	82	122	A
15098	Bn-N13-p59131422	Bn-C3-p56849312	Bn-scaff_26642_1-p10994	C3	82	122	A
15099	Bn-N13-p59138966	Bn-C3-p56850569	Bn-scaff_26642_1-p11119	C3	82	122	A
15100	Bn-N13-p59139337	Bn-C3-p56850940	Bn-scaff_26642_1-p11156	C3	82	122	A
15101	Bn-N13-p59139415	Bn-C3-p56851018	Bn-scaff_26642_1-p11164	C3	82	122	A
15102	Bn-N13-p59160525	Bn-C3-p56856842	Bn-scaff_26642_1-p11747	C3	82	122	A
15103	Bn-N13-p59168359	Bn-C3-p56859830	Bn-scaff_26642_1-p12045	C3	82	122	A
15104	Bn-N13-p59168415	Bn-C3-p56859886	Bn-scaff_26642_1-p12051	C3	82	122	A
15105	Bn-N13-p59177721	Bn-C3-p56868339	Bn-scaff_26642_1-p12896	C3	82	122	A
15106	Bn-N13-p59180659	Bn-C3-p56872774	Bn-scaff_26642_1-p13340	C3	82	122	A
15107	Bn-N13-p59182158	Bn-C3-p56874282	Bn-scaff_26642_1-p13491	C3	82	122	A
15108	Bn-N13-p59182283	Bn-C3-p56874407	Bn-scaff_26642_1-p13503	C3	82	122	A
15109	Bn-N13-p59187480	Bn-C3-p56880872	Bn-scaff_26642_1-p14150	C3	82	122	A
15110	Bn-N13-p59203646	Bn-Scaffold00887-p2	Bn-scaff_18206_1-p51441	C3	82	122	A
15111	Bn-N13-p59203938	Bn-Scaffold00887-p2	Bn-scaff_18206_1-p51471	C3	82	122	A
15112	Bn-N13-p59252214	Bn-Scaffold00887-p5	Bn-scaff_18206_1-p53812	C3	82	122	A
15113	Bn-N13-p59252278	Bn-Scaffold00887-p5	Bn-scaff_18206_1-p53819	C3	82	122	A
15114	Bn-N13-p59254002	Bn-Scaffold00887-p5	Bn-scaff_18206_1-p53991	C3	82	122	A
15115	Bn-N13-p59260795	Bn-C3-p56937971	Bn-scaff_17042_1-p2407	C3	82	122	A
15116	Bn-N13-p59261366	Bn-C3-p56938546	Bn-scaff_17042_1-p2982	C3	82	122	A
15117	Bn-N13-p59261419	Bn-C3-p56938599	Bn-scaff_17042_1-p3035	C3	82	122	A
15118	Bn-N13-p59261853	Bn-C3-p56939031	Bn-scaff_17042_1-p3467	C3	82	122	A
15119	Bn-N13-p59269796	Bn-C3-p56941408	Bn-scaff_17042_1-p5844	C3	82	122	A
15120	Bn-N13-p59269805	Bn-C3-p56941417	Bn-scaff_17042_1-p5853	C3	82	122	A
15121	Bn-N13-p59269978	Bn-C3-p56941590	Bn-scaff_17042_1-p6026	C3	82	122	A
15122	Bn-N13-p59278951	Bn-C3-p56950388	Bn-scaff_17042_1-p14824	C3	82	122	A
15123	Bn-N13-p59279070	Bn-C3-p56950507	Bn-scaff_17042_1-p14943	C3	82	122	A
15124	Bn-N13-p59279240	Bn-C3-p56950677	Bn-scaff_17042_1-p15113	C3	82	122	A
15125	Bn-N13-p59279629	Bn-C3-p56951066	Bn-scaff_17042_1-p15502	C3	82	122	A
15126	Bn-N13-p59280789	Bn-C3-p56951922	Bn-scaff_17042_1-p16358	C3	82	122	A
15127	Bn-N13-p59281599	Bn-C3-p56962524	Bn-scaff_17042_1-p26960	C3	82	122	A
15128	Bn-N13-p59281703	Bn-C3-p56962628	Bn-scaff_17042_1-p27064	C3	82	122	A
15129	Bn-N13-p59283369	Bn-C3-p56962933	Bn-scaff_17042_1-p27369	C3	82	122	A
15130	Bn-N13-p59290123	Bn-C3-p56966232	Bn-scaff_17042_1-p30668	C3	82	122	A
15131	Bn-N13-p59308929	Bn-C3-p56984894	Bn-scaff_17042_1-p49330	C3	82	122	A
15132	Bn-N13-p59310901	Bn-C3-p56986757	Bn-scaff_17042_1-p51193	C3	82	122	A
15133	Bn-N13-p59310956	Bn-C3-p56986812	Bn-scaff_17042_1-p51248	C3	82	122	A
15134	Bn-N13-p59311373	Bn-C3-p56987229	Bn-scaff_17042_1-p51665	C3	82	122	A
15135	Bn-N13-p59312403	Bn-C3-p56988259	Bn-scaff_17042_1-p52695	C3	82	122	A
15136	Bn-N13-p59322702	Bn-C3-p56999193	Bn-scaff_17042_1-p63629	C3	82	122	A

15137	Bn-N13-p59322773	Bn-C3-p56999265	Bn-scaff_17042_1-p63701	C3	82	122	A
15138	Bn-N15-p8772295	Bn-Scaffold00870-p2	Bn-scaff_15712_5-p112392	C3	82	122	A
15139	Bn-N9-p7300617	Bn-Scaffold00883-p2	Bn-scaff_21386_1-p27085	C3	82	122	A
15140	Bn-Scaffold05385-p748	Bn-C3-p56934992	Bn-scaff_18669_1-p41959	C3	82	122	A
15141	Bn-Scaffold05385-p817	Bn-C3-p56934923	Bn-scaff_18669_1-p41890	C3	82	122	A
15142	Bn-Scaffold14478-p150	Bn-C3-p56742096	Bn-scaff_26642_1-p2725	C3	82	122	A
15143	Bn-N13-p59403363	Bn-C3-p57082676	Bn-scaff_17042_1-p147112	C3	83	122.8	A
15144	Bn-N13-p59452352	Bn-C3-p57115810	Bn-scaff_17042_1-p180246	C3	83	122.8	A
15145	Bn-N13-p59452493	Bn-C3-p57115957	Bn-scaff_17042_1-p180393	C3	83	122.8	A
15146	Bn-N13-p59513152	Bn-A08-p11679083	Bn-A08-p11212494	C3	83	122.8	A
15147	Bn-N13-p59561211	Bn-C3-p57234174	Bn-scaff_17042_1-p298610	C3	83	122.8	A
15148	Bn-N13-p59579439	Bn-C3-p57254974	Bn-scaff_17042_1-p319410	C3	83	122.8	A
15149	Bn-N13-p59679488	Bn-C3-p57354163	Bn-scaff_17042_1-p418599	C3	84	123.6	A
15150	Bn-N13-p59742276	Bn-C3-p57404345	Bn-scaff_17042_1-p468781	C3	85	124.4	A
15151	Bn-N13-p59748038	Bn-C3-p57413190	Bn-scaff_17042_1-p477626	C3	85	124.4	A
15152	Bn-N13-p59750323	Bn-C3-p57419806	Bn-scaff_17042_1-p484242	C3	85	124.4	A
15153	Bn-N13-p59751310	Bn-C3-p57420792	Bn-scaff_17042_1-p485228	C3	85	124.4	A
15154	Bn-N13-p59768266	Bn-C3-p57435791	Bn-scaff_17042_1-p500227	C3	85	124.4	A
15155	Bn-N13-p59782623	Bn-C3-p57446705	Bn-scaff_17042_1-p511141	C3	85	124.4	A
15156	Bn-N13-p59782666	Bn-C3-p57446748	Bn-scaff_17042_1-p511184	C3	85	124.4	A
15157	Bn-N13-p59792507	Bn-C3-p57452414	Bn-scaff_17042_1-p516850	C3	85	124.4	A
15158	Bn-N13-p59792881	Bn-C3-p57452788	Bn-scaff_17042_1-p517224	C3	85	124.4	A
15159	Bn-N13-p59908230	Bn-C3-p57577875	Bn-scaff_17042_1-p642311	C3	85	124.4	A
15160	Bn-N13-p60001509	Bn-C3-p57629947	Bn-scaff_17042_1-p694383	C3	85	124.4	A
15161	Bn-N13-p60348841	Bn-C3-p57889296	Bn-scaff_17457_1-p290434	C3	86	127.6	A
15162	Bn-N13-p60355266	Bn-C3-p57896585	Bn-scaff_17457_1-p283145	C3	86	127.6	A
15163	Bn-N13-p60355479	Bn-C3-p57896798	Bn-scaff_17457_1-p282932	C3	86	127.6	A
15164	Bn-N13-p60391765	Bn-C3-p57925565	Bn-scaff_17457_1-p254165	C3	86	127.6	A
15165	Bn-N13-p60392106	Bn-C3-p57925906	Bn-scaff_17457_1-p253824	C3	86	127.6	A
15166	Bn-N13-p60395711	Bn-C3-p57929477	Bn-scaff_17457_1-p250253	C3	86	127.6	A
15167	Bn-N13-p60398721	Bn-C3-p57934401	Bn-scaff_17457_1-p245329	C3	86	127.6	A
15168	Bn-N13-p60399522	Bn-C3-p57935204	Bn-scaff_17457_1-p244526	C3	86	127.6	A
15169	Bn-N13-p60413363	Bn-C3-p57953500	Bn-scaff_17457_1-p226230	C3	86	127.6	A
15170	Bn-N13-p60413818	Bn-C3-p57953956	Bn-scaff_17457_1-p225774	C3	86	127.6	A
15171	Bn-N13-p60430225	Bn-C3-p57972076	Bn-scaff_17457_1-p207654	C3	86	127.6	A
15172	Bn-N13-p60430817	Bn-C3-p57972675	Bn-scaff_17457_1-p207055	C3	86	127.6	A
15173	Bn-N13-p60431013	Bn-C3-p57972870	Bn-scaff_17457_1-p206860	C3	86	127.6	A
15174	Bn-N13-p60431130	Bn-C3-p57972987	Bn-scaff_17457_1-p206743	C3	86	127.6	A
15175	Bn-N13-p60441858	Bn-C3-p57983388	Bn-scaff_17457_1-p196342	C3	86	127.6	A
15176	Bn-N13-p60446052	Bn-C3-p57988336	Bn-scaff_17457_1-p191394	C3	86	127.6	A
15177	Bn-N13-p60446640	Bn-C3-p57988937	Bn-scaff_17457_1-p190793	C3	86	127.6	A
15178	Bn-N13-p60446877	Bn-C3-p57989172	Bn-scaff_17457_1-p190558	C3	86	127.6	A
15179	Bn-N13-p60447993	Bn-C3-p57990289	Bn-scaff_17457_1-p189441	C3	86	127.6	A
15180	Bn-N13-p60448265	Bn-C3-p57990783	Bn-scaff_17457_1-p188947	C3	86	127.6	A
15181	Bn-N13-p60449945	Bn-C3-p57992396	Bn-scaff_17457_1-p187334	C3	86	127.6	A
15182	Bn-N13-p60449963	Bn-C3-p57992414	Bn-scaff_17457_1-p187316	C3	86	127.6	A
15183	Bn-N13-p60451738	Bn-C3-p57994128	Bn-scaff_17457_1-p185602	C3	86	127.6	A
15184	Bn-N13-p60452395	Bn-C3-p57994789	Bn-scaff_17457_1-p184941	C3	86	127.6	A
15185	Bn-N13-p60452447	Bn-C3-p57994841	Bn-scaff_17457_1-p184889	C3	86	127.6	A

15186	Bn-N13-p60452515	Bn-C3-p57994909	Bn-scaff_17457_1-p184821	C3	86	127.6	A
15187	Bn-N13-p60453039	Bn-C3-p57995435	Bn-scaff_17457_1-p184295	C3	86	127.6	A
15188	Bn-N13-p60453306	Bn-C3-p57995704	Bn-scaff_17457_1-p184026	C3	86	127.6	A
15189	Bn-N13-p60453307	Bn-C3-p57995705	Bn-scaff_17457_1-p184025	C3	86	127.6	A
15190	Bn-N13-p60453806	Bn-C3-p57996230	Bn-scaff_17457_1-p183500	C3	86	127.6	A
15191	Bn-N13-p60458891	Bn-C3-p58001158	Bn-scaff_17457_1-p178572	C3	86	127.6	A
15192	Bn-N13-p60458997	Bn-C3-p58001264	Bn-scaff_17457_1-p178466	C3	86	127.6	A
15193	Bn-N13-p60459108	Bn-C3-p58001375	Bn-scaff_17457_1-p178355	C3	86	127.6	A
15194	Bn-N13-p60459171	Bn-C3-p58001438	Bn-scaff_17457_1-p178292	C3	86	127.6	A
15195	Bn-N13-p60459918	Bn-C3-p58002163	Bn-scaff_17457_1-p177567	C3	86	127.6	A
15196	Bn-N13-p60460820	Bn-C3-p58003068	Bn-scaff_17457_1-p176662	C3	86	127.6	A
15197	Bn-N13-p60757762	Bn-C3-p58308144	Bn-scaff_24108_1-p128315	C3	86	127.6	A
15198	Bn-N13-p60925823	Bn-C3-p58469061	Bn-scaff_24108_1-p289232	C3	86	127.6	A
15199	Bn-N13-p60946117	Bn-C3-p58483863	Bn-scaff_24108_1-p304034	C3	86	127.6	A
15200	Bn-N13-p60946270	Bn-C3-p58484016	Bn-scaff_24108_1-p304187	C3	86	127.6	A
15201	Bn-N13-p60947743	Bn-C3-p58485490	Bn-scaff_24108_1-p305661	C3	86	127.6	A
15202	Bn-N13-p60956631	Bn-C3-p58494404	Bn-scaff_24108_1-p314575	C3	86	127.6	A
15203	Bn-N13-p60956721	Bn-C3-p58494494	Bn-scaff_24108_1-p314665	C3	86	127.6	A
15204	Bn-N13-p60982141	Bn-Scaffold01035-p1	Bn-scaff_19101_1-p10495	C3	86	127.6	A
15205	Bn-N13-p60984039	Bn-Scaffold01035-p1	Bn-scaff_19101_1-p13013	C3	86	127.6	A
15206	Bn-N13-p60990571	Bn-Scaffold01035-p1	Bn-scaff_19101_1-p15803	C3	86	127.6	A
15207	Bn-N13-p60994757	Bn-Scaffold01035-p2	Bn-scaff_19101_1-p20325	C3	86	127.6	A
15208	Bn-N13-p60994984	Bn-Scaffold01035-p2	Bn-scaff_19101_1-p20551	C3	86	127.6	A
15209	Bn-N13-p61366416	Bn-C3-p58859031	Bn-scaff_15794_3-p533873	C3	86	127.6	A
15210	Bn-N13-p61370630	Bn-C3-p58865068	Bn-scaff_15794_3-p527836	C3	86	127.6	A
15211	Bn-N13-p61382437	Bn-C1-p35355251	Bn-C13931552-p379	C3	86	127.6	A
15212	Bn-N14-p51586867	Bn-C4-p50199545	Bn-scaff_16888_1-p122213	C3	86	127.6	A
15213	Bn-N8-p11352102	Bn-A08-p13019549	Bn-A08-p11679083	C3	86	127.6	A
15214	Bn-Scaffold19092-p341	Bn-Scaffold01035-p1	Bn-scaff_19101_1-p16263	C3	86	127.6	A
15215	Bn-N13-p61395213	Bn-C3-p58884782	Bn-scaff_15794_3-p508122	C3	87	129.2	A
15216	Bn-N13-p61429791	Bn-C3-p58917709	Bn-scaff_15794_3-p475195	C3	87	129.2	A
15217	Bn-N13-p61469287	Bn-C3-p58963367	Bn-scaff_15794_3-p429537	C3	87	129.2	A
15218	Bn-N13-p61469453	Bn-C3-p58963533	Bn-scaff_15794_3-p429371	C3	87	129.2	A
15219	Bn-N13-p61470238	Bn-C3-p58964318	Bn-scaff_15794_3-p428586	C3	87	129.2	A
15220	Bn-N13-p61573914	Bn-C3-p59065023	Bn-scaff_15794_3-p327881	C3	87	129.2	A
15221	Bn-N13-p61583531	Bn-C3-p59073916	Bn-scaff_15794_3-p318988	C3	87	129.2	A
15222	Bn-N13-p61608619	Bn-C3-p59100759	Bn-scaff_15794_3-p292145	C3	87	129.2	A
15223	Bn-N13-p61613068	Bn-C3-p59105185	Bn-scaff_15794_3-p287719	C3	87	129.2	A
15224	Bn-N13-p61614309	Bn-C3-p59106426	Bn-scaff_15794_3-p286478	C3	87	129.2	A
15225	Bn-N13-p61614437	Bn-C3-p59106554	Bn-scaff_15794_3-p286350	C3	87	129.2	A
15226	Bn-N13-p61629476	Bn-C3-p59125945	Bn-scaff_15794_3-p266959	C3	87	129.2	A
15227	Bn-N13-p61631680	Bn-C3-p59128137	Bn-scaff_15794_3-p264767	C3	87	129.2	A
15228	Bn-N13-p61720359	Bn-C3-p59246536	Bn-scaff_15794_3-p146368	C3	88	130	A
15229	Bn-N13-p61770498	Bn-C3-p59317009	Bn-scaff_15794_3-p75895	C3	88	130	A
15230	Bn-N13-p61770685	Bn-C3-p59317195	Bn-scaff_15794_3-p75709	C3	88	130	A
15231	Bn-N13-p61796270	Bn-C3-p59332099	Bn-scaff_15794_3-p60805	C3	88	130	A
15232	Bn-N13-p61843983	Bn-C3-p59383724	Bn-scaff_15794_3-p9180	C3	88	130	A
15233	Bn-N13-p62015568	Bn-C3-p59522708	Bn-scaff_15794_1-p437864	C3	88	130	A
15234	Bn-N13-p62023981	Bn-C3-p59528363	Bn-scaff_15794_1-p432209	C3	88	130	A

15235	Bn-N13-p62315723	Bn-C3-p59782211	Bn-scaff_15794_1-p178361	C3	88	130	A
15236	Bn-N13-p62353372	Bn-C3-p59803384	Bn-scaff_15794_1-p157188	C3	89	130.8	A
15237	Bn-N13-p62791225	Bn-C3-p60269254	Bn-scaff_16996_1-p230771	C3	90	134.8	0
15238	Bn-N13-p63010986	Bn-A08-p17190098	Bn-A08-p13019549	C3	90	134.8	A
15239	Bn-N13-p63041416	Bn-C3-p60521177	Bn-scaff_17821_1-p21053	C3	90	134.8	A
15240	Bn-N13-p63044909	Bn-C3-p60524225	Bn-scaff_17821_1-p24101	C3	90	134.8	A
15241	Bn-N13-p63051506	Bn-C3-p60530925	Bn-scaff_17821_1-p30801	C3	90	134.8	A
15242	Bn-N13-p63142532	Bn-C3-p60627378	Bn-scaff_17821_1-p127254	C3	91	136.4	A
15243	Bn-N13-p63305448	Bn-Scaffold00911-p5	Bn-scaff_16712_1-p53301	C3	91	136.4	A
15244	Bn-N13-p63335542	Bn-C3-p60769039	Bn-scaff_17119_1-p103521	C3	91	136.4	A
15245	Bn-N13-p63335650	Bn-C3-p60769147	Bn-scaff_17119_1-p103629	C3	91	136.4	A
15246	Bn-N13-p63366840	Bn-C3-p60814408	Bn-scaff_17119_1-p148890	C3	91	136.4	A
15247	Bn-N13-p63388579	Bn-C3-p60849232	Bn-scaff_17119_1-p183714	C3	91	136.4	A
15248	Bn-N13-p63459192	Bn-C3-p60900950	Bn-scaff_17119_1-p235432	C3	91	136.4	A
15249	Bn-N13-p63459304	Bn-C3-p60901054	Bn-scaff_17119_1-p235536	C3	91	136.4	A
15250	Bn-N13-p63470097	Bn-C3-p60913516	Bn-scaff_17119_1-p247998	C3	91	136.4	A
15251	Bn-N13-p63497119	Bn-C3-p60951833	Bn-scaff_17119_1-p286315	C3	91	136.4	A
15252	Bn-N13-p63499103	Bn-C3-p60953805	Bn-scaff_17119_1-p288287	C3	91	136.4	A
15253	Bn-N13-p63505621	Bn-C3-p60964073	Bn-scaff_17119_1-p298555	C3	91	136.4	A
15254	Bn-N13-p63505927	Bn-C3-p60964379	Bn-scaff_17119_1-p298861	C3	91	136.4	A
15255	Bn-N13-p63506126	Bn-C3-p60964578	Bn-scaff_17119_1-p299060	C3	91	136.4	A
15256	Bn-N13-p63506858	Bn-C3-p60966124	Bn-scaff_17119_1-p300606	C3	91	136.4	A
15257	Bn-N13-p63537936	Bn-C3-p60989500	Bn-scaff_17119_1-p323982	C3	91	136.4	A
15258	Bn-N13-p63620459	Bn-C3-p61076096	Bn-scaff_17119_1-p410578	C3	91	136.4	A
15259	Bn-N17-p33528155	Bn-C3-p60985249	Bn-scaff_17119_1-p319731	C3	91	136.4	A
15260	Bn-N11-p5752034	Bn-C3-p61960156	Bn-scaff_23761_1-p800341	C3	92	137.2	A
15261	Bn-N12-p7014512	Bn-C3-p61121871	Bn-scaff_17119_1-p456353	C3	92	137.2	A
15262	Bn-N13-p63750898	Bn-C3-p61187054	Bn-scaff_23761_1-p27239	C3	92	137.2	A
15263	Bn-N13-p64037256	Bn-C3-p61409443	Bn-scaff_23761_1-p249628	C3	92	137.2	A
15264	Bn-N13-p64078469	Bn-C3-p61441419	Bn-scaff_23761_1-p281604	C3	92	137.2	0
15265	Bn-N13-p64261352	Bn-C3-p61608883	Bn-scaff_23761_1-p449068	C3	92	137.2	A
15266	Bn-N13-p64337717	Bn-C3-p61689950	Bn-scaff_23761_1-p530135	C3	92	137.2	A
15267	Bn-N13-p64389081	Bn-C3-p61741150	Bn-scaff_23761_1-p581335	C3	92	137.2	A
15268	Bn-N13-p64412087	Bn-C3-p61764134	Bn-scaff_23761_1-p604319	C3	92	137.2	A
15269	Bn-N13-p64489323	Bn-C3-p61834381	Bn-scaff_23761_1-p674566	C3	92	137.2	A
15270	Bn-N13-p64490604	Bn-C3-p61835667	Bn-scaff_23761_1-p675852	C3	92	137.2	A
15271	Bn-N13-p64531617	Bn-C3-p61881006	Bn-scaff_23761_1-p721191	C3	92	137.2	A
15272	Bn-N13-p64557861	Bn-C3-p61898397	Bn-scaff_23761_1-p738582	C3	92	137.2	A
15273	Bn-N13-p64557864	Bn-C3-p61898400	Bn-scaff_23761_1-p738585	C3	92	137.2	A
15274	Bn-N13-p64582781	Bn-C3-p61923967	Bn-scaff_23761_1-p764152	C3	92	137.2	A
15275	Bn-N13-p64649039	Bn-C3-p61987479	Bn-scaff_16676_1-p6894	C3	92	137.2	0
15276	Bn-N13-p64746883	Bn-C3-p62078517	Bn-scaff_18559_1-p191163	C3	92	137.2	A
15277	Bn-N13-p64764597	Bn-C3-p62094052	Bn-scaff_18559_1-p175628	C3	92	137.2	A
15278	Bn-N13-p64883740	Bn-C3-p62245741	Bn-scaff_18559_1-p23939	C3	92	137.2	A
15279	Bn-N13-p64918430	Bn-C3-p62216251	Bn-scaff_18559_1-p53429	C3	92	137.2	0
15280	Bn-N13-p64950919	Bn-C3-p62182650	Bn-scaff_18559_1-p87030	C3	92	137.2	A
15281	Bn-N13-p64959504	Bn-C3-p62175957	Bn-scaff_18559_1-p93723	C3	92	137.2	A
15282	Bn-N13-p64961290	Bn-C3-p62173585	Bn-scaff_18559_1-p96095	C3	92	137.2	A
15283	Bn-N13-p64961476	Bn-C3-p62173399	Bn-scaff_18559_1-p96281	C3	92	137.2	A

15284	Bn-N13-p64973694	Bn-C3-p62276431	Bn-scaff_19047_1-p387569	C3	92	137.2	A
15285	Bn-N13-p64974722	Bn-C3-p62277467	Bn-scaff_19047_1-p386533	C3	92	137.2	A
15286	Bn-N13-p64985652	Bn-C3-p62290489	Bn-scaff_19047_1-p373511	C3	92	137.2	A
15287	Bn-N19-p10243752	Bn-C3-p61844923	Bn-scaff_23761_1-p685108	C3	92	137.2	A
15288	Bn-N13-p65014421	Bn-C3-p62366802	Bn-scaff_19047_1-p297198	C3	93	140.4	A
15289	Bn-N13-p65066133	Bn-C3-p62436275	Bn-scaff_19047_1-p227725	C3	93	140.4	A
15290	Bn-N13-p65068547	Bn-C3-p62438461	Bn-scaff_19047_1-p225539	C3	93	140.4	A
15291	Bn-N13-p65106050	Bn-C3-p62439609	Bn-scaff_19047_1-p224391	C3	93	140.4	A
15292	Bn-N13-p65133374	Bn-C3-p62467612	Bn-scaff_19047_1-p196388	C3	93	140.4	A
15293	Bn-N13-p65508748	Bn-C3-p62758976	Bn-scaff_16148_1-p94877	C3	94	141.2	A
15294	Bn-N13-p65681849	Bn-C3-p62901691	Bn-scaff_16148_1-p237592	C3	94	141.2	A
15295	Bn-N13-p65737377	Bn-C3-p62959088	Bn-scaff_16148_1-p294989	C3	94	141.2	A
15296	Bn-N13-p65817586	Bn-C3-p63029580	Bn-scaff_16148_1-p365481	C3	94	141.2	A
15297	Bn-N13-p65434365	Bn-C3-p62727808	Bn-scaff_16148_1-p63709	C3	95	142	A
15298	Bn-N13-p66035170	Bn-C3-p63240373	Bn-scaff_23098_1-p232984	C3	95	142	A
15299	Bn-N13-p66055782	Bn-C3-p63263376	Bn-scaff_23098_1-p209981	C3	95	142	A
15300	Bn-N13-p66155080	Bn-C3-p63366148	Bn-scaff_23098_1-p107209	C3	95	142	A
15301	Bn-N13-p66236799	Bn-C3-p63430819	Bn-scaff_23098_1-p42538	C3	95	142	A
15302	Bn-N13-p66244128	Bn-C3-p63440867	Bn-scaff_23098_1-p32490	C3	95	142	A
15303	Bn-N13-p66313465	Bn-C3-p63485745	Bn-scaff_16755_1-p149895	C3	95	142	A
15304	Bn-N13-p66315241	Bn-C3-p63487521	Bn-scaff_16755_1-p149717	C3	95	142	A
15305	Bn-N13-p66321332	Bn-C3-p63490784	Bn-scaff_16755_1-p149391	C3	95	142	A
15306	Bn-N13-p66333400	Bn-C3-p63495801	Bn-scaff_16755_1-p148889	C3	95	142	A
15307	Bn-N13-p66347180	Bn-C3-p63501680	Bn-scaff_16755_1-p148301	C3	95	142	A
15308	Bn-N13-p66347559	Bn-C3-p63502075	Bn-scaff_16755_1-p148262	C3	95	142	A
15309	Bn-N13-p66347602	Bn-C3-p63502118	Bn-scaff_16755_1-p148257	C3	95	142	A
15310	Bn-N13-p66347929	Bn-C3-p63503444	Bn-scaff_16755_1-p148125	C3	95	142	A
15311	Bn-N13-p66348564	Bn-C3-p63506132	Bn-scaff_16755_1-p147856	C3	95	142	A
15312	Bn-N13-p66348634	Bn-C3-p63506204	Bn-scaff_16755_1-p147849	C3	95	142	A
15313	Bn-N13-p66353265	Bn-C3-p63506541	Bn-scaff_16755_1-p147815	C3	95	142	A
15314	Bn-N13-p66353349	Bn-C3-p63506626	Bn-scaff_16755_1-p147807	C3	95	142	A
15315	Bn-N13-p66353791	Bn-C3-p63506790	Bn-scaff_16755_1-p147790	C3	95	142	A
15316	Bn-N13-p66353846	Bn-C3-p63506844	Bn-scaff_16755_1-p147785	C3	95	142	A
15317	Bn-N13-p66354009	Bn-C3-p63507006	Bn-scaff_16755_1-p147769	C3	95	142	A
15318	Bn-N13-p66355185	Bn-C3-p63508169	Bn-scaff_16755_1-p147652	C3	95	142	A
15319	Bn-N13-p66355303	Bn-C3-p63508287	Bn-scaff_16755_1-p147640	C3	95	142	A
15320	Bn-N13-p66355876	Bn-C3-p63508860	Bn-scaff_16755_1-p147583	C3	95	142	A
15321	Bn-N13-p66356029	Bn-C3-p63509014	Bn-scaff_16755_1-p147568	C3	95	142	A
15322	Bn-N13-p66356205	Bn-C3-p63509190	Bn-scaff_16755_1-p147550	C3	95	142	A
15323	Bn-N13-p66356330	Bn-C3-p63509315	Bn-scaff_16755_1-p147538	C3	95	142	A
15324	Bn-N13-p66357302	Bn-C3-p63510291	Bn-scaff_16755_1-p147440	C3	95	142	A
15325	Bn-N13-p66357780	Bn-C3-p63510771	Bn-scaff_16755_1-p147392	C3	95	142	A
15326	Bn-N13-p66363420	Bn-C3-p63516414	Bn-scaff_16755_1-p146828	C3	95	142	A
15327	Bn-N13-p66381612	Bn-C3-p63523351	Bn-scaff_16755_1-p146134	C3	95	142	A
15328	Bn-N13-p66382053	Bn-C3-p63523792	Bn-scaff_16755_1-p146090	C3	95	142	A
15329	Bn-N13-p66383011	Bn-C3-p63524761	Bn-scaff_16755_1-p145993	C3	95	142	A
15330	Bn-N2-p10212435	Bn-C3-p63556608	Bn-scaff_16755_1-p142808	C3	95	142	A
15331	Bn-N2-p10212859	Bn-C3-p63557075	Bn-scaff_16755_1-p142762	C3	95	142	A
15332	Bn-N8-p1376618	Bn-C3-p63169111	Bn-scaff_23098_1-p304246	C3	95	142	A

15333	Bn-N13-p66466754	Bn-C3-p63620341	Bn-scaff_16755_1-p136435	C3	96	142.8	A
15334	Bn-N13-p66497258	Bn-C3-p63645270	Bn-scaff_16755_1-p133942	C3	96	142.8	A
15335	Bn-N13-p66540056	Bn-C3-p63693019	Bn-scaff_16755_1-p129167	C3	96	142.8	A
15336	Bn-N13-p66541393	Bn-C3-p63694386	Bn-scaff_16755_1-p129031	C3	96	142.8	0
15337	Bn-N13-p66632864	Bn-C3-p63774943	Bn-scaff_16755_1-p120975	C3	96	142.8	A
15338	Bn-N13-p66633418	Bn-C3-p63775495	Bn-scaff_16755_1-p120920	C3	96	142.8	A
15339	Bn-N13-p66634448	Bn-C3-p63776522	Bn-scaff_16755_1-p120817	C3	96	142.8	0
15340	Bn-N13-p66641183	Bn-C3-p63783277	Bn-scaff_16755_1-p120141	C3	96	142.8	A
15341	Bn-N13-p66649843	Bn-C3-p63789588	Bn-scaff_16755_1-p119510	C3	96	142.8	A
15342	Bn-N13-p66650888	Bn-C3-p63790627	Bn-scaff_16755_1-p119406	C3	96	142.8	A
15343	Bn-N13-p66653654	Bn-C3-p63793551	Bn-scaff_16755_1-p119114	C3	96	142.8	A
15344	Bn-N13-p66658070	Bn-C3-p63800115	Bn-scaff_16755_1-p118458	C3	96	142.8	A
15345	Bn-N13-p66892279	Bn-C3-p64050436	Bn-scaff_16755_1-p934260	C3	97	144.4	A
15346	Bn-N13-p66899527	Bn-C3-p64056775	Bn-scaff_16755_1-p927921	C3	97	144.4	A
15347	Bn-N13-p66900298	Bn-C3-p64057546	Bn-scaff_16755_1-p927150	C3	97	144.4	A
15348	Bn-N13-p66917610	Bn-C3-p64080980	Bn-scaff_16755_1-p903716	C3	97	144.4	A
15349	Bn-N13-p66918351	Bn-C3-p64081721	Bn-scaff_16755_1-p902975	C3	97	144.4	A
15350	Bn-N13-p66920796	Bn-C3-p64089947	Bn-scaff_16755_1-p894749	C3	97	144.4	A
15351	Bn-N13-p66937567	Bn-C3-p64106691	Bn-scaff_16755_1-p878005	C3	98	146	A
15352	Bn-N13-p67617287	Bn-C3-p64833746	Bn-scaff_16755_1-p150950	C3	99	146.8	A
15353	Bn-N13-p67617863	Bn-C3-p64834234	Bn-scaff_16755_1-p150462	C3	99	146.8	A
15354	Bn-N13-p67617970	Bn-C3-p64834341	Bn-scaff_16755_1-p150355	C3	99	146.8	A
15355	Bn-N13-p67629738	Bn-C3-p64844928	Bn-scaff_16755_1-p139768	C3	99	146.8	A
15356	Bn-N13-p67629830	Bn-C3-p64845020	Bn-scaff_16755_1-p139676	C3	99	146.8	A
15357	Bn-N13-p67655591	Bn-C3-p64856594	Bn-scaff_16755_1-p128102	C3	99	146.8	A
15358	Bn-N13-p67655843	Bn-C3-p64856858	Bn-scaff_16755_1-p127838	C3	99	146.8	A
15359	Bn-N13-p67666172	Bn-C3-p64867275	Bn-scaff_16755_1-p117421	C3	99	146.8	A
15360	Bn-N13-p67667405	Bn-C3-p64868267	Bn-scaff_16755_1-p116429	C3	99	146.8	A
15361	Bn-N13-p67668850	Bn-C3-p64869737	Bn-scaff_16755_1-p114959	C3	99	146.8	A
15362	Bn-N13-p67690930	Bn-C3-p64897433	Bn-scaff_16755_1-p87263	C3	99	146.8	A
15363	Bn-N13-p67738801	Bn-C3-p64942860	Bn-scaff_16755_1-p41836	C3	99	146.8	A
15364	Bn-N13-p67752105	Bn-C3-p64959444	Bn-scaff_16755_1-p25252	C3	99	146.8	A
15365	Bn-N13-p67764811	Bn-C3-p64972155	Bn-scaff_16755_1-p12541	C3	99	146.8	A
15366	Bn-N14-p10483	Bn-C4-p146560	Bn-scaff_22933_1-p110027	C4	1	0	B
15367	Bn-N14-p11335	Bn-C4-p147412	Bn-scaff_22933_1-p109175	C4	1	0	B
15368	Bn-N14-p24794	Bn-C4-p160885	Bn-scaff_22933_1-p95702	C4	1	0	B
15369	Bn-N14-p36142	Bn-C4-p172277	Bn-scaff_22933_1-p84310	C4	1	0	B
15370	Bn-N14-p39678	Bn-C4-p175788	Bn-scaff_22933_1-p80799	C4	1	0	B
15371	Bn-N14-p41306	Bn-C4-p180003	Bn-scaff_22933_1-p76584	C4	1	0	B
15372	Bn-N14-p44189	Bn-C4-p185707	Bn-scaff_22933_1-p70880	C4	1	0	B
15373	Bn-N14-p52428	Bn-C4-p194572	Bn-scaff_22933_1-p62015	C4	1	0	B
15374	Bn-Scaffold01076-p16442	Bn-C4-p53523	Bn-scaff_26877_1-p81778	C4	1	0	B
15375	Bn-Scaffold01076-p16673	Bn-C4-p53292	Bn-scaff_26877_1-p82009	C4	1	0	B
15376	Bn-Scaffold01899-p3196	Bn-Scaffold01447-p3:	Bn-scaff_17668_1-p3205	C4	1	0	B
15377	Bn-Scaffold01899-p327	Bn-Scaffold01447-p4:	Bn-scaff_17668_1-p419	C4	1	0	B
15378	Bn-Scaffold01899-p613	Bn-Scaffold01447-p7(	Bn-scaff_17668_1-p705	C4	1	0	B
15379	Bn-Scaffold03303-p2863	Bn-C4-p85511	Bn-scaff_26877_1-p49790	C4	1	0	B
15380	Bn-N12-p41445677	Bn-C4-p411358	Bn-scaff_15838_1-p96143	C4	2	1.7	B
15381	Bn-N12-p44819916	Bn-C4-p404389	Bn-scaff_15838_1-p103112	C4	2	1.7	B

15382	Bn-N14-p115390	Bn-C4-p264643	Bn-scaff_23534_1-p39080	C4	2	1.7	B
15383	Bn-N14-p117159	Bn-C4-p266411	Bn-scaff_23534_1-p37312	C4	2	1.7	B
15384	Bn-N14-p117784	Bn-C4-p267035	Bn-scaff_23534_1-p36688	C4	2	1.7	B
15385	Bn-N14-p234119	Bn-Scaffold01067-p1	Bn-scaff_19673_1-p11357	C4	2	1.7	B
15386	Bn-N14-p236792	Bn-C4-p415192	Bn-scaff_15838_1-p92309	C4	2	1.7	B
15387	Bn-N14-p312910	Bn-C4-p504277	Bn-scaff_15838_1-p3224	C4	2	1.7	B
15388	Bn-N14-p381430	Bn-Scaffold00802-p7	Bn-scaff_17712_1-p78048	C4	3	2.5	B
15389	Bn-N14-p399194	Bn-Scaffold00802-p5	Bn-scaff_17712_1-p59419	C4	3	2.5	B
15390	Bn-N14-p447277	Bn-C4-p544296	Bn-scaff_15832_1-p62268	C4	3	2.5	B
15391	Bn-N14-p607913	Bn-C4-p686637	Bn-scaff_16935_1-p10207	C4	4	3.3	B
15392	Bn-N14-p622065	Bn-C4-p700701	Bn-scaff_16935_1-p11613	C4	4	3.3	B
15393	Bn-N5-p533849	Bn-A05-p458357	Bn-A05-p458357	C4	4	3.3	B
15394	Bn-N14-p1029011	Bn-C4-p1163754	Bn-scaff_24859_1-p42833	C4	5	7.5	A
15395	Bn-N14-p1029165	Bn-C4-p1163908	Bn-scaff_24859_1-p42987	C4	5	7.5	A
15396	Bn-N14-p1241143	Bn-C13488683-p154	Bn-C13488683-p154	C4	5	7.5	A
15397	Bn-N14-p896458	Bn-C4-p983004	Bn-scaff_23946_1-p35627	C4	5	7.5	A
15398	Bn-N14-p905788	Bn-C4-p992655	Bn-scaff_23946_1-p25976	C4	5	7.5	A
15399	Bn-N14-p907544	Bn-C4-p994410	Bn-scaff_23946_1-p24221	C4	5	7.5	A
15400	Bn-N14-p916428	Bn-C4-p1030247	Bn-scaff_23974_1-p11517	C4	5	7.5	A
15401	Bn-N14-p976811	Bn-C4-p1105230	Bn-scaff_23974_1-p86500	C4	5	7.5	A
15402	Bn-N14-p1263905	Bn-C4-p1378830	Bn-scaff_16027_1-p18199	C4	6	9.5	A
15403	Bn-N14-p1400503	Bn-C4-p1516144	Bn-scaff_16027_1-p31931	C4	7	10.5	A
15404	Bn-N14-p1400554	Bn-C4-p1516195	Bn-scaff_16027_1-p31936	C4	7	10.5	A
15405	Bn-N14-p1400645	Bn-C4-p1516286	Bn-scaff_16027_1-p31945	C4	7	10.5	A
15406	Bn-N14-p1408322	Bn-C4-p1523498	Bn-scaff_16027_1-p32666	C4	7	10.5	A
15407	Bn-N14-p1408383	Bn-C4-p1523559	Bn-scaff_16027_1-p32672	C4	7	10.5	A
15408	Bn-N14-p1408638	Bn-C4-p1523814	Bn-scaff_16027_1-p32698	C4	7	10.5	A
15409	Bn-N14-p1410454	Bn-C4-p1525630	Bn-scaff_16027_1-p32879	C4	7	10.5	A
15410	Bn-N14-p1410844	Bn-C4-p1526020	Bn-scaff_16027_1-p32918	C4	7	10.5	A
15411	Bn-N14-p1434382	Bn-C4-p1549566	Bn-scaff_16027_1-p35273	C4	7	10.5	A
15412	Bn-N14-p1434779	Bn-C4-p1549963	Bn-scaff_16027_1-p35313	C4	7	10.5	A
15413	Bn-N14-p1437480	Bn-C4-p1552664	Bn-scaff_16027_1-p35583	C4	7	10.5	A
15414	Bn-N14-p1438018	Bn-C4-p1553202	Bn-scaff_16027_1-p35637	C4	7	10.5	A
15415	Bn-N14-p1438079	Bn-C4-p1553263	Bn-scaff_16027_1-p35643	C4	7	10.5	A
15416	Bn-N14-p1438880	Bn-C4-p1554064	Bn-scaff_16027_1-p35723	C4	7	10.5	A
15417	Bn-N14-p1439083	Bn-C4-p1554267	Bn-scaff_16027_1-p35743	C4	7	10.5	A
15418	Bn-N14-p1439555	Bn-C4-p1554739	Bn-scaff_16027_1-p35790	C4	7	10.5	A
15419	Bn-N14-p1446468	Bn-C4-p1561652	Bn-scaff_16027_1-p36482	C4	7	10.5	A
15420	Bn-N14-p1448745	Bn-C4-p1563929	Bn-scaff_16027_1-p36709	C4	7	10.5	A
15421	Bn-N14-p1463653	Bn-C4-p1583238	Bn-scaff_16027_1-p38640	C4	7	10.5	A
15422	Bn-N14-p1485582	Bn-C1-p31929709	Bn-scaff_17517_1-p12836	C4	8	12.5	A
15423	Bn-N14-p1704243	Bn-C4-p1782015	Bn-scaff_16027_1-p58518	C4	9	15.8	A
15424	Bn-N14-p1852009	Bn-Scaffold01231-p7	Bn-scaff_25686_1-p7882	C4	9	15.8	A
15425	Bn-N14-p1853283	Bn-Scaffold01231-p9	Bn-scaff_25686_1-p9156	C4	9	15.8	A
15426	Bn-N14-p2007730	Bn-C4-p2034100	Bn-scaff_21925_1-p15490	C4	10	17.4	A
15427	Bn-N14-p2010244	Bn-C13536262-p60	Bn-C13536262-p60	C4	10	17.4	A
15428	Bn-N14-p2399700	Bn-C4-p2325430	Bn-scaff_27469_1-p10105	C4	10	17.4	A
15429	Bn-N14-p2427396	Bn-C4-p2352891	Bn-scaff_27469_1-p73595	C4	10	17.4	A
15430	Bn-N5-p1644124	Bn-C4-p2352663	Bn-scaff_27469_1-p73823	C4	10	17.4	A

15431	Bn-N14-p2638346	Bn-C4-p2539802	Bn-scaff_28382_1-p28399	C4	11	25.6	A
15432	Bn-N14-p2647358	Bn-C4-p2550141	Bn-scaff_28382_1-p38738	C4	11	25.6	A
15433	Bn-N14-p2647806	Bn-C4-p2550590	Bn-scaff_28382_1-p39187	C4	11	25.6	A
15434	Bn-N14-p2653314	Bn-C4-p2556057	Bn-scaff_28382_1-p44654	C4	11	25.6	A
15435	Bn-N14-p2754388	Bn-C4-p2657428	Bn-scaff_28382_1-p14602	C4	11	25.6	A
15436	Bn-N14-p2820751	Bn-C4-p2719780	Bn-scaff_16214_1-p40152	C4	11	25.6	A
15437	Bn-N14-p2822964	Bn-C4-p2721988	Bn-scaff_16214_1-p42360	C4	11	25.6	A
15438	Bn-N14-p2845922	Bn-C4-p2748814	Bn-scaff_16214_1-p69186	C4	11	25.6	A
15439	Bn-N14-p2973517	Bn-C4-p2852349	Bn-scaff_16214_1-p17272	C4	12	26.4	A
15440	Bn-N14-p3075237	Bn-C4-p2943481	Bn-scaff_16214_1-p26385	C4	12	26.4	A
15441	Bn-N14-p3117929	Bn-C4-p2980416	Bn-scaff_16214_1-p30078	C4	12	26.4	A
15442	Bn-N14-p3157610	Bn-C4-p3027207	Bn-scaff_16214_1-p34757	C4	12	26.4	A
15443	Bn-N14-p3157789	Bn-C4-p3027386	Bn-scaff_16214_1-p34775	C4	12	26.4	A
15444	Bn-N14-p3164941	Bn-scaffold18260-p2	Bn-scaffold18260-p26	C4	12	26.4	A
15445	Bn-N14-p2983403	Bn-C4-p2875587	Bn-scaff_16214_1-p19595	C4	13	27.2	A
15446	Bn-N14-p3318213	Bn-C4-p3296035	Bn-scaff_23229_1-p41596	C4	13	27.2	A
15447	Bn-N14-p3510738	Bn-C4-p3457408	Bn-scaff_16534_1-p13421	C4	13	27.2	A
15448	Bn-N14-p3514032	Bn-C4-p3460456	Bn-scaff_16534_1-p13725	C4	13	27.2	A
15449	Bn-N14-p3515546	Bn-C4-p3461970	Bn-scaff_16534_1-p13877	C4	13	27.2	A
15450	Bn-N14-p3527590	Bn-C4-p3473159	Bn-scaff_16534_1-p14996	C4	13	27.2	A
15451	Bn-N14-p3528691	Bn-C4-p3474262	Bn-scaff_16534_1-p15106	C4	13	27.2	A
15452	Bn-N14-p3533133	Bn-C4-p3479169	Bn-scaff_16534_1-p15597	C4	13	27.2	A
15453	Bn-N14-p3546668	Bn-C4-p3495665	Bn-scaff_16534_1-p17246	C4	13	27.2	A
15454	Bn-N14-p3602663	Bn-C4-p3533159	Bn-scaff_16534_1-p20996	C4	13	27.2	A
15455	Bn-N14-p3663301	Bn-A02-p8967830	Bn-A02-p8967830	C4	13	27.2	A
15456	Bn-N14-p3670545	Bn-C4-p3582812	Bn-scaff_16534_1-p25961	C4	13	27.2	A
15457	Bn-N14-p3691513	Bn-C4-p3600297	Bn-scaff_16534_1-p27709	C4	13	27.2	A
15458	Bn-N14-p3692751	Bn-C4-p3601551	Bn-scaff_16534_1-p27835	C4	13	27.2	A
15459	Bn-N14-p3692891	Bn-C4-p3601694	Bn-scaff_16534_1-p27849	C4	14	28	A
15460	Bn-N14-p3694425	Bn-C4-p3603227	Bn-scaff_16534_1-p28002	C4	14	28	A
15461	Bn-N14-p3695427	Bn-C4-p3604229	Bn-scaff_16534_1-p28103	C4	14	28	A
15462	Bn-N14-p3697000	Bn-C4-p3605802	Bn-scaff_16534_1-p28260	C4	14	28	A
15463	Bn-N14-p3702046	Bn-C4-p3611763	Bn-scaff_16534_1-p28856	C4	14	28	A
15464	Bn-N14-p3702163	Bn-C4-p3611880	Bn-scaff_16534_1-p28868	C4	14	28	A
15465	Bn-N14-p3828796	Bn-C4-p3722891	Bn-scaff_16534_1-p39969	C4	15	28.8	A
15466	Bn-N14-p3931013	Bn-Scaffold01730-p5	Bn-scaff_28277_1-p5229	C4	16	29.6	A
15467	Bn-N14-p3985681	Bn-C4-p3809977	Bn-scaff_16534_1-p48677	C4	17	30.4	A
15468	Bn-N14-p4161722	Bn-C4-p3989532	Bn-scaff_16534_1-p66633	C4	17	30.4	A
15469	Bn-N14-p4209836	Bn-C4-p4030999	Bn-scaff_16534_1-p70780	C4	17	30.4	A
15470	Bn-N14-p4220883	Bn-C4-p4041903	Bn-scaff_16534_1-p71870	C4	17	30.4	A
15471	Bn-N14-p4221392	Bn-C4-p4042412	Bn-scaff_16534_1-p71921	C4	17	30.4	A
15472	Bn-N14-p4235731	Bn-C4-p4055551	Bn-scaff_16534_1-p73235	C4	17	30.4	0
15473	Bn-N14-p4291107	Bn-C4-p4130322	Bn-scaff_16534_1-p80712	C4	18	31.2	A
15474	Bn-N14-p4291329	Bn-C4-p4130544	Bn-scaff_16534_1-p80734	C4	18	31.2	A
15475	Bn-N14-p4292122	Bn-C4-p4131272	Bn-scaff_16534_1-p80807	C4	18	31.2	A
15476	Bn-N14-p4638550	Bn-C4-p4469621	Bn-scaff_16534_1-p11464	C4	18	31.2	A
15477	Bn-N14-p4655307	Bn-C4-p4486064	Bn-scaff_16534_1-p11628	C4	18	31.2	A
15478	Bn-N14-p4667673	Bn-C4-p4490346	Bn-scaff_16534_1-p11671	C4	18	31.2	A
15479	Bn-N5-p2794879	Bn-A05-p2604531	Bn-A05-p2604531	C4	18	31.2	A

15480	Bn-N5-p2961556	Bn-A05-p2765988	Bn-A05-p2765988	C4	18	31.2	0
15481	Bn-N14-p4781873	Bn-C4-p4585856	Bn-scaff_16534_1-p126265	C4	19	32	A
15482	Bn-N14-p4802956	Bn-C4-p4611071	Bn-scaff_16534_1-p128787	C4	19	32	A
15483	Bn-N14-p4843401	Bn-C4-p4651335	Bn-scaff_16534_1-p132813	C4	19	32	A
15484	Bn-N14-p4878672	Bn-C4-p4681049	Bn-scaff_16534_1-p135785	C4	20	32.8	A
15485	Bn-N14-p4941392	Bn-C4-p4743409	Bn-scaff_16534_1-p142021	C4	20	32.8	A
15486	Bn-N14-p4943104	Bn-C4-p4745104	Bn-scaff_16534_1-p142190	C4	20	32.8	A
15487	Bn-N14-p4950271	Bn-C4-p4752723	Bn-scaff_16534_1-p142952	C4	20	32.8	A
15488	Bn-N14-p4950802	Bn-C4-p4753253	Bn-scaff_16534_1-p143005	C4	20	32.8	A
15489	Bn-N14-p4990468	Bn-C4-p4791516	Bn-scaff_16534_1-p146831	C4	20	32.8	A
15490	Bn-N14-p4996982	Bn-C4-p4798042	Bn-scaff_16534_1-p147484	C4	20	32.8	A
15491	Bn-N14-p5005474	Bn-C4-p4805705	Bn-scaff_16534_1-p148250	C4	20	32.8	A
15492	Bn-N14-p5019622	Bn-C4-p4822834	Bn-scaff_16534_1-p149963	C4	20	32.8	A
15493	Bn-N14-p5020134	Bn-C4-p4823397	Bn-scaff_16534_1-p150019	C4	20	32.8	A
15494	Bn-N14-p5119858	Bn-C4-p4930312	Bn-scaff_16534_1-p160711	C4	20	32.8	A
15495	Bn-N5-p3270165	Bn-C4-p4782065	Bn-scaff_16534_1-p145886	C4	20	32.8	A
15496	Bn-N5-p3300147	Bn-C4-p4830978	Bn-scaff_16534_1-p150778	C4	20	32.8	A
15497	Bn-N14-p5170819	Bn-C4-p4976476	Bn-scaff_16534_1-p165327	C4	21	33.6	A
15498	Bn-N14-p5357044	Bn-C4-p5169938	Bn-scaff_16534_1-p184674	C4	21	33.6	A
15499	Bn-N14-p5375971	Bn-C4-p5185543	Bn-scaff_16534_1-p186234	C4	21	33.6	A
15500	Bn-N14-p5407191	Bn-C4-p5214684	Bn-scaff_16534_1-p189148	C4	21	33.6	A
15501	Bn-N14-p5411047	Bn-C4-p5218715	Bn-scaff_16534_1-p189551	C4	21	33.6	A
15502	Bn-N14-p5413492	Bn-C4-p5221161	Bn-scaff_16534_1-p189796	C4	21	33.6	A
15503	Bn-N14-p5416485	Bn-C4-p5224421	Bn-scaff_16534_1-p190122	C4	21	33.6	A
15504	Bn-N14-p5739858	Bn-C4-p5643468	Bn-scaff_16534_1-p232027	C4	21	33.6	A
15505	Bn-N14-p5855559	Bn-C4-p5759134	Bn-scaff_16534_1-p243593	C4	22	34.4	A
15506	Bn-N14-p5855583	Bn-C4-p5759158	Bn-scaff_16534_1-p243596	C4	22	34.4	A
15507	Bn-N14-p5858050	Bn-C4-p5762590	Bn-scaff_16534_1-p243939	C4	22	34.4	A
15508	Bn-N14-p5892118	Bn-C4-p5811379	Bn-scaff_16534_1-p248818	C4	22	34.4	A
15509	Bn-N14-p5909381	Bn-C4-p5813523	Bn-scaff_16534_1-p249032	C4	22	34.4	A
15510	Bn-N14-p5927524	Bn-C4-p5867711	Bn-scaff_16534_1-p254451	C4	22	34.4	A
15511	Bn-N14-p5927581	Bn-C4-p5867768	Bn-scaff_16534_1-p254457	C4	22	34.4	A
15512	Bn-N14-p5940050	Bn-C4-p5872332	Bn-scaff_16534_1-p254913	C4	22	34.4	A
15513	Bn-N14-p5940340	Bn-C4-p5872625	Bn-scaff_16534_1-p254942	C4	22	34.4	A
15514	Bn-N14-p5967577	Bn-C4-p5893156	Bn-scaff_16534_1-p256995	C4	22	34.4	A
15515	Bn-N14-p5978180	Bn-C4-p5903753	Bn-scaff_16534_1-p258055	C4	22	34.4	A
15516	Bn-N14-p6055307	Bn-Scaffold00722-p1(	Bn-scaff_19937_1-p107033	C4	22	34.4	A
15517	Bn-N14-p6129221	Bn-C4-p6060302	Bn-scaff_15908_1-p138105	C4	22	34.4	A
15518	Bn-N14-p6268431	Bn-C4-p6202228	Bn-scaff_15908_1-p280031	C4	22	34.4	A
15519	Bn-N14-p6285013	Bn-C4-p6223243	Bn-scaff_15908_1-p301046	C4	22	34.4	A
15520	Bn-N14-p6320272	Bn-C4-p6259059	Bn-scaff_15908_1-p336862	C4	22	34.4	A
15521	Bn-N14-p6329016	Bn-Scaffold01108-p2!	Bn-scaff_20063_1-p25913	C4	22	34.4	A
15522	Bn-N14-p6345297	Bn-Scaffold01108-p1:	Bn-scaff_20063_1-p13531	C4	22	34.4	A
15523	Bn-N14-p6389298	Bn-C4-p6324219	Bn-scaff_15908_1-p402022	C4	22	34.4	A
15524	Bn-N14-p6534594	Bn-C4-p6446959	Bn-scaff_15908_1-p524762	C4	22	34.4	A
15525	Bn-N14-p6548718	Bn-C4-p6457604	Bn-scaff_15908_1-p535407	C4	22	34.4	A
15526	Bn-N14-p6555691	Bn-C4-p6464150	Bn-scaff_15908_1-p541953	C4	22	34.4	A
15527	Bn-N14-p6560064	Bn-C4-p6468538	Bn-scaff_15908_1-p546341	C4	22	34.4	A
15528	Bn-N14-p6569553	Bn-A05-p3833308	Bn-A05-p3833308	C4	22	34.4	0

15529	Bn-N14-p6611111	Bn-C4-p6514655	Bn-scaff_15908_1-p59245	C4	22	34.4	A
15530	Bn-N14-p6627524	Bn-C4-p6531073	Bn-scaff_15908_1-p60887	C4	22	34.4	A
15531	Bn-N14-p6630571	Bn-C4-p6534838	Bn-scaff_15908_1-p61264	C4	22	34.4	A
15532	Bn-N5-p3693963	Bn-A05-p3454754	Bn-A05-p3454754	C4	22	34.4	0
15533	Bn-N14-p6788085	Bn-C4-p6677657	Bn-scaff_15908_1-p75546	C4	23	35.2	A
15534	Bn-N14-p6805980	Bn-C4-p6694171	Bn-scaff_15908_1-p77197	C4	23	35.2	A
15535	Bn-N14-p6806200	Bn-C4-p6694392	Bn-scaff_15908_1-p77219	C4	23	35.2	A
15536	Bn-N14-p6841370	Bn-C4-p6720984	Bn-scaff_15908_1-p79878	C4	23	35.2	A
15537	Bn-N14-p6863945	Bn-C4-p6744897	Bn-scaff_15908_1-p82270	C4	23	35.2	A
15538	Bn-N14-p6872606	Bn-C4-p6755347	Bn-scaff_15908_1-p83315	C4	23	35.2	A
15539	Bn-N14-p6895761	Bn-C4-p6776768	Bn-scaff_15908_1-p85457	C4	24	36	A
15540	Bn-N14-p6923580	Bn-C4-p6808204	Bn-scaff_15908_1-p88600	C4	24	36	A
15541	Bn-N14-p6942892	Bn-C4-p6825623	Bn-scaff_15908_1-p90342	C4	24	36	A
15542	Bn-N14-p6962280	Bn-C4-p6845299	Bn-scaff_15908_1-p92310	C4	24	36	A
15543	Bn-N14-p6973617	Bn-C4-p6861018	Bn-scaff_15908_1-p93882	C4	24	36	A
15544	Bn-N14-p6996175	Bn-C4-p6881080	Bn-scaff_15908_1-p95888	C4	24	36	A
15545	Bn-N14-p7004029	Bn-C4-p6888979	Bn-scaff_15908_1-p96678	C4	24	36	A
15546	Bn-N14-p7055048	Bn-C4-p6946857	Bn-scaff_15908_1-p10246	C4	24	36	A
15547	Bn-N14-p7062808	Bn-C4-p6955139	Bn-scaff_15908_1-p10329	C4	24	36	A
15548	Bn-N14-p7083428	Bn-C4-p6970594	Bn-scaff_15908_1-p10483	C4	24	36	A
15549	Bn-N14-p7083582	Bn-C4-p6970748	Bn-scaff_15908_1-p10485	C4	24	36	A
15550	Bn-N14-p7161002	Bn-C4-p7020247	Bn-scaff_15908_1-p10980	C4	24	36	A
15551	Bn-N14-p7193956	Bn-C4-p7046654	Bn-scaff_15908_1-p11244	C4	24	36	B
15552	Bn-N14-p7199337	Bn-C4-p7051935	Bn-scaff_15908_1-p11297	C4	24	36	A
15553	Bn-N14-p7388271	Bn-A05-p4304172	Bn-A05-p4304172	C4	25	37.6	A
15554	Bn-N14-p7398428	Bn-C4-p7245916	Bn-scaff_17983_1-p52117	C4	25	37.6	A
15555	Bn-N14-p7399250	Bn-C4-p7246745	Bn-scaff_17983_1-p52946	C4	25	37.6	A
15556	Bn-N14-p7430388	Bn-C4-p7315642	Bn-scaff_19248_1-p5520	C4	25	37.6	A
15557	Bn-N14-p7437300	Bn-C4-p7322826	Bn-scaff_19248_1-p12704	C4	25	37.6	A
15558	Bn-N14-p7445424	Bn-C4-p7330751	Bn-scaff_19248_1-p20629	C4	25	37.6	A
15559	Bn-N14-p7446052	Bn-C4-p7331381	Bn-scaff_19248_1-p21259	C4	25	37.6	A
15560	Bn-N14-p7523416	Bn-C4-p7367643	Bn-scaff_19248_1-p57521	C4	25	37.6	A
15561	Bn-N14-p7523659	Bn-C4-p7367882	Bn-scaff_19248_1-p57760	C4	25	37.6	A
15562	Bn-N14-p7524072	Bn-C4-p7368295	Bn-scaff_19248_1-p58173	C4	25	37.6	A
15563	Bn-N14-p7530935	Bn-C4-p7375040	Bn-scaff_19248_1-p64918	C4	25	37.6	A
15564	Bn-N14-p7532637	Bn-C4-p7376612	Bn-scaff_19248_1-p66490	C4	25	37.6	A
15565	Bn-N14-p7532739	Bn-C4-p7376714	Bn-scaff_19248_1-p66592	C4	25	37.6	A
15566	Bn-N14-p7540654	Bn-C4-p7383762	Bn-scaff_19248_1-p73640	C4	25	37.6	A
15567	Bn-N14-p7541003	Bn-C4-p7384110	Bn-scaff_19248_1-p73988	C4	25	37.6	A
15568	Bn-N14-p7546246	Bn-C4-p7389054	Bn-scaff_19248_1-p78932	C4	25	37.6	A
15569	Bn-N5-p4557642	Bn-A05-p4290068	Bn-A05-p4290068	C4	25	37.6	A
15570	Bn-N14-p7653870	Bn-C4-p7497891	Bn-scaff_19248_1-p18776	C4	26	39.2	A
15571	Bn-N14-p7657388	Bn-C4-p7507288	Bn-scaff_19248_1-p19716	C4	26	39.2	A
15572	Bn-N14-p7666768	Bn-C4-p7517266	Bn-scaff_19248_1-p20714	C4	26	39.2	A
15573	Bn-N14-p7668614	Bn-C4-p7519106	Bn-scaff_19248_1-p20898	C4	26	39.2	A
15574	Bn-N14-p7670761	Bn-C4-p7521262	Bn-scaff_19248_1-p21114	C4	26	39.2	A
15575	Bn-N14-p7701278	Bn-C4-p7545980	Bn-scaff_19248_1-p23585	C4	26	39.2	A
15576	Bn-N14-p7730982	Bn-C4-p7579770	Bn-scaff_19248_1-p26964	C4	26	39.2	A
15577	Bn-N14-p7770015	Bn-C4-p7623710	Bn-scaff_19248_1-p31358	C4	26	39.2	A

15578	Bn-N14-p7774115	Bn-Scaffold02122-p1	Bn-scaff_23535_1-p1899	C4	26	39.2	A
15579	Bn-N14-p7775784	Bn-C4-p7629204	Bn-scaff_19248_1-p31908	C4	26	39.2	A
15580	Bn-N15-p43392984	Bn-C5-p41588691	Bn-scaff_21369_1-p19063	C4	26	39.2	0
15581	Bn-N14-p8042737	Bn-C4-p7925803	Bn-scaff_18440_1-p77767	C4	27	40	A
15582	Bn-N14-p8124321	Bn-C4-p7984168	Bn-scaff_18440_1-p13613	C4	27	40	A
15583	Bn-N14-p8380248	Bn-C4-p8236479	Bn-scaff_23907_1-p16039	C4	27	40	A
15584	Bn-N14-p8419333	Bn-C4-p8271696	Bn-scaff_23907_1-p19560	C4	27	40	A
15585	Bn-N14-p8430411	Bn-C4-p8272875	Bn-scaff_23907_1-p19678	C4	27	40	A
15586	Bn-N14-p8430723	Bn-C4-p8273187	Bn-scaff_23907_1-p19709	C4	27	40	A
15587	Bn-N14-p9513241	Bn-C4-p9098489	Bn-scaff_23907_1-p10224	C4	27	40	A
15588	Bn-N5-p4975572	Bn-A05-p4715599	Bn-A05-p4715599	C4	27	40	A
15589	Bn-N5-p5315855	Bn-C4-p8509311	Bn-scaff_23907_1-p43322	C4	27	40	A
15590	Bn-N14-p8955502	Bn-C4-p8618530	Bn-scaff_23907_1-p54244	C4	28	44	A
15591	Bn-N14-p9067753	Bn-C4-p8723983	Bn-scaff_23907_1-p64789	C4	29	44.8	A
15592	Bn-N14-p9072254	Bn-C4-p8729868	Bn-scaff_23907_1-p65378	C4	29	44.8	A
15593	Bn-N14-p9072476	Bn-C4-p8730090	Bn-scaff_23907_1-p65400	C4	29	44.8	A
15594	Bn-N14-p9073231	Bn-C4-p8730845	Bn-scaff_23907_1-p65475	C4	29	44.8	A
15595	Bn-N14-p9075485	Bn-C4-p8733879	Bn-scaff_23907_1-p65779	C4	29	44.8	A
15596	Bn-N14-p9089341	Bn-C4-p8747808	Bn-scaff_23907_1-p67172	C4	29	44.8	A
15597	Bn-N14-p9093283	Bn-C4-p8751748	Bn-scaff_23907_1-p67566	C4	29	44.8	A
15598	Bn-N14-p9097334	Bn-C4-p8755799	Bn-scaff_23907_1-p67971	C4	29	44.8	A
15599	Bn-N5-p5540139	Bn-A05-p5264904	Bn-A05-p5264904	C4	29	44.8	A
15600	Bn-N14-p9174618	Bn-C4-p8822093	Bn-scaff_23907_1-p74600	C4	30	45.6	A
15601	Bn-N14-p9401986	Bn-C4-p9007623	Bn-scaff_23907_1-p93153	C4	31	46.4	A
15602	Bn-N14-p9455169	Bn-C4-p9045684	Bn-scaff_23907_1-p96959	C4	31	46.4	A
15603	Bn-N14-p9473219	Bn-C4-p9056617	Bn-scaff_23907_1-p98052	C4	31	46.4	A
15604	Bn-N14-p10251001	Bn-C4-p9777719	Bn-scaff_16447_1-p40324	C4	32	48	A
15605	Bn-N14-p10765256	Bn-C4-p10291436	Bn-scaff_17869_1-p11037	C4	32	48	A
15606	Bn-N14-p10770634	Bn-C4-p10296784	Bn-scaff_17869_1-p11572	C4	32	48	A
15607	Bn-N14-p10770705	Bn-C4-p10296855	Bn-scaff_17869_1-p11579	C4	32	48	A
15608	Bn-N14-p10819976	Bn-C4-p10349525	Bn-scaff_17869_1-p16846	C4	32	48	A
15609	Bn-N14-p10828169	Bn-C4-p10355474	Bn-scaff_17869_1-p17441	C4	32	48	A
15610	Bn-N14-p10828226	Bn-C4-p10355538	Bn-scaff_17869_1-p17448	C4	32	48	A
15611	Bn-N14-p10861042	Bn-C4-p10388581	Bn-scaff_17869_1-p20752	C4	32	48	A
15612	Bn-N5-p6538816	Bn-C4-p10592314	Bn-scaff_17869_1-p41125	C4	32	48	A
15613	Bn-N14-p11501551	Bn-C4-p10995381	Bn-scaff_17869_1-p81432	C4	33	48.8	A
15614	Bn-N14-p11566886	Bn-C4-p11050321	Bn-scaff_16095_1-p30079	C4	33	48.8	A
15615	Bn-N14-p11568815	Bn-C4-p11052101	Bn-scaff_16095_1-p31859	C4	33	48.8	A
15616	Bn-N14-p11569630	Bn-C4-p11052867	Bn-scaff_16095_1-p32625	C4	33	48.8	A
15617	Bn-N14-p11578725	Bn-C4-p11061485	Bn-scaff_16095_1-p41243	C4	33	48.8	A
15618	Bn-N14-p11581097	Bn-C4-p11063857	Bn-scaff_16095_1-p43615	C4	33	48.8	A
15619	Bn-N14-p11603534	Bn-C4-p11086769	Bn-scaff_16095_1-p66527	C4	33	48.8	A
15620	Bn-N14-p11609787	Bn-C4-p11092789	Bn-scaff_16095_1-p72547	C4	33	48.8	A
15621	Bn-N14-p11621202	Bn-C4-p11103009	Bn-scaff_16095_1-p82767	C4	33	48.8	A
15622	Bn-N14-p11655914	Bn-C4-p11140464	Bn-scaff_16095_1-p12022	C4	33	48.8	A
15623	Bn-N14-p11690848	Bn-C4-p11174326	Bn-scaff_21982_1-p77327	C4	33	48.8	A
15624	Bn-N14-p11698330	Bn-C4-p11181808	Bn-scaff_21982_1-p69845	C4	33	48.8	A
15625	Bn-N14-p11698566	Bn-C4-p11182044	Bn-scaff_21982_1-p69609	C4	33	48.8	A
15626	Bn-N9-p21108808	Bn-C8-p6244520	Bn-scaff_15896_1-p55043	C4	33	48.8	A

15627	Bn-Scaffold02855-p724	Bn-C4-p11200378	Bn-scaff_21982_1-p51275	C4	33	48.8	A
15628	Bn-N14-p12025135	Bn-C4-p11427976	Bn-scaff_18754_1-p233662	C4	34	51.2	A
15629	Bn-N14-p12040078	Bn-C4-p11441811	Bn-scaff_18754_1-p219827	C4	34	51.2	A
15630	Bn-N14-p12040205	Bn-C4-p11441939	Bn-scaff_18754_1-p219699	C4	34	51.2	A
15631	Bn-N14-p12049336	Bn-C4-p11450778	Bn-scaff_18754_1-p210860	C4	34	51.2	A
15632	Bn-N14-p12052353	Bn-C4-p11453800	Bn-scaff_18754_1-p207838	C4	34	51.2	A
15633	Bn-N14-p12058442	Bn-C4-p11458454	Bn-scaff_18754_1-p203184	C4	34	51.2	A
15634	Bn-N14-p12066833	Bn-C4-p11466156	Bn-scaff_18754_1-p195482	C4	34	51.2	A
15635	Bn-N14-p12073361	Bn-C4-p11472012	Bn-scaff_18754_1-p189626	C4	34	51.2	0
15636	Bn-N14-p12124603	Bn-C4-p11522713	Bn-scaff_18754_1-p138925	C4	34	51.2	A
15637	Bn-N14-p12124886	Bn-C4-p11522996	Bn-scaff_18754_1-p138642	C4	34	51.2	A
15638	Bn-N14-p12124910	Bn-C4-p11523020	Bn-scaff_18754_1-p138618	C4	34	51.2	A
15639	Bn-N14-p12124994	Bn-C4-p11523104	Bn-scaff_18754_1-p138534	C4	34	51.2	A
15640	Bn-N14-p12245645	Bn-C4-p11633548	Bn-scaff_18754_1-p28090	C4	34	51.2	A
15641	Bn-N14-p12273979	Bn-C9-p7599925	Bn-scaff_17084_1-p817929	C4	34	51.2	A
15642	Bn-N14-p12538710	Bn-C4-p11933784	Bn-scaff_23699_1-p272047	C4	34	51.2	A
15643	Bn-N14-p12562719	Bn-C4-p11958057	Bn-scaff_23699_1-p296320	C4	34	51.2	A
15644	Bn-N14-p12563399	Bn-C4-p11958737	Bn-scaff_23699_1-p297000	C4	34	51.2	A
15645	Bn-Scaffold01030-p12840	Bn-C4-p11597197	Bn-scaff_18754_1-p64441	C4	34	51.2	A
15646	Bn-N14-p12698070	Bn-C4-p12096888	Bn-scaff_23699_1-p435151	C4	35	52.8	A
15647	Bn-N14-p13122718	Bn-C4-p12774669	Bn-scaff_17346_1-p75859	C4	35	52.8	A
15648	Bn-N14-p13139068	Bn-C4-p12799638	Bn-scaff_17346_1-p100828	C4	35	52.8	A
15649	Bn-N14-p13141588	Bn-C4-p12802019	Bn-scaff_17346_1-p103209	C4	35	52.8	A
15650	Bn-N14-p13242488	Bn-C4-p12937344	Bn-scaff_23613_1-p35766	C4	35	52.8	A
15651	Bn-N14-p13301244	Bn-C4-p12991242	Bn-scaff_16576_1-p18033	C4	35	52.8	A
15652	Bn-N14-p13387927	Bn-C4-p13070378	Bn-scaff_16576_1-p97169	C4	35	52.8	A
15653	Bn-N14-p13532337	Bn-C4-p13165388	Bn-scaff_16576_1-p192179	C4	35	52.8	A
15654	Bn-N14-p13532618	Bn-C4-p13165659	Bn-scaff_16576_1-p192450	C4	35	52.8	A
15655	Bn-N14-p13539678	Bn-C4-p13175517	Bn-scaff_16576_1-p202308	C4	35	52.8	A
15656	Bn-N14-p13551997	Bn-C4-p13199353	Bn-scaff_16576_1-p226144	C4	35	52.8	A
15657	Bn-N14-p13573262	Bn-C4-p13205358	Bn-scaff_16576_1-p232149	C4	35	52.8	A
15658	Bn-N14-p13576377	Bn-C4-p13208478	Bn-scaff_16576_1-p235269	C4	35	52.8	A
15659	Bn-N14-p13576493	Bn-C4-p13208594	Bn-scaff_16576_1-p235385	C4	35	52.8	A
15660	Bn-N14-p13578035	Bn-C4-p13209958	Bn-scaff_16576_1-p236749	C4	35	52.8	A
15661	Bn-N14-p13582859	Bn-C4-p13215483	Bn-scaff_16576_1-p242274	C4	35	52.8	A
15662	Bn-N14-p13582934	Bn-C4-p13215558	Bn-scaff_16576_1-p242349	C4	35	52.8	A
15663	Bn-N14-p13587095	Bn-C4-p13219671	Bn-scaff_16576_1-p246462	C4	35	52.8	A
15664	Bn-N14-p13605295	Bn-C4-p13229576	Bn-scaff_16576_1-p256367	C4	35	52.8	A
15665	Bn-N14-p13611524	Bn-C4-p13234579	Bn-scaff_16576_1-p261370	C4	35	52.8	A
15666	Bn-N14-p13612121	Bn-C4-p13235341	Bn-scaff_16576_1-p262132	C4	35	52.8	A
15667	Bn-N14-p13612219	Bn-C4-p13235439	Bn-scaff_16576_1-p262230	C4	35	52.8	A
15668	Bn-N14-p13612346	Bn-C4-p13235566	Bn-scaff_16576_1-p262357	C4	35	52.8	A
15669	Bn-N14-p13612612	Bn-C4-p13235832	Bn-scaff_16576_1-p262623	C4	35	52.8	A
15670	Bn-N14-p13614036	Bn-C4-p13237267	Bn-scaff_16576_1-p264058	C4	35	52.8	A
15671	Bn-N14-p13617629	Bn-C4-p13240560	Bn-scaff_16576_1-p267351	C4	35	52.8	A
15672	Bn-N14-p13642444	Bn-C4-p13245363	Bn-scaff_16576_1-p272154	C4	35	52.8	A
15673	Bn-N14-p13643548	Bn-C4-p13246472	Bn-scaff_16576_1-p273263	C4	35	52.8	A
15674	Bn-N14-p13659873	Bn-C4-p13260738	Bn-scaff_16576_1-p287529	C4	35	52.8	A
15675	Bn-N14-p13662505	Bn-C4-p13262486	Bn-scaff_16576_1-p289277	C4	35	52.8	A

15676	Bn-N14-p13699883	Bn-C4-p13291503	Bn-scaff_16576_1-p31829	C4	35	52.8	A
15677	Bn-N14-p13708463	Bn-C4-p13297580	Bn-scaff_16576_1-p32437	C4	35	52.8	A
15678	Bn-N14-p13711161	Bn-C4-p13300271	Bn-scaff_16576_1-p32706	C4	35	52.8	A
15679	Bn-N14-p13713489	Bn-C4-p13306795	Bn-scaff_16576_1-p33358	C4	35	52.8	A
15680	Bn-N14-p13800238	Bn-Scaffold01754-p3	Bn-scaff_17112_1-p3928	C4	35	52.8	A
15681	Bn-N14-p13815298	Bn-C4-p13415173	Bn-scaff_16576_1-p44196	C4	35	52.8	A
15682	Bn-N14-p13815523	Bn-C4-p13416015	Bn-scaff_16576_1-p44280	C4	35	52.8	A
15683	Bn-N14-p13861752	Bn-C4-p13473593	Bn-scaff_16576_1-p50038	C4	35	52.8	A
15684	Bn-N14-p13861845	Bn-C4-p13473686	Bn-scaff_16576_1-p50047	C4	35	52.8	A
15685	Bn-N14-p13862021	Bn-C4-p13473861	Bn-scaff_16576_1-p50065	C4	35	52.8	A
15686	Bn-N14-p13954029	Bn-C4-p13552370	Bn-scaff_16576_1-p57916	C4	35	52.8	A
15687	Bn-N14-p13954844	Bn-C4-p13553206	Bn-scaff_16576_1-p57999	C4	35	52.8	A
15688	Bn-N14-p13954981	Bn-C4-p13553342	Bn-scaff_16576_1-p58013	C4	35	52.8	A
15689	Bn-N14-p13956548	Bn-C4-p13555744	Bn-scaff_16576_1-p58253	C4	35	52.8	A
15690	Bn-N14-p14177260	Bn-C4-p13776522	Bn-scaff_19821_1-p21800	C4	35	52.8	A
15691	Bn-N14-p14227972	Bn-C4-p13826610	Bn-scaff_19821_1-p26809	C4	35	52.8	A
15692	Bn-N14-p14232259	Bn-C4-p13830924	Bn-scaff_19821_1-p27240	C4	35	52.8	A
15693	Bn-N14-p14263973	Bn-C4-p13946976	Bn-scaff_19821_1-p38845	C4	35	52.8	A
15694	Bn-N14-p14323767	Bn-C4-p13982785	Bn-scaff_24119_1-p1067	C4	35	52.8	0
15695	Bn-N14-p14324080	Bn-C4-p13983098	Bn-scaff_24119_1-p1380	C4	35	52.8	0
15696	Bn-N14-p14339409	Bn-C4-p12604727	Bn-scaff_15993_1-p3906	C4	35	52.8	A
15697	Bn-N14-p14442968	Bn-C4-p13991681	Bn-scaff_15607_1-p13090	C4	35	52.8	A
15698	Bn-N14-p14448639	Bn-C4-p13996806	Bn-scaff_15607_1-p7965	C4	35	52.8	A
15699	Bn-N14-p14507468	Bn-Scaffold00898-p6	Bn-scaff_24123_1-p6612	C4	35	52.8	A
15700	Bn-N14-p14520766	Bn-Scaffold00898-p3	Bn-scaff_24123_1-p35437	C4	35	52.8	A
15701	Bn-N14-p14637980	Bn-C4-p14208047	Bn-scaff_17592_1-p88812	C4	35	52.8	A
15702	Bn-N14-p14643534	Bn-C4-p14213560	Bn-scaff_17592_1-p88261	C4	35	52.8	A
15703	Bn-N14-p15391415	Bn-C4-p14854578	Bn-scaff_17686_1-p10509	C4	35	52.8	A
15704	Bn-N14-p15398837	Bn-C4-p14862008	Bn-scaff_17686_1-p11252	C4	35	52.8	A
15705	Bn-N5-p8263026	Bn-C4-p12666174	Bn-scaff_15993_1-p65353	C4	35	52.8	A
15706	Bn-Scaffold01079-p1021	Bn-C4-p13850588	Bn-scaff_19821_1-p29206	C4	35	52.8	A
15707	Bn-Scaffold01079-p1238	Bn-C4-p13850805	Bn-scaff_19821_1-p29228	C4	35	52.8	A
15708	Bn-Scaffold01079-p1915	Bn-C4-p13851481	Bn-scaff_19821_1-p29296	C4	35	52.8	A
15709	Bn-Scaffold01079-p19780	Bn-C4-p13883000	Bn-scaff_19821_1-p32448	C4	35	52.8	A
15710	Bn-Scaffold01079-p3364	Bn-C4-p13852931	Bn-scaff_19821_1-p29441	C4	35	52.8	A
15711	Bn-Scaffold02092-p2921	Bn-C4-p13917973	Bn-scaff_19821_1-p35945	C4	35	52.8	A
15712	Bn-N11-p6400646	Bn-C4-p15360346	Bn-scaff_18712_1-p32875	C4	36	54.4	0
15713	Bn-N11-p6401642	Bn-C4-p15361342	Bn-scaff_18712_1-p32974	C4	36	54.4	0
15714	Bn-N14-p15885493	Bn-C4-p15358035	Bn-scaff_18712_1-p32644	C4	36	54.4	A
15715	Bn-N14-p15986748	Bn-C4-p15452632	Bn-scaff_18712_1-p42103	C4	36	54.4	A
15716	Bn-N14-p16004439	Bn-C4-p15457165	Bn-scaff_18712_1-p42557	C4	36	54.4	A
15717	Bn-N14-p16007877	Bn-C4-p15460599	Bn-scaff_18712_1-p42900	C4	36	54.4	A
15718	Bn-N14-p16008439	Bn-C4-p15461182	Bn-scaff_18712_1-p42958	C4	36	54.4	A
15719	Bn-N14-p16010740	Bn-C4-p15463307	Bn-scaff_18712_1-p43171	C4	36	54.4	A
15720	Bn-N14-p16012603	Bn-C4-p15465162	Bn-scaff_18712_1-p43356	C4	36	54.4	A
15721	Bn-N14-p16013762	Bn-C4-p15466312	Bn-scaff_18712_1-p43471	C4	36	54.4	A
15722	Bn-N14-p16015565	Bn-C4-p15468092	Bn-scaff_18712_1-p43649	C4	36	54.4	A
15723	Bn-N14-p16016211	Bn-C4-p15468738	Bn-scaff_18712_1-p43714	C4	36	54.4	A
15724	Bn-N14-p16020286	Bn-C4-p15472817	Bn-scaff_18712_1-p44122	C4	36	54.4	A

15725	Bn-N14-p16020368	Bn-C4-p15472895	Bn-scaff_18712_1-p441302	C4	36	54.4	A
15726	Bn-N14-p16020676	Bn-C4-p15473203	Bn-scaff_18712_1-p441610	C4	36	54.4	A
15727	Bn-N14-p16020781	Bn-C4-p15473308	Bn-scaff_18712_1-p441715	C4	36	54.4	A
15728	Bn-N14-p16021484	Bn-C4-p15474011	Bn-scaff_18712_1-p442418	C4	36	54.4	A
15729	Bn-N14-p16021485	Bn-C4-p15474012	Bn-scaff_18712_1-p442419	C4	36	54.4	A
15730	Bn-N14-p16022125	Bn-C4-p15474655	Bn-scaff_18712_1-p443062	C4	36	54.4	A
15731	Bn-N14-p16065483	Bn-C4-p15514194	Bn-scaff_18712_1-p482601	C4	36	54.4	A
15732	Bn-N14-p16088000	Bn-C4-p15545272	Bn-scaff_18712_1-p513679	C4	36	54.4	A
15733	Bn-N14-p16094893	Bn-C4-p15552147	Bn-scaff_18712_1-p520554	C4	36	54.4	A
15734	Bn-N14-p16129373	Bn-C4-p15588181	Bn-scaff_18712_1-p556588	C4	36	54.4	A
15735	Bn-N14-p16143942	Bn-C4-p15603645	Bn-scaff_18712_1-p572052	C4	36	54.4	A
15736	Bn-N14-p16183214	Bn-C4-p15633748	Bn-scaff_18712_1-p602155	C4	36	54.4	A
15737	Bn-N14-p16207491	Bn-C4-p15653487	Bn-scaff_18712_1-p621894	C4	36	54.4	A
15738	Bn-N14-p16211733	Bn-C4-p15657704	Bn-scaff_18712_1-p626111	C4	36	54.4	A
15739	Bn-N14-p16212480	Bn-C4-p15658451	Bn-scaff_18712_1-p626858	C4	36	54.4	A
15740	Bn-N14-p16212994	Bn-C4-p15658965	Bn-scaff_18712_1-p627372	C4	36	54.4	A
15741	Bn-N14-p16213293	Bn-C4-p15659264	Bn-scaff_18712_1-p627671	C4	36	54.4	A
15742	Bn-N14-p16213552	Bn-C4-p15659523	Bn-scaff_18712_1-p627930	C4	36	54.4	A
15743	Bn-N14-p16213653	Bn-C4-p15659624	Bn-scaff_18712_1-p628031	C4	36	54.4	A
15744	Bn-N14-p16214513	Bn-C4-p15660483	Bn-scaff_18712_1-p628890	C4	36	54.4	A
15745	Bn-N14-p16219561	Bn-C13734643-p116	Bn-C13734643-p116	C4	36	54.4	A
15746	Bn-N14-p16221929	Bn-C4-p15676237	Bn-scaff_27513_1-p8405	C4	36	54.4	A
15747	Bn-N14-p16236249	Bn-C4-p15686608	Bn-scaff_27513_1-p18776	C4	36	54.4	A
15748	Bn-N14-p16236258	Bn-C4-p15686617	Bn-scaff_27513_1-p18785	C4	36	54.4	A
15749	Bn-N14-p16278667	Bn-C4-p15723567	Bn-scaff_27513_1-p55735	C4	36	54.4	A
15750	Bn-N14-p16284511	Bn-C4-p15729405	Bn-scaff_27513_1-p61573	C4	36	54.4	A
15751	Bn-N14-p16285112	Bn-C4-p15730006	Bn-scaff_27513_1-p62174	C4	36	54.4	A
15752	Bn-N14-p16285613	Bn-C4-p15730507	Bn-scaff_27513_1-p62675	C4	36	54.4	A
15753	Bn-N14-p16455837	Bn-C4-p15864796	Bn-scaff_18505_1-p216926	C4	36	54.4	A
15754	Bn-N14-p16456313	Bn-C4-p15865273	Bn-scaff_18505_1-p216449	C4	36	54.4	A
15755	Bn-N14-p16472342	Bn-C4-p15888283	Bn-scaff_18505_1-p193439	C4	36	54.4	A
15756	Bn-N14-p16559320	Bn-C4-p15990255	Bn-scaff_18505_1-p91467	C4	36	54.4	A
15757	Bn-N14-p16559975	Bn-C4-p15990894	Bn-scaff_18505_1-p90828	C4	36	54.4	A
15758	Bn-N14-p16561590	Bn-C4-p15992374	Bn-scaff_18505_1-p89348	C4	36	54.4	A
15759	Bn-N14-p16568604	Bn-C4-p15995777	Bn-scaff_18505_1-p85945	C4	36	54.4	A
15760	Bn-N14-p16569370	Bn-C4-p15996543	Bn-scaff_18505_1-p85179	C4	36	54.4	A
15761	Bn-N14-p16578833	Bn-C4-p16005261	Bn-scaff_18505_1-p76461	C4	36	54.4	A
15762	Bn-N14-p16585673	Bn-C4-p16011961	Bn-scaff_18505_1-p69761	C4	36	54.4	A
15763	Bn-N14-p16585764	Bn-C4-p16012053	Bn-scaff_18505_1-p69669	C4	36	54.4	A
15764	Bn-N14-p16658386	Bn-C4-p16115670	Bn-scaff_16312_1-p33849	C4	36	54.4	A
15765	Bn-N14-p16658568	Bn-C4-p16115861	Bn-scaff_16312_1-p34040	C4	36	54.4	A
15766	Bn-N14-p16658729	Bn-C4-p16116022	Bn-scaff_16312_1-p34201	C4	36	54.4	A
15767	Bn-N14-p16710177	Bn-C4-p16153358	Bn-scaff_16312_1-p71537	C4	36	54.4	A
15768	Bn-N14-p16712478	Bn-C4-p16155644	Bn-scaff_16312_1-p73823	C4	36	54.4	A
15769	Bn-N14-p16712636	Bn-C4-p16155802	Bn-scaff_16312_1-p73981	C4	36	54.4	A
15770	Bn-N14-p16724716	Bn-C4-p16163917	Bn-scaff_16312_1-p82096	C4	36	54.4	A
15771	Bn-N14-p16724776	Bn-C4-p16163976	Bn-scaff_16312_1-p82155	C4	36	54.4	A
15772	Bn-N14-p16726421	Bn-C4-p16171037	Bn-scaff_16312_1-p89216	C4	36	54.4	A
15773	Bn-N14-p16726656	Bn-C4-p16177205	Bn-scaff_16312_1-p95384	C4	36	54.4	A

15774	Bn-N14-p16777647	Bn-C6-p24687736	Bn-scaff_16510_1-p362449	C4	36	54.4	A
15775	Bn-N14-p16793188	Bn-C4-p16201907	Bn-scaff_16312_1-p120086	C4	36	54.4	A
15776	Bn-N14-p16793302	Bn-C4-p16202019	Bn-scaff_16312_1-p120198	C4	36	54.4	A
15777	Bn-N14-p16793308	Bn-C4-p16202025	Bn-scaff_16312_1-p120204	C4	36	54.4	A
15778	Bn-N14-p16793466	Bn-C4-p16202199	Bn-scaff_16312_1-p120378	C4	36	54.4	A
15779	Bn-N14-p16934876	Bn-C4-p16843545	Bn-scaff_19253_1-p75285	C4	36	54.4	A
15780	Bn-N14-p16945898	Bn-C4-p16836323	Bn-scaff_19253_1-p82507	C4	36	54.4	A
15781	Bn-N14-p17413444	Bn-C4-p16502322	Bn-scaff_19253_1-p416508	C4	36	54.4	A
15782	Bn-N14-p23420054	Bn-C4-p16199426	Bn-scaff_16312_1-p117605	C4	36	54.4	A
15783	Bn-N7-p12122754	Bn-C4-p15948605	Bn-scaff_18505_1-p133117	C4	36	54.4	A
15784	Bn-N7-p12277971	Bn-C4-p15559020	Bn-scaff_18712_1-p527427	C4	36	54.4	A
15785	Bn-N9-p23609949	Bn-A07-p10468928	Bn-A07-p10468928	C4	36	54.4	A
15786	Bn-N13-p62149093	Bn-C4-p17637885	Bn-scaff_16511_1-p718956	C4	37	55.2	A
15787	Bn-N14-p16579507	Bn-C4-p16005894	Bn-scaff_18505_1-p75828	C4	37	55.2	A
15788	Bn-N14-p16959217	Bn-C4-p16826959	Bn-scaff_19253_1-p91871	C4	37	55.2	A
15789	Bn-N14-p16960929	Bn-C4-p16825244	Bn-scaff_19253_1-p93586	C4	37	55.2	A
15790	Bn-N14-p16962342	Bn-C4-p16823837	Bn-scaff_19253_1-p94993	C4	37	55.2	A
15791	Bn-N14-p16962797	Bn-C4-p16823384	Bn-scaff_19253_1-p95446	C4	37	55.2	A
15792	Bn-N14-p16969695	Bn-C4-p16816474	Bn-scaff_19253_1-p102356	C4	37	55.2	A
15793	Bn-N14-p16979069	Bn-C4-p16807471	Bn-scaff_19253_1-p111359	C4	37	55.2	A
15794	Bn-N14-p16979209	Bn-C4-p16807331	Bn-scaff_19253_1-p111499	C4	37	55.2	A
15795	Bn-N14-p16979769	Bn-C4-p16806748	Bn-scaff_19253_1-p112082	C4	37	55.2	A
15796	Bn-N14-p16979899	Bn-C4-p16806618	Bn-scaff_19253_1-p112212	C4	37	55.2	A
15797	Bn-N14-p16980362	Bn-C4-p16806128	Bn-scaff_19253_1-p112702	C4	37	55.2	A
15798	Bn-N14-p16980447	Bn-C4-p16806043	Bn-scaff_19253_1-p112787	C4	37	55.2	A
15799	Bn-N14-p16980895	Bn-C4-p16805595	Bn-scaff_19253_1-p113235	C4	37	55.2	A
15800	Bn-N14-p16983776	Bn-C4-p16802704	Bn-scaff_19253_1-p116126	C4	37	55.2	A
15801	Bn-N14-p16985652	Bn-C4-p16800862	Bn-scaff_19253_1-p117968	C4	37	55.2	A
15802	Bn-N14-p16986751	Bn-C4-p16799764	Bn-scaff_19253_1-p119066	C4	37	55.2	A
15803	Bn-N14-p16997608	Bn-C4-p16796642	Bn-scaff_19253_1-p122188	C4	37	55.2	A
15804	Bn-N14-p16997939	Bn-C4-p16796311	Bn-scaff_19253_1-p122519	C4	37	55.2	A
15805	Bn-N14-p16999146	Bn-C4-p16794457	Bn-scaff_19253_1-p124373	C4	37	55.2	A
15806	Bn-N14-p16999634	Bn-C4-p16793919	Bn-scaff_19253_1-p124911	C4	37	55.2	A
15807	Bn-N14-p16999771	Bn-C4-p16793782	Bn-scaff_19253_1-p125048	C4	37	55.2	A
15808	Bn-N14-p16999901	Bn-C4-p16793651	Bn-scaff_19253_1-p125179	C4	37	55.2	A
15809	Bn-N14-p17000059	Bn-C4-p16793493	Bn-scaff_19253_1-p125337	C4	37	55.2	A
15810	Bn-N14-p17000210	Bn-C4-p16793341	Bn-scaff_19253_1-p125489	C4	37	55.2	A
15811	Bn-N14-p17000722	Bn-C4-p16792829	Bn-scaff_19253_1-p126001	C4	37	55.2	A
15812	Bn-N14-p17000939	Bn-C4-p16792611	Bn-scaff_19253_1-p126219	C4	37	55.2	A
15813	Bn-N14-p17001462	Bn-C4-p16786468	Bn-scaff_19253_1-p132362	C4	37	55.2	A
15814	Bn-N14-p17001531	Bn-C4-p16786399	Bn-scaff_19253_1-p132431	C4	37	55.2	A
15815	Bn-N14-p17001607	Bn-C4-p16786323	Bn-scaff_19253_1-p132507	C4	37	55.2	0
15816	Bn-N14-p17008686	Bn-C4-p16785652	Bn-scaff_19253_1-p133178	C4	37	55.2	A
15817	Bn-N14-p17019024	Bn-C4-p16776660	Bn-scaff_19253_1-p142170	C4	37	55.2	A
15818	Bn-N14-p17019268	Bn-C4-p16776416	Bn-scaff_19253_1-p142414	C4	37	55.2	A
15819	Bn-N14-p17019354	Bn-C4-p16776330	Bn-scaff_19253_1-p142500	C4	37	55.2	A
15820	Bn-N14-p17022665	Bn-C4-p16773700	Bn-scaff_19253_1-p145130	C4	37	55.2	A
15821	Bn-N14-p17022991	Bn-C4-p16773374	Bn-scaff_19253_1-p145456	C4	37	55.2	A
15822	Bn-N14-p17033874	Bn-C4-p16766786	Bn-scaff_19253_1-p152044	C4	37	55.2	A

15823	Bn-N14-p17045179	Bn-C4-p16756596	Bn-scaff_19253_1-p16223[illegible]	C4	37	55.2	A
15824	Bn-N14-p17045732	Bn-C4-p16756043	Bn-scaff_19253_1-p16278[illegible]	C4	37	55.2	A
15825	Bn-N14-p17046025	Bn-C4-p16755750	Bn-scaff_19253_1-p16308[illegible]	C4	37	55.2	A
15826	Bn-N14-p17046093	Bn-C4-p16755682	Bn-scaff_19253_1-p16314[illegible]	C4	37	55.2	A
15827	Bn-N14-p17046197	Bn-C4-p16755578	Bn-scaff_19253_1-p16325[illegible]	C4	37	55.2	A
15828	Bn-N14-p17046272	Bn-C4-p16755503	Bn-scaff_19253_1-p16332[illegible]	C4	37	55.2	A
15829	Bn-N14-p17046378	Bn-C4-p16755397	Bn-scaff_19253_1-p16343[illegible]	C4	37	55.2	A
15830	Bn-N14-p17050381	Bn-C4-p16751399	Bn-scaff_19253_1-p16743[illegible]	C4	37	55.2	A
15831	Bn-N14-p17051078	Bn-C4-p16750702	Bn-scaff_19253_1-p16812[illegible]	C4	37	55.2	A
15832	Bn-N14-p17053605	Bn-C4-p16748190	Bn-scaff_19253_1-p17064[illegible]	C4	37	55.2	A
15833	Bn-N14-p17053795	Bn-C4-p16747846	Bn-scaff_19253_1-p17098[illegible]	C4	37	55.2	A
15834	Bn-N14-p17054057	Bn-C4-p16747581	Bn-scaff_19253_1-p17124[illegible]	C4	37	55.2	A
15835	Bn-N14-p17064594	Bn-C4-p16738324	Bn-scaff_19253_1-p18050[illegible]	C4	37	55.2	A
15836	Bn-N14-p17078326	Bn-C4-p16723798	Bn-scaff_19253_1-p19503[illegible]	C4	37	55.2	A
15837	Bn-N14-p17078954	Bn-C4-p16723171	Bn-scaff_19253_1-p19565[illegible]	C4	37	55.2	A
15838	Bn-N14-p17080641	Bn-C4-p16721411	Bn-scaff_19253_1-p19741[illegible]	C4	37	55.2	A
15839	Bn-N14-p17083337	Bn-C4-p16718701	Bn-scaff_19253_1-p20012[illegible]	C4	37	55.2	A
15840	Bn-N14-p17085182	Bn-C4-p16716860	Bn-scaff_19253_1-p20197[illegible]	C4	37	55.2	A
15841	Bn-N14-p17114838	Bn-C4-p16694872	Bn-scaff_19253_1-p22395[illegible]	C4	37	55.2	A
15842	Bn-N14-p17115818	Bn-C4-p16693892	Bn-scaff_19253_1-p22493[illegible]	C4	37	55.2	A
15843	Bn-N14-p17116066	Bn-C4-p16693644	Bn-scaff_19253_1-p22518[illegible]	C4	37	55.2	A
15844	Bn-N14-p17119125	Bn-C4-p16690556	Bn-scaff_19253_1-p22827[illegible]	C4	37	55.2	A
15845	Bn-N14-p17119421	Bn-C4-p16690261	Bn-scaff_19253_1-p22856[illegible]	C4	37	55.2	A
15846	Bn-N14-p17130896	Bn-C4-p16681019	Bn-scaff_19253_1-p23781[illegible]	C4	37	55.2	A
15847	Bn-N14-p17162376	Bn-C4-p16669951	Bn-scaff_19253_1-p24887[illegible]	C4	37	55.2	A
15848	Bn-N14-p17165103	Bn-C4-p16665673	Bn-scaff_19253_1-p25315[illegible]	C4	37	55.2	A
15849	Bn-N14-p17169171	Bn-C4-p16661604	Bn-scaff_19253_1-p25722[illegible]	C4	37	55.2	A
15850	Bn-N14-p17170762	Bn-C4-p16660013	Bn-scaff_19253_1-p25881[illegible]	C4	37	55.2	A
15851	Bn-N14-p17171150	Bn-C4-p16659625	Bn-scaff_19253_1-p25920[illegible]	C4	37	55.2	A
15852	Bn-N14-p17181646	Bn-C4-p16649600	Bn-scaff_19253_1-p26923[illegible]	C4	37	55.2	A
15853	Bn-N14-p17191193	Bn-C4-p16641477	Bn-scaff_19253_1-p27735[illegible]	C4	37	55.2	A
15854	Bn-N14-p17199195	Bn-C4-p16633426	Bn-scaff_19253_1-p28540[illegible]	C4	37	55.2	A
15855	Bn-N14-p17204440	Bn-C4-p16628199	Bn-scaff_19253_1-p29063[illegible]	C4	37	55.2	A
15856	Bn-N14-p17205601	Bn-C4-p16627033	Bn-scaff_19253_1-p29179[illegible]	C4	37	55.2	A
15857	Bn-N14-p17240222	Bn-C4-p16599464	Bn-scaff_19253_1-p31936[illegible]	C4	37	55.2	A
15858	Bn-N14-p17241301	Bn-C4-p16598384	Bn-scaff_19253_1-p32044[illegible]	C4	37	55.2	A
15859	Bn-N14-p17276146	Bn-C4-p16553663	Bn-scaff_19253_1-p36516[illegible]	C4	37	55.2	A
15860	Bn-N14-p17284104	Bn-C4-p16545626	Bn-scaff_19253_1-p37320[illegible]	C4	37	55.2	A
15861	Bn-N14-p17284656	Bn-C4-p16545068	Bn-scaff_19253_1-p37376[illegible]	C4	37	55.2	A
15862	Bn-N14-p17285599	Bn-C4-p16544126	Bn-scaff_19253_1-p37470[illegible]	C4	37	55.2	A
15863	Bn-N14-p17337319	Bn-C4-p16531637	Bn-scaff_19253_1-p38719[illegible]	C4	37	55.2	A
15864	Bn-N14-p17338834	Bn-C4-p16530123	Bn-scaff_19253_1-p38870[illegible]	C4	37	55.2	A
15865	Bn-N14-p17339276	Bn-C4-p16529681	Bn-scaff_19253_1-p38914[illegible]	C4	37	55.2	A
15866	Bn-N14-p17704257	Bn-C4-p18414636	Bn-scaff_16511_1-p14957[illegible]	C4	37	55.2	A
15867	Bn-N14-p18019485	Bn-C4-p18107415	Bn-scaff_16511_1-p11884[illegible]	C4	37	55.2	A
15868	Bn-N14-p18095856	Bn-C4-p18032512	Bn-scaff_16511_1-p11135[illegible]	C4	37	55.2	A
15869	Bn-N14-p18210737	Bn-C4-p17918050	Bn-scaff_16511_1-p99912[illegible]	C4	37	55.2	A
15870	Bn-N14-p18988486	Bn-C4-p17585088	Bn-scaff_16511_1-p66615[illegible]	C4	37	55.2	A
15871	Bn-N14-p19984061	Bn-Scaffold00956-p1[illegible]	Bn-scaff_17316_1-p17060	C4	37	55.2	A

15872	Bn-N14-p20172626	Bn-C4-p19967554	Bn-scaff_19575_1-p230391	C4	37	55.2	A
15873	Bn-N14-p20176250	Bn-C4-p19963924	Bn-scaff_19575_1-p226761	C4	37	55.2	A
15874	Bn-N14-p20338984	Bn-C4-p19826533	Bn-scaff_19575_1-p89370	C4	37	55.2	A
15875	Bn-N14-p20483435	Bn-C4-p19725981	Bn-scaff_16503_1-p358771	C4	37	55.2	A
15876	Bn-N14-p21108524	Bn-Scaffold01030-p4	Bn-scaff_26053_1-p4397	C4	37	55.2	A
15877	Bn-N14-p21524098	Bn-C4-p20755264	Bn-scaff_19170_1-p22650	C4	37	55.2	A
15878	Bn-N14-p21898086	Bn-C4-p21240817	Bn-scaff_19170_1-p50820	C4	37	55.2	A
15879	Bn-N14-p22963483	Bn-Scaffold00829-p4	Bn-scaff_21138_1-p40760	C4	37	55.2	A
15880	Bn-N14-p22998171	Bn-Scaffold00829-p1	Bn-scaff_21138_1-p17277	C4	37	55.2	A
15881	Bn-N14-p23841616	Bn-C4-p27014771	Bn-scaff_18275_1-p75186	C4	37	55.2	A
15882	Bn-N14-p23920417	Bn-C4-p22455371	Bn-scaff_15911_1-p82783	C4	37	55.2	A
15883	Bn-N14-p23929487	Bn-C4-p22469696	Bn-scaff_15911_1-p81350	C4	37	55.2	A
15884	Bn-N14-p24105436	Bn-C4-p22659437	Bn-scaff_15911_1-p62376	C4	37	55.2	A
15885	Bn-N14-p55051244	Bn-C4-p17637862	Bn-scaff_16511_1-p71893	C4	37	55.2	A
15886	Bn-Scaffold39853-p196	Bn-C4-p17638028	Bn-scaff_16511_1-p71909	C4	37	55.2	A
15887	Bn-N14-p23279842	Bn-C4-p22906600	Bn-scaff_15911_1-p37660	C4	38	56	A
15888	Bn-N14-p23280154	Bn-C4-p22906912	Bn-scaff_15911_1-p37629	C4	38	56	A
15889	Bn-N14-p23281898	Bn-C4-p22908571	Bn-scaff_15911_1-p37463	C4	38	56	A
15890	Bn-N14-p23282715	Bn-C4-p22909386	Bn-scaff_15911_1-p37381	C4	38	56	A
15891	Bn-N14-p23288680	Bn-C4-p22915365	Bn-scaff_15911_1-p36783	C4	38	56	A
15892	Bn-N14-p23288747	Bn-C4-p22915432	Bn-scaff_15911_1-p36777	C4	38	56	A
15893	Bn-N14-p23292421	Bn-C4-p22919749	Bn-scaff_15911_1-p36345	C4	38	56	A
15894	Bn-N14-p23292734	Bn-C4-p22920062	Bn-scaff_15911_1-p36314	C4	38	56	A
15895	Bn-N14-p23292889	Bn-C4-p22920217	Bn-scaff_15911_1-p36298	C4	38	56	A
15896	Bn-N14-p23293268	Bn-C4-p22920594	Bn-scaff_15911_1-p36260	C4	38	56	A
15897	Bn-N14-p23293416	Bn-C4-p22920742	Bn-scaff_15911_1-p36246	C4	38	56	A
15898	Bn-N14-p23293688	Bn-C4-p22921014	Bn-scaff_15911_1-p36218	C4	38	56	A
15899	Bn-N14-p23295250	Bn-C4-p22922767	Bn-scaff_15911_1-p36043	C4	38	56	A
15900	Bn-N14-p23295305	Bn-C4-p22922822	Bn-scaff_15911_1-p36038	C4	38	56	A
15901	Bn-N14-p23295653	Bn-C4-p22923170	Bn-scaff_15911_1-p36003	C4	38	56	A
15902	Bn-N14-p23296336	Bn-C4-p22923853	Bn-scaff_15911_1-p35935	C4	38	56	A
15903	Bn-N14-p23296791	Bn-C4-p22924308	Bn-scaff_15911_1-p35889	C4	38	56	A
15904	Bn-N14-p23297333	Bn-C4-p22924850	Bn-scaff_15911_1-p35835	C4	38	56	A
15905	Bn-N14-p23297662	Bn-C4-p22925179	Bn-scaff_15911_1-p35802	C4	38	56	A
15906	Bn-N14-p23298371	Bn-C4-p22925889	Bn-scaff_15911_1-p35731	C4	38	56	A
15907	Bn-N14-p23298595	Bn-C4-p22926113	Bn-scaff_15911_1-p35709	C4	38	56	A
15908	Bn-N14-p23300851	Bn-C4-p22928387	Bn-scaff_15911_1-p35481	C4	38	56	A
15909	Bn-N14-p23300907	Bn-C4-p22928443	Bn-scaff_15911_1-p35476	C4	38	56	A
15910	Bn-N14-p23301128	Bn-C4-p22928664	Bn-scaff_15911_1-p35453	C4	38	56	A
15911	Bn-N14-p23306086	Bn-C4-p22933463	Bn-scaff_15911_1-p34974	C4	38	56	A
15912	Bn-N14-p23306130	Bn-C4-p22933507	Bn-scaff_15911_1-p34969	C4	38	56	A
15913	Bn-N14-p23306263	Bn-C4-p22933640	Bn-scaff_15911_1-p34956	C4	38	56	A
15914	Bn-N14-p23306334	Bn-C4-p22933711	Bn-scaff_15911_1-p34949	C4	38	56	A
15915	Bn-N14-p23306428	Bn-C4-p22933805	Bn-scaff_15911_1-p34939	C4	38	56	A
15916	Bn-N14-p23307015	Bn-C4-p22934392	Bn-scaff_15911_1-p34881	C4	38	56	A
15917	Bn-N14-p23307093	Bn-C4-p22934470	Bn-scaff_15911_1-p34873	C4	38	56	A
15918	Bn-N14-p23307296	Bn-C4-p22934673	Bn-scaff_15911_1-p34853	C4	38	56	A
15919	Bn-N14-p23307373	Bn-C4-p22934750	Bn-scaff_15911_1-p34845	C4	38	56	A
15920	Bn-N14-p23307615	Bn-C4-p22934992	Bn-scaff_15911_1-p34821	C4	38	56	A

15921	Bn-N14-p23307911	Bn-C4-p22935288	Bn-scaff_15911_1-p34791[illegible]	C4	38	56	A
15922	Bn-N14-p23307947	Bn-C4-p22935324	Bn-scaff_15911_1-p34787[illegible]	C4	38	56	A
15923	Bn-N14-p23308371	Bn-C4-p22935748	Bn-scaff_15911_1-p34745[illegible]	C4	38	56	A
15924	Bn-N14-p23308671	Bn-C4-p22936047	Bn-scaff_15911_1-p34715[illegible]	C4	38	56	A
15925	Bn-N14-p23308791	Bn-C4-p22936167	Bn-scaff_15911_1-p34703[illegible]	C4	38	56	A
15926	Bn-N14-p23308917	Bn-C4-p22936293	Bn-scaff_15911_1-p34691[illegible]	C4	38	56	A
15927	Bn-N14-p23309192	Bn-C4-p22936568	Bn-scaff_15911_1-p34663[illegible]	C4	38	56	A
15928	Bn-N14-p23317900	Bn-C4-p22943230	Bn-scaff_15911_1-p33997[illegible]	C4	38	56	A
15929	Bn-N14-p23318445	Bn-C4-p22943775	Bn-scaff_15911_1-p33942[illegible]	C4	38	56	A
15930	Bn-N14-p23321839	Bn-C4-p22947170	Bn-scaff_15911_1-p33603[illegible]	C4	38	56	A
15931	Bn-N14-p23326045	Bn-C4-p22951245	Bn-scaff_15911_1-p33195[illegible]	C4	38	56	A
15932	Bn-N14-p23327141	Bn-C4-p22952341	Bn-scaff_15911_1-p33086[illegible]	C4	38	56	A
15933	Bn-N14-p23337731	Bn-C4-p22956036	Bn-scaff_15911_1-p32716[illegible]	C4	38	56	A
15934	Bn-N14-p23337800	Bn-C4-p22956105	Bn-scaff_15911_1-p32709[illegible]	C4	38	56	A
15935	Bn-N14-p23342981	Bn-C4-p22960410	Bn-scaff_15911_1-p32279[illegible]	C4	38	56	A
15936	Bn-N14-p23343089	Bn-C4-p22960518	Bn-scaff_15911_1-p32268[illegible]	C4	38	56	A
15937	Bn-N14-p23345957	Bn-C4-p22965520	Bn-scaff_15911_1-p31768[illegible]	C4	38	56	A
15938	Bn-N14-p23345961	Bn-C4-p22965524	Bn-scaff_15911_1-p31767[illegible]	C4	38	56	A
15939	Bn-N14-p23346305	Bn-C4-p22965867	Bn-scaff_15911_1-p31733[illegible]	C4	38	56	A
15940	Bn-N14-p23346356	Bn-C4-p22965918	Bn-scaff_15911_1-p31728[illegible]	C4	38	56	0
15941	Bn-N14-p23346836	Bn-C4-p22966399	Bn-scaff_15911_1-p31680[illegible]	C4	38	56	A
15942	Bn-N14-p23346973	Bn-C4-p22966536	Bn-scaff_15911_1-p31666[illegible]	C4	38	56	A
15943	Bn-N14-p23347109	Bn-C4-p22966672	Bn-scaff_15911_1-p31653[illegible]	C4	38	56	A
15944	Bn-N14-p23349342	Bn-C4-p22968890	Bn-scaff_15911_1-p31431[illegible]	C4	38	56	A
15945	Bn-N14-p23349404	Bn-C4-p22968952	Bn-scaff_15911_1-p31425[illegible]	C4	38	56	A
15946	Bn-N14-p23349589	Bn-C4-p22969137	Bn-scaff_15911_1-p31406[illegible]	C4	38	56	A
15947	Bn-N14-p23350047	Bn-C4-p22969595	Bn-scaff_15911_1-p31360[illegible]	C4	38	56	A
15948	Bn-N14-p23351091	Bn-C4-p22970656	Bn-scaff_15911_1-p31254[illegible]	C4	38	56	A
15949	Bn-N14-p23379738	Bn-C4-p22982767	Bn-scaff_15911_1-p30043[illegible]	C4	38	56	A
15950	Bn-N14-p23383208	Bn-C4-p22991638	Bn-scaff_15911_1-p29156[illegible]	C4	38	56	A
15951	Bn-N14-p23384132	Bn-C4-p22992564	Bn-scaff_15911_1-p29063[illegible]	C4	38	56	A
15952	Bn-N14-p23403120	Bn-C4-p23011217	Bn-scaff_15911_1-p27198[illegible]	C4	38	56	A
15953	Bn-N14-p23431745	Bn-C4-p23064151	Bn-scaff_15911_1-p21905[illegible]	C4	38	56	A
15954	Bn-N14-p23472560	Bn-C4-p23105762	Bn-scaff_15911_1-p17744[illegible]	C4	38	56	A
15955	Bn-N14-p23539176	Bn-C4-p23192544	Bn-scaff_15911_1-p90659	C4	38	56	A
15956	Bn-N14-p23553430	Bn-C4-p23206965	Bn-scaff_15911_1-p76238	C4	38	56	A
15957	Bn-N14-p23621496	Bn-C4-p23267650	Bn-scaff_15911_1-p15553	C4	38	56	A
15958	Bn-N14-p23666236	Bn-C4-p25106318	Bn-scaff_19929_1-p59215	C4	38	56	A
15959	Bn-N14-p23694794	Bn-Scaffold00858-p1[illegible]	Bn-scaff_25432_1-p16404	C4	38	56	A
15960	Bn-N14-p23713448	Bn-Scaffold00858-p3[illegible]	Bn-scaff_25432_1-p34840	C4	38	56	A
15961	Bn-N14-p23722740	Bn-Scaffold00858-p4[illegible]	Bn-scaff_25432_1-p42030	C4	38	56	A
15962	Bn-N14-p24146699	Bn-C4-p22698660	Bn-scaff_15911_1-p58454[illegible]	C4	38	56	A
15963	Bn-N14-p24147247	Bn-C4-p22699208	Bn-scaff_15911_1-p58399[illegible]	C4	38	56	A
15964	Bn-N14-p24150211	Bn-C4-p22700645	Bn-scaff_15911_1-p58255[illegible]	C4	38	56	A
15965	Bn-N14-p24150604	Bn-C4-p22701039	Bn-scaff_15911_1-p58216[illegible]	C4	38	56	A
15966	Bn-N14-p24151061	Bn-C4-p22701496	Bn-scaff_15911_1-p58170[illegible]	C4	38	56	A
15967	Bn-N14-p24151339	Bn-C4-p22701774	Bn-scaff_15911_1-p58142[illegible]	C4	38	56	A
15968	Bn-N14-p24151458	Bn-C4-p22701893	Bn-scaff_15911_1-p58131[illegible]	C4	38	56	A
15969	Bn-N14-p24151780	Bn-C4-p22702215	Bn-scaff_15911_1-p58098[illegible]	C4	38	56	A

15970	Bn-N14-p24153004	Bn-C4-p22703439	Bn-scaff_15911_1-p57976[illegible]	C4	38	56	A
15971	Bn-N14-p24161115	Bn-C4-p22711361	Bn-scaff_15911_1-p57184[illegible]	C4	38	56	A
15972	Bn-N14-p24161715	Bn-C4-p22711960	Bn-scaff_15911_1-p57124[illegible]	C4	38	56	A
15973	Bn-N14-p24162511	Bn-C4-p22712756	Bn-scaff_15911_1-p57044[illegible]	C4	38	56	A
15974	Bn-N14-p24162635	Bn-C4-p22712880	Bn-scaff_15911_1-p57032[illegible]	C4	38	56	A
15975	Bn-N14-p24163148	Bn-C4-p22713393	Bn-scaff_15911_1-p56981[illegible]	C4	38	56	A
15976	Bn-N14-p24163190	Bn-C4-p22713435	Bn-scaff_15911_1-p56976[illegible]	C4	38	56	A
15977	Bn-N14-p24163482	Bn-C4-p22713813	Bn-scaff_15911_1-p56939[illegible]	C4	38	56	A
15978	Bn-N14-p24163638	Bn-C4-p22713969	Bn-scaff_15911_1-p56923[illegible]	C4	38	56	A
15979	Bn-N14-p24163640	Bn-C4-p22713971	Bn-scaff_15911_1-p56923[illegible]	C4	38	56	A
15980	Bn-N14-p24163760	Bn-C4-p22714087	Bn-scaff_15911_1-p56911[illegible]	C4	38	56	A
15981	Bn-N14-p24164694	Bn-C4-p22715021	Bn-scaff_15911_1-p56818[illegible]	C4	38	56	A
15982	Bn-N14-p24171692	Bn-C4-p22722359	Bn-scaff_15911_1-p56084[illegible]	C4	38	56	A
15983	Bn-N14-p24171743	Bn-C4-p22722410	Bn-scaff_15911_1-p56079[illegible]	C4	38	56	A
15984	Bn-N14-p24171801	Bn-C4-p22722468	Bn-scaff_15911_1-p56073[illegible]	C4	38	56	A
15985	Bn-N14-p24172674	Bn-C4-p22723341	Bn-scaff_15911_1-p55986[illegible]	C4	38	56	A
15986	Bn-N14-p24176645	Bn-C4-p22727326	Bn-scaff_15911_1-p55587[illegible]	C4	38	56	A
15987	Bn-N14-p24186788	Bn-C4-p22737181	Bn-scaff_15911_1-p54602[illegible]	C4	38	56	A
15988	Bn-N14-p24186849	Bn-C4-p22737242	Bn-scaff_15911_1-p545961	C4	38	56	A
15989	Bn-N14-p24214934	Bn-C4-p22766221	Bn-scaff_15911_1-p51698[illegible]	C4	38	56	A
15990	Bn-N14-p24215016	Bn-C4-p22766303	Bn-scaff_15911_1-p51690[illegible]	C4	38	56	A
15991	Bn-N14-p24215443	Bn-C4-p22766730	Bn-scaff_15911_1-p51647[illegible]	C4	38	56	A
15992	Bn-N14-p24220511	Bn-C4-p22772316	Bn-scaff_15911_1-p51088[illegible]	C4	38	56	A
15993	Bn-N14-p24224374	Bn-C4-p22835619	Bn-scaff_15911_1-p44758[illegible]	C4	38	56	A
15994	Bn-N14-p24234797	Bn-C4-p22846163	Bn-scaff_15911_1-p43704[illegible]	C4	38	56	A
15995	Bn-N14-p24235170	Bn-C4-p22846535	Bn-scaff_15911_1-p43666[illegible]	C4	38	56	A
15996	Bn-N14-p24235595	Bn-C4-p22846960	Bn-scaff_15911_1-p43624[illegible]	C4	38	56	A
15997	Bn-N14-p24236752	Bn-C4-p22847833	Bn-scaff_15911_1-p43537[illegible]	C4	38	56	A
15998	Bn-N14-p24237087	Bn-C4-p22848168	Bn-scaff_15911_1-p43503[illegible]	C4	38	56	A
15999	Bn-N14-p24238001	Bn-C4-p22849082	Bn-scaff_15911_1-p43412[illegible]	C4	38	56	A
16000	Bn-N14-p24238357	Bn-C4-p22849438	Bn-scaff_15911_1-p43376[illegible]	C4	38	56	A
16001	Bn-N14-p24238465	Bn-C4-p22849546	Bn-scaff_15911_1-p43365[illegible]	C4	38	56	A
16002	Bn-N14-p24238641	Bn-C4-p22849722	Bn-scaff_15911_1-p433481	C4	38	56	A
16003	Bn-N14-p24240888	Bn-C4-p22851987	Bn-scaff_15911_1-p43121[illegible]	C4	38	56	A
16004	Bn-N14-p24240962	Bn-C4-p22852061	Bn-scaff_15911_1-p43114[illegible]	C4	38	56	A
16005	Bn-N14-p24268745	Bn-Scaffold01065-p1[illegible]	Bn-scaff_23070_1-p11258	C4	38	56	A
16006	Bn-N14-p24298595	Bn-C4-p23641667	Bn-scaff_20567_1-p35836[illegible]	C4	38	56	A
16007	Bn-N14-p24317441	Bn-C4-p23620276	Bn-scaff_20567_1-p33697[illegible]	C4	38	56	A
16008	Bn-N14-p24319025	Bn-scaffold14071-p6[illegible]	Bn-scaffold14071-p64	C4	38	56	A
16009	Bn-N14-p24328201	Bn-C4-p23610524	Bn-scaff_20567_1-p32722[illegible]	C4	38	56	A
16010	Bn-N14-p24437679	Bn-C4-p23518584	Bn-scaff_20567_1-p23528[illegible]	C4	38	56	A
16011	Bn-N14-p24438244	Bn-C4-p23518019	Bn-scaff_20567_1-p23471[illegible]	C4	38	56	A
16012	Bn-N14-p24581079	Bn-C4-p23351708	Bn-scaff_20567_1-p68406	C4	38	56	A
16013	Bn-N14-p24581293	Bn-C4-p23351494	Bn-scaff_20567_1-p68192	C4	38	56	A
16014	Bn-N14-p24584841	Bn-C4-p23347946	Bn-scaff_20567_1-p64644	C4	38	56	A
16015	Bn-N14-p24647785	Bn-C4-p23673917	Bn-scaff_15579_1-p18265	C4	38	56	A
16016	Bn-N14-p24647866	Bn-C4-p23673836	Bn-scaff_15579_1-p18184	C4	38	56	A
16017	Bn-N14-p24648378	Bn-C4-p23673324	Bn-scaff_15579_1-p17672	C4	38	56	A
16018	Bn-N14-p24695024	Bn-Scaffold01061-p9[illegible]	Bn-scaff_24587_1-p9788	C4	38	56	A

16019	Bn-N14-p24724212	Bn-C4-p23693010	Bn-scaff_16531_1-p14958	C4	38	56	A
16020	Bn-N14-p24748274	Bn-C4-p23717471	Bn-scaff_16531_1-p39419	C4	38	56	A
16021	Bn-N14-p24751499	Bn-C4-p23720709	Bn-scaff_16531_1-p42657	C4	38	56	A
16022	Bn-N14-p24751616	Bn-C4-p23720826	Bn-scaff_16531_1-p42774	C4	38	56	A
16023	Bn-N14-p24769111	Bn-C4-p23739413	Bn-scaff_16531_1-p61361	C4	38	56	A
16024	Bn-N14-p24784338	Bn-C4-p23754521	Bn-scaff_16531_1-p76469	C4	38	56	A
16025	Bn-N14-p24784615	Bn-C4-p23754799	Bn-scaff_16531_1-p76747	C4	38	56	A
16026	Bn-N14-p24787570	Bn-C4-p23763926	Bn-scaff_16531_1-p85874	C4	38	56	A
16027	Bn-N14-p24788528	Bn-C4-p23764849	Bn-scaff_16531_1-p86797	C4	38	56	0
16028	Bn-N14-p24789242	Bn-C4-p23766986	Bn-scaff_16531_1-p88934	C4	38	56	A
16029	Bn-N14-p24837712	Bn-C4-p23850443	Bn-scaff_16531_1-p172391	C4	38	56	A
16030	Bn-N14-p24843887	Bn-C4-p23856738	Bn-scaff_16531_1-p178686	C4	38	56	A
16031	Bn-N14-p24844481	Bn-C4-p23857334	Bn-scaff_16531_1-p179282	C4	38	56	A
16032	Bn-N14-p24864466	Bn-C4-p23880904	Bn-scaff_16531_1-p202852	C4	38	56	A
16033	Bn-N14-p24874997	Bn-C4-p23886943	Bn-scaff_16531_1-p208891	C4	38	56	A
16034	Bn-N14-p24877881	Bn-C4-p23888659	Bn-scaff_16531_1-p210607	C4	38	56	A
16035	Bn-N14-p24880404	Bn-C4-p23896048	Bn-scaff_16531_1-p217996	C4	38	56	A
16036	Bn-N14-p24884503	Bn-C4-p23900320	Bn-scaff_16531_1-p222268	C4	38	56	A
16037	Bn-N14-p24890774	Bn-C4-p23906605	Bn-scaff_16531_1-p228553	C4	38	56	A
16038	Bn-N14-p24894360	Bn-C4-p23908564	Bn-scaff_16531_1-p230512	C4	38	56	A
16039	Bn-N14-p24894532	Bn-C4-p23908736	Bn-scaff_16531_1-p230684	C4	38	56	A
16040	Bn-N14-p24903237	Bn-C4-p23909735	Bn-scaff_16531_1-p231683	C4	38	56	A
16041	Bn-N14-p24911327	Bn-C4-p23936137	Bn-scaff_16531_1-p258085	C4	38	56	A
16042	Bn-N14-p24930052	Bn-C4-p23956155	Bn-scaff_16531_1-p278103	C4	38	56	A
16043	Bn-N14-p24935128	Bn-C4-p23962723	Bn-scaff_16531_1-p284671	C4	38	56	A
16044	Bn-N14-p24941020	Bn-C4-p23964327	Bn-scaff_16531_1-p286275	C4	38	56	A
16045	Bn-N14-p24969045	Bn-C4-p24017284	Bn-scaff_16531_1-p339232	C4	38	56	A
16046	Bn-N14-p24969256	Bn-C4-p24017498	Bn-scaff_16531_1-p339446	C4	38	56	A
16047	Bn-N14-p24973810	Bn-C4-p24022000	Bn-scaff_16531_1-p343948	C4	38	56	A
16048	Bn-N14-p24975819	Bn-C4-p24024000	Bn-scaff_16531_1-p345948	C4	38	56	A
16049	Bn-N14-p24975886	Bn-C4-p24024067	Bn-scaff_16531_1-p346015	C4	38	56	A
16050	Bn-N14-p24982507	Bn-C4-p24026484	Bn-scaff_16531_1-p348432	C4	38	56	A
16051	Bn-N14-p24997242	Bn-C4-p24046134	Bn-scaff_16531_1-p368082	C4	38	56	A
16052	Bn-N14-p24998856	Bn-C4-p24047716	Bn-scaff_16531_1-p369664	C4	38	56	A
16053	Bn-N14-p25004010	Bn-C4-p24053791	Bn-scaff_16531_1-p375739	C4	38	56	A
16054	Bn-N14-p25004409	Bn-C4-p24054190	Bn-scaff_16531_1-p376138	C4	38	56	A
16055	Bn-N14-p25010070	Bn-C4-p24055408	Bn-scaff_16531_1-p377356	C4	38	56	A
16056	Bn-N14-p25010151	Bn-C4-p24055489	Bn-scaff_16531_1-p377437	C4	38	56	A
16057	Bn-N14-p25010441	Bn-C4-p24055776	Bn-scaff_16531_1-p377724	C4	38	56	A
16058	Bn-N14-p25011921	Bn-C4-p24057356	Bn-scaff_16531_1-p379304	C4	38	56	A
16059	Bn-N14-p25127896	Bn-C4-p24106942	Bn-scaff_16531_1-p428890	C4	38	56	A
16060	Bn-N14-p25134707	Bn-C4-p24113376	Bn-scaff_16531_1-p435324	C4	38	56	A
16061	Bn-N14-p25171734	Bn-C4-p24179689	Bn-scaff_16531_1-p501637	C4	38	56	A
16062	Bn-N14-p25172169	Bn-C4-p24180124	Bn-scaff_16531_1-p502072	C4	38	56	A
16063	Bn-N14-p25175186	Bn-C4-p24189731	Bn-scaff_16531_1-p511679	C4	38	56	A
16064	Bn-N14-p25184167	Bn-C4-p24199859	Bn-scaff_16531_1-p521807	C4	38	56	A
16065	Bn-N14-p25187916	Bn-C4-p24203605	Bn-scaff_16531_1-p525553	C4	38	56	A
16066	Bn-N14-p25188499	Bn-C4-p24204188	Bn-scaff_16531_1-p526136	C4	38	56	A
16067	Bn-N14-p25188585	Bn-C4-p24204274	Bn-scaff_16531_1-p526222	C4	38	56	A

16068	Bn-N14-p25189037	Bn-C4-p24204729	Bn-scaff_16531_1-p526677	C4	38	56	A
16069	Bn-N14-p25209628	Bn-C4-p24224621	Bn-scaff_16531_1-p546569	C4	38	56	A
16070	Bn-N14-p25212838	Bn-C4-p24227832	Bn-scaff_16531_1-p549780	C4	38	56	A
16071	Bn-N14-p25215170	Bn-C4-p24230164	Bn-scaff_16531_1-p552112	C4	38	56	A
16072	Bn-N14-p25215186	Bn-C4-p24230180	Bn-scaff_16531_1-p552128	C4	38	56	A
16073	Bn-N14-p25299904	Bn-C4-p24318463	Bn-scaff_16531_1-p640411	C4	38	56	A
16074	Bn-N14-p25300425	Bn-C4-p24318985	Bn-scaff_16531_1-p640933	C4	38	56	A
16075	Bn-N14-p25301724	Bn-C4-p24320299	Bn-scaff_16531_1-p642247	C4	38	56	A
16076	Bn-N14-p25302193	Bn-C4-p24320768	Bn-scaff_16531_1-p642716	C4	38	56	A
16077	Bn-N14-p25303069	Bn-C4-p24321644	Bn-scaff_16531_1-p643592	C4	38	56	A
16078	Bn-N14-p25504838	Bn-C4-p24521826	Bn-scaff_16217_1-p525178	C4	38	56	A
16079	Bn-N14-p25632832	Bn-C4-p24618602	Bn-scaff_16217_1-p428402	C4	38	56	A
16080	Bn-N14-p25939610	Bn-C4-p24925754	Bn-scaff_16217_1-p121250	C4	38	56	A
16081	Bn-N14-p25944039	Bn-C4-p24930180	Bn-scaff_16217_1-p116824	C4	38	56	A
16082	Bn-N14-p25946120	Bn-C4-p24932255	Bn-scaff_16217_1-p114749	C4	38	56	A
16083	Bn-N14-p26256069	Bn-C4-p25441556	Bn-scaff_16714_1-p248892	C4	38	56	A
16084	Bn-N14-p26256181	Bn-C4-p25441668	Bn-scaff_16714_1-p248780	C4	38	56	A
16085	Bn-N14-p26349802	Bn-C4-p25518742	Bn-scaff_16714_1-p171706	C4	38	56	A
16086	Bn-N14-p26711450	Bn-C4-p25819989	Bn-scaff_16625_1-p64201	C4	38	56	A
16087	Bn-N14-p26742663	Bn-C4-p25835775	Bn-scaff_16625_1-p79987	C4	38	56	A
16088	Bn-N14-p27024594	Bn-C4-p26159245	Bn-scaff_16309_1-p215682	C4	38	56	A
16089	Bn-N14-p27050582	Bn-C4-p26187502	Bn-scaff_16309_1-p187425	C4	38	56	A
16090	Bn-N14-p27050747	Bn-C4-p26187667	Bn-scaff_16309_1-p187260	C4	38	56	A
16091	Bn-N14-p27080051	Bn-C4-p26210377	Bn-scaff_16309_1-p164550	C4	38	56	A
16092	Bn-N14-p27103748	Bn-C4-p26230116	Bn-scaff_16309_1-p144811	C4	38	56	A
16093	Bn-N14-p27138485	Bn-C4-p26258566	Bn-scaff_16309_1-p116361	C4	38	56	A
16094	Bn-N14-p27186348	Bn-C4-p26307409	Bn-scaff_16309_1-p67518	C4	38	56	A
16095	Bn-N14-p27195333	Bn-C4-p26311822	Bn-scaff_16309_1-p63105	C4	38	56	A
16096	Bn-N14-p27195964	Bn-C4-p26312389	Bn-scaff_16309_1-p62538	C4	38	56	A
16097	Bn-N14-p27197840	Bn-C4-p26313576	Bn-scaff_16309_1-p61351	C4	38	56	A
16098	Bn-N14-p27212474	Bn-C3-p59864118	Bn-scaff_15794_1-p96454	C4	38	56	A
16099	Bn-N14-p27336499	Bn-C4-p26494263	Bn-scaff_17335_1-p119237	C4	38	56	A
16100	Bn-N14-p27477205	Bn-C4-p26594467	Bn-scaff_17335_1-p219441	C4	38	56	A
16101	Bn-N17-p19896381	Bn-Scaffold03128-p1(	Bn-scaff_25881_1-p1019	C4	38	56	A
16102	Bn-N4-p388282	Bn-A04-p375518	Bn-A04-p375518	C4	38	56	A
16103	Bn-N4-p410415	Bn-C4-p24934702	Bn-scaff_16217_1-p112302	C4	38	56	A
16104	Bn-Scaffold01178-p16096	Bn-C4-p25066664	Bn-scaff_19929_1-p19561	C4	38	56	A
16105	Bn-Scaffold19001-p382	Bn-Scaffold06436-p39	Bn-scaff_26618_1-p398	C4	38	56	A
16106	Bn-Scaffold21129-p434	Bn-Scaffold02166-p83	Bn-scaff_18396_1-p834	C4	38	56	A
16107	Bn-N14-p28855843	Bn-C4-p27836422	Bn-scaff_24602_1-p69684	C4	39	62.5	A
16108	Bn-N14-p28865280	Bn-C4-p27847059	Bn-scaff_24602_1-p80321	C4	39	62.5	A
16109	Bn-N14-p28868011	Bn-C4-p27847647	Bn-scaff_24602_1-p80909	C4	39	62.5	A
16110	Bn-N14-p28871962	Bn-C4-p27849241	Bn-scaff_24602_1-p82503	C4	39	62.5	A
16111	Bn-N14-p28872904	Bn-C4-p27850262	Bn-scaff_24602_1-p83524	C4	39	62.5	A
16112	Bn-N14-p28873040	Bn-C4-p27850398	Bn-scaff_24602_1-p83660	C4	39	62.5	A
16113	Bn-N14-p28884995	Bn-C4-p27861187	Bn-scaff_24602_1-p94449	C4	39	62.5	A
16114	Bn-N14-p28885004	Bn-C4-p27861196	Bn-scaff_24602_1-p94458	C4	39	62.5	A
16115	Bn-N14-p29608726	Bn-Scaffold00866-p24	Bn-scaff_19551_1-p24484	C4	40	63.3	A
16116	Bn-N14-p30168156	Bn-C4-p28995713	Bn-scaff_26946_1-p96772	C4	40	63.3	A

16117	Bn-N14-p30168669	Bn-C4-p28996226	Bn-scaff_26946_1-p97285	C4	40	63.3	A
16118	Bn-N14-p30173775	Bn-C4-p29001345	Bn-scaff_26946_1-p10240	C4	40	63.3	A
16119	Bn-N14-p30232271	Bn-C4-p29193627	Bn-scaff_17180_1-p85281	C4	40	63.3	A
16120	Bn-N14-p30256932	Bn-C4-p29170755	Bn-scaff_17180_1-p62409	C4	40	63.3	A
16121	Bn-N14-p30259347	Bn-C4-p29167906	Bn-scaff_17180_1-p59560	C4	40	63.3	A
16122	Bn-N14-p30263361	Bn-C4-p29163892	Bn-scaff_17180_1-p55546	C4	40	63.3	A
16123	Bn-N14-p30295147	Bn-C4-p29126166	Bn-scaff_17180_1-p17820	C4	40	63.3	A
16124	Bn-N14-p30306841	Bn-C4-p29115112	Bn-scaff_17180_1-p6766	C4	40	63.3	A
16125	Bn-N14-p30376657	Bn-C4-p29053363	Bn-scaff_26946_1-p15442	C4	40	63.3	A
16126	Bn-N14-p30398392	Bn-Scaffold00982-p1	Bn-scaff_27684_1-p15376	C4	40	63.3	A
16127	Bn-N14-p30399593	Bn-Scaffold00982-p1	Bn-scaff_27684_1-p14175	C4	40	63.3	A
16128	Bn-N14-p30399940	Bn-Scaffold00982-p1	Bn-scaff_27684_1-p13829	C4	40	63.3	A
16129	Bn-N14-p30558755	Bn-C4-p29362065	Bn-scaff_17180_1-p25371	C4	40	63.3	A
16130	Bn-N14-p30567592	Bn-C4-p29371377	Bn-scaff_17180_1-p26303	C4	40	63.3	A
16131	Bn-N14-p32618352	Bn-C4-p31572599	Bn-scaff_27914_1-p19775	C4	40	63.3	A
16132	Bn-N14-p32718256	Bn-Scaffold00576-p1	Bn-scaff_15712_8-p35754	C4	40	63.3	A
16133	Bn-N14-p32735582	Bn-Scaffold00576-p1	Bn-scaff_15712_8-p32715	C4	40	63.3	A
16134	Bn-N4-p3469581	Bn-A04-p3551969	Bn-A04-p3551969	C4	40	63.3	A
16135	Bn-N14-p21949894	Bn-C4-p21291779	Bn-scaff_19170_1-p55916	C4	41	64.9	A
16136	Bn-N14-p21951727	Bn-C4-p21293612	Bn-scaff_19170_1-p56099	C4	41	64.9	A
16137	Bn-N14-p33090231	Bn-C4-p31821483	Bn-scaff_22814_1-p16699	C4	41	64.9	A
16138	Bn-N14-p33091968	Bn-C4-p31823220	Bn-scaff_22814_1-p16872	C4	41	64.9	A
16139	Bn-N14-p33093018	Bn-C4-p31824273	Bn-scaff_22814_1-p16978	C4	41	64.9	A
16140	Bn-N14-p33107650	Bn-C4-p31838825	Bn-scaff_22148_1-p5629	C4	41	64.9	A
16141	Bn-N14-p33123634	Bn-C4-p31855037	Bn-scaff_22148_1-p21841	C4	41	64.9	A
16142	Bn-N14-p33125139	Bn-C4-p31856370	Bn-scaff_22148_1-p23174	C4	41	64.9	A
16143	Bn-N14-p33125510	Bn-C4-p31856734	Bn-scaff_22148_1-p23538	C4	41	64.9	A
16144	Bn-N14-p33152499	Bn-C4-p31899760	Bn-scaff_22148_1-p66564	C4	41	64.9	A
16145	Bn-N14-p33273034	Bn-C4-p32007448	Bn-scaff_22148_1-p17425	C4	41	64.9	A
16146	Bn-N14-p33277768	Bn-C4-p32020685	Bn-scaff_22148_1-p18748	C4	41	64.9	A
16147	Bn-N14-p33278794	Bn-C4-p32021745	Bn-scaff_22148_1-p18854	C4	41	64.9	A
16148	Bn-N14-p33317601	Bn-Scaffold02190-p6	Bn-scaff_27761_1-p615	C4	41	64.9	A
16149	Bn-N14-p33323692	Bn-C4-p32060253	Bn-scaff_22148_1-p22705	C4	41	64.9	A
16150	Bn-N14-p33324949	Bn-Scaffold08322-p1	Bn-scaff_18295_1-p189	C4	41	64.9	A
16151	Bn-N14-p33328357	Bn-C4-p32065014	Bn-scaff_22148_1-p23181	C4	41	64.9	A
16152	Bn-N14-p33329209	Bn-C4-p32065866	Bn-scaff_22148_1-p23267	C4	41	64.9	A
16153	Bn-N14-p33329895	Bn-C4-p32066552	Bn-scaff_22148_1-p23335	C4	41	64.9	A
16154	Bn-N14-p33330055	Bn-C4-p32066712	Bn-scaff_22148_1-p23351	C4	41	64.9	A
16155	Bn-N14-p33331178	Bn-C4-p32067833	Bn-scaff_22148_1-p23463	C4	41	64.9	A
16156	Bn-N14-p33337218	Bn-C4-p32073890	Bn-scaff_22148_1-p24069	C4	41	64.9	A
16157	Bn-N14-p33346175	Bn-C4-p32083972	Bn-scaff_22148_1-p25077	C4	41	64.9	A
16158	Bn-N14-p33346244	Bn-C4-p32084041	Bn-scaff_22148_1-p25084	C4	41	64.9	A
16159	Bn-N14-p33346921	Bn-C4-p32084717	Bn-scaff_22148_1-p25152	C4	41	64.9	A
16160	Bn-N14-p33348022	Bn-C4-p32085816	Bn-scaff_22148_1-p25262	C4	41	64.9	A
16161	Bn-N14-p33352051	Bn-C4-p32090175	Bn-scaff_22148_1-p25697	C4	41	64.9	A
16162	Bn-N14-p33356582	Bn-C4-p32094492	Bn-scaff_22148_1-p26129	C4	41	64.9	A
16163	Bn-N14-p33360372	Bn-C4-p32100202	Bn-scaff_22148_1-p26700	C4	41	64.9	A
16164	Bn-N14-p33360454	Bn-C4-p32100284	Bn-scaff_22148_1-p26708	C4	41	64.9	A
16165	Bn-N14-p33361214	Bn-C4-p32101110	Bn-scaff_22148_1-p26791	C4	41	64.9	A

16166	Bn-N14-p33361793	Bn-C4-p32101688	Bn-scaff_22148_1-p268492	C4	41	64.9	A
16167	Bn-N14-p33362151	Bn-C4-p32102046	Bn-scaff_22148_1-p268850	C4	41	64.9	A
16168	Bn-N14-p33362327	Bn-C4-p32102222	Bn-scaff_22148_1-p269026	C4	41	64.9	A
16169	Bn-N14-p33364863	Bn-C4-p32104778	Bn-scaff_22148_1-p271582	C4	41	64.9	A
16170	Bn-N14-p33365995	Bn-C4-p32105912	Bn-scaff_22148_1-p272716	C4	41	64.9	A
16171	Bn-N14-p33376307	Bn-C4-p32116760	Bn-scaff_22148_1-p283564	C4	41	64.9	A
16172	Bn-N14-p33381822	Bn-C4-p32121819	Bn-scaff_22148_1-p288623	C4	41	64.9	A
16173	Bn-N14-p33382178	Bn-C4-p32122175	Bn-scaff_22148_1-p288979	C4	41	64.9	A
16174	Bn-N14-p33383660	Bn-C4-p32123659	Bn-scaff_22148_1-p290463	C4	41	64.9	A
16175	Bn-N14-p33384447	Bn-C4-p32123868	Bn-scaff_22148_1-p290672	C4	41	64.9	A
16176	Bn-N14-p33384515	Bn-C4-p32123936	Bn-scaff_22148_1-p290740	C4	41	64.9	A
16177	Bn-N14-p33385733	Bn-C4-p32125170	Bn-scaff_22148_1-p291974	C4	41	64.9	A
16178	Bn-N14-p33386006	Bn-C4-p32125443	Bn-scaff_22148_1-p292247	C4	41	64.9	A
16179	Bn-N14-p33387590	Bn-C4-p32127031	Bn-scaff_22148_1-p293835	C4	41	64.9	A
16180	Bn-N14-p33387938	Bn-C4-p32127379	Bn-scaff_22148_1-p294183	C4	41	64.9	A
16181	Bn-N14-p33406093	Bn-C4-p32154515	Bn-scaff_22148_1-p321319	C4	41	64.9	A
16182	Bn-N14-p33406528	Bn-C4-p32154954	Bn-scaff_22148_1-p321758	C4	41	64.9	A
16183	Bn-N14-p33407141	Bn-C4-p32155574	Bn-scaff_22148_1-p322378	C4	41	64.9	A
16184	Bn-N14-p33411124	Bn-C4-p32159454	Bn-scaff_22148_1-p326258	C4	41	64.9	A
16185	Bn-N14-p33453163	Bn-C4-p32186988	Bn-scaff_22148_1-p353792	C4	41	64.9	A
16186	Bn-N14-p33454602	Bn-C4-p32188394	Bn-scaff_22148_1-p355198	C4	41	64.9	A
16187	Bn-N14-p33455611	Bn-C4-p32189404	Bn-scaff_22148_1-p356208	C4	41	64.9	A
16188	Bn-N14-p33455690	Bn-C4-p32189484	Bn-scaff_22148_1-p356288	C4	41	64.9	A
16189	Bn-N14-p33519568	Bn-C4-p32217097	Bn-scaff_22148_1-p383901	C4	41	64.9	A
16190	Bn-N14-p33537807	Bn-C4-p32233262	Bn-scaff_22148_1-p400066	C4	41	64.9	A
16191	Bn-N14-p33537880	Bn-C4-p32233336	Bn-scaff_22148_1-p400140	C4	41	64.9	A
16192	Bn-N14-p33559092	Bn-C4-p32269978	Bn-scaff_18776_1-p32882	C4	41	64.9	A
16193	Bn-N14-p33562766	Bn-C4-p32270651	Bn-scaff_18776_1-p33555	C4	41	64.9	A
16194	Bn-N14-p33565629	Bn-C4-p32273460	Bn-scaff_18776_1-p36364	C4	41	64.9	A
16195	Bn-N14-p33565720	Bn-C4-p32273551	Bn-scaff_18776_1-p36455	C4	41	64.9	A
16196	Bn-N14-p33583689	Bn-C4-p32291019	Bn-scaff_18776_1-p53923	C4	41	64.9	A
16197	Bn-N14-p33591755	Bn-C4-p32299911	Bn-scaff_18776_1-p62815	C4	41	64.9	A
16198	Bn-N14-p33593687	Bn-C4-p32301852	Bn-scaff_18776_1-p64756	C4	41	64.9	A
16199	Bn-N14-p33598015	Bn-C4-p32307061	Bn-scaff_18776_1-p69965	C4	41	64.9	A
16200	Bn-N14-p33599423	Bn-C4-p32309236	Bn-scaff_18776_1-p72140	C4	41	64.9	A
16201	Bn-N14-p33607156	Bn-C4-p32317310	Bn-scaff_18776_1-p80214	C4	41	64.9	A
16202	Bn-N14-p33608851	Bn-C4-p32319001	Bn-scaff_18776_1-p81905	C4	41	64.9	A
16203	Bn-N14-p33668919	Bn-C4-p32381435	Bn-scaff_18776_1-p144339	C4	41	64.9	A
16204	Bn-N14-p33768393	Bn-C4-p32483550	Bn-scaff_18776_1-p246454	C4	41	64.9	A
16205	Bn-N14-p33797799	Bn-C4-p32512958	Bn-scaff_18776_1-p275862	C4	41	64.9	A
16206	Bn-N14-p33839938	Bn-C4-p32555657	Bn-scaff_18776_1-p318561	C4	41	64.9	A
16207	Bn-N16-p39098235	Bn-C6-p83153	Bn-scaff_21018_1-p83153	C4	41	64.9	A
16208	Bn-N16-p39098299	Bn-C6-p83089	Bn-scaff_21018_1-p83089	C4	41	64.9	A
16209	Bn-N14-p34266628	Bn-C4-p32902510	Bn-scaff_15779_1-p41353	C4	42	68.1	A
16210	Bn-N14-p34319192	Bn-C4-p32955161	Bn-scaff_15779_1-p94004	C4	43	68.9	A
16211	Bn-N14-p34361941	Bn-C4-p33010519	Bn-scaff_15779_1-p149362	C4	43	68.9	A
16212	Bn-N14-p34552788	Bn-C4-p33179791	Bn-scaff_15779_1-p318634	C4	43	68.9	A
16213	Bn-N14-p34566954	Bn-C4-p33261161	Bn-scaff_15779_1-p400004	C4	43	68.9	A
16214	Bn-N14-p34596321	Bn-C4-p33284113	Bn-scaff_27765_1-p403263	C4	43	68.9	A

16215	Bn-N14-p34628372	Bn-C4-p33318023	Bn-scaff_27765_1-p437173	C4	43	68.9	A
16216	Bn-N14-p34761339	Bn-C4-p33481551	Bn-scaff_20191_1-p157818	C4	43	68.9	A
16217	Bn-N14-p34775832	Bn-C4-p33492715	Bn-scaff_20191_1-p146654	C4	43	68.9	A
16218	Bn-N14-p34791653	Bn-C4-p33502894	Bn-scaff_20191_1-p136475	C4	43	68.9	A
16219	Bn-N14-p34797881	Bn-C4-p33508420	Bn-scaff_20191_1-p130949	C4	43	68.9	A
16220	Bn-N14-p34798276	Bn-C4-p33508815	Bn-scaff_20191_1-p130554	C4	43	68.9	A
16221	Bn-N14-p34822898	Bn-C4-p33536017	Bn-scaff_20191_1-p103352	C4	43	68.9	A
16222	Bn-N14-p34866303	Bn-C4-p33576249	Bn-scaff_20191_1-p63120	C4	43	68.9	A
16223	Bn-N11-p14979759	Bn-C4-p33645090	Bn-scaff_26139_1-p376956	C4	44	69.7	A
16224	Bn-N14-p34993877	Bn-C4-p33688222	Bn-scaff_26139_1-p333824	C4	44	69.7	A
16225	Bn-N14-p35026618	Bn-C4-p33709393	Bn-scaff_26139_1-p312653	C4	44	69.7	A
16226	Bn-N14-p35030868	Bn-C4-p33714421	Bn-scaff_26139_1-p307625	C4	44	69.7	A
16227	Bn-N14-p35041965	Bn-C4-p33725598	Bn-scaff_26139_1-p296448	C4	44	69.7	A
16228	Bn-N14-p35044005	Bn-C4-p33727675	Bn-scaff_26139_1-p294371	C4	44	69.7	A
16229	Bn-N14-p35044828	Bn-C4-p33728498	Bn-scaff_26139_1-p293548	C4	44	69.7	A
16230	Bn-N14-p35046129	Bn-C4-p33729799	Bn-scaff_26139_1-p292247	C4	44	69.7	A
16231	Bn-N14-p35047680	Bn-C4-p33731349	Bn-scaff_26139_1-p290697	C4	44	69.7	A
16232	Bn-N14-p35048210	Bn-C4-p33731881	Bn-scaff_26139_1-p290165	C4	44	69.7	A
16233	Bn-N14-p35048899	Bn-C4-p33732571	Bn-scaff_26139_1-p289475	C4	44	69.7	A
16234	Bn-N14-p35049543	Bn-C4-p33733215	Bn-scaff_26139_1-p288831	C4	44	69.7	A
16235	Bn-N14-p35049976	Bn-C4-p33733647	Bn-scaff_26139_1-p288399	C4	44	69.7	A
16236	Bn-N14-p35050157	Bn-C4-p33733828	Bn-scaff_26139_1-p288218	C4	44	69.7	A
16237	Bn-N14-p35051170	Bn-C4-p33734841	Bn-scaff_26139_1-p287205	C4	44	69.7	A
16238	Bn-N14-p35054795	Bn-C4-p33738435	Bn-scaff_26139_1-p283611	C4	44	69.7	A
16239	Bn-N14-p35063782	Bn-C4-p33751717	Bn-scaff_26139_1-p270329	C4	44	69.7	A
16240	Bn-N14-p35108758	Bn-C4-p33807455	Bn-scaff_26139_1-p214591	C4	44	69.7	A
16241	Bn-N14-p35113990	Bn-C4-p33812360	Bn-scaff_26139_1-p209686	C4	44	69.7	A
16242	Bn-N14-p35216250	Bn-C4-p33915482	Bn-scaff_26139_1-p106564	C4	44	69.7	A
16243	Bn-N14-p35570898	Bn-C4-p34311901	Bn-scaff_20814_1-p248864	C4	44	69.7	A
16244	Bn-N14-p35571935	Bn-C4-p34312939	Bn-scaff_20814_1-p249902	C4	44	69.7	A
16245	Bn-N14-p35572851	Bn-C4-p34313855	Bn-scaff_20814_1-p250818	C4	44	69.7	A
16246	Bn-N14-p35572880	Bn-C4-p34313884	Bn-scaff_20814_1-p250847	C4	44	69.7	A
16247	Bn-N14-p35771457	Bn-C4-p34512563	Bn-scaff_18062_1-p257773	C4	44	69.7	A
16248	Bn-N14-p35775427	Bn-C4-p34516531	Bn-scaff_18062_1-p253805	C4	44	69.7	A
16249	Bn-N14-p35783321	Bn-C4-p34524170	Bn-scaff_18062_1-p246166	C4	44	69.7	A
16250	Bn-N14-p35784191	Bn-C4-p34525040	Bn-scaff_18062_1-p245296	C4	44	69.7	A
16251	Bn-N14-p35784810	Bn-C4-p34526071	Bn-scaff_18062_1-p244265	C4	44	69.7	A
16252	Bn-N14-p35797312	Bn-C4-p34536713	Bn-scaff_18062_1-p233623	C4	44	69.7	A
16253	Bn-N14-p35800955	Bn-C4-p34540355	Bn-scaff_18062_1-p229981	C4	44	69.7	A
16254	Bn-N14-p35812516	Bn-C4-p34550996	Bn-scaff_18062_1-p219340	C4	44	69.7	A
16255	Bn-N14-p35813441	Bn-C4-p34551921	Bn-scaff_18062_1-p218415	C4	44	69.7	A
16256	Bn-N14-p35813548	Bn-C4-p34552028	Bn-scaff_18062_1-p218308	C4	44	69.7	A
16257	Bn-N14-p35814967	Bn-C4-p34553447	Bn-scaff_18062_1-p216889	C4	44	69.7	A
16258	Bn-N14-p35817474	Bn-C4-p34555954	Bn-scaff_18062_1-p214382	C4	44	69.7	A
16259	Bn-N14-p35818075	Bn-C4-p34556558	Bn-scaff_18062_1-p213778	C4	44	69.7	A
16260	Bn-N14-p35854258	Bn-C4-p34585864	Bn-scaff_18062_1-p184472	C4	44	69.7	A
16261	Bn-N14-p35913495	Bn-C4-p34648580	Bn-scaff_18062_1-p121756	C4	44	69.7	A
16262	Bn-N14-p35918739	Bn-C4-p34655781	Bn-scaff_18062_1-p114555	C4	44	69.7	A
16263	Bn-N14-p36002379	Bn-C4-p34724554	Bn-scaff_18062_1-p45782	C4	44	69.7	A

16264	Bn-N14-p36094198	Bn-C4-p34813310	Bn-scaff_16394_1-p42875	C4	44	69.7	A
16265	Bn-N14-p36112194	Bn-C4-p34831216	Bn-scaff_16394_1-p60781	C4	44	69.7	A
16266	Bn-N14-p36131309	Bn-C4-p34850087	Bn-scaff_16394_1-p79652	C4	44	69.7	A
16267	Bn-N14-p36135311	Bn-C4-p34854090	Bn-scaff_16394_1-p83655	C4	44	69.7	A
16268	Bn-N14-p36159565	Bn-C4-p34882787	Bn-scaff_16394_1-p112352	C4	44	69.7	A
16269	Bn-N14-p36172097	Bn-C4-p34894452	Bn-scaff_16394_1-p124017	C4	44	69.7	0
16270	Bn-N14-p36256336	Bn-C4-p35007222	Bn-scaff_16394_1-p236787	C4	44	69.7	A
16271	Bn-N14-p36271017	Bn-C4-p35012816	Bn-scaff_16394_1-p242381	C4	44	69.7	A
16272	Bn-N14-p36333242	Bn-C4-p35079332	Bn-scaff_16394_1-p308897	C4	44	69.7	A
16273	Bn-N14-p36431589	Bn-C4-p35172328	Bn-scaff_16394_1-p401893	C4	44	69.7	A
16274	Bn-N14-p36446482	Bn-C4-p35187743	Bn-scaff_16394_1-p417308	C4	44	69.7	A
16275	Bn-N14-p36447114	Bn-C4-p35188374	Bn-scaff_16394_1-p417939	C4	44	69.7	A
16276	Bn-N14-p36448234	Bn-C4-p35189495	Bn-scaff_16394_1-p419060	C4	44	69.7	A
16277	Bn-N14-p36509474	Bn-C4-p35224795	Bn-scaff_16394_1-p454360	C4	44	69.7	A
16278	Bn-N14-p36509637	Bn-C4-p35224958	Bn-scaff_16394_1-p454523	C4	44	69.7	A
16279	Bn-N14-p36520049	Bn-C4-p35241348	Bn-scaff_16394_1-p470913	C4	44	69.7	A
16280	Bn-N14-p36530401	Bn-C4-p35244661	Bn-scaff_16394_1-p474226	C4	44	69.7	A
16281	Bn-N14-p36562854	Bn-C4-p35287024	Bn-scaff_16394_1-p516589	C4	44	69.7	A
16282	Bn-N14-p36567228	Bn-C4-p35291435	Bn-scaff_16394_1-p521000	C4	44	69.7	A
16283	Bn-N14-p36579933	Bn-C4-p35301683	Bn-scaff_16394_1-p531248	C4	44	69.7	A
16284	Bn-N14-p36621625	Bn-C4-p35336566	Bn-scaff_16394_1-p566131	C4	44	69.7	A
16285	Bn-N14-p36622497	Bn-C4-p35338027	Bn-scaff_16394_1-p567592	C4	44	69.7	A
16286	Bn-N14-p36856271	Bn-C4-p35526020	Bn-scaff_16394_1-p755585	C4	44	69.7	A
16287	Bn-N14-p36868136	Bn-C4-p35536923	Bn-scaff_16394_1-p766488	C4	44	69.7	A
16288	Bn-N14-p36869331	Bn-C4-p35538156	Bn-scaff_16394_1-p767721	C4	44	69.7	A
16289	Bn-N14-p36869442	Bn-C4-p35538267	Bn-scaff_16394_1-p767832	C4	44	69.7	A
16290	Bn-N14-p36869973	Bn-C4-p35544460	Bn-scaff_16394_1-p774025	C4	44	69.7	A
16291	Bn-N14-p36870097	Bn-C4-p35544584	Bn-scaff_16394_1-p774149	C4	44	69.7	A
16292	Bn-N14-p36870661	Bn-C4-p35545146	Bn-scaff_16394_1-p774711	C4	44	69.7	A
16293	Bn-N14-p36938797	Bn-C4-p35612817	Bn-scaff_16394_1-p842382	C4	44	69.7	A
16294	Bn-N14-p36944054	Bn-C4-p35618071	Bn-scaff_16394_1-p847636	C4	44	69.7	A
16295	Bn-N14-p36948349	Bn-C4-p35622380	Bn-scaff_16394_1-p851945	C4	44	69.7	A
16296	Bn-N14-p36949850	Bn-C4-p35623866	Bn-scaff_16394_1-p853431	C4	44	69.7	A
16297	Bn-N14-p36952859	Bn-C4-p35626697	Bn-scaff_16394_1-p856262	C4	44	69.7	A
16298	Bn-N14-p37061907	Bn-C4-p35734578	Bn-scaff_16394_1-p964143	C4	44	69.7	A
16299	Bn-N14-p37090984	Bn-C4-p35757534	Bn-scaff_16394_1-p987099	C4	44	69.7	A
16300	Bn-N14-p37098352	Bn-C4-p35770526	Bn-scaff_16394_1-p100009	C4	44	69.7	A
16301	Bn-N14-p37098753	Bn-C4-p35770881	Bn-scaff_16394_1-p100044	C4	44	69.7	A
16302	Bn-N14-p37100791	Bn-C4-p35772938	Bn-scaff_16394_1-p100250	C4	44	69.7	A
16303	Bn-N14-p37150145	Bn-C4-p35816192	Bn-scaff_16394_1-p104575	C4	44	69.7	A
16304	Bn-N14-p37198523	Bn-C4-p35855442	Bn-scaff_16394_1-p108500	C4	44	69.7	A
16305	Bn-N14-p37204192	Bn-C4-p35861331	Bn-scaff_16394_1-p109089	C4	44	69.7	A
16306	Bn-N14-p37249942	Bn-C4-p35891934	Bn-scaff_16394_1-p112149	C4	44	69.7	A
16307	Bn-N14-p37253746	Bn-C4-p35899932	Bn-scaff_16394_1-p112949	C4	44	69.7	A
16308	Bn-N14-p37262447	Bn-C4-p35902151	Bn-scaff_16394_1-p113171	C4	44	69.7	A
16309	Bn-N14-p37265012	Bn-C4-p35904807	Bn-scaff_16394_1-p113437	C4	44	69.7	A
16310	Bn-N14-p37524020	Bn-C4-p36112518	Bn-scaff_16394_1-p134208	C4	44	69.7	A
16311	Bn-N14-p38072540	Bn-C8-p34552026	Bn-scaff_16197_1-p184940	C4	44	69.7	A
16312	Bn-N14-p38110449	Bn-C4-p36715657	Bn-scaff_16394_1-p194522	C4	44	69.7	A

16313	Bn-N4-p7181355	Bn-Scaffold000104-p1	Bn-Scaffold000104-p11633	C4	44	69.7	A
16314	Bn-N4-p7563178	Bn-A04-p5919648	Bn-A04-p5919648	C4	44	69.7	A
16315	Bn-N4-p7586869	Bn-A04-p5938245	Bn-A04-p5938245	C4	44	69.7	A
16316	Bn-Scaffold02245-p2695	Bn-C4-p35691184	Bn-scaff_16394_1-p92074	C4	44	69.7	A
16317	Bn-Scaffold04476-p1399	Bn-C4-p34843574	Bn-scaff_16394_1-p73139	C4	44	69.7	A
16318	Bn-N14-p38673794	Bn-C4-p37078717	Bn-scaff_16876_1-p11805	C4	45	72.1	A
16319	Bn-N14-p38674272	Bn-C4-p37079195	Bn-scaff_16876_1-p11800	C4	45	72.1	A
16320	Bn-N14-p38675107	Bn-C4-p37080030	Bn-scaff_16876_1-p11792	C4	45	72.1	A
16321	Bn-N14-p38676008	Bn-C4-p37080930	Bn-scaff_16876_1-p11783	C4	45	72.1	A
16322	Bn-N14-p38689073	Bn-C4-p37096716	Bn-scaff_16876_1-p11625	C4	45	72.1	A
16323	Bn-N14-p38738442	Bn-C4-p37142354	Bn-scaff_16876_1-p11168	C4	46	72.9	A
16324	Bn-N14-p38738756	Bn-C4-p37142668	Bn-scaff_16876_1-p11165	C4	46	72.9	A
16325	Bn-N14-p38754206	Bn-C4-p37144266	Bn-scaff_16876_1-p11149	C4	46	72.9	A
16326	Bn-N14-p38754431	Bn-C4-p37145905	Bn-scaff_16876_1-p11133	C4	46	72.9	A
16327	Bn-N14-p38762596	Bn-C4-p37166003	Bn-scaff_16876_1-p10932	C4	46	72.9	A
16328	Bn-N14-p38763055	Bn-C4-p37166461	Bn-scaff_16876_1-p10927	C4	46	72.9	A
16329	Bn-N14-p38765746	Bn-C4-p37169163	Bn-scaff_16876_1-p10900	C4	46	72.9	A
16330	Bn-N14-p38774958	Bn-C4-p37178428	Bn-scaff_16876_1-p10808	C4	46	72.9	A
16331	Bn-N14-p38779897	Bn-C4-p37190876	Bn-scaff_16876_1-p10683	C4	46	72.9	A
16332	Bn-N14-p38793804	Bn-C4-p37204924	Bn-scaff_16876_1-p10543	C4	46	72.9	A
16333	Bn-N14-p38809929	Bn-C4-p37221049	Bn-scaff_16876_1-p10381	C4	46	72.9	A
16334	Bn-N14-p38828017	Bn-C4-p37241957	Bn-scaff_16876_1-p10172	C4	46	72.9	A
16335	Bn-N14-p38828221	Bn-C4-p37242161	Bn-scaff_16876_1-p10170	C4	46	72.9	A
16336	Bn-N14-p38828319	Bn-C4-p37242259	Bn-scaff_16876_1-p10169	C4	46	72.9	A
16337	Bn-N14-p39108843	Bn-C4-p37526124	Bn-scaff_16876_1-p73312	C4	46	72.9	A
16338	Bn-N14-p39128286	Bn-C4-p37546330	Bn-scaff_16876_1-p71291	C4	46	72.9	A
16339	Bn-N14-p39128403	Bn-C4-p37546447	Bn-scaff_16876_1-p71280	C4	46	72.9	A
16340	Bn-N14-p39130054	Bn-C4-p37550038	Bn-scaff_16876_1-p70921	C4	46	72.9	A
16341	Bn-N14-p39141372	Bn-C4-p37568011	Bn-scaff_16876_1-p69123	C4	46	72.9	A
16342	Bn-N14-p39158140	Bn-C4-p37578178	Bn-scaff_16876_1-p68107	C4	46	72.9	A
16343	Bn-N14-p39158216	Bn-C4-p37578254	Bn-scaff_16876_1-p68099	C4	46	72.9	A
16344	Bn-N14-p39161445	Bn-C4-p37581442	Bn-scaff_16876_1-p67780	C4	46	72.9	A
16345	Bn-N14-p39161895	Bn-C4-p37581891	Bn-scaff_16876_1-p67735	C4	46	72.9	A
16346	Bn-N14-p39162592	Bn-C4-p37582192	Bn-scaff_16876_1-p67705	C4	46	72.9	A
16347	Bn-N14-p39185674	Bn-C4-p37595206	Bn-scaff_16876_1-p66404	C4	46	72.9	A
16348	Bn-N14-p39187236	Bn-C4-p37596761	Bn-scaff_16876_1-p66248	C4	46	72.9	A
16349	Bn-N14-p39187807	Bn-C4-p37597332	Bn-scaff_16876_1-p66191	C4	46	72.9	A
16350	Bn-N14-p39191639	Bn-C4-p37601161	Bn-scaff_16876_1-p65808	C4	46	72.9	A
16351	Bn-N14-p39191695	Bn-C4-p37601217	Bn-scaff_16876_1-p65803	C4	46	72.9	A
16352	Bn-N14-p39225498	Bn-C4-p37631867	Bn-scaff_16876_1-p62738	C4	46	72.9	A
16353	Bn-N14-p39231784	Bn-C4-p37638152	Bn-scaff_16876_1-p62109	C4	46	72.9	A
16354	Bn-N14-p39250478	Bn-C4-p37655408	Bn-scaff_16876_1-p60384	C4	46	72.9	A
16355	Bn-N14-p39255011	Bn-C4-p37660500	Bn-scaff_16876_1-p59874	C4	46	72.9	A
16356	Bn-N14-p39280689	Bn-C4-p37687948	Bn-scaff_16876_1-p57130	C4	46	72.9	A
16357	Bn-N14-p39376416	Bn-C4-p37773469	Bn-scaff_16876_1-p48577	C4	46	72.9	A
16358	Bn-N14-p39411280	Bn-C4-p37792914	Bn-scaff_16876_1-p46633	C4	46	72.9	A
16359	Bn-N14-p39437206	Bn-C4-p37814558	Bn-scaff_16876_1-p44469	C4	46	72.9	A
16360	Bn-N14-p39459086	Bn-C4-p37842307	Bn-scaff_16876_1-p41694	C4	46	72.9	A
16361	Bn-N14-p39474803	Bn-C4-p37861825	Bn-scaff_16876_1-p39742	C4	46	72.9	A

16362	Bn-N14-p39497259	Bn-C4-p37891314	Bn-scaff_16876_1-p36793	C4	46	72.9	A
16363	Bn-N14-p39596025	Bn-C4-p38002086	Bn-scaff_16876_1-p25716	C4	47	73.7	A
16364	Bn-N14-p39683412	Bn-Scaffold04300-p9	Bn-scaff_22668_1-p95	C4	47	73.7	A
16365	Bn-N14-p39839969	Bn-A04-p8178053	Bn-A04-p8178053	C4	47	73.7	A
16366	Bn-N14-p40463269	Bn-C4-p38898227	Bn-scaff_17525_1-p11706	C4	48	76.1	A
16367	Bn-N14-p40736885	Bn-C4-p39135450	Bn-scaff_16694_1-p23603	C4	48	76.1	A
16368	Bn-N14-p40995320	Bn-C4-p39390001	Bn-scaff_19208_1-p18413	C4	48	76.1	A
16369	Bn-N14-p40995385	Bn-C4-p39390068	Bn-scaff_19208_1-p18480	C4	48	76.1	A
16370	Bn-N14-p41042088	Bn-C4-p39450486	Bn-scaff_19208_1-p78898	C4	48	76.1	A
16371	Bn-N14-p41042905	Bn-C4-p39451325	Bn-scaff_19208_1-p79737	C4	48	76.1	A
16372	Bn-N14-p41042914	Bn-C4-p39451334	Bn-scaff_19208_1-p79746	C4	48	76.1	A
16373	Bn-N14-p41043822	Bn-C4-p39452243	Bn-scaff_19208_1-p80655	C4	48	76.1	A
16374	Bn-N14-p41045666	Bn-C4-p39454123	Bn-scaff_19208_1-p82535	C4	48	76.1	A
16375	Bn-N14-p41062739	Bn-C4-p39465402	Bn-scaff_19208_1-p93814	C4	48	76.1	A
16376	Bn-N14-p41063438	Bn-C4-p39466086	Bn-scaff_19208_1-p94498	C4	48	76.1	A
16377	Bn-N14-p41063441	Bn-C4-p39466089	Bn-scaff_19208_1-p94501	C4	48	76.1	A
16378	Bn-N14-p41063493	Bn-C4-p39466141	Bn-scaff_19208_1-p94553	C4	48	76.1	A
16379	Bn-N14-p41195848	Bn-C4-p39604628	Bn-scaff_19208_1-p23304	C4	49	76.9	A
16380	Bn-N14-p41213068	Bn-C4-p39622062	Bn-scaff_19208_1-p25047	C4	49	76.9	A
16381	Bn-N14-p41213656	Bn-C4-p39622651	Bn-scaff_19208_1-p25106	C4	49	76.9	A
16382	Bn-N14-p41213715	Bn-C4-p39622711	Bn-scaff_19208_1-p25112	C4	49	76.9	A
16383	Bn-N14-p41274894	Bn-C4-p39650512	Bn-scaff_19208_1-p27892	C4	49	76.9	A
16384	Bn-N14-p41287587	Bn-C4-p39665345	Bn-scaff_19208_1-p29375	C4	49	76.9	A
16385	Bn-N14-p41289699	Bn-C4-p39667482	Bn-scaff_19208_1-p29589	C4	49	76.9	A
16386	Bn-N14-p41319075	Bn-C4-p39696180	Bn-scaff_19208_1-p32459	C4	49	76.9	A
16387	Bn-N14-p41342718	Bn-C2-p43597965	Bn-scaff_15712_6-p76491	C4	49	76.9	A
16388	Bn-N14-p41342725	Bn-C2-p43597958	Bn-scaff_15712_6-p76490	C4	49	76.9	A
16389	Bn-N14-p41343562	Bn-C2-p43597118	Bn-scaff_15712_6-p76406	C4	49	76.9	A
16390	Bn-N14-p41343645	Bn-C2-p43597035	Bn-scaff_15712_6-p76398	C4	49	76.9	A
16391	Bn-N14-p41345251	Bn-C2-p43595268	Bn-scaff_15712_6-p76221	C4	49	76.9	A
16392	Bn-N14-p41353186	Bn-C4-p39718487	Bn-scaff_19208_1-p34689	C4	49	76.9	A
16393	Bn-N14-p41434641	Bn-C4-p39813609	Bn-scaff_19208_1-p44202	C4	49	76.9	A
16394	Bn-N14-p41476290	Bn-C4-p39848436	Bn-scaff_19208_1-p47684	C4	49	76.9	A
16395	Bn-N14-p41491123	Bn-C4-p39862037	Bn-scaff_19208_1-p49044	C4	49	76.9	A
16396	Bn-N14-p41522347	Bn-C4-p39895860	Bn-scaff_19208_1-p52427	C4	49	76.9	A
16397	Bn-N14-p41883467	Bn-C4-p40183165	Bn-scaff_25960_1-p10431	C4	50	78.5	A
16398	Bn-N14-p41883607	Bn-C4-p40183305	Bn-scaff_25960_1-p10445	C4	50	78.5	A
16399	Bn-N14-p41886677	Bn-C4-p40190600	Bn-scaff_25960_1-p11175	C4	50	78.5	A
16400	Bn-N14-p41910764	Bn-C4-p40219218	Bn-scaff_25960_1-p14036	C4	50	78.5	A
16401	Bn-N14-p42004330	Bn-C4-p40287319	Bn-scaff_25960_1-p20846	C4	50	78.5	A
16402	Bn-N14-p42084675	Bn-C4-p40367606	Bn-scaff_19043_1-p44083	C4	50	78.5	A
16403	Bn-N14-p42093520	Bn-C4-p40377229	Bn-scaff_19043_1-p43120	C4	50	78.5	A
16404	Bn-N14-p42454700	Bn-C4-p40754289	Bn-scaff_19043_1-p54147	C4	50	78.5	A
16405	Bn-N14-p42455210	Bn-C4-p40754799	Bn-scaff_19043_1-p53637	C4	50	78.5	A
16406	Bn-N14-p42455218	Bn-C4-p40754807	Bn-scaff_19043_1-p53629	C4	50	78.5	A
16407	Bn-N14-p42455381	Bn-C4-p40754969	Bn-scaff_19043_1-p53467	C4	50	78.5	A
16408	Bn-N14-p42457420	Bn-C4-p40756183	Bn-scaff_19043_1-p52253	C4	50	78.5	A
16409	Bn-N14-p42458119	Bn-C4-p40756882	Bn-scaff_19043_1-p51554	C4	50	78.5	A
16410	Bn-N14-p42458212	Bn-C4-p40756975	Bn-scaff_19043_1-p51461	C4	50	78.5	A

16411	Bn-N14-p42459501	Bn-C4-p40758261	Bn-scaff_19043_1-p50175	C4	50	78.5	A
16412	Bn-N14-p42466868	Bn-C4-p40766874	Bn-scaff_19043_1-p41562	C4	50	78.5	A
16413	Bn-N14-p42466873	Bn-C4-p40766879	Bn-scaff_19043_1-p41557	C4	50	78.5	A
16414	Bn-N14-p42469509	Bn-C4-p40771202	Bn-scaff_19043_1-p37234	C4	50	78.5	A
16415	Bn-N14-p42472286	Bn-C4-p40775133	Bn-scaff_19043_1-p33303	C4	50	78.5	A
16416	Bn-N14-p42479411	Bn-C4-p40784085	Bn-scaff_19043_1-p24351	C4	50	78.5	A
16417	Bn-N14-p42481533	Bn-C4-p40786080	Bn-scaff_19043_1-p22356	C4	50	78.5	A
16418	Bn-N14-p42484271	Bn-C4-p40790258	Bn-scaff_19043_1-p18178	C4	50	78.5	A
16419	Bn-N14-p42484363	Bn-C4-p40790350	Bn-scaff_19043_1-p18086	C4	50	78.5	A
16420	Bn-N14-p42490266	Bn-C4-p40795693	Bn-scaff_19043_1-p12743	C4	50	78.5	A
16421	Bn-N14-p42535990	Bn-Scaffold00871-p1	Bn-scaff_23472_1-p11655	C4	50	78.5	A
16422	Bn-N14-p42537426	Bn-Scaffold00871-p1	Bn-scaff_23472_1-p19183	C4	50	78.5	A
16423	Bn-N14-p42549404	Bn-Scaffold00871-p2	Bn-scaff_23472_1-p29382	C4	50	78.5	A
16424	Bn-N14-p42568996	Bn-Scaffold00871-p5	Bn-scaff_23472_1-p57276	C4	50	78.5	A
16425	Bn-N14-p42614749	Bn-C4-p40850782	Bn-scaff_15798_1-p42247	C4	50	78.5	A
16426	Bn-N14-p42654524	Bn-C4-p40889880	Bn-scaff_15798_1-p81345	C4	50	78.5	A
16427	Bn-N14-p42671389	Bn-C4-p40902082	Bn-scaff_15798_1-p93547	C4	50	78.5	A
16428	Bn-N14-p42671643	Bn-C4-p40902336	Bn-scaff_15798_1-p93801	C4	50	78.5	A
16429	Bn-N14-p42677349	Bn-C4-p40902764	Bn-scaff_15798_1-p94229	C4	50	78.5	A
16430	Bn-N14-p42677721	Bn-C4-p40903136	Bn-scaff_15798_1-p94601	C4	50	78.5	A
16431	Bn-N14-p42677901	Bn-C4-p40903316	Bn-scaff_15798_1-p94781	C4	50	78.5	A
16432	Bn-N14-p42678020	Bn-C4-p40903435	Bn-scaff_15798_1-p94900	C4	50	78.5	A
16433	Bn-N14-p42679026	Bn-C4-p40904460	Bn-scaff_15798_1-p95925	C4	50	78.5	A
16434	Bn-N14-p42679276	Bn-C4-p40904733	Bn-scaff_15798_1-p96198	C4	50	78.5	A
16435	Bn-N14-p42680483	Bn-C4-p40905944	Bn-scaff_15798_1-p97409	C4	50	78.5	A
16436	Bn-N14-p42681016	Bn-C4-p40906477	Bn-scaff_15798_1-p97942	C4	50	78.5	A
16437	Bn-N14-p42681721	Bn-C4-p40907145	Bn-scaff_15798_1-p98610	C4	50	78.5	A
16438	Bn-N14-p42682619	Bn-C4-p40911683	Bn-scaff_15798_1-p10314	C4	50	78.5	A
16439	Bn-N14-p42683738	Bn-C4-p40912813	Bn-scaff_15798_1-p10427	C4	50	78.5	A
16440	Bn-N14-p42683917	Bn-C4-p40912992	Bn-scaff_15798_1-p10445	C4	50	78.5	A
16441	Bn-N14-p42684088	Bn-C4-p40913133	Bn-scaff_15798_1-p10459	C4	50	78.5	A
16442	Bn-N14-p42685580	Bn-C4-p40914625	Bn-scaff_15798_1-p10609	C4	50	78.5	A
16443	Bn-N14-p42685710	Bn-C4-p40914749	Bn-scaff_15798_1-p10621	C4	50	78.5	A
16444	Bn-N14-p42685972	Bn-C4-p40915011	Bn-scaff_15798_1-p10647	C4	50	78.5	A
16445	Bn-N14-p42686278	Bn-C4-p40915317	Bn-scaff_15798_1-p10678	C4	50	78.5	A
16446	Bn-N14-p42689321	Bn-C4-p40918199	Bn-scaff_15798_1-p10966	C4	50	78.5	A
16447	Bn-N14-p42690138	Bn-C4-p40919019	Bn-scaff_15798_1-p11048	C4	50	78.5	A
16448	Bn-N14-p42691301	Bn-C4-p40920196	Bn-scaff_15798_1-p11166	C4	50	78.5	A
16449	Bn-N14-p42692319	Bn-C4-p40921113	Bn-scaff_15798_1-p11257	C4	50	78.5	A
16450	Bn-N14-p42692696	Bn-C4-p40921518	Bn-scaff_15798_1-p11298	C4	50	78.5	A
16451	Bn-N14-p42693558	Bn-C4-p40922380	Bn-scaff_15798_1-p11384	C4	50	78.5	A
16452	Bn-N14-p42694671	Bn-C4-p40923493	Bn-scaff_15798_1-p11495	C4	50	78.5	A
16453	Bn-N14-p42695411	Bn-C4-p40924232	Bn-scaff_15798_1-p11569	C4	50	78.5	A
16454	Bn-N14-p42697166	Bn-C4-p40925635	Bn-scaff_15798_1-p11710	C4	50	78.5	A
16455	Bn-N14-p42698096	Bn-C4-p40926489	Bn-scaff_15798_1-p11795	C4	50	78.5	A
16456	Bn-N14-p42698158	Bn-C4-p40926551	Bn-scaff_15798_1-p11801	C4	50	78.5	A
16457	Bn-N14-p42698293	Bn-C4-p40929937	Bn-scaff_15798_1-p12140	C4	50	78.5	A
16458	Bn-N14-p42716069	Bn-C4-p40940728	Bn-scaff_15798_1-p13219	C4	50	78.5	A
16459	Bn-N14-p42719750	Bn-C4-p40944149	Bn-scaff_15798_1-p13561	C4	50	78.5	A

16460	Bn-N14-p42719980	Bn-C4-p40944379	Bn-scaff_15798_1-p13584	C4	50	78.5	A
16461	Bn-N14-p42720782	Bn-C4-p40945168	Bn-scaff_15798_1-p13663	C4	50	78.5	A
16462	Bn-N14-p42728754	Bn-C4-p40960666	Bn-scaff_15798_1-p15213	C4	50	78.5	A
16463	Bn-N14-p42731495	Bn-C4-p40963389	Bn-scaff_15798_1-p15485	C4	50	78.5	A
16464	Bn-N14-p42734664	Bn-C4-p40966505	Bn-scaff_15798_1-p15797	C4	50	78.5	A
16465	Bn-N14-p42737460	Bn-C4-p40969306	Bn-scaff_15798_1-p16077	C4	50	78.5	A
16466	Bn-N14-p42737521	Bn-C4-p40969367	Bn-scaff_15798_1-p16083	C4	50	78.5	A
16467	Bn-N14-p42737581	Bn-C4-p40969427	Bn-scaff_15798_1-p16089	C4	50	78.5	A
16468	Bn-N14-p42739245	Bn-C4-p40971126	Bn-scaff_15798_1-p16259	C4	50	78.5	A
16469	Bn-N14-p42741190	Bn-C4-p40973053	Bn-scaff_15798_1-p16451	C4	50	78.5	A
16470	Bn-N14-p42742052	Bn-C4-p40973915	Bn-scaff_15798_1-p16538	C4	50	78.5	A
16471	Bn-N14-p42742468	Bn-C4-p40974332	Bn-scaff_15798_1-p16579	C4	50	78.5	A
16472	Bn-N14-p42753685	Bn-C4-p40981589	Bn-scaff_15798_1-p17305	C4	50	78.5	A
16473	Bn-N14-p42754426	Bn-C4-p40986906	Bn-scaff_15798_1-p17837	C4	50	78.5	A
16474	Bn-N14-p42756074	Bn-C4-p40987810	Bn-scaff_15798_1-p17927	C4	50	78.5	A
16475	Bn-N14-p42756371	Bn-C4-p40988075	Bn-scaff_15798_1-p17954	C4	50	78.5	A
16476	Bn-N14-p42782252	Bn-C4-p41011093	Bn-scaff_15798_1-p20255	C4	50	78.5	A
16477	Bn-N14-p42787377	Bn-C4-p41014727	Bn-scaff_15798_1-p20619	C4	50	78.5	A
16478	Bn-N14-p42790596	Bn-C4-p41017947	Bn-scaff_15798_1-p20941	C4	50	78.5	A
16479	Bn-N14-p42804364	Bn-C4-p41032177	Bn-scaff_15798_1-p22364	C4	50	78.5	A
16480	Bn-N14-p42808134	Bn-C4-p41035934	Bn-scaff_15798_1-p22739	C4	50	78.5	A
16481	Bn-N14-p42808185	Bn-C4-p41035985	Bn-scaff_15798_1-p22745	C4	50	78.5	0
16482	Bn-N14-p42815111	Bn-C4-p41044290	Bn-scaff_15798_1-p23575	C4	50	78.5	A
16483	Bn-N14-p42815226	Bn-C4-p41044405	Bn-scaff_15798_1-p23587	C4	50	78.5	A
16484	Bn-N14-p42821707	Bn-C4-p41050266	Bn-scaff_15798_1-p24173	C4	50	78.5	A
16485	Bn-N14-p42821785	Bn-C4-p41050344	Bn-scaff_15798_1-p24180	C4	50	78.5	A
16486	Bn-N14-p42822509	Bn-C4-p41050952	Bn-scaff_15798_1-p24241	C4	50	78.5	A
16487	Bn-N14-p42830818	Bn-C4-p41054441	Bn-scaff_15798_1-p24590	C4	50	78.5	A
16488	Bn-N14-p42842424	Bn-C4-p41059804	Bn-scaff_15798_1-p25126	C4	50	78.5	A
16489	Bn-N14-p42853513	Bn-C4-p41069664	Bn-scaff_15798_1-p26112	C4	50	78.5	A
16490	Bn-N14-p42859360	Bn-C4-p41076790	Bn-scaff_15798_1-p26825	C4	50	78.5	A
16491	Bn-N14-p42860099	Bn-C4-p41077529	Bn-scaff_15798_1-p26899	C4	50	78.5	A
16492	Bn-N14-p42861433	Bn-C4-p41079802	Bn-scaff_15798_1-p27126	C4	50	78.5	A
16493	Bn-N14-p42862236	Bn-C4-p41080607	Bn-scaff_15798_1-p27207	C4	50	78.5	A
16494	Bn-N14-p42862785	Bn-C4-p41081160	Bn-scaff_15798_1-p27262	C4	50	78.5	A
16495	Bn-N14-p42862897	Bn-C4-p41081272	Bn-scaff_15798_1-p27273	C4	50	78.5	A
16496	Bn-N14-p42862998	Bn-C4-p41081373	Bn-scaff_15798_1-p27283	C4	50	78.5	A
16497	Bn-N14-p42863049	Bn-C4-p41081424	Bn-scaff_15798_1-p27288	C4	50	78.5	A
16498	Bn-N14-p42871439	Bn-C4-p41089576	Bn-scaff_15798_1-p28104	C4	50	78.5	A
16499	Bn-N14-p42871515	Bn-C4-p41089652	Bn-scaff_15798_1-p28111	C4	50	78.5	A
16500	Bn-N14-p42883238	Bn-C4-p41090280	Bn-scaff_15798_1-p28174	C4	50	78.5	A
16501	Bn-N14-p42883498	Bn-C4-p41090542	Bn-scaff_15798_1-p28200	C4	50	78.5	A
16502	Bn-N14-p42883635	Bn-C4-p41090678	Bn-scaff_15798_1-p28214	C4	50	78.5	A
16503	Bn-N14-p42884041	Bn-C4-p41091088	Bn-scaff_15798_1-p28255	C4	50	78.5	A
16504	Bn-N14-p42885306	Bn-C4-p41092350	Bn-scaff_15798_1-p28381	C4	50	78.5	A
16505	Bn-N14-p43075462	Bn-C4-p41265037	Bn-scaff_15798_1-p45650	C4	50	78.5	A
16506	Bn-N14-p43463240	Bn-C4-p41624810	Bn-scaff_15798_1-p81627	C4	50	78.5	A
16507	Bn-N14-p43671853	Bn-C4-p41824617	Bn-scaff_15798_1-p10160	C4	50	78.5	A
16508	Bn-N14-p43678169	Bn-C4-p41830932	Bn-scaff_15798_1-p10223	C4	50	78.5	A

16509	Bn-N14-p43689542	Bn-C4-p41842194	Bn-scaff_15798_1-p10336	C4	50	78.5	A
16510	Bn-N14-p43715940	Bn-C4-p41865803	Bn-scaff_15798_1-p10572	C4	50	78.5	A
16511	Bn-N14-p43717061	Bn-C4-p41866919	Bn-scaff_15798_1-p10583	C4	50	78.5	A
16512	Bn-N14-p43735194	Bn-C4-p41890313	Bn-scaff_15798_1-p10817	C4	50	78.5	A
16513	Bn-N14-p43735273	Bn-C4-p41890392	Bn-scaff_15798_1-p10818	C4	50	78.5	A
16514	Bn-N14-p43794760	Bn-Scaffold01446-p5	Bn-scaff_23213_1-p5752	C4	50	78.5	A
16515	Bn-N14-p43799090	Bn-C4-p41942993	Bn-scaff_15852_1-p69100	C4	50	78.5	A
16516	Bn-N14-p43816626	Bn-C13785341-p27	Bn-C13785341-p27	C4	50	78.5	A
16517	Bn-N14-p43820354	Bn-Scaffold01390-p7	Bn-scaff_17482_1-p7299	C4	50	78.5	A
16518	Bn-N14-p43820891	Bn-Scaffold01390-p6	Bn-scaff_17482_1-p6769	C4	50	78.5	A
16519	Bn-N14-p43821028	Bn-Scaffold01390-p6	Bn-scaff_17482_1-p6632	C4	50	78.5	A
16520	Bn-N14-p43856450	Bn-C4-p41971272	Bn-scaff_15852_1-p66272	C4	50	78.5	A
16521	Bn-N14-p43885529	Bn-C4-p41994459	Bn-scaff_15852_1-p63953	C4	50	78.5	A
16522	Bn-N14-p43891793	Bn-C4-p42000715	Bn-scaff_15852_1-p63328	C4	50	78.5	A
16523	Bn-N4-p11529941	Bn-A04-p9543062	Bn-A04-p9543062	C4	50	78.5	A
16524	Bn-Scaffold07944-p658	Bn-C4-p41090027	Bn-scaff_15798_1-p28149	C4	50	78.5	A
16525	Bn-Scaffold74248-p85	Bn-C13715031-p207	Bn-C13715031-p207	C4	50	78.5	A
16526	Bn-N14-p43994865	Bn-C4-p42101528	Bn-scaff_15852_1-p53246	C4	51	80.9	A
16527	Bn-N14-p43995922	Bn-C4-p42102585	Bn-scaff_15852_1-p53141	C4	51	80.9	A
16528	Bn-N14-p44004905	Bn-C4-p42111561	Bn-scaff_15852_1-p52243	C4	51	80.9	A
16529	Bn-N14-p44044697	Bn-C4-p42149406	Bn-scaff_15852_1-p48458	C4	51	80.9	A
16530	Bn-N14-p44045212	Bn-C4-p42149920	Bn-scaff_15852_1-p48407	C4	51	80.9	A
16531	Bn-N14-p44301058	Bn-C4-p42370006	Bn-scaff_15852_1-p26398	C4	52	83.3	A
16532	Bn-N14-p44301162	Bn-C4-p42370110	Bn-scaff_15852_1-p26388	C4	52	83.3	A
16533	Bn-N14-p44301865	Bn-C4-p42375286	Bn-scaff_15852_1-p25870	C4	52	83.3	A
16534	Bn-N14-p44414316	Bn-C4-p42498181	Bn-scaff_15852_1-p13581	C4	53	84.1	A
16535	Bn-N14-p44419616	Bn-C4-p42503280	Bn-scaff_15852_1-p13071	C4	53	84.1	A
16536	Bn-N14-p44423471	Bn-C4-p42506502	Bn-scaff_15852_1-p12749	C4	53	84.1	A
16537	Bn-N14-p44426914	Bn-C4-p42510364	Bn-scaff_15852_1-p12363	C4	53	84.1	A
16538	Bn-N14-p44431274	Bn-C4-p42512800	Bn-scaff_15852_1-p12119	C4	53	84.1	A
16539	Bn-N14-p44443050	Bn-C4-p42517957	Bn-scaff_15852_1-p11603	C4	53	84.1	A
16540	Bn-N14-p44449010	Bn-C4-p42521450	Bn-scaff_15852_1-p11254	C4	53	84.1	A
16541	Bn-N14-p44460709	Bn-C4-p42529992	Bn-scaff_15852_1-p10400	C4	53	84.1	A
16542	Bn-N14-p44461538	Bn-C4-p42530823	Bn-scaff_15852_1-p10317	C4	53	84.1	A
16543	Bn-N14-p44848820	Bn-C4-p42826203	Bn-scaff_24979_1-p7134	C4	53	84.1	A
16544	Bn-N14-p44860481	Bn-C4-p42832172	Bn-scaff_24979_1-p13103	C4	53	84.1	A
16545	Bn-N14-p44928010	Bn-C4-p42859410	Bn-scaff_24979_1-p40341	C4	53	84.1	A
16546	Bn-N14-p44928827	Bn-C4-p42860194	Bn-scaff_24979_1-p41125	C4	53	84.1	A
16547	Bn-N14-p44933125	Bn-C4-p42865613	Bn-scaff_24979_1-p46544	C4	53	84.1	A
16548	Bn-N14-p44933518	Bn-C4-p42866006	Bn-scaff_24979_1-p46937	C4	53	84.1	A
16549	Bn-N14-p44949073	Bn-C4-p42873271	Bn-scaff_24979_1-p54202	C4	53	84.1	A
16550	Bn-Scaffold05210-p1208	Bn-C4-p42514663	Bn-scaff_15852_1-p11933	C4	53	84.1	A
16551	Bn-N14-p45065343	Bn-C4-p42981603	Bn-scaff_24979_1-p16253	C4	54	88.1	B
16552	Bn-N14-p45065949	Bn-C4-p42982202	Bn-scaff_24979_1-p16313	C4	54	88.1	B
16553	Bn-N14-p45066472	Bn-C4-p42982737	Bn-scaff_24979_1-p16366	C4	54	88.1	B
16554	Bn-N14-p45081499	Bn-C4-p42988919	Bn-scaff_24979_1-p16985	C4	54	88.1	B
16555	Bn-N14-p45094756	Bn-C4-p42991320	Bn-scaff_24979_1-p17225	C4	54	88.1	B
16556	Bn-N14-p45099262	Bn-C4-p42995863	Bn-scaff_24979_1-p17679	C4	54	88.1	B
16557	Bn-N14-p45100357	Bn-C4-p42998533	Bn-scaff_24979_1-p17946	C4	54	88.1	B

16558	Bn-N14-p45100435	Bn-C4-p42998610	Bn-scaff_24979_1-p179541	C4	54	88.1	B
16559	Bn-N14-p45100470	Bn-C4-p42998645	Bn-scaff_24979_1-p179576	C4	54	88.1	B
16560	Bn-N14-p45183001	Bn-C4-p43069822	Bn-scaff_21956_1-p47467	C4	54	88.1	B
16561	Bn-N14-p45183244	Bn-C4-p43070065	Bn-scaff_21956_1-p47710	C4	54	88.1	B
16562	Bn-N14-p45186037	Bn-C4-p43072860	Bn-scaff_21956_1-p50505	C4	54	88.1	B
16563	Bn-N14-p45186756	Bn-C4-p43073575	Bn-scaff_21956_1-p51220	C4	54	88.1	B
16564	Bn-N14-p45288673	Bn-C4-p43144448	Bn-scaff_21956_1-p122093	C4	54	88.1	B
16565	Bn-N14-p45381776	Bn-C4-p43233403	Bn-scaff_21956_1-p211048	C4	54	88.1	B
16566	Bn-N14-p45403403	Bn-C4-p43254489	Bn-scaff_21956_1-p232134	C4	54	88.1	B
16567	Bn-N14-p45403614	Bn-C4-p43254775	Bn-scaff_21956_1-p232420	C4	54	88.1	B
16568	Bn-N14-p45430684	Bn-C4-p43276316	Bn-scaff_21956_1-p253961	C4	54	88.1	B
16569	Bn-N14-p45457184	Bn-C4-p43299061	Bn-scaff_21956_1-p276706	C4	54	88.1	B
16570	Bn-N14-p46250560	Bn-C4-p43300310	Bn-scaff_21956_1-p277955	C4	54	88.1	B
16571	Bn-N14-p46277339	Bn-C4-p44089443	Bn-scaff_20079_1-p654045	C4	55	89.7	B
16572	Bn-N14-p46311853	Bn-C4-p44119685	Bn-scaff_20079_1-p623803	C4	55	89.7	B
16573	Bn-N14-p46362217	Bn-C4-p44236133	Bn-scaff_20079_1-p507355	C4	55	89.7	B
16574	Bn-N14-p46367045	Bn-C4-p44240981	Bn-scaff_20079_1-p502507	C4	55	89.7	B
16575	Bn-N14-p46389017	Bn-C4-p44247047	Bn-scaff_20079_1-p496441	C4	56	90.5	B
16576	Bn-N14-p46396137	Bn-C4-p44250883	Bn-scaff_20079_1-p492605	C4	56	90.5	B
16577	Bn-N14-p46398344	Bn-C4-p44254130	Bn-scaff_20079_1-p489358	C4	56	90.5	B
16578	Bn-N14-p46424789	Bn-C4-p44277591	Bn-scaff_20079_1-p465897	C4	56	90.5	B
16579	Bn-N14-p46600583	Bn-C4-p44453984	Bn-scaff_20079_1-p289504	C4	56	90.5	B
16580	Bn-N11-p35137108	Bn-C1-p32366214	Bn-scaff_17517_1-p564869	C4	57	93.7	B
16581	Bn-N4-p14969070	Bn-A04-p12917949	Bn-A04-p12917949	C4	57	93.7	B
16582	Bn-N4-p15011729	Bn-C4-p44869640	Bn-scaff_16976_1-p24933	C4	57	93.7	B
16583	Bn-N14-p47057666	Bn-Scaffold01016-p2	Bn-scaff_16804_3-p29132	C4	58	94.5	B
16584	Bn-N14-p48462237	Bn-C4-p46385001	Bn-scaff_16517_1-p680544	C4	59	97.7	B
16585	Bn-N14-p48823749	Bn-C4-p46683666	Bn-scaff_16804_2-p210553	C4	59	97.7	B
16586	Bn-N14-p48884509	Bn-C4-p46740867	Bn-scaff_16804_2-p153352	C4	59	97.7	B
16587	Bn-N4-p15824941	Bn-C4-p46266278	Bn-scaff_16517_1-p561821	C4	59	97.7	B
16588	Bn-N4-p16097093	Bn-C4-p46728467	Bn-scaff_16804_2-p165752	C4	59	97.7	B
16589	Bn-N14-p49662304	Bn-C4-p47419520	Bn-scaff_21186_1-p220377	C4	60	100.1	B
16590	Bn-N14-p49752851	Bn-A04-p14630533	Bn-A04-p14630533	C4	60	100.1	B
16591	Bn-N14-p49752941	Bn-C4-p47486007	Bn-scaff_21186_1-p153890	C4	60	100.1	B
16592	Bn-N14-p49753343	Bn-C4-p47486377	Bn-scaff_21186_1-p153520	C4	60	100.1	B
16593	Bn-N14-p49753696	Bn-A04-p14631343	Bn-A04-p14631343	C4	60	100.1	B
16594	Bn-N14-p50016367	Bn-C4-p47693740	Bn-scaff_15585_1-p37231	C4	61	100.9	0
16595	Bn-N14-p50059672	Bn-Scaffold01527-p4	Bn-scaff_17319_1-p4691	C4	61	100.9	B
16596	Bn-N2-p8682855	Bn-A04-p15287200	Bn-A04-p15287200	C4	62	101.7	B
16597	Bn-Scaffold00208b-p15947	Bn-A04-p15150564	Bn-A04-p15150564	C4	62	101.7	B
16598	Bn-N14-p50447926	Bn-C4-p48677273	Bn-scaff_15585_1-p102076	C4	63	102.5	B
16599	Bn-N14-p50455921	Bn-C4-p48685268	Bn-scaff_15585_1-p102875	C4	63	102.5	B
16600	Bn-N14-p50457083	Bn-C4-p48686430	Bn-scaff_15585_1-p102992	C4	63	102.5	B
16601	Bn-N14-p50461414	Bn-C4-p48690761	Bn-scaff_15585_1-p103425	C4	63	102.5	B
16602	Bn-N7-p8648385	Bn-C4-p48686993	Bn-scaff_15585_1-p103048	C4	63	102.5	B
16603	Bn-Scaffold01385-p601	Bn-C4-p48865332	Bn-scaff_20804_1-p111983	C4	64	103.3	B
16604	Bn-N14-p50751772	Bn-C4-p48943622	Bn-scaff_20804_1-p33693	C4	65	104.1	B
16605	Bn-N14-p50755510	Bn-C4-p48947328	Bn-scaff_20804_1-p29987	C4	65	104.1	B
16606	Bn-N14-p50883109	Bn-C4-p49082294	Bn-scaff_16888_1-p104880	C4	66	104.9	B

16607	Bn-N14-p51104872	Bn-C4-p49723853	Bn-scaff_16888_1-p746439	C4	67	109.8	B
16608	Bn-N14-p51105249	Bn-A04-p16109558	Bn-A04-p16109558	C4	67	109.8	B
16609	Bn-N14-p51106258	Bn-C4-p49725241	Bn-scaff_16888_1-p747827	C4	67	109.8	B
16610	Bn-N14-p51120661	Bn-C4-p49741457	Bn-scaff_16888_1-p764043	C4	67	109.8	B
16611	Bn-N14-p51259960	Bn-C4-p49871832	Bn-scaff_16888_1-p894418	C4	67	109.8	B
16612	Bn-N14-p51803213	Bn-C4-p50397026	Bn-scaff_16888_1-p141961	C4	68	112.2	B
16613	Bn-N14-p52137515	Bn-C4-p50780868	Bn-scaff_16888_1-p180345	C4	69	116.2	B
16614	Bn-N14-p52138256	Bn-C4-p50781609	Bn-scaff_16888_1-p180419	C4	69	116.2	B
16615	Bn-N14-p52368863	Bn-C4-p51022991	Bn-scaff_20270_1-p39619	C4	70	117.8	B
16616	Bn-N14-p52488607	Bn-C4-p51151118	Bn-scaff_20270_1-p167746	C4	71	118.6	B
16617	Bn-N4-p19429574	Bn-A04-p17475728	Bn-A04-p17475728	C4	72	120.2	B
16618	Bn-N14-p53076599	Bn-C4-p51704384	Bn-scaff_20270_1-p721012	C4	73	122.6	B
16619	Bn-N14-p53148205	Bn-C4-p51764862	Bn-scaff_20270_1-p781490	C4	73	122.6	B
16620	Bn-N14-p53341832	Bn-C4-p52006616	Bn-scaff_26787_1-p10517	C4	73	122.6	B
16621	Bn-N14-p53793394	Bn-C4-p52507724	Bn-scaff_18903_1-p350780	C4	74	131.6	B
16622	Bn-N14-p53794309	Bn-C4-p52508596	Bn-scaff_18903_1-p351652	C4	74	131.6	B
16623	Bn-N14-p53977786	Bn-C4-p52713866	Bn-scaff_18903_1-p556922	C4	75	132.4	B
16624	Bn-N14-p53977941	Bn-C4-p52714021	Bn-scaff_18903_1-p557077	C4	75	132.4	B
16625	Bn-N14-p54056004	Bn-C4-p52800188	Bn-scaff_18903_1-p643244	C4	76	133.2	B
16626	Bn-N14-p54152768	Bn-C4-p52912186	Bn-scaff_18903_1-p755242	C4	77	134.8	B
16627	Bn-N14-p54268611	Bn-C4-p53005001	Bn-scaff_18903_1-p848057	C4	77	134.8	B
16628	Bn-N14-p54436284	Bn-C4-p53155836	Bn-scaff_18903_1-p998892	C4	77	134.8	B
16629	Bn-N14-p54447761	Bn-C4-p53167590	Bn-scaff_18903_1-p101064	C4	77	134.8	B
16630	Bn-N14-p54571000	Bn-C4-p53316094	Bn-scaff_27676_1-p110403	C4	78	135.6	B
16631	Bn-N14-p54751503	Bn-C4-p53484289	Bn-scaff_20817_1-p15523	C4	78	135.6	B
16632	Bn-N14-p54753191	Bn-C4-p53485962	Bn-scaff_20817_1-p17196	C4	78	135.6	B
16633	Bn-N4-p21004588	Bn-A04-p18961945	Bn-A04-p18961945	C4	78	135.6	B
16634	Bn-N4-p21004590	Bn-A04-p18961947	Bn-A04-p18961947	C4	78	135.6	B
16635	Bn-N4-p21004948	Bn-A04-p18962305	Bn-A04-p18962305	C4	78	135.6	B
16636	Bn-N4-p21005021	Bn-A04-p18962378	Bn-A04-p18962378	C4	78	135.6	B
16637	Bn-N4-p21005085	Bn-A04-p18962442	Bn-A04-p18962442	C4	78	135.6	B
16638	Bn-N10-p260628	Bn-A10-p4639202	Bn-A10-p4639202	C5	1	0	B
16639	Bn-N15-p1003087	Bn-C5-p899151	Bn-scaff_16414_1-p111651	C5	1	0	B
16640	Bn-N15-p1004798	Bn-C5-p900972	Bn-scaff_16414_1-p111469	C5	1	0	B
16641	Bn-N15-p1095506	Bn-C5-p980991	Bn-scaff_16414_1-p103467	C5	1	0	B
16642	Bn-N15-p1140446	Bn-C5-p1019111	Bn-scaff_16414_1-p996557	C5	1	0	B
16649	Bn-N15-p484356	Bn-C5-p404945	Bn-scaff_16414_1-p161072	C5	1	0	B
16650	Bn-N15-p53329	Bn-C5-p160625	Bn-scaff_23107_1-p47992	C5	1	0	B
16651	Bn-N15-p53524	Bn-C5-p160429	Bn-scaff_23107_1-p48188	C5	1	0	B
16652	Bn-N15-p565708	Bn-C5-p475900	Bn-scaff_16414_1-p153976	C5	1	0	B
16653	Bn-N15-p600737	Bn-C5-p519060	Bn-scaff_16414_1-p149660	C5	1	0	B
16654	Bn-N15-p630981	Bn-C5-p545683	Bn-scaff_16414_1-p146998	C5	1	0	B
16655	Bn-N15-p631168	Bn-C5-p545870	Bn-scaff_16414_1-p146979	C5	1	0	B
16656	Bn-N15-p651472	Bn-C5-p564738	Bn-scaff_16414_1-p145093	C5	1	0	B
16657	Bn-N15-p663780	Bn-C5-p594321	Bn-scaff_16414_1-p142134	C5	1	0	B
16643	Bn-N15-p1174899	Bn-C5-p1056773	Bn-scaff_16414_1-p958895	C5	2	0.8	B
16644	Bn-N15-p1181724	Bn-C5-p1063975	Bn-scaff_16414_1-p951693	C5	2	0.8	B
16645	Bn-N15-p1197211	Bn-C5-p1082969	Bn-scaff_16414_1-p932699	C5	2	0.8	B
16646	Bn-N15-p1240984	Bn-C5-p1131076	Bn-scaff_16414_1-p884592	C5	2	0.8	B

16647	Bn-N15-p1251075	Bn-C5-p1141171	Bn-scaff_16414_1-p87449	C5	2	0.8	B
16648	Bn-N15-p1261974	Bn-C5-p1152076	Bn-scaff_16414_1-p86359	C5	2	0.8	B
16658	Bn-N15-p1342122	Bn-C5-p1238391	Bn-scaff_16414_1-p77727	C5	3	2.4	B
16659	Bn-N15-p1344027	Bn-C5-p1240787	Bn-scaff_16414_1-p77488	C5	3	2.4	B
16660	Bn-N15-p1348947	Bn-C5-p1242251	Bn-scaff_16414_1-p77341	C5	3	2.4	B
16661	Bn-N15-p1350052	Bn-C5-p1243352	Bn-scaff_16414_1-p77231	C5	3	2.4	B
16662	Bn-N15-p1473353	Bn-C5-p1364481	Bn-scaff_16414_1-p65118	C5	4	3.2	B
16663	Bn-N15-p1474088	Bn-C5-p1365250	Bn-scaff_16414_1-p65041	C5	4	3.2	B
16664	Bn-N15-p1478245	Bn-C5-p1368336	Bn-scaff_16414_1-p64733	C5	4	3.2	B
16665	Bn-N15-p1578974	Bn-C5-p1474081	Bn-scaff_16414_1-p54158	C5	5	5.6	B
16666	Bn-N15-p1578989	Bn-C5-p1474096	Bn-scaff_16414_1-p54157	C5	5	5.6	B
16667	Bn-N15-p1580556	Bn-C5-p1475284	Bn-scaff_16414_1-p54038	C5	5	5.6	B
16668	Bn-N15-p1581612	Bn-C5-p1476340	Bn-scaff_16414_1-p53932	C5	5	5.6	B
16669	Bn-N15-p1582286	Bn-C5-p1477129	Bn-scaff_16414_1-p53853	C5	5	5.6	B
16670	Bn-N15-p1788350	Bn-C5-p1705838	Bn-scaff_16414_1-p30983	C5	6	10.8	B
16671	Bn-N15-p1788428	Bn-C5-p1705915	Bn-scaff_16414_1-p30975	C5	6	10.8	0
16672	Bn-N15-p1999936	Bn-C5-p1917307	Bn-scaff_16414_1-p98361	C5	7	17.8	B
16673	Bn-N15-p2012720	Bn-C5-p1924097	Bn-scaff_16414_1-p91571	C5	7	17.8	B
16674	Bn-N15-p2055863	Bn-C5-p1965064	Bn-scaff_16414_1-p50604	C5	7	17.8	0
16675	Bn-N15-p2078366	Bn-C5-p2025645	Bn-scaff_20901_1-p22038	C5	7	17.8	B
16676	Bn-N15-p2117649	Bn-C5-p2069364	Bn-scaff_20901_1-p21601	C5	8	18.6	B
16677	Bn-N15-p2117883	Bn-C5-p2069598	Bn-scaff_20901_1-p21598	C5	8	18.6	B
16678	Bn-N15-p2143706	Bn-C5-p2104134	Bn-scaff_20901_1-p21253	C5	8	18.6	B
16679	Bn-N15-p2193139	Bn-C5-p2151026	Bn-scaff_20901_1-p20784	C5	9	20.2	B
16680	Bn-N15-p2229669	Bn-C5-p2189242	Bn-scaff_20901_1-p20402	C5	10	21.8	B
16681	Bn-N15-p2241803	Bn-C5-p2199834	Bn-scaff_20901_1-p20296	C5	11	22.6	B
16682	Bn-N15-p2243150	Bn-C5-p2201182	Bn-scaff_20901_1-p20282	C5	11	22.6	B
16683	Bn-N15-p2256152	Bn-C5-p2214096	Bn-scaff_20901_1-p20153	C5	11	22.6	B
16684	Bn-N15-p2257895	Bn-C5-p2215919	Bn-scaff_20901_1-p20135	C5	11	22.6	B
16685	Bn-N15-p2505335	Bn-C5-p2468868	Bn-scaff_20901_1-p17605	C5	12	23.4	B
16686	Bn-N15-p2529890	Bn-C5-p2493209	Bn-scaff_20901_1-p17362	C5	12	23.4	B
16687	Bn-N10-p2866782	Bn-A10-p1125100	Bn-A10-p1125100	C5	13	24.2	B
16688	Bn-N15-p2559070	Bn-C5-p2523891	Bn-scaff_20901_1-p17055	C5	13	24.2	B
16689	Bn-N15-p2578161	Bn-C5-p2548160	Bn-scaff_20901_1-p16813	C5	13	24.2	B
16690	Bn-N15-p2750972	Bn-C5-p2691551	Bn-scaff_20901_1-p15379	C5	14	29.1	B
16691	Bn-N15-p2960014	Bn-C5-p2918201	Bn-scaff_20901_1-p13112	C5	15	33.1	B
16692	Bn-N15-p2961100	Bn-C5-p2919330	Bn-scaff_20901_1-p13101	C5	16	33.9	B
16693	Bn-N15-p2970279	Bn-C5-p2928560	Bn-scaff_20901_1-p13009	C5	17	34.7	B
16694	Bn-N15-p2987065	Bn-C5-p2949790	Bn-scaff_20901_1-p12796	C5	17	34.7	B
16695	Bn-N15-p3017053	Bn-C5-p2982006	Bn-scaff_20901_1-p12474	C5	18	36.3	B
16696	Bn-N15-p3192152	Bn-C5-p3126241	Bn-scaff_20901_1-p11032	C5	19	38.7	B
16697	Bn-N15-p3192573	Bn-C5-p3126661	Bn-scaff_20901_1-p11028	C5	19	38.7	B
16698	Bn-N15-p3365432	Bn-C5-p3281408	Bn-scaff_20901_1-p94805	C5	20	43.6	B
16699	Bn-N15-p3367671	Bn-C5-p3283654	Bn-scaff_20901_1-p94581	C5	20	43.6	B
16700	Bn-N15-p3394001	Bn-C5-p3317866	Bn-scaff_20901_1-p91159	C5	20	43.6	B
16701	Bn-N15-p3408060	Bn-C5-p3327713	Bn-scaff_20901_1-p90175	C5	20	43.6	B
16702	Bn-N15-p3409513	Bn-C5-p3329166	Bn-scaff_20901_1-p90029	C5	20	43.6	B
16703	Bn-N15-p3416186	Bn-C5-p3335116	Bn-scaff_20901_1-p89434	C5	20	43.6	B
16704	Bn-N15-p3419558	Bn-C5-p3338515	Bn-scaff_20901_1-p89095	C5	20	43.6	B

16705	Bn-N15-p3433436	Bn-C5-p3347330	Bn-scaff_20901_1-p88213!	C5	20	43.6	B
16706	Bn-N15-p3436207	Bn-C5-p3350096	Bn-scaff_20901_1-p87936!	C5	20	43.6	B
16707	Bn-N15-p3663901	Bn-C5-p3568847	Bn-scaff_20901_1-p66061	C5	21	46	B
16708	Bn-N15-p3663991	Bn-C5-p3568937	Bn-scaff_20901_1-p66052	C5	21	46	B
16709	Bn-N15-p3664172	Bn-C5-p3569117	Bn-scaff_20901_1-p66034	C5	21	46	B
16710	Bn-N15-p3668206	Bn-C5-p3572995	Bn-scaff_20901_1-p65647	C5	21	46	B
16711	Bn-N15-p3671214	Bn-C5-p3575973	Bn-scaff_20901_1-p65349	C5	21	46	B
16712	Bn-N15-p3671327	Bn-C5-p3576086	Bn-scaff_20901_1-p65337	C5	21	46	B
16713	Bn-N15-p3676679	Bn-C5-p3582195	Bn-scaff_20901_1-p64727	C5	21	46	B
16714	Bn-N6-p3577649	Bn-A06-p3651473	Bn-A06-p3651473	C5	22	50	A
16715	Bn-N15-p3730704	Bn-C5-p3633992	Bn-scaff_20901_1-p59547	C5	23	50.8	A
16716	Bn-N15-p3742162	Bn-C5-p3646026	Bn-scaff_20901_1-p58343	C5	23	50.8	A
16717	Bn-N15-p3967277	Bn-C5-p3848820	Bn-scaff_20901_1-p38064	C5	23	50.8	A
16718	Bn-N15-p3972135	Bn-C5-p3853699	Bn-scaff_20901_1-p37576	C5	23	50.8	A
16719	Bn-N15-p3984454	Bn-C5-p3860455	Bn-scaff_20901_1-p36901	C5	23	50.8	A
16720	Bn-N15-p4343868	Bn-A06-p3651074	Bn-A06-p3651074	C5	23	50.8	A
16721	Bn-N15-p4402394	Bn-C5-p4204839	Bn-scaff_20901_1-p24626	C5	23	50.8	A
16722	Bn-N15-p4419019	Bn-C5-p4277649	Bn-scaff_15838_2-p14257	C5	23	50.8	A
16723	Bn-N15-p4436641	Bn-C5-p4304416	Bn-scaff_15838_2-p11581	C5	23	50.8	A
16724	Bn-Scaffold09732-p367	Bn-C5-p3864433	Bn-scaff_20901_1-p36503	C5	23	50.8	A
16725	Bn-N15-p4454878	Bn-C5-p4318623	Bn-scaff_15838_2-p10160	C5	24	51.6	A
16726	Bn-N15-p4607710	Bn-C5-p4463720	Bn-scaff_19641_1-p18803	C5	24	51.6	A
16727	Bn-N15-p4623425	Bn-C5-p4481554	Bn-scaff_19641_1-p17020	C5	24	51.6	A
16728	Bn-N15-p5195547	Bn-C5-p5091946	Bn-scaff_21496_1-p44009	C5	25	55.6	A
16729	Bn-N15-p5195642	Bn-C5-p5092041	Bn-scaff_21496_1-p44018	C5	25	55.6	A
16730	Bn-N15-p5206904	Bn-C5-p5098626	Bn-scaff_21496_1-p44677	C5	25	55.6	A
16731	Bn-N15-p5209042	Bn-C5-p5105635	Bn-scaff_21496_1-p45378	C5	25	55.6	A
16732	Bn-N15-p5212596	Bn-C5-p5109191	Bn-scaff_21496_1-p45733	C5	25	55.6	A
16733	Bn-N15-p5224172	Bn-C5-p5134205	Bn-scaff_15712_10-p1412	C5	25	55.6	0
16734	Bn-N15-p5254445	Bn-C5-p5169621	Bn-scaff_15712_10-p4953	C5	25	55.6	0
16735	Bn-N15-p5337228	Bn-C5-p5294065	Bn-scaff_15712_10-p1739	C5	25	55.6	A
16736	Bn-N15-p5362797	Bn-C5-p5319666	Bn-scaff_15712_10-p1995	C5	25	55.6	A
16737	Bn-N15-p5531125	Bn-C5-p5497239	Bn-scaff_15712_10-p3771	C5	26	56.4	0
16738	Bn-N15-p5645612	Bn-C5-p5640950	Bn-scaff_15712_10-p5208	C5	26	56.4	A
16739	Bn-N15-p5743185	Bn-C5-p5718467	Bn-scaff_15712_10-p5983	C5	26	56.4	A
16740	Bn-N15-p6313834	Bn-C5-p6633826	Bn-scaff_18181_1-p15901	C5	26	56.4	A
16741	Bn-N15-p6339812	Bn-C5-p6620121	Bn-scaff_18181_1-p16038	C5	26	56.4	A
16742	Bn-N15-p6353706	Bn-C5-p6608933	Bn-scaff_18181_1-p16150	C5	26	56.4	A
16743	Bn-N15-p6436743	Bn-C5-p6559632	Bn-scaff_18181_1-p16643	C5	26	56.4	A
16744	Bn-N15-p6460541	Bn-C5-p6532161	Bn-scaff_18181_1-p16918	C5	26	56.4	A
16745	Bn-N15-p6475053	Bn-C5-p6516481	Bn-scaff_18181_1-p17074	C5	26	56.4	A
16746	Bn-N15-p6549784	Bn-C5-p6449493	Bn-scaff_18181_1-p17744	C5	26	56.4	A
16747	Bn-N15-p6634826	Bn-C5-p6302618	Bn-scaff_15712_10-p1182	C5	26	56.4	A
16748	Bn-N15-p6805908	Bn-C5-p6782897	Bn-scaff_18181_1-p14410	C5	26	56.4	A
16749	Bn-N15-p6826134	Bn-C5-p6816359	Bn-scaff_18181_1-p14076	C5	26	56.4	A
16750	Bn-N15-p6936454	Bn-C5-p6935002	Bn-scaff_18181_1-p12889	C5	26	56.4	A
16751	Bn-N4-p10933835	Bn-C8-p930824	Bn-scaff_18607_1-p24546	C5	26	56.4	A
16752	Bn-N15-p7309887	Bn-C5-p7266769	Bn-scaff_18181_1-p95719	C5	27	57.2	A
16753	Bn-N15-p7313800	Bn-C5-p7270686	Bn-scaff_18181_1-p95327	C5	27	57.2	A

16754	Bn-N6-p5524811	Bn-A06-p5930501	Bn-A06-p5930501	C5	27	57.2	A
16755	Bn-N6-p5545126	Bn-C5-p7271891	Bn-scaff_18181_1-p952071	C5	27	57.2	A
16756	Bn-N15-p7646696	Bn-C5-p7597925	Bn-scaff_18181_1-p626037	C5	28	58	A
16757	Bn-N15-p7709114	Bn-C5-p7651051	Bn-scaff_18181_1-p572911	C5	28	58	A
16758	Bn-N15-p7760016	Bn-C5-p7694117	Bn-scaff_18181_1-p529845	C5	28	58	A
16759	Bn-N15-p7760230	Bn-C5-p7694331	Bn-scaff_18181_1-p529631	C5	28	58	A
16760	Bn-N15-p7767721	Bn-C5-p7702503	Bn-scaff_18181_1-p521459	C5	28	58	A
16761	Bn-N15-p7769154	Bn-C5-p7703967	Bn-scaff_18181_1-p519995	C5	28	58	A
16762	Bn-N15-p8187090	Bn-C5-p8083741	Bn-scaff_18181_1-p140221	C5	28	58	A
16763	Bn-N15-p8225544	Bn-C5-p8110034	Bn-scaff_18181_1-p113928	C5	28	58	A
16764	Bn-N15-p8676373	Bn-C5-p8585240	Bn-scaff_16045_1-p361179	C5	28	58	A
16765	Bn-N15-p8816261	Bn-C5-p8638347	Bn-scaff_16082_1-p579183	C5	28	58	A
16766	Bn-N15-p8856315	Bn-C5-p8660551	Bn-scaff_16082_1-p556979	C5	28	58	A
16767	Bn-N15-p8869053	Bn-C5-p8673689	Bn-scaff_16082_1-p543841	C5	28	58	A
16768	Bn-N15-p8898796	Bn-C5-p8699470	Bn-scaff_16082_1-p518060	C5	28	58	A
16769	Bn-N6-p6017594	Bn-C5-p8028419	Bn-scaff_18181_1-p195543	C5	28	58	A
16770	Bn-N6-p6074760	Bn-A06-p6486764	Bn-A06-p6486764	C5	28	58	A
16771	Bn-N6-p6216436	Bn-A06-p6617274	Bn-A06-p6617274	C5	28	58	0
16772	Bn-N15-p8958496	Bn-C5-p8762652	Bn-scaff_16082_1-p454878	C5	29	58.8	A
16773	Bn-N15-p8962076	Bn-C5-p8767284	Bn-scaff_16082_1-p450246	C5	30	59.6	A
16774	Bn-N15-p9073426	Bn-C5-p8864297	Bn-scaff_16082_1-p353233	C5	30	59.6	A
16775	Bn-N15-p9365310	Bn-C5-p9147526	Bn-scaff_16082_1-p70004	C5	30	59.6	A
16776	Bn-N15-p9543487	Bn-C5-p9313359	Bn-scaff_22183_1-p701401	C5	30	59.6	A
16777	Bn-N15-p9583241	Bn-C5-p9350979	Bn-scaff_22183_1-p663781	C5	30	59.6	A
16778	Bn-N15-p9594601	Bn-C5-p9369572	Bn-scaff_22183_1-p645188	C5	30	59.6	A
16779	Bn-N15-p9594650	Bn-C5-p9369622	Bn-scaff_22183_1-p645138	C5	30	59.6	A
16780	Bn-N15-p9595787	Bn-C5-p9370759	Bn-scaff_22183_1-p644001	C5	31	60.4	A
16781	Bn-N15-p9598282	Bn-C5-p9373228	Bn-scaff_22183_1-p641532	C5	31	60.4	A
16782	Bn-N15-p9598337	Bn-C5-p9373283	Bn-scaff_22183_1-p641477	C5	31	60.4	A
16783	Bn-N15-p9600997	Bn-C5-p9378958	Bn-scaff_22183_1-p635802	C5	31	60.4	A
16784	Bn-N15-p9603869	Bn-C5-p9383094	Bn-scaff_22183_1-p631666	C5	31	60.4	A
16785	Bn-N15-p9606148	Bn-C5-p9385388	Bn-scaff_22183_1-p629372	C5	31	60.4	A
16786	Bn-N15-p9895813	Bn-C5-p9649370	Bn-scaff_22183_1-p365390	C5	31	60.4	A
16787	Bn-N6-p7254556	Bn-A06-p7556709	Bn-A06-p7556709	C5	31	60.4	A
16788	Bn-N15-p10102610	Bn-C5-p9837078	Bn-scaff_22183_1-p177682	C5	32	62	A
16789	Bn-N15-p10113280	Bn-C5-p9844538	Bn-scaff_22183_1-p170222	C5	32	62	A
16790	Bn-N15-p10118146	Bn-C5-p9849982	Bn-scaff_22183_1-p164778	C5	32	62	A
16791	Bn-N15-p10180486	Bn-C5-p9931056	Bn-scaff_22183_1-p83704	C5	32	62	A
16792	Bn-N15-p10181012	Bn-C5-p9931535	Bn-scaff_22183_1-p83225	C5	32	62	A
16793	Bn-N15-p10181209	Bn-C5-p9931733	Bn-scaff_22183_1-p83027	C5	32	62	A
16794	Bn-N15-p10183677	Bn-C5-p9934261	Bn-scaff_22183_1-p80499	C5	32	62	A
16795	Bn-N15-p10184315	Bn-C5-p9934915	Bn-scaff_22183_1-p79845	C5	32	62	A
16796	Bn-N15-p10195097	Bn-C5-p9937768	Bn-scaff_22183_1-p76992	C5	32	62	A
16797	Bn-N15-p10196520	Bn-C5-p9939193	Bn-scaff_22183_1-p75567	C5	32	62	A
16798	Bn-N15-p10225427	Bn-C5-p9953442	Bn-scaff_22183_1-p61318	C5	32	62	A
16799	Bn-N15-p10244602	Bn-C5-p9973220	Bn-scaff_22183_1-p41540	C5	32	62	A
16800	Bn-N15-p43065023	Bn-C5-p9855083	Bn-scaff_22183_1-p159677	C5	32	62	A
16801	Bn-N12-p17420648	Bn-C2-p47082473	Bn-scaff_17109_1-p945730	C5	33	64.4	A
16802	Bn-N15-p11363353	Bn-C5-p10853650	Bn-scaff_16093_1-p5778	C5	33	64.4	A

16803	Bn-N15-p11405952	Bn-C5-p10892255	Bn-scaff_16093_1-p44383	C5	33	64.4	A
16804	Bn-N15-p11423131	Bn-C5-p10878617	Bn-scaff_16093_1-p30745	C5	33	64.4	A
16805	Bn-N15-p11642651	Bn-C5-p11141339	Bn-scaff_20125_1-p66442	C5	33	64.4	A
16806	Bn-N15-p11643096	Bn-C5-p11141779	Bn-scaff_20125_1-p66398	C5	33	64.4	A
16807	Bn-N15-p11643360	Bn-C5-p11142035	Bn-scaff_20125_1-p66372	C5	33	64.4	A
16808	Bn-N15-p11683388	Bn-C5-p11201856	Bn-scaff_20125_1-p60390	C5	33	64.4	A
16809	Bn-N15-p11712309	Bn-C5-p11218902	Bn-scaff_20125_1-p58685	C5	33	64.4	A
16810	Bn-N15-p11725763	Bn-C5-p11234041	Bn-scaff_20125_1-p57171	C5	33	64.4	A
16811	Bn-N15-p11730021	Bn-C5-p11238261	Bn-scaff_20125_1-p56749	C5	33	64.4	A
16812	Bn-N15-p11731057	Bn-C5-p11239297	Bn-scaff_20125_1-p56646	C5	33	64.4	A
16813	Bn-N15-p11755153	Bn-C5-p11272563	Bn-scaff_20125_1-p53319	C5	33	64.4	A
16814	Bn-N15-p11761322	Bn-C5-p11283526	Bn-scaff_20125_1-p52223	C5	33	64.4	A
16815	Bn-N15-p11923031	Bn-C5-p11427017	Bn-scaff_20125_1-p37874	C5	33	64.4	A
16816	Bn-N15-p11931783	Bn-C5-p11435673	Bn-scaff_20125_1-p37008	C5	33	64.4	A
16817	Bn-N15-p11938560	Bn-C5-p11442159	Bn-scaff_20125_1-p36360	C5	33	64.4	A
16818	Bn-N15-p11939672	Bn-C5-p11443269	Bn-scaff_20125_1-p36249	C5	33	64.4	A
16819	Bn-N15-p11962553	Bn-C5-p11453335	Bn-scaff_20125_1-p35242	C5	33	64.4	A
16820	Bn-N15-p11962944	Bn-C5-p11453727	Bn-scaff_20125_1-p35203	C5	33	64.4	A
16821	Bn-N15-p11963132	Bn-C5-p11453915	Bn-scaff_20125_1-p35184	C5	33	64.4	A
16822	Bn-N15-p11964127	Bn-C5-p11454907	Bn-scaff_20125_1-p35085	C5	33	64.4	A
16823	Bn-N15-p11964238	Bn-C5-p11455033	Bn-scaff_20125_1-p35072	C5	33	64.4	A
16824	Bn-N15-p11969707	Bn-C5-p11460963	Bn-scaff_20125_1-p34479	C5	33	64.4	A
16825	Bn-N15-p11973629	Bn-C13729753-p243	Bn-C13729753-p243	C5	33	64.4	A
16826	Bn-N15-p11991759	Bn-C5-p11483587	Bn-scaff_20125_1-p32217	C5	33	64.4	A
16827	Bn-N15-p12017040	Bn-C5-p11499577	Bn-scaff_20125_1-p30618	C5	33	64.4	A
16828	Bn-N15-p12020198	Bn-C5-p11504340	Bn-scaff_20125_1-p30141	C5	33	64.4	A
16829	Bn-N15-p12028191	Bn-C5-p11511073	Bn-scaff_20125_1-p29468	C5	33	64.4	A
16830	Bn-N15-p12049752	Bn-C5-p11528507	Bn-scaff_20125_1-p27725	C5	33	64.4	A
16831	Bn-N15-p12099898	Bn-C5-p11575678	Bn-scaff_20125_1-p23008	C5	33	64.4	A
16832	Bn-N15-p12100993	Bn-C5-p11576773	Bn-scaff_20125_1-p22898	C5	33	64.4	A
16833	Bn-N15-p12125297	Bn-C5-p11604912	Bn-scaff_20125_1-p20084	C5	33	64.4	A
16834	Bn-N15-p12127258	Bn-C5-p11606871	Bn-scaff_20125_1-p19888	C5	33	64.4	A
16835	Bn-N15-p12151683	Bn-C5-p11628586	Bn-scaff_20125_1-p17717	C5	33	64.4	A
16836	Bn-N15-p12164836	Bn-C5-p11638839	Bn-scaff_20125_1-p16692	C5	33	64.4	A
16837	Bn-N15-p12167422	Bn-C5-p11641600	Bn-scaff_20125_1-p16415	C5	33	64.4	A
16838	Bn-N15-p12174673	Bn-C5-p11653109	Bn-scaff_20125_1-p15265	C5	33	64.4	A
16839	Bn-N15-p12175939	Bn-C5-p11654365	Bn-scaff_20125_1-p15139	C5	33	64.4	A
16840	Bn-N15-p12191352	Bn-C5-p11667279	Bn-scaff_20125_1-p13848	C5	33	64.4	A
16841	Bn-N15-p12197331	Bn-C5-p11689323	Bn-scaff_20125_1-p11643	C5	33	64.4	A
16842	Bn-N15-p12198739	Bn-C5-p11690926	Bn-scaff_20125_1-p11483	C5	33	64.4	A
16843	Bn-N15-p12212743	Bn-C5-p11695279	Bn-scaff_20125_1-p11048	C5	33	64.4	A
16844	Bn-N15-p12212940	Bn-C5-p11695452	Bn-scaff_20125_1-p11030	C5	33	64.4	A
16845	Bn-N15-p12236658	Bn-C5-p11717389	Bn-scaff_20125_1-p88370	C5	33	64.4	A
16846	Bn-N15-p12236723	Bn-C5-p11717454	Bn-scaff_20125_1-p88305	C5	33	64.4	A
16847	Bn-N15-p12236937	Bn-C5-p11717667	Bn-scaff_20125_1-p88092	C5	33	64.4	A
16848	Bn-N15-p12238056	Bn-C5-p11719138	Bn-scaff_20125_1-p86621	C5	33	64.4	A
16849	Bn-N15-p12245840	Bn-C5-p11737052	Bn-scaff_20125_1-p68707	C5	33	64.4	A
16850	Bn-N15-p12245925	Bn-C5-p11737137	Bn-scaff_20125_1-p68622	C5	33	64.4	A
16851	Bn-N15-p12253667	Bn-C5-p11738207	Bn-scaff_20125_1-p67552	C5	33	64.4	A

16852	Bn-N15-p12254318	Bn-C5-p11739069	Bn-scaff_20125_1-p66690	C5	33	64.4	A
16853	Bn-N15-p12315210	Bn-C5-p11787483	Bn-scaff_20125_1-p18276	C5	33	64.4	A
16854	Bn-N15-p12316521	Bn-C5-p11788967	Bn-scaff_20125_1-p16792	C5	33	64.4	A
16855	Bn-N15-p12320389	Bn-C5-p11792944	Bn-scaff_20125_1-p12815	C5	33	64.4	A
16856	Bn-N15-p12322969	Bn-C5-p11799582	Bn-scaff_20125_1-p6177	C5	33	64.4	A
16857	Bn-N15-p12332157	Bn-C5-p11802834	Bn-scaff_20125_1-p2925	C5	33	64.4	A
16858	Bn-N15-p12333616	Bn-C5-p11804896	Bn-scaff_20125_1-p863	C5	33	64.4	A
16859	Bn-N15-p12349467	Bn-C5-p11913724	Bn-scaff_21821_1-p11287	C5	33	64.4	A
16860	Bn-N15-p12350631	Bn-C5-p11914873	Bn-scaff_21821_1-p11172	C5	33	64.4	A
16861	Bn-N15-p12404427	Bn-C5-p11954757	Bn-scaff_21821_1-p71840	C5	33	64.4	A
16862	Bn-N15-p12421464	Bn-C5-p11971422	Bn-scaff_21821_1-p55175	C5	33	64.4	A
16863	Bn-N15-p12432276	Bn-C5-p11983986	Bn-scaff_21821_1-p42611	C5	33	64.4	A
16864	Bn-N15-p12433264	Bn-C5-p11984966	Bn-scaff_21821_1-p41631	C5	33	64.4	A
16865	Bn-N15-p12465509	Bn-C5-p12016210	Bn-scaff_21821_1-p10387	C5	33	64.4	A
16866	Bn-N15-p12506202	Bn-C5-p11869380	Bn-scaff_16792_1-p21478	C5	33	64.4	A
16867	Bn-N15-p12506319	Bn-C5-p11869263	Bn-scaff_16792_1-p21595	C5	33	64.4	A
16868	Bn-N15-p12519197	Bn-C5-p11856330	Bn-scaff_16792_1-p34528	C5	33	64.4	A
16869	Bn-N15-p12519746	Bn-C5-p11855781	Bn-scaff_16792_1-p35077	C5	33	64.4	A
16870	Bn-N15-p12605534	Bn-C5-p12045521	Bn-scaff_21338_1-p18825	C5	33	64.4	A
16871	Bn-N15-p12609502	Bn-C5-p12049155	Bn-scaff_21338_1-p22459	C5	33	64.4	A
16872	Bn-N15-p12609922	Bn-C5-p12049575	Bn-scaff_21338_1-p22879	C5	33	64.4	A
16873	Bn-N15-p12617616	Bn-C5-p12057261	Bn-scaff_21338_1-p30565	C5	33	64.4	A
16874	Bn-N15-p12638866	Bn-C5-p12077670	Bn-scaff_21338_1-p50974	C5	33	64.4	A
16875	Bn-N15-p12648591	Bn-C5-p12087295	Bn-scaff_21338_1-p60599	C5	33	64.4	A
16876	Bn-N15-p12649209	Bn-C5-p12087913	Bn-scaff_21338_1-p61217	C5	33	64.4	A
16877	Bn-N15-p12655095	Bn-C5-p12093984	Bn-scaff_21338_1-p67288	C5	33	64.4	A
16878	Bn-N15-p12658841	Bn-C5-p12097730	Bn-scaff_21338_1-p71034	C5	33	64.4	A
16879	Bn-N15-p12658900	Bn-C5-p12097789	Bn-scaff_21338_1-p71093	C5	33	64.4	A
16880	Bn-N15-p12660631	Bn-C5-p12099520	Bn-scaff_21338_1-p72824	C5	33	64.4	A
16881	Bn-N15-p12668894	Bn-C5-p12107728	Bn-scaff_21338_1-p81032	C5	33	64.4	A
16882	Bn-N15-p12726882	Bn-C5-p12165683	Bn-scaff_21338_1-p13898	C5	33	64.4	A
16883	Bn-N15-p12746611	Bn-C5-p12183631	Bn-scaff_21338_1-p15693	C5	33	64.4	A
16884	Bn-N15-p12808370	Bn-C5-p12242189	Bn-scaff_21338_1-p21549	C5	33	64.4	A
16885	Bn-N15-p12967600	Bn-C5-p12390260	Bn-scaff_21338_1-p36356	C5	33	64.4	A
16886	Bn-N15-p13085344	Bn-C5-p12494615	Bn-scaff_21338_1-p46791	C5	33	64.4	A
16887	Bn-N15-p13101931	Bn-C5-p12510203	Bn-scaff_21338_1-p48350	C5	33	64.4	A
16888	Bn-N15-p13109507	Bn-C5-p12518365	Bn-scaff_21338_1-p49166	C5	33	64.4	A
16889	Bn-N15-p13109761	Bn-C5-p12518618	Bn-scaff_21338_1-p49192	C5	33	64.4	A
16890	Bn-N15-p13110422	Bn-C5-p12519279	Bn-scaff_21338_1-p49258	C5	33	64.4	A
16891	Bn-N15-p13110649	Bn-C5-p12519506	Bn-scaff_21338_1-p49281	C5	33	64.4	A
16892	Bn-N15-p13110967	Bn-C5-p12519824	Bn-scaff_21338_1-p49312	C5	33	64.4	A
16893	Bn-N15-p13111416	Bn-C5-p12520274	Bn-scaff_21338_1-p49357	C5	33	64.4	A
16894	Bn-N15-p13111585	Bn-C5-p12520443	Bn-scaff_21338_1-p49374	C5	33	64.4	A
16895	Bn-N15-p13111661	Bn-C5-p12520519	Bn-scaff_21338_1-p49382	C5	33	64.4	A
16896	Bn-N15-p13111733	Bn-C5-p12520591	Bn-scaff_21338_1-p49389	C5	33	64.4	A
16897	Bn-N15-p13112634	Bn-C5-p12521491	Bn-scaff_21338_1-p49479	C5	33	64.4	A
16898	Bn-N15-p13113191	Bn-C5-p12522049	Bn-scaff_21338_1-p49535	C5	33	64.4	A
16899	Bn-N15-p13119574	Bn-C5-p12540693	Bn-scaff_21338_1-p51399	C5	33	64.4	A
16900	Bn-N15-p13119696	Bn-C5-p12540815	Bn-scaff_21338_1-p51411	C5	33	64.4	A

16901	Bn-N15-p13119712	Bn-C5-p12540831	Bn-scaff_21338_1-p514135	C5	33	64.4	A
16902	Bn-N15-p13119991	Bn-C5-p12541107	Bn-scaff_21338_1-p514411	C5	33	64.4	A
16903	Bn-N15-p13120078	Bn-C5-p12541208	Bn-scaff_21338_1-p514512	C5	33	64.4	A
16904	Bn-N15-p13121951	Bn-C5-p12543864	Bn-scaff_21338_1-p517168	C5	33	64.4	A
16905	Bn-N15-p13122465	Bn-C5-p12544378	Bn-scaff_21338_1-p517682	C5	33	64.4	A
16906	Bn-N15-p13122467	Bn-C5-p12544380	Bn-scaff_21338_1-p517684	C5	33	64.4	A
16907	Bn-N15-p13122674	Bn-C5-p12544587	Bn-scaff_21338_1-p517891	C5	33	64.4	A
16908	Bn-N15-p13122701	Bn-C5-p12544614	Bn-scaff_21338_1-p517918	C5	33	64.4	A
16909	Bn-N15-p13122755	Bn-C5-p12544668	Bn-scaff_21338_1-p517972	C5	33	64.4	A
16910	Bn-N15-p13128140	Bn-C5-p12550020	Bn-scaff_21338_1-p523324	C5	33	64.4	A
16911	Bn-N15-p13171329	Bn-C5-p12565079	Bn-scaff_21338_1-p538383	C5	33	64.4	A
16912	Bn-N15-p13171482	Bn-C5-p12565232	Bn-scaff_21338_1-p538536	C5	33	64.4	A
16913	Bn-N15-p13171977	Bn-C5-p12567231	Bn-scaff_21338_1-p540535	C5	33	64.4	A
16914	Bn-N15-p13172216	Bn-C5-p12567472	Bn-scaff_21338_1-p540776	C5	33	64.4	A
16915	Bn-N15-p13172884	Bn-C5-p12568141	Bn-scaff_21338_1-p541445	C5	33	64.4	A
16916	Bn-N15-p13173340	Bn-C5-p12568597	Bn-scaff_21338_1-p541901	C5	33	64.4	A
16917	Bn-N15-p13173458	Bn-C5-p12568715	Bn-scaff_21338_1-p542019	C5	33	64.4	A
16918	Bn-N15-p13181652	Bn-C5-p12573466	Bn-scaff_21338_1-p546770	C5	33	64.4	A
16919	Bn-N15-p13192092	Bn-C5-p12582209	Bn-scaff_21338_1-p555513	C5	33	64.4	A
16920	Bn-N15-p13203413	Bn-C5-p12593486	Bn-scaff_21338_1-p566790	C5	33	64.4	A
16921	Bn-N15-p13204443	Bn-C5-p12594515	Bn-scaff_21338_1-p567819	C5	33	64.4	A
16922	Bn-N15-p13220916	Bn-Scaffold01260-p29	Bn-scaff_16688_1-p2939	C5	33	64.4	A
16923	Bn-N15-p13224008	Bn-Scaffold01260-p59	Bn-scaff_16688_1-p5925	C5	33	64.4	A
16924	Bn-N15-p13242244	Bn-Scaffold01428-p5	Bn-scaff_16688_1-p23455	C5	33	64.4	A
16925	Bn-N15-p13264206	Bn-C5-p12631621	Bn-scaff_21338_1-p604925	C5	33	64.4	A
16926	Bn-N15-p13264656	Bn-C5-p12632069	Bn-scaff_21338_1-p605373	C5	33	64.4	A
16927	Bn-N15-p13265135	Bn-C5-p12632539	Bn-scaff_21338_1-p605843	C5	33	64.4	A
16928	Bn-N15-p13265406	Bn-C5-p12632810	Bn-scaff_21338_1-p606114	C5	33	64.4	A
16929	Bn-N15-p13266153	Bn-C5-p12633142	Bn-scaff_21338_1-p606446	C5	33	64.4	A
16930	Bn-N15-p13266246	Bn-C5-p12633235	Bn-scaff_21338_1-p606539	C5	33	64.4	A
16931	Bn-N15-p13267341	Bn-C5-p12634320	Bn-scaff_21338_1-p607624	C5	33	64.4	A
16932	Bn-N15-p13267595	Bn-C5-p12634574	Bn-scaff_21338_1-p607878	C5	33	64.4	A
16933	Bn-N15-p13272274	Bn-C5-p12639221	Bn-scaff_21338_1-p612525	C5	33	64.4	A
16934	Bn-N15-p13273572	Bn-C5-p12640231	Bn-scaff_21338_1-p613535	C5	33	64.4	A
16935	Bn-N15-p13274687	Bn-C5-p12642223	Bn-scaff_21338_1-p615527	C5	33	64.4	A
16936	Bn-N15-p13279856	Bn-C5-p12647346	Bn-scaff_21338_1-p620650	C5	33	64.4	A
16937	Bn-N15-p13279948	Bn-C5-p12647437	Bn-scaff_21338_1-p620741	C5	33	64.4	A
16938	Bn-N15-p13280022	Bn-C5-p12647511	Bn-scaff_21338_1-p620815	C5	33	64.4	A
16939	Bn-N15-p13294341	Bn-C5-p12648691	Bn-scaff_21338_1-p621995	C5	33	64.4	A
16940	Bn-N15-p13302012	Bn-C5-p12662942	Bn-scaff_21338_1-p636246	C5	33	64.4	A
16941	Bn-N15-p13302072	Bn-C5-p12663002	Bn-scaff_21338_1-p636306	C5	33	64.4	A
16942	Bn-N15-p13302096	Bn-C5-p12663026	Bn-scaff_21338_1-p636330	C5	33	64.4	A
16943	Bn-N15-p13304519	Bn-C5-p12669287	Bn-scaff_21338_1-p642591	C5	33	64.4	A
16944	Bn-N15-p13339354	Bn-C5-p12695917	Bn-scaff_21338_1-p669221	C5	33	64.4	A
16945	Bn-N15-p13344624	Bn-C5-p12699202	Bn-scaff_21338_1-p672506	C5	33	64.4	A
16946	Bn-N15-p13349000	Bn-C5-p12710432	Bn-scaff_21338_1-p683736	C5	33	64.4	A
16947	Bn-N15-p13349571	Bn-C5-p12710946	Bn-scaff_21338_1-p684250	C5	33	64.4	A
16948	Bn-N15-p13349922	Bn-C5-p12711297	Bn-scaff_21338_1-p684601	C5	33	64.4	A
16949	Bn-N15-p13358719	Bn-C5-p12714046	Bn-scaff_21338_1-p687350	C5	33	64.4	A

16950	Bn-N15-p13441585	Bn-C5-p12808830	Bn-scaff_21338_1-p782134	C5	33	64.4	A
16951	Bn-N15-p13894583	Bn-C5-p13242841	Bn-scaff_21338_1-p121614	C5	33	64.4	A
16952	Bn-N19-p20858476	Bn-C5-p10933516	Bn-scaff_20125_1-p872243	C5	33	64.4	A
16953	Bn-N6-p8470186	Bn-A06-p8791868	Bn-A06-p8791868	C5	33	64.4	A
16954	Bn-N7-p10191283	Bn-C5-p11738738	Bn-scaff_20125_1-p67021	C5	33	64.4	A
16955	Bn-Scaffold01365-p5237	Bn-Scaffold16914-p28	Bn-scaff_25690_1-p287	C5	33	64.4	A
16956	Bn-Scaffold38891-p122	Bn-C9-p22585767	Bn-scaff_18424_1-p885488	C5	33	64.4	A
16957	Bn-N10-p1848892	Bn-A10-p2022381	Bn-A10-p2022381	C5	34	65.2	A
16958	Bn-N14-p4133434	Bn-C5-p12902867	Bn-scaff_21338_1-p876171	C5	34	65.2	A
16959	Bn-N15-p13496739	Bn-C5-p12859344	Bn-scaff_21338_1-p832648	C5	34	65.2	A
16960	Bn-N15-p13496909	Bn-C5-p12859514	Bn-scaff_21338_1-p832818	C5	34	65.2	A
16961	Bn-N15-p13496990	Bn-C5-p12859595	Bn-scaff_21338_1-p832899	C5	34	65.2	A
16962	Bn-N15-p13555299	Bn-C5-p12913746	Bn-scaff_21338_1-p887050	C5	34	65.2	A
16963	Bn-N15-p13603528	Bn-C5-p12948066	Bn-scaff_21338_1-p921370	C5	34	65.2	A
16964	Bn-N15-p13715582	Bn-C5-p13063800	Bn-scaff_21338_1-p103710	C5	34	65.2	A
16965	Bn-N15-p13721828	Bn-C5-p13065823	Bn-scaff_21338_1-p103912	C5	34	65.2	A
16966	Bn-N15-p13723909	Bn-C5-p13067331	Bn-scaff_21338_1-p104063	C5	34	65.2	A
16967	Bn-N15-p13725069	Bn-C5-p13068486	Bn-scaff_21338_1-p104179	C5	34	65.2	A
16968	Bn-N15-p13731105	Bn-C5-p13074983	Bn-scaff_21338_1-p104828	C5	34	65.2	A
16969	Bn-N15-p13734031	Bn-C5-p13079747	Bn-scaff_21338_1-p105305	C5	34	65.2	A
16970	Bn-N15-p13734355	Bn-C5-p13080070	Bn-scaff_21338_1-p105337	C5	34	65.2	A
16971	Bn-N15-p13738528	Bn-C5-p13083887	Bn-scaff_21338_1-p105719	C5	34	65.2	A
16972	Bn-N15-p13739893	Bn-C5-p13085248	Bn-scaff_21338_1-p105855	C5	34	65.2	A
16973	Bn-N15-p13740089	Bn-C5-p13085444	Bn-scaff_21338_1-p105874	C5	34	65.2	A
16974	Bn-N15-p13740936	Bn-C5-p13086292	Bn-scaff_21338_1-p105959	C5	34	65.2	A
16975	Bn-N15-p13741280	Bn-C5-p13086636	Bn-scaff_21338_1-p105994	C5	34	65.2	A
16976	Bn-N15-p13741357	Bn-C5-p13086713	Bn-scaff_21338_1-p106001	C5	34	65.2	A
16977	Bn-N15-p13741962	Bn-C5-p13087035	Bn-scaff_21338_1-p106033	C5	34	65.2	A
16978	Bn-N15-p13742052	Bn-C5-p13087125	Bn-scaff_21338_1-p106042	C5	34	65.2	A
16979	Bn-N15-p13742187	Bn-C5-p13087260	Bn-scaff_21338_1-p106056	C5	34	65.2	A
16980	Bn-N15-p13742244	Bn-C5-p13087317	Bn-scaff_21338_1-p106062	C5	34	65.2	A
16981	Bn-N15-p13742258	Bn-C5-p13087331	Bn-scaff_21338_1-p106063	C5	34	65.2	A
16982	Bn-N15-p13743155	Bn-C5-p13088228	Bn-scaff_21338_1-p106153	C5	34	65.2	A
16983	Bn-N15-p13743283	Bn-C5-p13088341	Bn-scaff_21338_1-p106164	C5	34	65.2	A
16984	Bn-N15-p13744215	Bn-C5-p13089274	Bn-scaff_21338_1-p106257	C5	34	65.2	A
16985	Bn-N15-p13744448	Bn-C5-p13089507	Bn-scaff_21338_1-p106281	C5	34	65.2	A
16986	Bn-N15-p13744513	Bn-C5-p13089572	Bn-scaff_21338_1-p106287	C5	34	65.2	A
16987	Bn-N15-p13745112	Bn-C5-p13090170	Bn-scaff_21338_1-p106347	C5	34	65.2	A
16988	Bn-N15-p13746273	Bn-C5-p13091330	Bn-scaff_21338_1-p106463	C5	34	65.2	A
16989	Bn-N15-p13756648	Bn-C5-p13105618	Bn-scaff_21338_1-p107892	C5	34	65.2	A
16990	Bn-N15-p13758412	Bn-C5-p13107361	Bn-scaff_21338_1-p108066	C5	34	65.2	A
16991	Bn-N15-p13758776	Bn-C5-p13107721	Bn-scaff_21338_1-p108102	C5	34	65.2	A
16992	Bn-N15-p13767304	Bn-C5-p13111998	Bn-scaff_21338_1-p108530	C5	34	65.2	A
16993	Bn-N15-p13767664	Bn-C5-p13112369	Bn-scaff_21338_1-p108567	C5	34	65.2	A
16994	Bn-N15-p13801125	Bn-C5-p13141618	Bn-scaff_21338_1-p111492	C5	34	65.2	A
16995	Bn-N15-p13810049	Bn-C5-p13146736	Bn-scaff_21338_1-p112004	C5	34	65.2	A
16996	Bn-N15-p13810165	Bn-C5-p13146852	Bn-scaff_21338_1-p112015	C5	34	65.2	A
16997	Bn-N15-p13810329	Bn-C5-p13147016	Bn-scaff_21338_1-p112032	C5	34	65.2	A
16998	Bn-N15-p13826354	Bn-C5-p13162437	Bn-scaff_21338_1-p113574	C5	34	65.2	A

16999	Bn-N15-p13833175	Bn-C5-p13180628	Bn-scaff_21338_1-p115393	C5	34	65.2	A
17000	Bn-N15-p13833504	Bn-C5-p13180957	Bn-scaff_21338_1-p115426	C5	34	65.2	A
17001	Bn-N15-p13863790	Bn-C5-p13220154	Bn-scaff_21338_1-p119345	C5	34	65.2	A
17002	Bn-N15-p13863845	Bn-C5-p13220209	Bn-scaff_21338_1-p119351	C5	34	65.2	A
17003	Bn-N15-p13863925	Bn-C5-p13220327	Bn-scaff_21338_1-p119363	C5	34	65.2	A
17004	Bn-N15-p13876547	Bn-C5-p13224046	Bn-scaff_21338_1-p119735	C5	34	65.2	A
17005	Bn-N15-p13876671	Bn-C5-p13224170	Bn-scaff_21338_1-p119747	C5	34	65.2	A
17006	Bn-N15-p13876765	Bn-C5-p13224264	Bn-scaff_21338_1-p119756	C5	34	65.2	A
17007	Bn-N15-p13879950	Bn-C5-p13226091	Bn-scaff_21338_1-p119939	C5	34	65.2	A
17008	Bn-N15-p13885042	Bn-C5-p13231806	Bn-scaff_21338_1-p120511	C5	34	65.2	A
17009	Bn-N15-p13911277	Bn-C5-p13265976	Bn-scaff_16268_1-p643306	C5	34	65.2	A
17010	Bn-N15-p13913585	Bn-C5-p13268274	Bn-scaff_16268_1-p641008	C5	34	65.2	A
17011	Bn-N15-p13913710	Bn-C5-p13268399	Bn-scaff_16268_1-p640883	C5	34	65.2	A
17012	Bn-N15-p13914504	Bn-C5-p13269194	Bn-scaff_16268_1-p640088	C5	34	65.2	A
17013	Bn-N15-p13915020	Bn-C5-p13269710	Bn-scaff_16268_1-p639572	C5	34	65.2	A
17014	Bn-N15-p13927228	Bn-C5-p13273970	Bn-scaff_16268_1-p635312	C5	34	65.2	A
17015	Bn-N15-p13942325	Bn-C5-p13299824	Bn-scaff_16268_1-p609458	C5	34	65.2	A
17016	Bn-N15-p13942820	Bn-C5-p13300319	Bn-scaff_16268_1-p608963	C5	34	65.2	A
17017	Bn-N15-p13943562	Bn-C5-p13301061	Bn-scaff_16268_1-p608221	C5	34	65.2	A
17018	Bn-N15-p13955862	Bn-C5-p13306824	Bn-scaff_16268_1-p602458	C5	34	65.2	A
17019	Bn-N15-p13956454	Bn-C5-p13307416	Bn-scaff_16268_1-p601866	C5	34	65.2	A
17020	Bn-N15-p13957794	Bn-C5-p13308754	Bn-scaff_16268_1-p600528	C5	34	65.2	A
17021	Bn-N15-p13958409	Bn-C5-p13309205	Bn-scaff_16268_1-p600077	C5	34	65.2	A
17022	Bn-N15-p13958889	Bn-C5-p13309688	Bn-scaff_16268_1-p599594	C5	34	65.2	A
17023	Bn-N15-p13960812	Bn-C5-p13320485	Bn-scaff_16268_1-p588797	C5	34	65.2	A
17024	Bn-N15-p13967076	Bn-C5-p13333971	Bn-scaff_16268_1-p575311	C5	34	65.2	A
17025	Bn-N15-p13967116	Bn-C5-p13334011	Bn-scaff_16268_1-p575271	C5	34	65.2	A
17026	Bn-N15-p13968655	Bn-C5-p13335530	Bn-scaff_16268_1-p573752	C5	34	65.2	A
17027	Bn-N15-p13970534	Bn-C5-p13338135	Bn-scaff_16268_1-p571147	C5	34	65.2	A
17028	Bn-N15-p13970692	Bn-C5-p13338293	Bn-scaff_16268_1-p570989	C5	34	65.2	A
17029	Bn-N15-p13970914	Bn-C5-p13338516	Bn-scaff_16268_1-p570766	C5	34	65.2	A
17030	Bn-N15-p13978014	Bn-C5-p13344436	Bn-scaff_16268_1-p564846	C5	34	65.2	A
17031	Bn-N15-p13981662	Bn-C5-p13347244	Bn-scaff_16268_1-p562038	C5	34	65.2	A
17032	Bn-N15-p13995607	Bn-C5-p13360945	Bn-scaff_16268_1-p548337	C5	34	65.2	A
17033	Bn-N15-p13995676	Bn-C5-p13361013	Bn-scaff_16268_1-p548269	C5	34	65.2	A
17034	Bn-N15-p14002109	Bn-C5-p13366095	Bn-scaff_16268_1-p543187	C5	34	65.2	A
17035	Bn-N15-p14018708	Bn-C5-p13375414	Bn-scaff_16268_1-p533868	C5	34	65.2	A
17036	Bn-N15-p14019003	Bn-C5-p13375736	Bn-scaff_16268_1-p533546	C5	34	65.2	A
17037	Bn-N15-p14020208	Bn-C5-p13376943	Bn-scaff_16268_1-p532339	C5	34	65.2	A
17038	Bn-N15-p14036427	Bn-C5-p13402172	Bn-scaff_16268_1-p507110	C5	34	65.2	A
17039	Bn-N15-p14085329	Bn-C5-p13444644	Bn-scaff_16268_1-p464638	C5	34	65.2	A
17040	Bn-N15-p14093903	Bn-C5-p13452836	Bn-scaff_16268_1-p456446	C5	34	65.2	A
17041	Bn-N15-p14101227	Bn-C5-p13464998	Bn-scaff_16268_1-p444284	C5	34	65.2	A
17042	Bn-N15-p14109032	Bn-C5-p13471280	Bn-scaff_16268_1-p438002	C5	34	65.2	A
17043	Bn-N15-p14151046	Bn-C5-p13509321	Bn-scaff_16268_1-p399961	C5	34	65.2	A
17044	Bn-N15-p14171725	Bn-C5-p13519545	Bn-scaff_16268_1-p389737	C5	34	65.2	A
17045	Bn-N15-p14180361	Bn-C5-p13530951	Bn-scaff_16268_1-p378331	C5	34	65.2	A
17046	Bn-N15-p14181357	Bn-C5-p13531947	Bn-scaff_16268_1-p377335	C5	34	65.2	A
17047	Bn-N15-p14181618	Bn-C5-p13532209	Bn-scaff_16268_1-p377073	C5	34	65.2	A

17048	Bn-N15-p14244657	Bn-C5-p13588762	Bn-scaff_16268_1-p32052(	C5	34	65.2	A
17049	Bn-N15-p14254544	Bn-C5-p13608787	Bn-scaff_16268_1-p30049!	C5	34	65.2	A
17050	Bn-N15-p14255205	Bn-C5-p13609458	Bn-scaff_16268_1-p29982	C5	34	65.2	A
17051	Bn-N15-p14301787	Bn-C5-p13646286	Bn-scaff_16268_1-p26299	C5	34	65.2	A
17052	Bn-N15-p14303441	Bn-C5-p13647937	Bn-scaff_16268_1-p26134!	C5	34	65.2	A
17053	Bn-N15-p14305482	Bn-C5-p13649981	Bn-scaff_16268_1-p25930	C5	34	65.2	A
17054	Bn-N15-p14305988	Bn-C5-p13650486	Bn-scaff_16268_1-p25879	C5	34	65.2	A
17055	Bn-N15-p14320859	Bn-C5-p13662038	Bn-scaff_16268_1-p24724	C5	34	65.2	A
17056	Bn-N15-p14448479	Bn-C5-p13784445	Bn-scaff_16268_1-p12483	C5	34	65.2	A
17057	Bn-N15-p14555102	Bn-C5-p13899325	Bn-scaff_16268_1-p9957	C5	34	65.2	A
17058	Bn-N15-p14950656	Bn-Scaffold01011-p1!	Bn-scaff_18147_1-p15922	C5	34	65.2	A
17059	Bn-N15-p14953343	Bn-Scaffold01011-p1	Bn-scaff_18147_1-p18617	C5	34	65.2	A
17060	Bn-N7-p17394595	Bn-A07-p14766907	Bn-A07-p14766907	C5	34	65.2	A
17061	Bn-N9-p26149095	Bn-C5-p13821921	Bn-scaff_16268_1-p87361	C5	34	65.2	A
17062	Bn-N9-p26384425	Bn-Scaffold12442-p3	Bn-scaff_27567_1-p347	C5	34	65.2	A
17063	Bn-Scaffold05475-p764	Bn-C5-p13301602	Bn-scaff_16268_1-p60768(	C5	34	65.2	A
17064	Bn-N15-p15332549	Bn-C5-p14153917	Bn-scaff_18338_1-p89113	C5	35	66	A
17065	Bn-N15-p15923332	Bn-C5-p14722118	Bn-scaff_18338_1-p32293	C5	36	70	A
17066	Bn-N15-p16004336	Bn-C5-p14800814	Bn-scaff_18338_1-p24423	C5	36	70	A
17067	Bn-N15-p16277863	Bn-C5-p17041590	Bn-scaff_17869_1-p84375	C5	36	70	A
17068	Bn-N15-p16373321	Bn-C5-p17124560	Bn-scaff_17869_1-p92672	C5	36	70	A
17069	Bn-N15-p16377040	Bn-C5-p17128294	Bn-scaff_17869_1-p93045	C5	36	70	A
17070	Bn-N15-p16510269	Bn-C3-p51009888	Bn-scaff_18849_1-p23304(	C5	36	70	A
17071	Bn-N15-p16510311	Bn-C5-p17254362	Bn-scaff_17869_1-p10565	C5	36	70	A
17072	Bn-N15-p16737031	Bn-C5-p17470423	Bn-scaff_17869_1-p12725	C5	36	70	A
17073	Bn-N16-p15485187	Bn-C6-p23268058	Bn-scaff_15818_1-p74676	C5	37	70.8	A
17074	Bn-N16-p15485278	Bn-C6-p23268034	Bn-scaff_15818_1-p74700	C5	37	70.8	A
17075	Bn-N15-p17515855	Bn-C5-p18008781	Bn-scaff_16062_1-p14800(	C5	38	71.6	A
17076	Bn-N15-p17650426	Bn-C5-p18150666	Bn-scaff_16062_1-p28988!	C5	38	71.6	A
17077	Bn-N15-p17655644	Bn-C5-p18155864	Bn-scaff_16062_1-p29508	C5	38	71.6	A
17078	Bn-N15-p17655934	Bn-C5-p18156155	Bn-scaff_16062_1-p29537	C5	38	71.6	A
17079	Bn-N15-p17656078	Bn-C5-p18156299	Bn-scaff_16062_1-p29551	C5	38	71.6	A
17080	Bn-N15-p17656250	Bn-C5-p18156471	Bn-scaff_16062_1-p29569(	C5	38	71.6	A
17081	Bn-N15-p17769317	Bn-C5-p18238783	Bn-scaff_18461_1-p25071!	C5	38	71.6	A
17082	Bn-N15-p18376044	Bn-C5-p18812982	Bn-scaff_23195_1-p70804	C5	39	74	A
17083	Bn-N15-p18620814	Bn-C5-p19070681	Bn-scaff_16181_1-p44437	C5	39	74	A
17084	Bn-N15-p19119534	Bn-C5-p19562440	Bn-scaff_16478_1-p44722	C5	39	74	A
17085	Bn-N15-p19178909	Bn-C5-p19613384	Bn-scaff_16478_1-p49816	C5	39	74	A
17086	Bn-N10-p6933483	Bn-A02-p22682931	Bn-A02-p22682931	C5	40	74.8	A
17087	Bn-N15-p19745473	Bn-C5-p20180980	Bn-scaff_16478_1-p10657	C5	40	74.8	A
17088	Bn-N15-p20672947	Bn-C5-p21007718	Bn-scaff_18784_1-p12894	C5	40	74.8	A
17089	Bn-N15-p20815916	Bn-C5-p21144897	Bn-scaff_18795_1-p8136	C5	40	74.8	A
17090	Bn-N15-p21082325	Bn-C5-p21395896	Bn-scaff_27050_1-p16324	C5	40	74.8	A
17091	Bn-N15-p21083084	Bn-C5-p21396605	Bn-scaff_27050_1-p17033	C5	40	74.8	A
17092	Bn-N15-p21235432	Bn-C5-p21526948	Bn-scaff_27050_1-p14737	C5	40	74.8	A
17093	Bn-N15-p22549562	Bn-C5-p23059026	Bn-scaff_18371_1-p77708	C5	40	74.8	A
17094	Bn-N15-p23839011	Bn-C5-p24339872	Bn-scaff_16454_1-p88040(	C5	40	74.8	A
17095	Bn-N15-p24967534	Bn-C5-p25639711	Bn-scaff_15969_1-p19115	C5	40	74.8	A
17096	Bn-N15-p27876235	Bn-C5-p27193178	Bn-scaff_22099_1-p60859	C5	40	74.8	A

17097	Bn-N15-p28958518	Bn-C5-p27910762	Bn-scaff_15648_1-p24578	C5	40	74.8	A
17098	Bn-N15-p29351053	Bn-A05-p14253804	Bn-A05-p14253804	C5	40	74.8	A
17099	Bn-N15-p30248036	Bn-C5-p28949066	Bn-scaff_15705_2-p73782	C5	40	74.8	A
17100	Bn-N15-p33357321	Bn-C5-p31906444	Bn-scaff_16818_1-p28332	C5	40	74.8	A
17101	Bn-N15-p33944731	Bn-C5-p32224954	Bn-scaff_19224_1-p29858	C5	40	74.8	A
17102	Bn-N15-p33944976	Bn-C5-p32225199	Bn-scaff_19224_1-p29613	C5	40	74.8	A
17103	Bn-N15-p34023223	Bn-Scaffold000409-p	Bn-Scaffold000409-p12061	C5	40	74.8	A
17104	Bn-N15-p34023986	Bn-C5-p32331722	Bn-scaff_18589_1-p76811	C5	40	74.8	A
17105	Bn-N15-p34024010	Bn-Scaffold000409-p	Bn-Scaffold000409-p11274	C5	40	74.8	A
17106	Bn-N18-p3963219	Bn-C8-p2995705	Bn-scaff_17807_1-p46285	C5	40	74.8	A
17107	Bn-N19-p34238278	Bn-C9-p32716516	Bn-scaff_17726_1-p46071	C5	40	74.8	A
17108	Bn-N5-p12804242	Bn-A05-p12867516	Bn-A05-p12867516	C5	40	74.8	A
17109	Bn-N5-p13052478	Bn-A05-p13098758	Bn-A05-p13098758	C5	40	74.8	A
17110	Bn-N5-p13423832	Bn-C5-p27466822	Bn-scaff_15648_1-p46851	C5	40	74.8	A
17111	Bn-N5-p13478231	Bn-A05-p13809267	Bn-A05-p13809267	C5	40	74.8	A
17112	Bn-N5-p14880812	Bn-A05-p14347292	Bn-A05-p14347292	C5	40	74.8	A
17113	Bn-N6-p8777357	Bn-A02-p14941595	Bn-A02-p14941595	C5	40	74.8	A
17114	Bn-N7-p3168349	Bn-C5-p26333005	Bn-scaff_15712_4-p50204	C5	40	74.8	A
17115	Bn-N9-p21837615	Bn-C5-p21529816	Bn-scaff_27050_1-p15024	C5	40	74.8	A
17116	Bn-N5-p16808652	Bn-A05-p16509283	Bn-A05-p16509283	C5	41	75.6	A
17117	Bn-N15-p34045905	Bn-C5-p32349790	Bn-scaff_18589_1-p94879	C5	42	76.4	A
17118	Bn-N15-p35139549	Bn-C5-p33507842	Bn-scaff_15695_2-p44895	C5	42	76.4	A
17119	Bn-N15-p35269811	Bn-C5-p33616353	Bn-scaff_18762_1-p59746	C5	42	76.4	A
17120	Bn-N15-p35781864	Bn-Scaffold01052-p2	Bn-scaff_19676_1-p2428	C5	42	76.4	A
17121	Bn-N15-p36409108	Bn-C5-p34272719	Bn-scaff_25491_1-p7540	C5	42	76.4	A
17122	Bn-N15-p36486274	Bn-C5-p34346809	Bn-scaff_25491_1-p81630	C5	42	76.4	A
17123	Bn-N15-p36557068	Bn-A05-p17442162	Bn-A05-p17442162	C5	42	76.4	A
17124	Bn-N15-p37050786	Bn-C5-p34900340	Bn-scaff_16263_1-p26046	C5	42	76.4	A
17125	Bn-N18-p22865650	Bn-C5-p33602621	Bn-scaff_18762_1-p73478	C5	42	76.4	A
17126	Bn-N5-p16779461	Bn-A05-p16477848	Bn-A05-p16477848	C5	42	76.4	A
17127	Bn-N5-p17595916	Bn-A05-p17108506	Bn-A05-p17108506	C5	42	76.4	A
17128	Bn-N5-p18843295	Bn-A05-p17830597	Bn-A05-p17830597	C5	42	76.4	A
17129	Bn-N15-p42180350	Bn-C5-p40472703	Bn-scaff_23408_1-p27292	C5	43	77.2	A
17130	Bn-N17-p14787539	Bn-C9-p12064049	Bn-scaff_15701_1-p10521	C5	43	77.2	A
17131	Bn-N19-p12253667	Bn-C4-p25796641	Bn-scaff_16625_1-p40853	C5	43	77.2	A
17132	Bn-N4-p407412	Bn-C5-p37278022	Bn-scaff_16770_1-p98279	C5	43	77.2	A
17133	Bn-N15-p40027257	Bn-C5-p38042588	Bn-scaff_16770_1-p21823	C5	44	78.9	A
17134	Bn-N15-p40027801	Bn-C5-p38043152	Bn-scaff_16770_1-p21766	C5	44	78.9	A
17135	Bn-N15-p40164773	Bn-C5-p38182976	Bn-scaff_16770_1-p77845	C5	44	78.9	A
17136	Bn-N15-p40584373	Bn-C5-p38719470	Bn-scaff_18826_1-p10379	C5	44	78.9	A
17137	Bn-N5-p14782981	Bn-Scaffold000390-p	Bn-Scaffold000390-p7718	C5	44	78.9	A
17138	Bn-N15-p40606936	Bn-C5-p38756827	Bn-scaff_18826_1-p10006	C5	45	79.7	A
17139	Bn-N15-p40754671	Bn-C5-p38895000	Bn-scaff_18826_1-p86243	C5	46	80.5	A
17140	Bn-N15-p40756846	Bn-C5-p38906181	Bn-scaff_18826_1-p85125	C5	46	80.5	A
17141	Bn-N15-p40768170	Bn-C5-p38919532	Bn-scaff_18826_1-p83790	C5	46	80.5	A
17142	Bn-N15-p40774124	Bn-C5-p38928177	Bn-scaff_18826_1-p82926	C5	46	80.5	A
17143	Bn-N15-p40781284	Bn-C5-p38930617	Bn-scaff_18826_1-p82682	C5	46	80.5	A
17144	Bn-N15-p40782160	Bn-C5-p38931385	Bn-scaff_18826_1-p82605	C5	46	80.5	A
17145	Bn-N15-p40782514	Bn-C5-p38931727	Bn-scaff_18826_1-p82571	C5	46	80.5	A

17146	Bn-N15-p40785191	Bn-C5-p38932351	Bn-scaff_18826_1-p825088	C5	46	80.5	A
17147	Bn-N15-p40789172	Bn-C5-p38934694	Bn-scaff_18826_1-p822745	C5	46	80.5	A
17148	Bn-N15-p40804122	Bn-C5-p38939191	Bn-scaff_18826_1-p818248	C5	46	80.5	A
17149	Bn-N15-p40819935	Bn-C5-p38948228	Bn-scaff_18826_1-p809211	C5	46	80.5	A
17150	Bn-N15-p40820441	Bn-C5-p38948555	Bn-scaff_18826_1-p808884	C5	46	80.5	A
17151	Bn-N13-p52508672	Bn-C5-p40011643	Bn-scaff_21124_1-p254105	C5	47	81.4	A
17152	Bn-N15-p40858129	Bn-C5-p38976125	Bn-scaff_18826_1-p781314	C5	47	81.4	A
17153	Bn-N15-p40862517	Bn-C5-p38980482	Bn-scaff_18826_1-p776957	C5	47	81.4	A
17154	Bn-N15-p41096119	Bn-C5-p39195649	Bn-scaff_18826_1-p561790	C5	47	81.4	A
17155	Bn-N15-p41173358	Bn-C5-p39287384	Bn-scaff_18826_1-p470055	C5	47	81.4	A
17156	Bn-N15-p41276501	Bn-C5-p39386403	Bn-scaff_18826_1-p371036	C5	47	81.4	A
17157	Bn-N15-p41423143	Bn-C5-p39492823	Bn-scaff_18826_1-p264616	C5	47	81.4	A
17158	Bn-N15-p41460871	Bn-C5-p39524866	Bn-scaff_18826_1-p232573	C5	47	81.4	A
17159	Bn-N15-p41579668	Bn-C5-p39659643	Bn-scaff_18826_1-p97796	C5	47	81.4	A
17160	Bn-N15-p41596839	Bn-C5-p39681541	Bn-scaff_18826_1-p75898	C5	47	81.4	A
17161	Bn-N15-p41597514	Bn-C5-p39682207	Bn-scaff_18826_1-p75232	C5	47	81.4	A
17162	Bn-N15-p41635680	Bn-C5-p39739994	Bn-scaff_18826_1-p17445	C5	47	81.4	A
17163	Bn-N15-p41694030	Bn-C5-p39932480	Bn-scaff_21124_1-p174942	C5	47	81.4	A
17164	Bn-N15-p41724790	Bn-C5-p39973522	Bn-scaff_21124_1-p215984	C5	47	81.4	A
17165	Bn-N15-p41725892	Bn-C5-p39974427	Bn-scaff_21124_1-p216889	C5	47	81.4	A
17166	Bn-N15-p41749822	Bn-C5-p40000143	Bn-scaff_21124_1-p242605	C5	47	81.4	A
17167	Bn-N15-p41749935	Bn-C5-p40000256	Bn-scaff_21124_1-p242718	C5	47	81.4	A
17168	Bn-N15-p41750526	Bn-C5-p40008035	Bn-scaff_21124_1-p250497	C5	47	81.4	A
17169	Bn-N15-p41753284	Bn-C5-p40011501	Bn-scaff_21124_1-p253963	C5	47	81.4	A
17170	Bn-N15-p41894670	Bn-C5-p40149982	Bn-scaff_21124_1-p392444	C5	47	81.4	A
17171	Bn-N15-p42026036	Bn-C5-p40296587	Bn-scaff_23408_1-p96806	C5	47	81.4	A
17172	Bn-N15-p42026241	Bn-C5-p40296757	Bn-scaff_23408_1-p96976	C5	47	81.4	A
17173	Bn-N15-p42026294	Bn-C5-p40296810	Bn-scaff_23408_1-p97029	C5	47	81.4	A
17174	Bn-N15-p42124264	Bn-C5-p40402543	Bn-scaff_23408_1-p202762	C5	47	81.4	A
17175	Bn-N15-p42126239	Bn-C5-p40404524	Bn-scaff_23408_1-p204743	C5	47	81.4	A
17176	Bn-N15-p42126242	Bn-C5-p40404527	Bn-scaff_23408_1-p204746	C5	47	81.4	A
17177	Bn-N15-p42127251	Bn-C5-p40405075	Bn-scaff_23408_1-p205294	C5	47	81.4	A
17178	Bn-N15-p42135774	Bn-C5-p40418632	Bn-scaff_23408_1-p218851	C5	47	81.4	A
17179	Bn-N15-p42154733	Bn-C5-p40443037	Bn-scaff_23408_1-p243256	C5	47	81.4	A
17180	Bn-N15-p42171845	Bn-C5-p40460006	Bn-scaff_23408_1-p260225	C5	47	81.4	A
17181	Bn-N15-p42178210	Bn-C5-p40471518	Bn-scaff_23408_1-p271737	C5	47	81.4	A
17182	Bn-N15-p42181102	Bn-C5-p40473455	Bn-scaff_23408_1-p273674	C5	47	81.4	A
17183	Bn-N15-p42199846	Bn-C5-p40491449	Bn-scaff_23408_1-p291668	C5	47	81.4	A
17184	Bn-N15-p42199898	Bn-C5-p40491500	Bn-scaff_23408_1-p291719	C5	47	81.4	A
17185	Bn-N15-p42203662	Bn-C5-p40493442	Bn-scaff_23408_1-p293661	C5	47	81.4	A
17186	Bn-N15-p42204450	Bn-C5-p40494232	Bn-scaff_23408_1-p294451	C5	47	81.4	A
17187	Bn-N15-p42212348	Bn-C5-p40497152	Bn-scaff_23408_1-p297371	C5	47	81.4	A
17188	Bn-N15-p42213859	Bn-C5-p40498562	Bn-scaff_23408_1-p298781	C5	47	81.4	A
17189	Bn-N15-p42214024	Bn-C5-p40498727	Bn-scaff_23408_1-p298946	C5	47	81.4	A
17190	Bn-N15-p42239111	Bn-C5-p40511571	Bn-scaff_21369_1-p126775	C5	47	81.4	A
17191	Bn-N15-p42240027	Bn-C5-p40512520	Bn-scaff_21369_1-p126680	C5	47	81.4	A
17192	Bn-N15-p42245115	Bn-C5-p40515741	Bn-scaff_21369_1-p126358	C5	47	81.4	A
17193	Bn-N15-p42245474	Bn-C5-p40516098	Bn-scaff_21369_1-p126323	C5	47	81.4	A
17194	Bn-N15-p42245663	Bn-C5-p40516293	Bn-scaff_21369_1-p126303	C5	47	81.4	A

17195	Bn-N15-p42330001	Bn-C5-p40527713	Bn-scaff_21369_1-p125161	C5	47	81.4	A
17196	Bn-N15-p42356495	Bn-C5-p40542130	Bn-scaff_21369_1-p123719	C5	47	81.4	A
17197	Bn-N15-p42356856	Bn-C5-p40542490	Bn-scaff_21369_1-p123683	C5	47	81.4	A
17198	Bn-N15-p42381930	Bn-C5-p40566422	Bn-scaff_21369_1-p121290	C5	47	81.4	A
17199	Bn-N15-p42382656	Bn-C5-p40567613	Bn-scaff_21369_1-p121171	C5	47	81.4	A
17200	Bn-N15-p42399398	Bn-C5-p40585446	Bn-scaff_21369_1-p119388	C5	47	81.4	A
17201	Bn-N17-p33255785	Bn-C5-p39977666	Bn-scaff_21124_1-p220128	C5	47	81.4	A
17202	Bn-Scaffold04597-p26	Bn-C5-p40529305	Bn-scaff_21369_1-p125002	C5	47	81.4	A
17203	Bn-Scaffold10591-p109	Bn-C5-p40297153	Bn-scaff_23408_1-p97372	C5	47	81.4	A
17204	Unknown	Bn-C5-p40010033	Bn-scaff_21124_1-p252495	C5	47	81.4	A
17205	Bn-N15-p43202247	Bn-C5-p41398445	Bn-scaff_21369_1-p380883	C5	48	84	A
17206	Bn-N15-p43474159	Bn-C5-p41676458	Bn-scaff_21369_1-p102870	C5	48	84	A
17207	Bn-N5-p22366288	Bn-A05-p21326775	Bn-A05-p21326775	C5	49	85.7	A
17208	Bn-N15-p44281647	Bn-C5-p42447050	Bn-scaff_17441_1-p400751	C5	50	90	A
17209	Bn-N15-p44451287	Bn-C5-p42616169	Bn-scaff_17441_1-p569870	C5	51	91.7	A
17210	Bn-N15-p44461286	Bn-C5-p42619917	Bn-scaff_17441_1-p573618	C5	51	91.7	A
17211	Bn-N15-p44462703	Bn-C5-p42621337	Bn-scaff_17441_1-p575038	C5	51	91.7	A
17212	Bn-N15-p44730765	Bn-C5-p42877318	Bn-scaff_17441_1-p831019	C5	52	93.4	A
17213	Bn-N15-p44959556	Bn-C5-p43091364	Bn-scaff_17441_1-p104506	C5	53	96	A
17214	Bn-N15-p45002883	Bn-C5-p43140924	Bn-scaff_17441_1-p109462	C5	54	96.9	A
17215	Bn-N15-p45051720	Bn-C5-p43192758	Bn-scaff_17441_1-p114645	C5	55	97.8	A
17216	Bn-N15-p45051725	Bn-C5-p43192763	Bn-scaff_17441_1-p114646	C5	55	97.8	A
17217	Bn-N15-p45097379	Bn-C5-p43244070	Bn-scaff_17441_1-p119777	C5	55	97.8	A
17218	Bn-N15-p45110303	Bn-C5-p43257952	Bn-scaff_17441_1-p121165	C5	55	97.8	A
17219	Bn-N15-p45153698	Bn-C5-p43293633	Bn-scaff_17441_3-p28950	C5	55	97.8	A
17220	Bn-N15-p45156044	Bn-C5-p43295979	Bn-scaff_17441_3-p31296	C5	55	97.8	A
17221	Bn-N15-p45246777	Bn-C7-p46793910	Bn-scaff_16110_1-p153390	C5	55	97.8	A
17222	Bn-N15-p45264309	Bn-C5-p43408775	Bn-scaff_17441_3-p144092	C5	55	97.8	A
17223	Bn-N15-p45274243	Bn-C5-p43418714	Bn-scaff_17441_3-p154031	C5	56	99.5	A
17224	Bn-N15-p45276903	Bn-C5-p43421374	Bn-scaff_17441_3-p156691	C5	56	99.5	A
17225	Bn-N15-p45286728	Bn-C5-p43430640	Bn-scaff_17441_3-p165957	C5	56	99.5	A
17226	Bn-N15-p45298286	Bn-C5-p43447851	Bn-scaff_17441_3-p183168	C5	56	99.5	A
17227	Bn-N5-p23545128	Bn-C5-p43439212	Bn-scaff_17441_3-p174529	C5	56	99.5	A
17228	Bn-N15-p45401184	Bn-C5-p43590291	Bn-scaff_17441_3-p325608	C5	57	102	A
17229	Bn-N15-p45493170	Bn-C5-p43663437	Bn-scaff_17441_3-p398754	C5	57	102	A
17230	Bn-N15-p45574314	Bn-Scaffold01223-p9	Bn-scaff_29028_1-p9948	C5	57	102	0
17231	Bn-N15-p45577256	Bn-Scaffold01223-p1	Bn-scaff_29028_1-p11999	C5	57	102	A
17232	Bn-N15-p45581218	Bn-Scaffold01223-p1	Bn-scaff_29028_1-p15505	C5	57	102	A
17233	Bn-N15-p45584381	Bn-Scaffold01223-p1	Bn-scaff_29028_1-p18552	C5	57	102	A
17234	Bn-N15-p45592595	Bn-Scaffold03352-p9	Bn-scaff_18022_1-p991	C5	57	102	A
17235	Bn-N15-p45596602	Bn-Scaffold01895-p3	Bn-scaff_19134_1-p3309	C5	57	102	A
17236	Bn-N15-p45654698	Bn-C5-p43777858	Bn-scaff_20219_1-p52642	C5	57	102	A
17237	Bn-N15-p45655924	Bn-C5-p43779089	Bn-scaff_20219_1-p53873	C5	57	102	A
17238	Bn-N15-p45690850	Bn-C5-p43853330	Bn-scaff_20219_1-p128114	C5	57	102	A
17239	Bn-N15-p45711204	Bn-C5-p43873789	Bn-scaff_20219_1-p148573	C5	57	102	A
17240	Bn-N15-p45713405	Bn-C5-p43876003	Bn-scaff_20219_1-p150787	C5	57	102	A
17241	Bn-N15-p45782711	Bn-C5-p43945444	Bn-scaff_20219_1-p220228	C5	57	102	A
17242	Bn-N5-p24193947	Bn-A05-p23102053	Bn-A05-p23102053	C5	58	106.1	A
17243	Bn-N15-p46312622	Bn-C5-p44529363	Bn-scaff_23186_1-p121500	C5	59	108.6	A

17244	Bn-N15-p46323012	Bn-C5-p44539746	Bn-scaff_23186_1-p111117	C5	59	108.6	A
17245	Bn-N15-p46329966	Bn-C5-p44548511	Bn-scaff_23186_1-p102352	C5	59	108.6	A
17246	Bn-N15-p46330126	Bn-C5-p44548671	Bn-scaff_23186_1-p102192	C5	59	108.6	A
17247	Bn-N15-p46330933	Bn-C5-p44549478	Bn-scaff_23186_1-p101385	C5	59	108.6	A
17248	Bn-N15-p46331224	Bn-C5-p44549769	Bn-scaff_23186_1-p101094	C5	59	108.6	A
17249	Bn-N15-p46452855	Bn-Scaffold00944-p4	Bn-scaff_16526_1-p42180	C5	59	108.6	A
17250	Bn-Scaffold04778-p477	Bn-C5-p44642089	Bn-scaff_23186_1-p8774	C5	59	108.6	A
17251	Bn-N15-p46521594	Bn-C5-p44682176	Bn-scaff_20270_1-p104830	C5	60	111.9	A
17252	Bn-N15-p46540304	Bn-C5-p44704155	Bn-scaff_20270_1-p107028	C5	60	111.9	A
17253	Bn-N15-p46664287	Bn-C5-p44842156	Bn-scaff_20270_1-p120828	C5	60	111.9	A
17254	Bn-N15-p46667298	Bn-C5-p44845361	Bn-scaff_20270_1-p121149	C5	60	111.9	A
17255	Bn-N15-p46667440	Bn-C5-p44845502	Bn-scaff_20270_1-p121163	C5	60	111.9	A
17256	Bn-N15-p46773853	Bn-C5-p44957070	Bn-scaff_20270_1-p132320	C5	60	111.9	A
17257	Bn-N15-p46774545	Bn-C5-p44957762	Bn-scaff_20270_1-p132389	C5	60	111.9	A
17258	Bn-N15-p46774915	Bn-C5-p44958133	Bn-scaff_20270_1-p132426	C5	60	111.9	A
17259	Bn-N15-p46775034	Bn-C5-p44958262	Bn-scaff_20270_1-p132439	C5	60	111.9	A
17260	Bn-N15-p46775096	Bn-C5-p44958324	Bn-scaff_20270_1-p132445	C5	60	111.9	A
17261	Bn-N15-p46775125	Bn-C5-p44958353	Bn-scaff_20270_1-p132448	C5	60	111.9	A
17262	Bn-N15-p46778809	Bn-C5-p44966276	Bn-scaff_20270_1-p133240	C5	60	111.9	A
17263	Bn-N15-p46778915	Bn-C5-p44966399	Bn-scaff_20270_1-p133252	C5	60	111.9	A
17264	Bn-N15-p46779895	Bn-C5-p44967395	Bn-scaff_20270_1-p133352	C5	60	111.9	A
17265	Bn-N15-p46779948	Bn-C5-p44967448	Bn-scaff_20270_1-p133357	C5	60	111.9	A
17266	Bn-N15-p46878841	Bn-C5-p45068199	Bn-scaff_20270_1-p143432	C5	60	111.9	A
17267	Bn-N15-p46890058	Bn-C5-p45074869	Bn-scaff_20270_1-p144099	C5	60	111.9	A
17268	Bn-N15-p46895185	Bn-C5-p45080007	Bn-scaff_20270_1-p144613	C5	60	111.9	A
17269	Bn-N15-p47239484	Bn-C5-p45479754	Bn-scaff_20376_1-p336606	C5	61	118.6	B
17270	Bn-N15-p47247220	Bn-C5-p45482267	Bn-scaff_20376_1-p339119	C5	61	118.6	B
17271	Bn-N15-p47395762	Bn-Scaffold000191-p4	Bn-Scaffold000191-p47497	C5	62	119.4	B
17272	Bn-N15-p47396521	Bn-C5-p45603880	Bn-scaff_15676_1-p92150	C5	62	119.4	B
17273	Bn-N5-p25032882	Bn-Scaffold000191-p5	Bn-Scaffold000191-p54987	C5	62	119.4	B
17274	Bn-N15-p47567740	Bn-C5-p45810409	Bn-scaff_15676_1-p298679	C5	63	121.9	B
17275	Bn-N15-p47606924	Bn-C5-p45853238	Bn-scaff_15676_1-p341508	C5	63	121.9	B
17276	Bn-N15-p47608007	Bn-C5-p45854218	Bn-scaff_15676_1-p342488	C5	63	121.9	B
17277	Bn-N15-p47816877	Bn-C5-p46075640	Bn-scaff_21634_1-p19901	C5	63	121.9	B
17278	Bn-N15-p47823236	Bn-C5-p46081705	Bn-scaff_21634_1-p25966	C5	63	121.9	B
17279	Bn-N15-p48099448	Bn-C5-p46328665	Bn-scaff_24251_1-p65315	C5	64	123.6	B
17280	Bn-N15-p48107618	Bn-C5-p46336013	Bn-scaff_24251_1-p57967	C5	64	123.6	B
17281	Bn-N15-p48109457	Bn-C5-p46337851	Bn-scaff_24251_1-p56129	C5	64	123.6	B
17282	Bn-N15-p48268203	Bn-C5-p46513474	Bn-scaff_15856_1-p119395	C5	65	124.4	B
17283	Bn-N15-p48278032	Bn-C5-p46523272	Bn-scaff_15856_1-p129193	C5	65	124.4	B
17284	Bn-N2-p11049107	Bn-A05-p23928643	Bn-A05-p23928643	C5	65	124.4	B
17285	Bn-N5-p25534920	Bn-C5-p46472983	Bn-scaff_15856_1-p78904	C5	65	124.4	B
17286	Bn-N5-p25536705	Bn-C5-p46474769	Bn-scaff_15856_1-p80690	C5	65	124.4	B
17287	Bn-N5-p25695378	Bn-C5-p46825911	Bn-scaff_24641_1-p76675	C5	65	124.4	B
17288	Bn-Scaffold01727-p3326	Bn-C5-p46739370	Bn-scaff_20809_1-p64287	C5	65	124.4	B
17289	Bn-N16-p37379113	Bn-C6-p1757213	Bn-scaff_17799_1-p118098	C6	1	0	B
17290	Bn-N16-p37381701	Bn-C6-p1754625	Bn-scaff_17799_1-p117839	C6	1	0	B
17291	Bn-N16-p37400006	Bn-C6-p1741522	Bn-scaff_17799_1-p116529	C6	1	0	B
17292	Bn-N16-p37411388	Bn-C6-p1729175	Bn-scaff_17799_1-p115294	C6	1	0	B

17293	Bn-N16-p37478151	Bn-C6-p1646773	Bn-scaff_17799_1-p10705	C6	1	0	B
17294	Bn-N16-p37264967	Bn-C6-p1869417	Bn-scaff_17799_1-p12931	C6	2	1	B
17295	Bn-N16-p37278150	Bn-C6-p1854873	Bn-scaff_17799_1-p12786	C6	2	1	B
17296	Bn-N16-p37226193	Bn-C8-p138535	Bn-scaff_18503_1-p17982	C6	3	2.9	B
17297	Bn-N16-p37226753	Bn-A07-p20952117	Bn-A07-p20952117	C6	3	2.9	0
17298	Bn-N16-p37234103	Bn-C6-p1898891	Bn-scaff_17799_1-p13226	C6	3	2.9	B
17299	Bn-N16-p37027942	Bn-C6-p2065308	Bn-scaff_17799_1-p14890	C6	4	7.6	A
17300	Bn-N16-p37078831	Bn-C6-p2012564	Bn-scaff_17799_1-p14363	C6	4	7.6	A
17301	Bn-N16-p37117974	Bn-C6-p1975362	Bn-scaff_17799_1-p13991	C6	4	7.6	A
17302	Bn-N16-p37148391	Bn-C6-p1944065	Bn-scaff_17799_1-p13678	C6	4	7.6	A
17303	Bn-N16-p36961661	Bn-C6-p2132472	Bn-scaff_17799_1-p15562	C6	5	9.5	A
17304	Bn-N16-p36969033	Bn-C6-p2128581	Bn-scaff_17799_1-p15523	C6	5	9.5	A
17305	Bn-Scaffold35958-p94	Bn-C6-p2073633	Bn-scaff_17799_1-p14974	C6	5	9.5	A
17306	Bn-N16-p36892483	Bn-C6-p2206123	Bn-scaff_17799_1-p16298	C6	6	10.4	A
17307	Bn-N16-p36836129	Bn-C6-p2264074	Bn-scaff_17799_1-p16878	C6	7	12.3	A
17308	Bn-N16-p36841519	Bn-C6-p2259256	Bn-scaff_17799_1-p16830	C6	7	12.3	A
17309	Bn-N16-p36862481	Bn-C6-p2238419	Bn-scaff_17799_1-p16621	C6	7	12.3	A
17310	Bn-N16-p36864678	Bn-C6-p2235196	Bn-scaff_17799_1-p16589	C6	7	12.3	A
17311	Bn-N16-p36426332	Bn-C6-p2673885	Bn-scaff_17799_1-p20976	C6	8	13.2	A
17312	Bn-N16-p36459858	Bn-C6-p2655690	Bn-scaff_17799_1-p20794	C6	8	13.2	A
17313	Bn-N16-p36460086	Bn-C6-p2655455	Bn-scaff_17799_1-p20792	C6	8	13.2	A
17314	Bn-N16-p36461998	Bn-C6-p2648683	Bn-scaff_17799_1-p20724	C6	8	13.2	A
17315	Bn-N16-p36464799	Bn-C6-p2646816	Bn-scaff_17799_1-p20705	C6	8	13.2	A
17316	Bn-N16-p36571755	Bn-C6-p2537226	Bn-scaff_17799_1-p19609	C6	8	13.2	A
17317	Bn-N16-p36571872	Bn-C6-p2537108	Bn-scaff_17799_1-p19608	C6	8	13.2	A
17318	Bn-N16-p36576679	Bn-C6-p2530307	Bn-scaff_17799_1-p19540	C6	8	13.2	A
17319	Bn-N16-p36577713	Bn-C6-p2529274	Bn-scaff_17799_1-p19530	C6	8	13.2	A
17320	Bn-N16-p36577835	Bn-C6-p2529151	Bn-scaff_17799_1-p19529	C6	8	13.2	A
17321	Bn-N16-p36578522	Bn-C6-p2528465	Bn-scaff_17799_1-p19522	C6	8	13.2	A
17322	Bn-N16-p36579597	Bn-C6-p2527410	Bn-scaff_17799_1-p19511	C6	8	13.2	A
17323	Bn-N16-p36580980	Bn-C6-p2523692	Bn-scaff_17799_1-p19474	C6	8	13.2	A
17324	Bn-N16-p36581043	Bn-C6-p2523629	Bn-scaff_17799_1-p19473	C6	8	13.2	A
17325	Bn-N16-p36581844	Bn-C6-p2522828	Bn-scaff_17799_1-p19465	C6	8	13.2	A
17326	Bn-N16-p36582062	Bn-C6-p2522610	Bn-scaff_17799_1-p19463	C6	8	13.2	A
17327	Bn-N16-p36582108	Bn-C6-p2522564	Bn-scaff_17799_1-p19463	C6	8	13.2	A
17328	Bn-N7-p23409205	Bn-C6-p2492581	Bn-scaff_17799_1-p19163	C6	8	13.2	A
17329	Bn-N16-p36396201	Bn-C6-p2701748	Bn-scaff_17799_1-p21255	C6	9	14.1	A
17330	Bn-N16-p36107127	Bn-C6-p2939450	Bn-scaff_17799_1-p23632	C6	10	15	A
17331	Bn-N16-p36109202	Bn-C6-p2935044	Bn-scaff_17799_1-p23588	C6	10	15	A
17332	Bn-N16-p36110171	Bn-C6-p2934075	Bn-scaff_17799_1-p23578	C6	10	15	A
17333	Bn-N16-p36123758	Bn-C6-p2923022	Bn-scaff_17799_1-p23467	C6	10	15	A
17334	Bn-N16-p36124060	Bn-C6-p2922703	Bn-scaff_17799_1-p23464	C6	10	15	A
17335	Bn-N16-p36129018	Bn-C6-p2920369	Bn-scaff_17799_1-p23441	C6	10	15	A
17336	Bn-N16-p36179856	Bn-C6-p2876973	Bn-scaff_17799_1-p23007	C6	10	15	A
17337	Bn-N16-p36205249	Bn-C6-p2860461	Bn-scaff_17799_1-p22842	C6	10	15	A
17338	Bn-N16-p36205344	Bn-C6-p2860366	Bn-scaff_17799_1-p22841	C6	10	15	A
17339	Bn-N16-p36207990	Bn-C6-p2855077	Bn-scaff_17799_1-p22788	C6	10	15	A
17340	Bn-N16-p36208279	Bn-C6-p2854788	Bn-scaff_17799_1-p22785	C6	10	15	A
17341	Bn-N16-p24384425	Bn-C3-p893601	Bn-scaff_16614_1-p13356	C6	11	15.9	A

17342	Bn-N16-p35542405	Bn-C6-p3542831	Bn-scaff_20294_1-p35584[illegible]	C6	11	15.9	A
17343	Bn-N16-p35653973	Bn-C6-p3423197	Bn-scaff_20294_1-p23621[illegible]	C6	11	15.9	A
17344	Bn-N16-p35833910	Bn-C8-p39357201	Bn-scaff_20947_1-p32721[illegible]	C6	11	15.9	A
17345	Bn-N16-p35856689	Bn-C8-p39383516	Bn-scaff_20947_1-p30089[illegible]	C6	11	15.9	A
17346	Bn-N16-p35856785	Bn-C8-p39383612	Bn-scaff_20947_1-p30080[illegible]	C6	11	15.9	A
17347	Bn-N16-p35856786	Bn-C8-p39383613	Bn-scaff_20947_1-p30079[illegible]	C6	11	15.9	A
17348	Bn-N16-p35858490	Bn-C8-p39385333	Bn-scaff_20947_1-p29907[illegible]	C6	11	15.9	A
17349	Bn-N16-p35863396	Bn-C6-p3217147	Bn-scaff_20294_1-p30161	C6	11	15.9	A
17350	Bn-N16-p35980032	Bn-C6-p3073254	Bn-scaff_17799_1-p24970[illegible]	C6	11	15.9	A
17351	Bn-N16-p35981416	Bn-C6-p3071869	Bn-scaff_17799_1-p24956[illegible]	C6	11	15.9	A
17352	Bn-N16-p35982250	Bn-C6-p3071036	Bn-scaff_17799_1-p24948[illegible]	C6	11	15.9	A
17353	Bn-N16-p36057997	Bn-C6-p3003102	Bn-scaff_17799_1-p24268[illegible]	C6	11	15.9	A
17354	Bn-N16-p36088321	Bn-C6-p2967479	Bn-scaff_17799_1-p23912[illegible]	C6	11	15.9	A
17355	Bn-N16-p36088396	Bn-C6-p2967403	Bn-scaff_17799_1-p23911[illegible]	C6	11	15.9	A
17356	Bn-N16-p36092931	Bn-C6-p2957110	Bn-scaff_17799_1-p23808[illegible]	C6	11	15.9	A
17357	Bn-N16-p36093017	Bn-C6-p2957024	Bn-scaff_17799_1-p23807[illegible]	C6	11	15.9	A
17358	Bn-N16-p36097989	Bn-C6-p2951747	Bn-scaff_17799_1-p23755[illegible]	C6	11	15.9	A
17359	Bn-N16-p36101888	Bn-C6-p2946481	Bn-scaff_17799_1-p23702[illegible]	C6	11	15.9	A
17360	Bn-N16-p34731578	Bn-C6-p4212013	Bn-scaff_16397_1-p16560[illegible]	C6	12	18.4	A
17361	Bn-N16-p34805078	Bn-C6-p4148970	Bn-scaff_16397_1-p10256[illegible]	C6	12	18.4	A
17362	Bn-N16-p34805288	Bn-C6-p4148760	Bn-scaff_16397_1-p10235[illegible]	C6	12	18.4	A
17363	Bn-N16-p34806338	Bn-C6-p4147713	Bn-scaff_16397_1-p10130[illegible]	C6	12	18.4	A
17364	Bn-N16-p34806408	Bn-C6-p4147644	Bn-scaff_16397_1-p10124[illegible]	C6	12	18.4	A
17365	Bn-N16-p34807656	Bn-C6-p4146350	Bn-scaff_16397_1-p99946	C6	12	18.4	A
17366	Bn-N16-p34877783	Bn-C6-p4079018	Bn-scaff_16397_1-p32614	C6	12	18.4	A
17367	Bn-N16-p34891914	Bn-C6-p4069182	Bn-scaff_16397_1-p22778	C6	12	18.4	A
17368	Bn-N16-p34891916	Bn-C6-p4069180	Bn-scaff_16397_1-p22776	C6	12	18.4	A
17369	Bn-N16-p34892731	Bn-C6-p4068365	Bn-scaff_16397_1-p21961	C6	12	18.4	A
17370	Bn-N16-p34978840	Bn-C6-p3981943	Bn-scaff_25466_1-p22109[illegible]	C6	12	18.4	A
17371	Bn-N16-p34978995	Bn-C6-p3981788	Bn-scaff_25466_1-p22094[illegible]	C6	12	18.4	A
17372	Bn-N16-p35064155	Bn-C6-p3916820	Bn-scaff_25466_1-p15597[illegible]	C6	12	18.4	A
17373	Bn-N16-p35139350	Bn-C6-p3835667	Bn-scaff_25466_1-p74820	C6	12	18.4	A
17374	Bn-N16-p35181258	Bn-C6-p3794685	Bn-scaff_25466_1-p33838	C6	12	18.4	A
17375	Bn-N16-p35189312	Bn-C6-p3790193	Bn-scaff_25466_1-p29346	C6	12	18.4	A
17376	Bn-N16-p35189536	Bn-C6-p3789968	Bn-scaff_25466_1-p29121	C6	12	18.4	A
17377	Bn-N16-p35196824	Bn-C6-p3776436	Bn-scaff_25466_1-p15589	C6	12	18.4	A
17378	Bn-N16-p35197051	Bn-C6-p3776209	Bn-scaff_25466_1-p15362	C6	12	18.4	A
17379	Bn-N16-p35197325	Bn-C6-p3775935	Bn-scaff_25466_1-p15088	C6	12	18.4	A
17380	Bn-N16-p35197956	Bn-C6-p3775263	Bn-scaff_25466_1-p14416	C6	12	18.4	A
17381	Bn-N16-p35198348	Bn-C6-p3774871	Bn-scaff_25466_1-p14024	C6	12	18.4	A
17382	Bn-N16-p35198946	Bn-C6-p3774273	Bn-scaff_25466_1-p13426	C6	12	18.4	A
17383	Bn-N16-p35199215	Bn-C6-p3774004	Bn-scaff_25466_1-p13157	C6	12	18.4	A
17384	Bn-N16-p35199270	Bn-C6-p3773949	Bn-scaff_25466_1-p13102	C6	12	18.4	A
17385	Bn-N16-p35199676	Bn-C6-p3773549	Bn-scaff_25466_1-p12702	C6	12	18.4	A
17386	Bn-N16-p35199751	Bn-C6-p3773475	Bn-scaff_25466_1-p12628	C6	12	18.4	A
17387	Bn-N16-p35199801	Bn-C6-p3773425	Bn-scaff_25466_1-p12578	C6	12	18.4	A
17388	Bn-N16-p35200337	Bn-C6-p3772889	Bn-scaff_25466_1-p12042	C6	12	18.4	A
17389	Bn-N16-p35200531	Bn-C6-p3772695	Bn-scaff_25466_1-p11848	C6	12	18.4	A
17390	Bn-N16-p35202969	Bn-C6-p3770257	Bn-scaff_25466_1-p9410	C6	12	18.4	A

17391	Bn-N16-p35203580	Bn-C6-p3769647	Bn-scaff_25466_1-p8800	C6	12	18.4	A
17392	Bn-N16-p35221748	Bn-Scaffold00722-p19	Bn-scaff_19937_1-p19550	C6	12	18.4	A
17393	Bn-N13-p56022446	Bn-C6-p4669299	Bn-scaff_16397_1-p622895	C6	13	20.4	A
17394	Bn-N16-p34266718	Bn-C6-p4683276	Bn-scaff_16397_1-p636872	C6	13	20.4	A
17395	Bn-N16-p34271704	Bn-C6-p4679242	Bn-scaff_16397_1-p632838	C6	13	20.4	A
17396	Bn-N16-p34275981	Bn-C6-p4673178	Bn-scaff_16397_1-p626774	C6	13	20.4	A
17397	Bn-N16-p34278502	Bn-C6-p4672841	Bn-scaff_16397_1-p626437	C6	13	20.4	A
17398	Bn-N16-p34279147	Bn-C6-p4672196	Bn-scaff_16397_1-p625792	C6	13	20.4	A
17399	Bn-N16-p34314910	Bn-C6-p4653774	Bn-scaff_16397_1-p607370	C6	13	20.4	A
17400	Bn-N16-p34321442	Bn-C6-p4644500	Bn-scaff_16397_1-p598096	C6	13	20.4	A
17401	Bn-N16-p34321551	Bn-C6-p4644390	Bn-scaff_16397_1-p597986	C6	13	20.4	A
17402	Bn-N16-p34322327	Bn-C6-p4643614	Bn-scaff_16397_1-p597210	C6	13	20.4	A
17403	Bn-N16-p34325308	Bn-C6-p4630755	Bn-scaff_16397_1-p584351	C6	13	20.4	A
17404	Bn-N16-p34363788	Bn-C6-p4614793	Bn-scaff_16397_1-p568389	C6	13	20.4	A
17405	Bn-N16-p34363874	Bn-C6-p4614707	Bn-scaff_16397_1-p568303	C6	13	20.4	A
17406	Bn-N16-p34365778	Bn-C6-p4614642	Bn-scaff_16397_1-p568238	C6	13	20.4	A
17407	Bn-N16-p34365814	Bn-C6-p4614606	Bn-scaff_16397_1-p568202	C6	13	20.4	A
17408	Bn-N16-p34365883	Bn-C6-p4614537	Bn-scaff_16397_1-p568133	C6	13	20.4	A
17409	Bn-N16-p34366619	Bn-C6-p4613787	Bn-scaff_16397_1-p567383	C6	13	20.4	A
17410	Bn-N16-p34379635	Bn-C6-p4607533	Bn-scaff_16397_1-p561129	C6	13	20.4	A
17411	Bn-N16-p34380443	Bn-C6-p4606676	Bn-scaff_16397_1-p560272	C6	13	20.4	A
17412	Bn-N16-p34391990	Bn-C6-p4593725	Bn-scaff_16397_1-p547321	C6	13	20.4	A
17413	Bn-N16-p34394987	Bn-C6-p4598057	Bn-scaff_16397_1-p551653	C6	13	20.4	A
17414	Bn-N16-p34399704	Bn-C6-p4589279	Bn-scaff_16397_1-p542875	C6	13	20.4	A
17415	Bn-N16-p34402313	Bn-C6-p4588747	Bn-scaff_16397_1-p542343	C6	13	20.4	A
17416	Bn-N16-p34430583	Bn-C6-p4561340	Bn-scaff_16397_1-p514936	C6	13	20.4	A
17417	Bn-N16-p34431758	Bn-C6-p4560164	Bn-scaff_16397_1-p513760	C6	13	20.4	A
17418	Bn-N16-p34432497	Bn-C6-p4559425	Bn-scaff_16397_1-p513021	C6	13	20.4	A
17419	Bn-N16-p34433129	Bn-C6-p4558793	Bn-scaff_16397_1-p512389	C6	13	20.4	A
17420	Bn-N16-p34433811	Bn-C6-p4558111	Bn-scaff_16397_1-p511707	C6	13	20.4	A
17421	Bn-N7-p21871184	Bn-C6-p4648594	Bn-scaff_16397_1-p602190	C6	13	20.4	A
17422	Bn-Scaffold04730-p1550	Bn-C6-p4667680	Bn-scaff_16397_1-p621276	C6	13	20.4	A
17423	Bn-N12-p38695869	Bn-C6-p4731663	Bn-scaff_16397_1-p685259	C6	14	21.3	A
17424	Bn-N13-p30264654	Bn-C4-p44674985	Bn-scaff_20079_1-p68503	C6	14	21.3	A
17425	Bn-N14-p53435181	Bn-Scaffold15792-p99	Bn-scaff_20041_1-p99	C6	14	21.3	A
17426	Bn-N16-p33058389	Bn-C6-p5764339	Bn-scaff_23957_1-p432810	C6	14	21.3	A
17427	Bn-N16-p33074879	Bn-C6-p5748601	Bn-scaff_23957_1-p448548	C6	14	21.3	A
17428	Bn-N16-p33104039	Bn-C6-p5716501	Bn-scaff_23957_1-p480648	C6	14	21.3	A
17429	Bn-N16-p33113386	Bn-C6-p5708291	Bn-scaff_23957_1-p488858	C6	14	21.3	A
17430	Bn-N16-p33228385	Bn-C6-p5603145	Bn-scaff_23957_1-p594004	C6	14	21.3	A
17431	Bn-N16-p33247608	Bn-C6-p5582306	Bn-scaff_23957_1-p614843	C6	14	21.3	A
17432	Bn-N16-p33287593	Bn-C6-p5538880	Bn-scaff_23957_1-p658269	C6	14	21.3	A
17433	Bn-N16-p33296645	Bn-C6-p5531291	Bn-scaff_23957_1-p665858	C6	14	21.3	A
17434	Bn-N16-p33309147	Bn-C6-p5520593	Bn-scaff_23957_1-p676556	C6	14	21.3	A
17435	Bn-N16-p33321250	Bn-C6-p5508303	Bn-scaff_23957_1-p688846	C6	14	21.3	A
17436	Bn-N16-p33375246	Bn-C6-p5450449	Bn-scaff_16874_1-p41770	C6	14	21.3	A
17437	Bn-N16-p33515781	Bn-C6-p5319778	Bn-scaff_16874_1-p172441	C6	14	21.3	A
17438	Bn-N16-p33516652	Bn-C6-p5318907	Bn-scaff_16874_1-p173312	C6	14	21.3	A
17439	Bn-N16-p33516884	Bn-C6-p5318675	Bn-scaff_16874_1-p173544	C6	14	21.3	A

17440	Bn-N16-p33635183	Bn-C6-p5207050	Bn-scaff_16874_1-p28516	C6	14	21.3	A
17441	Bn-N16-p33641000	Bn-C6-p5200159	Bn-scaff_16874_1-p29206	C6	14	21.3	A
17442	Bn-N16-p33678195	Bn-C6-p5166880	Bn-scaff_16874_1-p32533	C6	14	21.3	A
17443	Bn-N16-p33678484	Bn-C6-p5166587	Bn-scaff_16874_1-p32563	C6	14	21.3	A
17444	Bn-N16-p33777516	Bn-C6-p5080628	Bn-scaff_16874_1-p41159	C6	14	21.3	A
17445	Bn-N16-p33791331	Bn-C6-p5066818	Bn-scaff_16874_1-p42540	C6	14	21.3	A
17446	Bn-N16-p33973257	Bn-C6-p4875301	Bn-scaff_16874_1-p61691	C6	14	21.3	A
17447	Bn-N16-p33989464	Bn-C4-p15786184	Bn-scaff_18505_1-p29553	C6	14	21.3	A
17448	Bn-N16-p34003814	Bn-C6-p4847689	Bn-scaff_16874_1-p64453	C6	14	21.3	A
17449	Bn-N16-p34077984	Bn-Scaffold00877-p3	Bn-scaff_16397_1-p80914	C6	14	21.3	A
17450	Bn-N16-p34078056	Bn-Scaffold00877-p3	Bn-scaff_16397_1-p80907	C6	14	21.3	A
17451	Bn-N16-p34080981	Bn-Scaffold00877-p3	Bn-scaff_16397_1-p80571	C6	14	21.3	A
17452	Bn-N16-p34175832	Bn-C6-p4768703	Bn-scaff_16397_1-p72229	C6	14	21.3	A
17453	Bn-N16-p34189362	Bn-C6-p4756815	Bn-scaff_16397_1-p71041	C6	14	21.3	A
17454	Bn-N16-p34221929	Bn-C6-p4731246	Bn-scaff_16397_1-p68484	C6	14	21.3	A
17455	Bn-N16-p34222052	Bn-C6-p4731122	Bn-scaff_16397_1-p68471	C6	14	21.3	A
17456	Bn-N16-p34222226	Bn-C6-p4730945	Bn-scaff_16397_1-p68454	C6	14	21.3	A
17457	Bn-N16-p34223082	Bn-C6-p4723869	Bn-scaff_16397_1-p67746	C6	14	21.3	A
17458	Bn-N16-p34228682	Bn-C6-p4723099	Bn-scaff_16397_1-p67669	C6	14	21.3	A
17459	Bn-N16-p34228800	Bn-C6-p4722981	Bn-scaff_16397_1-p67657	C6	14	21.3	A
17460	Bn-N16-p34230055	Bn-C6-p4721703	Bn-scaff_16397_1-p67529	C6	14	21.3	A
17461	Bn-N16-p34231159	Bn-C6-p4715888	Bn-scaff_16397_1-p66948	C6	14	21.3	A
17462	Bn-N16-p34231736	Bn-C6-p4715327	Bn-scaff_16397_1-p66892	C6	14	21.3	A
17463	Bn-N16-p34231867	Bn-C6-p4715180	Bn-scaff_16397_1-p66877	C6	14	21.3	A
17464	Bn-Scaffold31219-p228	Bn-C6-p4689586	Bn-scaff_16397_1-p64318	C6	14	21.3	A
17465	Bn-N16-p32865132	Bn-C6-p5954272	Bn-scaff_23957_1-p24287	C6	15	22.2	A
17466	Bn-N16-p32870253	Bn-C6-p5949689	Bn-scaff_23957_1-p24746	C6	15	22.2	A
17467	Bn-N16-p32870321	Bn-C6-p5949621	Bn-scaff_23957_1-p24752	C6	15	22.2	A
17468	Bn-N16-p32875155	Bn-C6-p5944743	Bn-scaff_23957_1-p25240	C6	15	22.2	A
17469	Bn-N16-p32879378	Bn-C6-p5939294	Bn-scaff_23957_1-p25785	C6	15	22.2	A
17470	Bn-N16-p32879591	Bn-C6-p5939081	Bn-scaff_23957_1-p25806	C6	15	22.2	A
17471	Bn-N16-p32976736	Bn-C6-p5850163	Bn-scaff_23957_1-p34698	C6	15	22.2	A
17472	Bn-N16-p32988742	Bn-C6-p5835757	Bn-scaff_23957_1-p36139	C6	15	22.2	A
17473	Bn-N16-p32991253	Bn-C6-p5833245	Bn-scaff_23957_1-p36390	C6	15	22.2	A
17474	Bn-N16-p33014710	Bn-C6-p5805284	Bn-scaff_23957_1-p39186	C6	15	22.2	A
17475	Bn-N16-p33016371	Bn-C6-p5803621	Bn-scaff_23957_1-p39352	C6	15	22.2	A
17476	Bn-N16-p33026351	Bn-C6-p5793583	Bn-scaff_23957_1-p40356	C6	15	22.2	A
17477	Bn-N16-p33038840	Bn-C6-p5780077	Bn-scaff_23957_1-p41707	C6	15	22.2	A
17478	Bn-N7-p20973254	Bn-C6-p5951789	Bn-scaff_23957_1-p24536	C6	15	22.2	A
17479	Bn-N16-p32047782	Bn-C6-p6726844	Bn-scaff_18807_1-p52959	C6	16	23.1	A
17480	Bn-N16-p32051414	Bn-C6-p6716876	Bn-scaff_18807_1-p51962	C6	16	23.1	A
17481	Bn-N16-p32057485	Bn-C6-p6710937	Bn-scaff_18807_1-p51368	C6	16	23.1	A
17482	Bn-N16-p32057542	Bn-C6-p6710880	Bn-scaff_18807_1-p51363	C6	16	23.1	A
17483	Bn-N16-p32059542	Bn-C6-p6708880	Bn-scaff_18807_1-p51163	C6	16	23.1	A
17484	Bn-N16-p32067096	Bn-C6-p6701326	Bn-scaff_18807_1-p50407	C6	16	23.1	A
17485	Bn-N16-p32071709	Bn-C6-p6696712	Bn-scaff_18807_1-p49946	C6	16	23.1	A
17486	Bn-N16-p32072163	Bn-C6-p6696241	Bn-scaff_18807_1-p49899	C6	16	23.1	A
17487	Bn-N16-p32072399	Bn-C6-p6696003	Bn-scaff_18807_1-p49875	C6	16	23.1	A
17488	Bn-N16-p32085273	Bn-C6-p6682811	Bn-scaff_18807_1-p48556	C6	16	23.1	A

17489	Bn-N16-p32093799	Bn-C6-p6677984	Bn-scaff_18807_1-p48073(	C6	16	23.1	A
17490	Bn-N16-p32210087	Bn-C6-p6570259	Bn-scaff_18807_1-p37301	C6	16	23.1	A
17491	Bn-N16-p32219753	Bn-C6-p6560609	Bn-scaff_18807_1-p36336	C6	16	23.1	A
17492	Bn-N16-p32225956	Bn-C6-p6556079	Bn-scaff_18807_1-p35883	C6	16	23.1	A
17493	Bn-N16-p32239741	Bn-C6-p6548736	Bn-scaff_18807_1-p35148	C6	16	23.1	A
17494	Bn-N16-p32307340	Bn-C6-p6478587	Bn-scaff_18807_1-p28133	C6	16	23.1	A
17495	Bn-N16-p32392145	Bn-C6-p6394491	Bn-scaff_18807_1-p19724	C6	16	23.1	A
17496	Bn-N16-p32432044	Bn-C6-p6352181	Bn-scaff_18807_1-p15493	C6	16	23.1	A
17497	Bn-N16-p32435901	Bn-C6-p6348493	Bn-scaff_18807_1-p15124	C6	16	23.1	A
17498	Bn-N16-p32437746	Bn-C6-p6347428	Bn-scaff_18807_1-p15018(	C6	16	23.1	A
17499	Bn-N16-p32448966	Bn-C6-p6336806	Bn-scaff_18807_1-p13955	C6	16	23.1	A
17500	Bn-N16-p32453710	Bn-C6-p6332312	Bn-scaff_18807_1-p13506	C6	16	23.1	A
17501	Bn-N16-p32457318	Bn-C6-p6327267	Bn-scaff_18807_1-p13001	C6	16	23.1	A
17502	Bn-N16-p32724861	Bn-C6-p6073958	Bn-scaff_23957_1-p12319	C6	16	23.1	A
17503	Bn-N16-p32724904	Bn-C6-p6073915	Bn-scaff_23957_1-p12323	C6	16	23.1	A
17504	Bn-N16-p32724991	Bn-C6-p6073828	Bn-scaff_23957_1-p12332	C6	16	23.1	A
17505	Bn-N16-p31856094	Bn-C6-p6875935	Bn-scaff_18807_1-p67868	C6	17	23.1	A
17506	Bn-N16-p31856398	Bn-C6-p6875631	Bn-scaff_18807_1-p67838	C6	17	23.1	A
17507	Bn-N16-p31856558	Bn-C6-p6875471	Bn-scaff_18807_1-p67822	C6	17	23.1	A
17508	Bn-N16-p31889066	Bn-C6-p6856103	Bn-scaff_18807_1-p65885	C6	17	23.1	A
17509	Bn-N16-p31947860	Bn-C6-p6813270	Bn-scaff_18807_1-p61602	C6	17	23.1	A
17510	Bn-N16-p31952282	Bn-C6-p6808848	Bn-scaff_18807_1-p61160(	C6	17	23.1	A
17511	Bn-N16-p32037489	Bn-C6-p6737089	Bn-scaff_18807_1-p53984	C6	17	23.1	A
17512	Bn-N16-p32037751	Bn-C6-p6736827	Bn-scaff_18807_1-p53957	C6	17	23.1	A
17513	Bn-N16-p31622907	Bn-C6-p7166070	Bn-scaff_16547_1-p36616	C6	18	23.1	A
17514	Bn-N16-p31623301	Bn-C6-p7165676	Bn-scaff_16547_1-p36655	C6	18	23.1	A
17515	Bn-N16-p31623306	Bn-C6-p7165671	Bn-scaff_16547_1-p36656(	C6	18	23.1	A
17516	Bn-N16-p31714014	Bn-C6-p7065345	Bn-scaff_23821_1-p22581	C6	18	23.1	A
17517	Bn-N16-p31736231	Bn-C6-p7042410	Bn-scaff_23821_1-p45516	C6	18	23.1	A
17518	Bn-N16-p31736327	Bn-C6-p7042314	Bn-scaff_23821_1-p45612	C6	18	23.1	A
17519	Bn-N16-p31736372	Bn-C6-p7042269	Bn-scaff_23821_1-p45657	C6	18	23.1	A
17520	Bn-N16-p31739292	Bn-C6-p7038833	Bn-scaff_23821_1-p49093	C6	18	23.1	A
17521	Bn-N16-p31748471	Bn-C6-p7026780	Bn-scaff_23821_1-p61146	C6	18	23.1	A
17522	Bn-N16-p31749420	Bn-C6-p7025830	Bn-scaff_23821_1-p62096	C6	18	23.1	A
17523	Bn-N16-p31801439	Bn-C6-p6965797	Bn-scaff_23821_1-p12212	C6	18	23.1	A
17524	Bn-N16-p31801710	Bn-C6-p6965529	Bn-scaff_23821_1-p12239	C6	18	23.1	A
17525	Bn-N16-p31818630	Bn-C6-p6953460	Bn-scaff_18807_1-p75621	C6	18	23.1	A
17526	Bn-Scaffold01026-p24098	Bn-Scaffold01658-p2	Bn-scaff_27401_1-p2581	C6	18	23.1	A
17527	Bn-Scaffold06576-p1055	Bn-C6-p7022920	Bn-scaff_23821_1-p65006	C6	18	23.1	A
17528	Bn-N16-p30681660	Bn-C6-p7863103	Bn-scaff_17436_1-p26077	C6	19	23.1	A
17529	Bn-N16-p31194408	Bn-C6-p7500464	Bn-scaff_16547_1-p31767	C6	19	23.1	A
17530	Bn-N16-p31195743	Bn-C6-p7499131	Bn-scaff_16547_1-p33100	C6	19	23.1	A
17531	Bn-N16-p31212289	Bn-C6-p7472616	Bn-scaff_16547_1-p59615	C6	19	23.1	A
17532	Bn-N16-p31341400	Bn-C6-p7352687	Bn-scaff_16547_1-p17954	C6	19	23.1	A
17533	Bn-N17-p1142550	Bn-C8-p4452345	Bn-scaff_25981_1-p15671	C6	19	23.1	A
17534	Bn-N16-p28249935	Bn-C6-p10407391	Bn-scaff_15892_1-p61080(	C6	20	24.8	A
17535	Bn-N16-p28253247	Bn-C6-p10406104	Bn-scaff_15892_1-p61208	C6	20	24.8	A
17536	Bn-N16-p28270084	Bn-C6-p10394225	Bn-scaff_15892_1-p62396	C6	20	24.8	A
17537	Bn-N16-p28379530	Bn-C6-p10297400	Bn-scaff_15892_1-p72079	C6	20	24.8	A

17538	Bn-N16-p28753227	Bn-C6-p9905086	Bn-scaff_15892_1-p111310	C6	20	24.8	A
17539	Bn-N16-p28755140	Bn-C6-p9903167	Bn-scaff_15892_1-p111502	C6	20	24.8	A
17540	Bn-N16-p28782898	Bn-C6-p9877037	Bn-scaff_15892_1-p114115	C6	20	24.8	A
17541	Bn-N16-p28811029	Bn-C6-p9847363	Bn-scaff_15892_1-p117082	C6	20	24.8	A
17542	Bn-N16-p28827187	Bn-C6-p9824085	Bn-scaff_15892_1-p119410	C6	20	24.8	A
17543	Bn-N16-p28827395	Bn-C6-p9823874	Bn-scaff_15892_1-p119431	C6	20	24.8	A
17544	Bn-N16-p28829618	Bn-C6-p9821507	Bn-scaff_15892_1-p119668	C6	20	24.8	A
17545	Bn-N16-p28830199	Bn-C6-p9820926	Bn-scaff_15892_1-p119726	C6	20	24.8	A
17546	Bn-N16-p28861968	Bn-C6-p9780284	Bn-scaff_15892_1-p123790	C6	20	24.8	A
17547	Bn-N16-p28874023	Bn-C6-p9719487	Bn-scaff_15892_1-p129870	C6	20	24.8	A
17548	Bn-N16-p28877955	Bn-C6-p9715551	Bn-scaff_15892_1-p130264	C6	20	24.8	A
17549	Bn-N16-p28972912	Bn-C6-p9566503	Bn-scaff_15892_1-p145168	C6	20	24.8	A
17550	Bn-N16-p29493889	Bn-C6-p9022311	Bn-scaff_15743_1-p638252	C6	20	24.8	A
17551	Bn-N16-p29624848	Bn-C6-p8947537	Bn-scaff_16116_1-p7960	C6	20	24.8	A
17552	Bn-N16-p29666154	Bn-C6-p8905311	Bn-scaff_16116_1-p50186	C6	20	24.8	A
17553	Bn-N16-p29714155	Bn-C6-p8844625	Bn-scaff_16116_1-p110872	C6	20	24.8	A
17554	Bn-N16-p29717745	Bn-C6-p8841514	Bn-scaff_16116_1-p113983	C6	20	24.8	A
17555	Bn-N16-p29996709	Bn-C6-p8504762	Bn-scaff_16116_1-p450735	C6	20	24.8	A
17556	Bn-N16-p30026697	Bn-C6-p8469110	Bn-scaff_16116_1-p486387	C6	20	24.8	A
17557	Bn-N16-p30027373	Bn-C6-p8468434	Bn-scaff_16116_1-p487063	C6	20	24.8	A
17558	Bn-N16-p30177574	Bn-C6-p8350120	Bn-scaff_16116_1-p605377	C6	20	24.8	A
17559	Bn-N7-p18936843	Bn-C6-p9847477	Bn-scaff_15892_1-p117071	C6	20	24.8	A
17560	Bn-N7-p18997633	Bn-C6-p9674510	Bn-scaff_15892_1-p134368	C6	20	24.8	A
17561	Bn-Scaffold01203-p17282	Bn-C6-p9506114	Bn-scaff_15892_1-p151207	C6	20	24.8	A
17562	Bn-N16-p26901876	Bn-C6-p11765338	Bn-scaff_16064_1-p9281	C6	21	25.6	A
17563	Bn-N16-p26903998	Bn-C6-p11763214	Bn-scaff_16064_1-p7157	C6	21	25.6	A
17564	Bn-N16-p26911758	Bn-C6-p11728830	Bn-scaff_16865_1-p134152	C6	21	25.6	0
17565	Bn-N16-p26938271	Bn-C6-p11708937	Bn-scaff_16865_1-p114259	C6	21	25.6	A
17566	Bn-N16-p26940011	Bn-C6-p11706984	Bn-scaff_16865_1-p112306	C6	21	25.6	A
17567	Bn-N16-p26949095	Bn-C6-p11690429	Bn-scaff_16865_1-p95751	C6	21	25.6	A
17568	Bn-N16-p26949760	Bn-C6-p11689762	Bn-scaff_16865_1-p95084	C6	21	25.6	A
17569	Bn-N16-p26950109	Bn-C6-p11689405	Bn-scaff_16865_1-p94727	C6	21	25.6	A
17570	Bn-N16-p26975842	Bn-C6-p11635510	Bn-scaff_16865_1-p40832	C6	21	25.6	A
17571	Bn-N16-p27504203	Bn-C6-p11189141	Bn-scaff_16357_1-p170851	C6	21	25.6	A
17572	Bn-N16-p27801073	Bn-C6-p10905640	Bn-scaff_15892_1-p112551	C6	21	25.6	A
17573	Bn-N16-p27803391	Bn-C6-p10903298	Bn-scaff_15892_1-p114893	C6	21	25.6	A
17574	Bn-N16-p27803483	Bn-C6-p10903206	Bn-scaff_15892_1-p114985	C6	21	25.6	A
17575	Bn-N16-p27803646	Bn-C6-p10903020	Bn-scaff_15892_1-p115171	C6	21	25.6	A
17576	Bn-N16-p27811565	Bn-C6-p10895432	Bn-scaff_15892_1-p122759	C6	21	25.6	A
17577	Bn-N16-p27841691	Bn-C6-p10861337	Bn-scaff_15892_1-p156854	C6	21	25.6	A
17578	Bn-N16-p27970169	Bn-C6-p10707434	Bn-scaff_15892_1-p310757	C6	21	25.6	A
17579	Bn-N16-p27970848	Bn-C6-p10706757	Bn-scaff_15892_1-p311434	C6	21	25.6	A
17580	Bn-N16-p27986404	Bn-C6-p10691298	Bn-scaff_15892_1-p326893	C6	21	25.6	A
17581	Bn-N16-p28005575	Bn-C6-p10672225	Bn-scaff_15892_1-p345966	C6	21	25.6	A
17582	Bn-N16-p28005633	Bn-C6-p10672167	Bn-scaff_15892_1-p346024	C6	21	25.6	A
17583	Bn-N16-p28007579	Bn-C6-p10652767	Bn-scaff_15892_1-p365424	C6	21	25.6	A
17584	Bn-N16-p28007738	Bn-C6-p10652607	Bn-scaff_15892_1-p365584	C6	21	25.6	A
17585	Bn-N16-p28012074	Bn-C6-p10650807	Bn-scaff_15892_1-p367384	C6	21	25.6	A
17586	Bn-N16-p28012664	Bn-C6-p10650217	Bn-scaff_15892_1-p367974	C6	21	25.6	A

17587	Bn-N16-p28013312	Bn-C6-p10649531	Bn-scaff_15892_1-p36866(	C6	21	25.6	A
17588	Bn-N16-p28013335	Bn-C6-p10649508	Bn-scaff_15892_1-p36868:	C6	21	25.6	A
17589	Bn-N16-p28048993	Bn-C6-p10613932	Bn-scaff_15892_1-p40425!	C6	21	25.6	A
17590	Bn-N16-p28077787	Bn-C6-p10586855	Bn-scaff_15892_1-p43133(	C6	21	25.6	A
17591	Bn-N16-p28091061	Bn-C6-p10571438	Bn-scaff_15892_1-p44675:	C6	21	25.6	A
17592	Bn-N16-p28092087	Bn-C6-p10570417	Bn-scaff_15892_1-p44777	C6	21	25.6	A
17593	Bn-N16-p28204416	Bn-C6-p10464352	Bn-scaff_15892_1-p55383!	C6	21	25.6	A
17594	Bn-N1-p27170805	Bn-Scaffold01463-p2:	Bn-scaff_16865_1-p16443:	C6	21	25.6	A
17595	Bn-N7-p18505098	Bn-A07-p15833211	Bn-A07-p15833211	C6	21	25.6	A
17596	Bn-N10-p7349599	Bn-C6-p11874851	Bn-scaff_16064_1-p11879	C6	22	26.4	A
17597	Bn-N16-p26712994	Bn-C2-p41605604	Bn-scaff_18360_1-p13419:	C6	22	26.4	A
17598	Bn-N16-p26506737	Bn-C6-p12136654	Bn-scaff_16064_1-p38059	C6	23	27.2	A
17599	Bn-N16-p26507287	Bn-C6-p12136102	Bn-scaff_16064_1-p38004!	C6	23	27.2	A
17600	Bn-N16-p26556755	Bn-C6-p12097890	Bn-scaff_16064_1-p34183:	C6	23	27.2	A
17601	Bn-N16-p26607578	Bn-C6-p12006047	Bn-scaff_16064_1-p24999(	C6	23	27.2	A
17602	Bn-N16-p26622358	Bn-C6-p11989808	Bn-scaff_16064_1-p23375:	C6	23	27.2	A
17603	Bn-N16-p26668123	Bn-C6-p11959643	Bn-scaff_16064_1-p20358(	C6	23	27.2	A
17604	Bn-N7-p16962092	Bn-A07-p14325380	Bn-A07-p14325380	C6	23	27.2	A
17605	Bn-N7-p17088805	Bn-A07-p14450900	Bn-A07-p14450900	C6	23	27.2	A
17606	Bn-Scaffold04089-p307	Bn-C6-p12022953	Bn-scaff_16064_1-p26689(	C6	23	27.2	A
17607	Bn-N16-p24349364	Bn-C6-p14338971	Bn-scaff_15818_1-p31722(	C6	24	27.2	A
17608	Bn-N16-p24365807	Bn-C6-p14335268	Bn-scaff_15818_1-p31759	C6	24	27.2	A
17609	Bn-N16-p24390486	Bn-C6-p14307703	Bn-scaff_15818_1-p32035:	C6	24	27.2	A
17610	Bn-N16-p24390854	Bn-C6-p14307337	Bn-scaff_15818_1-p32039(	C6	24	27.2	A
17611	Bn-N16-p24391758	Bn-C6-p14306456	Bn-scaff_15818_1-p32047!	C6	24	27.2	A
17612	Bn-N16-p24934318	Bn-C6-p13695614	Bn-scaff_15746_1-p18932(	C6	24	27.2	A
17613	Bn-N16-p24948872	Bn-C6-p13682667	Bn-scaff_15746_1-p17637:	C6	24	27.2	A
17614	Bn-N16-p24982013	Bn-C6-p13658301	Bn-scaff_15746_1-p15200	C6	24	27.2	A
17615	Bn-N16-p24990506	Bn-C6-p13651736	Bn-scaff_15746_1-p14544:	C6	24	27.2	A
17616	Bn-N16-p24991840	Bn-C6-p13650402	Bn-scaff_15746_1-p14410!	C6	24	27.2	A
17617	Bn-N16-p25006344	Bn-C6-p13634652	Bn-scaff_15746_1-p12835!	C6	24	27.2	A
17618	Bn-N16-p25016933	Bn-Scaffold01287-p8(	Bn-scaff_25878_1-p861	C6	24	27.2	0
17619	Bn-N16-p25017773	Bn-Scaffold01287-p1:	Bn-scaff_25878_1-p1701	C6	24	27.2	A
17620	Bn-N16-p25024704	Bn-Scaffold01287-p8:	Bn-scaff_25878_1-p8784	C6	24	27.2	A
17621	Bn-N16-p25029295	Bn-Scaffold01287-p1:	Bn-scaff_25878_1-p13369	C6	24	27.2	A
17622	Bn-N16-p25029487	Bn-Scaffold01287-p1:	Bn-scaff_25878_1-p13561	C6	24	27.2	A
17623	Bn-N16-p25048942	Bn-C6-p13608058	Bn-scaff_15746_1-p10176	C6	24	27.2	A
17624	Bn-N16-p25051731	Bn-C6-p13605275	Bn-scaff_15746_1-p98981	C6	24	27.2	A
17625	Bn-N16-p25054013	Bn-C6-p13602990	Bn-scaff_15746_1-p96696	C6	24	27.2	A
17626	Bn-N16-p25061741	Bn-C6-p13590982	Bn-scaff_15746_1-p84688	C6	24	27.2	A
17627	Bn-N16-p25061921	Bn-C6-p13590802	Bn-scaff_15746_1-p84508	C6	24	27.2	A
17628	Bn-N16-p25062166	Bn-C6-p13590557	Bn-scaff_15746_1-p84263	C6	24	27.2	A
17629	Bn-N16-p25181898	Bn-C6-p13479387	Bn-scaff_18439_1-p26808	C6	24	27.2	A
17630	Bn-N16-p25185083	Bn-C6-p13476220	Bn-scaff_18439_1-p29975	C6	24	27.2	A
17631	Bn-N16-p25188785	Bn-C6-p13467949	Bn-scaff_18439_1-p38246	C6	24	27.2	A
17632	Bn-N16-p25203124	Bn-C6-p13454162	Bn-scaff_18439_1-p52033	C6	24	27.2	A
17633	Bn-N16-p25205859	Bn-C6-p13450604	Bn-scaff_18439_1-p55591	C6	24	27.2	A
17634	Bn-N16-p25354786	Bn-C6-p13302645	Bn-scaff_18439_1-p20355(	C6	24	27.2	A
17635	Bn-N16-p25354957	Bn-C6-p13297744	Bn-scaff_18439_1-p20845:	C6	24	27.2	A

17636	Bn-N16-p25355050	Bn-C6-p13297651	Bn-scaff_18439_1-p208544	C6	24	27.2	A
17637	Bn-N16-p25355439	Bn-C6-p13286373	Bn-scaff_18439_1-p219822	C6	24	27.2	A
17638	Bn-N16-p25377554	Bn-C6-p13268248	Bn-scaff_18439_1-p237947	C6	24	27.2	A
17639	Bn-N16-p25378553	Bn-C6-p13267235	Bn-scaff_18439_1-p238960	C6	24	27.2	A
17640	Bn-N16-p25404861	Bn-C6-p13244141	Bn-scaff_18439_1-p262054	C6	24	27.2	A
17641	Bn-N16-p25447383	Bn-C6-p13198149	Bn-scaff_16064_1-p144209	C6	24	27.2	A
17642	Bn-N16-p25464384	Bn-C6-p13174110	Bn-scaff_16064_1-p141805	C6	24	27.2	A
17643	Bn-N16-p25616979	Bn-C6-p13020752	Bn-scaff_16064_1-p126469	C6	24	27.2	A
17644	Bn-N16-p25714009	Bn-C6-p12901951	Bn-scaff_16064_1-p114589	C6	24	27.2	A
17645	Bn-N16-p25715364	Bn-C6-p12900614	Bn-scaff_16064_1-p114455	C6	24	27.2	A
17646	Bn-N16-p25715478	Bn-C6-p12900500	Bn-scaff_16064_1-p114444	C6	24	27.2	A
17647	Bn-N16-p25717978	Bn-C6-p12898307	Bn-scaff_16064_1-p114225	C6	24	27.2	A
17648	Bn-N16-p25723027	Bn-C6-p12893191	Bn-scaff_16064_1-p113713	C6	24	27.2	A
17649	Bn-N16-p25727530	Bn-C6-p12885449	Bn-scaff_16064_1-p112939	C6	24	27.2	A
17650	Bn-N16-p25727742	Bn-C6-p12885237	Bn-scaff_16064_1-p112918	C6	24	27.2	A
17651	Bn-N16-p25727806	Bn-C6-p12885173	Bn-scaff_16064_1-p112911	C6	24	27.2	A
17652	Bn-N16-p25734987	Bn-C6-p12882571	Bn-scaff_16064_1-p112651	C6	24	27.2	A
17653	Bn-N16-p25737054	Bn-C6-p12880581	Bn-scaff_16064_1-p112452	C6	24	27.2	A
17654	Bn-N16-p25737294	Bn-C6-p12880340	Bn-scaff_16064_1-p112428	C6	24	27.2	A
17655	Bn-N16-p25801519	Bn-C6-p12832117	Bn-scaff_16064_1-p107606	C6	24	27.2	A
17656	Bn-N16-p25801921	Bn-C6-p12831714	Bn-scaff_16064_1-p107565	C6	24	27.2	A
17657	Bn-N16-p25802131	Bn-C6-p12831504	Bn-scaff_16064_1-p107544	C6	24	27.2	A
17658	Bn-N16-p25803859	Bn-C6-p12829777	Bn-scaff_16064_1-p107372	C6	24	27.2	A
17659	Bn-N16-p25824407	Bn-C6-p12812169	Bn-scaff_16064_1-p105611	C6	24	27.2	A
17660	Bn-N16-p25832743	Bn-C6-p12803831	Bn-scaff_16064_1-p104777	C6	24	27.2	A
17661	Bn-N16-p25947286	Bn-C6-p12694461	Bn-scaff_16064_1-p938404	C6	24	27.2	A
17662	Bn-N16-p25947560	Bn-C6-p12694187	Bn-scaff_16064_1-p938130	C6	24	27.2	A
17663	Bn-N7-p17076360	Bn-C6-p13909457	Bn-scaff_15746_1-p403163	C6	24	27.2	A
17664	Bn-N16-p24223842	Bn-C6-p14478368	Bn-scaff_15818_1-p303287	C6	25	28	A
17665	Bn-N16-p24225027	Bn-C6-p14477183	Bn-scaff_15818_1-p303405	C6	25	28	A
17666	Bn-N16-p24225935	Bn-C6-p14476275	Bn-scaff_15818_1-p303496	C6	25	28	A
17667	Bn-N16-p24241484	Bn-C6-p14461069	Bn-scaff_15818_1-p305017	C6	25	28	A
17668	Bn-N16-p24248478	Bn-C6-p14454040	Bn-scaff_15818_1-p305719	C6	25	28	A
17669	Bn-N16-p24262480	Bn-C6-p14438913	Bn-scaff_15818_1-p307232	C6	25	28	A
17670	Bn-N16-p24265414	Bn-C6-p14435965	Bn-scaff_15818_1-p307527	C6	25	28	A
17671	Bn-N16-p23582092	Bn-C6-p15140213	Bn-scaff_15818_1-p237102	C6	26	28.8	A
17672	Bn-N16-p23582282	Bn-C6-p15140023	Bn-scaff_15818_1-p237121	C6	26	28.8	A
17673	Bn-N16-p23583439	Bn-C6-p15136920	Bn-scaff_15818_1-p237431	C6	26	28.8	A
17674	Bn-N16-p23585506	Bn-C6-p15134852	Bn-scaff_15818_1-p237638	C6	26	28.8	A
17675	Bn-N16-p23589490	Bn-C6-p15130890	Bn-scaff_15818_1-p238034	C6	26	28.8	A
17676	Bn-N16-p23590126	Bn-C6-p15130253	Bn-scaff_15818_1-p238098	C6	26	28.8	A
17677	Bn-N16-p23590785	Bn-C6-p15129594	Bn-scaff_15818_1-p238164	C6	26	28.8	A
17678	Bn-N16-p23591081	Bn-C6-p15129298	Bn-scaff_15818_1-p238194	C6	26	28.8	A
17679	Bn-N16-p23591386	Bn-C6-p15128993	Bn-scaff_15818_1-p238224	C6	26	28.8	A
17680	Bn-N16-p23591790	Bn-C6-p15128589	Bn-scaff_15818_1-p238265	C6	26	28.8	A
17681	Bn-N16-p23592063	Bn-C6-p15128316	Bn-scaff_15818_1-p238292	C6	26	28.8	A
17682	Bn-N16-p23604110	Bn-C6-p15116045	Bn-scaff_15818_1-p239519	C6	26	28.8	A
17683	Bn-N16-p23604112	Bn-C6-p15116043	Bn-scaff_15818_1-p239519	C6	26	28.8	A
17684	Bn-N16-p23604169	Bn-C6-p15115986	Bn-scaff_15818_1-p239525	C6	26	28.8	A

17685	Bn-N16-p23604400	Bn-C6-p15115498	Bn-scaff_15818_1-p239574	C6	26	28.8	A
17686	Bn-N16-p23604618	Bn-C6-p15115279	Bn-scaff_15818_1-p239596	C6	26	28.8	A
17687	Bn-N16-p23605780	Bn-C6-p15114123	Bn-scaff_15818_1-p239711	C6	26	28.8	A
17688	Bn-N16-p23608476	Bn-C6-p15111468	Bn-scaff_15818_1-p239977	C6	26	28.8	A
17689	Bn-N16-p23608518	Bn-C6-p15111414	Bn-scaff_15818_1-p239982	C6	26	28.8	A
17690	Bn-N16-p23613945	Bn-C6-p15106472	Bn-scaff_15818_1-p240476	C6	26	28.8	A
17691	Bn-N16-p23701362	Bn-C6-p14996206	Bn-scaff_15818_1-p251503	C6	26	28.8	A
17692	Bn-N16-p23702580	Bn-C6-p14994988	Bn-scaff_15818_1-p251625	C6	26	28.8	A
17693	Bn-N16-p23715816	Bn-C6-p14982648	Bn-scaff_15818_1-p252859	C6	26	28.8	A
17694	Bn-N16-p23719462	Bn-C6-p14978990	Bn-scaff_15818_1-p253224	C6	26	28.8	A
17695	Bn-N16-p22236701	Bn-C6-p16414063	Bn-scaff_15763_1-p148162	C6	27	29.6	A
17696	Bn-N16-p22247194	Bn-C6-p16403568	Bn-scaff_15763_1-p149211	C6	27	29.6	A
17697	Bn-N16-p22247397	Bn-C6-p16403336	Bn-scaff_15763_1-p149234	C6	27	29.6	A
17698	Bn-N16-p22251077	Bn-C6-p16399651	Bn-scaff_15763_1-p149603	C6	27	29.6	A
17699	Bn-N16-p22935958	Bn-C6-p15821098	Bn-scaff_15818_1-p169014	C6	27	29.6	A
17700	Bn-N16-p23229642	Bn-C6-p15489096	Bn-scaff_15818_1-p202214	C6	27	29.6	A
17701	Bn-N16-p23234255	Bn-C6-p15482089	Bn-scaff_15818_1-p202915	C6	27	29.6	A
17702	Bn-N16-p23235018	Bn-C6-p15481327	Bn-scaff_15818_1-p202991	C6	27	29.6	A
17703	Bn-N7-p16149816	Bn-A07-p13512124	Bn-A07-p13512124	C6	27	29.6	A
17704	Bn-N14-p16531771	Bn-C6-p17291390	Bn-scaff_15763_1-p604295	C6	28	30.4	A
17705	Bn-N16-p20940109	Bn-C9-p32988625	Bn-scaff_16619_1-p24599	C6	28	30.4	A
17706	Bn-N16-p21115094	Bn-C6-p17662536	Bn-scaff_15763_1-p233149	C6	28	30.4	A
17707	Bn-N16-p21115944	Bn-C6-p17661686	Bn-scaff_15763_1-p233999	C6	28	30.4	A
17708	Bn-N16-p21136579	Bn-C6-p17639810	Bn-scaff_15763_1-p255875	C6	28	30.4	A
17709	Bn-N16-p21399223	Bn-C6-p17341700	Bn-scaff_15763_1-p553985	C6	28	30.4	A
17710	Bn-N16-p21418501	Bn-C6-p17321934	Bn-scaff_15763_1-p573751	C6	28	30.4	A
17711	Bn-N16-p21434209	Bn-C6-p17307362	Bn-scaff_15763_1-p588323	C6	28	30.4	A
17712	Bn-N16-p21434765	Bn-C6-p17306811	Bn-scaff_15763_1-p588874	C6	28	30.4	A
17713	Bn-N16-p21439649	Bn-C6-p17300591	Bn-scaff_15763_1-p595094	C6	28	30.4	A
17714	Bn-N16-p21439871	Bn-C6-p17300370	Bn-scaff_15763_1-p595315	C6	28	30.4	A
17715	Bn-N16-p21440758	Bn-C6-p17300107	Bn-scaff_15763_1-p595578	C6	28	30.4	A
17716	Bn-N16-p21441577	Bn-C6-p17299289	Bn-scaff_15763_1-p596396	C6	28	30.4	A
17717	Bn-N16-p21444319	Bn-C6-p17291488	Bn-scaff_15763_1-p604197	C6	28	30.4	A
17718	Bn-N16-p21453022	Bn-C6-p17272689	Bn-scaff_15763_1-p622996	C6	28	30.4	A
17719	Bn-N16-p21455487	Bn-C6-p17259598	Bn-scaff_15763_1-p636087	C6	28	30.4	A
17720	Bn-N16-p21455926	Bn-C6-p17259159	Bn-scaff_15763_1-p636526	C6	28	30.4	A
17721	Bn-N16-p21462230	Bn-C6-p17252874	Bn-scaff_15763_1-p642811	C6	28	30.4	A
17722	Bn-N16-p21463043	Bn-C6-p17251293	Bn-scaff_15763_1-p644392	C6	28	30.4	A
17723	Bn-N16-p21463144	Bn-C6-p17251207	Bn-scaff_15763_1-p644478	C6	28	30.4	A
17724	Bn-N16-p21463335	Bn-C6-p17251016	Bn-scaff_15763_1-p644669	C6	28	30.4	A
17725	Bn-N16-p21463413	Bn-C6-p17250938	Bn-scaff_15763_1-p644747	C6	28	30.4	A
17726	Bn-N16-p21464650	Bn-C6-p17250456	Bn-scaff_15763_1-p645229	C6	28	30.4	A
17727	Bn-N16-p21471244	Bn-C6-p17238022	Bn-scaff_15763_1-p657663	C6	28	30.4	A
17728	Bn-N16-p21475637	Bn-C6-p17233628	Bn-scaff_15763_1-p662057	C6	28	30.4	A
17729	Bn-N16-p21481815	Bn-C6-p17227450	Bn-scaff_15763_1-p668235	C6	28	30.4	A
17730	Bn-N16-p21489137	Bn-C6-p17220138	Bn-scaff_15763_1-p675547	C6	28	30.4	A
17731	Bn-N16-p21491184	Bn-C6-p17218091	Bn-scaff_15763_1-p677594	C6	28	30.4	A
17732	Bn-N16-p21494908	Bn-C6-p17214367	Bn-scaff_15763_1-p681318	C6	28	30.4	A
17733	Bn-N16-p21495113	Bn-C6-p17214162	Bn-scaff_15763_1-p681523	C6	28	30.4	A

17734	Bn-N16-p21495841	Bn-C6-p17213434	Bn-scaff_15763_1-p682251	C6	28	30.4	A
17735	Bn-N16-p21520633	Bn-C6-p17188731	Bn-scaff_15763_1-p706954	C6	28	30.4	A
17736	Bn-N16-p21543346	Bn-C6-p17181946	Bn-scaff_15763_1-p713739	C6	28	30.4	A
17737	Bn-N16-p21560116	Bn-C6-p17166376	Bn-scaff_15763_1-p729309	C6	28	30.4	A
17738	Bn-N16-p21930568	Bn-C6-p16776341	Bn-scaff_15763_1-p111934	C6	28	30.4	A
17739	Bn-N16-p22155542	Bn-C6-p16494238	Bn-scaff_15763_1-p140144	C6	28	30.4	A
17740	Bn-N16-p22158665	Bn-C6-p16491697	Bn-scaff_15763_1-p140398	C6	28	30.4	A
17741	Bn-N16-p22164779	Bn-C6-p16483793	Bn-scaff_15763_1-p141189	C6	28	30.4	A
17742	Bn-N16-p22166829	Bn-C6-p16481744	Bn-scaff_15763_1-p141394	C6	28	30.4	A
17743	Bn-N16-p22172293	Bn-C6-p16476902	Bn-scaff_15763_1-p141878	C6	28	30.4	A
17744	Bn-N16-p22173070	Bn-C6-p16476142	Bn-scaff_15763_1-p141954	C6	28	30.4	A
17745	Bn-N16-p22178337	Bn-C6-p16471315	Bn-scaff_15763_1-p142437	C6	28	30.4	A
17746	Bn-N16-p22189626	Bn-C6-p16461597	Bn-scaff_15763_1-p143408	C6	28	30.4	A
17747	Bn-N16-p22208754	Bn-C6-p16439278	Bn-scaff_15763_1-p145640	C6	28	30.4	A
17748	Bn-N16-p22215782	Bn-C6-p16432279	Bn-scaff_15763_1-p146340	C6	28	30.4	A
17749	Bn-N16-p20370684	Bn-C6-p18415817	Bn-scaff_18206_3-p934892	C6	29	31.2	A
17750	Bn-N16-p20713802	Bn-C6-p18054156	Bn-scaff_18806_1-p248783	C6	29	31.2	A
17751	Bn-N15-p3458353	Bn-C6-p19799516	Bn-scaff_18206_1-p142860	C6	30	32	A
17752	Bn-N16-p17911957	Bn-C6-p20863048	Bn-scaff_15818_2-p121258	C6	30	32	A
17753	Bn-N16-p17912806	Bn-C6-p20862094	Bn-scaff_15818_2-p121354	C6	30	32	A
17754	Bn-N16-p17920643	Bn-C6-p20851952	Bn-scaff_15818_2-p122368	C6	30	32	A
17755	Bn-N16-p17925231	Bn-C6-p20850773	Bn-scaff_15818_2-p122486	C6	30	32	A
17756	Bn-N16-p18124929	Bn-C6-p20690013	Bn-scaff_15818_2-p138562	C6	30	32	A
17757	Bn-N16-p18151563	Bn-C6-p20645598	Bn-scaff_15818_2-p143003	C6	30	32	A
17758	Bn-N16-p18153316	Bn-C6-p20643834	Bn-scaff_15818_2-p143180	C6	30	32	A
17759	Bn-N16-p18230161	Bn-C6-p20586831	Bn-scaff_15818_2-p148880	C6	30	32	A
17760	Bn-N16-p18258149	Bn-C6-p20553015	Bn-scaff_15818_2-p152261	C6	30	32	A
17761	Bn-N16-p18269244	Bn-C6-p20541081	Bn-scaff_15818_2-p153455	C6	30	32	A
17762	Bn-N16-p18290575	Bn-C6-p20520802	Bn-scaff_15818_2-p155483	C6	30	32	A
17763	Bn-N16-p18290700	Bn-C6-p20520677	Bn-scaff_15818_2-p155495	C6	30	32	A
17764	Bn-N16-p18290855	Bn-C6-p20520522	Bn-scaff_15818_2-p155511	C6	30	32	A
17765	Bn-N16-p18290857	Bn-C6-p20520520	Bn-scaff_15818_2-p155511	C6	30	32	A
17766	Bn-N16-p18296980	Bn-C6-p20509199	Bn-scaff_15818_2-p156643	C6	30	32	A
17767	Bn-N16-p18314791	Bn-C6-p20488745	Bn-scaff_15818_2-p158688	C6	30	32	A
17768	Bn-N16-p18359857	Bn-C6-p20437672	Bn-scaff_15818_2-p163796	C6	30	32	A
17769	Bn-N16-p18533861	Bn-C6-p20258502	Bn-scaff_15818_2-p181713	C6	30	32	A
17770	Bn-N16-p18579305	Bn-C6-p20201146	Bn-scaff_15818_2-p187448	C6	30	32	A
17771	Bn-N16-p18579410	Bn-C6-p20201041	Bn-scaff_15818_2-p187459	C6	30	32	A
17772	Bn-N16-p18579587	Bn-C6-p20200864	Bn-scaff_15818_2-p187477	C6	30	32	A
17773	Bn-N16-p18579798	Bn-C6-p20200653	Bn-scaff_15818_2-p187498	C6	30	32	A
17774	Bn-N16-p18579895	Bn-C6-p20200556	Bn-scaff_15818_2-p187507	C6	30	32	A
17775	Bn-N16-p18591377	Bn-C6-p20173017	Bn-scaff_15818_2-p190261	C6	30	32	A
17776	Bn-N16-p18594827	Bn-C6-p20171956	Bn-scaff_15818_2-p190367	C6	30	32	A
17777	Bn-N16-p18595265	Bn-C6-p20171519	Bn-scaff_15818_2-p190411	C6	30	32	A
17778	Bn-N16-p18595397	Bn-C6-p20171386	Bn-scaff_15818_2-p190424	C6	30	32	A
17779	Bn-N16-p18595578	Bn-C6-p20171205	Bn-scaff_15818_2-p190442	C6	30	32	A
17780	Bn-N16-p18600461	Bn-C6-p20168261	Bn-scaff_15818_2-p190737	C6	30	32	A
17781	Bn-N16-p18601779	Bn-C6-p20166936	Bn-scaff_15818_2-p190869	C6	30	32	A
17782	Bn-N16-p18612417	Bn-C6-p20154739	Bn-scaff_15818_2-p192089	C6	30	32	A

17783	Bn-N16-p18632204	Bn-C6-p20134680	Bn-scaff_15818_2-p194095	C6	30	32	A
17784	Bn-N16-p18633058	Bn-C6-p20134066	Bn-scaff_15818_2-p194156	C6	30	32	A
17785	Bn-N16-p18633803	Bn-C6-p20133317	Bn-scaff_15818_2-p194231	C6	30	32	A
17786	Bn-N16-p18633919	Bn-C6-p20133201	Bn-scaff_15818_2-p194243	C6	30	32	A
17787	Bn-N16-p18639940	Bn-C6-p20132260	Bn-scaff_15818_2-p194337	C6	30	32	A
17788	Bn-N16-p18641888	Bn-C6-p20130612	Bn-scaff_15818_2-p194502	C6	30	32	A
17789	Bn-N16-p18642345	Bn-C6-p20130136	Bn-scaff_15818_2-p194549	C6	30	32	A
17790	Bn-N16-p18642459	Bn-C6-p20130022	Bn-scaff_15818_2-p194561	C6	30	32	A
17791	Bn-N16-p18642661	Bn-C6-p20129818	Bn-scaff_15818_2-p194581	C6	30	32	A
17792	Bn-N16-p18657885	Bn-C6-p20115071	Bn-scaff_15818_2-p196056	C6	30	32	A
17793	Bn-N16-p18658117	Bn-C6-p20114840	Bn-scaff_15818_2-p196079	C6	30	32	A
17794	Bn-N16-p18688564	Bn-C6-p20083017	Bn-scaff_15818_2-p199261	C6	30	32	A
17795	Bn-N16-p18692575	Bn-C6-p20080460	Bn-scaff_15818_2-p199517	C6	30	32	A
17796	Bn-N16-p18692931	Bn-C6-p20079956	Bn-scaff_15818_2-p199567	C6	30	32	A
17797	Bn-N16-p18697754	Bn-C6-p20073365	Bn-scaff_15818_2-p200226	C6	30	32	A
17798	Bn-N16-p18752191	Bn-C6-p20014914	Bn-scaff_15818_2-p206072	C6	30	32	A
17799	Bn-N16-p18770663	Bn-C6-p19994899	Bn-scaff_15818_2-p208073	C6	30	32	A
17800	Bn-N16-p18791155	Bn-C6-p19966707	Bn-scaff_15818_2-p210892	C6	30	32	A
17801	Bn-N16-p18812393	Bn-C6-p19927452	Bn-scaff_18206_1-p14924	C6	30	32	A
17802	Bn-N16-p18842678	Bn-C6-p19880141	Bn-scaff_18206_1-p62235	C6	30	32	A
17803	Bn-N16-p18883634	Bn-C6-p19826961	Bn-scaff_18206_1-p115415	C6	30	32	A
17804	Bn-N16-p18899625	Bn-C5-p3370152	Bn-scaff_20901_1-p859313	C6	30	32	A
17805	Bn-N16-p18962676	Bn-C6-p19756206	Bn-scaff_18206_1-p186170	C6	30	32	A
17806	Bn-N16-p18963035	Bn-C6-p19755867	Bn-scaff_18206_1-p186509	C6	30	32	A
17807	Bn-N16-p19046965	Bn-C6-p19662115	Bn-scaff_18206_1-p280261	C6	30	32	A
17808	Bn-N16-p19181031	Bn-C6-p19506663	Bn-scaff_18206_1-p435713	C6	30	32	A
17809	Bn-N16-p19181239	Bn-C6-p19506451	Bn-scaff_18206_1-p435925	C6	30	32	A
17810	Bn-N16-p19239572	Bn-C6-p19449260	Bn-scaff_18206_2-p33548	C6	30	32	A
17811	Bn-N16-p19340248	Bn-C6-p19368689	Bn-scaff_18206_2-p114119	C6	30	32	A
17812	Bn-N16-p19340327	Bn-C6-p19368611	Bn-scaff_18206_2-p114197	C6	30	32	A
17813	Bn-N16-p19341290	Bn-C6-p19364522	Bn-scaff_18206_2-p118286	C6	30	32	A
17814	Bn-N16-p19341391	Bn-C6-p19364421	Bn-scaff_18206_2-p118387	C6	30	32	A
17815	Bn-N16-p19345448	Bn-C6-p19351297	Bn-scaff_18206_2-p131511	C6	30	32	A
17816	Bn-N16-p19392228	Bn-C6-p19321019	Bn-scaff_18206_3-p29690	C6	30	32	A
17817	Bn-N16-p19427067	Bn-C6-p19300068	Bn-scaff_18206_3-p50641	C6	30	32	A
17818	Bn-N16-p19434634	Bn-C6-p19290604	Bn-scaff_18206_3-p60105	C6	30	32	A
17819	Bn-N16-p19434772	Bn-C6-p19290466	Bn-scaff_18206_3-p60243	C6	30	32	A
17820	Bn-N16-p19435201	Bn-C6-p19290036	Bn-scaff_18206_3-p60673	C6	30	32	A
17821	Bn-N16-p19435339	Bn-C6-p19289898	Bn-scaff_18206_3-p60811	C6	30	32	A
17822	Bn-N16-p19436500	Bn-C6-p19288736	Bn-scaff_18206_3-p61973	C6	30	32	A
17823	Bn-N16-p19437108	Bn-C6-p19288127	Bn-scaff_18206_3-p62582	C6	30	32	A
17824	Bn-N16-p19437281	Bn-C6-p19287954	Bn-scaff_18206_3-p62755	C6	30	32	A
17825	Bn-N16-p19445992	Bn-C6-p19281341	Bn-scaff_18206_3-p69368	C6	30	32	A
17826	Bn-N16-p19455828	Bn-C6-p19271160	Bn-scaff_18206_3-p79549	C6	30	32	A
17827	Bn-N16-p19466937	Bn-C6-p19250883	Bn-scaff_18206_3-p99826	C6	30	32	A
17828	Bn-N16-p19913264	Bn-C6-p18857603	Bn-scaff_18206_3-p493106	C6	30	32	A
17829	Bn-N16-p21855976	Bn-C5-p35980653	Bn-scaff_18189_1-p150741	C6	30	32	A
17830	Bn-N9-p26159948	Bn-A09-p23708692	Bn-A09-p23708692	C6	30	32	A
17831	Bn-Scaffold01272-p10588	Bn-C6-p19488326	Bn-scaff_18206_1-p454050	C6	30	32	A

17832	Bn-Scaffold01272-p8936	Bn-C6-p19486674	Bn-scaff_18206_1-p455702	C6	30	32	A
17833	Bn-N16-p15871048	Bn-C6-p22863673	Bn-scaff_15818_1-p479061	C6	31	32.8	A
17834	Bn-N16-p15871657	Bn-C6-p22862818	Bn-scaff_15818_1-p479916	C6	31	32.8	A
17835	Bn-N16-p15883463	Bn-C6-p22847659	Bn-scaff_15818_1-p495075	C6	31	32.8	A
17836	Bn-N16-p15883481	Bn-C6-p22847641	Bn-scaff_15818_1-p495093	C6	31	32.8	A
17837	Bn-N16-p15884773	Bn-C6-p22846435	Bn-scaff_15818_1-p496299	C6	31	32.8	A
17838	Bn-N16-p15884907	Bn-C6-p22839591	Bn-scaff_15818_1-p503143	C6	31	32.8	A
17839	Bn-N16-p15891607	Bn-C6-p22830908	Bn-scaff_15818_1-p511826	C6	31	32.8	A
17840	Bn-N16-p15892638	Bn-C6-p22830286	Bn-scaff_15818_1-p512448	C6	31	32.8	A
17841	Bn-N16-p15893174	Bn-C6-p22826720	Bn-scaff_15818_1-p516014	C6	31	32.8	A
17842	Bn-N16-p15893451	Bn-C6-p22826448	Bn-scaff_15818_1-p516286	C6	31	32.8	A
17843	Bn-N16-p15980464	Bn-C6-p22740087	Bn-scaff_15818_1-p602647	C6	31	32.8	A
17844	Bn-N16-p16051335	Bn-C6-p22674840	Bn-scaff_15818_1-p667894	C6	31	32.8	A
17845	Bn-N16-p16055999	Bn-C6-p22664529	Bn-scaff_15818_1-p678205	C6	31	32.8	A
17846	Bn-N16-p16105376	Bn-C6-p22629367	Bn-scaff_15818_1-p713367	C6	31	32.8	A
17847	Bn-N16-p16899526	Bn-C6-p21899627	Bn-scaff_15818_2-p176007	C6	31	32.8	A
17848	Bn-N16-p16905392	Bn-C6-p21892846	Bn-scaff_15818_2-p182788	C6	31	32.8	A
17849	Bn-N16-p16906503	Bn-C6-p21891735	Bn-scaff_15818_2-p183899	C6	31	32.8	A
17850	Bn-N16-p16906822	Bn-C6-p21891416	Bn-scaff_15818_2-p184218	C6	31	32.8	A
17851	Bn-N16-p16906891	Bn-C6-p21891347	Bn-scaff_15818_2-p184287	C6	31	32.8	A
17852	Bn-N16-p16907324	Bn-C6-p21890910	Bn-scaff_15818_2-p184724	C6	31	32.8	A
17853	Bn-N16-p16907549	Bn-C6-p21890685	Bn-scaff_15818_2-p184949	C6	31	32.8	A
17854	Bn-N16-p16907656	Bn-C6-p21890578	Bn-scaff_15818_2-p185056	C6	31	32.8	A
17855	Bn-N16-p16908329	Bn-C6-p21889893	Bn-scaff_15818_2-p185741	C6	31	32.8	A
17856	Bn-N16-p17029052	Bn-C6-p21786722	Bn-scaff_15818_2-p288912	C6	31	32.8	A
17857	Bn-N16-p17029188	Bn-C6-p21786627	Bn-scaff_15818_2-p289007	C6	31	32.8	A
17858	Bn-N16-p12557485	Bn-C6-p26089476	Bn-scaff_18439_1-p304224	C6	32	33.6	A
17859	Bn-N16-p12565450	Bn-C6-p26081519	Bn-scaff_18439_1-p312181	C6	32	33.6	A
17860	Bn-N16-p12565529	Bn-C6-p26081440	Bn-scaff_18439_1-p312260	C6	32	33.6	A
17861	Bn-N16-p12566451	Bn-C6-p26080518	Bn-scaff_18439_1-p313182	C6	32	33.6	A
17862	Bn-N16-p12567298	Bn-C6-p26079671	Bn-scaff_18439_1-p314029	C6	32	33.6	A
17863	Bn-N16-p12567855	Bn-C6-p26079114	Bn-scaff_18439_1-p314586	C6	32	33.6	A
17864	Bn-N16-p12568812	Bn-C6-p26078157	Bn-scaff_18439_1-p315543	C6	32	33.6	A
17865	Bn-N16-p12568896	Bn-C6-p26078073	Bn-scaff_18439_1-p315627	C6	32	33.6	A
17866	Bn-N16-p12568931	Bn-C6-p26078038	Bn-scaff_18439_1-p315662	C6	32	33.6	A
17867	Bn-N16-p12569130	Bn-C6-p26077839	Bn-scaff_18439_1-p315861	C6	32	33.6	A
17868	Bn-N16-p12569285	Bn-C6-p26077684	Bn-scaff_18439_1-p316016	C6	32	33.6	A
17869	Bn-N16-p12570382	Bn-C6-p26076587	Bn-scaff_18439_1-p317113	C6	32	33.6	A
17870	Bn-N16-p12582608	Bn-C6-p26064500	Bn-scaff_18439_1-p329200	C6	32	33.6	A
17871	Bn-N16-p12584254	Bn-C6-p26062854	Bn-scaff_18439_1-p330846	C6	32	33.6	A
17872	Bn-N16-p12663412	Bn-C6-p25972850	Bn-scaff_18439_1-p420850	C6	32	33.6	A
17873	Bn-N16-p12819330	Bn-C6-p25818706	Bn-scaff_18439_1-p574994	C6	32	33.6	A
17874	Bn-N16-p12836084	Bn-C6-p25802989	Bn-scaff_18439_1-p590711	C6	32	33.6	A
17875	Bn-N16-p12877758	Bn-C6-p25765624	Bn-scaff_18439_1-p628076	C6	32	33.6	A
17876	Bn-N16-p12878069	Bn-C6-p25765314	Bn-scaff_18439_1-p628386	C6	32	33.6	A
17877	Bn-N16-p12878408	Bn-C6-p25764965	Bn-scaff_18439_1-p628735	C6	32	33.6	A
17878	Bn-N16-p12882603	Bn-C6-p25761846	Bn-scaff_18439_1-p631854	C6	32	33.6	A
17879	Bn-N16-p12883486	Bn-C6-p25760960	Bn-scaff_18439_1-p632740	C6	32	33.6	A
17880	Bn-N16-p12884195	Bn-C6-p25760251	Bn-scaff_18439_1-p633449	C6	32	33.6	A

17881	Bn-N16-p12884254	Bn-C6-p25760192	Bn-scaff_18439_1-p633508	C6	32	33.6	A
17882	Bn-N16-p12884720	Bn-C6-p25759727	Bn-scaff_18439_1-p633973	C6	32	33.6	A
17883	Bn-N16-p12887195	Bn-C6-p25754041	Bn-scaff_18439_1-p639659	C6	32	33.6	A
17884	Bn-N16-p12891758	Bn-C6-p25749907	Bn-scaff_18439_1-p643793	C6	32	33.6	A
17885	Bn-N16-p13206497	Bn-C6-p25441474	Bn-scaff_18439_1-p952226	C6	32	33.6	A
17886	Bn-N16-p13300635	Bn-C6-p25343334	Bn-scaff_22133_1-p31674	C6	32	33.6	A
17887	Bn-N16-p13874466	Bn-C6-p24650619	Bn-scaff_16510_1-p325332	C6	32	33.6	A
17888	Bn-N16-p13921356	Bn-C6-p24605112	Bn-scaff_16510_1-p279825	C6	32	33.6	A
17889	Bn-N16-p13921874	Bn-C6-p24604594	Bn-scaff_16510_1-p279307	C6	32	33.6	A
17890	Bn-N16-p13964534	Bn-C6-p24567794	Bn-scaff_16510_1-p242507	C6	32	33.6	A
17891	Bn-N16-p13967767	Bn-C6-p24564565	Bn-scaff_16510_1-p239278	C6	32	33.6	A
17892	Bn-N16-p13972792	Bn-C6-p24559527	Bn-scaff_16510_1-p234240	C6	32	33.6	A
17893	Bn-N16-p14099650	Bn-C6-p24414191	Bn-scaff_16510_1-p88904	C6	32	33.6	A
17894	Bn-N16-p14100407	Bn-C6-p24413425	Bn-scaff_16510_1-p88138	C6	32	33.6	A
17895	Bn-N16-p14551636	Bn-C6-p24007689	Bn-scaff_21352_1-p81801	C6	32	33.6	A
17896	Bn-N16-p14586882	Bn-C6-p23971396	Bn-scaff_21352_1-p118094	C6	32	33.6	A
17897	Bn-N16-p14586894	Bn-C6-p23971384	Bn-scaff_21352_1-p118106	C6	32	33.6	A
17898	Bn-N16-p14588689	Bn-C6-p23969581	Bn-scaff_21352_1-p119909	C6	32	33.6	A
17899	Bn-N16-p14592295	Bn-C6-p23965978	Bn-scaff_21352_1-p123512	C6	32	33.6	A
17900	Bn-N16-p14630612	Bn-C6-p23930592	Bn-scaff_21352_1-p158898	C6	32	33.6	A
17901	Bn-N16-p14650305	Bn-C6-p23912225	Bn-scaff_21352_1-p177265	C6	32	33.6	A
17902	Bn-N16-p14658626	Bn-C6-p23903898	Bn-scaff_21352_1-p185592	C6	32	33.6	A
17903	Bn-N16-p14710047	Bn-C6-p23832076	Bn-scaff_21352_1-p257414	C6	32	33.6	A
17904	Bn-N16-p14711426	Bn-C6-p23830694	Bn-scaff_21352_1-p258796	C6	32	33.6	A
17905	Bn-N16-p14711919	Bn-C6-p23830202	Bn-scaff_21352_1-p259288	C6	32	33.6	A
17906	Bn-N16-p14727491	Bn-C6-p23810715	Bn-scaff_17867_1-p73100	C6	32	33.6	A
17907	Bn-N16-p14727779	Bn-C6-p23810427	Bn-scaff_17867_1-p72812	C6	32	33.6	A
17908	Bn-N16-p14728019	Bn-C6-p23810187	Bn-scaff_17867_1-p72572	C6	32	33.6	A
17909	Bn-N16-p14728372	Bn-C6-p23809830	Bn-scaff_17867_1-p72215	C6	32	33.6	A
17910	Bn-N16-p14728636	Bn-C6-p23809566	Bn-scaff_17867_1-p71951	C6	32	33.6	A
17911	Bn-N16-p14730279	Bn-C6-p23807922	Bn-scaff_17867_1-p70307	C6	32	33.6	A
17912	Bn-N16-p14731102	Bn-C6-p23807068	Bn-scaff_17867_1-p69453	C6	32	33.6	A
17913	Bn-N16-p14734459	Bn-C6-p23803665	Bn-scaff_17867_1-p66050	C6	32	33.6	A
17914	Bn-N16-p14734882	Bn-C6-p23803242	Bn-scaff_17867_1-p65627	C6	32	33.6	A
17915	Bn-N16-p14741738	Bn-C6-p23796609	Bn-scaff_17867_1-p58994	C6	32	33.6	A
17916	Bn-N16-p14744268	Bn-C6-p23794095	Bn-scaff_17867_1-p56480	C6	32	33.6	A
17917	Bn-N16-p14746136	Bn-C6-p23792234	Bn-scaff_17867_1-p54619	C6	32	33.6	A
17918	Bn-N16-p14746649	Bn-C6-p23791721	Bn-scaff_17867_1-p54106	C6	32	33.6	A
17919	Bn-N16-p14749175	Bn-C6-p23788400	Bn-scaff_17867_1-p50785	C6	32	33.6	A
17920	Bn-N16-p14750738	Bn-C6-p23786825	Bn-scaff_17867_1-p49210	C6	32	33.6	A
17921	Bn-N16-p14758775	Bn-C6-p23779457	Bn-scaff_17867_1-p41842	C6	32	33.6	A
17922	Bn-N16-p14759335	Bn-C6-p23778896	Bn-scaff_17867_1-p41281	C6	32	33.6	A
17923	Bn-N16-p14759401	Bn-C6-p23778831	Bn-scaff_17867_1-p41216	C6	32	33.6	A
17924	Bn-N16-p14760044	Bn-C6-p23778190	Bn-scaff_17867_1-p40575	C6	32	33.6	A
17925	Bn-N16-p14760101	Bn-C6-p23778133	Bn-scaff_17867_1-p40518	C6	32	33.6	A
17926	Bn-N16-p14762060	Bn-C6-p23776183	Bn-scaff_17867_1-p38568	C6	32	33.6	A
17927	Bn-N16-p14762165	Bn-C6-p23776078	Bn-scaff_17867_1-p38463	C6	32	33.6	A
17928	Bn-N16-p14762577	Bn-C6-p23775666	Bn-scaff_17867_1-p38051	C6	32	33.6	A
17929	Bn-N16-p14763179	Bn-C6-p23775062	Bn-scaff_17867_1-p37447	C6	32	33.6	A

17930	Bn-N16-p14763447	Bn-C6-p23774794	Bn-scaff_17867_1-p37179	C6	32	33.6	A
17931	Bn-N16-p14763759	Bn-C6-p23774482	Bn-scaff_17867_1-p36867	C6	32	33.6	A
17932	Bn-N16-p14764755	Bn-C6-p23773486	Bn-scaff_17867_1-p35871	C6	32	33.6	A
17933	Bn-N16-p14766047	Bn-C6-p23772193	Bn-scaff_17867_1-p34578	C6	32	33.6	A
17934	Bn-N16-p14766447	Bn-C6-p23771793	Bn-scaff_17867_1-p34178	C6	32	33.6	A
17935	Bn-N16-p14766499	Bn-C6-p23771741	Bn-scaff_17867_1-p34126	C6	32	33.6	A
17936	Bn-N16-p14766839	Bn-C6-p23771401	Bn-scaff_17867_1-p33786	C6	32	33.6	A
17937	Bn-N16-p14767144	Bn-C6-p23770638	Bn-scaff_17867_1-p33023	C6	32	33.6	A
17938	Bn-N16-p14772844	Bn-C6-p23764946	Bn-scaff_17867_1-p27331	C6	32	33.6	A
17939	Bn-N16-p14791476	Bn-C6-p23747830	Bn-scaff_17867_1-p10215	C6	32	33.6	A
17940	Bn-N16-p14792910	Bn-C6-p23746396	Bn-scaff_17867_1-p8781	C6	32	33.6	A
17941	Bn-N16-p14793428	Bn-C6-p23745880	Bn-scaff_17867_1-p8265	C6	32	33.6	A
17942	Bn-N16-p14804372	Bn-Scaffold00955-p34	Bn-scaff_28522_1-p34164	C6	32	33.6	A
17943	Bn-N16-p14805398	Bn-Scaffold00955-p27	Bn-scaff_28522_1-p27206	C6	32	33.6	A
17944	Bn-N16-p14805867	Bn-Scaffold00955-p26	Bn-scaff_28522_1-p26738	C6	32	33.6	A
17945	Bn-N16-p15305037	Bn-Scaffold00667-p48	Bn-scaff_16903_1-p230137	C6	32	33.6	A
17946	Bn-N16-p15715221	Bn-C6-p23044779	Bn-scaff_15818_1-p297955	C6	32	33.6	A
17947	Bn-N16-p15773416	Bn-C6-p22997170	Bn-scaff_15818_1-p345564	C6	32	33.6	A
17948	Bn-N16-p15779978	Bn-C6-p22990529	Bn-scaff_15818_1-p352205	C6	32	33.6	A
17949	Bn-N16-p15844116	Bn-C6-p22889506	Bn-scaff_15818_1-p453228	C6	32	33.6	A
17950	Bn-N16-p15844318	Bn-C6-p22889305	Bn-scaff_15818_1-p453429	C6	32	33.6	A
17951	Bn-N16-p15844395	Bn-C6-p22889228	Bn-scaff_15818_1-p453506	C6	32	33.6	A
17952	Bn-N16-p15846358	Bn-C6-p22887535	Bn-scaff_15818_1-p455199	C6	32	33.6	A
17953	Bn-N16-p15849625	Bn-C6-p22875041	Bn-scaff_15818_1-p467693	C6	32	33.6	A
17954	Bn-N16-p15850330	Bn-C6-p22874334	Bn-scaff_15818_1-p468400	C6	32	33.6	A
17955	Bn-N16-p15851302	Bn-C6-p22873359	Bn-scaff_15818_1-p469375	C6	32	33.6	A
17956	Bn-N16-p15858421	Bn-C6-p22871628	Bn-scaff_15818_1-p471106	C6	32	33.6	A
17957	Bn-N16-p24904539	Bn-Scaffold00667-p42	Bn-scaff_16903_1-p224220	C6	32	33.6	A
17958	Bn-Scaffold01360-p7768	Bn-C6-p23873209	Bn-scaff_21352_1-p216281	C6	32	33.6	A
17959	Bn-Scaffold03518-p2013	Bn-C6-p23825595	Bn-scaff_21352_1-p263895	C6	32	33.6	A
17960	Bn-N12-p29932440	Bn-C2-p37162793	Bn-scaff_16545_1-p874419	C6	33	34.4	A
17961	Bn-N15-p49842655	Bn-C6-p26606137	Bn-scaff_17044_1-p462747	C6	33	34.4	A
17962	Bn-N16-p10004183	Bn-C6-p28653710	Bn-scaff_17984_1-p942824	C6	33	34.4	A
17963	Bn-N16-p10094412	Bn-C6-p28787698	Bn-scaff_17984_1-p107681	C6	33	34.4	A
17964	Bn-N16-p10512357	Bn-C6-p28351952	Bn-scaff_17984_1-p641066	C6	33	34.4	A
17965	Bn-N16-p10604608	Bn-C6-p28263334	Bn-scaff_17984_1-p552448	C6	33	34.4	A
17966	Bn-N16-p10633517	Bn-C6-p28228580	Bn-scaff_17984_1-p517694	C6	33	34.4	A
17967	Bn-N16-p10806519	Bn-C6-p28084180	Bn-scaff_17984_1-p373294	C6	33	34.4	A
17968	Bn-N16-p10970549	Bn-C6-p27933876	Bn-scaff_17984_1-p222990	C6	33	34.4	A
17969	Bn-N16-p10971267	Bn-C6-p27933157	Bn-scaff_17984_1-p222271	C6	33	34.4	A
17970	Bn-N16-p11028244	Bn-C6-p27832623	Bn-scaff_17984_1-p121737	C6	33	34.4	A
17971	Bn-N16-p11028490	Bn-C6-p27832868	Bn-scaff_17984_1-p121982	C6	33	34.4	A
17972	Bn-N16-p11195711	Bn-C6-p27705154	Bn-scaff_16297_1-p99269	C6	33	34.4	A
17973	Bn-N16-p11207071	Bn-C6-p27685736	Bn-scaff_16297_1-p79851	C6	33	34.4	A
17974	Bn-N16-p12222813	Bn-Scaffold00534-p23	Bn-scaff_17088_2-p232944	C6	33	34.4	A
17975	Bn-N16-p12471912	Bn-Scaffold00534-p11	Bn-scaff_17088_2-p119991	C6	33	34.4	A
17976	Bn-N16-p12471913	Bn-Scaffold00534-p11	Bn-scaff_17088_2-p119992	C6	33	34.4	A
17977	Bn-N16-p12476861	Bn-Scaffold00534-p12	Bn-scaff_17088_2-p124900	C6	33	34.4	A
17978	Bn-N16-p12509480	Bn-Scaffold00534-p16	Bn-scaff_17088_2-p163374	C6	33	34.4	A

17979	Bn-N16-p12518273	Bn-Scaffold00534-p1(	Bn-scaff_17088_2-p16576{	C6	33	34.4	A
17980	Bn-N16-p12527060	Bn-C6-p26115765	Bn-scaff_18439_1-p27793!	C6	33	34.4	A
17981	Bn-N16-p14559385	Bn-C6-p23994353	Bn-scaff_21352_1-p95137	C6	33	34.4	A
17982	Bn-N16-p29572196	Bn-Scaffold01163-p1!	Bn-scaff_27218_1-p19974	C6	33	34.4	A
17983	Bn-N16-p8018792	Bn-C6-p31402078	Bn-scaff_23199_1-p46390:	C6	33	34.4	A
17984	Bn-N16-p8281226	Bn-C6-p31162179	Bn-scaff_17917_1-p19148⁄	C6	33	34.4	A
17985	Bn-N16-p8284167	Bn-C6-p31159239	Bn-scaff_17917_1-p18854⁄	C6	33	34.4	A
17986	Bn-N16-p8285149	Bn-C6-p31157988	Bn-scaff_17917_1-p18729:	C6	33	34.4	A
17987	Bn-N16-p8369721	Bn-C6-p31079375	Bn-scaff_17917_1-p10868(	C6	33	34.4	A
17988	Bn-N16-p8374990	Bn-C6-p31074106	Bn-scaff_17917_1-p10341:	C6	33	34.4	A
17989	Bn-N16-p8405929	Bn-C6-p31047517	Bn-scaff_17917_1-p76822	C6	33	34.4	A
17990	Bn-N16-p8407140	Bn-C6-p31043805	Bn-scaff_17917_1-p73110	C6	33	34.4	A
17991	Bn-N16-p8418570	Bn-C6-p31037793	Bn-scaff_17917_1-p67098	C6	33	34.4	A
17992	Bn-N16-p9229439	Bn-C6-p30125093	Bn-scaff_18002_1-p24084:	C6	33	34.4	A
17993	Bn-N16-p9230206	Bn-C6-p30124326	Bn-scaff_18002_1-p24161(	C6	33	34.4	A
17994	Bn-N16-p9942476	Bn-C6-p28598432	Bn-scaff_17984_1-p88754(	C6	33	34.4	A
17995	Bn-N16-p9970902	Bn-C6-p28621056	Bn-scaff_17984_1-p91017(	C6	33	34.4	A
17996	Bn-N16-p9971193	Bn-C6-p28621347	Bn-scaff_17984_1-p91046:	C6	33	34.4	A
17997	Bn-N16-p9971444	Bn-C6-p28621598	Bn-scaff_17984_1-p91071:	C6	33	34.4	A
17998	Bn-N16-p9971613	Bn-C6-p28621767	Bn-scaff_17984_1-p91088:	C6	33	34.4	A
17999	Bn-N18-p831461	Bn-C8-p667281	Bn-scaff_18412_1-p29779	C6	33	34.4	A
18000	Bn-N2-p24295397	Bn-C1-p32179166	Bn-scaff_17517_1-p37782:	C6	33	34.4	A
18001	Bn-N6-p204046	Bn-A06-p288377	Bn-A06-p288377	C6	33	34.4	A
18002	Bn-N13-p15464313	Bn-C6-p32039066	Bn-scaff_18936_1-p12654(	C6	34	35.2	A
18003	Bn-N16-p5360756	Bn-C6-p34220372	Bn-scaff_20773_1-p48124⁄	C6	34	35.2	A
18004	Bn-N16-p5368962	Bn-C6-p34211357	Bn-scaff_20773_1-p47222!	C6	34	35.2	A
18005	Bn-N16-p5376590	Bn-C6-p34203712	Bn-scaff_20773_1-p46458⁄	C6	34	35.2	A
18006	Bn-N16-p5376707	Bn-C6-p34203595	Bn-scaff_20773_1-p46446;	C6	34	35.2	A
18007	Bn-N16-p5377598	Bn-C6-p34202725	Bn-scaff_20773_1-p46359;	C6	34	35.2	A
18008	Bn-N16-p5377649	Bn-C6-p34202674	Bn-scaff_20773_1-p46354(	C6	34	35.2	A
18009	Bn-N16-p5378103	Bn-C6-p34202223	Bn-scaff_20773_1-p46309!	C6	34	35.2	A
18010	Bn-N16-p5379018	Bn-C6-p34201306	Bn-scaff_20773_1-p46217{	C6	34	35.2	A
18011	Bn-N16-p5409695	Bn-C6-p34185647	Bn-scaff_20773_1-p44651!	C6	34	35.2	A
18012	Bn-N16-p5421873	Bn-C6-p34184191	Bn-scaff_20773_1-p44506:	C6	34	35.2	A
18013	Bn-N16-p5432612	Bn-C6-p34170392	Bn-scaff_20773_1-p43126⁄	C6	34	35.2	A
18014	Bn-N16-p5450230	Bn-C6-p34151782	Bn-scaff_20773_1-p41265⁄	C6	34	35.2	A
18015	Bn-N16-p5462199	Bn-C6-p34140911	Bn-scaff_20773_1-p40178:	C6	34	35.2	A
18016	Bn-N16-p5504548	Bn-C6-p34088688	Bn-scaff_20773_1-p34956(	C6	34	35.2	A
18017	Bn-N16-p5505143	Bn-C6-p34088093	Bn-scaff_20773_1-p34896!	C6	34	35.2	A
18018	Bn-N16-p5513483	Bn-C6-p34079996	Bn-scaff_20773_1-p34086{	C6	34	35.2	A
18019	Bn-N16-p5518436	Bn-C6-p34078833	Bn-scaff_20773_1-p33970!	C6	34	35.2	A
18020	Bn-N16-p5750855	Bn-C6-p33892228	Bn-scaff_20773_1-p15310(	C6	34	35.2	A
18021	Bn-N16-p5961851	Bn-C6-p33675607	Bn-scaff_16647_1-p49992(	C6	34	35.2	A
18022	Bn-N16-p5965814	Bn-C6-p33671566	Bn-scaff_16647_1-p49587!	C6	34	35.2	A
18023	Bn-N16-p5973313	Bn-C6-p33661687	Bn-scaff_16647_1-p48600(	C6	34	35.2	A
18024	Bn-N16-p5990387	Bn-C6-p33639959	Bn-scaff_16647_1-p46427:	C6	34	35.2	A
18025	Bn-N16-p5992011	Bn-C6-p33638337	Bn-scaff_16647_1-p46265(	C6	34	35.2	A
18026	Bn-N16-p5994276	Bn-C6-p33636137	Bn-scaff_16647_1-p46045(	C6	34	35.2	A
18027	Bn-N16-p6022253	Bn-C6-p33613699	Bn-scaff_16647_1-p43801:	C6	34	35.2	A

18028	Bn-N16-p6022389	Bn-C6-p33613563	Bn-scaff_16647_1-p437876	C6	34	35.2	A
18029	Bn-N16-p6025296	Bn-C6-p33610655	Bn-scaff_16647_1-p434968	C6	34	35.2	A
18030	Bn-N16-p6027109	Bn-C6-p33608842	Bn-scaff_16647_1-p433155	C6	34	35.2	A
18031	Bn-N16-p6027206	Bn-C6-p33608745	Bn-scaff_16647_1-p433058	C6	34	35.2	A
18032	Bn-N16-p6029087	Bn-C6-p33606864	Bn-scaff_16647_1-p431177	C6	34	35.2	A
18033	Bn-N16-p6029102	Bn-C6-p33606849	Bn-scaff_16647_1-p431162	C6	34	35.2	A
18034	Bn-N16-p6031090	Bn-C6-p33604860	Bn-scaff_16647_1-p429173	C6	34	35.2	A
18035	Bn-N16-p6081277	Bn-C6-p33523056	Bn-scaff_16647_1-p347369	C6	34	35.2	A
18036	Bn-N16-p6177452	Bn-C6-p33415644	Bn-scaff_16647_1-p239957	C6	34	35.2	A
18037	Bn-N16-p6210755	Bn-C6-p33388569	Bn-scaff_16647_1-p212882	C6	34	35.2	A
18038	Bn-N16-p6231887	Bn-C6-p33372585	Bn-scaff_16647_1-p196898	C6	34	35.2	A
18039	Bn-N16-p6237595	Bn-C6-p33366205	Bn-scaff_16647_1-p190518	C6	34	35.2	A
18040	Bn-N16-p6237846	Bn-C6-p33365954	Bn-scaff_16647_1-p190267	C6	34	35.2	A
18041	Bn-N16-p6237999	Bn-C6-p33365801	Bn-scaff_16647_1-p190114	C6	34	35.2	A
18042	Bn-N16-p6238628	Bn-C6-p33365174	Bn-scaff_16647_1-p189487	C6	34	35.2	A
18043	Bn-N16-p6238725	Bn-C6-p33365074	Bn-scaff_16647_1-p189387	C6	34	35.2	A
18044	Bn-N16-p6310119	Bn-C6-p33345282	Bn-scaff_16647_1-p169595	C6	34	35.2	A
18045	Bn-N16-p6310570	Bn-C6-p33344831	Bn-scaff_16647_1-p169144	C6	34	35.2	A
18046	Bn-N16-p6310638	Bn-C6-p33344763	Bn-scaff_16647_1-p169076	C6	34	35.2	A
18047	Bn-N16-p6310832	Bn-C6-p33344569	Bn-scaff_16647_1-p168882	C6	34	35.2	A
18048	Bn-N16-p6317069	Bn-C6-p33338334	Bn-scaff_16647_1-p162647	C6	34	35.2	A
18049	Bn-N16-p6441178	Bn-C6-p33243341	Bn-scaff_16647_1-p67654	C6	34	35.2	A
18050	Bn-N16-p6473703	Bn-C6-p33210318	Bn-scaff_16647_1-p34631	C6	34	35.2	A
18051	Bn-N16-p6474896	Bn-C6-p33209125	Bn-scaff_16647_1-p33438	C6	34	35.2	A
18052	Bn-N16-p6475555	Bn-C6-p33208466	Bn-scaff_16647_1-p32779	C6	34	35.2	A
18053	Bn-N16-p6475892	Bn-C6-p33208129	Bn-scaff_16647_1-p32442	C6	34	35.2	A
18054	Bn-N16-p6506342	Bn-C6-p33176916	Bn-scaff_16647_1-p1229	C6	34	35.2	A
18055	Bn-N16-p6535816	Bn-C6-p32829560	Bn-scaff_18936_1-p205595	C6	34	35.2	A
18056	Bn-N16-p6537147	Bn-C6-p32828229	Bn-scaff_18936_1-p205462	C6	34	35.2	A
18057	Bn-N16-p6539573	Bn-C6-p32825803	Bn-scaff_18936_1-p205219	C6	34	35.2	A
18058	Bn-N16-p6548610	Bn-C6-p32816681	Bn-scaff_18936_1-p204307	C6	34	35.2	A
18059	Bn-N16-p6565691	Bn-C6-p32803653	Bn-scaff_18936_1-p203004	C6	34	35.2	A
18060	Bn-N16-p6568241	Bn-C6-p32801091	Bn-scaff_18936_1-p202748	C6	34	35.2	A
18061	Bn-N16-p6569484	Bn-C6-p32799843	Bn-scaff_18936_1-p202623	C6	34	35.2	A
18062	Bn-N16-p6660672	Bn-C6-p32730898	Bn-scaff_18936_1-p195729	C6	34	35.2	A
18063	Bn-N16-p6679197	Bn-C6-p32706490	Bn-scaff_18936_1-p193288	C6	34	35.2	A
18064	Bn-N16-p6680464	Bn-C6-p32705230	Bn-scaff_18936_1-p193162	C6	34	35.2	A
18065	Bn-N16-p6711794	Bn-C6-p32662561	Bn-scaff_18936_1-p188895	C6	34	35.2	A
18066	Bn-N16-p6849884	Bn-C6-p32533596	Bn-scaff_18936_1-p175999	C6	34	35.2	A
18067	Bn-N16-p6851002	Bn-C6-p32532507	Bn-scaff_18936_1-p175890	C6	34	35.2	A
18068	Bn-N16-p6862181	Bn-C6-p32520681	Bn-scaff_18936_1-p174707	C6	34	35.2	A
18069	Bn-N16-p6876898	Bn-C6-p32506209	Bn-scaff_18936_1-p173260	C6	34	35.2	A
18070	Bn-N16-p6879888	Bn-C5-p37643549	Bn-scaff_16770_1-p617272	C6	34	35.2	A
18071	Bn-N16-p6880042	Bn-A01-p6512377	Bn-A01-p6512377	C6	34	35.2	A
18072	Bn-N16-p6906540	Bn-C6-p32480351	Bn-scaff_18936_1-p170674	C6	34	35.2	A
18073	Bn-N16-p6919339	Bn-C6-p32461159	Bn-scaff_18936_1-p168755	C6	34	35.2	A
18074	Bn-N16-p6929192	Bn-C6-p32449064	Bn-scaff_18936_1-p167545	C6	34	35.2	A
18075	Bn-N16-p6996021	Bn-C6-p32398271	Bn-scaff_18936_1-p162466	C6	34	35.2	A
18076	Bn-N16-p7000569	Bn-C6-p32392125	Bn-scaff_18936_1-p161852	C6	34	35.2	A

18077	Bn-N16-p7002374	Bn-C6-p32383738	Bn-scaff_18936_1-p161013	C6	34	35.2	A
18078	Bn-N16-p7010383	Bn-C6-p32376465	Bn-scaff_18936_1-p160286	C6	34	35.2	A
18079	Bn-N16-p7014671	Bn-C6-p32370395	Bn-scaff_18936_1-p159679	C6	34	35.2	A
18080	Bn-N16-p7015902	Bn-C6-p32369431	Bn-scaff_18936_1-p159582	C6	34	35.2	A
18081	Bn-N16-p7016752	Bn-C6-p32368577	Bn-scaff_18936_1-p159497	C6	34	35.2	A
18082	Bn-N16-p7019489	Bn-C6-p32365842	Bn-scaff_18936_1-p159223	C6	34	35.2	A
18083	Bn-N16-p7026583	Bn-C6-p32356901	Bn-scaff_18936_1-p158329	C6	34	35.2	A
18084	Bn-N16-p7030553	Bn-C6-p32352953	Bn-scaff_18936_1-p157934	C6	34	35.2	A
18085	Bn-N16-p7031121	Bn-C6-p32352385	Bn-scaff_18936_1-p157878	C6	34	35.2	A
18086	Bn-N16-p7053123	Bn-C6-p32345806	Bn-scaff_18936_1-p157220	C6	34	35.2	A
18087	Bn-N16-p7053240	Bn-C6-p32345689	Bn-scaff_18936_1-p157208	C6	34	35.2	A
18088	Bn-N16-p7074642	Bn-C6-p32335199	Bn-scaff_18936_1-p156159	C6	34	35.2	A
18089	Bn-N16-p7079593	Bn-C6-p32330798	Bn-scaff_18936_1-p155719	C6	34	35.2	A
18090	Bn-N16-p7080179	Bn-C6-p32330364	Bn-scaff_18936_1-p155675	C6	34	35.2	A
18091	Bn-N16-p7080491	Bn-C6-p32330051	Bn-scaff_18936_1-p155644	C6	34	35.2	A
18092	Bn-N16-p7080556	Bn-C6-p32329986	Bn-scaff_18936_1-p155638	C6	34	35.2	A
18093	Bn-N16-p7081019	Bn-C6-p32329523	Bn-scaff_18936_1-p155591	C6	34	35.2	A
18094	Bn-N16-p7081574	Bn-C6-p32328968	Bn-scaff_18936_1-p155536	C6	34	35.2	A
18095	Bn-N16-p7083574	Bn-C6-p32327465	Bn-scaff_18936_1-p155386	C6	34	35.2	A
18096	Bn-N16-p7086408	Bn-C6-p32324933	Bn-scaff_18936_1-p155132	C6	34	35.2	A
18097	Bn-N16-p7086873	Bn-C6-p32324467	Bn-scaff_18936_1-p155086	C6	34	35.2	A
18098	Bn-N16-p7091057	Bn-C6-p32315934	Bn-scaff_18936_1-p154232	C6	34	35.2	A
18099	Bn-N16-p7113011	Bn-C6-p32295105	Bn-scaff_18936_1-p152150	C6	34	35.2	A
18100	Bn-N16-p7113484	Bn-C6-p32294634	Bn-scaff_18936_1-p152102	C6	34	35.2	A
18101	Bn-N16-p7587999	Bn-C6-p31826197	Bn-scaff_23199_1-p39784	C6	34	35.2	A
18102	Bn-N16-p7783189	Bn-C6-p31616363	Bn-scaff_23199_1-p249618	C6	34	35.2	A
18103	Bn-N18-p29352777	Bn-C6-p33399352	Bn-scaff_16647_1-p223665	C6	34	35.2	A
18104	Bn-Scaffold12448-p133	Bn-C6-p33340548	Bn-scaff_16647_1-p164861	C6	34	35.2	A
18105	Bn-Scaffold12448-p603	Bn-C6-p33340078	Bn-scaff_16647_1-p164391	C6	34	35.2	A
18106	Bn-N16-p4535779	Bn-C6-p35031306	Bn-scaff_18344_1-p341469	C6	35	36	A
18107	Bn-N16-p4535925	Bn-C6-p35031160	Bn-scaff_18344_1-p341615	C6	35	36	A
18108	Bn-N16-p4537277	Bn-C6-p25431867	Bn-scaff_18439_1-p961833	C6	35	36	A
18109	Bn-N16-p4572026	Bn-C6-p34985528	Bn-scaff_18344_1-p387247	C6	35	36	A
18110	Bn-N16-p4572727	Bn-C6-p34984827	Bn-scaff_18344_1-p387948	C6	35	36	A
18111	Bn-N16-p4573134	Bn-C6-p34984419	Bn-scaff_18344_1-p388356	C6	35	36	A
18112	Bn-N16-p4573804	Bn-C6-p34983747	Bn-scaff_18344_1-p389028	C6	35	36	A
18113	Bn-N16-p4575468	Bn-C6-p34982083	Bn-scaff_18344_1-p390692	C6	35	36	A
18114	Bn-N16-p4576401	Bn-C6-p34981150	Bn-scaff_18344_1-p391625	C6	35	36	A
18115	Bn-N16-p4576432	Bn-C6-p34981119	Bn-scaff_18344_1-p391656	C6	35	36	A
18116	Bn-N16-p4582839	Bn-C6-p34975755	Bn-scaff_18344_1-p397020	C6	35	36	A
18117	Bn-N16-p4583683	Bn-C6-p34974911	Bn-scaff_18344_1-p397864	C6	35	36	A
18118	Bn-N16-p4583960	Bn-C6-p34974634	Bn-scaff_18344_1-p398141	C6	35	36	A
18119	Bn-N16-p4675427	Bn-C6-p34878714	Bn-scaff_18344_1-p494061	C6	35	36	A
18120	Bn-N16-p4681200	Bn-C6-p34872563	Bn-scaff_18344_1-p500212	C6	35	36	A
18121	Bn-N16-p4693158	Bn-C6-p34860482	Bn-scaff_18344_1-p512293	C6	35	36	A
18122	Bn-N16-p5292603	Bn-C6-p34277940	Bn-scaff_20773_1-p538812	C6	35	36	A
18123	Bn-N13-p4778478	Bn-C6-p35554964	Bn-scaff_27872_1-p67199	C6	36	36.8	A
18124	Bn-N13-p4784500	Bn-C6-p35547681	Bn-scaff_27872_1-p59916	C6	36	36.8	A
18125	Bn-N13-p4797513	Bn-C6-p35545799	Bn-scaff_27872_1-p58034	C6	36	36.8	A

18126	Bn-N13-p4797602	Bn-C6-p35545710	Bn-scaff_27872_1-p57945	C6	36	36.8	A
18127	Bn-N13-p4798066	Bn-C6-p35545586	Bn-scaff_27872_1-p57821	C6	36	36.8	A
18128	Bn-N13-p4799229	Bn-C6-p35544422	Bn-scaff_27872_1-p56657	C6	36	36.8	A
18129	Bn-N13-p4805341	Bn-C6-p35536531	Bn-scaff_27872_1-p48766	C6	36	36.8	A
18130	Bn-N13-p4805578	Bn-C6-p35536294	Bn-scaff_27872_1-p48529	C6	36	36.8	A
18131	Bn-N13-p4805588	Bn-C6-p35536284	Bn-scaff_27872_1-p48519	C6	36	36.8	A
18132	Bn-N13-p4805773	Bn-C6-p35536099	Bn-scaff_27872_1-p48334	C6	36	36.8	A
18133	Bn-N13-p4805811	Bn-C6-p35536061	Bn-scaff_27872_1-p48296	C6	36	36.8	A
18134	Bn-N13-p4806786	Bn-C6-p35535087	Bn-scaff_27872_1-p47322	C6	36	36.8	A
18135	Bn-N13-p4807089	Bn-C6-p35534815	Bn-scaff_27872_1-p47050	C6	36	36.8	A
18136	Bn-N13-p4814849	Bn-C6-p35527071	Bn-scaff_27872_1-p39306	C6	36	36.8	A
18137	Bn-N13-p4851419	Bn-C6-p35497970	Bn-scaff_27872_1-p10205	C6	36	36.8	A
18138	Bn-N13-p5154425	Bn-C6-p35283366	Bn-scaff_18344_1-p89409	C6	36	36.8	A
18139	Bn-N13-p5163983	Bn-C6-p35273250	Bn-scaff_18344_1-p99525	C6	36	36.8	A
18140	Bn-N13-p5188686	Bn-C6-p35253462	Bn-scaff_18344_1-p119313	C6	36	36.8	A
18141	Bn-N13-p5199414	Bn-Scaffold02889-p4	Bn-scaff_19646_1-p410	C6	36	36.8	A
18142	Bn-N13-p5206344	Bn-C6-p35224894	Bn-scaff_18344_1-p147881	C6	36	36.8	A
18143	Bn-N16-p4260159	Bn-C6-p35715999	Bn-scaff_16485_1-p630261	C6	36	36.8	A
18144	Bn-N16-p4277496	Bn-C6-p35699225	Bn-scaff_16485_1-p647035	C6	36	36.8	A
18145	Bn-N16-p4293233	Bn-C6-p35678144	Bn-scaff_16485_1-p668116	C6	36	36.8	A
18146	Bn-N16-p4300629	Bn-C6-p35669647	Bn-scaff_16485_1-p676613	C6	36	36.8	A
18147	Bn-N16-p4375847	Bn-C6-p35599090	Bn-scaff_16485_1-p747170	C6	36	36.8	A
18148	Bn-N16-p4413345	Bn-C6-p35145770	Bn-scaff_18344_1-p227005	C6	36	36.8	A
18149	Bn-N16-p4426311	Bn-C6-p35135834	Bn-scaff_18344_1-p236941	C6	36	36.8	A
18150	Bn-N16-p4430829	Bn-C6-p35123985	Bn-scaff_18344_1-p248790	C6	36	36.8	A
18151	Bn-N16-p4480900	Bn-C6-p35081297	Bn-scaff_18344_1-p291478	C6	36	36.8	A
18152	Bn-N6-p1597694	Bn-C6-p35134812	Bn-scaff_18344_1-p237963	C6	36	36.8	A
18153	Bn-Scaffold13401-p294	Bn-C6-p35224359	Bn-scaff_18344_1-p148416	C6	36	36.8	A
18154	Bn-Scaffold13401-p743	Bn-C6-p35223910	Bn-scaff_18344_1-p148865	C6	36	36.8	A
18155	Bn-Scaffold29785-p370	Bn-C6-p35223705	Bn-scaff_18344_1-p149070	C6	36	36.8	A
18156	Bn-N16-p4216788	Bn-C6-p35758398	Bn-scaff_16485_1-p587862	C6	37	38.5	A
18157	Bn-N16-p3333079	Bn-C6-p36636683	Bn-scaff_16984_1-p171996	C6	38	39.3	A
18158	Bn-N16-p3367847	Bn-C6-p36609795	Bn-scaff_16984_1-p145108	C6	38	39.3	A
18159	Bn-N16-p3372250	Bn-C6-p36605554	Bn-scaff_16984_1-p140867	C6	38	39.3	A
18160	Bn-N16-p3372904	Bn-C6-p36604914	Bn-scaff_16984_1-p140227	C6	38	39.3	A
18161	Bn-N16-p3372999	Bn-C6-p36604819	Bn-scaff_16984_1-p140132	C6	38	39.3	A
18162	Bn-N16-p3373709	Bn-C6-p36603608	Bn-scaff_16984_1-p138921	C6	38	39.3	A
18163	Bn-N16-p3373821	Bn-C6-p36603495	Bn-scaff_16984_1-p138808	C6	38	39.3	A
18164	Bn-N16-p3373880	Bn-C6-p36603436	Bn-scaff_16984_1-p138749	C6	38	39.3	A
18165	Bn-N16-p3381841	Bn-C6-p36595360	Bn-scaff_16984_1-p130673	C6	38	39.3	A
18166	Bn-N16-p3382589	Bn-C6-p36594610	Bn-scaff_16984_1-p129923	C6	38	39.3	A
18167	Bn-N16-p3390217	Bn-C6-p36591265	Bn-scaff_16984_1-p126578	C6	38	39.3	A
18168	Bn-N16-p3395956	Bn-C6-p36586040	Bn-scaff_16984_1-p121353	C6	38	39.3	A
18169	Bn-N16-p3401499	Bn-C6-p36580623	Bn-scaff_16984_1-p115936	C6	38	39.3	A
18170	Bn-N16-p3437561	Bn-Scaffold01113-p2	Bn-scaff_25107_1-p20683	C6	38	39.3	A
18171	Bn-N16-p3439756	Bn-Scaffold01113-p2	Bn-scaff_25107_1-p22878	C6	38	39.3	A
18172	Bn-N16-p3439775	Bn-Scaffold01113-p2	Bn-scaff_25107_1-p22897	C6	38	39.3	A
18173	Bn-N16-p3443806	Bn-Scaffold01113-p2	Bn-scaff_25107_1-p26913	C6	38	39.3	A
18174	Bn-N16-p3491307	Bn-C6-p36487162	Bn-scaff_16984_1-p22475	C6	38	39.3	A

18175	Bn-N16-p3491373	Bn-C6-p36487096	Bn-scaff_16984_1-p22409	C6	38	39.3	A
18176	Bn-N16-p3491401	Bn-C6-p36487068	Bn-scaff_16984_1-p22381	C6	38	39.3	A
18177	Bn-N16-p3491913	Bn-C6-p36486556	Bn-scaff_16984_1-p21869	C6	38	39.3	A
18178	Bn-N16-p4186556	Bn-C6-p35788379	Bn-scaff_16485_1-p557881	C6	38	39.3	A
18179	Bn-N16-p4196289	Bn-C6-p35778686	Bn-scaff_16485_1-p56757	C6	38	39.3	A
18180	Bn-N16-p4197137	Bn-C6-p35777838	Bn-scaff_16485_1-p56842	C6	38	39.3	A
18181	Bn-N16-p4200300	Bn-C6-p35774991	Bn-scaff_16485_1-p57126	C6	38	39.3	A
18182	Bn-N16-p4200421	Bn-C6-p35774870	Bn-scaff_16485_1-p57139	C6	38	39.3	A
18183	Bn-N16-p4200588	Bn-C6-p35774716	Bn-scaff_16485_1-p57154	C6	38	39.3	A
18184	Bn-N16-p4200640	Bn-C6-p35774664	Bn-scaff_16485_1-p57159	C6	38	39.3	A
18185	Bn-Scaffold08921-p450	Bn-C6-p36623492	Bn-scaff_16984_1-p15880	C6	38	39.3	A
18186	Bn-N16-p2016003	Bn-Scaffold01133-p2	Bn-scaff_16294_1-p20697	C6	39	41.8	A
18187	Bn-N16-p2731042	Bn-C6-p37323511	Bn-scaff_18140_1-p18386	C6	39	41.8	A
18188	Bn-N16-p2734761	Bn-C6-p37319126	Bn-scaff_18140_1-p17947	C6	39	41.8	A
18189	Bn-N16-p2735237	Bn-A06-p2405022	Bn-A06-p2405022	C6	39	41.8	A
18190	Bn-N16-p2772361	Bn-C6-p37234352	Bn-scaff_18140_1-p94701	C6	39	41.8	A
18191	Bn-N16-p2785867	Bn-C6-p37221188	Bn-scaff_18140_1-p81537	C6	39	41.8	A
18192	Bn-N16-p2788784	Bn-C6-p37219724	Bn-scaff_18140_1-p80073	C6	39	41.8	A
18193	Bn-N16-p2794844	Bn-C6-p37214618	Bn-scaff_18140_1-p74967	C6	39	41.8	A
18194	Bn-N16-p2833301	Bn-C6-p37179170	Bn-scaff_18140_1-p39519	C6	39	41.8	A
18195	Bn-N16-p2843278	Bn-C6-p37167579	Bn-scaff_18140_1-p27928	C6	39	41.8	A
18196	Bn-N16-p2843593	Bn-C6-p37167264	Bn-scaff_18140_1-p27613	C6	39	41.8	A
18197	Bn-N16-p2855685	Bn-C6-p37147850	Bn-scaff_18140_1-p8199	C6	39	41.8	A
18198	Bn-N16-p2873079	Bn-C6-p37129523	Bn-scaff_19911_1-p98995	C6	39	41.8	A
18199	Bn-N16-p2879840	Bn-C6-p37123936	Bn-scaff_19911_1-p93408	C6	39	41.8	A
18200	Bn-N16-p2879869	Bn-C6-p37123907	Bn-scaff_19911_1-p93379	C6	39	41.8	A
18201	Bn-N16-p2880604	Bn-C6-p37123172	Bn-scaff_19911_1-p92644	C6	39	41.8	A
18202	Bn-N16-p2887517	Bn-C6-p37116221	Bn-scaff_19911_1-p85693	C6	39	41.8	A
18203	Bn-N16-p2888923	Bn-C6-p37114804	Bn-scaff_19911_1-p84276	C6	39	41.8	A
18204	Bn-N16-p2889163	Bn-C6-p37114564	Bn-scaff_19911_1-p84036	C6	39	41.8	A
18205	Bn-N16-p2893919	Bn-C6-p37109801	Bn-scaff_19911_1-p79273	C6	39	41.8	A
18206	Bn-N16-p2914576	Bn-C6-p37089679	Bn-scaff_19911_1-p59151	C6	39	41.8	A
18207	Bn-N16-p2920805	Bn-C6-p37084600	Bn-scaff_19911_1-p54072	C6	39	41.8	A
18208	Bn-N16-p2943834	Bn-C6-p37061494	Bn-scaff_19911_1-p30966	C6	39	41.8	A
18209	Bn-N16-p2986768	Bn-C6-p37009308	Bn-scaff_24680_1-p45076	C6	39	41.8	A
18210	Bn-N16-p2991677	Bn-C6-p37004401	Bn-scaff_24680_1-p40169	C6	39	41.8	A
18211	Bn-N16-p3038737	Bn-C6-p36958007	Bn-scaff_16553_1-p6126	C6	39	41.8	A
18212	Bn-N16-p3038796	Bn-C6-p36957948	Bn-scaff_16553_1-p6185	C6	39	41.8	A
18213	Bn-N16-p3039332	Bn-C6-p36957390	Bn-scaff_16553_1-p6743	C6	39	41.8	A
18214	Bn-N16-p3050081	Bn-C6-p36948435	Bn-scaff_16553_1-p15698	C6	39	41.8	A
18215	Bn-N16-p3136944	Bn-C6-p36885678	Bn-scaff_16553_1-p78455	C6	39	41.8	0
18216	Bn-N16-p3196094	Bn-C6-p36789360	Bn-scaff_16984_1-p32467	C6	39	41.8	A
18217	Bn-N16-p3205436	Bn-Scaffold04014-p5	Bn-scaff_25413_1-p554	C6	39	41.8	A
18218	Bn-N16-p3212298	Bn-C6-p36776853	Bn-scaff_16984_1-p31216	C6	39	41.8	A
18219	Bn-N16-p3213227	Bn-C6-p36775900	Bn-scaff_16984_1-p31121	C6	39	41.8	A
18220	Bn-N16-p3227628	Bn-C6-p36763915	Bn-scaff_16984_1-p29922	C6	39	41.8	A
18221	Bn-N16-p3227774	Bn-C6-p36763769	Bn-scaff_16984_1-p29908	C6	39	41.8	A
18222	Bn-N16-p3228780	Bn-C6-p36759263	Bn-scaff_16984_1-p29457	C6	39	41.8	A
18223	Bn-N16-p3260870	Bn-C6-p36713260	Bn-scaff_16984_1-p24857	C6	39	41.8	A

18224	Bn-N16-p3265295	Bn-C6-p36709905	Bn-scaff_16984_1-p245218	C6	39	41.8	A
18225	Bn-Scaffold04331-p1656	Bn-Scaffold05929-p3(	Bn-scaff_28752_1-p303	C6	39	41.8	A
18226	Bn-Scaffold07954-p394	Bn-C6-p36797012	Bn-scaff_16984_1-p332325	C6	39	41.8	A
18227	Bn-N16-p1680978	Bn-C6-p38304975	Bn-scaff_22526_1-p99200	C6	40	42.6	A
18228	Bn-N16-p1965975	Bn-C6-p38041715	Bn-scaff_16670_1-p51961	C6	40	42.6	A
18229	Bn-N16-p1977431	Bn-C6-p38030144	Bn-scaff_16670_1-p40390	C6	40	42.6	A
18230	Bn-N16-p1977496	Bn-C6-p38030080	Bn-scaff_16670_1-p40326	C6	40	42.6	A
18231	Bn-N16-p1977625	Bn-C6-p38029952	Bn-scaff_16670_1-p40198	C6	40	42.6	A
18232	Bn-N16-p1981693	Bn-C6-p38027684	Bn-scaff_16670_1-p37930	C6	40	42.6	A
18233	Bn-N16-p1986025	Bn-C6-p38023403	Bn-scaff_16670_1-p33649	C6	40	42.6	A
18234	Bn-N16-p1986319	Bn-C6-p38023110	Bn-scaff_16670_1-p33356	C6	40	42.6	A
18235	Bn-N16-p1986440	Bn-C6-p38022989	Bn-scaff_16670_1-p33235	C6	40	42.6	A
18236	Bn-N16-p1990563	Bn-C6-p38011985	Bn-scaff_16670_1-p22231	C6	40	42.6	A
18237	Bn-N16-p1990685	Bn-C6-p38011863	Bn-scaff_16670_1-p22109	C6	40	42.6	A
18238	Bn-N16-p2003770	Bn-Scaffold04711-p8	Bn-scaff_16275_1-p819	C6	40	42.6	A
18239	Bn-N16-p2005164	Bn-Scaffold02378-p1!	Bn-scaff_25869_1-p1568	C6	40	42.6	A
18240	Bn-N16-p2005710	Bn-Scaffold02378-p1(	Bn-scaff_25869_1-p1017	C6	40	42.6	A
18241	Bn-N16-p2016001	Bn-Scaffold01133-p2(	Bn-scaff_16294_1-p20699	C6	40	42.6	A
18242	Bn-N16-p2030018	Bn-C6-p37981243	Bn-scaff_18140_1-p84159	C6	40	42.6	A
18243	Bn-N16-p2039718	Bn-C6-p37967166	Bn-scaff_18140_1-p82751	C6	40	42.6	A
18244	Bn-N16-p2039814	Bn-C6-p37967070	Bn-scaff_18140_1-p82741	C6	40	42.6	A
18245	Bn-N16-p2044386	Bn-C6-p37962886	Bn-scaff_18140_1-p82323	C6	40	42.6	A
18246	Bn-N16-p2044438	Bn-C6-p37962834	Bn-scaff_18140_1-p82318	C6	40	42.6	A
18247	Bn-N16-p2044578	Bn-C6-p37962694	Bn-scaff_18140_1-p82304	C6	40	42.6	A
18248	Bn-N16-p2044777	Bn-C6-p37962495	Bn-scaff_18140_1-p82284	C6	40	42.6	A
18249	Bn-N16-p2045723	Bn-C6-p37961549	Bn-scaff_18140_1-p82189	C6	40	42.6	A
18250	Bn-N16-p2050834	Bn-C6-p37956446	Bn-scaff_18140_1-p81679	C6	40	42.6	A
18251	Bn-N16-p2051129	Bn-C6-p37956151	Bn-scaff_18140_1-p81650(	C6	40	42.6	A
18252	Bn-N16-p2051281	Bn-C6-p37955999	Bn-scaff_18140_1-p81634	C6	40	42.6	A
18253	Bn-N16-p2069274	Bn-C6-p37938798	Bn-scaff_18140_1-p79914	C6	40	42.6	A
18254	Bn-N16-p2071853	Bn-C6-p37936390	Bn-scaff_18140_1-p79673	C6	40	42.6	A
18255	Bn-N16-p2073896	Bn-C6-p37934347	Bn-scaff_18140_1-p79469	C6	40	42.6	A
18256	Bn-N16-p2085089	Bn-C6-p37920042	Bn-scaff_18140_1-p78039	C6	40	42.6	A
18257	Bn-N16-p2086711	Bn-C6-p37918329	Bn-scaff_18140_1-p77867	C6	40	42.6	A
18258	Bn-N16-p2087431	Bn-C6-p37917615	Bn-scaff_18140_1-p77796	C6	40	42.6	A
18259	Bn-N16-p2093907	Bn-C6-p37911094	Bn-scaff_18140_1-p77144	C6	40	42.6	A
18260	Bn-N16-p2099175	Bn-C6-p37907487	Bn-scaff_18140_1-p76783	C6	40	42.6	A
18261	Bn-N16-p250141	Bn-C6-p39699513	Bn-scaff_18362_1-p90481	C6	40	42.6	A
18262	Bn-N16-p2570678	Bn-C6-p37475224	Bn-scaff_18140_1-p33557	C6	40	42.6	A
18263	Bn-N16-p310895	Bn-C6-p39637971	Bn-scaff_18362_1-p28939	C6	40	42.6	A
18264	Bn-N16-p494510	Bn-C6-p39372573	Bn-scaff_18549_1-p45173	C6	40	42.6	A
18265	Bn-N6-p2439044	Bn-A06-p2437284	Bn-A06-p2437284	C6	40	42.6	A
18266	Bn-N6-p9070049	Bn-Scaffold000946-p!	Bn-Scaffold000946-p999	C6	40	42.6	A
18267	Bn-Scaffold05553-p259	Bn-C6-p38007676	Bn-scaff_16670_1-p17922	C6	40	42.6	A
18268	Bn-Scaffold28989-p391	Bn-C5-p21938374	Bn-scaff_16759_1-p28657	C6	40	42.6	A
18269	Bn-Scaffold27754-p161	Bn-Scaffold08858-p3(	Bn-scaff_24413_1-p361	C7	1	0	A
18270	Bn-N12-p2864606	Bn-C4-p46360789	Bn-scaff_16517_1-p65633	C7	2	0.8	A
18271	Bn-N17-p394283	Bn-A07-p160648	Bn-A07-p160648	C7	2	0.8	A
18272	Bn-N17-p399808	Bn-C7-p451640	Bn-scaff_15627_1-p48223	C7	2	0.8	A

18273	Bn-N17-p443163	Bn-C7-p481019	Bn-scaff_15627_1-p452856	C7	2	0.8	A
18274	Bn-Scaffold00913-p87718	Bn-Scaffold00285-p8	Bn-scaff_15712_9-p369079	C7	2	0.8	A
18275	Bn-N15-p48513500	Bn-C2-p33034990	Bn-scaff_16328_1-p971878	C7	3	2.4	A
18276	Bn-N17-p1329229	Bn-C7-p1192208	Bn-scaff_15762_1-p258234	C7	3	2.4	A
18277	Bn-N17-p1329727	Bn-C7-p1192705	Bn-scaff_15762_1-p258731	C7	3	2.4	A
18278	Bn-N17-p1705847	Bn-C7-p1599797	Bn-scaff_15762_1-p665823	C7	3	2.4	A
18279	Bn-N17-p1764860	Bn-C7-p1651197	Bn-scaff_15762_1-p717223	C7	3	2.4	A
18280	Bn-N17-p1809139	Bn-C7-p1688673	Bn-scaff_15762_1-p754699	C7	3	2.4	A
18281	Bn-N17-p2990837	Bn-C7-p2510290	Bn-scaff_17168_1-p53199	C7	3	2.4	A
18282	Bn-N17-p936603	Bn-C7-p919437	Bn-scaff_15627_1-p14438	C7	3	2.4	A
18283	Bn-N9-p18462085	Bn-A06-p19968113	Bn-A06-p19968113	C7	3	2.4	A
18284	Bn-N17-p20884658	Bn-C7-p6033307	Bn-scaff_20084_1-p106565	C7	4	4	A
18285	Bn-N17-p4867251	Bn-C7-p4356314	Bn-scaff_17084_1-p161742	C7	4	4	A
18286	Bn-N17-p5435326	Bn-C7-p4922734	Bn-scaff_17461_1-p357866	C7	4	4	A
18287	Bn-N17-p5456908	Bn-C7-p4951917	Bn-scaff_17461_1-p387049	C7	4	4	A
18288	Bn-N17-p5494379	Bn-C7-p4994044	Bn-scaff_17461_1-p429176	C7	4	4	A
18289	Bn-N17-p5533500	Bn-C7-p5038825	Bn-scaff_17461_1-p473957	C7	4	4	A
18290	Bn-N17-p5534520	Bn-C7-p5039845	Bn-scaff_17461_1-p474977	C7	4	4	A
18291	Bn-N17-p5538654	Bn-C7-p5043983	Bn-scaff_17461_1-p479115	C7	4	4	A
18292	Bn-N17-p5665709	Bn-C7-p5191505	Bn-scaff_17461_1-p626637	C7	4	4	A
18293	Bn-N17-p5669172	Bn-C7-p5194981	Bn-scaff_17461_1-p630113	C7	4	4	A
18294	Bn-N17-p5669996	Bn-C7-p5195765	Bn-scaff_17461_1-p630897	C7	4	4	A
18295	Bn-N17-p5701947	Bn-C7-p5230170	Bn-scaff_17461_1-p665302	C7	4	4	A
18296	Bn-N17-p5702298	Bn-C7-p5230522	Bn-scaff_17461_1-p665654	C7	4	4	A
18297	Bn-N17-p5703621	Bn-C7-p5231846	Bn-scaff_17461_1-p666978	C7	4	4	A
18298	Bn-N17-p5704602	Bn-C7-p5232827	Bn-scaff_17461_1-p667959	C7	4	4	A
18299	Bn-N17-p5725964	Bn-C7-p5242231	Bn-scaff_17461_1-p677363	C7	4	4	A
18300	Bn-N17-p5789982	Bn-C7-p5312126	Bn-scaff_17461_1-p747258	C7	4	4	A
18301	Bn-N17-p5926520	Bn-C7-p5363794	Bn-scaff_17461_1-p798926	C7	4	4	A
18302	Bn-N17-p5929259	Bn-C7-p5366593	Bn-scaff_17461_1-p801725	C7	4	4	A
18303	Bn-N17-p5935668	Bn-C7-p5373077	Bn-scaff_17461_1-p808209	C7	4	4	A
18304	Bn-N17-p6073081	Bn-C7-p5480187	Bn-scaff_17461_1-p915319	C7	4	4	A
18305	Bn-N17-p6097704	Bn-C7-p5497802	Bn-scaff_17461_1-p932934	C7	4	4	A
18306	Bn-N17-p6102888	Bn-C7-p5511638	Bn-scaff_17461_1-p946770	C7	4	4	A
18307	Bn-N17-p6457841	Bn-Scaffold13826-p3	Bn-scaff_19692_1-p343	C7	4	4	A
18308	Bn-N17-p6623126	Bn-C7-p6012892	Bn-scaff_20084_1-p86150	C7	4	4	A
18309	Bn-N17-p6626813	Bn-C6-p37024908	Bn-scaff_24680_1-p60676	C7	4	4	A
18310	Bn-N17-p6634264	Bn-C7-p6023903	Bn-scaff_20084_1-p97161	C7	4	4	A
18311	Bn-N17-p6646717	Bn-C7-p6031291	Bn-scaff_20084_1-p104549	C7	4	4	A
18312	Bn-N17-p6735256	Bn-C7-p6114822	Bn-scaff_20084_1-p188080	C7	4	4	A
18313	Bn-N17-p6735334	Bn-C7-p6114900	Bn-scaff_20084_1-p188158	C7	4	4	A
18314	Bn-N17-p6735420	Bn-C7-p6114986	Bn-scaff_20084_1-p188244	C7	4	4	A
18315	Bn-N17-p6735663	Bn-C7-p6115229	Bn-scaff_20084_1-p188487	C7	4	4	A
18316	Bn-N17-p6736868	Bn-C7-p6116434	Bn-scaff_20084_1-p189692	C7	4	4	A
18317	Bn-N17-p6736927	Bn-C7-p6116493	Bn-scaff_20084_1-p189751	C7	4	4	A
18318	Bn-N17-p6775669	Bn-C7-p6159573	Bn-scaff_20084_1-p232831	C7	4	4	A
18319	Bn-N13-p65607830	Bn-C7-p7986051	Bn-scaff_22310_1-p159878	C7	5	4.8	A
18320	Bn-N17-p10157609	Bn-C7-p9654338	Bn-scaff_20288_1-p344108	C7	5	4.8	A
18321	Bn-N17-p10287846	Bn-C7-p9793336	Bn-scaff_20288_1-p483106	C7	5	4.8	A

18322	Bn-N17-p10288222	Bn-C7-p9793712	Bn-scaff_20288_1-p483482	C7	5	4.8	A
18323	Bn-N17-p10448599	Bn-C7-p9950759	Bn-scaff_20329_1-p20588	C7	5	4.8	A
18324	Bn-N17-p10453629	Bn-C7-p9955708	Bn-scaff_20329_1-p25537	C7	5	4.8	A
18325	Bn-N17-p10453648	Bn-C7-p9955727	Bn-scaff_20329_1-p25556	C7	5	4.8	A
18326	Bn-N17-p10500058	Bn-C7-p10001648	Bn-scaff_20329_1-p71477	C7	5	4.8	A
18327	Bn-N17-p10537269	Bn-C7-p10037542	Bn-scaff_20329_1-p107371	C7	5	4.8	A
18328	Bn-N17-p10539461	Bn-C7-p10039737	Bn-scaff_20329_1-p109566	C7	5	4.8	A
18329	Bn-N17-p10625213	Bn-Scaffold00709-p1	Bn-scaff_17484_1-p134541	C7	5	4.8	A
18330	Bn-N17-p10708040	Bn-C7-p10147451	Bn-scaff_15766_1-p21074	C7	5	4.8	A
18331	Bn-N17-p10725449	Bn-C7-p10168385	Bn-scaff_15766_1-p42008	C7	5	4.8	A
18332	Bn-N17-p10737634	Bn-C7-p10181148	Bn-scaff_15766_1-p54771	C7	5	4.8	A
18333	Bn-N17-p10783065	Bn-C7-p10213511	Bn-scaff_15766_1-p87134	C7	5	4.8	A
18334	Bn-N17-p10783235	Bn-C7-p10213681	Bn-scaff_15766_1-p87304	C7	5	4.8	A
18335	Bn-N17-p10783337	Bn-C7-p10213783	Bn-scaff_15766_1-p87406	C7	5	4.8	A
18336	Bn-N17-p10784603	Bn-C7-p10215060	Bn-scaff_15766_1-p88683	C7	5	4.8	A
18337	Bn-N17-p10790342	Bn-C7-p10220521	Bn-scaff_15766_1-p94144	C7	5	4.8	A
18338	Bn-N17-p10825672	Bn-C7-p10254856	Bn-scaff_15766_1-p128479	C7	5	4.8	A
18339	Bn-N17-p10828453	Bn-C7-p10257634	Bn-scaff_15766_1-p131257	C7	5	4.8	A
18340	Bn-N17-p11094889	Bn-C7-p10480157	Bn-scaff_16721_1-p25103	C7	5	4.8	A
18341	Bn-N17-p11125266	Bn-C7-p10521530	Bn-scaff_16721_1-p66476	C7	5	4.8	A
18342	Bn-N17-p11233718	Bn-C7-p10582728	Bn-scaff_16721_1-p127674	C7	5	4.8	A
18343	Bn-N17-p11320279	Bn-C7-p10688589	Bn-scaff_16721_1-p233535	C7	5	4.8	A
18344	Bn-N17-p11323284	Bn-C7-p10689830	Bn-scaff_16721_1-p234776	C7	5	4.8	A
18345	Bn-N17-p11323676	Bn-C7-p10690230	Bn-scaff_16721_1-p235176	C7	5	4.8	A
18346	Bn-N17-p11343279	Bn-C7-p10696403	Bn-scaff_16721_1-p241349	C7	5	4.8	A
18347	Bn-N17-p11344083	Bn-C7-p10697207	Bn-scaff_16721_1-p242153	C7	5	4.8	A
18348	Bn-N17-p11344351	Bn-C7-p10697472	Bn-scaff_16721_1-p242418	C7	5	4.8	A
18349	Bn-N17-p11345003	Bn-C7-p10698123	Bn-scaff_16721_1-p243069	C7	5	4.8	A
18350	Bn-N17-p11345322	Bn-C7-p10698442	Bn-scaff_16721_1-p243388	C7	5	4.8	A
18351	Bn-N17-p11360394	Bn-C7-p10715818	Bn-scaff_16721_1-p260764	C7	5	4.8	A
18352	Bn-N17-p11375596	Bn-C7-p10735851	Bn-scaff_16721_1-p280797	C7	5	4.8	A
18353	Bn-N17-p11398731	Bn-C7-p10771174	Bn-scaff_16721_1-p316120	C7	5	4.8	A
18354	Bn-N17-p11398898	Bn-C7-p10771340	Bn-scaff_16721_1-p316286	C7	5	4.8	A
18355	Bn-N17-p11399017	Bn-C7-p10771459	Bn-scaff_16721_1-p316405	C7	5	4.8	A
18356	Bn-N17-p11399466	Bn-C7-p10778485	Bn-scaff_16721_1-p323431	C7	5	4.8	A
18357	Bn-N17-p11399606	Bn-C7-p10778625	Bn-scaff_16721_1-p323571	C7	5	4.8	A
18358	Bn-N17-p11399704	Bn-C7-p10778723	Bn-scaff_16721_1-p323669	C7	5	4.8	A
18359	Bn-N17-p11399717	Bn-C7-p10778736	Bn-scaff_16721_1-p323682	C7	5	4.8	A
18360	Bn-N17-p11399783	Bn-C7-p10778803	Bn-scaff_16721_1-p323749	C7	5	4.8	A
18361	Bn-N17-p11399805	Bn-C7-p10778825	Bn-scaff_16721_1-p323771	C7	5	4.8	A
18362	Bn-N17-p11401983	Bn-C7-p10785661	Bn-scaff_16721_1-p330607	C7	5	4.8	A
18363	Bn-N17-p11403086	Bn-C7-p10786764	Bn-scaff_16721_1-p331710	C7	5	4.8	A
18364	Bn-N17-p11457720	Bn-C7-p10823948	Bn-scaff_16721_1-p368894	C7	5	4.8	A
18365	Bn-N17-p11461087	Bn-C7-p10832797	Bn-scaff_16721_1-p377743	C7	5	4.8	A
18366	Bn-N17-p11481662	Bn-C7-p10856625	Bn-scaff_16721_1-p401571	C7	5	4.8	A
18367	Bn-N17-p11487023	Bn-C7-p10861446	Bn-scaff_16721_1-p406392	C7	5	4.8	A
18368	Bn-N17-p11487192	Bn-C7-p10861615	Bn-scaff_16721_1-p406561	C7	5	4.8	A
18369	Bn-N17-p11542786	Bn-C7-p10895428	Bn-scaff_16721_1-p440374	C7	5	4.8	A
18370	Bn-N17-p11543114	Bn-C7-p10895755	Bn-scaff_16721_1-p440701	C7	5	4.8	A

18371	Bn-N17-p11563748	Bn-C7-p10917603	Bn-scaff_16721_1-p46254[illegible]	C7	5	4.8	A
18372	Bn-N17-p11614094	Bn-C7-p10965591	Bn-scaff_16721_1-p51053[illegible]	C7	5	4.8	A
18373	Bn-N17-p6826021	Bn-C7-p6238607	Bn-scaff_20084_1-p31186[illegible]	C7	5	4.8	A
18374	Bn-N17-p6826755	Bn-C7-p6239327	Bn-scaff_20084_1-p31258[illegible]	C7	5	4.8	A
18375	Bn-N17-p6840154	Bn-C7-p6252053	Bn-scaff_20084_1-p32531[illegible]	C7	5	4.8	A
18376	Bn-N17-p6849857	Bn-C7-p6260834	Bn-scaff_20084_1-p33409[illegible]	C7	5	4.8	A
18377	Bn-N17-p6857255	Bn-C7-p6271231	Bn-scaff_20084_1-p34448[illegible]	C7	5	4.8	A
18378	Bn-N17-p6857722	Bn-C7-p6271698	Bn-scaff_20084_1-p34495[illegible]	C7	5	4.8	A
18379	Bn-N17-p6898963	Bn-C7-p6319443	Bn-scaff_20084_1-p39270[illegible]	C7	5	4.8	A
18380	Bn-N17-p6906010	Bn-C7-p6326506	Bn-scaff_20084_1-p39976[illegible]	C7	5	4.8	A
18381	Bn-N17-p6907462	Bn-C7-p6327958	Bn-scaff_20084_1-p40121[illegible]	C7	5	4.8	A
18382	Bn-N17-p7230640	Bn-C7-p6722731	Bn-scaff_22301_1-p84996	C7	5	4.8	0
18383	Bn-N17-p7259703	Bn-C7-p6746452	Bn-scaff_22301_1-p61275	C7	5	4.8	A
18384	Bn-N17-p7307144	Bn-C7-p6791103	Bn-scaff_22301_1-p16624	C7	5	4.8	0
18385	Bn-N17-p7315544	Bn-C7-p6799503	Bn-scaff_22301_1-p8224	C7	5	4.8	A
18386	Bn-N17-p7316254	Bn-C7-p6800213	Bn-scaff_22301_1-p7514	C7	5	4.8	A
18387	Bn-N17-p7342769	Bn-C7-p6822927	Bn-scaff_28403_1-p15101	C7	5	4.8	A
18388	Bn-N17-p7348719	Bn-C7-p6828532	Bn-scaff_28403_1-p20706	C7	5	4.8	A
18389	Bn-N17-p7359849	Bn-C7-p6839365	Bn-scaff_28403_1-p31539	C7	5	4.8	A
18390	Bn-N17-p7443564	Bn-C7-p6931260	Bn-scaff_28403_1-p12343[illegible]	C7	5	4.8	A
18391	Bn-N17-p7472611	Bn-C7-p6962262	Bn-scaff_28403_1-p15443[illegible]	C7	5	4.8	A
18392	Bn-N17-p7477602	Bn-C7-p6967279	Bn-scaff_28403_1-p15945[illegible]	C7	5	4.8	A
18393	Bn-N17-p7482259	Bn-C7-p6971947	Bn-scaff_28403_1-p16412[illegible]	C7	5	4.8	A
18394	Bn-N17-p7607630	Bn-C7-p7109300	Bn-scaff_28403_1-p30147[illegible]	C7	5	4.8	A
18395	Bn-N17-p7620741	Bn-C7-p7120163	Bn-scaff_28403_1-p31233[illegible]	C7	5	4.8	A
18396	Bn-N17-p7643675	Bn-C7-p7142617	Bn-scaff_28403_1-p33479[illegible]	C7	5	4.8	A
18397	Bn-N17-p7755910	Bn-C7-p7250494	Bn-scaff_22310_1-p89543[illegible]	C7	5	4.8	A
18398	Bn-N17-p7797137	Bn-C7-p7293023	Bn-scaff_22310_1-p85290[illegible]	C7	5	4.8	A
18399	Bn-N17-p7820027	Bn-C3-p33121964	Bn-scaff_18855_1-p84064[illegible]	C7	5	4.8	A
18400	Bn-N17-p7820364	Bn-C3-p33121627	Bn-scaff_18855_1-p84098[illegible]	C7	5	4.8	A
18401	Bn-N17-p7845475	Bn-C7-p7330397	Bn-scaff_22310_1-p81553[illegible]	C7	5	4.8	A
18402	Bn-N17-p7888782	Bn-C7-p7370944	Bn-scaff_22310_1-p77498[illegible]	C7	5	4.8	A
18403	Bn-N17-p7890626	Bn-C7-p7372789	Bn-scaff_22310_1-p77314[illegible]	C7	5	4.8	A
18404	Bn-N17-p7942321	Bn-C7-p7422283	Bn-scaff_22310_1-p72364[illegible]	C7	5	4.8	A
18405	Bn-N17-p7946170	Bn-C7-p7426144	Bn-scaff_22310_1-p71978[illegible]	C7	5	4.8	A
18406	Bn-N17-p7977228	Bn-C7-p7460673	Bn-scaff_22310_1-p68525[illegible]	C7	5	4.8	A
18407	Bn-N17-p8000987	Bn-C7-p7492762	Bn-scaff_22310_1-p65316[illegible]	C7	5	4.8	A
18408	Bn-N17-p8006877	Bn-C7-p7498659	Bn-scaff_22310_1-p64727[illegible]	C7	5	4.8	A
18409	Bn-N17-p8014894	Bn-C7-p7506591	Bn-scaff_22310_1-p63933[illegible]	C7	5	4.8	A
18410	Bn-N17-p8028317	Bn-C7-p7519847	Bn-scaff_22310_1-p62608[illegible]	C7	5	4.8	A
18411	Bn-N17-p8089654	Bn-C7-p7577872	Bn-scaff_22310_1-p56805[illegible]	C7	5	4.8	A
18412	Bn-N17-p8335083	Bn-C7-p7809977	Bn-scaff_22310_1-p33595[illegible]	C7	5	4.8	A
18413	Bn-N17-p8831048	Bn-C7-p8208626	Bn-scaff_22441_1-p36733[illegible]	C7	5	4.8	A
18414	Bn-N17-p8957285	Bn-C7-p8342916	Bn-scaff_22441_1-p23304[illegible]	C7	5	4.8	A
18415	Bn-N17-p9303806	Bn-C7-p8719053	Bn-scaff_16975_1-p11144[illegible]	C7	5	4.8	A
18416	Bn-N17-p9450353	Bn-C7-p8919518	Bn-scaff_23771_1-p88926	C7	5	4.8	A
18417	Bn-N17-p9483475	Bn-C7-p8955042	Bn-scaff_23771_1-p12445[illegible]	C7	5	4.8	A
18418	Bn-N7-p3298796	Bn-C7-p7282336	Bn-scaff_22310_1-p86359[illegible]	C7	5	4.8	A
18419	Bn-Scaffold06874-p1127	Bn-C7-p10780448	Bn-scaff_16721_1-p32539[illegible]	C7	5	4.8	A

18420	Bn-Scaffold06874-p1134	Bn-C7-p10780455	Bn-scaff_16721_1-p325401	C7	5	4.8	A
18421	Bn-Scaffold06874-p34	Bn-C7-p10779355	Bn-scaff_16721_1-p324301	C7	5	4.8	A
18422	Bn-Scaffold06874-p899	Bn-C7-p10780220	Bn-scaff_16721_1-p325166	C7	5	4.8	A
18423	Bn-Scaffold07857-p1124	Bn-C7-p10780706	Bn-scaff_16721_1-p325652	C7	5	4.8	A
18424	Bn-Scaffold14049-p118	Bn-C7-p10720977	Bn-scaff_16721_1-p265923	C7	5	4.8	A
18425	Bn-Scaffold14049-p591	Bn-C7-p10721482	Bn-scaff_16721_1-p266428	C7	5	4.8	A
18426	Bn-Scaffold47787-p255	Bn-C7-p10779012	Bn-scaff_16721_1-p323958	C7	5	4.8	A
18427	Bn-N17-p11683264	Bn-C7-p11487715	Bn-scaff_16721_1-p103266	C7	6	5.6	A
18428	Bn-N17-p11685519	Bn-C7-p11489963	Bn-scaff_16721_1-p103490	C7	6	5.6	A
18429	Bn-N17-p11685681	Bn-C7-p11490125	Bn-scaff_16721_1-p103507	C7	6	5.6	A
18430	Bn-N17-p11685956	Bn-C7-p11490401	Bn-scaff_16721_1-p103534	C7	6	5.6	A
18431	Bn-N17-p11697861	Bn-C7-p11498734	Bn-scaff_16721_1-p104368	C7	6	5.6	A
18432	Bn-N17-p11698708	Bn-C7-p11499581	Bn-scaff_16721_1-p104452	C7	6	5.6	A
18433	Bn-N17-p11728748	Bn-C7-p11529357	Bn-scaff_16721_1-p107430	C7	6	5.6	A
18434	Bn-N17-p11815565	Bn-C7-p11618444	Bn-scaff_16721_1-p116339	C7	6	5.6	A
18435	Bn-N17-p11816869	Bn-C7-p11619749	Bn-scaff_16721_1-p116469	C7	6	5.6	A
18436	Bn-N17-p11817076	Bn-C7-p11619956	Bn-scaff_16721_1-p116490	C7	6	5.6	A
18437	Bn-N17-p11817315	Bn-C7-p11620195	Bn-scaff_16721_1-p116514	C7	6	5.6	A
18438	Bn-N17-p11817770	Bn-C7-p11620650	Bn-scaff_16721_1-p116559	C7	6	5.6	A
18439	Bn-N17-p11817828	Bn-C7-p11620708	Bn-scaff_16721_1-p116565	C7	6	5.6	A
18440	Bn-N17-p11818597	Bn-C7-p11621477	Bn-scaff_16721_1-p116642	C7	6	5.6	A
18441	Bn-N17-p11824865	Bn-C7-p11625456	Bn-scaff_16721_1-p117040	C7	6	5.6	A
18442	Bn-N17-p11827490	Bn-C7-p11628092	Bn-scaff_16721_1-p117303	C7	6	5.6	A
18443	Bn-N17-p11829187	Bn-C7-p11629787	Bn-scaff_16721_1-p117473	C7	6	5.6	A
18444	Bn-N17-p11829988	Bn-C7-p11630588	Bn-scaff_16721_1-p117553	C7	6	5.6	A
18445	Bn-N17-p11837406	Bn-C7-p11637946	Bn-scaff_16721_1-p118289	C7	6	5.6	A
18446	Bn-N17-p11838326	Bn-C7-p11638883	Bn-scaff_16721_1-p118382	C7	6	5.6	A
18447	Bn-N17-p11838697	Bn-C7-p11639254	Bn-scaff_16721_1-p118420	C7	6	5.6	A
18448	Bn-N17-p11839795	Bn-C7-p11640333	Bn-scaff_16721_1-p118527	C7	6	5.6	A
18449	Bn-N17-p11840877	Bn-C7-p11641415	Bn-scaff_16721_1-p118636	C7	6	5.6	A
18450	Bn-N17-p11842169	Bn-C7-p11642666	Bn-scaff_16721_1-p118761	C7	6	5.6	A
18451	Bn-N17-p11842848	Bn-C7-p11643345	Bn-scaff_16721_1-p118829	C7	6	5.6	A
18452	Bn-N17-p11844942	Bn-C7-p11645475	Bn-scaff_16721_1-p119042	C7	6	5.6	A
18453	Bn-N17-p11847287	Bn-C7-p11647815	Bn-scaff_16721_1-p119276	C7	6	5.6	A
18454	Bn-N17-p11848655	Bn-C7-p11649172	Bn-scaff_16721_1-p119411	C7	6	5.6	A
18455	Bn-N17-p11879914	Bn-C7-p11703576	Bn-scaff_16721_1-p124852	C7	6	5.6	A
18456	Bn-N17-p11897242	Bn-C7-p11706153	Bn-scaff_16721_1-p125109	C7	6	5.6	A
18457	Bn-N17-p11899878	Bn-C7-p11708795	Bn-scaff_16721_1-p125374	C7	6	5.6	A
18458	Bn-N17-p11900645	Bn-C7-p11709556	Bn-scaff_16721_1-p125450	C7	6	5.6	A
18459	Bn-N17-p11901857	Bn-C7-p11710765	Bn-scaff_16721_1-p125571	C7	6	5.6	A
18460	Bn-N17-p11901870	Bn-C7-p11710778	Bn-scaff_16721_1-p125572	C7	6	5.6	A
18461	Bn-N17-p11901974	Bn-C7-p11710882	Bn-scaff_16721_1-p125582	C7	6	5.6	A
18462	Bn-N17-p11902262	Bn-C7-p11711170	Bn-scaff_16721_1-p125611	C7	6	5.6	A
18463	Bn-N17-p11902536	Bn-C7-p11711444	Bn-scaff_16721_1-p125639	C7	6	5.6	A
18464	Bn-N17-p11903474	Bn-C7-p11712382	Bn-scaff_16721_1-p125732	C7	6	5.6	A
18465	Bn-N17-p11903817	Bn-C7-p11712725	Bn-scaff_16721_1-p125767	C7	6	5.6	A
18466	Bn-N17-p11909341	Bn-C7-p11718201	Bn-scaff_16721_1-p126314	C7	6	5.6	A
18467	Bn-N17-p11909868	Bn-C7-p11718728	Bn-scaff_16721_1-p126367	C7	6	5.6	A
18468	Bn-N17-p11910515	Bn-C7-p11719283	Bn-scaff_16721_1-p126422	C7	6	5.6	A

18469	Bn-N17-p11911324	Bn-C7-p11720091	Bn-scaff_16721_1-p126503	C7	6	5.6	A
18470	Bn-N17-p11911614	Bn-C7-p11720381	Bn-scaff_16721_1-p126532	C7	6	5.6	A
18471	Bn-N17-p11917739	Bn-C7-p11727235	Bn-scaff_16721_1-p127218	C7	6	5.6	A
18472	Bn-N17-p11918437	Bn-C7-p11727933	Bn-scaff_16721_1-p127287	C7	6	5.6	A
18473	Bn-N17-p11920174	Bn-C7-p11729670	Bn-scaff_16721_1-p127461	C7	6	5.6	A
18474	Bn-N17-p11920478	Bn-C7-p11729974	Bn-scaff_16721_1-p127492	C7	6	5.6	A
18475	Bn-N17-p11920927	Bn-C7-p11730498	Bn-scaff_16721_1-p127544	C7	6	5.6	A
18476	Bn-N17-p11921242	Bn-C7-p11730813	Bn-scaff_16721_1-p127575	C7	6	5.6	A
18477	Bn-N17-p11923070	Bn-C7-p11732288	Bn-scaff_16721_1-p127723	C7	6	5.6	A
18478	Bn-N17-p11937042	Bn-C7-p11741635	Bn-scaff_16721_1-p128658	C7	6	5.6	0
18479	Bn-N17-p11937302	Bn-C7-p11741897	Bn-scaff_16721_1-p128684	C7	6	5.6	A
18480	Bn-N17-p11937751	Bn-C7-p11742346	Bn-scaff_16721_1-p128729	C7	6	5.6	A
18481	Bn-N17-p11938429	Bn-C7-p11743023	Bn-scaff_16721_1-p128796	C7	6	5.6	A
18482	Bn-N17-p11956059	Bn-C7-p11760566	Bn-scaff_16721_1-p130551	C7	6	5.6	A
18483	Bn-N17-p11956535	Bn-C7-p11761073	Bn-scaff_16721_1-p130601	C7	6	5.6	A
18484	Bn-N17-p11956783	Bn-C7-p11761321	Bn-scaff_16721_1-p130626	C7	6	5.6	A
18485	Bn-N17-p11957082	Bn-C7-p11761607	Bn-scaff_16721_1-p130655	C7	6	5.6	A
18486	Bn-N17-p11976352	Bn-C1-p20445815	Bn-scaff_19392_1-p14147	C7	6	5.6	A
18487	Bn-N17-p11995546	Bn-C7-p11790905	Bn-scaff_16721_1-p133585	C7	6	5.6	A
18488	Bn-N17-p12001901	Bn-C7-p11802806	Bn-scaff_16721_1-p134775	C7	6	5.6	A
18489	Bn-N17-p12041034	Bn-C7-p11850761	Bn-scaff_16721_1-p139570	C7	6	5.6	A
18490	Bn-N17-p12044788	Bn-C7-p11854763	Bn-scaff_16721_1-p139970	C7	6	5.6	A
18491	Bn-N17-p12045364	Bn-C7-p11855339	Bn-scaff_16721_1-p140028	C7	6	5.6	A
18492	Bn-N17-p12045971	Bn-C7-p11855946	Bn-scaff_16721_1-p140089	C7	6	5.6	A
18493	Bn-N17-p12046412	Bn-C7-p11856387	Bn-scaff_16721_1-p140133	C7	6	5.6	A
18494	Bn-N17-p12054726	Bn-C7-p11862707	Bn-scaff_16721_1-p140765	C7	6	5.6	A
18495	Bn-N17-p12133717	Bn-C7-p11948869	Bn-scaff_16721_1-p149381	C7	6	5.6	A
18496	Bn-N17-p12177885	Bn-C7-p11976260	Bn-scaff_16721_1-p152120	C7	6	5.6	A
18497	Bn-N17-p12202027	Bn-C7-p12000465	Bn-scaff_16721_1-p154541	C7	6	5.6	A
18498	Bn-N17-p12202270	Bn-C7-p12000708	Bn-scaff_16721_1-p154565	C7	6	5.6	A
18499	Bn-N17-p12202719	Bn-C7-p12001157	Bn-scaff_16721_1-p154610	C7	6	5.6	A
18500	Bn-N17-p12204374	Bn-C7-p12002812	Bn-scaff_16721_1-p154775	C7	6	5.6	A
18501	Bn-N17-p12208227	Bn-C7-p12006685	Bn-scaff_16721_1-p155163	C7	6	5.6	A
18502	Bn-N17-p12209091	Bn-C7-p12015320	Bn-scaff_16721_1-p156026	C7	6	5.6	A
18503	Bn-N17-p12212464	Bn-C7-p12018689	Bn-scaff_16721_1-p156363	C7	6	5.6	A
18504	Bn-N17-p12259198	Bn-C7-p12072579	Bn-scaff_16721_1-p161752	C7	6	5.6	A
18505	Bn-N17-p12259371	Bn-C7-p12072752	Bn-scaff_16721_1-p161769	C7	6	5.6	A
18506	Bn-N17-p12260414	Bn-C7-p12073795	Bn-scaff_16721_1-p161874	C7	6	5.6	A
18507	Bn-N17-p12261526	Bn-C7-p12074905	Bn-scaff_16721_1-p161985	C7	6	5.6	A
18508	Bn-N17-p12266427	Bn-C7-p12079142	Bn-scaff_16721_1-p162408	C7	6	5.6	A
18509	Bn-N17-p12275369	Bn-C7-p12097726	Bn-scaff_16721_1-p164267	C7	6	5.6	A
18510	Bn-N17-p12285647	Bn-C7-p12109723	Bn-scaff_16721_1-p165466	C7	6	5.6	A
18511	Bn-N17-p12292070	Bn-C7-p12113374	Bn-scaff_16721_1-p165832	C7	6	5.6	A
18512	Bn-N17-p12300977	Bn-C7-p12122489	Bn-scaff_16721_1-p166743	C7	6	5.6	A
18513	Bn-N17-p12301588	Bn-C7-p12123100	Bn-scaff_16721_1-p166804	C7	6	5.6	A
18514	Bn-N17-p12301887	Bn-C7-p12123399	Bn-scaff_16721_1-p166834	C7	6	5.6	A
18515	Bn-N17-p12342416	Bn-C7-p12165812	Bn-scaff_16721_1-p171075	C7	6	5.6	A
18516	Bn-N17-p12347916	Bn-C7-p12178729	Bn-scaff_16721_1-p172367	C7	6	5.6	A
18517	Bn-N17-p12349429	Bn-C7-p12179970	Bn-scaff_16721_1-p172491	C7	6	5.6	A

18518	Bn-N17-p12366578	Bn-C7-p12224985	Bn-scaff_16721_1-p176993	C7	6	5.6	A
18519	Bn-N17-p12366711	Bn-C7-p12225118	Bn-scaff_16721_1-p177006	C7	6	5.6	A
18520	Bn-N17-p12366928	Bn-C7-p12225335	Bn-scaff_16721_1-p177028	C7	6	5.6	A
18521	Bn-N17-p12367073	Bn-C7-p12225480	Bn-scaff_16721_1-p177042	C7	6	5.6	A
18522	Bn-N17-p12367479	Bn-C7-p12227004	Bn-scaff_16721_1-p177195	C7	6	5.6	A
18523	Bn-N17-p12368191	Bn-C7-p12227725	Bn-scaff_16721_1-p177267	C7	6	5.6	A
18524	Bn-N17-p12368524	Bn-C7-p12228063	Bn-scaff_16721_1-p177300	C7	6	5.6	A
18525	Bn-N17-p12368592	Bn-C7-p12228131	Bn-scaff_16721_1-p177307	C7	6	5.6	A
18526	Bn-N17-p12368826	Bn-C7-p12228365	Bn-scaff_16721_1-p177331	C7	6	5.6	A
18527	Bn-N17-p12368965	Bn-C7-p12228504	Bn-scaff_16721_1-p177345	C7	6	5.6	A
18528	Bn-N17-p12369008	Bn-C7-p12228547	Bn-scaff_16721_1-p177349	C7	6	5.6	A
18529	Bn-N17-p12369657	Bn-C7-p12229196	Bn-scaff_16721_1-p177414	C7	6	5.6	A
18530	Bn-N17-p12369824	Bn-C7-p12229363	Bn-scaff_16721_1-p177430	C7	6	5.6	A
18531	Bn-N17-p12370670	Bn-C7-p12230213	Bn-scaff_16721_1-p177515	C7	6	5.6	A
18532	Bn-N17-p12371551	Bn-C7-p12231055	Bn-scaff_16721_1-p177600	C7	6	5.6	A
18533	Bn-N17-p12402184	Bn-C7-p12245400	Bn-scaff_16721_1-p179034	C7	6	5.6	A
18534	Bn-N17-p12421868	Bn-C7-p12268682	Bn-scaff_16721_1-p181362	C7	6	5.6	A
18535	Bn-N17-p12423230	Bn-C7-p12275385	Bn-scaff_16721_1-p182033	C7	6	5.6	A
18536	Bn-N17-p12428642	Bn-C7-p12280814	Bn-scaff_16721_1-p182576	C7	6	5.6	A
18537	Bn-N17-p12428993	Bn-C7-p12281165	Bn-scaff_16721_1-p182611	C7	6	5.6	A
18538	Bn-N17-p12429013	Bn-C7-p12281185	Bn-scaff_16721_1-p182613	C7	6	5.6	A
18539	Bn-N17-p12429186	Bn-C7-p12281358	Bn-scaff_16721_1-p182630	C7	6	5.6	A
18540	Bn-N17-p12429374	Bn-C7-p12281546	Bn-scaff_16721_1-p182649	C7	6	5.6	A
18541	Bn-N17-p12443058	Bn-C7-p12300699	Bn-scaff_16721_1-p184564	C7	6	5.6	A
18542	Bn-N17-p12443088	Bn-C7-p12300729	Bn-scaff_16721_1-p184567	C7	6	5.6	A
18543	Bn-N17-p12443346	Bn-C7-p12300987	Bn-scaff_16721_1-p184593	C7	6	5.6	A
18544	Bn-N17-p12444317	Bn-C7-p12301957	Bn-scaff_16721_1-p184690	C7	6	5.6	A
18545	Bn-N17-p12444528	Bn-C7-p12302176	Bn-scaff_16721_1-p184712	C7	6	5.6	A
18546	Bn-N17-p12464968	Bn-C7-p12356302	Bn-scaff_16721_1-p190124	C7	6	5.6	A
18547	Bn-N17-p12465121	Bn-C7-p12356455	Bn-scaff_16721_1-p190140	C7	6	5.6	A
18548	Bn-N17-p12487241	Bn-C7-p12381817	Bn-scaff_16721_1-p192676	C7	6	5.6	A
18549	Bn-N17-p12487551	Bn-C7-p12382127	Bn-scaff_16721_1-p192707	C7	6	5.6	A
18550	Bn-N17-p12491044	Bn-C7-p12385617	Bn-scaff_16721_1-p193056	C7	6	5.6	A
18551	Bn-N17-p12491084	Bn-C7-p12385657	Bn-scaff_16721_1-p193060	C7	6	5.6	A
18552	Bn-N17-p12491168	Bn-C7-p12385740	Bn-scaff_16721_1-p193068	C7	6	5.6	A
18553	Bn-N17-p12492153	Bn-C7-p12386743	Bn-scaff_16721_1-p193168	C7	6	5.6	A
18554	Bn-N17-p12492583	Bn-C7-p12387173	Bn-scaff_16721_1-p193211	C7	6	5.6	A
18555	Bn-N17-p12492731	Bn-C7-p12387329	Bn-scaff_16721_1-p193227	C7	6	5.6	A
18556	Bn-N17-p12506186	Bn-C7-p12394600	Bn-scaff_16721_1-p193954	C7	6	5.6	A
18557	Bn-N17-p12508353	Bn-C7-p12396771	Bn-scaff_16721_1-p194171	C7	6	5.6	A
18558	Bn-N17-p12508717	Bn-C7-p12397136	Bn-scaff_16721_1-p194208	C7	6	5.6	A
18559	Bn-N17-p12509592	Bn-C7-p12398007	Bn-scaff_16721_1-p194295	C7	6	5.6	A
18560	Bn-N17-p12511743	Bn-C7-p12400163	Bn-scaff_16721_1-p194510	C7	6	5.6	A
18561	Bn-N17-p12511832	Bn-C7-p12400252	Bn-scaff_16721_1-p194519	C7	6	5.6	A
18562	Bn-N17-p12511891	Bn-C7-p12400311	Bn-scaff_16721_1-p194525	C7	6	5.6	A
18563	Bn-N17-p12512813	Bn-C7-p12401233	Bn-scaff_16721_1-p194617	C7	6	5.6	A
18564	Bn-N17-p12513084	Bn-C7-p12401504	Bn-scaff_16721_1-p194645	C7	6	5.6	A
18565	Bn-N17-p12513960	Bn-C7-p12402380	Bn-scaff_16721_1-p194732	C7	6	5.6	A
18566	Bn-N17-p12529389	Bn-C7-p12429550	Bn-scaff_16721_1-p197449	C7	6	5.6	A

18567	Bn-N17-p12530302	Bn-C7-p12434509	Bn-scaff_16721_1-p197945	C7	6	5.6	A
18568	Bn-N17-p12530416	Bn-C7-p12434623	Bn-scaff_16721_1-p197956	C7	6	5.6	A
18569	Bn-N17-p12531746	Bn-C7-p12450535	Bn-scaff_16721_1-p199548	C7	6	5.6	A
18570	Bn-N17-p12531851	Bn-C7-p12450640	Bn-scaff_16721_1-p199558	C7	6	5.6	A
18571	Bn-N17-p12531992	Bn-C7-p12450781	Bn-scaff_16721_1-p199572	C7	6	5.6	A
18572	Bn-N17-p12532251	Bn-C7-p12451041	Bn-scaff_16721_1-p199598	C7	6	5.6	A
18573	Bn-N17-p12532609	Bn-C7-p12451399	Bn-scaff_16721_1-p199634	C7	6	5.6	A
18574	Bn-N17-p12532676	Bn-C7-p12451466	Bn-scaff_16721_1-p199641	C7	6	5.6	A
18575	Bn-N17-p12533173	Bn-C7-p12451963	Bn-scaff_16721_1-p199690	C7	6	5.6	A
18576	Bn-N17-p12533419	Bn-C7-p12452209	Bn-scaff_16721_1-p199715	C7	6	5.6	A
18577	Bn-N17-p12533572	Bn-C7-p12452362	Bn-scaff_16721_1-p199730	C7	6	5.6	A
18578	Bn-N17-p12533584	Bn-C7-p12452374	Bn-scaff_16721_1-p199732	C7	6	5.6	A
18579	Bn-N17-p12533674	Bn-C7-p12452464	Bn-scaff_16721_1-p199741	C7	6	5.6	A
18580	Bn-N17-p12533988	Bn-C7-p12452785	Bn-scaff_16721_1-p199773	C7	6	5.6	A
18581	Bn-N17-p12534060	Bn-C7-p12452857	Bn-scaff_16721_1-p199780	C7	6	5.6	A
18582	Bn-N17-p12534734	Bn-C7-p12465718	Bn-scaff_16721_1-p201066	C7	6	5.6	A
18583	Bn-N17-p12534828	Bn-C7-p12465812	Bn-scaff_16721_1-p201075	C7	6	5.6	A
18584	Bn-N17-p12535526	Bn-C7-p12466500	Bn-scaff_16721_1-p201144	C7	6	5.6	A
18585	Bn-N17-p12551684	Bn-C7-p12484790	Bn-scaff_16721_1-p202973	C7	6	5.6	A
18586	Bn-N17-p12551740	Bn-C7-p12484846	Bn-scaff_16721_1-p202979	C7	6	5.6	A
18587	Bn-N17-p12551924	Bn-C7-p12485026	Bn-scaff_16721_1-p202997	C7	6	5.6	A
18588	Bn-N17-p12552206	Bn-C7-p12485308	Bn-scaff_16721_1-p203025	C7	6	5.6	A
18589	Bn-N17-p12566719	Bn-C7-p12501335	Bn-scaff_16721_1-p204628	C7	6	5.6	A
18590	Bn-N17-p12574022	Bn-C7-p12508706	Bn-scaff_16721_1-p205365	C7	6	5.6	A
18591	Bn-N17-p12577509	Bn-C7-p12512146	Bn-scaff_16721_1-p205709	C7	6	5.6	A
18592	Bn-N17-p12577643	Bn-C7-p12512280	Bn-scaff_16721_1-p205722	C7	6	5.6	A
18593	Bn-N17-p12578138	Bn-C7-p12512780	Bn-scaff_16721_1-p205772	C7	6	5.6	A
18594	Bn-N17-p12578254	Bn-C7-p12512896	Bn-scaff_16721_1-p205784	C7	6	5.6	A
18595	Bn-N17-p12578677	Bn-C7-p12513319	Bn-scaff_16721_1-p205826	C7	6	5.6	A
18596	Bn-N17-p12578846	Bn-C7-p12513514	Bn-scaff_16721_1-p205846	C7	6	5.6	A
18597	Bn-N17-p12579364	Bn-C7-p12514021	Bn-scaff_16721_1-p205896	C7	6	5.6	A
18598	Bn-N17-p12579422	Bn-C7-p12514079	Bn-scaff_16721_1-p205902	C7	6	5.6	A
18599	Bn-N17-p12579858	Bn-C7-p12514520	Bn-scaff_16721_1-p205946	C7	6	5.6	A
18600	Bn-N17-p12592802	Bn-Scaffold01276-p15	Bn-scaff_28889_1-p15875	C7	6	5.6	A
18601	Bn-N17-p12593060	Bn-Scaffold01276-p15	Bn-scaff_28889_1-p15617	C7	6	5.6	A
18602	Bn-N17-p12593322	Bn-Scaffold01276-p15	Bn-scaff_28889_1-p15349	C7	6	5.6	A
18603	Bn-N17-p12593674	Bn-Scaffold01276-p15	Bn-scaff_28889_1-p15002	C7	6	5.6	A
18604	Bn-N17-p12621977	Bn-C7-p12552987	Bn-scaff_16721_1-p209793	C7	6	5.6	A
18605	Bn-N17-p12626437	Bn-C7-p12557460	Bn-scaff_16721_1-p210240	C7	6	5.6	A
18606	Bn-N17-p12626439	Bn-C7-p12557462	Bn-scaff_16721_1-p210240	C7	6	5.6	A
18607	Bn-N17-p12627697	Bn-C7-p12558720	Bn-scaff_16721_1-p210366	C7	6	5.6	A
18608	Bn-N17-p12629061	Bn-C7-p12559466	Bn-scaff_16721_1-p210441	C7	6	5.6	A
18609	Bn-N17-p12629123	Bn-C7-p12559528	Bn-scaff_16721_1-p210447	C7	6	5.6	A
18610	Bn-N17-p12634227	Bn-C7-p12565005	Bn-scaff_16721_1-p210995	C7	6	5.6	A
18611	Bn-N17-p12638362	Bn-C7-p12569131	Bn-scaff_16721_1-p211407	C7	6	5.6	A
18612	Bn-N17-p12657214	Bn-C7-p12589255	Bn-scaff_16721_1-p213420	C7	6	5.6	A
18613	Bn-N17-p12681770	Bn-C7-p12611045	Bn-scaff_16721_1-p215599	C7	6	5.6	A
18614	Bn-N17-p12683140	Bn-C7-p12612415	Bn-scaff_16721_1-p215736	C7	6	5.6	A
18615	Bn-N17-p12760587	Bn-C7-p12635611	Bn-scaff_16721_1-p218055	C7	6	5.6	A

18616	Bn-N17-p12773120	Bn-C7-p12644504	Bn-scaff_16721_1-p21894!	C7	6	5.6	A
18617	Bn-N17-p12773802	Bn-C7-p12645186	Bn-scaff_16721_1-p21901:	C7	6	5.6	A
18618	Bn-N17-p12794877	Bn-C7-p12667790	Bn-scaff_16721_1-p22127:	C7	6	5.6	A
18619	Bn-N17-p12795484	Bn-C7-p12668405	Bn-scaff_16721_1-p22133!	C7	6	5.6	A
18620	Bn-N17-p12817797	Bn-Scaffold01432-p5!	Bn-scaff_18464_1-p5547	C7	6	5.6	A
18621	Bn-N17-p12820118	Bn-Scaffold01432-p3:	Bn-scaff_18464_1-p3239	C7	6	5.6	A
18622	Bn-N17-p12820471	Bn-Scaffold01432-p2!	Bn-scaff_18464_1-p2880	C7	6	5.6	0
18623	Bn-N17-p12820838	Bn-Scaffold01432-p2!	Bn-scaff_18464_1-p2513	C7	6	5.6	A
18624	Bn-N17-p12820981	Bn-Scaffold01432-p2:	Bn-scaff_18464_1-p2370	C7	6	5.6	0
18625	Bn-N17-p12821366	Bn-Scaffold01432-p1!	Bn-scaff_18464_1-p1985	C7	6	5.6	A
18626	Bn-N17-p12830432	Bn-C7-p12684624	Bn-scaff_16200_1-p22642!	C7	6	5.6	A
18627	Bn-N17-p12868967	Bn-C7-p12756852	Bn-scaff_16200_1-p29865:	C7	6	5.6	A
18628	Bn-N17-p12869222	Bn-C7-p12757060	Bn-scaff_16200_1-p29886:	C7	6	5.6	A
18629	Bn-N17-p13096896	Bn-C7-p12953790	Bn-scaff_16200_1-p49559:	C7	6	5.6	A
18630	Bn-N17-p13099370	Bn-C7-p12956862	Bn-scaff_16200_1-p49866:	C7	6	5.6	A
18631	Bn-N17-p13103750	Bn-C7-p12961322	Bn-scaff_16200_1-p50312:	C7	6	5.6	A
18632	Bn-N17-p13103809	Bn-C7-p12961381	Bn-scaff_16200_1-p50318:	C7	6	5.6	A
18633	Bn-N17-p13111630	Bn-C7-p12970788	Bn-scaff_16200_1-p51258!	C7	6	5.6	A
18634	Bn-N17-p13112248	Bn-C7-p12971406	Bn-scaff_16200_1-p51320:	C7	6	5.6	A
18635	Bn-N17-p13112675	Bn-C7-p12971833	Bn-scaff_16200_1-p51363:	C7	6	5.6	A
18636	Bn-N17-p13112883	Bn-C7-p12972041	Bn-scaff_16200_1-p51384:	C7	6	5.6	A
18637	Bn-N17-p13119971	Bn-C7-p12976512	Bn-scaff_16200_1-p51831:	C7	6	5.6	A
18638	Bn-N17-p13125113	Bn-C7-p12984403	Bn-scaff_16200_1-p52620:	C7	6	5.6	A
18639	Bn-N17-p13125223	Bn-C7-p12984513	Bn-scaff_16200_1-p52631:	C7	6	5.6	A
18640	Bn-N17-p13125904	Bn-C7-p12989484	Bn-scaff_16200_1-p53128!	C7	6	5.6	A
18641	Bn-N17-p13126459	Bn-C7-p12990039	Bn-scaff_16200_1-p53184(	C7	6	5.6	A
18642	Bn-N17-p13133635	Bn-C7-p12990275	Bn-scaff_16200_1-p53207(	C7	6	5.6	A
18643	Bn-N17-p13138926	Bn-C7-p12991416	Bn-scaff_16200_1-p53321:	C7	6	5.6	A
18644	Bn-N17-p13139280	Bn-C7-p12992041	Bn-scaff_16200_1-p53384:	C7	6	5.6	A
18645	Bn-N17-p13139870	Bn-C7-p12992633	Bn-scaff_16200_1-p53443:	C7	6	5.6	A
18646	Bn-N17-p13140459	Bn-C7-p12993221	Bn-scaff_16200_1-p53502:	C7	6	5.6	A
18647	Bn-N17-p13142337	Bn-C7-p12993483	Bn-scaff_16200_1-p53528:	C7	6	5.6	A
18648	Bn-N17-p13142366	Bn-C7-p12993512	Bn-scaff_16200_1-p53531:	C7	6	5.6	A
18649	Bn-N17-p13143312	Bn-C7-p12994462	Bn-scaff_16200_1-p53626:	C7	6	5.6	A
18650	Bn-N17-p13144118	Bn-C7-p12990901	Bn-scaff_16200_1-p53270:	C7	6	5.6	A
18651	Bn-N17-p13157083	Bn-C7-p12995305	Bn-scaff_16200_1-p53710(	C7	6	5.6	A
18652	Bn-N17-p13157155	Bn-C7-p12995377	Bn-scaff_16200_1-p53717!	C7	6	5.6	A
18653	Bn-N17-p13157262	Bn-C7-p12995483	Bn-scaff_16200_1-p53728:	C7	6	5.6	A
18654	Bn-N17-p13167194	Bn-C7-p12998751	Bn-scaff_16200_1-p54055:	C7	6	5.6	A
18655	Bn-N17-p13167483	Bn-C7-p12999040	Bn-scaff_16200_1-p54084:	C7	6	5.6	A
18656	Bn-N17-p13167687	Bn-C7-p12999244	Bn-scaff_16200_1-p54104!	C7	6	5.6	A
18657	Bn-N17-p13167765	Bn-C7-p12999322	Bn-scaff_16200_1-p54112:	C7	6	5.6	A
18658	Bn-N17-p13168472	Bn-C7-p13014200	Bn-scaff_16200_1-p55600:	C7	6	5.6	A
18659	Bn-N17-p13169897	Bn-C7-p13015648	Bn-scaff_16200_1-p55744!	C7	6	5.6	A
18660	Bn-N17-p13170323	Bn-C7-p13023756	Bn-scaff_16200_1-p56555:	C7	6	5.6	A
18661	Bn-N17-p13170755	Bn-C7-p13024188	Bn-scaff_16200_1-p56598!	C7	6	5.6	A
18662	Bn-N17-p13170806	Bn-C7-p13024239	Bn-scaff_16200_1-p56604(	C7	6	5.6	A
18663	Bn-N17-p13170816	Bn-C7-p13024249	Bn-scaff_16200_1-p56605(	C7	6	5.6	A
18664	Bn-N17-p13170871	Bn-C7-p13024304	Bn-scaff_16200_1-p56610!	C7	6	5.6	A

18665	Bn-N17-p13171652	Bn-C7-p13025108	Bn-scaff_16200_1-p566909	C7	6	5.6	A
18666	Bn-N17-p13176015	Bn-C7-p13029440	Bn-scaff_16200_1-p571241	C7	6	5.6	A
18667	Bn-N17-p13176130	Bn-C7-p13029555	Bn-scaff_16200_1-p571356	C7	6	5.6	A
18668	Bn-N17-p13177845	Bn-C7-p13030191	Bn-scaff_16200_1-p571992	C7	6	5.6	A
18669	Bn-N17-p13203152	Bn-C7-p13050049	Bn-scaff_16200_1-p591850	C7	6	5.6	A
18670	Bn-N17-p13219366	Bn-C7-p13057461	Bn-scaff_16200_1-p599262	C7	6	5.6	A
18671	Bn-N17-p13220309	Bn-C7-p13058586	Bn-scaff_16200_1-p600387	C7	6	5.6	A
18672	Bn-N17-p13220689	Bn-C7-p13063858	Bn-scaff_16200_1-p605659	C7	6	5.6	A
18673	Bn-N17-p13220902	Bn-C7-p13064070	Bn-scaff_16200_1-p605871	C7	6	5.6	A
18674	Bn-N17-p13221101	Bn-C7-p13064269	Bn-scaff_16200_1-p606070	C7	6	5.6	A
18675	Bn-N17-p13222490	Bn-C7-p13065669	Bn-scaff_16200_1-p607470	C7	6	5.6	A
18676	Bn-N17-p13223587	Bn-C7-p13066762	Bn-scaff_16200_1-p608563	C7	6	5.6	A
18677	Bn-N17-p13224555	Bn-C7-p13067731	Bn-scaff_16200_1-p609532	C7	6	5.6	A
18678	Bn-N17-p13225533	Bn-C7-p13068706	Bn-scaff_16200_1-p610507	C7	6	5.6	A
18679	Bn-N17-p13225988	Bn-C7-p13069163	Bn-scaff_16200_1-p610964	C7	6	5.6	A
18680	Bn-N17-p13226158	Bn-C7-p13069333	Bn-scaff_16200_1-p611134	C7	6	5.6	A
18681	Bn-N17-p13226815	Bn-C7-p13069990	Bn-scaff_16200_1-p611791	C7	6	5.6	A
18682	Bn-N17-p13226869	Bn-C7-p13070044	Bn-scaff_16200_1-p611845	C7	6	5.6	A
18683	Bn-N17-p13227679	Bn-C7-p13070860	Bn-scaff_16200_1-p612661	C7	6	5.6	A
18684	Bn-N17-p13228071	Bn-C7-p13071252	Bn-scaff_16200_1-p613053	C7	6	5.6	A
18685	Bn-N17-p13228125	Bn-C7-p13071306	Bn-scaff_16200_1-p613107	C7	6	5.6	A
18686	Bn-N17-p13230415	Bn-C7-p13074296	Bn-scaff_16200_1-p616097	C7	6	5.6	A
18687	Bn-N17-p13230711	Bn-C7-p13074593	Bn-scaff_16200_1-p616394	C7	6	5.6	A
18688	Bn-N17-p13230871	Bn-C7-p13074751	Bn-scaff_16200_1-p616552	C7	6	5.6	A
18689	Bn-N17-p13231027	Bn-C7-p13074907	Bn-scaff_16200_1-p616708	C7	6	5.6	A
18690	Bn-N17-p13231162	Bn-C7-p13075042	Bn-scaff_16200_1-p616843	C7	6	5.6	A
18691	Bn-N17-p13231332	Bn-C7-p13075212	Bn-scaff_16200_1-p617013	C7	6	5.6	A
18692	Bn-N17-p13231792	Bn-C7-p13075672	Bn-scaff_16200_1-p617473	C7	6	5.6	A
18693	Bn-N17-p13232488	Bn-C7-p13076368	Bn-scaff_16200_1-p618169	C7	6	5.6	A
18694	Bn-N17-p13236594	Bn-C7-p13080378	Bn-scaff_16200_1-p622179	C7	6	5.6	A
18695	Bn-N17-p13239477	Bn-C7-p13083248	Bn-scaff_16200_1-p625049	C7	6	5.6	A
18696	Bn-N17-p13239600	Bn-C7-p13083371	Bn-scaff_16200_1-p625172	C7	6	5.6	A
18697	Bn-N17-p13249755	Bn-C7-p13095927	Bn-scaff_16200_1-p637728	C7	6	5.6	A
18698	Bn-N17-p13278121	Bn-C7-p13114958	Bn-scaff_16200_1-p656759	C7	6	5.6	A
18699	Bn-N17-p13278291	Bn-C7-p13115130	Bn-scaff_16200_1-p656931	C7	6	5.6	A
18700	Bn-N17-p13280953	Bn-C7-p13117583	Bn-scaff_16200_1-p659384	C7	6	5.6	A
18701	Bn-N17-p13281112	Bn-C7-p13117742	Bn-scaff_16200_1-p659543	C7	6	5.6	A
18702	Bn-N17-p13293704	Bn-Scaffold12203-p34	Bn-scaff_25833_1-p346	C7	6	5.6	A
18703	Bn-N17-p13322608	Bn-C7-p13135120	Bn-scaff_16371_1-p7515	C7	6	5.6	A
18704	Bn-N17-p13334224	Bn-C7-p13141634	Bn-scaff_16371_1-p14029	C7	6	5.6	A
18705	Bn-N17-p13383538	Bn-Scaffold00890-p23	Bn-scaff_25766_1-p23436	C7	6	5.6	A
18706	Bn-N17-p13385722	Bn-Scaffold00890-p21	Bn-scaff_25766_1-p21259	C7	6	5.6	A
18707	Bn-N17-p13414623	Bn-C7-p13214811	Bn-scaff_16371_1-p87206	C7	6	5.6	A
18708	Bn-N17-p13417825	Bn-C7-p13217975	Bn-scaff_16371_1-p90370	C7	6	5.6	A
18709	Bn-N17-p13429797	Bn-C7-p13229422	Bn-scaff_16371_1-p101817	C7	6	5.6	A
18710	Bn-N17-p13430177	Bn-C7-p13229802	Bn-scaff_16371_1-p102197	C7	6	5.6	A
18711	Bn-N17-p13445907	Bn-C7-p13245474	Bn-scaff_16371_1-p117869	C7	6	5.6	A
18712	Bn-N17-p13456094	Bn-C7-p13257448	Bn-scaff_16371_1-p129843	C7	6	5.6	A
18713	Bn-N17-p13456859	Bn-C7-p13258186	Bn-scaff_16371_1-p130581	C7	6	5.6	A

18714	Bn-N17-p13457017	Bn-C7-p13258346	Bn-scaff_16371_1-p130741	C7	6	5.6	A
18715	Bn-N17-p13457124	Bn-C7-p13258456	Bn-scaff_16371_1-p130851	C7	6	5.6	A
18716	Bn-N17-p13469160	Bn-C7-p13273673	Bn-scaff_15712_7-p3111	C7	6	5.6	A
18717	Bn-N17-p13469500	Bn-C7-p13274013	Bn-scaff_15712_7-p3451	C7	6	5.6	A
18718	Bn-N17-p13469541	Bn-C7-p13274054	Bn-scaff_15712_7-p3492	C7	6	5.6	A
18719	Bn-N17-p13469635	Bn-C7-p13274148	Bn-scaff_15712_7-p3586	C7	6	5.6	A
18720	Bn-N17-p13477702	Bn-C7-p13282128	Bn-scaff_15712_7-p11566	C7	6	5.6	A
18721	Bn-N17-p13477823	Bn-C7-p13282248	Bn-scaff_15712_7-p11686	C7	6	5.6	A
18722	Bn-N17-p13706399	Bn-C7-p13459031	Bn-scaff_16141_1-p29826	C7	6	5.6	A
18723	Bn-N17-p13778453	Bn-C7-p13540144	Bn-scaff_15847_1-p205021	C7	6	5.6	A
18724	Bn-N17-p13804761	Bn-C7-p13569212	Bn-scaff_15847_1-p175953	C7	6	5.6	A
18725	Bn-N17-p13832490	Bn-C7-p13585608	Bn-scaff_15847_1-p159557	C7	6	5.6	A
18726	Bn-N17-p13833241	Bn-C7-p13586208	Bn-scaff_15847_1-p158957	C7	6	5.6	A
18727	Bn-N17-p13852505	Bn-C7-p13602670	Bn-scaff_15847_1-p142495	C7	6	5.6	A
18728	Bn-N17-p13875407	Bn-C7-p13623621	Bn-scaff_15847_1-p121544	C7	6	5.6	A
18729	Bn-N17-p13897680	Bn-C7-p13650770	Bn-scaff_15847_1-p94395	C7	6	5.6	A
18730	Bn-N17-p13901889	Bn-C7-p13654989	Bn-scaff_15847_1-p90176	C7	6	5.6	A
18731	Bn-N17-p13901899	Bn-C7-p13654999	Bn-scaff_15847_1-p90166	C7	6	5.6	A
18732	Bn-N17-p14326597	Bn-C7-p14117291	Bn-scaff_17584_1-p428409	C7	6	5.6	A
18733	Bn-N18-p17339248	Bn-C7-p12200141	Bn-scaff_16721_1-p174508	C7	6	5.6	A
18734	Bn-N7-p4845267	Bn-A07-p3262752	Bn-A07-p3262752	C7	6	5.6	A
18735	Bn-Scaffold01814-p2311	Bn-Scaffold01171-p14	Bn-scaff_19975_1-p14174	C7	6	5.6	A
18736	Bn-Scaffold05240-p739	Bn-C7-p11475164	Bn-scaff_16721_1-p102011	C7	6	5.6	A
18737	Bn-Scaffold84368-p133	Bn-Scaffold07345-p4	Bn-scaff_24158_1-p427	C7	6	5.6	A
18738	Bn-N15-p29561828	Bn-Scaffold05955-p3	Bn-scaff_21791_1-p338	C7	7	6.4	A
18739	Bn-N17-p14892900	Bn-C7-p14676153	Bn-scaff_15626_1-p108422	C7	7	6.4	A
18740	Bn-N17-p14901473	Bn-C7-p14686580	Bn-scaff_15626_1-p107379	C7	7	6.4	A
18741	Bn-N17-p14994397	Bn-C7-p14771226	Bn-scaff_15626_1-p989150	C7	7	6.4	A
18742	Bn-N17-p15066827	Bn-C7-p14867470	Bn-scaff_15626_1-p892906	C7	7	6.4	A
18743	Bn-N17-p15077493	Bn-C7-p14869154	Bn-scaff_15626_1-p891222	C7	7	6.4	A
18744	Bn-N17-p15162474	Bn-C7-p14943850	Bn-scaff_15626_1-p816526	C7	7	6.4	A
18745	Bn-N17-p15187885	Bn-C7-p14973350	Bn-scaff_15626_1-p787026	C7	7	6.4	A
18746	Bn-N17-p15220865	Bn-C7-p14997610	Bn-scaff_15626_1-p762766	C7	7	6.4	A
18747	Bn-N17-p15220871	Bn-C7-p14997616	Bn-scaff_15626_1-p762760	C7	7	6.4	A
18748	Bn-N17-p15245657	Bn-C7-p15017209	Bn-scaff_15626_1-p743167	C7	7	6.4	A
18749	Bn-N17-p15249404	Bn-C7-p15024804	Bn-scaff_15626_1-p735572	C7	7	6.4	A
18750	Bn-N17-p15249513	Bn-C7-p15024912	Bn-scaff_15626_1-p735464	C7	7	6.4	A
18751	Bn-N17-p15265903	Bn-C7-p15031119	Bn-scaff_15626_1-p729257	C7	7	6.4	A
18752	Bn-N17-p15274346	Bn-C7-p15044322	Bn-scaff_15626_1-p716054	C7	7	6.4	A
18753	Bn-N17-p15293711	Bn-C7-p15068180	Bn-scaff_15626_1-p692196	C7	7	6.4	A
18754	Bn-N17-p15321537	Bn-C7-p15088328	Bn-scaff_15626_1-p672048	C7	7	6.4	A
18755	Bn-N17-p15328837	Bn-C7-p15096207	Bn-scaff_15626_1-p664169	C7	7	6.4	A
18756	Bn-N17-p15344777	Bn-C7-p15108390	Bn-scaff_15626_1-p651986	C7	7	6.4	A
18757	Bn-N17-p15360195	Bn-C7-p15128633	Bn-scaff_15626_1-p631743	C7	7	6.4	A
18758	Bn-N17-p15379148	Bn-C7-p15149316	Bn-scaff_15626_1-p611060	C7	7	6.4	A
18759	Bn-N17-p15402984	Bn-C7-p15174347	Bn-scaff_15626_1-p586029	C7	7	6.4	A
18760	Bn-N17-p15419727	Bn-C7-p15188411	Bn-scaff_15626_1-p571965	C7	7	6.4	A
18761	Bn-N17-p15420121	Bn-C7-p15188826	Bn-scaff_15626_1-p571550	C7	7	6.4	A
18762	Bn-N17-p15420213	Bn-C7-p15188918	Bn-scaff_15626_1-p571458	C7	7	6.4	A

18763	Bn-N17-p15440338	Bn-C7-p15211633	Bn-scaff_15626_1-p54874[illegible]	C7	7	6.4	A
18764	Bn-N17-p15466582	Bn-C7-p15231934	Bn-scaff_15626_1-p52844[illegible]	C7	7	6.4	A
18765	Bn-N17-p16181660	Bn-C7-p15950544	Bn-scaff_17580_1-p28133[illegible]	C7	7	6.4	A
18766	Bn-N17-p16376658	Bn-C7-p16148753	Bn-scaff_17580_1-p83128	C7	7	6.4	A
18767	Bn-N7-p6495019	Bn-A07-p4923886	Bn-A07-p4923886	C7	7	6.4	A
18768	Bn-N7-p6679232	Bn-C7-p15059747	Bn-scaff_15626_1-p70062[illegible]	C7	7	6.4	A
18769	Bn-N17-p16953516	Bn-C7-p16589778	Bn-scaff_17326_1-p35779[illegible]	C7	8	7.2	A
18770	Bn-N17-p17061241	Bn-C7-p16768494	Bn-scaff_17326_1-p53651[illegible]	C7	8	7.2	A
18771	Bn-N17-p17734925	Bn-C7-p17496540	Bn-scaff_17942_1-p61731	C7	8	7.2	A
18772	Bn-N19-p46943258	Bn-C4-p33915659	Bn-scaff_26139_1-p10638[illegible]	C7	8	7.2	A
18773	Bn-N8-p14762789	Bn-A08-p15031530	Bn-A08-p15031530	C7	8	7.2	A
18774	Bn-N8-p14762917	Bn-A08-p15031658	Bn-A08-p15031658	C7	8	7.2	A
18775	Bn-N13-p53096085	Bn-A07-p6906296	Bn-A07-p6906296	C7	9	8	A
18776	Bn-N16-p2932119	Bn-C6-p37073204	Bn-scaff_19911_1-p42676	C7	9	8	A
18777	Bn-N17-p18926136	Bn-C7-p18639853	Bn-scaff_16541_1-p52632[illegible]	C7	10	9.6	A
18778	Bn-N17-p19627529	Bn-C7-p19034209	Bn-scaff_22536_1-p31931[illegible]	C7	10	9.6	A
18779	Bn-N17-p20296411	Bn-C7-p20026038	Bn-scaff_16450_1-p10648[illegible]	C7	10	9.6	A
18780	Bn-N17-p20382472	Bn-C7-p20104233	Bn-scaff_16450_1-p28287	C7	10	9.6	A
18781	Bn-N17-p20395324	Bn-C7-p20117250	Bn-scaff_16450_1-p15270	C7	10	9.6	A
18782	Bn-N17-p20446675	Bn-C7-p20307138	Bn-scaff_19106_1-p60538[illegible]	C7	11	12.8	A
18783	Bn-N17-p20449703	Bn-Scaffold01176-p1[illegible]	Bn-scaff_18032_1-p13360	C7	11	12.8	A
18784	Bn-N17-p20460226	Bn-C7-p20319105	Bn-scaff_19106_1-p59341[illegible]	C7	11	12.8	A
18785	Bn-N17-p20464948	Bn-C7-p20324758	Bn-scaff_19106_1-p58776[illegible]	C7	11	12.8	A
18786	Bn-N17-p20479172	Bn-C7-p20343772	Bn-scaff_19106_1-p56875[illegible]	C7	11	12.8	A
18787	Bn-N17-p20479337	Bn-C7-p20343966	Bn-scaff_19106_1-p56855[illegible]	C7	11	12.8	A
18788	Bn-N17-p20486312	Bn-C7-p20349908	Bn-scaff_19106_1-p56261[illegible]	C7	11	12.8	A
18789	Bn-N17-p20486601	Bn-C7-p20350206	Bn-scaff_19106_1-p56231[illegible]	C7	11	12.8	A
18790	Bn-N17-p20588814	Bn-C7-p20441937	Bn-scaff_19106_1-p47058[illegible]	C7	11	12.8	A
18791	Bn-N17-p20634441	Bn-C7-p20472212	Bn-scaff_19106_1-p44031[illegible]	C7	11	12.8	A
18792	Bn-N17-p20693023	Bn-C7-p20528165	Bn-scaff_19106_1-p38435[illegible]	C7	11	12.8	A
18793	Bn-N17-p20740731	Bn-C7-p20601747	Bn-scaff_19106_1-p31077[illegible]	C7	11	12.8	A
18794	Bn-N17-p20740787	Bn-C7-p20601803	Bn-scaff_19106_1-p31072[illegible]	C7	11	12.8	A
18795	Bn-N17-p20743080	Bn-C7-p20608523	Bn-scaff_19106_1-p30400[illegible]	C7	11	12.8	A
18796	Bn-N17-p20767020	Bn-C7-p20632772	Bn-scaff_19106_1-p27975[illegible]	C7	11	12.8	A
18797	Bn-N17-p20834925	Bn-C7-p20706445	Bn-scaff_19106_1-p20607[illegible]	C7	11	12.8	A
18798	Bn-Scaffold01793-p508	Bn-C13835305-p333	Bn-C13835305-p333	C7	11	12.8	A
18799	Bn-Scaffold03614-p555	Bn-C7-p20449477	Bn-scaff_19106_1-p46304[illegible]	C7	11	12.8	A
18800	Bn-N17-p20998852	Bn-C7-p20921325	Bn-scaff_23400_1-p61356[illegible]	C7	12	13.6	A
18801	Bn-N17-p21003816	Bn-C7-p20926302	Bn-scaff_23400_1-p60858[illegible]	C7	12	13.6	A
18802	Bn-N17-p21053844	Bn-C7-p20952295	Bn-scaff_23400_1-p58259[illegible]	C7	12	13.6	A
18803	Bn-N17-p21103623	Bn-C7-p20978539	Bn-scaff_23400_1-p55634[illegible]	C7	12	13.6	A
18804	Bn-N17-p21154167	Bn-C7-p21026228	Bn-scaff_23400_1-p50865[illegible]	C7	12	13.6	A
18805	Bn-N17-p21264164	Bn-C7-p21135074	Bn-scaff_23400_1-p39981[illegible]	C7	13	14.4	A
18806	Bn-N17-p21281634	Bn-C7-p21153628	Bn-scaff_23400_1-p38125[illegible]	C7	13	14.4	A
18807	Bn-N17-p21322697	Bn-C7-p21201324	Bn-scaff_23400_1-p33356[illegible]	C7	13	14.4	A
18808	Bn-N17-p21335090	Bn-C7-p21212846	Bn-scaff_23400_1-p32203[illegible]	C7	13	14.4	A
18809	Bn-N17-p21357159	Bn-C7-p21244493	Bn-scaff_23400_1-p29039[illegible]	C7	13	14.4	A
18810	Bn-N17-p21558184	Bn-C7-p21433530	Bn-scaff_23400_1-p10135[illegible]	C7	13	14.4	A
18811	Bn-Scaffold02002-p3583	Bn-C7-p22036199	Bn-scaff_16100_1-p39885[illegible]	C7	13	14.4	A

18812	Bn-N17-p21604862	Bn-C7-p21817897	Bn-scaff_16100_1-p61716(	C7	14	15.2	A
18813	Bn-N17-p21871187	Bn-C7-p21662918	Bn-scaff_16100_1-p77213(	C7	14	15.2	A
18814	Bn-N11-p13539403	Bn-C1-p12163415	Bn-scaff_17369_1-p69072(	C7	15	16	A
18815	Bn-N17-p21712618	Bn-C7-p21544993	Bn-scaff_16100_1-p89006(	C7	15	16	A
18816	Bn-N17-p22812520	Bn-C7-p22549306	Bn-scaff_18501_1-p11415(	C7	15	16	A
18817	Bn-N17-p22814363	Bn-C7-p22551125	Bn-scaff_18501_1-p11596(	C7	15	16	A
18818	Bn-N17-p22820651	Bn-C7-p22557413	Bn-scaff_18501_1-p12225(	C7	15	16	A
18819	Bn-N17-p22826272	Bn-C7-p22566443	Bn-scaff_18501_1-p13128(	C7	15	16	A
18820	Bn-N17-p22965853	Bn-C7-p22699864	Bn-scaff_18501_1-p26470(	C7	15	16	A
18821	Bn-N17-p23066790	Bn-C7-p22804489	Bn-scaff_18501_1-p36933(	C7	15	16	A
18822	Bn-N17-p23125248	Bn-C7-p22856868	Bn-scaff_18501_1-p42171(	C7	15	16	A
18823	Bn-N6-p7749669	Bn-C7-p22771768	Bn-scaff_18501_1-p33661(	C7	15	16	A
18824	Bn-N7-p10817178	Bn-C7-p22771305	Bn-scaff_18501_1-p33614(	C7	15	16	A
18825	Bn-N7-p10817318	Bn-C7-p22771490	Bn-scaff_18501_1-p33633(	C7	15	16	A
18826	Bn-N7-p10825813	Bn-C7-p22781037	Bn-scaff_18501_1-p34588(	C7	15	16	A
18827	Bn-N17-p23162790	Bn-C7-p22905190	Bn-scaff_18501_1-p47003(	C7	16	16.8	A
18828	Bn-N17-p23208449	Bn-C7-p22965833	Bn-scaff_18501_1-p53067(	C7	16	16.8	A
18829	Bn-N17-p23223580	Bn-C7-p22995749	Bn-scaff_18501_1-p56059(	C7	16	16.8	A
18830	Bn-N17-p23258840	Bn-C7-p22993737	Bn-scaff_18501_1-p55858(	C7	16	16.8	A
18831	Bn-N17-p23287902	Bn-C7-p23039714	Bn-scaff_18501_1-p60455(	C7	16	16.8	A
18832	Bn-N17-p23330927	Bn-C9-p7109786	Bn-scaff_17423_1-p72277(	C7	16	16.8	A
18833	Bn-N17-p23333108	Bn-C7-p23089472	Bn-scaff_18501_1-p65431(	C7	16	16.8	0
18834	Bn-N17-p23335166	Bn-C7-p23091556	Bn-scaff_18501_1-p65640(	C7	16	16.8	A
18835	Bn-N17-p23338843	Bn-C7-p23094809	Bn-scaff_18501_1-p65965(	C7	16	16.8	A
18836	Bn-N17-p23346406	Bn-C7-p23101151	Bn-scaff_18501_1-p66599(	C7	16	16.8	A
18837	Bn-N17-p23357236	Bn-C7-p23111426	Bn-scaff_18501_1-p67627(	C7	16	16.8	A
18838	Bn-N17-p23390541	Bn-C7-p23138311	Bn-scaff_18501_1-p70315(	C7	16	16.8	A
18839	Bn-N17-p23390693	Bn-C7-p23138463	Bn-scaff_18501_1-p70330(	C7	16	16.8	A
18840	Bn-N17-p23390710	Bn-C7-p23138480	Bn-scaff_18501_1-p70332(	C7	16	16.8	A
18841	Bn-N17-p23425438	Bn-C7-p23174070	Bn-scaff_18501_1-p73891(	C7	16	16.8	A
18842	Bn-N17-p23425642	Bn-C7-p23174273	Bn-scaff_18501_1-p73911(	C7	16	16.8	A
18843	Bn-N17-p23451905	Bn-C7-p23203733	Bn-scaff_18501_1-p76857(	C7	16	16.8	A
18844	Bn-N17-p23476000	Bn-C7-p23215872	Bn-scaff_18501_1-p78071(	C7	16	16.8	A
18845	Bn-N17-p23476075	Bn-C7-p23215947	Bn-scaff_18501_1-p78079(	C7	16	16.8	A
18846	Bn-N17-p23476259	Bn-C7-p23216131	Bn-scaff_18501_1-p78097(	C7	16	16.8	A
18847	Bn-N17-p23498463	Bn-C7-p23217487	Bn-scaff_18501_1-p78233(	C7	16	16.8	A
18848	Bn-N17-p23498633	Bn-C7-p23217654	Bn-scaff_18501_1-p78249(	C7	16	16.8	A
18849	Bn-N17-p23522278	Bn-C7-p23224953	Bn-scaff_18501_1-p78979(	C7	16	16.8	A
18850	Bn-N17-p23522425	Bn-C7-p23225100	Bn-scaff_18501_1-p78994(	C7	16	16.8	A
18851	Bn-N17-p23523325	Bn-C7-p23225990	Bn-scaff_18501_1-p79083(	C7	16	16.8	A
18852	Bn-N17-p23524424	Bn-C7-p23227053	Bn-scaff_18501_1-p79189(	C7	16	16.8	A
18853	Bn-N17-p23532357	Bn-C7-p23230383	Bn-scaff_18501_1-p79522(	C7	16	16.8	A
18854	Bn-N17-p23543071	Bn-C7-p23245479	Bn-scaff_18501_1-p81032(	C7	16	16.8	A
18855	Bn-N17-p23543797	Bn-C7-p23246210	Bn-scaff_18501_1-p81105(	C7	16	16.8	A
18856	Bn-N17-p23558606	Bn-C7-p23273074	Bn-scaff_18501_1-p83791(	C7	16	16.8	A
18857	Bn-N17-p23558671	Bn-C7-p23273141	Bn-scaff_18501_1-p83798(	C7	16	16.8	A
18858	Bn-N17-p23558984	Bn-C7-p23273455	Bn-scaff_18501_1-p83829(	C7	16	16.8	A
18859	Bn-N17-p23565569	Bn-C7-p23274248	Bn-scaff_18501_1-p83909(	C7	16	16.8	A
18860	Bn-N17-p23578164	Bn-C7-p23284855	Bn-scaff_18501_1-p84969(	C7	16	16.8	A

18861	Bn-N17-p23578234	Bn-C7-p23284925	Bn-scaff_18501_1-p849769	C7	16	16.8	A
18862	Bn-N17-p23579031	Bn-C7-p23285714	Bn-scaff_18501_1-p850558	C7	16	16.8	A
18863	Bn-N17-p23580284	Bn-Scaffold01437-p85	Bn-scaff_26974_1-p8519	C7	16	16.8	A
18864	Bn-N17-p23596617	Bn-C7-p23314228	Bn-scaff_18501_1-p879072	C7	16	16.8	A
18865	Bn-N17-p23615957	Bn-C7-p23335651	Bn-scaff_18501_1-p900495	C7	16	16.8	A
18866	Bn-N17-p23681480	Bn-C7-p23374752	Bn-scaff_18501_1-p939596	C7	16	16.8	A
18867	Bn-N17-p23684490	Bn-C7-p23377794	Bn-scaff_18501_1-p942638	C7	16	16.8	A
18868	Bn-N17-p23684552	Bn-C7-p23377856	Bn-scaff_18501_1-p942700	C7	16	16.8	A
18869	Bn-N17-p23697062	Bn-C7-p23385716	Bn-scaff_18501_1-p950560	C7	16	16.8	A
18870	Bn-N17-p23697213	Bn-C7-p23385867	Bn-scaff_18501_1-p950711	C7	16	16.8	A
18871	Bn-N17-p23697217	Bn-C7-p23385871	Bn-scaff_18501_1-p950715	C7	16	16.8	A
18872	Bn-N17-p23697604	Bn-C7-p23386227	Bn-scaff_18501_1-p951071	C7	16	16.8	A
18873	Bn-N17-p23722896	Bn-C7-p23394861	Bn-scaff_18501_1-p959705	C7	16	16.8	A
18874	Bn-N17-p23729795	Bn-C7-p23398764	Bn-scaff_18501_1-p963608	C7	16	16.8	A
18875	Bn-N17-p23733382	Bn-C7-p23415521	Bn-scaff_18501_1-p980365	C7	16	16.8	A
18876	Bn-N17-p23735385	Bn-C7-p23417650	Bn-scaff_18501_1-p982494	C7	16	16.8	A
18877	Bn-N17-p23736074	Bn-C7-p23418335	Bn-scaff_18501_1-p983179	C7	16	16.8	A
18878	Bn-N17-p24193173	Bn-C7-p23829640	Bn-scaff_18202_1-p168334	C7	17	20	A
18879	Bn-N17-p24209828	Bn-C7-p23847404	Bn-scaff_18202_1-p166557	C7	17	20	A
18880	Bn-N17-p24216333	Bn-C7-p23853656	Bn-scaff_18202_1-p165932	C7	17	20	A
18881	Bn-N17-p24217075	Bn-C7-p23854398	Bn-scaff_18202_1-p165858	C7	17	20	A
18882	Bn-N17-p24222777	Bn-C7-p23860013	Bn-scaff_18202_1-p165297	C7	17	20	A
18883	Bn-N17-p24271722	Bn-C7-p23897491	Bn-scaff_18202_1-p161549	C7	18	20.8	A
18884	Bn-N17-p24272850	Bn-C7-p23898603	Bn-scaff_18202_1-p161438	C7	18	20.8	A
18885	Bn-N17-p24278329	Bn-C7-p23904082	Bn-scaff_18202_1-p160890	C7	18	20.8	A
18886	Bn-N17-p24280899	Bn-C7-p23906651	Bn-scaff_18202_1-p160633	C7	18	20.8	A
18887	Bn-N17-p24742395	Bn-C7-p24424315	Bn-scaff_18202_1-p108860	C7	19	24.8	A
18888	Bn-N17-p24742496	Bn-C7-p24424357	Bn-scaff_18202_1-p108862	C7	19	24.8	A
18889	Bn-N17-p21261593	Bn-C2-p44217918	Bn-scaff_15712_2-p15006	C7	20	25.6	A
18890	Bn-N17-p24838256	Bn-C7-p24526764	Bn-scaff_18202_1-p986219	C7	20	25.6	A
18891	Bn-N17-p24848719	Bn-C7-p24535996	Bn-scaff_18202_1-p976987	C7	20	25.6	A
18892	Bn-N7-p11585318	Bn-A07-p10100811	Bn-A07-p10100811	C7	20	25.6	A
18893	Bn-N17-p24950440	Bn-C7-p24641573	Bn-scaff_18202_1-p871410	C7	21	26.4	A
18894	Bn-N17-p24950641	Bn-C7-p24641774	Bn-scaff_18202_1-p871209	C7	21	26.4	A
18895	Bn-N17-p24951017	Bn-C7-p24642151	Bn-scaff_18202_1-p870832	C7	21	26.4	A
18896	Bn-N17-p24951184	Bn-C7-p24642318	Bn-scaff_18202_1-p870665	C7	21	26.4	A
18897	Bn-N17-p24977581	Bn-C7-p24666607	Bn-scaff_18202_1-p846376	C7	21	26.4	A
18898	Bn-N17-p25262220	Bn-C7-p24943849	Bn-scaff_18202_1-p569134	C7	22	28	A
18899	Bn-N17-p25469565	Bn-C7-p25156894	Bn-scaff_18202_1-p356089	C7	22	28	A
18900	Bn-N17-p25492931	Bn-C7-p25187654	Bn-scaff_18202_1-p325329	C7	22	28	A
18901	Bn-N17-p25499645	Bn-C7-p25190397	Bn-scaff_18202_1-p322586	C7	22	28	A
18902	Bn-N17-p25521776	Bn-C7-p25203849	Bn-scaff_18202_1-p309134	C7	22	28	A
18903	Bn-N17-p25570616	Bn-C7-p25260097	Bn-scaff_18202_1-p252886	C7	22	28	A
18904	Bn-N17-p25591469	Bn-C7-p25296943	Bn-scaff_18202_1-p216040	C7	22	28	A
18905	Bn-N17-p25591573	Bn-C7-p25297047	Bn-scaff_18202_1-p215936	C7	22	28	A
18906	Bn-N17-p25621099	Bn-C7-p25330352	Bn-scaff_18202_1-p182631	C7	22	28	A
18907	Bn-Scaffold07004-p1172	Bn-C7-p25200453	Bn-scaff_18202_1-p312530	C7	22	28	A
18908	Bn-N17-p25650924	Bn-C7-p25372185	Bn-scaff_18202_1-p140798	C7	23	28.8	A
18909	Bn-N17-p25651501	Bn-C7-p25372745	Bn-scaff_18202_1-p140238	C7	23	28.8	A

18910	Bn-N17-p25676105	Bn-C7-p25396539	Bn-scaff_18202_1-p116444	C7	23	28.8	A
18911	Bn-N17-p25771233	Bn-Scaffold00928-p13	Bn-scaff_22821_1-p13976	C7	23	28.8	A
18912	Bn-N17-p25966569	Bn-C7-p25603027	Bn-scaff_15712_1-p89945	C7	23	28.8	A
18913	Bn-N17-p26086727	Bn-C7-p25717968	Bn-scaff_15712_1-p204886	C7	23	28.8	A
18914	Bn-N17-p26161842	Bn-C7-p25791596	Bn-scaff_15712_1-p278514	C7	23	28.8	A
18915	Bn-N17-p26695844	Bn-C7-p26355002	Bn-scaff_15712_1-p841920	C7	24	29.6	A
18916	Bn-N17-p26837319	Bn-C7-p28271314	Bn-scaff_19724_1-p462268	C7	24	29.6	A
18917	Bn-N17-p26932446	Bn-C7-p28175965	Bn-scaff_19724_1-p557617	C7	24	29.6	A
18918	Bn-N17-p27196862	Bn-C7-p27858122	Bn-scaff_15715_1-p260062	C7	24	29.6	A
18919	Bn-N17-p28546575	Bn-C7-p26910065	Bn-scaff_23839_1-p144529	C7	24	29.6	A
18920	Bn-N17-p28550890	Bn-C7-p26914435	Bn-scaff_23839_1-p148899	C7	24	29.6	A
18921	Bn-N17-p28929164	Bn-C7-p28483795	Bn-scaff_19724_1-p249787	C7	24	29.6	A
18922	Bn-N17-p28963023	Bn-C7-p28517187	Bn-scaff_19724_1-p216395	C7	24	29.6	A
18923	Bn-N17-p28987722	Bn-C7-p28543449	Bn-scaff_19724_1-p190133	C7	24	29.6	A
18924	Bn-N17-p29012517	Bn-C7-p28565936	Bn-scaff_19724_1-p167646	C7	24	29.6	A
18925	Bn-N17-p29013806	Bn-C7-p28567226	Bn-scaff_19724_1-p166356	C7	24	29.6	A
18926	Bn-N17-p29013976	Bn-C7-p28567396	Bn-scaff_19724_1-p166186	C7	24	29.6	A
18927	Bn-N17-p29166869	Bn-C7-p28711238	Bn-scaff_19724_1-p22344	C7	24	29.6	A
18928	Bn-N17-p29227345	Bn-C7-p28777401	Bn-scaff_15754_1-p43720	C7	24	29.6	A
18929	Bn-N17-p29234411	Bn-C7-p28781506	Bn-scaff_15754_1-p47825	C7	24	29.6	A
18930	Bn-N17-p29426444	Bn-C7-p28973784	Bn-scaff_15754_1-p240103	C7	25	30.4	A
18931	Bn-N17-p29427271	Bn-C7-p28974611	Bn-scaff_15754_1-p240930	C7	25	30.4	A
18932	Bn-N17-p29428452	Bn-C7-p28975792	Bn-scaff_15754_1-p242111	C7	25	30.4	A
18933	Bn-N17-p29429440	Bn-C7-p28976780	Bn-scaff_15754_1-p243099	C7	25	30.4	A
18934	Bn-N17-p29480268	Bn-C7-p29019778	Bn-scaff_15754_1-p286097	C7	25	30.4	A
18935	Bn-N17-p29596837	Bn-C7-p29183954	Bn-scaff_15754_1-p450273	C7	25	30.4	A
18936	Bn-N17-p29599182	Bn-C7-p29186311	Bn-scaff_15754_1-p452630	C7	25	30.4	A
18937	Bn-N17-p29606684	Bn-C7-p29198835	Bn-scaff_15754_1-p465154	C7	25	30.4	A
18938	Bn-N17-p29621509	Bn-C7-p29212411	Bn-scaff_15754_1-p478730	C7	25	30.4	A
18939	Bn-N17-p29660365	Bn-C7-p29243322	Bn-scaff_15754_1-p509641	C7	25	30.4	A
18940	Bn-N17-p29669691	Bn-C7-p29252969	Bn-scaff_15754_1-p519288	C7	25	30.4	A
18941	Bn-N17-p29713676	Bn-C7-p29288755	Bn-scaff_15754_1-p555074	C7	25	30.4	A
18942	Bn-N17-p6586079	Bn-C7-p5977453	Bn-scaff_20084_1-p50711	C7	25	30.4	A
18943	Bn-N17-p29828465	Bn-C7-p29385521	Bn-scaff_15754_1-p651840	C7	26	32.8	A
18944	Bn-N17-p29860763	Bn-C7-p29422151	Bn-scaff_15754_1-p688470	C7	27	33.6	A
18945	Bn-N17-p29882929	Bn-C7-p29425346	Bn-scaff_15754_1-p691665	C7	27	33.6	A
18946	Bn-N17-p29883169	Bn-C7-p29425573	Bn-scaff_15754_1-p691892	C7	27	33.6	A
18947	Bn-N17-p29883425	Bn-C7-p29425829	Bn-scaff_15754_1-p692148	C7	27	33.6	A
18948	Bn-N17-p29884193	Bn-C7-p29426597	Bn-scaff_15754_1-p692916	C7	27	33.6	A
18949	Bn-N17-p29987636	Bn-C7-p29536265	Bn-scaff_15754_1-p802584	C7	27	33.6	A
18950	Bn-N17-p30386159	Bn-C7-p29874882	Bn-scaff_15754_1-p114120	C7	27	33.6	A
18951	Bn-N17-p30386363	Bn-C7-p29875087	Bn-scaff_15754_1-p114140	C7	27	33.6	A
18952	Bn-N17-p30386601	Bn-C7-p29875325	Bn-scaff_15754_1-p114164	C7	27	33.6	A
18953	Bn-N17-p30386777	Bn-C7-p29875501	Bn-scaff_15754_1-p114182	C7	27	33.6	A
18954	Bn-N17-p30393583	Bn-C7-p29880989	Bn-scaff_15754_1-p114730	C7	27	33.6	A
18955	Bn-N17-p30467558	Bn-C7-p29959038	Bn-scaff_15754_1-p122535	C7	27	33.6	A
18956	Bn-N17-p30593263	Bn-C7-p30190054	Bn-scaff_15754_1-p145637	C7	27	33.6	A
18957	Bn-N17-p30614817	Bn-C7-p30171372	Bn-scaff_15754_1-p143769	C7	27	33.6	A
18958	Bn-N17-p30616667	Bn-C7-p30169524	Bn-scaff_15754_1-p143584	C7	27	33.6	A

18959	Bn-N17-p30622148	Bn-C7-p30163892	Bn-scaff_15754_1-p14302	C7	27	33.6	A
18960	Bn-N17-p30622321	Bn-C7-p30163719	Bn-scaff_15754_1-p14300	C7	27	33.6	0
18961	Bn-N17-p30630717	Bn-C7-p30160580	Bn-scaff_15754_1-p14268	C7	27	33.6	0
18962	Bn-N17-p30632168	Bn-C7-p30159138	Bn-scaff_15754_1-p14254	C7	27	33.6	A
18963	Bn-N17-p30641361	Bn-C7-p30150223	Bn-scaff_15754_1-p14165	C7	27	33.6	A
18964	Bn-N17-p30654212	Bn-C7-p30137439	Bn-scaff_15754_1-p14037	C7	27	33.6	A
18965	Bn-N17-p30729383	Bn-C7-p30277405	Bn-scaff_16130_1-p80124	C7	27	33.6	A
18966	Bn-N17-p30730291	Bn-C7-p30279569	Bn-scaff_16130_1-p82288	C7	27	33.6	A
18967	Bn-N17-p30740705	Bn-C7-p30295414	Bn-scaff_16130_1-p98133	C7	27	33.6	A
18968	Bn-N17-p30744956	Bn-C7-p30299979	Bn-scaff_16130_1-p10269	C7	27	33.6	A
18969	Bn-N17-p30745755	Bn-C7-p30300777	Bn-scaff_16130_1-p10349	C7	27	33.6	A
18970	Bn-N17-p30745864	Bn-C7-p30306406	Bn-scaff_16130_1-p10912	C7	27	33.6	A
18971	Bn-N17-p30745877	Bn-C7-p30306419	Bn-scaff_16130_1-p10913	C7	27	33.6	A
18972	Bn-N17-p30746948	Bn-C7-p30307490	Bn-scaff_16130_1-p11020	C7	27	33.6	A
18973	Bn-N17-p30748215	Bn-C7-p30308757	Bn-scaff_16130_1-p11147	C7	27	33.6	A
18974	Bn-N17-p30750352	Bn-C7-p30310903	Bn-scaff_16130_1-p11362	C7	27	33.6	A
18975	Bn-N17-p30750543	Bn-C7-p30311105	Bn-scaff_16130_1-p11382	C7	27	33.6	A
18976	Bn-N17-p30752421	Bn-C7-p30312963	Bn-scaff_16130_1-p11568	C7	27	33.6	A
18977	Bn-N17-p30752582	Bn-C7-p30313123	Bn-scaff_16130_1-p11584	C7	27	33.6	A
18978	Bn-N17-p30807820	Bn-C7-p30371485	Bn-scaff_16130_1-p17420	C7	27	33.6	A
18979	Bn-N17-p30809215	Bn-C7-p30372882	Bn-scaff_16130_1-p17560	C7	27	33.6	A
18980	Bn-N17-p30833449	Bn-C7-p30414030	Bn-scaff_16130_1-p21674	C7	27	33.6	A
18981	Bn-N17-p30849010	Bn-C7-p30422102	Bn-scaff_16130_1-p22482	C7	27	33.6	A
18982	Bn-N17-p30860786	Bn-C7-p30434384	Bn-scaff_16130_1-p23710	C7	27	33.6	A
18983	Bn-N17-p30862260	Bn-C7-p30439287	Bn-scaff_16130_1-p24200	C7	27	33.6	A
18984	Bn-N17-p30881825	Bn-C7-p30455816	Bn-scaff_16130_1-p25853	C7	27	33.6	A
18985	Bn-N17-p30882700	Bn-C7-p30457730	Bn-scaff_16130_1-p26044	C7	27	33.6	A
18986	Bn-N17-p30883115	Bn-C7-p30458146	Bn-scaff_16130_1-p26086	C7	27	33.6	A
18987	Bn-N17-p30883426	Bn-C7-p30463363	Bn-scaff_16130_1-p26608	C7	27	33.6	A
18988	Bn-N17-p30883478	Bn-C7-p30463415	Bn-scaff_16130_1-p26613	C7	27	33.6	A
18989	Bn-N17-p30883676	Bn-C7-p30463613	Bn-scaff_16130_1-p26633	C7	27	33.6	A
18990	Bn-N17-p30895184	Bn-C7-p30471200	Bn-scaff_16130_1-p27391	C7	27	33.6	A
18991	Bn-N17-p30896208	Bn-C7-p30472019	Bn-scaff_16130_1-p27473	C7	27	33.6	A
18992	Bn-N17-p30900473	Bn-C7-p30477993	Bn-scaff_16130_1-p28071	C7	27	33.6	A
18993	Bn-N17-p30900678	Bn-C7-p30478195	Bn-scaff_16130_1-p28091	C7	27	33.6	A
18994	Bn-N17-p30900704	Bn-C7-p30478221	Bn-scaff_16130_1-p28094	C7	27	33.6	A
18995	Bn-N17-p30910178	Bn-C7-p30483463	Bn-scaff_16130_1-p28618	C7	27	33.6	A
18996	Bn-N6-p23983877	Bn-A06-p24167592	Bn-A06-p24167592	C7	27	33.6	A
18997	Bn-N6-p24026977	Bn-A06-p24207308	Bn-A06-p24207308	C7	27	33.6	A
18998	Bn-N17-p31158770	Bn-C7-p30740034	Bn-scaff_16130_1-p54275	C7	28	34.4	A
18999	Bn-N17-p31283084	Bn-C7-p30856330	Bn-scaff_16130_1-p65904	C7	29	35.2	A
19000	Bn-N17-p31283633	Bn-C7-p30856880	Bn-scaff_16130_1-p65959	C7	29	35.2	A
19001	Bn-N17-p31289112	Bn-C7-p30862263	Bn-scaff_16130_1-p66498	C7	29	35.2	A
19002	Bn-N17-p31291104	Bn-C7-p30864255	Bn-scaff_16130_1-p66697	C7	29	35.2	A
19003	Bn-N17-p31292625	Bn-C7-p30870707	Bn-scaff_16130_1-p67342	C7	29	35.2	A
19004	Bn-N17-p31307076	Bn-C7-p30960914	Bn-scaff_16130_1-p76363	C7	29	35.2	A
19005	Bn-N17-p31307499	Bn-C7-p30961337	Bn-scaff_16130_1-p76405	C7	29	35.2	A
19006	Bn-N17-p31310421	Bn-C7-p30964230	Bn-scaff_16130_1-p76694	C7	29	35.2	A
19007	Bn-N17-p31344456	Bn-C7-p30997405	Bn-scaff_16130_1-p80012	C7	29	35.2	A

19008	Bn-N17-p31348421	Bn-C7-p31001375	Bn-scaff_16130_1-p80409	C7	29	35.2	A
19009	Bn-N17-p31357476	Bn-C7-p31008257	Bn-scaff_16130_1-p81097	C7	29	35.2	A
19010	Bn-N17-p31454113	Bn-C7-p31107417	Bn-scaff_16130_1-p91013	C7	29	35.2	A
19011	Bn-N17-p31477122	Bn-C7-p31114127	Bn-scaff_16130_1-p91684	C7	29	35.2	A
19012	Bn-N17-p31512414	Bn-A06-p23442751	Bn-A06-p23442751	C7	29	35.2	A
19013	Bn-N17-p31567626	Bn-C7-p31180043	Bn-scaff_16130_1-p98276	C7	29	35.2	A
19014	Bn-N17-p31568212	Bn-C7-p31180629	Bn-scaff_16130_1-p98334	C7	29	35.2	A
19015	Bn-N17-p31594710	Bn-C7-p31206698	Bn-scaff_16130_1-p10094	C7	29	35.2	A
19016	Bn-N17-p31598705	Bn-C7-p31210726	Bn-scaff_16130_1-p10134	C7	29	35.2	A
19017	Bn-N17-p31602535	Bn-C7-p31217908	Bn-scaff_16130_1-p10206	C7	29	35.2	A
19018	Bn-N17-p31607140	Bn-C7-p31223544	Bn-scaff_16130_1-p10262	C7	29	35.2	A
19019	Bn-N17-p31608834	Bn-C7-p31225521	Bn-scaff_16130_1-p10282	C7	29	35.2	A
19020	Bn-N17-p31612361	Bn-C7-p31228520	Bn-scaff_16130_1-p10312	C7	29	35.2	A
19021	Bn-N17-p31612486	Bn-C7-p31228645	Bn-scaff_16130_1-p10313	C7	29	35.2	A
19022	Bn-N17-p31617606	Bn-C7-p31232110	Bn-scaff_16130_1-p10348	C7	29	35.2	A
19023	Bn-N17-p31618998	Bn-C7-p31233552	Bn-scaff_16130_1-p10362	C7	29	35.2	A
19024	Bn-N17-p31619494	Bn-C7-p31234048	Bn-scaff_16130_1-p10367	C7	29	35.2	A
19025	Bn-N17-p31621488	Bn-C7-p31236409	Bn-scaff_16130_1-p10391	C7	29	35.2	A
19026	Bn-N17-p31621760	Bn-C7-p31236681	Bn-scaff_16130_1-p10394	C7	29	35.2	A
19027	Bn-N17-p31621812	Bn-C7-p31236733	Bn-scaff_16130_1-p10394	C7	29	35.2	A
19028	Bn-N17-p31621859	Bn-C7-p31236780	Bn-scaff_16130_1-p10394	C7	29	35.2	A
19029	Bn-N17-p31624844	Bn-C7-p31239807	Bn-scaff_16130_1-p10425	C7	29	35.2	A
19030	Bn-N17-p31632419	Bn-C7-p31247947	Bn-scaff_16130_1-p10506	C7	29	35.2	A
19031	Bn-N17-p31676667	Bn-C7-p31288691	Bn-scaff_16130_1-p10914	C7	30	36	A
19032	Bn-N17-p31741179	Bn-C7-p31511074	Bn-scaff_16130_1-p13137	C7	30	36	A
19033	Bn-N17-p31864486	Bn-C7-p31607628	Bn-scaff_16130_1-p14103	C7	30	36	A
19034	Bn-N17-p32114150	Bn-C7-p32009812	Bn-scaff_16130_1-p18125	C7	30	36	A
19035	Bn-N17-p32114447	Bn-C7-p32010109	Bn-scaff_16130_1-p18128	C7	30	36	A
19036	Bn-N17-p32127743	Bn-C7-p32028131	Bn-scaff_16130_1-p18308	C7	30	36	A
19037	Bn-N17-p32128048	Bn-C7-p32028438	Bn-scaff_16130_1-p18311	C7	30	36	A
19038	Bn-N17-p32140884	Bn-C7-p32040137	Bn-scaff_16130_1-p18428	C7	30	36	A
19039	Bn-N17-p32140925	Bn-C7-p32040178	Bn-scaff_16130_1-p18428	C7	30	36	A
19040	Bn-N17-p32143309	Bn-C7-p32042568	Bn-scaff_16130_1-p18452	C7	30	36	A
19041	Bn-N17-p32144412	Bn-C7-p32043694	Bn-scaff_16130_1-p18464	C7	30	36	A
19042	Bn-N17-p32150924	Bn-C7-p32050128	Bn-scaff_16130_1-p18528	C7	30	36	A
19043	Bn-N17-p32158946	Bn-C7-p32058077	Bn-scaff_16130_1-p18607	C7	30	36	A
19044	Bn-N17-p32188185	Bn-C7-p32098446	Bn-scaff_16130_1-p19011	C7	30	36	A
19045	Bn-N17-p32249529	Bn-C7-p32157646	Bn-scaff_16130_1-p19603	C7	30	36	A
19046	Bn-N17-p32310007	Bn-C7-p32195207	Bn-scaff_16130_1-p19979	C7	30	36	A
19047	Bn-N17-p32312272	Bn-C7-p32198596	Bn-scaff_16130_1-p20013	C7	30	36	0
19048	Bn-N17-p32442285	Bn-C7-p32318633	Bn-scaff_16130_1-p21213	C7	30	36	A
19049	Bn-N17-p32458006	Bn-C7-p32327234	Bn-scaff_16130_1-p21299	C7	30	36	A
19050	Bn-N17-p32459458	Bn-C7-p32328524	Bn-scaff_16130_1-p21312	C7	30	36	A
19051	Bn-N17-p32462469	Bn-C7-p32335876	Bn-scaff_16130_1-p21385	C7	30	36	A
19052	Bn-N17-p32463501	Bn-C7-p32336857	Bn-scaff_16130_1-p21395	C7	30	36	A
19053	Bn-N17-p32463616	Bn-C7-p32336972	Bn-scaff_16130_1-p21396	C7	30	36	A
19054	Bn-N17-p32464886	Bn-C7-p32338239	Bn-scaff_16130_1-p21409	C7	30	36	A
19055	Bn-N17-p32465591	Bn-C7-p32338944	Bn-scaff_16130_1-p21416	C7	30	36	A
19056	Bn-N17-p32548092	Bn-C7-p32421757	Bn-scaff_16130_1-p22244	C7	30	36	A

19057	Bn-N17-p32567869	Bn-C7-p32445509	Bn-scaff_16130_1-p224822	C7	30	36	A
19058	Bn-N17-p32571279	Bn-C7-p32448937	Bn-scaff_16130_1-p225165	C7	30	36	A
19059	Bn-N17-p32574237	Bn-C7-p32451816	Bn-scaff_16130_1-p225453	C7	30	36	A
19060	Bn-N17-p32625526	Bn-C7-p32493517	Bn-scaff_16130_1-p229623	C7	30	36	A
19061	Bn-N17-p32628266	Bn-C7-p32496277	Bn-scaff_16130_1-p229899	C7	30	36	A
19062	Bn-N17-p32628866	Bn-C7-p32496877	Bn-scaff_16130_1-p229959	C7	30	36	A
19063	Bn-N17-p32631108	Bn-C7-p32503043	Bn-scaff_16130_1-p230576	C7	30	36	A
19064	Bn-N17-p32763644	Bn-C7-p32630456	Bn-scaff_16130_1-p243317	C7	30	36	A
19065	Bn-Scaffold04041-p1201	Bn-C7-p32008065	Bn-scaff_16130_1-p181078	C7	30	36	A
19066	Bn-Scaffold04041-p2064	Bn-C7-p32008707	Bn-scaff_16130_1-p181142	C7	30	36	A
19067	Bn-N17-p32855427	Bn-C7-p32722910	Bn-scaff_16130_1-p252562	C7	31	36.8	A
19068	Bn-N17-p32858341	Bn-C7-p32725773	Bn-scaff_16130_1-p252849	C7	31	36.8	A
19069	Bn-N17-p32858554	Bn-C7-p32725987	Bn-scaff_16130_1-p252870	C7	31	36.8	A
19070	Bn-N17-p32868953	Bn-C7-p32737499	Bn-scaff_16130_1-p254021	C7	31	36.8	A
19071	Bn-N17-p32873533	Bn-C7-p32752055	Bn-scaff_16130_1-p255477	C7	31	36.8	A
19072	Bn-N17-p32874158	Bn-C7-p32752674	Bn-scaff_16130_1-p255539	C7	31	36.8	A
19073	Bn-N17-p32875151	Bn-C7-p32753313	Bn-scaff_16130_1-p255603	C7	31	36.8	A
19074	Bn-N17-p32875434	Bn-C7-p32753596	Bn-scaff_16130_1-p255631	C7	31	36.8	A
19075	Bn-N17-p32876145	Bn-C7-p32754394	Bn-scaff_16130_1-p255711	C7	31	36.8	A
19076	Bn-N17-p32876920	Bn-C7-p32755169	Bn-scaff_16130_1-p255788	C7	31	36.8	A
19077	Bn-N17-p32877663	Bn-C7-p32755912	Bn-scaff_16130_1-p255863	C7	31	36.8	A
19078	Bn-N17-p32877875	Bn-C7-p32756124	Bn-scaff_16130_1-p255884	C7	31	36.8	A
19079	Bn-N17-p32879006	Bn-C7-p32757257	Bn-scaff_16130_1-p255997	C7	31	36.8	A
19080	Bn-N17-p32879373	Bn-C7-p32757615	Bn-scaff_16130_1-p256033	C7	31	36.8	A
19081	Bn-N17-p32880785	Bn-Scaffold03171-p1	Bn-scaff_26297_1-p1200	C7	31	36.8	A
19082	Bn-N17-p32904927	Bn-C7-p32763711	Bn-scaff_17740_1-p101783	C7	31	36.8	A
19083	Bn-N17-p32906727	Bn-C7-p32764098	Bn-scaff_17740_1-p101745	C7	31	36.8	A
19084	Bn-N17-p32907342	Bn-C7-p32764714	Bn-scaff_17740_1-p101683	C7	31	36.8	A
19085	Bn-N17-p32910304	Bn-C7-p32768157	Bn-scaff_17740_1-p101339	C7	31	36.8	A
19086	Bn-N17-p32912312	Bn-C7-p32768654	Bn-scaff_17740_1-p101289	C7	31	36.8	A
19087	Bn-N17-p32912431	Bn-C7-p32768772	Bn-scaff_17740_1-p101277	C7	31	36.8	A
19088	Bn-N17-p32913069	Bn-C7-p32769166	Bn-scaff_17740_1-p101238	C7	31	36.8	A
19089	Bn-N17-p32914041	Bn-C7-p32770140	Bn-scaff_17740_1-p101140	C7	31	36.8	A
19090	Bn-N17-p32914449	Bn-C7-p32770548	Bn-scaff_17740_1-p101100	C7	31	36.8	A
19091	Bn-N17-p32915657	Bn-C7-p32771757	Bn-scaff_17740_1-p100979	C7	31	36.8	A
19092	Bn-N17-p32915856	Bn-C7-p32771956	Bn-scaff_17740_1-p100959	C7	31	36.8	A
19093	Bn-N17-p32917036	Bn-C7-p32773140	Bn-scaff_17740_1-p100840	C7	31	36.8	A
19094	Bn-N17-p32917245	Bn-C7-p32773349	Bn-scaff_17740_1-p100820	C7	31	36.8	A
19095	Bn-N17-p32917505	Bn-C7-p32773610	Bn-scaff_17740_1-p100793	C7	31	36.8	A
19096	Bn-N17-p32917513	Bn-C7-p32773618	Bn-scaff_17740_1-p100793	C7	31	36.8	A
19097	Bn-N17-p32917583	Bn-C7-p32773688	Bn-scaff_17740_1-p100786	C7	31	36.8	A
19098	Bn-N17-p32917687	Bn-C7-p32773789	Bn-scaff_17740_1-p100776	C7	31	36.8	A
19099	Bn-N17-p32964494	Bn-C7-p32825653	Bn-scaff_17740_1-p955896	C7	31	36.8	A
19100	Bn-N17-p32999742	Bn-C7-p32859944	Bn-scaff_17740_1-p921605	C7	31	36.8	A
19101	Bn-N17-p33042618	Bn-C7-p32901065	Bn-scaff_17740_1-p880484	C7	31	36.8	A
19102	Bn-N17-p33058869	Bn-C7-p32918433	Bn-scaff_17740_1-p863116	C7	31	36.8	A
19103	Bn-N17-p33074632	Bn-C7-p32933505	Bn-scaff_17740_1-p848044	C7	31	36.8	A
19104	Bn-N17-p33074646	Bn-C7-p32933519	Bn-scaff_17740_1-p848030	C7	31	36.8	A
19105	Bn-N17-p33076217	Bn-C7-p32935143	Bn-scaff_17740_1-p846406	C7	31	36.8	A

19106	Bn-N17-p33077610	Bn-C7-p32936536	Bn-scaff_17740_1-p845013	C7	31	36.8	A
19107	Bn-N17-p33077740	Bn-C7-p32936666	Bn-scaff_17740_1-p844883	C7	31	36.8	A
19108	Bn-N17-p33083327	Bn-C7-p32942255	Bn-scaff_17740_1-p839294	C7	31	36.8	A
19109	Bn-N17-p33083608	Bn-C7-p32942535	Bn-scaff_17740_1-p839014	C7	31	36.8	A
19110	Bn-N17-p33088497	Bn-C7-p32947424	Bn-scaff_17740_1-p834125	C7	31	36.8	A
19111	Bn-N17-p33120233	Bn-C7-p32970776	Bn-scaff_17740_1-p810773	C7	31	36.8	A
19112	Bn-N17-p33137490	Bn-C7-p32988248	Bn-scaff_17740_1-p793301	C7	31	36.8	A
19113	Bn-N17-p33139902	Bn-C7-p32990661	Bn-scaff_17740_1-p790888	C7	31	36.8	A
19114	Bn-N17-p33143932	Bn-C7-p32994727	Bn-scaff_17740_1-p786822	C7	31	36.8	A
19115	Bn-N17-p33147488	Bn-C7-p32998283	Bn-scaff_17740_1-p783266	C7	31	36.8	A
19116	Bn-N17-p33161211	Bn-C7-p33022693	Bn-scaff_17740_1-p758856	C7	31	36.8	A
19117	Bn-N6-p22195373	Bn-A06-p22505810	Bn-A06-p22505810	C7	31	36.8	A
19118	Bn-N17-p33478895	Bn-C7-p33351378	Bn-scaff_16514_1-p47789	C7	32	40.8	A
19119	Bn-N17-p33481196	Bn-C7-p33353637	Bn-scaff_16514_1-p50048	C7	32	40.8	A
19120	Bn-N17-p33487356	Bn-C7-p33359797	Bn-scaff_16514_1-p56208	C7	32	40.8	A
19121	Bn-N17-p33489635	Bn-C7-p33362072	Bn-scaff_16514_1-p58483	C7	32	40.8	A
19122	Bn-N17-p33489722	Bn-C7-p33362159	Bn-scaff_16514_1-p58570	C7	32	40.8	A
19123	Bn-N17-p33493690	Bn-C7-p33366127	Bn-scaff_16514_1-p62538	C7	32	40.8	A
19124	Bn-N17-p33496913	Bn-C7-p33368923	Bn-scaff_16514_1-p65334	C7	32	40.8	A
19125	Bn-N17-p33511379	Bn-C7-p33390160	Bn-scaff_16514_1-p86571	C7	32	40.8	A
19126	Bn-N17-p33526639	Bn-C4-p21161191	Bn-scaff_19170_1-p428577	C7	32	40.8	A
19127	Bn-N17-p33535686	Bn-C7-p33428334	Bn-scaff_16514_1-p124745	C7	32	40.8	A
19128	Bn-N17-p33535852	Bn-C7-p33428500	Bn-scaff_16514_1-p124911	C7	32	40.8	A
19129	Bn-N17-p33536080	Bn-C7-p33428728	Bn-scaff_16514_1-p125139	C7	32	40.8	A
19130	Bn-N17-p33544027	Bn-C7-p33436596	Bn-scaff_16514_1-p133007	C7	32	40.8	A
19131	Bn-N17-p33554700	Bn-Scaffold00285-p4	Bn-scaff_15712_9-p686324	C7	32	40.8	A
19132	Bn-N17-p33554848	Bn-C4-p38768147	Bn-scaff_17525_1-p247145	C7	32	40.8	A
19133	Bn-N17-p33560200	Bn-C7-p33464779	Bn-scaff_16514_1-p161190	C7	32	40.8	A
19134	Bn-N17-p34061229	Bn-C7-p35151417	Bn-scaff_17972_1-p279228	C7	33	42.4	A
19135	Bn-N17-p34091420	Bn-C7-p35181585	Bn-scaff_17972_1-p249060	C7	33	42.4	A
19136	Bn-N17-p34191431	Bn-C7-p35282361	Bn-scaff_17972_1-p148284	C7	33	42.4	A
19137	Bn-N17-p34535913	Bn-C7-p33900798	Bn-scaff_21711_1-p54607	C7	34	43.2	A
19138	Bn-N17-p34551543	Bn-C7-p33915927	Bn-scaff_21711_1-p39478	C7	35	45.6	A
19139	Bn-N17-p34555421	Bn-C7-p33919816	Bn-scaff_21711_1-p35589	C7	36	46.4	A
19140	Bn-N17-p34556010	Bn-C7-p33920406	Bn-scaff_21711_1-p34999	C7	36	46.4	A
19141	Bn-N17-p34556721	Bn-C7-p33921117	Bn-scaff_21711_1-p34288	C7	36	46.4	A
19142	Bn-N17-p34556921	Bn-C7-p33921322	Bn-scaff_21711_1-p34083	C7	36	46.4	A
19143	Bn-N17-p34562507	Bn-C7-p33926974	Bn-scaff_21711_1-p28431	C7	36	46.4	A
19144	Bn-N17-p34563605	Bn-C7-p33928106	Bn-scaff_21711_1-p27299	C7	36	46.4	A
19145	Bn-N17-p34565463	Bn-C7-p33929129	Bn-scaff_21711_1-p26276	C7	36	46.4	A
19146	Bn-N17-p34577778	Bn-C7-p33937062	Bn-scaff_21711_1-p18343	C7	36	46.4	A
19147	Bn-N17-p34739460	Bn-C7-p34076365	Bn-scaff_18520_1-p120861	C7	36	46.4	A
19148	Bn-N17-p34823384	Bn-C7-p34157673	Bn-scaff_18520_1-p202169	C7	36	46.4	A
19149	Bn-N17-p34894091	Bn-C7-p34207857	Bn-scaff_18520_1-p252353	C7	36	46.4	A
19150	Bn-N17-p34923804	Bn-C7-p34236190	Bn-scaff_18520_1-p280686	C7	36	46.4	A
19151	Bn-N17-p34923878	Bn-C7-p34236264	Bn-scaff_18520_1-p280760	C7	36	46.4	A
19152	Bn-N17-p34924112	Bn-C7-p34236502	Bn-scaff_18520_1-p280998	C7	36	46.4	A
19153	Bn-N17-p34924307	Bn-C7-p34236697	Bn-scaff_18520_1-p281193	C7	36	46.4	A
19154	Bn-N17-p34948756	Bn-C7-p34256752	Bn-scaff_18520_1-p301248	C7	36	46.4	A

19155	Bn-N17-p34970606	Bn-C7-p34279739	Bn-scaff_18520_1-p324235	C7	36	46.4	A
19156	Bn-N17-p34977895	Bn-C7-p34286079	Bn-scaff_18520_1-p330575	C7	36	46.4	A
19157	Bn-N17-p34983468	Bn-C14051598-p519	Bn-C14051598-p519	C7	36	46.4	A
19158	Bn-N17-p34990613	Bn-C7-p34303169	Bn-scaff_18520_1-p347665	C7	36	46.4	A
19159	Bn-N17-p34994041	Bn-C7-p34306595	Bn-scaff_18520_1-p351091	C7	36	46.4	A
19160	Bn-N17-p35006656	Bn-C7-p34318900	Bn-scaff_18520_1-p363396	C7	36	46.4	A
19161	Bn-N17-p35044257	Bn-C7-p34360472	Bn-scaff_18520_1-p404968	C7	36	46.4	A
19162	Bn-N17-p35044942	Bn-C7-p34361110	Bn-scaff_18520_1-p405606	C7	36	46.4	A
19163	Bn-N17-p35053487	Bn-C7-p34370069	Bn-scaff_18520_1-p414565	C7	36	46.4	A
19164	Bn-N17-p35053579	Bn-C7-p34370160	Bn-scaff_18520_1-p414656	C7	36	46.4	A
19165	Bn-N17-p35087608	Bn-C7-p34406432	Bn-scaff_18520_1-p450928	C7	36	46.4	A
19166	Bn-N17-p35120304	Bn-C7-p34441835	Bn-scaff_18520_1-p486331	C7	37	47.2	A
19167	Bn-N17-p35120540	Bn-C7-p34442071	Bn-scaff_18520_1-p486567	C7	37	47.2	A
19168	Bn-N17-p35149009	Bn-C7-p34462713	Bn-scaff_18520_1-p507209	C7	37	47.2	A
19169	Bn-N6-p21104213	Bn-C7-p34446691	Bn-scaff_18520_1-p491187	C7	37	47.2	A
19170	Bn-N17-p35240900	Bn-C7-p34559064	Bn-scaff_18520_1-p603560	C7	38	48	A
19171	Bn-N17-p35258259	Bn-C7-p34575923	Bn-scaff_18520_1-p620419	C7	38	48	A
19172	Bn-N17-p35314465	Bn-C7-p34643554	Bn-scaff_18520_1-p688050	C7	38	48	A
19173	Bn-N17-p35332960	Bn-C7-p34660606	Bn-scaff_18520_1-p705102	C7	38	48	A
19174	Bn-N17-p35348429	Bn-C7-p34675969	Bn-scaff_18520_1-p720465	C7	38	48	A
19175	Bn-N17-p35360870	Bn-C7-p34697519	Bn-scaff_18520_1-p742015	C7	38	48	A
19176	Bn-N17-p35384507	Bn-C7-p34724160	Bn-scaff_18520_1-p768656	C7	38	48	A
19177	Bn-N17-p36190094	Bn-A06-p16314064	Bn-A06-p16314064	C7	38	48	A
19178	Bn-N6-p20981584	Bn-A06-p21278243	Bn-A06-p21278243	C7	38	48	A
19179	Bn-N13-p6404488	Bn-C7-p35946965	Bn-scaff_17291_1-p239559	C7	39	48.8	A
19180	Bn-N16-p10828673	Bn-C6-p28063382	Bn-scaff_17984_1-p352496	C7	39	48.8	A
19181	Bn-N17-p35433082	Bn-C7-p34769795	Bn-scaff_18520_1-p814291	C7	39	48.8	A
19182	Bn-N17-p35435979	Bn-C7-p34772846	Bn-scaff_18520_1-p817342	C7	39	48.8	A
19183	Bn-N17-p35436255	Bn-C7-p34773122	Bn-scaff_18520_1-p817618	C7	39	48.8	A
19184	Bn-N17-p35450441	Bn-Scaffold01327-p4	Bn-scaff_16818_2-p4788	C7	39	48.8	A
19185	Bn-N17-p35476841	Bn-C7-p35318878	Bn-scaff_17291_1-p867646	C7	39	48.8	A
19186	Bn-N17-p35499700	Bn-C7-p35342226	Bn-scaff_17291_1-p844298	C7	39	48.8	A
19187	Bn-N17-p35510582	Bn-C7-p35347572	Bn-scaff_17291_1-p838952	C7	39	48.8	A
19188	Bn-N17-p35510858	Bn-C7-p35347848	Bn-scaff_17291_1-p838676	C7	39	48.8	A
19189	Bn-N17-p35512142	Bn-C7-p35349121	Bn-scaff_17291_1-p837403	C7	39	48.8	A
19190	Bn-N17-p35512379	Bn-C7-p35349360	Bn-scaff_17291_1-p837164	C7	39	48.8	A
19191	Bn-N17-p35538508	Bn-C7-p35371442	Bn-scaff_17291_1-p815082	C7	39	48.8	A
19192	Bn-N17-p35635010	Bn-C7-p35468329	Bn-scaff_17291_1-p718195	C7	39	48.8	A
19193	Bn-N17-p35638776	Bn-C7-p35480557	Bn-scaff_17291_1-p705967	C7	39	48.8	A
19194	Bn-N17-p35702184	Bn-C7-p35541918	Bn-scaff_17291_1-p644606	C7	39	48.8	A
19195	Bn-N17-p35702311	Bn-C7-p35542044	Bn-scaff_17291_1-p644480	C7	39	48.8	A
19196	Bn-N17-p35733288	Bn-C7-p35566891	Bn-scaff_17291_1-p619633	C7	39	48.8	A
19197	Bn-N17-p35740514	Bn-C7-p35572312	Bn-scaff_17291_1-p614212	C7	39	48.8	A
19198	Bn-N17-p35774165	Bn-C7-p35691416	Bn-scaff_17291_1-p495108	C7	39	48.8	A
19199	Bn-N17-p35806298	Bn-C7-p35710304	Bn-scaff_17291_1-p476220	C7	39	48.8	A
19200	Bn-N17-p35808235	Bn-C7-p35712242	Bn-scaff_17291_1-p474282	C7	39	48.8	A
19201	Bn-N17-p35814298	Bn-C7-p35718853	Bn-scaff_17291_1-p467671	C7	39	48.8	A
19202	Bn-N17-p35814515	Bn-C7-p35719069	Bn-scaff_17291_1-p467455	C7	39	48.8	A
19203	Bn-N17-p35814953	Bn-C7-p35719505	Bn-scaff_17291_1-p467019	C7	39	48.8	A

19204	Bn-N17-p35815013	Bn-C7-p35719565	Bn-scaff_17291_1-p46695	C7	39	48.8	A
19205	Bn-N17-p35864789	Bn-C7-p35762591	Bn-scaff_17291_1-p42393	C7	39	48.8	A
19206	Bn-N17-p35921416	Bn-C7-p35810605	Bn-scaff_17291_1-p37591	C7	39	48.8	A
19207	Bn-N17-p35934717	Bn-C7-p35821775	Bn-scaff_17291_1-p36474	C7	39	48.8	A
19208	Bn-N17-p35938997	Bn-C7-p35826102	Bn-scaff_17291_1-p36042	C7	39	48.8	A
19209	Bn-N17-p35939145	Bn-C7-p35826250	Bn-scaff_17291_1-p36027	C7	39	48.8	A
19210	Bn-N17-p35940398	Bn-C7-p35827521	Bn-scaff_17291_1-p35900	C7	39	48.8	A
19211	Bn-N17-p35940593	Bn-C7-p35827716	Bn-scaff_17291_1-p35880	C7	39	48.8	A
19212	Bn-N17-p35940754	Bn-C7-p35827877	Bn-scaff_17291_1-p35864	C7	39	48.8	A
19213	Bn-N17-p35940836	Bn-C7-p35827959	Bn-scaff_17291_1-p35856	C7	39	48.8	A
19214	Bn-N17-p35941130	Bn-C7-p35828156	Bn-scaff_17291_1-p35836	C7	39	48.8	A
19215	Bn-N17-p35943915	Bn-C7-p35830887	Bn-scaff_17291_1-p35563	C7	39	48.8	A
19216	Bn-N17-p35946783	Bn-C7-p35833757	Bn-scaff_17291_1-p35276	C7	39	48.8	A
19217	Bn-N17-p35948025	Bn-C7-p35835033	Bn-scaff_17291_1-p35149	C7	39	48.8	A
19218	Bn-N17-p35955147	Bn-C7-p35841323	Bn-scaff_17291_1-p34520	C7	39	48.8	A
19219	Bn-N17-p35960712	Bn-C7-p35852973	Bn-scaff_17291_1-p33355	C7	39	48.8	A
19220	Bn-N17-p35978279	Bn-C7-p35861147	Bn-scaff_17291_1-p32537	C7	39	48.8	A
19221	Bn-N17-p36004078	Bn-C7-p35881245	Bn-scaff_17291_1-p30527	C7	39	48.8	A
19222	Bn-N17-p36060839	Bn-C7-p35933033	Bn-scaff_17291_1-p25349	C7	39	48.8	A
19223	Bn-N17-p36064701	Bn-C7-p35936902	Bn-scaff_17291_1-p24962	C7	39	48.8	A
19224	Bn-N17-p36093081	Bn-C7-p35960306	Bn-scaff_17291_1-p22621	C7	39	48.8	A
19225	Bn-N17-p36103147	Bn-C7-p35970372	Bn-scaff_17291_1-p21615	C7	39	48.8	A
19226	Bn-N17-p36107048	Bn-C7-p35974273	Bn-scaff_17291_1-p21225	C7	39	48.8	A
19227	Bn-N17-p36128109	Bn-C7-p35994163	Bn-scaff_17291_1-p19236	C7	39	48.8	A
19228	Bn-N17-p36128275	Bn-C7-p35994329	Bn-scaff_17291_1-p19219	C7	39	48.8	A
19229	Bn-N17-p36130053	Bn-C7-p35996107	Bn-scaff_17291_1-p19041	C7	39	48.8	A
19230	Bn-N17-p36132457	Bn-C7-p35998511	Bn-scaff_17291_1-p18801	C7	39	48.8	A
19231	Bn-N17-p36163436	Bn-C7-p36030987	Bn-scaff_17291_1-p15553	C7	39	48.8	A
19232	Bn-N17-p36165022	Bn-C7-p36032572	Bn-scaff_17291_1-p15395	C7	39	48.8	A
19233	Bn-N17-p36165357	Bn-C7-p36032907	Bn-scaff_17291_1-p15361	C7	39	48.8	A
19234	Bn-N17-p36165526	Bn-C7-p36033076	Bn-scaff_17291_1-p15344	C7	39	48.8	A
19235	Bn-N17-p36165590	Bn-C7-p36033140	Bn-scaff_17291_1-p15338	C7	39	48.8	A
19236	Bn-N17-p36165634	Bn-C7-p36033184	Bn-scaff_17291_1-p15334	C7	39	48.8	A
19237	Bn-N17-p36166166	Bn-C7-p36033716	Bn-scaff_17291_1-p15280	C7	39	48.8	A
19238	Bn-N17-p36166449	Bn-C7-p36033999	Bn-scaff_17291_1-p15252	C7	39	48.8	A
19239	Bn-N17-p36280693	Bn-C7-p36123359	Bn-scaff_17291_1-p63165	C7	39	48.8	A
19240	Bn-N6-p20388013	Bn-C7-p35883234	Bn-scaff_17291_1-p30329	C7	39	48.8	A
19241	Bn-N6-p20805203	Bn-C7-p34769109	Bn-scaff_18520_1-p81360	C7	39	48.8	A
19242	Bn-Scaffold01500-p4241	Bn-C7-p35379240	Bn-scaff_17291_1-p80728	C7	39	48.8	A
19243	Unknown	Bn-C7-p34769541	Bn-scaff_18520_1-p81403	C7	39	48.8	A
19244	Bn-N17-p36391568	Bn-C7-p36225591	Bn-scaff_15705_1-p38968	C7	40	49.6	A
19245	Bn-N17-p36829930	Bn-C7-p36618847	Bn-scaff_15705_1-p43222	C7	41	50.4	A
19246	Bn-N17-p36830418	Bn-C7-p36619337	Bn-scaff_15705_1-p43271	C7	41	50.4	A
19247	Bn-N17-p36857699	Bn-C7-p36646529	Bn-scaff_15705_1-p45990	C7	41	50.4	A
19248	Bn-N17-p36857876	Bn-C7-p36646706	Bn-scaff_15705_1-p46008	C7	41	50.4	A
19249	Bn-N17-p36872295	Bn-C7-p36667587	Bn-scaff_15705_1-p48096	C7	41	50.4	A
19250	Bn-N17-p36940096	Bn-C7-p36747264	Bn-scaff_15705_1-p56064	C7	41	50.4	A
19251	Bn-N17-p36940603	Bn-C7-p36747785	Bn-scaff_15705_1-p56116	C7	41	50.4	A
19252	Bn-N6-p20021777	Bn-C7-p36343912	Bn-scaff_15705_1-p15728	C7	41	50.4	A

19253	Bn-N6-p20022540	Bn-C7-p36343153	Bn-scaff_15705_1-p15653(	C7	41	50.4	A
19254	Bn-N17-p36957581	Bn-C7-p36763950	Bn-scaff_15705_1-p57732	C7	42	51.2	A
19255	Bn-N17-p37149977	Bn-C7-p36960533	Bn-scaff_15705_1-p77391(	C7	42	51.2	A
19256	Bn-N17-p37614576	Bn-C7-p37401578	Bn-scaff_15705_1-p12149	C7	43	52	A
19257	Bn-N17-p37701535	Bn-C7-p37495325	Bn-scaff_15705_1-p13087(	C7	43	52	A
19258	Bn-N17-p37711634	Bn-C7-p37537988	Bn-scaff_15705_1-p13513	C7	43	52	A
19259	Bn-N17-p37722774	Bn-C7-p37511435	Bn-scaff_15705_1-p13248	C7	43	52	A
19260	Bn-N17-p37792652	Bn-C7-p37590413	Bn-scaff_15705_1-p14037	C7	43	52	A
19261	Bn-N17-p37909489	Bn-C7-p37720330	Bn-scaff_15705_1-p15337(	C7	44	52.8	A
19262	Bn-N17-p38021731	Bn-C7-p37815425	Bn-scaff_15705_1-p16288(	C7	44	52.8	A
19263	Bn-N17-p38021782	Bn-C7-p37815476	Bn-scaff_15705_1-p16288	C7	44	52.8	A
19264	Bn-N17-p38162987	Bn-C7-p37978513	Bn-scaff_15705_1-p17918	C7	44	52.8	A
19265	Bn-N17-p38173935	Bn-C7-p37983337	Bn-scaff_15705_1-p17967	C7	44	52.8	A
19266	Bn-N17-p38619798	Bn-C7-p38434890	Bn-scaff_15705_1-p22482	C7	45	54.4	A
19267	Bn-N17-p38649931	Bn-C7-p38454535	Bn-scaff_15705_1-p22679	C7	46	56.8	A
19268	Bn-N17-p38658044	Bn-C7-p38461116	Bn-scaff_15705_1-p22744	C7	46	56.8	A
19269	Bn-N17-p38664474	Bn-C7-p38466443	Bn-scaff_15705_1-p22798	C7	46	56.8	A
19270	Bn-N17-p38706970	Bn-C7-p38504198	Bn-scaff_15705_1-p23175	C7	46	56.8	A
19271	Bn-N17-p38887023	Bn-C7-p38690796	Bn-scaff_15705_1-p25041	C7	46	56.8	A
19272	Bn-N17-p38887343	Bn-C7-p38691115	Bn-scaff_15705_1-p25044	C7	46	56.8	A
19273	Bn-N17-p38967842	Bn-C7-p38776912	Bn-scaff_15705_1-p25902	C7	46	56.8	A
19274	Bn-N17-p39004381	Bn-C7-p38813167	Bn-scaff_15705_1-p26265	C7	46	56.8	A
19275	Bn-N17-p39029937	Bn-C7-p38829862	Bn-scaff_15705_1-p26432	C7	46	56.8	A
19276	Bn-N17-p39087186	Bn-C7-p38889204	Bn-scaff_15705_1-p27025	C7	46	56.8	A
19277	Bn-N17-p39091932	Bn-C7-p38892255	Bn-scaff_15705_1-p27056	C7	46	56.8	A
19278	Bn-N17-p39419198	Bn-C7-p39215963	Bn-scaff_15705_3-p12932	C7	47	58.4	A
19279	Bn-N17-p39425879	Bn-C7-p39218523	Bn-scaff_15705_3-p13188	C7	47	58.4	A
19280	Bn-N15-p41349137	Bn-C7-p39332602	Bn-scaff_15705_3-p24596(	C7	48	59.2	A
19281	Bn-N15-p41349430	Bn-C7-p39332857	Bn-scaff_15705_3-p24621	C7	48	59.2	A
19282	Bn-N17-p39530753	Bn-C7-p39321626	Bn-scaff_15705_3-p23498	C7	48	59.2	A
19283	Bn-N17-p39542139	Bn-C7-p39339665	Bn-scaff_15705_3-p25302	C7	48	59.2	A
19284	Bn-N17-p39558857	Bn-C7-p39347992	Bn-scaff_15705_3-p26135(	C7	48	59.2	A
19285	Bn-N17-p39660615	Bn-C7-p39438047	Bn-scaff_15705_3-p35140	C7	48	59.2	A
19286	Bn-N17-p39754792	Bn-C7-p39523483	Bn-scaff_15705_3-p43684	C7	49	60	A
19287	Bn-N17-p39758705	Bn-C7-p39527403	Bn-scaff_15705_3-p44076	C7	49	60	A
19288	Bn-N17-p39899760	Bn-Scaffold00945-p1	Bn-scaff_15705_3-p60183	C7	49	60	A
19289	Bn-N17-p39912815	Bn-Scaffold00945-p3(	Bn-scaff_15705_3-p61661	C7	49	60	A
19290	Bn-N17-p39912876	Bn-Scaffold00945-p3(	Bn-scaff_15705_3-p61667	C7	49	60	A
19291	Bn-N17-p39936507	Bn-C7-p39719246	Bn-scaff_16069_1-p9308	C7	49	60	A
19292	Bn-N17-p39944819	Bn-C7-p39727260	Bn-scaff_16069_1-p17322	C7	49	60	A
19293	Bn-N17-p39960778	Bn-C7-p39752297	Bn-scaff_16069_1-p42359	C7	49	60	A
19294	Bn-N17-p39963594	Bn-C7-p39754929	Bn-scaff_16069_1-p44991	C7	49	60	A
19295	Bn-N17-p40165190	Bn-C7-p39971347	Bn-scaff_16069_1-p26140	C7	50	60.8	A
19296	Bn-N17-p40167703	Bn-C7-p39973848	Bn-scaff_16069_1-p26391(	C7	50	60.8	A
19297	Bn-N17-p40289009	Bn-C7-p40009397	Bn-scaff_16069_1-p29945	C7	50	60.8	A
19298	Bn-N17-p40297671	Bn-C7-p40016600	Bn-scaff_16069_1-p30666	C7	50	60.8	A
19299	Bn-N17-p40301735	Bn-C7-p40020698	Bn-scaff_16069_1-p31076(	C7	50	60.8	A
19300	Bn-N17-p40301789	Bn-C7-p40020752	Bn-scaff_16069_1-p31081	C7	50	60.8	A
19301	Bn-N17-p40329509	Bn-C7-p40052088	Bn-scaff_16069_1-p34215(	C7	50	60.8	A

19302	Bn-N17-p40348972	Bn-C7-p40071000	Bn-scaff_16069_1-p361062	C7	50	60.8	A
19303	Bn-N17-p40353497	Bn-C7-p40076061	Bn-scaff_16069_1-p366123	C7	50	60.8	A
19304	Bn-N17-p40376819	Bn-C7-p40106937	Bn-scaff_16069_1-p396999	C7	50	60.8	A
19305	Bn-N17-p40429889	Bn-C7-p40156764	Bn-scaff_16069_1-p446826	C7	50	60.8	A
19306	Bn-N17-p40513097	Bn-C7-p40204518	Bn-scaff_16069_1-p494580	C7	50	60.8	A
19307	Bn-N17-p40523111	Bn-C7-p40239515	Bn-scaff_16069_1-p529577	C7	50	60.8	A
19308	Bn-N17-p40523121	Bn-C7-p40239525	Bn-scaff_16069_1-p529587	C7	50	60.8	A
19309	Bn-N17-p40523211	Bn-C7-p40239615	Bn-scaff_16069_1-p529677	C7	50	60.8	A
19310	Bn-N17-p40523801	Bn-C7-p40240206	Bn-scaff_16069_1-p530268	C7	50	60.8	A
19311	Bn-N17-p40523987	Bn-C7-p40240386	Bn-scaff_16069_1-p530448	C7	50	60.8	A
19312	Bn-N17-p40524032	Bn-C7-p40240431	Bn-scaff_16069_1-p530493	C7	50	60.8	A
19313	Bn-N17-p40541840	Bn-C7-p40260107	Bn-scaff_16069_1-p550169	C7	50	60.8	A
19314	Bn-N17-p40625710	Bn-C7-p40335930	Bn-scaff_16069_1-p625992	C7	50	60.8	A
19315	Bn-N17-p40628259	Bn-C7-p40338503	Bn-scaff_16069_1-p628565	C7	50	60.8	A
19316	Bn-N17-p40667082	Bn-C7-p40395039	Bn-scaff_16069_1-p685101	C7	50	60.8	A
19317	Bn-N3-p22906612	Bn-C7-p40192253	Bn-scaff_16069_1-p482315	C7	50	60.8	A
19318	Bn-N3-p22911707	Bn-C7-p40191859	Bn-scaff_16069_1-p481921	C7	50	60.8	A
19319	Bn-N3-p22915811	Bn-C7-p40193960	Bn-scaff_16069_1-p484022	C7	50	60.8	A
19320	Bn-N17-p40699585	Bn-C7-p40436327	Bn-scaff_16069_1-p726389	C7	51	61.6	A
19321	Bn-N17-p40699735	Bn-C7-p40436476	Bn-scaff_16069_1-p726538	C7	51	61.6	A
19322	Bn-N18-p17960198	Bn-A08-p2997093	Bn-A08-p2997093	C7	51	61.6	0
19323	Bn-N17-p40703416	Bn-C7-p40440151	Bn-scaff_16069_1-p730213	C7	52	62.4	A
19324	Bn-N17-p40728835	Bn-C7-p40462785	Bn-scaff_16069_1-p752847	C7	52	62.4	A
19325	Bn-N17-p40730683	Bn-C7-p40464118	Bn-scaff_16069_1-p754180	C7	52	62.4	A
19326	Bn-N17-p40742876	Bn-C7-p40472112	Bn-scaff_16069_1-p762174	C7	52	62.4	A
19327	Bn-N17-p40763976	Bn-C7-p40486517	Bn-scaff_16069_1-p776579	C7	52	62.4	A
19328	Bn-N17-p40768959	Bn-C7-p40491376	Bn-scaff_16069_1-p781438	C7	52	62.4	A
19329	Bn-N17-p40796148	Bn-C7-p40519998	Bn-scaff_16069_1-p810060	C7	52	62.4	A
19330	Bn-N17-p40798468	Bn-C7-p40522319	Bn-scaff_16069_1-p812381	C7	52	62.4	A
19331	Bn-N17-p40835434	Bn-C7-p40559702	Bn-scaff_16069_1-p849764	C7	52	62.4	A
19332	Bn-N17-p40835732	Bn-C7-p40560013	Bn-scaff_16069_1-p850075	C7	52	62.4	A
19333	Bn-N17-p40836329	Bn-C7-p40560639	Bn-scaff_16069_1-p850701	C7	52	62.4	A
19334	Bn-N17-p40836384	Bn-C7-p40560694	Bn-scaff_16069_1-p850756	C7	52	62.4	A
19335	Bn-N17-p40836508	Bn-C7-p40560818	Bn-scaff_16069_1-p850880	C7	52	62.4	A
19336	Bn-N17-p40851667	Bn-C7-p40578173	Bn-scaff_16069_1-p868235	C7	52	62.4	A
19337	Bn-N17-p40851668	Bn-C7-p40578174	Bn-scaff_16069_1-p868236	C7	52	62.4	A
19338	Bn-N17-p40931408	Bn-C7-p40649292	Bn-scaff_16069_1-p939354	C7	52	62.4	A
19339	Bn-N17-p40934729	Bn-C7-p40652623	Bn-scaff_16069_1-p942685	C7	52	62.4	A
19340	Bn-N17-p40947395	Bn-C7-p40665356	Bn-scaff_16069_1-p955418	C7	52	62.4	A
19341	Bn-N17-p40947545	Bn-C7-p40665506	Bn-scaff_16069_1-p955568	C7	52	62.4	A
19342	Bn-N17-p40952035	Bn-C7-p40669994	Bn-scaff_16069_1-p960056	C7	52	62.4	A
19343	Bn-N17-p41041578	Bn-C7-p40754615	Bn-scaff_16069_1-p104467	C7	52	62.4	A
19344	Bn-N17-p41041896	Bn-C7-p40754937	Bn-scaff_16069_1-p104499	C7	52	62.4	A
19345	Bn-N17-p41047184	Bn-C7-p40760226	Bn-scaff_16069_1-p105028	C7	52	62.4	A
19346	Bn-N15-p4027205	Bn-C8-p21229919	Bn-scaff_16231_1-p139872	C7	53	63.2	A
19347	Bn-N17-p41097896	Bn-C7-p40811527	Bn-scaff_16069_1-p110158	C7	53	63.2	A
19348	Bn-N17-p41102350	Bn-C7-p40814950	Bn-scaff_16069_1-p110501	C7	53	63.2	A
19349	Bn-N17-p41108562	Bn-C7-p40821591	Bn-scaff_16069_1-p111165	C7	53	63.2	A
19350	Bn-N17-p41126890	Bn-C7-p40829659	Bn-scaff_16069_1-p111972	C7	53	63.2	A

19351	Bn-N17-p41136512	Bn-C7-p40836937	Bn-scaff_16069_1-p112699	C7	53	63.2 A
19352	Bn-N17-p41136575	Bn-C7-p40836999	Bn-scaff_16069_1-p112706	C7	53	63.2 A
19353	Bn-N17-p41145589	Bn-C7-p40841397	Bn-scaff_16069_1-p113145	C7	53	63.2 A
19354	Bn-N17-p41146328	Bn-C7-p40841866	Bn-scaff_16069_1-p113192	C7	53	63.2 A
19355	Bn-N17-p41146382	Bn-C7-p40841920	Bn-scaff_16069_1-p113198	C7	53	63.2 A
19356	Bn-N17-p41146589	Bn-C7-p40842094	Bn-scaff_16069_1-p113215	C7	53	63.2 A
19357	Bn-N17-p41146700	Bn-C7-p40842205	Bn-scaff_16069_1-p113226	C7	53	63.2 A
19358	Bn-N17-p41146969	Bn-C7-p40842474	Bn-scaff_16069_1-p113253	C7	53	63.2 A
19359	Bn-N17-p41147275	Bn-C7-p40842780	Bn-scaff_16069_1-p113284	C7	53	63.2 A
19360	Bn-N17-p41147455	Bn-C7-p40842960	Bn-scaff_16069_1-p113302	C7	53	63.2 A
19361	Bn-N17-p41147549	Bn-C7-p40843054	Bn-scaff_16069_1-p113311	C7	53	63.2 A
19362	Bn-N17-p41147657	Bn-C7-p40843162	Bn-scaff_16069_1-p113322	C7	53	63.2 A
19363	Bn-N17-p41147853	Bn-C7-p40843358	Bn-scaff_16069_1-p113342	C7	53	63.2 A
19364	Bn-N17-p41148732	Bn-C7-p40844238	Bn-scaff_16069_1-p113430	C7	53	63.2 A
19365	Bn-N17-p41150536	Bn-C7-p40846041	Bn-scaff_16069_1-p113610	C7	53	63.2 A
19366	Bn-N17-p41152267	Bn-C7-p40847546	Bn-scaff_16069_1-p113760	C7	53	63.2 A
19367	Bn-N17-p41200308	Bn-C7-p40874326	Bn-scaff_16069_1-p116438	C7	53	63.2 A
19368	Bn-N17-p41225757	Bn-C7-p40898427	Bn-scaff_16069_1-p118848	C7	53	63.2 A
19369	Bn-N17-p41249846	Bn-C7-p40914414	Bn-scaff_16069_1-p120447	C7	53	63.2 A
19370	Bn-N17-p41249847	Bn-C7-p40914415	Bn-scaff_16069_1-p120447	C7	53	63.2 A
19371	Bn-N17-p41301381	Bn-C7-p40969083	Bn-scaff_16069_1-p125914	C7	53	63.2 A
19372	Bn-N17-p41311087	Bn-C7-p40978679	Bn-scaff_16069_1-p126874	C7	53	63.2 A
19373	Bn-N17-p41312282	Bn-C7-p40979874	Bn-scaff_16069_1-p126993	C7	53	63.2 A
19374	Bn-N17-p41395137	Bn-C7-p41070068	Bn-scaff_16069_1-p136013	C7	53	63.2 A
19375	Bn-N17-p41410764	Bn-C7-p41085526	Bn-scaff_16069_1-p137558	C7	53	63.2 A
19376	Bn-N17-p41428728	Bn-C7-p41103982	Bn-scaff_16069_1-p139404	C7	53	63.2 A
19377	Bn-N17-p41432680	Bn-C7-p41107855	Bn-scaff_16069_1-p139791	C7	53	63.2 A
19378	Bn-N17-p41436401	Bn-C7-p41111414	Bn-scaff_16069_1-p140147	C7	53	63.2 A
19379	Bn-N17-p41436538	Bn-C7-p41111551	Bn-scaff_16069_1-p140161	C7	53	63.2 A
19380	Bn-N17-p41442893	Bn-C7-p41117906	Bn-scaff_16069_1-p140796	C7	53	63.2 A
19381	Bn-N17-p41443480	Bn-C7-p41118493	Bn-scaff_16069_1-p140855	C7	53	63.2 A
19382	Bn-N17-p41443773	Bn-C7-p41118786	Bn-scaff_16069_1-p140884	C7	53	63.2 A
19383	Bn-N17-p41466400	Bn-C7-p41143826	Bn-scaff_16069_1-p143388	C7	53	63.2 A
19384	Bn-N17-p41468417	Bn-C7-p41145858	Bn-scaff_16069_1-p143592	C7	53	63.2 A
19385	Bn-N17-p41475196	Bn-C7-p41154228	Bn-scaff_16069_1-p144429	C7	53	63.2 A
19386	Bn-N17-p41489839	Bn-C7-p41166207	Bn-scaff_16069_1-p145626	C7	53	63.2 A
19387	Bn-N17-p41493500	Bn-C7-p41174778	Bn-scaff_16069_1-p146484	C7	53	63.2 A
19388	Bn-N17-p41498245	Bn-C7-p41179368	Bn-scaff_16069_1-p146943	C7	53	63.2 A
19389	Bn-N17-p41518880	Bn-C7-p41193506	Bn-scaff_16069_1-p148356	C7	53	63.2 A
19390	Bn-N17-p41558908	Bn-C7-p41236646	Bn-scaff_16069_1-p152670	C7	53	63.2 A
19391	Bn-N17-p41561703	Bn-C7-p41239452	Bn-scaff_16069_1-p152951	C7	53	63.2 A
19392	Bn-N17-p41562092	Bn-C7-p41239841	Bn-scaff_16069_1-p152990	C7	53	63.2 A
19393	Bn-N17-p41562426	Bn-C7-p41240175	Bn-scaff_16069_1-p153023	C7	53	63.2 A
19394	Bn-N17-p41562835	Bn-C7-p41240583	Bn-scaff_16069_1-p153064	C7	53	63.2 A
19395	Bn-N17-p41563485	Bn-C7-p41241233	Bn-scaff_16069_1-p153129	C7	53	63.2 A
19396	Bn-N17-p41563725	Bn-C7-p41241473	Bn-scaff_16069_1-p153153	C7	53	63.2 A
19397	Bn-N17-p41563966	Bn-C7-p41241714	Bn-scaff_16069_1-p153177	C7	53	63.2 A
19398	Bn-N17-p41564118	Bn-C7-p41241866	Bn-scaff_16069_1-p153192	C7	53	63.2 A
19399	Bn-N17-p41564253	Bn-C7-p41242001	Bn-scaff_16069_1-p153206	C7	53	63.2 A

19400	Bn-N17-p41568697	Bn-C7-p41245814	Bn-scaff_16069_1-p153587	C7	53	63.2	A
19401	Bn-N17-p41618379	Bn-C7-p41289225	Bn-scaff_16069_1-p157928	C7	53	63.2	A
19402	Bn-N17-p41683627	Bn-C7-p41361394	Bn-scaff_16069_1-p165145	C7	53	63.2	A
19403	Bn-N17-p41797339	Bn-C7-p41492914	Bn-scaff_16069_1-p178297	C7	53	63.2	A
19404	Bn-N17-p41798847	Bn-A03-p23704255	Bn-A03-p23704255	C7	53	63.2	A
19405	Bn-N3-p23427253	Bn-C7-p40814779	Bn-scaff_16069_1-p110484	C7	53	63.2	A
19406	Bn-N3-p23820444	Bn-C7-p41273964	Bn-scaff_16069_1-p156402	C7	53	63.2	A
19407	Bn-N14-p46530372	Bn-C7-p41737753	Bn-scaff_16069_1-p202781	C7	54	66.4	A
19408	Bn-N17-p42018719	Bn-C7-p41694692	Bn-scaff_16069_1-p198475	C7	54	66.4	A
19409	Bn-N17-p42151468	Bn-C7-p41915528	Bn-scaff_16069_1-p220559	C7	54	66.4	A
19410	Bn-N3-p24244876	Bn-A03-p23980725	Bn-A03-p23980725	C7	54	66.4	A
19411	Unknown	Bn-C7-p41751734	Bn-scaff_16069_1-p204179	C7	54	66.4	A
19412	Bn-N17-p42170025	Bn-C7-p41932287	Bn-scaff_16069_1-p222234	C7	55	67.2	A
19413	Bn-N17-p42170392	Bn-C7-p41932648	Bn-scaff_16069_1-p222271	C7	55	67.2	A
19414	Bn-N13-p34985838	Bn-C3-p33278730	Bn-scaff_18855_1-p683881	C7	56	68	A
19415	Bn-N17-p42295766	Bn-C7-p42054639	Bn-scaff_16069_1-p234470	C7	56	68	A
19416	Bn-N17-p42617115	Bn-C7-p42315790	Bn-scaff_16069_1-p260585	C7	56	68	A
19417	Bn-N17-p42620912	Bn-C7-p42316312	Bn-scaff_16069_1-p260637	C7	56	68	A
19418	Bn-N17-p42680766	Bn-C7-p42376099	Bn-scaff_16069_1-p266616	C7	56	68	A
19419	Bn-N17-p42697915	Bn-C7-p42390302	Bn-scaff_16069_1-p268036	C7	56	68	A
19420	Bn-N17-p42839390	Bn-C7-p42530734	Bn-scaff_16069_1-p282079	C7	56	68	A
19421	Bn-N17-p42928058	Bn-C7-p42607119	Bn-scaff_16069_1-p289718	C7	56	68	A
19422	Bn-N14-p30966081	Bn-C7-p42702907	Bn-scaff_16069_1-p299296	C7	57	69.6	A
19423	Bn-N17-p42975451	Bn-C7-p42655999	Bn-scaff_16069_1-p294606	C7	57	69.6	A
19424	Bn-N17-p43032301	Bn-C7-p42706539	Bn-scaff_16069_1-p299660	C7	57	69.6	A
19425	Bn-N17-p43059081	Bn-C7-p42734095	Bn-scaff_16069_1-p302415	C7	57	69.6	A
19426	Bn-N17-p43097901	Bn-C7-p42781002	Bn-scaff_16069_1-p307106	C7	57	69.6	A
19427	Bn-N17-p43120635	Bn-C7-p42803742	Bn-scaff_16069_1-p309380	C7	57	69.6	A
19428	Bn-N17-p43144989	Bn-C7-p42827731	Bn-scaff_16069_1-p311779	C7	57	69.6	A
19429	Bn-N7-p15087771	Bn-C7-p42701979	Bn-scaff_16069_1-p299204	C7	57	69.6	A
19430	Bn-N7-p4599791	Bn-C7-p42704926	Bn-scaff_16069_1-p299498	C7	57	69.6	A
19431	Bn-Scaffold03128-p2586	Bn-C7-p42703122	Bn-scaff_16069_1-p299318	C7	57	69.6	A
19432	Unknown	Bn-C7-p42694655	Bn-scaff_16069_1-p298471	C7	57	69.6	A
19433	Unknown	Bn-C7-p42704047	Bn-scaff_16069_1-p299410	C7	57	69.6	A
19434	Unknown	Bn-C7-p42704858	Bn-scaff_16069_1-p299492	C7	57	69.6	A
19435	Unknown	Bn-C7-p42704977	Bn-scaff_16069_1-p299503	C7	57	69.6	A
19436	Unknown	Bn-C7-p42808806	Bn-scaff_16069_1-p309886	C7	57	69.6	A
19437	Bn-N17-p43158306	Bn-C7-p42845207	Bn-scaff_16069_1-p313526	C7	58	71.2	A
19438	Bn-N17-p43161574	Bn-C7-p42848477	Bn-scaff_16069_1-p313853	C7	58	71.2	A
19439	Bn-N17-p43197545	Bn-C7-p42879788	Bn-scaff_16069_1-p316985	C7	58	71.2	A
19440	Bn-N17-p43260732	Bn-C7-p42942159	Bn-scaff_16069_1-p323222	C7	59	72	A
19441	Bn-N17-p43323463	Bn-C7-p43017377	Bn-scaff_16069_1-p330743	C7	59	72	A
19442	Bn-N17-p43328478	Bn-C7-p43022994	Bn-scaff_16069_1-p331305	C7	59	72	A
19443	Bn-N17-p43330199	Bn-C7-p43026892	Bn-scaff_16069_1-p331695	C7	59	72	A
19444	Bn-N17-p43330234	Bn-C7-p43026927	Bn-scaff_16069_1-p331698	C7	59	72	A
19445	Bn-N17-p43361323	Bn-C7-p43061099	Bn-scaff_16069_1-p335116	C7	59	72	A
19446	Bn-N17-p43361660	Bn-C7-p43061436	Bn-scaff_16069_1-p335149	C7	59	72	A
19447	Bn-N17-p43362298	Bn-C7-p43062074	Bn-scaff_16069_1-p335213	C7	59	72	A
19448	Bn-N17-p43362541	Bn-C7-p43062317	Bn-scaff_16069_1-p335237	C7	59	72	A

19449	Bn-N17-p43362985	Bn-C7-p43062761	Bn-scaff_16069_1-p335282	C7	59	72	A
19450	Bn-N17-p43386199	Bn-C7-p43085517	Bn-scaff_16069_1-p337557	C7	59	72	A
19451	Bn-N17-p43457031	Bn-C7-p43156370	Bn-scaff_16069_1-p344643	C7	60	72.8	A
19452	Bn-N17-p43466400	Bn-C7-p43164661	Bn-scaff_16069_1-p345472	C7	61	73.6	A
19453	Bn-N17-p43478738	Bn-C7-p43180538	Bn-scaff_16069_1-p347060	C7	61	73.6	A
19454	Bn-N17-p43488328	Bn-C7-p43189278	Bn-scaff_16069_1-p347934	C7	61	73.6	A
19455	Bn-N17-p43494874	Bn-C7-p43196271	Bn-scaff_16069_1-p348633	C7	61	73.6	A
19456	Bn-N17-p43517333	Bn-C7-p43220784	Bn-scaff_16069_1-p351084	C7	61	73.6	A
19457	Bn-N17-p43576257	Bn-C7-p43275713	Bn-scaff_16069_1-p356577	C7	61	73.6	A
19458	Bn-N17-p43612998	Bn-C7-p43312579	Bn-scaff_16069_1-p360264	C7	61	73.6	A
19459	Bn-N17-p43618094	Bn-C7-p43318344	Bn-scaff_16069_1-p360840	C7	61	73.6	A
19460	Bn-N17-p43639910	Bn-C7-p43341125	Bn-scaff_16069_1-p363118	C7	61	73.6	A
19461	Bn-N17-p43647488	Bn-C7-p43348448	Bn-scaff_16069_1-p363851	C7	61	73.6	A
19462	Bn-N17-p43648388	Bn-C7-p43349196	Bn-scaff_16069_1-p363925	C7	61	73.6	A
19463	Bn-N17-p43648724	Bn-C7-p43349532	Bn-scaff_16069_1-p363959	C7	61	73.6	A
19464	Bn-N17-p43662882	Bn-C7-p43363689	Bn-scaff_16069_1-p365375	C7	61	73.6	A
19465	Bn-N17-p43766966	Bn-C7-p43480644	Bn-scaff_16069_1-p377070	C7	61	73.6	A
19466	Bn-N17-p43775738	Bn-C7-p43490432	Bn-scaff_16069_1-p378049	C7	61	73.6	A
19467	Bn-N17-p43942226	Bn-C7-p43636639	Bn-scaff_16069_1-p392670	C7	61	73.6	A
19468	Bn-N17-p43880846	Bn-C7-p43570972	Bn-scaff_16069_1-p386103	C7	62	76	A
19469	Bn-N17-p43903640	Bn-C7-p43592897	Bn-scaff_16069_1-p388295	C7	62	76	A
19470	Bn-N17-p43924735	Bn-C7-p43616248	Bn-scaff_16069_1-p390631	C7	62	76	A
19471	Bn-N17-p43927473	Bn-C7-p43618951	Bn-scaff_16069_1-p390901	C7	62	76	A
19472	Bn-N17-p43953157	Bn-C7-p43647710	Bn-scaff_16069_1-p393777	C7	62	76	A
19473	Bn-N17-p43960610	Bn-C7-p43655170	Bn-scaff_16069_1-p394523	C7	63	76.8	A
19474	Bn-N17-p43962944	Bn-C7-p43661735	Bn-scaff_16069_1-p395179	C7	63	76.8	A
19475	Bn-N17-p44060606	Bn-C7-p43755372	Bn-scaff_16069_1-p404543	C7	63	76.8	A
19476	Bn-N17-p44061276	Bn-C7-p43756016	Bn-scaff_16069_1-p404607	C7	63	76.8	A
19477	Bn-N17-p44134171	Bn-C7-p43829463	Bn-scaff_16069_1-p411952	C7	63	76.8	A
19478	Bn-N17-p44318806	Bn-C7-p44016812	Bn-scaff_16069_1-p430687	C7	64	77.6	A
19479	Bn-N17-p44340801	Bn-C7-p44037265	Bn-scaff_16069_1-p432732	C7	64	77.6	A
19480	Bn-N17-p44353390	Bn-C7-p44051043	Bn-scaff_16069_1-p434110	C7	64	77.6	A
19481	Bn-N17-p44520374	Bn-C7-p44188357	Bn-scaff_16069_1-p447841	C7	64	77.6	A
19482	Bn-N17-p44530761	Bn-C7-p44192837	Bn-scaff_16069_1-p448289	C7	64	77.6	A
19483	Bn-N17-p44571237	Bn-C3-p16597390	Bn-scaff_22466_1-p129181	C7	65	82.5	A
19484	Bn-N17-p44591616	Bn-A03-p26506472	Bn-A03-p26506472	C7	65	82.5	A
19485	Bn-N17-p44595392	Bn-C7-p44256784	Bn-scaff_16069_1-p454684	C7	65	82.5	A
19486	Bn-N17-p44600378	Bn-C7-p44263544	Bn-scaff_16069_1-p455360	C7	65	82.5	A
19487	Bn-N17-p44617975	Bn-C7-p44276916	Bn-scaff_16069_1-p456697	C7	65	82.5	A
19488	Bn-N17-p44650324	Bn-C7-p44315218	Bn-scaff_16069_1-p460528	C7	66	84.1	A
19489	Bn-N17-p44650632	Bn-C7-p44315526	Bn-scaff_16069_1-p460558	C7	66	84.1	A
19490	Bn-N17-p44654596	Bn-C7-p44319581	Bn-scaff_16069_1-p460964	C7	67	84.9	A
19491	Bn-N17-p44662598	Bn-C7-p44330685	Bn-scaff_16069_1-p462074	C7	67	84.9	A
19492	Bn-N17-p44662640	Bn-C7-p44330765	Bn-scaff_16069_1-p462082	C7	67	84.9	A
19493	Bn-N17-p44662693	Bn-C7-p44330818	Bn-scaff_16069_1-p462088	C7	67	84.9	A
19494	Bn-N17-p44662753	Bn-C7-p44330914	Bn-scaff_16069_1-p462097	C7	67	84.9	A
19495	Bn-N17-p44666731	Bn-C7-p44334900	Bn-scaff_16069_1-p462496	C7	67	84.9	A
19496	Bn-N17-p44686294	Bn-C7-p44352522	Bn-scaff_16069_1-p464258	C7	67	84.9	A
19497	Bn-N17-p44688243	Bn-C7-p44355380	Bn-scaff_16069_1-p464544	C7	67	84.9	A

19498	Bn-N17-p44689015	Bn-C7-p44356152	Bn-scaff_16069_1-p464621	C7	67	84.9	A
19499	Bn-N17-p44689056	Bn-C7-p44356193	Bn-scaff_16069_1-p464625	C7	67	84.9	A
19500	Bn-N17-p44689272	Bn-C7-p44356395	Bn-scaff_16069_1-p464645	C7	67	84.9	A
19501	Bn-N17-p44691641	Bn-C7-p44358766	Bn-scaff_16069_1-p464882	C7	67	84.9	A
19502	Bn-N17-p44710279	Bn-C7-p44376147	Bn-scaff_16069_1-p466620	C7	67	84.9	A
19503	Bn-N17-p44719200	Bn-C7-p44380330	Bn-scaff_16069_1-p467039	C7	67	84.9	A
19504	Bn-N17-p44719591	Bn-C7-p44380715	Bn-scaff_16069_1-p467077	C7	67	84.9	A
19505	Bn-N17-p44732624	Bn-C7-p44396107	Bn-scaff_16069_1-p468616	C7	67	84.9	A
19506	Bn-N17-p44765368	Bn-C7-p44455514	Bn-scaff_16069_1-p474557	C7	67	84.9	A
19507	Bn-N17-p44779800	Bn-C7-p44469333	Bn-scaff_16069_1-p475939	C7	67	84.9	A
19508	Bn-N17-p44822991	Bn-C7-p44512932	Bn-scaff_16069_1-p480299	C7	67	84.9	A
19509	Bn-N17-p44823139	Bn-C7-p44513079	Bn-scaff_16069_1-p480314	C7	67	84.9	A
19510	Bn-N17-p44823201	Bn-C7-p44513141	Bn-scaff_16069_1-p480320	C7	67	84.9	A
19511	Bn-N17-p44825295	Bn-C7-p44515236	Bn-scaff_16069_1-p480529	C7	67	84.9	A
19512	Bn-N17-p44825348	Bn-C7-p44515289	Bn-scaff_16069_1-p480535	C7	67	84.9	A
19513	Bn-N17-p44825382	Bn-C7-p44515323	Bn-scaff_16069_1-p480538	C7	67	84.9	A
19514	Bn-N17-p44829847	Bn-C7-p44518965	Bn-scaff_16069_1-p480902	C7	67	84.9	A
19515	Bn-N17-p44831329	Bn-C7-p44520417	Bn-scaff_16069_1-p481047	C7	67	84.9	A
19516	Bn-N17-p44833461	Bn-C7-p44523688	Bn-scaff_16069_1-p481375	C7	67	84.9	A
19517	Bn-N17-p44833634	Bn-C7-p44523861	Bn-scaff_16069_1-p481392	C7	67	84.9	A
19518	Bn-N17-p44833737	Bn-C7-p44523964	Bn-scaff_16069_1-p481402	C7	67	84.9	A
19519	Bn-N17-p44833794	Bn-C7-p44524021	Bn-scaff_16069_1-p481408	C7	67	84.9	A
19520	Bn-N17-p44837224	Bn-C7-p44525147	Bn-scaff_16069_1-p481520	C7	67	84.9	A
19521	Bn-N17-p44876223	Bn-C7-p44564475	Bn-scaff_16069_1-p485453	C7	67	84.9	A
19522	Bn-N17-p44929801	Bn-C7-p44614170	Bn-scaff_16110_1-p371364	C7	67	84.9	A
19523	Bn-N17-p44940369	Bn-C7-p44627064	Bn-scaff_16110_1-p370075	C7	67	84.9	A
19524	Bn-N17-p44940531	Bn-C7-p44627226	Bn-scaff_16110_1-p370059	C7	67	84.9	A
19525	Bn-N17-p44942794	Bn-C7-p44629485	Bn-scaff_16110_1-p369833	C7	67	84.9	A
19526	Bn-N17-p44943257	Bn-C7-p44629948	Bn-scaff_16110_1-p369786	C7	67	84.9	A
19527	Bn-N17-p44943396	Bn-C7-p44630087	Bn-scaff_16110_1-p369772	C7	67	84.9	A
19528	Bn-N17-p45050998	Bn-C7-p44736664	Bn-scaff_16110_1-p359115	C7	67	84.9	A
19529	Bn-N17-p45059487	Bn-C7-p44743743	Bn-scaff_16110_1-p358407	C7	67	84.9	A
19530	Bn-N17-p45059536	Bn-C7-p44743792	Bn-scaff_16110_1-p358402	C7	67	84.9	A
19531	Bn-N17-p45080325	Bn-C7-p44764164	Bn-scaff_16110_1-p356365	C7	67	84.9	A
19532	Bn-N17-p45086126	Bn-C7-p44770127	Bn-scaff_16110_1-p355768	C7	67	84.9	A
19533	Bn-N17-p45130513	Bn-C7-p44805008	Bn-scaff_16110_1-p352280	C7	67	84.9	A
19534	Bn-N17-p45141247	Bn-C7-p44812418	Bn-scaff_16110_1-p351539	C7	67	84.9	A
19535	Bn-N17-p45141426	Bn-C7-p44812572	Bn-scaff_16110_1-p351524	C7	67	84.9	A
19536	Bn-N17-p45156671	Bn-C7-p44820268	Bn-scaff_16110_1-p350754	C7	67	84.9	A
19537	Bn-N17-p45159960	Bn-C7-p44823594	Bn-scaff_16110_1-p350422	C7	67	84.9	A
19538	Bn-N17-p45160012	Bn-C7-p44823646	Bn-scaff_16110_1-p350417	C7	67	84.9	A
19539	Bn-N17-p45160580	Bn-C7-p44824200	Bn-scaff_16110_1-p350361	C7	67	84.9	A
19540	Bn-N17-p45166365	Bn-C7-p44829573	Bn-scaff_16110_1-p349824	C7	67	84.9	A
19541	Bn-N17-p45168335	Bn-C7-p44830774	Bn-scaff_16110_1-p349704	C7	67	84.9	A
19542	Bn-N17-p45269514	Bn-C7-p44959238	Bn-scaff_16110_1-p336857	C7	67	84.9	A
19543	Bn-N17-p45270881	Bn-C7-p44960401	Bn-scaff_16110_1-p336741	C7	67	84.9	A
19544	Bn-N17-p45290844	Bn-C7-p44981372	Bn-scaff_16110_1-p334644	C7	67	84.9	A
19545	Bn-N17-p45308585	Bn-C7-p45004328	Bn-scaff_16110_1-p332348	C7	67	84.9	A
19546	Bn-N17-p45432838	Bn-C7-p45128410	Bn-scaff_16110_1-p319940	C7	67	84.9	A

19547	Bn-N17-p45461078	Bn-C7-p45080858	Bn-scaff_16110_1-p324695	C7	67	84.9	A
19548	Bn-N17-p45472534	Bn-C7-p45159580	Bn-scaff_16110_1-p316823	C7	67	84.9	A
19549	Bn-N17-p45472597	Bn-C7-p45159643	Bn-scaff_16110_1-p316817	C7	67	84.9	A
19550	Bn-N3-p27098073	Bn-C7-p44529761	Bn-scaff_16069_1-p481982	C7	67	84.9	A
19551	Bn-Scaffold10054-p623	Bn-C7-p45129145	Bn-scaff_16110_1-p319867	C7	67	84.9	A
19552	Bn-Scaffold10054-p703	Bn-C7-p45129065	Bn-scaff_16110_1-p319875	C7	67	84.9	A
19553	Bn-Scaffold25230-p297	Bn-C7-p44741770	Bn-scaff_16110_1-p358604	C7	67	84.9	A
19554	Bn-Scaffold40474-p232	Bn-C7-p45129854	Bn-scaff_16110_1-p319796	C7	67	84.9	A
19555	Bn-Scaffold56235-p204	Bn-C7-p45128577	Bn-scaff_16110_1-p319923	C7	67	84.9	A
19556	Bn-N17-p45568856	Bn-C7-p45252338	Bn-scaff_16110_1-p307547	C7	68	86.5	A
19557	Bn-N17-p45590687	Bn-C7-p45273083	Bn-scaff_16110_1-p305473	C7	68	86.5	A
19558	Bn-N17-p45605723	Bn-C7-p45281229	Bn-scaff_16110_1-p304658	C7	68	86.5	A
19559	Bn-N17-p45637187	Bn-C7-p45308778	Bn-scaff_16110_1-p301903	C7	68	86.5	A
19560	Bn-N17-p45684541	Bn-C7-p45354442	Bn-scaff_16110_1-p297337	C7	68	86.5	A
19561	Bn-N17-p45692454	Bn-C7-p45362361	Bn-scaff_16110_1-p296545	C7	68	86.5	A
19562	Bn-N11-p4826468	Bn-C7-p45389243	Bn-scaff_16110_1-p293857	C7	69	87.3	0
19563	Bn-N17-p45707681	Bn-C7-p45377611	Bn-scaff_16110_1-p295020	C7	69	87.3	A
19564	Bn-N13-p51226502	Bn-A03-p11122273	Bn-A03-p11122273	C7	70	88.1	A
19565	Bn-N17-p45782916	Bn-C7-p45437529	Bn-scaff_16110_1-p289028	C7	70	88.1	A
19566	Bn-N17-p45884797	Bn-C7-p45533873	Bn-scaff_16110_1-p279394	C7	70	88.1	A
19567	Bn-N17-p46039061	Bn-C7-p45671980	Bn-scaff_16110_1-p265583	C7	71	91.3	A
19568	Bn-N17-p46086387	Bn-C7-p45715611	Bn-scaff_16110_1-p261220	C7	71	91.3	A
19569	Bn-N17-p46100692	Bn-C7-p45729422	Bn-scaff_16110_1-p259839	C7	72	92.1	A
19570	Bn-N17-p46118744	Bn-C7-p45747454	Bn-scaff_16110_1-p258036	C7	72	92.1	A
19571	Bn-N17-p46122548	Bn-C7-p45751307	Bn-scaff_16110_1-p257650	C7	73	92.9	A
19572	Bn-N17-p46127095	Bn-C7-p45751444	Bn-scaff_16110_1-p257637	C7	73	92.9	A
19573	Bn-N17-p46132089	Bn-C7-p45756232	Bn-scaff_16110_1-p257158	C7	73	92.9	A
19574	Bn-N17-p46158475	Bn-C7-p45786964	Bn-scaff_16110_1-p254085	C7	73	92.9	A
19575	Bn-N17-p46171749	Bn-C7-p45795281	Bn-scaff_16110_1-p253253	C7	73	92.9	A
19576	Bn-N17-p46174370	Bn-C7-p45797827	Bn-scaff_16110_1-p252998	C7	73	92.9	A
19577	Bn-N17-p46182022	Bn-C7-p45803891	Bn-scaff_16110_1-p252392	C7	73	92.9	A
19578	Bn-N17-p46183133	Bn-C7-p45805099	Bn-scaff_16110_1-p252271	C7	73	92.9	A
19579	Bn-N17-p46197946	Bn-C7-p45821943	Bn-scaff_16110_1-p250587	C7	73	92.9	A
19580	Bn-N17-p46213262	Bn-C7-p45838616	Bn-scaff_16110_1-p248920	C7	73	92.9	A
19581	Bn-N17-p46213552	Bn-C7-p45838909	Bn-scaff_16110_1-p248890	C7	73	92.9	A
19582	Bn-N17-p46279913	Bn-C7-p45888485	Bn-scaff_16110_1-p243933	C7	73	92.9	A
19583	Bn-N17-p46286447	Bn-C7-p45895017	Bn-scaff_16110_1-p243279	C7	73	92.9	A
19584	Bn-N17-p46286518	Bn-C7-p45895088	Bn-scaff_16110_1-p243272	C7	73	92.9	A
19585	Bn-N17-p46435680	Bn-C7-p46046384	Bn-scaff_16110_1-p228143	C7	73	92.9	A
19586	Bn-N17-p46435741	Bn-C7-p46046445	Bn-scaff_16110_1-p228137	C7	73	92.9	A
19587	Bn-N17-p46442219	Bn-C7-p46052547	Bn-scaff_16110_1-p227526	C7	73	92.9	A
19588	Bn-N17-p46543863	Bn-C7-p46159412	Bn-scaff_16110_1-p216840	C7	74	92.9	A
19589	Bn-N17-p46681732	Bn-C7-p46283570	Bn-scaff_16110_1-p204424	C7	75	94.5	A
19590	Bn-N17-p46700265	Bn-C7-p46297766	Bn-scaff_16110_1-p203005	C7	75	94.5	A
19591	Bn-N17-p46702285	Bn-C7-p46299784	Bn-scaff_16110_1-p202803	C7	75	94.5	A
19592	Bn-N17-p46743171	Bn-C7-p46337232	Bn-scaff_16110_1-p199058	C7	76	95.3	A
19593	Bn-N17-p46746858	Bn-C7-p46340927	Bn-scaff_16110_1-p198688	C7	76	95.3	A
19594	Bn-N17-p46765619	Bn-C7-p46356119	Bn-scaff_16110_1-p197169	C7	76	95.3	A
19595	Bn-N17-p46790427	Bn-C7-p46379355	Bn-scaff_16110_1-p194846	C7	76	95.3	A

19596	Bn-N17-p46809360	Bn-C7-p46387231	Bn-scaff_16110_1-p19405	C7	76	95.3	A
19597	Bn-N17-p46809475	Bn-C7-p46387346	Bn-scaff_16110_1-p19404	C7	76	95.3	A
19598	Bn-N17-p46809543	Bn-C7-p46387414	Bn-scaff_16110_1-p19404	C7	76	95.3	A
19599	Bn-N17-p46809724	Bn-C7-p46387601	Bn-scaff_16110_1-p19402	C7	76	95.3	A
19600	Bn-N17-p46811171	Bn-C7-p46388726	Bn-scaff_16110_1-p19390	C7	76	95.3	A
19601	Bn-N17-p46811650	Bn-C7-p46389205	Bn-scaff_16110_1-p19386	C7	76	95.3	A
19602	Bn-N17-p46811870	Bn-C7-p46389425	Bn-scaff_16110_1-p19383	C7	76	95.3	A
19603	Bn-N3-p29421015	Bn-C7-p46337400	Bn-scaff_16110_1-p19904	C7	76	95.3	A
19604	Bn-N3-p29480332	Bn-A03-p29053385	Bn-A03-p29053385	C7	77	96.1	A
19605	Bn-N17-p47049196	Bn-C7-p46637900	Bn-scaff_16110_1-p16899	C7	78	96.9	A
19606	Bn-N17-p47352443	Bn-C7-p46876015	Bn-scaff_16110_1-p14518	C7	79	97.7	A
19607	Bn-N17-p47408849	Bn-C7-p46938772	Bn-scaff_16110_1-p13890	C7	79	97.7	A
19608	Bn-N17-p47411006	Bn-C7-p46939380	Bn-scaff_16110_1-p13884	C7	79	97.7	A
19609	Bn-N3-p30295442	Bn-A03-p29834625	Bn-A03-p29834625	C7	79	97.7	A
19610	Bn-N17-p47594347	Bn-C7-p47122817	Bn-scaff_16110_1-p12049	C7	80	98.5	A
19611	Bn-N17-p47602177	Bn-C7-p47134619	Bn-scaff_16110_1-p11931	C7	80	98.5	A
19612	Bn-N17-p47613654	Bn-C7-p47145991	Bn-scaff_16110_1-p11818	C7	80	98.5	A
19613	Bn-N17-p47937586	Bn-C7-p47427771	Bn-scaff_16110_1-p90004	C7	80	98.5	A
19614	Bn-N17-p48184413	Bn-C7-p47656501	Bn-scaff_16110_1-p67131	C7	81	100.9	A
19615	Bn-N17-p48184736	Bn-C7-p47656824	Bn-scaff_16110_1-p67099	C7	81	100.9	A
19616	Bn-N17-p48205783	Bn-C7-p47677801	Bn-scaff_16110_1-p65001	C7	81	100.9	A
19617	Bn-N17-p48207044	Bn-C7-p47679060	Bn-scaff_16110_1-p64875	C7	81	100.9	A
19618	Bn-N17-p48264289	Bn-C7-p47733563	Bn-scaff_16110_1-p59425	C7	81	100.9	A
19619	Bn-N17-p48287526	Bn-C7-p47752337	Bn-scaff_16110_1-p57547	C7	81	100.9	A
19620	Bn-N17-p48308176	Bn-C7-p47773033	Bn-scaff_16110_1-p55478	C7	81	100.9	A
19621	Bn-N17-p48312502	Bn-C7-p47777341	Bn-scaff_16110_1-p55047	C7	81	100.9	A
19622	Bn-N17-p48392184	Bn-C7-p47856008	Bn-scaff_16110_1-p47180	C7	81	100.9	A
19623	Bn-N17-p48616786	Bn-A06-p26066533	Bn-A06-p26066533	C7	81	100.9	A
19624	Bn-N3-p31490325	Bn-A03-p30925102	Bn-A03-p30925102	C7	81	100.9	A
19625	Bn-N6-p25702938	Bn-C7-p47917291	Bn-scaff_16110_1-p41052	C7	81	100.9	A
19626	Bn-N17-p48696724	Bn-C7-p48192206	Bn-scaff_16110_1-p13561	C7	82	101.7	A
19627	Bn-N17-p48719259	Bn-C7-p48216215	Bn-scaff_16110_1-p11160	C7	82	101.7	A
19628	Bn-N6-p26002769	Bn-A06-p26213014	Bn-A06-p26213014	C7	82	101.7	A
19629	Bn-N18-p1454470	Bn-C8-p1210965	Bn-scaff_20796_1-p34573	C8	1	0	B
19630	Bn-N18-p22925019	Bn-A06-p23744370	Bn-A06-p23744370	C8	1	0	B
19631	Bn-N17-p22207347	Bn-C8-p4498643	Bn-scaff_25981_1-p11041	C8	2	1.6	B
19632	Bn-N18-p11557732	Bn-C8-p10037042	Bn-scaff_16142_1-p18354	C8	2	1.6	0
19633	Bn-N18-p4585694	Bn-C8-p3381353	Bn-scaff_21003_1-p63112	C8	2	1.6	B
19634	Bn-N18-p5618557	Bn-C8-p4368350	Bn-scaff_25981_1-p24070	C8	2	1.6	B
19635	Bn-N18-p5911035	Bn-C8-p4750298	Bn-scaff_16158_1-p14114	C8	2	1.6	B
19636	Bn-N8-p3797452	Bn-C8-p4417313	Bn-scaff_25981_1-p19174	C8	2	1.6	B
19637	Bn-N8-p3927842	Bn-A08-p4096136	Bn-A08-p4096136	C8	2	1.6	B
19638	Bn-N18-p12616173	Bn-A01-p10059043	Bn-A01-p10059043	C8	3	2.4	B
19639	Bn-N18-p14716087	Bn-C2-p38035448	Bn-scaff_16545_1-p1764	C8	3	2.4	B
19640	Bn-N18-p14716285	Bn-C2-p38035254	Bn-scaff_16545_1-p1958	C8	3	2.4	B
19641	Bn-N18-p14723087	Bn-C2-p38030203	Bn-scaff_16545_1-p7009	C8	3	2.4	B
19642	Bn-N18-p14742193	Bn-C2-p38011155	Bn-scaff_16545_1-p26057	C8	3	2.4	B
19643	Bn-N18-p14743532	Bn-C2-p38009817	Bn-scaff_16545_1-p27395	C8	3	2.4	B
19644	Bn-N18-p14744576	Bn-C2-p38008773	Bn-scaff_16545_1-p28439	C8	3	2.4	B

19645	Bn-N18-p14744642	Bn-C2-p38008707	Bn-scaff_16545_1-p28505	C8	3	2.4	B
19646	Bn-N18-p14745198	Bn-C2-p38008152	Bn-scaff_16545_1-p29060	C8	3	2.4	B
19647	Bn-N18-p14745831	Bn-C2-p38007519	Bn-scaff_16545_1-p29693	C8	3	2.4	B
19648	Bn-N18-p14752395	Bn-C2-p38000955	Bn-scaff_16545_1-p36257	C8	3	2.4	B
19649	Bn-N18-p14774528	Bn-C2-p37980423	Bn-scaff_16545_1-p56789	C8	3	2.4	0
19650	Bn-N18-p14795944	Bn-C2-p37959986	Bn-scaff_16545_1-p77226	C8	3	2.4	B
19651	Bn-N18-p14796221	Bn-C2-p37959709	Bn-scaff_16545_1-p77503	C8	3	2.4	B
19652	Bn-N18-p14796278	Bn-C2-p37959652	Bn-scaff_16545_1-p77560	C8	3	2.4	B
19653	Bn-N18-p14799018	Bn-C2-p37956909	Bn-scaff_16545_1-p80303	C8	3	2.4	B
19654	Bn-N18-p14799598	Bn-C2-p37956329	Bn-scaff_16545_1-p80883	C8	3	2.4	B
19655	Bn-N18-p14801105	Bn-C2-p37954831	Bn-scaff_16545_1-p82381	C8	3	2.4	B
19656	Bn-N18-p14801540	Bn-C2-p37954396	Bn-scaff_16545_1-p82816	C8	3	2.4	B
19657	Bn-N18-p14803714	Bn-C2-p37952243	Bn-scaff_16545_1-p84969	C8	3	2.4	B
19658	Bn-N18-p14804387	Bn-C2-p37951570	Bn-scaff_16545_1-p85642	C8	3	2.4	B
19659	Bn-N18-p14809041	Bn-C2-p37946916	Bn-scaff_16545_1-p90296	C8	3	2.4	B
19660	Bn-N18-p14809151	Bn-C2-p37946806	Bn-scaff_16545_1-p90406	C8	3	2.4	B
19661	Bn-N18-p14809219	Bn-C2-p37946738	Bn-scaff_16545_1-p90474	C8	3	2.4	B
19662	Bn-N18-p14813433	Bn-C2-p37942370	Bn-scaff_16545_1-p94842	C8	3	2.4	B
19663	Bn-N18-p14813858	Bn-C2-p37941945	Bn-scaff_16545_1-p95267	C8	3	2.4	B
19664	Bn-N18-p14814832	Bn-C2-p37940971	Bn-scaff_16545_1-p96241	C8	3	2.4	B
19665	Bn-N18-p14817467	Bn-C2-p37938313	Bn-scaff_16545_1-p98899	C8	3	2.4	B
19666	Bn-N18-p14889203	Bn-C2-p37864604	Bn-scaff_16545_1-p17260	C8	3	2.4	B
19667	Bn-N18-p14913707	Bn-C2-p37838880	Bn-scaff_16545_1-p19833	C8	3	2.4	B
19668	Bn-N18-p14916259	Bn-C2-p37836275	Bn-scaff_16545_1-p20093	C8	3	2.4	B
19669	Bn-N18-p14926340	Bn-C2-p37826435	Bn-scaff_16545_1-p21077	C8	3	2.4	B
19670	Bn-N18-p14970660	Bn-C2-p37790536	Bn-scaff_16545_1-p24667	C8	3	2.4	B
19671	Bn-N18-p14980417	Bn-C2-p37780758	Bn-scaff_16545_1-p25645	C8	3	2.4	B
19672	Bn-N18-p14996479	Bn-C2-p37764146	Bn-scaff_16545_1-p27306	C8	3	2.4	B
19673	Bn-N18-p15030014	Bn-C2-p37728619	Bn-scaff_16545_1-p30859	C8	3	2.4	B
19674	Bn-N18-p15030575	Bn-C2-p37728058	Bn-scaff_16545_1-p30915	C8	3	2.4	B
19675	Bn-N18-p2430358	Bn-C4-p9667002	Bn-scaff_16447_1-p51395	C8	3	2.4	0
19676	Bn-N18-p27636217	Bn-C3-p50050568	Bn-scaff_19740_1-p98443	C8	3	2.4	B
19677	Bn-N18-p3847174	Bn-A02-p17333549	Bn-A02-p17333549	C8	3	2.4	B
19678	Bn-N18-p5814844	Bn-A07-p1041609	Bn-A07-p1041609	C8	3	2.4	B
19679	Bn-N18-p5816288	Bn-A07-p1040165	Bn-A07-p1040165	C8	3	2.4	B
19680	Bn-N10-p4906330	Bn-C4-p30803760	Bn-scaff_18839_1-p19905	C8	4	3.2	B
19681	Bn-N18-p1550138	Bn-C8-p1324560	Bn-scaff_20796_1-p14816	C8	4	3.2	B
19682	Bn-N18-p1621343	Bn-C8-p1390828	Bn-scaff_26293_1-p13544	C8	4	3.2	B
19683	Bn-N18-p1633794	Bn-C8-p1408838	Bn-scaff_26293_1-p11743	C8	4	3.2	B
19684	Bn-N18-p1782299	Bn-C8-p1588704	Bn-scaff_15705_1-p29713	C8	4	3.2	B
19685	Bn-N18-p1843932	Bn-C8-p1650985	Bn-scaff_15705_1-p29090	C8	4	3.2	B
19686	Bn-N18-p1864156	Bn-C8-p1656778	Bn-scaff_15705_1-p29032	C8	4	3.2	B
19687	Bn-N18-p1864352	Bn-C8-p1656973	Bn-scaff_15705_1-p29030	C8	4	3.2	B
19688	Bn-N18-p1865587	Bn-C8-p1657787	Bn-scaff_15705_1-p29022	C8	4	3.2	B
19689	Bn-N18-p58598	Bn-Scaffold01256-p5	Bn-scaff_16130_3-p5466	C8	4	3.2	B
19690	Bn-Scaffold01052-p44529	Bn-C8-p1665283	Bn-scaff_16022_1-p84943	C8	4	3.2	B
19691	Bn-Scaffold01526-p60	Bn-Scaffold01118-p2	Bn-scaff_18935_1-p25523	C8	4	3.2	B
19692	Bn-Scaffold01526-p82	Bn-Scaffold01118-p2	Bn-scaff_18935_1-p25545	C8	4	3.2	B
19693	Bn-N18-p10112858	Bn-C8-p8635399	Bn-scaff_17591_1-p46152	C8	5	4	B

19694	Bn-N18-p10131363	Bn-C8-p8665798	Bn-scaff_17591_1-p431125	C8	5	4	B
19695	Bn-N18-p10363857	Bn-C8-p8943749	Bn-scaff_17591_1-p153174	C8	5	4	B
19696	Bn-N18-p10444900	Bn-C8-p9021022	Bn-scaff_17591_1-p75901	C8	5	4	B
19697	Bn-N18-p10567847	Bn-C8-p9121565	Bn-scaff_16142_1-p109901	C8	5	4	B
19698	Bn-N18-p11326372	Bn-C8-p9801765	Bn-scaff_16142_1-p418818	C8	5	4	B
19699	Bn-N18-p11452153	Bn-C8-p9924715	Bn-scaff_16142_1-p295868	C8	5	4	B
19700	Bn-N18-p11464139	Bn-C8-p9935772	Bn-scaff_16142_1-p284811	C8	5	4	B
19701	Bn-N18-p11469959	Bn-C8-p9940050	Bn-scaff_16142_1-p280533	C8	5	4	B
19702	Bn-N18-p11470496	Bn-C8-p9940587	Bn-scaff_16142_1-p279996	C8	5	4	B
19703	Bn-N18-p11477138	Bn-C8-p9947353	Bn-scaff_16142_1-p273230	C8	5	4	B
19704	Bn-N18-p11504718	Bn-C8-p9971222	Bn-scaff_16142_1-p249361	C8	5	4	B
19705	Bn-N18-p11540071	Bn-C8-p10017108	Bn-scaff_16142_1-p203475	C8	5	4	B
19706	Bn-N18-p11540311	Bn-C8-p10017348	Bn-scaff_16142_1-p203235	C8	5	4	B
19707	Bn-N18-p11577254	Bn-C8-p10053621	Bn-scaff_16142_1-p166962	C8	5	4	B
19708	Bn-N18-p11606839	Bn-C8-p10084490	Bn-scaff_16142_1-p136093	C8	5	4	B
19709	Bn-N18-p11642900	Bn-C8-p10130115	Bn-scaff_16142_1-p90468	C8	5	4	B
19710	Bn-N18-p11643445	Bn-C8-p10130729	Bn-scaff_16142_1-p89854	C8	5	4	B
19711	Bn-N18-p11644546	Bn-C8-p10131761	Bn-scaff_16142_1-p88822	C8	5	4	B
19712	Bn-N18-p11669928	Bn-C8-p10156513	Bn-scaff_16142_1-p64070	C8	5	4	B
19713	Bn-N18-p11669985	Bn-C8-p10156570	Bn-scaff_16142_1-p64013	C8	5	4	B
19714	Bn-N18-p11711613	Bn-Scaffold00974-p25	Bn-scaff_24134_1-p25752	C8	5	4	B
19715	Bn-N18-p11762355	Bn-C8-p10190515	Bn-scaff_16142_1-p30068	C8	5	4	B
19716	Bn-N18-p11820051	Bn-C8-p12798373	Bn-scaff_17637_1-p236365	C8	5	4	B
19717	Bn-N18-p11832715	Bn-C8-p12804119	Bn-scaff_17637_1-p230619	C8	5	4	B
19718	Bn-N18-p11834490	Bn-C8-p12805891	Bn-scaff_17637_1-p228847	C8	5	4	B
19719	Bn-N18-p11878421	Bn-C8-p12846583	Bn-scaff_17637_1-p188155	C8	5	4	B
19720	Bn-N18-p11878797	Bn-C8-p12846960	Bn-scaff_17637_1-p187778	C8	5	4	B
19721	Bn-N18-p11884389	Bn-C8-p12852563	Bn-scaff_17637_1-p182175	C8	5	4	B
19722	Bn-N18-p11888061	Bn-C8-p12856222	Bn-scaff_17637_1-p178516	C8	5	4	B
19723	Bn-N18-p11917503	Bn-C8-p12890137	Bn-scaff_17637_1-p144601	C8	5	4	B
19724	Bn-N18-p11920467	Bn-C8-p12893087	Bn-scaff_17637_1-p141651	C8	5	4	B
19725	Bn-N18-p11920660	Bn-C8-p12893280	Bn-scaff_17637_1-p141458	C8	5	4	B
19726	Bn-N18-p11921199	Bn-C8-p12893819	Bn-scaff_17637_1-p140919	C8	5	4	B
19727	Bn-N18-p11933789	Bn-C8-p12907225	Bn-scaff_17637_1-p127513	C8	5	4	B
19728	Bn-N18-p11953486	Bn-C8-p12927297	Bn-scaff_17637_1-p107441	C8	5	4	B
19729	Bn-N18-p11978595	Bn-C8-p12950067	Bn-scaff_17637_1-p84671	C8	5	4	B
19730	Bn-N18-p11996804	Bn-C8-p12966573	Bn-scaff_17637_1-p68165	C8	5	4	B
19731	Bn-N18-p12031207	Bn-C8-p13000659	Bn-scaff_17637_1-p34079	C8	5	4	B
19732	Bn-N18-p12101444	Bn-Scaffold01323-p1	Bn-scaff_20563_1-p1677	C8	5	4	B
19733	Bn-N18-p12102343	Bn-Scaffold01323-p7	Bn-scaff_20563_1-p778	C8	5	4	B
19734	Bn-N18-p12124596	Bn-Scaffold01006-p1	Bn-scaff_15714_1-p322615	C8	5	4	B
19735	Bn-N18-p12126747	Bn-Scaffold01006-p1	Bn-scaff_15714_1-p322400	C8	5	4	B
19736	Bn-N18-p12128481	Bn-Scaffold01006-p1	Bn-scaff_15714_1-p322227	C8	5	4	B
19737	Bn-N18-p12129533	Bn-Scaffold01006-p1	Bn-scaff_15714_1-p322122	C8	5	4	B
19738	Bn-N18-p12134428	Bn-Scaffold01006-p5	Bn-scaff_15714_1-p321634	C8	5	4	B
19739	Bn-N18-p12154172	Bn-C8-p10241765	Bn-scaff_16287_1-p21083	C8	5	4	B
19740	Bn-N18-p12245666	Bn-C8-p10333450	Bn-scaff_16287_1-p112768	C8	5	4	B
19741	Bn-N18-p12292047	Bn-C8-p10367290	Bn-scaff_16287_1-p146608	C8	5	4	B
19742	Bn-N18-p12292558	Bn-C8-p10367801	Bn-scaff_16287_1-p147119	C8	5	4	B

19743	Bn-N18-p12295116	Bn-C8-p10370741	Bn-scaff_16287_1-p15005	C8	5	4	B
19744	Bn-N18-p12308252	Bn-C8-p10382405	Bn-scaff_16287_1-p16172	C8	5	4	B
19745	Bn-N18-p12318458	Bn-C8-p10391397	Bn-scaff_16287_1-p17071	C8	5	4	B
19746	Bn-N18-p12318778	Bn-C8-p10391716	Bn-scaff_16287_1-p17103	C8	5	4	B
19747	Bn-N18-p12321472	Bn-C8-p10394349	Bn-scaff_16287_1-p17366	C8	5	4	B
19748	Bn-N18-p12335043	Bn-C8-p10404157	Bn-scaff_16287_1-p18347	C8	5	4	B
19749	Bn-N18-p12338842	Bn-C8-p10407960	Bn-scaff_16287_1-p18727	C8	5	4	B
19750	Bn-N18-p12349404	Bn-C8-p10428577	Bn-scaff_16287_1-p20789	C8	5	4	B
19751	Bn-N18-p12354832	Bn-C8-p10433986	Bn-scaff_16287_1-p21330	C8	5	4	B
19752	Bn-N18-p12362961	Bn-C8-p10442257	Bn-scaff_16287_1-p22157	C8	5	4	B
19753	Bn-N18-p12365002	Bn-C8-p10444299	Bn-scaff_16287_1-p22361	C8	5	4	B
19754	Bn-N18-p12365202	Bn-C8-p10444499	Bn-scaff_16287_1-p22381	C8	5	4	B
19755	Bn-N18-p12390539	Bn-C8-p4375385	Bn-scaff_25981_1-p23367	C8	5	4	B
19756	Bn-N18-p12411797	Bn-C8-p10503347	Bn-scaff_16287_1-p28266	C8	5	4	B
19757	Bn-N18-p12412595	Bn-C8-p10504132	Bn-scaff_16287_1-p28345	C8	5	4	B
19758	Bn-N18-p12413769	Bn-C8-p10505307	Bn-scaff_16287_1-p28462	C8	5	4	B
19759	Bn-N18-p12421848	Bn-C8-p10514746	Bn-scaff_16287_1-p29406	C8	5	4	B
19760	Bn-N18-p12421864	Bn-C8-p10514762	Bn-scaff_16287_1-p29408	C8	5	4	B
19761	Bn-N18-p12421997	Bn-C8-p10514895	Bn-scaff_16287_1-p29421	C8	5	4	B
19762	Bn-N18-p12431932	Bn-C8-p10524729	Bn-scaff_16287_1-p30404	C8	5	4	B
19763	Bn-N18-p12433017	Bn-C8-p10525885	Bn-scaff_16287_1-p30520	C8	5	4	B
19764	Bn-N18-p12434603	Bn-C8-p10527273	Bn-scaff_16287_1-p30659	C8	5	4	B
19765	Bn-N18-p12442346	Bn-C8-p10564375	Bn-scaff_16287_1-p34369	C8	5	4	B
19766	Bn-N18-p12444183	Bn-C8-p10567973	Bn-scaff_16287_1-p34729	C8	5	4	B
19767	Bn-N18-p12444412	Bn-C8-p10568202	Bn-scaff_16287_1-p34752	C8	5	4	B
19768	Bn-N18-p12445329	Bn-C8-p10569119	Bn-scaff_16287_1-p34843	C8	5	4	B
19769	Bn-N18-p12446132	Bn-C8-p10569922	Bn-scaff_16287_1-p34924	C8	5	4	B
19770	Bn-N18-p12449250	Bn-C8-p10572608	Bn-scaff_16287_1-p35192	C8	5	4	B
19771	Bn-N18-p12462117	Bn-C8-p10587267	Bn-scaff_16287_1-p36658	C8	5	4	B
19772	Bn-N18-p12492541	Bn-C8-p10615077	Bn-scaff_16287_1-p39439	C8	5	4	B
19773	Bn-N18-p12514226	Bn-C8-p10636833	Bn-scaff_16287_1-p41615	C8	5	4	B
19774	Bn-N18-p12533954	Bn-C8-p10650524	Bn-scaff_16287_1-p42984	C8	5	4	0
19775	Bn-N18-p12534945	Bn-C8-p10651535	Bn-scaff_16287_1-p43085	C8	5	4	B
19776	Bn-N18-p12536864	Bn-C8-p10654532	Bn-scaff_16287_1-p43385	C8	5	4	B
19777	Bn-N18-p12537020	Bn-C8-p10654688	Bn-scaff_16287_1-p43400	C8	5	4	B
19778	Bn-N18-p12537592	Bn-C8-p10655260	Bn-scaff_16287_1-p43457	C8	5	4	B
19779	Bn-N18-p12539856	Bn-C8-p10657491	Bn-scaff_16287_1-p43680	C8	5	4	B
19780	Bn-N18-p12542661	Bn-C8-p10660295	Bn-scaff_16287_1-p43961	C8	5	4	B
19781	Bn-N18-p12544944	Bn-C8-p10662578	Bn-scaff_16287_1-p44189	C8	5	4	B
19782	Bn-N18-p12544988	Bn-C8-p10662622	Bn-scaff_16287_1-p44194	C8	5	4	B
19783	Bn-N18-p12547543	Bn-C8-p10665178	Bn-scaff_16287_1-p44449	C8	5	4	B
19784	Bn-N18-p12555189	Bn-C8-p10672839	Bn-scaff_16287_1-p45215	C8	5	4	B
19785	Bn-N18-p12555369	Bn-C8-p10673020	Bn-scaff_16287_1-p45233	C8	5	4	B
19786	Bn-N18-p12586007	Bn-C8-p10703980	Bn-scaff_16287_1-p48329	C8	5	4	B
19787	Bn-N18-p12598153	Bn-C8-p10722403	Bn-scaff_16287_1-p50172	C8	5	4	B
19788	Bn-N18-p12599437	Bn-C8-p10723687	Bn-scaff_16287_1-p50300	C8	5	4	B
19789	Bn-N18-p12652333	Bn-Scaffold01008-p1	Bn-scaff_24300_1-p1264	C8	5	4	B
19790	Bn-N18-p12664102	Bn-Scaffold01008-p1	Bn-scaff_24300_1-p13018	C8	5	4	B
19791	Bn-N18-p12719480	Bn-C8-p10791739	Bn-scaff_16287_1-p57105	C8	5	4	B

19792	Bn-N18-p14009354	Bn-C8-p11980815	Bn-scaff_18310_1-p11717[illegible]	C8	5	4	B
19793	Bn-N18-p14321263	Bn-Scaffold10685-p2[illegible]	Bn-scaff_23765_1-p208	C8	5	4	B
19794	Bn-N18-p15042805	Bn-C8-p13041985	Bn-scaff_21250_1-p7148	C8	5	4	B
19795	Bn-N18-p15043093	Bn-C8-p13042273	Bn-scaff_21250_1-p7436	C8	5	4	B
19796	Bn-N18-p15043853	Bn-C8-p13043033	Bn-scaff_21250_1-p8196	C8	5	4	B
19797	Bn-N18-p15079623	Bn-C8-p13080234	Bn-scaff_21250_1-p45397	C8	5	4	B
19798	Bn-N18-p15081011	Bn-C8-p13081622	Bn-scaff_21250_1-p46785	C8	5	4	B
19799	Bn-N18-p15185956	Bn-C8-p13199719	Bn-scaff_21250_1-p16488[illegible]	C8	5	4	B
19800	Bn-N18-p15285903	Bn-C8-p13304944	Bn-scaff_21250_1-p27010[illegible]	C8	5	4	B
19801	Bn-N18-p15303981	Bn-C8-p13316252	Bn-scaff_21250_1-p28141[illegible]	C8	5	4	B
19802	Bn-N18-p15467301	Bn-C8-p13483761	Bn-scaff_21250_1-p44892[illegible]	C8	5	4	B
19803	Bn-N18-p2105542	Bn-C8-p1918709	Bn-scaff_16022_1-p59600[illegible]	C8	5	4	B
19804	Bn-N18-p2171631	Bn-C8-p2003438	Bn-scaff_16022_1-p51127[illegible]	C8	5	4	B
19805	Bn-N18-p2174497	Bn-C8-p2006186	Bn-scaff_16022_1-p50852[illegible]	C8	5	4	B
19806	Bn-N18-p2175055	Bn-C8-p2006834	Bn-scaff_16022_1-p50788[illegible]	C8	5	4	B
19807	Bn-N18-p2175168	Bn-C8-p2006947	Bn-scaff_16022_1-p50776[illegible]	C8	5	4	B
19808	Bn-N18-p2180278	Bn-C8-p2012054	Bn-scaff_16022_1-p50266[illegible]	C8	5	4	B
19809	Bn-N18-p2180635	Bn-C8-p2012410	Bn-scaff_16022_1-p50230[illegible]	C8	5	4	B
19810	Bn-N18-p2188436	Bn-C8-p2018275	Bn-scaff_16022_1-p49643[illegible]	C8	5	4	B
19811	Bn-N18-p2194763	Bn-C8-p2021334	Bn-scaff_16022_1-p49338[illegible]	C8	5	4	B
19812	Bn-N18-p2245596	Bn-C8-p2038984	Bn-scaff_16022_1-p47573[illegible]	C8	5	4	B
19813	Bn-N18-p2321606	Bn-C8-p2086141	Bn-scaff_16022_1-p42857[illegible]	C8	5	4	B
19814	Bn-N18-p2339914	Bn-C8-p2101714	Bn-scaff_16022_1-p41300[illegible]	C8	5	4	B
19815	Bn-N18-p2345243	Bn-C8-p2105778	Bn-scaff_16022_1-p40893[illegible]	C8	5	4	B
19816	Bn-N18-p3783652	Bn-C8-p2705228	Bn-scaff_27765_1-p58899	C8	5	4	B
19817	Bn-N18-p4039298	Bn-C8-p2897423	Bn-scaff_17807_1-p36457[illegible]	C8	5	4	B
19818	Bn-N18-p4500990	Bn-C8-p3278915	Bn-scaff_21003_1-p73355[illegible]	C8	5	4	B
19819	Bn-N18-p4513495	Bn-C8-p3327086	Bn-scaff_21003_1-p68538[illegible]	C8	5	4	B
19820	Bn-N18-p4536937	Bn-C8-p3357712	Bn-scaff_21003_1-p65476[illegible]	C8	5	4	B
19821	Bn-N18-p4537067	Bn-C8-p3357842	Bn-scaff_21003_1-p65463[illegible]	C8	5	4	B
19822	Bn-N18-p4537418	Bn-C8-p3358193	Bn-scaff_21003_1-p65428[illegible]	C8	5	4	B
19823	Bn-N18-p4540791	Bn-C8-p3361564	Bn-scaff_21003_1-p65091[illegible]	C8	5	4	B
19824	Bn-N18-p4549254	Bn-C8-p3362177	Bn-scaff_21003_1-p65029[illegible]	C8	5	4	B
19825	Bn-N18-p4549303	Bn-C8-p3362226	Bn-scaff_21003_1-p65024[illegible]	C8	5	4	B
19826	Bn-N18-p4552442	Bn-C8-p3365379	Bn-scaff_21003_1-p64709[illegible]	C8	5	4	B
19827	Bn-N18-p4553422	Bn-C8-p3366359	Bn-scaff_21003_1-p64611[illegible]	C8	5	4	B
19828	Bn-N18-p4554315	Bn-C8-p3367252	Bn-scaff_21003_1-p64522[illegible]	C8	5	4	B
19829	Bn-N18-p4578521	Bn-C8-p3375738	Bn-scaff_21003_1-p63673[illegible]	C8	5	4	B
19830	Bn-N18-p4697479	Bn-C8-p3554751	Bn-scaff_21003_1-p45772[illegible]	C8	5	4	B
19831	Bn-N18-p4964195	Bn-C8-p3686118	Bn-scaff_21003_1-p32635[illegible]	C8	5	4	B
19832	Bn-N18-p5005949	Bn-C8-p3727014	Bn-scaff_21003_1-p28546[illegible]	C8	5	4	B
19833	Bn-N18-p5045431	Bn-C8-p3767506	Bn-scaff_21003_1-p24496[illegible]	C8	5	4	B
19834	Bn-N18-p5145350	Bn-C8-p3833562	Bn-scaff_21003_1-p17891[illegible]	C8	5	4	B
19835	Bn-N18-p5346941	Bn-C8-p4105279	Bn-scaff_16677_1-p29691[illegible]	C8	5	4	B
19836	Bn-N18-p5618697	Bn-C8-p4368490	Bn-scaff_25981_1-p24056[illegible]	C8	5	4	B
19837	Bn-N18-p5655056	Bn-C8-p4421180	Bn-scaff_25981_1-p18787[illegible]	C8	5	4	B
19838	Bn-N18-p5852673	Bn-C8-p4677650	Bn-scaff_16158_1-p68492	C8	5	4	B
19839	Bn-N18-p5853205	Bn-C8-p4678192	Bn-scaff_16158_1-p69034	C8	5	4	B
19840	Bn-N18-p5948320	Bn-C8-p4780661	Bn-scaff_16158_1-p17150[illegible]	C8	5	4	B

19841	Bn-N18-p6031234	Bn-C8-p4836388	Bn-scaff_16158_1-p22723(	C8	5	4	B
19842	Bn-N18-p6309372	Bn-C8-p5125053	Bn-scaff_23993_1-p82034	C8	5	4	B
19843	Bn-N18-p8723533	Bn-C8-p7434793	Bn-scaff_16766_1-p15059-	C8	5	4	B
19844	Bn-N18-p8742917	Bn-C8-p7454684	Bn-scaff_16766_1-p17048!	C8	5	4	B
19845	Bn-N18-p8762388	Bn-C8-p7463260	Bn-scaff_16766_1-p17906:	C8	5	4	B
19846	Bn-N18-p8783024	Bn-C8-p7484075	Bn-scaff_16766_1-p19987(	C8	5	4	B
19847	Bn-N18-p8788652	Bn-C8-p7489704	Bn-scaff_16766_1-p20550!	C8	5	4	B
19848	Bn-N18-p8795016	Bn-C8-p7496069	Bn-scaff_16766_1-p21187(	C8	5	4	B
19849	Bn-N18-p8807682	Bn-C8-p7508722	Bn-scaff_16766_1-p22452:	C8	5	4	B
19850	Bn-N18-p9718013	Bn-C8-p8246482	Bn-scaff_17591_1-p85044:	C8	5	4	B
19851	Bn-N18-p9806718	Bn-C8-p8338846	Bn-scaff_17591_1-p75807	C8	5	4	B
19852	Bn-N18-p9949799	Bn-C8-p8478811	Bn-scaff_17591_1-p61811:	C8	5	4	B
19853	Bn-N18-p9998787	Bn-C8-p8528389	Bn-scaff_17591_1-p56853-	C8	5	4	B
19854	Bn-Scaffold02916-p2904	Bn-C8-p13447647	Bn-scaff_21250_1-p41281(	C8	5	4	B
19855	Bn-Scaffold06402-p808	Bn-C8-p4371136	Bn-scaff_25981_1-p23792:	C8	5	4	B
19856	Bn-N18-p15662944	Bn-C8-p13665548	Bn-scaff_24208_1-p74641	C8	6	4.8	B
19857	Bn-N18-p15850482	Bn-C8-p13822836	Bn-scaff_19104_1-p66262-	C8	6	4.8	B
19858	Bn-N18-p15856486	Bn-C8-p13828712	Bn-scaff_19104_1-p65674t	C8	6	4.8	B
19859	Bn-N18-p15858004	Bn-C8-p13830053	Bn-scaff_19104_1-p65540	C8	6	4.8	B
19860	Bn-N18-p15962349	Bn-C8-p13936030	Bn-scaff_19104_1-p54943(	C8	6	4.8	B
19861	Bn-N18-p16637821	Bn-C8-p14639728	Bn-scaff_17257_1-p15416!	C8	6	4.8	B
19862	Bn-N18-p16834441	Bn-C8-p14807279	Bn-scaff_26506_1-p29218!	C8	6	4.8	B
19863	Bn-N18-p17144319	Bn-C8-p15137164	Bn-scaff_20497_1-p37597	C8	6	4.8	B
19864	Bn-N18-p17145783	Bn-C8-p15138630	Bn-scaff_20497_1-p39063	C8	6	4.8	B
19865	Bn-N18-p17150411	Bn-C8-p15143257	Bn-scaff_20497_1-p43690	C8	6	4.8	B
19866	Bn-N18-p17173466	Bn-C8-p15171163	Bn-scaff_20497_1-p71596	C8	6	4.8	B
19867	Bn-N18-p17173626	Bn-C8-p15171323	Bn-scaff_20497_1-p71756	C8	6	4.8	B
19868	Bn-N18-p17192531	Bn-C8-p15189523	Bn-scaff_20497_1-p89956	C8	6	4.8	B
19869	Bn-N18-p17192582	Bn-C8-p15189574	Bn-scaff_20497_1-p90007	C8	6	4.8	B
19870	Bn-N18-p17193291	Bn-C8-p15190642	Bn-scaff_20497_1-p91075	C8	6	4.8	B
19871	Bn-N18-p17256451	Bn-C8-p15247158	Bn-scaff_18275_1-p10677:	C8	6	4.8	B
19872	Bn-N18-p17377487	Bn-C8-p15352984	Bn-scaff_18275_1-p11735-	C8	6	4.8	B
19873	Bn-N18-p17483229	Bn-C8-p15382325	Bn-scaff_18275_1-p12028!	C8	6	4.8	B
19874	Bn-N18-p17490884	Bn-C8-p15389959	Bn-scaff_18275_1-p12105:	C8	6	4.8	B
19875	Bn-N18-p17523000	Bn-A03-p6934438	Bn-A03-p6934438	C8	6	4.8	B
19876	Bn-N18-p17530072	Bn-A03-p6909237	Bn-A03-p6909237	C8	6	4.8	B
19877	Bn-N18-p17531568	Bn-A03-p6907743	Bn-A03-p6907743	C8	6	4.8	B
19878	Bn-N18-p19251163	Bn-C8-p17004422	Bn-scaff_15699_1-p12341	C8	7	5.6	B
19879	Bn-N8-p10038893	Bn-A08-p10394354	Bn-A08-p10394354	C8	7	5.6	B
19880	Bn-N8-p14403087	Bn-A08-p14694395	Bn-A08-p14694395	C8	7	5.6	B
19881	Bn-N18-p20024510	Bn-C8-p17859005	Bn-scaff_19242_1-p42133!	C8	8	7.2	B
19882	Bn-N18-p20049772	Bn-C8-p17890231	Bn-scaff_19242_1-p39010!	C8	8	7.2	B
19883	Bn-N18-p20934069	Bn-Scaffold01219-p8	Bn-scaff_26859_1-p8813	C8	9	8.8	B
19884	Bn-N18-p20996128	Bn-Scaffold006825-p	Bn-Scaffold006825-p88	C8	9	8.8	B
19885	Bn-N8-p20539877	Bn-A08-p20480348	Bn-A08-p20480348	C8	9	8.8	B
19886	Bn-N18-p21210849	Bn-C8-p18806260	Bn-scaff_17227_1-p29995(	C8	10	9.6	B
19887	Bn-N18-p21530494	Bn-C8-p19133238	Bn-scaff_17227_1-p62693-	C8	10	9.6	B
19888	Bn-N6-p3405585	Bn-C8-p19207955	Bn-scaff_17227_1-p70165:	C8	11	11.2	0
19889	Bn-N8-p20305257	Bn-C8-p19206552	Bn-scaff_17227_1-p70024t	C8	11	11.2	B

19890	Bn-N8-p20308092	Bn-A05-p8327216	Bn-A05-p8327216	C8	11	11.2	B
19891	Bn-N18-p22264975	Bn-C8-p19887099	Bn-scaff_17227_1-p13807	C8	12	15.2	B
19892	Bn-N18-p22441567	Bn-C8-p20069131	Bn-scaff_21786_1-p12735	C8	13	16	B
19893	Bn-N18-p22442183	Bn-C8-p20069746	Bn-scaff_21786_1-p12797	C8	13	16	B
19894	Bn-N18-p22462785	Bn-C8-p20091110	Bn-scaff_21786_1-p14933	C8	14	17.6	B
19895	Bn-N18-p22471550	Bn-C8-p20099875	Bn-scaff_21786_1-p15810	C8	14	17.6	B
19896	Bn-N18-p22473927	Bn-C8-p20102242	Bn-scaff_21786_1-p16047	C8	14	17.6	B
19897	Bn-N18-p22475276	Bn-C8-p20103593	Bn-scaff_21786_1-p16182	C8	14	17.6	B
19898	Bn-N18-p22552608	Bn-Scaffold00957-p1	Bn-scaff_16962_1-p57843	C8	14	17.6	B
19899	Bn-N18-p22559135	Bn-Scaffold00957-p3	Bn-scaff_16962_1-p56507	C8	14	17.6	B
19900	Bn-N18-p22568653	Bn-C8-p20108734	Bn-scaff_16962_1-p55675	C8	14	17.6	B
19901	Bn-N18-p22570835	Bn-C8-p20110448	Bn-scaff_16962_1-p55504	C8	14	17.6	B
19902	Bn-N18-p22571459	Bn-C8-p20111072	Bn-scaff_16962_1-p55441	C8	14	17.6	B
19903	Bn-N18-p22581894	Bn-C8-p20121992	Bn-scaff_16962_1-p54349	C8	14	17.6	B
19904	Bn-N18-p22582574	Bn-C8-p20122673	Bn-scaff_16962_1-p54281	C8	14	17.6	B
19905	Bn-N18-p22599399	Bn-C8-p20146343	Bn-scaff_16962_1-p51914	C8	14	17.6	B
19906	Bn-N18-p22601888	Bn-C8-p20148817	Bn-scaff_16962_1-p51667	C8	14	17.6	B
19907	Bn-N18-p22610611	Bn-C8-p20158547	Bn-scaff_16962_1-p50694	C8	14	17.6	B
19908	Bn-N18-p22612739	Bn-C8-p20160685	Bn-scaff_16962_1-p50480	C8	14	17.6	B
19909	Bn-N18-p22620163	Bn-C8-p20171144	Bn-scaff_16962_1-p49434	C8	14	17.6	B
19910	Bn-N18-p22663905	Bn-C8-p20211048	Bn-scaff_16962_1-p45444	C8	14	17.6	B
19911	Bn-N18-p22715061	Bn-Scaffold00758-p6	Bn-scaff_16962_1-p35904	C8	14	17.6	B
19912	Bn-N18-p22722325	Bn-Scaffold00758-p6	Bn-scaff_16962_1-p35191	C8	14	17.6	B
19913	Bn-N18-p22796016	Bn-C8-p20354193	Bn-scaff_16231_1-p22744	C8	14	17.6	B
19914	Bn-Scaffold02060-p1367	Bn-C8-p20321716	Bn-scaff_16231_1-p23069	C8	14	17.6	B
19915	Bn-Scaffold02060-p4380	Bn-C8-p20324746	Bn-scaff_16231_1-p23039	C8	14	17.6	B
19916	Bn-N18-p22891395	Bn-C8-p20415408	Bn-scaff_16231_1-p22132	C8	15	20	B
19917	Bn-N18-p23909536	Bn-C8-p21353685	Bn-scaff_16231_1-p12749	C8	16	24.9	B
19918	Bn-N18-p23927638	Bn-C8-p21371789	Bn-scaff_16231_1-p12568	C8	17	28.9	B
19919	Bn-N18-p23928188	Bn-C8-p21372339	Bn-scaff_16231_1-p12563	C8	17	28.9	B
19920	Bn-N18-p23948767	Bn-C8-p21392769	Bn-scaff_16231_1-p12358	C8	17	28.9	B
19921	Bn-N18-p23952842	Bn-C8-p21396843	Bn-scaff_16231_1-p12318	C8	17	28.9	B
19922	Bn-N18-p23995115	Bn-C8-p21439595	Bn-scaff_16231_1-p11890	C8	18	30.5	B
19923	Bn-N18-p23995226	Bn-C8-p21439737	Bn-scaff_16231_1-p11889	C8	18	30.5	B
19924	Bn-N18-p23997136	Bn-C8-p21441651	Bn-scaff_16231_1-p11869	C8	18	30.5	B
19925	Bn-N18-p23997311	Bn-C8-p21441825	Bn-scaff_16231_1-p11868	C8	18	30.5	B
19926	Bn-N18-p24003222	Bn-C8-p21443364	Bn-scaff_16231_1-p11852	C8	18	30.5	B
19927	Bn-N18-p24003766	Bn-C8-p21443882	Bn-scaff_16231_1-p11847	C8	18	30.5	B
19928	Bn-N18-p24004612	Bn-C8-p21444732	Bn-scaff_16231_1-p11839	C8	18	30.5	0
19929	Bn-N18-p24007532	Bn-C8-p21447656	Bn-scaff_16231_1-p11809	C8	18	30.5	B
19930	Bn-N18-p24011108	Bn-C8-p21451543	Bn-scaff_16231_1-p11771	C8	18	30.5	B
19931	Bn-N18-p24011236	Bn-C8-p21451671	Bn-scaff_16231_1-p11769	C8	18	30.5	B
19932	Bn-N18-p24011698	Bn-C8-p21452133	Bn-scaff_16231_1-p11765	C8	18	30.5	B
19933	Bn-N18-p24181807	Bn-C8-p21602272	Bn-scaff_16231_1-p10263	C8	18	30.5	B
19934	Bn-N18-p24193993	Bn-C8-p21614432	Bn-scaff_16231_1-p10142	C8	18	30.5	B
19935	Bn-N18-p24196802	Bn-C8-p21617252	Bn-scaff_16231_1-p10113	C8	18	30.5	B
19936	Bn-N18-p24233226	Bn-C8-p21645686	Bn-scaff_16231_1-p98296	C8	18	30.5	B
19937	Bn-N18-p24402776	Bn-C8-p21778490	Bn-scaff_16231_1-p85015	C8	18	30.5	B
19938	Bn-N18-p24433335	Bn-C8-p21833006	Bn-scaff_16231_1-p79564	C8	19	32.1	B

19939	Bn-N18-p24433445	Bn-C8-p21833129	Bn-scaff_16231_1-p79551{	C8	19	32.1	B
19940	Bn-N18-p24433501	Bn-C8-p21833185	Bn-scaff_16231_1-p795462	C8	19	32.1	B
19941	Bn-N18-p24433888	Bn-C8-p21833572	Bn-scaff_16231_1-p795075	C8	19	32.1	B
19942	Bn-N18-p24436113	Bn-C8-p21835830	Bn-scaff_16231_1-p792817	C8	19	32.1	B
19943	Bn-N18-p24447388	Bn-C8-p21842387	Bn-scaff_16231_1-p78626(	C8	19	32.1	B
19944	Bn-N18-p24502165	Bn-C8-p36014854	Bn-scaff_16197_1-p38657{	C8	19	32.1	B
19945	Bn-N18-p24481618	Bn-C8-p21881626	Bn-scaff_16231_1-p747021	C8	20	32.9	B
19946	Bn-N18-p24521394	Bn-C8-p21912885	Bn-scaff_16231_1-p715762	C8	20	32.9	B
19947	Bn-N18-p24561072	Bn-C8-p21950048	Bn-scaff_16231_1-p678599	C8	20	32.9	B
19948	Bn-N18-p24582718	Bn-C8-p21965925	Bn-scaff_16231_1-p662722	C8	20	32.9	B
19949	Bn-N18-p24988414	Bn-C8-p22331853	Bn-scaff_16231_1-p29679{	C8	21	34.5	B
19950	Bn-N18-p25074603	Bn-C8-p22423587	Bn-scaff_16231_1-p20506(	C8	21	34.5	B
19951	Bn-N8-p18545244	Bn-A08-p18789560	Bn-A08-p18789560	C8	21	34.5	B
19952	Bn-N18-p26041888	Bn-Scaffold01074-p6	Bn-scaff_17565_1-p6212	C8	22	37.7	B
19953	Bn-N8-p18101702	Bn-A08-p18356257	Bn-A08-p18356257	C8	22	37.7	B
19954	Bn-N18-p26132328	Bn-C8-p23430903	Bn-scaff_15923_1-p489042	C8	23	40.1	B
19955	Bn-N18-p26587389	Bn-C8-p23873394	Bn-scaff_15923_1-p46551	C8	24	42.5	B
19956	Bn-N18-p26913742	Bn-C8-p24127988	Bn-scaff_17807_1-p19205:	C8	25	43.3	B
19957	Bn-N18-p26946587	Bn-C8-p24155362	Bn-scaff_17807_1-p16467{	C8	25	43.3	B
19958	Bn-N18-p27842113	Bn-C8-p24973155	Bn-scaff_16174_1-p65301!	C8	25	43.3	B
19959	Bn-N8-p14464609	Bn-C4-p25880197	Bn-scaff_16625_1-p12440{	C8	25	43.3	B
19960	Bn-N18-p28274042	Bn-C8-p25393055	Bn-scaff_16174_1-p107291	C8	26	45.7	B
19961	Bn-N18-p28274891	Bn-C8-p25393902	Bn-scaff_16174_1-p10737(	C8	26	45.7	B
19962	Bn-N18-p28379898	Bn-C8-p25505275	Bn-scaff_16174_1-p11851:	C8	26	45.7	B
19963	Bn-N18-p28398253	Bn-C8-p25525144	Bn-scaff_16174_1-p12050(	C8	26	45.7	B
19964	Bn-N18-p28430362	Bn-C8-p25557305	Bn-scaff_16174_1-p12371(	C8	26	45.7	B
19965	Bn-N18-p28440677	Bn-C8-p25567799	Bn-scaff_16174_1-p12476!	C8	26	45.7	B
19966	Bn-N18-p28448210	Bn-C8-p25575372	Bn-scaff_16174_1-p12552:	C8	26	45.7	B
19967	Bn-N18-p28458828	Bn-C8-p25585274	Bn-scaff_16174_1-p12651:	C8	26	45.7	B
19968	Bn-N18-p28484962	Bn-C8-p25614364	Bn-scaff_16174_1-p129422	C8	26	45.7	B
19969	Bn-N18-p28498480	Bn-C8-p25629017	Bn-scaff_16174_1-p130887	C8	26	45.7	B
19970	Bn-N18-p28498553	Bn-C8-p25629089	Bn-scaff_16174_1-p13089	C8	26	45.7	B
19971	Bn-N18-p28498849	Bn-C8-p25629385	Bn-scaff_16174_1-p13092	C8	26	45.7	B
19972	Bn-N18-p28501268	Bn-C8-p25631766	Bn-scaff_16174_1-p131162	C8	26	45.7	B
19973	Bn-N18-p28509221	Bn-C8-p25641089	Bn-scaff_16174_1-p13209	C8	26	45.7	B
19974	Bn-N18-p28521872	Bn-C8-p25655339	Bn-scaff_16174_1-p13351{	C8	26	45.7	B
19975	Bn-N18-p28522018	Bn-C8-p25655485	Bn-scaff_16174_1-p13353	C8	26	45.7	B
19976	Bn-N18-p28529029	Bn-C8-p25662404	Bn-scaff_16174_1-p13422(	C8	26	45.7	B
19977	Bn-N18-p28566956	Bn-C8-p25697764	Bn-scaff_16174_1-p137762	C8	26	45.7	B
19978	Bn-N18-p28574552	Bn-C8-p25706196	Bn-scaff_16174_1-p13860!	C8	26	45.7	B
19979	Bn-N18-p28644980	Bn-C8-p25765120	Bn-scaff_16174_1-p14449{	C8	26	45.7	B
19980	Bn-N18-p28707191	Bn-C8-p25825655	Bn-scaff_16174_1-p150551	C8	26	45.7	B
19981	Bn-N18-p28753715	Bn-C8-p25871286	Bn-scaff_16174_1-p15511	C8	26	45.7	B
19982	Bn-N18-p28768179	Bn-C5-p41908886	Bn-scaff_18901_1-p13731	C8	26	45.7	B
19983	Bn-N6-p9312634	Bn-C8-p25353647	Bn-scaff_16174_1-p10335(	C8	26	45.7	B
19984	Bn-N18-p28819264	Bn-C8-p25926212	Bn-scaff_16770_1-p113742	C8	27	46.5	B
19985	Bn-N18-p29793921	Bn-C8-p26871466	Bn-scaff_16770_1-p20826{	C8	27	46.5	B
19986	Bn-N18-p30058864	Bn-C8-p27022493	Bn-scaff_16770_1-p223371	C8	27	46.5	B
19987	Bn-N18-p30465380	Bn-C8-p27775136	Bn-scaff_16770_1-p29863!	C8	28	47.3	B

19988	Bn-N18-p30465673	Bn-C8-p27774843	Bn-scaff_16770_1-p29860(	C8	28	47.3	B
19989	Bn-N18-p30466185	Bn-C8-p27774331	Bn-scaff_16770_1-p29855(	C8	28	47.3	B
19990	Bn-N18-p30466252	Bn-C8-p27774264	Bn-scaff_16770_1-p29854(	C8	28	47.3	B
19991	Bn-N18-p30466743	Bn-C8-p27773773	Bn-scaff_16770_1-p29849(	C8	28	47.3	B
19992	Bn-N18-p30502022	Bn-C8-p27735461	Bn-scaff_16770_1-p29466(	C8	29	48.1	B
19993	Bn-N18-p30628492	Bn-C8-p27594044	Bn-scaff_16770_1-p28052(	C8	29	48.1	B
19994	Bn-N18-p30754016	Bn-C3-p51141028	Bn-scaff_18849_1-p10190(	C8	29	48.1	B
19995	Bn-N18-p31188241	Bn-C8-p28178287	Bn-scaff_16770_1-p33895(	C8	30	48.9	B
19996	Bn-N18-p31877221	Bn-C8-p28860028	Bn-scaff_16770_1-p40712(	C8	31	54.6	A
19997	Bn-N18-p31905866	Bn-C8-p28886198	Bn-scaff_16770_1-p40974(	C8	31	54.6	A
19998	Bn-N18-p31979864	Bn-C8-p28977192	Bn-scaff_16770_1-p41884(	C8	31	54.6	A
19999	Bn-N18-p31996276	Bn-C8-p28993631	Bn-scaff_16770_1-p42048(	C8	31	54.6	A
20000	Bn-N18-p32039448	Bn-C8-p29035752	Bn-scaff_16770_1-p42469(	C8	31	54.6	A
20001	Bn-N18-p32039661	Bn-C8-p29035965	Bn-scaff_16770_1-p42471(	C8	31	54.6	A
20002	Bn-N18-p32059058	Bn-C8-p29061976	Bn-scaff_16770_1-p42731(	C8	31	54.6	A
20003	Bn-N18-p32059164	Bn-C8-p29062082	Bn-scaff_16770_1-p42732(	C8	31	54.6	A
20004	Bn-N18-p32059180	Bn-C8-p29062098	Bn-scaff_16770_1-p42733(	C8	31	54.6	A
20005	Bn-N18-p32059344	Bn-C8-p29062262	Bn-scaff_16770_1-p42734(	C8	31	54.6	A
20006	Bn-N18-p32059519	Bn-C8-p29062437	Bn-scaff_16770_1-p42736(	C8	31	54.6	A
20007	Bn-N18-p32066808	Bn-C8-p29068278	Bn-scaff_16770_1-p42794(	C8	31	54.6	A
20008	Bn-N18-p32071215	Bn-C8-p29072685	Bn-scaff_16770_1-p42839(	C8	31	54.6	A
20009	Bn-N18-p32082324	Bn-C4-p41118959	Bn-scaff_15798_1-p31042(	C8	31	54.6	A
20010	Bn-N18-p32083896	Bn-C8-p29085318	Bn-scaff_16770_1-p42965(	C8	31	54.6	A
20011	Bn-N18-p32084088	Bn-C8-p29085510	Bn-scaff_16770_1-p42967(	C8	31	54.6	A
20012	Bn-N18-p32115345	Bn-C8-p29120618	Bn-scaff_16770_1-p43318(	C8	32	55.4	A
20013	Bn-N18-p32130575	Bn-C8-p29126962	Bn-scaff_16770_1-p43381(	C8	32	55.4	A
20014	Bn-N18-p32150422	Bn-C8-p29141982	Bn-scaff_16770_1-p43531(	C8	32	55.4	A
20015	Bn-N18-p32167058	Bn-C8-p29163634	Bn-scaff_16770_1-p43748(	C8	32	55.4	A
20016	Bn-N18-p32167463	Bn-C8-p29164039	Bn-scaff_16770_1-p43752(	C8	32	55.4	A
20017	Bn-N18-p32167804	Bn-C8-p29164408	Bn-scaff_16770_1-p43756(	C8	32	55.4	A
20018	Bn-N18-p32168140	Bn-C8-p29164744	Bn-scaff_16770_1-p43759(	C8	32	55.4	A
20019	Bn-N18-p32168216	Bn-C8-p29164820	Bn-scaff_16770_1-p43760(	C8	32	55.4	A
20020	Bn-N18-p32184486	Bn-C8-p29172648	Bn-scaff_16770_1-p43838(	C8	32	55.4	A
20021	Bn-N18-p32191439	Bn-C8-p29179644	Bn-scaff_16770_1-p43908(	C8	32	55.4	A
20022	Bn-N18-p32234174	Bn-C8-p29194390	Bn-scaff_17854_1-p11838	C8	32	55.4	A
20023	Bn-N18-p32234609	Bn-C8-p29194825	Bn-scaff_17854_1-p12273	C8	32	55.4	A
20024	Bn-N18-p32357196	Bn-C8-p29299889	Bn-scaff_28637_1-p15131	C8	33	56.2	A
20025	Bn-N18-p32383799	Bn-C8-p29323029	Bn-scaff_28637_1-p38271	C8	33	56.2	A
20026	Bn-N18-p32416574	Bn-C8-p29395056	Bn-scaff_16200_1-p15598(	C8	33	56.2	A
20027	Bn-N18-p32416902	Bn-C8-p29395378	Bn-scaff_16200_1-p15566(	C8	33	56.2	A
20028	Bn-N18-p32478650	Bn-C8-p29450647	Bn-scaff_16200_1-p10039(	C8	33	56.2	A
20029	Bn-N18-p32488398	Bn-C8-p29459988	Bn-scaff_16200_1-p91053	C8	33	56.2	A
20030	Bn-N18-p32579095	Bn-C8-p29537277	Bn-scaff_16200_1-p13764	C8	33	56.2	A
20031	Bn-N18-p32901400	Bn-C8-p29872044	Bn-scaff_16361_1-p23612(	C8	33	56.2	0
20032	Bn-N18-p32912430	Bn-C8-p29875796	Bn-scaff_16361_1-p23987(	C8	33	56.2	A
20033	Bn-N18-p32957483	Bn-C8-p29954237	Bn-scaff_16361_1-p31831(	C8	33	56.2	A
20034	Bn-N18-p32958087	Bn-C8-p29954846	Bn-scaff_16361_1-p31892(	C8	33	56.2	A
20035	Bn-N18-p32958308	Bn-C8-p29955105	Bn-scaff_16361_1-p31918(	C8	33	56.2	A
20036	Bn-N18-p32966327	Bn-C8-p29966197	Bn-scaff_16361_1-p33027(	C8	33	56.2	A

20037	Bn-N18-p33027806	Bn-C8-p30046187	Bn-scaff_16361_1-p41026	C8	33	56.2	A
20038	Bn-N18-p33034502	Bn-C8-p30051454	Bn-scaff_16361_1-p41553	C8	33	56.2	A
20039	Bn-N18-p33042017	Bn-C8-p30059011	Bn-scaff_16361_1-p42309	C8	33	56.2	A
20040	Bn-N18-p33081369	Bn-C8-p30070434	Bn-scaff_16361_1-p43451	C8	33	56.2	A
20041	Bn-N18-p33087108	Bn-C8-p30075230	Bn-scaff_16361_1-p43930	C8	33	56.2	A
20042	Bn-N18-p33100705	Bn-C8-p30086971	Bn-scaff_16361_1-p45105	C8	33	56.2	A
20043	Bn-N18-p33127147	Bn-C8-p30121016	Bn-scaff_16361_1-p48509	C8	33	56.2	A
20044	Bn-N18-p33155237	Bn-C8-p30142368	Bn-scaff_16361_1-p50644	C8	33	56.2	A
20045	Bn-N18-p33228387	Bn-C8-p30182424	Bn-scaff_16361_1-p54650	C8	33	56.2	A
20046	Bn-N9-p30443354	Bn-C8-p29862284	Bn-scaff_16361_1-p22636	C8	33	56.2	A
20047	Bn-N9-p30613806	Bn-C8-p30144240	Bn-scaff_16361_1-p50831	C8	33	56.2	A
20048	Unknown	Bn-C8-p29702388	Bn-scaff_16361_1-p66467	C8	33	56.2	A
20049	Bn-N18-p33471666	Bn-C8-p30430808	Bn-scaff_16361_1-p79488	C8	34	57.8	A
20050	Bn-N18-p33602937	Bn-C8-p30537649	Bn-scaff_16361_1-p90172	C8	34	57.8	A
20051	Bn-N18-p33631903	Bn-C8-p30565985	Bn-scaff_16361_1-p93006	C8	34	57.8	A
20052	Bn-N18-p33632414	Bn-C8-p30566471	Bn-scaff_16361_1-p93055	C8	34	57.8	A
20053	Bn-N18-p33632491	Bn-C8-p30566539	Bn-scaff_16361_1-p93061	C8	34	57.8	A
20054	Bn-N18-p33632592	Bn-C8-p30566640	Bn-scaff_16361_1-p93071	C8	34	57.8	A
20055	Bn-N18-p33632730	Bn-C8-p30566778	Bn-scaff_16361_1-p93085	C8	34	57.8	A
20056	Bn-N18-p33635254	Bn-C8-p30569313	Bn-scaff_16361_1-p93339	C8	34	57.8	A
20057	Bn-Scaffold05866-p383	Bn-C8-p30395520	Bn-scaff_16361_1-p75959	C8	34	57.8	A
20058	Bn-N18-p33709219	Bn-C8-p30643825	Bn-scaff_16361_1-p10079	C8	35	58.6	0
20059	Bn-N18-p33748571	Bn-C8-p30692226	Bn-scaff_16361_1-p10563	C8	35	58.6	A
20060	Bn-N18-p33748922	Bn-C8-p30692578	Bn-scaff_16361_1-p10566	C8	35	58.6	A
20061	Bn-N18-p33767888	Bn-C8-p30708636	Bn-scaff_16361_1-p10727	C8	35	58.6	A
20062	Bn-N9-p31117314	Bn-C8-p30689454	Bn-scaff_16361_1-p10535	C8	35	58.6	A
20063	Bn-N18-p34020085	Bn-C8-p30959960	Bn-scaff_16361_1-p13240	C8	36	59.4	A
20064	Bn-N18-p34046220	Bn-C8-p30979331	Bn-scaff_16361_1-p13434	C8	36	59.4	A
20065	Bn-N18-p34081920	Bn-C8-p31013961	Bn-scaff_16361_1-p13780	C8	36	59.4	A
20066	Bn-N18-p34090563	Bn-C8-p31015767	Bn-scaff_16361_1-p13798	C8	36	59.4	A
20067	Bn-N18-p34090875	Bn-C8-p31016076	Bn-scaff_16361_1-p13801	C8	36	59.4	A
20068	Bn-N18-p34091077	Bn-C8-p31016278	Bn-scaff_16361_1-p13803	C8	36	59.4	A
20069	Bn-N18-p34091158	Bn-C8-p31016359	Bn-scaff_16361_1-p13804	C8	36	59.4	A
20070	Bn-N18-p34098757	Bn-C8-p31023043	Bn-scaff_16361_1-p13871	C8	37	60.2	A
20071	Bn-N18-p34102368	Bn-C8-p31026633	Bn-scaff_16361_1-p13907	C8	37	60.2	A
20072	Bn-N18-p34122556	Bn-C8-p31038829	Bn-scaff_16361_1-p14029	C8	38	61	A
20073	Bn-N18-p34167166	Bn-C8-p31102519	Bn-scaff_16361_1-p14665	C8	38	61	A
20074	Bn-N18-p34168513	Bn-C8-p31103866	Bn-scaff_16361_1-p14679	C8	38	61	A
20075	Bn-N18-p34197768	Bn-C8-p31132777	Bn-scaff_16361_1-p14968	C8	38	61	A
20076	Bn-N18-p34198577	Bn-C8-p31133587	Bn-scaff_16361_1-p14976	C8	38	61	A
20077	Bn-N18-p34204500	Bn-C8-p31139510	Bn-scaff_16361_1-p15035	C8	38	61	A
20078	Bn-N18-p34205080	Bn-C8-p31140090	Bn-scaff_16361_1-p15041	C8	38	61	A
20079	Bn-N18-p34205104	Bn-C8-p31140114	Bn-scaff_16361_1-p15041	C8	38	61	A
20080	Bn-N18-p34206598	Bn-C8-p31141608	Bn-scaff_16361_1-p15056	C8	38	61	A
20081	Bn-N18-p34209643	Bn-C8-p31144653	Bn-scaff_16361_1-p15087	C8	38	61	A
20082	Bn-N18-p34222271	Bn-C8-p31156506	Bn-scaff_16361_1-p15205	C8	38	61	A
20083	Bn-N18-p34223157	Bn-C8-p31157199	Bn-scaff_16361_1-p15212	C8	38	61	A
20084	Bn-N18-p34223464	Bn-C8-p31157506	Bn-scaff_16361_1-p15215	C8	38	61	A
20085	Bn-N18-p34248117	Bn-C8-p31184710	Bn-scaff_16361_1-p15487	C8	38	61	A

20086	Bn-N18-p34264845	Bn-C8-p31203317	Bn-scaff_16361_1-p15673[illegible]	C8	38	61	A
20087	Bn-N18-p34266593	Bn-C8-p31205064	Bn-scaff_16361_1-p15691[illegible]	C8	38	61	A
20088	Bn-N18-p34267253	Bn-C8-p31205725	Bn-scaff_16361_1-p15698[illegible]	C8	38	61	A
20089	Bn-N18-p34268387	Bn-C8-p31206859	Bn-scaff_16361_1-p15709[illegible]	C8	38	61	A
20090	Bn-N18-p34647062	Bn-C8-p31579687	Bn-scaff_16361_1-p19437[illegible]	C8	39	62.6	A
20091	Bn-N18-p34672855	Bn-A09-p28985128	Bn-A09-p28985128	C8	39	62.6	A
20092	Bn-N18-p34675526	Bn-A09-p28987525	Bn-A09-p28987525	C8	39	62.6	A
20093	Bn-N18-p34679616	Bn-C8-p31629832	Bn-scaff_16361_1-p19939[illegible]	C8	39	62.6	A
20094	Bn-N18-p34729491	Bn-C8-p31683741	Bn-scaff_16361_1-p20478[illegible]	C8	39	62.6	A
20095	Bn-N18-p34734011	Bn-C8-p31688245	Bn-scaff_16361_1-p20523[illegible]	C8	39	62.6	A
20096	Bn-N18-p34762202	Bn-C8-p31716178	Bn-scaff_16361_1-p20802[illegible]	C8	39	62.6	A
20097	Bn-N18-p34936297	Bn-C8-p31892623	Bn-scaff_16361_1-p22567[illegible]	C8	39	62.6	A
20098	Bn-N18-p34942668	Bn-C8-p31898993	Bn-scaff_16361_1-p22630[illegible]	C8	39	62.6	A
20099	Bn-N18-p34948312	Bn-C8-p31904870	Bn-scaff_16361_1-p22689[illegible]	C8	39	62.6	A
20100	Bn-N18-p34952204	Bn-C8-p31909051	Bn-scaff_16361_1-p22731[illegible]	C8	39	62.6	A
20101	Bn-N18-p34972083	Bn-C8-p31926949	Bn-scaff_16361_1-p22910[illegible]	C8	39	62.6	A
20102	Bn-N18-p34972849	Bn-C8-p31927716	Bn-scaff_16361_1-p22917[illegible]	C8	39	62.6	A
20103	Bn-N18-p35040928	Bn-C8-p31986390	Bn-scaff_16361_1-p23504[illegible]	C8	39	62.6	A
20104	Bn-N18-p35044131	Bn-C8-p31989502	Bn-scaff_16361_1-p23535[illegible]	C8	39	62.6	A
20105	Bn-N18-p35044616	Bn-C8-p31989994	Bn-scaff_16361_1-p23540[illegible]	C8	39	62.6	A
20106	Bn-N18-p35053164	Bn-C8-p31993294	Bn-scaff_16361_1-p23573[illegible]	C8	39	62.6	A
20107	Bn-N18-p35065613	Bn-C8-p32002263	Bn-scaff_16361_1-p23663[illegible]	C8	39	62.6	A
20108	Bn-N18-p35115181	Bn-Scaffold03985-p7[illegible]	Bn-scaff_22788_1-p739	C8	39	62.6	A
20109	Bn-N18-p35141385	Bn-C8-p32089936	Bn-scaff_16361_1-p24883[illegible]	C8	39	62.6	A
20110	Bn-N18-p35166068	Bn-C8-p32120186	Bn-scaff_16361_1-p25186[illegible]	C8	39	62.6	A
20111	Bn-N18-p35171156	Bn-C8-p32125534	Bn-scaff_16361_1-p25239[illegible]	C8	39	62.6	A
20112	Bn-N18-p35178875	Bn-C8-p32130915	Bn-scaff_16361_1-p25293[illegible]	C8	39	62.6	A
20113	Bn-N18-p35182353	Bn-C8-p32134392	Bn-scaff_16361_1-p25328[illegible]	C8	39	62.6	A
20114	Bn-N18-p35182456	Bn-C8-p32134496	Bn-scaff_16361_1-p25329[illegible]	C8	39	62.6	A
20115	Bn-N18-p35398843	Bn-C8-p32351611	Bn-scaff_16361_1-p27500[illegible]	C8	39	62.6	A
20116	Bn-N18-p35399382	Bn-C8-p32352150	Bn-scaff_16361_1-p27505[illegible]	C8	39	62.6	A
20117	Bn-N18-p35401975	Bn-C8-p32354743	Bn-scaff_16361_1-p27531[illegible]	C8	39	62.6	A
20118	Bn-N18-p35442223	Bn-C8-p32394987	Bn-scaff_16361_1-p27934[illegible]	C8	39	62.6	A
20119	Bn-N9-p32274681	Bn-A09-p29420099	Bn-A09-p29420099	C8	39	62.6	A
20120	Bn-N18-p35707246	Bn-C8-p32722330	Bn-scaff_16361_1-p31207[illegible]	C8	40	65.8	A
20121	Bn-N18-p35932322	Bn-C8-p32927370	Bn-scaff_16361_1-p33258[illegible]	C8	40	65.8	A
20122	Bn-N18-p35967462	Bn-C8-p32955768	Bn-scaff_16361_1-p33541[illegible]	C8	40	65.8	A
20123	Bn-N18-p35967497	Bn-C8-p32955803	Bn-scaff_16361_1-p33542[illegible]	C8	40	65.8	A
20124	Bn-N18-p35967970	Bn-C8-p32956270	Bn-scaff_16361_1-p33547[illegible]	C8	40	65.8	A
20125	Bn-N18-p35968663	Bn-C8-p32956964	Bn-scaff_16361_1-p33553[illegible]	C8	40	65.8	A
20126	Bn-N18-p36010220	Bn-C8-p32998968	Bn-scaff_16361_1-p33973[illegible]	C8	40	65.8	0
20127	Bn-N18-p36010384	Bn-C8-p32999132	Bn-scaff_16361_1-p33975[illegible]	C8	40	65.8	A
20128	Bn-N18-p36181089	Bn-C8-p33159725	Bn-scaff_16197_1-p32417[illegible]	C8	40	65.8	A
20129	Bn-N9-p32861877	Bn-A09-p30007298	Bn-A09-p30007298	C8	40	65.8	A
20130	Bn-N14-p28247635	Bn-C8-p34023225	Bn-scaff_16197_1-p23782[illegible]	C8	41	66.6	A
20131	Bn-N18-p36370965	Bn-C8-p33331256	Bn-scaff_16197_1-p30701[illegible]	C8	41	66.6	A
20132	Bn-N18-p36379175	Bn-C8-p33339466	Bn-scaff_16197_1-p30619[illegible]	C8	41	66.6	A
20133	Bn-N18-p36379604	Bn-C8-p33339895	Bn-scaff_16197_1-p30615[illegible]	C8	41	66.6	A
20134	Bn-N18-p36380037	Bn-C8-p33340328	Bn-scaff_16197_1-p30611[illegible]	C8	41	66.6	A

20135	Bn-N18-p36409361	Bn-C8-p33369838	Bn-scaff_16197_1-p303159	C8	41	66.6	A
20136	Bn-N18-p36411039	Bn-C8-p33375379	Bn-scaff_16197_1-p302605	C8	41	66.6	A
20137	Bn-N18-p36412313	Bn-C8-p33375950	Bn-scaff_16197_1-p302548	C8	41	66.6	A
20138	Bn-N18-p36413917	Bn-C8-p33377550	Bn-scaff_16197_1-p302388	C8	41	66.6	A
20139	Bn-N18-p36415320	Bn-C8-p33378914	Bn-scaff_16197_1-p302251	C8	41	66.6	A
20140	Bn-N18-p36415923	Bn-C8-p33379515	Bn-scaff_16197_1-p302191	C8	41	66.6	A
20141	Bn-N18-p36416686	Bn-C8-p33380274	Bn-scaff_16197_1-p302115	C8	41	66.6	A
20142	Bn-N18-p36418108	Bn-C8-p33381481	Bn-scaff_16197_1-p301995	C8	41	66.6	A
20143	Bn-N18-p36418297	Bn-C8-p33381670	Bn-scaff_16197_1-p301976	C8	41	66.6	A
20144	Bn-N18-p36446352	Bn-C8-p33425319	Bn-scaff_16197_1-p297611	C8	41	66.6	A
20145	Bn-N18-p36513244	Bn-C8-p33503993	Bn-scaff_16197_1-p289743	C8	41	66.6	A
20146	Bn-N18-p36519307	Bn-C8-p33510060	Bn-scaff_16197_1-p289137	C8	41	66.6	A
20147	Bn-N18-p36519429	Bn-C8-p33510182	Bn-scaff_16197_1-p289125	C8	41	66.6	A
20148	Bn-N18-p36520261	Bn-C8-p33510966	Bn-scaff_16197_1-p289046	C8	41	66.6	A
20149	Bn-N18-p36524368	Bn-C8-p33518339	Bn-scaff_16197_1-p288309	C8	41	66.6	A
20150	Bn-N18-p36525722	Bn-C8-p33519688	Bn-scaff_16197_1-p288174	C8	41	66.6	A
20151	Bn-N18-p36526942	Bn-C8-p33520874	Bn-scaff_16197_1-p288055	C8	41	66.6	A
20152	Bn-N18-p36529355	Bn-C8-p33528513	Bn-scaff_16197_1-p287291	C8	41	66.6	A
20153	Bn-N18-p36529560	Bn-C8-p33528700	Bn-scaff_16197_1-p287273	C8	41	66.6	A
20154	Bn-N18-p36529701	Bn-C8-p33528841	Bn-scaff_16197_1-p287259	C8	41	66.6	A
20155	Bn-N18-p36579593	Bn-C8-p33605268	Bn-scaff_16197_1-p279616	C8	41	66.6	A
20156	Bn-N18-p36771850	Bn-C8-p33779671	Bn-scaff_16197_1-p262176	C8	41	66.6	A
20157	Bn-N18-p36772697	Bn-C8-p33780518	Bn-scaff_16197_1-p262091	C8	41	66.6	A
20158	Bn-N18-p36778925	Bn-C8-p33786595	Bn-scaff_16197_1-p261483	C8	41	66.6	A
20159	Bn-N18-p36790258	Bn-C8-p33797928	Bn-scaff_16197_1-p260350	C8	41	66.6	A
20160	Bn-N18-p36791593	Bn-C8-p33799289	Bn-scaff_16197_1-p260214	C8	41	66.6	A
20161	Bn-N18-p36800943	Bn-C8-p33808382	Bn-scaff_16197_1-p259305	C8	41	66.6	A
20162	Bn-N18-p36807575	Bn-C8-p33818814	Bn-scaff_16197_1-p258261	C8	41	66.6	A
20163	Bn-N18-p36809130	Bn-C8-p33820360	Bn-scaff_16197_1-p258107	C8	41	66.6	A
20164	Bn-N18-p36813322	Bn-C8-p33824682	Bn-scaff_16197_1-p257675	C8	41	66.6	A
20165	Bn-N18-p36826543	Bn-C8-p33835870	Bn-scaff_16197_1-p255656	C8	41	66.6	A
20166	Bn-N18-p36841798	Bn-C8-p33852062	Bn-scaff_16197_1-p254937	C8	41	66.6	A
20167	Bn-N18-p36846248	Bn-C8-p33855545	Bn-scaff_16197_1-p254588	C8	41	66.6	A
20168	Bn-N18-p36851124	Bn-C8-p33860069	Bn-scaff_16197_1-p254136	C8	41	66.6	A
20169	Bn-N18-p36851565	Bn-C8-p33860509	Bn-scaff_16197_1-p254092	C8	41	66.6	A
20170	Bn-N18-p36867777	Bn-C8-p33876206	Bn-scaff_16197_1-p252522	C8	41	66.6	A
20171	Bn-N18-p36869632	Bn-C8-p33878054	Bn-scaff_16197_1-p252337	C8	41	66.6	A
20172	Bn-N18-p36871060	Bn-C8-p33879464	Bn-scaff_16197_1-p252196	C8	41	66.6	A
20173	Bn-N18-p36871339	Bn-C8-p33879750	Bn-scaff_16197_1-p252168	C8	41	66.6	A
20174	Bn-N18-p36871452	Bn-C8-p33879863	Bn-scaff_16197_1-p252156	C8	41	66.6	A
20175	Bn-N18-p36872112	Bn-C8-p33880522	Bn-scaff_16197_1-p252091	C8	41	66.6	A
20176	Bn-N18-p36933433	Bn-C8-p33932831	Bn-scaff_16197_1-p246860	C8	41	66.6	A
20177	Bn-N18-p37026923	Bn-C8-p34035010	Bn-scaff_16197_1-p236642	C8	41	66.6	A
20178	Bn-N18-p37027751	Bn-C8-p34035854	Bn-scaff_16197_1-p236557	C8	41	66.6	A
20179	Bn-N18-p37027803	Bn-C8-p34035906	Bn-scaff_16197_1-p236552	C8	41	66.6	A
20180	Bn-N18-p37044506	Bn-C8-p34044914	Bn-scaff_16197_1-p235651	C8	41	66.6	A
20181	Bn-N18-p37044597	Bn-C8-p34045005	Bn-scaff_16197_1-p235642	C8	41	66.6	A
20182	Bn-N18-p37057565	Bn-C8-p34049125	Bn-scaff_16197_1-p235230	C8	41	66.6	A
20183	Bn-N18-p37061656	Bn-C8-p34052524	Bn-scaff_16197_1-p234890	C8	41	66.6	A

20184	Bn-N18-p37072046	Bn-C8-p34065565	Bn-scaff_16197_1-p233586	C8	41	66.6	A
20185	Bn-N18-p37074230	Bn-C8-p34067738	Bn-scaff_16197_1-p233369	C8	41	66.6	A
20186	Bn-N18-p37082859	Bn-C8-p34070317	Bn-scaff_16197_1-p233111	C8	41	66.6	A
20187	Bn-N18-p37083110	Bn-C8-p34070559	Bn-scaff_16197_1-p233087	C8	41	66.6	A
20188	Bn-N18-p37083187	Bn-C8-p34070636	Bn-scaff_16197_1-p233079	C8	41	66.6	A
20189	Bn-N18-p37084121	Bn-C8-p34071568	Bn-scaff_16197_1-p232986	C8	41	66.6	A
20190	Bn-N18-p37102630	Bn-C8-p34091188	Bn-scaff_16197_1-p231024	C8	41	66.6	A
20191	Bn-N18-p37102952	Bn-C8-p34091723	Bn-scaff_16197_1-p230970	C8	41	66.6	A
20192	Bn-N18-p37107178	Bn-C8-p34095954	Bn-scaff_16197_1-p230547	C8	41	66.6	A
20193	Bn-N18-p37108675	Bn-C8-p34097452	Bn-scaff_16197_1-p230398	C8	41	66.6	A
20194	Bn-N18-p37145001	Bn-C8-p34128629	Bn-scaff_16197_1-p227280	C8	41	66.6	A
20195	Bn-N18-p37161528	Bn-C8-p34143460	Bn-scaff_16197_1-p225797	C8	41	66.6	A
20196	Bn-N9-p33228783	Bn-C8-p33381033	Bn-scaff_16197_1-p302039	C8	41	66.6	A
20197	Bn-N9-p33228962	Bn-C8-p33381213	Bn-scaff_16197_1-p302021	C8	41	66.6	A
20198	Bn-N9-p33811840	Bn-C8-p34063483	Bn-scaff_16197_1-p233794	C8	41	66.6	A
20199	Bn-N18-p37282326	Bn-C8-p34257170	Bn-scaff_16197_1-p214426	C8	42	67.4	A
20200	Bn-N18-p37364480	Bn-C8-p34343539	Bn-scaff_16197_1-p205789	C8	42	67.4	A
20201	Bn-N18-p37375149	Bn-C8-p34350580	Bn-scaff_16197_1-p205085	C8	42	67.4	A
20202	Bn-N18-p37377078	Bn-C8-p34352503	Bn-scaff_16197_1-p204892	C8	42	67.4	A
20203	Bn-N18-p37451933	Bn-C8-p34426456	Bn-scaff_16197_1-p197497	C8	43	68.2	A
20204	Bn-N18-p37515324	Bn-C8-p34488468	Bn-scaff_16197_1-p191296	C8	43	68.2	A
20205	Bn-N18-p37647240	Bn-C8-p34624073	Bn-scaff_16197_1-p177735	C8	43	68.2	A
20206	Bn-N18-p37656162	Bn-C8-p34632650	Bn-scaff_16197_1-p176878	C8	43	68.2	A
20207	Bn-N18-p37701779	Bn-C8-p34675874	Bn-scaff_16197_1-p172555	C8	43	68.2	A
20208	Bn-N18-p37727610	Bn-C8-p34706164	Bn-scaff_16197_1-p169526	C8	43	68.2	A
20209	Bn-N18-p37760132	Bn-C8-p34744368	Bn-scaff_16197_1-p165706	C8	43	68.2	A
20210	Bn-N18-p37762885	Bn-C6-p37358876	Bn-scaff_18140_1-p219225	C8	43	68.2	A
20211	Bn-N18-p37787284	Bn-C8-p34766209	Bn-scaff_16197_1-p163522	C8	43	68.2	A
20212	Bn-N18-p37852502	Bn-C8-p34839054	Bn-scaff_16197_1-p156237	C8	43	68.2	A
20213	Bn-N18-p37960338	Bn-C8-p34954896	Bn-scaff_16197_1-p144653	C8	43	68.2	0
20214	Bn-N18-p37991717	Bn-C8-p34987665	Bn-scaff_16197_1-p141376	C8	43	68.2	0
20215	Bn-N18-p38045592	Bn-C8-p35034416	Bn-scaff_16197_1-p136701	C8	43	68.2	0
20216	Bn-N18-p38265033	Bn-C8-p35234890	Bn-scaff_16197_1-p116654	C8	43	68.2	A
20217	Bn-N18-p38270960	Bn-C8-p35240864	Bn-scaff_16197_1-p116056	C8	43	68.2	0
20218	Bn-N18-p38310987	Bn-C8-p35281399	Bn-scaff_16197_1-p112003	C8	43	68.2	0
20219	Bn-N18-p38327971	Bn-C8-p35294638	Bn-scaff_16197_1-p110679	C8	43	68.2	0
20220	Bn-N18-p38328032	Bn-C8-p35294699	Bn-scaff_16197_1-p110673	C8	43	68.2	0
20221	Bn-N18-p38408096	Bn-C8-p35385336	Bn-scaff_16197_1-p101609	C8	43	68.2	A
20222	Bn-N18-p38409630	Bn-C8-p35386871	Bn-scaff_16197_1-p101456	C8	43	68.2	A
20223	Bn-N18-p38416954	Bn-C8-p35393124	Bn-scaff_16197_1-p100830	C8	43	68.2	0
20224	Bn-N18-p38409041	Bn-C8-p35386282	Bn-scaff_16197_1-p101515	C8	44	71.9	A
20225	Bn-N18-p38410398	Bn-C8-p35387639	Bn-scaff_16197_1-p101379	C8	44	71.9	A
20226	Bn-N18-p38458732	Bn-C8-p35426805	Bn-scaff_16197_1-p974627	C8	44	71.9	A
20227	Bn-N18-p38459102	Bn-C8-p35427148	Bn-scaff_16197_1-p974284	C8	44	71.9	A
20228	Bn-N18-p38535983	Bn-C8-p35500290	Bn-scaff_16197_1-p901142	C8	44	71.9	A
20229	Bn-N18-p38595607	Bn-C8-p35557727	Bn-scaff_16197_1-p843705	C8	44	71.9	A
20230	Bn-N18-p38602261	Bn-C8-p35564321	Bn-scaff_16197_1-p837111	C8	44	71.9	A
20231	Bn-N18-p38602525	Bn-C8-p35564585	Bn-scaff_16197_1-p836847	C8	44	71.9	A
20232	Bn-N18-p38602737	Bn-C8-p35564798	Bn-scaff_16197_1-p836634	C8	44	71.9	A

20233	Bn-N18-p38650144	Bn-C8-p35616220	Bn-scaff_16197_1-p785212	C8	44	71.9	0
20234	Bn-N18-p38650559	Bn-C8-p35616635	Bn-scaff_16197_1-p784797	C8	44	71.9	0
20235	Bn-N18-p38654705	Bn-C8-p35620644	Bn-scaff_16197_1-p780788	C8	44	71.9	A
20236	Bn-N18-p38705645	Bn-C8-p35661459	Bn-scaff_16197_1-p739973	C8	44	71.9	A
20237	Bn-N18-p38728965	Bn-C8-p35676792	Bn-scaff_16197_1-p724640	C8	44	71.9	0
20238	Bn-N18-p38767644	Bn-C8-p35704424	Bn-scaff_16197_1-p697008	C8	44	71.9	A
20239	Bn-N18-p38774778	Bn-C8-p35711815	Bn-scaff_16197_1-p689617	C8	44	71.9	A
20240	Bn-N18-p38898780	Bn-C8-p35834430	Bn-scaff_16197_1-p567002	C8	44	71.9	A
20241	Bn-N18-p38946203	Bn-C8-p35894667	Bn-scaff_16197_1-p506765	C8	44	71.9	A
20242	Bn-N18-p39028104	Bn-C8-p35976978	Bn-scaff_16197_1-p424454	C8	44	71.9	A
20243	Bn-N18-p39033688	Bn-C8-p35982715	Bn-scaff_16197_1-p418717	C8	44	71.9	A
20244	Bn-N18-p39033918	Bn-C8-p35982945	Bn-scaff_16197_1-p418487	C8	44	71.9	A
20245	Bn-N18-p39034050	Bn-C8-p35983077	Bn-scaff_16197_1-p418355	C8	44	71.9	A
20246	Bn-N18-p39087567	Bn-C8-p36028942	Bn-scaff_16197_1-p372490	C8	44	71.9	A
20247	Bn-N18-p39237257	Bn-C8-p36175531	Bn-scaff_16197_1-p225901	C8	44	71.9	A
20248	Bn-N18-p39241000	Bn-C8-p36179273	Bn-scaff_16197_1-p222159	C8	44	71.9	A
20249	Bn-N18-p39251304	Bn-C8-p36191258	Bn-scaff_16197_1-p210174	C8	44	71.9	A
20250	Bn-N18-p39869059	Bn-C8-p36742396	Bn-scaff_16445_1-p260110	C8	45	72.8	A
20251	Bn-N18-p40195326	Bn-C8-p37081224	Bn-scaff_16445_1-p226227	C8	45	72.8	0
20252	Bn-N18-p40232028	Bn-C8-p37116379	Bn-scaff_16445_1-p222712	C8	45	72.8	A
20253	Bn-N18-p40316184	Bn-C8-p37224819	Bn-scaff_16445_1-p211868	C8	45	72.8	0
20254	Bn-N18-p40316962	Bn-C8-p37225597	Bn-scaff_16445_1-p211790	C8	45	72.8	0
20255	Bn-N9-p36095294	Bn-A09-p32978334	Bn-A09-p32978334	C8	45	72.8	0
20256	Bn-N16-p9083293	Bn-C2-p16737048	Bn-scaff_16565_1-p41717	C8	46	74.7	0
20257	Bn-N9-p36764678	Bn-A09-p33660289	Bn-A09-p33660289	C8	46	74.7	0
20258	Bn-N18-p41190194	Bn-C8-p38051720	Bn-scaff_16445_1-p129178	C8	47	78.9	0
20259	Bn-N18-p41334426	Bn-A09-p34247590	Bn-A09-p34247590	C8	47	78.9	0
20260	Bn-N18-p41556749	Bn-C8-p38410249	Bn-scaff_16445_1-p933254	C8	47	78.9	0
20261	Bn-N18-p41557308	Bn-C8-p38410804	Bn-scaff_16445_1-p932699	C8	47	78.9	0
20262	Bn-N18-p41557858	Bn-C8-p38411352	Bn-scaff_16445_1-p932151	C8	47	78.9	0
20263	Bn-N9-p37552473	Bn-C9-p11708712	Bn-scaff_21656_1-p18965	C8	47	78.9	A
20264	Bn-N18-p41663055	Bn-C8-p38486825	Bn-scaff_16445_1-p856678	C8	48	83.1	A
20265	Bn-N18-p41704316	Bn-C8-p38543169	Bn-scaff_16445_1-p800334	C8	49	84.8	A
20266	Bn-N18-p41737543	Bn-C8-p38576855	Bn-scaff_16445_1-p766648	C8	49	84.8	0
20267	Bn-N18-p41770907	Bn-C8-p38615813	Bn-scaff_16445_1-p727690	C8	50	85.6	A
20268	Bn-N18-p41771120	Bn-C8-p38616026	Bn-scaff_16445_1-p727477	C8	50	85.6	A
20269	Bn-N18-p41906145	Bn-C8-p38749358	Bn-scaff_16445_1-p594145	C8	50	85.6	A
20270	Bn-N18-p42467540	Bn-C8-p39312052	Bn-scaff_16445_1-p31451	C8	51	88.1	A
20271	Bn-N18-p43035968	Bn-C8-p39889339	Bn-scaff_21269_1-p204828	C8	52	91.5	0
20272	Bn-N18-p43996802	Bn-C8-p40757738	Bn-scaff_16389_1-p660552	C8	53	98.3	0
20273	Bn-N18-p44094835	Bn-C8-p40864361	Bn-scaff_16389_1-p767175	C8	53	98.3	A
20274	Bn-N18-p44424911	Bn-C8-p41145335	Bn-scaff_16021_1-p613351	C8	54	100	A
20275	Bn-N18-p44455906	Bn-C8-p41172920	Bn-scaff_16021_1-p585766	C8	54	100	A
20276	Bn-N1-p4936980	Bn-C9-p11056	Bn-scaff_20131_1-p73163	C9	1	0	A
20277	Bn-N4-p5589	Bn-C9-p7599	Bn-scaff_20131_1-p76620	C9	1	0	A
20278	Bn-N19-p55940966	Bn-A09-p281844	Bn-A09-p281844	C9	2	4.9	A
20279	Bn-N9-p761934	Bn-C9-p1244493	Bn-scaff_15783_1-p273092	C9	2	4.9	A
20280	Bn-N9-p1081812	Bn-C9-p1600561	Bn-scaff_17526_1-p217919	C9	3	6.5	A
20281	Bn-N9-p1081882	Bn-A01-p26921609	Bn-A01-p26921609	C9	3	6.5	A

20282	Bn-N19-p1843248	Bn-C9-p1872500	Bn-scaff_17526_1-p190725	C9	4	10.6	A
20283	Bn-N19-p1924999	Bn-C9-p1945397	Bn-scaff_17526_1-p183436	C9	4	10.6	A
20284	Bn-N19-p2074812	Bn-C9-p2112499	Bn-scaff_17526_1-p166726	C9	4	10.6	A
20285	Bn-N19-p2092209	Bn-C9-p2129884	Bn-scaff_17526_1-p164987	C9	4	10.6	A
20286	Bn-N9-p1369397	Bn-A09-p1252150	Bn-A09-p1252150	C9	4	10.6	A
20287	Bn-N9-p1471037	Bn-C9-p2061433	Bn-scaff_17526_1-p171832	C9	4	10.6	A
20288	Bn-N19-p2260255	Bn-C9-p2304853	Bn-scaff_17526_1-p147490	C9	5	12.2	A
20289	Bn-N19-p2266494	Bn-C9-p2309674	Bn-scaff_17526_1-p147008	C9	5	12.2	A
20290	Bn-N19-p2267198	Bn-C9-p2310379	Bn-scaff_17526_1-p146938	C9	5	12.2	A
20291	Bn-N19-p2462824	Bn-C9-p2515276	Bn-scaff_17526_1-p126448	C9	5	12.2	A
20292	Bn-N19-p2579440	Bn-C9-p2635986	Bn-scaff_17526_1-p114377	C9	5	12.2	A
20293	Bn-N19-p2637647	Bn-C9-p2688672	Bn-scaff_17526_1-p109108	C9	6	13.8	A
20294	Bn-N19-p2759450	Bn-C9-p2803983	Bn-scaff_17526_1-p97577	C9	6	13.8	A
20295	Bn-N19-p2767307	Bn-C9-p2810282	Bn-scaff_17526_1-p96947	C9	6	13.8	A
20296	Bn-N19-p2784290	Bn-C9-p2827141	Bn-scaff_17526_1-p95261	C9	6	13.8	A
20297	Bn-N19-p2867698	Bn-C9-p2900723	Bn-scaff_17526_1-p87903	C9	6	13.8	A
20298	Bn-N19-p2887095	Bn-C9-p2919255	Bn-scaff_17526_1-p86050	C9	6	13.8	A
20299	Bn-N19-p2887140	Bn-C9-p2919300	Bn-scaff_17526_1-p86045	C9	6	13.8	A
20300	Bn-N17-p11449356	Bn-C7-p10815743	Bn-scaff_16721_1-p36068	C9	7	23.7	A
20301	Bn-N19-p4207026	Bn-C9-p4177835	Bn-scaff_19783_1-p327125	C9	8	24.5	A
20302	Bn-N19-p4207653	Bn-C9-p4178485	Bn-scaff_19783_1-p327775	C9	8	24.5	A
20303	Bn-N9-p3154514	Bn-A09-p2730673	Bn-A09-p2730673	C9	8	24.5	A
20304	Bn-N9-p3154616	Bn-C9-p4273944	Bn-scaff_19783_1-p423234	C9	8	24.5	A
20305	Bn-N19-p5017304	Bn-A09-p3233005	Bn-A09-p3233005	C9	9	25.3	A
20306	Bn-N19-p5017427	Bn-A09-p3233129	Bn-A09-p3233129	C9	9	25.3	A
20307	Bn-Scaffold19050-p415	Bn-C3-p50595364	Bn-scaff_20790_1-p284462	C9	9	25.3	A
20308	Bn-N9-p3763762	Bn-C9-p5218392	Bn-scaff_17190_1-p23794	C9	10	26.1	A
20309	Bn-N19-p5638360	Bn-C9-p5641324	Bn-scaff_17190_1-p660881	C9	11	27.7	A
20310	Bn-N19-p6041483	Bn-C9-p6099851	Bn-scaff_17190_1-p111940	C9	12	28.5	A
20311	Bn-N19-p6041900	Bn-C9-p6100268	Bn-scaff_17190_1-p111982	C9	12	28.5	A
20312	Bn-N19-p6258583	Bn-C9-p6285792	Bn-scaff_17297_1-p143112	C9	13	29.3	A
20313	Bn-N19-p6275644		Bn-Scaffold000149-p12172	C9	13	29.3	A
20314	Bn-N19-p6308938	Bn-C9-p6344887	Bn-scaff_17297_1-p202207	C9	13	29.3	A
20315	Bn-N19-p6321757	Bn-C9-p6357193	Bn-scaff_17297_1-p214513	C9	13	29.3	A
20316	Bn-N19-p6771134	Bn-C9-p6763573	Bn-scaff_17423_1-p37655	C9	14	30.1	A
20317	Bn-N16-p9970383	Bn-C6-p28620536	Bn-scaff_17984_1-p90965	C9	15	31.7	A
20318	Bn-N19-p7631927	Bn-C9-p7657260	Bn-scaff_17084_1-p76059	C9	15	31.7	A
20319	Bn-N11-p8300623	Bn-C1-p7069116	Bn-scaff_17731_1-p60165	C9	16	37.4	A
20320	Bn-N19-p10437358	Bn-C9-p10305984	Bn-scaff_17487_1-p178218	C9	16	37.4	A
20321	Bn-N19-p10533617	Bn-C9-p10411803	Bn-scaff_17487_1-p188800	C9	16	37.4	A
20322	Bn-N19-p10637780	Bn-C9-p10495311	Bn-scaff_20428_1-p430947	C9	16	37.4	A
20323	Bn-N19-p10740365	Bn-C9-p10604344	Bn-scaff_20428_1-p321914	C9	16	37.4	A
20324	Bn-N19-p10984798	Bn-C9-p10819458	Bn-scaff_20428_1-p106800	C9	16	37.4	A
20325	Bn-N19-p18873118	Bn-C9-p9127095	Bn-scaff_17487_1-p603292	C9	16	37.4	A
20326	Bn-N19-p48466239	Bn-C9-p9081079	Bn-scaff_17487_1-p557276	C9	16	37.4	A
20327	Bn-N19-p48467337	Bn-C9-p9084097	Bn-scaff_17487_1-p560294	C9	16	37.4	A
20328	Bn-N19-p48467492	Bn-C9-p9084252	Bn-scaff_17487_1-p560449	C9	16	37.4	A
20329	Bn-N19-p48468781	Bn-C9-p9085174	Bn-scaff_17487_1-p561371	C9	16	37.4	A
20330	Bn-N19-p8369331	Bn-C9-p8182477	Bn-scaff_23207_1-p153689	C9	16	37.4	A

20331	Bn-N19-p8421768	Bn-C9-p8152011	Bn-scaff_23207_1-p123223	C9	16	37.4	A
20332	Bn-N19-p8926497	Bn-C9-p8900660	Bn-scaff_17487_1-p376857	C9	16	37.4	A
20333	Bn-N19-p8953864	Bn-C9-p8927815	Bn-scaff_17487_1-p404012	C9	16	37.4	A
20334	Bn-N19-p8959462	Bn-C9-p8940014	Bn-scaff_17487_1-p416211	C9	16	37.4	A
20335	Bn-N19-p9033070	Bn-C9-p8988339	Bn-scaff_17487_1-p464536	C9	16	37.4	A
20336	Bn-N19-p9111348	Bn-C9-p9011639	Bn-scaff_17487_1-p487836	C9	16	37.4	A
20337	Bn-N19-p9114750	Bn-C9-p9014834	Bn-scaff_17487_1-p491031	C9	16	37.4	A
20338	Bn-N19-p9139196	Bn-C9-p9035400	Bn-scaff_17487_1-p511597	C9	16	37.4	A
20339	Bn-N19-p9140138	Bn-C9-p9036338	Bn-scaff_17487_1-p512535	C9	16	37.4	A
20340	Bn-N19-p9161418	Bn-C9-p9051190	Bn-scaff_17487_1-p527387	C9	16	37.4	A
20341	Bn-N19-p9173532	Bn-C9-p9069110	Bn-scaff_17487_1-p545307	C9	16	37.4	A
20342	Bn-N19-p9199533	Bn-C9-p9089532	Bn-scaff_17487_1-p565729	C9	16	37.4	A
20343	Bn-N19-p9237653	Bn-C9-p9138867	Bn-scaff_17487_1-p615064	C9	16	37.4	A
20344	Bn-N19-p9239505	Bn-C9-p9140719	Bn-scaff_17487_1-p616916	C9	16	37.4	A
20345	Bn-N19-p9345298	Bn-C9-p9247059	Bn-scaff_17487_1-p723256	C9	16	37.4	A
20346	Bn-N19-p9495480	Bn-C9-p13326808	Bn-scaff_25991_1-p10879	C9	16	37.4	A
20347	Bn-N19-p9495974	Bn-C9-p13327302	Bn-scaff_25991_1-p10385	C9	16	37.4	A
20348	Bn-N19-p9526061	Bn-C9-p9456671	Bn-scaff_17487_1-p932868	C9	16	37.4	A
20349	Bn-N19-p9894363	Bn-C9-p9782048	Bn-scaff_17487_1-p125824	C9	16	37.4	A
20350	Bn-N9-p6887304	Bn-C9-p10592427	Bn-scaff_20428_1-p333831	C9	16	37.4	A
20351	Bn-N19-p10836063	Bn-C9-p10670207	Bn-scaff_20428_1-p256051	C9	17	39	A
20352	Bn-N19-p11073861	Bn-C9-p10892372	Bn-scaff_20428_1-p33886	C9	17	39	A
20353	Bn-N19-p11436491	Bn-C9-p11335462	Bn-scaff_17683_1-p28746	C9	18	39.8	A
20354	Bn-N19-p11475691	Bn-C9-p11415019	Bn-scaff_21656_1-p312658	C9	18	39.8	A
20355	Bn-N19-p12291662	Bn-C9-p12100951	Bn-scaff_15701_1-p101523	C9	18	39.8	A
20356	Bn-N19-p13102887	Bn-C9-p12901367	Bn-scaff_15701_1-p214816	C9	18	39.8	A
20357	Bn-N19-p13216109	Bn-C9-p12992624	Bn-scaff_15701_1-p123559	C9	18	39.8	A
20358	Bn-N19-p13218037	Bn-C9-p12994563	Bn-scaff_15701_1-p121620	C9	18	39.8	A
20359	Bn-N19-p21638623	Bn-C9-p20770540	Bn-scaff_15650_1-p655781	C9	18	39.8	0
20360	Bn-N16-p31557075	Bn-C6-p7216717	Bn-scaff_16547_1-p315514	C9	19	40.6	A
20361	Bn-N19-p13406984	Bn-C9-p13245838	Bn-scaff_28725_1-p72570	C9	19	40.6	A
20362	Bn-N19-p13439313	Bn-C9-p13220878	Bn-scaff_28725_1-p97530	C9	19	40.6	A
20363	Bn-N19-p13466145	Bn-C9-p13197926	Bn-scaff_28725_1-p120482	C9	19	40.6	A
20364	Bn-N19-p13503872	Bn-C9-p13167774	Bn-scaff_28725_1-p150634	C9	19	40.6	A
20365	Bn-N19-p13530254	Bn-C9-p13143843	Bn-scaff_28725_1-p174565	C9	19	40.6	A
20366	Bn-N19-p13610375	Bn-C9-p13395671	Bn-scaff_20554_1-p57885	C9	19	40.6	A
20367	Bn-N19-p14457407	Bn-C9-p14056527	Bn-scaff_22835_1-p716171	C9	19	40.6	A
20368	Bn-N19-p14558751	Bn-C9-p14133649	Bn-scaff_22835_1-p639049	C9	19	40.6	A
20369	Bn-N19-p14563403	Bn-C9-p14138735	Bn-scaff_22835_1-p633963	C9	19	40.6	A
20370	Bn-N19-p14563984	Bn-C9-p14141466	Bn-scaff_22835_1-p631232	C9	19	40.6	A
20371	Bn-N19-p14588041	Bn-C9-p14160911	Bn-scaff_22835_1-p611787	C9	19	40.6	A
20372	Bn-N19-p14652066	Bn-C9-p14243116	Bn-scaff_22835_1-p529582	C9	19	40.6	A
20373	Bn-N19-p14655500	Bn-C9-p14238190	Bn-scaff_22835_1-p534508	C9	19	40.6	A
20374	Bn-N19-p14656684	Bn-C9-p14237006	Bn-scaff_22835_1-p535692	C9	19	40.6	A
20375	Bn-N19-p14685129	Bn-C9-p14225149	Bn-scaff_22835_1-p547549	C9	19	40.6	A
20376	Bn-N19-p14696834	Bn-C9-p14204960	Bn-scaff_22835_1-p567738	C9	19	40.6	A
20377	Bn-N19-p14699007	Bn-C9-p14202590	Bn-scaff_22835_1-p570108	C9	19	40.6	A
20378	Bn-N19-p14703374	Bn-C9-p14194903	Bn-scaff_22835_1-p577795	C9	19	40.6	A
20379	Bn-N19-p14705341	Bn-C9-p14192958	Bn-scaff_22835_1-p579740	C9	19	40.6	A

20380	Bn-N19-p14730872	Bn-C9-p14172872	Bn-scaff_22835_1-p59982	C9	19	40.6	A
20381	Bn-N19-p15047605	Bn-C9-p14608467	Bn-scaff_22835_1-p16423	C9	19	40.6	A
20382	Bn-N9-p9082262	Bn-A09-p8634971	Bn-A09-p8634971	C9	19	40.6	A
20383	Bn-N9-p9082898	Bn-A09-p8635608	Bn-A09-p8635608	C9	19	40.6	A
20384	Bn-N9-p9178305	Bn-C9-p14338666	Bn-scaff_22835_1-p43403	C9	19	40.6	A
20385	Bn-N19-p15841037	Bn-C9-p15329223	Bn-scaff_23618_1-p12015	C9	20	41.4	A
20386	Bn-N19-p16138141	Bn-C9-p15611432	Bn-scaff_16708_1-p14187	C9	20	41.4	A
20387	Bn-N18-p9793216	Bn-C6-p32810747	Bn-scaff_18936_1-p20371	C9	21	43	A
20388	Bn-N19-p16349352	Bn-C9-p15762391	Bn-scaff_18839_1-p10929	C9	21	43	A
20389	Bn-N19-p16373749	Bn-C9-p15784039	Bn-scaff_16912_1-p13193	C9	21	43	A
20390	Bn-N19-p16395667	Bn-C9-p15799250	Bn-scaff_16912_1-p28404	C9	21	43	A
20391	Bn-N19-p16397482	Bn-C9-p15801062	Bn-scaff_16912_1-p30216	C9	21	43	A
20392	Bn-N19-p16397536	Bn-C9-p15801116	Bn-scaff_16912_1-p30270	C9	21	43	A
20393	Bn-N19-p16421082	Bn-C9-p15811601	Bn-scaff_16912_1-p40755	C9	21	43	A
20394	Bn-N19-p16438243	Bn-C9-p15830262	Bn-scaff_16912_1-p59416	C9	21	43	A
20395	Bn-N19-p16438850	Bn-C9-p15830863	Bn-scaff_16912_1-p60017	C9	21	43	A
20396	Bn-N19-p16443905	Bn-C9-p15835144	Bn-scaff_16912_1-p64298	C9	21	43	A
20397	Bn-N19-p16467200	Bn-C9-p15864136	Bn-scaff_16912_1-p93290	C9	21	43	A
20398	Bn-N19-p16467464	Bn-C9-p15864400	Bn-scaff_16912_1-p93554	C9	21	43	A
20399	Bn-N19-p16467791	Bn-C9-p15864727	Bn-scaff_16912_1-p93881	C9	21	43	A
20400	Bn-N19-p16468181	Bn-C9-p15865155	Bn-scaff_16912_1-p94309	C9	21	43	A
20401	Bn-N19-p16474321	Bn-C9-p15866850	Bn-scaff_16912_1-p96004	C9	21	43	A
20402	Bn-N19-p16477525	Bn-C9-p15869844	Bn-scaff_16912_1-p98998	C9	21	43	A
20403	Bn-N19-p16478088	Bn-C9-p15870407	Bn-scaff_16912_1-p99561	C9	21	43	A
20404	Bn-N19-p16500967	Bn-C9-p15880473	Bn-scaff_16912_1-p10962	C9	21	43	A
20405	Bn-N19-p16501609	Bn-C9-p15881113	Bn-scaff_16912_1-p11026	C9	21	43	A
20406	Bn-N19-p16502738	Bn-C9-p15881640	Bn-scaff_16912_1-p11079	C9	21	43	A
20407	Bn-N19-p16504495	Bn-C9-p15883415	Bn-scaff_16912_1-p11256	C9	21	43	A
20408	Bn-N19-p16510456	Bn-C9-p15888708	Bn-scaff_16912_1-p11786	C9	21	43	A
20409	Bn-N19-p16510645	Bn-C9-p15888898	Bn-scaff_16912_1-p11805	C9	21	43	A
20410	Bn-N19-p16515518	Bn-C9-p15891248	Bn-scaff_16912_1-p12040	C9	21	43	A
20411	Bn-N19-p16551146	Bn-C9-p15949589	Bn-scaff_16912_1-p17874	C9	21	43	A
20412	Bn-N19-p16551313	Bn-C9-p15949748	Bn-scaff_16912_1-p17890	C9	21	43	A
20413	Bn-N19-p16554732	Bn-C9-p15953170	Bn-scaff_16912_1-p18232	C9	21	43	A
20414	Bn-N19-p16569159	Bn-C9-p15958341	Bn-scaff_16912_1-p18749	C9	21	43	A
20415	Bn-N19-p16571976	Bn-C9-p15961137	Bn-scaff_16912_1-p19029	C9	21	43	A
20416	Bn-N19-p16592624	Bn-C9-p15971997	Bn-scaff_16912_1-p20115	C9	21	43	A
20417	Bn-N19-p16675148	Bn-C9-p16049190	Bn-scaff_20836_1-p19986	C9	21	43	A
20418	Bn-N19-p16675645	Bn-C9-p16050250	Bn-scaff_20836_1-p19880	C9	21	43	A
20419	Bn-N19-p16676055	Bn-C9-p16050668	Bn-scaff_20836_1-p19839	C9	21	43	A
20420	Bn-N19-p16676506	Bn-C9-p16051119	Bn-scaff_20836_1-p19794	C9	21	43	A
20421	Bn-N19-p16677059	Bn-C9-p16051672	Bn-scaff_20836_1-p19738	C9	21	43	A
20422	Bn-N19-p16677852	Bn-C9-p16052458	Bn-scaff_20836_1-p19660	C9	21	43	A
20423	Bn-N19-p16713161	Bn-C9-p16114373	Bn-scaff_20836_1-p13468	C9	21	43	A
20424	Bn-N19-p16713395	Bn-C9-p16114607	Bn-scaff_20836_1-p13445	C9	21	43	A
20425	Bn-N19-p16732025	Bn-C9-p16120596	Bn-scaff_20836_1-p12846	C9	21	43	A
20426	Bn-N19-p16738850	Bn-C9-p16123434	Bn-scaff_20836_1-p12562	C9	21	43	A
20427	Bn-N19-p16760127	Bn-C9-p16143647	Bn-scaff_20836_1-p10541	C9	21	43	A
20428	Bn-N19-p16797621	Bn-C4-p10597497	Bn-scaff_17869_1-p41643	C9	21	43	A

20429	Bn-N19-p16797740	Bn-C4-p10597377	Bn-scaff_17869_1-p416319	C9	21	43	A
20430	Bn-N19-p16798012	Bn-C4-p10597115	Bn-scaff_17869_1-p416057	C9	21	43	A
20431	Bn-N19-p16799498	Bn-C9-p16181002	Bn-scaff_20836_1-p68057	C9	21	43	A
20432	Bn-N19-p16800611	Bn-C9-p16182168	Bn-scaff_20836_1-p66891	C9	21	43	A
20433	Bn-N19-p16834690	Bn-C9-p16229753	Bn-scaff_20836_1-p19306	C9	21	43	A
20434	Bn-N19-p16856792	Bn-C9-p16268779	Bn-scaff_17174_1-p503683	C9	21	43	A
20435	Bn-N19-p16872436	Bn-C9-p16308289	Bn-scaff_17174_1-p464173	C9	21	43	A
20436	Bn-N19-p16881511	Bn-C9-p16309189	Bn-scaff_17174_1-p463273	C9	21	43	A
20437	Bn-N19-p16959792	Bn-C9-p16372270	Bn-scaff_17174_1-p400192	C9	21	43	A
20438	Bn-N19-p16980858	Bn-C9-p16450572	Bn-scaff_17174_1-p321890	C9	21	43	A
20439	Bn-N19-p17010571	Bn-C9-p16480880	Bn-scaff_17174_1-p291582	C9	21	43	A
20440	Bn-N19-p17014016	Bn-C9-p16484245	Bn-scaff_17174_1-p288217	C9	21	43	A
20441	Bn-N19-p17015439	Bn-C9-p16485693	Bn-scaff_17174_1-p286769	C9	21	43	A
20442	Bn-N19-p17017350	Bn-C9-p16486571	Bn-scaff_17174_1-p285891	C9	21	43	A
20443	Bn-N19-p17019233	Bn-C9-p16488454	Bn-scaff_17174_1-p284008	C9	21	43	A
20444	Bn-N19-p17024768	Bn-C9-p16494424	Bn-scaff_17174_1-p278038	C9	21	43	A
20445	Bn-N19-p17025956	Bn-C9-p16495632	Bn-scaff_17174_1-p276830	C9	21	43	A
20446	Bn-N19-p17025957	Bn-C9-p16495633	Bn-scaff_17174_1-p276829	C9	21	43	A
20447	Bn-N19-p17030708	Bn-C9-p16500381	Bn-scaff_17174_1-p272081	C9	21	43	A
20448	Bn-N19-p17031324	Bn-C9-p16500979	Bn-scaff_17174_1-p271483	C9	21	43	A
20449	Bn-N19-p17034594	Bn-C9-p16513016	Bn-scaff_17174_1-p259446	C9	21	43	A
20450	Bn-N19-p17035808	Bn-C9-p16513836	Bn-scaff_17174_1-p258626	C9	21	43	A
20451	Bn-N19-p17041261	Bn-C9-p16521142	Bn-scaff_17174_1-p251320	C9	21	43	A
20452	Bn-N19-p17073408	Bn-C9-p16546910	Bn-scaff_17174_1-p225552	C9	21	43	A
20453	Bn-N19-p17076658	Bn-C9-p16550163	Bn-scaff_17174_1-p222299	C9	21	43	A
20454	Bn-N19-p17082722	Bn-C9-p16556233	Bn-scaff_17174_1-p216229	C9	21	43	A
20455	Bn-Scaffold08272-p447	Bn-C9-p16369162	Bn-scaff_17174_1-p403300	C9	21	43	A
20456	Bn-Scaffold41822-p217	Bn-C9-p16282834	Bn-scaff_17174_1-p489628	C9	21	43	A
20457	Bn-N19-p17203737	Bn-C9-p16683551	Bn-scaff_17174_1-p88911	C9	22	43.8	A
20458	Bn-N19-p17203746	Bn-C9-p16658238	Bn-scaff_17174_1-p114224	C9	22	43.8	A
20459	Bn-N19-p17246017	Bn-C9-p16708079	Bn-scaff_17174_1-p64383	C9	22	43.8	A
20460	Bn-N19-p17268150	Bn-C9-p16730589	Bn-scaff_17174_1-p41873	C9	22	43.8	A
20461	Bn-N19-p17271414	Bn-C9-p16733852	Bn-scaff_17174_1-p38610	C9	22	43.8	A
20462	Bn-N19-p17331108	Bn-C9-p16787519	Bn-scaff_15847_1-p273947	C9	22	43.8	A
20463	Bn-N19-p17339410	Bn-C9-p16796332	Bn-scaff_15847_1-p265134	C9	22	43.8	A
20464	Bn-N19-p17503726	Bn-C9-p16896412	Bn-scaff_18100_1-p96955	C9	22	43.8	A
20465	Bn-N19-p17509770	Bn-C9-p16918000	Bn-scaff_18100_1-p118543	C9	22	43.8	A
20466	Bn-N19-p17522476	Bn-C9-p16927992	Bn-scaff_18100_1-p128535	C9	22	43.8	A
20467	Bn-N19-p17533617	Bn-A09-p14287005	Bn-A09-p14287005	C9	22	43.8	A
20468	Bn-N19-p17534089	Bn-C9-p16941688	Bn-scaff_18100_1-p142231	C9	22	43.8	A
20469	Bn-N19-p17604293	Bn-C9-p17010294	Bn-scaff_18100_1-p210837	C9	22	43.8	A
20470	Bn-N19-p17620995	Bn-C9-p17023495	Bn-scaff_18100_1-p224038	C9	22	43.8	A
20471	Bn-N19-p17659842	Bn-C9-p17070755	Bn-scaff_18100_1-p271298	C9	22	43.8	A
20472	Bn-N19-p17724780	Bn-C9-p17131633	Bn-scaff_18100_1-p332176	C9	22	43.8	A
20473	Bn-N19-p17733319	Bn-C9-p17144109	Bn-scaff_18100_1-p344652	C9	22	43.8	A
20474	Bn-N12-p9239751	Bn-C9-p17359012	Bn-scaff_18100_1-p559555	C9	23	45.4	A
20475	Bn-N15-p39449991	Bn-C9-p14463354	Bn-scaff_22835_1-p309344	C9	23	45.4	A
20476	Bn-N19-p17936845	Bn-C9-p17340499	Bn-scaff_18100_1-p541042	C9	23	45.4	A
20477	Bn-N19-p17940483	Bn-C9-p17343895	Bn-scaff_18100_1-p544438	C9	23	45.4	A

20478	Bn-N19-p17949506	Bn-C9-p17368320	Bn-scaff_18100_1-p56886:	C9	23	45.4	A
20479	Bn-N19-p17951963	Bn-C9-p17376550	Bn-scaff_18100_1-p57709:	C9	23	45.4	A
20480	Bn-N19-p17969011	Bn-C9-p17396619	Bn-scaff_18100_1-p59716:	C9	23	45.4	A
20481	Bn-N19-p18130241	Bn-C9-p17501705	Bn-scaff_18100_1-p70224{	C9	23	45.4	A
20482	Bn-N19-p18175714	Bn-C9-p17560891	Bn-scaff_18100_1-p76143	C9	23	45.4	A
20483	Bn-N19-p18177911	Bn-C9-p17561845	Bn-scaff_18100_1-p76238	C9	23	45.4	A
20484	Bn-N19-p18373321	Bn-C9-p17786656	Bn-scaff_18100_1-p98719	C9	23	45.4	A
20485	Bn-N19-p18461234	Bn-C9-p17884066	Bn-scaff_18100_1-p10846(	C9	23	45.4	A
20486	Bn-N19-p18524162	Bn-C9-p17950764	Bn-scaff_18100_1-p11513(	C9	23	45.4	A
20487	Bn-N19-p18524517	Bn-C9-p17951119	Bn-scaff_18100_1-p11516	C9	23	45.4	A
20488	Bn-N19-p18527163	Bn-C9-p17953765	Bn-scaff_18100_1-p11543(	C9	23	45.4	A
20489	Bn-N19-p18527324	Bn-C9-p17953926	Bn-scaff_18100_1-p11544	C9	23	45.4	A
20490	Bn-N19-p18527781	Bn-C9-p17954383	Bn-scaff_18100_1-p11549:	C9	23	45.4	A
20491	Bn-N19-p18528045	Bn-C9-p17954647	Bn-scaff_18100_1-p11551	C9	23	45.4	A
20492	Bn-N19-p18601945	Bn-C9-p18028972	Bn-scaff_18100_1-p12295	C9	23	45.4	A
20493	Bn-N19-p18611527	Bn-C9-p18040107	Bn-scaff_18100_1-p12406	C9	23	45.4	A
20494	Bn-N19-p18612076	Bn-C9-p18040658	Bn-scaff_18100_1-p12412(	C9	23	45.4	A
20495	Bn-N19-p18621036	Bn-C9-p18045288	Bn-scaff_18100_1-p12458:	C9	23	45.4	A
20496	Bn-N19-p18634092	Bn-C9-p18054523	Bn-scaff_18100_1-p12550	C9	23	45.4	A
20497	Bn-N19-p18634224	Bn-C9-p18054634	Bn-scaff_18100_1-p12551	C9	23	45.4	A
20498	Bn-N19-p18634448	Bn-C9-p18054882	Bn-scaff_18100_1-p12554:	C9	23	45.4	A
20499	Bn-N19-p18634563	Bn-C9-p18054997	Bn-scaff_18100_1-p12555	C9	23	45.4	A
20500	Bn-N19-p18634627	Bn-C9-p18055061	Bn-scaff_18100_1-p12556(	C9	23	45.4	A
20501	Bn-N19-p18634831	Bn-C9-p18055265	Bn-scaff_18100_1-p12558(	C9	23	45.4	A
20502	Bn-N19-p18634885	Bn-C9-p18055319	Bn-scaff_18100_1-p12558	C9	23	45.4	A
20503	Bn-N19-p18635430	Bn-C9-p18055864	Bn-scaff_18100_1-p12564(	C9	23	45.4	A
20504	Bn-N19-p18635565	Bn-C9-p18055999	Bn-scaff_18100_1-p12565	C9	23	45.4	A
20505	Bn-N19-p18643686	Bn-C9-p18056865	Bn-scaff_18100_1-p12574(	C9	23	45.4	A
20506	Bn-N19-p18643877	Bn-C9-p18057056	Bn-scaff_18100_1-p12575	C9	23	45.4	A
20507	Bn-N19-p18644190	Bn-C9-p18057369	Bn-scaff_18100_1-p12579	C9	23	45.4	A
20508	Bn-N19-p18649535	Bn-C9-p18063614	Bn-scaff_18100_1-p12641	C9	23	45.4	A
20509	Bn-N19-p18683742	Bn-C9-p18109590	Bn-scaff_18100_1-p13101:	C9	23	45.4	A
20510	Bn-N19-p18728249	Bn-C9-p18135326	Bn-scaff_18100_1-p13358	C9	23	45.4	A
20511	Bn-N19-p18728254	Bn-C9-p18135332	Bn-scaff_18100_1-p13358	C9	23	45.4	A
20512	Bn-N19-p18728334	Bn-C9-p18135411	Bn-scaff_18100_1-p13359	C9	23	45.4	A
20513	Bn-N19-p18730167	Bn-C9-p18137253	Bn-scaff_18100_1-p13377	C9	23	45.4	A
20514	Bn-N19-p18742248	Bn-C9-p18145448	Bn-scaff_18100_1-p13459	C9	23	45.4	A
20515	Bn-N19-p18756344	Bn-C9-p18163556	Bn-scaff_18100_1-p13640	C9	23	45.4	A
20516	Bn-N19-p19798107	Bn-Scaffold00722-p5:	Bn-scaff_19937_1-p51016	C9	23	45.4	A
20517	Bn-N19-p19839447	Bn-C9-p20421889	Bn-scaff_20202_1-p14298	C9	23	45.4	A
20518	Bn-N19-p19839538	Bn-C9-p20421980	Bn-scaff_20202_1-p14307	C9	23	45.4	A
20519	Bn-N19-p19839861	Bn-C9-p20422304	Bn-scaff_20202_1-p14340	C9	23	45.4	A
20520	Bn-N19-p19839966	Bn-C9-p20422409	Bn-scaff_20202_1-p14350	C9	23	45.4	A
20521	Bn-N19-p19840132	Bn-C9-p20422575	Bn-scaff_20202_1-p14367:	C9	23	45.4	A
20522	Bn-N19-p19844342	Bn-C9-p20422784	Bn-scaff_20202_1-p14388	C9	23	45.4	A
20523	Bn-N19-p19844740	Bn-C9-p20423180	Bn-scaff_20202_1-p14427	C9	23	45.4	A
20524	Bn-N19-p19844901	Bn-C9-p20423878	Bn-scaff_20202_1-p14497	C9	23	45.4	A
20525	Bn-N19-p19847558	Bn-C9-p20426998	Bn-scaff_20202_1-p14809	C9	23	45.4	A
20526	Bn-N19-p19852375	Bn-C9-p20429808	Bn-scaff_20202_1-p15090	C9	23	45.4	A

20527	Bn-N19-p19852435	Bn-C9-p20429868	Bn-scaff_20202_1-p15096[illegible]	C9	23	45.4	A
20528	Bn-N19-p19855414	Bn-C9-p20436346	Bn-scaff_20202_1-p15744[illegible]	C9	23	45.4	A
20529	Bn-N19-p19855477	Bn-C9-p20436409	Bn-scaff_20202_1-p15750[illegible]	C9	23	45.4	A
20530	Bn-N19-p19893124	Bn-C9-p20458241	Bn-scaff_20202_1-p17933[illegible]	C9	23	45.4	A
20531	Bn-N19-p19893293	Bn-C9-p20458411	Bn-scaff_20202_1-p17950[illegible]	C9	23	45.4	A
20532	Bn-N19-p19904451	Bn-C9-p20458789	Bn-scaff_20202_1-p17988[illegible]	C9	23	45.4	A
20533	Bn-N19-p19905117	Bn-C9-p20459413	Bn-scaff_20202_1-p18051[illegible]	C9	23	45.4	A
20534	Bn-N19-p19906156	Bn-C9-p20460495	Bn-scaff_20202_1-p18159[illegible]	C9	23	45.4	A
20535	Bn-N19-p19907044	Bn-C9-p20461383	Bn-scaff_20202_1-p18248[illegible]	C9	23	45.4	A
20536	Bn-N19-p19907096	Bn-C9-p20462002	Bn-scaff_20202_1-p18309[illegible]	C9	23	45.4	A
20537	Bn-N19-p19907243	Bn-C9-p20462149	Bn-scaff_20202_1-p18324[illegible]	C9	23	45.4	A
20538	Bn-N19-p19907637	Bn-C9-p20462543	Bn-scaff_20202_1-p18364[illegible]	C9	23	45.4	A
20539	Bn-N19-p19919302	Bn-C9-p20468742	Bn-scaff_20202_1-p18983[illegible]	C9	23	45.4	A
20540	Bn-N19-p19919663	Bn-C9-p20469125	Bn-scaff_20202_1-p19022[illegible]	C9	23	45.4	A
20541	Bn-N19-p19936356	Bn-C9-p20489885	Bn-scaff_20202_1-p21098[illegible]	C9	23	45.4	A
20542	Bn-N19-p19942305	Bn-Scaffold00893-p1[illegible]	Bn-scaff_20168_1-p11649	C9	23	45.4	A
20543	Bn-N19-p19989760	Bn-C9-p18322922	Bn-scaff_17888_1-p8897	C9	23	45.4	A
20544	Bn-N19-p20019402	Bn-C9-p18349295	Bn-scaff_17888_1-p35270	C9	23	45.4	A
20545	Bn-N19-p20049810	Bn-C9-p18379397	Bn-scaff_17888_1-p65372	C9	23	45.4	A
20546	Bn-N19-p20135499	Bn-C9-p18484125	Bn-scaff_17888_1-p17010[illegible]	C9	23	45.4	A
20547	Bn-N19-p20135597	Bn-C9-p18484223	Bn-scaff_17888_1-p17019[illegible]	C9	23	45.4	A
20548	Bn-N19-p20138873	Bn-C9-p18487495	Bn-scaff_17888_1-p17347[illegible]	C9	23	45.4	A
20549	Bn-N19-p20139547	Bn-C9-p18497427	Bn-scaff_17888_1-p18340[illegible]	C9	23	45.4	A
20550	Bn-N19-p20146493	Bn-C9-p18505590	Bn-scaff_17888_1-p19156[illegible]	C9	23	45.4	A
20551	Bn-N19-p20154419	Bn-C9-p18508394	Bn-scaff_17888_1-p19436[illegible]	C9	23	45.4	A
20552	Bn-N19-p20154637	Bn-C9-p18508522	Bn-scaff_17888_1-p19449[illegible]	C9	23	45.4	A
20553	Bn-N19-p20154737	Bn-C9-p18508623	Bn-scaff_17888_1-p19459[illegible]	C9	23	45.4	A
20554	Bn-N19-p20180618	Bn-C9-p18536073	Bn-scaff_17888_1-p22204[illegible]	C9	23	45.4	A
20555	Bn-N19-p20192773	Bn-C9-p18542759	Bn-scaff_17888_1-p22873[illegible]	C9	23	45.4	A
20556	Bn-N19-p20195841	Bn-C9-p18545814	Bn-scaff_17888_1-p23178[illegible]	C9	23	45.4	A
20557	Bn-N9-p13517032	Bn-A09-p15362032	Bn-A09-p15362032	C9	23	45.4	A
20558	Bn-Scaffold01603-p1955	Bn-C9-p17312207	Bn-scaff_18100_1-p51275[illegible]	C9	23	45.4	A
20559	Bn-Scaffold01603-p2399	Bn-C9-p17312647	Bn-scaff_18100_1-p51319[illegible]	C9	23	45.4	A
20560	Bn-N13-p36788068	Bn-C9-p21057756	Bn-scaff_15650_1-p36856[illegible]	C9	24	46.2	0
20561	Bn-N19-p19118690	Bn-C9-p18692647	Bn-scaff_17888_1-p37862[illegible]	C9	24	46.2	A
20562	Bn-N19-p19124800	Bn-C9-p18704680	Bn-scaff_17888_1-p39065[illegible]	C9	24	46.2	A
20563	Bn-N19-p19137768	Bn-C9-p18711785	Bn-scaff_17888_1-p39776[illegible]	C9	24	46.2	A
20564	Bn-N19-p19174637	Bn-C9-p18751779	Bn-scaff_17888_1-p43775[illegible]	C9	24	46.2	A
20565	Bn-N19-p19252401	Bn-C9-p18946969	Bn-scaff_17801_1-p23357[illegible]	C9	24	46.2	A
20566	Bn-N19-p19252669	Bn-C9-p18947237	Bn-scaff_17801_1-p23331[illegible]	C9	24	46.2	A
20567	Bn-N19-p19253553	Bn-C9-p18948123	Bn-scaff_17801_1-p23242[illegible]	C9	24	46.2	A
20568	Bn-N19-p19253730	Bn-C9-p18948302	Bn-scaff_17801_1-p23224[illegible]	C9	24	46.2	A
20569	Bn-N19-p19255926	Bn-C9-p18950415	Bn-scaff_17801_1-p23013[illegible]	C9	24	46.2	A
20570	Bn-N19-p19256385	Bn-C9-p18950873	Bn-scaff_17801_1-p22967[illegible]	C9	24	46.2	A
20571	Bn-N19-p19257114	Bn-C9-p18951603	Bn-scaff_17801_1-p22894[illegible]	C9	24	46.2	A
20572	Bn-N19-p19264557	Bn-C9-p18959549	Bn-scaff_17801_1-p22099[illegible]	C9	24	46.2	A
20573	Bn-N19-p19264684	Bn-C9-p18959676	Bn-scaff_17801_1-p22087[illegible]	C9	24	46.2	A
20574	Bn-N19-p19264747	Bn-C9-p18959739	Bn-scaff_17801_1-p22080[illegible]	C9	24	46.2	A
20575	Bn-N19-p19272234	Bn-C9-p18977492	Bn-scaff_17801_1-p20305[illegible]	C9	24	46.2	A

20576	Bn-N19-p19274787	Bn-C9-p18979950	Bn-scaff_17801_1-p20059[illegible]	C9	24	46.2	A
20577	Bn-N19-p19276862	Bn-C9-p18982095	Bn-scaff_17801_1-p19845[illegible]	C9	24	46.2	A
20578	Bn-N19-p19288379	Bn-C9-p18989116	Bn-scaff_17801_1-p19143[illegible]	C9	24	46.2	A
20579	Bn-N19-p19288455	Bn-C9-p18989192	Bn-scaff_17801_1-p19135[illegible]	C9	24	46.2	A
20580	Bn-N19-p19289735	Bn-C9-p18990488	Bn-scaff_17801_1-p19005[illegible]	C9	24	46.2	A
20581	Bn-N19-p19289810	Bn-C9-p18990563	Bn-scaff_17801_1-p18998[illegible]	C9	24	46.2	A
20582	Bn-N19-p19290064	Bn-C9-p18990817	Bn-scaff_17801_1-p18973[illegible]	C9	24	46.2	A
20583	Bn-N19-p19294893	Bn-C9-p18995470	Bn-scaff_17801_1-p18507[illegible]	C9	24	46.2	A
20584	Bn-N19-p19300295	Bn-C9-p19000663	Bn-scaff_17801_1-p17988[illegible]	C9	24	46.2	A
20585	Bn-N19-p19301530	Bn-C9-p19002489	Bn-scaff_17801_1-p17805[illegible]	C9	24	46.2	A
20586	Bn-N19-p19301666	Bn-C9-p19002624	Bn-scaff_17801_1-p17792[illegible]	C9	24	46.2	A
20587	Bn-N19-p19679079	Bn-C9-p20034740	Bn-scaff_20903_1-p27902[illegible]	C9	24	46.2	A
20588	Bn-N19-p19835397	Bn-C9-p20417502	Bn-scaff_20202_1-p13859[illegible]	C9	24	46.2	A
20589	Bn-N19-p20200233	Bn-C9-p18550405	Bn-scaff_17888_1-p23638[illegible]	C9	24	46.2	A
20590	Bn-N19-p20321461	Bn-C9-p18635660	Bn-scaff_17888_1-p32163[illegible]	C9	24	46.2	A
20591	Bn-N19-p20667465	Bn-C9-p19664943	Bn-scaff_17996_1-p90674	C9	24	46.2	A
20592	Bn-N19-p20699676	Bn-C9-p19695172	Bn-scaff_17996_1-p60445	C9	24	46.2	A
20593	Bn-N19-p20748918	Bn-C9-p19708859	Bn-scaff_17996_1-p46758	C9	24	46.2	A
20594	Bn-N19-p20751394	Bn-C9-p19709044	Bn-scaff_17996_1-p46573	C9	24	46.2	A
20595	Bn-N19-p20753587	Bn-C9-p19711271	Bn-scaff_17996_1-p44346	C9	24	46.2	A
20596	Bn-N19-p20754517	Bn-C9-p19712201	Bn-scaff_17996_1-p43416	C9	24	46.2	A
20597	Bn-N19-p20756435	Bn-C9-p19714120	Bn-scaff_17996_1-p41497	C9	24	46.2	A
20598	Bn-N19-p20757318	Bn-C9-p19714999	Bn-scaff_17996_1-p40618	C9	24	46.2	A
20599	Bn-N19-p20757841	Bn-C9-p19715522	Bn-scaff_17996_1-p40095	C9	24	46.2	A
20600	Bn-N19-p20765421	Bn-C9-p19721861	Bn-scaff_17996_1-p33756	C9	24	46.2	A
20601	Bn-N19-p20765505	Bn-C9-p19721945	Bn-scaff_17996_1-p33672	C9	24	46.2	A
20602	Bn-N19-p20793001	Bn-C9-p19735711	Bn-scaff_17996_1-p19906	C9	24	46.2	A
20603	Bn-N19-p20795194	Bn-C9-p19739135	Bn-scaff_17996_1-p16482	C9	24	46.2	A
20604	Bn-N19-p20820427	Bn-Scaffold01130-p1[illegible]	Bn-scaff_15789_1-p13861	C9	24	46.2	A
20605	Bn-N19-p20843144	Bn-Scaffold01356-p3[illegible]	Bn-scaff_21906_1-p3534	C9	24	46.2	A
20606	Bn-N19-p21046147	Bn-C9-p20031187	Bn-scaff_20903_1-p27547[illegible]	C9	24	46.2	A
20607	Bn-N19-p21075198	Bn-C9-p20056535	Bn-scaff_20903_1-p30081[illegible]	C9	24	46.2	A
20608	Bn-N19-p21082371	Bn-C9-p20065981	Bn-scaff_20903_1-p31026[illegible]	C9	24	46.2	A
20609	Bn-N19-p21083707	Bn-C9-p20077419	Bn-scaff_20903_1-p32170[illegible]	C9	24	46.2	A
20610	Bn-N19-p21106733	Bn-C9-p20097157	Bn-scaff_20903_1-p34144[illegible]	C9	24	46.2	A
20611	Bn-N19-p21117338	Bn-C9-p20106801	Bn-scaff_20903_1-p35108[illegible]	C9	24	46.2	A
20612	Bn-N19-p21159907	Bn-C9-p20134786	Bn-scaff_20903_1-p37907[illegible]	C9	24	46.2	A
20613	Bn-N19-p21161317	Bn-C9-p20136375	Bn-scaff_20903_1-p38065[illegible]	C9	24	46.2	A
20614	Bn-N19-p21213676	Bn-C9-p20173445	Bn-scaff_20903_1-p41772[illegible]	C9	24	46.2	A
20615	Bn-N19-p21257997	Bn-C9-p20209272	Bn-scaff_20903_1-p45355[illegible]	C9	24	46.2	A
20616	Bn-N19-p21259964	Bn-C9-p20211253	Bn-scaff_20903_1-p45553[illegible]	C9	24	46.2	A
20617	Bn-N19-p21260985	Bn-C9-p20212272	Bn-scaff_20903_1-p45655[illegible]	C9	24	46.2	A
20618	Bn-N19-p21276754	Bn-C9-p20226448	Bn-scaff_20903_1-p47073[illegible]	C9	24	46.2	A
20619	Bn-N19-p21277057	Bn-C9-p20226146	Bn-scaff_20903_1-p47043[illegible]	C9	24	46.2	A
20620	Bn-N19-p21305423	Bn-C9-p20272922	Bn-scaff_20903_1-p51720[illegible]	C9	24	46.2	A
20621	Bn-N19-p21338800	Bn-C9-p20517683	Bn-scaff_15650_1-p90863[illegible]	C9	24	46.2	A
20622	Bn-N19-p21536094	Bn-C9-p20675871	Bn-scaff_15650_1-p75045[illegible]	C9	24	46.2	A
20623	Bn-N19-p21603035	Bn-C9-p20742917	Bn-scaff_15650_1-p68340[illegible]	C9	24	46.2	A
20624	Bn-N19-p21622805	Bn-C9-p20759364	Bn-scaff_15650_1-p66695[illegible]	C9	24	46.2	A

20625	Bn-N19-p21651158	Bn-C9-p20783308	Bn-scaff_15650_1-p643013	C9	24	46.2	A
20626	Bn-N19-p21770982	Bn-C9-p20898088	Bn-scaff_15650_1-p528233	C9	24	46.2	A
20627	Bn-N19-p21771296	Bn-C9-p20898415	Bn-scaff_15650_1-p527906	C9	24	46.2	A
20628	Bn-N19-p21831693	Bn-C9-p20945402	Bn-scaff_15650_1-p480919	C9	24	46.2	A
20629	Bn-N19-p21954228	Bn-C9-p21072398	Bn-scaff_15650_1-p353923	C9	24	46.2	A
20630	Bn-N19-p22227324	Bn-C9-p21381260	Bn-scaff_15650_1-p45061	C9	24	46.2	A
20631	Bn-N19-p22377955	Bn-C9-p21557434	Bn-scaff_23293_1-p215229	C9	24	46.2	A
20632	Bn-N19-p22378853	Bn-C9-p21558339	Bn-scaff_23293_1-p214324	C9	24	46.2	A
20633	Bn-N19-p22381852	Bn-C9-p21561350	Bn-scaff_23293_1-p211313	C9	24	46.2	A
20634	Bn-N19-p22401623	Bn-C9-p21575423	Bn-scaff_23293_1-p197240	C9	24	46.2	A
20635	Bn-N19-p22411879	Bn-C9-p21584405	Bn-scaff_23293_1-p188258	C9	24	46.2	A
20636	Bn-N19-p22425619	Bn-C9-p21596782	Bn-scaff_23293_1-p175881	C9	24	46.2	A
20637	Bn-N19-p22447022	Bn-C9-p21626786	Bn-scaff_23293_1-p145877	C9	24	46.2	A
20638	Bn-N19-p22448529	Bn-C9-p21628307	Bn-scaff_23293_1-p144356	C9	24	46.2	A
20639	Bn-N19-p22590767	Bn-C9-p21799060	Bn-scaff_17109_1-p406092	C9	24	46.2	A
20640	Bn-N19-p22632186	Bn-C9-p21831374	Bn-scaff_17109_1-p373778	C9	24	46.2	A
20641	Bn-N19-p23405095	Bn-C9-p22592360	Bn-scaff_18424_1-p878895	C9	24	46.2	A
20642	Bn-N19-p23618646	Bn-Scaffold01146-p1	Bn-scaff_28511_1-p19968	C9	24	46.2	A
20643	Bn-N19-p23711044	Bn-C9-p22936724	Bn-scaff_18424_1-p534531	C9	24	46.2	A
20644	Bn-N9-p12319883	Bn-C9-p18865725	Bn-scaff_17801_1-p314822	C9	24	46.2	A
20645	Bn-Scaffold10980-p367	Bn-C9-p18999982	Bn-scaff_17801_1-p180565	C9	24	46.2	A
20646	Bn-N14-p48894342	Bn-C5-p39799740	Bn-scaff_21124_1-p42202	C9	25	49.4	A
20647	Bn-N15-p2800417	Bn-C9-p35893531	Bn-scaff_17367_1-p502775	C9	25	49.4	A
20648	Bn-N19-p25177594	Bn-C9-p24331999	Bn-scaff_19691_1-p105535	C9	25	49.4	A
20649	Bn-N19-p25595655	Bn-C9-p29139348	Bn-scaff_17339_1-p393814	C9	25	49.4	A
20650	Bn-N19-p25689081	Bn-C9-p29038526	Bn-scaff_17339_1-p292992	C9	25	49.4	A
20651	Bn-N19-p25699435	Bn-C9-p29027837	Bn-scaff_17339_1-p282303	C9	25	49.4	A
20652	Bn-N19-p25824310	Bn-C9-p28908514	Bn-scaff_17339_1-p162980	C9	25	49.4	A
20653	Bn-N19-p25930611	Bn-C9-p28783569	Bn-scaff_17339_1-p38035	C9	25	49.4	A
20654	Bn-N19-p25937026	Bn-C9-p28777175	Bn-scaff_17339_1-p31641	C9	25	49.4	A
20655	Bn-N19-p25937089	Bn-C9-p28777112	Bn-scaff_17339_1-p31578	C9	25	49.4	A
20656	Bn-N19-p25939913	Bn-C9-p28774286	Bn-scaff_17339_1-p28752	C9	25	49.4	A
20657	Bn-N19-p25945474	Bn-C9-p28769337	Bn-scaff_17339_1-p23803	C9	25	49.4	A
20658	Bn-N19-p25945623	Bn-C9-p28769188	Bn-scaff_17339_1-p23654	C9	25	49.4	A
20659	Bn-N19-p26036141	Bn-C9-p28693866	Bn-scaff_17048_1-p337574	C9	25	49.4	A
20660	Bn-N19-p26086119	Bn-C9-p28655872	Bn-scaff_17048_1-p299580	C9	25	49.4	A
20661	Bn-N19-p26090115	Bn-C9-p28651876	Bn-scaff_17048_1-p295584	C9	25	49.4	A
20662	Bn-N19-p26090431	Bn-C9-p28651560	Bn-scaff_17048_1-p295268	C9	25	49.4	A
20663	Bn-N19-p26090708	Bn-C9-p28651283	Bn-scaff_17048_1-p294991	C9	25	49.4	A
20664	Bn-N19-p26117805	Bn-C9-p28631683	Bn-scaff_17048_1-p275391	C9	25	49.4	A
20665	Bn-N19-p26179560	Bn-C9-p28572482	Bn-scaff_17048_1-p216190	C9	25	49.4	A
20666	Bn-N19-p26189233	Bn-C9-p28562794	Bn-scaff_17048_1-p206502	C9	25	49.4	A
20667	Bn-N19-p26407845	Bn-C9-p28348122	Bn-scaff_16569_1-p674986	C9	25	49.4	A
20668	Bn-N19-p26731550	Bn-C9-p28084365	Bn-scaff_16569_1-p411229	C9	25	49.4	A
20669	Bn-N19-p28243443	Bn-C9-p26734173	Bn-scaff_19661_1-p165828	C9	25	49.4	A
20670	Bn-N19-p29780258	Bn-C9-p25056292	Bn-scaff_16793_1-p547539	C9	25	49.4	A
20671	Bn-N19-p31682054	Bn-C9-p29877786	Bn-scaff_18326_1-p422399	C9	25	49.4	A
20672	Bn-N19-p34436709	Bn-Scaffold00626-p1	Bn-scaff_28396_1-p123622	C9	25	49.4	A
20673	Bn-N19-p34471881	Bn-Scaffold00626-p1	Bn-scaff_28396_1-p165153	C9	25	49.4	A

20674	Bn-N19-p34549996	Bn-C9-p32830070	Bn-scaff_20666_1-p54157	C9	25	49.4	A
20675	Bn-N19-p34572560	Bn-C9-p32852566	Bn-scaff_20666_1-p76653	C9	25	49.4	A
20676	Bn-N19-p34587566	Bn-C9-p32868053	Bn-scaff_20666_1-p92140	C9	25	49.4	A
20677	Bn-N19-p34896485	Bn-C9-p33119374	Bn-scaff_16619_1-p155348	C9	25	49.4	A
20678	Bn-N19-p34953645	Bn-C9-p33172961	Bn-scaff_16619_1-p208935	C9	25	49.4	A
20679	Bn-N19-p35321037	Bn-C9-p33652076	Bn-scaff_17740_1-p411790	C9	25	49.4	A
20680	Bn-N19-p35978472	Bn-C9-p25604412	Bn-scaff_25543_1-p6077	C9	25	49.4	A
20681	Bn-N19-p36134261	Bn-C9-p34499534	Bn-scaff_20619_1-p693773	C9	25	49.4	A
20682	Bn-N19-p36254206	Bn-C9-p34626454	Bn-scaff_20619_1-p566853	C9	25	49.4	A
20683	Bn-N19-p36289818	Bn-C9-p34657145	Bn-scaff_20619_1-p536162	C9	25	49.4	A
20684	Bn-N19-p36292983	Bn-C9-p34660311	Bn-scaff_20619_1-p532996	C9	25	49.4	A
20685	Bn-N19-p36298172	Bn-C9-p34665294	Bn-scaff_20619_1-p528013	C9	25	49.4	A
20686	Bn-N19-p36302197	Bn-C9-p34669330	Bn-scaff_20619_1-p523977	C9	25	49.4	A
20687	Bn-N19-p36305438	Bn-C9-p34672542	Bn-scaff_20619_1-p520765	C9	25	49.4	A
20688	Bn-N19-p36317922	Bn-C9-p34773377	Bn-scaff_20619_1-p419930	C9	25	49.4	A
20689	Bn-N19-p36391787	Bn-C9-p34849287	Bn-scaff_20619_1-p344020	C9	25	49.4	A
20690	Bn-N19-p36431233	Bn-C9-p34886376	Bn-scaff_20619_1-p306931	C9	25	49.4	A
20691	Bn-N19-p36491917	Bn-C5-p5592482	Bn-scaff_15712_10-p47239	C9	25	49.4	A
20692	Bn-N19-p36513440	Bn-C9-p34945993	Bn-scaff_20619_1-p247314	C9	25	49.4	A
20693	Bn-N19-p36521263	Bn-C9-p34954690	Bn-scaff_20619_1-p238617	C9	25	49.4	A
20694	Bn-N19-p36524717	Bn-C9-p34958144	Bn-scaff_20619_1-p235163	C9	25	49.4	A
20695	Bn-N19-p36525079	Bn-C9-p34958506	Bn-scaff_20619_1-p234801	C9	25	49.4	A
20696	Bn-N19-p36525351	Bn-C9-p34958778	Bn-scaff_20619_1-p234529	C9	25	49.4	A
20697	Bn-N19-p36527111	Bn-C9-p34960568	Bn-scaff_20619_1-p232739	C9	25	49.4	A
20698	Bn-N19-p36528580	Bn-C9-p34962039	Bn-scaff_20619_1-p231268	C9	25	49.4	A
20699	Bn-N19-p36546445	Bn-C9-p34980860	Bn-scaff_20619_1-p212447	C9	25	49.4	A
20700	Bn-N19-p36546537	Bn-C9-p34980952	Bn-scaff_20619_1-p212355	C9	25	49.4	A
20701	Bn-N19-p36547604	Bn-C9-p34982019	Bn-scaff_20619_1-p211288	C9	25	49.4	A
20702	Bn-N19-p36547615	Bn-C9-p34982030	Bn-scaff_20619_1-p211277	C9	25	49.4	A
20703	Bn-N19-p36555884	Bn-C9-p34990301	Bn-scaff_20619_1-p203006	C9	25	49.4	A
20704	Bn-N19-p36593634	Bn-C9-p35021744	Bn-scaff_20619_1-p171563	C9	25	49.4	A
20705	Bn-N19-p36596203	Bn-C9-p35024213	Bn-scaff_20619_1-p169094	C9	25	49.4	A
20706	Bn-N19-p36596594	Bn-C9-p35024622	Bn-scaff_20619_1-p168685	C9	25	49.4	A
20707	Bn-N19-p36597394	Bn-C9-p35025420	Bn-scaff_20619_1-p167887	C9	25	49.4	A
20708	Bn-N19-p36597641	Bn-C9-p35025667	Bn-scaff_20619_1-p167640	C9	25	49.4	A
20709	Bn-N19-p36597679	Bn-C9-p35025705	Bn-scaff_20619_1-p167602	C9	25	49.4	A
20710	Bn-N19-p36597812	Bn-C9-p35025838	Bn-scaff_20619_1-p167469	C9	25	49.4	A
20711	Bn-N19-p36598396	Bn-C9-p35026422	Bn-scaff_20619_1-p166885	C9	25	49.4	A
20712	Bn-N19-p36601160	Bn-C9-p35029152	Bn-scaff_20619_1-p164155	C9	25	49.4	A
20713	Bn-N19-p36606040	Bn-C9-p35034031	Bn-scaff_20619_1-p159276	C9	25	49.4	A
20714	Bn-N19-p36607349	Bn-C9-p35035337	Bn-scaff_20619_1-p157970	C9	25	49.4	A
20715	Bn-N19-p36609219	Bn-C9-p35037210	Bn-scaff_20619_1-p156097	C9	25	49.4	A
20716	Bn-N19-p36620351	Bn-C9-p35048544	Bn-scaff_20619_1-p144763	C9	25	49.4	A
20717	Bn-N19-p36623092	Bn-C9-p35056079	Bn-scaff_20619_1-p137228	C9	25	49.4	A
20718	Bn-N19-p36624211	Bn-C9-p35057198	Bn-scaff_20619_1-p136109	C9	25	49.4	A
20719	Bn-N19-p36624312	Bn-C9-p35057299	Bn-scaff_20619_1-p136008	C9	25	49.4	A
20720	Bn-N19-p36625542	Bn-C9-p35058529	Bn-scaff_20619_1-p134778	C9	25	49.4	A
20721	Bn-N19-p36645943	Bn-C9-p35080430	Bn-scaff_20619_1-p112877	C9	25	49.4	A
20722	Bn-N19-p36765267	Bn-C9-p35242412	Bn-scaff_16246_2-p49006	C9	25	49.4	A

20723	Bn-N19-p36797800	Bn-C9-p35275804	Bn-scaff_16246_2-p82398	C9	25	49.4	A
20724	Bn-N19-p36942034	Bn-C9-p35400643	Bn-scaff_17367_1-p9887	C9	25	49.4	A
20725	Bn-N19-p36964112	Bn-C9-p35433556	Bn-scaff_17367_1-p42800	C9	25	49.4	A
20726	Bn-N19-p36964234	Bn-C9-p35433589	Bn-scaff_17367_1-p42833	C9	25	49.4	A
20727	Bn-N19-p36973732	Bn-C9-p35454497	Bn-scaff_17367_1-p63741	C9	25	49.4	A
20728	Bn-N19-p36975076	Bn-C9-p35455822	Bn-scaff_17367_1-p65066	C9	25	49.4	A
20729	Bn-N19-p37071656	Bn-C9-p35522874	Bn-scaff_17367_1-p13211[illegible]	C9	25	49.4	A
20730	Bn-N19-p37150513	Bn-C9-p35570137	Bn-scaff_17367_1-p17938[illegible]	C9	25	49.4	A
20731	Bn-N19-p37157330	Bn-C9-p35572431	Bn-scaff_17367_1-p18167[illegible]	C9	25	49.4	A
20732	Bn-N19-p37181808	Bn-C9-p35581596	Bn-scaff_17367_1-p19084[illegible]	C9	25	49.4	A
20733	Bn-N19-p37192932	Bn-C9-p35592508	Bn-scaff_17367_1-p20175[illegible]	C9	25	49.4	A
20734	Bn-N19-p37194094	Bn-C9-p35593673	Bn-scaff_17367_1-p20291[illegible]	C9	25	49.4	A
20735	Bn-N19-p37196006	Bn-C9-p35595594	Bn-scaff_17367_1-p20483[illegible]	C9	25	49.4	A
20736	Bn-N19-p37218201	Bn-C9-p35622370	Bn-scaff_17367_1-p23161[illegible]	C9	25	49.4	A
20737	Bn-N19-p37237183	Bn-C9-p35643039	Bn-scaff_17367_1-p25228[illegible]	C9	25	49.4	A
20738	Bn-N19-p37239210	Bn-C9-p35645028	Bn-scaff_17367_1-p25427[illegible]	C9	25	49.4	A
20739	Bn-N19-p37239335	Bn-C9-p35645153	Bn-scaff_17367_1-p25439[illegible]	C9	25	49.4	A
20740	Bn-N19-p37241112	Bn-C9-p35646927	Bn-scaff_17367_1-p25617[illegible]	C9	25	49.4	A
20741	Bn-N19-p37285169	Bn-C9-p35701030	Bn-scaff_17367_1-p31027[illegible]	C9	25	49.4	A
20742	Bn-N19-p37288812	Bn-C9-p35709442	Bn-scaff_17367_1-p31868[illegible]	C9	25	49.4	A
20743	Bn-N19-p37294200	Bn-C9-p35714268	Bn-scaff_17367_1-p32351[illegible]	C9	25	49.4	A
20744	Bn-N19-p37294435	Bn-C9-p35714503	Bn-scaff_17367_1-p32374[illegible]	C9	25	49.4	A
20745	Bn-N19-p37294603	Bn-C9-p35714672	Bn-scaff_17367_1-p32391[illegible]	C9	25	49.4	A
20746	Bn-N19-p37312410	Bn-C9-p35736310	Bn-scaff_17367_1-p34555[illegible]	C9	25	49.4	A
20747	Bn-N19-p37312602	Bn-C9-p35736501	Bn-scaff_17367_1-p34574[illegible]	C9	25	49.4	A
20748	Bn-N19-p37313953	Bn-C9-p35737852	Bn-scaff_17367_1-p34709[illegible]	C9	25	49.4	A
20749	Bn-N19-p37320797	Bn-A04-p18256793	Bn-A04-p18256793	C9	25	49.4	A
20750	Bn-N19-p37323286	Bn-C9-p35742560	Bn-scaff_17367_1-p35180[illegible]	C9	25	49.4	A
20751	Bn-N19-p37323977	Bn-C9-p35743305	Bn-scaff_17367_1-p35254[illegible]	C9	25	49.4	A
20752	Bn-N19-p37325200	Bn-C9-p35744473	Bn-scaff_17367_1-p35371[illegible]	C9	25	49.4	A
20753	Bn-N19-p37338574	Bn-C9-p35750111	Bn-scaff_17367_1-p35935[illegible]	C9	25	49.4	A
20754	Bn-N19-p37368179	Bn-C9-p35767554	Bn-scaff_17367_1-p37679[illegible]	C9	25	49.4	A
20755	Bn-N19-p37369771	Bn-C9-p35769148	Bn-scaff_17367_1-p37839[illegible]	C9	25	49.4	A
20756	Bn-N19-p37369963	Bn-C9-p35769340	Bn-scaff_17367_1-p37858[illegible]	C9	25	49.4	A
20757	Bn-N19-p37370225	Bn-C9-p35769602	Bn-scaff_17367_1-p37884[illegible]	C9	25	49.4	A
20758	Bn-N19-p37370238	Bn-C9-p35769615	Bn-scaff_17367_1-p37885[illegible]	C9	25	49.4	A
20759	Bn-N19-p37371368	Bn-C9-p35770743	Bn-scaff_17367_1-p37998[illegible]	C9	25	49.4	A
20760	Bn-N19-p37372927	Bn-C9-p35771771	Bn-scaff_17367_1-p38101[illegible]	C9	25	49.4	A
20761	Bn-N19-p37426097	Bn-C9-p35808133	Bn-scaff_17367_1-p41737[illegible]	C9	25	49.4	A
20762	Bn-N19-p37444943	Bn-C9-p41767147	Bn-scaff_15808_1-p62181[illegible]	C9	25	49.4	0
20763	Bn-N19-p37524108	Bn-C9-p35966410	Bn-scaff_17367_1-p57565[illegible]	C9	25	49.4	A
20764	Bn-N19-p37524898	Bn-C9-p35967245	Bn-scaff_17367_1-p57648[illegible]	C9	25	49.4	A
20765	Bn-N19-p37538284	Bn-C9-p35981738	Bn-scaff_17367_1-p59098[illegible]	C9	25	49.4	A
20766	Bn-N19-p37647614	Bn-C9-p36087400	Bn-scaff_17367_1-p69664[illegible]	C9	25	49.4	A
20767	Bn-N19-p37647679	Bn-C9-p36087465	Bn-scaff_17367_1-p69670[illegible]	C9	25	49.4	A
20768	Bn-N19-p37650739	Bn-C9-p36091224	Bn-scaff_17367_1-p70046[illegible]	C9	25	49.4	A
20769	Bn-N19-p37651208	Bn-C9-p36091693	Bn-scaff_17367_1-p70093[illegible]	C9	25	49.4	A
20770	Bn-N19-p37652808	Bn-C9-p36093292	Bn-scaff_17367_1-p70253[illegible]	C9	25	49.4	A
20771	Bn-N19-p37655026	Bn-C9-p36099903	Bn-scaff_17367_1-p70914[illegible]	C9	25	49.4	A

20772	Bn-N19-p37655407	Bn-C9-p36100321	Bn-scaff_17367_1-p70956!	C9	25	49.4	A
20773	Bn-N19-p37684257	Bn-C9-p36127939	Bn-scaff_17367_1-p73718:	C9	25	49.4	A
20774	Bn-N19-p37784810	Bn-C9-p36240924	Bn-scaff_17367_1-p85016	C9	25	49.4	A
20775	Bn-N19-p37908613	Bn-C9-p36373227	Bn-scaff_20972_1-p98600	C9	25	49.4	A
20776	Bn-Scaffold01071-p14399	Bn-C9-p34730248	Bn-scaff_20619_1-p46305	C9	25	49.4	A
20777	Bn-Scaffold01071-p21136	Bn-C9-p34736251	Bn-scaff_20619_1-p45705	C9	25	49.4	A
20778	Bn-Scaffold01071-p21458	Bn-C9-p34736573	Bn-scaff_20619_1-p45673	C9	25	49.4	A
20779	Bn-Scaffold01071-p27045	Bn-C9-p34742160	Bn-scaff_20619_1-p45114	C9	25	49.4	A
20780	Bn-Scaffold04087-p2080	Bn-C9-p28890972	Bn-scaff_17339_1-p14543	C9	25	49.4	A
20781	Bn-Scaffold09127-p905	Bn-C9-p28637746	Bn-scaff_17048_1-p28145	C9	25	49.4	A
20782	Bn-Scaffold23213-p219	Bn-C9-p35894386	Bn-scaff_17367_1-p50363	C9	25	49.4	A
20783	Bn-Scaffold82268-p102	Bn-C9-p35886517	Bn-scaff_17367_1-p49576	C9	25	49.4	A
20784	Bn-N10-p7602231	Bn-C9-p36661755	Bn-scaff_15930_1-p10496	C9	26	50.2	A
20785	Bn-N19-p37974163	Bn-C9-p36438578	Bn-scaff_20972_1-p16395	C9	26	50.2	A
20786	Bn-N19-p38170363	Bn-C7-p201797	Bn-scaff_15627_1-p73207	C9	26	50.2	A
20787	Bn-N19-p38254946	Bn-A10-p6012827	Bn-A10-p6012827	C9	26	50.2	A
20788	Bn-N19-p38504986	Bn-C9-p36846648	Bn-scaff_17910_1-p79832	C9	26	50.2	A
20789	Bn-N19-p38533778	Bn-C9-p36893596	Bn-scaff_17910_1-p12678	C9	26	50.2	A
20790	Bn-Scaffold01355-p330	Bn-Scaffold01305-p9	Bn-scaff_17910_2-p9166	C9	26	50.2	A
20791	Bn-N19-p38982545	Bn-C7-p13328709	Bn-scaff_15712_7-p58147	C9	27	55.9	B
20792	Bn-N19-p38983574	Bn-C7-p13327679	Bn-scaff_15712_7-p57117	C9	27	55.9	B
20793	Bn-N19-p39038724	Bn-A02-p20062658	Bn-A02-p20062658	C9	27	55.9	B
20794	Bn-N19-p39528164	Bn-C9-p37714282	Bn-scaff_17919_1-p17773	C9	27	55.9	B
20795	Bn-N19-p39607653	Bn-C9-p37794459	Bn-scaff_17919_1-p97553	C9	27	55.9	B
20796	Bn-N19-p40065238	Bn-C9-p38373521	Bn-scaff_16456_1-p45340	C9	27	55.9	B
20797	Bn-N19-p40101977	Bn-C9-p38420546	Bn-scaff_16456_1-p40637	C9	27	55.9	B
20798	Bn-N19-p40110552	Bn-C9-p38423087	Bn-scaff_16456_1-p40383	C9	27	55.9	B
20799	Bn-N19-p40250621	Bn-C9-p38596827	Bn-scaff_16456_1-p23009	C9	27	55.9	B
20800	Bn-N19-p40565817	Bn-C9-p38948894	Bn-scaff_19483_1-p12187	C9	27	55.9	B
20801	Bn-Scaffold01091-p14619	Bn-C9-p38477912	Bn-scaff_16456_1-p34901	C9	27	55.9	B
20802	Bn-Scaffold29888-p269	Bn-C9-p37797693	Bn-scaff_17919_1-p94319	C9	27	55.9	B
20803	Bn-N10-p10130407	Bn-C9-p41674407	Bn-scaff_15808_1-p52907	C9	28	56.7	B
20804	Bn-N13-p25811782	Bn-C3-p24550632	Bn-scaff_17298_1-p33495	C9	28	56.7	B
20805	Bn-N19-p42314902	Bn-C9-p40440348	Bn-scaff_15743_1-p26884	C9	28	56.7	B
20806	Bn-N19-p42315259	Bn-C9-p40440705	Bn-scaff_15743_1-p26527	C9	28	56.7	B
20807	Bn-N19-p42315271	Bn-C9-p40440717	Bn-scaff_15743_1-p26515	C9	28	56.7	B
20808	Bn-N19-p42548267	Bn-Scaffold01417-p3	Bn-scaff_27554_1-p3917	C9	28	56.7	B
20809	Bn-N19-p42548736	Bn-Scaffold01417-p3	Bn-scaff_27554_1-p3448	C9	28	56.7	B
20810	Bn-N19-p42577907	Bn-C9-p40681914	Bn-scaff_21276_1-p18827	C9	28	56.7	B
20811	Bn-N19-p42593645	Bn-C9-p40697745	Bn-scaff_21276_1-p20410	C9	28	56.7	B
20812	Bn-N19-p42700358	Bn-C9-p40786412	Bn-scaff_21276_1-p29277	C9	28	56.7	B
20813	Bn-N19-p42708708	Bn-C9-p40796028	Bn-scaff_21276_1-p30239	C9	28	56.7	B
20814	Bn-N19-p42709986	Bn-C9-p40797307	Bn-scaff_21276_1-p30367	C9	28	56.7	B
20815	Bn-N19-p42747765	Bn-C9-p40838718	Bn-scaff_21276_1-p34508	C9	28	56.7	B
20816	Bn-N19-p42748005	Bn-C9-p40838942	Bn-scaff_21276_1-p34530	C9	28	56.7	B
20817	Bn-N19-p42768868	Bn-C9-p40848530	Bn-scaff_21276_1-p35489	C9	28	56.7	B
20818	Bn-N19-p42792757	Bn-C9-p40871883	Bn-scaff_21276_1-p37824	C9	28	56.7	B
20819	Bn-N19-p42807804	Bn-C9-p40886521	Bn-scaff_21276_1-p39288	C9	28	56.7	B
20820	Bn-N19-p42968062	Bn-C9-p40997854	Bn-scaff_21276_1-p50421	C9	28	56.7	B

20821	Bn-N19-p42989152	Bn-C9-p41018061	Bn-scaff_21276_1-p524424	C9	28	56.7	B
20822	Bn-N19-p43411238	Bn-C9-p41385488	Bn-scaff_15808_1-p240156	C9	28	56.7	B
20823	Bn-N19-p43560390	Bn-C9-p41533626	Bn-scaff_15808_1-p388294	C9	28	56.7	B
20824	Bn-N19-p43564336	Bn-C9-p41535774	Bn-scaff_15808_1-p390442	C9	28	56.7	B
20825	Bn-N19-p43566204	Bn-C9-p41537412	Bn-scaff_15808_1-p392080	C9	28	56.7	B
20826	Bn-N19-p43567591	Bn-C9-p41538781	Bn-scaff_15808_1-p393449	C9	28	56.7	B
20827	Bn-N19-p43582202	Bn-C9-p41550174	Bn-scaff_15808_1-p404842	C9	28	56.7	B
20828	Bn-N19-p43583099	Bn-C9-p41551270	Bn-scaff_15808_1-p405938	C9	28	56.7	B
20829	Bn-N19-p43583445	Bn-C9-p41551616	Bn-scaff_15808_1-p406284	C9	28	56.7	B
20830	Bn-N19-p43583481	Bn-C9-p41551652	Bn-scaff_15808_1-p406320	C9	28	56.7	B
20831	Bn-N19-p43586216	Bn-C9-p41554370	Bn-scaff_15808_1-p409038	C9	28	56.7	B
20832	Bn-N19-p43587357	Bn-C9-p41555311	Bn-scaff_15808_1-p409979	C9	28	56.7	B
20833	Bn-N19-p43588828	Bn-C9-p41557115	Bn-scaff_15808_1-p411783	C9	28	56.7	B
20834	Bn-N19-p43588890	Bn-C9-p41557177	Bn-scaff_15808_1-p411845	C9	28	56.7	B
20835	Bn-N19-p43594190	Bn-C9-p41560035	Bn-scaff_15808_1-p414703	C9	28	56.7	B
20836	Bn-N19-p43596172	Bn-C9-p41562017	Bn-scaff_15808_1-p416685	C9	28	56.7	B
20837	Bn-N19-p43596343	Bn-C9-p41562188	Bn-scaff_15808_1-p416856	C9	28	56.7	B
20838	Bn-N19-p43597673	Bn-C9-p41563518	Bn-scaff_15808_1-p418186	C9	28	56.7	B
20839	Bn-N19-p43598178	Bn-C9-p41564019	Bn-scaff_15808_1-p418687	C9	28	56.7	B
20840	Bn-N19-p43599075	Bn-C9-p41564846	Bn-scaff_15808_1-p419514	C9	28	56.7	B
20841	Bn-N19-p43600343	Bn-C9-p41566132	Bn-scaff_15808_1-p420800	C9	28	56.7	B
20842	Bn-N19-p43604347	Bn-C9-p41570145	Bn-scaff_15808_1-p424813	C9	28	56.7	B
20843	Bn-N19-p43605045	Bn-C9-p41570844	Bn-scaff_15808_1-p425512	C9	28	56.7	B
20844	Bn-N19-p43605868	Bn-C9-p41571668	Bn-scaff_15808_1-p426336	C9	28	56.7	B
20845	Bn-N19-p43611388	Bn-C9-p41575402	Bn-scaff_15808_1-p430070	C9	28	56.7	B
20846	Bn-N19-p43611451	Bn-C9-p41575465	Bn-scaff_15808_1-p430133	C9	28	56.7	B
20847	Bn-N19-p43611965	Bn-C9-p41575979	Bn-scaff_15808_1-p430647	C9	28	56.7	B
20848	Bn-N19-p43612296	Bn-C9-p41576310	Bn-scaff_15808_1-p430978	C9	28	56.7	B
20849	Bn-N19-p43612640	Bn-C9-p41576654	Bn-scaff_15808_1-p431322	C9	28	56.7	B
20850	Bn-N19-p43612877	Bn-C9-p41576891	Bn-scaff_15808_1-p431559	C9	28	56.7	B
20851	Bn-N19-p43613843	Bn-C9-p41577857	Bn-scaff_15808_1-p432525	C9	28	56.7	B
20852	Bn-N19-p43613911	Bn-C9-p41577925	Bn-scaff_15808_1-p432593	C9	28	56.7	B
20853	Bn-N19-p43613941	Bn-C9-p41577955	Bn-scaff_15808_1-p432623	C9	28	56.7	B
20854	Bn-N19-p43614257	Bn-C9-p41578278	Bn-scaff_15808_1-p432946	C9	28	56.7	B
20855	Bn-N19-p43614433	Bn-C9-p41578455	Bn-scaff_15808_1-p433123	C9	28	56.7	B
20856	Bn-N19-p43614505	Bn-C9-p41578527	Bn-scaff_15808_1-p433195	C9	28	56.7	B
20857	Bn-N19-p43614806	Bn-C9-p41578828	Bn-scaff_15808_1-p433496	C9	28	56.7	B
20858	Bn-N19-p43615255	Bn-C9-p41579277	Bn-scaff_15808_1-p433945	C9	28	56.7	B
20859	Bn-N19-p43615653	Bn-C9-p41579674	Bn-scaff_15808_1-p434342	C9	28	56.7	B
20860	Bn-N19-p43618226	Bn-C9-p41582662	Bn-scaff_15808_1-p437330	C9	28	56.7	B
20861	Bn-N19-p43627062	Bn-C9-p41585717	Bn-scaff_15808_1-p440385	C9	28	56.7	B
20862	Bn-N19-p43631573	Bn-C9-p41587632	Bn-scaff_15808_1-p442300	C9	28	56.7	B
20863	Bn-N19-p43635224	Bn-C9-p41591331	Bn-scaff_15808_1-p445999	C9	28	56.7	B
20864	Bn-N19-p43636655	Bn-C9-p41592033	Bn-scaff_15808_1-p446701	C9	28	56.7	B
20865	Bn-N19-p43704435	Bn-C9-p41664997	Bn-scaff_15808_1-p519665	C9	28	56.7	B
20866	Bn-N19-p43758904	Bn-C9-p41720925	Bn-scaff_15808_1-p575593	C9	28	56.7	B
20867	Bn-N19-p43768862	Bn-C9-p41728414	Bn-scaff_15808_1-p583082	C9	28	56.7	B
20868	Bn-N19-p43776621	Bn-C9-p41731038	Bn-scaff_15808_1-p585706	C9	28	56.7	B
20869	Bn-N19-p43776753	Bn-C9-p41731170	Bn-scaff_15808_1-p585838	C9	28	56.7	B

20870	Bn-N19-p43877881	Bn-C9-p41791032	Bn-scaff_15808_1-p645700	C9	28	56.7	B
20871	Bn-N19-p43877887	Bn-C9-p41791039	Bn-scaff_15808_1-p645707	C9	28	56.7	B
20872	Bn-N19-p43878942	Bn-C9-p41792093	Bn-scaff_15808_1-p646761	C9	28	56.7	B
20873	Bn-N19-p43997828	Bn-C9-p41929425	Bn-scaff_15808_1-p784093	C9	28	56.7	B
20874	Bn-N19-p43999734	Bn-C9-p41931343	Bn-scaff_15808_1-p786011	C9	28	56.7	B
20875	Bn-N19-p44008971	Bn-C9-p41943194	Bn-scaff_15808_1-p797862	C9	28	56.7	B
20876	Bn-N19-p44031695	Bn-Scaffold01068-p98	Bn-scaff_23208_1-p9844	C9	28	56.7	B
20877	Bn-N19-p44048624	Bn-C9-p41964515	Bn-scaff_25457_1-p307463	C9	28	56.7	B
20878	Bn-N19-p44049315	Bn-C9-p41965209	Bn-scaff_25457_1-p306769	C9	28	56.7	B
20879	Bn-N19-p44049538	Bn-C9-p41965428	Bn-scaff_25457_1-p306550	C9	28	56.7	B
20880	Bn-N19-p44049587	Bn-C9-p41965477	Bn-scaff_25457_1-p306501	C9	28	56.7	B
20881	Bn-N19-p44049779	Bn-C9-p41965670	Bn-scaff_25457_1-p306308	C9	28	56.7	B
20882	Bn-N19-p44049836	Bn-C9-p41965727	Bn-scaff_25457_1-p306251	C9	28	56.7	B
20883	Bn-N19-p44050061	Bn-C9-p41965952	Bn-scaff_25457_1-p306026	C9	28	56.7	B
20884	Bn-N19-p44051435	Bn-C9-p41967004	Bn-scaff_25457_1-p304974	C9	28	56.7	B
20885	Bn-N19-p44063701	Bn-C9-p41974965	Bn-scaff_25457_1-p297013	C9	28	56.7	B
20886	Bn-N19-p44070789	Bn-C9-p41985175	Bn-scaff_25457_1-p286803	C9	28	56.7	B
20887	Bn-N19-p44100674	Bn-C9-p41988582	Bn-scaff_25457_1-p283396	C9	28	56.7	B
20888	Bn-N19-p44101433	Bn-C9-p41989341	Bn-scaff_25457_1-p282637	C9	28	56.7	B
20889	Bn-N19-p44101677	Bn-C9-p41989585	Bn-scaff_25457_1-p282393	C9	28	56.7	B
20890	Bn-N19-p44101755	Bn-C9-p41989663	Bn-scaff_25457_1-p282315	C9	28	56.7	B
20891	Bn-N19-p44101833	Bn-C9-p41989741	Bn-scaff_25457_1-p282237	C9	28	56.7	B
20892	Bn-N19-p44103682	Bn-C9-p41991584	Bn-scaff_25457_1-p280394	C9	28	56.7	B
20893	Bn-N19-p44104128	Bn-C9-p41992030	Bn-scaff_25457_1-p279948	C9	28	56.7	B
20894	Bn-N19-p44104785	Bn-C9-p41992687	Bn-scaff_25457_1-p279291	C9	28	56.7	B
20895	Bn-N19-p44105020	Bn-C9-p41992922	Bn-scaff_25457_1-p279056	C9	28	56.7	B
20896	Bn-N19-p44107438	Bn-C9-p41995340	Bn-scaff_25457_1-p276638	C9	28	56.7	B
20897	Bn-N19-p44107920	Bn-C9-p41995823	Bn-scaff_25457_1-p276155	C9	28	56.7	B
20898	Bn-N19-p44111244	Bn-C9-p42004846	Bn-scaff_25457_1-p267132	C9	28	56.7	B
20899	Bn-N19-p44116266	Bn-C9-p42010107	Bn-scaff_25457_1-p261871	C9	28	56.7	B
20900	Bn-N19-p44116561	Bn-C9-p42010408	Bn-scaff_25457_1-p261570	C9	28	56.7	B
20901	Bn-N19-p44119976	Bn-C9-p42013884	Bn-scaff_25457_1-p258094	C9	28	56.7	B
20902	Bn-N4-p8900853	Bn-Scaffold01848-p50	Bn-scaff_28191_1-p507	C9	28	56.7	B
20903	Bn-N19-p45447749	Bn-A10-p9271807	Bn-A10-p9271807	C9	29	58.3	B
20904	Bn-N19-p45517324	Bn-C9-p43400331	Bn-scaff_23622_1-p3989	C9	29	58.3	B
20905	Bn-N19-p45819748	Bn-C9-p43680237	Bn-scaff_15881_1-p99457	C9	29	58.3	B
20906	Bn-N19-p45833478	Bn-C9-p43699725	Bn-scaff_15881_1-p118945	C9	29	58.3	B
20907	Bn-N19-p45838088	Bn-C9-p43704304	Bn-scaff_15881_1-p123524	C9	29	58.3	B
20908	Bn-N19-p45851910	Bn-C9-p43720221	Bn-scaff_15881_1-p139441	C9	30	59.1	B
20909	Bn-N19-p45868247	Bn-C9-p43734709	Bn-scaff_15881_1-p153929	C9	30	59.1	B
20910	Bn-N19-p45868267	Bn-C9-p43734729	Bn-scaff_15881_1-p153949	C9	30	59.1	B
20911	Bn-N19-p45882587	Bn-C9-p43742134	Bn-scaff_15881_1-p161354	C9	30	59.1	B
20912	Bn-N19-p45888824	Bn-C9-p43748019	Bn-scaff_15881_1-p167239	C9	30	59.1	B
20913	Bn-N19-p45889088	Bn-C9-p43748281	Bn-scaff_15881_1-p167501	C9	30	59.1	B
20914	Bn-N19-p45906521	Bn-C9-p43762593	Bn-scaff_15881_1-p181813	C9	30	59.1	B
20915	Bn-N19-p45949923	Bn-Scaffold00916-p21	Bn-scaff_23903_1-p21341	C9	30	59.1	B
20916	Bn-N19-p45949959	Bn-Scaffold00916-p21	Bn-scaff_23903_1-p21377	C9	30	59.1	B
20917	Bn-N19-p45959052	Bn-Scaffold00916-p31	Bn-scaff_23903_1-p31457	C9	30	59.1	B
20918	Bn-N19-p45972796	Bn-Scaffold00916-p44	Bn-scaff_23903_1-p44421	C9	30	59.1	B

20919	Bn-N19-p45973494	Bn-Scaffold00916-p4!	Bn-scaff_23903_1-p45147	C9	30	59.1	B
20920	Bn-N19-p46003589	Bn-C9-p43801540	Bn-scaff_17799_1-p30506(	C9	30	59.1	B
20921	Bn-N19-p46008716	Bn-C9-p43801774	Bn-scaff_17799_1-p305037	C9	30	59.1	B
20922	Bn-N19-p46009047	Bn-C9-p43802105	Bn-scaff_17799_1-p30500‹	C9	30	59.1	B
20923	Bn-N19-p46009127	Bn-C9-p43802185	Bn-scaff_17799_1-p30499(	C9	30	59.1	B
20924	Bn-N19-p46009347	Bn-C9-p43802405	Bn-scaff_17799_1-p30497‹	C9	30	59.1	B
20925	Bn-N19-p46041580	Bn-C9-p43822424	Bn-scaff_17799_1-p30297:	C9	31	59.9	B
20926	Bn-N19-p46070104	Bn-C9-p43841254	Bn-scaff_17799_1-p30108!	C9	31	59.9	B
20927	Bn-N19-p46079636	Bn-C9-p43849825	Bn-scaff_17799_1-p30023:	C9	31	59.9	B
20928	Bn-N19-p46087296	Bn-C9-p43857488	Bn-scaff_17799_1-p29946(	C9	31	59.9	B
20929	Bn-N19-p46087319	Bn-C9-p43857511	Bn-scaff_17799_1-p29946:	C9	31	59.9	B
20930	Bn-N19-p46092475	Bn-C9-p43861926	Bn-scaff_17799_1-p29902:	C9	31	59.9	B
20931	Bn-N19-p46154952	Bn-C9-p43914358	Bn-scaff_17799_1-p29377!	C9	31	59.9	B
20932	Bn-N19-p46185029	Bn-Scaffold01002-p1!	Bn-scaff_17554_1-p19888	C9	31	59.9	B
20933	Bn-N19-p46190777	Bn-Scaffold01002-p2:	Bn-scaff_17554_1-p21274	C9	31	59.9	B
20934	Bn-N19-p46204439	Bn-Scaffold01002-p2{	Bn-scaff_17554_1-p28732	C9	31	59.9	B
20935	Bn-N19-p46217289	Bn-C9-p43934276	Bn-scaff_17799_1-p29178;	C9	31	59.9	B
20936	Bn-N19-p46247286	Bn-C9-p43966881	Bn-scaff_17799_1-p28852(	C9	31	59.9	B
20937	Bn-N19-p46257452	Bn-Scaffold01590-p4(	Bn-scaff_24451_1-p4603	C9	31	59.9	B
20938	Bn-N19-p46267871	Bn-C9-p43982840	Bn-scaff_17799_1-p28693(	C9	31	59.9	B
20939	Bn-N19-p46277198	Bn-C9-p43993177	Bn-scaff_17799_1-p28589;	C9	31	59.9	B
20940	Bn-N19-p46279746	Bn-C9-p43995714	Bn-scaff_17799_1-p28564:	C9	31	59.9	B
20941	Bn-N19-p46292769	Bn-C9-p44008794	Bn-scaff_17799_1-p28433!	C9	31	59.9	B
20942	Bn-N19-p46294444	Bn-C9-p44010466	Bn-scaff_17799_1-p28416{	C9	31	59.9	B
20943	Bn-N19-p46295211	Bn-C9-p44011252	Bn-scaff_17799_1-p28408!	C9	31	59.9	B
20944	Bn-N19-p46297441	Bn-C9-p44013483	Bn-scaff_17799_1-p28386(	C9	31	59.9	B
20945	Bn-N19-p46314383	Bn-C9-p44029876	Bn-scaff_17799_1-p28222;	C9	31	59.9	B
20946	Bn-N19-p46315260	Bn-C9-p44030754	Bn-scaff_17799_1-p28213!	C9	31	59.9	B
20947	Bn-N19-p46352856	Bn-C9-p44067046	Bn-scaff_17799_1-p27851(	C9	31	59.9	B
20948	Bn-N19-p46357631	Bn-C9-p44076536	Bn-scaff_17799_1-p277561	C9	31	59.9	B
20949	Bn-N19-p46357718	Bn-C9-p44076623	Bn-scaff_17799_1-p27755:	C9	31	59.9	B
20950	Bn-N19-p46357758	Bn-C9-p44076663	Bn-scaff_17799_1-p27754{	C9	31	59.9	B
20951	Bn-N19-p46358854	Bn-C9-p44077759	Bn-scaff_17799_1-p27743{	C9	31	59.9	B
20952	Bn-N19-p46359274	Bn-C9-p44078179	Bn-scaff_17799_1-p27739(	C9	31	59.9	B
20953	Bn-N19-p46367929	Bn-C9-p44087410	Bn-scaff_17799_1-p27647:	C9	31	59.9	B
20954	Bn-N10-p11698546	Bn-A10-p10083397	Bn-A10-p10083397	C9	32	63.1	B
20955	Bn-N19-p46571823	Bn-C9-p34098306	Bn-scaff_17275_1-p34341	C9	32	63.1	B
20956	Bn-N19-p46754810	Bn-C9-p44386081	Bn-scaff_17088_1-p14656‹	C9	32	63.1	B
20957	Bn-N19-p46949448	Bn-C9-p44566589	Bn-scaff_17088_1-p32706!	C9	32	63.1	B
20958	Bn-N19-p47334910	Bn-C9-p44973813	Bn-scaff_19899_1-p65159(	C9	33	63.9	B
20959	Bn-N19-p47451063	Bn-C9-p45066216	Bn-scaff_19899_1-p55918;	C9	33	63.9	B
20960	Bn-N19-p47611007	Bn-C9-p45267308	Bn-scaff_19899_1-p35809!	C9	34	67.9	B
20961	Bn-N19-p47678139	Bn-C9-p45342222	Bn-scaff_19899_1-p28318‹	C9	34	67.9	B
20962	Bn-N19-p47939658	Bn-C9-p45590669	Bn-scaff_19899_1-p34734	C9	35	68.7	B
20963	Bn-N19-p47957574	Bn-C9-p45609390	Bn-scaff_19899_1-p16013	C9	35	68.7	B
20964	Bn-N19-p47994995	Bn-Scaffold00852-p4{	Bn-scaff_21311_1-p48896	C9	35	68.7	B
20965	Bn-N19-p48005308	Bn-Scaffold00852-p3{	Bn-scaff_21311_1-p38653	C9	35	68.7	B
20966	Bn-N19-p48046580	Bn-Scaffold00915-p4:	Bn-scaff_15576_1-p47598	C9	35	68.7	B
20967	Bn-N19-p48064738	Bn-Scaffold01250-p1:	Bn-scaff_15576_1-p67692	C9	35	68.7	B

20968	Bn-N19-p48064948	Bn-Scaffold01250-p14	Bn-scaff_15576_1-p67905	C9	35	68.7	B
20969	Bn-N19-p48065074	Bn-Scaffold01250-p14	Bn-scaff_15576_1-p68031	C9	35	68.7	B
20970	Bn-N19-p48065160	Bn-Scaffold01250-p14	Bn-scaff_15576_1-p68117	C9	35	68.7	B
20971	Bn-N19-p48065521	Bn-Scaffold01250-p14	Bn-scaff_15576_1-p68473	C9	35	68.7	B
20972	Bn-N19-p48065671	Bn-Scaffold01250-p15	Bn-scaff_15576_1-p69050	C9	35	68.7	B
20973	Bn-N19-p48065728	Bn-Scaffold01250-p15	Bn-scaff_15576_1-p69107	C9	35	68.7	B
20974	Bn-N19-p48065816	Bn-Scaffold01250-p15	Bn-scaff_15576_1-p69195	C9	35	68.7	B
20975	Bn-N19-p48065968	Bn-Scaffold01250-p15	Bn-scaff_15576_1-p69347	C9	35	68.7	B
20976	Bn-N19-p48066243	Bn-Scaffold01250-p15	Bn-scaff_15576_1-p69622	C9	35	68.7	B
20977	Bn-N19-p48066498	Bn-Scaffold01250-p16	Bn-scaff_15576_1-p69876	C9	35	68.7	B
20978	Bn-N19-p48068773	Bn-C9-p45626480	Bn-scaff_15576_1-p74822	C9	35	68.7	B
20979	Bn-N19-p48068932	Bn-C9-p45626638	Bn-scaff_15576_1-p74980	C9	35	68.7	B
20980	Bn-N19-p48069234	Bn-C9-p45626944	Bn-scaff_15576_1-p75286	C9	35	68.7	B
20981	Bn-N19-p48107009	Bn-C9-p45663010	Bn-scaff_15576_1-p111352	C9	35	68.7	B
20982	Bn-N19-p48110932	Bn-C9-p45666924	Bn-scaff_15576_1-p115266	C9	35	68.7	B
20983	Bn-N19-p48227200	Bn-C9-p45792521	Bn-scaff_15576_1-p240863	C9	35	68.7	B
20984	Bn-N19-p48551956	Bn-C9-p46091296	Bn-scaff_15576_1-p539638	C9	35	68.7	B
20985	Bn-N19-p48552942	Bn-C9-p46092282	Bn-scaff_15576_1-p540624	C9	35	68.7	B
20986	Bn-N19-p48595649	Bn-C9-p46125433	Bn-scaff_15576_1-p573775	C9	35	68.7	B
20987	Bn-N19-p48600957	Bn-C9-p46130727	Bn-scaff_15576_1-p579069	C9	35	68.7	B
20988	Bn-N19-p48607852	Bn-C9-p46137404	Bn-scaff_15576_1-p585746	C9	35	68.7	B
20989	Bn-N19-p48607911	Bn-C9-p46137463	Bn-scaff_15576_1-p585805	C9	35	68.7	B
20990	Bn-N19-p48634847	Bn-C9-p46160117	Bn-scaff_15576_1-p608459	C9	35	68.7	B
20991	Bn-N19-p48636400	Bn-C9-p46161628	Bn-scaff_15576_1-p609970	C9	35	68.7	B
20992	Bn-N10-p12629368	Bn-C9-p46215031	Bn-scaff_15576_1-p663373	C9	36	70.3	B
20993	Bn-N19-p48639808	Bn-C9-p46165884	Bn-scaff_15576_1-p614226	C9	36	70.3	B
20994	Bn-N19-p48733846	Bn-C9-p46208645	Bn-scaff_15576_1-p656987	C9	36	70.3	B
20995	Bn-N19-p48742009	Bn-C9-p46212196	Bn-scaff_15576_1-p660538	C9	36	70.3	B
20996	Bn-N19-p48744959	Bn-C9-p46215140	Bn-scaff_15576_1-p663482	C9	36	70.3	B
20997	Bn-N19-p48757597	Bn-C9-p46219676	Bn-scaff_15576_1-p668018	C9	36	70.3	B
20998	Bn-N19-p48788170	Bn-C9-p46278900	Bn-scaff_15576_1-p727242	C9	36	70.3	B
20999	Bn-N19-p48800264	Bn-C9-p46291594	Bn-scaff_15576_1-p739936	C9	36	70.3	B
21000	Bn-N19-p48822467	Bn-C9-p46313422	Bn-scaff_15576_1-p761764	C9	36	70.3	B
21001	Bn-N19-p48833620	Bn-C9-p46324161	Bn-scaff_15576_1-p772503	C9	36	70.3	B
21002	Bn-N19-p48877654	Bn-C9-p46349494	Bn-scaff_15576_1-p797836	C9	36	70.3	B
21003	Bn-N19-p48895188	Bn-C9-p46366623	Bn-scaff_15576_1-p814965	C9	36	70.3	B
21004	Bn-N19-p48916872	Bn-C9-p46388981	Bn-scaff_15576_1-p837323	C9	36	70.3	B
21005	Bn-N19-p48917458	Bn-C9-p46389460	Bn-scaff_15576_1-p837802	C9	36	70.3	B
21006	Bn-N19-p48935687	Bn-C9-p46407260	Bn-scaff_15576_1-p855602	C9	36	70.3	B
21007	Bn-N19-p48966796	Bn-C9-p46457508	Bn-scaff_15576_1-p905850	C9	37	71.1	B
21008	Bn-N19-p48992662	Bn-C13791383-p286	Bn-C13791383-p286	C9	37	71.1	B
21009	Bn-N19-p48993248	Bn-Scaffold06309-p24	Bn-scaff_24057_1-p246	C9	37	71.1	B
21010	Bn-N19-p49007221	Bn-C9-p46484322	Bn-scaff_16962_1-p17444	C9	37	71.1	B
21011	Bn-N19-p49007991	Bn-C9-p46485092	Bn-scaff_16962_1-p18214	C9	37	71.1	B
21012	Bn-N19-p49008972	Bn-C9-p46486073	Bn-scaff_16962_1-p19195	C9	37	71.1	B
21013	Bn-N19-p49011102	Bn-C9-p46488203	Bn-scaff_16962_1-p21325	C9	37	71.1	B
21014	Bn-N19-p49011827	Bn-C9-p46488928	Bn-scaff_16962_1-p22050	C9	37	71.1	B
21015	Bn-N19-p49137317	Bn-A10-p11238287	Bn-A10-p11238287	C9	38	71.9	B
21016	Bn-N19-p49157514	Bn-C9-p46830604	Bn-scaff_27705_1-p129876	C9	38	71.9	B

21017	Bn-N19-p49165590	Bn-C9-p46838649	Bn-scaff_27705_1-p121831	C9	38	71.9	B
21018	Bn-N19-p49176335	Bn-C9-p46849393	Bn-scaff_27705_1-p111087	C9	38	71.9	B
21019	Bn-N19-p49299421	Bn-C9-p47004610	Bn-scaff_17028_1-p9587	C9	39	72.7	B
21020	Bn-N19-p49725586	Bn-C9-p47391900	Bn-scaff_17028_1-p396877	C9	40	75.9	B
21021	Bn-N19-p49750038	Bn-C9-p47427011	Bn-scaff_17028_1-p431988	C9	40	75.9	B
21022	Bn-N19-p49750055	Bn-C9-p47427028	Bn-scaff_17028_1-p432005	C9	40	75.9	B
21023	Bn-N19-p49764122	Bn-C9-p47445602	Bn-scaff_17028_1-p450579	C9	40	75.9	B
21024	Bn-N19-p49794501	Bn-A10-p12810049	Bn-A10-p12810049	C9	40	75.9	B
21025	Bn-N19-p49796719	Bn-C9-p47457064	Bn-scaff_21841_1-p173	C9	40	75.9	B
21026	Bn-N19-p49806198	Bn-C9-p47466846	Bn-scaff_21841_1-p9955	C9	40	75.9	B
21027	Bn-N19-p49954038	Bn-A10-p12901822	Bn-A10-p12901822	C9	40	75.9	B
21028	Bn-N19-p49960557	Bn-C2-p5599700	Bn-scaff_28612_1-p31093	C9	40	75.9	B
21029	Bn-N19-p49966534	Bn-C9-p47645007	Bn-scaff_21841_1-p188116	C9	40	75.9	B
21030	Bn-N10-p13354467	Bn-A10-p12958087	Bn-A10-p12958087	C9	41	79.9	B
21031	Bn-N18-p23623944	Bn-C7-p2721181	Bn-scaff_17708_1-p157593	C9	41	79.9	B
21032	Bn-N19-p50008223	Bn-A10-p12957851	Bn-A10-p12957851	C9	41	79.9	B
21033	Bn-N19-p50090437	Bn-C9-p47786366	Bn-scaff_21841_1-p329475	C9	41	79.9	B
21034	Bn-N19-p50272436	Bn-C9-p47931746	Bn-scaff_16362_1-p713218	C9	42	80.7	B
21035	Bn-N19-p50302870	Bn-C9-p47989399	Bn-scaff_16362_1-p655565	C9	42	80.7	B
21036	Bn-N19-p50322354	Bn-C9-p48010977	Bn-scaff_16362_1-p633987	C9	42	80.7	B
21037	Bn-N19-p50323780	Bn-C9-p48012403	Bn-scaff_16362_1-p632561	C9	42	80.7	B
21038	Bn-N19-p50334243	Bn-C9-p48023151	Bn-scaff_16362_1-p621813	C9	42	80.7	B
21039	Bn-N19-p50336988	Bn-C9-p48028842	Bn-scaff_16362_1-p616122	C9	42	80.7	B
21040	Bn-N19-p50337104	Bn-C9-p48028958	Bn-scaff_16362_1-p616006	C9	42	80.7	B
21041	Bn-N19-p50378008	Bn-C9-p48071992	Bn-scaff_16362_1-p572972	C9	42	80.7	B
21042	Bn-N19-p50378596	Bn-C9-p48072577	Bn-scaff_16362_1-p572387	C9	42	80.7	B
21043	Bn-N19-p50382502	Bn-C9-p48076502	Bn-scaff_16362_1-p568462	C9	42	80.7	B
21044	Bn-N19-p50415964	Bn-C9-p48102883	Bn-scaff_16362_1-p542081	C9	42	80.7	B
21045	Bn-N19-p50416376	Bn-C9-p48103298	Bn-scaff_16362_1-p541666	C9	42	80.7	B
21046	Bn-N19-p50461247	Bn-C9-p48154593	Bn-scaff_16362_1-p490371	C9	42	80.7	B
21047	Bn-N19-p50485351	Bn-C9-p48179385	Bn-scaff_16362_1-p465579	C9	42	80.7	B
21048	Bn-N19-p50523609	Bn-C9-p48219394	Bn-scaff_16362_1-p425570	C9	42	80.7	B
21049	Bn-N19-p50524074	Bn-C9-p48219859	Bn-scaff_16362_1-p425105	C9	42	80.7	B
21050	Bn-N19-p50540424	Bn-C9-p48236162	Bn-scaff_16362_1-p408802	C9	43	81.5	B
21051	Bn-N19-p50540818	Bn-C9-p48240465	Bn-scaff_16362_1-p404499	C9	43	81.5	B
21052	Bn-N19-p50540900	Bn-C9-p48240547	Bn-scaff_16362_1-p404417	C9	43	81.5	B
21053	Bn-N19-p50541155	Bn-C9-p48240802	Bn-scaff_16362_1-p404162	C9	43	81.5	B
21054	Bn-N19-p50542128	Bn-C9-p48241774	Bn-scaff_16362_1-p403190	C9	43	81.5	B
21055	Bn-N19-p50553776	Bn-C9-p48259350	Bn-scaff_16362_1-p385614	C9	43	81.5	B
21056	Bn-N19-p50557941	Bn-C9-p48263502	Bn-scaff_16362_1-p381462	C9	43	81.5	B
21057	Bn-N19-p50558425	Bn-C9-p48263982	Bn-scaff_16362_1-p380982	C9	43	81.5	B
21058	Bn-N2-p2679970	Bn-A02-p3607564	Bn-A02-p3607564	C9	44	84.7	B
21059	Bn-N19-p51469262	Bn-C9-p49082646	Bn-scaff_22082_1-p439197	C9	45	85.5	B
21060	Bn-N19-p51542682	Bn-C9-p49140588	Bn-scaff_22082_1-p381255	C9	45	85.5	B
21061	Bn-N19-p51542690	Bn-C9-p49140596	Bn-scaff_22082_1-p381247	C9	45	85.5	B
21062	Bn-N19-p51542741	Bn-C9-p49140647	Bn-scaff_22082_1-p381196	C9	45	85.5	B
21063	Bn-N19-p51603180	Bn-C9-p49199485	Bn-scaff_22082_1-p322358	C9	45	85.5	B
21064	Bn-N19-p51634525	Bn-C9-p49220061	Bn-scaff_22082_1-p301782	C9	45	85.5	B
21065	Bn-N19-p51639075	Bn-C9-p49225293	Bn-scaff_22082_1-p296550	C9	45	85.5	B

21066	Bn-N19-p51668675	Bn-C9-p49259370	Bn-scaff_22082_1-p262473	C9	45	85.5	B
21067	Bn-N14-p27271279	Bn-C2-p20262917	Bn-scaff_18702_1-p603145	C9	46	86.3	B
21068	Bn-N17-p43902457	Bn-C9-p3523301	Bn-scaff_17526_1-p256458	C9	46	86.3	B
21069	Bn-N19-p51827918	Bn-C9-p49406262	Bn-scaff_22082_1-p115581	C9	46	86.3	B
21070	Bn-N19-p51918660	Bn-C3-p58636673	Bn-scaff_24108_1-p456844	C9	46	86.3	B
21071	Bn-N19-p52346260	Bn-C9-p49881005	Bn-scaff_17109_1-p195993	C9	47	87.1	B
21072	Bn-N19-p52357194	Bn-C9-p49891963	Bn-scaff_17109_1-p194897	C9	47	87.1	B
21073	Bn-N19-p52358519	Bn-C9-p49893288	Bn-scaff_17109_1-p194764	C9	47	87.1	B
21074	Bn-N19-p52361347	Bn-C9-p49896159	Bn-scaff_17109_1-p194477	C9	47	87.1	B
21075	Bn-N19-p52362886	Bn-C9-p49904292	Bn-scaff_17109_1-p193664	C9	47	87.1	B
21076	Bn-N19-p52423130	Bn-C9-p49978999	Bn-scaff_17109_1-p186193	C9	48	87.9	B
21077	Bn-N10-p14852555	Bn-A10-p14394392	Bn-A10-p14394392	C9	49	89.6	0
21078	Bn-N12-p1896153	Bn-C9-p50637907	Bn-scaff_17109_2-p361161	C9	49	89.6	B
21079	Bn-N19-p53035406	Bn-C9-p50573150	Bn-scaff_17109_2-p425918	C9	49	89.6	B
21080	Bn-N19-p53090532	Bn-C9-p50628723	Bn-scaff_17109_2-p370345	C9	49	89.6	B
21081	Bn-N19-p53092083	Bn-C9-p50630273	Bn-scaff_17109_2-p368795	C9	49	89.6	B
21082	Bn-N19-p53094461	Bn-C9-p50635066	Bn-scaff_17109_2-p364002	C9	49	89.6	B
21083	Bn-N19-p53103773	Bn-C9-p50642546	Bn-scaff_17109_2-p356522	C9	49	89.6	B
21084	Bn-N19-p53103971	Bn-C9-p50642743	Bn-scaff_17109_2-p356325	C9	49	89.6	B
21085	Bn-N19-p53678595	Bn-C9-p51113926	Bn-scaff_17750_1-p208247	C9	50	90.4	B
21086	Bn-N10-p15646007	Bn-C9-p51214841	Bn-scaff_17750_1-p198156	C9	51	92.9	B
21087	Bn-N19-p53833236	Bn-C9-p51273237	Bn-scaff_17750_1-p192316	C9	51	92.9	B
21088	Bn-N19-p53910139	Bn-C9-p51356975	Bn-scaff_17750_1-p183942	C9	51	92.9	B
21089	Bn-N19-p53925611	Bn-C9-p51383692	Bn-scaff_17750_1-p181271	C9	51	92.9	B
21090	Bn-N19-p53936034	Bn-C9-p51384906	Bn-scaff_17750_1-p181149	C9	51	92.9	B
21091	Bn-N19-p53936660	Bn-C9-p51385531	Bn-scaff_17750_1-p181087	C9	51	92.9	B
21092	Bn-N19-p53936769	Bn-C9-p51385640	Bn-scaff_17750_1-p181076	C9	51	92.9	B
21093	Bn-N19-p53945924	Bn-C9-p51394423	Bn-scaff_17750_1-p180198	C9	52	93.7	B
21094	Bn-N10-p15906678	Bn-C9-p51570685	Bn-scaff_17750_1-p162571	C9	53	94.5	B
21095	Bn-N10-p15911145	Bn-C9-p51575163	Bn-scaff_17750_1-p162124	C9	53	94.5	B
21096	Bn-N19-p54445865	Bn-C9-p51869548	Bn-scaff_17750_1-p132685	C9	53	94.5	B
21097	Bn-N10-p16263606	Bn-C9-p52003103	Bn-scaff_17750_1-p119330	C9	54	95.3	B
21098	Bn-N19-p54520183	Bn-C9-p51943965	Bn-scaff_17750_1-p125243	C9	54	95.3	B
21099	Bn-N19-p54525486	Bn-C9-p51952893	Bn-scaff_17750_1-p124351	C9	54	95.3	B
21100	Bn-N19-p54610222	Bn-C9-p52030051	Bn-scaff_17750_1-p116635	C9	55	96.1	B
21101	Bn-N19-p54610640	Bn-C9-p52030469	Bn-scaff_17750_1-p116593	C9	55	96.1	B
21102	Bn-N19-p54665155	Bn-C9-p52088507	Bn-scaff_17750_1-p110789	C9	55	96.1	B
21103	Bn-N19-p54667995	Bn-C9-p52091347	Bn-scaff_17750_1-p110505	C9	55	96.1	B
21104	Bn-N19-p54800819	Bn-C9-p52227361	Bn-scaff_17750_1-p969043	C9	56	96.9	B
21105	Bn-N19-p54986241	Bn-C9-p52417079	Bn-scaff_17750_1-p779325	C9	57	98.6	B
21106	Bn-N19-p55129650	Bn-C9-p52563612	Bn-scaff_17750_1-p632792	C9	57	98.6	B
21107	Bn-N19-p55168628	Bn-C9-p52609055	Bn-scaff_17750_1-p587349	C9	57	98.6	B
21108	Bn-N19-p55178201	Bn-C9-p52618709	Bn-scaff_17750_1-p577695	C9	57	98.6	B
21109	Bn-N10-p17228691	Bn-C9-p53257045	Bn-scaff_28053_1-p60542	C9	58	105.6	B
21110	Bn-N10-p17259635	Bn-C9-p53208843	Bn-scaff_28053_1-p12340	C9	58	105.6	B
21111	Bn-N10-p17313543	Bn-C9-p53429774	Bn-scaff_21636_1-p15905	C9	58	105.6	B
21112	Bn-N10-p17375888	Bn-C9-p53487568	Bn-scaff_21636_1-p73699	C9	58	105.6	B
21113	Bn-N10-p17411781	Bn-C9-p53532302	Bn-scaff_21636_1-p118433	C9	58	105.6	B
21114	Bn-N10-p17428951	Bn-C9-p53551129	Bn-scaff_21636_1-p137260	C9	58	105.6	B

21115	Bn-N10-p17430622	Bn-C9-p53552820	Bn-scaff_21636_1-p138951	C9	58	105.6	B
21116	Bn-N10-p17483021	Bn-C9-p53637373	Bn-scaff_21636_1-p223504	C9	58	105.6	B
21117	Bn-N10-p17483861	Bn-C9-p53640926	Bn-scaff_21636_1-p227057	C9	58	105.6	B
21118	Unknown	Bn-C9-p53587592	Bn-scaff_21636_1-p173723	C9	58	105.6	B

SG_320	SG_65	SG_132	SG_221	SG_258	SG_9	SG_155	SG_324	SG_90	SG_137	SG_229	SG_259	SG_18	SG_159	SG_224	SG_103	SG_150	SG_232	SG_261	SG_39	SG_201	SG_29	SG_107	SG_166	SG_233	SG_262	SG_54	SG_215	SG_33	SG_108	SG_171	SG_241	SG_265	SG_67	SG_24	SG_42	SG_118	SG_172	SG_243	SG_267	SG_121	SG_290	SG_57	SG_119	SG_208	SG_246
A	0	A	B	B	A	0	B	A	B	B	B	B	B	B	B	A	A	A	B	B	B	A	B	B	A	A	B	B	A	B	B	A	B	B	0	B	B	A	0	B	B	B	B	B	B
A	0	A	B	B	A	0	B	A	B	B	B	B	B	B	B	A	A	A	B	B	B	A	B	B	A	A	B	B	A	B	B	A	B	B	0	B	B	A	0	B	B	B	B	B	B
A	0	A	B	B	A	0	B	A	B	B	B	B	B	B	B	A	A	A	B	B	B	A	B	B	A	A	B	B	A	B	B	A	B	B	0	B	B	A	0	B	B	B	B	B	B
A	0	A	B	B	A	0	B	A	B	B	B	B	B	B	B	A	A	A	B	B	B	A	B	B	A	A	B	B	A	B	B	A	B	B	0	B	B	A	0	B	B	B	B	B	B
A	0	A	B	B	A	0	B	A	B	B	B	B	B	B	B	A	A	A	B	B	B	A	B	B	A	A	B	B	A	B	B	A	B	B	0	B	B	A	0	B	B	B	B	B	B
A	A	A	B	B	A	A	B	A	B	B	B	B	B	B	B	A	A	A	B	B	B	A	B	B	A	A	B	B	A	B	B	A	B	B	A	B	B	A	A	B	B	B	B	B	B
A	0	A	B	B	A	0	B	A	B	B	B	B	B	B	B	A	A	A	B	B	B	A	B	B	A	A	B	B	A	B	B	A	B	B	0	B	B	A	0	B	B	B	B	B	B
A	B	A	B	B	A	B	B	A	B	B	B	B	B	B	B	A	A	A	B	B	B	A	B	B	A	A	B	B	A	B	B	A	B	B	0	B	B	A	B	B	B	B	B	B	B
A	0	A	B	B	A	0	B	A	B	B	B	B	B	B	B	A	A	A	B	B	B	A	B	B	A	A	B	B	A	B	B	A	B	B	0	B	B	A	0	B	B	B	B	B	B
A	0	A	B	B	A	0	B	A	B	B	B	B	B	B	B	A	A	A	B	B	B	A	B	B	A	A	B	B	A	B	B	B	B	B	0	B	B	A	0	B	B	B	B	B	B
0	A	A	B	B	A	0	B	A	B	B	B	B	B	B	B	A	A	A	B	B	B	A	B	B	A	A	B	B	A	B	B	B	B	B	A	B	B	A	A	B	B	B	B	B	B
A	A	A	B	B	A	0	B	A	B	B	B	B	B	B	B	A	A	A	B	B	B	A	B	B	A	A	B	B	A	B	B	B	B	B	A	B	B	A	A	B	B	B	B	B	B
A	0	A	B	B	A	0	B	A	B	B	B	B	B	B	B	A	A	A	B	B	B	A	B	B	A	A	B	B	A	B	B	B	B	B	0	B	B	A	0	B	B	B	B	B	B
A	0	A	B	B	A	0	B	A	B	B	B	B	B	B	B	A	A	A	B	B	B	A	B	B	A	A	B	B	A	B	B	B	B	B	0	B	B	A	0	B	B	B	B	B	B
A	0	A	B	B	A	0	B	A	B	B	B	B	B	B	B	A	A	A	B	B	B	A	B	B	A	A	B	B	A	B	B	B	B	B	0	B	B	A	0	B	B	B	B	B	B
A	0	A	B	B	A	0	B	A	B	B	B	B	B	B	B	A	A	A	B	B	B	A	B	B	A	A	B	B	A	B	B	B	B	B	0	B	B	A	0	B	B	B	B	B	B
A	0	A	0	B	A	B	B	A	B	B	B	B	B	B	B	A	A	A	0	B	B	A	B	B	A	A	B	B	A	B	B	B	B	B	0	B	B	A	0	B	B	B	B	B	B
A	0	A	B	B	A	0	B	A	B	B	B	B	B	B	B	A	A	A	B	B	B	A	B	0	A	A	B	B	A	B	B	B	B	B	0	B	B	A	0	B	B	B	B	B	B
A	0	A	B	B	A	0	B	A	B	B	B	B	B	B	B	A	A	A	B	B	B	A	B	B	A	A	B	B	A	B	B	B	B	B	0	B	B	A	0	B	B	B	B	B	B
A	0	A	B	B	A	0	B	A	B	B	B	B	B	B	B	A	A	A	B	B	B	A	B	B	A	A	B	B	A	B	B	B	B	B	0	B	B	A	0	B	B	B	B	B	B
A	0	A	B	B	A	0	B	A	B	B	B	B	B	B	B	A	A	A	B	B	B	A	B	B	A	A	B	B	A	B	B	B	B	B	0	B	B	A	0	B	B	B	B	B	B
A	0	A	B	B	A	0	B	A	B	B	B	B	B	B	B	A	A	A	B	B	B	A	B	B	A	A	B	B	A	B	B	B	B	B	0	B	B	A	0	B	B	B	B	B	B
A	0	A	B	B	A	0	B	A	B	B	B	B	B	B	B	A	A	A	B	B	B	A	B	B	A	A	B	B	A	B	B	B	B	B	0	B	B	A	0	B	B	B	B	B	B
A	0	A	B	B	A	0	B	A	B	B	B	B	B	B	B	A	A	A	B	B	B	A	B	B	A	A	B	B	A	B	B	B	B	B	0	B	B	A	0	B	B	B	B	B	B
A	0	A	B	B	A	0	B	A	B	B	B	B	B	B	B	A	A	A	B	B	B	A	B	B	A	A	B	B	A	B	B	B	B	B	A	B	B	A	0	B	B	B	B	B	B
A	0	A	B	B	A	0	B	A	B	B	B	B	B	B	B	A	A	A	B	B	B	A	B	B	A	A	B	B	A	B	B	B	B	B	0	B	B	A	0	B	B	B	B	B	B
A	0	A	B	B	A	0	B	A	B	B	B	B	B	B	B	A	A	A	B	B	B	A	B	B	A	A	B	B	A	B	B	B	B	B	0	B	B	A	0	B	B	B	B	B	B
A	A	0	B	B	0	0	B	A	B	B	B	B	B	B	B	0	A	0	B	B	B	A	B	B	0	A	B	B	A	B	B	B	B	B	A	B	B	A	A	B	B	B	B	B	B
A	A	A	B	B	A	0	B	A	B	B	B	B	B	B	B	A	A	A	B	B	B	A	B	B	A	A	B	B	A	B	B	B	B	B	A	B	B	A	B	B	B	B	B	B	B
A	0	A	B	B	A	0	B	A	B	B	B	B	B	B	B	A	A	A	B	B	B	A	B	B	A	0	B	B	A	B	B	B	B	B	0	B	B	A	B	B	B	B	B	B	B
A	0	A	B	B	A	0	B	A	B	B	B	B	B	B	B	A	A	A	B	B	B	A	B	B	A	0	B	B	A	B	B	B	B	B	0	B	B	A	B	B	B	B	B	B	B
A	0	A	B	B	A	0	B	A	B	B	B	B	B	B	B	A	A	A	B	B	B	A	B	B	A	0	B	B	A	B	B	B	B	B	0	B	B	A	B	B	B	B	B	B	B
A	B	A	B	B	A	B	B	B	B	B	B	B	B	B	B	A	A	A	B	B	B	A	B	B	A	B	B	B	A	B	B	B	B	B	B	B	B	A	B	B	B	B	B	B	B
A	B	A	B	B	A	B	B	B	B	B	B	B	B	B	B	A	A	A	B	B	B	A	B	B	A	B	B	B	A	B	B	B	B	B	B	B	B	A	B	B	B	B	B	B	B
A	0	A	B	B	A	0	B	0	B	B	B	B	B	B	B	A	A	A	B	B	B	A	B	B	A	0	B	B	A	B	B	B	B	B	0	B	B	A	B	B	B	B	B	B	B
A	B	A	B	B	A	B	B	B	B	B	B	B	B	B	B	A	A	A	B	B	B	A	B	B	A	B	B	B	A	B	B	B	B	B	B	B	B	A	B	B	B	B	B	B	B
A	B	A	B	B	A	B	B	B	B	B	B	B	B	B	B	A	A	A	B	B	B	A	B	B	A	B	B	B	A	B	B	B	B	B	B	B	B	A	B	B	B	B	B	B	B
A	0	A	B	B	A	0	B	0	B	B	B	B	B	B	B	A	A	A	B	B	B	A	B	B	A	0	B	B	A	B	B	B	B	B	0	B	B	A	B	B	B	B	B	B	B
A	0	A	B	B	A	0	B	0	B	B	B	B	B	B	B	A	A	A	B	B	B	A	B	B	A	0	B	B	A	B	B	B	B	B	0	B	B	A	B	B	B	B	B	B	B
A	0	A	B	B	A	0	B	0	B	B	B	B	B	B	B	A	A	A	B	B	B	A	B	B	A	0	B	B	A	B	B	B	B	B	0	B	B	A	B	B	B	B	B	B	B
A	A	A	B	B	A	A	B	A	B	B	B	B	B	B	B	A	A	A	B	B	B	A	B	B	A	A	B	B	A	B	B	B	B	B	A	B	B	A	B	B	B	B	B	B	B
A	0	A	B	B	A	B	B	0	B	B	B	B	B	B	B	A	A	A	B	B	B	A	B	B	A	0	B	B	A	B	B	B	B	B	0	B	B	A	B	B	B	B	B	B	B
A	A	A	B	B	A	B	B	A	B	B	B	B	B	B	B	A	A	A	B	B	B	A	B	B	A	A	B	B	A	B	B	B	B	B	A	B	B	A	B	B	B	B	B	B	B
A	0	A	B	B	A	B	B	0	B	B	B	B	B	B	B	A	A	A	B	B	B	A	B	B	A	0	B	B	A	B	B	B	B	B	0	B	B	A	B	B	B	B	B	B	B
A	0	A	B	B	A	B	B	0	B	B	B	B	B	B	B	A	A	A	B	B	B	A	B	B	A	0	B	B	A	B	B	B	B	B	0	B	B	A	B	B	B	B	B	B	B

A	A	A	B	B	A	B	B	A	B	B	B	B	B	B	B	A	A	A	B	B	B	A	B	B	A	A	B	B	A	B	B	B	B	B	A	B	B	A	B	B	B	B	B	B	B
A	A	A	0	0	A	B	0	A	B	B	B	0	0	0	B	A	A	A	0	0	0	A	0	B	A	A	0	B	A	0	0	0	0	B	A	0	0	A	B	0	0	0	0	0	B
A	0	A	B	B	A	B	B	0	B	B	B	B	B	B	B	A	A	A	B	B	B	A	B	B	A	0	B	B	A	B	B	B	B	B	0	B	B	A	B	B	B	B	B	B	B
A	0	A	B	B	A	0	B	A	B	B	B	B	B	B	B	A	A	A	B	B	B	A	B	B	A	0	B	B	A	B	B	B	B	B	0	B	B	A	B	B	B	B	B	B	B
A	0	A	0	B	A	0	B	A	B	B	B	B	B	B	B	A	A	0	B	B	B	A	B	B	0	0	B	B	A	B	B	B	B	B	0	B	B	A	B	B	B	B	B	B	B
A	A	A	B	B	A	0	B	A	B	B	B	B	B	B	B	A	A	A	B	B	B	A	B	B	A	A	B	B	A	B	B	B	B	B	A	B	B	A	B	B	B	B	B	B	B
A	A	A	B	B	A	A	B	A	B	B	B	B	B	B	B	A	A	A	B	B	B	A	B	B	A	A	B	B	A	B	B	B	B	B	A	B	B	A	B	B	B	B	B	B	B
A	0	A	B	B	A	0	B	0	B	B	B	B	B	B	B	A	A	A	B	B	B	A	B	B	A	0	B	B	A	B	B	B	B	B	0	B	B	A	B	B	B	B	B	B	B
A	0	A	B	B	A	0	B	0	B	B	B	B	B	B	B	A	A	A	B	B	B	A	B	B	A	0	B	B	A	B	B	B	B	B	0	B	B	A	B	B	B	B	B	B	B
A	0	A	B	B	A	0	B	0	B	B	B	B	B	B	B	A	A	A	B	B	B	A	B	B	A	0	B	B	A	B	B	B	B	B	0	B	B	A	B	B	B	B	B	B	B
A	0	A	B	B	A	0	B	0	B	B	B	B	B	B	B	A	A	A	B	B	B	A	B	B	A	0	B	B	A	B	B	B	B	B	0	B	B	A	B	B	B	B	B	B	B
A	0	A	B	B	A	0	B	0	B	B	B	B	B	B	B	A	A	A	B	B	B	A	B	B	A	0	B	B	A	B	B	B	B	B	0	B	B	A	B	B	B	B	B	B	B
A	B	A	B	B	A	B	B	B	B	B	B	B	B	B	B	A	A	A	B	B	B	A	B	B	A	B	B	B	A	B	B	B	B	B	B	B	B	A	B	B	B	B	B	B	B
A	A	A	B	B	A	B	B	A	B	B	B	B	B	B	B	A	A	A	B	B	B	A	B	B	A	A	B	B	A	B	B	B	B	B	A	B	B	A	B	B	B	B	B	B	B
A	0	A	B	B	A	B	B	0	B	B	B	B	B	B	B	A	A	A	B	B	B	A	B	B	A	0	B	B	A	B	B	B	B	B	0	B	B	A	B	B	B	B	B	B	B
A	0	A	B	B	A	B	B	0	B	B	B	B	B	B	B	A	A	A	B	B	B	A	B	B	A	0	B	B	A	B	B	B	B	B	0	B	B	A	B	B	B	B	B	B	B
A	0	A	B	B	A	B	B	0	B	B	B	B	B	B	B	A	A	A	B	B	B	A	B	B	A	0	B	B	A	B	B	B	B	B	0	B	B	A	B	B	B	B	B	B	B
A	0	A	B	B	A	B	B	0	B	B	B	B	B	B	B	A	A	A	B	B	B	A	B	B	A	0	B	B	A	B	B	B	B	B	0	B	B	A	B	B	B	B	B	B	B
A	B	A	B	B	A	B	B	B	B	B	B	B	B	B	B	A	A	A	0	B	B	A	B	B	A	B	B	B	A	B	B	B	B	B	B	B	B	A	B	B	B	B	B	B	B
A	0	A	B	B	A	0	B	0	B	B	B	B	B	B	B	A	A	A	B	B	B	A	B	B	A	0	B	B	A	B	B	B	B	B	0	B	B	B	B	B	B	B	B	B	B
A	0	A	B	B	A	0	B	0	B	B	B	B	B	B	B	A	A	A	B	B	B	A	B	B	A	0	B	B	A	B	B	B	B	B	0	B	B	B	B	B	B	B	B	B	B
A	B	A	B	B	A	B	B	B	B	B	B	B	B	B	B	A	A	A	0	B	B	A	B	B	A	B	B	B	A	B	B	B	B	B	B	B	B	B	B	B	B	B	B	B	B
A	0	A	B	B	A	0	B	0	B	B	B	B	B	B	B	A	A	A	B	B	B	A	B	B	A	0	B	B	A	B	B	B	B	B	0	B	B	B	B	B	B	B	B	B	B
A	0	A	B	B	A	0	B	0	B	B	B	B	B	B	B	A	A	A	B	B	B	A	B	B	A	0	B	B	A	B	B	B	B	B	0	B	B	B	B	B	B	B	B	B	B
A	0	A	B	B	A	0	B	0	B	B	B	B	B	B	B	A	A	A	B	B	B	A	B	B	A	0	B	B	A	B	B	B	B	B	0	B	B	B	B	B	B	B	B	B	B
A	0	A	B	B	A	0	B	0	B	B	B	B	B	B	B	A	A	A	B	B	B	A	B	B	A	0	B	B	A	B	B	B	B	B	0	B	B	B	B	B	B	B	B	B	B
A	B	A	B	B	A	B	B	B	B	B	B	B	B	B	B	A	A	A	0	B	B	A	B	B	A	B	B	B	A	B	B	B	B	B	B	B	B	B	B	B	B	B	B	B	B
A	0	A	B	B	A	0	B	0	B	B	B	B	B	B	B	A	A	B	B	B	B	A	B	B	A	0	B	B	A	B	B	B	B	B	0	B	B	B	B	B	B	B	B	B	B
A	A	A	B	B	A	0	B	A	B	B	B	B	B	B	B	A	A	B	B	B	B	A	B	B	A	0	B	B	A	B	B	B	B	B	A	B	B	B	B	B	B	B	B	B	B
A	B	A	B	B	A	B	B	B	B	B	B	B	B	B	B	A	A	B	0	B	B	A	B	B	A	B	B	B	A	B	B	B	B	B	B	B	B	B	B	B	B	B	B	B	B
A	0	A	B	B	A	0	B	0	B	B	B	B	B	B	B	A	A	B	B	B	B	A	B	B	A	0	B	B	A	B	B	B	B	B	0	B	B	B	B	B	B	B	B	B	B
A	0	A	B	B	A	0	B	0	B	B	B	B	B	B	B	A	A	B	B	B	B	A	B	B	A	0	B	B	A	B	B	B	B	B	0	B	B	B	B	B	B	B	B	B	B
0	0	0	B	0	0	0	B	0	B	B	B	B	B	B	B	0	0	B	B	0	B	A	B	B	0	A	B	B	A	B	0	B	0	B	0	B	B	0	B	B	0	0	B	B	B
A	0	A	B	B	A	0	B	0	B	B	B	B	B	B	B	A	A	B	B	B	B	A	B	B	A	0	B	B	A	B	B	B	B	B	0	B	B	B	B	B	B	B	B	B	B
A	A	A	B	B	A	0	B	A	B	B	B	B	B	B	B	A	A	B	B	B	B	A	B	B	A	A	B	B	A	B	B	B	B	B	A	B	B	B	B	B	B	B	B	B	B
A	0	0	B	B	A	0	B	0	B	B	B	B	B	B	B	A	A	B	B	B	B	A	B	B	A	0	B	B	A	B	B	B	B	B	0	B	B	B	B	B	B	B	B	B	B
A	0	A	B	B	A	0	B	0	B	B	B	B	B	B	B	A	A	B	B	B	B	A	B	B	B	0	B	B	A	B	B	B	B	B	0	B	B	B	B	B	B	B	B	B	B
A	0	A	B	B	A	0	B	0	B	B	B	B	B	B	B	A	A	B	B	B	B	A	B	B	B	0	B	B	A	B	B	B	B	B	0	B	B	B	B	B	B	B	B	B	B
A	A	A	B	B	A	A	B	A	B	B	B	B	B	B	B	A	A	B	B	B	B	A	B	B	B	A	B	B	A	B	B	B	B	B	A	B	B	B	B	B	B	B	B	B	B
A	0	A	B	B	A	0	B	0	B	B	B	B	B	B	B	A	A	B	B	B	B	A	B	B	B	0	B	B	A	B	B	B	B	B	0	B	B	B	B	B	B	B	B	B	B
A	A	A	B	B	A	A	B	A	B	B	B	B	B	B	B	A	A	B	B	B	B	A	B	B	B	A	B	B	A	B	B	B	B	B	A	B	B	B	B	B	B	B	B	B	B
A	0	A	B	B	A	0	B	0	B	B	B	B	B	B	B	A	A	B	B	B	B	A	B	B	B	0	B	B	A	B	B	B	B	B	0	B	B	B	B	B	B	B	B	B	B
A	0	A	B	B	A	0	B	0	B	B	B	B	B	B	B	A	A	B	B	B	B	A	B	B	B	0	B	B	A	B	B	B	B	B	0	B	B	B	B	B	B	B	B	B	B
A	A	A	B	B	A	0	B	A	B	B	B	B	B	B	B	A	A	B	B	B	B	A	B	B	B	A	B	B	A	B	B	B	B	B	A	B	B	B	B	B	B	B	B	B	B
A	0	A	B	B	A	0	B	0	B	B	B	B	B	B	B	A	A	B	B	B	B	A	B	B	B	0	B	B	A	B	B	B	B	B	0	B	B	B	B	B	B	B	B	B	B
A	0	A	B	B	A	0	B	0	B	B	B	B	B	B	B	A	A	B	B	B	B	A	B	B	B	0	B	B	A	B	B	B	B	B	0	B	B	B	B	B	B	B	B	B	B
A	0	A	B	B	A	0	B	0	B	B	B	B	B	B	B	A	A	B	B	B	B	A	B	B	B	0	B	B	A	B	B	B	B	B	0	B	B	B	B	B	B	B	B	B	B
A	0	A	B	B	A	0	B	0	B	B	B	B	B	B	B	A	A	B	B	B	B	A	B	B	B	0	B	B	A	B	B	B	B	B	0	B	B	B	B	B	B	B	B	B	B
A	0	A	B	B	A	0	B	0	B	B	B	B	B	B	B	A	A	B	B	B	B	A	B	B	B	0	B	B	A	B	B	B	B	B	0	B	B	B	B	B	B	B	B	B	B

A	0	A	B	B	A	0	B	0	B	B	B	B	B	B	B	A	A	B	B	B	B	A	B	B	B	0	B	B	A	B	B	B	B	B	0	B	B	B	B	B	B	B	B	B	B
A	0	A	B	B	A	0	B	0	B	B	B	B	B	B	B	A	A	B	B	B	B	A	B	B	B	0	B	B	A	B	B	B	B	B	0	B	B	B	B	B	B	B	B	B	B
A	A	A	B	B	A	A	B	A	B	B	B	B	0	B	B	A	A	B	B	B	0	A	0	B	B	A	B	0	A	0	B	B	B	B	A	B	B	B	B	B	B	0	B	B	B
A	0	A	B	B	A	0	B	0	B	B	B	B	B	B	B	A	A	B	B	B	B	A	B	B	B	0	B	B	A	B	B	B	B	B	0	B	B	B	B	B	B	B	B	B	B
A	0	A	B	B	A	0	B	0	B	B	B	B	B	B	B	A	A	B	B	B	B	A	B	B	B	0	B	B	A	B	B	B	B	B	0	B	B	B	B	B	B	B	B	B	B
A	B	A	B	B	A	B	B	B	B	B	B	B	B	B	B	A	A	B	B	B	B	A	B	B	B	B	B	B	A	B	A	B	B	B	B	B	B	B	B	B	B	B	B	B	B
A	0	A	B	B	A	0	B	0	B	B	B	B	B	B	B	A	A	B	B	B	B	A	B	B	B	0	B	B	A	B	A	B	B	B	0	B	B	B	B	B	B	B	B	B	B
A	A	A	B	B	A	0	B	A	B	B	B	B	B	B	B	A	A	B	B	B	B	A	B	B	B	A	B	B	A	B	A	B	B	B	A	B	B	B	B	B	B	B	B	B	B
A	0	A	B	B	A	0	B	0	B	B	B	B	B	B	B	A	A	B	B	B	B	A	B	B	B	0	B	B	A	B	A	B	B	B	0	B	B	B	B	B	B	B	B	B	B
A	B	A	B	B	A	0	B	B	B	B	B	B	0	B	B	A	A	B	B	B	0	A	0	B	B	B	0	B	A	0	A	B	B	B	B	0	B	B	B	B	B	0	B	B	B
A	0	A	0	0	A	0	0	0	B	0	B	B	0	0	B	A	A	0	B	B	0	A	0	B	0	0	0	0	A	0	A	B	B	B	0	0	0	0	B	B	0	0	0	0	0
A	0	A	B	B	A	0	B	0	B	B	B	B	B	B	B	A	A	B	B	B	B	A	B	B	B	0	B	B	A	B	A	B	B	B	0	B	B	B	B	B	B	B	B	B	B
A	0	A	B	B	A	0	B	0	B	B	B	B	B	B	B	A	A	B	B	B	B	A	B	B	B	0	B	B	A	B	A	B	B	B	0	B	B	B	B	B	B	B	B	B	B
A	A	A	B	B	A	0	B	A	B	B	B	B	B	B	B	A	A	B	B	B	B	A	B	B	B	A	B	B	A	B	A	B	B	B	A	B	B	B	B	B	B	B	B	B	B
A	0	A	B	B	A	0	B	0	B	B	B	B	B	B	B	A	A	B	B	B	B	A	B	B	B	0	B	B	A	B	A	B	B	B	0	B	B	B	B	B	B	B	B	B	B
A	0	A	B	B	A	0	B	0	B	B	B	B	B	B	B	A	A	B	B	B	B	A	B	B	B	0	B	B	A	B	A	B	B	B	0	B	B	B	B	B	B	B	B	B	B
A	0	A	B	B	A	0	B	0	B	B	B	B	B	B	B	A	A	B	B	B	B	A	B	B	B	0	B	B	A	B	A	B	B	B	0	B	B	B	B	B	B	B	B	B	B
A	0	A	B	B	A	B	B	0	B	B	B	B	B	B	B	A	A	B	B	B	B	A	B	B	B	0	B	B	A	B	A	B	B	B	0	B	B	B	B	B	B	B	B	B	B
A	A	A	B	B	A	0	B	A	B	B	B	B	B	B	B	A	0	B	B	B	B	A	B	B	B	A	B	B	A	B	A	B	B	B	A	B	B	B	B	B	B	B	B	B	B
A	A	A	B	B	A	A	B	A	B	B	B	B	B	B	B	A	A	B	B	B	B	A	B	B	B	A	B	B	A	B	A	B	B	B	A	B	B	B	B	B	B	B	B	B	B
A	0	A	B	B	A	0	B	0	B	B	B	B	B	B	B	A	A	B	B	B	B	A	B	B	B	0	B	B	A	B	A	B	B	B	0	B	B	B	B	B	B	B	B	B	B
A	0	A	B	B	A	0	B	0	B	B	B	B	B	B	B	A	A	B	B	B	B	A	B	B	B	0	B	B	A	B	A	B	B	B	0	B	B	B	B	B	B	B	B	B	B
A	0	A	B	B	A	0	B	0	B	B	B	B	B	B	B	A	A	B	B	B	B	A	B	B	B	0	B	B	A	B	A	B	B	B	0	B	B	B	B	B	B	B	B	B	B
A	0	A	B	B	A	0	B	0	B	B	B	B	B	B	B	A	A	B	B	B	B	0	B	B	B	0	B	B	0	B	A	B	B	B	0	B	B	B	B	B	B	B	B	B	B
A	0	A	B	B	A	0	B	0	B	B	B	B	B	B	B	A	A	B	B	B	B	0	B	B	B	0	B	B	0	B	A	B	B	B	0	B	B	B	B	B	B	B	B	B	B
A	0	A	B	B	A	0	B	0	B	B	B	B	B	B	B	A	A	B	B	B	B	0	B	B	B	0	B	B	0	B	A	B	B	B	0	B	B	B	B	B	B	B	B	B	B
A	0	A	B	B	A	0	B	0	B	B	B	B	B	B	B	A	A	B	B	B	B	A	B	B	B	0	B	B	A	B	A	B	B	B	A	B	B	B	B	B	B	B	B	B	B
A	0	A	B	B	A	0	B	0	B	B	B	B	B	B	B	A	0	B	B	B	B	0	B	B	B	0	B	B	0	B	0	B	B	B	0	B	B	B	B	B	B	B	B	B	B
A	0	A	B	B	A	0	B	0	B	B	B	B	B	B	B	A	A	B	B	B	B	0	B	B	B	0	B	B	0	B	A	B	B	B	0	B	B	B	B	B	B	B	B	B	B
A	0	A	B	B	A	0	B	0	B	B	B	B	B	B	B	A	A	B	B	B	B	0	B	B	B	0	B	B	0	B	A	B	B	B	0	B	B	B	B	B	B	B	B	B	B
A	0	A	B	B	A	0	B	0	B	B	B	B	B	B	B	A	A	B	B	B	B	0	B	B	B	0	B	B	0	B	A	B	B	B	0	B	B	B	B	B	B	B	B	B	B
A	0	A	B	B	A	0	B	0	B	B	B	B	B	B	B	A	A	B	B	B	B	0	B	B	B	0	B	B	0	B	A	B	B	B	0	B	B	B	B	B	B	B	B	B	B
A	0	A	B	B	A	0	B	0	B	B	B	B	B	B	B	A	A	B	B	B	B	0	B	B	B	0	B	B	0	B	A	B	B	B	0	B	B	B	B	B	B	B	B	B	B
A	0	A	B	B	A	0	B	0	B	B	B	B	B	B	B	A	A	B	B	B	B	0	B	B	B	0	B	B	0	B	A	B	B	B	0	B	B	B	B	B	B	B	B	B	B
A	B	A	B	B	A	B	B	B	B	B	B	B	B	B	B	A	A	B	B	B	B	B	B	B	B	B	B	B	B	B	A	B	B	B	B	B	B	B	B	B	B	B	B	B	B
A	0	A	B	B	A	0	B	0	B	B	B	B	B	B	B	A	A	B	B	B	B	0	B	B	B	0	B	B	0	B	A	B	B	B	0	B	B	B	B	A	B	B	B	B	B
A	0	A	B	B	A	0	B	0	B	B	B	B	B	B	B	A	A	B	B	B	B	0	B	B	B	0	A	B	0	B	A	B	B	B	0	B	B	B	B	A	B	B	B	B	B
A	A	0	B	B	0	A	B	A	B	B	B	B	B	B	B	0	A	B	B	B	B	A	B	B	B	A	A	B	A	B	A	B	B	B	A	B	B	B	B	0	B	B	B	B	B
A	A	A	B	B	A	A	B	A	B	B	B	B	B	B	B	A	A	B	B	B	B	A	B	B	B	A	A	B	A	B	A	B	B	B	A	B	B	B	B	A	B	B	B	B	B
A	0	A	B	B	A	0	B	0	B	B	B	B	B	B	B	A	A	B	B	B	B	0	B	B	B	0	A	B	0	B	A	B	B	B	0	B	B	B	B	A	B	B	B	B	B
A	0	A	B	B	A	0	B	0	B	B	B	B	B	B	B	A	A	B	B	B	B	0	B	B	B	0	A	B	0	B	A	B	B	B	0	B	B	B	B	A	B	B	B	B	B
A	0	A	B	B	A	0	B	0	B	B	B	B	B	B	B	A	A	B	B	B	B	0	B	B	B	0	A	B	0	B	A	B	B	B	0	B	B	B	B	A	B	B	B	B	B
A	0	A	B	B	A	0	B	0	B	B	B	B	B	B	B	A	A	B	B	B	B	0	B	B	B	0	A	B	0	B	A	B	B	B	0	B	B	B	B	A	B	B	B	B	B
A	0	A	B	B	A	0	B	0	B	B	B	B	B	B	B	A	A	B	B	B	B	0	B	B	B	0	A	B	0	B	A	B	B	B	0	B	B	B	B	A	B	B	B	B	B
A	0	A	B	B	A	0	B	A	B	B	B	B	B	B	B	A	A	B	B	B	B	A	B	B	B	A	A	B	A	B	A	B	B	B	A	B	B	B	B	A	B	B	B	B	B
A	0	A	B	B	A	0	B	0	B	B	B	B	B	B	B	A	A	B	B	B	B	0	B	B	B	0	A	B	0	B	A	B	B	B	0	B	B	B	B	A	B	B	B	B	B
A	0	A	B	B	A	0	B	0	B	B	B	B	B	B	B	A	A	B	B	B	B	0	B	B	B	0	A	B	0	B	A	B	B	B	0	B	B	B	B	A	B	B	B	B	B
A	0	A	B	B	A	0	B	0	B	B	B	B	B	B	B	A	A	B	B	B	B	0	B	B	B	0	A	B	0	B	A	B	B	B	0	B	B	B	B	A	B	B	B	B	B

A	0	A	B	B	A	0	B	0	B	B	B	B	B	B	B	A	A	B	B	B	B	0	B	B	B	0	A	B	0	B	A	B	B	B	0	B	B	B	B	A	B	B	B	B	B
A	0	A	B	B	A	0	B	0	B	B	B	B	B	B	B	A	A	B	B	B	B	0	B	B	B	0	A	B	0	B	A	B	B	B	0	B	B	B	B	A	B	B	B	B	B
A	0	A	B	B	A	0	B	0	B	B	B	B	B	B	B	A	A	B	B	B	B	0	B	B	B	0	A	B	0	B	A	B	B	B	0	B	B	B	B	A	B	B	B	B	B
A	0	A	B	B	A	0	B	0	B	B	B	B	B	B	B	A	A	B	B	B	B	0	B	B	B	0	A	B	0	B	A	B	B	B	0	B	B	B	B	A	B	B	B	B	B
A	A	A	B	B	A	A	B	A	B	B	B	B	B	B	B	A	A	B	B	B	B	A	B	B	B	B	A	B	A	B	A	B	B	B	A	B	B	B	B	A	B	B	B	B	B
A	0	A	B	B	A	0	B	0	B	B	B	B	B	B	B	A	A	B	B	B	B	0	B	B	B	B	A	B	0	B	A	B	B	B	0	B	B	B	B	A	B	B	B	B	B
A	A	A	B	B	A	A	B	A	B	B	B	B	B	B	B	A	A	B	B	B	B	A	B	B	B	B	A	B	A	B	A	B	B	B	A	B	B	B	B	A	B	B	B	B	B
A	A	A	B	B	A	A	B	A	B	B	B	B	B	B	B	A	A	B	B	B	B	A	B	B	B	B	A	B	A	B	A	B	B	B	A	B	B	B	B	A	B	B	B	B	B
A	0	A	B	B	A	0	B	0	B	B	B	B	B	B	B	A	A	B	B	B	B	0	B	B	B	B	A	B	0	B	A	B	B	B	0	B	B	B	B	A	B	B	B	B	B
A	0	A	B	B	A	0	B	0	B	B	B	B	B	B	A	A	A	B	B	B	B	0	B	B	B	B	A	B	0	B	A	B	B	B	0	B	B	B	B	A	B	B	B	B	B
A	0	A	B	B	A	0	B	0	B	B	B	B	B	B	A	A	A	B	B	B	B	0	B	B	B	B	A	B	0	B	A	B	B	B	0	B	B	B	B	A	B	B	B	B	B
A	0	A	B	B	A	0	B	0	B	B	B	B	B	B	A	A	A	B	B	B	B	0	B	B	B	B	A	B	0	B	A	B	B	B	0	B	B	B	B	A	B	B	B	B	B
A	0	A	B	B	A	0	B	0	B	B	B	B	B	B	A	A	A	B	B	B	B	0	B	B	B	B	A	B	0	B	A	B	B	B	0	B	B	B	B	A	B	B	B	B	B
A	0	A	B	B	A	0	B	0	B	B	B	B	B	B	A	A	A	B	B	B	B	0	B	B	B	B	A	B	0	B	A	B	B	B	0	B	B	B	B	A	B	B	B	B	B
A	0	A	B	B	A	0	B	0	B	B	B	B	B	B	A	A	A	B	B	B	B	0	B	B	B	B	A	0	0	B	A	B	B	B	0	B	B	B	B	A	B	B	B	B	B
A	0	A	B	B	A	0	B	A	B	B	B	B	B	B	A	A	A	B	B	B	B	A	B	B	B	B	A	B	A	B	A	B	B	B	A	B	B	B	B	A	B	B	B	B	B
A	0	A	B	B	A	0	B	0	B	B	B	B	B	B	A	A	A	B	B	B	B	0	B	B	B	B	A	B	0	B	A	B	B	B	0	B	B	B	B	A	B	B	B	B	B
A	0	A	B	B	A	0	B	0	B	B	B	B	B	B	A	A	A	B	B	B	B	0	B	B	B	B	A	B	0	B	A	B	B	B	0	B	B	B	B	A	B	B	B	B	B
A	0	A	B	B	A	0	B	0	B	B	B	B	B	B	A	A	A	B	B	B	B	0	B	B	B	B	A	B	0	B	A	B	B	B	0	B	B	B	B	A	B	B	B	B	B
A	0	A	B	B	A	0	B	0	B	B	B	B	B	B	A	A	A	B	B	B	B	0	B	B	B	B	A	B	0	B	A	B	B	B	0	B	B	B	B	A	B	B	B	B	B
A	0	A	B	B	A	0	B	A	B	B	B	B	B	B	A	A	A	B	B	B	B	A	B	B	B	B	A	B	A	B	A	B	B	B	A	B	B	B	B	A	B	B	B	B	B
A	0	A	B	B	A	0	B	0	B	B	B	B	B	B	A	A	A	B	B	B	B	0	B	B	B	B	A	B	0	B	A	B	B	B	0	B	B	B	B	A	B	B	B	B	B
A	0	A	B	B	A	0	B	A	B	B	B	B	B	B	A	A	A	B	B	B	B	A	B	B	B	B	A	B	A	B	A	B	B	B	A	B	B	B	B	A	B	B	B	B	B
A	0	A	B	B	A	0	B	A	B	B	B	B	B	B	A	A	A	B	B	B	B	A	B	B	B	B	A	B	A	B	A	B	B	B	A	B	B	B	B	A	B	B	B	B	B
A	0	A	B	B	A	0	B	A	B	B	B	B	B	B	A	A	A	B	B	B	B	A	B	B	B	B	A	B	A	B	A	B	B	B	A	B	B	B	B	A	B	B	B	B	B
A	0	A	B	B	A	0	B	0	B	B	B	B	B	B	A	A	A	B	B	B	B	0	B	B	B	B	A	B	0	B	A	B	B	B	0	B	B	B	B	A	B	B	B	B	B
0	0	0	B	B	0	0	B	0	B	B	B	B	B	B	0	0	0	B	B	B	B	0	B	B	B	B	0	B	0	B	0	B	B	B	0	B	B	B	B	0	B	B	B	B	B
A	0	A	B	B	A	0	B	0	B	B	B	B	B	B	A	A	A	B	B	B	B	0	B	B	B	B	A	B	0	B	A	B	B	B	0	B	B	B	B	A	B	B	B	B	B
A	0	A	B	B	A	0	B	0	B	B	B	B	B	B	A	A	A	B	B	B	B	0	B	B	B	B	A	B	0	B	A	B	B	B	0	B	B	B	B	A	B	B	B	B	B
A	0	A	B	B	A	0	B	0	B	B	B	B	B	B	A	A	A	B	B	B	B	0	B	B	B	B	A	B	0	B	A	B	B	B	0	B	B	B	B	A	B	B	B	B	B
A	A	A	B	B	A	A	B	A	B	B	B	B	B	B	A	A	A	B	B	B	B	A	B	B	B	B	A	B	A	B	A	B	B	B	A	B	B	B	B	A	B	B	B	B	B
A	0	A	B	B	A	0	B	0	B	B	B	B	B	B	A	A	A	B	B	B	B	0	B	B	B	B	A	B	0	B	A	B	B	B	0	B	B	B	B	A	B	B	B	B	B
A	0	A	B	B	A	0	B	0	B	B	B	B	B	B	A	A	A	B	B	B	B	0	B	B	B	B	A	B	0	B	A	B	B	B	0	B	B	B	B	A	B	B	B	B	B
A	0	0	B	B	0	0	B	A	B	B	B	B	B	B	A	0	A	B	B	B	B	A	B	B	B	B	A	B	A	B	0	B	B	B	A	B	B	B	B	0	B	B	B	B	B
A	A	A	B	B	A	A	B	A	B	B	B	B	B	B	A	A	A	B	B	B	B	A	B	B	B	B	A	B	A	B	A	B	B	B	A	B	B	B	B	A	B	B	B	B	B
A	A	A	B	B	A	A	B	A	B	B	B	B	B	B	A	A	A	B	B	B	B	A	B	B	B	B	A	B	A	B	A	B	B	B	A	B	B	B	B	A	B	B	B	B	B
A	A	A	B	B	A	A	B	A	B	B	B	B	B	B	A	A	A	B	B	B	B	A	B	B	B	B	A	B	A	B	A	B	B	B	A	B	B	B	B	A	B	B	B	B	B
A	0	A	B	B	A	0	B	0	B	B	B	B	B	B	A	A	0	B	B	B	B	0	B	B	B	B	A	B	0	B	0	B	B	B	0	B	B	B	B	A	B	B	B	B	B
A	A	A	B	B	A	A	B	A	B	B	B	B	B	B	A	A	A	B	B	B	B	A	B	B	B	B	A	B	A	B	A	B	B	B	A	B	B	B	B	A	B	B	B	B	B
A	A	A	B	B	A	A	B	A	B	B	B	B	B	B	A	A	A	B	B	B	B	A	B	B	B	B	A	B	A	B	A	B	B	B	A	B	B	B	B	A	B	B	B	B	B
A	A	A	B	B	A	A	B	A	B	B	B	B	B	B	A	A	A	B	B	B	B	A	B	B	B	B	A	B	A	B	A	B	B	B	A	B	B	B	B	A	B	B	B	B	B
A	A	A	B	B	A	A	B	A	B	B	B	B	B	B	A	A	A	B	B	B	B	A	B	B	B	B	A	B	A	B	A	B	B	B	A	B	B	B	B	A	B	B	B	B	B
A	A	A	B	B	A	A	B	A	B	B	B	B	B	B	A	A	A	B	B	B	B	A	B	B	B	B	A	B	A	B	A	B	B	B	A	B	B	B	B	A	B	B	B	B	B
A	A	A	B	B	A	A	B	A	B	B	B	B	B	B	A	A	A	B	B	B	B	A	B	B	B	B	A	B	A	B	A	B	B	B	A	B	B	B	B	A	B	B	B	B	B
A	0	A	B	B	A	0	B	A	B	B	B	B	B	B	A	A	A	B	B	B	B	A	B	B	B	B	A	B	A	B	A	B	B	B	A	B	B	B	B	A	B	B	B	B	B
A	A	A	B	B	A	A	B	A	B	B	B	B	B	B	A	A	A	B	B	B	B	A	B	B	B	B	A	B	A	B	A	B	B	B	A	B	B	B	B	A	B	B	B	B	B
A	0	A	B	B	A	B	B	A	B	B	B	B	B	B	A	A	A	B	B	B	B	B	B	B	B	B	A	B	B	B	A	B	B	B	B	B	B	B	B	A	B	B	B	B	B
A	A	A	0	0	A	A	0	A	B	0	0	B	B	0	A	A	A	B	B	0	0	0	B	0	B	B	A	0	0	0	A	B	B	0	A	0	0	0	B	A	0	0	0	B	B
A	A	A	B	B	A	A	B	A	B	B	B	B	B	B	A	A	A	B	B	B	B	B	B	B	B	B	A	B	B	B	A	B	B	B	A	B	B	B	B	A	B	B	B	B	B

A	A	A	B	B	A	A	B	A	B	B	B	B	B	B	A	A	A	B	B	B	B	B	B	B	B	B	A	B	B	B	A	B	B	B	A	B	B	B	B	A	B	B	B	B	B
A	A	A	B	B	A	A	B	A	B	B	B	B	B	B	A	A	A	B	B	B	B	B	B	B	B	B	A	B	B	B	A	B	B	B	A	B	B	B	B	A	B	B	B	B	B
A	A	A	B	B	A	A	B	A	B	B	B	B	B	B	A	A	A	B	B	B	B	B	B	B	B	B	A	B	B	B	A	B	B	B	A	B	B	B	B	A	B	B	B	B	B
A	A	A	B	B	A	A	B	A	B	B	B	B	B	B	A	A	A	B	B	B	B	B	B	B	B	B	A	B	B	B	A	B	B	B	A	B	B	B	B	A	B	B	B	B	B
A	A	A	B	B	A	A	B	A	B	B	B	B	B	B	A	A	A	B	B	B	B	B	B	B	B	B	A	B	B	B	A	B	B	B	A	B	B	B	B	A	B	B	B	B	B
A	A	A	B	B	A	A	B	A	B	B	B	B	B	B	A	A	A	B	B	B	B	B	B	B	B	B	A	B	B	B	A	B	B	B	A	B	B	B	B	A	B	B	B	B	B
A	0	A	B	B	A	0	B	A	B	B	B	B	B	B	A	A	A	B	B	B	B	B	B	B	B	B	A	B	B	B	A	B	B	B	A	B	B	B	B	A	B	B	B	B	B
A	A	A	B	B	A	A	B	A	B	B	B	B	B	B	A	A	A	B	B	B	B	B	B	B	B	B	A	B	B	B	A	B	B	B	A	B	B	B	B	A	B	B	B	B	B
A	A	A	B	B	A	A	B	A	B	B	B	B	B	B	A	A	A	B	B	B	B	B	B	B	B	B	A	B	B	B	A	B	B	B	A	B	B	B	B	A	B	B	B	B	B
A	A	A	B	B	A	A	B	A	B	B	B	B	B	B	A	A	A	B	B	B	B	B	B	B	B	B	A	B	B	B	A	B	B	B	A	B	B	B	B	A	B	B	B	B	B
A	A	A	B	B	A	A	B	A	B	B	B	B	B	B	A	A	A	B	B	B	B	B	B	B	B	B	A	B	B	B	A	B	B	B	A	B	B	B	B	A	B	B	B	B	B
A	A	A	B	B	A	A	B	A	B	B	B	B	B	B	A	A	A	B	B	B	B	B	B	B	B	B	A	B	B	B	A	B	B	B	A	B	B	B	B	A	B	B	B	B	B
A	0	A	B	B	A	0	B	A	B	B	B	B	B	B	A	A	A	B	B	B	B	B	B	B	B	B	A	B	B	B	A	B	B	B	A	B	B	B	B	A	B	B	B	B	B
A	0	A	B	B	A	0	B	A	B	B	B	B	B	B	A	A	A	B	B	B	B	B	B	B	B	B	A	B	B	B	A	B	B	B	A	B	B	B	B	A	B	B	B	B	B
A	A	A	B	B	A	A	B	A	B	B	B	B	B	B	A	A	A	B	B	B	B	B	B	B	B	B	A	B	B	B	A	B	B	B	A	B	B	B	B	A	B	B	B	B	B
A	A	A	B	B	A	A	B	A	B	B	B	B	B	B	A	A	A	B	B	B	B	B	B	B	B	B	A	B	B	B	A	B	B	B	A	B	B	B	B	A	B	B	B	B	B
A	A	A	B	B	A	A	B	A	B	B	B	B	B	B	A	A	A	B	B	B	B	B	B	B	B	B	A	B	B	B	A	B	B	B	A	B	B	B	B	A	B	B	B	B	B
A	0	A	B	B	A	0	B	A	B	B	B	B	B	B	A	A	A	B	B	B	B	B	B	B	B	B	A	B	B	B	A	B	B	B	A	B	B	B	B	A	B	B	B	B	B
A	A	A	B	B	A	A	B	A	B	B	B	B	B	B	A	A	A	B	0	B	B	B	B	B	B	B	A	B	B	B	A	B	B	B	A	B	B	B	B	A	B	B	B	B	B
A	0	A	B	B	A	0	B	A	B	B	B	B	B	B	A	A	A	B	B	B	B	B	B	B	B	B	A	B	B	B	A	B	B	B	A	B	B	B	B	A	B	B	B	B	B
A	A	A	B	B	A	A	B	A	B	B	B	B	B	B	A	A	A	B	B	B	B	B	B	B	B	B	A	B	B	B	A	B	B	B	A	B	B	B	B	A	B	B	B	B	B
A	A	A	B	B	A	A	B	A	B	B	B	B	B	B	A	A	A	B	B	B	B	B	B	B	B	B	A	B	B	B	A	B	B	B	A	B	B	B	B	A	B	B	B	B	B
A	0	A	B	B	A	0	B	A	B	B	B	B	B	B	A	A	A	B	B	B	B	B	B	B	B	B	A	B	B	B	A	B	B	B	A	B	B	B	B	A	B	B	B	B	B
A	0	A	B	B	A	0	B	A	B	B	B	B	B	B	A	A	A	B	B	B	B	B	B	B	B	B	A	B	B	B	A	B	B	B	A	B	B	B	B	A	B	B	B	B	B
A	0	A	B	B	A	0	B	A	B	B	B	B	B	B	A	A	A	B	B	B	B	B	B	B	B	B	A	B	B	B	A	B	A	B	A	B	B	B	B	A	B	B	B	B	B
A	0	A	B	B	A	0	B	B	B	B	B	B	B	B	A	A	A	B	B	B	B	B	B	B	B	B	A	B	B	B	A	B	A	B	A	B	B	B	B	A	B	B	B	B	B
A	A	A	B	B	A	A	B	B	B	B	B	B	B	B	A	A	A	B	B	B	B	B	B	B	B	B	A	B	B	B	A	B	A	B	A	B	B	B	B	A	B	B	B	B	B
A	A	A	B	B	A	A	B	B	B	B	B	B	B	B	A	A	A	B	B	B	B	B	B	B	B	B	A	B	B	B	A	B	A	B	A	B	B	B	B	A	B	B	B	B	B
A	A	A	B	B	A	A	B	B	B	B	B	B	B	B	A	A	A	B	B	B	B	B	B	B	B	B	A	B	B	B	A	B	A	B	A	B	B	B	B	A	B	B	B	B	B
A	A	A	B	B	A	A	B	B	B	B	B	B	B	B	A	A	A	B	B	B	B	B	B	B	B	B	A	B	B	B	A	B	A	B	A	B	B	B	B	A	B	B	B	B	B
A	A	A	B	B	A	A	B	B	B	B	B	B	B	B	A	A	A	B	B	B	B	B	B	B	B	B	A	B	B	B	A	B	A	B	A	B	B	B	B	A	B	B	B	B	B
A	A	A	B	B	A	A	B	B	B	B	B	B	B	B	A	A	A	B	B	B	B	B	B	B	B	B	A	B	B	B	A	B	A	B	A	B	B	B	B	A	B	B	B	B	B
A	A	A	B	B	A	A	B	B	B	B	B	B	B	B	A	A	A	B	B	B	B	B	B	B	B	B	A	B	B	B	A	B	A	B	A	B	B	B	B	A	B	B	B	B	B
A	A	A	B	B	A	A	B	B	B	B	B	B	B	B	A	A	A	B	B	B	B	B	B	B	B	B	A	B	B	B	A	B	A	B	A	B	B	B	B	A	B	B	B	B	B
A	A	A	B	B	A	A	B	B	B	B	B	B	B	B	A	A	A	B	B	B	B	B	B	B	B	B	A	B	B	B	A	B	A	B	A	B	B	B	B	A	B	B	B	B	B
A	A	A	B	B	A	A	B	B	B	B	B	B	B	B	A	A	A	B	B	B	B	B	B	B	B	B	A	B	B	B	A	B	A	B	A	B	B	B	B	A	B	B	B	B	B
A	A	A	B	B	A	A	B	B	B	B	B	B	B	B	A	A	A	B	B	B	B	B	B	B	B	B	A	B	B	B	A	B	A	B	A	B	B	B	B	A	B	B	B	B	B
A	A	A	B	B	A	A	B	B	B	B	B	B	B	B	A	A	A	B	B	B	B	B	B	B	B	B	A	B	B	B	A	B	A	B	A	B	B	B	B	A	B	B	B	B	B
A	A	A	B	B	A	A	B	B	B	B	B	B	B	B	A	A	A	B	B	B	B	B	B	B	B	B	A	B	B	B	A	B	A	B	A	B	B	B	B	A	B	B	B	B	B
A	A	A	B	B	A	A	B	B	B	B	B	B	B	B	A	A	A	B	B	B	B	B	B	B	B	B	A	B	B	B	A	B	A	B	A	B	B	B	B	A	B	B	B	B	B
A	A	A	B	B	A	A	B	B	B	B	B	B	B	B	A	A	A	B	B	B	B	B	B	B	B	B	A	B	B	B	A	B	A	B	A	B	B	B	B	A	B	B	B	B	B
A	A	A	B	B	A	A	B	B	B	B	B	B	B	B	A	A	A	B	B	B	B	B	B	B	B	B	A	B	B	B	A	B	A	B	A	B	B	B	B	A	B	B	B	B	B
A	A	A	B	B	A	A	B	B	B	B	B	B	B	B	A	A	A	B	B	B	B	B	B	0	B	B	A	B	B	B	A	B	A	B	A	B	B	B	B	A	B	B	B	B	B
A	0	A	B	B	A	0	B	B	B	B	B	B	B	B	A	A	A	B	B	B	B	B	B	B	B	B	A	B	B	B	A	B	A	B	A	B	B	B	B	A	B	B	B	B	B
A	A	A	B	B	A	A	B	B	B	B	B	B	B	B	A	A	A	B	B	B	B	B	B	B	B	B	A	B	B	B	A	B	A	B	A	B	B	B	B	A	B	B	B	B	B
A	A	A	B	B	A	A	B	B	B	B	B	B	B	B	A	A	A	B	B	B	B	B	B	B	B	B	A	B	B	B	A	B	A	B	A	B	B	B	B	A	B	B	B	B	B
A	0	A	B	B	A	0	B	B	B	B	B	B	B	B	A	A	0	B	B	B	B	B	B	B	B	B	A	B	B	B	0	B	A	B	0	B	B	B	B	A	B	B	B	B	B
A	A	A	B	B	A	A	B	B	B	B	B	B	B	B	A	A	A	B	B	B	B	B	B	B	B	B	A	B	B	B	A	B	A	B	A	B	B	B	B	A	B	B	B	B	B

A	A	A	B	B	A	A	B	B	B	B	B	B	B	B	A	A	A	B	B	B	B	B	B	B	B	B	A	B	B	B	A	B	A	B	A	B	B	B	B	A	B	B	B	B	B
A	A	A	B	B	A	A	B	B	B	B	B	B	B	B	A	A	A	B	B	B	B	B	B	B	B	B	A	B	B	B	A	B	A	B	A	B	B	B	B	A	B	B	B	B	B
A	0	A	B	B	A	0	B	B	B	B	B	B	B	B	A	A	A	B	B	B	B	B	B	B	B	B	A	B	B	B	A	B	A	B	A	B	B	B	B	A	B	B	B	B	B
A	A	A	B	B	A	A	B	B	B	B	B	B	B	B	A	A	A	B	B	B	B	B	B	B	B	B	A	B	B	B	A	B	A	B	A	B	B	B	B	A	B	B	B	B	B
A	A	A	B	B	A	A	B	B	B	B	B	B	B	B	A	A	A	B	B	B	B	B	B	B	B	B	A	B	B	B	A	B	A	B	A	B	B	B	B	A	B	B	B	B	B
A	A	A	B	B	A	A	B	B	B	B	B	B	B	B	A	A	A	B	B	B	B	B	B	B	B	B	A	B	B	B	A	B	A	B	A	B	B	B	B	A	B	B	B	B	B
A	A	A	B	B	A	A	B	B	B	B	B	B	B	B	A	A	A	B	B	B	B	B	B	B	B	B	A	B	B	B	A	B	A	B	A	B	B	B	B	A	B	B	B	B	B
A	A	A	B	B	A	A	B	B	B	B	B	B	B	B	A	A	A	B	B	B	B	B	B	B	B	B	A	B	B	B	A	B	A	B	A	B	B	B	B	A	B	B	B	B	B
A	A	A	B	B	A	A	B	B	B	B	B	B	B	B	A	A	A	B	B	B	B	B	B	B	B	B	A	B	B	B	A	B	A	B	A	B	B	B	B	A	B	B	B	B	B
A	A	A	B	B	A	A	B	B	B	B	B	B	B	B	A	A	A	B	B	B	B	B	B	B	B	B	A	B	B	B	A	B	A	B	A	B	B	B	B	A	B	B	B	B	B
A	A	A	B	B	A	A	B	B	B	B	B	B	B	B	A	A	A	B	B	B	B	B	B	B	B	B	A	B	B	B	A	B	A	B	A	B	B	B	B	A	B	B	B	B	B
A	A	A	B	B	A	A	B	B	B	B	B	B	B	B	A	A	A	B	B	B	B	B	B	B	B	B	A	B	B	B	A	B	A	B	A	B	B	B	B	A	B	B	B	B	B
A	A	A	B	B	A	A	B	B	B	B	B	B	B	B	A	A	A	B	B	B	B	B	B	B	B	B	A	B	B	B	A	B	A	B	A	B	B	B	B	A	B	B	B	B	B
A	A	A	B	B	A	A	B	B	B	B	B	B	B	B	A	A	A	B	B	B	B	B	B	B	B	B	A	B	B	B	A	B	A	B	A	B	B	B	B	A	B	B	B	B	B
A	A	A	B	B	A	A	B	B	B	B	B	B	B	B	A	A	A	B	B	B	B	B	B	B	B	B	A	B	B	B	A	B	A	B	A	B	B	B	B	A	B	B	B	B	B
A	A	A	B	B	A	A	B	B	B	B	B	B	B	B	A	A	A	B	B	B	B	B	B	B	B	B	A	B	B	B	A	B	A	B	A	B	B	B	B	A	B	B	B	B	B
A	A	A	B	B	A	A	B	B	B	B	B	B	B	B	A	A	A	B	B	B	B	B	B	B	B	B	A	B	B	B	A	B	A	B	A	B	B	B	B	A	B	B	B	B	B
A	0	A	B	B	A	0	B	B	B	B	B	B	B	B	A	A	A	B	B	B	B	B	B	B	B	B	A	B	B	B	A	B	A	B	A	B	B	B	B	A	B	B	B	B	B
A	A	A	B	B	A	A	B	B	B	B	B	B	B	B	A	A	A	B	B	B	B	B	B	B	B	B	A	B	B	B	A	B	A	B	A	B	B	B	B	A	B	B	B	B	B
A	0	A	B	B	A	0	B	B	B	B	B	B	B	B	A	A	A	B	B	B	B	B	B	B	B	B	A	B	B	B	A	B	A	B	A	B	B	B	B	A	B	B	B	B	B
A	0	A	B	B	A	A	B	B	B	B	B	B	B	B	A	A	A	B	B	B	B	B	B	B	B	B	A	B	B	B	A	B	A	B	A	B	B	B	B	A	B	B	B	B	B
A	A	A	B	B	A	A	B	B	B	B	B	B	B	B	A	A	A	B	B	B	B	B	B	0	B	B	A	B	B	B	A	B	A	B	A	B	B	B	B	A	B	B	B	B	B
A	A	A	B	B	A	A	B	B	B	B	B	B	B	B	A	A	A	B	B	B	B	B	B	B	B	B	A	B	B	B	A	B	A	B	A	B	B	B	B	A	B	B	B	B	B
A	A	A	B	B	A	A	B	B	B	B	B	B	B	B	A	A	A	B	B	B	B	B	B	B	B	B	A	B	B	B	A	B	A	B	A	B	B	B	B	A	B	B	B	B	B
A	A	A	B	B	A	A	B	B	B	B	B	B	B	B	A	A	A	B	B	B	B	B	B	B	B	B	A	B	B	B	A	B	A	B	A	B	B	B	B	A	B	B	B	B	B
A	A	A	B	B	A	A	B	B	B	B	B	B	B	B	A	A	A	B	B	B	B	B	B	B	B	B	A	B	B	B	A	B	A	B	A	B	B	B	B	A	B	B	B	B	B
A	A	A	B	B	A	A	B	B	B	B	B	B	B	B	A	A	A	B	B	B	B	B	B	B	B	B	A	B	B	B	A	B	A	B	A	B	B	B	B	A	B	B	B	B	B
A	0	A	B	B	A	0	B	B	B	B	B	B	B	B	A	A	A	B	B	B	B	B	B	B	B	B	A	B	B	B	A	B	A	B	A	B	B	B	B	A	B	B	B	B	B
A	0	A	B	B	A	0	B	B	B	B	B	B	B	B	A	A	A	B	B	B	B	B	B	B	B	B	A	B	B	B	A	B	A	B	A	B	B	B	B	A	B	B	B	B	B
A	A	A	B	B	A	A	B	B	B	B	B	B	B	B	A	A	A	B	B	B	B	B	B	B	B	B	A	B	B	B	A	B	A	B	A	B	B	B	B	A	B	B	B	B	B
A	A	A	B	B	A	A	B	B	B	B	B	B	B	B	A	A	A	B	B	B	B	B	B	B	B	B	A	B	B	B	A	B	A	B	A	B	B	B	B	A	B	B	B	B	B
A	A	A	B	B	A	A	B	B	B	B	B	B	B	B	A	A	A	B	B	B	B	B	B	B	B	B	A	B	B	B	A	B	A	B	A	B	B	B	B	A	B	B	B	B	B
A	A	A	B	B	A	A	B	B	B	B	B	B	B	B	A	A	A	B	B	B	B	B	B	B	B	B	A	B	B	B	A	B	A	B	A	B	B	B	B	A	B	B	B	B	B
A	A	A	B	B	A	A	B	B	B	B	B	B	B	B	A	A	A	B	B	B	B	B	B	B	B	B	A	B	B	B	A	B	A	B	A	B	B	B	B	A	B	B	B	B	B
A	A	A	B	B	A	A	B	B	B	B	B	B	B	B	A	A	A	B	B	B	B	B	B	B	B	B	A	B	B	B	A	B	A	B	A	B	B	B	B	A	B	B	B	B	B
A	0	A	B	B	A	0	B	B	B	B	B	B	B	B	A	A	A	B	B	B	B	B	B	B	B	B	A	B	B	B	A	B	A	B	A	B	B	B	B	A	B	B	B	B	B
A	0	A	B	B	A	0	B	B	B	B	B	B	B	B	A	A	A	B	B	B	B	B	B	B	B	B	A	B	B	B	A	B	A	B	A	B	B	B	B	A	B	B	B	B	B
A	A	A	B	B	A	0	B	B	B	B	B	B	B	B	A	A	A	B	B	B	B	B	B	B	B	B	A	B	B	B	A	B	A	B	A	B	B	B	B	A	B	B	B	B	B
A	A	A	B	B	A	A	B	B	B	B	B	B	B	B	A	A	A	B	B	B	B	B	B	B	B	B	A	B	B	B	A	B	A	B	A	B	B	B	B	A	B	B	B	B	B
A	A	A	B	B	A	0	B	B	B	B	B	B	B	B	A	A	A	B	B	B	B	B	B	B	B	B	A	B	B	B	A	B	A	B	A	B	B	B	B	A	B	B	B	B	B
A	A	A	B	B	A	A	B	B	B	B	B	B	B	B	A	A	A	B	B	B	B	B	B	B	B	B	A	B	B	B	A	B	A	B	A	B	B	B	B	A	B	B	B	B	B
A	A	A	B	B	A	A	B	B	B	B	B	B	B	B	A	A	A	B	B	B	B	B	B	B	B	B	A	B	B	B	A	B	A	B	A	B	B	B	B	A	B	B	B	B	B
A	A	A	B	B	A	A	B	B	B	B	B	B	B	B	A	A	A	B	B	B	B	B	B	B	B	B	A	B	B	B	A	B	A	B	A	B	B	B	B	A	B	B	B	B	B
A	0	A	B	B	A	0	B	B	B	B	B	B	B	B	A	A	A	B	B	B	B	B	B	B	B	B	A	B	B	B	A	B	A	B	A	B	B	B	B	A	B	B	B	B	B
A	A	A	B	B	A	A	B	B	B	B	B	B	B	B	A	A	A	B	B	B	B	B	B	B	B	B	A	B	B	B	A	B	A	B	A	B	B	B	B	A	B	B	B	B	B
A	A	A	B	B	A	A	B	B	B	B	B	B	B	B	A	A	A	B	B	B	B	B	B	B	B	B	A	B	B	B	A	B	A	B	A	B	B	B	B	A	B	B	B	B	B
A	A	A	B	B	A	B	B	B	B	B	B	B	B	B	A	A	A	B	B	B	B	B	B	B	B	B	A	B	B	B	A	B	A	B	A	B	B	B	B	A	B	B	B	B	B
A	A	A	B	B	A	B	B	B	B	B	B	B	B	B	A	A	A	B	B	B	B	B	B	B	B	B	A	B	B	B	A	B	A	B	A	B	B	B	B	A	B	B	B	B	B
A	0	A	B	B	A	B	B	B	B	B	B	B	B	B	A	A	A	B	B	B	B	B	B	B	B	B	A	B	B	B	A	B	A	B	A	B	B	B	B	A	B	B	B	B	B

A	A	A	B	B	A	B	B	B	B	B	B	B	B	B	A	A	A	B	B	B	B	B	B	B	B	B	A	B	B	B	A	B	A	B	A	B	B	B	B	A	B	B	B	B	B
A	A	A	B	B	A	B	B	B	B	B	B	B	B	B	A	A	A	B	B	B	B	B	B	B	B	B	A	B	B	B	A	B	A	B	A	B	B	B	B	A	B	B	B	B	B
A	A	A	B	B	A	B	B	B	B	B	B	B	B	B	A	A	A	B	B	B	B	B	B	B	B	B	A	B	B	B	A	B	A	B	A	B	B	B	B	A	B	B	B	B	B
A	A	A	B	B	A	B	B	B	B	B	B	B	B	B	A	A	A	B	B	B	B	B	B	B	B	B	A	B	B	B	A	B	A	B	A	B	B	B	B	A	B	B	B	B	B
A	A	A	B	B	A	B	B	B	B	B	B	B	B	B	A	A	A	B	B	B	B	B	B	B	B	B	A	B	B	B	A	B	A	B	A	B	B	B	B	A	B	B	B	B	B
A	A	A	B	B	A	B	B	B	B	B	B	B	B	B	A	A	A	B	B	B	B	B	B	B	B	B	A	B	B	B	A	B	A	B	A	B	B	B	B	A	B	B	B	B	B
A	A	A	B	B	A	B	B	B	B	B	B	B	B	B	A	A	A	B	B	B	B	B	B	B	B	B	A	B	B	B	A	B	A	B	A	B	B	B	B	A	B	B	B	B	B
A	A	A	B	B	A	B	B	B	B	B	B	B	B	B	A	A	A	B	B	B	B	B	B	B	B	B	A	B	B	B	A	B	A	B	A	B	B	B	B	A	B	B	B	B	B
A	A	A	B	B	A	B	B	B	B	B	B	B	B	B	A	A	A	B	B	B	B	B	B	B	B	B	A	B	B	B	A	B	A	B	A	B	B	B	B	A	B	B	B	B	B
A	A	A	B	B	A	B	B	B	B	B	B	B	B	B	A	A	A	B	B	B	B	B	B	B	B	B	A	B	B	B	A	B	A	B	A	B	B	B	B	A	B	B	B	B	B
A	A	A	B	B	A	B	B	B	B	B	B	B	B	B	A	A	A	B	B	B	B	B	B	B	B	B	A	B	B	B	A	B	A	B	A	B	B	B	B	A	B	B	B	B	B
A	A	A	B	B	A	B	B	B	B	B	B	B	B	B	A	A	A	B	B	B	B	B	B	B	B	B	A	B	B	B	A	B	A	B	A	B	B	B	B	A	B	B	B	B	B
A	0	A	B	B	A	B	B	B	B	B	B	B	B	B	A	A	A	B	B	B	B	B	B	B	B	B	A	B	B	B	A	B	A	B	A	B	B	B	B	A	B	B	B	B	B
A	A	A	B	B	A	B	B	B	B	B	B	B	B	B	A	A	A	B	B	B	B	B	B	B	B	B	A	B	B	B	A	B	A	B	A	B	B	B	B	A	B	B	B	B	B
A	A	A	B	B	A	B	B	B	B	B	B	B	B	B	A	A	A	B	B	B	B	B	B	B	B	B	A	B	B	B	A	B	A	B	A	B	B	B	B	A	B	B	B	B	B
A	A	A	B	B	A	B	B	B	B	B	B	B	B	B	A	A	A	B	B	B	B	B	B	B	B	B	A	B	B	B	A	B	A	B	A	B	B	B	B	A	B	B	B	B	B
A	A	A	B	B	A	B	B	B	B	B	B	B	B	B	A	A	A	B	B	B	B	B	B	B	B	B	A	B	B	B	A	B	A	B	A	B	B	B	B	A	B	B	B	B	B
A	A	A	B	B	A	B	B	B	B	B	B	B	B	B	A	A	A	B	B	B	B	B	B	B	B	B	A	B	B	B	A	B	A	B	A	B	B	B	B	A	B	B	B	B	B
A	A	A	B	B	A	B	B	B	B	B	B	B	B	B	A	A	A	B	B	B	B	B	B	B	B	B	A	B	B	B	A	B	A	B	A	B	B	B	B	A	B	B	B	B	B
A	A	A	B	B	A	B	B	B	B	B	B	B	B	B	A	A	A	B	B	B	B	B	B	B	B	B	A	B	B	B	A	B	A	B	A	B	B	B	B	A	B	B	B	B	B
A	A	A	B	B	A	B	B	B	B	B	B	B	B	B	A	A	A	B	B	B	B	B	B	B	B	B	A	B	B	B	A	B	A	B	A	B	B	B	B	A	B	B	B	B	B
A	A	A	B	B	A	B	B	B	B	B	B	B	B	B	A	A	A	B	B	B	B	B	B	B	B	B	A	B	B	B	A	B	A	B	A	B	B	B	B	A	B	B	B	B	B
A	A	A	B	B	A	B	B	B	B	B	B	B	B	B	A	A	A	B	B	B	B	B	B	B	B	B	A	B	B	B	A	B	A	B	A	B	B	B	B	A	B	B	B	B	B
A	A	A	B	B	A	B	B	B	B	B	B	B	B	B	A	A	A	B	B	B	B	B	B	B	B	B	A	B	B	B	A	B	A	B	A	B	B	B	B	A	B	B	B	B	B
A	A	A	B	B	A	B	B	B	B	B	B	B	B	B	A	A	A	B	B	B	B	B	B	B	B	B	A	B	B	B	A	B	A	B	A	B	B	B	B	A	B	B	B	B	B
A	A	A	B	B	A	B	B	B	B	B	B	B	B	B	A	A	A	B	B	B	B	B	B	B	B	B	A	B	B	B	A	B	A	B	A	B	B	B	B	A	B	B	B	B	B
A	A	A	B	B	A	B	B	B	B	B	B	B	B	B	A	A	A	B	B	B	B	B	B	B	B	B	A	B	B	B	A	B	A	B	A	B	B	B	B	A	B	B	B	B	B
A	A	A	B	B	A	B	B	B	B	B	B	B	B	B	A	A	A	B	B	B	B	B	B	B	B	B	A	B	B	B	A	B	A	B	A	B	B	B	B	A	B	B	B	B	B
A	A	A	B	B	A	B	B	B	B	B	B	B	B	B	A	A	A	B	B	B	B	B	B	B	B	B	A	B	B	B	A	B	A	B	A	B	B	B	B	A	B	B	B	B	B
A	A	A	B	B	A	B	B	B	B	B	B	B	B	B	A	A	0	B	B	B	B	B	B	B	B	B	A	B	B	B	0	B	0	B	A	B	B	B	B	A	B	B	B	B	B
A	A	A	B	B	A	B	B	B	B	B	B	B	B	B	A	A	A	B	B	B	B	B	B	0	B	B	A	B	B	B	A	B	A	B	A	B	B	B	B	A	B	B	B	B	B
A	A	A	B	B	A	B	B	B	B	B	B	B	B	B	A	A	A	B	B	B	B	B	B	B	B	B	A	B	B	B	A	B	A	B	A	B	B	B	B	A	B	B	B	B	B
A	A	A	B	B	A	B	B	B	B	B	B	B	B	B	A	A	A	B	B	B	B	B	B	B	B	B	A	B	B	B	A	B	A	B	A	B	B	B	B	A	B	B	B	B	B
A	0	A	B	B	A	B	B	B	B	B	B	B	B	B	A	A	A	B	B	B	B	B	B	B	B	B	A	B	B	B	A	B	A	B	A	B	B	B	B	A	B	B	B	B	B
A	A	A	B	B	A	B	B	B	B	B	B	B	B	B	A	A	A	B	B	B	B	B	B	B	B	B	A	B	B	B	A	B	A	B	A	B	B	B	B	A	B	B	B	B	B
A	A	A	B	B	A	B	B	B	B	B	B	B	B	B	A	A	A	B	B	B	B	B	B	B	B	B	A	B	B	B	A	B	A	B	A	B	B	B	B	A	B	B	B	B	B
A	A	A	B	B	A	B	B	B	B	B	B	B	B	B	A	A	A	B	B	B	B	B	B	B	B	B	A	B	B	A	A	B	A	B	A	B	B	B	B	A	B	B	B	B	B
A	0	0	B	B	A	B	B	B	B	B	B	B	B	B	A	A	A	B	B	B	B	B	B	B	B	B	A	B	B	A	A	B	A	B	A	B	B	B	B	A	B	B	B	B	B
A	A	A	B	B	A	B	B	B	0	B	0	0	B	B	A	A	A	0	0	0	B	B	B	0	B	B	A	B	B	A	A	0	A	0	A	0	0	B	0	A	B	0	0	B	B
A	A	A	B	B	A	B	B	B	B	B	B	B	B	B	A	A	A	B	B	B	B	B	B	B	B	B	A	B	B	A	A	B	A	B	A	B	B	B	B	A	B	B	B	B	B
A	0	A	B	B	A	B	B	B	B	B	B	B	B	B	A	A	A	B	B	B	B	B	B	B	B	B	A	B	B	A	A	B	A	B	A	B	B	B	B	A	B	B	B	B	B
A	0	A	B	B	A	B	B	B	B	B	B	B	B	B	A	A	A	B	B	B	B	B	B	B	B	B	A	B	B	A	A	B	A	B	A	B	B	B	B	A	B	B	B	B	B
A	0	A	B	B	A	B	B	B	B	B	B	B	B	B	A	A	A	B	B	B	B	B	B	B	B	B	A	B	B	A	A	B	A	B	A	B	B	B	B	A	B	B	B	B	B
A	0	A	B	B	A	B	B	B	B	B	B	B	B	B	A	A	A	B	B	B	B	B	B	B	B	B	A	B	B	A	A	B	A	B	A	B	B	B	B	A	B	B	B	B	B
A	A	A	B	B	A	B	B	B	B	B	B	B	B	B	A	A	A	B	B	B	B	B	B	B	B	B	A	B	B	A	A	B	A	B	A	B	B	B	B	A	B	B	B	B	B
A	0	A	B	B	A	B	B	B	B	B	B	B	B	B	A	A	A	B	B	B	B	B	B	B	B	B	A	B	B	A	A	B	A	B	A	B	B	B	B	A	B	B	B	B	B
A	A	A	B	B	A	B	B	B	B	B	A	B	B	B	A	A	A	B	B	B	B	B	B	B	B	B	A	B	B	A	A	B	A	B	A	B	B	B	B	A	B	B	B	B	B
A	0	A	B	B	A	B	B	B	B	B	A	B	B	B	A	A	A	B	B	B	B	B	B	B	B	B	A	B	B	A	A	B	A	B	A	B	B	B	B	A	B	B	B	B	B
A	0	A	B	B	A	B	B	B	B	B	A	B	B	B	A	A	A	B	B	B	B	B	B	B	B	B	A	B	B	A	A	B	A	B	A	B	B	B	B	A	B	B	B	B	B

A	A	A	B	B	A	B	B	B	B	B	A	B	B	B	A	A	A	B	B	B	B	B	B	B	B	B	A	B	B	A	A	B	A	B	A	B	B	B	B	A	B	B	B	B	B
A	0	A	B	B	A	B	B	B	B	B	A	B	B	B	A	A	A	B	B	B	B	B	B	B	B	B	A	B	B	A	A	B	A	B	A	B	B	B	B	A	B	B	B	B	B
A	0	A	B	B	A	B	B	B	B	B	A	B	B	B	A	A	A	B	B	B	B	B	B	B	B	B	A	B	B	A	A	B	A	B	A	B	B	B	B	A	B	B	B	B	B
A	A	A	B	B	A	B	B	B	B	B	A	B	B	B	A	A	A	B	B	B	B	B	B	B	B	B	A	B	B	A	A	B	A	B	A	B	B	B	B	A	B	B	B	B	B
A	A	A	B	B	A	B	B	B	B	B	A	B	B	B	A	A	A	B	B	B	B	B	B	B	B	B	A	B	B	A	A	B	A	B	A	B	B	B	B	A	B	B	B	B	B
A	A	A	B	B	A	B	B	B	B	B	A	B	B	B	A	A	A	B	B	B	B	B	B	B	B	B	A	B	B	A	A	B	A	B	A	B	B	B	B	A	B	B	B	B	B
A	A	A	B	B	A	B	B	B	B	B	A	B	B	B	A	A	A	B	B	B	B	B	B	B	B	B	A	B	B	A	A	B	A	B	A	B	B	B	B	A	B	B	B	B	B
A	A	A	B	B	A	B	B	B	B	B	A	B	B	B	A	A	A	B	B	B	B	B	B	B	B	B	A	B	B	A	A	B	A	B	A	B	B	B	B	A	B	B	B	B	B
A	A	A	B	B	A	B	B	B	B	B	A	B	B	B	A	A	A	B	B	B	B	B	B	B	B	B	A	B	B	A	A	B	A	B	A	B	B	B	B	A	B	B	B	B	B
A	A	A	B	B	A	B	B	B	B	B	A	B	B	B	A	A	A	B	B	B	B	B	B	B	B	B	A	B	B	A	A	B	A	B	A	B	B	B	B	A	B	B	B	B	B
A	A	A	B	B	A	B	B	B	B	B	A	B	B	B	A	A	A	B	B	B	B	B	B	B	B	B	A	B	B	A	A	B	A	B	A	B	B	B	B	A	B	B	B	B	B
A	A	A	B	B	A	B	B	B	B	B	A	B	B	B	A	A	A	B	B	B	B	B	B	B	B	B	A	A	B	A	A	B	A	B	A	B	B	B	B	A	B	B	B	B	B
A	A	A	B	B	A	B	B	B	B	B	A	B	B	B	A	A	A	B	B	B	B	B	B	B	B	B	A	A	B	A	A	B	A	B	A	B	B	B	B	A	B	B	B	B	B
A	A	A	B	B	A	B	B	B	B	B	A	B	B	B	A	A	A	B	B	B	B	B	B	B	B	B	A	A	B	A	A	B	A	B	A	B	B	B	B	A	B	B	B	B	B
A	A	A	B	B	A	B	B	B	B	B	A	B	B	B	A	A	A	B	B	B	B	B	B	B	B	B	A	0	B	A	A	B	A	B	A	B	B	B	B	A	B	B	B	B	B
A	0	A	B	B	A	B	B	B	B	B	A	B	B	B	A	A	A	B	B	B	B	B	B	B	B	B	A	B	B	A	A	B	A	B	A	B	B	B	B	A	B	B	B	B	B
A	A	A	B	B	A	B	B	B	B	B	A	B	B	B	A	A	A	B	B	B	B	B	B	B	B	B	A	B	B	A	A	B	A	B	A	B	B	B	B	A	B	B	B	B	B
A	A	A	B	B	A	B	B	B	B	B	A	B	B	B	A	A	A	B	B	B	B	B	B	B	B	B	A	B	B	A	A	B	A	B	A	B	B	B	B	A	B	B	B	B	B
A	A	A	B	B	A	B	B	B	B	B	A	B	B	B	A	A	A	B	B	B	B	B	B	B	B	B	A	B	B	A	A	B	A	B	A	B	B	B	B	A	B	B	B	B	B
A	A	A	0	B	A	B	B	B	0	B	A	0	B	B	A	A	A	0	0	0	B	B	B	0	B	B	A	0	0	A	A	0	A	0	A	0	0	B	0	A	0	0	0	B	B
A	A	A	B	B	A	B	B	B	B	B	A	B	B	B	A	A	A	B	B	B	B	B	B	B	B	B	A	B	B	A	A	B	A	B	A	B	B	B	B	A	B	B	B	B	B
A	A	A	B	B	A	B	B	B	B	B	A	B	B	B	A	A	A	B	B	B	B	B	B	B	B	B	A	0	B	A	A	B	A	B	A	B	B	B	B	A	B	B	B	B	B
A	A	A	B	B	A	B	B	B	B	B	A	B	B	B	A	A	A	B	B	B	B	B	B	B	B	B	A	0	B	A	A	B	A	B	A	B	B	B	B	A	B	B	B	B	B
A	A	A	B	B	A	B	B	B	B	B	A	B	B	B	A	A	A	B	B	B	B	B	B	B	B	B	A	0	B	A	A	B	A	B	A	B	B	B	B	A	B	B	B	B	B
A	0	A	B	B	A	B	B	B	B	B	A	B	B	B	A	A	0	B	B	B	B	B	B	B	B	B	A	B	B	A	A	B	0	B	A	B	B	B	B	A	B	B	B	B	B
A	0	A	B	B	A	B	B	B	B	B	A	B	B	B	A	A	A	B	B	B	B	B	B	B	B	B	A	B	B	A	A	B	A	B	A	B	B	B	B	A	B	B	B	B	B
A	A	A	B	B	A	B	B	B	B	B	A	B	B	B	A	A	A	B	B	B	B	B	B	B	B	B	A	B	B	A	A	B	A	B	A	B	B	B	B	A	B	B	B	B	B
A	A	A	B	B	A	B	B	B	B	B	A	B	B	B	A	A	A	B	B	B	B	B	B	B	B	B	A	A	B	A	A	B	A	B	A	B	B	B	B	A	B	B	B	B	B
A	0	A	B	B	A	B	B	B	B	B	A	B	B	B	A	A	A	B	B	B	B	B	B	B	B	B	A	B	B	A	A	B	A	B	A	B	B	B	B	A	B	B	B	B	B
A	A	0	B	B	A	B	B	B	B	B	A	B	B	B	A	A	A	B	B	B	B	B	B	B	B	B	A	B	B	A	A	B	A	B	A	B	B	B	B	A	B	B	B	B	B
A	A	A	B	B	A	B	B	B	B	B	A	B	B	B	A	A	A	B	B	B	B	B	B	B	B	B	A	0	B	A	A	B	A	B	A	B	B	B	B	A	B	B	B	B	B
A	A	A	B	B	A	B	B	B	B	B	A	B	B	B	A	A	A	B	B	B	B	B	B	B	B	B	A	0	B	A	A	B	A	B	A	B	B	B	B	A	B	B	B	B	B
A	A	A	B	B	A	B	B	B	B	B	A	B	B	B	A	A	A	B	B	B	B	B	B	B	B	B	A	0	B	A	A	B	A	B	A	B	B	B	B	A	B	B	B	B	B
A	A	A	B	B	A	B	B	B	B	B	A	B	B	B	A	A	A	B	B	B	B	B	B	B	B	B	A	0	B	A	A	B	A	B	A	B	B	B	B	A	B	B	B	B	B
A	A	A	B	B	A	B	B	B	B	B	A	B	B	B	A	A	A	B	B	B	B	B	B	B	B	B	A	0	B	A	A	B	A	B	A	B	B	B	B	A	B	B	B	B	B
A	A	A	B	B	A	B	B	B	B	B	A	B	B	B	A	A	A	B	B	B	B	B	B	B	B	B	A	0	B	A	A	B	A	B	A	B	B	B	B	A	B	B	B	B	B
A	0	A	B	B	A	B	B	B	B	B	A	B	B	B	A	A	A	B	B	B	B	B	B	B	B	B	A	B	B	A	A	B	A	B	A	B	B	B	B	A	B	B	B	B	B
A	A	A	B	B	A	B	B	B	B	B	A	B	B	B	A	A	A	B	B	B	B	B	B	B	B	B	A	0	B	A	A	B	A	B	A	B	B	B	B	A	B	B	B	B	B
A	A	A	B	B	A	B	B	B	B	B	A	B	B	B	A	A	A	B	B	B	B	B	B	B	B	B	A	B	B	A	A	B	A	B	A	B	B	B	B	A	B	B	B	B	B
A	0	A	B	B	A	B	B	B	B	B	A	B	B	B	A	A	A	B	B	B	B	B	B	B	B	B	A	B	B	A	A	B	A	B	A	B	B	B	B	A	B	B	B	B	B
A	0	A	B	B	A	B	B	B	B	B	A	B	B	B	A	A	A	B	B	B	B	B	B	B	B	B	A	B	B	A	A	B	A	B	A	B	B	B	B	A	B	B	B	B	B
A	A	A	B	B	A	B	B	B	B	B	A	B	B	B	A	A	A	B	B	B	B	B	B	B	B	B	A	0	B	A	A	B	A	B	A	B	B	B	B	A	B	B	B	B	B
A	A	A	B	B	A	B	B	B	B	B	A	B	B	B	A	A	A	B	B	B	B	B	B	B	B	B	A	0	B	A	A	B	A	B	A	B	B	B	B	A	B	B	B	B	B
A	A	A	B	B	A	B	B	B	0	B	A	0	B	B	A	A	A	0	0	0	B	B	B	0	B	B	A	A	B	A	A	0	A	0	A	0	0	B	0	A	B	0	0	B	B
A	0	A	B	B	A	B	B	B	B	B	A	B	B	B	A	A	A	B	B	B	B	B	B	B	B	B	A	B	B	A	A	B	A	B	A	B	B	B	B	A	B	B	B	B	B
A	A	A	B	B	A	B	B	B	B	B	A	B	B	B	A	A	A	B	B	B	B	B	B	B	B	B	A	B	B	A	A	B	A	B	A	B	B	B	B	A	B	B	B	B	B
A	A	A	B	B	A	B	B	B	B	B	A	B	B	B	A	A	A	B	B	B	B	B	B	B	B	B	A	0	B	A	A	B	A	B	A	B	B	B	B	A	B	B	B	B	B
A	A	A	B	B	A	B	B	B	B	B	A	B	B	B	A	A	A	B	B	B	B	B	B	B	B	B	A	0	B	A	A	B	A	B	A	B	B	B	B	A	B	B	B	B	B
A	0	A	B	B	A	B	B	B	B	B	A	B	B	B	A	A	A	B	B	B	B	B	B	B	B	B	A	B	B	A	A	B	A	B	A	B	B	B	B	A	B	B	B	B	B

A	A	A	B	B	A	B	B	B	B	B	A	B	B	B	A	A	A	B	B	B	B	B	B	B	B	B	A	0	B	A	A	B	A	B	A	B	B	B	B	A	B	B	B	B	B
A	A	A	B	B	A	B	B	B	B	B	A	B	B	B	A	A	A	B	B	B	B	B	B	B	B	B	A	A	B	A	A	B	A	B	A	B	B	B	B	A	B	B	B	B	B
A	A	A	0	B	A	0	B	0	0	0	A	B	0	B	A	A	A	B	0	0	B	B	B	0	0	0	A	A	B	A	A	0	A	0	A	0	0	0	0	A	0	0	0	0	0
A	0	A	B	B	A	B	B	B	B	B	A	B	B	B	A	A	A	B	B	B	B	B	B	B	B	B	A	B	B	A	A	B	A	B	A	B	B	B	B	A	B	B	B	B	B
A	A	A	B	B	A	B	B	B	B	B	A	B	B	B	A	A	A	B	B	B	B	B	B	B	B	B	A	0	B	A	A	B	A	B	A	B	B	B	B	A	B	B	B	B	B
A	A	A	B	B	A	B	B	B	B	B	A	B	B	B	A	A	A	B	B	B	B	B	B	B	B	B	A	0	B	A	A	B	A	B	A	B	B	B	B	A	B	B	B	B	B
A	A	A	B	B	A	B	B	B	B	B	A	B	B	B	A	A	A	B	B	B	B	B	B	B	B	B	A	0	B	A	A	B	A	B	A	B	B	B	B	A	B	B	B	B	B
A	A	A	B	B	A	B	B	B	B	B	A	B	B	B	A	A	A	B	B	B	B	B	B	B	B	B	A	0	B	A	A	B	A	B	A	B	B	B	B	A	B	B	B	B	B
A	A	A	B	B	A	B	B	B	B	B	A	B	B	B	A	A	A	B	B	B	B	B	B	B	B	B	A	0	B	A	A	B	A	B	A	B	B	B	B	A	B	B	B	B	B
A	A	A	B	B	A	B	B	B	B	B	A	B	B	B	A	A	A	B	B	B	B	B	B	B	B	B	A	0	B	A	A	B	A	B	A	B	B	B	B	A	B	B	B	B	B
A	A	A	B	B	A	B	B	B	B	B	A	B	B	B	A	A	A	B	B	B	B	B	B	B	B	B	A	0	B	A	A	B	A	B	A	B	B	B	B	A	B	B	B	B	B
A	A	A	B	B	A	B	B	B	B	B	A	B	B	B	A	A	A	B	B	B	B	B	B	B	B	B	A	0	B	A	A	B	A	B	A	B	B	B	B	A	B	B	B	B	B
A	A	A	B	B	A	B	B	B	B	B	A	B	B	B	A	A	A	B	B	B	B	B	B	B	B	B	A	0	B	A	A	B	A	B	A	B	B	B	B	A	B	B	B	B	B
A	A	A	B	B	A	B	B	B	B	B	A	B	B	B	A	A	A	B	B	B	B	B	B	B	B	B	A	0	B	A	A	B	A	B	A	B	B	B	B	A	B	B	B	B	B
A	A	A	B	B	A	B	B	B	B	B	A	B	B	B	A	A	A	B	B	B	B	B	B	B	B	B	A	0	B	A	A	B	A	B	A	B	B	B	B	A	B	B	B	B	B
A	A	A	B	B	A	B	B	B	B	B	A	B	B	B	A	A	A	B	B	B	B	B	B	B	B	B	A	0	B	A	A	B	A	B	A	B	B	B	B	A	B	B	B	B	B
A	A	A	B	B	A	B	B	B	B	B	A	B	B	B	A	A	A	B	B	B	B	B	B	B	B	B	A	0	B	A	A	B	A	B	A	B	B	B	B	A	B	B	B	B	B
A	A	A	B	B	A	B	B	B	B	B	A	B	B	B	A	A	A	B	B	B	B	B	B	B	B	B	A	0	B	A	A	B	A	B	B	B	B	B	B	A	B	B	B	B	B
A	A	A	B	B	A	B	B	B	B	B	A	B	B	B	A	A	A	B	B	B	B	B	B	B	B	B	A	0	B	A	A	B	A	B	B	B	B	B	B	A	B	B	B	B	B
A	A	A	B	B	A	B	B	B	B	B	A	B	B	B	A	A	A	B	B	B	B	B	B	B	B	B	A	0	B	A	A	B	A	B	B	B	B	B	B	A	B	B	B	B	B
A	A	A	B	B	A	B	B	B	B	B	A	B	B	B	A	A	A	B	B	B	B	B	B	B	B	B	A	B	B	A	A	B	A	B	B	B	B	B	B	A	B	B	B	B	B
A	A	A	B	B	A	B	B	B	B	B	A	B	B	B	A	A	A	B	B	B	B	B	B	B	B	B	A	0	B	A	A	B	A	B	B	B	B	B	B	A	B	B	B	B	B
A	A	A	B	B	A	B	B	B	B	B	A	B	B	B	A	A	A	B	B	B	B	B	B	B	B	B	A	B	B	A	A	B	A	B	B	B	B	B	B	A	B	B	B	B	B
A	A	0	B	B	0	B	B	B	B	B	0	B	B	B	0	0	0	B	B	B	B	B	B	B	B	B	0	B	B	0	A	B	A	B	B	B	B	B	B	0	B	B	B	B	B
A	A	A	B	B	A	B	B	B	B	B	A	B	B	B	A	A	A	B	B	B	B	B	B	B	B	B	A	B	B	A	A	B	A	B	B	B	B	B	B	A	B	B	B	B	B
A	A	A	B	B	A	B	B	B	B	B	A	B	B	B	A	A	A	B	B	B	B	B	B	B	B	B	A	0	B	A	A	B	A	B	B	B	B	B	B	A	B	B	B	B	B
A	A	A	B	B	A	B	B	B	B	B	A	B	B	B	A	A	A	B	B	B	B	B	B	B	B	B	A	B	B	A	A	B	A	B	B	B	B	B	B	A	B	B	B	B	B
A	A	A	B	B	A	B	B	B	B	B	A	B	B	B	A	A	A	B	B	B	B	B	B	B	B	B	A	0	B	A	A	B	A	B	B	B	B	B	B	A	B	B	B	B	B
A	A	A	0	B	A	B	B	B	0	B	A	0	B	B	A	A	A	0	0	0	B	B	0	0	B	B	A	A	B	A	A	0	A	0	0	0	0	B	0	A	B	0	0	0	0
A	A	A	B	B	A	B	B	B	B	B	A	B	B	B	A	A	A	B	B	B	B	B	B	B	B	B	A	0	B	A	A	B	A	B	B	B	B	B	B	A	B	B	B	B	B
A	A	A	B	B	A	B	B	B	B	B	A	B	B	B	A	A	A	B	B	B	B	B	B	B	B	B	A	0	B	A	A	B	A	B	B	B	B	B	B	A	B	B	B	B	B
[truncated: 1,115,029 more chars]
